# The X Chromosome of Hemipteran Insects: Conservation, Dosage Compensation and Sex-Biased Expression

**DOI:** 10.1093/gbe/evv215

**Published:** 2015-11-10

**Authors:** Arka Pal, Beatriz Vicoso

**Affiliations:** ^1^Institute of Science and Technology Austria, Klosterneuburg, Austria; ^2^Center for Ecological Sciences, Indian Institute of Science, Bangalore, India

**Keywords:** Hemiptera, dosage compensation, X chromosome, sex-biased expression

## Abstract

Insects of the order Hemiptera (true bugs) use a wide range of mechanisms of sex determination, including genetic sex determination, paternal genome elimination, and haplodiploidy. Genetic sex determination, the prevalent mode, is generally controlled by a pair of XY sex chromosomes or by an XX/X0 system, but different configurations that include additional sex chromosomes are also present. Although this diversity of sex determining systems has been extensively studied at the cytogenetic level, only the X chromosome of the model pea aphid *Acyrthosiphon pisum* has been analyzed at the genomic level, and little is known about X chromosome biology in the rest of the order.

In this study, we take advantage of published DNA- and RNA-seq data from three additional Hemiptera species to perform a comparative analysis of the gene content and expression of the X chromosome throughout this clade. We find that, despite showing evidence of dosage compensation, the X chromosomes of these species show female-biased expression, and a deficit of male-biased genes, in direct contrast to the pea aphid X. We further detect an excess of shared gene content between these very distant species, suggesting that despite the diversity of sex determining systems, the same chromosomal element is used as the X throughout a large portion of the order.

## Introduction

Despite their independent origin from ancestral autosomes, the sex chromosomes of various clades have acquired a similar appearance and biology, and understanding the forces that drive this specialization has been a longstanding goal of evolutionary biology (Charlesworth et al. 2005). After originating from a pair of autosomes, the nonrecombining Y chromosome becomes gene poor and heterochromatic (Bachtrog 2013), while the recombining X remains gene rich and euchromatic (Vicoso and Charlesworth 2006). Because gene loss on the degenerating Y chromosome can lead to reduced expression in males, the X often evolves mechanisms of dosage compensation, which regulate the expression of X-linked genes to compensate for their haploidy in males (Charlesworth 1998). A well-described example of such a mechanism is the inactivation of the X chromosome in mammals (Lyon 1974). Even in the absence of a known molecular mechanism, the presence of dosage compensation can be inferred when X-linked genes have similar expression levels in males and females, despite being present in only one copy in males (Smith et al. 2014; Mahajan and Bachtrog 2015).

The peculiar selective regime that the X is under may further lead to the acquisition of specialized gene content: because X chromosomes are transmitted through females two-thirds of the time, they will accumulate an excess of dominant mutations that are beneficial to females (Rice 1984), potentially leading to the accumulation of genes with female functions on this chromosome. On the other hand, recessive male-beneficial mutations will be immediately under positive selection in males if they are on the male haploid X (Rice 1984), and this can lead to the masculinization of the X if beneficial mutations are on average recessive.

The recent widespread application of next-generation sequencing technologies to a variety of organisms has made it possible to test these theories, but uncovered inconsistent patterns between different species. Chromosome-wide dosage compensation is generally present in male-heterogametic species (species where males carry the Y; Meyer 2005; Julien et al. 2012; Mahajan and Bachtrog 2015; Lucchesi and Kuroda 2015) and absent in female-heterogametic species (where females carry the Y; Ellegren et al. 2007; Itoh et al. 2007; Vicoso and Bachtrog 2011; Adolfsson and Ellegren 2013; Vicoso et al. 2013), but there are multiple exceptions to this rule (Julien et al. 2012; Smith et al. 2014; Schultheiß et al. 2015). Why there should be such a rule, and what drives these exceptions, is at this point not entirely clear (Mank 2013), but likely involves an interplay between pressures to maintain ancestral gene expression and various forms of genetic sexual conflict (Wright et al. 2012; Mullon et al. 2015). Even in the presence of dosage compensation, the X often carries an excess of sex-biased genes, consistent with the idea of specialized gene content. The direction of the bias, however, varies between different clades, with flies and nematodes showing a feminized X (Parisi et al. 2003; Albritton et al. 2014; Vicoso and Bachtrog 2015), and mammals a masculinized X (Lercher et al. 2003).

Nevertheless, once they are fully differentiated, both the X and Y consistently show very specialized biologies. This is thought to prevent them from reverting to autosomes (Bull 1983; Pokorná and Kratochvíl 2009), so that species with fully differentiated sex chromosomes are not expected to experience sex chromosome turnover (the appearance of a new XY pair, and reversal of either the old Y or X to an autosome). Although this view of differentiated sex chromosomes as “evolutionary traps” is well supported by many vertebrate clades, such as eutherian mammals (Waters et al. 2007), snakes (Matsubara et al. 2006), and birds (Shetty et al. 1999), dipteran insects were recently shown to have experienced multiple instances of turnover involving the ancestral differentiated X (Vicoso and Bachtrog 2015). Similarly, the high rate of Y chromosome loss and gain observed in beetles may be suggestive of frequent sex chromosome turnover in this clade (Blackmon and Demuth 2014). Whether these groups of insects represent exceptional cases, or whether the frequency of turnover has been underestimated in clades with differentiated sex chromosomes, has yet to be determined.

Hemipteran insects provide an interesting model for studying sex chromosome evolution for several reasons. First, many Hemiptera have XY sex chromosomes, with the Y showing a typical reduction in size relative to the X (with many cases of XX/X0 systems; Halkka 1960; Manna 1982; Papeschi and Bressa 2006), suggesting extensive loss of gene content on this chromosome. Theory predicts that this should drive the evolution of dosage compensation in this clade (Charlesworth 1996), making them an ideal independent group in which to test for the presence of dosage compensation. Second, it is unclear if the variation in sex determining systems that is observed, with the presence of XY, X0, and a variety of neo-sex chromosomes (Papeschi and Bressa 2006), is indicative of frequent sex chromosome turnover, as observed in dipteran insects, or if the same chromosome is used throughout the clade to determine sex.

Finally, only one hemipteran X has been studied at the genomic level: That of *Acyrthosiphon pisum*, the pea aphid, an insect with a very unusual life cycle. In this species, each yearly sexual cycle of reproduction is followed by ten generations of female-only asexual reproduction. The formation of the asexual female-only progeny from the sexual population involves the elimination of male gametes that do not carry an X, so that unlike other sex chromosomes, the X chromosome of this species is transmitted half of the time by males and half of the time by females (Jaquiéry et al. 2012). In this case, only recessive male-beneficial mutations, but not dominant female-beneficial mutations, are expected to accumulate preferentially on the X (Jaquiéry et al. 2013). The excess of male-biased expression that was observed on this chromosome was consistent with this idea, and therefore suggested to be driven by this unusual life cycle (Jaquiéry et al. 2013). However, given the diversity of gene expression patterns on the X of different clades, confirming that the male bias is specific to the pea aphid, and not a general characteristic of hemipteran X chromosomes, is important to fully support this theory.

Here, we take advantage of publicly available data to investigate several aspects of the biology of the X chromosome of hemipteran insects. In particular, males and females of three Hemiptera species (*Halyomorpha halys*, the brown marmorated stink bug; *Homalodisca vitripennis*, the glassy-winged sharpshooter; and *Oncopeltus fasciatus*, the large milkweed bug) have been sequenced both at the DNA and RNA levels (Sparks et al. 2014). *Oncopeltus fasciatus* has been shown cytogenetically to possess a pair of differentiated X and Y chromosomes (Wolfe and John 1965). Although no direct information is available for the other two species, male heterogamety with a reduced or absent Y is likely, as it represents the prevalent situation in close relatives (Halkka 1960; Nuamah 1982; Rebagliati et al. 2005; Kerisew 2012). We use the male and female DNA-seq data to identify X-derived scaffolds, as X-derived sequences show a diagnostic reduced male genomic coverage (Vicoso and Bachtrog 2011). We combine these results with the male and female RNA-seq data, and find that X-linked genes are generally expressed at similar levels between the sexes, as predicted under dosage compensation. A slight deficit of male expression is found on the X chromosome of all three species, similar to Drosophila and other fly species (Vicoso and Bachtrog 2015). We compare these results with the patterns described for the pea aphid (Jaquiéry et al. 2013), and discuss them in view of the different life histories of these organisms. Finally, we compare the gene content on these X chromosomes, and find that the X is conserved between distant Hemiptera species, supporting the idea that differentiated sex chromosomes are extremely stable, in direct contrast with what has recently been observed for Dipteran insects (Vicoso and Bachtrog 2015).

## Methods

### Genomic and RNA-seq Data Sets

Published male and female genomic reads, male and female whole-body RNA-seq reads, and genomic scaffolds were obtained for several Hemiptera species from the National Center for Biotechnology Information website. Data sets for all the species used in the study have been obtained from http://www.ncbi.nlm.nih.gov/sra/ (last accessed November 27, 2015) and the genomic scaffolds from http://www.ncbi.nlm.nih.gov/nuccore/ (last accessed November 27, 2015). All the respective accession numbers have been listed in supplementary table S2, Supplementary Material online.

### Gene Annotation, Expression Estimation, and Detection of Sex-Biased Genes

For all the species, the male and female RNA reads were mapped to the genomic scaffolds using Bowtie2 (Langmead and Salzberg 2012) with default parameters. A GTF file with the locations of putative genes was obtained by running Cufflinks (Trapnell et al. 2010) on the combined male and female SAM files, followed by the estimation with Cuffdiff (Trapnell et al. 2013) of male and female FPKM (fragments per kilobase of transcript per million mapped reads, the expression value obtained after normalization of read counts by both transcript length and number of mapped reads in each RNA-seq library; Cuffdiff further normalizes FPKM values via the median of the geometric means of fragment counts). The final normalized gene expression file is provided in supplementary data S1, Supplementary Material online. Only genes with FPKM > 1 for both male and female were considered for further analysis. Furthermore, the Cuffdiff output was utilized to determine the sex bias of all the genes in the study. Genes that showed no significant difference between the male and female FPKM (*P* > 0.05) were considered to be unbiased, whereas genes with a significant difference (*P* < 0.05) were considered either male biased (if male FPKM > female FPKM) or female biased (if male FPKM < female FPKM).

### Locating Genes on the X and Autosomes

**Fig. 1.—:**
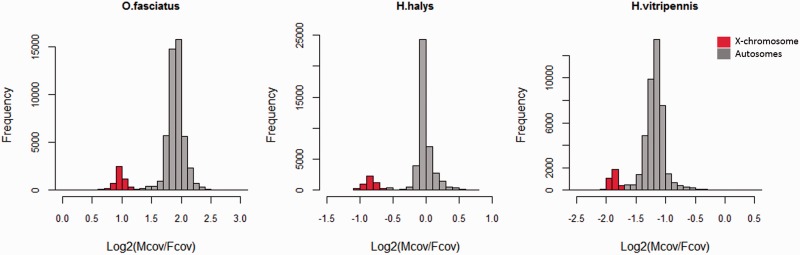
Frequency distribution of log2 of male to female ratio of genomic coverage. Genes were classified as X-linked if they were located on a scaffold that had reduced male/female coverage, as shown by the red bars, whereas the gray indicates autosomes. Data to generate this plot can be found in supplementary data S1, Supplementary Material online.

The genes detected in the previous section were assigned to the X and autosomes based on their genomic coverage patterns. For all the species except *A. pisum*, the male and female DNA reads were mapped separately to the genomic scaffolds using Bowtie2 (Langmead and Salzberg 2012) with default parameters. The resulting alignments were filtered to keep only uniquely mapped reads by selecting lines that did not match “XS:I,” as this is the Bowtie2 tag for the score of the second best alignment of the read, and is not present for reads that only have one match. This was followed by the estimation of male and female coverage from the filtered SAM files with SoapCoverage (http://soap.genomics.org.cn/soapaligner.html, last accessed November 27, 2015). The coverage values are provided in supplementary data S1, Supplementary Material online. Scaffolds with no coverage in either sex were excluded from further analyses. An R-script was used to assign genes to the X chromosomes and autosomes. First, a histogram for log2(male/female coverage) of all the genes was plotted and the highest frequency in the sample was considered to be the autosomal median, which we call “A_median.” Because males have only one copy of the X chromosome, X-linked genes are expected to show a 2-fold reduction in male coverage relative to female coverage. Therefore, the second peak of the distribution around A_median-1 should correspond to X-derived sequences (fig. 1), and all scaffolds having log2(male/female coverage) value less than A_median-0.5 were classified as X-linked. For *A. pisum*, scaffolds were assigned to either autosomes or to the X chromosome based on previously published autosomal (344 loci) and X-linked (52 loci) microsatellite markers (Jaquiéry et al. 2012, 2013). The sequences of the primers used to amplify the microsatellite markers were mapped to the genomic scaffold with BLAST (Altschul et al. 1990), thus assigning 50 scaffolds to the X and 189 to autosomes. In the eight cases when a particular primer mapped to more than one scaffold, the scaffold that matched both the forward and reverse sequence with the lowest *e*-value was accepted.

### Detection of Gene Orthology between the Species

The conservation of the X chromosome across several families of Hemiptera was examined by comparing the gene content of the X chromosome of the different species. Gene sequences for all the species except *A. pisum* were obtained from the genomic scaffolds and the cufflinks GTF file with the getfasta package of bedtools (Quinlan 2014), whereas the gene coding sequences for *A. pisum* were obtained from https://www.aphidbase.com/aphidbase/content/download/3250/33670/file/aphidbase_2.1b_mRNA.fasta.bz2 (last accessed November 27, 2015). Although even the model organism *A. pisum* has a high number of annotated genes (25,959 genes), the numbers detected for the other 3 species were much larger (>40,000 in each case), suggesting that the cufflinks annotations contain many fragmented genes. Direct comparisons between the species could therefore lead to a high extent of redundancy, as each gene might be counted more than once. Genes of all three species were therefore first mapped to the *A. pisum* gene sequences using BLAT with a translated database and query (Kent 2002), filtered for a minimum score of 50. One to one correspondence between *A. pisum* genes and the genes of each of the other species was ensured by keeping only reciprocal best hits. The relationship among the genes of other species was then extrapolated from their mutual 1:1 match with the pea aphid genes.

## Results

### Identification of X-Linked and Autosomal Genes

We analyzed the published genomes of representatives of three different families of Hemiptera in order to assess the presence and identity of their sex chromosomes, and combined this analysis with published X-linked and autosomal sequences of the model Hemiptera *A. pisum.* First, genes were identified by mapping available male and female RNA-seq reads of each species to the respective genomic scaffolds (see Methods). Second, we estimated the male and female genomic coverage of each scaffold to identify X-linked and autosomal genes in the species for which no linkage maps were available: In case of a fully differentiated Y chromosome, genomic reads map to X-derived scaffolds half as often as they do to autosomal scaffolds in male samples, but map at similar rates in female samples, as the X is present in only one copy in males but two copies in females; autosomal sequences have the same genomic coverage in both male and female samples (Vicoso and Bachtrog 2011). It should be noted that this approach only identifies the sex-specific portion of the X chromosome; pseudoautosomal sequences will be classified as autosomal, as, given the high rate of recombination in these regions, sex linkage is expected to decay very quickly. Figure 1 shows the distribution of male/female genomic coverage for the genes of each species. The presence of a secondary peak with reduced male/female coverage confirms the presence of differentiated sex chromosomes, which comprise between 8% and 11% of the total number of genes. A similar percentage (13%) of genes were X-linked in the published *A. pisum* genome, although it should be noted that in that case the assignment to the X was done using genetic markers (Jaquiéry et al. 2012), which may not cover the X and autosomes equally.

### Dosage Compensation Primarily through Upregulation of the X in Males

**Fig. 2.—:**
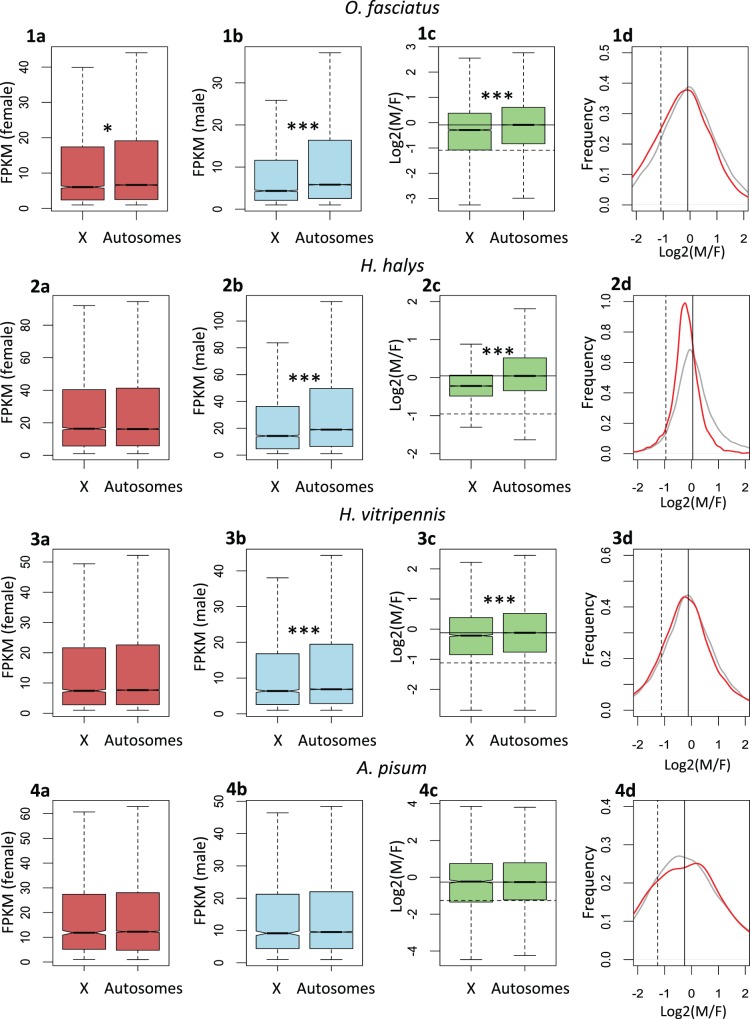
Assessing the extent and mechanism of dosage compensation in Hemiptera using RNA-seq: All boxplots and density plot represent whole-body analyses. (1*a*, 2*a*, 3*a*, 4*a*) Female expression (fragments per kilobase of transcript million mapped reads). (1*b*, 2*b*, 3*b*, 4*b*) Male expression. (1*c*, 2*c*, 3*c*, 4*c*) Log2 of male to female ratio of expression for the X and autosomes. The solid line is the autosomal median and the dashed line indicates a 2-fold reduction. (1*d*, 2*d*, 3*d*, 4*d*) The density plots show the distribution of the log2(M/F expression) for the X (denoted by the red line) and autosomes (denoted by the grey line). The solid line indicates the median for the autosomes, while the dashed line indicates a 2-fold reduction. Asterisks denote significant differences between expressions in X and autosomes: *P < 0.05, **P < 0.005, ***P < 0.001, estimated using Wilcoxon tests with Bonferroni correction. Data to generate this plot can be found in supplementary data S1, Supplementary Material online.

The independent evolution of mechanisms of dosage compensation has led to different fractions of X-linked genes being compensated in different organisms (Mank 2013), and to very different mechanisms of gene regulation to achieve dosage equalization (Disteche 2012). In order to investigate if and how dosage compensation operates in these Hemiptera species, we used published whole-body male and female RNA-seq data to obtain estimates of female and male gene expression for each of the species. We then compared the male to female ratio of expression levels of the X-linked genes with the autosomal genes (fig. 2; see also supplementary fig. S1, Supplementary Material online, for the same analysis using more stringent coverage limits, to ensure that scaffolds with intermediate coverage patterns, which are more likely to be misclassified as autosomal or X-linked, are not driving these patterns). If haploidy of the X in males resulted in a 2-fold reduction of expression, we would expect the ratio of male to female expression on the X (M/F_X_) to be half the ratio of male to female expression on the autosomes (M/F_A_) in the absence of dosage compensation. However, studies on both organisms that lack a global mechanism of dosage compensation and in Drosophila mutants for genes of the dosage compensation complex consistently find a higher value (0.5–0.8; Itoh et al. 2007; Ellegren et al. 2007; Mank and Ellegren 2009; Vicoso and Bachtrog 2011; Vicoso et al. 2013). This is thought to reflect a combination of general buffering mechanisms (Stenberg et al. 2009) as well as the equalization of expression between the sexes of specific dosage-sensitive genes on the sex chromosome (Mank and Ellegren 2009). In Hemiptera, we find that (M/F_X_)/(M/F_A_) values are above 0.8 in every species (fig. 2): 1.02 in *A. pisum*, 0.94 in *Ho. vitripennis*, 0.87 in *O. fasciatus,* and 0.83 in *Ha. halys.* This supports the presence of chromosome-wide dosage compensation, in this clade, consistent with what was previously found for the pea aphid *A. pisum* (Jaquiéry et al. 2013). Moreover, the density plot for the fold change between the male and female expression levels (fig. 2) depicts a single peak for X-linked genes in *Ho. vitripennis*, *O. fasciatus,* and *A. pisum*, with a distribution close to the autosomal distribution. This contrasts with species that lack complete dosage compensation, which often show a major peak of genes with strongly reduced expression in the heterogametic sex (Mank and Ellegren 2009; Vicoso et al. 2013), and further supports the presence of chromosome-wide mechanisms to equalize X-linked expression between the sexes in this clade. Interestingly, *Ha. halys* stands out from the other species, with a clear shift of the X chromosome toward female-biased expression (fig. 1 and supplementary table S3, Supplementary Material online). Whether this represents the absence or incompleteness of a global mechanism of dosage compensation, or the functional feminization of the X, is at this point unclear (see Discussion).

The expression levels of X-derived genes and autosomes within each sex can produce further insights into the mechanism of dosage compensation. Dosage compensation can be achieved by the downregulation of the expression levels of X-linked genes in female (as in primates; Disteche 2012) or upregulation of the same in males (as in Drosophila; Disteche 2012). If there was a downregulation of expression of the female X, both females and males should show generally lower levels of X-linked expression relative to autosomal expression; a simple upregulation of the male X should maintain similar levels of expression between the X and autosomes. For three of the species in our study (*Ha. halys*, *Ho. vitripennis,* and *A. pisum*), the female expression of the X chromosome is similar to or slightly higher than that of the autosomes, suggesting that no female X downregulation has occurred (although we cannot exclude the possibility that the ancestral level of expression of this chromosome was unusually high, and that downregulation brought it to similar expression levels as the other chromosomes), and that equalization of expression between the sexes was achieved through upregulation of the male X. Only *O. fasciatus* shows reduced levels of female expression on the X relative to the autosomes (fig. 2); while significant, this 10% decrease would be insufficient to fully equalize expression between the sexes after Y degeneration, so that even in this species, the primary mechanism of compensation seems to be an increase in male expression. Interestingly, there is a significant deficit of male expression level on the X compared with the autosomes in *O. fasciatus*, *Ha. halys,* and *Ho. vitripennis* (*P* < 0.001), suggesting that although dosage compensation is likely to occur through upregulation in males, this upregulation is not sufficient to fully reestablish a 1:1 sex ratio of expression (fig. 2).

### Conservation of Gene Content on the X Chromosome

**Fig. 3.—:**
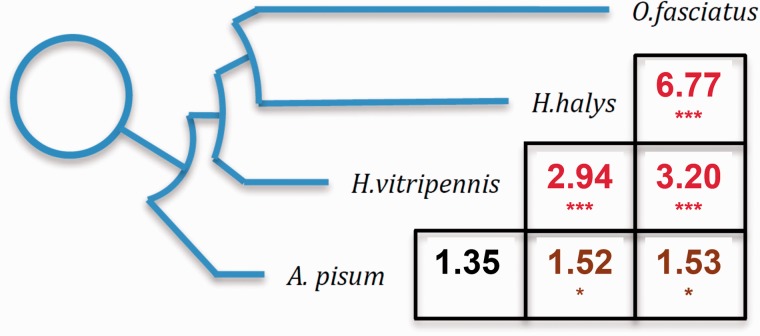
Conservation of gene content on the X. The topology of the phylogenetic tree is adapted from Bourgoin and Campbell (2002). The table indicates the observed/expected number of X-derived genes that are shared between the pairs of species. An excess of shared content indicates X chromosome conservation, while a deficit of shared X-linked genes suggests sex chromosome turnover. Asterisks denote significant excesses or deficits between the species under consideration: **P < 0.005 (denoted in brown), ***P < 0.001 (denoted in red), estimated using a chi-square test (Yates method). Data to generate this plot can be found in supplementary data S2, Supplementary Material online.

In order to test for the occurrence of sex chromosome turnover in Hemiptera, we compared the gene content of the X of the different species: Shared content indicates X chromosome conservation, while different X-linked gene sets (a deficit of shared X-linked genes) may suggest sex chromosome turnover. The four species used here cover a large fraction of the Hemiptera phylogeny (their relative topology is shown in fig. 3; Bourgoin and Campbell 2002; Wang et al. 2015): *Ha. halys* (family Pentatomidae) and *O. fasciatus* (family Lygaeidae) both fall in the suborder Heteroptera, while *Ho. vitripennis* (family Cicadellidae) and *A. pisum* (family Aphididae) are part of the paraphyletic Homoptera. The Cicadellidae are a closer outgroup to Heteroptera, and the Aphididae a basal outgroup to the other three lineages. Because *A. pisum* has a well-annotated genome, we mapped putative X-linked and autosomal genes from each species to *A. pisum* published gene sequences (and kept only reciprocal best hits). This 1:1 orthology was then used to find homologs between the genes of the 3 nonmodel species (the resulting numbers of 1:1 homologs with known chromosomal locations in both species were 1,614 for *Ha. halys–Ho. vitripennis*, 1,886 for *Ha. halys–O. fasciatus*, 2,800 for *Ho. vitripennis–O. fasciatus*, 3,397 for *O. fasciatus–A. pisum*, 1,973 for *Ha. halys–A. pisu*m, and 3,185 for *Ho. vitripennis–A. pisum*).

For each pair of species, we tested for an excess of shared X-linked genes using a 2 × 2 contingency table (X, Autosome/Species 1, Species 2). The observed numbers of shared X-linked genes between the different species, normalized by the respective expected numbers, are shown in figure 3 (specific gene numbers and *P* values obtained using chi-square tests with a Yate’s correction are provided in supplementary table S4, Supplementary Material online). Because applying a Yate’s correction can lead to inflated type II error rates, we also used a Monte Carlo approach to obtain significance values for our observations (supplementary fig. S2, Supplementary Material online); the resulting *P* values support the patterns shown in figure 3. We found a significant excess of shared X-linked genes for all comparisons between the three most closely related species, consistent with the idea that they share the same ancestral X chromosome, and that no turnover has occurred within this group. A small but significant excess of shared X-linked genes is also observed between *A. pisum* and *O. fasciatus* and *A. pisum* and *Ha. halys*, suggesting that *A. pisum* may also share the same X chromosome*.* The comparison between *A. pisum* and *Ho. vitripennis*, on the other hand, did not yield a significant excess or deficit of X-linked genes. A loss of power to detect chromosomal homologies is expected with increasing distance between species, as larger numbers of interchromosomal rearrangements become differentially fixed between them. Rates of protein-coding evolution can further differ between the X and autosomes (Meisel and Connallon 2013), potentially making it more difficult to detect conservation of gene content on this chromosome over long time distances. This is likely to account for the reduced enrichment of shared X-linked genes in comparisons involving *A. pisum*, because *A. pisum* represents a distant outgroup to the other 3 species (fig. 3).

### Sex-Biased Gene Expression on the X Chromosome

**Fig. 4.—:**
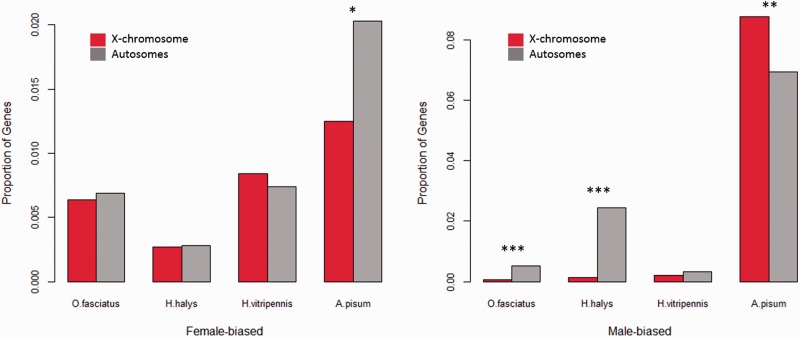
Proportion of male and female biased genes on the X and autosomes. Asterisks denote significant differences between proportions of biased genes on X and autosomes of the species: *P < 0.05, **P < 0.005, ***P < 0.001, estimated using a chi-square test (Yates method). Data to generate this plot can be found in supplementary table S1, Supplementary Material online.

Contrary to what was observed in other insects, the X chromosome of *A. pisum* was shown to harbor an excess of male-biased genes; this was suggested to be a consequence of its peculiar reproductive biology, which should promote the accumulation of male-beneficial mutations on this chromosome (Jaquiéry et al. 2013). In order to confirm that this excess of male-biased genes was a feature of only *A. pisum*, and not Hemiptera in general, we compared the proportion of significantly sex-biased genes found on the X and autosomes of each of the four species (fig. 4). As previously described, the X of *A. pisum* is masculinized, and shows both an excess of male-biased genes (*P* = 0.003) and a deficit of female-biased genes (*P* = 0.02) compared with the autosomes. The opposite pattern is found in the other Hemiptera: In *O. fasciatus* and *Ha. halys*, there is a significant deficit of male-biased genes on the X relative to the autosomes (*P* = 0.0001). The X chromosome of *Ho. vitripennis* also harbors a lower percentage of male-biased genes than do its autosomes, but the difference is not significant. No significant differences were detected between the proportion of female-biased genes on the X and autosomes of any of the three species (fig. 4).

## Discussion

Using published DNA and RNA-seq data, we were able to perform the first comparative genomic analysis of the X chromosome of several Hemiptera families. The karyotype of *O. fasciatus* had previously been described and consists of seven pairs of autosomes and a pair of sex chromosomes (Wolfe and John 1965). No direct evidence was available for the other two species. However, other described species in the genus Halyomorpha show either six or seven pairs of autosomes and a pair of sex chromosomes (Nuamah 1982; Rebagliati et al. 2005; Kerisew 2012), so that we expected *Ha. halys* to also possess a differentiated XY pair. Similarly, most members of the family Cicadellidae are XX-X0 (Halkka 1960), making this a likely configuration for *Ho. vitripennis.* Consistent with this cytogenetic literature, we find that a significant proportion of the genome has reduced coverage in males (8–11% of expressed genes were classified as X-linked) in all three species, as expected in the presence of a large X chromosome and a differentiated or absent Y.

We further found an excess of genes classified as X-linked in more than one species, suggesting that the same chromosomal element constitutes the X chromosome of these highly diverged clades: Heteroptera (*O. fasciatus* and *H*a*. halys*) and Cicadellidae (*H*o*. vitripennis*) are estimated to have split over 240 Ma (Li et al. 2012). The published genome of the pea aphid (International Aphid Genomics Consortium 2010) provides an even more distant outgroup; in this case, we find a significant enrichment of shared X-linked genes with only two species. This is most likely due to the occurrence of too many interchromosomal rearrangements since the species split for direct chromosome equivalences to be detected in the *A. pisum–Ho. vitripennis* comparison (if different chromosomes were X-linked, there should be a significant deficit of shared X-linked genes, which we do not observe).

Even if we do not consider this more distant outgroup, the conservation of the X over 240 Myr of evolution is in sharp contrast to what was found in Dipteran insects, where over a similar time span sex chromosome turnover has occurred multiple times (Vicoso and Bachtrog 2015). One possibility is that the difference is simply due to sampling, as although the available species were sampled widely from the Hemiptera phylogeny, they represent a much smaller proportion of families than was investigated for Diptera. However, it is also possible that the ancestral X of dipterans is unusually prone to reverting to an autosomal state. In particular, contrary to the hemipteran X, which contains ∼10% of genes, the ancestral X of Diptera is extremely small (∼100 genes, about 0.5% of the total). If there is a phenotypic cost to reverting X chromosomes to autosomes, this cost should become larger as the number of genes on the X increases, and larger X chromosomes are expected to be more stable. This is consistent with the reported stability of the gene-rich X chromosomes of nematodes (Albritton et al. 2014) and a variety of vertebrate species (Shetty et al. 1999; Matsubara et al. 2006; Waters et al. 2007; Rovatsos et al. 2014). It should also be noted that despite the frequent transitions of sex chromosomes in Diptera, once one of the large chromosomes became X-linked, no further turnover was observed (Vicoso and Bachtrog 2015), though again this could represent a sampling issue, as in most families only one species was investigated. On the other hand, further autosomes have fused to the X, creating a variety of neo-X and neo-Y chromosomes (Steinemann M and Steinemann S 1998; Schlötterer 2000; Bachtrog 2006; Vicoso and Bachtrog 2015). Our analysis does not allow us to test for this possibility, but the fact that a very similar percentage of genes are classified as X-linked in each of the species may be a first suggestion that fusions with large autosomes did not occur in these lineages.

The stability of the X throughout the clade is consistent with the idea that its unusual biology may promote its conservation: In every species examined, there was some evidence both of dosage compensation and of specialized sex-biased gene content. In particular, the ratio of male/female expression for the X chromosome was in every case higher than 80% of the value of the autosomes, an upper bound for species with no global mechanism of dosage compensation (Ellegren et al. 2007; Itoh et al. 2007; Mank and Ellegren 2009; Vicoso and Bachtrog 2011; Vicoso et al. 2013), with most studies using adult tissues finding values closer to 60–70% (Ellegren et al. 2007; Mank and Ellegren 2009; Vicoso and Bachtrog 2011; Vicoso et al. 2013; presumably because unequal expression between the sexes is more detrimental during early development). Interestingly, this value was only 83% for *Ha. halys*, and the distribution of expression of X-linked genes shows a strong shift toward female-biased expression (fig. 1 and supplementary table S3, Supplementary Material online). Although a deficit of male-biased genes, rather than a lack of fully compensated genes, seems to be primarily driving this pattern (supplementary table S3, Supplementary Material online), we can at this point not exclude the possibility that, in this species, dosage compensation is incomplete or localized rather than chromosome wide. *Halyomorpha halys* may therefore represent an interesting intermediate between the full dosage compensation of mammals and flies, and the few equalized genes of birds, schistosomes, and snakes, potentially yielding further information on what drives the evolution of one versus the other. Finally, the fact that female expression is similar on the X and autosomes in three of the four species, and shows only a small decrease (∼10%) in *O. fasciatus*, suggests that in this group downregulation of the female X is not the primary mechanism used to achieve dosage compensation. Instead, males seem to have upregulated their single X, similar to what has been described in other insects (Vicoso and Bachtrog 2015).

Despite the presence of some level of dosage compensation, and contrary to what was found in the pea aphid (Jaquiéry et al. 2013), the X chromosomes of the other three hemipteran species showed an excess of female-biased expression, and a deficit of male-biased genes (significant for only two of the species). A similar feminization of the X has been observed in many fly species (Sturgill et al. 2007; Vicoso and Bachtrog 2015), and found to be largely caused by a deficit of testis expression for X-linked genes. What drives this deficit is still a matter of controversy: It has been suggested that, similar to what happens in mammals (Lifschytz and Lindsley 1972), the X chromosome of flies may be inactivated during male meiosis (Vibranovski et al. 2009). However, unlike in mammals, there is no direct observation of an inactivated X in spermatocytes, and the expression of X-linked genes, although reduced, is still widespread during meiosis (Mikhaylova and Nurminsky 2011). An alternative hypothesis is that the absence of dosage compensation in the testis may instead be driving the apparent feminization of the X (Meiklejohn et al. 2011). In Hemiptera, cytogenetics do show evidence of inactivation of the X in spermatocytes of several species (Messthaler and Traut 2014), and this is likely to contribute to the observed feminization in this clade. Future studies looking at the expression of somatic and gonadal tissues will determine if a deficit of expression of X-linked genes in the testis is indeed causing the feminization of the X, and whether these patterns are consistent with full X-inactivation during male meiosis. More generally, whole-body comparisons between the sexes are limited, as differences in allometry between males and females combined with the relative nature of RNA-seq expression estimates can lead to biases in the analysis (Perry et al. 2014). Further comparisons using different male and female tissues will be required to confirm the extent and consistency of dosage compensation and of feminization of the X chromosome in Hemiptera.

Despite these caveats, the general female bias of the hemipteran X confirms that the masculizination of the X of the pea aphid *A. pisum* (Jaquiéry et al. 2013) is indeed specific to that species. Because the pea aphid X is transmitted half of the time through females (as opposed to two-thirds in other Hemiptera), this supports the hypothesis that sex-specific selection may drive patterns of gene expression on X chromosomes. Such an effect is expected if male-biased expression is driven to some extent by the accumulation of recessive mutations that are beneficial to males at the expense of females, followed by the modulation of expression to optimize the male benefit and reduce the female cost, as initially proposed by Rice (1984). The masculinization of the pea aphid X further shows that the accumulation of recessive male-beneficial mutations may be sufficient to masculinize the X, despite the potential effects of meiotic X-inactivation and/or lack of dosage compensation in the testis, as long as the X is not transmitted primarily through females. The feminization observed for other species is therefore likely to be influenced not only by their biology, but also by their female-biased selective regimes, and comparisons of further species with unusual life histories provide a promising approach to disentangling these parameters.

## Supplementary Material

Supplementary data S1–S3, figure S1 and S2, and tables S1–S4 are available at *Genome Biology and Evolution* online (http://www.gbe.oxfordjournals.org/).

Supplementary Data
