# Supplementary material for: The X Chromosome of Hemipteran Insects: Conservation, Dosage Compensation and Sex-Biased Expression
Source: Genome Biol Evol. 2015 Nov 10;7(12):3259–68. doi: 10.1093/gbe/evv215 (PMC4700948; doi:10.1093/gbe/evv215)
Supplement: Supplementary Data [file supp_7_12_3259__index.html]

The X chromosome of hemipteran insects: conservation, dosage compensation and sex-biased expression — The X Chromosome of Hemipteran Insects: Conservation, Dosage Compensation and Sex-Biased Expression — Supplementary Data 

# The X Chromosome of Hemipteran Insects: Conservation, Dosage Compensation and Sex-Biased Expression

## Supplementary Data

files

- Supplementary Data - zip file
