# Supplementary material for: The X Chromosome of Hemipteran Insects: Conservation, Dosage Compensation and Sex-Biased Expression
Source: Genome Biol Evol. 2015 Nov 10;7(12):3259–68. doi: 10.1093/gbe/evv215 (PMC4700948; doi:10.1093/gbe/evv215)
Supplement: Supplementary Data [file supp_evv215_suppl_data.zip › S4 Table (rev) Sheet1.pdf]

A.pisum* vs *H.halys

observed		*H.halys* X-Chromosome	Autosome	TOTAL	expected	X-Chromosome	Autosome	p-value
A.pisum	X-Chromosome	23	122	**145**	X-Chromosome	15.14	129.86	0.034
	Autosome	183	1645	**1828**	Autosome	190.86	1637.14	
	TOTAL	**206**	**1767**					

A.pisum* vs *H.vitripennis

observed		*H.vitripennis* X-Chromosome	Autosome	TOTAL	expected	X-Chromosome	Autosome	p-value
A.pisum	X-Chromosome	22	193	215	X-Chromosome	16.2	198.8	0.156
	Autosome	218	2752	2970	Autosome	223.8	2746.2	
	TOTAL	240	2945					

A.pisum* vs *O.fasciatus

observed		*O.fasciatus* X-Chromosome	Autosome	TOTAL	expected	X-Chromosome	Autosome	p-value
A.pisum	X-Chromosome	33	211	244	X-Chromosome	21.62	222.38	0.011
	Autosome	268	2885	3153	Autosome	279.38	2873.62	
	TOTAL	301	3096					

H.halys vs H.vitripennis

		H.vitripennis						
	**observed**	X-Chromosome	Autosome	**TOTAL**	**expected**	X-Chromosome	Autosome	**p-value**
H.halys	X-Chromosome	35	128	163	X-Chromosome	11.92	151.08	<0.0001
	Autosome	83	1368	1451	Autosome	106.08	1344.92	
	**TOTAL**	118	1496					

H.vitripennis vs O.fasciatus

		O.fasciatus						
	**observed**	X-Chromosome	Autosome	**TOTAL**	**expected**	X-Chromosome	Autosome	**p-value**
H.vitripenni	X-Chromosome	58	148	206	X-Chromosome	18.1	187.9	<0.0001
	Autosome	188	2406	2594	Autosome	227.9	2366.1	
	**TOTAL**	246	2554					

H.halys vs O.fasciatus

		O.fasciatus						
	**observed**	X-Chromosome	Autosome	**TOTAL**	**expected**	X-Chromosome	Autosome	**p-value**
H.halys	X-Chromosome	102	80	182	X-Chromosome	15.05	166.95	<0.0001
	Autosome	54	1650	1704	Autosome	140.95	1563.05	
	**TOTAL**	156	1730					
