# Supplementary material for: The X Chromosome of Hemipteran Insects: Conservation, Dosage Compensation and Sex-Biased Expression
Source: Genome Biol Evol. 2015 Nov 10;7(12):3259–68. doi: 10.1093/gbe/evv215 (PMC4700948; doi:10.1093/gbe/evv215)
Supplement: Supplementary Data [file supp_evv215_suppl_data.zip › S1 Data (rev) A.pisum (autosomes).pdf]

scaffold	gene	expF	expM	log2(fold_change)	p-value	significance
gi|320439876|ref|NW_003390392.1|	1020-3077	103747	152475	0.555509	0.7359	no
gi|320440089|ref|NW_003390179.1|	2198-2886	0.745279	203586	144979	0.375	no
gi|320441816|ref|NW_003388676.1|	49-796	0.329885	248648	291407	0.25645	no
gi|320442095|ref|NW_003388397.1|	13240-14349	283973	17518	-0.696918	0.6633	no
gi|320442095|ref|NW_003388397.1|	15653-17412	37877	372593	-0.0237208	0.9842	no
gi|320442095|ref|NW_003388397.1|	17514-18636	0.761384	105433	0.469637	1	no
gi|320442095|ref|NW_003388397.1|	19763-22146	238062	298975	0.328689	0.8041	no
gi|320442095|ref|NW_003388397.1|	22226-25203	308951	99391	-163619	0.2229	no
gi|320442095|ref|NW_003388397.1|	26404-27281	473506	0.724606	-270811	0.2696	no
gi|320442095|ref|NW_003388397.1|	2739-5186	212098	362299	0.772448	0.55875	no
gi|320442095|ref|NW_003388397.1|	30953-31489	15022	44952	-174062	0.4244	no
gi|320442095|ref|NW_003388397.1|	31845-32132	619212	163825	-191828	0.3826	no
gi|320442095|ref|NW_003388397.1|	37093-40149	287136	13761	-106115	0.41735	no
gi|320442095|ref|NW_003388397.1|	40654-45437	180684	426175	-208396	0.26535	no
gi|320442095|ref|NW_003388397.1|	45882-47851	710536	739719	0.0580694	0.97765	no
gi|320442095|ref|NW_003388397.1|	5315-6405	535158	48376	-0.145675	0.91085	no
gi|320442095|ref|NW_003388397.1|	6842-12143	115244	14188	0.299983	0.82195	no
gi|320442841|ref|NW_003387651.1|	8052-8454	287226	441298	0.619566	0.74255	no
gi|320442886|ref|NW_003387606.1|	3869-5093	102421	0.946917	-0.113204	1	no
gi|320442970|ref|NW_003387522.1|	[illegible]47055-14774[illegible]	11082	353272	167256	0.43145	no
gi|320442970|ref|NW_003387522.1|	[illegible]79597-18027[illegible]	189643	119086	265064	0.25515	no
gi|320442970|ref|NW_003387522.1|	[illegible]80403-18274[illegible]	34393	122848	183669	0.2767	no
gi|320442970|ref|NW_003387522.1|	[illegible]85769-18594[illegible]	562784	951609	0.757787	0.7189	no
gi|320442970|ref|NW_003387522.1|	[illegible]92658-19502[illegible]	775022	2816	-146059	0.50065	no
gi|320442970|ref|NW_003387522.1|	21585-25426	159104	320968	101246	0.6332	no
gi|320442970|ref|NW_003387522.1|	41387-41624	228856	26463	0.209532	0.9135	no
gi|320442970|ref|NW_003387522.1|	42201-42359	343734	240079	-0.517784	0.79215	no
gi|320443077|ref|NW_003387415.1|	11062-18152	153838	605694	-134475	0.3179	no
gi|320443077|ref|NW_003387415.1|	1420-2757	398288	0.425439	-322679	0.21755	no

gi|320443077|ref|NW_003387415.1|	19253-19812	212421	23086	0.120095	0.9529	no
gi|320443077|ref|NW_003387415.1|	30027-30320	343189	452518	0.398974	0.8397	no
gi|320443077|ref|NW_003387415.1|	30889-31531	37703	603446	0.678543	0.6745	no
gi|320443077|ref|NW_003387415.1|	3260-4224	122768	0.479662	-467777	0.14225	no
gi|320443077|ref|NW_003387415.1|	6499-7681	115881	0.370643	-496646	0.1415	no
gi|320443601|ref|NW_003386891.1|	1566-1934	474704	529522	-316427	0.2017	no
gi|320443601|ref|NW_003386891.1|	9589-10551	246211	545088	-217534	0.3353	no
gi|320443845|ref|NW_003386647.1|	3917-4135	668957	484112	-0.466574	0.8131	no
gi|320443967|ref|NW_003386529.1|	3813-5865	278658	423044	0.602314	0.64785	no
gi|320444560|ref|NW_003385937.1|	261-1217	117105	484896	204987	0.3545	no
gi|320444560|ref|NW_003385937.1|	3562-4880	0.311754	357077	351776	0.18305	no
gi|320444594|ref|NW_003385903.1|	11306-12206	139673	237998	0.768893	0.7255	no
gi|320444594|ref|NW_003385903.1|	12420-14545	0.62508	0.622985	-0.0048436	1	no
gi|320444594|ref|NW_003385903.1|	14771-15587	319779	229748	284491	0.22435	no
gi|320444594|ref|NW_003385903.1|	19253-23778	865329	231926	142234	0.28715	no
gi|320444594|ref|NW_003385903.1|	26191-27710	362838	228015	265173	0.0534	no
gi|320444594|ref|NW_003385903.1|	8112-8467	226005	310726	0.459285	0.82175	no
gi|320444666|ref|NW_003385831.1|	10923-16281	63568	157843	131212	0.32105	no
gi|320444666|ref|NW_003385831.1|	20013-20874	0.539579	55708	336798	0.19285	no
gi|320444666|ref|NW_003385831.1|	34435-37029	186001	124441	-0.57985	0.6488	no
gi|320444666|ref|NW_003385831.1|	38270-38986	400222	156109	-135825	0.5458	no
gi|320444666|ref|NW_003385831.1|	42217-44436	765584	11873	-268888	0.24905	no
gi|320444666|ref|NW_003385831.1|	4293-5445	846926	26145	162623	0.3218	no
gi|320444666|ref|NW_003385831.1|	460-4169	267156	148545	247515	0.17355	no
gi|320444666|ref|NW_003385831.1|	5597-6844	103428	238355	120448	0.4577	no
gi|320444666|ref|NW_003385831.1|	8219-8760	109519	114365	0.0624601	0.9726	no
gi|320444706|ref|NW_003385791.1|	1835-4045	291394	218259	-0.416927	0.7523	no
gi|320444706|ref|NW_003385791.1|	24060-25055	57767	138121	-206431	0.3356	no
gi|320444706|ref|NW_003385791.1|	35359-42366	411111	277731	-0.565839	0.73545	no
gi|320444706|ref|NW_003385791.1|	42517-43343	442965	235763	-0.909859	0.58075	no

gi|320444706|ref|NW_003385791.1|	4457-4662	591713	205251	-152751	0.49515	no
gi|320444706|ref|NW_003385791.1|	44956-45806	271874	130376	-106026	0.63465	no
gi|320444706|ref|NW_003385791.1|	45953-46838	545618	304237	-0.842695	0.6885	no
gi|320444706|ref|NW_003385791.1|	46950-49381	504139	481915	-0.0650431	0.95905	no
gi|320444706|ref|NW_003385791.1|	4777-6719	121615	634156	-0.93941	0.555	no
gi|320444706|ref|NW_003385791.1|	51585-56531	693215	851561	0.296807	0.50045	no
gi|320444706|ref|NW_003385791.1|	65762-66164	507432	378255	-374578	0.16075	no
gi|320444706|ref|NW_003385791.1|	66303-67331	958173	176614	-243969	0.2883	no
gi|320444706|ref|NW_003385791.1|	71257-72902	124498	308303	-201371	0.3633	no
gi|320444706|ref|NW_003385791.1|	74050-77101	248095	917623	-143492	0.2756	no
gi|320444706|ref|NW_003385791.1|	79080-84725	695521	356237	-0.965256	0.57005	no
gi|320444706|ref|NW_003385791.1|	845-1135	809478	265639	-160753	0.4467	no
gi|320444706|ref|NW_003385791.1|	84935-85512	140577	199503	-281688	0.1307	no
gi|320444706|ref|NW_003385791.1|	88841-92287	241744	290816	0.266628	0.84325	no
gi|320444706|ref|NW_003385791.1|	92445-94479	58541	729529	0.317518	0.81495	no
gi|320444706|ref|NW_003385791.1|	9392-9797	461953	21741	-108733	0.6029	no
gi|320444706|ref|NW_003385791.1|	94848-96990	312673	543061	0.79646	0.71155	no
gi|320444706|ref|NW_003385791.1|	9859-10340	114634	450531	-134734	0.5183	no
gi|320444861|ref|NW_003385636.1|	13190-15576	174021	790445	21834	0.16875	no
gi|320444878|ref|NW_003385619.1|	106298-10763[illegible]	107524	330606	162045	0.44585	no
gi|320444878|ref|NW_003385619.1|	90742-93032	22995	722305	165129	0.4482	no
gi|320444889|ref|NW_003385608.1|	3806-5000	0	244042	inf	0.0162	no
gi|320445004|ref|NW_003385493.1|	14106-15157	206921	0.286181	-285408	0.2709	no
gi|320445004|ref|NW_003385493.1|	15623-17241	501618	0.0849377	-920597	0.25045	no
gi|320445004|ref|NW_003385493.1|	17375-18477	412849	0.53916	-625876	0.12965	no
gi|320445004|ref|NW_003385493.1|	6518-7526	238101	194696	-0.290348	0.894	no
gi|320445004|ref|NW_003385493.1|	7731-13458	362845	266965	-376463	0.0735	no
gi|320445079|ref|NW_003385418.1|	132615-13315[illegible]	190456	267686	0.491084	0.81575	no
gi|320445079|ref|NW_003385418.1|	1525-1724	560006	651154	-310437	0.1937	no
gi|320445079|ref|NW_003385418.1|	180891-18117[illegible]	138567	191532	0.467006	0.8167	no

gi|320445079|ref|NW_003385418.1|	181270-18196[illegible]	111062	202296	0.86511	0.6867	no
gi|320445079|ref|NW_003385418.1|	182339-18449[illegible]	730723	202708	1472	0.39085	no
gi|320445079|ref|NW_003385418.1|	184595-18759[illegible]	512668	353308	278483	0.1469	no
gi|320445079|ref|NW_003385418.1|	27070-27811	180245	571623	-165683	0.43455	no
gi|320445079|ref|NW_003385418.1|	28632-28846	386856	138467	-148226	0.5084	no
gi|320445079|ref|NW_003385418.1|	29410-31939	382545	411078	0.103783	0.9616	no
gi|320445079|ref|NW_003385418.1|	42486-43029	108832	476701	213098	0.29365	no
gi|320445079|ref|NW_003385418.1|	50286-50977	0.370205	581602	397363	0.1967	no
gi|320445079|ref|NW_003385418.1|	72179-72664	132833	444022	174102	0.3346	no
gi|320445079|ref|NW_003385418.1|	76171-76647	0.687433	45893	273898	0.2661	no
gi|320445079|ref|NW_003385418.1|	88611-89518	0.753912	128174	408757	0.1636	no
gi|320445111|ref|NW_003385386.1|	4143-5119	79531	472019	-40746	0.0534	no
gi|320445111|ref|NW_003385386.1|	6964-8049	508513	481067	-340197	0.08225	no
gi|320445163|ref|NW_003385334.1|	26328-27313	245409	559735	-213237	0.34365	no
gi|320445163|ref|NW_003385334.1|	28404-30757	16016	178017	-316942	0.10015	no
gi|320445163|ref|NW_003385334.1|	5877-7596	0.454952	0.950447	106289	1	no
gi|320445278|ref|NW_003385293.1|	52446-53021	0.49435	802423	402076	0.1929	no
gi|320445363|ref|NW_003385208.1|	24646-29100	261968	949432	-146426	0.27605	no
gi|320445363|ref|NW_003385208.1|	29306-30695	394959	406468	0.041441	0.9814	no
gi|320445363|ref|NW_003385208.1|	30973-31584	519624	249321	-105946	0.63985	no
gi|320445363|ref|NW_003385208.1|	32053-33106	340584	177005	-0.944221	0.57105	no
gi|320445363|ref|NW_003385208.1|	36043-37129	164676	205933	-299938	0.21215	no
gi|320445363|ref|NW_003385208.1|	38757-39861	841282	154657	-244352	0.19285	no
gi|320445363|ref|NW_003385208.1|	41840-42762	172068	661019	-138022	0.52335	no
gi|320445363|ref|NW_003385208.1|	43584-44936	347597	15742	-11428	0.5843	no
gi|320445363|ref|NW_003385208.1|	45138-46009	204468	113354	-0.851045	0.7008	no
gi|320445363|ref|NW_003385208.1|	46083-46961	10506	108525	0.0468186	1	no
gi|320445370|ref|NW_003385201.1|	102061-10276[illegible]	143093	220136	0.621442	0.753	no
gi|320445370|ref|NW_003385201.1|	103024-10384[illegible]	257642	452629	0.81296	0.6889	no
gi|320445370|ref|NW_003385201.1|	104057-10756[illegible]	989296	155375	0.651278	0.617	no

gi|320445370|ref|NW_003385201.1|	11803-12613	13806	201823	-277414	0.234	no
gi|320445370|ref|NW_003385201.1|	124141-12471[illegible]	247175	167171	-0.564205	0.8006	no
gi|320445370|ref|NW_003385201.1|	133203-13384[illegible]	166536	708281	208849	0.3476	no
gi|320445370|ref|NW_003385201.1|	2161-2707	19407	218042	-31539	0.21205	no
gi|320445370|ref|NW_003385201.1|	81390-82442	163267	271544	-258798	0.2664	no
gi|320445383|ref|NW_003385188.1|	9659-10845	0.887637	369103	205598	0.35365	no
gi|320445414|ref|NW_003385157.1|	11214-12781	0.126723	308562	46058	0.1773	no
gi|320445414|ref|NW_003385157.1|	30012-30378	351587	688794	0.970188	0.6698	no
gi|320445414|ref|NW_003385157.1|	36835-37680	0.83062	342844	204529	0.3471	no
gi|320445414|ref|NW_003385157.1|	37782-38636	0.272843	281629	336765	0.22635	no
gi|320445414|ref|NW_003385157.1|	38740-43465	0.263066	592223	449264	0.0663	no
gi|320445414|ref|NW_003385157.1|	44969-45433	0.720963	432335	258415	0.27405	no
gi|320445414|ref|NW_003385157.1|	49988-54086	174726	669697	193841	0.25545	no
gi|320445414|ref|NW_003385157.1|	54664-56634	291833	147181	233438	0.1802	no
gi|320445451|ref|NW_003385120.1|	35210-35723	0.600511	306357	567288	0.15895	no
gi|320445451|ref|NW_003385120.1|	48362-49348	0	361798	inf	5.00E-05	yes
gi|320445451|ref|NW_003385120.1|	50300-51006	0	735292	inf	0.00935	no
gi|320445451|ref|NW_003385120.1|	52276-52956	189643	315836	405781	0.1454	no
gi|320445451|ref|NW_003385120.1|	53365-55372	428606	151435	-150095	0.2537	no
gi|320445451|ref|NW_003385120.1|	57438-62025	124073	133866	343153	0.08075	no
gi|320445451|ref|NW_003385120.1|	62310-63654	410994	184545	548871	0.0252	no
gi|320445451|ref|NW_003385120.1|	6517-6935	238613	17516	-376793	0.1698	no
gi|320445451|ref|NW_003385120.1|	7770-9305	240212	577987	-20552	0.23705	no
gi|320445489|ref|NW_003385082.1|	102704-10694[illegible]	113701	949389	306175	0.1072	no
gi|320445489|ref|NW_003385082.1|	28027-28525	189926	432549	118743	0.58175	no
gi|320445489|ref|NW_003385082.1|	36265-38627	166623	581661	180359	0.418	no
gi|320445489|ref|NW_003385082.1|	38756-41847	17726	111141	264844	0.1453	no
gi|320445489|ref|NW_003385082.1|	44963-46746	327101	126845	195525	0.3899	no
gi|320445489|ref|NW_003385082.1|	72600-73803	0.348815	241801	279329	0.2345	no
gi|320445491|ref|NW_003385080.1|	100738-10286[illegible]	793065	360573	-113715	0.60135	no

gi|320445491|ref|NW_003385080.1|	104876-105578	325387	345974	0.0885096	0.95615	no
gi|320445491|ref|NW_003385080.1|	106142-108891	83842	492163	-0.768537	0.62985	no
gi|320445491|ref|NW_003385080.1|	109231-111177	228851	306714	0.422486	0.7456	no
gi|320445491|ref|NW_003385080.1|	120369-121745	248554	174697	-0.508707	0.75555	no
gi|320445491|ref|NW_003385080.1|	121902-122890	0.672744	170359	134045	0.52435	no
gi|320445491|ref|NW_003385080.1|	123586-126574	404567	261417	-0.630026	0.76985	no
gi|320445491|ref|NW_003385080.1|	126688-127130	118671	840163	-0.498224	0.8017	no
gi|320445491|ref|NW_003385080.1|	130387-132165	0.109387	0.914388	306336	1	no
gi|320445491|ref|NW_003385080.1|	14325-15749	283945	0.591937	-22621	0.3296	no
gi|320445491|ref|NW_003385080.1|	150717-151619	273445	141332	-0.952166	0.6695	no
gi|320445491|ref|NW_003385080.1|	15847-29079	585471	608486	0.0556259	0.96465	no
gi|320445491|ref|NW_003385080.1|	171275-172612	100797	229737	-21334	0.1153	no
gi|320445491|ref|NW_003385080.1|	175728-176568	273581	729646	-19067	0.3941	no
gi|320445491|ref|NW_003385080.1|	176746-177989	224426	220704	-334605	0.09495	no
gi|320445491|ref|NW_003385080.1|	186027-187884	332984	116025	-152102	0.38695	no
gi|320445491|ref|NW_003385080.1|	191844-193547	26215	20015	-371124	0.0686	no
gi|320445491|ref|NW_003385080.1|	205416-206315	0.254335	157732	263267	0.26345	no
gi|320445491|ref|NW_003385080.1|	208006-208674	272793	348154	367384	0.1504	no
gi|320445491|ref|NW_003385080.1|	209692-210590	101887	131638	369153	0.1747	no
gi|320445491|ref|NW_003385080.1|	212317-212991	115328	228183	430638	0.16155	no
gi|320445491|ref|NW_003385080.1|	214615-215202	286728	595439	43762	0.07675	no
gi|320445491|ref|NW_003385080.1|	222010-224875	111648	304399	144701	0.38325	no
gi|320445491|ref|NW_003385080.1|	224996-234099	468049	197365	207614	0.1249	no
gi|320445491|ref|NW_003385080.1|	236309-237929	134059	0.593713	-117503	1	no
gi|320445491|ref|NW_003385080.1|	238291-239532	404435	324723	-0.316697	0.80965	no
gi|320445491|ref|NW_003385080.1|	244784-247170	0	117781	inf	5.00E-05	yes
gi|320445491|ref|NW_003385080.1|	248319-251012	0	335813	inf	5.00E-05	yes
gi|320445491|ref|NW_003385080.1|	36096-36820	203758	0.472722	-542972	0.1863	no
gi|320445491|ref|NW_003385080.1|	64119-64758	125207	227185	0.859551	0.6872	no
gi|320445491|ref|NW_003385080.1|	67398-74233	636574	460589	-0.466848	0.70815	no

gi|320445491|ref|NW_003385080.1|	75091-75810	767593	143236	-242195	0.28925	no
gi|320445491|ref|NW_003385080.1|	76236-77464	476039	106104	-21656	0.32675	no
gi|320445491|ref|NW_003385080.1|	77783-78288	493979	124208	-199169	0.30275	no
gi|320445491|ref|NW_003385080.1|	79814-80833	839957	133958	-264854	0.2403	no
gi|320445491|ref|NW_003385080.1|	81340-82256	495959	0.683837	-28585	0.25675	no
gi|320445491|ref|NW_003385080.1|	83098-83636	663395	148923	-21553	0.3524	no
gi|320445491|ref|NW_003385080.1|	87552-89140	948143	0.520826	-418623	0.1489	no
gi|320445495|ref|NW_003385076.1|	19368-19849	0	58569	inf	0.02915	no
gi|320445495|ref|NW_003385076.1|	47537-48658	133393	910376	277078	0.238	no
gi|320445498|ref|NW_003385073.1|	101259-10287	347837	386467	0.151931	0.90895	no
gi|320445498|ref|NW_003385073.1|	145-948	0.892109	100086	348788	0.19245	no
gi|320445498|ref|NW_003385073.1|	26183-29112	745425	157205	107651	0.52515	no
gi|320445498|ref|NW_003385073.1|	29889-30887	139423	162047	0.216941	0.91955	no
gi|320445498|ref|NW_003385073.1|	33320-34902	75187	76725	0.0292136	0.98875	no
gi|320445498|ref|NW_003385073.1|	35138-37660	981429	742194	-0.403087	0.80095	no
gi|320445498|ref|NW_003385073.1|	37966-39478	978163	661836	-0.563601	0.79995	no
gi|320445498|ref|NW_003385073.1|	77921-78428	196207	49349	-199128	0.37565	no
gi|320445498|ref|NW_003385073.1|	84390-84866	226853	734289	-162734	0.4335	no
gi|320445498|ref|NW_003385073.1|	85678-85920	103.17	42691	-127302	0.5423	no
gi|320445498|ref|NW_003385073.1|	88342-88742	77394	509391	-0.60345	0.75385	no
gi|320445498|ref|NW_003385073.1|	89502-89772	355106	274404	-0.371948	0.85085	no
gi|320445498|ref|NW_003385073.1|	91045-91270	366063	201326	-0.862558	0.67695	no
gi|320445498|ref|NW_003385073.1|	91543-91780	915425	216515	-207997	0.3339	no
gi|320445498|ref|NW_003385073.1|	92275-93137	511781	259564	-0.979436	0.64245	no
gi|320445498|ref|NW_003385073.1|	93527-94066	165322	144757	-0.19165	0.92115	no
gi|320445498|ref|NW_003385073.1|	94797-95124	762218	473057	-0.68819	0.7448	no
gi|320445498|ref|NW_003385073.1|	95897-97211	657111	521288	-0.334056	0.8747	no
gi|320445498|ref|NW_003385073.1|	99557-101122	0.634571	0.706335	0.154571	1	no
gi|320445503|ref|NW_003385068.1|	0-850	376229	286827	29305	0.03445	no
gi|320445503|ref|NW_003385068.1|	3190-3907	0.350347	359528	335925	0.22635	no

gi|320445509|ref|NW_003385062.1|	11301-12587	337314	110347	170989	0.43975	no
gi|320445509|ref|NW_003385062.1|	12894-13625	170248	536188	16551	0.44395	no
gi|320445509|ref|NW_003385062.1|	14183-14601	795376	700638	-0.182968	0.9246	no
gi|320445509|ref|NW_003385062.1|	14709-15925	0	608522	inf	0.0072	no
gi|320445509|ref|NW_003385062.1|	16039-16250	350614	29252	-0.261347	0.8975	no
gi|320445509|ref|NW_003385062.1|	16934-17256	633295	715511	0.176096	0.90555	no
gi|320445509|ref|NW_003385062.1|	19749-20026	188546	120218	-0.649269	0.7491	no
gi|320445509|ref|NW_003385062.1|	21638-22105	213688	379867	0.829985	0.69235	no
gi|320445509|ref|NW_003385062.1|	32816-33095	0	147332	inf	0.0312	no
gi|320445509|ref|NW_003385062.1|	6132-7766	137543	272078	0.984138	0.5664	no
gi|320445509|ref|NW_003385062.1|	8024-8266	35576	561724	0.658958	0.74665	no
gi|320445518|ref|NW_003385053.1|	85879-86472	282024	101917	-146842	0.49575	no
gi|320445518|ref|NW_003385053.1|	86538-87917	265609	0.102496	-469566	0.2894	no
gi|320445518|ref|NW_003385053.1|	95078-95742	275312	60868	114462	0.49305	no
gi|320445520|ref|NW_003385051.1|	14297-15865	113966	158571	0.47653	0.80835	no
gi|320445520|ref|NW_003385051.1|	16011-17763	211399	364225	0.784858	0.6952	no
gi|320445520|ref|NW_003385051.1|	2557-3324	0.952502	631504	2729	0.2519	no
gi|320445520|ref|NW_003385051.1|	31180-31765	965908	119078	0.301952	0.81505	no
gi|320445520|ref|NW_003385051.1|	40406-40991	336386	78084	121491	0.56365	no
gi|320445520|ref|NW_003385051.1|	45802-46363	206004	556451	143358	0.504	no
gi|320445520|ref|NW_003385051.1|	52849-53972	21675	413367	0.931391	0.58665	no
gi|320445520|ref|NW_003385051.1|	54198-55429	0.339003	117543	179382	1	no
gi|320445520|ref|NW_003385051.1|	57948-59945	21073	394079	0.903089	0.66	no
gi|320445520|ref|NW_003385051.1|	60856-61793	432998	111175	136039	0.52645	no
gi|320445520|ref|NW_003385051.1|	69859-71689	158729	398161	132678	0.52595	no
gi|320445520|ref|NW_003385051.1|	71929-75020	0.886302	173528	0.969299	0.645	no
gi|320445520|ref|NW_003385051.1|	76926-78160	0.506976	152352	158742	0.43945	no
gi|320445520|ref|NW_003385051.1|	7758-8784	0.213466	324598	392658	0.20525	no
gi|320445520|ref|NW_003385051.1|	83339-84017	266689	109202	203377	0.3511	no
gi|320445520|ref|NW_003385051.1|	88515-91767	173392	195601	0.173875	0.93215	no

gi|320445520|ref|NW_003385051.1|	9576-14129	430249	882297	103609	0.4244	no
gi|320445531|ref|NW_003385040.1|	86022-86869	0	949293	inf	0.0074	no
gi|320445531|ref|NW_003385040.1|	87013-88518	0	434391	inf	0.0071	no
gi|320445531|ref|NW_003385040.1|	94796-98890	139052	235708	0.761378	0.57225	no
gi|320445535|ref|NW_003385036.1|	35064-38078	570809	314175	-0.861442	0.694	no
gi|320445535|ref|NW_003385036.1|	39276-40990	490728	294037	-0.738925	0.7164	no
gi|320445535|ref|NW_003385036.1|	41140-42829	719732	735503	0.0312717	0.98715	no
gi|320445535|ref|NW_003385036.1|	43156-43377	39103	214795	-0.864314	0.67695	no
gi|320445535|ref|NW_003385036.1|	44196-45046	0.82386	18895	119754	0.56655	no
gi|320445535|ref|NW_003385036.1|	45196-46338	483979	644621	0.413507	0.84175	no
gi|320445535|ref|NW_003385036.1|	46441-48173	124025	172748	0.478035	0.81245	no
gi|320445535|ref|NW_003385036.1|	48277-49619	195194	446833	119483	0.3496	no
gi|320445535|ref|NW_003385036.1|	50914-53820	118147	138236	0.22655	0.85745	no
gi|320445550|ref|NW_003385021.1|	101028-10641	148836	313758	107593	0.42655	no
gi|320445550|ref|NW_003385021.1|	10144-11012	0.266803	238782	316185	0.24575	no
gi|320445550|ref|NW_003385021.1|	106568-10704	768105	153737	100109	0.6027	no
gi|320445550|ref|NW_003385021.1|	108846-11049	465548	938934	101209	0.638	no
gi|320445550|ref|NW_003385021.1|	113942-11857	13809	381469	146595	0.5144	no
gi|320445550|ref|NW_003385021.1|	118904-11973	282909	132283	222522	0.31775	no
gi|320445550|ref|NW_003385021.1|	14123-15274	0	242581	inf	0.0212	no
gi|320445550|ref|NW_003385021.1|	22294-23026	288842	111681	-137089	0.5301	no
gi|320445550|ref|NW_003385021.1|	24962-25329	221321	760869	-154042	0.47415	no
gi|320445550|ref|NW_003385021.1|	25435-26114	102636	337275	-160554	0.4486	no
gi|320445550|ref|NW_003385021.1|	27879-28850	208783	681083	-16161	0.45855	no
gi|320445550|ref|NW_003385021.1|	29312-30799	498853	384332	-0.37626	0.85245	no
gi|320445550|ref|NW_003385021.1|	30932-32315	0.73523	194071	140032	0.51875	no
gi|320445550|ref|NW_003385021.1|	32425-38876	138981	225086	0.695592	0.60575	no
gi|320445550|ref|NW_003385021.1|	40214-42340	0.356999	0.996252	148059	1	no
gi|320445550|ref|NW_003385021.1|	47272-48016	842835	107759	0.354481	0.7857	no
gi|320445550|ref|NW_003385021.1|	64412-65198	751054	71754	-0.0658587	0.95945	no

gi|320445550|ref|NW_003385021.1|	99527-100313	101162	113628	0.167645	0.9338	no
gi|320445574|ref|NW_003384997.1|	11298-13113	843914	468898	-0.847822	0.6989	no
gi|320445574|ref|NW_003384997.1|	13493-14898	220748	14936	-0.563602	0.7277	no
gi|320445574|ref|NW_003384997.1|	16362-18620	102612	150226	0.549935	0.65535	no
gi|320445574|ref|NW_003384997.1|	30781-33962	0.515532	460681	315963	0.19805	no
gi|320445574|ref|NW_003384997.1|	6082-7195	173069	173062	-5.85E+00	0.95935	no
gi|320445589|ref|NW_003384982.1|	100017-10055[illegible]	278186	22476	-0.307663	0.8817	no
gi|320445589|ref|NW_003384982.1|	10871-11354	0	67088	inf	0.02205	no
gi|320445589|ref|NW_003384982.1|	17410-18276	0.267649	644875	45906	0.17745	no
gi|320445589|ref|NW_003384982.1|	52736-54764	853757	0.590815	-717498	0.0627	no
gi|320445589|ref|NW_003384982.1|	55338-56623	823187	0.223134	-852717	0.1617	no
gi|320445589|ref|NW_003384982.1|	61755-68591	135475	258392	0.931537	0.48595	no
gi|320445589|ref|NW_003384982.1|	82522-83129	547747	512935	-0.0947339	0.96745	no
gi|320445589|ref|NW_003384982.1|	84656-85205	213638	111653	238578	0.30655	no
gi|320445589|ref|NW_003384982.1|	89678-90393	12313	336913	-186973	0.3846	no
gi|320445589|ref|NW_003384982.1|	95821-96304	133841	67088	232553	0.2622	no
gi|320445604|ref|NW_003384967.1|	172893-17338[illegible]	261719	306384	0.227323	0.89115	no
gi|320445604|ref|NW_003384967.1|	51630-54628	103829	721644	279707	0.2305	no
gi|320445604|ref|NW_003384967.1|	63630-64142	120516	206263	409718	0.16185	no
gi|320445604|ref|NW_003384967.1|	66675-67008	0	377835	inf	0.00815	no
gi|320445619|ref|NW_003384952.1|	116395-11827[illegible]	0.205204	171621	30641	0.2113	no
gi|320445619|ref|NW_003384952.1|	118580-12458[illegible]	0.467728	397452	308704	0.1018	no
gi|320445619|ref|NW_003384952.1|	18999-22153	647486	114816	0.826403	0.6226	no
gi|320445619|ref|NW_003384952.1|	33146-33596	917026	116828	0.349347	0.8628	no
gi|320445627|ref|NW_003384944.1|	15731-16483	0.980103	179132	0.870021	0.6867	no
gi|320445627|ref|NW_003384944.1|	28610-29056	272106	170408	-0.675174	0.74105	no
gi|320445627|ref|NW_003384944.1|	31750-32697	161278	474558	-176489	0.4186	no
gi|320445627|ref|NW_003384944.1|	45780-45979	597339	232555	-136098	0.5243	no
gi|320445627|ref|NW_003384944.1|	51469-52846	331115	354224	0.0973316	0.94135	no
gi|320445627|ref|NW_003384944.1|	53286-55195	66947	88903	0.409213	0.75995	no

gi|320445627|ref|NW_003384944.1|	56407-56670	959243	167115	0.80087	0.7044	no
gi|320445627|ref|NW_003384944.1|	57110-61933	849572	160229	0.915327	0.4867	no
gi|320445627|ref|NW_003384944.1|	62091-65940	0.186767	133986	284278	1	no
gi|320445627|ref|NW_003384944.1|	66959-74427	361579	218025	259211	0.06205	no
gi|320445627|ref|NW_003384944.1|	74673-81872	198676	50756	135316	0.3013	no
gi|320445651|ref|NW_003384920.1|	16484-16851	104836	532608	-0.976992	0.6644	no
gi|320445651|ref|NW_003384920.1|	17632-18851	17157	107063	-0.680338	0.73195	no
gi|320445651|ref|NW_003384920.1|	19190-20986	12975	0.753272	-0.784497	1	no
gi|320445651|ref|NW_003384920.1|	24139-27222	355517	414318	0.220819	0.9194	no
gi|320445651|ref|NW_003384920.1|	27588-29776	241964	669344	146796	0.49895	no
gi|320445657|ref|NW_003384914.1|	12173-12407	796727	22594	150379	0.35905	no
gi|320445657|ref|NW_003384914.1|	1476-1967	389681	204258	239003	0.28585	no
gi|320445657|ref|NW_003384914.1|	17431-17757	153589	14.89	32772	0.23025	no
gi|320445657|ref|NW_003384914.1|	57478-58378	279347	0	#NAME?	0.02105	no
gi|320445657|ref|NW_003384914.1|	61160-61913	127168	0	#NAME?	0.00665	no
gi|320445657|ref|NW_003384914.1|	63525-64876	272277	0	#NAME?	0.01345	no
gi|320445657|ref|NW_003384914.1|	89806-91249	796509	990623	363657	0.077	no
gi|320445657|ref|NW_003384914.1|	91766-92723	140331	484235	178687	0.41115	no
gi|320445671|ref|NW_003384900.1|	37333-38510	197147	0	#NAME?	0.02105	no
gi|320445678|ref|NW_003384893.1|	106587-10742[illegible]	604201	230951	193449	0.15745	no
gi|320445678|ref|NW_003384893.1|	111465-11172[illegible]	134903	309588	119843	0.59465	no
gi|320445678|ref|NW_003384893.1|	112044-11378[illegible]	437677	131307	158501	0.4775	no
gi|320445678|ref|NW_003384893.1|	115007-11664[illegible]	1564	854121	24492	0.2749	no
gi|320445678|ref|NW_003384893.1|	119272-12045[illegible]	0.178845	136329	293031	1	no
gi|320445678|ref|NW_003384893.1|	120572-12669[illegible]	339927	134695	19864	0.258	no
gi|320445678|ref|NW_003384893.1|	12469-12791	443307	306648	-0.531723	0.79285	no
gi|320445678|ref|NW_003384893.1|	159797-16145[illegible]	0.474797	338775	283494	0.23845	no
gi|320445678|ref|NW_003384893.1|	178145-17878[illegible]	209189	370049	0.822906	0.6933	no
gi|320445678|ref|NW_003384893.1|	186052-18741[illegible]	387139	0.312626	-695227	0.1105	no
gi|320445678|ref|NW_003384893.1|	194337-19495[illegible]	393886	0.297302	-372778	0.32305	no

gi|320445678|ref|NW_003384893.1|	202770-20478[illegible]	176911	321958	0.863849	0.4972	no
gi|320445678|ref|NW_003384893.1|	204940-20558[illegible]	662911	902349	0.444871	0.8256	no
gi|320445678|ref|NW_003384893.1|	218398-22190[illegible]	693778	855943	0.30304	0.8147	no
gi|320445678|ref|NW_003384893.1|	32695-33256	360508	142591	198378	0.3693	no
gi|320445678|ref|NW_003384893.1|	70486-71148	395127	323213	-361176	0.1612	no
gi|320445678|ref|NW_003384893.1|	77107-77319	45877	215375	-109092	0.5928	no
gi|320445678|ref|NW_003384893.1|	77835-78446	385239	167227	-120394	0.58525	no
gi|320445678|ref|NW_003384893.1|	80908-81180	101796	474262	-110192	0.59695	no
gi|320445685|ref|NW_003384886.1|	14844-16647	645855	157486	128594	0.4348	no
gi|320445685|ref|NW_003384886.1|	20283-20484	142973	490224	17777	0.3321	no
gi|320445688|ref|NW_003384883.1|	37515-37913	185749	643171	-153008	0.47585	no
gi|320445688|ref|NW_003384883.1|	39549-42683	314335	358212	0.188511	0.93015	no
gi|320445688|ref|NW_003384883.1|	44241-46965	874638	11892	0.443239	0.7182	no
gi|320445688|ref|NW_003384883.1|	48990-49508	708424	198276	-18371	0.40275	no
gi|320445688|ref|NW_003384883.1|	50945-51379	245977	434718	0.821554	0.69235	no
gi|320445688|ref|NW_003384883.1|	51481-55462	0.765785	299552	19678	0.37025	no
gi|320445688|ref|NW_003384883.1|	55634-56204	501542	440731	-0.186471	0.9206	no
gi|320445688|ref|NW_003384883.1|	57126-57611	796999	843642	0.0820531	0.95975	no
gi|320445688|ref|NW_003384883.1|	57766-59648	58412	564239	-0.0499584	0.97895	no
gi|320445702|ref|NW_003384869.1|	44005-44303	0	272283	inf	0.0186	no
gi|320445702|ref|NW_003384869.1|	44742-46018	0	146276	inf	0.0044	no
gi|320445702|ref|NW_003384869.1|	47095-47446	0	326975	inf	0.0088	no
gi|320445702|ref|NW_003384869.1|	49672-50058	0	191649	inf	0.01275	no
gi|320445702|ref|NW_003384869.1|	53878-55688	0.321492	231454	284788	0.24485	no
gi|320445704|ref|NW_003384867.1|	105410-10743[illegible]	765869	428599	-0.83747	0.70315	no
gi|320445704|ref|NW_003384867.1|	15608-17731	261918	698477	-190683	0.2826	no
gi|320445704|ref|NW_003384867.1|	17840-18679	545272	980851	-247487	0.15795	no
gi|320445704|ref|NW_003384867.1|	19045-19649	775704	92853	0.259442	0.89925	no
gi|320445704|ref|NW_003384867.1|	24507-26314	112737	463786	-128143	0.5619	no
gi|320445704|ref|NW_003384867.1|	26526-27517	0.446715	123414	146608	1	no

gi|320445704|ref|NW_003384867.1|	27635-33084	192877	304974	0.661009	0.6202	no
gi|320445704|ref|NW_003384867.1|	44543-46254	705569	93963	0.413306	0.7579	no
gi|320445704|ref|NW_003384867.1|	60056-61307	0.996975	0.461025	-111271	1	no
gi|320445704|ref|NW_003384867.1|	64261-68622	184854	808402	212869	0.11535	no
gi|320445704|ref|NW_003384867.1|	71545-77248	165092	239591	0.537299	0.6894	no
gi|320445704|ref|NW_003384867.1|	82045-87572	250902	411079	0.712293	0.69155	no
gi|320445704|ref|NW_003384867.1|	92696-95730	0.361742	160185	214671	0.3326	no
gi|320445704|ref|NW_003384867.1|	97352-98760	460513	701916	0.608056	0.65195	no
gi|320445704|ref|NW_003384867.1|	99446-101828	67443	178206	14018	0.2891	no
gi|320445709|ref|NW_003384862.1|	35715-36964	0	173221	inf	0.02205	no
gi|320445709|ref|NW_003384862.1|	46487-48021	0.649722	325366	232417	0.295	no
gi|320445709|ref|NW_003384862.1|	48674-51325	0.419103	126888	159818	1	no
gi|320445710|ref|NW_003384861.1|	152262-152888	0.431037	263668	261284	0.2636	no
gi|320445714|ref|NW_003384857.1|	15317-23084	0.156811	123777	630257	0.0612	no
gi|320445714|ref|NW_003384857.1|	48961-50162	0.349538	804421	784636	0.1183	no
gi|320445714|ref|NW_003384857.1|	53550-53780	0	151771	inf	0.007	no
gi|320445714|ref|NW_003384857.1|	54239-55270	0	186222	inf	0.0033	no
gi|320445714|ref|NW_003384857.1|	56035-56199	0	264522	inf	0.01575	no
gi|320445729|ref|NW_003384842.1|	13654-14480	171471	333997	0.961876	0.65585	no
gi|320445729|ref|NW_003384842.1|	17587-17925	168923	118657	-0.509578	0.8032	no
gi|320445729|ref|NW_003384842.1|	19117-20056	839547	893531	0.0899076	0.96445	no
gi|320445729|ref|NW_003384842.1|	20352-20925	248601	26896	0.113558	0.9296	no
gi|320445729|ref|NW_003384842.1|	21134-23935	64451	133754	105331	0.5315	no
gi|320445729|ref|NW_003384842.1|	24438-25168	307064	490522	0.675778	0.7404	no
gi|320445729|ref|NW_003384842.1|	25285-25951	90044	15.75	0.806645	0.7049	no
gi|320445729|ref|NW_003384842.1|	26393-30345	0.59017	0.95331	0.691815	1	no
gi|320445729|ref|NW_003384842.1|	30569-31308	469152	895171	0.932108	0.64665	no
gi|320445729|ref|NW_003384842.1|	34682-38261	131202	755605	-0.796089	0.53135	no
gi|320445729|ref|NW_003384842.1|	45809-46275	715162	619666	-0.20678	0.913	no
gi|320445729|ref|NW_003384842.1|	54968-55671	223679	175096	-0.353281	0.8706	no

gi|320445729|ref|NW_003384842.1|	57566-60758	463394	35361	-0.390081	0.77265	no
gi|320445729|ref|NW_003384842.1|	72257-76039	687918	408527	-0.751805	0.56725	no
gi|320445731|ref|NW_003384840.1|	25966-26406	0	688434	inf	0.02915	no
gi|320445731|ref|NW_003384840.1|	30009-30542	0	45371	inf	0.029	no
gi|320445731|ref|NW_003384840.1|	40962-41920	0	725365	inf	0.00885	no
gi|320445731|ref|NW_003384840.1|	51592-51876	13187	112326	-0.23143	0.8998	no
gi|320445731|ref|NW_003384840.1|	56017-56609	235664	510932	11164	0.5821	no
gi|320445731|ref|NW_003384840.1|	57146-57717	150026	30427	102014	0.5772	no
gi|320445731|ref|NW_003384840.1|	62570-66758	0.896425	627599	280759	0.13015	no
gi|320445731|ref|NW_003384840.1|	67629-68261	212308	4.62	112174	0.5821	no
gi|320445731|ref|NW_003384840.1|	74637-76671	159487	12366	-0.367057	0.7659	no
gi|320445731|ref|NW_003384840.1|	77013-79052	145947	561137	-137902	0.4089	no
gi|320445731|ref|NW_003384840.1|	79418-79858	119724	476608	-132883	0.51525	no
gi|320445731|ref|NW_003384840.1|	83944-84568	268596	88319	-160464	0.4554	no
gi|320445756|ref|NW_003384815.1|	109511-11023[illegible]	172677	141817	-0.284046	0.88515	no
gi|320445756|ref|NW_003384815.1|	65899-66782	0	430812	inf	0.0138	no
gi|320445778|ref|NW_003384793.1|	155583-15668[illegible]	0.38948	161748	205413	0.3018	no
gi|320445778|ref|NW_003384793.1|	157162-16117[illegible]	0.759096	271939	184093	0.393	no
gi|320445778|ref|NW_003384793.1|	161431-16318[illegible]	0.665383	270347	202256	0.34905	no
gi|320445778|ref|NW_003384793.1|	164034-16462[illegible]	286728	145624	234449	0.2843	no
gi|320445778|ref|NW_003384793.1|	25913-28896	245638	420742	0.7764	0.71875	no
gi|320445778|ref|NW_003384793.1|	29710-31400	0	137308	inf	1	no
gi|320445778|ref|NW_003384793.1|	32409-32687	699879	119032	0.766168	0.70645	no
gi|320445778|ref|NW_003384793.1|	3305-3794	523438	481461	-0.120601	0.9421	no
gi|320445778|ref|NW_003384793.1|	33106-34609	732176	564664	-0.374797	0.8573	no
gi|320445778|ref|NW_003384793.1|	35396-35858	581483	338894	-0.778902	0.70065	no
gi|320445778|ref|NW_003384793.1|	39153-40076	220909	16925	-0.384294	0.85015	no
gi|320445778|ref|NW_003384793.1|	41012-41491	475656	226927	-106769	0.59575	no
gi|320445778|ref|NW_003384793.1|	42739-44945	673878	884358	0.392144	0.7648	no
gi|320445778|ref|NW_003384793.1|	45152-46506	211203	11419	-0.887194	0.58055	no

gi|320445778|ref|NW_003384793.1|	65288-76427	0.324945	464619	383778	0.0673	no
gi|320445778|ref|NW_003384793.1|	77050-77560	121353	325706	474628	0.15365	no
gi|320445778|ref|NW_003384793.1|	78964-79326	120119	46235	526645	0.1692	no
gi|320445778|ref|NW_003384793.1|	82369-91729	0.536224	976579	418683	0.0494	no
gi|320445778|ref|NW_003384793.1|	93082-93675	225619	101917	-114649	0.5864	no
gi|320445778|ref|NW_003384793.1|	94168-94631	173735	723369	-126409	0.5383	no
gi|320445778|ref|NW_003384793.1|	95217-95948	166843	128219	-0.379888	0.85615	no
gi|320445779|ref|NW_003384792.1|	[illegible]11433-11356[illegible]	145017	984431	276307	0.04735	no
gi|320445779|ref|NW_003384792.1|	[illegible]15577-11745[illegible]	21136	166902	298123	0.03095	no
gi|320445779|ref|NW_003384792.1|	[illegible]93306-19375[illegible]	156165	777811	-100558	0.61475	no
gi|320445779|ref|NW_003384792.1|	[illegible]37576-23805[illegible]	166276	416138	-199844	0.3625	no
gi|320445779|ref|NW_003384792.1|	[illegible]39019-24090[illegible]	705807	670162	-0.0747645	0.97235	no
gi|320445779|ref|NW_003384792.1|	[illegible]42201-24338[illegible]	231029	307906	0.414418	0.8412	no
gi|320445779|ref|NW_003384792.1|	[illegible]43884-24409[illegible]	394029	246727	-0.675388	0.7459	no
gi|320445779|ref|NW_003384792.1|	[illegible]44256-24636[illegible]	17363	328237	0.918721	0.485	no
gi|320445779|ref|NW_003384792.1|	[illegible]51946-25226[illegible]	185454	0	#NAME?	0.02105	no
gi|320445779|ref|NW_003384792.1|	[illegible]52362-25280[illegible]	505077	0	#NAME?	0.0053	no
gi|320445779|ref|NW_003384792.1|	[illegible]54318-25533[illegible]	173406	0.149783	-685514	0.2606	no
gi|320445779|ref|NW_003384792.1|	[illegible]55443-25779[illegible]	274512	0	#NAME?	5.00E-05	yes
gi|320445779|ref|NW_003384792.1|	[illegible]65158-26647[illegible]	532935	0.108801	-561419	0.27235	no
gi|320445782|ref|NW_003384789.1|	33020-33949	0	167812	inf	0.0312	no
gi|320445782|ref|NW_003384789.1|	34081-34806	0	283072	inf	0.029	no
gi|320445785|ref|NW_003384786.1|	[illegible]17729-11789[illegible]	198994	109214	245637	0.2793	no
gi|320445789|ref|NW_003384782.1|	4248-6028	393284	167423	-123208	0.5573	no
gi|320445794|ref|NW_003384777.1|	[illegible]20381-12087[illegible]	0	436137	inf	0.0312	no
gi|320445794|ref|NW_003384777.1|	36470-37510	0.419501	17402	205251	0.3018	no
gi|320445794|ref|NW_003384777.1|	37620-40035	0.232244	1892	30262	0.2185	no
gi|320445794|ref|NW_003384777.1|	45733-49065	0.926294	438307	22424	0.3205	no
gi|320445801|ref|NW_003384770.1|	11651-13740	0.273095	438152	400396	0.17015	no
gi|320445801|ref|NW_003384770.1|	16633-17060	0.846759	672513	298954	0.2563	no

gi|320445801|ref|NW_003384770.1|	20014-20716	715851	484364	-0.563567	0.64975	no
gi|320445801|ref|NW_003384770.1|	22239-25592	67651	681444	0.010482	0.9939	no
gi|320445801|ref|NW_003384770.1|	36244-37564	222493	145811	-0.609663	0.70505	no
gi|320445801|ref|NW_003384770.1|	38523-39083	361586	237172	-0.608404	0.77835	no
gi|320445801|ref|NW_003384770.1|	41472-41890	530251	525479	-0.0130426	0.94	no
gi|320445801|ref|NW_003384770.1|	43547-44790	23.28	163786	-0.507283	0.7548	no
gi|320445801|ref|NW_003384770.1|	48144-56768	123895	205092	0.727151	0.585	no
gi|320445801|ref|NW_003384770.1|	59689-61303	298662	301544	0.0138563	0.99115	no
gi|320445801|ref|NW_003384770.1|	62503-62919	553276	447889	-0.30486	0.88665	no
gi|320445801|ref|NW_003384770.1|	66691-67557	497828	504845	0.0201928	0.9872	no
gi|320445801|ref|NW_003384770.1|	68000-68515	65605	240274	-144912	0.5024	no
gi|320445813|ref|NW_003384758.1|	13376-14326	118088	293338	13127	0.5464	no
gi|320445813|ref|NW_003384758.1|	17094-19396	0.979777	165285	0.754434	0.70755	no
gi|320445813|ref|NW_003384758.1|	23094-23829	472894	0.462629	-335359	0.23895	no
gi|320445813|ref|NW_003384758.1|	29585-30221	105109	0.858017	-361474	0.17525	no
gi|320445814|ref|NW_003384757.1|	106508-10704[illegible]	383317	140189	187076	0.3906	no
gi|320445814|ref|NW_003384757.1|	108525-10973[illegible]	180451	145547	-0.310121	0.8872	no
gi|320445814|ref|NW_003384757.1|	109963-11224[illegible]	275925	340932	0.305208	0.8177	no
gi|320445814|ref|NW_003384757.1|	120621-12183[illegible]	171919	312299	418312	0.06245	no
gi|320445814|ref|NW_003384757.1|	124180-12478[illegible]	0.461203	500348	343946	0.2167	no
gi|320445814|ref|NW_003384757.1|	125701-12644[illegible]	100334	962175	326149	0.2052	no
gi|320445814|ref|NW_003384757.1|	127040-12744[illegible]	277154	32289	354228	0.18835	no
gi|320445814|ref|NW_003384757.1|	129806-13044[illegible]	0	370926	inf	0.02915	no
gi|320445814|ref|NW_003384757.1|	131705-13232[illegible]	0.441033	152754	511418	0.1652	no
gi|320445814|ref|NW_003384757.1|	145933-14669[illegible]	0	85239	inf	0.0088	no
gi|320445814|ref|NW_003384757.1|	32649-32930	13582	577968	-123263	0.56145	no
gi|320445814|ref|NW_003384757.1|	34483-35211	342562	398652	0.218767	0.91095	no
gi|320445814|ref|NW_003384757.1|	37950-45528	247705	274537	0.148381	0.9117	no
gi|320445814|ref|NW_003384757.1|	46571-47433	342085	500588	-277266	0.1292	no
gi|320445814|ref|NW_003384757.1|	48896-49860	833894	223842	-189738	0.38335	no

gi|320445814|ref|NW_003384757.1|	50547-51887	314709	118803	-140545	0.4077	no
gi|320445814|ref|NW_003384757.1|	52105-53411	110337	153165	0.473173	0.81195	no
gi|320445814|ref|NW_003384757.1|	53588-55533	237868	211246	-0.171233	0.89425	no
gi|320445814|ref|NW_003384757.1|	72649-73680	129396	425232	-160547	0.4505	no
gi|320445814|ref|NW_003384757.1|	74277-75179	174701	558347	-164565	0.45395	no
gi|320445814|ref|NW_003384757.1|	76358-77089	749093	442938	-0.758042	0.70575	no
gi|320445814|ref|NW_003384757.1|	78192-79137	199787	160812	-0.313086	0.88705	no
gi|320445814|ref|NW_003384757.1|	91198-92807	442253	188076	-123355	0.5569	no
gi|320445814|ref|NW_003384757.1|	9370-9988	879799	597552	-0.558111	0.792	no
gi|320445817|ref|NW_003384754.1|	102473-10386	0.581594	252527	211835	0.34305	no
gi|320445817|ref|NW_003384754.1|	103972-10633	0.474948	165801	180361	0.41025	no
gi|320445817|ref|NW_003384754.1|	125267-12823	105123	158656	0.593817	0.64145	no
gi|320445817|ref|NW_003384754.1|	128520-13002	971797	351758	-146607	0.49785	no
gi|320445817|ref|NW_003384754.1|	133302-13417	203662	337639	0.729306	0.5827	no
gi|320445817|ref|NW_003384754.1|	134297-13672	173067	178.33	0.0432167	0.9734	no
gi|320445817|ref|NW_003384754.1|	13762-14895	15041	207016	378277	0.1466	no
gi|320445817|ref|NW_003384754.1|	147457-14959	327797	247244	-0.406866	0.8431	no
gi|320445817|ref|NW_003384754.1|	150662-15292	266958	588094	113943	0.5919	no
gi|320445817|ref|NW_003384754.1|	153023-15500	0.871979	331018	192454	0.38025	no
gi|320445817|ref|NW_003384754.1|	155847-15873	26736	39157	0.550488	0.80025	no
gi|320445817|ref|NW_003384754.1|	159179-16215	0.431122	198065	219981	0.31845	no
gi|320445817|ref|NW_003384754.1|	162312-16383	262722	420232	0.677651	0.7434	no
gi|320445817|ref|NW_003384754.1|	252-874	609594	147921	127891	0.5398	no
gi|320445817|ref|NW_003384754.1|	52946-56105	180063	163859	-0.136045	0.9141	no
gi|320445817|ref|NW_003384754.1|	56700-59594	55215	104697	0.923094	0.57275	no
gi|320445817|ref|NW_003384754.1|	59699-59955	148722	22628	0.605494	0.7597	no
gi|320445817|ref|NW_003384754.1|	65105-66928	130732	829429	-0.656422	0.6777	no
gi|320445817|ref|NW_003384754.1|	70797-71633	111004	773059	-38439	0.0619	no
gi|320445817|ref|NW_003384754.1|	71948-72345	186732	213323	-312986	0.0951	no
gi|320445817|ref|NW_003384754.1|	73354-73560	354349	281693	-365298	0.1627	no

gi|320445817|ref|NW_003384754.1|	74017-77169	122586	19257	-267034	0.05435	no
gi|320445817|ref|NW_003384754.1|	79635-81531	154452	375373	-204076	0.23255	no
gi|320445817|ref|NW_003384754.1|	82191-83258	322672	280757	-352268	0.0824	no
gi|320445817|ref|NW_003384754.1|	83363-88039	34652	269566	-0.362299	0.7871	no
gi|320445817|ref|NW_003384754.1|	88585-89537	235517	292533	0.312768	0.87535	no
gi|320445817|ref|NW_003384754.1|	89691-91528	421428	381752	-0.14265	0.9448	no
gi|320445817|ref|NW_003384754.1|	92213-94061	131844	714586	-0.883654	0.5768	no
gi|320445817|ref|NW_003384754.1|	96864-10131[illegible]	105387	101577	-0.0531274	0.96655	no
gi|320445819|ref|NW_003384752.1|	179990-18478[illegible]	0.481262	4408	319523	0.18995	no
gi|320445819|ref|NW_003384752.1|	206333-21123[illegible]	125956	106987	-0.235475	0.8575	no
gi|320445819|ref|NW_003384752.1|	212900-21339[illegible]	176006	82866	-108677	0.60325	no
gi|320445819|ref|NW_003384752.1|	214817-21655[illegible]	47422	173742	-144861	0.27565	no
gi|320445819|ref|NW_003384752.1|	90545-93873	441923	900642	102716	0.5359	no
gi|320445819|ref|NW_003384752.1|	95313-95826	54046	403102	-0.423044	0.8278	no
gi|320445820|ref|NW_003384751.1|	125253-12641[illegible]	0	240765	inf	0.0212	no
gi|320445820|ref|NW_003384751.1|	28754-29382	471763	670557	0.507297	0.8028	no
gi|320445824|ref|NW_003384747.1|	37093-38079	0.224846	419251	422081	0.1914	no
gi|320445829|ref|NW_003384742.1|	11813-13520	114666	20999	0.87289	0.59845	no
gi|320445829|ref|NW_003384742.1|	14709-16583	0.617866	0.789456	0.353566	1	no
gi|320445829|ref|NW_003384742.1|	38124-38743	298365	122195	-128789	0.5495	no
gi|320445829|ref|NW_003384742.1|	38880-41200	106845	559407	-0.933546	0.56605	no
gi|320445829|ref|NW_003384742.1|	5561-6584	0.21428	236957	346706	0.21395	no
gi|320445829|ref|NW_003384742.1|	8288-10244	12749	505962	198864	0.36325	no
gi|320445830|ref|NW_003384741.1|	112253-11356[illegible]	0.624661	187537	490796	0.13795	no
gi|320445832|ref|NW_003384739.1|	18602-24995	350725	283474	301481	0.02705	no
gi|320445832|ref|NW_003384739.1|	25379-27858	142892	651292	218839	0.3252	no
gi|320445832|ref|NW_003384739.1|	28205-28807	366989	55999	0.609664	0.7509	no
gi|320445832|ref|NW_003384739.1|	39946-41498	0	846976	inf	0.0055	no
gi|320445832|ref|NW_003384739.1|	42259-42924	0	374561	inf	0.00355	no
gi|320445832|ref|NW_003384739.1|	58290-59190	0	192499	inf	0.0294	no

gi|320445832|ref|NW_003384739.1|	59349-60986	0.120393	209491	412106	0.19215	no
gi|320445832|ref|NW_003384739.1|	81182-82387	0	120654	inf	1	no
gi|320445833|ref|NW_003384738.1|	101450-10491[illegible]	242787	93713	-137337	0.29675	no
gi|320445833|ref|NW_003384738.1|	10355-11333	204542	156923	-0.382338	0.8503	no
gi|320445833|ref|NW_003384738.1|	105467-10771[illegible]	225191	17061	-0.400447	0.7554	no
gi|320445833|ref|NW_003384738.1|	11495-14618	222042	298281	0.42584	0.84075	no
gi|320445833|ref|NW_003384738.1|	16548-19916	964124	203835	108011	0.41345	no
gi|320445833|ref|NW_003384738.1|	35302-35634	132241	313896	124712	0.545	no
gi|320445833|ref|NW_003384738.1|	39866-42528	45197	0.0971659	-886156	0.16165	no
gi|320445833|ref|NW_003384738.1|	43557-46916	432135	619683	0.520048	0.8181	no
gi|320445833|ref|NW_003384738.1|	47996-51254	138439	12728	-0.12124	0.9236	no
gi|320445833|ref|NW_003384738.1|	51548-52075	246335	18493	-0.413648	0.8474	no
gi|320445833|ref|NW_003384738.1|	55271-58881	518488	462089	315579	0.0701	no
gi|320445833|ref|NW_003384738.1|	82712-84315	814355	223238	-186707	0.39595	no
gi|320445833|ref|NW_003384738.1|	843-1335	776494	168893	112106	0.58125	no
gi|320445833|ref|NW_003384738.1|	85097-85572	207034	598841	-178963	0.4097	no
gi|320445833|ref|NW_003384738.1|	85699-86894	376344	438824	0.221587	0.86605	no
gi|320445833|ref|NW_003384738.1|	87227-92857	351229	214993	-0.708127	0.5929	no
gi|320445833|ref|NW_003384738.1|	8829-9818	604666	804284	0.411568	0.8478	no
gi|320445833|ref|NW_003384738.1|	92991-95028	56383	666268	-308109	0.1126	no
gi|320445833|ref|NW_003384738.1|	96264-97273	992622	161287	-262162	0.17045	no
gi|320445833|ref|NW_003384738.1|	97681-98208	326538	462324	-282027	0.2339	no
gi|320445833|ref|NW_003384738.1|	98369-100094	393081	112809	-180094	0.3185	no
gi|320445839|ref|NW_003384732.1|	105286-10553[illegible]	657962	306362	-110277	0.58975	no
gi|320445839|ref|NW_003384732.1|	106985-10758[illegible]	654474	253456	-13686	0.51635	no
gi|320445839|ref|NW_003384732.1|	108030-108814	406124	276635	-0.553939	0.72825	no
gi|320445839|ref|NW_003384732.1|	108997-109887	224314	147426	-0.605531	0.7909	no
gi|320445839|ref|NW_003384732.1|	110026-110262	657791	341571	-0.945444	0.64495	no
gi|320445839|ref|NW_003384732.1|	110521-110819	694722	581696	-0.25617	0.89695	no
gi|320445839|ref|NW_003384732.1|	117192-11814[illegible]	172893	140537	-0.298931	0.892	no

gi|320445839|ref|NW_003384732.1|	18506-12123	899229	78429	-0.1973	0.90205	no
gi|320445839|ref|NW_003384732.1|	21511-12375	568456	892218	0.650348	0.6233	no
gi|320445839|ref|NW_003384732.1|	27329-12932	460559	254244	-0.857175	0.68155	no
gi|320445839|ref|NW_003384732.1|	29473-13012	134763	556143	-12769	0.54145	no
gi|320445839|ref|NW_003384732.1|	30376-13072	20893	849109	-1299	0.53615	no
gi|320445839|ref|NW_003384732.1|	33032-13340	308874	822557	-190883	0.38725	no
gi|320445839|ref|NW_003384732.1|	36466-13845	452181	212882	-108685	0.41575	no
gi|320445839|ref|NW_003384732.1|	39697-14001	171659	689579	-131576	0.56365	no
gi|320445839|ref|NW_003384732.1|	24285-29594	256063	619748	127519	0.45455	no
gi|320445839|ref|NW_003384732.1|	29728-30611	0.781862	323109	204704	0.3471	no
gi|320445839|ref|NW_003384732.1|	32532-32952	525165	219829	206554	0.34445	no
gi|320445839|ref|NW_003384732.1|	33652-34365	70653	115981	0.715062	0.7339	no
gi|320445839|ref|NW_003384732.1|	38362-39477	0	265652	inf	0.0162	no
gi|320445839|ref|NW_003384732.1|	46075-48549	0.37687	189505	233009	0.295	no
gi|320445839|ref|NW_003384732.1|	64554-66958	0.155615	814944	571065	0.1419	no
gi|320445839|ref|NW_003384732.1|	7384-7850	0.715162	476666	273664	0.2671	no
gi|320445839|ref|NW_003384732.1|	87621-89286	873459	534102	-0.709624	0.74165	no
gi|320445839|ref|NW_003384732.1|	91545-93106	305513	54019	0.822232	0.6916	no
gi|320445846|ref|NW_003384725.1|	14116-14528	618879	144131	-210228	0.34485	no
gi|320445846|ref|NW_003384725.1|	15256-16226	227447	777156	-154925	0.48685	no
gi|320445846|ref|NW_003384725.1|	16823-20758	430979	260807	-0.724637	0.5905	no
gi|320445846|ref|NW_003384725.1|	20928-22036	0.773603	120489	0.639233	1	no
gi|320445846|ref|NW_003384725.1|	23184-26155	537224	496746	-0.113015	0.9349	no
gi|320445846|ref|NW_003384725.1|	26278-27950	145063	209256	0.528593	0.6806	no
gi|320445846|ref|NW_003384725.1|	32074-35181	507078	357046	-0.506097	0.7028	no
gi|320445846|ref|NW_003384725.1|	36616-38894	0.66086	0.576605	-0.196761	1	no
gi|320445846|ref|NW_003384725.1|	39545-40670	157454	919475	-0.77605	0.72195	no
gi|320445846|ref|NW_003384725.1|	41190-41952	128204	182413	38307	0.1755	no
gi|320445846|ref|NW_003384725.1|	43096-43583	0.659197	136659	437373	0.18695	no
gi|320445846|ref|NW_003384725.1|	9697-12880	469961	451963	-0.0563358	0.9689	no

<table>
<tr><td>gi|320445847|ref|NW_003384724.1|</td><td>25190-26898</td><td>0</td><td>271288</td><td>inf</td><td>0.0101</td><td>no</td></tr>
<tr><td>gi|320445851|ref|NW_003384720.1|</td><td>122894-12343</td><td>309152</td><td>371171</td><td>-638009</td><td>0.06375</td><td>no</td></tr>
<tr><td>gi|320445851|ref|NW_003384720.1|</td><td>125715-12594</td><td>855555</td><td>0</td><td>#NAME?</td><td>0.01335</td><td>no</td></tr>
<tr><td>gi|320445851|ref|NW_003384720.1|</td><td>126087-12639</td><td>132141</td><td>688863</td><td>-426172</td><td>0.14635</td><td>no</td></tr>
<tr><td>gi|320445851|ref|NW_003384720.1|</td><td>126620-12681</td><td>878035</td><td>0</td><td>#NAME?</td><td>0.0229</td><td>no</td></tr>
<tr><td>gi|320445851|ref|NW_003384720.1|</td><td>130545-13423</td><td>975559</td><td>174259</td><td>-580693</td><td>0.01735</td><td>no</td></tr>
<tr><td>gi|320445851|ref|NW_003384720.1|</td><td>139748-14014</td><td>117936</td><td>129286</td><td>-318936</td><td>0.25</td><td>no</td></tr>
<tr><td>gi|320445851|ref|NW_003384720.1|</td><td>158970-16252</td><td>380861</td><td>96289</td><td>133811</td><td>0.429</td><td>no</td></tr>
<tr><td>gi|320445851|ref|NW_003384720.1|</td><td>16359-16989</td><td>213368</td><td>841411</td><td>197947</td><td>0.37775</td><td>no</td></tr>
<tr><td>gi|320445851|ref|NW_003384720.1|</td><td>175594-17731</td><td>0.340755</td><td>213565</td><td>264787</td><td>0.26605</td><td>no</td></tr>
<tr><td>gi|320445851|ref|NW_003384720.1|</td><td>194215-19594</td><td>0.338024</td><td>219715</td><td>270044</td><td>0.2599</td><td>no</td></tr>
<tr><td>gi|320445851|ref|NW_003384720.1|</td><td>196727-19779</td><td>0.808836</td><td>545538</td><td>275376</td><td>0.2537</td><td>no</td></tr>
<tr><td>gi|320445851|ref|NW_003384720.1|</td><td>199514-20031</td><td>0</td><td>204258</td><td>inf</td><td>0.0312</td><td>no</td></tr>
<tr><td>gi|320445851|ref|NW_003384720.1|</td><td>204376-20465</td><td>243108</td><td>154878</td><td>267146</td><td>0.2703</td><td>no</td></tr>
<tr><td>gi|320445851|ref|NW_003384720.1|</td><td>209292-20995</td><td>0.790254</td><td>538688</td><td>276906</td><td>0.23485</td><td>no</td></tr>
<tr><td>gi|320445851|ref|NW_003384720.1|</td><td>211011-21202</td><td>129555</td><td>701319</td><td>243651</td><td>0.27935</td><td>no</td></tr>
<tr><td>gi|320445851|ref|NW_003384720.1|</td><td>217693-21831</td><td>174614</td><td>29657</td><td>0.764206</td><td>0.721</td><td>no</td></tr>
<tr><td>gi|320445851|ref|NW_003384720.1|</td><td>93936-95227</td><td>0.799323</td><td>410479</td><td>236046</td><td>0.2925</td><td>no</td></tr>
<tr><td>gi|320445862|ref|NW_003384709.1|</td><td>119375-12546</td><td>628781</td><td>477268</td><td>292417</td><td>0.03665</td><td>no</td></tr>
<tr><td>gi|320445862|ref|NW_003384709.1|</td><td>126325-12729</td><td>825956</td><td>432408</td><td>238826</td><td>0.18115</td><td>no</td></tr>
<tr><td>gi|320445862|ref|NW_003384709.1|</td><td>127514-12866</td><td>0.184719</td><td>15354</td><td>305521</td><td>0.2493</td><td>no</td></tr>
<tr><td>gi|320445862|ref|NW_003384709.1|</td><td>128801-12929</td><td>120286</td><td>648824</td><td>243136</td><td>0.29425</td><td>no</td></tr>
<tr><td>gi|320445862|ref|NW_003384709.1|</td><td>129773-13057</td><td>328845</td><td>854129</td><td>137705</td><td>0.4283</td><td>no</td></tr>
<tr><td>gi|320445862|ref|NW_003384709.1|</td><td>148854-14935</td><td>0</td><td>109499</td><td>inf</td><td>0.0142</td><td>no</td></tr>
<tr><td>gi|320445862|ref|NW_003384709.1|</td><td>149603-15084</td><td>0</td><td>467389</td><td>inf</td><td>0.00815</td><td>no</td></tr>
<tr><td>gi|320445864|ref|NW_003384707.1|</td><td>105472-10701</td><td>787204</td><td>368029</td><td>-109692</td><td>0.59905</td><td>no</td></tr>
<tr><td>gi|320445864|ref|NW_003384707.1|</td><td>112017-11301</td><td>205545</td><td>259551</td><td>-298536</td><td>0.21555</td><td>no</td></tr>
<tr><td>gi|320445864|ref|NW_003384707.1|</td><td>113438-11570</td><td>89088</td><td>174339</td><td>-235334</td><td>0.30405</td><td>no</td></tr>
<tr><td>gi|320445864|ref|NW_003384707.1|</td><td>115925-11684</td><td>312181</td><td>225424</td><td>-0.469738</td><td>0.766</td><td>no</td></tr>
<tr><td>gi|320445864|ref|NW_003384707.1|</td><td>117214-11963</td><td>208732</td><td>253721</td><td>0.281589</td><td>0.88865</td><td>no</td></tr>
</table>

gi|320445864|ref|NW_003384707.1|	19991-12119	171451	232842	0.441552	0.7845	no
gi|320445864|ref|NW_003384707.1|	12118-13915	26906	504371	-241537	0.1772	no
gi|320445864|ref|NW_003384707.1|	19082-21295	0.085323	167318	761544	0.1408	no
gi|320445864|ref|NW_003384707.1|	44370-45009	563431	357816	-0.655022	0.68185	no
gi|320445864|ref|NW_003384707.1|	50734-50924	461221	572257	0.311205	0.88145	no
gi|320445864|ref|NW_003384707.1|	55866-56820	986419	713126	-0.468044	0.8249	no
gi|320445864|ref|NW_003384707.1|	56951-58848	132018	240661	0.866274	0.67005	no
gi|320445864|ref|NW_003384707.1|	58973-60491	684142	130839	0.935426	0.6751	no
gi|320445864|ref|NW_003384707.1|	61387-63653	447886	215177	-105761	0.4235	no
gi|320445864|ref|NW_003384707.1|	65219-70071	19845	153082	-0.374476	0.78405	no
gi|320445864|ref|NW_003384707.1|	81624-82580	140526	145469	0.0498743	0.94	no
gi|320445864|ref|NW_003384707.1|	94490-95219	649554	374465	-0.794618	0.68955	no
gi|320445869|ref|NW_003384702.1|	26657-28814	0	20831	inf	0.0101	no
gi|320445869|ref|NW_003384702.1|	29011-29613	0.917474	183552	432238	0.1645	no
gi|320445869|ref|NW_003384702.1|	3296-4672	192333	637129	172798	0.4166	no
gi|320445869|ref|NW_003384702.1|	44112-45158	0	28792	inf	0.0162	no
gi|320445869|ref|NW_003384702.1|	50468-51965	0.267512	362715	376116	0.1747	no
gi|320445872|ref|NW_003384699.1|	79523-18265	0.815787	574546	281616	0.2282	no
gi|320445872|ref|NW_003384699.1|	84015-18422	264151	965804	-145156	0.493	no
gi|320445872|ref|NW_003384699.1|	85717-18596	985778	582025	-0.760183	0.7073	no
gi|320445872|ref|NW_003384699.1|	89642-19208	172503	554354	-163775	0.33975	no
gi|320445872|ref|NW_003384699.1|	19006-19551	232529	153087	-0.603056	0.7723	no
gi|320445872|ref|NW_003384699.1|	92190-19306	128498	614038	-106535	0.60615	no
gi|320445872|ref|NW_003384699.1|	93250-19654	447274	208564	-110067	0.61585	no
gi|320445872|ref|NW_003384699.1|	96726-19762	887495	314533	-149653	0.4769	no
gi|320445872|ref|NW_003384699.1|	63676-63852	545373	134227	-202257	0.3459	no
gi|320445875|ref|NW_003384696.1|	00451-10069	793444	204949	-195287	0.36425	no
gi|320445875|ref|NW_003384696.1|	02062-10224	377765	220252	-0.77833	0.70135	no
gi|320445875|ref|NW_003384696.1|	05838-10599	163073	707468	-120478	0.5746	no
gi|320445875|ref|NW_003384696.1|	08343-10868	108036	964704	-0.163359	0.93045	no

gi|320445875|ref|NW_003384696.1|	109059-10950	505339	304669	-0.73001	0.73085	no
gi|320445875|ref|NW_003384696.1|	110608-11097	154234	147133	-0.0679938	0.96345	no
gi|320445875|ref|NW_003384696.1|	115082-11541	281215	229894	-0.290706	0.88295	no
gi|320445875|ref|NW_003384696.1|	119084-11970	397954	390344	-0.0278576	0.95935	no
gi|320445875|ref|NW_003384696.1|	121442-12244	751456	80919	0.106789	0.9579	no
gi|320445875|ref|NW_003384696.1|	122655-12278	1362.76	1764.75	0.372931	0.84765	no
gi|320445875|ref|NW_003384696.1|	122898-12436	214591	389571	0.860299	0.5071	no
gi|320445875|ref|NW_003384696.1|	124598-12577	508629	118482	121998	0.36805	no
gi|320445875|ref|NW_003384696.1|	126558-12908	0.590333	865893	387459	0.1406	no
gi|320445875|ref|NW_003384696.1|	131319-13180	134351	538664	200338	0.3028	no
gi|320445875|ref|NW_003384696.1|	1505-2532	959384	972584	0.0197148	0.99075	no
gi|320445875|ref|NW_003384696.1|	19471-22436	770828	103456	0.424536	0.7438	no
gi|320445875|ref|NW_003384696.1|	23307-23581	247.97	244707	-0.0191095	0.9927	no
gi|320445875|ref|NW_003384696.1|	24100-24925	237603	202699	-0.229216	0.9137	no
gi|320445875|ref|NW_003384696.1|	2681-3400	526848	568169	0.108933	0.9317	no
gi|320445875|ref|NW_003384696.1|	27888-29334	161695	111414	-0.537344	0.8108	no
gi|320445875|ref|NW_003384696.1|	32686-33289	320259	105504	-160194	0.4592	no
gi|320445875|ref|NW_003384696.1|	34018-34160	484915	214868	-117428	0.5728	no
gi|320445875|ref|NW_003384696.1|	3553-4812	291809	480339	0.71903	0.57495	no
gi|320445875|ref|NW_003384696.1|	39691-39860	103157	631327	-0.708381	0.7359	no
gi|320445875|ref|NW_003384696.1|	43595-43794	448004	232555	-0.945941	0.6795	no
gi|320445875|ref|NW_003384696.1|	44127-44918	337212	206486	-0.707613	0.75295	no
gi|320445875|ref|NW_003384696.1|	52409-52710	152247	675621	-117212	0.5945	no
gi|320445875|ref|NW_003384696.1|	5522-5952	0	121661	inf	0.0186	no
gi|320445875|ref|NW_003384696.1|	6046-7815	0.660176	36022	244796	0.2781	no
gi|320445875|ref|NW_003384696.1|	60924-61344	44639	121485	-187753	0.3885	no
gi|320445875|ref|NW_003384696.1|	68251-68515	224917	63701	-1.82	0.4077	no
gi|320445875|ref|NW_003384696.1|	71443-72063	27572	101083	-144767	0.49345	no
gi|320445875|ref|NW_003384696.1|	818-1269	266329	36929	0.471544	0.8226	no
gi|320445875|ref|NW_003384696.1|	8294-8847	0	533919	inf	0.02205	no

gi|320445875|ref|NW_003384696.1|	94112-94316	335546	29.32	-0.194626	0.90745	no
gi|320445876|ref|NW_003384695.1|	102923-10703	474655	163706	178616	0.31685	no
gi|320445876|ref|NW_003384695.1|	40315-40977	252881	700295	-185243	0.394	no
gi|320445876|ref|NW_003384695.1|	52443-56215	433192	138229	167398	0.19905	no
gi|320445876|ref|NW_003384695.1|	61229-62282	371546	285492	-0.380092	0.85305	no
gi|320445876|ref|NW_003384695.1|	62385-63437	268668	100043	-142521	0.4954	no
gi|320445876|ref|NW_003384695.1|	63546-63948	95742	25217	-192475	0.39955	no
gi|320445876|ref|NW_003384695.1|	68963-69598	758654	0	#NAME?	0.01345	no
gi|320445876|ref|NW_003384695.1|	95555-96764	918662	829138	-0.147921	0.94415	no
gi|320445876|ref|NW_003384695.1|	97151-98741	199314	251364	0.334734	0.86335	no
gi|320445876|ref|NW_003384695.1|	99956-10065	662044	979984	0.565831	0.7793	no
gi|320445878|ref|NW_003384693.1|	38500-40630	178123	932027	23875	0.30665	no
gi|320445878|ref|NW_003384693.1|	42029-43679	310146	440076	0.504807	0.8016	no
gi|320445878|ref|NW_003384693.1|	43806-46510	479822	254564	-0.914473	0.49945	no
gi|320445878|ref|NW_003384693.1|	46686-47631	228328	159171	-0.520528	0.81605	no
gi|320445880|ref|NW_003384691.1|	18994-19376	0	251761	inf	0.0085	no
gi|320445882|ref|NW_003384689.1|	102721-10310	949396	553459	-0.778534	0.7042	no
gi|320445882|ref|NW_003384689.1|	103598-10396	306578	223251	-0.457586	0.819	no
gi|320445882|ref|NW_003384689.1|	123421-12411	281381	134841	-106127	0.6273	no
gi|320445882|ref|NW_003384689.1|	137785-14143	933088	127133	0.446251	0.7294	no
gi|320445882|ref|NW_003384689.1|	141559-14364	374645	286827	-0.385342	0.84775	no
gi|320445882|ref|NW_003384689.1|	144417-14542	106898	738603	-0.533361	0.7945	no
gi|320445882|ref|NW_003384689.1|	146115-14662	919721	575738	-0.675783	0.7421	no
gi|320445882|ref|NW_003384689.1|	146760-14776	657025	423675	-0.632988	0.7515	no
gi|320445882|ref|NW_003384689.1|	177309-17806	0.976332	401535	204008	0.34735	no
gi|320445882|ref|NW_003384689.1|	196916-19950	114803	721788	265242	0.25255	no
gi|320445882|ref|NW_003384689.1|	199556-20002	0	571999	inf	0.029	no
gi|320445882|ref|NW_003384689.1|	21293-23549	23215	106661	-112203	0.3825	no
gi|320445882|ref|NW_003384689.1|	237613-24694	969816	139335	38447	0.04835	no
gi|320445882|ref|NW_003384689.1|	23961-24364	304796	125462	-128059	0.53855	no

gi|320445882|ref|NW_003384689.1|	26325-26904	923717	297619	-495591	0.0643	no
gi|320445882|ref|NW_003384689.1|	27011-29180	537551	103513	-569852	0.04165	no
gi|320445882|ref|NW_003384689.1|	31369-31925	100378	17642	-583028	0.1126	no
gi|320445882|ref|NW_003384689.1|	35999-37377	611435	519079	-0.236245	0.86205	no
gi|320445882|ref|NW_003384689.1|	40599-43244	433452	683467	0.657001	0.62935	no
gi|320445882|ref|NW_003384689.1|	49947-50602	128526	602007	-10942	0.5924	no
gi|320445882|ref|NW_003384689.1|	52386-52649	274069	104447	-139178	0.51745	no
gi|320445882|ref|NW_003384689.1|	54998-55560	359436	312108	-0.203688	0.90765	no
gi|320445882|ref|NW_003384689.1|	56443-57502	676204	425165	-0.669437	0.74285	no
gi|320445882|ref|NW_003384689.1|	58191-59207	799946	59762	-0.420675	0.8365	no
gi|320445882|ref|NW_003384689.1|	59354-61495	106257	16186	0.607197	0.71115	no
gi|320445882|ref|NW_003384689.1|	71933-73642	414526	438553	0.0812863	0.9471	no
gi|320445882|ref|NW_003384689.1|	73819-75094	761307	888629	0.223102	0.86325	no
gi|320445882|ref|NW_003384689.1|	75271-78932	632919	112292	0.827162	0.5176	no
gi|320445882|ref|NW_003384689.1|	81725-84350	112977	127768	0.177497	0.8889	no
gi|320445891|ref|NW_003384680.1|	10061-13688	158199	140636	-0.169782	0.8984	no
gi|320445891|ref|NW_003384680.1|	20106-20674	221969	313658	0.498833	0.8103	no
gi|320445891|ref|NW_003384680.1|	22346-23886	104771	119657	0.191667	0.93225	no
gi|320445891|ref|NW_003384680.1|	24397-25544	185124	38468	105516	0.60365	no
gi|320445891|ref|NW_003384680.1|	25666-26263	325465	47275	0.538574	0.78955	no
gi|320445891|ref|NW_003384680.1|	27093-28431	811158	563201	-0.526332	0.7951	no
gi|320445891|ref|NW_003384680.1|	28792-30585	121334	73207	-0.728927	0.75075	no
gi|320445891|ref|NW_003384680.1|	31318-31582	16259	120515	-0.432026	0.82045	no
gi|320445891|ref|NW_003384680.1|	37495-38477	406891	171706	-12447	0.56165	no
gi|320445891|ref|NW_003384680.1|	39464-39967	559672	416848	-0.425061	0.8278	no
gi|320445891|ref|NW_003384680.1|	41474-41868	798898	262562	-160535	0.44495	no
gi|320445891|ref|NW_003384680.1|	5545-7242	634977	144695	-213369	0.32885	no
gi|320445891|ref|NW_003384680.1|	63645-64150	185242	372624	100831	0.5772	no
gi|320445891|ref|NW_003384680.1|	65310-66644	0.921636	0.426588	-111135	1	no
gi|320445891|ref|NW_003384680.1|	72463-73736	585499	925302	0.660258	0.75745	no

gi|320445891|ref|NW_003384680.1|	9126-9378	34.36	158249	-111853	0.5849	no
gi|320445892|ref|NW_003384679.1|	29165-29831	117449	587286	232203	0.3042	no
gi|320445892|ref|NW_003384679.1|	32931-33995	122204	366286	158368	0.4759	no
gi|320445892|ref|NW_003384679.1|	34681-35915	185891	58597	165637	0.42905	no
gi|320445892|ref|NW_003384679.1|	38041-38942	202856	891177	213526	0.3247	no
gi|320445892|ref|NW_003384679.1|	42896-44410	11878	53232	216401	0.3294	no
gi|320445892|ref|NW_003384679.1|	48592-48827	39258	272216	279369	0.2661	no
gi|320445892|ref|NW_003384679.1|	49019-49725	0.716975	12.5	412386	0.16135	no
gi|320445892|ref|NW_003384679.1|	50500-51707	0.521068	565923	344106	0.19	no
gi|320445892|ref|NW_003384679.1|	54534-56863	0.16119	0.956493	256899	1	no
gi|320445892|ref|NW_003384679.1|	57116-63889	14762	139304	-0.0836535	0.95225	no
gi|320445892|ref|NW_003384679.1|	65823-66540	17167	50334	-177003	0.41175	no
gi|320445892|ref|NW_003384679.1|	69712-70843	734887	267496	-145801	0.26775	no
gi|320445892|ref|NW_003384679.1|	89437-90403	0.924011	127566	0.465257	1	no
gi|320445892|ref|NW_003384679.1|	91779-93621	934758	973389	0.0584227	0.97815	no
gi|320445900|ref|NW_003384671.1|	7850-8171	558416	102978	-576094	0.2728	no
gi|320445901|ref|NW_003384670.1|	20036-21560	628574	646625	0.0408456	0.98335	no
gi|320445901|ref|NW_003384670.1|	22502-23540	147191	101761	-0.532511	0.7943	no
gi|320445901|ref|NW_003384670.1|	92176-92506	164005	173641	0.0823646	0.95905	no
gi|320445903|ref|NW_003384668.1|	17291-19336	365522	593107	0.698334	0.6053	no
gi|320445903|ref|NW_003384668.1|	1912-2425	288245	564342	-235265	0.2901	no
gi|320445903|ref|NW_003384668.1|	23004-24171	235494	218388	-0.108794	0.95955	no
gi|320445903|ref|NW_003384668.1|	24551-28438	475458	590218	0.31193	0.81175	no
gi|320445903|ref|NW_003384668.1|	2593-2803	595592	745099	-299882	0.2661	no
gi|320445903|ref|NW_003384668.1|	34010-34790	192156	355186	0.886298	0.69355	no
gi|320445903|ref|NW_003384668.1|	35344-36307	201801	393854	0.964725	0.5616	no
gi|320445903|ref|NW_003384668.1|	40069-40994	176214	590688	-157686	0.4754	no
gi|320445903|ref|NW_003384668.1|	41150-42461	105117	392044	-142291	0.50325	no
gi|320445903|ref|NW_003384668.1|	42597-44637	138386	443445	-164186	0.328	no
gi|320445903|ref|NW_003384668.1|	4419-6878	11003	514035	-109795	0.50335	no

gi|320445903|ref|NW_003384668.1|	45920-46459	308601	742342	-205559	0.35105	no
gi|320445903|ref|NW_003384668.1|	48662-51089	127805	575261	-115165	0.48885	no
gi|320445903|ref|NW_003384668.1|	8947-9873	293265	11797	-131379	0.54	no
gi|320445903|ref|NW_003384668.1|	9981-10587	771599	40031	-0.946734	0.64715	no
gi|320445904|ref|NW_003384667.1|	14201-14651	0	558741	inf	0.0294	no
gi|320445904|ref|NW_003384667.1|	15829-16886	0	170474	inf	0.029	no
gi|320445904|ref|NW_003384667.1|	17171-18187	0	373512	inf	0.01485	no
gi|320445904|ref|NW_003384667.1|	2205-2630	170946	124406	-0.458486	0.8239	no
gi|320445904|ref|NW_003384667.1|	23266-32783	248853	329717	0.405937	0.75425	no
gi|320445904|ref|NW_003384667.1|	3489-3719	325707	143783	-117969	0.59085	no
gi|320445904|ref|NW_003384667.1|	37726-39356	139148	960033	-0.53546	0.81475	no
gi|320445904|ref|NW_003384667.1|	42387-44674	33227	526442	0.663917	0.6292	no
gi|320445904|ref|NW_003384667.1|	49064-49329	455527	596008	0.387795	0.84345	no
gi|320445904|ref|NW_003384667.1|	50358-50587	304985	175705	-0.795584	0.715	no
gi|320445904|ref|NW_003384667.1|	56426-60525	247611	943448	192987	0.15175	no
gi|320445904|ref|NW_003384667.1|	6338-7135	0.300548	227032	291723	0.25645	no
gi|320445911|ref|NW_003384660.1|	134117-134935	231768	418131	0.851272	0.67435	no
gi|320445911|ref|NW_003384660.1|	217130-217365	510354	123735	-204425	0.35575	no
gi|320445911|ref|NW_003384660.1|	242103-245309	261276	143005	-0.869516	0.67505	no
gi|320445911|ref|NW_003384660.1|	245785-246390	455086	246975	-0.881776	0.6765	no
gi|320445911|ref|NW_003384660.1|	250204-254252	14604	734214	232984	0.18205	no
gi|320445911|ref|NW_003384660.1|	255166-258449	426303	212982	-100115	0.644	no
gi|320445911|ref|NW_003384660.1|	263414-264045	148985	578888	-136381	0.51765	no
gi|320445911|ref|NW_003384660.1|	265940-267839	10143	155536	0.616768	0.761	no
gi|320445911|ref|NW_003384660.1|	34571-36115	0.386854	547141	382205	0.17145	no
gi|320445916|ref|NW_003384655.1|	186-1014	569639	783279	0.459479	0.82355	no
gi|320445917|ref|NW_003384654.1|	107093-107789	0	450409	inf	0.0154	no
gi|320445917|ref|NW_003384654.1|	113509-115042	551389	246019	-11643	0.3767	no
gi|320445917|ref|NW_003384654.1|	116474-120667	152925	962002	-0.668707	0.6804	no
gi|320445917|ref|NW_003384654.1|	125019-126560	732834	400052	-0.873298	0.51595	no

gi|320445917|ref|NW_003384654.1|	55570-55874	36632	941315	46835	0.15465	no
gi|320445917|ref|NW_003384654.1|	77392-78318	537652	0.337056	-399561	0.21285	no
gi|320445917|ref|NW_003384654.1|	82397-82908	19954	227237	0.187517	0.9273	no
gi|320445917|ref|NW_003384654.1|	88535-88761	270175	198179	-0.447089	0.81985	no
gi|320445917|ref|NW_003384654.1|	94100-95672	353498	711453	100907	0.63135	no
gi|320445917|ref|NW_003384654.1|	95850-96906	0.20566	184902	316843	0.24575	no
gi|320445917|ref|NW_003384654.1|	97032-10036	142574	503215	181946	0.17645	no
gi|320445924|ref|NW_003384647.1|	11440-16148	846143	177125	106579	0.5088	no
gi|320445924|ref|NW_003384647.1|	17202-19275	146919	158843	34345	0.0813	no
gi|320445924|ref|NW_003384647.1|	19364-20475	0.963689	827229	310165	0.20895	no
gi|320445924|ref|NW_003384647.1|	26720-27987	0.654339	374517	251692	0.28175	no
gi|320445924|ref|NW_003384647.1|	67374-72053	201241	204498	334509	0.0865	no
gi|320445924|ref|NW_003384647.1|	81379-82076	109629	774084	281986	0.2457	no
gi|320445927|ref|NW_003384644.1|	108823-11011	223811	721111	-163398	0.32265	no
gi|320445927|ref|NW_003384644.1|	110241-11113	16632	352601	-223786	0.32205	no
gi|320445927|ref|NW_003384644.1|	112215-11341	202522	240838	0.249983	0.87375	no
gi|320445927|ref|NW_003384644.1|	113566-11508	190919	15.2	-0.328886	0.83935	no
gi|320445927|ref|NW_003384644.1|	116052-11823	24.11	21544	-0.162345	0.8999	no
gi|320445927|ref|NW_003384644.1|	141598-14227	11404	363219	16713	0.4315	no
gi|320445927|ref|NW_003384644.1|	2197-2839	190587	138172	-0.463982	0.8208	no
gi|320445927|ref|NW_003384644.1|	41828-43147	934397	104863	0.1664	0.9395	no
gi|320445927|ref|NW_003384644.1|	54362-60153	15944	495232	163509	0.22555	no
gi|320445927|ref|NW_003384644.1|	87504-92574	254413	165755	-0.618123	0.6414	no
gi|320445929|ref|NW_003384642.1|	45772-49648	0.741527	123265	0.733189	1	no
gi|320445930|ref|NW_003384641.1|	40425-40753	125.59	420751	-157768	0.47275	no
gi|320445930|ref|NW_003384641.1|	48636-50095	388909	179263	-111735	0.37725	no
gi|320445930|ref|NW_003384641.1|	55697-57248	214202	161493	-0.407506	0.7419	no
gi|320445938|ref|NW_003384633.1|	101776-10205	110971	212619	0.938094	0.66165	no
gi|320445938|ref|NW_003384633.1|	112586-11331	206371	447312	111604	0.58775	no
gi|320445938|ref|NW_003384633.1|	115960-11712	17157	459006	141972	0.4096	no

gi|320445938|ref|NW_003384633.1|	37112-37869	0.647153	266192	204029	0.30255	no
gi|320445938|ref|NW_003384633.1|	39291-39990	0.727728	273533	191024	0.3194	no
gi|320445938|ref|NW_003384633.1|	81946-82148	209932	523705	131883	0.5467	no
gi|320445943|ref|NW_003384628.1|	23072-24280	0	271848	inf	5.00E-05	yes
gi|320445947|ref|NW_003384624.1|	101899-10285[illegible]	604087	77911	0.367071	0.77995	no
gi|320445947|ref|NW_003384624.1|	109263-10972[illegible]	56927	486037	-0.228047	0.9166	no
gi|320445947|ref|NW_003384624.1|	122017-12240[illegible]	710442	533337	-0.413671	0.84605	no
gi|320445947|ref|NW_003384624.1|	13077-13943	10706	165825	0.631245	0.75205	no
gi|320445947|ref|NW_003384624.1|	21200-23776	0.432534	100717	121942	1	no
gi|320445947|ref|NW_003384624.1|	29041-30357	81206	617897	-0.394219	0.85185	no
gi|320445947|ref|NW_003384624.1|	57466-57850	949396	110692	0.221466	0.91375	no
gi|320445947|ref|NW_003384624.1|	5901-9617	756226	184207	128443	0.33185	no
gi|320445947|ref|NW_003384624.1|	80366-80862	153044	597834	-135613	0.51375	no
gi|320445947|ref|NW_003384624.1|	90338-90645	678561	608495	-0.157232	0.9383	no
gi|320445947|ref|NW_003384624.1|	9771-12916	614695	750225	0.287452	0.9019	no
gi|320445947|ref|NW_003384624.1|	97772-99170	0.435445	110923	134899	1	no
gi|320445947|ref|NW_003384624.1|	99466-10174[illegible]	268606	172489	-0.638988	0.6233	no
gi|320445948|ref|NW_003384623.1|	12123-15319	205168	275014	0.4227	0.8401	no
gi|320445948|ref|NW_003384623.1|	67732-72340	162063	229968	0.504878	0.7029	no
gi|320445948|ref|NW_003384623.1|	76198-76912	211519	217024	0.0370729	0.94	no
gi|320445953|ref|NW_003384618.1|	17646-18298	73619	449101	-0.713039	0.6673	no
gi|320445953|ref|NW_003384618.1|	20213-20453	950737	530891	-0.84063	0.68105	no
gi|320445953|ref|NW_003384618.1|	2554-2824	558024	11299	-230413	0.31135	no
gi|320445953|ref|NW_003384618.1|	4396-5042	295442	838092	-18177	0.41225	no
gi|320445953|ref|NW_003384618.1|	5174-7699	238772	83388	-151772	0.3957	no
gi|320445953|ref|NW_003384618.1|	57611-57847	657791	365969	-0.845908	0.6781	no
gi|320445953|ref|NW_003384618.1|	58632-62591	178984	271819	0.602816	0.6517	no
gi|320445953|ref|NW_003384618.1|	64080-64785	0.359246	442082	362127	0.20665	no
gi|320445953|ref|NW_003384618.1|	7963-14349	551374	805627	0.547081	0.67395	no
gi|320445957|ref|NW_003384614.1|	10597-11744	203637	166695	-0.288791	0.88625	no

gi|320445957|ref|NW_003384614.1|	12333-13893	203829	203831	8.79E-01	0.9754	no
gi|320445957|ref|NW_003384614.1|	15744-16298	578578	922753	0.673432	0.7435	no
gi|320445957|ref|NW_003384614.1|	16473-17986	132081	137776	0.0609012	1	no
gi|320445957|ref|NW_003384614.1|	18241-21054	490951	105227	109985	0.5022	no
gi|320445957|ref|NW_003384614.1|	32238-34066	937255	37651	-795961	0.0139	no
gi|320445957|ref|NW_003384614.1|	38397-39009	920375	0.606571	-105673	0.16165	no
gi|320445957|ref|NW_003384614.1|	4012-4683	154819	792035	-0.966948	0.64035	no
gi|320445957|ref|NW_003384614.1|	49307-50795	158966	148928	-0.0941071	0.96915	no
gi|320445957|ref|NW_003384614.1|	50903-51624	555962	332885	-0.739964	0.7049	no
gi|320445957|ref|NW_003384614.1|	51789-53081	23957	177338	-0.433944	0.8255	no
gi|320445957|ref|NW_003384614.1|	53254-55428	59187	28545	-105205	0.6213	no
gi|320445957|ref|NW_003384614.1|	55535-56885	116578	344837	156461	0.3677	no
gi|320445957|ref|NW_003384614.1|	59659-65210	183145	110981	-0.722676	0.5927	no
gi|320445957|ref|NW_003384614.1|	6573-6915	109526	622042	-0.816187	0.6873	no
gi|320445957|ref|NW_003384614.1|	68421-72787	641303	112119	0.80595	0.53135	no
gi|320445957|ref|NW_003384614.1|	73379-75350	314026	576185	-244628	0.1832	no
gi|320445957|ref|NW_003384614.1|	78518-78951	107076	109154	-32942	0.2428	no
gi|320445957|ref|NW_003384614.1|	81450-83209	420856	0.231425	-418471	0.16	no
gi|320445957|ref|NW_003384614.1|	84005-86191	397919	0.241451	-404268	0.179	no
gi|320445957|ref|NW_003384614.1|	9541-10427	544779	232301	-122967	0.56125	no
gi|320445963|ref|NW_003384608.1|	23228-29386	863175	320654	189329	0.1684	no
gi|320445963|ref|NW_003384608.1|	29500-30046	539082	247114	21966	0.32305	no
gi|320445963|ref|NW_003384608.1|	30168-31872	33321	160012	226368	0.1931	no
gi|320445963|ref|NW_003384608.1|	35795-37499	0.1149	744056	601696	0.156	no
gi|320445963|ref|NW_003384608.1|	57621-57977	237034	138154	-0.778818	0.6978	no
gi|320445963|ref|NW_003384608.1|	58288-58599	103649	889531	-0.220586	0.91025	no
gi|320445963|ref|NW_003384608.1|	59533-60291	161479	132848	-0.281575	0.88515	no
gi|320445963|ref|NW_003384608.1|	60454-63408	104889	131902	0.330595	0.799	no
gi|320445963|ref|NW_003384608.1|	63653-64933	0	134513	inf	1	no
gi|320445963|ref|NW_003384608.1|	67807-69170	0.598619	270229	217447	0.34115	no

gi|320445963|ref|NW_003384608.1|	75834-77157	0.31032	247741	2997	0.2201	no
gi|320445966|ref|NW_003384605.1|	101490-10460	251606	396893	0.657582	0.7611	no
gi|320445966|ref|NW_003384605.1|	104794-10600	896722	105212	0.230562	0.9148	no
gi|320445966|ref|NW_003384605.1|	110182-11113	0	146871	inf	0.0059	no
gi|320445966|ref|NW_003384605.1|	131745-13270	0.231637	239831	337208	0.2261	no
gi|320445966|ref|NW_003384605.1|	148626-14995	269301	865398	-163778	0.33715	no
gi|320445966|ref|NW_003384605.1|	153106-15368	733109	0.992064	-288552	0.2142	no
gi|320445966|ref|NW_003384605.1|	91492-91762	304377	22598	-0.429663	0.8252	no
gi|320445970|ref|NW_003384601.1|	26916-27785	772507	385128	-100421	0.62005	no
gi|320445970|ref|NW_003384601.1|	49752-52178	123241	234512	0.928174	0.4752	no
gi|320445970|ref|NW_003384601.1|	52390-53491	214462	269887	0.331636	0.86945	no
gi|320445970|ref|NW_003384601.1|	57384-59463	869492	740571	-0.231534	0.91725	no
gi|320445970|ref|NW_003384601.1|	61705-62265	981447	313904	-164459	0.42665	no
gi|320445970|ref|NW_003384601.1|	62837-63130	222063	517164	-210228	0.3574	no
gi|320445970|ref|NW_003384601.1|	63598-64474	86945	453572	-0.93877	0.6482	no
gi|320445970|ref|NW_003384601.1|	64681-66331	620292	29892	-105319	0.61265	no
gi|320445970|ref|NW_003384601.1|	66867-68488	462779	728897	0.655392	0.7585	no
gi|320445970|ref|NW_003384601.1|	72015-72808	18465	130939	-0.495894	0.81525	no
gi|320445970|ref|NW_003384601.1|	75583-76289	322639	343136	0.0888608	0.95615	no
gi|320445970|ref|NW_003384601.1|	77898-78293	695307	78366	0.172577	0.9237	no
gi|320445970|ref|NW_003384601.1|	78399-81100	171085	382547	116093	0.5809	no
gi|320445970|ref|NW_003384601.1|	81294-81737	866441	135953	0.649938	0.75	no
gi|320445970|ref|NW_003384601.1|	83591-84089	101294	203553	100686	0.6191	no
gi|320445970|ref|NW_003384601.1|	84213-86304	300081	811933	143601	0.50665	no
gi|320445970|ref|NW_003384601.1|	86665-89965	313824	157506	232738	0.2006	no
gi|320445972|ref|NW_003384599.1|	17256-17618	360356	21942	26062	0.2651	no
gi|320445972|ref|NW_003384599.1|	17738-19239	0.4	101984	135027	1	no
gi|320445972|ref|NW_003384599.1|	21480-21855	111065	944921	308879	0.24695	no
gi|320445972|ref|NW_003384599.1|	29766-31435	103228	583429	-0.823202	0.53495	no
gi|320445972|ref|NW_003384599.1|	46270-47345	0	166873	inf	0.029	no

gi|320445972|ref|NW_003384599.1|	5329-11375	661748	212495	168308	0.29	no
gi|320445973|ref|NW_003384598.1|	159931-16070	0.625597	386298	262641	0.23645	no
gi|320445973|ref|NW_003384598.1|	178058-18000	0.39342	589711	390586	0.1658	no
gi|320445978|ref|NW_003384593.1|	121150-12237	117904	30924	139112	0.40935	no
gi|320445978|ref|NW_003384593.1|	122581-12735	115742	198364	0.777239	0.5574	no
gi|320445978|ref|NW_003384593.1|	127958-12964	860812	202476	-208795	0.3426	no
gi|320445978|ref|NW_003384593.1|	130228-13072	14561	169627	-310167	0.2344	no
gi|320445978|ref|NW_003384593.1|	130799-13212	367927	144886	-13445	0.431	no
gi|320445978|ref|NW_003384593.1|	132673-13626	153725	166618	0.116192	0.93035	no
gi|320445978|ref|NW_003384593.1|	39090-39599	182664	224671	362055	0.1831	no
gi|320445978|ref|NW_003384593.1|	54028-54830	154905	939162	259999	0.1577	no
gi|320445978|ref|NW_003384593.1|	56186-57371	0.177714	123162	279293	1	no
gi|320445978|ref|NW_003384593.1|	57613-62566	178823	174615	328757	0.09845	no
gi|320445978|ref|NW_003384593.1|	65690-66991	148862	0	#NAME?	0.00295	no
gi|320445978|ref|NW_003384593.1|	71385-71784	15.56	0	#NAME?	0.015	no
gi|320445978|ref|NW_003384593.1|	80137-80828	0.370205	581602	397363	0.1967	no
gi|320445981|ref|NW_003384590.1|	101958-10504	372308	71188	-238679	0.2121	no
gi|320445981|ref|NW_003384590.1|	105998-10958	128564	275162	-222413	0.2155	no
gi|320445981|ref|NW_003384590.1|	10988-11232	207777	678373	170704	0.4296	no
gi|320445981|ref|NW_003384590.1|	113675-11404	228795	351456	394122	0.1691	no
gi|320445981|ref|NW_003384590.1|	119564-11977	12383	812722	27144	0.23475	no
gi|320445981|ref|NW_003384590.1|	160136-16348	460568	480039	0.0597369	0.96375	no
gi|320445981|ref|NW_003384590.1|	165345-16676	421303	201762	-10622	0.41025	no
gi|320445981|ref|NW_003384590.1|	167911-16873	580142	241657	-126345	0.45675	no
gi|320445981|ref|NW_003384590.1|	172419-17294	783696	238025	-171918	0.30185	no
gi|320445981|ref|NW_003384590.1|	173715-17502	793592	150022	-240323	0.20365	no
gi|320445981|ref|NW_003384590.1|	176202-17646	729097	890079	-303411	0.22095	no
gi|320445981|ref|NW_003384590.1|	178824-17947	636575	911312	-280431	0.12425	no
gi|320445981|ref|NW_003384590.1|	71307-72077	132603	433136	-161422	0.45035	no
gi|320445981|ref|NW_003384590.1|	72436-73973	191874	937799	-103281	0.52355	no

gi|320445981|ref|NW_003384590.1|	74633-78986	0.819525	720163	313546	0.1045	no
gi|320445981|ref|NW_003384590.1|	85111-86063	0.471035	471304	332275	0.19155	no
gi|320445981|ref|NW_003384590.1|	96501-101017	314539	69006	-218844	0.1074	no
gi|320445982|ref|NW_003384589.1|	30545-30841	785819	104517	0.411473	0.85	no
gi|320445983|ref|NW_003384588.1|	171585-17268[illegible]	0	174829	inf	0.02915	no
gi|320445983|ref|NW_003384588.1|	173421-17420[illegible]	0	294591	inf	0.0233	no
gi|320445983|ref|NW_003384588.1|	175500-17781[illegible]	0	782017	inf	0.00515	no
gi|320445986|ref|NW_003384585.1|	50592-52207	26909	0.851208	-166051	0.436	no
gi|320445986|ref|NW_003384585.1|	52378-56515	121086	0.242201	-232176	1	no
gi|320445986|ref|NW_003384585.1|	56851-58227	0.739743	0.822101	0.152292	1	no
gi|320445986|ref|NW_003384585.1|	59635-60648	325554	104981	-163278	0.44075	no
gi|320445986|ref|NW_003384585.1|	62152-64678	780825	339581	-120124	0.5945	no
gi|320445986|ref|NW_003384585.1|	82172-82897	0	283072	inf	0.029	no
gi|320445992|ref|NW_003384579.1|	16612-19685	0.773002	661077	309627	0.1981	no
gi|320445992|ref|NW_003384579.1|	661-1286	17285	202638	355131	0.185	no
gi|320445992|ref|NW_003384579.1|	8569-14446	687852	401749	254612	0.1056	no
gi|320445995|ref|NW_003384576.1|	100735-10158[illegible]	907515	935702	0.0441286	0.9734	no
gi|320445995|ref|NW_003384576.1|	101979-10343[illegible]	527077	491685	-0.100281	0.9611	no
gi|320445995|ref|NW_003384576.1|	103739-10468[illegible]	43525	282242	-0.624914	0.70105	no
gi|320445995|ref|NW_003384576.1|	104800-10594[illegible]	177915	100125	-0.829381	0.70275	no
gi|320445995|ref|NW_003384576.1|	129414-13026[illegible]	367638	159737	-120259	0.461	no
gi|320445995|ref|NW_003384576.1|	47779-48571	0	116595	inf	0.00785	no
gi|320445997|ref|NW_003384574.1|	12020-14977	16739	230575	0.462022	0.72295	no
gi|320445997|ref|NW_003384574.1|	15640-17215	780993	771222	-0.0181632	0.99195	no
gi|320445997|ref|NW_003384574.1|	1772-2687	0.745034	171206	120036	0.56655	no
gi|320445997|ref|NW_003384574.1|	18048-18732	225561	307997	0.4494	0.82105	no
gi|320445997|ref|NW_003384574.1|	26221-28765	116941	178685	0.61163	0.7571	no
gi|320445997|ref|NW_003384574.1|	37530-39334	165664	389719	-208775	0.2243	no
gi|320445997|ref|NW_003384574.1|	47048-48169	188465	308868	0.712697	0.58555	no
gi|320445997|ref|NW_003384574.1|	48334-50331	463606	457533	-0.0190253	0.9874	no

gi|320445997|ref|NW_003384574.1|	52200-53278	238343	146887	-0.698332	0.75375	no
gi|320445997|ref|NW_003384574.1|	596-1669	302227	177023	255023	0.2665	no
gi|320445997|ref|NW_003384574.1|	59880-60735	277851	710458	135444	0.423	no
gi|320445997|ref|NW_003384574.1|	63698-64267	563365	475966	-356514	0.15985	no
gi|320445997|ref|NW_003384574.1|	68142-69038	797129	600292	-0.40915	0.7512	no
gi|320445997|ref|NW_003384574.1|	69403-69711	230207	0	#NAME?	0.01755	no
gi|320445997|ref|NW_003384574.1|	70184-71026	139122	348333	132412	0.41595	no
gi|320445997|ref|NW_003384574.1|	9602-11469	376447	173699	-111586	0.39095	no
gi|320445998|ref|NW_003384573.1|	28256-31570	213745	314352	387842	0.06165	no
gi|320445998|ref|NW_003384573.1|	45817-46374	0	351818	inf	0.0312	no
gi|320445998|ref|NW_003384573.1|	47058-47445	518706	107571	437423	0.13705	no
gi|320445998|ref|NW_003384573.1|	59100-62020	182267	47802	471294	0.0377	no
gi|320445998|ref|NW_003384573.1|	66372-68887	199849	727917	518679	0.03	no
gi|320445999|ref|NW_003384572.1|	202195-20496[illegible]	0.798219	627568	297492	0.21275	no
gi|320445999|ref|NW_003384572.1|	25806-26133	0	157686	inf	0.0198	no
gi|320445999|ref|NW_003384572.1|	27485-27889	0	87392	inf	0.0233	no
gi|320445999|ref|NW_003384572.1|	30203-30817	0	663888	inf	0.0186	no
gi|320446002|ref|NW_003384569.1|	119730-12165[illegible]	0.0998098	431381	543364	0.16235	no
gi|320446002|ref|NW_003384569.1|	32348-36735	279608	379054	0.438997	0.7386	no
gi|320446002|ref|NW_003384569.1|	82724-83024	113764	109503	-0.0550689	0.93995	no
gi|320446016|ref|NW_003384555.1|	1932-4102	614027	287862	-109293	0.4168	no
gi|320446016|ref|NW_003384555.1|	21439-23134	810416	748605	-0.114459	0.9284	no
gi|320446016|ref|NW_003384555.1|	23285-26775	315721	204203	-0.628646	0.644	no
gi|320446016|ref|NW_003384555.1|	33758-39962	840692	478273	-0.813745	0.513	no
gi|320446016|ref|NW_003384555.1|	41817-43548	518997	730735	0.493624	0.8117	no
gi|320446016|ref|NW_003384555.1|	43748-45062	79792	401826	-0.989673	0.64275	no
gi|320446016|ref|NW_003384555.1|	46381-47898	316004	24723	-0.354092	0.8603	no
gi|320446016|ref|NW_003384555.1|	48057-51348	316405	192343	-0.718096	0.5932	no
gi|320446017|ref|NW_003384554.1|	14392-15800	0.287821	612927	77344	0.1181	no
gi|320446017|ref|NW_003384554.1|	1705-3598	203594	276714	0.442699	0.8265	no

gi|320446017|ref|NW_003384554.1|	24832-26187	0	178984	inf	0.004	no
gi|320446017|ref|NW_003384554.1|	36453-37367	0	126876	inf	0.0052	no
gi|320446017|ref|NW_003384554.1|	39254-39921	0	141169	inf	0.0075	no
gi|320446017|ref|NW_003384554.1|	4479-4698	252869	253836	0.00550201	0.9322	no
gi|320446017|ref|NW_003384554.1|	5538-6241	360773	419245	0.216703	0.91095	no
gi|320446017|ref|NW_003384554.1|	55694-60095	227228	228599	0.00867751	0.99415	no
gi|320446017|ref|NW_003384554.1|	64458-64914	745081	143772	0.948317	0.6466	no
gi|320446017|ref|NW_003384554.1|	8514-9498	189375	373647	-23415	0.29715	no
gi|320446020|ref|NW_003384551.1|	25584-25891	214282	137773	-0.637225	0.75745	no
gi|320446020|ref|NW_003384551.1|	30525-30780	180635	133565	-0.435537	0.8204	no
gi|320446020|ref|NW_003384551.1|	33175-34075	0.253952	174999	278472	0.2657	no
gi|320446020|ref|NW_003384551.1|	34298-38183	138693	229744	0.72813	0.73215	no
gi|320446020|ref|NW_003384551.1|	3472-4371	223814	403092	-247312	0.2785	no
gi|320446020|ref|NW_003384551.1|	40951-43155	0.942845	0.717771	-0.393496	1	no
gi|320446020|ref|NW_003384551.1|	45246-47541	306402	390591	0.350232	0.79385	no
gi|320446020|ref|NW_003384551.1|	61986-62389	0	332476	inf	0.0075	no
gi|320446020|ref|NW_003384551.1|	63462-63837	0	101761	inf	0.0233	no
gi|320446020|ref|NW_003384551.1|	65861-67153	0	126353	inf	0.0038	no
gi|320446022|ref|NW_003384549.1|	11185-11705	791612	417629	-0.922572	0.5646	no
gi|320446022|ref|NW_003384549.1|	137410-13826[illegible]	265948	192423	-0.466868	0.8279	no
gi|320446022|ref|NW_003384549.1|	140732-14099[illegible]	624123	486117	-0.360528	0.8591	no
gi|320446022|ref|NW_003384549.1|	141514-14202[illegible]	598453	0.803551	-289678	0.2695	no
gi|320446022|ref|NW_003384549.1|	142928-14363[illegible]	28501	175096	-0.702865	0.7496	no
gi|320446022|ref|NW_003384549.1|	25277-25543	476976	134757	-182356	0.4021	no
gi|320446022|ref|NW_003384549.1|	47505-51637	209574	20765	-0.0133098	0.99115	no
gi|320446026|ref|NW_003384545.1|	122542-12762[illegible]	0.209005	226941	344071	0.172	no
gi|320446026|ref|NW_003384545.1|	127761-12846[illegible]	0.361541	642524	415152	0.19215	no
gi|320446026|ref|NW_003384545.1|	27701-28334	23296	109462	-108965	0.60665	no
gi|320446026|ref|NW_003384545.1|	30638-36485	263082	318579	0.276141	0.8354	no
gi|320446026|ref|NW_003384545.1|	68628-70242	265246	724898	-187148	0.1634	no

gi|320446026|ref|NW_003384545.1|	70630-70871	122623	592074	-105038	0.6132	no
gi|320446026|ref|NW_003384545.1|	95474-96193	307386	866577	-182665	0.17955	no
gi|320446027|ref|NW_003384544.1|	101836-10612[illegible]	0.208219	259503	363958	0.16845	no
gi|320446028|ref|NW_003384543.1|	110827-11118[illegible]	408404	125383	-170366	0.417	no
gi|320446028|ref|NW_003384543.1|	11465-12571	608674	725911	0.254124	0.84775	no
gi|320446028|ref|NW_003384543.1|	16666-18938	597355	782431	0.389375	0.7728	no
gi|320446028|ref|NW_003384543.1|	19936-20445	182664	326794	0.839194	0.68815	no
gi|320446028|ref|NW_003384543.1|	20679-21605	0.488775	151675	163374	0.3325	no
gi|320446028|ref|NW_003384543.1|	21742-22292	106491	323194	160167	0.33275	no
gi|320446028|ref|NW_003384543.1|	26734-33747	440371	948862	110748	0.5002	no
gi|320446028|ref|NW_003384543.1|	34180-35942	150326	931593	-0.690324	0.66735	no
gi|320446028|ref|NW_003384543.1|	42311-43869	914791	585781	-0.643081	0.64295	no
gi|320446028|ref|NW_003384543.1|	44515-46920	377969	129788	-154211	0.2433	no
gi|320446028|ref|NW_003384543.1|	49445-50525	699349	1106	-266067	0.2474	no
gi|320446028|ref|NW_003384543.1|	51983-52883	170148	262498	-269641	0.2435	no
gi|320446028|ref|NW_003384543.1|	54748-55429	130563	981631	-373342	0.06825	no
gi|320446028|ref|NW_003384543.1|	57321-61149	432075	841471	-23603	0.08325	no
gi|320446029|ref|NW_003384542.1|	107154-10761[illegible]	0.73891	98366	373469	0.2083	no
gi|320446029|ref|NW_003384542.1|	120492-12125[illegible]	0.317501	32664	336287	0.22635	no
gi|320446029|ref|NW_003384542.1|	122889-12321[illegible]	0	173767	inf	0.02075	no
gi|320446029|ref|NW_003384542.1|	13800-14240	143668	635478	-117683	0.58015	no
gi|320446029|ref|NW_003384542.1|	16136-18419	362585	138049	-139314	0.5063	no
gi|320446029|ref|NW_003384542.1|	23022-24006	405804	311372	-0.382142	0.85295	no
gi|320446029|ref|NW_003384542.1|	27493-29401	857514	527405	-0.701248	0.74415	no
gi|320446029|ref|NW_003384542.1|	29722-32283	652986	745019	0.190224	0.92805	no
gi|320446029|ref|NW_003384542.1|	32431-33044	231723	347903	0.586282	0.7841	no
gi|320446029|ref|NW_003384542.1|	34252-37092	185923	120441	-0.626373	0.632	no
gi|320446029|ref|NW_003384542.1|	38122-38870	329243	225615	-0.545294	0.79685	no
gi|320446029|ref|NW_003384542.1|	42041-45065	326736	342666	0.0686796	0.9725	no
gi|320446031|ref|NW_003384540.1|	10155-11421	430646	444172	0.0446168	0.9702	no

gi|320446031|ref|NW_003384540.1|	11748-17527	128133	310961	12791	0.34375	no
gi|320446031|ref|NW_003384540.1|	149788-15239	952963	188298	0.982526	0.4351	no
gi|320446031|ref|NW_003384540.1|	153106-15558	194871	196525	0.0121987	0.9925	no
gi|320446031|ref|NW_003384540.1|	155705-15622	947776	875262	-0.114831	0.9501	no
gi|320446031|ref|NW_003384540.1|	156359-15826	980334	810131	-0.275119	0.89945	no
gi|320446031|ref|NW_003384540.1|	166090-17230	496769	854612	0.782695	0.5507	no
gi|320446031|ref|NW_003384540.1|	173994-17523	502768	471134	-0.0937555	0.94295	no
gi|320446031|ref|NW_003384540.1|	177380-17827	798062	514332	-0.6338	0.753	no
gi|320446031|ref|NW_003384540.1|	180046-18045	996183	119541	-305891	0.2561	no
gi|320446031|ref|NW_003384540.1|	180961-18295	326787	221165	-0.563227	0.7837	no
gi|320446031|ref|NW_003384540.1|	18121-22819	110778	127103	0.198326	0.8785	no
gi|320446031|ref|NW_003384540.1|	183477-18426	286655	205202	-0.482274	0.8289	no
gi|320446031|ref|NW_003384540.1|	185615-18649	211105	0.726829	-153828	0.4983	no
gi|320446031|ref|NW_003384540.1|	186542-18867	435941	130347	-174177	0.4188	no
gi|320446031|ref|NW_003384540.1|	189669-19212	199171	116794	-409196	0.0648	no
gi|320446031|ref|NW_003384540.1|	193195-19383	303189	169588	-416011	0.14855	no
gi|320446031|ref|NW_003384540.1|	196071-19633	650362	516494	-365442	0.1742	no
gi|320446031|ref|NW_003384540.1|	199017-20137	162732	112507	-0.532482	0.6772	no
gi|320446031|ref|NW_003384540.1|	201501-20188	571885	0.707001	-633787	0.2642	no
gi|320446031|ref|NW_003384540.1|	202945-20604	243596	0.987458	-462463	0.04265	no
gi|320446031|ref|NW_003384540.1|	209325-20967	252743	740508	-177109	0.4112	no
gi|320446031|ref|NW_003384540.1|	210472-21077	162069	347244	-222259	0.271	no
gi|320446031|ref|NW_003384540.1|	211555-21207	125246	600686	-106008	0.61005	no
gi|320446031|ref|NW_003384540.1|	212472-21728	570806	109366	0.938096	0.4701	no
gi|320446031|ref|NW_003384540.1|	32842-33081	852834	105298	0.304146	0.8781	no
gi|320446031|ref|NW_003384540.1|	40907-41508	183987	873347	224695	0.3318	no
gi|320446031|ref|NW_003384540.1|	6208-7587	172646	389485	-214818	0.35015	no
gi|320446031|ref|NW_003384540.1|	7731-10065	162414	297495	-244874	0.1736	no
gi|320446032|ref|NW_003384539.1|	28269-28643	142998	497052	-152453	0.3543	no
gi|320446032|ref|NW_003384539.1|	33024-33498	886803	374524	-124356	0.44255	no

gi|320446032|ref|NW_003384539.1|	33893-34512	131632	298037	-214294	0.337	no
gi|320446032|ref|NW_003384539.1|	35818-36096	746537	119032	-264887	0.24575	no
gi|320446032|ref|NW_003384539.1|	36541-37485	0.714532	164321	120145	0.56655	no
gi|320446032|ref|NW_003384539.1|	37667-38868	260406	81169	-168176	0.3099	no
gi|320446032|ref|NW_003384539.1|	38982-40179	143907	119212	-0.271601	0.9004	no
gi|320446032|ref|NW_003384539.1|	48708-55657	345301	311838	-0.147055	0.90755	no
gi|320446032|ref|NW_003384539.1|	59651-61263	476837	855605	-247848	0.1822	no
gi|320446032|ref|NW_003384539.1|	61400-65275	617954	889047	-279717	0.04355	no
gi|320446032|ref|NW_003384539.1|	65388-66690	0.632862	0.878475	0.473109	1	no
gi|320446032|ref|NW_003384539.1|	67179-67965	400665	33878	-356398	0.0876	no
gi|320446032|ref|NW_003384539.1|	6770-11123	215125	361516	0.748881	0.574	no
gi|320446038|ref|NW_003384533.1|	30580-31607	0.852786	383139	216761	0.3495	no
gi|320446038|ref|NW_003384533.1|	35797-40701	990233	896135	-0.144052	0.9109	no
gi|320446038|ref|NW_003384533.1|	50246-54322	16738	38935	121794	0.366	no
gi|320446039|ref|NW_003384532.1|	28650-29512	0	982636	inf	0.0075	no
gi|320446039|ref|NW_003384532.1|	43617-46377	227237	140119	262438	0.1472	no
gi|320446039|ref|NW_003384532.1|	49541-51434	0.203594	306514	723411	0.1181	no
gi|320446039|ref|NW_003384532.1|	51665-52649	0	264666	inf	0.02075	no
gi|320446046|ref|NW_003384525.1|	101006-10241[illegible]	10048	907594	317514	0.18625	no
gi|320446046|ref|NW_003384525.1|	103309-10436[illegible]	0.205409	213092	33749	0.2261	no
gi|320446046|ref|NW_003384525.1|	105856-10650[illegible]	0.412318	505202	361503	0.20665	no
gi|320446046|ref|NW_003384525.1|	110605-11103[illegible]	260067	199965	-0.379135	0.8526	no
gi|320446046|ref|NW_003384525.1|	111628-11228[illegible]	516058	243502	-10836	0.58675	no
gi|320446046|ref|NW_003384525.1|	112615-11301[illegible]	356088	152056	-122763	0.55785	no
gi|320446046|ref|NW_003384525.1|	113177-11381[illegible]	880752	256795	-177812	0.408	no
gi|320446046|ref|NW_003384525.1|	118991-12041[illegible]	284424	118585	-126212	0.5602	no
gi|320446046|ref|NW_003384525.1|	120815-12149[illegible]	187967	128332	-0.5506	0.80065	no
gi|320446046|ref|NW_003384525.1|	121649-12233[illegible]	29746	203154	-0.550117	0.7899	no
gi|320446046|ref|NW_003384525.1|	123583-12546[illegible]	369367	200225	-0.883433	0.66795	no
gi|320446046|ref|NW_003384525.1|	126758-12722[illegible]	777287	518329	-0.584577	0.78725	no

gi|320446046|ref|NW_003384525.1|	127336-12858	466173	207866	-116522	0.5727	no
gi|320446046|ref|NW_003384525.1|	130125-13060	400006	713011	0.833903	0.6789	no
gi|320446046|ref|NW_003384525.1|	131224-13251	812231	751579	-0.111967	0.9548	no
gi|320446046|ref|NW_003384525.1|	133505-13412	477751	973888	10275	0.6019	no
gi|320446046|ref|NW_003384525.1|	135416-13656	35251	758178	110487	0.59605	no
gi|320446046|ref|NW_003384525.1|	136960-13754	341149	956381	148718	0.47815	no
gi|320446046|ref|NW_003384525.1|	138568-13977	691908	205066	156744	0.4895	no
gi|320446046|ref|NW_003384525.1|	150955-15560	132299	110865	-0.254995	0.84875	no
gi|320446046|ref|NW_003384525.1|	156455-15680	755354	391573	-0.94787	0.6452	no
gi|320446046|ref|NW_003384525.1|	156962-16214	762606	289129	-139922	0.29075	no
gi|320446046|ref|NW_003384525.1|	162863-16328	641047	231847	-146726	0.5001	no
gi|320446046|ref|NW_003384525.1|	163399-16361	386856	276934	-0.482255	0.81355	no
gi|320446046|ref|NW_003384525.1|	163723-16493	243898	189476	-0.364258	0.816	no
gi|320446046|ref|NW_003384525.1|	165058-16683	111865	786874	-0.507555	0.81725	no
gi|320446046|ref|NW_003384525.1|	168360-17201	364349	317832	-351898	0.08825	no
gi|320446046|ref|NW_003384525.1|	174773-17725	389972	174749	-115809	0.3858	no
gi|320446046|ref|NW_003384525.1|	177471-17773	64497	142413	-217916	0.32575	no
gi|320446046|ref|NW_003384525.1|	180491-18278	303862	171977	-0.821199	0.68815	no
gi|320446046|ref|NW_003384525.1|	185535-18688	362062	0.419045	-311106	0.2265	no
gi|320446046|ref|NW_003384525.1|	189990-19194	590131	239999	-129801	0.5441	no
gi|320446046|ref|NW_003384525.1|	192766-19383	135643	854285	-0.66702	0.75625	no
gi|320446046|ref|NW_003384525.1|	193952-19509	238004	154541	-0.622996	0.68945	no
gi|320446046|ref|NW_003384525.1|	195445-19782	145351	171705	0.240388	0.85205	no
gi|320446046|ref|NW_003384525.1|	20536-21052	114121	45902	-131393	0.43735	no
gi|320446046|ref|NW_003384525.1|	212317-21371	120994	921555	-0.39279	0.8565	no
gi|320446046|ref|NW_003384525.1|	21383-21866	147225	983958	-0.581357	0.7801	no
gi|320446046|ref|NW_003384525.1|	214646-21488	199182	22594	0.181857	0.91575	no
gi|320446046|ref|NW_003384525.1|	216950-21905	164172	24101	0.553891	0.6694	no
gi|320446046|ref|NW_003384525.1|	2176-3168	272136	157148	-0.792201	0.7263	no
gi|320446046|ref|NW_003384525.1|	227929-22868	412439	201904	-435244	0.06835	no

gi|320446046|ref|NW_003384525.1|	233369-23390	211421	0.749201	-481863	0.19595	no
gi|320446046|ref|NW_003384525.1|	234707-23533	142242	175779	-301652	0.2159	no
gi|320446046|ref|NW_003384525.1|	23514-24181	234359	151823	-0.626331	0.76885	no
gi|320446046|ref|NW_003384525.1|	236602-23771	22014	251805	-312804	0.2073	no
gi|320446046|ref|NW_003384525.1|	238852-24053	114473	235839	-227913	0.31305	no
gi|320446046|ref|NW_003384525.1|	240657-24110	303204	464749	-270576	0.2324	no
gi|320446046|ref|NW_003384525.1|	24431-24710	254039	736662	-178597	0.4171	no
gi|320446046|ref|NW_003384525.1|	25022-26026	305214	28823	-0.082599	0.9464	no
gi|320446046|ref|NW_003384525.1|	31071-35759	299356	639593	109529	0.5068	no
gi|320446046|ref|NW_003384525.1|	3876-6632	227598	11695	-428252	0.05025	no
gi|320446046|ref|NW_003384525.1|	39612-41495	839803	299795	-148608	0.49685	no
gi|320446046|ref|NW_003384525.1|	41668-42937	357557	792912	-217294	0.218	no
gi|320446046|ref|NW_003384525.1|	44515-45766	0.166163	138307	305721	1	no
gi|320446046|ref|NW_003384525.1|	50197-58711	163449	575702	181649	0.16865	no
gi|320446046|ref|NW_003384525.1|	59444-61223	174906	373135	109312	0.4014	no
gi|320446046|ref|NW_003384525.1|	63150-63402	406073	534092	0.395351	0.84215	no
gi|320446046|ref|NW_003384525.1|	7195-11823	318426	186152	-0.774473	0.5626	no
gi|320446046|ref|NW_003384525.1|	82694-83161	178786	321462	0.846416	0.5209	no
gi|320446046|ref|NW_003384525.1|	96507-99656	151146	15632	0.0485511	0.9692	no
gi|320446047|ref|NW_003384524.1|	15244-15980	394418	537918	0.44766	0.78595	no
gi|320446050|ref|NW_003384521.1|	153952-15474	0	270667	inf	0.02915	no
gi|320446050|ref|NW_003384521.1|	15851-18955	274685	559777	102707	0.44785	no
gi|320446050|ref|NW_003384521.1|	19148-19387	111239	210597	0.920817	0.67085	no
gi|320446050|ref|NW_003384521.1|	19916-20617	717378	628996	-0.189681	0.8768	no
gi|320446050|ref|NW_003384521.1|	20861-23107	150221	304577	-230221	0.1948	no
gi|320446050|ref|NW_003384521.1|	23240-25608	0.791209	375641	224722	0.31565	no
gi|320446050|ref|NW_003384521.1|	26576-27286	639873	121551	-239622	0.30465	no
gi|320446050|ref|NW_003384521.1|	34401-38145	247178	306925	0.312336	0.81345	no
gi|320446050|ref|NW_003384521.1|	40108-41188	231784	176959	-0.389365	0.85955	no
gi|320446051|ref|NW_003384520.1|	105167-10824	186119	67143	-147092	0.4053	no

gi|320446051|ref|NW_003384520.1|	108761-10915	171604	112747	-0.605993	0.75665	no
gi|320446051|ref|NW_003384520.1|	109549-11032	465748	511364	0.134801	0.94325	no
gi|320446051|ref|NW_003384520.1|	112057-11243	230538	122195	-0.915817	0.6649	no
gi|320446051|ref|NW_003384520.1|	115196-11840	348278	710098	102778	0.4435	no
gi|320446051|ref|NW_003384520.1|	120058-12164	151066	972921	-0.634785	0.78175	no
gi|320446051|ref|NW_003384520.1|	124194-12471	826494	634484	-0.381421	0.84645	no
gi|320446051|ref|NW_003384520.1|	124826-12525	160884	134503	-0.258389	0.89715	no
gi|320446051|ref|NW_003384520.1|	126251-12697	401242	245888	-0.706469	0.75555	no
gi|320446051|ref|NW_003384520.1|	131725-13349	782739	104437	0.416023	0.85225	no
gi|320446051|ref|NW_003384520.1|	133639-13521	102339	188104	0.878178	0.5938	no
gi|320446051|ref|NW_003384520.1|	138256-13876	126107	257985	103264	0.62665	no
gi|320446051|ref|NW_003384520.1|	38813-43224	0.0404071	15279	52408	0.1671	no
gi|320446051|ref|NW_003384520.1|	43324-53478	0.391236	23268	257223	0.1538	no
gi|320446051|ref|NW_003384520.1|	53598-58242	574071	267228	221877	0.1016	no
gi|320446051|ref|NW_003384520.1|	69543-69928	933611	750054	-0.315827	0.88405	no
gi|320446051|ref|NW_003384520.1|	70505-71844	0.30582	106169	179561	1	no
gi|320446051|ref|NW_003384520.1|	72115-73202	753051	397676	-0.921153	0.65475	no
gi|320446051|ref|NW_003384520.1|	74284-74754	633439	563299	-0.169304	0.9324	no
gi|320446051|ref|NW_003384520.1|	74867-75790	29209	17602	-0.730675	0.7446	no
gi|320446051|ref|NW_003384520.1|	76146-77134	405889	136288	-157443	0.35365	no
gi|320446051|ref|NW_003384520.1|	78169-78642	159973	324881	-229985	0.3178	no
gi|320446051|ref|NW_003384520.1|	79474-79909	734598	378731	-0.955781	0.66475	no
gi|320446051|ref|NW_003384520.1|	80221-81041	713135	394902	-0.852682	0.5006	no
gi|320446051|ref|NW_003384520.1|	83872-86792	392189	271962	-0.528145	0.69605	no
gi|320446051|ref|NW_003384520.1|	87065-89421	169481	705512	-126438	0.44875	no
gi|320446051|ref|NW_003384520.1|	94614-95224	103299	213373	-227538	0.3199	no
gi|320446051|ref|NW_003384520.1|	95329-96161	877018	0.778135	-349451	0.2064	no
gi|320446051|ref|NW_003384520.1|	98731-99061	149096	385868	-195006	0.3973	no
gi|320446052|ref|NW_003384519.1|	137865-14055	516186	365484	282385	0.14215	no
gi|320446052|ref|NW_003384519.1|	141538-14207	0.559959	376921	275087	0.26605	no

gi|320446052|ref|NW_003384519.1|	l54061-15434(	138567	618796	215888	0.33535	no
gi|320446052|ref|NW_003384519.1|	l54653-15526(	0.905367	522149	252789	0.25095	no
gi|320446052|ref|NW_003384519.1|	l61637-16190!	124141	363601	155037	0.4865	no
gi|320446052|ref|NW_003384519.1|	l75307-17576!	107142	477075	-116724	0.47925	no
gi|320446052|ref|NW_003384519.1|	l78241-17931!	86535	487284	-0.82852	0.6862	no
gi|320446052|ref|NW_003384519.1|	l79450-18127(	316135	276452	-0.193515	0.8811	no
gi|320446052|ref|NW_003384519.1|	l83264-18562!	184698	283364	0.617488	0.6306	no
gi|320446052|ref|NW_003384519.1|	6303-6613	191589	112077	-409545	0.30775	no
gi|320446052|ref|NW_003384519.1|	67452-67930	0	100218	inf	0.0186	no
gi|320446052|ref|NW_003384519.1|	81638-86096	243739	173479	-0.490583	0.8184	no
gi|320446052|ref|NW_003384519.1|	87689-92421	44389	298543	-0.572263	0.66255	no
gi|320446052|ref|NW_003384519.1|	93309-93892	34794	166826	-106049	0.62075	no
gi|320446052|ref|NW_003384519.1|	95029-96033	44794	280645	-0.674557	0.6868	no
gi|320446052|ref|NW_003384519.1|	96923-100014	264709	26525	0.00294641	0.9977	no
gi|320446054|ref|NW_003384517.1|	77956-79250	0	121692	inf	1	no
gi|320446056|ref|NW_003384515.1|	13206-15512	0	335624	inf	0.00795	no
gi|320446056|ref|NW_003384515.1|	32770-35494	0.745815	331651	215277	0.32645	no
gi|320446056|ref|NW_003384515.1|	38707-39785	0.400577	277146	279049	0.2345	no
gi|320446056|ref|NW_003384515.1|	39973-41852	164263	150259	-0.128552	0.9444	no
gi|320446056|ref|NW_003384515.1|	42009-43511	133228	157489	0.241349	0.8982	no
gi|320446056|ref|NW_003384515.1|	46871-48571	103692	240668	121473	0.56165	no
gi|320446056|ref|NW_003384515.1|	48927-51364	216127	567311	-192967	0.2729	no
gi|320446056|ref|NW_003384515.1|	57547-57735	776413	120292	-269028	0.28955	no
gi|320446056|ref|NW_003384515.1|	59749-60571	460374	237337	-0.955871	0.65245	no
gi|320446056|ref|NW_003384515.1|	60686-63151	317849	173353	-0.874629	0.5064	no
gi|320446056|ref|NW_003384515.1|	64019-66125	100217	211435	-224484	0.09995	no
gi|320446056|ref|NW_003384515.1|	66247-67126	710673	151704	-222793	0.21815	no
gi|320446056|ref|NW_003384515.1|	69534-70015	67432	225265	-158181	0.49165	no
gi|320446056|ref|NW_003384515.1|	70154-70427	137576	265996	-237075	0.29115	no
gi|320446056|ref|NW_003384515.1|	70565-77472	187899	103358	-0.862306	0.51815	no

gi|320446056|ref|NW_003384515.1|	81777-83892	355049	175859	-10136	0.5432	no
gi|320446057|ref|NW_003384514.1|	85802-88241	0.459428	294094	267836	0.2451	no
gi|320446057|ref|NW_003384514.1|	99175-102967	0.0948802	511303	575193	0.13865	no
gi|320446058|ref|NW_003384513.1|	40385-40573	291155	186453	267895	0.26485	no
gi|320446061|ref|NW_003384510.1|	12747-14200	126117	166652	0.402067	0.8613	no
gi|320446061|ref|NW_003384510.1|	15332-17020	769017	102475	0.414178	0.7571	no
gi|320446061|ref|NW_003384510.1|	19783-24141	98633	376267	193161	0.15385	no
gi|320446061|ref|NW_003384510.1|	28813-29043	253798	346143	0.447691	0.82185	no
gi|320446061|ref|NW_003384510.1|	3411-4810	303099	345583	0.189242	0.8828	no
gi|320446061|ref|NW_003384510.1|	35595-35839	380925	65649	0.785264	0.69545	no
gi|320446061|ref|NW_003384510.1|	41798-43013	361768	148103	203347	0.36635	no
gi|320446061|ref|NW_003384510.1|	4971-6534	278361	477487	0.778507	0.54535	no
gi|320446061|ref|NW_003384510.1|	53017-54302	0.803894	20082	132083	0.5464	no
gi|320446061|ref|NW_003384510.1|	54881-55292	731721	138785	0.923485	0.6558	no
gi|320446061|ref|NW_003384510.1|	61091-62285	0.528136	23184	213415	0.34635	no
gi|320446061|ref|NW_003384510.1|	77140-77754	546693	724242	0.40574	0.74345	no
gi|320446061|ref|NW_003384510.1|	77909-78797	0.775869	213787	146229	0.50355	no
gi|320446061|ref|NW_003384510.1|	78897-79525	167261	221575	0.405692	0.8438	no
gi|320446061|ref|NW_003384510.1|	8032-9915	104361	188514	0.853088	0.5156	no
gi|320446062|ref|NW_003384509.1|	13434-14079	46891	896046	-238767	0.30525	no
gi|320446062|ref|NW_003384509.1|	14809-14989	725114	521879	-0.474492	0.8141	no
gi|320446062|ref|NW_003384509.1|	15172-15816	36284	252601	-38444	0.13945	no
gi|320446062|ref|NW_003384509.1|	22452-22898	186587	0	#NAME?	0.00975	no
gi|320446067|ref|NW_003384504.1|	1359-1834	138023	598841	211726	0.29385	no
gi|320446067|ref|NW_003384504.1|	24799-25327	742291	115217	-268764	0.227	no
gi|320446067|ref|NW_003384504.1|	27289-28435	759844	205385	-188737	0.37515	no
gi|320446067|ref|NW_003384504.1|	29134-29842	103524	292915	-182141	0.40235	no
gi|320446067|ref|NW_003384504.1|	76312-78007	173413	877397	233902	0.2965	no
gi|320446068|ref|NW_003384503.1|	13211-14025	0	581326	inf	0.0109	no
gi|320446068|ref|NW_003384503.1|	14138-19111	698025	19708	-18245	0.2916	no

gi|320446068|ref|NW_003384503.1|	19252-20860	68845	0.513306	-374546	0.16595	no
gi|320446068|ref|NW_003384503.1|	21608-22697	672203	0.547252	-361862	0.1956	no
gi|320446068|ref|NW_003384503.1|	27265-27797	225436	493051	112902	0.5922	no
gi|320446068|ref|NW_003384503.1|	29682-30313	723644	0	#NAME?	0.01065	no
gi|320446068|ref|NW_003384503.1|	3021-3663	319026	112794	-149999	0.49615	no
gi|320446068|ref|NW_003384503.1|	30515-31575	654918	0.141552	-553191	0.2748	no
gi|320446068|ref|NW_003384503.1|	48832-53321	43236	813869	0.912562	0.59	no
gi|320446068|ref|NW_003384503.1|	5212-5947	101334	370103	-145313	0.48565	no
gi|320446068|ref|NW_003384503.1|	53764-54952	728105	708438	-0.0395054	0.97595	no
gi|320446068|ref|NW_003384503.1|	55983-57362	295121	553479	0.907221	0.66415	no
gi|320446068|ref|NW_003384503.1|	58338-63758	270106	918888	176636	0.3146	no
gi|320446068|ref|NW_003384503.1|	64450-65214	0.3193	37224	354325	0.2165	no
gi|320446068|ref|NW_003384503.1|	65780-69728	13634	187686	0.461112	0.82115	no
gi|320446068|ref|NW_003384503.1|	69877-70506	229.73	145421	-730356	0.1125	no
gi|320446068|ref|NW_003384503.1|	768-2793	211204	460287	-219803	0.2119	no
gi|320446068|ref|NW_003384503.1|	7914-8734	441739	595329	-289144	0.11655	no
gi|320446068|ref|NW_003384503.1|	84223-91083	105466	295488	-183561	0.2951	no
gi|320446068|ref|NW_003384503.1|	9442-9751	509288	677851	-290944	0.2321	no
gi|320446069|ref|NW_003384502.1|	103037-103718	0	413318	inf	0.0198	no
gi|320446069|ref|NW_003384502.1|	106760-107943	0	185128	inf	0.02205	no
gi|320446069|ref|NW_003384502.1|	115752-116827	0	194685	inf	0.0233	no
gi|320446069|ref|NW_003384502.1|	118868-121647	0	205371	inf	5.00E-05	yes
gi|320446069|ref|NW_003384502.1|	121916-122679	0.319904	197432	262565	0.26355	no
gi|320446069|ref|NW_003384502.1|	123130-124504	0	164706	inf	0.0198	no
gi|320446069|ref|NW_003384502.1|	125930-128355	0	828698	inf	0.0039	no
gi|320446069|ref|NW_003384502.1|	62226-62531	363214	140039	194694	0.3158	no
gi|320446072|ref|NW_003384499.1|	1378-2560	606144	731402	359293	0.07335	no
gi|320446072|ref|NW_003384499.1|	45-979	338223	47.99	382669	0.0635	no
gi|320446072|ref|NW_003384499.1|	4868-5360	129416	909421	281294	0.2258	no
gi|320446072|ref|NW_003384499.1|	59458-60411	752605	746562	-0.0116316	0.98655	no

gi|320446072|ref|NW_003384499.1|	62139-64423	807182	112686	0.481337	0.7612	no
gi|320446073|ref|NW_003384498.1|	129317-12964	717255	735271	-328614	0.09155	no
gi|320446073|ref|NW_003384498.1|	151003-15213	131756	299787	118607	0.57085	no
gi|320446073|ref|NW_003384498.1|	152236-16441	751323	136841	0.864996	0.52975	no
gi|320446073|ref|NW_003384498.1|	167576-16825	158593	592867	-141955	0.50315	no
gi|320446073|ref|NW_003384498.1|	199461-19983	268121	0.730959	-519695	0.27925	no
gi|320446073|ref|NW_003384498.1|	201449-20560	116715	280412	-205737	0.2421	no
gi|320446073|ref|NW_003384498.1|	277227-27835	113313	24847	113276	0.5865	no
gi|320446073|ref|NW_003384498.1|	278506-27992	647279	258569	-132384	0.3191	no
gi|320446073|ref|NW_003384498.1|	280121-28177	524571	335345	-0.645494	0.62265	no
gi|320446073|ref|NW_003384498.1|	284710-28917	907309	578382	-0.649573	0.61605	no
gi|320446073|ref|NW_003384498.1|	289808-29007	715464	218.98	-170808	0.32515	no
gi|320446073|ref|NW_003384498.1|	298729-29924	0	556985	inf	0.0233	no
gi|320446073|ref|NW_003384498.1|	302803-30357	75491	282673	-141717	0.40215	no
gi|320446073|ref|NW_003384498.1|	304751-30542	615082	472104	-0.381676	0.8429	no
gi|320446073|ref|NW_003384498.1|	308264-30921	732148	374821	-0.965934	0.64565	no
gi|320446073|ref|NW_003384498.1|	309358-31502	167522	267007	0.672529	0.61125	no
gi|320446078|ref|NW_003384493.1|	125748-12694	157008	150396	-0.0620772	0.97825	no
gi|320446078|ref|NW_003384493.1|	127694-12894	122478	941439	-0.379581	0.8608	no
gi|320446078|ref|NW_003384493.1|	130127-13036	541711	146388	-188773	0.39685	no
gi|320446078|ref|NW_003384493.1|	131731-13263	118643	521913	-118475	0.5701	no
gi|320446078|ref|NW_003384493.1|	132745-13337	195809	781499	-132513	0.5274	no
gi|320446078|ref|NW_003384493.1|	137271-13798	274404	108293	-134136	0.5353	no
gi|320446078|ref|NW_003384493.1|	139972-14496	936047	121701	-294323	0.12075	no
gi|320446078|ref|NW_003384493.1|	74509-75023	89768	924083	0.0418213	0.97395	no
gi|320446078|ref|NW_003384493.1|	81109-82802	439915	430429	329048	0.09375	no
gi|320446078|ref|NW_003384493.1|	85540-86632	0.985071	222235	449572	0.1334	no
gi|320446078|ref|NW_003384493.1|	89698-89887	0	181837	inf	0.01195	no
gi|320446078|ref|NW_003384493.1|	90062-90272	297796	201177	275607	0.2483	no
gi|320446080|ref|NW_003384491.1|	101105-10703	764085	147993	0.953722	0.54745	no

<table>
<tr><td>gi|320446080|ref|NW_003384491.1|</td><td>110536-11252</td><td>117326</td><td>204526</td><td>0.801764</td><td>0.63645</td><td>no</td></tr>
<tr><td>gi|320446080|ref|NW_003384491.1|</td><td>114221-11507</td><td>41333</td><td>38235</td><td>-0.112401</td><td>0.92695</td><td>no</td></tr>
<tr><td>gi|320446080|ref|NW_003384491.1|</td><td>145660-14812</td><td>419634</td><td>125899</td><td>-173687</td><td>0.19435</td><td>no</td></tr>
<tr><td>gi|320446080|ref|NW_003384491.1|</td><td>18880-20012</td><td>319978</td><td>208548</td><td>-0.617596</td><td>0.75235</td><td>no</td></tr>
<tr><td>gi|320446080|ref|NW_003384491.1|</td><td>20156-23750</td><td>201953</td><td>107212</td><td>-0.91355</td><td>0.48635</td><td>no</td></tr>
<tr><td>gi|320446080|ref|NW_003384491.1|</td><td>24143-24443</td><td>246489</td><td>109503</td><td>-117055</td><td>0.5587</td><td>no</td></tr>
<tr><td>gi|320446080|ref|NW_003384491.1|</td><td>27773-30090</td><td>154824</td><td>956315</td><td>-0.69507</td><td>0.68015</td><td>no</td></tr>
<tr><td>gi|320446080|ref|NW_003384491.1|</td><td>30346-31745</td><td>232038</td><td>199491</td><td>-0.218035</td><td>0.859</td><td>no</td></tr>
<tr><td>gi|320446080|ref|NW_003384491.1|</td><td>31857-33521</td><td>164184</td><td>208033</td><td>0.341505</td><td>0.78615</td><td>no</td></tr>
<tr><td>gi|320446080|ref|NW_003384491.1|</td><td>33972-34463</td><td>324734</td><td>695347</td><td>109847</td><td>0.5887</td><td>no</td></tr>
<tr><td>gi|320446080|ref|NW_003384491.1|</td><td>36190-37315</td><td>132793</td><td>0.656768</td><td>-101572</td><td>1</td><td>no</td></tr>
<tr><td>gi|320446080|ref|NW_003384491.1|</td><td>38196-38877</td><td>52982</td><td>10333</td><td>-235825</td><td>0.30845</td><td>no</td></tr>
<tr><td>gi|320446080|ref|NW_003384491.1|</td><td>39309-40527</td><td>635454</td><td>392958</td><td>-0.693413</td><td>0.736</td><td>no</td></tr>
<tr><td>gi|320446080|ref|NW_003384491.1|</td><td>44629-45519</td><td>265568</td><td>284195</td><td>-322413</td><td>0.18365</td><td>no</td></tr>
<tr><td>gi|320446080|ref|NW_003384491.1|</td><td>45908-50311</td><td>256268</td><td>348687</td><td>-287765</td><td>0.14595</td><td>no</td></tr>
<tr><td>gi|320446080|ref|NW_003384491.1|</td><td>50441-57066</td><td>778961</td><td>466442</td><td>-0.739853</td><td>0.56325</td><td>no</td></tr>
<tr><td>gi|320446080|ref|NW_003384491.1|</td><td>56-1167</td><td>102151</td><td>0.400272</td><td>-467358</td><td>0.14225</td><td>no</td></tr>
<tr><td>gi|320446080|ref|NW_003384491.1|</td><td>57430-59184</td><td>152229</td><td>290225</td><td>0.930927</td><td>0.45735</td><td>no</td></tr>
<tr><td>gi|320446080|ref|NW_003384491.1|</td><td>59384-61623</td><td>266122</td><td>265667</td><td>-0.00246593</td><td>0.9976</td><td>no</td></tr>
<tr><td>gi|320446083|ref|NW_003384488.1|</td><td>1032-5281</td><td>117286</td><td>191314</td><td>-261602</td><td>0.14935</td><td>no</td></tr>
<tr><td>gi|320446083|ref|NW_003384488.1|</td><td>11074-11510</td><td>138133</td><td>0.538711</td><td>-46804</td><td>0.28625</td><td>no</td></tr>
<tr><td>gi|320446083|ref|NW_003384488.1|</td><td>134807-13592</td><td>115248</td><td>15957</td><td>0.46945</td><td>0.8172</td><td>no</td></tr>
<tr><td>gi|320446083|ref|NW_003384488.1|</td><td>14571-15435</td><td>596073</td><td>0.554474</td><td>-674823</td><td>0.1105</td><td>no</td></tr>
<tr><td>gi|320446083|ref|NW_003384488.1|</td><td>148715-15065</td><td>232045</td><td>129966</td><td>-0.836265</td><td>0.51505</td><td>no</td></tr>
<tr><td>gi|320446083|ref|NW_003384488.1|</td><td>150763-15095</td><td>597631</td><td>246784</td><td>-127601</td><td>0.5576</td><td>no</td></tr>
<tr><td>gi|320446083|ref|NW_003384488.1|</td><td>152441-15274</td><td>535734</td><td>261016</td><td>-103738</td><td>0.6124</td><td>no</td></tr>
<tr><td>gi|320446083|ref|NW_003384488.1|</td><td>152978-15672</td><td>202878</td><td>180335</td><td>-0.169938</td><td>0.8988</td><td>no</td></tr>
<tr><td>gi|320446083|ref|NW_003384488.1|</td><td>157169-15768</td><td>0</td><td>403102</td><td>inf</td><td>0.0312</td><td>no</td></tr>
<tr><td>gi|320446083|ref|NW_003384488.1|</td><td>157733-15977</td><td>811234</td><td>130718</td><td>0.688262</td><td>0.66885</td><td>no</td></tr>
<tr><td>gi|320446083|ref|NW_003384488.1|</td><td>159910-16125</td><td>381238</td><td>857665</td><td>-215221</td><td>0.2272</td><td>no</td></tr>
</table>

gi|320446083|ref|NW_003384488.1|	163562-17055	132787	862667	-0.622243	0.64855	no
gi|320446083|ref|NW_003384488.1|	18335-18517	125518	0	#NAME?	0.02105	no
gi|320446083|ref|NW_003384488.1|	19004-20374	361417	16528	-445068	0.05255	no
gi|320446083|ref|NW_003384488.1|	22066-23291	457018	169092	-143444	0.40985	no
gi|320446083|ref|NW_003384488.1|	24035-24618	28995	130844	-114796	0.58445	no
gi|320446083|ref|NW_003384488.1|	24815-25364	737052	162077	-218509	0.1946	no
gi|320446083|ref|NW_003384488.1|	7556-8563	503066	0.15112	-505698	0.28295	no
gi|320446085|ref|NW_003384486.1|	100497-10090	367691	303153	304348	0.2183	no
gi|320446085|ref|NW_003384486.1|	105818-10625	349395	269355	-0.375348	0.85655	no
gi|320446085|ref|NW_003384486.1|	108442-11424	5488	167856	161287	0.2282	no
gi|320446085|ref|NW_003384486.1|	12539-13851	120694	21869	0.857533	0.5998	no
gi|320446085|ref|NW_003384486.1|	14497-15246	591487	966054	402968	0.05655	no
gi|320446085|ref|NW_003384486.1|	17549-18130	437382	41769	325547	0.1824	no
gi|320446085|ref|NW_003384486.1|	19489-21930	306008	321074	339127	0.0861	no
gi|320446085|ref|NW_003384486.1|	22289-23740	165194	113.18	277638	0.1586	no
gi|320446085|ref|NW_003384486.1|	392-1764	0.296939	195929	272209	0.2412	no
gi|320446085|ref|NW_003384486.1|	41978-45285	101623	756926	-0.425005	0.7378	no
gi|320446085|ref|NW_003384486.1|	45411-46742	0.924161	0.962421	0.0585246	1	no
gi|320446085|ref|NW_003384486.1|	46845-51257	211682	188396	-0.168135	0.9022	no
gi|320446085|ref|NW_003384486.1|	55089-57584	165028	200792	0.282986	0.8248	no
gi|320446085|ref|NW_003384486.1|	59506-62069	674173	32054	224931	0.22735	no
gi|320446085|ref|NW_003384486.1|	6957-7402	343564	347422	0.016112	0.98915	no
gi|320446085|ref|NW_003384486.1|	9309-12358	323832	31867	-0.0231843	0.98935	no
gi|320446087|ref|NW_003384484.1|	1430-4811	0.0536391	161346	491073	0.1739	no
gi|320446087|ref|NW_003384484.1|	40891-42560	765087	138483	0.856009	0.70325	no
gi|320446087|ref|NW_003384484.1|	45356-46131	281519	133058	224075	0.3136	no
gi|320446087|ref|NW_003384484.1|	51926-52811	389727	572682	0.555269	0.78695	no
gi|320446087|ref|NW_003384484.1|	5212-6901	464343	226309	228503	0.19585	no
gi|320446087|ref|NW_003384484.1|	52911-53611	617247	235743	193329	0.3759	no
gi|320446087|ref|NW_003384484.1|	53783-54018	235548	692914	155666	0.47215	no

gi|320446087|ref|NW_003384484.1|	54641-54818	0	105813	inf	0.02915	no
gi|320446087|ref|NW_003384484.1|	55576-57748	112397	713748	266681	0.0498	no
gi|320446087|ref|NW_003384484.1|	7047-7422	666388	312551	222966	0.31365	no
gi|320446087|ref|NW_003384484.1|	7853-8075	48066	452762	323567	0.2322	no
gi|320446087|ref|NW_003384484.1|	8240-9000	0	419123	inf	0.0212	no
gi|320446090|ref|NW_003384481.1|	11273-11922	937015	388402	-127052	0.54745	no
gi|320446090|ref|NW_003384481.1|	12513-12877	355924	178109	-0.99881	0.6416	no
gi|320446090|ref|NW_003384481.1|	13333-14494	198733	16926	-0.231592	0.9169	no
gi|320446090|ref|NW_003384481.1|	43307-44781	0	123128	inf	1	no
gi|320446090|ref|NW_003384481.1|	44915-47597	93793	113482	359684	0.0113	no
gi|320446090|ref|NW_003384481.1|	49947-50885	0.720636	911351	366066	0.18195	no
gi|320446090|ref|NW_003384481.1|	58623-59482	405982	331574	302984	0.10395	no
gi|320446090|ref|NW_003384481.1|	60545-60943	0.977624	643171	271785	0.2673	no
gi|320446090|ref|NW_003384481.1|	8067-8348	117711	260086	-217819	0.3173	no
gi|320446091|ref|NW_003384480.1|	185199-18654	0.152633	137773	317415	1	no
gi|320446091|ref|NW_003384480.1|	190178-19128	0	174236	inf	0.02915	no
gi|320446091|ref|NW_003384480.1|	55229-55702	0	464116	inf	0.0312	no
gi|320446092|ref|NW_003384479.1|	106645-11003	403285	26.64	-0.598205	0.65725	no
gi|320446092|ref|NW_003384479.1|	110801-11153	464287	200465	-121167	0.45075	no
gi|320446092|ref|NW_003384479.1|	111722-11217	175763	182861	0.0571105	0.97365	no
gi|320446092|ref|NW_003384479.1|	118186-11884	118538	350147	156261	0.491	no
gi|320446092|ref|NW_003384479.1|	119593-12150	683967	77124	0.173253	0.9375	no
gi|320446092|ref|NW_003384479.1|	121676-12265	771674	167686	11197	0.6055	no
gi|320446092|ref|NW_003384479.1|	131722-13418	124503	197754	0.667519	0.60395	no
gi|320446092|ref|NW_003384479.1|	135392-13603	125514	313118	131886	0.5489	no
gi|320446092|ref|NW_003384479.1|	136111-13840	451907	378533	-0.255607	0.9049	no
gi|320446092|ref|NW_003384479.1|	138641-14047	390064	139573	-148269	0.4895	no
gi|320446092|ref|NW_003384479.1|	140601-14104	111742	529565	-107729	0.60185	no
gi|320446092|ref|NW_003384479.1|	141222-14208	377386	311382	-0.277352	0.8607	no
gi|320446092|ref|NW_003384479.1|	145327-14768	102547	140415	0.453416	0.71455	no

gi|320446092|ref|NW_003384479.1|	147903-15012	298427	55419	0.893	0.5038	no
gi|320446092|ref|NW_003384479.1|	161274-16249	138549	116229	-0.253419	0.90845	no
gi|320446092|ref|NW_003384479.1|	164634-16562	756411	84517	0.160072	0.94065	no
gi|320446092|ref|NW_003384479.1|	43138-46026	0.954136	271226	150723	0.4773	no
gi|320446092|ref|NW_003384479.1|	62755-64007	398398	138173	-152773	0.4793	no
gi|320446092|ref|NW_003384479.1|	67143-68584	559881	126463	-21464	0.33765	no
gi|320446092|ref|NW_003384479.1|	69671-70900	662386	270886	-128999	0.53645	no
gi|320446092|ref|NW_003384479.1|	71280-72292	847531	43547	-0.960691	0.6386	no
gi|320446092|ref|NW_003384479.1|	82850-83152	232919	334986	-279766	0.12765	no
gi|320446092|ref|NW_003384479.1|	87300-87913	155968	12101	-0.366123	0.85995	no
gi|320446092|ref|NW_003384479.1|	97111-97952	167222	364233	11231	0.5875	no
gi|320446092|ref|NW_003384479.1|	99683-10608	119214	280578	123485	0.35755	no
gi|320446095|ref|NW_003384476.1|	104307-10824	174986	105259	-0.733295	0.5764	no
gi|320446095|ref|NW_003384476.1|	118434-11918	296319	0.676844	-213026	0.27995	no
gi|320446095|ref|NW_003384476.1|	128754-12927	297189	478977	0.688576	0.7415	no
gi|320446095|ref|NW_003384476.1|	131368-13189	462869	19448	-125098	0.5558	no
gi|320446095|ref|NW_003384476.1|	132365-13369	200601	353526	0.817486	0.6838	no
gi|320446095|ref|NW_003384476.1|	133925-13442	0	503649	inf	0.029	no
gi|320446095|ref|NW_003384476.1|	142542-14358	436676	15385	181689	0.41995	no
gi|320446095|ref|NW_003384476.1|	143767-14565	795938	860584	0.112659	0.9587	no
gi|320446095|ref|NW_003384476.1|	148066-14865	131611	764378	-0.783923	0.7016	no
gi|320446095|ref|NW_003384476.1|	149275-14955	452417	197315	-119716	0.56355	no
gi|320446095|ref|NW_003384476.1|	156164-15765	835248	48706	-0.778104	0.71935	no
gi|320446095|ref|NW_003384476.1|	159276-16055	341521	119045	-152046	0.3696	no
gi|320446095|ref|NW_003384476.1|	161399-16869	300696	296121	-0.0221185	0.9866	no
gi|320446095|ref|NW_003384476.1|	16610-17681	565507	16766	-175401	0.4202	no
gi|320446095|ref|NW_003384476.1|	17765-19704	663622	359062	-0.886126	0.68235	no
gi|320446095|ref|NW_003384476.1|	4791-5281	215119	915887	-123189	0.55575	no
gi|320446095|ref|NW_003384476.1|	6818-7369	849313	429655	-0.983118	0.651	no
gi|320446098|ref|NW_003384473.1|	116285-12237	778566	318.35	203173	0.187	no

gi|320446098|ref|NW_003384473.1|	159590-16437[illegible]	153229	974485	-0.652981	0.62115	no
gi|320446098|ref|NW_003384473.1|	165645-166334	132741	35806	-189035	0.29685	no
gi|320446098|ref|NW_003384473.1|	173587-174264	129825	0	#NAME?	0.00615	no
gi|320446098|ref|NW_003384473.1|	175295-177568	136637	0.693614	-430007	0.0713	no
gi|320446098|ref|NW_003384473.1|	179367-180150	144869	105774	-377569	0.17315	no
gi|320446098|ref|NW_003384473.1|	180279-181465	142022	159945	0.171461	0.92275	no
gi|320446098|ref|NW_003384473.1|	181577-182335	871989	221413	-197757	0.3758	no
gi|320446098|ref|NW_003384473.1|	187306-188407	272951	520882	0.932313	0.4674	no
gi|320446098|ref|NW_003384473.1|	188549-191060	0.741484	13983	0.915191	1	no
gi|320446098|ref|NW_003384473.1|	191537-19182[illegible]	237132	27559	0.216833	0.9155	no
gi|320446098|ref|NW_003384473.1|	192055-192319	379378	516494	0.445117	0.8267	no
gi|320446098|ref|NW_003384473.1|	193790-19577[illegible]	29966	525727	0.810988	0.69615	no
gi|320446098|ref|NW_003384473.1|	196501-197230	405338	0.462629	-31312	0.255	no
gi|320446098|ref|NW_003384473.1|	197953-198919	157082	169024	0.105714	0.9627	no
gi|320446099|ref|NW_003384472.1|	113040-114535	0	121095	inf	1	no
gi|320446099|ref|NW_003384472.1|	127799-130177	113402	152858	0.430746	0.73555	no
gi|320446099|ref|NW_003384472.1|	130281-130820	192876	178162	-0.114482	0.9554	no
gi|320446100|ref|NW_003384471.1|	10126-11712	0	147784	inf	0.02075	no
gi|320446100|ref|NW_003384471.1|	16406-17136	852956	560597	-0.605508	0.76445	no
gi|320446100|ref|NW_003384471.1|	23313-23758	109316	622249	-0.812938	0.69235	no
gi|320446100|ref|NW_003384471.1|	23888-24957	971768	574192	-0.759079	0.70975	no
gi|320446100|ref|NW_003384471.1|	29582-31777	912733	106955	0.228743	0.88985	no
gi|320446100|ref|NW_003384471.1|	35114-36968	542101	114058	107314	0.6296	no
gi|320446100|ref|NW_003384471.1|	37099-37399	170646	292009	0.775006	0.7047	no
gi|320446100|ref|NW_003384471.1|	39454-45108	114897	360447	164944	0.2149	no
gi|320446100|ref|NW_003384471.1|	56095-57072	0.682726	314262	220259	0.33335	no
gi|320446100|ref|NW_003384471.1|	6374-6739	235829	13857	25548	0.2437	no
gi|320446100|ref|NW_003384471.1|	65168-65630	145371	580961	199871	0.3149	no
gi|320446100|ref|NW_003384471.1|	79444-79695	948876	160197	0.755553	0.7072	no
gi|320446100|ref|NW_003384471.1|	8779-9342	102392	141777	379146	0.1742	no

gi|320446101|ref|NW_003384470.1|	83330-86577	0	262539	inf	0.00565	no
gi|320446102|ref|NW_003384469.1|	108481-10989	211903	204938	-0.0482163	0.9672	no
gi|320446102|ref|NW_003384469.1|	110028-11173	162715	702157	-121248	0.45735	no
gi|320446102|ref|NW_003384469.1|	111975-11375	144391	495294	-154363	0.35245	no
gi|320446102|ref|NW_003384469.1|	113990-11446	897149	184259	-228361	0.33675	no
gi|320446102|ref|NW_003384469.1|	115186-11628	0.58422	525681	31696	0.2098	no
gi|320446102|ref|NW_003384469.1|	119197-12104	104768	299328	-18074	0.4163	no
gi|320446102|ref|NW_003384469.1|	122958-12330	276051	341823	-301361	0.24005	no
gi|320446102|ref|NW_003384469.1|	124878-12617	104717	154162	-276398	0.23525	no
gi|320446102|ref|NW_003384469.1|	126339-12677	180383	434718	-205292	0.341	no
gi|320446102|ref|NW_003384469.1|	127960-12912	117747	0.878573	-374438	0.16005	no
gi|320446102|ref|NW_003384469.1|	129723-13284	450238	0.735994	-261292	0.255	no
gi|320446102|ref|NW_003384469.1|	146799-14774	453818	263646	-0.783515	0.6929	no
gi|320446102|ref|NW_003384469.1|	157073-15754	217322	420873	-236838	0.28585	no
gi|320446102|ref|NW_003384469.1|	158403-15874	366001	264695	-0.467514	0.8144	no
gi|320446102|ref|NW_003384469.1|	159028-16235	250363	28754	0.199739	0.88025	no
gi|320446102|ref|NW_003384469.1|	167379-16966	120371	116248	-0.0502847	0.9674	no
gi|320446102|ref|NW_003384469.1|	170949-17149	707407	476701	-0.569457	0.78385	no
gi|320446102|ref|NW_003384469.1|	171626-17299	125487	560263	-116337	0.59125	no
gi|320446102|ref|NW_003384469.1|	173911-17445	268705	188406	-0.51218	0.8088	no
gi|320446102|ref|NW_003384469.1|	183397-18436	122431	430534	-150778	0.47815	no
gi|320446102|ref|NW_003384469.1|	185050-18665	122848	803599	-0.612328	0.78435	no
gi|320446102|ref|NW_003384469.1|	186991-18900	109286	14712	0.428886	0.7915	no
gi|320446102|ref|NW_003384469.1|	189074-19151	124698	0.641081	-428179	0.072	no
gi|320446102|ref|NW_003384469.1|	192040-19339	961201	0.417201	-452602	0.1704	no
gi|320446102|ref|NW_003384469.1|	193500-19419	982433	0.745999	-371911	0.17125	no
gi|320446102|ref|NW_003384469.1|	194386-19632	910173	131043	-27961	0.2344	no
gi|320446102|ref|NW_003384469.1|	196472-19826	684259	0.302655	-44988	0.169	no
gi|320446102|ref|NW_003384469.1|	204621-20519	0	45805	inf	0.00445	no
gi|320446102|ref|NW_003384469.1|	205628-20966	887885	858346	327312	0.02075	no

gi|320446102|ref|NW_003384469.1|	215038-21545	621633	293292	-108372	0.6145	no
gi|320446102|ref|NW_003384469.1|	241323-24183	714608	193489	-18849	0.4124	no
gi|320446102|ref|NW_003384469.1|	265911-26647	173551	551702	-16534	0.43	no
gi|320446102|ref|NW_003384469.1|	267794-26807	351654	168489	-106151	0.6053	no
gi|320446102|ref|NW_003384469.1|	58290-58862	104715	0.674273	-395699	0.2196	no
gi|320446102|ref|NW_003384469.1|	79483-79933	106986	0.507946	-439661	0.29665	no
gi|320446102|ref|NW_003384469.1|	86215-86758	157806	110375	280618	0.1333	no
gi|320446102|ref|NW_003384469.1|	94398-94679	27164	124263	219363	0.32355	no
gi|320446104|ref|NW_003384467.1|	23859-24282	0	108418	inf	0.0212	no
gi|320446104|ref|NW_003384467.1|	320071-32094	15759	379838	126921	0.54565	no
gi|320446104|ref|NW_003384467.1|	44260-44982	277412	189841	-0.547239	0.79035	no
gi|320446104|ref|NW_003384467.1|	96188-97431	0.837411	162624	0.957534	0.66275	no
gi|320446104|ref|NW_003384467.1|	98368-101624	12289	652447	24085	0.2933	no
gi|320446107|ref|NW_003384464.1|	12724-12951	101935	122644	0.266818	0.89555	no
gi|320446107|ref|NW_003384464.1|	17590-18501	117413	172201	0.552502	0.7983	no
gi|320446107|ref|NW_003384464.1|	19708-21007	539451	847889	0.652384	0.7573	no
gi|320446107|ref|NW_003384464.1|	21163-23475	211263	811126	194089	0.38775	no
gi|320446107|ref|NW_003384464.1|	23876-24445	155931	455568	154675	0.4874	no
gi|320446107|ref|NW_003384464.1|	33397-33637	204774	115411	-414918	0.16395	no
gi|320446107|ref|NW_003384464.1|	35512-38051	23439	319768	377004	0.06395	no
gi|320446107|ref|NW_003384464.1|	38321-38739	17675	642252	186143	0.32465	no
gi|320446107|ref|NW_003384464.1|	46323-47208	34296	232652	-0.559866	0.72525	no
gi|320446107|ref|NW_003384464.1|	56608-57465	868884	106513	0.293794	0.8877	no
gi|320446107|ref|NW_003384464.1|	64652-67614	589122	701277	0.251418	0.84905	no
gi|320446107|ref|NW_003384464.1|	72964-73753	152446	146516	-0.0572441	0.9269	no
gi|320446107|ref|NW_003384464.1|	74919-77343	678447	139435	103929	0.5326	no
gi|320446107|ref|NW_003384464.1|	80554-82044	147953	748141	233817	0.29095	no
gi|320446109|ref|NW_003384462.1|	121765-12409	0.887393	461806	237964	0.2893	no
gi|320446109|ref|NW_003384462.1|	127777-12902	0	311495	inf	0.0133	no
gi|320446109|ref|NW_003384462.1|	134455-13544	150446	992479	-0.600138	0.77965	no

gi|320446109|ref|NW_003384462.1|	136124-13671[illegible]	467481	285138	-0.713248	0.72555	no
gi|320446109|ref|NW_003384462.1|	136845-14056[illegible]	133602	156166	0.225135	0.86025	no
gi|320446109|ref|NW_003384462.1|	140842-14173[illegible]	724145	374127	-0.952749	0.64775	no
gi|320446109|ref|NW_003384462.1|	18302-18861	554367	227362	-128585	0.56115	no
gi|320446109|ref|NW_003384462.1|	20224-20848	476542	206078	-120941	0.56585	no
gi|320446109|ref|NW_003384462.1|	21218-25036	112559	160522	0.51209	0.698	no
gi|320446109|ref|NW_003384462.1|	25161-26006	232574	21523	-0.111808	0.9583	no
gi|320446109|ref|NW_003384462.1|	34110-34705	0	170766	inf	5.00E-05	yes
gi|320446109|ref|NW_003384462.1|	35522-35951	0	179413	inf	5.00E-05	yes
gi|320446109|ref|NW_003384462.1|	41044-42891	101562	350217	-153604	0.49535	no
gi|320446109|ref|NW_003384462.1|	44934-45484	356744	359105	-331241	0.1797	no
gi|320446109|ref|NW_003384462.1|	46294-46818	694304	116688	-257291	0.23895	no
gi|320446109|ref|NW_003384462.1|	47890-48539	179255	305173	-255431	0.26865	no
gi|320446109|ref|NW_003384462.1|	48872-49097	320305	862826	-18923	0.3088	no
gi|320446109|ref|NW_003384462.1|	49219-50457	340002	10039	-175993	0.3107	no
gi|320446109|ref|NW_003384462.1|	50783-51037	731398	173785	-207336	0.33435	no
gi|320446109|ref|NW_003384462.1|	52040-52453	181124	418392	-211405	0.34815	no
gi|320446109|ref|NW_003384462.1|	52599-52987	134122	101586	-0.400841	0.8389	no
gi|320446109|ref|NW_003384462.1|	53342-55268	233555	204558	-0.191252	0.8795	no
gi|320446109|ref|NW_003384462.1|	62296-64301	0.85818	239376	147992	0.4792	no
gi|320446109|ref|NW_003384462.1|	75705-76839	300485	468202	0.639837	0.7483	no
gi|320446109|ref|NW_003384462.1|	7636-8055	0.879494	929872	340229	0.21725	no
gi|320446109|ref|NW_003384462.1|	77648-78904	0.330697	172048	237923	0.2609	no
gi|320446109|ref|NW_003384462.1|	79015-79560	0	437392	inf	0.029	no
gi|320446109|ref|NW_003384462.1|	79678-81790	0.809242	420272	237668	0.28875	no
gi|320446109|ref|NW_003384462.1|	85943-86687	165912	136403	-0.282543	0.88515	no
gi|320446109|ref|NW_003384462.1|	86822-88005	837021	209812	-199617	0.3671	no
gi|320446109|ref|NW_003384462.1|	88301-88488	697236	185088	-191344	0.30655	no
gi|320446109|ref|NW_003384462.1|	88696-89754	246192	581755	-20813	0.35545	no
gi|320446110|ref|NW_003384461.1|	52613-54300	139495	18615	0.416255	0.83195	no

gi|320446110|ref|NW_003384461.1|	55945-58606	16695	320779	0.942162	0.65445	no
gi|320446113|ref|NW_003384458.1|	1070-2651	0.376214	113427	159214	1	no
gi|320446113|ref|NW_003384458.1|	11331-14805	0.312592	554025	41476	0.1333	no
gi|320446113|ref|NW_003384458.1|	145224-14616	114974	0.660952	-412062	0.17975	no
gi|320446113|ref|NW_003384458.1|	147494-15060	298935	836146	148392	0.3725	no
gi|320446113|ref|NW_003384458.1|	151484-15188	0.962399	126713	371879	0.20845	no
gi|320446113|ref|NW_003384458.1|	15158-15756	0	377215	inf	0.029	no
gi|320446113|ref|NW_003384458.1|	152028-15225	124981	209852	0.747666	0.70725	no
gi|320446113|ref|NW_003384458.1|	152552-15357	0.861495	267916	163686	0.44625	no
gi|320446113|ref|NW_003384458.1|	153967-15490	989094	114815	0.215128	0.91825	no
gi|320446113|ref|NW_003384458.1|	156855-15753	0.377603	231991	261913	0.26355	no
gi|320446113|ref|NW_003384458.1|	15881-18523	0.490783	121972	463532	0.06545	no
gi|320446113|ref|NW_003384458.1|	3993-8773	0.37126	291238	297169	0.21365	no
gi|320446113|ref|NW_003384458.1|	54177-58999	0.625363	553967	314703	0.0912	no
gi|320446113|ref|NW_003384458.1|	64018-64347	300394	223453	-0.426883	0.8374	no
gi|320446113|ref|NW_003384458.1|	65405-66743	734634	510069	-0.526332	0.80185	no
gi|320446113|ref|NW_003384458.1|	67763-68209	427595	216883	-0.979327	0.64865	no
gi|320446113|ref|NW_003384458.1|	68487-70218	473866	793594	0.743921	0.7317	no
gi|320446113|ref|NW_003384458.1|	71951-72467	368515	159659	-452865	0.1718	no
gi|320446113|ref|NW_003384458.1|	76949-77293	364623	350801	-337768	0.2086	no
gi|320446113|ref|NW_003384458.1|	77880-80461	402119	597995	-274941	0.1554	no
gi|320446113|ref|NW_003384458.1|	82914-83873	265892	820964	-169545	0.44625	no
gi|320446113|ref|NW_003384458.1|	92600-95006	202113	166096	303879	0.1045	no
gi|320446113|ref|NW_003384458.1|	98141-98644	310929	204255	271572	0.2541	no
gi|320446116|ref|NW_003384455.1|	116913-11786	184735	194464	0.0740445	0.97365	no
gi|320446116|ref|NW_003384455.1|	119653-12218	34471	312499	-0.141532	0.9431	no
gi|320446116|ref|NW_003384455.1|	122723-12400	137712	105669	-0.382104	0.8616	no
gi|320446116|ref|NW_003384455.1|	127310-12806	261361	111958	-122309	0.5656	no
gi|320446116|ref|NW_003384455.1|	142368-14573	684262	761326	0.153966	0.89775	no
gi|320446116|ref|NW_003384455.1|	149670-15056	0.515665	15986	16323	0.3325	no

gi|320446116|ref|NW_003384455.1|	150639-153157	146364	149214	0.0278252	0.9828	no
gi|320446116|ref|NW_003384455.1|	155094-156955	252237	133826	-0.914424	0.58895	no
gi|320446116|ref|NW_003384455.1|	158444-159925	350879	184606	-0.926528	0.4629	no
gi|320446116|ref|NW_003384455.1|	160717-165460	919947	119361	0.375712	0.816	no
gi|320446116|ref|NW_003384455.1|	19652-24234	110979	109254	-0.0225915	0.98365	no
gi|320446116|ref|NW_003384455.1|	3093-3336	119331	643007	-0.892064	0.66175	no
gi|320446116|ref|NW_003384455.1|	37789-38708	368855	255674	-0.528747	0.679	no
gi|320446116|ref|NW_003384455.1|	67432-71059	346248	202251	-0.775659	0.55885	no
gi|320446116|ref|NW_003384455.1|	71333-71714	611552	323449	-0.918939	0.6626	no
gi|320446116|ref|NW_003384455.1|	72013-72302	14666	8042	-0.866848	0.6867	no
gi|320446116|ref|NW_003384455.1|	72496-73740	210818	105602	-0.99736	0.5254	no
gi|320446116|ref|NW_003384455.1|	74793-75558	267706	122395	-112911	0.6036	no
gi|320446116|ref|NW_003384455.1|	75689-78582	118725	985235	-0.269086	0.82705	no
gi|320446116|ref|NW_003384455.1|	78755-79450	183504	200601	0.128518	0.9267	no
gi|320446116|ref|NW_003384455.1|	79588-80881	344655	53484	0.633955	0.6227	no
gi|320446118|ref|NW_003384453.1|	18612-18972	12163	182414	390665	0.20015	no
gi|320446118|ref|NW_003384453.1|	34298-37450	115702	554245	226011	0.3263	no
gi|320446118|ref|NW_003384453.1|	39058-40072	15173	43437	151742	0.4721	no
gi|320446118|ref|NW_003384453.1|	40866-41814	142104	359513	13391	0.52035	no
gi|320446118|ref|NW_003384453.1|	58135-58597	0.726854	532548	287317	0.25915	no
gi|320446118|ref|NW_003384453.1|	9612-11047	0.140671	552349	861712	0.14075	no
gi|320446120|ref|NW_003384451.1|	103365-10659[illegible]	131669	123572	-0.091565	0.94295	no
gi|320446120|ref|NW_003384451.1|	122780-12608[illegible]	785775	138366	-250562	0.15205	no
gi|320446120|ref|NW_003384451.1|	34500-34978	25921	186769	-0.472862	0.81885	no
gi|320446120|ref|NW_003384451.1|	47904-49002	234772	257271	0.132031	0.9415	no
gi|320446120|ref|NW_003384451.1|	49154-50783	108976	294957	143649	0.49395	no
gi|320446120|ref|NW_003384451.1|	53922-55101	0.894223	756005	307969	0.20715	no
gi|320446120|ref|NW_003384451.1|	74800-75650	0	245636	inf	0.02915	no
gi|320446120|ref|NW_003384451.1|	80159-81123	192954	613967	-165202	0.22265	no
gi|320446120|ref|NW_003384451.1|	81269-83176	621817	339851	-0.87159	0.5129	no

gi|320446120|ref|NW_003384451.1|	86494-87900	778444	620974	-0.326061	0.87715	no
gi|320446120|ref|NW_003384451.1|	90350-93908	161993	60758	-141478	0.422	no
gi|320446120|ref|NW_003384451.1|	94391-98301	505083	124056	129641	0.4495	no
gi|320446120|ref|NW_003384451.1|	99900-102532	154186	954246	-0.692235	0.57475	no
gi|320446121|ref|NW_003384450.1|	141379-14187	909255	130378	-280199	0.2208	no
gi|320446121|ref|NW_003384450.1|	14238-15307	384658	938313	128649	0.54095	no
gi|320446121|ref|NW_003384450.1|	146012-14700	492037	0.308937	-399338	0.21285	no
gi|320446121|ref|NW_003384450.1|	148899-14950	497952	0.92144	-243404	0.2454	no
gi|320446121|ref|NW_003384450.1|	156237-15656	251614	191578	-37152	0.2186	no
gi|320446121|ref|NW_003384450.1|	15851-17118	0.817924	0.907921	0.1506	1	no
gi|320446121|ref|NW_003384450.1|	158724-16005	147462	309276	-225337	0.3178	no
gi|320446121|ref|NW_003384450.1|	25322-30909	306445	108248	-15013	0.2712	no
gi|320446121|ref|NW_003384450.1|	33386-34385	517179	778654	-273161	0.1287	no
gi|320446121|ref|NW_003384450.1|	44996-46472	640658	127659	-232726	0.20965	no
gi|320446121|ref|NW_003384450.1|	5334-6654	0.933536	41043	213636	0.3341	no
gi|320446121|ref|NW_003384450.1|	69774-70934	711831	391988	-0.860724	0.67595	no
gi|320446121|ref|NW_003384450.1|	8062-8704	0.828638	225587	144487	0.37505	no
gi|320446121|ref|NW_003384450.1|	89099-89456	495841	193847	196697	0.38785	no
gi|320446124|ref|NW_003384447.1|	101178-10186	117497	284.67	127667	0.34155	no
gi|320446124|ref|NW_003384447.1|	10520-12037	25017	0	#NAME?	0.01335	no
gi|320446124|ref|NW_003384447.1|	111442-11240	313734	108763	179357	0.1756	no
gi|320446124|ref|NW_003384447.1|	115055-11854	100335	424013	207929	0.2	no
gi|320446124|ref|NW_003384447.1|	122013-12279	516377	0	#NAME?	5.00E-05	yes
gi|320446124|ref|NW_003384447.1|	122908-12380	438778	0	#NAME?	5.00E-05	yes
gi|320446124|ref|NW_003384447.1|	125336-12631	626418	0	#NAME?	5.00E-05	yes
gi|320446124|ref|NW_003384447.1|	133955-13590	799005	206998	137334	0.4289	no
gi|320446124|ref|NW_003384447.1|	136127-13796	0.94703	263963	147885	0.47925	no
gi|320446124|ref|NW_003384447.1|	141502-14260	155433	228623	0.556675	0.7789	no
gi|320446124|ref|NW_003384447.1|	143047-14382	0.625597	236071	191591	0.3194	no
gi|320446124|ref|NW_003384447.1|	144361-14542	0.815679	126942	0.638098	1	no

gi|320446124|ref|NW_003384447.1|	146464-14768[illegible]	102837	261447	134615	0.5199	no
gi|320446124|ref|NW_003384447.1|	149200-15024[illegible]	357784	172149	-105543	0.5228	no
gi|320446124|ref|NW_003384447.1|	150423-15092[illegible]	35196	215294	-0.709104	0.7426	no
gi|320446124|ref|NW_003384447.1|	151630-15403[illegible]	173051	18125	0.0667799	0.95885	no
gi|320446124|ref|NW_003384447.1|	154081-15554[illegible]	350241	227234	-0.624168	0.6229	no
gi|320446124|ref|NW_003384447.1|	155708-15640[illegible]	328181	199348	-0.719204	0.72925	no
gi|320446124|ref|NW_003384447.1|	156793-15936[illegible]	426784	343606	-0.312752	0.88255	no
gi|320446124|ref|NW_003384447.1|	159483-16015[illegible]	461312	0.52456	-313656	0.255	no
gi|320446124|ref|NW_003384447.1|	161577-16242[illegible]	526924	228931	-120268	0.56745	no
gi|320446124|ref|NW_003384447.1|	168472-16951[illegible]	15236	461794	-172217	0.43045	no
gi|320446124|ref|NW_003384447.1|	18917-19259	95835	710905	-0.430897	0.8211	no
gi|320446124|ref|NW_003384447.1|	20815-21961	337296	118096	-151405	0.37045	no
gi|320446124|ref|NW_003384447.1|	23124-25638	334709	227573	-0.556577	0.67485	no
gi|320446124|ref|NW_003384447.1|	27730-28748	0.215649	163932	292634	0.2564	no
gi|320446124|ref|NW_003384447.1|	30253-32631	322882	603736	0.902913	0.4996	no
gi|320446124|ref|NW_003384447.1|	32869-33099	198808	266264	-622238	0.2658	no
gi|320446124|ref|NW_003384447.1|	34190-39249	101438	174463	0.782326	0.5604	no
gi|320446124|ref|NW_003384447.1|	39970-40865	409404	634682	0.632508	0.7509	no
gi|320446124|ref|NW_003384447.1|	42668-43393	448046	518965	0.21199	0.91125	no
gi|320446124|ref|NW_003384447.1|	43515-44593	52075	101158	0.95795	0.6474	no
gi|320446124|ref|NW_003384447.1|	47015-47834	315243	38165	0.275786	0.86535	no
gi|320446124|ref|NW_003384447.1|	57575-57745	996178	233902	123143	0.553	no
gi|320446124|ref|NW_003384447.1|	68363-70049	152387	130394	-0.224859	0.8902	no
gi|320446124|ref|NW_003384447.1|	70166-72361	122272	142407	0.219926	0.85875	no
gi|320446124|ref|NW_003384447.1|	73027-75026	103332	101411	-0.0270704	0.99005	no
gi|320446124|ref|NW_003384447.1|	7457-9778	210351	0.0564788	-521894	0.2779	no
gi|320446124|ref|NW_003384447.1|	80427-80895	170268	99333	-0.777459	0.70505	no
gi|320446124|ref|NW_003384447.1|	84963-87323	500339	205158	-128617	0.5411	no
gi|320446124|ref|NW_003384447.1|	88213-88547	246207	15947	-0.626583	0.7521	no
gi|320446124|ref|NW_003384447.1|	88726-89300	743659	502903	-0.564359	0.78255	no

gi|320446124|ref|NW_003384447.1|	89654-90088	196782	119547	-0.719014	0.72495	no
gi|320446124|ref|NW_003384447.1|	91112-92385	31064	665766	109977	0.40835	no
gi|320446124|ref|NW_003384447.1|	96284-99503	23469	912445	195898	0.151	no
gi|320446125|ref|NW_003384446.1|	101201-10170[illegible]	106092	112934	0.0901543	0.96035	no
gi|320446125|ref|NW_003384446.1|	103472-10368[illegible]	250149	122941	-102481	0.6194	no
gi|320446125|ref|NW_003384446.1|	3352-4135	0.308232	190393	26269	0.26355	no
gi|320446125|ref|NW_003384446.1|	47162-49822	0	773103	inf	0.0033	no
gi|320446125|ref|NW_003384446.1|	49943-52041	0	132711	inf	1	no
gi|320446125|ref|NW_003384446.1|	54802-56990	0	241205	inf	0.00815	no
gi|320446125|ref|NW_003384446.1|	59705-60332	0	321479	inf	0.0294	no
gi|320446125|ref|NW_003384446.1|	67654-71931	129415	838013	269496	0.0514	no
gi|320446125|ref|NW_003384446.1|	76981-77384	180973	439119	-204309	0.3476	no
gi|320446125|ref|NW_003384446.1|	83732-84398	587244	373728	-0.651972	0.7495	no
gi|320446125|ref|NW_003384446.1|	85281-86110	312773	175947	-0.829977	0.6797	no
gi|320446125|ref|NW_003384446.1|	86528-86837	878083	790826	-0.150997	0.92095	no
gi|320446125|ref|NW_003384446.1|	87766-88883	574277	106023	-243737	0.28185	no
gi|320446125|ref|NW_003384446.1|	89883-91239	346356	0.941188	-18797	0.3833	no
gi|320446125|ref|NW_003384446.1|	94813-95184	113717	743508	-0.613025	0.7625	no
gi|320446125|ref|NW_003384446.1|	95669-98314	28.57	247066	-0.209605	0.87475	no
gi|320446128|ref|NW_003384443.1|	12784-13799	147206	448781	-171375	0.43515	no
gi|320446128|ref|NW_003384443.1|	14057-14449	252358	79586	-166489	0.43935	no
gi|320446128|ref|NW_003384443.1|	14570-15351	163961	955375	-0.779217	0.7031	no
gi|320446128|ref|NW_003384443.1|	18387-18777	204092	804191	-134361	0.53335	no
gi|320446128|ref|NW_003384443.1|	18929-19450	818177	314176	-138084	0.51275	no
gi|320446128|ref|NW_003384443.1|	21384-22050	48937	165508	-156402	0.34175	no
gi|320446128|ref|NW_003384443.1|	26196-28914	306523	243162	-0.334079	0.79875	no
gi|320446128|ref|NW_003384443.1|	2761-3882	18675	461785	-201581	0.36905	no
gi|320446128|ref|NW_003384443.1|	30111-31299	370253	95768	-19509	0.26475	no
gi|320446128|ref|NW_003384443.1|	34349-34978	171121	378095	114373	0.58825	no
gi|320446128|ref|NW_003384443.1|	39569-40855	0	122608	inf	1	no

gi|320446128|ref|NW_003384443.1|	43036-45145	324125	139476	-453846	0.04005	no
gi|320446128|ref|NW_003384443.1|	4494-8247	117037	483384	-127573	0.4569	no
gi|320446128|ref|NW_003384443.1|	52169-52639	344872	352062	0.0297676	0.9873	no
gi|320446128|ref|NW_003384443.1|	57381-61317	60184	132124	113444	0.37965	no
gi|320446128|ref|NW_003384443.1|	8354-12347	132911	927227	-0.519464	0.68995	no
gi|320446129|ref|NW_003384442.1|	114246-11452	113077	153316	-288273	0.2169	no
gi|320446129|ref|NW_003384442.1|	118141-11863	405175	339254	-35781	0.1608	no
gi|320446129|ref|NW_003384442.1|	121678-12189	646287	3.12	-437256	0.2989	no
gi|320446129|ref|NW_003384442.1|	126382-12654	338289	242698	-380102	0.21715	no
gi|320446129|ref|NW_003384442.1|	137192-13761	293365	570621	-236209	0.29	no
gi|320446129|ref|NW_003384442.1|	172251-17355	325788	178877	-0.864962	0.50385	no
gi|320446129|ref|NW_003384442.1|	177310-17747	240132	338553	-282637	0.21425	no
gi|320446129|ref|NW_003384442.1|	177747-17831	756714	10228	-288723	0.2142	no
gi|320446129|ref|NW_003384442.1|	180548-18237	38263	162924	-123176	0.55735	no
gi|320446129|ref|NW_003384442.1|	186517-18719	131001	210284	-263917	0.2463	no
gi|320446129|ref|NW_003384442.1|	18676-19469	281515	62352	-217471	0.33495	no
gi|320446129|ref|NW_003384442.1|	187763-18985	471142	800962	-255636	0.175	no
gi|320446129|ref|NW_003384442.1|	190055-19059	195356	338181	-253024	0.26105	no
gi|320446129|ref|NW_003384442.1|	190781-19326	530331	140204	-191937	0.15805	no
gi|320446129|ref|NW_003384442.1|	193628-19383	837552	160967	-237941	0.3071	no
gi|320446129|ref|NW_003384442.1|	194534-19548	514665	160368	-168225	0.3289	no
gi|320446129|ref|NW_003384442.1|	195626-19660	797713	262062	-160596	0.37065	no
gi|320446129|ref|NW_003384442.1|	196900-19832	105272	410791	-135765	0.3099	no
gi|320446129|ref|NW_003384442.1|	198490-20444	341185	22773	-0.583229	0.66255	no
gi|320446129|ref|NW_003384442.1|	204739-20506	273388	232285	-0.235056	0.85215	no
gi|320446129|ref|NW_003384442.1|	205517-20690	732233	784827	0.100072	0.9416	no
gi|320446129|ref|NW_003384442.1|	207195-20790	391854	608995	0.636114	0.75185	no
gi|320446129|ref|NW_003384442.1|	208144-21401	237155	385271	0.700045	0.59975	no
gi|320446129|ref|NW_003384442.1|	21258-22762	808749	221065	-187122	0.1677	no
gi|320446129|ref|NW_003384442.1|	217349-21811	229896	170792	-0.428737	0.84185	no

gi|320446129|ref|NW_003384442.1|	220694-22122	181526	992284	-0.87135	0.67275	no
gi|320446129|ref|NW_003384442.1|	221995-22251	270645	102769	-139699	0.50875	no
gi|320446129|ref|NW_003384442.1|	27130-27565	26119	541045	-227128	0.3139	no
gi|320446129|ref|NW_003384442.1|	28946-31689	108333	554853	-0.965295	0.5535	no
gi|320446129|ref|NW_003384442.1|	32327-35250	678031	171598	133961	0.43355	no
gi|320446129|ref|NW_003384442.1|	49042-49460	353501	350319	-333497	0.1893	no
gi|320446129|ref|NW_003384442.1|	58238-58532	102021	140966	-285544	0.23	no
gi|320446129|ref|NW_003384442.1|	7428-7877	191098	100476	-0.927459	0.46825	no
gi|320446129|ref|NW_003384442.1|	9341-11666	658107	688858	0.0658853	0.96275	no
gi|320446130|ref|NW_003384441.1|	24087-24373	0	372047	inf	0.0133	no
gi|320446130|ref|NW_003384441.1|	28252-28714	508797	677788	373567	0.1477	no
gi|320446131|ref|NW_003384440.1|	104009-10682	654093	233148	-148825	0.49535	no
gi|320446131|ref|NW_003384440.1|	13384-13901	0	676339	inf	0.02075	no
gi|320446131|ref|NW_003384440.1|	24426-24678	312363	237374	292587	0.2577	no
gi|320446131|ref|NW_003384440.1|	24854-25289	163244	108209	272872	0.23745	no
gi|320446131|ref|NW_003384440.1|	33318-35401	0.365309	554234	392331	0.16595	no
gi|320446131|ref|NW_003384440.1|	38440-38917	136954	68584	232418	0.2622	no
gi|320446131|ref|NW_003384440.1|	39086-39546	0	634339	inf	0.02915	no
gi|320446131|ref|NW_003384440.1|	43146-43439	201876	116362	252708	0.27435	no
gi|320446131|ref|NW_003384440.1|	47908-51704	361561	341593	-340389	0.0956	no
gi|320446131|ref|NW_003384440.1|	53019-53262	596656	443453	-375004	0.2178	no
gi|320446131|ref|NW_003384440.1|	53433-59027	227015	100826	-117092	0.3825	no
gi|320446131|ref|NW_003384440.1|	60601-61237	121927	171603	-282887	0.2382	no
gi|320446131|ref|NW_003384440.1|	62084-62892	324249	222764	-0.541587	0.7906	no
gi|320446131|ref|NW_003384440.1|	63559-63820	953435	890079	-342113	0.1949	no
gi|320446131|ref|NW_003384440.1|	66516-67503	33682	775374	120292	0.5616	no
gi|320446131|ref|NW_003384440.1|	69577-69930	139913	12419	-0.171989	0.92285	no
gi|320446131|ref|NW_003384440.1|	70789-78472	912964	158528	0.796108	0.54895	no
gi|320446131|ref|NW_003384440.1|	80146-80877	919341	0.233125	-530143	0.27765	no
gi|320446131|ref|NW_003384440.1|	8206-10795	101801	250409	-202339	0.22665	no

gi|320446132|ref|NW_003384439.1|	161811-16263	373417	157961	-124122	0.5486	no
gi|320446132|ref|NW_003384439.1|	163507-16434	252501	135065	-0.902635	0.6711	no
gi|320446132|ref|NW_003384439.1|	166073-17138	157774	292521	0.890679	0.5068	no
gi|320446132|ref|NW_003384439.1|	189199-18980	134738	146313	344083	0.1992	no
gi|320446132|ref|NW_003384439.1|	243682-24504	0	323047	inf	0.01195	no
gi|320446132|ref|NW_003384439.1|	72976-73758	197629	762935	-137316	0.5144	no
gi|320446134|ref|NW_003384437.1|	103761-10444	150374	513328	177133	0.4276	no
gi|320446134|ref|NW_003384437.1|	112445-11325	0.591611	589301	331628	0.1936	no
gi|320446134|ref|NW_003384437.1|	116618-11756	0.238852	296601	363434	0.2061	no
gi|320446134|ref|NW_003384437.1|	121528-12234	0.288719	654862	450345	0.17645	no
gi|320446134|ref|NW_003384437.1|	126997-12769	0.3686	130939	51507	0.1703	no
gi|320446134|ref|NW_003384437.1|	178797-17944	284499	429026	391457	0.1355	no
gi|320446134|ref|NW_003384437.1|	186481-18708	0	831412	inf	0.0133	no
gi|320446134|ref|NW_003384437.1|	209214-21018	183296	127018	611472	0.05735	no
gi|320446134|ref|NW_003384437.1|	219921-22132	202859	244622	359201	0.0719	no
gi|320446134|ref|NW_003384437.1|	227532-22805	114575	666517	586228	0.13835	no
gi|320446134|ref|NW_003384437.1|	245345-24633	0.447307	29349	271398	0.24135	no
gi|320446134|ref|NW_003384437.1|	256375-25717	120649	223683	421257	0.15425	no
gi|320446134|ref|NW_003384437.1|	264641-26526	0.436535	266913	26122	0.2636	no
gi|320446134|ref|NW_003384437.1|	270321-27152	0.347379	638169	419936	0.16305	no
gi|320446134|ref|NW_003384437.1|	281791-28330	0.263748	990449	523085	0.14525	no
gi|320446134|ref|NW_003384437.1|	304899-30603	0.564667	129821	784491	0.11565	no
gi|320446134|ref|NW_003384437.1|	311070-31253	172107	1114.71	601722	0.00865	no
gi|320446134|ref|NW_003384437.1|	72328-72691	477504	179168	190772	0.39635	no
gi|320446134|ref|NW_003384437.1|	91424-91951	114575	38527	174958	0.3346	no
gi|320446134|ref|NW_003384437.1|	95795-96512	0.350347	311591	31528	0.24575	no
gi|320446134|ref|NW_003384437.1|	99858-100698	0.558328	499231	316052	0.20765	no
gi|320446135|ref|NW_003384436.1|	105171-10708	0.100824	0.70279	280126	1	no
gi|320446135|ref|NW_003384436.1|	108247-10935	0	215419	inf	0.0198	no
gi|320446135|ref|NW_003384436.1|	142531-14300	141325	117802	-0.262649	0.90315	no

gi|320446135|ref|NW_003384436.1|	168944-16951	401234	745853	0.894449	0.65925	no
gi|320446135|ref|NW_003384436.1|	174439-17800	588179	149007	134105	0.4395	no
gi|320446135|ref|NW_003384436.1|	181857-18337	316004	265543	-0.250998	0.89965	no
gi|320446135|ref|NW_003384436.1|	185106-18611	889848	494908	-0.846398	0.68105	no
gi|320446135|ref|NW_003384436.1|	206439-20687	992914	602978	-0.719563	0.73145	no
gi|320446137|ref|NW_003384434.1|	17500-20325	0.260627	227698	312707	0.2129	no
gi|320446137|ref|NW_003384434.1|	4698-5434	134844	13852	0.0388015	1	no
gi|320446138|ref|NW_003384433.1|	5211-5967	0	288913	inf	0.02915	no
gi|320446138|ref|NW_003384433.1|	80802-81326	462869	0.77792	-257291	0.30045	no
gi|320446138|ref|NW_003384433.1|	82007-82793	275898	0.420844	-271277	0.28435	no
gi|320446139|ref|NW_003384432.1|	17373-19052	163646	309242	0.918159	0.6565	no
gi|320446139|ref|NW_003384432.1|	21360-22609	482822	877655	0.862161	0.682	no
gi|320446139|ref|NW_003384432.1|	24433-25866	0.986339	254598	136807	0.517	no
gi|320446139|ref|NW_003384432.1|	26096-33275	194389	108686	248315	0.18005	no
gi|320446139|ref|NW_003384432.1|	34022-35425	751539	463902	26259	0.15525	no
gi|320446139|ref|NW_003384432.1|	44333-45399	304775	224872	-0.438638	0.82465	no
gi|320446139|ref|NW_003384432.1|	49679-50326	450287	557433	0.307952	0.8821	no
gi|320446139|ref|NW_003384432.1|	57700-59729	338678	702024	105161	0.6295	no
gi|320446139|ref|NW_003384432.1|	60644-62015	13374	670866	232659	0.2931	no
gi|320446139|ref|NW_003384432.1|	6325-7457	790534	0.651711	-360052	0.1859	no
gi|320446139|ref|NW_003384432.1|	63415-66513	257578	208139	-0.307462	0.8166	no
gi|320446139|ref|NW_003384432.1|	77640-79094	0.415431	279131	274826	0.2505	no
gi|320446139|ref|NW_003384432.1|	79188-80618	0	235598	inf	0.0138	no
gi|320446142|ref|NW_003384429.1|	100488-10098	377129	842302	115928	0.58175	no
gi|320446142|ref|NW_003384429.1|	10927-12261	75267	319941	-123421	0.5544	no
gi|320446142|ref|NW_003384429.1|	111093-11212	254869	293627	0.204226	0.92015	no
gi|320446142|ref|NW_003384429.1|	112276-11381	311867	488048	0.646095	0.7515	no
gi|320446142|ref|NW_003384429.1|	115932-11672	48346	892148	0.883886	0.6668	no
gi|320446142|ref|NW_003384429.1|	12810-13576	445337	283608	-0.651	0.7504	no
gi|320446142|ref|NW_003384429.1|	13773-14643	744693	622578	-0.258391	0.89805	no

gi|320446142|ref|NW_003384429.1|	14776-16985	940456	110378	0.231018	0.92045	no
gi|320446142|ref|NW_003384429.1|	35577-36493	0.247979	188055	292286	0.25645	no
gi|320446142|ref|NW_003384429.1|	76907-78228	870497	931258	341927	0.08635	no
gi|320446142|ref|NW_003384429.1|	99942-100432	130375	697819	242019	0.24925	no
gi|320446144|ref|NW_003384427.1|	105386-107528	504454	0.308733	-403029	0.1693	no
gi|320446144|ref|NW_003384427.1|	108586-108974	0	893955	inf	0.0039	no
gi|320446144|ref|NW_003384427.1|	140847-14397[illegible]	116622	497467	-122916	0.4645	no
gi|320446144|ref|NW_003384427.1|	23936-27695	928958	727196	-0.353268	0.7798	no
gi|320446144|ref|NW_003384427.1|	42273-42729	111762	842803	-0.407164	0.8413	no
gi|320446145|ref|NW_003384426.1|	23912-26548	175718	677668	194732	0.389	no
gi|320446152|ref|NW_003384419.1|	107144-108152	283973	135834	-106391	0.59105	no
gi|320446152|ref|NW_003384419.1|	120378-12129[illegible]	249446	154756	-0.688729	0.7318	no
gi|320446152|ref|NW_003384419.1|	131185-134788	102711	655584	-0.647734	0.61175	no
gi|320446152|ref|NW_003384419.1|	135029-143518	964855	987732	0.0338067	0.97985	no
gi|320446152|ref|NW_003384419.1|	143673-146155	991394	314723	-165538	0.32275	no
gi|320446152|ref|NW_003384419.1|	148848-149289	101713	311121	-170896	0.30495	no
gi|320446152|ref|NW_003384419.1|	149915-150965	192673	104582	-0.881512	0.68475	no
gi|320446152|ref|NW_003384419.1|	15029-16054	123967	443186	-148397	0.4936	no
gi|320446152|ref|NW_003384419.1|	151336-153049	60526	326044	-0.89249	0.6646	no
gi|320446152|ref|NW_003384419.1|	154141-154627	152185	911391	-0.73968	0.553	no
gi|320446152|ref|NW_003384419.1|	154793-15573[illegible]	146738	346798	124085	0.44455	no
gi|320446152|ref|NW_003384419.1|	156583-158891	854823	15458	0.854658	0.60215	no
gi|320446152|ref|NW_003384419.1|	160145-160401	565143	641127	0.181995	0.92595	no
gi|320446152|ref|NW_003384419.1|	160624-161932	213969	247889	0.21229	0.86785	no
gi|320446152|ref|NW_003384419.1|	168105-173654	11177	671918	-0.734173	0.57135	no
gi|320446152|ref|NW_003384419.1|	173865-178251	421545	411594	-0.0344659	0.9794	no
gi|320446152|ref|NW_003384419.1|	17612-18111	567728	126781	-216286	0.2791	no
gi|320446152|ref|NW_003384419.1|	19305-20648	167593	571266	17692	0.40495	no
gi|320446152|ref|NW_003384419.1|	21132-21716	385518	554588	0.524618	0.7878	no
gi|320446152|ref|NW_003384419.1|	22157-22610	452712	7025	0.633903	0.75235	no

gi|320446152|ref|NW_003384419.1|	22992-24146	165342	152715	-0.114609	0.949	no
gi|320446152|ref|NW_003384419.1|	25034-25712	152394	234004	0.61873	0.753	no
gi|320446152|ref|NW_003384419.1|	26202-27694	188004	700277	189716	0.38485	no
gi|320446152|ref|NW_003384419.1|	28743-29322	122185	181878	0.573909	0.7834	no
gi|320446152|ref|NW_003384419.1|	29620-31466	754331	121191	0.684016	0.76325	no
gi|320446152|ref|NW_003384419.1|	35723-36735	139082	136648	-0.0254753	0.9891	no
gi|320446152|ref|NW_003384419.1|	36820-38324	0.532072	136894	468529	0.14115	no
gi|320446152|ref|NW_003384419.1|	38625-43247	0	420222	inf	0.00365	no
gi|320446152|ref|NW_003384419.1|	45423-46057	211258	977063	220944	0.325	no
gi|320446152|ref|NW_003384419.1|	49832-50238	28139	927217	172034	0.4259	no
gi|320446152|ref|NW_003384419.1|	50409-50615	128854	44266	178046	0.3321	no
gi|320446152|ref|NW_003384419.1|	50724-51106	106683	12588	356065	0.21025	no
gi|320446152|ref|NW_003384419.1|	51990-52676	261996	143119	244959	0.28195	no
gi|320446152|ref|NW_003384419.1|	57223-59228	151612	867072	251577	0.06815	no
gi|320446152|ref|NW_003384419.1|	59384-61740	0.556981	627739	349447	0.1667	no
gi|320446152|ref|NW_003384419.1|	61973-63096	271888	804353	156482	0.23455	no
gi|320446152|ref|NW_003384419.1|	66222-67623	17373	28163	0.696956	0.73065	no
gi|320446152|ref|NW_003384419.1|	84197-86393	266795	990937	189306	0.41085	no
gi|320446152|ref|NW_003384419.1|	86804-87611	0.885863	182573	104332	0.5772	no
gi|320446152|ref|NW_003384419.1|	89262-90077	122308	144085	0.236401	0.91135	no
gi|320446152|ref|NW_003384419.1|	94139-95377	740597	199612	143044	0.52015	no
gi|320446152|ref|NW_003384419.1|	95868-96303	0.81622	48694	257671	0.27405	no
gi|320446152|ref|NW_003384419.1|	98843-99736	975293	137925	0.499974	0.8121	no
gi|320446153|ref|NW_003384418.1|	29396-32057	294945	215797	-0.450774	0.7335	no
gi|320446153|ref|NW_003384418.1|	32201-36389	657379	310811	-108069	0.5059	no
gi|320446153|ref|NW_003384418.1|	46060-46695	379327	630711	405547	0.0679	no
gi|320446154|ref|NW_003384417.1|	23725-24348	0	271508	inf	0.005	no
gi|320446154|ref|NW_003384417.1|	42007-44296	0	286768	inf	0.0074	no
gi|320446154|ref|NW_003384417.1|	46247-48695	0	218347	inf	0.0072	no
gi|320446154|ref|NW_003384417.1|	48831-52696	0.0929718	601917	601663	0.13595	no

gi|320446154|ref|NW_003384417.1|	79018-79833	0	660391	inf	0.00945	no
gi|320446158|ref|NW_003384413.1|	112255-11261[illegible]	119376	934787	296913	0.2563	no
gi|320446158|ref|NW_003384413.1|	118782-12177[illegible]	110708	223648	101447	0.43785	no
gi|320446158|ref|NW_003384413.1|	12648-15961	97039	294129	159981	0.22545	no
gi|320446161|ref|NW_003384410.1|	16387-17182	0	331382	inf	0.0198	no
gi|320446161|ref|NW_003384410.1|	20301-25264	0.392577	234495	590044	0.04165	no
gi|320446161|ref|NW_003384410.1|	31562-31797	0	64342	inf	0.0142	no
gi|320446161|ref|NW_003384410.1|	32556-37102	767174	242312	165924	0.218	no
gi|320446161|ref|NW_003384410.1|	43089-43654	0	378219	inf	0.0294	no
gi|320446161|ref|NW_003384410.1|	54128-55765	0.120393	142454	356467	0.2162	no
gi|320446161|ref|NW_003384410.1|	56204-57395	0.353196	159119	217156	0.2872	no
gi|320446161|ref|NW_003384410.1|	57552-58105	211034	320351	0.602181	0.7533	no
gi|320446161|ref|NW_003384410.1|	60286-60841	0	389257	inf	0.0294	no
gi|320446163|ref|NW_003384408.1|	11486-14381	0.317208	164083	237092	0.29145	no
gi|320446163|ref|NW_003384408.1|	29587-29982	228458	125386	245637	0.1676	no
gi|320446163|ref|NW_003384408.1|	35537-37653	331981	278546	306874	0.1093	no
gi|320446163|ref|NW_003384408.1|	37822-39611	391005	464576	357065	0.0794	no
gi|320446163|ref|NW_003384408.1|	44831-45014	110953	109714	330572	0.2232	no
gi|320446163|ref|NW_003384408.1|	51253-51805	0.529198	821084	395565	0.19695	no
gi|320446163|ref|NW_003384408.1|	52427-53780	452982	109048	126744	0.5578	no
gi|320446163|ref|NW_003384408.1|	54593-55698	504577	110152	112635	0.59915	no
gi|320446163|ref|NW_003384408.1|	5610-5802	878035	545242	263455	0.27175	no
gi|320446163|ref|NW_003384408.1|	57823-59738	301391	151965	233403	0.1802	no
gi|320446163|ref|NW_003384408.1|	59904-66369	425214	292734	278333	0.0428	no
gi|320446163|ref|NW_003384408.1|	6000-7002	330227	282886	309869	0.1004	no
gi|320446163|ref|NW_003384408.1|	70978-71577	138734	407589	15548	0.491	no
gi|320446163|ref|NW_003384408.1|	74445-75410	0	191606	inf	0.029	no
gi|320446163|ref|NW_003384408.1|	83952-85139	0	17207	inf	0.0233	no
gi|320446163|ref|NW_003384408.1|	86349-86895	0.539082	101753	423842	0.18965	no
gi|320446166|ref|NW_003384405.1|	108346-10967[illegible]	0.153886	138891	317402	1	no

gi|320446166|ref|NW_003384405.1|	110045-11211	0.0922259	167252	41807	0.1906	no
gi|320446166|ref|NW_003384405.1|	127425-12800	0	465508	inf	0.0233	no
gi|320446166|ref|NW_003384405.1|	133685-13462	0.23919	84154	51368	0.16485	no
gi|320446166|ref|NW_003384405.1|	135490-13580	653276	115858	0.826592	0.70205	no
gi|320446166|ref|NW_003384405.1|	147302-14952	339233	0	#NAME?	0.007	no
gi|320446166|ref|NW_003384405.1|	15330-15645	334496	344865	0.044042	0.97985	no
gi|320446166|ref|NW_003384405.1|	162337-16321	322557	140868	-119521	0.4576	no
gi|320446166|ref|NW_003384405.1|	163358-16388	510542	572472	0.165176	0.93035	no
gi|320446166|ref|NW_003384405.1|	164114-16521	582204	349262	-0.737215	0.71755	no
gi|320446166|ref|NW_003384405.1|	167738-16897	29148	817931	-183335	0.28475	no
gi|320446166|ref|NW_003384405.1|	169685-17018	101294	203553	100686	0.6191	no
gi|320446166|ref|NW_003384405.1|	170333-17056	0	34101	inf	0.02915	no
gi|320446166|ref|NW_003384405.1|	20231-20674	866441	109808	0.341816	0.8614	no
gi|320446166|ref|NW_003384405.1|	21623-22087	937252	148915	0.667984	0.7409	no
gi|320446166|ref|NW_003384405.1|	27457-34266	354195	86364	128588	0.3156	no
gi|320446166|ref|NW_003384405.1|	34383-36314	118423	301776	134953	0.4395	no
gi|320446166|ref|NW_003384405.1|	36812-39620	603403	181523	158896	0.35155	no
gi|320446166|ref|NW_003384405.1|	89176-89433	499632	111823	-215965	0.337	no
gi|320446167|ref|NW_003384404.1|	36136-37491	301449	460543	0.611423	0.768	no
gi|320446167|ref|NW_003384404.1|	40523-42411	234842	804168	177581	0.42785	no
gi|320446175|ref|NW_003384396.1|	1544-4070	907525	159758	0.815875	0.5239	no
gi|320446175|ref|NW_003384396.1|	243504-24681	268726	306884	0.191553	0.92675	no
gi|320446175|ref|NW_003384396.1|	248570-24939	876205	37443	-122657	0.3404	no
gi|320446175|ref|NW_003384396.1|	249555-25031	648393	200017	-169675	0.4287	no
gi|320446175|ref|NW_003384396.1|	251315-25368	0.394495	170771	211398	0.3527	no
gi|320446175|ref|NW_003384396.1|	256620-25710	538526	162525	-172835	0.42475	no
gi|320446175|ref|NW_003384396.1|	257270-25791	162372	116158	-0.483217	0.8116	no
gi|320446175|ref|NW_003384396.1|	258091-25878	404582	402045	-0.00907819	0.96375	no
gi|320446175|ref|NW_003384396.1|	260870-26259	578504	65564	0.180575	0.93355	no
gi|320446175|ref|NW_003384396.1|	264829-26625	38487	176008	-112873	0.38435	no

gi|320446175|ref|NW_003384396.1|	271963-273674	331629	453889	0.45277	0.8268	no
gi|320446175|ref|NW_003384396.1|	278018-280813	652613	621956	-0.0694137	0.9768	no
gi|320446175|ref|NW_003384396.1|	281608-281853	239205	151198	-0.661808	0.7467	no
gi|320446175|ref|NW_003384396.1|	283741-284442	543468	173345	-164855	0.43885	no
gi|320446175|ref|NW_003384396.1|	301221-301773	952556	121378	0.349626	0.8644	no
gi|320446175|ref|NW_003384396.1|	313249-321290	26091	200982	-0.376489	0.77425	no
gi|320446175|ref|NW_003384396.1|	323593-324130	995812	374027	-141273	0.40105	no
gi|320446175|ref|NW_003384396.1|	332543-333155	536141	303285	-0.821936	0.6936	no
gi|320446175|ref|NW_003384396.1|	333270-334719	152952	724835	-107736	0.62375	no
gi|320446175|ref|NW_003384396.1|	340818-341449	229863	162089	-0.503992	0.81125	no
gi|320446175|ref|NW_003384396.1|	341920-342948	210798	220767	0.0666648	0.9756	no
gi|320446175|ref|NW_003384396.1|	344888-345074	562461	506476	-347319	0.161	no
gi|320446175|ref|NW_003384396.1|	346487-347837	682817	494126	-378855	0.0633	no
gi|320446175|ref|NW_003384396.1|	347949-348145	239651	496971	-559163	0.2751	no
gi|320446175|ref|NW_003384396.1|	348858-349582	873743	496358	-413776	0.0554	no
gi|320446175|ref|NW_003384396.1|	350194-350651	89038	493791	-417245	0.12545	no
gi|320446175|ref|NW_003384396.1|	353934-354637	487043	754641	0.63174	0.61845	no
gi|320446175|ref|NW_003384396.1|	355462-355688	135088	452982	174556	0.415	no
gi|320446175|ref|NW_003384396.1|	358219-358649	609595	663608	0.122481	0.95685	no
gi|320446175|ref|NW_003384396.1|	4387-7998	414368	581288	0.488341	0.7113	no
gi|320446176|ref|NW_003384395.1|	162020-162739	244234	214854	-0.184909	0.9166	no
gi|320446176|ref|NW_003384395.1|	329387-330305	222527	0.340935	-270642	0.2844	no
gi|320446176|ref|NW_003384395.1|	381513-383611	209267	992169	-107669	0.5248	no
gi|320446176|ref|NW_003384395.1|	384954-386260	158313	435205	-186302	0.40905	no
gi|320446176|ref|NW_003384395.1|	388182-388945	703789	241306	-154428	0.47215	no
gi|320446176|ref|NW_003384395.1|	399656-403060	434228	223085	-428278	0.04745	no
gi|320446176|ref|NW_003384395.1|	404569-405130	182144	0.341895	-573538	0.2744	no
gi|320446176|ref|NW_003384395.1|	405286-409728	127949	803274	-0.6716	0.60385	no
gi|320446176|ref|NW_003384395.1|	410545-411482	404132	497796	-30212	0.1045	no
gi|320446176|ref|NW_003384395.1|	411632-418235	461042	347323	-0.408621	0.75225	no

gi|320446176|ref|NW_003384395.1|	418319-41926	170525	114391	-389794	0.1554	no
gi|320446176|ref|NW_003384395.1|	427324-43027	266976	121533	-113536	0.3896	no
gi|320446176|ref|NW_003384395.1|	431543-43766	221825	83738	-472739	0.02285	no
gi|320446176|ref|NW_003384395.1|	441466-44185	277985	0	#NAME?	5.00E-05	yes
gi|320446177|ref|NW_003384394.1|	112193-11345	471581	806552	-254767	0.1662	no
gi|320446177|ref|NW_003384394.1|	113561-11429	324918	463529	-280934	0.23445	no
gi|320446177|ref|NW_003384394.1|	115231-11651	269851	847111	-167154	0.3162	no
gi|320446177|ref|NW_003384394.1|	118073-11990	11231	479866	-122678	0.5814	no
gi|320446177|ref|NW_003384394.1|	127345-12819	0.829259	275737	505533	0.14635	no
gi|320446177|ref|NW_003384394.1|	129806-13181	934463	904309	-0.0473221	0.9815	no
gi|320446177|ref|NW_003384394.1|	131927-13363	126047	0.638324	-0.981596	1	no
gi|320446177|ref|NW_003384394.1|	133892-13531	0.713466	109067	0.612292	1	no
gi|320446177|ref|NW_003384394.1|	137388-13905	615073	535216	-0.200637	0.9219	no
gi|320446177|ref|NW_003384394.1|	140923-14301	0.637221	0.698503	0.132471	1	no
gi|320446177|ref|NW_003384394.1|	146494-14753	0.840047	101636	0.274872	1	no
gi|320446177|ref|NW_003384394.1|	147698-14793	789703	355868	-114997	0.58305	no
gi|320446177|ref|NW_003384394.1|	15701-18360	175442	583713	-158766	0.3554	no
gi|320446177|ref|NW_003384394.1|	158073-15842	373017	137341	-144148	0.40935	no
gi|320446177|ref|NW_003384394.1|	171060-17218	167883	392294	122448	0.4658	no
gi|320446177|ref|NW_003384394.1|	172339-17436	348669	933208	142034	0.5263	no
gi|320446177|ref|NW_003384394.1|	176482-17708	741911	8173	0.139619	0.93935	no
gi|320446177|ref|NW_003384394.1|	178369-17924	816756	145143	0.8295	0.6916	no
gi|320446177|ref|NW_003384394.1|	181731-18244	110432	109619	-0.010654	0.98685	no
gi|320446177|ref|NW_003384394.1|	183398-18418	277409	61349	114503	0.5772	no
gi|320446177|ref|NW_003384394.1|	18969-19338	115086	451258	-135069	0.52435	no
gi|320446177|ref|NW_003384394.1|	20424-21386	239243	529056	-217699	0.3347	no
gi|320446177|ref|NW_003384394.1|	205215-20567	796287	120562	0.59841	0.75855	no
gi|320446177|ref|NW_003384394.1|	255765-25722	579226	325934	-0.829548	0.6922	no
gi|320446177|ref|NW_003384394.1|	25791-26340	363185	792374	-219645	0.33105	no
gi|320446177|ref|NW_003384394.1|	26512-29578	703383	504322	-0.479965	0.8333	no

gi|320446177|ref|NW_003384394.1|	265969-269224	188866	899657	225201	0.09955	no
gi|320446177|ref|NW_003384394.1|	269324-269828	687821	154956	117176	0.49	no
gi|320446177|ref|NW_003384394.1|	272267-273249	185362	382436	104488	0.5243	no
gi|320446177|ref|NW_003384394.1|	273660-273890	465296	74554	0.680137	0.73	no
gi|320446177|ref|NW_003384394.1|	278901-279607	483958	68137	0.493556	0.7005	no
gi|320446177|ref|NW_003384394.1|	291957-292340	102605	74105	285247	0.25935	no
gi|320446177|ref|NW_003384394.1|	30628-31076	539534	409707	-0.397122	0.84605	no
gi|320446177|ref|NW_003384394.1|	31189-34034	258629	3525	0.446739	0.8309	no
gi|320446177|ref|NW_003384394.1|	34383-35721	146927	818236	-0.844508	0.70115	no
gi|320446177|ref|NW_003384394.1|	62202-62665	796287	144674	-246048	0.24235	no
gi|320446177|ref|NW_003384394.1|	96591-97033	221519	10502	-439869	0.20495	no
gi|320446179|ref|NW_003384392.1|	101443-103015	0.126249	0.966171	2936	1	no
gi|320446179|ref|NW_003384392.1|	104884-105565	0.378443	361653	325646	0.2305	no
gi|320446179|ref|NW_003384392.1|	112595-113093	126617	466475	188133	0.3216	no
gi|320446179|ref|NW_003384392.1|	138849-139264	179351	242759	375867	0.17525	no
gi|320446179|ref|NW_003384392.1|	140276-140923	409352	345608	307772	0.19705	no
gi|320446179|ref|NW_003384392.1|	142307-143140	0.279627	826992	48863	0.17455	no
gi|320446179|ref|NW_003384392.1|	143441-145169	203494	358223	41378	0.05275	no
gi|320446179|ref|NW_003384392.1|	156674-157155	67432	112633	0.740122	0.7137	no
gi|320446179|ref|NW_003384392.1|	163832-166024	164716	269601	0.710842	0.59225	no
gi|320446179|ref|NW_003384392.1|	166718-166923	460221	205251	-116494	0.58455	no
gi|320446179|ref|NW_003384392.1|	167161-168488	200969	156692	-0.359042	0.8743	no
gi|320446179|ref|NW_003384392.1|	168944-169230	474264	0	#NAME?	0.01	no
gi|320446179|ref|NW_003384392.1|	172563-176040	154304	154117	-0.0017493	0.99805	no
gi|320446179|ref|NW_003384392.1|	176316-177311	182188	598526	-160594	0.45495	no
gi|320446179|ref|NW_003384392.1|	184760-185133	33713	808608	126214	0.55325	no
gi|320446179|ref|NW_003384392.1|	190334-191154	118375	119066	-331353	0.195	no
gi|320446179|ref|NW_003384392.1|	195471-198153	436551	257323	-0.76257	0.56605	no
gi|320446179|ref|NW_003384392.1|	199083-201952	16978	734432	-120897	0.48225	no
gi|320446179|ref|NW_003384392.1|	202475-204011	232243	821199	-149983	0.36665	no

gi|320446179|ref|NW_003384392.1|	205052-205488	601285	210097	-151699	0.4823	no
gi|320446179|ref|NW_003384392.1|	205669-206433	507687	164223	-162828	0.33555	no
gi|320446179|ref|NW_003384392.1|	206671-208265	775037	265323	-154651	0.25205	no
gi|320446179|ref|NW_003384392.1|	23851-25668	128027	899452	281259	0.225	no
gi|320446179|ref|NW_003384392.1|	289533-290298	404746	566075	0.483975	0.70805	no
gi|320446179|ref|NW_003384392.1|	96510-101228	222073	160281	28515	0.13955	no
gi|320446180|ref|NW_003384391.1|	153849-155237	131767	630567	225866	0.31015	no
gi|320446180|ref|NW_003384391.1|	155300-157511	0.426831	571908	374405	0.1602	no
gi|320446180|ref|NW_003384391.1|	288152-288723	0	574732	inf	0.02075	no
gi|320446180|ref|NW_003384391.1|	294127-298614	0.476217	33626	281989	0.22445	no
gi|320446180|ref|NW_003384391.1|	304758-306719	143744	116582	-0.302159	0.85215	no
gi|320446180|ref|NW_003384391.1|	306984-313541	154799	103083	-0.586594	0.6621	no
gi|320446180|ref|NW_003384391.1|	333141-337286	154867	1314.84	308578	0.2778	no
gi|320446180|ref|NW_003384391.1|	343166-343801	0.840875	400408	225151	0.2747	no
gi|320446180|ref|NW_003384391.1|	343943-350221	0.334952	136947	203159	1	no
gi|320446180|ref|NW_003384391.1|	350389-351321	484563	668408	0.464044	0.823	no
gi|320446180|ref|NW_003384391.1|	351566-351990	188929	596442	165854	0.44395	no
gi|320446180|ref|NW_003384391.1|	352369-354401	352201	147377	206504	0.1274	no
gi|320446180|ref|NW_003384391.1|	355490-360651	180177	293484	0.70387	0.59825	no
gi|320446180|ref|NW_003384391.1|	366971-368068	489676	596468	0.284617	0.888	no
gi|320446180|ref|NW_003384391.1|	374498-375283	159697	212904	0.414863	0.84715	no
gi|320446180|ref|NW_003384391.1|	392352-393580	11901	601257	23369	0.28335	no
gi|320446180|ref|NW_003384391.1|	393769-395348	113032	917494	302096	0.20415	no
gi|320446182|ref|NW_003384389.1|	101813-107910	0.632603	153161	127568	0.54075	no
gi|320446182|ref|NW_003384389.1|	112235-113068	0.847306	155372	0.874772	0.68225	no
gi|320446182|ref|NW_003384389.1|	116651-117020	920687	255713	147374	0.47875	no
gi|320446182|ref|NW_003384389.1|	130732-136914	326853	112552	178388	0.31165	no
gi|320446182|ref|NW_003384389.1|	137487-138521	11623	276109	124826	0.43635	no
gi|320446182|ref|NW_003384389.1|	139137-140362	208046	236493	0.184896	0.90565	no
gi|320446182|ref|NW_003384389.1|	140889-141084	24526	508377	105159	0.5868	no

gi|320446182|ref|NW_003384389.1|	141254-14181	72534	734556	0.0182149	0.97265	no
gi|320446182|ref|NW_003384389.1|	142441-14435	188984	172375	-0.132711	0.91845	no
gi|320446182|ref|NW_003384389.1|	144530-14504	517109	375199	-0.462813	0.83645	no
gi|320446182|ref|NW_003384389.1|	145424-14581	615632	471577	-0.384573	0.8476	no
gi|320446182|ref|NW_003384389.1|	147574-14835	955518	930812	-0.0377931	0.9834	no
gi|320446182|ref|NW_003384389.1|	150207-15112	596905	428635	-0.477753	0.8148	no
gi|320446182|ref|NW_003384389.1|	153613-15446	115906	117712	0.0223063	0.9884	no
gi|320446182|ref|NW_003384389.1|	222622-22346	252082	142551	249951	0.2727	no
gi|320446182|ref|NW_003384389.1|	22330-23703	192841	165879	310465	0.1951	no
gi|320446182|ref|NW_003384389.1|	24129-26647	206979	131143	266359	0.1449	no
gi|320446182|ref|NW_003384389.1|	31723-33166	0.139738	106832	293454	1	no
gi|320446182|ref|NW_003384389.1|	75693-76071	654886	714824	0.126343	0.93285	no
gi|320446184|ref|NW_003384387.1|	100695-10202	789863	365561	-111149	0.59735	no
gi|320446184|ref|NW_003384387.1|	103110-10512	39734	296887	-0.420458	0.8357	no
gi|320446184|ref|NW_003384387.1|	105532-10641	861379	3236	-141244	0.50075	no
gi|320446184|ref|NW_003384387.1|	108018-10867	919448	735477	-0.322087	0.8691	no
gi|320446184|ref|NW_003384387.1|	110266-11062	96696	394174	-129462	0.55035	no
gi|320446184|ref|NW_003384387.1|	113323-11370	130977	142965	-319559	0.25	no
gi|320446184|ref|NW_003384387.1|	12328-12654	629714	168754	-189978	0.38205	no
gi|320446184|ref|NW_003384387.1|	126422-12786	0	194718	inf	0.0162	no
gi|320446184|ref|NW_003384387.1|	131572-13312	0.385675	43818	350607	0.1915	no
gi|320446184|ref|NW_003384387.1|	1319-2597	132724	359382	-188484	0.39315	no
gi|320446184|ref|NW_003384387.1|	14486-14986	408556	968647	-207649	0.3533	no
gi|320446184|ref|NW_003384387.1|	16417-16735	310306	842603	-188077	0.38335	no
gi|320446184|ref|NW_003384387.1|	169164-17142	638603	192935	-17268	0.2067	no
gi|320446184|ref|NW_003384387.1|	171581-17191	120.5	376772	-167727	0.44055	no
gi|320446184|ref|NW_003384387.1|	172468-17316	384872	138964	-146966	0.5115	no
gi|320446184|ref|NW_003384387.1|	173285-17707	387547	455371	0.232671	0.8609	no
gi|320446184|ref|NW_003384387.1|	17827-18358	271403	228273	-357161	0.1779	no
gi|320446184|ref|NW_003384387.1|	18470-21585	237893	359336	0.595023	0.6625	no

gi|320446184|ref|NW_003384387.1|	21696-23397	217606	186792	-0.22028	0.86205	no
gi|320446184|ref|NW_003384387.1|	29758-30644	207535	178693	-0.215869	0.91015	no
gi|320446184|ref|NW_003384387.1|	66853-67259	675336	735592	0.1233	0.95505	no
gi|320446184|ref|NW_003384387.1|	69129-70114	652923	435349	-0.584739	0.77725	no
gi|320446184|ref|NW_003384387.1|	73163-73721	504068	715632	0.505601	0.74755	no
gi|320446184|ref|NW_003384387.1|	753-980	930714	111494	-306137	0.23825	no
gi|320446184|ref|NW_003384387.1|	90881-94932	132659	535525	201323	0.3752	no
gi|320446184|ref|NW_003384387.1|	97219-100497	0.99819	186179	0.899305	0.67185	no
gi|320446185|ref|NW_003384386.1|	10618-10985	23297	913043	197054	0.3149	no
gi|320446185|ref|NW_003384386.1|	169364-16979	989934	433258	-11921	0.59645	no
gi|320446185|ref|NW_003384386.1|	170035-17136	154309	128555	-0.263434	0.88795	no
gi|320446185|ref|NW_003384386.1|	171656-17204	912631	667479	-0.451309	0.8389	no
gi|320446185|ref|NW_003384386.1|	172891-17474	0.728851	747365	335812	0.17545	no
gi|320446185|ref|NW_003384386.1|	176235-17689	0.396045	296945	290646	0.25825	no
gi|320446185|ref|NW_003384386.1|	177791-17961	0.424877	162822	193818	0.39545	no
gi|320446185|ref|NW_003384386.1|	179874-18181	0.297144	193341	270192	0.25845	no
gi|320446185|ref|NW_003384386.1|	181904-18342	0	155303	inf	0.02075	no
gi|320446185|ref|NW_003384386.1|	184392-18696	295439	97654	172482	0.2986	no
gi|320446185|ref|NW_003384386.1|	217251-21773	531332	39962	-0.410987	0.8413	no
gi|320446185|ref|NW_003384386.1|	219273-22007	871589	80493	-0.114785	0.9517	no
gi|320446185|ref|NW_003384386.1|	225813-22890	708546	850521	0.263486	0.86895	no
gi|320446185|ref|NW_003384386.1|	229009-22956	315588	603339	0.934924	0.65825	no
gi|320446185|ref|NW_003384386.1|	23552-26183	0.493036	3543	284521	0.2217	no
gi|320446185|ref|NW_003384386.1|	255784-25688	312667	175827	-0.83047	0.68355	no
gi|320446185|ref|NW_003384386.1|	258301-25932	0.851709	0.883069	0.0521652	1	no
gi|320446185|ref|NW_003384386.1|	27407-28868	0.413057	468969	350508	0.1915	no
gi|320446185|ref|NW_003384386.1|	29495-30857	224681	24.55	344977	0.08115	no
gi|320446185|ref|NW_003384386.1|	66957-67400	157535	120266	293249	0.2225	no
gi|320446188|ref|NW_003384383.1|	107256-10778	283634	164109	253255	0.27755	no
gi|320446188|ref|NW_003384383.1|	56051-58034	308972	363552	0.234682	0.90585	no

gi|320446188|ref|NW_003384383.1|	58171-59562	204439	294185	0.525054	0.7934	no
gi|320446188|ref|NW_003384383.1|	59775-60484	142492	219238	0.621614	0.75255	no
gi|320446188|ref|NW_003384383.1|	60768-63228	872257	799842	-0.125039	0.9552	no
gi|320446188|ref|NW_003384383.1|	64606-64963	198336	323079	-261799	0.2722	no
gi|320446188|ref|NW_003384383.1|	65096-65709	106949	574797	-0.895803	0.6625	no
gi|320446188|ref|NW_003384383.1|	65982-68081	307846	732677	125097	0.56705	no
gi|320446188|ref|NW_003384383.1|	70068-70753	825232	358561	-120258	0.55895	no
gi|320446188|ref|NW_003384383.1|	71420-71825	942762	310586	-16019	0.44055	no
gi|320446188|ref|NW_003384383.1|	72009-74436	135504	13602	0.00548393	0.99635	no
gi|320446188|ref|NW_003384383.1|	74741-75400	994735	135584	0.446808	0.8243	no
gi|320446188|ref|NW_003384383.1|	81514-84177	13206	372478	149596	0.2569	no
gi|320446188|ref|NW_003384383.1|	89274-90340	447003	127896	151662	0.47315	no
gi|320446189|ref|NW_003384382.1|	139060-13973[illegible]	0	676013	inf	0.0142	no
gi|320446189|ref|NW_003384382.1|	148782-14906[illegible]	0	969.46	inf	5.00E-05	yes
gi|320446189|ref|NW_003384382.1|	151386-15339[illegible]	0.381413	255002	938494	0.1028	no
gi|320446189|ref|NW_003384382.1|	16814-23495	146586	844059	25256	0.17105	no
gi|320446189|ref|NW_003384382.1|	73693-75361	0.235576	172198	286981	0.22425	no
gi|320446189|ref|NW_003384382.1|	78424-80113	0.812601	307133	191825	0.37835	no
gi|320446189|ref|NW_003384382.1|	80358-81727	0.446587	175763	197662	0.39	no
gi|320446189|ref|NW_003384382.1|	82923-85614	240494	849814	182115	0.274	no
gi|320446189|ref|NW_003384382.1|	86072-88189	439416	227724	237363	0.18475	no
gi|320446191|ref|NW_003384380.1|	43389-44069	352737	315836	-0.159416	0.9426	no
gi|320446191|ref|NW_003384380.1|	44769-48274	261119	103621	-13334	0.32235	no
gi|320446191|ref|NW_003384380.1|	48918-50349	705707	274637	-136154	0.5255	no
gi|320446191|ref|NW_003384380.1|	51109-54119	454265	403911	-349142	0.0881	no
gi|320446191|ref|NW_003384380.1|	54373-54765	514811	464251	-347106	0.1765	no
gi|320446191|ref|NW_003384380.1|	56807-57882	767837	597961	-368268	0.07095	no
gi|320446191|ref|NW_003384380.1|	60578-62477	176995	250625	0.501823	0.68535	no
gi|320446191|ref|NW_003384380.1|	73171-76684	127667	615169	22686	0.09655	no
gi|320446194|ref|NW_003384377.1|	244440-24625[illegible]	0.107164	141859	372657	0.21225	no

gi|320446194|ref|NW_003384377.1|	247607-25048	0.127651	334604	471217	0.1501	no
gi|320446194|ref|NW_003384377.1|	251156-25290	0	267707	inf	5.00E-05	yes
gi|320446194|ref|NW_003384377.1|	255630-25754	742104	152395	103812	0.52135	no
gi|320446194|ref|NW_003384377.1|	257986-25904	0.617732	156643	134243	0.5243	no
gi|320446194|ref|NW_003384377.1|	263052-26345	293287	707488	127039	0.5503	no
gi|320446194|ref|NW_003384377.1|	52099-52598	107238	676167	-0.665358	0.73545	no
gi|320446194|ref|NW_003384377.1|	60639-62010	199124	241512	0.278425	0.8219	no
gi|320446195|ref|NW_003384376.1|	20234-22881	0.209899	171092	302701	0.2185	no
gi|320446195|ref|NW_003384376.1|	23718-24241	290258	277046	325472	0.19755	no
gi|320446195|ref|NW_003384376.1|	31918-32358	127705	0.529565	-459186	0.29375	no
gi|320446195|ref|NW_003384376.1|	3416-4804	0.73204	0.610226	-0.262579	1	no
gi|320446195|ref|NW_003384376.1|	36075-36346	526975	319472	-404398	0.2172	no
gi|320446195|ref|NW_003384376.1|	36816-37062	404698	426454	-324638	0.24925	no
gi|320446195|ref|NW_003384376.1|	37663-37879	692974	334113	-437439	0.2989	no
gi|320446195|ref|NW_003384376.1|	38673-38999	476125	0.99267	-558388	0.2787	no
gi|320446195|ref|NW_003384376.1|	43230-43452	48066	0	#NAME?	0.0229	no
gi|320446195|ref|NW_003384376.1|	46825-47027	76975	436421	-41406	0.3074	no
gi|320446195|ref|NW_003384376.1|	47143-47778	33718	0.573374	-255597	0.30045	no
gi|320446195|ref|NW_003384376.1|	57227-58039	178563	623527	-151791	0.4837	no
gi|320446195|ref|NW_003384376.1|	60786-62128	129621	804723	-0.687736	0.74525	no
gi|320446195|ref|NW_003384376.1|	62408-62785	362274	481593	0.410733	0.8432	no
gi|320446195|ref|NW_003384376.1|	64-2011	0.59151	247428	206454	0.3416	no
gi|320446195|ref|NW_003384376.1|	93608-94190	678456	196799	-178554	0.41945	no
gi|320446195|ref|NW_003384376.1|	9598-10347	0.328604	225187	27767	0.2657	no
gi|320446196|ref|NW_003384375.1|	134418-13460	107933	367087	176599	0.40675	no
gi|320446196|ref|NW_003384375.1|	184402-18619	0.108473	128464	356596	1	no
gi|320446196|ref|NW_003384375.1|	186326-19283	164858	699378	208484	0.11975	no
gi|320446196|ref|NW_003384375.1|	192958-19316	671092	460742	277938	0.2661	no
gi|320446196|ref|NW_003384375.1|	205364-20582	0.732835	82952	350072	0.2178	no
gi|320446196|ref|NW_003384375.1|	211838-21280	115978	176114	0.602658	0.75905	no

gi|320446196|ref|NW_003384375.1|	213294-21355	145215	110532	-0.393727	0.8552	no
gi|320446196|ref|NW_003384375.1|	213850-21512	178044	15723	-0.179362	0.92355	no
gi|320446196|ref|NW_003384375.1|	216153-21663	806104	0.897773	-316654	0.25455	no
gi|320446196|ref|NW_003384375.1|	221289-22227	134549	136288	334045	0.1794	no
gi|320446196|ref|NW_003384375.1|	223723-22491	281673	170829	-0.721464	0.7203	no
gi|320446196|ref|NW_003384375.1|	226791-22783	0.625365	922464	388272	0.1702	no
gi|320446196|ref|NW_003384375.1|	227990-22868	0.371013	278746	290941	0.25645	no
gi|320446196|ref|NW_003384375.1|	229871-23108	0	239601	inf	0.0162	no
gi|320446196|ref|NW_003384375.1|	232172-23341	0.837411	2904	179403	0.4141	no
gi|320446196|ref|NW_003384375.1|	236939-23743	517663	866115	0.742546	0.7096	no
gi|320446196|ref|NW_003384375.1|	252635-25316	0	534307	inf	0.0233	no
gi|320446196|ref|NW_003384375.1|	63652-64615	10206	648418	26675	0.1436	no
gi|320446196|ref|NW_003384375.1|	76561-76762	285946	84675	156619	0.496	no
gi|320446196|ref|NW_003384375.1|	80299-80451	209.42	691811	172398	0.41665	no
gi|320446198|ref|NW_003384373.1|	103338-10397	0.421475	344024	302899	0.25005	no
gi|320446198|ref|NW_003384373.1|	105902-10633	165486	43853	140597	0.38125	no
gi|320446198|ref|NW_003384373.1|	147953-14886	149668	292318	0.965774	0.65575	no
gi|320446198|ref|NW_003384373.1|	78402-80886	0.375194	277759	288813	0.229	no
gi|320446198|ref|NW_003384373.1|	88210-90575	246413	331359	0.427319	0.7523	no
gi|320446198|ref|NW_003384373.1|	90678-91261	401098	350008	-0.196566	0.9289	no
gi|320446198|ref|NW_003384373.1|	91372-91589	785527	788029	0.00458694	0.97485	no
gi|320446198|ref|NW_003384373.1|	92054-92329	481178	260637	-0.884532	0.67055	no
gi|320446198|ref|NW_003384373.1|	94511-95169	283166	184812	-0.615587	0.77985	no
gi|320446198|ref|NW_003384373.1|	95685-96455	0.631442	259882	204113	0.30255	no
gi|320446198|ref|NW_003384373.1|	98571-99032	0	680451	inf	0.0233	no
gi|320446199|ref|NW_003384372.1|	100070-10162	146613	239458	-261417	0.2681	no
gi|320446199|ref|NW_003384372.1|	102790-10371	129525	353909	-187179	0.3846	no
gi|320446199|ref|NW_003384372.1|	109564-10996	117315	0.643171	-418904	0.3058	no
gi|320446199|ref|NW_003384372.1|	121124-12154	150238	105096	-0.515543	0.78985	no
gi|320446199|ref|NW_003384372.1|	129142-13032	747187	838375	0.166126	0.93375	no

gi|320446199|ref|NW_003384372.1|	12927-13433	178438	165052	-343443	0.2075	no
gi|320446199|ref|NW_003384372.1|	132121-13387	946358	589345	-0.683275	0.74685	no
gi|320446199|ref|NW_003384372.1|	134107-14092	137404	265427	0.949887	0.47645	no
gi|320446199|ref|NW_003384372.1|	144002-14662	721739	726682	0.00984738	0.9944	no
gi|320446199|ref|NW_003384372.1|	147833-14941	452129	227189	-0.992843	0.6442	no
gi|320446199|ref|NW_003384372.1|	149657-15065	221889	0.766349	-153376	0.49255	no
gi|320446199|ref|NW_003384372.1|	29798-30176	174636	16441	-0.0870606	0.96215	no
gi|320446199|ref|NW_003384372.1|	31963-32452	137403	126931	-0.114369	0.9536	no
gi|320446199|ref|NW_003384372.1|	32579-33237	837532	706634	-0.24518	0.9054	no
gi|320446199|ref|NW_003384372.1|	33346-33917	250044	169039	-0.564826	0.8006	no
gi|320446199|ref|NW_003384372.1|	3355-3999	226775	179627	-0.336254	0.8728	no
gi|320446199|ref|NW_003384372.1|	34153-34913	579114	112501	0.958024	0.6458	no
gi|320446199|ref|NW_003384372.1|	35689-37344	140263	270571	0.947875	0.5746	no
gi|320446199|ref|NW_003384372.1|	4212-5007	723894	331382	-112728	0.57635	no
gi|320446199|ref|NW_003384372.1|	48636-49989	452982	346018	-0.388606	0.84565	no
gi|320446199|ref|NW_003384372.1|	5157-7248	147312	58992	-132029	0.42305	no
gi|320446199|ref|NW_003384372.1|	52561-53167	304101	157045	-0.953375	0.65155	no
gi|320446199|ref|NW_003384372.1|	54767-55703	146948	515111	-151235	0.48555	no
gi|320446199|ref|NW_003384372.1|	59250-62279	863696	844582	-0.0322862	0.9792	no
gi|320446199|ref|NW_003384372.1|	62639-64293	136793	455406	-158677	0.4777	no
gi|320446199|ref|NW_003384372.1|	77850-78960	0.964791	614443	267099	0.25325	no
gi|320446199|ref|NW_003384372.1|	8160-12205	876929	266633	-171761	0.3189	no
gi|320446199|ref|NW_003384372.1|	92529-93138	0	458386	inf	0.02205	no
gi|320446199|ref|NW_003384372.1|	94450-95546	745173	380005	-0.971556	0.6432	no
gi|320446199|ref|NW_003384372.1|	96876-97583	135938	366893	-188952	0.3827	no
gi|320446202|ref|NW_003384369.1|	233552-23548	0.298546	152623	235394	0.30375	no
gi|320446202|ref|NW_003384369.1|	236506-23730	0	306387	inf	0.02205	no
gi|320446202|ref|NW_003384369.1|	237413-23923	0.848686	473072	247876	0.2735	no
gi|320446202|ref|NW_003384369.1|	253107-25391	190603	15111	-0.334972	0.87755	no
gi|320446202|ref|NW_003384369.1|	271456-27486	100983	185905	0.880448	0.66585	no

gi|320446202|ref|NW_003384369.1|	275231-28450	281739	559562	0.98994	0.42925	no
gi|320446202|ref|NW_003384369.1|	289483-28978	314051	0	#NAME?	0.01065	no
gi|320446202|ref|NW_003384369.1|	301108-30145	129719	421861	-162055	0.44045	no
gi|320446202|ref|NW_003384369.1|	311277-31228	0.435192	150353	178863	0.3323	no
gi|320446202|ref|NW_003384369.1|	312475-31388	100737	0.799904	-0.332698	1	no
gi|320446202|ref|NW_003384369.1|	314594-31542	619284	348445	-0.829672	0.6761	no
gi|320446202|ref|NW_003384369.1|	316803-31847	113155	418485	-143506	0.5093	no
gi|320446202|ref|NW_003384369.1|	319322-32178	696866	217414	164149	0.33965	no
gi|320446202|ref|NW_003384369.1|	322073-32303	0.466477	402433	310887	0.2064	no
gi|320446202|ref|NW_003384369.1|	323140-32410	324738	400258	0.301656	0.88095	no
gi|320446202|ref|NW_003384369.1|	328969-32963	31537	120934	19391	0.36605	no
gi|320446202|ref|NW_003384369.1|	333459-33496	400451	138312	-15337	0.38175	no
gi|320446202|ref|NW_003384369.1|	336219-33793	341458	152684	-116116	0.3641	no
gi|320446202|ref|NW_003384369.1|	340833-34420	13627	403148	-175709	0.314	no
gi|320446202|ref|NW_003384369.1|	344409-34583	300476	213728	-0.491476	0.6979	no
gi|320446202|ref|NW_003384369.1|	351284-35209	117294	261923	115902	0.5865	no
gi|320446202|ref|NW_003384369.1|	355665-35720	116056	37672	169867	0.43	no
gi|320446202|ref|NW_003384369.1|	357358-35791	0.521224	105545	433981	0.18235	no
gi|320446202|ref|NW_003384369.1|	361675-36225	303881	56371	-243048	0.28995	no
gi|320446202|ref|NW_003384369.1|	362576-36288	12192	336231	-185842	0.3124	no
gi|320446202|ref|NW_003384369.1|	364377-36482	210823	777811	-143854	0.4969	no
gi|320446202|ref|NW_003384369.1|	365302-36861	882385	513697	-0.780489	0.6355	no
gi|320446202|ref|NW_003384369.1|	372575-37314	158647	92199	253893	0.1534	no
gi|320446202|ref|NW_003384369.1|	377238-37747	82074	723701	31404	0.20855	no
gi|320446202|ref|NW_003384369.1|	377903-38054	152206	871306	251716	0.0645	no
gi|320446202|ref|NW_003384369.1|	381746-38375	102634	801869	296586	0.13705	no
gi|320446202|ref|NW_003384369.1|	387020-39150	252276	331686	371674	0.04905	no
gi|320446202|ref|NW_003384369.1|	400879-40124	116024	237251	-228993	0.3218	no
gi|320446202|ref|NW_003384369.1|	401348-40199	615841	123783	-231475	0.18345	no
gi|320446202|ref|NW_003384369.1|	402440-40294	370484	111787	-172866	0.43205	no

gi|320446202|ref|NW_003384369.1|	403873-40450	176281	672184	-139095	0.5103	no
gi|320446202|ref|NW_003384369.1|	405685-40854	182438	110008	-0.729799	0.5723	no
gi|320446203|ref|NW_003384368.1|	108161-10893	111669	325349	154276	0.2602	no
gi|320446203|ref|NW_003384368.1|	113568-11522	0.237566	0.826856	179931	1	no
gi|320446203|ref|NW_003384368.1|	115604-11976	363338	134342	188653	0.2937	no
gi|320446203|ref|NW_003384368.1|	56450-57518	0	238361	inf	0.02075	no
gi|320446203|ref|NW_003384368.1|	93297-95142	0	175324	inf	0.0138	no
gi|320446208|ref|NW_003384363.1|	10600-15811	188539	177224	-0.089291	0.94615	no
gi|320446208|ref|NW_003384363.1|	114309-11472	149514	406819	-187782	0.38275	no
gi|320446208|ref|NW_003384363.1|	119528-12020	727126	20891	-179932	0.4062	no
gi|320446208|ref|NW_003384363.1|	120485-12094	515103	195954	-139435	0.52395	no
gi|320446208|ref|NW_003384363.1|	124535-12558	597871	228119	-139005	0.5012	no
gi|320446208|ref|NW_003384363.1|	127346-12901	105714	130836	0.307599	1	no
gi|320446208|ref|NW_003384363.1|	132016-13426	79766	937624	0.233236	0.91865	no
gi|320446208|ref|NW_003384363.1|	17816-18294	197818	0	#NAME?	0.0099	no
gi|320446208|ref|NW_003384363.1|	34657-35154	381226	110641	153716	0.4778	no
gi|320446208|ref|NW_003384363.1|	49680-50432	882092	29109	-159946	0.4492	no
gi|320446208|ref|NW_003384363.1|	65237-66487	648673	466089	284504	0.1276	no
gi|320446208|ref|NW_003384363.1|	67703-69902	120304	653625	244178	0.2806	no
gi|320446208|ref|NW_003384363.1|	70069-71109	188776	841098	21556	0.33015	no
gi|320446208|ref|NW_003384363.1|	71246-73082	0.843383	374643	215126	0.3229	no
gi|320446208|ref|NW_003384363.1|	75421-75672	948876	48059	234052	0.29875	no
gi|320446208|ref|NW_003384363.1|	76525-77039	179536	843728	22325	0.3279	no
gi|320446208|ref|NW_003384363.1|	79833-81209	207128	44188	109313	0.59855	no
gi|320446209|ref|NW_003384362.1|	15054-17352	237239	137052	-411355	0.0532	no
gi|320446209|ref|NW_003384362.1|	159777-16053	129928	578909	215563	0.34965	no
gi|320446209|ref|NW_003384362.1|	163469-16413	140294	0.265766	-572215	0.27455	no
gi|320446209|ref|NW_003384362.1|	165125-16804	0.879584	90474	336261	0.0832	no
gi|320446209|ref|NW_003384362.1|	168623-17067	131753	139135	0.0786499	0.9465	no
gi|320446209|ref|NW_003384362.1|	177644-17903	833802	111779	-289906	0.22615	no

gi|320446209|ref|NW_003384362.1|	179571-18106	21889	298785	-287303	0.1203	no
gi|320446209|ref|NW_003384362.1|	184538-18770	284224	341604	0.265294	0.83865	no
gi|320446209|ref|NW_003384362.1|	188211-18935	260317	309082	0.247721	0.9006	no
gi|320446209|ref|NW_003384362.1|	19354-19566	131896	358958	-519944	0.2812	no
gi|320446209|ref|NW_003384362.1|	19850-20402	825549	464091	-415287	0.0722	no
gi|320446209|ref|NW_003384362.1|	20680-20988	336456	113886	-488475	0.28635	no
gi|320446209|ref|NW_003384362.1|	207922-20897	515406	128317	-2006	0.3431	no
gi|320446209|ref|NW_003384362.1|	21089-22921	393156	670142	-255256	0.16935	no
gi|320446209|ref|NW_003384362.1|	212233-21276	171298	384055	116481	0.5745	no
gi|320446209|ref|NW_003384362.1|	217941-21929	281352	126426	-115408	0.4851	no
gi|320446209|ref|NW_003384362.1|	23039-25846	164032	101344	-0.694721	0.58075	no
gi|320446209|ref|NW_003384362.1|	244320-24548	543447	313776	-0.792404	0.6987	no
gi|320446209|ref|NW_003384362.1|	246616-25149	179376	196605	0.132313	0.92105	no
gi|320446209|ref|NW_003384362.1|	252242-25299	169557	133426	-0.345728	0.79715	no
gi|320446209|ref|NW_003384362.1|	25947-28052	0.541515	138513	135495	1	no
gi|320446209|ref|NW_003384362.1|	263770-26412	203238	69039	-155769	0.35545	no
gi|320446209|ref|NW_003384362.1|	270225-27614	746627	930433	0.317514	0.84475	no
gi|320446209|ref|NW_003384362.1|	28153-34151	620707	660717	0.0901205	0.94305	no
gi|320446209|ref|NW_003384362.1|	285193-28547	156895	858645	-0.869663	0.6867	no
gi|320446209|ref|NW_003384362.1|	293299-29353	245796	354126	0.526802	0.81385	no
gi|320446209|ref|NW_003384362.1|	296956-29873	665536	100325	0.592088	0.78375	no
gi|320446209|ref|NW_003384362.1|	299602-30190	636239	825633	0.375931	0.86755	no
gi|320446209|ref|NW_003384362.1|	305635-31056	152514	18242	0.258319	0.84295	no
gi|320446209|ref|NW_003384362.1|	311270-31181	267048	252119	-0.082994	0.9267	no
gi|320446209|ref|NW_003384362.1|	312165-31512	101091	572312	-0.820782	0.6088	no
gi|320446209|ref|NW_003384362.1|	318305-32000	994234	587614	-0.758715	0.7305	no
gi|320446209|ref|NW_003384362.1|	320611-32236	104587	953184	-0.133878	0.95325	no
gi|320446209|ref|NW_003384362.1|	322481-32534	178312	896666	-0.991764	0.4367	no
gi|320446209|ref|NW_003384362.1|	325539-32995	254138	514099	101643	0.52095	no
gi|320446209|ref|NW_003384362.1|	34790-38519	680969	790875	0.215862	0.85875	no

gi|320446209|ref|NW_003384362.1|	51611-35501	239397	599177	132358	0.32575	no
gi|320446209|ref|NW_003384362.1|	56457-35740	214057	229731	0.101951	0.9526	no
gi|320446209|ref|NW_003384362.1|	57597-35807	345057	460647	0.416825	0.8285	no
gi|320446209|ref|NW_003384362.1|	63835-36453	146172	102379	-0.513746	0.8029	no
gi|320446209|ref|NW_003384362.1|	69406-36986	508797	823028	0.693851	0.73235	no
gi|320446209|ref|NW_003384362.1|	70205-37063	191183	165172	-0.210979	0.9174	no
gi|320446209|ref|NW_003384362.1|	86105-38649	34136	343093	0.00730625	0.98825	no
gi|320446209|ref|NW_003384362.1|	86655-38723	155954	112127	-0.47598	0.81815	no
gi|320446209|ref|NW_003384362.1|	87466-38805	4289	29047	-0.562255	0.7863	no
gi|320446209|ref|NW_003384362.1|	88876-39112	870628	969907	0.15579	0.94575	no
gi|320446209|ref|NW_003384362.1|	39288-41686	596097	158535	141118	0.2881	no
gi|320446209|ref|NW_003384362.1|	13209-41398	526879	978357	0.892889	0.6613	no
gi|320446209|ref|NW_003384362.1|	15661-41665	246345	742416	159155	0.44965	no
gi|320446209|ref|NW_003384362.1|	18567-42031	211818	411529	0.958166	0.64155	no
gi|320446209|ref|NW_003384362.1|	20445-42104	370955	116308	164863	0.43065	no
gi|320446209|ref|NW_003384362.1|	23520-42395	329464	709501	110668	0.59675	no
gi|320446209|ref|NW_003384362.1|	35199-43585	481971	268167	-0.845816	0.7088	no
gi|320446209|ref|NW_003384362.1|	43693-45845	100364	152338	0.602026	0.7194	no
gi|320446209|ref|NW_003384362.1|	39525-44046	947785	481203	-429984	0.0461	no
gi|320446209|ref|NW_003384362.1|	41634-44233	204201	149511	-377167	0.1652	no
gi|320446209|ref|NW_003384362.1|	42651-44332	971511	572011	-408612	0.0643	no
gi|320446209|ref|NW_003384362.1|	43855-44981	573049	186863	-161668	0.2332	no
gi|320446209|ref|NW_003384362.1|	51011-45205	815999	452795	-0.84971	0.51905	no
gi|320446209|ref|NW_003384362.1|	55048-45682	183271	51218	-183925	0.2778	no
gi|320446209|ref|NW_003384362.1|	58037-45905	541198	942439	-252168	0.1682	no
gi|320446209|ref|NW_003384362.1|	67746-46896	134234	871021	-0.623969	0.7733	no
gi|320446209|ref|NW_003384362.1|	69496-47053	648614	332724	-0.963033	0.64635	no
gi|320446209|ref|NW_003384362.1|	15886-51617	14666	4021	-186685	0.3124	no
gi|320446209|ref|NW_003384362.1|	77690-79151	839883	880513	0.068156	0.9752	no
gi|320446209|ref|NW_003384362.1|	8084-9052	0	779242	inf	0.0089	no

gi|320446213|ref|NW_003384358.1|	22381-23049	0	292343	inf	0.0294	no
gi|320446213|ref|NW_003384358.1|	27942-32049	110258	790181	-0.480623	0.7104	no
gi|320446213|ref|NW_003384358.1|	3007-5045	964157	83564	-0.206386	0.9276	no
gi|320446213|ref|NW_003384358.1|	32179-32652	139107	603351	-120513	0.57205	no
gi|320446213|ref|NW_003384358.1|	32813-33183	120118	545879	-113779	0.6047	no
gi|320446213|ref|NW_003384358.1|	33321-34782	229935	189502	-0.279016	0.82025	no
gi|320446213|ref|NW_003384358.1|	35834-37037	645308	495692	-0.380543	0.8508	no
gi|320446213|ref|NW_003384358.1|	37650-38496	691049	532457	-0.376122	0.84865	no
gi|320446213|ref|NW_003384358.1|	39409-39925	142651	798296	-0.837492	0.6868	no
gi|320446213|ref|NW_003384358.1|	44011-44633	204649	97628	-106779	0.6017	no
gi|320446213|ref|NW_003384358.1|	54525-55821	127289	640455	233099	0.2936	no
gi|320446213|ref|NW_003384358.1|	9497-13825	0.865758	762075	31379	0.106	no
gi|320446215|ref|NW_003384356.1|	101213-10433[illegible]	129224	805504	-0.681912	0.59735	no
gi|320446215|ref|NW_003384356.1|	104851-10627[illegible]	0.142573	237764	405976	0.1924	no
gi|320446215|ref|NW_003384356.1|	11250-12639	304265	11686	-138055	0.41835	no
gi|320446215|ref|NW_003384356.1|	123370-12399[illegible]	436535	0.29657	-387965	0.31815	no
gi|320446215|ref|NW_003384356.1|	126253-12753[illegible]	0	335016	inf	0.00935	no
gi|320446215|ref|NW_003384356.1|	12990-13465	131122	737035	-0.831103	0.6756	no
gi|320446215|ref|NW_003384356.1|	133575-13473[illegible]	0	593672	inf	0.0071	no
gi|320446215|ref|NW_003384356.1|	145538-14613[illegible]	140627	47648	176054	0.41855	no
gi|320446215|ref|NW_003384356.1|	1487-2779	127771	277091	11168	0.5844	no
gi|320446215|ref|NW_003384356.1|	14882-17547	169453	752111	-117187	0.48495	no
gi|320446215|ref|NW_003384356.1|	3418-5044	861556	802308	-0.102789	0.963	no
gi|320446215|ref|NW_003384356.1|	35249-35776	687448	577905	-0.250418	0.89755	no
gi|320446215|ref|NW_003384356.1|	38534-39865	454379	152918	-157114	0.3781	no
gi|320446215|ref|NW_003384356.1|	45765-51023	329207	286418	-0.200872	0.8778	no
gi|320446215|ref|NW_003384356.1|	51154-54201	276053	323091	0.226996	0.9149	no
gi|320446215|ref|NW_003384356.1|	5280-7230	253904	651802	-196178	0.2651	no
gi|320446215|ref|NW_003384356.1|	55334-56336	352242	304179	-0.211647	0.91585	no
gi|320446215|ref|NW_003384356.1|	56446-57020	694081	469377	-0.564359	0.78545	no

gi|320446215|ref|NW_003384356.1|	58233-58822	712858	386261	-0.884037	0.6734	no
gi|320446215|ref|NW_003384356.1|	69754-70270	202089	0	#NAME?	0.00615	no
gi|320446215|ref|NW_003384356.1|	71452-72405	644418	0.162296	-863323	0.2504	no
gi|320446215|ref|NW_003384356.1|	73304-74193	125.24	0	#NAME?	5.00E-05	yes
gi|320446215|ref|NW_003384356.1|	82136-82597	21895	194415	315046	0.21355	no
gi|320446215|ref|NW_003384356.1|	83803-84604	0.596843	28695	226538	0.2738	no
gi|320446215|ref|NW_003384356.1|	89209-90195	0.449691	155278	178785	0.3323	no
gi|320446215|ref|NW_003384356.1|	90359-92367	0.190384	185868	32873	0.20015	no
gi|320446215|ref|NW_003384356.1|	92989-94590	0.123568	111781	31773	1	no
gi|320446215|ref|NW_003384356.1|	94779-95187	185702	183652	330591	0.19575	no
gi|320446215|ref|NW_003384356.1|	97966-98763	334.81	139934	-12586	0.34815	no
gi|320446215|ref|NW_003384356.1|	99183-100312	0.566563	143851	134427	0.5243	no
gi|320446216|ref|NW_003384355.1|	241774-24393	23433	235147	0.00501649	0.9967	no
gi|320446216|ref|NW_003384355.1|	29244-30155	149889	499384	173625	0.4194	no
gi|320446217|ref|NW_003384354.1|	11867-12271	379041	124846	171972	0.43725	no
gi|320446217|ref|NW_003384354.1|	122654-12368	0.851709	264921	163713	0.44625	no
gi|320446217|ref|NW_003384354.1|	123848-12451	117719	12307	0.0641286	0.9742	no
gi|320446217|ref|NW_003384354.1|	124623-12699	166232	165801	-0.00374072	0.98075	no
gi|320446217|ref|NW_003384354.1|	128751-13062	525507	251343	-106405	0.61505	no
gi|320446217|ref|NW_003384354.1|	130811-13124	74276	200186	-189155	0.3915	no
gi|320446217|ref|NW_003384354.1|	131384-13256	232006	124913	-0.893236	0.6896	no
gi|320446217|ref|NW_003384354.1|	135543-13597	155786	168453	0.112785	0.95485	no
gi|320446217|ref|NW_003384354.1|	136083-13905	358131	200297	248358	0.17875	no
gi|320446217|ref|NW_003384354.1|	15874-16465	141788	576322	202314	0.348	no
gi|320446217|ref|NW_003384354.1|	20526-20986	102597	326929	167199	0.43305	no
gi|320446217|ref|NW_003384354.1|	21129-23595	181547	781854	210656	0.3496	no
gi|320446217|ref|NW_003384354.1|	24630-25552	0	169492	inf	0.0312	no
gi|320446217|ref|NW_003384354.1|	27455-27843	412682	291213	281897	0.24265	no
gi|320446217|ref|NW_003384354.1|	32260-33425	145231	553418	193002	0.3768	no
gi|320446217|ref|NW_003384354.1|	42437-43125	111792	330828	156526	0.491	no

gi|320446217|ref|NW_003384354.1|	78377-79168	0	250286	inf	0.029	no
gi|320446217|ref|NW_003384354.1|	8527-10823	908933	290372	167566	0.3483	no
gi|320446217|ref|NW_003384354.1|	96616-97291	0.38356	680439	414894	0.19215	no
gi|320446218|ref|NW_003384353.1|	17382-18827	334818	306397	-0.127976	0.91935	no
gi|320446218|ref|NW_003384353.1|	19982-22489	430075	369384	-0.219469	0.8734	no
gi|320446218|ref|NW_003384353.1|	45462-48146	558164	36593	-0.609121	0.7822	no
gi|320446218|ref|NW_003384353.1|	49005-55982	17769	125622	-0.500271	0.70845	no
gi|320446218|ref|NW_003384353.1|	56280-60210	634785	59456	-0.0944473	0.937	no
gi|320446218|ref|NW_003384353.1|	69961-70665	0	36916	inf	0.02205	no
gi|320446218|ref|NW_003384353.1|	71668-72105	0.808904	482757	257726	0.27405	no
gi|320446218|ref|NW_003384353.1|	74682-76140	0.414071	268633	269769	0.2599	no
gi|320446218|ref|NW_003384353.1|	78855-80482	134233	789922	-0.764963	0.56865	no
gi|320446218|ref|NW_003384353.1|	80584-81337	580407	250314	-121333	0.46645	no
gi|320446218|ref|NW_003384353.1|	89051-91502	217808	56481	137471	0.30725	no
gi|320446219|ref|NW_003384352.1|	100227-10222[illegible]	137698	220045	-264564	0.13585	no
gi|320446219|ref|NW_003384352.1|	103235-10382[illegible]	513258	772523	-273203	0.2441	no
gi|320446219|ref|NW_003384352.1|	104664-10632[illegible]	101724	279959	-186137	0.4021	no
gi|320446219|ref|NW_003384352.1|	107549-11049[illegible]	643316	281137	-119426	0.3776	no
gi|320446219|ref|NW_003384352.1|	13080-15982	885851	928848	0.068378	0.95435	no
gi|320446219|ref|NW_003384352.1|	18549-20226	596967	236325	-133688	0.52585	no
gi|320446219|ref|NW_003384352.1|	21329-23154	658145	796589	359736	0.0805	no
gi|320446219|ref|NW_003384352.1|	27572-28510	144127	197183	377412	0.15155	no
gi|320446219|ref|NW_003384352.1|	30595-31298	577236	209622	186056	0.3916	no
gi|320446219|ref|NW_003384352.1|	58586-59388	137032	70386	236078	0.18695	no
gi|320446219|ref|NW_003384352.1|	60980-61985	0	333313	inf	0.0186	no
gi|320446219|ref|NW_003384352.1|	62175-62813	0	313118	inf	0.0294	no
gi|320446219|ref|NW_003384352.1|	71697-71922	0	316369	inf	0.0294	no
gi|320446219|ref|NW_003384352.1|	78722-79189	263549	19943	-0.402188	0.84695	no
gi|320446219|ref|NW_003384352.1|	79294-80197	404496	400722	-0.0135255	0.9754	no
gi|320446219|ref|NW_003384352.1|	80377-83419	264521	609498	120424	0.5836	no

gi|320446219|ref|NW_003384352.1|	8210-8595	28323	688123	-204124	0.35235	no
gi|320446219|ref|NW_003384352.1|	83474-89019	376872	268524	-0.489023	0.71185	no
gi|320446219|ref|NW_003384352.1|	8804-11365	319238	380112	0.251792	0.90595	no
gi|320446219|ref|NW_003384352.1|	98894-99850	468419	888976	-239758	0.178	no
gi|320446223|ref|NW_003384348.1|	103458-10532[illegible]	144787	670291	-111108	0.4833	no
gi|320446223|ref|NW_003384348.1|	111447-11369[illegible]	0.418779	0.759941	0.859698	1	no
gi|320446223|ref|NW_003384348.1|	113908-11856[illegible]	14965	203199	0.441298	0.746	no
gi|320446223|ref|NW_003384348.1|	12938-13471	191002	177703	-0.104114	0.95895	no
gi|320446223|ref|NW_003384348.1|	15292-15616	202702	392816	0.954491	0.6353	no
gi|320446223|ref|NW_003384348.1|	162829-16505[illegible]	41642	294754	-0.498529	0.7078	no
gi|320446223|ref|NW_003384348.1|	165578-16799[illegible]	142824	652304	-113062	0.49755	no
gi|320446223|ref|NW_003384348.1|	171429-17292[illegible]	18162	715007	-13449	0.4097	no
gi|320446223|ref|NW_003384348.1|	20460-22975	347886	413589	0.249585	0.90355	no
gi|320446223|ref|NW_003384348.1|	23972-24424	189429	957264	-0.984671	0.64015	no
gi|320446223|ref|NW_003384348.1|	25142-25716	376787	214572	-0.812287	0.7041	no
gi|320446223|ref|NW_003384348.1|	25821-26858	143164	436654	-171311	0.43525	no
gi|320446223|ref|NW_003384348.1|	37342-40525	117919	440354	-142107	0.4027	no
gi|320446223|ref|NW_003384348.1|	41858-44386	117758	843076	-0.482085	0.769	no
gi|320446223|ref|NW_003384348.1|	44500-46859	131103	226889	0.791288	0.5367	no
gi|320446223|ref|NW_003384348.1|	51171-51734	819133	117571	0.521366	0.79715	no
gi|320446223|ref|NW_003384348.1|	54907-55509	183495	404438	114018	0.58825	no
gi|320446223|ref|NW_003384348.1|	60896-61724	484193	127283	-192754	0.25875	no
gi|320446223|ref|NW_003384348.1|	78612-78887	312766	306631	-335051	0.2399	no
gi|320446223|ref|NW_003384348.1|	98056-99489	128224	528781	-127792	0.55925	no
gi|320446227|ref|NW_003384344.1|	125666-12643[illegible]	3.46	539452	0.640721	0.7482	no
gi|320446227|ref|NW_003384344.1|	158308-15905[illegible]	0	538411	inf	0.0138	no
gi|320446227|ref|NW_003384344.1|	173943-17525[illegible]	0	109202	inf	1	no
gi|320446227|ref|NW_003384344.1|	19336-27580	267554	130008	22807	0.0925	no
gi|320446227|ref|NW_003384344.1|	193957-19795[illegible]	19267	395197	103644	0.6255	no
gi|320446227|ref|NW_003384344.1|	198230-19860[illegible]	800783	119645	0.579273	0.78145	no

gi|320446227|ref|NW_003384344.1|	198711-19974	172037	132132	-0.380736	0.8694	no
gi|320446227|ref|NW_003384344.1|	204935-20646	273304	280604	0.0380274	0.9784	no
gi|320446227|ref|NW_003384344.1|	207242-21468	133392	214125	0.682782	0.6083	no
gi|320446227|ref|NW_003384344.1|	215451-21697	534354	280771	-0.9284	0.4828	no
gi|320446227|ref|NW_003384344.1|	217077-21949	236997	400204	-256606	0.1632	no
gi|320446227|ref|NW_003384344.1|	220444-23084	116665	111759	-0.0619802	0.96395	no
gi|320446227|ref|NW_003384344.1|	250758-25228	0.654257	100104	0.613575	1	no
gi|320446227|ref|NW_003384344.1|	265404-26876	349284	238277	-0.551764	0.6852	no
gi|320446227|ref|NW_003384344.1|	269261-27172	348877	200836	-0.796703	0.5461	no
gi|320446227|ref|NW_003384344.1|	271780-27327	371683	370388	-0.00503595	0.99685	no
gi|320446227|ref|NW_003384344.1|	273538-27709	112837	209466	0.89248	0.4978	no
gi|320446227|ref|NW_003384344.1|	277480-28110	0.348958	491844	381708	0.14275	no
gi|320446227|ref|NW_003384344.1|	27762-28151	112866	202105	0.840493	0.67625	no
gi|320446227|ref|NW_003384344.1|	28789-29341	158759	356993	116905	0.5745	no
gi|320446227|ref|NW_003384344.1|	290840-29208	568311	338524	25745	0.15165	no
gi|320446227|ref|NW_003384344.1|	78409-78712	738944	94915	0.361172	0.85715	no
gi|320446228|ref|NW_003384343.1|	11268-11658	102046	938223	652264	0.15425	no
gi|320446228|ref|NW_003384343.1|	11759-13755	0	104924	inf	0.0038	no
gi|320446228|ref|NW_003384343.1|	13886-14269	0	153023	inf	0.0186	no
gi|320446228|ref|NW_003384343.1|	14412-15407	0	130448	inf	0.00525	no
gi|320446228|ref|NW_003384343.1|	16035-16356	0	360422	inf	0.0114	no
gi|320446228|ref|NW_003384343.1|	20205-20705	0	117922	inf	0.01275	no
gi|320446228|ref|NW_003384343.1|	20916-21169	0	293143	inf	0.02205	no
gi|320446228|ref|NW_003384343.1|	27345-27810	0.718052	234471	502918	0.17225	no
gi|320446228|ref|NW_003384343.1|	32019-32362	108777	441391	-130124	0.5497	no
gi|320446228|ref|NW_003384343.1|	42184-43182	0.221306	305749	378823	0.2072	no
gi|320446228|ref|NW_003384343.1|	52884-54254	287051	252672	313789	0.0265	no
gi|320446228|ref|NW_003384343.1|	54611-55182	150026	60854	202014	0.34805	no
gi|320446229|ref|NW_003384342.1|	108887-11027	248033	0.405425	-261302	0.26915	no
gi|320446229|ref|NW_003384342.1|	11094-11408	185454	434431	-209386	0.35775	no

gi|320446229|ref|NW_003384342.1|	112030-11275	905293	23825	-192591	0.37865	no
gi|320446229|ref|NW_003384342.1|	113381-11500	103293	363668	-150604	0.48315	no
gi|320446229|ref|NW_003384342.1|	116182-11716	498648	172159	-153428	0.4736	no
gi|320446229|ref|NW_003384342.1|	118709-12286	984505	11949	0.279418	0.8285	no
gi|320446229|ref|NW_003384342.1|	124681-12641	184152	121164	-0.603931	0.70835	no
gi|320446229|ref|NW_003384342.1|	140744-14266	209725	215817	336324	0.0898	no
gi|320446229|ref|NW_003384342.1|	14083-17423	11576	150163	0.375388	0.76635	no
gi|320446229|ref|NW_003384342.1|	146768-14695	0	78.19	inf	0.02915	no
gi|320446229|ref|NW_003384342.1|	159309-16152	404466	147372	-145655	0.27635	no
gi|320446229|ref|NW_003384342.1|	164133-16430	112303	491744	-119141	0.57625	no
gi|320446229|ref|NW_003384342.1|	164459-16577	331711	864851	-19394	0.2624	no
gi|320446229|ref|NW_003384342.1|	170046-17188	212027	724105	-154997	0.36565	no
gi|320446229|ref|NW_003384342.1|	173362-17437	608484	225243	-143373	0.4935	no
gi|320446229|ref|NW_003384342.1|	17796-18739	16696	213915	0.357534	0.8577	no
gi|320446229|ref|NW_003384342.1|	181579-18346	583058	712938	0.290137	0.8929	no
gi|320446229|ref|NW_003384342.1|	183747-18599	79568	520267	-0.612936	0.782	no
gi|320446229|ref|NW_003384342.1|	18906-20413	96874	226085	122268	0.4566	no
gi|320446229|ref|NW_003384342.1|	23492-24557	349896	379922	0.118777	0.92605	no
gi|320446229|ref|NW_003384342.1|	41516-42519	124443	103288	-359074	0.07695	no
gi|320446229|ref|NW_003384342.1|	44589-45015	408349	174512	-45484	0.0446	no
gi|320446229|ref|NW_003384342.1|	54576-57497	680421	295138	-120504	0.37805	no
gi|320446229|ref|NW_003384342.1|	59277-59749	272328	46587	-254735	0.26715	no
gi|320446229|ref|NW_003384342.1|	60301-61507	399897	168744	-124479	0.5546	no
gi|320446229|ref|NW_003384342.1|	61607-64708	221391	117342	-0.915876	0.48855	no
gi|320446229|ref|NW_003384342.1|	66878-68249	579541	412841	-0.489325	0.8088	no
gi|320446229|ref|NW_003384342.1|	72933-73827	179386	215407	358592	0.1566	no
gi|320446229|ref|NW_003384342.1|	74090-74465	166597	363431	-219661	0.33515	no
gi|320446229|ref|NW_003384342.1|	78614-79441	256771	0.392287	-27105	0.2844	no
gi|320446229|ref|NW_003384342.1|	80956-81665	142492	341037	125904	0.5573	no
gi|320446229|ref|NW_003384342.1|	81864-82475	134386	36486	144096	0.5076	no

gi|320446229|ref|NW_003384342.1|	83169-83753	0.963794	358851	189659	0.32105	no
gi|320446229|ref|NW_003384342.1|	85287-85698	914651	241365	-1922	0.39955	no
gi|320446229|ref|NW_003384342.1|	99734-100642	200744	0.864777	-121496	0.5719	no
gi|320446230|ref|NW_003384341.1|	107725-10865	48666	755154	0.633856	0.756	no
gi|320446230|ref|NW_003384341.1|	116295-11744	22142	447341	101459	0.6106	no
gi|320446230|ref|NW_003384341.1|	132546-13321	227851	392948	0.78625	0.5545	no
gi|320446230|ref|NW_003384341.1|	135507-14580	11179	364704	170593	0.19975	no
gi|320446230|ref|NW_003384341.1|	145885-14805	113152	352257	163837	0.43565	no
gi|320446230|ref|NW_003384341.1|	19474-19770	117873	176294	0.580754	0.7884	no
gi|320446230|ref|NW_003384341.1|	23253-23856	320259	682673	109195	0.59235	no
gi|320446230|ref|NW_003384341.1|	7123-10358	264375	731744	146875	0.36775	no
gi|320446231|ref|NW_003384340.1|	30167-38018	331873	247468	-0.423389	0.7437	no
gi|320446231|ref|NW_003384340.1|	38180-40756	21843	498549	-213136	0.2413	no
gi|320446231|ref|NW_003384340.1|	43265-43825	20662	313904	0.603341	0.7533	no
gi|320446231|ref|NW_003384340.1|	44126-44493	594073	380435	-396492	0.1705	no
gi|320446231|ref|NW_003384340.1|	56976-60786	330408	627663	0.92574	0.56855	no
gi|320446231|ref|NW_003384340.1|	61741-62638	135205	15.82	0.226599	0.91605	no
gi|320446231|ref|NW_003384340.1|	63060-64540	474542	480734	0.0187014	0.9885	no
gi|320446231|ref|NW_003384340.1|	66649-68444	16662	128138	-0.378866	0.81955	no
gi|320446231|ref|NW_003384340.1|	74825-77260	223995	578537	-195298	0.27625	no
gi|320446231|ref|NW_003384340.1|	80164-81942	3391	236217	-0.5216	0.79805	no
gi|320446231|ref|NW_003384340.1|	82193-86457	678482	155122	119302	0.36665	no
gi|320446231|ref|NW_003384340.1|	93774-96871	465807	235011	-0.987005	0.6513	no
gi|320446234|ref|NW_003384337.1|	101076-10138	155473	489242	165388	0.4378	no
gi|320446234|ref|NW_003384337.1|	108471-10870	859111	324378	191676	0.31725	no
gi|320446234|ref|NW_003384337.1|	110064-11178	444477	220629	231144	0.19435	no
gi|320446234|ref|NW_003384337.1|	11136-11493	867721	726927	-0.255421	0.8898	no
gi|320446234|ref|NW_003384337.1|	113207-11341	262984	410503	0.642419	0.7586	no
gi|320446234|ref|NW_003384337.1|	113978-11432	308502	871292	-182405	0.4058	no
gi|320446234|ref|NW_003384337.1|	115290-11583	376187	144928	-137612	0.5274	no

gi|320446234|ref|NW_003384337.1|	116397-11692	231435	350064	0.597014	0.7533	no
gi|320446234|ref|NW_003384337.1|	126166-12726	10614	340069	-164206	0.4384	no
gi|320446234|ref|NW_003384337.1|	12918-14072	165342	152715	-0.114609	0.949	no
gi|320446234|ref|NW_003384337.1|	136637-13720	41367	988705	-206487	0.3467	no
gi|320446234|ref|NW_003384337.1|	1437-1700	249403	167115	-0.577642	0.79305	no
gi|320446234|ref|NW_003384337.1|	154404-15472	142491	25554	0.842672	0.68145	no
gi|320446234|ref|NW_003384337.1|	161154-16960	535244	162.33	160066	0.3063	no
gi|320446234|ref|NW_003384337.1|	172833-17824	802412	214827	142076	0.2878	no
gi|320446234|ref|NW_003384337.1|	29582-32705	449926	375915	-0.259281	0.90635	no
gi|320446234|ref|NW_003384337.1|	5108-9049	193513	149794	-0.369452	0.78105	no
gi|320446234|ref|NW_003384337.1|	80368-81413	281414	193141	-0.543042	0.7382	no
gi|320446234|ref|NW_003384337.1|	83012-84214	523764	387277	-0.435552	0.8311	no
gi|320446234|ref|NW_003384337.1|	912-1322	137884	121261	-350727	0.2335	no
gi|320446235|ref|NW_003384336.1|	1190-2098	928441	148742	0.679926	0.75065	no
gi|320446235|ref|NW_003384336.1|	158595-15915	281577	373462	0.407431	0.852	no
gi|320446235|ref|NW_003384336.1|	28207-28656	276286	816051	-175943	0.4113	no
gi|320446235|ref|NW_003384336.1|	2826-4199	964203	117455	0.2847	0.8965	no
gi|320446235|ref|NW_003384336.1|	31052-32654	203738	124589	-0.709542	0.66935	no
gi|320446235|ref|NW_003384336.1|	41570-44194	367329	196914	-0.89951	0.4976	no
gi|320446235|ref|NW_003384336.1|	48427-54194	134206	252214	0.910202	0.67165	no
gi|320446235|ref|NW_003384336.1|	5529-13014	467786	110144	123547	0.35295	no
gi|320446235|ref|NW_003384336.1|	55811-59668	160731	48292	158714	0.23835	no
gi|320446235|ref|NW_003384336.1|	61077-61532	598562	134395	11669	0.5759	no
gi|320446235|ref|NW_003384336.1|	65897-66056	101307	920915	-0.137588	0.91165	no
gi|320446235|ref|NW_003384336.1|	66869-67116	49931	673656	0.432075	0.83255	no
gi|320446236|ref|NW_003384335.1|	103312-10409	26152	380109	-278243	0.22635	no
gi|320446236|ref|NW_003384335.1|	125527-12596	0.812547	754195	321442	0.23205	no
gi|320446236|ref|NW_003384335.1|	44960-45457	514656	646823	0.329762	0.8878	no
gi|320446236|ref|NW_003384335.1|	67440-69386	232797	247572	0.0887775	0.94475	no
gi|320446236|ref|NW_003384335.1|	813-2702	387765	327164	-0.245168	0.90305	no

gi|320446236|ref|NW_003384335.1|	82219-83003	581496	291417	-0.996686	0.55035	no
gi|320446236|ref|NW_003384335.1|	83472-83928	543909	267714	-102267	0.63345	no
gi|320446236|ref|NW_003384335.1|	84182-85601	658685	360609	-0.869152	0.5051	no
gi|320446236|ref|NW_003384335.1|	85827-86981	123088	113264	-0.120002	0.9558	no
gi|320446236|ref|NW_003384335.1|	91715-92306	203229	112063	-0.858801	0.67565	no
gi|320446236|ref|NW_003384335.1|	92428-93260	367782	233441	-0.655794	0.748	no
gi|320446236|ref|NW_003384335.1|	95467-96147	56893	103553	-245789	0.30345	no
gi|320446236|ref|NW_003384335.1|	98348-98926	980261	265275	-188567	0.3935	no
gi|320446237|ref|NW_003384334.1|	110124-11051	0.998622	144409	385408	0.20765	no
gi|320446237|ref|NW_003384334.1|	116893-11948	214525	109901	235699	0.17975	no
gi|320446237|ref|NW_003384334.1|	15856-16510	24558	172787	-0.507205	0.8105	no
gi|320446237|ref|NW_003384334.1|	17319-17784	129249	334959	-19481	0.38445	no
gi|320446237|ref|NW_003384334.1|	182590-18465	331289	484685	0.548957	0.80155	no
gi|320446237|ref|NW_003384334.1|	18490-19353	110261	83301	-0.404517	0.8418	no
gi|320446237|ref|NW_003384334.1|	185548-18718	313694	344296	0.13429	0.9469	no
gi|320446237|ref|NW_003384334.1|	188499-18909	471329	383199	-0.29864	0.8822	no
gi|320446237|ref|NW_003384334.1|	195239-19634	754263	22759	-172863	0.4204	no
gi|320446237|ref|NW_003384334.1|	197098-19761	140259	314176	-215845	0.32745	no
gi|320446237|ref|NW_003384334.1|	20221-21658	0.421309	1952	2212	0.3332	no
gi|320446237|ref|NW_003384334.1|	21776-25058	132917	89869	275729	0.1275	no
gi|320446237|ref|NW_003384334.1|	26518-31280	0.7082	216684	161336	0.45975	no
gi|320446237|ref|NW_003384334.1|	40686-42828	0.177002	203764	352506	0.18205	no
gi|320446237|ref|NW_003384334.1|	43757-44506	0.328604	270225	303974	0.24995	no
gi|320446237|ref|NW_003384334.1|	64790-66496	18359	139023	292076	0.2187	no
gi|320446237|ref|NW_003384334.1|	66780-70405	129422	421458	170331	0.43375	no
gi|320446237|ref|NW_003384334.1|	6895-13705	90845	120352	0.405776	0.7612	no
gi|320446237|ref|NW_003384334.1|	73015-73454	320684	127638	199283	0.38735	no
gi|320446238|ref|NW_003384333.1|	118948-11960	160657	218912	0.446365	0.8222	no
gi|320446238|ref|NW_003384333.1|	12219-14365	203126	322263	39878	0.05935	no
gi|320446238|ref|NW_003384333.1|	125132-12617	103971	503248	227509	0.3193	no

gi|320446238|ref|NW_003384333.1|	1255-2416	275309	128713	222504	0.1016	no
gi|320446238|ref|NW_003384333.1|	179269-17965	102605	336841	-160697	0.44055	no
gi|320446238|ref|NW_003384333.1|	185101-18724	159385	0.49423	-168926	0.43275	no
gi|320446238|ref|NW_003384333.1|	187459-18985	148313	0.926577	-0.678659	0.7326	no
gi|320446238|ref|NW_003384333.1|	201223-20141	188.56	0	#NAME?	0.01	no
gi|320446238|ref|NW_003384333.1|	203600-20430	597613	0.246107	-792378	0.2504	no
gi|320446238|ref|NW_003384333.1|	206226-20698	72711	0.220591	-836466	0.25045	no
gi|320446238|ref|NW_003384333.1|	208802-20922	262582	983447	190508	0.39665	no
gi|320446238|ref|NW_003384333.1|	216045-21681	252106	778225	162616	0.43385	no
gi|320446238|ref|NW_003384333.1|	243742-24432	432496	91098	107473	0.60185	no
gi|320446238|ref|NW_003384333.1|	258687-25942	200272	640218	16766	0.4319	no
gi|320446238|ref|NW_003384333.1|	279116-28056	985613	434193	-118269	0.5843	no
gi|320446238|ref|NW_003384333.1|	280812-28185	271663	187832	-0.532371	0.7903	no
gi|320446238|ref|NW_003384333.1|	282392-28282	227563	100428	-118011	0.568	no
gi|320446238|ref|NW_003384333.1|	284294-28720	660262	180387	144998	0.2646	no
gi|320446238|ref|NW_003384333.1|	287335-29045	183146	30483	0.735012	0.5752	no
gi|320446238|ref|NW_003384333.1|	28811-30943	338076	376144	347587	0.0826	no
gi|320446238|ref|NW_003384333.1|	41577-42099	902809	395373	-11912	0.4724	no
gi|320446238|ref|NW_003384333.1|	44471-45122	608192	497079	-0.291052	0.88085	no
gi|320446238|ref|NW_003384333.1|	46797-50577	472675	515027	0.123799	0.9253	no
gi|320446238|ref|NW_003384333.1|	57100-57386	215575	143307	605478	0.1594	no
gi|320446238|ref|NW_003384333.1|	58582-59425	19445	487262	464723	0.06425	no
gi|320446238|ref|NW_003384333.1|	60457-64053	617548	789387	367611	0.00995	no
gi|320446238|ref|NW_003384333.1|	64901-68496	32343	0.843387	-526112	0.03385	no
gi|320446238|ref|NW_003384333.1|	78280-79043	147156	0.438738	-506784	0.1891	no
gi|320446238|ref|NW_003384333.1|	80998-83809	436303	100729	-543677	0.04185	no
gi|320446239|ref|NW_003384332.1|	110395-11076	299223	225629	-37292	0.17055	no
gi|320446239|ref|NW_003384332.1|	111535-11318	400521	248925	-400809	0.05635	no
gi|320446239|ref|NW_003384332.1|	1766-3290	458336	655732	0.516703	0.80805	no
gi|320446239|ref|NW_003384332.1|	81641-85881	0.210663	448382	441172	0.1339	no

gi|320446239|ref|NW_003384332.1|	86240-87103	161358	203625	0.335651	0.86355	no
gi|320446240|ref|NW_003384331.1|	13924-15035	597487	204139	177257	0.4386	no
gi|320446240|ref|NW_003384331.1|	3395-4078	11303	257215	118627	0.56815	no
gi|320446240|ref|NW_003384331.1|	35154-36221	426171	15582	187038	0.4038	no
gi|320446240|ref|NW_003384331.1|	36681-36943	110886	19363	0.804229	0.7139	no
gi|320446240|ref|NW_003384331.1|	37171-37535	355924	619508	0.799556	0.704	no
gi|320446240|ref|NW_003384331.1|	37800-38620	173231	813616	223165	0.31415	no
gi|320446240|ref|NW_003384331.1|	59270-59704	524752	179321	-154909	0.4626	no
gi|320446240|ref|NW_003384331.1|	5956-6177	195515	214795	0.135686	0.9142	no
gi|320446240|ref|NW_003384331.1|	60817-61708	736276	156073	-223802	0.21615	no
gi|320446242|ref|NW_003384329.1|	12402-15831	12627	188129	0.575209	0.6613	no
gi|320446242|ref|NW_003384329.1|	15996-17382	313858	234322	-0.421618	0.7426	no
gi|320446242|ref|NW_003384329.1|	17585-18293	246316	165985	-0.569458	0.7943	no
gi|320446242|ref|NW_003384329.1|	19338-20827	142686	449239	-166729	0.4666	no
gi|320446242|ref|NW_003384329.1|	20999-27580	135336	952386	-0.506924	0.69965	no
gi|320446242|ref|NW_003384329.1|	54961-55514	527584	434254	-0.280862	0.8972	no
gi|320446242|ref|NW_003384329.1|	56604-57348	109502	138677	0.340766	0.8685	no
gi|320446242|ref|NW_003384329.1|	70777-78182	144537	190189	0.395988	0.83525	no
gi|320446242|ref|NW_003384329.1|	78367-80298	166191	839424	-0.985368	0.54725	no
gi|320446242|ref|NW_003384329.1|	80402-82187	78403	675133	-0.215737	0.9207	no
gi|320446242|ref|NW_003384329.1|	84025-86262	841312	957233	0.186229	0.8916	no
gi|320446243|ref|NW_003384328.1|	100921-10135[illegible]	325019	102355	-166694	0.4416	no
gi|320446243|ref|NW_003384328.1|	103950-10516[illegible]	0.34314	237917	279359	0.2345	no
gi|320446243|ref|NW_003384328.1|	107207-10820[illegible]	130571	0.917246	-383138	0.16365	no
gi|320446243|ref|NW_003384328.1|	111763-11215[illegible]	385669	193335	-0.996263	0.63785	no
gi|320446243|ref|NW_003384328.1|	114392-11798[illegible]	295155	110122	-142237	0.289	no
gi|320446243|ref|NW_003384328.1|	120148-12217[illegible]	196512	186884	-0.0724718	0.9545	no
gi|320446243|ref|NW_003384328.1|	122792-12377[illegible]	159088	517846	-161923	0.4536	no
gi|320446243|ref|NW_003384328.1|	13084-13825	200272	274379	0.454206	0.82095	no
gi|320446243|ref|NW_003384328.1|	135638-13600[illegible]	20169	619508	-170294	0.4225	no

gi|320446243|ref|NW_003384328.1|	136407-13698	39548	334343	-0.242277	0.89835	no
gi|320446243|ref|NW_003384328.1|	137734-13947	176962	125588	-0.494747	0.7532	no
gi|320446243|ref|NW_003384328.1|	14154-15306	12888	5229	202051	0.3475	no
gi|320446243|ref|NW_003384328.1|	142835-14468	436575	179773	-128005	0.33755	no
gi|320446243|ref|NW_003384328.1|	146986-14869	285518	142921	-0.998359	0.4308	no
gi|320446243|ref|NW_003384328.1|	15468-17888	0.463424	161802	180382	0.41025	no
gi|320446243|ref|NW_003384328.1|	32873-35969	32146	556798	-252941	0.18255	no
gi|320446243|ref|NW_003384328.1|	42321-42643	253318	715511	-18239	0.39175	no
gi|320446243|ref|NW_003384328.1|	4330-4984	120777	356544	156173	0.491	no
gi|320446243|ref|NW_003384328.1|	62847-63680	112974	427273	191917	0.39735	no
gi|320446243|ref|NW_003384328.1|	68252-69025	125584	323084	136325	0.52475	no
gi|320446243|ref|NW_003384328.1|	7309-10561	106273	395115	18945	0.38625	no
gi|320446243|ref|NW_003384328.1|	82215-84734	654653	490798	-0.415602	0.754	no
gi|320446243|ref|NW_003384328.1|	86676-94556	118408	111.04	-0.0926877	0.96465	no
gi|320446244|ref|NW_003384327.1|	117344-12004	452031	464213	0.0383662	0.9849	no
gi|320446244|ref|NW_003384327.1|	120152-12099	19658	131424	-0.580892	0.7933	no
gi|320446244|ref|NW_003384327.1|	121142-12149	12396	113078	-0.132564	0.94085	no
gi|320446244|ref|NW_003384327.1|	134059-13442	931879	380435	-129249	0.55035	no
gi|320446244|ref|NW_003384327.1|	135967-13758	0	357507	inf	0.0069	no
gi|320446244|ref|NW_003384327.1|	142771-14362	111326	579281	-0.942452	0.6524	no
gi|320446244|ref|NW_003384327.1|	159677-15996	109892	842444	-0.383433	0.8553	no
gi|320446244|ref|NW_003384327.1|	166211-16702	32312	322916	-0.000909206	0.96375	no
gi|320446244|ref|NW_003384327.1|	20026-20319	605627	168078	147263	0.49745	no
gi|320446244|ref|NW_003384327.1|	29310-34090	0.259882	267835	336541	0.17525	no
gi|320446244|ref|NW_003384327.1|	64774-65555	0	566856	inf	5.00E-05	yes
gi|320446244|ref|NW_003384327.1|	66204-67001	0.300548	321972	67432	0.15265	no
gi|320446244|ref|NW_003384327.1|	77078-78229	165884	11363	27761	0.23855	no
gi|320446244|ref|NW_003384327.1|	85805-87324	538999	173708	-163362	0.4454	no
gi|320446244|ref|NW_003384327.1|	99453-100892	140203	14421	336258	0.1731	no
gi|320446246|ref|NW_003384325.1|	171059-17132	831645	373179	548776	0.1165	no

gi|320446246|ref|NW_003384325.1|	174228-17880	0.388441	561746	717608	0.0466	no
gi|320446246|ref|NW_003384325.1|	179885-18456	0.417669	21354	5676	0.0421	no
gi|320446246|ref|NW_003384325.1|	223764-22467	150112	189697	0.337664	0.8633	no
gi|320446246|ref|NW_003384325.1|	233477-23389	0	586584	inf	0.0312	no
gi|320446246|ref|NW_003384325.1|	234767-23503	0	166639	inf	0.0312	no
gi|320446246|ref|NW_003384325.1|	46761-49598	0.518849	122393	123813	1	no
gi|320446253|ref|NW_003384318.1|	100794-10454	326727	561545	0.781317	0.7317	no
gi|320446253|ref|NW_003384318.1|	106447-10682	147105	24439	-258959	0.1417	no
gi|320446253|ref|NW_003384318.1|	107201-10866	73928	114204	-269451	0.2524	no
gi|320446253|ref|NW_003384318.1|	109436-11108	559856	215584	-13768	0.5163	no
gi|320446253|ref|NW_003384318.1|	1226-2458	0.338662	22311	271983	0.2412	no
gi|320446253|ref|NW_003384318.1|	168500-17337	711736	604454	-0.235709	0.8568	no
gi|320446253|ref|NW_003384318.1|	175583-17598	13615	31997	-208919	0.3529	no
gi|320446253|ref|NW_003384318.1|	184774-18548	242742	914478	-14084	0.5197	no
gi|320446253|ref|NW_003384318.1|	189928-19271	355764	302243	-0.235211	0.8593	no
gi|320446253|ref|NW_003384318.1|	192921-19355	188733	111278	-0.762177	0.7125	no
gi|320446253|ref|NW_003384318.1|	194570-19561	166558	100772	-0.724933	0.7278	no
gi|320446253|ref|NW_003384318.1|	221097-22132	38.66	351409	-0.13769	0.9382	no
gi|320446253|ref|NW_003384318.1|	232385-23324	174804	0.925567	-423926	0.16175	no
gi|320446253|ref|NW_003384318.1|	246689-24705	0	325278	inf	0.00915	no
gi|320446253|ref|NW_003384318.1|	260954-26170	391286	156446	-132256	0.5185	no
gi|320446253|ref|NW_003384318.1|	283243-28379	891456	920062	0.0455679	0.9741	no
gi|320446253|ref|NW_003384318.1|	285480-28613	16028	245716	0.616401	0.75305	no
gi|320446253|ref|NW_003384318.1|	314536-31516	240774	350705	0.542577	0.80195	no
gi|320446253|ref|NW_003384318.1|	325050-33079	317141	469359	0.565567	0.66315	no
gi|320446253|ref|NW_003384318.1|	353973-35431	666139	138504	105603	0.594	no
gi|320446253|ref|NW_003384318.1|	354457-35506	864663	228452	140168	0.5085	no
gi|320446253|ref|NW_003384318.1|	360529-36162	273901	419759	0.615907	0.7616	no
gi|320446253|ref|NW_003384318.1|	361766-36297	0.519464	156056	158697	0.43945	no
gi|320446253|ref|NW_003384318.1|	363208-36704	283269	762885	142929	0.2861	no

gi|320446253|ref|NW_003384318.1|	368525-370314	115129	174783	0.602313	0.7175	no
gi|320446253|ref|NW_003384318.1|	371449-37499[illegible]	875606	885597	0.0163669	0.98905	no
gi|320446253|ref|NW_003384318.1|	378048-37844[illegible]	107428	124701	0.215105	0.8938	no
gi|320446253|ref|NW_003384318.1|	408349-408786	323562	965514	157726	0.49045	no
gi|320446253|ref|NW_003384318.1|	409048-411535	185848	511866	146164	0.2785	no
gi|320446253|ref|NW_003384318.1|	417186-41831[illegible]	122928	455797	-143135	0.2895	no
gi|320446253|ref|NW_003384318.1|	418458-418846	608705	0.677239	-648994	0.26605	no
gi|320446253|ref|NW_003384318.1|	419541-421875	108544	0.673572	-40103	0.07605	no
gi|320446253|ref|NW_003384318.1|	422297-427168	320295	169282	-0.919974	0.4918	no
gi|320446254|ref|NW_003384317.1|	24009-26329	218546	231674	0.0841564	0.9642	no
gi|320446254|ref|NW_003384317.1|	26825-27690	241267	516703	10987	0.5963	no
gi|320446254|ref|NW_003384317.1|	27912-28464	687957	571189	-0.268352	0.88575	no
gi|320446254|ref|NW_003384317.1|	30797-31040	14039	133036	-0.0776195	0.9157	no
gi|320446254|ref|NW_003384317.1|	31622-32115	386823	388381	0.00579577	0.94	no
gi|320446254|ref|NW_003384317.1|	34793-38956	311021	472182	-271959	0.16075	no
gi|320446254|ref|NW_003384317.1|	43245-43852	679025	92144	0.440425	0.8291	no
gi|320446254|ref|NW_003384317.1|	4369-7129	1423.71	853232	-0.738642	0.35605	no
gi|320446254|ref|NW_003384317.1|	44274-47453	162216	143509	-0.176778	0.8925	no
gi|320446254|ref|NW_003384317.1|	48267-49522	993063	516643	-0.942718	0.653	no
gi|320446254|ref|NW_003384317.1|	55845-57685	694056	101856	0.553406	0.8062	no
gi|320446258|ref|NW_003384313.1|	22876-23152	0	865149	inf	0.007	no
gi|320446258|ref|NW_003384313.1|	28062-29244	0	135902	inf	1	no
gi|320446258|ref|NW_003384313.1|	29434-30502	0	18508	inf	0.0039	no
gi|320446258|ref|NW_003384313.1|	30731-32831	0	156558	inf	5.00E-05	yes
gi|320446258|ref|NW_003384313.1|	35048-37703	0	87701	inf	5.00E-05	yes
gi|320446258|ref|NW_003384313.1|	37865-39599	0	156835	inf	5.00E-05	yes
gi|320446258|ref|NW_003384313.1|	52361-52775	378491	886381	122767	0.58315	no
gi|320446258|ref|NW_003384313.1|	62551-63865	422428	104258	130337	0.5495	no
gi|320446258|ref|NW_003384313.1|	64363-65901	0.518181	162188	164614	0.4448	no
gi|320446258|ref|NW_003384313.1|	67207-68732	497235	22751	219393	0.2112	no

gi|320446258|ref|NW_003384313.1|	70405-76289	113504	584013	236325	0.1862	no
gi|320446268|ref|NW_003384303.1|	109332-109715	541022	528626	-0.0334396	0.9848	no
gi|320446268|ref|NW_003384303.1|	12317-12992	882189	287878	-161563	0.45385	no
gi|320446268|ref|NW_003384303.1|	13954-14605	648739	524695	-0.306159	0.8756	no
gi|320446268|ref|NW_003384303.1|	146486-146994	366603	778739	108692	0.59775	no
gi|320446268|ref|NW_003384303.1|	147488-147731	210584	33259	0.659346	0.74255	no
gi|320446268|ref|NW_003384303.1|	148763-149015	218654	989059	-114452	0.58475	no
gi|320446268|ref|NW_003384303.1|	149876-150852	432977	629359	0.539593	0.79045	no
gi|320446268|ref|NW_003384303.1|	151007-151975	714153	906465	0.344018	0.871	no
gi|320446268|ref|NW_003384303.1|	152441-154721	108935	302423	14731	0.26315	no
gi|320446268|ref|NW_003384303.1|	176460-177640	314223	183321	-0.777416	0.6357	no
gi|320446268|ref|NW_003384303.1|	19601-19961	243259	793106	170502	0.3381	no
gi|320446268|ref|NW_003384303.1|	200937-201273	142773	101777	283361	0.2595	no
gi|320446268|ref|NW_003384303.1|	22898-23149	0	280344	inf	0.0233	no
gi|320446268|ref|NW_003384303.1|	24529-25277	0.329243	406106	362463	0.2063	no
gi|320446268|ref|NW_003384303.1|	2879-3114	353322	989878	-183566	0.41545	no
gi|320446268|ref|NW_003384303.1|	29809-30254	0.780827	108894	380177	0.20225	no
gi|320446268|ref|NW_003384303.1|	30398-30801	285746	351295	361988	0.18535	no
gi|320446268|ref|NW_003384303.1|	32239-37120	0	333258	inf	0.00375	no
gi|320446268|ref|NW_003384303.1|	42283-42995	0	384965	inf	0.0033	no
gi|320446268|ref|NW_003384303.1|	44083-44319	386936	319613	636809	0.1544	no
gi|320446268|ref|NW_003384303.1|	45171-45425	304749	150613	562708	0.16245	no
gi|320446268|ref|NW_003384303.1|	47900-48668	0.316905	486889	72634	0.1408	no
gi|320446268|ref|NW_003384303.1|	49122-50935	10696	947173	646848	0.0468	no
gi|320446268|ref|NW_003384303.1|	51936-56061	0.52054	655345	69761	0.0401	no
gi|320446268|ref|NW_003384303.1|	56502-57851	0	168362	inf	0.0198	no
gi|320446268|ref|NW_003384303.1|	58366-60739	0	137782	inf	1	no
gi|320446268|ref|NW_003384303.1|	6167-7192	0.427473	177274	205208	0.3018	no
gi|320446268|ref|NW_003384303.1|	7328-8493	163385	113199	-0.529412	0.7925	no
gi|320446268|ref|NW_003384303.1|	8609-8838	257733	135157	-0.93124	0.67975	no

gi|320446271|ref|NW_003384300.1|	103948-10598	0.749692	490173	270892	0.2424	no
gi|320446271|ref|NW_003384300.1|	163526-16453	0.657879	348464	240512	0.2994	no
gi|320446271|ref|NW_003384300.1|	259621-25982	495321	348309	-0.507995	0.79695	no
gi|320446273|ref|NW_003384298.1|	125270-12573	0.726854	484134	273567	0.2671	no
gi|320446273|ref|NW_003384298.1|	152498-15293	397317	126558	167144	0.4402	no
gi|320446273|ref|NW_003384298.1|	35961-38763	0	298648	inf	0.00615	no
gi|320446273|ref|NW_003384298.1|	39178-41535	0	277631	inf	0.0074	no
gi|320446273|ref|NW_003384298.1|	94111-96829	0	19472	inf	0.0072	no
gi|320446275|ref|NW_003384296.1|	102372-10427	107129	300101	148609	0.3969	no
gi|320446275|ref|NW_003384296.1|	120906-12189	238654	746313	164486	0.3546	no
gi|320446275|ref|NW_003384296.1|	142364-14557	120172	780982	-0.621735	0.62675	no
gi|320446275|ref|NW_003384296.1|	145847-14641	349049	269709	-0.372024	0.84975	no
gi|320446275|ref|NW_003384296.1|	148695-15081	153754	117244	-0.391103	0.75255	no
gi|320446275|ref|NW_003384296.1|	150958-15205	922185	924363	0.00340291	0.99745	no
gi|320446275|ref|NW_003384296.1|	165109-16550	504716	106115	107208	0.59155	no
gi|320446275|ref|NW_003384296.1|	21381-21762	278954	147661	-0.917733	0.6564	no
gi|320446275|ref|NW_003384296.1|	248612-24904	162509	538711	172899	0.33745	no
gi|320446275|ref|NW_003384296.1|	26126-26901	137631	600908	-119559	0.56535	no
gi|320446275|ref|NW_003384296.1|	27097-29524	388033	390317	0.00846779	0.99495	no
gi|320446275|ref|NW_003384296.1|	2841-3146	562982	700195	-300726	0.2205	no
gi|320446275|ref|NW_003384296.1|	29638-29986	157743	213639	0.437599	0.82665	no
gi|320446275|ref|NW_003384296.1|	30755-33294	915588	141721	0.630287	0.6149	no
gi|320446275|ref|NW_003384296.1|	3306-3557	638.91	376462	-0.763107	0.54525	no
gi|320446275|ref|NW_003384296.1|	396-611	976875	374057	-138492	0.5203	no
gi|320446275|ref|NW_003384296.1|	40054-40326	1097.41	219741	-232022	0.21325	no
gi|320446275|ref|NW_003384296.1|	40434-41582	131294	42269	-163513	0.45605	no
gi|320446275|ref|NW_003384296.1|	41682-44987	162813	565948	-152447	0.23915	no
gi|320446275|ref|NW_003384296.1|	47716-63775	152365	148911	-0.0330786	0.97915	no
gi|320446275|ref|NW_003384296.1|	63899-64816	141141	238998	-256206	0.2661	no
gi|320446275|ref|NW_003384296.1|	65236-66761	863619	163807	-23984	0.2839	no

gi|320446275|ref|NW_003384296.1|	71877-75172	285109	56509	0.986966	0.46525	no
gi|320446275|ref|NW_003384296.1|	75377-77904	0.662676	113145	0.771794	1	no
gi|320446275|ref|NW_003384296.1|	78236-82832	576521	823762	0.514855	0.6882	no
gi|320446275|ref|NW_003384296.1|	8278-8832	130443	543005	-126438	0.46015	no
gi|320446281|ref|NW_003384290.1|	12726-16558	0.18765	308635	403979	0.15915	no
gi|320446281|ref|NW_003384290.1|	129422-13185[illegible]	100812	629276	264203	0.05385	no
gi|320446281|ref|NW_003384290.1|	132618-13371[illegible]	4.69	205582	213206	0.34515	no
gi|320446281|ref|NW_003384290.1|	134692-13567[illegible]	406347	134066	172216	0.43365	no
gi|320446281|ref|NW_003384290.1|	157610-15798[illegible]	364383	90351	131009	0.54185	no
gi|320446281|ref|NW_003384290.1|	16678-17140	0	484134	inf	0.0312	no
gi|320446281|ref|NW_003384290.1|	189548-19183[illegible]	43263	127753	156215	0.2451	no
gi|320446281|ref|NW_003384290.1|	192676-19490[illegible]	963908	99727	0.0490886	0.9836	no
gi|320446281|ref|NW_003384290.1|	195023-19610[illegible]	641685	123474	0.944273	0.65145	no
gi|320446281|ref|NW_003384290.1|	197615-19782[illegible]	833829	52157	-0.676891	0.7419	no
gi|320446281|ref|NW_003384290.1|	198046-19827[illegible]	541584	498399	-0.119883	0.9468	no
gi|320446281|ref|NW_003384290.1|	201719-20215[illegible]	382365	231241	-0.725552	0.73165	no
gi|320446281|ref|NW_003384290.1|	206567-20676[illegible]	171568	84675	-101877	0.6135	no
gi|320446281|ref|NW_003384290.1|	231191-23176[illegible]	830508	501514	-0.727704	0.66	no
gi|320446281|ref|NW_003384290.1|	259660-26008[illegible]	229696	270213	0.234369	0.9105	no
gi|320446281|ref|NW_003384290.1|	267607-26845[illegible]	386355	0.379717	-334693	0.239	no
gi|320446281|ref|NW_003384290.1|	272038-27254[illegible]	788796	0	#NAME?	0.01755	no
gi|320446281|ref|NW_003384290.1|	276294-27654[illegible]	356845	0	#NAME?	0.02105	no
gi|320446281|ref|NW_003384290.1|	279680-28068[illegible]	483072	0.1517	-499295	0.28085	no
gi|320446281|ref|NW_003384290.1|	283769-28647[illegible]	193042	112012	-0.785264	0.5409	no
gi|320446281|ref|NW_003384290.1|	289241-29141[illegible]	186361	182295	-0.0318245	0.98045	no
gi|320446281|ref|NW_003384290.1|	291590-29201[illegible]	521385	622029	-30673	0.2043	no
gi|320446281|ref|NW_003384290.1|	296353-30054[illegible]	0.59656	665276	347921	0.0756	no
gi|320446281|ref|NW_003384290.1|	305253-30607[illegible]	373417	118471	166567	0.4326	no
gi|320446281|ref|NW_003384290.1|	318290-31888[illegible]	370955	534388	0.526643	0.7877	no
gi|320446281|ref|NW_003384290.1|	319274-32308[illegible]	278331	145603	238716	0.1931	no

gi|320446281|ref|NW_003384290.1|	324768-32602	235025	104477	-116963	0.4664	no
gi|320446281|ref|NW_003384290.1|	326667-32820	400295	125245	-167631	0.34245	no
gi|320446282|ref|NW_003384289.1|	108121-10874	423439	415199	-335028	0.17665	no
gi|320446282|ref|NW_003384289.1|	110546-11134	188673	267379	-281893	0.23285	no
gi|320446282|ref|NW_003384289.1|	124554-12702	70915	90569	367486	0.0781	no
gi|320446282|ref|NW_003384289.1|	127143-12923	218358	145.02	273149	0.04625	no
gi|320446282|ref|NW_003384289.1|	131699-13243	269688	283965	339635	0.17965	no
gi|320446282|ref|NW_003384289.1|	132647-13288	83914	558128	273361	0.12905	no
gi|320446282|ref|NW_003384289.1|	155185-15962	128843	868491	-0.569028	0.66045	no
gi|320446282|ref|NW_003384289.1|	163205-16426	925469	0.284464	-502387	0.1888	no
gi|320446282|ref|NW_003384289.1|	167225-16841	100978	110502	-31919	0.18785	no
gi|320446282|ref|NW_003384289.1|	169143-17002	172543	216061	-299744	0.21795	no
gi|320446282|ref|NW_003384289.1|	171841-17599	216788	409544	-240419	0.20135	no
gi|320446282|ref|NW_003384289.1|	176398-17923	739644	44441	-0.73494	0.75015	no
gi|320446282|ref|NW_003384289.1|	41388-47616	0.8728	370986	208764	0.2227	no
gi|320446282|ref|NW_003384289.1|	60278-62334	0.0926819	38786	538711	0.16615	no
gi|320446282|ref|NW_003384289.1|	65178-66889	0.114355	119445	338475	1	no
gi|320446282|ref|NW_003384289.1|	67180-68815	0	134264	inf	1	no
gi|320446282|ref|NW_003384289.1|	68928-71302	0.0788989	198314	465164	0.17395	no
gi|320446282|ref|NW_003384289.1|	73077-74829	0.222526	488216	445548	0.15925	no
gi|320446284|ref|NW_003384287.1|	100212-10058	678259	155249	119468	0.56875	no
gi|320446284|ref|NW_003384287.1|	101029-10820	13759	335744	128699	0.33615	no
gi|320446284|ref|NW_003384287.1|	111135-11277	806061	946232	0.231305	0.91605	no
gi|320446284|ref|NW_003384287.1|	115713-11679	219017	151544	-0.531308	0.79315	no
gi|320446284|ref|NW_003384287.1|	117169-12017	475018	374687	-0.342296	0.87605	no
gi|320446284|ref|NW_003384287.1|	120321-12137	117513	118337	0.0100811	0.9929	no
gi|320446284|ref|NW_003384287.1|	155908-15627	44444	266439	-0.738184	0.71935	no
gi|320446284|ref|NW_003384287.1|	156911-15712	584356	21939	-141335	0.5162	no
gi|320446284|ref|NW_003384287.1|	158008-15940	166328	189536	0.188446	0.91155	no
gi|320446284|ref|NW_003384287.1|	20459-25000	248445	329311	0.406522	0.76175	no

gi|320446284|ref|NW_003384287.1|	26324-30887	686184	78361	0.191539	0.8823	no
gi|320446284|ref|NW_003384287.1|	2844-3337	857459	155352	-246452	0.15865	no
gi|320446284|ref|NW_003384287.1|	32020-32618	278217	251477	-0.145783	0.94615	no
gi|320446284|ref|NW_003384287.1|	33593-34035	427215	115522	-188679	0.38715	no
gi|320446284|ref|NW_003384287.1|	4037-4692	22492	820919	-14541	0.496	no
gi|320446284|ref|NW_003384287.1|	49730-50337	226342	248789	0.136419	0.9491	no
gi|320446284|ref|NW_003384287.1|	5091-5314	850959	890904	-325575	0.19455	no
gi|320446284|ref|NW_003384287.1|	5541-6326	184266	505911	-186484	0.3949	no
gi|320446284|ref|NW_003384287.1|	61317-61661	945318	263101	-184518	0.31335	no
gi|320446284|ref|NW_003384287.1|	69872-70339	306287	42735	0.480536	0.82605	no
gi|320446284|ref|NW_003384287.1|	7266-7936	65948	158756	-205452	0.35005	no
gi|320446284|ref|NW_003384287.1|	8085-14047	875042	953932	0.124534	0.92675	no
gi|320446284|ref|NW_003384287.1|	90388-91539	368631	114907	-168171	0.3264	no
gi|320446284|ref|NW_003384287.1|	93139-93775	168175	371807	114459	0.58815	no
gi|320446284|ref|NW_003384287.1|	98868-99966	626059	825977	0.399803	0.8464	no
gi|320446285|ref|NW_003384286.1|	24498-25315	0	149584	inf	0.0048	no
gi|320446285|ref|NW_003384286.1|	34132-35251	0	211573	inf	0.0198	no
gi|320446285|ref|NW_003384286.1|	42783-46033	0.0559701	430607	626557	0.15735	no
gi|320446286|ref|NW_003384285.1|	128068-13152[illegible]	219764	428298	0.962656	0.66165	no
gi|320446286|ref|NW_003384285.1|	135151-13684[illegible]	356319	301256	-0.242176	0.84775	no
gi|320446286|ref|NW_003384285.1|	136960-13737[illegible]	735383	181892	-201541	0.28795	no
gi|320446286|ref|NW_003384285.1|	138592-13909[illegible]	478255	25497	-0.907453	0.6798	no
gi|320446286|ref|NW_003384285.1|	78458-83302	673632	0.410292	-403724	0.06165	no
gi|320446288|ref|NW_003384283.1|	100295-10067[illegible]	182191	101916	-0.838074	0.61655	no
gi|320446288|ref|NW_003384283.1|	115586-12009[illegible]	160366	611294	-139143	0.28945	no
gi|320446288|ref|NW_003384283.1|	120479-12104[illegible]	350208	834676	-206892	0.35195	no
gi|320446288|ref|NW_003384283.1|	124995-12637[illegible]	251292	0.410695	-261323	0.26915	no
gi|320446288|ref|NW_003384283.1|	127292-12790[illegible]	359301	0.914455	-197421	0.30275	no
gi|320446288|ref|NW_003384283.1|	130721-13248[illegible]	396623	0.460451	-310665	0.2151	no
gi|320446288|ref|NW_003384283.1|	134656-13556[illegible]	730959	156344	-222506	0.3081	no

gi|320446288|ref|NW_003384283.1|	37495-13829	995354	310671	-167982	0.4274	no
gi|320446288|ref|NW_003384283.1|	38398-14088	472745	225352	-106888	0.6147	no
gi|320446288|ref|NW_003384283.1|	41509-14249	519917	327802	-0.665455	0.74715	no
gi|320446288|ref|NW_003384283.1|	42625-14309	922283	520316	-0.825822	0.6898	no
gi|320446288|ref|NW_003384283.1|	43242-14480	469936	388774	-0.273533	0.8952	no
gi|320446288|ref|NW_003384283.1|	45647-14940	150614	12521	-0.266505	0.84145	no
gi|320446288|ref|NW_003384283.1|	50270-15048	463124	516386	0.15705	0.9317	no
gi|320446288|ref|NW_003384283.1|	50947-15190	283375	286973	0.0181984	0.99125	no
gi|320446288|ref|NW_003384283.1|	52053-15351	163579	201618	0.301637	0.85245	no
gi|320446288|ref|NW_003384283.1|	53896-15442	533681	759965	0.509956	0.7512	no
gi|320446288|ref|NW_003384283.1|	55026-15690	173608	325759	0.907967	0.48625	no
gi|320446288|ref|NW_003384283.1|	03317-20378	113967	778727	27725	0.241	no
gi|320446288|ref|NW_003384283.1|	05789-21116	142255	102431	28481	0.1488	no
gi|320446288|ref|NW_003384283.1|	12384-21368	0	132261	inf	1	no
gi|320446288|ref|NW_003384283.1|	14574-21741	0.324405	0.997656	162074	1	no
gi|320446288|ref|NW_003384283.1|	17599-21958	0.192887	578332	490607	0.1502	no
gi|320446288|ref|NW_003384283.1|	23153-22423	0.795488	412822	237561	0.29845	no
gi|320446288|ref|NW_003384283.1|	24450-22550	434534	656786	391788	0.0644	no
gi|320446288|ref|NW_003384283.1|	29486-23059	308381	389598	36592	0.0726	no
gi|320446288|ref|NW_003384283.1|	30784-23143	285861	208554	286703	0.22225	no
gi|320446288|ref|NW_003384283.1|	32812-23367	0	61183	inf	0.00945	no
gi|320446288|ref|NW_003384283.1|	34750-23512	109148	135817	363731	0.2158	no
gi|320446288|ref|NW_003384283.1|	35989-23622	370797	60839	403629	0.19465	no
gi|320446288|ref|NW_003384283.1|	38887-23936	0	160029	inf	0.0114	no
gi|320446288|ref|NW_003384283.1|	41369-24288	0.529151	735944	379784	0.1717	no
gi|320446288|ref|NW_003384283.1|	47614-24779	0	300238	inf	0.0113	no
gi|320446288|ref|NW_003384283.1|	49619-25024	130627	278092	441203	0.1574	no
gi|320446288|ref|NW_003384283.1|	50348-25141	0.407347	37333	651805	0.1182	no
gi|320446288|ref|NW_003384283.1|	51512-25243	0.97755	749949	626148	0.1028	no
gi|320446288|ref|NW_003384283.1|	58975-26110	685773	370325	243299	0.1917	no

gi|320446288|ref|NW_003384283.1|	264271-26452	301059	381614	3664	0.2104	no
gi|320446288|ref|NW_003384283.1|	286734-29000	212602	152584	-0.478553	0.71465	no
gi|320446288|ref|NW_003384283.1|	290300-29084	321449	216745	-0.568592	0.79695	no
gi|320446288|ref|NW_003384283.1|	291035-29270	680793	441268	-0.62556	0.7689	no
gi|320446288|ref|NW_003384283.1|	296551-30144	564993	17.2	160611	0.2247	no
gi|320446288|ref|NW_003384283.1|	301618-30352	212364	197367	-0.105665	0.95675	no
gi|320446288|ref|NW_003384283.1|	337117-33738	812952	137732	0.760619	0.7071	no
gi|320446288|ref|NW_003384283.1|	35244-37283	261956	802556	161528	0.4676	no
gi|320446288|ref|NW_003384283.1|	357293-35922	485212	297268	-0.706851	0.59845	no
gi|320446288|ref|NW_003384283.1|	359397-36132	29227	227385	-0.362163	0.7808	no
gi|320446288|ref|NW_003384283.1|	361609-36410	523976	508796	-0.0424121	0.9737	no
gi|320446288|ref|NW_003384283.1|	364315-36565	0.457486	169415	188877	0.388	no
gi|320446288|ref|NW_003384283.1|	366409-36680	784704	463665	-0.759064	0.72955	no
gi|320446288|ref|NW_003384283.1|	37491-38165	269099	314736	0.226007	0.9003	no
gi|320446288|ref|NW_003384283.1|	38278-44556	333946	130338	-135735	0.31375	no
gi|320446288|ref|NW_003384283.1|	44702-45995	189879	465078	-202954	0.37305	no
gi|320446288|ref|NW_003384283.1|	46224-48810	839853	43125	-0.961611	0.66945	no
gi|320446288|ref|NW_003384283.1|	53222-55667	196254	103508	239895	0.07695	no
gi|320446288|ref|NW_003384283.1|	68133-68841	264165	817721	163017	0.3427	no
gi|320446288|ref|NW_003384283.1|	81538-85613	562474	362688	268887	0.0527	no
gi|320446288|ref|NW_003384283.1|	91250-91829	219933	78373	18333	0.27845	no
gi|320446288|ref|NW_003384283.1|	93160-93505	606273	473112	-0.357786	0.79085	no
gi|320446288|ref|NW_003384283.1|	98376-98822	567535	314997	-0.849372	0.6936	no
gi|320446290|ref|NW_003384281.1|	110371-11089	467529	157088	-157348	0.49785	no
gi|320446290|ref|NW_003384281.1|	111828-11230	753249	502949	-0.582715	0.78725	no
gi|320446290|ref|NW_003384281.1|	113126-11381	0	330828	inf	0.02915	no
gi|320446290|ref|NW_003384281.1|	114036-11512	0.197244	122848	263882	1	no
gi|320446290|ref|NW_003384281.1|	116224-11720	911531	114858	0.333489	0.8748	no
gi|320446290|ref|NW_003384281.1|	14849-16438	710582	174349	12949	0.4305	no
gi|320446290|ref|NW_003384281.1|	17497-18141	107203	154367	0.526026	0.80145	no

gi|320446290|ref|NW_003384281.1|	18254-19944	0.696036	169616	128504	0.54245	no
gi|320446290|ref|NW_003384281.1|	194090-19530	202452	249813	-301866	0.21	no
gi|320446290|ref|NW_003384281.1|	196729-19734	287411	210185	-377339	0.15885	no
gi|320446290|ref|NW_003384281.1|	20111-21683	0.757496	158101	106153	0.59685	no
gi|320446290|ref|NW_003384281.1|	204159-20441	647885	586285	-346606	0.18685	no
gi|320446290|ref|NW_003384281.1|	204900-20603	208462	104045	-43245	0.1193	no
gi|320446290|ref|NW_003384281.1|	217064-21845	952478	386808	-130007	0.5443	no
gi|320446290|ref|NW_003384281.1|	218559-21905	129416	389752	-173138	0.42225	no
gi|320446290|ref|NW_003384281.1|	220227-22146	81035	37465	-1113	0.5978	no
gi|320446290|ref|NW_003384281.1|	222542-22336	372782	0.197123	-424117	0.3017	no
gi|320446290|ref|NW_003384281.1|	226239-22723	379205	120172	166405	0.43615	no
gi|320446290|ref|NW_003384281.1|	227410-22791	187222	455917	128402	0.55245	no
gi|320446290|ref|NW_003384281.1|	228245-22923	603065	194377	168847	0.44295	no
gi|320446290|ref|NW_003384281.1|	23633-24279	45137	726346	0.686347	0.7364	no
gi|320446290|ref|NW_003384281.1|	239099-24242	606186	794553	0.390384	0.8109	no
gi|320446290|ref|NW_003384281.1|	242575-24380	101395	0.937552	-0.113019	1	no
gi|320446290|ref|NW_003384281.1|	243976-24591	236289	184103	-0.360037	0.7835	no
gi|320446290|ref|NW_003384281.1|	247035-24849	131015	728557	-0.846621	0.7015	no
gi|320446290|ref|NW_003384281.1|	250825-25189	972935	813232	-0.258676	0.8996	no
gi|320446290|ref|NW_003384281.1|	287595-28913	154978	0.988143	-0.649274	0.75415	no
gi|320446290|ref|NW_003384281.1|	289273-29041	111811	103252	-0.114894	1	no
gi|320446290|ref|NW_003384281.1|	295705-29590	179588	0	#NAME?	0.0111	no
gi|320446290|ref|NW_003384281.1|	31920-33499	0.753549	270879	184588	0.402	no
gi|320446290|ref|NW_003384281.1|	35674-36809	0.375189	19487	237683	0.2616	no
gi|320446290|ref|NW_003384281.1|	36997-38058	0.408828	155522	192755	0.3182	no
gi|320446290|ref|NW_003384281.1|	40410-41218	16802	344271	103491	0.65005	no
gi|320446290|ref|NW_003384281.1|	43166-43627	328425	520059	0.663113	0.76485	no
gi|320446290|ref|NW_003384281.1|	43946-44447	375782	881385	122988	0.5545	no
gi|320446290|ref|NW_003384281.1|	44608-44904	196455	188887	-0.0566756	0.9604	no
gi|320446290|ref|NW_003384281.1|	45268-45899	340538	665722	0.967102	0.64755	no

gi|320446290|ref|NW_003384281.1|	46124-46903	558897	9375	0.746236	0.72085	no
gi|320446290|ref|NW_003384281.1|	52894-53135	119017	148018	0.314614	0.8768	no
gi|320446290|ref|NW_003384281.1|	94021-94463	0	630122	inf	0.029	no
gi|320446291|ref|NW_003384280.1|	29260-23003	0.941883	969251	336325	0.19055	no
gi|320446291|ref|NW_003384280.1|	79174-27963	146567	180543	362271	0.1801	no
gi|320446292|ref|NW_003384279.1|	66944-67572	0.428875	699711	402813	0.1928	no
gi|320446292|ref|NW_003384279.1|	89034-89423	0	175157	inf	0.0142	no
gi|320446292|ref|NW_003384279.1|	89539-90125	0	454268	inf	0.0233	no
gi|320446296|ref|NW_003384275.1|	04519-10583	690322	359374	-0.941785	0.6468	no
gi|320446296|ref|NW_003384275.1|	06611-10962	131772	571458	21166	0.11975	no
gi|320446296|ref|NW_003384275.1|	10446-11150	676204	157311	121809	0.5728	no
gi|320446296|ref|NW_003384275.1|	13205-11355	350242	430299	0.296985	0.88335	no
gi|320446296|ref|NW_003384275.1|	32604-13296	200455	107776	-0.895235	0.65945	no
gi|320446296|ref|NW_003384275.1|	33281-13465	10486	369305	-150557	0.4925	no
gi|320446296|ref|NW_003384275.1|	38722-13960	802963	0.535272	-390699	0.1688	no
gi|320446296|ref|NW_003384275.1|	39846-14009	312363	197812	-398103	0.31545	no
gi|320446296|ref|NW_003384275.1|	40909-14122	771047	661909	-354211	0.178	no
gi|320446296|ref|NW_003384275.1|	60061-60343	154653	887267	-0.801599	0.71405	no
gi|320446296|ref|NW_003384275.1|	61434-61876	126582	840163	-0.591334	0.7553	no
gi|320446296|ref|NW_003384275.1|	62025-62716	170294	111263	-0.614058	0.7696	no
gi|320446296|ref|NW_003384275.1|	64920-65727	212607	162287	-0.38964	0.85735	no
gi|320446296|ref|NW_003384275.1|	66264-67756	776187	69094	-0.167845	0.8977	no
gi|320446296|ref|NW_003384275.1|	70566-71065	883133	507126	-0.800287	0.7019	no
gi|320446296|ref|NW_003384275.1|	72938-74269	662315	454477	-0.543312	0.67895	no
gi|320446296|ref|NW_003384275.1|	76875-77582	178866	141865	-0.334358	0.8702	no
gi|320446296|ref|NW_003384275.1|	78277-80548	30587	41831	0.451655	0.7309	no
gi|320446296|ref|NW_003384275.1|	85107-85539	256503	822243	-164134	0.4428	no
gi|320446296|ref|NW_003384275.1|	97026-97550	983597	505648	-0.959934	0.6448	no
gi|320446298|ref|NW_003384273.1|	09528-11041	207216	249794	0.269601	0.88585	no
gi|320446298|ref|NW_003384273.1|	11936-11281	10506	180875	0.783784	0.7178	no

gi|320446298|ref|NW_003384273.1|	11935-12861	0	353909	inf	0.01575	no
gi|320446298|ref|NW_003384273.1|	15158-15582	0	681647	inf	0.029	no
gi|320446298|ref|NW_003384273.1|	19972-20433	0.729833	160392	445789	0.1782	no
gi|320446298|ref|NW_003384273.1|	24341-29843	594271	562523	-0.0792099	0.9496	no
gi|320446298|ref|NW_003384273.1|	30131-31527	101779	0.808086	-0.332859	1	no
gi|320446298|ref|NW_003384273.1|	32400-33863	0.687307	0.573322	-0.26161	1	no
gi|320446298|ref|NW_003384273.1|	38391-38646	800817	318648	-132951	0.44455	no
gi|320446298|ref|NW_003384273.1|	43503-44202	131719	149.2	0.179784	0.8915	no
gi|320446298|ref|NW_003384273.1|	50270-54329	242258	550163	118331	0.38285	no
gi|320446298|ref|NW_003384273.1|	57101-60083	471183	152037	-163186	0.2299	no
gi|320446298|ref|NW_003384273.1|	63793-64491	145858	598043	-128624	0.5425	no
gi|320446298|ref|NW_003384273.1|	65695-66669	0	236638	inf	0.02205	no
gi|320446298|ref|NW_003384273.1|	7172-8337	0.544616	226398	205555	0.3467	no
gi|320446298|ref|NW_003384273.1|	78086-78918	537527	332653	262961	0.25875	no
gi|320446298|ref|NW_003384273.1|	79781-80935	0.918566	458146	231835	0.31475	no
gi|320446298|ref|NW_003384273.1|	8593-9887	0.637646	2987	222787	0.3341	no
gi|320446298|ref|NW_003384273.1|	87199-87690	103915	317252	161023	0.44375	no
gi|320446298|ref|NW_003384273.1|	88196-90009	0.64176	0.968784	0.594141	1	no
gi|320446298|ref|NW_003384273.1|	90144-91535	116822	486927	205939	0.3549	no
gi|320446300|ref|NW_003384271.1|	126518-12674[illegible]	0	327292	inf	0.0056	no
gi|320446300|ref|NW_003384271.1|	173393-17617[illegible]	105307	907221	-0.21508	0.85765	no
gi|320446300|ref|NW_003384271.1|	176354-18160[illegible]	236533	77317	-161318	0.2328	no
gi|320446300|ref|NW_003384271.1|	181761-18202[illegible]	680862	288069	-124095	0.5412	no
gi|320446300|ref|NW_003384271.1|	182465-18346[illegible]	150291	564906	-141168	0.51325	no
gi|320446300|ref|NW_003384271.1|	183593-18460[illegible]	533687	258415	-104631	0.53685	no
gi|320446300|ref|NW_003384271.1|	197218-19764[illegible]	256419	735125	151949	0.49225	no
gi|320446300|ref|NW_003384271.1|	200927-20166[illegible]	0	438645	inf	0.0212	no
gi|320446300|ref|NW_003384271.1|	202262-20287[illegible]	0.900611	33615	190013	0.321	no
gi|320446300|ref|NW_003384271.1|	203962-20481[illegible]	870284	263895	160041	0.47115	no
gi|320446300|ref|NW_003384271.1|	205013-20810[illegible]	230277	49132	109329	0.61245	no

gi|320446300|ref|NW_003384271.1|	22654-22475	658602	604149	-0.124502	0.92545	no
gi|320446300|ref|NW_003384271.1|	29644-23202	159004	101126	-0.652915	0.59765	no
gi|320446300|ref|NW_003384271.1|	98052-101992	0.364358	325172	315777	0.19035	no
gi|320446308|ref|NW_003384263.1|	06737-10752	150211	288278	0.940474	0.6668	no
gi|320446308|ref|NW_003384263.1|	27847-12880	0	288576	inf	0.0154	no
gi|320446308|ref|NW_003384263.1|	76834-17878	22438	144769	-0.632198	0.61475	no
gi|320446308|ref|NW_003384263.1|	79901-18058	37264	178138	-106479	0.5988	no
gi|320446308|ref|NW_003384263.1|	80875-18125	227896	255921	0.167324	0.93655	no
gi|320446308|ref|NW_003384263.1|	85082-18653	542285	937453	0.789694	0.71545	no
gi|320446308|ref|NW_003384263.1|	95412-19588	0.706624	240316	508785	0.1653	no
gi|320446308|ref|NW_003384263.1|	96007-19693	220909	472208	44179	0.06105	no
gi|320446308|ref|NW_003384263.1|	3090-4233	241723	124921	236959	0.29265	no
gi|320446308|ref|NW_003384263.1|	42732-43094	360356	783643	112077	0.5798	no
gi|320446308|ref|NW_003384263.1|	60630-61100	985349	262873	141566	0.50065	no
gi|320446308|ref|NW_003384263.1|	85644-86029	524501	412874	-0.345244	0.8622	no
gi|320446308|ref|NW_003384263.1|	9419-10008	237619	321885	0.437891	0.8282	no
gi|320446309|ref|NW_003384262.1|	51557-52086	0.569125	689126	359795	0.207	no
gi|320446309|ref|NW_003384262.1|	60506-63915	0.159497	476065	489955	0.14665	no
gi|320446310|ref|NW_003384261.1|	10740-11236	44638	256215	-0.800919	0.70965	no
gi|320446310|ref|NW_003384261.1|	15653-17564	443095	16145	-145653	0.4899	no
gi|320446310|ref|NW_003384261.1|	75753-17628	284562	267993	-0.0865474	0.9267	no
gi|320446310|ref|NW_003384261.1|	17926-19687	608335	238828	-13489	0.52825	no
gi|320446310|ref|NW_003384261.1|	20344-22514	48843	35298	-0.468565	0.8181	no
gi|320446310|ref|NW_003384261.1|	22750-24227	0.815467	170077	106049	0.59685	no
gi|320446310|ref|NW_003384261.1|	26854-30633	695167	102634	0.562085	0.6548	no
gi|320446310|ref|NW_003384261.1|	8853-10656	324004	11324	-151663	0.38425	no
gi|320446313|ref|NW_003384258.1|	01281-10404	808057	886722	0.134026	0.93565	no
gi|320446313|ref|NW_003384258.1|	04148-10464	160587	301014	-241545	0.2888	no
gi|320446313|ref|NW_003384258.1|	05233-10589	141589	670353	-107872	0.6032	no
gi|320446313|ref|NW_003384258.1|	06337-10706	305154	986847	-162864	0.23155	no

gi|320446313|ref|NW_003384258.1|	108089-11021	544396	271284	-100485	0.4506	no
gi|320446313|ref|NW_003384258.1|	110490-11266	231406	534185	-211502	0.23975	no
gi|320446314|ref|NW_003384257.1|	10224-12567	104077	223571	110308	0.5909	no
gi|320446314|ref|NW_003384257.1|	115734-11875	714747	0.592897	-359158	0.1557	no
gi|320446314|ref|NW_003384257.1|	121383-12309	308131	0.556286	-246965	0.27715	no
gi|320446314|ref|NW_003384257.1|	123542-12516	499675	110261	-218007	0.3262	no
gi|320446314|ref|NW_003384257.1|	12658-14607	0.49235	171626	180151	0.4139	no
gi|320446314|ref|NW_003384257.1|	133122-13410	473438	0.311372	-392646	0.2204	no
gi|320446315|ref|NW_003384256.1|	127753-12805	332525	237288	-380875	0.2211	no
gi|320446315|ref|NW_003384256.1|	131570-13181	202349	106614	-0.924456	0.68005	no
gi|320446315|ref|NW_003384256.1|	131927-13239	868676	0.964493	-317098	0.25	no
gi|320446315|ref|NW_003384256.1|	133000-13382	952771	178599	-241541	0.2749	no
gi|320446315|ref|NW_003384256.1|	135163-13587	709484	266869	-141064	0.52035	no
gi|320446315|ref|NW_003384256.1|	136444-13767	545195	720791	0.40281	0.762	no
gi|320446315|ref|NW_003384256.1|	140347-14082	890204	107905	0.27756	0.86375	no
gi|320446315|ref|NW_003384256.1|	153040-15822	340605	672902	0.982297	0.556	no
gi|320446315|ref|NW_003384256.1|	158478-15950	809124	986094	0.285364	0.89275	no
gi|320446315|ref|NW_003384256.1|	159680-16037	113772	105316	-0.111433	0.9555	no
gi|320446315|ref|NW_003384256.1|	161473-16194	28265	376967	0.415423	0.82245	no
gi|320446315|ref|NW_003384256.1|	162341-16468	130373	103863	-0.327973	0.7876	no
gi|320446315|ref|NW_003384256.1|	170610-17119	336386	13014	-137005	0.52745	no
gi|320446315|ref|NW_003384256.1|	179499-18091	213678	237566	0.152887	0.93385	no
gi|320446315|ref|NW_003384256.1|	222049-22272	574047	235024	-128836	0.53325	no
gi|320446315|ref|NW_003384256.1|	267002-26731	168594	0	#NAME?	0.0229	no
gi|320446315|ref|NW_003384256.1|	334672-33551	752497	0.575104	-370979	0.1713	no
gi|320446315|ref|NW_003384256.1|	336542-33705	891567	359233	-131142	0.5307	no
gi|320446315|ref|NW_003384256.1|	340866-34214	435764	403159	-0.112197	0.95615	no
gi|320446315|ref|NW_003384256.1|	349581-35019	265303	300264	0.178593	0.9203	no
gi|320446315|ref|NW_003384256.1|	352162-35322	530833	211822	-13254	0.51805	no
gi|320446315|ref|NW_003384256.1|	356199-35661	256102	844778	-160008	0.44035	no

gi|320446315|ref|NW_003384256.1|	357249-35826[illegible]	116749	388453	-15876	0.45445	no
gi|320446315|ref|NW_003384256.1|	358935-36042[illegible]	209657	943783	-11515	0.4837	no
gi|320446315|ref|NW_003384256.1|	360722-36337[illegible]	261831	26977	0.0430913	0.9719	no
gi|320446316|ref|NW_003384255.1|	34494-35383	335695	192119	251678	0.26885	no
gi|320446316|ref|NW_003384255.1|	40966-41307	137855	518839	191213	0.3744	no
gi|320446316|ref|NW_003384255.1|	44409-51330	532487	198677	189961	0.16065	no
gi|320446317|ref|NW_003384254.1|	102066-10283[illegible]	107546	164893	0.616572	0.7663	no
gi|320446317|ref|NW_003384254.1|	103864-10405[illegible]	409063	633095	0.6301	0.7604	no
gi|320446317|ref|NW_003384254.1|	106286-10671[illegible]	221189	219548	-0.0107437	0.98315	no
gi|320446317|ref|NW_003384254.1|	1136-2121	0.450291	139934	163581	1	no
gi|320446317|ref|NW_003384254.1|	126434-12674[illegible]	856719	154445	0.850203	0.67995	no
gi|320446317|ref|NW_003384254.1|	138163-13925[illegible]	110457	0.955485	-353111	0.1722	no
gi|320446317|ref|NW_003384254.1|	140737-14155[illegible]	489147	0.593342	-304333	0.19695	no
gi|320446317|ref|NW_003384254.1|	142115-14263[illegible]	83213	159659	-238181	0.30695	no
gi|320446317|ref|NW_003384254.1|	14307-15725	128424	396606	162679	0.4421	no
gi|320446317|ref|NW_003384254.1|	144829-14741[illegible]	0.646866	115481	0.836116	1	no
gi|320446317|ref|NW_003384254.1|	17752-18319	354169	239327	-0.565456	0.79225	no
gi|320446317|ref|NW_003384254.1|	19042-20291	216438	138577	-0.643263	0.75275	no
gi|320446317|ref|NW_003384254.1|	20491-21686	123103	158464	0.364286	0.85115	no
gi|320446317|ref|NW_003384254.1|	26734-27185	304376	354114	0.218358	0.89885	no
gi|320446317|ref|NW_003384254.1|	29035-30217	106967	0.988381	-0.114022	1	no
gi|320446317|ref|NW_003384254.1|	3275-4447	234236	137334	-0.770273	0.70725	no
gi|320446317|ref|NW_003384254.1|	34380-34960	243678	164893	-0.563443	0.8006	no
gi|320446317|ref|NW_003384254.1|	5977-8578	171185	174402	0.026859	0.9825	no
gi|320446317|ref|NW_003384254.1|	84056-84373	14815	116742	-0.343729	0.86405	no
gi|320446317|ref|NW_003384254.1|	91152-91610	960584	885294	-0.117754	0.94635	no
gi|320446317|ref|NW_003384254.1|	93644-94354	46213	656376	0.506222	0.80025	no
gi|320446317|ref|NW_003384254.1|	9370-12074	0.95691	105073	0.134939	1	no
gi|320446317|ref|NW_003384254.1|	95784-99141	17837	597393	174381	0.43895	no
gi|320446317|ref|NW_003384254.1|	99442-100291	165041	208177	0.334993	0.86355	no

gi|320446319|ref|NW_003384252.1|	100532-10205	263911	278954	0.0799773	0.95025	no
gi|320446319|ref|NW_003384252.1|	112872-11416	0.318823	132755	205794	1	no
gi|320446319|ref|NW_003384252.1|	115104-11719	0.54678	13985	135485	1	no
gi|320446319|ref|NW_003384252.1|	117373-12005	17931	337315	0.911639	0.6614	no
gi|320446319|ref|NW_003384252.1|	126814-12881	0.57212	212778	189496	0.38675	no
gi|320446319|ref|NW_003384252.1|	129669-13021	272936	275852	0.0153302	0.9925	no
gi|320446319|ref|NW_003384252.1|	130495-13705	214414	455375	108666	0.409	no
gi|320446319|ref|NW_003384252.1|	152080-15269	670176	303285	-114386	0.58335	no
gi|320446319|ref|NW_003384252.1|	168198-17117	0.123178	11195	318404	1	no
gi|320446319|ref|NW_003384252.1|	186514-18729	497713	123802	131465	0.529	no
gi|320446319|ref|NW_003384252.1|	190225-19069	14189	378412	141519	0.3757	no
gi|320446319|ref|NW_003384252.1|	195997-19645	452712	110393	128598	0.5406	no
gi|320446319|ref|NW_003384252.1|	197239-19861	0	133591	inf	1	no
gi|320446319|ref|NW_003384252.1|	199046-20351	390841	927198	12463	0.46675	no
gi|320446319|ref|NW_003384252.1|	2571-3983	0.717105	328851	219718	0.3241	no
gi|320446319|ref|NW_003384252.1|	90755-91310	440484	173396	-134502	0.53415	no
gi|320446326|ref|NW_003384245.1|	109514-10995	0	249941	inf	0.0089	no
gi|320446326|ref|NW_003384245.1|	110961-11209	581543	623585	0.100701	0.9595	no
gi|320446326|ref|NW_003384245.1|	115563-11624	33909	643037	0.923233	0.65595	no
gi|320446326|ref|NW_003384245.1|	116430-11806	338794	480016	0.502676	0.80665	no
gi|320446326|ref|NW_003384245.1|	124020-12541	502058	164788	-160724	0.36985	no
gi|320446326|ref|NW_003384245.1|	125725-12647	590341	157333	-190772	0.38855	no
gi|320446326|ref|NW_003384245.1|	132482-13337	456848	120242	-192577	0.2649	no
gi|320446326|ref|NW_003384245.1|	175972-17808	107727	469708	212439	0.33395	no
gi|320446326|ref|NW_003384245.1|	180896-18168	302705	478032	0.659197	0.74895	no
gi|320446326|ref|NW_003384245.1|	193269-19366	893966	222037	131251	0.53075	no
gi|320446326|ref|NW_003384245.1|	244136-24566	128843	823708	-0.645413	0.7678	no
gi|320446326|ref|NW_003384245.1|	24651-25044	23092	197938	-354427	0.1819	no
gi|320446326|ref|NW_003384245.1|	30432-31032	184481	594164	-163454	0.4494	no
gi|320446326|ref|NW_003384245.1|	33637-35403	198441	119027	258451	0.2673	no

gi|320446326|ref|NW_003384245.1|	35635-37610	282264	845397	-173934	0.31985	no
gi|320446326|ref|NW_003384245.1|	38069-40054	371307	199054	-0.899455	0.48845	no
gi|320446326|ref|NW_003384245.1|	44666-47938	461186	214916	222035	0.2301	no
gi|320446326|ref|NW_003384245.1|	48375-49227	136864	621554	218313	0.32535	no
gi|320446326|ref|NW_003384245.1|	50182-50993	293235	543995	0.891536	0.67	no
gi|320446326|ref|NW_003384245.1|	51304-51709	942762	869642	-0.116472	0.9456	no
gi|320446326|ref|NW_003384245.1|	53738-54190	454631	100765	114822	0.58355	no
gi|320446326|ref|NW_003384245.1|	59560-63125	0.861469	216285	132806	0.5228	no
gi|320446327|ref|NW_003384244.1|	72733-75796	0	550751	inf	0.0039	no
gi|320446327|ref|NW_003384244.1|	76002-77064	0	155336	inf	0.0294	no
gi|320446327|ref|NW_003384244.1|	89394-92992	0	312478	inf	0.0048	no
gi|320446328|ref|NW_003384243.1|	100822-10506[illegible]	871927	849355	-0.0378401	0.97545	no
gi|320446328|ref|NW_003384243.1|	105219-11039[illegible]	177653	166342	-0.0949081	0.9426	no
gi|320446328|ref|NW_003384243.1|	111580-11605[illegible]	116235	186763	0.684167	0.60975	no
gi|320446328|ref|NW_003384243.1|	116678-11749[illegible]	463536	318576	-0.541045	0.78275	no
gi|320446328|ref|NW_003384243.1|	119098-12090[illegible]	198505	687764	-152919	0.3691	no
gi|320446328|ref|NW_003384243.1|	121221-12213[illegible]	849372	275522	-162423	0.4362	no
gi|320446328|ref|NW_003384243.1|	122256-12275[illegible]	118998	335766	-182541	0.39515	no
gi|320446328|ref|NW_003384243.1|	123091-12374[illegible]	488877	166458	-155431	0.486	no
gi|320446328|ref|NW_003384243.1|	124976-12542[illegible]	263191	462827	-250756	0.2604	no
gi|320446328|ref|NW_003384243.1|	131094-13199[illegible]	126785	297059	122837	0.56205	no
gi|320446328|ref|NW_003384243.1|	132297-13416[illegible]	454211	117987	137719	0.5369	no
gi|320446328|ref|NW_003384243.1|	136970-14608[illegible]	271293	244012	-0.152896	0.90445	no
gi|320446328|ref|NW_003384243.1|	14042-15920	0.719088	0.715953	-0.00630312	1	no
gi|320446328|ref|NW_003384243.1|	148870-14907[illegible]	349446	0	#NAME?	0.00635	no
gi|320446328|ref|NW_003384243.1|	150534-16322[illegible]	419232	693087	-259664	0.17485	no
gi|320446328|ref|NW_003384243.1|	16036-17297	148088	0.57073	-137557	0.52485	no
gi|320446328|ref|NW_003384243.1|	18011-20518	653655	695188	0.0888747	0.9695	no
gi|320446328|ref|NW_003384243.1|	24627-25606	79437	176305	447212	0.0433	no
gi|320446328|ref|NW_003384243.1|	25714-27059	0.152083	200643	37217	0.21225	no

gi|320446328|ref|NW_003384243.1|	27390-29757	0.237474	309496	370408	0.18025	no
gi|320446328|ref|NW_003384243.1|	30241-30855	142229	148771	338681	0.08405	no
gi|320446328|ref|NW_003384243.1|	38805-43595	137065	819907	25806	0.15805	no
gi|320446328|ref|NW_003384243.1|	50232-51488	231488	152549	272027	0.2406	no
gi|320446328|ref|NW_003384243.1|	54954-55233	0	220999	inf	0.02205	no
gi|320446328|ref|NW_003384243.1|	56622-57638	0	627501	inf	0.0069	no
gi|320446328|ref|NW_003384243.1|	63838-65712	0.205955	473674	452349	0.15425	no
gi|320446328|ref|NW_003384243.1|	67045-67924	0	325079	inf	0.0154	no
gi|320446328|ref|NW_003384243.1|	72206-73335	41548	117696	-181971	0.38725	no
gi|320446328|ref|NW_003384243.1|	73522-77317	173958	623664	184203	0.1713	no
gi|320446328|ref|NW_003384243.1|	77540-78163	21716	165266	292796	0.22765	no
gi|320446328|ref|NW_003384243.1|	80180-80750	396218	969609	129111	0.4403	no
gi|320446328|ref|NW_003384243.1|	82318-82926	270895	833317	162113	0.3404	no
gi|320446328|ref|NW_003384243.1|	84026-84664	506238	113576	116578	0.49985	no
gi|320446328|ref|NW_003384243.1|	97573-98786	569656	191487	-157284	0.45635	no
gi|320446328|ref|NW_003384243.1|	99039-100682	551449	27536	-100191	0.61635	no
gi|320446329|ref|NW_003384242.1|	102860-10397	190992	145456	-0.392929	0.84975	no
gi|320446329|ref|NW_003384242.1|	104818-10517	132957	315339	-207598	0.3596	no
gi|320446329|ref|NW_003384242.1|	109548-11054	13261	13741	0.0512941	1	no
gi|320446329|ref|NW_003384242.1|	114454-11554	412285	244569	-0.7534	0.7142	no
gi|320446329|ref|NW_003384242.1|	116645-11843	149885	143005	-0.0677975	0.9559	no
gi|320446329|ref|NW_003384242.1|	118635-11948	668984	405597	-0.721925	0.5656	no
gi|320446329|ref|NW_003384242.1|	129161-13022	917639	218623	125244	0.5673	no
gi|320446329|ref|NW_003384242.1|	130726-13209	0.297725	206781	279605	0.2345	no
gi|320446329|ref|NW_003384242.1|	132589-13542	330639	502978	0.605236	0.78215	no
gi|320446329|ref|NW_003384242.1|	31497-31817	643151	746999	353788	0.18285	no
gi|320446329|ref|NW_003384242.1|	39411-40612	489353	247141	233639	0.18055	no
gi|320446329|ref|NW_003384242.1|	41241-43638	142849	116149	302342	0.03205	no
gi|320446329|ref|NW_003384242.1|	43713-44421	0	292915	inf	0.029	no
gi|320446329|ref|NW_003384242.1|	4468-5095	236474	204578	-353096	0.1711	no

gi|320446329|ref|NW_003384242.1|	48871-51490	110076	953488	-0.207212	0.87065	no
gi|320446329|ref|NW_003384242.1|	51817-52246	11745	13331	0.182744	0.92445	no
gi|320446329|ref|NW_003384242.1|	53146-53884	271976	390956	-27984	0.23725	no
gi|320446329|ref|NW_003384242.1|	54973-55827	237374	375506	-266025	0.25505	no
gi|320446329|ref|NW_003384242.1|	59756-61413	37509	801491	-222648	0.22	no
gi|320446329|ref|NW_003384242.1|	62876-64341	300551	215603	-0.479228	0.6958	no
gi|320446329|ref|NW_003384242.1|	64583-66065	703903	357625	-0.97693	0.6407	no
gi|320446329|ref|NW_003384242.1|	6482-8461	62904	141703	-215028	0.33725	no
gi|320446329|ref|NW_003384242.1|	66238-68324	847967	298932	-150419	0.49195	no
gi|320446329|ref|NW_003384242.1|	71314-73071	0	262623	inf	0.0101	no
gi|320446329|ref|NW_003384242.1|	79660-81176	610101	502173	-0.280864	0.828	no
gi|320446329|ref|NW_003384242.1|	8663-10031	140054	32079	-212629	0.35135	no
gi|320446329|ref|NW_003384242.1|	87735-89085	211961	0.105133	-433351	0.29815	no
gi|320446329|ref|NW_003384242.1|	92839-94003	127214	0.377731	-175183	1	no
gi|320446329|ref|NW_003384242.1|	94969-96180	172977	0.359765	-226545	0.2663	no
gi|320446329|ref|NW_003384242.1|	97849-98674	343523	0.787181	-212564	0.35435	no
gi|320446330|ref|NW_003384241.1|	102469-10463	269333	994551	-143727	0.415	no
gi|320446330|ref|NW_003384241.1|	109807-11142	249657	131371	-0.926301	0.5789	no
gi|320446330|ref|NW_003384241.1|	11546-14335	892018	531053	-0.748215	0.64875	no
gi|320446330|ref|NW_003384241.1|	119641-12041	762312	160553	-224733	0.2045	no
gi|320446330|ref|NW_003384241.1|	120802-12132	299053	669782	-215864	0.3343	no
gi|320446330|ref|NW_003384241.1|	122318-12361	0.159562	110733	279489	1	no
gi|320446330|ref|NW_003384241.1|	124478-12703	92382	940021	0.0250813	0.9866	no
gi|320446330|ref|NW_003384241.1|	16903-18741	283233	464422	0.713445	0.5855	no
gi|320446330|ref|NW_003384241.1|	206071-20721	250263	310431	-30111	0.1063	no
gi|320446330|ref|NW_003384241.1|	208670-21241	171825	468191	-187577	0.30005	no
gi|320446330|ref|NW_003384241.1|	212533-21554	162309	905286	-0.8423	0.5083	no
gi|320446330|ref|NW_003384241.1|	215612-21609	22753	939233	-12765	0.5436	no
gi|320446330|ref|NW_003384241.1|	216531-22105	398931	30196	-0.401785	0.76625	no
gi|320446330|ref|NW_003384241.1|	24773-28733	869763	31709	-145573	0.3878	no

gi|320446330|ref|NW_003384241.1|	31461-33271	868027	365847	-12465	0.5687	no
gi|320446330|ref|NW_003384241.1|	4137-4572	195893	270522	-285625	0.2386	no
gi|320446330|ref|NW_003384241.1|	6648-7187	826611	111351	-289209	0.21375	no
gi|320446330|ref|NW_003384241.1|	7348-8335	943095	31015	-160444	0.4514	no
gi|320446330|ref|NW_003384241.1|	8523-10165	10317	475957	-111612	0.6124	no
gi|320446332|ref|NW_003384239.1|	100632-10140	161018	433136	-189433	0.38965	no
gi|320446332|ref|NW_003384239.1|	102268-10315	64755	285479	-118161	0.55955	no
gi|320446332|ref|NW_003384239.1|	108324-10931	104563	368801	-150345	0.47705	no
gi|320446332|ref|NW_003384239.1|	109830-11079	348412	224448	-0.634416	0.7531	no
gi|320446332|ref|NW_003384239.1|	110897-11282	377936	159467	-124489	0.55545	no
gi|320446332|ref|NW_003384239.1|	112933-11462	602817	379358	-0.66816	0.74285	no
gi|320446332|ref|NW_003384239.1|	115277-11699	93644	100199	0.0976059	0.96245	no
gi|320446332|ref|NW_003384239.1|	117153-11749	268262	226536	-0.243904	0.9074	no
gi|320446332|ref|NW_003384239.1|	50161-51807	240447	475452	0.983585	0.44945	no
gi|320446332|ref|NW_003384239.1|	51921-52808	310824	642327	104721	0.60725	no
gi|320446332|ref|NW_003384239.1|	53433-53780	211736	172014	-0.299739	0.88215	no
gi|320446332|ref|NW_003384239.1|	55400-55598	38172	522892	0.454	0.8261	no
gi|320446332|ref|NW_003384239.1|	59499-59963	77864	61968	-0.329433	0.8778	no
gi|320446332|ref|NW_003384239.1|	61326-63064	156097	308932	0.984852	0.4402	no
gi|320446332|ref|NW_003384239.1|	63197-63406	182156	265767	0.544989	0.7964	no
gi|320446332|ref|NW_003384239.1|	63805-65290	621188	873159	0.491215	0.81265	no
gi|320446332|ref|NW_003384239.1|	65679-66490	938352	664883	-0.497029	0.8065	no
gi|320446332|ref|NW_003384239.1|	66594-67169	69209	10699	0.62844	0.75835	no
gi|320446332|ref|NW_003384239.1|	67994-68196	489841	392779	-0.318596	0.87115	no
gi|320446332|ref|NW_003384239.1|	68352-69233	653571	882248	0.432841	0.82905	no
gi|320446332|ref|NW_003384239.1|	70983-75055	774007	434087	-0.834361	0.6157	no
gi|320446332|ref|NW_003384239.1|	76545-77660	157326	743825	-108072	0.6195	no
gi|320446332|ref|NW_003384239.1|	78022-78450	101139	33476	-159514	0.443	no
gi|320446332|ref|NW_003384239.1|	82766-84814	17408	311648	-248176	0.1734	no
gi|320446332|ref|NW_003384239.1|	90789-91145	112279	406335	-146635	0.5029	no

gi|320446332|ref|NW_003384239.1|	93342-94337	422143	245549	-0.781721	0.69315	no
gi|320446333|ref|NW_003384238.1|	109281-11071	0.281811	362313	368444	0.1784	no
gi|320446333|ref|NW_003384238.1|	117150-11769	0	696716	inf	0.0212	no
gi|320446333|ref|NW_003384238.1|	117811-11853	0.344651	731269	440719	0.18645	no
gi|320446333|ref|NW_003384238.1|	121376-12470	0.108941	240039	446165	0.15895	no
gi|320446333|ref|NW_003384238.1|	124830-12897	133853	127868	325592	0.09825	no
gi|320446333|ref|NW_003384238.1|	130493-13088	192834	533337	-185424	0.3906	no
gi|320446333|ref|NW_003384238.1|	131300-13170	431465	12981	-173284	0.41885	no
gi|320446333|ref|NW_003384238.1|	131864-13330	0.841218	185135	113802	0.58155	no
gi|320446333|ref|NW_003384238.1|	133477-13382	187784	191684	0.0296615	0.97335	no
gi|320446333|ref|NW_003384238.1|	145257-14825	966402	769724	-0.328282	0.7841	no
gi|320446333|ref|NW_003384238.1|	148797-14946	100404	553207	-0.859929	0.67535	no
gi|320446333|ref|NW_003384238.1|	150654-15143	139724	980114	-0.511562	0.80855	no
gi|320446333|ref|NW_003384238.1|	152591-15463	92211	201383	-2195	0.33955	no
gi|320446333|ref|NW_003384238.1|	155546-15734	744633	210515	-182261	0.409	no
gi|320446333|ref|NW_003384238.1|	162196-16266	443401	0	#NAME?	0.0071	no
gi|320446333|ref|NW_003384238.1|	167619-16784	558076	0	#NAME?	0.02015	no
gi|320446333|ref|NW_003384238.1|	169461-17053	184265	0.138573	-7055	0.26105	no
gi|320446333|ref|NW_003384238.1|	1723-3636	0.100583	166868	737417	0.1408	no
gi|320446333|ref|NW_003384238.1|	172446-17293	205891	0	#NAME?	0.01075	no
gi|320446333|ref|NW_003384238.1|	173032-17367	582875	113325	-568465	0.1295	no
gi|320446333|ref|NW_003384238.1|	175013-17551	201859	380344	-240797	0.2782	no
gi|320446333|ref|NW_003384238.1|	179771-18092	332131	0.76687	-21147	0.3516	no
gi|320446333|ref|NW_003384238.1|	183210-18382	105848	0.599037	-41432	0.21025	no
gi|320446333|ref|NW_003384238.1|	185778-18647	124514	325295	-193648	0.3754	no
gi|320446333|ref|NW_003384238.1|	189615-19004	160884	280214	-252142	0.2864	no
gi|320446333|ref|NW_003384238.1|	191125-19183	190763	241626	-298093	0.2137	no
gi|320446333|ref|NW_003384238.1|	194490-19474	462775	781714	-25656	0.29355	no
gi|320446333|ref|NW_003384238.1|	194906-19606	293941	114536	-135972	0.5087	no
gi|320446333|ref|NW_003384238.1|	197262-19751	537696	160197	-174695	0.413	no

gi|320446333|ref|NW_003384238.1|	199805-20020	198996	43696	-218717	0.33435	no
gi|320446333|ref|NW_003384238.1|	201733-20208	125559	40885	-161872	0.4405	no
gi|320446333|ref|NW_003384238.1|	203201-20363	823661	163731	-233072	0.25625	no
gi|320446333|ref|NW_003384238.1|	203785-20458	107432	245957	-212694	0.34475	no
gi|320446333|ref|NW_003384238.1|	205015-20600	126977	430827	-155939	0.47025	no
gi|320446333|ref|NW_003384238.1|	206916-20708	139607	285499	-228981	0.26805	no
gi|320446333|ref|NW_003384238.1|	207601-20827	353822	137893	-135947	0.5366	no
gi|320446333|ref|NW_003384238.1|	209874-21827	137247	676641	230161	0.22245	no
gi|320446333|ref|NW_003384238.1|	219213-22063	428079	149719	18063	0.4218	no
gi|320446333|ref|NW_003384238.1|	223160-22381	240419	60337	132749	0.41875	no
gi|320446333|ref|NW_003384238.1|	4046-5186	0.373115	116285	163997	1	no
gi|320446333|ref|NW_003384238.1|	72388-77380	189771	199028	0.0687112	0.96075	no
gi|320446334|ref|NW_003384237.1|	148914-14912	0	41.54	inf	0.029	no
gi|320446334|ref|NW_003384237.1|	15725-16918	112258	0.488587	-784399	0.1293	no
gi|320446334|ref|NW_003384237.1|	17923-19111	983208	0.368339	-806032	0.1105	no
gi|320446334|ref|NW_003384237.1|	19251-24170	220468	502151	-213438	0.1144	no
gi|320446334|ref|NW_003384237.1|	24229-28256	522426	203101	-136303	0.308	no
gi|320446334|ref|NW_003384237.1|	28400-28991	179598	320179	-248782	0.27615	no
gi|320446334|ref|NW_003384237.1|	31671-32992	149228	431638	-178963	0.4215	no
gi|320446334|ref|NW_003384237.1|	35250-36269	40921	17861	-119603	0.56885	no
gi|320446334|ref|NW_003384237.1|	38886-39352	107274	524333	-103275	0.61245	no
gi|320446334|ref|NW_003384237.1|	39829-44041	255424	158035	-0.692649	0.6088	no
gi|320446334|ref|NW_003384237.1|	44605-46387	187659	239411	0.351374	0.77975	no
gi|320446334|ref|NW_003384237.1|	46803-48780	157925	291836	0.885918	0.4959	no
gi|320446334|ref|NW_003384237.1|	54410-55057	0.818704	529561	269338	0.244	no
gi|320446334|ref|NW_003384237.1|	55131-57668	0.806417	281639	180425	0.4009	no
gi|320446334|ref|NW_003384237.1|	58905-61227	18599	101613	-0.872137	0.6738	no
gi|320446334|ref|NW_003384237.1|	64060-65210	387486	0.127812	-492205	0.28715	no
gi|320446334|ref|NW_003384237.1|	82644-84109	50778	0.1908	-473407	0.1968	no
gi|320446334|ref|NW_003384237.1|	84285-84500	814062	0	#NAME?	0.01585	no

gi|320446334|ref|NW_003384237.1|	9798-15588	482963	439978	-0.134484	0.9162	no
gi|320446337|ref|NW_003384234.1|	106504-110297	156034	132105	-0.240176	0.8531	no
gi|320446337|ref|NW_003384234.1|	111063-111987	392156	287315	-0.448796	0.81835	no
gi|320446337|ref|NW_003384234.1|	116620-117069	230239	510032	114746	0.575	no
gi|320446337|ref|NW_003384234.1|	126521-127139	347521	289813	-0.261979	0.9041	no
gi|320446337|ref|NW_003384234.1|	153679-154640	15456	611492	198416	0.2607	no
gi|320446337|ref|NW_003384234.1|	155699-157363	199619	37002	0.890355	0.49495	no
gi|320446337|ref|NW_003384234.1|	157527-159577	71.88	227189	166023	0.2091	no
gi|320446337|ref|NW_003384234.1|	165161-165623	399769	154923	-136762	0.5216	no
gi|320446337|ref|NW_003384234.1|	168679-169048	230172	376048	-261372	0.27635	no
gi|320446337|ref|NW_003384234.1|	169163-174431	164277	749272	-113257	0.39415	no
gi|320446337|ref|NW_003384234.1|	177140-179228	0.819727	603576	288032	0.22325	no
gi|320446337|ref|NW_003384234.1|	183322-184080	103347	0.221413	-554461	0.2748	no
gi|320446337|ref|NW_003384234.1|	188636-189451	611542	0.400237	-393352	0.22035	no
gi|320446337|ref|NW_003384234.1|	189619-190048	838927	166638	-233183	0.25625	no
gi|320446337|ref|NW_003384234.1|	190198-193291	235427	177412	-37301	0.06995	no
gi|320446337|ref|NW_003384234.1|	193509-195100	332135	388061	0.224514	0.8623	no
gi|320446337|ref|NW_003384234.1|	195239-198291	838076	37252	-116976	0.4682	no
gi|320446337|ref|NW_003384234.1|	198449-199443	229148	614669	-18984	0.4054	no
gi|320446337|ref|NW_003384234.1|	201636-201978	164289	0	#NAME?	0.02015	no
gi|320446337|ref|NW_003384234.1|	204404-205499	149208	394028	-192095	0.38145	no
gi|320446337|ref|NW_003384234.1|	212783-214144	313334	884995	-182396	0.29225	no
gi|320446337|ref|NW_003384234.1|	220091-220698	0.452683	430005	324778	0.2316	no
gi|320446337|ref|NW_003384234.1|	239659-240905	459208	144774	-166534	0.33265	no
gi|320446337|ref|NW_003384234.1|	241267-242284	107962	373042	-153312	0.47525	no
gi|320446337|ref|NW_003384234.1|	242777-243821	193863	663021	-154791	0.4811	no
gi|320446337|ref|NW_003384234.1|	244068-245010	224998	604636	-189577	0.3998	no
gi|320446337|ref|NW_003384234.1|	258570-258901	103606	383157	-14351	0.514	no
gi|320446337|ref|NW_003384234.1|	260061-260498	242671	482757	0.992293	0.6698	no
gi|320446337|ref|NW_003384234.1|	276994-279190	0.943804	0.838246	-0.171113	1	no

gi|320446337|ref|NW_003384234.1|	284262-28648[illegible]	164175	101514	-0.693561	0.67415	no
gi|320446337|ref|NW_003384234.1|	287036-28767[illegible]	348107	82745	-207278	0.35	no
gi|320446337|ref|NW_003384234.1|	288565-28896[illegible]	586574	385902	-0.604079	0.75475	no
gi|320446337|ref|NW_003384234.1|	289662-294179	670701	381928	-0.812369	0.53805	no
gi|320446337|ref|NW_003384234.1|	294446-295527	459029	966619	107436	0.6144	no
gi|320446337|ref|NW_003384234.1|	308650-310090	309591	421566	0.445392	0.73415	no
gi|320446337|ref|NW_003384234.1|	389766-393310	493769	801369	0.698632	0.6676	no
gi|320446337|ref|NW_003384234.1|	405164-40615[illegible]	269456	217105	-0.311656	0.87755	no
gi|320446337|ref|NW_003384234.1|	407717-408195	272852	364428	0.417513	0.82245	no
gi|320446337|ref|NW_003384234.1|	451463-452418	515977	161853	-167263	0.43305	no
gi|320446337|ref|NW_003384234.1|	497084-499807	162789	144563	315062	0.09665	no
gi|320446337|ref|NW_003384234.1|	50906-52528	0.60848	4997	303778	0.2154	no
gi|320446337|ref|NW_003384234.1|	53453-56884	0.422398	738751	412841	0.07235	no
gi|320446337|ref|NW_003384234.1|	74012-74502	541056	82866	-270693	0.25185	no
gi|320446337|ref|NW_003384234.1|	85252-85681	838927	55546	-0.594863	0.7629	no
gi|320446337|ref|NW_003384234.1|	91105-92124	40921	148842	-145906	0.49285	no
gi|320446337|ref|NW_003384234.1|	92462-92674	194977	43075	-217838	0.3274	no
gi|320446337|ref|NW_003384234.1|	92850-106401	713356	485452	-0.555295	0.6745	no
gi|320446338|ref|NW_003384233.1|	50378-52627	846354	292429	-153317	0.48905	no
gi|320446338|ref|NW_003384233.1|	55861-56842	119968	171932	-280273	0.2331	no
gi|320446338|ref|NW_003384233.1|	56964-57832	122729	165311	-289223	0.2115	no
gi|320446339|ref|NW_003384232.1|	15403-16069	313197	800845	135445	0.5128	no
gi|320446339|ref|NW_003384232.1|	16227-18194	136425	231003	0.759805	0.7103	no
gi|320446339|ref|NW_003384232.1|	18452-19858	0	387608	inf	5.00E-05	yes
gi|320446339|ref|NW_003384232.1|	19961-30398	0.0165384	938576	914851	0.14075	no
gi|320446340|ref|NW_003384231.1|	10701-10971	253647	22598	31553	0.2374	no
gi|320446340|ref|NW_003384231.1|	117670-118569	381502	508247	0.413839	0.8392	no
gi|320446340|ref|NW_003384231.1|	16552-19397	0.387944	53327	378095	0.1469	no
gi|320446340|ref|NW_003384231.1|	20119-23207	100553	195217	427905	0.0456	no
gi|320446340|ref|NW_003384231.1|	25572-26500	0.487363	184855	192333	0.3194	no

gi|320446340|ref|NW_003384231.1|	47409-50936	297306	186136	264633	0.16305	no
gi|320446340|ref|NW_003384231.1|	53067-53792	13786	448197	170093	0.4397	no
gi|320446340|ref|NW_003384231.1|	53940-54548	0.451491	337003	289999	0.25825	no
gi|320446340|ref|NW_003384231.1|	55547-56398	274174	471624	0.782548	0.7032	no
gi|320446340|ref|NW_003384231.1|	63131-64660	326117	381046	0.224577	0.90695	no
gi|320446340|ref|NW_003384231.1|	99916-102108	551929	136004	130109	0.4239	no
gi|320446341|ref|NW_003384230.1|	13606-14443	103806	499741	-105464	0.42205	no
gi|320446341|ref|NW_003384230.1|	15105-15985	717425	366059	-0.970753	0.4577	no
gi|320446341|ref|NW_003384230.1|	16460-17562	225898	242622	-321889	0.18435	no
gi|320446341|ref|NW_003384230.1|	17771-18286	500984	320366	-396697	0.1359	no
gi|320446341|ref|NW_003384230.1|	1814-5194	306376	258986	-356436	0.0788	no
gi|320446341|ref|NW_003384230.1|	18533-19348	273738	400237	-277387	0.24175	no
gi|320446341|ref|NW_003384230.1|	19758-20244	178652	265454	-275062	0.2477	no
gi|320446341|ref|NW_003384230.1|	31149-32108	135278	29941	114619	0.4846	no
gi|320446341|ref|NW_003384230.1|	37574-40103	130948	11356	-0.205536	0.86865	no
gi|320446341|ref|NW_003384230.1|	40963-43815	935023	468749	-0.996186	0.5356	no
gi|320446341|ref|NW_003384230.1|	47170-48289	420183	0.661165	-266794	0.2599	no
gi|320446341|ref|NW_003384230.1|	48541-49641	62461	405292	-0.623995	0.75805	no
gi|320446341|ref|NW_003384230.1|	57025-60244	791724	35596	-115328	0.4757	no
gi|320446341|ref|NW_003384230.1|	60759-61465	258111	686272	-191114	0.38935	no
gi|320446341|ref|NW_003384230.1|	72500-74936	621074	12208	0.974996	0.5539	no
gi|320446341|ref|NW_003384230.1|	75889-76518	299462	407179	0.443292	0.8196	no
gi|320446341|ref|NW_003384230.1|	76634-77206	134633	842841	-0.675701	0.7437	no
gi|320446341|ref|NW_003384230.1|	78648-79616	195816	168571	-0.216146	0.9188	no
gi|320446341|ref|NW_003384230.1|	79958-82558	206222	226818	0.137332	0.9175	no
gi|320446341|ref|NW_003384230.1|	82707-83228	185843	278831	0.585308	0.6587	no
gi|320446341|ref|NW_003384230.1|	83328-85195	14789	821647	-0.847933	0.5942	no
gi|320446341|ref|NW_003384230.1|	85682-87433	734819	643626	-0.191166	0.93115	no
gi|320446341|ref|NW_003384230.1|	87870-89059	513209	551936	0.104954	0.957	no
gi|320446341|ref|NW_003384230.1|	90184-91643	197213	261705	0.408193	0.74235	no

gi|320446342|ref|NW_003384229.1|	15707-16448	834469	580769	279903	0.131	no
gi|320446342|ref|NW_003384229.1|	17728-17981	154258	742628	226729	0.31925	no
gi|320446342|ref|NW_003384229.1|	18734-19277	0.544159	586709	343054	0.2167	no
gi|320446342|ref|NW_003384229.1|	19447-19977	107781	599187	24749	0.28255	no
gi|320446342|ref|NW_003384229.1|	28508-29050	709634	408261	252434	0.26985	no
gi|320446342|ref|NW_003384229.1|	29148-30387	0.16815	244896	386435	0.19995	no
gi|320446342|ref|NW_003384229.1|	47111-48688	163512	0.787585	-105389	0.59185	no
gi|320446342|ref|NW_003384229.1|	50723-52886	857858	275463	168305	0.33945	no
gi|320446342|ref|NW_003384229.1|	54245-55116	424869	6399	0.590829	0.76085	no
gi|320446342|ref|NW_003384229.1|	70349-70720	386637	106322	145938	0.51335	no
gi|320446343|ref|NW_003384228.1|	100416-10116	417371	62802	0.589479	0.6292	no
gi|320446343|ref|NW_003384228.1|	102033-10797	103704	336673	169888	0.21695	no
gi|320446343|ref|NW_003384228.1|	110637-11175	919371	293896	-164534	0.2077	no
gi|320446343|ref|NW_003384228.1|	56543-57305	128204	329662	136255	0.52575	no
gi|320446343|ref|NW_003384228.1|	57409-64625	125683	516833	203991	0.23935	no
gi|320446343|ref|NW_003384228.1|	64812-66697	593287	819878	0.46668	0.83365	no
gi|320446343|ref|NW_003384228.1|	67226-68403	770666	105566	0.453964	0.8322	no
gi|320446343|ref|NW_003384228.1|	71398-71849	12936	116352	-0.152898	0.9352	no
gi|320446343|ref|NW_003384228.1|	71961-72379	441876	583865	0.401995	0.8535	no
gi|320446343|ref|NW_003384228.1|	76752-77288	116838	11238	-0.0561247	0.9731	no
gi|320446343|ref|NW_003384228.1|	94978-98896	18006	323901	-247486	0.18115	no
gi|320446343|ref|NW_003384228.1|	99465-100118	431791	192424	-448797	0.13065	no
gi|320446344|ref|NW_003384227.1|	189969-19620	275596	508526	0.883768	0.5899	no
gi|320446344|ref|NW_003384227.1|	264032-26459	792693	61213	-0.372924	0.7585	no
gi|320446344|ref|NW_003384227.1|	264734-26530	230709	189854	-0.281189	0.89485	no
gi|320446344|ref|NW_003384227.1|	265412-26868	265393	291082	0.133297	0.91975	no
gi|320446345|ref|NW_003384226.1|	138295-13852	808584	203767	133345	0.38395	no
gi|320446345|ref|NW_003384226.1|	145184-14541	872575	219577	133137	0.38395	no
gi|320446345|ref|NW_003384226.1|	157918-15864	310186	306661	-0.0164867	0.95935	no
gi|320446345|ref|NW_003384226.1|	158703-15935	689285	524695	-0.393621	0.84195	no

gi|320446345|ref|NW_003384226.1|	16965-17379	450584	220108	228834	0.31475	no
gi|320446345|ref|NW_003384226.1|	179071-18051	292966	950217	169753	0.44245	no
gi|320446345|ref|NW_003384226.1|	181657-18244	0.913026	229833	133186	0.52445	no
gi|320446345|ref|NW_003384226.1|	361035-36294	151055	266744	0.82038	0.6915	no
gi|320446345|ref|NW_003384226.1|	365740-36859	128919	342417	140929	0.51355	no
gi|320446345|ref|NW_003384226.1|	374771-37506	173681	210401	0.276704	0.8864	no
gi|320446345|ref|NW_003384226.1|	376755-38158	282942	221315	-0.354404	0.79185	no
gi|320446345|ref|NW_003384226.1|	381794-38222	303417	106007	-151714	0.4803	no
gi|320446345|ref|NW_003384226.1|	403493-40520	130541	486395	-142431	0.5277	no
gi|320446345|ref|NW_003384226.1|	405418-41047	174507	118571	-0.55753	0.6738	no
gi|320446345|ref|NW_003384226.1|	411292-41680	304726	148991	-103229	0.44655	no
gi|320446345|ref|NW_003384226.1|	417251-41887	476547	483574	0.0211179	0.988	no
gi|320446345|ref|NW_003384226.1|	421498-42207	150654	164445	-319556	0.2143	no
gi|320446345|ref|NW_003384226.1|	423286-42480	841364	201136	-206456	0.34765	no
gi|320446345|ref|NW_003384226.1|	426261-42831	898028	393895	-118895	0.58715	no
gi|320446345|ref|NW_003384226.1|	430912-43158	185.43	693236	-141946	0.2846	no
gi|320446345|ref|NW_003384226.1|	432903-43329	969055	431091	-116859	0.5906	no
gi|320446355|ref|NW_003384216.1|	159186-15964	0	699669	inf	0.0233	no
gi|320446355|ref|NW_003384216.1|	160059-16099	0.241242	232957	327151	0.23045	no
gi|320446355|ref|NW_003384216.1|	17240-22811	12965	620408	22586	0.1965	no
gi|320446355|ref|NW_003384216.1|	204166-20482	118538	215475	0.862172	0.6872	no
gi|320446355|ref|NW_003384216.1|	23002-24187	231029	147795	-0.644476	0.7522	no
gi|320446355|ref|NW_003384216.1|	247929-24867	993527	0.680716	-386743	0.1659	no
gi|320446355|ref|NW_003384216.1|	262709-26392	0.515233	0.952626	0.886684	1	no
gi|320446355|ref|NW_003384216.1|	271401-27437	586379	302062	-0.956988	0.6657	no
gi|320446355|ref|NW_003384216.1|	275250-27592	653525	52456	-0.317136	0.8796	no
gi|320446355|ref|NW_003384216.1|	277512-27791	133348	131736	-0.0175539	0.97265	no
gi|320446355|ref|NW_003384216.1|	279024-28430	709338	111817	0.656592	0.61315	no
gi|320446356|ref|NW_003384215.1|	101303-10198	11404	311331	144891	0.50735	no
gi|320446356|ref|NW_003384215.1|	102630-10587	117811	294276	13207	0.54445	no

gi|320446356|ref|NW_003384215.1|	109290-10948[illegible]	439018	370765	307815	0.21415	no
gi|320446356|ref|NW_003384215.1|	17258-17596	295616	237313	-0.316933	0.87995	no
gi|320446356|ref|NW_003384215.1|	19484-21727	17482	155448	-0.169437	0.89545	no
gi|320446356|ref|NW_003384215.1|	22632-23659	127918	103153	-0.310434	1	no
gi|320446356|ref|NW_003384215.1|	23802-24812	102402	147533	0.526791	0.8051	no
gi|320446356|ref|NW_003384215.1|	24918-28882	828103	161543	0.964034	0.45795	no
gi|320446356|ref|NW_003384215.1|	32436-34847	151236	279444	0.885757	0.50265	no
gi|320446356|ref|NW_003384215.1|	34968-36316	124372	13481	0.116265	0.9559	no
gi|320446356|ref|NW_003384215.1|	36471-37458	107782	883927	-0.286121	0.8934	no
gi|320446356|ref|NW_003384215.1|	37630-38670	0.839003	116013	0.467545	1	no
gi|320446356|ref|NW_003384215.1|	38774-40747	0	121876	inf	1	no
gi|320446356|ref|NW_003384215.1|	46655-47431	159232	139245	-0.193512	0.92275	no
gi|320446356|ref|NW_003384215.1|	70553-72659	236335	673319	-181147	0.30375	no
gi|320446356|ref|NW_003384215.1|	7123-7473	121936	691853	-0.81759	0.7124	no
gi|320446356|ref|NW_003384215.1|	73900-75144	140545	127651	-0.138833	0.94905	no
gi|320446356|ref|NW_003384215.1|	75327-80720	122675	119873	-0.0333307	0.97845	no
gi|320446356|ref|NW_003384215.1|	81462-82277	136869	700415	-0.966512	0.64055	no
gi|320446356|ref|NW_003384215.1|	82998-83387	19495	538945	-185489	0.3906	no
gi|320446356|ref|NW_003384215.1|	84056-85044	31619	805336	-197313	0.24595	no
gi|320446356|ref|NW_003384215.1|	86537-87036	252324	338084	0.422105	0.82245	no
gi|320446356|ref|NW_003384215.1|	89112-91669	109019	715835	271505	0.2434	no
gi|320446358|ref|NW_003384213.1|	111760-11267[illegible]	248711	274326	0.141425	0.93535	no
gi|320446358|ref|NW_003384213.1|	114732-11600[illegible]	0.973018	0.450081	-111228	1	no
gi|320446358|ref|NW_003384213.1|	131905-13330[illegible]	199276	439431	114087	0.37565	no
gi|320446358|ref|NW_003384213.1|	133713-13499[illegible]	707427	134996	0.932263	0.6662	no
gi|320446358|ref|NW_003384213.1|	135133-13616[illegible]	720317	673673	-0.0965829	0.9623	no
gi|320446358|ref|NW_003384213.1|	136291-13850[illegible]	205086	286242	0.481001	0.81735	no
gi|320446358|ref|NW_003384213.1|	149280-14952[illegible]	295734	228653	-0.371141	0.85455	no
gi|320446358|ref|NW_003384213.1|	154889-15584[illegible]	159929	973776	-0.715766	0.74255	no
gi|320446358|ref|NW_003384213.1|	156017-15700[illegible]	10.93	739521	-0.563636	0.7871	no

gi|320446358|ref|NW_003384213.1|	157616-15851	204702	12341	-0.730062	0.7278	no
gi|320446358|ref|NW_003384213.1|	158633-15888	256244	608195	-207491	0.2864	no
gi|320446358|ref|NW_003384213.1|	160261-16052	344483	134757	-135407	0.51455	no
gi|320446358|ref|NW_003384213.1|	160904-16115	426649	579282	-288071	0.2132	no
gi|320446358|ref|NW_003384213.1|	17534-18968	957358	704459	-0.442543	0.8428	no
gi|320446358|ref|NW_003384213.1|	182234-18287	0.832678	110492	373004	0.1757	no
gi|320446358|ref|NW_003384213.1|	183769-18432	260612	0.351818	-621093	0.26925	no
gi|320446358|ref|NW_003384213.1|	186104-18706	236595	368238	-268371	0.2499	no
gi|320446358|ref|NW_003384213.1|	190883-19176	256824	500341	-23598	0.3022	no
gi|320446358|ref|NW_003384213.1|	1916-8138	182908	331851	0.859415	0.51855	no
gi|320446358|ref|NW_003384213.1|	191955-19378	11611	610469	239442	0.2938	no
gi|320446358|ref|NW_003384213.1|	265312-26561	129315	106779	-0.276258	0.8776	no
gi|320446358|ref|NW_003384213.1|	30036-30737	0	321927	inf	0.02915	no
gi|320446358|ref|NW_003384213.1|	36513-38390	369014	529409	0.520707	0.6997	no
gi|320446358|ref|NW_003384213.1|	38490-40652	0.350328	152774	212463	0.3429	no
gi|320446358|ref|NW_003384213.1|	40809-43600	0.792284	0.692139	-0.194956	1	no
gi|320446358|ref|NW_003384213.1|	43840-45861	359095	835632	12185	0.3678	no
gi|320446358|ref|NW_003384213.1|	46004-49278	558612	728216	0.38252	0.7759	no
gi|320446358|ref|NW_003384213.1|	49612-51271	0.592663	165026	147741	0.5	no
gi|320446358|ref|NW_003384213.1|	55603-56306	342734	125773	-144626	0.5136	no
gi|320446358|ref|NW_003384213.1|	56736-57603	304637	496702	-261664	0.26835	no
gi|320446358|ref|NW_003384213.1|	57814-58045	624904	157389	-19893	0.3855	no
gi|320446358|ref|NW_003384213.1|	58240-58470	592195	798792	-289018	0.21255	no
gi|320446358|ref|NW_003384213.1|	59525-60143	879799	149388	-255811	0.2926	no
gi|320446358|ref|NW_003384213.1|	60296-60561	401935	340576	-356092	0.2316	no
gi|320446358|ref|NW_003384213.1|	66951-67336	482541	619311	-296191	0.2056	no
gi|320446358|ref|NW_003384213.1|	71718-72771	28898	442512	0.614746	0.7616	no
gi|320446358|ref|NW_003384213.1|	74229-75804	352706	368083	0.061564	0.9735	no
gi|320446358|ref|NW_003384213.1|	8367-9856	848042	115118	0.440899	0.8358	no
gi|320446358|ref|NW_003384213.1|	88123-91459	0.0544176	791746	718482	0.1408	no

gi|320446358|ref|NW_003384213.1|	91977-92358	0	105472	inf	0.02205	no
gi|320446358|ref|NW_003384213.1|	94775-95076	0	410198	inf	0.0101	no
gi|320446359|ref|NW_003384212.1|	105813-10641	741911	383502	-0.952011	0.5611	no
gi|320446359|ref|NW_003384212.1|	107819-10820	189879	968553	-0.971179	0.64875	no
gi|320446359|ref|NW_003384212.1|	108944-11480	280872	318799	0.182734	0.88915	no
gi|320446359|ref|NW_003384212.1|	117689-12001	291115	72823	13228	0.54605	no
gi|320446359|ref|NW_003384212.1|	121163-12191	105153	103586	-0.0216624	0.98605	no
gi|320446359|ref|NW_003384212.1|	134579-13897	333178	181866	-0.87342	0.51105	no
gi|320446359|ref|NW_003384212.1|	147536-14775	205833	258193	0.326975	0.85835	no
gi|320446359|ref|NW_003384212.1|	15287-16254	985033	114814	0.221054	0.8708	no
gi|320446359|ref|NW_003384212.1|	1589-8570	104049	950374	-0.130701	0.9183	no
gi|320446359|ref|NW_003384212.1|	163526-16537	429816	547888	0.350162	0.8704	no
gi|320446359|ref|NW_003384212.1|	165536-16702	630654	142744	117851	0.59455	no
gi|320446359|ref|NW_003384212.1|	168973-17023	654974	102239	0.642436	0.7677	no
gi|320446359|ref|NW_003384212.1|	171100-17329	378098	359794	-0.0715918	0.97275	no
gi|320446359|ref|NW_003384212.1|	173660-17482	525897	263851	-0.995054	0.6435	no
gi|320446359|ref|NW_003384212.1|	17437-19820	401484	291295	-0.462861	0.72975	no
gi|320446359|ref|NW_003384212.1|	174970-17578	550449	378309	-0.541045	0.7862	no
gi|320446359|ref|NW_003384212.1|	177207-17804	528662	153113	-178774	0.4074	no
gi|320446359|ref|NW_003384212.1|	180435-18167	0.168486	116847	279393	1	no
gi|320446359|ref|NW_003384212.1|	182317-18355	0.671261	162944	127943	0.55415	no
gi|320446359|ref|NW_003384212.1|	183687-18522	103398	0.898994	-0.201826	1	no
gi|320446359|ref|NW_003384212.1|	188962-19070	447115	0.934212	-225882	0.3189	no
gi|320446359|ref|NW_003384212.1|	197491-19771	298286	21.84	-0.449725	0.81845	no
gi|320446359|ref|NW_003384212.1|	21239-23698	0.682943	0.741907	0.119472	1	no
gi|320446359|ref|NW_003384212.1|	225761-23140	698511	371467	-0.911048	0.5917	no
gi|320446359|ref|NW_003384212.1|	25845-26709	0.268501	443579	404619	0.19255	no
gi|320446359|ref|NW_003384212.1|	28668-29507	763381	532737	-0.51898	0.6866	no
gi|320446359|ref|NW_003384212.1|	34434-35717	531583	502998	-0.0797426	0.9656	no
gi|320446359|ref|NW_003384212.1|	38703-39264	360508	591229	0.713687	0.72775	no

gi|320446359|ref|NW_003384212.1|	39949-40843	410026	441408	0.106398	0.9542	no
gi|320446359|ref|NW_003384212.1|	40978-41666	335376	458069	0.449787	0.81675	no
gi|320446359|ref|NW_003384212.1|	42381-47624	19879	540557	14432	0.3852	no
gi|320446359|ref|NW_003384212.1|	50263-52915	251358	175619	-0.517298	0.8015	no
gi|320446359|ref|NW_003384212.1|	53533-54885	258431	808091	-167719	0.32605	no
gi|320446359|ref|NW_003384212.1|	57357-57607	736701	405464	-418343	0.21005	no
gi|320446359|ref|NW_003384212.1|	59112-59675	271338	587856	-220655	0.31675	no
gi|320446359|ref|NW_003384212.1|	59824-62509	285167	149682	-0.929911	0.48415	no
gi|320446359|ref|NW_003384212.1|	74070-74435	0	100078	inf	0.02915	no
gi|320446359|ref|NW_003384212.1|	9081-9591	157759	895691	-0.816651	0.68705	no
gi|320446359|ref|NW_003384212.1|	99377-101129	808881	843142	0.0598481	0.9658	no
gi|320446360|ref|NW_003384211.1|	276879-277120	0	35788	inf	0.02075	no
gi|320446360|ref|NW_003384211.1|	314265-315119	0	227181	inf	0.0041	no
gi|320446360|ref|NW_003384211.1|	322381-323264	0	110575	inf	5.00E-05	yes
gi|320446360|ref|NW_003384211.1|	326273-327330	0	102142	inf	5.00E-05	yes
gi|320446369|ref|NW_003384202.1|	100405-102315	244855	187534	-0.38478	0.76725	no
gi|320446369|ref|NW_003384202.1|	103907-106771	246489	915292	-142922	0.27725	no
gi|320446369|ref|NW_003384202.1|	111824-112651	176887	372673	-224684	0.3241	no
gi|320446369|ref|NW_003384202.1|	11310-11854	13019	511826	-134689	0.51615	no
gi|320446369|ref|NW_003384202.1|	16183-19398	246897	126337	-0.966632	0.46295	no
gi|320446369|ref|NW_003384202.1|	47309-49262	0	287665	inf	0.0069	no
gi|320446369|ref|NW_003384202.1|	6173-7099	378801	198863	-0.929664	0.5711	no
gi|320446369|ref|NW_003384202.1|	75502-76332	394567	117105	-175247	0.2939	no
gi|320446369|ref|NW_003384202.1|	80318-81989	644128	296222	-112067	0.4	no
gi|320446369|ref|NW_003384202.1|	83702-87052	102929	106483	0.0489782	0.9679	no
gi|320446369|ref|NW_003384202.1|	8521-9893	697955	530348	-0.396194	0.85635	no
gi|320446369|ref|NW_003384202.1|	99683-100140	148397	493791	173444	0.33745	no
gi|320446372|ref|NW_003384199.1|	11038-12095	453955	11649	-196234	0.2665	no
gi|320446372|ref|NW_003384199.1|	12387-12580	154277	372743	-204927	0.3526	no
gi|320446372|ref|NW_003384199.1|	12963-13298	448637	801188	-248534	0.17755	no

gi|320446372|ref|NW_003384199.1|	129850-13134[illegible]	435052	221523	-0.973733	0.44995	no
gi|320446372|ref|NW_003384199.1|	146000-14623[illegible]	727725	152825	-225151	0.32725	no
gi|320446372|ref|NW_003384199.1|	205043-20705[illegible]	11296	469992	-12651	0.5758	no
gi|320446372|ref|NW_003384199.1|	208377-20943[illegible]	20566	142232	-0.532014	0.79845	no
gi|320446372|ref|NW_003384199.1|	211519-21240[illegible]	465522	231603	-10072	0.6155	no
gi|320446372|ref|NW_003384199.1|	212771-21359[illegible]	221555	929569	-125303	0.56035	no
gi|320446372|ref|NW_003384199.1|	215247-21575[illegible]	111145	455429	-128715	0.55115	no
gi|320446372|ref|NW_003384199.1|	216436-21966[illegible]	119689	663352	-0.851445	0.60935	no
gi|320446372|ref|NW_003384199.1|	220251-22219[illegible]	810766	27572	-155608	0.47585	no
gi|320446372|ref|NW_003384199.1|	222463-22521[illegible]	396247	716868	-246662	0.19895	no
gi|320446372|ref|NW_003384199.1|	226376-22953[illegible]	11827	8755	-0.433905	0.7334	no
gi|320446372|ref|NW_003384199.1|	229674-22998[illegible]	112286	422527	-141006	0.5126	no
gi|320446372|ref|NW_003384199.1|	23-351	151313	0	#NAME?	0.0229	no
gi|320446372|ref|NW_003384199.1|	231080-23158[illegible]	282048	740235	-192988	0.3705	no
gi|320446372|ref|NW_003384199.1|	232059-23300[illegible]	765409	610529	-0.32617	0.86975	no
gi|320446372|ref|NW_003384199.1|	2352-2675	251386	202928	-363087	0.22965	no
gi|320446372|ref|NW_003384199.1|	250776-25515[illegible]	202635	490057	-204786	0.2682	no
gi|320446372|ref|NW_003384199.1|	25257-26580	119473	14649	0.294116	0.89735	no
gi|320446372|ref|NW_003384199.1|	259554-26053[illegible]	529536	251077	-439853	0.0531	no
gi|320446372|ref|NW_003384199.1|	27115-30821	743821	608981	-0.288559	0.81435	no
gi|320446372|ref|NW_003384199.1|	30974-31684	216846	16774	-0.370439	0.86535	no
gi|320446372|ref|NW_003384199.1|	36603-40991	426624	688506	0.690502	0.67375	no
gi|320446372|ref|NW_003384199.1|	41107-43229	277261	636448	11988	0.5794	no
gi|320446372|ref|NW_003384199.1|	43640-49363	101462	534318	239677	0.1744	no
gi|320446372|ref|NW_003384199.1|	50396-52495	246277	107375	-451955	0.05245	no
gi|320446372|ref|NW_003384199.1|	53732-56207	219241	105234	-438085	0.05265	no
gi|320446372|ref|NW_003384199.1|	61524-64297	471428	634757	0.429165	0.7495	no
gi|320446372|ref|NW_003384199.1|	64412-71435	221228	512332	121155	0.4704	no
gi|320446372|ref|NW_003384199.1|	6622-8248	152168	331058	-220051	0.10565	no
gi|320446372|ref|NW_003384199.1|	76657-77183	356354	170057	-10673	0.61795	no

gi|320446372|ref|NW_003384199.1|	78734-79041	714275	11481	0.684703	0.7444	no
gi|320446372|ref|NW_003384199.1|	80832-81009	105755	895339	-0.240224	0.90345	no
gi|320446372|ref|NW_003384199.1|	87629-88084	396548	119462	-173094	0.41615	no
gi|320446376|ref|NW_003384195.1|	107748-10852	248856	0.640355	-195837	0.30295	no
gi|320446376|ref|NW_003384195.1|	23620-24520	228557	122499	-0.89978	0.6711	no
gi|320446376|ref|NW_003384195.1|	24594-28702	261413	521568	0.996526	0.65955	no
gi|320446376|ref|NW_003384195.1|	40887-41841	213724	307941	-279503	0.2446	no
gi|320446376|ref|NW_003384195.1|	42289-47167	537866	250232	221795	0.10045	no
gi|320446376|ref|NW_003384195.1|	49338-51322	858838	225409	139208	0.4118	no
gi|320446376|ref|NW_003384195.1|	51428-52563	168835	350767	10549	0.60745	no
gi|320446376|ref|NW_003384195.1|	53225-53921	109865	225204	10355	0.5772	no
gi|320446376|ref|NW_003384195.1|	55287-55690	76199	131736	0.789801	0.6964	no
gi|320446376|ref|NW_003384195.1|	56171-58330	447365	210543	223459	0.2115	no
gi|320446376|ref|NW_003384195.1|	76160-76767	0	737152	inf	0.0138	no
gi|320446376|ref|NW_003384195.1|	79862-80520	0	978417	inf	0.0085	no
gi|320446376|ref|NW_003384195.1|	93086-93996	0.250186	258678	337009	0.2261	no
gi|320446376|ref|NW_003384195.1|	94129-95700	0.252688	210957	306152	0.2113	no
gi|320446376|ref|NW_003384195.1|	96959-97641	0.377603	412429	34492	0.2167	no
gi|320446378|ref|NW_003384193.1|	111109-11355	0.535513	48455	649958	0.0613	no
gi|320446378|ref|NW_003384193.1|	114958-11873	0.190726	112456	588172	0.10305	no
gi|320446378|ref|NW_003384193.1|	62893-68019	0.551989	529288	326134	0.09265	no
gi|320446378|ref|NW_003384193.1|	80206-84124	366535	737596	100888	0.53045	no
gi|320446378|ref|NW_003384193.1|	96375-97286	0.499631	258302	237012	0.2616	no
gi|320446381|ref|NW_003384190.1|	40124-40506	853466	153854	0.850156	0.67095	no
gi|320446381|ref|NW_003384190.1|	6710-6856	0	313372	inf	0.0294	no
gi|320446384|ref|NW_003384187.1|	103497-10828	878148	134691	0.617123	0.6328	no
gi|320446384|ref|NW_003384187.1|	108450-10890	561152	547533	-0.0354452	0.9863	no
gi|320446384|ref|NW_003384187.1|	114160-11569	164236	897493	-0.871797	0.5829	no
gi|320446384|ref|NW_003384187.1|	116458-11888	430394	149732	-152327	0.26435	no
gi|320446384|ref|NW_003384187.1|	121084-12214	616228	781338	0.342482	0.86465	no

gi|320446384|ref|NW_003384187.1|	124499-124999	565693	968647	0.775951	0.704	no
gi|320446384|ref|NW_003384187.1|	125675-126494	289213	278286	-0.0555654	0.961	no
gi|320446384|ref|NW_003384187.1|	127137-12783[illegible]	132409	158305	0.257708	0.8994	no
gi|320446384|ref|NW_003384187.1|	17810-18505	110102	250751	118741	0.56815	no
gi|320446384|ref|NW_003384187.1|	218523-21955[illegible]	148674	954288	268227	0.2439	no
gi|320446384|ref|NW_003384187.1|	78705-79314	0.450305	100845	448509	0.17665	no
gi|320446385|ref|NW_003384186.1|	132715-133035	482363	933748	0.952914	0.6698	no
gi|320446385|ref|NW_003384186.1|	138422-14134[illegible]	830249	202694	128769	0.32515	no
gi|320446385|ref|NW_003384186.1|	150999-151489	130375	187539	384645	0.1766	no
gi|320446385|ref|NW_003384186.1|	162738-16321[illegible]	0	55485	inf	0.029	no
gi|320446385|ref|NW_003384186.1|	169295-169857	282414	104036	-144073	0.50045	no
gi|320446385|ref|NW_003384186.1|	172163-173728	888399	264875	-174589	0.42845	no
gi|320446385|ref|NW_003384186.1|	173829-17781[illegible]	608123	737844	0.278952	0.81965	no
gi|320446385|ref|NW_003384186.1|	239615-24046[illegible]	246013	855735	-15235	0.48375	no
gi|320446385|ref|NW_003384186.1|	256299-26065[illegible]	332069	332124	0.000239786	0.99915	no
gi|320446385|ref|NW_003384186.1|	78034-83359	115363	717443	263668	0.0537	no
gi|320446385|ref|NW_003384186.1|	83513-88486	256417	55382	111092	0.5033	no
gi|320446385|ref|NW_003384186.1|	89577-90416	447403	100008	116047	0.5767	no
gi|320446385|ref|NW_003384186.1|	97226-98307	279409	113233	-130309	0.42615	no
gi|320446386|ref|NW_003384185.1|	104387-113058	25201	239579	324895	0.0197	no
gi|320446386|ref|NW_003384185.1|	113143-11688[illegible]	177753	639508	184709	0.17025	no
gi|320446386|ref|NW_003384185.1|	117025-11764[illegible]	930963	21372	119893	0.58005	no
gi|320446386|ref|NW_003384185.1|	120201-12062[illegible]	102407	283464	146885	0.40465	no
gi|320446386|ref|NW_003384185.1|	130603-131167	102089	310333	160399	0.33275	no
gi|320446386|ref|NW_003384185.1|	180242-182805	111275	969722	-0.198483	0.87805	no
gi|320446386|ref|NW_003384185.1|	247804-249085	0.322788	134386	205773	1	no
gi|320446386|ref|NW_003384185.1|	251279-255897	103946	488009	223108	0.19335	no
gi|320446386|ref|NW_003384185.1|	260325-26111[illegible]	22177	187714	-0.240525	0.9144	no
gi|320446386|ref|NW_003384185.1|	263853-26640[illegible]	817174	993816	0.282336	0.863	no
gi|320446386|ref|NW_003384185.1|	266530-26794[illegible]	382934	567018	0.566299	0.66695	no

gi|320446386|ref|NW_003384185.1|	268191-27100[illegible]	797685	185076	121423	0.4794	no
gi|320446386|ref|NW_003384185.1|	272416-27357[illegible]	475583	937716	0.979453	0.6438	no
gi|320446386|ref|NW_003384185.1|	296960-29731[illegible]	190791	554714	153975	0.47205	no
gi|320446386|ref|NW_003384185.1|	299871-30057[illegible]	668959	520988	-0.360667	0.77	no
gi|320446386|ref|NW_003384185.1|	300803-30186[illegible]	847273	823594	-0.0408938	0.97585	no
gi|320446386|ref|NW_003384185.1|	302285-30264[illegible]	119376	202537	0.762675	0.71425	no
gi|320446386|ref|NW_003384185.1|	304461-30579[illegible]	610796	899291	0.558097	0.6774	no
gi|320446386|ref|NW_003384185.1|	6708-9102	211053	906026	210195	0.35955	no
gi|320446386|ref|NW_003384185.1|	94296-94550	274274	173785	266361	0.25345	no
gi|320446389|ref|NW_003384182.1|	11421-12860	0.280406	243598	311891	0.20585	no
gi|320446389|ref|NW_003384182.1|	119238-12307[illegible]	360056	28772	-0.323553	0.8095	no
gi|320446389|ref|NW_003384182.1|	123482-12533[illegible]	45405	170934	-140941	0.2862	no
gi|320446389|ref|NW_003384182.1|	131617-13379[illegible]	739397	198394	-189798	0.1629	no
gi|320446389|ref|NW_003384182.1|	133970-13414[illegible]	192356	47406	-202064	0.354	no
gi|320446389|ref|NW_003384182.1|	135132-13529[illegible]	437041	117848	-189085	0.3895	no
gi|320446389|ref|NW_003384182.1|	136514-13764[illegible]	732433	0.910393	-300814	0.2114	no
gi|320446389|ref|NW_003384182.1|	14077-14454	87824	287518	-161096	0.44495	no
gi|320446389|ref|NW_003384182.1|	150469-15089[illegible]	0.854729	101787	357394	0.2102	no
gi|320446389|ref|NW_003384182.1|	156698-15733[illegible]	0.838811	542123	26922	0.244	no
gi|320446389|ref|NW_003384182.1|	170874-17164[illegible]	0.938396	278993	157196	0.491	no
gi|320446389|ref|NW_003384182.1|	182402-18302[illegible]	178979	130491	-0.455842	0.8271	no
gi|320446389|ref|NW_003384182.1|	18495-25822	0.428247	182986	541715	0.0313	no
gi|320446389|ref|NW_003384182.1|	196146-19645[illegible]	174172	190531	0.129512	0.93985	no
gi|320446389|ref|NW_003384182.1|	209267-21430[illegible]	924343	199424	110934	0.40505	no
gi|320446389|ref|NW_003384182.1|	26152-31851	0.555858	125293	449444	0.0451	no
gi|320446389|ref|NW_003384182.1|	32493-34022	0.260893	653222	464604	0.15405	no
gi|320446389|ref|NW_003384182.1|	37063-37407	270091	701603	469914	0.15455	no
gi|320446389|ref|NW_003384182.1|	53360-55401	113077	481004	208875	0.2689	no
gi|320446389|ref|NW_003384182.1|	56364-59751	219501	726553	172684	0.29825	no
gi|320446389|ref|NW_003384182.1|	59950-61609	0.948261	297046	164733	0.431	no

gi|320446389|ref|NW_003384182.1|	62742-63424	339843	799081	123347	0.5531	no
gi|320446389|ref|NW_003384182.1|	64908-65537	273794	44208	0.691219	0.75235	no
gi|320446389|ref|NW_003384182.1|	67218-68273	803052	356007	-117359	0.5672	no
gi|320446389|ref|NW_003384182.1|	68415-68832	168729	76256	-114579	0.57895	no
gi|320446389|ref|NW_003384182.1|	71088-71983	161203	246821	-270734	0.23965	no
gi|320446389|ref|NW_003384182.1|	74623-76669	643037	116996	-245844	0.2819	no
gi|320446389|ref|NW_003384182.1|	83214-84105	154464	0.88678	-0.800619	0.7079	no
gi|320446390|ref|NW_003384181.1|	01957-10367	131241	476817	-146071	0.51505	no
gi|320446390|ref|NW_003384181.1|	03960-10631	269993	180158	-0.583658	0.65715	no
gi|320446390|ref|NW_003384181.1|	06778-10807	907788	530526	-0.774932	0.718	no
gi|320446390|ref|NW_003384181.1|	10600-11101	178476	100186	-0.833056	0.68695	no
gi|320446390|ref|NW_003384181.1|	12132-11246	188276	46903	-200509	0.3563	no
gi|320446390|ref|NW_003384181.1|	12684-11378	311584	0.80874	-194587	0.3836	no
gi|320446390|ref|NW_003384181.1|	14294-11484	822772	247052	-173568	0.30605	no
gi|320446390|ref|NW_003384181.1|	16533-11738	787062	25763	-161118	0.3615	no
gi|320446390|ref|NW_003384181.1|	30477-13068	607185	11.39	-241437	0.25395	no
gi|320446390|ref|NW_003384181.1|	31391-13159	757809	197296	-194148	0.38965	no
gi|320446390|ref|NW_003384181.1|	64424-16487	164425	443364	-189086	0.28055	no
gi|320446390|ref|NW_003384181.1|	76407-18044	91332	986954	0.111863	0.93015	no
gi|320446390|ref|NW_003384181.1|	81537-18327	0.896607	140501	0.648034	1	no
gi|320446390|ref|NW_003384181.1|	83382-18585	543908	437887	-0.312806	0.89065	no
gi|320446390|ref|NW_003384181.1|	86070-18689	719334	474673	-0.599727	0.76515	no
gi|320446390|ref|NW_003384181.1|	87979-18862	118712	529561	-11646	0.5775	no
gi|320446390|ref|NW_003384181.1|	49716-51888	0.174259	425574	46101	0.1563	no
gi|320446395|ref|NW_003384176.1|	12544-13130	584639	519163	-0.171358	0.93985	no
gi|320446395|ref|NW_003384176.1|	38692-14067	107482	224154	106039	0.5323	no
gi|320446395|ref|NW_003384176.1|	40793-14100	505206	552428	0.128913	0.94155	no
gi|320446395|ref|NW_003384176.1|	42073-14253	359026	430661	0.262465	0.8937	no
gi|320446395|ref|NW_003384176.1|	43045-14345	942762	68329	-0.464396	0.81965	no
gi|320446395|ref|NW_003384176.1|	43994-14423	280779	199554	-0.492657	0.7989	no

gi|320446395|ref|NW_003384176.1|	146129-14653[illegible]	468984	370887	-0.338558	0.8623	no
gi|320446395|ref|NW_003384176.1|	25118-26052	157032	333264	-223632	0.3227	no
gi|320446395|ref|NW_003384176.1|	28066-28418	168994	266603	-266421	0.13815	no
gi|320446395|ref|NW_003384176.1|	28569-29140	540094	121708	-214979	0.3398	no
gi|320446395|ref|NW_003384176.1|	30664-32630	126753	122366	-0.0508234	1	no
gi|320446395|ref|NW_003384176.1|	34454-35716	189039	524567	-184949	0.41035	no
gi|320446395|ref|NW_003384176.1|	36984-39913	159108	814486	-0.966041	0.45065	no
gi|320446395|ref|NW_003384176.1|	40575-43491	958646	188676	0.97684	0.5634	no
gi|320446395|ref|NW_003384176.1|	71949-75152	0.34114	421493	362707	0.1607	no
gi|320446395|ref|NW_003384176.1|	79672-80449	713666	598742	-357524	0.0779	no
gi|320446395|ref|NW_003384176.1|	84573-84843	279012	11299	-130413	0.5399	no
gi|320446395|ref|NW_003384176.1|	88278-88834	386872	987955	-196934	0.37635	no
gi|320446395|ref|NW_003384176.1|	90483-90720	41957	168401	-131701	0.5397	no
gi|320446395|ref|NW_003384176.1|	92215-93233	823779	953786	-311052	0.10515	no
gi|320446396|ref|NW_003384175.1|	131260-13189[illegible]	127069	288059	118075	0.56835	no
gi|320446396|ref|NW_003384175.1|	152768-15603[illegible]	350403	115928	172614	0.3116	no
gi|320446396|ref|NW_003384175.1|	156762-15823[illegible]	478387	617612	0.368525	0.85825	no
gi|320446396|ref|NW_003384175.1|	160511-16155[illegible]	543999	39059	-0.477951	0.81485	no
gi|320446396|ref|NW_003384175.1|	161783-16212[illegible]	135045	12278	-0.137363	0.9408	no
gi|320446396|ref|NW_003384175.1|	175300-17863[illegible]	658759	70739	0.102756	0.93795	no
gi|320446396|ref|NW_003384175.1|	179822-18111[illegible]	478449	369777	-0.371709	0.7795	no
gi|320446396|ref|NW_003384175.1|	181212-18177[illegible]	114325	84192	-0.441381	0.82615	no
gi|320446396|ref|NW_003384175.1|	21612-22481	0.799145	201734	133592	0.52445	no
gi|320446396|ref|NW_003384175.1|	23272-24830	10462	185497	0.826233	0.6042	no
gi|320446396|ref|NW_003384175.1|	24949-27025	138429	312695	117561	0.37185	no
gi|320446396|ref|NW_003384175.1|	41624-45598	979332	18291	0.901262	0.48865	no
gi|320446396|ref|NW_003384175.1|	46562-48111	0.642301	178714	147633	0.5	no
gi|320446396|ref|NW_003384175.1|	49317-51681	336896	743278	11416	0.4	no
gi|320446396|ref|NW_003384175.1|	54479-57337	251557	245533	-0.0349678	0.97745	no
gi|320446396|ref|NW_003384175.1|	57805-58368	430045	300844	-0.515471	0.8081	no

gi|320446396|ref|NW_003384175.1|	58510-61582	110638	390962	-150074	0.37405	no
gi|320446396|ref|NW_003384175.1|	61800-62951	110589	127674	0.207254	1	no
gi|320446396|ref|NW_003384175.1|	63167-64912	278329	879716	-166168	0.33705	no
gi|320446396|ref|NW_003384175.1|	66814-67435	240094	415199	-253173	0.26525	no
gi|320446396|ref|NW_003384175.1|	68996-69575	0	26455	inf	0.0077	no
gi|320446396|ref|NW_003384175.1|	76211-76718	263653	131597	-100251	0.623	no
gi|320446396|ref|NW_003384175.1|	77342-78754	121908	508224	-126226	0.55205	no
gi|320446396|ref|NW_003384175.1|	78863-80667	290449	15214	-0.932882	0.46935	no
gi|320446396|ref|NW_003384175.1|	81416-82128	481443	399492	-0.269197	0.86965	no
gi|320446396|ref|NW_003384175.1|	94044-94485	317853	42186	0.408401	0.8506	no
gi|320446398|ref|NW_003384173.1|	102409-10667[illegible]	870704	410328	-108541	0.5168	no
gi|320446398|ref|NW_003384173.1|	106794-10924[illegible]	528811	141342	-190357	0.1592	no
gi|320446398|ref|NW_003384173.1|	112597-11396[illegible]	416081	557335	0.421682	0.8314	no
gi|320446398|ref|NW_003384173.1|	114757-11694[illegible]	120982	180904	0.580432	0.7837	no
gi|320446398|ref|NW_003384173.1|	118084-11942[illegible]	217526	130823	-0.73358	0.65355	no
gi|320446398|ref|NW_003384173.1|	119472-12265[illegible]	166229	19522	0.231933	0.8604	no
gi|320446398|ref|NW_003384173.1|	123530-12439[illegible]	187355	202675	0.113396	0.94235	no
gi|320446398|ref|NW_003384173.1|	12496-12980	740011	476826	-0.634083	0.77945	no
gi|320446398|ref|NW_003384173.1|	125799-12637[illegible]	0.994406	3362	175741	0.33425	no
gi|320446398|ref|NW_003384173.1|	13106-13422	331841	213888	-0.633633	0.7624	no
gi|320446398|ref|NW_003384173.1|	141391-14228[illegible]	609201	240844	-133882	0.4289	no
gi|320446398|ref|NW_003384173.1|	14711-16597	868945	100464	0.209338	0.92445	no
gi|320446398|ref|NW_003384173.1|	165058-16901[illegible]	100302	771974	-0.377724	0.76605	no
gi|320446398|ref|NW_003384173.1|	17320-18809	153455	239594	0.642775	0.69855	no
gi|320446398|ref|NW_003384173.1|	182163-18376[illegible]	184945	141568	293632	0.2259	no
gi|320446398|ref|NW_003384173.1|	184882-18564[illegible]	22564	839805	189603	0.3831	no
gi|320446398|ref|NW_003384173.1|	185896-18643[illegible]	0	405811	inf	0.0294	no
gi|320446398|ref|NW_003384173.1|	187567-18795[illegible]	311223	544665	0.807418	0.69235	no
gi|320446398|ref|NW_003384173.1|	19220-20296	602296	527811	-0.19045	0.92335	no
gi|320446398|ref|NW_003384173.1|	195457-19595[illegible]	126162	845209	274403	0.23585	no

gi|320446398|ref|NW_003384173.1|	20556-21066	970826	732838	-0.405718	0.8377	no
gi|320446398|ref|NW_003384173.1|	207388-21424	697948	955346	0.452903	0.7274	no
gi|320446398|ref|NW_003384173.1|	21458-21745	277578	259389	-0.0977726	0.9548	no
gi|320446398|ref|NW_003384173.1|	216002-21689	256266	229532	-0.158947	0.9297	no
gi|320446398|ref|NW_003384173.1|	217552-21849	357771	460739	0.364913	0.85285	no
gi|320446398|ref|NW_003384173.1|	218901-21928	649973	31153	-106101	0.61065	no
gi|320446398|ref|NW_003384173.1|	24278-24567	167611	670167	-132253	0.52545	no
gi|320446398|ref|NW_003384173.1|	25720-26485	956093	764966	-0.321757	0.8747	no
gi|320446398|ref|NW_003384173.1|	28191-34488	187047	116664	-0.681034	0.6186	no
gi|320446398|ref|NW_003384173.1|	3514-5496	367115	196025	241674	0.1774	no
gi|320446398|ref|NW_003384173.1|	38351-43115	176599	306355	0.794726	0.55235	no
gi|320446398|ref|NW_003384173.1|	43219-44069	0.54924	170055	16305	0.3326	no
gi|320446398|ref|NW_003384173.1|	44199-45483	209212	346183	0.726572	0.7198	no
gi|320446398|ref|NW_003384173.1|	52000-52584	385518	339277	-0.184333	0.93305	no
gi|320446398|ref|NW_003384173.1|	5602-6753	265414	676672	-197172	0.2458	no
gi|320446398|ref|NW_003384173.1|	60652-61251	0	344883	inf	0.0294	no
gi|320446398|ref|NW_003384173.1|	63391-64541	0.184517	779651	5401	0.1625	no
gi|320446398|ref|NW_003384173.1|	7081-9144	190193	51518	-188432	0.2785	no
gi|320446398|ref|NW_003384173.1|	72968-76984	544475	347086	-0.649574	0.6229	no
gi|320446398|ref|NW_003384173.1|	77193-77596	180973	50185	-185044	0.3909	no
gi|320446398|ref|NW_003384173.1|	77854-84010	427459	283613	-0.591862	0.7157	no
gi|320446398|ref|NW_003384173.1|	85767-87191	354932	295968	-0.262099	0.90055	no
gi|320446398|ref|NW_003384173.1|	9223-9957	0	32447	inf	0.0233	no
gi|320446408|ref|NW_003384163.1|	102754-10298	38.66	162189	-125317	0.55535	no
gi|320446408|ref|NW_003384163.1|	103521-10386	270091	12278	-113736	0.5903	no
gi|320446408|ref|NW_003384163.1|	10837-13034	928146	27252	-176799	0.20395	no
gi|320446408|ref|NW_003384163.1|	13920-18118	157969	115367	-0.453414	0.73385	no
gi|320446408|ref|NW_003384163.1|	1886-3119	465194	950211	-229151	0.2074	no
gi|320446408|ref|NW_003384163.1|	20191-22978	0.198382	143265	285234	0.24445	no
gi|320446408|ref|NW_003384163.1|	26022-26681	159158	216935	0.446808	0.8222	no

gi|320446408|ref|NW_003384163.1|	33671-34220	106819	540255	233847	0.2622	no
gi|320446408|ref|NW_003384163.1|	36661-37555	0.768798	141251	0.877579	1	no
gi|320446408|ref|NW_003384163.1|	39830-43426	112464	80098	-0.489625	0.70165	no
gi|320446408|ref|NW_003384163.1|	44273-47930	215493	172879	-0.317885	0.81075	no
gi|320446408|ref|NW_003384163.1|	58046-59147	77986	148438	-239336	0.2913	no
gi|320446408|ref|NW_003384163.1|	63423-64019	885803	315991	-14871	0.48225	no
gi|320446408|ref|NW_003384163.1|	66067-67106	88205	348467	-133984	0.52615	no
gi|320446408|ref|NW_003384163.1|	69220-72673	414266	60165	0.538368	0.8124	no
gi|320446408|ref|NW_003384163.1|	76104-76690	670897	356925	-0.910472	0.66745	no
gi|320446408|ref|NW_003384163.1|	78881-82543	204346	785609	-137913	0.292	no
gi|320446408|ref|NW_003384163.1|	83772-84883	982963	186794	-239569	0.28995	no
gi|320446408|ref|NW_003384163.1|	85144-86253	695445	10698	-270059	0.2447	no
gi|320446408|ref|NW_003384163.1|	88985-89200	103115	238036	-211499	0.3412	no
gi|320446408|ref|NW_003384163.1|	89683-90224	607831	664054	-31943	0.18245	no
gi|320446408|ref|NW_003384163.1|	93693-94891	208623	960012	-111978	0.6185	no
gi|320446408|ref|NW_003384163.1|	95269-96828	61195	345884	-0.823124	0.6862	no
gi|320446408|ref|NW_003384163.1|	97879-98805	635407	320203	-0.988695	0.64135	no
gi|320446409|ref|NW_003384162.1|	100417-10280[illegible]	919248	126173	0.456876	0.78085	no
gi|320446409|ref|NW_003384162.1|	102929-10515[illegible]	380981	458335	0.266685	0.84245	no
gi|320446409|ref|NW_003384162.1|	12036-12664	3431	0.874639	-197187	0.30275	no
gi|320446409|ref|NW_003384162.1|	14237-15125	106035	16034	-272534	0.23215	no
gi|320446409|ref|NW_003384162.1|	152769-15369[illegible]	0	303782	inf	0.0154	no
gi|320446409|ref|NW_003384162.1|	157632-15807[illegible]	0	774582	inf	0.02205	no
gi|320446409|ref|NW_003384162.1|	17794-18286	711786	0.433058	-403881	0.30915	no
gi|320446409|ref|NW_003384162.1|	22903-23632	259822	234041	-347269	0.16745	no
gi|320446409|ref|NW_003384162.1|	26759-29650	456197	755025	-259506	0.1828	no
gi|320446409|ref|NW_003384162.1|	3412-6636	598445	501463	-0.255077	0.90975	no
gi|320446409|ref|NW_003384162.1|	51077-51866	155495	627924	-130821	0.53725	no
gi|320446409|ref|NW_003384162.1|	72222-72441	505739	190377	-140954	0.5162	no
gi|320446409|ref|NW_003384162.1|	73737-73921	647596	266972	-12784	0.5576	no

gi|320446409|ref|NW_003384162.1|	74076-74298	769056	301842	-467123	0.2924	no
gi|320446409|ref|NW_003384162.1|	76879-77340	233547	923469	-133857	0.52045	no
gi|320446409|ref|NW_003384162.1|	80105-80702	46495	220617	-107553	0.5978	no
gi|320446409|ref|NW_003384162.1|	96096-96513	213131	117317	-0.861332	0.68035	no
gi|320446409|ref|NW_003384162.1|	97961-100068	180312	974851	-0.887242	0.5899	no
gi|320446410|ref|NW_003384161.1|	16657-17231	0.495772	368796	289507	0.25825	no
gi|320446410|ref|NW_003384161.1|	167003-16740(	285746	188194	271941	0.2536	no
gi|320446410|ref|NW_003384161.1|	46128-46735	0.452683	399291	314086	0.24575	no
gi|320446410|ref|NW_003384161.1|	47002-48160	10975	342139	164036	0.4407	no
gi|320446410|ref|NW_003384161.1|	49022-49879	0	560594	inf	0.00935	no
gi|320446410|ref|NW_003384161.1|	94799-96984	675079	205942	160911	0.356	no
gi|320446410|ref|NW_003384161.1|	99555-102131	273938	428047	0.643919	0.7631	no
gi|320446411|ref|NW_003384160.1|	104787-10558(	121082	166272	0.457563	0.8212	no
gi|320446411|ref|NW_003384160.1|	10612-12341	305036	212431	-0.521989	0.7987	no
gi|320446411|ref|NW_003384160.1|	108923-11086:	0.989899	151823	0.617033	0.761	no
gi|320446411|ref|NW_003384160.1|	111313-11249!	231029	234008	0.018489	0.96875	no
gi|320446411|ref|NW_003384160.1|	114656-11555(	205013	123594	-0.730104	0.7278	no
gi|320446411|ref|NW_003384160.1|	116011-11748	177256	218015	0.298598	0.88265	no
gi|320446411|ref|NW_003384160.1|	118571-11932!	647153	33274	-0.959714	0.64695	no
gi|320446411|ref|NW_003384160.1|	120270-12119	268437	302919	0.174349	0.9249	no
gi|320446411|ref|NW_003384160.1|	126371-12968	191885	157227	-0.287395	0.88805	no
gi|320446411|ref|NW_003384160.1|	12760-14078	280578	173128	-0.696563	0.7296	no
gi|320446411|ref|NW_003384160.1|	132098-13436!	0.9947	167787	0.754298	0.70755	no
gi|320446411|ref|NW_003384160.1|	135545-14188:	22951	1.88	-0.287825	0.89515	no
gi|320446411|ref|NW_003384160.1|	14181-15672	107516	102788	-0.0648805	1	no
gi|320446411|ref|NW_003384160.1|	142077-14656:	142932	202963	0.505885	0.8119	no
gi|320446411|ref|NW_003384160.1|	146628-14971	0.768667	198456	136839	0.51535	no
gi|320446411|ref|NW_003384160.1|	150046-15070(	322073	191985	-0.746392	0.70585	no
gi|320446411|ref|NW_003384160.1|	15773-16415	0.414319	253786	26148	0.2636	no
gi|320446411|ref|NW_003384160.1|	162675-16405(	0.736514	0.818552	0.152362	1	no

gi|320446411|ref|NW_003384160.1|	170663-17183	0.359212	149347	205576	0.3018	no
gi|320446411|ref|NW_003384160.1|	174759-17650	512824	232941	-11385	0.584	no
gi|320446411|ref|NW_003384160.1|	176658-17808	156301	33571	110289	0.5866	no
gi|320446411|ref|NW_003384160.1|	183275-18401	0.670217	275437	203902	0.3026	no
gi|320446411|ref|NW_003384160.1|	184084-18559	0.926742	202542	112798	0.58355	no
gi|320446411|ref|NW_003384160.1|	185864-18667	501112	222764	-116962	0.5797	no
gi|320446411|ref|NW_003384160.1|	188732-19001	11276	391221	179473	0.40815	no
gi|320446411|ref|NW_003384160.1|	190871-19174	0.531086	201111	192098	0.3194	no
gi|320446411|ref|NW_003384160.1|	191861-19671	237247	309346	0.382831	0.8593	no
gi|320446411|ref|NW_003384160.1|	196833-19831	135257	17867	0.401594	0.84435	no
gi|320446411|ref|NW_003384160.1|	199179-20013	2795	273283	-0.0324487	0.96625	no
gi|320446411|ref|NW_003384160.1|	203205-20382	219386	149019	-0.557981	0.8006	no
gi|320446411|ref|NW_003384160.1|	20531-20888	18594	0.807697	-452488	0.2941	no
gi|320446411|ref|NW_003384160.1|	205571-20692	135773	104761	-0.374092	1	no
gi|320446411|ref|NW_003384160.1|	209950-21181	0.31083	151633	228638	0.3232	no
gi|320446411|ref|NW_003384160.1|	211955-21514	131524	14397	0.130442	0.9481	no
gi|320446411|ref|NW_003384160.1|	216650-21801	209146	166004	-0.333291	0.86405	no
gi|320446411|ref|NW_003384160.1|	220895-22325	206489	266151	0.366181	0.85995	no
gi|320446411|ref|NW_003384160.1|	224321-22619	102978	265544	136662	0.50875	no
gi|320446411|ref|NW_003384160.1|	228797-22962	14265	235372	0.722461	0.7364	no
gi|320446411|ref|NW_003384160.1|	230435-23152	138395	0.684065	-101658	1	no
gi|320446411|ref|NW_003384160.1|	232522-23485	160653	336467	106652	0.6157	no
gi|320446411|ref|NW_003384160.1|	236178-23749	109316	314369	152396	0.471	no
gi|320446411|ref|NW_003384160.1|	23661-25229	0.75977	184999	128388	0.54245	no
gi|320446411|ref|NW_003384160.1|	237594-23917	150486	0.959768	-0.648868	0.75415	no
gi|320446411|ref|NW_003384160.1|	246104-24752	0.995484	128467	0.367926	1	no
gi|320446411|ref|NW_003384160.1|	251439-25261	179798	224256	0.318773	0.87525	no
gi|320446411|ref|NW_003384160.1|	25432-26169	8411	230419	-186801	0.39455	no
gi|320446411|ref|NW_003384160.1|	256248-25697	170248	116563	-0.546538	0.8008	no
gi|320446411|ref|NW_003384160.1|	259085-26005	0.691116	270349	196783	0.3902	no

gi|320446411|ref|NW_003384160.1|	260182-26330	0.52516	228503	212138	0.3346	no
gi|320446411|ref|NW_003384160.1|	26274-27494	205675	142607	-0.528316	0.79675	no
gi|320446411|ref|NW_003384160.1|	269695-27392	0.761047	165771	112313	0.5953	no
gi|320446411|ref|NW_003384160.1|	274152-27495	177795	122134	-0.541754	0.7976	no
gi|320446411|ref|NW_003384160.1|	275113-27672	183338	119084	-0.622528	0.7553	no
gi|320446411|ref|NW_003384160.1|	27642-29289	0.956323	0.998498	0.062261	1	no
gi|320446411|ref|NW_003384160.1|	277158-27883	105201	146483	0.477586	0.80835	no
gi|320446411|ref|NW_003384160.1|	279063-28041	0.45461	105226	12108	1	no
gi|320446411|ref|NW_003384160.1|	281638-28363	0.575372	160484	147987	0.48685	no
gi|320446411|ref|NW_003384160.1|	283911-28618	19009	242292	0.350062	0.86255	no
gi|320446411|ref|NW_003384160.1|	286524-28738	190375	299456	0.653502	0.7402	no
gi|320446411|ref|NW_003384160.1|	289467-29152	0.462901	206633	21583	0.34035	no
gi|320446411|ref|NW_003384160.1|	293686-29465	0.231003	175403	292469	0.25645	no
gi|320446411|ref|NW_003384160.1|	29407-30448	0.628471	101388	0.689965	1	no
gi|320446411|ref|NW_003384160.1|	296641-29778	0	128924	inf	1	no
gi|320446411|ref|NW_003384160.1|	297898-30018	197004	320869	0.70376	0.73445	no
gi|320446411|ref|NW_003384160.1|	300630-30207	0.139854	174959	364502	0.20565	no
gi|320446411|ref|NW_003384160.1|	302124-30335	0.850069	129683	0.609337	1	no
gi|320446411|ref|NW_003384160.1|	303504-30869	244939	271696	0.149571	0.94655	no
gi|320446411|ref|NW_003384160.1|	310182-31586	189101	249812	0.401689	0.8553	no
gi|320446411|ref|NW_003384160.1|	318695-31976	0.805938	334531	20534	0.357	no
gi|320446411|ref|NW_003384160.1|	320338-32302	130554	148838	0.189092	0.92625	no
gi|320446411|ref|NW_003384160.1|	32385-33501	210807	238819	0.179991	0.92445	no
gi|320446411|ref|NW_003384160.1|	325040-32614	0.582875	174829	158469	0.491	no
gi|320446411|ref|NW_003384160.1|	326247-32695	431095	122801	-181169	0.4036	no
gi|320446411|ref|NW_003384160.1|	330119-33472	682825	118335	0.79329	0.53565	no
gi|320446411|ref|NW_003384160.1|	335840-34193	18425	841372	219108	0.221	no
gi|320446411|ref|NW_003384160.1|	33847-36683	34387	185927	-0.887128	0.6658	no
gi|320446411|ref|NW_003384160.1|	342695-34622	39482	153905	196277	0.2809	no
gi|320446411|ref|NW_003384160.1|	350767-35142	481971	793555	0.719383	0.723	no

gi|320446411|ref|NW_003384160.1|	36851-38060	103999	20428	0.973975	0.65545	no
gi|320446411|ref|NW_003384160.1|	370547-37244[illegible]	193414	446999	120858	0.56785	no
gi|320446411|ref|NW_003384160.1|	372857-381947	356316	956868	142516	0.2854	no
gi|320446411|ref|NW_003384160.1|	38302-41070	5.13	349197	-0.554917	0.8032	no
gi|320446411|ref|NW_003384160.1|	42160-43046	181593	142955	-0.345152	0.8574	no
gi|320446411|ref|NW_003384160.1|	43178-46556	102012	180278	0.821495	0.6921	no
gi|320446411|ref|NW_003384160.1|	46755-47550	0.603245	248536	204264	0.30255	no
gi|320446411|ref|NW_003384160.1|	48449-50258	150125	194245	0.371719	0.8547	no
gi|320446411|ref|NW_003384160.1|	50700-51959	197836	0.686198	-152761	0.48775	no
gi|320446411|ref|NW_003384160.1|	53630-54909	274896	190741	-0.527268	0.788	no
gi|320446411|ref|NW_003384160.1|	57452-58269	116083	279223	126626	0.55445	no
gi|320446411|ref|NW_003384160.1|	58404-61210	262553	275248	0.0681205	0.9749	no
gi|320446411|ref|NW_003384160.1|	65961-71429	174119	158148	-0.138804	0.9481	no
gi|320446411|ref|NW_003384160.1|	680-1413	373047	232216	-0.683889	0.7368	no
gi|320446411|ref|NW_003384160.1|	71617-74326	115961	142993	0.302305	0.88075	no
gi|320446411|ref|NW_003384160.1|	74577-84004	374456	355344	-0.0755833	0.9525	no
gi|320446411|ref|NW_003384160.1|	85729-87135	273897	290455	0.084684	0.96275	no
gi|320446411|ref|NW_003384160.1|	9741-10552	263911	221628	-0.251916	0.9028	no
gi|320446411|ref|NW_003384160.1|	97618-100624	10962	208632	0.928459	0.65545	no
gi|320446412|ref|NW_003384159.1|	128873-13081[illegible]	110674	540786	228874	0.2224	no
gi|320446412|ref|NW_003384159.1|	131273-132499	112436	485508	21104	0.23815	no
gi|320446412|ref|NW_003384159.1|	132952-13318[illegible]	517872	107949	105969	0.6052	no
gi|320446413|ref|NW_003384158.1|	139269-13948[illegible]	303443	475942	0.649358	0.7477	no
gi|320446413|ref|NW_003384158.1|	21401-24251	761497	350476	220241	0.2368	no
gi|320446413|ref|NW_003384158.1|	24404-26940	755428	173667	120096	0.4897	no
gi|320446413|ref|NW_003384158.1|	27106-30670	75021	130165	0.79497	0.531	no
gi|320446413|ref|NW_003384158.1|	33172-33471	573839	110439	0.94453	0.6699	no
gi|320446413|ref|NW_003384158.1|	36195-36757	25674	208072	-0.303224	0.8817	no
gi|320446413|ref|NW_003384158.1|	43376-44009	156718	432089	-185878	0.39055	no
gi|320446413|ref|NW_003384158.1|	44196-44503	607134	183697	-172469	0.41295	no

gi|320446413|ref|NW_003384158.1|	46317-50115	395458	683798	0.790046	0.54545	no
gi|320446413|ref|NW_003384158.1|	50287-51718	224415	588507	-193104	0.26015	no
gi|320446413|ref|NW_003384158.1|	51926-53502	516078	70935	0.45891	0.829	no
gi|320446413|ref|NW_003384158.1|	77207-79746	0.878964	214884	128968	0.5351	no
gi|320446413|ref|NW_003384158.1|	80120-80829	0.712462	219238	162161	0.33265	no
gi|320446416|ref|NW_003384155.1|	101958-10366[illegible]	202362	165724	-0.288151	0.82115	no
gi|320446416|ref|NW_003384155.1|	105905-10708[illegible]	75255	401992	-0.904619	0.49025	no
gi|320446416|ref|NW_003384155.1|	107497-10926[illegible]	0.330735	122867	189334	1	no
gi|320446416|ref|NW_003384155.1|	109997-11466[illegible]	140584	284436	101668	0.4493	no
gi|320446416|ref|NW_003384155.1|	114770-11774[illegible]	31545	38809	0.29898	0.822	no
gi|320446416|ref|NW_003384155.1|	120217-12068[illegible]	463294	516003	0.155452	0.9435	no
gi|320446416|ref|NW_003384155.1|	127095-12748[illegible]	19076	263918	-28536	0.2479	no
gi|320446416|ref|NW_003384155.1|	129769-13127[illegible]	218962	276839	-298356	0.10935	no
gi|320446416|ref|NW_003384155.1|	164597-16529[illegible]	821978	926648	0.172922	0.9004	no
gi|320446416|ref|NW_003384155.1|	183638-18468[illegible]	257353	347191	0.431977	0.72895	no
gi|320446416|ref|NW_003384155.1|	185122-19336[illegible]	108313	207621	0.938751	0.4859	no
gi|320446416|ref|NW_003384155.1|	210860-21245[illegible]	0.742495	354775	557838	0.0723	no
gi|320446416|ref|NW_003384155.1|	212739-21375[illegible]	108379	720454	605476	0.0855	no
gi|320446416|ref|NW_003384155.1|	216401-21858[illegible]	0.864156	645223	290044	0.22305	no
gi|320446416|ref|NW_003384155.1|	222195-22287[illegible]	134209	118065	-350682	0.0755	no
gi|320446416|ref|NW_003384155.1|	224255-22552[illegible]	892308	793671	-349093	0.08205	no
gi|320446416|ref|NW_003384155.1|	226357-22894[illegible]	867827	140288	-262901	0.2679	no
gi|320446416|ref|NW_003384155.1|	229059-23014[illegible]	158723	0.549154	-153123	0.4987	no
gi|320446416|ref|NW_003384155.1|	230506-23139[illegible]	166313	272313	-261056	0.17235	no
gi|320446416|ref|NW_003384155.1|	241208-24223[illegible]	0	177496	inf	0.029	no
gi|320446416|ref|NW_003384155.1|	248259-24907[illegible]	0	457184	inf	5.00E-05	yes
gi|320446416|ref|NW_003384155.1|	249391-25006[illegible]	0	254969	inf	0.0054	no
gi|320446416|ref|NW_003384155.1|	313149-31400[illegible]	355271	300881	-0.239725	0.9013	no
gi|320446416|ref|NW_003384155.1|	316139-31655[illegible]	291638	180998	-0.688203	0.73575	no
gi|320446416|ref|NW_003384155.1|	379390-37975[illegible]	123722	0	#NAME?	0.00325	no

gi|320446416|ref|NW_003384155.1|	382805-38367[illegible]	743521	0.182829	-866774	0.2504	no
gi|320446416|ref|NW_003384155.1|	384869-38619[illegible]	678656	0.107323	-930458	0.25045	no
gi|320446416|ref|NW_003384155.1|	387322-38753[illegible]	906196	0	#NAME?	0.01065	no
gi|320446416|ref|NW_003384155.1|	392196-39253[illegible]	231.15	0	#NAME?	5.00E-05	yes
gi|320446416|ref|NW_003384155.1|	57480-58332	0.821186	131845	0.68306	1	no
gi|320446416|ref|NW_003384155.1|	58892-64499	128781	297125	120615	0.5822	no
gi|320446416|ref|NW_003384155.1|	64788-65950	577342	103719	0.84518	0.52225	no
gi|320446416|ref|NW_003384155.1|	66185-68994	171772	153044	-0.166552	0.89665	no
gi|320446416|ref|NW_003384155.1|	70143-70929	337208	126253	-473925	0.135	no
gi|320446416|ref|NW_003384155.1|	71673-73046	987937	443032	-447894	0.04325	no
gi|320446416|ref|NW_003384155.1|	74267-74621	442301	493672	-31634	0.20875	no
gi|320446419|ref|NW_003384152.1|	124585-12574[illegible]	109158	995751	-0.132564	0.95055	no
gi|320446419|ref|NW_003384152.1|	13051-13283	533481	206772	-13674	0.51205	no
gi|320446419|ref|NW_003384152.1|	147535-14784[illegible]	280987	214653	-0.388496	0.8447	no
gi|320446419|ref|NW_003384152.1|	157788-15803[illegible]	569326	140172	-202205	0.35415	no
gi|320446419|ref|NW_003384152.1|	158178-15844[illegible]	325181	120515	-143203	0.49835	no
gi|320446419|ref|NW_003384152.1|	160115-16035[illegible]	386071	886906	-212201	0.35425	no
gi|320446419|ref|NW_003384152.1|	207580-20782[illegible]	315877	886906	-183251	0.41545	no
gi|320446419|ref|NW_003384152.1|	210262-21061[illegible]	578392	188003	-162129	0.44475	no
gi|320446419|ref|NW_003384152.1|	39958-40207	291964	123163	-124522	0.55615	no
gi|320446419|ref|NW_003384152.1|	40658-41523	426239	206681	-104425	0.52975	no
gi|320446419|ref|NW_003384152.1|	42161-42433	347596	142279	-128869	0.533	no
gi|320446419|ref|NW_003384152.1|	42643-43265	104502	798774	-0.387668	0.84645	no
gi|320446419|ref|NW_003384152.1|	44436-44792	199608	276308	0.469109	0.81695	no
gi|320446419|ref|NW_003384152.1|	45093-45346	401072	293143	-0.452258	0.8188	no
gi|320446419|ref|NW_003384152.1|	4515-4854	223658	117859	-0.924236	0.65435	no
gi|320446419|ref|NW_003384152.1|	45805-47396	709533	666927	-0.0893407	0.9667	no
gi|320446419|ref|NW_003384152.1|	49617-50823	452058	843721	0.90026	0.6649	no
gi|320446419|ref|NW_003384152.1|	51561-53059	201812	488817	127628	0.3323	no
gi|320446419|ref|NW_003384152.1|	75529-76632	406537	378329	-0.103745	0.9352	no

gi|320446419|ref|NW_003384152.1|	77090-79962	424928	662925	0.641627	0.63255	no
gi|320446420|ref|NW_003384151.1|	11442-11987	270382	218696	-0.306075	0.8817	no
gi|320446420|ref|NW_003384151.1|	122711-12346	27504	425907	0.630895	0.688	no
gi|320446420|ref|NW_003384151.1|	12624-14747	169844	411603	127704	0.54555	no
gi|320446420|ref|NW_003384151.1|	144158-14459	319771	277133	-0.20646	0.91575	no
gi|320446420|ref|NW_003384151.1|	14944-15711	1.27	479072	191541	0.3976	no
gi|320446420|ref|NW_003384151.1|	152909-15341	207019	208331	0.00911893	0.98845	no
gi|320446420|ref|NW_003384151.1|	155816-15610	185071	210599	0.186425	0.92405	no
gi|320446420|ref|NW_003384151.1|	16042-16978	313167	531727	0.763753	0.70715	no
gi|320446420|ref|NW_003384151.1|	162531-16732	784138	156736	0.999153	0.4533	no
gi|320446420|ref|NW_003384151.1|	167769-17271	102352	407609	199365	0.14445	no
gi|320446420|ref|NW_003384151.1|	2141-2805	353973	241327	-0.55265	0.78665	no
gi|320446420|ref|NW_003384151.1|	224672-22492	162202	14369	-0.174834	0.91405	no
gi|320446420|ref|NW_003384151.1|	23354-24062	35698	0	#NAME?	0.0229	no
gi|320446420|ref|NW_003384151.1|	33595-34141	592991	0.726806	-302837	0.25725	no
gi|320446420|ref|NW_003384151.1|	36110-36487	98802	503157	-0.973532	0.6644	no
gi|320446420|ref|NW_003384151.1|	36733-37732	26522	0.610709	-211863	0.3545	no
gi|320446420|ref|NW_003384151.1|	38182-40449	313117	141449	-114642	0.3836	no
gi|320446420|ref|NW_003384151.1|	53596-55375	113689	338868	157563	0.369	no
gi|320446420|ref|NW_003384151.1|	5409-7357	364551	315975	-0.206311	0.9165	no
gi|320446420|ref|NW_003384151.1|	55483-58387	720795	110053	0.610533	0.71455	no
gi|320446420|ref|NW_003384151.1|	60087-60538	106532	111293	0.0630796	0.9681	no
gi|320446420|ref|NW_003384151.1|	63573-65670	706995	999021	0.498816	0.82435	no
gi|320446420|ref|NW_003384151.1|	65774-67258	129743	209536	0.691544	0.66895	no
gi|320446420|ref|NW_003384151.1|	69287-69790	26118	133391	-0.969381	0.64725	no
gi|320446420|ref|NW_003384151.1|	69991-72596	215044	785978	-145207	0.40835	no
gi|320446421|ref|NW_003384150.1|	145055-14692	288513	301611	0.0640521	0.97305	no
gi|320446421|ref|NW_003384150.1|	149146-15041	278905	500795	0.844445	0.6817	no
gi|320446421|ref|NW_003384150.1|	152467-15294	741752	540637	-0.456276	0.8224	no
gi|320446421|ref|NW_003384150.1|	19742-21618	386724	845847	-219284	0.22865	no

gi|320446421|ref|NW_003384150.1|	21725-22973	153989	409201	-191195	0.1583	no
gi|320446421|ref|NW_003384150.1|	232181-23403	0.313526	131089	206389	1	no
gi|320446421|ref|NW_003384150.1|	235277-23825	123043	326882	140961	0.5135	no
gi|320446421|ref|NW_003384150.1|	23734-26097	221821	797334	-147614	0.25715	no
gi|320446421|ref|NW_003384150.1|	238415-23981	0.72762	131426	0.852994	1	no
gi|320446421|ref|NW_003384150.1|	240793-24259	142452	759356	-0.907623	0.5744	no
gi|320446421|ref|NW_003384150.1|	255045-25846	645571	761096	0.237504	0.8581	no
gi|320446421|ref|NW_003384150.1|	258634-26030	124773	113095	-0.141765	0.91875	no
gi|320446421|ref|NW_003384150.1|	26190-28583	0.156408	169276	343599	0.19545	no
gi|320446421|ref|NW_003384150.1|	268022-27030	0.412029	103537	132933	1	no
gi|320446421|ref|NW_003384150.1|	270428-27473	103598	322065	163636	0.45195	no
gi|320446421|ref|NW_003384150.1|	290533-29085	773746	859912	347426	0.17685	no
gi|320446421|ref|NW_003384150.1|	295495-29573	142304	202221	0.506955	0.8049	no
gi|320446421|ref|NW_003384150.1|	303733-30417	0.794633	158197	431529	0.18315	no
gi|320446421|ref|NW_003384150.1|	306106-31040	166165	239379	384861	0.06605	no
gi|320446421|ref|NW_003384150.1|	36813-37752	311832	120792	195369	0.36395	no
gi|320446421|ref|NW_003384150.1|	3775-4478	155132	278675	0.845082	0.69645	no
gi|320446421|ref|NW_003384150.1|	40842-45241	162094	265313	403279	0.05735	no
gi|320446421|ref|NW_003384150.1|	49307-49644	567061	128654	118192	0.5757	no
gi|320446421|ref|NW_003384150.1|	52597-53499	0.506379	348967	27848	0.2345	no
gi|320446421|ref|NW_003384150.1|	55517-56189	154468	263432	0.770121	0.72055	no
gi|320446421|ref|NW_003384150.1|	6826-7335	115687	101306	313043	0.0972	no
gi|320446421|ref|NW_003384150.1|	75515-76149	802781	143686	-248209	0.2898	no
gi|320446422|ref|NW_003384149.1|	11420-12868	939345	127772	0.443842	0.7394	no
gi|320446422|ref|NW_003384149.1|	120586-12140	212923	0.988903	-442836	0.15705	no
gi|320446422|ref|NW_003384149.1|	125577-12586	287888	263249	-3451	0.2357	no
gi|320446422|ref|NW_003384149.1|	131656-13196	31611	112975	-480635	0.2876	no
gi|320446422|ref|NW_003384149.1|	135770-13613	209673	152174	-378435	0.22325	no
gi|320446422|ref|NW_003384149.1|	136418-13767	169976	137372	-362917	0.15985	no
gi|320446422|ref|NW_003384149.1|	139751-14087	245786	28802	-309316	0.20235	no

gi|320446422|ref|NW_003384149.1|	16877-17078	714866	713053	-0.00366432	0.9624	no
gi|320446422|ref|NW_003384149.1|	17381-17709	514464	332687	-0.628906	0.76195	no
gi|320446422|ref|NW_003384149.1|	183203-18664	48394	463679	-0.0617033	0.9784	no
gi|320446422|ref|NW_003384149.1|	186771-18809	0.465052	107615	121042	1	no
gi|320446422|ref|NW_003384149.1|	188515-19071	0.428784	143627	1744	0.4239	no
gi|320446422|ref|NW_003384149.1|	190971-19449	181404	192533	0.0858995	0.9473	no
gi|320446422|ref|NW_003384149.1|	219012-22033	494692	332701	-0.572303	0.77975	no
gi|320446422|ref|NW_003384149.1|	221194-22299	646267	315172	-103599	0.62685	no
gi|320446422|ref|NW_003384149.1|	223113-22359	606888	225265	-14298	0.51015	no
gi|320446422|ref|NW_003384149.1|	224711-22582	405676	133725	-160106	0.45015	no
gi|320446422|ref|NW_003384149.1|	226381-22660	336462	120737	-147858	0.5084	no
gi|320446422|ref|NW_003384149.1|	227094-22743	28353	119464	-124692	0.5591	no
gi|320446422|ref|NW_003384149.1|	229664-23072	370188	156455	-124251	0.56195	no
gi|320446422|ref|NW_003384149.1|	231133-23341	118664	94909	-0.32227	0.8438	no
gi|320446422|ref|NW_003384149.1|	235460-23742	31255	544785	0.801601	0.69855	no
gi|320446422|ref|NW_003384149.1|	246144-24635	128854	362176	149095	0.36015	no
gi|320446422|ref|NW_003384149.1|	252010-25293	855356	387614	-11419	0.58315	no
gi|320446422|ref|NW_003384149.1|	253635-25392	169706	469117	-185502	0.4063	no
gi|320446422|ref|NW_003384149.1|	258934-25925	329221	18042	245423	0.2592	no
gi|320446422|ref|NW_003384149.1|	266015-26653	477127	220252	220671	0.3212	no
gi|320446422|ref|NW_003384149.1|	28467-29642	413094	435596	-324541	0.09265	no
gi|320446422|ref|NW_003384149.1|	290484-29138	0.504109	156344	163292	0.3325	no
gi|320446422|ref|NW_003384149.1|	293-809	231808	223523	-0.0525054	0.9785	no
gi|320446422|ref|NW_003384149.1|	293523-29516	384162	108628	-182232	0.40225	no
gi|320446422|ref|NW_003384149.1|	296423-29904	233406	264857	35043	0.0787	no
gi|320446422|ref|NW_003384149.1|	299156-29976	0	617437	inf	0.0162	no
gi|320446422|ref|NW_003384149.1|	301893-30209	0	680359	inf	0.0233	no
gi|320446422|ref|NW_003384149.1|	302217-30270	0.654298	249484	525286	0.1682	no
gi|320446422|ref|NW_003384149.1|	30827-31189	432428	391822	-346419	0.19495	no
gi|320446422|ref|NW_003384149.1|	31641-32149	250512	204931	-361167	0.18475	no

gi|320446422|ref|NW_003384149.1|	324789-325439	0.406427	282327	611823	0.1552	no
gi|320446422|ref|NW_003384149.1|	33156-35282	193672	174344	-347361	0.0811	no
gi|320446422|ref|NW_003384149.1|	332098-333411	36018	0	#NAME?	0.0111	no
gi|320446422|ref|NW_003384149.1|	333652-335449	121023	0.0752793	-732881	0.25985	no
gi|320446422|ref|NW_003384149.1|	394414-397361	197253	600042	160502	0.44225	no
gi|320446422|ref|NW_003384149.1|	412584-414638	265362	315157	0.248111	0.84735	no
gi|320446422|ref|NW_003384149.1|	415172-415804	636923	779625	0.291661	0.88535	no
gi|320446422|ref|NW_003384149.1|	416375-417239	375902	499026	0.408761	0.8428	no
gi|320446422|ref|NW_003384149.1|	417369-418405	233987	151559	-0.626544	0.7776	no
gi|320446422|ref|NW_003384149.1|	418583-419958	259137	167649	-0.628277	0.69835	no
gi|320446422|ref|NW_003384149.1|	420503-421944	0.559881	0.875514	0.645009	1	no
gi|320446422|ref|NW_003384149.1|	422671-423870	0.696191	361963	237829	0.2984	no
gi|320446422|ref|NW_003384149.1|	423987-426567	333203	100043	-173577	0.18815	no
gi|320446422|ref|NW_003384149.1|	427619-428411	320292	601681	-241232	0.2912	no
gi|320446422|ref|NW_003384149.1|	429315-430191	974838	108857	0.159205	0.93715	no
gi|320446422|ref|NW_003384149.1|	430871-431695	180656	926476	-0.96342	0.63875	no
gi|320446422|ref|NW_003384149.1|	433958-434328	171596	897335	-0.9353	0.6585	no
gi|320446422|ref|NW_003384149.1|	434452-434918	951166	37.18	-135517	0.4107	no
gi|320446422|ref|NW_003384149.1|	436724-440727	217198	106898	-102278	0.43405	no
gi|320446422|ref|NW_003384149.1|	440875-441519	490659	137527	-1835	0.41805	no
gi|320446422|ref|NW_003384149.1|	444216-445192	763407	187234	-202761	0.2573	no
gi|320446422|ref|NW_003384149.1|	446011-446949	336297	41425	-302116	0.1046	no
gi|320446422|ref|NW_003384149.1|	452031-456451	58061	130158	116462	0.3681	no
gi|320446422|ref|NW_003384149.1|	456799-457270	472493	763568	0.692465	0.7571	no
gi|320446423|ref|NW_003384148.1|	108274-109049	187679	0.85844	-112848	0.58825	no
gi|320446423|ref|NW_003384148.1|	122061-124899	0.907637	194847	110216	0.59615	no
gi|320446423|ref|NW_003384148.1|	129107-132578	750916	117839	0.650094	0.60835	no
gi|320446423|ref|NW_003384148.1|	26806-30318	308966	55483	0.844595	0.70865	no
gi|320446423|ref|NW_003384148.1|	30422-31013	330838	352197	0.0902579	0.9468	no
gi|320446423|ref|NW_003384148.1|	87891-89817	20461	113411	-0.851311	0.61175	no

gi|320446423|ref|NW_003384148.1|	91029-91867	420136	539382	0.36045	0.8574	no
gi|320446423|ref|NW_003384148.1|	92713-93966	145936	167947	0.202678	0.9301	no
gi|320446427|ref|NW_003384144.1|	138132-13881	113786	385734	176128	0.432	no
gi|320446427|ref|NW_003384144.1|	172962-17427	0.785187	686708	312859	0.2096	no
gi|320446427|ref|NW_003384144.1|	174714-17582	0.576895	532497	320639	0.20725	no
gi|320446427|ref|NW_003384144.1|	176086-17894	0.579031	701523	359878	0.15345	no
gi|320446427|ref|NW_003384144.1|	179045-18628	402195	375353	322228	0.02225	no
gi|320446427|ref|NW_003384144.1|	188516-18929	217349	298296	0.456729	0.81755	no
gi|320446427|ref|NW_003384144.1|	190584-19104	360482	336261	-0.100346	0.9259	no
gi|320446427|ref|NW_003384144.1|	191582-19268	254042	595107	122808	0.55255	no
gi|320446427|ref|NW_003384144.1|	194638-19526	10371	234943	-214217	0.32875	no
gi|320446427|ref|NW_003384144.1|	195774-19835	749726	59423	-0.335341	0.876	no
gi|320446427|ref|NW_003384144.1|	199346-20076	157228	158908	0.0153311	0.96375	no
gi|320446427|ref|NW_003384144.1|	201926-20247	535748	469614	-0.19008	0.92055	no
gi|320446427|ref|NW_003384144.1|	202537-20388	425436	152443	-148067	0.391	no
gi|320446427|ref|NW_003384144.1|	209027-21105	143237	162326	0.180486	0.8838	no
gi|320446427|ref|NW_003384144.1|	219792-22036	991545	737592	-0.426856	0.8334	no
gi|320446427|ref|NW_003384144.1|	233349-23441	214341	127292	-0.751768	0.73315	no
gi|320446427|ref|NW_003384144.1|	243472-24544	281133	107473	193465	0.395	no
gi|320446427|ref|NW_003384144.1|	245580-24732	0.669785	33432	231946	0.3062	no
gi|320446427|ref|NW_003384144.1|	248345-24946	208909	381348	0.868233	0.6648	no
gi|320446427|ref|NW_003384144.1|	25387-26058	0	366976	inf	0.0037	no
gi|320446427|ref|NW_003384144.1|	262145-26239	332873	210517	-0.661034	0.75185	no
gi|320446427|ref|NW_003384144.1|	262878-26359	451719	927321	103764	0.60145	no
gi|320446427|ref|NW_003384144.1|	268056-26874	0.375934	28233	290883	0.25645	no
gi|320446427|ref|NW_003384144.1|	28042-28363	0	247147	inf	0.0138	no
gi|320446427|ref|NW_003384144.1|	282165-28382	755953	11594	0.617004	0.78265	no
gi|320446427|ref|NW_003384144.1|	284003-28444	632911	367571	-0.783979	0.70065	no
gi|320446427|ref|NW_003384144.1|	286094-28811	107849	435435	-130849	0.5667	no
gi|320446427|ref|NW_003384144.1|	289054-29029	993074	408563	-128134	0.5454	no

gi|320446427|ref|NW_003384144.1|	290867-29133	777287	235604	-172208	0.4274	no
gi|320446427|ref|NW_003384144.1|	292362-29308	554825	0.711905	-296228	0.20855	no
gi|320446427|ref|NW_003384144.1|	293191-29422	654294	14591	-216486	0.3373	no
gi|320446427|ref|NW_003384144.1|	31867-32101	0	351463	inf	0.0233	no
gi|320446427|ref|NW_003384144.1|	33002-33623	0	12456	inf	0.0069	no
gi|320446427|ref|NW_003384144.1|	34835-35505	0	185216	inf	0.00565	no
gi|320446427|ref|NW_003384144.1|	48810-49382	209429	0	#NAME?	0.0071	no
gi|320446427|ref|NW_003384144.1|	51825-53052	325054	0	#NAME?	5.00E-05	yes
gi|320446427|ref|NW_003384144.1|	54854-55275	217773	0	#NAME?	0.0104	no
gi|320446427|ref|NW_003384144.1|	55959-57106	212893	0	#NAME?	0.00355	no
gi|320446428|ref|NW_003384143.1|	101494-10306	0.379032	77351	435103	0.1602	no
gi|320446428|ref|NW_003384143.1|	109027-11047	527945	253968	226619	0.1997	no
gi|320446428|ref|NW_003384143.1|	11234-11467	323433	101884	-166655	0.44295	no
gi|320446428|ref|NW_003384143.1|	11969-12365	247005	149437	-0.725006	0.7222	no
gi|320446428|ref|NW_003384143.1|	119764-12101	0.665962	124719	42271	0.15375	no
gi|320446428|ref|NW_003384143.1|	12535-16940	16874	171993	0.0275518	0.9829	no
gi|320446428|ref|NW_003384143.1|	127578-12961	403407	54.96	376807	0.06615	no
gi|320446428|ref|NW_003384143.1|	140414-14103	0.432126	323047	290222	0.25825	no
gi|320446428|ref|NW_003384143.1|	151222-15368	665052	1072.36	401118	0.02625	no
gi|320446428|ref|NW_003384143.1|	154752-15711	276638	508048	419889	0.0419	no
gi|320446428|ref|NW_003384143.1|	23835-24443	243805	137865	-0.822474	0.6903	no
gi|320446428|ref|NW_003384143.1|	3028-4010	949413	43707	-111917	0.5946	no
gi|320446428|ref|NW_003384143.1|	36546-42072	685653	808905	0.238492	0.85045	no
gi|320446428|ref|NW_003384143.1|	50024-51895	567416	1066.48	423231	0.0243	no
gi|320446428|ref|NW_003384143.1|	6235-7611	650974	411051	-0.663284	0.7474	no
gi|320446428|ref|NW_003384143.1|	62732-63928	0.527036	133947	134569	1	no
gi|320446428|ref|NW_003384143.1|	68434-69687	812596	437123	-0.894498	0.6681	no
gi|320446428|ref|NW_003384143.1|	69904-74173	111685	109242	-0.0319103	0.98015	no
gi|320446428|ref|NW_003384143.1|	74911-76874	125997	898895	-0.48716	0.75465	no
gi|320446428|ref|NW_003384143.1|	77028-78717	391209	522127	0.416459	0.75565	no

gi|320446428|ref|NW_003384143.1|	81527-82995	0	152272	inf	0.0198	no
gi|320446428|ref|NW_003384143.1|	83337-86221	0.445936	614515	378454	0.14865	no
gi|320446428|ref|NW_003384143.1|	87097-88386	0.160168	466823	486522	0.1722	no
gi|320446428|ref|NW_003384143.1|	89124-90792	0.117788	278797	456495	0.1774	no
gi|320446428|ref|NW_003384143.1|	91472-92952	0.677917	282784	206052	0.353	no
gi|320446430|ref|NW_003384141.1|	141172-14236[illegible]	162096	853318	-0.925697	0.4868	no
gi|320446430|ref|NW_003384141.1|	143475-14369[illegible]	192264	573499	-174523	0.42185	no
gi|320446430|ref|NW_003384141.1|	144573-14548[illegible]	762418	328716	-121374	0.3474	no
gi|320446430|ref|NW_003384141.1|	146044-14641[illegible]	703175	382663	-0.877809	0.68375	no
gi|320446430|ref|NW_003384141.1|	152104-15336[illegible]	152429	171699	0.17174	0.93785	no
gi|320446430|ref|NW_003384141.1|	155068-15585[illegible]	339055	190393	-0.832535	0.6776	no
gi|320446430|ref|NW_003384141.1|	156809-15729[illegible]	430237	309657	-0.474461	0.82985	no
gi|320446430|ref|NW_003384141.1|	1619-2669	0.8287	587381	282537	0.23875	no
gi|320446430|ref|NW_003384141.1|	165238-17665[illegible]	130344	17559	0.429886	0.74345	no
gi|320446430|ref|NW_003384141.1|	178403-17893[illegible]	856489	34565	-130913	0.53085	no
gi|320446430|ref|NW_003384141.1|	180777-18178[illegible]	391673	120282	-170323	0.42865	no
gi|320446430|ref|NW_003384141.1|	195865-19641[illegible]	369309	142378	-13751	0.5274	no
gi|320446430|ref|NW_003384141.1|	224766-22548[illegible]	634556	192911	-171781	0.427	no
gi|320446430|ref|NW_003384141.1|	226113-22755[illegible]	111514	339086	160443	0.4397	no
gi|320446430|ref|NW_003384141.1|	239456-24430[illegible]	716787	124748	0.799396	0.54125	no
gi|320446430|ref|NW_003384141.1|	244902-24643[illegible]	242069	496941	103765	0.4209	no
gi|320446430|ref|NW_003384141.1|	249198-25024[illegible]	0.834851	0.865863	0.0526193	1	no
gi|320446430|ref|NW_003384141.1|	251572-25186[illegible]	733464	626153	-0.22821	0.9122	no
gi|320446430|ref|NW_003384141.1|	254106-25697[illegible]	204046	223354	0.130437	0.92225	no
gi|320446430|ref|NW_003384141.1|	258916-26147[illegible]	148736	805837	-0.884192	0.60325	no
gi|320446430|ref|NW_003384141.1|	262029-26382[illegible]	230797	108947	-108299	0.52415	no
gi|320446430|ref|NW_003384141.1|	264006-26436[illegible]	344889	208723	-0.724544	0.7256	no
gi|320446430|ref|NW_003384141.1|	275087-27571[illegible]	222457	0.872527	-467219	0.1459	no
gi|320446430|ref|NW_003384141.1|	275778-27720[illegible]	219007	424929	-236568	0.1749	no
gi|320446430|ref|NW_003384141.1|	277335-27912[illegible]	522349	212265	-129915	0.538	no

gi|320446430|ref|NW_003384141.1|	279257-28034	359826	264049	-0.446494	0.7294	no
gi|320446430|ref|NW_003384141.1|	281853-28222	319544	173981	-0.877086	0.5	no
gi|320446430|ref|NW_003384141.1|	292290-29278	386823	819914	10838	0.59775	no
gi|320446430|ref|NW_003384141.1|	292907-29545	152955	172983	0.17752	0.9299	no
gi|320446430|ref|NW_003384141.1|	296046-29680	0.325444	223075	277705	0.2657	no
gi|320446430|ref|NW_003384141.1|	297155-29756	461924	365535	-0.337645	0.8623	no
gi|320446430|ref|NW_003384141.1|	307906-30841	3628	20289	-0.838478	0.6954	no
gi|320446430|ref|NW_003384141.1|	521503-52226	224783	154128	-0.54441	0.7934	no
gi|320446430|ref|NW_003384141.1|	64389-66001	0.245161	102366	206193	1	no
gi|320446430|ref|NW_003384141.1|	66981-68300	0	118917	inf	1	no
gi|320446430|ref|NW_003384141.1|	74010-75067	0	170474	inf	0.029	no
gi|320446430|ref|NW_003384141.1|	75206-76123	0	221927	inf	0.02915	no
gi|320446430|ref|NW_003384141.1|	82125-87572	181296	182828	333407	0.09285	no
gi|320446432|ref|NW_003384139.1|	103022-10409	334065	129478	-136742	0.4049	no
gi|320446432|ref|NW_003384139.1|	109970-11089	0	236612	inf	0.0233	no
gi|320446432|ref|NW_003384139.1|	111409-11296	0.128168	178311	379829	0.2072	no
gi|320446432|ref|NW_003384139.1|	113582-11758	416372	106556	-196626	0.3752	no
gi|320446432|ref|NW_003384139.1|	120705-12144	70793	0.230866	-493848	0.28695	no
gi|320446432|ref|NW_003384139.1|	91702-92461	228864	119341	-0.939402	0.66675	no
gi|320446432|ref|NW_003384139.1|	94196-94794	602803	722996	0.262302	0.89835	no
gi|320446432|ref|NW_003384139.1|	96029-96898	138518	623541	-115152	0.58005	no
gi|320446432|ref|NW_003384139.1|	97074-98295	0.513665	0.949763	0.88674	1	no
gi|320446432|ref|NW_003384139.1|	98720-99975	248266	252581	0.024861	0.97445	no
gi|320446433|ref|NW_003384138.1|	104560-10732	179883	246766	0.456084	0.81975	no
gi|320446433|ref|NW_003384138.1|	107498-10829	15986	787032	-102231	0.62405	no
gi|320446433|ref|NW_003384138.1|	110690-11126	274473	198957	-0.46421	0.82295	no
gi|320446433|ref|NW_003384138.1|	124244-12478	108832	51337	22379	0.2757	no
gi|320446433|ref|NW_003384138.1|	126087-12635	529974	370582	28058	0.23565	no
gi|320446433|ref|NW_003384138.1|	126735-12732	0	357885	inf	0.0294	no
gi|320446433|ref|NW_003384138.1|	130313-13073	0	855931	inf	0.02205	no

gi|320446433|ref|NW_003384138.1|	130843-13156	0	405024	inf	0.02075	no
gi|320446433|ref|NW_003384138.1|	131838-13275	0.741758	869384	355097	0.1803	no
gi|320446433|ref|NW_003384138.1|	132945-13485	0.20201	401304	43122	0.1643	no
gi|320446433|ref|NW_003384138.1|	134959-13543	0.701039	467637	273782	0.2661	no
gi|320446433|ref|NW_003384138.1|	146913-14716	617034	175886	151122	0.359	no
gi|320446433|ref|NW_003384138.1|	147274-14798	0.35698	488191	377353	0.20725	no
gi|320446433|ref|NW_003384138.1|	153557-15387	509878	251751	230377	0.32575	no
gi|320446433|ref|NW_003384138.1|	154126-15485	0.344651	35384	335989	0.22635	no
gi|320446433|ref|NW_003384138.1|	155117-15583	105104	263654	132683	0.52445	no
gi|320446433|ref|NW_003384138.1|	160768-16102	124945	33628	142837	0.5137	no
gi|320446433|ref|NW_003384138.1|	161143-16157	165486	767427	221332	0.27865	no
gi|320446433|ref|NW_003384138.1|	165919-16608	129302	451404	-151825	0.50405	no
gi|320446433|ref|NW_003384138.1|	166465-16752	425197	160529	-14053	0.4137	no
gi|320446433|ref|NW_003384138.1|	23174-24422	0	161831	inf	0.0233	no
gi|320446433|ref|NW_003384138.1|	24596-26322	0.339612	252272	289302	0.236	no
gi|320446433|ref|NW_003384138.1|	26681-27829	0	192132	inf	0.02205	no
gi|320446433|ref|NW_003384138.1|	48589-49405	135761	246.73	0.861864	0.52485	no
gi|320446433|ref|NW_003384138.1|	49712-50249	116465	0.746901	-396284	0.2196	no
gi|320446433|ref|NW_003384138.1|	51527-52328	954949	0.819858	-354198	0.19805	no
gi|320446433|ref|NW_003384138.1|	53497-54670	100794	0.748307	-375164	0.1658	no
gi|320446433|ref|NW_003384138.1|	55201-60810	118371	143007	-304916	0.1089	no
gi|320446433|ref|NW_003384138.1|	61095-76861	65735	245494	190095	0.24235	no
gi|320446433|ref|NW_003384138.1|	78086-78412	82938	212431	135689	0.4025	no
gi|320446433|ref|NW_003384138.1|	80955-81478	505048	101063	100076	0.5456	no
gi|320446433|ref|NW_003384138.1|	81597-83559	304913	134912	-117638	0.3604	no
gi|320446433|ref|NW_003384138.1|	85916-86309	833321	289.65	179737	0.3137	no
gi|320446442|ref|NW_003384129.1|	101361-10465	118053	260836	114371	0.3944	no
gi|320446442|ref|NW_003384129.1|	115004-11684	966869	27096	-183524	0.396	no
gi|320446442|ref|NW_003384129.1|	118032-12067	102851	263971	-19621	0.246	no
gi|320446442|ref|NW_003384129.1|	124314-12630	674334	476919	-0.499718	0.82175	no

gi|320446442|ref|NW_003384129.1|	130125-13072	0.915027	248245	143988	0.37565	no
gi|320446442|ref|NW_003384129.1|	132270-13307	473289	304811	-0.634808	0.74815	no
gi|320446442|ref|NW_003384129.1|	133182-13432	186764	194019	0.0549866	0.96075	no
gi|320446442|ref|NW_003384129.1|	134921-13633	0.429168	109341	134922	1	no
gi|320446442|ref|NW_003384129.1|	137070-13796	102506	282501	146254	0.49755	no
gi|320446442|ref|NW_003384129.1|	139233-14084	14699	340973	121394	0.5607	no
gi|320446442|ref|NW_003384129.1|	141445-14820	915833	705478	-0.376483	0.77525	no
gi|320446442|ref|NW_003384129.1|	148535-15167	0.29085	150509	23715	0.29145	no
gi|320446442|ref|NW_003384129.1|	151814-15929	102196	189663	0.892097	0.5091	no
gi|320446442|ref|NW_003384129.1|	23910-24048	471688	322463	-0.548699	0.79425	no
gi|320446442|ref|NW_003384129.1|	24278-26497	210961	210151	-0.00554728	0.99605	no
gi|320446442|ref|NW_003384129.1|	30105-30809	154803	172275	0.154275	0.9432	no
gi|320446442|ref|NW_003384129.1|	37894-38452	623589	112256	0.848126	0.6784	no
gi|320446442|ref|NW_003384129.1|	39075-44578	752707	106828	0.505124	0.6931	no
gi|320446442|ref|NW_003384129.1|	44715-48763	708072	234205	17258	0.19395	no
gi|320446442|ref|NW_003384129.1|	58420-62699	25453	958258	191258	0.2735	no
gi|320446442|ref|NW_003384129.1|	62840-68709	14735	153212	0.056289	0.96415	no
gi|320446442|ref|NW_003384129.1|	84682-87344	382436	281781	-0.440645	0.82915	no
gi|320446442|ref|NW_003384129.1|	90310-93786	676864	528186	-0.357822	0.8751	no
gi|320446442|ref|NW_003384129.1|	94604-96245	900381	994366	0.143242	0.94775	no
gi|320446442|ref|NW_003384129.1|	97394-98005	447952	72972	0.703999	0.7301	no
gi|320446442|ref|NW_003384129.1|	98591-99679	108866	112316	0.0450107	0.9829	no
gi|320446444|ref|NW_003384127.1|	102174-10397	540627	738206	0.449391	0.8317	no
gi|320446444|ref|NW_003384127.1|	10941-12387	503206	81.09	0.688375	0.60545	no
gi|320446444|ref|NW_003384127.1|	121330-12247	240135	511799	109173	0.59435	no
gi|320446444|ref|NW_003384127.1|	122862-12391	0.83074	114889	0.467768	1	no
gi|320446444|ref|NW_003384127.1|	124446-12594	119905	176017	0.553817	0.78345	no
gi|320446444|ref|NW_003384127.1|	126189-12686	693534	946277	0.448296	0.82415	no
gi|320446444|ref|NW_003384127.1|	12746-13873	140066	27.13	0.953786	0.5638	no
gi|320446444|ref|NW_003384127.1|	134325-13602	403248	754093	0.903074	0.67365	no

gi|320446444|ref|NW_003384127.1|	1405-4145	57263	588469	0.039363	0.9848	no
gi|320446444|ref|NW_003384127.1|	143170-14359	170145	19703	353358	0.1851	no
gi|320446444|ref|NW_003384127.1|	146571-14749	0	978854	inf	0.00715	no
gi|320446444|ref|NW_003384127.1|	151311-15184	0	189046	inf	0.0074	no
gi|320446444|ref|NW_003384127.1|	161205-16174	221132	115415	238386	0.30675	no
gi|320446444|ref|NW_003384127.1|	182839-18599	335539	134084	-132334	0.3295	no
gi|320446444|ref|NW_003384127.1|	186582-18702	71517	369127	-0.954169	0.66495	no
gi|320446444|ref|NW_003384127.1|	251156-25302	992827	612632	-0.696523	0.75225	no
gi|320446444|ref|NW_003384127.1|	253171-25506	294194	418618	-281306	0.13455	no
gi|320446444|ref|NW_003384127.1|	255230-25592	324368	402891	-300917	0.19685	no
gi|320446444|ref|NW_003384127.1|	256231-25782	968796	120994	-300125	0.21225	no
gi|320446444|ref|NW_003384127.1|	258047-25828	519116	701989	-288654	0.21315	no
gi|320446444|ref|NW_003384127.1|	258617-25962	869262	0.300324	-48552	0.1942	no
gi|320446444|ref|NW_003384127.1|	260342-26136	132517	0.590915	-448708	0.1728	no
gi|320446444|ref|NW_003384127.1|	262382-26307	591041	100935	-587176	0.1295	no
gi|320446444|ref|NW_003384127.1|	32342-35680	456813	0.798846	-251562	0.26935	no
gi|320446444|ref|NW_003384127.1|	35973-36476	136809	166739	-30365	0.2282	no
gi|320446444|ref|NW_003384127.1|	37800-39099	11741	242254	-227696	0.3143	no
gi|320446444|ref|NW_003384127.1|	40502-42460	127342	327813	-195776	0.3969	no
gi|320446444|ref|NW_003384127.1|	4308-5329	988196	593876	-0.734636	0.72615	no
gi|320446444|ref|NW_003384127.1|	43460-44543	935801	247981	-191597	0.37585	no
gi|320446444|ref|NW_003384127.1|	46544-47354	126311	343099	-188028	0.38615	no
gi|320446444|ref|NW_003384127.1|	48830-53166	120147	812451	-0.564445	0.66495	no
gi|320446444|ref|NW_003384127.1|	54271-54834	220142	414957	-24074	0.29145	no
gi|320446444|ref|NW_003384127.1|	55424-60236	271706	730764	-189457	0.16085	no
gi|320446444|ref|NW_003384127.1|	6492-6906	495642	255801	-0.954277	0.6548	no
gi|320446444|ref|NW_003384127.1|	71557-72905	131955	84256	-0.647199	0.76435	no
gi|320446444|ref|NW_003384127.1|	73023-73853	817519	105785	0.371805	0.77635	no
gi|320446444|ref|NW_003384127.1|	76187-77593	446884	168264	-140918	0.42595	no
gi|320446444|ref|NW_003384127.1|	78434-79228	229644	157682	-0.542379	0.79945	no

gi|320446444|ref|NW_003384127.1|	79906-86919	228276	470679	104396	0.423	no
gi|320446444|ref|NW_003384127.1|	90196-90699	198995	875381	-118475	0.56855	no
gi|320446444|ref|NW_003384127.1|	91034-91825	273415	104286	-139055	0.52155	no
gi|320446444|ref|NW_003384127.1|	92412-93718	315249	404794	0.360698	0.8628	no
gi|320446444|ref|NW_003384127.1|	98484-98809	154749	189981	0.29592	0.8809	no
gi|320446444|ref|NW_003384127.1|	99106-10024	800426	438342	-0.868711	0.67415	no
gi|320446447|ref|NW_003384124.1|	36310-13752	0.687678	274155	199519	0.3909	no
gi|320446447|ref|NW_003384124.1|	84115-18572	184541	179787	-0.0376529	0.97295	no
gi|320446447|ref|NW_003384124.1|	08100-20933	252729	327172	0.372463	0.8507	no
gi|320446447|ref|NW_003384124.1|	10521-21105	283634	915955	169124	0.43725	no
gi|320446447|ref|NW_003384124.1|	11841-21578	306972	200523	-0.61434	0.6459	no
gi|320446447|ref|NW_003384124.1|	16549-21693	358661	131446	-144814	0.4937	no
gi|320446447|ref|NW_003384124.1|	18759-21904	934013	371894	-132855	0.528	no
gi|320446447|ref|NW_003384124.1|	20161-22134	100269	563558	-0.831246	0.5327	no
gi|320446447|ref|NW_003384124.1|	23866-22556	752087	278219	-143468	0.37415	no
gi|320446447|ref|NW_003384124.1|	27924-22861	258581	126169	-103526	0.59645	no
gi|320446447|ref|NW_003384124.1|	28735-22938	484252	0.824675	-255386	0.23925	no
gi|320446447|ref|NW_003384124.1|	29560-23051	210205	20955	-0.0045037	0.95935	no
gi|320446447|ref|NW_003384124.1|	31085-23272	147873	669426	-114336	0.47315	no
gi|320446447|ref|NW_003384124.1|	34655-23510	135243	599717	-11732	0.58025	no
gi|320446447|ref|NW_003384124.1|	36203-23640	515417	160967	-167897	0.44125	no
gi|320446447|ref|NW_003384124.1|	36558-23718	241381	878894	-145755	0.4955	no
gi|320446447|ref|NW_003384124.1|	37438-23798	23428	969583	-127279	0.5445	no
gi|320446447|ref|NW_003384124.1|	41540-24333	248211	631544	134732	0.53515	no
gi|320446447|ref|NW_003384124.1|	43472-24730	264982	219267	-0.273202	0.83405	no
gi|320446447|ref|NW_003384124.1|	49217-24971	156018	878373	-0.828809	0.6859	no
gi|320446447|ref|NW_003384124.1|	50299-25053	112815	189796	0.750497	0.70725	no
gi|320446447|ref|NW_003384124.1|	51942-25218	219401	923289	-124871	0.5611	no
gi|320446447|ref|NW_003384124.1|	52768-25302	374836	316499	-0.244059	0.89945	no
gi|320446447|ref|NW_003384124.1|	56299-25683	224708	264664	0.236115	0.8911	no

gi|320446447|ref|NW_003384124.1|	256934-257448	221428	16071	-0.462375	0.82125	no
gi|320446447|ref|NW_003384124.1|	257579-25985	248431	51443	105013	0.62105	no
gi|320446447|ref|NW_003384124.1|	261639-264247	462258	750211	0.698598	0.75185	no
gi|320446447|ref|NW_003384124.1|	271348-27192	132256	517748	-135301	0.4354	no
gi|320446447|ref|NW_003384124.1|	272487-27338	327523	172774	-0.922707	0.55745	no
gi|320446447|ref|NW_003384124.1|	274096-274925	0.568679	195497	178146	0.3324	no
gi|320446447|ref|NW_003384124.1|	275103-275785	20013	11084	-0.852452	0.6844	no
gi|320446447|ref|NW_003384124.1|	277578-278608	877176	73994	-0.245458	0.85415	no
gi|320446447|ref|NW_003384124.1|	283003-283325	145658	511079	-483289	0.1429	no
gi|320446447|ref|NW_003384124.1|	284787-286226	633718	136415	-553777	0.0499	no
gi|320446447|ref|NW_003384124.1|	290096-293577	135164	0.472819	-151535	1	no
gi|320446447|ref|NW_003384124.1|	293699-294849	184517	0.127812	-385166	0.3186	no
gi|320446447|ref|NW_003384124.1|	298995-299346	402393	235249	254751	0.2859	no
gi|320446447|ref|NW_003384124.1|	306891-307108	157105	394014	132652	0.52405	no
gi|320446447|ref|NW_003384124.1|	315825-318175	263322	646222	12952	0.5521	no
gi|320446447|ref|NW_003384124.1|	331026-33331	937594	648723	-0.531361	0.819	no
gi|320446447|ref|NW_003384124.1|	333550-333909	146877	135646	-0.114768	0.94815	no
gi|320446447|ref|NW_003384124.1|	334080-337426	210325	371795	0.821889	0.53775	no
gi|320446448|ref|NW_003384123.1|	103426-10413	610719	835044	0.451344	0.8229	no
gi|320446448|ref|NW_003384123.1|	202626-203578	143666	300659	106542	0.6346	no
gi|320446448|ref|NW_003384123.1|	23555-23834	41.57	265198	-0.648471	0.74705	no
gi|320446448|ref|NW_003384123.1|	238877-239588	0.709484	29113	203682	0.3026	no
gi|320446448|ref|NW_003384123.1|	240599-244494	696183	455656	271041	0.0479	no
gi|320446448|ref|NW_003384123.1|	267572-268234	43464	511754	0.23563	0.90175	no
gi|320446448|ref|NW_003384123.1|	27466-28085	114081	16094	0.496467	0.80815	no
gi|320446448|ref|NW_003384123.1|	31787-37400	190759	106055	247498	0.188	no
gi|320446448|ref|NW_003384123.1|	42269-43126	116756	541908	-110738	0.5907	no
gi|320446448|ref|NW_003384123.1|	43848-44120	538773	186543	-153017	0.3708	no
gi|320446448|ref|NW_003384123.1|	44907-45601	308954	183433	-0.752139	0.7273	no
gi|320446448|ref|NW_003384123.1|	45719-47454	585127	418474	-0.483612	0.7142	no

gi|320446448|ref|NW_003384123.1|	49628-50119	20783	108648	-0.935741	0.64685	no
gi|320446448|ref|NW_003384123.1|	53658-54542	728612	233004	-16448	0.43795	no
gi|320446448|ref|NW_003384123.1|	54648-55651	182487	106326	-0.779301	0.72015	no
gi|320446448|ref|NW_003384123.1|	55796-57860	120882	100405	-0.267761	0.86595	no
gi|320446448|ref|NW_003384123.1|	90794-91660	856478	33165	-136876	0.51105	no
gi|320446449|ref|NW_003384122.1|	01288-10205	789303	324852	-12808	0.53645	no
gi|320446449|ref|NW_003384122.1|	05094-10641	260429	247516	-0.0733725	0.95045	no
gi|320446449|ref|NW_003384122.1|	19453-22110	255274	19928	-367918	0.0686	no
gi|320446449|ref|NW_003384122.1|	22223-22352	424416	0.329734	-700803	0.1105	no
gi|320446449|ref|NW_003384122.1|	31423-31804	0	541425	inf	0.00515	no
gi|320446449|ref|NW_003384122.1|	50198-51366	452387	144184	167229	0.45145	no
gi|320446449|ref|NW_003384122.1|	54537-56630	118086	0.950467	-0.313133	1	no
gi|320446449|ref|NW_003384122.1|	56760-59476	353704	893577	133705	0.4104	no
gi|320446449|ref|NW_003384122.1|	68204-68452	394311	415732	-324561	0.24925	no
gi|320446449|ref|NW_003384122.1|	78028-78313	28296	0	#NAME?	0.01755	no
gi|320446449|ref|NW_003384122.1|	84155-88537	156655	13676	-351787	0.0839	no
gi|320446449|ref|NW_003384122.1|	94628-94946	179651	210651	-309227	0.25455	no
gi|320446449|ref|NW_003384122.1|	99414-10037	340528	130388	-138496	0.40145	no
gi|320446450|ref|NW_003384121.1|	20055-22245	444938	12427	148181	0.3712	no
gi|320446450|ref|NW_003384121.1|	22813-22425	322735	321814	-0.00412181	0.9828	no
gi|320446453|ref|NW_003384118.1|	04600-10704	0.305454	591932	427641	0.1518	no
gi|320446453|ref|NW_003384118.1|	17331-11824	0	255701	inf	0.02205	no
gi|320446453|ref|NW_003384118.1|	25343-12634	215467	188349	-0.194058	0.9327	no
gi|320446453|ref|NW_003384118.1|	26461-12682	856784	877707	0.034808	0.9489	no
gi|320446453|ref|NW_003384118.1|	27042-12735	103649	667148	-0.635623	0.7537	no
gi|320446453|ref|NW_003384118.1|	27549-12925	388189	234426	-0.727625	0.57815	no
gi|320446453|ref|NW_003384118.1|	29459-12994	597861	43542	-0.457402	0.8372	no
gi|320446453|ref|NW_003384118.1|	30397-13174	157457	111441	-0.498673	0.8207	no
gi|320446453|ref|NW_003384118.1|	32094-13268	100077	419567	-125413	0.55695	no
gi|320446453|ref|NW_003384118.1|	33530-13508	153801	151564	-0.0211368	0.96625	no

gi|320446453|ref|NW_003384118.1|	135437-13665[illegible]	103257	381818	-143528	0.5053	no
gi|320446453|ref|NW_003384118.1|	137142-13741[illegible]	192471	459947	-20651	0.28725	no
gi|320446453|ref|NW_003384118.1|	140733-14099[illegible]	332658	158425	-107024	0.59325	no
gi|320446453|ref|NW_003384118.1|	141274-14173[illegible]	493027	17146	-15238	0.4762	no
gi|320446453|ref|NW_003384118.1|	143289-14723[illegible]	217748	136825	-0.670331	0.6145	no
gi|320446453|ref|NW_003384118.1|	168715-17150[illegible]	165318	402067	128219	0.54465	no
gi|320446453|ref|NW_003384118.1|	172641-17476[illegible]	205166	267602	0.383296	0.84975	no
gi|320446453|ref|NW_003384118.1|	176995-17754[illegible]	973376	473841	-103859	0.61425	no
gi|320446453|ref|NW_003384118.1|	177653-17880[illegible]	110469	573915	23772	0.28205	no
gi|320446453|ref|NW_003384118.1|	19683-21480	0.324168	158087	22859	0.3232	no
gi|320446453|ref|NW_003384118.1|	24287-25275	246673	0.929233	-140849	0.5136	no
gi|320446453|ref|NW_003384118.1|	32365-33535	0	262737	inf	0.01575	no
gi|320446453|ref|NW_003384118.1|	62993-65428	14575	258735	0.827976	0.5209	no
gi|320446453|ref|NW_003384118.1|	65807-66981	197777	573099	153491	0.4634	no
gi|320446453|ref|NW_003384118.1|	67422-69173	100203	120971	0.271738	0.9011	no
gi|320446453|ref|NW_003384118.1|	69572-77781	209481	147196	-0.509077	0.6992	no
gi|320446453|ref|NW_003384118.1|	80428-85062	103951	521162	-0.996104	0.4385	no
gi|320446453|ref|NW_003384118.1|	85282-87071	543063	923099	0.765366	0.72825	no
gi|320446453|ref|NW_003384118.1|	88186-89866	350428	128492	187448	0.40995	no
gi|320446453|ref|NW_003384118.1|	90868-92714	136199	0.365034	-189961	1	no
gi|320446454|ref|NW_003384117.1|	101743-10202[illegible]	0	195354	inf	0.02915	no
gi|320446454|ref|NW_003384117.1|	105131-10534[illegible]	514582	106505	437137	0.1793	no
gi|320446454|ref|NW_003384117.1|	107562-10887[illegible]	219037	104258	225091	0.3124	no
gi|320446454|ref|NW_003384117.1|	111072-11127[illegible]	579844	140846	128039	0.538	no
gi|320446454|ref|NW_003384117.1|	114237-11469[illegible]	223524	941957	207523	0.35195	no
gi|320446454|ref|NW_003384117.1|	51710-51919	0	835267	inf	0.0186	no
gi|320446454|ref|NW_003384117.1|	52231-53945	0.228245	731119	500145	0.15005	no
gi|320446454|ref|NW_003384117.1|	60895-63980	15987	234754	0.554248	0.6732	no
gi|320446454|ref|NW_003384117.1|	65812-66868	672508	179212	-190788	0.28335	no
gi|320446454|ref|NW_003384117.1|	67999-69034	223726	671184	-173695	0.43775	no

gi|320446454|ref|NW_003384117.1|	94099-97530	0.105599	646407	593577	0.1183	no
gi|320446455|ref|NW_003384116.1|	50864-52779	243122	602256	-201323	0.2483	no
gi|320446455|ref|NW_003384116.1|	52985-53835	247158	132265	-0.901998	0.6711	no
gi|320446455|ref|NW_003384116.1|	53956-57536	110984	127779	0.2033	0.87375	no
gi|320446455|ref|NW_003384116.1|	81212-85214	640568	391957	-0.708657	0.65355	no
gi|320446455|ref|NW_003384116.1|	85804-89776	299827	219672	-0.448779	0.73815	no
gi|320446457|ref|NW_003384114.1|	102336-105378	0.240474	452.71	108785	0.103	no
gi|320446457|ref|NW_003384114.1|	108000-108658	0.398825	477522	102256	0.14075	no
gi|320446457|ref|NW_003384114.1|	110757-111297	0	251628	inf	0.00505	no
gi|320446457|ref|NW_003384114.1|	116055-116280	0	166813	inf	0.00715	no
gi|320446457|ref|NW_003384114.1|	122907-123134	531837	139368	-193209	0.3906	no
gi|320446457|ref|NW_003384114.1|	12498-13688	109606	637133	-0.782655	0.7186	no
gi|320446457|ref|NW_003384114.1|	14723-15348	604976	176207	-17796	0.4202	no
gi|320446457|ref|NW_003384114.1|	16129-16927	171008	721117	-124576	0.5597	no
gi|320446457|ref|NW_003384114.1|	162032-162710	563901	333663	-0.757051	0.7105	no
gi|320446457|ref|NW_003384114.1|	163892-164112	497144	12.48	-199405	0.3966	no
gi|320446457|ref|NW_003384114.1|	166417-168700	201894	173137	-0.221686	0.8622	no
gi|320446457|ref|NW_003384114.1|	171432-175104	216026	194459	-0.15174	0.9071	no
gi|320446457|ref|NW_003384114.1|	175789-177220	381082	31387	-0.27993	0.89195	no
gi|320446457|ref|NW_003384114.1|	181265-181928	220759	940595	-123083	0.56145	no
gi|320446457|ref|NW_003384114.1|	182913-183244	592032	670525	0.179614	0.90555	no
gi|320446457|ref|NW_003384114.1|	184148-184838	601042	182452	-171995	0.31045	no
gi|320446457|ref|NW_003384114.1|	205889-206285	116587	487294	-125854	0.57395	no
gi|320446457|ref|NW_003384114.1|	207020-208509	144033	907837	-0.66589	0.77125	no
gi|320446457|ref|NW_003384114.1|	215290-215519	730245	378441	-0.948313	0.6443	no
gi|320446457|ref|NW_003384114.1|	21619-21858	926994	140398	-272304	0.25245	no
gi|320446457|ref|NW_003384114.1|	216447-222582	144685	17415	0.267418	0.84235	no
gi|320446457|ref|NW_003384114.1|	22485-22969	733344	356506	-104057	0.59835	no
gi|320446457|ref|NW_003384114.1|	24666-25110	86266	468639	-0.880314	0.6621	no
gi|320446457|ref|NW_003384114.1|	255026-257304	415516	216227	-0.942357	0.477	no

gi|320446457|ref|NW_003384114.1|	257648-25881	45371	11773	-194628	0.26695	no
gi|320446457|ref|NW_003384114.1|	259107-25929	155283	481169	-169028	0.42485	no
gi|320446457|ref|NW_003384114.1|	259702-26027	114189	337136	-176002	0.3072	no
gi|320446457|ref|NW_003384114.1|	262516-26377	335993	13368	-132965	0.4313	no
gi|320446457|ref|NW_003384114.1|	264232-26498	326701	0.671746	-228198	0.26535	no
gi|320446457|ref|NW_003384114.1|	265252-26604	324476	499692	-2699	0.25135	no
gi|320446457|ref|NW_003384114.1|	27749-30217	200278	775879	-13681	0.4295	no
gi|320446457|ref|NW_003384114.1|	278922-27985	7961	633977	299341	0.1128	no
gi|320446457|ref|NW_003384114.1|	285063-28536	153024	100622	271712	0.2502	no
gi|320446457|ref|NW_003384114.1|	286313-28662	337189	260659	295054	0.21785	no
gi|320446457|ref|NW_003384114.1|	302794-30310	720308	474567	271993	0.2551	no
gi|320446457|ref|NW_003384114.1|	304131-30437	136689	79919	254765	0.27945	no
gi|320446457|ref|NW_003384114.1|	30661-31360	487578	236233	-104542	0.52085	no
gi|320446457|ref|NW_003384114.1|	307246-30767	164732	120069	286567	0.22955	no
gi|320446457|ref|NW_003384114.1|	318650-31948	338922	580703	409877	0.05945	no
gi|320446457|ref|NW_003384114.1|	323041-32899	0.177054	663838	522857	0.07235	no
gi|320446457|ref|NW_003384114.1|	4914-6070	22181	261599	0.238034	0.88105	no
gi|320446457|ref|NW_003384114.1|	56624-57313	204504	17776	-352412	0.17115	no
gi|320446457|ref|NW_003384114.1|	6636-7682	270657	417484	0.625254	0.75345	no
gi|320446457|ref|NW_003384114.1|	70364-70977	160424	363029	-214373	0.3423	no
gi|320446457|ref|NW_003384114.1|	7963-9480	934846	933979	-0.00133838	0.9943	no
gi|320446457|ref|NW_003384114.1|	93310-102104	0.157707	177438	101359	0.0578	no
gi|320446457|ref|NW_003384114.1|	9608-11197	723048	685252	-0.0774574	0.96845	no
gi|320446458|ref|NW_003384113.1|	127061-13118	112437	340081	159677	0.2398	no
gi|320446458|ref|NW_003384113.1|	181581-18190	659889	487028	-0.43822	0.83705	no
gi|320446458|ref|NW_003384113.1|	193040-19364	633057	120087	0.923671	0.4723	no
gi|320446458|ref|NW_003384113.1|	197529-20295	409241	147285	184759	0.28565	no
gi|320446458|ref|NW_003384113.1|	208581-21019	0	188076	inf	0.0186	no
gi|320446458|ref|NW_003384113.1|	210319-21140	0	219662	inf	0.0198	no
gi|320446458|ref|NW_003384113.1|	212057-21334	0.481419	122493	134733	1	no

gi|320446458|ref|NW_003384113.1|	216070-21660	0.567268	381648	275014	0.26605	no
gi|320446458|ref|NW_003384113.1|	217456-21874	0.160473	189307	356033	0.21625	no
gi|320446458|ref|NW_003384113.1|	218957-22011	0.183914	152879	305529	0.2493	no
gi|320446458|ref|NW_003384113.1|	220525-22165	0.188854	143851	292923	0.2564	no
gi|320446458|ref|NW_003384113.1|	267380-26809	437246	206637	-108135	0.502	no
gi|320446458|ref|NW_003384113.1|	297591-29839	200297	135511	-0.56373	0.79425	no
gi|320446458|ref|NW_003384113.1|	298506-30005	135295	12996	-0.0580478	0.97945	no
gi|320446458|ref|NW_003384113.1|	300268-30111	41126	622544	0.598123	0.762	no
gi|320446458|ref|NW_003384113.1|	304802-30592	168836	357282	108144	0.5132	no
gi|320446458|ref|NW_003384113.1|	314126-31640	123327	924368	-0.415947	0.79695	no
gi|320446458|ref|NW_003384113.1|	316701-31787	21171	93244	-118301	0.59725	no
gi|320446458|ref|NW_003384113.1|	325393-32864	247865	161229	-0.620449	0.6418	no
gi|320446458|ref|NW_003384113.1|	329648-33090	414646	141541	-155066	0.36475	no
gi|320446458|ref|NW_003384113.1|	332439-33494	135486	348357	-195951	0.2527	no
gi|320446458|ref|NW_003384113.1|	335094-33637	0.810848	11252	0.472681	1	no
gi|320446458|ref|NW_003384113.1|	336497-33928	128136	106169	-0.27131	0.8319	no
gi|320446458|ref|NW_003384113.1|	36420-36878	0	339363	inf	0.00495	no
gi|320446458|ref|NW_003384113.1|	37768-38165	0	349074	inf	0.0071	no
gi|320446458|ref|NW_003384113.1|	51376-52347	0.458864	221748	227278	0.2738	no
gi|320446458|ref|NW_003384113.1|	55542-56950	0.14391	139983	328201	1	no
gi|320446459|ref|NW_003384112.1|	63093-63479	344248	143736	-126002	0.54865	no
gi|320446462|ref|NW_003384109.1|	10901-11184	221941	127572	252306	0.2746	no
gi|320446462|ref|NW_003384109.1|	154061-15441	166434	749821	-115033	0.56885	no
gi|320446462|ref|NW_003384109.1|	175408-17627	484073	573851	0.245454	0.90305	no
gi|320446462|ref|NW_003384109.1|	180559-18116	162537	548396	175445	0.2946	no
gi|320446462|ref|NW_003384109.1|	183099-18357	226853	546127	126748	0.561	no
gi|320446462|ref|NW_003384109.1|	184532-18526	780008	150941	0.95242	0.65325	no
gi|320446462|ref|NW_003384109.1|	204112-20438	270239	359877	0.413268	0.84485	no
gi|320446462|ref|NW_003384109.1|	216221-21644	192264	301842	0.650703	0.7584	no
gi|320446462|ref|NW_003384109.1|	218651-21986	125629	572726	-113325	0.6029	no

gi|320446462|ref|NW_003384109.1|	220021-22635	118763	803313	-0.564049	0.67155	no
gi|320446462|ref|NW_003384109.1|	234613-23525	260389	295391	0.181954	0.93145	no
gi|320446462|ref|NW_003384109.1|	236941-24100	132209	157646	0.25387	0.8458	no
gi|320446462|ref|NW_003384109.1|	242023-24227	678737	527657	-0.363253	0.85455	no
gi|320446462|ref|NW_003384109.1|	268773-26934	467794	28558	-0.711981	0.74465	no
gi|320446462|ref|NW_003384109.1|	287571-28854	185336	837236	-114644	0.6004	no
gi|320446462|ref|NW_003384109.1|	306721-31209	357495	237594	-0.589427	0.6609	no
gi|320446462|ref|NW_003384109.1|	323938-32601	270882	183181	-0.564391	0.66185	no
gi|320446462|ref|NW_003384109.1|	327949-32976	459928	767575	0.7389	0.7262	no
gi|320446462|ref|NW_003384109.1|	329892-33543	374965	545509	0.540846	0.7477	no
gi|320446462|ref|NW_003384109.1|	335625-33689	119071	339495	-181035	0.4056	no
gi|320446462|ref|NW_003384109.1|	338876-34120	273609	21.58	-0.342424	0.796	no
gi|320446462|ref|NW_003384109.1|	343825-34602	452795	298624	-0.60053	0.65995	no
gi|320446462|ref|NW_003384109.1|	355244-35678	175911	160142	-0.135494	0.9167	no
gi|320446462|ref|NW_003384109.1|	357711-35897	385404	137107	-149107	0.3811	no
gi|320446462|ref|NW_003384109.1|	38283-39418	0.750378	701533	322482	0.20275	no
gi|320446462|ref|NW_003384109.1|	80487-80900	543373	107587	0.985484	0.65125	no
gi|320446463|ref|NW_003384108.1|	104072-10956	109988	137955	0.32685	0.8089	no
gi|320446463|ref|NW_003384108.1|	111677-11239	100768	26393	138912	0.5234	no
gi|320446463|ref|NW_003384108.1|	11176-14000	443237	0.774472	-251679	0.27355	no
gi|320446463|ref|NW_003384108.1|	132226-13248	235121	279557	0.24974	0.8963	no
gi|320446463|ref|NW_003384108.1|	150988-15324	0.583971	675435	353185	0.162	no
gi|320446463|ref|NW_003384108.1|	157967-15934	0.294863	901186	493371	0.15025	no
gi|320446463|ref|NW_003384108.1|	17021-17967	403761	0.655468	-262291	0.2687	no
gi|320446463|ref|NW_003384108.1|	171057-17182	0.945397	302643	167863	0.42935	no
gi|320446463|ref|NW_003384108.1|	19083-21822	667212	0.565154	-356143	0.16665	no
gi|320446463|ref|NW_003384108.1|	22391-22764	134852	0.735098	-41973	0.30575	no
gi|320446463|ref|NW_003384108.1|	22885-23498	40106	0.605049	-272869	0.28405	no
gi|320446463|ref|NW_003384108.1|	23899-24187	148051	405801	-186725	0.3124	no
gi|320446463|ref|NW_003384108.1|	33549-34678	105758	392321	-143067	0.50175	no

gi|320446463|ref|NW_003384108.1|	34783-35247	360482	336261	-0.100346	0.9259	no
gi|320446463|ref|NW_003384108.1|	35826-37996	149146	858108	-0.797492	0.6314	no
gi|320446463|ref|NW_003384108.1|	49453-52087	251134	501301	-232471	0.2024	no
gi|320446463|ref|NW_003384108.1|	4969-6295	587986	10742	-245251	0.2838	no
gi|320446463|ref|NW_003384108.1|	52573-52982	30487	24369	-364507	0.1944	no
gi|320446463|ref|NW_003384108.1|	55359-55761	651045	630425	-336836	0.1758	no
gi|320446463|ref|NW_003384108.1|	55887-58916	129252	278712	-221334	0.2146	no
gi|320446463|ref|NW_003384108.1|	64946-67277	118362	320402	-188524	0.2664	no
gi|320446463|ref|NW_003384108.1|	68515-69541	115272	486898	-124335	0.5502	no
gi|320446463|ref|NW_003384108.1|	69663-71436	406049	22169	-0.873111	0.6704	no
gi|320446463|ref|NW_003384108.1|	78683-80045	404426	135233	-158043	0.47335	no
gi|320446463|ref|NW_003384108.1|	86244-86840	261079	243313	-0.101671	0.96235	no
gi|320446463|ref|NW_003384108.1|	87057-88664	167317	182355	0.124167	0.9181	no
gi|320446463|ref|NW_003384108.1|	89201-90079	196987	179066	-0.137606	0.94635	no
gi|320446463|ref|NW_003384108.1|	91364-93718	172827	216301	0.323708	0.80175	no
gi|320446463|ref|NW_003384108.1|	94320-95187	120251	183964	0.613368	0.7827	no
gi|320446464|ref|NW_003384107.1|	101651-10281[illegible]	564593	52995	-0.0913542	0.9629	no
gi|320446464|ref|NW_003384107.1|	102959-10323[illegible]	129918	889231	-0.546973	0.7903	no
gi|320446464|ref|NW_003384107.1|	104173-10573[illegible]	355427	389937	0.133685	0.92285	no
gi|320446464|ref|NW_003384107.1|	106045-10801[illegible]	35972	671086	0.899625	0.6752	no
gi|320446464|ref|NW_003384107.1|	108647-10983[illegible]	247238	342717	0.471122	0.81255	no
gi|320446464|ref|NW_003384107.1|	14220-16001	0.655049	129289	0.980924	1	no
gi|320446464|ref|NW_003384107.1|	16670-18674	772799	286082	-143367	0.5132	no
gi|320446464|ref|NW_003384107.1|	29594-30687	275498	667302	12763	0.5399	no
gi|320446464|ref|NW_003384107.1|	30906-32134	391032	108462	147183	0.5024	no
gi|320446464|ref|NW_003384107.1|	33973-35078	931527	76569	-0.282837	0.8945	no
gi|320446464|ref|NW_003384107.1|	35178-35925	112161	101719	-0.140974	0.9435	no
gi|320446464|ref|NW_003384107.1|	36697-37243	118598	872167	-0.443405	0.82615	no
gi|320446464|ref|NW_003384107.1|	37733-40602	794444	35826	-114894	0.4744	no
gi|320446464|ref|NW_003384107.1|	40990-41416	170145	281472	-259571	0.27695	no

gi|320446464|ref|NW_003384107.1|	49902-50332	150311	497706	-159458	0.4366	no
gi|320446464|ref|NW_003384107.1|	50982-53628	394074	22838	-0.787029	0.5556	no
gi|320446464|ref|NW_003384107.1|	57594-68903	41442	419014	0.0159057	0.98925	no
gi|320446464|ref|NW_003384107.1|	69121-70542	143755	385722	-189798	0.3925	no
gi|320446464|ref|NW_003384107.1|	73167-73606	44094	585006	-291406	0.22005	no
gi|320446464|ref|NW_003384107.1|	76106-76847	163556	457298	-183858	0.39955	no
gi|320446464|ref|NW_003384107.1|	84718-86055	352332	361623	0.0375538	0.97915	no
gi|320446464|ref|NW_003384107.1|	86173-87860	546355	32374	-0.755005	0.71365	no
gi|320446464|ref|NW_003384107.1|	90178-91286	464162	307915	-0.592093	0.7681	no
gi|320446464|ref|NW_003384107.1|	91435-92604	162685	150294	-0.11429	0.949	no
gi|320446464|ref|NW_003384107.1|	92810-93493	124333	257215	-227316	0.31195	no
gi|320446464|ref|NW_003384107.1|	9369-14083	165762	133247	-0.315013	0.8098	no
gi|320446464|ref|NW_003384107.1|	94215-94860	65812	308016	-109535	0.59825	no
gi|320446464|ref|NW_003384107.1|	94966-95791	10592	275513	-194278	0.37225	no
gi|320446464|ref|NW_003384107.1|	96305-96973	10522	186036	-249976	0.2746	no
gi|320446464|ref|NW_003384107.1|	97102-98199	0.783482	135561	0.79097	1	no
gi|320446464|ref|NW_003384107.1|	98614-99362	0	270737	inf	0.029	no
gi|320446464|ref|NW_003384107.1|	99695-101508	449232	491844	0.130741	0.94885	no
gi|320446472|ref|NW_003384099.1|	102669-10353[illegible]	0.799145	128376	0.683848	1	no
gi|320446472|ref|NW_003384099.1|	111482-11343[illegible]	934919	432247	-111298	0.62255	no
gi|320446472|ref|NW_003384099.1|	113632-11438[illegible]	155456	61.36	-134114	0.30815	no
gi|320446472|ref|NW_003384099.1|	114548-11512[illegible]	90184	36807	-129289	0.4418	no
gi|320446472|ref|NW_003384099.1|	117877-12089[illegible]	176286	113441	-0.635977	0.6105	no
gi|320446472|ref|NW_003384099.1|	12463-14955	844932	449115	-0.911751	0.6854	no
gi|320446472|ref|NW_003384099.1|	135902-13609[illegible]	122979	351942	-180501	0.4147	no
gi|320446472|ref|NW_003384099.1|	138265-14255[illegible]	351803	827875	-208729	0.12805	no
gi|320446472|ref|NW_003384099.1|	145156-14580[illegible]	382954	416145	-320201	0.18965	no
gi|320446472|ref|NW_003384099.1|	146500-14986[illegible]	535898	214212	-132292	0.33115	no
gi|320446472|ref|NW_003384099.1|	150848-15188[illegible]	673715	494874	-0.445077	0.82875	no
gi|320446472|ref|NW_003384099.1|	152190-15353[illegible]	426987	307065	-0.475646	0.80945	no

gi|320446472|ref|NW_003384099.1|	154031-15485	143622	493628	-154078	0.4731	no
gi|320446472|ref|NW_003384099.1|	16404-19057	150054	123858	-0.276803	0.82485	no
gi|320446472|ref|NW_003384099.1|	164518-16785	204824	358375	0.807084	0.55185	no
gi|320446472|ref|NW_003384099.1|	182775-18434	0.253828	309021	360578	0.18175	no
gi|320446472|ref|NW_003384099.1|	27404-27793	102605	606314	256296	0.27405	no
gi|320446472|ref|NW_003384099.1|	282332-28285	0	277587	inf	0.00565	no
gi|320446472|ref|NW_003384099.1|	41673-42855	48135	277982	252983	0.1529	no
gi|320446472|ref|NW_003384099.1|	48557-48848	822536	381711	221433	0.3288	no
gi|320446472|ref|NW_003384099.1|	51238-51817	195496	142196	286267	0.2405	no
gi|320446472|ref|NW_003384099.1|	52579-54118	388338	22599	254088	0.15605	no
gi|320446472|ref|NW_003384099.1|	54328-54827	138778	70575	234638	0.3031	no
gi|320446472|ref|NW_003384099.1|	5620-6707	198171	863917	-119778	0.5928	no
gi|320446472|ref|NW_003384099.1|	59690-61620	497871	621954	364296	0.07625	no
gi|320446472|ref|NW_003384099.1|	64595-70893	221803	31385	0.500795	0.7059	no
gi|320446472|ref|NW_003384099.1|	7040-7775	118224	370103	-167552	0.42655	no
gi|320446472|ref|NW_003384099.1|	75857-80152	623426	395765	-0.655573	0.68445	no
gi|320446472|ref|NW_003384099.1|	80965-85329	181041	108739	-0.735448	0.57385	no
gi|320446472|ref|NW_003384099.1|	8219-9285	629867	224872	-148594	0.47655	no
gi|320446472|ref|NW_003384099.1|	9970-11277	13858	590236	-123136	0.5709	no
gi|320446473|ref|NW_003384098.1|	164462-16808	122099	21307	0.803271	0.54	no
gi|320446473|ref|NW_003384098.1|	168259-16866	76199	752775	-0.0175539	0.95455	no
gi|320446473|ref|NW_003384098.1|	168879-16959	460202	266328	-0.789065	0.70115	no
gi|320446473|ref|NW_003384098.1|	173434-17411	415363	231991	-0.840303	0.6772	no
gi|320446473|ref|NW_003384098.1|	175934-17616	310238	250862	-0.306481	0.8712	no
gi|320446473|ref|NW_003384098.1|	176426-17680	98233	857789	-0.195585	0.91735	no
gi|320446473|ref|NW_003384098.1|	177381-17833	678266	435812	-0.638147	0.75615	no
gi|320446473|ref|NW_003384098.1|	180507-18118	262002	525709	-231724	0.3123	no
gi|320446473|ref|NW_003384098.1|	204853-20503	110953	0	#NAME?	0.0229	no
gi|320446473|ref|NW_003384098.1|	209771-21074	0	539974	inf	0.0101	no
gi|320446473|ref|NW_003384098.1|	213879-21567	238334	211319	-0.173562	0.9261	no

gi|320446473|ref|NW_003384098.1|	19772-22235	552489	706751	0.355257	0.87785	no
gi|320446473|ref|NW_003384098.1|	36767-23839	908787	127343	0.486708	0.82445	no
gi|320446473|ref|NW_003384098.1|	39850-24625	137216	14101	0.0393549	0.9753	no
gi|320446473|ref|NW_003384098.1|	49402-25003	169846	317625	0.903095	0.6877	no
gi|320446473|ref|NW_003384098.1|	51196-25159	38697	382043	-0.0184875	0.9157	no
gi|320446473|ref|NW_003384098.1|	52698-25343	169228	162235	-0.0608837	0.9269	no
gi|320446473|ref|NW_003384098.1|	54349-25479	233233	671304	152519	0.49185	no
gi|320446473|ref|NW_003384098.1|	55822-25648	43565	755859	0.794949	0.6935	no
gi|320446473|ref|NW_003384098.1|	57839-25884	0.437449	211568	227393	0.2733	no
gi|320446473|ref|NW_003384098.1|	63116-26351	0	853328	inf	0.02915	no
gi|320446473|ref|NW_003384098.1|	63627-26550	0.102727	429572	538601	0.16615	no
gi|320446473|ref|NW_003384098.1|	87800-28872	0	23695	inf	0.0233	no
gi|320446473|ref|NW_003384098.1|	90934-29163	0.362312	10896	491042	0.1712	no
gi|320446475|ref|NW_003384096.1|	11004-14014	16.48	170918	0.0525865	0.9686	no
gi|320446475|ref|NW_003384096.1|	13462-11419	0	106819	inf	0.00695	no
gi|320446475|ref|NW_003384096.1|	14704-15003	114768	736259	-0.640433	0.7525	no
gi|320446475|ref|NW_003384096.1|	59475-16042	308325	0.818204	-191392	0.3914	no
gi|320446475|ref|NW_003384096.1|	64892-16603	0.926639	218221	123571	0.562	no
gi|320446475|ref|NW_003384096.1|	78337-17870	230172	601677	-193565	0.38575	no
gi|320446475|ref|NW_003384096.1|	79215-17999	523994	359632	-0.543028	0.7816	no
gi|320446475|ref|NW_003384096.1|	80736-18107	217553	529669	-20382	0.35075	no
gi|320446475|ref|NW_003384096.1|	81410-18190	510148	427024	-0.256599	0.8975	no
gi|320446475|ref|NW_003384096.1|	82148-18284	293606	225676	-0.379629	0.84275	no
gi|320446475|ref|NW_003384096.1|	83343-18370	468783	535728	0.192581	0.9054	no
gi|320446475|ref|NW_003384096.1|	83810-18564	865549	588404	-0.556807	0.79355	no
gi|320446475|ref|NW_003384096.1|	85979-18797	852499	217078	134844	0.43055	no
gi|320446475|ref|NW_003384096.1|	91530-19184	497761	26736	242526	0.2902	no
gi|320446475|ref|NW_003384096.1|	93061-19346	937967	38325	203068	0.35	no
gi|320446475|ref|NW_003384096.1|	93602-19379	163507	264356	401506	0.17245	no
gi|320446475|ref|NW_003384096.1|	22320-22973	242126	192424	-0.331467	0.85735	no

gi|320446475|ref|NW_003384096.1|	23171-24342	468973	224966	-10598	0.60115	no
gi|320446475|ref|NW_003384096.1|	240671-241648	113788	769943	275841	0.2507	no
gi|320446475|ref|NW_003384096.1|	257468-258377	200446	0.863517	-453685	0.14905	no
gi|320446475|ref|NW_003384096.1|	259204-26196[illegible]	117536	0.700044	-406951	0.06735	no
gi|320446475|ref|NW_003384096.1|	263420-26500[illegible]	787125	938862	0.25432	0.9056	no
gi|320446475|ref|NW_003384096.1|	265867-26709[illegible]	194403	107604	-0.853318	0.70315	no
gi|320446475|ref|NW_003384096.1|	273617-27447[illegible]	263805	617628	-209466	0.34515	no
gi|320446475|ref|NW_003384096.1|	277177-27829[illegible]	120872	3055	-198424	0.36815	no
gi|320446475|ref|NW_003384096.1|	278633-28124[illegible]	231353	140804	-0.716403	0.58605	no
gi|320446475|ref|NW_003384096.1|	301812-30217[illegible]	593206	10067	0.76303	0.7116	no
gi|320446475|ref|NW_003384096.1|	302506-30958[illegible]	751827	21429	-181084	0.3045	no
gi|320446475|ref|NW_003384096.1|	312005-313088	20508	551069	-189588	0.4061	no
gi|320446475|ref|NW_003384096.1|	41821-42309	525391	263555	-0.995288	0.66985	no
gi|320446475|ref|NW_003384096.1|	44020-44939	641916	272356	-123689	0.54215	no
gi|320446475|ref|NW_003384096.1|	4449-6307	161189	558027	-153035	0.3648	no
gi|320446475|ref|NW_003384096.1|	48832-49482	0	442866	inf	0.0198	no
gi|320446475|ref|NW_003384096.1|	71456-73393	0.297493	51157	4104	0.1634	no
gi|320446475|ref|NW_003384096.1|	8853-10878	169718	79564	-109295	0.51155	no
gi|320446476|ref|NW_003384095.1|	23831-24947	24147	822598	-155359	0.34215	no
gi|320446476|ref|NW_003384095.1|	25743-28417	927169	304574	-160604	0.33165	no
gi|320446476|ref|NW_003384095.1|	29873-30809	149357	0.830824	-416808	0.16615	no
gi|320446476|ref|NW_003384095.1|	38136-39464	494239	139394	-182604	0.40155	no
gi|320446476|ref|NW_003384095.1|	39650-40867	429799	0.238396	-417223	0.20885	no
gi|320446476|ref|NW_003384095.1|	41983-42659	803666	0	#NAME?	0.01425	no
gi|320446476|ref|NW_003384095.1|	44866-45201	431382	0.931613	-553309	0.27565	no
gi|320446476|ref|NW_003384095.1|	47730-48368	158984	0	#NAME?	0.00735	no
gi|320446476|ref|NW_003384095.1|	6164-8346	0.780138	0.665345	-0.229626	1	no
gi|320446476|ref|NW_003384095.1|	81791-82303	0.602582	485324	300972	0.25005	no
gi|320446476|ref|NW_003384095.1|	90729-91640	0.999262	396063	19868	0.3909	no
gi|320446477|ref|NW_003384094.1|	104456-10627[illegible]	680232	740561	0.122593	0.95485	no

gi|320446477|ref|NW_003384094.1|	106380-10722[illegible]	362913	249616	-0.539915	0.78985	no
gi|320446477|ref|NW_003384094.1|	107682-10797[illegible]	792935	355682	-115661	0.5813	no
gi|320446477|ref|NW_003384094.1|	108179-10856[illegible]	15735	825748	-0.930206	0.65965	no
gi|320446477|ref|NW_003384094.1|	110061-11121[illegible]	104262	291452	-183889	0.40115	no
gi|320446477|ref|NW_003384094.1|	111457-11282[illegible]	0.893963	455315	234858	0.2924	no
gi|320446477|ref|NW_003384094.1|	115979-12120[illegible]	4533	53087	0.227893	0.8578	no
gi|320446477|ref|NW_003384094.1|	11662-16281	121011	243976	101159	0.6401	no
gi|320446477|ref|NW_003384094.1|	126854-12847[illegible]	0	144498	inf	0.02075	no
gi|320446477|ref|NW_003384094.1|	130477-13115[illegible]	0	286005	inf	0.0294	no
gi|320446477|ref|NW_003384094.1|	135848-13646[illegible]	362581	0	#NAME?	0.0042	no
gi|320446477|ref|NW_003384094.1|	137931-13884[illegible]	138665	0	#NAME?	0.00605	no
gi|320446477|ref|NW_003384094.1|	139866-14163[illegible]	295593	0.153087	-759312	0.16165	no
gi|320446477|ref|NW_003384094.1|	158065-15852[illegible]	952686	0	#NAME?	0.01755	no
gi|320446477|ref|NW_003384094.1|	159593-16124[illegible]	534523	0	#NAME?	0.0062	no
gi|320446477|ref|NW_003384094.1|	18744-22513	656548	28939	-118188	0.3814	no
gi|320446477|ref|NW_003384094.1|	23439-25401	127047	115833	-0.13331	1	no
gi|320446477|ref|NW_003384094.1|	25537-27619	109652	165722	0.595834	0.7667	no
gi|320446477|ref|NW_003384094.1|	27771-29562	0.650838	0.906808	0.4785	1	no
gi|320446477|ref|NW_003384094.1|	29691-30823	112933	104274	-0.115096	1	no
gi|320446477|ref|NW_003384094.1|	30958-31158	876589	910432	-326728	0.2478	no
gi|320446477|ref|NW_003384094.1|	31446-31938	265302	952727	-14775	0.48805	no
gi|320446477|ref|NW_003384094.1|	32062-32931	927008	273258	-176232	0.32385	no
gi|320446477|ref|NW_003384094.1|	37859-40239	291109	412006	0.501104	0.8143	no
gi|320446477|ref|NW_003384094.1|	42220-43131	199852	774906	195509	0.36345	no
gi|320446477|ref|NW_003384094.1|	43348-48384	759156	168403	114945	0.38025	no
gi|320446477|ref|NW_003384094.1|	53160-54220	225128	198173	-0.183983	0.92235	no
gi|320446477|ref|NW_003384094.1|	54720-56613	132336	141905	0.100713	0.95495	no
gi|320446477|ref|NW_003384094.1|	56878-59048	231132	236132	0.0308734	0.98095	no
gi|320446477|ref|NW_003384094.1|	61138-63925	112416	111377	330853	0.08085	no
gi|320446477|ref|NW_003384094.1|	9906-11414	294371	0.553247	-573357	0.0974	no

gi|320446478|ref|NW_003384093.1|	102359-104687	878905	968206	0.139606	0.94935	no
gi|320446478|ref|NW_003384093.1|	105533-106749	203072	139602	-0.54067	0.8138	no
gi|320446478|ref|NW_003384093.1|	111035-112429	151476	596981	-134333	0.54465	no
gi|320446478|ref|NW_003384093.1|	113415-114014	346834	156765	-114564	0.59555	no
gi|320446478|ref|NW_003384093.1|	117715-119211	211502	134961	-0.648126	0.6837	no
gi|320446478|ref|NW_003384093.1|	119397-124019	14193	630333	-117099	0.3682	no
gi|320446478|ref|NW_003384093.1|	130049-130547	113955	339254	-174803	0.41865	no
gi|320446478|ref|NW_003384093.1|	130776-132206	396939	844226	-223322	0.2126	no
gi|320446478|ref|NW_003384093.1|	135994-137649	221092	301186	0.446007	0.7246	no
gi|320446478|ref|NW_003384093.1|	140023-140558	396294	432121	0.124865	0.95655	no
gi|320446478|ref|NW_003384093.1|	146695-147806	427878	226821	-0.915646	0.5931	no
gi|320446478|ref|NW_003384093.1|	147967-150145	338756	163646	-104967	0.6117	no
gi|320446478|ref|NW_003384093.1|	150261-150497	889953	390367	-11889	0.56445	no
gi|320446478|ref|NW_003384093.1|	153187-153665	204639	637749	-168202	0.4277	no
gi|320446478|ref|NW_003384093.1|	153778-154295	177708	795693	-115923	0.5818	no
gi|320446478|ref|NW_003384093.1|	174423-177221	106625	225397	-2242	0.2031	no
gi|320446478|ref|NW_003384093.1|	180778-181578	212255	184788	-0.199929	0.92855	no
gi|320446478|ref|NW_003384093.1|	187254-188398	105869	122212	0.207101	0.9238	no
gi|320446478|ref|NW_003384093.1|	73939-76638	0.547916	105285	0.942279	1	no
gi|320446478|ref|NW_003384093.1|	76865-79810	0.871792	24378	148352	0.4853	no
gi|320446478|ref|NW_003384093.1|	79928-80714	401584	744895	0.891334	0.4781	no
gi|320446478|ref|NW_003384093.1|	81623-88386	721137	200379	147438	0.33475	no
gi|320446478|ref|NW_003384093.1|	96646-98795	687781	892072	0.375212	0.86775	no
gi|320446479|ref|NW_003384092.1|	37690-38100	919229	0	#NAME?	0.0229	no
gi|320446479|ref|NW_003384092.1|	45366-46190	200729	195151	328127	0.1836	no
gi|320446480|ref|NW_003384091.1|	185524-186587	0	169256	inf	0.029	no
gi|320446480|ref|NW_003384091.1|	21909-28549	301634	232491	-0.375625	0.7801	no
gi|320446480|ref|NW_003384091.1|	220848-222657	193018	395962	103663	0.61895	no
gi|320446480|ref|NW_003384091.1|	29817-30632	0.582421	360213	262872	0.23645	no
gi|320446480|ref|NW_003384091.1|	307771-308159	464267	954907	104041	0.6398	no

gi|320446480|ref|NW_003384091.1|	30820-33064	0.504036	199344	198366	0.3675	no
gi|320446480|ref|NW_003384091.1|	316538-31818	149638	574944	-137998	0.39475	no
gi|320446480|ref|NW_003384091.1|	319183-31983	190115	688805	-146471	0.48595	no
gi|320446480|ref|NW_003384091.1|	33195-33939	13273	204605	0.624347	0.75255	no
gi|320446480|ref|NW_003384091.1|	35472-36376	126216	208765	0.725988	0.73635	no
gi|320446480|ref|NW_003384091.1|	38035-38478	488357	418318	-354527	0.1628	no
gi|320446480|ref|NW_003384091.1|	39013-40224	242168	419726	-252849	0.1513	no
gi|320446480|ref|NW_003384091.1|	40526-45250	648038	183235	-182238	0.1768	no
gi|320446480|ref|NW_003384091.1|	46056-46822	104972	174528	-258848	0.25155	no
gi|320446480|ref|NW_003384091.1|	46972-47592	301979	475683	-266638	0.2448	no
gi|320446480|ref|NW_003384091.1|	47896-49079	0.890448	0.987351	0.149033	1	no
gi|320446480|ref|NW_003384091.1|	49389-49964	103814	46808	-114917	0.5851	no
gi|320446480|ref|NW_003384091.1|	58053-58551	427333	415587	-336214	0.09585	no
gi|320446481|ref|NW_003384090.1|	56549-57182	46592	316865	276571	0.23355	no
gi|320446483|ref|NW_003384088.1|	135277-13986	72876	149635	435986	0.0448	no
gi|320446483|ref|NW_003384088.1|	146199-14684	619975	450119	-0.461902	0.81485	no
gi|320446483|ref|NW_003384088.1|	152666-15382	60428	837236	0.470418	0.8212	no
gi|320446483|ref|NW_003384088.1|	168769-16907	306131	104173	-155516	0.4618	no
gi|320446483|ref|NW_003384088.1|	178680-17929	177326	270913	0.611421	0.75315	no
gi|320446483|ref|NW_003384088.1|	186227-18649	211989	673785	-165363	0.44385	no
gi|320446483|ref|NW_003384088.1|	186745-18833	0.994369	0.864875	-0.201289	1	no
gi|320446483|ref|NW_003384088.1|	189215-19145	0.505799	211803	206609	0.34155	no
gi|320446483|ref|NW_003384088.1|	191997-19515	156144	113238	-0.463524	0.8162	no
gi|320446483|ref|NW_003384088.1|	196151-19790	326252	172362	-0.920544	0.472	no
gi|320446483|ref|NW_003384088.1|	199739-20065	218865	120018	-418872	0.1411	no
gi|320446483|ref|NW_003384088.1|	204821-20757	47134	646587	-286585	0.14495	no
gi|320446483|ref|NW_003384088.1|	208976-21262	593332	522878	-350429	0.09525	no
gi|320446483|ref|NW_003384088.1|	213017-21326	623332	65649	-324716	0.1957	no
gi|320446483|ref|NW_003384088.1|	215128-21561	133335	0.891264	-390306	0.2253	no
gi|320446483|ref|NW_003384088.1|	218547-21899	225459	154916	-38633	0.16685	no

gi|320446483|ref|NW_003384088.1|	221047-22272	195207	113931	-409876	0.06425	no
gi|320446483|ref|NW_003384088.1|	228934-22937	448957	478641	-322956	0.1822	no
gi|320446483|ref|NW_003384088.1|	236659-24123	209126	264325	0.337942	0.804	no
gi|320446483|ref|NW_003384088.1|	257358-25780	245253	273053	0.15491	0.94025	no
gi|320446483|ref|NW_003384088.1|	289314-28970	402365	948135	-208534	0.33965	no
gi|320446483|ref|NW_003384088.1|	289831-29211	84095	431614	-0.962278	0.6614	no
gi|320446483|ref|NW_003384088.1|	292316-29316	11886	11.6	-0.0351485	0.9849	no
gi|320446483|ref|NW_003384088.1|	296454-30164	308526	388869	0.333892	0.79985	no
gi|320446483|ref|NW_003384088.1|	301758-30218	186287	128898	-0.531294	0.7937	no
gi|320446483|ref|NW_003384088.1|	306226-30664	364979	236838	-0.623915	0.63325	no
gi|320446483|ref|NW_003384088.1|	310960-31124	435324	13909	167586	0.34255	no
gi|320446483|ref|NW_003384088.1|	52568-53188	345744	392439	0.182762	0.93155	no
gi|320446483|ref|NW_003384088.1|	56922-57412	5215	872273	0.742114	0.7096	no
gi|320446483|ref|NW_003384088.1|	59173-61829	185419	236695	0.352245	0.7891	no
gi|320446483|ref|NW_003384088.1|	63029-66079	353691	909564	136269	0.40805	no
gi|320446483|ref|NW_003384088.1|	76830-77363	112354	423463	523611	0.14575	no
gi|320446483|ref|NW_003384088.1|	79335-79764	0	116646	inf	0.01575	no
gi|320446483|ref|NW_003384088.1|	82692-82918	0	339736	inf	0.029	no
gi|320446483|ref|NW_003384088.1|	83338-85045	193785	909427	22305	0.10125	no
gi|320446483|ref|NW_003384088.1|	85239-88600	388673	111019	151418	0.37855	no
gi|320446483|ref|NW_003384088.1|	91320-91666	532911	125519	123594	0.5782	no
gi|320446486|ref|NW_003384085.1|	106431-11025	963581	385886	-132023	0.32305	no
gi|320446486|ref|NW_003384085.1|	110338-11141	627882	235289	-141606	0.4206	no
gi|320446486|ref|NW_003384085.1|	112909-11395	106312	57654	-0.882813	0.6786	no
gi|320446486|ref|NW_003384085.1|	114355-11552	522527	436972	-0.257963	0.89935	no
gi|320446486|ref|NW_003384085.1|	115963-11734	0	24599	inf	0.0138	no
gi|320446486|ref|NW_003384085.1|	136109-13664	580471	219661	-140194	0.53195	no
gi|320446486|ref|NW_003384085.1|	138347-14098	308047	148546	-105224	0.43355	no
gi|320446486|ref|NW_003384085.1|	141296-14578	998646	140143	0.488854	0.7058	no
gi|320446486|ref|NW_003384085.1|	152718-15398	360936	193489	242244	0.2961	no

gi|320446486|ref|NW_003384085.1|	190972-19163	256623	133178	-759015	0.1122	no
gi|320446486|ref|NW_003384085.1|	204500-20474	108197	318809	155903	0.48565	no
gi|320446486|ref|NW_003384085.1|	214204-21470	493979	248416	-0.991691	0.66985	no
gi|320446486|ref|NW_003384085.1|	225130-22723	262153	344943	371788	0.06895	no
gi|320446486|ref|NW_003384085.1|	228153-23278	207182	15719	292354	0.12705	no
gi|320446486|ref|NW_003384085.1|	233573-23400	163244	302985	421414	0.1638	no
gi|320446486|ref|NW_003384085.1|	237300-23893	0.842153	996474	356468	0.16	no
gi|320446486|ref|NW_003384085.1|	247488-24886	0	175305	inf	0.02075	no
gi|320446486|ref|NW_003384085.1|	38949-45866	409326	182333	-44886	0.0436	no
gi|320446486|ref|NW_003384085.1|	46064-46294	422996	532528	-298971	0.2661	no
gi|320446486|ref|NW_003384085.1|	47322-48636	62582	173763	-184863	0.38715	no
gi|320446486|ref|NW_003384085.1|	49714-52185	454361	110692	-535922	0.03475	no
gi|320446486|ref|NW_003384085.1|	52533-54520	3468	0	#NAME?	0.00775	no
gi|320446486|ref|NW_003384085.1|	54960-56596	428907	0.0838558	-899854	0.2504	no
gi|320446486|ref|NW_003384085.1|	56715-58598	460868	0	#NAME?	5.00E-05	yes
gi|320446486|ref|NW_003384085.1|	64092-64269	528777	0	#NAME?	0.007	no
gi|320446486|ref|NW_003384085.1|	70880-71252	226086	665353	155725	0.3575	no
gi|320446486|ref|NW_003384085.1|	81110-81873	105568	329054	164015	0.4597	no
gi|320446486|ref|NW_003384085.1|	90121-90367	101175	511745	233858	0.29875	no
gi|320446486|ref|NW_003384085.1|	91827-93031	0.871137	736742	308019	0.2071	no
gi|320446486|ref|NW_003384085.1|	94193-94551	615873	305056	230837	0.316	no
gi|320446486|ref|NW_003384085.1|	95613-96111	126617	720916	250936	0.2524	no
gi|320446486|ref|NW_003384085.1|	99233-10094	87934	68151	295424	0.13075	no
gi|320446487|ref|NW_003384084.1|	100426-10087	580335	52071	-347833	0.16555	no
gi|320446487|ref|NW_003384084.1|	11180-11762	145383	327998	117382	0.56835	no
gi|320446487|ref|NW_003384084.1|	123263-12346	336026	227608	-0.562022	0.7856	no
gi|320446487|ref|NW_003384084.1|	128974-12954	151343	146601	3276	0.2059	no
gi|320446487|ref|NW_003384084.1|	131790-13209	0	355931	inf	0.00935	no
gi|320446487|ref|NW_003384084.1|	15167-15377	655151	0	#NAME?	0.02105	no
gi|320446487|ref|NW_003384084.1|	153819-15448	0.395127	565623	383945	0.20095	no

gi|320446487|ref|NW_003384084.1|	16651-17100	920955	0.510032	-417447	0.3058	no
gi|320446487|ref|NW_003384084.1|	168855-170294	855238	27283	-164832	0.44455	no
gi|320446487|ref|NW_003384084.1|	171771-172228	118717	246895	-226556	0.32425	no
gi|320446487|ref|NW_003384084.1|	173494-173911	888047	175975	-233526	0.25625	no
gi|320446487|ref|NW_003384084.1|	188097-188539	632911	997694	0.656594	0.74245	no
gi|320446487|ref|NW_003384084.1|	19929-20355	137818	168883	-635059	0.11065	no
gi|320446487|ref|NW_003384084.1|	21914-23243	226834	222828	-0.025701	0.98365	no
gi|320446487|ref|NW_003384084.1|	23954-25794	488994	506349	0.0503144	0.9693	no
gi|320446487|ref|NW_003384084.1|	26792-27089	103193	749019	-0.462266	0.8231	no
gi|320446487|ref|NW_003384084.1|	71517-72092	0.49435	10699	443579	0.18255	no
gi|320446487|ref|NW_003384084.1|	74322-74636	168594	423571	465097	0.17495	no
gi|320446487|ref|NW_003384084.1|	90444-92392	305435	522046	0.773311	0.70805	no
gi|320446487|ref|NW_003384084.1|	92527-93100	198881	26896	0.435486	0.8224	no
gi|320446487|ref|NW_003384084.1|	93215-93667	378859	352676	-0.103316	0.92585	no
gi|320446487|ref|NW_003384084.1|	9586-10847	271494	262536	-337034	0.08745	no
gi|320446487|ref|NW_003384084.1|	95997-96754	355934	288374	-0.303668	0.88005	no
gi|320446489|ref|NW_003384082.1|	100096-100510	0	654375	inf	0.0294	no
gi|320446489|ref|NW_003384082.1|	104863-105339	0	114733	inf	0.01485	no
gi|320446489|ref|NW_003384082.1|	130488-131055	106251	0	#NAME?	0.01425	no
gi|320446489|ref|NW_003384082.1|	131416-132327	424686	0.516604	-303927	0.197	no
gi|320446489|ref|NW_003384082.1|	85883-91752	128781	728283	249958	0.1682	no
gi|320446492|ref|NW_003384079.1|	105046-106060	559258	416235	-0.426116	0.82935	no
gi|320446492|ref|NW_003384079.1|	136024-137009	184619	435349	-208431	0.34885	no
gi|320446492|ref|NW_003384079.1|	151514-153254	560752	945102	0.753108	0.7291	no
gi|320446492|ref|NW_003384079.1|	229567-230611	0	14431	inf	0.0312	no
gi|320446492|ref|NW_003384079.1|	230723-231730	0.218724	22668	337347	0.2261	no
gi|320446492|ref|NW_003384079.1|	235184-236780	186033	440123	124235	0.54605	no
gi|320446492|ref|NW_003384079.1|	237174-238151	522015	235073	-115098	0.5829	no
gi|320446492|ref|NW_003384079.1|	238902-239402	144566	631727	-119436	0.56705	no
gi|320446492|ref|NW_003384079.1|	239581-242451	16139	483468	-173906	0.31485	no

gi|320446492|ref|NW_003384079.1|	47306-24828	163413	642534	-134668	0.5344	no
gi|320446492|ref|NW_003384079.1|	49537-25072	61939	343427	-0.850846	0.67715	no
gi|320446492|ref|NW_003384079.1|	67271-67915	19379	129107	-0.585921	0.76955	no
gi|320446496|ref|NW_003384075.1|	17318-11831	464135	210695	218254	0.34535	no
gi|320446496|ref|NW_003384075.1|	27266-12855	0.160779	265529	736765	0.1408	no
gi|320446496|ref|NW_003384075.1|	28777-12975	0	204542	inf	0.02915	no
gi|320446496|ref|NW_003384075.1|	29898-13133	0	253342	inf	0.0142	no
gi|320446496|ref|NW_003384075.1|	32377-13495	0	206032	inf	0.0072	no
gi|320446496|ref|NW_003384075.1|	39099-14048	0	69457	inf	0.00505	no
gi|320446496|ref|NW_003384075.1|	40589-14106	0	455535	inf	0.0312	no
gi|320446496|ref|NW_003384075.1|	51954-15319	0.168486	116847	279393	1	no
gi|320446496|ref|NW_003384075.1|	55479-15610	0	436264	inf	0.02205	no
gi|320446496|ref|NW_003384075.1|	91565-19255	591897	220602	-14239	0.41695	no
gi|320446496|ref|NW_003384075.1|	19378-20334	49184	517222	0.0725944	0.97	no
gi|320446496|ref|NW_003384075.1|	93846-19454	245848	123562	-0.992527	0.64855	no
gi|320446496|ref|NW_003384075.1|	97205-19839	359246	160675	-116083	0.48485	no
gi|320446496|ref|NW_003384075.1|	98543-20080	272098	339049	0.317365	0.81445	no
gi|320446496|ref|NW_003384075.1|	09013-20944	0	11769	inf	0.01575	no
gi|320446496|ref|NW_003384075.1|	10622-21114	230668	205493	31552	0.21245	no
gi|320446496|ref|NW_003384075.1|	12182-21287	0.3686	105759	484258	0.17265	no
gi|320446496|ref|NW_003384075.1|	22666-22598	386306	296621	-0.381124	0.7707	no
gi|320446496|ref|NW_003384075.1|	35117-23675	169033	0.168068	-333019	0.239	no
gi|320446496|ref|NW_003384075.1|	41301-24174	0	847304	inf	0.0198	no
gi|320446496|ref|NW_003384075.1|	57533-25787	141765	101085	2834	0.2595	no
gi|320446496|ref|NW_003384075.1|	64139-26608	0.49408	372005	291251	0.22715	no
gi|320446496|ref|NW_003384075.1|	26902-27449	128978	152174	0.238591	0.9045	no
gi|320446496|ref|NW_003384075.1|	75696-27606	306578	230949	-37306	0.17055	no
gi|320446496|ref|NW_003384075.1|	77646-28120	167579	0.817213	-435798	0.0499	no
gi|320446496|ref|NW_003384075.1|	28615-31992	151455	229552	0.599935	0.64105	no
gi|320446496|ref|NW_003384075.1|	99624-30147	973135	242813	131914	0.4409	no

gi|320446496|ref|NW_003384075.1|	304757-30559[illegible]	362314	881827	128326	0.53145	no
gi|320446496|ref|NW_003384075.1|	305703-30694[illegible]	0.837411	151008	0.850618	0.6824	no
gi|320446496|ref|NW_003384075.1|	311963-31255[illegible]	376032	286642	-0.391606	0.84175	no
gi|320446496|ref|NW_003384075.1|	36144-37577	197268	430859	-219487	0.2046	no
gi|320446496|ref|NW_003384075.1|	37791-38811	0.430198	13379	163689	1	no
gi|320446496|ref|NW_003384075.1|	38944-40613	0.588528	0.819423	0.477498	1	no
gi|320446496|ref|NW_003384075.1|	51651-53651	0.286871	120025	206486	1	no
gi|320446496|ref|NW_003384075.1|	55328-58305	0.430808	296882	278477	0.23705	no
gi|320446496|ref|NW_003384075.1|	74531-74920	410421	646735	3978	0.1633	no
gi|320446496|ref|NW_003384075.1|	82256-84444	0.0864156	162813	423578	0.19125	no
gi|320446496|ref|NW_003384075.1|	84561-90906	162932	970711	58967	0.03555	no
gi|320446497|ref|NW_003384074.1|	26602-27103	432149	197262	-113141	0.59465	no
gi|320446497|ref|NW_003384074.1|	279457-28105[illegible]	508864	397263	-0.357188	0.8623	no
gi|320446497|ref|NW_003384074.1|	291112-29163[illegible]	764868	316213	-127431	0.5429	no
gi|320446498|ref|NW_003384073.1|	100920-10157[illegible]	242715	203377	-0.255111	0.90895	no
gi|320446498|ref|NW_003384073.1|	102891-10496[illegible]	34012	557833	0.713788	0.73615	no
gi|320446498|ref|NW_003384073.1|	109073-10993[illegible]	0	315185	inf	0.02075	no
gi|320446498|ref|NW_003384073.1|	110329-11099[illegible]	117991	241327	103231	0.5772	no
gi|320446498|ref|NW_003384073.1|	12856-13355	315405	295823	-0.0924679	0.9267	no
gi|320446498|ref|NW_003384073.1|	13900-14603	198425	715183	-147221	0.48825	no
gi|320446498|ref|NW_003384073.1|	15878-16425	166597	362319	-220103	0.32835	no
gi|320446498|ref|NW_003384073.1|	17678-18234	47052	141136	-173717	0.4322	no
gi|320446498|ref|NW_003384073.1|	183961-18426[illegible]	0	263203	inf	0.0186	no
gi|320446498|ref|NW_003384073.1|	19420-20188	0	217361	inf	0.0312	no
gi|320446498|ref|NW_003384073.1|	216-562	133228	952213	283739	0.2595	no
gi|320446498|ref|NW_003384073.1|	23899-24517	21995	179266	-0.295076	0.8821	no
gi|320446498|ref|NW_003384073.1|	24879-25304	683783	226192	-159599	0.44495	no
gi|320446498|ref|NW_003384073.1|	25556-27971	418039	443267	0.0845403	0.96705	no
gi|320446498|ref|NW_003384073.1|	29626-33166	204232	145396	28317	0.12925	no
gi|320446498|ref|NW_003384073.1|	33547-34686	0.933819	284562	160753	0.4505	no

gi|320446498|ref|NW_003384073.1|	63343-36404	0	13152	inf	0.0075	no
gi|320446498|ref|NW_003384073.1|	4239-6184	0.592202	295883	232086	0.3062	no
gi|320446498|ref|NW_003384073.1|	7440-11981	670097	636668	-0.0738293	0.95545	no
gi|320446498|ref|NW_003384073.1|	94059-96354	394062	207019	-0.928662	0.4788	no
gi|320446498|ref|NW_003384073.1|	98501-10052	203434	146472	-0.473935	0.7066	no
gi|320446500|ref|NW_003384071.1|	1268-2196	143772	569689	198639	0.2605	no
gi|320446500|ref|NW_003384071.1|	51279-15211	413163	224654	-0.879004	0.5892	no
gi|320446500|ref|NW_003384071.1|	52606-15372	217731	806621	-143258	0.5254	no
gi|320446500|ref|NW_003384071.1|	53941-15590	286013	925603	-162761	0.35345	no
gi|320446500|ref|NW_003384071.1|	56215-15778	114973	571343	-100887	0.6404	no
gi|320446500|ref|NW_003384071.1|	59225-15994	265715	239205	-347356	0.16745	no
gi|320446500|ref|NW_003384071.1|	60792-16108	589364	10074	-254853	0.26745	no
gi|320446500|ref|NW_003384071.1|	61633-16207	461075	590036	-296613	0.2192	no
gi|320446500|ref|NW_003384071.1|	62921-16367	130178	401535	-169688	0.431	no
gi|320446500|ref|NW_003384071.1|	63917-16445	915082	123239	-289245	0.11385	no
gi|320446500|ref|NW_003384071.1|	67815-16887	402113	198648	-433932	0.06045	no
gi|320446500|ref|NW_003384071.1|	69945-17042	285393	226927	-365265	0.1833	no
gi|320446500|ref|NW_003384071.1|	71189-17201	394567	117105	-50744	0.0974	no
gi|320446500|ref|NW_003384071.1|	72413-17292	158308	122548	-369132	0.17245	no
gi|320446500|ref|NW_003384071.1|	73225-17383	508347	0.615861	-636707	0.17585	no
gi|320446500|ref|NW_003384071.1|	75577-17743	312751	233202	-0.423431	0.7433	no
gi|320446500|ref|NW_003384071.1|	78178-17880	106843	483993	-114243	0.49965	no
gi|320446500|ref|NW_003384071.1|	84457-18753	349715	88695	134268	0.4118	no
gi|320446500|ref|NW_003384071.1|	87716-19003	125582	115947	-0.115161	0.9245	no
gi|320446500|ref|NW_003384071.1|	90258-19053	223454	25294	0.178815	0.92505	no
gi|320446500|ref|NW_003384071.1|	92033-19247	112239	9041	-0.312024	0.8776	no
gi|320446500|ref|NW_003384071.1|	93109-19735	397656	235697	-0.754588	0.5717	no
gi|320446500|ref|NW_003384071.1|	17129-22215	514837	595374	0.20968	0.89595	no
gi|320446500|ref|NW_003384071.1|	27940-22829	256052	133302	238018	0.2526	no
gi|320446500|ref|NW_003384071.1|	33869-23613	0.497594	202601	20256	0.3488	no

gi|320446500|ref|NW_003384071.1|	2639-6956	103344	526781	234975	0.17965	no
gi|320446500|ref|NW_003384071.1|	40872-45039	154494	931389	259183	0.15605	no
gi|320446500|ref|NW_003384071.1|	61622-62426	118739	448594	191762	0.39735	no
gi|320446500|ref|NW_003384071.1|	7517-8482	0.925278	654655	282277	0.2389	no
gi|320446500|ref|NW_003384071.1|	90091-90326	78516	371204	224115	0.27445	no
gi|320446502|ref|NW_003384069.1|	118810-119679	319658	0	#NAME?	0.02015	no
gi|320446502|ref|NW_003384069.1|	119834-120112	233293	0	#NAME?	0.0229	no
gi|320446502|ref|NW_003384069.1|	123103-123842	107235	275437	-196098	0.37775	no
gi|320446502|ref|NW_003384069.1|	143773-144307	167988	263845	0.651334	0.74155	no
gi|320446502|ref|NW_003384069.1|	34817-36159	0.457486	0.847077	0.888765	1	no
gi|320446502|ref|NW_003384069.1|	36574-37216	124296	310182	131934	0.5489	no
gi|320446504|ref|NW_003384067.1|	208214-211308	136347	62735	-111994	0.50615	no
gi|320446504|ref|NW_003384067.1|	211452-216414	425144	375821	-0.177905	0.8897	no
gi|320446504|ref|NW_003384067.1|	308375-309153	152425	320177	-225115	0.30935	no
gi|320446504|ref|NW_003384067.1|	310633-311188	104877	566192	-0.889337	0.66355	no
gi|320446504|ref|NW_003384067.1|	312462-315200	110572	918741	-0.267258	0.8306	no
gi|320446504|ref|NW_003384067.1|	3157-3420	71258	696311	-335525	0.2086	no
gi|320446504|ref|NW_003384067.1|	324389-324889	119424	0.842302	-382561	0.2198	no
gi|320446504|ref|NW_003384067.1|	327402-331532	152496	0.545928	-480392	0.04165	no
gi|320446504|ref|NW_003384067.1|	339853-341342	296142	0	#NAME?	0.01	no
gi|320446504|ref|NW_003384067.1|	345299-346062	993582	0.8791	-349854	0.20615	no
gi|320446504|ref|NW_003384067.1|	362086-363789	5289	134501	134655	0.54605	no
gi|320446504|ref|NW_003384067.1|	366147-371750	234231	135307	-0.791697	0.54915	no
gi|320446504|ref|NW_003384067.1|	375772-376680	351302	0.864777	-202231	0.359	no
gi|320446504|ref|NW_003384067.1|	376814-377902	753051	150843	-23197	0.31335	no
gi|320446504|ref|NW_003384067.1|	380943-381390	108373	0	#NAME?	0.0178	no
gi|320446504|ref|NW_003384067.1|	443246-443685	24853	101046	-12984	0.5398	no
gi|320446504|ref|NW_003384067.1|	444472-445305	112692	242769	-221473	0.227	no
gi|320446509|ref|NW_003384062.1|	113595-114959	26914	218071	-0.303558	0.87905	no
gi|320446509|ref|NW_003384062.1|	115995-117118	228158	0.658227	-179337	0.4174	no

gi|320446509|ref|NW_003384062.1|	117607-11874	652229	477539	-0.449761	0.82545	no
gi|320446509|ref|NW_003384062.1|	11790-16159	189802	182347	-0.0578101	0.96645	no
gi|320446509|ref|NW_003384062.1|	119190-12820	15725	103412	-0.604652	0.64935	no
gi|320446509|ref|NW_003384062.1|	128401-12902	221503	708282	-164493	0.4441	no
gi|320446509|ref|NW_003384062.1|	129390-13131	172569	604969	-151224	0.36705	no
gi|320446509|ref|NW_003384062.1|	132533-13507	42044	231879	-0.858526	0.5235	no
gi|320446509|ref|NW_003384062.1|	136869-13738	413247	872415	107801	0.595	no
gi|320446509|ref|NW_003384062.1|	16352-23864	213085	299783	0.492492	0.70635	no
gi|320446509|ref|NW_003384062.1|	24404-25210	278057	180854	-0.620554	0.7786	no
gi|320446509|ref|NW_003384062.1|	25550-26421	135427	151748	0.16416	0.9401	no
gi|320446509|ref|NW_003384062.1|	36232-36753	116882	667624	-0.807949	0.6914	no
gi|320446509|ref|NW_003384062.1|	37802-40447	980343	440315	-115475	0.47505	no
gi|320446509|ref|NW_003384062.1|	41786-43887	850192	750825	-0.179313	0.93595	no
gi|320446509|ref|NW_003384062.1|	49293-50421	262802	798601	-171843	0.30125	no
gi|320446509|ref|NW_003384062.1|	50563-51264	188402	520036	-185713	0.3903	no
gi|320446509|ref|NW_003384062.1|	54518-57201	221289	102117	-111571	0.38705	no
gi|320446509|ref|NW_003384062.1|	74537-76260	737309	616143	-0.259004	0.90305	no
gi|320446509|ref|NW_003384062.1|	76583-79826	111079	729805	-0.606	0.62725	no
gi|320446509|ref|NW_003384062.1|	81372-81687	210733	711284	-156692	0.3434	no
gi|320446509|ref|NW_003384062.1|	82182-82727	248752	131218	-0.922746	0.6568	no
gi|320446509|ref|NW_003384062.1|	82900-83870	252719	348927	0.465392	0.81525	no
gi|320446509|ref|NW_003384062.1|	83972-85128	458285	203184	-117346	0.56065	no
gi|320446509|ref|NW_003384062.1|	85234-86361	302845	406295	0.423949	0.82855	no
gi|320446513|ref|NW_003384058.1|	106258-10717	0.997784	10661	341747	0.19055	no
gi|320446513|ref|NW_003384058.1|	10769-12917	199384	115106	-0.792584	0.5336	no
gi|320446513|ref|NW_003384058.1|	13074-14522	625951	347044	-0.850932	0.59245	no
gi|320446513|ref|NW_003384058.1|	16713-17710	15955	734744	-111869	0.6104	no
gi|320446513|ref|NW_003384058.1|	22858-27400	31.93	218392	-0.547988	0.68665	no
gi|320446513|ref|NW_003384058.1|	246374-24733	0	177795	inf	0.0294	no
gi|320446513|ref|NW_003384058.1|	28103-28529	110594	788121	-0.488791	0.80315	no

gi|320446513|ref|NW_003384058.1|	30271-30752	411335	810956	-234262	0.2913	no
gi|320446513|ref|NW_003384058.1|	32863-34697	14461	492789	-155313	0.3439	no
gi|320446513|ref|NW_003384058.1|	336040-33702	0.904203	109267	0.273145	1	no
gi|320446513|ref|NW_003384058.1|	35571-37203	6887	639113	-0.107803	0.9601	no
gi|320446513|ref|NW_003384058.1|	367956-36901	163531	0.706917	-120995	0.5719	no
gi|320446513|ref|NW_003384058.1|	39437-43783	521285	419611	-0.313021	0.8441	no
gi|320446513|ref|NW_003384058.1|	4067-4729	106684	24241	-213783	0.3262	no
gi|320446513|ref|NW_003384058.1|	54693-55559	918037	26532	-179082	0.31895	no
gi|320446513|ref|NW_003384058.1|	55966-58683	335884	126856	-140477	0.2917	no
gi|320446513|ref|NW_003384058.1|	60668-63263	381855	247278	-0.626892	0.6386	no
gi|320446513|ref|NW_003384058.1|	6280-6993	105979	217464	-228494	0.30205	no
gi|320446513|ref|NW_003384058.1|	69147-69994	157026	599953	-138808	0.3024	no
gi|320446513|ref|NW_003384058.1|	70797-74469	347114	396132	0.190571	0.8907	no
gi|320446513|ref|NW_003384058.1|	74981-77463	59709	178868	-173906	0.1984	no
gi|320446513|ref|NW_003384058.1|	78316-80428	593444	219545	-14346	0.50345	no
gi|320446513|ref|NW_003384058.1|	81304-81718	171222	356932	-226215	0.32245	no
gi|320446513|ref|NW_003384058.1|	82477-84608	12284	304303	-20132	0.2349	no
gi|320446513|ref|NW_003384058.1|	8265-8713	693687	204854	-175969	0.4319	no
gi|320446513|ref|NW_003384058.1|	85584-91055	153073	104769	-0.547001	0.6838	no
gi|320446513|ref|NW_003384058.1|	8880-9940	13917	509588	-144944	0.50015	no
gi|320446513|ref|NW_003384058.1|	91116-91717	0.459966	374292	302456	0.25005	no
gi|320446513|ref|NW_003384058.1|	91885-93834	0.689289	508013	288168	0.21545	no
gi|320446515|ref|NW_003384056.1|	107479-11004	105737	439744	-126575	0.34605	no
gi|320446515|ref|NW_003384056.1|	110175-11058	375187	618145	-260159	0.2537	no
gi|320446515|ref|NW_003384056.1|	111001-11279	136676	332496	-203935	0.22615	no
gi|320446515|ref|NW_003384056.1|	114501-11571	242168	0.479687	-233584	0.3092	no
gi|320446515|ref|NW_003384056.1|	116435-11830	0.931346	144236	0.631045	0.757	no
gi|320446515|ref|NW_003384056.1|	118452-11941	199482	57317	152271	0.3832	no
gi|320446515|ref|NW_003384056.1|	119542-12088	364135	684462	0.910498	0.49375	no
gi|320446515|ref|NW_003384056.1|	122841-12417	299085	154126	-0.956441	0.5699	no

gi|320446515|ref|NW_003384056.1|	132968-13797:	213398	268727	0.332592	0.8062	no
gi|320446515|ref|NW_003384056.1|	139324-14089:	158159	199447	0.33463	0.7933	no
gi|320446515|ref|NW_003384056.1|	144365-14592:	574571	266464	-110855	0.5962	no
gi|320446515|ref|NW_003384056.1|	148188-15204(	337208	226887	-0.571666	0.6703	no
gi|320446515|ref|NW_003384056.1|	152158-15335!	384492	448247	0.221341	0.9121	no
gi|320446515|ref|NW_003384056.1|	153465-15546:	24234	196372	-0.303443	0.812	no
gi|320446515|ref|NW_003384056.1|	159467-15971	0	378931	inf	0.0154	no
gi|320446515|ref|NW_003384056.1|	162114-16245:	0	117859	inf	0.02915	no
gi|320446515|ref|NW_003384056.1|	164981-16619(	0.866662	588808	276426	0.25045	no
gi|320446515|ref|NW_003384056.1|	168489-16894	448922	228968	235061	0.2891	no
gi|320446515|ref|NW_003384056.1|	177628-17815:	0	429226	inf	0.0294	no
gi|320446515|ref|NW_003384056.1|	178404-17996:	114827	324842	48222	0.0556	no
gi|320446515|ref|NW_003384056.1|	194235-19864:	0.0808916	269054	505576	0.14965	no
gi|320446515|ref|NW_003384056.1|	200420-20111:	0.7372	7806	340446	0.19655	no
gi|320446515|ref|NW_003384056.1|	202833-20345:	0.875302	594604	276408	0.2352	no
gi|320446515|ref|NW_003384056.1|	29002-29753	11784	762749	-0.627548	0.7642	no
gi|320446515|ref|NW_003384056.1|	33119-33778	318315	298286	-0.0937608	0.94765	no
gi|320446515|ref|NW_003384056.1|	34947-36453	757009	748047	-0.0171802	0.99105	no
gi|320446515|ref|NW_003384056.1|	36576-40379	969523	969815	0.000434388	0.9974	no
gi|320446515|ref|NW_003384056.1|	41402-41739	552884	147033	-191084	0.3679	no
gi|320446515|ref|NW_003384056.1|	42011-42698	724508	425891	-0.766518	0.64195	no
gi|320446515|ref|NW_003384056.1|	43499-45444	399736	308956	-0.371645	0.78115	no
gi|320446515|ref|NW_003384056.1|	54749-64276	0.108949	141501	702102	0.07175	no
gi|320446515|ref|NW_003384056.1|	64896-65289	0	791754	inf	0.029	no
gi|320446515|ref|NW_003384056.1|	72535-79028	0.0269625	799439	821189	0.1409	no
gi|320446515|ref|NW_003384056.1|	79184-79870	0.37428	511138	377152	0.2075	no
gi|320446517|ref|NW_003384054.1|	102626-10532!	187357	439574	-209161	0.24535	no
gi|320446517|ref|NW_003384054.1|	112086-11262!	231451	0.371171	-596248	0.26975	no
gi|320446517|ref|NW_003384054.1|	118894-11944	200482	0.711892	-481567	0.19595	no
gi|320446517|ref|NW_003384054.1|	144707-14799:	105123	344418	171209	0.4289	no

gi|320446517|ref|NW_003384054.1|	161984-162799	273738	132078	-10514	0.6324	no
gi|320446517|ref|NW_003384054.1|	164087-165778	310683	183222	-0.76185	0.5536	no
gi|320446517|ref|NW_003384054.1|	168296-16931	255118	627501	-202348	0.37615	no
gi|320446517|ref|NW_003384054.1|	169413-16978	12736	37823	-175157	0.4201	no
gi|320446517|ref|NW_003384054.1|	170616-17130	345097	230504	-390414	0.13225	no
gi|320446517|ref|NW_003384054.1|	171500-17172	360234	84934	-208452	0.2863	no
gi|320446517|ref|NW_003384054.1|	175507-176688	50506	272088	-421431	0.0595	no
gi|320446517|ref|NW_003384054.1|	192864-19392	430312	226755	-0.924251	0.65555	no
gi|320446517|ref|NW_003384054.1|	21415-22434	947644	105678	0.157255	0.9403	no
gi|320446517|ref|NW_003384054.1|	222572-22504	642114	548677	-0.226872	0.914	no
gi|320446517|ref|NW_003384054.1|	22566-23745	307613	136329	-117403	0.4785	no
gi|320446517|ref|NW_003384054.1|	225899-226627	342562	281401	-0.283733	0.88605	no
gi|320446517|ref|NW_003384054.1|	227247-228627	678185	266259	-134885	0.5231	no
gi|320446517|ref|NW_003384054.1|	232920-233459	606181	556757	-0.122703	0.9446	no
gi|320446517|ref|NW_003384054.1|	243116-244829	41112	819085	0.994454	0.64685	no
gi|320446517|ref|NW_003384054.1|	245866-24644	19774	668686	175772	0.42945	no
gi|320446517|ref|NW_003384054.1|	248331-24895	394895	685486	0.795656	0.69795	no
gi|320446517|ref|NW_003384054.1|	25521-26849	132827	184429	0.473516	0.83775	no
gi|320446517|ref|NW_003384054.1|	259662-26046	209637	370217	0.820478	0.68335	no
gi|320446517|ref|NW_003384054.1|	263639-263947	141666	227773	-263682	0.29155	no
gi|320446517|ref|NW_003384054.1|	264659-26561	0.701655	145271	104991	0.5772	no
gi|320446517|ref|NW_003384054.1|	265733-26726	0.391038	135984	179805	1	no
gi|320446517|ref|NW_003384054.1|	295455-295888	494196	327462	-0.593757	0.75475	no
gi|320446517|ref|NW_003384054.1|	299317-299897	584827	461701	-0.341051	0.8611	no
gi|320446517|ref|NW_003384054.1|	320292-32142	0.375607	733516	760946	0.1181	no
gi|320446517|ref|NW_003384054.1|	332456-33280	228023	113268	-100944	0.6073	no
gi|320446517|ref|NW_003384054.1|	33526-34566	0.209751	551064	471547	0.17835	no
gi|320446517|ref|NW_003384054.1|	335589-33579	82096	276214	-157153	0.47395	no
gi|320446517|ref|NW_003384054.1|	340330-34269	602732	88039	0.546625	0.80905	no
gi|320446517|ref|NW_003384054.1|	55665-58490	0.521254	541922	337803	0.1749	no

gi|320446517|ref|NW_003384054.1|	58588-63217	0.19201	212467	346798	0.17835	no
gi|320446518|ref|NW_003384053.1|	107968-10821(	361452	0	#NAME?	0.02105	no
gi|320446518|ref|NW_003384053.1|	134986-13643	0.693523	163895	124076	0.5617	no
gi|320446518|ref|NW_003384053.1|	153802-15514	107037	0.743184	-0.526317	1	no
gi|320446518|ref|NW_003384053.1|	155491-15775	0.58167	139198	125887	1	no
gi|320446518|ref|NW_003384053.1|	172984-17417	0.351724	731373	437809	0.1589	no
gi|320446518|ref|NW_003384053.1|	174749-17507(	0	187252	inf	0.0212	no
gi|320446518|ref|NW_003384053.1|	182916-18323	269151	163545	-0.718722	0.7146	no
gi|320446518|ref|NW_003384053.1|	185393-18651	359473	125762	-151519	0.37375	no
gi|320446518|ref|NW_003384053.1|	186664-18931	192408	106566	-0.852417	0.5008	no
gi|320446518|ref|NW_003384053.1|	190060-19180	472824	323082	-0.549406	0.67035	no
gi|320446518|ref|NW_003384053.1|	192267-19370(	112724	900886	-0.323384	0.8772	no
gi|320446518|ref|NW_003384053.1|	193920-19707	133893	192514	0.523886	0.6861	no
gi|320446518|ref|NW_003384053.1|	203310-20380	136388	782265	-0.80199	0.6995	no
gi|320446518|ref|NW_003384053.1|	206233-20732	104908	109577	0.062826	0.9763	no
gi|320446518|ref|NW_003384053.1|	217080-21787	0.898443	164541	0.872946	0.6867	no
gi|320446518|ref|NW_003384053.1|	220364-22091	576824	424644	-0.441878	0.82485	no
gi|320446518|ref|NW_003384053.1|	232445-23291(	718052	478512	-0.585532	0.7629	no
gi|320446518|ref|NW_003384053.1|	247166-24861(	402906	393989	-0.0322875	0.98065	no
gi|320446518|ref|NW_003384053.1|	248770-25531	104588	583117	-0.842854	0.5285	no
gi|320446518|ref|NW_003384053.1|	255474-25685(	63.47	405913	-0.644905	0.62565	no
gi|320446518|ref|NW_003384053.1|	256967-26036	117756	421202	-148322	0.38605	no
gi|320446518|ref|NW_003384053.1|	269825-27039	150463	271219	0.850055	0.6872	no
gi|320446518|ref|NW_003384053.1|	284276-28501(	267557	549814	10391	0.6048	no
gi|320446518|ref|NW_003384053.1|	286233-28828	465198	648912	0.480176	0.82485	no
gi|320446518|ref|NW_003384053.1|	73015-73540	634336	0	#NAME?	0.02105	no
gi|320446519|ref|NW_003384052.1|	10760-12881	142284	530653	-142293	0.39775	no
gi|320446519|ref|NW_003384052.1|	112537-11315	0.435424	355011	302737	0.25005	no
gi|320446519|ref|NW_003384052.1|	139082-14062(	0.257902	340842	372421	0.17955	no
gi|320446519|ref|NW_003384052.1|	17981-18887	780204	2255	-179072	0.41535	no

gi|320446519|ref|NW_003384052.1|	209618-20979	0	107107	inf	0.029	no
gi|320446519|ref|NW_003384052.1|	236166-23687	107774	736803	277327	0.2505	no
gi|320446519|ref|NW_003384052.1|	237951-23916	0.864885	187078	443499	0.13355	no
gi|320446519|ref|NW_003384052.1|	241862-24433	422472	106428	133295	0.4136	no
gi|320446519|ref|NW_003384052.1|	250246-25084	179869	203266	0.176426	0.9277	no
gi|320446519|ref|NW_003384052.1|	258833-25951	198112	286005	-27922	0.2361	no
gi|320446519|ref|NW_003384052.1|	267962-26827	438345	325823	-37499	0.17005	no
gi|320446519|ref|NW_003384052.1|	27036-27329	100938	594738	255879	0.27365	no
gi|320446519|ref|NW_003384052.1|	277225-27744	488437	136021	-184435	0.41505	no
gi|320446519|ref|NW_003384052.1|	279178-27940	719093	159758	-217029	0.32815	no
gi|320446519|ref|NW_003384052.1|	305256-30583	17446	426397	-203263	0.3598	no
gi|320446519|ref|NW_003384052.1|	306516-30704	180349	189635	-324949	0.20555	no
gi|320446519|ref|NW_003384052.1|	308750-30903	649993	715538	-318332	0.2123	no
gi|320446519|ref|NW_003384052.1|	310425-31092	34324	376446	-318871	0.1831	no
gi|320446519|ref|NW_003384052.1|	311133-31139	401839	910554	-21418	0.34705	no
gi|320446519|ref|NW_003384052.1|	311865-31212	274069	104447	-139178	0.51745	no
gi|320446519|ref|NW_003384052.1|	312386-31340	431799	121745	-18265	0.297	no
gi|320446519|ref|NW_003384052.1|	313532-31491	355232	105026	-175802	0.30925	no
gi|320446519|ref|NW_003384052.1|	315135-31640	63922	337713	-0.920517	0.4867	no
gi|320446519|ref|NW_003384052.1|	32986-34283	0.158961	154449	328038	0.23005	no
gi|320446519|ref|NW_003384052.1|	331243-33158	234354	27731	0.242814	0.9008	no
gi|320446519|ref|NW_003384052.1|	337288-33753	236074	298518	0.338579	0.85805	no
gi|320446519|ref|NW_003384052.1|	338808-33912	150523	183209	0.283508	0.8848	no
gi|320446519|ref|NW_003384052.1|	342693-34488	17292	28356	0.713553	0.72695	no
gi|320446519|ref|NW_003384052.1|	345076-34682	0.556314	193737	180012	0.4139	no
gi|320446519|ref|NW_003384052.1|	346942-34781	500592	146957	155369	0.47185	no
gi|320446519|ref|NW_003384052.1|	349551-35201	105429	337946	168052	0.2037	no
gi|320446519|ref|NW_003384052.1|	352423-35264	342151	460276	0.427866	0.8223	no
gi|320446519|ref|NW_003384052.1|	353186-35489	80157	218479	144659	0.3935	no
gi|320446519|ref|NW_003384052.1|	356135-35646	283282	405162	0.516261	0.801	no

gi|320446519|ref|NW_003384052.1|	357307-35758	404794	531232	0.392153	0.84275	no
gi|320446519|ref|NW_003384052.1|	357713-35871	151312	169687	0.165348	0.93905	no
gi|320446519|ref|NW_003384052.1|	359622-36014	258878	23716	-0.126412	0.95045	no
gi|320446519|ref|NW_003384052.1|	360283-36120	138461	151716	0.131888	0.95195	no
gi|320446519|ref|NW_003384052.1|	362308-36258	744848	189705	134874	0.52365	no
gi|320446519|ref|NW_003384052.1|	362701-36367	103104	838353	-0.298473	0.8848	no
gi|320446519|ref|NW_003384052.1|	364667-36517	725601	105503	0.540034	0.79305	no
gi|320446519|ref|NW_003384052.1|	37379-37931	264599	253465	325991	0.1972	no
gi|320446519|ref|NW_003384052.1|	388941-38963	185103	209882	0.181256	0.9349	no
gi|320446519|ref|NW_003384052.1|	433529-43751	0.76456	522593	277299	0.23555	no
gi|320446519|ref|NW_003384052.1|	463466-46402	179185	221311	0.30462	0.8838	no
gi|320446519|ref|NW_003384052.1|	535267-53703	417295	275379	-0.599648	0.77005	no
gi|320446519|ref|NW_003384052.1|	537136-53788	140481	156741	-316393	0.20015	no
gi|320446519|ref|NW_003384052.1|	538368-53866	447817	37451	-357984	0.1807	no
gi|320446519|ref|NW_003384052.1|	539099-54052	637804	0.984921	-269503	0.24605	no
gi|320446519|ref|NW_003384052.1|	544689-54547	328689	200581	-0.712537	0.74675	no
gi|320446519|ref|NW_003384052.1|	546588-54762	207621	878704	-12405	0.57385	no
gi|320446519|ref|NW_003384052.1|	87406-89930	648799	121017	0.899363	0.58475	no
gi|320446519|ref|NW_003384052.1|	91942-92474	169077	796467	-1086	0.60325	no
gi|320446525|ref|NW_003384046.1|	106631-10763	813498	505809	263638	0.14985	no
gi|320446525|ref|NW_003384046.1|	107829-10876	0.480424	14913	163419	0.3325	no
gi|320446525|ref|NW_003384046.1|	109067-10948	0	703901	inf	0.029	no
gi|320446525|ref|NW_003384046.1|	113416-11365	116081	219582	0.919627	0.67085	no
gi|320446525|ref|NW_003384046.1|	115101-11621	0.764835	436856	251394	0.28175	no
gi|320446525|ref|NW_003384046.1|	116977-11928	0.325333	158974	22888	0.3272	no
gi|320446525|ref|NW_003384046.1|	121019-12191	228557	682495	157827	0.4619	no
gi|320446525|ref|NW_003384046.1|	145552-14613	633562	224255	182358	0.3978	no
gi|320446525|ref|NW_003384046.1|	196830-19695	751471	1713.39	118906	0.5674	no
gi|320446525|ref|NW_003384046.1|	21275-22166	136443	39373	152891	0.35515	no
gi|320446525|ref|NW_003384046.1|	236769-23837	0.247136	180569	286918	0.22425	no

gi|320446525|ref|NW_003384046.1|	[illegible]38618-23954[illegible]	0.728939	620026	308846	0.2131	no
gi|320446525|ref|NW_003384046.1|	[illegible]43119-24516[illegible]	0.464942	311307	274322	0.2549	no
gi|320446525|ref|NW_003384046.1|	[illegible]48450-24938[illegible]	0.962218	265491	146423	0.49755	no
gi|320446525|ref|NW_003384046.1|	[illegible]49509-24993[illegible]	417531	176962	208349	0.35045	no
gi|320446525|ref|NW_003384046.1|	[illegible]72661-27339[illegible]	102561	145105	0.500615	0.80925	no
gi|320446525|ref|NW_003384046.1|	[illegible]81634-28239[illegible]	256895	858711	174099	0.40855	no
gi|320446525|ref|NW_003384046.1|	[illegible]22017-42319[illegible]	19177	335326	-251574	0.283	no
gi|320446525|ref|NW_003384046.1|	[illegible]30028-43061[illegible]	814661	486715	-0.743121	0.7051	no
gi|320446525|ref|NW_003384046.1|	[illegible]32274-43574[illegible]	270913	187712	-0.529306	0.6906	no
gi|320446525|ref|NW_003384046.1|	[illegible]49975-45027[illegible]	149068	179457	0.267662	0.8844	no
gi|320446525|ref|NW_003384046.1|	[illegible]51711-45310[illegible]	0.146028	131876	317487	1	no
gi|320446525|ref|NW_003384046.1|	[illegible]55817-45667[illegible]	380137	127068	174101	0.4199	no
gi|320446525|ref|NW_003384046.1|	[illegible]57493-45776[illegible]	785777	333917	-123463	0.55295	no
gi|320446525|ref|NW_003384046.1|	[illegible]57876-45870[illegible]	25808	17741	-0.540729	0.78805	no
gi|320446525|ref|NW_003384046.1|	[illegible]65550-46657[illegible]	127918	10.61	-0.269792	0.8989	no
gi|320446525|ref|NW_003384046.1|	[illegible]72536-47314[illegible]	0.453882	277137	261021	0.2636	no
gi|320446525|ref|NW_003384046.1|	[illegible]75447-47578[illegible]	136907	142181	337646	0.2184	no
gi|320446525|ref|NW_003384046.1|	[illegible]84580-48507[illegible]	649468	608429	-0.0941708	0.95275	no
gi|320446525|ref|NW_003384046.1|	[illegible]89439-49008[illegible]	0	617469	inf	0.0186	no
gi|320446525|ref|NW_003384046.1|	[illegible]90611-49102[illegible]	271686	956325	181556	0.39715	no
gi|320446525|ref|NW_003384046.1|	[illegible]96120-49691[illegible]	121518	354571	154491	0.4967	no
gi|320446525|ref|NW_003384046.1|	[illegible]97216-49782[illegible]	134738	518191	194333	0.3919	no
gi|320446525|ref|NW_003384046.1|	[illegible]97967-49878[illegible]	0.876657	522072	257416	0.28405	no
gi|320446525|ref|NW_003384046.1|	[illegible]99006-49944[illegible]	0.805292	961381	357753	0.2102	no
gi|320446525|ref|NW_003384046.1|	[illegible]00384-50154[illegible]	292667	136856	222533	0.317	no
gi|320446525|ref|NW_003384046.1|	[illegible]03837-50582[illegible]	230936	241544	0.0647928	0.973	no
gi|320446525|ref|NW_003384046.1|	[illegible]05940-50878[illegible]	543538	366337	-0.569208	0.79295	no
gi|320446525|ref|NW_003384046.1|	[illegible]09048-51058[illegible]	324112	414796	0.355909	0.8593	no
gi|320446525|ref|NW_003384046.1|	[illegible]10766-51139[illegible]	355967	390672	0.134217	0.95145	no
gi|320446525|ref|NW_003384046.1|	[illegible]13102-51812[illegible]	507055	266397	-0.928561	0.5654	no

gi|320446525|ref|NW_003384046.1|	518484-52054	990608	697388	-0.506353	0.8232	no
gi|320446525|ref|NW_003384046.1|	521114-52166	172506	112655	-0.61474	0.76075	no
gi|320446525|ref|NW_003384046.1|	525233-62611	19436	0	#NAME?	0.00395	no
gi|320446527|ref|NW_003384044.1|	105819-10682	12719	11363	-0.162645	0.9398	no
gi|320446527|ref|NW_003384044.1|	107605-10833	483993	210906	-119839	0.4551	no
gi|320446527|ref|NW_003384044.1|	109123-11138	565887	435634	-0.3774	0.8649	no
gi|320446527|ref|NW_003384044.1|	111492-11339	271996	334603	0.298865	0.81675	no
gi|320446527|ref|NW_003384044.1|	116478-12043	838503	456854	-0.876084	0.60245	no
gi|320446527|ref|NW_003384044.1|	120812-12173	583424	229142	-13483	0.42745	no
gi|320446527|ref|NW_003384044.1|	123996-12947	0.837073	218814	138628	0.5236	no
gi|320446527|ref|NW_003384044.1|	130341-13216	30977	153009	-101758	0.43635	no
gi|320446527|ref|NW_003384044.1|	134045-13787	422095	21778	-0.954695	0.4789	no
gi|320446527|ref|NW_003384044.1|	14535-15625	710911	244613	178276	0.28885	no
gi|320446527|ref|NW_003384044.1|	147425-14838	269439	118637	-118341	0.5968	no
gi|320446527|ref|NW_003384044.1|	149525-15025	167517	63191	-140651	0.5091	no
gi|320446527|ref|NW_003384044.1|	150605-15130	167378	146713	-0.190108	0.92665	no
gi|320446527|ref|NW_003384044.1|	151584-15180	418948	164173	-135156	0.52495	no
gi|320446527|ref|NW_003384044.1|	151925-15635	720354	102857	0.513864	0.6922	no
gi|320446527|ref|NW_003384044.1|	159530-16145	335961	207793	-0.69315	0.59565	no
gi|320446527|ref|NW_003384044.1|	172512-17285	399683	164473	204092	0.35555	no
gi|320446527|ref|NW_003384044.1|	174403-17757	136701	239802	0.810821	0.54135	no
gi|320446527|ref|NW_003384044.1|	18468-18768	379214	133838	18194	0.32625	no
gi|320446527|ref|NW_003384044.1|	194739-19500	382042	246317	-0.633216	0.69785	no
gi|320446527|ref|NW_003384044.1|	198100-19877	583012	450137	-0.373161	0.818	no
gi|320446527|ref|NW_003384044.1|	198911-19995	104745	130356	0.31557	1	no
gi|320446527|ref|NW_003384044.1|	200130-20281	0.206559	0.721644	180474	1	no
gi|320446527|ref|NW_003384044.1|	203758-20607	38286	433964	0.180758	0.89245	no
gi|320446527|ref|NW_003384044.1|	23447-24526	160041	512121	167804	0.4261	no
gi|320446527|ref|NW_003384044.1|	25112-26050	360318	207125	252316	0.2692	no
gi|320446527|ref|NW_003384044.1|	27210-27889	152054	228309	390834	0.16425	no

gi|320446527|ref|NW_003384044.1|	28863-30470	295265	344163	354301	0.07405	no
gi|320446527|ref|NW_003384044.1|	41446-42740	593011	129.99	113227	0.40065	no
gi|320446527|ref|NW_003384044.1|	43220-43427	183137	220971	0.270932	0.8966	no
gi|320446527|ref|NW_003384044.1|	53323-53803	270762	406968	0.587893	0.7533	no
gi|320446527|ref|NW_003384044.1|	61899-63431	364406	415475	0.189216	0.88035	no
gi|320446527|ref|NW_003384044.1|	72408-72865	207755	839445	-130738	0.53575	no
gi|320446527|ref|NW_003384044.1|	73070-77898	235858	147189	-0.680249	0.61475	no
gi|320446527|ref|NW_003384044.1|	78075-81756	138091	476314	-153564	0.3785	no
gi|320446527|ref|NW_003384044.1|	82128-83553	235477	131103	-0.844877	0.607	no
gi|320446527|ref|NW_003384044.1|	83684-90205	259297	146194	-0.826718	0.5345	no
gi|320446527|ref|NW_003384044.1|	90941-98633	846244	896397	0.0830634	0.9478	no
gi|320446528|ref|NW_003384043.1|	68943-69638	0.367008	325977	315089	0.24575	no
gi|320446529|ref|NW_003384042.1|	11153-13123	275296	121407	-118113	0.3568	no
gi|320446529|ref|NW_003384042.1|	129526-132501	147813	140799	-0.0701446	0.9555	no
gi|320446529|ref|NW_003384042.1|	132835-133459	246935	126591	-0.963963	0.6531	no
gi|320446529|ref|NW_003384042.1|	13361-14560	339756	109256	-163678	0.33905	no
gi|320446529|ref|NW_003384042.1|	135870-136517	798237	418074	-0.933057	0.58415	no
gi|320446529|ref|NW_003384042.1|	145763-147274	0.264576	193185	286823	0.22425	no
gi|320446529|ref|NW_003384042.1|	147383-150984	340901	859429	133402	0.4272	no
gi|320446529|ref|NW_003384042.1|	152968-154149	232006	234985	0.0184078	0.96875	no
gi|320446529|ref|NW_003384042.1|	164488-166079	255183	222251	312259	0.02595	no
gi|320446529|ref|NW_003384042.1|	16560-17195	202308	143343	-3819	0.17585	no
gi|320446529|ref|NW_003384042.1|	18851-26130	138212	7339	-0.913232	0.4996	no
gi|320446529|ref|NW_003384042.1|	216800-221147	301205	682995	118113	0.3761	no
gi|320446529|ref|NW_003384042.1|	223544-224328	110761	143597	0.37457	0.8571	no
gi|320446529|ref|NW_003384042.1|	228195-228848	69813	967619	0.470944	0.71665	no
gi|320446529|ref|NW_003384042.1|	7575-8633	779607	227026	-177989	0.40365	no
gi|320446529|ref|NW_003384042.1|	81762-82944	0	185321	inf	0.02205	no
gi|320446529|ref|NW_003384042.1|	85586-88034	106764	378113	182439	0.4024	no
gi|320446529|ref|NW_003384042.1|	8834-11052	128513	475156	-143544	0.3845	no

gi|320446529|ref|NW_003384042.1|	94319-94742	949123	228248	-205599	0.36125	no
gi|320446530|ref|NW_003384041.1|	100335-10161[illegible]	285122	166548	-0.775639	0.6362	no
gi|320446530|ref|NW_003384041.1|	102800-10530[illegible]	142186	195496	0.459359	0.7253	no
gi|320446530|ref|NW_003384041.1|	107330-10793[illegible]	139744	209521	-273762	0.13765	no
gi|320446530|ref|NW_003384041.1|	114275-11633[illegible]	658245	716759	-319906	0.112	no
gi|320446530|ref|NW_003384041.1|	129997-13546[illegible]	53159	327265	-0.699856	0.58525	no
gi|320446530|ref|NW_003384041.1|	137120-13996[illegible]	879003	0.767977	-351673	0.08175	no
gi|320446530|ref|NW_003384041.1|	14716-16418	415355	475878	0.196249	0.8787	no
gi|320446530|ref|NW_003384041.1|	157651-15807[illegible]	235991	0	#NAME?	0.0093	no
gi|320446530|ref|NW_003384041.1|	16536-17789	364839	323241	-0.174649	0.89115	no
gi|320446530|ref|NW_003384041.1|	17862-18856	329261	258161	-0.350961	0.8214	no
gi|320446530|ref|NW_003384041.1|	19195-20803	113103	158269	0.48475	0.83245	no
gi|320446530|ref|NW_003384041.1|	201033-20146[illegible]	222388	54577	-202672	0.35385	no
gi|320446530|ref|NW_003384041.1|	201997-20297[illegible]	152888	441137	-179317	0.4103	no
gi|320446530|ref|NW_003384041.1|	203222-20421[illegible]	892248	261914	-176835	0.4155	no
gi|320446530|ref|NW_003384041.1|	206226-20718[illegible]	154364	387388	-199448	0.36635	no
gi|320446530|ref|NW_003384041.1|	207557-20823[illegible]	114552	208456	-245819	0.2807	no
gi|320446530|ref|NW_003384041.1|	209310-21016[illegible]	815891	299456	-144603	0.4873	no
gi|320446530|ref|NW_003384041.1|	210449-21067[illegible]	479916	137235	-180613	0.41685	no
gi|320446530|ref|NW_003384041.1|	211657-21366[illegible]	228203	232075	0.0242746	0.983	no
gi|320446530|ref|NW_003384041.1|	215825-21800[illegible]	252155	163814	-0.622255	0.76215	no
gi|320446530|ref|NW_003384041.1|	21868-22671	202211	285961	-282197	0.22855	no
gi|320446530|ref|NW_003384041.1|	23232-23685	17354	200714	-311205	0.2341	no
gi|320446530|ref|NW_003384041.1|	23798-24053	301059	572422	-23949	0.2542	no
gi|320446530|ref|NW_003384041.1|	24975-25631	156273	0.819054	-425396	0.1551	no
gi|320446530|ref|NW_003384041.1|	26922-31530	838485	282934	-156732	0.2351	no
gi|320446530|ref|NW_003384041.1|	32341-33732	151869	182598	-305609	0.19625	no
gi|320446530|ref|NW_003384041.1|	34450-35165	394016	336913	-354781	0.1608	no
gi|320446530|ref|NW_003384041.1|	36590-39438	160166	275358	-254019	0.15815	no
gi|320446530|ref|NW_003384041.1|	48087-49620	282197	352748	0.321938	0.80015	no

gi|320446530|ref|NW_003384041.1|	54002-54542	554823	543961	-0.0285237	0.98915	no
gi|320446530|ref|NW_003384041.1|	54802-55235	388768	342198	-0.184079	0.88915	no
gi|320446530|ref|NW_003384041.1|	55419-56411	153913	128492	-0.260438	0.84555	no
gi|320446530|ref|NW_003384041.1|	56669-59134	648563	819199	0.336966	0.7969	no
gi|320446530|ref|NW_003384041.1|	73234-74089	36502	243693	-390484	0.07805	no
gi|320446530|ref|NW_003384041.1|	84746-85032	301804	179134	-0.752579	0.71085	no
gi|320446530|ref|NW_003384041.1|	86540-87638	234772	0.541624	-21159	0.3545	no
gi|320446530|ref|NW_003384041.1|	88154-91170	250611	950334	-139894	0.291	no
gi|320446530|ref|NW_003384041.1|	91306-93045	944934	554148	-0.769942	0.56345	no
gi|320446530|ref|NW_003384041.1|	9739-10047	30104	182218	-0.724287	0.71325	no
gi|320446532|ref|NW_003384039.1|	114470-11507[illegible]	503265	0	#NAME?	0.02105	no
gi|320446532|ref|NW_003384039.1|	122227-12267[illegible]	989222	0	#NAME?	0.01755	no
gi|320446532|ref|NW_003384039.1|	130673-13112[illegible]	211266	0.501786	-539584	0.27715	no
gi|320446532|ref|NW_003384039.1|	28236-29433	540528	119212	-218084	0.2301	no
gi|320446535|ref|NW_003384036.1|	11656-13010	0	261903	inf	0.01485	no
gi|320446535|ref|NW_003384036.1|	147595-14828[illegible]	181874	0	#NAME?	5.00E-05	yes
gi|320446535|ref|NW_003384036.1|	14845-15166	0	144169	inf	0.0233	no
gi|320446535|ref|NW_003384036.1|	56865-57635	0	779645	inf	0.0085	no
gi|320446535|ref|NW_003384036.1|	65541-66032	1593.79	0.434592	-118405	0.2504	no
gi|320446537|ref|NW_003384034.1|	135553-13622[illegible]	38356	209366	-0.873427	0.68265	no
gi|320446537|ref|NW_003384034.1|	152514-15329[illegible]	249316	299371	0.263962	0.88585	no
gi|320446537|ref|NW_003384034.1|	166913-16790[illegible]	425486	147712	-152632	0.36785	no
gi|320446537|ref|NW_003384034.1|	168050-16917[illegible]	0.380263	118481	163959	1	no
gi|320446537|ref|NW_003384034.1|	169657-17268[illegible]	13851	617425	-116566	0.49365	no
gi|320446537|ref|NW_003384034.1|	173662-17407[illegible]	344136	173334	-0.989425	0.63795	no
gi|320446537|ref|NW_003384034.1|	175288-17635[illegible]	276996	274714	-0.0119325	0.99295	no
gi|320446537|ref|NW_003384034.1|	180175-18100[illegible]	15923	162262	0.0272145	0.98925	no
gi|320446537|ref|NW_003384034.1|	181436-18363[illegible]	352319	389776	0.145765	0.9415	no
gi|320446537|ref|NW_003384034.1|	183753-18530[illegible]	781228	38308	-10281	0.6277	no
gi|320446537|ref|NW_003384034.1|	192398-19375[illegible]	482749	544759	0.174345	0.93185	no

gi|320446537|ref|NW_003384034.1|	193970-19707(	922815	797365	-0.210802	0.86405	no
gi|320446537|ref|NW_003384034.1|	198327-19948	104376	266393	-197015	0.3738	no
gi|320446537|ref|NW_003384034.1|	201696-20218	985108	219629	-216521	0.3437	no
gi|320446537|ref|NW_003384034.1|	202318-20349	947877	123935	-293511	0.2105	no
gi|320446537|ref|NW_003384034.1|	204273-20489(	101438	0.299518	-50818	0.28285	no
gi|320446537|ref|NW_003384034.1|	205159-20930	199053	0.151144	-704109	0.1123	no
gi|320446537|ref|NW_003384034.1|	212991-21718	268094	267073	-0.00550504	0.99665	no
gi|320446537|ref|NW_003384034.1|	217459-21772	512808	424122	-0.27394	0.8959	no
gi|320446537|ref|NW_003384034.1|	217859-21827	630198	219829	-151942	0.4883	no
gi|320446537|ref|NW_003384034.1|	221656-22366	126605	406255	168205	0.20465	no
gi|320446537|ref|NW_003384034.1|	235367-23943	193704	557812	152593	0.34575	no
gi|320446537|ref|NW_003384034.1|	239591-23994(	914067	509465	-0.843316	0.69485	no
gi|320446537|ref|NW_003384034.1|	245065-24526	522672	139533	-19053	0.3084	no
gi|320446537|ref|NW_003384034.1|	245843-24681	783265	71563	-0.130285	0.94765	no
gi|320446537|ref|NW_003384034.1|	247886-24834	208623	168561	-0.307628	0.8806	no
gi|320446537|ref|NW_003384034.1|	252768-25380	382222	378958	-0.0123718	0.99295	no
gi|320446537|ref|NW_003384034.1|	254543-25559(	914941	77644	-0.236804	0.90655	no
gi|320446537|ref|NW_003384034.1|	256042-26018	505233	116261	120235	0.3471	no
gi|320446537|ref|NW_003384034.1|	260381-26131(	0.24333	167812	278586	0.2657	no
gi|320446537|ref|NW_003384034.1|	263861-26533	0.818103	0.568736	-0.524524	1	no
gi|320446537|ref|NW_003384034.1|	265446-26703	214421	508301	124523	0.3418	no
gi|320446538|ref|NW_003384033.1|	126429-12728	513421	799735	0.639381	0.753	no
gi|320446542|ref|NW_003384029.1|	159362-16121	124442	0	#NAME?	0.00285	no
gi|320446542|ref|NW_003384029.1|	180097-18058	119101	752119	-0.663153	0.744	no
gi|320446542|ref|NW_003384029.1|	181178-18144(	181448	65946	-14602	0.50875	no
gi|320446542|ref|NW_003384029.1|	183715-18407	159117	111708	-0.510354	0.79925	no
gi|320446542|ref|NW_003384029.1|	184875-18551	133554	851943	-0.648596	0.74835	no
gi|320446542|ref|NW_003384029.1|	185811-19159	922024	13882	0.590338	0.6595	no
gi|320446542|ref|NW_003384029.1|	194597-19513(	0	45371	inf	0.029	no
gi|320446542|ref|NW_003384029.1|	196298-19702	0.682365	210224	162331	0.33265	no

gi|320446542|ref|NW_003384029.1|	199609-20030	108926	372226	177283	0.41715	no
gi|320446542|ref|NW_003384029.1|	204590-20630	0.457729	100401	445513	0.1421	no
gi|320446542|ref|NW_003384029.1|	222945-22391	435921	0.633565	-27825	0.26085	no
gi|320446542|ref|NW_003384029.1|	225330-22638	495395	199844	-13097	0.53035	no
gi|320446542|ref|NW_003384029.1|	226875-22889	179548	164754	-0.124054	0.94885	no
gi|320446542|ref|NW_003384029.1|	229001-23002	257462	192768	-0.417495	0.82905	no
gi|320446542|ref|NW_003384029.1|	230181-23363	555332	615752	0.148999	0.94585	no
gi|320446542|ref|NW_003384029.1|	237182-23768	16.85	501927	-17472	0.42395	no
gi|320446542|ref|NW_003384029.1|	254950-25521	298082	189381	-0.654418	0.74525	no
gi|320446542|ref|NW_003384029.1|	42466-43034	403581	579586	0.522165	0.78855	no
gi|320446544|ref|NW_003384027.1|	119674-12159	742988	615898	-0.270646	0.9033	no
gi|320446544|ref|NW_003384027.1|	122283-12263	116747	843723	-0.46855	0.81985	no
gi|320446544|ref|NW_003384027.1|	123036-12511	119242	761365	-0.647231	0.77475	no
gi|320446544|ref|NW_003384027.1|	126338-12762	306212	11014	-147519	0.3863	no
gi|320446544|ref|NW_003384027.1|	127752-12849	491101	168231	-154558	0.35145	no
gi|320446544|ref|NW_003384027.1|	129395-13123	225605	124881	-0.853245	0.61615	no
gi|320446544|ref|NW_003384027.1|	131733-13224	326908	0	#NAME?	0.00495	no
gi|320446544|ref|NW_003384027.1|	136628-13691	470684	0	#NAME?	0.01425	no
gi|320446544|ref|NW_003384027.1|	181719-18261	671939	0	#NAME?	5.00E-05	yes
gi|320446544|ref|NW_003384027.1|	244495-24496	623533	161831	-194598	0.3786	no
gi|320446544|ref|NW_003384027.1|	245304-24629	318432	681438	-222433	0.1946	no
gi|320446544|ref|NW_003384027.1|	256139-25703	138174	460144	17356	0.303	no
gi|320446544|ref|NW_003384027.1|	269514-27017	392397	481578	0.295454	0.8793	no
gi|320446544|ref|NW_003384027.1|	297649-29787	139612	988094	-0.498702	0.80705	no
gi|320446544|ref|NW_003384027.1|	302119-30273	426687	715189	0.745145	0.64675	no
gi|320446544|ref|NW_003384027.1|	313686-31395	509118	953612	0.905402	0.6647	no
gi|320446544|ref|NW_003384027.1|	322323-32433	216549	631202	154341	0.2508	no
gi|320446544|ref|NW_003384027.1|	363047-36345	18293	132751	285935	0.22965	no
gi|320446545|ref|NW_003384026.1|	87451-87800	0	849109	inf	0.0312	no
gi|320446547|ref|NW_003384024.1|	107061-10743	305216	221784	-37826	0.1695	no

gi|320446547|ref|NW_003384024.1|	108535-10930	630265	0.432347	-386569	0.22575	no
gi|320446547|ref|NW_003384024.1|	113859-11411	210741	763229	-146528	0.50875	no
gi|320446547|ref|NW_003384024.1|	118323-11940	100026	12457	-300534	0.2029	no
gi|320446547|ref|NW_003384024.1|	145157-14844	101316	739628	-0.453993	0.712	no
gi|320446547|ref|NW_003384024.1|	179180-18281	318538	604458	0.924175	0.4925	no
gi|320446547|ref|NW_003384024.1|	188517-18907	160131	153464	-0.0613485	0.9762	no
gi|320446547|ref|NW_003384024.1|	200103-20032	257291	290467	0.174972	0.91645	no
gi|320446547|ref|NW_003384024.1|	202158-20249	816463	620665	-0.395574	0.85635	no
gi|320446547|ref|NW_003384024.1|	261205-26266	826115	106236	0.362857	0.864	no
gi|320446547|ref|NW_003384024.1|	262884-26429	897723	226625	133597	0.3138	no
gi|320446547|ref|NW_003384024.1|	269812-27264	292866	650468	115124	0.6065	no
gi|320446547|ref|NW_003384024.1|	272829-27541	459602	586949	0.35285	0.8693	no
gi|320446547|ref|NW_003384024.1|	279465-28174	48667	171578	181785	0.29595	no
gi|320446547|ref|NW_003384024.1|	38464-38894	659698	884811	-289837	0.2165	no
gi|320446547|ref|NW_003384024.1|	40509-41550	163402	202775	-301047	0.21205	no
gi|320446547|ref|NW_003384024.1|	43959-47268	232209	453093	-235754	0.20205	no
gi|320446547|ref|NW_003384024.1|	65945-66429	0	802138	inf	0.0154	no
gi|320446547|ref|NW_003384024.1|	92444-93003	191697	0	#NAME?	0.007	no
gi|320446551|ref|NW_003384020.1|	110609-11191	158216	0.658856	-126386	0.5499	no
gi|320446551|ref|NW_003384020.1|	112635-11324	316878	153573	-1045	0.59635	no
gi|320446551|ref|NW_003384020.1|	200777-20176	223357	66335	157042	0.462	no
gi|320446551|ref|NW_003384020.1|	86322-88220	0	877182	inf	0.00485	no
gi|320446551|ref|NW_003384020.1|	88799-90192	0.291551	495108	107298	0.1184	no
gi|320446551|ref|NW_003384020.1|	97108-10226	0.308475	451.78	105162	0.05135	no
gi|320446552|ref|NW_003384019.1|	127953-13004	0.637565	292258	21966	0.3187	no
gi|320446552|ref|NW_003384019.1|	17149-17861	247801	0.968465	-135541	0.5288	no
gi|320446552|ref|NW_003384019.1|	27708-28269	46351	104335	-215138	0.2792	no
gi|320446553|ref|NW_003384018.1|	105128-10547	219075	201216	-0.122684	0.9473	no
gi|320446553|ref|NW_003384018.1|	106290-10676	35191	469416	0.415659	0.8285	no
gi|320446553|ref|NW_003384018.1|	106876-10759	14394	228159	0.664579	0.75865	no

gi|320446553|ref|NW_003384018.1|	108509-11105	372267	820888	114085	0.61445	no
gi|320446553|ref|NW_003384018.1|	115473-11662	920571	178551	-236619	0.2946	no
gi|320446553|ref|NW_003384018.1|	117616-11986	259772	193003	-0.428621	0.8337	no
gi|320446553|ref|NW_003384018.1|	120729-12388	677088	77297	0.191068	0.9033	no
gi|320446553|ref|NW_003384018.1|	151759-15439	468619	755434	0.688889	0.60775	no
gi|320446553|ref|NW_003384018.1|	169267-16991	123995	759576	-0.707015	0.72945	no
gi|320446553|ref|NW_003384018.1|	170438-17115	213712	158192	-0.433986	0.8408	no
gi|320446553|ref|NW_003384018.1|	172747-17318	462931	238304	-0.957992	0.65095	no
gi|320446553|ref|NW_003384018.1|	186298-18743	549086	259986	-10786	0.39975	no
gi|320446553|ref|NW_003384018.1|	200134-20211	102289	182345	0.834029	0.61725	no
gi|320446553|ref|NW_003384018.1|	204388-21465	38533	959624	131638	0.41075	no
gi|320446553|ref|NW_003384018.1|	214847-21742	162339	322064	0.98834	0.45265	no
gi|320446553|ref|NW_003384018.1|	222173-22408	151055	203568	-289149	0.11095	no
gi|320446553|ref|NW_003384018.1|	224401-22471	256411	215917	-0.24798	0.84055	no
gi|320446553|ref|NW_003384018.1|	224886-22564	227562	292301	0.361191	0.8734	no
gi|320446553|ref|NW_003384018.1|	225951-22654	853071	131622	0.625663	0.75815	no
gi|320446553|ref|NW_003384018.1|	228045-23010	125702	292683	121933	0.3462	no
gi|320446553|ref|NW_003384018.1|	230279-23087	23502	286642	0.286466	0.8804	no
gi|320446553|ref|NW_003384018.1|	232510-23759	10783	211994	0.97527	0.64705	no
gi|320446553|ref|NW_003384018.1|	239244-24001	0.312799	386298	362641	0.2063	no
gi|320446553|ref|NW_003384018.1|	2514-5531	419759	208235	-101135	0.45175	no
gi|320446553|ref|NW_003384018.1|	32032-33650	125466	787373	-0.672173	0.6127	no
gi|320446553|ref|NW_003384018.1|	34090-36847	758871	677.81	-0.162973	0.9208	no
gi|320446553|ref|NW_003384018.1|	384-660	595286	758902	-29716	0.2338	no
gi|320446553|ref|NW_003384018.1|	54207-55100	128328	636576	23105	0.31475	no
gi|320446553|ref|NW_003384018.1|	56367-56632	0	187317	inf	0.0294	no
gi|320446553|ref|NW_003384018.1|	57731-58172	158927	790987	231529	0.27165	no
gi|320446553|ref|NW_003384018.1|	58921-59421	0	105288	inf	0.01485	no
gi|320446553|ref|NW_003384018.1|	60733-61343	134738	143265	341046	0.1914	no
gi|320446553|ref|NW_003384018.1|	6922-7593	181913	818436	-11523	0.5826	no

gi|320446553|ref|NW_003384018.1|	70252-70801	0	540255	inf	0.02205	no
gi|320446553|ref|NW_003384018.1|	7965-8596	148985	138933	-0.10078	0.9594	no
gi|320446553|ref|NW_003384018.1|	99555-99878	487061	324685	-0.585061	0.77005	no
gi|320446558|ref|NW_003384013.1|	100235-10592[illegible]	189682	118325	264109	0.16445	no
gi|320446558|ref|NW_003384013.1|	106038-11338[illegible]	107311	324919	159829	0.23545	no
gi|320446558|ref|NW_003384013.1|	116603-11712[illegible]	127986	231705	0.8563	0.51315	no
gi|320446558|ref|NW_003384013.1|	118178-12034[illegible]	477701	872969	-245211	0.1977	no
gi|320446558|ref|NW_003384013.1|	120405-12080[illegible]	212834	191021	-347792	0.1777	no
gi|320446558|ref|NW_003384013.1|	122545-12342[illegible]	156857	234066	-274446	0.2471	no
gi|320446558|ref|NW_003384013.1|	123904-12505[illegible]	244633	320914	-293036	0.11425	no
gi|320446558|ref|NW_003384013.1|	125392-12579[illegible]	838189	10037	-306195	0.1925	no
gi|320446558|ref|NW_003384013.1|	126458-12902[illegible]	19258	450103	-209713	0.2409	no
gi|320446558|ref|NW_003384013.1|	129178-12989[illegible]	279125	0.954906	-154748	0.49795	no
gi|320446558|ref|NW_003384013.1|	133120-13407[illegible]	623864	150523	-205125	0.24625	no
gi|320446558|ref|NW_003384013.1|	134230-13551[illegible]	135183	949212	-0.510111	0.814	no
gi|320446558|ref|NW_003384013.1|	137034-13729[illegible]	198585	900216	-114141	0.58475	no
gi|320446558|ref|NW_003384013.1|	137534-13947[illegible]	108761	282613	137766	0.50715	no
gi|320446558|ref|NW_003384013.1|	139591-14243[illegible]	116428	149192	0.357731	0.8589	no
gi|320446558|ref|NW_003384013.1|	146564-14751[illegible]	156314	604636	-137031	0.5196	no
gi|320446558|ref|NW_003384013.1|	152465-15269[illegible]	325707	772166	-207659	0.35185	no
gi|320446558|ref|NW_003384013.1|	155545-15816[illegible]	860065	230761	-189805	0.16115	no
gi|320446558|ref|NW_003384013.1|	160905-16305[illegible]	432293	246217	-0.812082	0.6978	no
gi|320446558|ref|NW_003384013.1|	163539-16435[illegible]	996998	221628	-216945	0.3321	no
gi|320446558|ref|NW_003384013.1|	164864-16554[illegible]	637679	307338	-1053	0.6066	no
gi|320446558|ref|NW_003384013.1|	16504-19468	306713	259815	-0.239403	0.8616	no
gi|320446558|ref|NW_003384013.1|	166460-16889[illegible]	613685	112493	-244766	0.2805	no
gi|320446558|ref|NW_003384013.1|	169003-16972[illegible]	553693	0.947322	-254716	0.2901	no
gi|320446558|ref|NW_003384013.1|	178455-18005[illegible]	119685	188894	-26636	0.25865	no
gi|320446558|ref|NW_003384013.1|	180165-18088[illegible]	553693	0.473661	-354716	0.23295	no
gi|320446558|ref|NW_003384013.1|	182177-18372[illegible]	115438	0.624557	-420815	0.1376	no

gi|320446558|ref|NW_003384013.1|	190454-19265	0.257139	20935	302529	0.2187	no
gi|320446558|ref|NW_003384013.1|	193113-19494	0.530095	192066	185728	0.4025	no
gi|320446558|ref|NW_003384013.1|	199272-20243	374235	221449	-0.756971	0.72385	no
gi|320446558|ref|NW_003384013.1|	20107-21847	0.672902	0.781076	0.215066	1	no
gi|320446558|ref|NW_003384013.1|	217761-22493	28227	233722	-0.272285	0.8981	no
gi|320446558|ref|NW_003384013.1|	21996-22790	0	207476	inf	0.0312	no
gi|320446558|ref|NW_003384013.1|	225099-22560	74359	415432	-0.839893	0.6891	no
gi|320446558|ref|NW_003384013.1|	226246-22704	539066	59646	0.145964	0.94175	no
gi|320446558|ref|NW_003384013.1|	229700-23008	0	684459	inf	0.0312	no
gi|320446558|ref|NW_003384013.1|	23067-24689	243392	111797	-11224	0.50045	no
gi|320446558|ref|NW_003384013.1|	231607-23261	352603	204272	-0.787553	0.63355	no
gi|320446558|ref|NW_003384013.1|	233229-23758	229078	111422	-10398	0.43785	no
gi|320446558|ref|NW_003384013.1|	237654-24026	134951	382077	150143	0.4852	no
gi|320446558|ref|NW_003384013.1|	240547-24180	132149	309386	122724	0.5521	no
gi|320446558|ref|NW_003384013.1|	242776-24341	633775	919589	0.537018	0.79305	no
gi|320446558|ref|NW_003384013.1|	243625-24615	327206	207441	-0.657499	0.61945	no
gi|320446558|ref|NW_003384013.1|	248483-24916	981768	721751	-0.443881	0.8231	no
gi|320446558|ref|NW_003384013.1|	249266-25522	71362	419517	-0.766426	0.5471	no
gi|320446558|ref|NW_003384013.1|	25128-26188	131598	44589	-156137	0.25005	no
gi|320446558|ref|NW_003384013.1|	256121-25662	493979	331221	-0.576654	0.7893	no
gi|320446558|ref|NW_003384013.1|	259652-26132	101836	211878	-226494	0.32145	no
gi|320446558|ref|NW_003384013.1|	263703-26520	801266	102144	-297168	0.21755	no
gi|320446558|ref|NW_003384013.1|	266146-26678	188733	427991	-214069	0.3313	no
gi|320446558|ref|NW_003384013.1|	267095-26753	104688	427281	-129284	0.5394	no
gi|320446558|ref|NW_003384013.1|	267755-26812	589573	461899	-0.352093	0.8613	no
gi|320446558|ref|NW_003384013.1|	268326-27519	486159	483558	-0.00773785	0.99485	no
gi|320446558|ref|NW_003384013.1|	275570-27667	737459	658225	-0.163982	0.9355	no
gi|320446558|ref|NW_003384013.1|	277150-27747	196707	684943	-152199	0.4772	no
gi|320446558|ref|NW_003384013.1|	279872-28036	970618	736198	-0.398809	0.84215	no
gi|320446558|ref|NW_003384013.1|	280854-28150	937015	388402	-127052	0.54745	no

gi|320446558|ref|NW_003384013.1|	282709-28440	704247	530547	-0.408601	0.84995	no
gi|320446558|ref|NW_003384013.1|	42738-43707	602752	0.158816	-856806	0.2504	no
gi|320446558|ref|NW_003384013.1|	43981-45330	275797	0.210453	-703396	0.16165	no
gi|320446558|ref|NW_003384013.1|	45837-46159	253318	0	#NAME?	0.015	no
gi|320446558|ref|NW_003384013.1|	46500-47503	391359	0.303789	-700928	0.1617	no
gi|320446558|ref|NW_003384013.1|	54698-55407	153179	438476	-180465	0.4031	no
gi|320446558|ref|NW_003384013.1|	59662-61721	758741	509848	-0.573539	0.7897	no
gi|320446558|ref|NW_003384013.1|	65679-66393	412461	272486	-0.598075	0.79325	no
gi|320446558|ref|NW_003384013.1|	66756-72089	375666	427844	0.187635	0.88455	no
gi|320446558|ref|NW_003384013.1|	73092-73300	557236	193505	-152592	0.4952	no
gi|320446558|ref|NW_003384013.1|	84491-92192	123846	171933	0.473301	0.72115	no
gi|320446558|ref|NW_003384013.1|	92323-93426	583547	605866	0.0541501	0.9752	no
gi|320446558|ref|NW_003384013.1|	94230-95238	163831	143381	-0.192354	0.9279	no
gi|320446558|ref|NW_003384013.1|	97319-98519	265924	160079	-0.732234	0.65105	no
gi|320446560|ref|NW_003384011.1|	13001-15486	533289	223.89	20698	0.1171	no
gi|320446560|ref|NW_003384011.1|	162559-16279	637381	0	#NAME?	0.015	no
gi|320446560|ref|NW_003384011.1|	163903-16593	363067	0.0654286	-91161	0.2504	no
gi|320446560|ref|NW_003384011.1|	166967-16727	540231	0	#NAME?	0.00935	no
gi|320446560|ref|NW_003384011.1|	172179-17289	253822	0	#NAME?	0.0046	no
gi|320446560|ref|NW_003384011.1|	176534-17713	919933	0	#NAME?	0.0132	no
gi|320446560|ref|NW_003384011.1|	220869-22129	251678	833189	172706	0.42575	no
gi|320446560|ref|NW_003384011.1|	231345-23296	0	61287	inf	0.00595	no
gi|320446560|ref|NW_003384011.1|	286839-28709	374836	197812	-424406	0.30545	no
gi|320446560|ref|NW_003384011.1|	290749-29087	288561	746225	137074	0.3759	no
gi|320446560|ref|NW_003384011.1|	58449-64267	157158	938242	257774	0.16305	no
gi|320446564|ref|NW_003384007.1|	110485-11220	816163	126304	0.629973	0.77745	no
gi|320446564|ref|NW_003384007.1|	158045-15878	13849	254446	-244436	0.2837	no
gi|320446564|ref|NW_003384007.1|	161406-16227	268501	166342	-0.690775	0.7318	no
gi|320446564|ref|NW_003384007.1|	184695-18503	225231	365097	-262506	0.27165	no
gi|320446564|ref|NW_003384007.1|	186430-18713	715465	122298	-254848	0.29275	no

gi|320446564|ref|NW_003384007.1|	187570-19075	123728	280414	-214154	0.22375	no
gi|320446564|ref|NW_003384007.1|	193024-19408	139509	510809	-14495	0.50015	no
gi|320446564|ref|NW_003384007.1|	200818-20178	148248	367741	-201125	0.3572	no
gi|320446564|ref|NW_003384007.1|	205250-20569	393837	418318	0.0870021	0.93155	no
gi|320446564|ref|NW_003384007.1|	206331-21350	246809	130756	-0.916515	0.49185	no
gi|320446564|ref|NW_003384007.1|	213696-21713	743081	984391	0.405713	0.7455	no
gi|320446564|ref|NW_003384007.1|	232628-23293	372671	131602	-150173	0.49335	no
gi|320446564|ref|NW_003384007.1|	233059-23333	173798	142279	-0.28869	0.8765	no
gi|320446564|ref|NW_003384007.1|	234309-23461	507489	482586	-0.0725892	0.9708	no
gi|320446564|ref|NW_003384007.1|	246878-24820	0.929251	129021	0.47347	1	no
gi|320446564|ref|NW_003384007.1|	265596-26624	0.411325	252013	261515	0.2636	no
gi|320446564|ref|NW_003384007.1|	275702-27727	0.884407	0.878989	-0.00886621	1	no
gi|320446564|ref|NW_003384007.1|	277730-28034	817152	129067	0.659446	0.69135	no
gi|320446564|ref|NW_003384007.1|	282579-28319	837962	8087	-0.0512813	0.9743	no
gi|320446564|ref|NW_003384007.1|	284212-28507	595441	316671	-0.910972	0.65225	no
gi|320446564|ref|NW_003384007.1|	285837-28614	145286	105029	-0.468102	0.8111	no
gi|320446564|ref|NW_003384007.1|	286592-28688	220044	128151	-0.779947	0.70615	no
gi|320446564|ref|NW_003384007.1|	286963-28747	341118	200888	-408581	0.1676	no
gi|320446564|ref|NW_003384007.1|	289345-28973	17734	0.684459	-469541	0.28625	no
gi|320446564|ref|NW_003384007.1|	308829-30968	187353	840892	-115577	0.5886	no
gi|320446564|ref|NW_003384007.1|	42026-42642	216664	249219	0.201957	0.925	no
gi|320446564|ref|NW_003384007.1|	48562-48826	298082	327113	0.134078	0.9407	no
gi|320446564|ref|NW_003384007.1|	50504-50663	506533	122789	127745	0.39825	no
gi|320446564|ref|NW_003384007.1|	6772-7151	314714	220377	-0.514069	0.80205	no
gi|320446564|ref|NW_003384007.1|	69339-73101	13779	238733	0.792931	0.55125	no
gi|320446569|ref|NW_003384002.1|	124574-12545	191734	11576	-0.727968	0.73635	no
gi|320446569|ref|NW_003384002.1|	125680-12623	135928	952671	-0.512795	0.8029	no
gi|320446569|ref|NW_003384002.1|	128193-12947	156552	131027	-0.256783	0.9074	no
gi|320446569|ref|NW_003384002.1|	129689-13025	102696	145651	382606	0.17685	no
gi|320446569|ref|NW_003384002.1|	130367-13300	295083	208125	281826	0.1316	no

gi|320446569|ref|NW_003384002.1|	135139-13631(	55916	112483	-231356	0.2093	no
gi|320446569|ref|NW_003384002.1|	142579-14292:	533938	213271	-132398	0.522	no
gi|320446569|ref|NW_003384002.1|	146803-14834:	9701	845696	-0.197995	0.9269	no
gi|320446569|ref|NW_003384002.1|	149381-14998:	366989	279995	-0.390336	0.84175	no
gi|320446569|ref|NW_003384002.1|	154586-15525(	145522	429026	-17621	0.40895	no
gi|320446569|ref|NW_003384002.1|	155497-16337:	772868	14524	0.910143	0.49505	no
gi|320446569|ref|NW_003384002.1|	173703-17644(	212629	347018	0.706675	0.5951	no
gi|320446569|ref|NW_003384002.1|	176597-17796!	566177	610535	0.108822	0.95865	no
gi|320446569|ref|NW_003384002.1|	178123-17863!	638737	319505	-0.999385	0.66225	no
gi|320446571|ref|NW_003384000.1|	129367-13068:	326436	0.107909	-491891	0.28715	no
gi|320446571|ref|NW_003384000.1|	131167-13169:	574765	0.386492	-389446	0.31815	no
gi|320446571|ref|NW_003384000.1|	170036-17027:	78516	247469	165619	0.34325	no
gi|320446571|ref|NW_003384000.1|	174118-17543:	595081	663078	0.156093	0.9389	no
gi|320446571|ref|NW_003384000.1|	177182-17761!	131786	109154	-0.271829	0.892	no
gi|320446571|ref|NW_003384000.1|	177987-18078!	0.591666	739727	364414	0.1524	no
gi|320446571|ref|NW_003384000.1|	201548-20202·	0	504823	inf	0.0294	no
gi|320446571|ref|NW_003384000.1|	220289-22101·	275721	0.471787	-2547	0.30055	no
gi|320446571|ref|NW_003384000.1|	222147-22281	213361	185216	-352602	0.17115	no
gi|320446571|ref|NW_003384000.1|	223757-22427(	335365	434794	-294733	0.2205	no
gi|320446571|ref|NW_003384000.1|	225191-22965:	139553	128158	-0.122893	0.92445	no
gi|320446571|ref|NW_003384000.1|	45161-46129	0	206738	inf	0.02915	no
gi|320446571|ref|NW_003384000.1|	48109-49186	0	291344	inf	0.01575	no
gi|320446571|ref|NW_003384000.1|	55367-57374	0.380983	199256	238683	0.297	no
gi|320446571|ref|NW_003384000.1|	57918-59454	0.259489	523402	433417	0.15955	no
gi|320446571|ref|NW_003384000.1|	70524-71358	225571	71742	166924	0.4268	no
gi|320446571|ref|NW_003384000.1|	73503-74544	377082	91249	127493	0.55605	no
gi|320446571|ref|NW_003384000.1|	74709-75495	0.613106	652309	341135	0.1964	no
gi|320446571|ref|NW_003384000.1|	75634-77882	217981	590998	143895	0.50445	no
gi|320446571|ref|NW_003384000.1|	77990-79655	212463	821696	195139	0.3789	no
gi|320446571|ref|NW_003384000.1|	79813-80426	0.891244	605049	276316	0.2352	no

gi|320446571|ref|NW_003384000.1|	80614-82333	0.796166	300975	19185	0.37835	no
gi|320446577|ref|NW_003383994.1|	104310-10598	0.117134	122321	338445	1	no
gi|320446577|ref|NW_003383994.1|	106206-10811	141153	653595	221113	0.32015	no
gi|320446577|ref|NW_003383994.1|	121937-12303	0.974826	323864	173217	0.43075	no
gi|320446577|ref|NW_003383994.1|	124077-12586	0.32689	136631	206341	1	no
gi|320446577|ref|NW_003383994.1|	130934-13385	0.188763	158345	306842	0.21365	no
gi|320446577|ref|NW_003383994.1|	134875-13547	0	403398	inf	0.02915	no
gi|320446577|ref|NW_003383994.1|	140956-14114	283799	527914	0.895434	0.6728	no
gi|320446577|ref|NW_003383994.1|	141384-14344	0	347746	inf	0.0071	no
gi|320446577|ref|NW_003383994.1|	15294-16332	0.630821	276208	213045	0.3464	no
gi|320446577|ref|NW_003383994.1|	167427-16823	0	609621	inf	0.00935	no
gi|320446577|ref|NW_003383994.1|	183238-18403	0.604325	622429	336451	0.19285	no
gi|320446577|ref|NW_003383994.1|	197780-20079	0.425224	271816	267634	0.24465	no
gi|320446577|ref|NW_003383994.1|	200951-20190	0.467122	757603	401957	0.16715	no
gi|320446577|ref|NW_003383994.1|	202027-20277	0.655935	606857	320973	0.2068	no
gi|320446577|ref|NW_003383994.1|	208518-20966	0.552946	114907	105525	1	no
gi|320446577|ref|NW_003383994.1|	210519-21316	0.420321	469864	348268	0.1686	no
gi|320446577|ref|NW_003383994.1|	217078-21760	0	384055	inf	0.0312	no
gi|320446577|ref|NW_003383994.1|	219616-22179	0.086682	157263	418131	0.1906	no
gi|320446577|ref|NW_003383994.1|	227101-22852	0.427718	18823	213777	0.3451	no
gi|320446577|ref|NW_003383994.1|	228662-23034	0.232492	121402	238455	1	no
gi|320446577|ref|NW_003383994.1|	231808-23260	0.896851	164256	0.873003	0.6867	no
gi|320446577|ref|NW_003383994.1|	33615-34839	448946	345625	-0.377336	0.77015	no
gi|320446577|ref|NW_003383994.1|	34987-35779	137675	158.86	0.206496	0.879	no
gi|320446577|ref|NW_003383994.1|	36004-36662	538413	771862	0.519629	0.68215	no
gi|320446577|ref|NW_003383994.1|	38206-39698	20949	428569	103265	0.42905	no
gi|320446577|ref|NW_003383994.1|	45396-46005	180122	183354	0.0256585	0.9157	no
gi|320446577|ref|NW_003383994.1|	53419-55372	137537	178079	0.372696	0.8537	no
gi|320446577|ref|NW_003383994.1|	57888-58647	109597	773505	-0.502722	0.80655	no
gi|320446577|ref|NW_003383994.1|	62663-63356	121638	528794	-120182	0.5655	no

gi|320446577|ref|NW_003383994.1|	64131-67024	491408	229888	-109599	0.417	no
gi|320446577|ref|NW_003383994.1|	79526-80330	474954	154969	170612	0.42015	no
gi|320446577|ref|NW_003383994.1|	82095-82420	0	119988	inf	0.029	no
gi|320446577|ref|NW_003383994.1|	84074-84651	491529	352456	284209	0.2228	no
gi|320446577|ref|NW_003383994.1|	8471-10805	0.884432	196459	11514	0.57645	no
gi|320446577|ref|NW_003383994.1|	84839-85214	0	138104	inf	0.0212	no
gi|320446577|ref|NW_003383994.1|	85730-86028	578935	111389	0.944128	0.6699	no
gi|320446577|ref|NW_003383994.1|	86611-89242	408516	337569	304672	0.1188	no
gi|320446577|ref|NW_003383994.1|	92247-93152	100822	191088	0.922425	0.6847	no
gi|320446577|ref|NW_003383994.1|	93869-95111	0.335297	127901	193152	1	no
gi|320446577|ref|NW_003383994.1|	95292-96577	0.482336	312387	269522	0.2599	no
gi|320446577|ref|NW_003383994.1|	96821-97455	0.422516	488531	353137	0.2166	no
gi|320446579|ref|NW_003383992.1|	127723-12862	178303	263276	0.562243	0.7849	no
gi|320446579|ref|NW_003383992.1|	129583-13127	0.924869	0.804951	-0.200347	1	no
gi|320446579|ref|NW_003383992.1|	131519-13231	11958	123192	0.0429277	1	no
gi|320446579|ref|NW_003383992.1|	137625-13880	0.713865	0.989412	0.47092	1	no
gi|320446579|ref|NW_003383992.1|	143326-14366	555259	900541	0.69763	0.74425	no
gi|320446579|ref|NW_003383992.1|	169528-17003	332567	770982	-210888	0.3413	no
gi|320446579|ref|NW_003383992.1|	171119-17170	653549	52056	-365016	0.07305	no
gi|320446579|ref|NW_003383992.1|	172190-17348	427244	47749	-316152	0.10085	no
gi|320446579|ref|NW_003383992.1|	173699-17603	338176	168633	-100389	0.62495	no
gi|320446579|ref|NW_003383992.1|	180294-18115	346919	907055	-193534	0.25135	no
gi|320446579|ref|NW_003383992.1|	185027-18626	101192	198836	0.974481	0.6554	no
gi|320446579|ref|NW_003383992.1|	186479-19113	0.572771	31289	244963	0.28525	no
gi|320446579|ref|NW_003383992.1|	219334-21970	0	74778	inf	0.0312	no
gi|320446579|ref|NW_003383992.1|	228160-22958	0.284424	187759	272277	0.24095	no
gi|320446579|ref|NW_003383992.1|	239055-23961	0.51348	381466	289317	0.25825	no
gi|320446579|ref|NW_003383992.1|	245153-24715	162837	627855	-137492	0.40835	no
gi|320446579|ref|NW_003383992.1|	247577-24849	169858	611046	-147497	0.4943	no
gi|320446579|ref|NW_003383992.1|	250280-25061	402558	289401	-379805	0.1693	no

gi|320446579|ref|NW_003383992.1|	251288-253428	177187	543937	161816	0.46295	no
gi|320446579|ref|NW_003383992.1|	262234-26666[illegible]	530704	836151	0.655856	0.60695	no
gi|320446579|ref|NW_003383992.1|	275194-28173[illegible]	162123	0.918883	-414106	0.05215	no
gi|320446579|ref|NW_003383992.1|	284206-287667	487516	124058	-197443	0.1457	no
gi|320446579|ref|NW_003383992.1|	287946-288347	162645	310448	-238931	0.17895	no
gi|320446579|ref|NW_003383992.1|	290092-290789	369084	107373	-178132	0.4247	no
gi|320446579|ref|NW_003383992.1|	291842-293564	161182	537509	-158433	0.3408	no
gi|320446579|ref|NW_003383992.1|	295521-296077	684869	324614	-10771	0.50465	no
gi|320446579|ref|NW_003383992.1|	296702-30149[illegible]	723109	299941	-126954	0.33715	no
gi|320446579|ref|NW_003383992.1|	302375-30407[illegible]	363669	329582	-346391	0.07765	no
gi|320446579|ref|NW_003383992.1|	304318-304808	78225	130841	-257981	0.23895	no
gi|320446579|ref|NW_003383992.1|	305948-30785[illegible]	958398	0.632887	-392061	0.1387	no
gi|320446579|ref|NW_003383992.1|	311693-314088	367219	496448	0.435004	0.83495	no
gi|320446579|ref|NW_003383992.1|	366704-367195	40267	139069	-153379	0.47625	no
gi|320446579|ref|NW_003383992.1|	374382-37468[illegible]	300734	120647	-13177	0.5353	no
gi|320446579|ref|NW_003383992.1|	412793-413124	162809	957892	-0.765245	0.712	no
gi|320446579|ref|NW_003383992.1|	424196-42574[illegible]	0.515805	0.627867	0.283633	1	no
gi|320446579|ref|NW_003383992.1|	432424-433665	364159	259546	-0.48858	0.7003	no
gi|320446579|ref|NW_003383992.1|	433819-44643[illegible]	244441	412473	0.754816	0.6468	no
gi|320446579|ref|NW_003383992.1|	44648-44934	366477	27559	-0.411198	0.84435	no
gi|320446579|ref|NW_003383992.1|	446714-448135	0	108793	inf	0.00445	no
gi|320446579|ref|NW_003383992.1|	458563-46103[illegible]	521713	665336	0.350828	0.869	no
gi|320446579|ref|NW_003383992.1|	461929-466195	895821	492393	-0.863399	0.6065	no
gi|320446579|ref|NW_003383992.1|	468869-469858	0.671851	216538	168841	0.4292	no
gi|320446579|ref|NW_003383992.1|	470598-471587	0.447901	139203	163594	1	no
gi|320446579|ref|NW_003383992.1|	56928-57319	548056	146668	-190177	0.38435	no
gi|320446579|ref|NW_003383992.1|	58726-59019	0	155149	inf	0.029	no
gi|320446579|ref|NW_003383992.1|	60886-61405	29418	47432	0.689163	0.7415	no
gi|320446579|ref|NW_003383992.1|	67748-76199	13554	161446	0.252328	0.8488	no
gi|320446579|ref|NW_003383992.1|	76363-77176	794836	259028	-161755	0.35305	no

gi|320446579|ref|NW_003383992.1|	78028-83049	260897	163249	-0.676404	0.6124	no
gi|320446580|ref|NW_003383991.1|	10410-11882	31386	123325	-134766	0.5272	no
gi|320446580|ref|NW_003383991.1|	12089-12554	502636	239256	-107096	0.59545	no
gi|320446580|ref|NW_003383991.1|	13728-15687	177196	416356	-208946	0.2295	no
gi|320446580|ref|NW_003383991.1|	297076-29758	0	482022	inf	0.00515	no
gi|320446580|ref|NW_003383991.1|	359823-36146	791772	370412	55479	0.04115	no
gi|320446580|ref|NW_003383991.1|	371989-37381	989085	344428	512197	0.05625	no
gi|320446580|ref|NW_003383991.1|	391547-39308	0.777869	414796	24148	0.2843	no
gi|320446580|ref|NW_003383991.1|	428755-42980	0	329109	inf	0.01485	no
gi|320446580|ref|NW_003383991.1|	435486-43658	176488	130152	620448	0.0509	no
gi|320446580|ref|NW_003383991.1|	445664-44707	0.864199	182132	439748	0.07715	no
gi|320446580|ref|NW_003383991.1|	492744-49303	185071	210599	0.186425	0.92405	no
gi|320446580|ref|NW_003383991.1|	529170-52965	338452	271312	-0.318998	0.8817	no
gi|320446580|ref|NW_003383991.1|	612258-61297	174812	239205	0.452439	0.82655	no
gi|320446580|ref|NW_003383991.1|	613153-61394	336597	210049	-0.680293	0.7368	no
gi|320446580|ref|NW_003383991.1|	614153-61474	189571	385235	1023	0.60635	no
gi|320446580|ref|NW_003383991.1|	616582-61855	358893	730423	102518	0.6346	no
gi|320446580|ref|NW_003383991.1|	631286-63275	128794	108581	-0.246295	0.9137	no
gi|320446580|ref|NW_003383991.1|	632867-63396	187166	688212	-14434	0.50665	no
gi|320446580|ref|NW_003383991.1|	637806-63958	216868	166328	-0.382787	0.75645	no
gi|320446580|ref|NW_003383991.1|	640491-64401	50619	218386	-12128	0.3704	no
gi|320446580|ref|NW_003383991.1|	645274-64546	179739	293285	-261553	0.2669	no
gi|320446580|ref|NW_003383991.1|	648362-64908	208486	19022	-0.132281	0.9301	no
gi|320446580|ref|NW_003383991.1|	65068-65945	260428	307958	-308008	0.2026	no
gi|320446580|ref|NW_003383991.1|	671128-67175	603452	234372	-136444	0.51635	no
gi|320446580|ref|NW_003383991.1|	679013-67926	617034	156343	134129	0.38395	no
gi|320446580|ref|NW_003383991.1|	685718-68621	711786	30314	-123146	0.5516	no
gi|320446580|ref|NW_003383991.1|	731954-73648	993276	159737	0.685435	0.59735	no
gi|320446580|ref|NW_003383991.1|	756324-75840	0.547667	10824	0.982868	1	no
gi|320446580|ref|NW_003383991.1|	758638-75978	0.372291	167601	217053	0.2872	no

gi|320446580|ref|NW_003383991.1|	77741-77920	995678	306927	-169778	0.4409	no
gi|320446580|ref|NW_003383991.1|	8141-8287	287239	113953	-133381	0.52805	no
gi|320446582|ref|NW_003383989.1|	14705-16149	0.139623	203785	386744	0.19995	no
gi|320446582|ref|NW_003383989.1|	148691-15030	724803	111136	-270526	0.25245	no
gi|320446582|ref|NW_003383989.1|	164550-16613	0.998784	32228	5012	0.05975	no
gi|320446582|ref|NW_003383989.1|	170665-17365	155415	203623	0.389776	0.7645	no
gi|320446582|ref|NW_003383989.1|	17077-18593	0.131771	311567	456344	0.1776	no
gi|320446582|ref|NW_003383989.1|	175225-17592	112317	718146	-0.645225	0.7481	no
gi|320446582|ref|NW_003383989.1|	18693-20643	0	334134	inf	5.00E-05	yes
gi|320446582|ref|NW_003383989.1|	187477-18865	338728	111193	-160706	0.3443	no
gi|320446582|ref|NW_003383989.1|	189393-19075	422776	0.832936	-566554	0.0699	no
gi|320446582|ref|NW_003383989.1|	191439-19211	585651	0	#NAME?	5.00E-05	yes
gi|320446582|ref|NW_003383989.1|	193344-19513	141287	305877	111432	0.51555	no
gi|320446582|ref|NW_003383989.1|	195393-20234	142178	568807	-132169	0.32445	no
gi|320446582|ref|NW_003383989.1|	203516-20491	969159	108537	0.163375	0.9385	no
gi|320446582|ref|NW_003383989.1|	206653-20876	176706	159121	-0.151226	0.9045	no
gi|320446582|ref|NW_003383989.1|	208990-20937	861052	476582	-0.853376	0.6938	no
gi|320446582|ref|NW_003383989.1|	210473-21620	224896	222399	-0.0161091	0.98905	no
gi|320446582|ref|NW_003383989.1|	21926-22435	0.608879	833326	709658	0.14075	no
gi|320446582|ref|NW_003383989.1|	224865-22593	152571	164633	0.109772	0.9601	no
gi|320446582|ref|NW_003383989.1|	227813-22854	15507	18931	0.287829	0.8922	no
gi|320446582|ref|NW_003383989.1|	229080-23083	100269	36703	519395	0.0529	no
gi|320446582|ref|NW_003383989.1|	30904-37127	0.281784	136984	89252	0.048	no
gi|320446585|ref|NW_003383986.1|	109294-11013	219199	734406	-157759	0.47795	no
gi|320446585|ref|NW_003383986.1|	112813-11873	149345	832296	-0.843479	0.529	no
gi|320446585|ref|NW_003383986.1|	132396-13467	671102	347285	-0.95041	0.4759	no
gi|320446585|ref|NW_003383986.1|	135670-13636	130968	395313	-172814	0.3357	no
gi|320446585|ref|NW_003383986.1|	136864-14740	109668	157885	0.525734	0.691	no
gi|320446585|ref|NW_003383986.1|	150231-15325	153963	983218	-0.646998	0.61515	no
gi|320446585|ref|NW_003383986.1|	153394-15577	181129	0.934746	-0.954369	0.6521	no

gi|320446585|ref|NW_003383986.1|	155931-15729	183587	839659	-112859	0.4836	no
gi|320446585|ref|NW_003383986.1|	164432-16556	471608	209007	-117403	0.56065	no
gi|320446585|ref|NW_003383986.1|	192690-19854	46837	403971	-0.213397	0.86315	no
gi|320446585|ref|NW_003383986.1|	199286-20089	272524	324625	-306954	0.1056	no
gi|320446585|ref|NW_003383986.1|	203988-20513	22.4	294921	-29251	0.2288	no
gi|320446585|ref|NW_003383986.1|	205372-20731	123375	323407	-193163	0.2474	no
gi|320446585|ref|NW_003383986.1|	208202-21039	123007	894393	-0.459763	0.7685	no
gi|320446585|ref|NW_003383986.1|	210554-21175	233465	121985	-0.9365	0.5583	no
gi|320446585|ref|NW_003383986.1|	212939-21505	0.179832	357545	431341	0.1643	no
gi|320446585|ref|NW_003383986.1|	215163-21718	264449	250946	324632	0.09575	no
gi|320446585|ref|NW_003383986.1|	217350-21789	109866	777087	282233	0.2258	no
gi|320446585|ref|NW_003383986.1|	219416-21999	939265	678716	28532	0.11775	no
gi|320446585|ref|NW_003383986.1|	234504-23703	303962	347512	0.193173	0.8855	no
gi|320446585|ref|NW_003383986.1|	238549-23983	113612	921666	-0.301794	0.8956	no
gi|320446585|ref|NW_003383986.1|	240285-24163	255867	176624	-0.534716	0.74615	no
gi|320446585|ref|NW_003383986.1|	241782-24200	205833	387289	0.911938	0.684	no
gi|320446585|ref|NW_003383986.1|	242649-24621	150784	7895	-0.933471	0.4705	no
gi|320446585|ref|NW_003383986.1|	246326-24731	130759	342509	-193269	0.38325	no
gi|320446585|ref|NW_003383986.1|	248372-24877	578037	174784	-172559	0.42735	no
gi|320446585|ref|NW_003383986.1|	266203-26684	0	310182	inf	0.0294	no
gi|320446585|ref|NW_003383986.1|	268134-26875	0.870849	266258	161233	0.33275	no
gi|320446585|ref|NW_003383986.1|	273848-27565	0.53616	298839	247863	0.283	no
gi|320446585|ref|NW_003383986.1|	276496-27848	0.288835	67805	455307	0.1555	no
gi|320446585|ref|NW_003383986.1|	284426-28487	774091	421687	24456	0.28645	no
gi|320446585|ref|NW_003383986.1|	293593-29388	846006	392274	221312	0.3288	no
gi|320446585|ref|NW_003383986.1|	294952-29580	192234	107702	248611	0.27625	no
gi|320446585|ref|NW_003383986.1|	296017-29862	0.497816	129175	137565	1	no
gi|320446585|ref|NW_003383986.1|	298851-30097	543849	41612	293572	0.13	no
gi|320446585|ref|NW_003383986.1|	301639-30302	0.14679	163147	347435	0.21395	no
gi|320446585|ref|NW_003383986.1|	307879-30922	0.303346	15798	238071	0.2609	no

gi|320446585|ref|NW_003383986.1|	320074-32071	251027	241955	326882	0.191	no
gi|320446585|ref|NW_003383986.1|	324236-33104	246472	52554	109238	0.5125	no
gi|320446585|ref|NW_003383986.1|	331673-33232	16028	144699	-0.147532	0.9426	no
gi|320446585|ref|NW_003383986.1|	332420-33300	477881	420691	-0.18389	0.921	no
gi|320446585|ref|NW_003383986.1|	334366-33745	532527	604059	0.181834	0.9325	no
gi|320446585|ref|NW_003383986.1|	36744-37177	181205	764078	-124583	0.54495	no
gi|320446585|ref|NW_003383986.1|	38832-40135	569042	383977	-0.567517	0.78295	no
gi|320446585|ref|NW_003383986.1|	41399-42707	19509	862697	-117721	0.46765	no
gi|320446585|ref|NW_003383986.1|	51300-51611	86374	555957	-0.635623	0.75175	no
gi|320446585|ref|NW_003383986.1|	54548-55605	22595	170474	-0.406456	0.8455	no
gi|320446585|ref|NW_003383986.1|	56384-57663	108341	628324	-0.786003	0.7161	no
gi|320446585|ref|NW_003383986.1|	58401-61220	330459	151998	-112042	0.4035	no
gi|320446585|ref|NW_003383986.1|	62615-63130	435379	300343	-0.53566	0.8088	no
gi|320446585|ref|NW_003383986.1|	63248-64014	257659	172346	-0.580154	0.7955	no
gi|320446585|ref|NW_003383986.1|	64449-65251	244274	12072	-101683	0.63725	no
gi|320446585|ref|NW_003383986.1|	65389-67939	236921	220998	-0.100377	0.93885	no
gi|320446585|ref|NW_003383986.1|	77761-78763	118882	684403	-0.79661	0.6994	no
gi|320446585|ref|NW_003383986.1|	89751-90153	134039	693468	-0.95075	0.65945	no
gi|320446585|ref|NW_003383986.1|	90364-93600	372251	283555	-0.392649	0.7709	no
gi|320446586|ref|NW_003383985.1|	303885-30410	803683	890904	-317328	0.18845	no
gi|320446586|ref|NW_003383985.1|	305232-30610	0.52942	16404	163156	0.3326	no
gi|320446586|ref|NW_003383985.1|	307121-30875	0.604556	244045	20132	0.3519	no
gi|320446586|ref|NW_003383985.1|	332713-33413	485157	535418	0.142214	0.94435	no
gi|320446586|ref|NW_003383985.1|	343358-34410	67428	115275	0.773661	0.5528	no
gi|320446586|ref|NW_003383985.1|	354801-35505	832995	138754	0.736151	0.73415	no
gi|320446586|ref|NW_003383985.1|	355845-35662	39817	900766	117777	0.35635	no
gi|320446586|ref|NW_003383985.1|	359534-36316	763457	337646	214489	0.20085	no
gi|320446586|ref|NW_003383985.1|	368747-36897	301374	39023	-294916	0.2139	no
gi|320446586|ref|NW_003383985.1|	371282-37222	283431	673718	-207278	0.35905	no
gi|320446586|ref|NW_003383985.1|	374949-37546	691928	299432	-120839	0.5855	no

gi|320446586|ref|NW_003383985.1|	388357-38893[illegible]	396618	569957	0.523104	0.78855	no
gi|320446586|ref|NW_003383985.1|	389713-39222[illegible]	555869	429661	-0.371544	0.86795	no
gi|320446586|ref|NW_003383985.1|	392584-39376[illegible]	163842	132058	-0.311134	0.88965	no
gi|320446592|ref|NW_003383979.1|	107620-10864[illegible]	0.42965	178163	205196	0.3018	no
gi|320446592|ref|NW_003383979.1|	17831-17942	24859.9	2251.03	-346516	0.183	no
gi|320446592|ref|NW_003383979.1|	18089-18564	110418	0.921293	-358318	0.2326	no
gi|320446592|ref|NW_003383979.1|	18793-20076	13209	111777	-356283	0.15995	no
gi|320446592|ref|NW_003383979.1|	21060-22896	249852	550945	-218109	0.2173	no
gi|320446592|ref|NW_003383979.1|	24978-25805	423101	262048	-0.691172	0.5917	no
gi|320446592|ref|NW_003383979.1|	28042-28516	727456	268178	-143967	0.51765	no
gi|320446592|ref|NW_003383979.1|	28640-29109	240252	127226	-0.917153	0.65815	no
gi|320446592|ref|NW_003383979.1|	29666-30350	375934	436329	0.214935	0.911	no
gi|320446592|ref|NW_003383979.1|	30522-32065	280038	211841	-0.402641	0.75045	no
gi|320446592|ref|NW_003383979.1|	35122-37488	966181	37931	197301	0.283	no
gi|320446592|ref|NW_003383979.1|	39179-39393	22106	346167	0.647028	0.7584	no
gi|320446592|ref|NW_003383979.1|	39936-40149	562899	352467	264654	0.27135	no
gi|320446592|ref|NW_003383979.1|	42085-43139	103081	427723	205289	0.3538	no
gi|320446592|ref|NW_003383979.1|	43385-43785	677198	159185	123305	0.55085	no
gi|320446592|ref|NW_003383979.1|	46178-46869	592329	910334	0.619998	0.75555	no
gi|320446592|ref|NW_003383979.1|	52347-53526	339805	105345	163234	0.45015	no
gi|320446592|ref|NW_003383979.1|	53920-54252	440803	760959	0.787683	0.70455	no
gi|320446592|ref|NW_003383979.1|	54879-56133	364481	321779	-0.179774	0.92455	no
gi|320446592|ref|NW_003383979.1|	61280-62694	768863	171113	-216778	0.24775	no
gi|320446592|ref|NW_003383979.1|	65860-66515	334569	0	#NAME?	5.00E-05	yes
gi|320446592|ref|NW_003383979.1|	68704-70627	250368	230011	-67662	0.01995	no
gi|320446592|ref|NW_003383979.1|	71124-72421	275797	154449	-748034	0.04815	no
gi|320446592|ref|NW_003383979.1|	73761-74891	169779	101891	-0.73663	0.72685	no
gi|320446592|ref|NW_003383979.1|	75010-76368	901928	887333	-0.0235366	0.99055	no
gi|320446592|ref|NW_003383979.1|	84274-84543	743572	652495	-351043	0.20215	no
gi|320446592|ref|NW_003383979.1|	86829-88072	133986	0.46464	-484982	0.1595	no

gi|320446592|ref|NW_003383979.1|	90577-91603	0.640398	13279	105211	1	no
gi|320446592|ref|NW_003383979.1|	91923-96900	974987	160522	0.71932	0.58915	no
gi|320446593|ref|NW_003383978.1|	157520-157994	346407	143336	204886	0.3608	no
gi|320446593|ref|NW_003383978.1|	49653-50265	357427	181971	-0.973939	0.6699	no
gi|320446595|ref|NW_003383976.1|	114874-115237	370065	155798	-457003	0.20375	no
gi|320446595|ref|NW_003383976.1|	115392-115938	145552	0.726806	-432382	0.20465	no
gi|320446595|ref|NW_003383976.1|	116038-116462	128815	0.56804	-450316	0.29535	no
gi|320446595|ref|NW_003383976.1|	117427-117838	128051	120683	-340743	0.2365	no
gi|320446595|ref|NW_003383976.1|	118831-119762	320272	167338	-425846	0.0727	no
gi|320446595|ref|NW_003383976.1|	122748-123778	169913	0.293627	-585467	0.178	no
gi|320446595|ref|NW_003383976.1|	127613-128362	623138	0.449524	-379308	0.2213	no
gi|320446595|ref|NW_003383976.1|	128990-130738	567826	79718	0.489458	0.71385	no
gi|320446595|ref|NW_003383976.1|	208517-210218	932595	0.0801684	-686207	0.262	no
gi|320446595|ref|NW_003383976.1|	210807-211707	584089	0	#NAME?	0.0111	no
gi|320446595|ref|NW_003383976.1|	222046-225211	535626	0.805541	-27332	0.2467	no
gi|320446595|ref|NW_003383976.1|	244943-245472	113825	306278	142803	0.37565	no
gi|320446596|ref|NW_003383975.1|	119164-122140	0	258252	inf	5.00E-05	yes
gi|320446596|ref|NW_003383975.1|	207747-209470	620474	175206	149761	0.2637	no
gi|320446596|ref|NW_003383975.1|	20922-21706	461505	515259	348088	0.0787	no
gi|320446596|ref|NW_003383975.1|	209583-211481	0.507455	134407	140526	1	no
gi|320446596|ref|NW_003383975.1|	220948-221217	999976	254473	134755	0.5416	no
gi|320446596|ref|NW_003383975.1|	223701-223991	10187	40439	198902	0.3886	no
gi|320446596|ref|NW_003383975.1|	225458-225938	527985	103551	0.971769	0.5567	no
gi|320446596|ref|NW_003383975.1|	226328-226663	172553	124836	285493	0.22365	no
gi|320446596|ref|NW_003383975.1|	35153-37881	0.473848	643321	376304	0.14425	no
gi|320446596|ref|NW_003383975.1|	38902-40806	0.404504	114896	482803	0.1406	no
gi|320446596|ref|NW_003383975.1|	41089-43171	0.456884	208428	551158	0.0856	no
gi|320446596|ref|NW_003383975.1|	69911-72418	0.891348	138519	395795	0.06525	no
gi|320446596|ref|NW_003383975.1|	85941-86695	781066	713841	319209	0.0964	no
gi|320446596|ref|NW_003383975.1|	93098-93633	206519	683878	172746	0.3013	no

gi|320446598|ref|NW_003383973.1|	104503-10680	889103	614954	-0.531873	0.81145	no
gi|320446598|ref|NW_003383973.1|	107347-10997	137042	370139	-188848	0.27585	no
gi|320446598|ref|NW_003383973.1|	111675-11225	155076	426397	-18627	0.3959	no
gi|320446598|ref|NW_003383973.1|	112948-11346	208032	359233	-253382	0.26075	no
gi|320446598|ref|NW_003383973.1|	125776-12635	0.488739	109127	44808	0.1768	no
gi|320446598|ref|NW_003383973.1|	131942-13231	0	813209	inf	0.0294	no
gi|320446598|ref|NW_003383973.1|	207561-20965	0.815325	183267	11685	0.57305	no
gi|320446598|ref|NW_003383973.1|	413614-41766	428931	569576	0.409142	0.7942	no
gi|320446598|ref|NW_003383973.1|	418076-42525	206627	66467	-163632	0.2261	no
gi|320446598|ref|NW_003383973.1|	431307-43248	321838	336384	-325815	0.0976	no
gi|320446598|ref|NW_003383973.1|	433343-43371	112377	220529	-23493	0.25625	no
gi|320446598|ref|NW_003383973.1|	434563-43495	129821	196922	-272083	0.224	no
gi|320446598|ref|NW_003383973.1|	435957-43638	434232	497706	-31251	0.18895	no
gi|320446598|ref|NW_003383973.1|	461624-46243	0.878178	181024	104359	0.5772	no
gi|320446598|ref|NW_003383973.1|	469923-47073	0	24782	inf	0.00525	no
gi|320446598|ref|NW_003383973.1|	471061-47132	0	34433	inf	0.0162	no
gi|320446598|ref|NW_003383973.1|	472647-47330	0	200665	inf	0.0052	no
gi|320446598|ref|NW_003383973.1|	486231-48668	802397	0	#NAME?	0.0033	no
gi|320446598|ref|NW_003383973.1|	489133-48955	853109	0	#NAME?	0.004	no
gi|320446598|ref|NW_003383973.1|	60840-62417	63644	325535	-0.967213	0.4726	no
gi|320446598|ref|NW_003383973.1|	63124-64164	266383	150818	-0.8207	0.60335	no
gi|320446598|ref|NW_003383973.1|	65116-65449	714784	236147	-159782	0.448	no
gi|320446598|ref|NW_003383973.1|	65721-66966	380768	143525	-140761	0.29015	no
gi|320446598|ref|NW_003383973.1|	67995-73090	120823	766148	-0.657206	0.61475	no
gi|320446598|ref|NW_003383973.1|	75010-77108	154006	575079	-142115	0.38795	no
gi|320446598|ref|NW_003383973.1|	78287-79368	319324	303795	-0.0719268	0.9648	no
gi|320446598|ref|NW_003383973.1|	98094-102458	118515	549418	-110909	0.38475	no
gi|320446602|ref|NW_003383969.1|	138265-14076	0.298166	26031	312604	0.2129	no
gi|320446602|ref|NW_003383969.1|	141260-14372	0.0757803	232858	494149	0.171	no
gi|320446602|ref|NW_003383969.1|	216623-21806	381486	160248	-125133	0.46135	no

gi|320446602|ref|NW_003383969.1|	220010-22556	126139	181877	0.527955	0.69645	no
gi|320446602|ref|NW_003383969.1|	234768-23518	0	138204	inf	0.0138	no
gi|320446602|ref|NW_003383969.1|	241222-24206	140045	558645	199604	0.3775	no
gi|320446602|ref|NW_003383969.1|	255148-25606	0	628019	inf	0.0103	no
gi|320446602|ref|NW_003383969.1|	260354-26052	802163	599928	290282	0.22655	no
gi|320446602|ref|NW_003383969.1|	52122-52511	410421	114526	14805	0.50525	no
gi|320446602|ref|NW_003383969.1|	56963-58017	0	955247	inf	0.00565	no
gi|320446602|ref|NW_003383969.1|	59227-59534	0	183697	inf	0.0198	no
gi|320446602|ref|NW_003383969.1|	6430-7737	0	185815	inf	0.02075	no
gi|320446606|ref|NW_003383965.1|	13702-13990	169201	608702	1847	0.38425	no
gi|320446606|ref|NW_003383965.1|	143079-14570	0.281971	201922	284018	0.2382	no
gi|320446606|ref|NW_003383965.1|	146816-14818	0.1486	258025	411801	0.19225	no
gi|320446606|ref|NW_003383965.1|	6976-7341	235829	923798	196984	0.3149	no
gi|320446609|ref|NW_003383962.1|	141718-14326	0.51306	124911	128371	1	no
gi|320446609|ref|NW_003383962.1|	149804-15467	0.691397	29567	20964	0.35035	no
gi|320446609|ref|NW_003383962.1|	155745-15661	0.52859	291178	246168	0.2454	no
gi|320446610|ref|NW_003383961.1|	10360-11182	143867	751566	-0.936762	0.6576	no
gi|320446610|ref|NW_003383961.1|	11352-11954	825726	155553	-240826	0.3042	no
gi|320446610|ref|NW_003383961.1|	116612-12151	104806	673367	268368	0.1422	no
gi|320446610|ref|NW_003383961.1|	12963-14201	454458	128405	-182344	0.40535	no
gi|320446610|ref|NW_003383961.1|	14660-15822	147523	403772	-186933	0.4055	no
gi|320446610|ref|NW_003383961.1|	1553-3267	376605	770854	-228852	0.21145	no
gi|320446610|ref|NW_003383961.1|	16063-18196	34679	0.620375	-248285	0.27435	no
gi|320446610|ref|NW_003383961.1|	18980-19558	122533	132638	-32076	0.2204	no
gi|320446610|ref|NW_003383961.1|	19738-20753	887564	149594	-25688	0.26525	no
gi|320446610|ref|NW_003383961.1|	200-548	111735	136729	-303069	0.19405	no
gi|320446610|ref|NW_003383961.1|	36039-36541	115453	217502	-240821	0.1805	no
gi|320446610|ref|NW_003383961.1|	40933-47768	105329	39159	-142748	0.3985	no
gi|320446610|ref|NW_003383961.1|	500790-50127	0	74942	inf	0.02075	no
gi|320446610|ref|NW_003383961.1|	602971-60379	143622	256686	0.83773	0.6906	no

gi|320446610|ref|NW_003383961.1|	603911-60453[illegible]	0.431037	351558	302788	0.25005	no
gi|320446611|ref|NW_003383960.1|	126534-12742[illegible]	517246	607511	0.23206	0.856	no
gi|320446611|ref|NW_003383960.1|	128573-13230[illegible]	443812	509432	0.198941	0.88165	no
gi|320446611|ref|NW_003383960.1|	141290-14334[illegible]	0.740641	6199	306519	0.2012	no
gi|320446611|ref|NW_003383960.1|	25659-28963	0.274926	292296	341032	0.17845	no
gi|320446611|ref|NW_003383960.1|	29111-34009	0.289491	347254	35844	0.1624	no
gi|320446611|ref|NW_003383960.1|	38172-39645	203162	219911	0.114288	0.9295	no
gi|320446611|ref|NW_003383960.1|	407879-40829[illegible]	27035	142773	240082	0.2952	no
gi|320446611|ref|NW_003383960.1|	42413-42808	129128	78366	-0.720507	0.7286	no
gi|320446611|ref|NW_003383960.1|	42953-43234	33955	260086	-0.384637	0.84405	no
gi|320446611|ref|NW_003383960.1|	45812-46084	546222	363601	-0.58713	0.7663	no
gi|320446611|ref|NW_003383960.1|	48598-49148	228955	118505	-0.950121	0.6426	no
gi|320446611|ref|NW_003383960.1|	60339-66148	281222	163131	-0.785679	0.55265	no
gi|320446611|ref|NW_003383960.1|	68277-68619	219052	231044	0.0768974	0.9649	no
gi|320446611|ref|NW_003383960.1|	70031-72037	991115	186077	0.908774	0.58025	no
gi|320446611|ref|NW_003383960.1|	72575-73352	779111	119748	0.620105	0.7651	no
gi|320446611|ref|NW_003383960.1|	73476-80236	148173	362366	129017	0.33125	no
gi|320446611|ref|NW_003383960.1|	84737-85650	423432	0.343405	-362415	0.2229	no
gi|320446611|ref|NW_003383960.1|	99212-99529	0	137968	inf	0.02915	no
gi|320446613|ref|NW_003383958.1|	160329-16236[illegible]	0.374846	274497	287242	0.2416	no
gi|320446613|ref|NW_003383958.1|	28706-29396	0.742027	405449	244998	0.24845	no
gi|320446613|ref|NW_003383958.1|	30085-33420	566126	110042	0.958865	0.5651	no
gi|320446613|ref|NW_003383958.1|	33593-34189	885803	916374	0.0489507	0.97455	no
gi|320446613|ref|NW_003383958.1|	34351-39004	240618	644646	142176	0.39035	no
gi|320446616|ref|NW_003383955.1|	102876-10731[illegible]	790529	615386	-0.361325	0.7723	no
gi|320446616|ref|NW_003383955.1|	109587-11018[illegible]	96593	779774	-0.308862	0.88035	no
gi|320446616|ref|NW_003383955.1|	111295-11353[illegible]	278329	482681	0.79428	0.7084	no
gi|320446616|ref|NW_003383955.1|	11601-13556	255128	307857	0.271037	0.89165	no
gi|320446616|ref|NW_003383955.1|	127200-12775[illegible]	277084	529261	-238827	0.28085	no
gi|320446616|ref|NW_003383955.1|	13672-15066	0.291299	0.910649	164439	1	no

gi|320446616|ref|NW_003383955.1|	149404-15018	144342	358354	-201003	0.3532	no
gi|320446616|ref|NW_003383955.1|	153346-15390	169448	658895	-136272	0.5145	no
gi|320446616|ref|NW_003383955.1|	16441-17034	47004	35034	-0.424027	0.8285	no
gi|320446616|ref|NW_003383955.1|	17764-19387	620203	287757	-110789	0.5984	no
gi|320446616|ref|NW_003383955.1|	188215-18858	189919	804055	-124002	0.55525	no
gi|320446616|ref|NW_003383955.1|	189128-19025	815726	262707	-163463	0.4449	no
gi|320446616|ref|NW_003383955.1|	190522-19167	943099	333029	-150176	0.48815	no
gi|320446616|ref|NW_003383955.1|	191829-19496	0.349261	158753	218441	0.31795	no
gi|320446616|ref|NW_003383955.1|	195655-20157	139026	17425	0.325796	0.7993	no
gi|320446616|ref|NW_003383955.1|	20696-21104	324979	979475	-173027	0.41335	no
gi|320446616|ref|NW_003383955.1|	229035-23316	12291	158763	0.369272	0.7777	no
gi|320446616|ref|NW_003383955.1|	25136-26821	109422	210719	-237651	0.3024	no
gi|320446616|ref|NW_003383955.1|	307302-30789	0.475239	354073	621925	0.1578	no
gi|320446616|ref|NW_003383955.1|	310207-31077	0	153853	inf	0.00885	no
gi|320446616|ref|NW_003383955.1|	44437-44770	145874	302268	437303	0.18415	no
gi|320446616|ref|NW_003383955.1|	6772-11448	115887	104846	-0.144441	0.91345	no
gi|320446616|ref|NW_003383955.1|	94657-98161	0.877547	621191	282349	0.22555	no
gi|320446616|ref|NW_003383955.1|	98223-100194	0.777772	20336	138661	0.5052	no
gi|320446617|ref|NW_003383954.1|	128607-13196	399853	134181	-157528	0.24535	no
gi|320446617|ref|NW_003383954.1|	133459-13411	655606	149819	-212961	0.218	no
gi|320446617|ref|NW_003383954.1|	135375-13861	122819	138068	0.168844	0.8941	no
gi|320446617|ref|NW_003383954.1|	176393-17697	910503	356925	-135104	0.5227	no
gi|320446617|ref|NW_003383954.1|	284178-28489	947895	168118	-249526	0.2747	no
gi|320446617|ref|NW_003383954.1|	295685-29756	994029	442821	-116657	0.59365	no
gi|320446617|ref|NW_003383954.1|	308271-30956	668266	276058	-127545	0.5441	no
gi|320446617|ref|NW_003383954.1|	310325-31341	251207	190121	-0.401959	0.76125	no
gi|320446617|ref|NW_003383954.1|	318035-31830	0	898293	inf	0.00715	no
gi|320446617|ref|NW_003383954.1|	329971-33086	0	144782	inf	0.00565	no
gi|320446617|ref|NW_003383954.1|	596136-59739	501989	266617	-0.912891	0.485	no
gi|320446617|ref|NW_003383954.1|	602307-60637	405122	181916	-115508	0.5699	no

gi|320446617|ref|NW_003383954.1|	06563-60816	941875	422555	-11564	0.58995	no
gi|320446617|ref|NW_003383954.1|	15084-61592	0.279627	171168	593577	0.1585	no
gi|320446617|ref|NW_003383954.1|	56036-65659	103932	5262	233998	0.2622	no
gi|320446617|ref|NW_003383954.1|	56787-65732	0.545872	478143	313081	0.24575	no
gi|320446617|ref|NW_003383954.1|	83363-68399	979047	520999	241183	0.17535	no
gi|320446617|ref|NW_003383954.1|	79669-80662	320785	0	#NAME?	5.00E-05	yes
gi|320446617|ref|NW_003383954.1|	90737-94136	139521	0.0746194	-108686	0.16165	no
gi|320446618|ref|NW_003383953.1|	10105-11288	108635	308547	-181592	0.4008	no
gi|320446618|ref|NW_003383953.1|	05441-10598	273798	959189	-151322	0.48685	no
gi|320446618|ref|NW_003383953.1|	11638-11184	402655	29.32	-0.457661	0.81845	no
gi|320446618|ref|NW_003383953.1|	12802-11327	841246	420873	-0.999144	0.65	no
gi|320446618|ref|NW_003383953.1|	13745-11454	840038	721117	-0.220221	0.91375	no
gi|320446618|ref|NW_003383953.1|	15168-11557	147077	848828	-0.793023	0.6938	no
gi|320446618|ref|NW_003383953.1|	17966-11890	288665	315271	0.127195	0.9424	no
gi|320446618|ref|NW_003383953.1|	19032-11979	211537	160436	-0.398914	0.85165	no
gi|320446618|ref|NW_003383953.1|	21272-12390	253736	487135	0.940993	0.4896	no
gi|320446618|ref|NW_003383953.1|	24174-12559	624155	630874	0.0154483	0.9894	no
gi|320446618|ref|NW_003383953.1|	27270-12819	10.8	6.77	-0.673801	0.74425	no
gi|320446618|ref|NW_003383953.1|	31130-13179	155882	372072	125513	0.55735	no
gi|320446618|ref|NW_003383953.1|	38197-14008	591494	554424	-0.0933741	0.96245	no
gi|320446618|ref|NW_003383953.1|	40213-14140	108178	104471	-0.0503048	0.98085	no
gi|320446618|ref|NW_003383953.1|	41521-14331	367626	414299	0.172434	0.9366	no
gi|320446618|ref|NW_003383953.1|	43437-14454	135071	146932	0.121432	0.9404	no
gi|320446618|ref|NW_003383953.1|	46021-14648	500614	238333	-107072	0.59545	no
gi|320446618|ref|NW_003383953.1|	46960-14751	583903	501264	-0.220158	0.9126	no
gi|320446618|ref|NW_003383953.1|	47621-14803	161416	888142	-0.86192	0.6776	no
gi|320446618|ref|NW_003383953.1|	53689-15586	36674	130378	-149206	0.25605	no
gi|320446618|ref|NW_003383953.1|	56849-15720	223128	153462	-0.539988	0.79175	no
gi|320446618|ref|NW_003383953.1|	57354-15778	293624	111092	-472415	0.19795	no
gi|320446618|ref|NW_003383953.1|	58115-15842	33928	0	#NAME?	0.01335	no

gi|320446618|ref|NW_003383953.1|	158560-15938:	229795	0.592353	-195582	0.30295	no
gi|320446618|ref|NW_003383953.1|	160239-16082:	197578	22836	-311304	0.2045	no
gi|320446618|ref|NW_003383953.1|	161128-16403:	609515	108992	0.838488	0.5265	no
gi|320446618|ref|NW_003383953.1|	164154-16488	135654	185773	0.453614	0.8212	no
gi|320446618|ref|NW_003383953.1|	165209-16567	380063	582076	0.614968	0.77735	no
gi|320446618|ref|NW_003383953.1|	176117-18392	149906	223816	0.57825	0.6624	no
gi|320446618|ref|NW_003383953.1|	194034-19467:	918184	709952	-0.371062	0.85435	no
gi|320446618|ref|NW_003383953.1|	212632-21319:	412009	591229	0.521042	0.78855	no
gi|320446618|ref|NW_003383953.1|	260236-26079:	20849	112582	-0.889005	0.6703	no
gi|320446618|ref|NW_003383953.1|	318342-31915:	0.882773	323467	187351	0.39335	no
gi|320446618|ref|NW_003383953.1|	320457-32583	0.393704	294233	290178	0.21825	no
gi|320446618|ref|NW_003383953.1|	327314-33071:	0.853341	142896	40657	0.0586	no
gi|320446618|ref|NW_003383953.1|	351608-35404	152585	351785	120508	0.3642	no
gi|320446618|ref|NW_003383953.1|	356386-35753	405494	778811	0.941594	0.647	no
gi|320446618|ref|NW_003383953.1|	38186-39865	0.46756	162759	179951	0.426	no
gi|320446618|ref|NW_003383953.1|	394055-39435:	467288	88634	0.923549	0.66275	no
gi|320446618|ref|NW_003383953.1|	455021-45687	0.104315	232618	447895	0.1817	no
gi|320446618|ref|NW_003383953.1|	470779-47366	13994	180965	0.370904	0.77475	no
gi|320446618|ref|NW_003383953.1|	473834-47451	105262	564661	-0.898523	0.66185	no
gi|320446618|ref|NW_003383953.1|	47499-48173	0.768853	288508	190783	0.3194	no
gi|320446618|ref|NW_003383953.1|	476488-47730	320478	200427	-0.677144	0.76365	no
gi|320446618|ref|NW_003383953.1|	65397-68116	14267	740587	237599	0.30995	no
gi|320446618|ref|NW_003383953.1|	68470-69683	189885	118483	264148	0.25045	no
gi|320446618|ref|NW_003383953.1|	85788-86283	128002	0	#NAME?	0.0132	no
gi|320446618|ref|NW_003383953.1|	87828-89181	543579	0.209708	-469603	0.19975	no
gi|320446618|ref|NW_003383953.1|	89740-90544	801485	0.407813	-42967	0.20565	no
gi|320446618|ref|NW_003383953.1|	91058-92387	771543	0.321387	-458536	0.14885	no
gi|320446618|ref|NW_003383953.1|	92510-92979	16959	141363	-358458	0.177	no
gi|320446618|ref|NW_003383953.1|	94155-96480	742893	1973	-191277	0.38195	no
gi|320446618|ref|NW_003383953.1|	98050-101184	181034	952519	-0.926439	0.48225	no

gi|320446619|ref|NW_003383952.1|	101935-10227	179212	0.89455	-432436	0.2992	no
gi|320446619|ref|NW_003383952.1|	103068-10343	328148	0.765326	-542213	0.2771	no
gi|320446619|ref|NW_003383952.1|	11642-17212	726174	107.75	0.569295	0.72155	no
gi|320446619|ref|NW_003383952.1|	142968-14352	2614	141136	243276	0.2912	no
gi|320446619|ref|NW_003383952.1|	148037-14858	160724	111985	280064	0.25695	no
gi|320446619|ref|NW_003383952.1|	150625-15241	543063	260284	-106104	0.41515	no
gi|320446619|ref|NW_003383952.1|	168625-16894	518484	0	#NAME?	0.0074	no
gi|320446619|ref|NW_003383952.1|	171871-17357	335043	0.0801684	-87071	0.2504	no
gi|320446619|ref|NW_003383952.1|	176875-17735	439988	0	#NAME?	0.0049	no
gi|320446619|ref|NW_003383952.1|	179112-17997	539579	0	#NAME?	0.0132	no
gi|320446619|ref|NW_003383952.1|	180345-18191	745429	0	#NAME?	0.0062	no
gi|320446619|ref|NW_003383952.1|	184440-18645	998389	305013	-171073	0.4415	no
gi|320446619|ref|NW_003383952.1|	204829-20641	0.125033	165291	372463	0.21225	no
gi|320446619|ref|NW_003383952.1|	206860-20794	0.395877	246548	263874	0.2359	no
gi|320446619|ref|NW_003383952.1|	240090-24156	0.136682	247045	417588	0.1906	no
gi|320446619|ref|NW_003383952.1|	245906-24637	0	5144	inf	0.0294	no
gi|320446619|ref|NW_003383952.1|	248508-25158	0	137156	inf	1	no
gi|320446619|ref|NW_003383952.1|	251823-25295	0	209469	inf	0.0198	no
gi|320446619|ref|NW_003383952.1|	4247-5104	126.26	878264	-0.523666	0.69515	no
gi|320446619|ref|NW_003383952.1|	74588-75821	316332	156022	-101969	0.53505	no
gi|320446619|ref|NW_003383952.1|	79424-81337	160933	224361	0.479357	0.81295	no
gi|320446619|ref|NW_003383952.1|	81460-84593	249808	289926	0.214865	0.87425	no
gi|320446619|ref|NW_003383952.1|	85260-86420	653424	183349	-183342	0.30615	no
gi|320446619|ref|NW_003383952.1|	86492-86822	805116	67527	-357566	0.169	no
gi|320446619|ref|NW_003383952.1|	91357-91903	91644	109021	-307144	0.1952	no
gi|320446619|ref|NW_003383952.1|	92340-92554	386856	10385	-189729	0.3084	no
gi|320446619|ref|NW_003383952.1|	94014-95733	23.43	364338	-268501	0.13825	no
gi|320446619|ref|NW_003383952.1|	96580-97203	651479	177071	-187939	0.39695	no
gi|320446620|ref|NW_003383951.1|	101170-10173	35521	342863	-0.0510421	0.94535	no
gi|320446620|ref|NW_003383951.1|	101851-10233	108721	816939	-0.412334	0.8376	no

gi|320446620|ref|NW_003383951.1|	103498-10441	813549	951879	0.226548	0.9132	no
gi|320446620|ref|NW_003383951.1|	115131-11566	222549	52444	123666	0.5595	no
gi|320446620|ref|NW_003383951.1|	49606-49873	104829	149975	0.516685	0.80155	no
gi|320446620|ref|NW_003383951.1|	76789-77124	431382	838452	0.958763	0.6698	no
gi|320446620|ref|NW_003383951.1|	94736-94957	439908	920552	-225663	0.2695	no
gi|320446620|ref|NW_003383951.1|	95535-97305	758707	22212	-17722	0.41885	no
gi|320446620|ref|NW_003383951.1|	98370-99388	582252	223544	-138109	0.51125	no
gi|320446620|ref|NW_003383951.1|	99516-100864	864535	36862	-122979	0.56435	no
gi|320446622|ref|NW_003383949.1|	104443-10572	225736	311034	378437	0.06675	no
gi|320446622|ref|NW_003383949.1|	113498-11395	0	54974	inf	0.0294	no
gi|320446622|ref|NW_003383949.1|	133097-13477	107838	238283	114381	0.4906	no
gi|320446622|ref|NW_003383949.1|	142921-14461	0.230901	0.803859	179967	1	no
gi|320446622|ref|NW_003383949.1|	146752-14894	18138	530381	154802	0.48085	no
gi|320446622|ref|NW_003383949.1|	149498-15118	710568	185722	13861	0.4021	no
gi|320446622|ref|NW_003383949.1|	152069-15240	153765	498633	169725	0.4243	no
gi|320446622|ref|NW_003383949.1|	154425-15470	221941	311842	0.490635	0.8061	no
gi|320446622|ref|NW_003383949.1|	156111-16067	268829	11214	206054	0.25305	no
gi|320446622|ref|NW_003383949.1|	166620-16767	514777	390184	292213	0.11685	no
gi|320446622|ref|NW_003383949.1|	167824-16943	29505	136026	220485	0.3353	no
gi|320446622|ref|NW_003383949.1|	171717-17449	910582	575981	-0.660768	0.67465	no
gi|320446622|ref|NW_003383949.1|	174626-17640	0.654626	129206	0.980931	1	no
gi|320446622|ref|NW_003383949.1|	176518-17745	395224	143757	-145904	0.37905	no
gi|320446622|ref|NW_003383949.1|	179005-17972	138706	355952	135965	0.52575	no
gi|320446622|ref|NW_003383949.1|	180991-18207	797368	841359	0.0774763	0.97025	no
gi|320446622|ref|NW_003383949.1|	182178-18472	519489	397526	-0.386043	0.77545	no
gi|320446622|ref|NW_003383949.1|	184958-18661	59.58	363629	-0.712363	0.5937	no
gi|320446622|ref|NW_003383949.1|	189189-18992	151098	827907	-0.867941	0.677	no
gi|320446622|ref|NW_003383949.1|	190071-19214	117663	109643	-0.10185	0.94825	no
gi|320446622|ref|NW_003383949.1|	204554-20521	551785	58.17	0.0761688	0.94895	no
gi|320446622|ref|NW_003383949.1|	236050-23637	87318	59426	-0.555184	0.7917	no

gi|320446622|ref|NW_003383949.1|	240182-24264	160799	220354	0.454566	0.72615	no
gi|320446622|ref|NW_003383949.1|	243075-24508	274795	108943	198715	0.1461	no
gi|320446622|ref|NW_003383949.1|	256389-25665	267957	442749	0.724486	0.7289	no
gi|320446622|ref|NW_003383949.1|	280906-28137	283779	614919	111563	0.5931	no
gi|320446622|ref|NW_003383949.1|	50470-50649	622299	920781	0.565251	0.7972	no
gi|320446622|ref|NW_003383949.1|	51630-53913	329623	500429	0.602348	0.77365	no
gi|320446622|ref|NW_003383949.1|	54854-55186	881607	180728	103561	0.60415	no
gi|320446622|ref|NW_003383949.1|	56910-57466	784201	112909	0.525867	0.79795	no
gi|320446622|ref|NW_003383949.1|	57656-57919	164442	31334	0.930153	0.65565	no
gi|320446622|ref|NW_003383949.1|	58049-63639	545117	17332	16688	0.21445	no
gi|320446622|ref|NW_003383949.1|	7677-8259	140537	655995	-10992	0.6009	no
gi|320446623|ref|NW_003383948.1|	110233-11146	627786	258851	-127815	0.54615	no
gi|320446623|ref|NW_003383948.1|	16134-16792	917297	418545	218992	0.343	no
gi|320446623|ref|NW_003383948.1|	203828-20452	0	276987	inf	0.0294	no
gi|320446623|ref|NW_003383948.1|	20666-21897	135601	149279	346057	0.1655	no
gi|320446623|ref|NW_003383948.1|	24871-25840	0.230058	571737	463528	0.17455	no
gi|320446623|ref|NW_003383948.1|	25942-26244	0	430696	inf	0.0085	no
gi|320446623|ref|NW_003383948.1|	30042-32669	0.42331	537263	366584	0.1557	no
gi|320446623|ref|NW_003383948.1|	55305-58002	219344	368797	0.749628	0.72155	no
gi|320446623|ref|NW_003383948.1|	52264-65515	133278	527924	19859	0.3791	no
gi|320446623|ref|NW_003383948.1|	56300-65899	561842	195335	-152421	0.25845	no
gi|320446623|ref|NW_003383948.1|	60132-66057	842311	205657	-203412	0.36695	no
gi|320446623|ref|NW_003383948.1|	62362-66423	554697	211975	-138781	0.3009	no
gi|320446623|ref|NW_003383948.1|	65318-66827	404662	119753	-175666	0.1948	no
gi|320446623|ref|NW_003383948.1|	69104-66949	60591	143671	-207634	0.349	no
gi|320446623|ref|NW_003383948.1|	82355-68331	279886	235021	306988	0.2023	no
gi|320446623|ref|NW_003383948.1|	83802-68689	141908	278252	429337	0.0479	no
gi|320446623|ref|NW_003383948.1|	89522-69064	0	159646	inf	0.0041	no
gi|320446623|ref|NW_003383948.1|	91618-69438	139799	683888	229041	0.32165	no
gi|320446623|ref|NW_003383948.1|	713081-71392	501665	249212	-100935	0.6155	no

gi|320446623|ref|NW_003383948.1|	726335-72723	0.511755	458381	316302	0.20765	no
gi|320446623|ref|NW_003383948.1|	741600-74362	37715	664129	0.816324	0.7037	no
gi|320446623|ref|NW_003383948.1|	743829-74480	250333	314262	0.328122	0.8696	no
gi|320446623|ref|NW_003383948.1|	748236-74847	106728	179752	0.752067	0.70725	no
gi|320446623|ref|NW_003383948.1|	749065-74989	229015	314872	0.459323	0.81605	no
gi|320446623|ref|NW_003383948.1|	750063-75053	197855	155499	-0.347538	0.86345	no
gi|320446623|ref|NW_003383948.1|	752476-75546	225943	252572	-31612	0.10445	no
gi|320446623|ref|NW_003383948.1|	756056-75706	24247	0.754636	-500588	0.14305	no
gi|320446623|ref|NW_003383948.1|	767130-76749	498681	317242	-397446	0.18305	no
gi|320446623|ref|NW_003383948.1|	767661-76814	200003	463457	121241	0.57445	no
gi|320446623|ref|NW_003383948.1|	770291-77227	37656	686709	0.866818	0.6846	no
gi|320446623|ref|NW_003383948.1|	772863-77674	930521	366797	197887	0.15115	no
gi|320446623|ref|NW_003383948.1|	780062-78105	564339	389809	-0.533794	0.67585	no
gi|320446623|ref|NW_003383948.1|	797671-79857	980085	147227	0.587063	0.77925	no
gi|320446623|ref|NW_003383948.1|	799157-80088	444532	921669	105196	0.44275	no
gi|320446627|ref|NW_003383944.1|	100471-10116	0	329427	inf	0.02915	no
gi|320446627|ref|NW_003383944.1|	101699-10261	0	375568	inf	0.0186	no
gi|320446627|ref|NW_003383944.1|	125151-12553	207482	885081	209282	0.2973	no
gi|320446627|ref|NW_003383944.1|	126488-12695	504674	16813	173616	0.4212	no
gi|320446627|ref|NW_003383944.1|	135916-13624	464248	269972	253985	0.2862	no
gi|320446627|ref|NW_003383944.1|	142141-14377	0	151476	inf	0.0154	no
gi|320446627|ref|NW_003383944.1|	150955-15267	136855	524147	-13846	0.5386	no
gi|320446627|ref|NW_003383944.1|	153915-15447	224127	562908	-199334	0.3588	no
gi|320446627|ref|NW_003383944.1|	169402-17002	152012	531212	-151682	0.4708	no
gi|320446627|ref|NW_003383944.1|	172088-17258	939454	419707	-116244	0.5799	no
gi|320446627|ref|NW_003383944.1|	175982-17722	436754	174754	-13215	0.5308	no
gi|320446627|ref|NW_003383944.1|	177337-18012	435756	108435	131524	0.4304	no
gi|320446627|ref|NW_003383944.1|	200890-20133	115119	107107	-0.104082	0.9545	no
gi|320446627|ref|NW_003383944.1|	208372-21059	509362	710953	0.481062	0.8273	no
gi|320446627|ref|NW_003383944.1|	214750-21522	621103	322453	-0.945745	0.6651	no

gi|320446627|ref|NW_003383944.1|	216145-217265	190776	171711	-0.151897	0.9345	no
gi|320446627|ref|NW_003383944.1|	218363-219472	869306	176517	102187	0.6383	no
gi|320446627|ref|NW_003383944.1|	224845-225098	215962	195428	-0.144136	0.9302	no
gi|320446627|ref|NW_003383944.1|	227942-228512	150463	305122	101998	0.5772	no
gi|320446627|ref|NW_003383944.1|	231101-231734	338851	345671	0.0287491	0.95455	no
gi|320446627|ref|NW_003383944.1|	235188-235808	118166	713525	-0.727776	0.7268	no
gi|320446627|ref|NW_003383944.1|	237611-238342	578845	396313	-0.546538	0.7811	no
gi|320446627|ref|NW_003383944.1|	239475-239861	417271	479121	0.199407	0.89915	no
gi|320446627|ref|NW_003383944.1|	241417-242244	105561	372673	-15021	0.48415	no
gi|320446627|ref|NW_003383944.1|	297267-299467	455205	115675	134549	0.4124	no
gi|320446627|ref|NW_003383944.1|	2991-3258	157243	349942	115412	0.58205	no
gi|320446627|ref|NW_003383944.1|	307630-308027	216215	362002	0.743529	0.718	no
gi|320446627|ref|NW_003383944.1|	32169-33214	152172	936877	-0.699772	0.74475	no
gi|320446627|ref|NW_003383944.1|	33323-34640	468063	400725	-0.224091	0.90895	no
gi|320446627|ref|NW_003383944.1|	34783-35317	13439	71615	-0.908094	0.65865	no
gi|320446627|ref|NW_003383944.1|	35451-39581	17459	25416	0.541761	0.6812	no
gi|320446627|ref|NW_003383944.1|	65506-65713	315754	138107	212891	0.34245	no
gi|320446627|ref|NW_003383944.1|	74558-76119	649216	600408	320917	0.1045	no
gi|320446629|ref|NW_003383942.1|	153508-163264	212657	111252	238724	0.2084	no
gi|320446629|ref|NW_003383942.1|	164069-167334	412128	554692	375052	0.07185	no
gi|320446629|ref|NW_003383942.1|	177610-178650	154066	3599	-209788	0.34145	no
gi|320446629|ref|NW_003383942.1|	179478-180050	997282	134855	-28866	0.25525	no
gi|320446629|ref|NW_003383942.1|	180913-182264	137651	47268	-154208	0.4756	no
gi|320446629|ref|NW_003383942.1|	182432-182649	418948	164173	-135156	0.52495	no
gi|320446629|ref|NW_003383942.1|	183336-183970	24506	948326	-136968	0.51955	no
gi|320446629|ref|NW_003383942.1|	184399-184780	289683	843779	-177954	0.4166	no
gi|320446629|ref|NW_003383942.1|	184973-186032	120751	59452	-102223	0.62865	no
gi|320446629|ref|NW_003383942.1|	186156-187404	214985	246214	0.195679	0.8686	no
gi|320446629|ref|NW_003383942.1|	215633-216352	0	430569	inf	0.0154	no
gi|320446629|ref|NW_003383942.1|	30158-30707	186934	900425	-105385	0.6085	no

gi|320446629|ref|NW_003383942.1|	5319-5991	0	816639	inf	0.01195	no
gi|320446629|ref|NW_003383942.1|	60637-61036	9725	191982	0.981199	0.6434	no
gi|320446629|ref|NW_003383942.1|	62755-63610	550254	277435	-0.987948	0.55935	no
gi|320446629|ref|NW_003383942.1|	63990-64269	286371	334445	0.223881	0.86095	no
gi|320446629|ref|NW_003383942.1|	69170-71379	297526	383041	0.364483	0.78285	no
gi|320446629|ref|NW_003383942.1|	71789-72779	125246	988598	-0.341307	0.86865	no
gi|320446629|ref|NW_003383942.1|	73006-74759	84504	696996	-0.27787	0.89845	no
gi|320446629|ref|NW_003383942.1|	75296-76988	135541	790463	-0.777956	0.7317	no
gi|320446630|ref|NW_003383941.1|	100414-10210	0.231853	2.26	328505	0.20055	no
gi|320446630|ref|NW_003383941.1|	102998-10364	163349	58395	183789	0.40745	no
gi|320446630|ref|NW_003383941.1|	103936-10832	0.690559	109509	398714	0.05285	no
gi|320446630|ref|NW_003383941.1|	109328-11091	0.249696	321411	368618	0.17835	no
gi|320446630|ref|NW_003383941.1|	111427-11316	0.225349	204021	317848	0.20465	no
gi|320446630|ref|NW_003383941.1|	114215-11621	0.286871	313398	344952	0.18995	no
gi|320446630|ref|NW_003383941.1|	93914-95156	0.335297	174411	237898	0.2609	no
gi|320446630|ref|NW_003383941.1|	96576-97047	210312	607927	153137	0.4913	no
gi|320446630|ref|NW_003383941.1|	97144-98436	175685	720437	203588	0.34685	no
gi|320446630|ref|NW_003383941.1|	99200-100046	110568	418359	191981	0.39735	no
gi|320446631|ref|NW_003383940.1|	101360-10281	269146	201475	-373972	0.0681	no
gi|320446631|ref|NW_003383940.1|	104041-10503	218024	153667	-382661	0.14925	no
gi|320446631|ref|NW_003383940.1|	105183-10599	117294	141036	-3056	0.21005	no
gi|320446631|ref|NW_003383940.1|	1058-1606	167689	121016	-0.470593	0.7143	no
gi|320446631|ref|NW_003383940.1|	112741-11766	997443	300153	-173254	0.3215	no
gi|320446631|ref|NW_003383940.1|	124339-12481	129107	590011	-112975	0.5849	no
gi|320446631|ref|NW_003383940.1|	124957-12598	554311	19157	-153283	0.4713	no
gi|320446631|ref|NW_003383940.1|	126087-13010	535125	312162	-0.777582	0.7348	no
gi|320446631|ref|NW_003383940.1|	131750-13432	177491	212694	0.261034	0.8408	no
gi|320446631|ref|NW_003383940.1|	13505-13798	211969	607667	-18025	0.4199	no
gi|320446631|ref|NW_003383940.1|	135844-13735	168269	875293	-0.942935	0.55935	no
gi|320446631|ref|NW_003383940.1|	143437-14362	183486	133486	-0.458976	0.82275	no

gi|320446631|ref|NW_003383940.1|	147859-14846[illegible]	160558	52888	-160208	0.44925	no
gi|320446631|ref|NW_003383940.1|	151042-151258	639668	534581	-0.258914	0.8977	no
gi|320446631|ref|NW_003383940.1|	152506-152884	48025	786306	-261062	0.25405	no
gi|320446631|ref|NW_003383940.1|	15508-16061	121344	498324	-128395	0.54375	no
gi|320446631|ref|NW_003383940.1|	157099-157920	126819	237733	-241536	0.2876	no
gi|320446631|ref|NW_003383940.1|	158495-15936[illegible]	304155	624508	-228402	0.31935	no
gi|320446631|ref|NW_003383940.1|	159834-160365	593695	114137	-237896	0.29645	no
gi|320446631|ref|NW_003383940.1|	162541-163594	437599	121334	-185062	0.29325	no
gi|320446631|ref|NW_003383940.1|	16310-20478	298183	278745	-0.0972516	0.94165	no
gi|320446631|ref|NW_003383940.1|	163720-164160	606407	258194	-123183	0.5736	no
gi|320446631|ref|NW_003383940.1|	168785-170535	646177	256067	-133541	0.53105	no
gi|320446631|ref|NW_003383940.1|	171236-171728	252361	736198	-177732	0.4091	no
gi|320446631|ref|NW_003383940.1|	173094-173715	742109	533827	-0.47526	0.8068	no
gi|320446631|ref|NW_003383940.1|	174513-176889	313724	103468	-160031	0.2241	no
gi|320446631|ref|NW_003383940.1|	178233-179795	563501	21.68	-137806	0.2989	no
gi|320446631|ref|NW_003383940.1|	181040-18250[illegible]	60483	0.286661	-439911	0.14965	no
gi|320446631|ref|NW_003383940.1|	20655-21504	36584	946261	-19509	0.2448	no
gi|320446631|ref|NW_003383940.1|	221326-222204	0	416013	inf	0.01485	no
gi|320446631|ref|NW_003383940.1|	228171-228880	13893	535915	-137428	0.5139	no
gi|320446631|ref|NW_003383940.1|	229085-23156[illegible]	150546	152454	0.0181618	0.98045	no
gi|320446631|ref|NW_003383940.1|	231663-232270	140332	14743	0.0711912	0.9708	no
gi|320446631|ref|NW_003383940.1|	24683-26216	342017	714542	-225898	0.2119	no
gi|320446631|ref|NW_003383940.1|	247758-248554	117724	0.206752	-915329	0.2504	no
gi|320446631|ref|NW_003383940.1|	251505-252419	813284	0	#NAME?	5.00E-05	yes
gi|320446631|ref|NW_003383940.1|	259465-265294	0.422286	346659	303722	0.20735	no
gi|320446631|ref|NW_003383940.1|	270395-270844	230239	510032	114746	0.575	no
gi|320446631|ref|NW_003383940.1|	2971-3462	694931	347674	-0.999137	0.65365	no
gi|320446631|ref|NW_003383940.1|	3595-4318	380664	402612	0.080872	0.95815	no
gi|320446631|ref|NW_003383940.1|	4959-5690	306447	102575	-157896	0.47185	no
gi|320446631|ref|NW_003383940.1|	6017-7789	395332	413068	0.0633165	0.9739	no

gi|320446631|ref|NW_003383940.1|	63517-69637	42434	962534	118162	0.3543	no
gi|320446631|ref|NW_003383940.1|	70109-71325	395818	847158	109779	0.61115	no
gi|320446631|ref|NW_003383940.1|	72433-72904	147218	224466	0.608539	0.77545	no
gi|320446631|ref|NW_003383940.1|	73069-73333	406476	499278	0.296672	0.88185	no
gi|320446631|ref|NW_003383940.1|	73547-74195	44921	834215	0.893029	0.659	no
gi|320446631|ref|NW_003383940.1|	74325-75306	54325	640839	0.238347	0.9059	no
gi|320446631|ref|NW_003383940.1|	75891-77601	549275	621529	0.178292	0.93045	no
gi|320446631|ref|NW_003383940.1|	77751-78763	391168	345373	-0.179633	0.93075	no
gi|320446631|ref|NW_003383940.1|	78903-79406	621858	625272	0.00789854	0.9618	no
gi|320446631|ref|NW_003383940.1|	79515-80608	177106	25875	0.546945	0.78565	no
gi|320446631|ref|NW_003383940.1|	8120-9362	972362	872055	-0.157073	0.94195	no
gi|320446631|ref|NW_003383940.1|	82082-82980	560381	526551	-0.0898334	0.96275	no
gi|320446631|ref|NW_003383940.1|	84446-85123	610943	156342	-196633	0.3816	no
gi|320446631|ref|NW_003383940.1|	85305-86128	287244	138216	-105535	0.60015	no
gi|320446631|ref|NW_003383940.1|	86443-86694	126517	160197	0.340516	0.85835	no
gi|320446631|ref|NW_003383940.1|	91523-92254	238348	139875	-0.76893	0.71385	no
gi|320446631|ref|NW_003383940.1|	93180-93608	162665	58025	-148716	0.37895	no
gi|320446631|ref|NW_003383940.1|	94148-94924	103345	30848	-174422	0.32795	no
gi|320446631|ref|NW_003383940.1|	95181-97454	804916	483218	-0.736165	0.58485	no
gi|320446632|ref|NW_003383939.1|	141303-142159	0	18716	inf	0.0312	no
gi|320446632|ref|NW_003383939.1|	229255-22948[illegible]	291622	333.14	351396	0.16225	no
gi|320446632|ref|NW_003383939.1|	271361-273547	147057	271632	0.885277	0.66015	no
gi|320446632|ref|NW_003383939.1|	274498-275597	136792	175827	0.362175	0.85765	no
gi|320446632|ref|NW_003383939.1|	284660-28631[illegible]	203549	101917	-0.997985	0.55045	no
gi|320446632|ref|NW_003383939.1|	286518-287029	45.35	125792	-185007	0.40875	no
gi|320446632|ref|NW_003383939.1|	287887-289459	0.757496	0.878337	0.213536	1	no
gi|320446632|ref|NW_003383939.1|	292633-293477	970649	133543	-286165	0.2282	no
gi|320446632|ref|NW_003383939.1|	301944-302992	149533	861665	-0.795267	0.71275	no
gi|320446632|ref|NW_003383939.1|	305929-306655	515928	706283	0.453077	0.8232	no
gi|320446632|ref|NW_003383939.1|	307660-308564	984484	939444	-0.0675615	0.972	no

gi|320446632|ref|NW_003383939.1|	309286-31273	247973	392535	0.662639	0.6198	no
gi|320446632|ref|NW_003383939.1|	346929-34953	0	527966	inf	0.00545	no
gi|320446632|ref|NW_003383939.1|	393024-39679	158914	156637	-0.0208233	0.98715	no
gi|320446632|ref|NW_003383939.1|	397454-39807	261255	355011	0.442407	0.8232	no
gi|320446632|ref|NW_003383939.1|	398529-39978	132669	713201	-0.895447	0.6779	no
gi|320446632|ref|NW_003383939.1|	399941-40036	179493	135715	-0.403344	0.84645	no
gi|320446632|ref|NW_003383939.1|	400492-40165	15365	62092	-130717	0.5455	no
gi|320446632|ref|NW_003383939.1|	409017-41184	754652	281913	-142056	0.5261	no
gi|320446632|ref|NW_003383939.1|	412003-41241	215221	473676	-218385	0.31935	no
gi|320446632|ref|NW_003383939.1|	414413-41522	960994	140083	-277825	0.24105	no
gi|320446632|ref|NW_003383939.1|	416757-41819	11535	166193	-279508	0.2321	no
gi|320446632|ref|NW_003383939.1|	435702-43855	48852	119532	-203102	0.1387	no
gi|320446632|ref|NW_003383939.1|	439872-44259	374414	483455	-295318	0.1249	no
gi|320446632|ref|NW_003383939.1|	449539-44998	397378	558741	-283026	0.2274	no
gi|320446632|ref|NW_003383939.1|	457356-45864	158717	478386	-173021	0.43595	no
gi|320446632|ref|NW_003383939.1|	459642-46118	138189	619835	-115669	0.6022	no
gi|320446632|ref|NW_003383939.1|	461335-46335	501687	356463	-0.493032	0.81045	no
gi|320446632|ref|NW_003383939.1|	463488-46551	212147	182142	-0.219998	0.8645	no
gi|320446632|ref|NW_003383939.1|	51379-52738	0.150188	219031	386629	0.19995	no
gi|320446632|ref|NW_003383939.1|	76230-79860	144142	182248	0.338409	0.79825	no
gi|320446632|ref|NW_003383939.1|	98700-101153	486963	244425	-0.994418	0.63625	no
gi|320446635|ref|NW_003383936.1|	104480-10475	0	255577	inf	0.0198	no
gi|320446635|ref|NW_003383936.1|	134949-13598	231301	145373	-0.670015	0.7453	no
gi|320446635|ref|NW_003383936.1|	136686-13741	376818	375202	-0.00619946	0.96375	no
gi|320446635|ref|NW_003383936.1|	139374-14032	0.704585	113452	0.687234	1	no
gi|320446635|ref|NW_003383936.1|	140383-14261	109809	112008	0.0285961	1	no
gi|320446635|ref|NW_003383936.1|	142755-14382	100742	209082	10534	0.60035	no
gi|320446635|ref|NW_003383936.1|	143989-14705	990905	202848	103358	0.43605	no
gi|320446635|ref|NW_003383936.1|	151499-15429	285659	209785	-0.445379	0.73885	no
gi|320446635|ref|NW_003383936.1|	172234-17272	330836	252181	-0.391657	0.85305	no

gi|320446635|ref|NW_003383936.1|	173061-17383	532744	322498	-0.724151	0.71785	no
gi|320446635|ref|NW_003383936.1|	174547-17547	726845	584857	-0.313564	0.87515	no
gi|320446635|ref|NW_003383936.1|	175994-17949	140192	100254	-0.483733	0.71005	no
gi|320446635|ref|NW_003383936.1|	180036-18091	527763	109024	-227524	0.32785	no
gi|320446635|ref|NW_003383936.1|	181212-18198	728882	347778	-106752	0.6005	no
gi|320446635|ref|NW_003383936.1|	182867-18411	9379	151008	-263481	0.2582	no
gi|320446635|ref|NW_003383936.1|	184668-18543	664255	130178	-235125	0.30665	no
gi|320446635|ref|NW_003383936.1|	186825-18718	134638	0.797916	-40767	0.30905	no
gi|320446635|ref|NW_003383936.1|	187740-18809	343424	579552	-256698	0.26875	no
gi|320446635|ref|NW_003383936.1|	194260-19970	683359	900874	0.398681	0.7533	no
gi|320446635|ref|NW_003383936.1|	199813-20142	0.614688	111216	0.855442	1	no
gi|320446635|ref|NW_003383936.1|	203039-20456	240767	176548	-0.447577	0.72035	no
gi|320446635|ref|NW_003383936.1|	227857-22945	0.494271	206365	206182	0.3563	no
gi|320446635|ref|NW_003383936.1|	229538-23041	0.261834	180325	278387	0.2657	no
gi|320446635|ref|NW_003383936.1|	231244-23227	0	160501	inf	0.0294	no
gi|320446635|ref|NW_003383936.1|	232502-23337	0.526939	181429	17837	0.3324	no
gi|320446635|ref|NW_003383936.1|	234405-24227	332196	137.36	204786	0.22895	no
gi|320446635|ref|NW_003383936.1|	261524-26231	458165	0	#NAME?	0.01585	no
gi|320446635|ref|NW_003383936.1|	265989-26807	929526	127137	0.451815	0.76975	no
gi|320446635|ref|NW_003383936.1|	268680-27104	941539	291674	163127	0.36305	no
gi|320446635|ref|NW_003383936.1|	277171-27749	66496	327091	-102358	0.626	no
gi|320446635|ref|NW_003383936.1|	310048-31037	302626	107634	183053	0.32615	no
gi|320446635|ref|NW_003383936.1|	326786-32722	33017	293755	-0.168592	0.9371	no
gi|320446635|ref|NW_003383936.1|	339629-34018	315588	248434	-0.345184	0.85705	no
gi|320446635|ref|NW_003383936.1|	341233-34163	15.56	10239	-0.603764	0.7549	no
gi|320446635|ref|NW_003383936.1|	341744-34583	111268	159774	0.521993	0.69145	no
gi|320446635|ref|NW_003383936.1|	85333-86284	0.235847	19529	30497	0.2493	no
gi|320446635|ref|NW_003383936.1|	86412-90351	0.546684	663274	360082	0.07695	no
gi|320446636|ref|NW_003383935.1|	182633-18366	401521	461643	352323	0.07735	no
gi|320446636|ref|NW_003383935.1|	358742-36086	150992	158321	0.0683833	0.95935	no

gi|320446636|ref|NW_003383935.1|	361313-36307	353477	484729	0.455564	0.82665	no
gi|320446636|ref|NW_003383935.1|	381731-38281	277766	0.549154	-233859	0.30915	no
gi|320446636|ref|NW_003383935.1|	383150-38398	336668	0.19295	-412503	0.30705	no
gi|320446636|ref|NW_003383935.1|	384384-38476	258967	919102	-149447	0.4811	no
gi|320446636|ref|NW_003383935.1|	386300-38823	36245	126334	-152053	0.24165	no
gi|320446636|ref|NW_003383935.1|	400500-40100	307651	107284	180206	0.4196	no
gi|320446636|ref|NW_003383935.1|	402393-40507	352011	25705	-0.453572	0.7367	no
gi|320446636|ref|NW_003383935.1|	405917-40658	181338	671853	-143246	0.49465	no
gi|320446636|ref|NW_003383935.1|	407404-40840	164684	56129	-155288	0.47475	no
gi|320446636|ref|NW_003383935.1|	408728-40999	475822	167324	-150777	0.39265	no
gi|320446636|ref|NW_003383935.1|	410713-41165	286217	329099	0.201415	0.921	no
gi|320446636|ref|NW_003383935.1|	413796-41522	353741	462145	0.385651	0.8475	no
gi|320446636|ref|NW_003383935.1|	415344-41882	0.571849	14912	138277	0.50365	no
gi|320446636|ref|NW_003383935.1|	440583-44233	899449	409917	-113371	0.6047	no
gi|320446636|ref|NW_003383935.1|	442540-44343	153526	1763	0.199549	0.9123	no
gi|320446636|ref|NW_003383935.1|	70796-75553	447779	514865	0.201408	0.9059	no
gi|320446641|ref|NW_003383930.1|	118821-11916	0	159954	inf	0.0154	no
gi|320446641|ref|NW_003383930.1|	120069-12115	0.198872	123847	263864	1	no
gi|320446641|ref|NW_003383930.1|	121302-12762	146711	122755	306474	0.11465	no
gi|320446641|ref|NW_003383930.1|	129312-13063	912089	579545	-0.654255	0.75405	no
gi|320446641|ref|NW_003383930.1|	131784-13924	856717	146159	0.770653	0.5642	no
gi|320446641|ref|NW_003383930.1|	139565-13986	0	151109	inf	0.029	no
gi|320446641|ref|NW_003383930.1|	172945-17339	0	819414	inf	0.0198	no
gi|320446641|ref|NW_003383930.1|	187749-18806	0	137968	inf	0.02915	no
gi|320446641|ref|NW_003383930.1|	206093-20752	126498	152381	35905	0.1536	no
gi|320446641|ref|NW_003383930.1|	209180-20973	317519	678287	109505	0.5957	no
gi|320446641|ref|NW_003383930.1|	27815-29223	19284	206975	0.102055	0.93245	no
gi|320446641|ref|NW_003383930.1|	2887-4268	0.294606	143247	228164	0.2731	no
gi|320446641|ref|NW_003383930.1|	30062-30954	115671	152298	0.396869	0.85815	no
gi|320446641|ref|NW_003383930.1|	43920-44708	580343	251614	-12057	0.56725	no

gi|320446641|ref|NW_003383930.1|	67220-69369	0.352708	252241	283826	0.2383	no
gi|320446641|ref|NW_003383930.1|	70065-71962	802261	196068	128921	0.4468	no
gi|320446641|ref|NW_003383930.1|	73125-74251	227389	865974	192916	0.37375	no
gi|320446643|ref|NW_003383928.1|	16609-20743	852581	119981	0.492901	0.7013	no
gi|320446644|ref|NW_003383927.1|	10040-10716	267889	182796	-0.551397	0.79275	no
gi|320446644|ref|NW_003383927.1|	102604-10312	891567	263438	156305	0.47135	no
gi|320446644|ref|NW_003383927.1|	113126-11454	51413	228239	-117159	0.57655	no
gi|320446644|ref|NW_003383927.1|	12189-14964	31891	212194	-0.587769	0.65395	no
gi|320446644|ref|NW_003383927.1|	124149-12540	0.33005	240401	286469	0.22435	no
gi|320446644|ref|NW_003383927.1|	125782-12606	525863	46688	-0.171633	0.93045	no
gi|320446644|ref|NW_003383927.1|	128815-12981	224403	205647	-0.125916	0.9568	no
gi|320446644|ref|NW_003383927.1|	152638-15337	235041	229974	-0.0314398	0.9455	no
gi|320446644|ref|NW_003383927.1|	16047-16658	806313	395265	-102852	0.61525	no
gi|320446644|ref|NW_003383927.1|	164647-16793	453539	456495	0.00937459	0.9941	no
gi|320446644|ref|NW_003383927.1|	168080-16948	429612	128636	-173974	0.32435	no
gi|320446644|ref|NW_003383927.1|	169736-17036	635345	0.288059	-44631	0.29565	no
gi|320446644|ref|NW_003383927.1|	170849-17245	158937	102884	-0.627436	0.68855	no
gi|320446644|ref|NW_003383927.1|	174064-17700	178063	136708	-0.381297	0.7679	no
gi|320446644|ref|NW_003383927.1|	177317-17882	890518	147878	-259024	0.24675	no
gi|320446644|ref|NW_003383927.1|	17803-18323	287326	236394	-360342	0.17285	no
gi|320446644|ref|NW_003383927.1|	180979-18212	141315	734069	-0.944925	0.6594	no
gi|320446644|ref|NW_003383927.1|	183775-18473	0.696825	144288	105008	0.5772	no
gi|320446644|ref|NW_003383927.1|	184911-18888	133028	143622	0.110549	0.93465	no
gi|320446644|ref|NW_003383927.1|	18692-19386	467109	201022	-453833	0.0717	no
gi|320446644|ref|NW_003383927.1|	20086-20724	70706	227722	-495649	0.0708	no
gi|320446644|ref|NW_003383927.1|	20923-21996	177306	129631	-0.451837	0.83415	no
gi|320446644|ref|NW_003383927.1|	23278-25796	990541	722835	-0.45455	0.7745	no
gi|320446644|ref|NW_003383927.1|	248525-25141	65468	110186	0.75108	0.65865	no
gi|320446644|ref|NW_003383927.1|	251526-25174	479751	334113	-0.521948	0.79845	no
gi|320446644|ref|NW_003383927.1|	254395-25518	274403	104654	-139067	0.52155	no

gi|320446644|ref|NW_003383927.1|	26231-27243	630215	976054	0.631117	0.75975	no
gi|320446644|ref|NW_003383927.1|	265835-26754	114355	119445	0.0628237	1	no
gi|320446644|ref|NW_003383927.1|	267993-26951	0.654257	22751	1798	0.4139	no
gi|320446644|ref|NW_003383927.1|	271682-27242	165588	181524	0.132566	0.92665	no
gi|320446644|ref|NW_003383927.1|	272774-27500	143813	188926	0.393632	0.84455	no
gi|320446644|ref|NW_003383927.1|	27409-29957	148842	233419	0.649139	0.62	no
gi|320446644|ref|NW_003383927.1|	275127-27715	188681	532914	149796	0.4963	no
gi|320446644|ref|NW_003383927.1|	277270-27797	0.718492	466642	269927	0.244	no
gi|320446644|ref|NW_003383927.1|	278090-27901	443107	110328	131607	0.53825	no
gi|320446644|ref|NW_003383927.1|	279234-28081	125498	672333	242151	0.289	no
gi|320446644|ref|NW_003383927.1|	41131-41647	237751	359233	0.595467	0.7533	no
gi|320446644|ref|NW_003383927.1|	41708-42358	203214	193754	-0.0687733	0.92685	no
gi|320446644|ref|NW_003383927.1|	66249-67463	172447	0.597794	-152843	0.4931	no
gi|320446644|ref|NW_003383927.1|	72200-72577	131736	934434	-0.495485	0.806	no
gi|320446644|ref|NW_003383927.1|	76136-76562	18716	253325	0.436715	0.82445	no
gi|320446644|ref|NW_003383927.1|	8955-9877	245812	711867	-178787	0.42505	no
gi|320446644|ref|NW_003383927.1|	90038-94986	153951	789983	235935	0.19195	no
gi|320446644|ref|NW_003383927.1|	97091-98057	231003	110025	225185	0.31145	no
gi|320446647|ref|NW_003383924.1|	130925-13177	0	136044	inf	0.00595	no
gi|320446647|ref|NW_003383924.1|	133756-13409	0	255568	inf	0.01275	no
gi|320446647|ref|NW_003383924.1|	26176-29734	0.101563	241611	457224	0.1539	no
gi|320446647|ref|NW_003383924.1|	284942-28569	0	232383	inf	0.0047	no
gi|320446647|ref|NW_003383924.1|	34560-35504	0	197186	inf	0.029	no
gi|320446647|ref|NW_003383924.1|	376960-37813	0.180567	112602	264062	1	no
gi|320446647|ref|NW_003383924.1|	378454-37947	0	26691	inf	0.0154	no
gi|320446647|ref|NW_003383924.1|	380043-38457	0.157186	396274	465596	0.14445	no
gi|320446647|ref|NW_003383924.1|	389388-39208	630615	322817	235588	0.19885	no
gi|320446647|ref|NW_003383924.1|	405044-40535	150523	377196	132533	0.52025	no
gi|320446647|ref|NW_003383924.1|	410977-41220	0.680055	223998	171976	0.43855	no
gi|320446647|ref|NW_003383924.1|	439785-44071	881071	180582	103532	0.63425	no

gi|320446647|ref|NW_003383924.1|	65709-68743	118591	130593	0.139081	0.91055	no
gi|320446647|ref|NW_003383924.1|	68947-70219	127795	865191	-0.562735	0.6733	no
gi|320446647|ref|NW_003383924.1|	73348-74135	131273	735173	-0.836412	0.5318	no
gi|320446647|ref|NW_003383924.1|	7690-9531	241717	153788	-0.652378	0.7501	no
gi|320446649|ref|NW_003383922.1|	132627-13337	0.989655	180835	0.869673	0.6867	no
gi|320446649|ref|NW_003383922.1|	14586-15377	984294	396286	-131255	0.3153	no
gi|320446649|ref|NW_003383922.1|	153245-15450	142339	884034	-0.687158	0.75665	no
gi|320446649|ref|NW_003383922.1|	158322-15963	127319	741891	-0.779167	0.72425	no
gi|320446649|ref|NW_003383922.1|	159748-16045	676839	438476	-0.626313	0.75045	no
gi|320446649|ref|NW_003383922.1|	162555-16618	172129	19877	0.207609	0.87695	no
gi|320446649|ref|NW_003383922.1|	16529-18503	0.970538	0.609033	-0.672263	1	no
gi|320446649|ref|NW_003383922.1|	169891-17346	0.252917	215897	309361	0.2053	no
gi|320446649|ref|NW_003383922.1|	185705-18637	0.385296	315426	303326	0.25	no
gi|320446649|ref|NW_003383922.1|	186440-18843	0.576027	180749	164978	0.43955	no
gi|320446649|ref|NW_003383922.1|	21217-22845	327163	421666	0.36609	0.85745	no
gi|320446649|ref|NW_003383922.1|	225919-22627	126372	905065	284035	0.2595	no
gi|320446649|ref|NW_003383922.1|	22962-28652	155278	10144	-0.614235	0.64275	no
gi|320446649|ref|NW_003383922.1|	29328-37378	161927	616189	192803	0.27605	no
gi|320446649|ref|NW_003383922.1|	37568-40384	238652	307546	0.365896	0.7819	no
gi|320446649|ref|NW_003383922.1|	40499-41177	685773	390007	-0.814229	0.6926	no
gi|320446649|ref|NW_003383922.1|	411037-41216	132644	695403	-0.931633	0.66375	no
gi|320446649|ref|NW_003383922.1|	412335-41363	636443	309185	-104156	0.6163	no
gi|320446649|ref|NW_003383922.1|	414413-41700	537453	225273	-125446	0.5577	no
gi|320446649|ref|NW_003383922.1|	41468-42997	769636	662295	-0.216703	0.91875	no
gi|320446649|ref|NW_003383922.1|	417303-41829	289981	261914	-0.146866	0.93585	no
gi|320446649|ref|NW_003383922.1|	420918-42145	263181	603074	-212565	0.32455	no
gi|320446649|ref|NW_003383922.1|	43148-46212	560718	838348	0.580273	0.7173	no
gi|320446649|ref|NW_003383922.1|	433733-43523	317002	23437	-0.435706	0.7307	no
gi|320446649|ref|NW_003383922.1|	435375-43647	605792	568056	-0.0927885	0.9629	no
gi|320446649|ref|NW_003383922.1|	436617-43820	0.626093	174247	147668	0.5	no

gi|320446649|ref|NW_003383922.1|	438317-43965[illegible]	270347	10558	-135647	0.41885	no
gi|320446649|ref|NW_003383922.1|	445685-44713[illegible]	298952	661049	114484	0.3855	no
gi|320446649|ref|NW_003383922.1|	447781-45299[illegible]	880419	209476	125053	0.34965	no
gi|320446649|ref|NW_003383922.1|	453361-45558[illegible]	173358	106157	-0.707557	0.57935	no
gi|320446649|ref|NW_003383922.1|	457101-45968[illegible]	466661	239097	-0.964781	0.465	no
gi|320446649|ref|NW_003383922.1|	46364-47506	837655	20.37	128202	0.56805	no
gi|320446649|ref|NW_003383922.1|	464683-46931[illegible]	107404	84353	-0.34853	0.78765	no
gi|320446649|ref|NW_003383922.1|	470465-47559[illegible]	479638	111439	121624	0.34725	no
gi|320446649|ref|NW_003383922.1|	476077-47740[illegible]	816342	74855	-0.125075	0.95355	no
gi|320446649|ref|NW_003383922.1|	47831-48576	794822	127067	0.676886	0.74635	no
gi|320446649|ref|NW_003383922.1|	479102-47988[illegible]	490484	631267	0.364042	0.85595	no
gi|320446649|ref|NW_003383922.1|	479995-48195[illegible]	987057	681373	-0.534689	0.815	no
gi|320446649|ref|NW_003383922.1|	48709-50717	106615	136082	0.352068	0.8257	no
gi|320446649|ref|NW_003383922.1|	520221-52165[illegible]	0.140788	971567	943065	0.14075	no
gi|320446649|ref|NW_003383922.1|	52240-52688	0.770763	143397	421759	0.18995	no
gi|320446649|ref|NW_003383922.1|	55334-57866	0.293881	246327	306727	0.22375	no
gi|320446649|ref|NW_003383922.1|	5987-7346	0.60075	0.625802	0.05894	1	no
gi|320446649|ref|NW_003383922.1|	64988-67220	306775	168685	-0.862851	0.5103	no
gi|320446649|ref|NW_003383922.1|	68279-68558	300228	441997	-276395	0.2207	no
gi|320446649|ref|NW_003383922.1|	68756-70044	112224	289257	-195596	0.3822	no
gi|320446649|ref|NW_003383922.1|	8300-10771	123779	690507	-0.842042	0.6012	no
gi|320446652|ref|NW_003383919.1|	102633-10472[illegible]	574119	966239	0.751032	0.73075	no
gi|320446652|ref|NW_003383919.1|	106667-10861[illegible]	417747	335987	-0.314222	0.81005	no
gi|320446652|ref|NW_003383919.1|	108765-11021[illegible]	908086	739571	-0.29614	0.8219	no
gi|320446652|ref|NW_003383919.1|	111017-11248[illegible]	752675	124215	0.722741	0.59105	no
gi|320446652|ref|NW_003383919.1|	118180-11841[illegible]	606438	0	#NAME?	0.01585	no
gi|320446652|ref|NW_003383919.1|	128410-13068[illegible]	157108	170841	344283	0.0806	no
gi|320446652|ref|NW_003383919.1|	130943-13177[illegible]	0	390993	inf	0.0162	no
gi|320446652|ref|NW_003383919.1|	131977-13320[illegible]	0.847507	32912	195732	0.38315	no
gi|320446652|ref|NW_003383919.1|	24900-25359	147172	122471	305686	0.20905	no

gi|320446652|ref|NW_003383919.1|	26956-27308	140829	391573	147534	0.47825	no
gi|320446652|ref|NW_003383919.1|	321203-321534	444024	249052	248774	0.2893	no
gi|320446652|ref|NW_003383919.1|	321684-326446	119276	480359	200981	0.23515	no
gi|320446652|ref|NW_003383919.1|	337287-337924	0.838811	256795	16142	0.33275	no
gi|320446652|ref|NW_003383919.1|	415570-41588[illegible]	103649	667148	-0.635623	0.7537	no
gi|320446652|ref|NW_003383919.1|	4303-4607	164844	85895	238147	0.2873	no
gi|320446652|ref|NW_003383919.1|	53001-54985	149573	450817	-173023	0.3053	no
gi|320446652|ref|NW_003383919.1|	55108-55948	125624	422427	-157234	0.4696	no
gi|320446652|ref|NW_003383919.1|	56058-57446	180082	105772	-0.767688	0.6288	no
gi|320446652|ref|NW_003383919.1|	59534-59909	777452	726863	-0.0970717	0.94265	no
gi|320446652|ref|NW_003383919.1|	60925-61550	518551	381783	-0.441734	0.8264	no
gi|320446652|ref|NW_003383919.1|	620261-620576	167248	150878	317332	0.23215	no
gi|320446652|ref|NW_003383919.1|	64108-65290	171147	163083	-0.069628	0.9728	no
gi|320446652|ref|NW_003383919.1|	648940-64941[illegible]	625982	0.464116	-375356	0.323	no
gi|320446652|ref|NW_003383919.1|	65543-66838	100334	285158	150694	0.3646	no
gi|320446652|ref|NW_003383919.1|	67657-69964	325804	528781	0.698667	0.73685	no
gi|320446652|ref|NW_003383919.1|	70081-71260	105518	137568	0.382652	0.858	no
gi|320446652|ref|NW_003383919.1|	72419-72984	865197	893972	0.0472018	0.9738	no
gi|320446652|ref|NW_003383919.1|	73087-73893	621192	487697	-0.349053	0.86585	no
gi|320446652|ref|NW_003383919.1|	74475-76537	57274	463914	-0.304023	0.88785	no
gi|320446652|ref|NW_003383919.1|	77044-77908	671253	258754	-137527	0.51155	no
gi|320446652|ref|NW_003383919.1|	78532-79966	577231	313093	-0.882557	0.674	no
gi|320446652|ref|NW_003383919.1|	81031-81477	132166	671304	-0.977308	0.6433	no
gi|320446652|ref|NW_003383919.1|	83644-84352	421237	361262	-0.221586	0.92455	no
gi|320446652|ref|NW_003383919.1|	84729-85243	31718	273207	-0.215308	0.91545	no
gi|320446652|ref|NW_003383919.1|	86238-88335	359842	265563	-0.438312	0.7409	no
gi|320446652|ref|NW_003383919.1|	88477-89461	297589	155686	-425661	0.0727	no
gi|320446652|ref|NW_003383919.1|	99823-101901	28663	204406	-0.487755	0.70405	no
gi|320446653|ref|NW_003383918.1|	209713-210285	314144	539418	-254195	0.2551	no
gi|320446653|ref|NW_003383918.1|	266452-266708	416421	188567	-44649	0.29535	no

gi|320446653|ref|NW_003383918.1|	22322-32260	199747	283492	-281679	0.27485	no
gi|320446653|ref|NW_003383918.1|	34207-33525	7314	202281	-18543	0.38685	no
gi|320446653|ref|NW_003383918.1|	36606-33786	640465	318994	-100559	0.6187	no
gi|320446653|ref|NW_003383918.1|	42180-34479	767413	968004	0.335011	0.8336	no
gi|320446653|ref|NW_003383918.1|	44856-34522	339129	133071	197228	0.3851	no
gi|320446653|ref|NW_003383918.1|	52657-35604	667294	633996	-0.0738485	0.95455	no
gi|320446653|ref|NW_003383918.1|	58466-36222	530914	569049	0.100074	0.96435	no
gi|320446653|ref|NW_003383918.1|	62636-36355	443107	441311	-0.00585936	0.97895	no
gi|320446653|ref|NW_003383918.1|	91100-39204	0.237173	196369	304956	0.2493	no
gi|320446653|ref|NW_003383918.1|	94880-39724	0.238033	232665	328902	0.204	no
gi|320446653|ref|NW_003383918.1|	06567-40853	0.0973337	291816	490597	0.17435	no
gi|320446653|ref|NW_003383918.1|	10579-41168	0	161015	inf	0.029	no
gi|320446653|ref|NW_003383918.1|	14698-41749	0.986879	859853	312315	0.09835	no
gi|320446653|ref|NW_003383918.1|	19212-42003	163451	689929	-124434	0.5599	no
gi|320446653|ref|NW_003383918.1|	20408-42092	135781	237931	-251267	0.28675	no
gi|320446653|ref|NW_003383918.1|	21066-42174	103097	130285	-298426	0.2324	no
gi|320446653|ref|NW_003383918.1|	22812-42355	21611	136664	-398305	0.1576	no
gi|320446653|ref|NW_003383918.1|	24619-42576	299035	0.385933	-627582	0.11055	no
gi|320446653|ref|NW_003383918.1|	28423-42892	551147	167883	-503691	0.1613	no
gi|320446653|ref|NW_003383918.1|	89794-49054	558689	151407	-188361	0.2695	no
gi|320446653|ref|NW_003383918.1|	91834-49281	0.225146	155482	278782	0.2657	no
gi|320446653|ref|NW_003383918.1|	94149-49475	458737	248885	-0.88219	0.6765	no
gi|320446653|ref|NW_003383918.1|	94856-49631	176526	67104	-139541	0.38975	no
gi|320446653|ref|NW_003383918.1|	99887-50041	196719	610667	-168768	0.34365	no
gi|320446653|ref|NW_003383918.1|	00678-50569	396094	180859	-113097	0.39635	no
gi|320446653|ref|NW_003383918.1|	46038-54684	206701	152144	287982	0.22095	no
gi|320446653|ref|NW_003383918.1|	69757-57020	840607	106669	0.343633	0.86135	no
gi|320446653|ref|NW_003383918.1|	63426-63968	327523	183901	-0.832667	0.6954	no
gi|320446657|ref|NW_003383914.1|	04258-11043	176088	408066	12125	0.4546	no
gi|320446657|ref|NW_003383914.1|	10728-11923	123103	487582	198577	0.3726	no

gi|320446657|ref|NW_003383914.1|	111538-112759	171222	249313	0.542092	0.7916	no
gi|320446657|ref|NW_003383914.1|	117680-119480	0.431396	571035	372649	0.16865	no
gi|320446657|ref|NW_003383914.1|	12061-14157	371825	525083	0.497919	0.81925	no
gi|320446657|ref|NW_003383914.1|	126823-127534	0.709484	970433	377379	0.1769	no
gi|320446657|ref|NW_003383914.1|	130107-131339	420788	248944	-40792	0.0054	no
gi|320446657|ref|NW_003383914.1|	136725-137422	204641	399527	-235673	0.2825	no
gi|320446657|ref|NW_003383914.1|	139016-140482	274252	114388	-126157	0.5602	no
gi|320446657|ref|NW_003383914.1|	141346-141771	196588	339288	-253459	0.2857	no
gi|320446657|ref|NW_003383914.1|	143251-146071	644341	214266	173351	0.33165	no
gi|320446657|ref|NW_003383914.1|	146988-147323	153.86	284142	0.884999	0.59925	no
gi|320446657|ref|NW_003383914.1|	14927-15270	679853	12359	0.862261	0.6797	no
gi|320446657|ref|NW_003383914.1|	197734-198455	173738	404217	-210371	0.34495	no
gi|320446657|ref|NW_003383914.1|	206572-208435	0.207347	223975	343322	0.19545	no
gi|320446657|ref|NW_003383914.1|	21654-22017	632693	662141	0.0656335	0.97495	no
gi|320446657|ref|NW_003383914.1|	234783-236940	11064	287958	-194195	0.2413	no
gi|320446657|ref|NW_003383914.1|	237782-238692	667996	776034	-310565	0.0967	no
gi|320446657|ref|NW_003383914.1|	239064-239787	269925	426295	-266264	0.25385	no
gi|320446657|ref|NW_003383914.1|	242776-243889	484592	998432	-227903	0.20065	no
gi|320446657|ref|NW_003383914.1|	244541-251099	571065	503174	-0.182597	0.88345	no
gi|320446657|ref|NW_003383914.1|	251698-252521	861732	434392	-0.98824	0.6375	no
gi|320446657|ref|NW_003383914.1|	255648-257590	278824	352921	0.339991	0.7883	no
gi|320446657|ref|NW_003383914.1|	257739-258069	283282	231521	-0.291094	0.88295	no
gi|320446657|ref|NW_003383914.1|	276617-278298	263806	617637	-209465	0.2288	no
gi|320446657|ref|NW_003383914.1|	278405-281107	608815	50667	-0.264956	0.9036	no
gi|320446657|ref|NW_003383914.1|	28412-30131	178569	863323	-104851	0.5225	no
gi|320446657|ref|NW_003383914.1|	30696-31619	249627	138277	-0.852209	0.5328	no
gi|320446657|ref|NW_003383914.1|	365370-365930	862653	480008	-0.845723	0.6786	no
gi|320446657|ref|NW_003383914.1|	372864-373270	84417	101376	0.264106	0.9055	no
gi|320446657|ref|NW_003383914.1|	374086-376178	111788	23077	104569	0.4061	no
gi|320446657|ref|NW_003383914.1|	377965-378889	612744	116616	0.928409	0.65905	no

gi|320446657|ref|NW_003383914.1|	379101-38109[illegible]	758867	10918	0.524796	0.8126	no
gi|320446657|ref|NW_003383914.1|	385794-388754	210555	212139	0.0108092	0.9881	no
gi|320446657|ref|NW_003383914.1|	39665-40430	235836	502692	-223004	0.32375	no
gi|320446657|ref|NW_003383914.1|	400318-40248[illegible]	0.174892	213554	361007	0.18175	no
gi|320446657|ref|NW_003383914.1|	410759-415588	120842	302032	132158	0.3244	no
gi|320446657|ref|NW_003383914.1|	41154-42400	140267	277967	-233519	0.2977	no
gi|320446657|ref|NW_003383914.1|	416203-417837	732356	219678	158477	0.23845	no
gi|320446657|ref|NW_003383914.1|	45481-47716	165393	459363	-184819	0.28685	no
gi|320446657|ref|NW_003383914.1|	49-540	0	912643	inf	0.01575	no
gi|320446657|ref|NW_003383914.1|	49073-49891	234665	117475	-0.998252	0.65435	no
gi|320446657|ref|NW_003383914.1|	50317-56502	243315	115278	-10777	0.41765	no
gi|320446657|ref|NW_003383914.1|	5153-9344	853091	140058	0.715252	0.5791	no
gi|320446657|ref|NW_003383914.1|	56635-59789	763109	857078	0.167537	0.89215	no
gi|320446657|ref|NW_003383914.1|	60011-62562	219331	257041	0.228891	0.8598	no
gi|320446657|ref|NW_003383914.1|	62837-63668	878491	13.25	0.592899	0.7745	no
gi|320446657|ref|NW_003383914.1|	65082-71754	775894	210023	143662	0.28855	no
gi|320446657|ref|NW_003383914.1|	76171-76430	212.4	109266	-428086	0.14605	no
gi|320446657|ref|NW_003383914.1|	87198-87978	619858	212686	-154321	0.4842	no
gi|320446657|ref|NW_003383914.1|	88136-88819	11303	231493	-228766	0.30185	no
gi|320446657|ref|NW_003383914.1|	90740-91622	605575	934843	-269551	0.1436	no
gi|320446657|ref|NW_003383914.1|	9941-10439	151941	161146	0.0848604	0.9636	no
gi|320446660|ref|NW_003383911.1|	117183-121509	160862	32635	102059	0.63545	no
gi|320446660|ref|NW_003383911.1|	21258-21627	149612	902516	-0.729198	0.7282	no
gi|320446660|ref|NW_003383911.1|	215893-217698	233286	501821	-221686	0.2075	no
gi|320446660|ref|NW_003383911.1|	21809-23395	174917	695453	-133064	0.4125	no
gi|320446660|ref|NW_003383911.1|	221758-22228[illegible]	725644	358989	-101532	0.5214	no
gi|320446660|ref|NW_003383911.1|	223533-227285	207752	146735	-0.501651	0.7068	no
gi|320446660|ref|NW_003383911.1|	227416-227978	112966	554859	-102569	0.5991	no
gi|320446660|ref|NW_003383911.1|	228519-230273	223344	634626	-181529	0.299	no
gi|320446660|ref|NW_003383911.1|	230439-230835	395208	51978	0.395288	0.8506	no

gi|320446660|ref|NW_003383911.1|	230997-23138	260794	547567	-22518	0.3128	no
gi|320446660|ref|NW_003383911.1|	23709-25042	164508	11635	-0.49969	0.8246	no
gi|320446660|ref|NW_003383911.1|	25162-26783	0.974271	118658	0.28441	1	no
gi|320446660|ref|NW_003383911.1|	251715-25214	22138	217359	-334837	0.21	no
gi|320446660|ref|NW_003383911.1|	257501-25925	151164	116405	-0.376963	0.77485	no
gi|320446660|ref|NW_003383911.1|	259717-26730	49179	550719	0.163274	0.8956	no
gi|320446660|ref|NW_003383911.1|	268887-27015	472107	463092	-0.0278154	0.98405	no
gi|320446660|ref|NW_003383911.1|	26896-30059	380381	261979	-0.537996	0.80025	no
gi|320446660|ref|NW_003383911.1|	272486-27389	143421	129547	-0.146778	0.9346	no
gi|320446660|ref|NW_003383911.1|	282455-28298	130899	719754	245905	0.1663	no
gi|320446660|ref|NW_003383911.1|	288202-28864	780827	280012	184241	0.3904	no
gi|320446660|ref|NW_003383911.1|	288782-29060	102163	399662	19679	0.2767	no
gi|320446660|ref|NW_003383911.1|	290829-29218	142062	741974	238485	0.2046	no
gi|320446660|ref|NW_003383911.1|	292742-29308	838717	371708	214791	0.3307	no
gi|320446660|ref|NW_003383911.1|	295422-29719	0.109958	130208	35658	1	no
gi|320446660|ref|NW_003383911.1|	297601-29960	141123	102599	286199	0.0394	no
gi|320446660|ref|NW_003383911.1|	299785-30344	108455	104525	326868	0.08805	no
gi|320446660|ref|NW_003383911.1|	305021-30628	245377	195203	29919	0.2128	no
gi|320446660|ref|NW_003383911.1|	307252-30826	0.649437	673171	337371	0.19035	no
gi|320446660|ref|NW_003383911.1|	310777-31302	109045	182071	40615	0.06005	no
gi|320446660|ref|NW_003383911.1|	31157-31924	952502	104525	0.134052	0.9467	no
gi|320446660|ref|NW_003383911.1|	313715-31506	984104	109338	0.151918	0.94305	no
gi|320446660|ref|NW_003383911.1|	315362-31601	158885	55486	-151779	0.47455	no
gi|320446660|ref|NW_003383911.1|	316552-31675	505206	197296	-135651	0.52435	no
gi|320446660|ref|NW_003383911.1|	317602-32165	449685	396122	-0.182969	0.891	no
gi|320446660|ref|NW_003383911.1|	32172-37333	271978	276446	0.0235105	0.98555	no
gi|320446660|ref|NW_003383911.1|	322688-32385	12124	253263	106277	0.51	no
gi|320446660|ref|NW_003383911.1|	326949-33222	264329	241449	-0.130613	0.9504	no
gi|320446660|ref|NW_003383911.1|	361859-36359	0.564127	974314	41103	0.1442	no
gi|320446660|ref|NW_003383911.1|	368059-36893	0.785502	577039	287698	0.2364	no

gi|320446660|ref|NW_003383911.1|	370270-37178	276288	304681	346306	0.08465	no
gi|320446660|ref|NW_003383911.1|	394737-39612	0.583606	16217	147444	0.49665	no
gi|320446660|ref|NW_003383911.1|	413940-41446	0	144376	inf	0.0103	no
gi|320446660|ref|NW_003383911.1|	418009-41851	0.606766	529272	31248	0.2458	no
gi|320446660|ref|NW_003383911.1|	419275-42039	0.190346	184509	327699	0.23005	no
gi|320446660|ref|NW_003383911.1|	589365-58981	76094	354114	-110357	0.59295	no
gi|320446660|ref|NW_003383911.1|	599398-60004	0	448023	inf	0.0198	no
gi|320446661|ref|NW_003383910.1|	175777-17703	594671	240634	-130525	0.54005	no
gi|320446661|ref|NW_003383910.1|	178924-17979	0.265543	182829	278347	0.2657	no
gi|320446661|ref|NW_003383910.1|	180851-18140	138013	100253	-0.461166	0.8164	no
gi|320446661|ref|NW_003383910.1|	184180-18579	170263	159405	-0.0950682	0.93825	no
gi|320446661|ref|NW_003383910.1|	185900-18710	123066	146601	0.252465	0.90985	no
gi|320446661|ref|NW_003383910.1|	187285-18798	0.360773	246615	27731	0.2657	no
gi|320446661|ref|NW_003383910.1|	188218-18868	267842	260413	-0.0405804	0.9818	no
gi|320446661|ref|NW_003383910.1|	189998-19025	692435	839552	0.27794	0.8921	no
gi|320446661|ref|NW_003383910.1|	190925-19417	206884	406716	0.975204	0.64955	no
gi|320446661|ref|NW_003383910.1|	196466-20000	0.766334	214472	148474	0.4871	no
gi|320446661|ref|NW_003383910.1|	200242-20512	197296	394737	100053	0.45215	no
gi|320446661|ref|NW_003383910.1|	210081-21134	0.326853	192749	256001	0.25005	no
gi|320446661|ref|NW_003383910.1|	237137-23787	0	456422	inf	0.0162	no
gi|320446661|ref|NW_003383910.1|	241790-25114	101546	124934	0.299032	0.82215	no
gi|320446661|ref|NW_003383910.1|	260629-26080	150068	128994	310361	0.2384	no
gi|320446661|ref|NW_003383910.1|	40777-41206	218121	999827	-112538	0.58235	no
gi|320446661|ref|NW_003383910.1|	66211-66961	144306	561905	-136073	0.5137	no
gi|320446661|ref|NW_003383910.1|	78494-78850	374265	121901	170357	0.42695	no
gi|320446662|ref|NW_003383909.1|	306445-31459	0.61805	514109	305628	0.10955	no
gi|320446662|ref|NW_003383909.1|	325976-32646	719728	0	#NAME?	0.02105	no
gi|320446662|ref|NW_003383909.1|	327271-32920	238135	214431	-0.151262	0.94335	no
gi|320446662|ref|NW_003383909.1|	330262-33110	19413	133543	-0.539719	0.7943	no
gi|320446662|ref|NW_003383909.1|	377465-37814	377175	884017	-209309	0.3496	no

gi|320446662|ref|NW_003383909.1|	78661-37890	427171	415732	-336109	0.2398	no
gi|320446662|ref|NW_003383909.1|	79090-37972	80677	259875	-163434	0.4281	no
gi|320446662|ref|NW_003383909.1|	80247-38117	100344	641318	-0.645845	0.7544	no
gi|320446662|ref|NW_003383909.1|	82105-38373	223206	791881	182691	0.17995	no
gi|320446662|ref|NW_003383909.1|	85076-38547	456916	92.08	101096	0.6456	no
gi|320446662|ref|NW_003383909.1|	85878-38636	922875	154292	0.741458	0.7105	no
gi|320446662|ref|NW_003383909.1|	86821-38746	615504	156444	13458	0.52365	no
gi|320446662|ref|NW_003383909.1|	88300-38885	286673	562908	0.973491	0.66125	no
gi|320446662|ref|NW_003383909.1|	11558-41204	0	50696	inf	0.0054	no
gi|320446662|ref|NW_003383909.1|	12146-41240	0	26092	inf	0.0233	no
gi|320446662|ref|NW_003383909.1|	13533-41428	0	786666	inf	0.0114	no
gi|320446662|ref|NW_003383909.1|	15113-41898	0	106102	inf	5.00E-05	yes
gi|320446662|ref|NW_003383909.1|	19684-42133	0	232997	inf	5.00E-05	yes
gi|320446663|ref|NW_003383908.1|	01710-10230	0.471329	447066	324568	0.2316	no
gi|320446663|ref|NW_003383908.1|	73419-17461	0.351358	194832	247122	0.2454	no
gi|320446663|ref|NW_003383908.1|	77871-17827	87525	684735	296778	0.2191	no
gi|320446663|ref|NW_003383908.1|	91074-19127	142973	445658	164019	0.3438	no
gi|320446663|ref|NW_003383908.1|	92477-19304	200617	213585	34123	0.1933	no
gi|320446663|ref|NW_003383908.1|	94356-19464	0	168078	inf	0.02915	no
gi|320446663|ref|NW_003383908.1|	94816-19559	0.926386	572201	262684	0.267	no
gi|320446663|ref|NW_003383908.1|	95889-19772	17978	282961	397629	0.05345	no
gi|320446663|ref|NW_003383908.1|	98914-19934	250518	149312	257534	0.28495	no
gi|320446663|ref|NW_003383908.1|	99484-19992	812547	571034	281305	0.2243	no
gi|320446663|ref|NW_003383908.1|	22649-25044	376594	393885	0.0647635	0.9605	no
gi|320446663|ref|NW_003383908.1|	25449-30390	455368	381764	-0.254351	0.8745	no
gi|320446663|ref|NW_003383908.1|	61110-26212	239665	120435	-0.992762	0.65595	no
gi|320446663|ref|NW_003383908.1|	64406-26664	557214	365317	-0.609085	0.7791	no
gi|320446663|ref|NW_003383908.1|	68263-26947	207572	239844	0.208479	0.9183	no
gi|320446663|ref|NW_003383908.1|	72279-27259	663681	962497	0.536292	0.7996	no
gi|320446663|ref|NW_003383908.1|	73987-27465	193965	185216	-0.0665879	0.92685	no

gi|320446663|ref|NW_003383908.1|	311386-31398[illegible]	0.930004	439791	224151	0.31685	no
gi|320446663|ref|NW_003383908.1|	31309-32471	220373	921104	-125851	0.5765	no
gi|320446663|ref|NW_003383908.1|	318965-31964[illegible]	189643	543652	151939	0.4905	no
gi|320446663|ref|NW_003383908.1|	319857-32151[illegible]	118533	239287	101346	0.6064	no
gi|320446663|ref|NW_003383908.1|	35030-38157	245075	146079	257545	0.159	no
gi|320446663|ref|NW_003383908.1|	352488-35328[illegible]	57206	86836	0.602127	0.7682	no
gi|320446663|ref|NW_003383908.1|	353608-35646[illegible]	726175	216506	157602	0.3806	no
gi|320446663|ref|NW_003383908.1|	39377-39570	514256	319494	-0.686697	0.73895	no
gi|320446663|ref|NW_003383908.1|	41906-42619	155437	652391	-125252	0.54995	no
gi|320446663|ref|NW_003383908.1|	45812-46264	621328	216644	-152003	0.48255	no
gi|320446663|ref|NW_003383908.1|	56119-56881	185896	659325	-149543	0.4911	no
gi|320446663|ref|NW_003383908.1|	87058-87377	162045	188158	353748	0.2109	no
gi|320446663|ref|NW_003383908.1|	88843-89446	0.915027	49649	243988	0.24865	no
gi|320446663|ref|NW_003383908.1|	91570-92247	19092	151131	298476	0.2206	no
gi|320446663|ref|NW_003383908.1|	92340-93370	0.424782	117451	146726	1	no
gi|320446663|ref|NW_003383908.1|	96207-96931	0	449086	inf	0.0212	no
gi|320446664|ref|NW_003383907.1|	104582-10520[illegible]	302753	119215	-466651	0.16775	no
gi|320446664|ref|NW_003383907.1|	13086-14054	0.921488	11132	0.27268	1	no
gi|320446664|ref|NW_003383907.1|	172614-17389[illegible]	273324	490894	0.8448	0.6817	no
gi|320446664|ref|NW_003383907.1|	175674-17605[illegible]	949396	0.691823	-377853	0.32245	no
gi|320446664|ref|NW_003383907.1|	177835-17867[illegible]	6162	115582	-241448	0.29165	no
gi|320446664|ref|NW_003383907.1|	20074-20302	305401	329365	0.108981	0.94345	no
gi|320446664|ref|NW_003383907.1|	227118-22823[illegible]	113327	132975	-309126	0.20195	no
gi|320446664|ref|NW_003383907.1|	22879-26517	59009	673154	0.190003	0.9041	no
gi|320446664|ref|NW_003383907.1|	35044-36404	150054	12505	-0.262974	0.8986	no
gi|320446664|ref|NW_003383907.1|	42259-43977	124058	129981	0.0672875	0.9769	no
gi|320446664|ref|NW_003383907.1|	44235-48534	118334	184897	0.643852	0.61845	no
gi|320446664|ref|NW_003383907.1|	50194-50994	896851	184788	-2279	0.30265	no
gi|320446664|ref|NW_003383907.1|	51547-52217	465515	132297	-181505	0.4036	no
gi|320446664|ref|NW_003383907.1|	52372-53157	104418	147557	-282302	0.2279	no

gi|320446664|ref|NW_003383907.1|	54330-55000	143534	211675	-276147	0.23795	no
gi|320446664|ref|NW_003383907.1|	61542-61913	125088	0.743508	-407246	0.30905	no
gi|320446665|ref|NW_003383906.1|	109330-10960	196538	203409	0.0495769	0.9543	no
gi|320446665|ref|NW_003383906.1|	11308-14125	801297	721746	-0.150846	0.90675	no
gi|320446665|ref|NW_003383906.1|	118580-11965	220056	429074	0.963355	0.64315	no
gi|320446665|ref|NW_003383906.1|	120176-12072	0.527584	747487	382457	0.20105	no
gi|320446665|ref|NW_003383906.1|	122084-12236	142869	212493	0.572725	0.7884	no
gi|320446665|ref|NW_003383906.1|	124528-12644	326506	483206	0.565526	0.67005	no
gi|320446665|ref|NW_003383906.1|	126552-12820	184764	341028	0.884205	0.49665	no
gi|320446665|ref|NW_003383906.1|	128335-12882	265666	310815	0.226443	0.89115	no
gi|320446665|ref|NW_003383906.1|	129469-13079	169895	225174	0.406402	0.84205	no
gi|320446665|ref|NW_003383906.1|	130897-13158	676682	359329	-0.91317	0.66185	no
gi|320446665|ref|NW_003383906.1|	132353-13379	870706	292799	-157227	0.4678	no
gi|320446665|ref|NW_003383906.1|	134759-13522	777287	518329	-0.584577	0.78725	no
gi|320446665|ref|NW_003383906.1|	144750-14735	595581	125.27	107268	0.414	no
gi|320446665|ref|NW_003383906.1|	14593-16213	107247	932978	-0.201026	0.92845	no
gi|320446665|ref|NW_003383906.1|	147468-14923	405437	42208	0.058041	0.96325	no
gi|320446665|ref|NW_003383906.1|	151425-15373	583.86	682041	0.224236	0.9039	no
gi|320446665|ref|NW_003383906.1|	156400-16301	275797	34.05	0.304046	0.8171	no
gi|320446665|ref|NW_003383906.1|	165193-16592	0.339814	23267	277547	0.2657	no
gi|320446665|ref|NW_003383906.1|	16696-17466	0.947163	173255	0.871208	0.6867	no
gi|320446665|ref|NW_003383906.1|	169804-17055	296897	113022	-139336	0.51795	no
gi|320446665|ref|NW_003383906.1|	170770-17142	178828	60894	-15542	0.46615	no
gi|320446665|ref|NW_003383906.1|	174540-17505	163827	488559	-174557	0.42395	no
gi|320446665|ref|NW_003383906.1|	175204-17563	838927	55546	-0.594863	0.7629	no
gi|320446665|ref|NW_003383906.1|	175814-17605	360656	182177	-0.985288	0.66335	no
gi|320446665|ref|NW_003383906.1|	180073-18086	204737	682283	-158533	0.4575	no
gi|320446665|ref|NW_003383906.1|	188383-18907	223095	203154	-0.13508	0.9301	no
gi|320446665|ref|NW_003383906.1|	200021-20177	311741	814226	138508	0.52165	no
gi|320446665|ref|NW_003383906.1|	201874-20451	406311	553069	0.444875	0.8432	no

gi|320446665|ref|NW_003383906.1|	204739-20790	164143	942483	-0.800419	0.5405	no
gi|320446665|ref|NW_003383906.1|	208004-20831	252108	104173	-127506	0.53555	no
gi|320446665|ref|NW_003383906.1|	208415-21319	401332	46.13	0.200907	0.87875	no
gi|320446665|ref|NW_003383906.1|	22450-27749	398847	124894	-167513	0.21695	no
gi|320446665|ref|NW_003383906.1|	225442-22632	121739	165934	0.446816	0.8292	no
gi|320446665|ref|NW_003383906.1|	226569-22831	239842	382475	0.673279	0.61	no
gi|320446665|ref|NW_003383906.1|	228412-22886	121209	207067	0.772604	0.55325	no
gi|320446665|ref|NW_003383906.1|	229010-22971	230895	478432	105108	0.51265	no
gi|320446665|ref|NW_003383906.1|	230033-23280	186325	758028	202443	0.37525	no
gi|320446665|ref|NW_003383906.1|	233459-23441	454998	133852	155671	0.24655	no
gi|320446665|ref|NW_003383906.1|	237997-23838	13266	556232	206796	0.356	no
gi|320446665|ref|NW_003383906.1|	245525-24587	352569	85.76	12824	0.5547	no
gi|320446665|ref|NW_003383906.1|	253615-25552	120628	378385	164929	0.43795	no
gi|320446665|ref|NW_003383906.1|	255858-25664	427621	285162	273738	0.238	no
gi|320446665|ref|NW_003383906.1|	257954-26299	607648	186998	162171	0.2285	no
gi|320446665|ref|NW_003383906.1|	263303-26375	833489	352676	-124082	0.55135	no
gi|320446665|ref|NW_003383906.1|	266675-26742	794822	726097	-0.130469	0.95015	no
gi|320446665|ref|NW_003383906.1|	268670-27092	245283	172363	-0.508995	0.69505	no
gi|320446665|ref|NW_003383906.1|	271263-27184	11273	729507	-0.627876	0.7615	no
gi|320446665|ref|NW_003383906.1|	272534-27534	694416	370867	-0.904899	0.6899	no
gi|320446665|ref|NW_003383906.1|	275487-27620	386179	624436	0.693284	0.7342	no
gi|320446665|ref|NW_003383906.1|	276824-27713	12192	123285	0.0160535	0.9489	no
gi|320446665|ref|NW_003383906.1|	277621-27897	661414	123183	0.897174	0.6757	no
gi|320446665|ref|NW_003383906.1|	279879-28116	388344	373047	-0.0579812	0.96305	no
gi|320446665|ref|NW_003383906.1|	282165-28252	38438	454513	0.241788	0.9027	no
gi|320446665|ref|NW_003383906.1|	283298-29169	295722	870144	155701	0.23765	no
gi|320446665|ref|NW_003383906.1|	294779-29537	283575	140879	231265	0.3088	no
gi|320446665|ref|NW_003383906.1|	297584-29843	621653	208177	-490022	0.06235	no
gi|320446665|ref|NW_003383906.1|	298591-29921	399857	204578	-428876	0.13745	no
gi|320446665|ref|NW_003383906.1|	299340-29956	161.3	0	#NAME?	0.0074	no

gi|320446665|ref|NW_003383906.1|	30182-30793	851108	0.91215	-3222	0.1933	no
gi|320446665|ref|NW_003383906.1|	304028-30614	443947	702172	-266049	0.16685	no
gi|320446665|ref|NW_003383906.1|	306667-30765	383464	216821	-414451	0.0637	no
gi|320446665|ref|NW_003383906.1|	307866-30997	116426	57294	-102295	0.5167	no
gi|320446665|ref|NW_003383906.1|	310617-31183	130792	18017	0.462086	0.833	no
gi|320446665|ref|NW_003383906.1|	329006-33187	0	398413	inf	0.0048	no
gi|320446665|ref|NW_003383906.1|	333216-33509	0.102789	487142	556658	0.1613	no
gi|320446665|ref|NW_003383906.1|	33486-34022	300441	14984	-432559	0.17245	no
gi|320446665|ref|NW_003383906.1|	335290-33666	0.14769	174394	356171	0.2162	no
gi|320446665|ref|NW_003383906.1|	338879-34052	0	216645	inf	0.0142	no
gi|320446665|ref|NW_003383906.1|	342678-34511	140163	3062.24	444941	0.22855	no
gi|320446665|ref|NW_003383906.1|	360837-36142	221673	521965	-208641	0.33325	no
gi|320446665|ref|NW_003383906.1|	37947-38653	144471	0	#NAME?	5.00E-05	yes
gi|320446665|ref|NW_003383906.1|	39388-39886	576108	12722	-550094	0.13525	no
gi|320446665|ref|NW_003383906.1|	402878-40548	100943	471789	-109733	0.5025	no
gi|320446665|ref|NW_003383906.1|	41739-42189	267466	0	#NAME?	0.0086	no
gi|320446665|ref|NW_003383906.1|	419427-42176	61019	104816	0.78053	0.62755	no
gi|320446665|ref|NW_003383906.1|	422864-42387	145041	867642	-0.741288	0.7261	no
gi|320446665|ref|NW_003383906.1|	42808-43583	303415	0	#NAME?	0.004	no
gi|320446665|ref|NW_003383906.1|	432888-43602	296872	183177	-0.696605	0.7386	no
gi|320446665|ref|NW_003383906.1|	436986-43749	728119	447845	-0.701174	0.73395	no
gi|320446665|ref|NW_003383906.1|	438981-44074	297856	291997	-0.0286605	0.98235	no
gi|320446665|ref|NW_003383906.1|	442755-44530	582436	135784	122115	0.45945	no
gi|320446665|ref|NW_003383906.1|	44408-45851	167686	0	#NAME?	0.02015	no
gi|320446665|ref|NW_003383906.1|	457114-45743	648179	146345	117491	0.5759	no
gi|320446665|ref|NW_003383906.1|	46395-46683	431463	0	#NAME?	5.00E-05	yes
gi|320446665|ref|NW_003383906.1|	48121-48820	285633	124333	-784381	0.1123	no
gi|320446665|ref|NW_003383906.1|	49072-54997	809562	310481	193929	0.15435	no
gi|320446665|ref|NW_003383906.1|	503884-50510	120344	214556	0.834197	0.6803	no
gi|320446665|ref|NW_003383906.1|	522182-52509	239816	176021	287575	0.1267	no

gi|320446665|ref|NW_003383906.1|	51553-55391	714434	240311	507196	0.05335	no
gi|320446665|ref|NW_003383906.1|	58494-58763	230764	587246	134755	0.51615	no
gi|320446665|ref|NW_003383906.1|	59509-60389	0.261834	198357	292138	0.25645	no
gi|320446665|ref|NW_003383906.1|	60499-62732	0.337875	194539	25255	0.2807	no
gi|320446665|ref|NW_003383906.1|	63570-65093	235899	583321	130612	0.5448	no
gi|320446665|ref|NW_003383906.1|	65800-66650	13731	15116	0.138644	0.9242	no
gi|320446665|ref|NW_003383906.1|	68214-68494	182909	5836	-164807	0.44435	no
gi|320446665|ref|NW_003383906.1|	6982-11138	176875	115996	-0.608658	0.6464	no
gi|320446665|ref|NW_003383906.1|	70787-71527	234113	572723	129063	0.5372	no
gi|320446665|ref|NW_003383906.1|	74785-75006	244393	644386	139872	0.51885	no
gi|320446665|ref|NW_003383906.1|	77362-79457	208697	873495	206539	0.36775	no
gi|320446665|ref|NW_003383906.1|	87553-87964	137198	663754	-104754	0.61105	no
gi|320446666|ref|NW_003383905.1|	02016-1044	132545	382062	-17946	0.29035	no
gi|320446666|ref|NW_003383905.1|	04589-10611	221588	0.453279	-228941	0.3224	no
gi|320446666|ref|NW_003383905.1|	07628-10964	179951	20475	0.186263	0.92665	no
gi|320446666|ref|NW_003383905.1|	09749-11087	510478	392755	-0.378221	0.85655	no
gi|320446666|ref|NW_003383905.1|	11788-11439	159368	211738	0.409913	0.7528	no
gi|320446666|ref|NW_003383905.1|	52860-15427	0.285628	119081	205974	1	no
gi|320446666|ref|NW_003383905.1|	55579-15728	0.45804	199342	21217	0.34305	no
gi|320446666|ref|NW_003383905.1|	57465-15807	153507	0	#NAME?	0.00615	no
gi|320446666|ref|NW_003383905.1|	68088-16908	376975	2311	-402788	0.0672	no
gi|320446666|ref|NW_003383905.1|	75298-17622	154411	152108	-334361	0.1734	no
gi|320446666|ref|NW_003383905.1|	90316-19108	659318	0.430778	-393596	0.22035	no
gi|320446666|ref|NW_003383905.1|	94098-19505	284158	803774	-182183	0.42195	no
gi|320446666|ref|NW_003383905.1|	98409-20056	202279	227043	0.166621	0.9312	no
gi|320446666|ref|NW_003383905.1|	00841-20211	195355	124244	-0.652917	0.7491	no
gi|320446666|ref|NW_003383905.1|	05359-20697	330486	289619	-0.190437	0.92575	no
gi|320446666|ref|NW_003383905.1|	08744-20918	791139	787653	-0.00637111	0.9618	no
gi|320446666|ref|NW_003383905.1|	15814-21642	0.445622	272272	261116	0.2636	no
gi|320446666|ref|NW_003383905.1|	17456-21839	0.483176	133306	146412	1	no

gi|320446666|ref|NW_003383905.1|	222443-22303	140244	278802	0.991296	0.64595	no
gi|320446666|ref|NW_003383905.1|	223148-22471	277957	676821	128392	0.54375	no
gi|320446666|ref|NW_003383905.1|	225763-22669	340662	587342	0.785859	0.69865	no
gi|320446666|ref|NW_003383905.1|	231120-23404	439628	368786	-0.253499	0.90645	no
gi|320446666|ref|NW_003383905.1|	250345-25219	364876	58119	0.671603	0.74655	no
gi|320446666|ref|NW_003383905.1|	252463-25305	803463	832465	0.0511581	0.97245	no
gi|320446666|ref|NW_003383905.1|	253407-25615	678793	497835	-0.447304	0.8437	no
gi|320446666|ref|NW_003383905.1|	256650-25823	62424	495147	-0.334243	0.87345	no
gi|320446666|ref|NW_003383905.1|	258349-26012	173926	121918	-0.512554	0.7543	no
gi|320446666|ref|NW_003383905.1|	27442-28610	235241	288369	0.293773	0.8833	no
gi|320446666|ref|NW_003383905.1|	310504-31409	167892	356297	108555	0.41755	no
gi|320446666|ref|NW_003383905.1|	315985-31757	323886	10748	173051	0.42835	no
gi|320446666|ref|NW_003383905.1|	338108-33972	720606	458993	-0.650739	0.7561	no
gi|320446666|ref|NW_003383905.1|	339871-34107	471876	145377	-169861	0.4392	no
gi|320446666|ref|NW_003383905.1|	341309-34177	246125	192899	-367348	0.19395	no
gi|320446666|ref|NW_003383905.1|	344935-34616	931322	211367	-213953	0.32745	no
gi|320446666|ref|NW_003383905.1|	354836-35621	218199	137226	-0.669088	0.67915	no
gi|320446666|ref|NW_003383905.1|	359583-36005	438188	116029	-191706	0.3734	no
gi|320446666|ref|NW_003383905.1|	360154-36044	246761	460686	0.900671	0.6609	no
gi|320446666|ref|NW_003383905.1|	364330-36691	139495	219005	0.650747	0.615	no
gi|320446666|ref|NW_003383905.1|	368269-36938	133846	115171	-0.216795	0.92045	no
gi|320446666|ref|NW_003383905.1|	394997-39592	0.24263	217539	316445	0.24575	no
gi|320446666|ref|NW_003383905.1|	396917-39801	0.195643	460381	455653	0.1776	no
gi|320446666|ref|NW_003383905.1|	415617-41632	0	613935	inf	5.00E-05	yes
gi|320446666|ref|NW_003383905.1|	417424-41816	0	839143	inf	5.00E-05	yes
gi|320446666|ref|NW_003383905.1|	49722-50093	102345	148702	0.538978	0.79315	no
gi|320446666|ref|NW_003383905.1|	73466-73807	427351	626185	-277076	0.24475	no
gi|320446668|ref|NW_003383903.1|	104221-10551	17745	145332	-0.288062	0.89655	no
gi|320446668|ref|NW_003383903.1|	106516-10742	103188	485693	-108716	0.60275	no
gi|320446668|ref|NW_003383903.1|	112231-11295	165432	589733	-148811	0.48265	no

gi|320446668|ref|NW_003383903.1|	13159-13698	104704	593874	-0.818089	0.67995	no
gi|320446668|ref|NW_003383903.1|	139231-14164:	139538	633334	21823	0.33605	no
gi|320446668|ref|NW_003383903.1|	14064-15756	324371	225847	-0.522301	0.7917	no
gi|320446668|ref|NW_003383903.1|	142542-14302:	135902	635397	22251	0.27615	no
gi|320446668|ref|NW_003383903.1|	15963-17514	166744	107067	-0.639125	0.7528	no
gi|320446668|ref|NW_003383903.1|	18538-19095	729714	949908	0.380456	0.84945	no
gi|320446668|ref|NW_003383903.1|	1933-2516	918176	523377	-0.810921	0.68045	no
gi|320446668|ref|NW_003383903.1|	20229-23632	186433	268292	0.525148	0.80495	no
gi|320446668|ref|NW_003383903.1|	24296-32228	602835	130658	111596	0.4004	no
gi|320446668|ref|NW_003383903.1|	246278-24706:	89062	822105	-0.115487	0.94975	no
gi|320446668|ref|NW_003383903.1|	32781-32984	137038	51299	190436	0.319	no
gi|320446668|ref|NW_003383903.1|	33872-34359	125247	167517	0.41953	0.8288	no
gi|320446668|ref|NW_003383903.1|	35680-38357	912208	112024	0.296367	0.81515	no
gi|320446668|ref|NW_003383903.1|	387591-38842!	0.560182	327482	254745	0.25025	no
gi|320446668|ref|NW_003383903.1|	39838-41016	286455	297756	0.0558211	0.9711	no
gi|320446668|ref|NW_003383903.1|	41554-42176	783764	710022	-0.142556	0.9416	no
gi|320446668|ref|NW_003383903.1|	469009-47072!	121946	0.079363	-726356	0.26075	no
gi|320446668|ref|NW_003383903.1|	471621-47252:	73646	0	#NAME?	0.0099	no
gi|320446668|ref|NW_003383903.1|	474316-47535:	632397	0	#NAME?	0.00935	no
gi|320446668|ref|NW_003383903.1|	476842-47818!	530205	0	#NAME?	5.00E-05	yes
gi|320446668|ref|NW_003383903.1|	478455-47935	508911	0	#NAME?	0.0001	yes
gi|320446668|ref|NW_003383903.1|	481467-48267(	444739	0.120901	-852299	0.2504	no
gi|320446668|ref|NW_003383903.1|	487232-48774:	231808	0	#NAME?	0.00665	no
gi|320446668|ref|NW_003383903.1|	507226-50760	128748	0.703149	-419457	0.3058	no
gi|320446668|ref|NW_003383903.1|	59205-60300	131539	978277	-0.427172	0.8465	no
gi|320446668|ref|NW_003383903.1|	60390-61268	170722	596888	-151612	0.4766	no
gi|320446668|ref|NW_003383903.1|	62057-63374	102974	379064	-144176	0.50165	no
gi|320446668|ref|NW_003383903.1|	63576-64018	213608	115522	-0.886789	0.6659	no
gi|320446668|ref|NW_003383903.1|	64156-70530	303162	222317	-0.44747	0.7368	no
gi|320446668|ref|NW_003383903.1|	72285-74608	387968	259555	-0.579896	0.7795	no

gi|320446668|ref|NW_003383903.1|	82679-83360	677413	27124	-132047	0.43095	no
gi|320446668|ref|NW_003383903.1|	87752-88221	0	518329	inf	0.0294	no
gi|320446668|ref|NW_003383903.1|	89872-90502	409666	108513	140535	0.4106	no
gi|320446668|ref|NW_003383903.1|	91185-92054	181139	300767	0.731545	0.75065	no
gi|320446668|ref|NW_003383903.1|	92528-92990	101759	111351	0.12995	0.94705	no
gi|320446668|ref|NW_003383903.1|	96848-97332	124002	142157	0.19712	0.88035	no
gi|320446669|ref|NW_003383902.1|	171435-177629	291367	132966	-113178	0.405	no
gi|320446669|ref|NW_003383902.1|	186826-188465	216399	38492	-249107	0.1628	no
gi|320446669|ref|NW_003383902.1|	198191-198679	341504	219629	-395876	0.16775	no
gi|320446670|ref|NW_003383901.1|	103994-106004	641821	149191	-210501	0.11805	no
gi|320446670|ref|NW_003383901.1|	107604-113644	363335	167042	-112109	0.4872	no
gi|320446670|ref|NW_003383901.1|	119355-119838	247606	402528	-262089	0.24565	no
gi|320446670|ref|NW_003383901.1|	121540-122482	296176	543769	-244539	0.15765	no
gi|320446670|ref|NW_003383901.1|	123727-123959	861777	129232	-273735	0.26525	no
gi|320446670|ref|NW_003383901.1|	12389-12830	230444	21093	-0.127651	0.94865	no
gi|320446670|ref|NW_003383901.1|	13078-17077	247017	109835	-116927	0.3801	no
gi|320446670|ref|NW_003383901.1|	132123-132752	342242	0.872527	-197175	0.30275	no
gi|320446670|ref|NW_003383901.1|	133618-134504	492895	196562	-132629	0.52555	no
gi|320446670|ref|NW_003383901.1|	137245-138351	116307	161015	0.46926	0.8172	no
gi|320446670|ref|NW_003383901.1|	138966-140470	944427	471728	-100148	0.6399	no
gi|320446670|ref|NW_003383901.1|	179668-180320	0.4045	24797	261595	0.2636	no
gi|320446670|ref|NW_003383901.1|	183901-185019	0	185333	inf	0.0233	no
gi|320446670|ref|NW_003383901.1|	18719-19380	752486	296945	-134147	0.52445	no
gi|320446670|ref|NW_003383901.1|	19484-20145	277232	43192	0.639671	0.74565	no
gi|320446670|ref|NW_003383901.1|	202280-203400	0.381553	250963	271752	0.24125	no
gi|320446670|ref|NW_003383901.1|	204872-208897	0.934985	49867	241507	0.2984	no
gi|320446670|ref|NW_003383901.1|	21501-22602	409427	148438	-146375	0.4973	no
gi|320446670|ref|NW_003383901.1|	229054-230492	0	165783	inf	0.02075	no
gi|320446670|ref|NW_003383901.1|	23012-25942	108328	744148	-0.54175	0.7476	no
gi|320446670|ref|NW_003383901.1|	27604-27902	212276	123765	-410027	0.30775	no

gi|320446670|ref|NW_003383901.1|	29037-30068	827284	117305	-281811	0.22355	no
gi|320446670|ref|NW_003383901.1|	30333-31378	16051	446818	-184491	0.41555	no
gi|320446670|ref|NW_003383901.1|	323756-32444[illegible]	0.375105	461007	361942	0.20665	no
gi|320446670|ref|NW_003383901.1|	36112-37020	276023	639935	121314	0.5545	no
gi|320446670|ref|NW_003383901.1|	41876-43389	277369	725618	13874	0.52415	no
gi|320446670|ref|NW_003383901.1|	43752-44517	924224	458979	-100981	0.62005	no
gi|320446670|ref|NW_003383901.1|	44854-45688	107146	911317	-0.233554	0.9082	no
gi|320446670|ref|NW_003383901.1|	47679-51693	115651	194733	0.751726	0.56455	no
gi|320446670|ref|NW_003383901.1|	521612-52194[illegible]	0	156559	inf	0.0198	no
gi|320446670|ref|NW_003383901.1|	5270-7730	10012	953454	-0.0704947	0.95355	no
gi|320446670|ref|NW_003383901.1|	537839-53955[illegible]	242589	217309	-0.158766	0.89915	no
gi|320446670|ref|NW_003383901.1|	540624-54143[illegible]	401035	179012	-116367	0.4686	no
gi|320446670|ref|NW_003383901.1|	550477-55094[illegible]	545719	124413	-213302	0.34245	no
gi|320446670|ref|NW_003383901.1|	554160-55580[illegible]	427349	167845	-134829	0.29925	no
gi|320446670|ref|NW_003383901.1|	576391-57761[illegible]	856977	475358	-0.850244	0.6866	no
gi|320446670|ref|NW_003383901.1|	577756-57931[illegible]	409514	0.0890214	-552362	0.2748	no
gi|320446670|ref|NW_003383901.1|	580840-58195[illegible]	134303	0.531304	-133788	1	no
gi|320446670|ref|NW_003383901.1|	583053-58533[illegible]	10739	0.807247	-0.411774	1	no
gi|320446670|ref|NW_003383901.1|	586304-59627[illegible]	254777	559247	113425	0.3798	no
gi|320446670|ref|NW_003383901.1|	60323-60883	145151	917297	-0.662091	0.6114	no
gi|320446670|ref|NW_003383901.1|	562571-66319[illegible]	0	208111	inf	0.00565	no
gi|320446670|ref|NW_003383901.1|	564819-66514[illegible]	0	615455	inf	0.00575	no
gi|320446670|ref|NW_003383901.1|	69827-70455	120085	262392	-219426	0.31685	no
gi|320446670|ref|NW_003383901.1|	732465-73362[illegible]	621916	658935	0.0834163	0.9674	no
gi|320446670|ref|NW_003383901.1|	734653-73490[illegible]	131437	145506	0.146708	0.91235	no
gi|320446670|ref|NW_003383901.1|	735590-73590[illegible]	320329	304102	-0.0749999	0.9668	no
gi|320446670|ref|NW_003383901.1|	75033-81421	290954	26506	-0.134475	0.9176	no
gi|320446670|ref|NW_003383901.1|	8269-11645	143982	172501	0.260718	0.8412	no
gi|320446671|ref|NW_003383900.1|	119370-12025[illegible]	522857	0.900253	-253801	0.2934	no
gi|320446671|ref|NW_003383900.1|	133552-13467[illegible]	11395	512847	-11518	0.58445	no

gi|320446671|ref|NW_003383900.1|	135039-13540	249147	54207	-220044	0.33305	no
gi|320446671|ref|NW_003383900.1|	135799-13603	964073	233996	-204266	0.3476	no
gi|320446671|ref|NW_003383900.1|	136449-13809	23942	958239	-132109	0.43865	no
gi|320446671|ref|NW_003383900.1|	138256-13965	232038	191431	-0.277536	0.8853	no
gi|320446671|ref|NW_003383900.1|	139770-14011	496279	143128	-179384	0.40525	no
gi|320446671|ref|NW_003383900.1|	140485-14213	153663	33996	-217633	0.2012	no
gi|320446671|ref|NW_003383900.1|	150698-15123	42015	141477	-157034	0.47135	no
gi|320446671|ref|NW_003383900.1|	155073-15678	330956	111257	-157274	0.3682	no
gi|320446671|ref|NW_003383900.1|	156903-16139	228423	333247	0.544885	0.69015	no
gi|320446671|ref|NW_003383900.1|	161994-16430	38056	187722	-434145	0.0418	no
gi|320446671|ref|NW_003383900.1|	166006-16623	112483	262315	-542226	0.276	no
gi|320446671|ref|NW_003383900.1|	166402-16693	237478	136964	-0.793998	0.70275	no
gi|320446671|ref|NW_003383900.1|	167280-16774	319252	170285	-0.906742	0.6653	no
gi|320446671|ref|NW_003383900.1|	167901-16810	657442	910432	-285224	0.2745	no
gi|320446671|ref|NW_003383900.1|	168991-17055	508421	424433	-0.260488	0.901	no
gi|320446671|ref|NW_003383900.1|	170666-17181	129445	179323	0.470223	0.81195	no
gi|320446671|ref|NW_003383900.1|	171914-17435	13.44	157849	0.232012	0.85445	no
gi|320446671|ref|NW_003383900.1|	177166-17957	101103	168345	0.735594	0.7194	no
gi|320446671|ref|NW_003383900.1|	17919-20663	0.807129	371322	22018	0.3239	no
gi|320446671|ref|NW_003383900.1|	254708-25766	484105	563186	354022	0.0878	no
gi|320446671|ref|NW_003383900.1|	258456-25932	476478	289803	260459	0.2678	no
gi|320446671|ref|NW_003383900.1|	259444-26008	671049	399459	257356	0.26305	no
gi|320446671|ref|NW_003383900.1|	260352-26128	218682	160872	287901	0.21995	no
gi|320446671|ref|NW_003383900.1|	26041-28013	188501	29267	0.634703	0.6272	no
gi|320446671|ref|NW_003383900.1|	261533-26235	517921	272937	239777	0.3018	no
gi|320446671|ref|NW_003383900.1|	262662-26354	729738	245922	175275	0.4305	no
gi|320446671|ref|NW_003383900.1|	263663-26445	273908	835756	160939	0.44415	no
gi|320446671|ref|NW_003383900.1|	264631-26727	237985	91939	194981	0.24335	no
gi|320446671|ref|NW_003383900.1|	268008-26872	105322	840588	29966	0.2282	no
gi|320446671|ref|NW_003383900.1|	268827-26920	539514	707001	0.390053	0.85385	no

gi|320446671|ref|NW_003383900.1|	269585-27104	151826	201475	0.408181	0.84205	no
gi|320446671|ref|NW_003383900.1|	271827-27444	120391	0.643181	-0.904429	1	no
gi|320446671|ref|NW_003383900.1|	275002-27609	158351	0.958798	-0.723827	0.7278	no
gi|320446671|ref|NW_003383900.1|	277433-27838	260159	0.81595	-167284	0.4339	no
gi|320446671|ref|NW_003383900.1|	28754-29838	715939	688037	-0.0573511	0.97705	no
gi|320446671|ref|NW_003383900.1|	294131-29879	121805	303907	131905	0.5438	no
gi|320446671|ref|NW_003383900.1|	301155-30352	0.632374	104861	0.729626	1	no
gi|320446671|ref|NW_003383900.1|	307121-30821	0.785305	149459	0.928424	0.68415	no
gi|320446671|ref|NW_003383900.1|	308611-30969	0.598733	110471	0.883679	1	no
gi|320446671|ref|NW_003383900.1|	33145-35451	221654	154729	-0.518568	0.6873	no
gi|320446671|ref|NW_003383900.1|	35823-36831	611635	482967	-0.340746	0.86855	no
gi|320446671|ref|NW_003383900.1|	447130-44812	129719	135932	0.0674983	0.975	no
gi|320446671|ref|NW_003383900.1|	452092-45294	192234	15116	-0.346783	0.8574	no
gi|320446671|ref|NW_003383900.1|	468688-46911	503356	833189	0.727065	0.72515	no
gi|320446671|ref|NW_003383900.1|	471005-47374	235789	338958	0.523612	0.80515	no
gi|320446671|ref|NW_003383900.1|	475585-47840	280282	519352	0.889833	0.6777	no
gi|320446671|ref|NW_003383900.1|	478659-47966	37087	132647	183861	0.39855	no
gi|320446671|ref|NW_003383900.1|	482844-48320	711847	147133	104748	0.6031	no
gi|320446671|ref|NW_003383900.1|	483334-48413	533385	168952	166336	0.453	no
gi|320446671|ref|NW_003383900.1|	485919-48743	723048	102396	0.502001	0.81515	no
gi|320446671|ref|NW_003383900.1|	490015-49056	750047	541862	-0.469056	0.81625	no
gi|320446671|ref|NW_003383900.1|	491050-49163	159026	110918	-0.519776	0.80125	no
gi|320446671|ref|NW_003383900.1|	527916-52875	251248	211213	-0.250409	0.903	no
gi|320446671|ref|NW_003383900.1|	53154-53702	428599	144497	-156859	0.49785	no
gi|320446671|ref|NW_003383900.1|	550987-55448	108502	118568	344992	0.08575	no
gi|320446671|ref|NW_003383900.1|	562024-56248	0.741984	153075	436671	0.18695	no
gi|320446671|ref|NW_003383900.1|	567833-56946	434968	163142	190715	0.26205	no
gi|320446671|ref|NW_003383900.1|	569793-57042	380265	126443	173342	0.421	no
gi|320446671|ref|NW_003383900.1|	581430-58241	51577	789848	0.614847	0.76385	no
gi|320446671|ref|NW_003383900.1|	584407-58485	0.784236	885207	349666	0.2178	no

gi|320446671|ref|NW_003383900.1|	599973-600438	0.718052	76562	341447	0.21715	no
gi|320446671|ref|NW_003383900.1|	61985-62970	315204	450897	0.516513	0.79505	no
gi|320446671|ref|NW_003383900.1|	84110-85618	11934	129091	0.113312	1	no
gi|320446672|ref|NW_003383899.1|	128463-128703	0	600138	inf	0.0142	no
gi|320446672|ref|NW_003383899.1|	133261-133568	0	172216	inf	0.02205	no
gi|320446672|ref|NW_003383899.1|	136548-136822	0	402683	inf	0.0142	no
gi|320446672|ref|NW_003383899.1|	169601-169815	11053	588484	241256	0.2614	no
gi|320446672|ref|NW_003383899.1|	18214-19871	0	669287	inf	0.00465	no
gi|320446672|ref|NW_003383899.1|	303505-304019	598453	12455	105742	0.60675	no
gi|320446672|ref|NW_003383899.1|	36495-37262	0	283088	inf	0.02915	no
gi|320446672|ref|NW_003383899.1|	64463-66121	0.118616	175049	720532	0.1409	no
gi|320446672|ref|NW_003383899.1|	71305-72654	242458	294634	0.281186	0.88135	no
gi|320446673|ref|NW_003383898.1|	175340-176081	0.667575	205784	162413	0.33265	no
gi|320446673|ref|NW_003383898.1|	190282-190655	324768	536622	-259743	0.1544	no
gi|320446673|ref|NW_003383898.1|	207340-207569	343645	324378	-0.0832426	0.9498	no
gi|320446673|ref|NW_003383898.1|	207830-208273	307193	888925	-178901	0.40645	no
gi|320446673|ref|NW_003383898.1|	210688-210978	643431	199229	-169136	0.4331	no
gi|320446673|ref|NW_003383898.1|	213194-213423	644333	297346	-111566	0.595	no
gi|320446673|ref|NW_003383898.1|	213832-214399	131548	410274	-168093	0.43445	no
gi|320446673|ref|NW_003383898.1|	215322-215604	784474	214661	-186966	0.38535	no
gi|320446673|ref|NW_003383898.1|	216638-221429	237774	140858	-0.755344	0.57545	no
gi|320446673|ref|NW_003383898.1|	221776-222402	70259	275387	-135122	0.41705	no
gi|320446673|ref|NW_003383898.1|	229502-230237	844454	532023	-0.66653	0.74615	no
gi|320446673|ref|NW_003383898.1|	237020-237657	335524	31386	322563	0.1877	no
gi|320446673|ref|NW_003383898.1|	242830-243103	737016	250349	176417	0.414	no
gi|320446673|ref|NW_003383898.1|	252604-252857	617034	195428	166322	0.34255	no
gi|320446673|ref|NW_003383898.1|	254086-254422	142773	194302	37665	0.2027	no
gi|320446673|ref|NW_003383898.1|	275115-275854	737239	172148	122345	0.55865	no
gi|320446673|ref|NW_003383898.1|	279360-280075	1759	481304	145219	0.5064	no
gi|320446673|ref|NW_003383898.1|	319272-320362	335708	0	#NAME?	0.01065	no

gi|320446673|ref|NW_003383898.1|	323038-324679	228096	0.0835602	-477068	0.2879	no
gi|320446673|ref|NW_003383898.1|	344993-345560	11131	160691	0.529701	0.79385	no
gi|320446673|ref|NW_003383898.1|	352907-354274	134213	217499	0.696485	0.7311	no
gi|320446673|ref|NW_003383898.1|	354503-361480	268448	234403	-0.19565	0.9103	no
gi|320446673|ref|NW_003383898.1|	375107-376759	190589	116084	-0.715301	0.7219	no
gi|320446673|ref|NW_003383898.1|	396780-396947	535.68	507.83	-0.0770252	0.9669	no
gi|320446673|ref|NW_003383898.1|	398325-399037	424802	363175	-0.226129	0.90455	no
gi|320446673|ref|NW_003383898.1|	400726-402233	139339	14857	0.0925425	0.9686	no
gi|320446673|ref|NW_003383898.1|	402510-402927	523948	346085	-0.598298	0.7768	no
gi|320446673|ref|NW_003383898.1|	404270-407249	178347	318164	0.835081	0.69325	no
gi|320446673|ref|NW_003383898.1|	407530-409264	417744	624205	0.579401	0.66635	no
gi|320446673|ref|NW_003383898.1|	43300-44358	303636	215675	-0.493486	0.75725	no
gi|320446673|ref|NW_003383898.1|	50619-51058	505077	563733	0.158509	0.93985	no
gi|320446673|ref|NW_003383898.1|	70036-70720	150374	384996	135629	0.52585	no
gi|320446673|ref|NW_003383898.1|	81482-81686	201327	272257	0.435424	0.8294	no
gi|320446673|ref|NW_003383898.1|	88813-89311	392513	805729	-228437	0.3185	no
gi|320446673|ref|NW_003383898.1|	92612-93870	364705	120201	-160128	0.3511	no
gi|320446676|ref|NW_003383895.1|	209588-212352	0	181868	inf	0.0088	no
gi|320446676|ref|NW_003383895.1|	78020-78738	0.349625	215285	262236	0.26355	no
gi|320446676|ref|NW_003383895.1|	79577-81284	0.917326	127751	0.477827	1	no
gi|320446676|ref|NW_003383895.1|	81351-82424	221633	334531	0.593967	0.75935	no
gi|320446676|ref|NW_003383895.1|	86722-87714	0.892248	354354	198967	0.3909	no
gi|320446677|ref|NW_003383894.1|	10482-14256	316605	134547	208736	0.1222	no
gi|320446677|ref|NW_003383894.1|	107062-107492	242168	110601	-445257	0.2029	no
gi|320446677|ref|NW_003383894.1|	108073-109789	467271	36507	-0.356087	0.86285	no
gi|320446677|ref|NW_003383894.1|	112946-115447	170549	270485	-265657	0.1469	no
gi|320446677|ref|NW_003383894.1|	125007-125882	321936	283463	-0.183611	0.93635	no
gi|320446677|ref|NW_003383894.1|	127016-127377	870264	480893	-0.855738	0.693	no
gi|320446677|ref|NW_003383894.1|	14653-18025	199034	793984	199609	0.2446	no
gi|320446677|ref|NW_003383894.1|	154834-155812	0.681806	188308	146566	0.50355	no

gi|320446677|ref|NW_003383894.1|	156159-16031(	0.473268	283139	258078	0.2598	no
gi|320446677|ref|NW_003383894.1|	167847-16855	280833	267412	-339257	0.17665	no
gi|320446677|ref|NW_003383894.1|	169002-16985	232669	3767	-26268	0.25585	no
gi|320446677|ref|NW_003383894.1|	171001-17639(	220658	14795	-0.576703	0.66995	no
gi|320446677|ref|NW_003383894.1|	177570-17826(	460057	129237	-183179	0.261	no
gi|320446677|ref|NW_003383894.1|	178722-18010	114397	0.509396	-448911	0.1557	no
gi|320446677|ref|NW_003383894.1|	18261-19462	873844	169607	0.956746	0.6666	no
gi|320446677|ref|NW_003383894.1|	193090-19396	458668	166118	-146524	0.3797	no
gi|320446677|ref|NW_003383894.1|	200736-20331	561814	966062	0.782023	0.6256	no
gi|320446677|ref|NW_003383894.1|	204118-20551	536127	543605	0.0199842	0.9873	no
gi|320446677|ref|NW_003383894.1|	205728-20747	19798	170606	-0.214686	0.8646	no
gi|320446677|ref|NW_003383894.1|	207648-20900	15059	167322	0.152006	0.92965	no
gi|320446677|ref|NW_003383894.1|	216934-21744	178923	112128	-0.674189	0.73915	no
gi|320446677|ref|NW_003383894.1|	226846-22769	253475	11942	-108581	0.6222	no
gi|320446677|ref|NW_003383894.1|	233716-23427	164804	521673	-165953	0.43405	no
gi|320446677|ref|NW_003383894.1|	235079-23542(	26467	189216	-0.484164	0.816	no
gi|320446677|ref|NW_003383894.1|	236360-23670	14855	10524	-0.497259	0.80485	no
gi|320446677|ref|NW_003383894.1|	242394-24375(	0.59915	114428	0.933452	1	no
gi|320446677|ref|NW_003383894.1|	262529-26288	155686	983451	-0.662713	0.6752	no
gi|320446677|ref|NW_003383894.1|	263192-26438	951413	564376	-0.753415	0.5746	no
gi|320446677|ref|NW_003383894.1|	26321-28289	0.0973899	0.950642	328706	1	no
gi|320446677|ref|NW_003383894.1|	264686-26600	473865	266184	-0.832051	0.52635	no
gi|320446677|ref|NW_003383894.1|	266122-26634	100667	34513	-154438	0.4737	no
gi|320446677|ref|NW_003383894.1|	266910-26855(	538313	184019	-154859	0.2416	no
gi|320446677|ref|NW_003383894.1|	268689-27118	649659	271199	-126033	0.56845	no
gi|320446677|ref|NW_003383894.1|	271360-27180	309636	411402	0.409975	0.8506	no
gi|320446677|ref|NW_003383894.1|	273373-27383	806119	243977	-172425	0.42725	no
gi|320446677|ref|NW_003383894.1|	275994-27693	359295	181762	-0.983119	0.65565	no
gi|320446677|ref|NW_003383894.1|	278156-27861	523742	547533	0.0640905	0.94865	no
gi|320446677|ref|NW_003383894.1|	278982-28098	107688	963612	-0.160339	0.9446	no

gi|320446677|ref|NW_003383894.1|	281859-28842	924809	18551	100427	0.4559	no
gi|320446677|ref|NW_003383894.1|	288925-29059	155697	120706	-0.367251	0.81975	no
gi|320446677|ref|NW_003383894.1|	291621-29549	690189	113269	0.714696	0.56765	no
gi|320446677|ref|NW_003383894.1|	296311-29842	138.38	139317	0.00973212	0.9934	no
gi|320446677|ref|NW_003383894.1|	299789-30142	93827	520069	-0.8513	0.5311	no
gi|320446677|ref|NW_003383894.1|	307512-30784	111822	414809	-143068	0.5099	no
gi|320446677|ref|NW_003383894.1|	308576-31085	127.34	540228	-123705	0.3633	no
gi|320446677|ref|NW_003383894.1|	33470-33841	111442	182903	0.71478	0.66555	no
gi|320446677|ref|NW_003383894.1|	66925-71638	189162	281596	0.574007	0.6623	no
gi|320446677|ref|NW_003383894.1|	72037-75098	621028	939441	0.597145	0.7141	no
gi|320446677|ref|NW_003383894.1|	7467-7883	0	135545	inf	0.01485	no
gi|320446677|ref|NW_003383894.1|	78365-79034	194408	397768	103284	0.6013	no
gi|320446677|ref|NW_003383894.1|	80599-81821	171048	201623	0.237256	0.90115	no
gi|320446677|ref|NW_003383894.1|	83941-84637	183109	225204	0.298535	0.87685	no
gi|320446677|ref|NW_003383894.1|	85576-86759	373988	222154	-0.751432	0.7146	no
gi|320446677|ref|NW_003383894.1|	86977-88977	223759	186038	-0.266348	0.8372	no
gi|320446677|ref|NW_003383894.1|	9105-9665	0	146489	inf	0.0069	no
gi|320446677|ref|NW_003383894.1|	99573-99848	113077	306631	-520466	0.18485	no
gi|320446683|ref|NW_003383888.1|	130522-13070	697236	370176	-0.913436	0.67715	no
gi|320446683|ref|NW_003383888.1|	135409-13691	172244	0.737089	-122455	0.5498	no
gi|320446683|ref|NW_003383888.1|	137675-13957	101369	0.494593	-10353	1	no
gi|320446683|ref|NW_003383888.1|	139686-14114	0.969338	0.866269	-0.162184	1	no
gi|320446683|ref|NW_003383888.1|	142082-14238	108046	57874	-0.90066	0.6817	no
gi|320446683|ref|NW_003383888.1|	145866-14909	0.564382	122363	111643	1	no
gi|320446683|ref|NW_003383888.1|	149235-15019	0.930381	192645	105005	0.60605	no
gi|320446683|ref|NW_003383888.1|	150311-15308	126186	148524	0.235146	0.90465	no
gi|320446683|ref|NW_003383888.1|	153225-15497	637139	3659	-0.80016	0.69905	no
gi|320446683|ref|NW_003383888.1|	156207-15650	204969	107674	-0.928736	0.65095	no
gi|320446683|ref|NW_003383888.1|	159271-16264	0.914189	0.978058	0.0974276	1	no
gi|320446683|ref|NW_003383888.1|	163540-16660	208704	293527	0.492033	0.71125	no

gi|320446683|ref|NW_003383888.1|	282690-28343	466383	821559	0.816849	0.68855	no
gi|320446683|ref|NW_003383888.1|	283660-28480	25889	550779	108913	0.59945	no
gi|320446683|ref|NW_003383888.1|	287943-28834	571493	313656	-0.865551	0.68795	no
gi|320446683|ref|NW_003383888.1|	45482-47635	159267	570971	-147996	0.3725	no
gi|320446683|ref|NW_003383888.1|	48845-51654	0.524495	123718	123805	1	no
gi|320446683|ref|NW_003383888.1|	55474-55838	249147	387193	-268587	0.2709	no
gi|320446683|ref|NW_003383888.1|	58003-58633	281645	406198	-279362	0.23275	no
gi|320446683|ref|NW_003383888.1|	90989-92200	347684	623593	-24791	0.1687	no
gi|320446684|ref|NW_003383887.1|	12625-13197	344062	141597	-128088	0.5572	no
gi|320446684|ref|NW_003383887.1|	15221-15944	103817	947322	-0.132122	0.9467	no
gi|320446684|ref|NW_003383887.1|	16739-18155	32875	109249	-158937	0.4549	no
gi|320446684|ref|NW_003383887.1|	173867-17424	106683	139867	-29312	0.2676	no
gi|320446684|ref|NW_003383887.1|	19806-20177	113717	371754	-161302	0.44055	no
gi|320446684|ref|NW_003383887.1|	20534-20991	667785	0.987582	-275741	0.2827	no
gi|320446684|ref|NW_003383887.1|	211011-21171	0	522199	inf	0.01575	no
gi|320446684|ref|NW_003383887.1|	211771-21361	0	226042	inf	0.01195	no
gi|320446684|ref|NW_003383887.1|	214014-21612	0.451021	578299	700248	0.0854	no
gi|320446684|ref|NW_003383887.1|	216251-21781	241318	316.32	70343	0.02625	no
gi|320446684|ref|NW_003383887.1|	21777-22032	481694	763229	-265793	0.27005	no
gi|320446684|ref|NW_003383887.1|	220387-22050	924981	6235.36	275298	0.2494	no
gi|320446684|ref|NW_003383887.1|	229122-23064	353299	116303	504086	0.03225	no
gi|320446684|ref|NW_003383887.1|	2327-2561	517872	125522	127728	0.53825	no
gi|320446684|ref|NW_003383887.1|	233150-23788	120876	117419	328007	0.10875	no
gi|320446684|ref|NW_003383887.1|	23789-24141	128026	24994	-235678	0.25605	no
gi|320446684|ref|NW_003383887.1|	240785-24822	136313	215452	0.660441	0.61725	no
gi|320446684|ref|NW_003383887.1|	248423-24944	555012	486898	-0.188901	0.92475	no
gi|320446684|ref|NW_003383887.1|	249850-25198	899046	689338	-0.383182	0.864	no
gi|320446684|ref|NW_003383887.1|	252201-25261	233156	710513	-171436	0.42835	no
gi|320446684|ref|NW_003383887.1|	253434-25520	427442	351193	-0.283463	0.8862	no
gi|320446684|ref|NW_003383887.1|	255497-25651	107824	178835	0.729944	0.73635	no

gi|320446684|ref|NW_003383887.1|	262744-263347	128104	248245	-236748	0.2907	no
gi|320446684|ref|NW_003383887.1|	267190-269234	111956	331854	156761	0.4538	no
gi|320446684|ref|NW_003383887.1|	28924-29199	336825	766578	-213549	0.3483	no
gi|320446684|ref|NW_003383887.1|	290326-290767	15098	738254	-103217	0.60685	no
gi|320446684|ref|NW_003383887.1|	306017-307304	320946	23385	-0.456746	0.82535	no
gi|320446684|ref|NW_003383887.1|	307517-308154	419406	285328	-0.555727	0.79545	no
gi|320446684|ref|NW_003383887.1|	309292-311597	193225	124639	-0.632528	0.61655	no
gi|320446684|ref|NW_003383887.1|	312525-315652	193726	241152	0.315927	0.8116	no
gi|320446684|ref|NW_003383887.1|	325779-333602	198.48	1509.99	292747	0.07415	no
gi|320446684|ref|NW_003383887.1|	333734-335655	0.300318	809476	475243	0.1506	no
gi|320446684|ref|NW_003383887.1|	338268-338663	0	235098	inf	0.0085	no
gi|320446684|ref|NW_003383887.1|	340359-341175	523275	496456	656795	0.0264	no
gi|320446684|ref|NW_003383887.1|	344424-344610	102266	949643	321506	0.23275	no
gi|320446684|ref|NW_003383887.1|	351960-353919	234956	50577	442802	0.04345	no
gi|320446684|ref|NW_003383887.1|	356730-358630	383818	300626	296948	0.11705	no
gi|320446684|ref|NW_003383887.1|	358859-361430	209506	212006	333905	0.01695	no
gi|320446684|ref|NW_003383887.1|	365608-366161	633101	142378	116922	0.57475	no
gi|320446684|ref|NW_003383887.1|	366311-367028	294292	445815	0.599199	0.70565	no
gi|320446684|ref|NW_003383887.1|	367179-367381	559818	261853	-10962	0.5927	no
gi|320446684|ref|NW_003383887.1|	367486-368188	433849	395399	-0.133883	0.9404	no
gi|320446684|ref|NW_003383887.1|	36761-37779	258779	223544	-0.211162	0.91375	no
gi|320446684|ref|NW_003383887.1|	369834-370020	409063	443167	0.115526	0.9152	no
gi|320446684|ref|NW_003383887.1|	370301-370527	855555	198179	-211005	0.3412	no
gi|320446684|ref|NW_003383887.1|	372274-372688	0.901168	535398	257074	0.27405	no
gi|320446684|ref|NW_003383887.1|	372801-373040	888477	129598	0.544643	0.793	no
gi|320446684|ref|NW_003383887.1|	375456-375720	243886	309897	0.345582	0.85835	no
gi|320446684|ref|NW_003383887.1|	375937-376360	207081	159774	-0.374167	0.84995	no
gi|320446684|ref|NW_003383887.1|	381453-381720	88442	860576	-0.0394281	0.983	no
gi|320446684|ref|NW_003383887.1|	38401-44412	347631	321427	-0.113066	0.9617	no
gi|320446684|ref|NW_003383887.1|	402650-404344	717265	515519	-0.47648	0.8229	no

gi|320446684|ref|NW_003383887.1|	45604-47853	64524	140951	112728	0.4976	no
gi|320446684|ref|NW_003383887.1|	57541-60285	154027	401404	138187	0.3009	no
gi|320446684|ref|NW_003383887.1|	61018-63644	0.211743	108484	235709	1	no
gi|320446684|ref|NW_003383887.1|	64775-65449	153771	367192	125575	0.55735	no
gi|320446684|ref|NW_003383887.1|	68763-69471	999544	209922	107051	0.6143	no
gi|320446684|ref|NW_003383887.1|	69798-70179	783216	597677	-0.390044	0.86115	no
gi|320446684|ref|NW_003383887.1|	74278-74804	948362	47152	-100812	0.54335	no
gi|320446684|ref|NW_003383887.1|	7779-8242	579118	289348	-100105	0.59615	no
gi|320446684|ref|NW_003383887.1|	82998-83683	0	358561	inf	0.0233	no
gi|320446684|ref|NW_003383887.1|	90876-92203	0.154591	118055	293293	1	no
gi|320446685|ref|NW_003383886.1|	[illegible]00624-10179[illegible]	150869	124456	-359959	0.15755	no
gi|320446685|ref|NW_003383886.1|	[illegible]03615-10435[illegible]	859477	63899	-374959	0.06805	no
gi|320446685|ref|NW_003383886.1|	[illegible]07977-10917[illegible]	227908	680515	-174375	0.43785	no
gi|320446685|ref|NW_003383886.1|	[illegible]09291-11102[illegible]	155284	383993	-201575	0.2345	no
gi|320446685|ref|NW_003383886.1|	[illegible]12520-11322[illegible]	284985	170519	-0.740956	0.70585	no
gi|320446685|ref|NW_003383886.1|	11282-12545	262755	123041	-109458	0.50245	no
gi|320446685|ref|NW_003383886.1|	[illegible]13888-11452[illegible]	751242	198787	-191806	0.3882	no
gi|320446685|ref|NW_003383886.1|	[illegible]15041-11669[illegible]	110313	173398	-266944	0.25105	no
gi|320446685|ref|NW_003383886.1|	[illegible]17271-11770[illegible]	805292	0.534101	-391433	0.3159	no
gi|320446685|ref|NW_003383886.1|	[illegible]18529-11939[illegible]	670187	0.55361	-359762	0.17555	no
gi|320446685|ref|NW_003383886.1|	[illegible]19669-12283[illegible]	215109	147214	-0.547152	0.6766	no
gi|320446685|ref|NW_003383886.1|	[illegible]22909-12350[illegible]	140244	221774	0.661148	0.7397	no
gi|320446685|ref|NW_003383886.1|	12649-13195	754715	254382	-156894	0.4799	no
gi|320446685|ref|NW_003383886.1|	[illegible]28797-12998[illegible]	477816	150863	165871	0.4619	no
gi|320446685|ref|NW_003383886.1|	[illegible]30164-13083[illegible]	116379	44981	195049	0.3912	no
gi|320446685|ref|NW_003383886.1|	13303-14661	220972	119007	-0.892819	0.58135	no
gi|320446685|ref|NW_003383886.1|	[illegible]43237-14475[illegible]	567947	119406	107204	0.61905	no
gi|320446685|ref|NW_003383886.1|	[illegible]50421-15244[illegible]	0.470911	407231	311232	0.2085	no
gi|320446685|ref|NW_003383886.1|	[illegible]59191-16031[illegible]	380263	364658	326147	0.09755	no
gi|320446685|ref|NW_003383886.1|	[illegible]68681-17211[illegible]	359263	54332	0.596763	0.7928	no

gi|320446685|ref|NW_003383886.1|	172184-173557	979037	443032	-114395	0.59695	no
gi|320446685|ref|NW_003383886.1|	173791-174488	328887	199764	-0.719295	0.72925	no
gi|320446685|ref|NW_003383886.1|	174589-175850	691077	365267	-0.919893	0.66255	no
gi|320446685|ref|NW_003383886.1|	175959-177640	123732	121089	-0.0311483	0.9876	no
gi|320446685|ref|NW_003383886.1|	180595-180904	122932	101678	-0.273854	0.8778	no
gi|320446685|ref|NW_003383886.1|	182759-183439	758573	56954	-0.413491	0.84655	no
gi|320446685|ref|NW_003383886.1|	183624-187871	103465	809807	-0.353498	0.78165	no
gi|320446685|ref|NW_003383886.1|	205915-208219	269178	335947	0.319673	0.8786	no
gi|320446685|ref|NW_003383886.1|	208374-208648	753635	263293	-15172	0.47825	no
gi|320446685|ref|NW_003383886.1|	209651-210171	457376	106377	-210419	0.35565	no
gi|320446685|ref|NW_003383886.1|	221537-222865	293455	128671	-118945	0.57135	no
gi|320446685|ref|NW_003383886.1|	238578-240274	127082	0.804405	-0.65977	1	no
gi|320446685|ref|NW_003383886.1|	243258-245401	565515	12.7	116719	0.4661	no
gi|320446685|ref|NW_003383886.1|	245598-246430	101847	169244	0.732702	0.72895	no
gi|320446685|ref|NW_003383886.1|	250829-251511	278191	202764	-0.456273	0.83375	no
gi|320446685|ref|NW_003383886.1|	256417-256762	281675	313665	0.155192	0.94	no
gi|320446685|ref|NW_003383886.1|	258649-258911	520732	365563	-0.51042	0.8011	no
gi|320446685|ref|NW_003383886.1|	264129-264597	354724	66222	0.900612	0.67005	no
gi|320446685|ref|NW_003383886.1|	273494-274715	109753	930767	-0.237768	0.857	no
gi|320446685|ref|NW_003383886.1|	277999-278960	209336	465558	115314	0.5744	no
gi|320446685|ref|NW_003383886.1|	279083-279778	183504	501503	145045	0.5067	no
gi|320446685|ref|NW_003383886.1|	279984-280842	12199	35635	154653	0.34895	no
gi|320446685|ref|NW_003383886.1|	37088-38227	279772	187811	-0.574972	0.65595	no
gi|320446685|ref|NW_003383886.1|	42392-47013	280846	646642	120319	0.4701	no
gi|320446685|ref|NW_003383886.1|	52790-53987	122847	316278	136433	0.5174	no
gi|320446685|ref|NW_003383886.1|	6195-6974	302426	729333	1.27	0.3274	no
gi|320446685|ref|NW_003383886.1|	70756-72846	855236	336371	-134627	0.53755	no
gi|320446685|ref|NW_003383886.1|	73026-73381	665461	228956	-153928	0.46755	no
gi|320446685|ref|NW_003383886.1|	73502-74355	453654	208737	-111991	0.50045	no
gi|320446685|ref|NW_003383886.1|	74455-76466	239478	104044	-12027	0.48375	no

gi|320446685|ref|NW_003383886.1|	76583-80405	0.611562	0.823119	0.428601	1	no
gi|320446685|ref|NW_003383886.1|	81894-83630	347468	299943	-0.212194	0.87385	no
gi|320446685|ref|NW_003383886.1|	88260-89285	128242	192047	0.582593	0.7901	no
gi|320446685|ref|NW_003383886.1|	90890-95353	91394	228051	131918	0.3213	no
gi|320446685|ref|NW_003383886.1|	95769-96988	39461	499626	0.340421	0.8655	no
gi|320446685|ref|NW_003383886.1|	97146-98971	191074	384611	100927	0.62715	no
gi|320446686|ref|NW_003383885.1|	207464-21568	117953	926469	297353	0.1222	no
gi|320446686|ref|NW_003383885.1|	216034-21648	208106	144934	2.8	0.12705	no
gi|320446686|ref|NW_003383885.1|	216885-21724	835632	358335	210037	0.33595	no
gi|320446686|ref|NW_003383885.1|	217648-21807	165342	864902	238708	0.0834	no
gi|320446686|ref|NW_003383885.1|	245079-25017	0.0347265	214079	594596	0.1595	no
gi|320446686|ref|NW_003383885.1|	250728-25114	0.892381	100186	348887	0.218	no
gi|320446686|ref|NW_003383885.1|	359262-35972	214549	104867	228918	0.3256	no
gi|320446686|ref|NW_003383885.1|	362379-36375	0.147431	259091	745727	0.14075	no
gi|320446686|ref|NW_003383885.1|	366040-36834	0.89726	187903	106639	0.5943	no
gi|320446686|ref|NW_003383885.1|	369272-36967	0.933217	799688	309915	0.24695	no
gi|320446686|ref|NW_003383885.1|	370311-37104	0	442938	inf	0.0212	no
gi|320446686|ref|NW_003383885.1|	371458-37227	0	241696	inf	0.00425	no
gi|320446686|ref|NW_003383885.1|	414264-41474	136426	546642	200248	0.3028	no
gi|320446690|ref|NW_003383881.1|	10325-10596	0	357808	inf	5.00E-05	yes
gi|320446690|ref|NW_003383881.1|	106606-10706	0	59492	inf	0.029	no
gi|320446690|ref|NW_003383881.1|	112804-11372	0.247253	146602	588977	0.16015	no
gi|320446690|ref|NW_003383881.1|	116471-11668	0	570744	inf	0.0033	no
gi|320446690|ref|NW_003383881.1|	118238-11877	0.552829	126212	78348	0.14085	no
gi|320446690|ref|NW_003383881.1|	12136-12906	0	749326	inf	5.00E-05	yes
gi|320446690|ref|NW_003383881.1|	124930-12585	243709	149367	-0.706299	0.752	no
gi|320446690|ref|NW_003383881.1|	129952-13051	295185	756438	-196432	0.3756	no
gi|320446690|ref|NW_003383881.1|	163693-16453	0	50167	inf	0.0142	no
gi|320446690|ref|NW_003383881.1|	175799-17807	113887	111177	-0.0347478	0.97755	no
gi|320446690|ref|NW_003383881.1|	179380-18217	14706	276072	0.908637	0.49735	no

gi|320446690|ref|NW_003383881.1|	194229-195204	164297	126039	-370437	0.1536	no
gi|320446690|ref|NW_003383881.1|	20979-21200	0	116603	inf	0.0113	no
gi|320446690|ref|NW_003383881.1|	22072-24648	0	232153	inf	5.00E-05	yes
gi|320446690|ref|NW_003383881.1|	226450-227335	623563	178963	-180087	0.40975	no
gi|320446690|ref|NW_003383881.1|	228069-229168	566709	148777	-192946	0.3825	no
gi|320446690|ref|NW_003383881.1|	229395-235041	355492	65553	0.882845	0.6024	no
gi|320446690|ref|NW_003383881.1|	243972-244900	0	132759	inf	0.0059	no
gi|320446690|ref|NW_003383881.1|	249321-249780	0	63685	inf	0.02915	no
gi|320446690|ref|NW_003383881.1|	254511-255429	232417	988711	-12331	0.57235	no
gi|320446690|ref|NW_003383881.1|	35854-36204	479962	0	#NAME?	0.007	no
gi|320446690|ref|NW_003383881.1|	36345-36729	327014	0	#NAME?	0.01075	no
gi|320446690|ref|NW_003383881.1|	37332-39412	37871	0.446656	-640579	0.08505	no
gi|320446690|ref|NW_003383881.1|	39828-40089	532802	0	#NAME?	0.01335	no
gi|320446690|ref|NW_003383881.1|	42061-42583	768844	117438	-603272	0.11075	no
gi|320446690|ref|NW_003383881.1|	49541-49816	360884	153316	-455696	0.2937	no
gi|320446690|ref|NW_003383881.1|	6713-7671	0	28531	inf	5.00E-05	yes
gi|320446690|ref|NW_003383881.1|	76604-78844	117843	140992	0.258748	1	no
gi|320446690|ref|NW_003383881.1|	81770-82721	636786	716064	0.16928	0.9359	no
gi|320446690|ref|NW_003383881.1|	82866-84340	122616	179956	0.55349	0.78345	no
gi|320446690|ref|NW_003383881.1|	85936-88136	103924	449515	-120909	0.59455	no
gi|320446690|ref|NW_003383881.1|	90300-91622	116477	485151	-126354	0.5542	no
gi|320446690|ref|NW_003383881.1|	990-1612	0	286967	inf	0.0054	no
gi|320446691|ref|NW_003383880.1|	100749-102170	0	207697	inf	0.01575	no
gi|320446691|ref|NW_003383880.1|	102844-103554	0	291723	inf	0.029	no
gi|320446691|ref|NW_003383880.1|	10469-16028	31091	514764	0.727416	0.57925	no
gi|320446691|ref|NW_003383880.1|	19867-20650	705851	890618	0.335444	0.79665	no
gi|320446691|ref|NW_003383880.1|	26441-28254	485599	219839	-114331	0.39565	no
gi|320446691|ref|NW_003383880.1|	31761-32115	644495	106962	-259107	0.26405	no
gi|320446691|ref|NW_003383880.1|	78871-84957	778632	263635	508146	0.0149	no
gi|320446691|ref|NW_003383880.1|	86932-88258	0	322261	inf	0.00935	no

gi|320446691|ref|NW_003383880.1|	88657-89427	0	16026	inf	0.0052	no
gi|320446691|ref|NW_003383880.1|	95652-98437	0	226628	inf	0.0089	no
gi|320446691|ref|NW_003383880.1|	98546-100114	0	149761	inf	0.02075	no
gi|320446693|ref|NW_003383878.1|	162168-16239	791545	498399	-0.667371	0.7423	no
gi|320446693|ref|NW_003383878.1|	1727-3556	222361	213961	-0.0555558	0.97325	no
gi|320446693|ref|NW_003383878.1|	177931-17841	131348	658887	232664	0.2622	no
gi|320446693|ref|NW_003383878.1|	258509-25916	0	32467	inf	0.029	no
gi|320446693|ref|NW_003383878.1|	263487-26448	0	184162	inf	0.029	no
gi|320446693|ref|NW_003383878.1|	303056-30327	0	138847	inf	0.00695	no
gi|320446693|ref|NW_003383878.1|	303535-30399	0	688014	inf	0.00365	no
gi|320446693|ref|NW_003383878.1|	304113-30468	0	444464	inf	0.0044	no
gi|320446693|ref|NW_003383878.1|	341898-34363	270419	129475	22594	0.3284	no
gi|320446693|ref|NW_003383878.1|	344593-34496	452172	177427	197228	0.3876	no
gi|320446693|ref|NW_003383878.1|	347129-34956	47177	0.482114	-661257	0.06265	no
gi|320446693|ref|NW_003383878.1|	361377-36282	292966	17453	-0.747258	0.71555	no
gi|320446693|ref|NW_003383878.1|	372826-37561	243936	0.832512	-819482	0.036	no
gi|320446693|ref|NW_003383878.1|	387474-39456	571363	0.344974	-106937	0.0969	no
gi|320446693|ref|NW_003383878.1|	396041-40626	268675	925759	178477	0.18205	no
gi|320446693|ref|NW_003383878.1|	433451-43644	128632	646646	232972	0.31585	no
gi|320446694|ref|NW_003383877.1|	10317-11609	0.159713	133004	305791	1	no
gi|320446694|ref|NW_003383877.1|	13146-15502	0.0795687	344423	543584	0.16235	no
gi|320446694|ref|NW_003383877.1|	154725-15555	0	341856	inf	0.0022	no
gi|320446694|ref|NW_003383877.1|	15963-17030	0	19653	inf	0.0233	no
gi|320446694|ref|NW_003383877.1|	179393-18050	0	227333	inf	0.02075	no
gi|320446694|ref|NW_003383877.1|	182478-18437	0.202982	431517	4.41	0.15605	no
gi|320446694|ref|NW_003383877.1|	257295-25791	0	29657	inf	0.0312	no
gi|320446694|ref|NW_003383877.1|	264290-26510	0	839081	inf	0.0069	no
gi|320446694|ref|NW_003383877.1|	30553-31875	0.621211	100264	401258	0.158	no
gi|320446694|ref|NW_003383877.1|	33173-34992	0	105423	inf	0.0037	no
gi|320446694|ref|NW_003383877.1|	36744-37749	0	196958	inf	0.02915	no

gi|320446694|ref|NW_003383877.1|	39464-40799	0	127861	inf	1	no
gi|320446694|ref|NW_003383877.1|	44156-45026	0.797885	128178	0.683893	1	no
gi|320446694|ref|NW_003383877.1|	86531-88428	0	191113	inf	0.0133	no
gi|320446694|ref|NW_003383877.1|	90272-91008	0	253953	inf	0.0294	no
gi|320446695|ref|NW_003383876.1|	101469-10616	109595	46319	207942	0.22205	no
gi|320446695|ref|NW_003383876.1|	106332-10687	714128	543961	292925	0.2138	no
gi|320446695|ref|NW_003383876.1|	106989-10744	298032	327206	345666	0.1883	no
gi|320446695|ref|NW_003383876.1|	108549-11461	12975	401489	162963	0.2286	no
gi|320446695|ref|NW_003383876.1|	114842-11901	117866	148533	0.333638	0.802	no
gi|320446695|ref|NW_003383876.1|	120214-12151	904373	473496	-0.933565	0.6613	no
gi|320446695|ref|NW_003383876.1|	121798-12338	187272	173736	-0.10824	0.9532	no
gi|320446695|ref|NW_003383876.1|	125391-12596	745804	30258	-130148	0.5312	no
gi|320446695|ref|NW_003383876.1|	126315-12756	567187	35869	-0.661088	0.74705	no
gi|320446695|ref|NW_003383876.1|	127705-12969	347197	215193	-0.690119	0.74035	no
gi|320446695|ref|NW_003383876.1|	175373-17604	119172	681927	-0.805358	0.70015	no
gi|320446695|ref|NW_003383876.1|	179221-17999	156399	150227	-0.0580899	0.9269	no
gi|320446695|ref|NW_003383876.1|	181993-18297	0.452101	140487	163571	1	no
gi|320446695|ref|NW_003383876.1|	184052-18583	0.217505	0.984888	217891	1	no
gi|320446695|ref|NW_003383876.1|	189109-19027	398956	427188	0.0986405	0.9595	no
gi|320446695|ref|NW_003383876.1|	191182-19165	765089	116029	0.600786	0.7584	no
gi|320446695|ref|NW_003383876.1|	192803-19815	491429	168226	177535	0.18045	no
gi|320446695|ref|NW_003383876.1|	198880-20079	219402	0.561234	-528883	0.07075	no
gi|320446695|ref|NW_003383876.1|	202073-20265	103031	0.648954	-731074	0.16165	no
gi|320446695|ref|NW_003383876.1|	204429-20487	553797	157531	-181373	0.31385	no
gi|320446695|ref|NW_003383876.1|	231765-23649	157018	92872	-0.757615	0.56735	no
gi|320446695|ref|NW_003383876.1|	237196-23769	14561	254441	-25167	0.2864	no
gi|320446695|ref|NW_003383876.1|	240760-24244	236305	16047	-0.558341	0.6561	no
gi|320446695|ref|NW_003383876.1|	242550-24457	131206	164571	0.326872	0.79435	no
gi|320446695|ref|NW_003383876.1|	262902-26340	248743	333478	0.422936	0.82245	no
gi|320446695|ref|NW_003383876.1|	272015-27269	152394	106602	280636	0.25025	no

gi|320446695|ref|NW_003383876.1|	318919-31971	120219	309589	136468	0.52475	no
gi|320446695|ref|NW_003383876.1|	323433-32718	426898	144344	175754	0.3206	no
gi|320446695|ref|NW_003383876.1|	334140-33778	418157	150109	-147804	0.2651	no
gi|320446695|ref|NW_003383876.1|	337962-33897	392835	674875	-254123	0.151	no
gi|320446695|ref|NW_003383876.1|	340244-34806	701904	467074	-0.587623	0.72085	no
gi|320446695|ref|NW_003383876.1|	348321-35013	878743	82129	-0.0975505	0.9619	no
gi|320446695|ref|NW_003383876.1|	350263-35096	877031	499409	-0.812405	0.6931	no
gi|320446695|ref|NW_003383876.1|	356639-36303	207868	737136	-149566	0.27555	no
gi|320446695|ref|NW_003383876.1|	363141-36544	259767	809718	-168172	0.34675	no
gi|320446695|ref|NW_003383876.1|	372565-37357	30385	224958	-0.433704	0.82705	no
gi|320446695|ref|NW_003383876.1|	374792-37616	858855	894811	0.0591683	0.9774	no
gi|320446695|ref|NW_003383876.1|	376377-37742	168848	113867	-0.568385	0.7991	no
gi|320446695|ref|NW_003383876.1|	377568-37818	160424	172439	0.104197	0.95955	no
gi|320446695|ref|NW_003383876.1|	378446-38038	297668	477283	0.681138	0.7456	no
gi|320446695|ref|NW_003383876.1|	381004-38162	302488	234943	-0.364566	0.8498	no
gi|320446695|ref|NW_003383876.1|	381730-38238	10517	771462	-0.447057	0.82295	no
gi|320446695|ref|NW_003383876.1|	383540-38566	303771	666944	113458	0.6015	no
gi|320446695|ref|NW_003383876.1|	385931-38767	492806	113107	119859	0.58535	no
gi|320446695|ref|NW_003383876.1|	57443-61980	145127	288414	0.99083	0.64935	no
gi|320446695|ref|NW_003383876.1|	96797-101247	396328	319861	301268	0.1284	no
gi|320446697|ref|NW_003383874.1|	114777-12521	0.14886	176363	688845	0.0511	no
gi|320446697|ref|NW_003383874.1|	129005-12955	0	911233	inf	0.01485	no
gi|320446697|ref|NW_003383874.1|	129995-13146	0.13624	776651	583304	0.16095	no
gi|320446697|ref|NW_003383874.1|	1549-3302	0.333568	278798	306317	0.2139	no
gi|320446697|ref|NW_003383874.1|	179143-18028	205896	272222	0.402865	0.8422	no
gi|320446697|ref|NW_003383874.1|	202145-20317	511026	144235	149695	0.4894	no
gi|320446697|ref|NW_003383874.1|	205832-20622	395208	389835	330218	0.20015	no
gi|320446697|ref|NW_003383874.1|	217535-21789	118641	170365	384395	0.20775	no
gi|320446697|ref|NW_003383874.1|	218554-21948	0	234937	inf	0.0233	no
gi|320446697|ref|NW_003383874.1|	219743-22059	110387	87335	298399	0.2287	no

gi|320446697|ref|NW_003383874.1|	221143-22319	296908	266622	31667	0.0981	no
gi|320446697|ref|NW_003383874.1|	242477-24334	801676	268587	17443	0.4379	no
gi|320446697|ref|NW_003383874.1|	246424-24693	303383	610698	100932	0.60135	no
gi|320446697|ref|NW_003383874.1|	252411-25300	0.927389	628692	276111	0.2352	no
gi|320446697|ref|NW_003383874.1|	261414-26201	139485	441233	166143	0.433	no
gi|320446697|ref|NW_003383874.1|	262354-26326	100223	395491	198043	0.24795	no
gi|320446697|ref|NW_003383874.1|	296921-29767	297298	125393	-124546	0.5686	no
gi|320446697|ref|NW_003383874.1|	300709-30122	129003	433388	-157368	0.47025	no
gi|320446697|ref|NW_003383874.1|	305707-30791	6346	347098	-0.870504	0.68355	no
gi|320446697|ref|NW_003383874.1|	308339-31023	547036	371388	-0.55871	0.67585	no
gi|320446697|ref|NW_003383874.1|	318040-31930	0.489805	1246	134703	1	no
gi|320446697|ref|NW_003383874.1|	325877-32940	0.768429	566314	288162	0.2255	no
gi|320446697|ref|NW_003383874.1|	340310-34209	30569	722497	124092	0.568	no
gi|320446697|ref|NW_003383874.1|	342183-34949	103026	784195	-0.393726	0.76035	no
gi|320446697|ref|NW_003383874.1|	381608-38199	102046	301572	488521	0.16925	no
gi|320446697|ref|NW_003383874.1|	386980-38744	0	629375	inf	0.02915	no
gi|320446697|ref|NW_003383874.1|	387637-38785	505739	285565	249736	0.27555	no
gi|320446697|ref|NW_003383874.1|	388182-39253	0.90104	203039	449402	0.0411	no
gi|320446697|ref|NW_003383874.1|	433637-43528	657472	357795	-0.877796	0.6781	no
gi|320446697|ref|NW_003383874.1|	436982-43725	253647	968486	-138902	0.51745	no
gi|320446697|ref|NW_003383874.1|	437846-43906	123908	250568	-2306	0.3117	no
gi|320446697|ref|NW_003383874.1|	440586-44123	609641	110716	-246109	0.29955	no
gi|320446697|ref|NW_003383874.1|	448942-44937	233817	276503	-308001	0.22455	no
gi|320446697|ref|NW_003383874.1|	541938-54267	211118	252484	-306379	0.20495	no
gi|320446697|ref|NW_003383874.1|	581618-58195	370759	462464	0.318861	0.80955	no
gi|320446697|ref|NW_003383874.1|	583143-58400	623916	692324	0.150096	0.90895	no
gi|320446697|ref|NW_003383874.1|	585911-58818	490371	119998	129106	0.34055	no
gi|320446697|ref|NW_003383874.1|	590496-59100	111538	116321	0.0605714	0.9719	no
gi|320446697|ref|NW_003383874.1|	591891-59634	192205	284045	0.563477	0.67495	no
gi|320446697|ref|NW_003383874.1|	625727-62696	0	875492	inf	0.0048	no

gi|320446697|ref|NW_003383874.1|	63550-66412	0	992064	inf	0.00935	no
gi|320446698|ref|NW_003383873.1|	09198-10982	661439	194395	-176662	0.29655	no
gi|320446698|ref|NW_003383873.1|	23794-12885	139856	151857	0.118771	0.9557	no
gi|320446698|ref|NW_003383873.1|	50703-15117	633439	234708	-143234	0.51015	no
gi|320446698|ref|NW_003383873.1|	58172-15851	347037	288173	-0.268154	0.8925	no
gi|320446698|ref|NW_003383873.1|	82742-18314	0	707488	inf	0.0294	no
gi|320446698|ref|NW_003383873.1|	97573-19993	174886	349651	0.999498	0.63975	no
gi|320446698|ref|NW_003383873.1|	21139-21889	587061	507962	-0.208791	0.86815	no
gi|320446698|ref|NW_003383873.1|	11732-21231	385518	22836	-0.75549	0.70565	no
gi|320446698|ref|NW_003383873.1|	14234-21492	111062	338846	160927	0.46765	no
gi|320446698|ref|NW_003383873.1|	17884-21829	186643	110726	256864	0.2435	no
gi|320446698|ref|NW_003383873.1|	18559-21882	152188	258263	0.762982	0.70135	no
gi|320446698|ref|NW_003383873.1|	20743-22103	185071	157949	-0.228612	0.9056	no
gi|320446698|ref|NW_003383873.1|	47265-24878	117858	318759	143542	0.494	no
gi|320446698|ref|NW_003383873.1|	33230-36935	836432	935853	0.162035	0.8984	no
gi|320446698|ref|NW_003383873.1|	37043-38205	418891	504714	0.268891	0.89105	no
gi|320446698|ref|NW_003383873.1|	38531-39031	188564	336921	0.837352	0.69175	no
gi|320446698|ref|NW_003383873.1|	39131-40892	0.331819	100154	159375	1	no
gi|320446698|ref|NW_003383873.1|	41124-41734	166176	701082	-124506	0.556	no
gi|320446698|ref|NW_003383873.1|	48223-49563	16041	133653	-0.263268	0.902	no
gi|320446698|ref|NW_003383873.1|	49742-51372	217796	176848	-0.300467	0.87915	no
gi|320446698|ref|NW_003383873.1|	51699-52781	199342	0.689638	-153133	0.49255	no
gi|320446698|ref|NW_003383873.1|	54493-54805	514032	132382	136478	0.52185	no
gi|320446698|ref|NW_003383873.1|	97870-59853	770874	187699	-5.36	0.0852	no
gi|320446698|ref|NW_003383873.1|	98746-60017	257092	127615	-433241	0.06695	no
gi|320446698|ref|NW_003383873.1|	03087-60741	234579	458977	-235358	0.21635	no
gi|320446698|ref|NW_003383873.1|	61642-62048	30015	179262	-0.743611	0.7098	no
gi|320446698|ref|NW_003383873.1|	84712-68597	216562	762575	-150583	0.36365	no
gi|320446698|ref|NW_003383873.1|	86316-68792	0.739241	0.51442	-0.523099	1	no
gi|320446698|ref|NW_003383873.1|	88065-68970	138298	747028	-0.888551	0.5045	no

gi|320446698|ref|NW_003383873.1|	69374-69595	0	368221	inf	0.029	no
gi|320446698|ref|NW_003383873.1|	72558-72964	0.937967	111266	356833	0.2102	no
gi|320446698|ref|NW_003383873.1|	731165-73395	82062	166965	102476	0.4223	no
gi|320446698|ref|NW_003383873.1|	734820-73571	382078	368586	-0.0518656	0.971	no
gi|320446698|ref|NW_003383873.1|	73756-74281	0	201616	inf	0.0079	no
gi|320446698|ref|NW_003383873.1|	75592-76358	0	207252	inf	0.0055	no
gi|320446698|ref|NW_003383873.1|	766392-76788	604779	102788	-255673	0.2707	no
gi|320446698|ref|NW_003383873.1|	768323-76972	735826	260629	-149737	0.48865	no
gi|320446698|ref|NW_003383873.1|	7691-7996	163447	151709	-0.107512	0.94845	no
gi|320446698|ref|NW_003383873.1|	775256-77761	0.159137	555521	51255	0.1455	no
gi|320446698|ref|NW_003383873.1|	78309-81841	621861	928557	0.578399	0.655	no
gi|320446698|ref|NW_003383873.1|	82048-83482	302694	192748	-0.651146	0.6087	no
gi|320446698|ref|NW_003383873.1|	86131-86561	576192	370514	-0.63702	0.7649	no
gi|320446699|ref|NW_003383872.1|	10059-17287	755217	600529	-0.330657	0.7968	no
gi|320446699|ref|NW_003383872.1|	106283-11009	249362	133535	-0.901018	0.50355	no
gi|320446699|ref|NW_003383872.1|	110318-11402	194351	467864	126743	0.3446	no
gi|320446699|ref|NW_003383872.1|	114134-11662	412347	198972	227064	0.1989	no
gi|320446699|ref|NW_003383872.1|	116682-11740	0	331563	inf	0.0233	no
gi|320446699|ref|NW_003383872.1|	124547-12555	298238	169687	-0.813591	0.61295	no
gi|320446699|ref|NW_003383872.1|	129119-12954	758544	781106	0.042287	0.9623	no
gi|320446699|ref|NW_003383872.1|	129732-13083	136633	0.945681	-0.530885	1	no
gi|320446699|ref|NW_003383872.1|	1393-1617	697595	876676	-299227	0.20885	no
gi|320446699|ref|NW_003383872.1|	140827-14148	0.793935	351725	214736	0.2925	no
gi|320446699|ref|NW_003383872.1|	141990-14365	0.118366	0.988778	306239	1	no
gi|320446699|ref|NW_003383872.1|	143751-14622	0.52809	105374	0.996664	1	no
gi|320446699|ref|NW_003383872.1|	146562-14848	151711	13011	-0.221592	0.85855	no
gi|320446699|ref|NW_003383872.1|	148630-14926	802781	135065	0.75057	0.70725	no
gi|320446699|ref|NW_003383872.1|	149384-15358	150502	269378	0.839847	0.53825	no
gi|320446699|ref|NW_003383872.1|	154064-15610	777373	921018	0.244621	0.9142	no
gi|320446699|ref|NW_003383872.1|	187156-18751	12163	586898	227062	0.3083	no

gi|320446699|ref|NW_003383872.1|	198189-20183	0.247646	246072	331273	0.194	no
gi|320446699|ref|NW_003383872.1|	19885-20792	175913	0.692833	-134428	0.52925	no
gi|320446699|ref|NW_003383872.1|	2082-2748	242727	266948	-31847	0.19195	no
gi|320446699|ref|NW_003383872.1|	215904-21702	0.189917	315598	405465	0.1925	no
gi|320446699|ref|NW_003383872.1|	21886-23018	319978	143376	-115816	0.57985	no
gi|320446699|ref|NW_003383872.1|	221138-22164	0	500218	inf	0.029	no
gi|320446699|ref|NW_003383872.1|	25711-26442	108959	5595	-0.961575	0.6395	no
gi|320446699|ref|NW_003383872.1|	27058-30810	163611	150764	-0.117973	0.9321	no
gi|320446699|ref|NW_003383872.1|	31707-36206	13811	728829	-0.922164	0.48115	no
gi|320446699|ref|NW_003383872.1|	3282-7612	119911	223606	0.898999	0.50225	no
gi|320446699|ref|NW_003383872.1|	36795-37263	219929	245967	0.161429	0.93815	no
gi|320446699|ref|NW_003383872.1|	46911-48330	131167	624132	-107148	0.6265	no
gi|320446699|ref|NW_003383872.1|	49477-50382	148712	107704	-0.465453	0.82665	no
gi|320446699|ref|NW_003383872.1|	51119-53016	385898	45301	0.231324	0.9119	no
gi|320446699|ref|NW_003383872.1|	545-1058	774659	165272	-222872	0.1937	no
gi|320446699|ref|NW_003383872.1|	60655-61733	100144	0.831437	-0.268401	1	no
gi|320446699|ref|NW_003383872.1|	75021-80802	331017	311489	-0.0877235	0.94725	no
gi|320446699|ref|NW_003383872.1|	7818-9031	0.517869	11968	120852	1	no
gi|320446699|ref|NW_003383872.1|	89331-90605	129986	270562	105761	0.60215	no
gi|320446699|ref|NW_003383872.1|	96868-10607	51693	390251	-0.405565	0.753	no
gi|320446700|ref|NW_003383871.1|	0-618	0.4399	507919	352935	0.2166	no
gi|320446700|ref|NW_003383871.1|	10136-14006	333774	321015	-0.056228	0.96675	no
gi|320446700|ref|NW_003383871.1|	102571-10345	15776	0.712623	-446845	0.1724	no
gi|320446700|ref|NW_003383871.1|	103699-10479	688745	0	#NAME?	0.0086	no
gi|320446700|ref|NW_003383871.1|	105084-10731	130992	0.589807	-447309	0.06995	no
gi|320446700|ref|NW_003383871.1|	109279-11027	708181	0.305749	-45337	0.1993	no
gi|320446700|ref|NW_003383871.1|	112510-11267	1015.45	871477	-354252	0.162	no
gi|320446700|ref|NW_003383871.1|	113711-11452	753246	219021	-178205	0.41175	no
gi|320446700|ref|NW_003383871.1|	1149-3092	296448	175669	-0.754917	0.55895	no
gi|320446700|ref|NW_003383871.1|	115818-11695	429082	284252	-0.594084	0.76915	no

gi|320446700|ref|NW_003383871.1|	17120-11780	263737	128607	-103612	0.59645	no
gi|320446700|ref|NW_003383871.1|	18772-11925	272852	227767	-358249	0.1891	no
gi|320446700|ref|NW_003383871.1|	19330-12323	108616	243111	-215955	0.1117	no
gi|320446700|ref|NW_003383871.1|	23344-12437	76789	737558	-0.058142	0.96355	no
gi|320446700|ref|NW_003383871.1|	24500-12777	234174	339223	0.534654	0.6951	no
gi|320446700|ref|NW_003383871.1|	29245-13074	99709	867362	-0.201091	0.8783	no
gi|320446700|ref|NW_003383871.1|	34217-13503	0	396222	inf	0.0162	no
gi|320446700|ref|NW_003383871.1|	41068-14185	213069	105963	-432969	0.15925	no
gi|320446700|ref|NW_003383871.1|	43590-14496	563193	0.411765	-377374	0.193	no
gi|320446700|ref|NW_003383871.1|	46499-14732	11737	0.590386	-431327	0.1546	no
gi|320446700|ref|NW_003383871.1|	49779-15094	178484	107252	-0.734794	0.7411	no
gi|320446700|ref|NW_003383871.1|	54006-15653	220315	307775	0.482309	0.8138	no
gi|320446700|ref|NW_003383871.1|	57246-15764	294839	517146	0.810643	0.69235	no
gi|320446700|ref|NW_003383871.1|	58425-16034	180834	189081	0.0643321	0.9697	no
gi|320446700|ref|NW_003383871.1|	62378-16273	507654	260155	-0.964474	0.64295	no
gi|320446700|ref|NW_003383871.1|	63462-16640	105354	989271	-0.0908136	0.9451	no
gi|320446700|ref|NW_003383871.1|	67802-16951	245051	480813	0.972392	0.4651	no
gi|320446700|ref|NW_003383871.1|	71838-17259	610126	66495	0.124139	0.9213	no
gi|320446700|ref|NW_003383871.1|	00389-20385	261138	237503	-0.136869	0.9462	no
gi|320446700|ref|NW_003383871.1|	06841-20766	120848	286782	124676	0.5747	no
gi|320446700|ref|NW_003383871.1|	07857-20928	10222	227896	115669	0.48515	no
gi|320446700|ref|NW_003383871.1|	10177-21085	113281	386652	177113	0.4172	no
gi|320446700|ref|NW_003383871.1|	11066-21257	356618	459252	0.364907	0.85765	no
gi|320446700|ref|NW_003383871.1|	14617-21634	315911	275008	-0.200045	0.92065	no
gi|320446700|ref|NW_003383871.1|	21551-23736	60584	917981	0.599527	0.7877	no
gi|320446700|ref|NW_003383871.1|	18528-21881	854086	39591	-110921	0.5935	no
gi|320446700|ref|NW_003383871.1|	19525-21967	403532	132015	-161198	0.4411	no
gi|320446700|ref|NW_003383871.1|	23990-30486	404243	646046	0.676412	0.5915	no
gi|320446700|ref|NW_003383871.1|	31002-31627	151244	525685	179732	0.27865	no
gi|320446700|ref|NW_003383871.1|	32058-33117	0.409821	184238	216851	0.29245	no

gi|320446700|ref|NW_003383871.1|	5292-5893	156389	748583	-10629	0.60675	no
gi|320446700|ref|NW_003383871.1|	62345-63615	0	181064	inf	0.0198	no
gi|320446700|ref|NW_003383871.1|	63731-65045	0.156455	173763	34733	0.21395	no
gi|320446700|ref|NW_003383871.1|	65638-66814	197357	559464	150324	0.4681	no
gi|320446700|ref|NW_003383871.1|	66960-69884	190155	119767	-0.666945	0.6091	no
gi|320446700|ref|NW_003383871.1|	72016-72562	253369	138093	-0.875597	0.66995	no
gi|320446700|ref|NW_003383871.1|	80649-81960	119237	446495	-141712	0.51065	no
gi|320446700|ref|NW_003383871.1|	82292-82873	383924	131556	-154515	0.4796	no
gi|320446700|ref|NW_003383871.1|	83029-84308	118043	852725	-0.469165	0.82895	no
gi|320446700|ref|NW_003383871.1|	88281-91872	789427	158323	100399	0.44005	no
gi|320446702|ref|NW_003383869.1|	105985-10866	131088	173547	0.40479	0.83975	no
gi|320446702|ref|NW_003383869.1|	11067-11301	199182	326358	0.712372	0.73275	no
gi|320446702|ref|NW_003383869.1|	123474-12524	591515	636021	0.10466	0.93845	no
gi|320446702|ref|NW_003383869.1|	127107-13047	484446	37051	-0.386822	0.7688	no
gi|320446702|ref|NW_003383869.1|	136070-13669	898386	546784	260556	0.15025	no
gi|320446702|ref|NW_003383869.1|	138934-13959	0	698727	inf	0.0142	no
gi|320446702|ref|NW_003383869.1|	14039-15689	250502	312205	0.317669	0.80715	no
gi|320446702|ref|NW_003383869.1|	143132-14384	3518	375417	341567	0.16775	no
gi|320446702|ref|NW_003383869.1|	145095-15005	167702	120722	284772	0.12855	no
gi|320446702|ref|NW_003383869.1|	150728-15270	824957	230079	-184219	0.39365	no
gi|320446702|ref|NW_003383869.1|	154986-15538	592813	51978	-0.189674	0.9175	no
gi|320446702|ref|NW_003383869.1|	15988-17257	194289	962822	-101286	0.65405	no
gi|320446702|ref|NW_003383869.1|	162780-16471	297493	0.69131	-210545	0.34265	no
gi|320446702|ref|NW_003383869.1|	164846-16827	359263	336341	-0.0951146	0.96585	no
gi|320446702|ref|NW_003383869.1|	169595-17021	909761	529914	-0.77973	0.70465	no
gi|320446702|ref|NW_003383869.1|	171817-17342	0.986375	0.68639	-0.523109	1	no
gi|320446702|ref|NW_003383869.1|	17456-20287	202819	826916	-129438	0.3172	no
gi|320446702|ref|NW_003383869.1|	177605-17871	212497	0.401176	-240514	0.248	no
gi|320446702|ref|NW_003383869.1|	180381-18418	138532	144274	0.0585865	0.96415	no
gi|320446702|ref|NW_003383869.1|	184293-18712	190615	431054	117721	0.37935	no

gi|320446702|ref|NW_003383869.1|	20886-22395	543232	506749	-0.100299	0.9606	no
gi|320446702|ref|NW_003383869.1|	225932-22661[illegible]	79473	160161	101098	0.6336	no
gi|320446702|ref|NW_003383869.1|	22686-24392	332757	319593	-0.0582339	0.9763	no
gi|320446702|ref|NW_003383869.1|	230030-23537[illegible]	330012	157458	225437	0.22495	no
gi|320446702|ref|NW_003383869.1|	239273-24119[illegible]	377952	289256	-0.385854	0.7635	no
gi|320446702|ref|NW_003383869.1|	241309-24350[illegible]	431195	202802	-108826	0.4078	no
gi|320446702|ref|NW_003383869.1|	243739-24573[illegible]	162824	337.8	105286	0.5252	no
gi|320446702|ref|NW_003383869.1|	24716-26272	178891	355551	0.990978	0.64335	no
gi|320446702|ref|NW_003383869.1|	257272-25760[illegible]	122225	98751	-0.30767	0.88755	no
gi|320446702|ref|NW_003383869.1|	257812-25931[illegible]	371812	243546	-0.610379	0.6216	no
gi|320446702|ref|NW_003383869.1|	260120-26767[illegible]	168222	974295	-0.787933	0.56355	no
gi|320446702|ref|NW_003383869.1|	267797-26943[illegible]	119965	100202	-0.259713	1	no
gi|320446702|ref|NW_003383869.1|	269563-27150[illegible]	702828	144231	103714	0.52755	no
gi|320446702|ref|NW_003383869.1|	271629-27255[illegible]	369256	112025	160113	0.45345	no
gi|320446702|ref|NW_003383869.1|	272684-27349[illegible]	219545	237342	0.112456	0.9576	no
gi|320446702|ref|NW_003383869.1|	284102-28514[illegible]	0.210012	188753	316796	0.24575	no
gi|320446702|ref|NW_003383869.1|	286228-28779[illegible]	0.253067	255285	333452	0.19155	no
gi|320446702|ref|NW_003383869.1|	289130-29026[illegible]	244932	0.388463	-597846	0.11065	no
gi|320446702|ref|NW_003383869.1|	293359-29566[illegible]	110172	717791	-0.618116	0.6988	no
gi|320446702|ref|NW_003383869.1|	295876-29678[illegible]	260967	124528	-10674	0.62925	no
gi|320446702|ref|NW_003383869.1|	297577-29895[illegible]	131214	68613	-0.935368	0.6678	no
gi|320446702|ref|NW_003383869.1|	39064-40120	0	199125	inf	0.0233	no
gi|320446702|ref|NW_003383869.1|	5807-6801	0.444947	414902	322106	0.2068	no
gi|320446702|ref|NW_003383869.1|	60587-61821	152093	209777	0.463906	0.7709	no
gi|320446702|ref|NW_003383869.1|	64251-65309	10258	15608	0.605543	0.75905	no
gi|320446702|ref|NW_003383869.1|	68495-70990	112757	283716	133123	0.31425	no
gi|320446705|ref|NW_003383866.1|	102647-11767[illegible]	141447	894174	266029	0.0511	no
gi|320446705|ref|NW_003383866.1|	119169-12581[illegible]	132648	110326	-0.265831	0.844	no
gi|320446705|ref|NW_003383866.1|	129499-13026[illegible]	384612	241752	-0.669875	0.7467	no
gi|320446705|ref|NW_003383866.1|	146785-14767[illegible]	363186	214432	-0.760189	0.71275	no

gi|320446705|ref|NW_003383866.1|	188719-19337	148056	137098	-0.110943	0.9301	no
gi|320446705|ref|NW_003383866.1|	193830-19406	381427	216515	-0.81694	0.6868	no
gi|320446705|ref|NW_003383866.1|	195044-19587	273048	145185	-0.911259	0.67495	no
gi|320446705|ref|NW_003383866.1|	196640-19725	250743	956083	-1391	0.51605	no
gi|320446705|ref|NW_003383866.1|	198030-19865	474141	231442	-103466	0.6383	no
gi|320446705|ref|NW_003383866.1|	199914-20175	861775	285606	-159328	0.4567	no
gi|320446705|ref|NW_003383866.1|	202015-20384	117166	404528	-153424	0.49205	no
gi|320446705|ref|NW_003383866.1|	204074-20425	109075	251676	-211568	0.2824	no
gi|320446705|ref|NW_003383866.1|	204662-20504	862267	371708	-121396	0.5778	no
gi|320446705|ref|NW_003383866.1|	206621-20817	241505	149226	-0.694553	0.67505	no
gi|320446705|ref|NW_003383866.1|	243142-24385	176266	651073	188507	0.39435	no
gi|320446705|ref|NW_003383866.1|	306568-30873	0.872197	170404	0.966236	0.6469	no
gi|320446705|ref|NW_003383866.1|	312153-31379	132244	334713	133972	0.5184	no
gi|320446705|ref|NW_003383866.1|	38726-39457	234943	489563	-226274	0.3194	no
gi|320446705|ref|NW_003383866.1|	41891-42318	186287	504385	-188493	0.3747	no
gi|320446705|ref|NW_003383866.1|	44216-51736	110247	763251	-0.530508	0.69585	no
gi|320446705|ref|NW_003383866.1|	53153-53643	0.651875	135202	437438	0.18695	no
gi|320446705|ref|NW_003383866.1|	8678-9282	124113	263084	-223806	0.21655	no
gi|320446705|ref|NW_003383866.1|	89683-89830	64615.7	51471.6	-0.328108	0.7949	no
gi|320446706|ref|NW_003383865.1|	183937-18558	167238	989482	256477	0.2621	no
gi|320446706|ref|NW_003383865.1|	191192-19529	0.523772	516398	330147	0.1783	no
gi|320446706|ref|NW_003383865.1|	198730-20295	529168	152937	153114	0.2405	no
gi|320446706|ref|NW_003383865.1|	205269-20551	160152	283825	0.825554	0.68925	no
gi|320446706|ref|NW_003383865.1|	219806-22063	657304	844816	0.362078	0.8588	no
gi|320446706|ref|NW_003383865.1|	223934-22414	292178	21939	-0.41335	0.85515	no
gi|320446706|ref|NW_003383865.1|	230298-23064	804787	871292	0.11455	0.93815	no
gi|320446706|ref|NW_003383865.1|	304702-30581	122512	159035	0.376412	0.86045	no
gi|320446706|ref|NW_003383865.1|	38941-39721	0.309929	701865	450118	0.17655	no
gi|320446706|ref|NW_003383865.1|	553173-55461	449396	387471	310803	0.1026	no
gi|320446706|ref|NW_003383865.1|	55397-56564	0.724596	652654	317107	0.20995	no

gi|320446706|ref|NW_003383865.1|	;65672-56618:	426215	302285	282625	0.22045	no
gi|320446706|ref|NW_003383865.1|	;75065-57576	108462	724075	273895	0.13995	no
gi|320446706|ref|NW_003383865.1|	;07746-60834:	139485	107157	294154	0.22955	no
gi|320446706|ref|NW_003383865.1|	;12263-61251:	328593	291012	314671	0.2374	no
gi|320446706|ref|NW_003383865.1|	;41410-64159!	315118	454934	0.529764	0.7973	no
gi|320446706|ref|NW_003383865.1|	;76317-67719(	30.42	346751	0.188881	0.9061	no
gi|320446706|ref|NW_003383865.1|	;82776-68345	908263	904133	-0.00657494	0.98345	no
gi|320446706|ref|NW_003383865.1|	;84340-68461(	735578	100077	0.44416	0.83185	no
gi|320446706|ref|NW_003383865.1|	;89792-69067!	260621	269258	0.0470364	0.9614	no
gi|320446706|ref|NW_003383865.1|	;90846-69110	145215	18422	0.343238	0.8635	no
gi|320446706|ref|NW_003383865.1|	;91573-69692:	303882	950109	164458	0.35685	no
gi|320446706|ref|NW_003383865.1|	;98044-69845!	36586	603413	0.721851	0.7419	no
gi|320446706|ref|NW_003383865.1|	;98726-69987	166429	896616	242958	0.28505	no
gi|320446706|ref|NW_003383865.1|	'11510-71202	179536	401775	116212	0.57455	no
gi|320446706|ref|NW_003383865.1|	'16765-71728!	0.578586	350064	259701	0.2636	no
gi|320446709|ref|NW_003383862.1|	121474-12321:	164067	417138	134624	0.3021	no
gi|320446709|ref|NW_003383862.1|	12587-14183	434077	405603	-0.0978818	0.96025	no
gi|320446709|ref|NW_003383862.1|	131484-13224!	401399	251008	-0.677304	0.6693	no
gi|320446709|ref|NW_003383862.1|	132545-13408:	168668	107388	-0.651358	0.7732	no
gi|320446709|ref|NW_003383862.1|	134460-13467:	630809	215375	-155035	0.48685	no
gi|320446709|ref|NW_003383862.1|	134852-13532	25138	10715	-123023	0.55825	no
gi|320446709|ref|NW_003383862.1|	136563-13780:	316122	106122	-157476	0.34935	no
gi|320446709|ref|NW_003383862.1|	15104-19504	222826	859578	194771	0.2603	no
gi|320446709|ref|NW_003383862.1|	153277-15360!	562431	325683	-0.788206	0.7068	no
gi|320446709|ref|NW_003383862.1|	154196-15634	489638	205591	-125194	0.34965	no
gi|320446709|ref|NW_003383862.1|	159126-16192	107199	119046	0.151224	0.90565	no
gi|320446709|ref|NW_003383862.1|	165778-16595	545373	125838	120625	0.5702	no
gi|320446709|ref|NW_003383862.1|	166616-16694(	298191	868204	15418	0.3575	no
gi|320446709|ref|NW_003383862.1|	169586-17185:	531813	163558	162081	0.339	no
gi|320446709|ref|NW_003383862.1|	172627-17334!	312729	128398	203764	0.3482	no

gi|320446709|ref|NW_003383862.1|	173642-17414	805556	348963	211502	0.3433	no
gi|320446709|ref|NW_003383862.1|	175193-17953	845549	591766	280707	0.0421	no
gi|320446709|ref|NW_003383862.1|	181078-18292	756209	873123	352933	0.08985	no
gi|320446709|ref|NW_003383862.1|	183208-18617	2409	355829	388468	0.06225	no
gi|320446709|ref|NW_003383862.1|	186320-18680	200762	657463	503335	0.1467	no
gi|320446709|ref|NW_003383862.1|	186928-18834	526628	104244	430703	0.0474	no
gi|320446709|ref|NW_003383862.1|	197634-19905	498583	544422	0.126892	0.952	no
gi|320446709|ref|NW_003383862.1|	203137-20544	0.977884	233231	125402	0.54205	no
gi|320446709|ref|NW_003383862.1|	205605-20699	175995	224134	0.348828	0.8585	no
gi|320446709|ref|NW_003383862.1|	207119-20793	990115	760451	-0.380741	0.85145	no
gi|320446709|ref|NW_003383862.1|	208614-20946	126325	793592	-0.670673	0.75125	no
gi|320446709|ref|NW_003383862.1|	209648-21367	921913	17592	-238971	0.0801	no
gi|320446709|ref|NW_003383862.1|	224605-22501	111986	455207	20232	0.3493	no
gi|320446709|ref|NW_003383862.1|	228381-23408	209029	337023	0.689143	0.6065	no
gi|320446709|ref|NW_003383862.1|	234256-23455	306048	466298	0.607495	0.76105	no
gi|320446709|ref|NW_003383862.1|	235568-23623	331139	204243	-0.697147	0.7422	no
gi|320446709|ref|NW_003383862.1|	236609-23698	230538	790675	-154385	0.48085	no
gi|320446709|ref|NW_003383862.1|	238321-24177	10789	729148	-0.565272	0.65505	no
gi|320446709|ref|NW_003383862.1|	247005-25047	358393	116595	-162003	0.23395	no
gi|320446709|ref|NW_003383862.1|	250716-25164	396628	127537	-163687	0.3267	no
gi|320446709|ref|NW_003383862.1|	253103-25369	377063	13412	-149129	0.4959	no
gi|320446709|ref|NW_003383862.1|	317633-31788	594887	961691	401489	0.16395	no
gi|320446709|ref|NW_003383862.1|	318950-31974	0	187817	inf	0.00445	no
gi|320446709|ref|NW_003383862.1|	324835-32513	0	141197	inf	0.029	no
gi|320446709|ref|NW_003383862.1|	334577-33499	0	130261	inf	0.0186	no
gi|320446709|ref|NW_003383862.1|	336344-33762	0	514661	inf	0.00695	no
gi|320446709|ref|NW_003383862.1|	357696-35793	115.41	432669	-141543	0.49835	no
gi|320446709|ref|NW_003383862.1|	546626-54774	0	303797	inf	0.01485	no
gi|320446709|ref|NW_003383862.1|	622717-62411	0	126733	inf	0.0032	no
gi|320446709|ref|NW_003383862.1|	7431-8063	118892	10395	-0.193766	0.92305	no

gi|320446710|ref|NW_003383861.1|	134326-13706	48601	880591	0.857485	0.52145	no
gi|320446710|ref|NW_003383861.1|	137198-13899	617105	874907	0.503613	0.81715	no
gi|320446710|ref|NW_003383861.1|	139246-14234	877321	786398	-0.157845	0.89765	no
gi|320446710|ref|NW_003383861.1|	150318-15343	779664	115016	0.560908	0.65905	no
gi|320446710|ref|NW_003383861.1|	154476-15514	305367	400423	-293095	0.22285	no
gi|320446710|ref|NW_003383861.1|	157424-15782	807175	10879	-289134	0.2229	no
gi|320446710|ref|NW_003383861.1|	159351-15983	142697	0.90771	-397458	0.2192	no
gi|320446710|ref|NW_003383861.1|	162523-16307	41824	141136	-156724	0.49785	no
gi|320446710|ref|NW_003383861.1|	164532-16507	276653	694621	-199378	0.3735	no
gi|320446710|ref|NW_003383861.1|	165176-16557	9725	127988	-292569	0.2676	no
gi|320446710|ref|NW_003383861.1|	165813-16610	362986	682604	-24108	0.2955	no
gi|320446710|ref|NW_003383861.1|	166941-16717	503017	12199	-204385	0.35575	no
gi|320446710|ref|NW_003383861.1|	168347-17046	114011	319401	-183573	0.26005	no
gi|320446710|ref|NW_003383861.1|	170818-17186	16574	701992	-12394	0.56145	no
gi|320446710|ref|NW_003383861.1|	19099-19538	384821	319094	-359213	0.1743	no
gi|320446710|ref|NW_003383861.1|	20390-21197	460649	791151	-254164	0.1528	no
gi|320446710|ref|NW_003383861.1|	21306-22450	248886	537733	11114	0.3821	no
gi|320446710|ref|NW_003383861.1|	227094-22739	578935	495061	-354772	0.2006	no
gi|320446710|ref|NW_003383861.1|	227602-22825	359784	217919	-0.723338	0.72905	no
gi|320446710|ref|NW_003383861.1|	23252-24687	13645	234626	0.781986	0.6326	no
gi|320446710|ref|NW_003383861.1|	234874-23563	924224	611973	-0.594775	0.7648	no
gi|320446710|ref|NW_003383861.1|	235981-23725	828658	39457	-107049	0.6125	no
gi|320446710|ref|NW_003383861.1|	237747-23839	567646	414233	-0.45455	0.82135	no
gi|320446710|ref|NW_003383861.1|	240451-24088	106109	270522	-197172	0.38655	no
gi|320446710|ref|NW_003383861.1|	241102-24136	287028	728443	-19783	0.3966	no
gi|320446710|ref|NW_003383861.1|	243112-24578	304818	382394	0.327111	0.87555	no
gi|320446710|ref|NW_003383861.1|	245931-24773	0.431396	0.97677	117901	1	no
gi|320446710|ref|NW_003383861.1|	247975-24904	118876	104541	-0.185388	0.9318	no
gi|320446710|ref|NW_003383861.1|	24948-25344	128443	181923	0.502203	0.7983	no
gi|320446710|ref|NW_003383861.1|	249575-25270	758827	200417	140116	0.42555	no

gi|320446710|ref|NW_003383861.1|	253978-25677	311732	188451	-404805	0.05135	no
gi|320446710|ref|NW_003383861.1|	259383-25977	139061	0.65305	-441239	0.29665	no
gi|320446710|ref|NW_003383861.1|	267789-27437	624634	264572	-123935	0.47035	no
gi|320446710|ref|NW_003383861.1|	26863-27448	206637	461997	116079	0.6004	no
gi|320446710|ref|NW_003383861.1|	281420-28306	841556	728645	-0.207843	0.877	no
gi|320446710|ref|NW_003383861.1|	283291-28603	125861	185176	0.55707	0.65475	no
gi|320446710|ref|NW_003383861.1|	286503-28746	0.695867	128083	0.880192	1	no
gi|320446710|ref|NW_003383861.1|	309383-31530	667574	173828	138066	0.2967	no
gi|320446710|ref|NW_003383861.1|	316462-31710	307342	497458	0.69473	0.66105	no
gi|320446710|ref|NW_003383861.1|	320398-32171	0.622357	162012	138028	0.52065	no
gi|320446710|ref|NW_003383861.1|	321987-32359	124077	23937	-237392	0.3008	no
gi|320446710|ref|NW_003383861.1|	323841-32513	128637	42985	-15814	0.4744	no
gi|320446710|ref|NW_003383861.1|	325415-32839	232912	549939	-208244	0.25295	no
gi|320446710|ref|NW_003383861.1|	329314-33315	123034	652303	-0.91544	0.47175	no
gi|320446710|ref|NW_003383861.1|	374129-37531	160112	702756	213395	0.33605	no
gi|320446710|ref|NW_003383861.1|	387314-39094	258462	123817	226019	0.20345	no
gi|320446710|ref|NW_003383861.1|	391109-39355	183189	533032	154089	0.47905	no
gi|320446710|ref|NW_003383861.1|	39246-41824	135412	372336	-186267	0.28075	no
gi|320446710|ref|NW_003383861.1|	395739-39668	35526	98049	146463	0.491	no
gi|320446710|ref|NW_003383861.1|	43359-45206	900447	116739	-294736	0.21665	no
gi|320446710|ref|NW_003383861.1|	52624-55114	0.0748391	172487	452655	0.17575	no
gi|320446710|ref|NW_003383861.1|	95324-95553	0	270315	inf	0.0312	no
gi|320446710|ref|NW_003383861.1|	96894-97230	0	185049	inf	0.0162	no
gi|320446711|ref|NW_003383860.1|	12739-14238	0.801266	17643	113875	0.58155	no
gi|320446711|ref|NW_003383860.1|	14454-17205	234758	346858	0.563172	0.7855	no
gi|320446711|ref|NW_003383860.1|	17507-18918	137801	438836	-165083	0.4575	no
gi|320446711|ref|NW_003383860.1|	19112-20686	265988	168391	-0.659545	0.5988	no
gi|320446711|ref|NW_003383860.1|	245090-25139	258603	103686	200341	0.27325	no
gi|320446711|ref|NW_003383860.1|	257811-25889	0.793617	0.96102	0.276123	1	no
gi|320446711|ref|NW_003383860.1|	262363-26414	229564	38075	0.729947	0.72985	no

gi|320446711|ref|NW_003383860.1|	283590-28472[illegible]	207045	143376	-0.530133	0.79635	no
gi|320446711|ref|NW_003383860.1|	290268-29133[illegible]	0.813712	126641	0.638151	1	no
gi|320446711|ref|NW_003383860.1|	5727-9208	0.519863	378255	286316	0.22035	no
gi|320446711|ref|NW_003383860.1|	57302-57806	619658	332346	-0.898787	0.67215	no
gi|320446711|ref|NW_003383860.1|	62321-62915	703136	508245	-0.468278	0.81445	no
gi|320446711|ref|NW_003383860.1|	81092-82800	0.572939	223413	196326	0.3829	no
gi|320446711|ref|NW_003383860.1|	9365-12631	0.445397	179125	200781	0.3468	no
gi|320446712|ref|NW_003383859.1|	106859-10710[illegible]	985778	166293	0.75439	0.70725	no
gi|320446712|ref|NW_003383859.1|	10946-12289	167593	0.31737	-240073	0.2481	no
gi|320446712|ref|NW_003383859.1|	12567-13562	244399	0.920809	-140826	0.5136	no
gi|320446712|ref|NW_003383859.1|	157627-15850[illegible]	36976	0.361199	-667765	0.1618	no
gi|320446712|ref|NW_003383859.1|	15815-17084	326536	0.906185	-184937	0.40125	no
gi|320446712|ref|NW_003383859.1|	158962-16034[illegible]	25698	0.20517	-69687	0.1617	no
gi|320446712|ref|NW_003383859.1|	18955-19800	276873	0.380938	-28616	0.27055	no
gi|320446712|ref|NW_003383859.1|	22192-23682	134503	346015	13632	0.50875	no
gi|320446712|ref|NW_003383859.1|	23809-24435	129311	439447	176484	0.41855	no
gi|320446712|ref|NW_003383859.1|	24913-27663	244916	355904	0.539202	0.68675	no
gi|320446712|ref|NW_003383859.1|	31325-33480	729581	65011	-0.166384	0.9388	no
gi|320446712|ref|NW_003383859.1|	39669-40632	0.463911	176114	192459	0.3194	no
gi|320446712|ref|NW_003383859.1|	4481-4829	118308	512735	-120626	0.57095	no
gi|320446712|ref|NW_003383859.1|	50739-51561	690561	0.197781	-51258	0.28005	no
gi|320446712|ref|NW_003383859.1|	52114-52932	434565	0.19911	-444794	0.2969	no
gi|320446712|ref|NW_003383859.1|	5326-6439	269218	119812	-1168	0.56995	no
gi|320446712|ref|NW_003383859.1|	65074-66736	0	263491	inf	0.01035	no
gi|320446712|ref|NW_003383859.1|	6604-7967	224482	0.727541	-16255	0.44145	no
gi|320446712|ref|NW_003383859.1|	9030-9792	256408	0.8791	-154434	0.49795	no
gi|320446712|ref|NW_003383859.1|	91623-92844	0.342443	201825	255917	0.25005	no
gi|320446715|ref|NW_003383856.1|	40732-41039	57142	803673	-282987	0.22675	no
gi|320446715|ref|NW_003383856.1|	479069-47965[illegible]	0.953112	548665	252521	0.25095	no
gi|320446715|ref|NW_003383856.1|	483466-48418[illegible]	13985	717615	235933	0.30025	no

gi|320446715|ref|NW_003383856.1|	491525-49323	0.4568	254473	247788	0.28965	no
gi|320446715|ref|NW_003383856.1|	493321-49634	894215	119973	0.42402	0.7333	no
gi|320446715|ref|NW_003383856.1|	503037-50451	170026	239243	0.492721	0.69	no
gi|320446715|ref|NW_003383856.1|	505958-50650	873396	845946	-0.0460703	0.9733	no
gi|320446715|ref|NW_003383856.1|	509573-50978	350614	21939	-0.676384	0.7476	no
gi|320446715|ref|NW_003383856.1|	515785-51618	852842	936343	0.134758	0.94235	no
gi|320446715|ref|NW_003383856.1|	5203-5876	192648	394282	-228867	0.3102	no
gi|320446715|ref|NW_003383856.1|	559807-56046	143914	106236	-0.437936	0.82745	no
gi|320446715|ref|NW_003383856.1|	6081-6538	964579	197516	-228793	0.3365	no
gi|320446715|ref|NW_003383856.1|	517659-61857	297139	409711	0.463471	0.8166	no
gi|320446715|ref|NW_003383856.1|	543314-64377	226248	777658	-15407	0.4649	no
gi|320446715|ref|NW_003383856.1|	545401-64663	101599	124472	0.292937	0.8918	no
gi|320446715|ref|NW_003383856.1|	6715-7021	216092	57874	-190066	0.39405	no
gi|320446715|ref|NW_003383856.1|	573468-67381	135971	794504	-0.775169	0.70465	no
gi|320446715|ref|NW_003383856.1|	721298-72195	59964	0	#NAME?	0.01585	no
gi|320446715|ref|NW_003383856.1|	8789-9161	101739	221784	-219764	0.2745	no
gi|320446715|ref|NW_003383856.1|	9756-10118	216214	313457	-278612	0.2664	no
gi|320446720|ref|NW_003383851.1|	241045-24543	207574	674118	-162255	0.2256	no
gi|320446720|ref|NW_003383851.1|	245537-24650	224073	102052	-113466	0.6043	no
gi|320446720|ref|NW_003383851.1|	246756-25213	775571	39139	-0.986651	0.44565	no
gi|320446720|ref|NW_003383851.1|	252499-25297	286294	419283	-27715	0.2284	no
gi|320446720|ref|NW_003383851.1|	255425-25635	122017	269262	-2.18	0.3197	no
gi|320446720|ref|NW_003383851.1|	262242-26354	369531	27.84	-0.408539	0.75145	no
gi|320446720|ref|NW_003383851.1|	264265-26535	0.992021	192204	0.954195	0.66285	no
gi|320446720|ref|NW_003383851.1|	265463-26696	0.66509	120244	0.854349	1	no
gi|320446720|ref|NW_003383851.1|	268103-26961	849306	479854	-0.823689	0.6992	no
gi|320446720|ref|NW_003383851.1|	288001-28843	144953	32046	-217736	0.33555	no
gi|320446720|ref|NW_003383851.1|	288702-29046	17.93	655276	-14522	0.3886	no
gi|320446720|ref|NW_003383851.1|	293189-29420	139814	609486	-119785	0.5728	no
gi|320446720|ref|NW_003383851.1|	295154-29667	317569	181442	-0.80756	0.5239	no

gi|320446720|ref|NW_003383851.1|	367881-36822!	216073	149091	-0.535327	0.785	no
gi|320446720|ref|NW_003383851.1|	368657-36896!	207298	289098	0.479854	0.81485	no
gi|320446720|ref|NW_003383851.1|	398302-39955!	0.82918	218562	139828	0.51925	no
gi|320446720|ref|NW_003383851.1|	431662-43202(	130505	185853	0.510049	0.7998	no
gi|320446720|ref|NW_003383851.1|	450059-45073!	379287	103553	-187292	0.4074	no
gi|320446720|ref|NW_003383851.1|	450965-45359!	190092	306933	0.691226	0.60155	no
gi|320446720|ref|NW_003383851.1|	453999-45446!	423975	753934	0.83046	0.6789	no
gi|320446720|ref|NW_003383851.1|	463561-46437!	263453	844778	168102	0.43185	no
gi|320446720|ref|NW_003383851.1|	465559-46684!	16032	166879	0.0578437	0.9604	no
gi|320446720|ref|NW_003383851.1|	470774-47100(	270175	368048	0.445996	0.8236	no
gi|320446720|ref|NW_003383851.1|	476771-47846(	638472	107497	-257033	0.17615	no
gi|320446720|ref|NW_003383851.1|	479094-47952!	166246	550574	-159431	0.4527	no
gi|320446720|ref|NW_003383851.1|	480152-48073!	146622	231482	0.658798	0.7397	no
gi|320446722|ref|NW_003383849.1|	123734-12404!	177082	125275	282261	0.2595	no
gi|320446722|ref|NW_003383849.1|	13200-15341	262985	358317	-287567	0.12605	no
gi|320446722|ref|NW_003383849.1|	158826-16181!	280847	201492	-0.479058	0.71545	no
gi|320446722|ref|NW_003383849.1|	162412-16398!	307358	195435	-0.653236	0.6059	no
gi|320446722|ref|NW_003383849.1|	16868-17139	185696	207657	-316067	0.1997	no
gi|320446722|ref|NW_003383849.1|	17280-17563	443883	850477	-238383	0.3041	no
gi|320446722|ref|NW_003383849.1|	181206-18413(	403529	197856	-102822	0.4501	no
gi|320446722|ref|NW_003383849.1|	185651-18975!	820577	72418	-0.18029	0.88755	no
gi|320446722|ref|NW_003383849.1|	189994-19078!	140504	985487	-0.511703	0.8042	no
gi|320446722|ref|NW_003383849.1|	190941-19191!	148525	646811	-119929	0.5719	no
gi|320446722|ref|NW_003383849.1|	192093-19425!	7258	231858	-164633	0.4519	no
gi|320446722|ref|NW_003383849.1|	197743-19842!	113786	100964	314944	0.21015	no
gi|320446722|ref|NW_003383849.1|	201717-20264!	0.733162	219086	157929	0.491	no
gi|320446722|ref|NW_003383849.1|	202815-20404!	0.337984	515654	393138	0.1711	no
gi|320446722|ref|NW_003383849.1|	204186-20523!	103715	120487	353818	0.1784	no
gi|320446722|ref|NW_003383849.1|	213455-21572(	148815	893625	-0.735776	0.6604	no
gi|320446722|ref|NW_003383849.1|	217092-21904!	157002	0.889364	-0.819937	0.6872	no

gi|320446722|ref|NW_003383849.1|	219242-22366	628094	504662	-0.315662	0.79925	no
gi|320446722|ref|NW_003383849.1|	22618-22999	965609	210945	-219457	0.27455	no
gi|320446722|ref|NW_003383849.1|	233293-23390	0	393282	inf	0.02915	no
gi|320446722|ref|NW_003383849.1|	244170-24670	366968	16321	-116892	0.37965	no
gi|320446722|ref|NW_003383849.1|	246823-24758	116488	953853	-0.288337	0.8883	no
gi|320446722|ref|NW_003383849.1|	250091-25187	298174	271396	-0.135755	0.91615	no
gi|320446722|ref|NW_003383849.1|	283822-28553	45109	850894	-240636	0.2022	no
gi|320446722|ref|NW_003383849.1|	2843-5099	334028	617815	0.887205	0.6763	no
gi|320446722|ref|NW_003383849.1|	286552-28710	247211	816282	-15986	0.4481	no
gi|320446722|ref|NW_003383849.1|	289541-29148	122675	758671	-0.693301	0.66675	no
gi|320446722|ref|NW_003383849.1|	292885-29504	227546	190028	-0.259944	0.8412	no
gi|320446722|ref|NW_003383849.1|	33056-33920	322202	129377	-131638	0.54	no
gi|320446722|ref|NW_003383849.1|	34029-37270	108346	467228	-121345	0.47175	no
gi|320446722|ref|NW_003383849.1|	43645-47456	415254	105022	133862	0.4287	no
gi|320446722|ref|NW_003383849.1|	565-1308	731448	751654	0.0393131	0.98055	no
gi|320446722|ref|NW_003383849.1|	58554-59124	336033	644146	-238314	0.2958	no
gi|320446722|ref|NW_003383849.1|	60879-62004	54445	115591	-223577	0.2169	no
gi|320446722|ref|NW_003383849.1|	64467-65649	729156	189028	-194763	0.28605	no
gi|320446722|ref|NW_003383849.1|	65772-68718	209153	905137	-120835	0.35055	no
gi|320446722|ref|NW_003383849.1|	69667-73352	336023	210842	-0.672399	0.6117	no
gi|320446722|ref|NW_003383849.1|	7952-10926	153412	168847	0.138305	0.9143	no
gi|320446724|ref|NW_003383847.1|	248041-24976	0.227476	388099	409264	0.1683	no
gi|320446724|ref|NW_003383847.1|	266158-26720	187609	792742	207912	0.34205	no
gi|320446724|ref|NW_003383847.1|	270660-27150	809575	576036	283092	0.12795	no
gi|320446724|ref|NW_003383847.1|	423003-42345	0.751349	849599	349923	0.2178	no
gi|320446725|ref|NW_003383846.1|	107803-11068	114583	418268	-145389	0.3931	no
gi|320446725|ref|NW_003383846.1|	111165-11264	182597	874545	-106206	0.50475	no
gi|320446725|ref|NW_003383846.1|	118064-11895	282752	1948	-0.537542	0.7928	no
gi|320446725|ref|NW_003383846.1|	119502-12093	0.987163	156805	0.667615	0.7385	no
gi|320446725|ref|NW_003383846.1|	121051-12230	132538	126413	-0.068266	1	no

gi|320446725|ref|NW_003383846.1|	24428-12535	122371	135014	0.141851	1	no
gi|320446725|ref|NW_003383846.1|	25690-12602	126693	134173	340469	0.17295	no
gi|320446725|ref|NW_003383846.1|	35118-13578	158921	0	#NAME?	5.00E-05	yes
gi|320446725|ref|NW_003383846.1|	37016-13726	843381	0	#NAME?	0.0091	no
gi|320446725|ref|NW_003383846.1|	37469-13815	140985	0.253406	-911988	0.2504	no
gi|320446725|ref|NW_003383846.1|	38562-14012	113122	0.794035	-715447	0.0625	no
gi|320446725|ref|NW_003383846.1|	41428-14256	167519	499964	-174443	0.20165	no
gi|320446725|ref|NW_003383846.1|	45002-14564	843165	334776	-133262	0.4315	no
gi|320446725|ref|NW_003383846.1|	46071-14837	138735	119632	-0.213726	1	no
gi|320446725|ref|NW_003383846.1|	49997-15520	116112	168399	0.536367	0.68955	no
gi|320446725|ref|NW_003383846.1|	55493-15635	352984	280297	-0.332645	0.86005	no
gi|320446725|ref|NW_003383846.1|	56598-15741	131499	104414	-0.332728	0.8767	no
gi|320446725|ref|NW_003383846.1|	70663-17162	233313	22324	-0.0636717	0.97825	no
gi|320446725|ref|NW_003383846.1|	71823-17286	946231	726863	-0.380508	0.8573	no
gi|320446725|ref|NW_003383846.1|	74875-17542	415093	232578	-0.835722	0.7023	no
gi|320446725|ref|NW_003383846.1|	76871-17892	178974	1811	-330489	0.0921	no
gi|320446725|ref|NW_003383846.1|	79642-18005	919229	788198	-0.221866	0.91255	no
gi|320446725|ref|NW_003383846.1|	83543-18424	753199	169093	-215521	0.2271	no
gi|320446725|ref|NW_003383846.1|	84345-18999	125173	173678	0.472494	0.7248	no
gi|320446725|ref|NW_003383846.1|	99233-19973	0.633085	110258	412234	0.1927	no
gi|320446725|ref|NW_003383846.1|	99975-20123	0.825124	480803	254276	0.2786	no
gi|320446725|ref|NW_003383846.1|	15838-22261	402107	178706	215193	0.1145	no
gi|320446725|ref|NW_003383846.1|	33647-23389	350974	266072	292238	0.2577	no
gi|320446725|ref|NW_003383846.1|	42693-24317	464916	124326	141909	0.498	no
gi|320446725|ref|NW_003383846.1|	26062-29798	0.771185	266473	178884	0.40645	no
gi|320446725|ref|NW_003383846.1|	65183-26551	222012	574735	-194967	0.3873	no
gi|320446725|ref|NW_003383846.1|	67406-26850	156333	0.94676	-0.723555	0.7278	no
gi|320446725|ref|NW_003383846.1|	74519-27514	17285	41115	-207178	0.34395	no
gi|320446725|ref|NW_003383846.1|	75835-27836	808878	118134	-27755	0.24175	no
gi|320446725|ref|NW_003383846.1|	82709-28528	12343	277325	-215405	0.2135	no

gi|320446725|ref|NW_003383846.1|	286615-288054	162635	652843	-131683	0.55775	no
gi|320446725|ref|NW_003383846.1|	288309-289122	780225	481912	-0.695119	0.5891	no
gi|320446725|ref|NW_003383846.1|	293184-293531	0	860071	inf	0.0312	no
gi|320446725|ref|NW_003383846.1|	294434-295084	0.406427	525903	369373	0.2145	no
gi|320446725|ref|NW_003383846.1|	29942-30307	943317	846815	-0.155696	0.93115	no
gi|320446725|ref|NW_003383846.1|	31001-33993	165264	552034	173998	0.4322	no
gi|320446725|ref|NW_003383846.1|	315855-316420	100017	709963	282749	0.22575	no
gi|320446725|ref|NW_003383846.1|	322829-324343	673085	307461	219154	0.2173	no
gi|320446725|ref|NW_003383846.1|	352685-353594	0	569921	inf	0.00945	no
gi|320446725|ref|NW_003383846.1|	354492-355145	0	142944	inf	0.0079	no
gi|320446725|ref|NW_003383846.1|	369340-370127	0	231054	inf	0.0294	no
gi|320446725|ref|NW_003383846.1|	39119-46723	675922	127152	-24103	0.0749	no
gi|320446725|ref|NW_003383846.1|	48674-49209	0.55816	150303	475105	0.17525	no
gi|320446725|ref|NW_003383846.1|	52468-54799	0.0805181	191117	4569	0.1774	no
gi|320446725|ref|NW_003383846.1|	548228-551597	0.4846	312623	268956	0.25865	no
gi|320446725|ref|NW_003383846.1|	55579-57676	0.0906404	113813	365036	1	no
gi|320446725|ref|NW_003383846.1|	58316-60163	0.104703	408586	528626	0.16715	no
gi|320446725|ref|NW_003383846.1|	60286-63717	311518	901276	153265	0.36025	no
gi|320446725|ref|NW_003383846.1|	63877-64995	108989	714856	-0.608458	0.7721	no
gi|320446725|ref|NW_003383846.1|	69947-71932	377094	377261	0.000638551	0.99825	no
gi|320446725|ref|NW_003383846.1|	72072-73515	0.279477	0.874079	164503	1	no
gi|320446725|ref|NW_003383846.1|	74949-77881	957338	599253	-0.675864	0.6722	no
gi|320446725|ref|NW_003383846.1|	82553-86281	0.338166	371885	345905	0.16925	no
gi|320446725|ref|NW_003383846.1|	90838-93978	0.17427	194987	348398	0.1947	no
gi|320446725|ref|NW_003383846.1|	98164-98729	234112	446986	-23889	0.2941	no
gi|320446726|ref|NW_003383845.1|	106017-108089	0.734995	333237	218074	0.327	no
gi|320446726|ref|NW_003383845.1|	109912-110872	13975	594793	-123239	0.5609	no
gi|320446726|ref|NW_003383845.1|	111488-111918	342375	105071	-170421	0.42745	no
gi|320446726|ref|NW_003383845.1|	113101-114198	115564	718473	-0.685681	0.7406	no
gi|320446726|ref|NW_003383845.1|	135125-137574	105953	160759	0.601469	0.63355	no

gi|320446726|ref|NW_003383845.1|	21831-22994	0.363861	34032	322543	0.20625	no
gi|320446726|ref|NW_003383845.1|	226868-22883	5076	387936	-0.387871	0.8539	no
gi|320446726|ref|NW_003383845.1|	24976-32851	412986	61944	0.584872	0.65075	no
gi|320446726|ref|NW_003383845.1|	274278-27544	234487	26246	0.162592	0.9314	no
gi|320446726|ref|NW_003383845.1|	275801-27910	445815	139746	164829	0.34995	no
gi|320446726|ref|NW_003383845.1|	300741-30169	0.234862	259318	346484	0.21395	no
gi|320446726|ref|NW_003383845.1|	301786-30399	0.171426	12561	287329	1	no
gi|320446726|ref|NW_003383845.1|	304353-31597	493456	361384	287254	0.04195	no
gi|320446726|ref|NW_003383845.1|	316760-32998	60064	159744	141119	0.46125	no
gi|320446726|ref|NW_003383845.1|	339786-34013	156697	679287	-120589	0.56405	no
gi|320446726|ref|NW_003383845.1|	344032-34492	178843	299266	0.742733	0.70945	no
gi|320446726|ref|NW_003383845.1|	34684-35114	334024	226733	-0.558961	0.78595	no
gi|320446726|ref|NW_003383845.1|	348020-34944	0.707482	0.688301	-0.0396546	1	no
gi|320446726|ref|NW_003383845.1|	350443-35156	114079	0.921518	-0.307947	1	no
gi|320446726|ref|NW_003383845.1|	351674-35439	121993	142079	0.219891	0.9178	no
gi|320446726|ref|NW_003383845.1|	374521-37499	139655	885153	266406	0.24485	no
gi|320446726|ref|NW_003383845.1|	37658-37954	294682	125925	-12266	0.55545	no
gi|320446726|ref|NW_003383845.1|	6719-8162	0.558954	165104	156257	0.4962	no
gi|320446726|ref|NW_003383845.1|	8505-10301	0.648752	436898	275156	0.239	no
gi|320446726|ref|NW_003383845.1|	87150-91906	193707	190568	-0.0235701	0.985	no
gi|320446726|ref|NW_003383845.1|	93465-98412	339837	193148	-0.815139	0.54345	no
gi|320446727|ref|NW_003383844.1|	10653-11757	18652	363107	-236086	0.3074	no
gi|320446727|ref|NW_003383844.1|	109798-11367	285103	42538	0.577269	0.6675	no
gi|320446727|ref|NW_003383844.1|	116875-11953	578562	14416	131713	0.43995	no
gi|320446727|ref|NW_003383844.1|	119703-12192	450844	563966	0.322979	0.88105	no
gi|320446727|ref|NW_003383844.1|	12117-13282	174277	276709	-265494	0.2617	no
gi|320446727|ref|NW_003383844.1|	122051-12315	503997	375703	-0.423825	0.8296	no
gi|320446727|ref|NW_003383844.1|	123316-12508	253398	260922	0.042216	0.97745	no
gi|320446727|ref|NW_003383844.1|	126293-12747	177714	0.492649	-185093	0.4081	no
gi|320446727|ref|NW_003383844.1|	138599-13940	16.98	141181	-0.266287	0.90015	no

gi|320446727|ref|NW_003383844.1|	140168-140914	158654	996483	-0.670966	0.7494	no
gi|320446727|ref|NW_003383844.1|	141102-143787	546915	650706	0.250689	0.84485	no
gi|320446727|ref|NW_003383844.1|	156020-156412	121132	530573	-119096	0.5669	no
gi|320446727|ref|NW_003383844.1|	157269-159325	553311	747277	0.433554	0.74825	no
gi|320446727|ref|NW_003383844.1|	159408-160530	298843	263584	-0.181127	0.88285	no
gi|320446727|ref|NW_003383844.1|	160661-161839	392085	529757	0.434164	0.7337	no
gi|320446727|ref|NW_003383844.1|	18153-23009	133646	29976	116539	0.3847	no
gi|320446727|ref|NW_003383844.1|	23203-26066	484176	578109	0.255809	0.851	no
gi|320446727|ref|NW_003383844.1|	238581-239862	0.322788	111989	179469	1	no
gi|320446727|ref|NW_003383844.1|	240130-245030	224259	366361	403003	0.06005	no
gi|320446727|ref|NW_003383844.1|	246107-247105	132784	117713	-0.173804	0.93665	no
gi|320446727|ref|NW_003383844.1|	248276-249018	832827	84438	0.0198758	0.98375	no
gi|320446727|ref|NW_003383844.1|	249534-251405	588049	589575	0.00373794	0.9929	no
gi|320446727|ref|NW_003383844.1|	278755-281285	0	554715	inf	0.0057	no
gi|320446727|ref|NW_003383844.1|	284380-284930	0	933673	inf	0.0142	no
gi|320446727|ref|NW_003383844.1|	304208-310499	0	730362	inf	5.00E-05	yes
gi|320446727|ref|NW_003383844.1|	312052-313678	0	0.844535	inf	1	no
gi|320446727|ref|NW_003383844.1|	355346-356818	201962	25329	0.326707	0.79565	no
gi|320446727|ref|NW_003383844.1|	36362-37443	209557	455692	-220121	0.3281	no
gi|320446727|ref|NW_003383844.1|	379976-381931	0	259968	inf	0.0113	no
gi|320446727|ref|NW_003383844.1|	38583-39719	110558	129771	-309076	0.20195	no
gi|320446727|ref|NW_003383844.1|	39954-43578	166804	153303	-34437	0.0814	no
gi|320446727|ref|NW_003383844.1|	400319-400783	0	480372	inf	0.0312	no
gi|320446727|ref|NW_003383844.1|	401681-402841	0	177027	inf	0.0233	no
gi|320446727|ref|NW_003383844.1|	45905-46421	202089	546833	-188582	0.2949	no
gi|320446727|ref|NW_003383844.1|	46982-49094	174886	681844	-13589	0.30935	no
gi|320446727|ref|NW_003383844.1|	51951-54806	300797	313794	0.0610279	0.9647	no
gi|320446727|ref|NW_003383844.1|	54998-56456	285709	116088	-129933	0.4422	no
gi|320446727|ref|NW_003383844.1|	57179-57803	476542	206078	-120941	0.56585	no
gi|320446727|ref|NW_003383844.1|	59496-61474	0.387324	114777	156723	1	no

gi|320446727|ref|NW_003383844.1|	61770-62491	28493	903544	-165694	0.44665	no
gi|320446727|ref|NW_003383844.1|	67237-70049	942991	19955	108143	0.40465	no
gi|320446727|ref|NW_003383844.1|	70449-71635	163325	205468	0.331164	0.882	no
gi|320446727|ref|NW_003383844.1|	71769-73491	251989	13912	-0.857031	0.49605	no
gi|320446727|ref|NW_003383844.1|	75590-76645	156492	119618	-0.387651	0.857	no
gi|320446727|ref|NW_003383844.1|	85043-85549	0.615303	660207	342355	0.21675	no
gi|320446727|ref|NW_003383844.1|	95541-96048	209696	195751	-0.0992818	0.9379	no
gi|320446727|ref|NW_003383844.1|	97055-97551	382611	896751	122883	0.5545	no
gi|320446728|ref|NW_003383843.1|	148876-14958[illegible]	319268	145565	-11331	0.5884	no
gi|320446728|ref|NW_003383843.1|	221099-22158[illegible]	234675	348909	-274974	0.24095	no
gi|320446728|ref|NW_003383843.1|	222634-22485[illegible]	128125	438202	-154788	0.3518	no
gi|320446728|ref|NW_003383843.1|	247429-25118[illegible]	119984	594496	230883	0.18515	no
gi|320446728|ref|NW_003383843.1|	255713-25639[illegible]	419083	0.52001	-301063	0.25735	no
gi|320446728|ref|NW_003383843.1|	259235-25971[illegible]	67432	0	#NAME?	0.0229	no
gi|320446728|ref|NW_003383843.1|	267366-26811[illegible]	496764	0.453811	-34524	0.23645	no
gi|320446728|ref|NW_003383843.1|	273585-27622[illegible]	287474	264683	-0.119164	0.9275	no
gi|320446728|ref|NW_003383843.1|	288905-29306[illegible]	280232	269004	-0.0589958	0.97615	no
gi|320446728|ref|NW_003383843.1|	295827-29664[illegible]	442127	259429	-0.76912	0.5605	no
gi|320446728|ref|NW_003383843.1|	299784-30026[illegible]	113765	447254	-134689	0.5183	no
gi|320446728|ref|NW_003383843.1|	300385-30262[illegible]	218524	586595	-189736	0.2753	no
gi|320446728|ref|NW_003383843.1|	303372-30429[illegible]	332367	758376	-213179	0.20145	no
gi|320446729|ref|NW_003383842.1|	112612-11443[illegible]	114789	466554	-129887	0.56235	no
gi|320446729|ref|NW_003383842.1|	127028-12889[illegible]	0.207093	577297	480096	0.15285	no
gi|320446729|ref|NW_003383842.1|	133373-13376[illegible]	100323	764068	-0.392877	0.86355	no
gi|320446729|ref|NW_003383842.1|	138909-13949[illegible]	312793	163725	-0.933932	0.6614	no
gi|320446729|ref|NW_003383842.1|	139625-14070[illegible]	162427	985019	-0.721564	0.74945	no
gi|320446729|ref|NW_003383842.1|	141369-14853[illegible]	148829	223541	0.586882	0.659	no
gi|320446729|ref|NW_003383842.1|	149015-15063[illegible]	218895	236976	0.114504	0.9537	no
gi|320446729|ref|NW_003383842.1|	158486-16572[illegible]	491164	100775	103685	0.43225	no
gi|320446729|ref|NW_003383842.1|	183219-18434[illegible]	135823	457204	-157082	0.47625	no

gi|320446729|ref|NW_003383842.1|	186214-18721	188034	314011	0.73982	0.57545	no
gi|320446729|ref|NW_003383842.1|	193713-19564	258131	768251	157347	0.4705	no
gi|320446729|ref|NW_003383842.1|	198350-20008	812886	228801	149297	0.3825	no
gi|320446729|ref|NW_003383842.1|	201005-20445	414661	114315	146301	0.26775	no
gi|320446729|ref|NW_003383842.1|	225226-22683	0	102587	inf	1	no
gi|320446729|ref|NW_003383842.1|	233914-23543	0.131668	210603	399955	0.1967	no
gi|320446729|ref|NW_003383842.1|	242107-24532	635855	132976	106439	0.405	no
gi|320446729|ref|NW_003383842.1|	245448-24662	247593	328219	0.406687	0.7411	no
gi|320446729|ref|NW_003383842.1|	247857-24833	128923	138938	0.107936	0.93165	no
gi|320446729|ref|NW_003383842.1|	248454-25023	395282	177507	-115501	0.38355	no
gi|320446729|ref|NW_003383842.1|	251870-25329	397272	412638	0.0547506	0.96525	no
gi|320446729|ref|NW_003383842.1|	253409-25367	504759	605254	0.261944	0.89815	no
gi|320446729|ref|NW_003383842.1|	254500-25693	115991	960174	-0.272648	0.8273	no
gi|320446729|ref|NW_003383842.1|	256994-26000	0.0608558	123388	434166	1	no
gi|320446729|ref|NW_003383842.1|	261252-26149	175487	99777	250734	0.2728	no
gi|320446729|ref|NW_003383842.1|	262166-26395	0.43473	219565	233646	0.2995	no
gi|320446729|ref|NW_003383842.1|	264410-26588	258231	129447	232563	0.30245	no
gi|320446729|ref|NW_003383842.1|	266538-27156	65563	370876	249998	0.073	no
gi|320446729|ref|NW_003383842.1|	283327-28381	151.05	571035	-47253	0.0622	no
gi|320446729|ref|NW_003383842.1|	287069-29079	184701	639513	-153014	0.23985	no
gi|320446729|ref|NW_003383842.1|	290915-29163	416971	427994	-328428	0.17255	no
gi|320446729|ref|NW_003383842.1|	292321-29397	530213	660563	-30048	0.1211	no
gi|320446729|ref|NW_003383842.1|	297612-30271	144225	145238	0.0100973	0.9937	no
gi|320446729|ref|NW_003383842.1|	317913-31853	108856	147921	-287952	0.2407	no
gi|320446729|ref|NW_003383842.1|	319369-32264	13882	442755	-164863	0.34045	no
gi|320446729|ref|NW_003383842.1|	323777-32583	795753	432403	-0.879944	0.68585	no
gi|320446729|ref|NW_003383842.1|	326887-32752	828638	986944	0.252226	0.9041	no
gi|320446729|ref|NW_003383842.1|	327683-33044	115761	165536	0.515996	0.6903	no
gi|320446729|ref|NW_003383842.1|	67228-68004	187332	214222	0.193511	0.9123	no
gi|320446729|ref|NW_003383842.1|	68181-68528	291137	860071	-175917	0.4151	no

gi|320446729|ref|NW_003383842.1|	91646-92288	455751	0	#NAME?	0.02105	no
gi|320446729|ref|NW_003383842.1|	93266-94146	115207	0.180325	-599749	0.2675	no
gi|320446730|ref|NW_003383841.1|	102905-10338	302289	345643	0.193352	0.9292	no
gi|320446730|ref|NW_003383841.1|	103629-10524	346366	217285	-0.672711	0.60095	no
gi|320446730|ref|NW_003383841.1|	106952-10724	136292	37451	-186363	0.3124	no
gi|320446730|ref|NW_003383841.1|	108190-10921	423191	255772	-0.726449	0.65795	no
gi|320446730|ref|NW_003383841.1|	110267-11110	261549	103352	-133952	0.54265	no
gi|320446730|ref|NW_003383841.1|	118193-11911	0	371289	inf	0.0186	no
gi|320446730|ref|NW_003383841.1|	119464-12179	0	140461	inf	1	no
gi|320446730|ref|NW_003383841.1|	121846-12300	0.367027	635631	411423	0.165	no
gi|320446730|ref|NW_003383841.1|	126443-13364	0.823543	711536	311102	0.1045	no
gi|320446730|ref|NW_003383841.1|	133773-13487	0.583547	0.942458	0.69158	1	no
gi|320446730|ref|NW_003383841.1|	142674-14343	0.6386	262758	204075	0.30255	no
gi|320446730|ref|NW_003383841.1|	159775-16037	976395	129218	-291765	0.112	no
gi|320446730|ref|NW_003383841.1|	160534-16205	171169	457833	-190253	0.3979	no
gi|320446730|ref|NW_003383841.1|	166228-17161	151194	972196	-0.637081	0.63385	no
gi|320446730|ref|NW_003383841.1|	16915-20764	30443	471567	0.631351	0.63625	no
gi|320446730|ref|NW_003383841.1|	174883-17541	569125	191424	-157197	0.49165	no
gi|320446730|ref|NW_003383841.1|	184481-18503	297996	13408	-115221	0.58755	no
gi|320446730|ref|NW_003383841.1|	185725-18636	714744	143003	-232138	0.32125	no
gi|320446730|ref|NW_003383841.1|	186641-18821	118232	875094	-0.434111	0.8459	no
gi|320446730|ref|NW_003383841.1|	189304-19066	164913	104117	-0.663497	0.7669	no
gi|320446730|ref|NW_003383841.1|	190867-19328	618465	647798	0.0668499	0.97405	no
gi|320446730|ref|NW_003383841.1|	195360-19643	234895	172233	-0.447655	0.84535	no
gi|320446730|ref|NW_003383841.1|	196591-19731	73468	881524	-305904	0.10145	no
gi|320446730|ref|NW_003383841.1|	2-635	211782	374477	0.822298	0.6933	no
gi|320446730|ref|NW_003383841.1|	20894-22988	173403	256489	0.564771	0.6635	no
gi|320446730|ref|NW_003383841.1|	221086-22270	767239	686519	-0.160375	0.93895	no
gi|320446730|ref|NW_003383841.1|	223509-22621	539752	845021	0.64669	0.6837	no
gi|320446730|ref|NW_003383841.1|	234128-23839	200307	807055	-131147	0.424	no

gi|320446730|ref|NW_003383841.1|	238508-23947	191209	636116	-158779	0.4664	no
gi|320446730|ref|NW_003383841.1|	239775-24106	173923	321125	-243724	0.2852	no
gi|320446730|ref|NW_003383841.1|	29551-30913	808853	780192	-0.052048	0.98015	no
gi|320446730|ref|NW_003383841.1|	31014-33080	104158	417902	-131754	0.5568	no
gi|320446730|ref|NW_003383841.1|	35479-38186	293551	186041	-0.657991	0.74475	no
gi|320446730|ref|NW_003383841.1|	3595-5209	372103	494907	0.411455	0.76025	no
gi|320446730|ref|NW_003383841.1|	40161-41388	816889	247823	-172083	0.4244	no
gi|320446730|ref|NW_003383841.1|	43404-44083	760268	259442	-155109	0.48345	no
gi|320446730|ref|NW_003383841.1|	49649-53479	606916	325561	-0.898569	0.5045	no
gi|320446730|ref|NW_003383841.1|	53690-57342	111114	698052	-0.67064	0.59185	no
gi|320446730|ref|NW_003383841.1|	57686-59291	505148	149182	-175964	0.32665	no
gi|320446730|ref|NW_003383841.1|	59907-61343	0.983874	0.976803	-0.0104061	1	no
gi|320446730|ref|NW_003383841.1|	6033-6539	0.615303	412629	274548	0.2661	no
gi|320446730|ref|NW_003383841.1|	66836-67604	383455	934654	-203655	0.37445	no
gi|320446730|ref|NW_003383841.1|	68403-68757	113734	493672	-120404	0.57095	no
gi|320446730|ref|NW_003383841.1|	70734-73396	326809	13166	-131163	0.3227	no
gi|320446730|ref|NW_003383841.1|	78127-85034	270343	334186	0.305863	0.81795	no
gi|320446730|ref|NW_003383841.1|	8231-9319	532455	464332	-0.197503	0.8785	no
gi|320446730|ref|NW_003383841.1|	90267-90878	161263	577695	-148103	0.4905	no
gi|320446730|ref|NW_003383841.1|	95629-95958	285375	213738	-0.417013	0.82745	no
gi|320446731|ref|NW_003383840.1|	100243-10340	230771	330834	0.519648	0.80505	no
gi|320446731|ref|NW_003383840.1|	103898-10676	263379	233486	-0.173802	0.93315	no
gi|320446731|ref|NW_003383840.1|	107613-10860	355022	260559	-0.446299	0.82055	no
gi|320446731|ref|NW_003383840.1|	126996-12764	972497	555934	-0.806779	0.52105	no
gi|320446731|ref|NW_003383840.1|	13085-13330	580927	431995	-374927	0.2178	no
gi|320446731|ref|NW_003383840.1|	135764-13774	825968	723773	-0.190548	0.88805	no
gi|320446731|ref|NW_003383840.1|	141161-14199	123631	427974	-153045	0.24515	no
gi|320446731|ref|NW_003383840.1|	142320-14586	128462	631239	-102508	0.42525	no
gi|320446731|ref|NW_003383840.1|	14799-18441	201088	894178	-116919	0.37235	no
gi|320446731|ref|NW_003383840.1|	169075-17012	165132	34259	105287	0.60245	no

gi|320446731|ref|NW_003383840.1|	170862-17606	112355	130792	0.219215	0.86455	no
gi|320446731|ref|NW_003383840.1|	176184-17665	156079	614919	-134381	0.51865	no
gi|320446731|ref|NW_003383840.1|	177228-17897	195483	110476	-0.823315	0.6203	no
gi|320446731|ref|NW_003383840.1|	179985-18070	19309	528369	-186965	0.39195	no
gi|320446731|ref|NW_003383840.1|	180821-18189	145242	390746	-189416	0.39365	no
gi|320446731|ref|NW_003383840.1|	182358-18325	484697	175777	-146333	0.48775	no
gi|320446731|ref|NW_003383840.1|	18977-19832	0.817209	862297	339941	0.19145	no
gi|320446731|ref|NW_003383840.1|	191914-19341	668251	130103	-236073	0.29515	no
gi|320446731|ref|NW_003383840.1|	33710-36401	27966	230458	-0.279171	0.8325	no
gi|320446731|ref|NW_003383840.1|	59091-59560	0	989538	inf	0.01575	no
gi|320446731|ref|NW_003383840.1|	60409-62117	326575	877696	-189562	0.28315	no
gi|320446731|ref|NW_003383840.1|	67335-68755	113962	48503	208952	0.3431	no
gi|320446731|ref|NW_003383840.1|	6844-7353	791542	204247	-195436	0.38715	no
gi|320446731|ref|NW_003383840.1|	70145-80663	234964	247128	0.0728212	0.9531	no
gi|320446731|ref|NW_003383840.1|	87410-90265	0.966157	52224	243438	0.28775	no
gi|320446731|ref|NW_003383840.1|	95463-98099	407665	117364	152554	0.36875	no
gi|320446731|ref|NW_003383840.1|	98815-99749	410699	699855	0.768973	0.70905	no
gi|320446733|ref|NW_003383838.1|	136252-13798	0.2255	188452	3063	0.2113	no
gi|320446733|ref|NW_003383838.1|	199990-20020	467485	248642	241108	0.29015	no
gi|320446733|ref|NW_003383838.1|	233156-23337	0	374057	inf	0.0294	no
gi|320446733|ref|NW_003383838.1|	236725-23731	191152	550134	152506	0.4978	no
gi|320446733|ref|NW_003383838.1|	249879-25171	0.73796	39668	242636	0.2821	no
gi|320446733|ref|NW_003383838.1|	252521-25383	314368	185473	256068	0.2856	no
gi|320446733|ref|NW_003383838.1|	25974-27577	0.740323	231824	164681	0.43965	no
gi|320446733|ref|NW_003383838.1|	29842-30136	440089	153782	-151691	0.479	no
gi|320446733|ref|NW_003383838.1|	304778-30626	0.26965	778038	485068	0.1548	no
gi|320446733|ref|NW_003383838.1|	306420-30733	0.747233	961533	368571	0.1824	no
gi|320446733|ref|NW_003383838.1|	308965-31115	0.690617	283128	20355	0.3455	no
gi|320446733|ref|NW_003383838.1|	312723-31414	185032	662651	184048	0.38955	no
gi|320446733|ref|NW_003383838.1|	319219-32264	529655	0.666955	-298939	0.21205	no

gi|320446733|ref|NW_003383838.1|	325162-32568	935059	0.78544	-357348	0.2326	no
gi|320446733|ref|NW_003383838.1|	325826-32886	348812	83678	12624	0.44795	no
gi|320446733|ref|NW_003383838.1|	329480-32974	622108	37919	-0.714247	0.72565	no
gi|320446733|ref|NW_003383838.1|	329853-33093	720184	678214	-0.0866246	0.96215	no
gi|320446733|ref|NW_003383838.1|	331401-33178	10317	812687	-0.344258	0.86405	no
gi|320446733|ref|NW_003383838.1|	332174-33265	226853	119322	-0.926899	0.65635	no
gi|320446733|ref|NW_003383838.1|	333182-33341	314064	148482	-108077	0.59385	no
gi|320446733|ref|NW_003383838.1|	335369-33628	791208	340935	-121456	0.56115	no
gi|320446733|ref|NW_003383838.1|	337646-33825	112281	579155	-0.955098	0.645	no
gi|320446733|ref|NW_003383838.1|	339536-34142	141498	383142	-188483	0.2629	no
gi|320446733|ref|NW_003383838.1|	342252-34320	244938	406297	-259181	0.2558	no
gi|320446733|ref|NW_003383838.1|	344005-34541	273663	0.900652	-160336	0.43075	no
gi|320446733|ref|NW_003383838.1|	345945-34637	754331	114113	-272473	0.23625	no
gi|320446733|ref|NW_003383838.1|	349870-35298	28.34	82709	-177672	0.18315	no
gi|320446733|ref|NW_003383838.1|	353436-35472	197857	321125	-262325	0.1371	no
gi|320446733|ref|NW_003383838.1|	360176-36072	271228	219354	-0.306248	0.8817	no
gi|320446733|ref|NW_003383838.1|	360850-36143	190096	257508	0.437891	0.8224	no
gi|320446733|ref|NW_003383838.1|	368613-37031	0.230743	265094	352214	0.18305	no
gi|320446733|ref|NW_003383838.1|	371645-37326	522539	778081	0.574381	0.78575	no
gi|320446733|ref|NW_003383838.1|	373617-37435	210701	121417	-0.795226	0.7011	no
gi|320446733|ref|NW_003383838.1|	375389-37598	125536	100853	-0.315849	0.87935	no
gi|320446733|ref|NW_003383838.1|	377409-37763	614581	237575	-137122	0.51185	no
gi|320446733|ref|NW_003383838.1|	377988-37899	139984	483583	-153342	0.47275	no
gi|320446733|ref|NW_003383838.1|	394508-39515	578643	196927	-155501	0.48025	no
gi|320446733|ref|NW_003383838.1|	395606-39593	171517	705055	-128255	0.5413	no
gi|320446733|ref|NW_003383838.1|	397457-39790	544211	516388	-0.0757118	0.9446	no
gi|320446733|ref|NW_003383838.1|	398874-39914	322767	158087	-102977	0.60355	no
gi|320446733|ref|NW_003383838.1|	405479-40766	657432	887332	0.432632	0.843	no
gi|320446733|ref|NW_003383838.1|	407819-40888	1225	197701	0.690539	0.735	no
gi|320446733|ref|NW_003383838.1|	409226-40979	902776	440731	-103447	0.61455	no

gi|320446733|ref|NW_003383838.1|	412308-41335	231751	101594	-118976	0.59945	no
gi|320446733|ref|NW_003383838.1|	413870-41467	343793	174522	-0.978134	0.6699	no
gi|320446733|ref|NW_003383838.1|	426653-42747	103057	137757	-290325	0.22885	no
gi|320446733|ref|NW_003383838.1|	433699-43500	128155	307466	-205939	0.36135	no
gi|320446733|ref|NW_003383838.1|	435497-43613	300497	482768	-263795	0.25785	no
gi|320446733|ref|NW_003383838.1|	438725-43966	476355	246482	-0.950552	0.6493	no
gi|320446733|ref|NW_003383838.1|	439887-44148	237947	948593	-132678	0.42655	no
gi|320446733|ref|NW_003383838.1|	444591-44859	689742	415145	258949	0.12105	no
gi|320446733|ref|NW_003383838.1|	448704-44939	0.746916	433542	253715	0.25045	no
gi|320446733|ref|NW_003383838.1|	449497-45179	0.245063	193876	298391	0.2277	no
gi|320446733|ref|NW_003383838.1|	452165-45418	79941	319979	200097	0.27105	no
gi|320446733|ref|NW_003383838.1|	465503-46666	912604	746043	-0.290731	0.88855	no
gi|320446733|ref|NW_003383838.1|	466852-46873	327728	328346	0.00271953	0.98665	no
gi|320446733|ref|NW_003383838.1|	469774-47121	0	32155	inf	0.00945	no
gi|320446733|ref|NW_003383838.1|	474532-47477	166437	12631	-0.398	0.85515	no
gi|320446733|ref|NW_003383838.1|	475246-47598	0.999392	433601	211724	0.34675	no
gi|320446733|ref|NW_003383838.1|	483669-48477	23315	403452	0.79114	0.6971	no
gi|320446733|ref|NW_003383838.1|	484911-48556	179255	638089	-149018	0.4792	no
gi|320446733|ref|NW_003383838.1|	486405-48682	121953	34551	-181953	0.40625	no
gi|320446733|ref|NW_003383838.1|	488141-48895	108309	522958	-105038	0.61635	no
gi|320446733|ref|NW_003383838.1|	489645-49341	336821	249413	-0.433448	0.74745	no
gi|320446733|ref|NW_003383838.1|	502789-50318	284177	666671	-209174	0.345	no
gi|320446733|ref|NW_003383838.1|	506658-50749	588191	2119	-14729	0.49615	no
gi|320446733|ref|NW_003383838.1|	507941-51012	112629	399004	-14971	0.5096	no
gi|320446733|ref|NW_003383838.1|	510279-51071	224479	797735	-14926	0.4773	no
gi|320446733|ref|NW_003383838.1|	511667-51211	155489	929498	-0.742288	0.71	no
gi|320446733|ref|NW_003383838.1|	512351-51282	765089	464116	-0.721142	0.73035	no
gi|320446733|ref|NW_003383838.1|	518217-51879	726917	229598	-166268	0.43785	no
gi|320446733|ref|NW_003383838.1|	518929-52431	17417	190156	0.126692	0.9263	no
gi|320446733|ref|NW_003383838.1|	524576-52485	332912	652032	0.969803	0.6376	no

gi|320446733|ref|NW_003383838.1|	524967-52926:	286705	118759	205039	0.12805	no
gi|320446733|ref|NW_003383838.1|	5464-6930	150839	117247	-0.363453	0.8684	no
gi|320446733|ref|NW_003383838.1|	713692-71404:	835718	874582	0.0655773	0.97425	no
gi|320446733|ref|NW_003383838.1|	767577-76866(	173982	191538	0.138695	0.9526	no
gi|320446733|ref|NW_003383838.1|	304196-80496!	0.941883	258467	145636	0.5073	no
gi|320446733|ref|NW_003383838.1|	305478-80632	110027	113551	0.0454864	1	no
gi|320446733|ref|NW_003383838.1|	326962-82759(	300213	845485	149379	0.47765	no
gi|320446733|ref|NW_003383838.1|	327767-82801:	444238	604793	0.445107	0.8196	no
gi|320446733|ref|NW_003383838.1|	329036-83110:	0.826869	243519	155831	0.4665	no
gi|320446733|ref|NW_003383838.1|	331215-83504:	591755	151859	135966	0.28835	no
gi|320446733|ref|NW_003383838.1|	8361-10315	322042	263541	-0.28922	0.8212	no
gi|320446733|ref|NW_003383838.1|	353641-85482:	904824	482338	-422952	0.04635	no
gi|320446733|ref|NW_003383838.1|	354957-85587:	704047	831701	-308154	0.11015	no
gi|320446733|ref|NW_003383838.1|	358880-86113:	717431	13.38	-242276	0.07975	no
gi|320446733|ref|NW_003383838.1|	366097-86681:	667037	247373	189085	0.38845	no
gi|320446733|ref|NW_003383838.1|	368101-86996:	0.206839	108111	238594	1	no
gi|320446733|ref|NW_003383838.1|	370581-87207	307883	147992	226507	0.3304	no
gi|320446733|ref|NW_003383838.1|	373409-87406!	0.793935	459949	253438	0.25095	no
gi|320446734|ref|NW_003383837.1|	100062-10057(	957525	433917	218004	0.3256	no
gi|320446734|ref|NW_003383837.1|	101216-10152:	714275	321469	217013	0.3378	no
gi|320446734|ref|NW_003383837.1|	101659-10195:	573839	282233	229817	0.32575	no
gi|320446734|ref|NW_003383837.1|	108038-10884:	385223	142489	188709	0.3797	no
gi|320446734|ref|NW_003383837.1|	122838-12345!	261921	118628	-114268	0.58445	no
gi|320446734|ref|NW_003383837.1|	127882-12868:	418531	20532	-102746	0.60725	no
gi|320446734|ref|NW_003383837.1|	130773-13255:	72914	639661	-0.188889	0.88725	no
gi|320446734|ref|NW_003383837.1|	136619-13697!	212083	893937	-124638	0.55395	no
gi|320446734|ref|NW_003383837.1|	155094-15551(	156051	515901	-159685	0.4366	no
gi|320446734|ref|NW_003383837.1|	155720-15598-	569067	275464	-104674	0.6073	no
gi|320446734|ref|NW_003383837.1|	156110-15723:	209617	105551	-0.989817	0.65595	no
gi|320446734|ref|NW_003383837.1|	157490-16019	169987	624908	-144371	0.3927	no

gi|320446734|ref|NW_003383837.1|	160688-16089	349887	261853	-0.418132	0.8247	no
gi|320446734|ref|NW_003383837.1|	162686-16342	471953	484819	0.0388015	0.97215	no
gi|320446734|ref|NW_003383837.1|	163597-16411	108092	967444	-0.16001	0.9354	no
gi|320446734|ref|NW_003383837.1|	164292-16475	500614	476666	-0.0707184	0.9449	no
gi|320446734|ref|NW_003383837.1|	165036-16649	116842	148108	0.342089	0.8738	no
gi|320446734|ref|NW_003383837.1|	168478-17019	295778	601988	102522	0.4445	no
gi|320446734|ref|NW_003383837.1|	170854-17376	499125	702222	0.492528	0.82645	no
gi|320446734|ref|NW_003383837.1|	250316-25068	326158	0.760869	-542178	0.2771	no
gi|320446734|ref|NW_003383837.1|	254221-25503	582421	0	#NAME?	0.0132	no
gi|320446734|ref|NW_003383837.1|	258275-25869	421475	0	#NAME?	0.0059	no
gi|320446734|ref|NW_003383837.1|	320411-32101	104393	800619	-0.382833	0.8498	no
gi|320446734|ref|NW_003383837.1|	330836-33254	35426	294432	-0.266873	0.8945	no
gi|320446734|ref|NW_003383837.1|	332804-33349	260848	0.763448	-177261	0.3225	no
gi|320446734|ref|NW_003383837.1|	335988-33629	454018	315088	-0.526996	0.7946	no
gi|320446734|ref|NW_003383837.1|	336460-33667	562899	281973	-0.997316	0.66335	no
gi|320446734|ref|NW_003383837.1|	337002-33901	110112	124449	0.176579	0.91265	no
gi|320446734|ref|NW_003383837.1|	339276-34006	497328	674585	0.439803	0.73235	no
gi|320446734|ref|NW_003383837.1|	341196-34201	374693	0	#NAME?	0.01755	no
gi|320446734|ref|NW_003383837.1|	345529-34808	929788	0	#NAME?	5.00E-05	yes
gi|320446734|ref|NW_003383837.1|	41474-44051	194557	573843	156046	0.48005	no
gi|320446734|ref|NW_003383837.1|	51725-52747	171642	415193	127438	0.5407	no
gi|320446734|ref|NW_003383837.1|	6062-6579	123211	160332	0.379934	0.76505	no
gi|320446734|ref|NW_003383837.1|	83738-84118	323708	494901	0.612446	0.7426	no
gi|320446734|ref|NW_003383837.1|	93484-99789	225161	586452	138106	0.42645	no
gi|320446735|ref|NW_003383836.1|	13439-15852	24253	492368	-230036	0.2021	no
gi|320446735|ref|NW_003383836.1|	139890-14231	540662	466529	310917	0.1162	no
gi|320446735|ref|NW_003383836.1|	143850-14478	120449	980372	302491	0.21635	no
gi|320446735|ref|NW_003383836.1|	16064-17994	197157	416487	-2243	0.2056	no
gi|320446735|ref|NW_003383836.1|	166090-16636	760942	371253	228654	0.32635	no
gi|320446735|ref|NW_003383836.1|	175712-17630	233741	127678	577146	0.08545	no

gi|320446735|ref|NW_003383836.1|	176428-17809	16008	618992	527306	0.0173	no
gi|320446735|ref|NW_003383836.1|	18156-18949	290596	62352	-222051	0.3284	no
gi|320446735|ref|NW_003383836.1|	1882-2693	850381	116858	0.458577	0.8213	no
gi|320446735|ref|NW_003383836.1|	20944-22419	242312	89906	-143038	0.39935	no
gi|320446735|ref|NW_003383836.1|	22654-23207	474826	185092	-135916	0.5313	no
gi|320446735|ref|NW_003383836.1|	23880-25613	206195	118489	-0.799252	0.62365	no
gi|320446735|ref|NW_003383836.1|	25859-26899	167801	101512	-0.7251	0.7278	no
gi|320446735|ref|NW_003383836.1|	260487-26308	252639	267486	0.0823828	0.94685	no
gi|320446735|ref|NW_003383836.1|	265384-26642	25463	119778	-108804	0.63985	no
gi|320446735|ref|NW_003383836.1|	27048-27450	172336	107172	-0.685289	0.73655	no
gi|320446735|ref|NW_003383836.1|	27966-31448	445905	312692	-0.511993	0.6995	no
gi|320446735|ref|NW_003383836.1|	324439-32503	746748	294565	-134204	0.41245	no
gi|320446735|ref|NW_003383836.1|	385793-38645	0	294955	inf	0.0294	no
gi|320446735|ref|NW_003383836.1|	4292-5012	487465	952999	0.967174	0.6454	no
gi|320446735|ref|NW_003383836.1|	8775-9173	518141	115771	-216207	0.31925	no
gi|320446736|ref|NW_003383835.1|	108633-11102	200705	894286	-116627	0.49425	no
gi|320446736|ref|NW_003383835.1|	11612-11972	364889	951727	138309	0.51925	no
gi|320446736|ref|NW_003383835.1|	116583-11696	221526	172956	-0.35707	0.864	no
gi|320446736|ref|NW_003383835.1|	12176-13293	133998	251805	0.910094	0.6615	no
gi|320446736|ref|NW_003383835.1|	124613-12649	12107	686485	-0.818543	0.7149	no
gi|320446736|ref|NW_003383835.1|	126697-12825	154146	114322	-0.431189	0.8519	no
gi|320446736|ref|NW_003383835.1|	128379-12995	403998	439169	0.120427	0.95265	no
gi|320446736|ref|NW_003383835.1|	134271-13859	187398	142485	-0.395291	0.77315	no
gi|320446736|ref|NW_003383835.1|	139896-14064	317929	181524	-0.808541	0.7078	no
gi|320446736|ref|NW_003383835.1|	141555-14216	384234	178938	-110252	0.61645	no
gi|320446736|ref|NW_003383835.1|	142921-14372	761555	922109	0.275989	0.8315	no
gi|320446736|ref|NW_003383835.1|	143890-14525	810468	107186	0.40329	0.761	no
gi|320446736|ref|NW_003383835.1|	157654-15872	245851	10822	-118382	0.6042	no
gi|320446736|ref|NW_003383835.1|	159673-16149	713917	423178	-0.754491	0.7254	no
gi|320446736|ref|NW_003383835.1|	161828-16402	105211	130588	0.31173	0.84815	no

gi|320446736|ref|NW_003383835.1|	165004-16744	568934	430494	-0.402267	0.76075	no
gi|320446736|ref|NW_003383835.1|	168629-16954	384587	113885	-175573	0.2965	no
gi|320446736|ref|NW_003383835.1|	169699-17198	260844	121766	-109907	0.39475	no
gi|320446736|ref|NW_003383835.1|	172196-17256	126608	162642	-29606	0.22565	no
gi|320446736|ref|NW_003383835.1|	18183-18961	342177	341523	-0.00276357	0.96375	no
gi|320446736|ref|NW_003383835.1|	192759-19345	302656	129576	209805	0.2532	no
gi|320446736|ref|NW_003383835.1|	19569-19821	93709	158249	0.755941	0.7072	no
gi|320446736|ref|NW_003383835.1|	198089-19910	0	17861	inf	0.029	no
gi|320446736|ref|NW_003383835.1|	199370-20350	160129	618085	194857	0.25345	no
gi|320446736|ref|NW_003383835.1|	203624-20494	0.310035	215231	279538	0.2345	no
gi|320446736|ref|NW_003383835.1|	205070-20768	210772	113982	243505	0.07965	no
gi|320446736|ref|NW_003383835.1|	20959-21909	245624	241189	-0.0262861	0.9895	no
gi|320446736|ref|NW_003383835.1|	23004-25666	161319	156923	-0.0398569	0.9741	no
gi|320446736|ref|NW_003383835.1|	2345-3097	496585	203763	-128515	0.42965	no
gi|320446736|ref|NW_003383835.1|	239127-24099	968734	573757	-0.75566	0.5772	no
gi|320446736|ref|NW_003383835.1|	241910-24399	655428	311151	-107482	0.61815	no
gi|320446736|ref|NW_003383835.1|	244923-24821	198529	0.732881	-14377	0.501	no
gi|320446736|ref|NW_003383835.1|	251818-25317	0.755647	0.944522	0.321873	1	no
gi|320446736|ref|NW_003383835.1|	255184-25583	119818	486093	-130154	0.457	no
gi|320446736|ref|NW_003383835.1|	267429-27222	918099	186951	102594	0.4408	no
gi|320446736|ref|NW_003383835.1|	272724-27511	243109	449002	0.885122	0.67915	no
gi|320446736|ref|NW_003383835.1|	278445-27914	195648	156011	-0.326617	0.8748	no
gi|320446736|ref|NW_003383835.1|	28245-28706	109475	102068	-0.101075	0.9546	no
gi|320446736|ref|NW_003383835.1|	28858-29633	82266	512918	-0.681569	0.59515	no
gi|320446736|ref|NW_003383835.1|	30796-34438	346704	25543	-0.440776	0.74255	no
gi|320446736|ref|NW_003383835.1|	308160-30924	138557	630067	218503	0.31955	no
gi|320446736|ref|NW_003383835.1|	317470-31816	554101	143832	137617	0.51665	no
gi|320446736|ref|NW_003383835.1|	334806-33500	836939	62425	289893	0.2595	no
gi|320446736|ref|NW_003383835.1|	337223-33797	559714	133113	124989	0.54955	no
gi|320446736|ref|NW_003383835.1|	338260-33862	729777	341035	222439	0.31375	no

gi|320446736|ref|NW_003383835.1|	340342-34056	154375	516386	174201	0.415	no
gi|320446736|ref|NW_003383835.1|	342526-34380	144977	860685	256966	0.27295	no
gi|320446736|ref|NW_003383835.1|	343935-34712	371194	291545	297347	0.1263	no
gi|320446736|ref|NW_003383835.1|	35181-36662	251983	17142	-0.555793	0.66005	no
gi|320446736|ref|NW_003383835.1|	37003-37187	863462	734174	-0.23401	0.90415	no
gi|320446736|ref|NW_003383835.1|	3793-4322	569125	574272	0.0129883	0.9618	no
gi|320446736|ref|NW_003383835.1|	39068-39537	16959	895296	-0.921612	0.65485	no
gi|320446736|ref|NW_003383835.1|	420279-42056	954769	41174	-121342	0.5632	no
gi|320446736|ref|NW_003383835.1|	425859-42616	902202	0	#NAME?	0.0061	no
gi|320446736|ref|NW_003383835.1|	430803-43251	342832	397881	0.214835	0.91325	no
gi|320446736|ref|NW_003383835.1|	432638-43755	320118	365985	0.193181	0.88145	no
gi|320446736|ref|NW_003383835.1|	439438-44095	118317	319988	143536	0.494	no
gi|320446736|ref|NW_003383835.1|	446396-44707	807296	123272	0.61067	0.768	no
gi|320446736|ref|NW_003383835.1|	44723-48430	233288	925113	198752	0.2491	no
gi|320446736|ref|NW_003383835.1|	449443-45026	288225	1783	-0.692889	0.7311	no
gi|320446736|ref|NW_003383835.1|	450787-45113	132335	146212	34658	0.21815	no
gi|320446736|ref|NW_003383835.1|	452104-45298	523668	378682	-0.467667	0.8211	no
gi|320446736|ref|NW_003383835.1|	453553-45546	0.502617	196199	196479	0.38275	no
gi|320446736|ref|NW_003383835.1|	456434-45691	137487	413037	158698	0.33275	no
gi|320446736|ref|NW_003383835.1|	457330-45753	489841	174568	-148852	0.50605	no
gi|320446736|ref|NW_003383835.1|	460792-46183	48123	462921	-0.0559609	0.97355	no
gi|320446736|ref|NW_003383835.1|	469238-46954	147789	94915	-0.638828	0.74925	no
gi|320446736|ref|NW_003383835.1|	48537-50811	389017	10168	138614	0.3911	no
gi|320446736|ref|NW_003383835.1|	50954-51839	241631	449197	0.894545	0.5844	no
gi|320446736|ref|NW_003383835.1|	5110-6071	19538	947169	-104459	0.63145	no
gi|320446736|ref|NW_003383835.1|	528140-52836	197596	0	#NAME?	0.00645	no
gi|320446736|ref|NW_003383835.1|	529349-52961	108394	189381	0.805013	0.7139	no
gi|320446736|ref|NW_003383835.1|	53284-54556	144888	224769	0.633505	0.6944	no
gi|320446736|ref|NW_003383835.1|	540256-54525	606303	0.993481	-260947	0.14795	no
gi|320446736|ref|NW_003383835.1|	54759-55448	163603	137129	-0.254661	0.90015	no

gi|320446736|ref|NW_003383835.1|	56991-57998	481193	544031	0.177072	0.92885	no
gi|320446736|ref|NW_003383835.1|	58705-58953	262874	166293	-0.660647	0.74435	no
gi|320446736|ref|NW_003383835.1|	59635-65790	758155	133241	0.813469	0.5385	no
gi|320446736|ref|NW_003383835.1|	91589-92758	759195	0	#NAME?	0.0071	no
gi|320446736|ref|NW_003383835.1|	94686-97143	248359	0	#NAME?	5.00E-05	yes
gi|320446736|ref|NW_003383835.1|	9551-10157	295023	203234	-0.537685	0.80105	no
gi|320446738|ref|NW_003383833.1|	104830-105209	325566	639803	0.974678	0.6698	no
gi|320446738|ref|NW_003383833.1|	108376-109505	736532	588482	-0.323751	0.8681	no
gi|320446738|ref|NW_003383833.1|	112994-11338	622447	544665	-0.192582	0.9175	no
gi|320446738|ref|NW_003383833.1|	118214-11900	489595	189045	-137286	0.50605	no
gi|320446738|ref|NW_003383833.1|	120176-120577	105864	886994	-0.255214	0.89825	no
gi|320446738|ref|NW_003383833.1|	122289-12300	176266	192911	0.130182	0.9267	no
gi|320446738|ref|NW_003383833.1|	141345-141655	31351	212946	-0.558021	0.78485	no
gi|320446738|ref|NW_003383833.1|	143865-144909	160709	620535	-137286	0.53065	no
gi|320446738|ref|NW_003383833.1|	148888-149698	146873	141276	-0.0560508	0.9269	no
gi|320446738|ref|NW_003383833.1|	150771-151138	116485	684782	25555	0.27405	no
gi|320446738|ref|NW_003383833.1|	151254-153918	0.486336	262133	243027	0.2819	no
gi|320446738|ref|NW_003383833.1|	154051-154575	0	427856	inf	0.0294	no
gi|320446738|ref|NW_003383833.1|	156039-15665	489546	419326	-35453	0.1573	no
gi|320446738|ref|NW_003383833.1|	170151-17500	169395	117167	-385375	0.06295	no
gi|320446738|ref|NW_003383833.1|	181675-18483	17897	791313	214453	0.2159	no
gi|320446738|ref|NW_003383833.1|	185296-186158	861948	261418	160069	0.47115	no
gi|320446738|ref|NW_003383833.1|	198706-199418	0	314751	inf	0.02915	no
gi|320446738|ref|NW_003383833.1|	205654-206269	221658	602028	14415	0.50735	no
gi|320446738|ref|NW_003383833.1|	209261-211564	244826	996932	202574	0.23265	no
gi|320446738|ref|NW_003383833.1|	213407-214678	114067	587897	236568	0.2932	no
gi|320446738|ref|NW_003383833.1|	217962-219255	253704	627855	130729	0.3232	no
gi|320446738|ref|NW_003383833.1|	224376-224808	992914	198435	0.998923	0.5585	no
gi|320446738|ref|NW_003383833.1|	231977-232968	446715	0.154267	-485585	0.29035	no
gi|320446738|ref|NW_003383833.1|	233659-235877	14979	243518	-262084	0.1447	no

gi|320446738|ref|NW_003383833.1|	238974-239464	580168	113396	-235511	0.3057	no
gi|320446738|ref|NW_003383833.1|	243241-244049	185706	101256	-419694	0.1616	no
gi|320446738|ref|NW_003383833.1|	244583-246115	845942	0.905174	-322429	0.18885	no
gi|320446738|ref|NW_003383833.1|	250111-250874	319904	0.877476	-186621	0.4074	no
gi|320446738|ref|NW_003383833.1|	25154-25581	0.846759	61647	286401	0.25915	no
gi|320446738|ref|NW_003383833.1|	255291-25588	266464	728686	-187057	0.3957	no
gi|320446738|ref|NW_003383833.1|	256307-256734	931435	168128	-246989	0.24235	no
gi|320446738|ref|NW_003383833.1|	258012-26200	939208	251652	-190002	0.2716	no
gi|320446738|ref|NW_003383833.1|	262447-26338	118378	599876	-0.980666	0.64025	no
gi|320446738|ref|NW_003383833.1|	263965-26478	133957	120071	-0.157877	0.93935	no
gi|320446738|ref|NW_003383833.1|	264924-266515	12448	476377	193619	0.36895	no
gi|320446738|ref|NW_003383833.1|	267393-269638	0.923606	205105	115101	0.57645	no
gi|320446738|ref|NW_003383833.1|	28090-28906	0	219759	inf	0.0294	no
gi|320446738|ref|NW_003383833.1|	295233-295939	179244	367646	103639	0.60125	no
gi|320446738|ref|NW_003383833.1|	304797-305578	0.618722	10403	407156	0.16925	no
gi|320446738|ref|NW_003383833.1|	310504-31627	143227	108269	291825	0.1204	no
gi|320446738|ref|NW_003383833.1|	316330-317074	0.66365	227339	177635	0.333	no
gi|320446738|ref|NW_003383833.1|	325811-327728	208716	116812	-0.837353	0.6158	no
gi|320446738|ref|NW_003383833.1|	328986-329594	442462	407468	-0.118867	0.9554	no
gi|320446738|ref|NW_003383833.1|	329685-33137	174369	226618	0.37812	0.85035	no
gi|320446738|ref|NW_003383833.1|	332535-333423	150001	133617	-0.166874	0.9382	no
gi|320446738|ref|NW_003383833.1|	333615-33729	406321	664592	0.70985	0.6588	no
gi|320446738|ref|NW_003383833.1|	337536-341164	387424	333737	-0.215201	0.8724	no
gi|320446738|ref|NW_003383833.1|	350864-352859	0	180545	inf	0.0133	no
gi|320446738|ref|NW_003383833.1|	364325-365478	0	165619	inf	0.02915	no
gi|320446738|ref|NW_003383833.1|	36943-37827	338284	0	#NAME?	0.01755	no
gi|320446738|ref|NW_003383833.1|	40317-42186	449324	943026	-225239	0.2184	no
gi|320446738|ref|NW_003383833.1|	46067-46625	316991	73668	-210533	0.3424	no
gi|320446738|ref|NW_003383833.1|	49711-50751	295749	420549	-281403	0.1186	no
gi|320446738|ref|NW_003383833.1|	51028-51510	423205	538664	-29739	0.2193	no

gi|320446738|ref|NW_003383833.1|	54201-56465	790188	156751	-233372	0.3154	no
gi|320446738|ref|NW_003383833.1|	67805-69346	400667	800104	0.997783	0.64415	no
gi|320446738|ref|NW_003383833.1|	70215-70767	317519	285594	-0.152874	0.9226	no
gi|320446738|ref|NW_003383833.1|	72537-73052	357846	800915	116231	0.5814	no
gi|320446738|ref|NW_003383833.1|	74197-74546	652905	93402	0.51658	0.8037	no
gi|320446739|ref|NW_003383832.1|	101173-10315	511736	814625	0.670737	0.7543	no
gi|320446739|ref|NW_003383832.1|	104864-10695	143151	604224	-124439	0.45675	no
gi|320446739|ref|NW_003383832.1|	107050-10744	397979	134032	-157011	0.4633	no
gi|320446739|ref|NW_003383832.1|	107943-10912	31119	121457	-135735	0.42325	no
gi|320446739|ref|NW_003383832.1|	110167-11063	164487	476666	-178693	0.41895	no
gi|320446739|ref|NW_003383832.1|	110747-11177	230252	368403	-264386	0.25375	no
gi|320446739|ref|NW_003383832.1|	115445-11601	411264	440731	-322209	0.18925	no
gi|320446739|ref|NW_003383832.1|	125938-12920	154827	144516	-0.0994239	0.9358	no
gi|320446739|ref|NW_003383832.1|	134543-13620	220781	354474	0.683065	0.60375	no
gi|320446739|ref|NW_003383832.1|	138273-13949	158166	201445	0.348945	0.87775	no
gi|320446739|ref|NW_003383832.1|	140014-14052	549905	204931	-142404	0.5105	no
gi|320446739|ref|NW_003383832.1|	141331-14234	186172	137626	-0.435883	0.8441	no
gi|320446739|ref|NW_003383832.1|	146495-14791	127212	913722	-0.477409	0.82695	no
gi|320446739|ref|NW_003383832.1|	160876-16354	939395	566331	-405202	0.061	no
gi|320446739|ref|NW_003383832.1|	165474-16584	318407	520456	-261302	0.25765	no
gi|320446739|ref|NW_003383832.1|	167398-16762	115231	278736	-536949	0.27645	no
gi|320446739|ref|NW_003383832.1|	168328-16879	244138	813471	-158553	0.448	no
gi|320446739|ref|NW_003383832.1|	170225-17110	138988	505678	-145867	0.4919	no
gi|320446739|ref|NW_003383832.1|	180764-18123	0	163673	inf	0.0114	no
gi|320446739|ref|NW_003383832.1|	189004-18920	0	480063	inf	0.0294	no
gi|320446739|ref|NW_003383832.1|	207105-20823	0.376445	143376	19293	0.3182	no
gi|320446739|ref|NW_003383832.1|	208674-20943	0	35099	inf	0.0198	no
gi|320446739|ref|NW_003383832.1|	209631-21364	449355	848505	0.917069	0.4815	no
gi|320446739|ref|NW_003383832.1|	213759-21549	47355	76166	0.685631	0.7548	no
gi|320446739|ref|NW_003383832.1|	215639-21615	302104	46548	0.623677	0.77645	no

gi|320446739|ref|NW_003383832.1|	216946-21719	132843	136167	0.035661	0.9835	no
gi|320446739|ref|NW_003383832.1|	221278-22163	715684	73593	0.0402458	0.98395	no
gi|320446739|ref|NW_003383832.1|	227076-22781	410457	460839	-31549	0.1997	no
gi|320446739|ref|NW_003383832.1|	252943-25333	81887	118153	0.528951	0.73735	no
gi|320446739|ref|NW_003383832.1|	305122-30647	292144	928639	166844	0.21855	no
gi|320446739|ref|NW_003383832.1|	310666-31124	537613	165344	-17011	0.4287	no
gi|320446739|ref|NW_003383832.1|	311710-31212	981619	235731	-205802	0.36125	no
gi|320446739|ref|NW_003383832.1|	312415-31325	703732	425877	-0.72459	0.72285	no
gi|320446739|ref|NW_003383832.1|	313934-31446	106736	756184	-0.49724	0.80805	no
gi|320446739|ref|NW_003383832.1|	322948-32576	363022	614415	0.759156	0.57255	no
gi|320446739|ref|NW_003383832.1|	325906-32758	21084	173696	304234	0.10915	no
gi|320446739|ref|NW_003383832.1|	331503-33341	327902	638025	-236158	0.1929	no
gi|320446739|ref|NW_003383832.1|	335324-33792	135809	21971	0.694026	0.7361	no
gi|320446739|ref|NW_003383832.1|	338267-33890	21597	621822	-179626	0.4079	no
gi|320446739|ref|NW_003383832.1|	339303-34456	640853	404317	-0.664507	0.6925	no
gi|320446739|ref|NW_003383832.1|	345030-34802	575156	633113	0.138508	0.9502	no
gi|320446739|ref|NW_003383832.1|	360628-36096	138815	171103	362363	0.21615	no
gi|320446739|ref|NW_003383832.1|	37746-38172	255218	506649	0.989256	0.6698	no
gi|320446739|ref|NW_003383832.1|	413717-41461	0	476721	inf	0.0133	no
gi|320446739|ref|NW_003383832.1|	414714-41564	0	45054	inf	0.0133	no
gi|320446739|ref|NW_003383832.1|	42139-44307	296854	566568	0.932495	0.6536	no
gi|320446739|ref|NW_003383832.1|	51540-51983	551371	313738	-0.813462	0.69665	no
gi|320446739|ref|NW_003383832.1|	72181-72791	853339	762046	-0.163241	0.93405	no
gi|320446739|ref|NW_003383832.1|	80578-82314	179919	321088	0.83562	0.676	no
gi|320446739|ref|NW_003383832.1|	82495-90497	123825	345793	148161	0.3753	no
gi|320446739|ref|NW_003383832.1|	91668-92419	12766	181714	0.509362	0.8069	no
gi|320446739|ref|NW_003383832.1|	93924-97446	410702	113862	147113	0.38865	no
gi|320446740|ref|NW_003383831.1|	129268-13139	258278	559216	111448	0.604	no
gi|320446740|ref|NW_003383831.1|	184269-18498	193892	554618	-180569	0.4059	no
gi|320446740|ref|NW_003383831.1|	305552-30785	0.979777	609846	263792	0.2568	no

gi|320446740|ref|NW_003383831.1|	317055-31797	185712	121206	-0.615601	0.77985	no
gi|320446740|ref|NW_003383831.1|	320869-32136	513879	21501	-125702	0.55575	no
gi|320446740|ref|NW_003383831.1|	321520-32198	170268	141904	-358481	0.177	no
gi|320446740|ref|NW_003383831.1|	334771-33519	256419	14137	24629	0.2844	no
gi|320446740|ref|NW_003383831.1|	33597-35035	189431	84842	-115883	0.47305	no
gi|320446740|ref|NW_003383831.1|	340703-34195	222439	27174	0.288821	0.81545	no
gi|320446740|ref|NW_003383831.1|	389631-39184	255374	125764	-10219	0.42665	no
gi|320446740|ref|NW_003383831.1|	396378-39764	492186	261779	-0.910852	0.65955	no
gi|320446740|ref|NW_003383831.1|	397745-39908	182829	158685	-0.204329	0.9155	no
gi|320446740|ref|NW_003383831.1|	401684-40426	381257	165831	-120105	0.5633	no
gi|320446740|ref|NW_003383831.1|	405560-40596	790565	144282	-245399	0.2883	no
gi|320446740|ref|NW_003383831.1|	417170-41863	151454	315836	106029	0.5955	no
gi|320446740|ref|NW_003383831.1|	426024-42672	218787	174429	-0.326887	0.85735	no
gi|320446740|ref|NW_003383831.1|	42613-43878	172098	932405	-0.8842	0.69355	no
gi|320446740|ref|NW_003383831.1|	450798-45441	964406	898597	-0.101967	0.93385	no
gi|320446740|ref|NW_003383831.1|	454918-45552	761518	0.30405	-46465	0.2863	no
gi|320446740|ref|NW_003383831.1|	455704-45604	978503	362642	-143203	0.51425	no
gi|320446740|ref|NW_003383831.1|	456276-45723	222984	56112	-199056	0.37555	no
gi|320446740|ref|NW_003383831.1|	459649-46167	226417	217113	-0.0605334	0.97305	no
gi|320446740|ref|NW_003383831.1|	463363-46405	296164	278157	-0.0904958	0.94845	no
gi|320446740|ref|NW_003383831.1|	464244-46478	124376	153087	0.299646	0.88045	no
gi|320446740|ref|NW_003383831.1|	46429-47350	150164	33947	-214518	0.33745	no
gi|320446740|ref|NW_003383831.1|	465087-46714	526551	117267	115515	0.4731	no
gi|320446740|ref|NW_003383831.1|	490183-49047	340069	128151	-140798	0.5043	no
gi|320446740|ref|NW_003383831.1|	501660-50215	12847	602029	-109353	0.60115	no
gi|320446740|ref|NW_003383831.1|	502646-50416	144385	593349	-128296	0.5584	no
gi|320446740|ref|NW_003383831.1|	50299-51345	291477	734195	133278	0.51615	no
gi|320446740|ref|NW_003383831.1|	504431-50484	101623	609226	-0.738183	0.71335	no
gi|320446740|ref|NW_003383831.1|	504988-50523	166437	105259	-0.661034	0.74795	no
gi|320446740|ref|NW_003383831.1|	505354-50634	712856	50776	-0.489463	0.8088	no

gi|320446740|ref|NW_003383831.1|	506623-50808	133338	936425	-0.509847	0.8155	no
gi|320446740|ref|NW_003383831.1|	511900-51394	592157	348504	-0.764807	0.5667	no
gi|320446740|ref|NW_003383831.1|	526346-52649	424593	0	#NAME?	0.02105	no
gi|320446740|ref|NW_003383831.1|	538494-53965	236509	289902	-302826	0.2062	no
gi|320446740|ref|NW_003383831.1|	539858-54135	0.801901	0.557585	-0.524231	1	no
gi|320446740|ref|NW_003383831.1|	563294-56381	330894	74139	-215806	0.33995	no
gi|320446740|ref|NW_003383831.1|	564606-56666	913265	212329	-210473	0.1244	no
gi|320446740|ref|NW_003383831.1|	567990-57206	251243	284614	0.179919	0.89025	no
gi|320446740|ref|NW_003383831.1|	57059-59359	147109	193969	0.398944	0.8469	no
gi|320446740|ref|NW_003383831.1|	59525-60648	342236	197468	-0.793374	0.70115	no
gi|320446740|ref|NW_003383831.1|	98659-98975	348433	534721	-270402	0.26725	no
gi|320446741|ref|NW_003383830.1|	108628-10960	0.682726	141418	105059	0.5772	no
gi|320446741|ref|NW_003383830.1|	111292-11218	154464	407919	140101	0.50825	no
gi|320446741|ref|NW_003383830.1|	117661-11789	309549	365969	0.241555	0.89735	no
gi|320446741|ref|NW_003383830.1|	118276-11964	623572	639346	0.0360424	0.984	no
gi|320446741|ref|NW_003383830.1|	131047-13232	152439	258797	-255835	0.2703	no
gi|320446741|ref|NW_003383830.1|	134497-13580	528567	132968	-199101	0.2742	no
gi|320446741|ref|NW_003383830.1|	139202-13948	285719	280815	-334691	0.23995	no
gi|320446741|ref|NW_003383830.1|	147667-14837	58792	121568	104808	0.4246	no
gi|320446741|ref|NW_003383830.1|	153028-15414	379502	119935	-166185	0.2188	no
gi|320446741|ref|NW_003383830.1|	154311-15565	371571	144496	-136261	0.29615	no
gi|320446741|ref|NW_003383830.1|	155807-15989	232622	170665	-0.446827	0.84045	no
gi|320446741|ref|NW_003383830.1|	162882-16466	65378	235309	-147425	0.4964	no
gi|320446741|ref|NW_003383830.1|	164847-16615	753079	283143	-141127	0.51055	no
gi|320446741|ref|NW_003383830.1|	170272-17068	493215	278284	-0.825657	0.68735	no
gi|320446741|ref|NW_003383830.1|	172342-17507	105516	136597	0.372464	0.7682	no
gi|320446741|ref|NW_003383830.1|	198904-19921	172748	217935	365716	0.0782	no
gi|320446741|ref|NW_003383830.1|	203595-20383	0	392398	inf	0.02075	no
gi|320446741|ref|NW_003383830.1|	206451-20683	536449	534393	331639	0.1895	no
gi|320446741|ref|NW_003383830.1|	210257-21257	331707	789009	457206	0.0402	no

gi|320446741|ref|NW_003383830.1|	21292-24259	0.0617693	285009	552797	0.1624	no
gi|320446741|ref|NW_003383830.1|	26164-29070	0.126361	145744	352782	0.18205	no
gi|320446741|ref|NW_003383830.1|	269794-26999	0	697665	inf	0.02205	no
gi|320446741|ref|NW_003383830.1|	272016-27515	0	427413	inf	0.00485	no
gi|320446741|ref|NW_003383830.1|	275262-27615	0.257439	727159	481997	0.17075	no
gi|320446741|ref|NW_003383830.1|	276507-27755	228454	548594	458576	0.0498	no
gi|320446741|ref|NW_003383830.1|	277682-27869	28324	153103	575633	0.036	no
gi|320446741|ref|NW_003383830.1|	282663-29119	543034	396519	-0.453654	0.7275	no
gi|320446741|ref|NW_003383830.1|	291345-29181	567841	420873	-0.432103	0.85185	no
gi|320446741|ref|NW_003383830.1|	291958-29436	225433	407099	0.852679	0.68485	no
gi|320446741|ref|NW_003383830.1|	294525-29547	800759	731334	-0.130839	0.94765	no
gi|320446741|ref|NW_003383830.1|	295701-29677	183132	218582	0.255288	0.9129	no
gi|320446741|ref|NW_003383830.1|	297166-29977	179441	151226	-0.246801	0.8444	no
gi|320446741|ref|NW_003383830.1|	301436-30233	297571	105155	-482265	0.13505	no
gi|320446741|ref|NW_003383830.1|	304042-30475	282612	386602	0.452027	0.81645	no
gi|320446741|ref|NW_003383830.1|	318061-31889	314556	409193	-294247	0.2149	no
gi|320446741|ref|NW_003383830.1|	320079-32161	504839	0.990396	-234975	0.29595	no
gi|320446741|ref|NW_003383830.1|	322427-32263	818099	21939	-189878	0.39565	no
gi|320446741|ref|NW_003383830.1|	324165-32534	166502	682357	-128694	0.55865	no
gi|320446741|ref|NW_003383830.1|	345132-34775	141987	106106	-0.420246	0.7358	no
gi|320446741|ref|NW_003383830.1|	347864-35007	44368	291764	-0.604714	0.7677	no
gi|320446741|ref|NW_003383830.1|	352463-35325	482596	186402	-13724	0.50605	no
gi|320446741|ref|NW_003383830.1|	354720-35537	127624	706634	-0.852863	0.67865	no
gi|320446741|ref|NW_003383830.1|	359907-36198	461539	251674	-0.874893	0.50955	no
gi|320446741|ref|NW_003383830.1|	362140-36564	356617	444766	0.318669	0.88445	no
gi|320446741|ref|NW_003383830.1|	365783-36663	0.821186	244855	157614	0.491	no
gi|320446741|ref|NW_003383830.1|	375143-37632	232498	36437	0.648184	0.69415	no
gi|320446741|ref|NW_003383830.1|	376459-37905	428687	544052	0.343819	0.8704	no
gi|320446741|ref|NW_003383830.1|	379213-38008	828399	191322	120761	0.57725	no
gi|320446741|ref|NW_003383830.1|	381446-38490	129122	332812	-195596	0.2642	no

gi|320446741|ref|NW_003383830.1|	38364-39609	0	139119	inf	1	no
gi|320446741|ref|NW_003383830.1|	15423-41631	205951	319241	-268959	0.24	no
gi|320446741|ref|NW_003383830.1|	42491-43172	113533	258324	118607	0.56815	no
gi|320446741|ref|NW_003383830.1|	26780-42723	205454	212468	0.0484329	0.97905	no
gi|320446741|ref|NW_003383830.1|	34700-43618	0.135149	310076	4.52	0.17645	no
gi|320446741|ref|NW_003383830.1|	36917-43793	0.431298	193738	216735	0.29245	no
gi|320446741|ref|NW_003383830.1|	38451-44099	131055	167742	0.356069	0.7851	no
gi|320446741|ref|NW_003383830.1|	44857-45000	42141	876512	105655	0.41165	no
gi|320446741|ref|NW_003383830.1|	45023-46333	0.942225	196202	10582	0.59735	no
gi|320446741|ref|NW_003383830.1|	50204-50689	19925	444022	115605	0.5747	no
gi|320446741|ref|NW_003383830.1|	58244-58804	191124	153464	-0.316606	0.8782	no
gi|320446741|ref|NW_003383830.1|	60785-61843	116941	124864	0.0945814	0.9662	no
gi|320446741|ref|NW_003383830.1|	62107-62611	867521	664692	-0.384213	0.8462	no
gi|320446741|ref|NW_003383830.1|	63189-64019	851583	126863	0.575058	0.77985	no
gi|320446741|ref|NW_003383830.1|	64181-65194	0.651108	26995	205172	0.3468	no
gi|320446741|ref|NW_003383830.1|	66594-67407	496772	883506	0.830655	0.6844	no
gi|320446741|ref|NW_003383830.1|	71468-72195	274604	422934	0.62308	0.74755	no
gi|320446741|ref|NW_003383830.1|	72297-73058	449567	792656	0.81816	0.6881	no
gi|320446743|ref|NW_003383828.1|	07780-11199	0.0849013	109967	369514	1	no
gi|320446743|ref|NW_003383828.1|	14446-11628	0	102716	inf	1	no
gi|320446743|ref|NW_003383828.1|	17376-11875	0.443069	441114	331555	0.2043	no
gi|320446743|ref|NW_003383828.1|	11876-12743	518417	423117	-361499	0.0764	no
gi|320446743|ref|NW_003383828.1|	19804-12192	617457	680484	0.140222	0.9468	no
gi|320446743|ref|NW_003383828.1|	24972-12533	815394	380435	-109985	0.59205	no
gi|320446743|ref|NW_003383828.1|	26453-12751	265738	113107	-123232	0.54975	no
gi|320446743|ref|NW_003383828.1|	27953-12916	242168	155898	-0.635402	0.75455	no
gi|320446743|ref|NW_003383828.1|	29425-13018	354577	198901	-0.834049	0.6776	no
gi|320446743|ref|NW_003383828.1|	30388-13073	144598	170912	-308073	0.25595	no
gi|320446743|ref|NW_003383828.1|	13366-14785	250928	297206	-307774	0.10315	no
gi|320446743|ref|NW_003383828.1|	15097-18691	236114	1.16	-102536	0.6137	no

gi|320446743|ref|NW_003383828.1|	19711-20122	731721	120683	-260008	0.2918	no
gi|320446743|ref|NW_003383828.1|	20972-22114	874884	683298	-0.356578	0.85635	no
gi|320446743|ref|NW_003383828.1|	23676-24116	159631	741391	221549	0.27865	no
gi|320446743|ref|NW_003383828.1|	238185-239148	0.695867	112072	0.687547	1	no
gi|320446743|ref|NW_003383828.1|	243900-246162	174846	10286	-0.7654	0.5426	no
gi|320446743|ref|NW_003383828.1|	251816-253778	258003	324334	0.33009	0.7945	no
gi|320446743|ref|NW_003383828.1|	275390-275820	0	608307	inf	0.0294	no
gi|320446743|ref|NW_003383828.1|	307794-308135	827131	805095	-0.0389571	0.94	no
gi|320446743|ref|NW_003383828.1|	344434-345945	119059	183986	0.627919	0.759	no
gi|320446743|ref|NW_003383828.1|	346065-346650	3781	224125	-0.754464	0.7058	no
gi|320446743|ref|NW_003383828.1|	347656-348064	464255	42852	-0.115556	0.9242	no
gi|320446743|ref|NW_003383828.1|	34891-35967	0.602296	125008	105348	1	no
gi|320446743|ref|NW_003383828.1|	350871-351582	0.709484	58226	303682	0.21255	no
gi|320446743|ref|NW_003383828.1|	352423-352793	45759	127123	147409	0.50535	no
gi|320446743|ref|NW_003383828.1|	355172-355687	238564	400457	0.747274	0.7213	no
gi|320446743|ref|NW_003383828.1|	355875-356248	112377	264635	123566	0.5495	no
gi|320446743|ref|NW_003383828.1|	357246-357814	151343	115917	29372	0.23025	no
gi|320446743|ref|NW_003383828.1|	358391-358719	453939	782793	0.786131	0.70455	no
gi|320446743|ref|NW_003383828.1|	359145-359805	635148	167746	140111	0.5038	no
gi|320446743|ref|NW_003383828.1|	360253-361162	35078	146798	206519	0.3522	no
gi|320446743|ref|NW_003383828.1|	363503-364361	108436	373141	178288	0.42685	no
gi|320446743|ref|NW_003383828.1|	364475-365210	26703	246941	320909	0.1885	no
gi|320446743|ref|NW_003383828.1|	37015-38286	0.488858	101751	105756	1	no
gi|320446743|ref|NW_003383828.1|	408024-408367	815824	150073	0.879334	0.6695	no
gi|320446743|ref|NW_003383828.1|	409621-410245	0.866439	108927	365212	0.179	no
gi|320446743|ref|NW_003383828.1|	412142-412930	120865	4357	184994	0.4058	no
gi|320446743|ref|NW_003383828.1|	44919-45211	61122	286987	-109071	0.5972	no
gi|320446743|ref|NW_003383828.1|	464436-464669	242575	636772	139235	0.5063	no
gi|320446743|ref|NW_003383828.1|	469540-471042	146551	123212	-0.250263	0.91175	no
gi|320446743|ref|NW_003383828.1|	471873-472448	103814	802423	-0.37156	0.85835	no

gi|320446743|ref|NW_003383828.1|	472628-47394	766629	347525	-114141	0.58695	no
gi|320446743|ref|NW_003383828.1|	475165-47553	670303	292384	-119695	0.5758	no
gi|320446743|ref|NW_003383828.1|	476025-47650	933347	178253	-238849	0.3069	no
gi|320446743|ref|NW_003383828.1|	476632-47716	244022	100832	-127506	0.54375	no
gi|320446743|ref|NW_003383828.1|	477299-47763	352643	104632	-175288	0.41655	no
gi|320446743|ref|NW_003383828.1|	479307-48010	475331	213532	-115448	0.48675	no
gi|320446743|ref|NW_003383828.1|	480807-48204	235134	868094	-143756	0.379	no
gi|320446743|ref|NW_003383828.1|	482810-48341	27598	530246	0.9421	0.65765	no
gi|320446743|ref|NW_003383828.1|	483565-48500	224885	156288	-0.524979	0.7894	no
gi|320446743|ref|NW_003383828.1|	487057-49169	552361	714583	0.37149	0.7699	no
gi|320446743|ref|NW_003383828.1|	52562-53231	114312	418982	-144801	0.40835	no
gi|320446743|ref|NW_003383828.1|	53851-55146	215002	106105	-101886	0.53145	no
gi|320446743|ref|NW_003383828.1|	56921-58678	306076	199285	-0.619061	0.62485	no
gi|320446743|ref|NW_003383828.1|	59933-60474	191658	332027	-252917	0.26105	no
gi|320446743|ref|NW_003383828.1|	61795-65703	131393	196154	-274383	0.13485	no
gi|320446743|ref|NW_003383828.1|	67986-68442	0.745081	644497	31127	0.2468	no
gi|320446743|ref|NW_003383828.1|	85990-86152	671117	1319.33	42971	0.16495	no
gi|320446746|ref|NW_003383825.1|	105450-10822	444446	268883	-0.725026	0.73275	no
gi|320446746|ref|NW_003383825.1|	109096-10960	305503	286904	-0.090618	0.9267	no
gi|320446746|ref|NW_003383825.1|	115733-11665	146632	522437	183305	0.40295	no
gi|320446746|ref|NW_003383825.1|	118623-11933	0	29113	inf	0.029	no
gi|320446746|ref|NW_003383825.1|	119755-12342	0.688557	104626	392552	0.0612	no
gi|320446746|ref|NW_003383825.1|	125148-12574	303863	437212	0.524912	0.8066	no
gi|320446746|ref|NW_003383825.1|	125902-13109	226909	210684	-0.107034	0.93495	no
gi|320446746|ref|NW_003383825.1|	131481-13265	828804	738166	-348901	0.085	no
gi|320446746|ref|NW_003383825.1|	133266-13371	548965	374911	-387209	0.06305	no
gi|320446746|ref|NW_003383825.1|	140378-14147	0.77986	147088	423732	0.15265	no
gi|320446746|ref|NW_003383825.1|	143102-14350	187593	111266	256833	0.2435	no
gi|320446746|ref|NW_003383825.1|	151958-15332	774085	514884	-0.588245	0.66035	no
gi|320446746|ref|NW_003383825.1|	153509-15639	193737	886481	-112794	0.38445	no

gi|320446746|ref|NW_003383825.1|	[illegible]56525-16027[illegible]	338162	261868	-0.368875	0.7814	no
gi|320446746|ref|NW_003383825.1|	[illegible]62168-16308[illegible]	170106	137682	-0.305087	0.88755	no
gi|320446746|ref|NW_003383825.1|	[illegible]63257-16343[illegible]	90041	737109	-0.288703	0.88355	no
gi|320446746|ref|NW_003383825.1|	[illegible]63611-16423[illegible]	372003	169462	-113435	0.60015	no
gi|320446746|ref|NW_003383825.1|	[illegible]83248-18803[illegible]	485496	232322	22586	0.0982	no
gi|320446746|ref|NW_003383825.1|	[illegible]97630-20305[illegible]	204054	235174	0.204777	0.8776	no
gi|320446746|ref|NW_003383825.1|	[illegible]03179-20429[illegible]	101456	662644	-0.614543	0.76425	no
gi|320446746|ref|NW_003383825.1|	[illegible]04493-20553[illegible]	216582	177355	-0.288275	0.90175	no
gi|320446746|ref|NW_003383825.1|	22625-23888	0.98533	17089	0.794387	0.698	no
gi|320446746|ref|NW_003383825.1|	[illegible]60375-26174[illegible]	148862	186103	0.322118	0.8613	no
gi|320446746|ref|NW_003383825.1|	[illegible]61865-26269[illegible]	14265	333444	122496	0.56205	no
gi|320446746|ref|NW_003383825.1|	[illegible]63144-26352[illegible]	751029	105472	0.489925	0.8016	no
gi|320446746|ref|NW_003383825.1|	[illegible]64040-26449[illegible]	972664	447982	-11185	0.57645	no
gi|320446746|ref|NW_003383825.1|	[illegible]65613-26877[illegible]	230455	120469	238611	0.18025	no
gi|320446746|ref|NW_003383825.1|	[illegible]77395-27750[illegible]	0	30971.2	inf	0.01275	no
gi|320446746|ref|NW_003383825.1|	[illegible]19592-32009[illegible]	123932	224334	417803	0.16355	no
gi|320446746|ref|NW_003383825.1|	[illegible]48971-34927[illegible]	108046	520866	226926	0.3035	no
gi|320446746|ref|NW_003383825.1|	[illegible]49462-34967[illegible]	209474	623856	157444	0.496	no
gi|320446746|ref|NW_003383825.1|	[illegible]54763-35507[illegible]	118972	842819	282461	0.2315	no
gi|320446746|ref|NW_003383825.1|	[illegible]77178-37762[illegible]	8515	154276	0.857434	0.6695	no
gi|320446746|ref|NW_003383825.1|	47039-47807	665501	39125	-0.766349	0.70905	no
gi|320446746|ref|NW_003383825.1|	47963-50852	801808	193787	-204879	0.13485	no
gi|320446746|ref|NW_003383825.1|	51153-51829	386525	255915	-0.594899	0.7912	no
gi|320446746|ref|NW_003383825.1|	52447-60167	324608	146207	217124	0.10915	no
gi|320446746|ref|NW_003383825.1|	60280-61967	0.348737	291366	306262	0.2139	no
gi|320446746|ref|NW_003383825.1|	63454-65893	666171	247039	189077	0.29345	no
gi|320446746|ref|NW_003383825.1|	81406-93169	916211	820462	-0.159243	0.90715	no
gi|320446746|ref|NW_003383825.1|	97398-100541	133469	101045	292043	0.12095	no
gi|320446747|ref|NW_003383824.1|	[illegible]01905-10364[illegible]	414956	343673	-0.271921	0.8958	no
gi|320446747|ref|NW_003383824.1|	[illegible]04995-10657[illegible]	137843	165656	0.265168	0.88715	no

gi|320446747|ref|NW_003383824.1|	106825-10731	13086	612769	-109461	0.60115	no
gi|320446747|ref|NW_003383824.1|	107703-10811	781344	685126	-0.189589	0.9282	no
gi|320446747|ref|NW_003383824.1|	108453-10884	192834	133334	-0.532315	0.79485	no
gi|320446747|ref|NW_003383824.1|	112454-11431	23845	3468	0.540421	0.7969	no
gi|320446747|ref|NW_003383824.1|	116483-11724	286288	239976	-0.254578	0.90265	no
gi|320446747|ref|NW_003383824.1|	118645-11946	605273	693389	0.196079	0.92305	no
gi|320446747|ref|NW_003383824.1|	119833-12455	481894	10969	118665	0.359	no
gi|320446747|ref|NW_003383824.1|	125237-12567	409962	543397	0.406516	0.8535	no
gi|320446747|ref|NW_003383824.1|	12747-14849	0.271193	163959	259594	0.26755	no
gi|320446747|ref|NW_003383824.1|	132759-13630	211379	193157	-0.130058	0.92105	no
gi|320446747|ref|NW_003383824.1|	136585-13790	15375	106733	-0.526576	0.81245	no
gi|320446747|ref|NW_003383824.1|	138463-13965	14.23	295902	105618	0.52705	no
gi|320446747|ref|NW_003383824.1|	139943-14074	162979	209663	0.363387	0.8653	no
gi|320446747|ref|NW_003383824.1|	141105-14382	193099	219979	0.188023	0.888	no
gi|320446747|ref|NW_003383824.1|	144294-14533	173446	158935	-0.126051	0.9528	no
gi|320446747|ref|NW_003383824.1|	145653-14704	930551	636368	-0.548224	0.79405	no
gi|320446747|ref|NW_003383824.1|	158929-16081	100819	775237	-0.379057	0.7729	no
gi|320446747|ref|NW_003383824.1|	241085-24233	16236	13101	-0.309517	0.8909	no
gi|320446747|ref|NW_003383824.1|	256182-25878	183311	246156	0.425281	0.74665	no
gi|320446747|ref|NW_003383824.1|	270748-27163	183061	181174	-0.0149486	0.99275	no
gi|320446747|ref|NW_003383824.1|	300082-30079	744958	994694	0.417094	0.83555	no
gi|320446747|ref|NW_003383824.1|	301587-30353	47238	912522	0.949912	0.6638	no
gi|320446747|ref|NW_003383824.1|	303800-30469	356606	119352	174282	0.4199	no
gi|320446747|ref|NW_003383824.1|	305365-30598	114668	200677	0.807408	0.6924	no
gi|320446747|ref|NW_003383824.1|	307584-30962	105718	319717	159658	0.36905	no
gi|320446747|ref|NW_003383824.1|	347132-34835	188153	118602	-0.665782	0.7458	no
gi|320446747|ref|NW_003383824.1|	348915-35722	140449	128539	-0.127845	0.92475	no
gi|320446747|ref|NW_003383824.1|	357875-35901	169767	852755	-0.993354	0.6545	no
gi|320446747|ref|NW_003383824.1|	359376-36016	0.605409	20784	177949	0.3324	no
gi|320446747|ref|NW_003383824.1|	360297-36625	151812	125605	-0.273399	0.8383	no

gi|320446747|ref|NW_003383824.1|	367853-36833	606888	225265	-14298	0.51015	no
gi|320446747|ref|NW_003383824.1|	368615-36890	555156	204781	-143881	0.4908	no
gi|320446747|ref|NW_003383824.1|	374420-37536	212857	179509	-0.245829	0.9038	no
gi|320446747|ref|NW_003383824.1|	379124-37994	218678	534008	-203387	0.36075	no
gi|320446747|ref|NW_003383824.1|	380209-38248	54521	236408	-120553	0.573	no
gi|320446747|ref|NW_003383824.1|	383939-38561	18911	137378	-0.461075	0.77625	no
gi|320446747|ref|NW_003383824.1|	386453-38669	741595	233996	-166415	0.4438	no
gi|320446747|ref|NW_003383824.1|	406624-40732	836888	221313	140298	0.52035	no
gi|320446747|ref|NW_003383824.1|	84392-91398	107652	443737	-12786	0.32935	no
gi|320446747|ref|NW_003383824.1|	93450-94289	559253	596204	0.0923033	0.96385	no
gi|320446747|ref|NW_003383824.1|	97008-97614	816987	0	#NAME?	0.01345	no
gi|320446747|ref|NW_003383824.1|	99677-10174	692451	869366	0.328251	0.88435	no
gi|320446749|ref|NW_003383822.1|	19-1673	511487	202863	198774	0.2469	no
gi|320446749|ref|NW_003383822.1|	224654-22530	44495	0.275522	-40134	0.3092	no
gi|320446750|ref|NW_003383821.1|	20778-21683	504109	0	#NAME?	0.0132	no
gi|320446750|ref|NW_003383821.1|	260891-26364	167817	520698	163356	0.4525	no
gi|320446750|ref|NW_003383821.1|	269044-27001	894785	152056	0.764986	0.7174	no
gi|320446750|ref|NW_003383821.1|	273388-27584	0.532135	222971	206699	0.3463	no
gi|320446750|ref|NW_003383821.1|	276891-27814	216438	254058	0.231206	0.90345	no
gi|320446750|ref|NW_003383821.1|	278360-28123	530757	15242	152193	0.3775	no
gi|320446750|ref|NW_003383821.1|	282451-28415	621306	504719	-0.299823	0.88435	no
gi|320446750|ref|NW_003383821.1|	284300-28475	262972	599717	-213256	0.3409	no
gi|320446750|ref|NW_003383821.1|	284998-28535	173544	121155	-0.518455	0.79635	no
gi|320446750|ref|NW_003383821.1|	375981-37673	0.324819	356252	345519	0.2167	no
gi|320446750|ref|NW_003383821.1|	381165-38160	949367	246798	137829	0.50535	no
gi|320446750|ref|NW_003383821.1|	386563-38798	298645	454575	0.606086	0.77075	no
gi|320446750|ref|NW_003383821.1|	455044-45735	126941	829334	-0.614132	0.7047	no
gi|320446750|ref|NW_003383821.1|	57241-57956	0	818217	inf	0.0101	no
gi|320446750|ref|NW_003383821.1|	58509-59686	0.179225	596136	50558	0.17375	no
gi|320446750|ref|NW_003383821.1|	91916-92368	0.757718	604588	299622	0.2563	no

gi|320446751|ref|NW_003383820.1|	121396-12195	67238	235718	-151222	0.3555	no
gi|320446751|ref|NW_003383820.1|	122140-12417	692695	561446	-0.303074	0.89045	no
gi|320446751|ref|NW_003383820.1|	126604-12750	160959	968212	-0.733295	0.7303	no
gi|320446751|ref|NW_003383820.1|	13196-14225	0.425318	117597	146723	1	no
gi|320446751|ref|NW_003383820.1|	133362-13464	0.161549	21298	372068	0.21225	no
gi|320446751|ref|NW_003383820.1|	134743-13680	195412	122799	-0.670221	0.5909	no
gi|320446751|ref|NW_003383820.1|	136949-13775	400191	319424	-0.325215	0.84135	no
gi|320446751|ref|NW_003383820.1|	150708-15093	374943	445936	0.250167	0.899	no
gi|320446751|ref|NW_003383820.1|	155331-15729	624738	741843	0.247863	0.905	no
gi|320446751|ref|NW_003383820.1|	158253-15989	397298	393844	-0.0125993	0.98755	no
gi|320446751|ref|NW_003383820.1|	161649-16244	151352	145488	-0.05701	0.9269	no
gi|320446751|ref|NW_003383820.1|	168698-16909	5835	575945	-0.0188012	0.94	no
gi|320446751|ref|NW_003383820.1|	203959-20788	475205	146801	-169469	0.2145	no
gi|320446751|ref|NW_003383820.1|	208123-20922	115021	699317	-0.717874	0.5963	no
gi|320446751|ref|NW_003383820.1|	212743-21433	162469	759427	-109718	0.49875	no
gi|320446751|ref|NW_003383820.1|	221607-22220	103127	381184	-143586	0.4989	no
gi|320446751|ref|NW_003383820.1|	226937-22774	243809	18164	-0.424664	0.85055	no
gi|320446751|ref|NW_003383820.1|	232563-23316	240557	615861	-19657	0.36795	no
gi|320446751|ref|NW_003383820.1|	247584-24780	94551	445452	223611	0.2748	no
gi|320446751|ref|NW_003383820.1|	328278-32850	531837	557471	-325401	0.2479	no
gi|320446751|ref|NW_003383820.1|	37645-38056	0	844778	inf	0.0233	no
gi|320446751|ref|NW_003383820.1|	400865-40145	347829	796227	-212713	0.33485	no
gi|320446751|ref|NW_003383820.1|	408710-41236	171113	131816	-0.376426	0.77585	no
gi|320446751|ref|NW_003383820.1|	415997-41629	225551	108582	-105467	0.5945	no
gi|320446751|ref|NW_003383820.1|	416943-41737	302014	111092	-144286	0.49975	no
gi|320446751|ref|NW_003383820.1|	417547-41818	481779	876204	-245903	0.29655	no
gi|320446751|ref|NW_003383820.1|	418384-41895	246472	747947	-172042	0.42735	no
gi|320446751|ref|NW_003383820.1|	419421-42026	169449	305733	-24705	0.26845	no
gi|320446751|ref|NW_003383820.1|	457084-45726	925359	325578	-150701	0.50475	no
gi|320446751|ref|NW_003383820.1|	483435-48511	101836	4808	-108273	0.6225	no

gi|320446751|ref|NW_003383820.1|	485245-48701	136436	858397	-0.668511	0.6761	no
gi|320446751|ref|NW_003383820.1|	74604-79682	364788	347072	-0.0718234	0.95585	no
gi|320446751|ref|NW_003383820.1|	84670-85987	115455	56318	-103567	0.6345	no
gi|320446751|ref|NW_003383820.1|	94684-95179	960014	0	#NAME?	0.01585	no
gi|320446752|ref|NW_003383819.1|	100493-10107	0.49013	298435	260618	0.2636	no
gi|320446752|ref|NW_003383819.1|	102103-10395	0.626276	114926	419776	0.13105	no
gi|320446752|ref|NW_003383819.1|	11112-29582	907673	176319	0.95794	0.4641	no
gi|320446752|ref|NW_003383819.1|	1160-1419	373136	182111	-435681	0.2992	no
gi|320446752|ref|NW_003383819.1|	142387-14426	0.205204	271734	372706	0.17955	no
gi|320446752|ref|NW_003383819.1|	152231-15760	0.821495	333829	202279	0.36945	no
gi|320446752|ref|NW_003383819.1|	191091-19163	0	597648	inf	0.00385	no
gi|320446752|ref|NW_003383819.1|	194826-19511	822536	121095	387991	0.1688	no
gi|320446752|ref|NW_003383819.1|	211669-21209	0	22929	inf	0.00815	no
gi|320446752|ref|NW_003383819.1|	219962-22018	0	484112	inf	0.02205	no
gi|320446752|ref|NW_003383819.1|	224073-23038	0.111117	110983	664211	0.1028	no
gi|320446752|ref|NW_003383819.1|	231874-23344	0	0.969765	inf	1	no
gi|320446752|ref|NW_003383819.1|	29838-30280	949367	735143	-0.368941	0.85105	no
gi|320446752|ref|NW_003383819.1|	37316-39779	146948	134363	-0.129171	0.92125	no
gi|320446752|ref|NW_003383819.1|	379311-38270	235959	381665	0.69377	0.61245	no
gi|320446752|ref|NW_003383819.1|	384893-38554	761328	982864	0.368474	0.8525	no
gi|320446752|ref|NW_003383819.1|	391794-39253	640508	13852	-220913	0.328	no
gi|320446752|ref|NW_003383819.1|	397482-39869	103999	0.600825	-0.79156	1	no
gi|320446752|ref|NW_003383819.1|	399405-40101	0.492827	171473	179883	0.426	no
gi|320446752|ref|NW_003383819.1|	401758-40250	318128	28.32	-0.167788	0.93835	no
gi|320446752|ref|NW_003383819.1|	407455-40816	846074	118158	0.481858	0.81405	no
gi|320446752|ref|NW_003383819.1|	412213-41298	221834	847709	193409	0.3713	no
gi|320446752|ref|NW_003383819.1|	414386-41472	271941	158901	254676	0.24395	no
gi|320446752|ref|NW_003383819.1|	414836-41654	240145	820186	177204	0.4251	no
gi|320446752|ref|NW_003383819.1|	426062-42671	121928	249112	103076	0.5772	no
gi|320446752|ref|NW_003383819.1|	42663-43631	921488	318058	-153468	0.4747	no

gi|320446752|ref|NW_003383819.1|	427512-42855	0.630035	26135	205248	0.3468	no
gi|320446752|ref|NW_003383819.1|	45705-46594	413164	284621	-0.537669	0.7851	no
gi|320446752|ref|NW_003383819.1|	46707-50609	201554	541109	-189717	0.2986	no
gi|320446752|ref|NW_003383819.1|	516200-51787	0	106451	inf	1	no
gi|320446752|ref|NW_003383819.1|	522747-52377	0.214008	917061	542128	0.16265	no
gi|320446752|ref|NW_003383819.1|	524399-52595	0.127586	834294	603101	0.1585	no
gi|320446752|ref|NW_003383819.1|	52547-53681	676092	247107	-145209	0.49755	no
gi|320446752|ref|NW_003383819.1|	5709-6184	48308	138194	-180557	0.322	no
gi|320446752|ref|NW_003383819.1|	69797-70462	36493	80263	-218481	0.33415	no
gi|320446752|ref|NW_003383819.1|	74913-78429	450541	154372	-154525	0.26025	no
gi|320446752|ref|NW_003383819.1|	84571-85230	147221	130161	-0.177683	0.9314	no
gi|320446752|ref|NW_003383819.1|	85331-86768	112349	20496	0.867351	0.6722	no
gi|320446752|ref|NW_003383819.1|	86961-88400	743076	32155	-120847	0.5606	no
gi|320446752|ref|NW_003383819.1|	88866-90771	148566	612881	-127742	0.4338	no
gi|320446752|ref|NW_003383819.1|	92230-93157	124457	302919	-203864	0.34995	no
gi|320446753|ref|NW_003383818.1|	1006-1979	320334	0.315938	-334186	0.239	no
gi|320446753|ref|NW_003383818.1|	10281-11812	117221	163056	0.476138	0.80865	no
gi|320446753|ref|NW_003383818.1|	11956-12190	199182	276149	0.471364	0.82175	no
gi|320446753|ref|NW_003383818.1|	13422-14296	343584	254781	-0.431405	0.823	no
gi|320446753|ref|NW_003383818.1|	14792-17171	0.551004	120911	113381	1	no
gi|320446753|ref|NW_003383818.1|	210763-21345	874074	182261	106018	0.39875	no
gi|320446753|ref|NW_003383818.1|	214302-21603	112374	219135	0.963503	0.64715	no
gi|320446753|ref|NW_003383818.1|	219443-21974	263142	36194	0.459906	0.8193	no
gi|320446753|ref|NW_003383818.1|	256480-25703	243431	464091	-239103	0.294	no
gi|320446753|ref|NW_003383818.1|	26855-29459	584141	756448	0.372924	0.8677	no
gi|320446753|ref|NW_003383818.1|	273560-27376	109574	455216	-458921	0.293	no
gi|320446753|ref|NW_003383818.1|	29793-30633	893325	126728	0.504479	0.80925	no
gi|320446753|ref|NW_003383818.1|	302577-30350	146209	10083	-0.536107	0.7982	no
gi|320446753|ref|NW_003383818.1|	327166-32735	118726	266972	-215287	0.35185	no
gi|320446753|ref|NW_003383818.1|	356431-35684	627728	236838	-140624	0.5239	no

gi|320446753|ref|NW_003383818.1|	386313-38666	270622	754558	-184258	0.394	no
gi|320446753|ref|NW_003383818.1|	38982-39723	0	663083	inf	0.0109	no
gi|320446753|ref|NW_003383818.1|	390531-39121	456161	0.518884	-313606	0.255	no
gi|320446753|ref|NW_003383818.1|	391922-39250	874764	296001	-156329	0.4713	no
gi|320446753|ref|NW_003383818.1|	394107-40239	366844	385148	0.0702446	0.95285	no
gi|320446753|ref|NW_003383818.1|	407744-41095	0.170398	127115	289916	1	no
gi|320446753|ref|NW_003383818.1|	413363-41730	185117	120343	270064	0.04865	no
gi|320446753|ref|NW_003383818.1|	419114-41958	126688	187766	0.567662	0.78305	no
gi|320446753|ref|NW_003383818.1|	426566-42705	131839	352668	-19024	0.3915	no
gi|320446753|ref|NW_003383818.1|	429217-42990	151784	556315	-144805	0.49615	no
gi|320446753|ref|NW_003383818.1|	430583-43295	168765	149217	-0.1776	0.89125	no
gi|320446753|ref|NW_003383818.1|	433373-43483	0.825441	210388	134981	0.5192	no
gi|320446753|ref|NW_003383818.1|	454329-45537	0	266003	inf	0.0025	no
gi|320446753|ref|NW_003383818.1|	45562-47621	259082	348504	0.427764	0.83005	no
gi|320446753|ref|NW_003383818.1|	457501-45782	0	456499	inf	0.00845	no
gi|320446753|ref|NW_003383818.1|	457960-45877	0	430077	inf	5.00E-05	yes
gi|320446753|ref|NW_003383818.1|	462397-46311	121368	396294	170718	0.44825	no
gi|320446753|ref|NW_003383818.1|	463263-46423	115029	635263	246536	0.284	no
gi|320446753|ref|NW_003383818.1|	464362-46581	179725	595795	172903	0.4166	no
gi|320446753|ref|NW_003383818.1|	469486-47036	15759	162788	0.0468186	0.94	no
gi|320446753|ref|NW_003383818.1|	473840-47456	759777	17018	116341	0.5826	no
gi|320446753|ref|NW_003383818.1|	487183-48813	247293	253529	0.0359279	0.98825	no
gi|320446753|ref|NW_003383818.1|	489040-49098	18896	180702	-0.0644698	0.95815	no
gi|320446753|ref|NW_003383818.1|	49410-50537	130602	563571	-121251	0.57915	no
gi|320446753|ref|NW_003383818.1|	494423-49690	127509	434789	-155221	0.36265	no
gi|320446753|ref|NW_003383818.1|	499265-49991	422684	17161	-130044	0.5542	no
gi|320446753|ref|NW_003383818.1|	500278-50075	77844	723499	-342752	0.16695	no
gi|320446753|ref|NW_003383818.1|	504691-50490	850059	759333	-348476	0.2322	no
gi|320446753|ref|NW_003383818.1|	505059-50692	788405	267488	-155946	0.4692	no
gi|320446753|ref|NW_003383818.1|	520416-52430	768987	3028.02	197734	0.115	no

gi|320446753|ref|NW_003383818.1|	;24431-52581:	0.294606	143247	228164	0.2731	no
gi|320446753|ref|NW_003383818.1|	;40218-54055:	423036	167192	198266	0.24045	no
gi|320446753|ref|NW_003383818.1|	;47915-54856;	871654	923637	0.0835702	0.9464	no
gi|320446753|ref|NW_003383818.1|	;50388-55168:	0.159261	198947	364292	0.2061	no
gi|320446753|ref|NW_003383818.1|	;51865-55244;	773201	222435	152447	0.47485	no
gi|320446753|ref|NW_003383818.1|	;53130-55754:	466882	150709	169063	0.33265	no
gi|320446753|ref|NW_003383818.1|	;77265-57930:	113391	160777	0.503755	0.76665	no
gi|320446753|ref|NW_003383818.1|	8070-9808	12353	101674	-0.280908	1	no
gi|320446754|ref|NW_003383817.1|	100853-10325	178957	0.325978	-245677	0.29495	no
gi|320446754|ref|NW_003383817.1|	146171-14775;	124848	295351	124226	0.54615	no
gi|320446754|ref|NW_003383817.1|	148431-15069	0.58167	115998	0.995831	1	no
gi|320446754|ref|NW_003383817.1|	155935-15638!	0	249882	inf	0.0074	no
gi|320446754|ref|NW_003383817.1|	157519-15835	0.280091	520118	421487	0.1914	no
gi|320446754|ref|NW_003383817.1|	159568-16012:	0.527584	206449	529024	0.165	no
gi|320446754|ref|NW_003383817.1|	242851-24464(	230455	121354	-0.925263	0.58135	no
gi|320446754|ref|NW_003383817.1|	244748-24663(	132532	765027	-0.792762	0.5468	no
gi|320446754|ref|NW_003383817.1|	248430-25254(	840444	160658	0.934769	0.4778	no
gi|320446754|ref|NW_003383817.1|	252973-25468!	171416	115386	-0.57104	0.7225	no
gi|320446754|ref|NW_003383817.1|	256601-25872	212752	187092	-350735	0.07705	no
gi|320446754|ref|NW_003383817.1|	260769-26642:	117721	547753	-110378	0.39945	no
gi|320446754|ref|NW_003383817.1|	27899-30713	503847	163267	169617	0.3331	no
gi|320446754|ref|NW_003383817.1|	30887-32558	211575	327317	0.629518	0.76015	no
gi|320446754|ref|NW_003383817.1|	33778-34281	192776	0	#NAME?	0.01075	no
gi|320446754|ref|NW_003383817.1|	342171-34439	587427	47.74	-0.299211	0.82165	no
gi|320446754|ref|NW_003383817.1|	344506-34957	494373	716611	0.535588	0.6773	no
gi|320446754|ref|NW_003383817.1|	34548-39115	716712	0.16367	-545253	0.09735	no
gi|320446754|ref|NW_003383817.1|	39222-42268	360198	524685	0.542662	0.80115	no
gi|320446754|ref|NW_003383817.1|	42903-44852	241251	106408	-118093	0.48565	no
gi|320446754|ref|NW_003383817.1|	49303-49861	16629	0.3508	-556691	0.27475	no
gi|320446754|ref|NW_003383817.1|	88821-90277	35945	778378	111468	0.60245	no

gi|320446754|ref|NW_003383817.1|	91713-92198	332083	666033	100405	0.60135	no
gi|320446754|ref|NW_003383817.1|	92871-93557	673704	145674	111256	0.599	no
gi|320446754|ref|NW_003383817.1|	99251-100486	202587	0.117078	-4113	0.3071	no
gi|320446755|ref|NW_003383816.1|	0-308	177082	148052	306362	0.24875	no
gi|320446755|ref|NW_003383816.1|	130768-13318(	0.0773082	124161	400545	1	no
gi|320446755|ref|NW_003383816.1|	13956-14446	123856	218068	-250581	0.2872	no
gi|320446755|ref|NW_003383816.1|	150385-15098(	0.459966	405483	314004	0.24575	no
gi|320446755|ref|NW_003383816.1|	153323-15377(	154818	102851	27319	0.23745	no
gi|320446755|ref|NW_003383816.1|	154439-15597(	207432	211907	335272	0.08525	no
gi|320446755|ref|NW_003383816.1|	15750-16377	902902	175352	-236431	0.306	no
gi|320446755|ref|NW_003383816.1|	158731-15922!	282634	0	#NAME?	0.0063	no
gi|320446755|ref|NW_003383816.1|	16699-18698	155955	613805	-134528	0.41625	no
gi|320446755|ref|NW_003383816.1|	169450-16989'	387045	565678	0.547478	0.8002	no
gi|320446755|ref|NW_003383816.1|	187945-18842!	200762	626155	-168089	0.42785	no
gi|320446755|ref|NW_003383816.1|	188809-19057(	133309	429754	-163319	0.47285	no
gi|320446755|ref|NW_003383816.1|	191199-19170:	684044	125054	-245153	0.24515	no
gi|320446755|ref|NW_003383816.1|	194075-19467!	314845	133089	-124224	0.5692	no
gi|320446755|ref|NW_003383816.1|	195276-19601'	700954	457298	-0.616183	0.7724	no
gi|320446755|ref|NW_003383816.1|	19558-19822	325181	154948	-106946	0.59325	no
gi|320446755|ref|NW_003383816.1|	197874-19807:	448004	232555	-0.945941	0.6795	no
gi|320446755|ref|NW_003383816.1|	198771-19969(	367113	172143	-109261	0.50265	no
gi|320446755|ref|NW_003383816.1|	201652-20357.	540251	167379	-169051	0.42915	no
gi|320446755|ref|NW_003383816.1|	203742-20441:	388815	212143	-0.874047	0.68265	no
gi|320446755|ref|NW_003383816.1|	204675-20554.	346296	0.550183	-265402	0.22905	no
gi|320446755|ref|NW_003383816.1|	207992-20837.	341386	349668	-328735	0.20365	no
gi|320446755|ref|NW_003383816.1|	208529-20949:	609206	147096	-205017	0.24865	no
gi|320446755|ref|NW_003383816.1|	210945-21309(	418403	202198	-104912	0.42425	no
gi|320446755|ref|NW_003383816.1|	216064-21642:	113.83	255333	-215643	0.34085	no
gi|320446755|ref|NW_003383816.1|	217407-21782(	193489	697404	-147218	0.48965	no
gi|320446755|ref|NW_003383816.1|	218826-21954.	234249	550172	-209009	0.3455	no

gi|320446755|ref|NW_003383816.1|	219699-22201	275338	983669	-148496	0.3981	no
gi|320446755|ref|NW_003383816.1|	240732-24103	73264	188263	136157	0.52015	no
gi|320446755|ref|NW_003383816.1|	250659-25093	142869	212493	0.572725	0.7884	no
gi|320446755|ref|NW_003383816.1|	271524-27214	573342	269567	-108876	0.5848	no
gi|320446755|ref|NW_003383816.1|	276360-27776	750253	631523	-0.248543	0.90375	no
gi|320446755|ref|NW_003383816.1|	278450-28321	465818	785632	0.754087	0.5469	no
gi|320446755|ref|NW_003383816.1|	299861-30068	113928	881188	295133	0.2264	no
gi|320446755|ref|NW_003383816.1|	306882-30731	142618	107759	291759	0.1079	no
gi|320446755|ref|NW_003383816.1|	340089-34627	406795	0.0198823	-109986	0.2504	no
gi|320446755|ref|NW_003383816.1|	353972-35962	420434	0.109074	-119124	0.1124	no
gi|320446755|ref|NW_003383816.1|	360461-36211	197757	0	#NAME?	5.00E-05	yes
gi|320446755|ref|NW_003383816.1|	364632-36633	1109.64	0.479712	-111756	0.0976	no
gi|320446755|ref|NW_003383816.1|	369154-37181	2654.58	0.729043	-118302	0.2597	no
gi|320446755|ref|NW_003383816.1|	372334-37366	108214	10303	325111	0.18015	no
gi|320446755|ref|NW_003383816.1|	375380-37582	619272	205701	-159003	0.44495	no
gi|320446755|ref|NW_003383816.1|	375983-37698	0.664791	260222	196877	0.3902	no
gi|320446755|ref|NW_003383816.1|	381834-38821	563536	0.038461	-105169	0.16165	no
gi|320446755|ref|NW_003383816.1|	390086-39257	2728.63	0.574956	-122124	0.26475	no
gi|320446755|ref|NW_003383816.1|	398158-39997	363664	0.298087	-36088	0.19585	no
gi|320446755|ref|NW_003383816.1|	416211-41733	25211	190416	-0.404901	0.80525	no
gi|320446755|ref|NW_003383816.1|	420192-42213	18786	0.27572	-276839	0.26145	no
gi|320446755|ref|NW_003383816.1|	424955-42597	173183	0.448781	-194822	0.30305	no
gi|320446755|ref|NW_003383816.1|	427407-42763	315205	84934	-189187	0.3088	no
gi|320446755|ref|NW_003383816.1|	427991-42818	553465	228903	-127376	0.5576	no
gi|320446755|ref|NW_003383816.1|	429726-43066	135096	615673	-113375	0.58935	no
gi|320446755|ref|NW_003383816.1|	433774-43412	197179	119638	-0.72083	0.72485	no
gi|320446755|ref|NW_003383816.1|	455353-45614	230469	433067	0.910017	0.5718	no
gi|320446755|ref|NW_003383816.1|	45716-48643	354183	0.394398	-64887	0.06255	no
gi|320446755|ref|NW_003383816.1|	471009-47154	561769	211732	191419	0.3726	no
gi|320446755|ref|NW_003383816.1|	486851-48718	155942	652459	206488	0.3473	no

gi|320446755|ref|NW_003383816.1|	487476-48870	0.340714	106316	164172	1	no
gi|320446755|ref|NW_003383816.1|	491546-49912	459251	242955	240334	0.0788	no
gi|320446755|ref|NW_003383816.1|	4960-5565	100119	493949	-101928	0.59935	no
gi|320446755|ref|NW_003383816.1|	505417-51036	0.75249	152357	433964	0.04955	no
gi|320446755|ref|NW_003383816.1|	518459-51959	129844	0.129205	-99729	0.2504	no
gi|320446755|ref|NW_003383816.1|	520702-52216	993771	0.287821	-510967	0.1386	no
gi|320446755|ref|NW_003383816.1|	617-985	138938	136163	-0.0291086	0.96685	no
gi|320446755|ref|NW_003383816.1|	6362-7031	349934	318214	-0.137082	0.94275	no
gi|320446755|ref|NW_003383816.1|	71340-74838	164455	281857	0.777272	0.5592	no
gi|320446755|ref|NW_003383816.1|	75698-77726	338867	361053	0.0914926	0.9435	no
gi|320446755|ref|NW_003383816.1|	7656-9362	837629	631195	-0.408225	0.85185	no
gi|320446755|ref|NW_003383816.1|	79524-80853	138878	100701	-0.463733	0.8313	no
gi|320446755|ref|NW_003383816.1|	9631-13857	353041	230599	-0.614448	0.6394	no
gi|320446756|ref|NW_003383815.1|	106012-10701	328939	196958	-0.739934	0.71205	no
gi|320446756|ref|NW_003383815.1|	114634-11529	274678	0.80263	-177494	0.3225	no
gi|320446756|ref|NW_003383815.1|	13747-14083	0	129534	inf	0.0233	no
gi|320446756|ref|NW_003383815.1|	144618-14577	265805	168897	-0.65423	0.68665	no
gi|320446756|ref|NW_003383815.1|	146928-14732	77394	509391	-0.60345	0.75385	no
gi|320446756|ref|NW_003383815.1|	149067-14995	205638	318764	0.632383	0.74535	no
gi|320446756|ref|NW_003383815.1|	150129-15302	244618	360191	0.55823	0.67415	no
gi|320446756|ref|NW_003383815.1|	162199-16415	0.294037	512503	412349	0.1628	no
gi|320446756|ref|NW_003383815.1|	164209-16728	0.237595	311501	371266	0.17155	no
gi|320446756|ref|NW_003383815.1|	233075-23449	485157	456096	323282	0.0961	no
gi|320446756|ref|NW_003383815.1|	234775-23698	45267	144837	16779	0.3138	no
gi|320446756|ref|NW_003383815.1|	241580-24318	66629	755576	350335	0.08205	no
gi|320446756|ref|NW_003383815.1|	243293-24481	142961	66.86	222552	0.2277	no
gi|320446756|ref|NW_003383815.1|	295800-29748	128401	42747	173516	0.33585	no
gi|320446756|ref|NW_003383815.1|	299429-30483	313379	138373	214259	0.24545	no
gi|320446756|ref|NW_003383815.1|	304978-30709	197604	714333	185398	0.40205	no
gi|320446756|ref|NW_003383815.1|	307316-30933	199005	85911	211004	0.353	no

gi|320446756|ref|NW_003383815.1|	15677-31802	127551	85711	274841	0.23935	no
gi|320446756|ref|NW_003383815.1|	51935-35360	410823	890708	111643	0.6025	no
gi|320446756|ref|NW_003383815.1|	10561-41511	104435	582351	247928	0.0686	no
gi|320446756|ref|NW_003383815.1|	30835-43284	180254	111809	263293	0.257	no
gi|320446756|ref|NW_003383815.1|	11674-51201	180459	900541	-100281	0.6038	no
gi|320446756|ref|NW_003383815.1|	20890-52132	17376	767427	-117899	0.57495	no
gi|320446756|ref|NW_003383815.1|	41149-54390	0.534888	485938	318346	0.18805	no
gi|320446756|ref|NW_003383815.1|	72294-57312	277211	831064	158398	0.36465	no
gi|320446756|ref|NW_003383815.1|	57765-58373	496641	0.612733	-301887	0.25735	no
gi|320446756|ref|NW_003383815.1|	92938-59388	135189	834568	-0.695872	0.7395	no
gi|320446756|ref|NW_003383815.1|	02469-60290	140846	28.38	-231117	0.19165	no
gi|320446756|ref|NW_003383815.1|	03956-60519	43632	418998	-0.0584439	0.97595	no
gi|320446756|ref|NW_003383815.1|	05455-60600	102108	905798	-0.172835	0.92895	no
gi|320446756|ref|NW_003383815.1|	06195-60791	382418	37964	-0.0105158	0.9923	no
gi|320446756|ref|NW_003383815.1|	08352-61009	268447	576476	110262	0.6001	no
gi|320446756|ref|NW_003383815.1|	43795-64424	309636	565678	0.869406	0.6937	no
gi|320446756|ref|NW_003383815.1|	44313-64567	150188	511071	176676	0.40755	no
gi|320446756|ref|NW_003383815.1|	55244-65973	111858	106942	-0.0648468	0.95865	no
gi|320446756|ref|NW_003383815.1|	63334-66359	1152.21	117499	-329369	0.0967	no
gi|320446756|ref|NW_003383815.1|	65246-66554	416291	101623	-203436	0.35135	no
gi|320446756|ref|NW_003383815.1|	68544-66987	294655	109748	-142484	0.39375	no
gi|320446756|ref|NW_003383815.1|	70095-67120	131362	829096	-0.663935	0.76525	no
gi|320446756|ref|NW_003383815.1|	76197-67681	28885	505413	-251478	0.27215	no
gi|320446756|ref|NW_003383815.1|	76954-67734	256513	0	#NAME?	0.0104	no
gi|320446756|ref|NW_003383815.1|	77497-67782	518308	689874	-29094	0.221	no
gi|320446756|ref|NW_003383815.1|	80532-68130	167332	194911	-310182	0.1876	no
gi|320446756|ref|NW_003383815.1|	82201-68302	231094	568817	-202244	0.3636	no
gi|320446756|ref|NW_003383815.1|	85625-68831	116859	830946	-0.491943	0.76615	no
gi|320446756|ref|NW_003383815.1|	18664-71978	0.382418	34419	316998	0.20465	no
gi|320446756|ref|NW_003383815.1|	25608-72616	10331	523173	234031	0.2622	no

gi|320446756|ref|NW_003383815.1|	88402-92627	173393	213188	0.298076	0.88935	no
gi|320446756|ref|NW_003383815.1|	95354-97502	414649	492433	0.248039	0.90355	no
gi|320446757|ref|NW_003383814.1|	24692-12501	158324	183989	353867	0.2109	no
gi|320446757|ref|NW_003383814.1|	67989-16901	584897	0.295089	-763089	0.16165	no
gi|320446757|ref|NW_003383814.1|	69271-17098	475892	0.397347	-690409	0.1125	no
gi|320446757|ref|NW_003383814.1|	72375-17330	106707	0	#NAME?	5.00E-05	yes
gi|320446757|ref|NW_003383814.1|	79013-18021	361146	486082	-289331	0.11955	no
gi|320446757|ref|NW_003383814.1|	80969-18149	634336	0.775444	-303215	0.2571	no
gi|320446757|ref|NW_003383814.1|	83523-18501	334921	89424	141684	0.5091	no
gi|320446757|ref|NW_003383814.1|	86856-18794	516458	879666	0.768303	0.7104	no
gi|320446757|ref|NW_003383814.1|	89912-19029	417271	410675	-0.0229855	0.9157	no
gi|320446757|ref|NW_003383814.1|	91196-19487	489685	359806	-0.444636	0.8409	no
gi|320446757|ref|NW_003383814.1|	07417-20864	728124	469705	-0.63243	0.75895	no
gi|320446757|ref|NW_003383814.1|	09395-21062	237541	211787	-0.16556	0.93205	no
gi|320446757|ref|NW_003383814.1|	10785-21241	339037	412939	0.284486	0.8877	no
gi|320446757|ref|NW_003383814.1|	12542-21428	492479	662604	0.428084	0.84025	no
gi|320446757|ref|NW_003383814.1|	15411-21637	219244	114291	-0.939825	0.6721	no
gi|320446757|ref|NW_003383814.1|	17004-21881	109516	845287	-0.373627	0.86665	no
gi|320446757|ref|NW_003383814.1|	19997-22080	225791	19745	-0.1935	0.92965	no
gi|320446757|ref|NW_003383814.1|	23177-22492	167891	693786	-127496	0.43465	no
gi|320446757|ref|NW_003383814.1|	26042-22684	254559	183052	-0.475749	0.82595	no
gi|320446757|ref|NW_003383814.1|	32464-23333	335655	347594	0.0504236	0.96215	no
gi|320446757|ref|NW_003383814.1|	34208-24071	110586	511111	-111346	0.39955	no
gi|320446757|ref|NW_003383814.1|	42291-24346	0.718425	0.995647	0.470797	1	no
gi|320446757|ref|NW_003383814.1|	43794-24619	662279	511402	-0.372981	0.8649	no
gi|320446757|ref|NW_003383814.1|	46326-25059	100281	101523	0.017746	0.9885	no
gi|320446757|ref|NW_003383814.1|	65996-26648	760923	734422	-0.0511413	0.9831	no
gi|320446757|ref|NW_003383814.1|	78143-27999	249892	203893	-0.293492	0.8123	no
gi|320446757|ref|NW_003383814.1|	80253-28092	828283	736593	-0.169256	0.897	no
gi|320446757|ref|NW_003383814.1|	81082-28505	442494	712305	0.686838	0.59615	no

gi|320446757|ref|NW_003383814.1|	286628-28970	0.474857	19092	20074	0.34685	no
gi|320446757|ref|NW_003383814.1|	291115-29209	205094	346148	0.755099	0.70975	no
gi|320446757|ref|NW_003383814.1|	292371-29715	449225	507066	0.174734	0.91635	no
gi|320446757|ref|NW_003383814.1|	297625-29848	168076	244407	0.540177	0.8023	no
gi|320446757|ref|NW_003383814.1|	301656-30222	0	477306	inf	0.0233	no
gi|320446757|ref|NW_003383814.1|	302332-30455	916852	143375	0.645038	0.69365	no
gi|320446757|ref|NW_003383814.1|	305495-30598	348141	280497	-0.311686	0.8786	no
gi|320446757|ref|NW_003383814.1|	316211-31745	0.16815	174926	337893	0.2261	no
gi|320446757|ref|NW_003383814.1|	32944-35450	995777	441171	-117448	0.4693	no
gi|320446757|ref|NW_003383814.1|	374247-37486	0.436535	661352	724318	0.14075	no
gi|320446757|ref|NW_003383814.1|	375171-37561	128274	0	#NAME?	0.015	no
gi|320446757|ref|NW_003383814.1|	377499-37890	880795	0.400628	-77804	0.1293	no
gi|320446757|ref|NW_003383814.1|	38100-42941	441077	327677	-0.428756	0.7479	no
gi|320446757|ref|NW_003383814.1|	463049-46340	220316	27927	0.342089	0.8635	no
gi|320446757|ref|NW_003383814.1|	47247-50973	874903	720516	-0.280092	0.82335	no
gi|320446757|ref|NW_003383814.1|	61272-61783	278147	109561	-134412	0.52405	no
gi|320446759|ref|NW_003383812.1|	126395-12741	0.858208	133455	0.636953	1	no
gi|320446759|ref|NW_003383812.1|	13620-14232	629965	615669	-0.033117	0.9783	no
gi|320446759|ref|NW_003383812.1|	136230-13758	0.151265	10504	279579	1	no
gi|320446759|ref|NW_003383812.1|	14339-16196	0.520287	645387	363278	0.1685	no
gi|320446759|ref|NW_003383812.1|	16301-17054	0.652143	136332	438579	0.15665	no
gi|320446759|ref|NW_003383812.1|	181838-18475	227485	964695	-123762	0.3486	no
gi|320446759|ref|NW_003383812.1|	185559-18720	569836	191218	-157533	0.2358	no
gi|320446759|ref|NW_003383812.1|	187388-18818	664758	35271	-0.914348	0.65175	no
gi|320446759|ref|NW_003383812.1|	188313-18990	81414	34941	-122036	0.3628	no
gi|320446759|ref|NW_003383812.1|	203210-20397	141292	946784	-0.577577	0.7791	no
gi|320446759|ref|NW_003383812.1|	209536-21063	163771	917616	-0.835715	0.69905	no
gi|320446759|ref|NW_003383812.1|	210832-21568	123217	172611	0.486329	0.7139	no
gi|320446759|ref|NW_003383812.1|	21225-21880	172706	848283	-10257	0.6215	no
gi|320446759|ref|NW_003383812.1|	22502-24247	300685	101985	-15599	0.36315	no

gi|320446759|ref|NW_003383812.1|	241503-24225[illegible]	0.329885	497295	391407	0.2053	no
gi|320446759|ref|NW_003383812.1|	248477-25575[illegible]	301311	224527	-0.424361	0.7438	no
gi|320446759|ref|NW_003383812.1|	24920-26920	17021	926857	-0.876897	0.60105	no
gi|320446759|ref|NW_003383812.1|	256937-25853[illegible]	674187	162395	-205364	0.125	no
gi|320446759|ref|NW_003383812.1|	258599-25963[illegible]	105929	131806	0.315315	1	no
gi|320446759|ref|NW_003383812.1|	28843-31039	123931	114108	-0.119134	0.9262	no
gi|320446759|ref|NW_003383812.1|	296193-29665[illegible]	559254	293931	-0.928029	0.6658	no
gi|320446759|ref|NW_003383812.1|	337715-33839[illegible]	128957	621316	-10535	0.60905	no
gi|320446759|ref|NW_003383812.1|	339675-34038[illegible]	179623	736803	-128562	0.5455	no
gi|320446759|ref|NW_003383812.1|	340934-34160[illegible]	523402	327618	-0.675905	0.67525	no
gi|320446759|ref|NW_003383812.1|	343096-34491[illegible]	105623	929186	-0.184879	0.9316	no
gi|320446759|ref|NW_003383812.1|	345019-34657[illegible]	217389	214381	-0.0201071	0.98805	no
gi|320446759|ref|NW_003383812.1|	34588-36905	124021	0	#NAME?	5.00E-05	yes
gi|320446759|ref|NW_003383812.1|	369670-37157[illegible]	109145	148554	0.444747	0.74205	no
gi|320446759|ref|NW_003383812.1|	38575-40020	962601	0.290883	-504843	0.141	no
gi|320446759|ref|NW_003383812.1|	40987-41795	312458	0.202513	-726951	0.2606	no
gi|320446759|ref|NW_003383812.1|	4147-5112	589865	328924	-0.84263	0.5014	no
gi|320446759|ref|NW_003383812.1|	42304-43002	215141	0	#NAME?	0.0062	no
gi|320446759|ref|NW_003383812.1|	427300-42860[illegible]	0	152883	inf	0.0233	no
gi|320446759|ref|NW_003383812.1|	43285-45312	338114	0.131365	-800779	0.16165	no
gi|320446759|ref|NW_003383812.1|	446392-44712[illegible]	0	460839	inf	0.0162	no
gi|320446759|ref|NW_003383812.1|	463151-46542[illegible]	0.0826075	282537	509602	0.17115	no
gi|320446759|ref|NW_003383812.1|	465494-46627[illegible]	0	320755	inf	0.02205	no
gi|320446759|ref|NW_003383812.1|	466740-46771[illegible]	0.689234	222045	168778	0.4293	no
gi|320446759|ref|NW_003383812.1|	47600-47843	631753	0	#NAME?	0.01345	no
gi|320446759|ref|NW_003383812.1|	47944-50783	423833	0.045296	-98699	0.2504	no
gi|320446759|ref|NW_003383812.1|	544801-54568[illegible]	130714	10803	-0.274978	1	no
gi|320446759|ref|NW_003383812.1|	5582-6185	205881	148947	-0.467014	0.8305	no
gi|320446759|ref|NW_003383812.1|	591427-59206[illegible]	254138	115224	-114118	0.58445	no
gi|320446759|ref|NW_003383812.1|	592294-59622[illegible]	228155	488678	109887	0.62585	no

gi|320446759|ref|NW_003383812.1|	96995-59919	456366	147214	168965	0.30955	no
gi|320446759|ref|NW_003383812.1|	57067-65738	643151	10375	0.68988	0.74435	no
gi|320446759|ref|NW_003383812.1|	66278-66700	342562	539353	0.654866	0.7494	no
gi|320446759|ref|NW_003383812.1|	67139-66812	559876	190244	176467	0.43265	no
gi|320446759|ref|NW_003383812.1|	73247-67481	356557	923623	-194876	0.27205	no
gi|320446759|ref|NW_003383812.1|	75890-67939	68139	675364	-0.0128139	0.9917	no
gi|320446759|ref|NW_003383812.1|	80559-68089	414423	670525	-262774	0.25675	no
gi|320446759|ref|NW_003383812.1|	82674-68845	231088	723278	-167582	0.2194	no
gi|320446759|ref|NW_003383812.1|	72331-72729	273735	643171	-208951	0.34505	no
gi|320446759|ref|NW_003383812.1|	73733-74387	281814	164559	-0.77614	0.71385	no
gi|320446759|ref|NW_003383812.1|	75958-76542	481897	424096	-0.184333	0.921	no
gi|320446759|ref|NW_003383812.1|	68356-76938	832518	153446	0.882182	0.67705	no
gi|320446759|ref|NW_003383812.1|	76468-77685	255114	670159	-192857	0.3794	no
gi|320446760|ref|NW_003383811.1|	11584-15510	365283	187201	-0.964429	0.47525	no
gi|320446760|ref|NW_003383811.1|	21154-12203	208497	448763	110593	0.5893	no
gi|320446760|ref|NW_003383811.1|	63177-16442	348941	0.230512	-392007	0.22055	no
gi|320446760|ref|NW_003383811.1|	17094-19124	818016	386883	-108023	0.62365	no
gi|320446760|ref|NW_003383811.1|	94588-19525	194408	291697	0.585384	0.75905	no
gi|320446760|ref|NW_003383811.1|	20608-21734	20844	32802	0.654154	0.74215	no
gi|320446760|ref|NW_003383811.1|	21937-23339	665393	109542	0.719199	0.7357	no
gi|320446760|ref|NW_003383811.1|	25057-25540	716051	411473	-0.799262	0.71955	no
gi|320446760|ref|NW_003383811.1|	25718-26612	222952	157141	-0.504669	0.81945	no
gi|320446760|ref|NW_003383811.1|	27474-27881	126918	112571	-0.17305	0.8874	no
gi|320446760|ref|NW_003383811.1|	47373-34794	0	440731	inf	0.02915	no
gi|320446760|ref|NW_003383811.1|	35037-40944	220138	116955	240948	0.19445	no
gi|320446760|ref|NW_003383811.1|	72217-37244	184666	775394	-457385	0.14735	no
gi|320446760|ref|NW_003383811.1|	73760-37460	349434	114465	-493204	0.0976	no
gi|320446760|ref|NW_003383811.1|	76258-37656	240157	118644	-433927	0.2992	no
gi|320446760|ref|NW_003383811.1|	77287-37807	618417	211172	-487209	0.0664	no
gi|320446760|ref|NW_003383811.1|	79743-38107	174952	873719	-100172	0.66325	no

gi|320446760|ref|NW_003383811.1|	381284-38261	416252	342503	-0.281341	0.8902	no
gi|320446760|ref|NW_003383811.1|	383647-38662	71365	15914	-216492	0.34095	no
gi|320446760|ref|NW_003383811.1|	386980-38728	178.23	401513	-215023	0.33425	no
gi|320446760|ref|NW_003383811.1|	387433-38781	127325	622161	-103316	0.6462	no
gi|320446760|ref|NW_003383811.1|	394041-40306	129568	258081	0.994117	0.4545	no
gi|320446760|ref|NW_003383811.1|	403208-40429	197349	220684	0.161239	0.94175	no
gi|320446760|ref|NW_003383811.1|	404695-40547	247489	339689	0.456851	0.81605	no
gi|320446760|ref|NW_003383811.1|	405582-40649	946496	824171	-0.199651	0.92455	no
gi|320446760|ref|NW_003383811.1|	407946-40907	112933	860259	29293	0.21785	no
gi|320446760|ref|NW_003383811.1|	409230-41103	0.538212	0.974915	0.857101	1	no
gi|320446760|ref|NW_003383811.1|	411311-41273	0.282993	13766	228227	1	no
gi|320446760|ref|NW_003383811.1|	413752-41426	238564	600686	133224	0.52715	no
gi|320446760|ref|NW_003383811.1|	415344-41658	0.50445	151603	158751	0.43945	no
gi|320446760|ref|NW_003383811.1|	472801-47306	498987	246439	-101777	0.6129	no
gi|320446760|ref|NW_003383811.1|	477402-48305	23366	433124	0.890369	0.49525	no
gi|320446760|ref|NW_003383811.1|	483153-48659	8887	853492	-0.0583179	0.96425	no
gi|320446760|ref|NW_003383811.1|	499918-50038	450445	469416	-326241	0.1841	no
gi|320446760|ref|NW_003383811.1|	501050-50196	246891	442579	-247987	0.2861	no
gi|320446760|ref|NW_003383811.1|	502089-50289	288876	425276	-276398	0.24175	no
gi|320446760|ref|NW_003383811.1|	504205-50440	992471	475357	-438394	0.2989	no
gi|320446760|ref|NW_003383811.1|	56253-57271	107824	10432	-0.0476636	1	no
gi|320446760|ref|NW_003383811.1|	572438-57338	0.237173	261825	346459	0.21395	no
gi|320446760|ref|NW_003383811.1|	573519-57502	0.66614	194545	15462	0.48745	no
gi|320446760|ref|NW_003383811.1|	58395-59808	0	129438	inf	1	no
gi|320446760|ref|NW_003383811.1|	61957-63144	159607	725151	218376	0.3255	no
gi|320446760|ref|NW_003383811.1|	654236-65542	141873	5285	189731	0.3781	no
gi|320446760|ref|NW_003383811.1|	661963-66265	0.732434	325295	215098	0.2925	no
gi|320446760|ref|NW_003383811.1|	665297-66562	756565	587094	-0.365872	0.8566	no
gi|320446760|ref|NW_003383811.1|	7985-8886	329641	104844	-165265	0.4365	no
gi|320446760|ref|NW_003383811.1|	9088-10650	852248	398203	-109777	0.6012	no

gi|320446761|ref|NW_003383810.1|	114958-11610	977751	346963	-481661	0.04095	no
gi|320446761|ref|NW_003383810.1|	145522-14588	354953	239375	-0.568359	0.78355	no
gi|320446761|ref|NW_003383810.1|	147964-14914	46549	0.868455	-242223	0.28365	no
gi|320446761|ref|NW_003383810.1|	150383-15086	910747	178318	-23526	0.1754	no
gi|320446761|ref|NW_003383810.1|	150973-15245	0.815467	0.661409	-0.302084	1	no
gi|320446761|ref|NW_003383810.1|	152693-15345	140546	340956	-204338	0.27165	no
gi|320446761|ref|NW_003383810.1|	181085-18146	21956	143759	-0.610962	0.7677	no
gi|320446761|ref|NW_003383810.1|	310971-31505	0.394462	233189	256354	0.26255	no
gi|320446761|ref|NW_003383810.1|	353053-35430	148813	114698	-0.375659	0.8532	no
gi|320446761|ref|NW_003383810.1|	365417-36759	461549	136114	156026	0.34565	no
gi|320446761|ref|NW_003383810.1|	368048-36973	15112	275179	0.864679	0.67015	no
gi|320446761|ref|NW_003383810.1|	370011-37233	0.806335	140728	0.803454	1	no
gi|320446761|ref|NW_003383810.1|	373800-37487	34049	401861	0.239083	0.90165	no
gi|320446761|ref|NW_003383810.1|	380218-38043	799585	902106	0.174045	0.92885	no
gi|320446761|ref|NW_003383810.1|	387550-38785	738944	711863	-0.0538659	0.9157	no
gi|320446761|ref|NW_003383810.1|	389748-39237	585333	497624	-0.234202	0.9145	no
gi|320446761|ref|NW_003383810.1|	405160-40661	0.277409	115691	206018	1	no
gi|320446761|ref|NW_003383810.1|	505058-50924	775125	261614	175494	0.28235	no
gi|320446761|ref|NW_003383810.1|	509506-51026	553255	153922	147618	0.48245	no
gi|320446761|ref|NW_003383810.1|	512653-51327	65985	985961	0.579393	0.77875	no
gi|320446761|ref|NW_003383810.1|	513391-51481	212245	393315	0.889956	0.6677	no
gi|320446761|ref|NW_003383810.1|	515664-51655	284485	338496	0.250784	0.89745	no
gi|320446761|ref|NW_003383810.1|	521772-52359	0.42461	0.813601	0.938183	1	no
gi|320446761|ref|NW_003383810.1|	524838-52545	170684	110002	-0.6338	0.75805	no
gi|320446761|ref|NW_003383810.1|	525562-52623	557278	350811	-0.667702	0.6727	no
gi|320446761|ref|NW_003383810.1|	547982-54830	188794	939904	-100623	0.6585	no
gi|320446761|ref|NW_003383810.1|	580128-58039	214366	510864	-206906	0.28725	no
gi|320446761|ref|NW_003383810.1|	580398-68115	0	602303	inf	0.0133	no
gi|320446761|ref|NW_003383810.1|	770614-77108	0	464116	inf	0.0312	no
gi|320446761|ref|NW_003383810.1|	86671-86876	854697	246302	-179499	0.41475	no

gi|320446761|ref|NW_003383810.1|	87990-88791	128321	430425	-157593	0.46595	no
gi|320446761|ref|NW_003383810.1|	89356-90263	100522	502304	-100087	0.61965	no
gi|320446762|ref|NW_003383809.1|	10566-11812	21708	243221	0.164038	0.93105	no
gi|320446762|ref|NW_003383809.1|	11948-16199	535719	338898	-0.660623	0.62325	no
gi|320446762|ref|NW_003383809.1|	[illegible]93779-19421[illegible]	0	582521	inf	0.0294	no
gi|320446762|ref|NW_003383809.1|	[illegible]24997-22624[illegible]	0	147528	inf	0.0037	no
gi|320446762|ref|NW_003383809.1|	25426-26075	977755	388402	-133192	0.51805	no
gi|320446762|ref|NW_003383809.1|	26830-29502	616311	246761	-132054	0.54115	no
gi|320446762|ref|NW_003383809.1|	[illegible]80353-28496[illegible]	181232	240343	0.407255	0.8483	no
gi|320446762|ref|NW_003383809.1|	[illegible]86489-28751[illegible]	0.428015	177496	205205	0.3018	no
gi|320446762|ref|NW_003383809.1|	[illegible]93848-29455[illegible]	179244	245097	0.451431	0.82655	no
gi|320446762|ref|NW_003383809.1|	[illegible]96368-29758[illegible]	0	179701	inf	0.02205	no
gi|320446762|ref|NW_003383809.1|	29668-32725	227236	284321	0.32333	0.8766	no
gi|320446762|ref|NW_003383809.1|	[illegible]00498-30082[illegible]	0	500858	inf	0.0089	no
gi|320446762|ref|NW_003383809.1|	[illegible]06457-30692[illegible]	141325	570162	533429	0.14375	no
gi|320446762|ref|NW_003383809.1|	[illegible]20757-32103[illegible]	243108	201341	304997	0.24945	no
gi|320446762|ref|NW_003383809.1|	[illegible]25450-32765[illegible]	[illegible].0856696	801107	654707	0.1532	no
gi|320446762|ref|NW_003383809.1|	[illegible]28372-32885[illegible]	202296	540637	141819	0.50885	no
gi|320446762|ref|NW_003383809.1|	34761-35992	135601	188068	0.471889	0.80965	no
gi|320446762|ref|NW_003383809.1|	[illegible]60227-36058[illegible]	360356	141056	196877	0.3853	no
gi|320446762|ref|NW_003383809.1|	[illegible]66017-36675[illegible]	0.674219	323212	226119	0.2746	no
gi|320446762|ref|NW_003383809.1|	37214-39790	272496	15309	-0.831859	0.5332	no
gi|320446762|ref|NW_003383809.1|	39906-40452	646899	218042	-156894	0.48585	no
gi|320446762|ref|NW_003383809.1|	40749-42738	305991	110708	-146673	0.4036	no
gi|320446762|ref|NW_003383809.1|	43600-45117	146152	228916	-267458	0.24865	no
gi|320446762|ref|NW_003383809.1|	45853-46206	64869	223541	-153699	0.4766	no
gi|320446762|ref|NW_003383809.1|	46544-48545	318246	103296	-162335	0.3491	no
gi|320446762|ref|NW_003383809.1|	49236-53187	599411	975815	0.703064	0.57805	no
gi|320446762|ref|NW_003383809.1|	53430-53869	136291	207411	0.605808	0.7577	no
gi|320446762|ref|NW_003383809.1|	54089-54856	381001	370192	-0.04152	0.96585	no

gi|320446762|ref|NW_003383809.1|	56612-57869	412966	194799	-108404	0.5975	no
gi|320446762|ref|NW_003383809.1|	59846-61146	190215	0.880106	-111189	0.59005	no
gi|320446762|ref|NW_003383809.1|	6707-9508	273588	192588	-0.506489	0.69705	no
gi|320446762|ref|NW_003383809.1|	74675-76102	778884	334595	-121899	0.56415	no
gi|320446764|ref|NW_003383807.1|	100208-10056	297505	371541	0.320609	0.8746	no
gi|320446764|ref|NW_003383807.1|	102573-10364	44486	433632	-0.0368774	0.9794	no
gi|320446764|ref|NW_003383807.1|	107030-10760	741525	902726	0.283792	0.88905	no
gi|320446764|ref|NW_003383807.1|	114431-11654	810105	276292	177001	0.31365	no
gi|320446764|ref|NW_003383807.1|	117330-11803	472012	156335	172774	0.4197	no
gi|320446764|ref|NW_003383807.1|	120627-12141	986341	325784	172376	0.44115	no
gi|320446764|ref|NW_003383807.1|	123699-12504	500071	155735	163889	0.45945	no
gi|320446764|ref|NW_003383807.1|	144437-14492	0.661672	442423	274124	0.2661	no
gi|320446764|ref|NW_003383807.1|	145162-14642	0.327487	181758	247251	0.2454	no
gi|320446764|ref|NW_003383807.1|	147179-15007	0.0633702	243633	526476	0.1667	no
gi|320446764|ref|NW_003383807.1|	150354-15286	103762	781673	291328	0.22255	no
gi|320446764|ref|NW_003383807.1|	169024-16980	0	611309	inf	0.0109	no
gi|320446764|ref|NW_003383807.1|	172761-17484	347797	220895	266704	0.14795	no
gi|320446764|ref|NW_003383807.1|	177646-17835	106423	58226	245186	0.2909	no
gi|320446764|ref|NW_003383807.1|	178458-17879	0	187855	inf	0.01575	no
gi|320446764|ref|NW_003383807.1|	184748-18525	186557	541902	153841	0.4913	no
gi|320446764|ref|NW_003383807.1|	23904-24935	0	275668	inf	5.00E-05	yes
gi|320446764|ref|NW_003383807.1|	252798-25338	66.35	388446	-0.772383	0.62875	no
gi|320446764|ref|NW_003383807.1|	260785-26196	571697	383798	-0.574904	0.77945	no
gi|320446764|ref|NW_003383807.1|	268459-26910	45137	329649	286855	0.2147	no
gi|320446764|ref|NW_003383807.1|	269216-27691	0.836632	707125	30793	0.10785	no
gi|320446764|ref|NW_003383807.1|	281656-28206	347048	12981	-141873	0.5027	no
gi|320446764|ref|NW_003383807.1|	285134-28555	606858	192263	-165827	0.4522	no
gi|320446764|ref|NW_003383807.1|	287843-29358	118947	842118	-0.498229	0.69995	no
gi|320446764|ref|NW_003383807.1|	295252-29643	458986	133154	-178536	0.3031	no
gi|320446764|ref|NW_003383807.1|	297673-30135	107117	393618	-144432	0.40055	no

gi|320446764|ref|NW_003383807.1|	302086-30329	201969	125664	-0.684568	0.7631	no
gi|320446764|ref|NW_003383807.1|	308736-31013	189345	161893	-0.225971	0.9033	no
gi|320446764|ref|NW_003383807.1|	313168-31591	0.673144	1176	0.804904	1	no
gi|320446764|ref|NW_003383807.1|	316852-31893	164746	197911	0.264612	0.89195	no
gi|320446764|ref|NW_003383807.1|	319496-32413	807893	121742	0.59159	0.65025	no
gi|320446764|ref|NW_003383807.1|	339274-34040	0.376025	143219	192932	0.3182	no
gi|320446764|ref|NW_003383807.1|	36903-38585	192469	196534	0.0301534	0.9813	no
gi|320446764|ref|NW_003383807.1|	374144-37480	356441	539899	0.599029	0.763	no
gi|320446764|ref|NW_003383807.1|	434569-43525	0.371013	861579	785937	0.14075	no
gi|320446764|ref|NW_003383807.1|	46910-47992	586065	439989	-0.413593	0.74515	no
gi|320446764|ref|NW_003383807.1|	50082-55161	269372	272574	0.0170464	0.9926	no
gi|320446764|ref|NW_003383807.1|	551067-55306	0.383146	354003	32078	0.20395	no
gi|320446764|ref|NW_003383807.1|	556550-55713	27872	71577	-196125	0.37605	no
gi|320446764|ref|NW_003383807.1|	558625-56126	22773	569634	-199922	0.2703	no
gi|320446764|ref|NW_003383807.1|	573872-57509	102942	14275	0.471665	0.81615	no
gi|320446764|ref|NW_003383807.1|	595398-59623	545186	274436	-0.99028	0.55895	no
gi|320446764|ref|NW_003383807.1|	599666-60075	194816	980525	-0.990489	0.6608	no
gi|320446764|ref|NW_003383807.1|	600922-60129	786636	367549	-109776	0.59365	no
gi|320446764|ref|NW_003383807.1|	601492-60169	274076	51299	0.904358	0.68565	no
gi|320446764|ref|NW_003383807.1|	604119-61044	121571	138588	0.189	0.88625	no
gi|320446764|ref|NW_003383807.1|	629188-63010	124539	223213	0.841825	0.6905	no
gi|320446764|ref|NW_003383807.1|	82859-88434	112174	119118	0.0866579	0.9465	no
gi|320446764|ref|NW_003383807.1|	89768-90245	205432	146312	-0.489606	0.8108	no
gi|320446764|ref|NW_003383807.1|	97041-97532	149378	956102	-0.643728	0.75635	no
gi|320446766|ref|NW_003383805.1|	10034-11956	334155	288032	-0.214291	0.8699	no
gi|320446766|ref|NW_003383805.1|	103723-11125	142516	144859	0.023525	0.98655	no
gi|320446766|ref|NW_003383805.1|	112289-11607	472884	607297	0.360915	0.78395	no
gi|320446766|ref|NW_003383805.1|	12065-13090	141066	100455	-0.489816	0.82085	no
gi|320446766|ref|NW_003383805.1|	122480-12278	242236	227312	-0.0917412	0.95685	no
gi|320446766|ref|NW_003383805.1|	122931-12433	565106	604005	0.0960384	0.96165	no

gi|320446766|ref|NW_003383805.1|	124433-125184	360066	134603	-141955	0.5046	no
gi|320446766|ref|NW_003383805.1|	125446-125904	147782	737745	-100228	0.61475	no
gi|320446766|ref|NW_003383805.1|	126004-12623[illegible]	270175	141557	-0.932516	0.67975	no
gi|320446766|ref|NW_003383805.1|	127129-12748[illegible]	492849	172785	-151217	0.4751	no
gi|320446766|ref|NW_003383805.1|	127542-129455	563267	403849	-0.480001	0.72115	no
gi|320446766|ref|NW_003383805.1|	129770-13159[illegible]	459351	572122	0.316725	0.80935	no
gi|320446766|ref|NW_003383805.1|	137003-137687	0	410662	inf	0.0198	no
gi|320446766|ref|NW_003383805.1|	15088-15340	593491	178031	-17371	0.4041	no
gi|320446766|ref|NW_003383805.1|	156778-15724[illegible]	163173	804124	-102091	0.618	no
gi|320446766|ref|NW_003383805.1|	15776-16782	240909	128616	-0.905421	0.68445	no
gi|320446766|ref|NW_003383805.1|	17410-17763	64869	215262	-159143	0.4589	no
gi|320446766|ref|NW_003383805.1|	21363-21650	170817	136521	-0.323332	0.8612	no
gi|320446766|ref|NW_003383805.1|	223217-223765	0	541862	inf	0.02205	no
gi|320446766|ref|NW_003383805.1|	22460-22728	362897	148379	-129028	0.53155	no
gi|320446766|ref|NW_003383805.1|	22909-29327	231688	289216	0.319963	0.807	no
gi|320446766|ref|NW_003383805.1|	238270-23937[illegible]	195191	216156	0.147187	0.93445	no
gi|320446766|ref|NW_003383805.1|	240661-24124[illegible]	341149	527658	0.629202	0.7494	no
gi|320446766|ref|NW_003383805.1|	243405-244879	299729	445154	0.570645	0.7777	no
gi|320446766|ref|NW_003383805.1|	248569-24909[illegible]	401011	500851	0.320739	0.87715	no
gi|320446766|ref|NW_003383805.1|	254683-255777	491388	965797	0.974859	0.64595	no
gi|320446766|ref|NW_003383805.1|	256402-258461	0.185059	225882	360951	0.18175	no
gi|320446766|ref|NW_003383805.1|	29444-31055	45265	472074	0.0606178	0.96295	no
gi|320446766|ref|NW_003383805.1|	34776-37374	342805	229004	-0.582012	0.65765	no
gi|320446766|ref|NW_003383805.1|	358404-358784	178.04	275731	-269087	0.1344	no
gi|320446766|ref|NW_003383805.1|	371849-37224[illegible]	324774	733338	-214688	0.3378	no
gi|320446766|ref|NW_003383805.1|	375505-376129	346576	111871	-163134	0.45255	no
gi|320446766|ref|NW_003383805.1|	377184-379044	119445	332959	-184293	0.41235	no
gi|320446766|ref|NW_003383805.1|	379968-380354	844974	280628	-159025	0.47	no
gi|320446766|ref|NW_003383805.1|	380477-385095	271799	355896	0.388915	0.77015	no
gi|320446766|ref|NW_003383805.1|	394915-395359	235271	468639	0.994155	0.6698	no

gi|320446766|ref|NW_003383805.1|	456692-45821	170902	548552	168246	0.4287	no
gi|320446766|ref|NW_003383805.1|	467199-46852	0	129491	inf	1	no
gi|320446766|ref|NW_003383805.1|	468666-47036	0.694127	402749	253661	0.26725	no
gi|320446766|ref|NW_003383805.1|	49385-49956	100017	30427	-171683	0.4229	no
gi|320446766|ref|NW_003383805.1|	51488-52093	364069	246975	-0.559848	0.7893	no
gi|320446766|ref|NW_003383805.1|	518547-52091	0.711088	634389	315727	0.19825	no
gi|320446766|ref|NW_003383805.1|	52230-53059	398075	195497	-10259	0.60725	no
gi|320446766|ref|NW_003383805.1|	53383-53873	586687	261682	-116478	0.5824	no
gi|320446766|ref|NW_003383805.1|	55158-57209	511154	414849	-0.301172	0.8845	no
gi|320446766|ref|NW_003383805.1|	57691-59665	63085	79851	0.340015	0.87325	no
gi|320446766|ref|NW_003383805.1|	68318-68707	307816	309894	333163	0.1943	no
gi|320446766|ref|NW_003383805.1|	72460-73149	0.371825	162524	544988	0.16275	no
gi|320446766|ref|NW_003383805.1|	73598-74268	155172	100546	269591	0.25975	no
gi|320446766|ref|NW_003383805.1|	74392-74852	0.732835	975906	373518	0.2083	no
gi|320446766|ref|NW_003383805.1|	80597-82639	0.373601	475528	366996	0.1719	no
gi|320446766|ref|NW_003383805.1|	83231-84511	242323	156932	269514	0.24705	no
gi|320446766|ref|NW_003383805.1|	85939-87038	201.67	120374	-0.744475	0.57705	no
gi|320446766|ref|NW_003383805.1|	88150-89558	118006	279966	-207554	0.3505	no
gi|320446766|ref|NW_003383805.1|	90103-91059	236552	937465	-133532	0.5481	no
gi|320446766|ref|NW_003383805.1|	92852-93254	325523	126085	-136836	0.51635	no
gi|320446770|ref|NW_003383801.1|	125532-12790	340692	268775	297986	0.1315	no
gi|320446770|ref|NW_003383801.1|	128856-13393	135797	116985	31068	0.11505	no
gi|320446770|ref|NW_003383801.1|	134152-13568	0.260491	172115	272407	0.24095	no
gi|320446770|ref|NW_003383801.1|	143476-14553	611204	109991	0.847659	0.52155	no
gi|320446770|ref|NW_003383801.1|	166206-16756	180396	1171.06	269857	0.1172	no
gi|320446770|ref|NW_003383801.1|	176559-17896	0.622172	187894	491647	0.0602	no
gi|320446770|ref|NW_003383801.1|	196379-19666	560338	200351	-148377	0.4798	no
gi|320446772|ref|NW_003383799.1|	165599-16625	0.801398	546036	27684	0.23485	no
gi|320446772|ref|NW_003383799.1|	18946-20527	639564	453709	-0.495323	0.8186	no
gi|320446772|ref|NW_003383799.1|	204575-20499	312333	530395	-255795	0.25835	no

gi|320446772|ref|NW_003383799.1|	253671-25421!	428599	288993	-0.568592	0.7893	no
gi|320446772|ref|NW_003383799.1|	261529-26261(	0.792685	150843	0.928225	0.68415	no
gi|320446772|ref|NW_003383799.1|	262858-26541(	0.218508	152632	28043	0.2497	no
gi|320446772|ref|NW_003383799.1|	265624-26667	476503	859582	0.851151	0.6887	no
gi|320446772|ref|NW_003383799.1|	269440-27012	596223	458069	-0.380288	0.8429	no
gi|320446772|ref|NW_003383799.1|	270577-27177(	775409	537446	-0.528838	0.79875	no
gi|320446772|ref|NW_003383799.1|	288729-28926!	197759	925104	-109605	0.59855	no
gi|320446772|ref|NW_003383799.1|	290197-29125(	55732	613808	0.139282	0.94445	no
gi|320446772|ref|NW_003383799.1|	292000-29411	144303	168827	0.226446	0.8581	no
gi|320446772|ref|NW_003383799.1|	332242-33327	969725	499785	-42782	0.04595	no
gi|320446772|ref|NW_003383799.1|	336030-33675!	526481	51489	-335405	0.0929	no
gi|320446772|ref|NW_003383799.1|	336960-33879	388441	764926	-234431	0.20325	no
gi|320446772|ref|NW_003383799.1|	340694-34162	291156	451813	0.633931	0.75645	no
gi|320446772|ref|NW_003383799.1|	341727-34279	203674	295846	0.538589	0.79375	no
gi|320446772|ref|NW_003383799.1|	344050-34464	155539	220339	0.502456	0.81035	no
gi|320446772|ref|NW_003383799.1|	345216-34646	183865	317656	0.788815	0.63055	no
gi|320446772|ref|NW_003383799.1|	347124-34821(	317447	211424	273555	0.24185	no
gi|320446772|ref|NW_003383799.1|	349673-35028(	199181	583579	-177108	0.41095	no
gi|320446772|ref|NW_003383799.1|	351564-35673(	164054	148212	-0.146506	0.91255	no
gi|320446772|ref|NW_003383799.1|	361651-36306	625686	140273	-21572	0.23975	no
gi|320446772|ref|NW_003383799.1|	36713-37021	354164	159441	-11514	0.5853	no
gi|320446772|ref|NW_003383799.1|	372214-37364(	0	975072	inf	0.00615	no
gi|320446772|ref|NW_003383799.1|	374589-37596	0.148078	2777	422909	0.1913	no
gi|320446772|ref|NW_003383799.1|	378625-37945(	113354	29228	136652	0.52475	no
gi|320446772|ref|NW_003383799.1|	38374-39335	316329	171775	-0.88091	0.58785	no
gi|320446772|ref|NW_003383799.1|	394434-39482	451463	269473	-40664	0.17785	no
gi|320446772|ref|NW_003383799.1|	398698-40052(	497977	428188	-0.217834	0.9127	no
gi|320446772|ref|NW_003383799.1|	406222-40653!	230455	0	#NAME?	0.0178	no
gi|320446772|ref|NW_003383799.1|	408553-41056(	276212	0.996281	-147115	0.48285	no
gi|320446772|ref|NW_003383799.1|	412877-41781	142079	317417	115969	0.38915	no

gi|320446772|ref|NW_003383799.1|	421810-422737	236713	235604	-0.0067743	0.9956	no
gi|320446772|ref|NW_003383799.1|	426289-42676	237414	139761	-408637	0.1594	no
gi|320446772|ref|NW_003383799.1|	427984-428275	637465	131625	-559785	0.2784	no
gi|320446772|ref|NW_003383799.1|	458696-45929	83691	0.315167	-473089	0.2883	no
gi|320446772|ref|NW_003383799.1|	473894-474679	0	210796	inf	0.0312	no
gi|320446772|ref|NW_003383799.1|	48492-48812	38589	20.75	-0.895083	0.66805	no
gi|320446772|ref|NW_003383799.1|	48945-49908	10206	912589	-0.161387	0.9387	no
gi|320446772|ref|NW_003383799.1|	50353-53837	196846	426962	111704	0.4097	no
gi|320446772|ref|NW_003383799.1|	504440-507319	136587	923507	-0.564625	0.66065	no
gi|320446772|ref|NW_003383799.1|	507578-51052	207362	322138	0.635528	0.63295	no
gi|320446772|ref|NW_003383799.1|	510983-51207	0.790827	0.957691	0.276198	1	no
gi|320446772|ref|NW_003383799.1|	512191-51442	379644	200634	-0.920082	0.4906	no
gi|320446772|ref|NW_003383799.1|	515871-516438	145209	475234	-161142	0.35965	no
gi|320446772|ref|NW_003383799.1|	520510-521048	442263	446769	-330731	0.18255	no
gi|320446772|ref|NW_003383799.1|	521267-52208	942836	814981	-353217	0.07365	no
gi|320446772|ref|NW_003383799.1|	522218-524644	422872	731505	-253128	0.18275	no
gi|320446772|ref|NW_003383799.1|	526166-52665	277902	0.884846	-497301	0.1924	no
gi|320446772|ref|NW_003383799.1|	526839-52780	123275	0.160537	-626283	0.26445	no
gi|320446772|ref|NW_003383799.1|	528964-529315	231962	419199	-246818	0.29555	no
gi|320446772|ref|NW_003383799.1|	529994-53039	127091	0	#NAME?	0.01755	no
gi|320446772|ref|NW_003383799.1|	531582-533369	522013	128793	-201903	0.35445	no
gi|320446772|ref|NW_003383799.1|	534648-53739	861625	352801	-128821	0.4246	no
gi|320446772|ref|NW_003383799.1|	537737-540508	129762	104636	-0.31049	0.8079	no
gi|320446772|ref|NW_003383799.1|	53915-54971	0.41132	270241	271591	0.24135	no
gi|320446772|ref|NW_003383799.1|	545378-546165	673194	966227	0.521341	0.79585	no
gi|320446772|ref|NW_003383799.1|	558957-564128	154515	427482	146812	0.2715	no
gi|320446772|ref|NW_003383799.1|	564446-568057	234925	503643	11002	0.6175	no
gi|320446772|ref|NW_003383799.1|	571236-57243	10497	291052	14713	0.48805	no
gi|320446772|ref|NW_003383799.1|	574224-57442	290107	513385	0.823456	0.7024	no
gi|320446772|ref|NW_003383799.1|	585059-585494	546868	281343	-0.95886	0.65685	no

gi|320446772|ref|NW_003383799.1|	75585-76520	0.96497	599033	263408	0.2611	no
gi|320446774|ref|NW_003383797.1|	14641-31595	0.471551	207293	213619	0.34635	no
gi|320446774|ref|NW_003383797.1|	58997-36058	17.44	152552	-0.193099	0.87425	no
gi|320446774|ref|NW_003383797.1|	62048-36326	277923	117286	-124466	0.455	no
gi|320446774|ref|NW_003383797.1|	66552-37316	0.741209	391531	240117	0.1723	no
gi|320446774|ref|NW_003383797.1|	80870-38245	0	103033	inf	0.00515	no
gi|320446774|ref|NW_003383797.1|	86185-38775	0	12.06	inf	0.00445	no
gi|320446774|ref|NW_003383797.1|	87867-38840	0.561769	117209	438296	0.18685	no
gi|320446774|ref|NW_003383797.1|	88538-38905	0	555173	inf	0.0233	no
gi|320446774|ref|NW_003383797.1|	05209-40970	439467	112287	135336	0.4302	no
gi|320446774|ref|NW_003383797.1|	14196-41654	0.958441	490699	235608	0.29635	no
gi|320446774|ref|NW_003383797.1|	81891-48213	767903	140802	0.874667	0.68025	no
gi|320446774|ref|NW_003383797.1|	84443-48573	194115	332374	0.775894	0.6458	no
gi|320446774|ref|NW_003383797.1|	87088-48805	541201	103727	0.938556	0.47445	no
gi|320446774|ref|NW_003383797.1|	52502-55310	415082	594164	0.517463	0.7934	no
gi|320446774|ref|NW_003383797.1|	83012-58517	157811	397624	13332	0.52965	no
gi|320446774|ref|NW_003383797.1|	85325-58984	358266	136749	193242	0.286	no
gi|320446774|ref|NW_003383797.1|	91013-59142	0	676659	inf	0.0294	no
gi|320446774|ref|NW_003383797.1|	01713-60227	269738	103151	-470873	0.14175	no
gi|320446774|ref|NW_003383797.1|	02785-60323	135814	0	#NAME?	0.01345	no
gi|320446774|ref|NW_003383797.1|	03551-60413	563737	131556	-542127	0.15445	no
gi|320446774|ref|NW_003383797.1|	05840-60777	123326	1.04	-356783	0.08395	no
gi|320446774|ref|NW_003383797.1|	08024-61006	356104	394753	0.148652	0.9093	no
gi|320446774|ref|NW_003383797.1|	10882-61221	139909	124889	-0.163837	0.94215	no
gi|320446774|ref|NW_003383797.1|	31184-63146	471365	390707	-0.270757	0.89695	no
gi|320446774|ref|NW_003383797.1|	32900-63436	252929	185374	-0.448293	0.7202	no
gi|320446774|ref|NW_003383797.1|	36109-63661	520513	344809	-0.594136	0.79005	no
gi|320446774|ref|NW_003383797.1|	45827-64620	215806	11312	239005	0.25255	no
gi|320446774|ref|NW_003383797.1|	46849-64733	353321	280123	-0.33492	0.87165	no
gi|320446774|ref|NW_003383797.1|	47517-64796	197007	130994	-0.588743	0.77115	no

gi|320446774|ref|NW_003383797.1|	49224-64994	175174	239685	0.452358	0.82655	no
gi|320446774|ref|NW_003383797.1|	50051-65373	185537	394302	108759	0.4245	no
gi|320446774|ref|NW_003383797.1|	59290-65955	255323	54013	-224095	0.27085	no
gi|320446774|ref|NW_003383797.1|	68806-66923	0.846759	728556	310502	0.2468	no
gi|320446774|ref|NW_003383797.1|	78407-67933	122017	176703	385617	0.15425	no
gi|320446774|ref|NW_003383797.1|	83113-68350	436415	206668	-107838	0.60485	no
gi|320446774|ref|NW_003383797.1|	87284-68914	14091	411574	-177555	0.28925	no
gi|320446774|ref|NW_003383797.1|	89691-69180	206476	344452	0.738329	0.72525	no
gi|320446774|ref|NW_003383797.1|	93328-69412	129698	807743	-0.683184	0.73465	no
gi|320446774|ref|NW_003383797.1|	06733-70710	926253	226938	-202911	0.28795	no
gi|320446774|ref|NW_003383797.1|	56847-75807	191344	340536	-249029	0.2815	no
gi|320446774|ref|NW_003383797.1|	83701-78542	15149	100775	-0.588087	0.70985	no
gi|320446774|ref|NW_003383797.1|	93289-79612	216953	442651	102879	0.4451	no
gi|320446776|ref|NW_003383795.1|	11887-11244	10362	241354	454178	0.15685	no
gi|320446776|ref|NW_003383795.1|	60932-16355	403325	456277	34999	0.08505	no
gi|320446776|ref|NW_003383795.1|	63690-16531	0	0.927006	inf	1	no
gi|320446776|ref|NW_003383795.1|	65817-16654	103186	776911	291251	0.2307	no
gi|320446776|ref|NW_003383795.1|	70903-17114	752098	237245	165739	0.34325	no
gi|320446776|ref|NW_003383795.1|	75705-17619	193412	992528	235943	0.30355	no
gi|320446776|ref|NW_003383795.1|	81710-18210	0.988021	77967	298025	0.2563	no
gi|320446776|ref|NW_003383795.1|	59909-26029	816366	596442	286909	0.22655	no
gi|320446776|ref|NW_003383795.1|	31759-33228	173001	34895	101224	0.5772	no
gi|320446776|ref|NW_003383795.1|	37611-33832	176632	217464	0.300024	0.876	no
gi|320446776|ref|NW_003383795.1|	90501-39111	688967	195578	-181669	0.28235	no
gi|320446776|ref|NW_003383795.1|	03068-40345	135613	273784	-230839	0.3362	no
gi|320446776|ref|NW_003383795.1|	04776-40586	172005	533571	-16887	0.4377	no
gi|320446776|ref|NW_003383795.1|	08563-40916	547556	123804	-214495	0.35295	no
gi|320446776|ref|NW_003383795.1|	09276-41061	304715	930953	-171068	0.3209	no
gi|320446776|ref|NW_003383795.1|	20622-42125	211258	158054	290334	0.22835	no
gi|320446776|ref|NW_003383795.1|	32362-43254	665718	486855	287051	0.22825	no

gi|320446776|ref|NW_003383795.1|	464694-46542	274604	822372	158244	0.46395	no
gi|320446776|ref|NW_003383795.1|	465595-46611	472283	134828	15134	0.46915	no
gi|320446776|ref|NW_003383795.1|	60156-61497	167897	320058	425269	0.05705	no
gi|320446776|ref|NW_003383795.1|	91156-91375	151722	396618	470825	0.1533	no
gi|320446778|ref|NW_003383793.1|	100453-10080	105162	683646	-0.621295	0.75295	no
gi|320446778|ref|NW_003383793.1|	101032-10219	29015	100515	-152939	0.47805	no
gi|320446778|ref|NW_003383793.1|	102590-10349	533299	192499	-14701	0.4963	no
gi|320446778|ref|NW_003383793.1|	106004-10749	107175	1863	-252427	0.27355	no
gi|320446778|ref|NW_003383793.1|	126944-12735	64392	110726	-253989	0.26655	no
gi|320446778|ref|NW_003383793.1|	127592-12906	227889	369974	-262284	0.14215	no
gi|320446778|ref|NW_003383793.1|	129790-13015	109467	158621	-278683	0.28215	no
gi|320446778|ref|NW_003383793.1|	130330-13100	15833	131716	-358743	0.1853	no
gi|320446778|ref|NW_003383793.1|	131875-13227	295271	627312	-223478	0.32195	no
gi|320446778|ref|NW_003383793.1|	134765-13908	162378	10299	-0.656842	0.61315	no
gi|320446778|ref|NW_003383793.1|	139245-14876	615959	827125	0.425271	0.75325	no
gi|320446778|ref|NW_003383793.1|	149003-15033	119813	114112	-0.0703276	0.9731	no
gi|320446778|ref|NW_003383793.1|	170531-17135	503231	351833	-0.51633	0.6838	no
gi|320446778|ref|NW_003383793.1|	171581-17220	301726	439447	0.542451	0.788	no
gi|320446778|ref|NW_003383793.1|	173704-17698	316198	663481	106923	0.63515	no
gi|320446778|ref|NW_003383793.1|	177693-17946	0.549788	153186	147834	0.49985	no
gi|320446778|ref|NW_003383793.1|	179957-18078	0	236154	inf	0.029	no
gi|320446778|ref|NW_003383793.1|	180883-18199	0.386358	147098	192876	0.3182	no
gi|320446778|ref|NW_003383793.1|	182523-18348	143418	154882	0.110939	0.9576	no
gi|320446778|ref|NW_003383793.1|	189504-19070	104861	117513	0.164342	0.93995	no
gi|320446778|ref|NW_003383793.1|	193465-19692	20904	215713	0.045331	0.9805	no
gi|320446778|ref|NW_003383793.1|	206175-20814	243194	142433	-0.771823	0.7044	no
gi|320446778|ref|NW_003383793.1|	208306-20912	489147	0.395561	-36283	0.22245	no
gi|320446778|ref|NW_003383793.1|	210377-21790	194393	477046	129515	0.4399	no
gi|320446778|ref|NW_003383793.1|	219033-22251	249765	444131	0.830415	0.7016	no
gi|320446778|ref|NW_003383793.1|	224558-22545	637759	667954	0.0667376	0.9688	no

gi|320446778|ref|NW_003383793.1|	265882-267237	0.602897	0.942019	0.643844	1	no
gi|320446778|ref|NW_003383793.1|	270648-271728	259758	0.829497	-164686	0.43745	no
gi|320446778|ref|NW_003383793.1|	64054-68006	208829	419456	10062	0.6387	no
gi|320446778|ref|NW_003383793.1|	97238-97798	413241	209269	-0.981621	0.66985	no
gi|320446780|ref|NW_003383791.1|	116527-117604	130342	327415	132881	0.42385	no
gi|320446780|ref|NW_003383791.1|	120659-121680	0.426393	132625	16371	1	no
gi|320446780|ref|NW_003383791.1|	124790-125328	110566	409538	188909	0.32155	no
gi|320446780|ref|NW_003383791.1|	125496-126274	746569	747081	0.000988565	0.98345	no
gi|320446780|ref|NW_003383791.1|	126876-127205	901183	12.63	0.48696	0.805	no
gi|320446780|ref|NW_003383791.1|	127748-128100	896182	12497	0.47972	0.8118	no
gi|320446780|ref|NW_003383791.1|	137571-138290	153519	162334	0.0805516	0.9675	no
gi|320446780|ref|NW_003383791.1|	144541-145784	0.837411	302016	185062	0.4027	no
gi|320446780|ref|NW_003383791.1|	46670-49663	195798	924002	223853	0.1978	no
gi|320446780|ref|NW_003383791.1|	50716-52714	363782	887848	128724	0.55685	no
gi|320446780|ref|NW_003383791.1|	64215-66262	139714	389774	148017	0.48735	no
gi|320446781|ref|NW_003383790.1|	132-546	180234	142773	298578	0.2164	no
gi|320446781|ref|NW_003383790.1|	62174-62341	0	112851	inf	0.0312	no
gi|320446782|ref|NW_003383789.1|	147862-148575	247285	152225	262195	0.25635	no
gi|320446782|ref|NW_003383789.1|	15512-17246	270239	172519	-0.647479	0.75165	no
gi|320446782|ref|NW_003383789.1|	176564-176783	101148	57113	249736	0.2483	no
gi|320446782|ref|NW_003383789.1|	20179-20747	252238	340933	0.434702	0.82835	no
gi|320446782|ref|NW_003383789.1|	242833-243034	142973	111414	296212	0.21515	no
gi|320446782|ref|NW_003383789.1|	262202-263221	452285	130981	153405	0.48455	no
gi|320446782|ref|NW_003383789.1|	336691-337391	108926	173705	0.673294	0.73955	no
gi|320446782|ref|NW_003383789.1|	340698-341869	0.721497	287456	199428	0.3909	no
gi|320446782|ref|NW_003383789.1|	342114-343604	0.269005	37407	37976	0.17595	no
gi|320446782|ref|NW_003383789.1|	345992-352023	0.291119	257073	314249	0.18855	no
gi|320446782|ref|NW_003383789.1|	414765-415044	692833	147332	108849	0.5844	no
gi|320446782|ref|NW_003383789.1|	420675-421740	0.813712	182926	116867	0.5864	no
gi|320446782|ref|NW_003383789.1|	435262-438610	0.108207	325475	491068	0.14955	no

gi|320446782|ref|NW_003383789.1|	44944-44542	137487	45893	173898	0.3346	no
gi|320446782|ref|NW_003383789.1|	48459-45075	0.163613	650995	531429	0.14305	no
gi|320446782|ref|NW_003383789.1|	47407-48030	608047	206582	-155747	0.4802	no
gi|320446782|ref|NW_003383789.1|	78252-48357	543452	211039	195729	0.14475	no
gi|320446782|ref|NW_003383789.1|	49336-50330	8454	722236	-0.227164	0.9106	no
gi|320446782|ref|NW_003383789.1|	96189-49668	519575	912643	0.81272	0.68565	no
gi|320446782|ref|NW_003383789.1|	02035-50261	147039	696348	224361	0.327	no
gi|320446782|ref|NW_003383789.1|	09087-50951	33713	100428	157478	0.49045	no
gi|320446782|ref|NW_003383789.1|	55013-59471	0	165084	inf	0.00795	no
gi|320446782|ref|NW_003383789.1|	93602-59384	129.86	509971	-134847	0.52465	no
gi|320446782|ref|NW_003383789.1|	62986-63710	0	236361	inf	0.0312	no
gi|320446782|ref|NW_003383789.1|	68492-66938	357145	685532	0.940714	0.6434	no
gi|320446782|ref|NW_003383789.1|	69636-67036	0	283072	inf	0.029	no
gi|320446782|ref|NW_003383789.1|	88237-68904	234181	221251	-0.0819368	0.9499	no
gi|320446782|ref|NW_003383789.1|	18057-71955	186814	167014	-0.161638	0.93265	no
gi|320446782|ref|NW_003383789.1|	83444-84827	0	265571	inf	0.0142	no
gi|320446782|ref|NW_003383789.1|	54082-85885	290286	11417	-134629	0.3163	no
gi|320446782|ref|NW_003383789.1|	85884-92533	0	815069	inf	5.00E-05	yes
gi|320446782|ref|NW_003383789.1|	59589-86057	171569	8574	-100075	0.63745	no
gi|320446782|ref|NW_003383789.1|	60673-86238	745088	35491	-106996	0.42565	no
gi|320446782|ref|NW_003383789.1|	63247-86800	212601	300162	0.497593	0.71105	no
gi|320446782|ref|NW_003383789.1|	69949-87249	804669	700026	-0.200988	0.9274	no
gi|320446782|ref|NW_003383789.1|	83242-88543	732282	547071	-0.420671	0.8437	no
gi|320446782|ref|NW_003383789.1|	91574-89256	0.893429	154267	0.788008	0.71775	no
gi|320446782|ref|NW_003383789.1|	00060-90037	315249	288749	-0.126673	0.9477	no
gi|320446782|ref|NW_003383789.1|	00692-90631	108396	205692	0.924177	0.49065	no
gi|320446782|ref|NW_003383789.1|	08084-91196	263879	430955	0.707659	0.7477	no
gi|320446782|ref|NW_003383789.1|	13055-91355	114396	102761	-0.154743	0.9022	no
gi|320446782|ref|NW_003383789.1|	15329-91579	319816	0	#NAME?	0.0063	no
gi|320446782|ref|NW_003383789.1|	30649-93125	63257	435293	-0.539238	0.74145	no

gi|320446782|ref|NW_003383789.1|	931427-93183	161092	143573	-0.166108	0.9323	no
gi|320446782|ref|NW_003383789.1|	932341-93352	259463	194597	-0.415043	0.7953	no
gi|320446782|ref|NW_003383789.1|	934046-93521	442864	493466	0.156086	0.90705	no
gi|320446782|ref|NW_003383789.1|	936248-93667	301994	233954	-0.368291	0.85775	no
gi|320446782|ref|NW_003383789.1|	936950-94012	125086	545728	-119666	0.4773	no
gi|320446782|ref|NW_003383789.1|	941309-94196	250197	714719	-180762	0.4071	no
gi|320446782|ref|NW_003383789.1|	942385-94310	11.79	213571	-246478	0.2659	no
gi|320446782|ref|NW_003383789.1|	943518-94487	0.300108	281363	322888	0.2061	no
gi|320446782|ref|NW_003383789.1|	946519-94683	172748	333574	-237259	0.25565	no
gi|320446783|ref|NW_003383788.1|	133147-13427	395148	284093	-0.476032	0.7038	no
gi|320446783|ref|NW_003383788.1|	134874-13704	24.11	188883	-0.352143	0.7858	no
gi|320446783|ref|NW_003383788.1|	142899-14455	215729	358106	0.731164	0.57105	no
gi|320446783|ref|NW_003383788.1|	145495-14611	119695	144487	0.271571	0.89495	no
gi|320446783|ref|NW_003383788.1|	146219-15410	557943	148607	141331	0.28825	no
gi|320446783|ref|NW_003383788.1|	155206-15584	299247	323956	0.114463	0.95965	no
gi|320446783|ref|NW_003383788.1|	156145-15682	52982	697474	0.396637	0.84465	no
gi|320446783|ref|NW_003383788.1|	157615-15849	653571	720203	0.140059	0.94345	no
gi|320446783|ref|NW_003383788.1|	158711-15990	168827	149931	-0.171247	0.9372	no
gi|320446783|ref|NW_003383788.1|	160213-16183	767793	619158	-0.31041	0.88265	no
gi|320446783|ref|NW_003383788.1|	164271-16854	0.584601	154955	140633	0.50355	no
gi|320446783|ref|NW_003383788.1|	168650-17276	317806	746699	123238	0.45445	no
gi|320446783|ref|NW_003383788.1|	204502-20487	115086	752096	270821	0.2402	no
gi|320446783|ref|NW_003383788.1|	205988-20696	0.454537	125538	146566	1	no
gi|320446783|ref|NW_003383788.1|	267305-26792	0	590235	inf	0.0162	no
gi|320446783|ref|NW_003383788.1|	305839-30851	0.82894	0.965327	0.219749	1	no
gi|320446783|ref|NW_003383788.1|	310837-31408	296642	242706	-0.289513	0.89095	no
gi|320446783|ref|NW_003383788.1|	314343-31600	14204	0.823981	-0.785609	0.7091	no
gi|320446783|ref|NW_003383788.1|	316135-31731	124531	0.986324	-0.336374	1	no
gi|320446783|ref|NW_003383788.1|	330451-33396	190646	428993	117006	0.5952	no
gi|320446783|ref|NW_003383788.1|	334850-33564	0.604325	228224	191705	0.3194	no

gi|320446783|ref|NW_003383788.1|	347454-35016	0.953049	285413	158243	0.4666	no
gi|320446784|ref|NW_003383787.1|	10-1152	487702	375169	-0.378457	0.76545	no
gi|320446784|ref|NW_003383787.1|	102604-10602	14941	340253	118734	0.3788	no
gi|320446784|ref|NW_003383787.1|	106443-10780	163996	564215	178258	0.32075	no
gi|320446784|ref|NW_003383787.1|	107973-10988	100944	353923	180988	0.31865	no
gi|320446784|ref|NW_003383787.1|	110335-11249	15814	81467	236501	0.08955	no
gi|320446784|ref|NW_003383787.1|	114459-11572	171432	149747	312682	0.11745	no
gi|320446784|ref|NW_003383787.1|	117102-11906	0.293185	170343	253856	0.27905	no
gi|320446784|ref|NW_003383787.1|	119164-11955	104318	616013	256198	0.27405	no
gi|320446784|ref|NW_003383787.1|	123946-12533	0.583102	111397	0.933887	1	no
gi|320446784|ref|NW_003383787.1|	128418-12972	0.313783	0.871209	147325	1	no
gi|320446784|ref|NW_003383787.1|	130731-13182	0.196784	163421	305391	0.2493	no
gi|320446784|ref|NW_003383787.1|	136083-13666	0	165797	inf	0.0074	no
gi|320446784|ref|NW_003383787.1|	137851-14040	0.145296	563288	527681	0.1443	no
gi|320446784|ref|NW_003383787.1|	141462-14274	145673	455966	496813	0.0544	no
gi|320446784|ref|NW_003383787.1|	150278-15088	430034	404386	-0.0887153	0.967	no
gi|320446784|ref|NW_003383787.1|	172352-17282	210312	607927	153137	0.4913	no
gi|320446784|ref|NW_003383787.1|	184621-18529	0.385296	28914	290773	0.25645	no
gi|320446784|ref|NW_003383787.1|	187810-18847	474152	107738	11841	0.5689	no
gi|320446784|ref|NW_003383787.1|	189387-19003	736834	117061	0.667848	0.75125	no
gi|320446784|ref|NW_003383787.1|	192003-19282	100707	377761	190731	0.2565	no
gi|320446784|ref|NW_003383787.1|	192930-19750	229267	69321	159626	0.23295	no
gi|320446784|ref|NW_003383787.1|	214443-21622	0.218633	159915	287073	0.22425	no
gi|320446784|ref|NW_003383787.1|	220947-22182	0.791645	181707	119869	0.56655	no
gi|320446784|ref|NW_003383787.1|	270879-27128	653252	307572	-108671	0.59495	no
gi|320446784|ref|NW_003383787.1|	272644-27386	121321	639808	-0.923119	0.6723	no
gi|320446784|ref|NW_003383787.1|	275341-27679	221927	241022	0.119077	0.94835	no
gi|320446784|ref|NW_003383787.1|	277192-27795	858865	719928	-0.254578	0.89975	no
gi|320446784|ref|NW_003383787.1|	293049-29353	137403	118177	-0.217462	0.91625	no
gi|320446784|ref|NW_003383787.1|	304059-30601	0.392962	123285	164954	1	no

gi|320446784|ref|NW_003383787.1|	306363-30737(	0.217036	149972	278869	0.2657	no
gi|320446784|ref|NW_003383787.1|	308380-31015!	306881	633674	104606	0.63075	no
gi|320446784|ref|NW_003383787.1|	311563-31241(	245957	150441	-0.70921	0.7314	no
gi|320446784|ref|NW_003383787.1|	312623-31353!	0.248345	171206	278532	0.2657	no
gi|320446784|ref|NW_003383787.1|	313707-31452:	262089	140083	-0.903776	0.6711	no
gi|320446784|ref|NW_003383787.1|	317538-31849(	0.467122	257908	246498	0.2454	no
gi|320446784|ref|NW_003383787.1|	318577-32195:	139684	608826	212387	0.3532	no
gi|320446784|ref|NW_003383787.1|	322160-32314!	31353	556812	0.828586	0.68545	no
gi|320446784|ref|NW_003383787.1|	342282-34335!	487592	673243	0.465452	0.7198	no
gi|320446784|ref|NW_003383787.1|	345726-34961!	138218	228201	0.723355	0.6443	no
gi|320446784|ref|NW_003383787.1|	352791-35370:	874354	327183	-141812	0.50095	no
gi|320446784|ref|NW_003383787.1|	366601-36996(	100279	107106	0.0950241	0.9413	no
gi|320446784|ref|NW_003383787.1|	40119-41019	355532	612495	0.784718	0.69865	no
gi|320446784|ref|NW_003383787.1|	42047-42427	647417	707001	0.127019	0.93285	no
gi|320446784|ref|NW_003383787.1|	447112-44881!	0.114666	159689	379976	0.2072	no
gi|320446784|ref|NW_003383787.1|	449755-45136(	0.369621	171473	221387	0.3331	no
gi|320446784|ref|NW_003383787.1|	451438-45210(	0	292343	inf	0.0294	no
gi|320446784|ref|NW_003383787.1|	452780-45388:	0	283381	inf	0.01575	no
gi|320446784|ref|NW_003383787.1|	466288-46766!	0	21487	inf	0.01575	no
gi|320446784|ref|NW_003383787.1|	471879-47375(	341261	368939	0.112506	0.93295	no
gi|320446784|ref|NW_003383787.1|	474601-47516:	359436	520181	0.533277	0.7898	no
gi|320446784|ref|NW_003383787.1|	477013-47808:	289159	23.64	-0.290634	0.85705	no
gi|320446784|ref|NW_003383787.1|	484748-48648!	0.112374	140872	3648	1	no
gi|320446784|ref|NW_003383787.1|	487412-48793:	0.586379	512186	312676	0.2458	no
gi|320446784|ref|NW_003383787.1|	49237-52977	332194	107479	169395	0.3309	no
gi|320446784|ref|NW_003383787.1|	502927-50463:	50556	117609	121804	0.57785	no
gi|320446784|ref|NW_003383787.1|	505179-50546:	923092	912648	-0.0164172	0.9888	no
gi|320446784|ref|NW_003383787.1|	506626-50734:	161828	866348	-0.901444	0.6632	no
gi|320446784|ref|NW_003383787.1|	508007-50874:	182766	834352	-113127	0.5901	no
gi|320446784|ref|NW_003383787.1|	509972-51031(	270091	613902	-213736	0.3426	no

gi|320446784|ref|NW_003383787.1|	53136-55258	581354	152872	139484	0.4043	no
gi|320446784|ref|NW_003383787.1|	547710-549417	700607	281052	-131777	0.32975	no
gi|320446784|ref|NW_003383787.1|	551611-554039	107738	703972	-0.613934	0.6973	no
gi|320446784|ref|NW_003383787.1|	55613-60176	651095	382249	255357	0.0638	no
gi|320446784|ref|NW_003383787.1|	573746-576470	455882	233963	-0.96238	0.47295	no
gi|320446784|ref|NW_003383787.1|	579851-580254	142873	376387	-192444	0.391	no
gi|320446784|ref|NW_003383787.1|	582432-582714	896541	100175	0.160084	0.90985	no
gi|320446784|ref|NW_003383787.1|	587659-588804	280153	591122	-224469	0.1918	no
gi|320446784|ref|NW_003383787.1|	589149-591249	141171	460835	-161512	0.3313	no
gi|320446784|ref|NW_003383787.1|	595324-600725	666317	969403	386282	0.00845	no
gi|320446784|ref|NW_003383787.1|	600844-601439	233741	760368	170179	0.433	no
gi|320446784|ref|NW_003383787.1|	602277-602854	147459	106402	285114	0.23825	no
gi|320446784|ref|NW_003383787.1|	60340-61983	347652	287877	304973	0.1098	no
gi|320446784|ref|NW_003383787.1|	63422-63952	0.567268	534307	323557	0.23165	no
gi|320446784|ref|NW_003383787.1|	79461-80490	382786	32339	-0.243262	0.90075	no
gi|320446784|ref|NW_003383787.1|	87036-88405	327497	206781	-0.663383	0.7446	no
gi|320446784|ref|NW_003383787.1|	93530-95980	441908	345849	-0.353604	0.86975	no
gi|320446784|ref|NW_003383787.1|	96191-102457	822536	100203	0.284773	0.8267	no
gi|320446786|ref|NW_003383785.1|	106108-107139	190912	175958	-0.117673	0.9464	no
gi|320446786|ref|NW_003383785.1|	107250-108153	151686	121959	-0.314695	0.87815	no
gi|320446786|ref|NW_003383785.1|	108311-109487	157885	845412	-0.901151	0.6799	no
gi|320446786|ref|NW_003383785.1|	111118-113040	148169	440068	-175144	0.1963	no
gi|320446786|ref|NW_003383785.1|	113144-113857	635877	106316	-258039	0.15435	no
gi|320446786|ref|NW_003383785.1|	114015-114361	286.44	476107	-258887	0.1503	no
gi|320446786|ref|NW_003383785.1|	114524-114783	318601	437066	-286583	0.22345	no
gi|320446786|ref|NW_003383785.1|	117453-117661	278618	309608	-316977	0.1938	no
gi|320446786|ref|NW_003383785.1|	123635-124249	288903	193131	-0.581004	0.78555	no
gi|320446786|ref|NW_003383785.1|	125065-125500	979464	757462	-0.370819	0.8508	no
gi|320446786|ref|NW_003383785.1|	127457-128099	211303	262245	0.311604	0.8852	no
gi|320446786|ref|NW_003383785.1|	135125-135987	0.808076	190965	456267	0.15535	no

gi|320446786|ref|NW_003383785.1|	155261-15763	892392	60101	-0.570288	0.8034	no
gi|320446786|ref|NW_003383785.1|	168526-16997	0.975745	0.871935	-0.162283	1	no
gi|320446786|ref|NW_003383785.1|	186438-18796	130447	154233	0.241651	0.8982	no
gi|320446786|ref|NW_003383785.1|	191537-19373	292316	641957	113495	0.6014	no
gi|320446786|ref|NW_003383785.1|	200855-20175	330635	0.525773	-265273	0.22905	no
gi|320446786|ref|NW_003383785.1|	223269-22415	103608	124897	0.269601	1	no
gi|320446786|ref|NW_003383785.1|	236611-23722	0	246217	inf	0.00565	no
gi|320446786|ref|NW_003383785.1|	259516-26364	492272	342809	-0.52205	0.6922	no
gi|320446786|ref|NW_003383785.1|	264186-26450	183973	140101	-0.393022	0.8464	no
gi|320446786|ref|NW_003383785.1|	474716-47617	0.137574	143446	338223	0.2261	no
gi|320446786|ref|NW_003383785.1|	492517-49506	0.655523	376493	25219	0.26915	no
gi|320446786|ref|NW_003383785.1|	516340-51705	315312	148605	223663	0.3141	no
gi|320446786|ref|NW_003383785.1|	519202-51954	827131	116291	0.491558	0.805	no
gi|320446786|ref|NW_003383785.1|	554238-55448	985778	145506	0.561745	0.7963	no
gi|320446786|ref|NW_003383785.1|	555252-55566	267714	942924	181645	0.39715	no
gi|320446786|ref|NW_003383785.1|	556342-55676	435546	172755	198783	0.38185	no
gi|320446786|ref|NW_003383785.1|	557167-55842	0.331672	218562	272021	0.2412	no
gi|320446786|ref|NW_003383785.1|	558902-56113	0.0843842	129564	394055	1	no
gi|320446786|ref|NW_003383785.1|	561550-56175	285946	53479	0.903226	0.68565	no
gi|320446786|ref|NW_003383785.1|	566336-56970	630383	433051	278024	0.16105	no
gi|320446786|ref|NW_003383785.1|	579150-57999	275968	379717	0.460427	0.82065	no
gi|320446786|ref|NW_003383785.1|	581595-58354	224643	11746	-0.935458	0.5782	no
gi|320446786|ref|NW_003383785.1|	584142-58518	17431	595298	-154997	0.47975	no
gi|320446786|ref|NW_003383785.1|	585302-58651	142985	609135	-123102	0.56685	no
gi|320446786|ref|NW_003383785.1|	587801-58833	191621	720613	-141096	0.5053	no
gi|320446786|ref|NW_003383785.1|	588462-58955	232206	59921	-195427	0.3901	no
gi|320446786|ref|NW_003383785.1|	594502-59553	610554	334768	-0.866959	0.67555	no
gi|320446786|ref|NW_003383785.1|	595986-59747	152231	561993	-143764	0.5249	no
gi|320446786|ref|NW_003383785.1|	597574-59778	495321	193505	-135599	0.52435	no
gi|320446786|ref|NW_003383785.1|	598301-59863	100656	279484	-184859	0.3126	no

gi|320446786|ref|NW_003383785.1|	;99360-59971!	15067	73593	-103375	0.59645	no
gi|320446786|ref|NW_003383785.1|	;01624-60340	125632	181882	0.533793	0.7449	no
gi|320446786|ref|NW_003383785.1|	;09660-61477	771385	634895	-0.280933	0.8254	no
gi|320446786|ref|NW_003383785.1|	;14922-61672!	119483	854926	-0.482936	0.8336	no
gi|320446786|ref|NW_003383785.1|	75016-76077	776773	109431	0.494454	0.70385	no
gi|320446786|ref|NW_003383785.1|	79060-86616	262508	701491	141806	0.4247	no
gi|320446786|ref|NW_003383785.1|	87220-89360	101883	148965	0.548059	0.7371	no
gi|320446786|ref|NW_003383785.1|	89655-90758	108929	125212	0.200991	0.9246	no
gi|320446786|ref|NW_003383785.1|	90967-92532	345206	271939	-0.344178	0.788	no
gi|320446786|ref|NW_003383785.1|	94196-94717	274674	26705	-0.0406101	0.9818	no
gi|320446786|ref|NW_003383785.1|	97673-106004	201114	23998	0.254899	0.84385	no
gi|320446788|ref|NW_003383783.1|	l38447-13930	0.271964	22272	635567	0.1556	no
gi|320446788|ref|NW_003383783.1|	l39760-14011	0	11591	inf	0.0233	no
gi|320446788|ref|NW_003383783.1|	l41686-14340	0.227783	537721	788305	0.1183	no
gi|320446788|ref|NW_003383783.1|	15294-16648	105601	502854	225151	0.3215	no
gi|320446788|ref|NW_003383783.1|	l59082-16026	0	107263	inf	0.0049	no
gi|320446788|ref|NW_003383783.1|	l60383-16114!	0.636196	194162	493165	0.14685	no
gi|320446788|ref|NW_003383783.1|	l61303-16415	0	118404	inf	5.00E-05	yes
gi|320446788|ref|NW_003383783.1|	l68946-16933	0	260094	inf	0.0113	no
gi|320446788|ref|NW_003383783.1|	!00773-20433	532244	125199	123407	0.4698	no
gi|320446788|ref|NW_003383783.1|	!10300-21869	0.0413395	390069	988199	0.1181	no
gi|320446788|ref|NW_003383783.1|	!52748-25353	0	167446	inf	0.0077	no
gi|320446788|ref|NW_003383783.1|	!53869-25446	0	16.88	inf	0.0075	no
gi|320446788|ref|NW_003383783.1|	!95475-29609	0	233624	inf	0.00655	no
gi|320446788|ref|NW_003383783.1|	!96389-29710	0	390644	inf	0.00385	no
gi|320446788|ref|NW_003383783.1|	;12185-31328	210077	861676	-12857	0.5628	no
gi|320446788|ref|NW_003383783.1|	;13641-31440	222669	174528	-0.351439	0.8554	no
gi|320446788|ref|NW_003383783.1|	;15002-31564	186444	592166	-165467	0.4395	no
gi|320446788|ref|NW_003383783.1|	;17244-31862!	28724	521827	-246062	0.15895	no
gi|320446788|ref|NW_003383783.1|	;22588-32362	112285	644383	-0.801171	0.7022	no

gi|320446788|ref|NW_003383783.1|	24172-32446	131131	568121	-120674	0.57415	no
gi|320446788|ref|NW_003383783.1|	25556-32575	876589	682824	-0.360388	0.85575	no
gi|320446788|ref|NW_003383783.1|	26789-32768	107144	650377	-0.720198	0.7301	no
gi|320446788|ref|NW_003383783.1|	27828-33039	334487	328027	-0.0281337	0.98235	no
gi|320446788|ref|NW_003383783.1|	81035-38153	127537	380052	157528	0.4794	no
gi|320446788|ref|NW_003383783.1|	81716-38227	41824	846818	101772	0.60625	no
gi|320446788|ref|NW_003383783.1|	96533-39872	181287	48192	141052	0.5148	no
gi|320446788|ref|NW_003383783.1|	99772-40235	0.358759	43608	360351	0.1705	no
gi|320446788|ref|NW_003383783.1|	04205-41042	72209	473479	271305	0.05045	no
gi|320446788|ref|NW_003383783.1|	54359-45468	125347	48995	-135522	0.5377	no
gi|320446788|ref|NW_003383783.1|	56660-45707	117731	298852	-197799	0.38655	no
gi|320446788|ref|NW_003383783.1|	40920-54118	39748	235825	-0.753168	0.7079	no
gi|320446788|ref|NW_003383783.1|	57142-58163	158971	12026	-0.402605	0.8545	no
gi|320446788|ref|NW_003383783.1|	78725-57895	249962	157389	-0.667371	0.7476	no
gi|320446788|ref|NW_003383783.1|	86430-58711	216193	137207	-0.655964	0.75235	no
gi|320446788|ref|NW_003383783.1|	94147-59595	741314	10554	0.509639	0.8232	no
gi|320446788|ref|NW_003383783.1|	96115-59687	194146	33274	0.777252	0.7039	no
gi|320446788|ref|NW_003383783.1|	97729-59875	535699	122921	119824	0.58185	no
gi|320446788|ref|NW_003383783.1|	98925-60195	256364	695412	143967	0.2843	no
gi|320446788|ref|NW_003383783.1|	14437-61901	0.738208	647585	313297	0.0977	no
gi|320446788|ref|NW_003383783.1|	19972-62161	167713	350212	106223	0.60795	no
gi|320446788|ref|NW_003383783.1|	21747-62480	579023	183648	-165668	0.45565	no
gi|320446788|ref|NW_003383783.1|	25091-62544	371276	116639	-167044	0.4305	no
gi|320446788|ref|NW_003383783.1|	31336-63173	0.998622	150973	391821	0.1982	no
gi|320446788|ref|NW_003383783.1|	32663-63824	232976	146657	-0.667738	0.6218	no
gi|320446788|ref|NW_003383783.1|	46705-64733	214438	291546	0.443167	0.8278	no
gi|320446788|ref|NW_003383783.1|	58657-65952	180183	981101	-0.876986	0.67785	no
gi|320446788|ref|NW_003383783.1|	80564-68136	118948	653625	-0.863794	0.6787	no
gi|320446788|ref|NW_003383783.1|	90115-69130	70183	444768	-0.658068	0.61845	no
gi|320446788|ref|NW_003383783.1|	93022-69565	38.3	474663	0.309561	0.8156	no

gi|320446788|ref|NW_003383783.1|	97531-69957	419372	204744	-103441	0.4353	no
gi|320446788|ref|NW_003383783.1|	01449-70375	927198	567765	-0.707584	0.76075	no
gi|320446788|ref|NW_003383783.1|	04282-70516	24981	123671	-101432	0.64465	no
gi|320446788|ref|NW_003383783.1|	05700-70704	128212	529897	-127475	0.55415	no
gi|320446788|ref|NW_003383783.1|	08132-70892	142527	291487	-228973	0.30155	no
gi|320446788|ref|NW_003383783.1|	10225-71628	172702	103978	-0.732012	0.583	no
gi|320446788|ref|NW_003383783.1|	40737-74317	178736	76067	-123249	0.46875	no
gi|320446788|ref|NW_003383783.1|	44282-74596	834595	295408	-149837	0.2668	no
gi|320446788|ref|NW_003383783.1|	56521-75730	541382	36092	-0.584969	0.7225	no
gi|320446788|ref|NW_003383783.1|	59163-75972	495163	415145	-0.25429	0.90595	no
gi|320446788|ref|NW_003383783.1|	60725-76248	207105	323994	0.645603	0.61305	no
gi|320446788|ref|NW_003383783.1|	64451-76493	720011	38.77	-0.893078	0.6857	no
gi|320446788|ref|NW_003383783.1|	66829-76701	755529	467202	-0.693442	0.7374	no
gi|320446788|ref|NW_003383783.1|	67167-76815	196554	114158	-0.783899	0.721	no
gi|320446788|ref|NW_003383783.1|	69184-77220	83043	11824	0.509795	0.68165	no
gi|320446788|ref|NW_003383783.1|	74425-77461	0	137342	inf	0.0138	no
gi|320446788|ref|NW_003383783.1|	74811-77552	0	528369	inf	0.0186	no
gi|320446788|ref|NW_003383783.1|	76549-77759	0	263605	inf	5.00E-05	yes
gi|320446788|ref|NW_003383783.1|	81935-78539	136002	238236	0.808757	0.5387	no
gi|320446788|ref|NW_003383783.1|	85649-78682	965761	45856	-107456	0.60625	no
gi|320446788|ref|NW_003383783.1|	87355-78799	707777	481631	-0.555367	0.7776	no
gi|320446788|ref|NW_003383783.1|	88097-79080	100476	487158	-104438	0.5219	no
gi|320446788|ref|NW_003383783.1|	91476-79313	933783	329135	-150441	0.48875	no
gi|320446788|ref|NW_003383783.1|	96234-79747	167649	0.116274	-384984	0.3186	no
gi|320446788|ref|NW_003383783.1|	80961-81198	228856	312744	0.45054	0.82185	no
gi|320446788|ref|NW_003383783.1|	25001-82554	910772	325117	-148613	0.471	no
gi|320446788|ref|NW_003383783.1|	38022-83893	596027	119844	-231422	0.3117	no
gi|320446788|ref|NW_003383783.1|	39294-83997	280672	698981	-200556	0.365	no
gi|320446788|ref|NW_003383783.1|	40103-84122	821267	304136	-143314	0.5008	no
gi|320446788|ref|NW_003383783.1|	41954-84217	549094	201326	-144752	0.49575	no

gi|320446788|ref|NW_003383783.1|	342566-84276	85.89	971942	-314355	0.25345	no
gi|320446788|ref|NW_003383783.1|	346951-85117	633044	450626	-0.490377	0.7077	no
gi|320446788|ref|NW_003383783.1|	351385-85226	165.19	692304	-125465	0.3551	no
gi|320446788|ref|NW_003383783.1|	92692-93126	139387	119547	-0.221515	0.90985	no
gi|320446788|ref|NW_003383783.1|	97202-97471	128202	293623	119555	0.5697	no
gi|320446788|ref|NW_003383783.1|	99044-103834	900182	23741	139909	0.2939	no
gi|320446789|ref|NW_003383782.1|	127778-12829	177106	277587	0.648326	0.74155	no
gi|320446789|ref|NW_003383782.1|	133788-13440	13231	299518	117872	0.56835	no
gi|320446789|ref|NW_003383782.1|	158398-15930	326209	0.172955	-423733	0.3017	no
gi|320446789|ref|NW_003383782.1|	183994-18480	0.591611	223528	191773	0.3194	no
gi|320446789|ref|NW_003383782.1|	230332-23117	0	346745	inf	0.0154	no
gi|320446789|ref|NW_003383782.1|	274267-27631	0.653078	136646	106511	1	no
gi|320446789|ref|NW_003383782.1|	276527-27798	0.826789	124521	0.590796	1	no
gi|320446789|ref|NW_003383782.1|	2777-4545	275253	138046	-0.995608	0.63605	no
gi|320446789|ref|NW_003383782.1|	278102-27951	0.999698	109158	0.126853	1	no
gi|320446789|ref|NW_003383782.1|	279575-28161	0.841067	319366	192492	0.38025	no
gi|320446789|ref|NW_003383782.1|	342527-34502	0.298826	396543	37301	0.1684	no
gi|320446789|ref|NW_003383782.1|	34367-34706	978503	453302	-11101	0.5919	no
gi|320446789|ref|NW_003383782.1|	346684-34714	0	532548	inf	0.0294	no
gi|320446789|ref|NW_003383782.1|	349528-35123	0	151909	inf	0.0212	no
gi|320446789|ref|NW_003383782.1|	356084-35640	232288	0	#NAME?	0.0178	no
gi|320446789|ref|NW_003383782.1|	357567-35925	101134	0.0809349	-696529	0.26225	no
gi|320446789|ref|NW_003383782.1|	361209-36145	386071	0	#NAME?	0.02105	no
gi|320446789|ref|NW_003383782.1|	365210-36574	442263	297846	-0.57034	0.7893	no
gi|320446789|ref|NW_003383782.1|	365975-36714	286215	121745	-123324	0.4551	no
gi|320446789|ref|NW_003383782.1|	367341-37039	107.1	114146	-3.23	0.02185	no
gi|320446789|ref|NW_003383782.1|	370941-37138	101366	105913	-658055	0.16165	no
gi|320446789|ref|NW_003383782.1|	374645-37538	370084	391713	0.0819444	0.95815	no
gi|320446789|ref|NW_003383782.1|	379538-38020	688945	818436	-307345	0.1084	no
gi|320446789|ref|NW_003383782.1|	380285-38224	432234	388819	-0.152714	0.9076	no

gi|320446789|ref|NW_003383782.1|	444571-44649	0.600279	111586	0.894452	1	no
gi|320446789|ref|NW_003383782.1|	496055-49678	0	589758	inf	5.00E-05	yes
gi|320446789|ref|NW_003383782.1|	580635-58111	171858	0.45893	-52268	0.27835	no
gi|320446789|ref|NW_003383782.1|	582101-58330	11499	0.241555	-557302	0.18205	no
gi|320446789|ref|NW_003383782.1|	625677-62640	101944	186136	0.868576	0.6867	no
gi|320446790|ref|NW_003383781.1|	118332-11947	0	154709	inf	0.029	no
gi|320446790|ref|NW_003383781.1|	121468-12259	0	404952	inf	0.01195	no
gi|320446790|ref|NW_003383781.1|	131588-13275	236255	0.251821	-322987	0.2448	no
gi|320446790|ref|NW_003383781.1|	170683-17335	0	128167	inf	5.00E-05	yes
gi|320446790|ref|NW_003383781.1|	17160-17537	43912	28033	-0.647488	0.75125	no
gi|320446790|ref|NW_003383781.1|	175107-17774	0	175237	inf	5.00E-05	yes
gi|320446790|ref|NW_003383781.1|	198593-19969	155612	430838	146919	0.48355	no
gi|320446790|ref|NW_003383781.1|	199940-20765	376656	501323	0.412493	0.743	no
gi|320446790|ref|NW_003383781.1|	207809-21000	284153	174253	-0.705483	0.72735	no
gi|320446790|ref|NW_003383781.1|	211496-21182	34802	127204	-145203	0.4993	no
gi|320446790|ref|NW_003383781.1|	21240-21914	499754	288508	-0.792609	0.70115	no
gi|320446790|ref|NW_003383781.1|	217308-21773	37432	0	#NAME?	0.0063	no
gi|320446790|ref|NW_003383781.1|	220928-22291	387181	0.0673244	-916766	0.2504	no
gi|320446790|ref|NW_003383781.1|	33338-34582	217511	371348	0.771685	0.70345	no
gi|320446790|ref|NW_003383781.1|	380075-38264	320587	391777	0.289315	0.8285	no
gi|320446790|ref|NW_003383781.1|	383360-38479	418849	280342	-0.579241	0.66145	no
gi|320446790|ref|NW_003383781.1|	398980-39992	760256	276171	-146092	0.40605	no
gi|320446790|ref|NW_003383781.1|	400299-40266	734587	480824	-0.611423	0.64925	no
gi|320446790|ref|NW_003383781.1|	403385-40455	0.363078	238976	271852	0.24125	no
gi|320446790|ref|NW_003383781.1|	406482-40990	256064	358268	0.484534	0.71685	no
gi|320446790|ref|NW_003383781.1|	410946-41203	285367	146729	-0.959665	0.55395	no
gi|320446790|ref|NW_003383781.1|	417120-41760	117337	0	#NAME?	5.00E-05	yes
gi|320446790|ref|NW_003383781.1|	420911-42158	197394	0	#NAME?	0.00635	no
gi|320446790|ref|NW_003383781.1|	422442-42322	282554	0	#NAME?	0.00465	no
gi|320446790|ref|NW_003383781.1|	423463-42390	112438	0	#NAME?	5.00E-05	yes

gi|320446790|ref|NW_003383781.1|	424582-42518[illegible]	643378	773775	-305568	0.10385	no
gi|320446790|ref|NW_003383781.1|	426840-42731[illegible]	253376	187766	-375427	0.1938	no
gi|320446790|ref|NW_003383781.1|	427417-42764[illegible]	451407	516929	-312639	0.25385	no
gi|320446790|ref|NW_003383781.1|	427804-42911[illegible]	352024	61903	-250759	0.16435	no
gi|320446790|ref|NW_003383781.1|	433320-43493[illegible]	472473	160994	-155323	0.23455	no
gi|320446790|ref|NW_003383781.1|	52767-53568	954949	200865	107273	0.61365	no
gi|320446790|ref|NW_003383781.1|	55078-56017	143922	959719	273732	0.23945	no
gi|320446790|ref|NW_003383781.1|	6059-6755	16846	400363	-207302	0.3346	no
gi|320446790|ref|NW_003383781.1|	64547-66842	0	257344	inf	0.00885	no
gi|320446790|ref|NW_003383781.1|	86797-90875	0.043909	177053	865545	0.14075	no
gi|320446790|ref|NW_003383781.1|	92795-93661	0	202675	inf	0.0294	no
gi|320446790|ref|NW_003383781.1|	97049-97594	0.540765	364493	275282	0.26605	no
gi|320446791|ref|NW_003383780.1|	103242-10594[illegible]	408197	170371	-126059	0.3476	no
gi|320446791|ref|NW_003383780.1|	107543-10957[illegible]	24662	925609	-141381	0.41385	no
gi|320446791|ref|NW_003383780.1|	113598-11394[illegible]	291137	774064	-191117	0.3694	no
gi|320446791|ref|NW_003383780.1|	114410-11788[illegible]	303495	277998	-0.1266	0.92245	no
gi|320446791|ref|NW_003383780.1|	119430-11998[illegible]	0	813901	inf	0.01485	no
gi|320446791|ref|NW_003383780.1|	120400-12127[illegible]	0	548486	inf	0.00935	no
gi|320446791|ref|NW_003383780.1|	129580-13022[illegible]	141156	634156	-115438	0.37885	no
gi|320446791|ref|NW_003383780.1|	132128-13291[illegible]	703793	546129	-0.365911	0.85595	no
gi|320446791|ref|NW_003383780.1|	133432-13447[illegible]	323821	239865	-0.432976	0.793	no
gi|320446791|ref|NW_003383780.1|	134812-13573[illegible]	176727	226795	0.359867	0.87185	no
gi|320446791|ref|NW_003383780.1|	161982-16250[illegible]	0.580515	11316	428488	0.18435	no
gi|320446791|ref|NW_003383780.1|	1836-2581	0	21556	inf	0.0055	no
gi|320446791|ref|NW_003383780.1|	225307-22597[illegible]	579255	0.790296	-287373	0.2155	no
gi|320446791|ref|NW_003383780.1|	226084-22674[illegible]	103452	0	#NAME?	0.00975	no
gi|320446791|ref|NW_003383780.1|	309045-30929[illegible]	406073	118687	154735	0.46405	no
gi|320446791|ref|NW_003383780.1|	3641-4229	0	193646	inf	0.00765	no
gi|320446791|ref|NW_003383780.1|	52744-55562	12152	129682	0.0937796	0.94215	no
gi|320446791|ref|NW_003383780.1|	555306-55710[illegible]	0	87324	inf	0.00475	no

gi|320446791|ref|NW_003383780.1|	56331-57271	526966	413095	-0.351236	0.862	no
gi|320446791|ref|NW_003383780.1|	57860-58230	263115	13.46	-0.967009	0.64585	no
gi|320446791|ref|NW_003383780.1|	58918-60363	108816	659334	-0.722805	0.73935	no
gi|320446791|ref|NW_003383780.1|	593418-594664	0.500955	196893	197466	0.39	no
gi|320446791|ref|NW_003383780.1|	596532-597332	0.29895	266915	31584	0.24575	no
gi|320446791|ref|NW_003383780.1|	60938-61422	600009	222816	-142913	0.51015	no
gi|320446791|ref|NW_003383780.1|	614327-618079	340656	973757	151524	0.3814	no
gi|320446791|ref|NW_003383780.1|	618951-623025	0.263728	501557	424929	0.12945	no
gi|320446791|ref|NW_003383780.1|	63363-64209	212843	494425	-210597	0.35495	no
gi|320446791|ref|NW_003383780.1|	682225-682390	962596	196698	103098	0.60815	no
gi|320446791|ref|NW_003383780.1|	688238-688678	127705	688434	-0.891425	0.66475	no
gi|320446791|ref|NW_003383780.1|	79761-81609	194627	176459	-0.141379	0.91375	no
gi|320446791|ref|NW_003383780.1|	81787-86813	103545	631592	-0.713197	0.5754	no
gi|320446791|ref|NW_003383780.1|	90787-91665	239011	958638	-131802	0.5455	no
gi|320446791|ref|NW_003383780.1|	91719-93495	142388	442524	-1686	0.4543	no
gi|320446791|ref|NW_003383780.1|	95971-100991	162918	103503	-0.654472	0.6219	no
gi|320446794|ref|NW_003383777.1|	101091-101622	508881	34241	-0.571606	0.78605	no
gi|320446794|ref|NW_003383777.1|	103475-104723	149989	254306	0.761703	0.70845	no
gi|320446794|ref|NW_003383777.1|	11518-13685	293513	234668	-0.322808	0.8029	no
gi|320446794|ref|NW_003383777.1|	116933-117607	922624	980926	341033	0.08225	no
gi|320446794|ref|NW_003383777.1|	118643-122130	0.207179	318888	39441	0.16305	no
gi|320446794|ref|NW_003383777.1|	127378-128578	402385	327434	302455	0.1144	no
gi|320446794|ref|NW_003383777.1|	27397-28454	308114	235822	293616	0.2208	no
gi|320446794|ref|NW_003383777.1|	292261-292601	319274	207124	-0.624298	0.76255	no
gi|320446794|ref|NW_003383777.1|	86165-87058	256656	0.53048	-227447	0.2658	no
gi|320446794|ref|NW_003383777.1|	95138-95817	22808	129721	-0.814129	0.69605	no
gi|320446794|ref|NW_003383777.1|	96483-97436	258708	210985	-0.294185	0.882	no
gi|320446795|ref|NW_003383776.1|	153678-154577	203468	0.70103	-153725	0.4983	no
gi|320446795|ref|NW_003383776.1|	418940-419761	694623	0	#NAME?	5.00E-05	yes
gi|320446795|ref|NW_003383776.1|	436512-436908	93862	194918	-226768	0.3256	no

gi|320446795|ref|NW_003383776.1|	467694-46845	232087	198901	-354454	0.1637	no
gi|320446795|ref|NW_003383776.1|	469217-46971	448006	214259	-106417	0.59575	no
gi|320446795|ref|NW_003383776.1|	469899-47476	252413	128002	-0.979623	0.46895	no
gi|320446795|ref|NW_003383776.1|	510003-51102	0.43185	164138	192631	0.3182	no
gi|320446795|ref|NW_003383776.1|	511242-51488	11972	540034	-114854	0.4947	no
gi|320446795|ref|NW_003383776.1|	524273-53135	165378	706707	-122658	0.35695	no
gi|320446795|ref|NW_003383776.1|	531465-53382	190785	0.610501	-164388	0.4424	no
gi|320446795|ref|NW_003383776.1|	533934-53490	268801	301346	-315705	0.20365	no
gi|320446796|ref|NW_003383775.1|	11017-12775	299226	501746	0.745725	0.7209	no
gi|320446796|ref|NW_003383775.1|	114823-11552	0.718492	663123	320623	0.20695	no
gi|320446796|ref|NW_003383775.1|	121790-12316	0.593878	226865	19336	0.39555	no
gi|320446796|ref|NW_003383775.1|	134777-13584	423623	418656	330491	0.09335	no
gi|320446796|ref|NW_003383775.1|	135975-13731	0.306097	382552	364359	0.17615	no
gi|320446796|ref|NW_003383775.1|	138258-13977	459405	149706	17043	0.45085	no
gi|320446796|ref|NW_003383775.1|	145262-14599	269152	14286	240811	0.2843	no
gi|320446796|ref|NW_003383775.1|	151098-15173	0.847127	518506	261371	0.2368	no
gi|320446796|ref|NW_003383775.1|	16138-18787	265652	678921	135371	0.54055	no
gi|320446796|ref|NW_003383775.1|	164743-16526	0.574765	579738	333436	0.2265	no
gi|320446796|ref|NW_003383775.1|	166130-16812	133949	400307	157943	0.4666	no
gi|320446796|ref|NW_003383775.1|	169538-17019	15953	271782	0.768626	0.72055	no
gi|320446796|ref|NW_003383775.1|	180536-18098	151544	554205	187069	0.32465	no
gi|320446796|ref|NW_003383775.1|	181411-18177	477504	623191	0.384163	0.85105	no
gi|320446796|ref|NW_003383775.1|	182026-18319	0.361909	162991	217109	0.2872	no
gi|320446796|ref|NW_003383775.1|	18944-22858	155947	317831	102721	0.63135	no
gi|320446796|ref|NW_003383775.1|	190283-19179	119152	2854	126018	0.5476	no
gi|320446796|ref|NW_003383775.1|	192190-19273	0	402149	inf	0.0294	no
gi|320446796|ref|NW_003383775.1|	219279-22281	117718	229185	0.961172	0.65195	no
gi|320446796|ref|NW_003383775.1|	225964-22684	0.522048	197755	192146	0.3194	no
gi|320446796|ref|NW_003383775.1|	248412-25050	0.453933	443314	328778	0.1942	no
gi|320446796|ref|NW_003383775.1|	251172-25255	0.294863	163852	247428	0.24535	no

gi|320446796|ref|NW_003383775.1|	25144-30694	182544	449344	129958	0.33315	no
gi|320446796|ref|NW_003383775.1|	258037-25849	294344	0	#NAME?	0.007	no
gi|320446796|ref|NW_003383775.1|	264517-26628	136704	0	#NAME?	5.00E-05	yes
gi|320446796|ref|NW_003383775.1|	266410-26687	208235	0	#NAME?	0.0099	no
gi|320446796|ref|NW_003383775.1|	272754-27308	375493	0	#NAME?	0.0104	no
gi|320446796|ref|NW_003383775.1|	274744-27725	0	124566	inf	1	no
gi|320446796|ref|NW_003383775.1|	31362-33063	111681	165147	0.564364	0.7211	no
gi|320446796|ref|NW_003383775.1|	41462-42392	461661	251362	-0.877068	0.6646	no
gi|320446796|ref|NW_003383775.1|	418718-41998	0.492186	216253	213544	0.34635	no
gi|320446796|ref|NW_003383775.1|	429228-43124	187212	230784	0.301866	0.81295	no
gi|320446796|ref|NW_003383775.1|	432815-43422	114639	786587	-0.543424	0.79945	no
gi|320446796|ref|NW_003383775.1|	436103-43652	222012	123183	-0.849836	0.6764	no
gi|320446796|ref|NW_003383775.1|	440152-44088	142722	53514	-141522	0.5033	no
gi|320446796|ref|NW_003383775.1|	441540-44246	294117	131827	-115775	0.6059	no
gi|320446796|ref|NW_003383775.1|	448157-44867	112167	0	#NAME?	0.01335	no
gi|320446796|ref|NW_003383775.1|	449544-45064	112819	0	#NAME?	0.0061	no
gi|320446796|ref|NW_003383775.1|	451087-45227	157502	0.122652	-700465	0.26205	no
gi|320446796|ref|NW_003383775.1|	453988-45443	114628	0	#NAME?	0.01585	no
gi|320446796|ref|NW_003383775.1|	454558-45533	84144	0	#NAME?	0.0091	no
gi|320446796|ref|NW_003383775.1|	455463-45573	279012	0	#NAME?	0.02105	no
gi|320446796|ref|NW_003383775.1|	456589-45750	176065	0	#NAME?	0.00555	no
gi|320446796|ref|NW_003383775.1|	53184-54642	0.552095	307009	247529	0.28965	no
gi|320446796|ref|NW_003383775.1|	54778-56806	282389	49891	0.821095	0.6926	no
gi|320446796|ref|NW_003383775.1|	57826-58254	168565	446346	140486	0.38125	no
gi|320446796|ref|NW_003383775.1|	8720-9070	778316	506234	-0.620553	0.7546	no
gi|320446796|ref|NW_003383775.1|	9183-10566	279387	183857	-0.603685	0.7562	no
gi|320446796|ref|NW_003383775.1|	93763-94649	0.778255	125085	0.684595	1	no
gi|320446796|ref|NW_003383775.1|	96158-99329	109205	116566	0.0941008	1	no
gi|320446798|ref|NW_003383773.1|	101319-10185	610055	896281	0.555012	0.78255	no
gi|320446798|ref|NW_003383773.1|	102046-10362	147161	848841	-0.79383	0.72995	no

gi|320446798|ref|NW_003383773.1|	103857-10516	198421	190187	-0.0611477	0.96865	no
gi|320446798|ref|NW_003383773.1|	128287-12868	0	791754	inf	0.029	no
gi|320446798|ref|NW_003383773.1|	134824-13509	0	191683	inf	0.029	no
gi|320446798|ref|NW_003383773.1|	143528-14828	160561	115856	285114	0.1305	no
gi|320446798|ref|NW_003383773.1|	148808-15474	421018	343605	-0.293131	0.8174	no
gi|320446798|ref|NW_003383773.1|	155519-15707	166113	0.533327	-163907	0.4382	no
gi|320446798|ref|NW_003383773.1|	160896-16206	236255	105765	-115948	0.60585	no
gi|320446798|ref|NW_003383773.1|	163023-16667	669453	715122	0.0952048	0.9402	no
gi|320446798|ref|NW_003383773.1|	166966-17292	183681	163232	-0.170278	0.9028	no
gi|320446798|ref|NW_003383773.1|	173055-17336	867702	763038	-0.185445	0.9271	no
gi|320446798|ref|NW_003383773.1|	176862-17748	393886	350816	-0.167061	0.93515	no
gi|320446798|ref|NW_003383773.1|	243463-24699	0.3586	333337	321653	0.18105	no
gi|320446798|ref|NW_003383773.1|	252711-25448	110696	180177	0.702809	0.66875	no
gi|320446798|ref|NW_003383773.1|	254614-25563	300373	311395	0.0519902	0.97045	no
gi|320446798|ref|NW_003383773.1|	256065-25792	435162	368251	-0.240864	0.90635	no
gi|320446798|ref|NW_003383773.1|	258035-26075	25137	218378	-0.202984	0.917	no
gi|320446798|ref|NW_003383773.1|	264327-26468	205479	126136	-0.704014	0.7185	no
gi|320446798|ref|NW_003383773.1|	264893-26566	938396	0.21461	-545041	0.27645	no
gi|320446798|ref|NW_003383773.1|	266319-26687	467692	0.3508	-373684	0.32305	no
gi|320446798|ref|NW_003383773.1|	267748-26937	532009	0.589075	-317493	0.19585	no
gi|320446798|ref|NW_003383773.1|	270254-27104	760855	0.626817	-360151	0.1755	no
gi|320446798|ref|NW_003383773.1|	272122-27451	547936	109311	-232557	0.3061	no
gi|320446798|ref|NW_003383773.1|	275253-27603	119556	294591	-20209	0.3475	no
gi|320446798|ref|NW_003383773.1|	276944-27742	508701	220287	-120744	0.58275	no
gi|320446798|ref|NW_003383773.1|	277842-28014	141115	535238	-139862	0.4077	no
gi|320446798|ref|NW_003383773.1|	280912-28310	189153	446002	-208443	0.2336	no
gi|320446798|ref|NW_003383773.1|	294018-29448	157971	430661	-187504	0.37515	no
gi|320446798|ref|NW_003383773.1|	295178-29686	203852	75424	-143442	0.3861	no
gi|320446798|ref|NW_003383773.1|	297019-30214	224062	741365	172628	0.3183	no
gi|320446798|ref|NW_003383773.1|	302238-30358	0.15222	380505	464368	0.17455	no

gi|320446798|ref|NW_003383773.1|	354213-35666	34255	223271	-0.617519	0.7744	no
gi|320446798|ref|NW_003383773.1|	360099-36226	113503	134027	0.239791	1	no
gi|320446798|ref|NW_003383773.1|	374768-37563	21378	294342	0.461365	0.81105	no
gi|320446798|ref|NW_003383773.1|	382397-38390	264368	827295	164585	0.45575	no
gi|320446798|ref|NW_003383773.1|	394634-39496	144828	844255	254334	0.27405	no
gi|320446798|ref|NW_003383773.1|	403128-40368	368186	177453	-1053	0.5963	no
gi|320446798|ref|NW_003383773.1|	428711-42907	815394	144565	0.826151	0.68625	no
gi|320446798|ref|NW_003383773.1|	460190-46094	0.97259	200017	104022	0.5772	no
gi|320446798|ref|NW_003383773.1|	509538-50993	0.988021	714698	285472	0.25935	no
gi|320446798|ref|NW_003383773.1|	510271-51070	251678	666551	140514	0.5186	no
gi|320446798|ref|NW_003383773.1|	510776-51536	123987	919866	289123	0.12005	no
gi|320446798|ref|NW_003383773.1|	52600-53464	139621	744843	241542	0.17815	no
gi|320446798|ref|NW_003383773.1|	56723-57733	0	165598	inf	0.0294	no
gi|320446798|ref|NW_003383773.1|	58744-63652	462195	632071	377351	0.00705	no
gi|320446798|ref|NW_003383773.1|	563731-66486	0.376025	117179	163981	1	no
gi|320446798|ref|NW_003383773.1|	566830-67242	107053	752333	281305	0.12775	no
gi|320446798|ref|NW_003383773.1|	592515-69978	821941	492862	-0.737851	0.6521	no
gi|320446798|ref|NW_003383773.1|	700592-70135	77884	270323	-152664	0.3814	no
gi|320446798|ref|NW_003383773.1|	707275-70887	315329	220283	-0.517502	0.68115	no
gi|320446798|ref|NW_003383773.1|	710046-71097	329503	372638	0.177481	0.8896	no
gi|320446798|ref|NW_003383773.1|	712345-71286	53494	439063	-0.284951	0.8885	no
gi|320446798|ref|NW_003383773.1|	715306-71738	595885	351701	-0.760683	0.72285	no
gi|320446798|ref|NW_003383773.1|	71822-72279	0.741984	108634	387194	0.2072	no
gi|320446798|ref|NW_003383773.1|	718640-72119	589208	624987	0.085049	0.97165	no
gi|320446798|ref|NW_003383773.1|	721791-72285	368391	651141	0.82173	0.6921	no
gi|320446798|ref|NW_003383773.1|	725842-72669	525738	0.749824	-613165	0.12955	no
gi|320446798|ref|NW_003383773.1|	726828-72765	63478	0	#NAME?	5.00E-05	yes
gi|320446798|ref|NW_003383773.1|	730467-73092	681537	0	#NAME?	0.004	no
gi|320446798|ref|NW_003383773.1|	732937-73392	173362	124385	-0.478971	0.825	no
gi|320446798|ref|NW_003383773.1|	734554-73589	237678	124832	-0.929016	0.5672	no

gi|320446798|ref|NW_003383773.1|	'36898-73836'	177831	88437	-100778	0.6431	no
gi|320446798|ref|NW_003383773.1|	'55654-75668:	680509	0.587983	-353277	0.1982	no
gi|320446798|ref|NW_003383773.1|	'61415-76172(	127125	420117	172454	0.41845	no
gi|320446798|ref|NW_003383773.1|	'61857-76310:	13372	359392	142634	0.49655	no
gi|320446798|ref|NW_003383773.1|	'70240-77106!	200388	747822	18999	0.3825	no
gi|320446798|ref|NW_003383773.1|	'76269-77688:	223392	12738	251149	0.28115	no
gi|320446798|ref|NW_003383773.1|	'77005-77790!	0.50562	313608	263284	0.23605	no
gi|320446798|ref|NW_003383773.1|	'78807-78022'	142452	217769	0.612319	0.7623	no
gi|320446798|ref|NW_003383773.1|	'80603-78126:	199412	271782	0.446698	0.8278	no
gi|320446798|ref|NW_003383773.1|	'82199-78272(	525971	102107	0.957031	0.65185	no
gi|320446798|ref|NW_003383773.1|	'85217-78802!	905105	311798	510638	0.0204	no
gi|320446798|ref|NW_003383773.1|	'90693-79182:	350876	313511	-348438	0.0793	no
gi|320446798|ref|NW_003383773.1|	'92810-79384(	0.424782	234902	246726	0.2454	no
gi|320446798|ref|NW_003383773.1|	'93997-79633.	55158	35985	-0.616174	0.64685	no
gi|320446798|ref|NW_003383773.1|	79403-79816	0.905621	777014	310096	0.24695	no
gi|320446798|ref|NW_003383773.1|	80306-81555	283034	114326	201411	0.3583	no
gi|320446798|ref|NW_003383773.1|	303757-80410!	0	838398	inf	0.0312	no
gi|320446798|ref|NW_003383773.1|	317303-81771:	179351	0	#NAME?	0.0132	no
gi|320446798|ref|NW_003383773.1|	319476-82029(	46195	0	#NAME?	0.015	no
gi|320446798|ref|NW_003383773.1|	322784-82322(	158228	367571	-210591	0.3483	no
gi|320446798|ref|NW_003383773.1|	87811-94341	138041	137343	-0.00730666	0.9943	no
gi|320446798|ref|NW_003383773.1|	94638-95535	224491	133591	-0.748838	0.727	no
gi|320446798|ref|NW_003383773.1|	956197-95773!	0	251527	inf	0.01275	no
gi|320446798|ref|NW_003383773.1|	95648-96088	107751	360104	-158122	0.346	no
gi|320446798|ref|NW_003383773.1|	96943-98015	0	251194	inf	0.0154	no
gi|320446798|ref|NW_003383773.1|	98148-98788	0.416339	339975	30296	0.25005	no
gi|320446799|ref|NW_003383772.1|	105686-10636(	166886	310658	-242546	0.28695	no
gi|320446799|ref|NW_003383772.1|	112742-11524!	397567	631653	0.667932	0.6135	no
gi|320446799|ref|NW_003383772.1|	116544-11678:	105294	830359	-0.342612	0.86385	no
gi|320446799|ref|NW_003383772.1|	117804-11858:	419173	296165	-0.501145	0.75905	no

gi|320446799|ref|NW_003383772.1|	120618-121169	589211	429655	-0.455606	0.84225	no
gi|320446799|ref|NW_003383772.1|	14920-15362	118671	131276	0.145632	0.9398	no
gi|320446799|ref|NW_003383772.1|	154574-168965	809282	981925	0.27897	0.8329	no
gi|320446799|ref|NW_003383772.1|	170084-172282	220097	125392	-0.811696	0.52065	no
gi|320446799|ref|NW_003383772.1|	189871-190459	371714	145235	-13558	0.53165	no
gi|320446799|ref|NW_003383772.1|	19473-20467	0.667421	107567	0.688567	1	no
gi|320446799|ref|NW_003383772.1|	216090-216591	62004	734487	0.244377	0.87595	no
gi|320446799|ref|NW_003383772.1|	217358-217824	193094	204966	0.0860857	0.96445	no
gi|320446799|ref|NW_003383772.1|	217944-219354	231301	144738	-0.676324	0.68065	no
gi|320446799|ref|NW_003383772.1|	219602-220702	936915	634957	-0.561259	0.7845	no
gi|320446799|ref|NW_003383772.1|	223193-223571	764034	357412	-109605	0.59365	no
gi|320446799|ref|NW_003383772.1|	224695-225097	134039	441298	-160283	0.4413	no
gi|320446799|ref|NW_003383772.1|	225596-226119	539879	300458	-0.845473	0.70045	no
gi|320446799|ref|NW_003383772.1|	236242-238181	39223	980517	-200009	0.2788	no
gi|320446799|ref|NW_003383772.1|	239348-241560	663295	200763	-172416	0.19555	no
gi|320446799|ref|NW_003383772.1|	241812-247991	166859	304301	-245506	0.2019	no
gi|320446799|ref|NW_003383772.1|	24349-26426	207072	502342	127854	0.3408	no
gi|320446799|ref|NW_003383772.1|	271258-271785	486942	778245	0.676474	0.67085	no
gi|320446799|ref|NW_003383772.1|	322206-323513	110234	524654	-107113	0.62395	no
gi|320446799|ref|NW_003383772.1|	324867-325535	128603	797298	-0.689728	0.7357	no
gi|320446799|ref|NW_003383772.1|	326436-327490	654918	608675	-0.105642	0.95845	no
gi|320446799|ref|NW_003383772.1|	330535-331719	587076	863033	0.55587	0.78865	no
gi|320446799|ref|NW_003383772.1|	33135-35555	257973	188769	-0.450598	0.73245	no
gi|320446799|ref|NW_003383772.1|	337472-340818	276637	109655	19869	0.2555	no
gi|320446799|ref|NW_003383772.1|	348380-349650	713546	11252	0.657106	0.75945	no
gi|320446799|ref|NW_003383772.1|	350022-350313	143944	355386	130388	0.53965	no
gi|320446799|ref|NW_003383772.1|	360398-366959	214981	533598	131154	0.3245	no
gi|320446799|ref|NW_003383772.1|	36109-36872	639808	35099	-0.866207	0.67095	no
gi|320446799|ref|NW_003383772.1|	367069-367924	762728	806061	0.0797195	0.96705	no
gi|320446799|ref|NW_003383772.1|	373949-374720	0	367495	inf	0.02075	no

gi|320446799|ref|NW_003383772.1|	37958-38627	618216	365947	-0.756478	0.6445	no
gi|320446799|ref|NW_003383772.1|	40565-51876	194072	217698	0.16574	0.90015	no
gi|320446799|ref|NW_003383772.1|	52013-54478	491909	428097	-0.200453	0.92445	no
gi|320446799|ref|NW_003383772.1|	5655-6288	93184	518506	-0.84572	0.67155	no
gi|320446799|ref|NW_003383772.1|	57682-59101	168236	525063	-167992	0.45425	no
gi|320446799|ref|NW_003383772.1|	66791-67316	132634	193861	-277436	0.26095	no
gi|320446799|ref|NW_003383772.1|	89851-92393	321868	194168	-0.729164	0.7243	no
gi|320446799|ref|NW_003383772.1|	96723-97665	191081	0.659114	-153559	0.49865	no
gi|320446799|ref|NW_003383772.1|	99960-102645	15636	34653	-217382	0.21905	no
gi|320446801|ref|NW_003383770.1|	182126-18325	965319	694634	-0.474752	0.8178	no
gi|320446801|ref|NW_003383770.1|	204270-20483	0	203986	inf	0.00765	no
gi|320446801|ref|NW_003383770.1|	238600-23898	127299	486893	-138655	0.50975	no
gi|320446801|ref|NW_003383770.1|	251284-25257	0.159412	222365	712403	0.14075	no
gi|320446801|ref|NW_003383770.1|	254245-25489	0	139358	inf	0.0074	no
gi|320446801|ref|NW_003383770.1|	255432-25833	0	903321	inf	5.00E-05	yes
gi|320446801|ref|NW_003383770.1|	258431-26588	0	200448	inf	5.00E-05	yes
gi|320446801|ref|NW_003383770.1|	273491-27419	0	105608	inf	0.00915	no
gi|320446801|ref|NW_003383770.1|	279074-28456	314731	256978	-0.292477	0.8248	no
gi|320446801|ref|NW_003383770.1|	289301-29071	157495	696391	-117734	0.59145	no
gi|320446801|ref|NW_003383770.1|	31244-33356	0.269747	426545	398302	0.16995	no
gi|320446801|ref|NW_003383770.1|	313115-31396	115906	265802	-212454	0.33315	no
gi|320446801|ref|NW_003383770.1|	324560-32546	669947	484351	-0.467995	0.71815	no
gi|320446801|ref|NW_003383770.1|	326748-32830	935623	962878	0.0414258	0.984	no
gi|320446801|ref|NW_003383770.1|	356661-36186	293852	10.21	-152511	0.2613	no
gi|320446801|ref|NW_003383770.1|	365618-36718	758063	184588	-203801	0.3563	no
gi|320446801|ref|NW_003383770.1|	371207-37270	198589	140878	-0.495338	0.75465	no
gi|320446801|ref|NW_003383770.1|	373180-37404	496412	603504	0.281824	0.82305	no
gi|320446801|ref|NW_003383770.1|	375823-37665	620611	633274	0.0291412	0.9808	no
gi|320446801|ref|NW_003383770.1|	380886-38403	12807	269896	107547	0.41585	no
gi|320446801|ref|NW_003383770.1|	385331-38559	198585	162039	-0.293414	0.87275	no

gi|320446801|ref|NW_003383770.1|	385992-386620	17155	583093	176509	0.42935	no
gi|320446801|ref|NW_003383770.1|	497200-498315	279.35	106659	-138906	0.2993	no
gi|320446801|ref|NW_003383770.1|	499995-504322	618138	201537	-161688	0.2289	no
gi|320446801|ref|NW_003383770.1|	512206-516459	123553	868584	-0.508392	0.6813	no
gi|320446801|ref|NW_003383770.1|	519676-520950	122674	500541	-129327	0.3453	no
gi|320446801|ref|NW_003383770.1|	530011-535711	167653	300518	0.841972	0.52665	no
gi|320446801|ref|NW_003383770.1|	59570-61025	0.276727	2212	299882	0.2201	no
gi|320446801|ref|NW_003383770.1|	61971-64340	166076	276078	0.733233	0.72945	no
gi|320446801|ref|NW_003383770.1|	64508-67466	148736	229624	0.626515	0.75455	no
gi|320446801|ref|NW_003383770.1|	67582-68352	110502	134272	0.281084	0.89575	no
gi|320446803|ref|NW_003383768.1|	001205-100487	471192	15559	172337	0.33015	no
gi|320446803|ref|NW_003383768.1|	010703-101395	0	324913	inf	0.00595	no
gi|320446803|ref|NW_003383768.1|	014883-101558	0	370687	inf	0.02205	no
gi|320446803|ref|NW_003383768.1|	10653-11467	116686	340777	15462	0.4967	no
gi|320446803|ref|NW_003383768.1|	110221-111040	13593	208715	0.618664	0.77335	no
gi|320446803|ref|NW_003383768.1|	111550-112422	503739	967499	0.941583	0.6458	no
gi|320446803|ref|NW_003383768.1|	112645-113109	605609	984763	0.70139	0.65855	no
gi|320446803|ref|NW_003383768.1|	114667-115630	828208	176286	108985	0.6163	no
gi|320446803|ref|NW_003383768.1|	115910-117655	178846	498246	147814	0.48745	no
gi|320446803|ref|NW_003383768.1|	11598-13257	0.829728	247539	157694	0.46955	no
gi|320446803|ref|NW_003383768.1|	119384-123325	264088	917885	179729	0.3002	no
gi|320446803|ref|NW_003383768.1|	123467-130010	86131	305621	182714	0.1817	no
gi|320446803|ref|NW_003383768.1|	133486-136368	130057	793231	-0.713329	0.57075	no
gi|320446803|ref|NW_003383768.1|	137041-137940	134797	613401	-113589	0.58915	no
gi|320446803|ref|NW_003383768.1|	14154-15200	828627	102787	0.310868	0.8206	no
gi|320446803|ref|NW_003383768.1|	148693-150885	245177	221458	-346872	0.0146	no
gi|320446803|ref|NW_003383768.1|	15814-16329	190851	116133	-0.716673	0.7206	no
gi|320446803|ref|NW_003383768.1|	16431-17229	147007	107137	-0.456419	0.8289	no
gi|320446803|ref|NW_003383768.1|	18044-21410	381045	204342	-0.89898	0.50585	no
gi|320446803|ref|NW_003383768.1|	195352-203903	239415	990339	204841	0.2677	no

gi|320446803|ref|NW_003383768.1|	[illegible]12546-21307[illegible]	57667	170598	156478	0.4667	no
gi|320446803|ref|NW_003383768.1|	23377-23892	650086	336384	-0.950521	0.6695	no
gi|320446803|ref|NW_003383768.1|	[illegible]43718-24389[illegible]	422088	112463	141383	0.5125	no
gi|320446803|ref|NW_003383768.1|	26985-28483	113603	473948	-12612	0.5521	no
gi|320446803|ref|NW_003383768.1|	2779-4238	54199	532039	-0.0267346	0.98415	no
gi|320446803|ref|NW_003383768.1|	28680-29733	866941	115624	0.415437	0.8449	no
gi|320446803|ref|NW_003383768.1|	33641-34576	272604	16307	-0.741314	0.743	no
gi|320446803|ref|NW_003383768.1|	[illegible]80505-38167[illegible]	386262	619422	0.681343	0.6009	no
gi|320446803|ref|NW_003383768.1|	39052-39604	223851	113881	-0.975013	0.4613	no
gi|320446803|ref|NW_003383768.1|	39755-41176	250504	123629	-101882	0.5312	no
gi|320446803|ref|NW_003383768.1|	41282-42804	133255	139831	0.0694962	0.9573	no
gi|320446803|ref|NW_003383768.1|	[illegible]64559-46546[illegible]	326209	397797	0.286237	0.8871	no
gi|320446803|ref|NW_003383768.1|	[illegible]68293-47120[illegible]	611932	233726	-138856	0.5279	no
gi|320446803|ref|NW_003383768.1|	[illegible]78921-48113[illegible]	169111	709284	-125353	0.4541	no
gi|320446803|ref|NW_003383768.1|	[illegible]81594-48812[illegible]	449842	488952	0.120272	0.9225	no
gi|320446803|ref|NW_003383768.1|	[illegible]04474-51220[illegible]	27915	102603	187796	0.15315	no
gi|320446803|ref|NW_003383768.1|	[illegible]71426-57214[illegible]	519087	971005	0.903502	0.6557	no
gi|320446803|ref|NW_003383768.1|	[illegible]57558-66060[illegible]	169893	191195	349235	0.0896	no
gi|320446803|ref|NW_003383768.1|	[illegible]47804-74990[illegible]	350967	138546	-134097	0.3023	no
gi|320446803|ref|NW_003383768.1|	[illegible]50706-75149[illegible]	154606	160256	0.051786	0.9686	no
gi|320446803|ref|NW_003383768.1|	[illegible]52002-75244[illegible]	360749	239527	-0.590807	0.77265	no
gi|320446803|ref|NW_003383768.1|	[illegible]52695-75376[illegible]	13191	968612	-0.445564	0.8361	no
gi|320446803|ref|NW_003383768.1|	[illegible]54344-75498[illegible]	414319	214308	-0.951058	0.6641	no
gi|320446803|ref|NW_003383768.1|	[illegible]57913-75834[illegible]	508056	61647	0.279045	0.888	no
gi|320446803|ref|NW_003383768.1|	[illegible]59249-76016[illegible]	223182	223957	0.00500143	0.99485	no
gi|320446803|ref|NW_003383768.1|	[illegible]64319-76606[illegible]	219377	170718	-0.361798	0.7757	no
gi|320446803|ref|NW_003383768.1|	[illegible]66579-76774[illegible]	230306	841811	-145199	0.3734	no
gi|320446803|ref|NW_003383768.1|	77028-77491	434338	289348	-0.586015	0.75475	no
gi|320446803|ref|NW_003383768.1|	[illegible]77837-77862[illegible]	184939	126929	-0.543028	0.7976	no
gi|320446803|ref|NW_003383768.1|	[illegible]96143-79681[illegible]	153424	261707	0.770429	0.72055	no

gi|320446803|ref|NW_003383768.1|	301166-80161	0.780827	466686	257938	0.27405	no
gi|320446803|ref|NW_003383768.1|	302850-80344	324327	107.65	173082	0.32215	no
gi|320446803|ref|NW_003383768.1|	335882-83640	403669	271406	-0.572722	0.79225	no
gi|320446803|ref|NW_003383768.1|	339169-84029	113694	104966	-0.115233	1	no
gi|320446803|ref|NW_003383768.1|	340700-84279	319813	890589	-18444	0.30565	no
gi|320446803|ref|NW_003383768.1|	347086-84752	1288.81	165.56	-296062	0.03425	no
gi|320446803|ref|NW_003383768.1|	8643-9467	143378	453382	16609	0.44375	no
gi|320446803|ref|NW_003383768.1|	88120-92977	104046	216589	105773	0.43185	no
gi|320446803|ref|NW_003383768.1|	94186-94918	815554	339698	20584	0.3638	no
gi|320446803|ref|NW_003383768.1|	976222-97657	371881	218078	-0.769994	0.70635	no
gi|320446803|ref|NW_003383768.1|	984142-98835	951373	868282	-0.131847	0.9211	no
gi|320446803|ref|NW_003383768.1|	989984-99046	116864	734289	-0.670408	0.7344	no
gi|320446803|ref|NW_003383768.1|	994802-99713	126233	987906	-0.353638	0.7738	no
gi|320446803|ref|NW_003383768.1|	99826-100128	372671	957102	-196116	0.3827	no
gi|320446804|ref|NW_003383767.1|	106562-10673	528777	244183	220723	0.3343	no
gi|320446804|ref|NW_003383767.1|	113871-11472	0.817209	16871	104577	0.5772	no
gi|320446804|ref|NW_003383767.1|	118472-11885	205015	544391	140891	0.50975	no
gi|320446804|ref|NW_003383767.1|	135530-13966	233463	938288	-131509	0.32755	no
gi|320446804|ref|NW_003383767.1|	139886-14055	337462	211359	-0.675027	0.75985	no
gi|320446804|ref|NW_003383767.1|	14144-16124	174096	465331	141838	0.50705	no
gi|320446804|ref|NW_003383767.1|	141922-14614	290466	163107	-0.832553	0.5391	no
gi|320446804|ref|NW_003383767.1|	146347-14723	131.41	582509	-117373	0.38115	no
gi|320446804|ref|NW_003383767.1|	150838-15327	461725	19838	-121877	0.3613	no
gi|320446804|ref|NW_003383767.1|	153639-15446	171761	177116	0.0442857	0.94	no
gi|320446804|ref|NW_003383767.1|	155230-15685	596741	118658	-23303	0.29145	no
gi|320446804|ref|NW_003383767.1|	157497-15954	409826	0.84451	-227883	0.3129	no
gi|320446804|ref|NW_003383767.1|	159844-16084	572393	0.608358	-323402	0.2171	no
gi|320446804|ref|NW_003383767.1|	161048-16182	715462	0.640355	-348193	0.18405	no
gi|320446804|ref|NW_003383767.1|	162401-16271	493832	212259	-454013	0.2007	no
gi|320446804|ref|NW_003383767.1|	163982-16685	188432	636151	-15666	0.37565	no

gi|320446804|ref|NW_003383767.1|	67211-1688	137691	305723	-217114	0.338	no
gi|320446804|ref|NW_003383767.1|	16760-18064	142127	197291	0.473141	0.80915	no
gi|320446804|ref|NW_003383767.1|	69139-16968	443552	848513	-23861	0.29895	no
gi|320446804|ref|NW_003383767.1|	69957-17123	289218	7215	-200309	0.2454	no
gi|320446804|ref|NW_003383767.1|	71360-17311	237164	864548	-145587	0.39705	no
gi|320446804|ref|NW_003383767.1|	18372-20990	141636	217692	0.620097	0.7659	no
gi|320446804|ref|NW_003383767.1|	21107-22264	23805	152225	-0.645059	0.7522	no
gi|320446804|ref|NW_003383767.1|	21082-22390	280066	883131	165686	0.31775	no
gi|320446804|ref|NW_003383767.1|	25476-22882	449527	699821	0.638579	0.6265	no
gi|320446804|ref|NW_003383767.1|	29260-23018	95588	169009	-249973	0.27255	no
gi|320446804|ref|NW_003383767.1|	32343-23852	115721	373127	168901	0.21215	no
gi|320446804|ref|NW_003383767.1|	41701-24239	367008	451352	0.298443	0.87885	no
gi|320446804|ref|NW_003383767.1|	51115-25165	111632	450909	201408	0.3028	no
gi|320446804|ref|NW_003383767.1|	52203-25260	167076	245644	0.55607	0.7815	no
gi|320446804|ref|NW_003383767.1|	80133-28114	93086	116683	0.325958	0.8766	no
gi|320446804|ref|NW_003383767.1|	13578-31542	262573	141983	243493	0.16705	no
gi|320446804|ref|NW_003383767.1|	17628-31789	256404	179436	280698	0.2661	no
gi|320446804|ref|NW_003383767.1|	33581-34432	219339	0.943248	-121745	0.5719	no
gi|320446804|ref|NW_003383767.1|	59538-46177	114648	168266	0.553535	0.661	no
gi|320446804|ref|NW_003383767.1|	62118-46309	684573	8035	0.231094	0.907	no
gi|320446804|ref|NW_003383767.1|	63398-46478	642522	304329	-107811	0.60195	no
gi|320446804|ref|NW_003383767.1|	65183-46550	158324	817727	-0.953187	0.66405	no
gi|320446804|ref|NW_003383767.1|	27569-52791	451794	133295	-176105	0.40765	no
gi|320446804|ref|NW_003383767.1|	28072-52984	113404	506168	-116378	0.6096	no
gi|320446804|ref|NW_003383767.1|	30501-53092	609765	23034	-140449	0.5239	no
gi|320446804|ref|NW_003383767.1|	31063-53268	11779	743721	-0.663388	0.7636	no
gi|320446804|ref|NW_003383767.1|	34877-53853	195883	56624	-179051	0.32645	no
gi|320446804|ref|NW_003383767.1|	47830-54911	0	20101	inf	0.0154	no
gi|320446804|ref|NW_003383767.1|	92682-59344	186249	211375	0.182571	0.93065	no
gi|320446804|ref|NW_003383767.1|	00178-60746	210193	293561	0.481945	0.7127	no

gi|320446804|ref|NW_003383767.1|	73571-75334	694803	515506	-0.430615	0.75345	no
gi|320446804|ref|NW_003383767.1|	79209-81221	239344	668955	-18391	0.29105	no
gi|320446804|ref|NW_003383767.1|	81705-82459	178994	602303	-157135	0.4704	no
gi|320446804|ref|NW_003383767.1|	82585-85225	221738	146585	-0.597115	0.64755	no
gi|320446804|ref|NW_003383767.1|	86696-87006	557351	134492	-205106	0.35625	no
gi|320446804|ref|NW_003383767.1|	88685-89560	237494	345244	-27822	0.24055	no
gi|320446804|ref|NW_003383767.1|	9373-14009	145696	418889	152361	0.49805	no
gi|320446804|ref|NW_003383767.1|	94407-95092	0.75021	384173	235639	0.2621	no
gi|320446804|ref|NW_003383767.1|	96229-97722	241527	190325	297821	0.1127	no
gi|320446804|ref|NW_003383767.1|	99017-101667	971325	20701	109168	0.39535	no
gi|320446805|ref|NW_003383766.1|	155271-15567	0.942762	559055	256803	0.27405	no
gi|320446805|ref|NW_003383766.1|	156427-15691	0	577229	inf	0.02915	no
gi|320446805|ref|NW_003383766.1|	161995-16419	0.859752	755949	31363	0.189	no
gi|320446805|ref|NW_003383766.1|	175494-17716	0.825667	435197	239804	0.2863	no
gi|320446805|ref|NW_003383766.1|	214674-21580	0.750378	168888	117037	0.5787	no
gi|320446805|ref|NW_003383766.1|	240606-24258	146396	156186	0.093396	0.94095	no
gi|320446805|ref|NW_003383766.1|	244361-24729	145467	336062	-211389	0.23435	no
gi|320446805|ref|NW_003383766.1|	247596-24841	43382	0	#NAME?	0.01585	no
gi|320446805|ref|NW_003383766.1|	250331-25112	852158	0.417878	-434997	0.2064	no
gi|320446805|ref|NW_003383766.1|	252688-25425	26164	224428	-0.221327	0.86085	no
gi|320446805|ref|NW_003383766.1|	254402-25710	97702	463091	-107709	0.51015	no
gi|320446805|ref|NW_003383766.1|	259733-26014	81709	287721	-150583	0.485	no
gi|320446805|ref|NW_003383766.1|	261306-26220	249248	117423	-108587	0.62275	no
gi|320446805|ref|NW_003383766.1|	263187-26385	125857	429026	-155265	0.45835	no
gi|320446805|ref|NW_003383766.1|	266488-26800	12489	575979	-111657	0.62145	no
gi|320446805|ref|NW_003383766.1|	269898-27400	877092	357414	-129513	0.44465	no
gi|320446805|ref|NW_003383766.1|	274132-27819	233433	180093	-0.374269	0.78465	no
gi|320446805|ref|NW_003383766.1|	278286-27961	0.769431	0.641036	-0.263388	1	no
gi|320446805|ref|NW_003383766.1|	286805-28730	0.630809	169042	474403	0.1753	no
gi|320446805|ref|NW_003383766.1|	2895-3471	0.985871	500133	234284	0.2622	no

gi|320446805|ref|NW_003383766.1|	342071-34274	155526	583393	190731	0.39775	no
gi|320446805|ref|NW_003383766.1|	423075-42342	130581	849109	2701	0.2673	no
gi|320446805|ref|NW_003383766.1|	60329-64214	799799	185413	121304	0.3627	no
gi|320446805|ref|NW_003383766.1|	65196-72639	779505	302156	-136727	0.4328	no
gi|320446806|ref|NW_003383765.1|	1317-1955	125514	370049	155987	0.491	no
gi|320446806|ref|NW_003383765.1|	145430-14603	436968	0.31191	-713026	0.2628	no
gi|320446806|ref|NW_003383765.1|	146220-14669	725859	0.457226	-731064	0.2604	no
gi|320446806|ref|NW_003383765.1|	149996-15042	270.56	165902	-734947	0.1105	no
gi|320446806|ref|NW_003383765.1|	150618-15112	553773	0.825259	-60683	0.17675	no
gi|320446806|ref|NW_003383765.1|	163839-16668	743559	777308	0.0640396	0.9784	no
gi|320446806|ref|NW_003383765.1|	167951-16879	113705	476939	-125341	0.5466	no
gi|320446806|ref|NW_003383765.1|	171194-17154	666139	432824	-0.622041	0.75225	no
gi|320446806|ref|NW_003383765.1|	171890-17265	102563	197797	-237442	0.28195	no
gi|320446806|ref|NW_003383765.1|	174213-17463	518943	106959	-227852	0.3157	no
gi|320446806|ref|NW_003383765.1|	193091-19407	590104	329103	24795	0.15825	no
gi|320446806|ref|NW_003383765.1|	198748-19923	200762	536704	141864	0.50885	no
gi|320446806|ref|NW_003383765.1|	219287-22068	158621	582318	187622	0.29555	no
gi|320446806|ref|NW_003383765.1|	221654-22347	221075	546534	130577	0.32655	no
gi|320446806|ref|NW_003383765.1|	223659-22687	154027	388515	133479	0.32425	no
gi|320446806|ref|NW_003383765.1|	231731-23338	226485	131101	253319	0.2754	no
gi|320446806|ref|NW_003383765.1|	236718-23999	144928	180209	0.314335	0.8097	no
gi|320446806|ref|NW_003383765.1|	240104-24537	161927	214203	0.403634	0.8466	no
gi|320446806|ref|NW_003383765.1|	24301-26004	140273	192144	0.453947	0.78245	no
gi|320446806|ref|NW_003383765.1|	245709-24796	18121	419648	-211041	0.22915	no
gi|320446806|ref|NW_003383765.1|	26260-26884	64983	529914	-0.294304	0.8807	no
gi|320446806|ref|NW_003383765.1|	275216-27661	0.578107	572346	330748	0.20315	no
gi|320446806|ref|NW_003383765.1|	276738-27811	296417	211029	283174	0.1277	no
gi|320446806|ref|NW_003383765.1|	281298-28214	0	567757	inf	0.00935	no
gi|320446806|ref|NW_003383765.1|	283514-28434	0	65925	inf	0.0101	no
gi|320446806|ref|NW_003383765.1|	284460-28701	0.726791	129967	416046	0.0636	no

gi|320446806|ref|NW_003383765.1|	28522-29463	18896	924044	-103205	0.6368	no
gi|320446806|ref|NW_003383765.1|	292091-29473	0.139991	420572	490895	0.14955	no
gi|320446806|ref|NW_003383765.1|	295034-29605	0.646947	312961	227426	0.3239	no
gi|320446806|ref|NW_003383765.1|	297037-29799	0.696825	32064	220209	0.33335	no
gi|320446806|ref|NW_003383765.1|	298119-29903	0.743938	393206	240203	0.30005	no
gi|320446806|ref|NW_003383765.1|	299187-29948	372671	251239	275309	0.2329	no
gi|320446806|ref|NW_003383765.1|	31268-35396	189851	278566	0.553152	0.67865	no
gi|320446806|ref|NW_003383765.1|	315766-31633	754507	339976	-11501	0.58265	no
gi|320446806|ref|NW_003383765.1|	317006-31884	136964	844259	-0.69804	0.76	no
gi|320446806|ref|NW_003383765.1|	319057-31985	262881	155607	-0.756504	0.72835	no
gi|320446806|ref|NW_003383765.1|	322394-32294	394485	159707	-130454	0.54305	no
gi|320446806|ref|NW_003383765.1|	330421-33148	169049	295846	-251452	0.27475	no
gi|320446806|ref|NW_003383765.1|	334028-33960	20582	110323	-0.899643	0.50585	no
gi|320446806|ref|NW_003383765.1|	344083-34494	0	185403	inf	0.0312	no
gi|320446806|ref|NW_003383765.1|	375025-37602	0	303789	inf	0.0162	no
gi|320446806|ref|NW_003383765.1|	4786-5758	0.458241	23727	237235	0.2616	no
gi|320446806|ref|NW_003383765.1|	51486-53209	0.907458	552949	260724	0.25645	no
gi|320446806|ref|NW_003383765.1|	58778-59325	107482	471015	213168	0.29365	no
gi|320446806|ref|NW_003383765.1|	6946-7884	120106	36454	160177	0.45055	no
gi|320446806|ref|NW_003383765.1|	9851-10356	617473	0.414026	-389858	0.31815	no
gi|320446807|ref|NW_003383764.1|	064995-10655	0	32535	inf	0.0312	no
gi|320446807|ref|NW_003383764.1|	141593-14233	0	385739	inf	0.02075	no
gi|320446807|ref|NW_003383764.1|	16885-17250	589573	123173	106294	0.59345	no
gi|320446807|ref|NW_003383764.1|	215355-21692	0.883745	272285	162341	0.44515	no
gi|320446807|ref|NW_003383764.1|	224513-22810	226269	14425	267246	0.14865	no
gi|320446807|ref|NW_003383764.1|	375237-37598	0.332477	273329	303931	0.24995	no
gi|320446807|ref|NW_003383764.1|	45718-46579	207738	323107	0.637247	0.77965	no
gi|320446807|ref|NW_003383764.1|	474379-47596	263154	131653	232276	0.3159	no
gi|320446807|ref|NW_003383764.1|	481193-48182	0.424616	259875	261359	0.2636	no
gi|320446807|ref|NW_003383764.1|	527078-52839	0.623507	21641	179529	0.426	no

gi|320446807|ref|NW_003383764.1|	5588-9167	736752	254929	179084	0.18115	no
gi|320446807|ref|NW_003383764.1|	59559-56297	291402	681989	122674	0.4451	no
gi|320446807|ref|NW_003383764.1|	57655-58045	408183	12733	164129	0.44975	no
gi|320446807|ref|NW_003383764.1|	93272-79436	0	627168	inf	0.00695	no
gi|320446807|ref|NW_003383764.1|	80214-80957	405621	28244	-0.52219	0.8206	no
gi|320446807|ref|NW_003383764.1|	81820-82739	0.246891	357467	385587	0.20055	no
gi|320446807|ref|NW_003383764.1|	84194-84927	386613	355291	-0.121888	0.9588	no
gi|320446807|ref|NW_003383764.1|	27160-92817	0.870384	466093	24209	0.30235	no
gi|320446807|ref|NW_003383764.1|	31141-93180	0	270558	inf	0.0312	no
gi|320446807|ref|NW_003383764.1|	9551-9750	149335	697665	222398	0.27495	no
gi|320446807|ref|NW_003383764.1|	96153-98829	320095	877231	145446	0.27795	no
gi|320446809|ref|NW_003383762.1|	05955-10761	0.3566	0.661947	0.892409	1	no
gi|320446809|ref|NW_003383762.1|	12577-11325	646228	856159	0.405837	0.83935	no
gi|320446809|ref|NW_003383762.1|	15377-11569	77367	604937	-0.354935	0.86535	no
gi|320446809|ref|NW_003383762.1|	21993-12268	70339	935621	0.411598	0.83855	no
gi|320446809|ref|NW_003383762.1|	23498-12373	796727	200836	133386	0.38395	no
gi|320446809|ref|NW_003383762.1|	13-722	106869	267958	132616	0.52445	no
gi|320446809|ref|NW_003383762.1|	34043-14046	0.654738	805032	362006	0.07165	no
gi|320446809|ref|NW_003383762.1|	40707-14096	0	312685	inf	0.0198	no
gi|320446809|ref|NW_003383762.1|	46523-14720	600168	317583	240369	0.2848	no
gi|320446809|ref|NW_003383762.1|	77415-17957	0.876274	0.550272	-0.671238	1	no
gi|320446809|ref|NW_003383762.1|	82436-18386	0.845433	0.783379	-0.10998	1	no
gi|320446809|ref|NW_003383762.1|	92236-19272	791036	264501	-158047	0.48585	no
gi|320446809|ref|NW_003383762.1|	94711-19569	113481	219402	0.951118	0.66285	no
gi|320446809|ref|NW_003383762.1|	96598-19918	229411	911489	199029	0.2405	no
gi|320446809|ref|NW_003383762.1|	05864-20604	149978	587245	-135271	0.5174	no
gi|320446809|ref|NW_003383762.1|	06227-20685	173288	412155	125002	0.55745	no
gi|320446809|ref|NW_003383762.1|	27811-22917	134332	13476	0.00459294	1	no
gi|320446809|ref|NW_003383762.1|	29755-23091	0.913592	139241	0.607964	1	no
gi|320446809|ref|NW_003383762.1|	34316-23507	259855	111329	-122288	0.55725	no

gi|320446809|ref|NW_003383762.1|	56068-58608	410005	644375	0.652258	0.76015	no
gi|320446809|ref|NW_003383762.1|	59247-60406	310621	189874	-0.710111	0.72205	no
gi|320446809|ref|NW_003383762.1|	60927-61352	205135	107441	-0.933025	0.65065	no
gi|320446809|ref|NW_003383762.1|	62764-64059	138557	150316	0.117515	0.95665	no
gi|320446809|ref|NW_003383762.1|	64257-64938	144944	948048	-0.61246	0.6419	no
gi|320446809|ref|NW_003383762.1|	68555-69416	169967	29711	0.805738	0.71215	no
gi|320446809|ref|NW_003383762.1|	6895-7451	47052	17642	-141524	0.5105	no
gi|320446809|ref|NW_003383762.1|	69591-70010	879494	302208	17808	0.40875	no
gi|320446809|ref|NW_003383762.1|	78482-79094	402106	0.909856	-214386	0.27935	no
gi|320446809|ref|NW_003383762.1|	83574-83957	137908	139112	-330938	0.24265	no
gi|320446809|ref|NW_003383762.1|	93395-94592	117231	902607	-0.377188	0.7733	no
gi|320446809|ref|NW_003383762.1|	94785-95696	147391	179089	0.281031	0.8963	no
gi|320446809|ref|NW_003383762.1|	96361-97418	102705	468803	219048	0.32505	no
gi|320446809|ref|NW_003383762.1|	98982-100846	0.31083	469339	391643	0.16825	no
gi|320446811|ref|NW_003383760.1|	10585-13944	325182	21.84	-0.57427	0.66475	no
gi|320446811|ref|NW_003383760.1|	154321-155347	277506	0.147545	-42333	0.3017	no
gi|320446811|ref|NW_003383760.1|	161487-165452	488586	0.791665	-262565	0.2563	no
gi|320446811|ref|NW_003383760.1|	16156-17020	461822	127529	-185651	0.27495	no
gi|320446811|ref|NW_003383760.1|	18214-20604	953069	148567	0.640463	0.6144	no
gi|320446811|ref|NW_003383760.1|	22092-22578	615355	221211	-479792	0.1477	no
gi|320446811|ref|NW_003383760.1|	22765-23193	396128	278967	-38278	0.1701	no
gi|320446811|ref|NW_003383760.1|	23425-24020	981711	0.95046	-33686	0.1915	no
gi|320446811|ref|NW_003383760.1|	25902-27149	350321	0.231413	-392014	0.22055	no
gi|320446811|ref|NW_003383760.1|	263208-264144	313167	33233	0.085681	0.96115	no
gi|320446811|ref|NW_003383760.1|	28764-29197	658928	436616	-391569	0.1338	no
gi|320446811|ref|NW_003383760.1|	31104-37078	22.58	183177	-0.301812	0.8209	no
gi|320446811|ref|NW_003383760.1|	314682-319019	242703	108589	216161	0.2272	no
gi|320446811|ref|NW_003383760.1|	392085-392542	670573	376783	249027	0.2702	no
gi|320446811|ref|NW_003383760.1|	394959-395840	0.261428	702198	474739	0.17355	no
gi|320446811|ref|NW_003383760.1|	400709-401271	143774	242751	-256626	0.2721	no

gi|320446811|ref|NW_003383760.1|	401663-40304	158533	469048	-175698	0.4277	no
gi|320446811|ref|NW_003383760.1|	405097-40658	514918	366454	-381264	0.06055	no
gi|320446811|ref|NW_003383760.1|	407263-40754	868816	5836	-3896	0.18905	no
gi|320446811|ref|NW_003383760.1|	408920-41064	247418	0.78678	-497485	0.06545	no
gi|320446811|ref|NW_003383760.1|	413484-41399	207742	0.819726	-466351	0.19575	no
gi|320446811|ref|NW_003383760.1|	423631-42660	0.432069	24165	248358	0.276	no
gi|320446811|ref|NW_003383760.1|	431055-43236	0.472432	184722	528911	0.14275	no
gi|320446811|ref|NW_003383760.1|	432667-43437	0	223113	inf	0.01275	no
gi|320446811|ref|NW_003383760.1|	435596-43623	0.415327	42397	335164	0.22635	no
gi|320446811|ref|NW_003383760.1|	44219-44854	366683	14621	-132649	0.5392	no
gi|320446811|ref|NW_003383760.1|	447566-45224	485906	531735	0.130032	0.9126	no
gi|320446811|ref|NW_003383760.1|	464717-46588	156926	0.499924	-497223	0.16335	no
gi|320446811|ref|NW_003383760.1|	46504-46902	567022	135066	-206974	0.35125	no
gi|320446811|ref|NW_003383760.1|	466760-46819	915122	0.0978416	-654737	0.2643	no
gi|320446811|ref|NW_003383760.1|	468322-46899	180273	104683	-410609	0.1749	no
gi|320446811|ref|NW_003383760.1|	484815-49221	667949	315265	223876	0.14305	no
gi|320446811|ref|NW_003383760.1|	49243-49628	13637	137625	-330872	0.24265	no
gi|320446811|ref|NW_003383760.1|	493405-49369	0	208636	inf	0.02205	no
gi|320446811|ref|NW_003383760.1|	511144-51292	875098	929628	0.0872097	0.9671	no
gi|320446811|ref|NW_003383760.1|	524415-53098	320847	150246	222737	0.20175	no
gi|320446811|ref|NW_003383760.1|	532374-53308	372827	130882	181169	0.30825	no
gi|320446811|ref|NW_003383760.1|	54344-54566	48066	603683	-299315	0.2661	no
gi|320446811|ref|NW_003383760.1|	54820-55082	582151	528083	-346256	0.18685	no
gi|320446811|ref|NW_003383760.1|	564255-56577	472321	136329	-179268	0.3187	no
gi|320446811|ref|NW_003383760.1|	569570-56987	25863	593219	-212426	0.34865	no
gi|320446811|ref|NW_003383760.1|	578427-57920	499553	85689	-254345	0.15505	no
gi|320446811|ref|NW_003383760.1|	600910-60196	159525	133235	-0.259805	0.90715	no
gi|320446811|ref|NW_003383760.1|	604655-60513	547818	68584	0.324175	0.8588	no
gi|320446811|ref|NW_003383760.1|	606753-61506	349653	16376	-109434	0.4069	no
gi|320446811|ref|NW_003383760.1|	623462-62489	202735	616402	-171765	0.30665	no

gi|320446811|ref|NW_003383760.1|	25691-62698	258491	143953	-0.844519	0.603	no
gi|320446811|ref|NW_003383760.1|	27388-62822	0.84589	135728	0.682177	1	no
gi|320446811|ref|NW_003383760.1|	41756-64404	120841	152061	0.331531	0.79275	no
gi|320446811|ref|NW_003383760.1|	48001-64869	255801	449468	0.8132	0.68375	no
gi|320446811|ref|NW_003383760.1|	49067-64947	379041	374537	-0.0172449	0.9157	no
gi|320446811|ref|NW_003383760.1|	49941-65122	272545	511762	0.90898	0.6529	no
gi|320446811|ref|NW_003383760.1|	51916-65377	217849	521964	126062	0.3505	no
gi|320446811|ref|NW_003383760.1|	54413-65706	93445	27772	-175049	0.2948	no
gi|320446811|ref|NW_003383760.1|	57759-65828	812332	290613	-148297	0.36535	no
gi|320446811|ref|NW_003383760.1|	60429-66197	195856	129063	-0.601714	0.71155	no
gi|320446811|ref|NW_003383760.1|	62675-66484	0.349421	298668	30955	0.2198	no
gi|320446811|ref|NW_003383760.1|	66991-68100	282042	227333	-363303	0.07605	no
gi|320446811|ref|NW_003383760.1|	91283-69167	691615	324863	-109014	0.59375	no
gi|320446811|ref|NW_003383760.1|	91877-69273	423592	122233	-179304	0.2912	no
gi|320446811|ref|NW_003383760.1|	93387-69736	813502	396146	-103811	0.4326	no
gi|320446811|ref|NW_003383760.1|	09172-71092	111336	403236	18567	0.39145	no
gi|320446811|ref|NW_003383760.1|	13913-71418	262072	166639	266869	0.2703	no
gi|320446811|ref|NW_003383760.1|	44700-74625	139174	14389	0.0480812	0.98395	no
gi|320446811|ref|NW_003383760.1|	49175-74937	167957	285214	-255797	0.2739	no
gi|320446811|ref|NW_003383760.1|	50310-75095	473024	182009	-13779	0.53675	no
gi|320446811|ref|NW_003383760.1|	51038-75200	196892	134305	-0.551885	0.7988	no
gi|320446811|ref|NW_003383760.1|	75762-77068	329435	787707	-206426	0.23655	no
gi|320446811|ref|NW_003383760.1|	71180-77169	19749	224994	0.188111	0.92705	no
gi|320446811|ref|NW_003383760.1|	77966-77924	334069	293678	-0.185913	0.88375	no
gi|320446811|ref|NW_003383760.1|	80138-78232	131757	212306	0.688267	0.59285	no
gi|320446811|ref|NW_003383760.1|	82877-78390	0.854946	10341	0.27447	1	no
gi|320446811|ref|NW_003383760.1|	85753-79198	171825	274367	0.675165	0.6134	no
gi|320446811|ref|NW_003383760.1|	92147-79281	199205	133178	-390283	0.1738	no
gi|320446811|ref|NW_003383760.1|	94460-79490	492673	0	#NAME?	0.0071	no
gi|320446811|ref|NW_003383760.1|	96169-79841	84.3	0.469276	-748895	0.07005	no

gi|320446811|ref|NW_003383760.1|	300116-80137	235.06	206258	-683244	0.03335	no
gi|320446811|ref|NW_003383760.1|	83124-83944	288719	0.198443	-386287	0.3182	no
gi|320446812|ref|NW_003383759.1|	111284-11163	3341.35	1226.62	-144574	0.26555	no
gi|320446812|ref|NW_003383759.1|	117686-11828	1152.48	530549	-111918	0.38225	no
gi|320446812|ref|NW_003383759.1|	118452-12057	226935	716809	-166262	0.3442	no
gi|320446812|ref|NW_003383759.1|	121537-12339	249317	123718	-101093	0.43225	no
gi|320446812|ref|NW_003383759.1|	123751-12537	222383	727337	-161235	0.34305	no
gi|320446812|ref|NW_003383759.1|	130855-13167	234263	46116	0.977139	0.5564	no
gi|320446812|ref|NW_003383759.1|	138489-14183	429207	164936	194216	0.27485	no
gi|320446812|ref|NW_003383759.1|	143302-14368	424331	626005	0.560983	0.7984	no
gi|320446812|ref|NW_003383759.1|	148135-14869	262193	672353	135859	0.52655	no
gi|320446812|ref|NW_003383759.1|	175934-17656	214438	145773	-0.556833	0.8006	no
gi|320446812|ref|NW_003383759.1|	305628-30626	0	140143	inf	0.0089	no
gi|320446812|ref|NW_003383759.1|	310495-31131	637362	238932	-141551	0.5008	no
gi|320446812|ref|NW_003383759.1|	312050-31310	453004	256325	-0.821548	0.67795	no
gi|320446812|ref|NW_003383759.1|	315796-32082	11432	108912	-0.0699133	0.9573	no
gi|320446812|ref|NW_003383759.1|	321024-32195	726845	367624	-0.983415	0.63765	no
gi|320446812|ref|NW_003383759.1|	322324-32260	362187	17339	-106271	0.60525	no
gi|320446812|ref|NW_003383759.1|	322730-32382	644875	324604	-0.990341	0.63745	no
gi|320446812|ref|NW_003383759.1|	325122-32561	644706	345227	-0.901095	0.67195	no
gi|320446812|ref|NW_003383759.1|	326145-32861	107785	35269	-161168	0.32835	no
gi|320446812|ref|NW_003383759.1|	331037-33132	166047	265639	-264405	0.29095	no
gi|320446812|ref|NW_003383759.1|	332187-33244	224338	890079	-133367	0.52545	no
gi|320446812|ref|NW_003383759.1|	334968-33710	947953	531575	-0.834543	0.7086	no
gi|320446812|ref|NW_003383759.1|	337250-33928	421234	411292	-0.0344585	0.98615	no
gi|320446812|ref|NW_003383759.1|	47298-47841	496273	192.88	-136343	0.3161	no
gi|320446812|ref|NW_003383759.1|	48450-55326	129577	744334	-0.799793	0.6074	no
gi|320446812|ref|NW_003383759.1|	507444-50899	113304	221212	0.965235	0.56085	no
gi|320446812|ref|NW_003383759.1|	509081-51078	207102	448639	111521	0.5993	no
gi|320446812|ref|NW_003383759.1|	524421-52507	648739	938928	0.533377	0.7929	no

gi|320446812|ref|NW_003383759.1|	61015-61543	159878	998543	-0.679075	0.7401	no
gi|320446812|ref|NW_003383759.1|	92433-92873	319263	635478	0.993098	0.6801	no
gi|320446812|ref|NW_003383759.1|	93347-93931	240949	358851	0.574659	0.79895	no
gi|320446812|ref|NW_003383759.1|	97515-98483	391632	49299	0.332058	0.8644	no
gi|320446812|ref|NW_003383759.1|	99094-101248	102898	179793	0.805122	0.6377	no
gi|320446813|ref|NW_003383758.1|	128952-13036	0	119986	inf	1	no
gi|320446813|ref|NW_003383758.1|	154772-15606	52375	800268	0.611604	0.6515	no
gi|320446813|ref|NW_003383758.1|	156161-15640	932046	842069	-0.146461	0.94165	no
gi|320446813|ref|NW_003383758.1|	161213-16529	174228	277117	0.669518	0.61705	no
gi|320446813|ref|NW_003383758.1|	165395-16613	260354	290384	0.157489	0.94095	no
gi|320446813|ref|NW_003383758.1|	168500-17312	229241	167819	-0.449959	0.73435	no
gi|320446813|ref|NW_003383758.1|	181243-18205	203496	159825	-0.348507	0.8554	no
gi|320446813|ref|NW_003383758.1|	188965-19152	225352	409995	-245851	0.1863	no
gi|320446813|ref|NW_003383758.1|	191676-19229	658159	327841	-432737	0.0682	no
gi|320446813|ref|NW_003383758.1|	199869-20224	929783	329269	-149763	0.2711	no
gi|320446813|ref|NW_003383758.1|	203211-20507	214737	730171	-155626	0.36655	no
gi|320446813|ref|NW_003383758.1|	205311-20639	0	287639	inf	0.01575	no
gi|320446813|ref|NW_003383758.1|	211571-21203	726854	522865	28467	0.22305	no
gi|320446813|ref|NW_003383758.1|	216275-21656	0	382258	inf	0.01275	no
gi|320446813|ref|NW_003383758.1|	217268-22085	125893	111343	314475	0.10105	no
gi|320446813|ref|NW_003383758.1|	255132-25612	0.670072	126499	423867	0.16505	no
gi|320446813|ref|NW_003383758.1|	258397-25919	0.597901	718619	358725	0.182	no
gi|320446813|ref|NW_003383758.1|	259395-26181	0.154546	253605	403648	0.1668	no
gi|320446813|ref|NW_003383758.1|	261996-26321	0.170875	154028	317218	0.24015	no
gi|320446813|ref|NW_003383758.1|	263330-26504	0	222068	inf	0.01275	no
gi|320446813|ref|NW_003383758.1|	265188-26579	224563	762046	176276	0.4191	no
gi|320446813|ref|NW_003383758.1|	268443-26895	305503	696767	118949	0.57545	no
gi|320446813|ref|NW_003383758.1|	272582-27343	0.822521	490489	25761	0.28405	no
gi|320446813|ref|NW_003383758.1|	275388-27637	0.225146	248771	346589	0.21395	no
gi|320446813|ref|NW_003383758.1|	277789-28074	0.186194	125827	275657	1	no

gi|320446813|ref|NW_003383758.1|	281275-28260	0	202629	inf	0.0212	no
gi|320446813|ref|NW_003383758.1|	287926-28856	290023	188929	27036	0.2383	no
gi|320446813|ref|NW_003383758.1|	294855-29644	76101	225691	-175356	0.42185	no
gi|320446813|ref|NW_003383758.1|	297837-29849	20035	737148	-144249	0.5039	no
gi|320446813|ref|NW_003383758.1|	300828-30188	307558	291202	-0.0788387	0.94775	no
gi|320446813|ref|NW_003383758.1|	306357-30843	968594	187394	0.952109	0.57205	no
gi|320446813|ref|NW_003383758.1|	308828-30935	226907	915955	201317	0.35895	no
gi|320446813|ref|NW_003383758.1|	313350-31375	0	122434	inf	0.0162	no
gi|320446813|ref|NW_003383758.1|	316731-31721	135902	131618	327572	0.1941	no
gi|320446813|ref|NW_003383758.1|	317377-31919	0.106757	215706	433667	0.18255	no
gi|320446813|ref|NW_003383758.1|	326397-32690	121776	175652	0.528493	0.79815	no
gi|320446813|ref|NW_003383758.1|	329203-33063	807207	128918	0.675439	0.7555	no
gi|320446813|ref|NW_003383758.1|	330741-33280	181159	17922	-0.0155259	0.99085	no
gi|320446813|ref|NW_003383758.1|	333386-33443	326066	150791	-111261	0.4984	no
gi|320446813|ref|NW_003383758.1|	346086-34687	559989	0.835756	-606617	0.1294	no
gi|320446813|ref|NW_003383758.1|	347026-34765	677702	0.288059	-787814	0.2504	no
gi|320446813|ref|NW_003383758.1|	347881-34884	38897	0.482265	-633369	0.1106	no
gi|320446813|ref|NW_003383758.1|	349870-35027	121896	0.636738	-758073	0.2504	no
gi|320446813|ref|NW_003383758.1|	350665-35225	611755	0.43434	-381606	0.17455	no
gi|320446813|ref|NW_003383758.1|	353287-35388	515633	0	#NAME?	0.02105	no
gi|320446813|ref|NW_003383758.1|	354885-35652	505292	0.251212	-433014	0.15565	no
gi|320446813|ref|NW_003383758.1|	357994-35986	350766	0.718994	-228646	0.30855	no
gi|320446813|ref|NW_003383758.1|	362259-36292	871299	0.539899	-401241	0.21135	no
gi|320446813|ref|NW_003383758.1|	363821-36432	106092	334618	-166473	0.429	no
gi|320446813|ref|NW_003383758.1|	365426-37103	186226	439664	123934	0.3484	no
gi|320446813|ref|NW_003383758.1|	372810-37612	200787	271296	0.434199	0.74115	no
gi|320446813|ref|NW_003383758.1|	376548-37753	596636	228558	-138429	0.42465	no
gi|320446813|ref|NW_003383758.1|	379071-38114	140629	733274	-0.939471	0.47705	no
gi|320446813|ref|NW_003383758.1|	381239-38343	125396	689256	-0.863381	0.5972	no
gi|320446813|ref|NW_003383758.1|	397286-39797	0.746916	170866	451578	0.1565	no

gi|320446813|ref|NW_003383758.1|	99927-40040	203071	904374	215493	0.34195	no
gi|320446813|ref|NW_003383758.1|	00975-40146	0	107883	inf	0.01485	no
gi|320446813|ref|NW_003383758.1|	13513-41369	132938	745542	-0.834388	0.68655	no
gi|320446813|ref|NW_003383758.1|	31079-43325	0.261119	115394	214379	1	no
gi|320446813|ref|NW_003383758.1|	46354-47188	23121	232677	0.00912323	0.99485	no
gi|320446813|ref|NW_003383758.1|	63989-46466	0.763679	234513	161863	0.3327	no
gi|320446813|ref|NW_003383758.1|	95446-98262	0.849992	466117	245517	0.2744	no
gi|320446815|ref|NW_003383756.1|	20334-12372	828508	170134	103808	0.4285	no
gi|320446815|ref|NW_003383756.1|	17613-17921	141666	136664	-0.0518619	0.95435	no
gi|320446815|ref|NW_003383756.1|	33925-38635	119908	776425	-0.627007	0.6285	no
gi|320446815|ref|NW_003383756.1|	40799-41081	181.55	286215	-266519	0.25245	no
gi|320446815|ref|NW_003383756.1|	41211-41876	211895	454824	-221997	0.31975	no
gi|320446815|ref|NW_003383756.1|	42357-44695	553725	295811	-0.904491	0.5074	no
gi|320446815|ref|NW_003383756.1|	44873-45925	1.24	400171	169027	0.4289	no
gi|320446815|ref|NW_003383756.1|	47875-49128	162519	529149	-161886	0.46025	no
gi|320446815|ref|NW_003383756.1|	51800-57347	161047	147789	-0.12395	0.9261	no
gi|320446815|ref|NW_003383756.1|	57794-58683	0.258227	1601	263226	0.26355	no
gi|320446815|ref|NW_003383756.1|	91344-91778	105.77	353208	-158234	0.33765	no
gi|320446815|ref|NW_003383756.1|	95271-101517	513971	201662	-134975	0.30805	no
gi|320446816|ref|NW_003383755.1|	05211-10629	117196	386228	172053	0.31905	no
gi|320446816|ref|NW_003383755.1|	06479-10729	495913	761737	0.619205	0.7583	no
gi|320446816|ref|NW_003383755.1|	08052-10846	356953	825058	120876	0.5726	no
gi|320446816|ref|NW_003383755.1|	08600-11062	792015	848244	0.0989505	0.9616	no
gi|320446816|ref|NW_003383755.1|	12466-11280	551421	805095	0.546005	0.799	no
gi|320446816|ref|NW_003383755.1|	12947-11332	858319	562519	-0.609611	0.7537	no
gi|320446816|ref|NW_003383755.1|	46186-14657	330145	155765	-108373	0.5996	no
gi|320446816|ref|NW_003383755.1|	14715-15556	0.836108	153361	0.875172	0.68225	no
gi|320446816|ref|NW_003383755.1|	17119-18003	41635	448084	0.105973	0.9542	no
gi|320446816|ref|NW_003383755.1|	72496-17282	154749	999898	269185	0.26995	no
gi|320446816|ref|NW_003383755.1|	80121-18092	293491	191279	-0.617643	0.77445	no

gi|320446816|ref|NW_003383755.1|	18151-21852	189875	214642	0.176884	0.92795	no
gi|320446816|ref|NW_003383755.1|	182769-182979	297796	260785	-0.191465	0.9138	no
gi|320446816|ref|NW_003383755.1|	183167-183842	230136	261707	0.185467	0.9195	no
gi|320446816|ref|NW_003383755.1|	184765-185213	138737	117791	-0.23613	0.90805	no
gi|320446816|ref|NW_003383755.1|	191103-191440	367121	150073	-129059	0.542	no
gi|320446816|ref|NW_003383755.1|	213617-219420	290011	194311	-0.577735	0.6617	no
gi|320446816|ref|NW_003383755.1|	222313-223880	832623	289637	-152342	0.4762	no
gi|320446816|ref|NW_003383755.1|	270377-271464	35869	130273	-14612	0.3893	no
gi|320446816|ref|NW_003383755.1|	273439-275042	703306	455063	-0.628088	0.7608	no
gi|320446816|ref|NW_003383755.1|	275703-276484	55685	530764	-0.069218	0.9677	no
gi|320446816|ref|NW_003383755.1|	280509-282760	395131	290388	-0.444351	0.7413	no
gi|320446816|ref|NW_003383755.1|	284177-286353	975579	0	#NAME?	5.00E-05	yes
gi|320446816|ref|NW_003383755.1|	288698-290110	165524	932023	-0.828603	0.7128	no
gi|320446816|ref|NW_003383755.1|	326990-327849	189458	353927	0.90157	0.66455	no
gi|320446816|ref|NW_003383755.1|	37607-39157	0.51345	205366	19999	0.3909	no
gi|320446816|ref|NW_003383755.1|	399588-400142	754446	423576	-0.832797	0.602	no
gi|320446816|ref|NW_003383755.1|	401456-402532	13652	106951	-0.352163	0.8729	no
gi|320446816|ref|NW_003383755.1|	429178-429435	114621	316831	-185509	0.38955	no
gi|320446816|ref|NW_003383755.1|	445713-446265	793797	713986	-0.152874	0.9372	no
gi|320446816|ref|NW_003383755.1|	451109-451498	133387	107789	-0.307405	0.87425	no
gi|320446816|ref|NW_003383755.1|	451808-452527	317504	167108	-424792	0.14075	no
gi|320446816|ref|NW_003383755.1|	453115-454112	201919	321866	-264924	0.255	no
gi|320446816|ref|NW_003383755.1|	454245-457284	291887	190618	-0.614721	0.6388	no
gi|320446816|ref|NW_003383755.1|	463885-464500	13096	29657	-214268	0.337	no
gi|320446816|ref|NW_003383755.1|	466211-467092	627428	126035	-231562	0.3117	no
gi|320446816|ref|NW_003383755.1|	467248-468169	689277	152761	-21738	0.31905	no
gi|320446816|ref|NW_003383755.1|	468483-470942	115342	524634	-113653	0.48735	no
gi|320446816|ref|NW_003383755.1|	471171-472835	233873	12334	-0.923085	0.5777	no
gi|320446816|ref|NW_003383755.1|	473853-475370	107465	865868	-0.311646	0.8873	no
gi|320446816|ref|NW_003383755.1|	482305-482817	180775	323549	0.839793	0.69175	no

gi|320446816|ref|NW_003383755.1|	487370-48837	548232	227259	-127045	0.5387	no
gi|320446816|ref|NW_003383755.1|	490830-49167	102612	362474	-150124	0.48415	no
gi|320446816|ref|NW_003383755.1|	506938-50841	191326	764126	-132415	0.41885	no
gi|320446816|ref|NW_003383755.1|	511954-51281	107916	482803	-11604	0.58335	no
gi|320446816|ref|NW_003383755.1|	513401-51534	16257	101662	-0.677289	0.6839	no
gi|320446816|ref|NW_003383755.1|	516017-51753	171169	998075	254373	0.26435	no
gi|320446816|ref|NW_003383755.1|	518008-51827	529974	168446	16683	0.34255	no
gi|320446816|ref|NW_003383755.1|	68622-68989	815394	913043	0.163186	0.92935	no
gi|320446816|ref|NW_003383755.1|	71943-73011	222964	33651	0.593836	0.75935	no
gi|320446816|ref|NW_003383755.1|	75836-76150	168594	108608	-0.634427	0.75965	no
gi|320446816|ref|NW_003383755.1|	79993-80549	575081	564546	-0.0266743	0.96375	no
gi|320446816|ref|NW_003383755.1|	86502-87024	232983	313167	0.426708	0.82245	no
gi|320446816|ref|NW_003383755.1|	87151-87901	295171	247238	-0.255648	0.9026	no
gi|320446816|ref|NW_003383755.1|	91636-92050	27035	713864	140082	0.5187	no
gi|320446817|ref|NW_003383754.1|	134564-13498	603987	798869	0.40344	0.84235	no
gi|320446817|ref|NW_003383754.1|	136743-13704	206755	108582	-0.929136	0.65095	no
gi|320446817|ref|NW_003383754.1|	144614-14555	143108	213915	0.579926	0.7901	no
gi|320446817|ref|NW_003383754.1|	145673-14939	169471	189756	0.163107	0.9372	no
gi|320446817|ref|NW_003383754.1|	166972-16861	0.240444	0.92046	193666	1	no
gi|320446817|ref|NW_003383754.1|	169515-17038	212434	182829	-0.216526	0.91015	no
gi|320446817|ref|NW_003383754.1|	173385-17368	112775	120647	0.0973358	0.94075	no
gi|320446817|ref|NW_003383754.1|	176182-18015	0.541566	138999	135986	1	no
gi|320446817|ref|NW_003383754.1|	181822-18668	156812	432455	146351	0.2803	no
gi|320446817|ref|NW_003383754.1|	257861-26012	415479	521993	0.329255	0.8782	no
gi|320446817|ref|NW_003383754.1|	260929-26452	121035	100504	-0.26818	0.83735	no
gi|320446817|ref|NW_003383754.1|	265628-26783	812624	280561	-153427	0.49175	no
gi|320446817|ref|NW_003383754.1|	301426-30236	548582	510104	-0.104917	0.9557	no
gi|320446817|ref|NW_003383754.1|	32861-33828	108423	589198	-0.879845	0.66855	no
gi|320446817|ref|NW_003383754.1|	374993-37571	174812	263126	0.589943	0.75905	no
gi|320446817|ref|NW_003383754.1|	460789-46103	0	262596	inf	0.029	no

gi|320446817|ref|NW_003383754.1|	15226-51545	436287	247024	25013	0.27555	no
gi|320446817|ref|NW_003383754.1|	20503-52531	106819	58565	245487	0.1658	no
gi|320446817|ref|NW_003383754.1|	27932-52825	0	12.45	inf	0.029	no
gi|320446817|ref|NW_003383754.1|	29702-53024	269541	148995	246669	0.2808	no
gi|320446817|ref|NW_003383754.1|	90542-59710	0.533284	487825	319339	0.10115	no
gi|320446817|ref|NW_003383754.1|	65064-86681	0	830823	inf	0.0053	no
gi|320446817|ref|NW_003383754.1|	68518-87127	0	365462	inf	0.00655	no
gi|320446817|ref|NW_003383754.1|	84078-88492	0.554657	496017	316072	0.20765	no
gi|320446817|ref|NW_003383754.1|	85238-88628	0	229777	inf	0.0034	no
gi|320446817|ref|NW_003383754.1|	90624-89202	261267	100839	-137347	0.5208	no
gi|320446817|ref|NW_003383754.1|	95543-89655	129721	104583	-0.310758	1	no
gi|320446817|ref|NW_003383754.1|	97980-98182	489841	436421	-0.166593	0.92605	no
gi|320446818|ref|NW_003383753.1|	20368-20742	111717	321622	152552	0.4727	no
gi|320446818|ref|NW_003383753.1|	05759-20861	405631	855068	107587	0.50105	no
gi|320446818|ref|NW_003383753.1|	56007-25769	291016	696993	126005	0.556	no
gi|320446818|ref|NW_003383753.1|	68461-27060	618856	561309	-0.140808	0.9467	no
gi|320446818|ref|NW_003383753.1|	71320-27203	248839	656376	139931	0.50915	no
gi|320446818|ref|NW_003383753.1|	72288-27665	665167	106582	0.680169	0.5934	no
gi|320446818|ref|NW_003383753.1|	79457-28526	639417	24273	192452	0.15805	no
gi|320446818|ref|NW_003383753.1|	95937-29647	0	666075	inf	0.0154	no
gi|320446818|ref|NW_003383753.1|	31887-33162	146093	382933	13902	0.5048	no
gi|320446818|ref|NW_003383753.1|	3375-4041	0	640676	inf	0.0138	no
gi|320446818|ref|NW_003383753.1|	96909-97863	0	275526	inf	0.02075	no
gi|320446819|ref|NW_003383752.1|	45590-14616	0.49013	862145	413669	0.1922	no
gi|320446819|ref|NW_003383752.1|	59375-36354	978075	447101	-445127	0.04465	no
gi|320446819|ref|NW_003383752.1|	63907-36548	115677	779409	-389158	0.06585	no
gi|320446819|ref|NW_003383752.1|	24867-42845	886813	105768	0.254207	0.84235	no
gi|320446819|ref|NW_003383752.1|	30004-43249	336925	538606	0.6768	0.7574	no
gi|320446819|ref|NW_003383752.1|	40665-44312	773307	582406	-0.409016	0.8541	no
gi|320446819|ref|NW_003383752.1|	43247-44459	311489	298586	-0.0610346	0.9608	no

gi|320446819|ref|NW_003383752.1|	144753-44697(	0.170302	136676	30046	1	no
gi|320446819|ref|NW_003383752.1|	147180-44984!	929074	765414	-0.279553	0.8614	no
gi|320446819|ref|NW_003383752.1|	156212-45799!	0.326258	121217	189351	1	no
gi|320446819|ref|NW_003383752.1|	158838-46267	101602	85202	-0.253974	0.8432	no
gi|320446819|ref|NW_003383752.1|	171891-47224	305266	413966	-288248	0.23655	no
gi|320446819|ref|NW_003383752.1|	173458-47387	875275	0	#NAME?	0.0229	no
gi|320446819|ref|NW_003383752.1|	175518-47699(	909872	169941	-242063	0.27895	no
gi|320446819|ref|NW_003383752.1|	180977-48184	15667	438789	-183613	0.4024	no
gi|320446819|ref|NW_003383752.1|	533101-53495	187303	18999	0.0205491	0.98565	no
gi|320446819|ref|NW_003383752.1|	536805-53801	155202	20325	0.389108	0.84385	no
gi|320446819|ref|NW_003383752.1|	538137-54005	481365	468369	-0.0394843	0.98285	no
gi|320446819|ref|NW_003383752.1|	526131-62704(	0.250557	136436	576694	0.16195	no
gi|320446819|ref|NW_003383752.1|	538277-64107	145684	289325	0.989845	0.45745	no
gi|320446819|ref|NW_003383752.1|	542015-65210	255203	533446	10637	0.4167	no
gi|320446819|ref|NW_003383752.1|	552759-65319(	149621	880919	-0.764233	0.7056	no
gi|320446819|ref|NW_003383752.1|	572204-67317(	504065	616902	0.29143	0.88115	no
gi|320446819|ref|NW_003383752.1|	581475-68188	108675	777014	-0.484002	0.81505	no
gi|320446819|ref|NW_003383752.1|	591662-69365	364402	541635	-275014	0.15135	no
gi|320446819|ref|NW_003383752.1|	595715-69658	201179	145813	-378629	0.1473	no
gi|320446819|ref|NW_003383752.1|	89586-90117	0	532637	inf	0.0233	no
gi|320446820|ref|NW_003383751.1|	101434-10508	133533	106245	299213	0.11295	no
gi|320446820|ref|NW_003383751.1|	130425-13297	67053	762833	-313586	0.11935	no
gi|320446820|ref|NW_003383751.1|	134081-13585	274536	313626	-312988	0.09895	no
gi|320446820|ref|NW_003383751.1|	136425-13754	26991	198793	-376314	0.0765	no
gi|320446820|ref|NW_003383751.1|	144996-14537	109148	278781	467478	0.1748	no
gi|320446820|ref|NW_003383751.1|	163630-16384	102916	645482	-399495	0.22235	no
gi|320446820|ref|NW_003383751.1|	167374-16822	218628	451322	-227625	0.3077	no
gi|320446820|ref|NW_003383751.1|	168518-17043	16036	342337	-222782	0.20365	no
gi|320446820|ref|NW_003383751.1|	170549-17181	213073	886922	-126447	0.57355	no
gi|320446820|ref|NW_003383751.1|	172528-17317	239723	123783	-0.953561	0.6595	no

gi|320446820|ref|NW_003383751.1|	173541-17552	106209	356819	-157364	0.4754	no
gi|320446820|ref|NW_003383751.1|	175791-17684	435603	143437	-160259	0.45015	no
gi|320446820|ref|NW_003383751.1|	329896-33146	0.884407	0.615292	-0.523439	1	no
gi|320446820|ref|NW_003383751.1|	333191-33449	0.317026	0.990119	1643	1	no
gi|320446820|ref|NW_003383751.1|	336335-34125	305944	317674	0.0542798	0.9787	no
gi|320446820|ref|NW_003383751.1|	342861-34856	117858	40429	177835	0.18935	no
gi|320446820|ref|NW_003383751.1|	352506-35399	147717	420166	150812	0.4677	no
gi|320446820|ref|NW_003383751.1|	355565-35683	99114	39457	-132881	0.53195	no
gi|320446820|ref|NW_003383751.1|	356971-36078	239581	167017	-0.520519	0.69455	no
gi|320446820|ref|NW_003383751.1|	362841-36489	840621	397344	-108107	0.42285	no
gi|320446820|ref|NW_003383751.1|	367716-37023	848604	479334	-0.824061	0.708	no
gi|320446820|ref|NW_003383751.1|	370410-37104	215483	10058	-109923	0.60475	no
gi|320446820|ref|NW_003383751.1|	376637-38153	304227	263835	-0.205511	0.92395	no
gi|320446820|ref|NW_003383751.1|	391106-39167	180031	101423	-414979	0.16155	no
gi|320446820|ref|NW_003383751.1|	411342-41208	331825	0.682017	-228254	0.26535	no
gi|320446820|ref|NW_003383751.1|	412215-41411	812416	134487	-259475	0.2616	no
gi|320446820|ref|NW_003383751.1|	423265-42398	100768	166442	-259794	0.258	no
gi|320446820|ref|NW_003383751.1|	425755-42989	188403	589912	-167525	0.20475	no
gi|320446820|ref|NW_003383751.1|	430322-43169	0.888471	25713	15331	0.47295	no
gi|320446820|ref|NW_003383751.1|	431841-43251	0	313365	inf	0.029	no
gi|320446820|ref|NW_003383751.1|	436011-43853	0.886663	634786	283981	0.23225	no
gi|320446820|ref|NW_003383751.1|	438696-43987	0.894223	557708	26408	0.25195	no
gi|320446820|ref|NW_003383751.1|	457566-46083	111239	0.816941	-0.445361	1	no
gi|320446820|ref|NW_003383751.1|	461993-46297	296126	967797	-161344	0.32965	no
gi|320446820|ref|NW_003383751.1|	466979-46764	469113	994343	-223812	0.32385	no
gi|320446820|ref|NW_003383751.1|	472700-47324	0	231194	inf	0.00715	no
gi|320446820|ref|NW_003383751.1|	474979-47571	0	115657	inf	0.0074	no
gi|320446820|ref|NW_003383751.1|	476212-47873	136515	152562	0.160342	0.8993	no
gi|320446820|ref|NW_003383751.1|	513043-51573	673933	188841	148649	0.3908	no
gi|320446820|ref|NW_003383751.1|	538228-53905	11412	353059	162935	0.44665	no

gi|320446820|ref|NW_003383751.1|	555987-55819	347616	771271	114974	0.3908	no
gi|320446820|ref|NW_003383751.1|	572280-57381	534006	0.181173	-488141	0.19395	no
gi|320446820|ref|NW_003383751.1|	86853-87340	0.659197	180742	477708	0.17185	no
gi|320446820|ref|NW_003383751.1|	96004-96656	12135	826566	276796	0.2508	no
gi|320446820|ref|NW_003383751.1|	98423-99187	0.3193	262758	304075	0.24995	no
gi|320446820|ref|NW_003383751.1|	99293-10122	0	159094	inf	0.01485	no
gi|320446823|ref|NW_003383748.1|	21941-22406	14361	105273	28739	0.22955	no
gi|320446823|ref|NW_003383748.1|	250030-25237	119975	178688	0.574706	0.78285	no
gi|320446823|ref|NW_003383748.1|	258568-25973	772371	157388	-229497	0.2129	no
gi|320446823|ref|NW_003383748.1|	263266-26667	627356	80336	0.356762	0.82815	no
gi|320446823|ref|NW_003383748.1|	266833-27367	554513	111602	100907	0.4446	no
gi|320446823|ref|NW_003383748.1|	275347-27785	876492	280151	-164554	0.46505	no
gi|320446823|ref|NW_003383748.1|	279087-28301	552882	812364	0.555154	0.7388	no
gi|320446823|ref|NW_003383748.1|	285142-28752	438992	498975	-313716	0.1117	no
gi|320446823|ref|NW_003383748.1|	288465-28885	93367	0.680831	-377754	0.32245	no
gi|320446823|ref|NW_003383748.1|	291891-29690	851813	104236	0.291249	0.827	no
gi|320446823|ref|NW_003383748.1|	297069-29892	103335	84376	-0.292431	0.8961	no
gi|320446823|ref|NW_003383748.1|	305590-30770	350859	294974	-0.250303	0.8972	no
gi|320446823|ref|NW_003383748.1|	308115-30851	7.78	767927	-0.0188012	0.95455	no
gi|320446823|ref|NW_003383748.1|	310728-31098	115574	474748	-128359	0.5432	no
gi|320446823|ref|NW_003383748.1|	91612-92183	100017	338078	17571	0.33425	no
gi|320446825|ref|NW_003383746.1|	12351-13056	326914	736803	-214956	0.3386	no
gi|320446825|ref|NW_003383746.1|	124200-12472	532299	595109	0.160915	0.9403	no
gi|320446825|ref|NW_003383746.1|	132486-13273	0	478092	inf	0.01485	no
gi|320446825|ref|NW_003383746.1|	140445-14220	432783	626069	0.532676	0.79865	no
gi|320446825|ref|NW_003383746.1|	14278-18281	461266	304078	-0.601156	0.7968	no
gi|320446825|ref|NW_003383746.1|	143506-14671	731479	118178	0.692073	0.58115	no
gi|320446825|ref|NW_003383746.1|	147303-15084	302591	901839	15755	0.2364	no
gi|320446825|ref|NW_003383746.1|	162859-16386	227769	101379	-11678	0.59265	no
gi|320446825|ref|NW_003383746.1|	164039-16887	165177	871569	-0.922327	0.48545	no

gi|320446825|ref|NW_003383746.1|	72787-17415	156582	445355	-181389	0.41455	no
gi|320446825|ref|NW_003383746.1|	75042-17611	303313	116102	193651	0.38575	no
gi|320446825|ref|NW_003383746.1|	76587-17741	113544	23421	104454	0.6061	no
gi|320446825|ref|NW_003383746.1|	82630-18341	121957	293031	126468	0.55445	no
gi|320446825|ref|NW_003383746.1|	97845-19833	398499	164288	204358	0.34465	no
gi|320446825|ref|NW_003383746.1|	05468-20566	146098	637302	212504	0.2859	no
gi|320446825|ref|NW_003383746.1|	34851-23859	404892	350401	-353046	0.08465	no
gi|320446825|ref|NW_003383746.1|	39876-24028	928511	183652	-233795	0.25625	no
gi|320446825|ref|NW_003383746.1|	40897-24251	135965	0.596703	-451008	0.12855	no
gi|320446825|ref|NW_003383746.1|	44256-24520	279079	832269	-174555	0.42915	no
gi|320446825|ref|NW_003383746.1|	45380-24604	351538	159814	-113729	0.5884	no
gi|320446825|ref|NW_003383746.1|	46395-24687	207034	967358	-109775	0.5997	no
gi|320446825|ref|NW_003383746.1|	51358-25189	46693	159118	-155311	0.4701	no
gi|320446825|ref|NW_003383746.1|	53491-25577	126596	688576	-0.878541	0.58815	no
gi|320446825|ref|NW_003383746.1|	56477-25743	79742	647411	-0.300658	0.8835	no
gi|320446825|ref|NW_003383746.1|	59313-26020	311501	218148	-0.513933	0.82585	no
gi|320446825|ref|NW_003383746.1|	60324-26339	458987	391371	-0.229918	0.8624	no
gi|320446825|ref|NW_003383746.1|	67345-26987	492037	820733	0.738148	0.74135	no
gi|320446825|ref|NW_003383746.1|	74255-27465	410602	257268	-39964	0.1844	no
gi|320446825|ref|NW_003383746.1|	75391-27574	214832	0.822787	-470655	0.286	no
gi|320446825|ref|NW_003383746.1|	82153-28268	448142	267153	-406822	0.1465	no
gi|320446825|ref|NW_003383746.1|	86976-28784	874915	127783	-277545	0.24125	no
gi|320446825|ref|NW_003383746.1|	89334-28966	246207	562836	-212908	0.34005	no
gi|320446825|ref|NW_003383746.1|	90669-29184	0.535966	136186	134537	1	no
gi|320446825|ref|NW_003383746.1|	92019-29302	441352	65231	-27583	0.133	no
gi|320446825|ref|NW_003383746.1|	40178-34195	120717	129956	0.106393	1	no
gi|320446825|ref|NW_003383746.1|	46826-34751	148082	227583	0.619998	0.753	no
gi|320446825|ref|NW_003383746.1|	49002-35168	282527	799268	150029	0.5054	no
gi|320446825|ref|NW_003383746.1|	73931-37954	451714	103749	119961	0.3538	no
gi|320446825|ref|NW_003383746.1|	69170-46948	696688	14.57	106442	0.60185	no

gi|320446825|ref|NW_003383746.1|	52626-52901	108265	384822	182962	0.28025	no
gi|320446825|ref|NW_003383746.1|	53439-55614	140062	108112	294839	0.03525	no
gi|320446825|ref|NW_003383746.1|	56466-57806	0.305543	148504	228105	0.2731	no
gi|320446825|ref|NW_003383746.1|	57931-59200	0.489805	135928	147256	1	no
gi|320446825|ref|NW_003383746.1|	61827-63514	395236	461329	0.223083	0.91565	no
gi|320446825|ref|NW_003383746.1|	64520-65408	620696	195971	-166324	0.4377	no
gi|320446825|ref|NW_003383746.1|	76614-67693	643151	10375	0.68988	0.74435	no
gi|320446825|ref|NW_003383746.1|	91673-69400	102258	171443	0.745519	0.5549	no
gi|320446825|ref|NW_003383746.1|	94391-69719	106458	411056	194904	0.15355	no
gi|320446825|ref|NW_003383746.1|	75694-77698	13357	186286	0.479919	0.81075	no
gi|320446825|ref|NW_003383746.1|	77913-78526	311935	302524	-0.0441953	0.9454	no
gi|320446825|ref|NW_003383746.1|	79181-80494	0.469801	152182	169568	0.4291	no
gi|320446825|ref|NW_003383746.1|	84757-86636	164263	264743	0.688584	0.73215	no
gi|320446825|ref|NW_003383746.1|	98150-98475	108324	419957	195488	0.3755	no
gi|320446826|ref|NW_003383745.1|	04682-10873	988966	859208	-0.202913	0.87575	no
gi|320446826|ref|NW_003383745.1|	10679-11181	13088	336668	-195884	0.382	no
gi|320446826|ref|NW_003383745.1|	12024-11267	192789	903011	-10942	0.5957	no
gi|320446826|ref|NW_003383745.1|	25700-13576	150142	15491	0.0451056	0.97325	no
gi|320446826|ref|NW_003383745.1|	13827-14408	0.97196	32889	175864	0.33425	no
gi|320446826|ref|NW_003383745.1|	56895-15714	973213	153953	39836	0.1686	no
gi|320446826|ref|NW_003383745.1|	65520-16714	108976	210683	0.951062	0.6494	no
gi|320446826|ref|NW_003383745.1|	70057-17561	11581	6892	-0.748772	0.5688	no
gi|320446826|ref|NW_003383745.1|	78470-18096	521683	141461	-188277	0.1688	no
gi|320446826|ref|NW_003383745.1|	81461-18446	206984	204301	-0.0188237	0.9881	no
gi|320446826|ref|NW_003383745.1|	87510-18784	179894	342341	-239364	0.16355	no
gi|320446826|ref|NW_003383745.1|	98359-19913	186987	106918	-0.806428	0.7079	no
gi|320446826|ref|NW_003383745.1|	01011-20305	539928	155824	-179285	0.4129	no
gi|320446826|ref|NW_003383745.1|	07966-20927	298094	0.435604	-277468	0.2612	no
gi|320446826|ref|NW_003383745.1|	10820-21254	102295	182047	0.831573	0.68395	no
gi|320446826|ref|NW_003383745.1|	13171-21448	0.62524	575062	320124	0.20445	no

gi|320446826|ref|NW_003383745.1|	214862-21550[illegible]	125514	626236	231886	0.32485	no
gi|320446826|ref|NW_003383745.1|	216642-21689[illegible]	0	251022	inf	0.02915	no
gi|320446826|ref|NW_003383745.1|	218527-21930[illegible]	155535	896497	252706	0.27935	no
gi|320446826|ref|NW_003383745.1|	228141-22874[illegible]	0.912593	105233	352748	0.1833	no
gi|320446826|ref|NW_003383745.1|	240905-24117[illegible]	245672	89187	518203	0.16925	no
gi|320446826|ref|NW_003383745.1|	243950-24618[illegible]	0.2529	747195	488485	0.14835	no
gi|320446826|ref|NW_003383745.1|	246837-24753[illegible]	11058	105759	325762	0.20555	no
gi|320446826|ref|NW_003383745.1|	247642-25708[illegible]	181059	36402	100756	0.4373	no
gi|320446826|ref|NW_003383745.1|	264470-26509[illegible]	0.875302	445953	234904	0.2621	no
gi|320446826|ref|NW_003383745.1|	265639-26601[illegible]	244342	363431	-274915	0.25365	no
gi|320446826|ref|NW_003383745.1|	266408-26721[illegible]	199589	184149	-343809	0.1661	no
gi|320446826|ref|NW_003383745.1|	268718-27134[illegible]	250849	507057	-23066	0.2117	no
gi|320446826|ref|NW_003383745.1|	279583-28154[illegible]	126973	142325	348659	0.07825	no
gi|320446826|ref|NW_003383745.1|	284840-28536[illegible]	0.574765	50244	312791	0.2458	no
gi|320446826|ref|NW_003383745.1|	285933-28709[illegible]	0.182127	239739	371845	0.21225	no
gi|320446826|ref|NW_003383745.1|	289966-29078[illegible]	232167	538501	121379	0.5616	no
gi|320446826|ref|NW_003383745.1|	293436-29424[illegible]	132879	162287	0.288432	0.82835	no
gi|320446826|ref|NW_003383745.1|	297956-30234[illegible]	48972	90694	0.889049	0.49085	no
gi|320446826|ref|NW_003383745.1|	456324-45663[illegible]	522516	134492	136398	0.52185	no
gi|320446826|ref|NW_003383745.1|	460412-46089[illegible]	0.659197	149884	450699	0.17865	no
gi|320446826|ref|NW_003383745.1|	526311-52651[illegible]	205557	192371	322629	0.2	no
gi|320446826|ref|NW_003383745.1|	536078-53735[illegible]	164265	108328	-0.600618	0.7878	no
gi|320446826|ref|NW_003383745.1|	541486-54219[illegible]	898116	368402	-128562	0.5358	no
gi|320446826|ref|NW_003383745.1|	545275-54605[illegible]	625597	278993	-116501	0.58495	no
gi|320446826|ref|NW_003383745.1|	549058-55031[illegible]	977471	140204	0.520397	0.81165	no
gi|320446826|ref|NW_003383745.1|	550532-55145[illegible]	122727	220025	0.842215	0.6905	no
gi|320446826|ref|NW_003383745.1|	553024-55503[illegible]	171909	153661	-0.161899	0.90125	no
gi|320446826|ref|NW_003383745.1|	555742-55625[illegible]	430886	168746	-135246	0.5372	no
gi|320446826|ref|NW_003383745.1|	557378-55800[illegible]	222066	100586	-114256	0.5876	no
gi|320446826|ref|NW_003383745.1|	560906-56479[illegible]	166113	181184	0.125287	0.924	no

gi|320446826|ref|NW_003383745.1|	68833-56956	373795	430439	352549	0.1563	no
gi|320446826|ref|NW_003383745.1|	88287-58916	261834	288519	346194	0.16135	no
gi|320446826|ref|NW_003383745.1|	91072-59134	486216	665976	37758	0.17895	no
gi|320446826|ref|NW_003383745.1|	01176-60136	0	821199	inf	0.0233	no
gi|320446826|ref|NW_003383745.1|	29588-63468	722015	162682	117195	0.37495	no
gi|320446826|ref|NW_003383745.1|	55055-65535	146528	247095	0.75389	0.7062	no
gi|320446826|ref|NW_003383745.1|	60148-66089	264941	108915	203946	0.35735	no
gi|320446826|ref|NW_003383745.1|	62853-66381	395958	118959	158704	0.4462	no
gi|320446826|ref|NW_003383745.1|	71737-67235	148044	147921	-0.00120014	0.98955	no
gi|320446826|ref|NW_003383745.1|	87110-68802	0	224188	inf	0.02915	no
gi|320446826|ref|NW_003383745.1|	88475-69064	0	346895	inf	0.007	no
gi|320446827|ref|NW_003383744.1|	01684-10260	160245	39095	-203522	0.36095	no
gi|320446827|ref|NW_003383744.1|	05555-10866	11399	694371	-0.715126	0.66715	no
gi|320446827|ref|NW_003383744.1|	09034-11239	122618	163234	0.412763	0.751	no
gi|320446827|ref|NW_003383744.1|	24131-12665	129135	136894	34061	0.0164	no
gi|320446827|ref|NW_003383744.1|	26759-12899	109864	146272	373486	0.06775	no
gi|320446827|ref|NW_003383744.1|	29995-13382	178367	176085	330336	0.0941	no
gi|320446827|ref|NW_003383744.1|	33929-13565	198839	115893	254312	0.0635	no
gi|320446827|ref|NW_003383744.1|	35795-14064	12932	50971	197873	0.1497	no
gi|320446827|ref|NW_003383744.1|	41123-14388	793758	224216	149812	0.39915	no
gi|320446827|ref|NW_003383744.1|	44591-14655	771161	123867	0.683693	0.6672	no
gi|320446827|ref|NW_003383744.1|	47208-14853	589608	80785	0.454331	0.83415	no
gi|320446827|ref|NW_003383744.1|	49157-15009	552488	81193	0.555414	0.7865	no
gi|320446827|ref|NW_003383744.1|	50205-15411	574426	614354	0.0969483	0.933	no
gi|320446827|ref|NW_003383744.1|	56451-15716	59559	455402	-0.387178	0.8435	no
gi|320446827|ref|NW_003383744.1|	57577-15855	34275	378621	0.143598	0.9395	no
gi|320446827|ref|NW_003383744.1|	60443-16078	978503	906605	-0.110103	0.93915	no
gi|320446827|ref|NW_003383744.1|	61374-16168	522516	112077	110094	0.5827	no
gi|320446827|ref|NW_003383744.1|	61824-16279	345558	286252	-0.271641	0.88835	no
gi|320446827|ref|NW_003383744.1|	64206-16460	577439	253427	-11881	0.5767	no

gi|320446827|ref|NW_003383744.1|	165126-16707	393191	363216	-0.114403	0.95425	no
gi|320446827|ref|NW_003383744.1|	167191-16825	162351	0.701893	-120979	0.5719	no
gi|320446827|ref|NW_003383744.1|	168584-16927	44232	226626	-0.964777	0.65055	no
gi|320446827|ref|NW_003383744.1|	169482-17017	1843	125903	-0.54974	0.80065	no
gi|320446827|ref|NW_003383744.1|	186656-18710	0	799285	inf	0.00395	no
gi|320446827|ref|NW_003383744.1|	187641-18900	193692	165569	-0.226333	0.9033	no
gi|320446827|ref|NW_003383744.1|	198789-19926	611557	226927	-143026	0.51015	no
gi|320446827|ref|NW_003383744.1|	237994-24160	884455	734262	-0.268494	0.82565	no
gi|320446827|ref|NW_003383744.1|	245614-24656	194755	376894	-236943	0.29555	no
gi|320446827|ref|NW_003383744.1|	246852-24788	507821	0.29254	-411762	0.2107	no
gi|320446827|ref|NW_003383744.1|	251628-25253	17513	0.517356	-17592	0.32375	no
gi|320446827|ref|NW_003383744.1|	252693-25705	216596	678188	164668	0.3215	no
gi|320446827|ref|NW_003383744.1|	293727-29548	0.444758	139399	164813	1	no
gi|320446827|ref|NW_003383744.1|	361337-36791	117027	76625	271098	0.14365	no
gi|320446827|ref|NW_003383744.1|	388958-39637	158602	128004	-0.309217	0.81865	no
gi|320446827|ref|NW_003383744.1|	396538-39706	165588	307244	-243014	0.28215	no
gi|320446827|ref|NW_003383744.1|	397563-39830	700954	365839	-0.938111	0.6512	no
gi|320446827|ref|NW_003383744.1|	407438-40836	10.8	321575	-17478	0.41605	no
gi|320446827|ref|NW_003383744.1|	415687-41789	737133	430065	-0.777372	0.5628	no
gi|320446827|ref|NW_003383744.1|	420267-42436	846985	125924	0.572142	0.66095	no
gi|320446827|ref|NW_003383744.1|	427699-42806	210952	535728	-197734	0.3823	no
gi|320446827|ref|NW_003383744.1|	428417-42863	646287	18.72	-178759	0.4151	no
gi|320446827|ref|NW_003383744.1|	428803-43126	236101	43474	-244118	0.19	no
gi|320446827|ref|NW_003383744.1|	435076-43597	507903	0.699995	-285914	0.25675	no
gi|320446827|ref|NW_003383744.1|	471244-47146	479751	200468	-125891	0.55525	no
gi|320446827|ref|NW_003383744.1|	473633-47603	43693	163438	-141865	0.50405	no
gi|320446827|ref|NW_003383744.1|	476288-47828	335062	36048	0.105488	0.95915	no
gi|320446827|ref|NW_003383744.1|	523767-52482	131783	49841	-140276	0.5071	no
gi|320446827|ref|NW_003383744.1|	527632-52824	188633	128024	-0.559169	0.7932	no
gi|320446827|ref|NW_003383744.1|	530332-53206	114851	108216	-0.0858492	0.9677	no

gi|320446827|ref|NW_003383744.1|	51302-55204	0.99743	364438	186939	0.39335	no
gi|320446827|ref|NW_003383744.1|	57593-55802	0.835061	774209	321277	0.23205	no
gi|320446827|ref|NW_003383744.1|	61895-56223	27763	990595	183513	0.32615	no
gi|320446827|ref|NW_003383744.1|	68447-56936	0.248345	856031	510725	0.16865	no
gi|320446827|ref|NW_003383744.1|	75272-57661	0.152772	148504	328105	0.23005	no
gi|320446827|ref|NW_003383744.1|	79000-58077	0	106679	inf	1	no
gi|320446827|ref|NW_003383744.1|	06637-60753	76071	0.873703	-312213	0.2211	no
gi|320446827|ref|NW_003383744.1|	14534-61564	111788	33356	-174475	0.4196	no
gi|320446827|ref|NW_003383744.1|	16638-61736	341871	140424	-128366	0.54835	no
gi|320446827|ref|NW_003383744.1|	19937-62092	102881	169915	-259808	0.25585	no
gi|320446827|ref|NW_003383744.1|	21745-62224	208167	253563	-303733	0.21495	no
gi|320446827|ref|NW_003383744.1|	22724-62947	994208	110531	0.15283	0.91375	no
gi|320446827|ref|NW_003383744.1|	35356-64035	407662	194185	225198	0.23085	no
gi|320446827|ref|NW_003383744.1|	42024-64255	335975	904611	142894	0.4976	no
gi|320446827|ref|NW_003383744.1|	52327-65333	614819	13653	115098	0.59165	no
gi|320446827|ref|NW_003383744.1|	62687-66435	106083	303606	151701	0.4701	no
gi|320446827|ref|NW_003383744.1|	65782-66659	410529	402959	-0.0268497	0.97285	no
gi|320446827|ref|NW_003383744.1|	68134-66986	113204	599146	-0.917943	0.67485	no
gi|320446827|ref|NW_003383744.1|	73890-67472	700227	0.385273	-418387	0.2088	no
gi|320446827|ref|NW_003383744.1|	74783-67601	321407	0.46924	-2776	0.2612	no
gi|320446827|ref|NW_003383744.1|	76502-67893	858378	123096	-280183	0.2444	no
gi|320446827|ref|NW_003383744.1|	82458-68329	507534	290846	-0.80325	0.6999	no
gi|320446827|ref|NW_003383744.1|	93804-69495	818415	522338	-0.64785	0.62715	no
gi|320446827|ref|NW_003383744.1|	95982-69653	154888	864408	-0.841439	0.6854	no
gi|320446827|ref|NW_003383744.1|	96716-69873	917833	765696	-0.261461	0.8459	no
gi|320446827|ref|NW_003383744.1|	00742-70706	510132	718996	0.495113	0.70375	no
gi|320446827|ref|NW_003383744.1|	07634-70787	163151	100618	-0.697312	0.73515	no
gi|320446827|ref|NW_003383744.1|	10999-71217	188186	119227	-0.658445	0.76715	no
gi|320446827|ref|NW_003383744.1|	12960-71374	165238	840198	-0.975749	0.6476	no
gi|320446827|ref|NW_003383744.1|	14414-71545	143164	392988	-186511	0.39885	no

gi|320446827|ref|NW_003383744.1|	715872-71618	456603	169463	-142997	0.49235	no
gi|320446827|ref|NW_003383744.1|	76086-77440	134265	0.733329	-419447	0.14305	no
gi|320446827|ref|NW_003383744.1|	79220-79474	335224	772376	-211775	0.35425	no
gi|320446827|ref|NW_003383744.1|	79604-80019	152448	0.592094	-468635	0.28625	no
gi|320446827|ref|NW_003383744.1|	84581-85597	972907	31375	-163269	0.4444	no
gi|320446827|ref|NW_003383744.1|	87589-87857	362897	164865	-44602	0.29535	no
gi|320446827|ref|NW_003383744.1|	88204-88454	320305	202732	-39818	0.31545	no
gi|320446827|ref|NW_003383744.1|	88585-89405	202103	0.595329	-176333	0.32375	no
gi|320446827|ref|NW_003383744.1|	99697-10068	167054	623564	-14217	0.5151	no
gi|320446828|ref|NW_003383743.1|	101967-10522	0.670974	47711	282999	0.2228	no
gi|320446828|ref|NW_003383743.1|	150720-15200	0	134006	inf	1	no
gi|320446828|ref|NW_003383743.1|	215984-21633	257736	243135	-0.0841308	0.96425	no
gi|320446828|ref|NW_003383743.1|	222116-22405	295336	14.44	-103229	0.42135	no
gi|320446828|ref|NW_003383743.1|	224476-22535	186481	741588	-133034	0.54245	no
gi|320446828|ref|NW_003383743.1|	225700-22654	289766	702477	-204436	0.36245	no
gi|320446828|ref|NW_003383743.1|	228702-22884	271685	134954	-100946	0.59635	no
gi|320446828|ref|NW_003383743.1|	324665-32589	0.339003	0.940342	147189	1	no
gi|320446828|ref|NW_003383743.1|	337466-33822	324197	577827	0.833768	0.6908	no
gi|320446828|ref|NW_003383743.1|	338604-34044	1475	26429	0.841408	0.6806	no
gi|320446828|ref|NW_003383743.1|	340659-34085	522672	372088	-0.490261	0.80195	no
gi|320446828|ref|NW_003383743.1|	341784-34256	620997	383523	-0.695274	0.7312	no
gi|320446828|ref|NW_003383743.1|	380884-38297	163416	462313	150032	0.4839	no
gi|320446828|ref|NW_003383743.1|	385976-38701	0.845308	336006	199094	0.3909	no
gi|320446828|ref|NW_003383743.1|	390376-39633	100903	111654	0.146063	0.9095	no
gi|320446828|ref|NW_003383743.1|	396813-39859	0.98321	0.761012	-0.369581	1	no
gi|320446828|ref|NW_003383743.1|	400215-40070	329598	264501	-0.317436	0.8817	no
gi|320446828|ref|NW_003383743.1|	415569-41619	247562	106242	-122043	0.5671	no
gi|320446828|ref|NW_003383743.1|	447458-45133	0.602325	778301	369171	0.0714	no
gi|320446828|ref|NW_003383743.1|	455560-45600	158927	896452	249586	0.254	no
gi|320446828|ref|NW_003383743.1|	456278-45664	0	125383	inf	0.0198	no

gi|320446828|ref|NW_003383743.1|	480513-481067	157794	106472	275435	0.2516	no
gi|320446828|ref|NW_003383743.1|	488906-490058	32.22	471885	-277145	0.12755	no
gi|320446828|ref|NW_003383743.1|	491849-492635	269766	399802	-275435	0.2432	no
gi|320446828|ref|NW_003383743.1|	495406-496340	165276	165238	-332226	0.17835	no
gi|320446828|ref|NW_003383743.1|	497068-499963	425694	288254	-38844	0.05915	no
gi|320446828|ref|NW_003383743.1|	520033-521047	440017	118328	-189476	0.2774	no
gi|320446828|ref|NW_003383743.1|	521856-525463	39284	415996	0.082628	0.9516	no
gi|320446828|ref|NW_003383743.1|	526459-527694	0.506469	117078	120893	1	no
gi|320446828|ref|NW_003383743.1|	528036-529533	98712	116813	0.242901	0.8575	no
gi|320446828|ref|NW_003383743.1|	529741-533555	422922	325031	-0.379811	0.7757	no
gi|320446828|ref|NW_003383743.1|	537191-537923	611665	0.46534	-371639	0.22495	no
gi|320446828|ref|NW_003383743.1|	539783-540880	10577	0.813366	-370088	0.1662	no
gi|320446828|ref|NW_003383743.1|	543529-544913	25123	0.306164	-635856	0.11055	no
gi|320446828|ref|NW_003383743.1|	548957-551882	334999	149105	-448976	0.04065	no
gi|320446828|ref|NW_003383743.1|	552204-553269	35193	196997	-415905	0.06425	no
gi|320446828|ref|NW_003383743.1|	554229-554509	112032	7295	-394085	0.16995	no
gi|320446828|ref|NW_003383743.1|	555557-557275	257221	277399	-321298	0.094	no
gi|320446828|ref|NW_003383743.1|	557986-560729	418529	540747	-29523	0.13255	no
gi|320446828|ref|NW_003383743.1|	71361-72064	108232	369922	17731	0.41715	no
gi|320446829|ref|NW_003383742.1|	180515-183272	675831	0.0467617	-717519	0.2598	no
gi|320446829|ref|NW_003383742.1|	237715-239171	511525	0	#NAME?	0.007	no
gi|320446829|ref|NW_003383742.1|	242714-243613	0	262886	inf	0.02205	no
gi|320446829|ref|NW_003383742.1|	68486-68965	0.679508	453855	273967	0.2661	no
gi|320446830|ref|NW_003383741.1|	208917-209530	412446	506663	-302511	0.2069	no
gi|320446830|ref|NW_003383741.1|	264877-265104	106367	111494	-325401	0.2161	no
gi|320446830|ref|NW_003383741.1|	267721-273070	18807	501639	-190655	0.15305	no
gi|320446830|ref|NW_003383741.1|	325832-326409	0.983058	731512	289553	0.2284	no
gi|320446830|ref|NW_003383741.1|	35823-36441	4399	409323	321799	0.187	no
gi|320446830|ref|NW_003383741.1|	398003-399977	28922	691591	125775	0.3556	no
gi|320446830|ref|NW_003383741.1|	400174-400740	498641	188796	192076	0.3715	no

gi|320446830|ref|NW_003383741.1|	402252-40272	481203	229465	-106837	0.59575	no
gi|320446830|ref|NW_003383741.1|	407057-41155	110659	893303	-0.308894	0.7992	no
gi|320446830|ref|NW_003383741.1|	412234-41254	312674	170123	-0.878081	0.59605	no
gi|320446830|ref|NW_003383741.1|	413017-41383	355124	111128	-16761	0.30895	no
gi|320446830|ref|NW_003383741.1|	414552-41585	340483	196741	-0.791284	0.53145	no
gi|320446830|ref|NW_003383741.1|	426209-43357	197225	342381	0.795762	0.5439	no
gi|320446830|ref|NW_003383741.1|	433825-43447	245024	166843	-0.554429	0.79695	no
gi|320446830|ref|NW_003383741.1|	434985-43542	638525	635478	-0.00690192	0.95455	no
gi|320446830|ref|NW_003383741.1|	436356-43741	652543	253884	-13619	0.513	no
gi|320446830|ref|NW_003383741.1|	471083-47177	0	478433	inf	0.0212	no
gi|320446830|ref|NW_003383741.1|	472405-47322	0.287734	553786	426652	0.1888	no
gi|320446830|ref|NW_003383741.1|	474502-47526	0.32173	198532	262545	0.26355	no
gi|320446830|ref|NW_003383741.1|	475929-47656	125514	54084	210736	0.3494	no
gi|320446830|ref|NW_003383741.1|	478406-47914	0	319495	inf	0.0233	no
gi|320446830|ref|NW_003383741.1|	479697-48157	0.922295	228553	130923	0.5332	no
gi|320446830|ref|NW_003383741.1|	482349-48307	755162	446431	-0.758349	0.70575	no
gi|320446830|ref|NW_003383741.1|	488440-48894	110366	370117	-157625	0.47105	no
gi|320446830|ref|NW_003383741.1|	490806-49136	148909	416144	-183928	0.39955	no
gi|320446830|ref|NW_003383741.1|	491572-49191	145568	137611	-0.0810989	0.95815	no
gi|320446830|ref|NW_003383741.1|	494096-49473	295762	40232	0.443909	0.81955	no
gi|320446830|ref|NW_003383741.1|	494935-49562	445216	760217	0.771906	0.70305	no
gi|320446830|ref|NW_003383741.1|	495834-49693	582204	591059	0.0217769	0.98665	no
gi|320446830|ref|NW_003383741.1|	497692-49814	149641	154305	0.044282	0.97805	no
gi|320446830|ref|NW_003383741.1|	498270-49898	488468	310344	-0.654395	0.7453	no
gi|320446830|ref|NW_003383741.1|	513234-51417	166724	427236	135757	0.5197	no
gi|320446830|ref|NW_003383741.1|	518719-51942	142194	243102	0.773702	0.72055	no
gi|320446830|ref|NW_003383741.1|	519973-52142	0.417831	0.871223	106012	1	no
gi|320446830|ref|NW_003383741.1|	521534-52973	951503	879752	-0.113111	0.9351	no
gi|320446830|ref|NW_003383741.1|	52795-53900	0	188064	inf	0.0233	no
gi|320446830|ref|NW_003383741.1|	533579-53500	121789	426117	180686	0.31365	no

gi|320446830|ref|NW_003383741.1|	539706-54212	184682	111694	-0.725489	0.5724	no
gi|320446830|ref|NW_003383741.1|	54123-55011	0.775869	302865	196479	0.3902	no
gi|320446830|ref|NW_003383741.1|	550521-55330	0.462709	240222	237619	0.29235	no
gi|320446830|ref|NW_003383741.1|	555873-55690	0.632397	33518	240603	0.2993	no
gi|320446830|ref|NW_003383741.1|	571647-57258	287436	743571	-19507	0.3786	no
gi|320446830|ref|NW_003383741.1|	573688-57408	668302	775719	-310689	0.20555	no
gi|320446830|ref|NW_003383741.1|	588670-59007	169697	496938	-177182	0.4154	no
gi|320446830|ref|NW_003383741.1|	84911-86566	0.118867	273053	452177	0.17645	no
gi|320446830|ref|NW_003383741.1|	92837-93380	108288	927733	-0.223088	0.8602	no
gi|320446830|ref|NW_003383741.1|	95492-102895	26717	402038	0.589573	0.6441	no
gi|320446831|ref|NW_003383740.1|	108163-10967	0	56203	inf	0.00605	no
gi|320446831|ref|NW_003383740.1|	10952-11405	528165	110393	106359	0.59905	no
gi|320446831|ref|NW_003383740.1|	117419-11800	0	340701	inf	0.00485	no
gi|320446831|ref|NW_003383740.1|	118173-11865	0	777285	inf	0.02075	no
gi|320446831|ref|NW_003383740.1|	121244-12173	0	167708	inf	0.0088	no
gi|320446831|ref|NW_003383740.1|	124736-12514	0	112361	inf	0.0154	no
gi|320446831|ref|NW_003383740.1|	125342-12565	0	179323	inf	0.0198	no
gi|320446831|ref|NW_003383740.1|	127946-12824	0	215348	inf	0.0154	no
gi|320446831|ref|NW_003383740.1|	129610-13006	0	31969	inf	0.00615	no
gi|320446831|ref|NW_003383740.1|	13427-15666	299808	212769	-0.494752	0.70295	no
gi|320446831|ref|NW_003383740.1|	18679-27371	149633	146917	-0.0264299	0.9894	no
gi|320446831|ref|NW_003383740.1|	272014-27351	123992	168.43	0.441902	0.73545	no
gi|320446831|ref|NW_003383740.1|	28280-29115	450388	19358	-121824	0.56285	no
gi|320446831|ref|NW_003383740.1|	282839-28301	320865	393396	0.294013	0.88665	no
gi|320446831|ref|NW_003383740.1|	29839-30757	138461	289795	-225638	0.31225	no
gi|320446831|ref|NW_003383740.1|	31013-32541	391643	156167	-132645	0.4455	no
gi|320446831|ref|NW_003383740.1|	33387-35151	120324	103803	-0.213077	0.92625	no
gi|320446831|ref|NW_003383740.1|	35264-36540	154061	103519	-0.573612	0.79455	no
gi|320446831|ref|NW_003383740.1|	38608-41025	139219	739906	240999	0.2966	no
gi|320446831|ref|NW_003383740.1|	41971-43174	122085	290161	124897	0.5493	no

gi|320446831|ref|NW_003383740.1|	45452-48555	115326	682992	-0.755772	0.65035	no
gi|320446831|ref|NW_003383740.1|	48743-52623	76386	780903	0.0318338	0.98065	no
gi|320446831|ref|NW_003383740.1|	55065-58577	911451	148795	0.707092	0.5854	no
gi|320446831|ref|NW_003383740.1|	58686-59615	510993	385967	-0.404824	0.8494	no
gi|320446831|ref|NW_003383740.1|	60173-60592	615646	639287	0.054363	0.94885	no
gi|320446831|ref|NW_003383740.1|	60699-60964	321548	221374	-0.538548	0.7927	no
gi|320446831|ref|NW_003383740.1|	61113-62545	578196	431215	-0.423151	0.8388	no
gi|320446831|ref|NW_003383740.1|	63565-64870	915076	613228	-0.577469	0.7918	no
gi|320446831|ref|NW_003383740.1|	66001-67706	838199	101539	0.276671	0.901	no
gi|320446831|ref|NW_003383740.1|	72888-75921	108561	2994	146356	0.50185	no
gi|320446833|ref|NW_003383738.1|	0-1176	197357	721087	186937	0.38445	no
gi|320446833|ref|NW_003383738.1|	106292-10667	944101	137625	-27782	0.2822	no
gi|320446833|ref|NW_003383738.1|	13927-17629	328048	128748	-134936	0.3118	no
gi|320446833|ref|NW_003383738.1|	161768-16223	154841	751066	-104377	0.597	no
gi|320446833|ref|NW_003383738.1|	163022-16359	69209	100303	-27866	0.22085	no
gi|320446833|ref|NW_003383738.1|	164757-16601	44644	149108	-158211	0.47335	no
gi|320446833|ref|NW_003383738.1|	166649-16694	120024	640757	-0.905478	0.6817	no
gi|320446833|ref|NW_003383738.1|	167225-16873	411059	378052	-0.120762	0.9516	no
gi|320446833|ref|NW_003383738.1|	169488-17055	283093	419644	0.567894	0.78445	no
gi|320446833|ref|NW_003383738.1|	171123-17163	487103	57189	0.231511	0.9017	no
gi|320446833|ref|NW_003383738.1|	171733-17216	751555	940111	0.322953	0.8624	no
gi|320446833|ref|NW_003383738.1|	172584-17328	104181	100696	-0.0490852	0.9786	no
gi|320446833|ref|NW_003383738.1|	173506-17641	373043	724845	0.95833	0.6691	no
gi|320446833|ref|NW_003383738.1|	179311-18169	111724	561195	-0.993365	0.4524	no
gi|320446833|ref|NW_003383738.1|	18361-18623	498987	352055	-382513	0.2211	no
gi|320446833|ref|NW_003383738.1|	187083-19047	0.213673	418527	429184	0.15335	no
gi|320446833|ref|NW_003383738.1|	190583-19187	0.318222	298143	32279	0.20625	no
gi|320446833|ref|NW_003383738.1|	191975-19262	0.403543	247403	261607	0.2636	no
gi|320446833|ref|NW_003383738.1|	193667-19558	0.602781	105045	0.801298	1	no
gi|320446833|ref|NW_003383738.1|	195736-19694	0.5184	10782	105649	1	no

gi|320446833|ref|NW_003383738.1|	20249-20595	279778	519389	-24294	0.2994	no
gi|320446833|ref|NW_003383738.1|	22856-24174	498806	140667	-18262	0.40155	no
gi|320446833|ref|NW_003383738.1|	52611-25511	639644	0.311688	-43591	0.1477	no
gi|320446833|ref|NW_003383738.1|	2566-6895	111285	29148	138914	0.52435	no
gi|320446833|ref|NW_003383738.1|	25868-27544	983922	375119	-13912	0.51665	no
gi|320446833|ref|NW_003383738.1|	65245-26555	973952	979422	0.00808063	0.99335	no
gi|320446833|ref|NW_003383738.1|	66648-26686	684302	889867	0.378957	0.8473	no
gi|320446833|ref|NW_003383738.1|	67717-26916	190182	218061	0.197355	0.8722	no
gi|320446833|ref|NW_003383738.1|	72718-27316	0	136527	inf	0.0142	no
gi|320446833|ref|NW_003383738.1|	75393-27621	0.870625	159556	0.873939	0.6867	no
gi|320446833|ref|NW_003383738.1|	05911-30916	127988	0	#NAME?	5.00E-05	yes
gi|320446833|ref|NW_003383738.1|	10116-31170	111867	0	#NAME?	0.00285	no
gi|320446833|ref|NW_003383738.1|	13770-31500	345741	0.233925	-72075	0.16165	no
gi|320446833|ref|NW_003383738.1|	15201-31555	235524	0	#NAME?	0.01335	no
gi|320446833|ref|NW_003383738.1|	19123-32370	649324	257827	-133254	0.3155	no
gi|320446833|ref|NW_003383738.1|	24661-32537	142492	170519	0.259044	0.89055	no
gi|320446833|ref|NW_003383738.1|	25877-32657	400904	273893	-0.549647	0.80485	no
gi|320446833|ref|NW_003383738.1|	27233-32783	903467	408553	-114495	0.4899	no
gi|320446833|ref|NW_003383738.1|	39827-34083	211068	222522	0.0762404	0.9721	no
gi|320446833|ref|NW_003383738.1|	41766-34295	276652	179444	-0.624536	0.6997	no
gi|320446833|ref|NW_003383738.1|	43590-34875	237092	263648	0.153162	0.90615	no
gi|320446833|ref|NW_003383738.1|	63847-36860	140739	123413	-0.189533	0.8864	no
gi|320446833|ref|NW_003383738.1|	68927-37487	205635	203536	-0.014801	0.99115	no
gi|320446833|ref|NW_003383738.1|	76514-38170	435898	276248	-0.658028	0.6194	no
gi|320446833|ref|NW_003383738.1|	11273-41296	0.924235	217189	123262	0.5519	no
gi|320446833|ref|NW_003383738.1|	29198-43380	210585	0.135302	-728208	0.1122	no
gi|320446833|ref|NW_003383738.1|	65723-46674	0	400365	inf	0.0133	no
gi|320446833|ref|NW_003383738.1|	98433-49911	11404	207554	0.863943	0.6867	no
gi|320446833|ref|NW_003383738.1|	03046-50482	119464	794002	-0.589367	0.788	no
gi|320446833|ref|NW_003383738.1|	05001-50657	213202	175537	-0.280442	0.8225	no

gi|320446833|ref|NW_003383738.1|	509510-51224	751234	101971	-28811	0.0378	no
gi|320446833|ref|NW_003383738.1|	512354-51638	313474	156252	-100447	0.4521	no
gi|320446833|ref|NW_003383738.1|	516547-51843	389806	381928	-0.0294526	0.9822	no
gi|320446833|ref|NW_003383738.1|	63466-65465	784558	114088	0.54019	0.8088	no
gi|320446833|ref|NW_003383738.1|	66253-66983	477655	607313	0.346471	0.86485	no
gi|320446833|ref|NW_003383738.1|	67365-67677	788182	496432	-0.666933	0.74425	no
gi|320446835|ref|NW_003383736.1|	107802-10811	0	144549	inf	0.02915	no
gi|320446835|ref|NW_003383736.1|	158773-15949	0	309107	inf	0.02915	no
gi|320446835|ref|NW_003383736.1|	184389-18538	0.669186	338947	234058	0.3042	no
gi|320446835|ref|NW_003383736.1|	215119-21562	0.611006	139353	451142	0.1786	no
gi|320446835|ref|NW_003383736.1|	227656-22789	0	301254	inf	0.029	no
gi|320446835|ref|NW_003383736.1|	228893-23155	316771	685863	-220745	0.2333	no
gi|320446835|ref|NW_003383736.1|	239070-24112	253809	704229	-184963	0.2956	no
gi|320446835|ref|NW_003383736.1|	242803-24442	158205	99093	-0.674938	0.7637	no
gi|320446835|ref|NW_003383736.1|	244534-24606	177887	260094	0.548074	0.65375	no
gi|320446835|ref|NW_003383736.1|	300948-30182	0.525299	112143	441605	0.15735	no
gi|320446835|ref|NW_003383736.1|	301929-30257	0.412318	589403	383742	0.20095	no
gi|320446835|ref|NW_003383736.1|	303302-30470	0.578602	723577	36445	0.17585	no
gi|320446835|ref|NW_003383736.1|	308631-30896	289655	15947	246088	0.25545	no
gi|320446835|ref|NW_003383736.1|	309075-30965	0	619833	inf	0.0212	no
gi|320446835|ref|NW_003383736.1|	356506-35676	499632	372743	-374461	0.2178	no
gi|320446835|ref|NW_003383736.1|	357814-35827	122069	143554	-308803	0.19375	no
gi|320446835|ref|NW_003383736.1|	361688-36314	116225	182731	-266913	0.25395	no
gi|320446835|ref|NW_003383736.1|	377922-37829	568584	104091	0.872402	0.6789	no
gi|320446835|ref|NW_003383736.1|	383096-38490	118256	464372	197337	0.36455	no
gi|320446835|ref|NW_003383736.1|	395530-39943	16741	240791	0.524399	0.6935	no
gi|320446835|ref|NW_003383736.1|	404732-40545	110513	337996	-170914	0.3386	no
gi|320446835|ref|NW_003383736.1|	405691-40596	491344	125175	-197279	0.38155	no
gi|320446835|ref|NW_003383736.1|	406313-40789	633925	312409	-102088	0.4435	no
gi|320446835|ref|NW_003383736.1|	447055-44768	0.423563	518506	361371	0.20675	no

gi|320446835|ref|NW_003383736.1|	47843-45206	0.549783	832024	391969	0.06175	no
gi|320446835|ref|NW_003383736.1|	17317-52050	450002	638204	0.504088	0.70025	no
gi|320446835|ref|NW_003383736.1|	23570-52403	215415	334959	0.636861	0.74235	no
gi|320446835|ref|NW_003383736.1|	24138-52701	469935	351243	-0.419989	0.7546	no
gi|320446835|ref|NW_003383736.1|	27188-52754	533029	218078	-128937	0.54315	no
gi|320446835|ref|NW_003383736.1|	27652-52903	439988	234322	-0.908969	0.6599	no
gi|320446835|ref|NW_003383736.1|	29250-53008	703732	309729	-118402	0.55935	no
gi|320446835|ref|NW_003383736.1|	30881-53182	160485	109057	-0.557355	0.79615	no
gi|320446835|ref|NW_003383736.1|	32587-53475	173657	147963	-0.231008	0.8537	no
gi|320446835|ref|NW_003383736.1|	34900-53601	769194	226311	-176504	0.41775	no
gi|320446835|ref|NW_003383736.1|	36166-53674	165701	49468	-174402	0.41025	no
gi|320446835|ref|NW_003383736.1|	43136-54504	746096	379507	-0.975235	0.6525	no
gi|320446835|ref|NW_003383736.1|	61731-56360	813027	208003	-19667	0.3742	no
gi|320446835|ref|NW_003383736.1|	65628-67470	0	131737	inf	1	no
gi|320446836|ref|NW_003383735.1|	39446-14027	500122	312386	-0.678953	0.6794	no
gi|320446836|ref|NW_003383735.1|	44385-14931	143882	191491	0.412393	0.843	no
gi|320446836|ref|NW_003383735.1|	49450-15022	186987	23522	0.331076	0.86375	no
gi|320446836|ref|NW_003383735.1|	50341-15505	134463	185713	0.46586	0.7286	no
gi|320446836|ref|NW_003383735.1|	72494-17526	612209	15021	129489	0.44065	no
gi|320446836|ref|NW_003383735.1|	75391-17586	414069	322453	-0.360783	0.85555	no
gi|320446836|ref|NW_003383735.1|	78949-17988	312721	182525	-0.776779	0.70675	no
gi|320446836|ref|NW_003383735.1|	80002-18287	204862	349039	0.768737	0.7089	no
gi|320446836|ref|NW_003383735.1|	34193-23729	162091	243172	0.585171	0.66115	no
gi|320446836|ref|NW_003383735.1|	38019-23871	261982	278506	0.088241	0.96515	no
gi|320446836|ref|NW_003383735.1|	39734-24022	139256	103568	-0.427164	0.7369	no
gi|320446836|ref|NW_003383735.1|	69466-27033	271186	107198	-1339	0.5473	no
gi|320446836|ref|NW_003383735.1|	72632-27331	979594	771645	-0.344247	0.8675	no
gi|320446836|ref|NW_003383735.1|	74015-27528	235599	124008	-0.925902	0.56765	no
gi|320446836|ref|NW_003383735.1|	75709-27791	926173	75404	-0.296639	0.8872	no
gi|320446836|ref|NW_003383735.1|	78427-28091	301735	143802	-10692	0.41245	no

gi|320446836|ref|NW_003383735.1|	282011-28239	356079	353501	-333241	0.19855	no
gi|320446836|ref|NW_003383735.1|	285993-28630	655204	0	#NAME?	0.007	no
gi|320446836|ref|NW_003383735.1|	286908-29102	205815	125198	-0.717132	0.58975	no
gi|320446836|ref|NW_003383735.1|	291274-29165	194225	0	#NAME?	0.01345	no
gi|320446836|ref|NW_003383735.1|	328621-32908	0	137702	inf	0.0109	no
gi|320446836|ref|NW_003383735.1|	330190-33065	0	729055	inf	0.02205	no
gi|320446836|ref|NW_003383735.1|	331822-33223	0	165786	inf	0.01275	no
gi|320446836|ref|NW_003383735.1|	333762-33411	0	237133	inf	0.0109	no
gi|320446836|ref|NW_003383735.1|	340832-34461	0.380484	216355	25075	0.2692	no
gi|320446836|ref|NW_003383735.1|	351962-35338	0	0.988207	inf	1	no
gi|320446836|ref|NW_003383735.1|	65377-67049	0.587299	147191	132552	0.525	no
gi|320446836|ref|NW_003383735.1|	70056-72418	150754	137383	318793	0.09315	no
gi|320446836|ref|NW_003383735.1|	72555-76005	38316	318363	305465	0.11685	no
gi|320446836|ref|NW_003383735.1|	83496-84483	0.898186	217105	127331	0.55415	no
gi|320446836|ref|NW_003383735.1|	84860-85884	107004	133122	0.315083	1	no
gi|320446836|ref|NW_003383735.1|	86154-87548	233039	263077	0.174909	0.9264	no
gi|320446836|ref|NW_003383735.1|	87686-88395	356231	682074	0.937116	0.65355	no
gi|320446837|ref|NW_003383734.1|	189156-19010	0.235847	260387	346474	0.21395	no
gi|320446837|ref|NW_003383734.1|	228485-23282	135518	859757	266544	0.1435	no
gi|320446837|ref|NW_003383734.1|	3036-3357	159548	113276	282778	0.2595	no
gi|320446837|ref|NW_003383734.1|	64577-65072	320005	514221	0.684295	0.7417	no
gi|320446837|ref|NW_003383734.1|	8217-8880	228644	295615	0.370621	0.8696	no
gi|320446837|ref|NW_003383734.1|	916-1109	0	798735	inf	0.02205	no
gi|320446839|ref|NW_003383732.1|	148005-14865	394235	442866	-315411	0.1915	no
gi|320446839|ref|NW_003383732.1|	149673-15180	24.67	695913	-182578	0.29845	no
gi|320446839|ref|NW_003383732.1|	153215-15468	342259	119914	-151309	0.3722	no
gi|320446839|ref|NW_003383732.1|	155619-15682	112898	373268	-159674	0.46305	no
gi|320446839|ref|NW_003383732.1|	171557-17197	74914	714054	-0.0692027	0.97595	no
gi|320446839|ref|NW_003383732.1|	172112-17365	150988	520626	-153612	0.49525	no
gi|320446839|ref|NW_003383732.1|	174648-17543	975793	417495	-122482	0.34965	no

gi|320446839|ref|NW_003383732.1|	175645-17812	276266	166205	-0.733092	0.575	no
gi|320446839|ref|NW_003383732.1|	190025-19210	420333	159348	-139935	0.50385	no
gi|320446839|ref|NW_003383732.1|	201476-20287	279647	273195	-0.0336781	0.9775	no
gi|320446839|ref|NW_003383732.1|	203155-20839	143734	251509	0.80721	0.55375	no
gi|320446839|ref|NW_003383732.1|	209620-21299	274432	782944	-180947	0.1707	no
gi|320446839|ref|NW_003383732.1|	219192-22021	193836	0.893052	-111802	0.58925	no
gi|320446839|ref|NW_003383732.1|	223752-22413	104318	0	#NAME?	0.0229	no
gi|320446839|ref|NW_003383732.1|	226468-22832	114534	130608	0.189466	1	no
gi|320446839|ref|NW_003383732.1|	240719-24268	311648	862369	146839	0.49725	no
gi|320446839|ref|NW_003383732.1|	272470-27338	0.498892	257927	237016	0.2616	no
gi|320446839|ref|NW_003383732.1|	286518-28917	948005	209422	-217848	0.19815	no
gi|320446839|ref|NW_003383732.1|	292586-29448	0.506236	112915	115736	1	no
gi|320446839|ref|NW_003383732.1|	294832-29698	0.528786	135279	135518	1	no
gi|320446839|ref|NW_003383732.1|	297104-29914	0.841067	0.521414	-0.689793	1	no
gi|320446839|ref|NW_003383732.1|	309371-31016	0.606497	229026	191694	0.3194	no
gi|320446839|ref|NW_003383732.1|	310499-31153	0.629252	159519	134201	0.5243	no
gi|320446839|ref|NW_003383732.1|	312187-31359	0.288806	130425	217504	1	no
gi|320446839|ref|NW_003383732.1|	315893-32053	619692	110499	0.83441	0.50035	no
gi|320446839|ref|NW_003383732.1|	322026-32468	258077	241556	-0.0954427	0.94325	no
gi|320446839|ref|NW_003383732.1|	339987-34188	401614	222524	-0.85185	0.5171	no
gi|320446839|ref|NW_003383732.1|	371913-37535	102448	108115	0.0776673	0.95055	no
gi|320446839|ref|NW_003383732.1|	375555-37618	705874	328652	-110285	0.50755	no
gi|320446839|ref|NW_003383732.1|	376782-37857	293208	14472	-101866	0.4255	no
gi|320446839|ref|NW_003383732.1|	380675-38337	102776	191505	0.897882	0.66445	no
gi|320446839|ref|NW_003383732.1|	383604-38583	110876	72064	-0.621596	0.6995	no
gi|320446839|ref|NW_003383732.1|	386066-38672	584788	210088	-147692	0.37525	no
gi|320446839|ref|NW_003383732.1|	387443-38841	468425	118319	-198514	0.25385	no
gi|320446839|ref|NW_003383732.1|	394642-39523	186993	0.31682	-588317	0.2696	no
gi|320446839|ref|NW_003383732.1|	396572-39726	5529	0	#NAME?	0.01585	no
gi|320446839|ref|NW_003383732.1|	398833-40570	358676	0.285324	-697394	0.04085	no

gi|320446839|ref|NW_003383732.1|	406319-41161	395317	208247	-0.924711	0.48435	no
gi|320446839|ref|NW_003383732.1|	412178-41270	189248	460442	-203919	0.2559	no
gi|320446839|ref|NW_003383732.1|	412829-41362	215.39	490143	-213568	0.1153	no
gi|320446839|ref|NW_003383732.1|	422144-42433	148483	855407	-0.795611	0.6296	no
gi|320446839|ref|NW_003383732.1|	425579-42615	514637	159165	-169303	0.4574	no
gi|320446839|ref|NW_003383732.1|	435645-43591	225636	185961	-0.278993	0.90205	no
gi|320446839|ref|NW_003383732.1|	438822-43921	11225	938223	-0.258717	0.89815	no
gi|320446839|ref|NW_003383732.1|	439541-44592	119463	256015	109966	0.41615	no
gi|320446839|ref|NW_003383732.1|	447146-44825	450492	759662	-256807	0.16045	no
gi|320446840|ref|NW_003383731.1|	189817-19009	0	19931	inf	0.02915	no
gi|320446840|ref|NW_003383731.1|	190864-19165	0.917991	819193	315765	0.20985	no
gi|320446840|ref|NW_003383731.1|	199516-20401	28011	255527	-0.132514	0.92365	no
gi|320446840|ref|NW_003383731.1|	204248-20652	90735	4894	-0.890645	0.68885	no
gi|320446840|ref|NW_003383731.1|	208358-20879	786057	411122	-0.935069	0.6747	no
gi|320446840|ref|NW_003383731.1|	210665-21116	563673	503649	-0.16244	0.9326	no
gi|320446840|ref|NW_003383731.1|	212713-21357	279686	107366	-138128	0.52955	no
gi|320446840|ref|NW_003383731.1|	215245-21624	772546	426946	-0.855565	0.67655	no
gi|320446840|ref|NW_003383731.1|	219572-22134	22197	128593	-0.787548	0.6386	no
gi|320446840|ref|NW_003383731.1|	231138-23371	354563	295099	-0.264844	0.8457	no
gi|320446840|ref|NW_003383731.1|	233978-23523	214323	709071	-159578	0.47785	no
gi|320446840|ref|NW_003383731.1|	252678-25523	378257	0.914615	-204813	0.3443	no
gi|320446840|ref|NW_003383731.1|	256465-25823	691381	29049	-125099	0.5556	no
gi|320446840|ref|NW_003383731.1|	258439-25994	558235	194089	-152415	0.4753	no
gi|320446840|ref|NW_003383731.1|	260256-26257	116502	497014	-1229	0.45235	no
gi|320446840|ref|NW_003383731.1|	266345-27206	35044	399373	35105	0.0934	no
gi|320446840|ref|NW_003383731.1|	278691-28165	141248	130795	-0.110926	0.92955	no
gi|320446840|ref|NW_003383731.1|	281767-28298	202452	102304	-0.984713	0.6611	no
gi|320446840|ref|NW_003383731.1|	286115-28749	267552	134502	-0.992189	0.5425	no
gi|320446840|ref|NW_003383731.1|	310584-31309	68958	108239	0.650432	0.68495	no
gi|320446840|ref|NW_003383731.1|	313441-31382	67893	132157	-236101	0.29595	no

gi|320446840|ref|NW_003383731.1|	314346-31538	154076	481502	-167803	0.43515	no
gi|320446840|ref|NW_003383731.1|	315520-31660	35629	164365	-111614	0.5973	no
gi|320446840|ref|NW_003383731.1|	318407-31978	146855	93758	-0.647384	0.76725	no
gi|320446840|ref|NW_003383731.1|	320594-32208	648097	811681	-299722	0.1229	no
gi|320446840|ref|NW_003383731.1|	322243-32274	104601	425008	-129934	0.43245	no
gi|320446840|ref|NW_003383731.1|	323022-32779	163751	210355	0.361322	0.78605	no
gi|320446840|ref|NW_003383731.1|	338683-33976	0.199814	193549	327597	0.23005	no
gi|320446840|ref|NW_003383731.1|	353883-35496	0.200051	119033	589486	0.16005	no
gi|320446840|ref|NW_003383731.1|	397110-39758	166276	786038	-108091	0.59915	no
gi|320446840|ref|NW_003383731.1|	431697-43204	670656	697034	0.0556567	0.9322	no
gi|320446840|ref|NW_003383731.1|	434013-43457	139469	11161	-0.321471	0.87925	no
gi|320446840|ref|NW_003383731.1|	437402-43832	390454	471208	0.27121	0.885	no
gi|320446840|ref|NW_003383731.1|	443314-45272	267241	207683	-0.363756	0.7755	no
gi|320446840|ref|NW_003383731.1|	454186-45624	0.739019	0.644325	-0.197824	1	no
gi|320446840|ref|NW_003383731.1|	459766-46016	15978	157537	-0.0203873	0.975	no
gi|320446840|ref|NW_003383731.1|	466948-46742	734384	18495	-19894	0.38485	no
gi|320446840|ref|NW_003383731.1|	468920-47356	465657	241443	-0.947587	0.4786	no
gi|320446840|ref|NW_003383731.1|	479396-48091	166569	355735	-222724	0.1886	no
gi|320446840|ref|NW_003383731.1|	494494-49808	0.653276	6294	326821	0.08885	no
gi|320446840|ref|NW_003383731.1|	524945-52678	220424	519308	123631	0.56925	no
gi|320446840|ref|NW_003383731.1|	527873-52819	179651	12639	-0.507309	0.8021	no
gi|320446840|ref|NW_003383731.1|	528466-53165	246952	522114	108013	0.41905	no
gi|320446840|ref|NW_003383731.1|	544448-54500	264599	214196	-0.304877	0.8817	no
gi|320446840|ref|NW_003383731.1|	618550-62125	102568	143817	380959	0.06615	no
gi|320446840|ref|NW_003383731.1|	622444-62440	185362	117675	266639	0.25365	no
gi|320446840|ref|NW_003383731.1|	625473-62638	323324	942997	154427	0.4628	no
gi|320446840|ref|NW_003383731.1|	633239-63694	199267	462556	121493	0.57995	no
gi|320446840|ref|NW_003383731.1|	640154-64551	842031	813491	-0.0497462	0.9687	no
gi|320446840|ref|NW_003383731.1|	652281-65256	182909	72.95	199579	0.36705	no
gi|320446840|ref|NW_003383731.1|	659086-65988	0.298422	266454	315846	0.24575	no

gi|320446840|ref|NW_003383731.1|	567414-667648	119509	652717	244934	0.2954	no
gi|320446840|ref|NW_003383731.1|	568232-666868	539534	302159	248552	0.27795	no
gi|320446840|ref|NW_003383731.1|	570579-670920	964986	885604	319808	0.18965	no
gi|320446840|ref|NW_003383731.1|	584990-685727	370084	253461	-0.546087	0.7905	no
gi|320446840|ref|NW_003383731.1|	586236-686727	246798	204258	-0.272936	0.89365	no
gi|320446840|ref|NW_003383731.1|	586994-687448	976754	849599	-0.201214	0.914	no
gi|320446840|ref|NW_003383731.1|	589549-690608	11475	104874	-0.129835	0.95015	no
gi|320446840|ref|NW_003383731.1|	591923-692523	401246	384643	-0.0609688	0.97735	no
gi|320446840|ref|NW_003383731.1|	598074-701884	599455	669287	34809	0.0127	no
gi|320446840|ref|NW_003383731.1|	703248-703900	997062	62.51	264833	0.14195	no
gi|320446841|ref|NW_003383730.1|	100376-100627	0	340418	inf	0.02075	no
gi|320446841|ref|NW_003383730.1|	10435-11513	280404	45729	0.705604	0.7283	no
gi|320446841|ref|NW_003383730.1|	179102-179938	758765	0	#NAME?	0.0091	no
gi|320446841|ref|NW_003383730.1|	185245-185528	288524	170095	-0.762346	0.7106	no
gi|320446841|ref|NW_003383730.1|	186214-188742	772784	817373	0.0809286	0.9718	no
gi|320446841|ref|NW_003383730.1|	188844-190630	249327	475771	0.932225	0.6646	no
gi|320446841|ref|NW_003383730.1|	190857-192181	511557	6995	0.451429	0.82495	no
gi|320446841|ref|NW_003383730.1|	192459-194290	539341	156218	153429	0.35705	no
gi|320446841|ref|NW_003383730.1|	200209-202723	2041.43	1002.41	-102611	0.40205	no
gi|320446841|ref|NW_003383730.1|	211938-214531	695938	219888	-166219	0.41605	no
gi|320446841|ref|NW_003383730.1|	214670-216144	0.953683	0.662995	-0.524512	1	no
gi|320446841|ref|NW_003383730.1|	216783-217860	696873	454632	-0.616196	0.7611	no
gi|320446841|ref|NW_003383730.1|	230920-234249	578132	812605	0.491157	0.76145	no
gi|320446841|ref|NW_003383730.1|	237154-238000	193494	323278	0.740487	0.70945	no
gi|320446841|ref|NW_003383730.1|	241299-241757	103447	393464	-139459	0.5113	no
gi|320446841|ref|NW_003383730.1|	252033-252319	150902	110236	-0.453018	0.81865	no
gi|320446841|ref|NW_003383730.1|	303453-304205	555391	246307	-117305	0.5738	no
gi|320446841|ref|NW_003383730.1|	307941-308509	252238	647772	13607	0.5265	no
gi|320446841|ref|NW_003383730.1|	309144-310833	220563	387958	0.814708	0.69365	no
gi|320446841|ref|NW_003383730.1|	311769-318561	197371	180751	-0.126905	0.92545	no

gi|320446841|ref|NW_003383730.1|	319350-32036	202364	422491	106197	0.52275	no
gi|320446841|ref|NW_003383730.1|	334318-34077	39593	176208	215396	0.10835	no
gi|320446841|ref|NW_003383730.1|	340922-34183	15284	350588	119775	0.46905	no
gi|320446841|ref|NW_003383730.1|	347152-34816	11937	250454	10691	0.63445	no
gi|320446841|ref|NW_003383730.1|	348863-34909	358527	50209	0.485863	0.8073	no
gi|320446841|ref|NW_003383730.1|	350503-35124	152043	226474	0.574859	0.79275	no
gi|320446841|ref|NW_003383730.1|	359791-36035	174066	798793	219819	0.2006	no
gi|320446841|ref|NW_003383730.1|	363233-37230	148005	606761	203548	0.26965	no
gi|320446841|ref|NW_003383730.1|	396734-39878	779384	802453	0.042083	0.98445	no
gi|320446841|ref|NW_003383730.1|	399418-40194	626407	468414	-0.419316	0.84395	no
gi|320446841|ref|NW_003383730.1|	403903-40487	222247	680174	-170819	0.44025	no
gi|320446841|ref|NW_003383730.1|	41237-43175	564905	400726	-0.495392	0.81065	no
gi|320446841|ref|NW_003383730.1|	418281-41865	124347	258748	105717	0.601	no
gi|320446841|ref|NW_003383730.1|	419986-42037	421954	761006	0.850822	0.6944	no
gi|320446841|ref|NW_003383730.1|	422529-42283	166262	711863	-122379	0.5567	no
gi|320446841|ref|NW_003383730.1|	429869-43649	0.422157	30.48	617394	0.032	no
gi|320446841|ref|NW_003383730.1|	436943-43793	0.89107	281576	498184	0.1036	no
gi|320446841|ref|NW_003383730.1|	441258-44185	0	377215	inf	0.029	no
gi|320446841|ref|NW_003383730.1|	44612-45224	312749	242628	-0.366256	0.8498	no
gi|320446841|ref|NW_003383730.1|	465371-46598	0	763976	inf	0.01485	no
gi|320446841|ref|NW_003383730.1|	466509-46796	111617	648467	253848	0.17755	no
gi|320446841|ref|NW_003383730.1|	468335-47923	308439	126056	203101	0.13825	no
gi|320446841|ref|NW_003383730.1|	480274-48135	100026	844307	30774	0.20715	no
gi|320446841|ref|NW_003383730.1|	483270-48576	32138	381058	0.245727	0.9088	no
gi|320446841|ref|NW_003383730.1|	48488-49907	0	208044	inf	0.01575	no
gi|320446841|ref|NW_003383730.1|	57978-58161	0	891425	inf	0.02915	no
gi|320446841|ref|NW_003383730.1|	58643-59065	0	108913	inf	0.0212	no
gi|320446841|ref|NW_003383730.1|	61829-62306	0	237758	inf	0.0079	no
gi|320446841|ref|NW_003383730.1|	65940-66253	169959	103984	593503	0.15995	no
gi|320446841|ref|NW_003383730.1|	69111-71194	0.0913273	163085	748036	0.1408	no

gi|320446841|ref|NW_003383730.1|	71427-74809	0.053622	139914	80275	0.14075	no
gi|320446841|ref|NW_003383730.1|	85251-85870	100918	441095	212791	0.35185	no
gi|320446841|ref|NW_003383730.1|	88866-89078	286731	574334	100219	0.59555	no
gi|320446841|ref|NW_003383730.1|	99877-10023	734386	957499	0.382731	0.85025	no
gi|320446842|ref|NW_003383729.1|	132086-13289	0	12824	inf	0.0054	no
gi|320446842|ref|NW_003383729.1|	136412-13665	0	243899	inf	0.0294	no
gi|320446842|ref|NW_003383729.1|	272317-27300	144695	149509	0.0472187	0.98015	no
gi|320446842|ref|NW_003383729.1|	284406-28459	553465	40058	-0.466402	0.81465	no
gi|320446842|ref|NW_003383729.1|	57058-57483	0	135715	inf	0.0138	no
gi|320446842|ref|NW_003383729.1|	83787-84289	436851	154761	182483	0.39295	no
gi|320446842|ref|NW_003383729.1|	87732-88096	106777	309754	-178541	0.429	no
gi|320446842|ref|NW_003383729.1|	89457-89741	285719	0	#NAME?	0.01755	no
gi|320446842|ref|NW_003383729.1|	93401-93768	465939	304348	-393635	0.18555	no
gi|320446844|ref|NW_003383727.1|	132627-13503	13986	213222	0.608377	0.63285	no
gi|320446844|ref|NW_003383727.1|	135154-13630	184315	10.98	-0.747303	0.735	no
gi|320446844|ref|NW_003383727.1|	136719-13930	157136	887198	-0.824689	0.50635	no
gi|320446844|ref|NW_003383727.1|	141143-14213	201022	647923	-163346	0.4546	no
gi|320446844|ref|NW_003383727.1|	144902-14556	593709	354253	-0.744975	0.6474	no
gi|320446844|ref|NW_003383727.1|	145925-14740	218641	123679	-0.821958	0.6118	no
gi|320446844|ref|NW_003383727.1|	150235-15081	199252	0	#NAME?	0.0076	no
gi|320446844|ref|NW_003383727.1|	152131-15348	176347	115136	-393701	0.14185	no
gi|320446844|ref|NW_003383727.1|	154143-15521	66809	0.980327	-276871	0.24185	no
gi|320446844|ref|NW_003383727.1|	156011-16111	193989	165018	-355528	0.07985	no
gi|320446844|ref|NW_003383727.1|	163877-16477	0.25357	143287	582038	0.1611	no
gi|320446844|ref|NW_003383727.1|	166009-16661	252835	82719	-161191	0.4547	no
gi|320446844|ref|NW_003383727.1|	215200-21946	402682	139738	179501	0.3142	no
gi|320446844|ref|NW_003383727.1|	219570-22202	380268	632034	0.732986	0.7375	no
gi|320446844|ref|NW_003383727.1|	222220-22347	479266	60162	0.328026	0.80165	no
gi|320446844|ref|NW_003383727.1|	246637-24869	112762	116042	0.0413586	0.97895	no
gi|320446844|ref|NW_003383727.1|	324807-32669	118514	116781	-0.0212445	0.99215	no

gi|320446844|ref|NW_003383727.1|	327454-32832	845744	837137	-0.0147585	0.9865	no
gi|320446844|ref|NW_003383727.1|	328661-33105	209296	107969	-0.954931	0.4537	no
gi|320446844|ref|NW_003383727.1|	331192-33217	901786	591607	-0.608146	0.76895	no
gi|320446844|ref|NW_003383727.1|	336562-33901	533819	447345	-0.254964	0.906	no
gi|320446844|ref|NW_003383727.1|	339994-34250	170317	181024	0.087963	0.9598	no
gi|320446844|ref|NW_003383727.1|	343564-34486	9791	416504	-123313	0.564	no
gi|320446844|ref|NW_003383727.1|	345465-34593	356147	223172	264761	0.25495	no
gi|320446844|ref|NW_003383727.1|	346539-34748	0.238852	444902	42193	0.1914	no
gi|320446844|ref|NW_003383727.1|	352320-35318	428239	20636	226867	0.31135	no
gi|320446844|ref|NW_003383727.1|	354750-35661	307063	163385	-0.91026	0.47535	no
gi|320446844|ref|NW_003383727.1|	358065-35948	666377	338106	-0.978862	0.46065	no
gi|320446844|ref|NW_003383727.1|	359690-36036	426723	105838	-201145	0.3624	no
gi|320446844|ref|NW_003383727.1|	360848-36184	168857	306936	-245979	0.28185	no
gi|320446844|ref|NW_003383727.1|	364480-36514	664017	186449	-183244	0.39295	no
gi|320446844|ref|NW_003383727.1|	366267-36819	527743	360955	-0.548015	0.7947	no
gi|320446844|ref|NW_003383727.1|	379456-38042	0.690174	412921	258084	0.28405	no
gi|320446844|ref|NW_003383727.1|	415565-41585	403751	310298	294212	0.218	no
gi|320446844|ref|NW_003383727.1|	416102-41678	0.37428	690036	420448	0.19175	no
gi|320446844|ref|NW_003383727.1|	417906-42284	0.0716962	276218	526777	0.1455	no
gi|320446844|ref|NW_003383727.1|	423401-42704	158586	24691	396064	0.05565	no
gi|320446844|ref|NW_003383727.1|	441261-44286	123207	102884	-0.260065	1	no
gi|320446844|ref|NW_003383727.1|	449209-45054	0.459563	180812	197615	0.39	no
gi|320446844|ref|NW_003383727.1|	452813-45373	147487	0.677968	-11213	0.5884	no
gi|320446844|ref|NW_003383727.1|	457357-45977	564091	474835	-0.248502	0.91105	no
gi|320446844|ref|NW_003383727.1|	462460-46364	566895	257837	-113662	0.5856	no
gi|320446844|ref|NW_003383727.1|	472601-47303	208765	276503	-291651	0.2371	no
gi|320446844|ref|NW_003383727.1|	477015-47754	75942	326447	-121805	0.45865	no
gi|320446844|ref|NW_003383727.1|	558757-55923	0	49924	inf	0.0294	no
gi|320446844|ref|NW_003383727.1|	560799-56184	188074	21673	0.204595	0.916	no
gi|320446844|ref|NW_003383727.1|	565955-56636	153199	151101	330204	0.0838	no

gi|320446844|ref|NW_003383727.1|	68315-68589	121554	170366	0.487039	0.81065	no
gi|320446844|ref|NW_003383727.1|	86504-87405	177499	332007	0.903404	0.6645	no
gi|320446844|ref|NW_003383727.1|	96849-97523	230656	629472	14484	0.49435	no
gi|320446844|ref|NW_003383727.1|	98035-98517	403052	583552	0.533896	0.79375	no
gi|320446844|ref|NW_003383727.1|	98740-10102	100241	210488	107026	0.5293	no
gi|320446845|ref|NW_003383726.1|	11094-11432	0	584155	inf	0.00715	no
gi|320446845|ref|NW_003383726.1|	22296-12304	0.66365	52288	297798	0.2201	no
gi|320446845|ref|NW_003383726.1|	12948-14550	0	339396	inf	5.00E-05	yes
gi|320446845|ref|NW_003383726.1|	78990-17947	198502	309696	0.641702	0.74155	no
gi|320446845|ref|NW_003383726.1|	79584-18646	106599	443035	205523	0.23375	no
gi|320446845|ref|NW_003383726.1|	86595-18729	110102	300902	145045	0.50735	no
gi|320446845|ref|NW_003383726.1|	21125-21714	0	106222	inf	0.00945	no
gi|320446845|ref|NW_003383726.1|	14694-21569	308615	852795	146639	0.48845	no
gi|320446845|ref|NW_003383726.1|	34071-23431	292534	20774	-0.493827	0.7989	no
gi|320446845|ref|NW_003383726.1|	34818-23557	355934	266192	-0.419145	0.8274	no
gi|320446845|ref|NW_003383726.1|	23871-26183	0	180376	inf	5.00E-05	yes
gi|320446845|ref|NW_003383726.1|	47878-24823	349314	536362	0.618686	0.7671	no
gi|320446845|ref|NW_003383726.1|	28273-28749	137487	45893	173898	0.3346	no
gi|320446845|ref|NW_003383726.1|	13706-31433	385988	116619	-172676	0.4381	no
gi|320446845|ref|NW_003383726.1|	22045-32304	154712	0.916064	-0.756061	0.71485	no
gi|320446845|ref|NW_003383726.1|	36044-36824	0	221194	inf	0.0059	no
gi|320446845|ref|NW_003383726.1|	98343-49950	274375	202749	-0.436451	0.82485	no
gi|320446845|ref|NW_003383726.1|	99568-50036	181298	145233	-0.319986	0.87815	no
gi|320446845|ref|NW_003383726.1|	01612-50227	198023	21596	0.125098	0.9286	no
gi|320446845|ref|NW_003383726.1|	76821-57743	495336	519504	0.0687272	0.96065	no
gi|320446845|ref|NW_003383726.1|	01195-60211	368181	456975	0.3117	0.8794	no
gi|320446845|ref|NW_003383726.1|	04553-60518	46592	288059	-0.693717	0.73585	no
gi|320446845|ref|NW_003383726.1|	55014-65607	0.20392	141047	27901	1	no
gi|320446845|ref|NW_003383726.1|	56483-65793	0.138591	366055	472316	0.1782	no
gi|320446845|ref|NW_003383726.1|	58686-66133	650958	145244	115784	0.492	no

gi|320446845|ref|NW_003383726.1|	561773-66279	537063	460254	-0.222662	0.9095	no
gi|320446845|ref|NW_003383726.1|	562984-66419	104321	144638	0.471417	0.81615	no
gi|320446845|ref|NW_003383726.1|	564472-66507	635434	246344	-136707	0.51635	no
gi|320446845|ref|NW_003383726.1|	565458-66571	25238	534047	-224056	0.27085	no
gi|320446845|ref|NW_003383726.1|	565942-66726	654078	140538	-22185	0.3247	no
gi|320446845|ref|NW_003383726.1|	567375-66808	932068	220588	-207908	0.33395	no
gi|320446845|ref|NW_003383726.1|	568798-66943	117723	286006	-204127	0.352	no
gi|320446845|ref|NW_003383726.1|	574359-67610	427009	110059	-195599	0.28105	no
gi|320446845|ref|NW_003383726.1|	576584-67692	842362	138787	-260157	0.2567	no
gi|320446845|ref|NW_003383726.1|	577051-67781	51088	142327	-184377	0.2756	no
gi|320446845|ref|NW_003383726.1|	577981-67879	0.876657	140558	0.681078	1	no
gi|320446845|ref|NW_003383726.1|	578908-68094	25246	210637	-0.261299	0.8382	no
gi|320446845|ref|NW_003383726.1|	586453-68839	258364	577616	116071	0.38065	no
gi|320446845|ref|NW_003383726.1|	589570-69161	0.465969	175494	191312	0.39055	no
gi|320446845|ref|NW_003383726.1|	592187-69298	0.597901	184788	162789	0.33265	no
gi|320446845|ref|NW_003383726.1|	594246-69487	235882	233237	-0.0162648	0.99195	no
gi|320446845|ref|NW_003383726.1|	73598-74641	0.208971	21673	337452	0.2261	no
gi|320446845|ref|NW_003383726.1|	750538-75218	572577	270688	224109	0.2046	no
gi|320446845|ref|NW_003383726.1|	754944-75522	307697	561629	0.868106	0.67485	no
gi|320446845|ref|NW_003383726.1|	9994-10528	0	297768	inf	0.0059	no
gi|320446846|ref|NW_003383725.1|	103776-10524	411713	150732	187227	0.4115	no
gi|320446846|ref|NW_003383725.1|	137917-14064	270879	553666	103137	0.6366	no
gi|320446846|ref|NW_003383725.1|	144634-14537	301002	251998	-0.25636	0.9026	no
gi|320446846|ref|NW_003383725.1|	150831-15189	284799	0.562848	-233913	0.30915	no
gi|320446846|ref|NW_003383725.1|	153275-15405	308232	0.846193	-186496	0.40745	no
gi|320446846|ref|NW_003383725.1|	168305-16873	164732	600347	186567	0.32465	no
gi|320446846|ref|NW_003383725.1|	177411-17849	119464	840381	281447	0.22295	no
gi|320446846|ref|NW_003383725.1|	179017-18051	173745	576172	172953	0.4166	no
gi|320446846|ref|NW_003383725.1|	180664-18102	120119	21942	419116	0.19035	no
gi|320446846|ref|NW_003383725.1|	182726-18380	0	194457	inf	0.0233	no

gi|320446846|ref|NW_003383725.1|	190722-19305	122212	926162	-0.400055	0.8053	no
gi|320446846|ref|NW_003383725.1|	196392-19864	819993	648233	-0.339099	0.87635	no
gi|320446846|ref|NW_003383725.1|	201354-20165	837224	199739	-20675	0.3383	no
gi|320446846|ref|NW_003383725.1|	203026-20434	118466	411179	-152664	0.4814	no
gi|320446846|ref|NW_003383725.1|	210684-21105	102345	914515	315956	0.2035	no
gi|320446846|ref|NW_003383725.1|	213480-21439	0.247979	205151	304839	0.2493	no
gi|320446846|ref|NW_003383725.1|	214545-21674	0.171775	125864	287327	1	no
gi|320446846|ref|NW_003383725.1|	216848-22512	0.797368	688657	311047	0.10705	no
gi|320446846|ref|NW_003383725.1|	226010-22735	173063	121753	281459	0.15615	no
gi|320446846|ref|NW_003383725.1|	227469-23108	192325	196549	335328	0.09715	no
gi|320446846|ref|NW_003383725.1|	265291-26566	575429	120335	106435	0.59255	no
gi|320446846|ref|NW_003383725.1|	270217-27167	88047	112844	0.357987	0.86425	no
gi|320446846|ref|NW_003383725.1|	271822-27311	117742	124778	0.0837376	0.96835	no
gi|320446846|ref|NW_003383725.1|	278901-27938	328369	0	#NAME?	0.0081	no
gi|320446846|ref|NW_003383725.1|	283530-28509	124316	0.440802	-813966	0.11235	no
gi|320446846|ref|NW_003383725.1|	285542-28667	108617	0.259827	-870749	0.16165	no
gi|320446846|ref|NW_003383725.1|	288145-29075	802941	724762	-0.147786	0.9493	no
gi|320446846|ref|NW_003383725.1|	291056-29138	195256	39833	10286	0.60405	no
gi|320446846|ref|NW_003383725.1|	304972-30520	171822	486566	150172	0.49765	no
gi|320446846|ref|NW_003383725.1|	311764-31364	575972	219347	192914	0.26405	no
gi|320446846|ref|NW_003383725.1|	341615-34265	0.628471	825586	37155	0.1823	no
gi|320446846|ref|NW_003383725.1|	343662-34841	148513	422607	150873	0.26305	no
gi|320446846|ref|NW_003383725.1|	348914-34917	25238	195817	-0.366087	0.85475	no
gi|320446846|ref|NW_003383725.1|	351318-35200	925514	42988	-110632	0.5906	no
gi|320446846|ref|NW_003383725.1|	377595-37830	932068	563724	-0.725447	0.7222	no
gi|320446846|ref|NW_003383725.1|	389369-39044	118451	106951	-0.147343	0.94575	no
gi|320446846|ref|NW_003383725.1|	39648-40654	304421	105919	-484504	0.086	no
gi|320446846|ref|NW_003383725.1|	397381-39796	0	121689	inf	0.0103	no
gi|320446846|ref|NW_003383725.1|	410443-41146	0.214008	399365	422197	0.1914	no
gi|320446846|ref|NW_003383725.1|	413905-41457	0	341711	inf	0.02915	no

gi|320446846|ref|NW_003383725.1|	54869-60538	311447	609244	-235389	0.0841	no
gi|320446846|ref|NW_003383725.1|	57718-55897	660099	0.915815	-284956	0.2221	no
gi|320446846|ref|NW_003383725.1|	60653-65374	0.564216	337209	257932	0.26535	no
gi|320446846|ref|NW_003383725.1|	11607-61677	41055	261251	-0.652121	0.77305	no
gi|320446846|ref|NW_003383725.1|	45593-74859	0.0609437	191739	497552	0.16815	no
gi|320446846|ref|NW_003383725.1|	74883-75851	191209	16698	-0.195469	0.9273	no
gi|320446846|ref|NW_003383725.1|	49592-75115	0	141583	inf	0.0198	no
gi|320446846|ref|NW_003383725.1|	76372-77391	926107	759094	-0.2869	0.8889	no
gi|320446846|ref|NW_003383725.1|	78367-79444	22.86	190067	-0.266319	0.90555	no
gi|320446846|ref|NW_003383725.1|	81793-84035	416244	667876	0.68215	0.61305	no
gi|320446847|ref|NW_003383724.1|	47242-14778	238681	182795	-370679	0.1766	no
gi|320446847|ref|NW_003383724.1|	49212-14971	126162	169042	-289982	0.2547	no
gi|320446847|ref|NW_003383724.1|	50294-15279	24226	317579	-293137	0.11545	no
gi|320446847|ref|NW_003383724.1|	55959-15624	599242	240968	-131429	0.53875	no
gi|320446847|ref|NW_003383724.1|	70750-71703	141113	0	#NAME?	0.0051	no
gi|320446848|ref|NW_003383723.1|	01222-10157	64013	499881	-0.356781	0.8613	no
gi|320446848|ref|NW_003383723.1|	05565-10629	447137	376684	-0.247363	0.89775	no
gi|320446848|ref|NW_003383723.1|	13003-11690	171229	361159	107671	0.4224	no
gi|320446848|ref|NW_003383723.1|	17009-11872	697092	692313	-0.00992424	0.99225	no
gi|320446848|ref|NW_003383723.1|	19110-12117	249146	199524	-0.320432	0.87785	no
gi|320446848|ref|NW_003383723.1|	15022-15975	635011	0.649184	-329008	0.2156	no
gi|320446848|ref|NW_003383723.1|	02/77	802071	112593	0.489312	0.70035	no
gi|320446848|ref|NW_003383723.1|	13371-21478	0.143056	504957	846343	0.14075	no
gi|320446848|ref|NW_003383723.1|	14920-21586	0	903768	inf	0.00685	no
gi|320446848|ref|NW_003383723.1|	16603-21785	0	918477	inf	0.0077	no
gi|320446848|ref|NW_003383723.1|	19345-21986	0	212069	inf	0.0071	no
gi|320446848|ref|NW_003383723.1|	23535-23800	158095	102173	-395171	0.1569	no
gi|320446848|ref|NW_003383723.1|	53194-25368	154729	237344	0.61723	0.76535	no
gi|320446848|ref|NW_003383723.1|	55628-25762	373992	448019	0.260553	0.8983	no
gi|320446848|ref|NW_003383723.1|	60591-26596	101707	237225	122184	0.36695	no

gi|320446848|ref|NW_003383723.1|	66130-26958	0.41964	102753	129195	1	no
gi|320446848|ref|NW_003383723.1|	84791-28512	15931	393985	13063	0.53205	no
gi|320446848|ref|NW_003383723.1|	89761-29025	20783	378095	0.863346	0.68	no
gi|320446848|ref|NW_003383723.1|	08812-30931	309829	220179	-0.492794	0.8074	no
gi|320446848|ref|NW_003383723.1|	10079-31287	129053	135751	0.072997	0.9575	no
gi|320446848|ref|NW_003383723.1|	22154-32308	313444	117302	-141798	0.38285	no
gi|320446848|ref|NW_003383723.1|	23273-32438	198067	111824	-0.824756	0.7193	no
gi|320446848|ref|NW_003383723.1|	24568-32592	167155	115034	-0.539122	0.8112	no
gi|320446848|ref|NW_003383723.1|	4721-5350	124063	785274	-0.659802	0.7485	no
gi|320446848|ref|NW_003383723.1|	71280-76240	614238	762967	0.312822	0.80445	no
gi|320446848|ref|NW_003383723.1|	85095-86465	206736	271679	0.394113	0.7547	no
gi|320446848|ref|NW_003383723.1|	87219-89255	248335	17973	-0.466457	0.7136	no
gi|320446848|ref|NW_003383723.1|	95659-96343	714275	182231	135122	0.52655	no
gi|320446848|ref|NW_003383723.1|	99705-100171	214549	381333	0.829746	0.69235	no
gi|320446849|ref|NW_003383722.1|	18183-22348	656286	9196	0.486683	0.7051	no
gi|320446849|ref|NW_003383722.1|	34815-23539	19774	46808	124315	0.55875	no
gi|320446849|ref|NW_003383722.1|	50800-25153	473839	440352	-0.105738	0.953	no
gi|320446849|ref|NW_003383722.1|	37592-33984	0.921771	719376	296426	0.21725	no
gi|320446849|ref|NW_003383722.1|	47264-55058	102355	103372	0.0142673	0.99145	no
gi|320446849|ref|NW_003383722.1|	51117-55308	180936	103094	-0.811516	0.62525	no
gi|320446849|ref|NW_003383722.1|	55791-55712	116954	662403	-0.820154	0.70445	no
gi|320446849|ref|NW_003383722.1|	08775-61033	178216	38079	109537	0.59825	no
gi|320446849|ref|NW_003383722.1|	11300-61178	0	710435	inf	0.0198	no
gi|320446849|ref|NW_003383722.1|	51148-65212	410744	0.945294	-21194	0.34725	no
gi|320446849|ref|NW_003383722.1|	60155-66248	475738	602314	0.340349	0.87645	no
gi|320446849|ref|NW_003383722.1|	71915-67259	380134	233498	-0.703098	0.72735	no
gi|320446849|ref|NW_003383722.1|	07237-70866	0.282047	254809	317541	0.20465	no
gi|320446849|ref|NW_003383722.1|	32239-73383	0.124205	207419	406176	0.1924	no
gi|320446850|ref|NW_003383721.1|	71261-27206	0.296325	305334	336514	0.22635	no
gi|320446850|ref|NW_003383721.1|	72140-27512	0.735576	638543	311784	0.19625	no

gi|320446850|ref|NW_003383721.1|	279029-27945	11164	397628	-148936	0.48175	no
gi|320446850|ref|NW_003383721.1|	281623-28219	551696	101707	-243945	0.24535	no
gi|320446850|ref|NW_003383721.1|	282380-28307	43946	200182	-113442	0.5854	no
gi|320446850|ref|NW_003383721.1|	283992-28579	473023	164777	-15214	0.4735	no
gi|320446850|ref|NW_003383721.1|	286882-28769	523275	31965	-0.711077	0.72355	no
gi|320446850|ref|NW_003383721.1|	290132-29226	374058	789116	107698	0.62305	no
gi|320446850|ref|NW_003383721.1|	294147-29668	270896	3631	0.422627	0.84325	no
gi|320446850|ref|NW_003383721.1|	306820-30744	465904	917111	-234486	0.3131	no
gi|320446850|ref|NW_003383721.1|	307624-31075	598025	160437	-18982	0.16705	no
gi|320446850|ref|NW_003383721.1|	312939-31709	155669	188764	0.278102	0.8315	no
gi|320446850|ref|NW_003383721.1|	317324-31755	205185	129232	-0.666958	0.74775	no
gi|320446850|ref|NW_003383721.1|	317906-31865	560805	248648	-11734	0.5738	no
gi|320446850|ref|NW_003383721.1|	319952-32057	405175	216794	-0.90222	0.68145	no
gi|320446850|ref|NW_003383721.1|	320685-32611	981659	797122	-0.300422	0.8204	no
gi|320446850|ref|NW_003383721.1|	341639-34196	264401	102216	-137111	0.4105	no
gi|320446850|ref|NW_003383721.1|	343634-34420	256532	577959	-21501	0.33555	no
gi|320446850|ref|NW_003383721.1|	344299-34473	827428	433048	-0.934107	0.6735	no
gi|320446850|ref|NW_003383721.1|	345121-34621	217669	168288	-0.371203	0.87185	no
gi|320446850|ref|NW_003383721.1|	358295-35965	149921	635114	208282	0.3405	no
gi|320446850|ref|NW_003383721.1|	360075-36612	406165	161034	198723	0.139	no
gi|320446850|ref|NW_003383721.1|	366499-36738	233118	802908	178417	0.4001	no
gi|320446850|ref|NW_003383721.1|	403715-40497	0.494591	160113	169478	0.4291	no
gi|320446850|ref|NW_003383721.1|	431338-43383	211233	876819	-126849	0.3256	no
gi|320446850|ref|NW_003383721.1|	435710-43608	170695	924101	-0.885299	0.5846	no
gi|320446850|ref|NW_003383721.1|	436955-44047	124941	143533	0.200141	0.8779	no
gi|320446850|ref|NW_003383721.1|	440583-44201	618951	469252	-0.399461	0.84795	no
gi|320446850|ref|NW_003383721.1|	442302-44876	266903	153275	-0.800193	0.5538	no
gi|320446850|ref|NW_003383721.1|	449100-45069	433122	955826	114197	0.5968	no
gi|320446850|ref|NW_003383721.1|	452769-45367	500372	82777	0.726229	0.72995	no
gi|320446850|ref|NW_003383721.1|	454233-45547	122627	132813	0.115118	0.9608	no

gi|320446850|ref|NW_003383721.1|	159867-46083	230687	493652	109756	0.59595	no
gi|320446850|ref|NW_003383721.1|	166800-467450	0.801398	245716	16164	0.33275	no
gi|320446850|ref|NW_003383721.1|	173075-47501	240828	313672	0.381253	0.77365	no
gi|320446850|ref|NW_003383721.1|	176333-47655	488787	398906	-0.293158	0.8828	no
gi|320446850|ref|NW_003383721.1|	177615-47792	101976	766199	-0.412433	0.84675	no
gi|320446850|ref|NW_003383721.1|	178044-47881	211929	151875	-0.480699	0.82185	no
gi|320446850|ref|NW_003383721.1|	180974-49006	128074	720486	-0.829935	0.5395	no
gi|320446850|ref|NW_003383721.1|	193184-49392	723346	735038	0.0231329	0.98585	no
gi|320446850|ref|NW_003383721.1|	194730-49539	807932	863839	0.0965288	0.94015	no
gi|320446850|ref|NW_003383721.1|	199616-50065	112845	110966	-0.0242241	0.98535	no
gi|320446850|ref|NW_003383721.1|	501684-50300	309207	157184	-0.976117	0.5589	no
gi|320446850|ref|NW_003383721.1|	509321-51219	230033	138013	-0.737042	0.573	no
gi|320446850|ref|NW_003383721.1|	529078-53205	450092	278456	262916	0.1619	no
gi|320446850|ref|NW_003383721.1|	534116-54144	135592	680471	232726	0.20215	no
gi|320446850|ref|NW_003383721.1|	541693-55080	121591	546325	216773	0.22665	no
gi|320446850|ref|NW_003383721.1|	554998-55820	685798	247878	185378	0.28305	no
gi|320446850|ref|NW_003383721.1|	590321-59090	11598	261688	-214796	0.3284	no
gi|320446850|ref|NW_003383721.1|	530730-63155	54299	0.785876	-278855	0.26055	no
gi|320446850|ref|NW_003383721.1|	532462-63352	165175	253884	-270175	0.23845	no
gi|320446850|ref|NW_003383721.1|	534185-63496	991772	170149	-254321	0.2663	no
gi|320446850|ref|NW_003383721.1|	536116-63659	164984	275358	-258294	0.2761	no
gi|320446850|ref|NW_003383721.1|	537219-63780	913047	13014	-281062	0.24885	no
gi|320446850|ref|NW_003383721.1|	538129-63925	999809	209007	-22581	0.2971	no
gi|320446850|ref|NW_003383721.1|	539894-64221	168685	407619	-204904	0.24025	no
gi|320446850|ref|NW_003383721.1|	542809-64499	234667	882194	-141145	0.41715	no
gi|320446850|ref|NW_003383721.1|	545128-64536	283004	764127	-188894	0.3088	no
gi|320446850|ref|NW_003383721.1|	546875-64737	337973	149458	-117716	0.57555	no
gi|320446850|ref|NW_003383721.1|	548006-64888	949974	690487	-0.460274	0.82205	no
gi|320446850|ref|NW_003383721.1|	549870-65048	424484	174153	-128535	0.5574	no
gi|320446850|ref|NW_003383721.1|	550720-65106	353524	238351	-0.568718	0.7833	no

gi|320446850|ref|NW_003383721.1|	i51278-65204ɛ	265206	184083	-0.526756	0.8074	no
gi|320446850|ref|NW_003383721.1|	i53203-65776(	276807	384418	0.473797	0.72255	no
gi|320446850|ref|NW_003383721.1|	'53965-75456⁄	0.92489	376236	202428	0.3028	no
gi|320446850|ref|NW_003383721.1|	'67993-76974⁄	0.89069	147336	0.726116	0.721	no
gi|320446850|ref|NW_003383721.1|	'70371-77125ɛ	466236	121328	137978	0.52135	no
gi|320446850|ref|NW_003383721.1|	'91791-79745:	780398	237257	160417	0.23535	no
gi|320446850|ref|NW_003383721.1|	301499-80240⁄	327671	555891	0.762554	0.7072	no
gi|320446850|ref|NW_003383721.1|	328978-83019(	137396	27388	0.995208	0.64705	no
gi|320446850|ref|NW_003383721.1|	333599-83397!	132503	722808	-0.874337	0.67995	no
gi|320446850|ref|NW_003383721.1|	368329-87044⁄	142738	901839	-0.662426	0.6885	no
gi|320446850|ref|NW_003383721.1|	371024-87231:	128256	100127	-0.357194	1	no
gi|320446850|ref|NW_003383721.1|	372418-87320:	552799	316194	-0.805943	0.6999	no
gi|320446850|ref|NW_003383721.1|	374312-87597	0.23607	0.986035	206242	1	no
gi|320446850|ref|NW_003383721.1|	908242-90846!	576157	222989	-136949	0.51185	no
gi|320446850|ref|NW_003383721.1|	914183-91460:	367615	636348	-253031	0.27455	no
gi|320446850|ref|NW_003383721.1|	914661-91523(	173023	0.334343	-569349	0.27345	no
gi|320446850|ref|NW_003383721.1|	915554-91653(	339076	0.312193	-676303	0.1617	no
gi|320446850|ref|NW_003383721.1|	916784-92195(	616226	908665	0.56029	0.65575	no
gi|320446850|ref|NW_003383721.1|	922218-92290(	26.83	992483	-143474	0.5071	no
gi|320446850|ref|NW_003383721.1|	923026-92538!	14461	887586	-0.704213	0.6708	no
gi|320446850|ref|NW_003383721.1|	926530-93147(	134674	732612	-0.878354	0.5045	no
gi|320446850|ref|NW_003383721.1|	931590-93307ɛ	0.538869	121765	117609	1	no
gi|320446850|ref|NW_003383721.1|	933250-93425!	493038	76875	-268111	0.13425	no
gi|320446850|ref|NW_003383721.1|	943328-94703:	107016	211053	0.979775	0.4599	no
gi|320446850|ref|NW_003383721.1|	950505-95120ɛ	122663	813828	-0.591901	0.76845	no
gi|320446850|ref|NW_003383721.1|	951713-95260:	206582	122743	-0.751073	0.72755	no
gi|320446850|ref|NW_003383721.1|	954871-95727	101834	553654	-0.879157	0.58275	no
gi|320446850|ref|NW_003383721.1|	963744-96499:	366641	277424	-0.402273	0.8433	no
gi|320446850|ref|NW_003383721.1|	969510-97143!	677504	145856	-221569	0.3261	no
gi|320446850|ref|NW_003383721.1|	973027-97378⁄	119723	42147	-15062	0.4816	no

gi|320446850|ref|NW_003383721.1|	975346-978509	186733	201522	-321196	0.0929	no
gi|320446850|ref|NW_003383721.1|	980656-981421	127479	415267	-161815	0.4522	no
gi|320446850|ref|NW_003383721.1|	982250-983305	123546	0.99682	-0.309649	1	no
gi|320446850|ref|NW_003383721.1|	984873-985451	190069	122021	-0.639392	0.75615	no
gi|320446850|ref|NW_003383721.1|	993097-994359	116711	147107	0.333922	0.8792	no
gi|320446851|ref|NW_003383720.1|	140505-143644	912327	27.47	159024	0.23255	no
gi|320446851|ref|NW_003383720.1|	146279-146727	308305	107548	180255	0.42155	no
gi|320446851|ref|NW_003383720.1|	149653-150383	204709	467164	119035	0.57025	no
gi|320446851|ref|NW_003383720.1|	153507-153782	168412	153316	-0.135493	0.93375	no
gi|320446851|ref|NW_003383720.1|	21029-21408	54261	348337	26825	0.2591	no
gi|320446851|ref|NW_003383720.1|	230768-231209	127141	110738	-0.199281	0.91625	no
gi|320446851|ref|NW_003383720.1|	231392-232147	354053	187032	-0.920678	0.67875	no
gi|320446851|ref|NW_003383720.1|	232612-235941	722665	473066	-0.611285	0.64345	no
gi|320446851|ref|NW_003383720.1|	23678-25178	0	176292	inf	0.0212	no
gi|320446851|ref|NW_003383720.1|	289761-294638	257722	44863	0.799708	0.546	no
gi|320446851|ref|NW_003383720.1|	29271-32524	0.950546	133747	381461	0.0587	no
gi|320446851|ref|NW_003383720.1|	297327-300732	152246	217105	0.511991	0.696	no
gi|320446851|ref|NW_003383720.1|	3138-4158	537748	208117	-136953	0.51185	no
gi|320446851|ref|NW_003383720.1|	319030-319690	168343	149491	-0.171346	0.93535	no
gi|320446851|ref|NW_003383720.1|	320462-321388	0.733162	134822	0.878855	1	no
gi|320446851|ref|NW_003383720.1|	323293-324371	197816	266822	0.431717	0.79135	no
gi|320446851|ref|NW_003383720.1|	325096-326078	160496	251315	0.646959	0.77895	no
gi|320446851|ref|NW_003383720.1|	327096-327602	504548	55705	0.142814	0.9496	no
gi|320446851|ref|NW_003383720.1|	339382-341540	127083	887311	-0.518254	0.69345	no
gi|320446851|ref|NW_003383720.1|	385905-387120	0.684886	0.831042	0.279058	1	no
gi|320446851|ref|NW_003383720.1|	387232-388017	0.30711	189717	262702	0.26355	no
gi|320446851|ref|NW_003383720.1|	397022-397608	364201	107077	-176608	0.4152	no
gi|320446851|ref|NW_003383720.1|	404421-404831	182023	0.600545	-492171	0.2886	no
gi|320446851|ref|NW_003383720.1|	418979-420670	193957	466238	-205659	0.2298	no
gi|320446851|ref|NW_003383720.1|	420819-424491	235527	101637	-121247	0.3578	no

gi|320446851|ref|NW_003383720.1|	429985-43029	101157	108608	0.102538	0.93965	no
gi|320446851|ref|NW_003383720.1|	432630-43396	22971	63149	-186298	0.27055	no
gi|320446851|ref|NW_003383720.1|	43522-44201	11404	311331	144891	0.50735	no
gi|320446851|ref|NW_003383720.1|	435440-43623	130867	459666	-150945	0.4812	no
gi|320446851|ref|NW_003383720.1|	437833-43924	443474	397604	-0.157519	0.9397	no
gi|320446851|ref|NW_003383720.1|	440085-44078	7937	567214	-0.484701	0.8155	no
gi|320446851|ref|NW_003383720.1|	456897-45803	0.373528	129346	179195	1	no
gi|320446851|ref|NW_003383720.1|	458174-45886	0.740411	278157	19095	0.3194	no
gi|320446851|ref|NW_003383720.1|	459149-45953	45.36	359748	-0.334434	0.87365	no
gi|320446851|ref|NW_003383720.1|	459672-46146	140563	128056	-0.134439	0.9527	no
gi|320446851|ref|NW_003383720.1|	461616-46318	798358	114609	0.521607	0.8054	no
gi|320446851|ref|NW_003383720.1|	463755-46605	151865	297514	0.970162	0.4611	no
gi|320446851|ref|NW_003383720.1|	46636-50050	0.318493	196812	262748	0.2525	no
gi|320446851|ref|NW_003383720.1|	471356-47179	158927	896452	249586	0.254	no
gi|320446851|ref|NW_003383720.1|	475880-47723	0	240951	inf	0.01485	no
gi|320446851|ref|NW_003383720.1|	479629-48031	0	361653	inf	0.0233	no
gi|320446851|ref|NW_003383720.1|	482342-48428	0.29697	489973	404431	0.16905	no
gi|320446851|ref|NW_003383720.1|	484994-48582	0.567722	936838	404454	0.17195	no
gi|320446851|ref|NW_003383720.1|	485990-48881	157679	293276	0.895264	0.4962	no
gi|320446851|ref|NW_003383720.1|	490378-49110	708233	648088	-0.128036	0.9212	no
gi|320446851|ref|NW_003383720.1|	491241-49211	529264	221564	-125626	0.45275	no
gi|320446851|ref|NW_003383720.1|	495725-49774	418153	206502	-101788	0.4457	no
gi|320446851|ref|NW_003383720.1|	497858-49923	384666	392553	0.0292809	0.9832	no
gi|320446851|ref|NW_003383720.1|	50173-51666	0	28922	inf	0.01195	no
gi|320446851|ref|NW_003383720.1|	515712-51658	0	125378	inf	0.00495	no
gi|320446851|ref|NW_003383720.1|	518856-51963	0	894887	inf	0.0069	no
gi|320446851|ref|NW_003383720.1|	6105-6402	116822	873856	-0.418844	0.82045	no
gi|320446851|ref|NW_003383720.1|	6453-6792	698931	997265	0.512827	0.80935	no
gi|320446852|ref|NW_003383719.1|	110169-11074	0.977478	363757	189584	0.32105	no
gi|320446852|ref|NW_003383719.1|	112970-11331	925505	688321	-0.427159	0.8408	no

gi|320446852|ref|NW_003383719.1|	13490-11511	110584	158999	0.523878	0.74325	no
gi|320446852|ref|NW_003383719.1|	15240-11564	277652	34043	0.29408	0.8836	no
gi|320446852|ref|NW_003383719.1|	16083-11919	427887	102879	126564	0.4462	no
gi|320446852|ref|NW_003383719.1|	25777-12629	21696	37035	0.771458	0.71835	no
gi|320446852|ref|NW_003383719.1|	32219-13496	235319	521054	114682	0.3991	no
gi|320446852|ref|NW_003383719.1|	37930-13861	450894	193407	-122115	0.5876	no
gi|320446852|ref|NW_003383719.1|	54134-15658	200745	261186	-294222	0.1215	no
gi|320446852|ref|NW_003383719.1|	68765-16901	209284	890153	-123333	0.5599	no
gi|320446852|ref|NW_003383719.1|	69240-17180	154076	106114	-0.538035	0.6708	no
gi|320446852|ref|NW_003383719.1|	72632-17367	237018	114563	-104885	0.6418	no
gi|320446852|ref|NW_003383719.1|	73883-17417	628542	16084	135554	0.523	no
gi|320446852|ref|NW_003383719.1|	75738-17681	100502	514525	235601	0.2928	no
gi|320446852|ref|NW_003383719.1|	07643-20830	248354	223055	-0.154998	0.9427	no
gi|320446852|ref|NW_003383719.1|	11646-21328	145261	768754	-0.918058	0.68565	no
gi|320446852|ref|NW_003383719.1|	25552-23186	843936	856146	0.020722	0.9883	no
gi|320446852|ref|NW_003383719.1|	33465-23446	219864	182273	-0.27051	0.88795	no
gi|320446852|ref|NW_003383719.1|	34683-23532	876449	0.851943	-336284	0.19155	no
gi|320446852|ref|NW_003383719.1|	35821-23669	847071	127586	-273101	0.2432	no
gi|320446852|ref|NW_003383719.1|	37200-23770	164972	245918	-274597	0.24815	no
gi|320446852|ref|NW_003383719.1|	49381-25042	475543	19843	-126094	0.4573	no
gi|320446852|ref|NW_003383719.1|	98035-29837	136907	106636	296142	0.2564	no
gi|320446852|ref|NW_003383719.1|	98536-30091	102234	38436	191059	0.3745	no
gi|320446852|ref|NW_003383719.1|	01001-30459	110423	481148	212344	0.34505	no
gi|320446852|ref|NW_003383719.1|	36492-33970	194363	50727	-193793	0.27975	no
gi|320446852|ref|NW_003383719.1|	41178-34151	396942	183791	-443279	0.20455	no
gi|320446852|ref|NW_003383719.1|	47554-35021	419635	189629	-446788	0.04015	no
gi|320446852|ref|NW_003383719.1|	52348-35264	675424	0	#NAME?	0.0086	no
gi|320446852|ref|NW_003383719.1|	53932-35464	598468	0.487196	-694063	0.16165	no
gi|320446852|ref|NW_003383719.1|	60001-36304	639806	421726	-0.60133	0.655	no
gi|320446852|ref|NW_003383719.1|	63594-36462	709724	171347	-205033	0.25715	no

gi|320446852|ref|NW_003383719.1|	366224-36669	261451	706813	-188714	0.38395	no
gi|320446852|ref|NW_003383719.1|	373343-37424	0	191088	inf	0.0294	no
gi|320446852|ref|NW_003383719.1|	374620-37614	0	200517	inf	0.0186	no
gi|320446852|ref|NW_003383719.1|	377547-38320	0.435925	467992	100682	0.03255	no
gi|320446852|ref|NW_003383719.1|	391907-39519	0.387167	425121	101007	0.06135	no
gi|320446852|ref|NW_003383719.1|	453701-45666	0.495606	389785	297541	0.21675	no
gi|320446852|ref|NW_003383719.1|	457857-45864	0.306553	589182	426451	0.1888	no
gi|320446852|ref|NW_003383719.1|	466719-46787	127765	101158	-0.336873	1	no
gi|320446852|ref|NW_003383719.1|	476961-48542	208601	221233	0.0848178	0.94735	no
gi|320446852|ref|NW_003383719.1|	485567-48635	133429	687077	-0.957533	0.64045	no
gi|320446852|ref|NW_003383719.1|	486576-48842	534318	401537	-0.412164	0.8482	no
gi|320446852|ref|NW_003383719.1|	494930-49517	450185	547075	0.281221	0.8855	no
gi|320446852|ref|NW_003383719.1|	49773-50712	167909	182016	0.11638	0.9405	no
gi|320446852|ref|NW_003383719.1|	53349-53841	258831	866115	174255	0.43025	no
gi|320446852|ref|NW_003383719.1|	53981-54471	195562	392523	100515	0.5772	no
gi|320446852|ref|NW_003383719.1|	565063-56576	153151	22925	0.581965	0.78675	no
gi|320446852|ref|NW_003383719.1|	59172-62516	787024	171611	112467	0.38265	no
gi|320446852|ref|NW_003383719.1|	69073-70786	380286	85487	116862	0.38325	no
gi|320446853|ref|NW_003383718.1|	101854-10234	340235	144438	-123608	0.5526	no
gi|320446853|ref|NW_003383718.1|	131801-13234	36772	134862	-144712	0.50315	no
gi|320446853|ref|NW_003383718.1|	141949-14227	459139	153324	-158235	0.4629	no
gi|320446853|ref|NW_003383718.1|	166159-16636	335546	335085	-0.00198136	0.9322	no
gi|320446853|ref|NW_003383718.1|	177796-17813	267462	547646	-228802	0.31905	no
gi|320446853|ref|NW_003383718.1|	180512-18084	15931	750449	-108601	0.5942	no
gi|320446853|ref|NW_003383718.1|	182099-18615	233935	313313	0.421495	0.7498	no
gi|320446853|ref|NW_003383718.1|	18566-21137	346768	163004	223287	0.2098	no
gi|320446853|ref|NW_003383718.1|	218060-21839	440803	123656	148812	0.4949	no
gi|320446853|ref|NW_003383718.1|	224738-22756	286137	103177	185034	0.26925	no
gi|320446853|ref|NW_003383718.1|	245739-24643	512354	198927	-13649	0.40545	no
gi|320446853|ref|NW_003383718.1|	276233-27673	0.615303	11141	417844	0.192	no

gi|320446853|ref|NW_003383718.1|	27984-30250	19943	823588	204604	0.36705	no
gi|320446853|ref|NW_003383718.1|	284654-285604	165324	391117	124231	0.55105	no
gi|320446853|ref|NW_003383718.1|	306048-30628[illegible]	376049	427042	0.183457	0.923	no
gi|320446853|ref|NW_003383718.1|	307762-308078	282065	320832	0.185795	0.9256	no
gi|320446853|ref|NW_003383718.1|	310633-31090[illegible]	233291	296757	0.347154	0.85835	no
gi|320446853|ref|NW_003383718.1|	315618-315798	362557	134197	188808	0.39265	no
gi|320446853|ref|NW_003383718.1|	325528-326277	591487	743118	0.329245	0.86825	no
gi|320446853|ref|NW_003383718.1|	329289-331164	0	150623	inf	0.01575	no
gi|320446853|ref|NW_003383718.1|	331274-33416[illegible]	313756	90347	152584	0.2581	no
gi|320446853|ref|NW_003383718.1|	33722-34569	165581	398703	126778	0.5457	no
gi|320446853|ref|NW_003383718.1|	347731-34864[illegible]	210596	535875	-197451	0.14755	no
gi|320446853|ref|NW_003383718.1|	34785-38230	0.262847	264795	333258	0.1846	no
gi|320446853|ref|NW_003383718.1|	348809-352759	138537	298864	-221271	0.21955	no
gi|320446853|ref|NW_003383718.1|	354177-357549	254979	492947	-237087	0.1994	no
gi|320446853|ref|NW_003383718.1|	369171-37001[illegible]	139351	262631	0.91431	0.67745	no
gi|320446853|ref|NW_003383718.1|	374072-37505[illegible]	156394	377186	127009	0.44745	no
gi|320446853|ref|NW_003383718.1|	38284-40302	24611	534695	111941	0.60015	no
gi|320446853|ref|NW_003383718.1|	386320-387655	168813	841753	231797	0.3067	no
gi|320446853|ref|NW_003383718.1|	389321-390439	0.191209	119143	263947	1	no
gi|320446853|ref|NW_003383718.1|	390740-393178	773722	777828	332956	0.0997	no
gi|320446853|ref|NW_003383718.1|	400326-40051[illegible]	304406	680986	116163	0.59405	no
gi|320446853|ref|NW_003383718.1|	407889-412214	257855	125.43	228225	0.2282	no
gi|320446853|ref|NW_003383718.1|	412373-413595	0.85524	249063	154211	0.48765	no
gi|320446853|ref|NW_003383718.1|	414324-416988	409912	601935	0.554293	0.8021	no
gi|320446853|ref|NW_003383718.1|	417964-41872[illegible]	32051	681302	108792	0.59715	no
gi|320446853|ref|NW_003383718.1|	418826-419379	195206	298995	0.61512	0.7726	no
gi|320446853|ref|NW_003383718.1|	419498-42124[illegible]	0.672009	483629	284735	0.2256	no
gi|320446853|ref|NW_003383718.1|	421820-42431[illegible]	231796	107579	221447	0.2029	no
gi|320446853|ref|NW_003383718.1|	42643-43487	748787	240377	168267	0.4446	no
gi|320446853|ref|NW_003383718.1|	43693-45298	271055	891661	171791	0.42755	no

gi|320446853|ref|NW_003383718.1|	47248-47810	564828	194201	178166	0.40165	no
gi|320446853|ref|NW_003383718.1|	49238-53815	0.854965	310259	185954	0.4009	no
gi|320446853|ref|NW_003383718.1|	[illegible]28626-52903[illegible]	0	142173	inf	0.01485	no
gi|320446853|ref|NW_003383718.1|	62659-63800	0.372702	116158	163999	1	no
gi|320446853|ref|NW_003383718.1|	[illegible]28370-63349[illegible]	745948	315477	208039	0.1278	no
gi|320446853|ref|NW_003383718.1|	63960-66187	643896	188026	154604	0.36645	no
gi|320446853|ref|NW_003383718.1|	[illegible]54071-66091[illegible]	119956	358555	157968	0.24355	no
gi|320446853|ref|NW_003383718.1|	[illegible]61092-66274[illegible]	700819	181082	136952	0.40715	no
gi|320446853|ref|NW_003383718.1|	[illegible]62875-66339[illegible]	221334	301423	0.445567	0.8364	no
gi|320446853|ref|NW_003383718.1|	66327-67605	0.485575	134768	147271	1	no
gi|320446853|ref|NW_003383718.1|	[illegible]63546-66403[illegible]	43838	516477	0.236522	0.90955	no
gi|320446853|ref|NW_003383718.1|	[illegible]64188-66496[illegible]	264898	23522	-0.171425	0.93445	no
gi|320446853|ref|NW_003383718.1|	[illegible]69285-67076[illegible]	101443	114725	0.177513	0.93205	no
gi|320446853|ref|NW_003383718.1|	[illegible]75835-67664[illegible]	0	202859	inf	0.0312	no
gi|320446853|ref|NW_003383718.1|	[illegible]77068-67802[illegible]	0.233238	209265	316546	0.24575	no
gi|320446853|ref|NW_003383718.1|	[illegible]91545-69386[illegible]	18608	790704	208722	0.35915	no
gi|320446853|ref|NW_003383718.1|	[illegible]95953-69787[illegible]	0	12524	inf	1	no
gi|320446853|ref|NW_003383718.1|	69854-70428	991545	472729	225326	0.3265	no
gi|320446853|ref|NW_003383718.1|	[illegible]99906-70044[illegible]	111992	414613	188837	0.32155	no
gi|320446853|ref|NW_003383718.1|	70694-72406	239982	233954	328522	0.0938	no
gi|320446853|ref|NW_003383718.1|	[illegible]13652-71413[illegible]	269728	630743	122555	0.5597	no
gi|320446853|ref|NW_003383718.1|	72637-73473	0	212591	inf	0.0294	no
gi|320446853|ref|NW_003383718.1|	73722-75168	0.278784	135634	22825	1	no
gi|320446853|ref|NW_003383718.1|	80000-80691	340589	809185	-207349	0.3474	no
gi|320446853|ref|NW_003383718.1|	84370-85865	234445	791775	-156609	0.35905	no
gi|320446853|ref|NW_003383718.1|	88773-90302	0.78268	535279	27738	0.24105	no
gi|320446853|ref|NW_003383718.1|	90881-95827	540847	370571	277646	0.0439	no
gi|320446853|ref|NW_003383718.1|	95886-96786	0.253952	157499	263271	0.26345	no
gi|320446853|ref|NW_003383718.1|	97994-99695	8.52	243712	151625	0.38045	no
gi|320446854|ref|NW_003383717.1|	[illegible]51385-15190[illegible]	0.596409	440503	288478	0.25835	no

gi|320446854|ref|NW_003383717.1|	152112-15311	0.220727	259217	355383	0.2164	no
gi|320446854|ref|NW_003383717.1|	154150-15737	0.169598	276766	402847	0.16935	no
gi|320446854|ref|NW_003383717.1|	157482-15829	379228	144329	192823	0.3733	no
gi|320446854|ref|NW_003383717.1|	203069-20470	0	753864	inf	5.00E-05	yes
gi|320446854|ref|NW_003383717.1|	257158-25747	497761	438471	313896	0.21225	no
gi|320446854|ref|NW_003383717.1|	31130-35269	324171	300781	321389	0.10575	no
gi|320446854|ref|NW_003383717.1|	405582-40598	0	945638	inf	0.02205	no
gi|320446854|ref|NW_003383717.1|	434592-43967	0.0696826	30021	542903	0.14555	no
gi|320446854|ref|NW_003383717.1|	439980-44102	0	203024	inf	0.0233	no
gi|320446854|ref|NW_003383717.1|	442292-44329	0.441455	115885	471429	0.14945	no
gi|320446854|ref|NW_003383717.1|	530203-53054	0	101085	inf	0.0294	no
gi|320446854|ref|NW_003383717.1|	536907-53763	0	734174	inf	0.01195	no
gi|320446854|ref|NW_003383717.1|	58906-59265	122398	718124	255266	0.27405	no
gi|320446854|ref|NW_003383717.1|	72835-73258	0.862839	912993	340344	0.21725	no
gi|320446854|ref|NW_003383717.1|	99364-10095	0	432122	inf	0.0074	no
gi|320446855|ref|NW_003383716.1|	116549-11983	0	799757	inf	5.00E-05	yes
gi|320446855|ref|NW_003383716.1|	120144-12088	0	110601	inf	0.0089	no
gi|320446855|ref|NW_003383716.1|	128202-12904	112036	308218	145999	0.49755	no
gi|320446855|ref|NW_003383716.1|	177898-17833	661943	877059	0.405968	0.8405	no
gi|320446855|ref|NW_003383716.1|	217824-21858	260601	132355	-429936	0.1452	no
gi|320446855|ref|NW_003383716.1|	224528-22519	869279	107738	-30123	0.2292	no
gi|320446855|ref|NW_003383716.1|	233286-23417	144607	0.355777	-534502	0.18625	no
gi|320446855|ref|NW_003383716.1|	234320-23477	764243	296275	-468903	0.13655	no
gi|320446855|ref|NW_003383716.1|	264791-26843	143508	3255	118153	0.5837	no
gi|320446855|ref|NW_003383716.1|	274162-27439	929871	534889	-0.797792	0.7031	no
gi|320446855|ref|NW_003383716.1|	281824-28218	352726	237932	-0.567999	0.78395	no
gi|320446855|ref|NW_003383716.1|	286077-28705	274572	121636	-117461	0.60165	no
gi|320446855|ref|NW_003383716.1|	297741-29840	647057	133926	104947	0.4232	no
gi|320446855|ref|NW_003383716.1|	298884-30459	322674	856287	140802	0.28095	no
gi|320446855|ref|NW_003383716.1|	311599-31278	123878	417018	175119	0.41195	no

gi|320446855|ref|NW_003383716.1|	313265-31593	0.833033	57234	278043	0.23025	no
gi|320446855|ref|NW_003383716.1|	317155-31740	0	383809	inf	0.0154	no
gi|320446855|ref|NW_003383716.1|	323131-32405	0.246891	136178	578548	0.1624	no
gi|320446855|ref|NW_003383716.1|	324348-32538	0	285579	inf	5.00E-05	yes
gi|320446855|ref|NW_003383716.1|	327065-33010	735017	177005	458987	0.04145	no
gi|320446855|ref|NW_003383716.1|	330780-33684	300998	162413	575377	0.033	no
gi|320446855|ref|NW_003383716.1|	349110-34949	187772	520189	147005	0.4943	no
gi|320446855|ref|NW_003383716.1|	351421-35519	448586	323627	285087	0.14335	no
gi|320446855|ref|NW_003383716.1|	57620-58015	893966	52244	-0.774955	0.70785	no
gi|320446856|ref|NW_003383715.1|	109920-11059	104859	370815	-149968	0.3931	no
gi|320446856|ref|NW_003383715.1|	111391-11262	134923	0.935701	-0.52802	1	no
gi|320446856|ref|NW_003383715.1|	112807-11867	350478	279227	-0.327886	0.80795	no
gi|320446856|ref|NW_003383715.1|	132621-13306	263191	462827	-250756	0.2604	no
gi|320446856|ref|NW_003383715.1|	139520-14010	335449	194686	-410687	0.15385	no
gi|320446856|ref|NW_003383715.1|	145472-14589	218819	0.578498	-524128	0.27815	no
gi|320446856|ref|NW_003383715.1|	146053-14795	728843	40274	-417769	0.0529	no
gi|320446856|ref|NW_003383715.1|	150440-15096	867879	155584	-24798	0.2991	no
gi|320446856|ref|NW_003383715.1|	151170-15347	329539	353029	-322259	0.0983	no
gi|320446856|ref|NW_003383715.1|	153835-15404	844348	352467	-458228	0.2936	no
gi|320446856|ref|NW_003383715.1|	154481-15511	459727	665722	-278779	0.2409	no
gi|320446856|ref|NW_003383715.1|	155739-16555	215574	257581	0.256843	0.841	no
gi|320446856|ref|NW_003383715.1|	176363-17675	133387	134736	-330741	0.24265	no
gi|320446856|ref|NW_003383715.1|	177612-17795	277903	0.860071	-501398	0.2859	no
gi|320446856|ref|NW_003383715.1|	179142-18134	164486	0.777193	-440355	0.06595	no
gi|320446856|ref|NW_003383715.1|	181583-18205	199356	275358	-285596	0.23685	no
gi|320446856|ref|NW_003383715.1|	185488-18612	126442	143343	-314093	0.2201	no
gi|320446856|ref|NW_003383715.1|	186259-18748	0.683499	0.82938	0.279095	1	no
gi|320446856|ref|NW_003383715.1|	187614-18789	309548	910683	-176514	0.4185	no
gi|320446856|ref|NW_003383715.1|	228935-23129	257924	60747	123587	0.36555	no
gi|320446856|ref|NW_003383715.1|	231555-24144	417619	603038	0.530062	0.68565	no

gi|320446856|ref|NW_003383715.1|	41660-24209	721538	138274	0.938383	0.6561	no
gi|320446856|ref|NW_003383715.1|	45861-24660	310697	409917	0.399822	0.80255	no
gi|320446856|ref|NW_003383715.1|	73333-27409	193038	110296	-0.807511	0.69605	no
gi|320446856|ref|NW_003383715.1|	27523-27927	758082	156057	104165	0.6027	no
gi|320446856|ref|NW_003383715.1|	93578-29463	0.206415	110771	906782	0.14075	no
gi|320446856|ref|NW_003383715.1|	57785-36024	0.605698	359551	256952	0.2674	no
gi|320446856|ref|NW_003383715.1|	89617-38979	410787	133.91	-161713	0.45235	no
gi|320446856|ref|NW_003383715.1|	28152-42936	0	190909	inf	0.0198	no
gi|320446856|ref|NW_003383715.1|	27149-53251	220289	391614	0.830033	0.7177	no
gi|320446856|ref|NW_003383715.1|	54102-65469	0	346683	inf	0.0294	no
gi|320446856|ref|NW_003383715.1|	89157-69401	142225	142789	332764	0.08775	no
gi|320446856|ref|NW_003383715.1|	03338-70587	237733	147604	-0.687609	0.59655	no
gi|320446856|ref|NW_003383715.1|	07403-70776	379433	103729	-187103	0.39845	no
gi|320446856|ref|NW_003383715.1|	17690-72181	183396	0.515995	-515146	0.0454	no
gi|320446856|ref|NW_003383715.1|	35759-74022	761561	449529	-0.760545	0.65175	no
gi|320446856|ref|NW_003383715.1|	40818-74109	161661	265198	0.714099	0.72495	no
gi|320446856|ref|NW_003383715.1|	41654-74352	105037	128466	0.290487	0.85455	no
gi|320446856|ref|NW_003383715.1|	46304-75214	484848	126623	138493	0.29915	no
gi|320446856|ref|NW_003383715.1|	57292-75852	169501	0.470171	-185004	0.4081	no
gi|320446856|ref|NW_003383715.1|	59921-76086	215577	0.330476	-270559	0.2844	no
gi|320446856|ref|NW_003383715.1|	96649-99801	242974	137954	250532	0.1716	no
gi|320446857|ref|NW_003383714.1|	04540-10059	153331	238536	0.637561	0.6958	no
gi|320446857|ref|NW_003383714.1|	06130-10075	146969	137164	-0.0996103	0.96465	no
gi|320446857|ref|NW_003383714.1|	00695-10101	210658	940791	-116296	0.56655	no
gi|320446857|ref|NW_003383714.1|	08520-10089	239773	703901	-176822	0.4192	no
gi|320446857|ref|NW_003383714.1|	09083-10096	9935	39272	-133902	0.519	no
gi|320446857|ref|NW_003383714.1|	16073-10165	313343	768429	-202776	0.35535	no
gi|320446857|ref|NW_003383714.1|	16870-10175	757623	320599	-124071	0.5602	no
gi|320446857|ref|NW_003383714.1|	17736-10183	492403	139682	-18177	0.4036	no
gi|320446857|ref|NW_003383714.1|	18484-10188	266766	584753	-218967	0.31545	no

gi|320446857|ref|NW_003383714.1|	019093-10207	0.366941	0.936328	135147	1	no
gi|320446857|ref|NW_003383714.1|	021321-10255	234067	427566	419115	0.0517	no
gi|320446857|ref|NW_003383714.1|	102907-10438	101688	348768	-154381	0.47735	no
gi|320446857|ref|NW_003383714.1|	029124-10293	159768	546668	177469	0.33265	no
gi|320446857|ref|NW_003383714.1|	034052-10350	0	148283	inf	0.0312	no
gi|320446857|ref|NW_003383714.1|	036688-10392	311129	153459	-101965	0.44215	no
gi|320446857|ref|NW_003383714.1|	056297-10577	56637	292606	-0.952784	0.47825	no
gi|320446857|ref|NW_003383714.1|	061102-10615	0	659692	inf	0.0233	no
gi|320446857|ref|NW_003383714.1|	064208-10644	219147	591781	143316	0.5029	no
gi|320446857|ref|NW_003383714.1|	065837-10662	582147	204317	181136	0.304	no
gi|320446857|ref|NW_003383714.1|	11593-12017	601136	102247	0.766296	0.7017	no
gi|320446857|ref|NW_003383714.1|	152853-15624	246114	329362	0.42035	0.84315	no
gi|320446857|ref|NW_003383714.1|	17315-17648	291749	132242	218039	0.27975	no
gi|320446857|ref|NW_003383714.1|	186822-18886	420534	482307	0.197732	0.9264	no
gi|320446857|ref|NW_003383714.1|	193102-19437	0	283454	inf	0.01485	no
gi|320446857|ref|NW_003383714.1|	19575-19832	0	186371	inf	0.0312	no
gi|320446857|ref|NW_003383714.1|	20298-21127	113736	781986	278146	0.25495	no
gi|320446857|ref|NW_003383714.1|	205055-20583	550729	20906	-139743	0.4071	no
gi|320446857|ref|NW_003383714.1|	206011-21039	14.59	839496	-0.797385	0.5518	no
gi|320446857|ref|NW_003383714.1|	210467-21313	284962	108514	-139288	0.2923	no
gi|320446857|ref|NW_003383714.1|	214060-21948	195394	397043	102291	0.44325	no
gi|320446857|ref|NW_003383714.1|	21463-21908	0	933373	inf	0.0154	no
gi|320446857|ref|NW_003383714.1|	22631-25373	181749	18722	336471	0.0852	no
gi|320446857|ref|NW_003383714.1|	240468-24136	923962	477432	-0.952538	0.4665	no
gi|320446857|ref|NW_003383714.1|	271257-27372	480667	55.42	0.205367	0.8778	no
gi|320446857|ref|NW_003383714.1|	275179-27675	31586	448284	0.50513	0.80275	no
gi|320446857|ref|NW_003383714.1|	276901-27765	129679	155569	0.262611	0.89055	no
gi|320446857|ref|NW_003383714.1|	285029-28601	158235	764872	-104878	0.62615	no
gi|320446857|ref|NW_003383714.1|	288122-28892	150597	105487	-0.513628	0.8133	no
gi|320446857|ref|NW_003383714.1|	293177-29765	173556	211014	0.28194	0.83175	no

gi|320446857|ref|NW_003383714.1|	299893-30098	89236	238049	-190637	0.15345	no
gi|320446857|ref|NW_003383714.1|	301104-30180	168101	231742	-285874	0.13235	no
gi|320446857|ref|NW_003383714.1|	302695-30440	825617	168245	-229491	0.08975	no
gi|320446857|ref|NW_003383714.1|	308617-31076	978765	0.922833	-340682	0.1708	no
gi|320446857|ref|NW_003383714.1|	310868-31228	315258	0.597408	-239975	0.292	no
gi|320446857|ref|NW_003383714.1|	313197-31432	132942	210399	-26596	0.2467	no
gi|320446857|ref|NW_003383714.1|	314793-31538	890638	540011	-0.721852	0.7207	no
gi|320446857|ref|NW_003383714.1|	315885-31754	116489	488186	-12547	0.5713	no
gi|320446857|ref|NW_003383714.1|	322842-32384	155294	140541	-0.14401	0.9473	no
gi|320446857|ref|NW_003383714.1|	32315-32731	258791	825058	-164922	0.43585	no
gi|320446857|ref|NW_003383714.1|	324064-32598	175707	223963	0.350093	0.7797	no
gi|320446857|ref|NW_003383714.1|	326588-32705	434644	41152	-0.0788708	0.9695	no
gi|320446857|ref|NW_003383714.1|	327272-32845	266291	639779	126457	0.3311	no
gi|320446857|ref|NW_003383714.1|	339561-34089	254291	14784	-0.782445	0.6396	no
gi|320446857|ref|NW_003383714.1|	343707-35095	296257	19252	-0.621838	0.63885	no
gi|320446857|ref|NW_003383714.1|	351630-35227	441042	0.556143	-630932	0.17825	no
gi|320446857|ref|NW_003383714.1|	358627-36050	369592	379226	0.037126	0.9842	no
gi|320446857|ref|NW_003383714.1|	361225-36589	460576	818417	0.829396	0.62275	no
gi|320446857|ref|NW_003383714.1|	375024-38128	700064	34235	-103201	0.42905	no
gi|320446857|ref|NW_003383714.1|	385376-39391	0.16257	414711	467298	0.06245	no
gi|320446857|ref|NW_003383714.1|	394676-39544	0.6386	197068	162571	0.33265	no
gi|320446857|ref|NW_003383714.1|	402739-40571	307383	107879	-151063	0.25435	no
gi|320446857|ref|NW_003383714.1|	451133-45457	284667	105648	-1.43	0.28555	no
gi|320446857|ref|NW_003383714.1|	456098-46073	394643	108944	-185696	0.1728	no
gi|320446857|ref|NW_003383714.1|	465466-46797	921059	153045	0.732592	0.56375	no
gi|320446857|ref|NW_003383714.1|	468141-47023	121458	339541	148313	0.25565	no
gi|320446857|ref|NW_003383714.1|	475883-47660	315312	695088	114041	0.5776	no
gi|320446857|ref|NW_003383714.1|	477946-47871	0.316905	304306	32634	0.23045	no
gi|320446857|ref|NW_003383714.1|	481335-48176	910171	548162	259039	0.2522	no
gi|320446857|ref|NW_003383714.1|	483763-48590	159218	925717	253957	0.2785	no

gi|320446857|ref|NW_003383714.1|	486019-48729	0.809292	550304	27655	0.25045	no
gi|320446857|ref|NW_003383714.1|	490836-49113	578935	297036	235917	0.2983	no
gi|320446857|ref|NW_003383714.1|	491249-49248	137883	827983	-0.73577	0.7395	no
gi|320446857|ref|NW_003383714.1|	492712-49332	531979	270913	-0.973541	0.6501	no
gi|320446857|ref|NW_003383714.1|	497242-49749	154258	117257	-0.395674	0.8552	no
gi|320446857|ref|NW_003383714.1|	497868-50040	969808	944252	-0.038527	0.9737	no
gi|320446857|ref|NW_003383714.1|	503091-50655	345854	800449	-211128	0.11625	no
gi|320446857|ref|NW_003383714.1|	507788-50802	348243	975918	-183526	0.41545	no
gi|320446857|ref|NW_003383714.1|	508169-51167	114562	107592	-0.0905599	0.9437	no
gi|320446857|ref|NW_003383714.1|	519566-52011	609163	127191	-225983	0.3261	no
gi|320446857|ref|NW_003383714.1|	520625-52080	995678	920781	-0.112821	0.94525	no
gi|320446857|ref|NW_003383714.1|	521250-52564	158232	73013	-111581	0.4013	no
gi|320446857|ref|NW_003383714.1|	52136-52724	381245	129098	-156226	0.49785	no
gi|320446857|ref|NW_003383714.1|	525893-52657	923401	573479	-0.687217	0.5909	no
gi|320446857|ref|NW_003383714.1|	52894-54262	96846	434618	-115594	0.59175	no
gi|320446857|ref|NW_003383714.1|	529168-53425	198186	149553	-0.406199	0.76445	no
gi|320446857|ref|NW_003383714.1|	536439-53879	160235	146377	-0.130508	0.91825	no
gi|320446857|ref|NW_003383714.1|	539773-54421	125873	138743	0.140441	0.9511	no
gi|320446857|ref|NW_003383714.1|	55691-60579	116408	995734	-0.225358	0.8646	no
gi|320446857|ref|NW_003383714.1|	61780-66034	190616	12611	-0.595978	0.6631	no
gi|320446857|ref|NW_003383714.1|	656889-65781	364995	536998	0.557041	0.78585	no
gi|320446857|ref|NW_003383714.1|	67166-69638	377207	152266	201316	0.2495	no
gi|320446857|ref|NW_003383714.1|	69833-70455	478967	100586	107044	0.59485	no
gi|320446857|ref|NW_003383714.1|	724370-72758	18549	531567	151891	0.26535	no
gi|320446857|ref|NW_003383714.1|	79942-80162	298286	34.32	0.202351	0.91445	no
gi|320446857|ref|NW_003383714.1|	804745-80600	23006	677377	-176398	0.2928	no
gi|320446857|ref|NW_003383714.1|	806287-80730	111709	133622	-306352	0.1937	no
gi|320446857|ref|NW_003383714.1|	807498-80849	402147	108962	-188389	0.2755	no
gi|320446857|ref|NW_003383714.1|	809974-81019	447429	935999	-225708	0.2695	no
gi|320446857|ref|NW_003383714.1|	810369-81324	124554	79919	-0.640164	0.6207	no

gi|320446857|ref|NW_003383714.1|	15677-81645	402907	829476	-228018	0.3139	no
gi|320446857|ref|NW_003383714.1|	19576-82154	538918	881729	-261166	0.1712	no
gi|320446857|ref|NW_003383714.1|	22052-82271	223153	667371	-174147	0.4184	no
gi|320446857|ref|NW_003383714.1|	23450-82830	366487	20.27	-0.854418	0.5238	no
gi|320446857|ref|NW_003383714.1|	89198-89026	50857	171669	175511	0.4257	no
gi|320446857|ref|NW_003383714.1|	90561-89091	890358	206983	121705	0.56105	no
gi|320446857|ref|NW_003383714.1|	93742-89400	652492	104425	0.678435	0.7414	no
gi|320446857|ref|NW_003383714.1|	01600-90264	0.831764	158164	0.927172	0.68415	no
gi|320446857|ref|NW_003383714.1|	76081-97667	0.932425	315991	176083	0.33425	no
gi|320446857|ref|NW_003383714.1|	76807-97840	0.37234	319537	310129	0.21295	no
gi|320446857|ref|NW_003383714.1|	97806-98674	120061	532668	-117246	0.57165	no
gi|320446857|ref|NW_003383714.1|	78474-97913	0	542338	inf	0.0162	no
gi|320446857|ref|NW_003383714.1|	79792-98288	187962	169869	-0.146019	0.9136	no
gi|320446857|ref|NW_003383714.1|	83630-98606	149022	343303	-211797	0.22655	no
gi|320446857|ref|NW_003383714.1|	86771-98770	660583	84507	-29666	0.10925	no
gi|320446857|ref|NW_003383714.1|	88539-98940	316994	421807	-29098	0.2257	no
gi|320446857|ref|NW_003383714.1|	90214-99297	33.07	457542	-285354	0.1388	no
gi|320446857|ref|NW_003383714.1|	93188-99497	403993	108476	-189695	0.2941	no
gi|320446857|ref|NW_003383714.1|	99328-100504	915018	360543	-134363	0.52415	no
gi|320446858|ref|NW_003383713.1|	55906-15629	0	800005	inf	0.029	no
gi|320446858|ref|NW_003383713.1|	23939-32505	0.382851	132529	179145	1	no
gi|320446858|ref|NW_003383713.1|	39869-34422	16788	427197	134747	0.54405	no
gi|320446858|ref|NW_003383713.1|	49479-34998	0	461678	inf	0.0294	no
gi|320446858|ref|NW_003383713.1|	54130-35501	103449	231603	448466	0.14465	no
gi|320446858|ref|NW_003383713.1|	59458-36039	382704	0.82504	-22137	0.33025	no
gi|320446858|ref|NW_003383713.1|	60594-36180	104106	132316	-2976	0.21725	no
gi|320446858|ref|NW_003383713.1|	64525-36539	0.534451	165567	163129	0.3326	no
gi|320446858|ref|NW_003383713.1|	70010-37106	88867	371587	-125795	0.5523	no
gi|320446858|ref|NW_003383713.1|	71971-37352	8044	115468	-280042	0.2406	no
gi|320446858|ref|NW_003383713.1|	76509-37845	563912	0.896611	-265291	0.2596	no

gi|320446858|ref|NW_003383713.1|	378897-37918	217662	695452	-164607	0.43885	no
gi|320446858|ref|NW_003383713.1|	381394-38240	699918	181344	-194846	0.37975	no
gi|320446858|ref|NW_003383713.1|	383073-38345	129483	1414	-319491	0.25	no
gi|320446858|ref|NW_003383713.1|	387240-38799	156213	200768	-295992	0.2073	no
gi|320446858|ref|NW_003383713.1|	388416-38953	305588	105786	-153043	0.47805	no
gi|320446858|ref|NW_003383713.1|	389658-39071	22912	471062	-228212	0.30855	no
gi|320446858|ref|NW_003383713.1|	395670-39872	0.23979	192811	300734	0.2173	no
gi|320446858|ref|NW_003383713.1|	398900-39962	0	260513	inf	0.0294	no
gi|320446858|ref|NW_003383713.1|	449729-45039	941754	374561	-133015	0.43155	no
gi|320446858|ref|NW_003383713.1|	451260-45168	243289	122201	-0.993413	0.6383	no
gi|320446858|ref|NW_003383713.1|	451820-45750	328119	301071	-0.124116	0.92815	no
gi|320446858|ref|NW_003383713.1|	457635-45803	464364	108245	-210095	0.3399	no
gi|320446858|ref|NW_003383713.1|	458363-45919	730662	251244	-154011	0.47055	no
gi|320446858|ref|NW_003383713.1|	460859-46126	952488	188194	-233948	0.25625	no
gi|320446858|ref|NW_003383713.1|	461412-46560	106636	15917	0.577871	0.6593	no
gi|320446858|ref|NW_003383713.1|	467647-46822	326528	333084	0.0286791	0.9882	no
gi|320446858|ref|NW_003383713.1|	468311-46868	312808	138882	-117142	0.57185	no
gi|320446858|ref|NW_003383713.1|	469221-46974	977101	657037	-0.572533	0.7749	no
gi|320446858|ref|NW_003383713.1|	470029-47059	163342	158614	-0.0423736	0.98045	no
gi|320446858|ref|NW_003383713.1|	470918-47583	208175	973813	-109608	0.4191	no
gi|320446858|ref|NW_003383713.1|	482745-48380	500589	141891	-181884	0.2941	no
gi|320446858|ref|NW_003383713.1|	484258-48448	172847	0	#NAME?	0.00665	no
gi|320446858|ref|NW_003383713.1|	496958-49744	344788	282443	-0.287749	0.8295	no
gi|320446858|ref|NW_003383713.1|	497806-50621	425482	58504	0.459435	0.71025	no
gi|320446858|ref|NW_003383713.1|	508816-50975	0.478381	247512	237127	0.2616	no
gi|320446858|ref|NW_003383713.1|	55324-56947	231056	0.677075	-177085	0.40925	no
gi|320446858|ref|NW_003383713.1|	57049-74321	269366	127265	22402	0.10185	no
gi|320446860|ref|NW_003383711.1|	188767-18978	222973	792846	-149176	0.50725	no
gi|320446860|ref|NW_003383711.1|	198217-19940	95764	442465	-111392	0.5957	no
gi|320446860|ref|NW_003383711.1|	203130-20622	159311	13079	-0.284596	0.82335	no

gi|320446860|ref|NW_003383711.1|	265570-26646	509437	0	#NAME?	0.0132	no
gi|320446860|ref|NW_003383711.1|	356369-35710	186898	258263	0.466595	0.8267	no
gi|320446860|ref|NW_003383711.1|	365885-37242	436212	83476	0.936332	0.46825	no
gi|320446860|ref|NW_003383711.1|	379540-38064	153667	144061	-0.0931262	0.96565	no
gi|320446860|ref|NW_003383711.1|	386258-38901	314496	355389	0.176358	0.93255	no
gi|320446860|ref|NW_003383711.1|	389626-39301	369288	93224	133596	0.42645	no
gi|320446860|ref|NW_003383711.1|	42354-43654	37409	25083	-0.576676	0.6552	no
gi|320446860|ref|NW_003383711.1|	428649-42985	180451	348832	0.950925	0.567	no
gi|320446860|ref|NW_003383711.1|	464195-47235	789481	285466	185434	0.1702	no
gi|320446860|ref|NW_003383711.1|	495135-49678	0.357608	124463	179926	1	no
gi|320446860|ref|NW_003383711.1|	497252-49903	0.544114	318398	254885	0.27815	no
gi|320446860|ref|NW_003383711.1|	499583-50056	0	25141	inf	0.0198	no
gi|320446860|ref|NW_003383711.1|	501914-50246	106491	111323	338595	0.1974	no
gi|320446860|ref|NW_003383711.1|	503919-50423	334496	862162	136597	0.38305	no
gi|320446860|ref|NW_003383711.1|	513129-51378	143577	163069	-313826	0.21325	no
gi|320446860|ref|NW_003383711.1|	513890-51439	741908	904732	-303568	0.2175	no
gi|320446860|ref|NW_003383711.1|	514754-51639	372563	708762	-239411	0.19465	no
gi|320446860|ref|NW_003383711.1|	560189-56046	245672	187762	29341	0.25735	no
gi|320446860|ref|NW_003383711.1|	566874-56744	0	640559	inf	0.0212	no
gi|320446860|ref|NW_003383711.1|	587403-58919	120172	979812	302741	0.08285	no
gi|320446860|ref|NW_003383711.1|	596748-59840	247.9	1960.15	298313	0.05815	no
gi|320446860|ref|NW_003383711.1|	701051-70289	136114	919319	275575	0.2293	no
gi|320446860|ref|NW_003383711.1|	705550-70626	0	312218	inf	0.02915	no
gi|320446860|ref|NW_003383711.1|	788352-79303	0.417669	555788	37341	0.0711	no
gi|320446860|ref|NW_003383711.1|	79537-81423	337355	0	#NAME?	0.0074	no
gi|320446860|ref|NW_003383711.1|	800211-80071	231732	251824	-320197	0.20285	no
gi|320446860|ref|NW_003383711.1|	81475-82953	339504	0	#NAME?	0.0104	no
gi|320446860|ref|NW_003383711.1|	828714-82898	471737	474262	-331423	0.1886	no
gi|320446860|ref|NW_003383711.1|	830805-83167	137649	291626	-22388	0.30355	no
gi|320446860|ref|NW_003383711.1|	832946-83460	110158	263858	-206174	0.35975	no

gi|320446860|ref|NW_003383711.1|	34972-83929	218715	130026	-0.750252	0.7142	no
gi|320446860|ref|NW_003383711.1|	39438-84090	0.686189	0.858598	0.323377	1	no
gi|320446860|ref|NW_003383711.1|	41031-84374	100151	490353	-103029	0.52555	no
gi|320446860|ref|NW_003383711.1|	52525-85326	636706	734499	0.206133	0.9187	no
gi|320446860|ref|NW_003383711.1|	53423-85499	569828	273975	-105648	0.42315	no
gi|320446860|ref|NW_003383711.1|	55042-85536	116062	269972	-210401	0.34925	no
gi|320446860|ref|NW_003383711.1|	56236-85810	133248	43192	-162527	0.3256	no
gi|320446860|ref|NW_003383711.1|	58253-85939	29314	131075	-11612	0.47395	no
gi|320446860|ref|NW_003383711.1|	71016-87359	0.071874	305774	873278	0.14075	no
gi|320446860|ref|NW_003383711.1|	89454-88997	146595	866777	-0.758102	0.70985	no
gi|320446860|ref|NW_003383711.1|	90674-89138	60055	555741	-0.111874	0.9532	no
gi|320446860|ref|NW_003383711.1|	92261-89716	848924	15992	0.91364	0.49145	no
gi|320446860|ref|NW_003383711.1|	03145-90361	0.692815	786038	350406	0.2175	no
gi|320446860|ref|NW_003383711.1|	11877-91518	449742	503557	0.163059	0.90235	no
gi|320446860|ref|NW_003383711.1|	15553-91603	394832	237044	-0.73608	0.72355	no
gi|320446860|ref|NW_003383711.1|	21312-92231	356983	254205	-0.489863	0.75705	no
gi|320446861|ref|NW_003383710.1|	54664-15495	396467	139732	181739	0.32625	no
gi|320446861|ref|NW_003383710.1|	68238-16909	0	198077	inf	0.00545	no
gi|320446861|ref|NW_003383710.1|	81435-18344	869102	100236	-311612	0.1241	no
gi|320446861|ref|NW_003383710.1|	83693-18420	942802	201551	-554774	0.11255	no
gi|320446861|ref|NW_003383710.1|	84866-18558	750946	23683	-498678	0.0657	no
gi|320446861|ref|NW_003383710.1|	90081-19097	644811	1763	-519277	0.0651	no
gi|320446861|ref|NW_003383710.1|	19520-21600	0.548855	389229	282612	0.2226	no
gi|320446861|ref|NW_003383710.1|	15557-21643	236017	270899	-312306	0.19605	no
gi|320446861|ref|NW_003383710.1|	22055-22397	0.803708	154066	0.938801	0.64715	no
gi|320446861|ref|NW_003383710.1|	24389-22682	926093	159273	0.782277	0.64405	no
gi|320446861|ref|NW_003383710.1|	27062-22779	30522	487654	0.676007	0.7404	no
gi|320446861|ref|NW_003383710.1|	27936-22867	347825	28178	-0.303792	0.8892	no
gi|320446861|ref|NW_003383710.1|	28837-23031	342476	201399	-0.765944	0.54595	no
gi|320446861|ref|NW_003383710.1|	67603-26820	0.9299	409716	213948	0.2929	no

gi|320446861|ref|NW_003383710.1|	93946-39669	277464	291317	0.0702913	0.96065	no
gi|320446861|ref|NW_003383710.1|	08250-40919	785985	279346	-149245	0.47905	no
gi|320446861|ref|NW_003383710.1|	33304-43568	308706	230937	-0.418734	0.75525	no
gi|320446861|ref|NW_003383710.1|	36137-43729	862513	24154	-183629	0.39885	no
gi|320446861|ref|NW_003383710.1|	37482-43794	732835	390363	-0.908674	0.6701	no
gi|320446861|ref|NW_003383710.1|	38159-44066	957777	938612	-0.0291607	0.9791	no
gi|320446861|ref|NW_003383710.1|	40977-44275	679081	183876	143708	0.38845	no
gi|320446861|ref|NW_003383710.1|	46859-44928	0.077061	181345	787852	0.14075	no
gi|320446861|ref|NW_003383710.1|	59318-46070	383256	268269	-0.514628	0.6864	no
gi|320446861|ref|NW_003383710.1|	60840-46134	656838	419587	-0.646567	0.77335	no
gi|320446861|ref|NW_003383710.1|	62222-46323	473139	227958	-10535	0.5289	no
gi|320446861|ref|NW_003383710.1|	64312-46458	570266	197838	-152732	0.4766	no
gi|320446861|ref|NW_003383710.1|	64731-46498	170.66	810995	-107336	0.61425	no
gi|320446861|ref|NW_003383710.1|	79892-48090	956189	931006	-0.0385052	0.98345	no
gi|320446861|ref|NW_003383710.1|	81660-48349	547173	28596	-0.936185	0.64675	no
gi|320446861|ref|NW_003383710.1|	84089-48578	611887	361737	-0.758325	0.7112	no
gi|320446861|ref|NW_003383710.1|	88405-48928	368857	290286	-0.345587	0.86085	no
gi|320446861|ref|NW_003383710.1|	89715-49110	231727	774282	-158149	0.3391	no
gi|320446861|ref|NW_003383710.1|	93701-49396	312062	900216	-179349	0.41695	no
gi|320446861|ref|NW_003383710.1|	54683-55519	211633	527514	-200429	0.3578	no
gi|320446861|ref|NW_003383710.1|	43342-64385	356627	10777	159547	0.444	no
gi|320446862|ref|NW_003383709.1|	27289-12927	38459	440896	0.197119	0.88	no
gi|320446862|ref|NW_003383709.1|	29532-13345	229542	49002	109408	0.41485	no
gi|320446862|ref|NW_003383709.1|	54991-15647	0.946802	488993	236868	0.29315	no
gi|320446862|ref|NW_003383709.1|	57223-15781	426536	285716	274384	0.24235	no
gi|320446862|ref|NW_003383709.1|	64450-16494	254151	280857	346608	0.18475	no
gi|320446862|ref|NW_003383709.1|	65135-16592	183932	101003	245715	0.2901	no
gi|320446862|ref|NW_003383709.1|	66723-17068	203905	168737	30488	0.11355	no
gi|320446862|ref|NW_003383709.1|	76064-17673	126952	46202	-145825	0.26255	no
gi|320446862|ref|NW_003383709.1|	77691-18028	286506	135264	-108278	0.40595	no

gi|320446862|ref|NW_003383709.1|	80587-18247	570783	444873	-0.359548	0.7901	no
gi|320446862|ref|NW_003383709.1|	87332-18961	109868	87644	-0.326044	0.83635	no
gi|320446862|ref|NW_003383709.1|	96567-19696	0	722047	inf	0.0294	no
gi|320446862|ref|NW_003383709.1|	98961-19959	301972	285328	-340372	0.1768	no
gi|320446862|ref|NW_003383709.1|	00983-20296	188496	363965	-237266	0.183	no
gi|320446862|ref|NW_003383709.1|	03563-20829	628779	787703	0.325099	0.7926	no
gi|320446862|ref|NW_003383709.1|	19711-22041	161319	318626	-233998	0.3002	no
gi|320446862|ref|NW_003383709.1|	20691-22158	112076	421241	-141176	0.50255	no
gi|320446862|ref|NW_003383709.1|	22788-22335	231066	693574	-173618	0.4234	no
gi|320446862|ref|NW_003383709.1|	24387-22484	521557	198307	-139509	0.52395	no
gi|320446862|ref|NW_003383709.1|	26259-22680	591151	434783	-0.443233	0.82485	no
gi|320446862|ref|NW_003383709.1|	27647-22882	183394	859648	-109312	0.61915	no
gi|320446862|ref|NW_003383709.1|	29317-22967	124755	650136	-0.940282	0.66535	no
gi|320446862|ref|NW_003383709.1|	30721-23150	433105	212306	-102858	0.6072	no
gi|320446862|ref|NW_003383709.1|	34562-23849	708046	671458	-0.0765453	0.9482	no
gi|320446862|ref|NW_003383709.1|	38644-24121	285717	102819	-147448	0.2659	no
gi|320446862|ref|NW_003383709.1|	45957-24735	248799	522583	-225125	0.1987	no
gi|320446862|ref|NW_003383709.1|	06762-30715	494011	649725	0.395288	0.85375	no
gi|320446862|ref|NW_003383709.1|	13280-31365	0	21079	inf	0.0109	no
gi|320446862|ref|NW_003383709.1|	15348-31965	0.0414596	154145	853836	0.14075	no
gi|320446862|ref|NW_003383709.1|	21759-32311	181031	125714	-0.526095	0.79675	no
gi|320446862|ref|NW_003383709.1|	29013-32953	0	505648	inf	0.02915	no
gi|320446862|ref|NW_003383709.1|	35465-33682	0.902733	626905	279588	0.23755	no
gi|320446862|ref|NW_003383709.1|	49161-34985	110102	175526	0.672838	0.73955	no
gi|320446862|ref|NW_003383709.1|	51151-35185	108002	246107	118822	0.56815	no
gi|320446862|ref|NW_003383709.1|	56429-35766	220352	340874	0.62943	0.7518	no
gi|320446862|ref|NW_003383709.1|	57779-35849	283202	363175	0.358834	0.85375	no
gi|320446862|ref|NW_003383709.1|	59963-36080	19413	610482	165293	0.4371	no
gi|320446862|ref|NW_003383709.1|	62600-36334	210286	182919	-352307	0.16215	no
gi|320446862|ref|NW_003383709.1|	64181-36515	57125	0.788794	-28564	0.2421	no

gi|320446862|ref|NW_003383709.1|	365965-36787	158293	351395	-217143	0.2084	no
gi|320446862|ref|NW_003383709.1|	371162-37237	34352	442814	-295562	0.10855	no
gi|320446862|ref|NW_003383709.1|	372532-37502	515247	323353	-0.672154	0.758	no
gi|320446862|ref|NW_003383709.1|	376205-37678	339228	229598	-0.563143	0.79225	no
gi|320446862|ref|NW_003383709.1|	377891-37970	757975	5653	-0.423134	0.8446	no
gi|320446862|ref|NW_003383709.1|	381404-38242	195242	459678	-208657	0.3595	no
gi|320446862|ref|NW_003383709.1|	385275-38686	121282	269686	-216901	0.3432	no
gi|320446862|ref|NW_003383709.1|	387766-38849	431691	803627	-24254	0.1642	no
gi|320446862|ref|NW_003383709.1|	393235-39347	235548	155906	272658	0.2459	no
gi|320446862|ref|NW_003383709.1|	393593-39685	142325	834731	255212	0.09815	no
gi|320446862|ref|NW_003383709.1|	398199-39859	993296	587745	-0.757033	0.7073	no
gi|320446862|ref|NW_003383709.1|	401013-40222	0.520532	120287	120842	1	no
gi|320446862|ref|NW_003383709.1|	402615-40337	416663	417572	0.0031434	0.96875	no
gi|320446862|ref|NW_003383709.1|	414315-42266	395097	828298	106794	0.41085	no
gi|320446862|ref|NW_003383709.1|	44477-46180	457614	0.4003	-68369	0.11235	no
gi|320446862|ref|NW_003383709.1|	46329-48087	135206	0.0771918	-745249	0.2584	no
gi|320446862|ref|NW_003383709.1|	48208-51880	75118	164912	-218746	0.2142	no
gi|320446862|ref|NW_003383709.1|	53407-54014	0	430005	inf	0.0233	no
gi|320446862|ref|NW_003383709.1|	545157-54532	0	133484	inf	0.0294	no
gi|320446862|ref|NW_003383709.1|	54997-55167	132824	30509	-212221	0.2824	no
gi|320446863|ref|NW_003383708.1|	016394-10197	322682	963471	157813	0.2415	no
gi|320446863|ref|NW_003383708.1|	021663-10229	824504	423642	-0.960683	0.4646	no
gi|320446863|ref|NW_003383708.1|	023120-10259	239006	152271	-0.650408	0.6165	no
gi|320446863|ref|NW_003383708.1|	103313-11160	208109	255321	0.294976	0.82285	no
gi|320446863|ref|NW_003383708.1|	170843-17191	221369	0.139224	-399097	0.31135	no
gi|320446863|ref|NW_003383708.1|	175716-17773	558168	0.5278	-340264	0.17325	no
gi|320446863|ref|NW_003383708.1|	187886-18822	126693	182549	-279498	0.28215	no
gi|320446863|ref|NW_003383708.1|	199643-20180	181672	134719	-375331	0.0763	no
gi|320446863|ref|NW_003383708.1|	218474-21988	422016	239365	-414001	0.0522	no
gi|320446863|ref|NW_003383708.1|	253120-25358	155457	126284	302208	0.10515	no

gi|320446863|ref|NW_003383708.1|	258440-25943	156143	129416	305107	0.20725	no
gi|320446863|ref|NW_003383708.1|	259592-26018	473928	43339	319292	0.18555	no
gi|320446863|ref|NW_003383708.1|	260298-26388	785804	108662	378954	0.0078	no
gi|320446863|ref|NW_003383708.1|	280563-28099	513006	619423	0.271951	0.9011	no
gi|320446863|ref|NW_003383708.1|	296471-29828	369715	605515	0.711747	0.60155	no
gi|320446863|ref|NW_003383708.1|	305323-30578	434158	798512	0.879093	0.68885	no
gi|320446863|ref|NW_003383708.1|	309117-31081	149676	288606	0.947262	0.64255	no
gi|320446863|ref|NW_003383708.1|	30991-31497	0	66846	inf	0.00385	no
gi|320446863|ref|NW_003383708.1|	313434-31493	0.133439	1299	328315	1	no
gi|320446863|ref|NW_003383708.1|	315040-31805	0.242548	17806	287602	0.2405	no
gi|320446863|ref|NW_003383708.1|	318356-31974	321261	114631	183518	0.405	no
gi|320446863|ref|NW_003383708.1|	320240-32096	940112	22872	128268	0.55515	no
gi|320446863|ref|NW_003383708.1|	322609-32461	360639	144771	200514	0.14135	no
gi|320446863|ref|NW_003383708.1|	33462-33963	0	579196	inf	0.00515	no
gi|320446863|ref|NW_003383708.1|	334947-33548	116094	632922	-0.875194	0.67525	no
gi|320446863|ref|NW_003383708.1|	3403-3734	118406	383157	-162774	0.44495	no
gi|320446863|ref|NW_003383708.1|	346180-34661	428719	429117	-332059	0.1744	no
gi|320446863|ref|NW_003383708.1|	348264-34973	159659	151784	-33949	0.17005	no
gi|320446863|ref|NW_003383708.1|	351043-35410	554361	404291	-0.455431	0.8376	no
gi|320446863|ref|NW_003383708.1|	354239-35838	842197	114899	0.448131	0.73055	no
gi|320446863|ref|NW_003383708.1|	360759-36259	187183	42002	-215592	0.2185	no
gi|320446863|ref|NW_003383708.1|	363447-36403	134931	22836	-256284	0.2722	no
gi|320446863|ref|NW_003383708.1|	364254-36749	137629	687555	-100124	0.43055	no
gi|320446863|ref|NW_003383708.1|	369058-37219	164678	939985	-0.80894	0.52945	no
gi|320446863|ref|NW_003383708.1|	374294-38576	75861	522954	-0.536674	0.8083	no
gi|320446863|ref|NW_003383708.1|	3839-7255	705548	426777	-0.725262	0.6537	no
gi|320446863|ref|NW_003383708.1|	394145-39795	788481	647665	-0.283828	0.8153	no
gi|320446863|ref|NW_003383708.1|	398187-40037	673697	699139	0.0534802	0.979	no
gi|320446863|ref|NW_003383708.1|	402098-40251	145618	900818	-0.692887	0.7256	no
gi|320446863|ref|NW_003383708.1|	402763-40307	421486	380127	-0.149001	0.94025	no

gi|320446863|ref|NW_003383708.1|	11453-41372	112955	157101	0.475948	0.7068	no
gi|320446863|ref|NW_003383708.1|	25001-42540	937967	803588	-0.223081	0.9045	no
gi|320446863|ref|NW_003383708.1|	19339-52083	21452	288992	0.429919	0.8274	no
gi|320446863|ref|NW_003383708.1|	21023-52168	15953	190248	0.254052	0.89055	no
gi|320446863|ref|NW_003383708.1|	21828-52241	384441	618165	0.685231	0.7312	no
gi|320446863|ref|NW_003383708.1|	31375-53209	630625	0.239685	-471757	0.2885	no
gi|320446863|ref|NW_003383708.1|	34167-53482	752486	0.539899	-38009	0.2213	no
gi|320446863|ref|NW_003383708.1|	37198-53886	647833	0.081999	-630387	0.2654	no
gi|320446863|ref|NW_003383708.1|	39923-54065	244666	953946	-135883	0.5307	no
gi|320446863|ref|NW_003383708.1|	40953-54120	288274	608195	-224484	0.27085	no
gi|320446863|ref|NW_003383708.1|	41710-54268	689234	269626	-135404	0.5203	no
gi|320446863|ref|NW_003383708.1|	43468-54400	201585	301537	-274098	0.24135	no
gi|320446863|ref|NW_003383708.1|	44191-54439	105256	38701	-476538	0.285	no
gi|320446863|ref|NW_003383708.1|	52994-55464	201595	361195	0.841318	0.5181	no
gi|320446863|ref|NW_003383708.1|	55829-55605	300689	216252	-0.47556	0.8136	no
gi|320446863|ref|NW_003383708.1|	58408-55865	128122	141912	0.147482	0.91235	no
gi|320446863|ref|NW_003383708.1|	58830-55902	399419	447274	0.163257	0.92095	no
gi|320446863|ref|NW_003383708.1|	65489-56590	275295	164244	-0.745139	0.7108	no
gi|320446863|ref|NW_003383708.1|	84483-58489	109763	35949	-161037	0.32515	no
gi|320446863|ref|NW_003383708.1|	87023-58722	511344	318651	-0.682316	0.73765	no
gi|320446863|ref|NW_003383708.1|	87916-58851	303578	109168	-147551	0.49225	no
gi|320446863|ref|NW_003383708.1|	94115-59595	158035	939697	-0.749981	0.63935	no
gi|320446863|ref|NW_003383708.1|	97092-59731	635423	490961	-0.372109	0.84685	no
gi|320446863|ref|NW_003383708.1|	97929-59916	121431	105266	-0.206092	0.92435	no
gi|320446863|ref|NW_003383708.1|	15967-61674	373285	256142	-0.543332	0.8137	no
gi|320446863|ref|NW_003383708.1|	17651-61834	223392	250227	0.163657	0.94185	no
gi|320446863|ref|NW_003383708.1|	19845-62141	110681	663917	-0.737336	0.5844	no
gi|320446863|ref|NW_003383708.1|	21913-62263	210655	952999	-114433	0.3962	no
gi|320446863|ref|NW_003383708.1|	63293-64015	416119	144754	179854	0.4026	no
gi|320446863|ref|NW_003383708.1|	43567-64571	64117	153429	-206313	0.1318	no

gi|320446863|ref|NW_003383708.1|	546042-64741	395277	30447	-0.376562	0.77255	no
gi|320446863|ref|NW_003383708.1|	547637-64865	405857	521903	0.362809	0.7744	no
gi|320446863|ref|NW_003383708.1|	65714-66083	920687	752096	-0.291793	0.87955	no
gi|320446863|ref|NW_003383708.1|	563515-67264	349173	612051	0.809709	0.62475	no
gi|320446863|ref|NW_003383708.1|	67040-67270	422996	266264	265414	0.27135	no
gi|320446863|ref|NW_003383708.1|	572971-67370	983488	208995	-223444	0.3076	no
gi|320446863|ref|NW_003383708.1|	575097-67565	405649	142183	-151249	0.4859	no
gi|320446863|ref|NW_003383708.1|	575776-67679	689196	729326	0.0816491	0.96455	no
gi|320446863|ref|NW_003383708.1|	577687-67929	100809	102661	0.0262686	0.9895	no
gi|320446863|ref|NW_003383708.1|	703227-70411	0	516652	inf	0.0109	no
gi|320446863|ref|NW_003383708.1|	722362-72327	0	42056	inf	5.00E-05	yes
gi|320446863|ref|NW_003383708.1|	319853-82063	463737	147557	-165203	0.3224	no
gi|320446863|ref|NW_003383708.1|	320815-82349	290486	118397	-129483	0.32245	no
gi|320446863|ref|NW_003383708.1|	323906-82446	31086	419746	0.433252	0.82425	no
gi|320446863|ref|NW_003383708.1|	334799-83529	286958	896751	-167806	0.43385	no
gi|320446863|ref|NW_003383708.1|	339262-84112	145501	528715	186146	0.3883	no
gi|320446863|ref|NW_003383708.1|	347630-84779	111853	680069	-0.71785	0.7371	no
gi|320446863|ref|NW_003383708.1|	378981-88219	169034	321726	0.928519	0.4916	no
gi|320446863|ref|NW_003383708.1|	390855-89254	431839	235199	-0.87661	0.5041	no
gi|320446863|ref|NW_003383708.1|	393859-89463	183532	747081	-129669	0.5415	no
gi|320446863|ref|NW_003383708.1|	394795-89752	553968	410716	-0.431659	0.8402	no
gi|320446863|ref|NW_003383708.1|	399362-90017	256708	0	#NAME?	0.0047	no
gi|320446863|ref|NW_003383708.1|	90716-92721	371878	345765	-0.105038	0.95685	no
gi|320446864|ref|NW_003383707.1|	158637-15951	31304	318827	0.026426	0.98465	no
gi|320446864|ref|NW_003383707.1|	161458-16360	147796	99412	-0.572118	0.7262	no
gi|320446864|ref|NW_003383707.1|	167437-16812	108781	107568	-0.0161662	0.9864	no
gi|320446864|ref|NW_003383707.1|	168227-16915	180585	232238	0.362927	0.8673	no
gi|320446864|ref|NW_003383707.1|	169411-17026	0.543928	149728	146086	0.3715	no
gi|320446864|ref|NW_003383707.1|	170980-17575	401403	879879	113225	0.49905	no
gi|320446864|ref|NW_003383707.1|	175894-17898	164146	701886	-122567	0.4804	no

gi|320446864|ref|NW_003383707.1|	181367-18170	724138	562836	-0.363549	0.8566	no
gi|320446864|ref|NW_003383707.1|	182359-18312	293202	100538	-154415	0.47205	no
gi|320446864|ref|NW_003383707.1|	185800-18646	190519	265179	-28449	0.22495	no
gi|320446864|ref|NW_003383707.1|	187634-18812	263363	387018	-276658	0.22855	no
gi|320446864|ref|NW_003383707.1|	191025-19148	335286	396613	-307959	0.1991	no
gi|320446864|ref|NW_003383707.1|	192537-19300	209079	384298	-244375	0.2809	no
gi|320446864|ref|NW_003383707.1|	196201-19674	390024	777087	-232741	0.30835	no
gi|320446864|ref|NW_003383707.1|	196855-19707	995001	985036	-333645	0.18605	no
gi|320446864|ref|NW_003383707.1|	198178-19891	940164	206977	-218344	0.3183	no
gi|320446864|ref|NW_003383707.1|	199041-20014	128085	389562	-171718	0.4283	no
gi|320446864|ref|NW_003383707.1|	202009-20411	451988	611691	0.43652	0.8383	no
gi|320446864|ref|NW_003383707.1|	204521-20551	274366	785741	-180398	0.27	no
gi|320446864|ref|NW_003383707.1|	205923-20707	278926	895648	-163888	0.32505	no
gi|320446864|ref|NW_003383707.1|	209370-21372	788602	121079	0.618574	0.61505	no
gi|320446864|ref|NW_003383707.1|	213856-21555	138068	128183	-0.107177	1	no
gi|320446864|ref|NW_003383707.1|	215788-21627	50099	383526	-0.385454	0.85545	no
gi|320446864|ref|NW_003383707.1|	217186-21891	108676	13402	0.302415	0.8946	no
gi|320446864|ref|NW_003383707.1|	219216-22479	624708	459843	-0.442041	0.7251	no
gi|320446864|ref|NW_003383707.1|	231468-23167	753708	683987	-0.140036	0.9357	no
gi|320446864|ref|NW_003383707.1|	233124-23331	572273	152513	-190777	0.30655	no
gi|320446864|ref|NW_003383707.1|	233451-23437	83576	305086	-145387	0.48855	no
gi|320446864|ref|NW_003383707.1|	234690-23493	396722	683162	-253783	0.2388	no
gi|320446864|ref|NW_003383707.1|	237721-23916	160832	573477	-148775	0.50925	no
gi|320446864|ref|NW_003383707.1|	239356-24021	164571	687066	-126019	0.55065	no
gi|320446864|ref|NW_003383707.1|	241924-24272	180329	123835	-0.542206	0.7976	no
gi|320446864|ref|NW_003383707.1|	242997-24368	0.373458	255025	277162	0.2657	no
gi|320446864|ref|NW_003383707.1|	243806-24823	562868	796737	0.501306	0.69115	no
gi|320446864|ref|NW_003383707.1|	24494-26641	128873	138569	0.104652	0.93585	no
gi|320446864|ref|NW_003383707.1|	256005-25708	220317	110858	-0.990867	0.65595	no
gi|320446864|ref|NW_003383707.1|	257219-25824	148861	117597	-0.340123	0.8579	no

gi|320446864|ref|NW_003383707.1|	58922-25984	855356	758376	-0.173612	0.9305	no
gi|320446864|ref|NW_003383707.1|	59969-26131	192798	185528	-0.055451	0.96035	no
gi|320446864|ref|NW_003383707.1|	61460-26313	124681	876177	-0.508947	0.8226	no
gi|320446864|ref|NW_003383707.1|	63285-26548	142864	834789	-0.775163	0.64305	no
gi|320446864|ref|NW_003383707.1|	26817-27604	152999	12603	-0.279755	0.88545	no
gi|320446864|ref|NW_003383707.1|	27959-30571	118565	634876	-0.90113	0.57895	no
gi|320446864|ref|NW_003383707.1|	82298-28360	662022	109404	-259722	0.2574	no
gi|320446864|ref|NW_003383707.1|	83750-28859	207902	246122	-307846	0.1151	no
gi|320446864|ref|NW_003383707.1|	89407-29453	818669	649163	-0.334699	0.7839	no
gi|320446864|ref|NW_003383707.1|	95563-29833	805596	263166	-161409	0.2299	no
gi|320446864|ref|NW_003383707.1|	98464-29975	0	12192	inf	1	no
gi|320446864|ref|NW_003383707.1|	07193-30836	420726	103407	-202455	0.25765	no
gi|320446864|ref|NW_003383707.1|	31910-32963	150683	52816	-151247	0.4803	no
gi|320446864|ref|NW_003383707.1|	83147-38357	26722	995412	-142466	0.4936	no
gi|320446864|ref|NW_003383707.1|	89757-39121	116121	60777	-0.93403	0.489	no
gi|320446864|ref|NW_003383707.1|	92222-39264	119952	410949	-154543	0.3445	no
gi|320446864|ref|NW_003383707.1|	92776-39365	365652	137675	-14092	0.3856	no
gi|320446864|ref|NW_003383707.1|	95616-39975	322175	374503	0.217133	0.87	no
gi|320446864|ref|NW_003383707.1|	99956-40074	26057	151504	-0.782315	0.72075	no
gi|320446864|ref|NW_003383707.1|	12387-41373	0.303073	178885	256129	0.25	no
gi|320446864|ref|NW_003383707.1|	13885-41461	406961	325103	-0.323993	0.86365	no
gi|320446864|ref|NW_003383707.1|	17949-41924	509635	25421	-100344	0.6151	no
gi|320446864|ref|NW_003383707.1|	19407-41970	379214	109503	-179203	0.4082	no
gi|320446864|ref|NW_003383707.1|	28151-42882	263792	127005	-105452	0.63255	no
gi|320446864|ref|NW_003383707.1|	43554-44411	0	697565	inf	0.0162	no
gi|320446864|ref|NW_003383707.1|	46490-49459	288252	245965	-0.228876	0.86585	no
gi|320446864|ref|NW_003383707.1|	65833-46731	0	113384	inf	1	no
gi|320446864|ref|NW_003383707.1|	71126-47149	448075	192458	-12192	0.5617	no
gi|320446864|ref|NW_003383707.1|	71596-47189	279503	179457	-0.639229	0.74855	no
gi|320446864|ref|NW_003383707.1|	50574-52615	318671	186405	-0.773625	0.55435	no

gi|320446864|ref|NW_003383707.1|	36845-53765	118115	263717	11588	0.5865	no
gi|320446864|ref|NW_003383707.1|	5476-6716	0	45436	inf	0.0088	no
gi|320446864|ref|NW_003383707.1|	55045-56346	348401	703432	101366	0.61895	no
gi|320446864|ref|NW_003383707.1|	64007-56607	0.18425	12209	27282	1	no
gi|320446864|ref|NW_003383707.1|	97579-60031	195915	197805	333577	0.0893	no
gi|320446864|ref|NW_003383707.1|	06627-60693	908036	875244	326886	0.19605	no
gi|320446864|ref|NW_003383707.1|	09676-61137	0.68987	0.4003	-0.785241	1	no
gi|320446864|ref|NW_003383707.1|	61354-62622	964215	657613	-0.552117	0.79015	no
gi|320446864|ref|NW_003383707.1|	15403-61629	409404	209798	23574	0.29395	no
gi|320446864|ref|NW_003383707.1|	25346-62576	713905	960603	375014	0.15245	no
gi|320446864|ref|NW_003383707.1|	63314-66182	163436	14291	-0.193614	0.8806	no
gi|320446864|ref|NW_003383707.1|	7731-8205	0	462375	inf	0.0312	no
gi|320446864|ref|NW_003383707.1|	87280-88111	323058	82618	135466	0.43955	no
gi|320446864|ref|NW_003383707.1|	88628-90139	926015	159148	0.781261	0.7343	no
gi|320446864|ref|NW_003383707.1|	91471-92337	10706	165825	0.631245	0.75205	no
gi|320446864|ref|NW_003383707.1|	92529-94580	0.371749	142604	193962	0.39415	no
gi|320446864|ref|NW_003383707.1|	95369-96288	0.987563	221289	116399	0.5864	no
gi|320446864|ref|NW_003383707.1|	96448-97811	0.598619	0.935409	0.64396	1	no
gi|320446864|ref|NW_003383707.1|	98000-10301	544198	782227	0.523457	0.68085	no
gi|320446865|ref|NW_003383706.1|	01971-10606	173641	930826	-0.899522	0.5035	no
gi|320446865|ref|NW_003383706.1|	06935-10898	267024	168717	-398429	0.06035	no
gi|320446865|ref|NW_003383706.1|	09180-11235	206845	365651	0.821919	0.69475	no
gi|320446865|ref|NW_003383706.1|	14720-11690	0.520359	369153	282664	0.22255	no
gi|320446865|ref|NW_003383706.1|	20183-12048	559006	167493	158316	0.4856	no
gi|320446865|ref|NW_003383706.1|	21428-12167	142304	179752	0.33703	0.85835	no
gi|320446865|ref|NW_003383706.1|	23127-12339	253647	193697	293291	0.25735	no
gi|320446865|ref|NW_003383706.1|	34564-13497	910116	282256	163288	0.4338	no
gi|320446865|ref|NW_003383706.1|	91205-19149	108831	417271	19389	0.3855	no
gi|320446865|ref|NW_003383706.1|	20275-22096	225063	153669	-0.550503	0.7972	no
gi|320446865|ref|NW_003383706.1|	28329-22855	450292	283113	265245	0.27135	no

gi|320446865|ref|NW_003383706.1|	240313-24056	118977	263994	114981	0.58485	no
gi|320446865|ref|NW_003383706.1|	240977-24227	494643	140631	150745	0.4927	no
gi|320446865|ref|NW_003383706.1|	244366-24634	155286	11504	288913	0.2274	no
gi|320446865|ref|NW_003383706.1|	248342-24955	103786	116324	348646	0.17115	no
gi|320446865|ref|NW_003383706.1|	249669-25243	792817	638332	300925	0.1326	no
gi|320446865|ref|NW_003383706.1|	257591-25975	271504	81276	-174007	0.32875	no
gi|320446865|ref|NW_003383706.1|	260901-26119	980198	128151	-293523	0.2165	no
gi|320446865|ref|NW_003383706.1|	261254-26213	856158	179778	-557359	0.06455	no
gi|320446865|ref|NW_003383706.1|	262938-26312	239626	0	#NAME?	0.01425	no
gi|320446865|ref|NW_003383706.1|	263246-26461	432046	113432	-525128	0.06045	no
gi|320446865|ref|NW_003383706.1|	265010-26624	184201	201574	0.130024	0.95255	no
gi|320446865|ref|NW_003383706.1|	284533-28501	127633	130177	0.028472	0.97865	no
gi|320446865|ref|NW_003383706.1|	287059-28744	928533	14222	0.6151	0.75745	no
gi|320446865|ref|NW_003383706.1|	304789-30538	336587	171083	-0.976282	0.6521	no
gi|320446865|ref|NW_003383706.1|	314933-31687	198096	0.759555	-138297	0.5241	no
gi|320446865|ref|NW_003383706.1|	319118-32092	0.322516	277125	31031	0.21295	no
gi|320446865|ref|NW_003383706.1|	327244-32881	0.630775	473951	290954	0.22715	no
gi|320446865|ref|NW_003383706.1|	342718-34438	0.1182	189253	400101	0.19615	no
gi|320446865|ref|NW_003383706.1|	348581-35110	0	375761	inf	0.0053	no
gi|320446865|ref|NW_003383706.1|	362378-36290	0	152659	inf	0.00815	no
gi|320446865|ref|NW_003383706.1|	367329-37102	512088	309892	25973	0.1758	no
gi|320446865|ref|NW_003383706.1|	371293-37271	0	118783	inf	1	no
gi|320446865|ref|NW_003383706.1|	375596-37808	224219	120059	242077	0.16945	no
gi|320446865|ref|NW_003383706.1|	385471-38604	42763	748137	0.806939	0.62385	no
gi|320446865|ref|NW_003383706.1|	386326-38760	292683	319772	0.127704	0.9209	no
gi|320446865|ref|NW_003383706.1|	388924-39173	897848	622931	-0.5274	0.7343	no
gi|320446865|ref|NW_003383706.1|	392054-39341	0.149522	155765	338094	0.2261	no
gi|320446865|ref|NW_003383706.1|	393835-39529	67466	47854	-0.495522	0.8079	no
gi|320446865|ref|NW_003383706.1|	413097-41454	978169	29136	-174728	0.428	no
gi|320446865|ref|NW_003383706.1|	415101-41566	761165	171431	-215058	0.34425	no

gi|320446865|ref|NW_003383706.1|	417272-42007	12204	56404	-111348	0.5048	no
gi|320446865|ref|NW_003383706.1|	420543-42305	419307	58521	0.480947	0.72325	no
gi|320446865|ref|NW_003383706.1|	426221-42869	246473	172134	-0.517902	0.6963	no
gi|320446865|ref|NW_003383706.1|	428882-43008	138951	0.481637	-152856	1	no
gi|320446865|ref|NW_003383706.1|	436351-43660	301059	152646	-0.979858	0.66335	no
gi|320446865|ref|NW_003383706.1|	443440-44465	498566	369514	-0.432157	0.8307	no
gi|320446865|ref|NW_003383706.1|	445620-44669	402969	223021	-0.853492	0.67395	no
gi|320446865|ref|NW_003383706.1|	449091-44957	197022	307481	0.642142	0.74155	no
gi|320446865|ref|NW_003383706.1|	450090-45128	193247	0.852391	-118086	0.57055	no
gi|320446865|ref|NW_003383706.1|	452550-45520	121459	103.14	-0.235868	0.8521	no
gi|320446865|ref|NW_003383706.1|	459109-46022	305242	0.396257	-294545	0.209	no
gi|320446865|ref|NW_003383706.1|	461532-46206	665514	0	#NAME?	0.02015	no
gi|320446865|ref|NW_003383706.1|	464943-46591	553649	0.796214	-279774	0.2518	no
gi|320446865|ref|NW_003383706.1|	476127-47706	0.967737	166867	0.786009	0.71775	no
gi|320446865|ref|NW_003383706.1|	477952-47883	209467	0.901623	-121613	0.5719	no
gi|320446865|ref|NW_003383706.1|	502600-50503	229052	674046	-176476	0.31285	no
gi|320446865|ref|NW_003383706.1|	506067-50665	177308	519163	-1772	0.4055	no
gi|320446865|ref|NW_003383706.1|	506893-50745	364571	450823	-301557	0.2217	no
gi|320446865|ref|NW_003383706.1|	510092-51182	107256	21229	-233695	0.31505	no
gi|320446865|ref|NW_003383706.1|	511949-51574	385404	210748	-0.87085	0.51455	no
gi|320446865|ref|NW_003383706.1|	520473-52205	129263	340532	-192445	0.3922	no
gi|320446865|ref|NW_003383706.1|	522790-52414	128575	10504	-0.291674	0.89425	no
gi|320446865|ref|NW_003383706.1|	525045-52608	246281	447362	-246079	0.29405	no
gi|320446865|ref|NW_003383706.1|	526234-52920	234209	219754	-0.0919079	0.9649	no
gi|320446865|ref|NW_003383706.1|	529353-53051	896291	367483	-128629	0.53505	no
gi|320446865|ref|NW_003383706.1|	530647-53131	220759	77935	-150213	0.4798	no
gi|320446865|ref|NW_003383706.1|	533597-53897	187155	103522	-0.854301	0.5194	no
gi|320446865|ref|NW_003383706.1|	5378-5849	469696	201084	-122393	0.5685	no
gi|320446865|ref|NW_003383706.1|	539196-54226	396294	519663	0.391006	0.76425	no
gi|320446865|ref|NW_003383706.1|	560410-56513	0.60116	547356	318666	0.09485	no

gi|320446865|ref|NW_003383706.1|	65613-56591	189607	474515	464537	0.17495	no
gi|320446865|ref|NW_003383706.1|	68343-57493	0.477616	500331	338896	0.08495	no
gi|320446865|ref|NW_003383706.1|	57443-57656	450319	21148	-109043	0.5928	no
gi|320446865|ref|NW_003383706.1|	75371-57676	0.87089	36302	205949	0.342	no
gi|320446865|ref|NW_003383706.1|	81697-58205	0	253487	inf	0.01195	no
gi|320446865|ref|NW_003383706.1|	91916-101077	119654	823317	-0.539354	0.68915	no
gi|320446867|ref|NW_003383704.1|	60214-16132	115248	5319	220642	0.32155	no
gi|320446867|ref|NW_003383704.1|	33114-23394	197045	232297	0.237443	0.89925	no
gi|320446867|ref|NW_003383704.1|	35761-23703	195355	135539	-0.527386	0.79675	no
gi|320446867|ref|NW_003383704.1|	39553-23972	0	865099	inf	0.0312	no
gi|320446867|ref|NW_003383704.1|	6560-7273	0	338277	inf	0.0233	no
gi|320446867|ref|NW_003383704.1|	65795-66494	149184	696266	-109939	0.5991	no
gi|320446867|ref|NW_003383704.1|	68020-68429	110862	48738	-118564	0.56715	no
gi|320446867|ref|NW_003383704.1|	69659-71056	0.581094	0.807398	0.47451	1	no
gi|320446867|ref|NW_003383704.1|	71604-74329	582858	473597	-0.299483	0.8937	no
gi|320446869|ref|NW_003383702.1|	17069-12787	0.367034	100755	47788	0.0387	no
gi|320446869|ref|NW_003383702.1|	28013-13042	0.154829	108114	28038	1	no
gi|320446869|ref|NW_003383702.1|	14754-14984	0	66566	inf	0.01485	no
gi|320446869|ref|NW_003383702.1|	15107-15332	0	805304	inf	0.01275	no
gi|320446869|ref|NW_003383702.1|	15438-15643	0	262722	inf	0.00715	no
gi|320446869|ref|NW_003383702.1|	38109-23855	0	572781	inf	0.0294	no
gi|320446869|ref|NW_003383702.1|	27403-28053	0.812855	30447	190523	0.321	no
gi|320446869|ref|NW_003383702.1|	2901-3613	109741	101689	-0.109936	0.9563	no
gi|320446869|ref|NW_003383702.1|	30027-31655	101784	125657	0.303973	0.8886	no
gi|320446869|ref|NW_003383702.1|	33150-33770	875302	267572	-170985	0.42435	no
gi|320446869|ref|NW_003383702.1|	3747-6598	483817	730445	0.594312	0.7932	no
gi|320446869|ref|NW_003383702.1|	00562-40205	0	233058	inf	0.01485	no
gi|320446869|ref|NW_003383702.1|	47125-48047	294974	372883	0.338134	0.8629	no
gi|320446869|ref|NW_003383702.1|	33620-53442	0.294772	263266	315885	0.24575	no
gi|320446869|ref|NW_003383702.1|	34502-53552	0.428015	162704	192652	0.3182	no

gi|320446869|ref|NW_003383702.1|	59727-56353	615766	284854	220977	0.2033	no
gi|320446869|ref|NW_003383702.1|	63654-57111	330502	105652	167659	0.32505	no
gi|320446869|ref|NW_003383702.1|	85232-58622	872248	447959	-0.961374	0.6385	no
gi|320446869|ref|NW_003383702.1|	86389-58837	114899	538595	-10931	0.62475	no
gi|320446869|ref|NW_003383702.1|	91238-59183	230601	218902	-0.0751129	0.92685	no
gi|320446869|ref|NW_003383702.1|	92455-59280	231962	586879	-198275	0.38205	no
gi|320446869|ref|NW_003383702.1|	01035-60166	0	321479	inf	0.0294	no
gi|320446869|ref|NW_003383702.1|	03667-60521	0.128363	205366	39999	0.1967	no
gi|320446869|ref|NW_003383702.1|	05612-60814	0.440247	691895	397417	0.13915	no
gi|320446869|ref|NW_003383702.1|	14888-61656	0.11689	113931	328494	1	no
gi|320446869|ref|NW_003383702.1|	22406-62342	0.216479	149594	278875	0.2657	no
gi|320446869|ref|NW_003383702.1|	24594-62519	0	168431	inf	0.0071	no
gi|320446869|ref|NW_003383702.1|	26819-62888	0.276527	0.835804	159575	1	no
gi|320446869|ref|NW_003383702.1|	41793-64217	305916	830188	-188163	0.38865	no
gi|320446869|ref|NW_003383702.1|	42330-64336	846369	204778	-204723	0.34845	no
gi|320446869|ref|NW_003383702.1|	43590-64553	850814	186219	-219184	0.3387	no
gi|320446869|ref|NW_003383702.1|	45819-64677	605582	0.482265	-365043	0.17335	no
gi|320446869|ref|NW_003383702.1|	46951-64731	244795	558541	-213184	0.3428	no
gi|320446869|ref|NW_003383702.1|	47478-64913	190455	513726	-189038	0.2655	no
gi|320446869|ref|NW_003383702.1|	49261-65119	935445	339932	-146041	0.5073	no
gi|320446869|ref|NW_003383702.1|	56255-65681	257506	22258	311165	0.2063	no
gi|320446869|ref|NW_003383702.1|	66359-68424	387349	694738	0.842835	0.69775	no
gi|320446869|ref|NW_003383702.1|	69316-66960	108831	43118	19862	0.3886	no
gi|320446869|ref|NW_003383702.1|	6699-7200	131524	377736	-179987	0.40655	no
gi|320446869|ref|NW_003383702.1|	72728-67582	0.295021	177412	258822	0.261	no
gi|320446869|ref|NW_003383702.1|	86037-68684	0	203207	inf	0.0312	no
gi|320446869|ref|NW_003383702.1|	89921-69042	551147	55821	0.0183732	0.99185	no
gi|320446869|ref|NW_003383702.1|	90618-70137	102515	161278	0.653721	0.61875	no
gi|320446869|ref|NW_003383702.1|	70218-70994	0.624441	257067	204151	0.30255	no
gi|320446869|ref|NW_003383702.1|	04552-70660	313717	253214	-0.309104	0.80685	no

gi|320446869|ref|NW_003383702.1|	'06908-71203:	250516	176465	-0.50552	0.70545	no
gi|320446869|ref|NW_003383702.1|	'12889-71368!	254108	289501	0.188126	0.9305	no
gi|320446869|ref|NW_003383702.1|	'15576-71801:	130408	128028	329536	0.08245	no
gi|320446869|ref|NW_003383702.1|	72925-73576	283823	220924	-0.361441	0.85525	no
gi|320446869|ref|NW_003383702.1|	74127-74449	411642	275983	-0.576811	0.78285	no
gi|320446869|ref|NW_003383702.1|	'44899-74550:	135092	366709	14407	0.5076	no
gi|320446869|ref|NW_003383702.1|	'55218-75549(	699879	208305	157352	0.4856	no
gi|320446869|ref|NW_003383702.1|	'55947-75628(	145874	132242	318039	0.23215	no
gi|320446869|ref|NW_003383702.1|	'58649-75921:	0.503005	407971	301982	0.25005	no
gi|320446869|ref|NW_003383702.1|	'72791-77450(	0.22902	358816	39697	0.1643	no
gi|320446869|ref|NW_003383702.1|	78982-79628	143618	949837	-0.596481	0.7725	no
gi|320446869|ref|NW_003383702.1|	'89883-79079:	0.250557	328136	371108	0.21225	no
gi|320446869|ref|NW_003383702.1|	'91205-79204(	0.844478	251655	157531	0.491	no
gi|320446869|ref|NW_003383702.1|	308319-81269(	0	285672	inf	0.0042	no
gi|320446869|ref|NW_003383702.1|	81305-82778	599942	44551	-0.429367	0.83315	no
gi|320446869|ref|NW_003383702.1|	319146-81961·	0	898727	inf	0.0212	no
gi|320446869|ref|NW_003383702.1|	84283-84491	619151	348309	249201	0.27555	no
gi|320446869|ref|NW_003383702.1|	84976-86215	53808	524778	-0.0361122	0.9817	no
gi|320446869|ref|NW_003383702.1|	87744-90013	921002	677596	-0.44278	0.84625	no
gi|320446869|ref|NW_003383702.1|	397393-89804:	0	278072	inf	0.0312	no
gi|320446869|ref|NW_003383702.1|	90727-92224	15382	166477	0.114083	0.9441	no
gi|320446869|ref|NW_003383702.1|	)22249-92320:	0.235517	406297	410863	0.19235	no
gi|320446869|ref|NW_003383702.1|	92359-92979	525181	832446	0.664541	0.7409	no
gi|320446869|ref|NW_003383702.1|	93574-94799	131308	243588	0.891491	0.58215	no
gi|320446874|ref|NW_003383697.1|	115799-11690·	0.776272	698524	316968	0.21685	no
gi|320446874|ref|NW_003383697.1|	127681-13151:	455051	117216	136507	0.42965	no
gi|320446874|ref|NW_003383697.1|	131658-13375'	184708	457923	130986	0.32005	no
gi|320446874|ref|NW_003383697.1|	133927-13717:	100947	2628	138036	0.5189	no
gi|320446874|ref|NW_003383697.1|	141004-14132:	654885	564044	-0.215434	0.9182	no
gi|320446874|ref|NW_003383697.1|	167873-16939:	224758	125156	-0.844647	0.51835	no

gi|320446874|ref|NW_003383697.1|	169530-17326	447799	163781	-145109	0.28835	no
gi|320446874|ref|NW_003383697.1|	173508-17522	204556	30239	-275802	0.13085	no
gi|320446874|ref|NW_003383697.1|	184180-18460	110781	57.94	-0.935076	0.55725	no
gi|320446874|ref|NW_003383697.1|	185390-18695	116.42	562882	-104843	0.437	no
gi|320446874|ref|NW_003383697.1|	187366-19111	480071	587945	0.292435	0.84925	no
gi|320446874|ref|NW_003383697.1|	193971-19559	473931	236807	-100096	0.6187	no
gi|320446874|ref|NW_003383697.1|	196177-19685	14256	28914	-230173	0.31465	no
gi|320446874|ref|NW_003383697.1|	217101-21825	457788	532787	0.218879	0.9108	no
gi|320446874|ref|NW_003383697.1|	218715-22181	200124	819052	203306	0.2284	no
gi|320446874|ref|NW_003383697.1|	221958-22239	239447	423652	0.823173	0.69235	no
gi|320446874|ref|NW_003383697.1|	224249-22506	255823	51144	0.999416	0.5448	no
gi|320446874|ref|NW_003383697.1|	225244-22841	943257	342695	18612	0.1679	no
gi|320446874|ref|NW_003383697.1|	231617-23575	0.432449	14532	174863	0.4194	no
gi|320446874|ref|NW_003383697.1|	241202-24240	17597	294697	0.743904	0.655	no
gi|320446874|ref|NW_003383697.1|	243092-24331	497144	37.44	-0.409083	0.8483	no
gi|320446874|ref|NW_003383697.1|	246962-24738	300568	119288	-133324	0.52555	no
gi|320446874|ref|NW_003383697.1|	266993-26740	540701	535398	-0.0142195	0.94	no
gi|320446874|ref|NW_003383697.1|	269055-26992	662814	730188	0.139662	0.94345	no
gi|320446874|ref|NW_003383697.1|	308913-30966	754325	137105	0.862021	0.68485	no
gi|320446874|ref|NW_003383697.1|	309818-31082	917452	158474	0.788537	0.71695	no
gi|320446874|ref|NW_003383697.1|	311386-31532	296441	227288	293871	0.1312	no
gi|320446874|ref|NW_003383697.1|	315988-31772	365217	361572	-0.0144707	0.9911	no
gi|320446874|ref|NW_003383697.1|	318152-32140	211567	555875	-192828	0.29195	no
gi|320446874|ref|NW_003383697.1|	326721-32783	192298	359436	0.902386	0.6655	no
gi|320446874|ref|NW_003383697.1|	343175-34377	330291	0.933317	-514522	0.13795	no
gi|320446874|ref|NW_003383697.1|	344913-34547	921524	380377	-127659	0.55345	no
gi|320446874|ref|NW_003383697.1|	345663-34962	152045	115614	-0.395184	0.7597	no
gi|320446874|ref|NW_003383697.1|	355914-35623	109336	26975	-201907	0.3638	no
gi|320446874|ref|NW_003383697.1|	357161-35788	317504	596816	-241142	0.2892	no
gi|320446874|ref|NW_003383697.1|	358325-35931	172671	526566	-171334	0.438	no

gi|320446874|ref|NW_003383697.1|	360437-36366	155083	193.74	0.321078	0.8404	no
gi|320446874|ref|NW_003383697.1|	365238-36622	153509	717098	-109808	0.60905	no
gi|320446874|ref|NW_003383697.1|	36539-37316	0.311644	256604	304157	0.24995	no
gi|320446874|ref|NW_003383697.1|	366415-36771	337315	161218	-106508	0.528	no
gi|320446874|ref|NW_003383697.1|	368998-36947	379364	165516	-119661	0.37145	no
gi|320446874|ref|NW_003383697.1|	374231-37506	0	69689	inf	0.0085	no
gi|320446874|ref|NW_003383697.1|	386113-38718	0.202452	252084	363826	0.2061	no
gi|320446874|ref|NW_003383697.1|	387378-38882	0.139392	232516	406011	0.1924	no
gi|320446874|ref|NW_003383697.1|	389519-39299	0.0520504	396928	625282	0.15535	no
gi|320446874|ref|NW_003383697.1|	408852-40988	0	255456	inf	0.0022	no
gi|320446874|ref|NW_003383697.1|	412548-41688	0.411462	21723	572232	0.0472	no
gi|320446874|ref|NW_003383697.1|	417566-41958	252214	872814	-153091	0.38105	no
gi|320446874|ref|NW_003383697.1|	421806-42218	751029	28126	-141697	0.51425	no
gi|320446874|ref|NW_003383697.1|	422678-42371	294017	624337	-22355	0.19345	no
gi|320446874|ref|NW_003383697.1|	424603-42610	135465	249349	-244168	0.28845	no
gi|320446874|ref|NW_003383697.1|	430610-43154	303288	276108	-0.135456	0.93315	no
gi|320446874|ref|NW_003383697.1|	437567-43975	248522	245927	-0.0151447	0.99145	no
gi|320446874|ref|NW_003383697.1|	440829-44179	20876	249761	0.258703	0.90585	no
gi|320446874|ref|NW_003383697.1|	441936-44361	0.937069	0.978571	0.0625221	1	no
gi|320446874|ref|NW_003383697.1|	443794-44982	289905	376975	0.37889	0.77315	no
gi|320446874|ref|NW_003383697.1|	450432-45231	183769	186035	-330425	0.0919	no
gi|320446874|ref|NW_003383697.1|	535465-53620	0.335108	229531	277599	0.2657	no
gi|320446874|ref|NW_003383697.1|	536882-53713	288274	202732	-38298	0.3201	no
gi|320446874|ref|NW_003383697.1|	537246-53831	135806	140212	-327586	0.18165	no
gi|320446874|ref|NW_003383697.1|	538446-53964	135555	305052	-215175	0.33735	no
gi|320446875|ref|NW_003383696.1|	150907-15135	450918	180736	-131898	0.54365	no
gi|320446875|ref|NW_003383696.1|	256471-25718	42216	168456	-132541	0.5185	no
gi|320446875|ref|NW_003383696.1|	257823-25853	131529	388963	-175768	0.40955	no
gi|320446875|ref|NW_003383696.1|	263247-26389	409352	195101	-106912	0.5988	no
gi|320446875|ref|NW_003383696.1|	265574-26688	788122	100652	0.352879	0.8674	no

gi|320446875|ref|NW_003383696.1|	267786-27159	727917	949425	0.383281	0.76545	no
gi|320446875|ref|NW_003383696.1|	271950-27566	139651	668492	-106285	0.41465	no
gi|320446875|ref|NW_003383696.1|	275886-27799	147619	420717	-181096	0.285	no
gi|320446875|ref|NW_003383696.1|	279208-28026	515578	201727	-135378	0.42795	no
gi|320446875|ref|NW_003383696.1|	282393-28731	291782	303174	0.0552553	0.965	no
gi|320446875|ref|NW_003383696.1|	287587-28963	120818	123158	0.0276753	1	no
gi|320446875|ref|NW_003383696.1|	294585-29536	900241	696362	-0.370473	0.77945	no
gi|320446875|ref|NW_003383696.1|	295538-29604	48978	852138	-252298	0.2726	no
gi|320446875|ref|NW_003383696.1|	296458-29798	250509	611135	-20353	0.239	no
gi|320446875|ref|NW_003383696.1|	299879-30330	39133	15.76	20098	0.263	no
gi|320446875|ref|NW_003383696.1|	303702-30442	312729	261552	-0.257812	0.9026	no
gi|320446875|ref|NW_003383696.1|	307064-30763	944685	30258	-164252	0.42735	no
gi|320446875|ref|NW_003383696.1|	308291-31059	216472	143126	-0.596894	0.6399	no
gi|320446875|ref|NW_003383696.1|	315076-31575	512403	735635	0.52171	0.6814	no
gi|320446875|ref|NW_003383696.1|	336967-33735	123804	14222	0.200063	0.91725	no
gi|320446875|ref|NW_003383696.1|	338111-34506	730661	146803	100661	0.4537	no
gi|320446875|ref|NW_003383696.1|	353332-35787	425959	230579	-0.885455	0.5124	no
gi|320446875|ref|NW_003383696.1|	361049-36148	51082	111209	-219955	0.32105	no
gi|320446875|ref|NW_003383696.1|	370730-37239	366165	285326	296205	0.12015	no
gi|320446875|ref|NW_003383696.1|	384751-38615	0.431731	709915	403944	0.16915	no
gi|320446875|ref|NW_003383696.1|	407251-40748	78516	32171	20347	0.30095	no
gi|320446875|ref|NW_003383696.1|	409192-41056	0.891601	120756	375955	0.15345	no
gi|320446875|ref|NW_003383696.1|	445509-44710	0	172221	inf	0.0162	no
gi|320446875|ref|NW_003383696.1|	449994-45042	0.831228	121126	386512	0.2072	no
gi|320446875|ref|NW_003383696.1|	456946-45756	130627	106503	302737	0.21565	no
gi|320446875|ref|NW_003383696.1|	464239-46479	0	713986	inf	0.0162	no
gi|320446875|ref|NW_003383696.1|	467811-46920	0	124137	inf	0.00525	no
gi|320446875|ref|NW_003383696.1|	568072-56830	300689	270315	-0.153632	0.9267	no
gi|320446875|ref|NW_003383696.1|	570071-57063	763545	872863	0.193042	0.8803	no
gi|320446875|ref|NW_003383696.1|	574210-57482	233545	46942	100718	0.64805	no

gi|320446875|ref|NW_003383696.1|	;33512-63520:	524347	0	#NAME?	5.00E-05	yes
gi|320446875|ref|NW_003383696.1|	;62350-66323:	0	195971	inf	0.0294	no
gi|320446875|ref|NW_003383696.1|	;88929-69027:	287919	0.526132	-245217	0.2944	no
gi|320446875|ref|NW_003383696.1|	90901-95443	404316	544006	375007	0.0282	no
gi|320446875|ref|NW_003383696.1|	95652-98180	266427	201516	291908	0.03415	no
gi|320446876|ref|NW_003383695.1|	l07911-11185!	0.681699	165418	127891	0.54315	no
gi|320446876|ref|NW_003383695.1|	l12140-11234:	371491	38701	0.0590457	0.9428	no
gi|320446876|ref|NW_003383695.1|	l13660-12076	68779	242474	181779	0.1781	no
gi|320446876|ref|NW_003383695.1|	l49708-15063	170331	119146	-0.515605	0.81415	no
gi|320446876|ref|NW_003383695.1|	l66943-16823:	0.800082	0.999397	0.320911	1	no
gi|320446876|ref|NW_003383695.1|	l69046-17112:	247656	204737	-0.274568	0.8935	no
gi|320446876|ref|NW_003383695.1|	l87826-18843	0	337003	inf	0.0294	no
gi|320446876|ref|NW_003383695.1|	l93545-19562:	237308	175756	-0.43319	0.7352	no
gi|320446876|ref|NW_003383695.1|	l96914-19766!	870705	204147	-209257	0.2399	no
gi|320446876|ref|NW_003383695.1|	l98080-20029!	110584	30276	-18689	0.39975	no
gi|320446876|ref|NW_003383695.1|	!00433-20106	37604	977063	-194436	0.3781	no
gi|320446876|ref|NW_003383695.1|	!42766-24366:	153526	211561	0.462583	0.81725	no
gi|320446876|ref|NW_003383695.1|	!44308-24732	818601	199258	-203852	0.2263	no
gi|320446876|ref|NW_003383695.1|	!53704-25395	55533	0	#NAME?	0.01345	no
gi|320446876|ref|NW_003383695.1|	l60526-46288!	211749	107972	-0.971696	0.44305	no
gi|320446876|ref|NW_003383695.1|	l63018-46450	433134	429667	-0.0115961	0.9919	no
gi|320446876|ref|NW_003383695.1|	l66310-46795	156598	840402	-0.897913	0.5745	no
gi|320446876|ref|NW_003383695.1|	l68085-47106:	115745	46055	-132952	0.43285	no
gi|320446876|ref|NW_003383695.1|	l71387-47398:	0.213981	134539	265246	1	no
gi|320446876|ref|NW_003383695.1|	l74901-47585	305095	832269	-187414	0.2536	no
gi|320446876|ref|NW_003383695.1|	l77556-47943	99645	108825	0.127138	0.95345	no
gi|320446876|ref|NW_003383695.1|	l79783-48072	119089	377939	-165581	0.44385	no
gi|320446876|ref|NW_003383695.1|	l81002-48146:	4379	291622	-0.586501	0.75475	no
gi|320446876|ref|NW_003383695.1|	l84339-48573!	122585	443944	-146533	0.274	no
gi|320446876|ref|NW_003383695.1|	l90969-49130	992356	191143	426765	0.06945	no

gi|320446876|ref|NW_003383695.1|	91485-49201	563591	697857	363021	0.07865	no
gi|320446876|ref|NW_003383695.1|	96155-49666	240204	644963	142495	0.50435	no
gi|320446876|ref|NW_003383695.1|	02152-50631	274865	862942	165054	0.3339	no
gi|320446876|ref|NW_003383695.1|	27248-53347	50845	483904	-0.0713831	0.95465	no
gi|320446876|ref|NW_003383695.1|	53633-54014	396972	984409	-201171	0.3524	no
gi|320446876|ref|NW_003383695.1|	41523-54206	165849	335077	101462	0.5772	no
gi|320446876|ref|NW_003383695.1|	44033-54448	306985	357022	0.217846	0.89885	no
gi|320446876|ref|NW_003383695.1|	45180-54690	272604	284753	0.0629072	0.9737	no
gi|320446876|ref|NW_003383695.1|	48756-54993	0.893276	940924	33969	0.1789	no
gi|320446876|ref|NW_003383695.1|	50136-55126	0.564038	546836	327724	0.2044	no
gi|320446876|ref|NW_003383695.1|	55113-55559	108842	258194	-207571	0.35565	no
gi|320446876|ref|NW_003383695.1|	52182-55368	0.133544	167145	364571	0.20565	no
gi|320446876|ref|NW_003383695.1|	54336-55533	154914	901958	254159	0.27255	no
gi|320446876|ref|NW_003383695.1|	57015-55864	387471	674186	0.799059	0.6993	no
gi|320446876|ref|NW_003383695.1|	58849-56024	116319	454548	196635	0.3625	no
gi|320446876|ref|NW_003383695.1|	65858-56815	0.0820052	166004	433936	0.18255	no
gi|320446876|ref|NW_003383695.1|	61091-63465	213027	39112	0.876574	0.6805	no
gi|320446876|ref|NW_003383695.1|	28639-62983	12349	0.366818	-175125	1	no
gi|320446876|ref|NW_003383695.1|	32747-63302	156306	687407	213679	0.23295	no
gi|320446876|ref|NW_003383695.1|	33967-63578	394252	223913	250575	0.07085	no
gi|320446876|ref|NW_003383695.1|	63881-67690	128895	227013	0.816575	0.5312	no
gi|320446876|ref|NW_003383695.1|	76290-77797	132704	276839	106083	0.6025	no
gi|320446876|ref|NW_003383695.1|	81718-82438	66156	100065	0.596991	0.7691	no
gi|320446876|ref|NW_003383695.1|	98945-99206	168253	409436	128301	0.5413	no
gi|320446878|ref|NW_003383693.1|	10795-11795	904982	426946	-108383	0.60295	no
gi|320446878|ref|NW_003383693.1|	1535-2127	612728	702532	0.197317	0.9156	no
gi|320446878|ref|NW_003383693.1|	54392-15517	0.308795	445045	384923	0.2006	no
gi|320446878|ref|NW_003383693.1|	15987-16211	372051	876676	-208538	0.2863	no
gi|320446878|ref|NW_003383693.1|	18366-20124	73144	115788	-265926	0.2485	no
gi|320446878|ref|NW_003383693.1|	20404-22017	832941	102292	-302552	0.21275	no

gi|320446878|ref|NW_003383693.1|	212424-21319	101791	32724	168473	0.4512	no
gi|320446878|ref|NW_003383693.1|	233542-23486	27852	161131	-0.789549	0.7015	no
gi|320446878|ref|NW_003383693.1|	235664-23657	203943	925851	-113931	0.597	no
gi|320446878|ref|NW_003383693.1|	237926-23829	157327	154371	-0.0273701	0.9726	no
gi|320446878|ref|NW_003383693.1|	238717-24408	143625	173855	0.275576	0.83875	no
gi|320446878|ref|NW_003383693.1|	247575-25255	130153	991423	-0.392637	0.7634	no
gi|320446878|ref|NW_003383693.1|	252641-25420	0.762632	18569	128383	0.54245	no
gi|320446878|ref|NW_003383693.1|	255526-25661	0.795488	137607	0.790646	1	no
gi|320446878|ref|NW_003383693.1|	31010-33327	794385	560208	-0.503874	0.8202	no
gi|320446878|ref|NW_003383693.1|	324169-32549	932675	152152	0.706069	0.7544	no
gi|320446878|ref|NW_003383693.1|	325627-32653	120446	882072	-0.449423	0.82435	no
gi|320446878|ref|NW_003383693.1|	327565-32797	18477	127937	279164	0.23095	no
gi|320446878|ref|NW_003383693.1|	330073-33067	182519	216657	0.24737	0.8906	no
gi|320446878|ref|NW_003383693.1|	33844-34167	125693	152196	0.276026	0.88365	no
gi|320446878|ref|NW_003383693.1|	34454-34631	528777	569761	0.107699	0.9162	no
gi|320446878|ref|NW_003383693.1|	3460-6895	266833	122111	-112774	0.40035	no
gi|320446878|ref|NW_003383693.1|	34983-36004	816336	66811	-0.289076	0.88745	no
gi|320446878|ref|NW_003383693.1|	355753-35654	272434	956064	18112	0.3944	no
gi|320446878|ref|NW_003383693.1|	36167-37725	599654	550279	-0.123967	0.9526	no
gi|320446878|ref|NW_003383693.1|	43556-44198	0	127175	inf	5.00E-05	yes
gi|320446878|ref|NW_003383693.1|	45344-50482	0.103245	605628	919622	0.1156	no
gi|320446878|ref|NW_003383693.1|	59974-60461	0	529002	inf	0.029	no
gi|320446878|ref|NW_003383693.1|	64742-67952	0.0567228	376868	605399	0.159	no
gi|320446878|ref|NW_003383693.1|	9140-10682	271212	125764	-110871	0.59775	no
gi|320446879|ref|NW_003383692.1|	10938-14406	245318	839877	177552	0.2876	no
gi|320446879|ref|NW_003383692.1|	14850-15319	423975	424088	0.000385191	0.94	no
gi|320446879|ref|NW_003383692.1|	260200-26084	186897	650087	-152354	0.47665	no
gi|320446879|ref|NW_003383692.1|	262743-26339	982445	362331	-143907	0.4949	no
gi|320446879|ref|NW_003383692.1|	264113-26487	241757	106081	-11884	0.5891	no
gi|320446879|ref|NW_003383692.1|	267039-26825	253497	156622	-0.694679	0.66455	no

gi|320446879|ref|NW_003383692.1|	270368-27132	958928	968471	0.0142853	0.99115	no
gi|320446879|ref|NW_003383692.1|	271548-27503	160712	268402	0.739919	0.72135	no
gi|320446879|ref|NW_003383692.1|	278670-28189	440218	671026	0.608149	0.7919	no
gi|320446879|ref|NW_003383692.1|	312461-31687	210219	86457	-128184	0.33235	no
gi|320446879|ref|NW_003383692.1|	318242-31844	167946	174568	-326613	0.2159	no
gi|320446879|ref|NW_003383692.1|	318639-31931	356529	362435	-329823	0.18195	no
gi|320446879|ref|NW_003383692.1|	326085-32716	201812	221199	-31896	0.18185	no
gi|320446879|ref|NW_003383692.1|	333512-33388	115782	680813	255585	0.27405	no
gi|320446879|ref|NW_003383692.1|	348676-34995	233076	853533	-144928	0.37595	no
gi|320446879|ref|NW_003383692.1|	350389-35077	289683	28126	-0.0425701	0.98115	no
gi|320446879|ref|NW_003383692.1|	350937-35514	105782	110942	0.0687124	0.9579	no
gi|320446879|ref|NW_003383692.1|	360663-36116	883133	0.845209	-338525	0.238	no
gi|320446879|ref|NW_003383692.1|	36773-37202	419463	666551	0.668171	0.75045	no
gi|320446879|ref|NW_003383692.1|	376816-37861	797063	142578	-248294	0.2832	no
gi|320446879|ref|NW_003383692.1|	412573-41488	267715	897498	-157672	0.3733	no
gi|320446879|ref|NW_003383692.1|	415595-41672	694872	377163	-0.88156	0.50175	no
gi|320446879|ref|NW_003383692.1|	423354-42656	343067	178358	-0.943712	0.4816	no
gi|320446879|ref|NW_003383692.1|	426817-43164	149152	141559	-0.0753841	0.97545	no
gi|320446879|ref|NW_003383692.1|	436627-43946	419297	235131	248742	0.1923	no
gi|320446879|ref|NW_003383692.1|	441027-44235	192886	802396	205657	0.1244	no
gi|320446879|ref|NW_003383692.1|	44193-44765	134633	131483	-0.0341548	0.98085	no
gi|320446879|ref|NW_003383692.1|	453837-45420	620755	230581	189318	0.2715	no
gi|320446879|ref|NW_003383692.1|	516709-51708	899013	229351	135114	0.4232	no
gi|320446879|ref|NW_003383692.1|	536158-53661	447049	44619	-0.00277369	0.94	no
gi|320446879|ref|NW_003383692.1|	539412-54003	261255	384595	0.557884	0.79045	no
gi|320446879|ref|NW_003383692.1|	542241-54411	525828	567661	0.110438	0.95745	no
gi|320446879|ref|NW_003383692.1|	546359-54773	2229	288989	0.374614	0.85035	no
gi|320446881|ref|NW_003383690.1|	106382-10743	740123	564594	-0.390552	0.7703	no
gi|320446881|ref|NW_003383690.1|	107596-10789	187711	936863	-10026	0.65305	no
gi|320446881|ref|NW_003383690.1|	108888-10986	939369	361058	-137946	0.29335	no

gi|320446881|ref|NW_003383690.1|	[illegible]11275-11235[illegible]	372329	895488	-205583	0.23415	no
gi|320446881|ref|NW_003383690.1|	[illegible]13784-11467[illegible]	560344	171545	-170772	0.31785	no
gi|320446881|ref|NW_003383690.1|	[illegible]53281-25496[illegible]	0.350428	284633	302192	0.2187	no
gi|320446881|ref|NW_003383690.1|	[illegible]05667-30681[illegible]	110589	29365	140889	0.50775	no
gi|320446881|ref|NW_003383690.1|	[illegible]06991-30782[illegible]	103806	636735	-0.705124	0.7316	no
gi|320446881|ref|NW_003383690.1|	[illegible]08008-30908[illegible]	0.804018	139061	0.790416	1	no
gi|320446881|ref|NW_003383690.1|	[illegible]09345-31039[illegible]	995663	120487	0.27515	0.89795	no
gi|320446881|ref|NW_003383690.1|	3746-4405	0	325403	inf	0.029	no
gi|320446881|ref|NW_003383690.1|	[illegible]31669-43387[illegible]	569703	169189	-175157	0.19615	no
gi|320446881|ref|NW_003383690.1|	[illegible]38331-44393[illegible]	148707	137025	-0.118038	0.93205	no
gi|320446881|ref|NW_003383690.1|	[illegible]45954-44692[illegible]	595578	784594	0.397655	0.75935	no
gi|320446881|ref|NW_003383690.1|	[illegible]66696-46892[illegible]	198352	588024	156781	0.24175	no
gi|320446881|ref|NW_003383690.1|	[illegible]69045-47749[illegible]	170741	707911	205176	0.26035	no
gi|320446881|ref|NW_003383690.1|	48077-51719	158493	381239	126627	0.5563	no
gi|320446881|ref|NW_003383690.1|	[illegible]05200-50763[illegible]	0.306703	139215	21824	1	no
gi|320446881|ref|NW_003383690.1|	[illegible]60923-66229[illegible]	642731	160535	-200133	0.2636	no
gi|320446881|ref|NW_003383690.1|	[illegible]72236-67252[illegible]	699155	184274	-192376	0.3689	no
gi|320446881|ref|NW_003383690.1|	[illegible]84413-68531[illegible]	113765	139381	-302894	0.2023	no
gi|320446881|ref|NW_003383690.1|	[illegible]00364-70111[illegible]	440329	405337	-344139	0.0804	no
gi|320446881|ref|NW_003383690.1|	[illegible]05461-70585[illegible]	161509	132643	-360599	0.22995	no
gi|320446881|ref|NW_003383690.1|	[illegible]20002-72056[illegible]	121906	802199	-392566	0.0623	no
gi|320446881|ref|NW_003383690.1|	78098-88345	211327	483503	119405	0.3476	no
gi|320446881|ref|NW_003383690.1|	[illegible]91689-79321[illegible]	0.131055	14583	347605	0.21395	no
gi|320446881|ref|NW_003383690.1|	[illegible]39763-84022[illegible]	130301	819819	-0.668477	0.74345	no
gi|320446881|ref|NW_003383690.1|	[illegible]46463-85042[illegible]	128652	916389	-0.489446	0.70565	no
gi|320446881|ref|NW_003383690.1|	[illegible]54244-85485[illegible]	352041	142489	201703	0.26445	no
gi|320446881|ref|NW_003383690.1|	[illegible]55314-85547[illegible]	485601	117848	127908	0.39825	no
gi|320446881|ref|NW_003383690.1|	[illegible]56574-85789[illegible]	624084	162456	138024	0.539	no
gi|320446881|ref|NW_003383690.1|	[illegible]60023-86138[illegible]	0.751607	365373	228132	0.31875	no
gi|320446881|ref|NW_003383690.1|	[illegible]63285-86410[illegible]	694112	11529	0.732026	0.7207	no

gi|320446881|ref|NW_003383690.1|	364518-86490	12588	199556	0.664741	0.7409	no
gi|320446881|ref|NW_003383690.1|	365072-86584	40815	667706	0.710116	0.7253	no
gi|320446881|ref|NW_003383690.1|	89125-90594	314623	323318	0.039329	0.97885	no
gi|320446881|ref|NW_003383690.1|	91116-92441	108413	14945	0.463128	0.83785	no
gi|320446884|ref|NW_003383687.1|	118930-12027	0.152772	413689	47591	0.1731	no
gi|320446884|ref|NW_003383687.1|	155509-15667	326359	180628	-0.853441	0.6148	no
gi|320446884|ref|NW_003383687.1|	171895-17262	172326	424608	1301	0.5466	no
gi|320446884|ref|NW_003383687.1|	173303-17484	309247	152367	-102121	0.6117	no
gi|320446884|ref|NW_003383687.1|	174970-17900	253047	186462	-0.440525	0.8345	no
gi|320446884|ref|NW_003383687.1|	17583-18296	130708	108732	-0.265573	0.89255	no
gi|320446884|ref|NW_003383687.1|	179159-17987	667037	384269	-0.795652	0.68955	no
gi|320446884|ref|NW_003383687.1|	19300-19515	217083	238036	0.132933	0.9142	no
gi|320446884|ref|NW_003383687.1|	196078-19635	411545	35016	-0.233034	0.9063	no
gi|320446884|ref|NW_003383687.1|	202264-20300	143812	155781	0.115332	0.95615	no
gi|320446884|ref|NW_003383687.1|	203628-20495	0.62064	129256	10584	1	no
gi|320446884|ref|NW_003383687.1|	205198-20686	212761	197481	-0.107519	0.954	no
gi|320446884|ref|NW_003383687.1|	20594-25241	160631	176782	0.138222	0.94535	no
gi|320446884|ref|NW_003383687.1|	213840-21673	39002	611756	0.64941	0.62755	no
gi|320446884|ref|NW_003383687.1|	25657-29555	250605	281167	0.166013	0.9005	no
gi|320446884|ref|NW_003383687.1|	256807-26003	176416	102491	-0.783482	0.54205	no
gi|320446884|ref|NW_003383687.1|	260914-26228	165848	452548	-187372	0.4091	no
gi|320446884|ref|NW_003383687.1|	263333-26411	0.613106	231464	191658	0.3194	no
gi|320446884|ref|NW_003383687.1|	264346-26530	181675	562642	-169107	0.4455	no
gi|320446884|ref|NW_003383687.1|	267198-26762	465933	0.570621	-635145	0.26565	no
gi|320446884|ref|NW_003383687.1|	274551-27616	287646	102218	-481457	0.0638	no
gi|320446884|ref|NW_003383687.1|	277262-27810	233339	267517	-312473	0.1947	no
gi|320446884|ref|NW_003383687.1|	278556-27940	606137	0.75822	-299896	0.2374	no
gi|320446884|ref|NW_003383687.1|	280216-28075	446528	52606	-308545	0.19665	no
gi|320446884|ref|NW_003383687.1|	287942-29056	571001	1070.83	422909	0.01845	no
gi|320446884|ref|NW_003383687.1|	317813-32215	197453	441555	448301	0.0427	no

gi|320446884|ref|NW_003383687.1|	324088-32601	0.0995154	104061	338636	1	no
gi|320446884|ref|NW_003383687.1|	326140-32653	0	187645	inf	0.01275	no
gi|320446884|ref|NW_003383687.1|	327535-32816	125001	102245	303202	0.11195	no
gi|320446884|ref|NW_003383687.1|	32862-38208	601847	654363	0.120694	0.92225	no
gi|320446884|ref|NW_003383687.1|	405764-40779	225.19	654723	153974	0.3312	no
gi|320446884|ref|NW_003383687.1|	422336-42311	110963	575411	237451	0.18325	no
gi|320446884|ref|NW_003383687.1|	423257-42456	0.472432	295118	264311	0.2664	no
gi|320446884|ref|NW_003383687.1|	432615-43692	18749	583935	1639	0.2292	no
gi|320446884|ref|NW_003383687.1|	450700-45187	0.357689	223083	26408	0.2359	no
gi|320446884|ref|NW_003383687.1|	479356-48119	270937	120473	-116924	0.4932	no
gi|320446884|ref|NW_003383687.1|	481315-48308	253067	145621	-0.797304	0.70065	no
gi|320446884|ref|NW_003383687.1|	483820-48495	124505	300448	-205101	0.35435	no
gi|320446884|ref|NW_003383687.1|	485137-48588	393561	224762	-0.808189	0.69385	no
gi|320446884|ref|NW_003383687.1|	486775-48703	667989	736879	-318032	0.225	no
gi|320446884|ref|NW_003383687.1|	487137-48775	221086	690608	-167867	0.43695	no
gi|320446884|ref|NW_003383687.1|	490122-49125	11306	221826	0.972344	0.6555	no
gi|320446884|ref|NW_003383687.1|	495899-49719	102217	137.88	0.43179	0.75055	no
gi|320446884|ref|NW_003383687.1|	497566-49810	276862	193162	-0.519359	0.6985	no
gi|320446884|ref|NW_003383687.1|	498488-49920	165722	975956	-0.763879	0.56205	no
gi|320446884|ref|NW_003383687.1|	499416-49969	310122	170501	-0.86306	0.58305	no
gi|320446884|ref|NW_003383687.1|	501709-50306	103129	653241	-0.658763	0.6214	no
gi|320446884|ref|NW_003383687.1|	503816-50417	158118	118966	-0.410458	0.83885	no
gi|320446884|ref|NW_003383687.1|	504935-50637	182567	283039	0.632575	0.75125	no
gi|320446884|ref|NW_003383687.1|	506498-50772	343111	394154	0.200086	0.87605	no
gi|320446884|ref|NW_003383687.1|	52399-52751	0	833135	inf	0.0312	no
gi|320446884|ref|NW_003383687.1|	52859-53686	0.285301	254987	315987	0.24575	no
gi|320446884|ref|NW_003383687.1|	570908-57442	133722	496581	189279	0.39805	no
gi|320446884|ref|NW_003383687.1|	67745-68712	18455	160835	31235	0.19655	no
gi|320446884|ref|NW_003383687.1|	735919-73693	0.43185	17906	205184	0.3018	no
gi|320446884|ref|NW_003383687.1|	74090-74936	0.27642	323278	354784	0.2164	no

gi|320446884|ref|NW_003383687.1|	75052-78257	0.795457	3775	224662	0.3176	no
gi|320446884|ref|NW_003383687.1|	756554-75778[illegible]	202587	761008	190937	0.3753	no
gi|320446884|ref|NW_003383687.1|	762126-76608[illegible]	579375	259488	21631	0.10755	no
gi|320446884|ref|NW_003383687.1|	800672-80098[illegible]	798809	284588	-148897	0.4842	no
gi|320446884|ref|NW_003383687.1|	801804-80552[illegible]	278662	179437	-0.635037	0.63395	no
gi|320446884|ref|NW_003383687.1|	810485-81072[illegible]	353322	247469	-0.513734	0.8044	no
gi|320446884|ref|NW_003383687.1|	811176-81366[illegible]	160761	637117	-133529	0.4314	no
gi|320446884|ref|NW_003383687.1|	817743-81843[illegible]	214719	202296	-0.0859804	0.96665	no
gi|320446884|ref|NW_003383687.1|	820140-82144[illegible]	901829	911417	0.0152583	0.9924	no
gi|320446884|ref|NW_003383687.1|	821677-82402[illegible]	102935	210022	102881	0.4149	no
gi|320446884|ref|NW_003383687.1|	833204-83384[illegible]	150616	116708	-0.367979	0.85545	no
gi|320446884|ref|NW_003383687.1|	834571-83571[illegible]	980084	96066	-0.0288788	0.9875	no
gi|320446884|ref|NW_003383687.1|	838467-84048[illegible]	168775	767018	-113777	0.49595	no
gi|320446884|ref|NW_003383687.1|	840648-84325[illegible]	242917	291133	0.261213	0.84755	no
gi|320446884|ref|NW_003383687.1|	844088-84646[illegible]	141819	127074	-0.158378	0.90305	no
gi|320446884|ref|NW_003383687.1|	846931-84945[illegible]	901441	650268	-0.471198	0.83045	no
gi|320446884|ref|NW_003383687.1|	851955-85331[illegible]	135289	104392	-0.374034	1	no
gi|320446884|ref|NW_003383687.1|	858386-85955[illegible]	382394	178723	-109734	0.5174	no
gi|320446884|ref|NW_003383687.1|	861420-86233[illegible]	0.500372	155207	163312	0.3325	no
gi|320446884|ref|NW_003383687.1|	864914-86850[illegible]	197726	0	#NAME?	5.00E-05	yes
gi|320446884|ref|NW_003383687.1|	877828-88220[illegible]	151287	108503	-380148	0.0663	no
gi|320446884|ref|NW_003383687.1|	897739-89836[illegible]	0.43322	382716	31431	0.24575	no
gi|320446884|ref|NW_003383687.1|	925780-92728[illegible]	144761	0.554109	-470736	0.1354	no
gi|320446884|ref|NW_003383687.1|	936712-93813[illegible]	870428	0.892363	-328602	0.18095	no
gi|320446884|ref|NW_003383687.1|	939970-94177[illegible]	27001	389719	-279251	0.13335	no
gi|320446884|ref|NW_003383687.1|	946695-94700[illegible]	127833	266859	-226011	0.3132	no
gi|320446884|ref|NW_003383687.1|	947117-94912[illegible]	17001	814667	-106134	0.52925	no
gi|320446884|ref|NW_003383687.1|	99450-99737	106761	177477	0.733251	0.7322	no
gi|320446886|ref|NW_003383685.1|	107504-11002[illegible]	115752	545862	-108442	0.50565	no
gi|320446886|ref|NW_003383685.1|	111157-11186[illegible]	577094	204622	-149584	0.37285	no

gi|320446886|ref|NW_003383685.1|	25533-12634	763738	195768	1358	0.5314	no
gi|320446886|ref|NW_003383685.1|	33014-13318	738869	180562	12891	0.54555	no
gi|320446886|ref|NW_003383685.1|	62179-16290	415269	615759	0.568318	0.78495	no
gi|320446886|ref|NW_003383685.1|	63340-16400	18528	204243	0.140581	0.9463	no
gi|320446886|ref|NW_003383685.1|	64103-16471	149386	113644	-0.394516	0.84465	no
gi|320446886|ref|NW_003383685.1|	65375-16596	114373	677763	-0.7549	0.7112	no
gi|320446886|ref|NW_003383685.1|	66159-16761	609298	413884	-0.557921	0.78465	no
gi|320446886|ref|NW_003383685.1|	68392-16916	144154	945995	-0.607709	0.77155	no
gi|320446886|ref|NW_003383685.1|	73446-17462	0	481836	inf	0.0088	no
gi|320446886|ref|NW_003383685.1|	85427-18625	0.284339	527841	421442	0.1914	no
gi|320446886|ref|NW_003383685.1|	14366-21523	294414	62645	108935	0.5902	no
gi|320446886|ref|NW_003383685.1|	2919-3149	380697	346143	-0.137272	0.94165	no
gi|320446886|ref|NW_003383685.1|	3418-3978	14.98	313904	106729	0.61945	no
gi|320446886|ref|NW_003383685.1|	62949-36353	41192	203031	230126	0.20875	no
gi|320446886|ref|NW_003383685.1|	68765-36948	0.348906	931033	473792	0.1736	no
gi|320446886|ref|NW_003383685.1|	70788-37278	162652	196818	3597	0.0696	no
gi|320446886|ref|NW_003383685.1|	72927-37541	735708	811626	346361	0.095	no
gi|320446886|ref|NW_003383685.1|	83954-38450	306324	873189	151124	0.366	no
gi|320446886|ref|NW_003383685.1|	88782-39013	165649	700662	208059	0.338	no
gi|320446886|ref|NW_003383685.1|	90328-39366	975333	438307	216798	0.11425	no
gi|320446886|ref|NW_003383685.1|	94652-39487	531837	808334	0.603967	0.76085	no
gi|320446886|ref|NW_003383685.1|	96159-39666	187222	117116	264512	0.26255	no
gi|320446886|ref|NW_003383685.1|	98775-40010	0.309183	311237	333148	0.19155	no
gi|320446886|ref|NW_003383685.1|	00244-40342	0.515707	937702	418451	0.06365	no
gi|320446886|ref|NW_003383685.1|	05523-40649	207335	294204	0.504853	0.82045	no
gi|320446886|ref|NW_003383685.1|	08778-40977	110508	427496	195176	0.38395	no
gi|320446886|ref|NW_003383685.1|	12119-41652	258791	889081	178053	0.29845	no
gi|320446886|ref|NW_003383685.1|	16867-42033	480494	238599	2312	0.20775	no
gi|320446886|ref|NW_003383685.1|	24100-42649	175102	131167	-0.416789	0.7404	no
gi|320446886|ref|NW_003383685.1|	26669-43484	363197	326185	-0.155063	0.90405	no

gi|320446886|ref|NW_003383685.1|	435635-43622	223792	102859	-112149	0.39895	no
gi|320446886|ref|NW_003383685.1|	43847-44964	132084	147107	0.155412	0.94225	no
gi|320446886|ref|NW_003383685.1|	45061-46192	120597	112218	-0.103891	0.9597	no
gi|320446886|ref|NW_003383685.1|	46565-48381	130244	141325	0.117804	0.9435	no
gi|320446886|ref|NW_003383685.1|	48725-49402	916415	677482	-0.435819	0.8311	no
gi|320446886|ref|NW_003383685.1|	67074-67878	237477	978751	204315	0.3569	no
gi|320446886|ref|NW_003383685.1|	68051-69054	0.659593	486062	288149	0.2363	no
gi|320446886|ref|NW_003383685.1|	7583-8403	11.26	654862	-0.781947	0.7024	no
gi|320446886|ref|NW_003383685.1|	89867-90826	0	499017	inf	0.01195	no
gi|320446888|ref|NW_003383683.1|	110030-11084	0	278754	inf	0.0233	no
gi|320446888|ref|NW_003383683.1|	136957-13881	0	225211	inf	0.01195	no
gi|320446888|ref|NW_003383683.1|	140547-14178	0	445778	inf	0.0113	no
gi|320446888|ref|NW_003383683.1|	173340-17496	389427	0.846949	-220101	0.3252	no
gi|320446888|ref|NW_003383683.1|	175068-17685	6847	0.605698	-34988	0.1634	no
gi|320446888|ref|NW_003383683.1|	178553-17925	654956	0.994665	-271911	0.26925	no
gi|320446888|ref|NW_003383683.1|	181169-18148	144709	2075	-280197	0.28215	no
gi|320446888|ref|NW_003383683.1|	185200-18615	424524	0.813709	-238326	0.30585	no
gi|320446888|ref|NW_003383683.1|	370455-37214	0	267268	inf	0.00945	no
gi|320446888|ref|NW_003383683.1|	39317-39849	146534	75854	-0.949934	0.6511	no
gi|320446889|ref|NW_003383682.1|	121707-12599	225043	484375	110592	0.6222	no
gi|320446889|ref|NW_003383682.1|	1234-1808	396618	358738	317711	0.195	no
gi|320446889|ref|NW_003383682.1|	138642-14031	199682	112846	249859	0.27635	no
gi|320446889|ref|NW_003383682.1|	316075-31695	0	611244	inf	0.0101	no
gi|320446889|ref|NW_003383682.1|	44297-45283	0.674537	263973	196842	0.3902	no
gi|320446889|ref|NW_003383682.1|	452217-45349	0	123071	inf	1	no
gi|320446889|ref|NW_003383682.1|	50525-51069	325474	201074	262711	0.25025	no
gi|320446889|ref|NW_003383682.1|	522408-52331	931211	260193	-183953	0.3954	no
gi|320446889|ref|NW_003383682.1|	526431-52685	612692	231399	-140478	0.5239	no
gi|320446889|ref|NW_003383682.1|	547427-55145	145322	143104	-0.0221879	0.98635	no
gi|320446889|ref|NW_003383682.1|	593465-59373	248283	537497	44362	0.18025	no

gi|320446889|ref|NW_003383682.1|	594915-59553	13231	146764	347151	0.19285	no
gi|320446889|ref|NW_003383682.1|	599726-60595	7.74	371454	226278	0.09745	no
gi|320446889|ref|NW_003383682.1|	606316-60734	445461	636382	383652	0.0668	no
gi|320446889|ref|NW_003383682.1|	607523-61027	282158	330037	354805	0.08065	no
gi|320446889|ref|NW_003383682.1|	610378-61060	141826	326665	120369	0.56965	no
gi|320446889|ref|NW_003383682.1|	610750-61250	643627	746645	353613	0.0855	no
gi|320446889|ref|NW_003383682.1|	612654-61302	0	20.19	inf	0.0133	no
gi|320446889|ref|NW_003383682.1|	671276-67152	328593	952026	817856	0.14075	no
gi|320446889|ref|NW_003383682.1|	671675-67211	0	384553	inf	0.00595	no
gi|320446889|ref|NW_003383682.1|	734438-73477	14077	237313	407538	0.19415	no
gi|320446892|ref|NW_003383679.1|	024707-10250	133877	26654	-232848	0.3013	no
gi|320446892|ref|NW_003383679.1|	038810-10398	901928	464054	-0.95872	0.645	no
gi|320446892|ref|NW_003383679.1|	042393-10427	571988	448668	-0.350338	0.8613	no
gi|320446892|ref|NW_003383679.1|	043721-10448	484613	295195	-0.715165	0.7269	no
gi|320446892|ref|NW_003383679.1|	087412-10880	662911	394778	-0.747774	0.70485	no
gi|320446892|ref|NW_003383679.1|	195802-11970	155812	937891	-0.732311	0.7405	no
gi|320446892|ref|NW_003383679.1|	197159-11983	383787	149819	-135708	0.41425	no
gi|320446892|ref|NW_003383679.1|	122482-12286	521589	479121	-0.122521	0.9242	no
gi|320446892|ref|NW_003383679.1|	123754-12571	216733	601684	-184884	0.2903	no
gi|320446892|ref|NW_003383679.1|	148536-14927	420572	457298	-320115	0.09905	no
gi|320446892|ref|NW_003383679.1|	153845-15411	96386	290546	-173006	0.41965	no
gi|320446892|ref|NW_003383679.1|	155586-16187	119088	262721	11415	0.4004	no
gi|320446892|ref|NW_003383679.1|	176586-17720	297792	316856	0.0895204	0.96605	no
gi|320446892|ref|NW_003383679.1|	18537-18854	181072	0	#NAME?	0.02105	no
gi|320446892|ref|NW_003383679.1|	28865-29520	321314	114929	-148325	0.49835	no
gi|320446892|ref|NW_003383679.1|	30695-32370	459483	922122	-231698	0.2106	no
gi|320446892|ref|NW_003383679.1|	32924-33235	82919	177906	-222059	0.3095	no
gi|320446892|ref|NW_003383679.1|	38305-41012	157699	129752	-0.281415	0.82385	no
gi|320446892|ref|NW_003383679.1|	465012-46541	193485	509391	-192538	0.3877	no
gi|320446892|ref|NW_003383679.1|	471493-47289	188531	191431	0.0220244	0.96875	no

gi|320446892|ref|NW_003383679.1|	474178-47520	0.851709	13246	0.637128	1	no
gi|320446892|ref|NW_003383679.1|	476970-48086	135861	815549	-0.736285	0.56775	no
gi|320446892|ref|NW_003383679.1|	496928-49720	0	156468	inf	0.0312	no
gi|320446892|ref|NW_003383679.1|	49894-52008	700596	664205	-0.0769554	0.9715	no
gi|320446892|ref|NW_003383679.1|	52387-54805	149978	458857	-170863	0.3222	no
gi|320446892|ref|NW_003383679.1|	57129-57432	314051	130508	-126686	0.54615	no
gi|320446892|ref|NW_003383679.1|	633299-63459	110959	0.990119	-0.164353	1	no
gi|320446892|ref|NW_003383679.1|	634699-63776	0.894447	191793	110048	0.59135	no
gi|320446892|ref|NW_003383679.1|	723869-72422	107.98	161087	0.57707	0.72555	no
gi|320446892|ref|NW_003383679.1|	724885-72605	0.722269	112602	0.640618	1	no
gi|320446892|ref|NW_003383679.1|	728544-72978	0.336635	0.933858	147202	1	no
gi|320446892|ref|NW_003383679.1|	729898-73247	204471	458685	116561	0.3818	no
gi|320446892|ref|NW_003383679.1|	738828-73911	515511	128797	-200091	0.3484	no
gi|320446892|ref|NW_003383679.1|	745080-74676	12787	0.728415	-0.811851	1	no
gi|320446892|ref|NW_003383679.1|	746875-74778	200446	0.51811	-195188	0.30305	no
gi|320446892|ref|NW_003383679.1|	747898-75015	242651	122639	-0.984469	0.6436	no
gi|320446892|ref|NW_003383679.1|	750251-75193	128401	0.487608	-139687	1	no
gi|320446892|ref|NW_003383679.1|	753951-75428	442597	101777	-212058	0.343	no
gi|320446892|ref|NW_003383679.1|	761671-76267	176813	0.458032	-194871	0.30305	no
gi|320446892|ref|NW_003383679.1|	763782-76613	129023	750658	-0.781401	0.63675	no
gi|320446892|ref|NW_003383679.1|	820058-82126	10791	133926	0.311617	0.8848	no
gi|320446892|ref|NW_003383679.1|	821603-82242	804283	375157	-110021	0.59365	no
gi|320446892|ref|NW_003383679.1|	822735-82524	187595	999528	-0.908305	0.4794	no
gi|320446892|ref|NW_003383679.1|	825365-83100	230617	160067	-0.526817	0.686	no
gi|320446892|ref|NW_003383679.1|	85210-86158	378944	555611	-276984	0.12605	no
gi|320446892|ref|NW_003383679.1|	852380-85495	144363	190598	372276	0.0747	no
gi|320446892|ref|NW_003383679.1|	933658-93464	53963	543473	0.0102399	0.98345	no
gi|320446892|ref|NW_003383679.1|	937278-93837	958652	501002	-0.936191	0.6523	no
gi|320446892|ref|NW_003383679.1|	939008-93968	26491	129162	-103632	0.59645	no
gi|320446892|ref|NW_003383679.1|	940441-94131	131941	76317	-0.789815	0.71145	no

gi|320446892|ref|NW_003383679.1|	44216-94724	737651	891987	0.274084	0.86695	no
gi|320446892|ref|NW_003383679.1|	48041-94936	55909	377339	-0.567218	0.78295	no
gi|320446892|ref|NW_003383679.1|	50897-95475	107319	332638	-168988	0.2034	no
gi|320446892|ref|NW_003383679.1|	72856-97335	231732	151095	-0.617006	0.7644	no
gi|320446892|ref|NW_003383679.1|	73505-97605	121064	779428	-0.635288	0.70225	no
gi|320446892|ref|NW_003383679.1|	77096-97896	136765	189902	0.473553	0.7112	no
gi|320446892|ref|NW_003383679.1|	79123-98084	155507	110664	-0.490796	0.75395	no
gi|320446892|ref|NW_003383679.1|	81621-98253	176325	11642	-0.598892	0.7855	no
gi|320446892|ref|NW_003383679.1|	83242-98354	157374	754357	-106087	0.62915	no
gi|320446892|ref|NW_003383679.1|	89738-99159	651967	423802	-0.621409	0.64435	no
gi|320446892|ref|NW_003383679.1|	91794-99298	143194	931195	-0.620821	0.7784	no
gi|320446892|ref|NW_003383679.1|	93393-99528	102105	0.711653	-0.520809	1	no
gi|320446892|ref|NW_003383679.1|	95680-99665	41.13	157759	-138247	0.415	no
gi|320446892|ref|NW_003383679.1|	97326-99761	125708	147437	0.230014	0.9029	no
gi|320446894|ref|NW_003383677.1|	07381-10799	0.446784	667228	390053	0.20695	no
gi|320446894|ref|NW_003383677.1|	73452-17402	0	43706	inf	0.02915	no
gi|320446894|ref|NW_003383677.1|	20682-21036	821415	403165	-102674	0.6286	no
gi|320446894|ref|NW_003383677.1|	21418-21757	489252	117859	-205352	0.353	no
gi|320446894|ref|NW_003383677.1|	22594-23801	192795	722455	-141609	0.52475	no
gi|320446894|ref|NW_003383677.1|	27914-22849	0	391474	inf	0.029	no
gi|320446894|ref|NW_003383677.1|	25219-25561	95835	977494	0.0285344	0.9489	no
gi|320446894|ref|NW_003383677.1|	54605-25522	109693	268233	-203191	0.34245	no
gi|320446894|ref|NW_003383677.1|	59140-26002	627428	216061	-153801	0.4778	no
gi|320446894|ref|NW_003383677.1|	26037-27985	140894	107157	-0.394889	0.80505	no
gi|320446894|ref|NW_003383677.1|	60588-26429	821369	357121	-120162	0.466	no
gi|320446894|ref|NW_003383677.1|	64894-26824	926655	614084	-0.593597	0.7189	no
gi|320446894|ref|NW_003383677.1|	70022-27240	146478	458026	164474	0.21555	no
gi|320446894|ref|NW_003383677.1|	73009-27340	141074	170001	0.269096	0.8286	no
gi|320446894|ref|NW_003383677.1|	81013-28335	140771	158027	0.166823	0.895	no
gi|320446894|ref|NW_003383677.1|	83577-28431	103271	13008	0.332972	0.87515	no

gi|320446894|ref|NW_003383677.1|	284509-28670	215048	43219	100701	0.62885	no
gi|320446894|ref|NW_003383677.1|	286966-28982	146268	347241	124733	0.3507	no
gi|320446894|ref|NW_003383677.1|	297411-29959	26438	0.120972	-77718	0.16165	no
gi|320446894|ref|NW_003383677.1|	305012-30550	451294	0	#NAME?	0.00405	no
gi|320446894|ref|NW_003383677.1|	306654-30702	526229	0	#NAME?	0.00645	no
gi|320446894|ref|NW_003383677.1|	307355-30758	487517	0	#NAME?	0.02105	no
gi|320446894|ref|NW_003383677.1|	307899-30858	18125	0	#NAME?	0.0061	no
gi|320446894|ref|NW_003383677.1|	309276-31215	114075	0	#NAME?	5.00E-05	yes
gi|320446894|ref|NW_003383677.1|	312526-31299	142459	0	#NAME?	0.0132	no
gi|320446894|ref|NW_003383677.1|	313120-31511	203414	0.0669062	-824806	0.25065	no
gi|320446894|ref|NW_003383677.1|	32650-33189	22043	148468	275177	0.2564	no
gi|320446894|ref|NW_003383677.1|	50575-52351	396496	362412	-0.129677	0.92025	no
gi|320446894|ref|NW_003383677.1|	53284-54966	614733	511637	-358677	0.07725	no
gi|320446894|ref|NW_003383677.1|	60936-61553	211696	191692	-0.143204	0.94515	no
gi|320446894|ref|NW_003383677.1|	61688-62245	20849	351818	0.754851	0.7213	no
gi|320446894|ref|NW_003383677.1|	64459-65270	67444	543995	-0.310097	0.87965	no
gi|320446894|ref|NW_003383677.1|	66246-66468	336462	181105	-0.893617	0.67955	no
gi|320446894|ref|NW_003383677.1|	69431-70192	738574	506419	-0.54441	0.79275	no
gi|320446894|ref|NW_003383677.1|	70885-72227	869223	434127	-100161	0.6248	no
gi|320446894|ref|NW_003383677.1|	73321-74309	103154	511078	-101318	0.6161	no
gi|320446894|ref|NW_003383677.1|	75356-76353	288076	122457	-123417	0.54965	no
gi|320446894|ref|NW_003383677.1|	76479-77528	122384	229499	-241485	0.2808	no
gi|320446894|ref|NW_003383677.1|	80128-83823	134118	907833	-0.563009	0.66505	no
gi|320446894|ref|NW_003383677.1|	83945-84381	203137	808066	-132991	0.5184	no
gi|320446894|ref|NW_003383677.1|	84566-85416	123579	340111	-186136	0.3879	no
gi|320446895|ref|NW_003383676.1|	137323-14041	566242	342328	-0.72604	0.74905	no
gi|320446895|ref|NW_003383676.1|	140534-14434	125485	723061	-0.795325	0.531	no
gi|320446895|ref|NW_003383676.1|	151192-15162	233817	331804	-281698	0.23965	no
gi|320446895|ref|NW_003383676.1|	188279-18922	0	179262	inf	0.0294	no
gi|320446895|ref|NW_003383676.1|	193650-19424	0.468757	130238	479616	0.1715	no

gi|320446895|ref|NW_003383676.1|	217241-218037	102369	0.620257	-404476	0.16015	no
gi|320446895|ref|NW_003383676.1|	218311-21897	277877	108223	-136044	0.5288	no
gi|320446895|ref|NW_003383676.1|	219739-22048	133252	0.912843	-386765	0.18835	no
gi|320446895|ref|NW_003383676.1|	318922-32233	222233	121992	-0.86529	0.51595	no
gi|320446895|ref|NW_003383676.1|	322476-323859	661707	296214	-115955	0.57705	no
gi|320446895|ref|NW_003383676.1|	325867-32608	759791	136021	-248178	0.2973	no
gi|320446895|ref|NW_003383676.1|	372520-373187	660111	141169	-222529	0.20235	no
gi|320446895|ref|NW_003383676.1|	38293-39078	429955	316194	-0.443373	0.82455	no
gi|320446895|ref|NW_003383676.1|	386443-386717	243108	263293	0.11507	0.9471	no
gi|320446895|ref|NW_003383676.1|	387383-387554	208562	265542	0.348461	0.8589	no
gi|320446895|ref|NW_003383676.1|	39218-40738	801301	191845	-20624	0.3515	no
gi|320446895|ref|NW_003383676.1|	42784-49451	394807	127013	168575	0.3236	no
gi|320446896|ref|NW_003383675.1|	268326-268879	184655	640703	-15271	0.4681	no
gi|320446896|ref|NW_003383675.1|	274329-27586	272883	117493	210622	0.35365	no
gi|320446896|ref|NW_003383675.1|	27581-27928	132335	103208	29633	0.2564	no
gi|320446896|ref|NW_003383675.1|	282702-284267	176411	123609	-0.513159	0.74935	no
gi|320446896|ref|NW_003383675.1|	284412-287015	145384	168281	0.210999	0.86605	no
gi|320446896|ref|NW_003383675.1|	287233-294847	118675	252357	108845	0.41975	no
gi|320446896|ref|NW_003383675.1|	295442-296485	710502	173384	128706	0.5496	no
gi|320446896|ref|NW_003383675.1|	297938-299864	19962	327014	0.712101	0.7271	no
gi|320446896|ref|NW_003383675.1|	301914-302979	162742	140712	-0.209845	0.91045	no
gi|320446896|ref|NW_003383675.1|	310791-311317	109205	695686	-0.650536	0.7438	no
gi|320446896|ref|NW_003383675.1|	328666-32891	665747	168414	133897	0.38395	no
gi|320446896|ref|NW_003383675.1|	332807-33474	417226	161358	195136	0.25285	no
gi|320446896|ref|NW_003383675.1|	339736-34063	179114	334971	0.903156	0.6645	no
gi|320446896|ref|NW_003383675.1|	341327-34213	0.879705	382811	212154	0.34675	no
gi|320446896|ref|NW_003383675.1|	350748-352035	0.320946	167036	237976	0.2609	no
gi|320446896|ref|NW_003383675.1|	352463-354095	0.845772	714798	30792	0.20125	no
gi|320446896|ref|NW_003383675.1|	360289-360680	304475	166668	245258	0.2888	no
gi|320446896|ref|NW_003383675.1|	370299-371137	112036	192636	0.781913	0.71785	no

gi|320446896|ref|NW_003383675.1|	71379-37158	268437	335085	0.319947	0.87555	no
gi|320446896|ref|NW_003383675.1|	74164-37451	648597	928095	0.516951	0.8037	no
gi|320446896|ref|NW_003383675.1|	74651-37533	679686	162394	125656	0.5607	no
gi|320446896|ref|NW_003383675.1|	75542-37638	915184	20413	115735	0.5941	no
gi|320446896|ref|NW_003383675.1|	84102-38461	480409	564342	0.232308	0.9017	no
gi|320446896|ref|NW_003383675.1|	86337-38704	177001	242116	0.451943	0.82655	no
gi|320446896|ref|NW_003383675.1|	90301-39069	699035	105025	0.587295	0.75475	no
gi|320446896|ref|NW_003383675.1|	03534-40547	0.297144	269297	317996	0.20475	no
gi|320446896|ref|NW_003383675.1|	14267-41491	0	388402	inf	0.0233	no
gi|320446896|ref|NW_003383675.1|	18732-41923	245259	452366	0.883185	0.6898	no
gi|320446896|ref|NW_003383675.1|	21730-42350	0.219629	160638	287067	0.22425	no
gi|320446896|ref|NW_003383675.1|	33784-43410	482363	217875	217531	0.33305	no
gi|320446896|ref|NW_003383675.1|	52966-45382	64543	536829	-0.265799	0.89205	no
gi|320446896|ref|NW_003383675.1|	54055-45806	996019	731614	-0.445091	0.73125	no
gi|320446896|ref|NW_003383675.1|	58250-46012	543024	610404	-315318	0.1112	no
gi|320446896|ref|NW_003383675.1|	60226-46399	203468	892482	-118891	0.3723	no
gi|320446896|ref|NW_003383675.1|	65846-46891	10893	229332	107403	0.41675	no
gi|320446896|ref|NW_003383675.1|	72420-47320	132782	190734	0.5225	0.81	no
gi|320446896|ref|NW_003383675.1|	75155-47568	29313	336248	0.197985	0.9257	no
gi|320446896|ref|NW_003383675.1|	77023-47757	127007	160647	0.338979	0.8629	no
gi|320446896|ref|NW_003383675.1|	89371-48998	203399	243214	0.257916	0.904	no
gi|320446896|ref|NW_003383675.1|	92960-49343	372662	460647	0.305794	0.88675	no
gi|320446896|ref|NW_003383675.1|	05403-50575	211736	430035	-229974	0.3221	no
gi|320446896|ref|NW_003383675.1|	07605-50827	549356	18728	-155254	0.4803	no
gi|320446896|ref|NW_003383675.1|	08575-50957	421588	137943	-161176	0.4302	no
gi|320446896|ref|NW_003383675.1|	10506-51121	682568	196481	-179658	0.40625	no
gi|320446896|ref|NW_003383675.1|	13883-51440	0.58836	671954	351359	0.21745	no
gi|320446896|ref|NW_003383675.1|	50411-55194	386639	730958	-240313	0.1905	no
gi|320446896|ref|NW_003383675.1|	56366-55681	400797	542862	0.437714	0.83595	no
gi|320446896|ref|NW_003383675.1|	57063-55754	821727	86873	0.0802497	0.95975	no

<table>
<tr><td>gi|320446896|ref|NW_003383675.1|</td><td>58313-55943</td><td>0.189491</td><td>406745</td><td>442393</td><td>0.18625</td><td>no</td></tr>
<tr><td>gi|320446896|ref|NW_003383675.1|</td><td>61142-56149</td><td>109784</td><td>78829</td><td>-0.477872</td><td>0.8296</td><td>no</td></tr>
<tr><td>gi|320446896|ref|NW_003383675.1|</td><td>06188-60860</td><td>860091</td><td>252136</td><td>155164</td><td>0.3755</td><td>no</td></tr>
<tr><td>gi|320446896|ref|NW_003383675.1|</td><td>26683-62748</td><td>149741</td><td>308514</td><td>104287</td><td>0.60105</td><td>no</td></tr>
<tr><td>gi|320446896|ref|NW_003383675.1|</td><td>82515-68332</td><td>118115</td><td>11563</td><td>-0.0306808</td><td>0.9869</td><td>no</td></tr>
<tr><td>gi|320446897|ref|NW_003383674.1|</td><td>00152-10071</td><td>460762</td><td>152151</td><td>172341</td><td>0.42205</td><td>no</td></tr>
<tr><td>gi|320446897|ref|NW_003383674.1|</td><td>02241-10269</td><td>530402</td><td>302294</td><td>-0.811135</td><td>0.69665</td><td>no</td></tr>
<tr><td>gi|320446897|ref|NW_003383674.1|</td><td>05187-10720</td><td>204691</td><td>483084</td><td>123883</td><td>0.3498</td><td>no</td></tr>
<tr><td>gi|320446897|ref|NW_003383674.1|</td><td>11366-11210</td><td>0.999392</td><td>228211</td><td>119124</td><td>0.56815</td><td>no</td></tr>
<tr><td>gi|320446897|ref|NW_003383674.1|</td><td>12187-11408</td><td>425494</td><td>30364</td><td>-0.486775</td><td>0.81565</td><td>no</td></tr>
<tr><td>gi|320446897|ref|NW_003383674.1|</td><td>14292-11600</td><td>0.455567</td><td>174482</td><td>193734</td><td>0.39545</td><td>no</td></tr>
<tr><td>gi|320446897|ref|NW_003383674.1|</td><td>16189-11771</td><td>219032</td><td>107633</td><td>-102502</td><td>0.52965</td><td>no</td></tr>
<tr><td>gi|320446897|ref|NW_003383674.1|</td><td>18573-12027</td><td>271719</td><td>237298</td><td>-0.195413</td><td>0.87755</td><td>no</td></tr>
<tr><td>gi|320446897|ref|NW_003383674.1|</td><td>21347-12279</td><td>180911</td><td>725426</td><td>-131838</td><td>0.5567</td><td>no</td></tr>
<tr><td>gi|320446897|ref|NW_003383674.1|</td><td>23145-12374</td><td>463694</td><td>282911</td><td>-0.712823</td><td>0.72555</td><td>no</td></tr>
<tr><td>gi|320446897|ref|NW_003383674.1|</td><td>27042-13130</td><td>165432</td><td>14046</td><td>-0.236079</td><td>0.8596</td><td>no</td></tr>
<tr><td>gi|320446897|ref|NW_003383674.1|</td><td>32073-13337</td><td>273713</td><td>977303</td><td>-148579</td><td>0.3792</td><td>no</td></tr>
<tr><td>gi|320446897|ref|NW_003383674.1|</td><td>00273-20234</td><td>818127</td><td>224415</td><td>-186615</td><td>0.39935</td><td>no</td></tr>
<tr><td>gi|320446897|ref|NW_003383674.1|</td><td>05419-20644</td><td>255836</td><td>0.442084</td><td>-253283</td><td>0.24045</td><td>no</td></tr>
<tr><td>gi|320446897|ref|NW_003383674.1|</td><td>10742-21137</td><td>3431</td><td>320701</td><td>-0.0974015</td><td>0.9476</td><td>no</td></tr>
<tr><td>gi|320446897|ref|NW_003383674.1|</td><td>23298-22839</td><td>106376</td><td>580928</td><td>-0.872748</td><td>0.60185</td><td>no</td></tr>
<tr><td>gi|320446897|ref|NW_003383674.1|</td><td>28892-23027</td><td>178392</td><td>798778</td><td>-115919</td><td>0.61045</td><td>no</td></tr>
<tr><td>gi|320446897|ref|NW_003383674.1|</td><td>30828-23211</td><td>185763</td><td>51.92</td><td>-18391</td><td>0.179</td><td>no</td></tr>
<tr><td>gi|320446897|ref|NW_003383674.1|</td><td>32307-23248</td><td>140885</td><td>724718</td><td>-0.959031</td><td>0.65955</td><td>no</td></tr>
<tr><td>gi|320446897|ref|NW_003383674.1|</td><td>59996-26129</td><td>269725</td><td>0</td><td>#NAME?</td><td>0.01065</td><td>no</td></tr>
<tr><td>gi|320446897|ref|NW_003383674.1|</td><td>76845-27749</td><td>52585</td><td>0.275522</td><td>-425441</td><td>0.3007</td><td>no</td></tr>
<tr><td>gi|320446897|ref|NW_003383674.1|</td><td>82292-28573</td><td>494769</td><td>219095</td><td>214673</td><td>0.24375</td><td>no</td></tr>
<tr><td>gi|320446897|ref|NW_003383674.1|</td><td>86454-28774</td><td>366993</td><td>719763</td><td>0.971769</td><td>0.6484</td><td>no</td></tr>
<tr><td>gi|320446897|ref|NW_003383674.1|</td><td>87848-28826</td><td>0.871093</td><td>633435</td><td>28623</td><td>0.25915</td><td>no</td></tr>
<tr><td>gi|320446897|ref|NW_003383674.1|</td><td>88632-28909</td><td>432578</td><td>336261</td><td>-0.36338</td><td>0.84975</td><td>no</td></tr>
</table>

gi|320446897|ref|NW_003383674.1|	302233-30271	100381	894507	-0.16632	0.93525	no
gi|320446897|ref|NW_003383674.1|	30478-31436	179842	286922	0.673928	0.6746	no
gi|320446897|ref|NW_003383674.1|	31580-31835	180635	228969	0.34207	0.8626	no
gi|320446897|ref|NW_003383674.1|	426239-42811	412162	545773	0.405089	0.84525	no
gi|320446897|ref|NW_003383674.1|	460730-46100	200752	127789	-0.651658	0.7491	no
gi|320446897|ref|NW_003383674.1|	526885-52808	134851	631259	-109507	0.61855	no
gi|320446897|ref|NW_003383674.1|	534341-53870	609215	10507	0.786324	0.539	no
gi|320446897|ref|NW_003383674.1|	543554-54414	175354	449441	-196406	0.3698	no
gi|320446897|ref|NW_003383674.1|	545343-54647	199259	978022	-102671	0.4425	no
gi|320446897|ref|NW_003383674.1|	547278-54985	169767	733675	-121035	0.48385	no
gi|320446897|ref|NW_003383674.1|	554422-55596	144315	519839	-147309	0.5104	no
gi|320446897|ref|NW_003383674.1|	556187-55694	102563	307685	-173699	0.41185	no
gi|320446897|ref|NW_003383674.1|	557192-55832	994251	688542	-0.530066	0.7943	no
gi|320446897|ref|NW_003383674.1|	55732-56034	31677	550334	0.79687	0.69275	no
gi|320446897|ref|NW_003383674.1|	559481-56133	106139	964454	-0.138164	0.94955	no
gi|320446897|ref|NW_003383674.1|	563322-56388	943844	168003	-249006	0.16835	no
gi|320446897|ref|NW_003383674.1|	565136-56536	97.03	229238	-208158	0.3338	no
gi|320446897|ref|NW_003383674.1|	565639-56643	354647	701734	-233739	0.3068	no
gi|320446897|ref|NW_003383674.1|	567165-56773	704207	305978	-120257	0.558	no
gi|320446897|ref|NW_003383674.1|	568658-57141	637943	227819	-148554	0.275	no
gi|320446897|ref|NW_003383674.1|	56924-59284	833898	204604	129489	0.4486	no
gi|320446897|ref|NW_003383674.1|	571813-57259	0.309929	531716	410065	0.19245	no
gi|320446897|ref|NW_003383674.1|	572723-57409	0.296678	123637	205914	1	no
gi|320446897|ref|NW_003383674.1|	582045-58248	324367	241547	-0.425324	0.837	no
gi|320446897|ref|NW_003383674.1|	587151-58960	110.76	105009	-0.0769308	0.9523	no
gi|320446897|ref|NW_003383674.1|	590589-59110	120933	730404	259448	0.2369	no
gi|320446897|ref|NW_003383674.1|	591631-59189	55443	31685	-0.807205	0.695	no
gi|320446897|ref|NW_003383674.1|	595941-59729	450964	375812	-0.263003	0.8978	no
gi|320446897|ref|NW_003383674.1|	597445-60041	12208	115951	-0.0743024	0.9505	no
gi|320446897|ref|NW_003383674.1|	500738-60493	123667	474703	-138136	0.42865	no

gi|320446897|ref|NW_003383674.1|	60183-60650	925983	132953	0.521863	0.7889	no
gi|320446897|ref|NW_003383674.1|	05338-60622	607974	608474	-332074	0.085	no
gi|320446897|ref|NW_003383674.1|	09492-61101	206425	351645	0.768503	0.5397	no
gi|320446897|ref|NW_003383674.1|	11123-61310	217371	229707	0.0796351	0.9509	no
gi|320446897|ref|NW_003383674.1|	62538-63324	0.306553	210422	277908	0.2657	no
gi|320446897|ref|NW_003383674.1|	66685-70191	175402	692278	198069	0.1508	no
gi|320446897|ref|NW_003383674.1|	88612-68904	108558	663608	-0.710062	0.7321	no
gi|320446897|ref|NW_003383674.1|	28319-72919	968786	0	#NAME?	0.007	no
gi|320446897|ref|NW_003383674.1|	80139-81460	138347	144599	0.0637643	0.9774	no
gi|320446897|ref|NW_003383674.1|	85515-85784	282044	212061	-0.411444	0.84535	no
gi|320446897|ref|NW_003383674.1|	88826-90165	15291	233572	0.611186	0.76235	no
gi|320446897|ref|NW_003383674.1|	93380-93839	662275	156763	124308	0.5498	no
gi|320446897|ref|NW_003383674.1|	95230-96009	279449	108665	195923	0.3624	no
gi|320446897|ref|NW_003383674.1|	98472-99537	528913	205439	195761	0.3851	no
gi|320446898|ref|NW_003383673.1|	16222-11715	0.241934	2503	337097	0.2261	no
gi|320446898|ref|NW_003383673.1|	22007-12388	174849	666642	193081	0.37015	no
gi|320446898|ref|NW_003383673.1|	24952-12532	175648	215639	0.295929	0.8833	no
gi|320446898|ref|NW_003383673.1|	40784-14156	0	675749	inf	0.01035	no
gi|320446898|ref|NW_003383673.1|	53200-15361	0	266775	inf	0.00845	no
gi|320446898|ref|NW_003383673.1|	54050-15523	0	191338	inf	0.00365	no
gi|320446898|ref|NW_003383673.1|	81016-18126	0	207866	inf	0.0312	no
gi|320446898|ref|NW_003383673.1|	86450-18783	0	408571	inf	0.00815	no
gi|320446898|ref|NW_003383673.1|	88617-18988	0.162796	169424	33795	0.2261	no
gi|320446898|ref|NW_003383673.1|	90172-19099	0	692232	inf	0.0114	no
gi|320446898|ref|NW_003383673.1|	98789-19903	0	259197	inf	0.029	no
gi|320446898|ref|NW_003383673.1|	01459-20216	0	144311	inf	0.00795	no
gi|320446898|ref|NW_003383673.1|	08863-20933	0	693563	inf	0.02205	no
gi|320446898|ref|NW_003383673.1|	27646-22866	0	653264	inf	0.00845	no
gi|320446898|ref|NW_003383673.1|	40917-24144	108846	269689	-201292	0.3489	no
gi|320446898|ref|NW_003383673.1|	51968-25314	132626	24839	-241669	0.2915	no

gi|320446898|ref|NW_003383673.1|	253295-254705	166652	588935	-150065	0.50645	no
gi|320446898|ref|NW_003383673.1|	261569-26899[illegible]	828506	873807	0.0768023	0.9527	no
gi|320446898|ref|NW_003383673.1|	270378-271999	132744	0.508533	-470617	0.13545	no
gi|320446898|ref|NW_003383673.1|	279812-28020[illegible]	58485	471577	-36325	0.16715	no
gi|320446898|ref|NW_003383673.1|	286192-28650[illegible]	129956	677851	-426091	0.1464	no
gi|320446898|ref|NW_003383673.1|	327971-32853[illegible]	232739	177785	-0.388578	0.8537	no
gi|320446898|ref|NW_003383673.1|	333498-33387[illegible]	873182	107224	0.296268	0.87775	no
gi|320446898|ref|NW_003383673.1|	333999-33455[illegible]	796231	39385	-101554	0.613	no
gi|320446898|ref|NW_003383673.1|	335311-33620[illegible]	337245	331656	-0.0241131	0.9851	no
gi|320446898|ref|NW_003383673.1|	420091-420619	0	652894	inf	0.02075	no
gi|320446898|ref|NW_003383673.1|	420827-421575	0.329243	564036	409856	0.19245	no
gi|320446898|ref|NW_003383673.1|	425581-42655[illegible]	419861	194821	-110776	0.50695	no
gi|320446898|ref|NW_003383673.1|	426718-42698[illegible]	71258	261117	-144836	0.48805	no
gi|320446898|ref|NW_003383673.1|	427879-42807[illegible]	439018	436194	-0.00930984	0.9322	no
gi|320446898|ref|NW_003383673.1|	428177-42887[illegible]	420204	307204	-0.451892	0.84555	no
gi|320446898|ref|NW_003383673.1|	430469-43088[illegible]	996183	537933	-0.888985	0.6616	no
gi|320446898|ref|NW_003383673.1|	432872-43504[illegible]	296241	316141	0.0937958	0.9632	no
gi|320446898|ref|NW_003383673.1|	43304-44286	158235	374631	12434	0.55105	no
gi|320446898|ref|NW_003383673.1|	435854-43808[illegible]	0.677105	124045	0.873407	1	no
gi|320446898|ref|NW_003383673.1|	438216-43893[illegible]	504119	605257	0.263783	0.83445	no
gi|320446898|ref|NW_003383673.1|	47105-47650	324459	236921	28683	0.22265	no
gi|320446898|ref|NW_003383673.1|	49300-49580	0	208637	inf	0.0038	no
gi|320446898|ref|NW_003383673.1|	50899-51233	0	272038	inf	0.0109	no
gi|320446898|ref|NW_003383673.1|	520046-52042[illegible]	0	128668	inf	0.0154	no
gi|320446898|ref|NW_003383673.1|	523721-524089	219985	680813	-169207	0.41855	no
gi|320446898|ref|NW_003383673.1|	525679-52735[illegible]	329117	351865	0.0964252	0.96045	no
gi|320446898|ref|NW_003383673.1|	53931-54155	0	789009	inf	0.0133	no
gi|320446898|ref|NW_003383673.1|	55004-55407	0	740229	inf	0.00515	no
gi|320446898|ref|NW_003383673.1|	5510-6132	17417	295842	0.764335	0.721	no
gi|320446898|ref|NW_003383673.1|	573867-57522[illegible]	0.301179	399482	705136	0.1181	no

gi|320446898|ref|NW_003383673.1|	580263-58071	0	286795	inf	0.00785	no
gi|320446898|ref|NW_003383673.1|	584391-58478	0	513283	inf	0.0059	no
gi|320446898|ref|NW_003383673.1|	585035-58739	0	466857	inf	0.00625	no
gi|320446898|ref|NW_003383673.1|	588846-58920	0	359788	inf	0.00845	no
gi|320446898|ref|NW_003383673.1|	60673-60929	565143	754267	-290547	0.2452	no
gi|320446898|ref|NW_003383673.1|	61083-61474	101492	133334	-292824	0.2676	no
gi|320446898|ref|NW_003383673.1|	62311-63300	985381	340274	-153398	0.46975	no
gi|320446898|ref|NW_003383673.1|	64304-64614	104503	448308	-122099	0.57555	no
gi|320446898|ref|NW_003383673.1|	66997-69742	598382	56381	-0.0858586	0.96855	no
gi|320446898|ref|NW_003383673.1|	7330-7833	310929	708642	118847	0.5755	no
gi|320446898|ref|NW_003383673.1|	73693-74156	723897	0.482246	-390794	0.3159	no
gi|320446898|ref|NW_003383673.1|	76574-77350	593219	0	#NAME?	0.01335	no
gi|320446898|ref|NW_003383673.1|	87458-88135	0	52114	inf	0.0162	no
gi|320446898|ref|NW_003383673.1|	88621-90236	0	102145	inf	1	no
gi|320446898|ref|NW_003383673.1|	90379-92231	0	581903	inf	0.0077	no
gi|320446898|ref|NW_003383673.1|	96954-99488	0.0734064	139977	757507	0.1408	no
gi|320446899|ref|NW_003383672.1|	103266-10449	137697	120243	-0.195551	0.881	no
gi|320446899|ref|NW_003383672.1|	105768-10659	247451	482585	0.963639	0.56185	no
gi|320446899|ref|NW_003383672.1|	115397-11830	385599	573503	0.572701	0.6663	no
gi|320446899|ref|NW_003383672.1|	119987-12089	199557	204622	0.0361636	0.98745	no
gi|320446899|ref|NW_003383672.1|	121957-12251	141156	218761	0.632066	0.76505	no
gi|320446899|ref|NW_003383672.1|	122763-12348	34819	381199	0.130673	0.9394	no
gi|320446899|ref|NW_003383672.1|	12633-14544	0	16145	inf	0.01485	no
gi|320446899|ref|NW_003383672.1|	129787-13267	916316	212169	12113	0.3473	no
gi|320446899|ref|NW_003383672.1|	204379-20483	193721	168561	-0.200713	0.9191	no
gi|320446899|ref|NW_003383672.1|	214067-21464	0	399007	inf	0.029	no
gi|320446899|ref|NW_003383672.1|	239065-23993	32169	0.738147	-212369	0.3545	no
gi|320446899|ref|NW_003383672.1|	247409-24778	245778	153501	-0.679102	0.7332	no
gi|320446899|ref|NW_003383672.1|	250683-25138	104402	664488	-0.651837	0.75295	no
gi|320446899|ref|NW_003383672.1|	255948-25719	614802	507127	-0.277772	0.89355	no

gi|320446899|ref|NW_003383672.1|	:57516-26052:	536111	885942	0.72468	0.65415	no
gi|320446899|ref|NW_003383672.1|	:74912-27702	307512	438463	0.511812	0.70215	no
gi|320446899|ref|NW_003383672.1|	:78490-28101!	198976	272813	0.455314	0.82005	no
gi|320446899|ref|NW_003383672.1|	:82897-28374(	522629	283878	-0.880513	0.66445	no
gi|320446899|ref|NW_003383672.1|	:86658-28752:	349052	406614	0.22022	0.91055	no
gi|320446899|ref|NW_003383672.1|	:89057-29037	920523	106138	0.205413	0.924	no
gi|320446899|ref|NW_003383672.1|	:95321-29638:	0	482694	inf	5.00E-05	yes
gi|320446899|ref|NW_003383672.1|	307463-31029:	38684	10434	-189044	0.1592	no
gi|320446899|ref|NW_003383672.1|	311373-31242:	139149	114889	-0.276394	0.89725	no
gi|320446899|ref|NW_003383672.1|	313822-31579:	128406	120729	-0.0889449	0.9439	no
gi|320446899|ref|NW_003383672.1|	316113-31639!	851714	343458	-131024	0.53535	no
gi|320446899|ref|NW_003383672.1|	321586-32473!	246699	209371	-0.236689	0.85825	no
gi|320446899|ref|NW_003383672.1|	325020-32548:	445198	345086	-0.367492	0.86815	no
gi|320446899|ref|NW_003383672.1|	331424-33205:	686201	303208	-117832	0.47175	no
gi|320446899|ref|NW_003383672.1|	348713-34903	287186	195658	-0.553651	0.7861	no
gi|320446899|ref|NW_003383672.1|	351759-35219(	29093	121126	-126416	0.5435	no
gi|320446899|ref|NW_003383672.1|	353140-35802:	381266	701981	0.880635	0.5993	no
gi|320446899|ref|NW_003383672.1|	358409-36263	105728	106298	0.00775919	0.9945	no
gi|320446899|ref|NW_003383672.1|	367159-37220:	715937	208969	-177655	0.3044	no
gi|320446899|ref|NW_003383672.1|	374628-37504!	775273	529782	-0.549306	0.8013	no
gi|320446899|ref|NW_003383672.1|	376008-37944!	653727	381116	-0.778459	0.5598	no
gi|320446899|ref|NW_003383672.1|	384683-38576!	277766	906104	170581	0.42795	no
gi|320446899|ref|NW_003383672.1|	387633-38956(	268375	810725	159496	0.4721	no
gi|320446899|ref|NW_003383672.1|	390214-39166	0.138591	0.963304	279716	1	no
gi|320446899|ref|NW_003383672.1|	393236-39437!	334693	824218	130019	0.54615	no
gi|320446899|ref|NW_003383672.1|	414215-41500(	972142	185629	0.93318	0.65495	no
gi|320446899|ref|NW_003383672.1|	443340-44570!	165285	147426	-0.164969	0.8979	no
gi|320446899|ref|NW_003383672.1|	445867-44627!	649958	42852	-0.600982	0.75445	no
gi|320446899|ref|NW_003383672.1|	47254-48705	0.832912	250867	159069	0.4452	no
gi|320446899|ref|NW_003383672.1|	49265-50276	0.652788	375882	252559	0.2798	no

gi|320446899|ref|NW_003383672.1|	54314-55026	495603	111374	116815	0.5718	no
gi|320446899|ref|NW_003383672.1|	55936-56846	250186	134513	242667	0.2869	no
gi|320446899|ref|NW_003383672.1|	57594-59960	506849	253795	232403	0.1985	no
gi|320446899|ref|NW_003383672.1|	62625-63216	15124	800447	-0.917963	0.6516	no
gi|320446899|ref|NW_003383672.1|	65727-67714	529833	409747	-0.370804	0.8587	no
gi|320446899|ref|NW_003383672.1|	74778-75953	15985	38208	125716	0.45585	no
gi|320446899|ref|NW_003383672.1|	77888-78635	122387	165916	0.438996	0.7449	no
gi|320446899|ref|NW_003383672.1|	79185-80313	105877	257909	128447	0.4347	no
gi|320446899|ref|NW_003383672.1|	82776-83276	628548	421151	-0.577686	0.79465	no
gi|320446899|ref|NW_003383672.1|	83525-87564	381489	85084	115725	0.4909	no
gi|320446899|ref|NW_003383672.1|	9404-11117	0.2284	206759	317832	0.20465	no
gi|320446899|ref|NW_003383672.1|	95106-95547	23839	58533	129593	0.5509	no
gi|320446899|ref|NW_003383672.1|	95673-96763	134283	347104	137009	0.41735	no
gi|320446900|ref|NW_003383671.1|	122266-122429	644955	562.63	312492	0.21675	no
gi|320446900|ref|NW_003383671.1|	126503-12666[illegible]	528822	768253	386073	0.17785	no
gi|320446900|ref|NW_003383671.1|	139407-14207[illegible]	200622	131929	-0.604717	0.63715	no
gi|320446900|ref|NW_003383671.1|	146224-147585	344818	16867	229029	0.32665	no
gi|320446900|ref|NW_003383671.1|	148429-149365	120449	365563	16017	0.45055	no
gi|320446900|ref|NW_003383671.1|	150629-152390	387122	102465	140427	0.5214	no
gi|320446900|ref|NW_003383671.1|	154038-155000	139365	304608	112808	0.5869	no
gi|320446900|ref|NW_003383671.1|	155528-155939	256102	132751	-0.948	0.6439	no
gi|320446900|ref|NW_003383671.1|	156259-16249[illegible]	573222	175451	-170803	0.1977	no
gi|320446900|ref|NW_003383671.1|	162677-163367	638143	146975	-211831	0.218	no
gi|320446900|ref|NW_003383671.1|	163544-163765	488787	12274	-19936	0.3966	no
gi|320446900|ref|NW_003383671.1|	166932-168505	181663	763587	-12504	0.4399	no
gi|320446900|ref|NW_003383671.1|	178939-17909[illegible]	332431	559854	407392	0.1937	no
gi|320446900|ref|NW_003383671.1|	19323-20158	0.281493	406519	385215	0.20055	no
gi|320446900|ref|NW_003383671.1|	199537-20321[illegible]	0.147162	274614	422193	0.16415	no
gi|320446900|ref|NW_003383671.1|	208088-210500	0.775213	671225	311413	0.1934	no
gi|320446900|ref|NW_003383671.1|	214438-215200	0.950716	478195	233051	0.3042	no

gi|320446900|ref|NW_003383671.1|	277333-27802	18632	407172	112786	0.58145	no
gi|320446900|ref|NW_003383671.1|	29150-29353	0	470241	inf	0.0294	no
gi|320446900|ref|NW_003383671.1|	293927-29440	940455	282799	158834	0.45175	no
gi|320446900|ref|NW_003383671.1|	296666-30205	169685	248563	0.550753	0.68175	no
gi|320446900|ref|NW_003383671.1|	303477-30468	974667	410225	-124849	0.5526	no
gi|320446900|ref|NW_003383671.1|	305582-30748	314433	544377	-253007	0.17735	no
gi|320446900|ref|NW_003383671.1|	308088-30903	294808	126939	-121565	0.4519	no
gi|320446900|ref|NW_003383671.1|	309350-31102	155464	903314	-0.783281	0.62645	no
gi|320446900|ref|NW_003383671.1|	311536-31346	0.399475	0.765802	0.938866	1	no
gi|320446900|ref|NW_003383671.1|	316094-31953	199826	124713	-0.680134	0.59975	no
gi|320446900|ref|NW_003383671.1|	320397-32355	115623	468967	202006	0.373	no
gi|320446900|ref|NW_003383671.1|	324217-32491	662044	201022	-171957	0.4269	no
gi|320446900|ref|NW_003383671.1|	327284-32782	382111	294242	-0.376987	0.84975	no
gi|320446900|ref|NW_003383671.1|	327985-32894	162592	24048	0.564656	0.7849	no
gi|320446900|ref|NW_003383671.1|	330117-33570	532911	477262	-0.159114	0.89965	no
gi|320446900|ref|NW_003383671.1|	336533-33776	84496	225012	141305	0.389	no
gi|320446900|ref|NW_003383671.1|	339079-34052	0.278324	0.870512	16451	1	no
gi|320446900|ref|NW_003383671.1|	344642-34508	0	622249	inf	0.029	no
gi|320446900|ref|NW_003383671.1|	345652-34657	0.98612	543931	246359	0.2902	no
gi|320446900|ref|NW_003383671.1|	350919-35461	271259	548347	101542	0.44475	no
gi|320446900|ref|NW_003383671.1|	355095-35666	381559	481686	0.336188	0.80085	no
gi|320446900|ref|NW_003383671.1|	388615-38900	405967	400003	-0.0213535	0.9157	no
gi|320446900|ref|NW_003383671.1|	434853-43595	0.193845	268359	379119	0.2072	no
gi|320446900|ref|NW_003383671.1|	449914-45020	209514	16084	294051	0.2567	no
gi|320446900|ref|NW_003383671.1|	451847-45492	11262	106931	324715	0.0917	no
gi|320446900|ref|NW_003383671.1|	457972-46306	205751	165561	-0.313536	0.8178	no
gi|320446900|ref|NW_003383671.1|	467292-46774	71681	455799	-0.653192	0.76455	no
gi|320446900|ref|NW_003383671.1|	468410-47118	763755	844729	0.145378	0.9261	no
gi|320446900|ref|NW_003383671.1|	479995-48028	30561	313076	0.0348241	0.97415	no
gi|320446900|ref|NW_003383671.1|	484258-48495	403709	250751	-0.687057	0.73675	no

gi|320446900|ref|NW_003383671.1|	485657-48683(	227133	242913	0.0969043	0.93315	no
gi|320446900|ref|NW_003383671.1|	487401-49002:	728509	909209	0.319665	0.8443	no
gi|320446900|ref|NW_003383671.1|	490201-49466	126425	246322	0.962262	0.47495	no
gi|320446900|ref|NW_003383671.1|	498512-49876:	221404	340418	0.620623	0.7541	no
gi|320446900|ref|NW_003383671.1|	500929-50261!	823643	345693	20694	0.26195	no
gi|320446900|ref|NW_003383671.1|	504053-50507	0.644476	341479	24056	0.2994	no
gi|320446900|ref|NW_003383671.1|	512097-51252!	151709	357077	123493	0.56345	no
gi|320446900|ref|NW_003383671.1|	530915-53412(	0.793853	471919	257159	0.26165	no
gi|320446900|ref|NW_003383671.1|	550304-55074!	156165	881519	249692	0.254	no
gi|320446900|ref|NW_003383671.1|	591895-59443:	190608	312364	0.71262	0.59125	no
gi|320446900|ref|NW_003383671.1|	594536-59503(	179858	103205	-0.801345	0.69615	no
gi|320446900|ref|NW_003383671.1|	595131-59541:	156895	858645	-0.869663	0.6867	no
gi|320446900|ref|NW_003383671.1|	595641-59695(	734657	542511	-0.437418	0.82985	no
gi|320446900|ref|NW_003383671.1|	658155-65933!	312647	162186	-0.946889	0.56115	no
gi|320446900|ref|NW_003383671.1|	660301-66489:	983697	238947	128041	0.34395	no
gi|320446900|ref|NW_003383671.1|	665303-66707!	417295	168287	201179	0.24095	no
gi|320446900|ref|NW_003383671.1|	66832-68215	0.147046	1.43	328167	0.23005	no
gi|320446900|ref|NW_003383671.1|	704579-70623	689426	266434	-137162	0.3065	no
gi|320446900|ref|NW_003383671.1|	707422-70801	152076	708146	-110268	0.58995	no
gi|320446900|ref|NW_003383671.1|	709830-71114	0.627566	0.762308	0.280607	1	no
gi|320446900|ref|NW_003383671.1|	86488-87208	0.34819	262075	291203	0.25645	no
gi|320446900|ref|NW_003383671.1|	88944-89459	178923	800915	216231	0.34165	no
gi|320446901|ref|NW_003383670.1|	102644-10355:	0.994842	480071	227071	0.3283	no
gi|320446901|ref|NW_003383670.1|	121471-12316!	336652	365696	0.11939	0.92905	no
gi|320446901|ref|NW_003383670.1|	123333-12467!	108272	667073	-0.698739	0.75065	no
gi|320446901|ref|NW_003383670.1|	125129-12900	705226	174383	130611	0.3194	no
gi|320446901|ref|NW_003383670.1|	133455-13403	15023	491997	-161045	0.44745	no
gi|320446901|ref|NW_003383670.1|	141284-14153!	210741	572422	-188032	0.30885	no
gi|320446901|ref|NW_003383670.1|	143230-14424(	0.653631	301087	220364	0.33335	no
gi|320446901|ref|NW_003383670.1|	144408-14537!	0	253426	inf	0.0198	no

gi|320446901|ref|NW_003383670.1|	146227-14778[illegible]	0.127586	0.887547	279835	1	no
gi|320446901|ref|NW_003383670.1|	147944-14866[illegible]	243233	19022	-0.354674	0.85525	no
gi|320446901|ref|NW_003383670.1|	152130-15468[illegible]	14118	823507	-0.777682	0.64165	no
gi|320446901|ref|NW_003383670.1|	166140-17069[illegible]	381793	211543	-0.851836	0.524	no
gi|320446901|ref|NW_003383670.1|	170882-17451[illegible]	0.396932	385381	327932	0.1841	no
gi|320446901|ref|NW_003383670.1|	174578-17711[illegible]	0.219932	199708	318276	0.20465	no
gi|320446901|ref|NW_003383670.1|	177863-17872[illegible]	32169	867323	-189103	0.3975	no
gi|320446901|ref|NW_003383670.1|	178851-18142[illegible]	139072	493304	-149529	0.37725	no
gi|320446901|ref|NW_003383670.1|	183010-18548[illegible]	162344	284763	-251122	0.16375	no
gi|320446901|ref|NW_003383670.1|	187335-18765[illegible]	592599	116742	-234373	0.2999	no
gi|320446901|ref|NW_003383670.1|	189167-19069[illegible]	201199	513384	135141	0.30215	no
gi|320446901|ref|NW_003383670.1|	190811-19425[illegible]	246922	657901	-190812	0.14905	no
gi|320446901|ref|NW_003383670.1|	203950-20466[illegible]	166035	724879	-119567	0.5672	no
gi|320446901|ref|NW_003383670.1|	222255-22254[illegible]	81496	130449	0.67868	0.7444	no
gi|320446901|ref|NW_003383670.1|	225260-22643[illegible]	556779	273803	-102397	0.6156	no
gi|320446901|ref|NW_003383670.1|	227180-22788[illegible]	249362	267958	0.103766	0.94455	no
gi|320446901|ref|NW_003383670.1|	228157-22844[illegible]	166047	106255	-0.644052	0.74915	no
gi|320446901|ref|NW_003383670.1|	239156-24303[illegible]	643958	303936	-10832	0.41845	no
gi|320446901|ref|NW_003383670.1|	245383-24653[illegible]	440033	307363	-0.517667	0.6841	no
gi|320446901|ref|NW_003383670.1|	250556-25361[illegible]	269789	255979	-0.0758038	0.95175	no
gi|320446901|ref|NW_003383670.1|	253765-26335[illegible]	197312	187104	-0.0766381	0.95505	no
gi|320446901|ref|NW_003383670.1|	264163-26540[illegible]	187023	195735	0.0656851	0.9777	no
gi|320446901|ref|NW_003383670.1|	265568-27533[illegible]	903126	114028	0.336385	0.80485	no
gi|320446901|ref|NW_003383670.1|	281987-28224[illegible]	594887	16971	151238	0.359	no
gi|320446901|ref|NW_003383670.1|	286451-28671[illegible]	140211	124611	-0.170167	0.91405	no
gi|320446901|ref|NW_003383670.1|	287853-28917[illegible]	452461	996396	113893	0.5936	no
gi|320446901|ref|NW_003383670.1|	291054-29219[illegible]	299818	127176	208467	0.3443	no
gi|320446901|ref|NW_003383670.1|	317338-32489[illegible]	102574	244852	125525	0.3514	no
gi|320446901|ref|NW_003383670.1|	326546-32998[illegible]	314195	12613	-131675	0.32785	no
gi|320446901|ref|NW_003383670.1|	330167-33302[illegible]	438751	534774	0.285526	0.8365	no

gi|320446901|ref|NW_003383670.1|	37528-33829	188743	438738	-210499	0.3467	no
gi|320446901|ref|NW_003383670.1|	38668-34309	141957	577279	-129811	0.3134	no
gi|320446901|ref|NW_003383670.1|	72351-72532	0	101.46	inf	0.0233	no
gi|320446902|ref|NW_003383669.1|	49598-14981	0	782119	inf	0.01485	no
gi|320446902|ref|NW_003383669.1|	82306-28359	192751	329927	409734	0.0601	no
gi|320446902|ref|NW_003383669.1|	39812-40267	254389	0.995515	-467545	0.1956	no
gi|320446902|ref|NW_003383669.1|	58222-45908	0.267225	294342	346137	0.21395	no
gi|320446902|ref|NW_003383669.1|	48532-50600	548766	0.064223	-973889	0.2504	no
gi|320446902|ref|NW_003383669.1|	85924-48676	139351	115021	-0.276831	1	no
gi|320446902|ref|NW_003383669.1|	16310-51954	106911	436831	203067	0.3567	no
gi|320446902|ref|NW_003383669.1|	22226-52303	0	344271	inf	0.02075	no
gi|320446902|ref|NW_003383669.1|	27969-52849	283634	160292	-0.823328	0.70045	no
gi|320446902|ref|NW_003383669.1|	28803-53004	252476	198445	-0.347411	0.86165	no
gi|320446902|ref|NW_003383669.1|	30167-53083	104503	68643	-0.606358	0.7697	no
gi|320446902|ref|NW_003383669.1|	31165-53246	142796	231242	0.695453	0.7311	no
gi|320446902|ref|NW_003383669.1|	32585-53387	160016	12548	-0.350763	0.87305	no
gi|320446902|ref|NW_003383669.1|	58129-58516	0	231483	inf	0.0101	no
gi|320446902|ref|NW_003383669.1|	72022-73605	0	217808	inf	0.01485	no
gi|320446902|ref|NW_003383669.1|	80685-80928	0	221727	inf	0.0312	no
gi|320446903|ref|NW_003383668.1|	00738-10139	0	273018	inf	0.0312	no
gi|320446903|ref|NW_003383668.1|	35260-13558	0	11161	inf	0.0294	no
gi|320446903|ref|NW_003383668.1|	50219-15080	0.484611	623196	368478	0.2145	no
gi|320446903|ref|NW_003383668.1|	50993-15161	0.43322	677112	396622	0.1969	no
gi|320446903|ref|NW_003383668.1|	52897-15499	0.362951	670946	420835	0.15605	no
gi|320446903|ref|NW_003383668.1|	56520-15727	0.322959	130634	533803	0.1682	no
gi|320446903|ref|NW_003383668.1|	79245-18441	0.684527	117491	0.779365	1	no
gi|320446903|ref|NW_003383668.1|	86196-18783	107969	400806	189229	0.3911	no
gi|320446903|ref|NW_003383668.1|	88035-19266	0.729307	217747	157805	0.46765	no
gi|320446903|ref|NW_003383668.1|	94386-19563	0.832453	138577	0.735248	1	no
gi|320446903|ref|NW_003383668.1|	10186-21122	125694	260711	105254	0.59815	no

gi|320446903|ref|NW_003383668.1|	21330-22490	0.151569	413604	477021	0.1509	no
gi|320446903|ref|NW_003383668.1|	25048-23163	135538	536512	198492	0.25485	no
gi|320446903|ref|NW_003383668.1|	31970-23554	106225	417638	197512	0.38005	no
gi|320446903|ref|NW_003383668.1|	39867-24077	526953	207546	197769	0.3832	no
gi|320446903|ref|NW_003383668.1|	46832-24905	144392	729091	233611	0.30545	no
gi|320446903|ref|NW_003383668.1|	53389-25438	221597	826587	189923	0.37765	no
gi|320446903|ref|NW_003383668.1|	54524-26084	0.998084	239018	125988	0.57065	no
gi|320446903|ref|NW_003383668.1|	61673-26370	0.940773	295244	164999	0.4269	no
gi|320446903|ref|NW_003383668.1|	83360-28395	27897	9455	176097	0.4153	no
gi|320446903|ref|NW_003383668.1|	02008-30238	32187	274228	309083	0.21245	no
gi|320446903|ref|NW_003383668.1|	49408-34957	0	169889	inf	0.0233	no
gi|320446903|ref|NW_003383668.1|	31127-43193	0.593693	163125	145819	0.375	no
gi|320446903|ref|NW_003383668.1|	57586-59909	573061	380869	-0.589394	0.66095	no
gi|320446903|ref|NW_003383668.1|	87037-58899	168974	431498	-196937	0.2514	no
gi|320446903|ref|NW_003383668.1|	91907-59972	261482	245143	-0.093086	0.94185	no
gi|320446903|ref|NW_003383668.1|	60070-60702	116769	53.13	-113606	0.37135	no
gi|320446903|ref|NW_003383668.1|	01914-60496	133648	673104	-0.989538	0.4438	no
gi|320446903|ref|NW_003383668.1|	09375-61124	855236	140034	0.711381	0.66015	no
gi|320446903|ref|NW_003383668.1|	11403-61505	236264	499094	107891	0.4194	no
gi|320446903|ref|NW_003383668.1|	61373-62873	811308	566919	-0.517107	0.6968	no
gi|320446903|ref|NW_003383668.1|	16599-61692	159548	720844	-114623	0.5825	no
gi|320446903|ref|NW_003383668.1|	18222-62007	281824	53826	-238842	0.1836	no
gi|320446903|ref|NW_003383668.1|	20183-62198	28851	219063	-0.397276	0.75585	no
gi|320446903|ref|NW_003383668.1|	22136-62455	352813	889506	-198783	0.28335	no
gi|320446903|ref|NW_003383668.1|	24940-62634	366659	179827	-434976	0.04825	no
gi|320446903|ref|NW_003383668.1|	70889-67492	0.355154	43197	360442	0.1529	no
gi|320446903|ref|NW_003383668.1|	68033-68408	888517	58149	-0.611645	0.75355	no
gi|320446903|ref|NW_003383668.1|	91156-69187	0.700694	383497	245236	0.24845	no
gi|320446903|ref|NW_003383668.1|	75582-76182	184481	218902	0.246815	0.8906	no
gi|320446903|ref|NW_003383668.1|	79440-80691	66465	979678	0.559712	0.79265	no

gi|320446903|ref|NW_003383668.1|	94046-94889	0	2293	inf	0.029	no
gi|320446903|ref|NW_003383668.1|	95860-97291	0.141141	12751	31754	1	no
gi|320446905|ref|NW_003383666.1|	102871-10318	474165	112975	-539131	0.27645	no
gi|320446905|ref|NW_003383666.1|	103354-10381	266756	384298	-279523	0.23555	no
gi|320446905|ref|NW_003383666.1|	103978-10724	600889	581775	-0.0466381	0.97265	no
gi|320446905|ref|NW_003383666.1|	113474-11694	240695	15853	-0.60245	0.64975	no
gi|320446905|ref|NW_003383666.1|	119850-12031	102177	680451	-0.586501	0.7614	no
gi|320446905|ref|NW_003383666.1|	120704-12166	123959	151727	0.291615	0.894	no
gi|320446905|ref|NW_003383666.1|	125618-12745	178772	344405	0.945985	0.4672	no
gi|320446905|ref|NW_003383666.1|	127576-13005	217903	230899	0.0835741	0.96665	no
gi|320446905|ref|NW_003383666.1|	130253-13433	806246	460121	-0.809206	0.62655	no
gi|320446905|ref|NW_003383666.1|	136600-13761	507212	35798	-0.502708	0.6975	no
gi|320446905|ref|NW_003383666.1|	138788-13909	112395	711744	-0.659144	0.75425	no
gi|320446905|ref|NW_003383666.1|	139251-14165	223513	163682	-0.449462	0.72745	no
gi|320446905|ref|NW_003383666.1|	141848-14643	285045	177623	-0.682367	0.6079	no
gi|320446905|ref|NW_003383666.1|	146866-14970	286762	0.904882	-498598	0.0419	no
gi|320446905|ref|NW_003383666.1|	152772-15724	975718	214181	11343	0.3928	no
gi|320446905|ref|NW_003383666.1|	158024-15867	359069	432898	0.269768	0.84115	no
gi|320446905|ref|NW_003383666.1|	160480-16111	339849	119858	-150357	0.50415	no
gi|320446905|ref|NW_003383666.1|	416174-41640	859111	486566	250172	0.2483	no
gi|320446905|ref|NW_003383666.1|	427250-42909	0.944086	137422	386355	0.07325	no
gi|320446905|ref|NW_003383666.1|	454036-45515	191209	0	#NAME?	0.0229	no
gi|320446905|ref|NW_003383666.1|	457703-45866	256208	0	#NAME?	0.02105	no
gi|320446905|ref|NW_003383666.1|	463314-46460	144288	0.44501	-169704	0.4422	no
gi|320446905|ref|NW_003383666.1|	466274-46795	14056	507226	185144	0.30505	no
gi|320446905|ref|NW_003383666.1|	489194-49126	422852	30777	286363	0.12825	no
gi|320446905|ref|NW_003383666.1|	493393-49443	417943	182053	212298	0.34635	no
gi|320446905|ref|NW_003383666.1|	506234-50699	418249	183091	213012	0.3453	no
gi|320446905|ref|NW_003383666.1|	541793-54243	205168	474919	121087	0.5726	no
gi|320446905|ref|NW_003383666.1|	546168-54724	281071	15001	241605	0.28425	no

gi|320446905|ref|NW_003383666.1|	49145-55450	16801	731667	212264	0.2298	no
gi|320446905|ref|NW_003383666.1|	56589-55702	478894	116504	12826	0.54065	no
gi|320446905|ref|NW_003383666.1|	57360-55874	102664	48902	225197	0.3215	no
gi|320446905|ref|NW_003383666.1|	60366-56247	269747	332454	0.301546	0.87795	no
gi|320446905|ref|NW_003383666.1|	63724-56404	497761	962497	0.95133	0.6698	no
gi|320446905|ref|NW_003383666.1|	60157-62761	121102	204042	0.752637	0.7046	no
gi|320446905|ref|NW_003383666.1|	25714-62628	0	501514	inf	0.02205	no
gi|320446905|ref|NW_003383666.1|	88855-68904	0	780313	inf	0.02205	no
gi|320446905|ref|NW_003383666.1|	79710-80379	342157	143197	-125666	0.56355	no
gi|320446905|ref|NW_003383666.1|	82118-82835	490486	33556	-0.547642	0.7879	no
gi|320446905|ref|NW_003383666.1|	84201-86863	152975	505263	-159819	0.34835	no
gi|320446905|ref|NW_003383666.1|	87336-87724	196024	474067	-204786	0.347	no
gi|320446905|ref|NW_003383666.1|	88510-89842	27084	608984	-215296	0.2196	no
gi|320446905|ref|NW_003383666.1|	90653-91130	23967	365781	-2712	0.2399	no
gi|320446905|ref|NW_003383666.1|	94281-94732	213063	0.505877	-539635	0.27715	no
gi|320446905|ref|NW_003383666.1|	94878-95538	234211	405837	-252884	0.2694	no
gi|320446905|ref|NW_003383666.1|	99451-99729	163305	401732	129866	0.54035	no
gi|320446906|ref|NW_003383665.1|	20289-12065	937567	841859	-0.155343	0.93115	no
gi|320446906|ref|NW_003383665.1|	57544-15856	259442	522917	101117	0.61065	no
gi|320446906|ref|NW_003383665.1|	77050-17772	266689	260005	-0.0366218	0.94545	no
gi|320446906|ref|NW_003383665.1|	78117-17932	127186	153387	0.270238	0.90375	no
gi|320446906|ref|NW_003383665.1|	79538-18247	175499	407352	121481	0.36085	no
gi|320446906|ref|NW_003383665.1|	86906-18752	482094	354141	-376692	0.1473	no
gi|320446906|ref|NW_003383665.1|	88606-18950	270913	150056	-0.85233	0.70195	no
gi|320446906|ref|NW_003383665.1|	89662-19098	342296	151211	-117868	0.5658	no
gi|320446906|ref|NW_003383665.1|	91517-19260	454725	20522	-114783	0.583	no
gi|320446906|ref|NW_003383665.1|	92978-19388	249534	573263	-212197	0.34205	no
gi|320446906|ref|NW_003383665.1|	97820-19858	104972	152712	-278112	0.241	no
gi|320446906|ref|NW_003383665.1|	19975-20348	134852	117616	-0.197295	0.91705	no
gi|320446906|ref|NW_003383665.1|	99807-20023	119662	226192	-240334	0.3061	no

gi|320446906|ref|NW_003383665.1|	01808-20254	194748	482946	-201168	0.3593	no
gi|320446906|ref|NW_003383665.1|	03979-20423	545353	546332	-331934	0.1886	no
gi|320446906|ref|NW_003383665.1|	09062-21173	387632	0.918932	-207666	0.34825	no
gi|320446906|ref|NW_003383665.1|	22583-22353	238205	911354	-138612	0.53565	no
gi|320446906|ref|NW_003383665.1|	24128-22479	865267	294955	-155265	0.4711	no
gi|320446906|ref|NW_003383665.1|	25045-22534	631223	306775	-104097	0.60965	no
gi|320446906|ref|NW_003383665.1|	25454-22928	50736	986373	0.959123	0.5651	no
gi|320446906|ref|NW_003383665.1|	47340-24806	451719	404217	-0.160295	0.9313	no
gi|320446906|ref|NW_003383665.1|	52672-25340	0	254446	inf	0.0294	no
gi|320446906|ref|NW_003383665.1|	64196-26510	599557	189422	-16623	0.43985	no
gi|320446906|ref|NW_003383665.1|	71310-27334	798321	113972	0.513633	0.81645	no
gi|320446906|ref|NW_003383665.1|	12703-31339	111062	227583	103504	0.5772	no
gi|320446906|ref|NW_003383665.1|	15582-31663	165537	228945	0.46785	0.81085	no
gi|320446906|ref|NW_003383665.1|	18869-31912	602118	228969	192703	0.3165	no
gi|320446906|ref|NW_003383665.1|	20733-32150	0.632624	282053	215655	0.29245	no
gi|320446906|ref|NW_003383665.1|	21614-32247	0.808076	352266	21241	0.34675	no
gi|320446906|ref|NW_003383665.1|	24119-32655	629886	155559	13043	0.43525	no
gi|320446906|ref|NW_003383665.1|	38625-34152	246865	491146	0.992427	0.6461	no
gi|320446906|ref|NW_003383665.1|	14278-41526	201555	262939	0.383554	0.84555	no
gi|320446906|ref|NW_003383665.1|	31684-43207	337721	633458	0.90742	0.6741	no
gi|320446906|ref|NW_003383665.1|	40369-44540	140467	639596	218693	0.2094	no
gi|320446906|ref|NW_003383665.1|	46815-44878	107005	48156	217003	0.32295	no
gi|320446906|ref|NW_003383665.1|	49434-45239	649291	147416	118295	0.484	no
gi|320446906|ref|NW_003383665.1|	53257-45371	200336	167889	-0.254912	0.89885	no
gi|320446906|ref|NW_003383665.1|	54385-45507	398088	304686	-0.385764	0.8598	no
gi|320446906|ref|NW_003383665.1|	59736-46075	181609	268929	0.566386	0.72085	no
gi|320446906|ref|NW_003383665.1|	66891-46736	171193	173746	0.021356	0.9822	no
gi|320446906|ref|NW_003383665.1|	67556-46931	355572	379228	0.0929226	0.96115	no
gi|320446906|ref|NW_003383665.1|	69975-47086	0.259418	232301	316264	0.24575	no
gi|320446906|ref|NW_003383665.1|	71091-47259	372744	323988	-0.202246	0.91945	no

gi|320446906|ref|NW_003383665.1|	473354-47362	786217	133311	0.761798	0.7071	no
gi|320446906|ref|NW_003383665.1|	473997-47521	118624	109662	-0.113335	0.9575	no
gi|320446906|ref|NW_003383665.1|	487181-49109	953767	145071	0.605054	0.641	no
gi|320446906|ref|NW_003383665.1|	492002-49246	441517	538873	0.287477	0.88735	no
gi|320446906|ref|NW_003383665.1|	494552-49563	103535	67506	-0.617037	0.7627	no
gi|320446906|ref|NW_003383665.1|	495970-49677	505528	653625	0.370672	0.85285	no
gi|320446906|ref|NW_003383665.1|	496984-49834	268901	249006	-0.110899	0.9539	no
gi|320446906|ref|NW_003383665.1|	498978-50038	404675	281153	-0.52541	0.79055	no
gi|320446906|ref|NW_003383665.1|	501893-50248	477881	291247	-0.714405	0.72555	no
gi|320446906|ref|NW_003383665.1|	504346-50612	383104	259243	-0.563427	0.7868	no
gi|320446906|ref|NW_003383665.1|	506841-50723	908489	862181	-0.075478	0.95645	no
gi|320446906|ref|NW_003383665.1|	522297-52272	388278	148361	-138797	0.51555	no
gi|320446906|ref|NW_003383665.1|	522862-52429	11846	56842	-105937	0.62245	no
gi|320446906|ref|NW_003383665.1|	524432-52502	998001	125535	0.330976	0.86855	no
gi|320446906|ref|NW_003383665.1|	529870-53349	240852	251408	0.0618801	0.9616	no
gi|320446906|ref|NW_003383665.1|	540656-54109	625333	425459	-0.555606	0.79755	no
gi|320446906|ref|NW_003383665.1|	541193-54812	324713	19479	-0.737249	0.5752	no
gi|320446906|ref|NW_003383665.1|	549770-55108	228927	209726	-0.126382	0.9196	no
gi|320446906|ref|NW_003383665.1|	551715-55247	152081	31943	107066	0.6273	no
gi|320446906|ref|NW_003383665.1|	553443-55702	106035	169583	-264447	0.1541	no
gi|320446906|ref|NW_003383665.1|	557899-55837	266395	327346	-302468	0.21195	no
gi|320446906|ref|NW_003383665.1|	68434-68653	217468	0	#NAME?	0.00735	no
gi|320446906|ref|NW_003383665.1|	68758-69396	262742	0	#NAME?	5.00E-05	yes
gi|320446906|ref|NW_003383665.1|	734413-73634	244389	0.20861	-687223	0.11055	no
gi|320446906|ref|NW_003383665.1|	737564-73813	168079	0.668686	-465167	0.1959	no
gi|320446906|ref|NW_003383665.1|	739150-73949	22347	0.854558	-470876	0.286	no
gi|320446906|ref|NW_003383665.1|	855767-85622	17281	134936	-0.356912	0.8597	no
gi|320446906|ref|NW_003383665.1|	861826-86207	599172	589449	-0.0236044	0.97795	no
gi|320446906|ref|NW_003383665.1|	862642-86482	330446	409259	0.3086	0.82005	no
gi|320446906|ref|NW_003383665.1|	866555-86805	588931	303646	-0.955708	0.4725	no

gi|320446906|ref|NW_003383665.1|	368198-86968	442934	287298	-0.624546	0.6288	no
gi|320446906|ref|NW_003383665.1|	369787-87087	0.594514	150843	134326	0.5243	no
gi|320446906|ref|NW_003383665.1|	374543-87805	443746	549258	0.307751	0.8139	no
gi|320446906|ref|NW_003383665.1|	378183-87997	525009	311337	-0.753863	0.5799	no
gi|320446906|ref|NW_003383665.1|	91135-92014	131121	361199	14619	0.50075	no
gi|320446906|ref|NW_003383665.1|	92134-97238	0.79707	148092	0.893712	0.6736	no
gi|320446906|ref|NW_003383665.1|	97387-10179	170194	442.9	13798	0.4822	no
gi|320446907|ref|NW_003383664.1|	000634-10009	125309	221729	-249862	0.2826	no
gi|320446907|ref|NW_003383664.1|	001119-10033	19755	65961	-158253	0.3578	no
gi|320446907|ref|NW_003383664.1|	004517-10104	126483	11199	-0.175583	0.8933	no
gi|320446907|ref|NW_003383664.1|	011761-10127	256432	105114	-128663	0.56385	no
gi|320446907|ref|NW_003383664.1|	013008-10139	297425	12855	-12102	0.5869	no
gi|320446907|ref|NW_003383664.1|	015934-10164	15811	917861	-0.784579	0.70875	no
gi|320446907|ref|NW_003383664.1|	021943-10225	33738	117665	-151969	0.48155	no
gi|320446907|ref|NW_003383664.1|	023475-10261	142732	679482	-10708	0.5249	no
gi|320446907|ref|NW_003383664.1|	026507-10281	111048	171722	395081	0.0713	no
gi|320446907|ref|NW_003383664.1|	029116-10295	17675	875798	230889	0.27165	no
gi|320446907|ref|NW_003383664.1|	029809-10333	406618	199508	22947	0.2101	no
gi|320446907|ref|NW_003383664.1|	038694-10393	112284	332239	156507	0.491	no
gi|320446907|ref|NW_003383664.1|	039738-10416	0.497871	236009	2245	0.3195	no
gi|320446907|ref|NW_003383664.1|	041799-10451	0.693121	205139	156542	0.4594	no
gi|320446907|ref|NW_003383664.1|	067190-10713	167624	168507	0.0075809	0.9903	no
gi|320446907|ref|NW_003383664.1|	077839-10784	516645	295391	-0.80655	0.6133	no
gi|320446907|ref|NW_003383664.1|	078656-10801	275643	0.547706	-233133	0.3072	no
gi|320446907|ref|NW_003383664.1|	080278-10812	114063	942258	-0.27564	0.89525	no
gi|320446907|ref|NW_003383664.1|	082119-10845	158719	259153	0.707333	0.59045	no
gi|320446907|ref|NW_003383664.1|	085811-10880	572334	352469	-0.699362	0.60235	no
gi|320446907|ref|NW_003383664.1|	18812-19423	0	568573	inf	5.00E-05	yes
gi|320446907|ref|NW_003383664.1|	224133-22444	354164	91109	136318	0.38305	no
gi|320446907|ref|NW_003383664.1|	224998-22599	11109	296194	473674	0.08675	no

gi|320446907|ref|NW_003383664.1|	226249-22654	194703	187255	326565	0.23045	no
gi|320446907|ref|NW_003383664.1|	227396-22758	685674	745486	0.120658	0.9442	no
gi|320446907|ref|NW_003383664.1|	227767-22818	0.871093	11517	372479	0.20835	no
gi|320446907|ref|NW_003383664.1|	230246-23143	407884	13151	168895	0.45115	no
gi|320446907|ref|NW_003383664.1|	241205-24168	201526	161599	300338	0.21645	no
gi|320446907|ref|NW_003383664.1|	26269-28085	0.640542	386784	259416	0.25605	no
gi|320446907|ref|NW_003383664.1|	336497-33877	187977	175523	-0.0988962	0.93855	no
gi|320446907|ref|NW_003383664.1|	339124-34052	169824	826879	-103829	0.64875	no
gi|320446907|ref|NW_003383664.1|	340894-34847	156754	11.7	-0.42199	0.749	no
gi|320446907|ref|NW_003383664.1|	348627-34962	678066	202279	-174508	0.32315	no
gi|320446907|ref|NW_003383664.1|	351601-35190	288123	810236	-183027	0.39095	no
gi|320446907|ref|NW_003383664.1|	355519-35688	253962	290507	-312797	0.1021	no
gi|320446907|ref|NW_003383664.1|	358414-36046	552672	114669	-226895	0.093	no
gi|320446907|ref|NW_003383664.1|	367888-37136	286189	773589	475653	0.03555	no
gi|320446907|ref|NW_003383664.1|	374109-37482	194597	747529	-138029	0.30345	no
gi|320446907|ref|NW_003383664.1|	375188-37740	973765	379912	-135791	0.3181	no
gi|320446907|ref|NW_003383664.1|	428840-43015	0.627566	119791	0.932684	1	no
gi|320446907|ref|NW_003383664.1|	432479-43395	775318	964536	0.315047	0.8833	no
gi|320446907|ref|NW_003383664.1|	444181-44770	114787	237709	105024	0.427	no
gi|320446907|ref|NW_003383664.1|	451535-45262	452077	306062	275919	0.1338	no
gi|320446907|ref|NW_003383664.1|	452928-45416	0.169331	117426	279384	1	no
gi|320446907|ref|NW_003383664.1|	45645-46282	0	542123	inf	0.0212	no
gi|320446907|ref|NW_003383664.1|	463417-46384	168565	446346	140486	0.38125	no
gi|320446907|ref|NW_003383664.1|	465992-46898	6601	427316	-0.627381	0.78025	no
gi|320446907|ref|NW_003383664.1|	469503-47146	224385	200659	-0.161234	0.90135	no
gi|320446907|ref|NW_003383664.1|	487427-48813	0	268505	inf	0.0294	no
gi|320446907|ref|NW_003383664.1|	494322-49897	871402	183889	107742	0.4163	no
gi|320446907|ref|NW_003383664.1|	499575-50131	212521	210336	-0.0149073	0.97935	no
gi|320446907|ref|NW_003383664.1|	521480-52214	43464	269344	-0.690369	0.7366	no
gi|320446907|ref|NW_003383664.1|	590210-59090	770717	626878	-0.298015	0.88415	no

gi|320446907|ref|NW_003383664.1|	518860-62014	146588	637131	-120211	0.5834	no
gi|320446907|ref|NW_003383664.1|	520758-62122	159908	629375	-134525	0.5185	no
gi|320446907|ref|NW_003383664.1|	521338-62185	894614	760869	-0.233617	0.90465	no
gi|320446907|ref|NW_003383664.1|	522662-62520	84856	185992	113215	0.50455	no
gi|320446907|ref|NW_003383664.1|	545691-64684	31862	190281	-0.743707	0.66155	no
gi|320446907|ref|NW_003383664.1|	549494-65230	305875	33672	0.138608	0.91925	no
gi|320446907|ref|NW_003383664.1|	568084-66841	484202	254408	-0.928466	0.65315	no
gi|320446907|ref|NW_003383664.1|	569862-67029	209732	183302	-0.194325	0.9207	no
gi|320446907|ref|NW_003383664.1|	570526-67216	306018	277563	-0.140801	0.9117	no
gi|320446907|ref|NW_003383664.1|	572945-67315	182156	303733	0.737634	0.70725	no
gi|320446907|ref|NW_003383664.1|	574439-67794	162187	345116	108942	0.41935	no
gi|320446907|ref|NW_003383664.1|	722687-72504	667285	937306	0.490218	0.71385	no
gi|320446907|ref|NW_003383664.1|	734046-73490	359395	875989	128534	0.32535	no
gi|320446907|ref|NW_003383664.1|	736524-73696	15165	307148	101819	0.61715	no
gi|320446907|ref|NW_003383664.1|	737100-73821	115775	333936	152825	0.4736	no
gi|320446907|ref|NW_003383664.1|	739705-74002	225103	612124	144324	0.4984	no
gi|320446907|ref|NW_003383664.1|	743531-74580	596234	0.693614	-310367	0.2097	no
gi|320446907|ref|NW_003383664.1|	746453-74760	340255	96066	-182452	0.2889	no
gi|320446907|ref|NW_003383664.1|	751223-75231	108611	218648	-231249	0.2953	no
gi|320446907|ref|NW_003383664.1|	753797-75467	228861	380418	-258881	0.25925	no
gi|320446907|ref|NW_003383664.1|	755543-75706	190473	407442	109701	0.39345	no
gi|320446907|ref|NW_003383664.1|	767086-76794	826931	49688	-0.734869	0.5709	no
gi|320446907|ref|NW_003383664.1|	768098-76913	586573	275195	-109185	0.5976	no
gi|320446907|ref|NW_003383664.1|	769547-77037	119422	105568	-0.1779	0.9303	no
gi|320446907|ref|NW_003383664.1|	770749-77408	196865	223677	0.184211	0.88845	no
gi|320446907|ref|NW_003383664.1|	778452-78221	237447	180553	-0.395181	0.7639	no
gi|320446907|ref|NW_003383664.1|	785084-78589	531518	54772	0.0433198	0.97625	no
gi|320446907|ref|NW_003383664.1|	786236-78720	0.462639	143705	163515	0.3325	no
gi|320446907|ref|NW_003383664.1|	787771-78966	0.508066	148733	154963	0.487	no
gi|320446907|ref|NW_003383664.1|	790808-79425	958866	130.89	0.448948	0.717	no

gi|320446907|ref|NW_003383664.1|	798117-80039	280314	363615	-294656	0.124	no
gi|320446907|ref|NW_003383664.1|	800503-80258	131024	127822	-335761	0.0904	no
gi|320446907|ref|NW_003383664.1|	803224-80628	232206	26462	0.188518	0.88815	no
gi|320446907|ref|NW_003383664.1|	810161-81113	16104	33669	438592	0.0692	no
gi|320446907|ref|NW_003383664.1|	811322-81495	719863	118114	0.714383	0.575	no
gi|320446907|ref|NW_003383664.1|	815109-81568	374631	333422	-0.168123	0.9371	no
gi|320446907|ref|NW_003383664.1|	815973-81633	338436	260155	-0.379511	0.8489	no
gi|320446907|ref|NW_003383664.1|	816589-81795	269618	197649	-0.447977	0.82355	no
gi|320446907|ref|NW_003383664.1|	818052-82148	113413	106281	-0.0937053	0.9404	no
gi|320446907|ref|NW_003383664.1|	821772-82426	199072	250367	0.330756	0.80075	no
gi|320446907|ref|NW_003383664.1|	824412-82486	223524	644497	152774	0.4918	no
gi|320446907|ref|NW_003383664.1|	827893-83018	336385	458163	0.445748	0.8316	no
gi|320446907|ref|NW_003383664.1|	8290-8656	585979	206638	181819	0.4077	no
gi|320446907|ref|NW_003383664.1|	831460-83321	234765	751698	-164299	0.3429	no
gi|320446907|ref|NW_003383664.1|	835769-83658	6965	0.79778	-312606	0.2257	no
gi|320446907|ref|NW_003383664.1|	836724-83729	331215	200606	-404533	0.15055	no
gi|320446907|ref|NW_003383664.1|	839297-84146	161691	49397	-171074	0.3186	no
gi|320446907|ref|NW_003383664.1|	841880-84246	198133	107946	-0.876151	0.6703	no
gi|320446907|ref|NW_003383664.1|	859500-86263	0.34902	321356	320279	0.19525	no
gi|320446907|ref|NW_003383664.1|	863567-86406	0.640009	428517	274319	0.2661	no
gi|320446907|ref|NW_003383664.1|	864397-86875	0.450411	668032	38906	0.06725	no
gi|320446907|ref|NW_003383664.1|	903703-90632	904195	715601	-0.337478	0.82845	no
gi|320446907|ref|NW_003383664.1|	906905-90795	0.206162	270891	371586	0.21225	no
gi|320446907|ref|NW_003383664.1|	909212-91050	0.800082	333132	205788	0.35365	no
gi|320446907|ref|NW_003383664.1|	910810-91273	177767	863268	-10421	0.53335	no
gi|320446907|ref|NW_003383664.1|	913443-91541	116935	319327	144932	0.4845	no
gi|320446907|ref|NW_003383664.1|	915464-91824	0.596782	213169	183672	0.39105	no
gi|320446907|ref|NW_003383664.1|	922014-92390	0.5096	120764	124475	1	no
gi|320446907|ref|NW_003383664.1|	926172-92872	283448	413763	0.545717	0.68275	no
gi|320446907|ref|NW_003383664.1|	928849-93214	332419	227489	-0.547202	0.68685	no

gi|320446907|ref|NW_003383664.1|	933811-935625	545151	283005	-0.945827	0.6567	no
gi|320446907|ref|NW_003383664.1|	938820-942717	208741	228349	0.129528	0.92425	no
gi|320446907|ref|NW_003383664.1|	947384-951608	199664	350663	0.812513	0.5465	no
gi|320446907|ref|NW_003383664.1|	952424-95384	179946	108166	-0.73432	0.63815	no
gi|320446907|ref|NW_003383664.1|	954234-954600	597699	313784	-0.929648	0.65765	no
gi|320446907|ref|NW_003383664.1|	954689-958775	122471	651839	-0.909849	0.47165	no
gi|320446907|ref|NW_003383664.1|	963946-968840	18978	121009	-0.649211	0.6295	no
gi|320446907|ref|NW_003383664.1|	969541-971527	786529	61833	-0.347123	0.79085	no
gi|320446907|ref|NW_003383664.1|	973203-973938	202669	64768	167616	0.4319	no
gi|320446907|ref|NW_003383664.1|	975944-978517	381119	404895	0.0873075	0.94875	no
gi|320446907|ref|NW_003383664.1|	978677-97937	561522	162988	-178457	0.2878	no
gi|320446907|ref|NW_003383664.1|	994913-99564	177773	491486	-185481	0.39055	no
gi|320446907|ref|NW_003383664.1|	995744-99890	301939	228916	-0.399443	0.7681	no
gi|320446908|ref|NW_003383663.1|	109204-112417	0.113331	305155	475092	0.15385	no
gi|320446908|ref|NW_003383663.1|	112802-113699	0	404288	inf	0.01485	no
gi|320446908|ref|NW_003383663.1|	113830-114309	0.679508	590011	311818	0.2459	no
gi|320446908|ref|NW_003383663.1|	11485-12382	153062	210933	0.462666	0.81725	no
gi|320446908|ref|NW_003383663.1|	116025-118061	224908	333318	0.567566	0.779	no
gi|320446908|ref|NW_003383663.1|	119207-119880	0	49294	inf	0.0212	no
gi|320446908|ref|NW_003383663.1|	150004-150597	188016	382189	102343	0.60635	no
gi|320446908|ref|NW_003383663.1|	155576-156630	0.818647	14863	418234	0.1552	no
gi|320446908|ref|NW_003383663.1|	15662-20875	189819	648294	177202	0.2985	no
gi|320446908|ref|NW_003383663.1|	159428-161721	0.0820052	240419	487369	0.1722	no
gi|320446908|ref|NW_003383663.1|	162198-163698	0.133439	250521	423068	0.1913	no
gi|320446908|ref|NW_003383663.1|	163838-167021	0.0572425	452363	630425	0.1565	no
gi|320446908|ref|NW_003383663.1|	22257-22905	408373	278072	-0.554429	0.79545	no
gi|320446908|ref|NW_003383663.1|	246615-248030	0.142332	31649	447483	0.1818	no
gi|320446908|ref|NW_003383663.1|	256867-257489	0.435424	251466	58518	0.1618	no
gi|320446908|ref|NW_003383663.1|	27642-30243	0	184368	inf	0.0103	no
gi|320446908|ref|NW_003383663.1|	31424-32013	522763	611581	0.226387	0.9033	no

gi|320446908|ref|NW_003383663.1|	33481-35579	0.634142	884737	380237	0.1449	no
gi|320446908|ref|NW_003383663.1|	67085-67625	329598	44405	0.430015	0.8246	no
gi|320446908|ref|NW_003383663.1|	75845-76398	12662	0.711892	-415271	0.21015	no
gi|320446908|ref|NW_003383663.1|	79610-84397	4.3	141501	17184	0.3401	no
gi|320446909|ref|NW_003383662.1|	100610-10144	0.562986	232297	20448	0.30225	no
gi|320446909|ref|NW_003383662.1|	105445-10646	107413	0.742345	-0.533002	1	no
gi|320446909|ref|NW_003383662.1|	10943-11237	102021	230672	-214494	0.32965	no
gi|320446909|ref|NW_003383662.1|	116016-11806	455141	745024	0.710973	0.7439	no
gi|320446909|ref|NW_003383662.1|	118825-11980	478554	487753	0.0274714	0.9804	no
gi|320446909|ref|NW_003383662.1|	122171-12442	860975	309212	-147738	0.5109	no
gi|320446909|ref|NW_003383662.1|	1227-2057	695459	267389	-137902	0.42255	no
gi|320446909|ref|NW_003383662.1|	124540-12510	258796	123431	-106812	0.61455	no
gi|320446909|ref|NW_003383662.1|	132943-13404	159871	593751	-142898	0.51085	no
gi|320446909|ref|NW_003383662.1|	168643-16891	312766	168647	-0.891074	0.6711	no
gi|320446909|ref|NW_003383662.1|	184818-18541	32813	571776	0.801183	0.69475	no
gi|320446909|ref|NW_003383662.1|	20441-22780	302399	912774	-172812	0.33775	no
gi|320446909|ref|NW_003383662.1|	209953-21054	467481	15841	-156124	0.49165	no
gi|320446909|ref|NW_003383662.1|	224131-22456	177164	149548	-0.244476	0.89865	no
gi|320446909|ref|NW_003383662.1|	227045-22899	345451	247716	-0.479797	0.815	no
gi|320446909|ref|NW_003383662.1|	229244-23117	322854	355957	0.14082	0.9143	no
gi|320446909|ref|NW_003383662.1|	22967-23472	185242	579637	-167619	0.43085	no
gi|320446909|ref|NW_003383662.1|	232224-23253	535706	149254	147825	0.49685	no
gi|320446909|ref|NW_003383662.1|	23686-24398	955805	605291	-0.659088	0.74785	no
gi|320446909|ref|NW_003383662.1|	249475-25029	465936	340202	-0.453743	0.8173	no
gi|320446909|ref|NW_003383662.1|	260528-26249	239158	135437	-0.820334	0.51415	no
gi|320446909|ref|NW_003383662.1|	264058-26549	0.982237	0.585117	-0.747346	1	no
gi|320446909|ref|NW_003383662.1|	26451-27032	340186	361779	0.088784	0.94685	no
gi|320446909|ref|NW_003383662.1|	266436-26742	11.63	355277	-171083	0.4236	no
gi|320446909|ref|NW_003383662.1|	267532-26914	149439	37507	-199433	0.3853	no
gi|320446909|ref|NW_003383662.1|	269319-26976	491176	0.510032	-658951	0.2638	no

gi|320446909|ref|NW_003383662.1|	273200-27373[illegible]	463648	0.380455	-692916	0.2619	no
gi|320446909|ref|NW_003383662.1|	276871-28105[illegible]	250826	122655	-435401	0.0424	no
gi|320446909|ref|NW_003383662.1|	281749-28436[illegible]	637096	336293	-0.921793	0.66545	no
gi|320446909|ref|NW_003383662.1|	29105-30537	222817	145045	-0.619358	0.6945	no
gi|320446909|ref|NW_003383662.1|	30685-33886	287889	516477	0.843192	0.5341	no
gi|320446909|ref|NW_003383662.1|	311281-31232[illegible]	438925	152454	-15256	0.3657	no
gi|320446909|ref|NW_003383662.1|	314713-31675[illegible]	205822	21532	0.0650828	0.97225	no
gi|320446909|ref|NW_003383662.1|	316987-31902[illegible]	169618	155548	-0.124925	0.92315	no
gi|320446909|ref|NW_003383662.1|	332014-33274[illegible]	37156	601417	0.694772	0.7342	no
gi|320446909|ref|NW_003383662.1|	333557-33426[illegible]	430185	563724	0.39003	0.84765	no
gi|320446909|ref|NW_003383662.1|	3369-5042	179588	980596	-0.872964	0.5913	no
gi|320446909|ref|NW_003383662.1|	341687-34565[illegible]	0.724025	281908	196111	0.368	no
gi|320446909|ref|NW_003383662.1|	354401-35699[illegible]	114608	150119	0.389396	0.8477	no
gi|320446909|ref|NW_003383662.1|	357194-35844[illegible]	115971	0.574605	-101312	1	no
gi|320446909|ref|NW_003383662.1|	36240-37992	0.667577	193737	153709	0.47245	no
gi|320446909|ref|NW_003383662.1|	364917-36527[illegible]	12087	709514	255337	0.27405	no
gi|320446909|ref|NW_003383662.1|	446937-44764[illegible]	920405	564131	261569	0.1424	no
gi|320446909|ref|NW_003383662.1|	447859-44857[illegible]	283202	14527	-0.963094	0.67005	no
gi|320446909|ref|NW_003383662.1|	448729-44968[illegible]	117759	130015	0.142843	1	no
gi|320446909|ref|NW_003383662.1|	449859-45066[illegible]	117498	18164	0.628447	0.75205	no
gi|320446909|ref|NW_003383662.1|	45432-46287	490325	119972	129088	0.54805	no
gi|320446909|ref|NW_003383662.1|	464996-46626[illegible]	0.32717	192933	255999	0.25005	no
gi|320446909|ref|NW_003383662.1|	548701-54987[illegible]	342711	899863	471464	0.04055	no
gi|320446909|ref|NW_003383662.1|	5499-6424	117476	47255	-131383	0.53505	no
gi|320446909|ref|NW_003383662.1|	551087-55137[illegible]	439568	519507	356299	0.18215	no
gi|320446909|ref|NW_003383662.1|	551633-55281[illegible]	0.178845	45856	468033	0.1765	no
gi|320446909|ref|NW_003383662.1|	555616-55673[illegible]	0.384159	598387	39613	0.16455	no
gi|320446909|ref|NW_003383662.1|	559732-56328[illegible]	0.050812	671939	704702	0.14095	no
gi|320446909|ref|NW_003383662.1|	593873-59421[illegible]	108777	450219	-127267	0.54865	no
gi|320446909|ref|NW_003383662.1|	598943-59914[illegible]	129785	380285	-177097	0.40895	no

gi|320446909|ref|NW_003383662.1|	99382-60001	124063	407179	-160733	0.442	no
gi|320446909|ref|NW_003383662.1|	01185-60152	907297	192981	-223312	0.3139	no
gi|320446909|ref|NW_003383662.1|	05650-60853	110168	158467	0.524476	0.68405	no
gi|320446909|ref|NW_003383662.1|	12576-61394	411951	360742	-0.191503	0.8788	no
gi|320446909|ref|NW_003383662.1|	22191-62582	228169	373814	0.712218	0.5982	no
gi|320446909|ref|NW_003383662.1|	47342-64769	0	121901	inf	0.02205	no
gi|320446909|ref|NW_003383662.1|	64880-68248	343099	137899	200692	0.1349	no
gi|320446909|ref|NW_003383662.1|	56396-65681	0	598884	inf	0.0059	no
gi|320446909|ref|NW_003383662.1|	60244-66066	0.883752	794057	648946	0.1545	no
gi|320446909|ref|NW_003383662.1|	68040-66841	800783	598224	-0.420727	0.82115	no
gi|320446909|ref|NW_003383662.1|	96511-70020	395611	632298	0.67652	0.77315	no
gi|320446909|ref|NW_003383662.1|	18945-71914	536873	29.32	-0.872698	0.67695	no
gi|320446909|ref|NW_003383662.1|	03725-80606	0.0803264	282633	845884	0.14075	no
gi|320446909|ref|NW_003383662.1|	11285-81197	0	20187	inf	0.0077	no
gi|320446909|ref|NW_003383662.1|	15656-81601	0	126897	inf	0.0198	no
gi|320446909|ref|NW_003383662.1|	8227-9306	0.800204	12457	0.638516	1	no
gi|320446909|ref|NW_003383662.1|	87789-88937	0.184921	345838	422511	0.19135	no
gi|320446909|ref|NW_003383662.1|	89066-90046	0.906632	672987	289199	0.23855	no
gi|320446909|ref|NW_003383662.1|	90308-91253	713525	326547	219426	0.2048	no
gi|320446909|ref|NW_003383662.1|	92746-100444	158261	296445	0.905464	0.57625	no
gi|320446911|ref|NW_003383660.1|	01584-10252	0	19746	inf	0.029	no
gi|320446911|ref|NW_003383660.1|	02609-10429	0	170196	inf	0.01575	no
gi|320446911|ref|NW_003383660.1|	05725-10723	238867	0.738237	-169405	0.43255	no
gi|320446911|ref|NW_003383660.1|	43514-14412	0.449126	426746	324819	0.2305	no
gi|320446911|ref|NW_003383660.1|	71197-17202	0	411221	inf	0.01575	no
gi|320446911|ref|NW_003383660.1|	73448-17452	0	250898	inf	0.0154	no
gi|320446911|ref|NW_003383660.1|	14740-21600	0	239938	inf	5.00E-05	yes
gi|320446911|ref|NW_003383660.1|	31594-23220	496641	0.612733	-301887	0.25735	no
gi|320446911|ref|NW_003383660.1|	69590-27009	0	585582	inf	0.0233	no
gi|320446911|ref|NW_003383660.1|	98831-29942	0	603541	inf	0.0212	no

gi|320446911|ref|NW_003383660.1|	299635-30055	172319	594072	178555	0.40895	no
gi|320446911|ref|NW_003383660.1|	300674-30101	137855	116291	307652	0.24865	no
gi|320446911|ref|NW_003383660.1|	301527-30288	0.15059	250983	40589	0.1924	no
gi|320446911|ref|NW_003383660.1|	304755-30533	294078	10611	185129	0.3948	no
gi|320446911|ref|NW_003383660.1|	308728-30963	125279	345407	146315	0.50075	no
gi|320446911|ref|NW_003383660.1|	310201-31068	531332	79924	0.589013	0.75135	no
gi|320446911|ref|NW_003383660.1|	310794-31376	104701	50808	227878	0.3077	no
gi|320446911|ref|NW_003383660.1|	322845-32334	189243	143686	292461	0.23085	no
gi|320446911|ref|NW_003383660.1|	338113-33892	0.884316	384774	212138	0.34675	no
gi|320446911|ref|NW_003383660.1|	339181-34458	0.751385	14878	0.98556	0.6451	no
gi|320446911|ref|NW_003383660.1|	344681-34661	0.495531	0.89818	0.858029	1	no
gi|320446911|ref|NW_003383660.1|	349098-35002	0.737435	491527	273668	0.25185	no
gi|320446911|ref|NW_003383660.1|	352371-35686	0.871424	13869	0.670421	1	no
gi|320446911|ref|NW_003383660.1|	357036-35906	0.469862	0.983057	106504	1	no
gi|320446911|ref|NW_003383660.1|	359935-36136	0.28323	0.984103	179683	1	no
gi|320446911|ref|NW_003383660.1|	361777-36298	0.864885	119922	0.471513	1	no
gi|320446911|ref|NW_003383660.1|	363094-36596	0.576831	111998	0.957258	1	no
gi|320446911|ref|NW_003383660.1|	374436-37540	0.458864	142552	163535	0.3325	no
gi|320446911|ref|NW_003383660.1|	383456-38368	0	339736	inf	0.029	no
gi|320446911|ref|NW_003383660.1|	384519-38609	0.631719	175798	147656	0.5	no
gi|320446911|ref|NW_003383660.1|	389384-39275	295673	260983	314188	0.1059	no
gi|320446911|ref|NW_003383660.1|	75015-75480	502636	38281	-0.392887	0.84605	no
gi|320446911|ref|NW_003383660.1|	97337-98362	0	147729	inf	0.0312	no
gi|320446912|ref|NW_003383659.1|	118495-11968	759372	0.734394	-337018	0.18585	no
gi|320446912|ref|NW_003383659.1|	120459-12283	141687	0.879353	-0.688191	0.73095	no
gi|320446912|ref|NW_003383659.1|	123991-12572	562997	682234	0.277139	0.89615	no
gi|320446912|ref|NW_003383659.1|	184677-18791	26982	143893	241492	0.1777	no
gi|320446912|ref|NW_003383659.1|	205991-20724	249244	0	#NAME?	0.01585	no
gi|320446912|ref|NW_003383659.1|	227542-22894	188207	0.50291	-190395	0.39305	no
gi|320446912|ref|NW_003383659.1|	249607-25201	783685	0.704348	-347592	0.1686	no

gi|320446912|ref|NW_003383659.1|	[illegible]59792-26007[illegible]	115472	353598	161456	0.4473	no
gi|320446912|ref|NW_003383659.1|	[illegible]61981-26263[illegible]	241555	630808	138485	0.51085	no
gi|320446912|ref|NW_003383659.1|	[illegible]62939-26427[illegible]	0.306375	138268	217409	1	no
gi|320446912|ref|NW_003383659.1|	[illegible]64756-26848[illegible]	498015	191516	19432	0.29205	no
gi|320446912|ref|NW_003383659.1|	[illegible]72921-27346[illegible]	291145	388544	0.41634	0.8459	no
gi|320446912|ref|NW_003383659.1|	[illegible]73750-27423[illegible]	85376	439258	-0.958762	0.65515	no
gi|320446912|ref|NW_003383659.1|	[illegible]74740-27495[illegible]	516116	43075	-0.260845	0.9004	no
gi|320446912|ref|NW_003383659.1|	[illegible]75419-27569[illegible]	501881	39934	-0.32973	0.87255	no
gi|320446912|ref|NW_003383659.1|	[illegible]76573-27720[illegible]	256041	290142	0.180382	0.9203	no
gi|320446912|ref|NW_003383659.1|	[illegible]80768-28119[illegible]	482112	275287	-0.808433	0.7032	no
gi|320446912|ref|NW_003383659.1|	[illegible]83461-28602[illegible]	173106	40476	-209652	0.2379	no
gi|320446912|ref|NW_003383659.1|	[illegible]87809-28808[illegible]	274363	7295	-191111	0.392	no
gi|320446912|ref|NW_003383659.1|	[illegible]94955-29748[illegible]	69022	257259	-474576	0.0372	no
gi|320446912|ref|NW_003383659.1|	[illegible]99078-30031[illegible]	846656	140911	-258699	0.26575	no
gi|320446912|ref|NW_003383659.1|	[illegible]00540-30307[illegible]	178222	100922	-0.820442	0.524	no
gi|320446912|ref|NW_003383659.1|	[illegible]07582-30923[illegible]	220461	0.330742	-93806	0.1293	no
gi|320446912|ref|NW_003383659.1|	[illegible]11637-31257[illegible]	183916	318022	0.790083	0.6294	no
gi|320446912|ref|NW_003383659.1|	[illegible]16637-31892[illegible]	661718	515384	-0.360569	0.78205	no
gi|320446912|ref|NW_003383659.1|	[illegible]19850-32325[illegible]	657633	162973	130928	0.44825	no
gi|320446912|ref|NW_003383659.1|	[illegible]31883-33667[illegible]	718245	178083	1.31	0.42605	no
gi|320446912|ref|NW_003383659.1|	[illegible]37946-33920[illegible]	115517	732652	266502	0.24535	no
gi|320446912|ref|NW_003383659.1|	[illegible]40187-34260[illegible]	0.618749	448264	285692	0.23655	no
gi|320446912|ref|NW_003383659.1|	[illegible]51984-35440[illegible]	843045	0.594085	-382687	0.14235	no
gi|320446912|ref|NW_003383659.1|	[illegible]60298-36084[illegible]	283947	491288	0.790951	0.71615	no
gi|320446912|ref|NW_003383659.1|	[illegible]61144-36199[illegible]	525197	304261	-0.787549	0.69225	no
gi|320446912|ref|NW_003383659.1|	[illegible]62126-36234[illegible]	692974	50117	-0.467501	0.8131	no
gi|320446912|ref|NW_003383659.1|	[illegible]62478-36267[illegible]	312327	388777	0.315884	0.87555	no
gi|320446912|ref|NW_003383659.1|	[illegible]62834-36408[illegible]	31602	323032	0.0316592	0.97785	no
gi|320446912|ref|NW_003383659.1|	[illegible]64223-36461[illegible]	89876	918969	0.0320801	0.96245	no
gi|320446912|ref|NW_003383659.1|	[illegible]67744-36862[illegible]	139204	128421	-0.116317	0.9565	no

gi|320446912|ref|NW_003383659.1|	370649-371322	963241	525709	-0.873632	0.67505	no
gi|320446912|ref|NW_003383659.1|	410739-41181	0	152252	inf	0.0294	no
gi|320446912|ref|NW_003383659.1|	416006-41749	0	234164	inf	0.01485	no
gi|320446912|ref|NW_003383659.1|	418485-42032	0	190287	inf	0.0142	no
gi|320446912|ref|NW_003383659.1|	42287-42732	312331	674103	110989	0.59635	no
gi|320446912|ref|NW_003383659.1|	427826-42875	0	202522	inf	0.029	no
gi|320446912|ref|NW_003383659.1|	428982-43013	0	280581	inf	0.0186	no
gi|320446912|ref|NW_003383659.1|	430255-43109	0	289425	inf	0.02205	no
gi|320446912|ref|NW_003383659.1|	441077-44169	0	385542	inf	0.02915	no
gi|320446912|ref|NW_003383659.1|	441858-44225	0	969649	inf	0.02205	no
gi|320446912|ref|NW_003383659.1|	443634-44543	0	157287	inf	0.01575	no
gi|320446912|ref|NW_003383659.1|	481510-48231	0	389432	inf	0.0212	no
gi|320446912|ref|NW_003383659.1|	483069-48470	0	368705	inf	0.00845	no
gi|320446912|ref|NW_003383659.1|	505832-50648	401643	0.54728	-287556	0.2697	no
gi|320446912|ref|NW_003383659.1|	506907-50771	713687	163406	-212683	0.3306	no
gi|320446912|ref|NW_003383659.1|	83653-84450	120219	433424	185011	0.4058	no
gi|320446912|ref|NW_003383659.1|	88034-92550	709485	265811	190556	0.1599	no
gi|320446914|ref|NW_003383657.1|	102984-10856	428427	274433	-0.642598	0.7175	no
gi|320446914|ref|NW_003383657.1|	108799-11198	238209	108123	-113955	0.3856	no
gi|320446914|ref|NW_003383657.1|	112818-11369	1072.6	423.04	-134225	0.4216	no
gi|320446914|ref|NW_003383657.1|	114271-11511	150998	365415	-204692	0.3538	no
gi|320446914|ref|NW_003383657.1|	125099-12650	221811	118085	-0.9095	0.5739	no
gi|320446914|ref|NW_003383657.1|	129032-13196	108088	891002	-0.278701	0.8201	no
gi|320446914|ref|NW_003383657.1|	14016-14987	0.229432	205909	316587	0.24575	no
gi|320446914|ref|NW_003383657.1|	142478-14284	326158	334782	0.0376547	0.9829	no
gi|320446914|ref|NW_003383657.1|	157820-15847	480839	0	#NAME?	0.02015	no
gi|320446914|ref|NW_003383657.1|	15983-17416	126815	166468	0.39252	0.8431	no
gi|320446914|ref|NW_003383657.1|	176835-17778	517416	0	#NAME?	0.01	no
gi|320446914|ref|NW_003383657.1|	181267-18211	884543	0.190163	-553962	0.2748	no
gi|320446914|ref|NW_003383657.1|	349574-35130	0.112901	21229	423291	0.19125	no

gi|320446914|ref|NW_003383657.1|	35192-36092	73646	0	#NAME?	0.0099	no
gi|320446914|ref|NW_003383657.1|	353408-35455[illegible]	0	192757	inf	0.02205	no
gi|320446914|ref|NW_003383657.1|	36241-36641	183811	0	#NAME?	0.01335	no
gi|320446914|ref|NW_003383657.1|	39282-40212	745947	502724	-389124	0.06	no
gi|320446914|ref|NW_003383657.1|	42728-44072	400339	525308	0.391941	0.76985	no
gi|320446914|ref|NW_003383657.1|	44182-44759	688141	385706	248673	0.2768	no
gi|320446914|ref|NW_003383657.1|	442313-44281[illegible]	120722	536182	215104	0.334	no
gi|320446914|ref|NW_003383657.1|	49911-50133	48066	332026	278821	0.2661	no
gi|320446914|ref|NW_003383657.1|	50986-51201	162812	272041	0.740615	0.70725	no
gi|320446914|ref|NW_003383657.1|	545232-54571[illegible]	744595	0.452187	-404146	0.30915	no
gi|320446914|ref|NW_003383657.1|	54820-55713	282322	778037	14625	0.4862	no
gi|320446914|ref|NW_003383657.1|	59939-60828	167848	816508	228231	0.2123	no
gi|320446914|ref|NW_003383657.1|	62314-63263	520317	200724	194775	0.3873	no
gi|320446914|ref|NW_003383657.1|	64687-65152	359026	172264	226247	0.31905	no
gi|320446914|ref|NW_003383657.1|	68595-69147	423358	221336	238628	0.28735	no
gi|320446914|ref|NW_003383657.1|	72879-74405	573993	325544	-0.818178	0.5359	no
gi|320446914|ref|NW_003383657.1|	74518-76909	284144	929141	-161265	0.3672	no
gi|320446914|ref|NW_003383657.1|	82129-82531	382968	441298	0.204529	0.89915	no
gi|320446914|ref|NW_003383657.1|	83999-85114	249419	212521	-0.230966	0.9032	no
gi|320446914|ref|NW_003383657.1|	98940-10174[illegible]	0.98381	559238	250701	0.272	no
gi|320446915|ref|NW_003383656.1|	105510-11027[illegible]	218507	110571	233923	0.0858	no
gi|320446915|ref|NW_003383656.1|	110383-11227[illegible]	437728	113524	137489	0.5371	no
gi|320446915|ref|NW_003383656.1|	113739-11557[illegible]	0.210846	0.881512	206379	1	no
gi|320446915|ref|NW_003383656.1|	121276-12176[illegible]	103915	460667	214832	0.3321	no
gi|320446915|ref|NW_003383656.1|	134513-13695[illegible]	121693	791023	-0.621458	0.70545	no
gi|320446915|ref|NW_003383656.1|	140460-14109[illegible]	237893	119272	-0.996059	0.6478	no
gi|320446915|ref|NW_003383656.1|	141863-14297[illegible]	107058	158461	0.565731	0.675	no
gi|320446915|ref|NW_003383656.1|	147345-15035[illegible]	164074	798746	-103854	0.41285	no
gi|320446915|ref|NW_003383656.1|	153204-15465[illegible]	318236	240435	-0.404448	0.84295	no
gi|320446915|ref|NW_003383656.1|	164796-16506[illegible]	964645	408691	-123899	0.55395	no

gi|320446915|ref|NW_003383656.1|	166330-16845	232542	126666	-0.876464	0.49515	no
gi|320446915|ref|NW_003383656.1|	169262-17333	151842	210435	0.470805	0.72585	no
gi|320446915|ref|NW_003383656.1|	173501-17770	371197	182692	-102277	0.4446	no
gi|320446915|ref|NW_003383656.1|	178004-17829	619212	177477	-18028	0.40755	no
gi|320446915|ref|NW_003383656.1|	178615-17905	563192	11903	-22423	0.3257	no
gi|320446915|ref|NW_003383656.1|	187920-18914	562178	212631	-140267	0.50175	no
gi|320446915|ref|NW_003383656.1|	189352-19020	492712	0.565049	-31243	0.20145	no
gi|320446915|ref|NW_003383656.1|	192938-19313	585857	156063	-190842	0.30655	no
gi|320446915|ref|NW_003383656.1|	220406-22097	550096	0	#NAME?	0.02105	no
gi|320446915|ref|NW_003383656.1|	238417-23903	533359	241414	-11436	0.57915	no
gi|320446915|ref|NW_003383656.1|	241246-24262	399812	0.925667	-211076	0.3338	no
gi|320446915|ref|NW_003383656.1|	295580-29621	721846	4.62	-0.643799	0.74315	no
gi|320446915|ref|NW_003383656.1|	299769-30081	709624	678259	-0.0652171	0.9725	no
gi|320446915|ref|NW_003383656.1|	308957-30924	532659	708731	-29099	0.23355	no
gi|320446915|ref|NW_003383656.1|	314479-31586	190827	662785	179627	0.4011	no
gi|320446915|ref|NW_003383656.1|	50207-50711	520513	706235	-288171	0.2206	no
gi|320446915|ref|NW_003383656.1|	53389-54598	310265	817122	-192488	0.2671	no
gi|320446915|ref|NW_003383656.1|	54921-60169	300244	192898	-0.638297	0.63545	no
gi|320446915|ref|NW_003383656.1|	61892-67769	735404	814229	0.146898	0.90705	no
gi|320446915|ref|NW_003383656.1|	68841-69473	250523	0	#NAME?	0.0062	no
gi|320446915|ref|NW_003383656.1|	70380-70827	774091	0.514253	-391195	0.3159	no
gi|320446915|ref|NW_003383656.1|	71418-72159	704291	411569	-409697	0.0545	no
gi|320446915|ref|NW_003383656.1|	74960-75368	445685	367303	-360098	0.17405	no
gi|320446915|ref|NW_003383656.1|	75878-76754	376762	290286	-36981	0.0727	no
gi|320446915|ref|NW_003383656.1|	76906-78589	116567	170429	0.54801	0.7895	no
gi|320446915|ref|NW_003383656.1|	85452-85829	10978	790675	284847	0.25935	no
gi|320446916|ref|NW_003383655.1|	160148-16113	608706	294247	227321	0.18825	no
gi|320446916|ref|NW_003383655.1|	171656-17310	277409	169679	261272	0.14965	no
gi|320446916|ref|NW_003383655.1|	317527-31900	302379	101721	-157173	0.24215	no
gi|320446916|ref|NW_003383655.1|	323098-32489	0.758335	0.528298	-0.521485	1	no

gi|320446916|ref|NW_003383655.1|	325078-32713	863364	450655	-0.937945	0.48335	no
gi|320446916|ref|NW_003383655.1|	327322-33226	258051	331391	0.360877	0.87055	no
gi|320446916|ref|NW_003383655.1|	334700-33616	151084	57286	-13991	0.5246	no
gi|320446916|ref|NW_003383655.1|	336285-33672	162509	754195	-110751	0.59685	no
gi|320446916|ref|NW_003383655.1|	337325-34048	367702	440528	0.260695	0.84855	no
gi|320446916|ref|NW_003383655.1|	354810-35561	186357	101604	-419705	0.1616	no
gi|320446916|ref|NW_003383655.1|	358522-35929	143887	0.42922	-506708	0.1891	no
gi|320446916|ref|NW_003383655.1|	361605-36208	123259	137168	-316768	0.20075	no
gi|320446916|ref|NW_003383655.1|	364178-36481	674359	0.573374	-355597	0.23275	no
gi|320446916|ref|NW_003383655.1|	365192-36622	612849	189916	-169017	0.43165	no
gi|320446916|ref|NW_003383655.1|	369998-37119	147878	219638	-275121	0.232	no
gi|320446916|ref|NW_003383655.1|	383795-38726	443028	256582	-0.787975	0.56215	no
gi|320446916|ref|NW_003383655.1|	387914-38832	203247	974761	-106011	0.5932	no
gi|320446916|ref|NW_003383655.1|	388821-38953	105979	132894	364842	0.1823	no
gi|320446916|ref|NW_003383655.1|	390569-39103	283779	113523	200015	0.35895	no
gi|320446916|ref|NW_003383655.1|	393024-39551	134021	240019	0.840687	0.5198	no
gi|320446916|ref|NW_003383655.1|	395770-39697	891321	969182	0.120822	0.95365	no
gi|320446916|ref|NW_003383655.1|	397237-40101	748387	573799	-0.383239	0.8166	no
gi|320446916|ref|NW_003383655.1|	403132-40554	329296	44523	0.435167	0.74315	no
gi|320446916|ref|NW_003383655.1|	424219-42877	221758	492143	115009	0.39065	no
gi|320446916|ref|NW_003383655.1|	429010-43011	151897	172531	0.183761	0.931	no
gi|320446916|ref|NW_003383655.1|	433289-43350	758608	114226	0.590465	0.7642	no
gi|320446916|ref|NW_003383655.1|	449779-45022	635707	22675	183467	0.3906	no
gi|320446916|ref|NW_003383655.1|	635270-63787	0	14002	inf	5.00E-05	yes
gi|320446916|ref|NW_003383655.1|	638706-64008	0	104727	inf	0.00355	no
gi|320446916|ref|NW_003383655.1|	642152-64315	0	136181	inf	0.0059	no
gi|320446916|ref|NW_003383655.1|	682884-68342	552829	558461	0.0146227	0.9618	no
gi|320446916|ref|NW_003383655.1|	734741-73529	105517	284757	143226	0.37565	no
gi|320446916|ref|NW_003383655.1|	735421-73725	0.632537	359951	250858	0.2743	no
gi|320446916|ref|NW_003383655.1|	790521-79358	160702	1077.46	274517	0.07205	no

gi|320446916|ref|NW_003383655.1|	83755-84713	280273	0	#NAME?	0.02015	no
gi|320446917|ref|NW_003383654.1|	128908-12955	366658	388402	0.0831154	0.9574	no
gi|320446917|ref|NW_003383654.1|	134268-13464	113043	739281	270925	0.2673	no
gi|320446917|ref|NW_003383654.1|	176176-17644	267957	442749	404641	0.1943	no
gi|320446917|ref|NW_003383654.1|	178077-17973	0.830896	56187	27575	0.24075	no
gi|320446917|ref|NW_003383654.1|	180213-18129	199342	355853	415797	0.0635	no
gi|320446917|ref|NW_003383654.1|	200771-20403	1673	826893	230526	0.1876	no
gi|320446917|ref|NW_003383654.1|	218436-21950	0	506563	inf	0.0085	no
gi|320446917|ref|NW_003383654.1|	219629-22437	149491	204956	377718	0.0683	no
gi|320446917|ref|NW_003383654.1|	240555-24095	11489	630425	-0.865861	0.68035	no
gi|320446917|ref|NW_003383654.1|	241275-24190	145818	43732	-173741	0.4106	no
gi|320446917|ref|NW_003383654.1|	251710-25276	0.410819	170474	205298	0.3018	no
gi|320446917|ref|NW_003383654.1|	254773-25512	113734	411393	-146708	0.5029	no
gi|320446917|ref|NW_003383654.1|	25494-26130	210219	134423	267681	0.2521	no
gi|320446917|ref|NW_003383654.1|	257468-25788	172068	478162	-184741	0.39335	no
gi|320446917|ref|NW_003383654.1|	261089-26156	409278	182214	-116745	0.58395	no
gi|320446917|ref|NW_003383654.1|	266145-26719	268011	156832	-0.773076	0.70675	no
gi|320446917|ref|NW_003383654.1|	269838-27049	6472	0	#NAME?	0.015	no
gi|320446917|ref|NW_003383654.1|	274620-27739	943815	12077	-296624	0.1122	no
gi|320446917|ref|NW_003383654.1|	277562-27841	18918	433894	-212434	0.346	no
gi|320446917|ref|NW_003383654.1|	286923-28819	0.811628	270305	17357	0.43075	no
gi|320446917|ref|NW_003383654.1|	312519-31430	0.325838	559912	410298	0.1634	no
gi|320446917|ref|NW_003383654.1|	314529-31703	0.66969	327416	228956	0.3109	no
gi|320446917|ref|NW_003383654.1|	328809-32977	207052	444684	110279	0.5937	no
gi|320446917|ref|NW_003383654.1|	336840-33721	333194	508804	0.610748	0.7426	no
gi|320446917|ref|NW_003383654.1|	349650-35081	0.363469	339958	322545	0.20625	no
gi|320446917|ref|NW_003383654.1|	352086-35349	806586	670485	-0.266622	0.8986	no
gi|320446917|ref|NW_003383654.1|	357261-35945	431415	289005	-0.577983	0.7852	no
gi|320446917|ref|NW_003383654.1|	359630-35987	526461	133036	-198451	0.3855	no
gi|320446917|ref|NW_003383654.1|	359987-36070	197008	986674	-0.99761	0.63915	no

gi|320446917|ref|NW_003383654.1|	37856-38697	501665	32781	270806	0.2508	no
gi|320446917|ref|NW_003383654.1|	59113-61014	0	162412	inf	0.01485	no
gi|320446917|ref|NW_003383654.1|	61735-63398	0	156339	inf	0.0212	no
gi|320446917|ref|NW_003383654.1|	67960-68346	208635	25325	360151	0.1803	no
gi|320446918|ref|NW_003383653.1|	)06968-10080	0.201004	125155	263841	1	no
gi|320446918|ref|NW_003383653.1|	100854-10531	611566	538394	-0.183846	0.88675	no
gi|320446918|ref|NW_003383653.1|	)29370-10297	498084	227216	-445425	0.17375	no
gi|320446918|ref|NW_003383653.1|	)36938-10374	667647	41206	-0.696229	0.7371	no
gi|320446918|ref|NW_003383653.1|	106206-10746	115179	281941	129151	0.44575	no
gi|320446918|ref|NW_003383653.1|	109024-11037	299064	137038	-112588	0.4961	no
gi|320446918|ref|NW_003383653.1|	111240-11164	554309	24369	-118564	0.57675	no
gi|320446918|ref|NW_003383653.1|	112519-11319	490373	179015	-14538	0.37115	no
gi|320446918|ref|NW_003383653.1|	120851-12145	522563	128547	-202331	0.3729	no
gi|320446918|ref|NW_003383653.1|	124029-12503	24838	903262	-145933	0.5212	no
gi|320446918|ref|NW_003383653.1|	125465-12764	197432	785515	-132964	0.43235	no
gi|320446918|ref|NW_003383653.1|	128575-12936	315378	137415	-119854	0.58705	no
gi|320446918|ref|NW_003383653.1|	131528-13241	191675	107054	-0.840317	0.69895	no
gi|320446918|ref|NW_003383653.1|	132537-13482	246976	287324	0.218307	0.91265	no
gi|320446918|ref|NW_003383653.1|	137984-13855	994406	36982	-142701	0.5105	no
gi|320446918|ref|NW_003383653.1|	142378-14277	197604	51978	139529	0.3813	no
gi|320446918|ref|NW_003383653.1|	163336-16797	0.843693	937342	347379	0.07495	no
gi|320446918|ref|NW_003383653.1|	173624-17435	0.341182	327015	326074	0.2305	no
gi|320446918|ref|NW_003383653.1|	187905-18837	628449	419283	-0.583872	0.78605	no
gi|320446918|ref|NW_003383653.1|	188497-18934	123983	796131	-0.63906	0.76735	no
gi|320446918|ref|NW_003383653.1|	189493-19056	0.805938	334531	20534	0.357	no
gi|320446918|ref|NW_003383653.1|	197874-19850	360255	495629	-286169	0.2233	no
gi|320446918|ref|NW_003383653.1|	209785-21000	52003	0	#NAME?	0.02105	no
gi|320446918|ref|NW_003383653.1|	228513-22921	33767	843711	-200079	0.36335	no
gi|320446918|ref|NW_003383653.1|	233419-23450	167854	423631	-198633	0.3745	no
gi|320446918|ref|NW_003383653.1|	234760-23625	428359	13.31	163562	0.4632	no

gi|320446918|ref|NW_003383653.1|	243476-24631	0.905897	696493	294269	0.21545	no
gi|320446918|ref|NW_003383653.1|	272860-27319	0	918956	inf	0.0312	no
gi|320446918|ref|NW_003383653.1|	310449-31146	0.651947	450487	278866	0.24975	no
gi|320446918|ref|NW_003383653.1|	321704-32490	121309	844403	-0.522685	0.6751	no
gi|320446918|ref|NW_003383653.1|	325023-32618	601018	933722	0.635584	0.76165	no
gi|320446918|ref|NW_003383653.1|	327093-33299	756143	738527	-0.0340087	0.97895	no
gi|320446918|ref|NW_003383653.1|	333678-33475	118311	929525	-0.348015	0.8684	no
gi|320446918|ref|NW_003383653.1|	448913-45045	248346	189742	293362	0.03635	no
gi|320446918|ref|NW_003383653.1|	450564-45658	279764	130099	221733	0.2359	no
gi|320446918|ref|NW_003383653.1|	456768-46004	814653	237611	154434	0.2416	no
gi|320446918|ref|NW_003383653.1|	532624-53382	0.349176	266253	293077	0.22645	no
gi|320446918|ref|NW_003383653.1|	601795-60386	285432	212578	289677	0.12555	no
gi|320446918|ref|NW_003383653.1|	604007-60442	0.896755	769723	310155	0.24695	no
gi|320446918|ref|NW_003383653.1|	606767-60789	303526	203598	274583	0.23485	no
gi|320446918|ref|NW_003383653.1|	608697-60965	162817	146089	316553	0.1928	no
gi|320446918|ref|NW_003383653.1|	624291-62459	708328	614986	311806	0.21435	no
gi|320446918|ref|NW_003383653.1|	675051-67602	0	30175	inf	0.0212	no
gi|320446918|ref|NW_003383653.1|	676134-67705	0.245454	321575	371163	0.21225	no
gi|320446918|ref|NW_003383653.1|	787829-79163	803995	407124	-0.981719	0.5555	no
gi|320446918|ref|NW_003383653.1|	796579-79770	159531	512847	-163723	0.4464	no
gi|320446918|ref|NW_003383653.1|	797837-79817	445761	130426	-177304	0.4107	no
gi|320446918|ref|NW_003383653.1|	800341-80079	697465	128386	-244164	0.28995	no
gi|320446918|ref|NW_003383653.1|	804578-80511	114981	368242	-164267	0.34045	no
gi|320446918|ref|NW_003383653.1|	81819-83010	187194	258262	0.464301	0.7752	no
gi|320446918|ref|NW_003383653.1|	824177-82558	219305	106256	-104539	0.5151	no
gi|320446918|ref|NW_003383653.1|	827467-82857	561506	187639	-158134	0.4643	no
gi|320446918|ref|NW_003383653.1|	828801-83115	231514	0.891761	-137637	0.5062	no
gi|320446918|ref|NW_003383653.1|	831351-83167	115857	128119	-317678	0.1939	no
gi|320446918|ref|NW_003383653.1|	92128-95450	104409	542961	-0.943328	0.5788	no
gi|320446918|ref|NW_003383653.1|	99179-10069	195059	125085	-0.641005	0.6301	no

gi|320446919|ref|NW_003383652.1|	01393-10193	273798	59027	110826	0.5856	no
gi|320446919|ref|NW_003383652.1|	06091-10789	290449	427192	0.5566	0.7899	no
gi|320446919|ref|NW_003383652.1|	09555-11096	103615	126985	0.293417	0.89435	no
gi|320446919|ref|NW_003383652.1|	62534-16303	185242	409886	446774	0.1616	no
gi|320446919|ref|NW_003383652.1|	65899-16632	0.842826	295705	513278	0.1677	no
gi|320446919|ref|NW_003383652.1|	66452-17277	0.693	431327	595978	0.02295	no
gi|320446919|ref|NW_003383652.1|	42169-24241	0	336828	inf	0.0198	no
gi|320446919|ref|NW_003383652.1|	91430-29250	0.402009	111249	146849	1	no
gi|320446919|ref|NW_003383652.1|	95223-29578	202059	664598	-160422	0.4485	no
gi|320446919|ref|NW_003383652.1|	99678-30061	361864	249597	-0.535844	0.79005	no
gi|320446919|ref|NW_003383652.1|	01743-30273	381729	139567	-145159	0.4836	no
gi|320446919|ref|NW_003383652.1|	02937-50654	0.44999	114752	467249	0.05685	no
gi|320446919|ref|NW_003383652.1|	07232-50793	0	371455	inf	0.02205	no
gi|320446919|ref|NW_003383652.1|	18267-51856	113764	608352	-0.903066	0.6817	no
gi|320446919|ref|NW_003383652.1|	30883-53120	648179	731726	0.174911	0.90555	no
gi|320446919|ref|NW_003383652.1|	88941-89397	253328	228053	-0.151637	0.9403	no
gi|320446920|ref|NW_003383651.1|	33612-13476	110108	0.762757	-0.529625	1	no
gi|320446920|ref|NW_003383651.1|	1862-2883	0	19301	inf	0.02915	no
gi|320446920|ref|NW_003383651.1|	187-1258	626097	656668	0.0687765	0.9716	no
gi|320446920|ref|NW_003383651.1|	03686-20475	506737	277621	245381	0.16565	no
gi|320446920|ref|NW_003383651.1|	16041-21629	129762	738977	250966	0.283	no
gi|320446920|ref|NW_003383651.1|	17385-22148	253419	248066	329113	0.09915	no
gi|320446920|ref|NW_003383651.1|	30426-23092	0	774037	inf	0.0154	no
gi|320446920|ref|NW_003383651.1|	00197-30133	0	565124	inf	5.00E-05	yes
gi|320446920|ref|NW_003383651.1|	03321-30443	0	399373	inf	5.00E-05	yes
gi|320446920|ref|NW_003383651.1|	12464-41322	0.319904	605458	756425	0.14075	no
gi|320446920|ref|NW_003383651.1|	15382-41749	0.270035	665613	462346	0.1551	no
gi|320446920|ref|NW_003383651.1|	41595-44250	252055	885952	181349	0.38485	no
gi|320446920|ref|NW_003383651.1|	43030-44381	429955	716707	0.7372	0.7144	no
gi|320446920|ref|NW_003383651.1|	45346-44633	46905	539936	0.203045	0.92065	no

gi|320446920|ref|NW_003383651.1|	48561-44979	135058	117078	311582	0.1963	no
gi|320446920|ref|NW_003383651.1|	453449-45387	421413	217594	236833	0.2931	no
gi|320446920|ref|NW_003383651.1|	456099-45685	227811	379228	0.735229	0.72655	no
gi|320446920|ref|NW_003383651.1|	460052-46046	0.888047	879876	330859	0.22995	no
gi|320446920|ref|NW_003383651.1|	461029-46203	0.879458	2886	171438	0.43925	no
gi|320446920|ref|NW_003383651.1|	481707-48367	0.0972216	460948	556718	0.1613	no
gi|320446920|ref|NW_003383651.1|	502162-50265	127075	382987	159161	0.33275	no
gi|320446920|ref|NW_003383651.1|	527841-52835	15222	204247	-289777	0.2394	no
gi|320446920|ref|NW_003383651.1|	542090-54238	17841	101623	-0.811966	0.69115	no
gi|320446920|ref|NW_003383651.1|	549194-55022	107912	277913	-195715	0.37835	no
gi|320446920|ref|NW_003383651.1|	551172-55144	426599	638943	-273912	0.25955	no
gi|320446920|ref|NW_003383651.1|	551739-55203	356821	21595	-0.724503	0.7257	no
gi|320446920|ref|NW_003383651.1|	552166-55303	181713	680666	-141664	0.51045	no
gi|320446920|ref|NW_003383651.1|	578516-58107	738192	449738	-0.714912	0.5965	no
gi|320446920|ref|NW_003383651.1|	581598-58202	108558	774209	-0.48767	0.80315	no
gi|320446920|ref|NW_003383651.1|	582278-58466	125592	15894	0.339739	0.862	no
gi|320446920|ref|NW_003383651.1|	584817-58750	193024	260108	0.430331	0.82945	no
gi|320446920|ref|NW_003383651.1|	587688-58906	302345	288235	-339088	0.0873	no
gi|320446920|ref|NW_003383651.1|	589275-59111	312715	594284	-239562	0.19215	no
gi|320446920|ref|NW_003383651.1|	591266-59246	137172	26817	-235477	0.3074	no
gi|320446920|ref|NW_003383651.1|	592709-59357	164047	55534	-156266	0.46855	no
gi|320446920|ref|NW_003383651.1|	511841-61347	144369	139841	327596	0.1821	no
gi|320446920|ref|NW_003383651.1|	513589-62330	890183	277485	164024	0.2217	no
gi|320446920|ref|NW_003383651.1|	523462-62536	152236	311258	10318	0.61705	no
gi|320446920|ref|NW_003383651.1|	527422-62832	100671	156116	0.632959	0.75205	no
gi|320446920|ref|NW_003383651.1|	537209-63799	802868	996053	0.31106	0.87785	no
gi|320446920|ref|NW_003383651.1|	538340-63882	890044	760331	-354918	0.0823	no
gi|320446920|ref|NW_003383651.1|	539444-64095	359628	45217	-299157	0.11915	no
gi|320446920|ref|NW_003383651.1|	541775-64369	694381	162977	-209106	0.12285	no
gi|320446920|ref|NW_003383651.1|	559190-66009	300669	0.690813	-212181	0.3545	no

gi|320446920|ref|NW_003383651.1|	561931-66237	949367	420082	-11763	0.5679	no
gi|320446920|ref|NW_003383651.1|	564728-66496	706644	494939	-0.513734	0.807	no
gi|320446920|ref|NW_003383651.1|	570718-67264	399712	271671	-0.557098	0.7833	no
gi|320446920|ref|NW_003383651.1|	68937-69563	0	380854	inf	0.02915	no
gi|320446920|ref|NW_003383651.1|	69656-70401	0	249596	inf	0.0294	no
gi|320446920|ref|NW_003383651.1|	706885-71003	759671	136257	0.842886	0.5118	no
gi|320446920|ref|NW_003383651.1|	710138-71216	642232	37543	-0.774549	0.7221	no
gi|320446920|ref|NW_003383651.1|	712277-71298	331991	270946	-0.293139	0.89325	no
gi|320446920|ref|NW_003383651.1|	713489-72002	212522	160428	-0.40569	0.76105	no
gi|320446920|ref|NW_003383651.1|	720123-72221	250337	10287	-128304	0.45935	no
gi|320446920|ref|NW_003383651.1|	722659-72320	162229	0.728986	-4476	0.20195	no
gi|320446920|ref|NW_003383651.1|	729415-73044	106464	147178	0.467203	0.82325	no
gi|320446920|ref|NW_003383651.1|	731860-73620	548088	434402	-0.335375	0.79805	no
gi|320446920|ref|NW_003383651.1|	750948-75305	0.991187	308015	163577	0.43705	no
gi|320446920|ref|NW_003383651.1|	754169-75531	111075	333389	158568	0.4452	no
gi|320446920|ref|NW_003383651.1|	755532-75635	144113	297167	104408	0.60105	no
gi|320446920|ref|NW_003383651.1|	756519-75694	417531	663608	0.668449	0.75045	no
gi|320446920|ref|NW_003383651.1|	757116-75902	277085	309442	0.159343	0.90435	no
gi|320446921|ref|NW_003383650.1|	105002-10668	653679	698905	0.0965134	0.9641	no
gi|320446921|ref|NW_003383650.1|	116919-11867	155154	0.694726	-115919	0.57025	no
gi|320446921|ref|NW_003383650.1|	119354-12197	0.495519	168146	176271	0.41045	no
gi|320446921|ref|NW_003383650.1|	142162-14524	135144	100383	289294	0.0364	no
gi|320446921|ref|NW_003383650.1|	146803-14799	193852	146576	291862	0.21565	no
gi|320446921|ref|NW_003383650.1|	149532-14971	0	112766	inf	0.0198	no
gi|320446921|ref|NW_003383650.1|	158057-15858	158835	74803	223557	0.19965	no
gi|320446921|ref|NW_003383650.1|	173572-17589	833313	683394	-0.286141	0.9006	no
gi|320446921|ref|NW_003383650.1|	176540-17724	822784	244596	-175012	0.4252	no
gi|320446921|ref|NW_003383650.1|	178767-18070	113972	131272	0.203883	0.89995	no
gi|320446921|ref|NW_003383650.1|	180969-18131	109526	977494	-0.164111	0.9304	no
gi|320446921|ref|NW_003383650.1|	182313-18301	290375	186274	-0.640492	0.77205	no

gi|320446921|ref|NW_003383650.1|	86964-18936	0	246113	inf	5.00E-05	yes
gi|320446921|ref|NW_003383650.1|	99278-20021	0	346517	inf	0.01575	no
gi|320446921|ref|NW_003383650.1|	71539-27273	367777	194234	-0.921037	0.65605	no
gi|320446923|ref|NW_003383648.1|	02348-10254	798838	496971	-400667	0.3133	no
gi|320446923|ref|NW_003383648.1|	10635-11091	21653	766578	-149806	0.49855	no
gi|320446923|ref|NW_003383648.1|	13569-11743	492751	36115	-0.448259	0.84105	no
gi|320446923|ref|NW_003383648.1|	17541-11903	170682	160723	-0.0867321	0.9559	no
gi|320446923|ref|NW_003383648.1|	20156-12082	54.45	632237	-31064	0.1029	no
gi|320446923|ref|NW_003383648.1|	25492-12576	601473	153316	-529392	0.27765	no
gi|320446923|ref|NW_003383648.1|	54641-15879	225505	903865	-131898	0.32235	no
gi|320446923|ref|NW_003383648.1|	16563-17576	0.651108	133475	435753	0.1602	no
gi|320446923|ref|NW_003383648.1|	26451-26826	222129	196253	314324	0.2106	no
gi|320446923|ref|NW_003383648.1|	26932-27927	0.444361	537139	359549	0.182	no
gi|320446923|ref|NW_003383648.1|	90853-29495	527864	34419	270497	0.1686	no
gi|320446923|ref|NW_003383648.1|	97043-29722	102.59	790099	-0.376781	0.85085	no
gi|320446923|ref|NW_003383648.1|	97604-29777	108583	237916	113165	0.58325	no
gi|320446923|ref|NW_003383648.1|	30545-33441	0.974325	272779	148526	0.4962	no
gi|320446923|ref|NW_003383648.1|	36148-33666	0	553374	inf	0.0233	no
gi|320446923|ref|NW_003383648.1|	46814-34790	394028	695338	0.819415	0.6918	no
gi|320446923|ref|NW_003383648.1|	54005-35440	5835	703933	0.270705	0.88835	no
gi|320446923|ref|NW_003383648.1|	74506-37486	134638	143625	0.0932247	0.9533	no
gi|320446923|ref|NW_003383648.1|	87545-38968	0.265921	247373	321762	0.2069	no
gi|320446923|ref|NW_003383648.1|	89806-39071	0.504109	191088	192243	0.3194	no
gi|320446923|ref|NW_003383648.1|	91987-39319	967679	377372	196339	0.2648	no
gi|320446923|ref|NW_003383648.1|	96430-39888	887826	564379	266832	0.17615	no
gi|320446923|ref|NW_003383648.1|	00237-40449	791955	321253	202022	0.1387	no
gi|320446923|ref|NW_003383648.1|	07096-40738	105751	12174	0.203139	0.9082	no
gi|320446923|ref|NW_003383648.1|	09566-41005	375742	392343	0.0623731	0.9758	no
gi|320446923|ref|NW_003383648.1|	92613-49298	163079	456521	-183681	0.40515	no
gi|320446923|ref|NW_003383648.1|	93550-49778	177178	779739	213779	0.21965	no

gi|320446923|ref|NW_003383648.1|	497909-49909	124926	148412	0.248528	0.89605	no
gi|320446923|ref|NW_003383648.1|	504178-50475	195496	231482	0.243761	0.8906	no
gi|320446923|ref|NW_003383648.1|	513986-51475	675639	253531	-141409	0.4098	no
gi|320446923|ref|NW_003383648.1|	516054-51672	258734	158059	-0.711005	0.7403	no
gi|320446923|ref|NW_003383648.1|	517216-51789	237363	146612	-0.695088	0.73995	no
gi|320446923|ref|NW_003383648.1|	518059-51891	159431	156227	-0.029282	0.98735	no
gi|320446924|ref|NW_003383647.1|	108129-10948	0.150859	178094	356137	0.2162	no
gi|320446924|ref|NW_003383647.1|	130324-13600	662673	151242	119049	0.3737	no
gi|320446924|ref|NW_003383647.1|	156982-15918	114173	569097	-100447	0.53785	no
gi|320446924|ref|NW_003383647.1|	160088-16089	594739	183832	-169387	0.4288	no
gi|320446924|ref|NW_003383647.1|	161288-16270	727735	297454	-129075	0.54275	no
gi|320446924|ref|NW_003383647.1|	181385-18245	482986	380082	297626	0.10935	no
gi|320446924|ref|NW_003383647.1|	182667-18341	13068	10524	300957	0.21835	no
gi|320446924|ref|NW_003383647.1|	185982-18985	514717	293725	251262	0.1885	no
gi|320446924|ref|NW_003383647.1|	192276-19310	398075	0.390993	-334782	0.239	no
gi|320446924|ref|NW_003383647.1|	195640-19633	183901	301533	0.713388	0.7371	no
gi|320446924|ref|NW_003383647.1|	197461-19900	424889	161207	-139817	0.50285	no
gi|320446924|ref|NW_003383647.1|	205797-20631	0.582457	626334	342671	0.2167	no
gi|320446924|ref|NW_003383647.1|	262693-26539	472194	146324	163172	0.3442	no
gi|320446924|ref|NW_003383647.1|	267507-26883	0.308335	139142	217399	1	no
gi|320446924|ref|NW_003383647.1|	272407-27266	308517	234514	292625	0.25735	no
gi|320446924|ref|NW_003383647.1|	272813-27324	0.81622	703358	310723	0.2468	no
gi|320446924|ref|NW_003383647.1|	273378-27472	273503	147711	-0.888783	0.66985	no
gi|320446924|ref|NW_003383647.1|	281670-28320	0.259889	0.903793	17981	1	no
gi|320446924|ref|NW_003383647.1|	285161-28546	186335	119638	26827	0.26995	no
gi|320446924|ref|NW_003383647.1|	288404-29151	687224	503963	287446	0.15135	no
gi|320446924|ref|NW_003383647.1|	291628-29270	0.402009	111249	146849	1	no
gi|320446924|ref|NW_003383647.1|	298624-29931	58976	344975	254829	0.2669	no
gi|320446924|ref|NW_003383647.1|	348605-35134	881819	130772	0.568494	0.65455	no
gi|320446924|ref|NW_003383647.1|	353685-35398	166262	130508	-0.349322	0.8618	no

gi|320446924|ref|NW_003383647.1|	56854-35761	192671	114495	-0.750861	0.7294	no
gi|320446924|ref|NW_003383647.1|	63916-36723	0.821297	137108	406127	0.0584	no
gi|320446924|ref|NW_003383647.1|	75650-38023	957466	449013	222946	0.10315	no
gi|320446924|ref|NW_003383647.1|	94614-39556	10421	0.490245	-440984	0.1495	no
gi|320446924|ref|NW_003383647.1|	07014-40744	131188	108679	-359348	0.23	no
gi|320446924|ref|NW_003383647.1|	10185-41129	661012	496464	-373491	0.0635	no
gi|320446924|ref|NW_003383647.1|	12788-41394	110348	0.636997	-0.792703	1	no
gi|320446924|ref|NW_003383647.1|	19651-42082	422075	261357	-40134	0.05755	no
gi|320446924|ref|NW_003383647.1|	25804-42708	779777	836751	-322019	0.1036	no
gi|320446924|ref|NW_003383647.1|	27997-42876	138086	170467	-3018	0.12165	no
gi|320446924|ref|NW_003383647.1|	29245-43497	310672	320416	0.0445571	0.9738	no
gi|320446924|ref|NW_003383647.1|	43270-44419	0.184719	12795	279217	1	no
gi|320446924|ref|NW_003383647.1|	37384-44194	126379	483494	-138618	0.2883	no
gi|320446924|ref|NW_003383647.1|	44536-46953	527484	681578	0.369752	0.78475	no
gi|320446924|ref|NW_003383647.1|	47506-44876	0.823513	148533	0.850919	0.6824	no
gi|320446924|ref|NW_003383647.1|	48971-45068	843937	841812	-0.00363816	0.99465	no
gi|320446924|ref|NW_003383647.1|	52239-45481	807052	172656	109717	0.52025	no
gi|320446924|ref|NW_003383647.1|	55755-45633	289138	326228	0.174121	0.92035	no
gi|320446924|ref|NW_003383647.1|	62002-46360	259303	146069	-0.827983	0.6926	no
gi|320446924|ref|NW_003383647.1|	69574-47558	251316	876551	180233	0.3192	no
gi|320446924|ref|NW_003383647.1|	77355-47765	196455	113332	252829	0.27435	no
gi|320446924|ref|NW_003383647.1|	47887-53326	619299	185607	158354	0.24295	no
gi|320446924|ref|NW_003383647.1|	40933-54134	307911	105196	177249	0.2839	no
gi|320446924|ref|NW_003383647.1|	44622-54531	0.371013	43079	353744	0.2165	no
gi|320446924|ref|NW_003383647.1|	47733-54845	139562	954906	277445	0.25515	no
gi|320446924|ref|NW_003383647.1|	48575-54914	199456	195539	329331	0.19885	no
gi|320446924|ref|NW_003383647.1|	49492-54973	103889	919086	314516	0.21435	no
gi|320446924|ref|NW_003383647.1|	50913-55141	246989	368483	389908	0.1671	no
gi|320446924|ref|NW_003383647.1|	51528-55470	166512	212014	367047	0.0714	no
gi|320446924|ref|NW_003383647.1|	64947-56576	377271	139611	-143418	0.4945	no

gi|320446924|ref|NW_003383647.1|	67684-56860	247616	0.682852	-185846	0.4076	no
gi|320446924|ref|NW_003383647.1|	72580-57416	375099	113094	-172975	0.4234	no
gi|320446924|ref|NW_003383647.1|	57502-62262	307644	38.08	0.307774	0.81795	no
gi|320446925|ref|NW_003383646.1|	04306-10501	247801	52055	439278	0.06735	no
gi|320446925|ref|NW_003383646.1|	05306-10555	632584	660811	338491	0.1885	no
gi|320446925|ref|NW_003383646.1|	37652-13805	279965	492116	0.813751	0.69235	no
gi|320446925|ref|NW_003383646.1|	43253-14376	237463	187907	-0.337684	0.8667	no
gi|320446925|ref|NW_003383646.1|	44127-14441	155359	127572	-0.284299	0.8765	no
gi|320446925|ref|NW_003383646.1|	44530-14565	249567	333841	0.419735	0.7388	no
gi|320446925|ref|NW_003383646.1|	45795-15272	854361	192227	116989	0.38645	no
gi|320446925|ref|NW_003383646.1|	02177-20338	0	936337	inf	0.00655	no
gi|320446925|ref|NW_003383646.1|	43525-24670	652526	621548	-0.0701701	0.9576	no
gi|320446925|ref|NW_003383646.1|	46855-24742	860124	307706	-148299	0.4755	no
gi|320446925|ref|NW_003383646.1|	48163-24910	233006	187241	-0.315468	0.88645	no
gi|320446925|ref|NW_003383646.1|	52605-25835	193634	155411	-0.317244	0.8122	no
gi|320446925|ref|NW_003383646.1|	61929-26258	216887	492552	-21386	0.32635	no
gi|320446925|ref|NW_003383646.1|	66339-26763	353484	277233	-0.350543	0.7825	no
gi|320446925|ref|NW_003383646.1|	76208-27672	307317	186041	-0.724108	0.7292	no
gi|320446925|ref|NW_003383646.1|	76880-27729	263848	104611	198725	0.385	no
gi|320446925|ref|NW_003383646.1|	85255-28598	247645	155382	-0.672454	0.7578	no
gi|320446925|ref|NW_003383646.1|	92914-29318	43306	766578	-249806	0.294	no
gi|320446925|ref|NW_003383646.1|	99569-30096	155711	242633	-268202	0.25305	no
gi|320446925|ref|NW_003383646.1|	08624-40999	0.446194	11363	13486	1	no
gi|320446925|ref|NW_003383646.1|	63521-46385	289655	131328	218077	0.27975	no
gi|320446925|ref|NW_003383646.1|	66324-46810	152647	16786	345899	0.0812	no
gi|320446925|ref|NW_003383646.1|	69475-47010	0.426735	609297	383573	0.20095	no
gi|320446925|ref|NW_003383646.1|	74956-47540	356127	125956	-149948	0.47755	no
gi|320446925|ref|NW_003383646.1|	92932-49438	570565	0.870512	-271246	0.2332	no
gi|320446925|ref|NW_003383646.1|	96992-49747	806104	314221	-135919	0.51645	no
gi|320446925|ref|NW_003383646.1|	97577-49792	965392	20304	-224935	0.33005	no

gi|320446925|ref|NW_003383646.1|	99031-50105	100248	35055	-151588	0.2594	no
gi|320446925|ref|NW_003383646.1|	05883-50671	131017	19582	-274216	0.23855	no
gi|320446925|ref|NW_003383646.1|	09283-51067	903808	151905	-257285	0.2677	no
gi|320446925|ref|NW_003383646.1|	11018-51275	258461	0.704361	-187556	0.38335	no
gi|320446925|ref|NW_003383646.1|	14448-51467	465296	159758	-154226	0.48725	no
gi|320446925|ref|NW_003383646.1|	14784-51500	418557	876676	-225531	0.26955	no
gi|320446925|ref|NW_003383646.1|	18915-52014	918074	235787	-196113	0.37395	no
gi|320446925|ref|NW_003383646.1|	20605-52181	10606	253116	-2067	0.3508	no
gi|320446925|ref|NW_003383646.1|	26259-53039	223054	144634	-0.624991	0.6419	no
gi|320446925|ref|NW_003383646.1|	33755-53531	141407	381074	-18917	0.39445	no
gi|320446925|ref|NW_003383646.1|	35737-53689	0.912604	252896	147048	0.5005	no
gi|320446925|ref|NW_003383646.1|	37567-53837	0.881236	201823	119549	0.56655	no
gi|320446925|ref|NW_003383646.1|	38911-53991	722727	408544	-0.822959	0.6889	no
gi|320446925|ref|NW_003383646.1|	56189-55645	117.85	609272	-0.951791	0.64475	no
gi|320446925|ref|NW_003383646.1|	58799-55955	484438	0	#NAME?	0.01585	no
gi|320446925|ref|NW_003383646.1|	60569-56260	103312	0.0655009	-730128	0.26075	no
gi|320446925|ref|NW_003383646.1|	65135-56600	412891	0.183394	-781467	0.2504	no
gi|320446925|ref|NW_003383646.1|	66723-56920	537278	0	#NAME?	5.00E-05	yes
gi|320446925|ref|NW_003383646.1|	85214-58715	127847	0.276363	-553171	0.1298	no
gi|320446925|ref|NW_003383646.1|	88023-58853	208743	0	#NAME?	0.0086	no
gi|320446925|ref|NW_003383646.1|	89715-59042	162841	0.242116	-607162	0.2675	no
gi|320446925|ref|NW_003383646.1|	92275-59439	608831	0.249851	-460691	0.1669	no
gi|320446925|ref|NW_003383646.1|	95190-59605	351288	0	#NAME?	0.01755	no
gi|320446925|ref|NW_003383646.1|	96660-59767	477479	0	#NAME?	0.01	no
gi|320446925|ref|NW_003383646.1|	00284-60533	269838	218485	-0.304559	0.8908	no
gi|320446925|ref|NW_003383646.1|	05672-60625	218691	0	#NAME?	0.0062	no
gi|320446925|ref|NW_003383646.1|	93565-94891	0.309466	110643	515999	0.146	no
gi|320446925|ref|NW_003383646.1|	95185-104141	109698	818897	290014	0.1286	no
gi|320446927|ref|NW_003383644.1|	03864-10413	0	27731	inf	0.02075	no
gi|320446927|ref|NW_003383644.1|	09639-11040	0.639808	701981	345572	0.19115	no

gi|320446927|ref|NW_003383644.1|	11721-13786	599468	385965	-0.635212	0.76415	no
gi|320446927|ref|NW_003383644.1|	117578-120159	690579	425632	262373	0.17275	no
gi|320446927|ref|NW_003383644.1|	1577-1990	679216	16138	-207341	0.3539	no
gi|320446927|ref|NW_003383644.1|	20928-21474	485174	654125	0.431064	0.8213	no
gi|320446927|ref|NW_003383644.1|	271663-273017	392234	0	#NAME?	0.00975	no
gi|320446927|ref|NW_003383644.1|	279507-280304	480877	0	#NAME?	0.015	no
gi|320446927|ref|NW_003383644.1|	3247-3655	779949	183652	-208641	0.349	no
gi|320446927|ref|NW_003383644.1|	335845-336329	133335	757575	250633	0.2524	no
gi|320446927|ref|NW_003383644.1|	336567-337275	0	439372	inf	0.0154	no
gi|320446927|ref|NW_003383644.1|	343026-343938	0	240732	inf	0.0233	no
gi|320446927|ref|NW_003383644.1|	348802-349835	0.634776	453437	283658	0.2453	no
gi|320446927|ref|NW_003383644.1|	350035-353080	0.180163	172158	325636	0.20355	no
gi|320446927|ref|NW_003383644.1|	353224-354970	134046	157156	35514	0.07895	no
gi|320446927|ref|NW_003383644.1|	372730-373067	127589	156222	0.292102	0.8801	no
gi|320446927|ref|NW_003383644.1|	398410-399272	107916	501372	221598	0.3363	no
gi|320446927|ref|NW_003383644.1|	403983-405988	0.28606	0.930906	170232	1	no
gi|320446927|ref|NW_003383644.1|	406286-407667	0.736514	16371	115236	0.5753	no
gi|320446927|ref|NW_003383644.1|	409484-413914	151645	170126	0.165913	0.90105	no
gi|320446927|ref|NW_003383644.1|	415777-416332	943894	460031	-103689	0.61435	no
gi|320446927|ref|NW_003383644.1|	416529-417332	0.892109	204258	11951	0.56655	no
gi|320446927|ref|NW_003383644.1|	417474-419145	117542	278219	124305	0.54615	no
gi|320446927|ref|NW_003383644.1|	4178-8265	491945	409402	-0.26498	0.83745	no
gi|320446927|ref|NW_003383644.1|	41912-42333	696874	161238	121022	0.5616	no
gi|320446927|ref|NW_003383644.1|	420363-422664	120898	798313	-0.598759	0.70895	no
gi|320446927|ref|NW_003383644.1|	42485-48074	115347	346484	158681	0.24235	no
gi|320446927|ref|NW_003383644.1|	425555-426080	135702	304364	-215657	0.32775	no
gi|320446927|ref|NW_003383644.1|	431847-433405	433792	0.887547	-228911	0.3085	no
gi|320446927|ref|NW_003383644.1|	434716-435588	177634	383349	-221218	0.32145	no
gi|320446927|ref|NW_003383644.1|	436879-437595	709166	177724	-199648	0.25205	no
gi|320446927|ref|NW_003383644.1|	438121-438527	656577	216351	-160159	0.4624	no

gi|320446927|ref|NW_003383644.1|	38716-43933	25779	573358	-216869	0.33785	no
gi|320446927|ref|NW_003383644.1|	40520-44259	137587	767763	-0.841609	0.6016	no
gi|320446927|ref|NW_003383644.1|	42703-44435	201168	142518	-0.497261	0.76475	no
gi|320446927|ref|NW_003383644.1|	44483-44526	271244	228472	-0.247573	0.9115	no
gi|320446927|ref|NW_003383644.1|	45437-44782	174097	133606	-0.381912	0.76475	no
gi|320446927|ref|NW_003383644.1|	76883-48453	201355	861462	-122488	0.3686	no
gi|320446927|ref|NW_003383644.1|	84706-48580	174527	434292	-200672	0.37	no
gi|320446927|ref|NW_003383644.1|	98689-49951	399269	612098	-270553	0.13995	no
gi|320446927|ref|NW_003383644.1|	99648-50071	244704	747549	-17108	0.4449	no
gi|320446927|ref|NW_003383644.1|	00815-50298	540517	215605	-132595	0.33045	no
gi|320446927|ref|NW_003383644.1|	05523-50683	382313	351631	-0.120691	0.92705	no
gi|320446927|ref|NW_003383644.1|	07000-50775	194146	221826	0.192289	0.9195	no
gi|320446927|ref|NW_003383644.1|	07858-50815	275037	188887	-0.542102	0.78845	no
gi|320446927|ref|NW_003383644.1|	08613-50900	549828	340416	-0.691684	0.73755	no
gi|320446927|ref|NW_003383644.1|	17446-51781	471658	307933	-0.615127	0.7677	no
gi|320446927|ref|NW_003383644.1|	51779-54166	0.705799	109513	0.633769	1	no
gi|320446927|ref|NW_003383644.1|	26163-52712	0.466477	563406	35943	0.182	no
gi|320446927|ref|NW_003383644.1|	33900-53458	0.37264	687103	420467	0.19175	no
gi|320446927|ref|NW_003383644.1|	49891-55029	19248	196406	335106	0.1987	no
gi|320446927|ref|NW_003383644.1|	68-1392	187571	710262	-140101	0.5393	no
gi|320446927|ref|NW_003383644.1|	8477-9802	1239	161277	0.380361	0.84275	no
gi|320446927|ref|NW_003383644.1|	90676-92458	229119	442341	427099	0.05155	no
gi|320446927|ref|NW_003383644.1|	92623-93478	245163	121846	231325	0.30485	no
gi|320446927|ref|NW_003383644.1|	93729-96272	0.14624	357523	461163	0.15625	no
gi|320446927|ref|NW_003383644.1|	98456-98710	304749	32826	342914	0.2211	no
gi|320446928|ref|NW_003383643.1|	51540-10524	0.750558	448375	257867	0.28405	no
gi|320446928|ref|NW_003383643.1|	81396-28379	0.311804	244932	297367	0.225	no
gi|320446928|ref|NW_003383643.1|	01937-40295	0.8637	313355	18592	0.405	no
gi|320446928|ref|NW_003383643.1|	48319-44916	137534	738084	2424	0.28715	no
gi|320446928|ref|NW_003383643.1|	49736-45017	0.812547	700324	31075	0.2468	no

gi|320446928|ref|NW_003383643.1|	455180-45663	0.41441	201638	228264	0.32355	no
gi|320446928|ref|NW_003383643.1|	457244-45831	0.607355	378126	263826	0.2664	no
gi|320446928|ref|NW_003383643.1|	463271-46363	360356	705279	0.968769	0.6698	no
gi|320446928|ref|NW_003383643.1|	510095-51473	34323	15497	-114718	0.39425	no
gi|320446928|ref|NW_003383643.1|	514830-51669	124027	100843	-0.298552	1	no
gi|320446928|ref|NW_003383643.1|	517066-51911	315812	0.583063	-243734	0.27115	no
gi|320446928|ref|NW_003383643.1|	524005-52440	151616	749074	-101724	0.60985	no
gi|320446928|ref|NW_003383643.1|	526560-52707	457671	155668	-155584	0.47685	no
gi|320446928|ref|NW_003383643.1|	527609-53031	43524	860731	-233818	0.0885	no
gi|320446928|ref|NW_003383643.1|	531054-53206	194831	344065	-250147	0.27455	no
gi|320446928|ref|NW_003383643.1|	536819-53791	527504	678581	-295859	0.1153	no
gi|320446928|ref|NW_003383643.1|	575377-57692	0	152482	inf	0.02075	no
gi|320446928|ref|NW_003383643.1|	577031-57832	0	232974	inf	0.01575	no
gi|320446928|ref|NW_003383643.1|	592064-59247	179351	100656	248858	0.25415	no
gi|320446928|ref|NW_003383643.1|	599320-60440	872894	312079	183803	0.1725	no
gi|320446928|ref|NW_003383643.1|	604528-60806	357839	636651	0.831191	0.6066	no
gi|320446928|ref|NW_003383643.1|	608370-60884	130612	128501	-0.0235173	0.9784	no
gi|320446928|ref|NW_003383643.1|	623203-62423	147929	102263	-0.532625	0.7943	no
gi|320446928|ref|NW_003383643.1|	627557-62864	192226	113818	-0.75608	0.7365	no
gi|320446928|ref|NW_003383643.1|	636687-63868	744571	136489	-244763	0.07455	no
gi|320446928|ref|NW_003383643.1|	639324-64070	306537	64461	-224956	0.09585	no
gi|320446928|ref|NW_003383643.1|	641580-64221	423563	201641	-107079	0.5979	no
gi|320446928|ref|NW_003383643.1|	644544-64500	145967	194415	-290843	0.25265	no
gi|320446928|ref|NW_003383643.1|	645531-64593	161092	187269	-310471	0.19355	no
gi|320446928|ref|NW_003383643.1|	646104-64673	210677	204578	-336431	0.1831	no
gi|320446928|ref|NW_003383643.1|	647032-64773	765755	124592	-261967	0.27575	no
gi|320446928|ref|NW_003383643.1|	648385-64960	702008	11872	-256392	0.2656	no
gi|320446928|ref|NW_003383643.1|	649946-65095	520886	0.749861	-279627	0.2518	no
gi|320446928|ref|NW_003383643.1|	651089-65205	300715	0.479016	-265025	0.2292	no
gi|320446928|ref|NW_003383643.1|	656960-65734	10.49	206437	-234524	0.25625	no

gi|320446928|ref|NW_003383643.1|	557457-657748	185071	315899	0.771388	0.70475	no
gi|320446928|ref|NW_003383643.1|	557934-659107	215987	123471	-0.806778	0.7243	no
gi|320446928|ref|NW_003383643.1|	560101-662259	851313	869552	0.0305842	0.9879	no
gi|320446928|ref|NW_003383643.1|	598061-699178	191426	0.397586	-226744	0.2663	no
gi|320446928|ref|NW_003383643.1|	741799-748022	185414	936137	-0.985956	0.4616	no
gi|320446928|ref|NW_003383643.1|	748223-750913	304521	220945	-0.462853	0.7263	no
gi|320446928|ref|NW_003383643.1|	756393-756704	172748	422527	129038	0.53655	no
gi|320446928|ref|NW_003383643.1|	766343-766613	131897	355112	-189306	0.38485	no
gi|320446928|ref|NW_003383643.1|	769397-769983	148556	35368	125144	0.564	no
gi|320446928|ref|NW_003383643.1|	779455-785125	475001	731611	0.623148	0.6277	no
gi|320446928|ref|NW_003383643.1|	786536-787993	163001	547304	-157447	0.4865	no
gi|320446928|ref|NW_003383643.1|	853459-854240	154681	191075	0.304848	0.876	no
gi|320446928|ref|NW_003383643.1|	877247-878098	109669	282975	136751	0.5247	no
gi|320446929|ref|NW_003383642.1|	102252-102918	234898	480507	103253	0.5994	no
gi|320446929|ref|NW_003383642.1|	103390-107045	184535	34078	0.884946	0.50555	no
gi|320446929|ref|NW_003383642.1|	110258-120088	128368	997877	295857	0.12355	no
gi|320446929|ref|NW_003383642.1|	130893-131482	237619	193131	-0.299075	0.8821	no
gi|320446929|ref|NW_003383642.1|	141705-142785	109898	235024	-222528	0.3168	no
gi|320446929|ref|NW_003383642.1|	143625-144962	444244	180812	-129687	0.5378	no
gi|320446929|ref|NW_003383642.1|	157071-160180	0	394162	inf	0.005	no
gi|320446929|ref|NW_003383642.1|	187564-188560	102069	797003	-0.356885	0.8657	no
gi|320446929|ref|NW_003383642.1|	191858-194100	251517	327682	0.381641	0.8527	no
gi|320446929|ref|NW_003383642.1|	194974-195841	21378	220757	0.0463276	0.9545	no
gi|320446929|ref|NW_003383642.1|	195943-197910	369017	467192	0.340331	0.87285	no
gi|320446929|ref|NW_003383642.1|	198099-201621	811135	105242	0.375693	0.762	no
gi|320446929|ref|NW_003383642.1|	201811-204751	132161	223131	0.755597	0.55615	no
gi|320446929|ref|NW_003383642.1|	218278-222592	111694	105144	323474	0.09325	no
gi|320446929|ref|NW_003383642.1|	237635-240871	0.168638	373492	446908	0.1588	no
gi|320446929|ref|NW_003383642.1|	267513-268760	0.667279	196701	155964	0.49625	no
gi|320446929|ref|NW_003383642.1|	268956-271170	477567	12974	144185	0.38105	no

gi|320446929|ref|NW_003383642.1|	271787-272278	298755	54324	0.862625	0.68715	no
gi|320446929|ref|NW_003383642.1|	277418-277769	167528	234752	0.486731	0.80045	no
gi|320446929|ref|NW_003383642.1|	292406-293677	267243	397961	0.574476	0.65515	no
gi|320446929|ref|NW_003383642.1|	304661-305230	412464	0	#NAME?	0.0042	no
gi|320446929|ref|NW_003383642.1|	306522-306973	684846	101175	-275892	0.2827	no
gi|320446929|ref|NW_003383642.1|	307998-308222	102314	0	#NAME?	0.01	no
gi|320446929|ref|NW_003383642.1|	309490-309729	852834	233996	-518771	0.28125	no
gi|320446929|ref|NW_003383642.1|	311914-312818	169129	0	#NAME?	0.00365	no
gi|320446929|ref|NW_003383642.1|	317428-318261	101677	52438	-0.955303	0.6479	no
gi|320446929|ref|NW_003383642.1|	319395-321360	175606	0.61218	-152031	0.4762	no
gi|320446929|ref|NW_003383642.1|	321539-323320	696017	174249	-199797	0.3673	no
gi|320446929|ref|NW_003383642.1|	325945-326691	214844	203826	-339788	0.1715	no
gi|320446929|ref|NW_003383642.1|	329436-329990	168314	283924	-256758	0.2638	no
gi|320446929|ref|NW_003383642.1|	330701-331612	849372	0.688806	-362423	0.19555	no
gi|320446929|ref|NW_003383642.1|	332253-333004	2946	134603	-113005	0.58845	no
gi|320446929|ref|NW_003383642.1|	333435-333851	11601	117865	-329903	0.24275	no
gi|320446929|ref|NW_003383642.1|	336340-337162	198536	8109	-129181	0.54995	no
gi|320446929|ref|NW_003383642.1|	35381-36364	0.225748	280604	363575	0.2061	no
gi|320446929|ref|NW_003383642.1|	364806-365094	0	216427	inf	0.0198	no
gi|320446929|ref|NW_003383642.1|	413072-416161	0.0591282	471334	631676	0.1549	no
gi|320446929|ref|NW_003383642.1|	47291-47597	630269	682913	0.115734	0.956	no
gi|320446929|ref|NW_003383642.1|	52820-54983	122551	15575	0.345845	0.78305	no
gi|320446929|ref|NW_003383642.1|	55323-55934	134386	334455	131543	0.5489	no
gi|320446929|ref|NW_003383642.1|	585376-586514	108443	673336	-0.68754	0.75335	no
gi|320446929|ref|NW_003383642.1|	586756-587852	529465	285004	-0.893553	0.66555	no
gi|320446929|ref|NW_003383642.1|	589246-595897	441814	980728	115041	0.3787	no
gi|320446929|ref|NW_003383642.1|	609718-610800	0.593816	0.958798	0.691211	1	no
gi|320446929|ref|NW_003383642.1|	614016-614729	0.70653	386602	245203	0.24845	no
gi|320446929|ref|NW_003383642.1|	642483-642994	241867	568092	123191	0.5597	no
gi|320446929|ref|NW_003383642.1|	671829-672319	306381	697819	118752	0.58945	no

gi|320446929|ref|NW_003383642.1|	86463-68679	222012	680103	161512	0.45435	no
gi|320446929|ref|NW_003383642.1|	86986-69117	760404	364283	226022	0.0971	no
gi|320446929|ref|NW_003383642.1|	39958-74137	200279	208742	0.0597121	0.96895	no
gi|320446929|ref|NW_003383642.1|	47770-74887	658317	62993	-0.0635901	0.97325	no
gi|320446929|ref|NW_003383642.1|	49143-74941	357171	166959	-109713	0.60235	no
gi|320446929|ref|NW_003383642.1|	49934-75025	233887	906499	-136744	0.50975	no
gi|320446929|ref|NW_003383642.1|	65095-76980	158746	107221	-0.566137	0.67025	no
gi|320446929|ref|NW_003383642.1|	85492-78623	120639	123947	0.0390229	0.98275	no
gi|320446929|ref|NW_003383642.1|	94465-79511	977755	998748	0.030648	0.98285	no
gi|320446929|ref|NW_003383642.1|	95766-79788	173788	134366	-0.371168	0.7623	no
gi|320446929|ref|NW_003383642.1|	98020-79900	426071	480104	0.172252	0.9309	no
gi|320446929|ref|NW_003383642.1|	99201-80036	61608	750426	0.284594	0.83305	no
gi|320446929|ref|NW_003383642.1|	03112-80348	114398	254245	115216	0.57355	no
gi|320446929|ref|NW_003383642.1|	03705-80507	207857	381545	0.876262	0.66785	no
gi|320446929|ref|NW_003383642.1|	05178-80728	541805	201581	189551	0.27905	no
gi|320446929|ref|NW_003383642.1|	09900-81154	113805	200121	-250762	0.2813	no
gi|320446929|ref|NW_003383642.1|	13982-81475	772612	500041	-0.627698	0.62455	no
gi|320446929|ref|NW_003383642.1|	16039-81802	486483	192111	-134045	0.3101	no
gi|320446929|ref|NW_003383642.1|	18134-81942	611562	284053	-110634	0.39965	no
gi|320446929|ref|NW_003383642.1|	19588-82060	243372	314057	0.367859	0.8201	no
gi|320446929|ref|NW_003383642.1|	23189-82558	109435	283815	137488	0.515	no
gi|320446929|ref|NW_003383642.1|	25752-82993	990838	78639	-0.333404	0.798	no
gi|320446929|ref|NW_003383642.1|	82751-83467	0	148904	inf	0.00575	no
gi|320446929|ref|NW_003383642.1|	30098-83042	300394	165161	-0.862982	0.67695	no
gi|320446929|ref|NW_003383642.1|	30785-83105	687882	265996	-137075	0.5148	no
gi|320446929|ref|NW_003383642.1|	32166-83296	817766	292089	-148528	0.39545	no
gi|320446929|ref|NW_003383642.1|	81901-88262	0.693531	854286	362269	0.17685	no
gi|320446929|ref|NW_003383642.1|	94355-96177	0	970742	inf	0.00375	no
gi|320446929|ref|NW_003383642.1|	98576-99135	0	13292	inf	0.0113	no
gi|320446929|ref|NW_003383642.1|	99849-100518	14775	198884	0.428772	0.84305	no

gi|320446930|ref|NW_003383641.1|	106818-10923	307405	687119	116042	0.38935	no
gi|320446930|ref|NW_003383641.1|	256310-25691	55196	561437	0.0245622	0.96685	no
gi|320446930|ref|NW_003383641.1|	270861-27472	757303	625455	304596	0.0685	no
gi|320446930|ref|NW_003383641.1|	288708-29519	261331	881831	-15673	0.24535	no
gi|320446930|ref|NW_003383641.1|	305214-30577	824018	939011	0.188467	0.92275	no
gi|320446930|ref|NW_003383641.1|	307179-30826	396808	274577	-0.531232	0.7993	no
gi|320446930|ref|NW_003383641.1|	308395-31066	720096	392856	-0.874187	0.6905	no
gi|320446930|ref|NW_003383641.1|	310819-31273	44204	609259	0.462881	0.8236	no
gi|320446930|ref|NW_003383641.1|	313834-31510	51756	437234	-0.24332	0.8547	no
gi|320446930|ref|NW_003383641.1|	315215-31740	282116	429338	0.605827	0.77755	no
gi|320446930|ref|NW_003383641.1|	319539-32102	0	159736	inf	0.02075	no
gi|320446930|ref|NW_003383641.1|	323082-32426	0.535966	581887	344053	0.1902	no
gi|320446930|ref|NW_003383641.1|	326830-32816	184664	111113	258905	0.253	no
gi|320446930|ref|NW_003383641.1|	328536-32899	146567	439158	158318	0.3575	no
gi|320446930|ref|NW_003383641.1|	329462-33040	40836	106048	13768	0.51405	no
gi|320446930|ref|NW_003383641.1|	333154-33538	25366	365862	0.528405	0.79945	no
gi|320446930|ref|NW_003383641.1|	344222-34487	280489	709847	133956	0.5227	no
gi|320446930|ref|NW_003383641.1|	346326-34779	218161	293847	0.429672	0.8274	no
gi|320446930|ref|NW_003383641.1|	350793-35264	0.73156	145655	0.993503	0.65565	no
gi|320446930|ref|NW_003383641.1|	353651-35433	0.75021	230504	161942	0.3327	no
gi|320446930|ref|NW_003383641.1|	355991-35922	0.225376	279786	363392	0.17845	no
gi|320446930|ref|NW_003383641.1|	359541-36012	0	38833	inf	0.029	no
gi|320446930|ref|NW_003383641.1|	399376-40375	0.203694	470679	453027	0.135	no
gi|320446930|ref|NW_003383641.1|	404249-40797	0.386365	101732	471867	0.0618	no
gi|320446930|ref|NW_003383641.1|	411399-41274	212724	0.738553	-152621	0.4824	no
gi|320446930|ref|NW_003383641.1|	446845-44706	0	386059	inf	0.02915	no
gi|320446930|ref|NW_003383641.1|	490309-49059	108831	403362	188999	0.3886	no
gi|320446930|ref|NW_003383641.1|	506086-50636	461889	162066	181096	0.32625	no
gi|320446930|ref|NW_003383641.1|	590575-59243	0.20773	0.651442	164893	1	no
gi|320446930|ref|NW_003383641.1|	648002-64876	0.642238	396328	262551	0.23645	no

gi|320446931|ref|NW_003383640.1|	202116-20333	0	215206	inf	0.0154	no
gi|320446931|ref|NW_003383640.1|	229455-23059	167531	206279	0.300166	0.87725	no
gi|320446931|ref|NW_003383640.1|	230934-23210	10834	212692	0.973193	0.65545	no
gi|320446931|ref|NW_003383640.1|	233977-23461	290023	310182	0.0969473	0.94585	no
gi|320446931|ref|NW_003383640.1|	234871-23767	0.722303	148691	436357	0.0552	no
gi|320446931|ref|NW_003383640.1|	240048-24175	253125	107353	208444	0.3535	no
gi|320446931|ref|NW_003383640.1|	243514-24435	140278	212245	0.597439	0.75905	no
gi|320446931|ref|NW_003383640.1|	244983-24632	703386	171994	128997	0.5642	no
gi|320446931|ref|NW_003383640.1|	261341-26203	0	128964	inf	0.0072	no
gi|320446931|ref|NW_003383640.1|	265140-26644	0	524654	inf	0.0089	no
gi|320446931|ref|NW_003383640.1|	307006-30759	330838	416232	0.331266	0.86195	no
gi|320446931|ref|NW_003383640.1|	335467-33640	843143	0.166165	-566509	0.2736	no
gi|320446931|ref|NW_003383640.1|	350893-35130	149315	209149	0.486176	0.80865	no
gi|320446931|ref|NW_003383640.1|	369703-37264	232831	102605	-118218	0.48325	no
gi|320446931|ref|NW_003383640.1|	372797-37345	475057	191985	-130711	0.55845	no
gi|320446931|ref|NW_003383640.1|	373839-37603	0.515851	14399	148094	0.48685	no
gi|320446931|ref|NW_003383640.1|	376200-38191	66552	427119	-0.639843	0.61275	no
gi|320446931|ref|NW_003383640.1|	395735-39630	495772	469377	-0.0789324	0.95685	no
gi|320446931|ref|NW_003383640.1|	416081-41648	273401	229834	-0.250427	0.90115	no
gi|320446933|ref|NW_003383638.1|	191770-19262	533049	492231	-0.114934	0.92805	no
gi|320446933|ref|NW_003383638.1|	193050-19342	198754	238527	0.263166	0.8924	no
gi|320446933|ref|NW_003383638.1|	193650-19803	137025	27133	0.985609	0.46	no
gi|320446933|ref|NW_003383638.1|	205618-20732	120.17	682669	-0.815815	0.5413	no
gi|320446933|ref|NW_003383638.1|	207385-21066	109282	636321	-0.780234	0.63985	no
gi|320446933|ref|NW_003383638.1|	212318-21316	433269	159481	-144188	0.38955	no
gi|320446933|ref|NW_003383638.1|	215241-21573	228736	808528	-150031	0.48525	no
gi|320446933|ref|NW_003383638.1|	215898-21896	40879	156024	-138959	0.2965	no
gi|320446933|ref|NW_003383638.1|	221221-22232	677679	455694	-0.572536	0.78385	no
gi|320446933|ref|NW_003383638.1|	222474-22458	337005	15987	-107587	0.40905	no
gi|320446933|ref|NW_003383638.1|	233721-23568	246681	172672	-0.514618	0.6853	no

gi|320446933|ref|NW_003383638.1|	237104-24289	131291	877448	-0.581384	0.6585	no
gi|320446933|ref|NW_003383638.1|	248970-253564	197423	241549	0.291022	0.8295	no
gi|320446933|ref|NW_003383638.1|	254110-254869	109597	773505	-0.502722	0.80655	no
gi|320446933|ref|NW_003383638.1|	255626-255807	939236	579774	-0.695997	0.7327	no
gi|320446933|ref|NW_003383638.1|	256030-258775	887488	549715	-0.691045	0.66895	no
gi|320446933|ref|NW_003383638.1|	432632-434647	107144	0.330611	-501827	0.1467	no
gi|320446933|ref|NW_003383638.1|	434873-435740	330887	0.729065	-550415	0.12955	no
gi|320446933|ref|NW_003383638.1|	488332-490528	516378	504477	-0.0336382	0.9865	no
gi|320446933|ref|NW_003383638.1|	492830-493620	557006	554096	-0.0075556	0.9943	no
gi|320446933|ref|NW_003383638.1|	546919-549159	378781	55222	0.543876	0.80615	no
gi|320446933|ref|NW_003383638.1|	550046-550450	459614	115198	132562	0.52805	no
gi|320446933|ref|NW_003383638.1|	558272-558665	803201	725774	-0.146239	0.936	no
gi|320446933|ref|NW_003383638.1|	571779-572149	263115	30659	0.220618	0.91165	no
gi|320446933|ref|NW_003383638.1|	610748-611423	360547	157024	-452113	0.13945	no
gi|320446933|ref|NW_003383638.1|	734630-734944	842971	543039	-0.634427	0.75175	no
gi|320446936|ref|NW_003383635.1|	104582-105894	0.783728	326403	205823	0.35365	no
gi|320446936|ref|NW_003383635.1|	106212-106664	0.757718	604588	299622	0.2563	no
gi|320446936|ref|NW_003383635.1|	121484-123625	354189	735168	105355	0.62935	no
gi|320446936|ref|NW_003383635.1|	125738-126244	307651	949048	162518	0.4475	no
gi|320446936|ref|NW_003383635.1|	16512-17896	822741	561301	-0.551664	0.7947	no
gi|320446936|ref|NW_003383635.1|	18021-19884	117151	686376	-0.771302	0.7371	no
gi|320446936|ref|NW_003383635.1|	180532-181380	0.275517	125106	550487	0.1627	no
gi|320446936|ref|NW_003383635.1|	21323-23477	897061	539994	-0.732264	0.7412	no
gi|320446936|ref|NW_003383635.1|	215839-216282	110274	941215	-0.2285	0.9063	no
gi|320446936|ref|NW_003383635.1|	216556-217467	174871	843787	-105134	0.6253	no
gi|320446936|ref|NW_003383635.1|	217525-217982	378412	256771	-0.559473	0.7955	no
gi|320446936|ref|NW_003383635.1|	218141-219050	44702	308171	-0.536608	0.78835	no
gi|320446936|ref|NW_003383635.1|	219244-219627	954745	556449	-0.778867	0.7042	no
gi|320446936|ref|NW_003383635.1|	221096-222038	764325	708547	-0.109323	0.9569	no
gi|320446936|ref|NW_003383635.1|	222543-224439	209323	927808	-117383	0.48225	no

gi|320446936|ref|NW_003383635.1|	225310-22622	176845	188873	-3227	0.19625	no
gi|320446936|ref|NW_003383635.1|	235535-23664	131815	375703	-181085	0.4085	no
gi|320446936|ref|NW_003383635.1|	254332-25498	411614	142944	-152585	0.49265	no
gi|320446936|ref|NW_003383635.1|	255092-25945	22997	125562	-0.873053	0.5133	no
gi|320446936|ref|NW_003383635.1|	260861-26206	77058	764795	-0.0108715	0.9901	no
gi|320446936|ref|NW_003383635.1|	263729-26407	229577	149091	-0.62279	0.7523	no
gi|320446936|ref|NW_003383635.1|	30393-31249	815891	336888	-127611	0.54055	no
gi|320446936|ref|NW_003383635.1|	311662-31280	0.186351	116158	263999	1	no
gi|320446936|ref|NW_003383635.1|	312881-31390	0	2828	inf	0.0212	no
gi|320446936|ref|NW_003383635.1|	313951-31465	0	269607	inf	0.0294	no
gi|320446936|ref|NW_003383635.1|	3359-5984	133454	532778	-132474	0.42975	no
gi|320446936|ref|NW_003383635.1|	394170-39461	0	167327	inf	0.01035	no
gi|320446936|ref|NW_003383635.1|	395858-39691	0	152006	inf	0.0053	no
gi|320446936|ref|NW_003383635.1|	40554-41716	555487	719218	-294925	0.1206	no
gi|320446936|ref|NW_003383635.1|	42048-42303	722541	381614	-424289	0.2079	no
gi|320446936|ref|NW_003383635.1|	449585-45016	0.481897	190843	862945	0.14075	no
gi|320446936|ref|NW_003383635.1|	47517-47812	186.34	165138	-349619	0.1687	no
gi|320446936|ref|NW_003383635.1|	51996-54216	272071	284808	338794	0.08475	no
gi|320446936|ref|NW_003383635.1|	56728-57951	119612	177724	0.571276	0.7847	no
gi|320446936|ref|NW_003383635.1|	58096-60425	128146	41298	168828	0.2086	no
gi|320446936|ref|NW_003383635.1|	62064-62601	166379	29876	0.844519	0.69175	no
gi|320446936|ref|NW_003383635.1|	649184-65316	0.315323	592798	423264	0.07095	no
gi|320446936|ref|NW_003383635.1|	653578-66440	260385	838418	500895	0.25595	no
gi|320446936|ref|NW_003383635.1|	69964-70799	0	367803	inf	0.0212	no
gi|320446936|ref|NW_003383635.1|	722623-72364	0.426932	147545	178907	0.3323	no
gi|320446936|ref|NW_003383635.1|	724472-72517	724624	495273	-0.549009	0.7943	no
gi|320446936|ref|NW_003383635.1|	727164-72888	974207	150675	0.629142	0.69405	no
gi|320446936|ref|NW_003383635.1|	729291-73042	168647	155726	-0.115006	0.949	no
gi|320446936|ref|NW_003383635.1|	731814-73289	701843	735296	0.0671767	0.97175	no
gi|320446936|ref|NW_003383635.1|	814674-81736	110075	427828	195855	0.3683	no

gi|320446936|ref|NW_003383635.1|	317437-818905	109523	47585	211927	0.3328	no
gi|320446936|ref|NW_003383635.1|	8325-10521	229788	438414	-238994	0.1812	no
gi|320446936|ref|NW_003383635.1|	98820-99771	0.943387	488225	237163	0.2987	no
gi|320446938|ref|NW_003383633.1|	134331-135457	0	198124	inf	0.0038	no
gi|320446938|ref|NW_003383633.1|	136589-136954	0	769832	inf	0.0312	no
gi|320446938|ref|NW_003383633.1|	138320-138474	316947	782661	462607	0.17445	no
gi|320446938|ref|NW_003383633.1|	154622-15588	0.164542	14839	317286	0.24015	no
gi|320446938|ref|NW_003383633.1|	156324-156858	335975	727458	443644	0.07615	no
gi|320446938|ref|NW_003383633.1|	164261-165284	599983	236957	-13403	0.5133	no
gi|320446938|ref|NW_003383633.1|	165525-16741	459473	362943	-0.340237	0.87055	no
gi|320446938|ref|NW_003383633.1|	175888-17638	386823	189875	22953	0.30985	no
gi|320446938|ref|NW_003383633.1|	264652-265215	409566	276638	-0.566097	0.7893	no
gi|320446938|ref|NW_003383633.1|	269695-270110	627728	142103	117872	0.5697	no
gi|320446938|ref|NW_003383633.1|	272789-27325	506728	916268	0.854558	0.6766	no
gi|320446938|ref|NW_003383633.1|	275199-27825	18564	690871	189591	0.40995	no
gi|320446938|ref|NW_003383633.1|	287289-287710	348437	806189	121022	0.5726	no
gi|320446938|ref|NW_003383633.1|	29524-35724	19518	319194	0.709625	0.59785	no
gi|320446938|ref|NW_003383633.1|	356635-358897	0.333039	168528	233922	0.29945	no
gi|320446938|ref|NW_003383633.1|	35854-38602	221607	164249	-0.432118	0.83045	no
gi|320446938|ref|NW_003383633.1|	359539-361224	128047	46196	18511	0.3902	no
gi|320446938|ref|NW_003383633.1|	361738-36433	193073	116395	259182	0.1464	no
gi|320446938|ref|NW_003383633.1|	366292-368815	132767	303961	119499	0.57305	no
gi|320446938|ref|NW_003383633.1|	369554-37217	138037	726985	-0.925061	0.5863	no
gi|320446938|ref|NW_003383633.1|	372280-374399	463137	307478	-0.590954	0.6625	no
gi|320446938|ref|NW_003383633.1|	38789-39878	128509	643021	-0.998936	0.63795	no
gi|320446938|ref|NW_003383633.1|	40344-43129	109195	504132	-111504	0.49935	no
gi|320446938|ref|NW_003383633.1|	409833-411914	0	446416	inf	0.00565	no
gi|320446938|ref|NW_003383633.1|	422093-425340	156561	547503	180615	0.4203	no
gi|320446938|ref|NW_003383633.1|	425490-426387	0.765311	474599	263259	0.2665	no
gi|320446938|ref|NW_003383633.1|	480939-481338	29175	703933	127071	0.5503	no

gi|320446938|ref|NW_003383633.1|	481686-48496	183243	47771	138238	0.53145	no
gi|320446938|ref|NW_003383633.1|	485430-48847	150296	542243	185113	0.40345	no
gi|320446938|ref|NW_003383633.1|	494133-49488	0.650888	379228	254258	0.25045	no
gi|320446938|ref|NW_003383633.1|	498211-49935	0.185124	243631	371813	0.21225	no
gi|320446938|ref|NW_003383633.1|	500363-50362	41825	569464	0.44524	0.8418	no
gi|320446938|ref|NW_003383633.1|	509707-51338	104884	161196	-270191	0.13645	no
gi|320446938|ref|NW_003383633.1|	514721-51837	246849	722031	154843	0.35315	no
gi|320446938|ref|NW_003383633.1|	518911-52203	184188	842304	-112877	0.3821	no
gi|320446938|ref|NW_003383633.1|	535300-53574	313694	676923	110963	0.59635	no
gi|320446938|ref|NW_003383633.1|	575107-57563	397088	534307	0.42821	0.8214	no
gi|320446938|ref|NW_003383633.1|	580543-58093	102605	101052	329992	0.22995	no
gi|320446938|ref|NW_003383633.1|	621681-62431	0.140809	250858	415506	0.16045	no
gi|320446938|ref|NW_003383633.1|	626456-62822	0.110173	115113	338521	1	no
gi|320446938|ref|NW_003383633.1|	90295-90890	0	411866	inf	0.02915	no
gi|320446942|ref|NW_003383629.1|	101958-10481	342926	390626	0.187892	0.89055	no
gi|320446942|ref|NW_003383629.1|	104954-10548	86507	761867	-0.183277	0.88135	no
gi|320446942|ref|NW_003383629.1|	105599-10772	350902	549896	0.648091	0.6297	no
gi|320446942|ref|NW_003383629.1|	125080-12627	0.175132	194234	347128	0.21395	no
gi|320446942|ref|NW_003383629.1|	126758-12720	157535	575187	186836	0.32465	no
gi|320446942|ref|NW_003383629.1|	143524-14391	422999	0.677239	-596484	0.27105	no
gi|320446942|ref|NW_003383629.1|	145872-14851	221369	336491	392604	0.0537	no
gi|320446942|ref|NW_003383629.1|	150038-15078	598458	204997	177628	0.41975	no
gi|320446942|ref|NW_003383629.1|	151580-15223	12021	245716	103144	0.5772	no
gi|320446942|ref|NW_003383629.1|	152357-15287	0	942528	inf	0.0138	no
gi|320446942|ref|NW_003383629.1|	158103-15852	260084	200628	294747	0.23055	no
gi|320446942|ref|NW_003383629.1|	160287-16101	752115	292551	195966	0.3734	no
gi|320446942|ref|NW_003383629.1|	161163-16257	962557	123776	0.362791	0.87005	no
gi|320446942|ref|NW_003383629.1|	163686-16437	148082	505741	-154993	0.47175	no
gi|320446942|ref|NW_003383629.1|	174560-17744	37706	726256	0.945686	0.4714	no
gi|320446942|ref|NW_003383629.1|	180443-18188	0.418522	0.872648	10601	1	no

gi|320446942|ref|NW_003383629.1|	82987-18433	157457	375325	125319	0.46235	no
gi|320446942|ref|NW_003383629.1|	84451-18698	39358	129079	171352	0.20435	no
gi|320446942|ref|NW_003383629.1|	87382-19264	259671	221227	-0.231155	0.85955	no
gi|320446942|ref|NW_003383629.1|	93960-19523	175819	414524	123736	0.46485	no
gi|320446942|ref|NW_003383629.1|	95466-19734	471109	349047	288929	0.1264	no
gi|320446942|ref|NW_003383629.1|	97880-19958	483238	382946	298633	0.11925	no
gi|320446942|ref|NW_003383629.1|	11149-21579	601516	327604	244528	0.14915	no
gi|320446942|ref|NW_003383629.1|	89923-29036	0	142983	inf	0.0133	no
gi|320446942|ref|NW_003383629.1|	09497-31055	306621	378908	362732	0.0727	no
gi|320446942|ref|NW_003383629.1|	13827-31470	100275	581463	253572	0.165	no
gi|320446942|ref|NW_003383629.1|	22141-32303	128328	318288	13105	0.5464	no
gi|320446942|ref|NW_003383629.1|	29827-33237	133636	321865	126815	0.33845	no
gi|320446942|ref|NW_003383629.1|	33158-33492	427192	344348	-0.311018	0.8135	no
gi|320446942|ref|NW_003383629.1|	35473-33913	239028	367224	0.619484	0.6414	no
gi|320446942|ref|NW_003383629.1|	43976-34501	196921	10863	-0.858197	0.6938	no
gi|320446942|ref|NW_003383629.1|	67314-36791	150178	586565	-135631	0.51675	no
gi|320446942|ref|NW_003383629.1|	68030-36868	2427	275522	0.182993	0.9196	no
gi|320446942|ref|NW_003383629.1|	70120-37142	30881	178056	-0.794387	0.6424	no
gi|320446942|ref|NW_003383629.1|	00033-40085	0	101376	inf	0.0072	no
gi|320446942|ref|NW_003383629.1|	50455-51807	123926	146926	-307632	0.19775	no
gi|320446942|ref|NW_003383629.1|	53214-55351	13.31	23525	-250025	0.15615	no
gi|320446942|ref|NW_003383629.1|	37086-53823	26003	114493	213851	0.3363	no
gi|320446942|ref|NW_003383629.1|	55512-56265	107604	312892	-178199	0.40235	no
gi|320446942|ref|NW_003383629.1|	80332-58140	0.806902	34888	211227	0.34635	no
gi|320446942|ref|NW_003383629.1|	58348-58862	143629	522308	-145937	0.49165	no
gi|320446942|ref|NW_003383629.1|	60383-62380	155174	534345	-153804	0.35675	no
gi|320446942|ref|NW_003383629.1|	21843-62279	296466	130913	-450119	0.07265	no
gi|320446942|ref|NW_003383629.1|	63708-64916	867554	45709	-0.924473	0.66165	no
gi|320446942|ref|NW_003383629.1|	49517-65037	229188	114529	-100082	0.6395	no
gi|320446942|ref|NW_003383629.1|	50745-65484	925331	931961	0.0103004	0.99295	no

gi|320446942|ref|NW_003383629.1|	70116-67087	0	26373	inf	0.029	no
gi|320446942|ref|NW_003383629.1|	67339-75722	246009	205631	-0.258653	0.84535	no
gi|320446942|ref|NW_003383629.1|	85136-68616	176963	0	#NAME?	5.00E-05	yes
gi|320446942|ref|NW_003383629.1|	00904-70151	620176	165859	-190272	0.25705	no
gi|320446942|ref|NW_003383629.1|	04583-70955	172726	740921	-122109	0.35515	no
gi|320446942|ref|NW_003383629.1|	15209-71573	796775	459417	-0.794367	0.70255	no
gi|320446942|ref|NW_003383629.1|	18795-72197	0.458718	360899	297592	0.21675	no
gi|320446942|ref|NW_003383629.1|	22245-72272	174656	179555	0.0399069	0.9809	no
gi|320446942|ref|NW_003383629.1|	34964-73520	438802	738631	0.751285	0.71415	no
gi|320446942|ref|NW_003383629.1|	35414-73615	605592	152077	132838	0.5351	no
gi|320446942|ref|NW_003383629.1|	36948-73771	156457	402895	136464	0.3956	no
gi|320446942|ref|NW_003383629.1|	50973-75220	844957	171935	-229701	0.2186	no
gi|320446942|ref|NW_003383629.1|	52805-75414	198065	52895	-190477	0.39745	no
gi|320446942|ref|NW_003383629.1|	57081-75959	147555	559322	-139951	0.4067	no
gi|320446942|ref|NW_003383629.1|	59719-76235	107629	747036	-0.526815	0.7435	no
gi|320446942|ref|NW_003383629.1|	81359-78292	39178	997278	134795	0.3232	no
gi|320446942|ref|NW_003383629.1|	84036-78813	646488	688045	0.0898795	0.94355	no
gi|320446942|ref|NW_003383629.1|	88299-78927	750998	424254	-0.823881	0.6876	no
gi|320446942|ref|NW_003383629.1|	89670-79092	0.827553	0.688857	-0.264646	1	no
gi|320446942|ref|NW_003383629.1|	91038-79188	142802	396796	-184755	0.3931	no
gi|320446942|ref|NW_003383629.1|	05409-80719	100182	477903	-106783	0.6277	no
gi|320446942|ref|NW_003383629.1|	07320-81310	275241	34633	0.33145	0.8023	no
gi|320446942|ref|NW_003383629.1|	13281-81398	106.38	954414	-0.15654	0.9031	no
gi|320446942|ref|NW_003383629.1|	57832-85847	652671	426979	-0.61219	0.70715	no
gi|320446942|ref|NW_003383629.1|	86095-86515	175055	809898	-1112	0.59665	no
gi|320446942|ref|NW_003383629.1|	75716-87624	210576	199081	-0.0809879	0.9689	no
gi|320446942|ref|NW_003383629.1|	76349-87700	679592	381358	-0.833521	0.6805	no
gi|320446942|ref|NW_003383629.1|	78269-88164	0.375712	356688	324696	0.18575	no
gi|320446942|ref|NW_003383629.1|	85331-88718	250355	247157	-0.0185484	0.98405	no
gi|320446942|ref|NW_003383629.1|	89685-89551	165928	591962	183495	0.28195	no

gi|320446942|ref|NW_003383629.1|	91582-92208	219829	111327	-0.981582	0.64645	no
gi|320446942|ref|NW_003383629.1|	92397-93375	204542	282462	0.465659	0.81185	no
gi|320446942|ref|NW_003383629.1|	93482-94140	191436	116866	-0.712	0.73185	no
gi|320446942|ref|NW_003383629.1|	95848-96921	346553	291321	-0.25047	0.8395	no
gi|320446942|ref|NW_003383629.1|	98634-10112	229349	373063	0.701875	0.59945	no
gi|320446944|ref|NW_003383627.1|	101639-10237	15569	486705	-167756	0.43145	no
gi|320446944|ref|NW_003383627.1|	105831-10762	111584	422638	-140063	0.5361	no
gi|320446944|ref|NW_003383627.1|	111430-11338	833104	164684	0.983134	0.5476	no
gi|320446944|ref|NW_003383627.1|	120911-12342	0.148362	538841	518267	0.1474	no
gi|320446944|ref|NW_003383627.1|	181142-18190	0	517039	inf	5.00E-05	yes
gi|320446944|ref|NW_003383627.1|	189001-19049	199772	12119	-0.721078	0.65435	no
gi|320446944|ref|NW_003383627.1|	192016-19268	178448	127005	-0.490614	0.81545	no
gi|320446944|ref|NW_003383627.1|	193230-19510	485179	604321	0.316798	0.8805	no
gi|320446944|ref|NW_003383627.1|	249467-25048	0	194472	inf	0.02915	no
gi|320446944|ref|NW_003383627.1|	268208-27007	423245	270443	-0.646169	0.62275	no
gi|320446944|ref|NW_003383627.1|	270368-27177	690181	519499	-0.409852	0.84695	no
gi|320446944|ref|NW_003383627.1|	271955-27277	121888	10.77	-0.178529	0.93015	no
gi|320446944|ref|NW_003383627.1|	284634-28567	216793	188817	-0.199332	0.92515	no
gi|320446944|ref|NW_003383627.1|	289972-29076	121299	251928	105444	0.6279	no
gi|320446944|ref|NW_003383627.1|	290906-29393	249356	504458	101653	0.44965	no
gi|320446944|ref|NW_003383627.1|	297078-29768	419887	251221	-0.741046	0.73545	no
gi|320446944|ref|NW_003383627.1|	298329-29994	271143	135148	-100452	0.55525	no
gi|320446944|ref|NW_003383627.1|	301298-30173	34127	134678	-13414	0.5238	no
gi|320446944|ref|NW_003383627.1|	323677-32436	299424	460024	0.619519	0.7485	no
gi|320446944|ref|NW_003383627.1|	327908-32865	14.73	560845	-139308	0.50935	no
gi|320446944|ref|NW_003383627.1|	337138-33810	164237	718523	-119267	0.5805	no
gi|320446944|ref|NW_003383627.1|	339101-34501	136875	225552	0.720605	0.5917	no
gi|320446944|ref|NW_003383627.1|	356364-35787	0	0.920645	inf	1	no
gi|320446944|ref|NW_003383627.1|	40372-42212	606773	54885	-0.144746	0.9172	no
gi|320446944|ref|NW_003383627.1|	42356-43472	371788	199016	-0.901597	0.5909	no

gi|320446944|ref|NW_003383627.1|	43946-44219	783694	398994	-0.973922	0.4567	no
gi|320446944|ref|NW_003383627.1|	460333-46263(	0	281662	inf	5.00E-05	yes
gi|320446944|ref|NW_003383627.1|	482442-48835	150315	662232	213935	0.1151	no
gi|320446944|ref|NW_003383627.1|	489616-49098	0.896338	19713	113703	0.58645	no
gi|320446944|ref|NW_003383627.1|	491064-49203	0.690174	111171	0.687751	1	no
gi|320446944|ref|NW_003383627.1|	492107-49344(	0.45873	0.955523	105865	1	no
gi|320446944|ref|NW_003383627.1|	493571-50320	257728	16844	-0.613615	0.63875	no
gi|320446944|ref|NW_003383627.1|	503363-50416	117877	532908	-114532	0.3822	no
gi|320446944|ref|NW_003383627.1|	504404-50682(	403582	286129	-0.496196	0.71165	no
gi|320446944|ref|NW_003383627.1|	508299-50952	275387	241947	-0.18677	0.87685	no
gi|320446944|ref|NW_003383627.1|	510614-51210	200409	279618	0.480509	0.7083	no
gi|320446944|ref|NW_003383627.1|	515361-51765	215167	248794	0.209492	0.87005	no
gi|320446944|ref|NW_003383627.1|	519818-52073	352649	179767	-0.972109	0.54995	no
gi|320446944|ref|NW_003383627.1|	534109-53455	342076	242702	-0.495128	0.807	no
gi|320446944|ref|NW_003383627.1|	53544-57372	703062	141998	101415	0.5439	no
gi|320446944|ref|NW_003383627.1|	545927-54662	229471	290336	-298251	0.1299	no
gi|320446944|ref|NW_003383627.1|	548418-54862	757809	0	#NAME?	0.02015	no
gi|320446944|ref|NW_003383627.1|	549111-54953	313593	57585	-244513	0.285	no
gi|320446944|ref|NW_003383627.1|	555078-55917	886278	599054	-0.565072	0.65075	no
gi|320446944|ref|NW_003383627.1|	559614-56471	447696	136391	160716	0.22275	no
gi|320446944|ref|NW_003383627.1|	57994-58501	404677	789584	0.964321	0.55055	no
gi|320446944|ref|NW_003383627.1|	58771-62853	257478	265305	0.0432027	0.97505	no
gi|320446944|ref|NW_003383627.1|	506254-60690	568998	279559	229666	0.30625	no
gi|320446944|ref|NW_003383627.1|	64070-65409	111624	721951	-0.628679	0.7723	no
gi|320446944|ref|NW_003383627.1|	546215-64695	167886	213876	367122	0.1632	no
gi|320446944|ref|NW_003383627.1|	547091-64931(	0.678467	532682	297292	0.21695	no
gi|320446944|ref|NW_003383627.1|	549451-65551	159264	391273	461869	0.03885	no
gi|320446944|ref|NW_003383627.1|	559377-66758	188228	209203	0.152426	0.9087	no
gi|320446944|ref|NW_003383627.1|	568617-66896	670656	139407	105566	0.594	no
gi|320446944|ref|NW_003383627.1|	569565-67052	245579	255031	0.054482	0.9809	no

gi|320446944|ref|NW_003383627.1|	[illegible]73378-67462[illegible]	633915	254554	-131632	0.5364	no
gi|320446944|ref|NW_003383627.1|	[illegible]74878-67570[illegible]	514412	196469	-138862	0.51645	no
gi|320446944|ref|NW_003383627.1|	[illegible]75851-67830[illegible]	99093	468436	-108093	0.63055	no
gi|320446944|ref|NW_003383627.1|	[illegible]78687-68001[illegible]	927548	772726	-0.263465	0.9005	no
gi|320446944|ref|NW_003383627.1|	[illegible]80728-68193[illegible]	864885	899413	0.0564755	0.97835	no
gi|320446944|ref|NW_003383627.1|	68855-69473	813815	448164	-0.860673	0.60995	no
gi|320446944|ref|NW_003383627.1|	[illegible]92160-69280[illegible]	106687	586664	-0.862783	0.67395	no
gi|320446944|ref|NW_003383627.1|	69695-71518	329487	281413	-0.227532	0.9111	no
gi|320446944|ref|NW_003383627.1|	[illegible]00634-70311[illegible]	244638	199767	-0.292331	0.8218	no
gi|320446944|ref|NW_003383627.1|	[illegible]04495-70634[illegible]	523841	737368	0.493257	0.81695	no
gi|320446944|ref|NW_003383627.1|	[illegible]09941-71022[illegible]	488271	467762	-0.0619063	0.9735	no
gi|320446944|ref|NW_003383627.1|	[illegible]11373-71435[illegible]	11566	178495	0.625985	0.6233	no
gi|320446944|ref|NW_003383627.1|	[illegible]15737-71796[illegible]	174032	470414	143458	0.2792	no
gi|320446944|ref|NW_003383627.1|	71654-74474	280716	378707	0.431971	0.8438	no
gi|320446944|ref|NW_003383627.1|	[illegible]22295-72323[illegible]	523085	303192	-0.786812	0.5237	no
gi|320446944|ref|NW_003383627.1|	[illegible]23708-72691[illegible]	346009	733901	108477	0.6289	no
gi|320446944|ref|NW_003383627.1|	[illegible]27807-73038[illegible]	170203	267013	-267227	0.1444	no
gi|320446944|ref|NW_003383627.1|	[illegible]34744-73534[illegible]	157656	125738	-364828	0.1947	no
gi|320446944|ref|NW_003383627.1|	[illegible]56164-75913[illegible]	221266	280509	0.342268	0.804	no
gi|320446944|ref|NW_003383627.1|	77473-79685	128049	327655	135548	0.5188	no
gi|320446944|ref|NW_003383627.1|	80146-90937	240075	439664	0.872914	0.48805	no
gi|320446944|ref|NW_003383627.1|	[illegible]14479-81572[illegible]	0.335631	39572	355953	0.18235	no
gi|320446944|ref|NW_003383627.1|	[illegible]16919-81791[illegible]	0	2143	inf	0.0233	no
gi|320446944|ref|NW_003383627.1|	[illegible]18624-81983[illegible]	0	18043	inf	0.02205	no
gi|320446944|ref|NW_003383627.1|	97607-98425	11009	159288	-278897	0.2355	no
gi|320446944|ref|NW_003383627.1|	99498-10144[illegible]	300577	654835	-219853	0.22525	no
gi|320446945|ref|NW_003383626.1|	[illegible]06974-10840[illegible]	513754	311909	-0.719955	0.5785	no
gi|320446945|ref|NW_003383626.1|	[illegible]09203-10954[illegible]	354413	367582	-326929	0.21795	no
gi|320446945|ref|NW_003383626.1|	[illegible]11257-11555[illegible]	303592	465264	-270601	0.1609	no
gi|320446945|ref|NW_003383626.1|	[illegible]32230-13456[illegible]	0.321305	213096	272949	0.256	no

gi|320446945|ref|NW_003383626.1|	134946-13591	0.23132	191606	305019	0.2493	no
gi|320446945|ref|NW_003383626.1|	136061-13742	0.297988	196613	272203	0.2412	no
gi|320446945|ref|NW_003383626.1|	137544-13813	284357	642059	1175	0.5728	no
gi|320446945|ref|NW_003383626.1|	140837-14145	481416	862176	0.840698	0.6755	no
gi|320446945|ref|NW_003383626.1|	143797-14774	154643	496653	168329	0.45425	no
gi|320446945|ref|NW_003383626.1|	14664-15594	0.485959	301634	263389	0.23605	no
gi|320446945|ref|NW_003383626.1|	147883-15022	0.881492	38043	210961	0.33765	no
gi|320446945|ref|NW_003383626.1|	150350-15115	117909	32402	145841	0.50225	no
gi|320446945|ref|NW_003383626.1|	151305-15948	327008	877054	142334	0.2726	no
gi|320446945|ref|NW_003383626.1|	160113-16256	827463	482789	-0.777302	0.56425	no
gi|320446945|ref|NW_003383626.1|	170689-17110	198257	267699	0.433239	0.82585	no
gi|320446945|ref|NW_003383626.1|	188991-18992	0.726845	551437	292348	0.2303	no
gi|320446945|ref|NW_003383626.1|	198395-19955	121091	973197	-0.315285	0.8124	no
gi|320446945|ref|NW_003383626.1|	200818-20121	359825	153585	-122825	0.55785	no
gi|320446945|ref|NW_003383626.1|	225981-22652	163248	113675	279978	0.25695	no
gi|320446945|ref|NW_003383626.1|	29205-30458	116085	345097	157182	0.4696	no
gi|320446945|ref|NW_003383626.1|	326623-32767	0.20566	284464	378991	0.2072	no
gi|320446945|ref|NW_003383626.1|	334395-33581	426636	276698	-0.624695	0.75445	no
gi|320446945|ref|NW_003383626.1|	337616-34060	312847	0.943497	-172937	0.4277	no
gi|320446945|ref|NW_003383626.1|	340763-34236	296346	0.945154	-164866	0.44225	no
gi|320446945|ref|NW_003383626.1|	343033-34596	269056	0.83108	-169485	0.43245	no
gi|320446945|ref|NW_003383626.1|	348525-34969	397674	0.751471	-24038	0.2918	no
gi|320446945|ref|NW_003383626.1|	350175-35084	196199	214035	0.125529	0.9286	no
gi|320446945|ref|NW_003383626.1|	353656-35434	0	280527	inf	0.0294	no
gi|320446945|ref|NW_003383626.1|	35601-39179	0.302865	377915	364131	0.1627	no
gi|320446945|ref|NW_003383626.1|	369475-37018	0	269607	inf	0.0294	no
gi|320446945|ref|NW_003383626.1|	381247-38409	0.388241	42061	343746	0.1722	no
gi|320446945|ref|NW_003383626.1|	388557-38989	0.610533	169567	147371	0.4972	no
gi|320446945|ref|NW_003383626.1|	413117-41364	565424	0.380455	-389353	0.31815	no
gi|320446945|ref|NW_003383626.1|	480094-48580	0.0616492	103896	739685	0.1181	no

gi|320446945|ref|NW_003383626.1|	485895-48790	0.0953533	332466	512378	0.1685	no
gi|320446945|ref|NW_003383626.1|	487984-48866	0.375105	384173	335639	0.22635	no
gi|320446945|ref|NW_003383626.1|	490282-49140	0	249849	inf	0.0212	no
gi|320446945|ref|NW_003383626.1|	501993-50288	0	177622	inf	0.0312	no
gi|320446945|ref|NW_003383626.1|	524519-52684	774452	0.112636	-610344	0.1794	no
gi|320446945|ref|NW_003383626.1|	530802-53576	265882	254495	-0.0631507	0.96095	no
gi|320446945|ref|NW_003383626.1|	536474-53748	19198	5125	-190533	0.3822	no
gi|320446945|ref|NW_003383626.1|	540368-54240	129035	352148	-187351	0.2639	no
gi|320446945|ref|NW_003383626.1|	542916-54842	202635	143328	-0.499564	0.71345	no
gi|320446945|ref|NW_003383626.1|	550764-55134	304748	698262	-212578	0.34045	no
gi|320446945|ref|NW_003383626.1|	551701-55239	622569	112602	-2467	0.15625	no
gi|320446945|ref|NW_003383626.1|	552888-55317	783583	236454	-172853	0.4276	no
gi|320446945|ref|NW_003383626.1|	554428-55600	195394	315733	-262961	0.13895	no
gi|320446945|ref|NW_003383626.1|	556164-55696	268579	0.409929	-27119	0.2844	no
gi|320446945|ref|NW_003383626.1|	56131-56843	276121	116216	-12485	0.57365	no
gi|320446945|ref|NW_003383626.1|	56949-57164	271354	408062	0.588612	0.7597	no
gi|320446945|ref|NW_003383626.1|	57306-59498	171615	12457	-0.462222	0.71735	no
gi|320446945|ref|NW_003383626.1|	592421-59291	196289	700307	183501	0.39645	no
gi|320446945|ref|NW_003383626.1|	597554-59857	705331	428413	-0.719298	0.72235	no
gi|320446945|ref|NW_003383626.1|	59894-60456	924264	971004	0.0711715	0.9678	no
gi|320446945|ref|NW_003383626.1|	60605-65963	251637	196381	-0.357689	0.7905	no
gi|320446945|ref|NW_003383626.1|	617637-61784	469764	125657	-190245	0.3084	no
gi|320446945|ref|NW_003383626.1|	66312-69002	155354	778111	-0.997509	0.558	no
gi|320446945|ref|NW_003383626.1|	69103-70400	0.476883	0.992884	105799	1	no
gi|320446945|ref|NW_003383626.1|	70588-74256	230532	19.95	-0.20858	0.8757	no
gi|320446945|ref|NW_003383626.1|	74483-75654	459954	416186	-0.144261	0.91015	no
gi|320446945|ref|NW_003383626.1|	83535-83985	175763	0.507946	-511281	0.28255	no
gi|320446945|ref|NW_003383626.1|	89605-90734	679876	130774	-23782	0.2945	no
gi|320446945|ref|NW_003383626.1|	91447-99969	189759	110517	-0.779898	0.55205	no
gi|320446946|ref|NW_003383625.1|	10150-14513	102192	149982	0.553496	0.67065	no

gi|320446946|ref|NW_003383625.1|	140031-14404	183846	485045	139962	0.38125	no
gi|320446946|ref|NW_003383625.1|	14625-16321	635412	459315	-0.468207	0.72605	no
gi|320446946|ref|NW_003383625.1|	146796-14894	520516	190818	-144775	0.5023	no
gi|320446946|ref|NW_003383625.1|	149143-14968	716396	371171	-0.948673	0.65585	no
gi|320446946|ref|NW_003383625.1|	510528-15112	384612	241752	-0.669875	0.7467	no
gi|320446946|ref|NW_003383625.1|	151286-15241	75373	443652	-0.764621	0.7142	no
gi|320446946|ref|NW_003383625.1|	516786-15175	154103	228224	-275537	0.24405	no
gi|320446946|ref|NW_003383625.1|	152648-15295	702467	790826	0.170931	0.90555	no
gi|320446946|ref|NW_003383625.1|	154632-15860	11111	202658	0.867056	0.5076	no
gi|320446946|ref|NW_003383625.1|	160026-16383	628655	159781	-197617	0.14995	no
gi|320446946|ref|NW_003383625.1|	164268-16548	122187	620454	-0.977699	0.6598	no
gi|320446946|ref|NW_003383625.1|	165803-16629	0.644706	776761	359076	0.207	no
gi|320446946|ref|NW_003383625.1|	168264-16993	0.588118	0.900743	0.61501	1	no
gi|320446946|ref|NW_003383625.1|	170370-17196	0.248592	103785	206175	1	no
gi|320446946|ref|NW_003383625.1|	171-1324	0	242059	inf	0.0212	no
gi|320446946|ref|NW_003383625.1|	172131-17349	0.14939	404634	475947	0.1731	no
gi|320446946|ref|NW_003383625.1|	175286-17576	958681	187463	0.967482	0.6392	no
gi|320446946|ref|NW_003383625.1|	184111-18484	136748	11702	-354669	0.18975	no
gi|320446946|ref|NW_003383625.1|	185333-18785	680073	402722	-0.755904	0.72575	no
gi|320446946|ref|NW_003383625.1|	188542-18923	409903	381724	-0.102754	0.95015	no
gi|320446946|ref|NW_003383625.1|	189562-19495	546735	403709	-0.437527	0.7197	no
gi|320446946|ref|NW_003383625.1|	195058-19638	222083	282466	0.346976	0.7751	no
gi|320446946|ref|NW_003383625.1|	197631-20389	103568	980946	-0.0783325	0.95435	no
gi|320446946|ref|NW_003383625.1|	205112-20847	770714	309151	-131788	0.4223	no
gi|320446946|ref|NW_003383625.1|	209012-20934	742421	360846	-104085	0.61165	no
gi|320446946|ref|NW_003383625.1|	213731-21407	430326	720433	-257849	0.26945	no
gi|320446946|ref|NW_003383625.1|	214817-21578	253756	143318	-0.824216	0.67975	no
gi|320446946|ref|NW_003383625.1|	216158-21705	136028	618893	-113614	0.58915	no
gi|320446946|ref|NW_003383625.1|	217201-21886	286647	211086	-0.441446	0.7289	no
gi|320446946|ref|NW_003383625.1|	219135-22213	542988	303275	-0.840293	0.53585	no

gi|320446946|ref|NW_003383625.1|	230618-23299	399644	368742	-343803	0.08295	no
gi|320446946|ref|NW_003383625.1|	233081-23362	927983	110341	-307213	0.1952	no
gi|320446946|ref|NW_003383625.1|	240080-24624	274204	0.996789	-478182	0.03535	no
gi|320446946|ref|NW_003383625.1|	24161-25024	215143	166602	-0.368893	0.84595	no
gi|320446946|ref|NW_003383625.1|	252365-25305	14438	531028	-144301	0.4921	no
gi|320446946|ref|NW_003383625.1|	253208-25556	478315	378464	-0.337803	0.8726	no
gi|320446946|ref|NW_003383625.1|	258369-26085	20515	17.57	-0.223562	0.8616	no
gi|320446946|ref|NW_003383625.1|	264390-26494	368186	177453	-1053	0.5963	no
gi|320446946|ref|NW_003383625.1|	267009-26825	108112	117676	0.122289	0.95405	no
gi|320446946|ref|NW_003383625.1|	273803-27717	163531	363502	11524	0.39375	no
gi|320446946|ref|NW_003383625.1|	296983-30091	314908	866969	-186088	0.1717	no
gi|320446946|ref|NW_003383625.1|	302092-30409	449686	212163	-108374	0.41425	no
gi|320446946|ref|NW_003383625.1|	308202-31031	509466	844574	0.729238	0.58205	no
gi|320446946|ref|NW_003383625.1|	320855-32415	121464	455247	-141581	0.2814	no
gi|320446946|ref|NW_003383625.1|	324258-32479	840766	430215	-0.966648	0.46435	no
gi|320446946|ref|NW_003383625.1|	342341-34299	0	144371	inf	0.0075	no
gi|320446946|ref|NW_003383625.1|	345840-34661	109277	214222	-235081	0.2941	no
gi|320446946|ref|NW_003383625.1|	346844-34788	376615	101264	-189497	0.3903	no
gi|320446946|ref|NW_003383625.1|	348016-34909	299721	0.829497	-185331	0.4028	no
gi|320446946|ref|NW_003383625.1|	349747-35462	701557	165499	123819	0.34775	no
gi|320446946|ref|NW_003383625.1|	355182-35574	969844	172407	0.829993	0.6844	no
gi|320446946|ref|NW_003383625.1|	362104-36277	0.388815	50384	369581	0.2145	no
gi|320446946|ref|NW_003383625.1|	364284-36475	986134	231777	-208905	0.22355	no
gi|320446946|ref|NW_003383625.1|	364987-36640	37718	484625	-296031	0.1188	no
gi|320446946|ref|NW_003383625.1|	367008-36732	669608	526627	-366846	0.18225	no
gi|320446946|ref|NW_003383625.1|	375161-37560	174192	0	#NAME?	5.00E-05	yes
gi|320446946|ref|NW_003383625.1|	375862-37618	256411	0	#NAME?	5.00E-05	yes
gi|320446946|ref|NW_003383625.1|	376700-37722	181676	0	#NAME?	5.00E-05	yes
gi|320446946|ref|NW_003383625.1|	377348-37786	288662	0	#NAME?	5.00E-05	yes
gi|320446946|ref|NW_003383625.1|	378175-37910	713706	0	#NAME?	5.00E-05	yes

gi|320446946|ref|NW_003383625.1|	379632-38068	273499	0	#NAME?	5.00E-05	yes
gi|320446946|ref|NW_003383625.1|	388563-38884	329479	105.9	-163749	0.3254	no
gi|320446946|ref|NW_003383625.1|	398498-40267	376407	27.76	-0.439283	0.73975	no
gi|320446946|ref|NW_003383625.1|	406291-40706	560948	681546	0.280944	0.8287	no
gi|320446946|ref|NW_003383625.1|	428022-43045	229574	270765	0.238083	0.8589	no
gi|320446946|ref|NW_003383625.1|	430577-43244	142894	104635	-0.449581	0.7783	no
gi|320446946|ref|NW_003383625.1|	433500-43406	570012	264753	-110634	0.6254	no
gi|320446946|ref|NW_003383625.1|	447145-44769	245.77	0	#NAME?	5.00E-05	yes
gi|320446946|ref|NW_003383625.1|	459470-46155	273242	266844	-0.0341839	0.98285	no
gi|320446946|ref|NW_003383625.1|	461828-46248	277877	297614	0.0989937	0.94585	no
gi|320446946|ref|NW_003383625.1|	471127-47182	254161	471486	0.891474	0.669	no
gi|320446946|ref|NW_003383625.1|	472351-47330	39871	971116	12843	0.5377	no
gi|320446946|ref|NW_003383625.1|	475040-47592	445118	13885	164127	0.44375	no
gi|320446946|ref|NW_003383625.1|	476615-47891	155357	702051	217599	0.33865	no
gi|320446946|ref|NW_003383625.1|	479055-47950	310978	407947	37135	0.17585	no
gi|320446946|ref|NW_003383625.1|	498060-49978	0.681969	150387	11409	0.58155	no
gi|320446946|ref|NW_003383625.1|	512857-51380	190273	508691	141872	0.4971	no
gi|320446946|ref|NW_003383625.1|	517516-51815	165728	507571	16148	0.4473	no
gi|320446946|ref|NW_003383625.1|	520357-52084	0	849782	inf	0.0212	no
gi|320446946|ref|NW_003383625.1|	522501-52345	0.704585	486222	278677	0.24985	no
gi|320446946|ref|NW_003383625.1|	524129-52457	0.764188	863508	349821	0.2178	no
gi|320446946|ref|NW_003383625.1|	533118-53556	762599	777528	0.0279699	0.9874	no
gi|320446946|ref|NW_003383625.1|	539314-53959	827115	278181	-489399	0.19465	no
gi|320446946|ref|NW_003383625.1|	54482-57502	187234	54.1	153079	0.26425	no
gi|320446946|ref|NW_003383625.1|	545177-54592	856033	31586	-143838	0.4915	no
gi|320446946|ref|NW_003383625.1|	546884-54878	206297	366538	-249269	0.1662	no
gi|320446946|ref|NW_003383625.1|	550036-55029	20821	754267	-14649	0.50875	no
gi|320446946|ref|NW_003383625.1|	551026-55171	187986	428071	-21347	0.33795	no
gi|320446946|ref|NW_003383625.1|	552145-55301	851077	238044	-183806	0.39985	no
gi|320446946|ref|NW_003383625.1|	565909-56699	0.198171	123417	263872	1	no

gi|320446946|ref|NW_003383625.1|	68818-57047	0.711695	148627	106236	0.59685	no
gi|320446946|ref|NW_003383625.1|	77974-57839	0	852059	inf	0.02205	no
gi|320446946|ref|NW_003383625.1|	82071-58257	183302	368877	100892	0.5772	no
gi|320446946|ref|NW_003383625.1|	82857-58444	0.124388	147136	356424	0.2162	no
gi|320446946|ref|NW_003383625.1|	91674-59307	274836	17185	26445	0.25665	no
gi|320446946|ref|NW_003383625.1|	96477-59890	169612	157201	32123	0.08995	no
gi|320446946|ref|NW_003383625.1|	00705-60422	184872	736556	-132766	0.3026	no
gi|320446946|ref|NW_003383625.1|	08104-60850	765936	126085	-260283	0.2918	no
gi|320446946|ref|NW_003383625.1|	12674-61336	231544	0	#NAME?	0.0071	no
gi|320446946|ref|NW_003383625.1|	70437-71322	961326	34003	-149937	0.48415	no
gi|320446946|ref|NW_003383625.1|	13519-71398	281528	11266	-132131	0.5362	no
gi|320446946|ref|NW_003383625.1|	15054-71549	397317	790987	0.993364	0.65895	no
gi|320446946|ref|NW_003383625.1|	15755-71612	465939	98913	108602	0.5997	no
gi|320446946|ref|NW_003383625.1|	71585-71805	104.4	46.8	-115754	0.58005	no
gi|320446946|ref|NW_003383625.1|	17265-71778	295177	396552	0.425934	0.8285	no
gi|320446946|ref|NW_003383625.1|	18113-71900	0.512532	211876	20475	0.30225	no
gi|320446946|ref|NW_003383625.1|	71907-73119	226368	104226	-111895	0.48635	no
gi|320446946|ref|NW_003383625.1|	20485-72130	523275	799125	0.610851	0.76615	no
gi|320446946|ref|NW_003383625.1|	25219-72638	236765	126179	-0.907987	0.66295	no
gi|320446946|ref|NW_003383625.1|	26561-72895	234178	35973	0.619309	0.7676	no
gi|320446946|ref|NW_003383625.1|	73258-73921	165569	833098	-0.990878	0.6448	no
gi|320446946|ref|NW_003383625.1|	37473-73784	137277	426234	163456	0.4429	no
gi|320446946|ref|NW_003383625.1|	41721-74200	348259	139.09	199779	0.3638	no
gi|320446946|ref|NW_003383625.1|	43501-74389	816366	381991	222625	0.3194	no
gi|320446946|ref|NW_003383625.1|	43993-74419	0	100526	inf	0.0138	no
gi|320446946|ref|NW_003383625.1|	44307-74474	15098	769894	23503	0.30515	no
gi|320446946|ref|NW_003383625.1|	44897-74732	115433	585748	234322	0.29615	no
gi|320446946|ref|NW_003383625.1|	74632-75322	333912	354768	0.0874078	0.9574	no
gi|320446946|ref|NW_003383625.1|	49174-74999	838719	300152	183943	0.4114	no
gi|320446946|ref|NW_003383625.1|	50432-75066	381427	185241	227992	0.31425	no

gi|320446946|ref|NW_003383625.1|	750847-75124	127764	795112	263768	0.2609	no
gi|320446946|ref|NW_003383625.1|	752112-75312	0	211298	inf	0.0233	no
gi|320446946|ref|NW_003383625.1|	760204-76198	470976	0.686674	-277795	0.2276	no
gi|320446946|ref|NW_003383625.1|	762070-76346	0.728877	0.607618	-0.26251	1	no
gi|320446946|ref|NW_003383625.1|	76479-76981	124815	104568	-0.255342	0.90355	no
gi|320446946|ref|NW_003383625.1|	770041-77095	0.249078	257554	33702	0.2261	no
gi|320446946|ref|NW_003383625.1|	771411-77198	451388	311902	278865	0.241	no
gi|320446946|ref|NW_003383625.1|	772154-77279	494782	342415	279088	0.2321	no
gi|320446946|ref|NW_003383625.1|	773151-77600	244947	181121	288641	0.12455	no
gi|320446946|ref|NW_003383625.1|	791513-79210	190622	29047	0.60767	0.7533	no
gi|320446946|ref|NW_003383625.1|	793785-79483	0.618487	484752	297043	0.2282	no
gi|320446946|ref|NW_003383625.1|	807964-80828	0	463432	inf	0.00845	no
gi|320446946|ref|NW_003383625.1|	808616-80960	0.893429	160438	416652	0.1574	no
gi|320446946|ref|NW_003383625.1|	812084-81333	0.16682	601673	517262	0.1702	no
gi|320446946|ref|NW_003383625.1|	813604-81645	0.840223	317581	524021	0.03885	no
gi|320446946|ref|NW_003383625.1|	822056-82274	338341	205331	-0.720525	0.72915	no
gi|320446946|ref|NW_003383625.1|	823127-82360	342386	594394	0.795796	0.7043	no
gi|320446946|ref|NW_003383625.1|	827352-82793	0.97196	361779	189614	0.32105	no
gi|320446946|ref|NW_003383625.1|	829420-83111	17377	459759	14037	0.50955	no
gi|320446946|ref|NW_003383625.1|	832238-83254	87086	616424	282341	0.2339	no
gi|320446946|ref|NW_003383625.1|	843539-84533	0.97064	119466	362153	0.15415	no
gi|320446946|ref|NW_003383625.1|	846179-84699	0.583428	84192	385106	0.1759	no
gi|320446946|ref|NW_003383625.1|	847870-85507	125972	130269	337032	0.08785	no
gi|320446946|ref|NW_003383625.1|	862106-86239	640564	191129	157713	0.4856	no
gi|320446946|ref|NW_003383625.1|	890929-89805	51427	301483	255148	0.0635	no
gi|320446946|ref|NW_003383625.1|	927571-92789	151313	225053	389466	0.20015	no
gi|320446946|ref|NW_003383625.1|	93372-93636	541968	137732	134558	0.38395	no
gi|320446946|ref|NW_003383625.1|	966944-96995	43832	140457	168007	0.3338	no
gi|320446946|ref|NW_003383625.1|	974279-97734	119344	563268	22387	0.3291	no
gi|320446948|ref|NW_003383623.1|	109446-11307	253417	42767	0.754985	0.7353	no

gi|320446948|ref|NW_003383623.1|	140006-14081	0	531491	inf	5.00E-05	yes
gi|320446948|ref|NW_003383623.1|	163871-16420	915002	1140.02	0.317206	0.8109	no
gi|320446948|ref|NW_003383623.1|	175443-18390	100678	236869	123434	0.5247	no
gi|320446948|ref|NW_003383623.1|	199973-20137	226325	110173	-103863	0.5287	no
gi|320446948|ref|NW_003383623.1|	204464-20466	399419	496971	0.31526	0.88145	no
gi|320446948|ref|NW_003383623.1|	205440-20562	91286	70479	-0.3732	0.85085	no
gi|320446948|ref|NW_003383623.1|	214368-21581	626747	464652	-0.431732	0.84295	no
gi|320446948|ref|NW_003383623.1|	216325-21903	628572	773409	0.299154	0.89385	no
gi|320446948|ref|NW_003383623.1|	273926-27566	978309	111754	351389	0.08975	no
gi|320446948|ref|NW_003383623.1|	275794-28723	737639	284843	194918	0.14855	no
gi|320446948|ref|NW_003383623.1|	303448-30447	0.213196	221042	337406	0.2261	no
gi|320446948|ref|NW_003383623.1|	32590-33631	358228	110078	-170235	0.312	no
gi|320446948|ref|NW_003383623.1|	35087-35365	396598	119032	-173633	0.42015	no
gi|320446948|ref|NW_003383623.1|	35493-37097	752111	205917	-186888	0.39265	no
gi|320446948|ref|NW_003383623.1|	361346-36737	0.786157	479543	260877	0.14475	no
gi|320446948|ref|NW_003383623.1|	37948-39890	128536	0.96502	-0.413543	1	no
gi|320446948|ref|NW_003383623.1|	43645-47276	905842	31884	-150642	0.26	no
gi|320446948|ref|NW_003383623.1|	47745-49342	508117	45705	-0.152809	0.9385	no
gi|320446948|ref|NW_003383623.1|	479364-48011	231958	0.895662	-469476	0.1703	no
gi|320446948|ref|NW_003383623.1|	49699-51115	135788	685291	-0.986568	0.6611	no
gi|320446948|ref|NW_003383623.1|	508565-50883	285133	32973	-311228	0.25445	no
gi|320446948|ref|NW_003383623.1|	515477-51618	116821	242116	-227052	0.31245	no
gi|320446948|ref|NW_003383623.1|	522203-52386	284278	14842	-0.937621	0.64625	no
gi|320446948|ref|NW_003383623.1|	52658-52870	344078	43075	0.324118	0.86335	no
gi|320446948|ref|NW_003383623.1|	528756-52906	178569	0	#NAME?	0.0229	no
gi|320446948|ref|NW_003383623.1|	533193-53801	50798	195949	-13743	0.39495	no
gi|320446948|ref|NW_003383623.1|	53364-54576	20736	131781	-0.653999	0.7491	no
gi|320446948|ref|NW_003383623.1|	55999-58314	137934	24922	0.853439	0.6774	no
gi|320446948|ref|NW_003383623.1|	569374-56967	417135	486682	-309946	0.2255	no
gi|320446948|ref|NW_003383623.1|	570212-57129	318195	261454	-0.283355	0.88315	no

gi|320446948|ref|NW_003383623.1|	71520-57245	817885	365051	-11638	0.5748	no
gi|320446948|ref|NW_003383623.1|	73039-57379	227811	17846	-0.352234	0.8554	no
gi|320446948|ref|NW_003383623.1|	74724-57727	147256	132904	-0.147938	0.90965	no
gi|320446948|ref|NW_003383623.1|	77972-58446	415758	18779	-114663	0.3897	no
gi|320446948|ref|NW_003383623.1|	84779-58570	248632	746833	-173515	0.4394	no
gi|320446948|ref|NW_003383623.1|	93270-59656	171011	0.424438	-201047	0.35995	no
gi|320446948|ref|NW_003383623.1|	29754-63028	450873	227562	-0.98646	0.66985	no
gi|320446948|ref|NW_003383623.1|	50794-65158	245631	303979	0.30748	0.8941	no
gi|320446948|ref|NW_003383623.1|	52090-65339	498273	384038	-0.375687	0.7725	no
gi|320446948|ref|NW_003383623.1|	53505-65552	124738	342688	-186394	0.27065	no
gi|320446948|ref|NW_003383623.1|	55740-65615	170383	710513	-126185	0.5516	no
gi|320446948|ref|NW_003383623.1|	56595-65864	109726	102472	-0.0986804	0.967	no
gi|320446948|ref|NW_003383623.1|	59664-66046	448426	268969	-0.737431	0.6431	no
gi|320446948|ref|NW_003383623.1|	60635-66102	878787	301947	-154122	0.47555	no
gi|320446948|ref|NW_003383623.1|	64530-66494	208293	149426	-0.479185	0.8146	no
gi|320446948|ref|NW_003383623.1|	66153-66761	923248	613026	-0.59077	0.78225	no
gi|320446948|ref|NW_003383623.1|	67939-66931	930445	297496	-164505	0.4381	no
gi|320446948|ref|NW_003383623.1|	70956-67118	443197	222989	-0.99098	0.66335	no
gi|320446948|ref|NW_003383623.1|	71308-67287	114051	546595	-106114	0.6222	no
gi|320446948|ref|NW_003383623.1|	73521-67463	519798	506441	-0.0375569	0.9806	no
gi|320446948|ref|NW_003383623.1|	75355-67919	570587	713596	0.322662	0.8387	no
gi|320446948|ref|NW_003383623.1|	79315-68201	882797	0.908549	-328045	0.0993	no
gi|320446948|ref|NW_003383623.1|	68599-69140	197134	140189	-0.491806	0.8104	no
gi|320446948|ref|NW_003383623.1|	91703-69308	149167	26672	-248352	0.28635	no
gi|320446948|ref|NW_003383623.1|	05148-70575	0	306367	inf	0.0312	no
gi|320446948|ref|NW_003383623.1|	24092-72720	345587	827376	12595	0.4396	no
gi|320446948|ref|NW_003383623.1|	28217-72903	988409	559387	-0.82126	0.6879	no
gi|320446948|ref|NW_003383623.1|	35568-73703	15221	108668	-0.486134	0.83165	no
gi|320446948|ref|NW_003383623.1|	74133-77566	41158	885949	110605	0.5109	no
gi|320446948|ref|NW_003383623.1|	44550-74574	659179	425322	-395405	0.05695	no

gi|320446948|ref|NW_003383623.1|	747058-74735	791211	495061	-399839	0.1826	no
gi|320446948|ref|NW_003383623.1|	747446-74786	149514	697404	-110021	0.59335	no
gi|320446948|ref|NW_003383623.1|	748090-74921	832751	36415	-119335	0.3652	no
gi|320446948|ref|NW_003383623.1|	750052-75086	396731	100229	-530679	0.1125	no
gi|320446948|ref|NW_003383623.1|	752513-75273	890264	656691	-376095	0.21745	no
gi|320446948|ref|NW_003383623.1|	752873-75371	141437	0	#NAME?	0.00635	no
gi|320446948|ref|NW_003383623.1|	755314-75566	190791	0.827931	-452634	0.2941	no
gi|320446948|ref|NW_003383623.1|	756919-75767	143748	134349	-341949	0.1837	no
gi|320446948|ref|NW_003383623.1|	758398-76191	378536	176322	-110222	0.4181	no
gi|320446948|ref|NW_003383623.1|	762969-76542	243043	238684	-0.0261118	0.98445	no
gi|320446948|ref|NW_003383623.1|	774310-77914	214497	305874	0.511982	0.70165	no
gi|320446948|ref|NW_003383623.1|	782718-78311	439931	443788	0.0125928	0.99245	no
gi|320446948|ref|NW_003383623.1|	783306-78418	0.261428	360101	378392	0.2072	no
gi|320446948|ref|NW_003383623.1|	798360-79868	160788	933748	-0.784051	0.70035	no
gi|320446948|ref|NW_003383623.1|	826455-82665	139955	567347	201927	0.301	no
gi|320446948|ref|NW_003383623.1|	832410-83269	217662	13909	267586	0.2703	no
gi|320446948|ref|NW_003383623.1|	838957-84052	189943	616664	169892	0.429	no
gi|320446948|ref|NW_003383623.1|	842215-84262	185702	489737	139902	0.38125	no
gi|320446948|ref|NW_003383623.1|	846995-84822	371782	738322	0.989792	0.6399	no
gi|320446948|ref|NW_003383623.1|	855245-85695	0.458663	287439	264775	0.26025	no
gi|320446948|ref|NW_003383623.1|	859345-86101	0	10682	inf	1	no
gi|320446948|ref|NW_003383623.1|	861346-86287	0.261703	23661	317651	0.20465	no
gi|320446948|ref|NW_003383623.1|	863027-86643	324107	192537	257059	0.16905	no
gi|320446948|ref|NW_003383623.1|	895135-89551	305614	28593	-341798	0.2095	no
gi|320446948|ref|NW_003383623.1|	89559-90703	612928	501712	-0.288857	0.88445	no
gi|320446948|ref|NW_003383623.1|	91764-92187	949123	912993	-0.0559912	0.96245	no
gi|320446948|ref|NW_003383623.1|	96319-97121	0.297895	184149	2628	0.26355	no
gi|320446949|ref|NW_003383622.1|	133796-13672	851915	0.175158	-560398	0.1295	no
gi|320446949|ref|NW_003383622.1|	149316-15047	348676	0	#NAME?	0.01335	no
gi|320446949|ref|NW_003383622.1|	169754-17028	166682	0	#NAME?	0.0099	no

gi|320446949|ref|NW_003383622.1|	230354-23103	0.765396	443934	253607	0.25045	no
gi|320446949|ref|NW_003383622.1|	231142-23191	315132	994399	165787	0.43005	no
gi|320446949|ref|NW_003383622.1|	237362-23814	0.30767	190054	262696	0.26355	no
gi|320446949|ref|NW_003383622.1|	272565-27314	680372	151289	115291	0.57435	no
gi|320446949|ref|NW_003383622.1|	273822-27543	489255	680966	0.476998	0.81825	no
gi|320446949|ref|NW_003383622.1|	290274-29062	530761	320887	-0.725997	0.72435	no
gi|320446949|ref|NW_003383622.1|	337204-33915	0.885712	143396	401703	0.0682	no
gi|320446949|ref|NW_003383622.1|	343420-34422	0.890539	570938	268058	0.2604	no
gi|320446949|ref|NW_003383622.1|	34762-35455	77406	276987	-148263	0.49425	no
gi|320446949|ref|NW_003383622.1|	36638-37831	810655	232079	-180447	0.4076	no
gi|320446949|ref|NW_003383622.1|	383050-38413	178564	0.823731	-111619	0.58925	no
gi|320446949|ref|NW_003383622.1|	38491-39021	453815	190824	-124986	0.5558	no
gi|320446949|ref|NW_003383622.1|	386860-38782	0.46455	32064	278705	0.2345	no
gi|320446949|ref|NW_003383622.1|	39149-40027	499034	271313	-0.879181	0.66455	no
gi|320446949|ref|NW_003383622.1|	392364-39354	0.356555	111193	164087	1	no
gi|320446949|ref|NW_003383622.1|	395605-39979	86184	463592	242736	0.07825	no
gi|320446949|ref|NW_003383622.1|	403508-40401	366603	131156	183899	0.3975	no
gi|320446949|ref|NW_003383622.1|	433660-43394	263741	77224	154993	0.46595	no
gi|320446949|ref|NW_003383622.1|	54941-55327	625906	109513	0.807089	0.6926	no
gi|320446949|ref|NW_003383622.1|	562634-56843	760907	220723	153645	0.24775	no
gi|320446949|ref|NW_003383622.1|	7092-8861	0	30657	inf	0.00815	no
gi|320446949|ref|NW_003383622.1|	757481-75819	212845	363912	0.773787	0.7039	no
gi|320446951|ref|NW_003383620.1|	15622-16417	54292	186402	177961	0.4186	no
gi|320446951|ref|NW_003383620.1|	18549-19089	219732	703079	167794	0.4461	no
gi|320446951|ref|NW_003383620.1|	335988-33643	201264	411402	103146	0.61975	no
gi|320446951|ref|NW_003383620.1|	37375-37947	698098	707986	0.020293	0.97265	no
gi|320446951|ref|NW_003383620.1|	406328-40669	0	907751	inf	0.029	no
gi|320446951|ref|NW_003383620.1|	416961-41794	0	170582	inf	0.0294	no
gi|320446951|ref|NW_003383620.1|	418118-41902	0.25281	243918	327027	0.23045	no
gi|320446951|ref|NW_003383620.1|	420317-42398	0.442257	532991	359116	0.152	no

gi|320446951|ref|NW_003383620.1|	428262-42973	0.13613	27445	433348	0.1826	no
gi|320446951|ref|NW_003383620.1|	429856-43136	0	15712	inf	0.02075	no
gi|320446951|ref|NW_003383620.1|	432421-43264	425479	193029	218166	0.32455	no
gi|320446951|ref|NW_003383620.1|	706767-70716	0	110453	inf	0.02075	no
gi|320446951|ref|NW_003383620.1|	709470-70979	0	157686	inf	0.0198	no
gi|320446951|ref|NW_003383620.1|	713470-71416	0	7806	inf	0.01195	no
gi|320446951|ref|NW_003383620.1|	738079-73865	314579	0.332506	-65639	0.26405	no
gi|320446951|ref|NW_003383620.1|	742668-74360	35261	0	#NAME?	5.00E-05	yes
gi|320446951|ref|NW_003383620.1|	744732-74998	115429	834624	-0.467813	0.72305	no
gi|320446951|ref|NW_003383620.1|	753533-75428	457381	403048	-0.182446	0.9251	no
gi|320446951|ref|NW_003383620.1|	755128-75577	574471	363173	-0.661576	0.7448	no
gi|320446951|ref|NW_003383620.1|	756532-75785	651073	333608	-0.964666	0.64935	no
gi|320446951|ref|NW_003383620.1|	758379-75875	67426	294039	-11973	0.5758	no
gi|320446951|ref|NW_003383620.1|	759605-76010	573917	0.854048	-274845	0.28295	no
gi|320446951|ref|NW_003383620.1|	761587-76262	528318	116872	-217648	0.3228	no
gi|320446951|ref|NW_003383620.1|	771600-77280	433331	0.841155	-236503	0.29435	no
gi|320446951|ref|NW_003383620.1|	816000-81676	0	750009	inf	0.0101	no
gi|320446951|ref|NW_003383620.1|	826215-83011	0	750874	inf	5.00E-05	yes
gi|320446953|ref|NW_003383618.1|	100320-10097	125608	263294	-225419	0.21105	no
gi|320446953|ref|NW_003383618.1|	103078-10607	922583	128149	0.474067	0.70985	no
gi|320446953|ref|NW_003383618.1|	106471-10836	233992	283639	0.277598	0.8875	no
gi|320446953|ref|NW_003383618.1|	108586-11148	68944	92408	0.422593	0.7915	no
gi|320446953|ref|NW_003383618.1|	113963-11440	127705	370695	-178451	0.40555	no
gi|320446953|ref|NW_003383618.1|	114521-11569	992059	449931	-114072	0.5882	no
gi|320446953|ref|NW_003383618.1|	163340-11644	0	204276	inf	0.004	no
gi|320446953|ref|NW_003383618.1|	173686-11808	16554	249525	0.592	0.6584	no
gi|320446953|ref|NW_003383618.1|	192647-11932	162763	149388	-0.123708	0.952	no
gi|320446953|ref|NW_003383618.1|	201588-12023	127441	123479	-0.0455564	0.9804	no
gi|320446953|ref|NW_003383618.1|	202735-12049	543895	331612	-0.713831	0.5917	no
gi|320446953|ref|NW_003383618.1|	212420-12126	124571	297346	-206675	0.35055	no

gi|320446953|ref|NW_003383618.1|	213087-12138	497343	128044	-195761	0.24475	no
gi|320446953|ref|NW_003383618.1|	215719-12182	935957	256501	-186748	0.4001	no
gi|320446953|ref|NW_003383618.1|	12169-13821	223704	0.331668	-939764	0.1293	no
gi|320446953|ref|NW_003383618.1|	220514-12226	442414	704853	-2.65	0.1656	no
gi|320446953|ref|NW_003383618.1|	223808-12246	470473	124494	-191803	0.26255	no
gi|320446953|ref|NW_003383618.1|	225198-12320	218109	133522	-0.707972	0.5975	no
gi|320446953|ref|NW_003383618.1|	232365-12341	104067	332284	-164702	0.45835	no
gi|320446953|ref|NW_003383618.1|	234305-12350	82512	379323	-112118	0.50695	no
gi|320446953|ref|NW_003383618.1|	237315-12385	101962	584301	-0.803243	0.55425	no
gi|320446953|ref|NW_003383618.1|	238945-12436	160122	276351	0.787328	0.5568	no
gi|320446953|ref|NW_003383618.1|	244220-12449	501883	833331	0.731539	0.7247	no
gi|320446953|ref|NW_003383618.1|	246949-12480	656278	770601	0.231679	0.9112	no
gi|320446953|ref|NW_003383618.1|	257919-12584	521224	562908	0.110995	0.94785	no
gi|320446953|ref|NW_003383618.1|	272495-12731	245691	234372	-0.0680465	0.9733	no
gi|320446953|ref|NW_003383618.1|	284346-12854	36617	886485	127558	0.55605	no
gi|320446953|ref|NW_003383618.1|	286010-12870	714793	749454	0.0683152	0.9602	no
gi|320446953|ref|NW_003383618.1|	287612-12936	100354	512518	-0.969415	0.5607	no
gi|320446953|ref|NW_003383618.1|	295174-12981	337017	218054	-0.628133	0.6359	no
gi|320446953|ref|NW_003383618.1|	309192-13096	281176	129701	-111629	0.58805	no
gi|320446953|ref|NW_003383618.1|	311429-13127	0.159412	121692	293241	1	no
gi|320446953|ref|NW_003383618.1|	312833-13141	158662	209219	0.39906	0.8445	no
gi|320446953|ref|NW_003383618.1|	316037-13166	225046	837375	-142628	0.49505	no
gi|320446953|ref|NW_003383618.1|	317112-13201	161533	203973	0.33655	0.7983	no
gi|320446953|ref|NW_003383618.1|	326390-13269	185571	84756	-113058	0.58925	no
gi|320446953|ref|NW_003383618.1|	334603-13350	191736	457226	-206814	0.34935	no
gi|320446953|ref|NW_003383618.1|	336431-13367	459139	511079	-316731	0.21935	no
gi|320446953|ref|NW_003383618.1|	336931-13379	129408	198886	-270191	0.23985	no
gi|320446953|ref|NW_003383618.1|	347179-13485	176455	112357	-0.651213	0.75415	no
gi|320446953|ref|NW_003383618.1|	348662-13519	402184	300829	-0.418915	0.84735	no
gi|320446953|ref|NW_003383618.1|	352233-13526	296632	117001	-134215	0.5266	no

gi|320446953|ref|NW_003383618.1|	355272-13557	151709	128325	-0.241506	0.90405	no
gi|320446953|ref|NW_003383618.1|	356195-13588	349687	49352	0.497045	0.8159	no
gi|320446953|ref|NW_003383618.1|	359089-13595	301808	401429	0.411511	0.8506	no
gi|320446953|ref|NW_003383618.1|	359660-13624	76796	404837	-0.923691	0.6803	no
gi|320446953|ref|NW_003383618.1|	363914-13643	152417	134503	-0.180386	0.92765	no
gi|320446953|ref|NW_003383618.1|	385100-13873	713169	130532	-244984	0.07325	no
gi|320446953|ref|NW_003383618.1|	388523-13888	93459	108308	-31092	0.2016	no
gi|320446953|ref|NW_003383618.1|	396198-13997	154856	194373	-299403	0.11595	no
gi|320446953|ref|NW_003383618.1|	402454-14052	493728	333917	-0.564226	0.6738	no
gi|320446953|ref|NW_003383618.1|	421870-14224	0	431073	inf	0.02915	no
gi|320446953|ref|NW_003383618.1|	545369-15459	0.902983	27573	161049	0.33275	no
gi|320446953|ref|NW_003383618.1|	556620-15575	0.247253	221608	316395	0.24575	no
gi|320446953|ref|NW_003383618.1|	16334-16766	115013	0	#NAME?	5.00E-05	yes
gi|320446953|ref|NW_003383618.1|	18723-19068	0	810302	inf	0.004	no
gi|320446953|ref|NW_003383618.1|	19333-19670	0	385961	inf	0.0069	no
gi|320446953|ref|NW_003383618.1|	21073-26319	0.0673461	424638	59785	0.11825	no
gi|320446953|ref|NW_003383618.1|	26681-27687	0.657025	287494	212951	0.3464	no
gi|320446953|ref|NW_003383618.1|	306393-31066	711281	957935	0.429509	0.7359	no
gi|320446953|ref|NW_003383618.1|	320014-32080	785623	684672	-0.198424	0.92055	no
gi|320446953|ref|NW_003383618.1|	33974-34969	0.888723	322283	185852	0.405	no
gi|320446953|ref|NW_003383618.1|	37116-39352	902459	24134	141914	0.4192	no
gi|320446953|ref|NW_003383618.1|	43681-45349	106009	138578	370844	0.14605	no
gi|320446953|ref|NW_003383618.1|	578456-57933	162337	703266	-120685	0.5672	no
gi|320446953|ref|NW_003383618.1|	58752-60168	214402	0.0993176	-443213	0.2969	no
gi|320446953|ref|NW_003383618.1|	60563-61546	776574	0	#NAME?	5.00E-05	yes
gi|320446953|ref|NW_003383618.1|	608858-60985	0.221306	101661	884351	0.14085	no
gi|320446953|ref|NW_003383618.1|	62693-63289	293714	0	#NAME?	0.0053	no
gi|320446953|ref|NW_003383618.1|	769913-77036	693687	30728	-117473	0.57405	no
gi|320446953|ref|NW_003383618.1|	77369-79218	149644	0.145744	-100039	0.16165	no
gi|320446953|ref|NW_003383618.1|	7860-9970	124216	0.439555	-814259	0.08495	no

gi|320446953|ref|NW_003383618.1|	796165-80070	764146	134194	0.812403	0.54415	no
gi|320446953|ref|NW_003383618.1|	327414-82769	209964	743947	-149687	0.4986	no
gi|320446953|ref|NW_003383618.1|	341618-84293	0	140795	inf	0.0044	no
gi|320446953|ref|NW_003383618.1|	84314-91107	691491	524.64	-0.398383	0.1318	no
gi|320446953|ref|NW_003383618.1|	93101-97263	214977	97228	-114474	0.47435	no
gi|320446953|ref|NW_003383618.1|	975005-97546	145967	199275	377105	0.175	no
gi|320446953|ref|NW_003383618.1|	97686-98183	149314	289368	-236737	0.1882	no
gi|320446953|ref|NW_003383618.1|	986052-98935	0.881486	45086	235467	0.29615	no
gi|320446953|ref|NW_003383618.1|	98783-98972	662197	351942	-0.911921	0.6772	no
gi|320446953|ref|NW_003383618.1|	990115-99223	0.269173	388087	384977	0.17355	no
gi|320446954|ref|NW_003383617.1|	110832-11301	251727	114689	-113413	0.37955	no
gi|320446954|ref|NW_003383617.1|	113147-11466	264161	202071	-0.386558	0.7601	no
gi|320446954|ref|NW_003383617.1|	115841-11881	193864	714238	-144056	0.4154	no
gi|320446954|ref|NW_003383617.1|	121133-12132	654026	305026	-110042	0.59055	no
gi|320446954|ref|NW_003383617.1|	122366-12404	204275	536369	-192921	0.26335	no
gi|320446954|ref|NW_003383617.1|	134274-13636	0.0908356	519588	583797	0.16095	no
gi|320446954|ref|NW_003383617.1|	145359-14595	544219	275192	-0.983751	0.6623	no
gi|320446954|ref|NW_003383617.1|	148328-14959	165221	567451	-154183	0.4869	no
gi|320446954|ref|NW_003383617.1|	150594-15317	152633	203692	0.416323	0.7422	no
gi|320446954|ref|NW_003383617.1|	159961-16047	218436	15471	-0.497641	0.8083	no
gi|320446954|ref|NW_003383617.1|	167117-16750	185707	203172	0.129674	0.949	no
gi|320446954|ref|NW_003383617.1|	168583-16943	329008	641409	0.96312	0.6412	no
gi|320446954|ref|NW_003383617.1|	169626-17353	33087	762314	120412	0.46885	no
gi|320446954|ref|NW_003383617.1|	2134-2780	369303	123758	506658	0.05395	no
gi|320446954|ref|NW_003383617.1|	246984-24752	165322	445405	142984	0.50885	no
gi|320446954|ref|NW_003383617.1|	262077-26236	13187	112326	-0.23143	0.8998	no
gi|320446954|ref|NW_003383617.1|	276477-27986	310711	723265	121895	0.46465	no
gi|320446954|ref|NW_003383617.1|	295356-30013	456751	100395	113621	0.37225	no
gi|320446954|ref|NW_003383617.1|	300237-30082	235664	351266	0.575829	0.79895	no
gi|320446954|ref|NW_003383617.1|	401533-40369	148889	336104	117467	0.57095	no

gi|320446954|ref|NW_003383617.1|	04970-40662	0.952275	165718	0.799282	0.6976	no
gi|320446954|ref|NW_003383617.1|	77680-48155	407488	509742	0.323008	0.8075	no
gi|320446954|ref|NW_003383617.1|	81806-48365	326269	247765	-0.397088	0.7594	no
gi|320446954|ref|NW_003383617.1|	87046-49198	320328	477658	0.576431	0.66565	no
gi|320446954|ref|NW_003383617.1|	92113-49275	166536	283312	0.766561	0.72055	no
gi|320446954|ref|NW_003383617.1|	93812-49531	8.4	472835	-0.829052	0.6887	no
gi|320446954|ref|NW_003383617.1|	95514-49590	28.96	164102	-0.819475	0.69	no
gi|320446954|ref|NW_003383617.1|	96045-49709	145738	273524	0.908285	0.6615	no
gi|320446954|ref|NW_003383617.1|	98214-49942	221023	980588	-117248	0.3804	no
gi|320446954|ref|NW_003383617.1|	99607-50064	388312	102507	-192149	0.1529	no
gi|320446954|ref|NW_003383617.1|	00777-50128	14152	288841	-229266	0.31865	no
gi|320446954|ref|NW_003383617.1|	03522-50379	397252	948524	-20663	0.34845	no
gi|320446954|ref|NW_003383617.1|	05629-50711	146959	328088	-216326	0.3404	no
gi|320446954|ref|NW_003383617.1|	11849-51564	1239.96	377887	-171426	0.30045	no
gi|320446954|ref|NW_003383617.1|	18705-52013	578196	225408	-135902	0.5196	no
gi|320446954|ref|NW_003383617.1|	20838-52163	300548	0.825569	-186413	0.40745	no
gi|320446954|ref|NW_003383617.1|	21817-52199	115413	15802	-286863	0.27425	no
gi|320446954|ref|NW_003383617.1|	25649-52701	255683	181726	-0.492592	0.69145	no
gi|320446954|ref|NW_003383617.1|	27230-52910	188902	143104	-0.400575	0.74205	no
gi|320446954|ref|NW_003383617.1|	29896-53040	797091	123372	-269172	0.2269	no
gi|320446954|ref|NW_003383617.1|	31438-53187	121336	268198	-217763	0.33635	no
gi|320446954|ref|NW_003383617.1|	32644-53326	202354	74694	-143782	0.4935	no
gi|320446954|ref|NW_003383617.1|	39098-53956	218184	187766	-353854	0.2042	no
gi|320446954|ref|NW_003383617.1|	40058-54039	167743	362642	-220964	0.34435	no
gi|320446954|ref|NW_003383617.1|	40570-54211	8093	160843	-233102	0.29035	no
gi|320446954|ref|NW_003383617.1|	44454-54596	145289	0.734804	-0.983493	0.65595	no
gi|320446954|ref|NW_003383617.1|	46160-54723	262243	0.418656	-264707	0.22925	no
gi|320446954|ref|NW_003383617.1|	50092-55146	296939	123744	-12628	0.5602	no
gi|320446954|ref|NW_003383617.1|	51846-55232	233727	100965	-121098	0.56255	no
gi|320446954|ref|NW_003383617.1|	53379-55513	755093	208826	-185435	0.40165	no

gi|320446954|ref|NW_003383617.1|	555349-55560[illegible]	331165	152646	-111736	0.5849	no
gi|320446954|ref|NW_003383617.1|	558018-55842[illegible]	839895	0.615145	-377121	0.32265	no
gi|320446954|ref|NW_003383617.1|	560022-56073[illegible]	141012	361707	1359	0.5258	no
gi|320446954|ref|NW_003383617.1|	561178-56383[illegible]	216089	745152	178591	0.4358	no
gi|320446954|ref|NW_003383617.1|	565659-56623[illegible]	248601	57154	120102	0.5735	no
gi|320446954|ref|NW_003383617.1|	575118-57585[illegible]	168891	208183	0.301761	0.876	no
gi|320446954|ref|NW_003383617.1|	529605-63079[illegible]	106516	258372	127838	0.5446	no
gi|320446954|ref|NW_003383617.1|	718976-71989[illegible]	16961	661019	-135946	0.5287	no
gi|320446954|ref|NW_003383617.1|	722500-72347[illegible]	543754	107706	-233585	0.1932	no
gi|320446954|ref|NW_003383617.1|	723689-72489[illegible]	445661	112667	-198388	0.25455	no
gi|320446954|ref|NW_003383617.1|	726490-72915[illegible]	523326	371478	-0.494433	0.709	no
gi|320446954|ref|NW_003383617.1|	733361-73594[illegible]	632492	296234	-109431	0.6155	no
gi|320446954|ref|NW_003383617.1|	736381-73875[illegible]	520246	506332	-0.0391098	0.9845	no
gi|320446954|ref|NW_003383617.1|	739858-74095[illegible]	137109	0.813366	-0.753351	1	no
gi|320446954|ref|NW_003383617.1|	741141-74200[illegible]	187058	0.919819	-102406	0.59645	no
gi|320446954|ref|NW_003383617.1|	743087-74355[illegible]	391035	605631	-269079	0.2519	no
gi|320446954|ref|NW_003383617.1|	743837-74417[illegible]	438103	115522	-19231	0.38325	no
gi|320446954|ref|NW_003383617.1|	744299-74494[illegible]	529617	0.83229	-266979	0.22755	no
gi|320446954|ref|NW_003383617.1|	747420-74988[illegible]	659882	158909	-205401	0.36055	no
gi|320446954|ref|NW_003383617.1|	750095-75050[illegible]	111986	116877	0.0616782	0.9645	no
gi|320446954|ref|NW_003383617.1|	751312-75151[illegible]	479632	427492	-0.166032	0.92605	no
gi|320446954|ref|NW_003383617.1|	768730-76908[illegible]	753352	89947	0.255752	0.9	no
gi|320446954|ref|NW_003383617.1|	769488-77060[illegible]	190561	40901	110188	0.594	no
gi|320446954|ref|NW_003383617.1|	770740-77271[illegible]	126097	104829	305543	0.1947	no
gi|320446954|ref|NW_003383617.1|	773266-77478[illegible]	381243	157251	204429	0.37105	no
gi|320446954|ref|NW_003383617.1|	775232-77800[illegible]	239655	160039	27394	0.14115	no
gi|320446954|ref|NW_003383617.1|	779253-78308[illegible]	174983	925906	-0.918281	0.48045	no
gi|320446954|ref|NW_003383617.1|	784504-78820[illegible]	117922	135034	0.195495	0.8818	no
gi|320446954|ref|NW_003383617.1|	788400-79063[illegible]	101059	0.822863	-0.296472	1	no
gi|320446954|ref|NW_003383617.1|	791781-79206[illegible]	20373	144492	-38176	0.3201	no

gi|320446954|ref|NW_003383617.1|	793124-794379	148959	113661	-0.390176	0.8547	no
gi|320446954|ref|NW_003383617.1|	794554-798420	258855	352919	0.447193	0.7377	no
gi|320446954|ref|NW_003383617.1|	799966-800717	392799	0.448676	-313005	0.255	no
gi|320446954|ref|NW_003383617.1|	830108-832025	0.200688	230826	352378	0.18205	no
gi|320446954|ref|NW_003383617.1|	85505-88186	0.0689936	467612	940463	0.14075	no
gi|320446955|ref|NW_003383616.1|	177605-179250	272974	929321	-155451	0.36115	no
gi|320446955|ref|NW_003383616.1|	306229-307050	23058	198111	-0.218958	0.91015	no
gi|320446955|ref|NW_003383616.1|	316316-317351	0.210536	174661	305242	0.2493	no
gi|320446955|ref|NW_003383616.1|	449495-450691	404061	146124	-146738	0.50005	no
gi|320446955|ref|NW_003383616.1|	462109-465623	118365	140423	0.246543	1	no
gi|320446955|ref|NW_003383616.1|	472272-476693	314422	152426	-10446	0.6304	no
gi|320446955|ref|NW_003383616.1|	476813-478702	561239	66144	0.236997	0.9127	no
gi|320446955|ref|NW_003383616.1|	480280-480850	389419	0	#NAME?	0.0047	no
gi|320446955|ref|NW_003383616.1|	481834-483147	908281	0.108701	-63847	0.268	no
gi|320446955|ref|NW_003383616.1|	489957-490193	100603	0	#NAME?	0.00975	no
gi|320446955|ref|NW_003383616.1|	514130-517737	17265	192241	0.155068	0.90605	no
gi|320446955|ref|NW_003383616.1|	520780-524669	458573	244686	-0.906221	0.49805	no
gi|320446955|ref|NW_003383616.1|	530644-531525	14.64	306086	-22579	0.3122	no
gi|320446955|ref|NW_003383616.1|	535788-536130	177979	177726	-332398	0.23995	no
gi|320446955|ref|NW_003383616.1|	558729-560605	629455	215046	-487139	0.03355	no
gi|320446955|ref|NW_003383616.1|	562475-563622	75901	269276	-481696	0.04075	no
gi|320446955|ref|NW_003383616.1|	566544-569127	119455	286192	-538334	0.0258	no
gi|320446955|ref|NW_003383616.1|	569615-570562	96055	0.490922	-761222	0.1106	no
gi|320446955|ref|NW_003383616.1|	572050-572892	706738	0.382784	-75285	0.16165	no
gi|320446955|ref|NW_003383616.1|	573069-573429	425704	317242	-374619	0.19165	no
gi|320446955|ref|NW_003383616.1|	576039-577158	315137	0	#NAME?	5.00E-05	yes
gi|320446955|ref|NW_003383616.1|	581612-581994	257107	0	#NAME?	5.00E-05	yes
gi|320446955|ref|NW_003383616.1|	587509-593230	116749	604977	237347	0.184	no
gi|320446955|ref|NW_003383616.1|	593376-594021	0.82265	448023	244522	0.2485	no
gi|320446955|ref|NW_003383616.1|	594206-595650	0.972531	260728	142273	0.4994	no

gi|320446955|ref|NW_003383616.1|	96932-59775	828774	0.982345	-307668	0.2237	no
gi|320446955|ref|NW_003383616.1|	99846-60052	653525	0.786839	-30541	0.1963	no
gi|320446955|ref|NW_003383616.1|	01951-60220	301059	0	#NAME?	0.0229	no
gi|320446955|ref|NW_003383616.1|	16028-62039	616198	908657	0.560344	0.6623	no
gi|320446955|ref|NW_003383616.1|	25956-62674	184602	14782	-0.320576	0.87815	no
gi|320446955|ref|NW_003383616.1|	27094-62820	154721	107101	-0.530692	0.7917	no
gi|320446955|ref|NW_003383616.1|	29390-63048	781667	41928	-0.898641	0.66545	no
gi|320446955|ref|NW_003383616.1|	31236-63194	320608	0.730794	-213327	0.27995	no
gi|320446955|ref|NW_003383616.1|	34270-63549	160622	248814	-269053	0.2499	no
gi|320446955|ref|NW_003383616.1|	37951-63847	391556	78544	-231765	0.30855	no
gi|320446955|ref|NW_003383616.1|	38624-63898	359485	121155	-156908	0.463	no
gi|320446955|ref|NW_003383616.1|	47512-64835	0.279627	653901	45475	0.1778	no
gi|320446955|ref|NW_003383616.1|	49277-64956	443883	212619	226002	0.2734	no
gi|320446955|ref|NW_003383616.1|	57618-65850	119149	128465	0.108609	0.96	no
gi|320446955|ref|NW_003383616.1|	97339-79843	531318	449408	-0.241551	0.9048	no
gi|320446955|ref|NW_003383616.1|	52473-85277	620264	313681	-0.983584	0.6411	no
gi|320446957|ref|NW_003383614.1|	38995-13966	0.38793	291053	290742	0.25645	no
gi|320446957|ref|NW_003383614.1|	86838-18712	448271	314837	281216	0.2312	no
gi|320446957|ref|NW_003383614.1|	98408-19933	0.487363	50415	337078	0.1919	no
gi|320446957|ref|NW_003383614.1|	91666-29272	143612	184459	0.361125	0.85765	no
gi|320446957|ref|NW_003383614.1|	33384-33421	162347	158614	-0.0335614	0.98535	no
gi|320446957|ref|NW_003383614.1|	41240-34217	259988	620026	-206804	0.35895	no
gi|320446957|ref|NW_003383614.1|	47577-34843	130965	713461	-0.876273	0.6739	no
gi|320446957|ref|NW_003383614.1|	51733-35576	103924	501703	-105062	0.41795	no
gi|320446957|ref|NW_003383614.1|	55876-36404	188994	258756	0.45325	0.7979	no
gi|320446957|ref|NW_003383614.1|	68366-37034	752272	903721	0.264623	0.843	no
gi|320446957|ref|NW_003383614.1|	84925-38530	318248	194757	261345	0.2648	no
gi|320446957|ref|NW_003383614.1|	92715-39299	0	193426	inf	0.02915	no
gi|320446957|ref|NW_003383614.1|	96643-39704	285746	188194	271941	0.2536	no
gi|320446957|ref|NW_003383614.1|	99970-40313	126534	143996	350843	0.07435	no

gi|320446957|ref|NW_003383614.1|	04463-40481	0	114478	inf	0.0233	no
gi|320446957|ref|NW_003383614.1|	06132-41218	38579	113497	48787	0.02905	no
gi|320446957|ref|NW_003383614.1|	19160-41993	0	12561	inf	0.00715	no
gi|320446957|ref|NW_003383614.1|	20054-42108	0	102897	inf	0.00565	no
gi|320446957|ref|NW_003383614.1|	21487-42462	0.05807	128322	778776	0.14075	no
gi|320446957|ref|NW_003383614.1|	30584-43120	0.442171	450397	334852	0.2264	no
gi|320446957|ref|NW_003383614.1|	43008-44377	0.315721	346509	345617	0.2167	no
gi|320446957|ref|NW_003383614.1|	50476-45256	0	160278	inf	5.00E-05	yes
gi|320446957|ref|NW_003383614.1|	58450-45892	0	138713	inf	0.00935	no
gi|320446957|ref|NW_003383614.1|	64815-47407	0.0747981	284084	85691	0.10285	no
gi|320446957|ref|NW_003383614.1|	45157-54635	0	327434	inf	0.0133	no
gi|320446957|ref|NW_003383614.1|	49778-55082	0	143785	inf	0.0312	no
gi|320446957|ref|NW_003383614.1|	51389-55278	0	130868	inf	1	no
gi|320446957|ref|NW_003383614.1|	52920-55776	0.109831	520558	55667	0.11595	no
gi|320446957|ref|NW_003383614.1|	58863-55951	161417	384849	12535	0.5574	no
gi|320446957|ref|NW_003383614.1|	63201-56358	213366	76927	185016	0.325	no
gi|320446957|ref|NW_003383614.1|	68867-56930	326488	703358	110723	0.5964	no
gi|320446957|ref|NW_003383614.1|	70195-57394	177879	159139	316132	0.1045	no
gi|320446957|ref|NW_003383614.1|	79315-58225	0.685992	11335	404645	0.0626	no
gi|320446957|ref|NW_003383614.1|	83060-58553	0.677156	184965	477162	0.05515	no
gi|320446957|ref|NW_003383614.1|	86315-58722	410978	118475	-179448	0.29	no
gi|320446957|ref|NW_003383614.1|	87451-58933	704952	356041	-0.985484	0.64815	no
gi|320446957|ref|NW_003383614.1|	89603-59031	252924	487196	-237613	0.30805	no
gi|320446957|ref|NW_003383614.1|	97268-59776	10166	382987	-140839	0.4984	no
gi|320446957|ref|NW_003383614.1|	99262-59977	900766	644963	-0.481938	0.8116	no
gi|320446957|ref|NW_003383614.1|	46490-64698	158271	110258	-0.521521	0.7989	no
gi|320446957|ref|NW_003383614.1|	58346-65857	296097	133132	-115321	0.58465	no
gi|320446957|ref|NW_003383614.1|	60455-66074	427043	955645	-215983	0.33985	no
gi|320446957|ref|NW_003383614.1|	60945-66182	143786	504142	-151202	0.4809	no
gi|320446957|ref|NW_003383614.1|	62968-66356	289838	120392	-126751	0.55375	no

gi|320446957|ref|NW_003383614.1|	564244-66501[illegible]	485737	124699	-196172	0.25065	no
gi|320446957|ref|NW_003383614.1|	574005-67517[illegible]	849576	219179	136729	0.3981	no
gi|320446957|ref|NW_003383614.1|	577047-67814[illegible]	116844	323496	146916	0.4885	no
gi|320446957|ref|NW_003383614.1|	581180-68181[illegible]	0	318389	inf	0.0294	no
gi|320446957|ref|NW_003383614.1|	582199-68313[illegible]	484563	350914	-0.465567	0.8218	no
gi|320446957|ref|NW_003383614.1|	584485-68470[illegible]	542708	612093	0.173575	0.9247	no
gi|320446957|ref|NW_003383614.1|	68998-73445	0.0400617	101833	798977	0.14075	no
gi|320446957|ref|NW_003383614.1|	728332-72954[illegible]	155361	0.718077	-111341	0.5929	no
gi|320446957|ref|NW_003383614.1|	730950-73354[illegible]	50771	374664	-0.43841	0.84425	no
gi|320446957|ref|NW_003383614.1|	742189-74375[illegible]	140027	407356	-178134	0.4204	no
gi|320446957|ref|NW_003383614.1|	74720-77414	0.0686288	158248	452723	0.17575	no
gi|320446957|ref|NW_003383614.1|	750795-75105[illegible]	609903	221064	-146412	0.49945	no
gi|320446957|ref|NW_003383614.1|	754935-75587[illegible]	143718	528762	-144255	0.5032	no
gi|320446957|ref|NW_003383614.1|	756743-75760[illegible]	21179	822205	-136507	0.53795	no
gi|320446957|ref|NW_003383614.1|	757743-75912[illegible]	19427	736061	-140017	0.3998	no
gi|320446957|ref|NW_003383614.1|	759427-76349[illegible]	189741	209565	0.143361	0.9126	no
gi|320446957|ref|NW_003383614.1|	77518-79387	0	316742	inf	0.00845	no
gi|320446957|ref|NW_003383614.1|	80318-81503	0	29559	inf	0.0138	no
gi|320446958|ref|NW_003383613.1|	112103-11320[illegible]	560783	1236.68	114096	0.46045	no
gi|320446958|ref|NW_003383613.1|	122433-12396[illegible]	111966	216.53	0.951506	0.4714	no
gi|320446958|ref|NW_003383613.1|	13013-14219	139095	0.361595	-194362	1	no
gi|320446958|ref|NW_003383613.1|	131224-13481[illegible]	11629	238823	103822	0.42565	no
gi|320446958|ref|NW_003383613.1|	15417-17787	13438	209722	0.642163	0.74925	no
gi|320446958|ref|NW_003383613.1|	172012-17263[illegible]	326765	0.292254	-680489	0.2611	no
gi|320446958|ref|NW_003383613.1|	175518-17580[illegible]	277578	0	#NAME?	0.01755	no
gi|320446958|ref|NW_003383613.1|	182119-18263[illegible]	164741	0	#NAME?	0.0093	no
gi|320446958|ref|NW_003383613.1|	183754-18449[illegible]	100932	253461	132838	0.52445	no
gi|320446958|ref|NW_003383613.1|	185674-18712[illegible]	0.832227	260304	164515	0.43965	no
gi|320446958|ref|NW_003383613.1|	187228-19011[illegible]	0.635372	213183	174642	0.4199	no
gi|320446958|ref|NW_003383613.1|	191155-19184[illegible]	0.743649	35552	225724	0.2746	no

gi|320446958|ref|NW_003383613.1|	19511-21524	132894	119153	-0.157458	1	no
gi|320446958|ref|NW_003383613.1|	00066-20177	0.45804	271105	256531	0.2801	no
gi|320446958|ref|NW_003383613.1|	01917-20308	0.901872	162475	0.849226	0.6824	no
gi|320446958|ref|NW_003383613.1|	05075-20567	0.922405	281446	160939	0.33275	no
gi|320446958|ref|NW_003383613.1|	12083-21278	130432	0.742909	-413397	0.16175	no
gi|320446958|ref|NW_003383613.1|	19373-22238	227683	740592	-162027	0.3588	no
gi|320446958|ref|NW_003383613.1|	22527-22331	708933	169239	125534	0.56075	no
gi|320446958|ref|NW_003383613.1|	25544-22602	86668	249554	152578	0.46595	no
gi|320446958|ref|NW_003383613.1|	24895-27285	18012	0.492125	-187186	0.3894	no
gi|320446958|ref|NW_003383613.1|	27590-29468	225999	0.429572	-239535	0.29255	no
gi|320446958|ref|NW_003383613.1|	86056-28692	304637	10302	-156417	0.49025	no
gi|320446958|ref|NW_003383613.1|	18884-32059	122609	191497	-267867	0.25325	no
gi|320446958|ref|NW_003383613.1|	27810-32885	0.20923	144663	278953	0.2657	no
gi|320446958|ref|NW_003383613.1|	28979-32970	832237	474603	-0.810273	0.69315	no
gi|320446958|ref|NW_003383613.1|	29911-33039	640604	22076	-153695	0.49675	no
gi|320446958|ref|NW_003383613.1|	30658-33114	19407	849782	-119141	0.56935	no
gi|320446958|ref|NW_003383613.1|	31290-33186	333126	121032	-146068	0.4903	no
gi|320446958|ref|NW_003383613.1|	32817-33613	138068	133365	-0.0499961	0.96585	no
gi|320446958|ref|NW_003383613.1|	37007-33769	0	384996	inf	0.02205	no
gi|320446958|ref|NW_003383613.1|	37830-33866	113544	136622	0.266936	1	no
gi|320446958|ref|NW_003383613.1|	39595-34142	0.211905	155033	287109	0.22425	no
gi|320446958|ref|NW_003383613.1|	41603-34179	0	601461	inf	0.0312	no
gi|320446958|ref|NW_003383613.1|	42110-34277	0	478379	inf	0.0154	no
gi|320446958|ref|NW_003383613.1|	42965-34330	135971	211868	39618	0.1951	no
gi|320446958|ref|NW_003383613.1|	34495-36919	138773	291253	439147	0.0474	no
gi|320446958|ref|NW_003383613.1|	45842-34607	159345	552299	179329	0.42565	no
gi|320446958|ref|NW_003383613.1|	46628-34688	609499	366879	258961	0.24715	no
gi|320446958|ref|NW_003383613.1|	47489-34795	0.726854	484134	273567	0.2671	no
gi|320446958|ref|NW_003383613.1|	49467-35012	0.790254	269344	176906	0.33305	no
gi|320446958|ref|NW_003383613.1|	50619-35144	255475	761181	157506	0.4619	no

gi|320446958|ref|NW_003383613.1|	353428-35612	0.54903	652172	35703	0.1599	no
gi|320446958|ref|NW_003383613.1|	357363-35815	0.904867	248536	145768	0.5073	no
gi|320446958|ref|NW_003383613.1|	360196-36125	0.410319	241215	25555	0.2501	no
gi|320446958|ref|NW_003383613.1|	37054-44615	0.46063	48446	33947	0.09095	no
gi|320446958|ref|NW_003383613.1|	371304-37154	381427	336801	314242	0.2375	no
gi|320446958|ref|NW_003383613.1|	380293-38057	107787	261811	128034	0.55125	no
gi|320446958|ref|NW_003383613.1|	380835-38234	0	514362	inf	0.00785	no
gi|320446958|ref|NW_003383613.1|	383672-38615	390549	639937	0.712425	0.74355	no
gi|320446958|ref|NW_003383613.1|	386287-38767	0.438083	101443	121139	1	no
gi|320446958|ref|NW_003383613.1|	399538-40092	0.877686	315013	184363	0.40205	no
gi|320446958|ref|NW_003383613.1|	402883-40366	0	316757	inf	0.02205	no
gi|320446958|ref|NW_003383613.1|	406395-40684	0	106669	inf	0.01575	no
gi|320446958|ref|NW_003383613.1|	415904-41669	0.304892	100468	504229	0.17405	no
gi|320446958|ref|NW_003383613.1|	416804-41749	0.743649	533281	28422	0.2255	no
gi|320446958|ref|NW_003383613.1|	420842-42142	0.483251	186453	52699	0.16805	no
gi|320446958|ref|NW_003383613.1|	422805-42320	0	945638	inf	0.02205	no
gi|320446958|ref|NW_003383613.1|	424117-42465	163762	27953	409333	0.16205	no
gi|320446958|ref|NW_003383613.1|	425535-42599	0	118039	inf	0.0138	no
gi|320446958|ref|NW_003383613.1|	427152-42821	0.811754	10388	367773	0.17305	no
gi|320446958|ref|NW_003383613.1|	429004-42958	242306	393597	0.699892	0.74085	no
gi|320446958|ref|NW_003383613.1|	431359-43210	0.985812	562968	251367	0.2811	no
gi|320446958|ref|NW_003383613.1|	435309-43592	26462	8087	161168	0.4438	no
gi|320446958|ref|NW_003383613.1|	445277-44618	0	225831	inf	0.02915	no
gi|320446958|ref|NW_003383613.1|	447484-44793	152188	111293	287043	0.22955	no
gi|320446958|ref|NW_003383613.1|	45636-46128	0.647079	476363	288005	0.25835	no
gi|320446958|ref|NW_003383613.1|	50979-51969	0.894614	139022	0.635973	1	no
gi|320446958|ref|NW_003383613.1|	512299-51264	0	97106	inf	0.0294	no
gi|320446958|ref|NW_003383613.1|	553980-55516	0.713865	766794	342512	0.1904	no
gi|320446958|ref|NW_003383613.1|	596076-59761	843989	135466	-26393	0.25255	no
gi|320446958|ref|NW_003383613.1|	598397-59883	190712	42186	-217656	0.3197	no

gi|320446958|ref|NW_003383613.1|	99443-60098	118635	349811	-176188	0.4114	no
gi|320446958|ref|NW_003383613.1|	60111-61794	944193	25889	145518	0.3979	no
gi|320446958|ref|NW_003383613.1|	39007-64021	393869	685636	-25222	0.1662	no
gi|320446958|ref|NW_003383613.1|	40525-64107	272389	504238	-243349	0.28385	no
gi|320446958|ref|NW_003383613.1|	43412-64382	371404	146921	198398	0.3874	no
gi|320446958|ref|NW_003383613.1|	45533-64566	0	622706	inf	0.0294	no
gi|320446958|ref|NW_003383613.1|	49179-64968	487103	383983	297875	0.2131	no
gi|320446958|ref|NW_003383613.1|	50073-65463	142728	925801	-0.624495	0.6284	no
gi|320446958|ref|NW_003383613.1|	54739-65736	312674	858007	-18656	0.30395	no
gi|320446958|ref|NW_003383613.1|	60004-66179	185132	263981	-281005	0.1233	no
gi|320446958|ref|NW_003383613.1|	69212-66972	128794	231295	-247726	0.16795	no
gi|320446958|ref|NW_003383613.1|	71459-67513	106813	946322	-0.174688	0.8916	no
gi|320446958|ref|NW_003383613.1|	75242-67740	397878	137285	-153516	0.2468	no
gi|320446958|ref|NW_003383613.1|	78042-68014	579606	374876	-0.62866	0.7668	no
gi|320446958|ref|NW_003383613.1|	81384-68223	199498	169246	-355918	0.15545	no
gi|320446958|ref|NW_003383613.1|	82364-68310	498922	461732	-343369	0.0886	no
gi|320446958|ref|NW_003383613.1|	68432-68919	197759	440835	11565	0.5747	no
gi|320446958|ref|NW_003383613.1|	85354-68638	207882	64518	-168799	0.4467	no
gi|320446958|ref|NW_003383613.1|	70670-71094	601136	124969	10558	0.59955	no
gi|320446958|ref|NW_003383613.1|	71245-72193	757888	915124	0.271984	0.89575	no
gi|320446958|ref|NW_003383613.1|	95592-95986	109848	137845	0.327536	0.86395	no
gi|320446959|ref|NW_003383612.1|	44857-14542	0.504476	375026	289413	0.25825	no
gi|320446959|ref|NW_003383612.1|	45517-14608	750131	111566	0.572678	0.7791	no
gi|320446959|ref|NW_003383612.1|	46556-14880	652971	78878	0.272604	0.90455	no
gi|320446959|ref|NW_003383612.1|	48976-14933	100447	65416	-0.618717	0.75295	no
gi|320446959|ref|NW_003383612.1|	50537-15385	123315	640205	-0.945739	0.57035	no
gi|320446959|ref|NW_003383612.1|	54559-15690	926931	741974	-0.321094	0.886	no
gi|320446959|ref|NW_003383612.1|	58061-15878	411074	703504	0.77516	0.70275	no
gi|320446959|ref|NW_003383612.1|	58897-16251	313877	24752	-0.342651	0.8714	no
gi|320446959|ref|NW_003383612.1|	63466-16432	217922	0.562368	-195423	0.30295	no

gi|320446959|ref|NW_003383612.1|	168881-16938	138778	338084	-203733	0.34245	no
gi|320446959|ref|NW_003383612.1|	169559-17085	604052	176513	-17749	0.40465	no
gi|320446959|ref|NW_003383612.1|	171317-17325	613737	193229	-166731	0.44035	no
gi|320446959|ref|NW_003383612.1|	174444-17494	114368	340433	-174824	0.41865	no
gi|320446959|ref|NW_003383612.1|	178778-18088	107171	345548	168897	0.34125	no
gi|320446959|ref|NW_003383612.1|	181026-18266	192629	410603	109192	0.59335	no
gi|320446959|ref|NW_003383612.1|	183323-18437	390754	796499	102741	0.62155	no
gi|320446959|ref|NW_003383612.1|	184543-18531	283091	474718	0.745803	0.711	no
gi|320446959|ref|NW_003383612.1|	187705-18894	354174	362584	0.0338591	0.97945	no
gi|320446959|ref|NW_003383612.1|	192590-19292	646512	108437	0.746104	0.72825	no
gi|320446959|ref|NW_003383612.1|	213735-21465	0	238984	inf	0.00365	no
gi|320446959|ref|NW_003383612.1|	215020-21556	0	178067	inf	0.0089	no
gi|320446959|ref|NW_003383612.1|	216206-21720	0	569427	inf	5.00E-05	yes
gi|320446959|ref|NW_003383612.1|	242497-24323	168891	185052	0.131836	0.92665	no
gi|320446959|ref|NW_003383612.1|	278563-28208	0.0513689	226425	546199	0.1647	no
gi|320446959|ref|NW_003383612.1|	284279-28509	144359	238132	0.722095	0.7364	no
gi|320446959|ref|NW_003383612.1|	298536-29985	660982	408233	-0.695216	0.59775	no
gi|320446959|ref|NW_003383612.1|	301176-30282	246079	103106	-1255	0.45815	no
gi|320446959|ref|NW_003383612.1|	307817-30835	798617	317433	-133105	0.4202	no
gi|320446959|ref|NW_003383612.1|	322037-32253	0	715957	inf	0.02075	no
gi|320446959|ref|NW_003383612.1|	323480-32395	0	594394	inf	0.02915	no
gi|320446959|ref|NW_003383612.1|	381441-38192	400006	490195	0.293335	0.88365	no
gi|320446959|ref|NW_003383612.1|	382745-38337	46824	839388	0.84209	0.67545	no
gi|320446959|ref|NW_003383612.1|	385173-38535	539664	667431	0.306558	0.8815	no
gi|320446959|ref|NW_003383612.1|	388599-38903	322117	587511	0.867032	0.69375	no
gi|320446959|ref|NW_003383612.1|	389887-39067	172295	542711	16553	0.3299	no
gi|320446959|ref|NW_003383612.1|	390794-39110	159374	535266	174784	0.41165	no
gi|320446959|ref|NW_003383612.1|	400909-40380	0.953058	0.977091	0.0359279	1	no
gi|320446959|ref|NW_003383612.1|	454960-45844	975238	109241	0.163689	0.89655	no
gi|320446959|ref|NW_003383612.1|	461229-46230	100742	390286	195386	0.38395	no

gi|320446959|ref|NW_003383612.1|	476693-47734	368417	975507	140481	0.50395	no
gi|320446959|ref|NW_003383612.1|	47793-48340	435302	543479	0.320205	0.89005	no
gi|320446959|ref|NW_003383612.1|	503129-50354	0.910116	522474	584317	0.1602	no
gi|320446959|ref|NW_003383612.1|	568882-56983	259794	0.814828	-16728	0.4339	no
gi|320446959|ref|NW_003383612.1|	580146-58236	0.34026	379935	348104	0.1832	no
gi|320446959|ref|NW_003383612.1|	582562-58397	0.142814	476325	505974	0.17375	no
gi|320446959|ref|NW_003383612.1|	58731-61709	1415	606452	20996	0.35835	no
gi|320446959|ref|NW_003383612.1|	62098-64528	615083	141204	119892	0.46735	no
gi|320446959|ref|NW_003383612.1|	64787-66510	128178	853121	-0.58733	0.7966	no
gi|320446959|ref|NW_003383612.1|	69428-74725	316004	153435	-104231	0.43975	no
gi|320446959|ref|NW_003383612.1|	78778-79340	294224	714381	-204215	0.12985	no
gi|320446959|ref|NW_003383612.1|	79661-80305	37521	384515	0.0353433	0.98695	no
gi|320446959|ref|NW_003383612.1|	81455-81645	645709	343354	-0.911187	0.6772	no
gi|320446959|ref|NW_003383612.1|	82566-82949	190949	208668	0.128024	0.9495	no
gi|320446959|ref|NW_003383612.1|	84039-85233	38.73	471001	0.282279	0.8293	no
gi|320446959|ref|NW_003383612.1|	85347-85786	376803	319094	-0.23983	0.90615	no
gi|320446959|ref|NW_003383612.1|	86509-87675	87045	167106	0.940929	0.66685	no
gi|320446959|ref|NW_003383612.1|	88578-93398	126599	133783	0.0796285	0.9504	no
gi|320446959|ref|NW_003383612.1|	93863-95435	338348	974954	-17951	0.30345	no
gi|320446959|ref|NW_003383612.1|	96062-97420	0.300643	0.93953	164389	1	no
gi|320446959|ref|NW_003383612.1|	98740-10241	16365	143663	-0.187926	0.8874	no
gi|320446960|ref|NW_003383611.1|	111445-11285	0	109802	inf	1	no
gi|320446960|ref|NW_003383611.1|	155384-15717	466734	461255	-0.0170353	0.99045	no
gi|320446960|ref|NW_003383611.1|	162349-16423	474215	105696	115631	0.3889	no
gi|320446960|ref|NW_003383611.1|	164388-16473	92017	854558	-0.106721	0.94115	no
gi|320446960|ref|NW_003383611.1|	166541-16774	447408	579086	0.372186	0.77375	no
gi|320446960|ref|NW_003383611.1|	188992-19068	927411	215508	121646	0.46115	no
gi|320446960|ref|NW_003383611.1|	193114-19442	454141	268492	-0.758258	0.561	no
gi|320446960|ref|NW_003383611.1|	205434-21130	533188	608759	0.191226	0.87925	no
gi|320446960|ref|NW_003383611.1|	218040-21834	486208	57874	-307059	0.2208	no

gi|320446960|ref|NW_003383611.1|	!19284-22470!	439244	307757	-0.513232	0.7564	no
gi|320446960|ref|NW_003383611.1|	!24809-22760!	144422	967864	-0.577417	0.6455	no
gi|320446960|ref|NW_003383611.1|	!27908-22878!	0	255173	inf	0.0233	no
gi|320446960|ref|NW_003383611.1|	!35282-23839!	300485	115505	-137934	0.3053	no
gi|320446960|ref|NW_003383611.1|	!56193-25710!	149668	275122	0.878311	0.6632	no
gi|320446960|ref|NW_003383611.1|	!58816-25942!	178249	423534	124859	0.55745	no
gi|320446960|ref|NW_003383611.1|	!59543-26081!	490279	0.793671	-262699	0.2571	no
gi|320446960|ref|NW_003383611.1|	!62004-26260!	688105	839986	0.287735	0.88815	no
gi|320446960|ref|NW_003383611.1|	!63462-26410!	330653	101277	161491	0.4351	no
gi|320446960|ref|NW_003383611.1|	!64886-26584!	0.938141	242779	137177	0.52455	no
gi|320446960|ref|NW_003383611.1|	26538-27908	156168	112597	-0.471926	0.83455	no
gi|320446960|ref|NW_003383611.1|	!66181-26661!	65594	434718	-0.593484	0.7539	no
gi|320446960|ref|NW_003383611.1|	!71858-27254!	708015	114517	0.693713	0.7302	no
gi|320446960|ref|NW_003383611.1|	!79782-28046!	353378	405529	0.198592	0.9294	no
gi|320446960|ref|NW_003383611.1|	28661-30232	328494	474654	0.531008	0.7951	no
gi|320446960|ref|NW_003383611.1|	!89187-28991!	238348	46625	0.968036	0.6561	no
gi|320446960|ref|NW_003383611.1|	!91664-29242!	289557	617655	109295	0.59695	no
gi|320446960|ref|NW_003383611.1|	!92689-29471!	132076	394751	157957	0.4666	no
gi|320446960|ref|NW_003383611.1|	!94822-29789!	214354	261154	0.284901	0.82815	no
gi|320446960|ref|NW_003383611.1|	!98132-29896!	670494	233441	-152217	0.37145	no
gi|320446960|ref|NW_003383611.1|	!99735-30051!	458695	214813	-109445	0.5007	no
gi|320446960|ref|NW_003383611.1|	!01962-30400!	268505	213362	-0.33164	0.8002	no
gi|320446960|ref|NW_003383611.1|	30514-33143	120542	99976	-0.269887	0.8281	no
gi|320446960|ref|NW_003383611.1|	!11942-31242!	272852	0.455535	-590441	0.26955	no
gi|320446960|ref|NW_003383611.1|	!12595-31313!	521319	14938	-512511	0.1576	no
gi|320446960|ref|NW_003383611.1|	!13736-31416!	963683	327462	-487916	0.1337	no
gi|320446960|ref|NW_003383611.1|	!14858-31507!	412629	18.72	-446219	0.13995	no
gi|320446960|ref|NW_003383611.1|	!25451-32738!	220275	664045	-172995	0.3185	no
gi|320446960|ref|NW_003383611.1|	!29945-33356!	194707	363313	0.899908	0.6717	no
gi|320446960|ref|NW_003383611.1|	33284-33684	580455	114613	0.981512	0.65125	no

gi|320446960|ref|NW_003383611.1|	334675-335167	129416	216529	0.742546	0.71745	no
gi|320446960|ref|NW_003383611.1|	335661-337175	11878	725056	-0.712122	0.73935	no
gi|320446960|ref|NW_003383611.1|	337335-339163	572143	442953	-0.369224	0.85835	no
gi|320446960|ref|NW_003383611.1|	340788-341252	144193	177738	0.301753	0.88425	no
gi|320446960|ref|NW_003383611.1|	344137-346170	807264	216034	-190178	0.39245	no
gi|320446960|ref|NW_003383611.1|	346698-348837	134733	550405	-129154	0.43045	no
gi|320446960|ref|NW_003383611.1|	358218-358777	25905	349788	0.433252	0.82835	no
gi|320446960|ref|NW_003383611.1|	366394-367534	578328	607265	0.0704389	0.9709	no
gi|320446960|ref|NW_003383611.1|	368210-371637	391208	680499	0.798659	0.61775	no
gi|320446960|ref|NW_003383611.1|	372325-382324	112149	132366	0.239118	0.85215	no
gi|320446960|ref|NW_003383611.1|	385276-391103	218762	139982	-0.644124	0.6298	no
gi|320446960|ref|NW_003383611.1|	38759-40065	0.630497	0.984634	0.643098	1	no
gi|320446960|ref|NW_003383611.1|	392006-395959	271859	500354	0.88009	0.5143	no
gi|320446960|ref|NW_003383611.1|	396167-399488	534792	186655	-15186	0.2594	no
gi|320446960|ref|NW_003383611.1|	399616-404302	457197	346225	-0.401104	0.7667	no
gi|320446960|ref|NW_003383611.1|	40254-40584	745478	115761	0.634906	0.75705	no
gi|320446960|ref|NW_003383611.1|	405412-406309	81123	666197	-0.284163	0.8243	no
gi|320446960|ref|NW_003383611.1|	419978-420728	721528	404571	-0.834661	0.67315	no
gi|320446960|ref|NW_003383611.1|	431462-432363	136928	873703	-0.6482	0.7529	no
gi|320446960|ref|NW_003383611.1|	432483-433870	0.29307	173046	256184	0.25	no
gi|320446960|ref|NW_003383611.1|	436882-437100	411666	225919	-0.86567	0.67695	no
gi|320446960|ref|NW_003383611.1|	438998-440249	648034	530179	-0.289591	0.883	no
gi|320446960|ref|NW_003383611.1|	441771-447877	26412	41131	0.639029	0.6262	no
gi|320446960|ref|NW_003383611.1|	457398-458235	10.1	108052	0.0973653	0.96045	no
gi|320446960|ref|NW_003383611.1|	458387-459733	0.607784	316523	238068	0.2973	no
gi|320446960|ref|NW_003383611.1|	460760-464600	831804	187409	117188	0.3771	no
gi|320446960|ref|NW_003383611.1|	465654-468252	322808	131964	20314	0.1367	no
gi|320446960|ref|NW_003383611.1|	468356-469645	206617	563522	144751	0.26405	no
gi|320446960|ref|NW_003383611.1|	470346-471184	117638	265838	117619	0.5956	no
gi|320446960|ref|NW_003383611.1|	471344-473758	257134	540817	107262	0.4311	no

gi|320446960|ref|NW_003383611.1|	473918-47510(	301289	581909	0.949644	0.46935	no
gi|320446960|ref|NW_003383611.1|	507394-50962:	378713	115299	-171572	0.20105	no
gi|320446960|ref|NW_003383611.1|	509791-51095	347487	894915	-195714	0.2611	no
gi|320446960|ref|NW_003383611.1|	511585-52145	139714	840021	-0.733982	0.5818	no
gi|320446960|ref|NW_003383611.1|	536156-53692	0.629091	302093	226365	0.2738	no
gi|320446960|ref|NW_003383611.1|	54740-57702	141.03	573654	-129775	0.3169	no
gi|320446960|ref|NW_003383611.1|	567859-56927	464787	134733	-178646	0.3172	no
gi|320446960|ref|NW_003383611.1|	514520-61550	391068	107706	-186031	0.28215	no
gi|320446960|ref|NW_003383611.1|	527129-62753(	28872	950351	-160314	0.44455	no
gi|320446960|ref|NW_003383611.1|	527671-62822(	46146	166319	-147225	0.4949	no
gi|320446960|ref|NW_003383611.1|	528407-63146	358666	276697	-0.374335	0.7824	no
gi|320446960|ref|NW_003383611.1|	70470-71303	296557	0.582645	-566955	0.1334	no
gi|320446960|ref|NW_003383611.1|	72390-73321	101905	0.167338	-592831	0.26995	no
gi|320446960|ref|NW_003383611.1|	74620-76088	177975	209374	-308752	0.2026	no
gi|320446960|ref|NW_003383611.1|	77580-79289	103975	382737	-144181	0.2811	no
gi|320446960|ref|NW_003383611.1|	79416-80076	174666	946953	-0.883232	0.66985	no
gi|320446960|ref|NW_003383611.1|	80201-80916	8795	818217	-0.1042	0.95645	no
gi|320446960|ref|NW_003383611.1|	81107-86405	559896	123051	113602	0.38045	no
gi|320446961|ref|NW_003383610.1|	142854-14334	554447	25029	-114745	0.60205	no
gi|320446961|ref|NW_003383610.1|	144446-14676	210654	124997	-0.752982	0.54855	no
gi|320446961|ref|NW_003383610.1|	147996-14855(	489162	269728	-0.858807	0.6914	no
gi|320446961|ref|NW_003383610.1|	148726-14898	493325	191485	-13653	0.52545	no
gi|320446961|ref|NW_003383610.1|	149871-15281	842211	10118	0.264678	0.8298	no
gi|320446961|ref|NW_003383610.1|	154660-15546	315406	631839	100235	0.5535	no
gi|320446961|ref|NW_003383610.1|	157174-15779	235729	31733	0.428857	0.8428	no
gi|320446961|ref|NW_003383610.1|	160709-16113	532435	556679	0.0642396	0.9732	no
gi|320446961|ref|NW_003383610.1|	162059-16308(	0.213196	26525	36371	0.2061	no
gi|320446961|ref|NW_003383610.1|	193205-19341	281449	246727	-0.189961	0.9138	no
gi|320446961|ref|NW_003383610.1|	235400-23574(	694074	810487	0.223699	0.896	no
gi|320446961|ref|NW_003383610.1|	237556-23863	721896	430079	-0.747189	0.7143	no

gi|320446961|ref|NW_003383610.1|	238906-23959	933645	969094	0.0537623	0.97345	no
gi|320446961|ref|NW_003383610.1|	239711-23991	349887	392779	0.166831	0.92095	no
gi|320446961|ref|NW_003383610.1|	240109-24044	118406	258631	112715	0.58455	no
gi|320446961|ref|NW_003383610.1|	240977-24348	815991	197921	12783	0.4517	no
gi|320446961|ref|NW_003383610.1|	245921-24802	133536	608845	-113308	0.3903	no
gi|320446961|ref|NW_003383610.1|	255882-25645	109385	0	#NAME?	0.01	no
gi|320446961|ref|NW_003383610.1|	263270-26486	934286	104013	-31671	0.19815	no
gi|320446961|ref|NW_003383610.1|	265286-26986	82738	0.952112	-311935	0.1004	no
gi|320446961|ref|NW_003383610.1|	282706-28660	418632	196913	223381	0.2157	no
gi|320446961|ref|NW_003383610.1|	287197-28804	0.551935	208845	191986	0.3194	no
gi|320446961|ref|NW_003383610.1|	366146-36768	141955	628342	214612	0.3275	no
gi|320446961|ref|NW_003383610.1|	369891-37104	569511	370334	270103	0.1423	no
gi|320446961|ref|NW_003383610.1|	371747-37324	215177	190508	314626	0.02395	no
gi|320446961|ref|NW_003383610.1|	373791-37476	113788	254552	448355	0.13305	no
gi|320446961|ref|NW_003383610.1|	374890-37602	90246	755154	306484	0.11095	no
gi|320446961|ref|NW_003383610.1|	377087-37973	294347	406894	378906	0.06795	no
gi|320446961|ref|NW_003383610.1|	380385-39005	287824	535442	421747	0.00335	no
gi|320446961|ref|NW_003383610.1|	42328-44206	0.205454	15035	287144	0.22425	no
gi|320446961|ref|NW_003383610.1|	45771-46609	252082	269691	0.0974152	0.9543	no
gi|320446961|ref|NW_003383610.1|	46727-48259	409956	191897	-109514	0.3978	no
gi|320446961|ref|NW_003383610.1|	49058-49524	429097	476666	0.151674	0.92995	no
gi|320446961|ref|NW_003383610.1|	53535-53946	283542	663754	-209484	0.3489	no
gi|320446961|ref|NW_003383610.1|	54135-55263	277927	549856	-233758	0.18155	no
gi|320446961|ref|NW_003383610.1|	55374-55977	183005	713704	-135849	0.52225	no
gi|320446961|ref|NW_003383610.1|	562917-56459	186765	0.568876	-171504	0.42195	no
gi|320446961|ref|NW_003383610.1|	56379-62387	60794	686204	0.174707	0.8915	no
gi|320446961|ref|NW_003383610.1|	604736-60637	0.120566	310486	468664	0.1765	no
gi|320446961|ref|NW_003383610.1|	614583-62001	168995	140722	30578	0.10915	no
gi|320446961|ref|NW_003383610.1|	639954-64014	669551	312125	-110107	0.59055	no
gi|320446961|ref|NW_003383610.1|	642910-64370	180973	14498	-0.319929	0.87815	no

gi|320446961|ref|NW_003383610.1|	65220-65629	166293	170583	0.0367506	0.97655	no
gi|320446961|ref|NW_003383610.1|	65848-66431	144975	294399	102197	0.5772	no
gi|320446961|ref|NW_003383610.1|	59778-66227	0	297407	inf	0.007	no
gi|320446961|ref|NW_003383610.1|	64841-66606	0	711609	inf	0.00765	no
gi|320446961|ref|NW_003383610.1|	67094-66833	0	301425	inf	0.0142	no
gi|320446961|ref|NW_003383610.1|	69123-67080	0	464502	inf	0.007	no
gi|320446961|ref|NW_003383610.1|	72879-67466	0	136046	inf	5.00E-05	yes
gi|320446961|ref|NW_003383610.1|	75220-67712	0.101065	174002	742767	0.14075	no
gi|320446961|ref|NW_003383610.1|	91760-69300	0.16682	161989	327953	0.23005	no
gi|320446961|ref|NW_003383610.1|	57550-75939	272059	0.145834	-422152	0.2071	no
gi|320446961|ref|NW_003383610.1|	76876-78248	208.6	148081	-0.494351	0.70205	no
gi|320446961|ref|NW_003383610.1|	79531-81143	479289	281505	-0.767734	0.56395	no
gi|320446961|ref|NW_003383610.1|	95135-96678	0.516199	125668	128362	1	no
gi|320446963|ref|NW_003383608.1|	65320-10656	0	621316	inf	0.00915	no
gi|320446963|ref|NW_003383608.1|	70732-10738	0.705091	206668	487336	0.04665	no
gi|320446963|ref|NW_003383608.1|	19043-11198	0.3193	853962	474119	0.1736	no
gi|320446963|ref|NW_003383608.1|	29675-13601	153116	284381	0.893197	0.5083	no
gi|320446963|ref|NW_003383608.1|	36736-13719	353134	414804	0.232213	0.9143	no
gi|320446963|ref|NW_003383608.1|	51159-15186	716975	0.245097	-48705	0.28945	no
gi|320446963|ref|NW_003383608.1|	67309-16843	266184	0.789872	-175273	0.42385	no
gi|320446963|ref|NW_003383608.1|	88486-18985	114624	765088	-0.583212	0.79055	no
gi|320446963|ref|NW_003383608.1|	93834-39608	317799	0	#NAME?	0.00735	no
gi|320446963|ref|NW_003383608.1|	17839-51990	1474	488359	17282	0.4152	no
gi|320446963|ref|NW_003383608.1|	80968-58153	255222	206888	-0.302901	0.8817	no
gi|320446963|ref|NW_003383608.1|	40803-64144	0	370926	inf	0.02915	no
gi|320446963|ref|NW_003383608.1|	56522-65944	0.565447	140542	131354	1	no
gi|320446963|ref|NW_003383608.1|	83632-68471	538223	993079	0.883705	0.67925	no
gi|320446963|ref|NW_003383608.1|	88294-68915	0.808076	185403	11981	0.56655	no
gi|320446963|ref|NW_003383608.1|	93049-69959	670862	299946	216062	0.10975	no
gi|320446963|ref|NW_003383608.1|	00689-70216	0.408391	0.851741	106046	1	no

gi|320446963|ref|NW_003383608.1|	03220-70360	515852	128675	131871	0.54905	no
gi|320446963|ref|NW_003383608.1|	04827-70540	182386	750199	204028	0.2346	no
gi|320446963|ref|NW_003383608.1|	06420-70709	765396	383872	232635	0.3122	no
gi|320446963|ref|NW_003383608.1|	07206-70786	11038	233805	108283	0.60715	no
gi|320446963|ref|NW_003383608.1|	08036-71133	8476	161305	0.928333	0.4676	no
gi|320446963|ref|NW_003383608.1|	26977-72723	304749	270332	314903	0.2374	no
gi|320446963|ref|NW_003383608.1|	54837-75592	151143	21742	0.524571	0.81445	no
gi|320446963|ref|NW_003383608.1|	89460-79221	648551	724234	0.159236	0.941	no
gi|320446963|ref|NW_003383608.1|	93096-79388	670762	606993	-0.144122	0.94045	no
gi|320446963|ref|NW_003383608.1|	94013-79621	180548	245983	0.446179	0.82895	no
gi|320446963|ref|NW_003383608.1|	01446-80228	224073	250427	0.160425	0.9302	no
gi|320446963|ref|NW_003383608.1|	02600-80350	353934	174227	-102251	0.60725	no
gi|320446963|ref|NW_003383608.1|	05186-80680	499675	161151	-163258	0.4454	no
gi|320446963|ref|NW_003383608.1|	17895-82218	604041	145247	-205614	0.1272	no
gi|320446963|ref|NW_003383608.1|	24014-82498	373203	572504	-27046	0.13125	no
gi|320446963|ref|NW_003383608.1|	25234-82620	193512	333961	-253468	0.26695	no
gi|320446963|ref|NW_003383608.1|	26370-82769	279803	0.431638	-269652	0.2729	no
gi|320446963|ref|NW_003383608.1|	38425-84028	0.624344	239301	193841	0.37225	no
gi|320446963|ref|NW_003383608.1|	41790-84309	0.792564	0.660079	-0.263889	1	no
gi|320446963|ref|NW_003383608.1|	46751-85783	290512	164662	250284	0.128	no
gi|320446963|ref|NW_003383608.1|	13225-91372	0	458533	inf	0.0294	no
gi|320446964|ref|NW_003383607.1|	10702-11115	741984	54317	-0.449984	0.8245	no
gi|320446964|ref|NW_003383607.1|	11463-11380	544147	162013	-174789	0.4224	no
gi|320446964|ref|NW_003383607.1|	14078-11434	415822	140822	-156209	0.4787	no
gi|320446964|ref|NW_003383607.1|	23965-12706	167751	104542	-0.682237	0.5956	no
gi|320446964|ref|NW_003383607.1|	27383-12905	223953	599007	141938	0.49845	no
gi|320446964|ref|NW_003383607.1|	55010-15566	0.403543	439827	344614	0.2167	no
gi|320446964|ref|NW_003383607.1|	57391-15861	0.511588	201019	197428	0.39	no
gi|320446964|ref|NW_003383607.1|	58699-15923	223984	527689	12363	0.5595	no
gi|320446964|ref|NW_003383607.1|	59381-16007	146487	575522	19741	0.3911	no

gi|320446964|ref|NW_003383607.1|	166727-16743	0.358488	269607	291086	0.25645	no
gi|320446964|ref|NW_003383607.1|	167699-16944	179202	66304	188751	0.3853	no
gi|320446964|ref|NW_003383607.1|	169541-16983	207558	252357	360388	0.21625	no
gi|320446964|ref|NW_003383607.1|	170114-17081	511601	289657	250126	0.27525	no
gi|320446964|ref|NW_003383607.1|	174873-17517	187959	132711	28198	0.2595	no
gi|320446964|ref|NW_003383607.1|	183284-18437	0	192204	inf	0.0233	no
gi|320446964|ref|NW_003383607.1|	184633-18696	104574	584039	248155	0.27105	no
gi|320446964|ref|NW_003383607.1|	187041-19307	134381	719592	-0.90108	0.4935	no
gi|320446964|ref|NW_003383607.1|	198151-19991	0.110752	146569	372618	0.21225	no
gi|320446964|ref|NW_003383607.1|	201300-20199	0	276406	inf	0.0294	no
gi|320446964|ref|NW_003383607.1|	202111-20557	10465	103597	330734	0.09655	no
gi|320446964|ref|NW_003383607.1|	205779-20894	161319	187755	354086	0.07935	no
gi|320446964|ref|NW_003383607.1|	210593-21235	155052	354851	119446	0.5616	no
gi|320446964|ref|NW_003383607.1|	215448-21594	128941	345227	142083	0.3757	no
gi|320446964|ref|NW_003383607.1|	218968-22007	446357	253887	250792	0.1603	no
gi|320446964|ref|NW_003383607.1|	220513-22277	0.75082	937458	364221	0.15315	no
gi|320446964|ref|NW_003383607.1|	227426-22891	351387	278129	298462	0.1104	no
gi|320446964|ref|NW_003383607.1|	229289-23240	146384	179339	361486	0.0719	no
gi|320446964|ref|NW_003383607.1|	240573-24281	438359	611876	0.481127	0.81935	no
gi|320446964|ref|NW_003383607.1|	367007-36747	0	619666	inf	0.02915	no
gi|320446964|ref|NW_003383607.1|	50947-61654	257147	447595	0.799603	0.52585	no
gi|320446964|ref|NW_003383607.1|	514263-52304	963588	656406	-0.553828	0.6757	no
gi|320446964|ref|NW_003383607.1|	543221-54419	227575	461966	434337	0.05965	no
gi|320446965|ref|NW_003383606.1|	107873-10865	0	319602	inf	0.02205	no
gi|320446965|ref|NW_003383606.1|	109791-11031	171298	422461	130231	0.5489	no
gi|320446965|ref|NW_003383606.1|	176404-18212	840436	748567	-0.167006	0.8956	no
gi|320446965|ref|NW_003383606.1|	182475-18437	0.606755	155074	135377	0.5185	no
gi|320446965|ref|NW_003383606.1|	184519-18488	15143	311956	104269	0.60145	no
gi|320446965|ref|NW_003383606.1|	188641-19172	202289	227064	0.166675	0.9007	no
gi|320446965|ref|NW_003383606.1|	191902-19314	497123	260339	-0.933209	0.47405	no

gi|320446965|ref|NW_003383606.1|	207971-20830	963419	186323	-237036	0.29495	no
gi|320446965|ref|NW_003383606.1|	222137-22235	308749	129096	-125799	0.5611	no
gi|320446965|ref|NW_003383606.1|	222466-22285	112866	336841	-174447	0.4204	no
gi|320446965|ref|NW_003383606.1|	225104-22704	278652	108536	-136029	0.4295	no
gi|320446965|ref|NW_003383606.1|	231555-23214	413635	222944	-0.891679	0.6819	no
gi|320446965|ref|NW_003383606.1|	232340-23303	266191	79739	-17391	0.4246	no
gi|320446965|ref|NW_003383606.1|	236510-23716	86551	185652	-222095	0.2131	no
gi|320446965|ref|NW_003383606.1|	247993-24963	334942	685194	-228933	0.20795	no
gi|320446965|ref|NW_003383606.1|	252648-25473	764409	212775	-184502	0.17515	no
gi|320446965|ref|NW_003383606.1|	257101-25766	126119	477306	-14018	0.50315	no
gi|320446965|ref|NW_003383606.1|	258357-26237	690837	199678	-179067	0.29355	no
gi|320446965|ref|NW_003383606.1|	323001-32460	233922	548318	122898	0.55925	no
gi|320446965|ref|NW_003383606.1|	39064-43117	274019	246584	-0.152193	0.90745	no
gi|320446965|ref|NW_003383606.1|	408195-41069	290199	161109	247293	0.1687	no
gi|320446965|ref|NW_003383606.1|	410834-41973	316265	234062	288769	0.0826	no
gi|320446965|ref|NW_003383606.1|	423051-42695	141179	398746	-182399	0.3184	no
gi|320446965|ref|NW_003383606.1|	430611-43139	285613	463752	-262264	0.2621	no
gi|320446965|ref|NW_003383606.1|	43231-43685	826484	249882	-172574	0.42725	no
gi|320446965|ref|NW_003383606.1|	433532-43449	274893	478371	-252267	0.2738	no
gi|320446965|ref|NW_003383606.1|	45103-45835	445156	302471	-0.557517	0.72255	no
gi|320446965|ref|NW_003383606.1|	476510-47685	903588	124408	-286058	0.22175	no
gi|320446965|ref|NW_003383606.1|	500704-50340	485487	10846	115966	0.4911	no
gi|320446965|ref|NW_003383606.1|	505087-50636	173856	68768	-133808	0.5472	no
gi|320446965|ref|NW_003383606.1|	507570-50838	0	240142	inf	0.029	no
gi|320446965|ref|NW_003383606.1|	509203-50990	317924	873966	-186303	0.40235	no
gi|320446965|ref|NW_003383606.1|	510011-51076	752866	515977	-0.545085	0.79275	no
gi|320446965|ref|NW_003383606.1|	512597-51723	778322	147417	0.921461	0.47595	no
gi|320446965|ref|NW_003383606.1|	518466-51872	0	281644	inf	0.0198	no
gi|320446965|ref|NW_003383606.1|	520271-52116	280614	179293	267566	0.2398	no
gi|320446965|ref|NW_003383606.1|	521797-52266	318151	231835	286531	0.22205	no

gi|320446965|ref|NW_003383606.1|	28281-53479	402688	158287	197481	0.24335	no
gi|320446965|ref|NW_003383606.1|	34904-53641	0.531234	156998	156332	0.4962	no
gi|320446965|ref|NW_003383606.1|	37358-54027	138003	33434	127661	0.33465	no
gi|320446965|ref|NW_003383606.1|	51797-55514	471154	750067	0.670822	0.6754	no
gi|320446965|ref|NW_003383606.1|	55312-57026	0.342368	132714	527663	0.144	no
gi|320446965|ref|NW_003383606.1|	55283-55624	138982	495651	183442	0.40295	no
gi|320446965|ref|NW_003383606.1|	60819-56139	293244	165344	-0.826629	0.696	no
gi|320446965|ref|NW_003383606.1|	62341-56673	173482	134817	-0.363788	0.7864	no
gi|320446965|ref|NW_003383606.1|	73690-57449	0	285961	inf	0.0233	no
gi|320446965|ref|NW_003383606.1|	75734-57678	135492	659013	-103983	0.4392	no
gi|320446965|ref|NW_003383606.1|	77092-57730	287078	986907	-154046	0.47375	no
gi|320446965|ref|NW_003383606.1|	58444-58841	0	129286	inf	0.0162	no
gi|320446965|ref|NW_003383606.1|	86078-58919	0.93556	114079	360806	0.07425	no
gi|320446965|ref|NW_003383606.1|	97688-59812	0	815096	inf	0.02205	no
gi|320446965|ref|NW_003383606.1|	98292-59900	0.351072	312218	315272	0.24575	no
gi|320446965|ref|NW_003383606.1|	99267-60086	0	103183	inf	1	no
gi|320446965|ref|NW_003383606.1|	04002-60479	0.603245	165691	145768	0.375	no
gi|320446965|ref|NW_003383606.1|	61933-62435	0	246781	inf	0.00795	no
gi|320446965|ref|NW_003383606.1|	44173-64471	0.547596	479594	313063	0.24575	no
gi|320446965|ref|NW_003383606.1|	59100-65962	350647	510536	0.541993	0.792	no
gi|320446965|ref|NW_003383606.1|	64063-66521	0	355572	inf	0.01275	no
gi|320446965|ref|NW_003383606.1|	65643-66819	248178	560855	117625	0.5877	no
gi|320446965|ref|NW_003383606.1|	69345-67305	49185	495548	0.0108066	0.99295	no
gi|320446965|ref|NW_003383606.1|	73342-67473	327429	116261	-149381	0.3879	no
gi|320446965|ref|NW_003383606.1|	89332-68978	333396	156185	-109398	0.59935	no
gi|320446965|ref|NW_003383606.1|	89930-69202	170587	128492	-0.408826	0.7446	no
gi|320446965|ref|NW_003383606.1|	96106-69938	919345	127443	0.47118	0.71205	no
gi|320446965|ref|NW_003383606.1|	00871-70151	108966	281984	-195019	0.2655	no
gi|320446965|ref|NW_003383606.1|	02301-70346	142954	451359	-16632	0.443	no
gi|320446965|ref|NW_003383606.1|	05179-70558	113131	248469	-218686	0.34545	no

gi|320446965|ref|NW_003383606.1|	712006-71383	541757	18985	-151279	0.2589	no
gi|320446967|ref|NW_003383604.1|	103563-10515	585054	320472	-0.868373	0.67265	no
gi|320446967|ref|NW_003383604.1|	108483-10967	405327	219864	-0.882475	0.67035	no
gi|320446967|ref|NW_003383604.1|	110500-11298	638114	644897	0.0152544	0.9932	no
gi|320446967|ref|NW_003383604.1|	113089-11685	496729	166129	174177	0.3329	no
gi|320446967|ref|NW_003383604.1|	127803-12803	0	243979	inf	0.0312	no
gi|320446967|ref|NW_003383604.1|	130889-13263	0.895417	136419	392934	0.07585	no
gi|320446967|ref|NW_003383604.1|	133783-13691	0.11638	585964	565389	0.14035	no
gi|320446967|ref|NW_003383604.1|	143882-14411	398363	702926	414122	0.1912	no
gi|320446967|ref|NW_003383604.1|	148769-14993	0.545203	177534	502516	0.1465	no
gi|320446967|ref|NW_003383604.1|	176360-17673	976698	355446	-145828	0.5081	no
gi|320446967|ref|NW_003383604.1|	178969-17945	15.94	843642	-0.917947	0.655	no
gi|320446967|ref|NW_003383604.1|	182781-18506	280454	0.172729	-73431	0.1105	no
gi|320446967|ref|NW_003383604.1|	185188-18548	589364	125925	-554853	0.27565	no
gi|320446967|ref|NW_003383604.1|	186980-18744	266756	0.480372	-579523	0.27135	no
gi|320446967|ref|NW_003383604.1|	224655-22813	596161	140607	-208404	0.1293	no
gi|320446967|ref|NW_003383604.1|	229106-23322	71648	969739	0.43667	0.73305	no
gi|320446967|ref|NW_003383604.1|	233395-23391	65605	600686	-0.127195	0.94155	no
gi|320446967|ref|NW_003383604.1|	234114-23524	985352	48547	-102126	0.62015	no
gi|320446967|ref|NW_003383604.1|	291835-29228	0	777811	inf	0.02205	no
gi|320446967|ref|NW_003383604.1|	319277-32307	553175	726278	0.392786	0.76415	no
gi|320446967|ref|NW_003383604.1|	337651-33915	0.662999	11987	0.854394	1	no
gi|320446967|ref|NW_003383604.1|	349845-35005	105184	21939	-226135	0.32675	no
gi|320446967|ref|NW_003383604.1|	355947-35730	0.75497	146796	0.959317	0.66275	no
gi|320446967|ref|NW_003383604.1|	358159-35930	327978	277489	-0.24117	0.84415	no
gi|320446967|ref|NW_003383604.1|	359903-36108	188186	157728	-0.254723	0.9093	no
gi|320446967|ref|NW_003383604.1|	363101-36361	121353	21578	0.830349	0.68905	no
gi|320446967|ref|NW_003383604.1|	369273-37065	837432	469451	-0.834997	0.6912	no
gi|320446967|ref|NW_003383604.1|	373203-37397	756318	475582	-0.669297	0.7435	no
gi|320446967|ref|NW_003383604.1|	374075-37465	969223	491997	-0.97818	0.6452	no

gi|320446967|ref|NW_003383604.1|	78224-38159	107792	712556	-0.597176	0.6323	no
gi|320446967|ref|NW_003383604.1|	82758-38350	125112	428668	-154529	0.4718	no
gi|320446967|ref|NW_003383604.1|	85782-38617	653093	33508	-0.962784	0.64685	no
gi|320446967|ref|NW_003383604.1|	86300-39068	585226	321569	-0.863863	0.59325	no
gi|320446967|ref|NW_003383604.1|	97033-39935	12124	581178	-106081	0.5123	no
gi|320446967|ref|NW_003383604.1|	00207-40324	21689	126372	-0.779289	0.70265	no
gi|320446967|ref|NW_003383604.1|	04996-40547	240914	129704	-0.893301	0.6614	no
gi|320446967|ref|NW_003383604.1|	13287-41421	255497	203052	-0.331451	0.88235	no
gi|320446967|ref|NW_003383604.1|	44359-44474	708113	492205	-384665	0.1517	no
gi|320446967|ref|NW_003383604.1|	92202-49882	240214	446497	421626	0.055	no
gi|320446967|ref|NW_003383604.1|	01917-50517	200893	105381	239112	0.18175	no
gi|320446967|ref|NW_003383604.1|	50379-51227	0	20851	inf	0.0294	no
gi|320446967|ref|NW_003383604.1|	07221-50803	176555	707585	200279	0.34935	no
gi|320446967|ref|NW_003383604.1|	45605-54593	453939	293547	-395083	0.16335	no
gi|320446967|ref|NW_003383604.1|	52486-55311	207945	0.294397	-61423	0.2676	no
gi|320446967|ref|NW_003383604.1|	53331-55442	11962	0.542865	-446172	0.1724	no
gi|320446967|ref|NW_003383604.1|	54565-55505	173434	0.430021	-533384	0.277	no
gi|320446967|ref|NW_003383604.1|	55582-55624	198023	0.539899	-519683	0.18895	no
gi|320446967|ref|NW_003383604.1|	55648-56174	114953	347843	159739	0.33275	no
gi|320446967|ref|NW_003383604.1|	56533-55733	260766	142489	-419383	0.14095	no
gi|320446967|ref|NW_003383604.1|	58845-56010	888527	182634	-228246	0.29745	no
gi|320446967|ref|NW_003383604.1|	78944-57967	543702	151235	147591	0.48345	no
gi|320446967|ref|NW_003383604.1|	58022-58440	203263	12845	-0.662135	0.75075	no
gi|320446967|ref|NW_003383604.1|	90790-59143	123995	309457	131946	0.5489	no
gi|320446967|ref|NW_003383604.1|	93373-59528	777272	344776	214917	0.24355	no
gi|320446967|ref|NW_003383604.1|	99938-60086	806479	439858	244733	0.16825	no
gi|320446967|ref|NW_003383604.1|	05473-60747	325674	300569	32062	0.098	no
gi|320446967|ref|NW_003383604.1|	60678-61713	865357	379365	-118971	0.5725	no
gi|320446967|ref|NW_003383604.1|	62707-64119	111868	827109	-0.435653	0.84685	no
gi|320446967|ref|NW_003383604.1|	76098-67719	0.585572	256685	213208	0.3464	no

gi|320446967|ref|NW_003383604.1|	77501-67925	0.111116	208962	42331	0.19125	no
gi|320446967|ref|NW_003383604.1|	83734-68510	0.298251	196785	272202	0.2412	no
gi|320446967|ref|NW_003383604.1|	85545-68932	0.0952014	383105	533061	0.14435	no
gi|320446967|ref|NW_003383604.1|	89426-69246	0.120194	348761	48588	0.1543	no
gi|320446967|ref|NW_003383604.1|	92680-69326	0	855113	inf	0.0142	no
gi|320446967|ref|NW_003383604.1|	75008-75447	120256	159547	-291406	0.21225	no
gi|320446967|ref|NW_003383604.1|	76-334	58086	147376	134324	0.38395	no
gi|320446967|ref|NW_003383604.1|	83705-78465	237507	0	#NAME?	0.0229	no
gi|320446967|ref|NW_003383604.1|	87263-87595	102854	28536	-184975	0.3126	no
gi|320446967|ref|NW_003383604.1|	93419-94029	359301	0.609637	-255917	0.30045	no
gi|320446968|ref|NW_003383603.1|	02910-10049	339731	869785	135626	0.31455	no
gi|320446968|ref|NW_003383603.1|	05147-10082	174161	214879	0.303097	0.8157	no
gi|320446968|ref|NW_003383603.1|	08924-10098	154395	141438	-0.126453	0.95385	no
gi|320446968|ref|NW_003383603.1|	11009-10116	673688	609637	-0.144132	0.94205	no
gi|320446968|ref|NW_003383603.1|	12732-10140	407776	350811	-0.21708	0.91365	no
gi|320446968|ref|NW_003383603.1|	33820-10352	137798	204981	389486	0.0705	no
gi|320446968|ref|NW_003383603.1|	03881-10466	773918	333058	-121641	0.4811	no
gi|320446968|ref|NW_003383603.1|	11967-11239	254028	151315	-0.747427	0.71385	no
gi|320446968|ref|NW_003383603.1|	12621-11333	390216	121304	-168564	0.43135	no
gi|320446968|ref|NW_003383603.1|	13704-11774	17788	187868	0.0788107	0.95	no
gi|320446968|ref|NW_003383603.1|	31951-13631	980639	57388	-0.772973	0.5422	no
gi|320446968|ref|NW_003383603.1|	36671-13764	113634	125538	0.14373	1	no
gi|320446968|ref|NW_003383603.1|	38371-13927	0.25281	174227	278484	0.2657	no
gi|320446968|ref|NW_003383603.1|	03110-20379	0	462987	inf	0.0154	no
gi|320446968|ref|NW_003383603.1|	07006-20809	217733	0.410913	-240565	0.248	no
gi|320446968|ref|NW_003383603.1|	08176-21003	763781	369103	-104914	0.4355	no
gi|320446968|ref|NW_003383603.1|	34694-23514	343885	270735	297689	0.1175	no
gi|320446968|ref|NW_003383603.1|	39405-23969	261194	172472	272317	0.24005	no
gi|320446968|ref|NW_003383603.1|	40145-24564	471111	111222	456124	0.03675	no
gi|320446968|ref|NW_003383603.1|	45907-24757	0.825667	135486	403644	0.1363	no

gi|320446968|ref|NW_003383603.1|	55404-26141	660563	409729	-0.689025	0.58695	no
gi|320446968|ref|NW_003383603.1|	61474-26416	797066	539656	275927	0.15855	no
gi|320446968|ref|NW_003383603.1|	64295-26491	0	706552	inf	0.0138	no
gi|320446968|ref|NW_003383603.1|	65062-26585	0	355823	inf	0.02075	no
gi|320446968|ref|NW_003383603.1|	67948-26892	0.227575	25141	346563	0.21395	no
gi|320446968|ref|NW_003383603.1|	27336-30339	140221	404928	-179197	0.30525	no
gi|320446968|ref|NW_003383603.1|	94745-29542	0	28477	inf	0.0294	no
gi|320446968|ref|NW_003383603.1|	30594-31629	0.422125	364774	311126	0.2064	no
gi|320446968|ref|NW_003383603.1|	51040-35280	0.884271	0.846903	-0.0622925	1	no
gi|320446968|ref|NW_003383603.1|	62502-36305	267048	324153	0.279576	0.88095	no
gi|320446968|ref|NW_003383603.1|	66737-37526	0.101778	130476	700222	0.0854	no
gi|320446968|ref|NW_003383603.1|	37309-38764	513328	205043	199797	0.14115	no
gi|320446968|ref|NW_003383603.1|	89963-39039	250518	608307	127988	0.55025	no
gi|320446968|ref|NW_003383603.1|	98598-39889	155763	112353	-0.471311	0.8098	no
gi|320446968|ref|NW_003383603.1|	99779-40106	817634	167991	103886	0.64165	no
gi|320446968|ref|NW_003383603.1|	01340-40196	121919	233715	0.938832	0.6576	no
gi|320446968|ref|NW_003383603.1|	02570-40361	314235	782134	131557	0.53385	no
gi|320446968|ref|NW_003383603.1|	05056-40588	112974	38843	178166	0.42685	no
gi|320446968|ref|NW_003383603.1|	06095-40820	721248	29.56	203508	0.2524	no
gi|320446968|ref|NW_003383603.1|	14104-41867	0.583468	435851	290111	0.22045	no
gi|320446968|ref|NW_003383603.1|	84896-48680	141153	773069	245334	0.2813	no
gi|320446968|ref|NW_003383603.1|	33952-53478	140982	11246	-0.326091	0.8726	no
gi|320446968|ref|NW_003383603.1|	65325-56694	0	101998	inf	1	no
gi|320446968|ref|NW_003383603.1|	67058-56798	0	251362	inf	0.02205	no
gi|320446968|ref|NW_003383603.1|	68106-57374	0.68718	877682	367494	0.0691	no
gi|320446968|ref|NW_003383603.1|	57106-59488	525866	279355	-0.912594	0.49285	no
gi|320446968|ref|NW_003383603.1|	74997-57854	0.713301	880519	362577	0.07	no
gi|320446968|ref|NW_003383603.1|	97073-59984	684598	32608	225189	0.2325	no
gi|320446968|ref|NW_003383603.1|	59829-61228	0.290047	15113	238143	0.2609	no
gi|320446968|ref|NW_003383603.1|	99947-60147	195973	490668	132409	0.52595	no

gi|320446968|ref|NW_003383603.1|	;03200-60454!	288958	950414	17177	0.42675	no
gi|320446968|ref|NW_003383603.1|	;05738-60781:	192621	499046	13734	0.5278	no
gi|320446968|ref|NW_003383603.1|	;09517-61019:	52982	111079	106801	0.60655	no
gi|320446968|ref|NW_003383603.1|	61360-62596	188893	666687	-150248	0.50785	no
gi|320446968|ref|NW_003383603.1|	;15587-61716!	501247	444656	-0.17283	0.9324	no
gi|320446968|ref|NW_003383603.1|	;17498-61837(	477226	602405	0.336059	0.8668	no
gi|320446968|ref|NW_003383603.1|	;19988-62023!	126517	280344	114787	0.58485	no
gi|320446968|ref|NW_003383603.1|	;20343-62119'	845815	208406	130098	0.5518	no
gi|320446968|ref|NW_003383603.1|	;37472-64220!	54347	155144	151333	0.25225	no
gi|320446968|ref|NW_003383603.1|	;46467-64887(	252292	11871	-108766	0.3994	no
gi|320446968|ref|NW_003383603.1|	64836-68771	525384	331675	-0.663603	0.615	no
gi|320446968|ref|NW_003383603.1|	;49553-65089!	182994	0.847077	-111123	0.59005	no
gi|320446968|ref|NW_003383603.1|	;53183-65470!	708246	136821	-237196	0.2882	no
gi|320446968|ref|NW_003383603.1|	;59440-66081'	202512	903528	-116437	0.46985	no
gi|320446968|ref|NW_003383603.1|	;61618-66191!	564639	237189	-125129	0.54955	no
gi|320446968|ref|NW_003383603.1|	;70585-67122!	0.412318	589403	383742	0.20095	no
gi|320446968|ref|NW_003383603.1|	;72050-67250(	0	243814	inf	0.0089	no
gi|320446968|ref|NW_003383603.1|	;77645-67790(	0	195817	inf	0.0294	no
gi|320446968|ref|NW_003383603.1|	;78558-67970(	0.369843	435499	355769	0.18235	no
gi|320446968|ref|NW_003383603.1|	;80350-68130(	0.234862	4376	421973	0.1914	no
gi|320446968|ref|NW_003383603.1|	;83444-68571:	0.662157	129411	428865	0.06795	no
gi|320446968|ref|NW_003383603.1|	69162-71723	187189	694337	-143079	0.4013	no
gi|320446968|ref|NW_003383603.1|	'07938-70832:	318248	208668	-0.608942	0.7652	no
gi|320446968|ref|NW_003383603.1|	'08837-71043:	782489	950669	0.280872	0.89635	no
gi|320446968|ref|NW_003383603.1|	'11556-71436!	176742	260779	0.56118	0.78875	no
gi|320446968|ref|NW_003383603.1|	'15966-71763!	348409	201578	-0.789441	0.54285	no
gi|320446968|ref|NW_003383603.1|	'19068-71955(	429922	282799	-0.604302	0.77615	no
gi|320446968|ref|NW_003383603.1|	'19707-72427(	151842	157051	0.0486645	0.96955	no
gi|320446968|ref|NW_003383603.1|	72093-73435	731978	127062	-252627	0.2708	no
gi|320446968|ref|NW_003383603.1|	'25448-72682(	0.593878	123744	105913	1	no

gi|320446968|ref|NW_003383603.1|	'27024-72935:	807562	623971	-0.372093	0.78445	no
gi|320446968|ref|NW_003383603.1|	'29516-73003'	935059	12567	0.426516	0.83145	no
gi|320446968|ref|NW_003383603.1|	'30180-73102	0.554657	190776	178221	0.3324	no
gi|320446968|ref|NW_003383603.1|	'31164-73784	144514	243714	0.753978	0.57105	no
gi|320446968|ref|NW_003383603.1|	'37951-74499	0.917549	128652	0.487613	1	no
gi|320446968|ref|NW_003383603.1|	'48771-74949	120139	51692	-12167	0.56035	no
gi|320446968|ref|NW_003383603.1|	74992-77028	756252	402596	-0.909535	0.5012	no
gi|320446968|ref|NW_003383603.1|	'67583-76776	132194	162789	-302158	0.26075	no
gi|320446968|ref|NW_003383603.1|	77456-79934	266348	23541	-0.178138	0.89465	no
gi|320446968|ref|NW_003383603.1|	'93748-79495'	977595	304017	-168508	0.2089	no
gi|320446968|ref|NW_003383603.1|	80085-82430	151169	893441	-0.758718	0.64505	no
gi|320446968|ref|NW_003383603.1|	317306-81871	343946	328961	-0.0642659	0.96085	no
gi|320446968|ref|NW_003383603.1|	318832-82105	662171	444346	-0.575521	0.7949	no
gi|320446968|ref|NW_003383603.1|	321560-82267	0.770951	106739	0.469379	1	no
gi|320446968|ref|NW_003383603.1|	323233-82358	285383	160307	-0.832057	0.67715	no
gi|320446968|ref|NW_003383603.1|	325136-82539	240847	248049	0.04251	0.95445	no
gi|320446968|ref|NW_003383603.1|	83135-83736	487564	935729	-238143	0.29725	no
gi|320446968|ref|NW_003383603.1|	85771-86743	458241	506176	-31784	0.0963	no
gi|320446968|ref|NW_003383603.1|	359782-86047	488671	23517	-105516	0.5088	no
gi|320446968|ref|NW_003383603.1|	87014-87270	178466	16971	-339451	0.16755	no
gi|320446968|ref|NW_003383603.1|	87400-87953	274344	391541	-280875	0.2279	no
gi|320446968|ref|NW_003383603.1|	378837-87915	114323	108485	324631	0.1872	no
gi|320446968|ref|NW_003383603.1|	88168-89369	725291	323465	-116495	0.37855	no
gi|320446968|ref|NW_003383603.1|	383834-88497	0	245758	inf	0.0212	no
gi|320446968|ref|NW_003383603.1|	385337-88664	629321	838673	373624	0.06915	no
gi|320446968|ref|NW_003383603.1|	390245-89294	0.273625	630948	452725	0.14465	no
gi|320446968|ref|NW_003383603.1|	394169-89478	109413	222977	102711	0.6219	no
gi|320446968|ref|NW_003383603.1|	900408-90344	837737	321942	194223	0.1453	no
gi|320446968|ref|NW_003383603.1|	90124-92900	482162	380592	-0.341272	0.79925	no
gi|320446968|ref|NW_003383603.1|	907514-91225	16634	249537	0.585116	0.6663	no

gi|320446968|ref|NW_003383603.1|	93027-94189	947059	118608	0.324674	0.8102	no
gi|320446968|ref|NW_003383603.1|	939913-94051[illegible]	148784	100853	-0.560961	0.7853	no
gi|320446968|ref|NW_003383603.1|	981091-98179[illegible]	431095	439626	33502	0.0878	no
gi|320446968|ref|NW_003383603.1|	984251-98510[illegible]	604164	273978	218105	0.3344	no
gi|320446971|ref|NW_003383600.1|	106870-107435	559833	0	#NAME?	0.02105	no
gi|320446971|ref|NW_003383600.1|	109577-110625	394602	0	#NAME?	0.01335	no
gi|320446971|ref|NW_003383600.1|	17153-18046	0.256656	229875	316294	0.24575	no
gi|320446971|ref|NW_003383600.1|	20291-21519	0	17684	inf	0.02205	no
gi|320446971|ref|NW_003383600.1|	207336-20858[illegible]	0	132674	inf	0.0054	no
gi|320446971|ref|NW_003383600.1|	210687-21094[illegible]	0	270332	inf	0.0233	no
gi|320446971|ref|NW_003383600.1|	21626-22508	0	197755	inf	0.0294	no
gi|320446971|ref|NW_003383600.1|	218154-21852[illegible]	0	413997	inf	0.00785	no
gi|320446971|ref|NW_003383600.1|	227645-229534	489808	106684	-219887	0.3178	no
gi|320446971|ref|NW_003383600.1|	237977-238629	60675	0	#NAME?	0.01585	no
gi|320446971|ref|NW_003383600.1|	25934-26821	0.777061	142739	0.877283	0.68225	no
gi|320446971|ref|NW_003383600.1|	29584-29957	112377	102914	319502	0.23215	no
gi|320446971|ref|NW_003383600.1|	30386-31599	0.345246	382974	347155	0.18895	no
gi|320446971|ref|NW_003383600.1|	32140-33234	0.196555	258453	37169	0.21225	no
gi|320446971|ref|NW_003383600.1|	324459-32506[illegible]	0.457514	279275	26098	0.2636	no
gi|320446971|ref|NW_003383600.1|	346633-347465	0	447428	inf	0.01485	no
gi|320446971|ref|NW_003383600.1|	347940-348449	0	694438	inf	0.02075	no
gi|320446971|ref|NW_003383600.1|	353315-354009	0	251278	inf	0.0312	no
gi|320446971|ref|NW_003383600.1|	359269-35978[illegible]	0	642841	inf	0.0198	no
gi|320446971|ref|NW_003383600.1|	383250-383814	163342	0	#NAME?	0.0085	no
gi|320446971|ref|NW_003383600.1|	69376-70348	0.229121	253088	346546	0.21395	no
gi|320446971|ref|NW_003383600.1|	71291-72476	0.533143	184744	179293	0.4162	no
gi|320446975|ref|NW_003383596.1|	11291-11967	110982	731185	-0.602023	0.7637	no
gi|320446975|ref|NW_003383596.1|	118634-123935	952592	280026	-17663	0.3139	no
gi|320446975|ref|NW_003383596.1|	124049-12649[illegible]	108583	619432	-0.809787	0.61485	no
gi|320446975|ref|NW_003383596.1|	12471-13126	602464	437824	-0.460526	0.81485	no

gi|320446975|ref|NW_003383596.1|	140627-14277	0.617239	516787	306567	0.20565	no
gi|320446975|ref|NW_003383596.1|	15930-17729	361525	124805	-153442	0.3851	no
gi|320446975|ref|NW_003383596.1|	190781-19137	472625	39382	305877	0.20945	no
gi|320446975|ref|NW_003383596.1|	196271-19688	400019	555252	3795	0.07455	no
gi|320446975|ref|NW_003383596.1|	202754-20426	135358	197478	386684	0.0682	no
gi|320446975|ref|NW_003383596.1|	20459-21230	252106	194556	-0.373841	0.84555	no
gi|320446975|ref|NW_003383596.1|	218-1111	274622	134388	-103104	0.638	no
gi|320446975|ref|NW_003383596.1|	25166-25576	367691	424414	0.206977	0.8989	no
gi|320446975|ref|NW_003383596.1|	2562-7902	176833	128757	-0.457742	0.7341	no
gi|320446975|ref|NW_003383596.1|	272178-27280	144729	136039	-0.0893338	0.96385	no
gi|320446975|ref|NW_003383596.1|	273182-27429	105524	557869	-0.919568	0.6664	no
gi|320446975|ref|NW_003383596.1|	275364-27704	631644	358316	-0.817879	0.697	no
gi|320446975|ref|NW_003383596.1|	28124-28960	309126	309224	0.000453694	0.96375	no
gi|320446975|ref|NW_003383596.1|	283483-28485	0.742347	835275	349209	0.1758	no
gi|320446975|ref|NW_003383596.1|	285896-28685	0.468419	420243	316535	0.20765	no
gi|320446975|ref|NW_003383596.1|	29481-32148	215112	329688	0.616016	0.7673	no
gi|320446975|ref|NW_003383596.1|	32566-33906	32082	806164	132931	0.5337	no
gi|320446975|ref|NW_003383596.1|	333742-33419	225405	449787	0.996725	0.6698	no
gi|320446975|ref|NW_003383596.1|	336718-33724	4553	382848	-0.250046	0.8978	no
gi|320446975|ref|NW_003383596.1|	344710-34506	276229	310726	0.169779	0.9306	no
gi|320446975|ref|NW_003383596.1|	35090-35548	369455	639379	0.791273	0.7045	no
gi|320446975|ref|NW_003383596.1|	35885-36967	777433	107584	0.468667	0.82095	no
gi|320446975|ref|NW_003383596.1|	382971-38789	67113	929862	0.470424	0.7027	no
gi|320446975|ref|NW_003383596.1|	388308-39070	698362	491991	-0.505343	0.70735	no
gi|320446975|ref|NW_003383596.1|	394905-39549	496996	31.39	-0.662929	0.7676	no
gi|320446975|ref|NW_003383596.1|	403638-40488	543295	273957	-0.987789	0.44985	no
gi|320446975|ref|NW_003383596.1|	406238-40668	435369	175572	-131018	0.53485	no
gi|320446975|ref|NW_003383596.1|	408072-41170	331723	266572	-0.315451	0.8124	no
gi|320446975|ref|NW_003383596.1|	413777-41552	592036	918039	-268906	0.1561	no
gi|320446975|ref|NW_003383596.1|	416876-41816	0.480049	0.888353	0.887952	1	no

gi|320446975|ref|NW_003383596.1|	18343-41948	165487	180298	-319827	0.1896	no
gi|320446975|ref|NW_003383596.1|	38027-44026	240615	252775	0.0711287	0.9575	no
gi|320446975|ref|NW_003383596.1|	40451-44073	465303	257007	-0.856362	0.67	no
gi|320446975|ref|NW_003383596.1|	40943-44217	340371	177016	-0.943223	0.65175	no
gi|320446975|ref|NW_003383596.1|	55282-45553	235121	931857	-133522	0.52545	no
gi|320446975|ref|NW_003383596.1|	64493-46463	430859	85465	-233381	0.25915	no
gi|320446975|ref|NW_003383596.1|	85277-48832	383392	347652	-0.141178	0.94945	no
gi|320446975|ref|NW_003383596.1|	88627-49008	207375	221021	0.0919416	0.961	no
gi|320446975|ref|NW_003383596.1|	97721-50179	594163	124782	107048	0.40245	no
gi|320446975|ref|NW_003383596.1|	01952-50502	148812	378751	-197417	0.25905	no
gi|320446975|ref|NW_003383596.1|	07351-50841	103874	549429	-0.918823	0.6591	no
gi|320446975|ref|NW_003383596.1|	09214-51077	549451	22222	-1306	0.5347	no
gi|320446975|ref|NW_003383596.1|	11099-51271	106259	900986	-0.238006	0.9127	no
gi|320446975|ref|NW_003383596.1|	13823-51724	751874	422273	-0.832316	0.607	no
gi|320446975|ref|NW_003383596.1|	21913-52383	267919	657503	-202672	0.25055	no
gi|320446975|ref|NW_003383596.1|	25862-52764	708262	142704	101067	0.5313	no
gi|320446975|ref|NW_003383596.1|	59696-56024	702994	984131	0.485339	0.80995	no
gi|320446975|ref|NW_003383596.1|	68997-56973	12659	152901	0.27244	0.8972	no
gi|320446975|ref|NW_003383596.1|	70809-57121	0.942762	621173	272003	0.2673	no
gi|320446975|ref|NW_003383596.1|	72403-57332	46978	801197	0.770171	0.7009	no
gi|320446975|ref|NW_003383596.1|	73457-57424	249213	534264	110018	0.50835	no
gi|320446975|ref|NW_003383596.1|	11318-61526	168789	570031	-156611	0.2289	no
gi|320446975|ref|NW_003383596.1|	23863-62441	142448	334589	123196	0.56995	no
gi|320446975|ref|NW_003383596.1|	26071-62643	585979	244904	20633	0.35305	no
gi|320446975|ref|NW_003383596.1|	30114-63034	274806	618673	117076	0.5684	no
gi|320446975|ref|NW_003383596.1|	30803-63321	232671	622792	142046	0.5221	no
gi|320446975|ref|NW_003383596.1|	33928-63413	205557	769485	190436	0.392	no
gi|320446975|ref|NW_003383596.1|	35134-63658	240949	778292	169158	0.2145	no
gi|320446975|ref|NW_003383596.1|	42253-64284	242372	611581	-198661	0.3741	no
gi|320446975|ref|NW_003383596.1|	45245-64660	602897	533811	-0.175583	0.9316	no

gi|320446975|ref|NW_003383596.1|	;46701-64939	226977	670811	-175857	0.3206	no
gi|320446975|ref|NW_003383596.1|	;50130-65105	383466	440679	-31213	0.10225	no
gi|320446975|ref|NW_003383596.1|	;51236-65512	28793	155347	-0.890226	0.5096	no
gi|320446975|ref|NW_003383596.1|	;55341-65674	235579	177729	-0.406532	0.73725	no
gi|320446975|ref|NW_003383596.1|	;57200-66272	0.542144	218762	533454	0.02795	no
gi|320446975|ref|NW_003383596.1|	;62846-66407	186639	345919	421211	0.05795	no
gi|320446975|ref|NW_003383596.1|	;68013-66991	974427	357587	-144626	0.2849	no
gi|320446975|ref|NW_003383596.1|	;72241-67322	171108	442543	-195102	0.15305	no
gi|320446975|ref|NW_003383596.1|	;80641-68558	591745	895074	391896	0.00675	no
gi|320446975|ref|NW_003383596.1|	;85709-68942	843968	716883	308648	0.0286	no
gi|320446975|ref|NW_003383596.1|	;92994-69358	0	139425	inf	5.00E-05	yes
gi|320446975|ref|NW_003383596.1|	;97517-69829	0.310499	191761	262665	0.26355	no
gi|320446975|ref|NW_003383596.1|	'00096-70471	0.231096	101692	545957	0.07235	no
gi|320446975|ref|NW_003383596.1|	'14097-71665	351494	338044	-0.0562878	0.9675	no
gi|320446975|ref|NW_003383596.1|	'17027-72003	368178	10339	-18323	0.1759	no
gi|320446975|ref|NW_003383596.1|	'20237-72648	433.14	350794	-0.304213	0.8535	no
gi|320446975|ref|NW_003383596.1|	'30406-73098	0	590396	inf	0.0154	no
gi|320446975|ref|NW_003383596.1|	'36636-73792	845.84	2332.7	146354	0.43705	no
gi|320446975|ref|NW_003383596.1|	'57958-76194	703.62	3892.09	246768	0.07715	no
gi|320446975|ref|NW_003383596.1|	77254-77945	518288	0.505741	-335728	0.2389	no
gi|320446975|ref|NW_003383596.1|	'73615-77522	771135	109033	0.499708	0.81395	no
gi|320446975|ref|NW_003383596.1|	'77838-78166	583174	128371	-218361	0.1958	no
gi|320446975|ref|NW_003383596.1|	'81938-78464	437243	243089	-416888	0.04535	no
gi|320446975|ref|NW_003383596.1|	'90869-79193	144608	352198	-203769	0.36285	no
gi|320446975|ref|NW_003383596.1|	'97322-79913	26774	0.820773	-170578	0.4281	no
gi|320446975|ref|NW_003383596.1|	300007-80045	855189	0	#NAME?	0.02105	no
gi|320446975|ref|NW_003383596.1|	300580-80099	349307	303153	-352638	0.1907	no
gi|320446975|ref|NW_003383596.1|	301486-81065	230218	527193	-21266	0.1194	no
gi|320446975|ref|NW_003383596.1|	311395-81245	265416	0.84729	-496926	0.1292	no
gi|320446975|ref|NW_003383596.1|	312588-81465	917146	115727	-630836	0.0338	no

gi|320446975|ref|NW_003383596.1|	316151-81665	230907	0.836546	-478672	0.19555	no
gi|320446975|ref|NW_003383596.1|	316765-81741	50397	0.830373	-592343	0.11065	no
gi|320446975|ref|NW_003383596.1|	319553-82224	345203	115089	-490662	0.03915	no
gi|320446975|ref|NW_003383596.1|	328180-82868	360493	331989	-0.118838	0.9553	no
gi|320446975|ref|NW_003383596.1|	330147-83218	224409	166944	-0.426765	0.7368	no
gi|320446975|ref|NW_003383596.1|	333205-83444	0.671261	116388	0.793999	1	no
gi|320446975|ref|NW_003383596.1|	335733-83686	509907	287702	-0.825657	0.68895	no
gi|320446975|ref|NW_003383596.1|	337063-83829	138852	904182	-0.618858	0.777	no
gi|320446975|ref|NW_003383596.1|	340328-84210	118138	403855	-154857	0.48435	no
gi|320446975|ref|NW_003383596.1|	8657-11166	153622	10937	-0.490164	0.6983	no
gi|320446975|ref|NW_003383596.1|	381387-88202	535273	232216	-12048	0.45695	no
gi|320446975|ref|NW_003383596.1|	928082-92985	0.547291	282118	236592	0.2919	no
gi|320446975|ref|NW_003383596.1|	930063-93125	0.176969	196244	347108	0.21395	no
gi|320446975|ref|NW_003383596.1|	932374-93302	612559	105667	0.786607	0.7018	no
gi|320446977|ref|NW_003383594.1|	157765-15794	704427	405842	25264	0.2719	no
gi|320446977|ref|NW_003383594.1|	190233-19296	0.0676383	141796	438983	0.1819	no
gi|320446977|ref|NW_003383594.1|	223128-22524	0.629075	714711	350606	0.16505	no
gi|320446977|ref|NW_003383594.1|	268595-26983	126364	0	#NAME?	0.00465	no
gi|320446977|ref|NW_003383594.1|	395201-39552	321575	2075	-395398	0.2231	no
gi|320446977|ref|NW_003383594.1|	482424-48312	653556	0	#NAME?	0.01345	no
gi|320446977|ref|NW_003383594.1|	499659-50007	131924	0	#NAME?	0.01585	no
gi|320446977|ref|NW_003383594.1|	500607-50139	396359	0.209308	-424311	0.3017	no
gi|320446977|ref|NW_003383594.1|	502468-50347	417742	0.151895	-478147	0.2877	no
gi|320446977|ref|NW_003383594.1|	503778-50441	6727	0	#NAME?	0.015	no
gi|320446977|ref|NW_003383594.1|	506182-50676	588156	0.331594	-414871	0.3068	no
gi|320446977|ref|NW_003383594.1|	518289-51889	450305	366709	-0.29627	0.8822	no
gi|320446977|ref|NW_003383594.1|	519119-51977	208364	122858	-0.76211	0.70925	no
gi|320446977|ref|NW_003383594.1|	519915-52146	103004	143295	-284563	0.216	no
gi|320446977|ref|NW_003383594.1|	521725-52199	311054	164865	-0.91588	0.66675	no
gi|320446977|ref|NW_003383594.1|	523564-52520	794597	23463	-175983	0.42075	no

gi|320446977|ref|NW_003383594.1|	25353-52631	997409	192124	-237615	0.29605	no
gi|320446977|ref|NW_003383594.1|	28408-53295	203536	890853	-119202	0.3758	no
gi|320446977|ref|NW_003383594.1|	33613-53424	162565	5526	-155671	0.46945	no
gi|320446977|ref|NW_003383594.1|	43496-54476	0.984372	102435	0.0574391	1	no
gi|320446977|ref|NW_003383594.1|	63016-56376	174519	287621	-260114	0.1681	no
gi|320446977|ref|NW_003383594.1|	69188-57226	844058	334994	-133321	0.3131	no
gi|320446977|ref|NW_003383594.1|	22184-62303	165041	155187	-0.0888147	0.9658	no
gi|320446977|ref|NW_003383594.1|	24106-62485	995475	863888	-0.204541	0.9171	no
gi|320446977|ref|NW_003383594.1|	25097-62563	60046	662044	0.140861	0.93715	no
gi|320446977|ref|NW_003383594.1|	25790-62777	244561	498093	102622	0.43265	no
gi|320446978|ref|NW_003383593.1|	02095-10024	0	212238	inf	0.0101	no
gi|320446978|ref|NW_003383593.1|	02744-10030	0	977494	inf	0.0294	no
gi|320446978|ref|NW_003383593.1|	05324-10075	0	435545	inf	0.0053	no
gi|320446978|ref|NW_003383593.1|	21780-10221	0	100763	inf	0.00325	no
gi|320446978|ref|NW_003383593.1|	64760-10660	0.631678	569952	317358	0.20995	no
gi|320446978|ref|NW_003383593.1|	12687-12890	137038	166722	36048	0.18	no
gi|320446978|ref|NW_003383593.1|	58786-15903	103889	787788	292277	0.2294	no
gi|320446978|ref|NW_003383593.1|	57468-25884	117.99	248635	-224656	0.10045	no
gi|320446978|ref|NW_003383593.1|	59025-26056	400911	550474	-286453	0.12345	no
gi|320446978|ref|NW_003383593.1|	61429-26295	316333	58422	-243686	0.17405	no
gi|320446978|ref|NW_003383593.1|	63230-26365	520169	286612	-0.859881	0.688	no
gi|320446978|ref|NW_003383593.1|	68335-26904	320608	194878	-0.718236	0.72925	no
gi|320446978|ref|NW_003383593.1|	83424-28376	844617	173421	103791	0.6039	no
gi|320446978|ref|NW_003383593.1|	88339-28879	135059	35125	-526495	0.0854	no
gi|320446978|ref|NW_003383593.1|	98002-29859	155966	160089	-660621	0.1123	no
gi|320446978|ref|NW_003383593.1|	09594-31036	695887	130178	-241836	0.2893	no
gi|320446978|ref|NW_003383593.1|	24386-32486	138023	829164	258675	0.2369	no
gi|320446978|ref|NW_003383593.1|	26278-32893	397819	227723	251709	0.1765	no
gi|320446978|ref|NW_003383593.1|	35410-33574	221298	145264	-0.607321	0.7142	no
gi|320446978|ref|NW_003383593.1|	35860-33823	515144	412628	-0.320131	0.8123	no

gi|320446978|ref|NW_003383593.1|	33671-33898	443197	278736	265288	0.27135	no
gi|320446978|ref|NW_003383593.1|	339382-33980	101047	978944	-336765	0.1737	no
gi|320446978|ref|NW_003383593.1|	340042-34056	282568	271406	-338008	0.1826	no
gi|320446978|ref|NW_003383593.1|	341260-34199	654757	597933	-34529	0.08275	no
gi|320446978|ref|NW_003383593.1|	348612-35171	359886	69606	-237026	0.21075	no
gi|320446978|ref|NW_003383593.1|	363862-36788	0.401131	262078	270785	0.2478	no
gi|320446978|ref|NW_003383593.1|	453639-45584	231029	2138.18	321024	0.113	no
gi|320446978|ref|NW_003383593.1|	461481-46299	163247	304904	0.901295	0.4859	no
gi|320446978|ref|NW_003383593.1|	471569-47182	587802	503203	309774	0.2126	no
gi|320446978|ref|NW_003383593.1|	476966-47778	0.861732	270508	497229	0.1488	no
gi|320446978|ref|NW_003383593.1|	512762-51484	228195	110151	227114	0.3228	no
gi|320446978|ref|NW_003383593.1|	524433-52558	0	116309	inf	0.00445	no
gi|320446978|ref|NW_003383593.1|	525836-52712	0	255641	inf	0.01485	no
gi|320446978|ref|NW_003383593.1|	539715-54229	122238	738387	259469	0.25515	no
gi|320446978|ref|NW_003383593.1|	554245-55488	251643	713319	150317	0.47755	no
gi|320446978|ref|NW_003383593.1|	556756-56126	128462	72521	-0.824868	0.522	no
gi|320446978|ref|NW_003383593.1|	561886-56418	159013	165924	-326055	0.0874	no
gi|320446978|ref|NW_003383593.1|	564506-56525	194632	180835	-342801	0.17185	no
gi|320446978|ref|NW_003383593.1|	565416-56653	150373	263584	-251222	0.28025	no
gi|320446978|ref|NW_003383593.1|	567907-56923	554496	513291	-0.1114	0.95345	no
gi|320446978|ref|NW_003383593.1|	578473-57968	727249	88832	0.28863	0.88985	no
gi|320446978|ref|NW_003383593.1|	586294-58670	367691	666937	0.859054	0.6944	no
gi|320446978|ref|NW_003383593.1|	586831-59060	10266	418713	202809	0.13995	no
gi|320446978|ref|NW_003383593.1|	600544-60385	216078	725012	-157547	0.23415	no
gi|320446978|ref|NW_003383593.1|	605619-60640	895507	445045	-100875	0.62005	no
gi|320446978|ref|NW_003383593.1|	607536-60864	287837	144423	-0.994949	0.5444	no
gi|320446978|ref|NW_003383593.1|	609966-61421	546223	700155	0.358183	0.8273	no
gi|320446978|ref|NW_003383593.1|	616067-62035	71592	115699	0.692507	0.58785	no
gi|320446978|ref|NW_003383593.1|	620593-62319	157185	219617	0.482525	0.8145	no
gi|320446978|ref|NW_003383593.1|	623527-62588	0.955726	100088	0.0665957	1	no

gi|320446978|ref|NW_003383593.1|	26004-62747	123213	0.57102	-110955	1	no
gi|320446978|ref|NW_003383593.1|	27581-62951	416736	525703	0.335114	0.8726	no
gi|320446978|ref|NW_003383593.1|	29947-63070	224357	285707	0.34874	0.8581	no
gi|320446978|ref|NW_003383593.1|	30874-63200	264692	235653	-0.167654	0.932	no
gi|320446978|ref|NW_003383593.1|	37422-63851	197707	20522	0.0538059	0.9611	no
gi|320446978|ref|NW_003383593.1|	39090-64352	969168	571647	-0.761623	0.5531	no
gi|320446978|ref|NW_003383593.1|	43706-64679	343776	276378	-0.314827	0.8113	no
gi|320446978|ref|NW_003383593.1|	76875-67972	238957	157992	272503	0.1416	no
gi|320446978|ref|NW_003383593.1|	80024-68378	183397	173555	324235	0.10375	no
gi|320446978|ref|NW_003383593.1|	85385-68746	0.642071	0.959704	0.579855	1	no
gi|320446978|ref|NW_003383593.1|	87565-69003	128136	132127	0.0442505	0.97215	no
gi|320446978|ref|NW_003383593.1|	91054-69220	343202	38727	0.174282	0.89205	no
gi|320446978|ref|NW_003383593.1|	92715-69456	667614	65424	-0.0291957	0.98765	no
gi|320446978|ref|NW_003383593.1|	94732-69523	195551	0.845209	-453209	0.20425	no
gi|320446978|ref|NW_003383593.1|	95355-69697	99647	126861	-297358	0.209	no
gi|320446978|ref|NW_003383593.1|	98908-69953	723644	0.578888	-364392	0.2217	no
gi|320446978|ref|NW_003383593.1|	99653-70264	778485	128566	-259816	0.13785	no
gi|320446978|ref|NW_003383593.1|	70942-71152	238237	856864	184667	0.4072	no
gi|320446978|ref|NW_003383593.1|	19115-72971	255406	863082	17567	0.29555	no
gi|320446978|ref|NW_003383593.1|	30212-73298	146716	855069	254301	0.0649	no
gi|320446978|ref|NW_003383593.1|	66933-76832	170116	122223	-0.477005	0.83145	no
gi|320446978|ref|NW_003383593.1|	68975-77046	0.268791	0.934439	179762	1	no
gi|320446978|ref|NW_003383593.1|	71827-77394	237265	849492	-148183	0.39225	no
gi|320446978|ref|NW_003383593.1|	74085-78119	215634	245425	0.186693	0.88605	no
gi|320446978|ref|NW_003383593.1|	82673-78786	130713	236805	0.857291	0.52275	no
gi|320446978|ref|NW_003383593.1|	88030-78965	887101	143776	0.69665	0.76275	no
gi|320446978|ref|NW_003383593.1|	89824-79123	446122	729913	0.710285	0.7321	no
gi|320446978|ref|NW_003383593.1|	92123-79313	284317	37793	0.410617	0.7371	no
gi|320446978|ref|NW_003383593.1|	94101-79459	165198	238303	0.5286	0.8012	no
gi|320446978|ref|NW_003383593.1|	94738-79566	115363	143863	0.318505	0.8808	no

gi|320446978|ref|NW_003383593.1|	799057-79956	243552	449342	0.883588	0.6898	no
gi|320446978|ref|NW_003383593.1|	812117-81675	583521	211573	-146363	0.26975	no
gi|320446978|ref|NW_003383593.1|	817996-81899	597527	259886	-120112	0.56645	no
gi|320446978|ref|NW_003383593.1|	819110-81944	198471	367582	-243279	0.3041	no
gi|320446978|ref|NW_003383593.1|	820146-82067	259252	341343	-292506	0.20825	no
gi|320446978|ref|NW_003383593.1|	823538-82455	402034	765827	-239223	0.1703	no
gi|320446978|ref|NW_003383593.1|	828568-82921	281737	219948	-0.35719	0.86805	no
gi|320446978|ref|NW_003383593.1|	830252-83148	34478	21082	-0.709668	0.57675	no
gi|320446978|ref|NW_003383593.1|	832100-83409	29507	130689	-117492	0.36365	no
gi|320446978|ref|NW_003383593.1|	834656-83545	390712	288949	-0.435291	0.82245	no
gi|320446978|ref|NW_003383593.1|	836011-83623	405263	566227	-283941	0.27475	no
gi|320446978|ref|NW_003383593.1|	837608-83851	933998	330545	-149857	0.48425	no
gi|320446978|ref|NW_003383593.1|	851489-85301	365817	490668	0.423626	0.83115	no
gi|320446978|ref|NW_003383593.1|	866547-86737	116776	0.391639	-489807	0.19365	no
gi|320446978|ref|NW_003383593.1|	868577-86974	795348	0.50098	-398876	0.1806	no
gi|320446978|ref|NW_003383593.1|	870335-87114	643997	0.201138	-50008	0.28045	no
gi|320446978|ref|NW_003383593.1|	871934-87233	186732	0.646432	-485232	0.28715	no
gi|320446978|ref|NW_003383593.1|	895259-89601	128692	694862	575473	0.103	no
gi|320446978|ref|NW_003383593.1|	902890-90335	0	554583	inf	0.0054	no
gi|320446978|ref|NW_003383593.1|	912699-91420	106414	194241	0.868155	0.6722	no
gi|320446978|ref|NW_003383593.1|	914629-91700	315596	986063	16436	0.32175	no
gi|320446978|ref|NW_003383593.1|	918748-91907	921533	287874	164333	0.4341	no
gi|320446978|ref|NW_003383593.1|	923975-92612	264808	57891	112839	0.6008	no
gi|320446978|ref|NW_003383593.1|	926667-92750	224073	443064	0.983547	0.64705	no
gi|320446978|ref|NW_003383593.1|	932107-93240	196455	113332	252829	0.27435	no
gi|320446978|ref|NW_003383593.1|	935937-93628	705137	15284	-220589	0.3144	no
gi|320446978|ref|NW_003383593.1|	938553-93896	438612	153786	-151202	0.47295	no
gi|320446978|ref|NW_003383593.1|	939467-94082	268289	753615	-183189	0.2827	no
gi|320446978|ref|NW_003383593.1|	941375-94434	588044	33251	-0.822524	0.53875	no
gi|320446978|ref|NW_003383593.1|	952951-95667	374882	349369	322025	0.0818	no

gi|320446978|ref|NW_003383593.1|	980369-98097	273052	185231	-388178	0.15915	no
gi|320446978|ref|NW_003383593.1|	981893-98223	439472	459478	-32577	0.21085	no
gi|320446978|ref|NW_003383593.1|	983601-98550	368098	697832	-239914	0.1934	no
gi|320446980|ref|NW_003383591.1|	012402-10145	128355	249329	0.95791	0.46345	no
gi|320446980|ref|NW_003383591.1|	021633-10222	196172	265181	0.43486	0.83305	no
gi|320446980|ref|NW_003383591.1|	030192-10304	0	721713	inf	0.0072	no
gi|320446980|ref|NW_003383591.1|	030609-10313	0	737102	inf	0.01195	no
gi|320446980|ref|NW_003383591.1|	032570-10328	0	253358	inf	0.01575	no
gi|320446980|ref|NW_003383591.1|	063143-10639	261826	142327	-0.879398	0.68145	no
gi|320446980|ref|NW_003383591.1|	068164-10684	173793	121948	-0.511106	0.8032	no
gi|320446980|ref|NW_003383591.1|	079065-10820	936493	15894	0.763146	0.55095	no
gi|320446980|ref|NW_003383591.1|	156480-11573	0	358093	inf	5.00E-05	yes
gi|320446980|ref|NW_003383591.1|	170926-11721	179415	223786	0.318814	0.87525	no
gi|320446980|ref|NW_003383591.1|	182451-11833	0.786723	307019	19644	0.3902	no
gi|320446980|ref|NW_003383591.1|	197349-11976	213064	226794	-323183	0.1805	no
gi|320446980|ref|NW_003383591.1|	203076-12032	522672	46511	-0.168333	0.92605	no
gi|320446980|ref|NW_003383591.1|	203899-12044	164397	129901	-0.339776	0.7892	no
gi|320446980|ref|NW_003383591.1|	207684-12083	465875	289963	-4006	0.0606	no
gi|320446980|ref|NW_003383591.1|	208656-12088	874235	101173	-31112	0.1992	no
gi|320446980|ref|NW_003383591.1|	216914-12173	117996	0	#NAME?	0.0178	no
gi|320446980|ref|NW_003383591.1|	226102-12269	910008	0	#NAME?	0.00615	no
gi|320446980|ref|NW_003383591.1|	281960-12826	0.341182	233582	277531	0.2657	no
gi|320446980|ref|NW_003383591.1|	283944-12849	0.653631	135489	105163	1	no
gi|320446980|ref|NW_003383591.1|	285537-12866	0.987377	204983	105383	0.60035	no
gi|320446980|ref|NW_003383591.1|	292365-12928	181285	293773	0.696438	0.6025	no
gi|320446980|ref|NW_003383591.1|	295229-12962	757409	11.54	0.607499	0.77345	no
gi|320446980|ref|NW_003383591.1|	300452-13038	192428	415052	110898	0.61105	no
gi|320446980|ref|NW_003383591.1|	131308-13270	279889	213679	-0.389408	0.7547	no
gi|320446980|ref|NW_003383591.1|	136832-13833	176496	802832	-113647	0.4814	no
gi|320446980|ref|NW_003383591.1|	140253-14047	803683	415755	-0.950892	0.6443	no

gi|320446980|ref|NW_003383591.1|	140613-14152	918875	359533	-135374	0.5245	no
gi|320446980|ref|NW_003383591.1|	142171-14308	865384	323888	-141785	0.501	no
gi|320446980|ref|NW_003383591.1|	145360-15033	841011	564155	-0.576034	0.651	no
gi|320446980|ref|NW_003383591.1|	159692-16008	282641	271919	-0.0557957	0.97645	no
gi|320446980|ref|NW_003383591.1|	160458-16108	317368	507291	0.676657	0.7644	no
gi|320446980|ref|NW_003383591.1|	190762-19357	326519	230494	-0.502436	0.701	no
gi|320446980|ref|NW_003383591.1|	194989-19649	557369	138097	-201295	0.27215	no
gi|320446980|ref|NW_003383591.1|	197119-19745	577155	136911	-207572	0.33955	no
gi|320446980|ref|NW_003383591.1|	212042-21268	786881	130324	-259405	0.14855	no
gi|320446980|ref|NW_003383591.1|	236237-23750	683741	813235	0.250221	0.90645	no
gi|320446980|ref|NW_003383591.1|	245063-24589	0.566768	175368	162956	0.3326	no
gi|320446980|ref|NW_003383591.1|	254229-25461	155612	612748	-134458	0.5144	no
gi|320446980|ref|NW_003383591.1|	255907-25924	133409	498968	-141884	0.40545	no
gi|320446980|ref|NW_003383591.1|	261888-26291	126586	163112	-295618	0.22165	no
gi|320446980|ref|NW_003383591.1|	263051-26620	172042	391619	-213524	0.23865	no
gi|320446980|ref|NW_003383591.1|	267188-26809	160834	136834	-0.233145	0.90995	no
gi|320446980|ref|NW_003383591.1|	318895-32001	437796	579884	0.405507	0.84395	no
gi|320446980|ref|NW_003383591.1|	53849-57383	439898	165343	191022	0.2934	no
gi|320446980|ref|NW_003383591.1|	565211-56566	153492	765048	231738	0.27165	no
gi|320446980|ref|NW_003383591.1|	58219-59230	478711	159374	173519	0.4237	no
gi|320446980|ref|NW_003383591.1|	59642-60757	613956	796955	0.376365	0.85355	no
gi|320446980|ref|NW_003383591.1|	698047-69847	0	109632	inf	0.0162	no
gi|320446980|ref|NW_003383591.1|	706344-70746	0.191426	702403	519744	0.16715	no
gi|320446980|ref|NW_003383591.1|	761606-76220	185478	534388	152664	0.4978	no
gi|320446980|ref|NW_003383591.1|	76833-77488	401643	492552	0.294362	0.8793	no
gi|320446980|ref|NW_003383591.1|	774431-77715	108526	0.710964	-0.61019	1	no
gi|320446980|ref|NW_003383591.1|	777263-77809	169745	11672	-0.540317	0.7982	no
gi|320446980|ref|NW_003383591.1|	778286-77920	0.737435	169492	120063	0.56655	no
gi|320446980|ref|NW_003383591.1|	779615-78133	0.910518	0.554798	-0.714725	1	no
gi|320446980|ref|NW_003383591.1|	789679-79221	0.807119	205006	134481	0.51765	no

gi|320446980|ref|NW_003383591.1|	'94041-79836'	156738	213716	0.447343	0.8308	no
gi|320446980|ref|NW_003383591.1|	300186-80095:	828614	161736	0.964865	0.647	no
gi|320446980|ref|NW_003383591.1|	302563-80514:	115146	673659	254856	0.2667	no
gi|320446980|ref|NW_003383591.1|	306209-80673(	687448	354448	236625	0.2927	no
gi|320446980|ref|NW_003383591.1|	328792-83066'	288161	136278	-108032	0.60075	no
gi|320446980|ref|NW_003383591.1|	331542-83382:	759246	558763	-0.44233	0.84025	no
gi|320446980|ref|NW_003383591.1|	336386-83758;	0	169434	inf	0.0233	no
gi|320446980|ref|NW_003383591.1|	343590-84468:	335316	900886	142582	0.50035	no
gi|320446980|ref|NW_003383591.1|	361583-86206(	684772	823008	0.265282	0.88685	no
gi|320446980|ref|NW_003383591.1|	363610-86436(	393561	494476	0.329314	0.8644	no
gi|320446980|ref|NW_003383591.1|	365937-86633!	670194	113477	0.759744	0.70415	no
gi|320446980|ref|NW_003383591.1|	367126-86951(	109638	271216	130669	0.31495	no
gi|320446980|ref|NW_003383591.1|	371471-87190;	728014	965514	0.407331	0.8392	no
gi|320446980|ref|NW_003383591.1|	372084-87348!	608054	714133	0.231993	0.9134	no
gi|320446980|ref|NW_003383591.1|	373630-87413·	105342	581605	-0.856966	0.6721	no
gi|320446980|ref|NW_003383591.1|	375021-87590·	114673	735971	-0.639806	0.7514	no
gi|320446980|ref|NW_003383591.1|	376017-87660(	123562	547204	-117509	0.56975	no
gi|320446980|ref|NW_003383591.1|	378249-87858·	373864	298116	-0.326639	0.87305	no
gi|320446980|ref|NW_003383591.1|	378919-87940:	153336	178253	-31047	0.23425	no
gi|320446980|ref|NW_003383591.1|	382841-88395:	346928	0.266848	-370055	0.22495	no
gi|320446980|ref|NW_003383591.1|	384057-88468!	471763	0.583093	-301626	0.25735	no
gi|320446980|ref|NW_003383591.1|	387581-88788;	428565	0	#NAME?	0.00975	no
gi|320446980|ref|NW_003383591.1|	389367-89041:	104099	100772	-0.0468608	1	no
gi|320446980|ref|NW_003383591.1|	391323-89171:	196024	203172	-327026	0.19005	no
gi|320446980|ref|NW_003383591.1|	391938-89307(	673095	0.776926	-311496	0.2149	no
gi|320446980|ref|NW_003383591.1|	394032-89451(	429742	104773	-20362	0.35405	no
gi|320446980|ref|NW_003383591.1|	915038-91733;	294218	273839	-0.103556	0.9566	no
gi|320446980|ref|NW_003383591.1|	94643-95099	305483	376783	0.302639	0.8863	no
gi|320446980|ref|NW_003383591.1|	984913-98742:	0.519267	647646	364066	0.15375	no
gi|320446980|ref|NW_003383591.1|	989513-99001!	100476	740343	-0.440582	0.72195	no

gi|320446980|ref|NW_003383591.1|	)91867-99305(	455909	255477	-0.835552	0.5169	no
gi|320446980|ref|NW_003383591.1|	)93875-99589!	236379	21364	-0.145922	0.91085	no
gi|320446980|ref|NW_003383591.1|	)96207-99843	271416	384746	0.503398	0.69915	no
gi|320446981|ref|NW_003383590.1|	)56223-10594	144211	612631	-12351	0.33845	no
gi|320446981|ref|NW_003383590.1|	l58058-15893(	525299	633063	0.269211	0.8967	no
gi|320446981|ref|NW_003383590.1|	l59252-15991!	157685	510608	169517	0.443	no
gi|320446981|ref|NW_003383590.1|	l60247-16133	611458	846287	0.468893	0.8238	no
gi|320446981|ref|NW_003383590.1|	l61504-16429	196597	49703	133809	0.32055	no
gi|320446981|ref|NW_003383590.1|	!40156-24053(	1119.41	110375	-334225	0.108	no
gi|320446981|ref|NW_003383590.1|	!41141-24243!	155635	264523	-25567	0.2706	no
gi|320446981|ref|NW_003383590.1|	!76319-27893(	131826	167519	0.345689	0.779	no
gi|320446981|ref|NW_003383590.1|	!80171-28057	653252	492116	-0.408642	0.84605	no
gi|320446981|ref|NW_003383590.1|	!81124-28181(	542706	153341	-182342	0.2767	no
gi|320446981|ref|NW_003383590.1|	!83898-28415	156031	558134	-148315	0.4935	no
gi|320446981|ref|NW_003383590.1|	!89600-29346	424.65	342142	-363361	0.04595	no
gi|320446981|ref|NW_003383590.1|	!95406-29650	751697	283059	-473098	0.03055	no
gi|320446981|ref|NW_003383590.1|	!96643-29764!	385454	166444	-453345	0.0655	no
gi|320446981|ref|NW_003383590.1|	314359-31526	737734	0	#NAME?	5.00E-05	yes
gi|320446981|ref|NW_003383590.1|	316625-31817	525487	0.445777	-688119	0.1122	no
gi|320446981|ref|NW_003383590.1|	l10197-41193!	363065	740733	102873	0.63035	no
gi|320446981|ref|NW_003383590.1|	l13744-41402	177553	425239	-206191	0.28725	no
gi|320446981|ref|NW_003383590.1|	l83600-48565(	232471	205592	314466	0.10035	no
gi|320446981|ref|NW_003383590.1|	l85806-48767	13461	173911	369148	0.06985	no
gi|320446981|ref|NW_003383590.1|	l87932-49006	409682	388345	324476	0.0954	no
gi|320446981|ref|NW_003383590.1|	l90183-49204(	478664	0.652638	-287466	0.21235	no
gi|320446981|ref|NW_003383590.1|	l92736-49439	201647	0.0825704	-461006	0.28735	no
gi|320446981|ref|NW_003383590.1|	l96099-49680	320608	0.730794	-213327	0.27995	no
gi|320446981|ref|NW_003383590.1|	500877-50152	45355	0	#NAME?	0.02105	no
gi|320446981|ref|NW_003383590.1|	536456-54206(	847595	288161	176543	0.19395	no
gi|320446981|ref|NW_003383590.1|	542281-54256	114309	196057	0.778336	0.72195	no

gi|320446981|ref|NW_003383590.1|	42698-54287	562784	951609	0.757787	0.7189	no
gi|320446981|ref|NW_003383590.1|	44215-54454	240315	427476	0.830914	0.6841	no
gi|320446981|ref|NW_003383590.1|	45162-54595	108584	147051	0.437501	0.83895	no
gi|320446981|ref|NW_003383590.1|	72780-57333	573347	299045	238288	0.2869	no
gi|320446981|ref|NW_003383590.1|	75282-57580	0	434249	inf	0.0056	no
gi|320446981|ref|NW_003383590.1|	92122-59243	239881	981832	203315	0.3563	no
gi|320446981|ref|NW_003383590.1|	92560-59602	198773	14016	-0.504044	0.69565	no
gi|320446981|ref|NW_003383590.1|	96953-59817	584459	142607	-203505	0.25725	no
gi|320446981|ref|NW_003383590.1|	01000-60323	411795	111896	-187976	0.15675	no
gi|320446981|ref|NW_003383590.1|	04728-60722	197313	228113	0.209262	0.8711	no
gi|320446981|ref|NW_003383590.1|	07941-61414	0.961275	253642	139977	0.51855	no
gi|320446981|ref|NW_003383590.1|	14302-61685	0.801888	132395	0.723375	1	no
gi|320446981|ref|NW_003383590.1|	21873-62286	740018	619489	-0.256482	0.8982	no
gi|320446981|ref|NW_003383590.1|	24621-62551	180207	0.709424	-134494	0.52925	no
gi|320446981|ref|NW_003383590.1|	25700-62671	112281	283512	-198563	0.3695	no
gi|320446981|ref|NW_003383590.1|	27596-62826	113534	400423	-150353	0.4748	no
gi|320446981|ref|NW_003383590.1|	29122-63149	884495	38597	-119637	0.59405	no
gi|320446981|ref|NW_003383590.1|	31997-63596	269699	19987	-0.432292	0.74885	no
gi|320446981|ref|NW_003383590.1|	45985-64853	300263	380689	0.342387	0.7931	no
gi|320446981|ref|NW_003383590.1|	56200-65690	108462	741374	-0.54892	0.7892	no
gi|320446981|ref|NW_003383590.1|	66120-66731	375171	253978	-0.562847	0.65505	no
gi|320446981|ref|NW_003383590.1|	66727-66939	45877	251271	-0.868527	0.67695	no
gi|320446981|ref|NW_003383590.1|	70423-67127	0.545687	262854	226812	0.2738	no
gi|320446981|ref|NW_003383590.1|	74228-67497	0.655935	337143	236174	0.262	no
gi|320446981|ref|NW_003383590.1|	75941-67624	841631	24542	-177794	0.40785	no
gi|320446981|ref|NW_003383590.1|	77640-67819	133113	538657	-130522	0.5321	no
gi|320446981|ref|NW_003383590.1|	78393-68132	937871	563844	-0.734094	0.6503	no
gi|320446981|ref|NW_003383590.1|	83097-68683	428964	404982	-0.0829955	0.95035	no
gi|320446981|ref|NW_003383590.1|	88089-68865	136072	627684	-111626	0.3822	no
gi|320446981|ref|NW_003383590.1|	91886-69738	223547	108543	-104232	0.4366	no

gi|320446981|ref|NW_003383590.1|	;97490-69778:	226197	171112	-0.402642	0.84545	no
gi|320446981|ref|NW_003383590.1|	'06008-70696	313841	598038	-239172	0.1666	no
gi|320446981|ref|NW_003383590.1|	'07680-70985	304397	535557	-250684	0.178	no
gi|320446981|ref|NW_003383590.1|	'10469-71090:	205915	272885	-291569	0.2371	no
gi|320446981|ref|NW_003383590.1|	'11028-71130	397252	790437	-232933	0.3012	no
gi|320446981|ref|NW_003383590.1|	'18558-71921	465536	379636	-36162	0.15775	no
gi|320446981|ref|NW_003383590.1|	'30279-73090	298715	232113	-0.363939	0.8498	no
gi|320446981|ref|NW_003383590.1|	'38689-74034	404097	107038	-191658	0.28205	no
gi|320446981|ref|NW_003383590.1|	'43313-74448:	126533	225441	-248869	0.27455	no
gi|320446981|ref|NW_003383590.1|	'44677-74565	408533	282088	-0.534309	0.789	no
gi|320446981|ref|NW_003383590.1|	'45730-74867	156069	189087	0.276863	0.823	no
gi|320446981|ref|NW_003383590.1|	'48868-75113	13621	395361	153734	0.24245	no
gi|320446981|ref|NW_003383590.1|	'73385-77430:	197802	818243	204847	0.35645	no
gi|320446981|ref|NW_003383590.1|	'88872-78932	976754	199906	-228868	0.3365	no
gi|320446981|ref|NW_003383590.1|	'90052-79128	523351	292695	-0.838378	0.68265	no
gi|320446981|ref|NW_003383590.1|	'92887-79436	839269	649371	-0.370089	0.86285	no
gi|320446981|ref|NW_003383590.1|	82636-83027	111641	186668	0.741607	0.7067	no
gi|320446981|ref|NW_003383590.1|	330506-83089	0	152201	inf	0.0186	no
gi|320446981|ref|NW_003383590.1|	397481-89876	104706	141957	0.439115	0.8385	no
gi|320446981|ref|NW_003383590.1|	)26356-92722:	123119	994949	-0.307355	0.8804	no
gi|320446981|ref|NW_003383590.1|	)56141-95839	125447	682936	244467	0.28615	no
gi|320446981|ref|NW_003383590.1|	)76558-97735:	25986	124486	-438368	0.147	no
gi|320446981|ref|NW_003383590.1|	)77559-97846:	103808	10469	-330972	0.1951	no
gi|320446981|ref|NW_003383590.1|	)81776-98396:	218968	295928	-28874	0.1272	no
gi|320446982|ref|NW_003383589.1|	.82333-18372:	0.292309	111683	193385	1	no
gi|320446982|ref|NW_003383589.1|	!57195-25871	158249	696983	213893	0.3248	no
gi|320446982|ref|NW_003383589.1|	!73003-27418	0.888572	369487	205596	0.35365	no
gi|320446982|ref|NW_003383589.1|	!83710-28391	268437	251314	-0.0950908	0.91535	no
gi|320446982|ref|NW_003383589.1|	!89056-28930	131437	12472	-0.0756846	0.9157	no
gi|320446982|ref|NW_003383589.1|	377556-37840:	717516	493633	-0.539573	0.79485	no

gi|320446982|ref|NW_003383589.1|	60623-60913	269826	969581	184533	0.3873	no
gi|320446982|ref|NW_003383589.1|	43876-64446	339357	440679	0.376925	0.8616	no
gi|320446982|ref|NW_003383589.1|	44608-64789	448008	615108	0.457317	0.84455	no
gi|320446982|ref|NW_003383589.1|	48031-64848	894097	29746	-158774	0.443	no
gi|320446982|ref|NW_003383589.1|	50930-65130	130226	0	#NAME?	0.02015	no
gi|320446982|ref|NW_003383589.1|	53744-65402	396598	297579	-373633	0.2183	no
gi|320446982|ref|NW_003383589.1|	56111-65648	131736	0.718796	-419592	0.3058	no
gi|320446982|ref|NW_003383589.1|	58751-65930	181034	107731	-407075	0.1595	no
gi|320446982|ref|NW_003383589.1|	61075-66144	117196	153065	-29367	0.2676	no
gi|320446982|ref|NW_003383589.1|	80260-68152	360586	193303	-0.899475	0.65675	no
gi|320446982|ref|NW_003383589.1|	90499-69212	0.242343	421666	412098	0.1648	no
gi|320446982|ref|NW_003383589.1|	32365-73276	379041	87392	120515	0.5728	no
gi|320446982|ref|NW_003383589.1|	73910-77515	0.167815	325887	427943	0.1888	no
gi|320446982|ref|NW_003383589.1|	77165-77858	555095	0.0989031	-91325	0.2504	no
gi|320446982|ref|NW_003383589.1|	84037-78493	290021	0.176827	-735768	0.26155	no
gi|320446982|ref|NW_003383589.1|	61479-86195	0	135656	inf	0.00935	no
gi|320446982|ref|NW_003383589.1|	62139-86343	0	146318	inf	0.0037	no
gi|320446982|ref|NW_003383589.1|	63609-86395	0	755707	inf	0.0048	no
gi|320446982|ref|NW_003383589.1|	74572-87554	0	15818	inf	0.0312	no
gi|320446982|ref|NW_003383589.1|	98089-89897	0	358467	inf	0.0162	no
gi|320446982|ref|NW_003383589.1|	00946-90292	0.387546	14254	520086	0.1036	no
gi|320446982|ref|NW_003383589.1|	03769-90724	0.417176	871936	43855	0.0663	no
gi|320446982|ref|NW_003383589.1|	10753-91148	0.341182	443806	370131	0.21225	no
gi|320446982|ref|NW_003383589.1|	18557-91920	52585	24797	-108449	0.5867	no
gi|320446982|ref|NW_003383589.1|	24370-92501	492403	251427	-0.969698	0.65035	no
gi|320446982|ref|NW_003383589.1|	34198-93476	100601	407971	201982	0.3028	no
gi|320446986|ref|NW_003383585.1|	09113-11306	0.272533	181544	605775	0.0719	no
gi|320446986|ref|NW_003383585.1|	13519-11888	0.296257	213333	617011	0.051	no
gi|320446986|ref|NW_003383585.1|	19000-11931	668992	196142	487376	0.1041	no
gi|320446986|ref|NW_003383585.1|	21665-12223	101489	332577	50343	0.1505	no

gi|320446986|ref|NW_003383585.1|	127875-12825	105488	761006	285082	0.25935	no
gi|320446986|ref|NW_003383585.1|	174862-17700	0.708006	778007	345795	0.165	no
gi|320446986|ref|NW_003383585.1|	291479-29214	120533	161759	0.424422	0.83755	no
gi|320446986|ref|NW_003383585.1|	313240-31385	0	393282	inf	0.02915	no
gi|320446986|ref|NW_003383585.1|	344391-34493	0	479594	inf	0.02915	no
gi|320446986|ref|NW_003383585.1|	357399-36132	0.548016	383587	280726	0.23475	no
gi|320446986|ref|NW_003383585.1|	363324-36464	0	358685	inf	5.00E-05	yes
gi|320446986|ref|NW_003383585.1|	368062-37082	0.0667551	158334	112118	0.14075	no
gi|320446986|ref|NW_003383585.1|	38782-39274	388247	125587	169364	0.42645	no
gi|320446986|ref|NW_003383585.1|	435854-43786	200403	216582	0.112013	0.9302	no
gi|320446986|ref|NW_003383585.1|	440403-44124	289851	182116	-0.67045	0.7647	no
gi|320446986|ref|NW_003383585.1|	451380-45355	0	262775	inf	5.00E-05	yes
gi|320446986|ref|NW_003383585.1|	456415-45791	0.133439	113013	972609	0.14075	no
gi|320446986|ref|NW_003383585.1|	470951-47176	233775	461833	0.982248	0.64705	no
gi|320446986|ref|NW_003383585.1|	476665-47740	132991	318884	126171	0.5573	no
gi|320446986|ref|NW_003383585.1|	488887-49036	216759	470933	111943	0.58505	no
gi|320446986|ref|NW_003383585.1|	504533-50674	0.513496	507653	330542	0.1887	no
gi|320446986|ref|NW_003383585.1|	536207-53669	528018	266043	-0.988927	0.6543	no
gi|320446986|ref|NW_003383585.1|	537647-53820	154503	107812	-0.519115	0.79815	no
gi|320446986|ref|NW_003383585.1|	538918-54372	520158	767416	0.561061	0.65245	no
gi|320446986|ref|NW_003383585.1|	548393-54901	140048	0.297302	-555785	0.27475	no
gi|320446986|ref|NW_003383585.1|	551411-55260	270545	0.487081	-579557	0.1295	no
gi|320446986|ref|NW_003383585.1|	556313-55681	291219	212034	-377974	0.1743	no
gi|320446986|ref|NW_003383585.1|	565965-56679	398747	156656	-134788	0.52025	no
gi|320446986|ref|NW_003383585.1|	569397-57005	639616	163439	-196845	0.3815	no
gi|320446986|ref|NW_003383585.1|	570335-57140	563484	264526	-109097	0.5977	no
gi|320446986|ref|NW_003383585.1|	571656-57225	781943	623819	-0.325935	0.8691	no
gi|320446986|ref|NW_003383585.1|	573571-57444	847071	565025	-0.584169	0.77915	no
gi|320446986|ref|NW_003383585.1|	574585-57583	414998	333918	-0.313608	0.8728	no
gi|320446986|ref|NW_003383585.1|	577317-58086	158041	195472	0.306663	0.82065	no

gi|320446986|ref|NW_003383585.1|	;80983-58143:	634276	396198	-0.67889	0.75225	no
gi|320446986|ref|NW_003383585.1|	;82681-58325	164077	141204	-0.216591	0.91375	no
gi|320446986|ref|NW_003383585.1|	;83947-58550	431894	246219	-0.810732	0.53455	no
gi|320446986|ref|NW_003383585.1|	;86655-58720	583966	260094	-116685	0.6031	no
gi|320446986|ref|NW_003383585.1|	;88772-58904	147751	807839	-0.871026	0.67925	no
gi|320446986|ref|NW_003383585.1|	;89234-59040	183157	854376	-110014	0.6225	no
gi|320446986|ref|NW_003383585.1|	;90818-59168	0.799145	421807	240006	0.30005	no
gi|320446986|ref|NW_003383585.1|	;94025-59431	419028	134033	167747	0.34255	no
gi|320446986|ref|NW_003383585.1|	;95235-59609	217922	299367	710196	0.0575	no
gi|320446986|ref|NW_003383585.1|	;96257-59831	0.463664	297522	600377	0.0856	no
gi|320446986|ref|NW_003383585.1|	;98825-59979	0.460116	295398	600452	0.1183	no
gi|320446986|ref|NW_003383585.1|	;04672-60518	604667	475.98	629862	0.04675	no
gi|320446986|ref|NW_003383585.1|	;12646-61368	0.422654	9788	453346	0.1558	no
gi|320446986|ref|NW_003383585.1|	;15478-61594	212835	132444	263758	0.2635	no
gi|320446986|ref|NW_003383585.1|	;16567-61939	39761	0	#NAME?	0.0057	no
gi|320446986|ref|NW_003383585.1|	;19516-62036	903305	0	#NAME?	0.0074	no
gi|320446986|ref|NW_003383585.1|	;36571-63808	0.527497	452122	64214	0.103	no
gi|320446986|ref|NW_003383585.1|	;38364-64002	0	346554	inf	0.0069	no
gi|320446986|ref|NW_003383585.1|	81563-83870	130322	184789	382573	0.0667	no
gi|320446986|ref|NW_003383585.1|	88661-90636	552888	973897	-250515	0.18915	no
gi|320446986|ref|NW_003383585.1|	91758-93229	428834	111081	-19488	0.276	no
gi|320446986|ref|NW_003383585.1|	96897-97812	362583	121556	-157669	0.3443	no
gi|320446986|ref|NW_003383585.1|	97987-101622	638753	307353	-105536	0.42945	no
gi|320446987|ref|NW_003383584.1|	229722-23054	144113	336789	122465	0.56205	no
gi|320446987|ref|NW_003383584.1|	265463-26578	176866	518749	-176955	0.41835	no
gi|320446987|ref|NW_003383584.1|	267578-26839	43382	357797	-0.277958	0.8869	no
gi|320446987|ref|NW_003383584.1|	268660-27026	49391	429616	-0.2012	0.92135	no
gi|320446987|ref|NW_003383584.1|	270377-27191	362171	197929	-0.871688	0.6711	no
gi|320446987|ref|NW_003383584.1|	314128-31762	439311	238655	-0.880318	0.6859	no
gi|320446987|ref|NW_003383584.1|	320450-32083	144439	677239	-109272	0.5974	no

gi|320446987|ref|NW_003383584.1|	21036-32171	26491	129162	-103632	0.59645	no
gi|320446987|ref|NW_003383584.1|	22387-32319	998734	302734	-172205	0.4183	no
gi|320446987|ref|NW_003383584.1|	24888-42520	514032	661909	36867	0.18015	no
gi|320446987|ref|NW_003383584.1|	33353-43389	218349	154477	282269	0.24425	no
gi|320446987|ref|NW_003383584.1|	48112-44863	0.574765	143002	463692	0.1772	no
gi|320446987|ref|NW_003383584.1|	63257-56414	0	121695	inf	0.00505	no
gi|320446987|ref|NW_003383584.1|	47984-65055	0.144735	171876	356989	0.18215	no
gi|320446987|ref|NW_003383584.1|	50666-65200	0.459563	233992	234812	0.3039	no
gi|320446987|ref|NW_003383584.1|	68460-69742	193488	727239	191019	0.37525	no
gi|320446987|ref|NW_003383584.1|	07990-70827	0	527541	inf	0.0088	no
gi|320446987|ref|NW_003383584.1|	23533-72390	0	219373	inf	0.0109	no
gi|320446987|ref|NW_003383584.1|	25861-72693	100502	236403	123402	0.56205	no
gi|320446987|ref|NW_003383584.1|	27477-73064	0.344976	13671	198655	1	no
gi|320446987|ref|NW_003383584.1|	31178-73449	966553	199.64	104648	0.5244	no
gi|320446987|ref|NW_003383584.1|	36080-73672	450287	130997	154061	0.46135	no
gi|320446987|ref|NW_003383584.1|	39439-74040	415772	163366	197424	0.1465	no
gi|320446987|ref|NW_003383584.1|	41519-74280	0.803894	10041	0.320829	1	no
gi|320446987|ref|NW_003383584.1|	42922-74457	118867	0.49646	-125959	1	no
gi|320446987|ref|NW_003383584.1|	44926-74598	0.614731	0.992052	0.690459	1	no
gi|320446987|ref|NW_003383584.1|	69649-77033	527474	0	#NAME?	0.0178	no
gi|320446987|ref|NW_003383584.1|	71816-77228	299164	522317	-251794	0.27615	no
gi|320446987|ref|NW_003383584.1|	76818-77713	181072	530646	-177074	0.41835	no
gi|320446987|ref|NW_003383584.1|	84976-78575	56096	128302	-212836	0.3468	no
gi|320446987|ref|NW_003383584.1|	89647-79026	531979	180608	-15585	0.486	no
gi|320446987|ref|NW_003383584.1|	90794-79194	129021	753277	-0.776352	0.7196	no
gi|320446987|ref|NW_003383584.1|	93921-79708	162416	861929	-0.91405	0.4716	no
gi|320446987|ref|NW_003383584.1|	97280-79763	140829	166627	-307925	0.25595	no
gi|320446987|ref|NW_003383584.1|	97794-79891	861416	371081	-121498	0.56445	no
gi|320446987|ref|NW_003383584.1|	00323-80152	127055	313701	-201799	0.3599	no
gi|320446987|ref|NW_003383584.1|	01771-80419	779384	328698	-124557	0.57815	no

gi|320446987|ref|NW_003383584.1|	32753-83420	968748	595322	-0.702452	0.60255	no
gi|320446987|ref|NW_003383584.1|	34350-84058	151783	0.768318	-0.982233	0.63775	no
gi|320446987|ref|NW_003383584.1|	40725-85179	159332	125984	-0.338792	0.8025	no
gi|320446987|ref|NW_003383584.1|	71661-87793	85204	219492	136518	0.30975	no
gi|320446987|ref|NW_003383584.1|	80236-88172	215893	197009	-0.132053	0.9434	no
gi|320446987|ref|NW_003383584.1|	82061-88284	0.916331	230646	133174	0.52445	no
gi|320446987|ref|NW_003383584.1|	88119-88843	103649	122311	0.238846	0.9026	no
gi|320446987|ref|NW_003383584.1|	96527-89745	11663	147466	0.338438	0.87375	no
gi|320446987|ref|NW_003383584.1|	98449-89959	148752	180298	0.277468	0.88505	no
gi|320446988|ref|NW_003383583.1|	12933-10132	502584	73779	0.553846	0.7953	no
gi|320446988|ref|NW_003383583.1|	13400-10144	264286	26103	-0.0178818	0.9875	no
gi|320446988|ref|NW_003383583.1|	14637-10156	174963	144273	-0.278251	0.89765	no
gi|320446988|ref|NW_003383583.1|	15777-10172	20285	680161	-157647	0.3399	no
gi|320446988|ref|NW_003383583.1|	41581-10437	192381	988449	-0.960725	0.5722	no
gi|320446988|ref|NW_003383583.1|	61024-10615	148718	997038	-0.576858	0.7806	no
gi|320446988|ref|NW_003383583.1|	65311-10656	176866	10375	-0.769552	0.71025	no
gi|320446988|ref|NW_003383583.1|	66306-10682	856537	133014	0.634988	0.68875	no
gi|320446988|ref|NW_003383583.1|	70168-10756	346319	54598	0.656745	0.68815	no
gi|320446988|ref|NW_003383583.1|	81700-10826	826422	987311	0.256626	0.8996	no
gi|320446988|ref|NW_003383583.1|	83268-10838	236964	288927	0.286033	0.8804	no
gi|320446988|ref|NW_003383583.1|	84254-10854	164091	252626	0.622507	0.75905	no
gi|320446988|ref|NW_003383583.1|	85667-10859	151313	978491	-0.628906	0.75975	no
gi|320446988|ref|NW_003383583.1|	86520-10868	206755	36194	-25141	0.2402	no
gi|320446988|ref|NW_003383583.1|	88795-10893	681695	214111	-167077	0.31385	no
gi|320446988|ref|NW_003383583.1|	91987-10932	176018	843935	-106052	0.62465	no
gi|320446988|ref|NW_003383583.1|	94801-10954	222608	278746	0.324447	0.86375	no
gi|320446988|ref|NW_003383583.1|	96308-10977	430065	513941	0.257048	0.90095	no
gi|320446988|ref|NW_003383583.1|	12703-11407	759198	258476	-155445	0.4693	no
gi|320446988|ref|NW_003383583.1|	14851-11552	112756	530357	-108817	0.6027	no
gi|320446988|ref|NW_003383583.1|	23125-12817	144671	140607	-0.0411106	0.9768	no

gi|320446988|ref|NW_003383583.1|	138930-139827	229593	105466	-11223	0.58925	no
gi|320446988|ref|NW_003383583.1|	211161-21157[illegible]	114888	291933	-197652	0.38655	no
gi|320446988|ref|NW_003383583.1|	211681-21368[illegible]	380983	219182	-0.797597	0.69845	no
gi|320446988|ref|NW_003383583.1|	216845-21811[illegible]	311113	283997	-0.13156	0.9456	no
gi|320446988|ref|NW_003383583.1|	219474-22060[illegible]	565298	469749	-0.267122	0.89325	no
gi|320446988|ref|NW_003383583.1|	220862-22208[illegible]	869699	744953	-0.223368	0.91385	no
gi|320446988|ref|NW_003383583.1|	350757-35174[illegible]	0	125564	inf	5.00E-05	yes
gi|320446988|ref|NW_003383583.1|	376276-37719[illegible]	0	114049	inf	0.00565	no
gi|320446988|ref|NW_003383583.1|	430908-43455[illegible]	28413	366122	0.36577	0.78975	no
gi|320446988|ref|NW_003383583.1|	436744-43907[illegible]	328671	411097	0.322836	0.8056	no
gi|320446988|ref|NW_003383583.1|	440429-44106[illegible]	652288	509865	-0.355394	0.77415	no
gi|320446988|ref|NW_003383583.1|	447126-44965[illegible]	428905	31811	-0.431132	0.74555	no
gi|320446988|ref|NW_003383583.1|	449780-45023[illegible]	160479	101589	-0.659642	0.75705	no
gi|320446988|ref|NW_003383583.1|	450369-45131[illegible]	119.13	51966	-11969	0.37435	no
gi|320446988|ref|NW_003383583.1|	451832-45393[illegible]	467453	276796	-0.755998	0.5642	no
gi|320446988|ref|NW_003383583.1|	454860-45750[illegible]	308749	310819	0.00964379	0.99415	no
gi|320446988|ref|NW_003383583.1|	458973-45935[illegible]	138688	839203	-0.724753	0.7282	no
gi|320446988|ref|NW_003383583.1|	460180-46166[illegible]	310098	170606	-0.862054	0.4922	no
gi|320446988|ref|NW_003383583.1|	463264-46425[illegible]	112273	201597	-247746	0.2849	no
gi|320446988|ref|NW_003383583.1|	466218-46676[illegible]	592991	145361	-202837	0.36235	no
gi|320446988|ref|NW_003383583.1|	467463-46788[illegible]	888047	0.586584	-392023	0.3159	no
gi|320446988|ref|NW_003383583.1|	48233-49128	332641	0.705202	-223786	0.34035	no
gi|320446988|ref|NW_003383583.1|	491710-49251[illegible]	0	149859	inf	0.0052	no
gi|320446988|ref|NW_003383583.1|	526598-52700[illegible]	223972	922717	-127936	0.5365	no
gi|320446988|ref|NW_003383583.1|	527304-52843[illegible]	397149	136859	-153699	0.367	no
gi|320446988|ref|NW_003383583.1|	538773-54029[illegible]	604731	164565	-187763	0.3811	no
gi|320446988|ref|NW_003383583.1|	540681-54112[illegible]	847839	0.512134	-40492	0.30905	no
gi|320446988|ref|NW_003383583.1|	541562-54710[illegible]	759893	630474	-0.269359	0.8352	no
gi|320446988|ref|NW_003383583.1|	548110-54853[illegible]	503356	55546	0.142102	0.9301	no
gi|320446988|ref|NW_003383583.1|	553959-55449[illegible]	0	33546	inf	0.0048	no

gi|320446988|ref|NW_003383583.1|	55216-55616	0.705567	129837	420177	0.16465	no
gi|320446988|ref|NW_003383583.1|	56362-55734	224846	201862	-0.155569	0.9298	no
gi|320446988|ref|NW_003383583.1|	58150-55878	417356	0.567962	-287741	0.2697	no
gi|320446988|ref|NW_003383583.1|	59239-56005	548565	496107	-0.145011	0.9411	no
gi|320446988|ref|NW_003383583.1|	60801-56166	32169	239898	-0.423249	0.82905	no
gi|320446988|ref|NW_003383583.1|	03799-60466	186173	238044	0.354585	0.85775	no
gi|320446988|ref|NW_003383583.1|	31154-63189	268618	528941	0.977549	0.6471	no
gi|320446988|ref|NW_003383583.1|	19784-72037	708938	960536	0.438181	0.8292	no
gi|320446988|ref|NW_003383583.1|	23274-72536	344804	354462	0.039854	0.98395	no
gi|320446988|ref|NW_003383583.1|	25518-72602	36406	529272	0.539834	0.79355	no
gi|320446988|ref|NW_003383583.1|	26176-72756	322377	7329	118487	0.5762	no
gi|320446988|ref|NW_003383583.1|	39435-74007	250414	105073	2069	0.3418	no
gi|320446988|ref|NW_003383583.1|	41391-74332	119067	421945	182528	0.3929	no
gi|320446988|ref|NW_003383583.1|	43988-74491	0.490908	220025	216414	0.29245	no
gi|320446988|ref|NW_003383583.1|	45903-74706	108923	135839	364051	0.15995	no
gi|320446988|ref|NW_003383583.1|	63549-76810	0.0390148	163934	539295	0.16595	no
gi|320446988|ref|NW_003383583.1|	73339-77475	0.571256	0.793875	0.474776	1	no
gi|320446988|ref|NW_003383583.1|	75540-77751	0.485828	115171	124526	1	no
gi|320446988|ref|NW_003383583.1|	79312-78036	0.619244	128471	105287	1	no
gi|320446988|ref|NW_003383583.1|	80710-78154	168055	154109	-0.124977	0.93295	no
gi|320446988|ref|NW_003383583.1|	81784-78439	0.426161	287799	275559	0.2381	no
gi|320446988|ref|NW_003383583.1|	84530-78531	0.305997	231054	291664	0.25645	no
gi|320446988|ref|NW_003383583.1|	89649-79099	121776	0.317087	-194128	1	no
gi|320446988|ref|NW_003383583.1|	00886-80143	218306	301648	0.466515	0.82615	no
gi|320446988|ref|NW_003383583.1|	03721-80409	102345	141267	0.464978	0.81645	no
gi|320446988|ref|NW_003383583.1|	04958-80523	163305	163668	0.00320687	0.9489	no
gi|320446988|ref|NW_003383583.1|	05415-80734	298194	616705	104833	0.62145	no
gi|320446988|ref|NW_003383583.1|	07681-80795	94273	135245	0.520656	0.80155	no
gi|320446988|ref|NW_003383583.1|	08411-80932	400892	466299	0.218042	0.91295	no
gi|320446988|ref|NW_003383583.1|	09447-81063	550914	230314	20637	0.22755	no

gi|320446988|ref|NW_003383583.1|	15543-81613	629054	129.42	104081	0.42155	no
gi|320446988|ref|NW_003383583.1|	16346-81654	390409	728956	0.900847	0.6684	no
gi|320446988|ref|NW_003383583.1|	16696-81953	10381	277076	141634	0.2811	no
gi|320446988|ref|NW_003383583.1|	19777-82253	85786	309115	184933	0.1641	no
gi|320446988|ref|NW_003383583.1|	73986-87488	0.51098	158435	163255	0.3325	no
gi|320446988|ref|NW_003383583.1|	76697-87830	0.490321	145018	156443	0.4962	no
gi|320446988|ref|NW_003383583.1|	25795-92612	0	609938	inf	0.00605	no
gi|320446988|ref|NW_003383583.1|	27744-92803	0	439589	inf	0.0101	no
gi|320446988|ref|NW_003383583.1|	33099-93369	0	107437	inf	0.0101	no
gi|320446988|ref|NW_003383583.1|	35800-93626	0	111351	inf	0.01485	no
gi|320446988|ref|NW_003383583.1|	38051-93870	0	846356	inf	0.01195	no
gi|320446988|ref|NW_003383583.1|	95797-97161	912085	134996	-275625	0.24515	no
gi|320446989|ref|NW_003383582.1|	22525-23386	24281	0.185693	-703076	0.2592	no
gi|320446989|ref|NW_003383582.1|	60285-36118	0	296186	inf	0.02075	no
gi|320446989|ref|NW_003383582.1|	22063-52404	0.771107	578004	290608	0.2226	no
gi|320446989|ref|NW_003383582.1|	25062-52642	13457	446526	-159154	0.4596	no
gi|320446989|ref|NW_003383582.1|	26551-52927	105855	388816	-144493	0.39045	no
gi|320446989|ref|NW_003383582.1|	31689-53222	314591	642756	-229113	0.3077	no
gi|320446989|ref|NW_003383582.1|	32280-53690	899632	780827	-0.204333	0.87075	no
gi|320446989|ref|NW_003383582.1|	41195-54213	714532	279346	-135494	0.5203	no
gi|320446989|ref|NW_003383582.1|	43846-54513	0.640673	0.778038	0.280253	1	no
gi|320446989|ref|NW_003383582.1|	46348-54820	133358	68974	-0.951179	0.5494	no
gi|320446989|ref|NW_003383582.1|	48824-55244	193651	111057	-0.802158	0.54085	no
gi|320446989|ref|NW_003383582.1|	52617-55371	356786	163282	-11277	0.4951	no
gi|320446989|ref|NW_003383582.1|	59647-56120	179162	142434	-0.330971	0.86465	no
gi|320446989|ref|NW_003383582.1|	61816-56211	0	134981	inf	0.0294	no
gi|320446989|ref|NW_003383582.1|	66037-56648	112.93	183811	0.702792	0.5779	no
gi|320446989|ref|NW_003383582.1|	68038-56894	708677	897218	0.340331	0.78935	no
gi|320446989|ref|NW_003383582.1|	69713-57092	189304	0.119318	-398782	0.31135	no
gi|320446989|ref|NW_003383582.1|	74642-57526	952136	178822	-573457	0.09725	no

gi|320446989|ref|NW_003383582.1|	75351-57595	656672	489106	-0.425025	0.79175	no
gi|320446989|ref|NW_003383582.1|	76358-57831	219931	24369	0.147996	0.9107	no
gi|320446989|ref|NW_003383582.1|	62923-63778	408604	0	#NAME?	0.01585	no
gi|320446989|ref|NW_003383582.1|	63971-64875	429134	0	#NAME?	0.01065	no
gi|320446989|ref|NW_003383582.1|	65231-65997	731626	0	#NAME?	0.0111	no
gi|320446989|ref|NW_003383582.1|	60291-66300	170256	164178	326949	0.092	no
gi|320446989|ref|NW_003383582.1|	64346-66676	17039	962654	249817	0.1511	no
gi|320446989|ref|NW_003383582.1|	11407-71958	911065	484004	-0.912535	0.49795	no
gi|320446989|ref|NW_003383582.1|	42709-74322	118472	835477	281805	0.2258	no
gi|320446989|ref|NW_003383582.1|	97272-79791	206159	898137	212318	0.3436	no
gi|320446989|ref|NW_003383582.1|	12813-81656	100643	107998	342369	0.0886	no
gi|320446989|ref|NW_003383582.1|	22904-82327	224753	661588	155759	0.3575	no
gi|320446989|ref|NW_003383582.1|	62860-86341	0.524386	460031	313303	0.24575	no
gi|320446989|ref|NW_003383582.1|	27961-92951	149389	157214	0.0736563	0.9616	no
gi|320446989|ref|NW_003383582.1|	29719-93216	687582	41614	-0.724464	0.7296	no
gi|320446989|ref|NW_003383582.1|	32483-93393	834285	54121	-0.624352	0.7707	no
gi|320446989|ref|NW_003383582.1|	53475-95477	175187	121579	-0.527004	0.7964	no
gi|320446989|ref|NW_003383582.1|	63393-96582	974225	16874	0.792476	0.53065	no
gi|320446989|ref|NW_003383582.1|	67343-96804	682568	270161	-133715	0.5251	no
gi|320446990|ref|NW_003383581.1|	07687-10086	0.235517	195022	304973	0.2493	no
gi|320446990|ref|NW_003383581.1|	08746-10095	0	217922	inf	0.0294	no
gi|320446990|ref|NW_003383581.1|	09755-10113	0	149761	inf	0.02075	no
gi|320446990|ref|NW_003383581.1|	11552-10138	0.244471	0.967054	198393	1	no
gi|320446990|ref|NW_003383581.1|	15181-10160	250186	234535	322873	0.18315	no
gi|320446990|ref|NW_003383581.1|	57529-10579	184.76	109218	-0.758442	0.54355	no
gi|320446990|ref|NW_003383581.1|	60727-10614	897741	678165	-0.404663	0.7478	no
gi|320446990|ref|NW_003383581.1|	61514-10617	147.52	117028	-0.334056	0.8709	no
gi|320446990|ref|NW_003383581.1|	62032-10626	148077	151208	0.0301853	0.98515	no
gi|320446990|ref|NW_003383581.1|	62938-10643	134515	178652	0.409383	0.85365	no
gi|320446990|ref|NW_003383581.1|	65054-10698	983259	791874	-0.312301	0.80415	no

gi|320446990|ref|NW_003383581.1|	109860-11114!	0.37234	207267	24768	0.28595	no
gi|320446990|ref|NW_003383581.1|	130194-11303:	283799	41.06	0.532864	0.7967	no
gi|320446990|ref|NW_003383581.1|	131844-11321:	452733	202289	215968	0.28415	no
gi|320446990|ref|NW_003383581.1|	133044-11333(	135492	103299	-0.391384	0.8552	no
gi|320446990|ref|NW_003383581.1|	138063-11384:	892381	165012	0.886836	0.66835	no
gi|320446990|ref|NW_003383581.1|	144971-11455:	101788	343836	175615	0.33425	no
gi|320446990|ref|NW_003383581.1|	147600-11482(	315214	580622	0.881269	0.66995	no
gi|320446990|ref|NW_003383581.1|	149059-11493:	150523	323311	110294	0.5932	no
gi|320446990|ref|NW_003383581.1|	149983-11505:	195704	448639	119688	0.58635	no
gi|320446990|ref|NW_003383581.1|	157192-11578!	954946	108468	0.183773	0.92995	no
gi|320446990|ref|NW_003383581.1|	157974-11629:	384788	638908	0.731543	0.65555	no
gi|320446990|ref|NW_003383581.1|	163180-11650!	140736	57849	20393	0.1257	no
gi|320446990|ref|NW_003383581.1|	168264-11686:	27035	594886	113778	0.57505	no
gi|320446990|ref|NW_003383581.1|	170019-11727(	371805	678345	0.867472	0.6987	no
gi|320446990|ref|NW_003383581.1|	195516-11957:	401424	107688	-189827	0.3084	no
gi|320446990|ref|NW_003383581.1|	195844-11969:	147933	118753	-0.316982	0.88155	no
gi|320446990|ref|NW_003383581.1|	204757-12076:	216142	262979	0.282966	0.83175	no
gi|320446990|ref|NW_003383581.1|	210866-12133:	165971	421496	134459	0.523	no
gi|320446990|ref|NW_003383581.1|	217284-12177(	132833	39962	158901	0.33275	no
gi|320446990|ref|NW_003383581.1|	197674-19810:	379272	189697	-0.999533	0.6377	no
gi|320446990|ref|NW_003383581.1|	199598-20017!	540682	232754	-121598	0.5656	no
gi|320446990|ref|NW_003383581.1|	202506-20282(	964726	414999	-121701	0.57555	no
gi|320446990|ref|NW_003383581.1|	203468-20428:	321441	100398	-167882	0.4314	no
gi|320446990|ref|NW_003383581.1|	204646-20521!	603606	13599	-21501	0.3525	no
gi|320446990|ref|NW_003383581.1|	234589-23500:	313864	296047	-340624	0.19615	no
gi|320446990|ref|NW_003383581.1|	236216-23776:	141414	0	#NAME?	0.02105	no
gi|320446990|ref|NW_003383581.1|	27723-28619	0.76647	334473	212559	0.34655	no
gi|320446990|ref|NW_003383581.1|	299363-30085(	16179	637429	-134379	0.5506	no
gi|320446990|ref|NW_003383581.1|	301574-30215:	245065	198957	-0.300712	0.8817	no
gi|320446990|ref|NW_003383581.1|	302722-30344:	861628	330251	-13835	0.5064	no

gi|320446990|ref|NW_003383581.1|	303602-304284	166145	386652	-210334	0.33345	no
gi|320446990|ref|NW_003383581.1|	305807-306582	312799	0.85844	-186544	0.4074	no
gi|320446990|ref|NW_003383581.1|	306971-307515	75944	0.73118	-337664	0.2381	no
gi|320446990|ref|NW_003383581.1|	31573-31972	0.9725	639939	271816	0.2673	no
gi|320446990|ref|NW_003383581.1|	361978-36479[illegible]	757867	200915	-191536	0.39795	no
gi|320446990|ref|NW_003383581.1|	36465-36868	571493	752775	0.397484	0.8489	no
gi|320446990|ref|NW_003383581.1|	367160-36806[illegible]	169637	279173	-260322	0.2461	no
gi|320446990|ref|NW_003383581.1|	403741-40606[illegible]	170062	299623	0.817091	0.6981	no
gi|320446990|ref|NW_003383581.1|	406297-407194	127552	210933	0.725701	0.73635	no
gi|320446990|ref|NW_003383581.1|	472289-474434	11487	480872	206565	0.35065	no
gi|320446990|ref|NW_003383581.1|	53036-53596	191124	139513	-377604	0.19055	no
gi|320446990|ref|NW_003383581.1|	54822-56193	25262	0.928892	-144339	0.4847	no
gi|320446990|ref|NW_003383581.1|	56683-57631	170525	21244	-300486	0.21655	no
gi|320446990|ref|NW_003383581.1|	58333-59173	837492	0.768048	-344681	0.20675	no
gi|320446990|ref|NW_003383581.1|	59291-60767	202671	586287	-178946	0.2841	no
gi|320446990|ref|NW_003383581.1|	70754-77903	959323	207672	111422	0.5913	no
gi|320446990|ref|NW_003383581.1|	79172-79667	422406	664202	0.652991	0.76905	no
gi|320446990|ref|NW_003383581.1|	857019-857618	277467	533001	0.941821	0.65765	no
gi|320446990|ref|NW_003383581.1|	88942-89653	210717	284579	0.433527	0.74555	no
gi|320446990|ref|NW_003383581.1|	974271-974767	127537	341619	142147	0.3757	no
gi|320446990|ref|NW_003383581.1|	979593-98014[illegible]	214299	397366	0.89084	0.68975	no
gi|320446990|ref|NW_003383581.1|	984296-985015	139562	262599	0.911952	0.6862	no
gi|320446990|ref|NW_003383581.1|	985932-98795[illegible]	37694	133409	182345	0.28425	no
gi|320446990|ref|NW_003383581.1|	991082-99164[illegible]	152233	582866	193689	0.3925	no
gi|320446990|ref|NW_003383581.1|	992074-993667	174015	588115	175689	0.4171	no
gi|320446990|ref|NW_003383581.1|	993985-99879[illegible]	265787	142466	242227	0.1949	no
gi|320446991|ref|NW_003383580.1|	12710-13196	132334	247757	422666	0.1635	no
gi|320446991|ref|NW_003383580.1|	128117-129347	363098	243555	-0.57611	0.653	no
gi|320446991|ref|NW_003383580.1|	129533-132619	156855	201975	0.364749	0.77355	no
gi|320446991|ref|NW_003383580.1|	141720-14242[illegible]	451695	286764	-0.655484	0.67565	no

gi|320446991|ref|NW_003383580.1|	142853-14738	194034	712413	-144553	0.28315	no
gi|320446991|ref|NW_003383580.1|	147820-14830	26099	357803	-286676	0.2202	no
gi|320446991|ref|NW_003383580.1|	148429-15025	55234	651287	0.237736	0.9119	no
gi|320446991|ref|NW_003383580.1|	185677-18681	276105	73647	-190652	0.2632	no
gi|320446991|ref|NW_003383580.1|	187823-19220	928172	835208	-0.152257	0.9084	no
gi|320446991|ref|NW_003383580.1|	192441-19396	900779	217908	-204746	0.35815	no
gi|320446991|ref|NW_003383580.1|	194105-19509	172671	495591	-18008	0.42105	no
gi|320446991|ref|NW_003383580.1|	195251-20044	252916	132591	-0.931673	0.49345	no
gi|320446991|ref|NW_003383580.1|	200980-20221	135804	746831	-0.862668	0.5137	no
gi|320446991|ref|NW_003383580.1|	202350-20513	178022	21229	0.253987	0.8432	no
gi|320446991|ref|NW_003383580.1|	220225-22091	193773	114517	-0.7588	0.711	no
gi|320446991|ref|NW_003383580.1|	221828-22318	115542	937877	-0.300941	0.8941	no
gi|320446991|ref|NW_003383580.1|	252184-25374	0.12749	266065	438333	0.18195	no
gi|320446991|ref|NW_003383580.1|	27499-29469	0.778221	264519	176512	0.40585	no
gi|320446991|ref|NW_003383580.1|	281043-28245	0	145614	inf	0.00445	no
gi|320446991|ref|NW_003383580.1|	283843-28413	0	127029	inf	0.0312	no
gi|320446991|ref|NW_003383580.1|	32822-34862	317913	593434	0.900458	0.6663	no
gi|320446991|ref|NW_003383580.1|	350131-35039	196295	160214	-0.293024	0.87275	no
gi|320446991|ref|NW_003383580.1|	35659-36705	707872	561443	-0.334348	0.86735	no
gi|320446991|ref|NW_003383580.1|	37179-38207	653687	116271	-249111	0.1813	no
gi|320446991|ref|NW_003383580.1|	40527-40732	177514	451553	-197497	0.3781	no
gi|320446991|ref|NW_003383580.1|	42607-43880	499301	141052	149825	0.26935	no
gi|320446991|ref|NW_003383580.1|	45480-46631	171966	241687	0.491014	0.7078	no
gi|320446991|ref|NW_003383580.1|	50113-51553	266164	83729	165341	0.4446	no
gi|320446991|ref|NW_003383580.1|	51895-54844	161666	347746	110502	0.5992	no
gi|320446991|ref|NW_003383580.1|	54947-55777	14193	175657	0.307578	0.8759	no
gi|320446991|ref|NW_003383580.1|	555433-55609	196199	160526	-0.289508	0.8821	no
gi|320446991|ref|NW_003383580.1|	599900-60127	0.593356	0.824246	0.474178	1	no
gi|320446991|ref|NW_003383580.1|	604829-60765	107384	28657	-190582	0.264	no
gi|320446991|ref|NW_003383580.1|	608288-60919	100671	329577	-161097	0.4532	no

gi|320446991|ref|NW_003383580.1|	10059-61205	12158	97463	-0.318976	0.8414	no
gi|320446991|ref|NW_003383580.1|	12539-61408	116323	0.898994	-0.371751	1	no
gi|320446991|ref|NW_003383580.1|	16745-61986	846975	909675	0.103032	0.9374	no
gi|320446991|ref|NW_003383580.1|	30974-63214	171907	927794	-0.889751	0.69065	no
gi|320446991|ref|NW_003383580.1|	63647-64306	278526	271169	-0.0386193	0.94545	no
gi|320446991|ref|NW_003383580.1|	36563-63903	276116	12961	-109109	0.4072	no
gi|320446991|ref|NW_003383580.1|	39362-64014	402169	488303	0.279973	0.88935	no
gi|320446991|ref|NW_003383580.1|	41131-64152	119835	131281	0.131616	0.9447	no
gi|320446991|ref|NW_003383580.1|	41875-64263	57474	67879	0.240057	0.9063	no
gi|320446991|ref|NW_003383580.1|	43534-64453	19762	13653	-0.533514	0.78905	no
gi|320446991|ref|NW_003383580.1|	47258-64816	676505	708084	0.0658191	0.97205	no
gi|320446991|ref|NW_003383580.1|	49101-65004	163649	252007	0.622855	0.7845	no
gi|320446991|ref|NW_003383580.1|	56664-66403	420699	241211	-0.80249	0.536	no
gi|320446991|ref|NW_003383580.1|	67273-66806	814381	0.828455	-329721	0.21545	no
gi|320446992|ref|NW_003383579.1|	13068-13470	0.95742	630425	27191	0.2673	no
gi|320446992|ref|NW_003383579.1|	64050-16597	0.99928	153251	393886	0.06865	no
gi|320446992|ref|NW_003383579.1|	1897-2280	636497	17389	144995	0.492	no
gi|320446992|ref|NW_003383579.1|	20287-22763	0.527147	73105	379369	0.1413	no
gi|320446992|ref|NW_003383579.1|	66686-26700	63819	102978	0.690275	0.74435	no
gi|320446992|ref|NW_003383579.1|	93873-29471	0	190776	inf	0.0312	no
gi|320446992|ref|NW_003383579.1|	15286-31580	0	587188	inf	0.02205	no
gi|320446992|ref|NW_003383579.1|	68126-37029	244089	316303	0.373899	0.85625	no
gi|320446992|ref|NW_003383579.1|	72550-37348	0.483176	133306	146412	1	no
gi|320446992|ref|NW_003383579.1|	66421-56763	152064	706823	-110526	0.6118	no
gi|320446992|ref|NW_003383579.1|	72803-57353	752115	725526	-0.0519261	0.97555	no
gi|320446992|ref|NW_003383579.1|	73869-57569	838077	524815	-0.675275	0.7587	no
gi|320446992|ref|NW_003383579.1|	76865-57725	109848	590766	-0.894856	0.65745	no
gi|320446992|ref|NW_003383579.1|	79285-58375	134211	154222	0.200503	0.8799	no
gi|320446992|ref|NW_003383579.1|	96923-59715	872575	139.98	40038	0.164	no
gi|320446992|ref|NW_003383579.1|	97641-60142	97947	278854	150944	0.26125	no

gi|320446992|ref|NW_003383579.1|	503592-60449	458493	0.877585	-238529	0.30565	no
gi|320446992|ref|NW_003383579.1|	517737-61834	180597	765917	208442	0.3494	no
gi|320446992|ref|NW_003383579.1|	528111-62832	238237	894119	190807	0.393	no
gi|320446992|ref|NW_003383579.1|	580007-68076	877012	287228	171153	0.43955	no
gi|320446992|ref|NW_003383579.1|	763827-76442	963347	11822	0.295349	0.88515	no
gi|320446992|ref|NW_003383579.1|	779976-78046	661672	172545	138278	0.50425	no
gi|320446992|ref|NW_003383579.1|	819415-82039	0	476399	inf	5.00E-05	yes
gi|320446992|ref|NW_003383579.1|	82202-84426	154429	154555	0.00117811	0.99725	no
gi|320446992|ref|NW_003383579.1|	84637-85116	122311	453855	-143026	0.5042	no
gi|320446992|ref|NW_003383579.1|	863214-86707	400441	141765	182384	0.31	no
gi|320446992|ref|NW_003383579.1|	87136-88592	19908	12973	-0.617839	0.703	no
gi|320446992|ref|NW_003383579.1|	906218-91315	825899	827884	0.00346392	0.9972	no
gi|320446992|ref|NW_003383579.1|	914958-91563	157972	499424	-166133	0.43905	no
gi|320446992|ref|NW_003383579.1|	915761-91723	888432	209038	-20875	0.35	no
gi|320446992|ref|NW_003383579.1|	918233-91875	879569	315192	-148057	0.49115	no
gi|320446992|ref|NW_003383579.1|	920164-92063	0.709449	804124	350265	0.2175	no
gi|320446992|ref|NW_003383579.1|	923068-92402	140526	161632	0.201877	0.91225	no
gi|320446992|ref|NW_003383579.1|	929095-92949	1945	575945	156616	0.3575	no
gi|320446992|ref|NW_003383579.1|	969102-96972	131295	267572	102711	0.5772	no
gi|320446993|ref|NW_003383578.1|	006388-10085	369693	15599	-124488	0.55615	no
gi|320446993|ref|NW_003383578.1|	009195-10100	374693	0.990556	-19194	0.39075	no
gi|320446993|ref|NW_003383578.1|	028813-10297	617227	10724	0.796972	0.70115	no
gi|320446993|ref|NW_003383578.1|	070366-10707	187593	105085	248587	0.25415	no
gi|320446993|ref|NW_003383578.1|	070930-10711	108394	223814	104602	0.6033	no
gi|320446993|ref|NW_003383578.1|	078799-10792	318843	128107	200644	0.35755	no
gi|320446993|ref|NW_003383578.1|	080758-10858	171182	903048	239927	0.18025	no
gi|320446993|ref|NW_003383578.1|	129633-13201	0.627961	0.657685	0.0667207	1	no
gi|320446993|ref|NW_003383578.1|	133209-13481	184945	128698	-0.523109	0.7957	no
gi|320446993|ref|NW_003383578.1|	134914-13903	865009	105594	0.287742	0.81945	no
gi|320446993|ref|NW_003383578.1|	170793-17155	483515	141441	154857	0.4736	no

gi|320446993|ref|NW_003383578.1|	201622-20181	113519	469257	204744	0.34935	no
gi|320446993|ref|NW_003383578.1|	204037-20420	292278	1820.33	263878	0.2541	no
gi|320446993|ref|NW_003383578.1|	259702-26137	0.234267	138631	256502	1	no
gi|320446993|ref|NW_003383578.1|	266561-26772	0.364253	126179	179245	1	no
gi|320446993|ref|NW_003383578.1|	269462-27154	0	254274	inf	0.00815	no
gi|320446993|ref|NW_003383578.1|	275126-27634	0.343489	440589	36811	0.1785	no
gi|320446993|ref|NW_003383578.1|	278427-27882	42168	306145	285999	0.1276	no
gi|320446993|ref|NW_003383578.1|	298313-29953	0	189953	inf	0.0198	no
gi|320446993|ref|NW_003383578.1|	336884-33851	579128	0.587823	-330043	0.1919	no
gi|320446993|ref|NW_003383578.1|	340385-34304	725245	155913	-221773	0.32665	no
gi|320446993|ref|NW_003383578.1|	343316-34390	22523	389372	-253218	0.2707	no
gi|320446993|ref|NW_003383578.1|	344128-34442	125731	264442	-224932	0.31005	no
gi|320446993|ref|NW_003383578.1|	346507-34876	302681	675104	-216462	0.23595	no
gi|320446993|ref|NW_003383578.1|	349877-35217	432546	134132	-16892	0.2052	no
gi|320446993|ref|NW_003383578.1|	352313-35888	180936	149057	-0.279611	0.83245	no
gi|320446993|ref|NW_003383578.1|	358992-36065	0.591416	197618	174047	0.42395	no
gi|320446993|ref|NW_003383578.1|	360820-36233	96043	109795	0.193061	0.9307	no
gi|320446993|ref|NW_003383578.1|	362463-36318	199698	282829	0.502111	0.8176	no
gi|320446993|ref|NW_003383578.1|	364779-36867	677001	119915	0.824779	0.51995	no
gi|320446993|ref|NW_003383578.1|	371720-37447	159861	215954	0.433904	0.74355	no
gi|320446993|ref|NW_003383578.1|	374638-37615	100303	122984	0.294112	0.89215	no
gi|320446993|ref|NW_003383578.1|	376943-37916	251068	103347	-128059	0.46445	no
gi|320446993|ref|NW_003383578.1|	382475-38393	590603	362811	-0.702969	0.7325	no
gi|320446993|ref|NW_003383578.1|	384204-38855	227529	201801	-0.173118	0.897	no
gi|320446993|ref|NW_003383578.1|	390878-39344	0.723984	192179	140842	0.50385	no
gi|320446993|ref|NW_003383578.1|	393645-39479	395414	336335	-0.233467	0.85195	no
gi|320446993|ref|NW_003383578.1|	397452-40019	806808	142832	0.824021	0.63045	no
gi|320446993|ref|NW_003383578.1|	404138-40705	574055	407009	-0.496129	0.71025	no
gi|320446993|ref|NW_003383578.1|	407194-40885	165365	0.411133	-200797	0.359	no
gi|320446993|ref|NW_003383578.1|	418335-41924	358297	253874	-0.497044	0.75265	no

gi|320446993|ref|NW_003383578.1|	29138-43290	132817	98301	-0.434164	0.73385	no
gi|320446993|ref|NW_003383578.1|	37630-43856	339182	116152	-154604	0.347	no
gi|320446993|ref|NW_003383578.1|	45648-44666	839939	106959	0.348707	0.79145	no
gi|320446993|ref|NW_003383578.1|	46922-44771	521365	121608	122187	0.34965	no
gi|320446993|ref|NW_003383578.1|	49504-45194	441452	138081	-167674	0.2081	no
gi|320446993|ref|NW_003383578.1|	52355-45388	549181	168749	-17024	0.19675	no
gi|320446993|ref|NW_003383578.1|	60643-46112	381447	223627	-409232	0.1671	no
gi|320446993|ref|NW_003383578.1|	62163-46307	368092	0.85727	-542417	0.1126	no
gi|320446993|ref|NW_003383578.1|	64401-46510	603534	0.736803	-635601	0.11065	no
gi|320446993|ref|NW_003383578.1|	65286-46588	971745	516873	-0.910768	0.58515	no
gi|320446993|ref|NW_003383578.1|	76398-47838	222403	140981	-0.657679	0.6005	no
gi|320446993|ref|NW_003383578.1|	79167-48342	220097	273274	0.312207	0.8175	no
gi|320446993|ref|NW_003383578.1|	87681-48846	183142	925652	-0.984418	0.46785	no
gi|320446993|ref|NW_003383578.1|	90919-49176	627154	251705	-131708	0.4358	no
gi|320446993|ref|NW_003383578.1|	98718-50010	151869	466638	-170245	0.43815	no
gi|320446993|ref|NW_003383578.1|	00300-50095	528356	415187	-0.347749	0.8578	no
gi|320446993|ref|NW_003383578.1|	30722-53151	0	172205	inf	0.00595	no
gi|320446993|ref|NW_003383578.1|	60629-56164	0.652788	150353	120367	0.56645	no
gi|320446993|ref|NW_003383578.1|	87587-58946	515203	660673	0.358795	0.86475	no
gi|320446993|ref|NW_003383578.1|	89612-59334	134957	246234	0.867529	0.6794	no
gi|320446993|ref|NW_003383578.1|	00880-60127	29175	127988	21332	0.34385	no
gi|320446993|ref|NW_003383578.1|	04019-60485	505002	23347	220887	0.3284	no
gi|320446993|ref|NW_003383578.1|	29371-63109	181614	885309	228531	0.30915	no
gi|320446993|ref|NW_003383578.1|	36306-63663	583497	85013	0.542957	0.799	no
gi|320446993|ref|NW_003383578.1|	37628-63836	0	299545	inf	0.02915	no
gi|320446993|ref|NW_003383578.1|	38534-63903	187222	334618	0.837767	0.69175	no
gi|320446993|ref|NW_003383578.1|	49602-65122	207482	985278	224754	0.31895	no
gi|320446993|ref|NW_003383578.1|	82471-83274	148685	91916	-0.693869	0.73595	no
gi|320446993|ref|NW_003383578.1|	25920-83062	130234	100466	-0.374398	0.77775	no
gi|320446993|ref|NW_003383578.1|	31134-83185	104672	286472	-186941	0.3951	no

gi|320446993|ref|NW_003383578.1|	331983-834058	550347	319901	-0.782715	0.7115	no
gi|320446993|ref|NW_003383578.1|	366482-867001	458921	751007	-261135	0.27045	no
gi|320446993|ref|NW_003383578.1|	373544-876800	0.501734	463999	320913	0.1878	no
gi|320446993|ref|NW_003383578.1|	381928-882997	0	127443	inf	0.00445	no
gi|320446993|ref|NW_003383578.1|	385691-886341	141575	0	#NAME?	0.0086	no
gi|320446993|ref|NW_003383578.1|	392585-892760	0	942133	inf	0.02915	no
gi|320446993|ref|NW_003383578.1|	927675-932629	264601	768944	153906	0.368	no
gi|320446993|ref|NW_003383578.1|	933640-934259	658159	119215	0.857057	0.67795	no
gi|320446993|ref|NW_003383578.1|	935665-936160	108802	107129	-0.0223459	0.97505	no
gi|320446993|ref|NW_003383578.1|	938055-938928	661774	692611	0.0657063	0.96885	no
gi|320446993|ref|NW_003383578.1|	940411-942054	135465	127667	-0.0855295	0.9699	no
gi|320446993|ref|NW_003383578.1|	942146-943401	155936	468663	-173433	0.20405	no
gi|320446993|ref|NW_003383578.1|	944688-947421	49432	240669	-436032	0.039	no
gi|320446993|ref|NW_003383578.1|	952878-953871	308831	0.920809	-506777	0.09745	no
gi|320446993|ref|NW_003383578.1|	964856-965484	24017	367348	0.613092	0.77535	no
gi|320446993|ref|NW_003383578.1|	966758-967409	162185	19607	0.273733	0.8977	no
gi|320446993|ref|NW_003383578.1|	970430-972655	229831	417859	0.862447	0.51835	no
gi|320446994|ref|NW_003383577.1|	101130-102010	337244	178693	-0.916309	0.66255	no
gi|320446994|ref|NW_003383577.1|	102502-103040	608112	0.372307	-402977	0.30915	no
gi|320446994|ref|NW_003383577.1|	129304-130610	0	240688	inf	0.0186	no
gi|320446994|ref|NW_003383577.1|	131128-131748	0	416223	inf	0.0233	no
gi|320446994|ref|NW_003383577.1|	132245-133377	0	521369	inf	0.00815	no
gi|320446994|ref|NW_003383577.1|	233197-233900	266972	641198	-205785	0.35935	no
gi|320446994|ref|NW_003383577.1|	234089-237264	127424	557955	-119142	0.48715	no
gi|320446994|ref|NW_003383577.1|	238218-239354	37852	155726	-128136	0.44545	no
gi|320446994|ref|NW_003383577.1|	242423-244050	190376	464163	-203615	0.2363	no
gi|320446994|ref|NW_003383577.1|	244646-246445	150006	300735	-231845	0.1864	no
gi|320446994|ref|NW_003383577.1|	250000-250640	410336	139682	-155466	0.49165	no
gi|320446994|ref|NW_003383577.1|	250756-251900	118091	356595	-172754	0.1986	no
gi|320446994|ref|NW_003383577.1|	252098-254121	158403	732515	-111267	0.39615	no

gi|320446994|ref|NW_003383577.1|	256401-25686(	115.53	122471	0.0841714	0.94685	no
gi|320446994|ref|NW_003383577.1|	257054-25979!	170102	286134	0.750289	0.568	no
gi|320446994|ref|NW_003383577.1|	260855-26245!	623115	509748	-0.289714	0.8975	no
gi|320446994|ref|NW_003383577.1|	270027-27075(	225635	140424	-0.684194	0.75015	no
gi|320446994|ref|NW_003383577.1|	286502-28823:	0.449797	0.626512	0.478068	1	no
gi|320446994|ref|NW_003383577.1|	293394-29420:	648498	344271	-0.913556	0.6519	no
gi|320446994|ref|NW_003383577.1|	296929-30075!	639076	122015	0.933003	0.4654	no
gi|320446994|ref|NW_003383577.1|	300915-30367:	112149	173543	0.629878	0.62625	no
gi|320446994|ref|NW_003383577.1|	303744-30474:	300582	726744	127369	0.31995	no
gi|320446994|ref|NW_003383577.1|	319321-32090:	0.501619	226854	21771	0.34115	no
gi|320446994|ref|NW_003383577.1|	322168-32282	0.400699	382225	325383	0.2305	no
gi|320446994|ref|NW_003383577.1|	336954-33757:	13197	388409	155737	0.491	no
gi|320446994|ref|NW_003383577.1|	342563-34463!	407035	268572	-392177	0.0599	no
gi|320446994|ref|NW_003383577.1|	346250-34683:	116635	0.32889	-514826	0.2797	no
gi|320446994|ref|NW_003383577.1|	346940-34716(	497144	0	#NAME?	0.0229	no
gi|320446994|ref|NW_003383577.1|	350540-35158	179493	0.288621	-595861	0.18065	no
gi|320446994|ref|NW_003383577.1|	378593-37928!	0	325295	inf	0.02915	no
gi|320446994|ref|NW_003383577.1|	379339-38240!	0.119217	591849	563356	0.14075	no
gi|320446994|ref|NW_003383577.1|	382660-38335:	11082	302805	145017	0.50735	no
gi|320446994|ref|NW_003383577.1|	385698-38618	281348	0.875384	-50063	0.1913	no
gi|320446994|ref|NW_003383577.1|	388513-38904(	224708	0	#NAME?	0.007	no
gi|320446994|ref|NW_003383577.1|	393702-39479:	197707	300989	0.606347	0.76305	no
gi|320446994|ref|NW_003383577.1|	408622-40936:	0	344296	inf	0.02205	no
gi|320446994|ref|NW_003383577.1|	422783-42327:	169487	157009	-0.110329	0.95745	no
gi|320446994|ref|NW_003383577.1|	455203-45550	751835	470521	264577	0.2626	no
gi|320446994|ref|NW_003383577.1|	460054-46109(	0.418461	303792	285992	0.2244	no
gi|320446994|ref|NW_003383577.1|	469613-46992:	348344	168116	227087	0.2727	no
gi|320446994|ref|NW_003383577.1|	471940-47226	152444	14783	327759	0.23025	no
gi|320446994|ref|NW_003383577.1|	480561-48206:	106667	917857	310516	0.20085	no
gi|320446994|ref|NW_003383577.1|	490233-49160!	0.147949	113039	293365	1	no

gi|320446994|ref|NW_003383577.1|	;03797-50639!	0.999847	140197	38096	0.0628	no
gi|320446994|ref|NW_003383577.1|	;21109-52215	0.83074	139303	406768	0.16095	no
gi|320446994|ref|NW_003383577.1|	95647-96544	0	193355	inf	0.0294	no
gi|320446994|ref|NW_003383577.1|	98102-99644	271212	14373	-0.916061	0.658	no
gi|320446994|ref|NW_003383577.1|	99905-10062!	51803	141817	-186901	0.40155	no
gi|320446995|ref|NW_003383576.1|	17291-20164	889195	537026	259442	0.05965	no
gi|320446995|ref|NW_003383576.1|	173712-17590!	792993	147965	0.899873	0.58355	no
gi|320446995|ref|NW_003383576.1|	176040-17690!	0.532763	238413	21619	0.29245	no
gi|320446995|ref|NW_003383576.1|	177019-17929!	255958	207477	-0.302956	0.88225	no
gi|320446995|ref|NW_003383576.1|	184000-18455	833959	351818	-124515	0.5501	no
gi|320446995|ref|NW_003383576.1|	185033-18554:	606766	447845	-0.43814	0.82755	no
gi|320446995|ref|NW_003383576.1|	187226-18784	105576	507919	-105561	0.6065	no
gi|320446995|ref|NW_003383576.1|	189761-19588:	0.143309	450571	829648	0.08545	no
gi|320446995|ref|NW_003383576.1|	202502-20295!	670573	198307	-175766	0.4319	no
gi|320446995|ref|NW_003383576.1|	290929-29151:	0.966501	294399	160693	0.33275	no
gi|320446995|ref|NW_003383576.1|	333280-33371!	179568	595149	-159321	0.446	no
gi|320446995|ref|NW_003383576.1|	353019-35329	697708	214642	-170069	0.4211	no
gi|320446995|ref|NW_003383576.1|	357481-35870	753557	241705	-164047	0.219	no
gi|320446995|ref|NW_003383576.1|	378285-37979	0	193036	inf	5.00E-05	yes
gi|320446995|ref|NW_003383576.1|	393293-39451!	0	16538	inf	0.0233	no
gi|320446995|ref|NW_003383576.1|	;29707-53522:	624445	901274	0.52939	0.6695	no
gi|320446995|ref|NW_003383576.1|	;36498-53809(	32092	34101	0.0875973	0.94515	no
gi|320446995|ref|NW_003383576.1|	;39115-54006	520493	775214	0.574714	0.66185	no
gi|320446995|ref|NW_003383576.1|	;41501-54302:	300349	135909	-1144	0.5007	no
gi|320446995|ref|NW_003383576.1|	;46433-55338!	102443	663606	-0.626425	0.632	no
gi|320446995|ref|NW_003383576.1|	;53516-55417!	948305	150833	0.669527	0.74765	no
gi|320446995|ref|NW_003383576.1|	;57954-56177	216339	556274	13625	0.54105	no
gi|320446995|ref|NW_003383576.1|	;62402-56560!	113145	731648	-0.628948	0.60895	no
gi|320446995|ref|NW_003383576.1|	;67282-56771(	421413	33476	-0.332109	0.86235	no
gi|320446995|ref|NW_003383576.1|	;72200-57286:	161627	349364	-220987	0.32155	no

gi|320446995|ref|NW_003383576.1|	;73064-57342:	67746	393362	-0.784279	0.7061	no
gi|320446995|ref|NW_003383576.1|	;73912-57627!	538275	663206	0.301114	0.89235	no
gi|320446995|ref|NW_003383576.1|	;76388-57824!	105942	121603	0.198892	0.9261	no
gi|320446995|ref|NW_003383576.1|	;88624-58976!	0.186351	18069	327742	0.23005	no
gi|320446995|ref|NW_003383576.1|	;08097-61237!	955766	397425	-126598	0.45425	no
gi|320446995|ref|NW_003383576.1|	;13177-61373:	151612	352841	-21033	0.3433	no
gi|320446995|ref|NW_003383576.1|	63079-67295	144115	724097	232897	0.1782	no
gi|320446995|ref|NW_003383576.1|	;31821-63230	22753	402528	-24989	0.26245	no
gi|320446995|ref|NW_003383576.1|	;34454-63549	674557	0	#NAME?	0.0085	no
gi|320446995|ref|NW_003383576.1|	;35993-63696:	122097	127223	-32626	0.17665	no
gi|320446995|ref|NW_003383576.1|	;39557-64051:	128815	0	#NAME?	0.00495	no
gi|320446995|ref|NW_003383576.1|	;61900-66861!	397427	728141	0.87353	0.4896	no
gi|320446995|ref|NW_003383576.1|	;68752-66986:	192738	320218	0.732414	0.72655	no
gi|320446995|ref|NW_003383576.1|	;72759-67319	410699	523419	0.349885	0.8736	no
gi|320446995|ref|NW_003383576.1|	;90858-69192	352266	44815	0.347316	0.7891	no
gi|320446995|ref|NW_003383576.1|	'28206-72897	353608	184083	-0.941794	0.6794	no
gi|320446995|ref|NW_003383576.1|	'60114-76053	0.866948	515901	257308	0.27405	no
gi|320446995|ref|NW_003383576.1|	'70515-77462:	20.27	196223	-0.0468504	0.9733	no
gi|320446995|ref|NW_003383576.1|	94171-94830	0	271169	inf	0.0312	no
gi|320446997|ref|NW_003383574.1|	133013-13337!	204202	0.783643	-470366	0.286	no
gi|320446997|ref|NW_003383574.1|	144864-14561:	0	248176	inf	0.0294	no
gi|320446997|ref|NW_003383574.1|	177191-17771:	291229	352313	0.274705	0.88105	no
gi|320446997|ref|NW_003383574.1|	!18293-21945	0.182324	694722	525186	0.1668	no
gi|320446997|ref|NW_003383574.1|	!41614-24288:	0	136058	inf	1	no
gi|320446997|ref|NW_003383574.1|	!56735-25746!	0	25694	inf	0.0294	no
gi|320446997|ref|NW_003383574.1|	!60299-26301:	0.408615	214146	238978	0.28125	no
gi|320446997|ref|NW_003383574.1|	!71771-27244	0	604566	inf	0.01485	no
gi|320446997|ref|NW_003383574.1|	;10538-31227:	449572	520254	0.210664	0.87305	no
gi|320446997|ref|NW_003383574.1|	;12998-31463!	322937	62503	0.952675	0.47035	no
gi|320446997|ref|NW_003383574.1|	;23491-32391	0.879494	871755	330918	0.22995	no

gi|320446997|ref|NW_003383574.1|	;22063-52276ε	0.359246	270161	291078	0.25645	no
gi|320446998|ref|NW_003383573.1|	ι07481-10969ː	264769	155268	587388	0.02625	no
gi|320446998|ref|NW_003383573.1|	10858-11074	0	534581	inf	0.0198	no
gi|320446998|ref|NW_003383573.1|	ι10526-11270ι	0.867711	314243	517852	0.0495	no
gi|320446998|ref|NW_003383573.1|	ι13382-11465ι	129986	356241	477643	0.06135	no
gi|320446998|ref|NW_003383573.1|	ι16164-11778ι	0.366675	48484	372493	0.18185	no
gi|320446998|ref|NW_003383573.1|	ι17893-11993ˈ	0.279891	917477	503474	0.14635	no
gi|320446998|ref|NW_003383573.1|	ι21680-12260ι	0	20281	inf	0.029	no
gi|320446998|ref|NW_003383573.1|	ι24440-12530ː	0.808076	185403	452003	0.15405	no
gi|320446998|ref|NW_003383573.1|	ι25576-12717ː	0.62011	146708	456428	0.1329	no
gi|320446998|ref|NW_003383573.1|	ι29319-12955ι	0	600138	inf	0.0142	no
gi|320446998|ref|NW_003383573.1|	ι29684-13026ː	147039	24538	406074	0.16485	no
gi|320446998|ref|NW_003383573.1|	ι31356-13215ι	0	13486	inf	0.00615	no
gi|320446998|ref|NW_003383573.1|	15829-16330	0.626303	17208	478007	0.17185	no
gi|320446998|ref|NW_003383573.1|	17309-17822	120102	298295	463441	0.1513	no
gi|320446998|ref|NW_003383573.1|	ι74076-17452ι	354551	143397	-130597	0.53385	no
gi|320446998|ref|NW_003383573.1|	ι75307-17555ː	380925	415777	0.126301	0.9416	no
gi|320446998|ref|NW_003383573.1|	ι75809-17648ι	117106	921815	-0.34526	0.7901	no
gi|320446998|ref|NW_003383573.1|	ι76748-18014ι	132561	183221	0.466928	0.72115	no
gi|320446998|ref|NW_003383573.1|	18035-18689	0.402591	658235	403122	0.19275	no
gi|320446998|ref|NW_003383573.1|	ι86576-18832ι	189397	4035	109116	0.5975	no
gi|320446998|ref|NW_003383573.1|	ι95489-19590ˈ	707001	467092	-0.598005	0.7539	no
gi|320446998|ref|NW_003383573.1|	21780-22832	0.82667	132914	400704	0.15805	no
gi|320446998|ref|NW_003383573.1|	ː23010-22838ˈ	120431	437067	185964	0.1695	no
gi|320446998|ref|NW_003383573.1|	23159-24260	0.584895	809661	379107	0.1764	no
gi|320446998|ref|NW_003383573.1|	ː66439-26672ː	310718	992223	167506	0.43325	no
gi|320446998|ref|NW_003383573.1|	ː91286-29343ˈ	239591	251979	0.0727323	0.952	no
gi|320446998|ref|NW_003383573.1|	ː95754-29618ː	421413	446346	0.0829289	0.93155	no
gi|320446998|ref|NW_003383573.1|	ː98786-29941ˈ	46824	243133	-0.945502	0.6683	no
gi|320446998|ref|NW_003383573.1|	ι01364-30242ι	183085	121012	-0.597362	0.7801	no

gi|320446998|ref|NW_003383573.1|	302595-30510[illegible]	225855	154646	-0.546422	0.6746	no
gi|320446998|ref|NW_003383573.1|	305280-30569[illegible]	184694	156916	-0.235144	0.90845	no
gi|320446998|ref|NW_003383573.1|	306156-30636[illegible]	45877	466646	0.0245573	0.95445	no
gi|320446998|ref|NW_003383573.1|	306593-30709[illegible]	47094	344809	-0.449747	0.8393	no
gi|320446998|ref|NW_003383573.1|	316581-31688[illegible]	828354	833386	0.00873697	0.99135	no
gi|320446998|ref|NW_003383573.1|	326007-32690[illegible]	749441	94.57	0.335567	0.79715	no
gi|320446998|ref|NW_003383573.1|	327971-33019[illegible]	301676	750213	13143	0.32965	no
gi|320446998|ref|NW_003383573.1|	382584-38547[illegible]	0.190899	244639	367977	0.18115	no
gi|320446998|ref|NW_003383573.1|	385577-38774[illegible]	0.262609	158804	259625	0.26755	no
gi|320446998|ref|NW_003383573.1|	4363-4854	0.649468	478051	287983	0.25835	no
gi|320446998|ref|NW_003383573.1|	45528-46018	195562	475389	460341	0.1534	no
gi|320446998|ref|NW_003383573.1|	46162-46786	0	114815	inf	0.0088	no
gi|320446998|ref|NW_003383573.1|	472639-47980[illegible]	319211	573816	0.846075	0.5047	no
gi|320446998|ref|NW_003383573.1|	485138-48789[illegible]	57546	284311	230468	0.2208	no
gi|320446998|ref|NW_003383573.1|	49271-49696	0	961317	inf	0.02075	no
gi|320446998|ref|NW_003383573.1|	499973-50056[illegible]	381245	157822	204951	0.2552	no
gi|320446998|ref|NW_003383573.1|	500783-50179[illegible]	0.434072	299944	278869	0.2345	no
gi|320446998|ref|NW_003383573.1|	50277-50553	238114	531232	447962	0.17995	no
gi|320446998|ref|NW_003383573.1|	515022-51531[illegible]	209514	308277	387911	0.2002	no
gi|320446998|ref|NW_003383573.1|	523294-52368[illegible]	0	123203	inf	0.0154	no
gi|320446998|ref|NW_003383573.1|	523988-52430[illegible]	0	175131	inf	0.0198	no
gi|320446998|ref|NW_003383573.1|	524374-52620[illegible]	101714	125503	-301873	0.20755	no
gi|320446998|ref|NW_003383573.1|	526490-52807[illegible]	563484	156822	-184525	0.3918	no
gi|320446998|ref|NW_003383573.1|	528360-52907[illegible]	174812	167444	-0.0621339	0.9269	no
gi|320446998|ref|NW_003383573.1|	529212-53036[illegible]	22142	357873	0.692659	0.7313	no
gi|320446998|ref|NW_003383573.1|	530572-53348[illegible]	0.568197	238313	206839	0.3441	no
gi|320446998|ref|NW_003383573.1|	535976-53764[illegible]	0.822218	228964	147752	0.4855	no
gi|320446998|ref|NW_003383573.1|	537753-54222[illegible]	585984	119753	103113	0.4292	no
gi|320446998|ref|NW_003383573.1|	561010-56139[illegible]	412682	151024	519361	0.10305	no
gi|320446998|ref|NW_003383573.1|	562795-56809[illegible]	199963	820261	535828	0.0226	no

gi|320446998|ref|NW_003383573.1|	;69320-57144	0.536066	193227	517174	0.07285	no
gi|320446998|ref|NW_003383573.1|	;73246-57443	105738	205207	427852	0.1265	no
gi|320446998|ref|NW_003383573.1|	;78419-57947	0.413335	138631	506779	0.15005	no
gi|320446998|ref|NW_003383573.1|	;79669-58323	14206	106142	290143	0.12175	no
gi|320446998|ref|NW_003383573.1|	;00825-60238	0.382758	230762	25919	0.2676	no
gi|320446998|ref|NW_003383573.1|	;02515-60320	0	280527	inf	0.0294	no
gi|320446998|ref|NW_003383573.1|	;05331-60743	0.632105	699232	346754	0.16425	no
gi|320446998|ref|NW_003383573.1|	;08156-60863	0	783099	inf	0.02075	no
gi|320446998|ref|NW_003383573.1|	;12135-61333	105079	30349	153017	0.4736	no
gi|320446998|ref|NW_003383573.1|	;13514-61396	0.764188	177781	454003	0.17855	no
gi|320446998|ref|NW_003383573.1|	;18277-62006	0.326468	141005	543266	0.11605	no
gi|320446998|ref|NW_003383573.1|	;20771-62160	0.562048	869691	395174	0.1649	no
gi|320446998|ref|NW_003383573.1|	;43053-64479	0.672902	282749	539298	0.07225	no
gi|320446998|ref|NW_003383573.1|	;46770-64716	496648	143671	153247	0.4911	no
gi|320446998|ref|NW_003383573.1|	;75226-67632	35175	825034	12299	0.5634	no
gi|320446998|ref|NW_003383573.1|	;76513-67893	100179	209865	106688	0.59375	no
gi|320446998|ref|NW_003383573.1|	;83425-68398	20912	352841	0.754685	0.7213	no
gi|320446998|ref|NW_003383573.1|	;84306-68531	349054	602941	0.788567	0.6976	no
gi|320446998|ref|NW_003383573.1|	;93160-69730	226829	294657	0.377431	0.77755	no
gi|320446998|ref|NW_003383573.1|	'06457-71480	0.103986	13972	7.07	0.0855	no
gi|320446998|ref|NW_003383573.1|	'20060-72193	0	201074	inf	0.01275	no
gi|320446998|ref|NW_003383573.1|	73544-74133	190096	933465	229587	0.32095	no
gi|320446998|ref|NW_003383573.1|	'35761-73654	0	211172	inf	0.0312	no
gi|320446998|ref|NW_003383573.1|	'39903-74113	0	117426	inf	1	no
gi|320446998|ref|NW_003383573.1|	'42643-74418	0	0.898994	inf	1	no
gi|320446998|ref|NW_003383573.1|	74297-74874	294917	458858	395966	0.14535	no
gi|320446998|ref|NW_003383573.1|	'44918-74541	270776	988213	186772	0.2696	no
gi|320446998|ref|NW_003383573.1|	'46102-74727	864872	342087	19838	0.24945	no
gi|320446998|ref|NW_003383573.1|	75219-75940	0.694952	106999	394453	0.16525	no
gi|320446998|ref|NW_003383573.1|	76249-76945	0.732434	250227	177247	0.33305	no

gi|320446998|ref|NW_003383573.1|	77809-78455	0.820672	251427	161526	0.33275	no
gi|320446998|ref|NW_003383573.1|	300998-80130	272411	466797	-254492	0.2943	no
gi|320446998|ref|NW_003383573.1|	301459-80204	99525	160515	-263235	0.2747	no
gi|320446998|ref|NW_003383573.1|	302573-80294	115782	226938	-235104	0.2561	no
gi|320446998|ref|NW_003383573.1|	304360-80482	720963	0	#NAME?	0.0229	no
gi|320446998|ref|NW_003383573.1|	306823-80730	67432	0.901062	-290373	0.2695	no
gi|320446998|ref|NW_003383573.1|	80819-81870	268997	170278	266223	0.2488	no
gi|320446998|ref|NW_003383573.1|	313967-81479	367782	0.194534	-424076	0.3017	no
gi|320446998|ref|NW_003383573.1|	321577-82220	727265	0.872527	-305921	0.19555	no
gi|320446998|ref|NW_003383573.1|	82422-83847	0.85112	473155	247488	0.2843	no
gi|320446998|ref|NW_003383573.1|	331258-83172	630935	467637	-0.432103	0.82755	no
gi|320446998|ref|NW_003383573.1|	332297-83367	177383	287486	0.696626	0.73065	no
gi|320446998|ref|NW_003383573.1|	336977-83795	135995	0.782543	-0.79731	1	no
gi|320446998|ref|NW_003383573.1|	340317-84159	145255	0.78392	-0.889805	0.6744	no
gi|320446998|ref|NW_003383573.1|	346796-84811	125164	0.543009	-120477	1	no
gi|320446998|ref|NW_003383573.1|	85390-85812	0.866948	917158	340315	0.21725	no
gi|320446998|ref|NW_003383573.1|	354663-85535	263419	103896	-134222	0.54025	no
gi|320446998|ref|NW_003383573.1|	356848-85922	16411	116785	-0.490805	0.69575	no
gi|320446998|ref|NW_003383573.1|	85968-86506	110566	558461	233655	0.2622	no
gi|320446998|ref|NW_003383573.1|	360594-86206	0.545843	227676	206043	0.3563	no
gi|320446998|ref|NW_003383573.1|	369629-87055	356806	198863	-0.843364	0.5991	no
gi|320446998|ref|NW_003383573.1|	371094-87508	272847	283183	0.0536418	0.9711	no
gi|320446998|ref|NW_003383573.1|	383714-88418	985349	0.469416	-43917	0.2975	no
gi|320446998|ref|NW_003383573.1|	384650-88594	239797	0.776581	-162661	0.4414	no
gi|320446998|ref|NW_003383573.1|	394589-89820	82376	272135	172403	0.19085	no
gi|320446998|ref|NW_003383573.1|	902772-90397	382117	103552	143827	0.50255	no
gi|320446998|ref|NW_003383573.1|	905587-90607	328369	439258	0.419749	0.8285	no
gi|320446998|ref|NW_003383573.1|	906459-90710	285178	499374	0.808256	0.68375	no
gi|320446998|ref|NW_003383573.1|	907545-90806	40772	430605	0.0787847	0.94785	no
gi|320446998|ref|NW_003383573.1|	94406-95354	142104	522928	187967	0.3883	no

gi|320446999|ref|NW_003383572.1|	16555-11714	235664	223533	-0.0762476	0.92685	no
gi|320446999|ref|NW_003383572.1|	36274-13746	0.528136	378265	284042	0.2453	no
gi|320446999|ref|NW_003383572.1|	48617-14888	512808	375185	287111	0.22625	no
gi|320446999|ref|NW_003383572.1|	49054-14925	730491	500738	277712	0.26625	no
gi|320446999|ref|NW_003383572.1|	49703-15008	629401	123862	0.976685	0.6513	no
gi|320446999|ref|NW_003383572.1|	52572-15283	283692	306073	0.109551	0.94725	no
gi|320446999|ref|NW_003383572.1|	57459-15984	832823	932582	0.163221	0.9449	no
gi|320446999|ref|NW_003383572.1|	60069-16158	0.264161	128591	228329	1	no
gi|320446999|ref|NW_003383572.1|	61989-16360	0.734414	238509	169938	0.4284	no
gi|320446999|ref|NW_003383572.1|	66260-16808	395203	160201	-130271	0.31555	no
gi|320446999|ref|NW_003383572.1|	78777-18396	203899	108029	-0.916441	0.49035	no
gi|320446999|ref|NW_003383572.1|	44704-34554	306067	22967	-373621	0.15045	no
gi|320446999|ref|NW_003383572.1|	45753-34610	595507	103208	-252856	0.27385	no
gi|320446999|ref|NW_003383572.1|	46576-34799	164515	268382	-261585	0.26785	no
gi|320446999|ref|NW_003383572.1|	48121-34858	152018	0.482246	-497833	0.28625	no
gi|320446999|ref|NW_003383572.1|	49005-35008	240635	288975	-305783	0.20945	no
gi|320446999|ref|NW_003383572.1|	50283-35167	36977	467038	-298502	0.111	no
gi|320446999|ref|NW_003383572.1|	97115-59746	140829	833135	-0.757319	0.71275	no
gi|320446999|ref|NW_003383572.1|	99034-60119	105153	243954	121412	0.4791	no
gi|320446999|ref|NW_003383572.1|	15319-61840	171833	356313	105214	0.6217	no
gi|320446999|ref|NW_003383572.1|	19454-62097	248618	0.45502	-244993	0.29445	no
gi|320446999|ref|NW_003383572.1|	21101-62156	858195	1.43	-258529	0.2331	no
gi|320446999|ref|NW_003383572.1|	21676-62278	0.776272	161198	10542	0.606	no
gi|320446999|ref|NW_003383572.1|	84191-85487	978532	759712	-0.365166	0.7826	no
gi|320446999|ref|NW_003383572.1|	85680-87860	40175	216761	-0.890195	0.49675	no
gi|320446999|ref|NW_003383572.1|	88168-92614	258459	117852	-113296	0.39365	no
gi|320447000|ref|NW_003383571.1|	43428-14432	180306	0	#NAME?	0.00555	no
gi|320447000|ref|NW_003383571.1|	52417-15596	864846	0	#NAME?	5.00E-05	yes
gi|320447000|ref|NW_003383571.1|	98089-19987	305295	205078	-0.574029	0.78005	no
gi|320447000|ref|NW_003383571.1|	01403-20286	275147	143446	-0.939694	0.652	no

gi|320447000|ref|NW_003383571.1|	203367-20433	41752	144093	-153485	0.47385	no
gi|320447000|ref|NW_003383571.1|	207749-20861	194152	201422	-326889	0.1828	no
gi|320447000|ref|NW_003383571.1|	209556-21193	154353	43988	-181105	0.2919	no
gi|320447000|ref|NW_003383571.1|	220653-22100	383771	438636	0.192777	0.92295	no
gi|320447000|ref|NW_003383571.1|	221682-22305	80103	161758	101391	0.6491	no
gi|320447000|ref|NW_003383571.1|	234166-23458	227571	381809	0.746531	0.7216	no
gi|320447000|ref|NW_003383571.1|	234962-23585	129312	427574	172532	0.4311	no
gi|320447000|ref|NW_003383571.1|	235979-23976	14165	144045	0.0241862	0.9856	no
gi|320447000|ref|NW_003383571.1|	24197-29988	229898	39244	0.771478	0.55425	no
gi|320447000|ref|NW_003383571.1|	242865-24750	178055	234255	0.395756	0.84845	no
gi|320447000|ref|NW_003383571.1|	247859-24842	270399	221947	-0.284873	0.832	no
gi|320447000|ref|NW_003383571.1|	259024-25982	335144	327929	-0.0313962	0.97965	no
gi|320447000|ref|NW_003383571.1|	263626-26391	215.8	115269	-0.904691	0.6934	no
gi|320447000|ref|NW_003383571.1|	298167-29926	273901	0	#NAME?	0.0178	no
gi|320447000|ref|NW_003383571.1|	303070-30584	222346	99094	-116594	0.3719	no
gi|320447000|ref|NW_003383571.1|	306649-30695	116384	605083	-0.943683	0.64945	no
gi|320447000|ref|NW_003383571.1|	32340-34322	147812	165712	0.164915	0.8957	no
gi|320447000|ref|NW_003383571.1|	332927-33337	409167	236797	-0.789041	0.69945	no
gi|320447000|ref|NW_003383571.1|	333798-33872	124924	110201	-0.180912	0.88975	no
gi|320447000|ref|NW_003383571.1|	338882-33951	556143	349011	-0.672185	0.74595	no
gi|320447000|ref|NW_003383571.1|	340263-34412	470415	24123	-0.963522	0.6674	no
gi|320447000|ref|NW_003383571.1|	344640-34554	200744	0.518866	-195192	0.30305	no
gi|320447000|ref|NW_003383571.1|	346338-34857	623819	170619	-187035	0.3961	no
gi|320447000|ref|NW_003383571.1|	350142-35221	780932	179435	-212173	0.33785	no
gi|320447000|ref|NW_003383571.1|	353673-35729	214605	651536	-171976	0.18765	no
gi|320447000|ref|NW_003383571.1|	37197-40087	635612	622018	-0.0311889	0.9873	no
gi|320447000|ref|NW_003383571.1|	374899-37632	263404	338532	0.362012	0.77285	no
gi|320447000|ref|NW_003383571.1|	376756-37803	415554	185835	-116101	0.4932	no
gi|320447000|ref|NW_003383571.1|	379403-38080	626337	236167	-140713	0.2873	no
gi|320447000|ref|NW_003383571.1|	381522-38183	679271	201739	-17515	0.41045	no

gi|320447000|ref|NW_003383571.1|	402967-40591	806083	267952	173297	0.32325	no
gi|320447000|ref|NW_003383571.1|	406175-40848	261205	474311	0.860648	0.5173	no
gi|320447000|ref|NW_003383571.1|	40746-42334	798436	19965	-199971	0.3673	no
gi|320447000|ref|NW_003383571.1|	408613-40928	50546	121982	1271	0.53605	no
gi|320447000|ref|NW_003383571.1|	409609-40995	216073	254331	0.235191	0.90245	no
gi|320447000|ref|NW_003383571.1|	410209-41050	570472	122622	110399	0.60645	no
gi|320447000|ref|NW_003383571.1|	42553-44674	0.447433	118617	140656	1	no
gi|320447000|ref|NW_003383571.1|	441214-44176	846359	108294	0.355612	0.77795	no
gi|320447000|ref|NW_003383571.1|	452246-45295	790342	343841	-120073	0.56035	no
gi|320447000|ref|NW_003383571.1|	46529-47292	153554	263243	-254428	0.2691	no
gi|320447000|ref|NW_003383571.1|	468043-46862	72897	105245	0.529817	0.7972	no
gi|320447000|ref|NW_003383571.1|	470854-47312	124582	614491	-101964	0.5257	no
gi|320447000|ref|NW_003383571.1|	473307-47356	200919	145689	-0.463728	0.8145	no
gi|320447000|ref|NW_003383571.1|	530598-53177	305982	0.997743	-161671	0.4347	no
gi|320447000|ref|NW_003383571.1|	534668-53511	102398	156869	-270655	0.2243	no
gi|320447000|ref|NW_003383571.1|	550316-55075	201323	154889	-0.378275	0.8489	no
gi|320447000|ref|NW_003383571.1|	552658-55301	146877	478749	-161727	0.4427	no
gi|320447000|ref|NW_003383571.1|	553244-55356	949943	817727	-0.216221	0.91025	no
gi|320447000|ref|NW_003383571.1|	553839-55409	331165	209888	-0.65793	0.74515	no
gi|320447000|ref|NW_003383571.1|	554287-55660	186348	158367	-0.234724	0.9009	no
gi|320447000|ref|NW_003383571.1|	556842-55957	72315	868988	0.265041	0.86735	no
gi|320447000|ref|NW_003383571.1|	559699-56238	100607	136742	0.442724	0.7243	no
gi|320447000|ref|NW_003383571.1|	571707-57331	961716	506212	-0.925868	0.67255	no
gi|320447000|ref|NW_003383571.1|	592021-59241	19076	329897	-253167	0.28515	no
gi|320447000|ref|NW_003383571.1|	576661-67788	137256	130855	-0.0689033	1	no
gi|320447000|ref|NW_003383571.1|	714987-71678	108264	203642	0.911482	0.6627	no
gi|320447000|ref|NW_003383571.1|	719556-72067	0.572977	145456	134404	0.5243	no
gi|320447000|ref|NW_003383571.1|	725603-72617	40007	169039	-12429	0.557	no
gi|320447000|ref|NW_003383571.1|	726452-72713	341358	207105	-0.72092	0.72915	no
gi|320447000|ref|NW_003383571.1|	727873-72910	320443	339192	0.0820337	0.96435	no

gi|320447000|ref|NW_003383571.1|	745491-74664	215937	305544	0.500773	0.70175	no
gi|320447000|ref|NW_003383571.1|	768911-76989	293473	187069	-0.649655	0.75195	no
gi|320447000|ref|NW_003383571.1|	772085-77328	57974	608851	0.0706832	0.9718	no
gi|320447000|ref|NW_003383571.1|	774232-77538	203191	895648	21401	0.32805	no
gi|320447001|ref|NW_003383570.1|	02232-10033	0.941112	195513	105483	0.5998	no
gi|320447001|ref|NW_003383570.1|	04493-10171	529204	120798	11907	0.3813	no
gi|320447001|ref|NW_003383570.1|	92222-10926	152838	558741	187018	0.32465	no
gi|320447001|ref|NW_003383570.1|	22829-27325	102962	149751	0.540445	0.7931	no
gi|320447001|ref|NW_003383570.1|	31664-32444	619858	191418	-169521	0.4287	no
gi|320447001|ref|NW_003383570.1|	23945-32462	203004	0.513328	-530549	0.1862	no
gi|320447001|ref|NW_003383570.1|	32666-33304	251027	142326	-0.81864	0.69605	no
gi|320447001|ref|NW_003383570.1|	34872-36110	353467	0.700393	-233534	0.30715	no
gi|320447001|ref|NW_003383570.1|	39901-40357	104311	0.991533	-339509	0.2366	no
gi|320447001|ref|NW_003383570.1|	09337-41046	320693	0.522518	-261764	0.26885	no
gi|320447001|ref|NW_003383570.1|	11439-41265	515758	0.357594	-38503	0.16655	no
gi|320447001|ref|NW_003383570.1|	35720-43614	121953	0.57585	-440449	0.29665	no
gi|320447001|ref|NW_003383570.1|	15619-51901	752245	0.5225	-38477	0.07265	no
gi|320447001|ref|NW_003383570.1|	25660-52594	194017	55118	-181559	0.41575	no
gi|320447001|ref|NW_003383570.1|	55088-61705	183061	251415	0.457752	0.80235	no
gi|320447001|ref|NW_003383570.1|	82810-58324	823661	218308	-191569	0.39995	no
gi|320447001|ref|NW_003383570.1|	85830-58692	491961	122566	-200499	0.3431	no
gi|320447001|ref|NW_003383570.1|	88455-59172	499752	135934	-187831	0.39375	no
gi|320447001|ref|NW_003383570.1|	91902-59304	819041	219171	-190188	0.3808	no
gi|320447001|ref|NW_003383570.1|	94239-59452	179308	715538	-132534	0.52545	no
gi|320447001|ref|NW_003383570.1|	94765-59636	780765	301825	-137117	0.51925	no
gi|320447001|ref|NW_003383570.1|	96915-59887	104812	840021	-0.31931	0.88615	no
gi|320447001|ref|NW_003383570.1|	99440-60521	28823	103552	-147686	0.2793	no
gi|320447001|ref|NW_003383570.1|	27200-62751	348344	212946	-0.710024	0.73075	no
gi|320447001|ref|NW_003383570.1|	27671-62958	378973	257161	-0.559421	0.6703	no
gi|320447001|ref|NW_003383570.1|	30746-63188	384733	129346	-157262	0.35995	no

gi|320447001|ref|NW_003383570.1|	33693-63611	432134	204763	-107752	0.61055	no
gi|320447001|ref|NW_003383570.1|	36223-63660	858319	42189	-102465	0.59615	no
gi|320447001|ref|NW_003383570.1|	37914-63884	194104	0.502014	-195103	0.30305	no
gi|320447001|ref|NW_003383570.1|	39822-64240	133929	664107	-101198	0.4419	no
gi|320447001|ref|NW_003383570.1|	43206-64413	604836	130156	110563	0.60125	no
gi|320447001|ref|NW_003383570.1|	44377-64502	448138	110972	130818	0.5314	no
gi|320447001|ref|NW_003383570.1|	45242-64547	305401	247024	-0.306057	0.8712	no
gi|320447001|ref|NW_003383570.1|	45797-64989	244807	960949	197281	0.25795	no
gi|320447001|ref|NW_003383570.1|	50670-65137	136225	441175	-162657	0.44355	no
gi|320447001|ref|NW_003383570.1|	51888-65261	439241	156194	-149168	0.3612	no
gi|320447001|ref|NW_003383570.1|	56226-65725	275023	442084	-263716	0.13735	no
gi|320447001|ref|NW_003383570.1|	58008-65896	135842	226285	-258571	0.25855	no
gi|320447001|ref|NW_003383570.1|	60006-66068	109993	103553	-340898	0.2089	no
gi|320447001|ref|NW_003383570.1|	62795-66436	12994	149207	-312246	0.1999	no
gi|320447001|ref|NW_003383570.1|	64492-66474	662329	572422	-35324	0.17595	no
gi|320447001|ref|NW_003383570.1|	65031-66740	125391	368913	-176508	0.29725	no
gi|320447001|ref|NW_003383570.1|	68331-66953	820564	242048	-176132	0.415	no
gi|320447001|ref|NW_003383570.1|	67163-67360	460683	374198	-0.299972	0.889	no
gi|320447001|ref|NW_003383570.1|	72614-67338	104775	152432	-278106	0.241	no
gi|320447001|ref|NW_003383570.1|	73500-67485	307478	293073	-339115	0.0873	no
gi|320447001|ref|NW_003383570.1|	80730-68184	766573	344961	-115199	0.5861	no
gi|320447001|ref|NW_003383570.1|	83609-68573	356434	814397	11921	0.58415	no
gi|320447001|ref|NW_003383570.1|	87050-68815	253748	324234	0.353638	0.858	no
gi|320447001|ref|NW_003383570.1|	88794-69075	576622	709852	-302204	0.12055	no
gi|320447001|ref|NW_003383570.1|	90906-69157	467646	292343	-399968	0.13325	no
gi|320447001|ref|NW_003383570.1|	92016-69243	269026	473676	0.816151	0.69235	no
gi|320447001|ref|NW_003383570.1|	53890-75466	346646	302643	-0.195843	0.91565	no
gi|320447001|ref|NW_003383570.1|	65605-76712	170902	75883	215061	0.34215	no
gi|320447001|ref|NW_003383570.1|	70106-77122	0	15868	inf	0.029	no
gi|320447001|ref|NW_003383570.1|	74376-77561	278297	404634	0.539993	0.6784	no

gi|320447001|ref|NW_003383570.1|	775737-77667	114658	109209	-0.0702415	0.95785	no
gi|320447001|ref|NW_003383570.1|	79561-80024	234543	863221	-144205	0.26945	no
gi|320447001|ref|NW_003383570.1|	83155-85184	376309	524878	0.480064	0.8173	no
gi|320447001|ref|NW_003383570.1|	86744-87572	854458	783279	-0.125484	0.9507	no
gi|320447001|ref|NW_003383570.1|	88541-89139	148382	116308	-0.351367	0.86135	no
gi|320447001|ref|NW_003383570.1|	950440-95180	375135	23968	-0.646302	0.7527	no
gi|320447002|ref|NW_003383569.1|	107195-10775	306267	896516	154954	0.47675	no
gi|320447002|ref|NW_003383569.1|	165246-16570	144779	482246	173591	0.33745	no
gi|320447002|ref|NW_003383569.1|	267644-27261	0.82015	559516	277022	0.13455	no
gi|320447002|ref|NW_003383569.1|	281392-28228	0.256266	264845	336943	0.2261	no
gi|320447002|ref|NW_003383569.1|	310454-31121	0.647153	354922	245532	0.24845	no
gi|320447002|ref|NW_003383569.1|	328522-32924	0.693531	427143	262269	0.2367	no
gi|320447002|ref|NW_003383569.1|	483497-48417	265501	181217	-0.550995	0.79275	no
gi|320447002|ref|NW_003383569.1|	486573-48958	906098	914114	0.0127082	0.99115	no
gi|320447002|ref|NW_003383569.1|	502840-50459	102025	996423	-0.0340982	0.9872	no
gi|320447002|ref|NW_003383569.1|	505605-50750	871773	459265	-0.924626	0.6732	no
gi|320447002|ref|NW_003383569.1|	507606-50811	161111	581605	-146994	0.4821	no
gi|320447002|ref|NW_003383569.1|	508727-50913	530498	193092	-145806	0.5027	no
gi|320447002|ref|NW_003383569.1|	509402-51098	106.06	210839	-233067	0.08635	no
gi|320447002|ref|NW_003383569.1|	530896-53145	51348	0	#NAME?	0.0229	no
gi|320447002|ref|NW_003383569.1|	536412-53781	159116	221093	0.474577	0.8129	no
gi|320447002|ref|NW_003383569.1|	542019-54598	116328	918823	-0.340336	0.7918	no
gi|320447002|ref|NW_003383569.1|	567281-56784	665545	152151	11929	0.5637	no
gi|320447002|ref|NW_003383569.1|	604698-60521	123974	107069	-0.211496	0.91795	no
gi|320447002|ref|NW_003383569.1|	613586-62087	653192	106857	0.710101	0.587	no
gi|320447002|ref|NW_003383569.1|	624336-62619	0.103801	18808	417945	0.1906	no
gi|320447002|ref|NW_003383569.1|	651004-65199	0.224546	49624	446595	0.1818	no
gi|320447002|ref|NW_003383569.1|	654805-65536	0	345798	inf	0.0312	no
gi|320447002|ref|NW_003383569.1|	678790-67953	0	458178	inf	0.0162	no
gi|320447002|ref|NW_003383569.1|	729366-73055	16172	0.123162	-703679	0.26285	no

gi|320447002|ref|NW_003383569.1|	34979-73568	972021	0	#NAME?	0.0091	no
gi|320447002|ref|NW_003383569.1|	11438-81392	114453	206893	0.854129	0.50675	no
gi|320447002|ref|NW_003383569.1|	18997-82263	274622	414562	0.594142	0.65675	no
gi|320447002|ref|NW_003383569.1|	23473-82397	123932	498519	20081	0.3028	no
gi|320447002|ref|NW_003383569.1|	27276-82885	0.626558	444656	282717	0.2256	no
gi|320447002|ref|NW_003383569.1|	29500-82991	177609	938535	24017	0.25155	no
gi|320447002|ref|NW_003383569.1|	30992-83527	0.87625	937808	341988	0.08665	no
gi|320447002|ref|NW_003383569.1|	39150-84301	325222	234131	-0.474109	0.8306	no
gi|320447002|ref|NW_003383569.1|	45198-84606	10706	165825	0.631245	0.75205	no
gi|320447002|ref|NW_003383569.1|	47613-84791	230498	300945	0.384742	0.7532	no
gi|320447002|ref|NW_003383569.1|	49379-85111	148236	294072	0.988279	0.4366	no
gi|320447002|ref|NW_003383569.1|	52574-85616	137122	854805	-0.681794	0.58425	no
gi|320447002|ref|NW_003383569.1|	58300-85854	361452	789891	-219408	0.3338	no
gi|320447002|ref|NW_003383569.1|	58925-86013	282532	104544	-143431	0.38675	no
gi|320447002|ref|NW_003383569.1|	60246-86154	158662	0.660693	-12639	0.5499	no
gi|320447002|ref|NW_003383569.1|	87440-87902	290741	726201	132063	0.55025	no
gi|320447004|ref|NW_003383567.1|	05839-10095	253405	422193	0.736456	0.58725	no
gi|320447004|ref|NW_003383567.1|	13459-10137	245672	438112	0.834564	0.68535	no
gi|320447004|ref|NW_003383567.1|	14401-10146	336462	452762	0.428311	0.8223	no
gi|320447004|ref|NW_003383567.1|	02540-10276	366063	632739	0.789519	0.6951	no
gi|320447004|ref|NW_003383567.1|	35000-10367	142388	18998	0.416019	0.7958	no
gi|320447004|ref|NW_003383567.1|	47741-10484	21973	157643	284286	0.2293	no
gi|320447004|ref|NW_003383567.1|	63674-10647	485221	139228	-18012	0.2961	no
gi|320447004|ref|NW_003383567.1|	64867-10663	215033	411153	-238681	0.1758	no
gi|320447004|ref|NW_003383567.1|	66537-10667	364229	405132	-316838	0.2014	no
gi|320447004|ref|NW_003383567.1|	70736-10710	147116	145273	-334011	0.1763	no
gi|320447004|ref|NW_003383567.1|	71180-10746	118299	229019	-23689	0.08685	no
gi|320447004|ref|NW_003383567.1|	77934-10785	64493	192888	158054	0.4698	no
gi|320447004|ref|NW_003383567.1|	80238-10805	386603	754558	0.964779	0.6698	no
gi|320447004|ref|NW_003383567.1|	81749-10831	0.301988	157281	238078	0.2609	no

gi|320447004|ref|NW_003383567.1|	)84548-10852:	632203	700295	0.147574	0.93705	no
gi|320447004|ref|NW_003383567.1|	)85335-10862:	118921	147685	0.312518	0.8759	no
gi|320447004|ref|NW_003383567.1|	)87828-10881	427988	477582	0.158177	0.93505	no
gi|320447004|ref|NW_003383567.1|	)88909-10894	758389	154.64	10279	0.4206	no
gi|320447004|ref|NW_003383567.1|	)89987-10935	142603	187527	0.395098	0.7574	no
gi|320447004|ref|NW_003383567.1|	)94591-10951	694951	812358	0.225203	0.85455	no
gi|320447004|ref|NW_003383567.1|	112309-11163	199717	1084.86	244149	0.2311	no
gi|320447004|ref|NW_003383567.1|	11365-11865	38.97	176883	-113956	0.5935	no
gi|320447004|ref|NW_003383567.1|	118569-11975	212362	126332	257262	0.26465	no
gi|320447004|ref|NW_003383567.1|	189926-11941	510771	923757	0.854838	0.49725	no
gi|320447004|ref|NW_003383567.1|	200137-12025	0.625053	109109	0.803726	1	no
gi|320447004|ref|NW_003383567.1|	120111-12202	190881	133757	280887	0.1192	no
gi|320447004|ref|NW_003383567.1|	204305-12049	0.422516	40232	325126	0.2305	no
gi|320447004|ref|NW_003383567.1|	216310-12173	216997	115737	-0.906823	0.4997	no
gi|320447004|ref|NW_003383567.1|	219462-12197	384535	211916	-0.859627	0.6018	no
gi|320447004|ref|NW_003383567.1|	222070-12232	0.711607	406859	251538	0.28175	no
gi|320447004|ref|NW_003383567.1|	223441-12249	416169	277606	-0.58413	0.7213	no
gi|320447004|ref|NW_003383567.1|	252460-12544	0.19478	420999	44339	0.15705	no
gi|320447004|ref|NW_003383567.1|	257643-12588	149252	126536	-0.238209	0.90635	no
gi|320447004|ref|NW_003383567.1|	259089-12639	117767	0.902199	-0.384414	1	no
gi|320447004|ref|NW_003383567.1|	264002-12653	109824	130681	0.25086	1	no
gi|320447004|ref|NW_003383567.1|	323591-13241	0.456297	278559	260994	0.2636	no
gi|320447004|ref|NW_003383567.1|	132375-13297	417325	125738	-173075	0.4381	no
gi|320447004|ref|NW_003383567.1|	133719-13515	0.982237	126775	0.368131	1	no
gi|320447004|ref|NW_003383567.1|	145068-14540	964986	134182	0.475616	0.81225	no
gi|320447004|ref|NW_003383567.1|	150959-15161	0.79952	272399	176852	0.33305	no
gi|320447004|ref|NW_003383567.1|	511382-15128	0.399054	143368	5167	0.1437	no
gi|320447004|ref|NW_003383567.1|	15116-17345	317393	0.413482	-62623	0.085	no
gi|320447004|ref|NW_003383567.1|	152711-15342	0.355485	291723	303674	0.24995	no
gi|320447004|ref|NW_003383567.1|	528867-15296	458266	0.448676	-335244	0.23895	no

gi|320447004|ref|NW_003383567.1|	38835-15409	429308	0.935953	-21975	0.31795	no
gi|320447004|ref|NW_003383567.1|	41685-15447	436687	12548	-179914	0.41085	no
gi|320447004|ref|NW_003383567.1|	44926-15460	304553	210867	-0.530363	0.78905	no
gi|320447004|ref|NW_003383567.1|	46180-15488	266819	629425	-208376	0.2555	no
gi|320447004|ref|NW_003383567.1|	56691-16170	204476	418328	103271	0.44285	no
gi|320447004|ref|NW_003383567.1|	71622-15719	199608	146281	-0.448429	0.8163	no
gi|320447004|ref|NW_003383567.1|	73286-15748	705994	937542	0.409228	0.85045	no
gi|320447004|ref|NW_003383567.1|	75096-15762	12578	773511	-0.701406	0.75	no
gi|320447004|ref|NW_003383567.1|	76438-15796	269362	118136	-11891	0.3684	no
gi|320447004|ref|NW_003383567.1|	84735-15852	424736	301278	-0.495473	0.81625	no
gi|320447004|ref|NW_003383567.1|	85656-15868	230786	120574	-0.936637	0.6762	no
gi|320447004|ref|NW_003383567.1|	88676-15917	299168	235487	-0.345312	0.79685	no
gi|320447004|ref|NW_003383567.1|	03567-16046	0.398684	110342	146867	1	no
gi|320447004|ref|NW_003383567.1|	61833-16343	0.616034	111458	0.855412	1	no
gi|320447004|ref|NW_003383567.1|	22123-16229	450822	0	#NAME?	0.01585	no
gi|320447004|ref|NW_003383567.1|	67237-16750	129606	230811	0.832581	0.6876	no
gi|320447004|ref|NW_003383567.1|	94995-16956	349228	919368	139647	0.50695	no
gi|320447004|ref|NW_003383567.1|	01695-17040	102569	34705	175855	0.41225	no
gi|320447004|ref|NW_003383567.1|	10082-17107	0.3518	288783	303716	0.24995	no
gi|320447004|ref|NW_003383567.1|	42530-17456	192892	498668	137028	0.53065	no
gi|320447004|ref|NW_003383567.1|	45783-17467	0.659593	121516	0.881493	1	no
gi|320447004|ref|NW_003383567.1|	74881-17667	33605	777848	121081	0.57525	no
gi|320447004|ref|NW_003383567.1|	49116-17495	370992	54317	0.550016	0.79965	no
gi|320447004|ref|NW_003383567.1|	17660-18156	765222	0.854048	-316349	0.25455	no
gi|320447004|ref|NW_003383567.1|	77770-17936	0.12448	692045	911881	0.14075	no
gi|320447004|ref|NW_003383567.1|	23857-18251	0.637646	2987	222787	0.3341	no
gi|320447004|ref|NW_003383567.1|	32638-18331	373867	396187	0.0836558	0.94755	no
gi|320447004|ref|NW_003383567.1|	37902-18391	340714	425263	0.319793	0.8801	no
gi|320447004|ref|NW_003383567.1|	42988-18433	401378	22207	-0.853948	0.67525	no
gi|320447004|ref|NW_003383567.1|	49214-18498	252263	48621	0.946653	0.6572	no

gi|320447004|ref|NW_003383567.1|	350007-18505	287383	270545	-0.0871066	0.9267	no
gi|320447004|ref|NW_003383567.1|	354056-18581	28674	784706	145241	0.38565	no
gi|320447004|ref|NW_003383567.1|	900587-19019	105791	251873	125148	0.54915	no
gi|320447004|ref|NW_003383567.1|	919510-19204	474777	847613	0.836155	0.61055	no
gi|320447004|ref|NW_003383567.1|	192655-19287	325544	526006	0.692224	0.73035	no
gi|320447004|ref|NW_003383567.1|	927323-19286	0	139394	inf	1	no
gi|320447004|ref|NW_003383567.1|	947152-19507	249273	178967	-0.478029	0.7139	no
gi|320447004|ref|NW_003383567.1|	952815-19607	175545	117903	-0.574236	0.6672	no
gi|320447004|ref|NW_003383567.1|	962349-19639	528223	760398	-279632	0.1423	no
gi|320447004|ref|NW_003383567.1|	964828-19657	314277	89019	-181985	0.2757	no
gi|320447004|ref|NW_003383567.1|	966743-19693	158969	277387	0.803157	0.6179	no
gi|320447004|ref|NW_003383567.1|	970874-19724	220735	43349	-234825	0.1895	no
gi|320447004|ref|NW_003383567.1|	974237-19760	330349	487674	-2.76	0.1377	no
gi|320447004|ref|NW_003383567.1|	197710-19962	100225	230553	120186	0.5558	no
gi|320447004|ref|NW_003383567.1|	200229-20121	0.451496	545618	359511	0.182	no
gi|320447004|ref|NW_003383567.1|	205387-20669	140286	820529	-0.773742	0.7234	no
gi|320447004|ref|NW_003383567.1|	22876-23787	32476	137761	-123721	0.54925	no
gi|320447004|ref|NW_003383567.1|	229039-23131	371636	448896	0.272492	0.84075	no
gi|320447004|ref|NW_003383567.1|	231492-23256	423623	279104	-0.601979	0.77565	no
gi|320447004|ref|NW_003383567.1|	233436-23432	390117	235179	-0.730146	0.65465	no
gi|320447004|ref|NW_003383567.1|	257908-25882	646854	0.170223	-856987	0.2504	no
gi|320447004|ref|NW_003383567.1|	26755-27826	432209	194206	-115414	0.5003	no
gi|320447004|ref|NW_003383567.1|	268562-27121	120542	0.0486827	-795191	0.2504	no
gi|320447004|ref|NW_003383567.1|	271367-27317	193386	0.0748514	-469131	0.2894	no
gi|320447004|ref|NW_003383567.1|	274523-27555	254869	0	#NAME?	0.02015	no
gi|320447004|ref|NW_003383567.1|	275856-27727	299402	0	#NAME?	0.01425	no
gi|320447004|ref|NW_003383567.1|	278763-28366	259763	0.202733	-700147	0.0696	no
gi|320447004|ref|NW_003383567.1|	286457-28874	673753	110119	0.708769	0.65515	no
gi|320447004|ref|NW_003383567.1|	289407-29054	154672	18069	0.224312	0.91785	no
gi|320447004|ref|NW_003383567.1|	290875-29267	309329	220009	-0.491575	0.7117	no

gi|320447004|ref|NW_003383567.1|	292927-29315	451407	775394	-254143	0.2388	no
gi|320447004|ref|NW_003383567.1|	294131-29476	166427	116057	-0.520058	0.79855	no
gi|320447004|ref|NW_003383567.1|	296949-29726	625521	955163	-271124	0.23445	no
gi|320447004|ref|NW_003383567.1|	298633-29956	145578	167338	0.200972	0.9123	no
gi|320447004|ref|NW_003383567.1|	302829-30745	120637	763613	-0.659764	0.68895	no
gi|320447004|ref|NW_003383567.1|	307715-30856	240881	772234	-164121	0.4516	no
gi|320447004|ref|NW_003383567.1|	317644-31795	853443	369854	-120634	0.55975	no
gi|320447004|ref|NW_003383567.1|	324224-32525	211064	736806	-151833	0.4909	no
gi|320447004|ref|NW_003383567.1|	348476-34983	148158	914622	-0.69589	0.7562	no
gi|320447004|ref|NW_003383567.1|	350222-35054	319095	113276	182778	0.32625	no
gi|320447004|ref|NW_003383567.1|	350903-35278	117849	131418	0.157223	0.9216	no
gi|320447004|ref|NW_003383567.1|	353210-35487	357215	172916	-104672	0.41975	no
gi|320447004|ref|NW_003383567.1|	355666-35735	392289	110222	-18315	0.30425	no
gi|320447004|ref|NW_003383567.1|	357441-35873	179218	148799	-0.268352	0.90575	no
gi|320447004|ref|NW_003383567.1|	358854-36001	183948	194315	0.0790998	0.97345	no
gi|320447004|ref|NW_003383567.1|	364409-36524	807765	646736	-0.320759	0.80525	no
gi|320447004|ref|NW_003383567.1|	366079-36945	279637	189983	-0.557684	0.6755	no
gi|320447004|ref|NW_003383567.1|	38055-39107	268668	190081	-0.499208	0.8249	no
gi|320447004|ref|NW_003383567.1|	381633-38250	0.533606	422461	298497	0.2201	no
gi|320447004|ref|NW_003383567.1|	382963-38349	674123	120989	0.843797	0.68395	no
gi|320447004|ref|NW_003383567.1|	389476-39014	732063	28914	198173	0.3689	no
gi|320447004|ref|NW_003383567.1|	390381-39181	197763	108964	246201	0.2783	no
gi|320447004|ref|NW_003383567.1|	391989-39329	11044	799386	285563	0.14215	no
gi|320447004|ref|NW_003383567.1|	39661-42747	308974	279785	-0.143166	0.91615	no
gi|320447004|ref|NW_003383567.1|	406822-40711	232652	662808	151042	0.47435	no
gi|320447004|ref|NW_003383567.1|	4243-7868	402701	72031	0.838911	0.52565	no
gi|320447004|ref|NW_003383567.1|	431711-43196	178466	301707	0.757497	0.70215	no
gi|320447004|ref|NW_003383567.1|	433487-43398	436851	108751	131581	0.53935	no
gi|320447004|ref|NW_003383567.1|	434160-43548	293455	643356	113248	0.5861	no
gi|320447004|ref|NW_003383567.1|	43540-44176	542364	337487	-0.684432	0.6618	no

gi|320447004|ref|NW_003383567.1|	ı35790-44003ī	46265	245003	240481	0.2034	no
gi|320447004|ref|NW_003383567.1|	ı48955-44952ī	249321	539418	11134	0.5856	no
gi|320447004|ref|NW_003383567.1|	;05498-50596(	290741	484134	0.735671	0.7419	no
gi|320447004|ref|NW_003383567.1|	;06073-50807(	315022	116494	188673	0.25775	no
gi|320447004|ref|NW_003383567.1|	;22083-52234:	143514	16.39	0.191624	0.9157	no
gi|320447004|ref|NW_003383567.1|	;34033-53476(	135654	162551	0.260969	0.89055	no
gi|320447004|ref|NW_003383567.1|	;41235-54169:	519389	246895	-107291	0.59545	no
gi|320447004|ref|NW_003383567.1|	;44043-54912ī	515013	24621	-106472	0.51115	no
gi|320447004|ref|NW_003383567.1|	;49889-55046:	198881	30258	0.605411	0.7533	no
gi|320447004|ref|NW_003383567.1|	;51530-55320!	257873	456981	0.825474	0.68755	no
gi|320447004|ref|NW_003383567.1|	;55510-55653ι	70266	500406	-0.489729	0.8117	no
gi|320447004|ref|NW_003383567.1|	;57414-55992!	734069	419491	-0.807275	0.71725	no
gi|320447004|ref|NW_003383567.1|	;60330-56155!	815244	40044	-102565	0.6189	no
gi|320447004|ref|NW_003383567.1|	;61725-56461!	290037	192537	-0.591104	0.6503	no
gi|320447004|ref|NW_003383567.1|	;71150-57697!	131063	115431	-0.183227	0.93595	no
gi|320447004|ref|NW_003383567.1|	;78187-58014ı	803701	371052	220689	0.2252	no
gi|320447004|ref|NW_003383567.1|	;81193-58402ı	663062	178559	142919	0.4033	no
gi|320447004|ref|NW_003383567.1|	;85889-58625!	45759	822557	0.846059	0.7005	no
gi|320447004|ref|NW_003383567.1|	;91475-59187ı	9725	575945	-0.755767	0.7073	no
gi|320447004|ref|NW_003383567.1|	;92494-59285ī	358128	545292	0.606555	0.7426	no
gi|320447004|ref|NW_003383567.1|	;08646-60936:	667037	127289	0.932269	0.64925	no
gi|320447004|ref|NW_003383567.1|	;12274-61521!	0.685992	222341	169651	0.4143	no
gi|320447004|ref|NW_003383567.1|	;15351-61626:	0.997784	292318	155074	0.4964	no
gi|320447004|ref|NW_003383567.1|	;16884-61732:	240513	531824	114483	0.57505	no
gi|320447004|ref|NW_003383567.1|	;17901-61976:	280263	303821	0.116437	0.95195	no
gi|320447004|ref|NW_003383567.1|	;23693-62469ī	592861	31857	-0.896084	0.66545	no
gi|320447004|ref|NW_003383567.1|	;24885-62536ı	475656	317698	-0.582259	0.79225	no
gi|320447004|ref|NW_003383567.1|	;34911-63591:	132091	0.912537	-0.533575	1	no
gi|320447004|ref|NW_003383567.1|	;54202-65502:	546613	337919	-401577	0.06255	no
gi|320447004|ref|NW_003383567.1|	;59119-65976:	578643	196927	-155501	0.48025	no

gi|320447004|ref|NW_003383567.1|	60788-66133	300019	252869	-356859	0.1709	no
gi|320447004|ref|NW_003383567.1|	61468-66170	849013	254709	-505886	0.2856	no
gi|320447004|ref|NW_003383567.1|	62445-66295	407949	449342	-31825	0.18915	no
gi|320447004|ref|NW_003383567.1|	63084-66463	182863	149444	-0.291159	0.84975	no
gi|320447004|ref|NW_003383567.1|	65369-66597	459966	218337	-107497	0.5978	no
gi|320447004|ref|NW_003383567.1|	67430-66860	215527	199129	-0.114166	0.94875	no
gi|320447004|ref|NW_003383567.1|	69488-66976	192471	137984	-0.480141	0.80945	no
gi|320447004|ref|NW_003383567.1|	82254-68337	210568	304816	0.533651	0.7915	no
gi|320447004|ref|NW_003383567.1|	83587-68450	247253	0.681869	-185842	0.4076	no
gi|320447004|ref|NW_003383567.1|	88012-68963	169762	0.843933	-100831	0.60725	no
gi|320447004|ref|NW_003383567.1|	95902-69637	492675	89189	0.856231	0.6765	no
gi|320447004|ref|NW_003383567.1|	98459-69896	154915	0.415432	-522071	0.27835	no
gi|320447004|ref|NW_003383567.1|	01238-70182	285143	8369	155337	0.47655	no
gi|320447004|ref|NW_003383567.1|	03753-70582	229811	596304	13756	0.5152	no
gi|320447004|ref|NW_003383567.1|	06278-70719	417857	0	#NAME?	5.00E-05	yes
gi|320447004|ref|NW_003383567.1|	08139-71289	183086	126401	-0.534517	0.68825	no
gi|320447004|ref|NW_003383567.1|	13094-71704	640107	84209	0.395662	0.74855	no
gi|320447004|ref|NW_003383567.1|	18684-71949	526907	724095	0.458632	0.82205	no
gi|320447004|ref|NW_003383567.1|	19612-72009	122783	100218	-0.29298	0.88185	no
gi|320447004|ref|NW_003383567.1|	20724-72191	136.73	105946	-0.368001	0.7834	no
gi|320447004|ref|NW_003383567.1|	73011-73531	175914	393989	116329	0.5745	no
gi|320447004|ref|NW_003383567.1|	33004-73631	0.274389	314759	351995	0.1802	no
gi|320447004|ref|NW_003383567.1|	61913-76369	459129	275663	258594	0.15605	no
gi|320447004|ref|NW_003383567.1|	65435-76702	149046	210011	381664	0.06845	no
gi|320447004|ref|NW_003383567.1|	68878-77069	106487	115745	344219	0.16635	no
gi|320447004|ref|NW_003383567.1|	71736-77309	165354	148237	316428	0.19525	no
gi|320447004|ref|NW_003383567.1|	77390-77926	102664	694055	275712	0.23575	no
gi|320447004|ref|NW_003383567.1|	8002-11249	308703	234326	-0.397701	0.7679	no
gi|320447004|ref|NW_003383567.1|	03565-80683	0.555829	55982	333225	0.17735	no
gi|320447004|ref|NW_003383567.1|	80460-81950	0.53801	21509	199924	0.3909	no

gi|320447004|ref|NW_003383567.1|	82058-82832	0.313379	279498	315686	0.24575	no
gi|320447004|ref|NW_003383567.1|	354049-85581	208227	905554	-120128	0.47155	no
gi|320447004|ref|NW_003383567.1|	365942-86701	124474	444473	-148568	0.48845	no
gi|320447004|ref|NW_003383567.1|	921793-92365	0.725716	679151	322625	0.18405	no
gi|320447004|ref|NW_003383567.1|	924118-92883	0.263653	41416	397347	0.13305	no
gi|320447004|ref|NW_003383567.1|	929105-93141	0.892943	103702	353773	0.08015	no
gi|320447004|ref|NW_003383567.1|	932455-93279	138815	11707	307614	0.24865	no
gi|320447004|ref|NW_003383567.1|	992710-99356	0	867788	inf	0.00695	no
gi|320447004|ref|NW_003383567.1|	996915-99850	0.37399	51177	377442	0.181	no
gi|320447005|ref|NW_003383566.1|	100722-10213	254287	345376	0.441706	0.7222	no
gi|320447005|ref|NW_003383566.1|	102529-10390	967802	568236	-0.768223	0.5652	no
gi|320447005|ref|NW_003383566.1|	105574-10963	220137	341562	0.633744	0.6308	no
gi|320447005|ref|NW_003383566.1|	109742-11288	248454	208655	-0.251858	0.84975	no
gi|320447005|ref|NW_003383566.1|	114098-11514	215507	10705	-100945	0.6576	no
gi|320447005|ref|NW_003383566.1|	116921-11776	249596	419706	0.749788	0.71075	no
gi|320447005|ref|NW_003383566.1|	117910-12221	987953	186014	-240903	0.17955	no
gi|320447005|ref|NW_003383566.1|	122972-12444	599942	113747	-239899	0.2908	no
gi|320447005|ref|NW_003383566.1|	124656-12611	205319	296935	-278965	0.1299	no
gi|320447005|ref|NW_003383566.1|	142795-14306	253647	548809	111348	0.5873	no
gi|320447005|ref|NW_003383566.1|	146331-15121	428193	31518	-0.442088	0.8508	no
gi|320447005|ref|NW_003383566.1|	158284-16201	0	256427	inf	0.00425	no
gi|320447005|ref|NW_003383566.1|	182111-18240	0	130449	inf	0.0312	no
gi|320447005|ref|NW_003383566.1|	185785-18630	0	108909	inf	0.01275	no
gi|320447005|ref|NW_003383566.1|	193530-19500	158423	6456	-129507	0.5666	no
gi|320447005|ref|NW_003383566.1|	196816-19808	783687	509729	-0.620547	0.76045	no
gi|320447005|ref|NW_003383566.1|	200274-20138	234337	142283	-0.71982	0.75285	no
gi|320447005|ref|NW_003383566.1|	203368-20583	270443	121342	-447817	0.04665	no
gi|320447005|ref|NW_003383566.1|	22042-23617	667623	146357	113239	0.61255	no
gi|320447005|ref|NW_003383566.1|	25795-26884	948992	177857	-241568	0.28615	no
gi|320447005|ref|NW_003383566.1|	27524-29195	893316	237305	-191243	0.38315	no

gi|320447005|ref|NW_003383566.1|	29462-29912	343885	121907	-149614	0.48295	no
gi|320447005|ref|NW_003383566.1|	30631-31120	458009	350154	-0.387387	0.8463	no
gi|320447005|ref|NW_003383566.1|	309912-31076	233807	196822	-0.248427	0.9072	no
gi|320447005|ref|NW_003383566.1|	310922-31343	696383	476043	-0.548788	0.80155	no
gi|320447005|ref|NW_003383566.1|	314081-31436	213521	136521	267667	0.2703	no
gi|320447005|ref|NW_003383566.1|	314593-32068	253055	946291	-14191	0.29305	no
gi|320447005|ref|NW_003383566.1|	320796-32239	172264	250084	0.53779	0.67055	no
gi|320447005|ref|NW_003383566.1|	323541-32462	492042	32812	-0.584561	0.6465	no
gi|320447005|ref|NW_003383566.1|	325108-33098	242799	184819	-0.393645	0.76745	no
gi|320447005|ref|NW_003383566.1|	331194-33187	298504	117512	-134495	0.5345	no
gi|320447005|ref|NW_003383566.1|	333723-33629	432033	176426	-129208	0.3317	no
gi|320447005|ref|NW_003383566.1|	339749-34131	236973	204532	-0.212394	0.86545	no
gi|320447005|ref|NW_003383566.1|	34347-35110	537439	219369	-129274	0.43575	no
gi|320447005|ref|NW_003383566.1|	347607-35202	118683	107133	-0.147705	0.90585	no
gi|320447005|ref|NW_003383566.1|	352806-35492	125748	151013	0.264133	0.83375	no
gi|320447005|ref|NW_003383566.1|	360710-36349	136597	145511	0.0912052	0.9433	no
gi|320447005|ref|NW_003383566.1|	36410-37630	186821	855644	-112658	0.6148	no
gi|320447005|ref|NW_003383566.1|	371353-37194	289096	199038	-0.538505	0.80125	no
gi|320447005|ref|NW_003383566.1|	372922-37700	105628	112606	0.0922825	0.94335	no
gi|320447005|ref|NW_003383566.1|	377604-37992	599319	588783	-334752	0.09515	no
gi|320447005|ref|NW_003383566.1|	381896-38315	150762	55162	-145053	0.5153	no
gi|320447005|ref|NW_003383566.1|	38492-41861	123304	941636	-0.388976	0.76025	no
gi|320447005|ref|NW_003383566.1|	406393-40828	0	191342	inf	0.0133	no
gi|320447005|ref|NW_003383566.1|	447056-44876	100906	119766	0.247215	0.91325	no
gi|320447005|ref|NW_003383566.1|	449454-45031	484845	296645	-0.708789	0.7252	no
gi|320447005|ref|NW_003383566.1|	454325-45529	388977	331735	-0.229653	0.9042	no
gi|320447005|ref|NW_003383566.1|	46446-46923	30.13	0.914453	-504215	0.18885	no
gi|320447005|ref|NW_003383566.1|	47496-48027	130047	0	#NAME?	0.0111	no
gi|320447005|ref|NW_003383566.1|	486617-48775	0	193386	inf	0.02205	no
gi|320447005|ref|NW_003383566.1|	48829-49934	873306	0.268663	-502262	0.1888	no

gi|320447005|ref|NW_003383566.1|	08262-50864	536449	42189	-0.346576	0.8622	no
gi|320447005|ref|NW_003383566.1|	50870-52442	150237	0.527002	-483328	0.13385	no
gi|320447005|ref|NW_003383566.1|	52879-53984	834493	0.402995	-437207	0.15105	no
gi|320447005|ref|NW_003383566.1|	55193-55503	557351	224154	-463602	0.19765	no
gi|320447005|ref|NW_003383566.1|	08814-60956	349678	226043	-395136	0.1388	no
gi|320447005|ref|NW_003383566.1|	10092-61212	110518	105819	-0.0626841	0.97935	no
gi|320447005|ref|NW_003383566.1|	12242-61493	377919	115229	-171357	0.42565	no
gi|320447005|ref|NW_003383566.1|	15728-61805	330439	309454	-0.0946625	0.9631	no
gi|320447005|ref|NW_003383566.1|	19410-62041	218159	30147	0.466639	0.82035	no
gi|320447005|ref|NW_003383566.1|	21552-62308	0.388935	0.991903	135067	1	no
gi|320447005|ref|NW_003383566.1|	25644-62675	204069	30653	-273496	0.2454	no
gi|320447005|ref|NW_003383566.1|	37373-64226	293362	195339	273523	0.15285	no
gi|320447005|ref|NW_003383566.1|	31409-73348	0.367497	179435	228766	0.3272	no
gi|320447005|ref|NW_003383566.1|	73994-77403	488062	725627	0.572165	0.6631	no
gi|320447005|ref|NW_003383566.1|	60813-76164	0.282909	194534	278161	0.2657	no
gi|320447005|ref|NW_003383566.1|	77538-82727	121596	763577	-0.67125	0.61045	no
gi|320447005|ref|NW_003383566.1|	01297-80161	453725	104532	-54398	0.2767	no
gi|320447005|ref|NW_003383566.1|	04401-80510	117803	0.976383	-359279	0.19625	no
gi|320447005|ref|NW_003383566.1|	11677-82092	593565	492489	-0.269315	0.83515	no
gi|320447005|ref|NW_003383566.1|	22614-82389	199601	0.337241	-920913	0.1105	no
gi|320447005|ref|NW_003383566.1|	25548-82661	301291	0	#NAME?	5.00E-05	yes
gi|320447005|ref|NW_003383566.1|	82847-90042	795162	428309	-0.892598	0.4919	no
gi|320447005|ref|NW_003383566.1|	84569-88625	197891	316078	0.675578	0.7344	no
gi|320447005|ref|NW_003383566.1|	06098-90683	0	320725	inf	0.0233	no
gi|320447005|ref|NW_003383566.1|	92806-94684	10.17	121712	-306277	0.20375	no
gi|320447005|ref|NW_003383566.1|	97440-99960	242248	182099	-0.411764	0.7499	no
gi|320447006|ref|NW_003383565.1|	03143-10499	267874	977087	-145499	0.40095	no
gi|320447006|ref|NW_003383565.1|	07284-10761	376552	121948	-162658	0.4394	no
gi|320447006|ref|NW_003383565.1|	11144-11208	0.714532	312211	212745	0.34655	no
gi|320447006|ref|NW_003383565.1|	36308-13661	174172	100869	25339	0.27435	no

gi|320447006|ref|NW_003383565.1|	06311-30744	0	326214	inf	0.01485	no
gi|320447006|ref|NW_003383565.1|	08655-31268	0.0890462	274269	494489	0.1501	no
gi|320447006|ref|NW_003383565.1|	19908-32054	0	311643	inf	0.0294	no
gi|320447006|ref|NW_003383565.1|	54386-35534	0	307941	inf	5.00E-05	yes
gi|320447006|ref|NW_003383565.1|	56844-35831	0.136793	364208	805663	0.14075	no
gi|320447006|ref|NW_003383565.1|	58462-35951	0	309759	inf	5.00E-05	yes
gi|320447006|ref|NW_003383565.1|	02903-40449	0	291038	inf	5.00E-05	yes
gi|320447006|ref|NW_003383565.1|	06681-40716	0	55704	inf	0.0047	no
gi|320447006|ref|NW_003383565.1|	08797-41099	0	128456	inf	5.00E-05	yes
gi|320447006|ref|NW_003383565.1|	11139-41655	0.0325617	178408	909779	0.14075	no
gi|320447006|ref|NW_003383565.1|	19784-42341	0	450271	inf	5.00E-05	yes
gi|320447006|ref|NW_003383565.1|	05297-50617	0	320638	inf	5.00E-05	yes
gi|320447006|ref|NW_003383565.1|	07749-50810	0	789875	inf	0.005	no
gi|320447006|ref|NW_003383565.1|	12577-51332	0.66365	272807	203938	0.3026	no
gi|320447006|ref|NW_003383565.1|	20593-52089	360154	104173	15323	0.3578	no
gi|320447006|ref|NW_003383565.1|	64650-56562	273829	149672	245045	0.28045	no
gi|320447006|ref|NW_003383565.1|	66177-56663	0.741984	691307	321987	0.232	no
gi|320447006|ref|NW_003383565.1|	57083-58606	249004	328118	0.398047	0.84155	no
gi|320447006|ref|NW_003383565.1|	72371-57268	514032	176509	177981	0.41195	no
gi|320447006|ref|NW_003383565.1|	74904-57562	0	286472	inf	0.029	no
gi|320447006|ref|NW_003383565.1|	77466-57856	117796	43479	188403	0.38815	no
gi|320447006|ref|NW_003383565.1|	82806-58339	0.47004	445887	324582	0.2316	no
gi|320447006|ref|NW_003383565.1|	44297-64525	812962	0.961919	-30792	0.21435	no
gi|320447006|ref|NW_003383565.1|	46493-64754	370639	0.427209	-3117	0.2016	no
gi|320447006|ref|NW_003383565.1|	56446-65808	952467	151047	-265667	0.2501	no
gi|320447006|ref|NW_003383565.1|	58664-66047	331576	0.521653	-266818	0.255	no
gi|320447006|ref|NW_003383565.1|	60702-66139	62662	0	#NAME?	0.01065	no
gi|320447006|ref|NW_003383565.1|	81677-68223	157794	745301	223978	0.32725	no
gi|320447006|ref|NW_003383565.1|	02174-70436	0.689559	108267	0.650846	1	no
gi|320447006|ref|NW_003383565.1|	09759-71025	196967	10213	-0.947549	0.64825	no

gi|320447006|ref|NW_003383565.1|	746794-74726	216466	0.93174	-453807	0.20425	no
gi|320447006|ref|NW_003383565.1|	747392-75022	523546	356845	-0.553018	0.6789	no
gi|320447006|ref|NW_003383565.1|	753470-75600	657758	409815	-0.682584	0.61275	no
gi|320447006|ref|NW_003383565.1|	96228-97124	0	528116	inf	0.00935	no
gi|320447007|ref|NW_003383564.1|	114616-11647	0	196152	inf	0.0133	no
gi|320447007|ref|NW_003383564.1|	120391-12117	733064	163549	115771	0.5844	no
gi|320447007|ref|NW_003383564.1|	122340-12274	238122	288564	0.277189	0.88795	no
gi|320447007|ref|NW_003383564.1|	122957-12361	395127	457885	0.212669	0.91105	no
gi|320447007|ref|NW_003383564.1|	125277-12569	260084	630546	127762	0.55025	no
gi|320447007|ref|NW_003383564.1|	175085-17572	29215	14199	-104091	0.59645	no
gi|320447007|ref|NW_003383564.1|	183938-18556	352664	228513	-0.626019	0.76155	no
gi|320447007|ref|NW_003383564.1|	191306-19327	0.0972216	25081	468918	0.1765	no
gi|320447007|ref|NW_003383564.1|	204263-20642	0.437456	640992	38731	0.15345	no
gi|320447007|ref|NW_003383564.1|	206523-20749	0	253426	inf	0.0198	no
gi|320447007|ref|NW_003383564.1|	29566-29932	164074	107146	270715	0.243	no
gi|320447007|ref|NW_003383564.1|	385655-38607	0	69102	inf	0.029	no
gi|320447007|ref|NW_003383564.1|	629301-63098	0	365706	inf	0.00885	no
gi|320447007|ref|NW_003383564.1|	6752-7382	230437	377184	0.710897	0.73855	no
gi|320447007|ref|NW_003383564.1|	7699-8277	0	862145	inf	0.0142	no
gi|320447007|ref|NW_003383564.1|	785593-78766	0.0920247	801068	976569	0.14085	no
gi|320447007|ref|NW_003383564.1|	800123-80378	170222	105001	-0.697013	0.59665	no
gi|320447007|ref|NW_003383564.1|	805611-80632	181678	243598	-289881	0.22455	no
gi|320447007|ref|NW_003383564.1|	810404-81310	445362	141714	166993	0.33265	no
gi|320447010|ref|NW_003383561.1|	111781-11226	414908	0	#NAME?	0.0071	no
gi|320447010|ref|NW_003383561.1|	115529-11737	541315	0.291847	-753511	0.1293	no
gi|320447010|ref|NW_003383561.1|	117767-11986	189846	817401	-121572	0.464	no
gi|320447010|ref|NW_003383561.1|	119964-12089	162796	16925	0.0560911	0.97875	no
gi|320447010|ref|NW_003383561.1|	122029-12945	159253	182862	0.199434	0.8819	no
gi|320447010|ref|NW_003383561.1|	131757-13257	985014	617241	-0.674312	0.74255	no
gi|320447010|ref|NW_003383561.1|	140496-14125	103544	199644	-237475	0.28195	no

gi|320447010|ref|NW_003383561.1|	44725-24556	0.844478	290371	178176	0.41655	no
gi|320447010|ref|NW_003383561.1|	46392-24689	124372	541902	212338	0.29385	no
gi|320447010|ref|NW_003383561.1|	36201-43661	182023	6606	185965	0.32465	no
gi|320447010|ref|NW_003383561.1|	38406-43898	146622	462963	16588	0.433	no
gi|320447010|ref|NW_003383561.1|	41177-44203	215487	129782	-0.731509	0.7278	no
gi|320447010|ref|NW_003383561.1|	42986-54331	464248	799918	0.784958	0.70455	no
gi|320447010|ref|NW_003383561.1|	84015-92079	0.603446	291676	227307	0.19425	no
gi|320447010|ref|NW_003383561.1|	99621-10010	484841	0	#NAME?	0.00385	no
gi|320447011|ref|NW_003383560.1|	27952-10306	285684	17111	-0.739494	0.7209	no
gi|320447011|ref|NW_003383560.1|	50283-10507	539534	158761	155707	0.4756	no
gi|320447011|ref|NW_003383560.1|	54082-10551	380735	193172	-0.978896	0.55445	no
gi|320447011|ref|NW_003383560.1|	55321-10574	0.624089	0.435403	-0.519401	1	no
gi|320447011|ref|NW_003383560.1|	05663-10678	82618	276849	-157736	0.23155	no
gi|320447011|ref|NW_003383560.1|	57792-10630	258865	472097	0.86688	0.50795	no
gi|320447011|ref|NW_003383560.1|	63739-10679	52188	487176	-0.0992742	0.9512	no
gi|320447011|ref|NW_003383560.1|	75610-10763	0.335773	321964	658327	0.15405	no
gi|320447011|ref|NW_003383560.1|	38316-11406	110692	411588	189465	0.15075	no
gi|320447011|ref|NW_003383560.1|	45904-11473	224325	161944	285184	0.04	no
gi|320447011|ref|NW_003383560.1|	55795-11564	623914	130391	106342	0.6068	no
gi|320447011|ref|NW_003383560.1|	78203-11788	102013	147743	0.534337	0.79115	no
gi|320447011|ref|NW_003383560.1|	94972-11952	374943	262315	-0.515368	0.79855	no
gi|320447011|ref|NW_003383560.1|	95785-11964	102447	869855	-0.236029	0.90795	no
gi|320447011|ref|NW_003383560.1|	97627-11982	840395	969594	0.206313	0.91485	no
gi|320447011|ref|NW_003383560.1|	98321-11987	153984	221748	0.526145	0.7919	no
gi|320447011|ref|NW_003383560.1|	99361-12008	51439	941118	0.871511	0.68765	no
gi|320447011|ref|NW_003383560.1|	00949-12021	102477	203491	0.989672	0.65825	no
gi|320447011|ref|NW_003383560.1|	02711-12034	620614	0.219369	-814419	0.2504	no
gi|320447011|ref|NW_003383560.1|	03926-12047	653595	0.18895	-843425	0.2504	no
gi|320447011|ref|NW_003383560.1|	06744-12070	650351	0	#NAME?	0.00615	no
gi|320447011|ref|NW_003383560.1|	14302-12145	551817	0	#NAME?	0.01335	no

gi|320447011|ref|NW_003383560.1|	41002-14245	261194	714587	-186994	0.27915	no
gi|320447011|ref|NW_003383560.1|	14066-14162	654148	170404	138127	0.4106	no
gi|320447011|ref|NW_003383560.1|	21374-14218	292261	373749	0.354814	0.87245	no
gi|320447011|ref|NW_003383560.1|	42576-14376	329162	662323	-231319	0.1916	no
gi|320447011|ref|NW_003383560.1|	44675-14527	366011	124122	-156012	0.4979	no
gi|320447011|ref|NW_003383560.1|	47881-14908	31775	665632	-22551	0.19755	no
gi|320447011|ref|NW_003383560.1|	62493-15634	124355	137163	0.141425	1	no
gi|320447011|ref|NW_003383560.1|	56330-15708	558719	30555	-0.870713	0.602	no
gi|320447011|ref|NW_003383560.1|	72606-15768	249329	130184	-0.937505	0.65895	no
gi|320447011|ref|NW_003383560.1|	57342-15854	107687	288982	-18978	0.38515	no
gi|320447011|ref|NW_003383560.1|	80534-15815	415402	405887	-0.0334273	0.97885	no
gi|320447011|ref|NW_003383560.1|	84747-15898	56.35	73.53	0.383918	0.76085	no
gi|320447011|ref|NW_003383560.1|	58672-16074	202565	61654	-171612	0.31895	no
gi|320447011|ref|NW_003383560.1|	91534-15948	18.75	869121	-110926	0.39565	no
gi|320447011|ref|NW_003383560.1|	13968-16144	582439	12298	-224369	0.3086	no
gi|320447011|ref|NW_003383560.1|	20512-16207	317102	364917	-31193	0.19155	no
gi|320447011|ref|NW_003383560.1|	62549-16353	0.895801	139203	0.635941	1	no
gi|320447011|ref|NW_003383560.1|	28282-16311	100283	181673	-246467	0.07235	no
gi|320447011|ref|NW_003383560.1|	33462-16342	301403	129233	-122171	0.5771	no
gi|320447011|ref|NW_003383560.1|	34450-16358	735871	286246	-13622	0.5255	no
gi|320447011|ref|NW_003383560.1|	36513-16374	123949	373566	-173031	0.4223	no
gi|320447011|ref|NW_003383560.1|	63689-17055	605516	586786	-0.0453308	0.97165	no
gi|320447011|ref|NW_003383560.1|	37687-16390	60583	147085	-204226	0.26495	no
gi|320447011|ref|NW_003383560.1|	40278-16424	111654	341631	-170852	0.45165	no
gi|320447011|ref|NW_003383560.1|	42766-16439	152376	157257	0.0454852	0.982	no
gi|320447011|ref|NW_003383560.1|	44684-16466	646541	403717	-0.679398	0.7503	no
gi|320447011|ref|NW_003383560.1|	47110-16483	155859	79672	-0.968096	0.6626	no
gi|320447011|ref|NW_003383560.1|	51118-16520	105995	731125	-0.535807	0.79585	no
gi|320447011|ref|NW_003383560.1|	52179-16527	457514	279275	-0.712126	0.7256	no
gi|320447011|ref|NW_003383560.1|	52938-16542	0.158067	219415	379505	0.2072	no

gi|320447011|ref|NW_003383560.1|	;55017-16566:	0	16939	inf	0.0162	no
gi|320447011|ref|NW_003383560.1|	;56709-16583(	0.124296	129731	338367	1	no
gi|320447011|ref|NW_003383560.1|	;61195-16645!	219944	829058	-140759	0.28805	no
gi|320447011|ref|NW_003383560.1|	;67406-16682!	135224	231628	0.776455	0.7206	no
gi|320447011|ref|NW_003383560.1|	;69059-16707	179389	884002	-102097	0.5284	no
gi|320447011|ref|NW_003383560.1|	;70895-16712:	6237	906499	0.539453	0.7996	no
gi|320447011|ref|NW_003383560.1|	;73563-16751(	220435	101684	-111626	0.50145	no
gi|320447011|ref|NW_003383560.1|	;75526-16765!	115259	741372	-0.636612	0.7591	no
gi|320447011|ref|NW_003383560.1|	;77268-16792:	242945	100521	-127314	0.45455	no
gi|320447011|ref|NW_003383560.1|	;88532-16926(	10733	672622	-0.674185	0.5972	no
gi|320447011|ref|NW_003383560.1|	;93537-16956(	277763	404167	-278083	0.1395	no
gi|320447011|ref|NW_003383560.1|	'04739-17049!	347921	200246	-411892	0.30755	no
gi|320447011|ref|NW_003383560.1|	'07097-17075'	378088	917861	-204238	0.3516	no
gi|320447011|ref|NW_003383560.1|	171444-17370(	868473	13.93	0.68164	0.6748	no
gi|320447011|ref|NW_003383560.1|	'22480-17227:	681249	102636	-273065	0.26635	no
gi|320447011|ref|NW_003383560.1|	'22931-17232(	285737	106246	-142728	0.49845	no
gi|320447011|ref|NW_003383560.1|	'23729-17240	244978	315976	-295477	0.2099	no
gi|320447011|ref|NW_003383560.1|	'25452-17261:	118264	510608	-121172	0.56325	no
gi|320447011|ref|NW_003383560.1|	'26325-17274!	108923	0.880438	-0.307019	1	no
gi|320447011|ref|NW_003383560.1|	'29487-17301(	758573	232994	-1703	0.4246	no
gi|320447011|ref|NW_003383560.1|	'31022-17323:	32886	184792	-0.831571	0.6909	no
gi|320447011|ref|NW_003383560.1|	'32462-17362(	10862	938417	-0.210984	0.87155	no
gi|320447011|ref|NW_003383560.1|	'55478-17564!	315372	105919	-157409	0.34125	no
gi|320447011|ref|NW_003383560.1|	'56649-17583:	501238	324627	-0.626716	0.76115	no
gi|320447011|ref|NW_003383560.1|	'58491-17601:	125696	541614	-12146	0.58085	no
gi|320447011|ref|NW_003383560.1|	'60298-17652(	408206	766703	0.909372	0.5947	no
gi|320447011|ref|NW_003383560.1|	'65456-17665(	145918	216202	0.56722	0.7848	no
gi|320447011|ref|NW_003383560.1|	'66705-17671!	681946	503823	-0.43674	0.8269	no
gi|320447011|ref|NW_003383560.1|	'68340-17699!	181551	631709	-152304	0.35685	no
gi|320447011|ref|NW_003383560.1|	'70266-17721!	152161	879258	-0.791239	0.6205	no

gi|320447011|ref|NW_003383560.1|	774147-17744	240049	102521	-122741	0.5517	no
gi|320447011|ref|NW_003383560.1|	775055-17758	821793	621214	-0.403684	0.7562	no
gi|320447011|ref|NW_003383560.1|	781410-17850	330874	262633	-0.333232	0.8751	no
gi|320447011|ref|NW_003383560.1|	785266-17856	197286	769723	-135788	0.51545	no
gi|320447011|ref|NW_003383560.1|	794885-17952	848384	130426	-270149	0.24325	no
gi|320447011|ref|NW_003383560.1|	801251-18045	715468	652877	-0.132076	0.93345	no
gi|320447011|ref|NW_003383560.1|	811133-18115	116372	935976	-0.3142	0.87755	no
gi|320447011|ref|NW_003383560.1|	812604-18129	105868	748262	-0.500652	0.8128	no
gi|320447011|ref|NW_003383560.1|	813218-18134	111768	328402	-176697	0.40905	no
gi|320447011|ref|NW_003383560.1|	814869-18174	213502	92536	-120616	0.4863	no
gi|320447011|ref|NW_003383560.1|	823102-18259	281473	920824	-1612	0.2185	no
gi|320447011|ref|NW_003383560.1|	826584-18278	319485	87463	-1869	0.27815	no
gi|320447011|ref|NW_003383560.1|	842195-18425	106157	315976	-507024	0.13975	no
gi|320447011|ref|NW_003383560.1|	187598-18878	337657	504966	0.580626	0.7768	no
gi|320447011|ref|NW_003383560.1|	190615-19084	82074	206772	133304	0.38395	no
gi|320447011|ref|NW_003383560.1|	199302-19964	135971	141245	337683	0.2184	no
gi|320447011|ref|NW_003383560.1|	201127-20165	231435	77792	174902	0.43005	no
gi|320447011|ref|NW_003383560.1|	201793-20315	0	114328	inf	1	no
gi|320447011|ref|NW_003383560.1|	207217-21441	150327	601758	200108	0.2526	no
gi|320447011|ref|NW_003383560.1|	214538-21489	251117	8177	170321	0.3381	no
gi|320447011|ref|NW_003383560.1|	215536-21884	120731	116691	327283	0.08855	no
gi|320447011|ref|NW_003383560.1|	230182-23107	120664	256635	-223321	0.23285	no
gi|320447011|ref|NW_003383560.1|	232588-23282	96734	292775	-172423	0.4273	no
gi|320447011|ref|NW_003383560.1|	232930-23343	370484	289818	-0.354261	0.85555	no
gi|320447011|ref|NW_003383560.1|	234914-23521	261514	781885	-174186	0.29755	no
gi|320447011|ref|NW_003383560.1|	235562-23606	355625	11926	-157625	0.4678	no
gi|320447011|ref|NW_003383560.1|	236541-23743	608066	225329	-143219	0.39865	no
gi|320447011|ref|NW_003383560.1|	238058-23888	369019	15614	-124086	0.54915	no
gi|320447011|ref|NW_003383560.1|	239617-24049	755528	453728	-0.735659	0.5658	no
gi|320447011|ref|NW_003383560.1|	240688-24142	236447	115657	-103166	0.59645	no

gi|320447011|ref|NW_003383560.1|	244935-24645	0.92096	0.914958	-0.0094323	1	no
gi|320447011|ref|NW_003383560.1|	248528-24916	167762	256795	0.614198	0.75305	no
gi|320447011|ref|NW_003383560.1|	257324-25804	0.690706	354541	235981	0.262	no
gi|320447011|ref|NW_003383560.1|	328200-32869	194124	346446	0.835656	0.6919	no
gi|320447011|ref|NW_003383560.1|	372716-37315	186048	429117	-211623	0.33805	no
gi|320447011|ref|NW_003383560.1|	374152-37503	133535	414747	-168692	0.43525	no
gi|320447011|ref|NW_003383560.1|	375141-37588	113042	159442	-282576	0.22765	no
gi|320447011|ref|NW_003383560.1|	376059-37632	414739	115406	-184549	0.39	no
gi|320447011|ref|NW_003383560.1|	376453-37863	7447	525691	-0.502444	0.8176	no
gi|320447011|ref|NW_003383560.1|	393160-39345	362073	768908	-22354	0.20495	no
gi|320447011|ref|NW_003383560.1|	395213-39567	44519	133324	-173949	0.42785	no
gi|320447011|ref|NW_003383560.1|	396343-39694	449783	880169	-235338	0.30285	no
gi|320447011|ref|NW_003383560.1|	397276-39793	994735	406753	-129016	0.53465	no
gi|320447011|ref|NW_003383560.1|	398039-39893	252935	968212	-138537	0.5321	no
gi|320447011|ref|NW_003383560.1|	399063-40024	50149	239932	-106359	0.4081	no
gi|320447011|ref|NW_003383560.1|	404211-40462	249298	166379	-0.583401	0.6451	no
gi|320447011|ref|NW_003383560.1|	406079-40767	145641	658266	-114567	0.60635	no
gi|320447011|ref|NW_003383560.1|	408907-40972	466129	284804	-0.710759	0.6667	no
gi|320447011|ref|NW_003383560.1|	409879-41286	301806	212716	-0.504692	0.70355	no
gi|320447011|ref|NW_003383560.1|	413508-41491	100909	180283	0.837201	0.6797	no
gi|320447011|ref|NW_003383560.1|	431919-43241	0	645031	inf	0.02205	no
gi|320447011|ref|NW_003383560.1|	433882-43456	0.755206	824858	34492	0.19115	no
gi|320447011|ref|NW_003383560.1|	434664-43504	0	848402	inf	0.029	no
gi|320447011|ref|NW_003383560.1|	435289-43620	0.497421	651525	371128	0.17985	no
gi|320447011|ref|NW_003383560.1|	466621-46682	140929	247125	0.810272	0.7032	no
gi|320447011|ref|NW_003383560.1|	472539-48139	408951	26568	269969	0.05175	no
gi|320447011|ref|NW_003383560.1|	481691-48922	180335	178316	-0.0162456	0.99235	no
gi|320447011|ref|NW_003383560.1|	489371-49047	214921	321667	-274017	0.2408	no
gi|320447011|ref|NW_003383560.1|	490653-49755	339283	605273	0.835095	0.50785	no
gi|320447011|ref|NW_003383560.1|	501261-50325	605517	492595	302416	0.11985	no

gi|320447011|ref|NW_003383560.1|	04456-50501	255979	197105	294487	0.2269	no
gi|320447011|ref|NW_003383560.1|	08792-50949	57358	232842	202129	0.35615	no
gi|320447011|ref|NW_003383560.1|	12741-51299	58086	202642	180267	0.33195	no
gi|320447011|ref|NW_003383560.1|	58366-56106	137202	220498	0.684469	0.73915	no
gi|320447011|ref|NW_003383560.1|	61161-56533	124106	125837	0.0199848	1	no
gi|320447011|ref|NW_003383560.1|	56405-56861	745081	14873	0.997226	0.6433	no
gi|320447011|ref|NW_003383560.1|	66399-56955	133103	376368	14996	0.4888	no
gi|320447011|ref|NW_003383560.1|	72431-57322	150274	80493	242127	0.2874	no
gi|320447011|ref|NW_003383560.1|	75896-57698	0.593816	150668	134329	0.5243	no
gi|320447011|ref|NW_003383560.1|	58454-58983	398387	306278	-0.379329	0.84695	no
gi|320447011|ref|NW_003383560.1|	97822-59836	179573	14301	-0.328449	0.867	no
gi|320447011|ref|NW_003383560.1|	98428-60141	261458	810798	163277	0.2307	no
gi|320447011|ref|NW_003383560.1|	01527-60181	205634	368549	416371	0.1911	no
gi|320447011|ref|NW_003383560.1|	02386-60702	116015	78.14	275175	0.0469	no
gi|320447011|ref|NW_003383560.1|	61356-61971	398984	391318	-0.0279892	0.95935	no
gi|320447011|ref|NW_003383560.1|	15460-61586	0.923848	731071	298428	0.2563	no
gi|320447011|ref|NW_003383560.1|	16644-61760	0	225057	inf	0.0233	no
gi|320447011|ref|NW_003383560.1|	18354-61951	0.911618	947348	337739	0.1846	no
gi|320447011|ref|NW_003383560.1|	19669-62135	197618	297841	391376	0.055	no
gi|320447011|ref|NW_003383560.1|	62257-62863	136164	338724	131476	0.5489	no
gi|320447011|ref|NW_003383560.1|	63493-65630	505781	699559	0.467933	0.8327	no
gi|320447011|ref|NW_003383560.1|	38740-63955	168611	133853	-0.333044	0.87845	no
gi|320447011|ref|NW_003383560.1|	39965-64119	120954	307134	-197751	0.3873	no
gi|320447011|ref|NW_003383560.1|	41330-64193	139595	886213	-0.655519	0.74305	no
gi|320447011|ref|NW_003383560.1|	42473-64280	136182	293547	-221387	0.27355	no
gi|320447011|ref|NW_003383560.1|	43584-64399	21464	104575	-103739	0.6159	no
gi|320447011|ref|NW_003383560.1|	44214-64474	559959	603074	0.107013	0.9479	no
gi|320447011|ref|NW_003383560.1|	45510-65041	127519	106276	-0.262902	0.84065	no
gi|320447011|ref|NW_003383560.1|	54884-65786	156037	119826	-0.380947	0.7696	no
gi|320447011|ref|NW_003383560.1|	57989-65932	733303	509156	-0.526302	0.80185	no

gi|320447011|ref|NW_003383560.1|	59646-66063	178686	154267	-0.211992	0.9103	no
gi|320447011|ref|NW_003383560.1|	60865-66214	45061	161924	-147656	0.39775	no
gi|320447011|ref|NW_003383560.1|	66702-67629	195227	370235	0.923287	0.65215	no
gi|320447011|ref|NW_003383560.1|	73847-67467	498756	459855	-0.117156	0.9263	no
gi|320447011|ref|NW_003383560.1|	75416-67812	184707	315285	0.771422	0.5569	no
gi|320447011|ref|NW_003383560.1|	67899-72322	616456	187056	16014	0.22595	no
gi|320447011|ref|NW_003383560.1|	84668-68496	0	12271	inf	0.0312	no
gi|320447011|ref|NW_003383560.1|	94569-69506	0.635377	110641	412212	0.1927	no
gi|320447011|ref|NW_003383560.1|	02194-70515	841603	227126	143228	0.26795	no
gi|320447011|ref|NW_003383560.1|	05709-70871	450171	612464	0.444153	0.84505	no
gi|320447011|ref|NW_003383560.1|	10233-71129	632151	9027	0.513977	0.8049	no
gi|320447011|ref|NW_003383560.1|	11421-71532	17927	28313	0.659332	0.758	no
gi|320447011|ref|NW_003383560.1|	38242-74412	191574	886264	220984	0.3234	no
gi|320447011|ref|NW_003383560.1|	44331-74543	191686	545091	150775	0.2457	no
gi|320447011|ref|NW_003383560.1|	47506-74785	278555	581208	106109	0.4223	no
gi|320447011|ref|NW_003383560.1|	48551-74901	143032	414699	153572	0.48295	no
gi|320447011|ref|NW_003383560.1|	49276-75296	415388	142264	177603	0.3117	no
gi|320447011|ref|NW_003383560.1|	53586-75546	168677	382781	118225	0.36555	no
gi|320447011|ref|NW_003383560.1|	55583-76237	787306	185233	123434	0.3585	no
gi|320447011|ref|NW_003383560.1|	62630-76303	247647	514396	10546	0.6207	no
gi|320447011|ref|NW_003383560.1|	63817-76465	30708	902457	155524	0.4594	no
gi|320447011|ref|NW_003383560.1|	64773-76577	130.38	220095	0.755409	0.5645	no
gi|320447011|ref|NW_003383560.1|	66062-76662	0.5181	59464	352072	0.21745	no
gi|320447011|ref|NW_003383560.1|	66730-76710	21956	150947	278136	0.2313	no
gi|320447011|ref|NW_003383560.1|	11610-81945	718747	219794	161259	0.2349	no
gi|320447011|ref|NW_003383560.1|	23820-82536	173421	110803	-0.646288	0.69085	no
gi|320447011|ref|NW_003383560.1|	26484-82970	989324	782851	-0.337704	0.7803	no
gi|320447011|ref|NW_003383560.1|	34732-83568	314997	168318	-0.904153	0.5712	no
gi|320447011|ref|NW_003383560.1|	84054-84342	972907	365221	-141353	0.50515	no
gi|320447011|ref|NW_003383560.1|	85999-90207	518158	309251	-0.744614	0.7406	no

gi|320447011|ref|NW_003383560.1|	366105-86698	262241	242003	-0.115867	0.958	no
gi|320447011|ref|NW_003383560.1|	368433-86963	940828	495187	-0.925958	0.65675	no
gi|320447011|ref|NW_003383560.1|	370888-87159	488575	223497	-112832	0.4813	no
gi|320447011|ref|NW_003383560.1|	384323-88556	239976	349165	0.541018	0.66545	no
gi|320447011|ref|NW_003383560.1|	901645-90209	692455	252938	-145294	0.5064	no
gi|320447011|ref|NW_003383560.1|	90599-93285	773906	441646	-0.809268	0.54995	no
gi|320447011|ref|NW_003383560.1|	913261-91513	62778	659873	0.0719279	0.97335	no
gi|320447011|ref|NW_003383560.1|	916696-91713	922329	411194	-116546	0.6015	no
gi|320447011|ref|NW_003383560.1|	917795-92231	331093	513404	0.632859	0.69155	no
gi|320447011|ref|NW_003383560.1|	922478-92404	244042	267039	0.12992	0.9172	no
gi|320447011|ref|NW_003383560.1|	96238-98816	858567	611336	-0.489966	0.71345	no
gi|320447011|ref|NW_003383560.1|	966856-96926	512347	249887	228608	0.20795	no
gi|320447011|ref|NW_003383560.1|	972658-97527	538218	158321	155659	0.36215	no
gi|320447011|ref|NW_003383560.1|	986738-98785	576895	49256	-0.22801	0.9086	no
gi|320447011|ref|NW_003383560.1|	988560-99188	492299	114401	12165	0.4616	no
gi|320447011|ref|NW_003383560.1|	991988-99434	25438	804752	166156	0.4534	no
gi|320447011|ref|NW_003383560.1|	995410-99898	372354	170391	-112783	0.39915	no
gi|320447012|ref|NW_003383559.1|	055432-10559	161725	545104	175299	0.4189	no
gi|320447012|ref|NW_003383559.1|	058433-10595	0.187594	168888	317037	0.24015	no
gi|320447012|ref|NW_003383559.1|	064337-10657	19033	94585	231311	0.3025	no
gi|320447012|ref|NW_003383559.1|	078585-10827	211142	151131	-0.48241	0.7173	no
gi|320447012|ref|NW_003383559.1|	082882-10846	0.794025	102691	0.371046	1	no
gi|320447012|ref|NW_003383559.1|	084800-10855	129679	197795	0.609062	0.7753	no
gi|320447012|ref|NW_003383559.1|	112730-11146	121862	382227	164918	0.43795	no
gi|320447012|ref|NW_003383559.1|	115277-11169	269407	24872	320666	0.09915	no
gi|320447012|ref|NW_003383559.1|	117776-11200	12306	759566	-0.696114	0.66335	no
gi|320447012|ref|NW_003383559.1|	120230-11206	808904	482757	-0.744672	0.7078	no
gi|320447012|ref|NW_003383559.1|	121271-11248	31891	344383	0.110863	0.93575	no
gi|320447012|ref|NW_003383559.1|	125917-11284	244125	149525	-0.707231	0.5842	no
gi|320447012|ref|NW_003383559.1|	131515-11334	736804	423279	252226	0.17625	no

gi|320447012|ref|NW_003383559.1|	148851-114957	720836	364193	233696	0.307	no
gi|320447012|ref|NW_003383559.1|	167493-116777	6791	17339	135233	0.52355	no
gi|320447012|ref|NW_003383559.1|	214164-121431	171.53	332836	0.956345	0.67435	no
gi|320447012|ref|NW_003383559.1|	241042-124147	992914	493346	-100907	0.5965	no
gi|320447012|ref|NW_003383559.1|	271050-127239	880494	53761	-0.711754	0.73705	no
gi|320447012|ref|NW_003383559.1|	272902-127421	14094	138051	-0.0298848	0.98755	no
gi|320447012|ref|NW_003383559.1|	280659-128098	155925	110794	282896	0.2595	no
gi|320447012|ref|NW_003383559.1|	292840-129420	202169	235295	0.218907	0.8673	no
gi|320447012|ref|NW_003383559.1|	295328-129659	275356	30.36	0.140872	0.9106	no
gi|320447012|ref|NW_003383559.1|	297215-129781	24243	180115	-0.428652	0.8369	no
gi|320447012|ref|NW_003383559.1|	297914-130047	104126	199025	0.934618	0.45735	no
gi|320447012|ref|NW_003383559.1|	300649-130137	303652	116791	-137849	0.52865	no
gi|320447012|ref|NW_003383559.1|	302159-130468	12795	580397	-114047	0.4971	no
gi|320447012|ref|NW_003383559.1|	307389-130894	229072	854606	-142247	0.39635	no
gi|320447012|ref|NW_003383559.1|	309131-131004	237711	78528	-159793	0.4652	no
gi|320447012|ref|NW_003383559.1|	314130-131478	0.405462	171216	540011	0.16305	no
gi|320447012|ref|NW_003383559.1|	314969-131784	0.830676	159452	426269	0.0528	no
gi|320447012|ref|NW_003383559.1|	341434-134168	914248	868923	324857	0.19875	no
gi|320447012|ref|NW_003383559.1|	353374-135374	452172	18482	203118	0.3538	no
gi|320447012|ref|NW_003383559.1|	353921-135458	119368	271169	118377	0.56835	no
gi|320447012|ref|NW_003383559.1|	356595-135691	810224	250878	163059	0.44555	no
gi|320447012|ref|NW_003383559.1|	358474-135874	243108	573049	123706	0.54735	no
gi|320447012|ref|NW_003383559.1|	358862-136101	104718	523654	232211	0.29475	no
gi|320447012|ref|NW_003383559.1|	362183-136401	114746	712394	263423	0.2477	no
gi|320447012|ref|NW_003383559.1|	364140-136878	169198	164877	-0.0373188	0.97715	no
gi|320447012|ref|NW_003383559.1|	139550-141675	401837	448549	0.158655	0.94185	no
gi|320447012|ref|NW_003383559.1|	141726-142961	405175	234156	-0.791072	0.70145	no
gi|320447012|ref|NW_003383559.1|	163146-163501	24792	161539	-0.617991	0.76585	no
gi|320447012|ref|NW_003383559.1|	171166-176072	498517	852736	0.774457	0.53795	no
gi|320447012|ref|NW_003383559.1|	176283-177051	0.947163	173255	0.871208	0.6867	no

gi|320447012|ref|NW_003383559.1|	180014-18142	129409	0.599422	-111029	1	no
gi|320447012|ref|NW_003383559.1|	181628-18252	127552	105466	-0.274299	1	no
gi|320447012|ref|NW_003383559.1|	189109-18989	274403	0.627924	-212763	0.27995	no
gi|320447012|ref|NW_003383559.1|	193225-19404	719334	106802	0.570198	0.7804	no
gi|320447012|ref|NW_003383559.1|	205282-20726	357659	909913	134714	0.543	no
gi|320447012|ref|NW_003383559.1|	207664-21040	208925	220881	340221	0.08985	no
gi|320447012|ref|NW_003383559.1|	252517-25750	681621	959917	0.493939	0.6999	no
gi|320447012|ref|NW_003383559.1|	257779-25959	166752	737303	-117737	0.47555	no
gi|320447012|ref|NW_003383559.1|	261768-26368	163173	341934	-225461	0.19965	no
gi|320447012|ref|NW_003383559.1|	263911-26424	834935	154347	-243548	0.2705	no
gi|320447012|ref|NW_003383559.1|	266684-26880	0.627738	262759	20655	0.34635	no
gi|320447012|ref|NW_003383559.1|	269397-27007	189643	207105	0.127077	0.9267	no
gi|320447012|ref|NW_003383559.1|	279644-28137	316971	670098	108002	0.61355	no
gi|320447012|ref|NW_003383559.1|	283485-28397	398499	266413	-0.580912	0.79695	no
gi|320447012|ref|NW_003383559.1|	301580-30406	175513	324782	-243403	0.17365	no
gi|320447012|ref|NW_003383559.1|	305281-30634	470722	636986	-288554	0.1149	no
gi|320447012|ref|NW_003383559.1|	324920-32515	0	697855	inf	0.0133	no
gi|320447012|ref|NW_003383559.1|	325367-32661	0	522208	inf	0.00885	no
gi|320447012|ref|NW_003383559.1|	32927-33476	11216	331356	156282	0.4772	no
gi|320447012|ref|NW_003383559.1|	398849-39922	76846	862555	0.166645	0.9292	no
gi|320447012|ref|NW_003383559.1|	400294-40141	132942	223549	0.749792	0.70945	no
gi|320447012|ref|NW_003383559.1|	539014-54071	449948	118891	14018	0.5255	no
gi|320447012|ref|NW_003383559.1|	540848-54261	0.110534	146283	37262	0.21225	no
gi|320447012|ref|NW_003383559.1|	548287-54953	614197	154293	13289	0.54865	no
gi|320447012|ref|NW_003383559.1|	551408-55292	11878	440541	189099	0.39115	no
gi|320447012|ref|NW_003383559.1|	553024-55454	0.395005	21976	247598	0.28595	no
gi|320447012|ref|NW_003383559.1|	554668-55546	0.301084	289453	326509	0.23045	no
gi|320447012|ref|NW_003383559.1|	556935-55750	45536	12992	151255	0.47585	no
gi|320447012|ref|NW_003383559.1|	561068-56129	24033	36221	0.591809	0.7597	no
gi|320447012|ref|NW_003383559.1|	586853-58737	527741	622503	0.23825	0.9125	no

gi|320447012|ref|NW_003383559.1|	;00949-60162	113914	618812	-0.880379	0.49375	no
gi|320447012|ref|NW_003383559.1|	;02576-60448	100442	651031	-0.625565	0.64065	no
gi|320447012|ref|NW_003383559.1|	;24926-62555	476753	170772	-148117	0.5123	no
gi|320447012|ref|NW_003383559.1|	;25876-62776	210464	555423	-192191	0.2756	no
gi|320447012|ref|NW_003383559.1|	;27872-62845	288331	32535	0.174269	0.92035	no
gi|320447012|ref|NW_003383559.1|	63634-63880	101175	46.91	221305	0.33275	no
gi|320447012|ref|NW_003383559.1|	;36469-64075	108327	34423	166798	0.44315	no
gi|320447012|ref|NW_003383559.1|	;41221-64409	311184	617707	0.989155	0.4604	no
gi|320447012|ref|NW_003383559.1|	;44210-64532	0.382851	145782	192895	0.3182	no
gi|320447012|ref|NW_003383559.1|	;45434-64632	119149	927805	-0.360877	0.86485	no
gi|320447012|ref|NW_003383559.1|	64585-64862	0	210381	inf	0.0233	no
gi|320447012|ref|NW_003383559.1|	;46478-64862	397212	788303	0.988842	0.6495	no
gi|320447012|ref|NW_003383559.1|	;48787-65015	0	124285	inf	1	no
gi|320447012|ref|NW_003383559.1|	;51294-65291	271382	282034	0.0555431	0.96685	no
gi|320447012|ref|NW_003383559.1|	;54907-65600	200253	137231	-0.545222	0.8037	no
gi|320447012|ref|NW_003383559.1|	;77152-67797	606309	932682	0.621332	0.76575	no
gi|320447012|ref|NW_003383559.1|	'14603-71634	492153	740071	0.588557	0.78165	no
gi|320447012|ref|NW_003383559.1|	'21439-72168	337249	191904	25085	0.2746	no
gi|320447012|ref|NW_003383559.1|	'27569-72908	116231	23.33	100519	0.5417	no
gi|320447012|ref|NW_003383559.1|	'37207-73921	27351	729687	141569	0.2962	no
gi|320447012|ref|NW_003383559.1|	'54646-75518	371579	242743	-0.61424	0.77225	no
gi|320447012|ref|NW_003383559.1|	'55312-75607	214336	10091	-108681	0.6074	no
gi|320447012|ref|NW_003383559.1|	'56182-75713	117923	0.976451	-0.27223	1	no
gi|320447012|ref|NW_003383559.1|	'58292-75905	457727	298352	-0.617474	0.7051	no
gi|320447012|ref|NW_003383559.1|	;22970-82342	0	66033	inf	0.02915	no
gi|320447012|ref|NW_003383559.1|	;25570-82606	0	714909	inf	0.0035	no
gi|320447012|ref|NW_003383559.1|	;77500-87794	0	679767	inf	0.02915	no
gi|320447012|ref|NW_003383559.1|	91845-92370	230668	34895	0.597203	0.7533	no
gi|320447012|ref|NW_003383559.1|	;49955-95649	128374	950462	-0.433648	0.7465	no
gi|320447012|ref|NW_003383559.1|	;56761-95722	265679	334959	0.334298	0.87265	no

gi|320447012|ref|NW_003383559.1|	962347-96472	654249	957629	0.549626	0.80665	no
gi|320447012|ref|NW_003383559.1|	965039-96994	628565	138158	113619	0.387	no
gi|320447012|ref|NW_003383559.1|	970239-97118	284607	425466	0.58007	0.78445	no
gi|320447012|ref|NW_003383559.1|	976656-97872	0.18425	115664	26502	1	no
gi|320447013|ref|NW_003383558.1|	120061-12049	573947	717285	364356	0.15485	no
gi|320447013|ref|NW_003383558.1|	167870-16922	0	21008	inf	0.0162	no
gi|320447013|ref|NW_003383558.1|	173545-17707	117861	1287.82	100936	0.1279	no
gi|320447013|ref|NW_003383558.1|	214516-21554	169066	25441	0.58957	0.79225	no
gi|320447013|ref|NW_003383558.1|	221535-22201	341065	409981	0.26551	0.90175	no
gi|320447013|ref|NW_003383558.1|	223802-23048	281043	709707	133643	0.30235	no
gi|320447013|ref|NW_003383558.1|	232431-23287	596019	52071	-351681	0.1653	no
gi|320447013|ref|NW_003383558.1|	235071-23583	351765	369514	-325091	0.1823	no
gi|320447013|ref|NW_003383558.1|	237509-23820	277726	699173	-198994	0.37235	no
gi|320447013|ref|NW_003383558.1|	25060-25291	416603	393473	323952	0.2322	no
gi|320447013|ref|NW_003383558.1|	30050-31525	0.408391	179812	213846	0.3451	no
gi|320447013|ref|NW_003383558.1|	343533-34397	317853	580057	0.867833	0.69375	no
gi|320447013|ref|NW_003383558.1|	345277-34637	0.585572	148607	134358	0.5243	no
gi|320447013|ref|NW_003383558.1|	352843-35660	0.335001	35167	339199	0.17145	no
gi|320447013|ref|NW_003383558.1|	370813-37118	896933	133913	0.578223	0.7155	no
gi|320447013|ref|NW_003383558.1|	371307-37154	451259	498215	0.142815	0.939	no
gi|320447013|ref|NW_003383558.1|	40641-43154	268181	142813	-0.909083	0.48785	no
gi|320447013|ref|NW_003383558.1|	43958-44376	291638	110934	-139447	0.50635	no
gi|320447013|ref|NW_003383558.1|	45657-47036	65812	193717	-17644	0.1855	no
gi|320447013|ref|NW_003383558.1|	525299-52564	551421	366765	-0.588296	0.77175	no
gi|320447013|ref|NW_003383558.1|	52652-53534	0.783072	143822	0.877068	0.68225	no
gi|320447013|ref|NW_003383558.1|	552515-55479	231301	593903	136046	0.5279	no
gi|320447013|ref|NW_003383558.1|	555072-55649	241151	100546	205984	0.34535	no
gi|320447013|ref|NW_003383558.1|	562589-56403	271148	102669	-140108	0.2875	no
gi|320447013|ref|NW_003383558.1|	564154-56666	136507	827071	-0.722894	0.57525	no
gi|320447013|ref|NW_003383558.1|	569172-57150	105177	179925	0.77457	0.54045	no

gi|320447013|ref|NW_003383558.1|	71648-57498	631244	810778	0.361109	0.819	no
gi|320447013|ref|NW_003383558.1|	80358-58129	819053	780974	-0.0686814	0.9719	no
gi|320447013|ref|NW_003383558.1|	82979-58392	453818	23069	-0.97616	0.6426	no
gi|320447013|ref|NW_003383558.1|	84243-58527	105796	62896	-0.750245	0.7213	no
gi|320447013|ref|NW_003383558.1|	86319-58714	710848	449642	-0.660764	0.7474	no
gi|320447013|ref|NW_003383558.1|	89028-59098	374079	195773	-0.934159	0.474	no
gi|320447013|ref|NW_003383558.1|	91145-59323	180243	172721	-0.0614991	0.9593	no
gi|320447013|ref|NW_003383558.1|	94651-59549	439507	150229	-154872	0.35745	no
gi|320447013|ref|NW_003383558.1|	97048-59736	972269	627194	-0.632444	0.7537	no
gi|320447013|ref|NW_003383558.1|	99281-60825	577354	858612	0.572551	0.66715	no
gi|320447013|ref|NW_003383558.1|	08397-61286	796881	118581	0.573433	0.65695	no
gi|320447013|ref|NW_003383558.1|	61045-62275	0.678687	200021	155933	0.4963	no
gi|320447013|ref|NW_003383558.1|	17028-61743	341137	387022	0.182064	0.9305	no
gi|320447013|ref|NW_003383558.1|	26187-62735	117873	804118	-0.551759	0.7942	no
gi|320447013|ref|NW_003383558.1|	27852-63162	724758	987746	0.446641	0.7217	no
gi|320447013|ref|NW_003383558.1|	55119-65567	0	672353	inf	0.0212	no
gi|320447013|ref|NW_003383558.1|	84438-68598	179844	554014	162317	0.44555	no
gi|320447013|ref|NW_003383558.1|	86817-68782	305817	482967	0.659254	0.7474	no
gi|320447013|ref|NW_003383558.1|	88817-68950	225063	128058	-0.813537	0.69605	no
gi|320447013|ref|NW_003383558.1|	89759-69021	374101	298655	-0.324952	0.86235	no
gi|320447013|ref|NW_003383558.1|	90308-69171	158166	249759	0.659095	0.74115	no
gi|320447013|ref|NW_003383558.1|	17219-71916	287554	422295	0.554417	0.68065	no
gi|320447013|ref|NW_003383558.1|	19404-72075	681914	925993	0.44141	0.84235	no
gi|320447013|ref|NW_003383558.1|	21155-72355	303449	488967	0.688284	0.74695	no
gi|320447013|ref|NW_003383558.1|	23691-72390	619188	704933	-313482	0.25355	no
gi|320447013|ref|NW_003383558.1|	45669-74637	432856	271501	-0.67293	0.7697	no
gi|320447015|ref|NW_003383556.1|	22537-12338	0	303288	inf	0.0198	no
gi|320447015|ref|NW_003383556.1|	35663-23663	0.230372	143126	263525	0.26345	no
gi|320447015|ref|NW_003383556.1|	39052-24069	424686	489863	0.205984	0.87615	no
gi|320447015|ref|NW_003383556.1|	40918-24606	151209	288901	0.934027	0.6648	no

gi|320447015|ref|NW_003383556.1|	247140-25099	272378	229811	-0.245164	0.8571	no
gi|320447015|ref|NW_003383556.1|	262256-26330	189226	948982	-0.995661	0.65435	no
gi|320447015|ref|NW_003383556.1|	311777-31347	230113	544777	-207861	0.23625	no
gi|320447015|ref|NW_003383556.1|	317493-31931	139499	200327	-279982	0.1308	no
gi|320447015|ref|NW_003383556.1|	333565-33488	0	183279	inf	0.02075	no
gi|320447015|ref|NW_003383556.1|	337245-33894	0.345406	0.801684	121474	1	no
gi|320447015|ref|NW_003383556.1|	39592-40216	21661	114815	240614	0.2892	no
gi|320447015|ref|NW_003383556.1|	446427-44731	0.51804	303321	254971	0.25025	no
gi|320447015|ref|NW_003383556.1|	449994-45159	605039	130603	111009	0.61385	no
gi|320447015|ref|NW_003383556.1|	453110-45483	499102	710934	0.510383	0.812	no
gi|320447015|ref|NW_003383556.1|	455029-45536	832889	108065	0.375702	0.8528	no
gi|320447015|ref|NW_003383556.1|	455499-45576	541968	223814	204602	0.3003	no
gi|320447015|ref|NW_003383556.1|	456287-45832	318973	327143	0.0364853	0.98515	no
gi|320447015|ref|NW_003383556.1|	459737-46006	414423	383157	-0.113168	0.95525	no
gi|320447015|ref|NW_003383556.1|	488496-48928	0.302705	581952	426492	0.1888	no
gi|320447015|ref|NW_003383556.1|	490675-49123	408355	306884	-0.41213	0.8495	no
gi|320447015|ref|NW_003383556.1|	491576-49308	116964	125696	0.10388	0.9644	no
gi|320447015|ref|NW_003383556.1|	493377-49384	780394	851426	0.125679	0.9418	no
gi|320447015|ref|NW_003383556.1|	494030-49490	236017	343139	0.539902	0.7878	no
gi|320447015|ref|NW_003383556.1|	495128-49639	200546	289652	0.530389	0.74585	no
gi|320447015|ref|NW_003383556.1|	503428-50736	113954	219828	0.94792	0.47865	no
gi|320447015|ref|NW_003383556.1|	509388-50974	627793	73593	0.22928	0.896	no
gi|320447015|ref|NW_003383556.1|	509908-51096	175452	104205	-0.751662	0.72665	no
gi|320447015|ref|NW_003383556.1|	512585-51446	163964	699942	-122807	0.4554	no
gi|320447015|ref|NW_003383556.1|	518075-51898	820745	377199	-112161	0.5854	no
gi|320447015|ref|NW_003383556.1|	519695-52012	204981	103246	-0.989412	0.64015	no
gi|320447015|ref|NW_003383556.1|	523382-52430	148861	622668	-125743	0.5514	no
gi|320447015|ref|NW_003383556.1|	526566-52737	111429	382811	-154142	0.47255	no
gi|320447015|ref|NW_003383556.1|	580188-58067	193336	356506	-243912	0.28135	no
gi|320447015|ref|NW_003383556.1|	675782-67639	0.447952	36486	302593	0.25005	no

gi|320447015|ref|NW_003383556.1|	80606-68190	0.317922	231673	286535	0.22425	no
gi|320447015|ref|NW_003383556.1|	85497-69108	0.0315273	938882	82182	0.14075	no
gi|320447015|ref|NW_003383556.1|	91238-69589	0.0381244	304387	631905	0.1549	no
gi|320447015|ref|NW_003383556.1|	02541-70476	0.0847232	390244	552547	0.1624	no
gi|320447015|ref|NW_003383556.1|	05114-70627	0	264693	inf	0.01575	no
gi|320447015|ref|NW_003383556.1|	07141-70820	0	1546	inf	0.0294	no
gi|320447015|ref|NW_003383556.1|	17500-71894	0	145919	inf	0.02205	no
gi|320447015|ref|NW_003383556.1|	69904-77034	999458	674103	-389011	0.07755	no
gi|320447016|ref|NW_003383555.1|	95358-19623	265126	0	#NAME?	0.0229	no
gi|320447016|ref|NW_003383555.1|	93914-29476	0	187456	inf	0.0312	no
gi|320447016|ref|NW_003383555.1|	98624-29886	239205	172798	-0.469163	0.8145	no
gi|320447016|ref|NW_003383555.1|	87125-38797	16531	170599	0.0454382	0.94	no
gi|320447016|ref|NW_003383555.1|	53646-45575	0.090156	276732	493992	0.171	no
gi|320447016|ref|NW_003383555.1|	56305-45716	0	203625	inf	0.0294	no
gi|320447016|ref|NW_003383555.1|	30014-53024	0	348932	inf	0.00365	no
gi|320447016|ref|NW_003383555.1|	30412-53084	0	234332	inf	0.0069	no
gi|320447016|ref|NW_003383555.1|	81798-58328	0.538869	103032	0.935085	1	no
gi|320447016|ref|NW_003383555.1|	18826-61932	540551	290594	-0.895426	0.6869	no
gi|320447016|ref|NW_003383555.1|	31357-63189	324776	316614	-0.0367224	0.9845	no
gi|320447016|ref|NW_003383555.1|	69696-73494	0.331521	351345	340572	0.1727	no
gi|320447016|ref|NW_003383555.1|	85109-86434	0.154875	11827	29329	1	no
gi|320447017|ref|NW_003383554.1|	00639-10195	264746	0.864851	-161409	0.4349	no
gi|320447017|ref|NW_003383554.1|	28295-10288	340361	190824	-0.834824	0.6954	no
gi|320447017|ref|NW_003383554.1|	36586-10371	411522	100114	-203932	0.35505	no
gi|320447017|ref|NW_003383554.1|	39027-10403	698421	288989	-127308	0.54395	no
gi|320447017|ref|NW_003383554.1|	49094-10535	311858	628788	101168	0.44115	no
gi|320447017|ref|NW_003383554.1|	05320-10575	658928	218308	-159376	0.44495	no
gi|320447017|ref|NW_003383554.1|	70364-10720	0.476474	331668	279927	0.2545	no
gi|320447017|ref|NW_003383554.1|	10294-11233	242572	117125	-105037	0.60385	no
gi|320447017|ref|NW_003383554.1|	13065-11462	204292	115468	-0.823145	0.6863	no

gi|320447017|ref|NW_003383554.1|	117079-11775[illegible]	303429	181217	-0.74364	0.70585	no
gi|320447017|ref|NW_003383554.1|	118502-11980[illegible]	505342	383622	-0.397576	0.8438	no
gi|320447017|ref|NW_003383554.1|	119952-12053[illegible]	631774	0.986669	-267877	0.2271	no
gi|320447017|ref|NW_003383554.1|	121615-12200[illegible]	829929	680831	-0.285691	0.8832	no
gi|320447017|ref|NW_003383554.1|	123080-12365[illegible]	40007	60854	0.605099	0.75115	no
gi|320447017|ref|NW_003383554.1|	123941-12481[illegible]	477977	457071	-0.0645232	0.9688	no
gi|320447017|ref|NW_003383554.1|	126026-12737[illegible]	394349	516068	0.388087	0.84835	no
gi|320447017|ref|NW_003383554.1|	127770-12805[illegible]	219784	140407	-0.646468	0.75695	no
gi|320447017|ref|NW_003383554.1|	128154-13254[illegible]	661646	141833	110006	0.39845	no
gi|320447017|ref|NW_003383554.1|	141601-14239[illegible]	121737	167151	0.457388	0.8212	no
gi|320447017|ref|NW_003383554.1|	1882-2932	16574	358159	111168	0.5882	no
gi|320447017|ref|NW_003383554.1|	237002-23723[illegible]	444721	178296	-131862	0.5397	no
gi|320447017|ref|NW_003383554.1|	24159-24439	754498	7295	-337054	0.1972	no
gi|320447017|ref|NW_003383554.1|	260541-26103[illegible]	902588	776761	-0.216597	0.91275	no
gi|320447017|ref|NW_003383554.1|	26318-27262	154815	345075	-216557	0.3355	no
gi|320447017|ref|NW_003383554.1|	263337-26386[illegible]	804671	386492	-105796	0.6047	no
gi|320447017|ref|NW_003383554.1|	278736-27945[illegible]	100357	331563	-159779	0.44275	no
gi|320447017|ref|NW_003383554.1|	28592-29534	231686	313079	-288757	0.22945	no
gi|320447017|ref|NW_003383554.1|	292201-29256[illegible]	122398	398958	-161727	0.4405	no
gi|320447017|ref|NW_003383554.1|	301451-30170[illegible]	312363	197812	266283	0.27135	no
gi|320447017|ref|NW_003383554.1|	30210-31219	63266	135662	-222142	0.3116	no
gi|320447017|ref|NW_003383554.1|	321407-32168[illegible]	264648	306631	0.21243	0.91605	no
gi|320447017|ref|NW_003383554.1|	32714-34955	841317	270101	-163915	0.45705	no
gi|320447017|ref|NW_003383554.1|	330217-33138[illegible]	253065	0.626226	-201475	0.359	no
gi|320447017|ref|NW_003383554.1|	332751-33641[illegible]	678522	261489	-137564	0.3942	no
gi|320447017|ref|NW_003383554.1|	339504-34026[illegible]	118141	218965	-243174	0.2851	no
gi|320447017|ref|NW_003383554.1|	345892-35249[illegible]	166824	135501	-0.300026	0.8867	no
gi|320447017|ref|NW_003383554.1|	355963-35734[illegible]	0.73459	204109	147433	0.5	no
gi|320447017|ref|NW_003383554.1|	358449-35929[illegible]	139351	153361	0.138206	0.9242	no
gi|320447017|ref|NW_003383554.1|	360840-36141[illegible]	245765	432257	0.814613	0.69345	no

gi|320447017|ref|NW_003383554.1|	363143-36932	596026	147203	-201757	0.2469	no
gi|320447017|ref|NW_003383554.1|	369829-37070	345206	0.182829	-423889	0.3017	no
gi|320447017|ref|NW_003383554.1|	39552-39836	153849	561629	-477575	0.1663	no
gi|320447017|ref|NW_003383554.1|	414096-41549	115621	210864	0.866908	0.6722	no
gi|320447017|ref|NW_003383554.1|	41655-43120	137238	16218	0.240915	0.90115	no
gi|320447017|ref|NW_003383554.1|	416803-42126	455513	609973	0.421252	0.7972	no
gi|320447017|ref|NW_003383554.1|	46161-48544	14928	268803	0.848533	0.67435	no
gi|320447017|ref|NW_003383554.1|	49212-50174	116137	208416	0.843632	0.6905	no
gi|320447017|ref|NW_003383554.1|	504337-50580	0	171444	inf	0.0154	no
gi|320447017|ref|NW_003383554.1|	505907-50767	0	482848	inf	0.0054	no
gi|320447017|ref|NW_003383554.1|	507967-50911	0	690183	inf	0.0071	no
gi|320447017|ref|NW_003383554.1|	509443-51051	0	250898	inf	0.0154	no
gi|320447017|ref|NW_003383554.1|	510680-51270	0	1579	inf	0.0138	no
gi|320447017|ref|NW_003383554.1|	512866-51421	0	200643	inf	0.0212	no
gi|320447017|ref|NW_003383554.1|	514434-51523	0	225072	inf	0.0294	no
gi|320447017|ref|NW_003383554.1|	5170-5666	255074	298917	0.228828	0.89115	no
gi|320447017|ref|NW_003383554.1|	517354-51838	19212	118036	-0.70278	0.7316	no
gi|320447017|ref|NW_003383554.1|	523028-52494	0.100106	244238	460869	0.1773	no
gi|320447017|ref|NW_003383554.1|	526807-52744	0	339975	inf	0.029	no
gi|320447017|ref|NW_003383554.1|	52684-53760	0.602296	0.972284	0.690906	1	no
gi|320447017|ref|NW_003383554.1|	529429-52979	0	107146	inf	0.0233	no
gi|320447017|ref|NW_003383554.1|	540558-54386	960049	656176	-0.549026	0.736	no
gi|320447017|ref|NW_003383554.1|	545504-54604	243245	893538	-144481	0.49135	no
gi|320447017|ref|NW_003383554.1|	562602-56413	187989	149811	-0.327498	0.84235	no
gi|320447017|ref|NW_003383554.1|	564666-56813	190799	322772	0.758464	0.56695	no
gi|320447017|ref|NW_003383554.1|	572234-57510	711696	143412	-231109	0.31145	no
gi|320447017|ref|NW_003383554.1|	582-1511	0.72999	13425	0.878969	1	no
gi|320447017|ref|NW_003383554.1|	58467-60124	178049	173519	-0.0371834	0.97305	no
gi|320447017|ref|NW_003383554.1|	597260-59907	0	156792	inf	0.01575	no
gi|320447017|ref|NW_003383554.1|	60443-61578	225113	0.649568	-17931	0.4174	no

gi|320447017|ref|NW_003383554.1|	07503-60887	715803	300356	-125289	0.54645	no
gi|320447017|ref|NW_003383554.1|	09251-60996	151904	265789	-251481	0.27395	no
gi|320447017|ref|NW_003383554.1|	10206-61230	624077	100951	-262807	0.2504	no
gi|320447017|ref|NW_003383554.1|	12767-61468	395057	185689	-108917	0.4054	no
gi|320447017|ref|NW_003383554.1|	15207-61596	126923	713841	-0.830282	0.68875	no
gi|320447017|ref|NW_003383554.1|	16159-61932	128791	940884	-0.452945	0.72765	no
gi|320447017|ref|NW_003383554.1|	21920-62467	615121	202786	172101	0.31535	no
gi|320447017|ref|NW_003383554.1|	27349-62806	125348	833874	-0.588041	0.76945	no
gi|320447017|ref|NW_003383554.1|	33065-63336	507489	398134	-0.350123	0.86325	no
gi|320447017|ref|NW_003383554.1|	35865-63781	216381	287998	0.412484	0.84455	no
gi|320447017|ref|NW_003383554.1|	34132-73538	579287	0.574048	-333503	0.19925	no
gi|320447017|ref|NW_003383554.1|	37873-73831	346576	0.522897	-60505	0.2672	no
gi|320447017|ref|NW_003383554.1|	49704-75025	153938	0	#NAME?	0.0099	no
gi|320447017|ref|NW_003383554.1|	53175-75486	191806	0	#NAME?	5.00E-05	yes
gi|320447017|ref|NW_003383554.1|	57340-75832	270898	0.311782	-644107	0.17555	no
gi|320447017|ref|NW_003383554.1|	79-475	296406	51978	0.810326	0.69235	no
gi|320447017|ref|NW_003383554.1|	99293-80079	0.133228	176017	372374	0.21225	no
gi|320447017|ref|NW_003383554.1|	01241-80462	327302	5.48	0.743551	0.7394	no
gi|320447017|ref|NW_003383554.1|	05407-80726	0.625115	0.508229	-0.298642	1	no
gi|320447017|ref|NW_003383554.1|	09826-81064	123726	830678	-0.574781	0.78575	no
gi|320447017|ref|NW_003383554.1|	10772-81145	38807	174906	-114974	0.61355	no
gi|320447017|ref|NW_003383554.1|	21680-82256	0.259418	285909	34622	0.21395	no
gi|320447017|ref|NW_003383554.1|	22824-82376	334392	164778	-102101	0.60725	no
gi|320447017|ref|NW_003383554.1|	26552-82711	177605	960015	-0.887543	0.6653	no
gi|320447017|ref|NW_003383554.1|	51739-85204	0	261016	inf	0.0186	no
gi|320447017|ref|NW_003383554.1|	85596-86050	120216	349835	-178088	0.40635	no
gi|320447017|ref|NW_003383554.1|	57504-85810	0	116917	inf	0.0103	no
gi|320447017|ref|NW_003383554.1|	72496-87352	213196	501028	123271	0.5481	no
gi|320447017|ref|NW_003383554.1|	13824-91529	0.136793	18258	706039	0.1408	no
gi|320447017|ref|NW_003383554.1|	94764-95673	35078	0.51811	-275924	0.2218	no

gi|320447017|ref|NW_003383554.1|	92245-99340	264369	821035	-168704	0.3096	no
gi|320447018|ref|NW_003383553.1|	77993-17978	0.433335	935842	443271	0.1471	no
gi|320447018|ref|NW_003383553.1|	79958-18076	0	531987	inf	0.0142	no
gi|320447018|ref|NW_003383553.1|	85417-18648	0.406366	292334	616869	0.1182	no
gi|320447018|ref|NW_003383553.1|	93303-19437	0.803061	0.833386	0.0534761	1	no
gi|320447018|ref|NW_003383553.1|	94531-19577	0.668601	0.81153	0.279498	1	no
gi|320447018|ref|NW_003383553.1|	08483-20956	670581	555115	-0.272624	0.8334	no
gi|320447018|ref|NW_003383553.1|	10590-21177	276361	153474	-0.848556	0.5966	no
gi|320447018|ref|NW_003383553.1|	24908-22537	413131	171.89	-126512	0.34125	no
gi|320447018|ref|NW_003383553.1|	32000-23313	194086	600234	-169309	0.4503	no
gi|320447018|ref|NW_003383553.1|	33451-23434	109861	440096	-131978	0.5329	no
gi|320447018|ref|NW_003383553.1|	34884-23539	121353	366419	-172765	0.4225	no
gi|320447018|ref|NW_003383553.1|	36367-23677	510681	159165	-16819	0.436	no
gi|320447018|ref|NW_003383553.1|	36977-23802	258804	29728	0.199961	0.8683	no
gi|320447018|ref|NW_003383553.1|	38537-23954	610657	139.2	118873	0.3706	no
gi|320447018|ref|NW_003383553.1|	42111-24313	285323	108912	-138943	0.3923	no
gi|320447018|ref|NW_003383553.1|	43332-24463	549487	158714	-179166	0.3218	no
gi|320447018|ref|NW_003383553.1|	44962-24572	353903	0.882364	-200391	0.3624	no
gi|320447018|ref|NW_003383553.1|	90308-29147	151514	999847	-0.599675	0.77825	no
gi|320447018|ref|NW_003383553.1|	91745-29195	433406	38701	-0.163347	0.92615	no
gi|320447018|ref|NW_003383553.1|	92153-29318	148861	793776	-0.907164	0.6719	no
gi|320447018|ref|NW_003383553.1|	93778-29491	10164	443164	-119756	0.56835	no
gi|320447018|ref|NW_003383553.1|	95442-29645	281423	119675	-123362	0.54965	no
gi|320447018|ref|NW_003383553.1|	00982-30140	153851	28274	-244399	0.3016	no
gi|320447018|ref|NW_003383553.1|	01497-30227	938738	720549	-370355	0.0646	no
gi|320447018|ref|NW_003383553.1|	02685-30341	669348	892861	-290625	0.118	no
gi|320447018|ref|NW_003383553.1|	03504-30449	154321	648768	-125016	0.56685	no
gi|320447018|ref|NW_003383553.1|	04694-30588	194258	673195	-152888	0.48775	no
gi|320447018|ref|NW_003383553.1|	07993-30970	114123	492711	211015	0.3357	no
gi|320447018|ref|NW_003383553.1|	34868-37599	1432.75	4126.31	152607	0.02405	no

gi|320447018|ref|NW_003383553.1|	349631-350250	15357	0.894112	-41023	0.16025	no
gi|320447018|ref|NW_003383553.1|	396677-397720	110755	0.577946	-426029	0.17125	no
gi|320447018|ref|NW_003383553.1|	417788-418785	132958	352065	140487	0.5079	no
gi|320447018|ref|NW_003383553.1|	519600-529218	225315	27118	0.267309	0.833	no
gi|320447018|ref|NW_003383553.1|	534481-535040	134706	384767	-180776	0.40105	no
gi|320447018|ref|NW_003383553.1|	535912-539460	29644	962222	-16233	0.2286	no
gi|320447018|ref|NW_003383553.1|	560058-560557	567728	126781	-216286	0.2791	no
gi|320447018|ref|NW_003383553.1|	562807-563390	3866	294399	-0.393068	0.8414	no
gi|320447018|ref|NW_003383553.1|	563518-565840	152836	270969	-249578	0.173	no
gi|320447018|ref|NW_003383553.1|	571104-573891	30683	230149	-0.414874	0.7594	no
gi|320447018|ref|NW_003383553.1|	574043-575850	631064	872651	0.467618	0.7277	no
gi|320447018|ref|NW_003383553.1|	707182-707550	0	730959	inf	0.0312	no
gi|320447018|ref|NW_003383553.1|	707765-709260	0.4	222511	24758	0.28595	no
gi|320447018|ref|NW_003383553.1|	710374-713320	0.993766	581835	254963	0.2667	no
gi|320447018|ref|NW_003383553.1|	713447-721607	166492	997022	258217	0.16805	no
gi|320447018|ref|NW_003383553.1|	725736-725995	746272	351474	223564	0.19575	no
gi|320447018|ref|NW_003383553.1|	735583-736471	284485	224476	-0.341791	0.8735	no
gi|320447018|ref|NW_003383553.1|	736621-739340	246615	312376	0.341026	0.7973	no
gi|320447018|ref|NW_003383553.1|	747532-750222	646162	156103	127253	0.4553	no
gi|320447018|ref|NW_003383553.1|	752321-754090	103024	348903	175984	0.324	no
gi|320447018|ref|NW_003383553.1|	797240-798517	0.486042	966756	4314	0.16065	no
gi|320447018|ref|NW_003383553.1|	804088-806467	0.865864	198954	452215	0.05105	no
gi|320447018|ref|NW_003383553.1|	806823-808733	184397	295699	0.681314	0.59405	no
gi|320447018|ref|NW_003383553.1|	810280-811689	805211	759268	-0.084757	0.9689	no
gi|320447018|ref|NW_003383553.1|	811923-812162	852834	514792	-0.728276	0.72735	no
gi|320447018|ref|NW_003383553.1|	850572-856983	131138	783223	257833	0.15845	no
gi|320447018|ref|NW_003383553.1|	872611-873280	155526	185625	0.255236	0.89055	no
gi|320447018|ref|NW_003383553.1|	873476-873802	460766	168754	187281	0.4015	no
gi|320447018|ref|NW_003383553.1|	876058-878924	365604	138983	192656	0.2714	no
gi|320447018|ref|NW_003383553.1|	879059-884072	310775	175396	249668	0.18035	no

gi|320447018|ref|NW_003383553.1|	384352-88504[illegible]	185103	295858	399851	0.1501	no
gi|320447018|ref|NW_003383553.1|	385266-88848[illegible]	588532	474931	301252	0.1244	no
gi|320447018|ref|NW_003383553.1|	389355-89330[illegible]	490163	553407	349701	0.09335	no
gi|320447018|ref|NW_003383553.1|	393462-89711[illegible]	153181	253451	40484	0.0589	no
gi|320447018|ref|NW_003383553.1|	910451-91146[illegible]	329471	18573	-0.826941	0.6155	no
gi|320447018|ref|NW_003383553.1|	914487-91494[illegible]	167862	631848	-140962	0.5124	no
gi|320447018|ref|NW_003383553.1|	929334-93202[illegible]	44269	250725	-0.820191	0.5383	no
gi|320447019|ref|NW_003383552.1|	013551-10150[illegible]	0	158729	inf	0.02075	no
gi|320447019|ref|NW_003383552.1|	030201-10341[illegible]	0.320979	215039	274405	0.23505	no
gi|320447019|ref|NW_003383552.1|	107-1171	142572	281758	0.982773	0.65565	no
gi|320447019|ref|NW_003383552.1|	087779-10896[illegible]	212993	216716	0.0250047	0.98475	no
gi|320447019|ref|NW_003383552.1|	089820-10911[illegible]	533083	442263	-0.269454	0.8944	no
gi|320447019|ref|NW_003383552.1|	091693-10921[illegible]	793059	52841	-0.585773	0.7582	no
gi|320447019|ref|NW_003383552.1|	194517-11964[illegible]	0.298019	228532	293892	0.2298	no
gi|320447019|ref|NW_003383552.1|	209816-12107[illegible]	248055	16455	-0.592135	0.79185	no
gi|320447019|ref|NW_003383552.1|	212489-12129[illegible]	197818	12755	-0.633113	0.75335	no
gi|320447019|ref|NW_003383552.1|	213469-12148[illegible]	552253	549407	-0.00745469	0.98895	no
gi|320447019|ref|NW_003383552.1|	216874-12209[illegible]	235282	325774	0.469483	0.727	no
gi|320447019|ref|NW_003383552.1|	222847-12247[illegible]	257662	246449	-0.0641875	0.95985	no
gi|320447019|ref|NW_003383552.1|	246700-12477[illegible]	458601	242147	240057	0.2928	no
gi|320447019|ref|NW_003383552.1|	260305-12612[illegible]	0	191348	inf	0.029	no
gi|320447019|ref|NW_003383552.1|	262102-12625[illegible]	418966	745392	0.831166	0.6789	no
gi|320447019|ref|NW_003383552.1|	131954-13275[illegible]	818949	229495	-183531	0.29685	no
gi|320447019|ref|NW_003383552.1|	133582-13451[illegible]	55727	181374	-161941	0.34255	no
gi|320447019|ref|NW_003383552.1|	134825-13582[illegible]	752442	321036	-122885	0.557	no
gi|320447019|ref|NW_003383552.1|	13675-15044	133976	4239	166175	0.435	no
gi|320447019|ref|NW_003383552.1|	137926-13894[illegible]	738732	416757	-0.825843	0.5243	no
gi|320447019|ref|NW_003383552.1|	155127-15547[illegible]	486163	701603	-279272	0.2379	no
gi|320447019|ref|NW_003383552.1|	582809-15873[illegible]	182904	112828	262497	0.1489	no
gi|320447019|ref|NW_003383552.1|	594248-15950[illegible]	604164	132265	113042	0.58925	no

gi|320447019|ref|NW_003383552.1|	00691-16008	38172	570428	0.579531	0.79715	no
gi|320447019|ref|NW_003383552.1|	05515-16059	101759	111351	0.12995	0.94705	no
gi|320447019|ref|NW_003383552.1|	12847-16133	823023	139372	0.759937	0.7037	no
gi|320447019|ref|NW_003383552.1|	39990-16408	390184	101602	13807	0.5089	no
gi|320447019|ref|NW_003383552.1|	1659-4658	0.427378	20489	226127	0.3207	no
gi|320447019|ref|NW_003383552.1|	17116-20161	35432	142345	200626	0.2563	no
gi|320447019|ref|NW_003383552.1|	10564-18108	153842	212061	0.463025	0.8167	no
gi|320447019|ref|NW_003383552.1|	22218-24372	0.967419	435677	217105	0.32295	no
gi|320447019|ref|NW_003383552.1|	24633-31548	343522	245957	283993	0.04025	no
gi|320447019|ref|NW_003383552.1|	91785-29318	130074	0.200823	-269533	1	no
gi|320447019|ref|NW_003383552.1|	93400-29496	114654	168382	0.554449	0.78345	no
gi|320447019|ref|NW_003383552.1|	15041-31563	278217	282911	0.0241423	0.94	no
gi|320447019|ref|NW_003383552.1|	35993-36537	461088	563008	0.288113	0.89945	no
gi|320447019|ref|NW_003383552.1|	36754-37555	232769	120314	236984	0.2083	no
gi|320447019|ref|NW_003383552.1|	85290-38649	0	132991	inf	1	no
gi|320447019|ref|NW_003383552.1|	90816-39109	182909	10213	-0.840716	0.6853	no
gi|320447019|ref|NW_003383552.1|	39857-58137	206727	103328	232143	0.13755	no
gi|320447019|ref|NW_003383552.1|	98743-39904	277104	474575	-254572	0.2943	no
gi|320447019|ref|NW_003383552.1|	06310-40697	23818	32467	-287501	0.2289	no
gi|320447019|ref|NW_003383552.1|	10870-41356	624268	559874	316487	0.11455	no
gi|320447019|ref|NW_003383552.1|	16578-41744	0.796629	237677	157702	0.491	no
gi|320447019|ref|NW_003383552.1|	42379-44258	257708	112677	212838	0.34855	no
gi|320447019|ref|NW_003383552.1|	72958-47332	240238	862008	184324	0.3257	no
gi|320447019|ref|NW_003383552.1|	26221-52689	387048	765634	0.984142	0.6428	no
gi|320447019|ref|NW_003383552.1|	32518-53336	496742	125307	13349	0.5342	no
gi|320447019|ref|NW_003383552.1|	35078-53910	200248	513983	135993	0.54265	no
gi|320447019|ref|NW_003383552.1|	61573-56210	0.556372	973961	412974	0.1922	no
gi|320447019|ref|NW_003383552.1|	70057-57089	110161	989515	-0.154825	0.90845	no
gi|320447019|ref|NW_003383552.1|	71330-57162	74694	956753	0.357154	0.86105	no
gi|320447019|ref|NW_003383552.1|	72106-57268	43615	655995	0.58886	0.7631	no

gi|320447019|ref|NW_003383552.1|	572976-58283	146801	218373	0.572926	0.66375	no
gi|320447019|ref|NW_003383552.1|	588689-58902	204224	944589	-111239	0.59385	no
gi|320447019|ref|NW_003383552.1|	601776-60265	10997	432779	-134541	0.5248	no
gi|320447019|ref|NW_003383552.1|	602796-60449	183965	248186	0.43199	0.8267	no
gi|320447019|ref|NW_003383552.1|	607164-60776	319408	464265	0.53955	0.78955	no
gi|320447019|ref|NW_003383552.1|	608089-61087	127064	157715	0.311765	0.8031	no
gi|320447019|ref|NW_003383552.1|	611044-61131	503475	269514	-0.90156	0.65175	no
gi|320447019|ref|NW_003383552.1|	611894-61273	169449	103185	-0.715617	0.73465	no
gi|320447019|ref|NW_003383552.1|	613490-61680	0.273677	107201	196977	1	no
gi|320447019|ref|NW_003383552.1|	617005-61825	0.166326	126905	293166	1	no
gi|320447019|ref|NW_003383552.1|	619395-62010	103015	803826	-0.357893	0.7845	no
gi|320447019|ref|NW_003383552.1|	621191-62180	206058	12162	-0.760669	0.7156	no
gi|320447019|ref|NW_003383552.1|	625055-62630	201255	320724	0.67231	0.69245	no
gi|320447019|ref|NW_003383552.1|	6645-7133	394043	307481	-0.357858	0.85555	no
gi|320447019|ref|NW_003383552.1|	8967-10334	492114	403927	-0.284899	0.88535	no
gi|320447022|ref|NW_003383549.1|	102680-11037	1054	48091	21899	0.32505	no
gi|320447022|ref|NW_003383549.1|	13216-13682	79383	633966	-0.324425	0.88685	no
gi|320447022|ref|NW_003383549.1|	138177-14160	0.211464	56585	474194	0.14295	no
gi|320447022|ref|NW_003383549.1|	141879-14332	0	212792	inf	0.0186	no
gi|320447022|ref|NW_003383549.1|	144675-14764	0.677725	633178	322384	0.18325	no
gi|320447022|ref|NW_003383549.1|	16460-17965	147534	152499	0.0477548	0.9841	no
gi|320447022|ref|NW_003383549.1|	2-1852	867421	859362	-0.0134662	0.9928	no
gi|320447022|ref|NW_003383549.1|	20163-20801	460216	347276	-0.406228	0.85475	no
gi|320447022|ref|NW_003383549.1|	21385-21943	280615	185924	-0.593879	0.76855	no
gi|320447022|ref|NW_003383549.1|	227237-22824	415608	481129	-311073	0.10115	no
gi|320447022|ref|NW_003383549.1|	233669-23797	109812	510747	-110435	0.3878	no
gi|320447022|ref|NW_003383549.1|	238210-24118	514883	243706	-10791	0.4232	no
gi|320447022|ref|NW_003383549.1|	241434-24236	101758	250653	-202138	0.34865	no
gi|320447022|ref|NW_003383549.1|	24304-24852	278589	216745	-0.362141	0.8619	no
gi|320447022|ref|NW_003383549.1|	246374-24781	0.559417	0.971995	0.797025	1	no

gi|320447022|ref|NW_003383549.1|	48334-25022	0.204458	171002	306414	0.2113	no
gi|320447022|ref|NW_003383549.1|	50795-25167	0.260219	197157	292155	0.25645	no
gi|320447022|ref|NW_003383549.1|	52801-25352	174706	492453	-182688	0.40085	no
gi|320447022|ref|NW_003383549.1|	25328-25772	274483	192663	-0.510637	0.8031	no
gi|320447022|ref|NW_003383549.1|	53991-25437	868176	355446	-128836	0.5509	no
gi|320447022|ref|NW_003383549.1|	57601-26097	919274	744593	-0.304043	0.805	no
gi|320447022|ref|NW_003383549.1|	61104-26135	316292	160197	-0.981413	0.66335	no
gi|320447022|ref|NW_003383549.1|	61627-26443	459826	215778	-109154	0.60565	no
gi|320447022|ref|NW_003383549.1|	67991-26839	829631	36028	-120335	0.57905	no
gi|320447022|ref|NW_003383549.1|	68561-26934	956336	377131	-134245	0.3029	no
gi|320447022|ref|NW_003383549.1|	71083-27238	215578	935112	-1205	0.46025	no
gi|320447022|ref|NW_003383549.1|	73412-27541	510052	263757	-0.951432	0.47385	no
gi|320447022|ref|NW_003383549.1|	87073-28740	296016	114947	-136471	0.52835	no
gi|320447022|ref|NW_003383549.1|	87624-28864	10714	133288	0.315054	1	no
gi|320447022|ref|NW_003383549.1|	90194-29215	254536	156987	-0.697229	0.73305	no
gi|320447022|ref|NW_003383549.1|	92309-29452	133889	553479	-127444	0.4361	no
gi|320447022|ref|NW_003383549.1|	95564-29704	308882	753493	-203539	0.24775	no
gi|320447022|ref|NW_003383549.1|	98038-29878	288123	113453	-134459	0.539	no
gi|320447022|ref|NW_003383549.1|	00103-30130	342902	739758	-221267	0.2074	no
gi|320447022|ref|NW_003383549.1|	01440-30186	66894	229775	-154165	0.47925	no
gi|320447022|ref|NW_003383549.1|	15250-31880	661353	391545	-0.756241	0.7366	no
gi|320447022|ref|NW_003383549.1|	18932-31966	674219	108507	0.6865	0.7362	no
gi|320447022|ref|NW_003383549.1|	20155-32094	134395	104839	-0.358305	0.86025	no
gi|320447022|ref|NW_003383549.1|	21421-32201	236964	321029	0.438036	0.8282	no
gi|320447022|ref|NW_003383549.1|	22971-32427	0.632269	120678	0.932557	1	no
gi|320447022|ref|NW_003383549.1|	24380-32473	495841	565388	0.189364	0.9054	no
gi|320447022|ref|NW_003383549.1|	33756-33432	202382	341895	0.756472	0.72125	no
gi|320447022|ref|NW_003383549.1|	48505-34896	130834	338894	-194883	0.38445	no
gi|320447022|ref|NW_003383549.1|	53872-35480	0	956528	inf	0.007	no
gi|320447022|ref|NW_003383549.1|	88486-39227	147622	183375	0.312892	0.8139	no

gi|320447022|ref|NW_003383549.1|	392518-39342	805372	867309	0.10689	0.9592	no
gi|320447022|ref|NW_003383549.1|	393897-39521	104053	700773	-0.570297	0.7873	no
gi|320447022|ref|NW_003383549.1|	395515-39582	118972	164186	0.46471	0.81355	no
gi|320447022|ref|NW_003383549.1|	396371-39676	803201	989692	0.30122	0.87555	no
gi|320447022|ref|NW_003383549.1|	397072-39813	718059	283783	-133932	0.52445	no
gi|320447022|ref|NW_003383549.1|	398943-40047	571755	388631	-0.556997	0.7847	no
gi|320447022|ref|NW_003383549.1|	402144-40315	780326	359478	-111817	0.5916	no
gi|320447022|ref|NW_003383549.1|	403485-40413	764923	109706	-280167	0.26035	no
gi|320447022|ref|NW_003383549.1|	406085-40666	13196	496032	-141159	0.50725	no
gi|320447022|ref|NW_003383549.1|	406840-40900	664932	598259	-0.152438	0.94195	no
gi|320447022|ref|NW_003383549.1|	410087-41201	239685	487329	102375	0.62235	no
gi|320447022|ref|NW_003383549.1|	412645-41427	242169	261247	0.109399	0.9585	no
gi|320447022|ref|NW_003383549.1|	415488-41614	4045	275522	-0.553973	0.79545	no
gi|320447022|ref|NW_003383549.1|	422984-42372	123745	194726	0.654071	0.76015	no
gi|320447022|ref|NW_003383549.1|	423844-42486	115564	902269	-0.357064	0.864	no
gi|320447022|ref|NW_003383549.1|	425090-42819	232761	112206	-105269	0.42505	no
gi|320447022|ref|NW_003383549.1|	430606-43132	484979	217241	-115863	0.4703	no
gi|320447022|ref|NW_003383549.1|	450314-45324	155624	257226	0.724973	0.64735	no
gi|320447022|ref|NW_003383549.1|	453567-45419	297231	433125	0.543199	0.788	no
gi|320447022|ref|NW_003383549.1|	454321-45503	63324	962608	0.604196	0.76795	no
gi|320447022|ref|NW_003383549.1|	466237-46771	102263	104268	0.028017	0.9887	no
gi|320447022|ref|NW_003383549.1|	470036-47104	195836	345811	0.820337	0.68845	no
gi|320447022|ref|NW_003383549.1|	471239-47194	106645	461894	211474	0.3494	no
gi|320447022|ref|NW_003383549.1|	472301-47445	0.176354	399894	450307	0.15415	no
gi|320447022|ref|NW_003383549.1|	493490-49932	221344	228766	0.0475813	0.97155	no
gi|320447022|ref|NW_003383549.1|	525994-52740	190588	693989	-145747	0.27955	no
gi|320447022|ref|NW_003383549.1|	531568-53431	696501	205852	-175851	0.19625	no
gi|320447022|ref|NW_003383549.1|	535300-53552	769056	211289	-186387	0.38735	no
gi|320447022|ref|NW_003383549.1|	535658-53641	300565	738921	-202418	0.34995	no
gi|320447022|ref|NW_003383549.1|	546584-55058	854008	414832	-104172	0.42855	no

gi|320447022|ref|NW_003383549.1|	550703-55167	120941	488402	-130816	0.53715	no
gi|320447022|ref|NW_003383549.1|	551981-55372	118407	754659	-0.649859	0.7757	no
gi|320447022|ref|NW_003383549.1|	554737-55528	0.544159	403362	288997	0.25825	no
gi|320447022|ref|NW_003383549.1|	555849-55725	0.431363	169836	197717	0.39	no
gi|320447022|ref|NW_003383549.1|	561320-56174	765654	450355	-0.765631	0.70805	no
gi|320447022|ref|NW_003383549.1|	568057-56930	316332	161673	-0.968359	0.6431	no
gi|320447022|ref|NW_003383549.1|	569430-57046	78486	454559	-0.787968	0.70125	no
gi|320447022|ref|NW_003383549.1|	570944-57233	400536	0.202367	-762882	0.16165	no
gi|320447022|ref|NW_003383549.1|	573442-57382	792815	0	#NAME?	0.0035	no
gi|320447022|ref|NW_003383549.1|	575472-57793	333284	41261	362996	0.0721	no
gi|320447022|ref|NW_003383549.1|	582429-58314	0.709484	315391	21523	0.29245	no
gi|320447022|ref|NW_003383549.1|	596714-59694	404292	636772	397731	0.1964	no
gi|320447022|ref|NW_003383549.1|	534002-63493	267278	552996	104893	0.59785	no
gi|320447022|ref|NW_003383549.1|	538969-64046	720751	412655	-0.804565	0.5497	no
gi|320447022|ref|NW_003383549.1|	540814-64107	241161	85144	-150202	0.49855	no
gi|320447022|ref|NW_003383549.1|	543242-64422	35573	133714	-141163	0.3935	no
gi|320447022|ref|NW_003383549.1|	544328-64891	245593	626668	-19705	0.1446	no
gi|320447022|ref|NW_003383549.1|	64538-66826	0	258216	inf	0.00885	no
gi|320447022|ref|NW_003383549.1|	549561-65034	153555	147557	-0.0574816	0.9269	no
gi|320447022|ref|NW_003383549.1|	550804-65364	175361	128.18	-0.452152	0.71005	no
gi|320447022|ref|NW_003383549.1|	556883-66000	187112	323019	0.78772	0.7063	no
gi|320447022|ref|NW_003383549.1|	560322-66215	433588	552658	0.350062	0.8704	no
gi|320447022|ref|NW_003383549.1|	569083-66949	535429	471462	-0.183554	0.9175	no
gi|320447022|ref|NW_003383549.1|	700022-70031	372935	179605	-10541	0.53115	no
gi|320447022|ref|NW_003383549.1|	703539-70579	357714	208351	-0.779792	0.5578	no
gi|320447022|ref|NW_003383549.1|	706061-70732	406023	396846	-0.0329817	0.9804	no
gi|320447022|ref|NW_003383549.1|	721015-72666	246077	190112	294967	0.1351	no
gi|320447022|ref|NW_003383549.1|	726788-72762	0	291801	inf	0.02205	no
gi|320447022|ref|NW_003383549.1|	727738-73306	451022	213993	224629	0.0953	no
gi|320447022|ref|NW_003383549.1|	734127-73661	666068	386789	-0.784124	0.72195	no

gi|320447022|ref|NW_003383549.1|	736743-73762	55775	403846	-0.465811	0.70575	no
gi|320447022|ref|NW_003383549.1|	741585-74212	189774	116485	-0.704145	0.73	no
gi|320447022|ref|NW_003383549.1|	743545-74607	40332	609174	0.59493	0.65865	no
gi|320447022|ref|NW_003383549.1|	758317-75921	638725	103863	0.701412	0.7333	no
gi|320447022|ref|NW_003383549.1|	762603-76376	18193	214275	0.236081	0.90125	no
gi|320447022|ref|NW_003383549.1|	790043-79187	463012	133772	153065	0.2587	no
gi|320447022|ref|NW_003383549.1|	791926-79413	241711	655564	143945	0.2863	no
gi|320447022|ref|NW_003383549.1|	795327-79680	0.818103	104268	0.349945	1	no
gi|320447022|ref|NW_003383549.1|	797383-79922	725152	131818	0.862195	0.59395	no
gi|320447022|ref|NW_003383549.1|	802702-81238	168208	406563	127324	0.334	no
gi|320447022|ref|NW_003383549.1|	813388-81613	470263	191074	202259	0.25625	no
gi|320447022|ref|NW_003383549.1|	834257-83513	234385	634936	143773	0.27135	no
gi|320447022|ref|NW_003383549.1|	86290-86582	0	156538	inf	0.029	no
gi|320447022|ref|NW_003383549.1|	874591-87594	349057	109395	164801	0.2227	no
gi|320447023|ref|NW_003383548.1|	004784-10055	194406	10491	-0.889924	0.67815	no
gi|320447023|ref|NW_003383548.1|	010929-10123	167507	103336	-0.696877	0.75495	no
gi|320447023|ref|NW_003383548.1|	013185-10146	224607	727207	-162696	0.33445	no
gi|320447023|ref|NW_003383548.1|	020712-10219	218451	802526	-144469	0.37815	no
gi|320447023|ref|NW_003383548.1|	022359-10231	546941	132355	-204698	0.3508	no
gi|320447023|ref|NW_003383548.1|	023464-10250	587784	177776	-172523	0.42265	no
gi|320447023|ref|NW_003383548.1|	026798-10275	611004	667236	-319491	0.09935	no
gi|320447023|ref|NW_003383548.1|	077874-10797	306142	11856	-136858	0.4395	no
gi|320447023|ref|NW_003383548.1|	100246-11004	457713	120286	-192797	0.3906	no
gi|320447023|ref|NW_003383548.1|	100705-11013	388913	323047	-0.267704	0.89075	no
gi|320447023|ref|NW_003383548.1|	102570-11101	111824	132025	0.239592	0.86095	no
gi|320447023|ref|NW_003383548.1|	113220-11170	368965	625591	-256019	0.0633	no
gi|320447023|ref|NW_003383548.1|	169942-11703	0.95742	567383	25671	0.27405	no
gi|320447023|ref|NW_003383548.1|	171424-11718	114984	122656	0.0931812	0.93665	no
gi|320447023|ref|NW_003383548.1|	171981-11731	0.690492	203455	155901	0.4963	no
gi|320447023|ref|NW_003383548.1|	173347-11750	358075	314056	-0.189238	0.888	no

gi|320447023|ref|NW_003383548.1|	189365-11900	154812	732757	224282	0.20185	no
gi|320447023|ref|NW_003383548.1|	190173-11913	0	147335	inf	0.029	no
gi|320447023|ref|NW_003383548.1|	192252-11932	125073	590953	224027	0.3134	no
gi|320447023|ref|NW_003383548.1|	193907-11950	609364	180083	156328	0.4794	no
gi|320447023|ref|NW_003383548.1|	209250-12110	724904	0.678376	-341763	0.16715	no
gi|320447023|ref|NW_003383548.1|	122946-12312	340858	209.73	-0.700638	0.7282	no
gi|320447023|ref|NW_003383548.1|	233912-12342	198177	0	#NAME?	0.01755	no
gi|320447023|ref|NW_003383548.1|	237123-12375	232918	499764	-222051	0.326	no
gi|320447023|ref|NW_003383548.1|	388662-13892	913304	713137	-0.356917	0.77155	no
gi|320447023|ref|NW_003383548.1|	472992-14762	0.337726	165342	229153	0.313	no
gi|320447023|ref|NW_003383548.1|	491208-14930	102.11	175265	0.77941	0.55105	no
gi|320447023|ref|NW_003383548.1|	494004-14959	203442	282973	0.476049	0.71205	no
gi|320447023|ref|NW_003383548.1|	500329-15008	870408	797518	-0.126175	0.9186	no
gi|320447023|ref|NW_003383548.1|	501511-15018	138.49	138787	0.00308891	0.9957	no
gi|320447023|ref|NW_003383548.1|	503304-15043	408398	368228	-0.149376	0.9044	no
gi|320447023|ref|NW_003383548.1|	505891-15076	0.110389	789673	948251	0.14075	no
gi|320447023|ref|NW_003383548.1|	508196-15083	109566	92544	-0.243584	0.8983	no
gi|320447023|ref|NW_003383548.1|	509903-15101	568357	355132	-0.678442	0.7278	no
gi|320447023|ref|NW_003383548.1|	514528-15150	217682	24.8	0.188114	0.9254	no
gi|320447023|ref|NW_003383548.1|	527592-15281	139864	123237	-0.182591	0.9244	no
gi|320447023|ref|NW_003383548.1|	528474-15292	105568	242323	119876	0.58145	no
gi|320447023|ref|NW_003383548.1|	541222-15419	245201	661811	143246	0.40105	no
gi|320447023|ref|NW_003383548.1|	544400-15449	189571	192618	0.0229989	0.9157	no
gi|320447023|ref|NW_003383548.1|	548883-15495	263737	200628	-0.394579	0.8536	no
gi|320447023|ref|NW_003383548.1|	549916-15501	126935	511621	-131094	0.5394	no
gi|320447023|ref|NW_003383548.1|	551353-15515	809182	507671	-0.67257	0.7343	no
gi|320447023|ref|NW_003383548.1|	552911-15531	143819	692287	-105481	0.61125	no
gi|320447023|ref|NW_003383548.1|	554414-15546	738666	620315	-0.25192	0.89905	no
gi|320447023|ref|NW_003383548.1|	555283-15555	107558	552299	-0.961596	0.6418	no
gi|320447023|ref|NW_003383548.1|	556434-15585	167624	424724	13413	0.523	no

gi|320447023|ref|NW_003383548.1|	58716-15590	598823	284435	-107403	0.60665	no
gi|320447023|ref|NW_003383548.1|	60815-15614	152394	754014	-101514	0.6193	no
gi|320447023|ref|NW_003383548.1|	62175-15628	108868	424286	-135947	0.50945	no
gi|320447023|ref|NW_003383548.1|	63882-15642	111683	494293	-117597	0.59365	no
gi|320447023|ref|NW_003383548.1|	65212-15670	31.21	115199	-143788	0.41715	no
gi|320447023|ref|NW_003383548.1|	67814-15680	440857	916484	-226613	0.30775	no
gi|320447023|ref|NW_003383548.1|	17836-16185	992258	231191	-210163	0.2432	no
gi|320447023|ref|NW_003383548.1|	32880-16334	323159	351818	0.122583	0.95335	no
gi|320447023|ref|NW_003383548.1|	49184-16497	111274	48698	212974	0.29365	no
gi|320447023|ref|NW_003383548.1|	54219-16556	0.422717	156676	521195	0.1449	no
gi|320447023|ref|NW_003383548.1|	58320-16595	0.523223	169261	169375	0.4291	no
gi|320447023|ref|NW_003383548.1|	60764-16615	112974	271901	126709	0.55445	no
gi|320447023|ref|NW_003383548.1|	61802-16629	0.542864	213142	197315	0.39005	no
gi|320447023|ref|NW_003383548.1|	34529-23472	327013	711728	112198	0.58955	no
gi|320447023|ref|NW_003383548.1|	76721-27832	0.371247	0.774994	10618	1	no
gi|320447023|ref|NW_003383548.1|	90579-29504	215064	33501	0.639439	0.6256	no
gi|320447023|ref|NW_003383548.1|	07082-30862	0.38804	0.719741	0.891273	1	no
gi|320447023|ref|NW_003383548.1|	08744-31066	0.799898	313651	197127	0.3617	no
gi|320447023|ref|NW_003383548.1|	10882-31333	530572	29291	-0.85709	0.5283	no
gi|320447023|ref|NW_003383548.1|	51759-35196	184634	558825	-17242	0.424	no
gi|320447023|ref|NW_003383548.1|	52891-35525	134695	259998	0.948804	0.6353	no
gi|320447023|ref|NW_003383548.1|	55395-35600	339513	215003	-0.659111	0.7586	no
gi|320447023|ref|NW_003383548.1|	63913-36574	137135	15436	0.170701	0.9259	no
gi|320447023|ref|NW_003383548.1|	78846-37921	825402	692848	-0.252557	0.8898	no
gi|320447023|ref|NW_003383548.1|	82052-38657	479753	144851	159421	0.37905	no
gi|320447023|ref|NW_003383548.1|	00995-40467	421129	325881	295201	0.1397	no
gi|320447023|ref|NW_003383548.1|	18630-41957	0	377416	inf	0.01485	no
gi|320447023|ref|NW_003383548.1|	20082-42059	241033	566211	123211	0.5597	no
gi|320447023|ref|NW_003383548.1|	22368-42352	164625	316796	0.944371	0.6498	no
gi|320447023|ref|NW_003383548.1|	30229-43349	0.278465	610005	777518	0.0855	no

gi|320447023|ref|NW_003383548.1|	433632-43560	0	128721	inf	1	no
gi|320447023|ref|NW_003383548.1|	438779-44072	0.790065	537029	276496	0.2421	no
gi|320447023|ref|NW_003383548.1|	448319-44861	0	190544	inf	0.02205	no
gi|320447023|ref|NW_003383548.1|	459286-46026	0.912763	148096	402015	0.1596	no
gi|320447023|ref|NW_003383548.1|	460769-46153	19158	107293	248553	0.27745	no
gi|320447023|ref|NW_003383548.1|	46102-46863	0	312659	inf	0.0037	no
gi|320447023|ref|NW_003383548.1|	463809-46673	0.501312	151568	491811	0.0602	no
gi|320447023|ref|NW_003383548.1|	476413-47897	286465	486335	-255834	0.17135	no
gi|320447023|ref|NW_003383548.1|	480012-48027	794338	108026	-287838	0.23625	no
gi|320447023|ref|NW_003383548.1|	480450-48161	867652	463407	-0.904837	0.66445	no
gi|320447023|ref|NW_003383548.1|	484494-48525	637396	437123	-0.544149	0.79615	no
gi|320447023|ref|NW_003383548.1|	485374-48581	553797	420082	-0.398689	0.84605	no
gi|320447023|ref|NW_003383548.1|	485949-48719	51868	406162	-0.352788	0.86345	no
gi|320447023|ref|NW_003383548.1|	488681-49101	627143	443435	-0.500071	0.81755	no
gi|320447023|ref|NW_003383548.1|	492258-49873	597686	173643	153867	0.25745	no
gi|320447023|ref|NW_003383548.1|	498890-50256	373135	426022	0.19123	0.9319	no
gi|320447023|ref|NW_003383548.1|	504705-50650	237116	225266	-0.0739661	0.9691	no
gi|320447023|ref|NW_003383548.1|	511638-51322	137029	710754	-0.947055	0.67175	no
gi|320447023|ref|NW_003383548.1|	513515-51424	216251	845869	-13542	0.5214	no
gi|320447023|ref|NW_003383548.1|	519963-52055	334516	582495	-252176	0.2702	no
gi|320447023|ref|NW_003383548.1|	525807-53053	135667	178983	-292217	0.12685	no
gi|320447023|ref|NW_003383548.1|	531594-53378	128891	694171	-0.892792	0.58945	no
gi|320447023|ref|NW_003383548.1|	536322-53789	0	405169	inf	5.00E-05	yes
gi|320447023|ref|NW_003383548.1|	539525-54091	0.876167	730391	305939	0.20295	no
gi|320447023|ref|NW_003383548.1|	541862-54286	287698	366898	0.350823	0.85925	no
gi|320447023|ref|NW_003383548.1|	559654-56089	282484	135641	-105837	0.52285	no
gi|320447023|ref|NW_003383548.1|	561028-56494	161953	126203	-0.359827	0.7802	no
gi|320447023|ref|NW_003383548.1|	573294-57434	187378	820571	213068	0.3364	no
gi|320447023|ref|NW_003383548.1|	574399-57783	0.684896	427543	264211	0.253	no
gi|320447023|ref|NW_003383548.1|	578737-57934	0.910172	246975	144015	0.37565	no

gi|320447023|ref|NW_003383548.1|	80037-58063	234379	158827	-0.561388	0.8006	no
gi|320447023|ref|NW_003383548.1|	76875-67777	0.759569	122138	0.685264	1	no
gi|320447023|ref|NW_003383548.1|	10556-81288	265963	230684	-0.205312	0.8732	no
gi|320447023|ref|NW_003383548.1|	13004-81404	948596	48091	-0.980027	0.6369	no
gi|320447023|ref|NW_003383548.1|	15239-81607	217112	930706	-122204	0.58255	no
gi|320447023|ref|NW_003383548.1|	37012-83757	0	151288	inf	0.00845	no
gi|320447023|ref|NW_003383548.1|	43581-84595	616854	235771	193438	0.28365	no
gi|320447023|ref|NW_003383548.1|	46051-84753	0.134933	300204	447563	0.1818	no
gi|320447023|ref|NW_003383548.1|	47690-84920	0.131668	485303	52039	0.16715	no
gi|320447023|ref|NW_003383548.1|	51775-85233	819133	414957	-0.981134	0.651	no
gi|320447023|ref|NW_003383548.1|	53969-85493	231003	207294	-0.156232	0.9298	no
gi|320447023|ref|NW_003383548.1|	68325-86985	0	126628	inf	1	no
gi|320447023|ref|NW_003383548.1|	74425-87522	17937	225852	0.332434	0.8637	no
gi|320447023|ref|NW_003383548.1|	75766-87619	584543	608307	0.0574918	0.9487	no
gi|320447023|ref|NW_003383548.1|	78071-88095	413771	970026	122919	0.44915	no
gi|320447023|ref|NW_003383548.1|	88061-88959	0.25929	195675	623775	0.1182	no
gi|320447023|ref|NW_003383548.1|	96043-89972	524115	603277	0.202938	0.89755	no
gi|320447023|ref|NW_003383548.1|	01582-90222	107462	506384	-108553	0.5943	no
gi|320447023|ref|NW_003383548.1|	03518-90444	264848	904903	-154933	0.4884	no
gi|320447023|ref|NW_003383548.1|	65088-96583	524748	539428	0.0398078	0.9737	no
gi|320447023|ref|NW_003383548.1|	66046-96653	666677	935827	0.489255	0.8071	no
gi|320447023|ref|NW_003383548.1|	66927-96905	509263	19946	-135231	0.52845	no
gi|320447023|ref|NW_003383548.1|	69871-97129	371173	919032	-201391	0.2481	no
gi|320447023|ref|NW_003383548.1|	71406-97175	308502	435646	-282405	0.248	no
gi|320447023|ref|NW_003383548.1|	74557-97630	671563	202679	-172833	0.4314	no
gi|320447023|ref|NW_003383548.1|	85419-98637	0.234535	226594	327223	0.23045	no
gi|320447023|ref|NW_003383548.1|	87008-98835	242024	0.945359	-135621	0.50885	no
gi|320447023|ref|NW_003383548.1|	91088-99217	856159	358195	-125713	0.5528	no
gi|320447023|ref|NW_003383548.1|	94804-99735	691046	155993	117462	0.49495	no
gi|320447023|ref|NW_003383548.1|	99728-100414	0	306683	inf	0.029	no

gi|320447023|ref|NW_003383548.1|	97571-99853	599786	541425	-0.147686	0.94065	no
gi|320447023|ref|NW_003383548.1|	98683-99894	258325	145689	-0.826298	0.6903	no
gi|320447023|ref|NW_003383548.1|	99342-99983	236803	162837	-0.540265	0.79345	no
gi|320447025|ref|NW_003383546.1|	43517-10440	678456	295198	-120057	0.5587	no
gi|320447025|ref|NW_003383546.1|	44207-10456	159938	181819	0.184989	0.92285	no
gi|320447025|ref|NW_003383546.1|	46783-10470	120024	897059	-0.420051	0.82045	no
gi|320447025|ref|NW_003383546.1|	48396-10575	448719	782794	0.802819	0.5506	no
gi|320447025|ref|NW_003383546.1|	57648-10585	336776	481203	0.514856	0.79505	no
gi|320447025|ref|NW_003383546.1|	63851-10650	0.359212	323585	317124	0.20465	no
gi|320447025|ref|NW_003383546.1|	65102-10680	0.694984	216408	16387	0.4361	no
gi|320447025|ref|NW_003383546.1|	04852-11052	180341	827717	219841	0.3243	no
gi|320447025|ref|NW_003383546.1|	16319-11183	0.476497	0.863928	0.858442	1	no
gi|320447025|ref|NW_003383546.1|	33282-12347	236021	911434	-13727	0.4146	no
gi|320447025|ref|NW_003383546.1|	36744-12378	148075	460832	163791	0.34495	no
gi|320447025|ref|NW_003383546.1|	39602-12415	162402	986264	-0.719521	0.6595	no
gi|320447025|ref|NW_003383546.1|	46553-12487	112342	112256	-0.00111288	0.9955	no
gi|320447025|ref|NW_003383546.1|	55973-12562	0	158165	inf	0.0233	no
gi|320447025|ref|NW_003383546.1|	60149-12611	0	173535	inf	0.0294	no
gi|320447025|ref|NW_003383546.1|	65438-12659	164279	774729	223755	0.32725	no
gi|320447025|ref|NW_003383546.1|	30411-13623	394336	774176	0.973238	0.44115	no
gi|320447025|ref|NW_003383546.1|	19129-13195	0	877059	inf	0.0198	no
gi|320447025|ref|NW_003383546.1|	37153-13888	0.678313	125968	0.89304	1	no
gi|320447025|ref|NW_003383546.1|	46297-14795	110235	371308	-15699	0.47245	no
gi|320447025|ref|NW_003383546.1|	48074-14825	939236	579774	-0.695997	0.7327	no
gi|320447025|ref|NW_003383546.1|	48889-14998	401535	223676	-0.844116	0.61545	no
gi|320447025|ref|NW_003383546.1|	53846-15763	223031	474915	109042	0.6233	no
gi|320447025|ref|NW_003383546.1|	60416-16135	794963	432028	-0.879762	0.67055	no
gi|320447025|ref|NW_003383546.1|	61622-16219	144189	5043	-15156	0.4714	no
gi|320447025|ref|NW_003383546.1|	66474-16806	0	334086	inf	5.00E-05	yes
gi|320447025|ref|NW_003383546.1|	68694-16969	0	336774	inf	5.00E-05	yes

gi|320447025|ref|NW_003383546.1|	173556-17601	12.9	270266	-225492	0.1866	no
gi|320447025|ref|NW_003383546.1|	180853-18546	709667	702285	-0.0150845	0.9897	no
gi|320447025|ref|NW_003383546.1|	266059-26730	0	492428	inf	5.00E-05	yes
gi|320447025|ref|NW_003383546.1|	379927-38109	0.541702	125113	120766	1	no
gi|320447025|ref|NW_003383546.1|	418241-42474	274447	633702	120728	0.4821	no
gi|320447025|ref|NW_003383546.1|	476467-48036	187134	446821	125562	0.3508	no
gi|320447025|ref|NW_003383546.1|	482373-48354	0.543447	12551	12076	1	no
gi|320447025|ref|NW_003383546.1|	484294-48538	117933	0.952194	-0.30864	1	no
gi|320447025|ref|NW_003383546.1|	486543-48758	0.835885	288973	178956	0.42635	no
gi|320447025|ref|NW_003383546.1|	490380-49071	116699	122797	0.0734716	0.9526	no
gi|320447025|ref|NW_003383546.1|	493065-49326	160322	175882	0.133632	0.94675	no
gi|320447025|ref|NW_003383546.1|	493708-49593	212655	244198	0.199535	0.87355	no
gi|320447025|ref|NW_003383546.1|	518713-51937	431637	107017	-201197	0.3624	no
gi|320447025|ref|NW_003383546.1|	570679-57090	443197	195115	-118362	0.57115	no
gi|320447025|ref|NW_003383546.1|	591331-59173	476244	50185	0.0755555	0.93205	no
gi|320447025|ref|NW_003383546.1|	610425-61109	276787	133763	-10491	0.62485	no
gi|320447025|ref|NW_003383546.1|	612891-61441	367238	195198	-0.911777	0.48165	no
gi|320447025|ref|NW_003383546.1|	616007-61650	118574	962027	-0.301636	0.88195	no
gi|320447025|ref|NW_003383546.1|	667993-66902	23363	0	#NAME?	0.02105	no
gi|320447025|ref|NW_003383546.1|	689775-68998	0	692334	inf	0.0162	no
gi|320447025|ref|NW_003383546.1|	716082-71661	15217	265489	-251896	0.2722	no
gi|320447025|ref|NW_003383546.1|	718870-72212	184396	50806	14622	0.50255	no
gi|320447025|ref|NW_003383546.1|	800371-80100	0.422516	287371	276584	0.2657	no
gi|320447025|ref|NW_003383546.1|	801652-80265	109646	49997	-113294	0.5873	no
gi|320447025|ref|NW_003383546.1|	803477-80393	169949	132794	-0.355914	0.86035	no
gi|320447025|ref|NW_003383546.1|	804651-80772	174756	234413	0.423712	0.74555	no
gi|320447025|ref|NW_003383546.1|	846295-84737	0	360711	inf	0.0142	no
gi|320447025|ref|NW_003383546.1|	848075-85016	0.182063	457202	465032	0.15405	no
gi|320447025|ref|NW_003383546.1|	850252-85193	205584	398486	-236713	0.185	no
gi|320447025|ref|NW_003383546.1|	869015-86950	350686	0	#NAME?	0.0046	no

gi|320447025|ref|NW_003383546.1|	380588-88098	488812	385902	-0.341044	0.8622	no
gi|320447025|ref|NW_003383546.1|	381269-88247	13967	169434	0.278694	0.88505	no
gi|320447025|ref|NW_003383546.1|	383668-88500	199688	373264	0.902451	0.657	no
gi|320447025|ref|NW_003383546.1|	385594-88620	226342	522149	120596	0.573	no
gi|320447025|ref|NW_003383546.1|	389601-89157	388662	955243	129735	0.56335	no
gi|320447025|ref|NW_003383546.1|	900030-90125	0.170875	14218	30567	0.2493	no
gi|320447025|ref|NW_003383546.1|	916748-91698	15042	403317	142292	0.5139	no
gi|320447025|ref|NW_003383546.1|	917230-91774	119691	602663	233204	0.2622	no
gi|320447025|ref|NW_003383546.1|	921671-92218	0.590353	713794	359586	0.207	no
gi|320447025|ref|NW_003383546.1|	922321-92270	434088	163505	191328	0.39635	no
gi|320447025|ref|NW_003383546.1|	930509-93075	0	241682	inf	0.00435	no
gi|320447025|ref|NW_003383546.1|	930922-93118	0	43796	inf	0.0142	no
gi|320447025|ref|NW_003383546.1|	931976-93266	0	543902	inf	5.00E-05	yes
gi|320447025|ref|NW_003383546.1|	933383-93366	443883	396889	316049	0.2082	no
gi|320447025|ref|NW_003383546.1|	934052-93489	0	674789	inf	5.00E-05	yes
gi|320447025|ref|NW_003383546.1|	947374-94814	12.7	500848	-134239	0.52665	no
gi|320447025|ref|NW_003383546.1|	955314-95597	749005	131683	0.814025	0.6869	no
gi|320447026|ref|NW_003383545.1|	101631-10332	266082	20943	-0.345406	0.86385	no
gi|320447026|ref|NW_003383545.1|	025382-10258	163985	706417	210696	0.29675	no
gi|320447026|ref|NW_003383545.1|	028369-10306	0.407255	352521	31137	0.2085	no
gi|320447026|ref|NW_003383545.1|	103580-10518	86371	489407	-0.819512	0.7035	no
gi|320447026|ref|NW_003383545.1|	215139-12155	230172	601677	138628	0.38165	no
gi|320447026|ref|NW_003383545.1|	230740-12311	538053	592094	0.13808	0.9301	no
gi|320447026|ref|NW_003383545.1|	124683-12613	393274	11069	-182901	0.30355	no
gi|320447026|ref|NW_003383545.1|	126690-12759	128356	329577	-196146	0.3777	no
gi|320447026|ref|NW_003383545.1|	127935-12837	104222	319094	-170761	0.4274	no
gi|320447026|ref|NW_003383545.1|	129722-12999	737016	782342	-323583	0.2094	no
gi|320447026|ref|NW_003383545.1|	130573-13316	535268	240596	-115365	0.38275	no
gi|320447026|ref|NW_003383545.1|	134657-13758	230234	149982	-0.618309	0.6297	no
gi|320447026|ref|NW_003383545.1|	145580-14832	154089	59247	-137895	0.41015	no

gi|320447026|ref|NW_003383545.1|	48479-15014	175059	757536	-120846	0.45965	no
gi|320447026|ref|NW_003383545.1|	50726-15266	176305	303822	-253678	0.1564	no
gi|320447026|ref|NW_003383545.1|	52838-15351	133944	250692	-241765	0.1879	no
gi|320447026|ref|NW_003383545.1|	54251-15503	284577	136532	-105958	0.6242	no
gi|320447026|ref|NW_003383545.1|	57340-15856	785228	485295	-0.694251	0.7337	no
gi|320447026|ref|NW_003383545.1|	58939-15975	23588	11807	-0.998413	0.65435	no
gi|320447026|ref|NW_003383545.1|	62040-16275	154792	770087	-100724	0.622	no
gi|320447026|ref|NW_003383545.1|	62887-17382	242205	350336	0.532512	0.6726	no
gi|320447026|ref|NW_003383545.1|	76778-17808	179357	172539	-0.0559046	0.9812	no
gi|320447026|ref|NW_003383545.1|	15936-21654	216716	101101	-110001	0.5933	no
gi|320447026|ref|NW_003383545.1|	16774-21958	426649	486271	0.188711	0.92995	no
gi|320447026|ref|NW_003383545.1|	19651-22517	81018	663785	-0.287527	0.8251	no
gi|320447026|ref|NW_003383545.1|	25952-22647	234552	512186	112676	0.5922	no
gi|320447026|ref|NW_003383545.1|	28326-23274	972646	201781	105281	0.42845	no
gi|320447026|ref|NW_003383545.1|	33092-23357	270762	271312	0.00293015	0.9157	no
gi|320447026|ref|NW_003383545.1|	41834-24581	859464	425857	230886	0.08755	no
gi|320447026|ref|NW_003383545.1|	52423-25287	372541	168561	21778	0.32815	no
gi|320447026|ref|NW_003383545.1|	70407-27100	327237	982142	15856	0.44705	no
gi|320447026|ref|NW_003383545.1|	75220-27564	609765	138204	118047	0.5697	no
gi|320447026|ref|NW_003383545.1|	15317-31582	495727	747778	0.593067	0.75135	no
gi|320447026|ref|NW_003383545.1|	34228-35222	362632	0	#NAME?	5.00E-05	yes
gi|320447026|ref|NW_003383545.1|	21494-42185	0	201924	inf	0.01485	no
gi|320447026|ref|NW_003383545.1|	40923-44142	0.617473	111787	417823	0.192	no
gi|320447026|ref|NW_003383545.1|	96658-69921	0.435324	258476	256987	0.2576	no
gi|320447026|ref|NW_003383545.1|	02645-70375	154543	280823	0.861649	0.6737	no
gi|320447026|ref|NW_003383545.1|	10463-71153	0.803061	0.972284	0.275868	1	no
gi|320447026|ref|NW_003383545.1|	15660-71757	106682	0.491079	-444122	0.13245	no
gi|320447026|ref|NW_003383545.1|	21204-72167	151825	184259	-304261	0.22805	no
gi|320447026|ref|NW_003383545.1|	82434-82732	192978	259907	375148	0.20315	no
gi|320447026|ref|NW_003383545.1|	43293-84749	680752	110811	0.702906	0.5858	no

gi|320447026|ref|NW_003383545.1|	379609-88093	17005	171717	0.0140694	0.96375	no
gi|320447026|ref|NW_003383545.1|	381484-88219	634556	113335	0.836774	0.6821	no
gi|320447026|ref|NW_003383545.1|	382559-89176	360951	698839	0.953155	0.4609	no
gi|320447026|ref|NW_003383545.1|	392177-89259	166302	809898	-1038	0.60595	no
gi|320447026|ref|NW_003383545.1|	398039-89961	730061	481657	-0.60001	0.78165	no
gi|320447026|ref|NW_003383545.1|	399754-90044	997383	252337	-198279	0.37545	no
gi|320447026|ref|NW_003383545.1|	902027-90388	184994	586656	-165689	0.32835	no
gi|320447026|ref|NW_003383545.1|	904406-90529	290906	620746	-222848	0.3313	no
gi|320447026|ref|NW_003383545.1|	908543-91230	244211	170573	280419	0.1378	no
gi|320447026|ref|NW_003383545.1|	91610-93120	688437	239368	-152409	0.47545	no
gi|320447026|ref|NW_003383545.1|	921200-92169	569777	139942	129636	0.5308	no
gi|320447026|ref|NW_003383545.1|	925911-92772	171028	700065	-128867	0.43575	no
gi|320447026|ref|NW_003383545.1|	928351-92948	30646	101555	-159344	0.34325	no
gi|320447026|ref|NW_003383545.1|	931171-93139	744101	409116	-0.862991	0.67085	no
gi|320447026|ref|NW_003383545.1|	932582-93327	25802	133457	-0.951102	0.6577	no
gi|320447026|ref|NW_003383545.1|	934324-93629	362709	190347	-0.930184	0.4791	no
gi|320447026|ref|NW_003383545.1|	940002-94325	139366	939507	-0.5689	0.6502	no
gi|320447026|ref|NW_003383545.1|	94187-94462	481178	107321	-216464	0.3398	no
gi|320447026|ref|NW_003383545.1|	96577-96835	697031	257908	-143437	0.49155	no
gi|320447026|ref|NW_003383545.1|	98472-99182	152858	364653	-20676	0.3439	no
gi|320447026|ref|NW_003383545.1|	99612-101328	232496	833312	-148028	0.38225	no
gi|320447026|ref|NW_003383545.1|	997130-99867	0	287678	inf	0.01035	no
gi|320447028|ref|NW_003383543.1|	085581-10872	148661	366297	130098	0.52695	no
gi|320447028|ref|NW_003383543.1|	088097-10886	0	501514	inf	0.02205	no
gi|320447028|ref|NW_003383543.1|	109618-11103	0.702144	240168	17742	0.333	no
gi|320447028|ref|NW_003383543.1|	110906-11160	122661	236129	0.944898	0.4871	no
gi|320447028|ref|NW_003383543.1|	116712-11169	298082	791958	140971	0.4973	no
gi|320447028|ref|NW_003383543.1|	119029-11199	292226	378395	0.372806	0.8185	no
gi|320447028|ref|NW_003383543.1|	130701-11310	526147	113988	111535	0.605	no
gi|320447028|ref|NW_003383543.1|	139992-11413	255092	625251	129342	0.53475	no

gi|320447028|ref|NW_003383543.1|	142378-11445	790307	201045	134704	0.43215	no
gi|320447028|ref|NW_003383543.1|	166632-11682	0.830896	380089	21936	0.3188	no
gi|320447028|ref|NW_003383543.1|	172972-11750	26605	434936	0.709104	0.5999	no
gi|320447028|ref|NW_003383543.1|	181046-11826	134295	13032	-0.043356	0.98515	no
gi|320447028|ref|NW_003383543.1|	193306-11940	367283	157426	-122222	0.583	no
gi|320447028|ref|NW_003383543.1|	200659-12008	411821	115043	-183984	0.41505	no
gi|320447028|ref|NW_003383543.1|	203647-12095	903503	146566	0.697945	0.60025	no
gi|320447028|ref|NW_003383543.1|	214954-12163	940184	791886	-0.247651	0.907	no
gi|320447028|ref|NW_003383543.1|	219058-12199	940845	0.332796	-482125	0.1939	no
gi|320447028|ref|NW_003383543.1|	220512-12211	348339	443763	0.349297	0.8547	no
gi|320447028|ref|NW_003383543.1|	221823-12223	580515	156082	-189503	0.4022	no
gi|320447028|ref|NW_003383543.1|	223088-12240	62873	449717	-0.483422	0.8079	no
gi|320447028|ref|NW_003383543.1|	224684-12261	383034	514362	0.425313	0.83805	no
gi|320447028|ref|NW_003383543.1|	227263-12295	332664	674025	101874	0.63955	no
gi|320447028|ref|NW_003383543.1|	211111-21161	135844	910858	-0.576654	0.78015	no
gi|320447028|ref|NW_003383543.1|	216047-21721	101552	753861	-0.429855	0.8351	no
gi|320447028|ref|NW_003383543.1|	222609-22488	974292	18039	0.888691	0.59985	no
gi|320447028|ref|NW_003383543.1|	261324-26209	46902	652084	-284652	0.12055	no
gi|320447028|ref|NW_003383543.1|	262245-26246	639668	133645	-225891	0.33965	no
gi|320447028|ref|NW_003383543.1|	266475-26748	396024	721693	-245613	0.176	no
gi|320447028|ref|NW_003383543.1|	306032-30683	298422	163972	-0.863906	0.6829	no
gi|320447028|ref|NW_003383543.1|	307020-30857	12494	159639	-296835	0.2126	no
gi|320447028|ref|NW_003383543.1|	311101-31203	240204	267741	-316535	0.1886	no
gi|320447028|ref|NW_003383543.1|	312742-31304	252108	462992	-244498	0.303	no
gi|320447028|ref|NW_003383543.1|	331394-33259	370127	228735	-0.694339	0.57745	no
gi|320447028|ref|NW_003383543.1|	335711-33848	319414	320883	0.00662128	0.9919	no
gi|320447028|ref|NW_003383543.1|	338627-33908	155817	316026	10202	0.62915	no
gi|320447028|ref|NW_003383543.1|	345618-34726	513087	141852	-185481	0.3086	no
gi|320447028|ref|NW_003383543.1|	361611-36331	255426	149814	-0.769736	0.5472	no
gi|320447028|ref|NW_003383543.1|	363488-36994	363622	625778	0.783213	0.53165	no

gi|320447028|ref|NW_003383543.1|	373311-373650	167743	543963	-162468	0.4424	no
gi|320447028|ref|NW_003383543.1|	375084-375317	121288	229238	-240351	0.2777	no
gi|320447028|ref|NW_003383543.1|	375883-376324	794633	474592	-0.743602	0.7078	no
gi|320447028|ref|NW_003383543.1|	378149-378843	283208	105537	-142412	0.51965	no
gi|320447028|ref|NW_003383543.1|	383266-384297	0.212124	259978	102593	0.14075	no
gi|320447028|ref|NW_003383543.1|	386279-387514	0	286256	inf	5.00E-05	yes
gi|320447028|ref|NW_003383543.1|	399500-400061	231755	118246	-0.970811	0.6411	no
gi|320447028|ref|NW_003383543.1|	400392-400674	112068	128797	0.200726	0.90825	no
gi|320447028|ref|NW_003383543.1|	402450-403578	202301	191141	-0.0818668	0.9717	no
gi|320447028|ref|NW_003383543.1|	421041-421888	0	168974	inf	0.0048	no
gi|320447028|ref|NW_003383543.1|	582580-583062	0	763107	inf	0.02075	no
gi|320447028|ref|NW_003383543.1|	597105-598227	0	461271	inf	0.0114	no
gi|320447028|ref|NW_003383543.1|	598295-602220	0	656179	inf	5.00E-05	yes
gi|320447028|ref|NW_003383543.1|	663925-664572	0	33446	inf	0.029	no
gi|320447028|ref|NW_003383543.1|	870320-870688	115782	756459	270786	0.2673	no
gi|320447032|ref|NW_003383539.1|	10638-11121	568825	760331	-290329	0.21795	no
gi|320447032|ref|NW_003383539.1|	068154-107427	496541	254025	-0.966942	0.46165	no
gi|320447032|ref|NW_003383539.1|	079581-108001	307555	110115	-148183	0.48905	no
gi|320447032|ref|NW_003383539.1|	085003-108535	567018	419199	-375769	0.17485	no
gi|320447032|ref|NW_003383539.1|	086498-108697	834643	580145	-0.524745	0.81725	no
gi|320447032|ref|NW_003383539.1|	108356-110908	393151	912759	-210678	0.3508	no
gi|320447032|ref|NW_003383539.1|	132687-113447	241275	114174	224248	0.1025	no
gi|320447032|ref|NW_003383539.1|	134850-113597	227901	854746	190709	0.3753	no
gi|320447032|ref|NW_003383539.1|	136633-113704	191124	342311	0.840795	0.6919	no
gi|320447032|ref|NW_003383539.1|	141636-114189	274069	870388	-165481	0.438	no
gi|320447032|ref|NW_003383539.1|	114945-118479	0.920716	350728	192952	0.38555	no
gi|320447032|ref|NW_003383539.1|	1158-1673	339953	160183	-440754	0.17335	no
gi|320447032|ref|NW_003383539.1|	166714-116702	668992	113159	408022	0.15935	no
gi|320447032|ref|NW_003383539.1|	174007-117437	830489	232316	-183787	0.3134	no
gi|320447032|ref|NW_003383539.1|	11971-13466	100476	363285	-146768	0.49805	no

gi|320447032|ref|NW_003383539.1|	!36025-12391(	273227	303844	0.153228	0.9102	no
gi|320447032|ref|NW_003383539.1|	!39580-12435!	149547	217806	0.542445	0.68055	no
gi|320447032|ref|NW_003383539.1|	!43703-12461·	196521	168742	-0.219865	0.8642	no
gi|320447032|ref|NW_003383539.1|	!46477-12478·	442121	210916	-106777	0.4134	no
gi|320447032|ref|NW_003383539.1|	!47907-12495(	165713	87342	-0.923937	0.56085	no
gi|320447032|ref|NW_003383539.1|	l24878-12560	820489	0.468081	-413165	0.2103	no
gi|320447032|ref|NW_003383539.1|	!49700-12518	106408	76682	-0.472648	0.83	no
gi|320447032|ref|NW_003383539.1|	!52059-12568:	365929	329522	-0.151186	0.94355	no
gi|320447032|ref|NW_003383539.1|	!57725-12616	114587	366024	-164643	0.34745	no
gi|320447032|ref|NW_003383539.1|	l26144-12648:	760156	0	#NAME?	0.0053	no
gi|320447032|ref|NW_003383539.1|	!62044-12625:	139107	649763	-109821	0.60085	no
gi|320447032|ref|NW_003383539.1|	!65675-12660(	279175	114144	-129032	0.5413	no
gi|320447032|ref|NW_003383539.1|	!66140-12678(	236914	876235	-143497	0.3923	no
gi|320447032|ref|NW_003383539.1|	!68004-12689(	459187	207417	217538	0.33335	no
gi|320447032|ref|NW_003383539.1|	!69063-12724(	346014	882572	135089	0.4172	no
gi|320447032|ref|NW_003383539.1|	!72675-12744:	254228	515841	10208	0.62925	no
gi|320447032|ref|NW_003383539.1|	!78191-12832:	336778	715072	108629	0.5151	no
gi|320447032|ref|NW_003383539.1|	!83707-12842!	642898	577986	-0.153554	0.93135	no
gi|320447032|ref|NW_003383539.1|	!85512-12857!	239428	375544	0.64939	0.74765	no
gi|320447032|ref|NW_003383539.1|	14587-15209	522509	177505	-155759	0.486	no
gi|320447032|ref|NW_003383539.1|	15929-16567	351438	797028	-214057	0.3387	no
gi|320447032|ref|NW_003383539.1|	l61827-16449!	166196	413688	131565	0.3186	no
gi|320447032|ref|NW_003383539.1|	l66952-17134·	0.243551	346765	383166	0.1472	no
gi|320447032|ref|NW_003383539.1|	17959-19682	327819	199852	-0.713971	0.57905	no
gi|320447032|ref|NW_003383539.1|	20078-20873	123665	115984	-0.092518	0.96445	no
gi|320447032|ref|NW_003383539.1|	!02541-20347!	0	4971	inf	0.00935	no
gi|320447032|ref|NW_003383539.1|	!06749-20746(	0	359528	inf	0.02205	no
gi|320447032|ref|NW_003383539.1|	!08341-20879:	0.757718	130994	41117	0.19295	no
gi|320447032|ref|NW_003383539.1|	!11245-21836(	238417	125.49	239602	0.1855	no
gi|320447032|ref|NW_003383539.1|	!18518-21932!	0.58647	261923	215902	0.29245	no

gi|320447032|ref|NW_003383539.1|	22139-23382	0.502446	0.813121	0.694499	1	no
gi|320447032|ref|NW_003383539.1|	27237-27852	8423	602028	-0.484503	0.81505	no
gi|320447032|ref|NW_003383539.1|	3609-4571	262471	192384	-37701	0.15355	no
gi|320447032|ref|NW_003383539.1|	91456-39274	0	299819	inf	0.0133	no
gi|320447032|ref|NW_003383539.1|	42207-42927	616295	419319	-0.555573	0.651	no
gi|320447032|ref|NW_003383539.1|	26585-42780	0	534777	inf	0.00885	no
gi|320447032|ref|NW_003383539.1|	59230-45960	0	336241	inf	0.00695	no
gi|320447032|ref|NW_003383539.1|	17208-51795	1375.21	2002.37	0.542056	0.79445	no
gi|320447032|ref|NW_003383539.1|	37291-53867	0	163428	inf	0.0198	no
gi|320447032|ref|NW_003383539.1|	76471-57707	948132	113356	0.257697	0.8999	no
gi|320447032|ref|NW_003383539.1|	77830-57950	163646	183918	0.168482	0.88925	no
gi|320447032|ref|NW_003383539.1|	79725-58172	45535	673668	0.565061	0.67085	no
gi|320447032|ref|NW_003383539.1|	86376-58714	604386	534492	314463	0.09745	no
gi|320447032|ref|NW_003383539.1|	87410-58789	132833	710435	241909	0.24925	no
gi|320447032|ref|NW_003383539.1|	88490-58887	323708	438341	375929	0.17835	no
gi|320447032|ref|NW_003383539.1|	89700-59202	249964	223475	316032	0.1049	no
gi|320447032|ref|NW_003383539.1|	92163-59382	0.235905	147803	26474	0.2353	no
gi|320447032|ref|NW_003383539.1|	93974-59530	0.154733	161131	338038	0.2261	no
gi|320447032|ref|NW_003383539.1|	96187-60003	158664	109743	279008	0.1297	no
gi|320447032|ref|NW_003383539.1|	02626-60295	0	117419	inf	0.029	no
gi|320447032|ref|NW_003383539.1|	14690-61533	0.824637	505202	261503	0.23675	no
gi|320447032|ref|NW_003383539.1|	39782-64020	972127	251062	136883	0.5099	no
gi|320447032|ref|NW_003383539.1|	45134-64538	617034	332228	242875	0.26005	no
gi|320447032|ref|NW_003383539.1|	46547-65068	116702	396401	176414	0.426	no
gi|320447032|ref|NW_003383539.1|	50795-65237	0.375377	365649	328405	0.20425	no
gi|320447032|ref|NW_003383539.1|	53154-65414	201288	132843	272239	0.2435	no
gi|320447032|ref|NW_003383539.1|	57110-65764	0	450909	inf	0.029	no
gi|320447032|ref|NW_003383539.1|	59808-66317	134826	144866	342554	0.08275	no
gi|320447032|ref|NW_003383539.1|	64275-67708	301116	369665	361783	0.07285	no
gi|320447032|ref|NW_003383539.1|	67208-67334	151522	110403	286519	0.1252	no

gi|320447032|ref|NW_003383539.1|	89088-69033	21708	180678	305712	0.19945	no
gi|320447032|ref|NW_003383539.1|	00317-70160	0.321251	568456	414527	0.16075	no
gi|320447032|ref|NW_003383539.1|	7516-8155	296323	425971	-279834	0.24355	no
gi|320447032|ref|NW_003383539.1|	53222-75379	104112	569957	-0.869214	0.6793	no
gi|320447032|ref|NW_003383539.1|	67414-77604	464054	545229	0.232569	0.8596	no
gi|320447032|ref|NW_003383539.1|	81397-78212	275161	235428	-0.224995	0.90135	no
gi|320447032|ref|NW_003383539.1|	87773-78898	519464	432156	-0.265472	0.8933	no
gi|320447032|ref|NW_003383539.1|	90238-79352	23039	291299	0.338425	0.7949	no
gi|320447032|ref|NW_003383539.1|	04038-80438	86429	219251	-197893	0.3599	no
gi|320447032|ref|NW_003383539.1|	8275-8536	420633	142413	-156248	0.4787	no
gi|320447032|ref|NW_003383539.1|	33854-83425	270879	222858	-0.281522	0.8878	no
gi|320447032|ref|NW_003383539.1|	41767-84225	133335	579322	-120262	0.57215	no
gi|320447032|ref|NW_003383539.1|	46979-84720	429556	243283	-0.820208	0.6866	no
gi|320447032|ref|NW_003383539.1|	48106-84835	102517	431995	-124677	0.5539	no
gi|320447032|ref|NW_003383539.1|	49197-84965	517237	393464	-0.394594	0.84605	no
gi|320447032|ref|NW_003383539.1|	50362-85068	105229	48995	-110283	0.61045	no
gi|320447032|ref|NW_003383539.1|	50858-85107	619188	458207	-0.43438	0.8271	no
gi|320447032|ref|NW_003383539.1|	52138-85258	370206	193472	-0.936205	0.64955	no
gi|320447032|ref|NW_003383539.1|	52727-85447	737252	459019	-0.683602	0.75125	no
gi|320447032|ref|NW_003383539.1|	55291-85929	224483	22.52	0.00460217	0.99695	no
gi|320447032|ref|NW_003383539.1|	62685-86375	161767	131489	302295	0.2049	no
gi|320447032|ref|NW_003383539.1|	64598-86584	0.334301	255052	293157	0.22645	no
gi|320447032|ref|NW_003383539.1|	65953-87218	0.168845	396441	455334	0.07525	no
gi|320447032|ref|NW_003383539.1|	73262-87390	0.412318	280668	276703	0.2657	no
gi|320447032|ref|NW_003383539.1|	74132-87774	545649	25057	-112276	0.41115	no
gi|320447032|ref|NW_003383539.1|	87510-88119	171116	0.305591	-580723	0.27235	no
gi|320447032|ref|NW_003383539.1|	78107-87851	42497	609226	-280231	0.2339	no
gi|320447032|ref|NW_003383539.1|	84389-88524	183398	33903	-24355	0.27635	no
gi|320447032|ref|NW_003383539.1|	8986-9477	714415	0.869184	-303903	0.257	no
gi|320447032|ref|NW_003383539.1|	99175-90332	293089	428483	0.547899	0.8139	no

gi|320447032|ref|NW_003383539.1|	90255-91549	103617	0.442518	-454939	0.1697	no
gi|320447032|ref|NW_003383539.1|	903452-90413	336852	102228	-172033	0.43835	no
gi|320447032|ref|NW_003383539.1|	94065-95866	258672	126149	228593	0.31725	no
gi|320447032|ref|NW_003383539.1|	96967-98169	279341	895578	168079	0.4277	no
gi|320447032|ref|NW_003383539.1|	972322-97298	0	509468	inf	0.0212	no
gi|320447033|ref|NW_003383538.1|	130344-13123	129709	178693	0.462203	0.82325	no
gi|320447033|ref|NW_003383538.1|	131390-13522	0.328022	242699	28873	0.21525	no
gi|320447033|ref|NW_003383538.1|	153827-15433	0	488559	inf	0.029	no
gi|320447033|ref|NW_003383538.1|	194184-19837	0.0853307	20312	457313	0.15385	no
gi|320447033|ref|NW_003383538.1|	198536-20045	0.300318	216325	284864	0.24445	no
gi|320447033|ref|NW_003383538.1|	204420-20689	0.150883	105374	280402	1	no
gi|320447033|ref|NW_003383538.1|	206995-20774	0.329243	248176	291414	0.25645	no
gi|320447033|ref|NW_003383538.1|	209311-21071	0	271111	inf	0.0133	no
gi|320447033|ref|NW_003383538.1|	211730-21224	0.59236	397846	274767	0.26605	no
gi|320447033|ref|NW_003383538.1|	222228-22297	0	294977	inf	0.02915	no
gi|320447033|ref|NW_003383538.1|	234192-23623	0.279891	104111	189519	1	no
gi|320447033|ref|NW_003383538.1|	243669-24504	118359	0.616576	-0.940818	1	no
gi|320447033|ref|NW_003383538.1|	253363-25755	0.213489	39459	420813	0.14155	no
gi|320447033|ref|NW_003383538.1|	260776-26137	0	848734	inf	0.0133	no
gi|320447033|ref|NW_003383538.1|	277708-27941	0	143333	inf	0.0154	no
gi|320447033|ref|NW_003383538.1|	298362-29916	0	24724	inf	0.029	no
gi|320447033|ref|NW_003383538.1|	299278-30145	0.521163	642463	362381	0.16115	no
gi|320447033|ref|NW_003383538.1|	305633-30680	113636	237464	106329	0.5061	no
gi|320447033|ref|NW_003383538.1|	317550-31772	0	921386	inf	0.0312	no
gi|320447033|ref|NW_003383538.1|	325768-32713	0	207142	inf	0.0162	no
gi|320447033|ref|NW_003383538.1|	359334-36024	0.251304	43302	410693	0.19235	no
gi|320447033|ref|NW_003383538.1|	369489-37143	0.0987581	0.895048	317999	1	no
gi|320447033|ref|NW_003383538.1|	396456-39770	0	323346	inf	0.01275	no
gi|320447033|ref|NW_003383538.1|	416145-41864	0.821045	484816	25619	0.25795	no
gi|320447033|ref|NW_003383538.1|	418899-43204	354146	331668	322732	0.02315	no

gi|320447033|ref|NW_003383538.1|	34084-43459	128311	159847	0.31704	0.8762	no
gi|320447033|ref|NW_003383538.1|	39809-44059	471236	140733	-174349	0.29575	no
gi|320447033|ref|NW_003383538.1|	45330-44564	696688	190531	-187049	0.39255	no
gi|320447033|ref|NW_003383538.1|	46952-44764	508288	157112	-169386	0.30685	no
gi|320447033|ref|NW_003383538.1|	47903-44935	41475	164324	-13357	0.4438	no
gi|320447033|ref|NW_003383538.1|	49944-45052	634841	321438	-0.981856	0.5438	no
gi|320447033|ref|NW_003383538.1|	51811-45279	317748	17082	-0.895407	0.57755	no
gi|320447033|ref|NW_003383538.1|	54243-45852	241535	224317	-0.106691	0.93985	no
gi|320447033|ref|NW_003383538.1|	59355-46046	285578	124224	-120094	0.45665	no
gi|320447033|ref|NW_003383538.1|	70105-47030	731741	260462	-149026	0.50245	no
gi|320447033|ref|NW_003383538.1|	70851-47192	19675	131953	-0.576341	0.7988	no
gi|320447033|ref|NW_003383538.1|	72106-47240	229536	736259	-164043	0.44115	no
gi|320447033|ref|NW_003383538.1|	72607-47437	403184	43164	0.0983881	0.93875	no
gi|320447033|ref|NW_003383538.1|	54205-55466	144193	720558	-100081	0.61475	no
gi|320447033|ref|NW_003383538.1|	22382-62320	692925	337353	-103844	0.6095	no
gi|320447033|ref|NW_003383538.1|	30458-63151	100406	595231	-0.754326	0.7207	no
gi|320447033|ref|NW_003383538.1|	49095-65248	201235	153501	-0.39063	0.76785	no
gi|320447033|ref|NW_003383538.1|	65075-65688	0	169414	inf	0.00785	no
gi|320447033|ref|NW_003383538.1|	52712-65593	756778	608275	-0.315146	0.8449	no
gi|320447033|ref|NW_003383538.1|	57437-65844	196597	875378	-116726	0.5863	no
gi|320447033|ref|NW_003383538.1|	59650-66134	336417	411924	0.292126	0.88375	no
gi|320447033|ref|NW_003383538.1|	82171-68312	236177	0	#NAME?	0.0229	no
gi|320447033|ref|NW_003383538.1|	90241-69112	1571	306552	0.964444	0.6558	no
gi|320447033|ref|NW_003383538.1|	92428-69337	190542	312211	0.712413	0.7238	no
gi|320447033|ref|NW_003383538.1|	93474-69502	243333	0.445442	-244963	0.29445	no
gi|320447033|ref|NW_003383538.1|	95259-69606	147386	32402	113648	0.58145	no
gi|320447033|ref|NW_003383538.1|	96276-69974	140971	1315	-0.100341	1	no
gi|320447033|ref|NW_003383538.1|	03085-70519	161762	163004	0.0110359	0.97895	no
gi|320447033|ref|NW_003383538.1|	05414-70681	0.577118	12029	105958	1	no
gi|320447033|ref|NW_003383538.1|	84974-78551	436698	514923	0.237723	0.90105	no

gi|320447033|ref|NW_003383538.1|	788185-78908[illegible]	229593	281244	0.292741	0.87945	no
gi|320447033|ref|NW_003383538.1|	93646-95799	0.087993	147347	406569	0.1924	no
gi|320447033|ref|NW_003383538.1|	97560-100944	0	584793	inf	0.00365	no
gi|320447034|ref|NW_003383537.1|	[illegible]30294-23059[illegible]	389406	998693	135876	0.38385	no
gi|320447034|ref|NW_003383537.1|	35005-36558	11014	474841	21081	0.2548	no
gi|320447034|ref|NW_003383537.1|	[illegible]50288-35105[illegible]	0	460675	inf	0.01575	no
gi|320447034|ref|NW_003383537.1|	[illegible]51484-35227[illegible]	0	630148	inf	0.00935	no
gi|320447034|ref|NW_003383537.1|	38100-39143	238227	845246	182703	0.319	no
gi|320447034|ref|NW_003383537.1|	[illegible]33196-53371[illegible]	0	480549	inf	0.029	no
gi|320447034|ref|NW_003383537.1|	[illegible]55460-65867[illegible]	0	494554	inf	0.0047	no
gi|320447034|ref|NW_003383537.1|	[illegible]65432-66584[illegible]	267714	141439	240141	0.2952	no
gi|320447034|ref|NW_003383537.1|	[illegible]71640-67222[illegible]	191685	551611	152491	0.4978	no
gi|320447034|ref|NW_003383537.1|	[illegible]79748-68090[illegible]	109988	596853	244002	0.27885	no
gi|320447034|ref|NW_003383537.1|	[illegible]99706-70001[illegible]	535706	33295	263579	0.25845	no
gi|320447034|ref|NW_003383537.1|	[illegible]00706-70093[illegible]	0	356812	inf	0.02915	no
gi|320447034|ref|NW_003383537.1|	[illegible]01686-70193[illegible]	0	26032	inf	0.02915	no
gi|320447034|ref|NW_003383537.1|	[illegible]02984-70326[illegible]	0	407181	inf	0.0109	no
gi|320447034|ref|NW_003383537.1|	[illegible]66114-76866[illegible]	33504	331414	-0.0156974	0.9902	no
gi|320447034|ref|NW_003383537.1|	[illegible]68744-76929[illegible]	409703	686831	0.745379	0.63685	no
gi|320447034|ref|NW_003383537.1|	[illegible]71462-77366[illegible]	376755	669244	0.828906	0.7002	no
gi|320447034|ref|NW_003383537.1|	[illegible]73814-77495[illegible]	0.185736	141509	292957	0.2564	no
gi|320447034|ref|NW_003383537.1|	[illegible]90454-79099[illegible]	164279	143878	313063	0.2105	no
gi|320447034|ref|NW_003383537.1|	[illegible]92297-79267[illegible]	555323	203522	187378	0.3993	no
gi|320447034|ref|NW_003383537.1|	[illegible]46896-84758[illegible]	485495	969094	0.997179	0.6374	no
gi|320447034|ref|NW_003383537.1|	[illegible]49745-85042[illegible]	152394	364007	125616	0.55735	no
gi|320447034|ref|NW_003383537.1|	[illegible]86597-88695[illegible]	301341	40885	-288175	0.23655	no
gi|320447034|ref|NW_003383537.1|	[illegible]91585-89451[illegible]	133276	481152	-146986	0.3828	no
gi|320447034|ref|NW_003383537.1|	[illegible]96865-89921[illegible]	103782	161632	0.639154	0.74805	no
gi|320447035|ref|NW_003383536.1|	[illegible]02267-10330[illegible]	299571	233192	-0.361381	0.8213	no
gi|320447035|ref|NW_003383536.1|	[illegible]04456-10538[illegible]	181109	172143	-0.0732486	0.97255	no

gi|320447035|ref|NW_003383536.1|	06300-10768	0.146663	29545	433234	0.1826	no
gi|320447035|ref|NW_003383536.1|	07789-11052	234517	870593	521424	0.011	no
gi|320447035|ref|NW_003383536.1|	69229-11714	431248	150503	-151872	0.47765	no
gi|320447035|ref|NW_003383536.1|	72474-11743	834233	858009	0.0405426	0.9836	no
gi|320447035|ref|NW_003383536.1|	88429-11894	0	130534	inf	5.00E-05	yes
gi|320447035|ref|NW_003383536.1|	04479-12083	0	373217	inf	0.00475	no
gi|320447035|ref|NW_003383536.1|	18668-12189	0	231716	inf	0.0186	no
gi|320447035|ref|NW_003383536.1|	19355-12204	0	105762	inf	0.0059	no
gi|320447035|ref|NW_003383536.1|	20895-12217	0	182264	inf	0.005	no
gi|320447035|ref|NW_003383536.1|	22589-12229	0	113469	inf	0.02205	no
gi|320447035|ref|NW_003383536.1|	71334-12718	0	498519	inf	0.029	no
gi|320447035|ref|NW_003383536.1|	95575-12969	118985	1033	-0.203937	1	no
gi|320447035|ref|NW_003383536.1|	33506-14340	247175	902726	186875	0.40125	no
gi|320447035|ref|NW_003383536.1|	90741-14998	121522	158079	370135	0.0747	no
gi|320447035|ref|NW_003383536.1|	01686-15022	238278	345336	385728	0.1602	no
gi|320447035|ref|NW_003383536.1|	05148-15057	405954	315434	295795	0.22045	no
gi|320447035|ref|NW_003383536.1|	06224-15066	231229	819414	182527	0.397	no
gi|320447035|ref|NW_003383536.1|	10060-15104	0.875275	196689	449004	0.179	no
gi|320447035|ref|NW_003383536.1|	17730-15182	0.545872	956286	413081	0.1922	no
gi|320447035|ref|NW_003383536.1|	22557-15236	228932	686845	158507	0.44865	no
gi|320447035|ref|NW_003383536.1|	53520-15441	122081	595876	-103475	0.6167	no
gi|320447035|ref|NW_003383536.1|	00210-20110	0.515665	213146	204734	0.30225	no
gi|320447035|ref|NW_003383536.1|	07881-21193	0	39024	inf	0.0032	no
gi|320447035|ref|NW_003383536.1|	21240-22207	422157	939532	-216777	0.21685	no
gi|320447035|ref|NW_003383536.1|	12539-21350	0	239185	inf	0.02205	no
gi|320447035|ref|NW_003383536.1|	22757-26543	306031	153982	-0.990916	0.45995	no
gi|320447035|ref|NW_003383536.1|	27267-28514	343649	150418	-451388	0.06415	no
gi|320447035|ref|NW_003383536.1|	28620-30906	198307	0.631806	-497211	0.0614	no
gi|320447035|ref|NW_003383536.1|	27617-32854	145578	117137	-0.313601	0.87815	no
gi|320447035|ref|NW_003383536.1|	50298-35064	206188	12576	-0.713293	0.71965	no

gi|320447035|ref|NW_003383536.1|	37011-37513	68648	794718	0.211226	0.91205	no
gi|320447035|ref|NW_003383536.1|	418206-41870[illegible]	124815	878373	-0.506881	0.8069	no
gi|320447035|ref|NW_003383536.1|	441322-44173[illegible]	717404	355257	-101392	0.59615	no
gi|320447035|ref|NW_003383536.1|	444833-44508[illegible]	93709	158249	0.755941	0.7072	no
gi|320447035|ref|NW_003383536.1|	445866-44617[illegible]	249996	195178	-0.357117	0.8573	no
gi|320447035|ref|NW_003383536.1|	447299-44770[illegible]	188552	496938	13981	0.3813	no
gi|320447035|ref|NW_003383536.1|	448002-44915[illegible]	219977	139689	-0.655134	0.7491	no
gi|320447035|ref|NW_003383536.1|	52557-53660	291773	552011	0.919849	0.65065	no
gi|320447035|ref|NW_003383536.1|	54266-55045	959441	764915	-0.326894	0.80505	no
gi|320447035|ref|NW_003383536.1|	[illegible]43275-54679[illegible]	231674	0.503167	-552492	0.0499	no
gi|320447035|ref|NW_003383536.1|	[illegible]47826-54945[illegible]	139247	0.0842733	-736836	0.25985	no
gi|320447035|ref|NW_003383536.1|	[illegible]57659-55825[illegible]	467481	0	#NAME?	0.0229	no
gi|320447035|ref|NW_003383536.1|	56449-58427	245951	220778	-0.155774	0.9055	no
gi|320447035|ref|NW_003383536.1|	58555-59513	0.700683	241788	178691	0.41635	no
gi|320447035|ref|NW_003383536.1|	[illegible]90706-59163[illegible]	0	184855	inf	0.0294	no
gi|320447035|ref|NW_003383536.1|	59621-62201	261884	310436	0.245366	0.90865	no
gi|320447035|ref|NW_003383536.1|	[illegible]18492-62014[illegible]	150195	107868	-379951	0.08065	no
gi|320447035|ref|NW_003383536.1|	[illegible]20869-62186[illegible]	188605	0.459809	-535819	0.13425	no
gi|320447035|ref|NW_003383536.1|	[illegible]22019-62332[illegible]	20472	502793	129631	0.52765	no
gi|320447035|ref|NW_003383536.1|	[illegible]24477-62473[illegible]	308517	293143	324818	0.2305	no
gi|320447035|ref|NW_003383536.1|	[illegible]40539-64122[illegible]	231544	153015	-391954	0.1608	no
gi|320447035|ref|NW_003383536.1|	[illegible]51245-65599[illegible]	186366	423765	-21368	0.2548	no
gi|320447035|ref|NW_003383536.1|	[illegible]56641-66240[illegible]	393824	825785	106822	0.40335	no
gi|320447035|ref|NW_003383536.1|	[illegible]87096-68830[illegible]	320007	111527	-152071	0.36545	no
gi|320447035|ref|NW_003383536.1|	70888-72337	778666	256108	171768	0.3103	no
gi|320447035|ref|NW_003383536.1|	72449-73212	287914	10091	180936	0.3944	no
gi|320447035|ref|NW_003383536.1|	[illegible]25864-72758[illegible]	227783	808961	-149352	0.37705	no
gi|320447035|ref|NW_003383536.1|	[illegible]35769-73662[illegible]	466095	115076	130389	0.5331	no
gi|320447035|ref|NW_003383536.1|	[illegible]45864-74731[illegible]	515325	75506	0.551108	0.7915	no
gi|320447035|ref|NW_003383536.1|	74667-77073	0.388678	141128	186035	1	no

gi|320447035|ref|NW_003383536.1|	52271-76070	565715	739069	0.385633	0.80765	no
gi|320447035|ref|NW_003383536.1|	67027-76954	202632	599698	156537	0.2525	no
gi|320447035|ref|NW_003383536.1|	77177-81701	444568	161725	186306	0.3083	no
gi|320447035|ref|NW_003383536.1|	72112-77252	114558	160138	0.483232	0.69555	no
gi|320447035|ref|NW_003383536.1|	75662-77861	669175	51.95	-0.365261	0.78395	no
gi|320447035|ref|NW_003383536.1|	78733-77929	626901	636615	0.0221839	0.98735	no
gi|320447035|ref|NW_003383536.1|	79716-78504	834405	925619	0.14967	0.90545	no
gi|320447035|ref|NW_003383536.1|	89113-79027	763284	12591	-259982	0.2572	no
gi|320447035|ref|NW_003383536.1|	94994-79613	970098	258411	-190847	0.38205	no
gi|320447035|ref|NW_003383536.1|	99392-80245	0.418	434231	337689	0.1715	no
gi|320447035|ref|NW_003383536.1|	85856-86518	553178	161607	154667	0.4702	no
gi|320447035|ref|NW_003383536.1|	91844-93148	495867	196195	-133766	0.3034	no
gi|320447035|ref|NW_003383536.1|	20564-92122	0	334429	inf	0.0047	no
gi|320447035|ref|NW_003383536.1|	34221-93439	0	333717	inf	0.0072	no
gi|320447035|ref|NW_003383536.1|	96201-101783	374264	480714	0.361123	0.7794	no
gi|320447035|ref|NW_003383536.1|	91689-99208	0	118763	inf	0.0154	no
gi|320447035|ref|NW_003383536.1|	94398-99562	0	201019	inf	0.02075	no
gi|320447036|ref|NW_003383535.1|	08768-12095	255923	0.658107	-195932	0.30295	no
gi|320447036|ref|NW_003383535.1|	09804-12118	180661	0.198922	-318301	0.1942	no
gi|320447036|ref|NW_003383535.1|	1217-3462	0.335857	843862	465109	0.1446	no
gi|320447036|ref|NW_003383535.1|	13097-15361	0.582244	267058	219746	0.31865	no
gi|320447036|ref|NW_003383535.1|	03622-15083	337326	431661	0.355754	0.78385	no
gi|320447036|ref|NW_003383535.1|	12210-15130	707272	369614	-0.936245	0.6499	no
gi|320447036|ref|NW_003383535.1|	52601-15482	10548	753933	-0.484468	0.76285	no
gi|320447036|ref|NW_003383535.1|	54997-15648	302149	295984	-0.0297427	0.9817	no
gi|320447036|ref|NW_003383535.1|	64376-15664	0.459622	0.833543	0.858809	1	no
gi|320447036|ref|NW_003383535.1|	60035-16185	924108	688292	-0.42504	0.8436	no
gi|320447036|ref|NW_003383535.1|	62736-16296	697629	361851	-0.947066	0.64495	no
gi|320447036|ref|NW_003383535.1|	93341-16937	271226	0	#NAME?	0.00975	no
gi|320447036|ref|NW_003383535.1|	95384-16960	11141	0	#NAME?	0.0093	no

gi|320447036|ref|NW_003383535.1|	;97101-16989	23522	0	#NAME?	5.00E-05	yes
gi|320447036|ref|NW_003383535.1|	'39972-17412	0.321864	569527	414524	0.16075	no
gi|320447036|ref|NW_003383535.1|	'59048-17612	347908	19248	-0.853996	0.5216	no
gi|320447036|ref|NW_003383535.1|	'61887-17658	922884	813832	-0.181419	0.8859	no
gi|320447036|ref|NW_003383535.1|	'70574-17746	326712	141553	211525	0.24505	no
gi|320447036|ref|NW_003383535.1|	'75886-17769	238995	129421	-0.884904	0.5836	no
gi|320447036|ref|NW_003383535.1|	'80583-17814	109669	282975	136751	0.5247	no
gi|320447036|ref|NW_003383535.1|	'81575-17844	0	363012	inf	0.00465	no
gi|320447036|ref|NW_003383535.1|	307030-18076	3431	553938	0.691094	0.73085	no
gi|320447036|ref|NW_003383535.1|	!27979-22896	0.223653	139022	263597	1	no
gi|320447036|ref|NW_003383535.1|	111546-41205	185242	828053	216031	0.34175	no
gi|320447036|ref|NW_003383535.1|	122044-42553	177301	267314	0.59233	0.6604	no
gi|320447036|ref|NW_003383535.1|	127355-42847	391534	200994	-0.961985	0.5703	no
gi|320447036|ref|NW_003383535.1|	129587-43062	101184	48091	-107314	0.60225	no
gi|320447036|ref|NW_003383535.1|	134131-43488	834699	237798	-181152	0.2939	no
gi|320447036|ref|NW_003383535.1|	134938-43549	36596	635114	0.795327	0.6951	no
gi|320447036|ref|NW_003383535.1|	137661-43863	298668	840598	149287	0.47275	no
gi|320447036|ref|NW_003383535.1|	139567-44109	117221	205632	0.810833	0.61065	no
gi|320447036|ref|NW_003383535.1|	143187-44389	0	132898	inf	0.0071	no
gi|320447036|ref|NW_003383535.1|	146892-44961	0.609967	11319	421387	0.06405	no
gi|320447036|ref|NW_003383535.1|	149801-45126	0.137686	248841	417577	0.1906	no
gi|320447036|ref|NW_003383535.1|	153491-45387	10317	196399	425069	0.18455	no
gi|320447036|ref|NW_003383535.1|	170504-47093	357305	247695	-0.528592	0.80395	no
gi|320447036|ref|NW_003383535.1|	184355-48535	290674	689714	-207533	0.21275	no
gi|320447036|ref|NW_003383535.1|	185871-48745	311889	355898	0.190429	0.92455	no
gi|320447036|ref|NW_003383535.1|	195769-49669	294545	660075	116414	0.5674	no
gi|320447036|ref|NW_003383535.1|	196936-49778	110207	265377	126783	0.5544	no
gi|320447036|ref|NW_003383535.1|	197890-49863	300782	520889	-252967	0.2749	no
gi|320447036|ref|NW_003383535.1|	199515-50099	188916	661409	-151413	0.35705	no
gi|320447036|ref|NW_003383535.1|	;70924-57132	622453	649725	-326006	0.18375	no

gi|320447036|ref|NW_003383535.1|	81591-58415	522165	0.10132	-568752	0.1825	no
gi|320447036|ref|NW_003383535.1|	00003-70198	43599	918743	107537	0.62355	no
gi|320447036|ref|NW_003383535.1|	05023-70669	26311	123477	-109143	0.51545	no
gi|320447036|ref|NW_003383535.1|	07056-70849	0.279477	126256	217555	1	no
gi|320447036|ref|NW_003383535.1|	08615-70955	313167	28248	-0.148784	0.93585	no
gi|320447036|ref|NW_003383535.1|	10957-71144	113335	102495	-0.145035	0.9398	no
gi|320447036|ref|NW_003383535.1|	28288-82923	0.237173	540014	450899	0.17645	no
gi|320447036|ref|NW_003383535.1|	30113-83143	0.311179	270019	311725	0.20585	no
gi|320447036|ref|NW_003383535.1|	83229-88506	622384	115403	0.890801	0.68875	no
gi|320447036|ref|NW_003383535.1|	86945-89021	661655	742783	0.166863	0.91495	no
gi|320447036|ref|NW_003383535.1|	91551-89442	504987	254683	-0.987546	0.6512	no
gi|320447036|ref|NW_003383535.1|	95793-89897	240909	0.882496	-144883	0.49865	no
gi|320447037|ref|NW_003383534.1|	08593-10093	0	240671	inf	0.00615	no
gi|320447037|ref|NW_003383534.1|	10169-10106	0	271431	inf	0.0075	no
gi|320447037|ref|NW_003383534.1|	12203-10128	0	120408	inf	0.0072	no
gi|320447037|ref|NW_003383534.1|	16936-10172	0	459942	inf	0.00845	no
gi|320447037|ref|NW_003383534.1|	51690-10530	0	214646	inf	0.0162	no
gi|320447037|ref|NW_003383534.1|	53528-10554	0.0978418	225112	452405	0.17575	no
gi|320447037|ref|NW_003383534.1|	16312-11170	941976	115782	0.297647	0.8852	no
gi|320447037|ref|NW_003383534.1|	21246-11218	0	144264	inf	0.0079	no
gi|320447037|ref|NW_003383534.1|	25496-11267	0	547025	inf	0.0071	no
gi|320447037|ref|NW_003383534.1|	42146-11462	122385	530076	-120716	0.4819	no
gi|320447037|ref|NW_003383534.1|	47911-11483	58578	256067	-119384	0.57885	no
gi|320447037|ref|NW_003383534.1|	56542-11570	321126	777658	-204593	0.33915	no
gi|320447037|ref|NW_003383534.1|	60163-11636	160187	705462	-118312	0.3572	no
gi|320447037|ref|NW_003383534.1|	64967-11660	284978	23642	-0.269499	0.8243	no
gi|320447037|ref|NW_003383534.1|	19708-12068	883966	892099	0.0132121	0.99015	no
gi|320447037|ref|NW_003383534.1|	22512-12233	0	311254	inf	0.0198	no
gi|320447037|ref|NW_003383534.1|	23478-12251	0.3566	273053	29368	0.2302	no
gi|320447037|ref|NW_003383534.1|	48077-12488	182935	104654	-0.805705	0.7079	no

gi|320447037|ref|NW_003383534.1|	249266-12507	209261	213314	0.027675	0.9742	no
gi|320447037|ref|NW_003383534.1|	139156-14036	0.345954	131914	193094	1	no
gi|320447037|ref|NW_003383534.1|	151534-15178	331165	286211	-0.210471	0.91235	no
gi|320447037|ref|NW_003383534.1|	15654-16638	216429	902979	-126113	0.56405	no
gi|320447037|ref|NW_003383534.1|	163036-16331	177553	283492	0.675059	0.74435	no
gi|320447037|ref|NW_003383534.1|	170247-17282	116014	533573	-112054	0.49485	no
gi|320447037|ref|NW_003383534.1|	248160-24841	293901	223646	292781	0.25735	no
gi|320447037|ref|NW_003383534.1|	252212-25306	0.547458	30136	246067	0.2454	no
gi|320447037|ref|NW_003383534.1|	253214-25432	0.193401	22759	355677	0.21625	no
gi|320447037|ref|NW_003383534.1|	25386-26074	260848	178138	-0.550213	0.79275	no
gi|320447037|ref|NW_003383534.1|	257536-25801	138023	124375	317171	0.20745	no
gi|320447037|ref|NW_003383534.1|	258689-25983	0.185532	668225	51706	0.1702	no
gi|320447037|ref|NW_003383534.1|	259994-26100	0	241792	inf	0.0198	no
gi|320447037|ref|NW_003383534.1|	261242-26213	257833	184727	284088	0.22235	no
gi|320447037|ref|NW_003383534.1|	26497-26894	196559	581789	-17564	0.41375	no
gi|320447037|ref|NW_003383534.1|	26967-27879	823172	361098	-11888	0.5708	no
gi|320447037|ref|NW_003383534.1|	277287-27780	0	562485	inf	0.0233	no
gi|320447037|ref|NW_003383534.1|	279996-28042	0	718909	inf	0.02915	no
gi|320447037|ref|NW_003383534.1|	372832-37409	0	387702	inf	5.00E-05	yes
gi|320447037|ref|NW_003383534.1|	417291-41899	71238	0.080006	-647639	0.26705	no
gi|320447037|ref|NW_003383534.1|	482203-48289	0	423035	inf	0.0035	no
gi|320447037|ref|NW_003383534.1|	484030-48442	102046	750578	620071	0.158	no
gi|320447037|ref|NW_003383534.1|	605851-60649	250414	283981	0.181479	0.9196	no
gi|320447037|ref|NW_003383534.1|	606770-60782	268668	62884	122687	0.5526	no
gi|320447037|ref|NW_003383534.1|	608813-60933	553603	248142	-115768	0.59815	no
gi|320447037|ref|NW_003383534.1|	613468-61508	122137	331495	144049	0.48945	no
gi|320447037|ref|NW_003383534.1|	615207-61563	334024	331804	-0.00962244	0.9157	no
gi|320447037|ref|NW_003383534.1|	615784-61685	302227	432102	0.515742	0.79705	no
gi|320447037|ref|NW_003383534.1|	617102-61790	419273	719866	0.779836	0.6997	no
gi|320447037|ref|NW_003383534.1|	620912-62118	212114	150272	-0.497266	0.806	no

gi|320447037|ref|NW_003383534.1|	21351-62166	522642	228076	-119631	0.57035	no
gi|320447037|ref|NW_003383534.1|	23299-62375	443346	590196	0.412761	0.84875	no
gi|320447037|ref|NW_003383534.1|	24616-62727	167715	141979	-0.240337	0.8521	no
gi|320447037|ref|NW_003383534.1|	27975-63082	352247	790113	116547	0.38675	no
gi|320447037|ref|NW_003383534.1|	57623-65826	0.82265	364019	214566	0.29285	no
gi|320447037|ref|NW_003383534.1|	6786-14355	283899	388759	0.4535	0.7261	no
gi|320447037|ref|NW_003383534.1|	86186-78748	0.637646	221259	179491	0.42605	no
gi|320447037|ref|NW_003383534.1|	99406-80002	304797	295842	-0.0430203	0.94545	no
gi|320447037|ref|NW_003383534.1|	96276-89667	116091	241961	105951	0.60435	no
gi|320447037|ref|NW_003383534.1|	25846-92794	0.815325	265421	170283	0.42665	no
gi|320447037|ref|NW_003383534.1|	31686-93228	106362	783824	-0.440386	0.82885	no
gi|320447037|ref|NW_003383534.1|	33543-93418	164927	561336	-155489	0.4706	no
gi|320447037|ref|NW_003383534.1|	36046-93660	107831	589538	-0.871113	0.67925	no
gi|320447037|ref|NW_003383534.1|	37155-93811	913418	193958	-223553	0.3209	no
gi|320447037|ref|NW_003383534.1|	43839-94595	0.629746	294974	222774	0.31235	no
gi|320447037|ref|NW_003383534.1|	62901-96476	155129	201808	0.379513	0.8466	no
gi|320447037|ref|NW_003383534.1|	67787-96886	220842	138898	-0.66899	0.7454	no
gi|320447037|ref|NW_003383534.1|	84380-98752	795011	0.892771	-315461	0.1048	no
gi|320447038|ref|NW_003383533.1|	05268-10067	162049	375552	121258	0.56095	no
gi|320447038|ref|NW_003383533.1|	11958-10125	736474	735991	-0.000944628	0.97505	no
gi|320447038|ref|NW_003383533.1|	13576-10197	842955	525605	-0.681477	0.60215	no
gi|320447038|ref|NW_003383533.1|	20041-10205	175323	495584	-182281	0.40035	no
gi|320447038|ref|NW_003383533.1|	34385-10347	30283	663216	113097	0.57945	no
gi|320447038|ref|NW_003383533.1|	34959-10358	0.787948	50645	268425	0.2604	no
gi|320447038|ref|NW_003383533.1|	93137-10959	332209	231705	-384173	0.06115	no
gi|320447038|ref|NW_003383533.1|	04837-11071	191818	0.804507	-457549	0.05725	no
gi|320447038|ref|NW_003383533.1|	64576-11651	26462	209663	-0.335849	0.8742	no
gi|320447038|ref|NW_003383533.1|	66045-11663	32606	174602	-0.90107	0.6686	no
gi|320447038|ref|NW_003383533.1|	71761-11720	268962	0	#NAME?	0.02015	no
gi|320447038|ref|NW_003383533.1|	75214-11771	0.387102	452101	354586	0.18025	no

gi|320447038|ref|NW_003383533.1|	77263-11778	0.497203	30258	260541	0.2636	no
gi|320447038|ref|NW_003383533.1|	91432-11918	612275	469112	-0.384248	0.8476	no
gi|320447038|ref|NW_003383533.1|	25289-12261	199037	0.977483	-10259	0.59645	no
gi|320447038|ref|NW_003383533.1|	27211-12278	263154	179665	-0.5506	0.79275	no
gi|320447038|ref|NW_003383533.1|	51460-12519	444764	170216	-138567	0.52445	no
gi|320447038|ref|NW_003383533.1|	69818-12727	0.699964	173472	130935	0.5259	no
gi|320447038|ref|NW_003383533.1|	82012-12824	0	149772	inf	0.00945	no
gi|320447038|ref|NW_003383533.1|	23858-13253	180959	363909	100791	0.42525	no
gi|320447038|ref|NW_003383533.1|	25420-13257	169201	257007	0.603069	0.75565	no
gi|320447038|ref|NW_003383533.1|	26424-13279	786002	117412	0.578974	0.79015	no
gi|320447038|ref|NW_003383533.1|	28586-13293	161459	202415	0.326145	0.87925	no
gi|320447038|ref|NW_003383533.1|	48662-13508	0.338723	313221	320901	0.20395	no
gi|320447038|ref|NW_003383533.1|	55003-13561	0.742126	218458	155762	0.4963	no
gi|320447038|ref|NW_003383533.1|	60263-13734	212994	286989	0.430187	0.733	no
gi|320447038|ref|NW_003383533.1|	73658-13749	183492	116786	-0.651848	0.75415	no
gi|320447038|ref|NW_003383533.1|	52792-15407	201165	20101	-332304	0.08395	no
gi|320447038|ref|NW_003383533.1|	19109-20187	320462	15243	-1072	0.60365	no
gi|320447038|ref|NW_003383533.1|	20315-21153	0.840273	134845	0.682378	1	no
gi|320447038|ref|NW_003383533.1|	48696-24917	0	542624	inf	0.029	no
gi|320447038|ref|NW_003383533.1|	50546-25276	0.765198	178005	121801	0.56155	no
gi|320447038|ref|NW_003383533.1|	08496-30978	0.801602	300382	190584	0.39245	no
gi|320447038|ref|NW_003383533.1|	14076-31443	48073.9	49804.5	0.0510221	0.55075	no
gi|320447038|ref|NW_003383533.1|	19366-42041	0.206667	257253	36378	0.2061	no
gi|320447038|ref|NW_003383533.1|	21524-42393	0.388678	423383	344532	0.1825	no
gi|320447038|ref|NW_003383533.1|	29595-42986	44221	469405	-323583	0.1958	no
gi|320447038|ref|NW_003383533.1|	31733-43493	487337	476682	-335382	0.09955	no
gi|320447038|ref|NW_003383533.1|	37075-43829	137816	214989	-268041	0.2413	no
gi|320447038|ref|NW_003383533.1|	42187-44312	749996	183553	-203068	0.3586	no
gi|320447038|ref|NW_003383533.1|	43482-44550	148114	611864	-127543	0.4359	no
gi|320447038|ref|NW_003383533.1|	47740-44987	640904	209907	171157	0.32845	no

gi|320447038|ref|NW_003383533.1|	458311-461342	464734	120689	137682	0.4178	no
gi|320447038|ref|NW_003383533.1|	461521-463989	513921	596424	0.214792	0.9236	no
gi|320447038|ref|NW_003383533.1|	468368-471700	87012	787434	-0.144057	0.9067	no
gi|320447038|ref|NW_003383533.1|	472026-472397	147832	111526	-0.406574	0.8389	no
gi|320447038|ref|NW_003383533.1|	472524-475931	659671	781535	0.244563	0.8446	no
gi|320447038|ref|NW_003383533.1|	476384-477170	229915	967942	-124811	0.5617	no
gi|320447038|ref|NW_003383533.1|	477817-478220	240201	852916	-149376	0.4755	no
gi|320447038|ref|NW_003383533.1|	478455-478850	202104	316784	-267353	0.27125	no
gi|320447038|ref|NW_003383533.1|	484095-484305	357355	44706	0.323109	0.86335	no
gi|320447038|ref|NW_003383533.1|	586161-686888	102977	211467	103812	0.5772	no
gi|320447038|ref|NW_003383533.1|	701956-703749	0	173584	inf	0.01485	no
gi|320447038|ref|NW_003383533.1|	720993-721610	354839	206582	-74243	0.085	no
gi|320447038|ref|NW_003383533.1|	722031-722555	26615	0.38896	-609647	0.2673	no
gi|320447038|ref|NW_003383533.1|	724063-725371	165826	0.655213	-798349	0.09715	no
gi|320447038|ref|NW_003383533.1|	725908-726340	182034	767427	-124611	0.54495	no
gi|320447038|ref|NW_003383533.1|	726575-727024	383731	459029	0.258488	0.8937	no
gi|320447038|ref|NW_003383533.1|	727221-727704	535365	626155	0.225998	0.902	no
gi|320447038|ref|NW_003383533.1|	728017-734028	356395	773882	111864	0.512	no
gi|320447038|ref|NW_003383533.1|	735507-736670	916929	518043	-0.823739	0.534	no
gi|320447038|ref|NW_003383533.1|	742826-743829	30781	188349	-0.708631	0.66115	no
gi|320447038|ref|NW_003383533.1|	744562-746291	0.677405	0.629007	-0.106944	1	no
gi|320447038|ref|NW_003383533.1|	746466-748581	0.897722	125255	0.480532	1	no
gi|320447038|ref|NW_003383533.1|	748893-752561	629662	549312	-0.196951	0.87915	no
gi|320447038|ref|NW_003383533.1|	897184-898790	18387	588602	16786	0.4335	no
gi|320447038|ref|NW_003383533.1|	901984-902819	337791	735606	11228	0.586	no
gi|320447038|ref|NW_003383533.1|	915914-916421	182664	367644	100912	0.5772	no
gi|320447038|ref|NW_003383533.1|	918730-919188	0.73891	541013	287219	0.25915	no
gi|320447038|ref|NW_003383533.1|	920317-921809	81916	122315	0.578385	0.79025	no
gi|320447038|ref|NW_003383533.1|	924812-929920	148366	47777	168715	0.2159	no
gi|320447038|ref|NW_003383533.1|	930030-933210	131524	131972	0.00491136	1	no

gi|320447038|ref|NW_003383533.1|	)33406-93526(	103993	181177	0.800914	0.69955	no
gi|320447038|ref|NW_003383533.1|	)36414-94071!	248758	583485	122996	0.4532	no
gi|320447038|ref|NW_003383533.1|	)40839-94116:	785582	134947	0.780561	0.7204	no
gi|320447038|ref|NW_003383533.1|	)41341-94209(	460046	121601	140231	0.50595	no
gi|320447038|ref|NW_003383533.1|	)42222-94351:	264124	354459	0.424402	0.74	no
gi|320447038|ref|NW_003383533.1|	)48305-94853:	797755	136.58	0.775733	0.7087	no
gi|320447038|ref|NW_003383533.1|	)48778-94894(	313188	244825	-0.355279	0.85605	no
gi|320447038|ref|NW_003383533.1|	)49755-95002:	868989	116985	0.42891	0.8348	no
gi|320447038|ref|NW_003383533.1|	)60359-96108!	110409	121245	0.135076	0.9175	no
gi|320447038|ref|NW_003383533.1|	)91201-99739:	223843	496473	114923	0.49375	no
gi|320447039|ref|NW_003383532.1|	164456-16565!	802275	640773	-0.324283	0.8698	no
gi|320447039|ref|NW_003383532.1|	187868-19073:	845698	339899	200689	0.13785	no
gi|320447039|ref|NW_003383532.1|	192449-19379(	775623	295684	193063	0.26405	no
gi|320447039|ref|NW_003383532.1|	296758-29802!	0.814764	248725	16101	0.45045	no
gi|320447039|ref|NW_003383532.1|	298130-29916:	0.210536	218327	337435	0.2261	no
gi|320447039|ref|NW_003383532.1|	324510-32563!	0.566563	287702	234427	0.30415	no
gi|320447039|ref|NW_003383532.1|	344584-34613(	0	267869	inf	0.00935	no
gi|320447039|ref|NW_003383532.1|	352853-35400:	0.183513	165264	317081	0.24015	no
gi|320447039|ref|NW_003383532.1|	395802-39642:	131295	445953	176408	0.41855	no
gi|320447039|ref|NW_003383532.1|	433620-43516:	0.258495	0.898994	179817	1	no
gi|320447039|ref|NW_003383532.1|	592386-69291!	280885	793993	149915	0.49395	no
gi|320447039|ref|NW_003383532.1|	593386-69359:	114693	43075	190908	0.31725	no
gi|320447039|ref|NW_003383532.1|	747531-75047:	995599	452402	-113796	0.48585	no
gi|320447039|ref|NW_003383532.1|	750636-75654(	16799	148149	-0.181327	0.892	no
gi|320447039|ref|NW_003383532.1|	757584-75920:	40831	231733	-0.817202	0.52805	no
gi|320447039|ref|NW_003383532.1|	760400-76394(	378164	390826	0.0475139	0.9721	no
gi|320447039|ref|NW_003383532.1|	765590-76709:	173706	0.0922078	-755754	0.2504	no
gi|320447039|ref|NW_003383532.1|	768246-76948!	554895	0.116617	-557236	0.274	no
gi|320447039|ref|NW_003383532.1|	770067-77073!	888192	0	#NAME?	0.0111	no
gi|320447039|ref|NW_003383532.1|	771832-77225(	242745	0	#NAME?	0.0093	no

gi|320447039|ref|NW_003383532.1|	774131-77466	907629	0.381648	-457179	0.2946	no
gi|320447039|ref|NW_003383532.1|	774817-77508	228283	322829	-282198	0.27485	no
gi|320447039|ref|NW_003383532.1|	779372-77991	422984	370042	-351484	0.1662	no
gi|320447039|ref|NW_003383532.1|	796430-79673	119255	275167	-211567	0.33785	no
gi|320447039|ref|NW_003383532.1|	818739-81998	0.334964	127776	193154	1	no
gi|320447039|ref|NW_003383532.1|	825742-82736	0.607604	126861	106205	1	no
gi|320447039|ref|NW_003383532.1|	828471-82931	363515	269253	-0.43305	0.82255	no
gi|320447039|ref|NW_003383532.1|	835822-83780	10144	545637	-0.894607	0.68795	no
gi|320447039|ref|NW_003383532.1|	845871-84670	224073	712755	166944	0.4268	no
gi|320447039|ref|NW_003383532.1|	859610-86085	0.665962	265606	199578	0.3909	no
gi|320447039|ref|NW_003383532.1|	864745-86605	0.156746	206722	37212	0.21225	no
gi|320447039|ref|NW_003383532.1|	876514-87733	0.57942	159288	145895	0.375	no
gi|320447039|ref|NW_003383532.1|	888720-89154	722124	129589	0.843624	0.615	no
gi|320447039|ref|NW_003383532.1|	897527-89821	892379	101577	0.186848	0.92825	no
gi|320447039|ref|NW_003383532.1|	915250-91575	123932	498519	20081	0.3028	no
gi|320447041|ref|NW_003383530.1|	112147-11286	0.70653	579903	303699	0.21255	no
gi|320447041|ref|NW_003383530.1|	153440-15437	411288	20024	-103842	0.60885	no
gi|320447041|ref|NW_003383530.1|	183831-18532	0.941518	187035	0.990249	0.65565	no
gi|320447041|ref|NW_003383530.1|	185435-18701	0.501992	253216	233463	0.2995	no
gi|320447041|ref|NW_003383530.1|	187594-18917	0	375182	inf	0.00915	no
gi|320447041|ref|NW_003383530.1|	189913-19125	0.76455	445911	254407	0.27855	no
gi|320447041|ref|NW_003383530.1|	204168-20647	585101	118891	-229905	0.08715	no
gi|320447041|ref|NW_003383530.1|	206861-20871	135275	108773	-0.31457	1	no
gi|320447041|ref|NW_003383530.1|	208823-20907	474438	120148	-198141	0.3855	no
gi|320447041|ref|NW_003383530.1|	210032-21278	394192	436434	0.146863	0.91265	no
gi|320447041|ref|NW_003383530.1|	215555-21865	0	144205	inf	0.0114	no
gi|320447041|ref|NW_003383530.1|	219687-22179	0	716607	inf	0.0038	no
gi|320447041|ref|NW_003383530.1|	221917-22768	136218	798671	-0.770243	0.5555	no
gi|320447041|ref|NW_003383530.1|	227825-22905	245562	543934	-217459	0.20145	no
gi|320447041|ref|NW_003383530.1|	229233-23029	793369	225139	-181718	0.3887	no

gi|320447041|ref|NW_003383530.1|	230448-23146	20782	359024	-253318	0.2733	no
gi|320447041|ref|NW_003383530.1|	243006-24496	441779	157077	-149185	0.25875	no
gi|320447041|ref|NW_003383530.1|	264659-26502	271204	284838	0.0707641	0.9671	no
gi|320447041|ref|NW_003383530.1|	265325-26673	844032	107036	0.34272	0.7947	no
gi|320447041|ref|NW_003383530.1|	268985-27152	107714	736996	-0.547473	0.6782	no
gi|320447041|ref|NW_003383530.1|	271635-28029	294291	137173	-110125	0.41075	no
gi|320447041|ref|NW_003383530.1|	492830-49342	0	573283	inf	0.0154	no
gi|320447041|ref|NW_003383530.1|	504450-50507	0	349856	inf	0.029	no
gi|320447041|ref|NW_003383530.1|	514054-51850	118182	15822	0.420927	0.7491	no
gi|320447041|ref|NW_003383530.1|	519411-52050	728102	5175	-0.492583	0.809	no
gi|320447041|ref|NW_003383530.1|	522457-52293	212279	155457	-0.449449	0.8282	no
gi|320447041|ref|NW_003383530.1|	526389-52692	452339	228273	-0.986643	0.66985	no
gi|320447041|ref|NW_003383530.1|	527453-52841	279886	257557	-0.119946	0.9453	no
gi|320447041|ref|NW_003383530.1|	542809-54372	247253	255701	0.0484721	0.96125	no
gi|320447041|ref|NW_003383530.1|	545266-54628	0.864807	253988	155431	0.49635	no
gi|320447041|ref|NW_003383530.1|	547441-54890	0.411045	0.857218	106037	1	no
gi|320447041|ref|NW_003383530.1|	550281-55163	0.75497	0.629124	-0.263075	1	no
gi|320447041|ref|NW_003383530.1|	555386-55969	277575	481529	0.794748	0.72585	no
gi|320447041|ref|NW_003383530.1|	561969-56260	209189	142326	-0.555606	0.8006	no
gi|320447041|ref|NW_003383530.1|	60230-63967	72278	89361	0.30609	0.8074	no
gi|320447041|ref|NW_003383530.1|	636498-63693	0	261709	inf	0.0089	no
gi|320447041|ref|NW_003383530.1|	637104-63737	245672	287902	68727	0.1408	no
gi|320447041|ref|NW_003383530.1|	65595-65916	223367	154467	-0.532118	0.79095	no
gi|320447041|ref|NW_003383530.1|	69139-70392	87893	793724	-0.147111	0.9444	no
gi|320447041|ref|NW_003383530.1|	721940-72235	0	226057	inf	0.0113	no
gi|320447041|ref|NW_003383530.1|	725645-72719	656147	277425	-124192	0.5607	no
gi|320447041|ref|NW_003383530.1|	727946-72831	711847	309754	-120044	0.5758	no
gi|320447041|ref|NW_003383530.1|	730824-73141	950478	128754	-288404	0.25525	no
gi|320447041|ref|NW_003383530.1|	731863-73211	0	28285	inf	0.02205	no
gi|320447041|ref|NW_003383530.1|	733486-73570	0.254809	616467	459653	0.1546	no

gi|320447041|ref|NW_003383530.1|	753579-75470	0.189491	131208	279166	1	no
gi|320447041|ref|NW_003383530.1|	773281-77435	444327	614753	0.468384	0.8194	no
gi|320447041|ref|NW_003383530.1|	776572-77720	210219	343207	0.707187	0.7384	no
gi|320447041|ref|NW_003383530.1|	779986-78124	180996	0.342438	-240204	0.2481	no
gi|320447041|ref|NW_003383530.1|	781307-78242	0	146437	inf	0.0294	no
gi|320447041|ref|NW_003383530.1|	784766-78534	396618	234688	-0.757004	0.70565	no
gi|320447041|ref|NW_003383530.1|	21983-82351	0.520579	0.633622	0.283503	1	no
gi|320447041|ref|NW_003383530.1|	23696-82461	0.987563	37449	192298	0.39735	no
gi|320447041|ref|NW_003383530.1|	30349-83385	155004	151825	-0.0298926	0.9838	no
gi|320447041|ref|NW_003383530.1|	34700-83529	325465	66185	1024	0.607	no
gi|320447041|ref|NW_003383530.1|	83778-84602	114702	197123	0.781199	0.71785	no
gi|320447041|ref|NW_003383530.1|	58811-86122	186051	0.48718	-193317	0.36685	no
gi|320447041|ref|NW_003383530.1|	73750-87447	199492	0	#NAME?	0.0061	no
gi|320447041|ref|NW_003383530.1|	77307-87848	230141	0.622934	-52073	0.11265	no
gi|320447041|ref|NW_003383530.1|	00363-90259	499616	195025	-135716	0.51865	no
gi|320447041|ref|NW_003383530.1|	24379-92487	0	82281	inf	0.0212	no
gi|320447041|ref|NW_003383530.1|	33737-93687	29677	358089	0.270976	0.9014	no
gi|320447041|ref|NW_003383530.1|	38997-93948	164799	74942	-113686	0.58245	no
gi|320447041|ref|NW_003383530.1|	46076-94668	0.446784	363942	302606	0.25005	no
gi|320447041|ref|NW_003383530.1|	48988-94979	0	362663	inf	0.0154	no
gi|320447041|ref|NW_003383530.1|	49877-95188	0	305698	inf	0.00695	no
gi|320447041|ref|NW_003383530.1|	72546-97412	843346	350296	205438	0.2451	no
gi|320447041|ref|NW_003383530.1|	74371-97773	469735	501748	0.0951155	0.94105	no
gi|320447041|ref|NW_003383530.1|	78125-97910	83751	328235	-135138	0.5254	no
gi|320447041|ref|NW_003383530.1|	79223-98029	193656	614029	-165712	0.45505	no
gi|320447041|ref|NW_003383530.1|	81652-98214	270776	992528	-144792	0.49655	no
gi|320447041|ref|NW_003383530.1|	89280-99043	0	382198	inf	0.00935	no
gi|320447042|ref|NW_003383529.1|	05596-10060	277154	13403	227379	0.326	no
gi|320447042|ref|NW_003383529.1|	18371-10195	178655	0.371417	-226607	0.2663	no
gi|320447042|ref|NW_003383529.1|	21029-10220	113481	141044	0.313688	1	no

gi|320447042|ref|NW_003383529.1|	23623-10255	0.500529	153521	161691	0.4501	no
gi|320447042|ref|NW_003383529.1|	26331-10277	147303	266029	0.852801	0.6827	no
gi|320447042|ref|NW_003383529.1|	28677-10290	138815	810487	254563	0.27405	no
gi|320447042|ref|NW_003383529.1|	31690-10330	0.29307	519138	41468	0.16075	no
gi|320447042|ref|NW_003383529.1|	47325-10486	0	495031	inf	0.00695	no
gi|320447042|ref|NW_003383529.1|	48755-10495	0	520889	inf	0.01485	no
gi|320447042|ref|NW_003383529.1|	61294-10620	0.654666	150306	4521	0.15645	no
gi|320447042|ref|NW_003383529.1|	71740-10724	103817	161045	395534	0.1709	no
gi|320447042|ref|NW_003383529.1|	90316-10910	101944	111681	345354	0.199	no
gi|320447042|ref|NW_003383529.1|	07890-11085	0.862074	234372	144292	0.37505	no
gi|320447042|ref|NW_003383529.1|	78418-17868	107183	170288	-597595	0.26905	no
gi|320447042|ref|NW_003383529.1|	92259-19397	0.341444	435913	367432	0.18125	no
gi|320447042|ref|NW_003383529.1|	24426-24655	472511	810944	-254267	0.2388	no
gi|320447042|ref|NW_003383529.1|	58449-25899	0	362319	inf	0.0312	no
gi|320447042|ref|NW_003383529.1|	99235-30205	0.457511	200993	213527	0.33465	no
gi|320447042|ref|NW_003383529.1|	57894-55882	0	319748	inf	0.0212	no
gi|320447042|ref|NW_003383529.1|	62391-66287	0	67088	inf	0.02205	no
gi|320447042|ref|NW_003383529.1|	86623-68750	965788	151013	0.644895	0.7618	no
gi|320447042|ref|NW_003383529.1|	87828-68802	234245	583165	131588	0.5467	no
gi|320447042|ref|NW_003383529.1|	88221-68920	137339	273648	0.994581	0.5298	no
gi|320447042|ref|NW_003383529.1|	89500-69066	134919	140208	0.0554659	0.9782	no
gi|320447042|ref|NW_003383529.1|	95396-69676	157239	278183	0.823073	0.62265	no
gi|320447042|ref|NW_003383529.1|	96879-69853	0.948261	132021	0.477408	1	no
gi|320447042|ref|NW_003383529.1|	02108-70376	201647	317896	0.656723	0.6119	no
gi|320447042|ref|NW_003383529.1|	04117-70516	312451	33197	0.0874262	0.94595	no
gi|320447042|ref|NW_003383529.1|	06040-70941	136183	553506	-129888	0.44585	no
gi|320447042|ref|NW_003383529.1|	09518-71080	0.480505	100033	105786	1	no
gi|320447042|ref|NW_003383529.1|	11299-71697	204169	314618	0.623838	0.63765	no
gi|320447042|ref|NW_003383529.1|	33465-73467	206936	741264	18408	0.39165	no
gi|320447042|ref|NW_003383529.1|	36959-73818	0.339344	129425	19313	1	no

gi|320447042|ref|NW_003383529.1|	38400-74162	992649	635078	-0.64435	0.69845	no
gi|320447042|ref|NW_003383529.1|	44608-74533	147004	538294	-144939	0.49645	no
gi|320447042|ref|NW_003383529.1|	52311-75284	617946	298693	-104882	0.6348	no
gi|320447042|ref|NW_003383529.1|	58692-75967	134907	263973	0.96842	0.65555	no
gi|320447042|ref|NW_003383529.1|	60549-76192	310965	339411	0.126283	0.9488	no
gi|320447042|ref|NW_003383529.1|	62090-76503	170938	156568	-0.12668	0.92105	no
gi|320447042|ref|NW_003383529.1|	80799-78151	0	14295	inf	0.00765	no
gi|320447042|ref|NW_003383529.1|	87446-78844	110219	548226	231439	0.31475	no
gi|320447042|ref|NW_003383529.1|	47538-84815	0	302524	inf	0.0312	no
gi|320447043|ref|NW_003383528.1|	07330-10101	353852	184375	238142	0.18925	no
gi|320447043|ref|NW_003383528.1|	16227-10199	17096	717852	-12519	0.3416	no
gi|320447043|ref|NW_003383528.1|	21594-10224	321999	259468	301043	0.1993	no
gi|320447043|ref|NW_003383528.1|	37018-10393	197292	15493	-0.34872	0.7845	no
gi|320447043|ref|NW_003383528.1|	39638-10403	542614	208995	-137646	0.50605	no
gi|320447043|ref|NW_003383528.1|	40569-10418	484831	17541	-146675	0.40325	no
gi|320447043|ref|NW_003383528.1|	47090-10480	806388	916388	0.184485	0.93105	no
gi|320447043|ref|NW_003383528.1|	59107-10596	0.479212	324477	275938	0.26575	no
gi|320447043|ref|NW_003383528.1|	86929-11872	40748	130449	167868	0.34065	no
gi|320447043|ref|NW_003383528.1|	99028-11992	175487	177381	0.0154899	0.9322	no
gi|320447043|ref|NW_003383528.1|	99561-12005	463179	305658	-0.599654	0.7629	no
gi|320447043|ref|NW_003383528.1|	01075-12024	463677	241602	-0.940487	0.6448	no
gi|320447043|ref|NW_003383528.1|	03078-12037	724624	445746	-0.701012	0.72695	no
gi|320447043|ref|NW_003383528.1|	87650-18849	0	321729	inf	0.02075	no
gi|320447043|ref|NW_003383528.1|	55816-25667	0	558832	inf	0.00935	no
gi|320447043|ref|NW_003383528.1|	58204-25913	0	399917	inf	0.0138	no
gi|320447043|ref|NW_003383528.1|	32900-34016	0.383286	29189	292893	0.22655	no
gi|320447043|ref|NW_003383528.1|	48236-34954	0	206533	inf	0.0212	no
gi|320447043|ref|NW_003383528.1|	35045-37637	221959	103038	221481	0.20285	no
gi|320447043|ref|NW_003383528.1|	42728-44416	0.81316	574246	282006	0.2263	no
gi|320447043|ref|NW_003383528.1|	28742-43081	201644	240194	0.252389	0.83795	no

gi|320447043|ref|NW_003383528.1|	431800-43277	228191	154398	-0.563587	0.8004	no
gi|320447043|ref|NW_003383528.1|	432894-43566	660092	391408	-0.753994	0.7327	no
gi|320447043|ref|NW_003383528.1|	435808-44415	152934	192902	0.334956	0.7982	no
gi|320447043|ref|NW_003383528.1|	451660-45216	374444	334618	-0.162233	0.92225	no
gi|320447043|ref|NW_003383528.1|	45564-46888	108512	817877	291403	0.2122	no
gi|320447043|ref|NW_003383528.1|	466281-46738	854883	119691	0.485514	0.816	no
gi|320447043|ref|NW_003383528.1|	508930-51004	346137	718871	105439	0.61525	no
gi|320447043|ref|NW_003383528.1|	51713-52196	200762	805057	200361	0.3482	no
gi|320447043|ref|NW_003383528.1|	52320-52889	0.503005	509964	334175	0.2265	no
gi|320447043|ref|NW_003383528.1|	54647-55588	0.478381	561027	355184	0.1826	no
gi|320447043|ref|NW_003383528.1|	707944-70830	120119	313457	470573	0.1756	no
gi|320447043|ref|NW_003383528.1|	771021-77198	209925	214978	0.0343117	0.988	no
gi|320447043|ref|NW_003383528.1|	772157-77353	141658	114796	-0.303346	0.88745	no
gi|320447043|ref|NW_003383528.1|	849642-85174	333577	235222	281793	0.13155	no
gi|320447043|ref|NW_003383528.1|	851865-85311	182959	749895	203517	0.34725	no
gi|320447043|ref|NW_003383528.1|	853773-85565	0.613745	34221	247917	0.28405	no
gi|320447043|ref|NW_003383528.1|	857396-86115	722713	537979	-0.425874	0.74325	no
gi|320447043|ref|NW_003383528.1|	863701-87043	218119	577203	140396	0.28575	no
gi|320447043|ref|NW_003383528.1|	871091-87180	730716	104772	0.519867	0.67865	no
gi|320447043|ref|NW_003383528.1|	874303-87520	249162	327843	0.39592	0.80435	no
gi|320447043|ref|NW_003383528.1|	880013-88037	272875	449144	0.718937	0.7288	no
gi|320447043|ref|NW_003383528.1|	882547-88454	0.764555	539807	281975	0.2326	no
gi|320447043|ref|NW_003383528.1|	889749-89066	0.497421	257181	237024	0.2616	no
gi|320447043|ref|NW_003383528.1|	891502-89163	1257.26	1891.62	0.589338	0.7582	no
gi|320447043|ref|NW_003383528.1|	961310-96164	875246	170026	0.957994	0.65215	no
gi|320447043|ref|NW_003383528.1|	972701-97334	123695	70167	2504	0.28125	no
gi|320447043|ref|NW_003383528.1|	990392-99133	166021	261825	0.657238	0.7401	no
gi|320447044|ref|NW_003383527.1|	006529-10068	763233	171111	-215719	0.31945	no
gi|320447044|ref|NW_003383527.1|	008586-10092	138515	416145	-173489	0.41685	no
gi|320447044|ref|NW_003383527.1|	009799-10109	242447	105481	-120069	0.46065	no

gi|320447044|ref|NW_003383527.1|	11363-10186	32856	146122	-116899	0.38535	no
gi|320447044|ref|NW_003383527.1|	23102-10233	250.56	231635	-0.113302	0.9553	no
gi|320447044|ref|NW_003383527.1|	25770-10262	538608	48213	-0.159813	0.93315	no
gi|320447044|ref|NW_003383527.1|	39203-10521	242038	375477	0.633488	0.6112	no
gi|320447044|ref|NW_003383527.1|	53434-10544	0.458864	19007	205039	0.30225	no
gi|320447044|ref|NW_003383527.1|	56323-10570	314137	832732	-191547	0.38765	no
gi|320447044|ref|NW_003383527.1|	65670-10669	745417	500026	-0.576044	0.6701	no
gi|320447044|ref|NW_003383527.1|	69203-10701	665956	672785	0.0147183	0.98575	no
gi|320447044|ref|NW_003383527.1|	71129-10789	663739	797619	0.265084	0.84425	no
gi|320447044|ref|NW_003383527.1|	97567-10990	0.653244	208988	167773	0.44175	no
gi|320447044|ref|NW_003383527.1|	99278-11023	757566	424179	248523	0.06435	no
gi|320447044|ref|NW_003383527.1|	03018-11046	384186	232837	259944	0.0615	no
gi|320447044|ref|NW_003383527.1|	04840-11067	367293	237805	269478	0.0525	no
gi|320447044|ref|NW_003383527.1|	11749-11130	0	129846	inf	1	no
gi|320447044|ref|NW_003383527.1|	15283-11193	461886	692872	0.585054	0.7211	no
gi|320447044|ref|NW_003383527.1|	19653-11203	604324	777927	0.364312	0.8537	no
gi|320447044|ref|NW_003383527.1|	21420-11218	127764	517146	-130483	0.53775	no
gi|320447044|ref|NW_003383527.1|	21972-11227	256511	209954	-0.288946	0.895	no
gi|320447044|ref|NW_003383527.1|	16512-11741	354998	0.873703	-20226	0.359	no
gi|320447044|ref|NW_003383527.1|	58698-15932	991385	0.585929	-408065	0.21415	no
gi|320447044|ref|NW_003383527.1|	62571-16310	778921	0.3746	-437805	0.2975	no
gi|320447044|ref|NW_003383527.1|	25274-25904	512082	290142	-0.819618	0.6936	no
gi|320447044|ref|NW_003383527.1|	43997-34463	0.838811	31386	190371	0.321	no
gi|320447044|ref|NW_003383527.1|	39632-40339	321959	107622	174102	0.4155	no
gi|320447044|ref|NW_003383527.1|	52843-45483	0.86404	368192	209129	0.33915	no
gi|320447044|ref|NW_003383527.1|	54955-45576	116686	481097	20437	0.3579	no
gi|320447044|ref|NW_003383527.1|	55934-45693	0.665666	199251	158171	0.491	no
gi|320447044|ref|NW_003383527.1|	57968-45834	327443	12152	189188	0.39715	no
gi|320447044|ref|NW_003383527.1|	59001-46047	144066	462986	-163769	0.46695	no
gi|320447044|ref|NW_003383527.1|	62591-46318	134471	502954	-14188	0.4911	no

gi|320447044|ref|NW_003383527.1|	77239-47896	570547	114074	0.999555	0.4576	no
gi|320447044|ref|NW_003383527.1|	03926-50485	341127	108463	-16531	0.31815	no
gi|320447044|ref|NW_003383527.1|	19182-52018	422138	152631	-146767	0.38495	no
gi|320447044|ref|NW_003383527.1|	20978-52379	267317	138411	-0.949595	0.4781	no
gi|320447044|ref|NW_003383527.1|	28161-52912	268699	187068	-0.522428	0.81535	no
gi|320447044|ref|NW_003383527.1|	29226-53654	338364	83031	129507	0.3254	no
gi|320447044|ref|NW_003383527.1|	37137-53750	150516	983397	-0.614071	0.7574	no
gi|320447044|ref|NW_003383527.1|	38251-53941	228151	367963	0.689573	0.57775	no
gi|320447044|ref|NW_003383527.1|	39591-54018	518462	989931	0.93309	0.6487	no
gi|320447044|ref|NW_003383527.1|	40489-54092	296518	562143	0.922818	0.6661	no
gi|320447044|ref|NW_003383527.1|	56316-55651	139955	741916	24063	0.2614	no
gi|320447044|ref|NW_003383527.1|	80310-58148	0	897024	inf	0.00595	no
gi|320447044|ref|NW_003383527.1|	81665-58201	0	128184	inf	0.02205	no
gi|320447044|ref|NW_003383527.1|	85251-58660	211771	273104	0.366946	0.8553	no
gi|320447044|ref|NW_003383527.1|	88255-58936	55894	400272	-0.481711	0.8147	no
gi|320447044|ref|NW_003383527.1|	96337-60068	713507	70633	-0.0145853	0.9916	no
gi|320447044|ref|NW_003383527.1|	01245-60459	312919	515033	0.718877	0.59375	no
gi|320447044|ref|NW_003383527.1|	04832-60585	27821	415636	390107	0.06345	no
gi|320447044|ref|NW_003383527.1|	08043-60823	0	721754	inf	0.029	no
gi|320447044|ref|NW_003383527.1|	09434-61039	0	135771	inf	0.00625	no
gi|320447044|ref|NW_003383527.1|	75047-67573	11303	668759	256478	0.2841	no
gi|320447044|ref|NW_003383527.1|	08244-70905	191937	0.608578	-497905	0.14115	no
gi|320447044|ref|NW_003383527.1|	14371-71494	550096	811386	0.560705	0.7815	no
gi|320447044|ref|NW_003383527.1|	16050-71631	333325	978743	-176793	0.4163	no
gi|320447044|ref|NW_003383527.1|	18470-71873	358966	326248	-345981	0.2324	no
gi|320447044|ref|NW_003383527.1|	19266-71994	167633	676013	-131019	0.53405	no
gi|320447044|ref|NW_003383527.1|	22464-72305	518462	255466	-102111	0.5986	no
gi|320447044|ref|NW_003383527.1|	24066-72468	140768	62743	-116579	0.5764	no
gi|320447044|ref|NW_003383527.1|	24826-72507	820132	259197	-166181	0.4413	no
gi|320447044|ref|NW_003383527.1|	25172-72568	261526	957955	-144893	0.49055	no

gi|320447044|ref|NW_003383527.1|	725938-72739	715966	344549	-105518	0.61205	no
gi|320447044|ref|NW_003383527.1|	727573-72834	470068	236499	-0.991038	0.6556	no
gi|320447044|ref|NW_003383527.1|	728516-72965	0.930728	167601	0.848602	0.6824	no
gi|320447044|ref|NW_003383527.1|	729884-73093	192673	974193	-0.983873	0.65195	no
gi|320447044|ref|NW_003383527.1|	795172-79564	628449	46587	-0.431869	0.82755	no
gi|320447044|ref|NW_003383527.1|	325576-82804	298173	368904	0.307096	0.81985	no
gi|320447044|ref|NW_003383527.1|	332398-83364	344671	158983	-111634	0.507	no
gi|320447044|ref|NW_003383527.1|	341315-84153	744101	149035	100208	0.61185	no
gi|320447044|ref|NW_003383527.1|	344388-84554	0.548155	215191	197296	0.39005	no
gi|320447044|ref|NW_003383527.1|	346430-84753	0	320941	inf	0.0138	no
gi|320447044|ref|NW_003383527.1|	349565-85023	0.768853	288508	190783	0.3194	no
gi|320447044|ref|NW_003383527.1|	350438-85121	309361	169844	245685	0.28135	no
gi|320447044|ref|NW_003383527.1|	382570-88289	467775	110794	1244	0.55485	no
gi|320447044|ref|NW_003383527.1|	395739-89719	0	105877	inf	1	no
gi|320447044|ref|NW_003383527.1|	399362-90029	145161	233613	0.686474	0.7351	no
gi|320447044|ref|NW_003383527.1|	900872-90290	0.941297	190374	101611	0.6064	no
gi|320447044|ref|NW_003383527.1|	903416-90394	22617	951138	207225	0.35	no
gi|320447044|ref|NW_003383527.1|	911698-91267	565885	548621	327723	0.09175	no
gi|320447044|ref|NW_003383527.1|	922929-92424	0.313491	500485	399683	0.16555	no
gi|320447044|ref|NW_003383527.1|	963568-96417	180597	235902	370735	0.1763	no
gi|320447044|ref|NW_003383527.1|	978080-97855	0	162441	inf	0.0114	no
gi|320447044|ref|NW_003383527.1|	979508-98028	162055	455472	149088	0.3662	no
gi|320447044|ref|NW_003383527.1|	984502-98525	292337	102422	180883	0.3944	no
gi|320447044|ref|NW_003383527.1|	986421-98686	154818	97708	26579	0.24565	no
gi|320447044|ref|NW_003383527.1|	988066-99384	206084	31827	0.627017	0.63395	no
gi|320447044|ref|NW_003383527.1|	994018-99550	111637	542402	-104138	0.6298	no
gi|320447045|ref|NW_003383526.1|	100980-10298	133873	186705	0.479895	0.81075	no
gi|320447045|ref|NW_003383526.1|	019829-10200	126517	180221	0.510441	0.8049	no
gi|320447045|ref|NW_003383526.1|	058451-10629	123798	119584	-0.0499624	0.9705	no
gi|320447045|ref|NW_003383526.1|	067546-10690	289257	20.85	-0.4723	0.7067	no

gi|320447045|ref|NW_003383526.1|	)69194-10804!	197625	238202	0.269421	0.8331	no
gi|320447045|ref|NW_003383526.1|	)82010-10839(	937665	149178	-265203	0.24695	no
gi|320447045|ref|NW_003383526.1|	)84088-10848(	708004	0.968465	-286998	0.2564	no
gi|320447045|ref|NW_003383526.1|	)85261-10890:	154055	393875	-196763	0.2679	no
gi|320447045|ref|NW_003383526.1|	)89152-10897!	125514	398514	-165514	0.4351	no
gi|320447045|ref|NW_003383526.1|	)92720-10954:	575779	847754	0.55813	0.723	no
gi|320447045|ref|NW_003383526.1|	)97258-11082!	632539	213223	175313	0.19345	no
gi|320447045|ref|NW_003383526.1|	109679-11109!	275219	150124	-0.874421	0.5056	no
gi|320447045|ref|NW_003383526.1|	112094-11133!	953209	325163	-155163	0.248	no
gi|320447045|ref|NW_003383526.1|	113863-11168'	205693	166786	-0.302491	0.8145	no
gi|320447045|ref|NW_003383526.1|	111627-11264:	114587	31375	-186875	0.38495	no
gi|320447045|ref|NW_003383526.1|	119827-11209'	213127	551972	-194905	0.3869	no
gi|320447045|ref|NW_003383526.1|	123700-11254(	243839	114516	-109038	0.51455	no
gi|320447045|ref|NW_003383526.1|	126532-11278'	112847	698838	-0.691331	0.75305	no
gi|320447045|ref|NW_003383526.1|	129352-11306!	190215	132016	-0.526923	0.79675	no
gi|320447045|ref|NW_003383526.1|	130780-11320:	0.67327	105059	0.641942	1	no
gi|320447045|ref|NW_003383526.1|	132182-11341(	120841	11487	-0.0731068	0.9767	no
gi|320447045|ref|NW_003383526.1|	134241-11352!	0.205911	142403	278989	0.2657	no
gi|320447045|ref|NW_003383526.1|	135474-11371(	187156	319693	0.772448	0.54765	no
gi|320447045|ref|NW_003383526.1|	144567-11455:	0.458864	285104	263535	0.23605	no
gi|320447045|ref|NW_003383526.1|	167897-11691!	326692	100393	161966	0.2238	no
gi|320447045|ref|NW_003383526.1|	169543-11730:	119091	264089	114895	0.3859	no
gi|320447045|ref|NW_003383526.1|	173186-11737:	642898	686359	0.0943732	0.9555	no
gi|320447045|ref|NW_003383526.1|	174202-11755!	697913	103031	0.561959	0.7897	no
gi|320447045|ref|NW_003383526.1|	190824-11911(	0	315571	inf	0.01575	no
gi|320447045|ref|NW_003383526.1|	197286-11979(	427802	625993	0.549205	0.7289	no
gi|320447045|ref|NW_003383526.1|	213554-12146:	0.201004	431088	442268	0.18625	no
gi|320447045|ref|NW_003383526.1|	214825-12173'	16774	769239	219721	0.33845	no
gi|320447045|ref|NW_003383526.1|	225616-12285(	335381	168403	-0.993882	0.4547	no
gi|320447045|ref|NW_003383526.1|	229919-12306(	0.746916	20402	144969	0.375	no

gi|320447045|ref|NW_003383526.1|	23373-12430	870963	15018	-253592	0.2646	no
gi|320447045|ref|NW_003383526.1|	24724-12520	103115	229465	-216791	0.3437	no
gi|320447045|ref|NW_003383526.1|	26235-12702	208835	463752	-217094	0.3338	no
gi|320447045|ref|NW_003383526.1|	27632-12809	150184	381333	-197761	0.38055	no
gi|320447045|ref|NW_003383526.1|	29387-13557	107406	137248	0.353715	0.7975	no
gi|320447045|ref|NW_003383526.1|	14605-15655	358413	616034	-254054	0.15675	no
gi|320447045|ref|NW_003383526.1|	47071-14876	0	137121	inf	1	no
gi|320447045|ref|NW_003383526.1|	78845-14795	719568	0	#NAME?	0.01345	no
gi|320447045|ref|NW_003383526.1|	87769-14884	941754	0	#NAME?	0.00975	no
gi|320447045|ref|NW_003383526.1|	92489-15929	711933	85522	0.264555	0.86465	no
gi|320447045|ref|NW_003383526.1|	93575-15941	0	389257	inf	0.0294	no
[truncated: 406,858 more chars]
