# Supplementary material for: The X Chromosome of Hemipteran Insects: Conservation, Dosage Compensation and Sex-Biased Expression
Source: Genome Biol Evol. 2015 Nov 10;7(12):3259–68. doi: 10.1093/gbe/evv215 (PMC4700948; doi:10.1093/gbe/evv215)
Supplement: Supplementary Data [file supp_evv215_suppl_data.zip › S1 Data (rev) A.pisum (X-chromosome).pdf]

scaffold	gene	expF	expM	log2(fold_change)	p-value	significance
gi|320444995|ref|NW_003385502.1|	11415-15428	20903	359554	0.782498	0.5574	no
gi|320444995|ref|NW_003385502.1|	17344-17933	508505	366948	-0.470686	0.83665	no
gi|320444995|ref|NW_003385502.1|	21454-21888	223.02	0.543397	-868095	0.2504	no
gi|320444995|ref|NW_003385502.1|	28196-29509	122148	0.108701	-10134	0.2504	no
gi|320444995|ref|NW_003385502.1|	33045-34736	289816	143672	230957	0.18415	no
gi|320444995|ref|NW_003385502.1|	40525-41050	115334	659128	251474	0.25215	no
gi|320445051|ref|NW_003385446.1|	27685-29797	0.179832	169677	988193	0.1181	no
gi|320446069|ref|NW_003384502.1|	103037-103718	0	413318	inf	0.0198	no
gi|320446069|ref|NW_003384502.1|	106760-107943	0	185128	inf	0.02205	no
gi|320446069|ref|NW_003384502.1|	115752-116827	0	194685	inf	0.0233	no
gi|320446069|ref|NW_003384502.1|	118868-121647	0	205371	inf	5.00E-05	yes
gi|320446069|ref|NW_003384502.1|	121916-122679	0.319904	197432	262565	0.26355	no
gi|320446069|ref|NW_003384502.1|	123130-124504	0	164706	inf	0.0198	no
gi|320446069|ref|NW_003384502.1|	125930-128355	0	828698	inf	0.0039	no
gi|320446069|ref|NW_003384502.1|	62226-62531	363214	140039	194694	0.3158	no
gi|320446100|ref|NW_003384471.1|	10126-11712	0	147784	inf	0.02075	no
gi|320446100|ref|NW_003384471.1|	16406-17136	852956	560597	-0.605508	0.76445	no
gi|320446100|ref|NW_003384471.1|	23313-23758	109316	622249	-0.812938	0.69235	no
gi|320446100|ref|NW_003384471.1|	23888-24957	971768	574192	-0.759079	0.70975	no
gi|320446100|ref|NW_003384471.1|	29582-31777	912733	106955	0.228743	0.88985	no
gi|320446100|ref|NW_003384471.1|	35114-36968	542101	114058	107314	0.6296	no
gi|320446100|ref|NW_003384471.1|	37099-37399	170646	292009	0.775006	0.7047	no
gi|320446100|ref|NW_003384471.1|	39454-45108	114897	360447	164944	0.2149	no
gi|320446100|ref|NW_003384471.1|	56095-57072	0.682726	314262	220259	0.33335	no
gi|320446100|ref|NW_003384471.1|	6374-6739	235829	13857	25548	0.2437	no
gi|320446100|ref|NW_003384471.1|	65168-65630	145371	580961	199871	0.3149	no
gi|320446100|ref|NW_003384471.1|	79444-79695	948876	160197	0.755553	0.7072	no
gi|320446100|ref|NW_003384471.1|	8779-9342	102392	141777	379146	0.1742	no
gi|320446189|ref|NW_003384382.1|	139060-139738	0	676013	inf	0.0142	no

gi|320446189|ref|NW_003384382.1|	148782-149067	0	969.46	inf	5.00E-05	yes
gi|320446189|ref|NW_003384382.1|	151386-153391	0.381413	255002	938494	0.1028	no
gi|320446189|ref|NW_003384382.1|	16814-23495	146586	844059	25256	0.17105	no
gi|320446189|ref|NW_003384382.1|	73693-75361	0.235576	172198	286981	0.22425	no
gi|320446189|ref|NW_003384382.1|	78424-80113	0.812601	307133	191825	0.37835	no
gi|320446189|ref|NW_003384382.1|	80358-81727	0.446587	175763	197662	0.39	no
gi|320446189|ref|NW_003384382.1|	82923-85614	240494	849814	182115	0.274	no
gi|320446189|ref|NW_003384382.1|	86072-88189	439416	227724	237363	0.18475	no
gi|320446190|ref|NW_003384381.1|	116355-116680	170224	19998	-308951	0.25455	no
gi|320446190|ref|NW_003384381.1|	13312-13583	0	367392	inf	0.01485	no
gi|320446190|ref|NW_003384381.1|	13887-15745	0	300755	inf	5.00E-05	yes
gi|320446190|ref|NW_003384381.1|	18005-18238	0	433005	inf	0.02075	no
gi|320446190|ref|NW_003384381.1|	19669-21452	119937	111654	321868	0.1843	no
gi|320446190|ref|NW_003384381.1|	34758-38319	253171	165071	-0.617027	0.6381	no
gi|320446190|ref|NW_003384381.1|	394-904	0	398989	inf	0.0044	no
gi|320446190|ref|NW_003384381.1|	39676-41623	100557	316158	-166929	0.44585	no
gi|320446190|ref|NW_003384381.1|	42382-42763	193122	773464	-13201	0.54835	no
gi|320446190|ref|NW_003384381.1|	44159-44689	136144	228989	-257179	0.27625	no
gi|320446190|ref|NW_003384381.1|	47113-47714	119591	218337	-245349	0.28085	no
gi|320446190|ref|NW_003384381.1|	47838-48141	295578	474575	-263883	0.27115	no
gi|320446190|ref|NW_003384381.1|	50950-51924	10054	283966	-182398	0.39195	no
gi|320446190|ref|NW_003384381.1|	59653-61633	133474	923919	-0.530716	0.7322	no
gi|320446190|ref|NW_003384381.1|	62368-62782	811051	237955	-176911	0.4302	no
gi|320446190|ref|NW_003384381.1|	63487-64067	311908	461701	-275609	0.23435	no
gi|320446190|ref|NW_003384381.1|	71486-73434	312332	14425	-111451	0.39065	no
gi|320446190|ref|NW_003384381.1|	74635-75277	124296	338381	-187706	0.3907	no
gi|320446190|ref|NW_003384381.1|	75614-76517	131461	174227	-29156	0.21615	no
gi|320446190|ref|NW_003384381.1|	77590-80351	621314	448205	-0.471166	0.8294	no
gi|320446190|ref|NW_003384381.1|	81747-82646	206011	368041	-248478	0.27825	no
gi|320446190|ref|NW_003384381.1|	91736-94980	398741	972752	-203531	0.1325	no

gi|320446203|ref|NW_003384368.1|	108161-108936	111669	325349	154276	0.2602	no
gi|320446203|ref|NW_003384368.1|	113568-115224	0.237566	0.826856	179931	1	no
gi|320446203|ref|NW_003384368.1|	115604-119768	363338	134342	188653	0.2937	no
gi|320446203|ref|NW_003384368.1|	56450-57518	0	238361	inf	0.02075	no
gi|320446203|ref|NW_003384368.1|	93297-95142	0	175324	inf	0.0138	no
gi|320446230|ref|NW_003384341.1|	107725-108654	48666	755154	0.633856	0.756	no
gi|320446230|ref|NW_003384341.1|	116295-117445	22142	447341	101459	0.6106	no
gi|320446230|ref|NW_003384341.1|	132546-133212	227851	392948	0.78625	0.5545	no
gi|320446230|ref|NW_003384341.1|	135507-145809	11179	364704	170593	0.19975	no
gi|320446230|ref|NW_003384341.1|	145885-148059	113152	352257	163837	0.43565	no
gi|320446230|ref|NW_003384341.1|	19474-19770	117873	176294	0.580754	0.7884	no
gi|320446230|ref|NW_003384341.1|	23253-23856	320259	682673	109195	0.59235	no
gi|320446230|ref|NW_003384341.1|	7123-10358	264375	731744	146875	0.36775	no
gi|320446232|ref|NW_003384339.1|	109024-113054	831485	457552	-0.861756	0.5982	no
gi|320446232|ref|NW_003384339.1|	113668-114470	268105	122766	-112688	0.5885	no
gi|320446232|ref|NW_003384339.1|	114618-115960	396488	158827	-131982	0.5308	no
gi|320446232|ref|NW_003384339.1|	11484-12872	0.146408	122045	305935	1	no
gi|320446232|ref|NW_003384339.1|	116146-117180	147929	219134	0.566911	0.78485	no
gi|320446232|ref|NW_003384339.1|	15036-16482	0	280957	inf	0.0109	no
gi|320446232|ref|NW_003384339.1|	167691-167963	124141	458453	188479	0.38895	no
gi|320446232|ref|NW_003384339.1|	176088-176460	113043	162642	0.524825	0.79715	no
gi|320446232|ref|NW_003384339.1|	57291-57820	0.569125	765695	374995	0.208	no
gi|320446232|ref|NW_003384339.1|	58515-59152	0.419406	485057	353174	0.2166	no
gi|320446232|ref|NW_003384339.1|	59666-61027	269857	144723	242302	0.2927	no
gi|320446232|ref|NW_003384339.1|	88798-89298	0	800187	inf	0.0212	no
gi|320446296|ref|NW_003384275.1|	104519-105830	690322	359374	-0.941785	0.6468	no
gi|320446296|ref|NW_003384275.1|	106611-109625	131772	571458	21166	0.11975	no
gi|320446296|ref|NW_003384275.1|	110446-111505	676204	157311	121809	0.5728	no
gi|320446296|ref|NW_003384275.1|	113205-113555	350242	430299	0.296985	0.88335	no
gi|320446296|ref|NW_003384275.1|	132604-132969	200455	107776	-0.895235	0.65945	no

gi|320446296|ref|NW_003384275.1|	133281-134659	10486	369305	-150557	0.4925	no
gi|320446296|ref|NW_003384275.1|	138722-139609	802963	0.535272	-390699	0.1688	no
gi|320446296|ref|NW_003384275.1|	139846-140098	312363	197812	-398103	0.31545	no
gi|320446296|ref|NW_003384275.1|	140909-141221	771047	661909	-354211	0.178	no
gi|320446296|ref|NW_003384275.1|	60061-60343	154653	887267	-0.801599	0.71405	no
gi|320446296|ref|NW_003384275.1|	61434-61876	126582	840163	-0.591334	0.7553	no
gi|320446296|ref|NW_003384275.1|	62025-62716	170294	111263	-0.614058	0.7696	no
gi|320446296|ref|NW_003384275.1|	64920-65727	212607	162287	-0.38964	0.85735	no
gi|320446296|ref|NW_003384275.1|	66264-67756	776187	69094	-0.167845	0.8977	no
gi|320446296|ref|NW_003384275.1|	70566-71065	883133	507126	-0.800287	0.7019	no
gi|320446296|ref|NW_003384275.1|	72938-74269	662315	454477	-0.543312	0.67895	no
gi|320446296|ref|NW_003384275.1|	76875-77582	178866	141865	-0.334358	0.8702	no
gi|320446296|ref|NW_003384275.1|	78277-80548	30587	41831	0.451655	0.7309	no
gi|320446296|ref|NW_003384275.1|	85107-85539	256503	822243	-164134	0.4428	no
gi|320446296|ref|NW_003384275.1|	97026-97550	983597	505648	-0.959934	0.6448	no
gi|320446304|ref|NW_003384267.1|	62587-64476	163269	199143	0.286553	0.88075	no
gi|320446304|ref|NW_003384267.1|	7704-8932	748061	120251	0.684827	0.74655	no
gi|320446304|ref|NW_003384267.1|	93793-94745	6.83	276282	-130575	0.53615	no
gi|320446304|ref|NW_003384267.1|	9882-10326	705812	728994	0.0466228	0.96225	no
gi|320446370|ref|NW_003384201.1|	175538-176780	0.335297	162784	227944	0.2733	no
gi|320446382|ref|NW_003384189.1|	102804-103427	221503	354141	-264493	0.26075	no
gi|320446382|ref|NW_003384189.1|	104298-105194	843117	158435	-241184	0.27695	no
gi|320446382|ref|NW_003384189.1|	105477-105836	709907	175541	-201582	0.36	no
gi|320446382|ref|NW_003384189.1|	106808-108692	190376	620629	-161705	0.33675	no
gi|320446382|ref|NW_003384189.1|	110132-110517	16784	963372	-0.800923	0.69245	no
gi|320446382|ref|NW_003384189.1|	111059-112050	6254	308535	-101935	0.61945	no
gi|320446382|ref|NW_003384189.1|	113416-114271	217922	0.749824	-153919	0.4983	no
gi|320446382|ref|NW_003384189.1|	115284-117600	17274	117757	-0.552795	0.66015	no
gi|320446382|ref|NW_003384189.1|	120467-122365	571394	112053	0.971623	0.47115	no
gi|320446382|ref|NW_003384189.1|	122488-123097	121582	366709	-172923	0.431	no

gi|320446382|ref|NW_003384189.1|	123261-123857	340335	505586	-275093	0.2271	no
gi|320446382|ref|NW_003384189.1|	23441-26449	492932	872223	0.823309	0.613	no
gi|320446382|ref|NW_003384189.1|	26590-29632	464716	135939	154854	0.2412	no
gi|320446382|ref|NW_003384189.1|	32357-33140	0.308232	465406	39164	0.2053	no
gi|320446382|ref|NW_003384189.1|	36771-38191	341885	34645	0.0191356	0.98325	no
gi|320446382|ref|NW_003384189.1|	41366-42973	688952	120714	0.809117	0.7191	no
gi|320446382|ref|NW_003384189.1|	55802-56322	527741	315192	-0.743602	0.71075	no
gi|320446382|ref|NW_003384189.1|	65585-66069	400006	106952	141887	0.4983	no
gi|320446382|ref|NW_003384189.1|	70665-75589	446223	380624	-0.2294	0.88545	no
gi|320446382|ref|NW_003384189.1|	76529-79868	630634	126633	100578	0.5539	no
gi|320446382|ref|NW_003384189.1|	87765-91687	245312	278061	0.180786	0.89125	no
gi|320446382|ref|NW_003384189.1|	92555-93790	469328	458947	-0.0322693	0.9815	no
gi|320446382|ref|NW_003384189.1|	93899-96513	865434	48567	-0.833447	0.71395	no
gi|320446385|ref|NW_003384186.1|	132715-133035	482363	933748	0.952914	0.6698	no
gi|320446385|ref|NW_003384186.1|	138422-141340	830249	202694	128769	0.32515	no
gi|320446385|ref|NW_003384186.1|	150999-151489	130375	187539	384645	0.1766	no
gi|320446385|ref|NW_003384186.1|	162738-163212	0	55485	inf	0.029	no
gi|320446385|ref|NW_003384186.1|	169295-169857	282414	104036	-144073	0.50045	no
gi|320446385|ref|NW_003384186.1|	172163-173728	888399	264875	-174589	0.42845	no
gi|320446385|ref|NW_003384186.1|	173829-177810	608123	737844	0.278952	0.81965	no
gi|320446385|ref|NW_003384186.1|	239615-240461	246013	855735	-15235	0.48375	no
gi|320446385|ref|NW_003384186.1|	256299-260650	332069	332124	0.000239786	0.99915	no
gi|320446385|ref|NW_003384186.1|	78034-83359	115363	717443	263668	0.0537	no
gi|320446385|ref|NW_003384186.1|	83513-88486	256417	55382	111092	0.5033	no
gi|320446385|ref|NW_003384186.1|	89577-90416	447403	100008	116047	0.5767	no
gi|320446385|ref|NW_003384186.1|	97226-98307	279409	113233	-130309	0.42615	no
gi|320446454|ref|NW_003384117.1|	101743-102020	0	195354	inf	0.02915	no
gi|320446454|ref|NW_003384117.1|	105131-105349	514582	106505	437137	0.1793	no
gi|320446454|ref|NW_003384117.1|	107562-108876	219037	104258	225091	0.3124	no
gi|320446454|ref|NW_003384117.1|	111072-111278	579844	140846	128039	0.538	no

gi|320446454|ref|NW_003384117.1|	114237-114693	223524	941957	207523	0.35195	no
gi|320446454|ref|NW_003384117.1|	51710-51919	0	835267	inf	0.0186	no
gi|320446454|ref|NW_003384117.1|	52231-53945	0.228245	731119	500145	0.15005	no
gi|320446454|ref|NW_003384117.1|	60895-63980	15987	234754	0.554248	0.6732	no
gi|320446454|ref|NW_003384117.1|	65812-66868	672508	179212	-190788	0.28335	no
gi|320446454|ref|NW_003384117.1|	67999-69034	223726	671184	-173695	0.43775	no
gi|320446454|ref|NW_003384117.1|	94099-97530	0.105599	646407	593577	0.1183	no
gi|320446457|ref|NW_003384114.1|	102336-105378	0.240474	452.71	108785	0.103	no
gi|320446457|ref|NW_003384114.1|	108000-108658	0.398825	477522	102256	0.14075	no
gi|320446457|ref|NW_003384114.1|	110757-111297	0	251628	inf	0.00505	no
gi|320446457|ref|NW_003384114.1|	116055-116280	0	166813	inf	0.00715	no
gi|320446457|ref|NW_003384114.1|	122907-123134	531837	139368	-193209	0.3906	no
gi|320446457|ref|NW_003384114.1|	12498-13688	109606	637133	-0.782655	0.7186	no
gi|320446457|ref|NW_003384114.1|	14723-15348	604976	176207	-17796	0.4202	no
gi|320446457|ref|NW_003384114.1|	16129-16927	171008	721117	-124576	0.5597	no
gi|320446457|ref|NW_003384114.1|	162032-162716	563901	333663	-0.757051	0.7105	no
gi|320446457|ref|NW_003384114.1|	163892-164112	497144	12.48	-199405	0.3966	no
gi|320446457|ref|NW_003384114.1|	166417-168700	201894	173137	-0.221686	0.8622	no
gi|320446457|ref|NW_003384114.1|	171432-175104	216026	194459	-0.15174	0.9071	no
gi|320446457|ref|NW_003384114.1|	175789-177220	381082	31387	-0.27993	0.89195	no
gi|320446457|ref|NW_003384114.1|	181265-181928	220759	940595	-123083	0.56145	no
gi|320446457|ref|NW_003384114.1|	182913-183244	592032	670525	0.179614	0.90555	no
gi|320446457|ref|NW_003384114.1|	184148-184838	601042	182452	-171995	0.31045	no
gi|320446457|ref|NW_003384114.1|	205889-206285	116587	487294	-125854	0.57395	no
gi|320446457|ref|NW_003384114.1|	207020-208509	144033	907837	-0.66589	0.77125	no
gi|320446457|ref|NW_003384114.1|	215290-215519	730245	378441	-0.948313	0.6443	no
gi|320446457|ref|NW_003384114.1|	21619-21858	926994	140398	-272304	0.25245	no
gi|320446457|ref|NW_003384114.1|	216447-222583	144685	17415	0.267418	0.84235	no
gi|320446457|ref|NW_003384114.1|	22485-22969	733344	356506	-104057	0.59835	no
gi|320446457|ref|NW_003384114.1|	24666-25110	86266	468639	-0.880314	0.6621	no

gi|320446457|ref|NW_003384114.1|	255026-257304	415516	216227	-0.942357	0.477	no
gi|320446457|ref|NW_003384114.1|	257648-258817	45371	11773	-194628	0.26695	no
gi|320446457|ref|NW_003384114.1|	259107-259295	155283	481169	-169028	0.42485	no
gi|320446457|ref|NW_003384114.1|	259702-260274	114189	337136	-176002	0.3072	no
gi|320446457|ref|NW_003384114.1|	262516-263776	335993	13368	-132965	0.4313	no
gi|320446457|ref|NW_003384114.1|	264232-264984	326701	0.671746	-228198	0.26535	no
gi|320446457|ref|NW_003384114.1|	265252-266044	324476	499692	-2699	0.25135	no
gi|320446457|ref|NW_003384114.1|	27749-30217	200278	775879	-13681	0.4295	no
gi|320446457|ref|NW_003384114.1|	278922-279857	7961	633977	299341	0.1128	no
gi|320446457|ref|NW_003384114.1|	285063-285362	153024	100622	271712	0.2502	no
gi|320446457|ref|NW_003384114.1|	286313-286627	337189	260659	295054	0.21785	no
gi|320446457|ref|NW_003384114.1|	302794-303100	720308	474567	271993	0.2551	no
gi|320446457|ref|NW_003384114.1|	304131-304376	136689	79919	254765	0.27945	no
gi|320446457|ref|NW_003384114.1|	30661-31360	487578	236233	-104542	0.52085	no
gi|320446457|ref|NW_003384114.1|	307246-307679	164732	120069	286567	0.22955	no
gi|320446457|ref|NW_003384114.1|	318650-319483	338922	580703	409877	0.05945	no
gi|320446457|ref|NW_003384114.1|	323041-328994	0.177054	663838	522857	0.07235	no
gi|320446457|ref|NW_003384114.1|	4914-6070	22181	261599	0.238034	0.88105	no
gi|320446457|ref|NW_003384114.1|	56624-57313	204504	17776	-352412	0.17115	no
gi|320446457|ref|NW_003384114.1|	6636-7682	270657	417484	0.625254	0.75345	no
gi|320446457|ref|NW_003384114.1|	70364-70977	160424	363029	-214373	0.3423	no
gi|320446457|ref|NW_003384114.1|	7963-9480	934846	933979	-0.00133838	0.9943	no
gi|320446457|ref|NW_003384114.1|	93310-102104	0.157707	177438	101359	0.0578	no
gi|320446457|ref|NW_003384114.1|	9608-11197	723048	685252	-0.0774574	0.96845	no
gi|320446484|ref|NW_003384087.1|	145249-151122	0.0598557	797817	103804	0.1181	no
gi|320446484|ref|NW_003384087.1|	151729-158593	0.45816	703033	393967	0.0588	no
gi|320446484|ref|NW_003384087.1|	165376-166330	61064	599674	-0.0261447	0.98365	no
gi|320446484|ref|NW_003384087.1|	20189-21107	0	221608	inf	0.02915	no
gi|320446484|ref|NW_003384087.1|	35288-39136	317592	447834	0.495791	0.82245	no
gi|320446484|ref|NW_003384087.1|	43034-43451	710438	175975	-201334	0.28795	no

gi|320446484|ref|NW_003384087.1|	45527-47600	0.183649	198553	34345	0.19545	no
gi|320446484|ref|NW_003384087.1|	67708-72078	0.489693	814363	405572	0.0626	no
gi|320446484|ref|NW_003384087.1|	76886-77879	356428	2308	-0.626966	0.7505	no
gi|320446484|ref|NW_003384087.1|	78915-79167	218654	158249	-0.466452	0.8145	no
gi|320446484|ref|NW_003384087.1|	81578-82014	162509	96968	-0.744943	0.7099	no
gi|320446484|ref|NW_003384087.1|	83755-84975	0.171395	677384	530457	0.16735	no
gi|320446484|ref|NW_003384087.1|	85941-88159	0.0851078	43952	569049	0.15975	no
gi|320446484|ref|NW_003384087.1|	88620-91251	0	211596	inf	0.00915	no
gi|320446484|ref|NW_003384087.1|	98919-99643	0	378178	inf	0.0198	no
gi|320446640|ref|NW_003383931.1|	101287-101887	0	406533	inf	0.02915	no
gi|320446640|ref|NW_003383931.1|	106808-107250	870253	577612	-0.591334	0.7582	no
gi|320446640|ref|NW_003383931.1|	108431-108832	384959	57021	0.566787	0.7984	no
gi|320446640|ref|NW_003383931.1|	111732-112187	598562	34843	-0.780631	0.70065	no
gi|320446640|ref|NW_003383931.1|	112696-116857	275075	346042	0.331118	0.8761	no
gi|320446640|ref|NW_003383931.1|	118099-119174	321607	0.417182	-294655	0.209	no
gi|320446640|ref|NW_003383931.1|	125291-128027	33738	160318	-107344	0.6088	no
gi|320446640|ref|NW_003383931.1|	130189-131294	135848	214931	0.661881	0.7399	no
gi|320446640|ref|NW_003383931.1|	136668-139950	365523	751491	103979	0.5229	no
gi|320446640|ref|NW_003383931.1|	140165-141698	364125	151049	205251	0.36925	no
gi|320446640|ref|NW_003383931.1|	143992-144474	738929	0.897773	-304101	0.25695	no
gi|320446640|ref|NW_003383931.1|	155290-156219	997653	0.167812	-589362	0.27155	no
gi|320446640|ref|NW_003383931.1|	158706-159208	131055	0.836546	-396959	0.21945	no
gi|320446640|ref|NW_003383931.1|	159497-161893	663811	0.654356	-334263	0.1767	no
gi|320446640|ref|NW_003383931.1|	162067-163043	546918	141606	-194944	0.36465	no
gi|320446640|ref|NW_003383931.1|	165386-166489	0.194516	65972	50839	0.1716	no
gi|320446640|ref|NW_003383931.1|	204856-206600	0.782971	140224	416263	0.07085	no
gi|320446640|ref|NW_003383931.1|	215123-216903	12017	65447	244525	0.27655	no
gi|320446640|ref|NW_003383931.1|	228817-232269	535041	553029	336963	0.0956	no
gi|320446640|ref|NW_003383931.1|	22906-23354	231229	138276	-0.74177	0.7146	no
gi|320446640|ref|NW_003383931.1|	236317-236806	523438	131308	-199507	0.30275	no

gi|320446640|ref|NW_003383931.1|	240921-241770	247561	170327	-0.539476	0.78905	no
gi|320446640|ref|NW_003383931.1|	242893-244078	3732	172427	-111396	0.59745	no
gi|320446640|ref|NW_003383931.1|	245927-247264	428925	393531	-0.124249	0.9507	no
gi|320446640|ref|NW_003383931.1|	25698-28256	733742	110628	0.592368	0.71395	no
gi|320446640|ref|NW_003383931.1|	282971-284367	959631	0.606065	-398494	0.1544	no
gi|320446640|ref|NW_003383931.1|	284980-285719	268087	0.688593	-196098	0.30285	no
gi|320446640|ref|NW_003383931.1|	288080-288992	848117	0.515855	-403923	0.1605	no
gi|320446640|ref|NW_003383931.1|	289714-290268	631177	0	#NAME?	0.02015	no
gi|320446640|ref|NW_003383931.1|	290478-291236	15502	110707	-380764	0.1761	no
gi|320446640|ref|NW_003383931.1|	292622-293964	182994	105885	-0.789307	0.7077	no
gi|320446640|ref|NW_003383931.1|	29536-29771	39258	395951	333426	0.219	no
gi|320446640|ref|NW_003383931.1|	306482-307366	0.260219	156.65	923361	0.14075	no
gi|320446640|ref|NW_003383931.1|	309287-309549	0	492878	inf	0.01275	no
gi|320446640|ref|NW_003383931.1|	310080-310716	0	234525	inf	0.00565	no
gi|320446640|ref|NW_003383931.1|	311473-312062	0	898058	inf	5.00E-05	yes
gi|320446640|ref|NW_003383931.1|	313528-314043	0	391247	inf	5.00E-05	yes
gi|320446640|ref|NW_003383931.1|	315430-316155	0	119598	inf	5.00E-05	yes
gi|320446640|ref|NW_003383931.1|	46034-46862	626602	953642	392782	0.05795	no
gi|320446640|ref|NW_003383931.1|	47241-47898	0	544798	inf	0.0162	no
gi|320446640|ref|NW_003383931.1|	48260-53215	107248	33005	494366	0.0337	no
gi|320446640|ref|NW_003383931.1|	71828-72481	686024	137446	-231939	0.32125	no
gi|320446640|ref|NW_003383931.1|	73469-78300	506653	457737	-0.146479	0.90865	no
gi|320446640|ref|NW_003383931.1|	79889-80844	46907	598855	0.352402	0.86435	no
gi|320446640|ref|NW_003383931.1|	82332-82948	190134	240211	0.337291	0.87275	no
gi|320446675|ref|NW_003383896.1|	101482-102310	250641	939934	-141499	0.52405	no
gi|320446675|ref|NW_003383896.1|	104460-106034	0.882425	289422	171363	0.4185	no
gi|320446675|ref|NW_003383896.1|	130192-131237	198032	201789	0.027112	0.9889	no
gi|320446675|ref|NW_003383896.1|	1336-2206	0.531923	457778	310536	0.20645	no
gi|320446675|ref|NW_003383896.1|	137342-140753	288508	412579	0.516056	0.69815	no
gi|320446675|ref|NW_003383896.1|	146427-149131	181129	248832	0.458154	0.72585	no

gi|320446675|ref|NW_003383896.1|	152483-152928	437263	243714	-0.843312	0.6842	no
gi|320446675|ref|NW_003383896.1|	153626-154515	877973	426932	-104017	0.6131	no
gi|320446675|ref|NW_003383896.1|	156316-156949	571811	397522	-0.524505	0.7424	no
gi|320446675|ref|NW_003383896.1|	161388-162034	697571	279364	-13202	0.5356	no
gi|320446675|ref|NW_003383896.1|	164527-165363	23325	212591	-345572	0.1706	no
gi|320446675|ref|NW_003383896.1|	168038-168931	102662	123779	-305207	0.2101	no
gi|320446675|ref|NW_003383896.1|	175576-176641	569598	281424	-10172	0.6196	no
gi|320446675|ref|NW_003383896.1|	184918-185106	388206	421023	0.117075	0.9152	no
gi|320446675|ref|NW_003383896.1|	197482-200480	372564	324526	-0.199155	0.92795	no
gi|320446675|ref|NW_003383896.1|	200639-201841	209506	314662	0.586816	0.76675	no
gi|320446675|ref|NW_003383896.1|	201951-202785	366552	271456	-0.4333	0.82255	no
gi|320446675|ref|NW_003383896.1|	211356-211804	177276	133155	-0.412889	0.84205	no
gi|320446675|ref|NW_003383896.1|	211937-212374	283116	182375	-0.634489	0.759	no
gi|320446675|ref|NW_003383896.1|	212846-213789	407859	315935	-0.368441	0.76845	no
gi|320446675|ref|NW_003383896.1|	213977-214826	181545	115444	-0.653133	0.7611	no
gi|320446675|ref|NW_003383896.1|	214977-218259	342816	358314	0.0637896	0.96325	no
gi|320446675|ref|NW_003383896.1|	218692-219514	693438	917702	0.404258	0.76005	no
gi|320446675|ref|NW_003383896.1|	219636-220244	0.451491	27573	261049	0.2636	no
gi|320446675|ref|NW_003383896.1|	220701-228131	418129	100297	126226	0.44775	no
gi|320446675|ref|NW_003383896.1|	241693-242653	0.232916	610868	471298	0.17835	no
gi|320446675|ref|NW_003383896.1|	2425-3035	103299	249951	127482	0.559	no
gi|320446675|ref|NW_003383896.1|	246529-246800	100376	559075	247762	0.2884	no
gi|320446675|ref|NW_003383896.1|	25163-25511	24976	452916	0.858698	0.67375	no
gi|320446675|ref|NW_003383896.1|	258363-259502	280146	217302	295545	0.21605	no
gi|320446675|ref|NW_003383896.1|	263967-264375	371404	550955	0.568943	0.7984	no
gi|320446675|ref|NW_003383896.1|	265005-266552	102925	170035	0.724237	0.72135	no
gi|320446675|ref|NW_003383896.1|	32103-33254	552946	324292	-0.769846	0.5504	no
gi|320446675|ref|NW_003383896.1|	34841-35634	217947	105998	-103994	0.6273	no
gi|320446675|ref|NW_003383896.1|	36192-36789	102289	598816	-0.772465	0.6997	no
gi|320446675|ref|NW_003383896.1|	4091-4506	448377	177628	198608	0.38185	no

gi|320446675|ref|NW_003383896.1|	4652-5109	296793	691307	121987	0.5726	no
gi|320446675|ref|NW_003383896.1|	49044-49459	142584	266443	-241992	0.16545	no
gi|320446675|ref|NW_003383896.1|	52111-53903	385916	126872	-160491	0.36595	no
gi|320446675|ref|NW_003383896.1|	5454-6290	0.562048	270571	226724	0.2738	no
gi|320446675|ref|NW_003383896.1|	68397-69563	106993	131926	0.302212	0.8879	no
gi|320446675|ref|NW_003383896.1|	70439-71019	243678	290212	0.252132	0.9072	no
gi|320446675|ref|NW_003383896.1|	71468-73080	140967	272975	0.953407	0.5709	no
gi|320446675|ref|NW_003383896.1|	73909-79433	87733	612496	-0.518418	0.6899	no
gi|320446675|ref|NW_003383896.1|	8-954	997528	44408	215439	0.21955	no
gi|320446675|ref|NW_003383896.1|	82463-83859	127951	212123	-259262	0.25575	no
gi|320446675|ref|NW_003383896.1|	84624-85161	171925	0.746901	-452471	0.20425	no
gi|320446675|ref|NW_003383896.1|	94077-95468	335864	233319	-0.525572	0.8002	no
gi|320446675|ref|NW_003383896.1|	96741-98881	345516	241063	-0.51934	0.79415	no
gi|320446675|ref|NW_003383896.1|	99582-100406	123305	512519	-126655	0.54885	no
gi|320446681|ref|NW_003383890.1|	12995-14979	186242	263089	0.498368	0.69955	no
gi|320446681|ref|NW_003383890.1|	16032-16812	247943	14888	-0.735855	0.7278	no
gi|320446681|ref|NW_003383890.1|	17414-17760	666139	432824	-0.622041	0.75225	no
gi|320446681|ref|NW_003383890.1|	426916-427831	0.496689	136965	146339	1	no
gi|320446681|ref|NW_003383890.1|	428377-430784	0.310799	238722	294128	0.23405	no
gi|320446681|ref|NW_003383890.1|	430902-432744	0.105029	409848	528623	0.16715	no
gi|320446681|ref|NW_003383890.1|	443628-445236	504044	461975	-0.125734	0.9506	no
gi|320446681|ref|NW_003383890.1|	447660-448245	206637	357885	-252953	0.2723	no
gi|320446681|ref|NW_003383890.1|	448558-449195	209703	142664	-0.555727	0.8006	no
gi|320446681|ref|NW_003383890.1|	451196-451466	101459	11299	0.155299	0.90985	no
gi|320446681|ref|NW_003383890.1|	459672-465974	0.166868	14191	641012	0.0722	no
gi|320446681|ref|NW_003383890.1|	525201-526386	0	47048	inf	5.00E-05	yes
gi|320446709|ref|NW_003383862.1|	121474-123211	164067	417138	134624	0.3021	no
gi|320446709|ref|NW_003383862.1|	12587-14183	434077	405603	-0.0978818	0.96025	no
gi|320446709|ref|NW_003383862.1|	131484-132245	401399	251008	-0.677304	0.6693	no
gi|320446709|ref|NW_003383862.1|	132545-134081	168668	107388	-0.651358	0.7732	no

gi|320446709|ref|NW_003383862.1|	134460-134672	630809	215375	-155035	0.48685	no
gi|320446709|ref|NW_003383862.1|	134852-135324	25138	10715	-123023	0.55825	no
gi|320446709|ref|NW_003383862.1|	136563-137802	316122	106122	-157476	0.34935	no
gi|320446709|ref|NW_003383862.1|	15104-19504	222826	859578	194771	0.2603	no
gi|320446709|ref|NW_003383862.1|	153277-153608	562431	325683	-0.788206	0.7068	no
gi|320446709|ref|NW_003383862.1|	154196-156344	489638	205591	-125194	0.34965	no
gi|320446709|ref|NW_003383862.1|	159126-161927	107199	119046	0.151224	0.90565	no
gi|320446709|ref|NW_003383862.1|	165778-165954	545373	125838	120625	0.5702	no
gi|320446709|ref|NW_003383862.1|	166616-166946	298191	868204	15418	0.3575	no
gi|320446709|ref|NW_003383862.1|	169586-171852	531813	163558	162081	0.339	no
gi|320446709|ref|NW_003383862.1|	172627-173348	312729	128398	203764	0.3482	no
gi|320446709|ref|NW_003383862.1|	173642-174146	805556	348963	211502	0.3433	no
gi|320446709|ref|NW_003383862.1|	175193-179539	845549	591766	280707	0.0421	no
gi|320446709|ref|NW_003383862.1|	181078-182920	756209	873123	352933	0.08985	no
gi|320446709|ref|NW_003383862.1|	183208-186175	2409	355829	388468	0.06225	no
gi|320446709|ref|NW_003383862.1|	186320-186803	200762	657463	503335	0.1467	no
gi|320446709|ref|NW_003383862.1|	186928-188349	526628	104244	430703	0.0474	no
gi|320446709|ref|NW_003383862.1|	197634-199054	498583	544422	0.126892	0.952	no
gi|320446709|ref|NW_003383862.1|	203137-205443	0.977884	233231	125402	0.54205	no
gi|320446709|ref|NW_003383862.1|	205605-206991	175995	224134	0.348828	0.8585	no
gi|320446709|ref|NW_003383862.1|	207119-207934	990115	760451	-0.380741	0.85145	no
gi|320446709|ref|NW_003383862.1|	208614-209464	126325	793592	-0.670673	0.75125	no
gi|320446709|ref|NW_003383862.1|	209648-213670	921913	17592	-238971	0.0801	no
gi|320446709|ref|NW_003383862.1|	224605-225012	111986	455207	20232	0.3493	no
gi|320446709|ref|NW_003383862.1|	228381-234089	209029	337023	0.689143	0.6065	no
gi|320446709|ref|NW_003383862.1|	234256-234555	306048	466298	0.607495	0.76105	no
gi|320446709|ref|NW_003383862.1|	235568-236231	331139	204243	-0.697147	0.7422	no
gi|320446709|ref|NW_003383862.1|	236609-236986	230538	790675	-154385	0.48085	no
gi|320446709|ref|NW_003383862.1|	238321-241778	10789	729148	-0.565272	0.65505	no
gi|320446709|ref|NW_003383862.1|	247005-250470	358393	116595	-162003	0.23395	no

gi|320446709|ref|NW_003383862.1|	250716-251645	396628	127537	-163687	0.3267	no
gi|320446709|ref|NW_003383862.1|	253103-253695	377063	13412	-149129	0.4959	no
gi|320446709|ref|NW_003383862.1|	317633-317889	594887	961691	401489	0.16395	no
gi|320446709|ref|NW_003383862.1|	318950-319747	0	187817	inf	0.00445	no
gi|320446709|ref|NW_003383862.1|	324835-325139	0	141197	inf	0.029	no
gi|320446709|ref|NW_003383862.1|	334577-334992	0	130261	inf	0.0186	no
gi|320446709|ref|NW_003383862.1|	336344-337626	0	514661	inf	0.00695	no
gi|320446709|ref|NW_003383862.1|	357696-357937	115.41	432669	-141543	0.49835	no
gi|320446709|ref|NW_003383862.1|	546626-547746	0	303797	inf	0.01485	no
gi|320446709|ref|NW_003383862.1|	622717-624118	0	126733	inf	0.0032	no
gi|320446709|ref|NW_003383862.1|	7431-8063	118892	10395	-0.193766	0.92305	no
gi|320446737|ref|NW_003383834.1|	115456-116542	0	149.37	inf	5.00E-05	yes
gi|320446737|ref|NW_003383834.1|	130938-131649	0	339652	inf	0.0233	no
gi|320446737|ref|NW_003383834.1|	140671-141535	112771	443579	-134613	0.5247	no
gi|320446737|ref|NW_003383834.1|	143342-143863	818177	19636	-205891	0.3566	no
gi|320446737|ref|NW_003383834.1|	156608-156938	238553	0.964671	-462813	0.29365	no
gi|320446737|ref|NW_003383834.1|	214571-215341	126288	216568	0.778099	0.71785	no
gi|320446737|ref|NW_003383834.1|	246779-247580	268579	204964	-0.389975	0.84875	no
gi|320446737|ref|NW_003383834.1|	249457-254808	682724	298093	-119554	0.47905	no
gi|320446737|ref|NW_003383834.1|	258819-260892	798872	211363	-191824	0.38455	no
gi|320446737|ref|NW_003383834.1|	262910-263578	362425	690992	-239094	0.30085	no
gi|320446737|ref|NW_003383834.1|	264904-267839	315008	114912	-145486	0.2746	no
gi|320446737|ref|NW_003383834.1|	268629-269234	354967	19758	-0.84525	0.6966	no
gi|320446737|ref|NW_003383834.1|	271683-272958	142847	74334	-0.942373	0.6678	no
gi|320446737|ref|NW_003383834.1|	279489-280729	0.839912	116503	0.472053	1	no
gi|320446737|ref|NW_003383834.1|	280830-282774	148137	853739	252686	0.2677	no
gi|320446737|ref|NW_003383834.1|	284563-284997	245977	141283	252199	0.287	no
gi|320446737|ref|NW_003383834.1|	286226-286939	0.353265	241626	277396	0.2657	no
gi|320446737|ref|NW_003383834.1|	287099-287564	359026	291892	302328	0.21075	no
gi|320446737|ref|NW_003383834.1|	287710-289882	331093	207315	264652	0.15105	no

gi|320446737|ref|NW_003383834.1|	304693-305243	266227	466836	0.810261	0.6935	no
gi|320446737|ref|NW_003383834.1|	311195-312426	0	152806	inf	0.02915	no
gi|320446737|ref|NW_003383834.1|	324734-326307	0.126155	298413	456404	0.1776	no
gi|320446737|ref|NW_003383834.1|	327067-329874	0.262451	206356	297501	0.22485	no
gi|320446737|ref|NW_003383834.1|	350291-350690	301475	959909	-165107	0.4413	no
gi|320446737|ref|NW_003383834.1|	354129-354713	289138	130491	-114781	0.58445	no
gi|320446737|ref|NW_003383834.1|	357232-357825	799067	636982	-0.327065	0.86905	no
gi|320446737|ref|NW_003383834.1|	360205-361291	535691	288306	-0.893802	0.66555	no
gi|320446737|ref|NW_003383834.1|	365355-365986	168141	520999	-501224	0.03905	no
gi|320446737|ref|NW_003383834.1|	374718-375010	101.87	0	#NAME?	0.0081	no
gi|320446737|ref|NW_003383834.1|	387278-387975	807599	0.749114	-675231	0.1106	no
gi|320446737|ref|NW_003383834.1|	48447-49734	141216	157014	0.152984	0.94595	no
gi|320446737|ref|NW_003383834.1|	487884-492220	0.329169	478251	386087	0.14375	no
gi|320446737|ref|NW_003383834.1|	492356-493821	0.548951	15264	147538	0.49665	no
gi|320446737|ref|NW_003383834.1|	49837-50186	27422	220768	-0.312801	0.8806	no
gi|320446737|ref|NW_003383834.1|	50826-51152	875456	833843	-0.0702599	0.97245	no
gi|320446737|ref|NW_003383834.1|	52008-52804	171618	237765	0.470337	0.8293	no
gi|320446737|ref|NW_003383834.1|	54077-56085	239884	365098	0.60595	0.63895	no
gi|320446737|ref|NW_003383834.1|	93847-94113	105995	134757	0.346367	0.8581	no
gi|320446737|ref|NW_003383834.1|	95010-96486	149623	349881	122553	0.5462	no
gi|320446741|ref|NW_003383830.1|	108628-109605	0.682726	141418	105059	0.5772	no
gi|320446741|ref|NW_003383830.1|	111292-112183	154464	407919	140101	0.50825	no
gi|320446741|ref|NW_003383830.1|	117661-117897	309549	365969	0.241555	0.89735	no
gi|320446741|ref|NW_003383830.1|	118276-119648	623572	639346	0.0360424	0.984	no
gi|320446741|ref|NW_003383830.1|	131047-132323	152439	258797	-255835	0.2703	no
gi|320446741|ref|NW_003383830.1|	134497-135808	528567	132968	-199101	0.2742	no
gi|320446741|ref|NW_003383830.1|	139202-139486	285719	280815	-334691	0.23995	no
gi|320446741|ref|NW_003383830.1|	147667-148373	58792	121568	104808	0.4246	no
gi|320446741|ref|NW_003383830.1|	153028-154147	379502	119935	-166185	0.2188	no
gi|320446741|ref|NW_003383830.1|	154311-155650	371571	144496	-136261	0.29615	no

gi|320446741|ref|NW_003383830.1|	155807-159896	232622	170665	-0.446827	0.84045	no
gi|320446741|ref|NW_003383830.1|	162882-164666	65378	235309	-147425	0.4964	no
gi|320446741|ref|NW_003383830.1|	164847-166158	753079	283143	-141127	0.51055	no
gi|320446741|ref|NW_003383830.1|	170272-170687	493215	278284	-0.825657	0.68735	no
gi|320446741|ref|NW_003383830.1|	172342-175072	105516	136597	0.372464	0.7682	no
gi|320446741|ref|NW_003383830.1|	198904-199215	172748	217935	365716	0.0782	no
gi|320446741|ref|NW_003383830.1|	203595-203835	0	392398	inf	0.02075	no
gi|320446741|ref|NW_003383830.1|	206451-206832	536449	534393	331639	0.1895	no
gi|320446741|ref|NW_003383830.1|	210257-212578	331707	789009	457206	0.0402	no
gi|320446741|ref|NW_003383830.1|	21292-24259	0.0617693	285009	552797	0.1624	no
gi|320446741|ref|NW_003383830.1|	26164-29070	0.126361	145744	352782	0.18205	no
gi|320446741|ref|NW_003383830.1|	269794-269993	0	697665	inf	0.02205	no
gi|320446741|ref|NW_003383830.1|	272016-275150	0	427413	inf	0.00485	no
gi|320446741|ref|NW_003383830.1|	275262-276153	0.257439	727159	481997	0.17075	no
gi|320446741|ref|NW_003383830.1|	276507-277555	228454	548594	458576	0.0498	no
gi|320446741|ref|NW_003383830.1|	277682-278692	28324	153103	575633	0.036	no
gi|320446741|ref|NW_003383830.1|	282663-291194	543034	396519	-0.453654	0.7275	no
gi|320446741|ref|NW_003383830.1|	291345-291816	567841	420873	-0.432103	0.85185	no
gi|320446741|ref|NW_003383830.1|	291958-294364	225433	407099	0.852679	0.68485	no
gi|320446741|ref|NW_003383830.1|	294525-295477	800759	731334	-0.130839	0.94765	no
gi|320446741|ref|NW_003383830.1|	295701-296775	183132	218582	0.255288	0.9129	no
gi|320446741|ref|NW_003383830.1|	297166-299771	179441	151226	-0.246801	0.8444	no
gi|320446741|ref|NW_003383830.1|	301436-302335	297571	105155	-482265	0.13505	no
gi|320446741|ref|NW_003383830.1|	304042-304755	282612	386602	0.452027	0.81645	no
gi|320446741|ref|NW_003383830.1|	318061-318892	314556	409193	-294247	0.2149	no
gi|320446741|ref|NW_003383830.1|	320079-321618	504839	0.990396	-234975	0.29595	no
gi|320446741|ref|NW_003383830.1|	322427-322638	818099	21939	-189878	0.39565	no
gi|320446741|ref|NW_003383830.1|	324165-325343	166502	682357	-128694	0.55865	no
gi|320446741|ref|NW_003383830.1|	345132-347756	141987	106106	-0.420246	0.7358	no
gi|320446741|ref|NW_003383830.1|	347864-350077	44368	291764	-0.604714	0.7677	no

gi|320446741|ref|NW_003383830.1|	352463-353258	482596	186402	-13724	0.50605	no
gi|320446741|ref|NW_003383830.1|	354720-355378	127624	706634	-0.852863	0.67865	no
gi|320446741|ref|NW_003383830.1|	359907-361985	461539	251674	-0.874893	0.50955	no
gi|320446741|ref|NW_003383830.1|	362140-365640	356617	444766	0.318669	0.88445	no
gi|320446741|ref|NW_003383830.1|	365783-366635	0.821186	244855	157614	0.491	no
gi|320446741|ref|NW_003383830.1|	375143-376322	232498	36437	0.648184	0.69415	no
gi|320446741|ref|NW_003383830.1|	376459-379056	428687	544052	0.343819	0.8704	no
gi|320446741|ref|NW_003383830.1|	379213-380080	828399	191322	120761	0.57725	no
gi|320446741|ref|NW_003383830.1|	381446-384909	129122	332812	-195596	0.2642	no
gi|320446741|ref|NW_003383830.1|	38364-39609	0	139119	inf	1	no
gi|320446741|ref|NW_003383830.1|	415423-416314	205951	319241	-268959	0.24	no
gi|320446741|ref|NW_003383830.1|	42491-43172	113533	258324	118607	0.56815	no
gi|320446741|ref|NW_003383830.1|	426780-427231	205454	212468	0.0484329	0.97905	no
gi|320446741|ref|NW_003383830.1|	434700-436184	0.135149	310076	4.52	0.17645	no
gi|320446741|ref|NW_003383830.1|	436917-437935	0.431298	193738	216735	0.29245	no
gi|320446741|ref|NW_003383830.1|	438451-440991	131055	167742	0.356069	0.7851	no
gi|320446741|ref|NW_003383830.1|	444857-450001	42141	876512	105655	0.41165	no
gi|320446741|ref|NW_003383830.1|	45023-46333	0.942225	196202	10582	0.59735	no
gi|320446741|ref|NW_003383830.1|	50204-50689	19925	444022	115605	0.5747	no
gi|320446741|ref|NW_003383830.1|	58244-58804	191124	153464	-0.316606	0.8782	no
gi|320446741|ref|NW_003383830.1|	60785-61843	116941	124864	0.0945814	0.9662	no
gi|320446741|ref|NW_003383830.1|	62107-62611	867521	664692	-0.384213	0.8462	no
gi|320446741|ref|NW_003383830.1|	63189-64019	851583	126863	0.575058	0.77985	no
gi|320446741|ref|NW_003383830.1|	64181-65194	0.651108	26995	205172	0.3468	no
gi|320446741|ref|NW_003383830.1|	66594-67407	496772	883506	0.830655	0.6844	no
gi|320446741|ref|NW_003383830.1|	71468-72195	274604	422934	0.62308	0.74755	no
gi|320446741|ref|NW_003383830.1|	72297-73058	449567	792656	0.81816	0.6881	no
gi|320446753|ref|NW_003383818.1|	1006-1979	320334	0.315938	-334186	0.239	no
gi|320446753|ref|NW_003383818.1|	10281-11812	117221	163056	0.476138	0.80865	no
gi|320446753|ref|NW_003383818.1|	11956-12190	199182	276149	0.471364	0.82175	no

gi|320446753|ref|NW_003383818.1|	13422-14296	343584	254781	-0.431405	0.823	no
gi|320446753|ref|NW_003383818.1|	14792-17171	0.551004	120911	113381	1	no
gi|320446753|ref|NW_003383818.1|	210763-213450	874074	182261	106018	0.39875	no
gi|320446753|ref|NW_003383818.1|	214302-216039	112374	219135	0.963503	0.64715	no
gi|320446753|ref|NW_003383818.1|	219443-219744	263142	36194	0.459906	0.8193	no
gi|320446753|ref|NW_003383818.1|	256480-257032	243431	464091	-239103	0.294	no
gi|320446753|ref|NW_003383818.1|	26855-29459	584141	756448	0.372924	0.8677	no
gi|320446753|ref|NW_003383818.1|	273560-273760	109574	455216	-458921	0.293	no
gi|320446753|ref|NW_003383818.1|	29793-30633	893325	126728	0.504479	0.80925	no
gi|320446753|ref|NW_003383818.1|	302577-303505	146209	10083	-0.536107	0.7982	no
gi|320446753|ref|NW_003383818.1|	327166-327350	118726	266972	-215287	0.35185	no
gi|320446753|ref|NW_003383818.1|	356431-356846	627728	236838	-140624	0.5239	no
gi|320446753|ref|NW_003383818.1|	386313-386664	270622	754558	-184258	0.394	no
gi|320446753|ref|NW_003383818.1|	38982-39723	0	663083	inf	0.0109	no
gi|320446753|ref|NW_003383818.1|	390531-391210	456161	0.518884	-313606	0.255	no
gi|320446753|ref|NW_003383818.1|	391922-392503	874764	296001	-156329	0.4713	no
gi|320446753|ref|NW_003383818.1|	394107-402391	366844	385148	0.0702446	0.95285	no
gi|320446753|ref|NW_003383818.1|	407744-410950	0.170398	127115	289916	1	no
gi|320446753|ref|NW_003383818.1|	413363-417308	185117	120343	270064	0.04865	no
gi|320446753|ref|NW_003383818.1|	419114-419584	126688	187766	0.567662	0.78305	no
gi|320446753|ref|NW_003383818.1|	426566-427053	131839	352668	-19024	0.3915	no
gi|320446753|ref|NW_003383818.1|	429217-429908	151784	556315	-144805	0.49615	no
gi|320446753|ref|NW_003383818.1|	430583-432958	168765	149217	-0.1776	0.89125	no
gi|320446753|ref|NW_003383818.1|	433373-434835	0.825441	210388	134981	0.5192	no
gi|320446753|ref|NW_003383818.1|	454329-455376	0	266003	inf	0.0025	no
gi|320446753|ref|NW_003383818.1|	45562-47621	259082	348504	0.427764	0.83005	no
gi|320446753|ref|NW_003383818.1|	457501-457821	0	456499	inf	0.00845	no
gi|320446753|ref|NW_003383818.1|	457960-458778	0	430077	inf	5.00E-05	yes
gi|320446753|ref|NW_003383818.1|	462397-463119	121368	396294	170718	0.44825	no
gi|320446753|ref|NW_003383818.1|	463263-464232	115029	635263	246536	0.284	no

gi|320446753|ref|NW_003383818.1|	464362-465818	179725	595795	172903	0.4166	no
gi|320446753|ref|NW_003383818.1|	469486-470364	15759	162788	0.0468186	0.94	no
gi|320446753|ref|NW_003383818.1|	473840-474564	759777	17018	116341	0.5826	no
gi|320446753|ref|NW_003383818.1|	487183-488135	247293	253529	0.0359279	0.98825	no
gi|320446753|ref|NW_003383818.1|	489040-490981	18896	180702	-0.0644698	0.95815	no
gi|320446753|ref|NW_003383818.1|	49410-50537	130602	563571	-121251	0.57915	no
gi|320446753|ref|NW_003383818.1|	494423-496908	127509	434789	-155221	0.36265	no
gi|320446753|ref|NW_003383818.1|	499265-499915	422684	17161	-130044	0.5542	no
gi|320446753|ref|NW_003383818.1|	500278-500758	77844	723499	-342752	0.16695	no
gi|320446753|ref|NW_003383818.1|	504691-504900	850059	759333	-348476	0.2322	no
gi|320446753|ref|NW_003383818.1|	505059-506921	788405	267488	-155946	0.4692	no
gi|320446753|ref|NW_003383818.1|	520416-524309	768987	3028.02	197734	0.115	no
gi|320446753|ref|NW_003383818.1|	524431-525812	0.294606	143247	228164	0.2731	no
gi|320446753|ref|NW_003383818.1|	540218-540551	423036	167192	198266	0.24045	no
gi|320446753|ref|NW_003383818.1|	547915-548568	871654	923637	0.0835702	0.9464	no
gi|320446753|ref|NW_003383818.1|	550388-551683	0.159261	198947	364292	0.2061	no
gi|320446753|ref|NW_003383818.1|	551865-552448	773201	222435	152447	0.47485	no
gi|320446753|ref|NW_003383818.1|	553130-557543	466882	150709	169063	0.33265	no
gi|320446753|ref|NW_003383818.1|	577265-579301	113391	160777	0.503755	0.76665	no
gi|320446753|ref|NW_003383818.1|	8070-9808	12353	101674	-0.280908	1	no
gi|320446787|ref|NW_003383784.1|	101157-101654	190613	10213	242168	0.29225	no
gi|320446787|ref|NW_003383784.1|	104659-104904	0	323996	inf	0.02205	no
gi|320446787|ref|NW_003383784.1|	117914-118127	562899	31722	249454	0.27555	no
gi|320446787|ref|NW_003383784.1|	121610-122162	10584	856783	301705	0.21295	no
gi|320446787|ref|NW_003383784.1|	255618-256486	0.533606	146943	146141	0.3715	no
gi|320446787|ref|NW_003383784.1|	331640-332278	418379	313118	-0.418103	0.8285	no
gi|320446787|ref|NW_003383784.1|	339743-342356	525156	520579	-0.0126302	0.99335	no
gi|320446787|ref|NW_003383784.1|	342554-343722	126668	313444	130715	0.53575	no
gi|320446787|ref|NW_003383784.1|	371553-373733	0	296684	inf	0.0089	no
gi|320446787|ref|NW_003383784.1|	413736-414974	496537	630354	-297767	0.11615	no

gi|320446787|ref|NW_003383784.1|	416106-416781	529313	444903	-357256	0.0816	no
gi|320446787|ref|NW_003383784.1|	418560-418921	568089	394174	-384921	0.1693	no
gi|320446787|ref|NW_003383784.1|	419029-419333	64106	117664	-576771	0.2728	no
gi|320446787|ref|NW_003383784.1|	419407-419699	264862	156538	-0.758725	0.71155	no
gi|320446787|ref|NW_003383784.1|	422154-422714	413241	104635	-198162	0.30275	no
gi|320446787|ref|NW_003383784.1|	52285-52696	146344	603413	-127815	0.54135	no
gi|320446787|ref|NW_003383784.1|	54050-57214	437865	157134	-147849	0.49075	no
gi|320446787|ref|NW_003383784.1|	57329-58376	291118	10065	-153226	0.48175	no
gi|320446787|ref|NW_003383784.1|	59702-60820	726593	489809	-0.56893	0.78545	no
gi|320446787|ref|NW_003383784.1|	61802-62835	380866	585079	0.61935	0.7638	no
gi|320446787|ref|NW_003383784.1|	63194-66202	121712	227629	0.903218	0.4886	no
gi|320446787|ref|NW_003383784.1|	82279-83072	0.605409	893712	388383	0.17505	no
gi|320446787|ref|NW_003383784.1|	93417-94071	120777	106963	314669	0.21025	no
gi|320446787|ref|NW_003383784.1|	94221-94772	0.530821	358046	275385	0.26605	no
gi|320446787|ref|NW_003383784.1|	96092-96573	33716	856009	134419	0.5275	no
gi|320446787|ref|NW_003383784.1|	97157-97516	979182	239375	128962	0.53495	no
gi|320446787|ref|NW_003383784.1|	99281-99798	177708	636554	184077	0.396	no
gi|320446791|ref|NW_003383780.1|	103242-105941	408197	170371	-126059	0.3476	no
gi|320446791|ref|NW_003383780.1|	107543-109571	24662	925609	-141381	0.41385	no
gi|320446791|ref|NW_003383780.1|	113598-113945	291137	774064	-191117	0.3694	no
gi|320446791|ref|NW_003383780.1|	114410-117881	303495	277998	-0.1266	0.92245	no
gi|320446791|ref|NW_003383780.1|	119430-119985	0	813901	inf	0.01485	no
gi|320446791|ref|NW_003383780.1|	120400-121271	0	548486	inf	0.00935	no
gi|320446791|ref|NW_003383780.1|	129580-130226	141156	634156	-115438	0.37885	no
gi|320446791|ref|NW_003383780.1|	132128-132915	703793	546129	-0.365911	0.85595	no
gi|320446791|ref|NW_003383780.1|	133432-134470	323821	239865	-0.432976	0.793	no
gi|320446791|ref|NW_003383780.1|	134812-135735	176727	226795	0.359867	0.87185	no
gi|320446791|ref|NW_003383780.1|	161982-162505	0.580515	11316	428488	0.18435	no
gi|320446791|ref|NW_003383780.1|	1836-2581	0	21556	inf	0.0055	no
gi|320446791|ref|NW_003383780.1|	225307-225979	579255	0.790296	-287373	0.2155	no

gi|320446791|ref|NW_003383780.1|	226084-226743	103452	0	#NAME?	0.00975	no
gi|320446791|ref|NW_003383780.1|	309045-309297	406073	118687	154735	0.46405	no
gi|320446791|ref|NW_003383780.1|	3641-4229	0	193646	inf	0.00765	no
gi|320446791|ref|NW_003383780.1|	52744-55562	12152	129682	0.0937796	0.94215	no
gi|320446791|ref|NW_003383780.1|	555306-557103	0	87324	inf	0.00475	no
gi|320446791|ref|NW_003383780.1|	56331-57271	526966	413095	-0.351236	0.862	no
gi|320446791|ref|NW_003383780.1|	57860-58230	263115	13.46	-0.967009	0.64585	no
gi|320446791|ref|NW_003383780.1|	58918-60363	108816	659334	-0.722805	0.73935	no
gi|320446791|ref|NW_003383780.1|	593418-594664	0.500955	196893	197466	0.39	no
gi|320446791|ref|NW_003383780.1|	596532-597332	0.29895	266915	31584	0.24575	no
gi|320446791|ref|NW_003383780.1|	60938-61422	600009	222816	-142913	0.51015	no
gi|320446791|ref|NW_003383780.1|	614327-618079	340656	973757	151524	0.3814	no
gi|320446791|ref|NW_003383780.1|	618951-623025	0.263728	501557	424929	0.12945	no
gi|320446791|ref|NW_003383780.1|	63363-64209	212843	494425	-210597	0.35495	no
gi|320446791|ref|NW_003383780.1|	682225-682396	962596	196698	103098	0.60815	no
gi|320446791|ref|NW_003383780.1|	688238-688678	127705	688434	-0.891425	0.66475	no
gi|320446791|ref|NW_003383780.1|	79761-81609	194627	176459	-0.141379	0.91375	no
gi|320446791|ref|NW_003383780.1|	81787-86813	103545	631592	-0.713197	0.5754	no
gi|320446791|ref|NW_003383780.1|	90787-91665	239011	958638	-131802	0.5455	no
gi|320446791|ref|NW_003383780.1|	91719-93495	142388	442524	-1686	0.4543	no
gi|320446791|ref|NW_003383780.1|	95971-100991	162918	103503	-0.654472	0.6219	no
gi|320446811|ref|NW_003383760.1|	10585-13944	325182	21.84	-0.57427	0.66475	no
gi|320446811|ref|NW_003383760.1|	154321-155347	277506	0.147545	-42333	0.3017	no
gi|320446811|ref|NW_003383760.1|	161487-165452	488586	0.791665	-262565	0.2563	no
gi|320446811|ref|NW_003383760.1|	16156-17020	461822	127529	-185651	0.27495	no
gi|320446811|ref|NW_003383760.1|	18214-20604	953069	148567	0.640463	0.6144	no
gi|320446811|ref|NW_003383760.1|	22092-22578	615355	221211	-479792	0.1477	no
gi|320446811|ref|NW_003383760.1|	22765-23193	396128	278967	-38278	0.1701	no
gi|320446811|ref|NW_003383760.1|	23425-24020	981711	0.95046	-33686	0.1915	no
gi|320446811|ref|NW_003383760.1|	25902-27149	350321	0.231413	-392014	0.22055	no

gi|320446811|ref|NW_003383760.1|	263208-264144	313167	33233	0.085681	0.96115	no
gi|320446811|ref|NW_003383760.1|	28764-29197	658928	436616	-391569	0.1338	no
gi|320446811|ref|NW_003383760.1|	31104-37078	22.58	183177	-0.301812	0.8209	no
gi|320446811|ref|NW_003383760.1|	314682-319019	242703	108589	216161	0.2272	no
gi|320446811|ref|NW_003383760.1|	392085-392541	670573	376783	249027	0.2702	no
gi|320446811|ref|NW_003383760.1|	394959-395840	0.261428	702198	474739	0.17355	no
gi|320446811|ref|NW_003383760.1|	400709-401271	143774	242751	-256626	0.2721	no
gi|320446811|ref|NW_003383760.1|	401663-403048	158533	469048	-175698	0.4277	no
gi|320446811|ref|NW_003383760.1|	405097-406581	514918	366454	-381264	0.06055	no
gi|320446811|ref|NW_003383760.1|	407263-407543	868816	5836	-3896	0.18905	no
gi|320446811|ref|NW_003383760.1|	408920-410649	247418	0.78678	-497485	0.06545	no
gi|320446811|ref|NW_003383760.1|	413484-413992	207742	0.819726	-466351	0.19575	no
gi|320446811|ref|NW_003383760.1|	423631-426600	0.432069	24165	248358	0.276	no
gi|320446811|ref|NW_003383760.1|	431055-432362	0.472432	184722	528911	0.14275	no
gi|320446811|ref|NW_003383760.1|	432667-434377	0	223113	inf	0.01275	no
gi|320446811|ref|NW_003383760.1|	435596-436237	0.415327	42397	335164	0.22635	no
gi|320446811|ref|NW_003383760.1|	44219-44854	366683	14621	-132649	0.5392	no
gi|320446811|ref|NW_003383760.1|	447566-452246	485906	531735	0.130032	0.9126	no
gi|320446811|ref|NW_003383760.1|	464717-465888	156926	0.499924	-497223	0.16335	no
gi|320446811|ref|NW_003383760.1|	46504-46902	567022	135066	-206974	0.35125	no
gi|320446811|ref|NW_003383760.1|	466760-468194	915122	0.0978416	-654737	0.2643	no
gi|320446811|ref|NW_003383760.1|	468322-468997	180273	104683	-410609	0.1749	no
gi|320446811|ref|NW_003383760.1|	484815-492219	667949	315265	223876	0.14305	no
gi|320446811|ref|NW_003383760.1|	49243-49628	13637	137625	-330872	0.24265	no
gi|320446811|ref|NW_003383760.1|	493405-493690	0	208636	inf	0.02205	no
gi|320446811|ref|NW_003383760.1|	511144-512922	875098	929628	0.0872097	0.9671	no
gi|320446811|ref|NW_003383760.1|	524415-530987	320847	150246	222737	0.20175	no
gi|320446811|ref|NW_003383760.1|	532374-533080	372827	130882	181169	0.30825	no
gi|320446811|ref|NW_003383760.1|	54344-54566	48066	603683	-299315	0.2661	no
gi|320446811|ref|NW_003383760.1|	54820-55082	582151	528083	-346256	0.18685	no

gi|320446811|ref|NW_003383760.1|	564255-565773	472321	136329	-179268	0.3187	no
gi|320446811|ref|NW_003383760.1|	569570-569873	25863	593219	-212426	0.34865	no
gi|320446811|ref|NW_003383760.1|	578427-579203	499553	85689	-254345	0.15505	no
gi|320446811|ref|NW_003383760.1|	600910-601960	159525	133235	-0.259805	0.90715	no
gi|320446811|ref|NW_003383760.1|	604655-605132	547818	68584	0.324175	0.8588	no
gi|320446811|ref|NW_003383760.1|	606753-615066	349653	16376	-109434	0.4069	no
gi|320446811|ref|NW_003383760.1|	623462-624896	202735	616402	-171765	0.30665	no
gi|320446811|ref|NW_003383760.1|	625691-626984	258491	143953	-0.844519	0.603	no
gi|320446811|ref|NW_003383760.1|	627388-628222	0.84589	135728	0.682177	1	no
gi|320446811|ref|NW_003383760.1|	641756-644044	120841	152061	0.331531	0.79275	no
gi|320446811|ref|NW_003383760.1|	648001-648698	255801	449468	0.8132	0.68375	no
gi|320446811|ref|NW_003383760.1|	649067-649471	379041	374537	-0.0172449	0.9157	no
gi|320446811|ref|NW_003383760.1|	649941-651229	272545	511762	0.90898	0.6529	no
gi|320446811|ref|NW_003383760.1|	651916-653778	217849	521964	126062	0.3505	no
gi|320446811|ref|NW_003383760.1|	654413-657068	93445	27772	-175049	0.2948	no
gi|320446811|ref|NW_003383760.1|	657759-658280	812332	290613	-148297	0.36535	no
gi|320446811|ref|NW_003383760.1|	660429-661974	195856	129063	-0.601714	0.71155	no
gi|320446811|ref|NW_003383760.1|	662675-664842	0.349421	298668	30955	0.2198	no
gi|320446811|ref|NW_003383760.1|	66991-68100	282042	227333	-363303	0.07605	no
gi|320446811|ref|NW_003383760.1|	691283-691679	691615	324863	-109014	0.59375	no
gi|320446811|ref|NW_003383760.1|	691877-692730	423592	122233	-179304	0.2912	no
gi|320446811|ref|NW_003383760.1|	693387-697364	813502	396146	-103811	0.4326	no
gi|320446811|ref|NW_003383760.1|	709172-710923	111336	403236	18567	0.39145	no
gi|320446811|ref|NW_003383760.1|	713913-714180	262072	166639	266869	0.2703	no
gi|320446811|ref|NW_003383760.1|	744700-746257	139174	14389	0.0480812	0.98395	no
gi|320446811|ref|NW_003383760.1|	749175-749373	167957	285214	-255797	0.2739	no
gi|320446811|ref|NW_003383760.1|	750310-750955	473024	182009	-13779	0.53675	no
gi|320446811|ref|NW_003383760.1|	751038-752002	196892	134305	-0.551885	0.7988	no
gi|320446811|ref|NW_003383760.1|	75762-77068	329435	787707	-206426	0.23655	no
gi|320446811|ref|NW_003383760.1|	771180-771694	19749	224994	0.188111	0.92705	no

gi|320446811|ref|NW_003383760.1|	777966-779242	334069	293678	-0.185913	0.88375	no
gi|320446811|ref|NW_003383760.1|	780138-782320	131757	212306	0.688267	0.59285	no
gi|320446811|ref|NW_003383760.1|	782877-783902	0.854946	10341	0.27447	1	no
gi|320446811|ref|NW_003383760.1|	785753-791988	171825	274367	0.675165	0.6134	no
gi|320446811|ref|NW_003383760.1|	792147-792814	199205	133178	-390283	0.1738	no
gi|320446811|ref|NW_003383760.1|	794460-794901	492673	0	#NAME?	0.0071	no
gi|320446811|ref|NW_003383760.1|	796169-798412	84.3	0.469276	-748895	0.07005	no
gi|320446811|ref|NW_003383760.1|	800116-801373	235.06	206258	-683244	0.03335	no
gi|320446811|ref|NW_003383760.1|	83124-83944	288719	0.198443	-386287	0.3182	no
gi|320446822|ref|NW_003383749.1|	114843-115001	86145.1	77257.5	-0.157094	0.94625	no
gi|320446822|ref|NW_003383749.1|	13124-13702	637169	0.663189	-326419	0.24355	no
gi|320446822|ref|NW_003383749.1|	1532-1801	256404	114187	-116703	0.58095	no
gi|320446822|ref|NW_003383749.1|	163775-174896	0.123993	489309	530241	0.0586	no
gi|320446822|ref|NW_003383749.1|	195647-200985	0.165329	609285	52037	0.08605	no
gi|320446822|ref|NW_003383749.1|	203693-204324	336281	810444	-205289	0.3575	no
gi|320446822|ref|NW_003383749.1|	205836-206811	433563	173304	-132294	0.52555	no
gi|320446822|ref|NW_003383749.1|	206954-207470	124819	239489	-238181	0.305	no
gi|320446822|ref|NW_003383749.1|	210003-211643	432491	242496	-0.83471	0.6822	no
gi|320446822|ref|NW_003383749.1|	211873-213475	432171	2234	-0.951972	0.6495	no
gi|320446822|ref|NW_003383749.1|	213727-216210	840809	629167	-0.418334	0.85435	no
gi|320446822|ref|NW_003383749.1|	227256-227616	595985	325173	-0.874067	0.6698	no
gi|320446822|ref|NW_003383749.1|	230749-231308	336765	160903	-106555	0.6138	no
gi|320446822|ref|NW_003383749.1|	233688-235970	19045	880492	-111303	0.5107	no
gi|320446822|ref|NW_003383749.1|	237031-239376	967802	130107	0.426919	0.7991	no
gi|320446822|ref|NW_003383749.1|	248009-250330	389149	137244	-150358	0.25205	no
gi|320446822|ref|NW_003383749.1|	255322-256405	254857	509739	-232185	0.17865	no
gi|320446822|ref|NW_003383749.1|	288722-289299	141.56	495433	-151465	0.3891	no
gi|320446822|ref|NW_003383749.1|	291116-291696	750528	379254	-0.98474	0.5481	no
gi|320446822|ref|NW_003383749.1|	301485-303132	442299	599099	0.43777	0.8379	no
gi|320446822|ref|NW_003383749.1|	304980-305542	564828	312108	-0.855765	0.6689	no

gi|320446822|ref|NW_003383749.1|	305757-306733	432977	298946	-0.534407	0.78935	no
gi|320446822|ref|NW_003383749.1|	308512-308924	546069	240218	-118474	0.57675	no
gi|320446822|ref|NW_003383749.1|	313945-314719	125352	279498	115686	0.58685	no
gi|320446822|ref|NW_003383749.1|	368883-369773	0.257833	284195	346237	0.21395	no
gi|320446822|ref|NW_003383749.1|	377050-378419	0.744312	351527	223966	0.3197	no
gi|320446822|ref|NW_003383749.1|	451339-455156	36745	428617	0.222143	0.9172	no
gi|320446822|ref|NW_003383749.1|	456674-457046	124347	147856	-307211	0.25595	no
gi|320446822|ref|NW_003383749.1|	458920-460331	760778	259312	-155278	0.4687	no
gi|320446822|ref|NW_003383749.1|	465155-466007	410593	11301	-186126	0.402	no
gi|320446822|ref|NW_003383749.1|	4694-5604	272703	724298	-191267	0.39445	no
gi|320446822|ref|NW_003383749.1|	94871-95414	529467	563607	0.0901483	0.94565	no
gi|320446822|ref|NW_003383749.1|	9632-10174	655047	147121	-215459	0.3524	no
gi|320446825|ref|NW_003383746.1|	12351-13056	326914	736803	-214956	0.3386	no
gi|320446825|ref|NW_003383746.1|	124200-124724	532299	595109	0.160915	0.9403	no
gi|320446825|ref|NW_003383746.1|	132486-132734	0	478092	inf	0.01485	no
gi|320446825|ref|NW_003383746.1|	140445-142201	432783	626069	0.532676	0.79865	no
gi|320446825|ref|NW_003383746.1|	14278-18281	461266	304078	-0.601156	0.7968	no
gi|320446825|ref|NW_003383746.1|	143506-146717	731479	118178	0.692073	0.58115	no
gi|320446825|ref|NW_003383746.1|	147303-150845	302591	901839	15755	0.2364	no
gi|320446825|ref|NW_003383746.1|	162859-163865	227769	101379	-11678	0.59265	no
gi|320446825|ref|NW_003383746.1|	164039-168871	165177	871569	-0.922327	0.48545	no
gi|320446825|ref|NW_003383746.1|	172787-174154	156582	445355	-181389	0.41455	no
gi|320446825|ref|NW_003383746.1|	175042-176112	303313	116102	193651	0.38575	no
gi|320446825|ref|NW_003383746.1|	176587-177417	113544	23421	104454	0.6061	no
gi|320446825|ref|NW_003383746.1|	182630-183419	121957	293031	126468	0.55445	no
gi|320446825|ref|NW_003383746.1|	197845-198330	398499	164288	204358	0.34465	no
gi|320446825|ref|NW_003383746.1|	205468-205668	146098	637302	212504	0.2859	no
gi|320446825|ref|NW_003383746.1|	234851-238591	404892	350401	-353046	0.08465	no
gi|320446825|ref|NW_003383746.1|	239876-240284	928511	183652	-233795	0.25625	no
gi|320446825|ref|NW_003383746.1|	240897-242510	135965	0.596703	-451008	0.12855	no

gi|320446825|ref|NW_003383746.1|	244256-245205	279079	832269	-174555	0.42915	no
gi|320446825|ref|NW_003383746.1|	245380-246047	351538	159814	-113729	0.5884	no
gi|320446825|ref|NW_003383746.1|	246395-246870	207034	967358	-109775	0.5997	no
gi|320446825|ref|NW_003383746.1|	251358-251898	46693	159118	-155311	0.4701	no
gi|320446825|ref|NW_003383746.1|	253491-255779	126596	688576	-0.878541	0.58815	no
gi|320446825|ref|NW_003383746.1|	256477-257432	79742	647411	-0.300658	0.8835	no
gi|320446825|ref|NW_003383746.1|	259313-260204	311501	218148	-0.513933	0.82585	no
gi|320446825|ref|NW_003383746.1|	260324-263390	458987	391371	-0.229918	0.8624	no
gi|320446825|ref|NW_003383746.1|	267345-269878	492037	820733	0.738148	0.74135	no
gi|320446825|ref|NW_003383746.1|	274255-274653	410602	257268	-39964	0.1844	no
gi|320446825|ref|NW_003383746.1|	275391-275745	214832	0.822787	-470655	0.286	no
gi|320446825|ref|NW_003383746.1|	282153-282683	448142	267153	-406822	0.1465	no
gi|320446825|ref|NW_003383746.1|	286976-287848	874915	127783	-277545	0.24125	no
gi|320446825|ref|NW_003383746.1|	289334-289668	246207	562836	-212908	0.34005	no
gi|320446825|ref|NW_003383746.1|	290669-291849	0.535966	136186	134537	1	no
gi|320446825|ref|NW_003383746.1|	292019-293023	441352	65231	-27583	0.133	no
gi|320446825|ref|NW_003383746.1|	340178-341951	120717	129956	0.106393	1	no
gi|320446825|ref|NW_003383746.1|	346826-347517	148082	227583	0.619998	0.753	no
gi|320446825|ref|NW_003383746.1|	349002-351686	282527	799268	150029	0.5054	no
gi|320446825|ref|NW_003383746.1|	373931-379545	451714	103749	119961	0.3538	no
gi|320446825|ref|NW_003383746.1|	469170-469480	696688	14.57	106442	0.60185	no
gi|320446825|ref|NW_003383746.1|	52626-52901	108265	384822	182962	0.28025	no
gi|320446825|ref|NW_003383746.1|	53439-55614	140062	108112	294839	0.03525	no
gi|320446825|ref|NW_003383746.1|	56466-57806	0.305543	148504	228105	0.2731	no
gi|320446825|ref|NW_003383746.1|	57931-59200	0.489805	135928	147256	1	no
gi|320446825|ref|NW_003383746.1|	61827-63514	395236	461329	0.223083	0.91565	no
gi|320446825|ref|NW_003383746.1|	64520-65408	620696	195971	-166324	0.4377	no
gi|320446825|ref|NW_003383746.1|	676614-676934	643151	10375	0.68988	0.74435	no
gi|320446825|ref|NW_003383746.1|	691673-694004	102258	171443	0.745519	0.5549	no
gi|320446825|ref|NW_003383746.1|	694391-697194	106458	411056	194904	0.15355	no

gi|320446825|ref|NW_003383746.1|	75694-77698	13357	186286	0.479919	0.81075	no
gi|320446825|ref|NW_003383746.1|	77913-78526	311935	302524	-0.0441953	0.9454	no
gi|320446825|ref|NW_003383746.1|	79181-80494	0.469801	152182	169568	0.4291	no
gi|320446825|ref|NW_003383746.1|	84757-86636	164263	264743	0.688584	0.73215	no
gi|320446825|ref|NW_003383746.1|	98150-98475	108324	419957	195488	0.3755	no
gi|320446898|ref|NW_003383673.1|	116222-117155	0.241934	2503	337097	0.2261	no
gi|320446898|ref|NW_003383673.1|	122007-123883	174849	666642	193081	0.37015	no
gi|320446898|ref|NW_003383673.1|	124952-125329	175648	215639	0.295929	0.8833	no
gi|320446898|ref|NW_003383673.1|	140784-141568	0	675749	inf	0.01035	no
gi|320446898|ref|NW_003383673.1|	153200-153610	0	266775	inf	0.00845	no
gi|320446898|ref|NW_003383673.1|	154050-155239	0	191338	inf	0.00365	no
gi|320446898|ref|NW_003383673.1|	181016-181264	0	207866	inf	0.0312	no
gi|320446898|ref|NW_003383673.1|	186450-187833	0	408571	inf	0.00815	no
gi|320446898|ref|NW_003383673.1|	188617-189889	0.162796	169424	33795	0.2261	no
gi|320446898|ref|NW_003383673.1|	190172-190994	0	692232	inf	0.0114	no
gi|320446898|ref|NW_003383673.1|	198789-199034	0	259197	inf	0.029	no
gi|320446898|ref|NW_003383673.1|	201459-202166	0	144311	inf	0.00795	no
gi|320446898|ref|NW_003383673.1|	208863-209337	0	693563	inf	0.02205	no
gi|320446898|ref|NW_003383673.1|	227646-228667	0	653264	inf	0.00845	no
gi|320446898|ref|NW_003383673.1|	240917-241444	108846	269689	-201292	0.3489	no
gi|320446898|ref|NW_003383673.1|	251968-253145	132626	24839	-241669	0.2915	no
gi|320446898|ref|NW_003383673.1|	253295-254705	166652	588935	-150065	0.50645	no
gi|320446898|ref|NW_003383673.1|	261569-268993	828506	873807	0.0768023	0.9527	no
gi|320446898|ref|NW_003383673.1|	270378-271999	132744	0.508533	-470617	0.13545	no
gi|320446898|ref|NW_003383673.1|	279812-280201	58485	471577	-36325	0.16715	no
gi|320446898|ref|NW_003383673.1|	286192-286501	129956	677851	-426091	0.1464	no
gi|320446898|ref|NW_003383673.1|	327971-328538	232739	177785	-0.388578	0.8537	no
gi|320446898|ref|NW_003383673.1|	333498-333876	873182	107224	0.296268	0.87775	no
gi|320446898|ref|NW_003383673.1|	333999-334550	796231	39385	-101554	0.613	no
gi|320446898|ref|NW_003383673.1|	335311-336202	337245	331656	-0.0241131	0.9851	no

gi|320446898|ref|NW_003383673.1|	420091-420619	0	652894	inf	0.02075	no
gi|320446898|ref|NW_003383673.1|	420827-421575	0.329243	564036	409856	0.19245	no
gi|320446898|ref|NW_003383673.1|	425581-426552	419861	194821	-110776	0.50695	no
gi|320446898|ref|NW_003383673.1|	426718-426981	71258	261117	-144836	0.48805	no
gi|320446898|ref|NW_003383673.1|	427879-428071	439018	436194	-0.00930984	0.9322	no
gi|320446898|ref|NW_003383673.1|	428177-428870	420204	307204	-0.451892	0.84555	no
gi|320446898|ref|NW_003383673.1|	430469-430882	996183	537933	-0.888985	0.6616	no
gi|320446898|ref|NW_003383673.1|	432872-435044	296241	316141	0.0937958	0.9632	no
gi|320446898|ref|NW_003383673.1|	43304-44286	158235	374631	12434	0.55105	no
gi|320446898|ref|NW_003383673.1|	435854-438083	0.677105	124045	0.873407	1	no
gi|320446898|ref|NW_003383673.1|	438216-438930	504119	605257	0.263783	0.83445	no
gi|320446898|ref|NW_003383673.1|	47105-47650	324459	236921	28683	0.22265	no
gi|320446898|ref|NW_003383673.1|	49300-49580	0	208637	inf	0.0038	no
gi|320446898|ref|NW_003383673.1|	50899-51233	0	272038	inf	0.0109	no
gi|320446898|ref|NW_003383673.1|	520046-520424	0	128668	inf	0.0154	no
gi|320446898|ref|NW_003383673.1|	523721-524089	219985	680813	-169207	0.41855	no
gi|320446898|ref|NW_003383673.1|	525679-527350	329117	351865	0.0964252	0.96045	no
gi|320446898|ref|NW_003383673.1|	53931-54155	0	789009	inf	0.0133	no
gi|320446898|ref|NW_003383673.1|	55004-55407	0	740229	inf	0.00515	no
gi|320446898|ref|NW_003383673.1|	5510-6132	17417	295842	0.764335	0.721	no
gi|320446898|ref|NW_003383673.1|	573867-575223	0.301179	399482	705136	0.1181	no
gi|320446898|ref|NW_003383673.1|	580263-580711	0	286795	inf	0.00785	no
gi|320446898|ref|NW_003383673.1|	584391-584787	0	513283	inf	0.0059	no
gi|320446898|ref|NW_003383673.1|	585035-587390	0	466857	inf	0.00625	no
gi|320446898|ref|NW_003383673.1|	588846-589201	0	359788	inf	0.00845	no
gi|320446898|ref|NW_003383673.1|	60673-60929	565143	754267	-290547	0.2452	no
gi|320446898|ref|NW_003383673.1|	61083-61474	101492	133334	-292824	0.2676	no
gi|320446898|ref|NW_003383673.1|	62311-63300	985381	340274	-153398	0.46975	no
gi|320446898|ref|NW_003383673.1|	64304-64614	104503	448308	-122099	0.57555	no
gi|320446898|ref|NW_003383673.1|	66997-69742	598382	56381	-0.0858586	0.96855	no

gi|320446898|ref|NW_003383673.1|	7330-7833	310929	708642	118847	0.5755	no
gi|320446898|ref|NW_003383673.1|	73693-74156	723897	0.482246	-390794	0.3159	no
gi|320446898|ref|NW_003383673.1|	76574-77350	593219	0	#NAME?	0.01335	no
gi|320446898|ref|NW_003383673.1|	87458-88135	0	52114	inf	0.0162	no
gi|320446898|ref|NW_003383673.1|	88621-90236	0	102145	inf	1	no
gi|320446898|ref|NW_003383673.1|	90379-92231	0	581903	inf	0.0077	no
gi|320446898|ref|NW_003383673.1|	96954-99488	0.0734064	139977	757507	0.1408	no
gi|320446921|ref|NW_003383650.1|	105002-106683	653679	698905	0.0965134	0.9641	no
gi|320446921|ref|NW_003383650.1|	116919-118677	155154	0.694726	-115919	0.57025	no
gi|320446921|ref|NW_003383650.1|	119354-121973	0.495519	168146	176271	0.41045	no
gi|320446921|ref|NW_003383650.1|	142162-145244	135144	100383	289294	0.0364	no
gi|320446921|ref|NW_003383650.1|	146803-147996	193852	146576	291862	0.21565	no
gi|320446921|ref|NW_003383650.1|	149532-149714	0	112766	inf	0.0198	no
gi|320446921|ref|NW_003383650.1|	158057-158587	158835	74803	223557	0.19965	no
gi|320446921|ref|NW_003383650.1|	173572-175893	833313	683394	-0.286141	0.9006	no
gi|320446921|ref|NW_003383650.1|	176540-177247	822784	244596	-175012	0.4252	no
gi|320446921|ref|NW_003383650.1|	178767-180705	113972	131272	0.203883	0.89995	no
gi|320446921|ref|NW_003383650.1|	180969-181311	109526	977494	-0.164111	0.9304	no
gi|320446921|ref|NW_003383650.1|	182313-183019	290375	186274	-0.640492	0.77205	no
gi|320446921|ref|NW_003383650.1|	186964-189368	0	246113	inf	5.00E-05	yes
gi|320446921|ref|NW_003383650.1|	199278-200219	0	346517	inf	0.01575	no
gi|320446921|ref|NW_003383650.1|	271539-272738	367777	194234	-0.921037	0.65605	no
gi|320446936|ref|NW_003383635.1|	104582-105894	0.783728	326403	205823	0.35365	no
gi|320446936|ref|NW_003383635.1|	106212-106664	0.757718	604588	299622	0.2563	no
gi|320446936|ref|NW_003383635.1|	121484-123625	354189	735168	105355	0.62935	no
gi|320446936|ref|NW_003383635.1|	125738-126244	307651	949048	162518	0.4475	no
gi|320446936|ref|NW_003383635.1|	16512-17896	822741	561301	-0.551664	0.7947	no
gi|320446936|ref|NW_003383635.1|	18021-19884	117151	686376	-0.771302	0.7371	no
gi|320446936|ref|NW_003383635.1|	180532-181380	0.275517	125106	550487	0.1627	no
gi|320446936|ref|NW_003383635.1|	21323-23477	897061	539994	-0.732264	0.7412	no

gi|320446936|ref|NW_003383635.1|	215839-216282	110274	941215	-0.2285	0.9063	no
gi|320446936|ref|NW_003383635.1|	216556-217467	174871	843787	-105134	0.6253	no
gi|320446936|ref|NW_003383635.1|	217525-217982	378412	256771	-0.559473	0.7955	no
gi|320446936|ref|NW_003383635.1|	218141-219056	44702	308171	-0.536608	0.78835	no
gi|320446936|ref|NW_003383635.1|	219244-219627	954745	556449	-0.778867	0.7042	no
gi|320446936|ref|NW_003383635.1|	221096-222038	764325	708547	-0.109323	0.9569	no
gi|320446936|ref|NW_003383635.1|	222543-224439	209323	927808	-117383	0.48225	no
gi|320446936|ref|NW_003383635.1|	225310-226223	176845	188873	-3227	0.19625	no
gi|320446936|ref|NW_003383635.1|	235535-236641	131815	375703	-181085	0.4085	no
gi|320446936|ref|NW_003383635.1|	254332-254985	411614	142944	-152585	0.49265	no
gi|320446936|ref|NW_003383635.1|	255092-259458	22997	125562	-0.873053	0.5133	no
gi|320446936|ref|NW_003383635.1|	260861-262060	77058	764795	-0.0108715	0.9901	no
gi|320446936|ref|NW_003383635.1|	263729-264073	229577	149091	-0.62279	0.7523	no
gi|320446936|ref|NW_003383635.1|	30393-31249	815891	336888	-127611	0.54055	no
gi|320446936|ref|NW_003383635.1|	311662-312803	0.186351	116158	263999	1	no
gi|320446936|ref|NW_003383635.1|	312881-313900	0	2828	inf	0.0212	no
gi|320446936|ref|NW_003383635.1|	313951-314657	0	269607	inf	0.0294	no
gi|320446936|ref|NW_003383635.1|	3359-5984	133454	532778	-132474	0.42975	no
gi|320446936|ref|NW_003383635.1|	394170-394613	0	167327	inf	0.01035	no
gi|320446936|ref|NW_003383635.1|	395858-396915	0	152006	inf	0.0053	no
gi|320446936|ref|NW_003383635.1|	40554-41716	555487	719218	-294925	0.1206	no
gi|320446936|ref|NW_003383635.1|	42048-42303	722541	381614	-424289	0.2079	no
gi|320446936|ref|NW_003383635.1|	449585-450169	0.481897	190843	862945	0.14075	no
gi|320446936|ref|NW_003383635.1|	47517-47812	186.34	165138	-349619	0.1687	no
gi|320446936|ref|NW_003383635.1|	51996-54216	272071	284808	338794	0.08475	no
gi|320446936|ref|NW_003383635.1|	56728-57951	119612	177724	0.571276	0.7847	no
gi|320446936|ref|NW_003383635.1|	58096-60425	128146	41298	168828	0.2086	no
gi|320446936|ref|NW_003383635.1|	62064-62601	166379	29876	0.844519	0.69175	no
gi|320446936|ref|NW_003383635.1|	649184-653165	0.315323	592798	423264	0.07095	no
gi|320446936|ref|NW_003383635.1|	653578-664401	260385	838418	500895	0.25595	no

gi|320446936|ref|NW_003383635.1|	69964-70799	0	367803	inf	0.0212	no
gi|320446936|ref|NW_003383635.1|	722623-723649	0.426932	147545	178907	0.3323	no
gi|320446936|ref|NW_003383635.1|	724472-725173	724624	495273	-0.549009	0.7943	no
gi|320446936|ref|NW_003383635.1|	727164-728889	974207	150675	0.629142	0.69405	no
gi|320446936|ref|NW_003383635.1|	729291-730427	168647	155726	-0.115006	0.949	no
gi|320446936|ref|NW_003383635.1|	731814-732891	701843	735296	0.0671767	0.97175	no
gi|320446936|ref|NW_003383635.1|	814674-817362	110075	427828	195855	0.3683	no
gi|320446936|ref|NW_003383635.1|	817437-818905	109523	47585	211927	0.3328	no
gi|320446936|ref|NW_003383635.1|	8325-10521	229788	438414	-238994	0.1812	no
gi|320446936|ref|NW_003383635.1|	98820-99771	0.943387	488225	237163	0.2987	no
gi|320446953|ref|NW_003383618.1|	100320-100974	125608	263294	-225419	0.21105	no
gi|320446953|ref|NW_003383618.1|	103078-106075	922583	128149	0.474067	0.70985	no
gi|320446953|ref|NW_003383618.1|	106471-108365	233992	283639	0.277598	0.8875	no
gi|320446953|ref|NW_003383618.1|	108586-111489	68944	92408	0.422593	0.7915	no
gi|320446953|ref|NW_003383618.1|	113963-114403	127705	370695	-178451	0.40555	no
gi|320446953|ref|NW_003383618.1|	114521-115692	992059	449931	-114072	0.5882	no
gi|320446953|ref|NW_003383618.1|	1163340-1164433	0	204276	inf	0.004	no
gi|320446953|ref|NW_003383618.1|	1173686-1180852	16554	249525	0.592	0.6584	no
gi|320446953|ref|NW_003383618.1|	1192647-1193265	162763	149388	-0.123708	0.952	no
gi|320446953|ref|NW_003383618.1|	1201588-1202300	127441	123479	-0.0455564	0.9804	no
gi|320446953|ref|NW_003383618.1|	1202735-1204959	543895	331612	-0.713831	0.5917	no
gi|320446953|ref|NW_003383618.1|	1212420-1212649	124571	297346	-206675	0.35055	no
gi|320446953|ref|NW_003383618.1|	1213087-1213828	497343	128044	-195761	0.24475	no
gi|320446953|ref|NW_003383618.1|	1215719-1218298	935957	256501	-186748	0.4001	no
gi|320446953|ref|NW_003383618.1|	12169-13821	223704	0.331668	-939764	0.1293	no
gi|320446953|ref|NW_003383618.1|	1220514-1222603	442414	704853	-2.65	0.1656	no
gi|320446953|ref|NW_003383618.1|	1223808-1224621	470473	124494	-191803	0.26255	no
gi|320446953|ref|NW_003383618.1|	1225198-1232086	218109	133522	-0.707972	0.5975	no
gi|320446953|ref|NW_003383618.1|	1232365-1234157	104067	332284	-164702	0.45835	no
gi|320446953|ref|NW_003383618.1|	1234305-1235062	82512	379323	-112118	0.50695	no

gi|320446953|ref|NW_003383618.1|	1237315-1238560	101962	584301	-0.803243	0.55425	no
gi|320446953|ref|NW_003383618.1|	1238945-1243606	160122	276351	0.787328	0.5568	no
gi|320446953|ref|NW_003383618.1|	1244220-1244926	501883	833331	0.731539	0.7247	no
gi|320446953|ref|NW_003383618.1|	1246949-1248033	656278	770601	0.231679	0.9112	no
gi|320446953|ref|NW_003383618.1|	1257919-1258476	521224	562908	0.110995	0.94785	no
gi|320446953|ref|NW_003383618.1|	1272495-1273121	245691	234372	-0.0680465	0.9733	no
gi|320446953|ref|NW_003383618.1|	1284346-1285411	36617	886485	127558	0.55605	no
gi|320446953|ref|NW_003383618.1|	1286010-1287093	714793	749454	0.0683152	0.9602	no
gi|320446953|ref|NW_003383618.1|	1287612-1293636	100354	512518	-0.969415	0.5607	no
gi|320446953|ref|NW_003383618.1|	1295174-1298163	337017	218054	-0.628133	0.6359	no
gi|320446953|ref|NW_003383618.1|	1309192-1309656	281176	129701	-111629	0.58805	no
gi|320446953|ref|NW_003383618.1|	1311429-1312723	0.159412	121692	293241	1	no
gi|320446953|ref|NW_003383618.1|	1312833-1314132	158662	209219	0.39906	0.8445	no
gi|320446953|ref|NW_003383618.1|	1316037-1316669	225046	837375	-142628	0.49505	no
gi|320446953|ref|NW_003383618.1|	1317112-1320199	161533	203973	0.33655	0.7983	no
gi|320446953|ref|NW_003383618.1|	1326390-1326960	185571	84756	-113058	0.58925	no
gi|320446953|ref|NW_003383618.1|	1334603-1335080	191736	457226	-206814	0.34935	no
gi|320446953|ref|NW_003383618.1|	1336431-1336753	459139	511079	-316731	0.21935	no
gi|320446953|ref|NW_003383618.1|	1336931-1337988	129408	198886	-270191	0.23985	no
gi|320446953|ref|NW_003383618.1|	1347179-1348562	176455	112357	-0.651213	0.75415	no
gi|320446953|ref|NW_003383618.1|	1348662-1351918	402184	300829	-0.418915	0.84735	no
gi|320446953|ref|NW_003383618.1|	1352233-1352672	296632	117001	-134215	0.5266	no
gi|320446953|ref|NW_003383618.1|	1355272-1355700	151709	128325	-0.241506	0.90405	no
gi|320446953|ref|NW_003383618.1|	1356195-1358843	349687	49352	0.497045	0.8159	no
gi|320446953|ref|NW_003383618.1|	1359089-1359542	301808	401429	0.411511	0.8506	no
gi|320446953|ref|NW_003383618.1|	1359660-1362488	76796	404837	-0.923691	0.6803	no
gi|320446953|ref|NW_003383618.1|	1363914-1364341	152417	134503	-0.180386	0.92765	no
gi|320446953|ref|NW_003383618.1|	1385100-1387311	713169	130532	-244984	0.07325	no
gi|320446953|ref|NW_003383618.1|	1388523-1388875	93459	108308	-31092	0.2016	no
gi|320446953|ref|NW_003383618.1|	1396198-1399713	154856	194373	-299403	0.11595	no

gi|320446953|ref|NW_003383618.1|	1402454-140523[illegible]	493728	333917	-0.564226	0.6738	no
gi|320446953|ref|NW_003383618.1|	1421870-142244[illegible]	0	431073	inf	0.02915	no
gi|320446953|ref|NW_003383618.1|	1545369-154597[illegible]	0.902983	27573	161049	0.33275	no
gi|320446953|ref|NW_003383618.1|	1556620-155753[illegible]	0.247253	221608	316395	0.24575	no
gi|320446953|ref|NW_003383618.1|	16334-16766	115013	0	#NAME?	5.00E-05	yes
gi|320446953|ref|NW_003383618.1|	18723-19068	0	810302	inf	0.004	no
gi|320446953|ref|NW_003383618.1|	19333-19670	0	385961	inf	0.0069	no
gi|320446953|ref|NW_003383618.1|	21073-26319	0.0673461	424638	59785	0.11825	no
gi|320446953|ref|NW_003383618.1|	26681-27687	0.657025	287494	212951	0.3464	no
gi|320446953|ref|NW_003383618.1|	306393-310661	711281	957935	0.429509	0.7359	no
gi|320446953|ref|NW_003383618.1|	320014-320808	785623	684672	-0.198424	0.92055	no
gi|320446953|ref|NW_003383618.1|	33974-34969	0.888723	322283	185852	0.405	no
gi|320446953|ref|NW_003383618.1|	37116-39352	902459	24134	141914	0.4192	no
gi|320446953|ref|NW_003383618.1|	43681-45349	106009	138578	370844	0.14605	no
gi|320446953|ref|NW_003383618.1|	578456-579336	162337	703266	-120685	0.5672	no
gi|320446953|ref|NW_003383618.1|	58752-60168	214402	0.0993176	-443213	0.2969	no
gi|320446953|ref|NW_003383618.1|	60563-61546	776574	0	#NAME?	5.00E-05	yes
gi|320446953|ref|NW_003383618.1|	608858-609856	0.221306	101661	884351	0.14085	no
gi|320446953|ref|NW_003383618.1|	62693-63289	293714	0	#NAME?	0.0053	no
gi|320446953|ref|NW_003383618.1|	769913-770361	693687	30728	-117473	0.57405	no
gi|320446953|ref|NW_003383618.1|	77369-79218	149644	0.145744	-100039	0.16165	no
gi|320446953|ref|NW_003383618.1|	7860-9970	124216	0.439555	-814259	0.08495	no
gi|320446953|ref|NW_003383618.1|	796165-800706	764146	134194	0.812403	0.54415	no
gi|320446953|ref|NW_003383618.1|	827414-827692	209964	743947	-149687	0.4986	no
gi|320446953|ref|NW_003383618.1|	841618-842935	0	140795	inf	0.0044	no
gi|320446953|ref|NW_003383618.1|	84314-91107	691491	524.64	-0.398383	0.1318	no
gi|320446953|ref|NW_003383618.1|	93101-97263	214977	97228	-114474	0.47435	no
gi|320446953|ref|NW_003383618.1|	975005-975466	145967	199275	377105	0.175	no
gi|320446953|ref|NW_003383618.1|	97686-98183	149314	289368	-236737	0.1882	no
gi|320446953|ref|NW_003383618.1|	986052-989350	0.881486	45086	235467	0.29615	no

gi|320446953|ref|NW_003383618.1|	98783-98972	662197	351942	-0.911921	0.6772	no
gi|320446953|ref|NW_003383618.1|	990115-992231	0.269173	388087	384977	0.17355	no
gi|320446956|ref|NW_003383615.1|	102917-103508	45372	17.93	-133943	0.5432	no
gi|320446956|ref|NW_003383615.1|	111051-113295	0.084006	205207	461044	0.1773	no
gi|320446956|ref|NW_003383615.1|	118126-119386	362346	0	#NAME?	5.00E-05	yes
gi|320446956|ref|NW_003383615.1|	122161-123197	255067	0.29146	-645143	0.1772	no
gi|320446956|ref|NW_003383615.1|	124943-125308	224038	0	#NAME?	0.01335	no
gi|320446956|ref|NW_003383615.1|	126486-128160	152485	0.16332	-654483	0.176	no
gi|320446956|ref|NW_003383615.1|	130734-132783	243764	231013	-0.0775132	0.95285	no
gi|320446956|ref|NW_003383615.1|	13626-13969	299135	0.882782	-50826	0.2796	no
gi|320446956|ref|NW_003383615.1|	148117-153747	248644	110674	-116777	0.38185	no
gi|320446956|ref|NW_003383615.1|	165537-167490	0.196481	301364	393904	0.17095	no
gi|320446956|ref|NW_003383615.1|	167636-168847	0.345954	143906	205648	0.3018	no
gi|320446956|ref|NW_003383615.1|	245004-245463	0	93078	inf	0.0212	no
gi|320446956|ref|NW_003383615.1|	264223-265638	0.143056	248502	411861	0.19225	no
gi|320446956|ref|NW_003383615.1|	282885-283404	0.58836	909114	394969	0.19775	no
gi|320446956|ref|NW_003383615.1|	294023-294803	247943	382835	0.626715	0.74745	no
gi|320446956|ref|NW_003383615.1|	295390-296122	339814	651475	0.938965	0.65195	no
gi|320446956|ref|NW_003383615.1|	297910-298764	354696	506933	0.51521	0.7941	no
gi|320446956|ref|NW_003383615.1|	302382-303467	15891	494812	163867	0.4317	no
gi|320446956|ref|NW_003383615.1|	303975-307502	158905	503535	166393	0.22375	no
gi|320446956|ref|NW_003383615.1|	457165-458373	0.694043	288689	205642	0.35695	no
gi|320446956|ref|NW_003383615.1|	459681-460847	0	175901	inf	0.0233	no
gi|320446956|ref|NW_003383615.1|	460970-462014	0	158742	inf	0.0294	no
gi|320446956|ref|NW_003383615.1|	462893-465686	910414	217162	125418	0.332	no
gi|320446956|ref|NW_003383615.1|	539724-540225	41336	113321	-186698	0.3959	no
gi|320446956|ref|NW_003383615.1|	588206-588521	217422	646622	-174951	0.41955	no
gi|320446956|ref|NW_003383615.1|	588723-593738	384776	173592	-114832	0.39965	no
gi|320446956|ref|NW_003383615.1|	593963-595155	243451	212755	-0.194439	0.9008	no
gi|320446956|ref|NW_003383615.1|	595314-596311	265917	306143	0.203235	0.9202	no

gi|320446956|ref|NW_003383615.1|	599592-600487	0.767632	14104	0.877621	1	no
gi|320446956|ref|NW_003383615.1|	600905-602735	354495	522771	0.560412	0.67425	no
gi|320446956|ref|NW_003383615.1|	638532-639477	0.713525	147685	104948	0.5772	no
gi|320446956|ref|NW_003383615.1|	640108-647099	159839	723108	217759	0.2204	no
gi|320446956|ref|NW_003383615.1|	707922-708240	832927	273846	-160483	0.458	no
gi|320446956|ref|NW_003383615.1|	708738-713850	50652	192689	-139435	0.29755	no
gi|320446956|ref|NW_003383615.1|	713998-714715	508003	790962	-268316	0.1319	no
gi|320446956|ref|NW_003383615.1|	71469-72693	307263	520804	0.761265	0.71665	no
gi|320446956|ref|NW_003383615.1|	718581-724166	864171	579009	-0.577732	0.6488	no
gi|320446956|ref|NW_003383615.1|	725795-726380	129749	19521	-273262	0.25015	no
gi|320446956|ref|NW_003383615.1|	727211-727950	130692	390203	-174388	0.4157	no
gi|320446956|ref|NW_003383615.1|	728594-729260	211408	427117	-230732	0.2949	no
gi|320446956|ref|NW_003383615.1|	72909-75450	0.951385	618538	270076	0.23995	no
gi|320446956|ref|NW_003383615.1|	745185-750564	133833	694072	-0.947281	0.47195	no
gi|320446956|ref|NW_003383615.1|	752145-754485	34154	442179	-294935	0.12795	no
gi|320446956|ref|NW_003383615.1|	754585-755554	782197	365276	-109854	0.5978	no
gi|320446956|ref|NW_003383615.1|	755673-756260	669033	323608	-104783	0.6056	no
gi|320446956|ref|NW_003383615.1|	756449-756931	470228	224443	-106701	0.59575	no
gi|320446956|ref|NW_003383615.1|	757295-758126	119021	448163	-140912	0.50845	no
gi|320446956|ref|NW_003383615.1|	77209-79714	0.148689	0.778878	23891	1	no
gi|320446956|ref|NW_003383615.1|	787925-789499	230691	135064	-0.772322	0.6392	no
gi|320446956|ref|NW_003383615.1|	83073-88997	239056	210349	-0.18456	0.8894	no
gi|320446956|ref|NW_003383615.1|	90533-90979	396497	619666	-267775	0.2568	no
gi|320446956|ref|NW_003383615.1|	92084-95013	42721	113853	-190778	0.15555	no
gi|320446956|ref|NW_003383615.1|	96913-98097	889509	616452	-0.529019	0.7976	no
gi|320446963|ref|NW_003383608.1|	1065320-106560[illegible]	0	621316	inf	0.00915	no
gi|320446963|ref|NW_003383608.1|	1070732-107383[illegible]	0.705091	206668	487336	0.04665	no
gi|320446963|ref|NW_003383608.1|	1119043-111980[illegible]	0.3193	853962	474119	0.1736	no
gi|320446963|ref|NW_003383608.1|	129675-136015	153116	284381	0.893197	0.5083	no
gi|320446963|ref|NW_003383608.1|	136736-137190	353134	414804	0.232213	0.9143	no

gi|320446963|ref|NW_003383608.1|	151159-151865	716975	0.245097	-48705	0.28945	no
gi|320446963|ref|NW_003383608.1|	167309-168432	266184	0.789872	-175273	0.42385	no
gi|320446963|ref|NW_003383608.1|	188486-189855	114624	765088	-0.583212	0.79055	no
gi|320446963|ref|NW_003383608.1|	393834-396087	317799	0	#NAME?	0.00735	no
gi|320446963|ref|NW_003383608.1|	517839-519906	1474	488359	17282	0.4152	no
gi|320446963|ref|NW_003383608.1|	580968-581532	255222	206888	-0.302901	0.8817	no
gi|320446963|ref|NW_003383608.1|	640803-641440	0	370926	inf	0.02915	no
gi|320446963|ref|NW_003383608.1|	656522-659443	0.565447	140542	131354	1	no
gi|320446963|ref|NW_003383608.1|	683632-684714	538223	993079	0.883705	0.67925	no
gi|320446963|ref|NW_003383608.1|	688294-689156	0.808076	185403	11981	0.56655	no
gi|320446963|ref|NW_003383608.1|	693049-699597	670862	299946	216062	0.10975	no
gi|320446963|ref|NW_003383608.1|	700689-702164	0.408391	0.851741	106046	1	no
gi|320446963|ref|NW_003383608.1|	703220-703608	515852	128675	131871	0.54905	no
gi|320446963|ref|NW_003383608.1|	704827-705403	182386	750199	204028	0.2346	no
gi|320446963|ref|NW_003383608.1|	706420-707096	765396	383872	232635	0.3122	no
gi|320446963|ref|NW_003383608.1|	707206-707869	11038	233805	108283	0.60715	no
gi|320446963|ref|NW_003383608.1|	708036-711337	8476	161305	0.928333	0.4676	no
gi|320446963|ref|NW_003383608.1|	726977-727231	304749	270332	314903	0.2374	no
gi|320446963|ref|NW_003383608.1|	754837-755921	151143	21742	0.524571	0.81445	no
gi|320446963|ref|NW_003383608.1|	789460-792219	648551	724234	0.159236	0.941	no
gi|320446963|ref|NW_003383608.1|	793096-793885	670762	606993	-0.144122	0.94045	no
gi|320446963|ref|NW_003383608.1|	794013-796211	180548	245983	0.446179	0.82895	no
gi|320446963|ref|NW_003383608.1|	801446-802284	224073	250427	0.160425	0.9302	no
gi|320446963|ref|NW_003383608.1|	802600-803503	353934	174227	-102251	0.60725	no
gi|320446963|ref|NW_003383608.1|	805186-806806	499675	161151	-163258	0.4454	no
gi|320446963|ref|NW_003383608.1|	817895-822189	604041	145247	-205614	0.1272	no
gi|320446963|ref|NW_003383608.1|	824014-824982	373203	572504	-27046	0.13125	no
gi|320446963|ref|NW_003383608.1|	825234-826202	193512	333961	-253468	0.26695	no
gi|320446963|ref|NW_003383608.1|	826370-827691	279803	0.431638	-269652	0.2729	no
gi|320446963|ref|NW_003383608.1|	838425-840282	0.624344	239301	193841	0.37225	no

gi|320446963|ref|NW_003383608.1|	841790-843090	0.792564	0.660079	-0.263889	1	no
gi|320446963|ref|NW_003383608.1|	846751-857835	290512	164662	250284	0.128	no
gi|320446963|ref|NW_003383608.1|	913225-913728	0	458533	inf	0.0294	no
gi|320446966|ref|NW_003383605.1|	103751-104020	179483	228373	0.347548	0.8572	no
gi|320446966|ref|NW_003383605.1|	1038582-1039715	0.376025	195299	237678	0.2616	no
gi|320446966|ref|NW_003383605.1|	1039974-1040640	234898	186864	-0.330044	0.85735	no
gi|320446966|ref|NW_003383605.1|	1071591-1072665	163008	236681	-278393	0.2382	no
gi|320446966|ref|NW_003383605.1|	1114555-1115232	137462	130285	-339929	0.2003	no
gi|320446966|ref|NW_003383605.1|	1126085-1128668	110.47	172117	0.639729	0.6108	no
gi|320446966|ref|NW_003383605.1|	1138974-1142091	394652	917169	-210532	0.11695	no
gi|320446966|ref|NW_003383605.1|	1142212-1142947	165175	536649	-162195	0.2218	no
gi|320446966|ref|NW_003383605.1|	114414-115586	0.180182	199759	347073	0.21395	no
gi|320446966|ref|NW_003383605.1|	1155217-1155572	125559	98124	296625	0.2563	no
gi|320446966|ref|NW_003383605.1|	1156087-1158063	397463	212243	241682	0.17595	no
gi|320446966|ref|NW_003383605.1|	116019-119516	136053	172278	0.340572	0.7924	no
gi|320446966|ref|NW_003383605.1|	120006-120405	826625	428759	-0.947065	0.65885	no
gi|320446966|ref|NW_003383605.1|	13246-14409	318378	90878	151319	0.25935	no
gi|320446966|ref|NW_003383605.1|	136968-137324	154696	186102	0.266654	0.8317	no
gi|320446966|ref|NW_003383605.1|	140731-142192	296024	415373	0.48869	0.70105	no
gi|320446966|ref|NW_003383605.1|	142944-146782	31.66	336641	0.0885488	0.95015	no
gi|320446966|ref|NW_003383605.1|	14526-16241	0.228091	166774	287021	0.22425	no
gi|320446966|ref|NW_003383605.1|	147693-150569	108627	13042	0.263781	0.83135	no
gi|320446966|ref|NW_003383605.1|	151417-153999	244476	128088	-0.932562	0.47005	no
gi|320446966|ref|NW_003383605.1|	155006-155545	0.551074	779459	382216	0.20105	no
gi|320446966|ref|NW_003383605.1|	157387-159176	727704	469116	-0.633408	0.7704	no
gi|320446966|ref|NW_003383605.1|	161308-162752	672982	438622	-0.617589	0.6401	no
gi|320446966|ref|NW_003383605.1|	17338-19118	190087	340933	0.842827	0.52595	no
gi|320446966|ref|NW_003383605.1|	19361-20733	155893	211397	0.439398	0.78345	no
gi|320446966|ref|NW_003383605.1|	21959-24321	111875	151232	0.434872	0.723	no
gi|320446966|ref|NW_003383605.1|	222600-223381	0.309361	254767	304181	0.24995	no

gi|320446966|ref|NW_003383605.1|	228749-230002	0.331672	299084	317272	0.20465	no
gi|320446966|ref|NW_003383605.1|	230093-230708	132995	571927	210446	0.34945	no
gi|320446966|ref|NW_003383605.1|	264172-268486	290818	17466	-0.735563	0.5835	no
gi|320446966|ref|NW_003383605.1|	275326-282097	400141	295522	288469	0.0379	no
gi|320446966|ref|NW_003383605.1|	28763-29882	133695	105786	-0.337787	1	no
gi|320446966|ref|NW_003383605.1|	292676-293490	291714	7016	126609	0.54115	no
gi|320446966|ref|NW_003383605.1|	325182-326054	212101	109528	-0.953447	0.67015	no
gi|320446966|ref|NW_003383605.1|	32574-34585	133044	251827	0.920532	0.6565	no
gi|320446966|ref|NW_003383605.1|	330299-334059	259939	428489	0.721087	0.5854	no
gi|320446966|ref|NW_003383605.1|	337323-338830	13005	107044	-0.280858	0.8984	no
gi|320446966|ref|NW_003383605.1|	43225-43455	338397	335493	-0.0124347	0.9935	no
gi|320446966|ref|NW_003383605.1|	448243-449990	100468	241021	126243	0.5476	no
gi|320446966|ref|NW_003383605.1|	46353-46854	482253	600181	0.315607	0.8933	no
gi|320446966|ref|NW_003383605.1|	498486-501050	0.797066	409995	236283	0.28995	no
gi|320446966|ref|NW_003383605.1|	517482-518667	0.888572	184744	105596	0.5998	no
gi|320446966|ref|NW_003383605.1|	523377-524045	974262	186036	-238873	0.2922	no
gi|320446966|ref|NW_003383605.1|	525459-528478	167298	953552	-0.811038	0.5314	no
gi|320446966|ref|NW_003383605.1|	529822-530894	24207	237238	-0.0290896	0.96625	no
gi|320446966|ref|NW_003383605.1|	531007-535468	263531	480922	0.867832	0.70185	no
gi|320446966|ref|NW_003383605.1|	550252-552196	91845	351134	-138718	0.5223	no
gi|320446966|ref|NW_003383605.1|	552320-553545	107433	520284	-104607	0.6231	no
gi|320446966|ref|NW_003383605.1|	553888-554967	98025	193775	-233876	0.2909	no
gi|320446966|ref|NW_003383605.1|	555882-556434	635037	142797	-215287	0.3524	no
gi|320446966|ref|NW_003383605.1|	561084-561344	312062	54013	-253045	0.23925	no
gi|320446966|ref|NW_003383605.1|	575264-575983	118628	214854	-246502	0.2659	no
gi|320446966|ref|NW_003383605.1|	584263-586662	417231	111087	-190916	0.1533	no
gi|320446966|ref|NW_003383605.1|	586966-589044	0.274725	102203	189537	1	no
gi|320446966|ref|NW_003383605.1|	592416-593167	163666	246772	0.592418	0.75905	no
gi|320446966|ref|NW_003383605.1|	593799-594897	958652	379137	-133829	0.52485	no
gi|320446966|ref|NW_003383605.1|	597440-598028	176326	548665	-168425	0.43435	no

gi|320446966|ref|NW_003383605.1|	598495-599896	179521	653784	-145726	0.37045	no
gi|320446966|ref|NW_003383605.1|	604056-604563	551832	370117	-0.576247	0.78605	no
gi|320446966|ref|NW_003383605.1|	604787-605812	218011	112274	-0.957382	0.65965	no
gi|320446966|ref|NW_003383605.1|	608229-608787	291008	171892	-0.759557	0.7158	no
gi|320446966|ref|NW_003383605.1|	609811-612888	178728	235827	0.399968	0.75995	no
gi|320446966|ref|NW_003383605.1|	613986-618852	14939	130416	-0.195958	0.88315	no
gi|320446966|ref|NW_003383605.1|	620865-622616	267207	116318	-119988	0.5599	no
gi|320446966|ref|NW_003383605.1|	623375-626320	483222	949001	-23482	0.0872	no
gi|320446966|ref|NW_003383605.1|	626415-626777	504499	509368	0.0138571	0.98935	no
gi|320446966|ref|NW_003383605.1|	635466-637031	0.634571	123609	0.961926	1	no
gi|320446966|ref|NW_003383605.1|	642419-643841	0.284424	187759	272277	0.24095	no
gi|320446966|ref|NW_003383605.1|	644020-648235	131432	41557	166078	0.4585	no
gi|320446966|ref|NW_003383605.1|	660107-661547	103664	181088	0.804777	0.6203	no
gi|320446966|ref|NW_003383605.1|	663635-674569	790081	168827	109547	0.41245	no
gi|320446966|ref|NW_003383605.1|	676261-679759	533185	709889	0.412956	0.75385	no
gi|320446966|ref|NW_003383605.1|	711703-713546	419855	928903	-217629	0.23335	no
gi|320446966|ref|NW_003383605.1|	714157-718542	334634	181931	-0.879195	0.51605	no
gi|320446966|ref|NW_003383605.1|	719774-720487	441581	210215	-107081	0.49925	no
gi|320446966|ref|NW_003383605.1|	72075-72952	578729	833297	0.525943	0.7945	no
gi|320446966|ref|NW_003383605.1|	720831-721348	710832	278492	-135187	0.5172	no
gi|320446966|ref|NW_003383605.1|	722495-727790	190744	10163	-0.90832	0.4969	no
gi|320446966|ref|NW_003383605.1|	778240-778851	0.447952	456075	334786	0.2264	no
gi|320446966|ref|NW_003383605.1|	808254-812809	15624	148258	324628	0.0986	no
gi|320446966|ref|NW_003383605.1|	813014-813519	185242	567216	493642	0.1493	no
gi|320446966|ref|NW_003383605.1|	816841-819233	336433	267688	-0.329769	0.87245	no
gi|320446966|ref|NW_003383605.1|	822582-823179	46495	0.9455	-229793	0.2652	no
gi|320446966|ref|NW_003383605.1|	824087-824570	187378	894507	-106678	0.6087	no
gi|320446966|ref|NW_003383605.1|	824708-825586	10506	325575	-169015	0.4318	no
gi|320446966|ref|NW_003383605.1|	827515-829450	943171	207635	-218347	0.34195	no
gi|320446966|ref|NW_003383605.1|	830416-832187	0.329658	221976	275136	0.25035	no

gi|320446966|ref|NW_003383605.1|	874425-875102	0	531563	inf	5.00E-05	yes
gi|320446966|ref|NW_003383605.1|	911517-912692	0.179606	114126	931157	0.1408	no
gi|320446966|ref|NW_003383605.1|	913741-914339	0	105306	inf	5.00E-05	yes
gi|320446966|ref|NW_003383605.1|	974906-976019	0	452623	inf	0.0101	no
gi|320446976|ref|NW_003383595.1|	100535-101144	0	495057	inf	0.00385	no
gi|320446976|ref|NW_003383595.1|	1369-2129	0.64346	551478	309938	0.20675	no
gi|320446976|ref|NW_003383595.1|	137688-141010	0	184683	inf	5.00E-05	yes
gi|320446976|ref|NW_003383595.1|	144790-146219	0	138528	inf	0.00365	no
gi|320446976|ref|NW_003383595.1|	224034-226209	0.260985	163898	265076	0.26605	no
gi|320446976|ref|NW_003383595.1|	234292-234770	174625	26421	-272451	0.1404	no
gi|320446976|ref|NW_003383595.1|	236584-239485	651978	216812	-158838	0.4803	no
gi|320446976|ref|NW_003383595.1|	239618-240122	241667	706235	-17748	0.4093	no
gi|320446976|ref|NW_003383595.1|	242400-243164	670529	197068	-176661	0.409	no
gi|320446976|ref|NW_003383595.1|	246169-247455	224876	70221	-167915	0.3136	no
gi|320446976|ref|NW_003383595.1|	250051-251093	523076	925842	0.823745	0.69	no
gi|320446976|ref|NW_003383595.1|	2552-3240	105457	1230.68	354473	0.0828	no
gi|320446976|ref|NW_003383595.1|	354228-354639	439033	223263	-0.975586	0.6403	no
gi|320446976|ref|NW_003383595.1|	356139-357635	147248	135892	-0.11579	0.96075	no
gi|320446976|ref|NW_003383595.1|	586403-587970	126723	0.528963	-126045	1	no
gi|320446976|ref|NW_003383595.1|	588074-593104	0.668612	160232	126092	0.5472	no
gi|320446976|ref|NW_003383595.1|	647960-648573	222811	423534	0.926658	0.66535	no
gi|320446976|ref|NW_003383595.1|	660439-661706	147226	397215	143189	0.4952	no
gi|320446976|ref|NW_003383595.1|	662368-664801	122849	86323	281286	0.23345	no
gi|320446976|ref|NW_003383595.1|	678687-679326	0.834713	823545	330249	0.19365	no
gi|320446976|ref|NW_003383595.1|	681168-681762	234379	149297	267127	0.25245	no
gi|320446976|ref|NW_003383595.1|	682071-683381	0.62815	632207	333122	0.19305	no
gi|320446976|ref|NW_003383595.1|	691819-692069	960914	141912	0.56252	0.7963	no
gi|320446976|ref|NW_003383595.1|	693659-694127	212835	898727	207815	0.35135	no
gi|320446976|ref|NW_003383595.1|	695003-695704	0.724624	165916	451708	0.1565	no
gi|320446976|ref|NW_003383595.1|	696426-697255	511811	390993	293346	0.11665	no

gi|320446976|ref|NW_003383595.1|	699336-699808	139655	163054	354541	0.1844	no
gi|320446976|ref|NW_003383595.1|	711730-713683	0.0982406	109587	347961	1	no
gi|320446976|ref|NW_003383595.1|	716377-719491	0.820606	110666	0.431456	1	no
gi|320446976|ref|NW_003383595.1|	724333-727345	0.668449	365384	245052	0.27615	no
gi|320446976|ref|NW_003383595.1|	729667-731356	0.580429	113963	42953	0.1389	no
gi|320446976|ref|NW_003383595.1|	732453-740379	239128	225676	323839	0.1081	no
gi|320446976|ref|NW_003383595.1|	740497-747253	1242	15454	363724	0.07605	no
gi|320446976|ref|NW_003383595.1|	750704-753345	164831	123492	-0.416569	0.74365	no
gi|320446976|ref|NW_003383595.1|	753547-753754	505206	197296	-135651	0.52435	no
gi|320446976|ref|NW_003383595.1|	761387-762572	0.888572	147795	0.734036	0.73635	no
gi|320446976|ref|NW_003383595.1|	763024-763703	152054	155665	0.0338678	0.9157	no
gi|320446976|ref|NW_003383595.1|	763848-765649	905352	116387	0.362382	0.8672	no
gi|320446976|ref|NW_003383595.1|	766530-769237	118103	596286	-0.985972	0.55175	no
gi|320446976|ref|NW_003383595.1|	770349-770568	758608	158647	-225753	0.32855	no
gi|320446976|ref|NW_003383595.1|	770685-772049	131579	33126	133203	0.4285	no
gi|320446976|ref|NW_003383595.1|	773179-777439	127447	965703	-0.40024	0.7603	no
gi|320446976|ref|NW_003383595.1|	777603-779240	124487	133991	0.10614	0.93755	no
gi|320446976|ref|NW_003383595.1|	786347-787016	100703	843268	-0.256045	0.8406	no
gi|320446976|ref|NW_003383595.1|	809353-810031	304788	182003	-0.74384	0.70585	no
gi|320446976|ref|NW_003383595.1|	8695-9451	816976	1114.76	37703	0.0699	no
gi|320446976|ref|NW_003383595.1|	889014-890281	0.32717	11349	179446	1	no
gi|320446976|ref|NW_003383595.1|	909492-909977	929832	0	#NAME?	0.0178	no
gi|320446976|ref|NW_003383595.1|	911896-912872	546918	0	#NAME?	0.00975	no
gi|320446992|ref|NW_003383579.1|	13068-13470	0.95742	630425	27191	0.2673	no
gi|320446992|ref|NW_003383579.1|	164050-165974	0.99928	153251	393886	0.06865	no
gi|320446992|ref|NW_003383579.1|	1897-2280	636497	17389	144995	0.492	no
gi|320446992|ref|NW_003383579.1|	20287-22763	0.527147	73105	379369	0.1413	no
gi|320446992|ref|NW_003383579.1|	266686-267007	63819	102978	0.690275	0.74435	no
gi|320446992|ref|NW_003383579.1|	293873-294717	0	190776	inf	0.0312	no
gi|320446992|ref|NW_003383579.1|	315286-315808	0	587188	inf	0.02205	no

gi|320446992|ref|NW_003383579.1|	368126-370297	244089	316303	0.373899	0.85625	no
gi|320446992|ref|NW_003383579.1|	372550-373484	0.483176	133306	146412	1	no
gi|320446992|ref|NW_003383579.1|	566421-567633	152064	706823	-110526	0.6118	no
gi|320446992|ref|NW_003383579.1|	572803-573532	752115	725526	-0.0519261	0.97555	no
gi|320446992|ref|NW_003383579.1|	573869-575695	838077	524815	-0.675275	0.7587	no
gi|320446992|ref|NW_003383579.1|	576865-577259	109848	590766	-0.894856	0.65745	no
gi|320446992|ref|NW_003383579.1|	579285-583757	134211	154222	0.200503	0.8799	no
gi|320446992|ref|NW_003383579.1|	596923-597151	872575	139.98	40038	0.164	no
gi|320446992|ref|NW_003383579.1|	597641-601425	97947	278854	150944	0.26125	no
gi|320446992|ref|NW_003383579.1|	603592-604490	458493	0.877585	-238529	0.30565	no
gi|320446992|ref|NW_003383579.1|	617737-618345	180597	765917	208442	0.3494	no
gi|320446992|ref|NW_003383579.1|	628111-628321	238237	894119	190807	0.393	no
gi|320446992|ref|NW_003383579.1|	680007-680762	877012	287228	171153	0.43955	no
gi|320446992|ref|NW_003383579.1|	763827-764429	963347	11822	0.295349	0.88515	no
gi|320446992|ref|NW_003383579.1|	779976-780462	661672	172545	138278	0.50425	no
gi|320446992|ref|NW_003383579.1|	819415-820399	0	476399	inf	5.00E-05	yes
gi|320446992|ref|NW_003383579.1|	82202-84426	154429	154555	0.00117811	0.99725	no
gi|320446992|ref|NW_003383579.1|	84637-85116	122311	453855	-143026	0.5042	no
gi|320446992|ref|NW_003383579.1|	863214-867073	400441	141765	182384	0.31	no
gi|320446992|ref|NW_003383579.1|	87136-88592	19908	12973	-0.617839	0.703	no
gi|320446992|ref|NW_003383579.1|	906218-913154	825899	827884	0.00346392	0.9972	no
gi|320446992|ref|NW_003383579.1|	914958-915631	157972	499424	-166133	0.43905	no
gi|320446992|ref|NW_003383579.1|	915761-917231	888432	209038	-20875	0.35	no
gi|320446992|ref|NW_003383579.1|	918233-918753	879569	315192	-148057	0.49115	no
gi|320446992|ref|NW_003383579.1|	920164-920632	0.709449	804124	350265	0.2175	no
gi|320446992|ref|NW_003383579.1|	923068-924024	140526	161632	0.201877	0.91225	no
gi|320446992|ref|NW_003383579.1|	929095-929494	1945	575945	156616	0.3575	no
gi|320446992|ref|NW_003383579.1|	969102-969722	131295	267572	102711	0.5772	no
gi|320447013|ref|NW_003383558.1|	120061-120495	573947	717285	364356	0.15485	no
gi|320447013|ref|NW_003383558.1|	167870-169221	0	21008	inf	0.0162	no

gi|320447013|ref|NW_003383558.1|	173545-177073	117861	1287.82	100936	0.1279	no
gi|320447013|ref|NW_003383558.1|	214516-215540	169066	25441	0.58957	0.79225	no
gi|320447013|ref|NW_003383558.1|	221535-222013	341065	409981	0.26551	0.90175	no
gi|320447013|ref|NW_003383558.1|	223802-230485	281043	709707	133643	0.30235	no
gi|320447013|ref|NW_003383558.1|	232431-232875	596019	52071	-351681	0.1653	no
gi|320447013|ref|NW_003383558.1|	235071-235839	351765	369514	-325091	0.1823	no
gi|320447013|ref|NW_003383558.1|	237509-238206	277726	699173	-198994	0.37235	no
gi|320447013|ref|NW_003383558.1|	25060-25291	416603	393473	323952	0.2322	no
gi|320447013|ref|NW_003383558.1|	30050-31525	0.408391	179812	213846	0.3451	no
gi|320447013|ref|NW_003383558.1|	343533-343974	317853	580057	0.867833	0.69375	no
gi|320447013|ref|NW_003383558.1|	345277-346377	0.585572	148607	134358	0.5243	no
gi|320447013|ref|NW_003383558.1|	352843-356604	0.335001	35167	339199	0.17145	no
gi|320447013|ref|NW_003383558.1|	370813-371180	896933	133913	0.578223	0.7155	no
gi|320447013|ref|NW_003383558.1|	371307-371545	451259	498215	0.142815	0.939	no
gi|320447013|ref|NW_003383558.1|	40641-43154	268181	142813	-0.909083	0.48785	no
gi|320447013|ref|NW_003383558.1|	43958-44376	291638	110934	-139447	0.50635	no
gi|320447013|ref|NW_003383558.1|	45657-47036	65812	193717	-17644	0.1855	no
gi|320447013|ref|NW_003383558.1|	525299-525640	551421	366765	-0.588296	0.77175	no
gi|320447013|ref|NW_003383558.1|	52652-53534	0.783072	143822	0.877068	0.68225	no
gi|320447013|ref|NW_003383558.1|	552515-554793	231301	593903	136046	0.5279	no
gi|320447013|ref|NW_003383558.1|	555072-556497	241151	100546	205984	0.34535	no
gi|320447013|ref|NW_003383558.1|	562589-564033	271148	102669	-140108	0.2875	no
gi|320447013|ref|NW_003383558.1|	564154-566665	136507	827071	-0.722894	0.57525	no
gi|320447013|ref|NW_003383558.1|	569172-571509	105177	179925	0.77457	0.54045	no
gi|320447013|ref|NW_003383558.1|	571648-574984	631244	810778	0.361109	0.819	no
gi|320447013|ref|NW_003383558.1|	580358-581294	819053	780974	-0.0686814	0.9719	no
gi|320447013|ref|NW_003383558.1|	582979-583921	453818	23069	-0.97616	0.6426	no
gi|320447013|ref|NW_003383558.1|	584243-585276	105796	62896	-0.750245	0.7213	no
gi|320447013|ref|NW_003383558.1|	586319-587148	710848	449642	-0.660764	0.7474	no
gi|320447013|ref|NW_003383558.1|	589028-590982	374079	195773	-0.934159	0.474	no

gi|320447013|ref|NW_003383558.1|	591145-593234	180243	172721	-0.0614991	0.9593	no
gi|320447013|ref|NW_003383558.1|	594651-595497	439507	150229	-154872	0.35745	no
gi|320447013|ref|NW_003383558.1|	597048-597367	972269	627194	-0.632444	0.7537	no
gi|320447013|ref|NW_003383558.1|	599281-608254	577354	858612	0.572551	0.66715	no
gi|320447013|ref|NW_003383558.1|	608397-612867	796881	118581	0.573433	0.65695	no
gi|320447013|ref|NW_003383558.1|	61045-62275	0.678687	200021	155933	0.4963	no
gi|320447013|ref|NW_003383558.1|	617028-617432	341137	387022	0.182064	0.9305	no
gi|320447013|ref|NW_003383558.1|	626187-627353	117873	804118	-0.551759	0.7942	no
gi|320447013|ref|NW_003383558.1|	627852-631626	724758	987746	0.446641	0.7217	no
gi|320447013|ref|NW_003383558.1|	655119-655674	0	672353	inf	0.0212	no
gi|320447013|ref|NW_003383558.1|	684438-685987	179844	554014	162317	0.44555	no
gi|320447013|ref|NW_003383558.1|	686817-687825	305817	482967	0.659254	0.7474	no
gi|320447013|ref|NW_003383558.1|	688817-689502	225063	128058	-0.813537	0.69605	no
gi|320447013|ref|NW_003383558.1|	689759-690214	374101	298655	-0.324952	0.86235	no
gi|320447013|ref|NW_003383558.1|	690308-691717	158166	249759	0.659095	0.74115	no
gi|320447013|ref|NW_003383558.1|	717219-719162	287554	422295	0.554417	0.68065	no
gi|320447013|ref|NW_003383558.1|	719404-720753	681914	925993	0.44141	0.84235	no
gi|320447013|ref|NW_003383558.1|	721155-723559	303449	488967	0.688284	0.74695	no
gi|320447013|ref|NW_003383558.1|	723691-723904	619188	704933	-313482	0.25355	no
gi|320447013|ref|NW_003383558.1|	745669-746376	432856	271501	-0.67293	0.7697	no
gi|320447028|ref|NW_003383543.1|	1085581-1087292	148661	366297	130098	0.52695	no
gi|320447028|ref|NW_003383543.1|	1088097-1088672	0	501514	inf	0.02205	no
gi|320447028|ref|NW_003383543.1|	1109618-1110334	0.702144	240168	17742	0.333	no
gi|320447028|ref|NW_003383543.1|	1110906-1116052	122661	236129	0.944898	0.4871	no
gi|320447028|ref|NW_003383543.1|	1116712-1116976	298082	791958	140971	0.4973	no
gi|320447028|ref|NW_003383543.1|	1119029-1119969	292226	378395	0.372806	0.8185	no
gi|320447028|ref|NW_003383543.1|	1130701-1131026	526147	113988	111535	0.605	no
gi|320447028|ref|NW_003383543.1|	1139992-1141352	255092	625251	129342	0.53475	no
gi|320447028|ref|NW_003383543.1|	1142378-1144599	790307	201045	134704	0.43215	no
gi|320447028|ref|NW_003383543.1|	1166632-1168289	0.830896	380089	21936	0.3188	no

gi|320447028|ref|NW_003383543.1|	1172972-1175053	26605	434936	0.709104	0.5999	no
gi|320447028|ref|NW_003383543.1|	1181046-1182651	134295	13032	-0.043356	0.98515	no
gi|320447028|ref|NW_003383543.1|	1193306-1194033	367283	157426	-122222	0.583	no
gi|320447028|ref|NW_003383543.1|	1200659-1200884	411821	115043	-183984	0.41505	no
gi|320447028|ref|NW_003383543.1|	1203647-1209578	903503	146566	0.697945	0.60025	no
gi|320447028|ref|NW_003383543.1|	1214954-1216374	940184	791886	-0.247651	0.907	no
gi|320447028|ref|NW_003383543.1|	1219058-1219993	940845	0.332796	-482125	0.1939	no
gi|320447028|ref|NW_003383543.1|	1220512-1221134	348339	443763	0.349297	0.8547	no
gi|320447028|ref|NW_003383543.1|	1221823-1222346	580515	156082	-189503	0.4022	no
gi|320447028|ref|NW_003383543.1|	1223088-1224075	62873	449717	-0.483422	0.8079	no
gi|320447028|ref|NW_003383543.1|	1224684-1226197	383034	514362	0.425313	0.83805	no
gi|320447028|ref|NW_003383543.1|	1227263-1229578	332664	674025	101874	0.63955	no
gi|320447028|ref|NW_003383543.1|	211111-211616	135844	910858	-0.576654	0.78015	no
gi|320447028|ref|NW_003383543.1|	216047-217213	101552	753861	-0.429855	0.8351	no
gi|320447028|ref|NW_003383543.1|	222609-224888	974292	18039	0.888691	0.59985	no
gi|320447028|ref|NW_003383543.1|	261324-262092	46902	652084	-284652	0.12055	no
gi|320447028|ref|NW_003383543.1|	262245-262461	639668	133645	-225891	0.33965	no
gi|320447028|ref|NW_003383543.1|	266475-267486	396024	721693	-245613	0.176	no
gi|320447028|ref|NW_003383543.1|	306032-306833	298422	163972	-0.863906	0.6829	no
gi|320447028|ref|NW_003383543.1|	307020-308579	12494	159639	-296835	0.2126	no
gi|320447028|ref|NW_003383543.1|	311101-312032	240204	267741	-316535	0.1886	no
gi|320447028|ref|NW_003383543.1|	312742-313048	252108	462992	-244498	0.303	no
gi|320447028|ref|NW_003383543.1|	331394-332596	370127	228735	-0.694339	0.57745	no
gi|320447028|ref|NW_003383543.1|	335711-338482	319414	320883	0.00662128	0.9919	no
gi|320447028|ref|NW_003383543.1|	338627-339084	155817	316026	10202	0.62915	no
gi|320447028|ref|NW_003383543.1|	345618-347261	513087	141852	-185481	0.3086	no
gi|320447028|ref|NW_003383543.1|	361611-363313	255426	149814	-0.769736	0.5472	no
gi|320447028|ref|NW_003383543.1|	363488-369941	363622	625778	0.783213	0.53165	no
gi|320447028|ref|NW_003383543.1|	373311-373650	167743	543963	-162468	0.4424	no
gi|320447028|ref|NW_003383543.1|	375084-375317	121288	229238	-240351	0.2777	no

gi|320447028|ref|NW_003383543.1|	375883-376324	794633	474592	-0.743602	0.7078	no
gi|320447028|ref|NW_003383543.1|	378149-378843	283208	105537	-142412	0.51965	no
gi|320447028|ref|NW_003383543.1|	383266-384297	0.212124	259978	102593	0.14075	no
gi|320447028|ref|NW_003383543.1|	386279-387514	0	286256	inf	5.00E-05	yes
gi|320447028|ref|NW_003383543.1|	399500-400061	231755	118246	-0.970811	0.6411	no
gi|320447028|ref|NW_003383543.1|	400392-400674	112068	128797	0.200726	0.90825	no
gi|320447028|ref|NW_003383543.1|	402450-403578	202301	191141	-0.0818668	0.9717	no
gi|320447028|ref|NW_003383543.1|	421041-421888	0	168974	inf	0.0048	no
gi|320447028|ref|NW_003383543.1|	582580-583062	0	763107	inf	0.02075	no
gi|320447028|ref|NW_003383543.1|	597105-598227	0	461271	inf	0.0114	no
gi|320447028|ref|NW_003383543.1|	598295-602220	0	656179	inf	5.00E-05	yes
gi|320447028|ref|NW_003383543.1|	663925-664572	0	33446	inf	0.029	no
gi|320447028|ref|NW_003383543.1|	870320-870688	115782	756459	270786	0.2673	no
gi|320447038|ref|NW_003383533.1|	1005268-1006753	162049	375552	121258	0.56095	no
gi|320447038|ref|NW_003383533.1|	1011958-1012582	736474	735991	-0.000944628	0.97505	no
gi|320447038|ref|NW_003383533.1|	1013576-1019796	842955	525605	-0.681477	0.60215	no
gi|320447038|ref|NW_003383533.1|	1020041-1020522	175323	495584	-182281	0.40035	no
gi|320447038|ref|NW_003383533.1|	1034385-1034777	30283	663216	113097	0.57945	no
gi|320447038|ref|NW_003383533.1|	1034959-1035837	0.787948	50645	268425	0.2604	no
gi|320447038|ref|NW_003383533.1|	1093137-1095917	332209	231705	-384173	0.06115	no
gi|320447038|ref|NW_003383533.1|	1104837-1107122	191818	0.804507	-457549	0.05725	no
gi|320447038|ref|NW_003383533.1|	1164576-1165193	26462	209663	-0.335849	0.8742	no
gi|320447038|ref|NW_003383533.1|	1166045-1166382	32606	174602	-0.90107	0.6686	no
gi|320447038|ref|NW_003383533.1|	1171761-1172043	268962	0	#NAME?	0.02015	no
gi|320447038|ref|NW_003383533.1|	1175214-1177193	0.387102	452101	354586	0.18025	no
gi|320447038|ref|NW_003383533.1|	1177263-1177836	0.497203	30258	260541	0.2636	no
gi|320447038|ref|NW_003383533.1|	1191432-1191822	612275	469112	-0.384248	0.8476	no
gi|320447038|ref|NW_003383533.1|	1225289-1226118	199037	0.977483	-10259	0.59645	no
gi|320447038|ref|NW_003383533.1|	1227211-1227895	263154	179665	-0.5506	0.79275	no
gi|320447038|ref|NW_003383533.1|	1251460-1251957	444764	170216	-138567	0.52445	no

gi|320447038|ref|NW_003383533.1|	1269818-1272705	0.699964	173472	130935	0.5259	no
gi|320447038|ref|NW_003383533.1|	1282012-1282491	0	149772	inf	0.00945	no
gi|320447038|ref|NW_003383533.1|	1323858-1325315	180959	363909	100791	0.42525	no
gi|320447038|ref|NW_003383533.1|	1325420-1325708	169201	257007	0.603069	0.75565	no
gi|320447038|ref|NW_003383533.1|	1326424-1327969	786002	117412	0.578974	0.79015	no
gi|320447038|ref|NW_003383533.1|	1328586-1329365	161459	202415	0.326145	0.87925	no
gi|320447038|ref|NW_003383533.1|	1348662-1350890	0.338723	313221	320901	0.20395	no
gi|320447038|ref|NW_003383533.1|	1355003-1356148	0.742126	218458	155762	0.4963	no
gi|320447038|ref|NW_003383533.1|	1360263-1373410	212994	286989	0.430187	0.733	no
gi|320447038|ref|NW_003383533.1|	1373658-1374997	183492	116786	-0.651848	0.75415	no
gi|320447038|ref|NW_003383533.1|	152792-154076	201165	20101	-332304	0.08395	no
gi|320447038|ref|NW_003383533.1|	19109-20187	320462	15243	-1072	0.60365	no
gi|320447038|ref|NW_003383533.1|	20315-21153	0.840273	134845	0.682378	1	no
gi|320447038|ref|NW_003383533.1|	248696-249176	0	542624	inf	0.029	no
gi|320447038|ref|NW_003383533.1|	250546-252766	0.765198	178005	121801	0.56155	no
gi|320447038|ref|NW_003383533.1|	308496-309784	0.801602	300382	190584	0.39245	no
gi|320447038|ref|NW_003383533.1|	314076-314431	48073.9	49804.5	0.0510221	0.55075	no
gi|320447038|ref|NW_003383533.1|	419366-420418	0.206667	257253	36378	0.2061	no
gi|320447038|ref|NW_003383533.1|	421524-423930	0.388678	423383	344532	0.1825	no
gi|320447038|ref|NW_003383533.1|	429595-429868	44221	469405	-323583	0.1958	no
gi|320447038|ref|NW_003383533.1|	431733-434939	487337	476682	-335382	0.09955	no
gi|320447038|ref|NW_003383533.1|	437075-438290	137816	214989	-268041	0.2413	no
gi|320447038|ref|NW_003383533.1|	442187-443120	749996	183553	-203068	0.3586	no
gi|320447038|ref|NW_003383533.1|	443482-445506	148114	611864	-127543	0.4359	no
gi|320447038|ref|NW_003383533.1|	447740-449871	640904	209907	171157	0.32845	no
gi|320447038|ref|NW_003383533.1|	458311-461342	464734	120689	137682	0.4178	no
gi|320447038|ref|NW_003383533.1|	461521-463989	513921	596424	0.214792	0.9236	no
gi|320447038|ref|NW_003383533.1|	468368-471706	87012	787434	-0.144057	0.9067	no
gi|320447038|ref|NW_003383533.1|	472026-472397	147832	111526	-0.406574	0.8389	no
gi|320447038|ref|NW_003383533.1|	472524-475931	659671	781535	0.244563	0.8446	no

gi|320447038|ref|NW_003383533.1|	476384-477170	229915	967942	-124811	0.5617	no
gi|320447038|ref|NW_003383533.1|	477817-478226	240201	852916	-149376	0.4755	no
gi|320447038|ref|NW_003383533.1|	478455-478856	202104	316784	-267353	0.27125	no
gi|320447038|ref|NW_003383533.1|	484095-484305	357355	44706	0.323109	0.86335	no
gi|320447038|ref|NW_003383533.1|	686161-686888	102977	211467	103812	0.5772	no
gi|320447038|ref|NW_003383533.1|	701956-703749	0	173584	inf	0.01485	no
gi|320447038|ref|NW_003383533.1|	720993-721616	354839	206582	-74243	0.085	no
gi|320447038|ref|NW_003383533.1|	722031-722555	26615	0.38896	-609647	0.2673	no
gi|320447038|ref|NW_003383533.1|	724063-725371	165826	0.655213	-798349	0.09715	no
gi|320447038|ref|NW_003383533.1|	725908-726340	182034	767427	-124611	0.54495	no
gi|320447038|ref|NW_003383533.1|	726575-727024	383731	459029	0.258488	0.8937	no
gi|320447038|ref|NW_003383533.1|	727221-727704	535365	626155	0.225998	0.902	no
gi|320447038|ref|NW_003383533.1|	728017-734028	356395	773882	111864	0.512	no
gi|320447038|ref|NW_003383533.1|	735507-736670	916929	518043	-0.823739	0.534	no
gi|320447038|ref|NW_003383533.1|	742826-743829	30781	188349	-0.708631	0.66115	no
gi|320447038|ref|NW_003383533.1|	744562-746292	0.677405	0.629007	-0.106944	1	no
gi|320447038|ref|NW_003383533.1|	746466-748581	0.897722	125255	0.480532	1	no
gi|320447038|ref|NW_003383533.1|	748893-752561	629662	549312	-0.196951	0.87915	no
gi|320447038|ref|NW_003383533.1|	897184-898796	18387	588602	16786	0.4335	no
gi|320447038|ref|NW_003383533.1|	901984-902819	337791	735606	11228	0.586	no
gi|320447038|ref|NW_003383533.1|	915914-916423	182664	367644	100912	0.5772	no
gi|320447038|ref|NW_003383533.1|	918730-919188	0.73891	541013	287219	0.25915	no
gi|320447038|ref|NW_003383533.1|	920317-921809	81916	122315	0.578385	0.79025	no
gi|320447038|ref|NW_003383533.1|	924812-929926	148366	47777	168715	0.2159	no
gi|320447038|ref|NW_003383533.1|	930030-933216	131524	131972	0.00491136	1	no
gi|320447038|ref|NW_003383533.1|	933406-935264	103993	181177	0.800914	0.69955	no
gi|320447038|ref|NW_003383533.1|	936414-940719	248758	583485	122996	0.4532	no
gi|320447038|ref|NW_003383533.1|	940839-941162	785582	134947	0.780561	0.7204	no
gi|320447038|ref|NW_003383533.1|	941341-942090	460046	121601	140231	0.50595	no
gi|320447038|ref|NW_003383533.1|	942222-943518	264124	354459	0.424402	0.74	no

gi|320447038|ref|NW_003383533.1|	948305-948532	797755	136.58	0.775733	0.7087	no
gi|320447038|ref|NW_003383533.1|	948778-948940	313188	244825	-0.355279	0.85605	no
gi|320447038|ref|NW_003383533.1|	949755-950027	868989	116985	0.42891	0.8348	no
gi|320447038|ref|NW_003383533.1|	960359-961085	110409	121245	0.135076	0.9175	no
gi|320447038|ref|NW_003383533.1|	991201-997391	223843	496473	114923	0.49375	no
gi|320447082|ref|NW_003383514.1|	1008983-1009837	109137	150202	0.460762	0.81855	no
gi|320447082|ref|NW_003383514.1|	1038932-1040190	247537	114477	-111259	0.5953	no
gi|320447082|ref|NW_003383514.1|	1065773-1066481	0	244096	inf	0.0312	no
gi|320447082|ref|NW_003383514.1|	1124954-1129358	367512	225317	-0.705836	0.5951	no
gi|320447082|ref|NW_003383514.1|	1129493-1131015	142961	538163	-14095	0.53025	no
gi|320447082|ref|NW_003383514.1|	1132566-1134269	187415	464348	-201295	0.2396	no
gi|320447082|ref|NW_003383514.1|	1141215-1142564	14699	315679	-221919	0.32765	no
gi|320447082|ref|NW_003383514.1|	1156313-1157899	812113	117358	0.531161	0.81295	no
gi|320447082|ref|NW_003383514.1|	1158026-1158513	527357	396752	-0.410545	0.8413	no
gi|320447082|ref|NW_003383514.1|	1159462-1161488	263858	236588	-0.157383	0.93775	no
gi|320447082|ref|NW_003383514.1|	1161980-1162593	623871	605049	-0.0441953	0.97185	no
gi|320447082|ref|NW_003383514.1|	1163159-1163819	277877	108223	-136044	0.5288	no
gi|320447082|ref|NW_003383514.1|	1167396-1168127	408596	163188	-132415	0.5185	no
gi|320447082|ref|NW_003383514.1|	1171318-1171923	409577	123487	-172977	0.4381	no
gi|320447082|ref|NW_003383514.1|	1177317-1178590	29275	0.67705	-211233	0.3516	no
gi|320447082|ref|NW_003383514.1|	1190125-1192499	662751	470996	282918	0.1393	no
gi|320447082|ref|NW_003383514.1|	1205115-1205540	512837	28274	-0.859024	0.688	no
gi|320447082|ref|NW_003383514.1|	1207097-1209744	230889	0.635486	-186127	0.39215	no
gi|320447082|ref|NW_003383514.1|	1217292-1218689	261492	0.403699	-269542	0.2729	no
gi|320447082|ref|NW_003383514.1|	1252131-1253328	43874	24329	-0.850687	0.6792	no
gi|320447082|ref|NW_003383514.1|	1253455-1254192	538304	322587	-0.738732	0.7049	no
gi|320447082|ref|NW_003383514.1|	1256318-1257487	867652	275539	-165486	0.4434	no
gi|320447082|ref|NW_003383514.1|	125646-126743	0	488019	inf	0.0085	no
gi|320447082|ref|NW_003383514.1|	1261528-1263698	514596	152147	-175798	0.4173	no
gi|320447082|ref|NW_003383514.1|	1267016-1267634	21995	478042	111996	0.5821	no

gi|320447082|ref|NW_003383514.1|	1267852-1268334	134351	763107	250588	0.2524	no
gi|320447082|ref|NW_003383514.1|	1271028-1272244	0.688379	405681	255907	0.28065	no
gi|320447082|ref|NW_003383514.1|	1272351-1273037	336852	117562	180323	0.40235	no
gi|320447082|ref|NW_003383514.1|	1273865-1277131	43983	187692	209335	0.25195	no
gi|320447082|ref|NW_003383514.1|	1281235-1282336	0	441265	inf	5.00E-05	yes
gi|320447082|ref|NW_003383514.1|	1285134-1286271	0	598887	inf	5.00E-05	yes
gi|320447082|ref|NW_003383514.1|	128861-130671	0	283718	inf	0.0113	no
gi|320447082|ref|NW_003383514.1|	262507-263466	100292	611698	-0.713321	0.72885	no
gi|320447082|ref|NW_003383514.1|	264454-267109	495119	589546	0.251828	0.90905	no
gi|320447082|ref|NW_003383514.1|	271265-272141	131735	1.27	-0.0528051	1	no
gi|320447082|ref|NW_003383514.1|	291734-293819	153264	278092	0.859545	0.5075	no
gi|320447082|ref|NW_003383514.1|	295174-296829	0.594333	0.661947	0.155444	1	no
gi|320447082|ref|NW_003383514.1|	297919-299387	0.821422	0.951699	0.212382	1	no
gi|320447082|ref|NW_003383514.1|	306935-308163	134311	179198	0.41598	0.8514	no
gi|320447082|ref|NW_003383514.1|	312658-315106	23488	516577	-218487	0.23065	no
gi|320447082|ref|NW_003383514.1|	317326-319387	19872	593101	-174439	0.31365	no
gi|320447082|ref|NW_003383514.1|	323805-326037	209587	12091	-0.793612	0.5278	no
gi|320447082|ref|NW_003383514.1|	33363-34346	451496	530029	0.231357	0.90975	no
gi|320447082|ref|NW_003383514.1|	334581-344841	129089	471424	186866	0.1612	no
gi|320447082|ref|NW_003383514.1|	346215-348757	17849	273879	0.617692	0.6366	no
gi|320447082|ref|NW_003383514.1|	362845-363478	804771	123865	0.622124	0.7588	no
gi|320447082|ref|NW_003383514.1|	376255-376706	0.76094	607052	299597	0.2563	no
gi|320447082|ref|NW_003383514.1|	390815-391989	0	224256	inf	0.0154	no
gi|320447082|ref|NW_003383514.1|	393275-393481	135297	245475	0.859449	0.6872	no
gi|320447082|ref|NW_003383514.1|	396802-397432	132288	22631	0.774622	0.7091	no
gi|320447082|ref|NW_003383514.1|	397936-399578	117566	260524	114794	0.4998	no
gi|320447082|ref|NW_003383514.1|	399710-400772	571666	187816	171607	0.4355	no
gi|320447082|ref|NW_003383514.1|	66237-67526	0.961009	133378	0.472898	1	no
gi|320447082|ref|NW_003383514.1|	699096-699343	99862	252621	133897	0.52405	no
gi|320447082|ref|NW_003383514.1|	834417-834845	160137	390553	-203572	0.348	no

gi|320447082|ref|NW_003383514.1|	838706-838975	282044	489371	-252692	0.23925	no
gi|320447082|ref|NW_003383514.1|	839164-839824	635148	108223	-255308	0.2898	no
gi|320447082|ref|NW_003383514.1|	841901-842523	566052	0.295842	-425803	0.3007	no
gi|320447082|ref|NW_003383514.1|	845459-845941	114198	134666	-308408	0.1951	no
gi|320447082|ref|NW_003383514.1|	847467-847978	14512	162312	-316041	0.2241	no
gi|320447082|ref|NW_003383514.1|	995880-998929	34782	557672	0.681077	0.76065	no
gi|320447089|ref|NW_003383510.1|	1000845-1001347	131055	4601	-151016	0.487	no
gi|320447089|ref|NW_003383510.1|	1001981-1003417	261429	937731	-147918	0.3789	no
gi|320447089|ref|NW_003383510.1|	1003517-1005765	578489	415454	-0.477601	0.8292	no
gi|320447089|ref|NW_003383510.1|	1014297-1015979	104983	422304	200813	0.354	no
gi|320447089|ref|NW_003383510.1|	1087816-1088743	0.732102	74047	333832	0.1983	no
gi|320447089|ref|NW_003383510.1|	1122526-1128032	726669	301235	205152	0.1317	no
gi|320447089|ref|NW_003383510.1|	1177989-1179197	523655	1268.43	127635	0.5468	no
gi|320447089|ref|NW_003383510.1|	119197-119440	105292	155209	0.55981	0.7963	no
gi|320447089|ref|NW_003383510.1|	1202896-1203787	0.772318	283769	187745	0.38825	no
gi|320447089|ref|NW_003383510.1|	121805-123137	0	115386	inf	0.0057	no
gi|320447089|ref|NW_003383510.1|	1219197-1220257	0.613986	198173	169049	0.4292	no
gi|320447089|ref|NW_003383510.1|	1253687-1254433	727164	323857	2155	0.3416	no
gi|320447089|ref|NW_003383510.1|	1255781-1256428	736834	465456	265923	0.2592	no
gi|320447089|ref|NW_003383510.1|	1258097-1258532	244866	102798	206975	0.3535	no
gi|320447089|ref|NW_003383510.1|	1258592-1258903	103649	100072	-0.0506608	0.94	no
gi|320447089|ref|NW_003383510.1|	1293063-1295501	0	438665	inf	0.00565	no
gi|320447089|ref|NW_003383510.1|	1296125-1299476	0.162467	208352	368081	0.18105	no
gi|320447089|ref|NW_003383510.1|	1307993-1308511	0.590353	674139	35134	0.21745	no
gi|320447089|ref|NW_003383510.1|	1311543-1312195	0	330626	inf	0.029	no
gi|320447089|ref|NW_003383510.1|	1313002-1313284	224135	171729	293769	0.25735	no
gi|320447089|ref|NW_003383510.1|	1332807-1334512	16075	590846	187796	0.1562	no
gi|320447089|ref|NW_003383510.1|	1340097-1343627	421483	292065	279274	0.12215	no
gi|320447089|ref|NW_003383510.1|	1357202-1358109	0.251304	173208	2785	0.2657	no
gi|320447089|ref|NW_003383510.1|	1374050-1374779	953819	234041	-202696	0.2507	no

gi|320447089|ref|NW_003383510.1|	1381536-1382047	701414	198832	-181871	0.421	no
gi|320447089|ref|NW_003383510.1|	1386471-1387358	419613	140955	-157382	0.35125	no
gi|320447089|ref|NW_003383510.1|	1388271-1389297	0.853865	13279	0.63707	1	no
gi|320447089|ref|NW_003383510.1|	1390561-1391510	0.473016	16319	178659	0.3323	no
gi|320447089|ref|NW_003383510.1|	1392317-1395685	215447	158245	-0.445169	0.83085	no
gi|320447089|ref|NW_003383510.1|	1395786-1397879	698526	571547	-0.28944	0.82785	no
gi|320447089|ref|NW_003383510.1|	1399187-1400790	296129	231824	-0.353194	0.86105	no
gi|320447089|ref|NW_003383510.1|	1400980-1401705	172326	306661	0.83151	0.69095	no
gi|320447089|ref|NW_003383510.1|	1402401-1404023	827533	33878	-128847	0.55385	no
gi|320447089|ref|NW_003383510.1|	1404553-1410352	58529	380401	-0.621632	0.62715	no
gi|320447089|ref|NW_003383510.1|	1413645-1414595	0.944707	260745	14647	0.49755	no
gi|320447089|ref|NW_003383510.1|	1414773-1415898	474259	112964	125212	0.55805	no
gi|320447089|ref|NW_003383510.1|	1424148-1424421	319374	93881	-176634	0.4163	no
gi|320447089|ref|NW_003383510.1|	1432616-1432788	930711	123716	0.41063	0.8486	no
gi|320447089|ref|NW_003383510.1|	144766-145686	0.73959	305961	204855	0.3471	no
gi|320447089|ref|NW_003383510.1|	145831-147317	0.404799	140721	179756	1	no
gi|320447089|ref|NW_003383510.1|	1520719-1521379	170696	16504	-0.0486104	0.97965	no
gi|320447089|ref|NW_003383510.1|	1521598-1522481	547303	323109	-0.760319	0.7121	no
gi|320447089|ref|NW_003383510.1|	1522719-1523552	124272	10876	-0.192343	0.92765	no
gi|320447089|ref|NW_003383510.1|	1532588-1533341	0.326071	245844	291448	0.25645	no
gi|320447089|ref|NW_003383510.1|	155282-156494	0.3456	754743	444881	0.15925	no
gi|320447089|ref|NW_003383510.1|	1557802-1558601	188673	370217	-234945	0.28845	no
gi|320447089|ref|NW_003383510.1|	1562758-1564209	242933	3763	-26906	0.13665	no
gi|320447089|ref|NW_003383510.1|	1564805-1565127	506636	112437	-217183	0.3294	no
gi|320447089|ref|NW_003383510.1|	1569095-1569677	247152	590396	-206564	0.3454	no
gi|320447089|ref|NW_003383510.1|	1570702-1572882	152717	248246	-262102	0.14465	no
gi|320447089|ref|NW_003383510.1|	1579601-1580588	107782	139567	-294909	0.208	no
gi|320447089|ref|NW_003383510.1|	1595252-1595916	121924	697167	-0.806406	0.70015	no
gi|320447089|ref|NW_003383510.1|	1605365-1605835	19707	469416	-206977	0.34935	no
gi|320447089|ref|NW_003383510.1|	1617590-1618984	233039	111302	-10661	0.60515	no

gi|320447089|ref|NW_003383510.1|	1622174-1622842	116911	584685	-0.999683	0.6374	no
gi|320447089|ref|NW_003383510.1|	1694853-1695764	124908	154981	0.311231	0.8759	no
gi|320447089|ref|NW_003383510.1|	1701226-1707091	140261	228721	0.705472	0.5983	no
gi|320447089|ref|NW_003383510.1|	1841702-1842113	0	152663	inf	5.00E-05	yes
gi|320447089|ref|NW_003383510.1|	1842518-1843868	0	480458	inf	5.00E-05	yes
gi|320447089|ref|NW_003383510.1|	185700-186614	248711	330906	373388	0.07515	no
gi|320447089|ref|NW_003383510.1|	1935806-1936136	298191	771737	137187	0.38305	no
gi|320447089|ref|NW_003383510.1|	1957245-1957454	485748	22.78	-109244	0.5928	no
gi|320447089|ref|NW_003383510.1|	1966233-1966482	227083	615814	-188265	0.30885	no
gi|320447089|ref|NW_003383510.1|	1968636-1968885	291964	266853	-0.129746	0.942	no
gi|320447089|ref|NW_003383510.1|	1973202-1973567	471658	123173	138487	0.5147	no
gi|320447089|ref|NW_003383510.1|	197614-199262	788408	241134	-170911	0.43085	no
gi|320447089|ref|NW_003383510.1|	1979996-1980482	119101	11503	-0.0501759	0.97505	no
gi|320447089|ref|NW_003383510.1|	1986794-1987117	235675	101464	-121583	0.56575	no
gi|320447089|ref|NW_003383510.1|	1988726-1988961	78516	222722	150419	0.35905	no
gi|320447089|ref|NW_003383510.1|	199447-200797	287661	0.946198	-160416	0.43075	no
gi|320447089|ref|NW_003383510.1|	1996690-1997900	675303	648233	-0.0590209	0.97535	no
gi|320447089|ref|NW_003383510.1|	2002434-2003652	0	392958	inf	0.00945	no
gi|320447089|ref|NW_003383510.1|	201052-201897	249186	133328	-0.90224	0.6711	no
gi|320447089|ref|NW_003383510.1|	2027822-2028603	250582	144368	-0.795536	0.71815	no
gi|320447089|ref|NW_003383510.1|	203296-203823	286436	308216	0.105726	0.93055	no
gi|320447089|ref|NW_003383510.1|	2033102-2033490	639656	636605	-0.00689949	0.9935	no
gi|320447089|ref|NW_003383510.1|	2044269-2045903	141163	138558	-0.0268645	0.99065	no
gi|320447089|ref|NW_003383510.1|	2048885-2049083	122.15	118839	-0.0396476	0.974	no
gi|320447089|ref|NW_003383510.1|	2049193-2050700	0.530816	0.922796	0.797799	1	no
gi|320447089|ref|NW_003383510.1|	2051480-2051723	245682	66518	143695	0.49485	no
gi|320447089|ref|NW_003383510.1|	2052124-2052601	23967	516666	110818	0.6086	no
gi|320447089|ref|NW_003383510.1|	2057436-2060346	656093	18213	147299	0.39775	no
gi|320447089|ref|NW_003383510.1|	2067280-2068443	0.545791	352924	269294	0.25995	no
gi|320447089|ref|NW_003383510.1|	2074803-2076222	0.712863	208044	154519	0.48745	no

gi|320447089|ref|NW_003383510.1|	2080836-2081516	0	85431	inf	0.00945	no
gi|320447089|ref|NW_003383510.1|	2089979-2092628	559268	193419	179012	0.30455	no
gi|320447089|ref|NW_003383510.1|	209024-210693	0.823939	0.327769	-132986	1	no
gi|320447089|ref|NW_003383510.1|	2092818-2097091	63936	245186	193918	0.1468	no
gi|320447089|ref|NW_003383510.1|	2097232-2100994	107648	262171	128418	0.3322	no
gi|320447089|ref|NW_003383510.1|	2101308-2102148	809575	145929	0.850031	0.6845	no
gi|320447089|ref|NW_003383510.1|	2102757-2103177	70022	173549	130947	0.5319	no
gi|320447089|ref|NW_003383510.1|	211872-212969	274219	0.406683	-275335	0.22245	no
gi|320447089|ref|NW_003383510.1|	2131624-2132292	779409	983334	0.335301	0.8707	no
gi|320447089|ref|NW_003383510.1|	2133040-2134618	427329	118925	147663	0.50095	no
gi|320447089|ref|NW_003383510.1|	2138895-2139338	393837	732056	0.894357	0.67045	no
gi|320447089|ref|NW_003383510.1|	2153818-2156257	176114	844851	226219	0.3317	no
gi|320447089|ref|NW_003383510.1|	2157477-2159623	0.529893	345062	270308	0.2448	no
gi|320447089|ref|NW_003383510.1|	2168062-2168425	121763	241487	-233407	0.3078	no
gi|320447089|ref|NW_003383510.1|	2169783-2171076	0.797811	0.664397	-0.264002	1	no
gi|320447089|ref|NW_003383510.1|	2182278-2183702	124936	234802	0.910253	0.578	no
gi|320447089|ref|NW_003383510.1|	2183883-2184246	107438	186957	0.799201	0.697	no
gi|320447089|ref|NW_003383510.1|	2184649-2185121	118707	135102	0.186646	0.92165	no
gi|320447089|ref|NW_003383510.1|	2187673-2188144	420623	701455	0.737822	0.70715	no
gi|320447089|ref|NW_003383510.1|	2188883-2189404	9935	353448	-149102	0.471	no
gi|320447089|ref|NW_003383510.1|	2189635-2190473	271688	109803	-130704	0.55405	no
gi|320447089|ref|NW_003383510.1|	2191418-2193431	110112	807593	-0.447272	0.84095	no
gi|320447089|ref|NW_003383510.1|	2194637-2195397	0.32173	198532	262545	0.26355	no
gi|320447089|ref|NW_003383510.1|	2195956-2196467	495827	243468	-434803	0.1428	no
gi|320447089|ref|NW_003383510.1|	2196682-2197568	189375	0.178693	-672762	0.26225	no
gi|320447089|ref|NW_003383510.1|	2200147-2201095	348155	0.490245	-615008	0.1109	no
gi|320447089|ref|NW_003383510.1|	2202307-2202598	115155	394874	-486604	0.1421	no
gi|320447089|ref|NW_003383510.1|	2204721-2209107	0.243903	227715	322285	0.1934	no
gi|320447089|ref|NW_003383510.1|	222704-223167	108585	0.482246	-449291	0.2954	no
gi|320447089|ref|NW_003383510.1|	223679-224270	345016	60834	-250372	0.275	no

gi|320447089|ref|NW_003383510.1|	224429-224786	867721	323079	-142535	0.51425	no
gi|320447089|ref|NW_003383510.1|	2250948-2252107	584699	278483	225182	0.19685	no
gi|320447089|ref|NW_003383510.1|	2252368-2254604	244592	400272	0.710604	0.73455	no
gi|320447089|ref|NW_003383510.1|	2265613-2266102	248633	363284	0.54708	0.8044	no
gi|320447089|ref|NW_003383510.1|	228687-229303	163603	480423	-176783	0.40595	no
gi|320447089|ref|NW_003383510.1|	229618-230030	31854	480436	-272906	0.2388	no
gi|320447089|ref|NW_003383510.1|	230503-232453	188952	542025	-180159	0.2972	no
gi|320447089|ref|NW_003383510.1|	233128-234508	148906	604204	-130129	0.5622	no
gi|320447089|ref|NW_003383510.1|	2334768-2335194	0	129477	inf	0.01485	no
gi|320447089|ref|NW_003383510.1|	234686-235879	211475	158791	-0.413361	0.84545	no
gi|320447089|ref|NW_003383510.1|	236180-236779	258969	815177	-166759	0.44125	no
gi|320447089|ref|NW_003383510.1|	236906-237944	567739	188984	-158696	0.4493	no
gi|320447089|ref|NW_003383510.1|	238056-243713	14751	389251	139989	0.29095	no
gi|320447089|ref|NW_003383510.1|	2401373-2401928	0	237093	inf	0.00565	no
gi|320447089|ref|NW_003383510.1|	2404333-2405215	182717	269666	0.561566	0.78515	no
gi|320447089|ref|NW_003383510.1|	2405624-2405826	209932	436421	10558	0.5867	no
gi|320447089|ref|NW_003383510.1|	2413764-2414049	0	180818	inf	0.02915	no
gi|320447089|ref|NW_003383510.1|	2430816-2431888	0	149321	inf	0.0053	no
gi|320447089|ref|NW_003383510.1|	245479-246372	179659	53048	156204	0.4699	no
gi|320447089|ref|NW_003383510.1|	246495-248177	0.349943	0.974547	147761	1	no
gi|320447089|ref|NW_003383510.1|	248281-249210	802989	855841	0.0919624	0.96375	no
gi|320447089|ref|NW_003383510.1|	249449-250636	124139	270395	112312	0.5841	no
gi|320447089|ref|NW_003383510.1|	252821-254536	330732	54003	0.707377	0.7361	no
gi|320447089|ref|NW_003383510.1|	259352-259766	144187	0.594886	-459918	0.29375	no
gi|320447089|ref|NW_003383510.1|	261723-262836	480746	0.133124	-517443	0.279	no
gi|320447089|ref|NW_003383510.1|	262911-264544	0.965905	0.252103	-193787	1	no
gi|320447089|ref|NW_003383510.1|	264900-275117	451307	442269	-0.0291877	0.98145	no
gi|320447089|ref|NW_003383510.1|	316847-319360	249661	144365	-0.790245	0.54615	no
gi|320447089|ref|NW_003383510.1|	322332-323106	18176	171999	-340156	0.1761	no
gi|320447089|ref|NW_003383510.1|	323639-324435	273987	144727	-42427	0.14075	no

gi|320447089|ref|NW_003383510.1|	324890-325623	508701	0.232216	-445328	0.2969	no
gi|320447089|ref|NW_003383510.1|	328497-329298	101463	0	#NAME?	0.00615	no
gi|320447089|ref|NW_003383510.1|	331341-331843	349481	0	#NAME?	0.00605	no
gi|320447089|ref|NW_003383510.1|	405516-408824	274568	649132	124135	0.5826	no
gi|320447089|ref|NW_003383510.1|	411905-412523	26394	179266	-0.558111	0.79695	no
gi|320447089|ref|NW_003383510.1|	446683-447842	109631	265824	127782	0.5446	no
gi|320447089|ref|NW_003383510.1|	5247-5473	450292	594538	0.400908	0.84515	no
gi|320447089|ref|NW_003383510.1|	614850-626026	71277	515522	-0.467403	0.83335	no
gi|320447089|ref|NW_003383510.1|	626170-628371	118465	12.58	0.0866798	0.9437	no
gi|320447089|ref|NW_003383510.1|	628484-630157	275838	458428	0.732873	0.575	no
gi|320447089|ref|NW_003383510.1|	655472-656532	613986	402009	271095	0.1399	no
gi|320447089|ref|NW_003383510.1|	657964-658713	0	54045	inf	0.0138	no
gi|320447089|ref|NW_003383510.1|	663907-664375	212835	756823	183022	0.39645	no
gi|320447089|ref|NW_003383510.1|	670437-671570	225615	703075	163981	0.4397	no
gi|320447089|ref|NW_003383510.1|	685684-686249	966985	223493	120867	0.56095	no
gi|320447089|ref|NW_003383510.1|	691317-692631	0.93873	347525	188834	0.3877	no
gi|320447089|ref|NW_003383510.1|	913015-913327	325553	0	#NAME?	0.01335	no
gi|320447089|ref|NW_003383510.1|	915837-916406	905409	0.339976	-473506	0.2883	no
gi|320447089|ref|NW_003383510.1|	917904-918351	185782	205701	-317499	0.2193	no
gi|320447089|ref|NW_003383510.1|	918652-918898	370974	213227	-412085	0.30755	no
gi|320447089|ref|NW_003383510.1|	921144-921403	51665	364222	-38263	0.2211	no
gi|320447089|ref|NW_003383510.1|	921820-922672	123178	0.941749	-370926	0.179	no
gi|320447089|ref|NW_003383510.1|	922962-923217	632224	0	#NAME?	0.01425	no
gi|320447089|ref|NW_003383510.1|	925142-925740	250395	785865	-167185	0.4313	no
gi|320447089|ref|NW_003383510.1|	927709-929875	110999	237838	-222249	0.1889	no
gi|320447089|ref|NW_003383510.1|	936770-937321	313184	182603	-0.778299	0.7078	no
gi|320447089|ref|NW_003383510.1|	947539-948889	499622	483612	-0.0469859	0.97985	no
gi|320447089|ref|NW_003383510.1|	949482-949701	606887	666319	0.134785	0.9427	no
gi|320447089|ref|NW_003383510.1|	952516-954575	296094	374319	0.338213	0.868	no
gi|320447089|ref|NW_003383510.1|	958056-959092	0.421598	131157	163736	1	no

gi|320447089|ref|NW_003383510.1|	961389-964816	26803	445283	0.732328	0.5842	no
gi|320447089|ref|NW_003383510.1|	966639-967466	145503	294216	-230611	0.3071	no
gi|320447089|ref|NW_003383510.1|	967785-969448	183211	40319	-218397	0.21145	no
gi|320447089|ref|NW_003383510.1|	970516-970913	786238	129286	-260439	0.2918	no
gi|320447089|ref|NW_003383510.1|	971707-973440	416896	871013	-225892	0.2173	no
gi|320447089|ref|NW_003383510.1|	974387-974778	192834	0	#NAME?	0.01335	no
gi|320447089|ref|NW_003383510.1|	975533-976389	195814	0.56148	-512411	0.13835	no
gi|320447089|ref|NW_003383510.1|	977500-983046	147734	238421	0.690508	0.60475	no
gi|320447089|ref|NW_003383510.1|	983347-983716	207155	210587	0.0237089	0.97795	no
gi|320447089|ref|NW_003383510.1|	984769-985014	444238	237597	-0.902816	0.6667	no
gi|320447089|ref|NW_003383510.1|	986123-986544	174219	978944	-0.8316	0.68695	no
gi|320447089|ref|NW_003383510.1|	988542-989108	811909	617153	-0.39569	0.83965	no
gi|320447089|ref|NW_003383510.1|	994194-995291	276177	184363	-0.583047	0.7114	no
gi|320447104|ref|NW_003383505.1|	1000747-100202[illegible]	0.162482	101461	264257	1	no
gi|320447104|ref|NW_003383505.1|	1004287-100603[illegible]	0.223558	163487	287046	0.22425	no
gi|320447104|ref|NW_003383505.1|	1026477-102709[illegible]	306682	784595	-196672	0.381	no
gi|320447104|ref|NW_003383505.1|	1029059-103056[illegible]	677323	249349	-144168	0.5011	no
gi|320447104|ref|NW_003383505.1|	1030671-103369[illegible]	846182	34399	-12986	0.32845	no
gi|320447104|ref|NW_003383505.1|	1033842-103523[illegible]	101779	0.606065	-0.747896	1	no
gi|320447104|ref|NW_003383505.1|	1300903-130124[illegible]	703848	118657	0.753456	0.7121	no
gi|320447104|ref|NW_003383505.1|	1337181-134181[illegible]	622214	48872	-0.348402	0.7847	no
gi|320447104|ref|NW_003383505.1|	1342981-134450[illegible]	186242	948627	-0.973269	0.54605	no
gi|320447104|ref|NW_003383505.1|	1344773-134525[illegible]	203071	452187	115493	0.5747	no
gi|320447104|ref|NW_003383505.1|	1346006-134749[illegible]	254413	105758	-12664	0.4551	no
gi|320447104|ref|NW_003383505.1|	1374175-137447[illegible]	206755	735944	-149025	0.4975	no
gi|320447104|ref|NW_003383505.1|	1384905-138722[illegible]	364069	100532	146538	0.3714	no
gi|320447104|ref|NW_003383505.1|	1387359-138928[illegible]	0.30014	265017	314238	0.2138	no
gi|320447104|ref|NW_003383505.1|	1394014-139426[illegible]	46003	540452	0.232438	0.9074	no
gi|320447104|ref|NW_003383505.1|	1454024-145457[illegible]	699554	259081	-143303	0.3813	no
gi|320447104|ref|NW_003383505.1|	1456458-145717[illegible]	229334	737102	-163752	0.4552	no

gi|320447104|ref|NW_003383505.1|	1457478-1458221	664953	0.911096	-286758	0.2565	no
gi|320447104|ref|NW_003383505.1|	1458343-1458611	777636	131892	-255974	0.2668	no
gi|320447104|ref|NW_003383505.1|	1462297-1463743	119877	184075	-270319	0.2592	no
gi|320447104|ref|NW_003383505.1|	1464878-1465635	427121	687662	-263487	0.14775	no
gi|320447104|ref|NW_003383505.1|	1467037-1467716	646228	163449	133872	0.52845	no
gi|320447104|ref|NW_003383505.1|	1468310-1469296	78696	885085	0.169526	0.93695	no
gi|320447104|ref|NW_003383505.1|	1469500-1469826	921533	694869	-0.407295	0.84675	no
gi|320447104|ref|NW_003383505.1|	1470031-1471771	13458	421781	164803	0.43805	no
gi|320447104|ref|NW_003383505.1|	1472429-1473439	653631	767773	0.232204	0.90615	no
gi|320447104|ref|NW_003383505.1|	1476714-1477192	109141	118439	0.117953	0.94905	no
gi|320447104|ref|NW_003383505.1|	1477693-1479447	600028	58819	-0.0287473	0.98725	no
gi|320447104|ref|NW_003383505.1|	1479636-1481764	615173	821045	0.416468	0.8485	no
gi|320447104|ref|NW_003383505.1|	1482631-1482853	144198	271657	0.913737	0.67155	no
gi|320447104|ref|NW_003383505.1|	1483924-1484418	835054	21071	133532	0.5176	no
gi|320447104|ref|NW_003383505.1|	1497602-1498681	0	221458	inf	0.0198	no
gi|320447104|ref|NW_003383505.1|	1509329-1510005	0	313365	inf	0.029	no
gi|320447104|ref|NW_003383505.1|	1520714-1522664	0	439108	inf	0.00715	no
gi|320447104|ref|NW_003383505.1|	1523340-1524143	0.29737	510645	410199	0.19245	no
gi|320447104|ref|NW_003383505.1|	1779154-1779966	204908	683868	173874	0.41305	no
gi|320447104|ref|NW_003383505.1|	189001-189605	365037	866628	124737	0.54905	no
gi|320447104|ref|NW_003383505.1|	1984696-1985266	150463	915365	260494	0.26785	no
gi|320447104|ref|NW_003383505.1|	1986382-1991943	342157	270161	298109	0.1347	no
gi|320447104|ref|NW_003383505.1|	2010002-2010483	134864	360425	141819	0.3757	no
gi|320447104|ref|NW_003383505.1|	2012702-2013655	0.235189	162296	278673	0.2657	no
gi|320447104|ref|NW_003383505.1|	2016089-2016682	376032	0.955472	-197657	0.30275	no
gi|320447104|ref|NW_003383505.1|	2035758-2036183	683783	226192	-159599	0.44495	no
gi|320447104|ref|NW_003383505.1|	2105145-2106103	205067	245012	0.25676	0.8466	no
gi|320447104|ref|NW_003383505.1|	2106336-2108216	56431	522015	-0.112398	0.95795	no
gi|320447104|ref|NW_003383505.1|	2108329-2110255	24653	22404	-0.13801	0.9182	no
gi|320447104|ref|NW_003383505.1|	2110575-2110976	230976	221748	-0.058817	0.9729	no

gi|320447104|ref|NW_003383505.1|	2114465-2115660	105517	134085	0.34567	1	no
gi|320447104|ref|NW_003383505.1|	2115791-2117213	573115	477304	-0.263916	0.84225	no
gi|320447104|ref|NW_003383505.1|	2127201-2127551	181607	421861	-210598	0.3523	no
gi|320447104|ref|NW_003383505.1|	2147119-2149038	148333	139031	-0.0934332	0.94025	no
gi|320447104|ref|NW_003383505.1|	2149166-2155753	382514	631222	0.722637	0.56725	no
gi|320447104|ref|NW_003383505.1|	2155972-2156312	832889	810487	-0.0393351	0.94	no
gi|320447104|ref|NW_003383505.1|	2156542-2157123	826166	164445	-232883	0.30065	no
gi|320447104|ref|NW_003383505.1|	2157312-2157644	146934	570719	-136432	0.522	no
gi|320447104|ref|NW_003383505.1|	2171017-2172774	897158	532198	-0.753399	0.58395	no
gi|320447104|ref|NW_003383505.1|	2173626-2174687	901465	458082	-0.976665	0.4618	no
gi|320447104|ref|NW_003383505.1|	2180534-2183588	202934	19505	-0.057168	0.9651	no
gi|320447104|ref|NW_003383505.1|	2183787-2184042	481694	133565	-185057	0.3882	no
gi|320447104|ref|NW_003383505.1|	2186222-2186904	268098	108263	-130823	0.55405	no
gi|320447104|ref|NW_003383505.1|	2187113-2187460	423472	60205	-281431	0.22735	no
gi|320447104|ref|NW_003383505.1|	2216846-2217736	162435	461817	-181447	0.40305	no
gi|320447104|ref|NW_003383505.1|	2218197-2219137	138927	413095	-174979	0.4189	no
gi|320447104|ref|NW_003383505.1|	2220039-2220752	459244	169138	-144106	0.49235	no
gi|320447104|ref|NW_003383505.1|	2223262-2224645	186748	776284	-126644	0.4274	no
gi|320447104|ref|NW_003383505.1|	2227121-2231817	132426	464399	-151175	0.38855	no
gi|320447104|ref|NW_003383505.1|	2233389-2234655	37661	147679	-135061	0.427	no
gi|320447104|ref|NW_003383505.1|	2234801-2239214	118336	88802	-0.414225	0.7496	no
gi|320447104|ref|NW_003383505.1|	2241427-2244439	182304	896464	22979	0.1863	no
gi|320447104|ref|NW_003383505.1|	2244870-2245563	22116	251807	0.187226	0.9195	no
gi|320447104|ref|NW_003383505.1|	2246525-2247032	183944	452366	129822	0.549	no
gi|320447104|ref|NW_003383505.1|	2251960-2253675	364946	436789	0.259254	0.9003	no
gi|320447104|ref|NW_003383505.1|	2254800-2255613	61366	441753	-0.4742	0.8187	no
gi|320447104|ref|NW_003383505.1|	2261366-2261870	247863	103858	-125493	0.5496	no
gi|320447104|ref|NW_003383505.1|	2263646-2268933	103539	800281	-0.371598	0.7772	no
gi|320447104|ref|NW_003383505.1|	2269586-2270449	145222	499806	-153882	0.47195	no
gi|320447104|ref|NW_003383505.1|	236310-236942	0	124162	inf	0.00915	no

gi|320447104|ref|NW_003383505.1|	43925-47183	0.446576	335769	291049	0.21945	no
gi|320447104|ref|NW_003383505.1|	524935-527201	616571	523153	-0.237034	0.85845	no
gi|320447104|ref|NW_003383505.1|	528921-530755	103444	814204	-0.345382	0.79145	no
gi|320447104|ref|NW_003383505.1|	532942-535574	537185	485485	-0.145994	0.9127	no
gi|320447104|ref|NW_003383505.1|	540518-543263	102868	620191	-0.730009	0.64955	no
gi|320447104|ref|NW_003383505.1|	543385-545198	350829	25.71	-0.448435	0.72805	no
gi|320447104|ref|NW_003383505.1|	546501-547288	250918	525124	-225649	0.30825	no
gi|320447104|ref|NW_003383505.1|	548253-548931	304788	0.780015	-196623	0.30285	no
gi|320447104|ref|NW_003383505.1|	549982-551951	123614	251097	-229952	0.1776	no
gi|320447104|ref|NW_003383505.1|	555958-557110	412416	931017	-214722	0.2171	no
gi|320447104|ref|NW_003383505.1|	557529-558879	369417	872605	-208185	0.24825	no
gi|320447104|ref|NW_003383505.1|	559576-563519	445977	199087	-116357	0.38635	no
gi|320447104|ref|NW_003383505.1|	566737-573173	282716	241269	-0.228708	0.8679	no
gi|320447104|ref|NW_003383505.1|	584896-586119	0	189573	inf	0.0198	no
gi|320447104|ref|NW_003383505.1|	586951-589261	11468	282179	1299	0.3221	no
gi|320447104|ref|NW_003383505.1|	612372-613178	621192	995715	0.680694	0.7448	no
gi|320447104|ref|NW_003383505.1|	616940-620984	624644	595429	-0.0691047	0.95285	no
gi|320447104|ref|NW_003383505.1|	621465-622982	237003	183133	-0.372014	0.85585	no
gi|320447104|ref|NW_003383505.1|	624066-624720	644145	329117	-0.968785	0.65125	no
gi|320447104|ref|NW_003383505.1|	625865-626486	266286	160148	-0.733572	0.7291	no
gi|320447104|ref|NW_003383505.1|	626649-627386	477745	347933	-0.457429	0.77285	no
gi|320447104|ref|NW_003383505.1|	627497-628218	128566	199731	0.635545	0.7657	no
gi|320447104|ref|NW_003383505.1|	630402-632108	81468	114254	0.487943	0.8343	no
gi|320447104|ref|NW_003383505.1|	639919-644199	188139	120045	-0.648223	0.62	no
gi|320447104|ref|NW_003383505.1|	646438-650486	712497	111216	0.642412	0.61635	no
gi|320447104|ref|NW_003383505.1|	657495-658119	212278	129534	-0.712616	0.73235	no
gi|320447104|ref|NW_003383505.1|	697123-699171	567855	29217	-0.958713	0.6442	no
gi|320447104|ref|NW_003383505.1|	709299-709659	12163	237932	-235387	0.25605	no
gi|320447104|ref|NW_003383505.1|	749372-750970	0.49536	258519	238372	0.29705	no
gi|320447104|ref|NW_003383505.1|	826354-826673	518543	418129	-0.310516	0.87665	no

gi|320447105|ref|NW_003383504.1|	1007420-1011623	484791	391218	301254	0.1331	no
gi|320447105|ref|NW_003383504.1|	101140-110406	117142	19787	0.756293	0.56835	no
gi|320447105|ref|NW_003383504.1|	1021095-1021573	286495	350762	0.291982	0.8913	no
gi|320447105|ref|NW_003383504.1|	1023181-1024217	990755	115127	0.216624	0.9156	no
gi|320447105|ref|NW_003383504.1|	1027568-1027863	991168	914611	320596	0.1989	no
gi|320447105|ref|NW_003383504.1|	1041005-1042514	172244	0.276408	-263958	0.23025	no
gi|320447105|ref|NW_003383504.1|	1043962-1044671	356231	0.243598	-387024	0.3182	no
gi|320447105|ref|NW_003383504.1|	1053122-1053532	0	727567	inf	0.029	no
gi|320447105|ref|NW_003383504.1|	1057501-1060704	278598	20677	-0.430155	0.83915	no
gi|320447105|ref|NW_003383504.1|	110518-111307	670762	774439	0.207351	0.915	no
gi|320447105|ref|NW_003383504.1|	11236-11843	516059	476077	-0.116341	0.95865	no
gi|320447105|ref|NW_003383504.1|	1198220-1200150	0.796593	166595	106443	0.60035	no
gi|320447105|ref|NW_003383504.1|	1200218-1200781	0.511958	345798	275583	0.26575	no
gi|320447105|ref|NW_003383504.1|	120556-121428	230659	217231	-0.0865354	0.96895	no
gi|320447105|ref|NW_003383504.1|	1240018-1243536	0.565403	345238	261024	0.2487	no
gi|320447105|ref|NW_003383504.1|	1244255-1245558	0.158067	120678	293256	1	no
gi|320447105|ref|NW_003383504.1|	1246100-1248143	0.840137	442714	239768	0.2899	no
gi|320447105|ref|NW_003383504.1|	1248282-1250207	0.199738	132275	272736	1	no
gi|320447105|ref|NW_003383504.1|	1252001-1253748	0.669785	295446	214112	0.3332	no
gi|320447105|ref|NW_003383504.1|	1254093-1255229	0.374772	142748	192939	0.3182	no
gi|320447105|ref|NW_003383504.1|	1257531-1258085	0	390396	inf	0.0294	no
gi|320447105|ref|NW_003383504.1|	1291069-1293209	0.974531	216339	115051	0.57645	no
gi|320447105|ref|NW_003383504.1|	1294692-1295953	0.164542	239707	386474	0.19995	no
gi|320447105|ref|NW_003383504.1|	1322216-1324565	973954	131535	0.433518	0.7896	no
gi|320447105|ref|NW_003383504.1|	1325998-1326490	0	433058	inf	0.0312	no
gi|320447105|ref|NW_003383504.1|	13320-15177	296563	49528	0.739903	0.5777	no
gi|320447105|ref|NW_003383504.1|	1332607-1333322	619168	693078	0.162687	0.89745	no
gi|320447105|ref|NW_003383504.1|	1333925-1334171	809397	895554	0.145934	0.94275	no
gi|320447105|ref|NW_003383504.1|	1336014-1340443	177028	298074	0.751692	0.56845	no
gi|320447105|ref|NW_003383504.1|	1353864-1356801	164882	153248	-0.105562	0.9343	no

gi|320447105|ref|NW_003383504.1|	1361282-1361677	367519	13714	-142217	0.5025	no
gi|320447105|ref|NW_003383504.1|	1364265-1365568	0.316135	329123	338002	0.1918	no
gi|320447105|ref|NW_003383504.1|	1370020-1370522	474295	167309	-48252	0.1628	no
gi|320447105|ref|NW_003383504.1|	1372381-1373496	320408	265652	-35923	0.076	no
gi|320447105|ref|NW_003383504.1|	1374789-1376315	196125	37283	-239518	0.1697	no
gi|320447105|ref|NW_003383504.1|	1382182-1382500	0	179053	inf	0.02075	no
gi|320447105|ref|NW_003383504.1|	1384414-1384730	215696	192499	-0.164148	0.9313	no
gi|320447105|ref|NW_003383504.1|	1391385-1393241	624729	682062	0.12667	0.9523	no
gi|320447105|ref|NW_003383504.1|	1394285-1401543	176105	146544	-0.2651	0.8417	no
gi|320447105|ref|NW_003383504.1|	1404680-1409835	144386	778452	-0.89125	0.4997	no
gi|320447105|ref|NW_003383504.1|	1414217-1414679	0.726854	726201	332063	0.22995	no
gi|320447105|ref|NW_003383504.1|	1418987-1419515	0.570993	460866	30128	0.25005	no
gi|320447105|ref|NW_003383504.1|	1430225-1431127	470932	221594	-10876	0.50915	no
gi|320447105|ref|NW_003383504.1|	1431292-1433308	160151	157944	-0.0200237	0.9876	no
gi|320447105|ref|NW_003383504.1|	143340-143527	498026	61696	0.308957	0.8815	no
gi|320447105|ref|NW_003383504.1|	1433557-1435211	153446	306364	-232441	0.1741	no
gi|320447105|ref|NW_003383504.1|	1435412-1437040	775499	244566	-16649	0.43425	no
gi|320447105|ref|NW_003383504.1|	1440245-1442394	176354	203023	0.203171	0.92205	no
gi|320447105|ref|NW_003383504.1|	1444417-1444855	644233	480691	-0.422475	0.81865	no
gi|320447105|ref|NW_003383504.1|	1445214-1448588	817132	109056	0.416434	0.7403	no
gi|320447105|ref|NW_003383504.1|	1463004-1465508	0.669395	100779	391219	0.07165	no
gi|320447105|ref|NW_003383504.1|	1465609-1467207	0.12384	284371	452123	0.17645	no
gi|320447105|ref|NW_003383504.1|	1472659-1474882	0.169787	207361	361035	0.18175	no
gi|320447105|ref|NW_003383504.1|	1476155-1476787	0.424616	28875	276559	0.2657	no
gi|320447105|ref|NW_003383504.1|	1538652-1539985	0.153746	213486	379552	0.2072	no
gi|320447105|ref|NW_003383504.1|	158489-158862	640547	271986	-123577	0.55975	no
gi|320447105|ref|NW_003383504.1|	159011-159351	138815	189114	0.446092	0.82445	no
gi|320447105|ref|NW_003383504.1|	223906-225062	201645	101592	-0.989035	0.65595	no
gi|320447105|ref|NW_003383504.1|	228896-230383	0.26965	684299	466547	0.1507	no
gi|320447105|ref|NW_003383504.1|	30802-32344	114942	139238	0.276648	0.899	no

gi|320447105|ref|NW_003383504.1|	32463-33197	111691	12747	0.190655	0.9286	no
gi|320447105|ref|NW_003383504.1|	3540-3916	386466	354176	-0.125876	0.9495	no
gi|320447105|ref|NW_003383504.1|	38086-40412	161421	407371	133551	0.3134	no
gi|320447105|ref|NW_003383504.1|	561138-564757	134638	216335	0.684186	0.7472	no
gi|320447105|ref|NW_003383504.1|	567873-568611	36935	0.459949	-300544	0.25735	no
gi|320447105|ref|NW_003383504.1|	634163-634796	0	288059	inf	0.0312	no
gi|320447105|ref|NW_003383504.1|	643127-644702	0.3779	42943	350635	0.1915	no
gi|320447105|ref|NW_003383504.1|	646825-649938	0.11727	217309	421184	0.16265	no
gi|320447105|ref|NW_003383504.1|	755534-756021	131839	837587	266746	0.24485	no
gi|320447105|ref|NW_003383504.1|	768823-770120	155782	103701	-0.587093	0.7929	no
gi|320447105|ref|NW_003383504.1|	815651-816786	356429	181879	235129	0.30055	no
gi|320447105|ref|NW_003383504.1|	816890-819542	328162	697596	108798	0.6237	no
gi|320447105|ref|NW_003383504.1|	820685-826147	231135	123723	-0.901623	0.5002	no
gi|320447105|ref|NW_003383504.1|	826398-827239	863979	115021	-29091	0.2337	no
gi|320447105|ref|NW_003383504.1|	827733-829769	974599	823491	-0.243056	0.9088	no
gi|320447105|ref|NW_003383504.1|	834025-835813	728173	11584	0.669776	0.7676	no
gi|320447105|ref|NW_003383504.1|	835970-836581	851108	942555	0.147234	0.9423	no
gi|320447105|ref|NW_003383504.1|	836776-839204	113125	795327	-0.508293	0.75265	no
gi|320447105|ref|NW_003383504.1|	840294-840679	503521	309655	-0.701387	0.73145	no
gi|320447105|ref|NW_003383504.1|	840790-842670	666912	665033	-0.00407172	0.99315	no
gi|320447105|ref|NW_003383504.1|	842866-844142	10541	180032	0.772242	0.72725	no
gi|320447105|ref|NW_003383504.1|	844566-848262	31594	181173	-0.802284	0.54945	no
gi|320447105|ref|NW_003383504.1|	848502-850538	955857	307175	-163773	0.45305	no
gi|320447105|ref|NW_003383504.1|	852115-852348	323433	127354	-134462	0.5252	no
gi|320447105|ref|NW_003383504.1|	852492-852951	294344	342919	0.220363	0.89885	no
gi|320447105|ref|NW_003383504.1|	853077-854375	266803	991961	-142742	0.39315	no
gi|320447105|ref|NW_003383504.1|	86621-87378	42065	399287	-0.0751911	0.96155	no
gi|320447105|ref|NW_003383504.1|	879405-879852	0	719954	inf	0.0233	no
gi|320447105|ref|NW_003383504.1|	891941-892838	0	298822	inf	0.02075	no
gi|320447105|ref|NW_003383504.1|	892950-896314	0.97075	235784	460222	0.0431	no

gi|320447105|ref|NW_003383504.1|	902945-903835	0.257833	301957	354984	0.2164	no
gi|320447105|ref|NW_003383504.1|	910722-914589	0.882746	405502	552157	0.0306	no
gi|320447105|ref|NW_003383504.1|	91215-91840	0	469886	inf	0.0198	no
gi|320447105|ref|NW_003383504.1|	914773-916156	0.294092	592427	43323	0.15955	no
gi|320447105|ref|NW_003383504.1|	949428-951155	179873	339553	0.916655	0.4871	no
gi|320447105|ref|NW_003383504.1|	954222-954596	116186	752888	-0.625927	0.77615	no
gi|320447105|ref|NW_003383504.1|	957020-957873	847185	733399	-0.208079	0.91535	no
gi|320447105|ref|NW_003383504.1|	957990-960941	153469	257578	0.747061	0.56745	no
gi|320447105|ref|NW_003383504.1|	963689-964094	443098	223622	-0.986563	0.6358	no
gi|320447105|ref|NW_003383504.1|	965555-966732	591441	434682	-0.444273	0.82625	no
gi|320447105|ref|NW_003383504.1|	967612-969527	166769	194683	0.223271	0.86235	no
gi|320447105|ref|NW_003383504.1|	97010-98315	0	273762	inf	0.01485	no
gi|320447105|ref|NW_003383504.1|	970268-972157	346947	938819	143613	0.51375	no
gi|320447105|ref|NW_003383504.1|	972440-973865	907861	401196	214376	0.2267	no
gi|320447105|ref|NW_003383504.1|	977343-978032	252841	721198	151217	0.37565	no
gi|320447105|ref|NW_003383504.1|	978573-983334	104762	418057	199658	0.1391	no
gi|320447105|ref|NW_003383504.1|	9825-10554	191448	196594	0.0382718	0.98465	no
gi|320447105|ref|NW_003383504.1|	984264-984566	614907	0	#NAME?	0.0074	no
gi|320447105|ref|NW_003383504.1|	987872-988748	139639	0.362858	-526615	0.1857	no
gi|320447105|ref|NW_003383504.1|	992036-993187	104323	740509	-381639	0.063	no
gi|320447105|ref|NW_003383504.1|	994152-995402	47902	18459	-469769	0.04855	no
gi|320447145|ref|NW_003383500.1|	1030794-103128[illegible]	70401	0	#NAME?	0.02105	no
gi|320447145|ref|NW_003383500.1|	1119196-112028[illegible]	157427	626447	19925	0.36545	no
gi|320447145|ref|NW_003383500.1|	1122313-112266[illegible]	24951	731403	155157	0.3575	no
gi|320447145|ref|NW_003383500.1|	1131414-113553[illegible]	420011	267308	-0.65192	0.6298	no
gi|320447145|ref|NW_003383500.1|	1136431-113710[illegible]	240516	33868	0.493789	0.8199	no
gi|320447145|ref|NW_003383500.1|	1144192-114828[illegible]	101552	211437	105801	0.42245	no
gi|320447145|ref|NW_003383500.1|	1149029-115042[illegible]	120889	157846	0.384832	0.86605	no
gi|320447145|ref|NW_003383500.1|	1169766-117046[illegible]	329595	277752	-0.246899	0.9076	no
gi|320447145|ref|NW_003383500.1|	1187994-119201[illegible]	244503	466615	0.932382	0.4901	no

gi|320447145|ref|NW_003383500.1|	1193391-119399	206985	327505	0.661992	0.7655	no
gi|320447145|ref|NW_003383500.1|	1195382-119634	132033	655538	-101015	0.62465	no
gi|320447145|ref|NW_003383500.1|	1197506-119918	107539	276691	-195851	0.36755	no
gi|320447145|ref|NW_003383500.1|	1199918-120047	115016	529261	-111978	0.582	no
gi|320447145|ref|NW_003383500.1|	1200776-120179	752847	222983	-175542	0.4099	no
gi|320447145|ref|NW_003383500.1|	1201900-120266	353903	529418	0.581055	0.7803	no
gi|320447145|ref|NW_003383500.1|	1202832-120548	789311	105415	0.417408	0.7974	no
gi|320447145|ref|NW_003383500.1|	1208262-120881	36596	211705	253229	0.27045	no
gi|320447145|ref|NW_003383500.1|	1209658-121095	0.638853	166255	470177	0.14465	no
gi|320447145|ref|NW_003383500.1|	1212838-121355	0.3518	60163	409605	0.19245	no
gi|320447145|ref|NW_003383500.1|	1216163-121853	126238	176279	0.481714	0.81295	no
gi|320447145|ref|NW_003383500.1|	1233411-123448	0.202452	364122	416877	0.1921	no
gi|320447145|ref|NW_003383500.1|	1236750-123898	151588	305635	101165	0.6271	no
gi|320447145|ref|NW_003383500.1|	129861-130169	708328	216384	16111	0.45135	no
gi|320447145|ref|NW_003383500.1|	1353724-135443	0.705062	385821	245211	0.24845	no
gi|320447145|ref|NW_003383500.1|	1354522-135548	0.697786	12843	0.880123	1	no
gi|320447145|ref|NW_003383500.1|	167092-167962	34575	787378	118733	0.5656	no
gi|320447145|ref|NW_003383500.1|	252266-252937	193524	44882	121362	0.57245	no
gi|320447145|ref|NW_003383500.1|	253575-254816	43632	581941	0.415487	0.8343	no
gi|320447145|ref|NW_003383500.1|	29587-30461	0	236582	inf	0.02915	no
gi|320447145|ref|NW_003383500.1|	347644-348463	0	536695	inf	0.0133	no
gi|320447145|ref|NW_003383500.1|	534120-536874	0.334968	234085	280494	0.2417	no
gi|320447145|ref|NW_003383500.1|	538573-539427	0.81853	244079	157624	0.491	no
gi|320447145|ref|NW_003383500.1|	539892-540548	12021	546036	218344	0.33465	no
gi|320447145|ref|NW_003383500.1|	546245-547968	0.794025	339669	209687	0.33645	no
gi|320447145|ref|NW_003383500.1|	550297-551245	0.94736	21244	116507	0.5864	no
gi|320447145|ref|NW_003383500.1|	595978-596336	615873	722501	0.230368	0.896	no
gi|320447145|ref|NW_003383500.1|	627979-628172	317124	266245	-357422	0.1883	no
gi|320447145|ref|NW_003383500.1|	629312-630096	60611	274523	-446458	0.06535	no
gi|320447145|ref|NW_003383500.1|	642623-643380	296.72	0.221826	-103855	0.2504	no

gi|320447145|ref|NW_003383500.1|	695916-696660	0	318274	inf	0.0233	no
gi|320447145|ref|NW_003383500.1|	696818-698071	0.497508	138039	147228	1	no
gi|320447145|ref|NW_003383500.1|	705210-706734	0.261906	446262	409077	0.1683	no
gi|320447145|ref|NW_003383500.1|	708013-712296	112549	356074	166162	0.45455	no
gi|320447145|ref|NW_003383500.1|	713569-714845	113519	236293	105764	0.60415	no
gi|320447145|ref|NW_003383500.1|	945469-946452	225748	0.467673	-227114	0.26625	no
