# Supplementary material for: The X Chromosome of Hemipteran Insects: Conservation, Dosage Compensation and Sex-Biased Expression
Source: Genome Biol Evol. 2015 Nov 10;7(12):3259–68. doi: 10.1093/gbe/evv215 (PMC4700948; doi:10.1093/gbe/evv215)
Supplement: Supplementary Data [file supp_evv215_suppl_data.zip › S2 Data (rev) AP-HH (A).pdf]

AP	HH	gene	covF	covM
ACYPI000427-RA	ni|645902308|nb|KK922121.1|	113622-115049	nan	nan
ACYPI009967-RA	ni|645904065|nb|KK920366.1|	575237-575928	18	18
ACYPI065154-RA	ni|645904065|nb|KK920366.1|	63698-64092	18	18
ACYPI006033-RA	ni|645903557|nb|KK920873.1|	1172871-1173128	nan	nan
ACYPI53104-RA	ni|645903709|nb|KK920721.1|	90870-92494	nan	nan
ACYPI003938-RA	ni|645903857|nb|KK920573.1|	639110-639837	16	16
ACYPI006974-RA	ni|645904045|nb|KK920386.1|	69653-71370	19	21
ACYPI007586-RA	ni|645903970|nb|KK920461.1|	2342158-2342363	17	16
ACYPI003220-RA	ni|645904015|nb|KK920416.1|	916644-917264	18	16
ACYPI007117-RA	ni|645904015|nb|KK920416.1|	444779-447912	18	16
ACYPI000716-RA	ni|645904168|nb|KK920265.1|	509938-510710	16	16
ACYPI004513-RA	ni|645904168|nb|KK920265.1|	529291-529720	16	16
ACYPI010139-RA	ni|645903617|nb|KK920813.1|	388345-388778	nan	nan
ACYPI29050-RA	ni|645904009|nb|KK920422.1|	1363128-1363328	17	9.4
ACYPI006979-RA	ni|645904033|nb|KK920398.1|	96265-96779	14	16
ACYPI007210-RA	ni|645904262|nb|KK920227.1|	492439-492736	17	17
ACYPI005053-RA	ni|645900486|nb|KK923943.1|	49912-50218	nan	nan
ACYPI003213-RA	ni|645903644|nb|KK920786.1|	1109097-1109498	nan	nan
ACYPI003304-RA	ni|645903965|nb|KK920466.1|	1445001-1445241	17	16
ACYPI000201-RA	ni|645903731|nb|KK920699.1|	884810-885019	nan	nan
ACYPI004675-RA	ni|645904262|nb|KK920227.1|	732757-732976	17	17
ACYPI066907-RA	ni|645903811|nb|KK920619.1|	338524-339733	15	15
ACYPI072923-RA	ni|645903876|nb|KK920555.1|	174309-174787	14	15
ACYPI006131-RA	ni|645904100|nb|KK920331.1|	181368-184208	18	22
ACYPI003244-RA	ni|645903827|nb|KK920603.1|	1139888-1140413	17	18
ACYPI001807-RA	ni|645904053|nb|KK920378.1|	1862598-1863287	18	17
ACYPI004345-RA	ni|645903860|nb|KK920570.1|	263037-263422	17	17
ACYPI010028-RA	ni|645903548|nb|KK920882.1|	745298-745714	nan	nan
ACYPI006611-RA	ni|645903549|nb|KK920881.1|	5588-6823	nan	nan

ACYPI006649-RA	ni|645904039|nb|KK920392.1|	417356-417592	15	16
ACYPI009254-RA	ni|645903816|nb|KK920614.1|	202350-203080	14	15
ACYPI003976-RA	ni|645902649|nb|KK921780.1|	164145-166426	nan	nan
ACYPI006101-RA	ni|645903832|nb|KK920598.1|	923349-923848	18	8.8
ACYPI002250-RA	ni|645904065|nb|KK920366.1|	1184436-1184704	18	18
ACYPI065159-RA	ni|645904065|nb|KK920366.1|	1181080-1182159	18	18
ACYPI001710-RA	ni|645903763|nb|KK920667.1|	69903-70148	26	21
ACYPI085022-RA	ni|645903937|nb|KK920494.1|	288673-290802	11	12
ACYPI000030-RA	ni|645903923|nb|KK920508.1|	126453-126713	18	18
ACYPI001328-RA	ni|645898594|nb|KK925835.1|	2869-3716	nan	nan
ACYPI004428-RA	ni|645903663|nb|KK920767.1|	32890-33099	nan	nan
ACYPI000265-RA	ni|645904106|nb|KK920325.1|	994361-995652	17	17
ACYPI001324-RA	ni|645904107|nb|KK920324.1|	763473-764537	17	9.5
ACYPI002167-RA	ni|645903551|nb|KK920879.1|	586203-586761	nan	nan
ACYPI002547-RA	ni|645903714|nb|KK920716.1|	271011-271906	nan	nan
ACYPI002351-RA	ni|645903738|nb|KK920692.1|	1023137-1023627	18	17
ACYPI008351-RA	ni|645903540|nb|KK920890.1|	182497-182752	nan	nan
ACYPI000086-RA	ni|645902589|nb|KK921840.1|	118937-119181	nan	nan
ACYPI000446-RA	ni|645902779|nb|KK921650.1|	74199-74708	nan	nan
ACYPI001096-RA	ni|645903597|nb|KK920833.1|	929086-929543	nan	nan
ACYPI001146-RA	ni|645903800|nb|KK920630.1|	1043669-1044647	18	17
ACYPI002411-RA	ni|645904052|nb|KK920379.1|	512757-513063	18	16
ACYPI003035-RA	ni|645903679|nb|KK920751.1|	386971-387601	nan	nan
ACYPI003654-RA	ni|645903768|nb|KK920662.1|	1401054-1401653	18	17
ACYPI004313-RA	ni|645903857|nb|KK920573.1|	616828-617466	16	16
ACYPI007471-RA	ni|645903734|nb|KK920696.1|	425967-426404	nan	nan
ACYPI066985-RA	ni|645903800|nb|KK920630.1|	991301-991451	18	17
ACYPI007426-RA	ni|645903805|nb|KK920625.1|	256521-256690	12	13
ACYPI005793-RA	ni|645904015|nb|KK920416.1|	1315364-1316340	18	16
ACYPI006102-RA	ni|645903765|nb|KK920665.1|	592158-594403	17	15

ACYPI008172-RA	ni|645904156|nb|KK920277.1|	1047652-1048070	17	8.8
ACYPI002300-RA	ni|645902463|nb|KK921966.1|	262165-262901	nan	nan
ACYPI007522-RA	ni|645904260|nb|KK920228.1|	1347963-1348396	15	15
ACYPI009872-RA	ni|645903860|nb|KK920570.1|	181468-181760	17	17
ACYPI010167-RA	ni|645903477|nb|KK920953.1|	55620-56290	nan	nan
ACYPI007136-RA	ni|645903557|nb|KK920873.1|	724006-725057	nan	nan
ACYPI004157-RA	ni|645902172|nb|KK922257.1|	212265-212450	nan	nan
ACYPI009841-RA	ni|645903651|nb|KK920779.1|	588907-589975	nan	nan
ACYPI36355-RA	ni|645901996|nb|KK922433.1|	36734-38492	nan	nan
ACYPI36357-RA	ni|645901996|nb|KK922433.1|	34327-34840	nan	nan
ACYPI002040-RA	ni|645904245|nb|KK920234.1|	507990-508183	16	17
ACYPI002689-RA	ni|645904146|nb|KK920287.1|	967584-968143	16	18
ACYPI005864-RA	ni|645903588|nb|KK920842.1|	613875-614319	nan	nan
ACYPI080213-RA	ni|645904057|nb|KK920374.1|	1341602-1341761	16	15
ACYPI085715-RA	ni|645904057|nb|KK920374.1|	1352763-1353041	16	15
ACYPI009487-RA	ni|645899152|nb|KK925277.1|	43101-43681	nan	nan
ACYPI002739-RA	ni|645903831|nb|KK920599.1|	1307472-1307731	18	17
ACYPI002789-RA	ni|645903495|nb|KK920935.1|	471438-471750	nan	nan
ACYPI004656-RA	ni|645902669|nb|KK921760.1|	47359-47563	nan	nan
ACYPI072949-RA	ni|645903831|nb|KK920599.1|	1303806-1304472	18	17
ACYPI005456-RA	ni|645903981|nb|KK920450.1|	455935-456537	16	8.6
ACYPI001978-RA	ni|645902219|nb|KK922210.1|	189865-191931	nan	nan
ACYPI003867-RA	ni|645904240|nb|KK920239.1|	955121-955382	16	16
ACYPI005210-RA	ni|645904156|nb|KK920277.1|	2084859-2085654	17	8.8
ACYPI007707-RA	ni|645903598|nb|KK920832.1|	282275-283028	nan	nan
ACYPI008974-RA	ni|645903910|nb|KK920521.1|	323363-323549	19	17
ACYPI083423-RA	ni|645903594|nb|KK920836.1|	883003-883909	nan	nan
ACYPI002301-RA	ni|645903796|nb|KK920634.1|	942702-943875	18	17
ACYPI004180-RA	ni|645902060|nb|KK922369.1|	40231-40524	nan	nan
ACYPI005682-RA	ni|645902600|nb|KK921829.1|	137335-137614	nan	nan

ACYPI27242-RA	ni|645904153|nb|KK920280.1|	660052-660584	17	16
ACYPI001059-RA	ni|645904015|nb|KK920416.1|	187496-193131	18	16
ACYPI008110-RA	ni|645902638|nb|KK921791.1|	166061-166293	nan	nan
ACYPI009986-RA	ni|645903923|nb|KK920508.1|	777772-778297	18	18
ACYPI002612-RA	ni|645904281|nb|KK920220.1|	827541-827926	15	16
ACYPI004520-RA	ni|645904281|nb|KK920220.1|	831339-831560	15	16
ACYPI008955-RA	ni|645903728|nb|KK920702.1|	46399-46982	nan	nan
ACYPI000737-RA	ni|645903537|nb|KK920893.1|	508337-508812	nan	nan
ACYPI29847-RA	ni|645903739|nb|KK920691.1|	819840-820525	17	16
ACYPI007714-RA	ni|645903957|nb|KK920474.1|	928403-928902	18	17
ACYPI009613-RA	ni|645904122|nb|KK920309.1|	1169824-1170769	18	17
ACYPI004240-RA	ni|645904155|nb|KK920278.1|	691774-691900	18	9.4
ACYPI005422-RA	ni|645904164|nb|KK920269.1|	347242-347695	19	20
ACYPI009796-RA	ni|645904242|nb|KK920237.1|	716575-717075	15	16
ACYPI35323-RA	ni|645903831|nb|KK920599.1|	724480-725693	18	17
ACYPI27507-RA	ni|645904116|nb|KK920315.1|	1847470-1849096	19	18
ACYPI000504-RA	ni|645903702|nb|KK920728.1|	728664-728902	nan	nan
ACYPI008295-RA	ni|645903999|nb|KK920432.1|	233461-234006	17	15
ACYPI004947-RA	ni|645903836|nb|KK920594.1|	686180-687064	18	11
ACYPI009357-RA	ni|645903855|nb|KK920575.1|	75829-76211	16	17
ACYPI000271-RA	ni|645903949|nb|KK920482.1|	2716-3421	17	14
ACYPI002742-RA	ni|645903797|nb|KK920633.1|	412881-413784	17	8.9
ACYPI004045-RA	ni|645903837|nb|KK920593.1|	312634-312806	18	9.8
ACYPI088569-RA	ni|645903949|nb|KK920482.1|	315232-315404	17	14
ACYPI009848-RA	ni|645903749|nb|KK920681.1|	277623-278061	19	18
ACYPI000181-RA	ni|645903788|nb|KK920642.1|	321728-321979	16	15
ACYPI008558-RA	ni|645903984|nb|KK920447.1|	402221-403164	18	17
ACYPI003234-RA	ni|645900872|nb|KK923557.1|	33586-35038	nan	nan
ACYPI006272-RA	ni|645903566|nb|KK920864.1|	172135-173272	nan	nan
ACYPI006910-RA	ni|645904159|nb|KK920274.1|	1079126-1079292	15	17

ACYPI088980-RA	ni|645904132|nb|KK920301.1|	614949-616150	16	18
ACYPI001379-RA	ni|645903922|nb|KK920509.1|	463448-463682	15	16
ACYPI005627-RA	ni|645903709|nb|KK920721.1|	260922-262443	nan	nan
ACYPI007507-RA	ni|645904153|nb|KK920280.1|	3183413-3183965	17	16
ACYPI003483-RA	ni|645904021|nb|KK920410.1|	777693-778167	15	16
ACYPI007926-RA	ni|645903955|nb|KK920476.1|	559090-559345	18	17
ACYPI001508-RA	ni|645903890|nb|KK920541.1|	58112-58820	17	18
ACYPI009721-RA	ni|645904120|nb|KK920311.1|	999835-1000370	18	18
ACYPI53120-RA	ni|645902600|nb|KK921829.1|	154361-154889	nan	nan
ACYPI005509-RA	ni|645903970|nb|KK920461.1|	840452-841449	17	16
ACYPI000698-RA	ni|645904231|nb|KK920242.1|	630687-631223	16	17
ACYPI001356-RA	ni|645903634|nb|KK920796.1|	1113157-1113379	nan	nan
ACYPI002591-RA	ni|645904168|nb|KK920265.1|	1339397-1339804	16	16
ACYPI003241-RA	ni|645903966|nb|KK920465.1|	140514-141562	16	15
ACYPI007065-RA	ni|645903832|nb|KK920598.1|	1042906-1043137	18	8.8
ACYPI008933-RA	ni|645901860|nb|KK922569.1|	108241-108487	nan	nan
ACYPI009563-RA	ni|645903973|nb|KK920458.1|	1332539-1332972	20	18
ACYPI080332-RA	ni|645903973|nb|KK920458.1|	1335332-1335565	20	18
ACYPI003522-RA	ni|645903915|nb|KK920516.1|	209340-209601	17	17
ACYPI003669-RA	ni|645903731|nb|KK920699.1|	98773-99067	nan	nan
ACYPI002653-RA	ni|645901808|nb|KK922621.1|	145364-146379	nan	nan
ACYPI003777-RA	ni|645904166|nb|KK920267.1|	2650791-2651081	18	16
ACYPI005709-RA	ni|645903766|nb|KK920664.1|	810545-810795	18	15
ACYPI008927-RA	ni|645903716|nb|KK920714.1|	598067-598438	nan	nan
ACYPI010124-RA	ni|645903771|nb|KK920659.1|	84857-85258	16	15
ACYPI010192-RA	ni|645903935|nb|KK920496.1|	1067206-1067448	17	18
ACYPI007694-RA	ni|645903521|nb|KK920909.1|	510215-510402	nan	nan
ACYPI002108-RA	ni|645903637|nb|KK920793.1|	540911-541464	nan	nan
ACYPI002846-RA	ni|645904222|nb|KK920245.1|	946504-946672	17	12
ACYPI005425-RA	ni|645903734|nb|KK920696.1|	411790-412820	nan	nan

ACYPI007291-RA	ni|645903614|nb|KK920816.1|	849350-849641	nan	nan
ACYPI007741-RA	ni|645903822|nb|KK920608.1|	357400-357571	18	20
ACYPI005044-RA	ni|645904115|nb|KK920316.1|	1087675-1088263	17	18
ACYPI005692-RA	ni|645902698|nb|KK921731.1|	94775-96390	nan	nan
ACYPI008188-RA	ni|645904114|nb|KK920317.1|	1764971-1765254	18	18
ACYPI34905-RA	ni|645903849|nb|KK920581.1|	244165-244985	17	17
ACYPI008325-RA	ni|645904014|nb|KK920417.1|	822928-823625	19	18
ACYPI009957-RA	ni|645904116|nb|KK920315.1|	2552016-2552377	19	18
ACYPI007413-RA	ni|645904004|nb|KK920427.1|	957413-959059	16	16
ACYPI003759-RA	ni|645904112|nb|KK920319.1|	2208263-2208527	18	17
ACYPI001071-RA	ni|645903749|nb|KK920681.1|	180990-181603	19	18
ACYPI002134-RA	ni|645904138|nb|KK920295.1|	653486-653745	15	15
ACYPI005660-RA	ni|645904138|nb|KK920295.1|	471760-473136	15	15
ACYPI53153-RA	ni|645903644|nb|KK920786.1|	214279-214967	nan	nan
ACYPI003077-RA	ni|645904130|nb|KK920303.1|	691046-691282	18	17
ACYPI007561-RA	ni|645903496|nb|KK920934.1|	941503-941833	nan	nan
ACYPI008256-RA	ni|645904240|nb|KK920239.1|	388562-390169	16	16
ACYPI009408-RA	ni|645904240|nb|KK920239.1|	1132942-1134040	16	16
ACYPI083041-RA	ni|645903533|nb|KK920897.1|	188118-188373	nan	nan
ACYPI25975-RA	ni|645904094|nb|KK920337.1|	2092396-2096337	17	16
ACYPI002730-RA	ni|645904014|nb|KK920417.1|	1483679-1484832	19	18
ACYPI005062-RA	ni|645903492|nb|KK920938.1|	61847-62348	nan	nan
ACYPI003764-RA	ni|645903747|nb|KK920683.1|	773739-773928	19	18
ACYPI004436-RA	ni|645903491|nb|KK920939.1|	471174-471546	nan	nan
ACYPI006355-RA	ni|645904177|nb|KK920260.1|	1972497-1973357	18	17
ACYPI005858-RA	ni|645903848|nb|KK920582.1|	604272-604444	18	18
ACYPI002075-RA	ni|645903911|nb|KK920520.1|	143165-143371	16	17
ACYPI000310-RA	ni|645904171|nb|KK920262.1|	517579-519623	15	16
ACYPI002865-RA	ni|645904135|nb|KK920298.1|	549274-549511	14	15
ACYPI001196-RA	ni|645902531|nb|KK921898.1|	190966-191720	nan	nan

ACYPI006572-RA	ni|645903901|nb|KK920530.1|	580610-581184	17	17
ACYPI003888-RA	ni|645904130|nb|KK920303.1|	641810-642455	18	17
ACYPI007176-RA	ni|645904075|nb|KK920356.1|	739586-741800	16	17
ACYPI000702-RA	ni|645904076|nb|KK920355.1|	810963-811167	17	17
ACYPI004498-RA	ni|645903768|nb|KK920662.1|	1343180-1343435	18	17
ACYPI008937-RA	ni|645904100|nb|KK920331.1|	399596-399999	18	22
ACYPI010019-RA	ni|645904195|nb|KK920254.1|	2238338-2238818	18	17
ACYPI008472-RA	ni|645902346|nb|KK922083.1|	26162-28212	nan	nan
ACYPI061464-RA	ni|645904180|nb|KK920259.1|	567379-567596	17	17
ACYPI003205-RA	ni|645903732|nb|KK920698.1|	84319-85128	nan	nan
ACYPI003757-RA	ni|645903720|nb|KK920710.1|	122913-123283	nan	nan
ACYPI005180-RA	ni|645904052|nb|KK920379.1|	1255539-1255826	18	16
ACYPI49229-RA	ni|645904052|nb|KK920379.1|	1650698-1650922	18	16
ACYPI001405-RA	ni|645903518|nb|KK920912.1|	195769-196427	nan	nan
ACYPI002636-RA	ni|645903650|nb|KK920780.1|	1007873-1008356	nan	nan
ACYPI004549-RA	ni|645903973|nb|KK920458.1|	1471462-1471703	20	18
ACYPI005777-RA	ni|645904088|nb|KK920343.1|	1297357-1297871	17	18
ACYPI010176-RA	ni|645903650|nb|KK920780.1|	993985-994172	nan	nan
ACYPI001396-RA	ni|645904258|nb|KK920229.1|	1280567-1281094	16	16
ACYPI004883-RA	ni|645903716|nb|KK920714.1|	266928-268964	nan	nan
ACYPI006122-RA	ni|645904231|nb|KK920242.1|	622179-623197	16	17
ACYPI008005-RA	ni|645904136|nb|KK920297.1|	1673877-1674535	16	17
ACYPI005162-RA	ni|645904234|nb|KK920241.1|	817806-818049	13	15
ACYPI007068-RA	ni|645904234|nb|KK920241.1|	1118185-1118392	13	15
ACYPI002247-RA	ni|645902172|nb|KK922257.1|	165026-165677	nan	nan
ACYPI004126-RA	ni|645904065|nb|KK920366.1|	1614206-1615248	18	18
ACYPI004824-RA	ni|645904096|nb|KK920335.1|	2416798-2417710	18	17
ACYPI006059-RA	ni|645904044|nb|KK920387.1|	1355635-1356063	15	16
ACYPI007941-RA	ni|645904096|nb|KK920335.1|	2432498-2432922	18	17
ACYPI002837-RA	ni|645903673|nb|KK920757.1|	83429-86295	nan	nan

ACYPI000455-RA	ni|645904234|nb|KK920241.1|	67395-67714	13	15
ACYPI009317-RA	ni|645903525|nb|KK920905.1|	82010-82229	nan	nan
ACYPI000476-RA	ni|645903837|nb|KK920593.1|	581975-582221	18	9.8
ACYPI002544-RA	ni|645904028|nb|KK920403.1|	1831841-1832235	18	17
ACYPI004442-RA	ni|645904028|nb|KK920403.1|	1829708-1830314	18	17
ACYPI005751-RA	ni|645904240|nb|KK920239.1|	1219655-1220125	16	16
ACYPI006748-RA	ni|645904130|nb|KK920303.1|	1467726-1469517	18	17
ACYPI004810-RA	ni|645904116|nb|KK920315.1|	3097800-3098512	19	18
ACYPI006670-RA	ni|645904115|nb|KK920316.1|	281045-281594	17	18
ACYPI006712-RA	ni|645904248|nb|KK920232.1|	9345-9952	15	16
ACYPI007934-RA	ni|645903464|nb|KK920966.1|	477450-478748	nan	nan
ACYPI000889-RA	ni|645904258|nb|KK920229.1|	591162-591769	16	16
ACYPI000961-RA	ni|645903746|nb|KK920684.1|	834812-835345	15	15
ACYPI087743-RA	ni|645904115|nb|KK920316.1|	134721-135287	17	18
ACYPI000806-RA	ni|645902503|nb|KK921926.1|	37124-37276	nan	nan
ACYPI004608-RA	ni|645904244|nb|KK920235.1|	387642-387829	15	14
ACYPI008270-RA	ni|645901628|nb|KK922801.1|	9728-10461	nan	nan
ACYPI009503-RA	ni|645903936|nb|KK920495.1|	196417-197600	19	18
ACYPI38303-RA	ni|645903690|nb|KK920740.1|	998974-999823	nan	nan
ACYPI088955-RA	ni|645903773|nb|KK920657.1|	1564980-1565163	18	17
ACYPI001339-RA	ni|645904118|nb|KK920313.1|	759127-759934	17	17
ACYPI005787-RA	ni|645904115|nb|KK920316.1|	1802020-1802247	17	18
ACYPI008317-RA	ni|645902415|nb|KK922014.1|	18270-18456	nan	nan
ACYPI010190-RA	ni|645904174|nb|KK920261.1|	902505-902862	19	17
ACYPI000541-RA	ni|645903789|nb|KK920641.1|	752792-753539	17	17
ACYPI001827-RA	ni|645904195|nb|KK920254.1|	673514-673800	18	17
ACYPI005644-RA	ni|645902662|nb|KK921767.1|	422594-422809	nan	nan
ACYPI003991-RA	ni|645904125|nb|KK920307.1|	960143-960680	18	17
ACYPI005480-RA	ni|645904246|nb|KK920233.1|	1205132-1207608	16	17
ACYPI000034-RA	ni|645903679|nb|KK920751.1|	66250-66545	nan	nan

ACYPI000572-RA	ni|645902427|nb|KK922002.1|	143925-144072	nan	nan
ACYPI005672-RA	ni|645904009|nb|KK920422.1|	839049-840138	17	9.4
ACYPI006928-RA	ni|645904088|nb|KK920343.1|	1230362-1231781	17	18
ACYPI008793-RA	ni|645904170|nb|KK920263.1|	1927624-1928348	15	16
ACYPI009437-RA	ni|645904098|nb|KK920333.1|	119008-119192	16	8.8
ACYPI060526-RA	ni|645903567|nb|KK920863.1|	210532-210741	nan	nan
ACYPI46839-RA	ni|645903567|nb|KK920863.1|	232665-232868	nan	nan
ACYPI001736-RA	ni|645903578|nb|KK920852.1|	203808-205276	nan	nan
ACYPI002345-RA	ni|645903592|nb|KK920838.1|	341159-341340	nan	nan
ACYPI003573-RA	ni|645903786|nb|KK920644.1|	118074-118640	18	19
ACYPI004227-RA	ni|645899876|nb|KK924553.1|	4626-5541	nan	nan
ACYPI004872-RA	ni|645902471|nb|KK921958.1|	210443-211128	nan	nan
ACYPI005524-RA	ni|645904086|nb|KK920345.1|	410385-411669	14	15
ACYPI007386-RA	ni|645903938|nb|KK920493.1|	1639934-1640148	19	18
ACYPI009267-RA	ni|645902193|nb|KK922236.1|	54992-55570	nan	nan
ACYPI071995-RA	ni|645901786|nb|KK922643.1|	74287-74502	nan	nan
ACYPI000690-RA	ni|645903549|nb|KK920881.1|	463009-463704	nan	nan
ACYPI004485-RA	ni|645903470|nb|KK920960.1|	206988-207502	nan	nan
ACYPI008829-RA	ni|645903728|nb|KK920702.1|	609892-610489	nan	nan
ACYPI004054-RA	ni|645903496|nb|KK920934.1|	159765-160674	nan	nan
ACYPI008778-RA	ni|645903597|nb|KK920833.1|	235130-235288	nan	nan
ACYPI082847-RA	ni|645903848|nb|KK920582.1|	1046771-1047071	18	18
ACYPI001995-RA	ni|645904027|nb|KK920404.1|	611613-612299	17	17
ACYPI061470-RA	ni|645903798|nb|KK920632.1|	123621-123771	18	17
ACYPI000068-RA	ni|645903971|nb|KK920460.1|	1942428-1944356	19	18
ACYPI067466-RA	ni|645903501|nb|KK920929.1|	72804-74726	nan	nan
ACYPI004686-RA	ni|645904114|nb|KK920317.1|	1490002-1490945	18	18
ACYPI001408-RA	ni|645903637|nb|KK920793.1|	685066-685738	nan	nan
ACYPI002592-RA	ni|645903650|nb|KK920780.1|	794327-794821	nan	nan
ACYPI000303-RA	ni|645902364|nb|KK922065.1|	28028-29134	nan	nan

ACYPI002858-RA	ni|645904095|nb|KK920336.1|	398924-402649	16	19
ACYPI006026-RA	ni|645904228|nb|KK920243.1|	1273740-1274345	17	18
ACYPI007298-RA	ni|645903740|nb|KK920690.1|	398952-399831	18	10
ACYPI007924-RA	ni|645904116|nb|KK920315.1|	1098828-1100927	19	18
ACYPI005934-RA	ni|645904245|nb|KK920234.1|	497794-498277	16	17
ACYPI067829-RA	ni|645902130|nb|KK922299.1|	29268-30056	nan	nan
ACYPI003918-RA	ni|645903843|nb|KK920587.1|	968274-968543	18	16
ACYPI004218-RA	ni|645903827|nb|KK920603.1|	1090808-1091258	17	18
ACYPI004594-RA	ni|645903557|nb|KK920873.1|	777735-779356	nan	nan
ACYPI004868-RA	ni|645903751|nb|KK920679.1|	668456-669410	17	16
ACYPI005059-RA	ni|645903643|nb|KK920787.1|	109214-109468	nan	nan
ACYPI005317-RA	ni|645903643|nb|KK920787.1|	137981-138383	nan	nan
ACYPI007989-RA	ni|645904171|nb|KK920262.1|	722019-724441	15	16
ACYPI008388-RA	ni|645903934|nb|KK920497.1|	257626-257958	15	16
ACYPI007697-RA	ni|645904114|nb|KK920317.1|	1729093-1729808	18	18
ACYPI41445-RA	ni|645903479|nb|KK920951.1|	107173-107351	nan	nan
ACYPI002737-RA	ni|645901565|nb|KK922864.1|	28414-28977	nan	nan
ACYPI005326-RA	ni|645903925|nb|KK920506.1|	676544-677276	19	17
ACYPI009717-RA	ni|645904116|nb|KK920315.1|	1587396-1587626	19	18
ACYPI002878-RA	ni|645902395|nb|KK922034.1|	13334-13646	nan	nan
ACYPI003832-RA	ni|645903841|nb|KK920589.1|	1239705-1240393	17	17
ACYPI000754-RA	ni|645903923|nb|KK920508.1|	1127142-1127692	18	18
ACYPI36303-RA	ni|645904037|nb|KK920394.1|	1887698-1888521	18	17
ACYPI001984-RA	ni|645903598|nb|KK920832.1|	341205-341773	nan	nan
ACYPI004543-RA	ni|645903917|nb|KK920514.1|	566675-567505	19	17
ACYPI004559-RA	ni|645904079|nb|KK920352.1|	217402-217949	15	16
ACYPI005824-RA	ni|645904222|nb|KK920245.1|	692309-692579	17	12
ACYPI006136-RA	ni|645902523|nb|KK921906.1|	134669-135239	nan	nan
ACYPI007683-RA	ni|645903789|nb|KK920641.1|	777339-778494	17	17
ACYPI071352-RA	ni|645904152|nb|KK920281.1|	862607-863077	15	15

ACYPI001440-RA	ni|645903607|nb|KK920823.1|	314259-315418	nan	nan
ACYPI003689-RA	ni|645900910|nb|KK923519.1|	34359-35064	nan	nan
ACYPI006738-RA	ni|645904133|nb|KK920300.1|	2280485-2280994	18	18
ACYPI007182-RA	ni|645903697|nb|KK920733.1|	392830-393328	nan	nan
ACYPI009063-RA	ni|645902554|nb|KK921875.1|	21660-22323	nan	nan
ACYPI006784-RA	ni|645903854|nb|KK920576.1|	31398-31593	16	8.4
ACYPI003549-RA	ni|645903536|nb|KK920894.1|	858250-858529	nan	nan
ACYPI004497-RA	ni|645904134|nb|KK920299.1|	467117-469455	17	13
ACYPI004676-RA	ni|645903744|nb|KK920686.1|	709747-709948	17	8.6
ACYPI005514-RA	ni|645903547|nb|KK920883.1|	500931-501158	nan	nan
ACYPI003646-RA	ni|645903626|nb|KK920804.1|	336789-337409	nan	nan
ACYPI061611-RA	ni|645903626|nb|KK920804.1|	338135-338282	nan	nan
ACYPI000605-RA	ni|645904132|nb|KK920301.1|	71003-71455	16	18
ACYPI001507-RA	ni|645904262|nb|KK920227.1|	667170-667442	17	17
ACYPI003283-RA	ni|645903957|nb|KK920474.1|	846525-846814	18	17
ACYPI004533-RA	ni|645904262|nb|KK920227.1|	732425-732612	17	17
ACYPI005339-RA	ni|645904262|nb|KK920227.1|	656673-657184	17	17
ACYPI008826-RA	ni|645903834|nb|KK920596.1|	166448-167734	18	17
ACYPI000484-RA	ni|645904242|nb|KK920237.1|	53205-54736	15	16
ACYPI001336-RA	ni|645901664|nb|KK922765.1|	52325-53615	nan	nan
ACYPI003222-RA	ni|645903806|nb|KK920624.1|	586237-587179	16	9.3
ACYPI010188-RA	ni|645903806|nb|KK920624.1|	525233-526587	16	9.3
ACYPI010216-RA	ni|645904117|nb|KK920314.1|	227743-227969	15	15
ACYPI067812-RA	ni|645904243|nb|KK920236.1|	618218-619796	15	8.5
ACYPI082338-RA	ni|645904058|nb|KK920373.1|	1109523-1110556	16	15
ACYPI086092-RA	ni|645904151|nb|KK920282.1|	891454-893091	16	20
ACYPI084883-RA	ni|645904146|nb|KK920287.1|	87931-88150	16	18
ACYPI003993-RA	ni|645904139|nb|KK920294.1|	554250-554978	15	18
ACYPI009705-RA	ni|645903833|nb|KK920597.1|	225912-226898	17	15
ACYPI060550-RA	ni|645904159|nb|KK920274.1|	190468-190721	15	17

ACYPI000007-RA	ni|645904141|nb|KK920292.1|	555475-556035	14	14
ACYPI004453-RA	ni|645902649|nb|KK921780.1|	204894-206241	nan	nan
ACYPI008255-RA	ni|645904210|nb|KK920249.1|	795621-795857	16	16
ACYPI008888-RA	ni|645903511|nb|KK920919.1|	481925-482124	nan	nan
ACYPI48107-RA	ni|645904210|nb|KK920249.1|	809385-809692	16	16
ACYPI000848-RA	ni|645904177|nb|KK920260.1|	1202685-1203630	18	17
ACYPI001218-RA	ni|645904240|nb|KK920239.1|	1775246-1777473	16	16
ACYPI002808-RA	ni|645902404|nb|KK922025.1|	41336-42420	nan	nan
ACYPI006640-RA	ni|645904096|nb|KK920335.1|	1995033-1995242	18	17
ACYPI009711-RA	ni|645903757|nb|KK920673.1|	77025-77407	18	17
ACYPI082655-RA	ni|645903597|nb|KK920833.1|	788549-788978	nan	nan
ACYPI000474-RA	ni|645903650|nb|KK920780.1|	143175-143398	nan	nan
ACYPI001119-RA	ni|645902639|nb|KK921790.1|	162983-163480	nan	nan
ACYPI003014-RA	ni|645904118|nb|KK920313.1|	722742-723171	17	17
ACYPI008707-RA	ni|645904116|nb|KK920315.1|	420920-421158	19	18
ACYPI009338-RA	ni|645903472|nb|KK920958.1|	229918-230887	nan	nan
ACYPI50902-RA	ni|645904013|nb|KK920418.1|	716342-716903	19	18
ACYPI000023-RA	ni|645904072|nb|KK920359.1|	947375-948150	17	17
ACYPI001212-RA	ni|645904281|nb|KK920220.1|	992431-993452	15	16
ACYPI007158-RA	ni|645903494|nb|KK920936.1|	334594-334733	nan	nan
ACYPI007537-RA	ni|645903563|nb|KK920867.1|	699462-700637	nan	nan
ACYPI008785-RA	ni|645902580|nb|KK921849.1|	57156-57395	nan	nan
ACYPI000527-RA	ni|645904130|nb|KK920303.1|	486428-487041	18	17
ACYPI002352-RA	ni|645899094|nb|KK925335.1|	7387-8369	nan	nan
ACYPI003042-RA	ni|645904242|nb|KK920237.1|	790552-790887	15	16
ACYPI004235-RA	ni|645903848|nb|KK920582.1|	586066-586783	18	18
ACYPI006934-RA	ni|645903634|nb|KK920796.1|	1045632-1045976	nan	nan
ACYPI000598-RA	ni|645901961|nb|KK922468.1|	109885-111850	nan	nan
ACYPI001579-RA	ni|645900333|nb|KK924096.1|	21715-21953	nan	nan
ACYPI001617-RA	ni|645902779|nb|KK921650.1|	135035-135804	nan	nan

ACYPI002866-RA	ni|645903858|nb|KK920572.1|	639028-643519	16	16
ACYPI004093-RA	ni|645902226|nb|KK922203.1|	33938-34166	nan	nan
ACYPI006031-RA	ni|645902724|nb|KK921705.1|	54894-55158	nan	nan
ACYPI007307-RA	ni|645904098|nb|KK920333.1|	296655-296910	16	8.8
ACYPI007911-RA	ni|645902724|nb|KK921705.1|	80612-80844	nan	nan
ACYPI009786-RA	ni|645901288|nb|KK923141.1|	16166-16419	nan	nan
ACYPI24300-RA	ni|645902700|nb|KK921729.1|	73177-73385	nan	nan
ACYPI068779-RA	ni|645903733|nb|KK920697.1|	620918-621791	nan	nan
ACYPI001729-RA	ni|645904007|nb|KK920424.1|	755776-755927	18	17
ACYPI004908-RA	ni|645903971|nb|KK920460.1|	1573467-1573685	19	18
ACYPI006808-RA	ni|645903627|nb|KK920803.1|	520496-520725	nan	nan
ACYPI000052-RA	ni|645902360|nb|KK922069.1|	41628-41906	nan	nan
ACYPI001864-RA	ni|645902463|nb|KK921966.1|	60976-61400	nan	nan
ACYPI002416-RA	ni|645903702|nb|KK920728.1|	95833-97249	nan	nan
ACYPI003853-RA	ni|645901733|nb|KK922696.1|	24484-25097	nan	nan
ACYPI005784-RA	ni|645903985|nb|KK920446.1|	775004-775409	18	16
ACYPI007791-RA	ni|645903902|nb|KK920529.1|	684141-685269	19	17
ACYPI005474-RA	ni|645904006|nb|KK920425.1|	77400-77787	17	16
ACYPI007336-RA	ni|645902293|nb|KK922136.1|	86714-87314	nan	nan
ACYPI008861-RA	ni|645904231|nb|KK920242.1|	48525-49467	16	17
ACYPI002938-RA	ni|645903787|nb|KK920643.1|	1196813-1197302	19	18
ACYPI20476-RA	ni|645904168|nb|KK920265.1|	316824-317136	16	16
ACYPI20477-RA	ni|645904168|nb|KK920265.1|	308543-309824	16	16
ACYPI20759-RA	ni|645904168|nb|KK920265.1|	36921-37712	16	16
ACYPI008491-RA	ni|645904279|nb|KK920221.1|	2628854-2630142	16	16
ACYPI005969-RA	ni|645904007|nb|KK920424.1|	78405-78734	18	17
ACYPI31575-RA	ni|645903626|nb|KK920804.1|	93016-93141	nan	nan
ACYPI000585-RA	ni|645903954|nb|KK920477.1|	197722-197837	35	44
ACYPI34621-RA	ni|645903787|nb|KK920643.1|	814254-814958	19	18
ACYPI000953-RA	ni|645904246|nb|KK920233.1|	1237740-1237975	16	17

ACYPI002851-RA	ni|645903753|nb|KK920677.1|	538715-539315	17	9.8
ACYPI004812-RA	ni|645903634|nb|KK920796.1|	1224631-1225300	nan	nan
ACYPI001502-RA	ni|645903863|nb|KK920567.1|	155734-156144	15	9.2
ACYPI003428-RA	ni|645903690|nb|KK920740.1|	291212-292032	nan	nan
ACYPI009718-RA	ni|645903811|nb|KK920619.1|	38980-40053	15	15
ACYPI001480-RA	ni|645904262|nb|KK920227.1|	2432336-2433221	17	17
ACYPI002304-RA	ni|645903532|nb|KK920898.1|	268766-269396	nan	nan
ACYPI004992-RA	ni|645903612|nb|KK920818.1|	61000-61918	nan	nan
ACYPI005674-RA	ni|645904051|nb|KK920380.1|	480022-480316	16	17
ACYPI002110-RA	ni|645903634|nb|KK920796.1|	1217326-1217924	nan	nan
ACYPI007090-RA	ni|645903835|nb|KK920595.1|	610427-610893	16	8.8
ACYPI003242-RA	ni|645904157|nb|KK920276.1|	1456385-1457320	15	15
ACYPI003864-RA	ni|645903871|nb|KK920559.1|	798954-799442	18	9.4
ACYPI002900-RA	ni|645903999|nb|KK920432.1|	1120887-1121762	17	15
ACYPI008024-RA	ni|645903644|nb|KK920786.1|	346022-346513	nan	nan
ACYPI000080-RA	ni|645903724|nb|KK920706.1|	262716-263267	nan	nan
ACYPI001206-RA	ni|645903680|nb|KK920750.1|	1007743-1008436	nan	nan
ACYPI003099-RA	ni|645903680|nb|KK920750.1|	1003668-1003939	nan	nan
ACYPI005019-RA	ni|645903603|nb|KK920827.1|	332132-332554	nan	nan
ACYPI005619-RA	ni|645904015|nb|KK920416.1|	1253180-1255265	18	16
ACYPI005661-RA	ni|645903982|nb|KK920449.1|	56583-57174	16	14
ACYPI006280-RA	ni|645904171|nb|KK920262.1|	573805-574877	15	16
ACYPI007534-RA	ni|645904008|nb|KK920423.1|	276725-276966	16	9.5
ACYPI010029-RA	ni|645904122|nb|KK920309.1|	541252-543711	18	17
ACYPI001745-RA	ni|645904046|nb|KK920385.1|	1050279-1050433	17	8.4
ACYPI062376-RA	ni|645904189|nb|KK920256.1|	528381-528856	18	18
ACYPI003824-RA	ni|645904057|nb|KK920374.1|	84508-85245	16	15
ACYPI005074-RA	ni|645903504|nb|KK920926.1|	597383-597853	nan	nan
ACYPI008698-RA	ni|645904073|nb|KK920358.1|	1104368-1108545	16	18
ACYPI010049-RA	ni|645903594|nb|KK920836.1|	68071-68597	nan	nan

ACYPI000489-RA	ni|645902758|nb|KK921671.1|	220670-223892	nan	nan
ACYPI001732-RA	ni|645903688|nb|KK920742.1|	217204-218425	nan	nan
ACYPI004266-RA	ni|645903960|nb|KK920471.1|	963897-964137	16	8.6
ACYPI008075-RA	ni|645904168|nb|KK920265.1|	682992-683261	16	16
ACYPI003311-RA	ni|645904129|nb|KK920304.1|	994384-994837	16	17
ACYPI009707-RA	ni|645904129|nb|KK920304.1|	1130026-1130759	16	17
ACYPI006314-RA	ni|645904216|nb|KK920247.1|	329926-330200	15	16
ACYPI003657-RA	ni|645901672|nb|KK922757.1|	3755-4399	nan	nan
ACYPI003904-RA	ni|645904210|nb|KK920249.1|	2063110-2063469	16	16
ACYPI004955-RA	ni|645904210|nb|KK920249.1|	2011892-2012338	16	16
ACYPI005837-RA	ni|645903985|nb|KK920446.1|	1038427-1038967	18	16
ACYPI008512-RA	ni|645903704|nb|KK920726.1|	204369-205036	nan	nan
ACYPI003822-RA	ni|645902622|nb|KK921807.1|	138278-138478	nan	nan
ACYPI004910-RA	ni|645903808|nb|KK920622.1|	971282-971634	17	17
ACYPI005580-RA	ni|645903762|nb|KK920668.1|	884594-885578	17	17
ACYPI006247-RA	ni|645903988|nb|KK920443.1|	138738-139187	14	16
ACYPI008162-RA	ni|645903988|nb|KK920443.1|	248706-249243	14	16
ACYPI008800-RA	ni|645903820|nb|KK920610.1|	394818-395314	18	19
ACYPI000621-RA	ni|645903833|nb|KK920597.1|	238521-239619	17	15
ACYPI002506-RA	ni|645903858|nb|KK920572.1|	440371-440559	16	16
ACYPI003343-RA	ni|645903713|nb|KK920717.1|	199518-199736	nan	nan
ACYPI003925-RA	ni|645904122|nb|KK920309.1|	1507549-1507785	18	17
ACYPI004592-RA	ni|645904154|nb|KK920279.1|	317755-317942	14	14
ACYPI005264-RA	ni|645904154|nb|KK920279.1|	710537-711640	14	14
ACYPI009028-RA	ni|645904104|nb|KK920327.1|	289588-290609	15	15
ACYPI087473-RA	ni|645903891|nb|KK920540.1|	293681-293895	15	17
ACYPI001646-RA	ni|645903687|nb|KK920743.1|	43586-44176	nan	nan
ACYPI008302-RA	ni|645904112|nb|KK920319.1|	223064-223885	18	17
ACYPI001110-RA	ni|645904231|nb|KK920242.1|	456319-456444	16	17
ACYPI003425-RA	ni|645903511|nb|KK920919.1|	774604-776708	nan	nan

ACYPI000735-RA	ni|645903592|nb|KK920838.1|	763281-763503	nan	nan
ACYPI002246-RA	ni|645903742|nb|KK920688.1|	536200-537907	17	16
ACYPI005007-RA	ni|645901858|nb|KK922571.1|	142168-142762	nan	nan
ACYPI006058-RA	ni|645903568|nb|KK920862.1|	230542-230789	nan	nan
ACYPI007679-RA	ni|645904241|nb|KK920238.1|	116765-117564	18	17
ACYPI008763-RA	ni|645904271|nb|KK920224.1|	458452-459710	15	16
ACYPI000839-RA	ni|645903714|nb|KK920716.1|	204343-204510	nan	nan
ACYPI006557-RA	ni|645903965|nb|KK920466.1|	1174266-1174946	17	16
ACYPI008242-RA	ni|645904222|nb|KK920245.1|	2329941-2330336	17	12
ACYPI002445-RA	ni|645904262|nb|KK920227.1|	2516351-2516636	17	17
ACYPI003103-RA	ni|645903938|nb|KK920493.1|	495510-496133	19	18
ACYPI065095-RA	ni|645903922|nb|KK920509.1|	643536-644090	15	16
ACYPI089209-RA	ni|645903922|nb|KK920509.1|	504280-504778	15	16
ACYPI009548-RA	ni|645903704|nb|KK920726.1|	434282-434826	nan	nan
ACYPI071951-RA	ni|645903743|nb|KK920687.1|	545031-545451	19	18
ACYPI083523-RA	ni|645904262|nb|KK920227.1|	285265-285757	17	17
ACYPI000686-RA	ni|645903724|nb|KK920706.1|	159437-159822	nan	nan
ACYPI001081-RA	ni|645903557|nb|KK920873.1|	1071122-1071677	nan	nan
ACYPI002342-RA	ni|645903724|nb|KK920706.1|	151621-153446	nan	nan
ACYPI002979-RA	ni|645903743|nb|KK920687.1|	286000-286328	19	18
ACYPI005064-RA	ni|645903929|nb|KK920502.1|	1363339-1363518	17	18
ACYPI006142-RA	ni|645903879|nb|KK920552.1|	951007-951994	18	18
ACYPI23338-RA	ni|645903860|nb|KK920570.1|	155282-157970	17	17
ACYPI061620-RA	ni|645903917|nb|KK920514.1|	394530-394742	19	17
ACYPI002382-RA	ni|645904049|nb|KK920382.1|	518290-520008	18	18
ACYPI008717-RA	ni|645903744|nb|KK920686.1|	793998-794662	17	8.6
ACYPI007374-RA	ni|645904177|nb|KK920260.1|	2420978-2421580	18	17
ACYPI001090-RA	ni|645904153|nb|KK920280.1|	1494710-1495060	17	16
ACYPI073321-RA	ni|645902443|nb|KK921986.1|	12019-12412	nan	nan
ACYPI001359-RA	ni|645903654|nb|KK920776.1|	1200-2169	nan	nan

ACYPI003246-RA	ni|645904222|nb|KK920245.1|	1189060-1189599	17	12
ACYPI088026-RA	ni|645903588|nb|KK920842.1|	622246-623104	nan	nan
ACYPI002405-RA	ni|645903964|nb|KK920467.1|	637409-637559	15	8
ACYPI008746-RA	ni|645904056|nb|KK920375.1|	759901-760171	16	16
ACYPI009713-RA	ni|645904019|nb|KK920412.1|	726396-726638	19	9.4
ACYPI001596-RA	ni|645903557|nb|KK920873.1|	862467-862681	nan	nan
ACYPI003480-RA	ni|645904258|nb|KK920229.1|	731952-732355	16	16
ACYPI006207-RA	ni|645903674|nb|KK920756.1|	146009-146180	nan	nan
ACYPI008726-RA	ni|645903702|nb|KK920728.1|	321599-322061	nan	nan
ACYPI080006-RA	ni|645903996|nb|KK920435.1|	1111700-1111956	17	16
ACYPI000487-RA	ni|645904106|nb|KK920325.1|	677994-678427	17	17
ACYPI002373-RA	ni|645904155|nb|KK920278.1|	870870-871470	18	9.4
ACYPI002732-RA	ni|645903496|nb|KK920934.1|	335367-335584	nan	nan
ACYPI003255-RA	ni|645903965|nb|KK920466.1|	1675427-1676261	17	16
ACYPI009003-RA	ni|645904240|nb|KK920239.1|	1829221-1829755	16	16
ACYPI010211-RA	ni|645903891|nb|KK920540.1|	41989-42456	15	17
ACYPI087879-RA	ni|645904240|nb|KK920239.1|	1824204-1825051	16	16
ACYPI42350-RA	ni|645904136|nb|KK920297.1|	912875-913912	16	17
ACYPI005894-RA	ni|645903783|nb|KK920647.1|	1589483-1589714	17	17
ACYPI007763-RA	ni|645903866|nb|KK920564.1|	355348-355500	18	15
ACYPI006567-RA	ni|645904094|nb|KK920337.1|	1674256-1674606	17	16
ACYPI003377-RA	ni|645904078|nb|KK920353.1|	563997-564219	17	17
ACYPI003469-RA	ni|645903617|nb|KK920813.1|	565660-566135	nan	nan
ACYPI006027-RA	ni|645904057|nb|KK920374.1|	1242701-1243051	16	15
ACYPI007195-RA	ni|645903754|nb|KK920676.1|	112124-112395	14	8.8
ACYPI009151-RA	ni|645904159|nb|KK920274.1|	1063712-1064149	15	17
ACYPI009704-RA	ni|645904210|nb|KK920249.1|	367814-373916	16	16
ACYPI089560-RA	ni|645903722|nb|KK920708.1|	842682-842941	nan	nan
ACYPI007802-RA	ni|645903734|nb|KK920696.1|	910962-911422	nan	nan
ACYPI084662-RA	ni|645904135|nb|KK920298.1|	607439-607929	14	15

ACYPI004766-RA	ni|645903823|nb|KK920607.1|	360117-360659	16	17
ACYPI008535-RA	ni|645903552|nb|KK920878.1|	629324-631169	nan	nan
ACYPI005517-RA	ni|645903592|nb|KK920838.1|	573285-574973	nan	nan
ACYPI008053-RA	ni|645904088|nb|KK920343.1|	1517099-1520331	17	18
ACYPI009339-RA	ni|645904046|nb|KK920385.1|	1212651-1213385	17	8.4
ACYPI009928-RA	ni|645903609|nb|KK920821.1|	283028-283227	nan	nan
ACYPI001042-RA	ni|645902373|nb|KK922056.1|	185613-186176	nan	nan
ACYPI002287-RA	ni|645904118|nb|KK920313.1|	897231-898115	17	17
ACYPI003347-RA	ni|645903891|nb|KK920540.1|	121603-121814	15	17
ACYPI004209-RA	ni|645903557|nb|KK920873.1|	828896-829330	nan	nan
ACYPI007357-RA	ni|645903957|nb|KK920474.1|	574062-574529	18	17
ACYPI007778-RA	ni|645903891|nb|KK920540.1|	106464-107629	15	17
ACYPI009675-RA	ni|645904244|nb|KK920235.1|	538558-538709	15	14
ACYPI009896-RA	ni|645903622|nb|KK920808.1|	382634-383629	nan	nan
ACYPI52571-RA	ni|645903743|nb|KK920687.1|	924656-928329	19	18
ACYPI002538-RA	ni|645904278|nb|KK920222.1|	481380-482153	15	16
ACYPI003809-RA	ni|645903690|nb|KK920740.1|	229891-233078	nan	nan
ACYPI004435-RA	ni|645903597|nb|KK920833.1|	910585-911169	nan	nan
ACYPI006352-RA	ni|645902662|nb|KK921767.1|	340787-341003	nan	nan
ACYPI006535-RA	ni|645904076|nb|KK920355.1|	198158-198356	17	17
ACYPI009511-RA	ni|645903765|nb|KK920665.1|	1194221-1194721	17	15
ACYPI010117-RA	ni|645903563|nb|KK920867.1|	186680-187279	nan	nan
ACYPI001382-RA	ni|645903808|nb|KK920622.1|	617187-617566	17	17
ACYPI010152-RA	ni|645903798|nb|KK920632.1|	149817-150353	18	17
ACYPI002463-RA	ni|645903674|nb|KK920756.1|	560964-561467	nan	nan
ACYPI002870-RA	ni|645903999|nb|KK920432.1|	531549-532153	17	15
ACYPI004366-RA	ni|645904088|nb|KK920343.1|	274452-275280	17	18
ACYPI006693-RA	ni|645903999|nb|KK920432.1|	549019-549429	17	15
ACYPI009210-RA	ni|645903931|nb|KK920500.1|	473760-474018	16	18
ACYPI007100-RA	ni|645904231|nb|KK920242.1|	651668-652542	16	17

ACYPI008357-RA	ni|645903664|nb|KK920766.1|	200467-200729	nan	nan
ACYPI073873-RA	ni|645904243|nb|KK920236.1|	1733080-1733496	15	8.5
ACYPI37088-RA	ni|645903716|nb|KK920714.1|	418040-418587	nan	nan
ACYPI007666-RA	ni|645902589|nb|KK921840.1|	254022-254912	nan	nan
ACYPI007713-RA	ni|645903997|nb|KK920434.1|	203274-203542	15	16
ACYPI009612-RA	ni|645902683|nb|KK921746.1|	125609-125751	nan	nan
ACYPI001360-RA	ni|645904071|nb|KK920360.1|	730063-731046	17	17
ACYPI001392-RA	ni|645904074|nb|KK920357.1|	230139-230496	17	18
ACYPI005208-RA	ni|645901808|nb|KK922621.1|	104436-104610	nan	nan
ACYPI006413-RA	ni|645903743|nb|KK920687.1|	126791-129557	19	18
ACYPI001043-RA	ni|645902354|nb|KK922075.1|	45603-45806	nan	nan
ACYPI003742-RA	ni|645904116|nb|KK920315.1|	1788704-1788930	19	18
ACYPI005687-RA	ni|645902371|nb|KK922058.1|	113237-113981	nan	nan
ACYPI008632-RA	ni|645903862|nb|KK920568.1|	337828-338042	17	17
ACYPI002678-RA	ni|645901760|nb|KK922669.1|	93073-94496	nan	nan
ACYPI004278-RA	ni|645903650|nb|KK920780.1|	574199-577259	nan	nan
ACYPI006506-RA	ni|645904149|nb|KK920284.1|	504474-505015	16	16
ACYPI000423-RA	ni|645904210|nb|KK920249.1|	813969-814148	16	16
ACYPI003054-RA	ni|645903662|nb|KK920768.1|	373262-373655	nan	nan
ACYPI003596-RA	ni|645903831|nb|KK920599.1|	1505400-1505744	18	17
ACYPI004199-RA	ni|645903651|nb|KK920779.1|	244594-244999	nan	nan
ACYPI006737-RA	ni|645903727|nb|KK920703.1|	958477-958937	nan	nan
ACYPI008014-RA	ni|645903727|nb|KK920703.1|	888351-889177	nan	nan
ACYPI008596-RA	ni|645903496|nb|KK920934.1|	949022-949806	nan	nan
ACYPI071357-RA	ni|645903727|nb|KK920703.1|	952093-954572	nan	nan
ACYPI21591-RA	ni|645903662|nb|KK920768.1|	384806-385049	nan	nan
ACYPI000220-RA	ni|645903999|nb|KK920432.1|	1424063-1424766	17	15
ACYPI002122-RA	ni|645896935|nb|KK927494.1|	97-939	nan	nan
ACYPI006276-RA	ni|645904094|nb|KK920337.1|	1336781-1337401	17	16
ACYPI008000-RA	ni|645903466|nb|KK920964.1|	698959-700474	nan	nan

ACYPI002966-RA	ni|645904070|nb|KK920361.1|	384229-385310	17	16
ACYPI005542-RA	ni|645902591|nb|KK921838.1|	63791-63980	nan	nan
ACYPI006792-RA	ni|645904028|nb|KK920403.1|	413374-413757	18	17
ACYPI009286-RA	ni|645903879|nb|KK920552.1|	1696389-1697091	18	18
ACYPI001461-RA	ni|645904149|nb|KK920284.1|	216961-217463	16	16
ACYPI073515-RA	ni|645904149|nb|KK920284.1|	216446-216703	16	16
ACYPI083652-RA	ni|645904075|nb|KK920356.1|	715621-715925	16	17
ACYPI009409-RA	ni|645904240|nb|KK920239.1|	4415-5106	16	16
ACYPI087996-RA	ni|645903634|nb|KK920796.1|	1016809-1017378	nan	nan
ACYPI23573-RA	ni|645904254|nb|KK920230.1|	1035239-1035406	16	16
ACYPI001796-RA	ni|645903557|nb|KK920873.1|	1093184-1094901	nan	nan
ACYPI003679-RA	ni|645902449|nb|KK921980.1|	82936-83142	nan	nan
ACYPI005047-RA	ni|645903911|nb|KK920520.1|	543859-544272	16	17
ACYPI006948-RA	ni|645903911|nb|KK920520.1|	527512-527958	16	17
ACYPI009467-RA	ni|645903637|nb|KK920793.1|	780139-780405	nan	nan
ACYPI002210-RA	ni|645903720|nb|KK920710.1|	331001-331671	nan	nan
ACYPI004082-RA	ni|645903511|nb|KK920919.1|	538835-539076	nan	nan
ACYPI006681-RA	ni|645904258|nb|KK920229.1|	46202-46577	16	16
ACYPI005528-RA	ni|645903534|nb|KK920896.1|	579054-579287	nan	nan
ACYPI009950-RA	ni|645903783|nb|KK920647.1|	139010-139195	17	17
ACYPI010122-RA	ni|645903633|nb|KK920797.1|	570529-570684	nan	nan
ACYPI009753-RA	ni|645904115|nb|KK920316.1|	1474168-1474349	17	18
ACYPI000278-RA	ni|645904152|nb|KK920281.1|	260381-260684	15	15
ACYPI005309-RA	ni|645904002|nb|KK920429.1|	357420-357664	14	18
ACYPI006257-RA	ni|645902745|nb|KK921684.1|	116758-117211	nan	nan
ACYPI006656-RA	ni|645903957|nb|KK920474.1|	1448344-1449893	18	17
ACYPI003203-RA	ni|645901755|nb|KK922674.1|	8809-9287	nan	nan
ACYPI006860-RA	ni|645904228|nb|KK920243.1|	1219030-1219556	17	18
ACYPI004352-RA	ni|645903600|nb|KK920830.1|	403804-404402	nan	nan
ACYPI010073-RA	ni|645903496|nb|KK920934.1|	472271-473782	nan	nan

ACYPI29600-RA	ni|645904112|nb|KK920319.1|	2322643-2323547	18	17
ACYPI069530-RA	ni|645904123|nb|KK920308.1|	1089378-1089862	16	10
ACYPI000994-RA	ni|645903740|nb|KK920690.1|	354203-355126	18	10
ACYPI071217-RA	ni|645904012|nb|KK920419.1|	305201-305741	27	32
ACYPI22575-RA	ni|645904064|nb|KK920367.1|	634999-635783	17	9.9
ACYPI004768-RA	ni|645903981|nb|KK920450.1|	124918-125103	16	8.6
ACYPI005453-RA	ni|645902723|nb|KK921706.1|	115268-115819	nan	nan
ACYPI006674-RA	ni|645904152|nb|KK920281.1|	386092-387170	15	15
ACYPI000819-RA	ni|645903891|nb|KK920540.1|	90267-90459	15	17
ACYPI004238-RA	ni|645904094|nb|KK920337.1|	125980-126428	17	16
ACYPI004619-RA	ni|645903597|nb|KK920833.1|	258266-258724	nan	nan
ACYPI000627-RA	ni|645903728|nb|KK920702.1|	961199-961505	nan	nan
ACYPI003792-RA	ni|645904116|nb|KK920315.1|	2451604-2452325	19	18
ACYPI005155-RA	ni|645902293|nb|KK922136.1|	66072-66318	nan	nan
ACYPI008850-RA	ni|645903738|nb|KK920692.1|	914605-914994	18	17
ACYPI072156-RA	ni|645903472|nb|KK920958.1|	367392-367613	nan	nan
ACYPI087691-RA	ni|645904281|nb|KK920220.1|	825315-825573	15	16
ACYPI004416-RA	ni|645903944|nb|KK920487.1|	105834-106597	15	17
ACYPI005175-RA	ni|645902690|nb|KK921739.1|	292933-293317	nan	nan
ACYPI24155-RA	ni|645903960|nb|KK920471.1|	910992-911316	16	8.6
ACYPI000824-RA	ni|645903597|nb|KK920833.1|	349204-349726	nan	nan
ACYPI004781-RA	ni|645902092|nb|KK922337.1|	12651-13873	nan	nan
ACYPI006544-RA	ni|645904119|nb|KK920312.1|	514158-514550	19	19
ACYPI46242-RA	ni|645903664|nb|KK920766.1|	252319-252528	nan	nan
ACYPI001776-RA	ni|645903955|nb|KK920476.1|	514726-514963	18	17
ACYPI003031-RA	ni|645904100|nb|KK920331.1|	283814-284205	18	22
ACYPI001303-RA	ni|645904183|nb|KK920258.1|	873941-877047	25	35
ACYPI003950-RA	ni|645903469|nb|KK920961.1|	709058-709355	nan	nan
ACYPI002129-RA	ni|645903869|nb|KK920561.1|	444223-445565	17	15
ACYPI002738-RA	ni|645903896|nb|KK920535.1|	422979-424346	16	9.1

ACYPI004003-RA	ni|645904241|nb|KK920238.1|	1879707-1882732	18	17
ACYPI073116-RA	ni|645904174|nb|KK920261.1|	913928-914530	19	17
ACYPI001672-RA	ni|645904245|nb|KK920234.1|	1316654-1317159	16	17
ACYPI007348-RA	ni|645902473|nb|KK921956.1|	219352-219785	nan	nan
ACYPI37413-RA	ni|645904087|nb|KK920344.1|	914546-914941	16	16
ACYPI000610-RA	ni|645903970|nb|KK920461.1|	2124908-2125698	17	16
ACYPI008191-RA	ni|645904088|nb|KK920343.1|	1323348-1323905	17	18
ACYPI008619-RA	ni|645904088|nb|KK920343.1|	1433232-1433497	17	18
ACYPI010077-RA	ni|645904088|nb|KK920343.1|	1253879-1254536	17	18
ACYPI010087-RA	ni|645903788|nb|KK920642.1|	536483-536835	16	15
ACYPI086281-RA	ni|645904116|nb|KK920315.1|	2079937-2080922	19	18
ACYPI001113-RA	ni|645903530|nb|KK920900.1|	523489-523650	nan	nan
ACYPI005904-RA	ni|645903812|nb|KK920618.1|	791676-791954	20	18
ACYPI008128-RA	ni|645903854|nb|KK920576.1|	403864-404055	16	8.4
ACYPI009043-RA	ni|645903812|nb|KK920618.1|	794574-794914	20	18
ACYPI34776-RA	ni|645897392|nb|KK927037.1|	3550-3709	nan	nan
ACYPI003729-RA	ni|645902468|nb|KK921961.1|	44084-44541	nan	nan
ACYPI000886-RA	ni|645904014|nb|KK920417.1|	794089-794343	19	18
ACYPI001538-RA	ni|645904014|nb|KK920417.1|	479278-480177	19	18
ACYPI001812-RA	ni|645903860|nb|KK920570.1|	206810-207053	17	17
ACYPI001560-RA	ni|645904053|nb|KK920378.1|	1114687-1116998	18	17
ACYPI007326-RA	ni|645904053|nb|KK920378.1|	1386856-1387233	18	17
ACYPI000656-RA	ni|645904201|nb|KK920252.1|	347420-349693	17	16
ACYPI005655-RA	ni|645903873|nb|KK920558.1|	469213-470024	16	15
ACYPI008874-RA	ni|645903837|nb|KK920593.1|	552939-554801	18	9.8
ACYPI010059-RA	ni|645904219|nb|KK920246.1|	692992-694048	16	17
ACYPI000376-RA	ni|645903808|nb|KK920622.1|	452907-453185	17	17
ACYPI000821-RA	ni|645903622|nb|KK920808.1|	572228-572457	nan	nan
ACYPI001024-RA	ni|645903915|nb|KK920516.1|	1437030-1437551	17	17
ACYPI003357-RA	ni|645903622|nb|KK920808.1|	593426-593710	nan	nan

ACYPI005588-RA	ni|645903849|nb|KK920581.1|	60264-61423	17	17
ACYPI006841-RA	ni|645904106|nb|KK920325.1|	669062-669656	17	17
ACYPI007972-RA	ni|645904237|nb|KK920240.1|	824927-827638	16	15
ACYPI008384-RA	ni|645904009|nb|KK920422.1|	897228-897413	17	9.4
ACYPI087089-RA	ni|645904130|nb|KK920303.1|	778273-778986	18	17
ACYPI001474-RA	ni|645903795|nb|KK920635.1|	417028-418190	14	16
ACYPI002471-RA	ni|645904107|nb|KK920324.1|	685216-685440	17	9.5
ACYPI003359-RA	ni|645904045|nb|KK920386.1|	1350246-1351270	19	21
ACYPI003778-RA	ni|645903899|nb|KK920532.1|	378523-378730	15	16
ACYPI009686-RA	ni|645903962|nb|KK920469.1|	68256-68614	17	9.8
ACYPI001309-RA	ni|645904174|nb|KK920261.1|	1533967-1534548	19	17
ACYPI005116-RA	ni|645903787|nb|KK920643.1|	1199864-1200269	19	18
ACYPI51262-RA	ni|645904116|nb|KK920315.1|	1033913-1034077	19	18
ACYPI000592-RA	ni|645904246|nb|KK920233.1|	1091833-1092019	16	17
ACYPI001082-RA	ni|645896565|nb|KK927864.1|	3666-3907	nan	nan
ACYPI002162-RA	ni|645903491|nb|KK920939.1|	597383-597625	nan	nan
ACYPI002617-RA	ni|645903688|nb|KK920742.1|	1109903-1110303	nan	nan
ACYPI001704-RA	ni|645904271|nb|KK920224.1|	467005-467470	15	16
ACYPI005537-RA	ni|645903983|nb|KK920448.1|	241823-242393	18	17
ACYPI000626-RA	ni|645904155|nb|KK920278.1|	283747-283984	18	9.4
ACYPI001870-RA	ni|645904064|nb|KK920367.1|	544253-544556	17	9.9
ACYPI006763-RA	ni|645903789|nb|KK920641.1|	720826-721147	17	17
ACYPI007594-RA	ni|645904112|nb|KK920319.1|	2274504-2275518	18	17
ACYPI008203-RA	ni|645904144|nb|KK920289.1|	1052544-1052950	16	9
ACYPI009489-RA	ni|645903938|nb|KK920493.1|	1187032-1187400	19	18
ACYPI010132-RA	ni|645903863|nb|KK920567.1|	8773-9010	15	9.2
ACYPI002333-RA	ni|645902420|nb|KK922009.1|	93122-93831	nan	nan
ACYPI002477-RA	ni|645904118|nb|KK920313.1|	1620303-1621083	17	17
ACYPI004216-RA	ni|645904153|nb|KK920280.1|	788784-789127	17	16
ACYPI002140-RA	ni|645902758|nb|KK921671.1|	229334-229536	nan	nan

ACYPI004015-RA	ni|645902758|nb|KK921671.1|	201981-202814	nan	nan
ACYPI005189-RA	ni|645902758|nb|KK921671.1|	360126-360387	nan	nan
ACYPI006245-RA	ni|645904125|nb|KK920307.1|	2064819-2065058	18	17
ACYPI010179-RA	ni|645904116|nb|KK920315.1|	2067854-2068338	19	18
ACYPI003214-RA	ni|645903773|nb|KK920657.1|	1120081-1120789	18	17
ACYPI003413-RA	ni|645903817|nb|KK920613.1|	267579-267977	13	16
ACYPI56610-RA	ni|645897009|nb|KK927420.1|	980-1583	nan	nan
ACYPI001274-RA	ni|645903824|nb|KK920606.1|	54539-55657	19	17
ACYPI003160-RA	ni|645903655|nb|KK920775.1|	221657-222485	nan	nan
ACYPI009681-RA	ni|645903699|nb|KK920731.1|	171041-171293	nan	nan
ACYPI002289-RA	ni|645903566|nb|KK920864.1|	668231-668439	nan	nan
ACYPI004170-RA	ni|645902078|nb|KK922351.1|	57080-57324	nan	nan
ACYPI007346-RA	ni|645903961|nb|KK920470.1|	75335-75999	17	15
ACYPI007748-RA	ni|645904120|nb|KK920311.1|	757724-758236	18	18
ACYPI009639-RA	ni|645904077|nb|KK920354.1|	158206-159024	16	17
ACYPI31510-RA	ni|645903612|nb|KK920818.1|	127967-128460	nan	nan
ACYPI001886-RA	ni|645904086|nb|KK920345.1|	374776-374980	14	15
ACYPI002952-RA	ni|645904008|nb|KK920423.1|	1214544-1215178	16	9.5
ACYPI073693-RA	ni|645903728|nb|KK920702.1|	1089584-1090311	nan	nan
ACYPI002045-RA	ni|645904228|nb|KK920243.1|	1269442-1270971	17	18
ACYPI004034-RA	ni|645903544|nb|KK920886.1|	311834-312444	nan	nan
ACYPI000895-RA	ni|645904160|nb|KK920273.1|	1790699-1790875	18	18
ACYPI004714-RA	ni|645903773|nb|KK920657.1|	235958-236214	18	17
ACYPI006615-RA	ni|645904160|nb|KK920273.1|	1791608-1791910	18	18
ACYPI065331-RA	ni|645904156|nb|KK920277.1|	248842-249374	17	8.8
ACYPI002888-RA	ni|645903602|nb|KK920828.1|	640054-640255	nan	nan
ACYPI001198-RA	ni|645904022|nb|KK920409.1|	18476-18737	18	20
ACYPI001832-RA	ni|645903716|nb|KK920714.1|	373150-373390	nan	nan
ACYPI008969-RA	ni|645903773|nb|KK920657.1|	780212-780486	18	17
ACYPI002483-RA	ni|645904225|nb|KK920244.1|	647324-648019	17	17

ACYPI010153-RA	ni|645903491|nb|KK920939.1|	606494-606927	nan	nan
ACYPI000025-RA	ni|645903855|nb|KK920575.1|	1041320-1041814	16	17
ACYPI000365-RA	ni|645904112|nb|KK920319.1|	2214714-2215473	18	17
ACYPI001129-RA	ni|645904159|nb|KK920274.1|	240197-240382	15	17
ACYPI002062-RA	ni|645903611|nb|KK920819.1|	79801-80120	nan	nan
ACYPI004145-RA	ni|645903751|nb|KK920679.1|	659121-659902	17	16
ACYPI006740-RA	ni|645903751|nb|KK920679.1|	759317-760058	17	16
ACYPI007358-RA	ni|645903751|nb|KK920679.1|	742570-743052	17	16
ACYPI008225-RA	ni|645903617|nb|KK920813.1|	496192-496801	nan	nan
ACYPI009061-RA	ni|645903467|nb|KK920963.1|	102154-103009	nan	nan
ACYPI002517-RA	ni|645903774|nb|KK920656.1|	460176-460352	16	9.2
ACYPI004419-RA	ni|645904192|nb|KK920255.1|	410184-410972	15	16
ACYPI000099-RA	ni|645903870|nb|KK920560.1|	235406-236741	14	23
ACYPI002039-RA	ni|645903663|nb|KK920767.1|	239325-239480	nan	nan
ACYPI004927-RA	ni|645904001|nb|KK920430.1|	76550-77191	17	15
ACYPI005271-RA	ni|645903771|nb|KK920659.1|	28378-28555	16	15
ACYPI006518-RA	ni|645903979|nb|KK920452.1|	693623-694953	15	17
ACYPI072184-RA	ni|645903679|nb|KK920751.1|	63150-63420	nan	nan
ACYPI001742-RA	ni|645903698|nb|KK920732.1|	158467-158767	nan	nan
ACYPI004480-RA	ni|645903466|nb|KK920964.1|	57258-57432	nan	nan
ACYPI006852-RA	ni|645903466|nb|KK920964.1|	533802-533989	nan	nan
ACYPI008278-RA	ni|645904116|nb|KK920315.1|	1559610-1559981	19	18
ACYPI009960-RA	ni|645903823|nb|KK920607.1|	99711-100073	16	17
ACYPI000320-RA	ni|645903768|nb|KK920662.1|	1159763-1159985	18	17
ACYPI000252-RA	ni|645904030|nb|KK920401.1|	406005-408372	14	15
ACYPI003751-RA	ni|645901760|nb|KK922669.1|	114170-114664	nan	nan
ACYPI004712-RA	ni|645904142|nb|KK920291.1|	1498568-1500425	20	23
ACYPI001601-RA	ni|645902659|nb|KK921770.1|	163553-163945	nan	nan
ACYPI001835-RA	ni|645904064|nb|KK920367.1|	159366-159610	17	9.9
ACYPI007932-RA	ni|645904137|nb|KK920296.1|	1275581-1276237	17	17

ACYPI009806-RA	ni|645903829|nb|KK920601.1|	629199-629807	16	15
ACYPI001416-RA	ni|645903762|nb|KK920668.1|	130978-131264	17	17
ACYPI005899-RA	ni|645903635|nb|KK920795.1|	289480-289973	nan	nan
ACYPI000096-RA	ni|645904096|nb|KK920335.1|	177494-177915	18	17
ACYPI001850-RA	ni|645903921|nb|KK920510.1|	579588-579948	16	11
ACYPI006170-RA	ni|645904114|nb|KK920317.1|	1707595-1708268	18	18
ACYPI006965-RA	ni|645904117|nb|KK920314.1|	209720-209948	15	15
ACYPI001760-RA	ni|645904279|nb|KK920221.1|	303514-303727	16	16
ACYPI002754-RA	ni|645904119|nb|KK920312.1|	1546830-1550831	19	19
ACYPI003632-RA	ni|645902693|nb|KK921736.1|	119626-120102	nan	nan
ACYPI009334-RA	ni|645904119|nb|KK920312.1|	1987572-1987965	19	19
ACYPI003885-RA	ni|645903731|nb|KK920699.1|	680621-681065	nan	nan
ACYPI008166-RA	ni|645903821|nb|KK920609.1|	1054354-1054855	17	17
ACYPI008605-RA	ni|645903597|nb|KK920833.1|	369926-370350	nan	nan
ACYPI002662-RA	ni|645903774|nb|KK920656.1|	526446-526707	16	9.2
ACYPI001667-RA	ni|645904014|nb|KK920417.1|	1330327-1330589	19	18
ACYPI002426-RA	ni|645902373|nb|KK922056.1|	156186-156548	nan	nan
ACYPI002841-RA	ni|645904014|nb|KK920417.1|	1267411-1267664	19	18
ACYPI004063-RA	ni|645903635|nb|KK920795.1|	528552-529148	nan	nan
ACYPI006008-RA	ni|645903655|nb|KK920775.1|	22059-22780	nan	nan
ACYPI006262-RA	ni|645903728|nb|KK920702.1|	633118-633588	nan	nan
ACYPI009758-RA	ni|645904207|nb|KK920250.1|	562802-563314	18	20
ACYPI005219-RA	ni|645904139|nb|KK920294.1|	385140-388914	15	18
ACYPI006453-RA	ni|645904133|nb|KK920300.1|	97695-98050	18	18
ACYPI006823-RA	ni|645904010|nb|KK920421.1|	1030657-1031841	17	17
ACYPI068502-RA	ni|645903836|nb|KK920594.1|	139219-139673	18	11
ACYPI000564-RA	ni|645904029|nb|KK920402.1|	244073-245021	14	15
ACYPI001210-RA	ni|645903543|nb|KK920887.1|	553406-553699	nan	nan
ACYPI005607-RA	ni|645904027|nb|KK920404.1|	598107-599723	17	17
ACYPI008781-RA	ni|645904079|nb|KK920352.1|	110690-111591	15	16

ACYPI064034-RA	ni|645903935|nb|KK920496.1|	963918-964772	17	18
ACYPI000739-RA	ni|645903969|nb|KK920462.1|	418285-418808	14	16
ACYPI002084-RA	ni|645902715|nb|KK921714.1|	154324-154501	nan	nan
ACYPI003341-RA	ni|645903752|nb|KK920678.1|	1300203-1300430	18	16
ACYPI003960-RA	ni|645903858|nb|KK920572.1|	446872-447291	16	16
ACYPI005207-RA	ni|645904022|nb|KK920409.1|	209261-209846	18	20
ACYPI006229-RA	ni|645904279|nb|KK920221.1|	2631897-2632633	16	16
ACYPI007703-RA	ni|645903627|nb|KK920803.1|	82985-83485	nan	nan
ACYPI40062-RA	ni|645903762|nb|KK920668.1|	840971-841138	17	17
ACYPI50391-RA	ni|645898156|nb|KK926273.1|	6080-6266	nan	nan
ACYPI000329-RA	ni|645903754|nb|KK920676.1|	99757-100698	14	8.8
ACYPI001057-RA	ni|645903644|nb|KK920786.1|	188924-189137	nan	nan
ACYPI002306-RA	ni|645904058|nb|KK920373.1|	614672-615628	16	15
ACYPI002895-RA	ni|645903567|nb|KK920863.1|	14677-14843	nan	nan
ACYPI004185-RA	ni|645904045|nb|KK920386.1|	817728-819194	19	21
ACYPI005535-RA	ni|645904058|nb|KK920373.1|	336435-337268	16	15
ACYPI002835-RA	ni|645904265|nb|KK920226.1|	233761-233968	14	14
ACYPI072241-RA	ni|645902354|nb|KK922075.1|	112303-112676	nan	nan
ACYPI085401-RA	ni|645901587|nb|KK922842.1|	124-323	nan	nan
ACYPI004514-RA	ni|645901817|nb|KK922612.1|	72837-73613	nan	nan
ACYPI063189-RA	ni|645904133|nb|KK920300.1|	2162114-2162975	18	18
ACYPI000143-RA	ni|645904123|nb|KK920308.1|	633353-633523	16	10
ACYPI000149-RA	ni|645903979|nb|KK920452.1|	532494-536473	15	17
ACYPI001423-RA	ni|645903846|nb|KK920584.1|	736311-736504	17	10
ACYPI001462-RA	ni|645904159|nb|KK920274.1|	1051715-1052406	15	17
ACYPI003349-RA	ni|645904141|nb|KK920292.1|	313936-314558	14	14
ACYPI003961-RA	ni|645904083|nb|KK920348.1|	851600-852307	15	16
ACYPI005282-RA	ni|645901710|nb|KK922719.1|	73895-74944	nan	nan
ACYPI007166-RA	ni|645903680|nb|KK920750.1|	656416-657225	nan	nan
ACYPI009634-RA	ni|645904118|nb|KK920313.1|	320004-320166	17	17

ACYPI009679-RA	ni|645904053|nb|KK920378.1|	174667-174980	18	17
ACYPI080661-RA	ni|645903697|nb|KK920733.1|	56927-57419	nan	nan
ACYPI007014-RA	ni|645903650|nb|KK920780.1|	162023-163087	nan	nan
ACYPI008637-RA	ni|645904231|nb|KK920242.1|	1112045-1113932	16	17
ACYPI003387-RA	ni|645903545|nb|KK920885.1|	249192-249445	nan	nan
ACYPI004696-RA	ni|645903727|nb|KK920703.1|	338427-338812	nan	nan
ACYPI006602-RA	ni|645904192|nb|KK920255.1|	45063-46373	15	16
ACYPI24233-RA	ni|645903939|nb|KK920492.1|	162509-162691	15	16
ACYPI24234-RA	ni|645903688|nb|KK920742.1|	193095-193674	nan	nan
ACYPI003530-RA	ni|645904201|nb|KK920252.1|	290873-291099	17	16
ACYPI005478-RA	ni|645904201|nb|KK920252.1|	334053-334539	17	16
ACYPI002088-RA	ni|645904219|nb|KK920246.1|	342533-342770	16	17
ACYPI002656-RA	ni|645903538|nb|KK920892.1|	54690-55240	nan	nan
ACYPI005300-RA	ni|645903855|nb|KK920575.1|	1046028-1046940	16	17
ACYPI008930-RA	ni|645903987|nb|KK920444.1|	795163-797652	15	17
ACYPI52843-RA	ni|645903896|nb|KK920535.1|	459081-460504	16	9.1
ACYPI000304-RA	ni|645904106|nb|KK920325.1|	996965-998163	17	17
ACYPI000589-RA	ni|645904177|nb|KK920260.1|	1584120-1584841	18	17
ACYPI001460-RA	ni|645902696|nb|KK921733.1|	5333-5930	nan	nan
ACYPI002470-RA	ni|645903879|nb|KK920552.1|	1708908-1709530	18	18
ACYPI002859-RA	ni|645904271|nb|KK920224.1|	4150386-4150594	15	16
ACYPI003749-RA	ni|645904241|nb|KK920238.1|	2712552-2712842	18	17
ACYPI060717-RA	ni|645904088|nb|KK920343.1|	541953-545536	17	18
ACYPI080022-RA	ni|645902696|nb|KK921733.1|	7333-7769	nan	nan
ACYPI27999-RA	ni|645903936|nb|KK920495.1|	674426-674742	19	18
ACYPI006333-RA	ni|645904013|nb|KK920418.1|	1093625-1094463	19	18
ACYPI006577-RA	ni|645903869|nb|KK920561.1|	669371-669557	17	15
ACYPI007899-RA	ni|645904039|nb|KK920392.1|	218176-219136	15	16
ACYPI008877-RA	ni|645903869|nb|KK920561.1|	462134-462578	17	15
ACYPI009769-RA	ni|645903690|nb|KK920740.1|	203871-204642	nan	nan

ACYPI010095-RA	ni|645903977|nb|KK920454.1|	32080-32542	13	16
ACYPI002857-RA	ni|645903901|nb|KK920530.1|	479419-480429	17	17
ACYPI002950-RA	ni|645903773|nb|KK920657.1|	1078881-1079877	18	17
ACYPI004328-RA	ni|645904137|nb|KK920296.1|	1649706-1651088	17	17
ACYPI004979-RA	ni|645903534|nb|KK920896.1|	618842-619558	nan	nan
ACYPI007706-RA	ni|645903599|nb|KK920831.1|	114256-114454	nan	nan
ACYPI007769-RA	ni|645903534|nb|KK920896.1|	623885-624405	nan	nan
ACYPI009635-RA	ni|645904231|nb|KK920242.1|	280597-282308	16	17
ACYPI083537-RA	ni|645900840|nb|KK923589.1|	1025-1494	nan	nan
ACYPI010131-RA	ni|645904072|nb|KK920359.1|	421302-421700	17	17
ACYPI006270-RA	ni|645904153|nb|KK920280.1|	1738822-1740379	17	16
ACYPI009787-RA	ni|645903879|nb|KK920552.1|	1495229-1495809	18	18
ACYPI071107-RA	ni|645904153|nb|KK920280.1|	1821357-1822580	17	16
ACYPI009851-RA	ni|645904231|nb|KK920242.1|	388105-388739	16	17
ACYPI002147-RA	ni|645903613|nb|KK920817.1|	489615-490018	nan	nan
ACYPI002982-RA	ni|645904078|nb|KK920353.1|	1136941-1137323	17	17
ACYPI005204-RA	ni|645903592|nb|KK920838.1|	964818-965024	nan	nan
ACYPI060618-RA	ni|645903662|nb|KK920768.1|	259026-260190	nan	nan
ACYPI50297-RA	ni|645904152|nb|KK920281.1|	1072209-1072521	15	15
ACYPI000521-RA	ni|645903713|nb|KK920717.1|	446097-447247	nan	nan
ACYPI001086-RA	ni|645903634|nb|KK920796.1|	990855-991032	nan	nan
ACYPI002256-RA	ni|645903837|nb|KK920593.1|	390502-390731	18	9.8
ACYPI002404-RA	ni|645903597|nb|KK920833.1|	755945-756646	nan	nan
ACYPI002985-RA	ni|645904028|nb|KK920403.1|	1868123-1870213	18	17
ACYPI003133-RA	ni|645903749|nb|KK920681.1|	353834-354568	19	18
ACYPI004304-RA	ni|645903634|nb|KK920796.1|	987148-987910	nan	nan
ACYPI004377-RA	ni|645902706|nb|KK921723.1|	51035-51369	nan	nan
ACYPI005144-RA	ni|645904144|nb|KK920289.1|	673521-674551	16	9
ACYPI006881-RA	ni|645903491|nb|KK920939.1|	632013-632259	nan	nan
ACYPI006949-RA	ni|645903742|nb|KK920688.1|	522985-524102	17	16

ACYPI007422-RA	ni|645904155|nb|KK920278.1|	919335-919641	18	9.4
ACYPI061529-RA	ni|645903938|nb|KK920493.1|	720240-720787	19	18
ACYPI000104-RA	ni|645903915|nb|KK920516.1|	207249-208913	17	17
ACYPI22867-RA	ni|645903734|nb|KK920696.1|	708677-709201	nan	nan
ACYPI53200-RA	ni|645904135|nb|KK920298.1|	350799-351591	14	15
ACYPI008019-RA	ni|645904166|nb|KK920267.1|	2788074-2788701	18	16
ACYPI000430-RA	ni|645903743|nb|KK920687.1|	896515-897013	19	18
ACYPI001075-RA	ni|645900111|nb|KK924318.1|	2404-2587	nan	nan
ACYPI001193-RA	ni|645904028|nb|KK920403.1|	502305-502707	18	17
ACYPI002976-RA	ni|645903879|nb|KK920552.1|	257231-257595	18	18
ACYPI006797-RA	ni|645901733|nb|KK922696.1|	223835-227076	nan	nan
ACYPI007330-RA	ni|645902723|nb|KK921706.1|	142630-143014	nan	nan
ACYPI003117-RA	ni|645903639|nb|KK920791.1|	306704-308756	nan	nan
ACYPI004368-RA	ni|645903921|nb|KK920510.1|	428521-428770	16	11
ACYPI005195-RA	ni|645904201|nb|KK920252.1|	469930-471385	17	16
ACYPI005378-RA	ni|645903800|nb|KK920630.1|	1617453-1618121	18	17
ACYPI006767-RA	ni|645903984|nb|KK920447.1|	213395-214666	18	17
ACYPI007250-RA	ni|645904157|nb|KK920276.1|	630398-631174	15	15
ACYPI007433-RA	ni|645904068|nb|KK920363.1|	1109941-1110970	16	8.6
ACYPI009536-RA	ni|645904148|nb|KK920285.1|	1274335-1276011	16	16
ACYPI48345-RA	ni|645903981|nb|KK920450.1|	850506-850767	16	8.6
ACYPI56660-RA	ni|645901937|nb|KK922492.1|	4406-4714	nan	nan
ACYPI000035-RA	ni|645903788|nb|KK920642.1|	730081-730597	16	15
ACYPI001539-RA	ni|645904090|nb|KK920341.1|	254208-255472	15	17
ACYPI004043-RA	ni|645904136|nb|KK920297.1|	21127-21878	16	17
ACYPI004701-RA	ni|645904166|nb|KK920267.1|	2675049-2676056	18	16
ACYPI005987-RA	ni|645904111|nb|KK920320.1|	1519708-1520820	17	17
ACYPI007232-RA	ni|645904125|nb|KK920307.1|	618395-618993	18	17
ACYPI000298-RA	ni|645903931|nb|KK920500.1|	567663-571406	16	18
ACYPI004774-RA	ni|645903752|nb|KK920678.1|	90954-91173	18	16

ACYPI008539-RA	ni|645903847|nb|KK920583.1|	825801-825996	18	18
ACYPI001593-RA	ni|645904153|nb|KK920280.1|	1495836-1496308	17	16
ACYPI005775-RA	ni|645904088|nb|KK920343.1|	1595763-1596067	17	18
ACYPI006129-RA	ni|645904007|nb|KK920424.1|	214486-214810	18	17
ACYPI009257-RA	ni|645904207|nb|KK920250.1|	545992-547422	18	20
ACYPI010103-RA	ni|645903778|nb|KK920652.1|	238027-238475	12	13
ACYPI072994-RA	ni|645903860|nb|KK920570.1|	348064-348497	17	17
ACYPI000467-RA	ni|645904093|nb|KK920338.1|	23707-24122	13	15
ACYPI001501-RA	ni|645903562|nb|KK920868.1|	392843-393532	nan	nan
ACYPI001605-RA	ni|645896512|nb|KK927917.1|	992-1347	nan	nan
ACYPI002057-RA	ni|645904133|nb|KK920300.1|	2564518-2565376	18	18
ACYPI005434-RA	ni|645903851|nb|KK920579.1|	246228-247288	14	19
ACYPI008701-RA	ni|645904015|nb|KK920416.1|	1217734-1218076	18	16
ACYPI009090-RA	ni|645903669|nb|KK920761.1|	25222-26286	nan	nan
ACYPI088146-RA	ni|645903814|nb|KK920616.1|	453469-453922	17	16
ACYPI005666-RA	ni|645903616|nb|KK920814.1|	475113-475281	nan	nan
ACYPI062442-RA	ni|645904160|nb|KK920273.1|	2363982-2364169	18	18
ACYPI068391-RA	ni|645902439|nb|KK921990.1|	7385-7731	nan	nan
ACYPI001652-RA	ni|645904158|nb|KK920275.1|	1166500-1166931	15	8.3
ACYPI009439-RA	ni|645903925|nb|KK920506.1|	596336-597133	19	17
ACYPI000866-RA	ni|645903744|nb|KK920686.1|	718814-719189	17	8.6
ACYPI003225-RA	ni|645904070|nb|KK920361.1|	165378-165549	17	16
ACYPI004634-RA	ni|645903868|nb|KK920562.1|	441720-441967	16	8.7
ACYPI009065-RA	ni|645902755|nb|KK921674.1|	73065-77441	nan	nan
ACYPI009098-RA	ni|645902673|nb|KK921756.1|	13452-13943	nan	nan
ACYPI070418-RA	ni|645903592|nb|KK920838.1|	877950-878893	nan	nan
ACYPI000500-RA	ni|645903898|nb|KK920533.1|	483988-484252	17	16
ACYPI000532-RA	ni|645904077|nb|KK920354.1|	307038-307495	16	17
ACYPI003672-RA	ni|645903879|nb|KK920552.1|	1080299-1081130	18	18
ACYPI004282-RA	ni|645903953|nb|KK920478.1|	1626516-1626760	18	17

ACYPI006213-RA	ni|645903747|nb|KK920683.1|	973165-974284	19	18
ACYPI083213-RA	ni|645903736|nb|KK920694.1|	305120-305513	18	17
ACYPI000876-RA	ni|645904019|nb|KK920412.1|	181000-181322	19	9.4
ACYPI005350-RA	ni|645902772|nb|KK921657.1|	258437-260296	nan	nan
ACYPI007373-RA	ni|645903901|nb|KK920530.1|	1076969-1077983	17	17
ACYPI070323-RA	ni|645903952|nb|KK920479.1|	45914-46164	16	16
ACYPI45536-RA	ni|645904195|nb|KK920254.1|	2351366-2351531	18	17
ACYPI000257-RA	ni|645904112|nb|KK920319.1|	1677836-1678586	18	17
ACYPI001490-RA	ni|645903822|nb|KK920608.1|	144790-145265	18	20
ACYPI003333-RA	ni|645904106|nb|KK920325.1|	1022587-1022793	17	17
ACYPI004031-RA	ni|645901982|nb|KK922447.1|	193727-194176	nan	nan
ACYPI004733-RA	ni|645903822|nb|KK920608.1|	267288-267538	18	20
ACYPI005313-RA	ni|645904114|nb|KK920317.1|	1910124-1910376	18	18
ACYPI008493-RA	ni|645904174|nb|KK920261.1|	361095-361770	19	17
ACYPI000944-RA	ni|645903919|nb|KK920512.1|	1245222-1245590	17	15
ACYPI002533-RA	ni|645903512|nb|KK920918.1|	419909-420112	nan	nan
ACYPI002840-RA	ni|645903661|nb|KK920769.1|	281469-281921	nan	nan
ACYPI001189-RA	ni|645904038|nb|KK920393.1|	489586-489742	17	19
ACYPI001816-RA	ni|645903965|nb|KK920466.1|	263403-264022	17	16
ACYPI003232-RA	ni|645901733|nb|KK922696.1|	67070-67542	nan	nan
ACYPI007653-RA	ni|645903936|nb|KK920495.1|	972501-972783	19	18
ACYPI008002-RA	ni|645903607|nb|KK920823.1|	96658-96897	nan	nan
ACYPI009407-RA	ni|645903938|nb|KK920493.1|	545332-545510	19	18
ACYPI009554-RA	ni|645903843|nb|KK920587.1|	419452-419901	18	16
ACYPI000938-RA	ni|645904168|nb|KK920265.1|	1905788-1906395	16	16
ACYPI004755-RA	ni|645904112|nb|KK920319.1|	221742-221981	18	17
ACYPI005419-RA	ni|645903873|nb|KK920558.1|	662728-662833	16	15
ACYPI009174-RA	ni|645903919|nb|KK920512.1|	791168-791782	17	15
ACYPI084854-RA	ni|645903876|nb|KK920555.1|	294777-295109	14	15
ACYPI001106-RA	ni|645903504|nb|KK920926.1|	274425-274907	nan	nan

ACYPI004245-RA	ni|645904120|nb|KK920311.1|	731310-731957	18	18
ACYPI005925-RA	ni|645903696|nb|KK920734.1|	407629-408730	nan	nan
ACYPI005955-RA	ni|645904086|nb|KK920345.1|	418009-418377	14	15
ACYPI006829-RA	ni|645904120|nb|KK920311.1|	1031428-1031721	18	18
ACYPI060630-RA	ni|645903773|nb|KK920657.1|	1142933-1143509	18	17
ACYPI003478-RA	ni|645904013|nb|KK920418.1|	1265675-1265965	19	18
ACYPI005418-RA	ni|645902503|nb|KK921926.1|	132663-133214	nan	nan
ACYPI007287-RA	ni|645901591|nb|KK922838.1|	43492-43825	nan	nan
ACYPI52551-RA	ni|645902511|nb|KK921918.1|	108350-109032	nan	nan
ACYPI001483-RA	ni|645904115|nb|KK920316.1|	1772375-1773141	17	18
ACYPI002558-RA	ni|645903973|nb|KK920458.1|	1403221-1403443	20	18
ACYPI003568-RA	ni|645903727|nb|KK920703.1|	1085041-1085523	nan	nan
ACYPI004460-RA	ni|645904231|nb|KK920242.1|	388995-389151	16	17
ACYPI006375-RA	ni|645903929|nb|KK920502.1|	1431575-1432721	17	18
ACYPI007801-RA	ni|645903973|nb|KK920458.1|	1433224-1433756	20	18
ACYPI21355-RA	ni|645902771|nb|KK921658.1|	152022-152876	nan	nan
ACYPI55712-RA	ni|645903505|nb|KK920925.1|	98540-99214	nan	nan
ACYPI56745-RA	ni|645902694|nb|KK921735.1|	178952-179243	nan	nan
ACYPI007692-RA	ni|645903773|nb|KK920657.1|	417116-417575	18	17
ACYPI000295-RA	ni|645903717|nb|KK920713.1|	67347-67736	nan	nan
ACYPI002204-RA	ni|645903717|nb|KK920713.1|	42725-43374	nan	nan
ACYPI002237-RA	ni|645901808|nb|KK922621.1|	169276-169783	nan	nan
ACYPI005389-RA	ni|645904204|nb|KK920251.1|	782045-786457	15	16
ACYPI006714-RA	ni|645904152|nb|KK920281.1|	338801-339291	15	15
ACYPI067416-RA	ni|645903496|nb|KK920934.1|	915259-916095	nan	nan
ACYPI54756-RA	ni|645904137|nb|KK920296.1|	2405535-2405907	17	17
ACYPI005908-RA	ni|645903662|nb|KK920768.1|	210882-211773	nan	nan
ACYPI008444-RA	ni|645903779|nb|KK920651.1|	428101-428367	18	16
ACYPI010244-RA	ni|645904035|nb|KK920396.1|	684433-684719	16	16
ACYPI073204-RA	ni|645903768|nb|KK920662.1|	1058058-1058653	18	17

ACYPI084620-RA	ni|645903580|nb|KK920850.1|	180239-180662	nan	nan
ACYPI41460-RA	ni|645902699|nb|KK921730.1|	224995-225145	nan	nan
ACYPI005364-RA	ni|645903574|nb|KK920856.1|	95005-95530	nan	nan
ACYPI23394-RA	ni|645903887|nb|KK920544.1|	84001-85004	15	15
ACYPI001085-RA	ni|645904118|nb|KK920313.1|	733371-734277	17	17
ACYPI003577-RA	ni|645901994|nb|KK922435.1|	64579-64831	nan	nan
ACYPI004617-RA	ni|645903957|nb|KK920474.1|	976318-976658	18	17
ACYPI009049-RA	ni|645904061|nb|KK920370.1|	423445-423651	17	16
ACYPI073829-RA	ni|645904274|nb|KK920223.1|	622623-622814	14	17
ACYPI000227-RA	ni|645903988|nb|KK920443.1|	96980-98231	14	16
ACYPI001125-RA	ni|645901914|nb|KK922515.1|	54583-55440	nan	nan
ACYPI001932-RA	ni|645903591|nb|KK920839.1|	164055-165291	nan	nan
ACYPI005359-RA	ni|645903988|nb|KK920443.1|	505487-506255	14	16
ACYPI006584-RA	ni|645903773|nb|KK920657.1|	1196725-1197140	18	17
ACYPI001717-RA	ni|645904228|nb|KK920243.1|	710294-712388	17	18
ACYPI001756-RA	ni|645902394|nb|KK922035.1|	300175-301057	nan	nan
ACYPI007388-RA	ni|645903874|nb|KK920557.1|	512753-513426	18	18
ACYPI008275-RA	ni|645904274|nb|KK920223.1|	603859-604286	14	17
ACYPI009095-RA	ni|645903915|nb|KK920516.1|	1295916-1296209	17	17
ACYPI010151-RA	ni|645903929|nb|KK920502.1|	1319426-1320004	17	18
ACYPI000782-RA	ni|645902172|nb|KK922257.1|	29244-29389	nan	nan
ACYPI001813-RA	ni|645902756|nb|KK921673.1|	36146-36726	nan	nan
ACYPI001852-RA	ni|645904279|nb|KK920221.1|	1518632-1519343	16	16
ACYPI002118-RA	ni|645904076|nb|KK920355.1|	456636-456861	17	17
ACYPI002673-RA	ni|645903705|nb|KK920725.1|	449863-450660	nan	nan
ACYPI007139-RA	ni|645903971|nb|KK920460.1|	61534-62359	19	18
ACYPI008190-RA	ni|645901741|nb|KK922688.1|	52845-53508	nan	nan
ACYPI009014-RA	ni|645903734|nb|KK920696.1|	415403-415597	nan	nan
ACYPI001764-RA	ni|645903969|nb|KK920462.1|	266128-266480	14	16
ACYPI001777-RA	ni|645903650|nb|KK920780.1|	174435-174913	nan	nan

ACYPI003651-RA	ni|645902449|nb|KK921980.1|	100182-100441	nan	nan
ACYPI006221-RA	ni|645903820|nb|KK920610.1|	838691-841384	18	19
ACYPI008100-RA	ni|645902438|nb|KK921991.1|	157788-158064	nan	nan
ACYPI008606-RA	ni|645903532|nb|KK920898.1|	880246-880776	nan	nan
ACYPI009993-RA	ni|645903650|nb|KK920780.1|	341364-342209	nan	nan
ACYPI001635-RA	ni|645903970|nb|KK920461.1|	877939-879028	17	16
ACYPI008409-RA	ni|645904177|nb|KK920260.1|	1203793-1204261	18	17
ACYPI003210-RA	ni|645903759|nb|KK920671.1|	82411-82980	18	17
ACYPI005735-RA	ni|645903973|nb|KK920458.1|	1312921-1313506	20	18
ACYPI007036-RA	ni|645904168|nb|KK920265.1|	1885333-1887515	16	16
ACYPI007453-RA	ni|645902779|nb|KK921650.1|	36038-37050	nan	nan
ACYPI007611-RA	ni|645904222|nb|KK920245.1|	621357-622768	17	12
ACYPI081969-RA	ni|645902779|nb|KK921650.1|	30569-30788	nan	nan
ACYPI006807-RA	ni|645903780|nb|KK920650.1|	979338-980184	19	19
ACYPI007960-RA	ni|645904079|nb|KK920352.1|	313224-313632	15	16
ACYPI080807-RA	ni|645904262|nb|KK920227.1|	503562-504087	17	17
ACYPI38602-RA	ni|645904262|nb|KK920227.1|	710264-710776	17	17
ACYPI53596-RA	ni|645904114|nb|KK920317.1|	1751645-1751899	18	18
ACYPI004037-RA	ni|645903984|nb|KK920447.1|	764452-764682	18	17
ACYPI006164-RA	ni|645903768|nb|KK920662.1|	829449-831659	18	17
ACYPI50514-RA	ni|645904133|nb|KK920300.1|	1623860-1624336	18	18
ACYPI002475-RA	ni|645904136|nb|KK920297.1|	2012897-2013262	16	17
ACYPI006520-RA	ni|645903545|nb|KK920885.1|	239477-239988	nan	nan
ACYPI007301-RA	ni|645904065|nb|KK920366.1|	10037-10282	18	18
ACYPI007798-RA	ni|645902344|nb|KK922085.1|	187154-187280	nan	nan
ACYPI001846-RA	ni|645903853|nb|KK920577.1|	677892-678142	16	16
ACYPI002092-RA	ni|645903780|nb|KK920650.1|	175459-177094	19	19
ACYPI003010-RA	ni|645902664|nb|KK921765.1|	230670-231927	nan	nan
ACYPI003059-RA	ni|645903925|nb|KK920506.1|	706742-707595	19	17
ACYPI004974-RA	ni|645904132|nb|KK920301.1|	478994-479499	16	18

ACYPI005024-RA	ni|645903946|nb|KK920485.1|	417847-418050	16	14
ACYPI006498-RA	ni|645897725|nb|KK926704.1|	3473-3733	nan	nan
ACYPI008376-RA	ni|645904133|nb|KK920300.1|	2796953-2797695	18	18
ACYPI073612-RA	ni|645903840|nb|KK920590.1|	224391-225284	18	17
ACYPI083009-RA	ni|645903946|nb|KK920485.1|	568168-568417	16	14
ACYPI001730-RA	ni|645903885|nb|KK920546.1|	845631-846212	19	12
ACYPI005972-RA	ni|645903619|nb|KK920811.1|	600346-600751	nan	nan
ACYPI006200-RA	ni|645903855|nb|KK920575.1|	134274-134542	16	17
ACYPI006328-RA	ni|645903920|nb|KK920511.1|	934422-934617	20	20
ACYPI009568-RA	ni|645904231|nb|KK920242.1|	599775-600035	16	17
ACYPI000928-RA	ni|645903879|nb|KK920552.1|	1212873-1213647	18	18
ACYPI002255-RA	ni|645904015|nb|KK920416.1|	1231196-1233296	18	16
ACYPI007949-RA	ni|645904133|nb|KK920300.1|	3069084-3069244	18	18
ACYPI009162-RA	ni|645903984|nb|KK920447.1|	222678-222911	18	17
ACYPI001044-RA	ni|645903515|nb|KK920915.1|	733824-734944	nan	nan
ACYPI001902-RA	ni|645903610|nb|KK920820.1|	535344-535755	nan	nan
ACYPI003060-RA	ni|645901695|nb|KK922734.1|	10733-10961	nan	nan
ACYPI006871-RA	ni|645903858|nb|KK920572.1|	844912-845156	16	16
ACYPI009224-RA	ni|645898360|nb|KK926069.1|	8524-9062	nan	nan
ACYPI009633-RA	ni|645904010|nb|KK920421.1|	827123-827952	17	17
ACYPI000262-RA	ni|645903674|nb|KK920756.1|	567814-568257	nan	nan
ACYPI001914-RA	ni|645903511|nb|KK920919.1|	459064-459329	nan	nan
ACYPI002867-RA	ni|645903999|nb|KK920432.1|	506688-507466	17	15
ACYPI004787-RA	ni|645903983|nb|KK920448.1|	1061047-1061542	18	17
ACYPI006281-RA	ni|645904033|nb|KK920398.1|	154114-154355	14	16
ACYPI006639-RA	ni|645902538|nb|KK921891.1|	104472-105673	nan	nan
ACYPI008782-RA	ni|645903654|nb|KK920776.1|	631302-631455	nan	nan
ACYPI072178-RA	ni|645903496|nb|KK920934.1|	910043-910315	nan	nan
ACYPI000762-RA	ni|645903895|nb|KK920536.1|	945016-945176	17	16
ACYPI003318-RA	ni|645904150|nb|KK920283.1|	967770-968125	17	17

ACYPI004654-RA	ni|645903956|nb|KK920475.1|	801681-803612	20	20
ACYPI51366-RA	ni|645902664|nb|KK921765.1|	248185-248939	nan	nan
ACYPI52834-RA	ni|645903815|nb|KK920615.1|	276405-276656	19	17
ACYPI000071-RA	ni|645903843|nb|KK920587.1|	406702-406961	18	16
ACYPI003760-RA	ni|645902598|nb|KK921831.1|	93834-94461	nan	nan
ACYPI009460-RA	ni|645903688|nb|KK920742.1|	375787-375945	nan	nan
ACYPI000020-RA	ni|645904195|nb|KK920254.1|	171684-171968	18	17
ACYPI000633-RA	ni|645903918|nb|KK920513.1|	369839-370062	15	18
ACYPI002565-RA	ni|645904072|nb|KK920359.1|	1042977-1043231	17	17
ACYPI003625-RA	ni|645904160|nb|KK920273.1|	979490-979699	18	18
ACYPI003756-RA	ni|645903788|nb|KK920642.1|	534919-535323	16	15
ACYPI005614-RA	ni|645903491|nb|KK920939.1|	648313-649104	nan	nan
ACYPI007495-RA	ni|645904070|nb|KK920361.1|	183874-184096	17	16
ACYPI060544-RA	ni|645903491|nb|KK920939.1|	720202-720614	nan	nan
ACYPI004740-RA	ni|645903957|nb|KK920474.1|	1222746-1223539	18	17
ACYPI073759-RA	ni|645903866|nb|KK920564.1|	515014-515439	18	15
ACYPI000992-RA	ni|645904130|nb|KK920303.1|	737556-738080	18	17
ACYPI001609-RA	ni|645903788|nb|KK920642.1|	349959-350092	16	15
ACYPI001724-RA	ni|645903569|nb|KK920861.1|	219433-219752	nan	nan
ACYPI005391-RA	ni|645904177|nb|KK920260.1|	1495333-1496298	18	17
ACYPI005557-RA	ni|645903569|nb|KK920861.1|	255917-256907	nan	nan
ACYPI005641-RA	ni|645903506|nb|KK920924.1|	474615-474776	nan	nan
ACYPI003171-RA	ni|645904169|nb|KK920264.1|	645806-646818	16	9.5
ACYPI008211-RA	ni|645904268|nb|KK920225.1|	1076619-1077067	14	15
ACYPI008867-RA	ni|645900733|nb|KK923696.1|	11828-11969	nan	nan
ACYPI25794-RA	ni|645904241|nb|KK920238.1|	507167-507914	18	17
ACYPI064109-RA	ni|645904132|nb|KK920301.1|	454581-454781	16	18
ACYPI009196-RA	ni|645904010|nb|KK920421.1|	926770-928049	17	17
ACYPI000402-RA	ni|645904262|nb|KK920227.1|	1248071-1251182	17	17
ACYPI002296-RA	ni|645903944|nb|KK920487.1|	132787-133451	15	17

ACYPI003717-RA	ni|645904055|nb|KK920376.1|	655616-656570	17	17
ACYPI006306-RA	ni|645904027|nb|KK920404.1|	633549-634046	17	17
ACYPI010056-RA	ni|645904058|nb|KK920373.1|	313892-314368	16	15
ACYPI36103-RA	ni|645903572|nb|KK920858.1|	999029-999670	nan	nan
ACYPI46256-RA	ni|645903645|nb|KK920785.1|	220301-220528	nan	nan
ACYPI001975-RA	ni|645903854|nb|KK920576.1|	378827-379480	16	8.4
ACYPI009755-RA	ni|645904071|nb|KK920360.1|	709807-710539	17	17
ACYPI010201-RA	ni|645901835|nb|KK922594.1|	26833-27796	nan	nan
ACYPI072205-RA	ni|645904061|nb|KK920370.1|	789963-792181	17	16
ACYPI000758-RA	ni|645903895|nb|KK920536.1|	657557-660370	17	16
ACYPI002575-RA	ni|645904278|nb|KK920222.1|	1554186-1554452	15	16
ACYPI005173-RA	ni|645903811|nb|KK920619.1|	12571-13989	15	15
ACYPI006821-RA	ni|645903899|nb|KK920532.1|	279543-279817	15	16
ACYPI008362-RA	ni|645896832|nb|KK927597.1|	2087-2490	nan	nan
ACYPI008575-RA	ni|645903713|nb|KK920717.1|	399990-401389	nan	nan
ACYPI002955-RA	ni|645903798|nb|KK920632.1|	678375-678548	18	17
ACYPI004580-RA	ni|645904228|nb|KK920243.1|	1778676-1779205	17	18
ACYPI005014-RA	ni|645902350|nb|KK922079.1|	61214-61959	nan	nan
ACYPI005706-RA	ni|645904177|nb|KK920260.1|	2568717-2568910	18	17
ACYPI006125-RA	ni|645902441|nb|KK921988.1|	81551-81703	nan	nan
ACYPI006494-RA	ni|645904022|nb|KK920409.1|	663842-664394	18	20
ACYPI010118-RA	ni|645904262|nb|KK920227.1|	2612837-2613430	17	17
ACYPI35291-RA	ni|645903578|nb|KK920852.1|	262639-262841	nan	nan
ACYPI000717-RA	ni|645902035|nb|KK922394.1|	237948-239067	nan	nan
ACYPI000853-RA	ni|645903634|nb|KK920796.1|	618023-618626	nan	nan
ACYPI004035-RA	ni|645904132|nb|KK920301.1|	856026-856242	16	18
ACYPI004920-RA	ni|645904004|nb|KK920427.1|	814425-815530	16	16
ACYPI003705-RA	ni|645904171|nb|KK920262.1|	864052-864729	15	16
ACYPI006953-RA	ni|645904145|nb|KK920288.1|	1264246-1264639	16	9.3
ACYPI008159-RA	ni|645903852|nb|KK920578.1|	53771-54026	15	14

ACYPI008169-RA	ni|645902313|nb|KK922116.1|	126278-127422	nan	nan
ACYPI008816-RA	ni|645904145|nb|KK920288.1|	1303592-1303856	16	9.3
ACYPI45279-RA	ni|645903641|nb|KK920789.1|	37048-37929	nan	nan
ACYPI001585-RA	ni|645904225|nb|KK920244.1|	638078-638523	17	17
ACYPI009262-RA	ni|645904009|nb|KK920422.1|	1096853-1097117	17	9.4
ACYPI063239-RA	ni|645903740|nb|KK920690.1|	292545-293973	18	10
ACYPI55715-RA	ni|645904241|nb|KK920238.1|	1299935-1300383	18	17
ACYPI008524-RA	ni|645904115|nb|KK920316.1|	295134-295697	17	18
ACYPI002401-RA	ni|645903547|nb|KK920883.1|	649534-649957	nan	nan
ACYPI39748-RA	ni|645904177|nb|KK920260.1|	277788-278479	18	17
ACYPI001243-RA	ni|645903473|nb|KK920957.1|	540287-540462	nan	nan
ACYPI007241-RA	ni|645903664|nb|KK920766.1|	194474-194750	nan	nan
ACYPI009620-RA	ni|645903716|nb|KK920714.1|	555445-556307	nan	nan
ACYPI067597-RA	ni|645904180|nb|KK920259.1|	571708-571865	17	17
ACYPI001108-RA	ni|645903812|nb|KK920618.1|	740357-740592	20	18
ACYPI001804-RA	ni|645904106|nb|KK920325.1|	774579-776900	17	17
ACYPI002046-RA	ni|645903644|nb|KK920786.1|	897790-898036	nan	nan
ACYPI002999-RA	ni|645903812|nb|KK920618.1|	739434-740185	20	18
ACYPI005421-RA	ni|645904028|nb|KK920403.1|	1977265-1977844	18	17
ACYPI005571-RA	ni|645903879|nb|KK920552.1|	204141-204275	18	18
ACYPI006140-RA	ni|645904130|nb|KK920303.1|	684546-685003	18	17
ACYPI006274-RA	ni|645903960|nb|KK920471.1|	1068883-1069102	16	8.6
ACYPI006527-RA	ni|645903644|nb|KK920786.1|	834643-835099	nan	nan
ACYPI007445-RA	ni|645903650|nb|KK920780.1|	796964-797450	nan	nan
ACYPI008020-RA	ni|645904058|nb|KK920373.1|	1008378-1009294	16	15
ACYPI008168-RA	ni|645902668|nb|KK921761.1|	142400-145561	nan	nan
ACYPI008511-RA	ni|645903910|nb|KK920521.1|	352774-352900	19	17
ACYPI009564-RA	ni|645902471|nb|KK921958.1|	214168-214959	nan	nan
ACYPI072830-RA	ni|645903773|nb|KK920657.1|	1153809-1154029	18	17
ACYPI22631-RA	ni|645903956|nb|KK920475.1|	781402-785776	20	20

ACYPI56877-RA	ni|645904207|nb|KK920250.1|	794578-797254	18	20
ACYPI002367-RA	ni|645903619|nb|KK920811.1|	779382-779518	nan	nan
ACYPI005243-RA	ni|645903838|nb|KK920592.1|	211824-212061	16	16
ACYPI006124-RA	ni|645904121|nb|KK920310.1|	285995-286702	14	15
ACYPI007736-RA	ni|645904258|nb|KK920229.1|	1021535-1021889	16	16
ACYPI009004-RA	ni|645903957|nb|KK920474.1|	952969-953494	18	17
ACYPI002958-RA	ni|645904168|nb|KK920265.1|	352625-352925	16	16
ACYPI005677-RA	ni|645904007|nb|KK920424.1|	420823-421043	18	17
ACYPI010079-RA	ni|645904060|nb|KK920371.1|	179255-180872	15	16
ACYPI000855-RA	ni|645903910|nb|KK920521.1|	544035-544262	19	17
ACYPI001003-RA	ni|645904268|nb|KK920225.1|	305124-305324	14	15
ACYPI002621-RA	ni|645903780|nb|KK920650.1|	599152-601193	19	19
ACYPI007293-RA	ni|645904268|nb|KK920225.1|	275328-276253	14	15
ACYPI008327-RA	ni|645904130|nb|KK920303.1|	218328-220002	18	17
ACYPI009382-RA	ni|645903999|nb|KK920432.1|	1356881-1357073	17	15
ACYPI072080-RA	ni|645903680|nb|KK920750.1|	467277-468129	nan	nan
ACYPI000433-RA	ni|645902311|nb|KK922118.1|	36587-37174	nan	nan
ACYPI001277-RA	ni|645902313|nb|KK922116.1|	169757-170835	nan	nan
ACYPI001529-RA	ni|645903866|nb|KK920564.1|	115407-115862	18	15
ACYPI001901-RA	ni|645903780|nb|KK920650.1|	472506-473049	19	19
ACYPI002880-RA	ni|645904183|nb|KK920258.1|	419934-420233	25	35
ACYPI003790-RA	ni|645903717|nb|KK920713.1|	20131-20327	nan	nan
ACYPI005720-RA	ni|645903798|nb|KK920632.1|	63777-64298	18	17
ACYPI007110-RA	ni|645903713|nb|KK920717.1|	599877-600405	nan	nan
ACYPI008479-RA	ni|645904248|nb|KK920232.1|	709544-709774	15	16
ACYPI008848-RA	ni|645904050|nb|KK920381.1|	530500-531337	18	20
ACYPI001285-RA	ni|645903898|nb|KK920533.1|	489653-490437	17	16
ACYPI002557-RA	ni|645904130|nb|KK920303.1|	2292377-2292691	18	17
ACYPI005092-RA	ni|645903743|nb|KK920687.1|	915431-916235	19	18
ACYPI006374-RA	ni|645903561|nb|KK920869.1|	34861-35181	nan	nan

ACYPI001448-RA	ni|645903688|nb|KK920742.1|	444881-445193	nan	nan
ACYPI005897-RA	ni|645903738|nb|KK920692.1|	911121-912293	18	17
ACYPI001856-RA	ni|645904271|nb|KK920224.1|	2044972-2045379	15	16
ACYPI003711-RA	ni|645904271|nb|KK920224.1|	3629925-3630457	15	16
ACYPI009147-RA	ni|645904271|nb|KK920224.1|	2844807-2845651	15	16
ACYPI009325-RA	ni|645904014|nb|KK920417.1|	209646-210874	19	18
ACYPI002361-RA	ni|645903703|nb|KK920727.1|	464445-464826	nan	nan
ACYPI002482-RA	ni|645904102|nb|KK920329.1|	416843-417423	15	16
ACYPI003002-RA	ni|645903879|nb|KK920552.1|	376531-377201	18	18
ACYPI005545-RA	ni|645903865|nb|KK920565.1|	365577-366039	15	15
ACYPI008182-RA	ni|645902622|nb|KK921807.1|	44576-44957	nan	nan
ACYPI008652-RA	ni|645903807|nb|KK920623.1|	213172-213410	12	14
ACYPI009324-RA	ni|645903557|nb|KK920873.1|	705219-705432	nan	nan
ACYPI010069-RA	ni|645902622|nb|KK921807.1|	78163-78609	nan	nan
ACYPI087848-RA	ni|645904102|nb|KK920329.1|	423218-423763	15	16
ACYPI006310-RA	ni|645903849|nb|KK920581.1|	138521-138751	17	17
ACYPI008174-RA	ni|645903929|nb|KK920502.1|	1423681-1424121	17	18
ACYPI009237-RA	ni|645903694|nb|KK920736.1|	442129-442610	nan	nan
ACYPI45156-RA	ni|645904234|nb|KK920241.1|	210937-211652	13	15
ACYPI48166-RA	ni|645904071|nb|KK920360.1|	1222594-1223379	17	17
ACYPI000169-RA	ni|645903981|nb|KK920450.1|	740964-742702	16	8.6
ACYPI001614-RA	ni|645904023|nb|KK920408.1|	656810-657813	17	17
ACYPI003083-RA	ni|645903566|nb|KK920864.1|	767900-768374	nan	nan
ACYPI004129-RA	ni|645897631|nb|KK926798.1|	4349-4507	nan	nan
ACYPI007070-RA	ni|645904094|nb|KK920337.1|	66379-69115	17	16
ACYPI088345-RA	ni|645904023|nb|KK920408.1|	666362-666891	17	17
ACYPI35610-RA	ni|645903625|nb|KK920805.1|	215329-218791	nan	nan
ACYPI50527-RA	ni|645904013|nb|KK920418.1|	1384871-1385151	19	18
ACYPI001522-RA	ni|645903703|nb|KK920727.1|	329219-329707	nan	nan
ACYPI002157-RA	ni|645903931|nb|KK920500.1|	498842-499745	16	18

ACYPI004029-RA	ni|645903938|nb|KK920493.1|	446255-446423	19	18
ACYPI007834-RA	ni|645903713|nb|KK920717.1|	498638-502396	nan	nan
ACYPI000736-RA	ni|645903716|nb|KK920714.1|	674019-674789	nan	nan
ACYPI001164-RA	ni|645903921|nb|KK920510.1|	389127-389769	16	11
ACYPI005200-RA	ni|645901741|nb|KK922688.1|	42828-45563	nan	nan
ACYPI005353-RA	ni|645903592|nb|KK920838.1|	563792-564642	nan	nan
ACYPI005651-RA	ni|645904241|nb|KK920238.1|	41769-42131	18	17
ACYPI007627-RA	ni|645904245|nb|KK920234.1|	57388-57661	16	17
ACYPI007946-RA	ni|645903527|nb|KK920903.1|	515311-517771	nan	nan
ACYPI008968-RA	ni|645903805|nb|KK920625.1|	266220-266703	12	13
ACYPI41200-RA	ni|645904245|nb|KK920234.1|	52453-52736	16	17
ACYPI000767-RA	ni|645903597|nb|KK920833.1|	1034927-1035235	nan	nan
ACYPI008437-RA	ni|645903800|nb|KK920630.1|	1377677-1378069	18	17
ACYPI010036-RA	ni|645904019|nb|KK920412.1|	280048-280268	19	9.4
ACYPI45421-RA	ni|645904005|nb|KK920426.1|	177812-178022	17	16
ACYPI006263-RA	ni|645903634|nb|KK920796.1|	950381-950912	nan	nan
ACYPI56633-RA	ni|645903871|nb|KK920559.1|	275736-275988	18	9.4
ACYPI000814-RA	ni|645903895|nb|KK920536.1|	540572-540824	17	16
ACYPI002695-RA	ni|645902460|nb|KK921969.1|	14013-14243	nan	nan
ACYPI007245-RA	ni|645901030|nb|KK923399.1|	499-971	nan	nan
ACYPI44011-RA	ni|645903701|nb|KK920729.1|	179813-180573	nan	nan
ACYPI000759-RA	ni|645903891|nb|KK920540.1|	214051-214946	15	17
ACYPI005115-RA	ni|645903980|nb|KK920451.1|	230334-230699	11	14
ACYPI007054-RA	ni|645903891|nb|KK920540.1|	218032-218752	15	17
ACYPI082267-RA	ni|645902373|nb|KK922056.1|	77481-77714	nan	nan
ACYPI000014-RA	ni|645904195|nb|KK920254.1|	121981-122438	18	17
ACYPI001424-RA	ni|645904174|nb|KK920261.1|	1852450-1852684	19	17
ACYPI008222-RA	ni|645903674|nb|KK920756.1|	246220-248151	nan	nan
ACYPI46077-RA	ni|645903857|nb|KK920573.1|	545029-545350	16	16
ACYPI001511-RA	ni|645902373|nb|KK922056.1|	194351-196978	nan	nan

ACYPI002756-RA	ni|645904125|nb|KK920307.1|	2812631-2812847	18	17
ACYPI003401-RA	ni|645902394|nb|KK922035.1|	344370-344995	nan	nan
ACYPI004017-RA	ni|645903680|nb|KK920750.1|	544452-545887	nan	nan
ACYPI004349-RA	ni|645904116|nb|KK920315.1|	1821169-1823111	19	18
ACYPI005038-RA	ni|645904112|nb|KK920319.1|	232715-233665	18	17
ACYPI008129-RA	ni|645904166|nb|KK920267.1|	1937975-1938696	18	16
ACYPI009451-RA	ni|645903917|nb|KK920514.1|	749129-750041	19	17
ACYPI009725-RA	ni|645902726|nb|KK921703.1|	31110-31240	nan	nan
ACYPI001898-RA	ni|645903954|nb|KK920477.1|	238354-238779	35	44
ACYPI002963-RA	ni|645904225|nb|KK920244.1|	629765-630415	17	17
ACYPI004154-RA	ni|645904107|nb|KK920324.1|	299475-299869	17	9.5
ACYPI004450-RA	ni|645903871|nb|KK920559.1|	408493-409714	18	9.4
ACYPI004560-RA	ni|645904241|nb|KK920238.1|	134166-134460	18	17
ACYPI006790-RA	ni|645903627|nb|KK920803.1|	529579-530412	nan	nan
ACYPI007404-RA	ni|645904234|nb|KK920241.1|	187521-187724	13	15
ACYPI007466-RA	ni|645903964|nb|KK920467.1|	308537-308839	15	8
ACYPI008244-RA	ni|645902229|nb|KK922200.1|	198305-198659	nan	nan
ACYPI008884-RA	ni|645903836|nb|KK920594.1|	893375-893865	18	11
ACYPI009285-RA	ni|645904078|nb|KK920353.1|	960962-961144	17	17
ACYPI41599-RA	ni|645903536|nb|KK920894.1|	660492-662437	nan	nan
ACYPI000870-RA	ni|645903636|nb|KK920794.1|	239469-240653	nan	nan
ACYPI002907-RA	ni|645903491|nb|KK920939.1|	40510-43295	nan	nan
ACYPI007276-RA	ni|645903829|nb|KK920601.1|	387120-387358	16	15
ACYPI008625-RA	ni|645903680|nb|KK920750.1|	670844-671194	nan	nan
ACYPI008807-RA	ni|645901672|nb|KK922757.1|	58782-59091	nan	nan
ACYPI25558-RA	ni|645903984|nb|KK920447.1|	31528-32310	18	17
ACYPI31202-RA	ni|645901672|nb|KK922757.1|	34721-34882	nan	nan
ACYPI37793-RA	ni|645904271|nb|KK920224.1|	841398-841921	15	16
ACYPI000453-RA	ni|645903595|nb|KK920835.1|	903022-906579	nan	nan
ACYPI002346-RA	ni|645903914|nb|KK920517.1|	110775-110977	15	17

ACYPI008552-RA	ni|645904240|nb|KK920239.1|	1868699-1869848	16	16
ACYPI008944-RA	ni|645903957|nb|KK920474.1|	892583-892726	18	17
ACYPI064995-RA	ni|645904072|nb|KK920359.1|	1035310-1036437	17	17
ACYPI084988-RA	ni|645903970|nb|KK920461.1|	839323-839555	17	16
ACYPI001945-RA	ni|645903644|nb|KK920786.1|	164963-165304	nan	nan
ACYPI003839-RA	ni|645903644|nb|KK920786.1|	134294-134697	nan	nan
ACYPI004640-RA	ni|645903808|nb|KK920622.1|	988853-989136	17	17
ACYPI005773-RA	ni|645903644|nb|KK920786.1|	147865-148815	nan	nan
ACYPI007193-RA	ni|645904132|nb|KK920301.1|	481853-482184	16	18
ACYPI001622-RA	ni|645903655|nb|KK920775.1|	217072-217635	nan	nan
ACYPI004805-RA	ni|645904166|nb|KK920267.1|	2737685-2737853	18	16
ACYPI007971-RA	ni|645904118|nb|KK920313.1|	730336-730825	17	17
ACYPI008564-RA	ni|645903654|nb|KK920776.1|	55618-56120	nan	nan
ACYPI009308-RA	ni|645903775|nb|KK920655.1|	144433-144677	16	16
ACYPI009596-RA	ni|645903747|nb|KK920683.1|	14922-16502	19	18
ACYPI064059-RA	ni|645904120|nb|KK920311.1|	767819-768584	18	18
ACYPI001567-RA	ni|645903973|nb|KK920458.1|	1592954-1594013	20	18
ACYPI001746-RA	ni|645903803|nb|KK920627.1|	887915-889665	17	17
ACYPI002680-RA	ni|645903812|nb|KK920618.1|	550461-551001	20	18
ACYPI003001-RA	ni|645902269|nb|KK922160.1|	73465-73769	nan	nan
ACYPI067549-RA	ni|645904100|nb|KK920331.1|	279904-280673	18	22
ACYPI001584-RA	ni|645903626|nb|KK920804.1|	421339-422422	nan	nan
ACYPI006725-RA	ni|645903858|nb|KK920572.1|	894147-894397	16	16
ACYPI008369-RA	ni|645904111|nb|KK920320.1|	1511846-1512848	17	17
ACYPI008584-RA	ni|645904120|nb|KK920311.1|	719855-720350	18	18
ACYPI009821-RA	ni|645904004|nb|KK920427.1|	994362-994583	16	16
ACYPI064464-RA	ni|645904177|nb|KK920260.1|	1006492-1008125	18	17
ACYPI001591-RA	ni|645901190|nb|KK923239.1|	15775-15957	nan	nan
ACYPI002372-RA	ni|645903779|nb|KK920651.1|	368336-369036	18	16
ACYPI002600-RA	ni|645903498|nb|KK920932.1|	415503-416524	nan	nan

ACYPI002624-RA	ni|645904035|nb|KK920396.1|	641155-641400	16	16
ACYPI003661-RA	ni|645904035|nb|KK920396.1|	640519-640913	16	16
ACYPI004531-RA	ni|645903874|nb|KK920557.1|	851410-851698	18	18
ACYPI006900-RA	ni|645904258|nb|KK920229.1|	1082156-1082296	16	16
ACYPI008758-RA	ni|645904258|nb|KK920229.1|	1062965-1063748	16	16
ACYPI083436-RA	ni|645904262|nb|KK920227.1|	336091-336742	17	17
ACYPI000822-RA	ni|645903535|nb|KK920895.1|	451600-451735	nan	nan
ACYPI001194-RA	ni|645904022|nb|KK920409.1|	635284-636006	18	20
ACYPI002106-RA	ni|645904056|nb|KK920375.1|	657409-657827	16	16
ACYPI003448-RA	ni|645903590|nb|KK920840.1|	181592-181743	nan	nan
ACYPI005122-RA	ni|645903634|nb|KK920796.1|	1096273-1096492	nan	nan
ACYPI007009-RA	ni|645902760|nb|KK921669.1|	17306-18078	nan	nan
ACYPI009714-RA	ni|645902463|nb|KK921966.1|	303667-303869	nan	nan
ACYPI002137-RA	ni|645903920|nb|KK920511.1|	931872-932434	20	20
ACYPI004206-RA	ni|645904177|nb|KK920260.1|	2202705-2203229	18	17
ACYPI004809-RA	ni|645903650|nb|KK920780.1|	141367-142899	nan	nan
ACYPI006711-RA	ni|645904139|nb|KK920294.1|	500525-501582	15	18
ACYPI008022-RA	ni|645904058|nb|KK920373.1|	1239274-1239605	16	15
ACYPI009055-RA	ni|645904111|nb|KK920320.1|	281504-282092	17	17
ACYPI069860-RA	ni|645903920|nb|KK920511.1|	1112520-1112619	20	20
ACYPI004423-RA	ni|645903957|nb|KK920474.1|	844498-844636	18	17
ACYPI006340-RA	ni|645904119|nb|KK920312.1|	500249-501529	19	19
ACYPI008218-RA	ni|645904086|nb|KK920345.1|	347455-347600	14	15
ACYPI008248-RA	ni|645904057|nb|KK920374.1|	57181-57562	16	15
ACYPI008506-RA	ni|645904043|nb|KK920388.1|	756206-756420	16	9.1
ACYPI002874-RA	ni|645904105|nb|KK920326.1|	615540-615868	13	15
ACYPI010018-RA	ni|645903952|nb|KK920479.1|	1085468-1085697	16	16
ACYPI001400-RA	ni|645904157|nb|KK920276.1|	42098-42737	15	15
ACYPI004978-RA	ni|645903821|nb|KK920609.1|	769058-769332	17	17
ACYPI006354-RA	ni|645903536|nb|KK920894.1|	320223-320658	nan	nan

ACYPI007272-RA	ni|645903488|nb|KK920942.1|	89331-90743	nan	nan
ACYPI060811-RA	ni|645904053|nb|KK920378.1|	595396-596242	18	17
ACYPI002497-RA	ni|645904281|nb|KK920220.1|	1628644-1628950	15	16
ACYPI005073-RA	ni|645903547|nb|KK920883.1|	611079-611282	nan	nan
ACYPI005416-RA	ni|645904134|nb|KK920299.1|	441790-442267	17	13
ACYPI008368-RA	ni|645904279|nb|KK920221.1|	2642380-2643820	16	16
ACYPI008833-RA	ni|645903547|nb|KK920883.1|	608168-608673	nan	nan
ACYPI23453-RA	ni|645904012|nb|KK920419.1|	434518-435598	27	32
ACYPI000055-RA	ni|645903787|nb|KK920643.1|	1368639-1369809	19	18
ACYPI000431-RA	ni|645904053|nb|KK920378.1|	575073-575639	18	17
ACYPI001692-RA	ni|645903655|nb|KK920775.1|	267027-267260	nan	nan
ACYPI001971-RA	ni|645904144|nb|KK920289.1|	915192-916007	16	9
ACYPI002220-RA	ni|645902756|nb|KK921673.1|	274233-274678	nan	nan
ACYPI002324-RA	ni|645904265|nb|KK920226.1|	1381277-1381567	14	14
ACYPI002792-RA	ni|645901262|nb|KK923167.1|	33437-33676	nan	nan
ACYPI003562-RA	ni|645903763|nb|KK920667.1|	49159-49585	26	21
ACYPI004715-RA	ni|645901262|nb|KK923167.1|	56562-57239	nan	nan
ACYPI005512-RA	ni|645903627|nb|KK920803.1|	238941-239709	nan	nan
ACYPI006399-RA	ni|645903563|nb|KK920867.1|	634869-635472	nan	nan
ACYPI008279-RA	ni|645903923|nb|KK920508.1|	1343612-1344248	18	18
ACYPI009136-RA	ni|645904151|nb|KK920282.1|	442439-442616	16	20
ACYPI009523-RA	ni|645903731|nb|KK920699.1|	142800-144005	nan	nan
ACYPI071206-RA	ni|645903546|nb|KK920884.1|	138442-138845	nan	nan
ACYPI000076-RA	ni|645902589|nb|KK921840.1|	122803-123041	nan	nan
ACYPI002684-RA	ni|645904120|nb|KK920311.1|	282852-283120	18	18
ACYPI003008-RA	ni|645904065|nb|KK920366.1|	757969-759008	18	18
ACYPI003204-RA	ni|645901808|nb|KK922621.1|	50970-52099	nan	nan
ACYPI003233-RA	ni|645903678|nb|KK920752.1|	436682-436883	nan	nan
ACYPI005152-RA	ni|645903678|nb|KK920752.1|	435236-436098	nan	nan
ACYPI007640-RA	ni|645903780|nb|KK920650.1|	506717-509085	19	19

ACYPI010224-RA	ni|645904112|nb|KK920319.1|	2378044-2378321	18	17
ACYPI41167-RA	ni|645902238|nb|KK922191.1|	138899-139230	nan	nan
ACYPI000261-RA	ni|645903773|nb|KK920657.1|	1520215-1523516	18	17
ACYPI001532-RA	ni|645903773|nb|KK920657.1|	1509079-1509276	18	17
ACYPI003421-RA	ni|645903773|nb|KK920657.1|	1510837-1511913	18	17
ACYPI003481-RA	ni|645904210|nb|KK920249.1|	916557-917222	16	16
ACYPI004036-RA	ni|645903898|nb|KK920533.1|	466747-466992	17	16
ACYPI004169-RA	ni|645904120|nb|KK920311.1|	284791-284993	18	18
ACYPI007012-RA	ni|645904043|nb|KK920388.1|	547055-547301	16	9.1
ACYPI007485-RA	ni|645897959|nb|KK926470.1|	42725-43504	nan	nan
ACYPI007988-RA	ni|645902785|nb|KK921644.1|	105108-106086	nan	nan
ACYPI009378-RA	ni|645903557|nb|KK920873.1|	916386-916644	nan	nan
ACYPI009741-RA	ni|645904163|nb|KK920270.1|	337608-337857	16	16
ACYPI065310-RA	ni|645903841|nb|KK920589.1|	538381-539472	17	17
ACYPI002468-RA	ni|645904242|nb|KK920237.1|	826682-827255	15	16
ACYPI003786-RA	ni|645903741|nb|KK920689.1|	349057-349477	17	18
ACYPI004248-RA	ni|645903789|nb|KK920641.1|	487883-488098	17	17
ACYPI004749-RA	ni|645902496|nb|KK921933.1|	139905-140729	nan	nan
ACYPI006654-RA	ni|645904033|nb|KK920398.1|	443809-444125	14	16
ACYPI49091-RA	ni|645903996|nb|KK920435.1|	282643-282776	17	16
ACYPI000890-RA	ni|645902336|nb|KK922093.1|	77307-78304	nan	nan
ACYPI002781-RA	ni|645902336|nb|KK922093.1|	9899-10262	nan	nan
ACYPI007858-RA	ni|645903749|nb|KK920681.1|	132994-134621	19	18
ACYPI37835-RA	ni|645903598|nb|KK920832.1|	246380-246657	nan	nan
ACYPI004106-RA	ni|645904168|nb|KK920265.1|	2088813-2089040	16	16
ACYPI006039-RA	ni|645904145|nb|KK920288.1|	429623-430445	16	9.3
ACYPI009988-RA	ni|645904000|nb|KK920431.1|	357066-359305	18	19
ACYPI000235-RA	ni|645904112|nb|KK920319.1|	1717915-1718101	18	17
ACYPI000904-RA	ni|645903984|nb|KK920447.1|	214903-215208	18	17
ACYPI001700-RA	ni|645904065|nb|KK920366.1|	940193-941009	18	18

ACYPI002154-RA	ni|645903825|nb|KK920605.1|	1057642-1058639	19	17
ACYPI002951-RA	ni|645903594|nb|KK920836.1|	76411-78155	nan	nan
ACYPI003409-RA	ni|645903502|nb|KK920928.1|	240818-241179	nan	nan
ACYPI003886-RA	ni|645903745|nb|KK920685.1|	316489-319185	16	18
ACYPI004025-RA	ni|645903634|nb|KK920796.1|	954886-956206	nan	nan
ACYPI004880-RA	ni|645903568|nb|KK920862.1|	224022-224602	nan	nan
ACYPI005346-RA	ni|645904065|nb|KK920366.1|	18734-19163	18	18
ACYPI005668-RA	ni|645903648|nb|KK920782.1|	256402-256734	nan	nan
ACYPI005896-RA	ni|645903734|nb|KK920696.1|	278859-279218	nan	nan
ACYPI005927-RA	ni|645904038|nb|KK920393.1|	1017910-1018129	17	19
ACYPI006624-RA	ni|645904139|nb|KK920294.1|	204208-205253	15	18
ACYPI007220-RA	ni|645904038|nb|KK920393.1|	360832-361350	17	19
ACYPI007391-RA	ni|645903984|nb|KK920447.1|	502606-502828	18	17
ACYPI007584-RA	ni|645904106|nb|KK920325.1|	701620-701886	17	17
ACYPI007727-RA	ni|645903674|nb|KK920756.1|	694920-695445	nan	nan
ACYPI008033-RA	ni|645904138|nb|KK920295.1|	667482-668200	15	15
ACYPI008591-RA	ni|645904166|nb|KK920267.1|	2471076-2471954	18	16
ACYPI009105-RA	ni|645904038|nb|KK920393.1|	334054-334324	17	19
ACYPI009908-RA	ni|645903946|nb|KK920485.1|	582119-582401	16	14
ACYPI067763-RA	ni|645903564|nb|KK920866.1|	334772-335057	nan	nan
ACYPI52393-RA	ni|645903541|nb|KK920889.1|	271090-272483	nan	nan
ACYPI001600-RA	ni|645902338|nb|KK922091.1|	105712-105924	nan	nan
ACYPI001626-RA	ni|645903983|nb|KK920448.1|	770849-771385	18	17
ACYPI003488-RA	ni|645904135|nb|KK920298.1|	310414-311019	14	15
ACYPI064172-RA	ni|645904135|nb|KK920298.1|	308063-308336	14	15
ACYPI001217-RA	ni|645903780|nb|KK920650.1|	756372-757114	19	19
ACYPI002491-RA	ni|645903794|nb|KK920636.1|	416041-416238	25	34
ACYPI004395-RA	ni|645901755|nb|KK922674.1|	18471-19736	nan	nan
ACYPI005673-RA	ni|645903696|nb|KK920734.1|	559883-560274	nan	nan
ACYPI006887-RA	ni|645903898|nb|KK920533.1|	583449-583922	17	16

ACYPI006936-RA	ni|645903984|nb|KK920447.1|	9665-10132	18	17
ACYPI009401-RA	ni|645903898|nb|KK920533.1|	698160-698434	17	16
ACYPI009438-RA	ni|645903858|nb|KK920572.1|	374401-376611	16	16
ACYPI010075-RA	ni|645903975|nb|KK920456.1|	1089045-1089210	15	8.2
ACYPI23935-RA	ni|645903876|nb|KK920555.1|	72946-73761	14	15
ACYPI49333-RA	ni|645904244|nb|KK920235.1|	229483-230537	15	14
ACYPI001352-RA	ni|645902016|nb|KK922413.1|	79165-79360	nan	nan
ACYPI008880-RA	ni|645902576|nb|KK921853.1|	270272-270745	nan	nan
ACYPI009481-RA	ni|645903867|nb|KK920563.1|	1108061-1108370	18	18
ACYPI080140-RA	ni|645903867|nb|KK920563.1|	1044435-1044666	18	18
ACYPI000150-RA	ni|645903496|nb|KK920934.1|	195331-196100	nan	nan
ACYPI001458-RA	ni|645904150|nb|KK920283.1|	697879-699399	17	17
ACYPI005065-RA	ni|645904088|nb|KK920343.1|	211643-211811	17	18
ACYPI006043-RA	ni|645903898|nb|KK920533.1|	453573-453923	17	16
ACYPI006154-RA	ni|645904060|nb|KK920371.1|	430259-430449	15	16
ACYPI008418-RA	ni|645904079|nb|KK920352.1|	1102974-1104379	15	16
ACYPI46815-RA	ni|645903661|nb|KK920769.1|	729398-730003	nan	nan
ACYPI000479-RA	ni|645902233|nb|KK922196.1|	159856-160197	nan	nan
ACYPI002383-RA	ni|645904177|nb|KK920260.1|	2156654-2157402	18	17
ACYPI002595-RA	ni|645903923|nb|KK920508.1|	1117897-1118761	18	18
ACYPI004570-RA	ni|645904117|nb|KK920314.1|	256015-256460	15	15
ACYPI004938-RA	ni|645902681|nb|KK921748.1|	310537-311067	nan	nan
ACYPI006482-RA	ni|645904133|nb|KK920300.1|	2963047-2963435	18	18
ACYPI007060-RA	ni|645903965|nb|KK920466.1|	1741246-1741496	17	16
ACYPI007710-RA	ni|645903743|nb|KK920687.1|	423212-423721	19	18
ACYPI007807-RA	ni|645904222|nb|KK920245.1|	1639262-1640460	17	12
ACYPI21475-RA	ni|645903874|nb|KK920557.1|	919431-919924	18	18
ACYPI003771-RA	ni|645902229|nb|KK922200.1|	84208-85489	nan	nan
ACYPI005239-RA	ni|645904216|nb|KK920247.1|	446565-446795	15	16
ACYPI005325-RA	ni|645903627|nb|KK920803.1|	474225-474436	nan	nan

ACYPI005496-RA	ni|645903694|nb|KK920736.1|	422731-423446	nan	nan
ACYPI008431-RA	ni|645904171|nb|KK920262.1|	605547-605867	15	16
ACYPI009074-RA	ni|645903538|nb|KK920892.1|	285349-285585	nan	nan
ACYPI009659-RA	ni|645903472|nb|KK920958.1|	210696-211466	nan	nan
ACYPI089540-RA	ni|645902426|nb|KK922003.1|	197837-198248	nan	nan
ACYPI001292-RA	ni|645903731|nb|KK920699.1|	1221498-1222392	nan	nan
ACYPI001295-RA	ni|645903496|nb|KK920934.1|	847339-847712	nan	nan
ACYPI002350-RA	ni|645903983|nb|KK920448.1|	1024961-1025313	18	17
ACYPI003185-RA	ni|645903599|nb|KK920831.1|	216340-217609	nan	nan
ACYPI004334-RA	ni|645904258|nb|KK920229.1|	1594016-1594707	16	16
ACYPI008555-RA	ni|645903518|nb|KK920912.1|	96093-96497	nan	nan
ACYPI009525-RA	ni|645903949|nb|KK920482.1|	566371-567845	17	14
ACYPI26723-RA	ni|645904258|nb|KK920229.1|	1221512-1222476	16	16
ACYPI48246-RA	ni|645903747|nb|KK920683.1|	876580-876856	19	18
ACYPI009537-RA	ni|645904052|nb|KK920379.1|	572945-573618	18	16
ACYPI000463-RA	ni|645904192|nb|KK920255.1|	1230639-1230783	15	16
ACYPI005502-RA	ni|645904112|nb|KK920319.1|	2851364-2851641	18	17
ACYPI006680-RA	ni|645901967|nb|KK922462.1|	6398-6822	nan	nan
ACYPI009340-RA	ni|645903772|nb|KK920658.1|	139488-139671	15	16
ACYPI010180-RA	ni|645903854|nb|KK920576.1|	350740-350985	16	8.4
ACYPI080365-RA	ni|645903494|nb|KK920936.1|	437650-439455	nan	nan
ACYPI086093-RA	ni|645903495|nb|KK920935.1|	455897-456189	nan	nan
ACYPI29397-RA	ni|645903494|nb|KK920936.1|	405062-406234	nan	nan
ACYPI000600-RA	ni|645903696|nb|KK920734.1|	625575-625788	nan	nan
ACYPI002480-RA	ni|645903780|nb|KK920650.1|	251973-252387	19	19
ACYPI002650-RA	ni|645904021|nb|KK920410.1|	598172-598882	15	16
ACYPI006958-RA	ni|645903950|nb|KK920481.1|	689445-690534	15	15
ACYPI007402-RA	ni|645903812|nb|KK920618.1|	842794-843046	20	18
ACYPI008272-RA	ni|645902662|nb|KK921767.1|	276242-276971	nan	nan
ACYPI010149-RA	ni|645903795|nb|KK920635.1|	47902-48204	14	16

ACYPI55567-RA	ni|645901786|nb|KK922643.1|	62098-63715	nan	nan
ACYPI001020-RA	ni|645903495|nb|KK920935.1|	228461-228992	nan	nan
ACYPI001604-RA	ni|645904177|nb|KK920260.1|	1149866-1150800	18	17
ACYPI001665-RA	ni|645903466|nb|KK920964.1|	694000-694399	nan	nan
ACYPI003550-RA	ni|645904065|nb|KK920366.1|	1757004-1757181	18	18
ACYPI005499-RA	ni|645903612|nb|KK920818.1|	95659-96271	nan	nan
ACYPI007366-RA	ni|645904210|nb|KK920249.1|	1910079-1910246	16	16
ACYPI001145-RA	ni|645903662|nb|KK920768.1|	400678-400881	nan	nan
ACYPI004521-RA	ni|645903690|nb|KK920740.1|	569499-569954	nan	nan
ACYPI008315-RA	ni|645904028|nb|KK920403.1|	1957327-1957653	18	17
ACYPI009964-RA	ni|645903574|nb|KK920856.1|	47556-47803	nan	nan
ACYPI064435-RA	ni|645903662|nb|KK920768.1|	393288-393934	nan	nan
ACYPI067901-RA	ni|645901878|nb|KK922551.1|	10788-11135	nan	nan
ACYPI082299-RA	ni|645902029|nb|KK922400.1|	36963-38009	nan	nan
ACYPI50578-RA	ni|645902418|nb|KK922011.1|	70358-70727	nan	nan
ACYPI003075-RA	ni|645904228|nb|KK920243.1|	376485-376722	17	18
ACYPI005237-RA	ni|645901808|nb|KK922621.1|	146881-147793	nan	nan
ACYPI007190-RA	ni|645904262|nb|KK920227.1|	1223112-1223535	17	17
ACYPI004845-RA	ni|645903755|nb|KK920675.1|	110707-110922	18	16
ACYPI37546-RA	ni|645903782|nb|KK920648.1|	154534-155176	13	14
ACYPI000502-RA	ni|645903759|nb|KK920671.1|	525437-525857	18	17
ACYPI001088-RA	ni|645903861|nb|KK920569.1|	322258-322457	16	17
ACYPI003519-RA	ni|645902511|nb|KK921918.1|	156844-157080	nan	nan
ACYPI006827-RA	ni|645903797|nb|KK920633.1|	82754-83399	17	8.9
ACYPI089403-RA	ni|645903861|nb|KK920569.1|	346960-347337	16	17
ACYPI56637-RA	ni|645903973|nb|KK920458.1|	1225789-1226308	20	18
ACYPI000882-RA	ni|645903827|nb|KK920603.1|	437169-438833	17	18
ACYPI004822-RA	ni|645902290|nb|KK922139.1|	142812-143238	nan	nan
ACYPI006318-RA	ni|645903678|nb|KK920752.1|	366223-366478	nan	nan
ACYPI007240-RA	ni|645899821|nb|KK924608.1|	636-864	nan	nan

ACYPI007299-RA	ni|645904125|nb|KK920307.1|	240239-240645	18	17
ACYPI008113-RA	ni|645903813|nb|KK920617.1|	480728-481433	17	18
ACYPI009989-RA	ni|645904122|nb|KK920309.1|	167167-167773	18	17
ACYPI073296-RA	ni|645903678|nb|KK920752.1|	370664-371056	nan	nan
ACYPI005100-RA	ni|645903697|nb|KK920733.1|	120724-121715	nan	nan
ACYPI081400-RA	ni|645903665|nb|KK920765.1|	456905-457093	nan	nan
ACYPI21068-RA	ni|645904248|nb|KK920232.1|	1623680-1623843	15	16
ACYPI061977-RA	ni|645903773|nb|KK920657.1|	238203-240216	18	17
ACYPI56773-RA	ni|645904183|nb|KK920258.1|	923160-924975	25	35
ACYPI001755-RA	ni|645903702|nb|KK920728.1|	421398-422211	nan	nan
ACYPI005417-RA	ni|645902593|nb|KK921836.1|	52075-55794	nan	nan
ACYPI40226-RA	ni|645904119|nb|KK920312.1|	1078292-1078905	19	19
ACYPI001663-RA	ni|645904246|nb|KK920233.1|	224451-226144	16	17
ACYPI005463-RA	ni|645902384|nb|KK922045.1|	156766-156975	nan	nan
ACYPI008010-RA	ni|645903785|nb|KK920645.1|	255363-256690	15	16
ACYPI009208-RA	ni|645903743|nb|KK920687.1|	176528-176884	19	18
ACYPI009278-RA	ni|645903915|nb|KK920516.1|	1378687-1380438	17	17
ACYPI082601-RA	ni|645903548|nb|KK920882.1|	206915-207236	nan	nan
ACYPI001877-RA	ni|645904177|nb|KK920260.1|	1634882-1635268	18	17
ACYPI004421-RA	ni|645904246|nb|KK920233.1|	933495-935260	16	17
ACYPI005270-RA	ni|645904077|nb|KK920354.1|	705564-705709	16	17
ACYPI006978-RA	ni|645903938|nb|KK920493.1|	1462006-1465201	19	18
ACYPI061546-RA	ni|645902609|nb|KK921820.1|	121195-121412	nan	nan
ACYPI007020-RA	ni|645903919|nb|KK920512.1|	805405-805944	17	15
ACYPI008084-RA	ni|645903633|nb|KK920797.1|	373456-373931	nan	nan
ACYPI003151-RA	ni|645904014|nb|KK920417.1|	833759-833964	19	18
ACYPI005774-RA	ni|645903957|nb|KK920474.1|	1424751-1425165	18	17
ACYPI007909-RA	ni|645903911|nb|KK920520.1|	231431-231603	16	17
ACYPI067736-RA	ni|645904114|nb|KK920317.1|	2078039-2078737	18	18
ACYPI000413-RA	ni|645903620|nb|KK920810.1|	8735-8932	nan	nan

ACYPI000753-RA	ni|645904262|nb|KK920227.1|	2473960-2474135	17	17
ACYPI000896-RA	ni|645901860|nb|KK922569.1|	75991-76640	nan	nan
ACYPI002111-RA	ni|645901817|nb|KK922612.1|	91160-91639	nan	nan
ACYPI002791-RA	ni|645903654|nb|KK920776.1|	646197-646560	nan	nan
ACYPI004886-RA	ni|645903708|nb|KK920722.1|	27374-27583	nan	nan
ACYPI007249-RA	ni|645903654|nb|KK920776.1|	676053-676866	nan	nan
ACYPI008165-RA	ni|645903953|nb|KK920478.1|	1425661-1426058	18	17
ACYPI070244-RA	ni|645901817|nb|KK922612.1|	84775-85007	nan	nan
ACYPI089246-RA	ni|645903953|nb|KK920478.1|	1430144-1430687	18	17
ACYPI005622-RA	ni|645902471|nb|KK921958.1|	183292-184343	nan	nan
ACYPI009335-RA	ni|645903502|nb|KK920928.1|	284970-285155	nan	nan
ACYPI069554-RA	ni|645904019|nb|KK920412.1|	623072-623241	19	9.4
ACYPI085203-RA	ni|645904043|nb|KK920388.1|	547753-548066	16	9.1
ACYPI000694-RA	ni|645904064|nb|KK920367.1|	761472-761990	17	9.9
ACYPI001296-RA	ni|645903827|nb|KK920603.1|	233378-235221	17	18
ACYPI001437-RA	ni|645903716|nb|KK920714.1|	894373-894735	nan	nan
ACYPI001841-RA	ni|645904133|nb|KK920300.1|	997092-997404	18	18
ACYPI004343-RA	ni|645904122|nb|KK920309.1|	276961-277772	18	17
ACYPI005659-RA	ni|645903762|nb|KK920668.1|	528044-528549	17	17
ACYPI061215-RA	ni|645903993|nb|KK920438.1|	124966-126232	19	22
ACYPI56627-RA	ni|645903993|nb|KK920438.1|	132747-133864	19	22
ACYPI000618-RA	ni|645904174|nb|KK920261.1|	205017-205694	19	17
ACYPI001675-RA	ni|645903783|nb|KK920647.1|	1524293-1525615	17	17
ACYPI001786-RA	ni|645904046|nb|KK920385.1|	992328-992491	17	8.4
ACYPI002171-RA	ni|645904043|nb|KK920388.1|	835475-835772	16	9.1
ACYPI003039-RA	ni|645903783|nb|KK920647.1|	1560256-1560671	17	17
ACYPI003662-RA	ni|645904160|nb|KK920273.1|	751784-753300	18	18
ACYPI004420-RA	ni|645903566|nb|KK920864.1|	613351-613600	nan	nan
ACYPI005511-RA	ni|645902598|nb|KK921831.1|	55071-55206	nan	nan
ACYPI007776-RA	ni|645903879|nb|KK920552.1|	523748-525691	18	18

ACYPI065923-RA	ni|645904166|nb|KK920267.1|	2311041-2311466	18	16
ACYPI069326-RA	ni|645902644|nb|KK921785.1|	70423-71842	nan	nan
ACYPI35199-RA	ni|645901638|nb|KK922791.1|	5981-6551	nan	nan
ACYPI000091-RA	ni|645904122|nb|KK920309.1|	589900-590358	18	17
ACYPI000454-RA	ni|645903688|nb|KK920742.1|	78374-78772	nan	nan
ACYPI001153-RA	ni|645902718|nb|KK921711.1|	126947-127213	nan	nan
ACYPI003660-RA	ni|645904122|nb|KK920309.1|	597988-598717	18	17
ACYPI005360-RA	ni|645904065|nb|KK920366.1|	963245-963848	18	18
ACYPI005600-RA	ni|645903545|nb|KK920885.1|	58888-59114	nan	nan
ACYPI006254-RA	ni|645904160|nb|KK920273.1|	1906377-1906572	18	18
ACYPI007477-RA	ni|645903728|nb|KK920702.1|	519649-522439	nan	nan
ACYPI009370-RA	ni|645903749|nb|KK920681.1|	173250-173737	19	18
ACYPI009740-RA	ni|645904160|nb|KK920273.1|	1915958-1916555	18	18
ACYPI37245-RA	ni|645904122|nb|KK920309.1|	579566-580003	18	17
ACYPI001430-RA	ni|645904017|nb|KK920414.1|	95643-96303	17	17
ACYPI006703-RA	ni|645903699|nb|KK920731.1|	166653-168033	nan	nan
ACYPI007219-RA	ni|645903747|nb|KK920683.1|	752430-753512	19	18
ACYPI008562-RA	ni|645902469|nb|KK921960.1|	30157-30385	nan	nan
ACYPI072117-RA	ni|645904017|nb|KK920414.1|	97512-97714	17	17
ACYPI002551-RA	ni|645904228|nb|KK920243.1|	1621803-1621944	17	18
ACYPI006566-RA	ni|645904043|nb|KK920388.1|	546204-546451	16	9.1
ACYPI008886-RA	ni|645903539|nb|KK920891.1|	252857-253068	nan	nan
ACYPI000740-RA	ni|645904133|nb|KK920300.1|	936147-936443	18	18
ACYPI001124-RA	ni|645904133|nb|KK920300.1|	379734-380311	18	18
ACYPI002364-RA	ni|645903564|nb|KK920866.1|	320803-321054	nan	nan
ACYPI002711-RA	ni|645903597|nb|KK920833.1|	928664-929004	nan	nan
ACYPI003062-RA	ni|645904162|nb|KK920271.1|	612792-612986	15	18
ACYPI004629-RA	ni|645902463|nb|KK921966.1|	231132-232219	nan	nan
ACYPI004936-RA	ni|645903785|nb|KK920645.1|	450559-450956	15	16
ACYPI006545-RA	ni|645903774|nb|KK920656.1|	654437-655141	16	9.2

ACYPI006876-RA	ni|645903971|nb|KK920460.1|	1584455-1584602	19	18
ACYPI008708-RA	ni|645903896|nb|KK920535.1|	387555-388105	16	9.1
ACYPI010209-RA	ni|645903671|nb|KK920759.1|	772245-772536	nan	nan
ACYPI45753-RA	ni|645904133|nb|KK920300.1|	440125-440902	18	18
ACYPI000443-RA	ni|645902239|nb|KK922190.1|	60675-62288	nan	nan
ACYPI000513-RA	ni|645904098|nb|KK920333.1|	21662-24773	16	8.8
ACYPI001047-RA	ni|645904116|nb|KK920315.1|	466094-466760	19	18
ACYPI002631-RA	ni|645904098|nb|KK920333.1|	26137-26786	16	8.8
ACYPI002904-RA	ni|645903837|nb|KK920593.1|	949706-949945	18	9.8
ACYPI003499-RA	ni|645901914|nb|KK922515.1|	103453-103691	nan	nan
ACYPI003537-RA	ni|645903823|nb|KK920607.1|	34946-35223	16	17
ACYPI003571-RA	ni|645901808|nb|KK922621.1|	103745-104204	nan	nan
ACYPI004779-RA	ni|645903745|nb|KK920685.1|	24146-24563	16	18
ACYPI004832-RA	ni|645904091|nb|KK920340.1|	657636-657919	15	18
ACYPI005444-RA	ni|645903743|nb|KK920687.1|	1183192-1183357	19	18
ACYPI005521-RA	ni|645904134|nb|KK920299.1|	849449-850516	17	13
ACYPI006658-RA	ni|645903902|nb|KK920529.1|	1120723-1121108	19	17
ACYPI006682-RA	ni|645904278|nb|KK920222.1|	561997-563106	15	16
ACYPI006728-RA	ni|645903984|nb|KK920447.1|	24882-26004	18	17
ACYPI007303-RA	ni|645903773|nb|KK920657.1|	1419473-1419637	18	17
ACYPI007945-RA	ni|645903773|nb|KK920657.1|	1414144-1414902	18	17
ACYPI007967-RA	ni|645902344|nb|KK922085.1|	278849-279094	nan	nan
ACYPI009202-RA	ni|645903743|nb|KK920687.1|	1045386-1045619	19	18
ACYPI009906-RA	ni|645904156|nb|KK920277.1|	1567846-1568297	17	8.8
ACYPI072782-RA	ni|645904103|nb|KK920328.1|	53052-54088	15	19
ACYPI002842-RA	ni|645903833|nb|KK920597.1|	34353-34959	17	15
ACYPI005883-RA	ni|645903635|nb|KK920795.1|	326326-327821	nan	nan
ACYPI006954-RA	ni|645903834|nb|KK920596.1|	42082-43166	18	17
ACYPI009066-RA	ni|645904054|nb|KK920377.1|	604310-604521	16	16
ACYPI000112-RA	ni|645904174|nb|KK920261.1|	923528-923778	19	17

ACYPI005171-RA	ni|645902600|nb|KK921829.1|	76985-77420	nan	nan
ACYPI009436-RA	ni|645904237|nb|KK920240.1|	901127-901479	16	15
ACYPI008923-RA	ni|645903910|nb|KK920521.1|	634275-636995	19	17
ACYPI009474-RA	ni|645903975|nb|KK920456.1|	1111538-1111907	15	8.2
ACYPI067841-RA	ni|645904057|nb|KK920374.1|	1363239-1363915	16	15
ACYPI005594-RA	ni|645904123|nb|KK920308.1|	1606239-1606800	16	10
ACYPI006242-RA	ni|645903609|nb|KK920821.1|	384374-386063	nan	nan
ACYPI009033-RA	ni|645902589|nb|KK921840.1|	89658-89959	nan	nan
ACYPI001025-RA	ni|645904051|nb|KK920380.1|	503984-507038	16	17
ACYPI002276-RA	ni|645904053|nb|KK920378.1|	172377-172852	18	17
ACYPI005543-RA	ni|645903639|nb|KK920791.1|	413526-414440	nan	nan
ACYPI001166-RA	ni|645903841|nb|KK920589.1|	1018358-1019195	17	17
ACYPI001246-RA	ni|645903679|nb|KK920751.1|	545156-546016	nan	nan
ACYPI001798-RA	ni|645904052|nb|KK920379.1|	522385-524539	18	16
ACYPI008639-RA	ni|645903727|nb|KK920703.1|	581157-581355	nan	nan
ACYPI009457-RA	ni|645904049|nb|KK920382.1|	477429-477868	18	18
ACYPI089164-RA	ni|645903636|nb|KK920794.1|	205924-206110	nan	nan
ACYPI002937-RA	ni|645903697|nb|KK920733.1|	189565-189800	nan	nan
ACYPI002949-RA	ni|645904098|nb|KK920333.1|	321544-321965	16	8.8
ACYPI004239-RA	ni|645903808|nb|KK920622.1|	691045-692713	17	17
ACYPI006777-RA	ni|645904156|nb|KK920277.1|	1517327-1517575	17	8.8
ACYPI008640-RA	ni|645904098|nb|KK920333.1|	304677-305873	16	8.8
ACYPI009258-RA	ni|645903697|nb|KK920733.1|	167567-168183	nan	nan
ACYPI009973-RA	ni|645903804|nb|KK920626.1|	148489-149298	17	17
ACYPI061385-RA	ni|645904177|nb|KK920260.1|	277424-277679	18	17
ACYPI083672-RA	ni|645904133|nb|KK920300.1|	2795566-2795810	18	18
ACYPI002047-RA	ni|645904228|nb|KK920243.1|	802463-802870	17	18
ACYPI003006-RA	ni|645904278|nb|KK920222.1|	1348709-1349001	15	16
ACYPI008560-RA	ni|645904278|nb|KK920222.1|	1322795-1323147	15	16
ACYPI009292-RA	ni|645904258|nb|KK920229.1|	689516-689792	16	16

ACYPI003045-RA	ni|645904251|nb|KK920231.1|	586083-586627	16	16
ACYPI004195-RA	ni|645904114|nb|KK920317.1|	1648005-1649903	18	18
ACYPI004964-RA	ni|645904074|nb|KK920357.1|	1008029-1008284	17	18
ACYPI005458-RA	ni|645902779|nb|KK921650.1|	94716-96301	nan	nan
ACYPI005919-RA	ni|645903574|nb|KK920856.1|	164115-169273	nan	nan
ACYPI008653-RA	ni|645904146|nb|KK920287.1|	1462381-1462532	16	18
ACYPI068685-RA	ni|645904078|nb|KK920353.1|	931978-932360	17	17
ACYPI000582-RA	ni|645903541|nb|KK920889.1|	3564-3724	nan	nan
ACYPI003455-RA	ni|645903971|nb|KK920460.1|	1173108-1174341	19	18
ACYPI003942-RA	ni|645904024|nb|KK920407.1|	646272-646516	14	16
ACYPI000063-RA	ni|645904278|nb|KK920222.1|	543245-543785	15	16
ACYPI004899-RA	ni|645904160|nb|KK920273.1|	738414-738741	18	18
ACYPI006235-RA	ni|645903760|nb|KK920670.1|	549325-549548	18	19
ACYPI007418-RA	ni|645904160|nb|KK920273.1|	759761-760100	18	18
ACYPI007642-RA	ni|645901974|nb|KK922455.1|	40252-40780	nan	nan
ACYPI009680-RA	ni|645903788|nb|KK920642.1|	14105-14388	16	15
ACYPI009944-RA	ni|645904260|nb|KK920228.1|	665441-665829	15	15
ACYPI070297-RA	ni|645904219|nb|KK920246.1|	813898-814077	16	17
ACYPI082722-RA	ni|645901974|nb|KK922455.1|	35775-36152	nan	nan
ACYPI003754-RA	ni|645904033|nb|KK920398.1|	51728-52526	14	16
ACYPI003986-RA	ni|645902620|nb|KK921809.1|	209538-209802	nan	nan
ACYPI004024-RA	ni|645903743|nb|KK920687.1|	1269681-1270427	19	18
ACYPI005967-RA	ni|645902497|nb|KK921932.1|	182742-183088	nan	nan
ACYPI006066-RA	ni|645903811|nb|KK920619.1|	546584-546951	15	15
ACYPI066036-RA	ni|645904071|nb|KK920360.1|	1701562-1702383	17	17
ACYPI068671-RA	ni|645903811|nb|KK920619.1|	356216-356819	15	15
ACYPI084147-RA	ni|645904033|nb|KK920398.1|	119946-120331	14	16
ACYPI31758-RA	ni|645904262|nb|KK920227.1|	1392042-1392269	17	17
ACYPI39103-RA	ni|645902105|nb|KK922324.1|	131745-132491	nan	nan
ACYPI000575-RA	ni|645903787|nb|KK920643.1|	1376064-1377647	19	18

ACYPI001809-RA	ni|645904231|nb|KK920242.1|	924989-925233	16	17
ACYPI004747-RA	ni|645903981|nb|KK920450.1|	78644-79726	16	8.6
ACYPI005400-RA	ni|645903787|nb|KK920643.1|	1271270-1272930	19	18
ACYPI005676-RA	ni|645904052|nb|KK920379.1|	576008-576381	18	16
ACYPI006929-RA	ni|645903752|nb|KK920678.1|	73879-75440	18	16
ACYPI008148-RA	ni|645904231|nb|KK920242.1|	691139-691300	16	17
ACYPI008587-RA	ni|645904065|nb|KK920366.1|	1167172-1167532	18	18
ACYPI002245-RA	ni|645904088|nb|KK920343.1|	1235452-1236597	17	18
ACYPI006364-RA	ni|645903773|nb|KK920657.1|	1109229-1109505	18	17
ACYPI008243-RA	ni|645903518|nb|KK920912.1|	151403-152288	nan	nan
ACYPI000090-RA	ni|645903841|nb|KK920589.1|	1249445-1250199	17	17
ACYPI006822-RA	ni|645903923|nb|KK920508.1|	1014195-1015151	18	18
ACYPI000681-RA	ni|645904210|nb|KK920249.1|	882672-883125	16	16
ACYPI003186-RA	ni|645903999|nb|KK920432.1|	1196286-1196686	17	15
ACYPI003944-RA	ni|645904271|nb|KK920224.1|	844435-846478	15	16
ACYPI005138-RA	ni|645903699|nb|KK920731.1|	312038-312378	nan	nan
ACYPI005308-RA	ni|645903780|nb|KK920650.1|	689781-690381	19	19
ACYPI010101-RA	ni|645904160|nb|KK920273.1|	552094-552470	18	18
ACYPI000648-RA	ni|645904156|nb|KK920277.1|	163195-163420	17	8.8
ACYPI001631-RA	ni|645904121|nb|KK920310.1|	769101-769364	14	15
ACYPI005107-RA	ni|645901950|nb|KK922479.1|	75662-75848	nan	nan
ACYPI005253-RA	ni|645903495|nb|KK920935.1|	450806-451174	nan	nan
ACYPI23168-RA	ni|645903839|nb|KK920591.1|	61233-61447	15	8.5
ACYPI000398-RA	ni|645904243|nb|KK920236.1|	1494499-1494714	15	8.5
ACYPI002805-RA	ni|645904251|nb|KK920231.1|	1854506-1854709	16	16
ACYPI004255-RA	ni|645903879|nb|KK920552.1|	488487-489167	18	18
ACYPI005394-RA	ni|645901763|nb|KK922666.1|	44341-45141	nan	nan
ACYPI006268-RA	ni|645902395|nb|KK922034.1|	93398-94604	nan	nan
ACYPI007266-RA	ni|645903965|nb|KK920466.1|	1635166-1636273	17	16
ACYPI007901-RA	ni|645904019|nb|KK920412.1|	141997-142358	19	9.4

ACYPI009158-RA	ni|645903782|nb|KK920648.1|	143193-143403	13	14
ACYPI009249-RA	ni|645903665|nb|KK920765.1|	550156-550324	nan	nan
ACYPI009771-RA	ni|645903803|nb|KK920627.1|	830837-831067	17	17
ACYPI010205-RA	ni|645903633|nb|KK920797.1|	501209-502117	nan	nan
ACYPI080138-RA	ni|645904019|nb|KK920412.1|	144059-144390	19	9.4
ACYPI000249-RA	ni|645904262|nb|KK920227.1|	2592282-2593418	17	17
ACYPI000662-RA	ni|645903920|nb|KK920511.1|	663830-664865	20	20
ACYPI001061-RA	ni|645904241|nb|KK920238.1|	154535-154849	18	17
ACYPI001698-RA	ni|645903727|nb|KK920703.1|	242818-243047	nan	nan
ACYPI002549-RA	ni|645903577|nb|KK920853.1|	594400-594644	nan	nan
ACYPI002798-RA	ni|645902083|nb|KK922346.1|	90008-90229	nan	nan
ACYPI002806-RA	ni|645903925|nb|KK920506.1|	717164-717635	19	17
ACYPI003581-RA	ni|645904192|nb|KK920255.1|	342346-342688	15	16
ACYPI004142-RA	ni|645902394|nb|KK922035.1|	307797-308242	nan	nan
ACYPI006625-RA	ni|645904075|nb|KK920356.1|	535648-536240	16	17
ACYPI006785-RA	ni|645904216|nb|KK920247.1|	225407-225673	15	16
ACYPI007197-RA	ni|645903920|nb|KK920511.1|	306694-307562	20	20
ACYPI008488-RA	ni|645903865|nb|KK920565.1|	385463-386009	15	15
ACYPI009076-RA	ni|645901682|nb|KK922747.1|	71058-71247	nan	nan
ACYPI009528-RA	ni|645904009|nb|KK920422.1|	657532-658077	17	9.4
ACYPI009915-RA	ni|645903866|nb|KK920564.1|	529253-529783	18	15
ACYPI010231-RA	ni|645902785|nb|KK921644.1|	106287-107581	nan	nan
ACYPI067699-RA	ni|645903879|nb|KK920552.1|	1323044-1323694	18	18
ACYPI000259-RA	ni|645903529|nb|KK920901.1|	101501-103180	nan	nan
ACYPI001527-RA	ni|645901875|nb|KK922554.1|	41241-41460	nan	nan
ACYPI003417-RA	ni|645904007|nb|KK920424.1|	435437-437061	18	17
ACYPI004878-RA	ni|645904132|nb|KK920301.1|	576845-577012	16	18
ACYPI006635-RA	ni|645902105|nb|KK922324.1|	61899-65886	nan	nan
ACYPI006817-RA	ni|645901537|nb|KK922892.1|	78395-78995	nan	nan
ACYPI008495-RA	ni|645902463|nb|KK921966.1|	127148-127419	nan	nan

ACYPI009739-RA	ni|645903931|nb|KK920500.1|	530063-530717	16	18
ACYPI000505-RA	ni|645903500|nb|KK920930.1|	190218-190357	nan	nan
ACYPI000733-RA	ni|645903509|nb|KK920921.1|	174691-174885	nan	nan
ACYPI001338-RA	ni|645904078|nb|KK920353.1|	873078-873394	17	17
ACYPI002126-RA	ni|645903551|nb|KK920879.1|	486163-486583	nan	nan
ACYPI003058-RA	ni|645903516|nb|KK920914.1|	489975-490205	nan	nan
ACYPI004294-RA	ni|645903566|nb|KK920864.1|	646341-646947	nan	nan
ACYPI004372-RA	ni|645903566|nb|KK920864.1|	455311-455516	nan	nan
ACYPI004697-RA	ni|645904112|nb|KK920319.1|	457243-457446	18	17
ACYPI005940-RA	ni|645903499|nb|KK920931.1|	143518-144160	nan	nan
ACYPI006828-RA	ni|645904116|nb|KK920315.1|	424251-426274	19	18
ACYPI007260-RA	ni|645903832|nb|KK920598.1|	931194-931414	18	8.8
ACYPI008241-RA	ni|645903832|nb|KK920598.1|	1034509-1034878	18	8.8
ACYPI008811-RA	ni|645903566|nb|KK920864.1|	599818-600189	nan	nan
ACYPI009386-RA	ni|645903680|nb|KK920750.1|	441315-443132	nan	nan
ACYPI010127-RA	ni|645904174|nb|KK920261.1|	377187-377618	19	17
ACYPI064024-RA	ni|645903983|nb|KK920448.1|	646868-649196	18	17
ACYPI071272-RA	ni|645904145|nb|KK920288.1|	1224093-1224638	16	9.3
ACYPI086258-RA	ni|645903848|nb|KK920582.1|	95901-96402	18	18
ACYPI22226-RA	ni|645902614|nb|KK921815.1|	50461-51444	nan	nan
ACYPI25862-RA	ni|645903973|nb|KK920458.1|	1328392-1329063	20	18
ACYPI45465-RA	ni|645904078|nb|KK920353.1|	871568-871825	17	17
ACYPI000506-RA	ni|645903701|nb|KK920729.1|	222548-222958	nan	nan
ACYPI001686-RA	ni|645904125|nb|KK920307.1|	1973333-1973838	18	17
ACYPI002240-RA	ni|645901974|nb|KK922455.1|	9259-9744	nan	nan
ACYPI004120-RA	ni|645903568|nb|KK920862.1|	266556-267027	nan	nan
ACYPI006052-RA	ni|645902619|nb|KK921810.1|	251936-254055	nan	nan
ACYPI006151-RA	ni|645903597|nb|KK920833.1|	558940-559343	nan	nan
ACYPI008028-RA	ni|645903957|nb|KK920474.1|	896646-897817	18	17
ACYPI008231-RA	ni|645903831|nb|KK920599.1|	610607-611519	18	17

ACYPI008576-RA	ni|645904087|nb|KK920344.1|	779535-780301	16	16
ACYPI008730-RA	ni|645903831|nb|KK920599.1|	955318-956461	18	17
ACYPI009782-RA	ni|645903681|nb|KK920749.1|	232986-233688	nan	nan
ACYPI009904-RA	ni|645904125|nb|KK920307.1|	1975375-1975562	18	17
ACYPI010116-RA	ni|645904111|nb|KK920320.1|	1516205-1519339	17	17
ACYPI068591-RA	ni|645904207|nb|KK920250.1|	1107127-1108889	18	20
ACYPI23235-RA	ni|645903597|nb|KK920833.1|	519791-522166	nan	nan
ACYPI40836-RA	ni|645904087|nb|KK920344.1|	794162-794863	16	16
ACYPI001982-RA	ni|645903918|nb|KK920513.1|	303681-304599	15	18
ACYPI006619-RA	ni|645903695|nb|KK920735.1|	498936-499815	nan	nan
ACYPI008866-RA	ni|645903912|nb|KK920519.1|	278636-280118	17	18
ACYPI008953-RA	ni|645902075|nb|KK922354.1|	14125-14338	nan	nan
ACYPI006532-RA	ni|645904055|nb|KK920376.1|	670247-670824	17	17
ACYPI081961-RA	ni|645902085|nb|KK922344.1|	73863-75502	nan	nan
ACYPI000065-RA	ni|645903698|nb|KK920732.1|	147048-147807	nan	nan
ACYPI000222-RA	ni|645903607|nb|KK920823.1|	306997-307436	nan	nan
ACYPI002123-RA	ni|645904068|nb|KK920363.1|	287474-288119	16	8.6
ACYPI004694-RA	ni|645902546|nb|KK921883.1|	34416-34771	nan	nan
ACYPI004981-RA	ni|645903513|nb|KK920917.1|	413628-414737	nan	nan
ACYPI005363-RA	ni|645903958|nb|KK920473.1|	25119-26075	16	16
ACYPI006178-RA	ni|645903752|nb|KK920678.1|	1088651-1089251	18	16
ACYPI006875-RA	ni|645902316|nb|KK922113.1|	5977-6230	nan	nan
ACYPI007058-RA	ni|645903627|nb|KK920803.1|	131399-132040	nan	nan
ACYPI008050-RA	ni|645904251|nb|KK920231.1|	519172-519987	16	16
ACYPI084991-RA	ni|645902630|nb|KK921799.1|	63048-63443	nan	nan
ACYPI089177-RA	ni|645903837|nb|KK920593.1|	777960-778412	18	9.8
ACYPI23999-RA	ni|645904189|nb|KK920256.1|	267210-267474	18	18
ACYPI47651-RA	ni|645903961|nb|KK920470.1|	460205-460444	17	15
ACYPI000499-RA	ni|645903858|nb|KK920572.1|	822831-823007	16	16
ACYPI001294-RA	ni|645903965|nb|KK920466.1|	1834533-1835010	17	16

ACYPI002010-RA	ni|645903530|nb|KK920900.1|	410134-412502	nan	nan
ACYPI002397-RA	ni|645904262|nb|KK920227.1|	2407415-2408168	17	17
ACYPI002657-RA	ni|645904171|nb|KK920262.1|	732525-733189	15	16
ACYPI004409-RA	ni|645903699|nb|KK920731.1|	378448-378904	nan	nan
ACYPI004431-RA	ni|645904116|nb|KK920315.1|	501005-501621	19	18
ACYPI005832-RA	ni|645903592|nb|KK920838.1|	875477-875682	nan	nan
ACYPI006227-RA	ni|645904213|nb|KK920248.1|	1725719-1726188	16	19
ACYPI006348-RA	ni|645903606|nb|KK920824.1|	135270-136147	nan	nan
ACYPI008107-RA	ni|645904177|nb|KK920260.1|	2522848-2523442	18	17
ACYPI008366-RA	ni|645903952|nb|KK920479.1|	959333-960791	16	16
ACYPI008723-RA	ni|645904065|nb|KK920366.1|	1330772-1331603	18	18
ACYPI006271-RA	ni|645903562|nb|KK920868.1|	428334-428704	nan	nan
ACYPI061311-RA	ni|645903832|nb|KK920598.1|	225647-226085	18	8.8
ACYPI46291-RA	ni|645903637|nb|KK920793.1|	523514-524301	nan	nan
ACYPI002029-RA	ni|645904231|nb|KK920242.1|	154505-155068	16	17
ACYPI003916-RA	ni|645904262|nb|KK920227.1|	2280415-2280720	17	17
ACYPI004258-RA	ni|645902496|nb|KK921933.1|	43143-45569	nan	nan
ACYPI005273-RA	ni|645901688|nb|KK922741.1|	59584-60072	nan	nan
ACYPI007721-RA	ni|645903716|nb|KK920714.1|	677228-678538	nan	nan
ACYPI008065-RA	ni|645902496|nb|KK921933.1|	72732-75601	nan	nan
ACYPI40717-RA	ni|645903780|nb|KK920650.1|	847265-848343	19	19
ACYPI001310-RA	ni|645901903|nb|KK922526.1|	48739-49992	nan	nan
ACYPI004635-RA	ni|645903768|nb|KK920662.1|	1306710-1307624	18	17
ACYPI004687-RA	ni|645904077|nb|KK920354.1|	264566-265373	16	17
ACYPI005081-RA	ni|645903931|nb|KK920500.1|	593917-594619	16	18
ACYPI005233-RA	ni|645904213|nb|KK920248.1|	991614-991998	16	19
ACYPI010163-RA	ni|645904036|nb|KK920395.1|	1173974-1174601	21	18
ACYPI071399-RA	ni|645904077|nb|KK920354.1|	260882-261128	16	17
ACYPI087808-RA	ni|645904077|nb|KK920354.1|	222087-222245	16	17
ACYPI088277-RA	ni|645902312|nb|KK922117.1|	11287-12741	nan	nan

ACYPI001052-RA	ni|645903511|nb|KK920919.1|	163280-163457	nan	nan
ACYPI001697-RA	ni|645903673|nb|KK920757.1|	106093-106310	nan	nan
ACYPI003579-RA	ni|645904065|nb|KK920366.1|	1116200-1116384	18	18
ACYPI004896-RA	ni|645903738|nb|KK920692.1|	314062-314314	18	17
ACYPI005464-RA	ni|645900799|nb|KK923630.1|	6023-6272	nan	nan
ACYPI008037-RA	ni|645903983|nb|KK920448.1|	208182-208887	18	17
ACYPI061188-RA	ni|645903567|nb|KK920863.1|	143230-144218	nan	nan
ACYPI000229-RA	ni|645904189|nb|KK920256.1|	1514046-1514692	18	18
ACYPI001683-RA	ni|645903704|nb|KK920726.1|	404991-405503	nan	nan
ACYPI001960-RA	ni|645903713|nb|KK920717.1|	521722-523395	nan	nan
ACYPI002132-RA	ni|645904021|nb|KK920410.1|	753525-753751	15	16
ACYPI002370-RA	ni|645904262|nb|KK920227.1|	757744-757990	17	17
ACYPI003296-RA	ni|645903954|nb|KK920477.1|	86066-86415	35	44
ACYPI004006-RA	ni|645903973|nb|KK920458.1|	1492006-1492263	20	18
ACYPI005593-RA	ni|645902354|nb|KK922075.1|	125620-125890	nan	nan
ACYPI008675-RA	ni|645903953|nb|KK920478.1|	1661335-1662416	18	17
ACYPI004792-RA	ni|645904268|nb|KK920225.1|	868600-868959	14	15
ACYPI005371-RA	ni|645904268|nb|KK920225.1|	324463-324783	14	15
ACYPI006603-RA	ni|645904131|nb|KK920302.1|	492245-492558	15	16
ACYPI006694-RA	ni|645902349|nb|KK922080.1|	33226-33377	nan	nan
ACYPI007238-RA	ni|645904009|nb|KK920422.1|	706924-707051	17	9.4
ACYPI007669-RA	ni|645904268|nb|KK920225.1|	516702-516930	14	15
ACYPI085301-RA	ni|645904268|nb|KK920225.1|	317448-317732	14	15
ACYPI007773-RA	ni|645903917|nb|KK920514.1|	1338436-1340705	19	17
ACYPI27105-RA	ni|645904007|nb|KK920424.1|	20753-21466	18	17
ACYPI003852-RA	ni|645904005|nb|KK920426.1|	752171-752660	17	16
ACYPI004948-RA	ni|645901380|nb|KK923049.1|	59135-62089	nan	nan
ACYPI060086-RA	ni|645902728|nb|KK921701.1|	48241-50106	nan	nan
ACYPI000720-RA	ni|645903752|nb|KK920678.1|	652309-652693	18	16
ACYPI000776-RA	ni|645904118|nb|KK920313.1|	678422-679063	17	17

ACYPI000929-RA	ni|645903971|nb|KK920460.1|	1606345-1606702	19	18
ACYPI001378-RA	ni|645903752|nb|KK920678.1|	620577-620899	18	16
ACYPI003303-RA	ni|645903634|nb|KK920796.1|	1078869-1079269	nan	nan
ACYPI004900-RA	ni|645904100|nb|KK920331.1|	274672-275395	18	22
ACYPI005532-RA	ni|645904278|nb|KK920222.1|	1011353-1012442	15	16
ACYPI007084-RA	ni|645904008|nb|KK920423.1|	1363306-1363960	16	9.5
ACYPI008834-RA	ni|645902463|nb|KK921966.1|	161240-161656	nan	nan
ACYPI001220-RA	ni|645903635|nb|KK920795.1|	410452-411276	nan	nan
ACYPI007771-RA	ni|645903902|nb|KK920529.1|	249637-251076	19	17
ACYPI56678-RA	ni|645903557|nb|KK920873.1|	917401-917614	nan	nan
ACYPI000038-RA	ni|645903989|nb|KK920442.1|	731456-731831	17	20
ACYPI000051-RA	ni|645903759|nb|KK920671.1|	97725-98872	18	17
ACYPI002536-RA	ni|645903604|nb|KK920826.1|	121822-122270	nan	nan
ACYPI003033-RA	ni|645903747|nb|KK920683.1|	949998-950947	19	18
ACYPI004378-RA	ni|645903955|nb|KK920476.1|	173976-174869	18	17
ACYPI004950-RA	ni|645904090|nb|KK920341.1|	297359-298085	15	17
ACYPI005993-RA	ni|645903884|nb|KK920547.1|	431836-432278	16	19
ACYPI007184-RA	ni|645904087|nb|KK920344.1|	810877-812424	16	16
ACYPI007468-RA	ni|645903978|nb|KK920453.1|	528780-528974	16	17
ACYPI008114-RA	ni|645903699|nb|KK920731.1|	201054-201684	nan	nan
ACYPI008713-RA	ni|645904258|nb|KK920229.1|	241912-242535	16	16
ACYPI009867-RA	ni|645903868|nb|KK920562.1|	393543-393956	16	8.7
ACYPI088273-RA	ni|645903941|nb|KK920490.1|	609045-610870	18	18
ACYPI003322-RA	ni|645902615|nb|KK921814.1|	32483-34953	nan	nan
ACYPI003863-RA	ni|645903644|nb|KK920786.1|	716927-717168	nan	nan
ACYPI005247-RA	ni|645903644|nb|KK920786.1|	397188-398703	nan	nan
ACYPI005331-RA	ni|645904004|nb|KK920427.1|	840532-840922	16	16
ACYPI005368-RA	ni|645903961|nb|KK920470.1|	866911-867475	17	15
ACYPI005747-RA	ni|645904007|nb|KK920424.1|	332050-334718	18	17
ACYPI007878-RA	ni|645904240|nb|KK920239.1|	1875285-1876724	16	16

ACYPI009239-RA	ni|645904210|nb|KK920249.1|	1964765-1964940	16	16
ACYPI009513-RA	ni|645903494|nb|KK920936.1|	436158-436346	nan	nan
ACYPI069769-RA	ni|645903538|nb|KK920892.1|	249135-251291	nan	nan
ACYPI080823-RA	ni|645903534|nb|KK920896.1|	66163-66465	nan	nan
ACYPI46801-RA	ni|645904078|nb|KK920353.1|	522324-524679	17	17
ACYPI56554-RA	ni|645903711|nb|KK920719.1|	576566-577495	nan	nan
ACYPI001063-RA	ni|645904004|nb|KK920427.1|	1239613-1240863	16	16
ACYPI003590-RA	ni|645904036|nb|KK920395.1|	821842-822574	21	18
ACYPI082898-RA	ni|645904151|nb|KK920282.1|	1055601-1056480	16	20
ACYPI000192-RA	ni|645902694|nb|KK921735.1|	14577-15368	nan	nan
ACYPI001354-RA	ni|645903681|nb|KK920749.1|	216850-217555	nan	nan
ACYPI001487-RA	ni|645904254|nb|KK920230.1|	478767-478925	16	16
ACYPI002115-RA	ni|645903768|nb|KK920662.1|	1276766-1277523	18	17
ACYPI002590-RA	ni|645904009|nb|KK920422.1|	268830-268994	17	9.4
ACYPI003235-RA	ni|645903789|nb|KK920641.1|	461715-462182	17	17
ACYPI006488-RA	ni|645903753|nb|KK920677.1|	439386-440035	17	9.8
ACYPI006597-RA	ni|645903768|nb|KK920662.1|	1278799-1279602	18	17
ACYPI007401-RA	ni|645903829|nb|KK920601.1|	679214-679590	16	15
ACYPI007695-RA	ni|645903989|nb|KK920442.1|	295902-296254	17	20
ACYPI008463-RA	ni|645904004|nb|KK920427.1|	964748-965362	16	16
ACYPI010237-RA	ni|645904028|nb|KK920403.1|	1050661-1051730	18	17
ACYPI001920-RA	ni|645903973|nb|KK920458.1|	1513606-1514135	20	18
ACYPI002460-RA	ni|645904166|nb|KK920267.1|	965375-966678	18	16
ACYPI002758-RA	ni|645904265|nb|KK920226.1|	1357564-1358173	14	14
ACYPI004615-RA	ni|645904260|nb|KK920228.1|	1049634-1050848	15	15
ACYPI006251-RA	ni|645903821|nb|KK920609.1|	1085978-1086994	17	17
ACYPI008122-RA	ni|645902779|nb|KK921650.1|	60287-60820	nan	nan
ACYPI008481-RA	ni|645904016|nb|KK920415.1|	151231-151467	12	14
ACYPI069450-RA	ni|645904201|nb|KK920252.1|	1228739-1228838	17	16
ACYPI071169-RA	ni|645903774|nb|KK920656.1|	479324-480395	16	9.2

ACYPI080920-RA	ni|645903847|nb|KK920583.1|	58591-59777	18	18
ACYPI000496-RA	ni|645904015|nb|KK920416.1|	1300407-1300888	18	16
ACYPI003043-RA	ni|645903578|nb|KK920852.1|	227415-228057	nan	nan
ACYPI003470-RA	ni|645903733|nb|KK920697.1|	678373-678510	nan	nan
ACYPI003667-RA	ni|645903532|nb|KK920898.1|	530982-531254	nan	nan
ACYPI004137-RA	ni|645902350|nb|KK922079.1|	46646-47590	nan	nan
ACYPI004268-RA	ni|645904170|nb|KK920263.1|	744155-744693	15	16
ACYPI006069-RA	ni|645903723|nb|KK920707.1|	81759-82009	nan	nan
ACYPI006708-RA	ni|645902715|nb|KK921714.1|	34127-34575	nan	nan
ACYPI007952-RA	ni|645904120|nb|KK920311.1|	329033-329441	18	18
ACYPI008721-RA	ni|645904015|nb|KK920416.1|	1249980-1250325	18	16
ACYPI001771-RA	ni|645903919|nb|KK920512.1|	1260224-1260382	17	15
ACYPI002248-RA	ni|645903716|nb|KK920714.1|	763307-763747	nan	nan
ACYPI004333-RA	ni|645904096|nb|KK920335.1|	2213452-2213887	18	17
ACYPI005033-RA	ni|645903831|nb|KK920599.1|	1003530-1004000	18	17
ACYPI006266-RA	ni|645903674|nb|KK920756.1|	609789-610695	nan	nan
ACYPI006932-RA	ni|645903747|nb|KK920683.1|	98138-99138	19	18
ACYPI008581-RA	ni|645903625|nb|KK920805.1|	613413-613709	nan	nan
ACYPI007379-RA	ni|645903831|nb|KK920599.1|	356965-358299	18	17
ACYPI088100-RA	ni|645903843|nb|KK920587.1|	974231-974700	18	16
ACYPI25873-RA	ni|645903923|nb|KK920508.1|	1172449-1173015	18	18
ACYPI42284-RA	ni|645903541|nb|KK920889.1|	95139-95315	nan	nan
ACYPI001070-RA	ni|645903748|nb|KK920682.1|	807613-807753	17	18
ACYPI002986-RA	ni|645903725|nb|KK920705.1|	164986-165419	nan	nan
ACYPI004307-RA	ni|645904260|nb|KK920228.1|	915794-916419	15	15
ACYPI006238-RA	ni|645903710|nb|KK920720.1|	722916-723469	nan	nan
ACYPI007974-RA	ni|645904111|nb|KK920320.1|	438553-439390	17	17
ACYPI072244-RA	ni|645903895|nb|KK920536.1|	451323-452561	17	16
ACYPI080327-RA	ni|645904011|nb|KK920420.1|	284055-284433	14	16
ACYPI48827-RA	ni|645902705|nb|KK921724.1|	287562-288868	nan	nan

ACYPI48834-RA	ni|645903627|nb|KK920803.1|	347973-348960	nan	nan
ACYPI001691-RA	ni|645904163|nb|KK920270.1|	1217538-1218173	16	16
ACYPI001931-RA	ni|645903938|nb|KK920493.1|	532281-532615	19	18
ACYPI003462-RA	ni|645903938|nb|KK920493.1|	527354-527759	19	18
ACYPI004222-RA	ni|645904163|nb|KK920270.1|	1220871-1222568	16	16
ACYPI005529-RA	ni|645904163|nb|KK920270.1|	1185945-1186372	16	16
ACYPI001453-RA	ni|645903787|nb|KK920643.1|	489918-490969	19	18
ACYPI001759-RA	ni|645902468|nb|KK921961.1|	70634-70897	nan	nan
ACYPI002745-RA	ni|645902349|nb|KK922080.1|	30404-30925	nan	nan
ACYPI005826-RA	ni|645902468|nb|KK921961.1|	82852-83463	nan	nan
ACYPI006885-RA	ni|645903953|nb|KK920478.1|	1168857-1169879	18	17
ACYPI008055-RA	ni|645903614|nb|KK920816.1|	827187-827825	nan	nan
ACYPI010135-RA	ni|645903953|nb|KK920478.1|	1310910-1311870	18	17
ACYPI060796-RA	ni|645903953|nb|KK920478.1|	1152914-1153282	18	17
ACYPI082595-RA	ni|645904274|nb|KK920223.1|	754622-754880	14	17
ACYPI001204-RA	ni|645903885|nb|KK920546.1|	913844-914087	19	12
ACYPI001509-RA	ni|645904195|nb|KK920254.1|	652880-654017	18	17
ACYPI002138-RA	ni|645903924|nb|KK920507.1|	715949-716349	16	12
ACYPI003301-RA	ni|645903728|nb|KK920702.1|	999112-999576	nan	nan
ACYPI003398-RA	ni|645904251|nb|KK920231.1|	369602-369966	16	16
ACYPI003508-RA	ni|645904271|nb|KK920224.1|	1648410-1649755	15	16
ACYPI004013-RA	ni|645904009|nb|KK920422.1|	1179061-1179398	17	9.4
ACYPI004014-RA	ni|645903871|nb|KK920559.1|	760280-760484	18	9.4
ACYPI005457-RA	ni|645903547|nb|KK920883.1|	627388-627777	nan	nan
ACYPI007767-RA	ni|645904145|nb|KK920288.1|	903758-904512	16	9.3
ACYPI009012-RA	ni|645902671|nb|KK921758.1|	61708-61961	nan	nan
ACYPI39408-RA	ni|645903879|nb|KK920552.1|	1240265-1240971	18	18
ACYPI006010-RA	ni|645903512|nb|KK920918.1|	476158-476593	nan	nan
ACYPI006283-RA	ni|645903637|nb|KK920793.1|	189871-190363	nan	nan
ACYPI008396-RA	ni|645904116|nb|KK920315.1|	2470307-2470964	19	18

ACYPI45707-RA	ni|645903740|nb|KK920690.1|	183121-183357	18	10
ACYPI000114-RA	ni|645903743|nb|KK920687.1|	837160-837684	19	18
ACYPI002031-RA	ni|645904201|nb|KK920252.1|	384449-385682	17	16
ACYPI004098-RA	ni|645904102|nb|KK920329.1|	27343-28738	15	16
ACYPI004913-RA	ni|645904198|nb|KK920253.1|	288076-288524	11	14
ACYPI006036-RA	ni|645903938|nb|KK920493.1|	1191107-1192840	19	18
ACYPI006112-RA	ni|645904130|nb|KK920303.1|	387276-388223	18	17
ACYPI006225-RA	ni|645904025|nb|KK920406.1|	471446-472143	15	19
ACYPI006514-RA	ni|645903703|nb|KK920727.1|	257231-257612	nan	nan
ACYPI008390-RA	ni|645904201|nb|KK920252.1|	402088-402335	17	16
ACYPI062495-RA	ni|645903973|nb|KK920458.1|	1614504-1614703	20	18
ACYPI27183-RA	ni|645903821|nb|KK920609.1|	1125950-1126406	17	17
ACYPI43885-RA	ni|645904108|nb|KK920323.1|	357232-357525	14	15
ACYPI003636-RA	ni|645904125|nb|KK920307.1|	233077-233328	18	17
ACYPI004271-RA	ni|645903860|nb|KK920570.1|	234961-235920	17	17
ACYPI008080-RA	ni|645904049|nb|KK920382.1|	413680-413891	18	18
ACYPI009955-RA	ni|645904078|nb|KK920353.1|	352390-353002	17	17
ACYPI001438-RA	ni|645902035|nb|KK922394.1|	47759-47998	nan	nan
ACYPI003418-RA	ni|645903800|nb|KK920630.1|	1314426-1315385	18	17
ACYPI003510-RA	ni|645904231|nb|KK920242.1|	167347-168576	16	17
ACYPI003915-RA	ni|645903539|nb|KK920891.1|	57975-58318	nan	nan
ACYPI004393-RA	ni|645904042|nb|KK920389.1|	138625-138784	16	16
ACYPI006509-RA	ni|645904116|nb|KK920315.1|	2319988-2320692	19	18
ACYPI008386-RA	ni|645903968|nb|KK920463.1|	577927-578187	16	16
ACYPI065189-RA	ni|645903999|nb|KK920432.1|	1279878-1280184	17	15
ACYPI45255-RA	ni|645903539|nb|KK920891.1|	57041-57280	nan	nan
ACYPI001512-RA	ni|645904057|nb|KK920374.1|	56065-56342	16	15
ACYPI002263-RA	ni|645903768|nb|KK920662.1|	1150166-1150736	18	17
ACYPI003874-RA	ni|645903703|nb|KK920727.1|	203556-203898	nan	nan
ACYPI005865-RA	ni|645904052|nb|KK920379.1|	540363-541135	18	16

ACYPI006698-RA	ni|645903523|nb|KK920907.1|	177425-177891	nan	nan
ACYPI007733-RA	ni|645902285|nb|KK922144.1|	22629-23257	nan	nan
ACYPI008202-RA	ni|645904157|nb|KK920276.1|	475768-475998	15	15
ACYPI066811-RA	ni|645903552|nb|KK920878.1|	212369-212505	nan	nan
ACYPI33616-RA	ni|645902501|nb|KK921928.1|	89200-90034	nan	nan
ACYPI001885-RA	ni|645904130|nb|KK920303.1|	1432601-1433229	18	17
ACYPI010114-RA	ni|645904130|nb|KK920303.1|	1458370-1459773	18	17
ACYPI28157-RA	ni|645903971|nb|KK920460.1|	842233-843037	19	18
ACYPI000066-RA	ni|645903749|nb|KK920681.1|	222022-222532	19	18
ACYPI000667-RA	ni|645903597|nb|KK920833.1|	566971-569213	nan	nan
ACYPI001012-RA	ni|645901733|nb|KK922696.1|	16211-18451	nan	nan
ACYPI001163-RA	ni|645904258|nb|KK920229.1|	1479260-1479645	16	16
ACYPI000828-RA	ni|645904177|nb|KK920260.1|	782203-785242	18	17
ACYPI001011-RA	ni|645903724|nb|KK920706.1|	255608-256096	nan	nan
ACYPI001079-RA	ni|645903829|nb|KK920601.1|	81478-81647	16	15
ACYPI002023-RA	ni|645903887|nb|KK920544.1|	95258-96858	15	15
ACYPI002715-RA	ni|645903699|nb|KK920731.1|	207825-208680	nan	nan
ACYPI003518-RA	ni|645904106|nb|KK920325.1|	805788-806358	17	17
ACYPI006505-RA	ni|645904242|nb|KK920237.1|	789028-790134	15	16
ACYPI007179-RA	ni|645903496|nb|KK920934.1|	858330-858799	nan	nan
ACYPI007717-RA	ni|645903773|nb|KK920657.1|	223658-225109	18	17
ACYPI009500-RA	ni|645903812|nb|KK920618.1|	977459-978237	20	18
ACYPI010246-RA	ni|645903925|nb|KK920506.1|	1265816-1266334	19	17
ACYPI073870-RA	ni|645904059|nb|KK920372.1|	458491-461892	16	9
ACYPI088059-RA	ni|645904065|nb|KK920366.1|	1259587-1260269	18	18
ACYPI36232-RA	ni|645903506|nb|KK920924.1|	99654-100452	nan	nan
ACYPI52437-RA	ni|645903829|nb|KK920601.1|	89936-90553	16	15
ACYPI000165-RA	ni|645897572|nb|KK926857.1|	730-950	nan	nan
ACYPI006756-RA	ni|645903821|nb|KK920609.1|	1060752-1061343	17	17
ACYPI008618-RA	ni|645903830|nb|KK920600.1|	41407-42066	19	24

ACYPI071178-RA	ni|645903831|nb|KK920599.1|	411996-412310	18	17
ACYPI55194-RA	ni|645903922|nb|KK920509.1|	144063-144158	15	16
ACYPI000340-RA	ni|645903957|nb|KK920474.1|	962526-963350	18	17
ACYPI000691-RA	ni|645903988|nb|KK920443.1|	125895-127501	14	16
ACYPI000955-RA	ni|645904005|nb|KK920426.1|	657821-658181	17	16
ACYPI001266-RA	ni|645904077|nb|KK920354.1|	1521562-1521763	16	17
ACYPI001851-RA	ni|645903925|nb|KK920506.1|	571471-571673	19	17
ACYPI002179-RA	ni|645903466|nb|KK920964.1|	287340-288682	nan	nan
ACYPI002584-RA	ni|645904077|nb|KK920354.1|	1490297-1490968	16	17
ACYPI003718-RA	ni|645903639|nb|KK920791.1|	242254-242437	nan	nan
ACYPI003981-RA	ni|645903800|nb|KK920630.1|	1002143-1002970	18	17
ACYPI004727-RA	ni|645903597|nb|KK920833.1|	845219-846840	nan	nan
ACYPI005001-RA	ni|645903703|nb|KK920727.1|	301262-301530	nan	nan
ACYPI006403-RA	ni|645904142|nb|KK920291.1|	69328-71035	20	23
ACYPI006626-RA	ni|645904039|nb|KK920392.1|	666983-667491	15	16
ACYPI006716-RA	ni|645904075|nb|KK920356.1|	1017782-1018551	16	17
ACYPI006897-RA	ni|645904231|nb|KK920242.1|	339818-340689	16	17
ACYPI007006-RA	ni|645902768|nb|KK921661.1|	246442-247007	nan	nan
ACYPI007154-RA	ni|645903785|nb|KK920645.1|	420292-420574	15	16
ACYPI007994-RA	ni|645904246|nb|KK920233.1|	2171131-2171357	16	17
ACYPI008056-RA	ni|645904005|nb|KK920426.1|	649656-650128	17	16
ACYPI008281-RA	ni|645904271|nb|KK920224.1|	4107686-4108670	15	16
ACYPI008489-RA	ni|645903743|nb|KK920687.1|	1241440-1241806	19	18
ACYPI008578-RA	ni|645903597|nb|KK920833.1|	497897-498481	nan	nan
ACYPI008757-RA	ni|645903491|nb|KK920939.1|	495703-496133	nan	nan
ACYPI008831-RA	ni|645902620|nb|KK921809.1|	180239-181284	nan	nan
ACYPI009478-RA	ni|645903639|nb|KK920791.1|	746128-746832	nan	nan
ACYPI071869-RA	ni|645903822|nb|KK920608.1|	316857-319377	18	20
ACYPI086018-RA	ni|645903831|nb|KK920599.1|	699674-700060	18	17
ACYPI39630-RA	ni|645901662|nb|KK922767.1|	131-368	nan	nan

ACYPI001957-RA	ni|645904067|nb|KK920364.1|	240108-240759	17	14
ACYPI002580-RA	ni|645902673|nb|KK921756.1|	141570-142041	nan	nan
ACYPI003691-RA	ni|645904122|nb|KK920309.1|	863712-864135	18	17
ACYPI005617-RA	ni|645903662|nb|KK920768.1|	191428-192016	nan	nan
ACYPI006499-RA	ni|645903796|nb|KK920634.1|	710949-711345	18	17
ACYPI007533-RA	ni|645903569|nb|KK920861.1|	167593-168964	nan	nan
ACYPI008922-RA	ni|645903827|nb|KK920603.1|	205549-205711	17	18
ACYPI010026-RA	ni|645904122|nb|KK920309.1|	1120991-1121283	18	17
ACYPI010154-RA	ni|645904094|nb|KK920337.1|	75686-75854	17	16
ACYPI53701-RA	ni|645904028|nb|KK920403.1|	865669-865891	18	17
ACYPI000157-RA	ni|645903617|nb|KK920813.1|	290263-290735	nan	nan
ACYPI009032-RA	ni|645904015|nb|KK920416.1|	1373377-1374462	18	16
ACYPI20534-RA	ni|645902196|nb|KK922233.1|	154324-155563	nan	nan
ACYPI25192-RA	ni|645903849|nb|KK920581.1|	132069-132623	17	17
ACYPI003941-RA	ni|645903778|nb|KK920652.1|	211254-211895	12	13
ACYPI38389-RA	ni|645904078|nb|KK920353.1|	106813-107034	17	17
ACYPI000002-RA	ni|645903617|nb|KK920813.1|	121155-121965	nan	nan
ACYPI000193-RA	ni|645903511|nb|KK920919.1|	462549-463071	nan	nan
ACYPI000768-RA	ni|645903617|nb|KK920813.1|	105665-106351	nan	nan
ACYPI001254-RA	ni|645904119|nb|KK920312.1|	486359-487030	19	19
ACYPI001933-RA	ni|645903466|nb|KK920964.1|	261928-262349	nan	nan
ACYPI002198-RA	ni|645903860|nb|KK920570.1|	175134-175671	17	17
ACYPI003451-RA	ni|645903965|nb|KK920466.1|	1721255-1721440	17	16
ACYPI003780-RA	ni|645901736|nb|KK922693.1|	39046-39418	nan	nan
ACYPI003820-RA	ni|645901447|nb|KK922982.1|	162-556	nan	nan
ACYPI004039-RA	ni|645904114|nb|KK920317.1|	1779524-1779867	18	18
ACYPI004071-RA	ni|645902642|nb|KK921787.1|	136868-138509	nan	nan
ACYPI005055-RA	ni|645903803|nb|KK920627.1|	872855-873066	17	17
ACYPI006313-RA	ni|645903618|nb|KK920812.1|	303349-303582	nan	nan
ACYPI006405-RA	ni|645903938|nb|KK920493.1|	426643-427800	19	18

ACYPI006608-RA	ni|645902648|nb|KK921781.1|	4233-4966	nan	nan
ACYPI006957-RA	ni|645904171|nb|KK920262.1|	1446852-1447285	15	16
ACYPI007327-RA	ni|645904097|nb|KK920334.1|	241706-242276	15	17
ACYPI007389-RA	ni|645903519|nb|KK920911.1|	531644-531969	nan	nan
ACYPI007628-RA	ni|645904153|nb|KK920280.1|	1404610-1404953	17	16
ACYPI007886-RA	ni|645903639|nb|KK920791.1|	282456-282914	nan	nan
ACYPI009007-RA	ni|645903796|nb|KK920634.1|	582023-582518	18	17
ACYPI068681-RA	ni|645904150|nb|KK920283.1|	1041291-1041787	17	17
ACYPI072774-RA	ni|645903552|nb|KK920878.1|	216659-217157	nan	nan
ACYPI34559-RA	ni|645903639|nb|KK920791.1|	288867-289465	nan	nan
ACYPI38061-RA	ni|645903704|nb|KK920726.1|	209286-210380	nan	nan
ACYPI000119-RA	ni|645903743|nb|KK920687.1|	881121-881542	19	18
ACYPI000887-RA	ni|645903985|nb|KK920446.1|	1048896-1049470	18	16
ACYPI002036-RA	ni|645904204|nb|KK920251.1|	590620-590963	15	16
ACYPI002136-RA	ni|645904098|nb|KK920333.1|	1244288-1244519	16	8.8
ACYPI002292-RA	ni|645903779|nb|KK920651.1|	604892-605170	18	16
ACYPI004870-RA	ni|645903496|nb|KK920934.1|	591520-592676	nan	nan
ACYPI005367-RA	ni|645903803|nb|KK920627.1|	819586-820867	17	17
ACYPI005597-RA	ni|645903464|nb|KK920966.1|	444766-445195	nan	nan
ACYPI005988-RA	ni|645904088|nb|KK920343.1|	197068-197525	17	18
ACYPI007282-RA	ni|645904137|nb|KK920296.1|	1638944-1639499	17	17
ACYPI007473-RA	ni|645903464|nb|KK920966.1|	224393-224963	nan	nan
ACYPI082110-RA	ni|645903762|nb|KK920668.1|	865990-866960	17	17
ACYPI003298-RA	ni|645903679|nb|KK920751.1|	338350-338854	nan	nan
ACYPI004820-RA	ni|645904067|nb|KK920364.1|	260046-260291	17	14
ACYPI005778-RA	ni|645904118|nb|KK920313.1|	707702-708492	17	17
ACYPI009542-RA	ni|645904271|nb|KK920224.1|	4049223-4049732	15	16
ACYPI064196-RA	ni|645904104|nb|KK920327.1|	302549-302872	15	15
ACYPI066987-RA	ni|645903627|nb|KK920803.1|	501669-505207	nan	nan
ACYPI001575-RA	ni|645903857|nb|KK920573.1|	251012-251425	16	16

ACYPI004663-RA	ni|645904177|nb|KK920260.1|	995312-996716	18	17
ACYPI069348-RA	ni|645903915|nb|KK920516.1|	1036154-1036478	17	17
ACYPI009909-RA	ni|645903919|nb|KK920512.1|	1244584-1245065	17	15
ACYPI002072-RA	ni|645903617|nb|KK920813.1|	178440-179694	nan	nan
ACYPI004075-RA	ni|645903837|nb|KK920593.1|	73821-74327	18	9.8
ACYPI004270-RA	ni|645903770|nb|KK920660.1|	291833-292084	12	15
ACYPI006016-RA	ni|645904137|nb|KK920296.1|	2407211-2407362	17	17
ACYPI006812-RA	ni|645903746|nb|KK920684.1|	282653-283542	15	15
ACYPI007890-RA	ni|645904106|nb|KK920325.1|	2253517-2254727	17	17
ACYPI070145-RA	ni|645903495|nb|KK920935.1|	417822-418749	nan	nan
ACYPI084112-RA	ni|645901982|nb|KK922447.1|	95119-96148	nan	nan
ACYPI26223-RA	ni|645903868|nb|KK920562.1|	614213-614381	16	8.7
ACYPI26228-RA	ni|645903569|nb|KK920861.1|	43628-44350	nan	nan
ACYPI000514-RA	ni|645902454|nb|KK921975.1|	158491-159962	nan	nan
ACYPI000617-RA	ni|645903860|nb|KK920570.1|	154050-154973	17	17
ACYPI003125-RA	ni|645904166|nb|KK920267.1|	2779492-2780314	18	16
ACYPI004100-RA	ni|645904156|nb|KK920277.1|	228291-228504	17	8.8
ACYPI004627-RA	ni|645903910|nb|KK920521.1|	561599-562098	19	17
ACYPI004976-RA	ni|645902143|nb|KK922286.1|	184851-186239	nan	nan
ACYPI005041-RA	ni|645902172|nb|KK922257.1|	110453-111178	nan	nan
ACYPI007630-RA	ni|645904088|nb|KK920343.1|	99647-100036	17	18
ACYPI008516-RA	ni|645903594|nb|KK920836.1|	552289-552505	nan	nan
ACYPI008736-RA	ni|645904043|nb|KK920388.1|	330047-334814	16	9.1
ACYPI008771-RA	ni|645903989|nb|KK920442.1|	279222-279509	17	20
ACYPI008810-RA	ni|645903843|nb|KK920587.1|	403015-403161	18	16
ACYPI080720-RA	ni|645903871|nb|KK920559.1|	793232-793517	18	9.4
ACYPI000801-RA	ni|645903761|nb|KK920669.1|	283918-284158	18	18
ACYPI000878-RA	ni|645904028|nb|KK920403.1|	99330-99586	18	17
ACYPI002371-RA	ni|645903780|nb|KK920650.1|	594562-594738	19	19
ACYPI003025-RA	ni|645903920|nb|KK920511.1|	1292481-1292778	20	20

ACYPI004077-RA	ni|645904013|nb|KK920418.1|	1060029-1060248	19	18
ACYPI004942-RA	ni|645902662|nb|KK921767.1|	32427-32683	nan	nan
ACYPI007078-RA	ni|645904106|nb|KK920325.1|	583865-584052	17	17
ACYPI070419-RA	ni|645903747|nb|KK920683.1|	1135493-1138289	19	18
ACYPI080886-RA	ni|645903956|nb|KK920475.1|	218554-220052	20	20
ACYPI085126-RA	ni|645903957|nb|KK920474.1|	1445637-1445788	18	17
ACYPI000089-RA	ni|645904130|nb|KK920303.1|	1356279-1356683	18	17
ACYPI000219-RA	ni|645903690|nb|KK920740.1|	1034038-1034219	nan	nan
ACYPI001031-RA	ni|645904246|nb|KK920233.1|	1110042-1110497	16	17
ACYPI003044-RA	ni|645904177|nb|KK920260.1|	900620-900803	18	17
ACYPI003554-RA	ni|645904047|nb|KK920384.1|	614437-615833	15	16
ACYPI004693-RA	ni|645903762|nb|KK920668.1|	344765-345186	17	17
ACYPI004827-RA	ni|645903983|nb|KK920448.1|	716230-716994	18	17
ACYPI004851-RA	ni|645904088|nb|KK920343.1|	1482527-1483333	17	18
ACYPI006683-RA	ni|645904278|nb|KK920222.1|	1175767-1176278	15	16
ACYPI006857-RA	ni|645903780|nb|KK920650.1|	276650-277152	19	19
ACYPI008468-RA	ni|645903504|nb|KK920926.1|	341109-341299	nan	nan
ACYPI009253-RA	ni|645904061|nb|KK920370.1|	101849-102864	17	16
ACYPI009376-RA	ni|645903857|nb|KK920573.1|	547497-547693	16	16
ACYPI066981-RA	ni|645904140|nb|KK920293.1|	357688-358565	22	22
ACYPI082181-RA	ni|645904160|nb|KK920273.1|	1628541-1628832	18	18
ACYPI082950-RA	ni|645903983|nb|KK920448.1|	705762-707673	18	17
ACYPI31659-RA	ni|645904177|nb|KK920260.1|	901342-901899	18	17
ACYPI002101-RA	ni|645903885|nb|KK920546.1|	189634-190509	19	12
ACYPI003316-RA	ni|645904049|nb|KK920382.1|	141156-141604	18	18
ACYPI003670-RA	ni|645903833|nb|KK920597.1|	326172-326681	17	15
ACYPI003732-RA	ni|645903821|nb|KK920609.1|	1124171-1125294	17	17
ACYPI004308-RA	ni|645904260|nb|KK920228.1|	579285-579507	15	15
ACYPI004355-RA	ni|645903721|nb|KK920709.1|	870385-870646	nan	nan
ACYPI005606-RA	ni|645904251|nb|KK920231.1|	392565-392735	16	16

ACYPI007103-RA	ni|645904228|nb|KK920243.1|	1783701-1783830	17	18
ACYPI007716-RA	ni|645902470|nb|KK921959.1|	46818-46963	nan	nan
ACYPI007795-RA	ni|645903876|nb|KK920555.1|	292698-292896	14	15
ACYPI009312-RA	ni|645903470|nb|KK920960.1|	163104-163979	nan	nan
ACYPI085603-RA	ni|645903954|nb|KK920477.1|	249860-250558	35	44
ACYPI22584-RA	ni|645904195|nb|KK920254.1|	2298196-2298496	18	17
ACYPI25601-RA	ni|645903702|nb|KK920728.1|	93073-93541	nan	nan
ACYPI29303-RA	ni|645904112|nb|KK920319.1|	2181838-2182623	18	17
ACYPI56611-RA	ni|645904251|nb|KK920231.1|	352483-353339	16	16
ACYPI000941-RA	ni|645904067|nb|KK920364.1|	829093-829550	17	14
ACYPI009480-RA	ni|645904131|nb|KK920302.1|	558772-559055	15	16
ACYPI062389-RA	ni|645903987|nb|KK920444.1|	328008-331687	15	17
ACYPI083717-RA	ni|645903971|nb|KK920460.1|	2093327-2093575	19	18
ACYPI43876-RA	ni|645904037|nb|KK920394.1|	1477728-1478876	18	17
ACYPI000208-RA	ni|645903915|nb|KK920516.1|	1487401-1488626	17	17
ACYPI000636-RA	ni|645902394|nb|KK922035.1|	65973-66172	nan	nan
ACYPI001233-RA	ni|645902060|nb|KK922369.1|	9729-11162	nan	nan
ACYPI001969-RA	ni|645903800|nb|KK920630.1|	201061-201780	18	17
ACYPI006038-RA	ni|645903858|nb|KK920572.1|	908277-908695	16	16
ACYPI006940-RA	ni|645903679|nb|KK920751.1|	395581-396385	nan	nan
ACYPI007920-RA	ni|645903598|nb|KK920832.1|	290445-291398	nan	nan
ACYPI008556-RA	ni|645903829|nb|KK920601.1|	714638-715795	16	15
ACYPI008853-RA	ni|645904012|nb|KK920419.1|	241925-242178	27	32
ACYPI082435-RA	ni|645904207|nb|KK920250.1|	509855-510200	18	20
ACYPI30602-RA	ni|645903827|nb|KK920603.1|	1117281-1117930	17	18
ACYPI41722-RA	ni|645904177|nb|KK920260.1|	1166857-1168338	18	17
ACYPI001326-RA	ni|645903627|nb|KK920803.1|	174437-174606	nan	nan
ACYPI005093-RA	ni|645903975|nb|KK920456.1|	1140547-1141974	15	8.2
ACYPI006993-RA	ni|645903521|nb|KK920909.1|	22287-23846	nan	nan
ACYPI007164-RA	ni|645902426|nb|KK922003.1|	152396-152585	nan	nan

ACYPI34001-RA	ni|645903538|nb|KK920892.1|	234004-236988	nan	nan
ACYPI005018-RA	ni|645903748|nb|KK920682.1|	324107-324781	17	18
ACYPI005477-RA	ni|645902649|nb|KK921780.1|	264878-265128	nan	nan
ACYPI006660-RA	ni|645904222|nb|KK920245.1|	784017-784294	17	12
ACYPI006915-RA	ni|645903941|nb|KK920490.1|	933727-934420	18	18
ACYPI007340-RA	ni|645902779|nb|KK921650.1|	252092-252496	nan	nan
ACYPI007439-RA	ni|645903475|nb|KK920955.1|	380957-381220	nan	nan
ACYPI009274-RA	ni|645904053|nb|KK920378.1|	517838-518305	18	17
ACYPI068713-RA	ni|645903576|nb|KK920854.1|	61640-66745	nan	nan
ACYPI087360-RA	ni|645903475|nb|KK920955.1|	569995-570471	nan	nan
ACYPI088544-RA	ni|645904045|nb|KK920386.1|	561180-562312	19	21
ACYPI008852-RA	ni|645903916|nb|KK920515.1|	200513-201243	16	23
ACYPI009394-RA	ni|645904053|nb|KK920378.1|	594785-595281	18	17
ACYPI000678-RA	ni|645904254|nb|KK920230.1|	332320-333280	16	16
ACYPI000946-RA	ni|645904192|nb|KK920255.1|	1153192-1153392	15	16
ACYPI001255-RA	ni|645901950|nb|KK922479.1|	128801-129479	nan	nan
ACYPI004471-RA	ni|645904153|nb|KK920280.1|	2075696-2077098	17	16
ACYPI004811-RA	ni|645903774|nb|KK920656.1|	584313-584493	16	9.2
ACYPI007341-RA	ni|645903957|nb|KK920474.1|	1168310-1168709	18	17
ACYPI009981-RA	ni|645903953|nb|KK920478.1|	1411822-1412942	18	17
ACYPI000925-RA	ni|645904130|nb|KK920303.1|	612240-612473	18	17
ACYPI002526-RA	ni|645903890|nb|KK920541.1|	1013869-1016550	17	18
ACYPI006443-RA	ni|645903825|nb|KK920605.1|	1018135-1018727	19	17
ACYPI009781-RA	ni|645903498|nb|KK920932.1|	946482-947862	nan	nan
ACYPI009849-RA	ni|645902011|nb|KK922418.1|	23793-24187	nan	nan
ACYPI010128-RA	ni|645899372|nb|KK925057.1|	487-1146	nan	nan
ACYPI072935-RA	ni|645902600|nb|KK921829.1|	153672-154133	nan	nan
ACYPI39720-RA	ni|645902612|nb|KK921817.1|	58499-58928	nan	nan
ACYPI000440-RA	ni|645903964|nb|KK920467.1|	368833-370094	15	8
ACYPI000475-RA	ni|645903587|nb|KK920843.1|	222774-222914	nan	nan

ACYPI001040-RA	ni|645903538|nb|KK920892.1|	241491-243755	nan	nan
ACYPI001666-RA	ni|645903964|nb|KK920467.1|	363802-364164	15	8
ACYPI002513-RA	ni|645904128|nb|KK920305.1|	868231-868594	15	8.6
ACYPI003141-RA	ni|645901605|nb|KK922824.1|	8520-9309	nan	nan
ACYPI004374-RA	ni|645903491|nb|KK920939.1|	576190-576438	nan	nan
ACYPI005613-RA	ni|645903836|nb|KK920594.1|	169031-170158	18	11
ACYPI061196-RA	ni|645903874|nb|KK920557.1|	721664-722070	18	18
ACYPI064385-RA	ni|645904045|nb|KK920386.1|	21485-22136	19	21
ACYPI072792-RA	ni|645903679|nb|KK920751.1|	63523-63749	nan	nan
ACYPI000102-RA	ni|645904228|nb|KK920243.1|	803500-803990	17	18
ACYPI003903-RA	ni|645904061|nb|KK920370.1|	252640-252912	17	16
ACYPI004515-RA	ni|645904112|nb|KK920319.1|	2115216-2116748	18	17
ACYPI008308-RA	ni|645904009|nb|KK920422.1|	208078-208633	17	9.4
ACYPI089538-RA	ni|645904116|nb|KK920315.1|	942153-942330	19	18
ACYPI000082-RA	ni|645903923|nb|KK920508.1|	282216-282451	18	18
ACYPI003682-RA	ni|645901914|nb|KK922515.1|	117307-117808	nan	nan
ACYPI063806-RA	ni|645903832|nb|KK920598.1|	959378-959882	18	8.8
ACYPI069416-RA	ni|645903548|nb|KK920882.1|	256275-256851	nan	nan
ACYPI28737-RA	ni|645904258|nb|KK920229.1|	1088182-1088354	16	16
ACYPI009350-RA	ni|645903636|nb|KK920794.1|	224466-224736	nan	nan
ACYPI009859-RA	ni|645904123|nb|KK920308.1|	1644219-1645013	16	10
ACYPI066960-RA	ni|645903636|nb|KK920794.1|	196511-196815	nan	nan
ACYPI37669-RA	ni|645903748|nb|KK920682.1|	297954-298168	17	18
ACYPI003157-RA	ni|645903925|nb|KK920506.1|	1557437-1558088	19	17
ACYPI004165-RA	ni|645903953|nb|KK920478.1|	1851719-1852599	18	17
ACYPI005479-RA	ni|645903784|nb|KK920646.1|	340916-341776	17	19
ACYPI007342-RA	ni|645903784|nb|KK920646.1|	322383-322874	17	19
ACYPI007585-RA	ni|645904222|nb|KK920245.1|	680499-681176	17	12
ACYPI010047-RA	ni|645902673|nb|KK921756.1|	147732-149229	nan	nan
ACYPI36505-RA	ni|645903765|nb|KK920665.1|	534599-537011	17	15

ACYPI49270-RA	ni|645903975|nb|KK920456.1|	1234424-1234827	15	8.2
ACYPI000156-RA	ni|645903476|nb|KK920954.1|	175927-176047	nan	nan
ACYPI001415-RA	ni|645904268|nb|KK920225.1|	985511-986014	14	15
ACYPI002180-RA	ni|645904116|nb|KK920315.1|	677401-679525	19	18
ACYPI002839-RA	ni|645903476|nb|KK920954.1|	298788-298973	nan	nan
ACYPI006432-RA	ni|645904107|nb|KK920324.1|	26186-26600	17	9.5
ACYPI006867-RA	ni|645903967|nb|KK920464.1|	171857-172289	13	14
ACYPI008078-RA	ni|645903952|nb|KK920479.1|	456578-456926	16	16
ACYPI008876-RA	ni|645904146|nb|KK920287.1|	1227542-1227893	16	18
ACYPI085421-RA	ni|645903998|nb|KK920433.1|	271170-272288	17	11
ACYPI085777-RA	ni|645904071|nb|KK920360.1|	687116-688095	17	17
ACYPI088724-RA	ni|645903971|nb|KK920460.1|	762283-763116	19	18
ACYPI20976-RA	ni|645903798|nb|KK920632.1|	108188-109055	18	17
ACYPI001909-RA	ni|645904177|nb|KK920260.1|	1151321-1151755	18	17
ACYPI003798-RA	ni|645903538|nb|KK920892.1|	113270-114186	nan	nan
ACYPI005234-RA	ni|645902529|nb|KK921900.1|	52812-53954	nan	nan
ACYPI005705-RA	ni|645903642|nb|KK920788.1|	384543-384874	nan	nan
ACYPI006179-RA	ni|645903860|nb|KK920570.1|	143051-143482	17	17
ACYPI006377-RA	ni|645903680|nb|KK920750.1|	633144-633267	nan	nan
ACYPI008157-RA	ni|645903495|nb|KK920935.1|	254128-254414	nan	nan
ACYPI008262-RA	ni|645903680|nb|KK920750.1|	644973-645217	nan	nan
ACYPI009470-RA	ni|645903979|nb|KK920452.1|	494737-495943	15	17
ACYPI010042-RA	ni|645904144|nb|KK920289.1|	913890-914433	16	9
ACYPI010138-RA	ni|645904177|nb|KK920260.1|	1138601-1139757	18	17
ACYPI56642-RA	ni|645903836|nb|KK920594.1|	964993-965471	18	11
ACYPI001286-RA	ni|645904014|nb|KK920417.1|	1442671-1442982	19	18
ACYPI005802-RA	ni|645904062|nb|KK920369.1|	82197-82446	14	15
ACYPI008134-RA	ni|645902035|nb|KK922394.1|	198241-199816	nan	nan
ACYPI009795-RA	ni|645904014|nb|KK920417.1|	1416692-1417378	19	18
ACYPI22227-RA	ni|645904065|nb|KK920366.1|	743716-743908	18	18

ACYPI34506-RA	ni|645904262|nb|KK920227.1|	357004-357215	17	17
ACYPI36199-RA	ni|645904059|nb|KK920372.1|	714530-715541	16	9
ACYPI37890-RA	ni|645904074|nb|KK920357.1|	576939-579178	17	18
ACYPI43332-RA	ni|645904114|nb|KK920317.1|	2090564-2093986	18	18
ACYPI001007-RA	ni|645904097|nb|KK920334.1|	243359-243893	15	17
ACYPI001597-RA	ni|645901319|nb|KK923110.1|	127511-129267	nan	nan
ACYPI001706-RA	ni|645904241|nb|KK920238.1|	1387883-1388465	18	17
ACYPI002909-RA	ni|645903543|nb|KK920887.1|	611055-611757	nan	nan
ACYPI006500-RA	ni|645904171|nb|KK920262.1|	796596-797212	15	16
ACYPI006735-RA	ni|645903968|nb|KK920463.1|	608505-608646	16	16
ACYPI007353-RA	ni|645903949|nb|KK920482.1|	265249-265524	17	14
ACYPI008573-RA	ni|645904114|nb|KK920317.1|	441494-441665	18	18
ACYPI010142-RA	ni|645903664|nb|KK920766.1|	175448-175612	nan	nan
ACYPI067751-RA	ni|645901319|nb|KK923110.1|	129853-130551	nan	nan
ACYPI069389-RA	ni|645903965|nb|KK920466.1|	1738635-1739353	17	16
ACYPI29477-RA	ni|645904137|nb|KK920296.1|	2391514-2391814	17	17
ACYPI001132-RA	ni|645904096|nb|KK920335.1|	1556835-1557239	18	17
ACYPI003404-RA	ni|645901808|nb|KK922621.1|	116535-116930	nan	nan
ACYPI003908-RA	ni|645904228|nb|KK920243.1|	1591174-1591688	17	18
ACYPI004941-RA	ni|645903985|nb|KK920446.1|	848569-849261	18	16
ACYPI008454-RA	ni|645903731|nb|KK920699.1|	1196390-1196631	nan	nan
ACYPI009399-RA	ni|645904042|nb|KK920389.1|	694925-695154	16	16
ACYPI005272-RA	ni|645903495|nb|KK920935.1|	38185-38524	nan	nan
ACYPI005805-RA	ni|645903592|nb|KK920838.1|	739318-739591	nan	nan
ACYPI080648-RA	ni|645904008|nb|KK920423.1|	490787-491306	16	9.5
ACYPI004799-RA	ni|645903936|nb|KK920495.1|	964041-964281	19	18
ACYPI005167-RA	ni|645904146|nb|KK920287.1|	1377193-1377659	16	18
ACYPI006699-RA	ni|645903936|nb|KK920495.1|	1054006-1054287	19	18
ACYPI006741-RA	ni|645903544|nb|KK920886.1|	344250-344705	nan	nan
ACYPI007882-RA	ni|645903923|nb|KK920508.1|	258417-258686	18	18

ACYPI008557-RA	ni|645903874|nb|KK920557.1|	201467-202088	18	18
ACYPI073717-RA	ni|645903923|nb|KK920508.1|	254468-254869	18	18
ACYPI40278-RA	ni|645903857|nb|KK920573.1|	316508-316741	16	16
ACYPI42418-RA	ni|645904151|nb|KK920282.1|	1057191-1058196	16	20
ACYPI50334-RA	ni|645902703|nb|KK921726.1|	112875-113136	nan	nan
ACYPI000103-RA	ni|645903836|nb|KK920594.1|	719371-719729	18	11
ACYPI000178-RA	ni|645904160|nb|KK920273.1|	2096928-2097166	18	18
ACYPI000563-RA	ni|645902589|nb|KK921840.1|	97255-97493	nan	nan
ACYPI001017-RA	ni|645904014|nb|KK920417.1|	1286141-1286816	19	18
ACYPI001058-RA	ni|645902589|nb|KK921840.1|	86656-87401	nan	nan
ACYPI001767-RA	ni|645904201|nb|KK920252.1|	141257-142165	17	16
ACYPI002481-RA	ni|645904242|nb|KK920237.1|	75657-76274	15	16
ACYPI002596-RA	ni|645901830|nb|KK922599.1|	36884-37080	nan	nan
ACYPI002953-RA	ni|645903796|nb|KK920634.1|	904946-908161	18	17
ACYPI003379-RA	ni|645903961|nb|KK920470.1|	102563-102797	17	15
ACYPI003639-RA	ni|645904062|nb|KK920369.1|	141376-148496	14	15
ACYPI003912-RA	ni|645903981|nb|KK920450.1|	25428-25624	16	8.6
ACYPI004568-RA	ni|645904057|nb|KK920374.1|	166051-166772	16	15
ACYPI005315-RA	ni|645902449|nb|KK921980.1|	207290-207451	nan	nan
ACYPI005585-RA	ni|645904201|nb|KK920252.1|	183108-183483	17	16
ACYPI005852-RA	ni|645904116|nb|KK920315.1|	655723-656372	19	18
ACYPI006222-RA	ni|645904201|nb|KK920252.1|	250127-252110	17	16
ACYPI006977-RA	ni|645903466|nb|KK920964.1|	284805-286825	nan	nan
ACYPI007315-RA	ni|645903666|nb|KK920764.1|	77098-77333	nan	nan
ACYPI008799-RA	ni|645903655|nb|KK920775.1|	896317-896877	nan	nan
ACYPI008845-RA	ni|645901334|nb|KK923095.1|	46647-46940	nan	nan
ACYPI009279-RA	ni|645903681|nb|KK920749.1|	258016-258201	nan	nan
ACYPI009424-RA	ni|645904045|nb|KK920386.1|	671713-672080	19	21
ACYPI010058-RA	ni|645904045|nb|KK920386.1|	685895-686433	19	21
ACYPI010234-RA	ni|645903763|nb|KK920667.1|	373613-374536	26	21

ACYPI066741-RA	ni|645904171|nb|KK920262.1|	778712-778900	15	16
ACYPI069332-RA	ni|645903833|nb|KK920597.1|	122824-123405	17	15
ACYPI085620-RA	ni|645903833|nb|KK920597.1|	204997-205425	17	15
ACYPI54769-RA	ni|645904102|nb|KK920329.1|	200895-201281	15	16
ACYPI003164-RA	ni|645902662|nb|KK921767.1|	286650-286833	nan	nan
ACYPI000175-RA	ni|645903833|nb|KK920597.1|	253578-253870	17	15
ACYPI001465-RA	ni|645903899|nb|KK920532.1|	443478-444036	15	16
ACYPI001769-RA	ni|645903627|nb|KK920803.1|	365807-366954	nan	nan
ACYPI001934-RA	ni|645903796|nb|KK920634.1|	784931-786308	18	17
ACYPI002290-RA	ni|645902469|nb|KK921960.1|	16644-17355	nan	nan
ACYPI003282-RA	ni|645903966|nb|KK920465.1|	336558-337066	16	15
ACYPI003641-RA	ni|645903681|nb|KK920749.1|	520210-521217	nan	nan
ACYPI003821-RA	ni|645903923|nb|KK920508.1|	1141891-1142874	18	18
ACYPI004613-RA	ni|645903977|nb|KK920454.1|	228959-229861	13	16
ACYPI004940-RA	ni|645904045|nb|KK920386.1|	660891-661449	19	21
ACYPI005202-RA	ni|645903996|nb|KK920435.1|	1339129-1339390	17	16
ACYPI005586-RA	ni|645904112|nb|KK920319.1|	2574053-2574610	18	17
ACYPI006528-RA	ni|645904007|nb|KK920424.1|	443940-444217	18	17
ACYPI007094-RA	ni|645903765|nb|KK920665.1|	519942-520641	17	15
ACYPI008115-RA	ni|645904115|nb|KK920316.1|	1125426-1126130	17	18
ACYPI008403-RA	ni|645903833|nb|KK920597.1|	245191-246411	17	15
ACYPI008438-RA	ni|645903980|nb|KK920451.1|	84506-85063	11	14
ACYPI008607-RA	ni|645904251|nb|KK920231.1|	448430-448934	16	16
ACYPI000033-RA	ni|645903879|nb|KK920552.1|	1065460-1065856	18	18
ACYPI000844-RA	ni|645903892|nb|KK920539.1|	401026-401886	20	18
ACYPI002959-RA	ni|645903824|nb|KK920606.1|	1109982-1111715	19	17
ACYPI004646-RA	ni|645903938|nb|KK920493.1|	1460580-1461744	19	18
ACYPI005113-RA	ni|645903983|nb|KK920448.1|	879016-880592	18	17
ACYPI006290-RA	ni|645903968|nb|KK920463.1|	1007339-1008102	16	16
ACYPI006562-RA	ni|645902552|nb|KK921877.1|	387747-388300	nan	nan

ACYPI008261-RA	ni|645904262|nb|KK920227.1|	1163111-1163678	17	17
ACYPI008452-RA	ni|645903965|nb|KK920466.1|	375425-376062	17	16
ACYPI008999-RA	ni|645903855|nb|KK920575.1|	978731-979525	16	17
ACYPI010137-RA	ni|645903738|nb|KK920692.1|	312955-313469	18	17
ACYPI000111-RA	ni|645903549|nb|KK920881.1|	19337-19606	nan	nan
ACYPI000519-RA	ni|645904067|nb|KK920364.1|	1536829-1538669	17	14
ACYPI003061-RA	ni|645904043|nb|KK920388.1|	627384-628441	16	9.1
ACYPI004588-RA	ni|645904251|nb|KK920231.1|	538202-538573	16	16
ACYPI004977-RA	ni|645904028|nb|KK920403.1|	377163-379793	18	17
ACYPI005259-RA	ni|645903917|nb|KK920514.1|	728107-728820	19	17
ACYPI005997-RA	ni|645903806|nb|KK920624.1|	64708-65009	16	9.3
ACYPI007414-RA	ni|645904043|nb|KK920388.1|	630085-630715	16	9.1
ACYPI007764-RA	ni|645903789|nb|KK920641.1|	477941-478376	17	17
ACYPI008958-RA	ni|645903742|nb|KK920688.1|	499134-499927	17	16
ACYPI070374-RA	ni|645904242|nb|KK920237.1|	639850-640379	15	16
ACYPI071956-RA	ni|645903873|nb|KK920558.1|	438336-438560	16	15
ACYPI000550-RA	ni|645903812|nb|KK920618.1|	271009-272231	20	18
ACYPI001312-RA	ni|645902035|nb|KK922394.1|	208791-209270	nan	nan
ACYPI002996-RA	ni|645904244|nb|KK920235.1|	1110343-1110524	15	14
ACYPI003177-RA	ni|645903862|nb|KK920568.1|	174507-175633	17	17
ACYPI007077-RA	ni|645903506|nb|KK920924.1|	234869-235461	nan	nan
ACYPI008063-RA	ni|645904106|nb|KK920325.1|	1970771-1971261	17	17
ACYPI008179-RA	ni|645903506|nb|KK920924.1|	206225-206395	nan	nan
ACYPI009972-RA	ni|645904002|nb|KK920429.1|	392667-393196	14	18
ACYPI061797-RA	ni|645904166|nb|KK920267.1|	2784302-2785059	18	16
ACYPI068599-RA	ni|645901290|nb|KK923139.1|	43029-43492	nan	nan
ACYPI50290-RA	ni|645904174|nb|KK920261.1|	356416-357550	19	17
ACYPI001279-RA	ni|645904177|nb|KK920260.1|	986799-987029	18	17
ACYPI003134-RA	ni|645904136|nb|KK920297.1|	2097032-2097239	16	17
ACYPI003736-RA	ni|645904122|nb|KK920309.1|	1193064-1193353	18	17

ACYPI004008-RA	ni|645902788|nb|KK921641.1|	253252-254000	nan	nan
ACYPI005049-RA	ni|645904136|nb|KK920297.1|	1817981-1818200	16	17
ACYPI005949-RA	ni|645903931|nb|KK920500.1|	564017-564471	16	18
ACYPI007598-RA	ni|645903923|nb|KK920508.1|	43228-43568	18	18
ACYPI008815-RA	ni|645903496|nb|KK920934.1|	854265-854460	nan	nan
ACYPI008830-RA	ni|645901593|nb|KK922836.1|	1586-1846	nan	nan
ACYPI009250-RA	ni|645903968|nb|KK920463.1|	469656-471177	16	16
ACYPI068084-RA	ni|645902500|nb|KK921929.1|	54575-55422	nan	nan
ACYPI084955-RA	ni|645904157|nb|KK920276.1|	1433404-1434161	15	15
ACYPI39080-RA	ni|645904146|nb|KK920287.1|	434569-435645	16	18
ACYPI000027-RA	ni|645902384|nb|KK922045.1|	184957-185581	nan	nan
ACYPI004983-RA	ni|645903535|nb|KK920895.1|	192452-192719	nan	nan
ACYPI22332-RA	ni|645904268|nb|KK920225.1|	1100793-1101054	14	15
ACYPI000534-RA	ni|645902143|nb|KK922286.1|	173814-174760	nan	nan
ACYPI000538-RA	ni|645903755|nb|KK920675.1|	656301-657340	18	16
ACYPI001583-RA	ni|645903836|nb|KK920594.1|	260909-261504	18	11
ACYPI001926-RA	ni|645903539|nb|KK920891.1|	318809-319006	nan	nan
ACYPI002286-RA	ni|645904157|nb|KK920276.1|	992108-994731	15	15
ACYPI003560-RA	ni|645904068|nb|KK920363.1|	362858-363091	16	8.6
ACYPI005000-RA	ni|645904113|nb|KK920318.1|	446869-447110	14	15
ACYPI006521-RA	ni|645903780|nb|KK920650.1|	865015-865652	19	19
ACYPI006896-RA	ni|645903946|nb|KK920485.1|	33603-33801	16	14
ACYPI008627-RA	ni|645904076|nb|KK920355.1|	1204533-1205266	17	17
ACYPI008847-RA	ni|645904158|nb|KK920275.1|	979395-979785	15	8.3
ACYPI009259-RA	ni|645904128|nb|KK920305.1|	959376-960551	15	8.6
ACYPI010034-RA	ni|645903860|nb|KK920570.1|	217249-217482	17	17
ACYPI23068-RA	ni|645901569|nb|KK922860.1|	6146-7237	nan	nan
ACYPI56077-RA	ni|645903860|nb|KK920570.1|	209729-210132	17	17
ACYPI000183-RA	ni|645904065|nb|KK920366.1|	981607-981746	18	18
ACYPI002690-RA	ni|645904072|nb|KK920359.1|	1156789-1157004	17	17

ACYPI006718-RA	ni|645902469|nb|KK921960.1|	158898-159124	nan	nan
ACYPI008756-RA	ni|645904177|nb|KK920260.1|	2525990-2526624	18	17
ACYPI088085-RA	ni|645904122|nb|KK920309.1|	168771-169261	18	17
ACYPI52542-RA	ni|645904225|nb|KK920244.1|	615493-615858	17	17
ACYPI000387-RA	ni|645903771|nb|KK920659.1|	798050-798518	16	15
ACYPI000947-RA	ni|645904070|nb|KK920361.1|	558710-558940	17	16
ACYPI001046-RA	ni|645903837|nb|KK920593.1|	861735-861955	18	9.8
ACYPI001643-RA	ni|645903994|nb|KK920437.1|	232683-233854	15	20
ACYPI002599-RA	ni|645903699|nb|KK920731.1|	823030-825201	nan	nan
ACYPI002801-RA	ni|645904138|nb|KK920295.1|	871736-872408	15	15
ACYPI006013-RA	ni|645903665|nb|KK920765.1|	258979-259719	nan	nan
ACYPI006551-RA	ni|645904043|nb|KK920388.1|	528913-529749	16	9.1
ACYPI006668-RA	ni|645904070|nb|KK920361.1|	564335-564551	17	16
ACYPI007983-RA	ni|645903746|nb|KK920684.1|	949671-949930	15	15
ACYPI009020-RA	ni|645903768|nb|KK920662.1|	960888-961077	18	17
ACYPI068701-RA	ni|645902788|nb|KK921641.1|	279060-279276	nan	nan
ACYPI071965-RA	ni|645901688|nb|KK922741.1|	69373-69563	nan	nan
ACYPI39685-RA	ni|645904097|nb|KK920334.1|	713426-714218	15	17
ACYPI55143-RA	ni|645903564|nb|KK920866.1|	380885-381072	nan	nan
ACYPI55208-RA	ni|645904189|nb|KK920256.1|	1442314-1442520	18	18
ACYPI000164-RA	ni|645902751|nb|KK921678.1|	149947-150147	nan	nan
ACYPI002794-RA	ni|645904281|nb|KK920220.1|	1146197-1146455	15	16
ACYPI003947-RA	ni|645902571|nb|KK921858.1|	128717-129534	nan	nan
ACYPI004211-RA	ni|645903971|nb|KK920460.1|	32290-33116	19	18
ACYPI005221-RA	ni|645903953|nb|KK920478.1|	1394055-1394425	18	17
ACYPI005241-RA	ni|645902556|nb|KK921873.1|	49937-50656	nan	nan
ACYPI006305-RA	ni|645903603|nb|KK920827.1|	22406-23147	nan	nan
ACYPI006324-RA	ni|645904153|nb|KK920280.1|	2096918-2097247	17	16
ACYPI006593-RA	ni|645897070|nb|KK927359.1|	1338-2331	nan	nan
ACYPI007107-RA	ni|645904094|nb|KK920337.1|	1317258-1317470	17	16

ACYPI007192-RA	ni|645903541|nb|KK920889.1|	326333-326548	nan	nan
ACYPI007800-RA	ni|645904242|nb|KK920237.1|	1338734-1338925	15	16
ACYPI008195-RA	ni|645903999|nb|KK920432.1|	1241125-1241995	17	15
ACYPI008435-RA	ni|645903971|nb|KK920460.1|	1152948-1153305	19	18
ACYPI008980-RA	ni|645896873|nb|KK927556.1|	2477-4051	nan	nan
ACYPI063394-RA	ni|645904281|nb|KK920220.1|	1193080-1193831	15	16
ACYPI066904-RA	ni|645903774|nb|KK920656.1|	586900-587258	16	9.2
ACYPI081140-RA	ni|645903603|nb|KK920827.1|	66248-66959	nan	nan
ACYPI084470-RA	ni|645904281|nb|KK920220.1|	1137111-1137383	15	16
ACYPI25475-RA	ni|645904094|nb|KK920337.1|	799952-800421	17	16
ACYPI38188-RA	ni|645903618|nb|KK920812.1|	322903-323233	nan	nan
ACYPI42579-RA	ni|645903561|nb|KK920869.1|	80183-81266	nan	nan
ACYPI004234-RA	ni|645903907|nb|KK920524.1|	571143-571354	16	9
ACYPI005770-RA	ni|645902084|nb|KK922345.1|	118102-118554	nan	nan
ACYPI005973-RA	ni|645903938|nb|KK920493.1|	756987-757419	19	18
ACYPI006676-RA	ni|645903938|nb|KK920493.1|	737733-738151	19	18
ACYPI007324-RA	ni|645903984|nb|KK920447.1|	57630-58023	18	17
ACYPI008335-RA	ni|645902426|nb|KK922003.1|	95772-96580	nan	nan
ACYPI009034-RA	ni|645903484|nb|KK920946.1|	179069-180146	nan	nan
ACYPI009193-RA	ni|645903938|nb|KK920493.1|	596777-597180	19	18
ACYPI009626-RA	ni|645903625|nb|KK920805.1|	92414-92665	nan	nan
ACYPI067654-RA	ni|645902785|nb|KK921644.1|	25524-26177	nan	nan
ACYPI081137-RA	ni|645904156|nb|KK920277.1|	411435-411682	17	8.8
ACYPI000079-RA	ni|645904096|nb|KK920335.1|	2233322-2233597	18	17
ACYPI000109-RA	ni|645901808|nb|KK922621.1|	148239-149469	nan	nan
ACYPI002279-RA	ni|645904118|nb|KK920313.1|	341388-341918	17	17
ACYPI003183-RA	ni|645903688|nb|KK920742.1|	336727-337437	nan	nan
ACYPI003980-RA	ni|645903601|nb|KK920829.1|	93146-93593	nan	nan
ACYPI006273-RA	ni|645904088|nb|KK920343.1|	695-2196	17	18
ACYPI006616-RA	ni|645903846|nb|KK920584.1|	256698-256934	17	10

ACYPI007040-RA	ni|645904009|nb|KK920422.1|	1250919-1251405	17	9.4
ACYPI008142-RA	ni|645904122|nb|KK920309.1|	1068006-1068724	18	17
ACYPI009430-RA	ni|645902129|nb|KK922300.1|	139007-140643	nan	nan
ACYPI009856-RA	ni|645904169|nb|KK920264.1|	120999-121484	16	9.5
ACYPI000218-RA	ni|645902229|nb|KK922200.1|	21019-21430	nan	nan
ACYPI000787-RA	ni|645903716|nb|KK920714.1|	772982-773444	nan	nan
ACYPI000849-RA	ni|645904036|nb|KK920395.1|	1599328-1599778	21	18
ACYPI002674-RA	ni|645904037|nb|KK920394.1|	1132586-1133153	18	17
ACYPI002787-RA	ni|645903724|nb|KK920706.1|	273927-274285	nan	nan
ACYPI003996-RA	ni|645903617|nb|KK920813.1|	467778-469324	nan	nan
ACYPI004152-RA	ni|645903923|nb|KK920508.1|	1133407-1133757	18	18
ACYPI004385-RA	ni|645903727|nb|KK920703.1|	279408-280206	nan	nan
ACYPI004738-RA	ni|645904183|nb|KK920258.1|	969466-969957	25	35
ACYPI004966-RA	ni|645903957|nb|KK920474.1|	1149806-1150831	18	17
ACYPI005060-RA	ni|645902538|nb|KK921891.1|	151786-152139	nan	nan
ACYPI005936-RA	ni|645904045|nb|KK920386.1|	1178571-1179184	19	21
ACYPI006316-RA	ni|645904281|nb|KK920220.1|	816769-817407	15	16
ACYPI006664-RA	ni|645904070|nb|KK920361.1|	310719-311376	17	16
ACYPI008467-RA	ni|645902445|nb|KK921984.1|	210468-210982	nan	nan
ACYPI009488-RA	ni|645903822|nb|KK920608.1|	393376-393498	18	20
ACYPI010096-RA	ni|645904105|nb|KK920326.1|	60496-60640	13	15
ACYPI066875-RA	ni|645903620|nb|KK920810.1|	554716-555165	nan	nan
ACYPI086610-RA	ni|645903824|nb|KK920606.1|	279595-279772	19	17
ACYPI25540-RA	ni|645901733|nb|KK922696.1|	246594-247027	nan	nan
ACYPI000058-RA	ni|645903798|nb|KK920632.1|	767915-768764	18	17
ACYPI001740-RA	ni|645903768|nb|KK920662.1|	188788-189038	18	17
ACYPI001872-RA	ni|645902238|nb|KK922191.1|	50345-50581	nan	nan
ACYPI002041-RA	ni|645904107|nb|KK920324.1|	952646-953072	17	9.5
ACYPI006409-RA	ni|645903684|nb|KK920746.1|	238386-238931	nan	nan
ACYPI007734-RA	ni|645903783|nb|KK920647.1|	123344-123764	17	17

ACYPI089162-RA	ni|645903598|nb|KK920832.1|	328497-328978	nan	nan
ACYPI52139-RA	ni|645904153|nb|KK920280.1|	2320493-2321736	17	16
ACYPI000013-RA	ni|645904195|nb|KK920254.1|	110171-111932	18	17
ACYPI000061-RA	ni|645903749|nb|KK920681.1|	644306-645583	19	18
ACYPI001762-RA	ni|645903780|nb|KK920650.1|	579350-580736	19	19
ACYPI003380-RA	ni|645903822|nb|KK920608.1|	396084-396257	18	20
ACYPI005722-RA	ni|645901356|nb|KK923073.1|	6022-6956	nan	nan
ACYPI005867-RA	ni|645902172|nb|KK922257.1|	159028-159242	nan	nan
ACYPI006525-RA	ni|645903879|nb|KK920552.1|	1654115-1654401	18	18
ACYPI088840-RA	ni|645903965|nb|KK920466.1|	1492083-1493317	17	16
ACYPI54426-RA	ni|645904136|nb|KK920297.1|	1684525-1685868	16	17
ACYPI002433-RA	ni|645903768|nb|KK920662.1|	1317363-1317940	18	17
ACYPI003087-RA	ni|645903824|nb|KK920606.1|	9558-9703	19	17
ACYPI006909-RA	ni|645904091|nb|KK920340.1|	663578-663801	15	18
ACYPI007005-RA	ni|645904222|nb|KK920245.1|	631377-631689	17	12
ACYPI008769-RA	ni|645903760|nb|KK920670.1|	740770-741114	18	19
ACYPI009420-RA	ni|645904053|nb|KK920378.1|	185444-186415	18	17
ACYPI064853-RA	ni|645903596|nb|KK920834.1|	408754-409246	nan	nan
ACYPI000031-RA	ni|645903634|nb|KK920796.1|	572293-572511	nan	nan
ACYPI000348-RA	ni|645903765|nb|KK920665.1|	929864-930059	17	15
ACYPI000789-RA	ni|645903597|nb|KK920833.1|	695933-696325	nan	nan
ACYPI000979-RA	ni|645903748|nb|KK920682.1|	527271-527526	17	18
ACYPI001434-RA	ni|645904133|nb|KK920300.1|	1034586-1035086	18	18
ACYPI001613-RA	ni|645903965|nb|KK920466.1|	1075595-1075976	17	16
ACYPI002819-RA	ni|645904177|nb|KK920260.1|	1576006-1576179	18	17
ACYPI003557-RA	ni|645903992|nb|KK920439.1|	670143-670824	15	15
ACYPI004127-RA	ni|645903992|nb|KK920439.1|	683615-684039	15	15
ACYPI004158-RA	ni|645904014|nb|KK920417.1|	1288046-1288209	19	18
ACYPI004804-RA	ni|645903932|nb|KK920499.1|	174152-174303	16	9.1
ACYPI005103-RA	ni|645904012|nb|KK920419.1|	235441-235997	27	32

ACYPI006093-RA	ni|645904058|nb|KK920373.1|	1124013-1124171	16	15
ACYPI006903-RA	ni|645903762|nb|KK920668.1|	875636-876172	17	17
ACYPI007143-RA	ni|645903614|nb|KK920816.1|	891321-891648	nan	nan
ACYPI007943-RA	ni|645903721|nb|KK920709.1|	759956-761446	nan	nan
ACYPI008464-RA	ni|645903539|nb|KK920891.1|	292513-292654	nan	nan
ACYPI008544-RA	ni|645904098|nb|KK920333.1|	389548-390018	16	8.8
ACYPI008895-RA	ni|645902011|nb|KK922418.1|	94606-97129	nan	nan
ACYPI009045-RA	ni|645903521|nb|KK920909.1|	475818-476511	nan	nan
ACYPI060572-RA	ni|645904122|nb|KK920309.1|	298971-299710	18	17
ACYPI064056-RA	ni|645903985|nb|KK920446.1|	1030509-1033100	18	16
ACYPI069386-RA	ni|645903879|nb|KK920552.1|	1086942-1089661	18	18
ACYPI083882-RA	ni|645903734|nb|KK920696.1|	789175-789382	nan	nan
ACYPI26209-RA	ni|645904115|nb|KK920316.1|	1557721-1558398	17	18
ACYPI34963-RA	ni|645904094|nb|KK920337.1|	911749-911953	17	16
ACYPI34964-RA	ni|645904094|nb|KK920337.1|	1023933-1024133	17	16
ACYPI45053-RA	ni|645903760|nb|KK920670.1|	676641-677963	18	19
ACYPI001275-RA	ni|645903941|nb|KK920490.1|	252591-252742	18	18
ACYPI001871-RA	ni|645903644|nb|KK920786.1|	170154-170877	nan	nan
ACYPI004414-RA	ni|645902788|nb|KK921641.1|	205012-205419	nan	nan
ACYPI006018-RA	ni|645903491|nb|KK920939.1|	537069-537568	nan	nan
ACYPI006464-RA	ni|645904094|nb|KK920337.1|	8220-9045	17	16
ACYPI008220-RA	ni|645904077|nb|KK920354.1|	294671-295333	16	17
ACYPI008722-RA	ni|645903774|nb|KK920656.1|	157199-158161	16	9.2
ACYPI080668-RA	ni|645903491|nb|KK920939.1|	541848-542030	nan	nan
ACYPI083883-RA	ni|645903902|nb|KK920529.1|	1139340-1139682	19	17
ACYPI43494-RA	ni|645903491|nb|KK920939.1|	560529-560693	nan	nan
ACYPI45687-RA	ni|645904164|nb|KK920269.1|	296101-296712	19	20
ACYPI001849-RA	ni|645904149|nb|KK920284.1|	462643-463120	16	16
ACYPI002485-RA	ni|645904201|nb|KK920252.1|	437267-437522	17	16
ACYPI003528-RA	ni|645902649|nb|KK921780.1|	296040-296311	nan	nan

ACYPI003697-RA	ni|645903936|nb|KK920495.1|	566077-567182	19	18
ACYPI005035-RA	ni|645903549|nb|KK920881.1|	467638-467842	nan	nan
ACYPI006802-RA	ni|645903633|nb|KK920797.1|	598602-598999	nan	nan
ACYPI008186-RA	ni|645902011|nb|KK922418.1|	89405-91913	nan	nan
ACYPI008671-RA	ni|645904133|nb|KK920300.1|	1015481-1016086	18	18
ACYPI008804-RA	ni|645903702|nb|KK920728.1|	537841-538709	nan	nan
ACYPI009808-RA	ni|645904150|nb|KK920283.1|	310181-310941	17	17
ACYPI009855-RA	ni|645903890|nb|KK920541.1|	95532-96317	17	18
ACYPI081417-RA	ni|645903678|nb|KK920752.1|	369376-369608	nan	nan
ACYPI081909-RA	ni|645904116|nb|KK920315.1|	2115574-2116251	19	18
ACYPI082646-RA	ni|645903769|nb|KK920661.1|	956970-957512	22	24
ACYPI25950-RA	ni|645903774|nb|KK920656.1|	803601-805218	16	9.2
ACYPI000862-RA	ni|645904210|nb|KK920249.1|	887986-888169	16	16
ACYPI002150-RA	ni|645904225|nb|KK920244.1|	630516-630984	17	17
ACYPI002620-RA	ni|645901057|nb|KK923372.1|	22671-23217	nan	nan
ACYPI004168-RA	ni|645904228|nb|KK920243.1|	1582839-1583310	17	18
ACYPI004488-RA	ni|645903574|nb|KK920856.1|	19612-20166	nan	nan
ACYPI004665-RA	ni|645904125|nb|KK920307.1|	36652-37219	18	17
ACYPI005847-RA	ni|645903959|nb|KK920472.1|	917138-917370	15	15
ACYPI006701-RA	ni|645903669|nb|KK920761.1|	60069-60796	nan	nan
ACYPI008566-RA	ni|645904143|nb|KK920290.1|	604666-605675	15	7.9
ACYPI008963-RA	ni|645900068|nb|KK924361.1|	32900-34808	nan	nan
ACYPI082349-RA	ni|645903494|nb|KK920936.1|	434699-435178	nan	nan
ACYPI34041-RA	ni|645903500|nb|KK920930.1|	203018-203278	nan	nan
ACYPI56663-RA	ni|645904074|nb|KK920357.1|	1028780-1029133	17	18
ACYPI000032-RA	ni|645903782|nb|KK920648.1|	179359-179729	13	14
ACYPI000077-RA	ni|645903673|nb|KK920757.1|	187211-187723	nan	nan
ACYPI004286-RA	ni|645903760|nb|KK920670.1|	672622-673008	18	19
ACYPI004467-RA	ni|645903697|nb|KK920733.1|	207850-208582	nan	nan
ACYPI006216-RA	ni|645903876|nb|KK920555.1|	133590-133815	14	15

ACYPI007141-RA	ni|645903882|nb|KK920549.1|	701186-701692	16	16
ACYPI007939-RA	ni|645904098|nb|KK920333.1|	399665-399791	16	8.8
ACYPI008728-RA	ni|645903760|nb|KK920670.1|	705601-707676	18	19
ACYPI000509-RA	ni|645903662|nb|KK920768.1|	240183-240947	nan	nan
ACYPI000629-RA	ni|645904240|nb|KK920239.1|	654026-655526	16	16
ACYPI001107-RA	ni|645903903|nb|KK920528.1|	308925-309243	12	15
ACYPI001168-RA	ni|645903825|nb|KK920605.1|	1008582-1009330	19	17
ACYPI002448-RA	ni|645903524|nb|KK920906.1|	445143-445539	nan	nan
ACYPI002830-RA	ni|645904159|nb|KK920274.1|	181392-181831	15	17
ACYPI002930-RA	ni|645904159|nb|KK920274.1|	168652-168850	15	17
ACYPI003896-RA	ni|645904207|nb|KK920250.1|	1076650-1078867	18	20
ACYPI004440-RA	ni|645903879|nb|KK920552.1|	1099466-1100015	18	18
ACYPI004785-RA	ni|645903751|nb|KK920679.1|	643409-643953	17	16
ACYPI005570-RA	ni|645903977|nb|KK920454.1|	201631-202200	13	16
ACYPI006335-RA	ni|645902638|nb|KK921791.1|	115764-115998	nan	nan
ACYPI006688-RA	ni|645903604|nb|KK920826.1|	25905-26096	nan	nan
ACYPI006818-RA	ni|645904132|nb|KK920301.1|	249661-250513	16	18
ACYPI007382-RA	ni|645903925|nb|KK920506.1|	1189184-1189389	19	17
ACYPI010238-RA	ni|645903476|nb|KK920954.1|	300449-302305	nan	nan
ACYPI062520-RA	ni|645904115|nb|KK920316.1|	159450-160048	17	18
ACYPI064212-RA	ni|645902771|nb|KK921658.1|	44404-46394	nan	nan
ACYPI085389-RA	ni|645903955|nb|KK920476.1|	531055-531570	18	17
ACYPI49124-RA	ni|645904134|nb|KK920299.1|	393202-393792	17	13
ACYPI55202-RA	ni|645904029|nb|KK920402.1|	575031-575226	14	15
ACYPI000070-RA	ni|645904160|nb|KK920273.1|	612857-613080	18	18
ACYPI000591-RA	ni|645903981|nb|KK920450.1|	11143-11539	16	8.6
ACYPI001244-RA	ni|645903717|nb|KK920713.1|	112334-112905	nan	nan
ACYPI001765-RA	ni|645903957|nb|KK920474.1|	1047815-1048656	18	17
ACYPI006369-RA	ni|645903724|nb|KK920706.1|	23762-23905	nan	nan
ACYPI006744-RA	ni|645903544|nb|KK920886.1|	287707-288169	nan	nan

ACYPI009280-RA	ni|645903796|nb|KK920634.1|	819026-819611	18	17
ACYPI009293-RA	ni|645903944|nb|KK920487.1|	232200-232646	15	17
ACYPI009455-RA	ni|645904112|nb|KK920319.1|	2494607-2495327	18	17
ACYPI065062-RA	ni|645903969|nb|KK920462.1|	268977-269416	14	16
ACYPI000805-RA	ni|645901936|nb|KK922493.1|	42095-42241	nan	nan
ACYPI003545-RA	ni|645903981|nb|KK920450.1|	1135912-1136121	16	8.6
ACYPI003674-RA	ni|645904159|nb|KK920274.1|	672353-672558	15	17
ACYPI003966-RA	ni|645903861|nb|KK920569.1|	340700-340917	16	17
ACYPI007961-RA	ni|645903671|nb|KK920759.1|	1122835-1125211	nan	nan
ACYPI072921-RA	ni|645903748|nb|KK920682.1|	801623-802390	17	18
ACYPI087735-RA	ni|645903695|nb|KK920735.1|	179600-180348	nan	nan
ACYPI34996-RA	ni|645903747|nb|KK920683.1|	104035-104488	19	18
ACYPI54877-RA	ni|645902354|nb|KK922075.1|	138068-138259	nan	nan
ACYPI000901-RA	ni|645904067|nb|KK920364.1|	876361-876993	17	14
ACYPI001547-RA	ni|645903787|nb|KK920643.1|	476251-476586	19	18
ACYPI001612-RA	ni|645903551|nb|KK920879.1|	459721-460030	nan	nan
ACYPI001780-RA	ni|645904195|nb|KK920254.1|	370779-371520	18	17
ACYPI002098-RA	ni|645904246|nb|KK920233.1|	911766-912520	16	17
ACYPI002622-RA	ni|645903866|nb|KK920564.1|	374109-374600	18	15
ACYPI002989-RA	ni|645903946|nb|KK920485.1|	49525-49739	16	14
ACYPI003280-RA	ni|645903743|nb|KK920687.1|	547481-547680	19	18
ACYPI004089-RA	ni|645903970|nb|KK920461.1|	165284-165710	17	16
ACYPI004312-RA	ni|645904086|nb|KK920345.1|	395855-397009	14	15
ACYPI004530-RA	ni|645902172|nb|KK922257.1|	177555-178127	nan	nan
ACYPI004698-RA	ni|645904271|nb|KK920224.1|	452193-452584	15	16
ACYPI005067-RA	ni|645904104|nb|KK920327.1|	313298-313596	15	15
ACYPI005769-RA	ni|645903804|nb|KK920626.1|	618759-619033	17	17
ACYPI006028-RA	ni|645903622|nb|KK920808.1|	688236-689374	nan	nan
ACYPI006243-RA	ni|645904133|nb|KK920300.1|	2772885-2773556	18	18
ACYPI007079-RA	ni|645904166|nb|KK920267.1|	2277830-2278076	18	16

ACYPI007246-RA	ni|645904228|nb|KK920243.1|	715934-716163	17	18
ACYPI007905-RA	ni|645904057|nb|KK920374.1|	1370747-1370991	16	15
ACYPI008967-RA	ni|645904112|nb|KK920319.1|	434115-434313	18	17
ACYPI009565-RA	ni|645904170|nb|KK920263.1|	460380-460647	15	16
ACYPI009997-RA	ni|645902615|nb|KK921814.1|	92722-94146	nan	nan
ACYPI072101-RA	ni|645903688|nb|KK920742.1|	1099562-1100097	nan	nan
ACYPI080074-RA	ni|645904119|nb|KK920312.1|	604534-605729	19	19
ACYPI47548-RA	ni|645903999|nb|KK920432.1|	572824-574232	17	15
ACYPI000666-RA	ni|645904043|nb|KK920388.1|	77415-78131	16	9.1
ACYPI000921-RA	ni|645904015|nb|KK920416.1|	1411325-1411546	18	16
ACYPI001656-RA	ni|645904116|nb|KK920315.1|	2067259-2067672	19	18
ACYPI002524-RA	ni|645902078|nb|KK922351.1|	50868-51074	nan	nan
ACYPI003541-RA	ni|645903829|nb|KK920601.1|	414787-415281	16	15
ACYPI003975-RA	ni|645903911|nb|KK920520.1|	628403-629095	16	17
ACYPI004424-RA	ni|645903771|nb|KK920659.1|	59045-59718	16	15
ACYPI005699-RA	ni|645903814|nb|KK920616.1|	926439-927519	17	16
ACYPI005809-RA	ni|645904116|nb|KK920315.1|	650221-651171	19	18
ACYPI006388-RA	ni|645903902|nb|KK920529.1|	1243762-1244926	19	17
ACYPI006417-RA	ni|645904125|nb|KK920307.1|	2824394-2824691	18	17
ACYPI006956-RA	ni|645904122|nb|KK920309.1|	1250331-1251362	18	17
ACYPI007268-RA	ni|645903518|nb|KK920912.1|	160605-161040	nan	nan
ACYPI007996-RA	ni|645903557|nb|KK920873.1|	752108-752334	nan	nan
ACYPI008271-RA	ni|645903902|nb|KK920529.1|	1140000-1140811	19	17
ACYPI008947-RA	ni|645903496|nb|KK920934.1|	277533-277921	nan	nan
ACYPI009777-RA	ni|645903680|nb|KK920750.1|	158914-159126	nan	nan
ACYPI010168-RA	ni|645904004|nb|KK920427.1|	293331-293571	16	16
ACYPI088144-RA	ni|645904004|nb|KK920427.1|	279715-279852	16	16
ACYPI56670-RA	ni|645902703|nb|KK921726.1|	89253-89510	nan	nan
