# Supplementary material for: The X Chromosome of Hemipteran Insects: Conservation, Dosage Compensation and Sex-Biased Expression
Source: Genome Biol Evol. 2015 Nov 10;7(12):3259–68. doi: 10.1093/gbe/evv215 (PMC4700948; doi:10.1093/gbe/evv215)
Supplement: Supplementary Data [file supp_evv215_suppl_data.zip › S2 Data (rev) AP-HH (X).pdf]

| AP | HH | gene | covF | covM |
|---|---|---|---|---|
| ACYPI003600-RA | ni\|645903834\|nb\|KK920596.1\| | 292220-293215 | 18 | 17 |
| ACYPI003888-RA | ni\|645904130\|nb\|KK920303.1\| | 641810-642455 | 18 | 17 |
| ACYPI061470-RA | ni\|645903798\|nb\|KK920632.1\| | 123621-123771 | 18 | 17 |
| ACYPI006784-RA | ni\|645903854\|nb\|KK920576.1\| | 31398-31593 | 16 | 8.4 |
| ACYPI005743-RA | ni\|645904023\|nb\|KK920408.1\| | 727289-731538 | 17 | 17 |
| ACYPI007405-RA | ni\|645903622\|nb\|KK920808.1\| | 608084-608331 | nan | nan |
| ACYPI001729-RA | ni\|645904007\|nb\|KK920424.1\| | 755776-755927 | 18 | 17 |
| ACYPI004908-RA | ni\|645903971\|nb\|KK920460.1\| | 1573467-1573685 | 19 | 18 |
| ACYPI006808-RA | ni\|645903627\|nb\|KK920803.1\| | 520496-520725 | nan | nan |
| ACYPI007677-RA | ni\|645903866\|nb\|KK920564.1\| | 375640-375831 | 18 | 15 |
| ACYPI086926-RA | ni\|645903798\|nb\|KK920632.1\| | 1045434-1045719 | 18 | 17 |
| ACYPI003057-RA | ni\|645903751\|nb\|KK920679.1\| | 773272-773743 | 17 | 16 |
| ACYPI003598-RA | ni\|645903915\|nb\|KK920516.1\| | 1444625-1444844 | 17 | 17 |
| ACYPI001110-RA | ni\|645904231\|nb\|KK920242.1\| | 456319-456444 | 16 | 17 |
| ACYPI003425-RA | ni\|645903511\|nb\|KK920919.1\| | 774604-776708 | nan | nan |
| ACYPI007100-RA | ni\|645904231\|nb\|KK920242.1\| | 651668-652542 | 16 | 17 |
| ACYPI008357-RA | ni\|645903664\|nb\|KK920766.1\| | 200467-200729 | nan | nan |
| ACYPI073873-RA | ni\|645904243\|nb\|KK920236.1\| | 1733080-1733496 | 15 | 8.5 |
| ACYPI37088-RA | ni\|645903716\|nb\|KK920714.1\| | 418040-418587 | nan | nan |
| ACYPI001611-RA | ni\|645902717\|nb\|KK921712.1\| | 10272-10529 | nan | nan |
| ACYPI003643-RA | ni\|645903740\|nb\|KK920690.1\| | 533460-533662 | 18 | 10 |
| ACYPI006233-RA | ni\|645903820\|nb\|KK920610.1\| | 304701-304902 | 18 | 19 |
| ACYPI007099-RA | ni\|645904045\|nb\|KK920386.1\| | 197113-197533 | 19 | 21 |
| ACYPI41578-RA | ni\|645904129\|nb\|KK920304.1\| | 1294829-1295273 | 16 | 17 |
| ACYPI001182-RA | ni\|645903716\|nb\|KK920714.1\| | 905157-905464 | nan | nan |
| ACYPI004986-RA | ni\|645903716\|nb\|KK920714.1\| | 622792-623290 | nan | nan |
| ACYPI005626-RA | ni\|645903539\|nb\|KK920891.1\| | 144225-144461 | nan | nan |
| ACYPI005729-RA | ni\|645903906\|nb\|KK920525.1\| | 370642-371179 | 18 | 22 |
| ACYPI006964-RA | ni\|645903831\|nb\|KK920599.1\| | 1563083-1563812 | 18 | 17 |

| | | | | |
|---|---|---|---|---|
| ACYPI009396-RA | ni\|645903470\|nb\|KK920960.1\| | 111484-112130 | nan | nan |
| ACYPI003290-RA | ni\|645904186\|nb\|KK920257.1\| | 69588-69888 | 15 | 16 |
| ACYPI009861-RA | ni\|645904245\|nb\|KK920234.1\| | 311780-312543 | 16 | 17 |
| ACYPI088713-RA | ni\|645904186\|nb\|KK920257.1\| | 113567-114079 | 15 | 16 |
| ACYPI006129-RA | ni\|645904007\|nb\|KK920424.1\| | 214486-214810 | 18 | 17 |
| ACYPI009257-RA | ni\|645904207\|nb\|KK920250.1\| | 545992-547422 | 18 | 20 |
| ACYPI010103-RA | ni\|645903778\|nb\|KK920652.1\| | 238027-238475 | 12 | 13 |
| ACYPI072994-RA | ni\|645903860\|nb\|KK920570.1\| | 348064-348497 | 17 | 17 |
| ACYPI007185-RA | ni\|645903578\|nb\|KK920852.1\| | 259655-259892 | nan | nan |
| ACYPI007681-RA | ni\|645903752\|nb\|KK920678.1\| | 405416-405704 | 18 | 16 |
| ACYPI009068-RA | ni\|645903740\|nb\|KK920690.1\| | 273330-275198 | 18 | 10 |
| ACYPI009619-RA | ni\|645904133\|nb\|KK920300.1\| | 2854802-2855298 | 18 | 18 |
| ACYPI073714-RA | ni\|645904201\|nb\|KK920252.1\| | 354533-355894 | 17 | 16 |
| ACYPI000227-RA | ni\|645903988\|nb\|KK920443.1\| | 96980-98231 | 14 | 16 |
| ACYPI001125-RA | ni\|645901914\|nb\|KK922515.1\| | 54583-55440 | nan | nan |
| ACYPI001932-RA | ni\|645903591\|nb\|KK920839.1\| | 164055-165291 | nan | nan |
| ACYPI005359-RA | ni\|645903988\|nb\|KK920443.1\| | 505487-506255 | 14 | 16 |
| ACYPI006584-RA | ni\|645903773\|nb\|KK920657.1\| | 1196725-1197140 | 18 | 17 |
| ACYPI004037-RA | ni\|645903984\|nb\|KK920447.1\| | 764452-764682 | 18 | 17 |
| ACYPI006164-RA | ni\|645903768\|nb\|KK920662.1\| | 829449-831659 | 18 | 17 |
| ACYPI50514-RA | ni\|645904133\|nb\|KK920300.1\| | 1623860-1624336 | 18 | 18 |
| ACYPI000704-RA | ni\|645903661\|nb\|KK920769.1\| | 654640-655669 | nan | nan |
| ACYPI003251-RA | ni\|645903661\|nb\|KK920769.1\| | 396573-397204 | nan | nan |
| ACYPI004501-RA | ni\|645903971\|nb\|KK920460.1\| | 698189-698696 | 19 | 18 |
| ACYPI006418-RA | ni\|645902600\|nb\|KK921829.1\| | 36785-38549 | nan | nan |
| ACYPI001585-RA | ni\|645904225\|nb\|KK920244.1\| | 638078-638523 | 17 | 17 |
| ACYPI009262-RA | ni\|645904009\|nb\|KK920422.1\| | 1096853-1097117 | 17 | 9.4 |
| ACYPI063239-RA | ni\|645903740\|nb\|KK920690.1\| | 292545-293973 | 18 | 10 |
| ACYPI55715-RA | ni\|645904241\|nb\|KK920238.1\| | 1299935-1300383 | 18 | 17 |
| ACYPI002361-RA | ni\|645903703\|nb\|KK920727.1\| | 464445-464826 | nan | nan |

| | | | | |
|---|---|---|---|---|
| ACYPI002482-RA | ni\|645904102\|nb\|KK920329.1\| | 416843-417423 | 15 | 16 |
| ACYPI003002-RA | ni\|645903879\|nb\|KK920552.1\| | 376531-377201 | 18 | 18 |
| ACYPI005545-RA | ni\|645903865\|nb\|KK920565.1\| | 365577-366039 | 15 | 15 |
| ACYPI008182-RA | ni\|645902622\|nb\|KK921807.1\| | 44576-44957 | nan | nan |
| ACYPI008652-RA | ni\|645903807\|nb\|KK920623.1\| | 213172-213410 | 12 | 14 |
| ACYPI009324-RA | ni\|645903557\|nb\|KK920873.1\| | 705219-705432 | nan | nan |
| ACYPI010069-RA | ni\|645902622\|nb\|KK921807.1\| | 78163-78609 | nan | nan |
| ACYPI087848-RA | ni\|645904102\|nb\|KK920329.1\| | 423218-423763 | 15 | 16 |
| ACYPI004718-RA | ni\|645903541\|nb\|KK920889.1\| | 128640-129120 | nan | nan |
| ACYPI006620-RA | ni\|645904016\|nb\|KK920415.1\| | 71503-73329 | 12 | 14 |
| ACYPI008482-RA | ni\|645903629\|nb\|KK920801.1\| | 127081-127314 | nan | nan |
| ACYPI31697-RA | ni\|645904007\|nb\|KK920424.1\| | 679512-679714 | 18 | 17 |
| ACYPI000014-RA | ni\|645904195\|nb\|KK920254.1\| | 121981-122438 | 18 | 17 |
| ACYPI001424-RA | ni\|645904174\|nb\|KK920261.1\| | 1852450-1852684 | 19 | 17 |
| ACYPI008222-RA | ni\|645903674\|nb\|KK920756.1\| | 246220-248151 | nan | nan |
| ACYPI46077-RA | ni\|645903857\|nb\|KK920573.1\| | 545029-545350 | 16 | 16 |
| ACYPI005622-RA | ni\|645902471\|nb\|KK921958.1\| | 183292-184343 | nan | nan |
| ACYPI009335-RA | ni\|645903502\|nb\|KK920928.1\| | 284970-285155 | nan | nan |
| ACYPI069554-RA | ni\|645904019\|nb\|KK920412.1\| | 623072-623241 | 19 | 9.4 |
| ACYPI085203-RA | ni\|645904043\|nb\|KK920388.1\| | 547753-548066 | 16 | 9.1 |
| ACYPI000681-RA | ni\|645904210\|nb\|KK920249.1\| | 882672-883125 | 16 | 16 |
| ACYPI003186-RA | ni\|645903999\|nb\|KK920432.1\| | 1196286-1196686 | 17 | 15 |
| ACYPI003944-RA | ni\|645904271\|nb\|KK920224.1\| | 844435-846478 | 15 | 16 |
| ACYPI005138-RA | ni\|645903699\|nb\|KK920731.1\| | 312038-312378 | nan | nan |
| ACYPI005308-RA | ni\|645903780\|nb\|KK920650.1\| | 689781-690381 | 19 | 19 |
| ACYPI010101-RA | ni\|645904160\|nb\|KK920273.1\| | 552094-552470 | 18 | 18 |
| ACYPI000065-RA | ni\|645903698\|nb\|KK920732.1\| | 147048-147807 | nan | nan |
| ACYPI000222-RA | ni\|645903607\|nb\|KK920823.1\| | 306997-307436 | nan | nan |
| ACYPI002123-RA | ni\|645904068\|nb\|KK920363.1\| | 287474-288119 | 16 | 8.6 |
| ACYPI004694-RA | ni\|645902546\|nb\|KK921883.1\| | 34416-34771 | nan | nan |

| | | | | |
|---|---|---|---|---|
| ACYPI004981-RA | ni\|645903513\|nb\|KK920917.1\| | 413628-414737 | nan | nan |
| ACYPI005363-RA | ni\|645903958\|nb\|KK920473.1\| | 25119-26075 | 16 | 16 |
| ACYPI006178-RA | ni\|645903752\|nb\|KK920678.1\| | 1088651-1089251 | 18 | 16 |
| ACYPI006875-RA | ni\|645902316\|nb\|KK922113.1\| | 5977-6230 | nan | nan |
| ACYPI007058-RA | ni\|645903627\|nb\|KK920803.1\| | 131399-132040 | nan | nan |
| ACYPI008050-RA | ni\|645904251\|nb\|KK920231.1\| | 519172-519987 | 16 | 16 |
| ACYPI084991-RA | ni\|645902630\|nb\|KK921799.1\| | 63048-63443 | nan | nan |
| ACYPI089177-RA | ni\|645903837\|nb\|KK920593.1\| | 777960-778412 | 18 | 9.8 |
| ACYPI23999-RA | ni\|645904189\|nb\|KK920256.1\| | 267210-267474 | 18 | 18 |
| ACYPI47651-RA | ni\|645903961\|nb\|KK920470.1\| | 460205-460444 | 17 | 15 |
| ACYPI002850-RA | ni\|645904028\|nb\|KK920403.1\| | 1725856-1726547 | 18 | 17 |
| ACYPI007248-RA | ni\|645903773\|nb\|KK920657.1\| | 1227279-1227687 | 18 | 17 |
| ACYPI008429-RA | ni\|645903741\|nb\|KK920689.1\| | 233027-233229 | 17 | 18 |
| ACYPI009072-RA | ni\|645903755\|nb\|KK920675.1\| | 733067-733703 | 18 | 16 |
| ACYPI007773-RA | ni\|645903917\|nb\|KK920514.1\| | 1338436-1340705 | 19 | 17 |
| ACYPI27105-RA | ni\|645904007\|nb\|KK920424.1\| | 20753-21466 | 18 | 17 |
| ACYPI000837-RA | ni\|645903564\|nb\|KK920866.1\| | 7905-11322 | nan | nan |
| ACYPI001120-RA | ni\|645903503\|nb\|KK920927.1\| | 37321-38592 | nan | nan |
| ACYPI001316-RA | ni\|645904011\|nb\|KK920420.1\| | 171865-172077 | 14 | 16 |
| ACYPI002722-RA | ni\|645903910\|nb\|KK920521.1\| | 371370-372329 | 19 | 17 |
| ACYPI003198-RA | ni\|645904237\|nb\|KK920240.1\| | 1203459-1204047 | 16 | 15 |
| ACYPI003565-RA | ni\|645904057\|nb\|KK920374.1\| | 1266431-1267005 | 16 | 15 |
| ACYPI005123-RA | ni\|645904237\|nb\|KK920240.1\| | 1198842-1199030 | 16 | 15 |
| ACYPI005492-RA | ni\|645903742\|nb\|KK920688.1\| | 498277-498503 | 17 | 16 |
| ACYPI005516-RA | ni\|645904065\|nb\|KK920366.1\| | 972657-973459 | 18 | 18 |
| ACYPI005583-RA | ni\|645903846\|nb\|KK920584.1\| | 55085-55966 | 17 | 10 |
| ACYPI006555-RA | ni\|645904019\|nb\|KK920412.1\| | 597169-597333 | 19 | 9.4 |
| ACYPI006803-RA | ni\|645903959\|nb\|KK920472.1\| | 718521-718767 | 15 | 15 |
| ACYPI007021-RA | ni\|645904246\|nb\|KK920233.1\| | 231517-231963 | 16 | 17 |
| ACYPI008427-RA | ni\|645903496\|nb\|KK920934.1\| | 311412-312191 | nan | nan |

| | | | | |
|---|---|---|---|---|
| ACYPI009070-RA | ni\|645904106\|nb\|KK920325.1\| | 917795-921017 | 17 | 17 |
| ACYPI009533-RA | ni\|645902742\|nb\|KK921687.1\| | 63946-64188 | nan | nan |
| ACYPI48369-RA | ni\|645904052\|nb\|KK920379.1\| | 2054940-2056756 | 18 | 16 |
| ACYPI005028-RA | ni\|645903866\|nb\|KK920564.1\| | 507344-508540 | 18 | 15 |
| ACYPI005554-RA | ni\|645904026\|nb\|KK920405.1\| | 403919-405645 | 17 | 19 |
| ACYPI38268-RA | ni\|645897346\|nb\|KK927083.1\| | 41626-47887 | nan | nan |
| ACYPI006010-RA | ni\|645903512\|nb\|KK920918.1\| | 476158-476593 | nan | nan |
| ACYPI006283-RA | ni\|645903637\|nb\|KK920793.1\| | 189871-190363 | nan | nan |
| ACYPI008396-RA | ni\|645904116\|nb\|KK920315.1\| | 2470307-2470964 | 19 | 18 |
| ACYPI45707-RA | ni\|645903740\|nb\|KK920690.1\| | 183121-183357 | 18 | 10 |
| ACYPI003298-RA | ni\|645903679\|nb\|KK920751.1\| | 338350-338854 | nan | nan |
| ACYPI004820-RA | ni\|645904067\|nb\|KK920364.1\| | 260046-260291 | 17 | 14 |
| ACYPI005778-RA | ni\|645904118\|nb\|KK920313.1\| | 707702-708492 | 17 | 17 |
| ACYPI009542-RA | ni\|645904271\|nb\|KK920224.1\| | 4049223-4049732 | 15 | 16 |
| ACYPI064196-RA | ni\|645904104\|nb\|KK920327.1\| | 302549-302872 | 15 | 15 |
| ACYPI066987-RA | ni\|645903627\|nb\|KK920803.1\| | 501669-505207 | nan | nan |
| ACYPI001326-RA | ni\|645903627\|nb\|KK920803.1\| | 174437-174606 | nan | nan |
| ACYPI005093-RA | ni\|645903975\|nb\|KK920456.1\| | 1140547-1141974 | 15 | 8.2 |
| ACYPI006993-RA | ni\|645903521\|nb\|KK920909.1\| | 22287-23846 | nan | nan |
| ACYPI007164-RA | ni\|645902426\|nb\|KK922003.1\| | 152396-152585 | nan | nan |
| ACYPI34001-RA | ni\|645903538\|nb\|KK920892.1\| | 234004-236988 | nan | nan |
| ACYPI000102-RA | ni\|645904228\|nb\|KK920243.1\| | 803500-803990 | 17 | 18 |
| ACYPI003903-RA | ni\|645904061\|nb\|KK920370.1\| | 252640-252912 | 17 | 16 |
| ACYPI004515-RA | ni\|645904112\|nb\|KK920319.1\| | 2115216-2116748 | 18 | 17 |
| ACYPI008308-RA | ni\|645904009\|nb\|KK920422.1\| | 208078-208633 | 17 | 9.4 |
| ACYPI089538-RA | ni\|645904116\|nb\|KK920315.1\| | 942153-942330 | 19 | 18 |
| ACYPI001030-RA | ni\|645903934\|nb\|KK920497.1\| | 244198-244576 | 15 | 16 |
| ACYPI001668-RA | ni\|645903847\|nb\|KK920583.1\| | 645752-646613 | 18 | 18 |
| ACYPI001797-RA | ni\|645904041\|nb\|KK920390.1\| | 136452-138459 | 20 | 33 |
| ACYPI002929-RA | ni\|645904222\|nb\|KK920245.1\| | 415542-416016 | 17 | 12 |

| | | | | |
|---|---|---|---|---|
| ACYPI004850-RA | ni\|645904045\|nb\|KK920386.1\| | 1238286-1238462 | 19 | 21 |
| ACYPI006758-RA | ni\|645903633\|nb\|KK920797.1\| | 573722-574669 | nan | nan |
| ACYPI007368-RA | ni\|645903795\|nb\|KK920635.1\| | 147135-147517 | 14 | 16 |
| ACYPI009885-RA | ni\|645903608\|nb\|KK920822.1\| | 283435-283713 | nan | nan |
| ACYPI072215-RA | ni\|645903905\|nb\|KK920526.1\| | 76972-78488 | 15 | 8.3 |
| ACYPI087592-RA | ni\|645903795\|nb\|KK920635.1\| | 93841-94075 | 14 | 16 |
| ACYPI29851-RA | ni\|645903985\|nb\|KK920446.1\| | 148456-148691 | 18 | 16 |
| ACYPI56793-RA | ni\|645903865\|nb\|KK920565.1\| | 236202-236438 | 15 | 15 |
| ACYPI000534-RA | ni\|645902143\|nb\|KK922286.1\| | 173814-174760 | nan | nan |
| ACYPI000538-RA | ni\|645903755\|nb\|KK920675.1\| | 656301-657340 | 18 | 16 |
| ACYPI001583-RA | ni\|645903836\|nb\|KK920594.1\| | 260909-261504 | 18 | 11 |
| ACYPI001926-RA | ni\|645903539\|nb\|KK920891.1\| | 318809-319006 | nan | nan |
| ACYPI002286-RA | ni\|645904157\|nb\|KK920276.1\| | 992108-994731 | 15 | 15 |
| ACYPI003560-RA | ni\|645904068\|nb\|KK920363.1\| | 362858-363091 | 16 | 8.6 |
| ACYPI005000-RA | ni\|645904113\|nb\|KK920318.1\| | 446869-447110 | 14 | 15 |
| ACYPI006521-RA | ni\|645903780\|nb\|KK920650.1\| | 865015-865652 | 19 | 19 |
| ACYPI006896-RA | ni\|645903946\|nb\|KK920485.1\| | 33603-33801 | 16 | 14 |
| ACYPI008627-RA | ni\|645904076\|nb\|KK920355.1\| | 1204533-1205266 | 17 | 17 |
| ACYPI008847-RA | ni\|645904158\|nb\|KK920275.1\| | 979395-979785 | 15 | 8.3 |
| ACYPI009259-RA | ni\|645904128\|nb\|KK920305.1\| | 959376-960551 | 15 | 8.6 |
| ACYPI010034-RA | ni\|645903860\|nb\|KK920570.1\| | 217249-217482 | 17 | 17 |
| ACYPI23068-RA | ni\|645901569\|nb\|KK922860.1\| | 6146-7237 | nan | nan |
| ACYPI56077-RA | ni\|645903860\|nb\|KK920570.1\| | 209729-210132 | 17 | 17 |
| ACYPI000079-RA | ni\|645904096\|nb\|KK920335.1\| | 2233322-2233597 | 18 | 17 |
| ACYPI000109-RA | ni\|645901808\|nb\|KK922621.1\| | 148239-149469 | nan | nan |
| ACYPI002279-RA | ni\|645904118\|nb\|KK920313.1\| | 341388-341918 | 17 | 17 |
| ACYPI003183-RA | ni\|645903688\|nb\|KK920742.1\| | 336727-337437 | nan | nan |
| ACYPI003980-RA | ni\|645903601\|nb\|KK920829.1\| | 93146-93593 | nan | nan |
| ACYPI006273-RA | ni\|645904088\|nb\|KK920343.1\| | 695-2196 | 17 | 18 |
| ACYPI006616-RA | ni\|645903846\|nb\|KK920584.1\| | 256698-256934 | 17 | 10 |

| | | | | |
|---|---|---|---|---|
| ACYPI007040-RA | ni\|645904009\|nb\|KK920422.1\| | 1250919-1251405 | 17 | 9.4 |
| ACYPI008142-RA | ni\|645904122\|nb\|KK920309.1\| | 1068006-1068724 | 18 | 17 |
| ACYPI009430-RA | ni\|645902129\|nb\|KK922300.1\| | 139007-140643 | nan | nan |
| ACYPI009856-RA | ni\|645904169\|nb\|KK920264.1\| | 120999-121484 | 16 | 9.5 |
| ACYPI000218-RA | ni\|645902229\|nb\|KK922200.1\| | 21019-21430 | nan | nan |
| ACYPI000787-RA | ni\|645903716\|nb\|KK920714.1\| | 772982-773444 | nan | nan |
| ACYPI000849-RA | ni\|645904036\|nb\|KK920395.1\| | 1599328-1599778 | 21 | 18 |
| ACYPI002674-RA | ni\|645904037\|nb\|KK920394.1\| | 1132586-1133153 | 18 | 17 |
| ACYPI002787-RA | ni\|645903724\|nb\|KK920706.1\| | 273927-274285 | nan | nan |
| ACYPI003996-RA | ni\|645903617\|nb\|KK920813.1\| | 467778-469324 | nan | nan |
| ACYPI004152-RA | ni\|645903923\|nb\|KK920508.1\| | 1133407-1133757 | 18 | 18 |
| ACYPI004385-RA | ni\|645903727\|nb\|KK920703.1\| | 279408-280206 | nan | nan |
| ACYPI004738-RA | ni\|645904183\|nb\|KK920258.1\| | 969466-969957 | 25 | 35 |
| ACYPI004966-RA | ni\|645903957\|nb\|KK920474.1\| | 1149806-1150831 | 18 | 17 |
| ACYPI005060-RA | ni\|645902538\|nb\|KK921891.1\| | 151786-152139 | nan | nan |
| ACYPI005936-RA | ni\|645904045\|nb\|KK920386.1\| | 1178571-1179184 | 19 | 21 |
| ACYPI006316-RA | ni\|645904281\|nb\|KK920220.1\| | 816769-817407 | 15 | 16 |
| ACYPI006664-RA | ni\|645904070\|nb\|KK920361.1\| | 310719-311376 | 17 | 16 |
| ACYPI008467-RA | ni\|645902445\|nb\|KK921984.1\| | 210468-210982 | nan | nan |
| ACYPI009488-RA | ni\|645903822\|nb\|KK920608.1\| | 393376-393498 | 18 | 20 |
| ACYPI010096-RA | ni\|645904105\|nb\|KK920326.1\| | 60496-60640 | 13 | 15 |
| ACYPI066875-RA | ni\|645903620\|nb\|KK920810.1\| | 554716-555165 | nan | nan |
| ACYPI086610-RA | ni\|645903824\|nb\|KK920606.1\| | 279595-279772 | 19 | 17 |
| ACYPI25540-RA | ni\|645901733\|nb\|KK922696.1\| | 246594-247027 | nan | nan |
| ACYPI002433-RA | ni\|645903768\|nb\|KK920662.1\| | 1317363-1317940 | 18 | 17 |
| ACYPI003087-RA | ni\|645903824\|nb\|KK920606.1\| | 9558-9703 | 19 | 17 |
| ACYPI006909-RA | ni\|645904091\|nb\|KK920340.1\| | 663578-663801 | 15 | 18 |
| ACYPI007005-RA | ni\|645904222\|nb\|KK920245.1\| | 631377-631689 | 17 | 12 |
| ACYPI008769-RA | ni\|645903760\|nb\|KK920670.1\| | 740770-741114 | 18 | 19 |
| ACYPI009420-RA | ni\|645904053\|nb\|KK920378.1\| | 185444-186415 | 18 | 17 |

| | | | | |
|---|---|---|---|---|
| ACYPI064853-RA | ni\|645903596\|nb\|KK920834.1\| | 408754-409246 | nan | nan |
