# Supplementary material for: The X Chromosome of Hemipteran Insects: Conservation, Dosage Compensation and Sex-Biased Expression
Source: Genome Biol Evol. 2015 Nov 10;7(12):3259–68. doi: 10.1093/gbe/evv215 (PMC4700948; doi:10.1093/gbe/evv215)
Supplement: Supplementary Data [file supp_evv215_suppl_data.zip › S2 Data (rev) AP-HV (A).pdf]

AP	HV	gene	covF	covM
ACYPI000427-RA	gi|646779006|gb|KK961585.1|	202694-213347	20	9.9
ACYPI001670-RA	gi|646745543|gb|KK962350.1|	694900-695263	21	10
ACYPI003555-RA	gi|646778803|gb|KK961591.1|	580793-581684	15	7.8
ACYPI065154-RA	gi|646776762|gb|KK961659.1|	699217-699426	21	8.9
ACYPI006033-RA	gi|646778983|gb|KK961586.1|	297548-299398	20	9.9
ACYPI53104-RA	gi|646767850|gb|KK961823.1|	847900-852526	21	9.8
ACYPI006974-RA	gi|646694989|gb|KK966396.1|	9127-12831	12	9.8
ACYPI007586-RA	gi|646782357|gb|KK961494.1|	6680439-6681649	21	9.2
ACYPI007765-RA	gi|646777065|gb|KK961647.1|	579470-580885	20	7.9
ACYPI003220-RA	gi|646779038|gb|KK961584.1|	2320231-2320857	21	6.9
ACYPI007117-RA	gi|646748129|gb|KK962169.1|	1016679-1021280	21	9.8
ACYPI008697-RA	gi|646777729|gb|KK961621.1|	674935-676655	21	9.8
ACYPI004513-RA	gi|646780147|gb|KK961556.1|	1827963-1828393	18	7.5
ACYPI29050-RA	gi|646770723|gb|KK961775.1|	384002-384548	20	8.8
ACYPI008304-RA	gi|646776368|gb|KK961679.1|	926583-930114	21	9.7
ACYPI006979-RA	gi|646777089|gb|KK961646.1|	425760-428811	20	9.4
ACYPI007210-RA	gi|646766985|gb|KK961844.1|	185288-192663	21	9.7
ACYPI004073-RA	gi|646775807|gb|KK961708.1|	774589-775300	21	8.6
ACYPI005053-RA	gi|646747884|gb|KK962186.1|	871535-872155	22	11
ACYPI008267-RA	gi|646767265|gb|KK961836.1|	899006-901026	22	9.9
ACYPI003815-RA	gi|646745997|gb|KK962319.1|	522269-522522	21	8.5
ACYPI33986-RA	gi|646747424|gb|KK962220.1|	1412228-1418228	22	5.7
ACYPI56628-RA	gi|646750341|gb|KK962044.1|	489726-490876	19	5.6
ACYPI003304-RA	gi|646751569|gb|KK961985.1|	313648-314234	21	9.2
ACYPI000201-RA	gi|646778029|gb|KK961613.1|	2338609-2338874	21	8.6
ACYPI006131-RA	gi|646778517|gb|KK961599.1|	700522-703862	20	9.6
ACYPI003244-RA	gi|646776429|gb|KK961676.1|	2429979-2431841	20	9.9
ACYPI088574-RA	gi|646779298|gb|KK961577.1|	342713-351888	20	9.1
ACYPI001807-RA	gi|646781659|gb|KK961513.1|	5267786-5268047	22	9.8

ACYPI006279-RA	gi|646640511|gb|KK968988.1|	35927-36632	21	10
ACYPI010028-RA	gi|646747482|gb|KK962215.1|	284871-285500	21	9.2
ACYPI31612-RA	gi|646746974|gb|KK962252.1|	1269536-1273215	23	6.1
ACYPI006611-RA	gi|646776184|gb|KK961688.1|	914142-920365	21	9.6
ACYPI006649-RA	gi|646777802|gb|KK961619.1|	91410-96661	24	9
ACYPI009254-RA	gi|646781893|gb|KK961506.1|	1553013-1556132	19	8.5
ACYPI003976-RA	gi|646773721|gb|KK961747.1|	3070756-3087407	21	9.2
ACYPI53798-RA	gi|646769102|gb|KK961799.1|	2305770-2308233	20	8.3
ACYPI006101-RA	gi|646779869|gb|KK961563.1|	327235-331078	17	8.8
ACYPI001710-RA	gi|646777182|gb|KK961642.1|	2323435-2330458	20	9
ACYPI085022-RA	gi|646750600|gb|KK962030.1|	1121578-1123517	23	8.6
ACYPI35733-RA	gi|646742902|gb|KK962544.1|	228959-229907	18	7.1
ACYPI000030-RA	gi|646776588|gb|KK961666.1|	2584306-2591169	20	5.8
ACYPI004428-RA	gi|646751012|gb|KK962011.1|	205541-207542	22	5.6
ACYPI009462-RA	gi|646771092|gb|KK961771.1|	1163884-1164640	23	9.7
ACYPI061325-RA	gi|646780723|gb|KK961542.1|	1737731-1740817	21	9.4
ACYPI32342-RA	gi|646780723|gb|KK961542.1|	1896610-1903730	21	9.4
ACYPI000265-RA	gi|646595672|gb|KK977392.1|	665-1940	21	8.2
ACYPI003206-RA	gi|646766426|gb|KK961862.1|	316962-317482	20	7.1
ACYPI065077-RA	gi|646780222|gb|KK961554.1|	3518572-3520165	20	9.7
ACYPI069803-RA	gi|646769275|gb|KK961796.1|	2079979-2087886	22	5.6
ACYPI005814-RA	gi|646777108|gb|KK961645.1|	1198488-1200408	17	6.5
ACYPI007278-RA	gi|646780147|gb|KK961556.1|	2320679-2321695	18	7.5
ACYPI008351-RA	gi|646746868|gb|KK962259.1|	620512-623808	21	10
ACYPI000086-RA	gi|646738524|gb|KK962968.1|	61726-63075	24	11
ACYPI000446-RA	gi|646781421|gb|KK961521.1|	1473544-1476975	21	8.7
ACYPI001096-RA	gi|646769102|gb|KK961799.1|	1906841-1907065	20	8.3
ACYPI001146-RA	gi|646776024|gb|KK961697.1|	1100000-1100423	22	9.2
ACYPI002411-RA	gi|646778948|gb|KK961587.1|	1260121-1261019	20	7.3
ACYPI066985-RA	gi|646740694|gb|KK962742.1|	92818-93987	20	7.9

ACYPI21107-RA	gi|646749755|gb|KK962075.1|	489313-490584	22	9.2
ACYPI007426-RA	gi|646741594|gb|KK962653.1|	259680-264345	17	6.6
ACYPI005793-RA	gi|646769102|gb|KK961799.1|	180565-181419	20	8.3
ACYPI002300-RA	gi|646781732|gb|KK961511.1|	2969307-2971241	22	9.8
ACYPI007522-RA	gi|646775723|gb|KK961712.1|	1905555-1905868	21	9.5
ACYPI008001-RA	gi|646777313|gb|KK961637.1|	1860377-1860852	20	8.2
ACYPI009872-RA	gi|646775471|gb|KK961725.1|	1403012-1403250	20	10
ACYPI003422-RA	gi|646698808|gb|KK966113.1|	31629-34291	23	6.5
ACYPI005250-RA	gi|646765880|gb|KK961885.1|	900418-901413	23	10
ACYPI007136-RA	gi|646765880|gb|KK961885.1|	914787-922715	23	10
ACYPI004157-RA	gi|646746355|gb|KK962294.1|	1011490-1024685	22	10
ACYPI006090-RA	gi|646769102|gb|KK961799.1|	355238-358125	20	8.3
ACYPI007973-RA	gi|646769102|gb|KK961799.1|	322512-322982	20	8.3
ACYPI009841-RA	gi|646775944|gb|KK961701.1|	56031-56296	22	9.3
ACYPI36355-RA	gi|646776024|gb|KK961697.1|	1632771-1634123	22	9.2
ACYPI002040-RA	gi|646732591|gb|KK963652.1|	829-1456	17	7.3
ACYPI002689-RA	gi|646749794|gb|KK962073.1|	646060-646346	20	8.4
ACYPI064572-RA	gi|646766137|gb|KK961874.1|	393581-394611	20	9
ACYPI009487-RA	gi|646780752|gb|KK961541.1|	3351133-3351684	21	8
ACYPI002789-RA	gi|646775488|gb|KK961724.1|	354096-355626	19	7.8
ACYPI004656-RA	gi|646775488|gb|KK961724.1|	666126-667962	19	7.8
ACYPI005456-RA	gi|646780925|gb|KK961536.1|	949915-950232	22	9.9
ACYPI001978-RA	gi|646780858|gb|KK961538.1|	2482212-2492121	21	10
ACYPI003867-RA	gi|646778948|gb|KK961587.1|	1819307-1819795	20	7.3
ACYPI008974-RA	gi|646750204|gb|KK962051.1|	64911-65267	19	7.5
ACYPI060585-RA	gi|646780270|gb|KK961553.1|	2583802-2585072	22	8.6
ACYPI083423-RA	gi|646770641|gb|KK961776.1|	946840-948042	20	9.1
ACYPI002301-RA	gi|646781043|gb|KK961532.1|	2294310-2296497	26	11
ACYPI27242-RA	gi|646738652|gb|KK962955.1|	129394-130166	19	8.8
ACYPI003266-RA	gi|646777955|gb|KK961615.1|	638449-638730	21	10

ACYPI001059-RA	gi|646758845|gb|KK961921.1|	1390254-1396147	22	9.5
ACYPI001660-RA	gi|646712499|gb|KK965372.1|	33689-34165	14	6.5
ACYPI002612-RA	gi|646779298|gb|KK961577.1|	2973102-2974775	20	9.1
ACYPI004520-RA	gi|646779298|gb|KK961577.1|	2976283-2980139	20	9.1
ACYPI008955-RA	gi|646776389|gb|KK961678.1|	2209118-2211754	22	9.5
ACYPI005299-RA	gi|646742413|gb|KK962582.1|	832617-841435	23	10
ACYPI004437-RA	gi|646776389|gb|KK961678.1|	2082231-2082627	22	9.5
ACYPI006356-RA	gi|646781421|gb|KK961521.1|	1630913-1631321	21	8.7
ACYPI007547-RA	gi|646780311|gb|KK961552.1|	1447646-1448282	21	9.7
ACYPI000737-RA	gi|646748200|gb|KK962164.1|	474820-476214	19	7.7
ACYPI009613-RA	gi|646779741|gb|KK961566.1|	3087859-3088087	20	8.5
ACYPI35323-RA	gi|646767813|gb|KK961824.1|	234889-241469	17	6.3
ACYPI003236-RA	gi|646780311|gb|KK961552.1|	1346051-1346654	21	9.7
ACYPI009357-RA	gi|646768406|gb|KK961812.1|	258091-258331	20	9.6
ACYPI000271-RA	gi|646778448|gb|KK961601.1|	1740135-1741627	19	8.3
ACYPI000860-RA	gi|646780222|gb|KK961554.1|	3738423-3750138	20	9.7
ACYPI002742-RA	gi|646776351|gb|KK961680.1|	2481434-2482398	22	10
ACYPI009848-RA	gi|646748407|gb|KK962151.1|	233521-235352	22	9.1
ACYPI000181-RA	gi|646781564|gb|KK961516.1|	637656-637956	23	9.4
ACYPI008558-RA	gi|646781421|gb|KK961521.1|	4773198-4780250	21	8.7
ACYPI003234-RA	gi|646748955|gb|KK962119.1|	669317-676548	19	9.9
ACYPI004340-RA	gi|646781083|gb|KK961531.1|	2327159-2328393	19	8.5
ACYPI006272-RA	gi|646750354|gb|KK962043.1|	198445-198952	20	9.2
ACYPI006910-RA	gi|646776389|gb|KK961678.1|	1059735-1059932	22	9.5
ACYPI009023-RA	gi|646768631|gb|KK961807.1|	735501-741815	19	9.2
ACYPI001379-RA	gi|646738604|gb|KK962960.1|	159271-161115	19	8.4
ACYPI38317-RA	gi|646561369|gb|KK983956.1|	5134-6081	17	7.4
ACYPI005627-RA	gi|646777089|gb|KK961646.1|	757626-761528	20	9.4
ACYPI007507-RA	gi|646775635|gb|KK961716.1|	2367226-2367574	22	10
ACYPI003483-RA	gi|646782276|gb|KK961497.1|	1548160-1551014	21	9.7

ACYPI007926-RA	gi|646730603|gb|KK963922.1|	93526-93748	18	8.3
ACYPI001508-RA	gi|646778948|gb|KK961587.1|	1870106-1870370	20	7.3
ACYPI53120-RA	gi|646779976|gb|KK961560.1|	1397731-1398391	16	7.9
ACYPI003559-RA	gi|646777416|gb|KK961632.1|	808295-808939	22	8.8
ACYPI005509-RA	gi|646781421|gb|KK961521.1|	4436012-4437649	21	8.7
ACYPI000698-RA	gi|646746243|gb|KK962302.1|	220034-222108	12	7.7
ACYPI001356-RA	gi|646776219|gb|KK961687.1|	1973493-1973897	21	9.9
ACYPI003241-RA	gi|646780723|gb|KK961542.1|	1959785-1960954	21	9.4
ACYPI007065-RA	gi|646782334|gb|KK961495.1|	7711720-7712181	21	9
ACYPI008933-RA	gi|646780925|gb|KK961536.1|	1887988-1888248	22	9.9
ACYPI36116-RA	gi|646750702|gb|KK962025.1|	471993-472681	12	6.5
ACYPI001791-RA	gi|646775901|gb|KK961703.1|	294861-295278	20	9.6
ACYPI003522-RA	gi|646743394|gb|KK962507.1|	418259-418636	20	8.8
ACYPI003669-RA	gi|646773721|gb|KK961747.1|	1007516-1007922	21	9.2
ACYPI002653-RA	gi|646782087|gb|KK961501.1|	1763854-1768289	20	8.7
ACYPI003687-RA	gi|646776409|gb|KK961677.1|	40185-54017	17	8.4
ACYPI008600-RA	gi|646781043|gb|KK961532.1|	3003655-3006976	26	11
ACYPI010124-RA	gi|646781564|gb|KK961516.1|	730325-730644	23	9.4
ACYPI002108-RA	gi|646544887|gb|KK987173.1|	256-865	14	6
ACYPI54601-RA	gi|646749573|gb|KK962085.1|	286841-287824	23	10
ACYPI002846-RA	gi|646780010|gb|KK961559.1|	2614772-2615721	22	10
ACYPI005044-RA	gi|646780147|gb|KK961556.1|	2209925-2210176	18	7.5
ACYPI005692-RA	gi|646782276|gb|KK961497.1|	4909580-4909813	21	9.7
ACYPI008188-RA	gi|646782276|gb|KK961497.1|	4729408-4733508	21	9.7
ACYPI28869-RA	gi|646782276|gb|KK961497.1|	4685936-4687052	21	9.7
ACYPI008325-RA	gi|646772786|gb|KK961754.1|	357201-358960	22	9.6
ACYPI007413-RA	gi|646782334|gb|KK961495.1|	6585760-6587408	21	9
ACYPI001071-RA	gi|646760564|gb|KK961914.1|	16075-16612	21	8.8
ACYPI005660-RA	gi|646777729|gb|KK961621.1|	1881764-1888857	21	9.8
ACYPI001190-RA	gi|646780978|gb|KK961534.1|	1910695-1912865	20	8

ACYPI003077-RA	gi|646751856|gb|KK961977.1|	104921-105121	18	8.5
ACYPI007561-RA	gi|646780358|gb|KK961551.1|	2280632-2284883	21	8.8
ACYPI008256-RA	gi|646746339|gb|KK962295.1|	245856-252050	20	8.2
ACYPI007392-RA	gi|646747914|gb|KK962184.1|	874016-876573	23	10
ACYPI083041-RA	gi|646779498|gb|KK961572.1|	1200915-1201372	22	5.6
ACYPI002730-RA	gi|646781873|gb|KK961507.1|	1771011-1773062	20	9.1
ACYPI003764-RA	gi|646770323|gb|KK961780.1|	218387-218816	19	7.1
ACYPI005306-RA	gi|646775451|gb|KK961726.1|	1211785-1213304	20	5.6
ACYPI005858-RA	gi|646781344|gb|KK961523.1|	661397-672200	21	8.9
ACYPI005292-RA	gi|646576893|gb|KK981008.1|	13725-13998	8.2	3.8
ACYPI008132-RA	gi|646630825|gb|KK970725.1|	60-1211	22	6.2
ACYPI000310-RA	gi|646775842|gb|KK961706.1|	1806878-1810297	22	9
ACYPI002865-RA	gi|646775258|gb|KK961737.1|	657982-658236	19	9.8
ACYPI39790-RA	gi|646747094|gb|KK962244.1|	222205-225192	20	10
ACYPI006572-RA	gi|646741719|gb|KK962642.1|	303527-304437	21	8.9
ACYPI000930-RA	gi|646732431|gb|KK963675.1|	257184-258001	24	9.6
ACYPI003888-RA	gi|646751458|gb|KK961989.1|	565234-565510	21	5.1
ACYPI007680-RA	gi|646762797|gb|KK961906.1|	636444-637871	19	5.6
ACYPI000702-RA	gi|646751103|gb|KK962006.1|	587949-591119	20	8.3
ACYPI010019-RA	gi|646779936|gb|KK961561.1|	1521301-1521777	19	9.6
ACYPI008472-RA	gi|646781243|gb|KK961526.1|	1249478-1252833	21	8.9
ACYPI003757-RA	gi|646781893|gb|KK961506.1|	370852-371343	19	8.5
ACYPI062526-RA	gi|646741778|gb|KK962637.1|	184131-185071	19	6.1
ACYPI081347-RA	gi|646750557|gb|KK962032.1|	595091-596039	20	6.1
ACYPI009496-RA	gi|646768717|gb|KK961805.1|	218756-218970	20	8
ACYPI24660-RA	gi|646738532|gb|KK962967.1|	35877-37853	12	9.3
ACYPI001405-RA	gi|646780627|gb|KK961545.1|	1261614-1262532	20	7.7
ACYPI002636-RA	gi|646762191|gb|KK961910.1|	768673-769023	18	7.1
ACYPI004549-RA	gi|646781732|gb|KK961511.1|	1664817-1668346	22	9.8
ACYPI001396-RA	gi|646764075|gb|KK961900.1|	652769-656504	18	6.5

ACYPI004883-RA	gi|646780222|gb|KK961554.1|	1181800-1185558	20	9.7
ACYPI006122-RA	gi|646781313|gb|KK961524.1|	1713596-1714426	19	8.5
ACYPI006781-RA	gi|646748580|gb|KK962141.1|	431095-435122	20	5.6
ACYPI008005-RA	gi|646738812|gb|KK962939.1|	198961-200077	20	8.1
ACYPI007068-RA	gi|646745372|gb|KK962364.1|	169035-170654	23	10
ACYPI008935-RA	gi|646745372|gb|KK962364.1|	231280-233646	23	10
ACYPI002247-RA	gi|646782334|gb|KK961495.1|	2443819-2444685	21	9
ACYPI004126-RA	gi|646781243|gb|KK961526.1|	4103877-4107442	21	8.9
ACYPI004824-RA	gi|646743164|gb|KK962524.1|	64291-67104	19	7.1
ACYPI006059-RA	gi|646781243|gb|KK961526.1|	4078636-4082723	21	8.9
ACYPI002837-RA	gi|646781183|gb|KK961528.1|	3597736-3600845	20	9.5
ACYPI000455-RA	gi|646776148|gb|KK961690.1|	1761896-1762566	21	9.4
ACYPI005566-RA	gi|646781421|gb|KK961521.1|	4948889-4949336	21	8.7
ACYPI009317-RA	gi|646781421|gb|KK961521.1|	1809600-1809864	21	8.7
ACYPI000476-RA	gi|646780222|gb|KK961554.1|	94940-95790	20	9.7
ACYPI002544-RA	gi|646782168|gb|KK961499.1|	5396962-5397413	21	9.4
ACYPI004442-RA	gi|646776510|gb|KK961671.1|	875906-876449	20	7.8
ACYPI006748-RA	gi|646781601|gb|KK961515.1|	3502615-3503549	19	8.6
ACYPI006712-RA	gi|646781421|gb|KK961521.1|	451694-452266	21	8.7
ACYPI006053-RA	gi|646746432|gb|KK962289.1|	159675-160772	14	7.8
ACYPI007934-RA	gi|646782357|gb|KK961494.1|	8662262-8664381	21	9.2
ACYPI004537-RA	gi|646782043|gb|KK961502.1|	3316555-3317209	20	9.2
ACYPI000961-RA	gi|646781421|gb|KK961521.1|	918407-918938	21	8.7
ACYPI087743-RA	gi|646781421|gb|KK961521.1|	417491-418092	21	8.7
ACYPI065346-RA	gi|646775368|gb|KK961731.1|	1351821-1360271	17	8.4
ACYPI082935-RA	gi|646564073|gb|KK983396.1|	4085-4753	4.5	5.2
ACYPI006386-RA	gi|646776762|gb|KK961659.1|	1286762-1289278	21	8.9
ACYPI009503-RA	gi|646776762|gb|KK961659.1|	1278958-1284746	21	8.9
ACYPI010145-RA	gi|646758845|gb|KK961921.1|	1141558-1141957	22	9.5
ACYPI38303-RA	gi|646758845|gb|KK961921.1|	1042669-1042911	22	9.5

ACYPI000918-RA	gi|646767439|gb|KK961832.1|	17249-20743	23	10
ACYPI001339-RA	gi|646775723|gb|KK961712.1|	1181440-1181891	21	9.5
ACYPI003855-RA	gi|646780889|gb|KK961537.1|	4076145-4076560	22	10
ACYPI005787-RA	gi|646776219|gb|KK961687.1|	1625147-1625384	21	9.9
ACYPI007646-RA	gi|646747482|gb|KK962215.1|	547666-549340	21	9.2
ACYPI008317-RA	gi|646775576|gb|KK961719.1|	1132574-1134047	20	8.6
ACYPI009551-RA	gi|646768669|gb|KK961806.1|	316739-317160	18	9.1
ACYPI010190-RA	gi|646750267|gb|KK962048.1|	490400-490683	16	8.2
ACYPI001827-RA	gi|646780441|gb|KK961549.1|	2099232-2099803	21	9.5
ACYPI005644-RA	gi|646753890|gb|KK961949.1|	744826-745365	20	8.9
ACYPI000199-RA	gi|646766451|gb|KK961861.1|	44377-45280	19	8.9
ACYPI003531-RA	gi|646782043|gb|KK961502.1|	3482110-3485209	20	9.2
ACYPI003991-RA	gi|646776904|gb|KK961654.1|	1541135-1547468	21	9.3
ACYPI001216-RA	gi|646750389|gb|KK962041.1|	421687-421872	21	9.5
ACYPI005672-RA	gi|646747471|gb|KK962216.1|	712268-712957	24	6.4
ACYPI008793-RA	gi|646776429|gb|KK961676.1|	1359768-1360536	20	9.9
ACYPI060526-RA	gi|646780406|gb|KK961550.1|	589392-597180	22	5.9
ACYPI001736-RA	gi|646738604|gb|KK962960.1|	28697-32181	19	8.4
ACYPI002345-RA	gi|646764801|gb|KK961898.1|	322441-322718	20	9.7
ACYPI004227-RA	gi|646781344|gb|KK961523.1|	243344-243909	21	8.9
ACYPI004872-RA	gi|646782168|gb|KK961499.1|	7298011-7298940	21	9.4
ACYPI005524-RA	gi|646776647|gb|KK961663.1|	2188197-2190437	23	9.2
ACYPI009267-RA	gi|646769102|gb|KK961799.1|	1848323-1855596	20	8.3
ACYPI004108-RA	gi|646780953|gb|KK961535.1|	4335589-4336024	20	9.8
ACYPI071995-RA	gi|646745171|gb|KK962378.1|	721517-721903	19	8.1
ACYPI004485-RA	gi|646749616|gb|KK962083.1|	1156594-1157883	22	10
ACYPI000807-RA	gi|646769001|gb|KK961801.1|	323682-324208	20	8.5
ACYPI004054-RA	gi|646764801|gb|KK961898.1|	628095-628569	20	9.7
ACYPI008778-RA	gi|646501544|gb|KK994170.1|	1242-2507	20	8.3
ACYPI065650-RA	gi|646778840|gb|KK961590.1|	1141803-1143071	20	9.9

ACYPI007675-RA	gi|646781873|gb|KK961507.1|	4637236-4639260	20	9.1
ACYPI010223-RA	gi|646748086|gb|KK962172.1|	949892-950239	20	10
ACYPI000068-RA	gi|646775987|gb|KK961699.1|	249584-250665	19	7.5
ACYPI007981-RA	gi|646777207|gb|KK961641.1|	589995-590360	20	8.5
ACYPI51589-RA	gi|646751628|gb|KK961983.1|	683735-684914	18	7.6
ACYPI001009-RA	gi|646779262|gb|KK961578.1|	2213172-2217946	20	8.1
ACYPI067466-RA	gi|646732119|gb|KK963722.1|	68693-70735	20	9.4
ACYPI004686-RA	gi|646777665|gb|KK961623.1|	1956668-1957068	21	9.7
ACYPI007803-RA	gi|646777665|gb|KK961623.1|	2286062-2289263	21	9.7
ACYPI001408-RA	gi|646749532|gb|KK962087.1|	209132-209467	14	6
ACYPI002592-RA	gi|646776588|gb|KK961666.1|	2333642-2334573	20	5.8
ACYPI004303-RA	gi|646776514|gb|KK961670.1|	903227-909723	20	9.6
ACYPI006921-RA	gi|646779898|gb|KK961562.1|	2429549-2431550	19	9.3
ACYPI000303-RA	gi|646781118|gb|KK961530.1|	4711619-4713254	21	10
ACYPI007298-RA	gi|646777288|gb|KK961638.1|	1509819-1512984	22	6.1
ACYPI49591-RA	gi|646775383|gb|KK961730.1|	583468-586028	19	8.7
ACYPI005934-RA	gi|646779337|gb|KK961576.1|	419527-419980	18	9
ACYPI002810-RA	gi|646745372|gb|KK962364.1|	894991-895620	23	10
ACYPI003918-RA	gi|646750341|gb|KK962044.1|	885961-886389	19	5.6
ACYPI004218-RA	gi|646779337|gb|KK961576.1|	967751-968216	18	9
ACYPI005317-RA	gi|646750600|gb|KK962030.1|	1446627-1447523	23	8.6
ACYPI005982-RA	gi|646781212|gb|KK961527.1|	2286713-2289432	21	10
ACYPI006773-RA	gi|646776822|gb|KK961657.1|	1538043-1539284	19	7.9
ACYPI007845-RA	gi|646777955|gb|KK961615.1|	705608-706507	21	10
ACYPI007989-RA	gi|646720990|gb|KK965002.1|	73604-74083	20	8.4
ACYPI008388-RA	gi|646766482|gb|KK961860.1|	398798-399806	17	6.7
ACYPI007697-RA	gi|646780827|gb|KK961539.1|	3648534-3649122	20	9.4
ACYPI000081-RA	gi|646777495|gb|KK961629.1|	1861075-1861463	19	8.7
ACYPI002737-RA	gi|646771352|gb|KK961768.1|	127433-127823	21	9.6
ACYPI001211-RA	gi|646745434|gb|KK962359.1|	138459-139791	22	10

ACYPI002878-RA	gi|646778029|gb|KK961613.1|	453892-455464	21	8.6
ACYPI009532-RA	gi|646782276|gb|KK961497.1|	2860084-2870284	21	9.7
ACYPI000754-RA	gi|646779539|gb|KK961571.1|	1476637-1476921	15	7.5
ACYPI000829-RA	gi|646776456|gb|KK961674.1|	309923-310206	18	7.9
ACYPI004632-RA	gi|646775451|gb|KK961726.1|	2326009-2326304	20	5.6
ACYPI003872-RA	gi|646738028|gb|KK963018.1|	516618-517119	22	7.1
ACYPI008346-RA	gi|646765902|gb|KK961884.1|	677299-678989	22	9.2
ACYPI005824-RA	gi|646777125|gb|KK961644.1|	2232728-2233437	20	10
ACYPI25923-RA	gi|646728843|gb|KK964153.1|	85183-86129	13	5
ACYPI071352-RA	gi|646743501|gb|KK962498.1|	353080-353332	19	9.5
ACYPI001440-RA	gi|646777313|gb|KK961637.1|	726388-729786	20	8.2
ACYPI005615-RA	gi|646782127|gb|KK961500.1|	2709931-2710151	22	9.6
ACYPI007182-RA	gi|646781443|gb|KK961520.1|	2942491-2943215	20	8.3
ACYPI006784-RA	gi|646767307|gb|KK961835.1|	322881-327857	20	9
ACYPI003549-RA	gi|646777631|gb|KK961624.1|	1806372-1810062	20	9.3
ACYPI004190-RA	gi|646781659|gb|KK961513.1|	3464204-3476907	22	9.8
ACYPI005514-RA	gi|646781043|gb|KK961532.1|	332887-333200	26	11
ACYPI000540-RA	gi|646776389|gb|KK961678.1|	1320601-1322300	22	9.5
ACYPI003646-RA	gi|646776389|gb|KK961678.1|	1305035-1305529	22	9.5
ACYPI001390-RA	gi|646766985|gb|KK961844.1|	212494-214239	21	9.7
ACYPI001507-RA	gi|646766985|gb|KK961844.1|	215457-221179	21	9.7
ACYPI003283-RA	gi|646768534|gb|KK961809.1|	339483-342446	21	9.8
ACYPI005339-RA	gi|646768294|gb|KK961814.1|	133164-134676	23	10
ACYPI000484-RA	gi|646744140|gb|KK962454.1|	286280-288386	21	9.3
ACYPI001336-RA	gi|646769832|gb|KK961787.1|	381980-385374	15	9.8
ACYPI005141-RA	gi|646746974|gb|KK962252.1|	536137-540254	23	6.1
ACYPI005785-RA	gi|646770047|gb|KK961784.1|	167561-169527	21	9.1
ACYPI010188-RA	gi|646746974|gb|KK962252.1|	709420-712207	23	6.1
ACYPI010216-RA	gi|646745778|gb|KK962333.1|	345179-351942	17	7.7
ACYPI082338-RA	gi|646776952|gb|KK961652.1|	1459298-1470166	19	7.7

ACYPI003993-RA	gi|646775765|gb|KK961710.1|	2033052-2033718	21	6
ACYPI060550-RA	gi|646781659|gb|KK961513.1|	5847286-5848312	22	9.8
ACYPI000664-RA	gi|646750185|gb|KK962052.1|	340664-341625	18	7.1
ACYPI004453-RA	gi|646780925|gb|KK961536.1|	1357413-1360757	22	9.9
ACYPI006370-RA	gi|646781243|gb|KK961526.1|	4125546-4127408	21	8.9
ACYPI008255-RA	gi|646777017|gb|KK961649.1|	902831-903859	18	8
ACYPI008888-RA	gi|646750795|gb|KK962021.1|	618720-619779	19	8.9
ACYPI002882-RA	gi|646768582|gb|KK961808.1|	235534-235766	21	6
ACYPI001218-RA	gi|646777765|gb|KK961620.1|	1001671-1008255	21	8.7
ACYPI002808-RA	gi|646776647|gb|KK961663.1|	2271830-2274478	23	9.2
ACYPI003382-RA	gi|646750428|gb|KK962039.1|	659764-664813	21	7.8
ACYPI007198-RA	gi|646782127|gb|KK961500.1|	6433692-6436313	22	9.6
ACYPI008498-RA	gi|646745186|gb|KK962377.1|	703333-703725	21	9.8
ACYPI009711-RA	gi|646742842|gb|KK962548.1|	276634-282758	22	10
ACYPI082655-RA	gi|646750002|gb|KK962062.1|	353610-354217	24	12
ACYPI000474-RA	gi|646767850|gb|KK961823.1|	1181490-1184713	21	9.8
ACYPI001119-RA	gi|646745091|gb|KK962384.1|	517591-518107	19	7.9
ACYPI008707-RA	gi|646768294|gb|KK961814.1|	419566-421241	23	10
ACYPI009338-RA	gi|646776588|gb|KK961666.1|	981015-981822	20	5.8
ACYPI50902-RA	gi|646781628|gb|KK961514.1|	1417682-1421156	23	9
ACYPI000023-RA	gi|646779785|gb|KK961565.1|	3459246-3460123	18	8.9
ACYPI001212-RA	gi|646775723|gb|KK961712.1|	1678105-1678926	21	9.5
ACYPI004351-RA	gi|646738229|gb|KK962998.1|	162168-164516	22	8.9
ACYPI007158-RA	gi|646778517|gb|KK961599.1|	540293-540893	20	9.6
ACYPI008785-RA	gi|646780889|gb|KK961537.1|	4035156-4037119	22	10
ACYPI000527-RA	gi|646751544|gb|KK961986.1|	1295621-1295843	22	6
ACYPI004516-RA	gi|646745372|gb|KK962364.1|	462957-466027	23	10
ACYPI007083-RA	gi|646775765|gb|KK961710.1|	1486279-1486802	21	6
ACYPI002352-RA	gi|646741656|gb|KK962648.1|	158226-160455	24	9.7
ACYPI003042-RA	gi|646781732|gb|KK961511.1|	3714803-3715407	22	9.8

ACYPI004235-RA	gi|646781344|gb|KK961523.1|	587351-588847	21	8.9
ACYPI008043-RA	gi|646780858|gb|KK961538.1|	3948303-3948707	21	10
ACYPI002456-RA	gi|646782276|gb|KK961497.1|	6277666-6282973	21	9.7
ACYPI004364-RA	gi|646746828|gb|KK962262.1|	126747-128797	20	9.2
ACYPI006934-RA	gi|646767196|gb|KK961838.1|	1107352-1107584	21	9.7
ACYPI000598-RA	gi|646781510|gb|KK961518.1|	184400-187619	17	7.8
ACYPI001293-RA	gi|646781443|gb|KK961520.1|	1480704-1485431	20	8.3
ACYPI001579-RA	gi|646782357|gb|KK961494.1|	2963857-2964531	21	9.2
ACYPI001617-RA	gi|646768494|gb|KK961810.1|	474833-475486	21	10
ACYPI002866-RA	gi|646742413|gb|KK962582.1|	575185-579755	23	10
ACYPI004093-RA	gi|646782357|gb|KK961494.1|	2810374-2810643	21	9.2
ACYPI007307-RA	gi|646782357|gb|KK961494.1|	2769530-2773845	21	9.2
ACYPI007911-RA	gi|646782357|gb|KK961494.1|	2864684-2864942	21	9.2
ACYPI009786-RA	gi|646782357|gb|KK961494.1|	2947856-2948116	21	9.2
ACYPI001572-RA	gi|646768088|gb|KK961818.1|	890070-890671	16	8.7
ACYPI006024-RA	gi|646768088|gb|KK961818.1|	1119828-1121305	16	8.7
ACYPI009772-RA	gi|646778112|gb|KK961611.1|	3051534-3053207	22	10
ACYPI063759-RA	gi|646743598|gb|KK962492.1|	423179-424158	17	8.9
ACYPI001091-RA	gi|646749945|gb|KK962065.1|	1300714-1302205	22	6.1
ACYPI002987-RA	gi|646743352|gb|KK962510.1|	516250-516623	20	5.4
ACYPI004908-RA	gi|646766540|gb|KK961858.1|	1265743-1266136	21	8.5
ACYPI006808-RA	gi|646747802|gb|KK962192.1|	207843-209426	19	7.8
ACYPI000052-RA	gi|646775807|gb|KK961708.1|	2100664-2101889	21	8.6
ACYPI001864-RA	gi|646775807|gb|KK961708.1|	1998340-2011589	21	8.6
ACYPI006256-RA	gi|646775658|gb|KK961715.1|	834512-836360	19	8.6
ACYPI003853-RA	gi|646766382|gb|KK961864.1|	34358-34968	22	9.7
ACYPI005917-RA	gi|646741540|gb|KK962657.1|	403975-404317	19	7
ACYPI007791-RA	gi|646781282|gb|KK961525.1|	3620595-3621917	22	9.6
ACYPI008314-RA	gi|646768717|gb|KK961805.1|	1169909-1170265	20	8
ACYPI008861-RA	gi|646685323|gb|KK967172.1|	15217-15509	11	6.4

ACYPI006765-RA	gi|646781313|gb|KK961524.1|	514821-516711	19	8.5
ACYPI20477-RA	gi|646769001|gb|KK961801.1|	682974-688284	20	8.5
ACYPI008491-RA	gi|646747201|gb|KK962236.1|	177019-178705	18	7.3
ACYPI42894-RA	gi|646778903|gb|KK961588.1|	2169225-2171291	23	10
ACYPI56225-RA	gi|646629125|gb|KK971064.1|	11847-12920	15	5.8
ACYPI000585-RA	gi|646781772|gb|KK961510.1|	4238842-4240612	20	9.2
ACYPI004250-RA	gi|646780222|gb|KK961554.1|	269962-274301	20	9.7
ACYPI34621-RA	gi|646781118|gb|KK961530.1|	6295386-6296813	21	10
ACYPI000953-RA	gi|646775507|gb|KK961723.1|	1640561-1641654	20	9.8
ACYPI006675-RA	gi|646749173|gb|KK962106.1|	77815-78901	21	5.7
ACYPI009192-RA	gi|646775723|gb|KK961712.1|	1820124-1820817	21	9.5
ACYPI009220-RA	gi|646776429|gb|KK961676.1|	1037995-1038322	20	9.9
ACYPI073835-RA	gi|646725370|gb|KK964662.1|	39440-40173	21	11
ACYPI001502-RA	gi|646746651|gb|KK962274.1|	19285-19984	16	6.6
ACYPI003428-RA	gi|646781690|gb|KK961512.1|	4507501-4509485	21	8.8
ACYPI007814-RA	gi|646745091|gb|KK962384.1|	422584-422797	19	7.9
ACYPI009718-RA	gi|646763796|gb|KK961901.1|	485985-486726	14	6.4
ACYPI001480-RA	gi|646776280|gb|KK961684.1|	823608-824198	22	9.3
ACYPI002304-RA	gi|646732431|gb|KK963675.1|	282660-283322	24	9.6
ACYPI004182-RA	gi|646776024|gb|KK961697.1|	2703197-2703698	22	9.2
ACYPI004992-RA	gi|646749188|gb|KK962105.1|	342343-342850	18	7.9
ACYPI005674-RA	gi|646736283|gb|KK963203.1|	80665-81078	24	9.7
ACYPI006120-RA	gi|646782357|gb|KK961494.1|	6325792-6326798	21	9.2
ACYPI000872-RA	gi|646782276|gb|KK961497.1|	6436723-6437127	21	9.7
ACYPI002110-RA	gi|646741767|gb|KK962638.1|	332202-334645	21	10
ACYPI007090-RA	gi|646778903|gb|KK961588.1|	3763475-3766883	23	10
ACYPI087259-RA	gi|646782276|gb|KK961497.1|	1195075-1200768	21	9.7
ACYPI003242-RA	gi|646778274|gb|KK961606.1|	1540844-1542907	18	7.5
ACYPI007529-RA	gi|646779375|gb|KK961575.1|	914355-915245	22	9.3
ACYPI007655-RA	gi|646779539|gb|KK961571.1|	459883-460620	15	7.5

ACYPI008372-RA	gi|646758845|gb|KK961921.1|	1824003-1835961	22	9.5
ACYPI002900-RA	gi|646778983|gb|KK961586.1|	3162080-3163259	20	9.9
ACYPI004212-RA	gi|646776219|gb|KK961687.1|	2152082-2157103	21	9.9
ACYPI008024-RA	gi|646766382|gb|KK961864.1|	1216679-1217538	22	9.7
ACYPI000080-RA	gi|646743195|gb|KK962522.1|	250620-251412	14	6.4
ACYPI001206-RA	gi|646777416|gb|KK961632.1|	1542675-1542970	22	8.8
ACYPI003099-RA	gi|646777416|gb|KK961632.1|	1492866-1494040	22	8.8
ACYPI005019-RA	gi|646777065|gb|KK961647.1|	990932-993461	20	7.9
ACYPI005619-RA	gi|646782357|gb|KK961494.1|	3452828-3456929	21	9.2
ACYPI007534-RA	gi|646750081|gb|KK962058.1|	417054-421332	19	8.6
ACYPI010029-RA	gi|646769275|gb|KK961796.1|	2451712-2452216	22	5.6
ACYPI003963-RA	gi|646755567|gb|KK961936.1|	982966-985260	20	8.6
ACYPI062376-RA	gi|646782288|gb|KK961496.1|	1471608-1472973	21	9.7
ACYPI062987-RA	gi|646725599|gb|KK964628.1|	64656-65345	12	8.2
ACYPI005074-RA	gi|646778149|gb|KK961610.1|	2302044-2304193	19	8.9
ACYPI008698-RA	gi|646780978|gb|KK961534.1|	2915836-2920336	20	8
ACYPI010049-RA	gi|646742413|gb|KK962582.1|	127362-127636	23	10
ACYPI000047-RA	gi|646771951|gb|KK961762.1|	59483-59825	21	8.3
ACYPI003613-RA	gi|646777474|gb|KK961630.1|	1263850-1264681	21	9.3
ACYPI004266-RA	gi|646778565|gb|KK961598.1|	1496804-1497159	19	7.8
ACYPI008075-RA	gi|646750889|gb|KK962017.1|	1634077-1635940	22	6.1
ACYPI009949-RA	gi|646766511|gb|KK961859.1|	836677-836948	17	8.5
ACYPI009707-RA	gi|646741656|gb|KK962648.1|	661141-669664	24	9.7
ACYPI001589-RA	gi|646776148|gb|KK961690.1|	886138-888420	21	9.4
ACYPI006314-RA	gi|646776952|gb|KK961652.1|	1685567-1686316	19	7.7
ACYPI001148-RA	gi|646750100|gb|KK962057.1|	467178-467744	16	8.2
ACYPI003657-RA	gi|646776647|gb|KK961663.1|	768432-774690	23	9.2
ACYPI003904-RA	gi|646750100|gb|KK962057.1|	278314-278701	16	8.2
ACYPI008512-RA	gi|646749036|gb|KK962114.1|	10465-11041	20	7.5
ACYPI25456-RA	gi|646771568|gb|KK961766.1|	622892-630199	18	7.4

ACYPI002344-RA	gi|646746991|gb|KK962251.1|	366687-370251	22	9.2
ACYPI003822-RA	gi|646739361|gb|KK962890.1|	129238-130324	22	9.6
ACYPI005580-RA	gi|646777363|gb|KK961635.1|	1885460-1885947	18	9.2
ACYPI008162-RA	gi|646781083|gb|KK961531.1|	2092356-2092959	19	8.5
ACYPI008800-RA	gi|646766158|gb|KK961873.1|	1042121-1042559	22	5.8
ACYPI002506-RA	gi|646776998|gb|KK961650.1|	155617-156732	20	9.6
ACYPI003343-RA	gi|646778149|gb|KK961610.1|	269958-270647	19	8.9
ACYPI003925-RA	gi|646766650|gb|KK961854.1|	1469365-1476563	22	10
ACYPI005264-RA	gi|646740304|gb|KK962788.1|	600228-607968	24	9.6
ACYPI007148-RA	gi|646781873|gb|KK961507.1|	2026607-2028522	20	9.1
ACYPI009039-RA	gi|646731892|gb|KK963756.1|	232410-233048	22	9.8
ACYPI008269-RA	gi|646780311|gb|KK961552.1|	1625822-1635441	21	9.7
ACYPI001646-RA	gi|646781118|gb|KK961530.1|	186752-190040	21	10
ACYPI008302-RA	gi|646772786|gb|KK961754.1|	520437-521419	22	9.6
ACYPI001110-RA	gi|646781849|gb|KK961508.1|	2570548-2571249	17	8
ACYPI001535-RA	gi|646778767|gb|KK961592.1|	778804-786113	21	5.4
ACYPI005365-RA	gi|646748310|gb|KK962157.1|	61750-65290	23	6.3
ACYPI000735-RA	gi|646744179|gb|KK962451.1|	13535-16722	24	11
ACYPI005007-RA	gi|646776351|gb|KK961680.1|	151273-151552	22	10
ACYPI006058-RA	gi|646777313|gb|KK961637.1|	1737743-1738177	20	8.2
ACYPI007679-RA	gi|646749512|gb|KK962088.1|	483441-483797	18	7.2
ACYPI008763-RA	gi|646781659|gb|KK961513.1|	5765849-5768012	22	9.8
ACYPI060982-RA	gi|646775842|gb|KK961706.1|	2320186-2320490	22	9
ACYPI006557-RA	gi|646738524|gb|KK962968.1|	127285-127568	24	11
ACYPI007242-RA	gi|646765705|gb|KK961893.1|	652533-652976	22	9.7
ACYPI008242-RA	gi|646782357|gb|KK961494.1|	8255281-8258040	21	9.2
ACYPI084157-RA	gi|646782127|gb|KK961500.1|	2673116-2676517	22	9.6
ACYPI002445-RA	gi|646777065|gb|KK961647.1|	779402-779706	20	7.9
ACYPI086747-RA	gi|646777065|gb|KK961647.1|	780115-781798	20	7.9
ACYPI009548-RA	gi|646742541|gb|KK962572.1|	504419-505534	19	10

ACYPI071951-RA	gi|646768494|gb|KK961810.1|	1387747-1388176	21	10
ACYPI083523-RA	gi|646748129|gb|KK962169.1|	533550-536495	21	9.8
ACYPI000434-RA	gi|646748185|gb|KK962165.1|	690134-690393	22	9.9
ACYPI000686-RA	gi|646780311|gb|KK961552.1|	3429318-3430008	21	9.7
ACYPI001081-RA	gi|646751289|gb|KK961996.1|	851263-852771	24	9.1
ACYPI002342-RA	gi|646778767|gb|KK961592.1|	1756085-1757360	21	5.4
ACYPI002979-RA	gi|646750600|gb|KK962030.1|	995287-1000766	23	8.6
ACYPI004205-RA	gi|646772574|gb|KK961756.1|	1029566-1031856	21	8
ACYPI006142-RA	gi|646755567|gb|KK961936.1|	598856-599852	20	8.6
ACYPI008021-RA	gi|646755567|gb|KK961936.1|	273383-273637	20	8.6
ACYPI008592-RA	gi|646770251|gb|KK961781.1|	697741-698014	20	9.6
ACYPI009892-RA	gi|646766624|gb|KK961855.1|	1033069-1035164	21	10
ACYPI23338-RA	gi|646780222|gb|KK961554.1|	1257230-1261312	20	9.7
ACYPI008716-RA	gi|646776514|gb|KK961670.1|	2077778-2079273	20	9.6
ACYPI002382-RA	gi|646768631|gb|KK961807.1|	1304186-1308383	19	9.2
ACYPI008717-RA	gi|646745387|gb|KK962363.1|	370653-370892	20	8.6
ACYPI007374-RA	gi|646738455|gb|KK962975.1|	420513-425076	23	9.5
ACYPI55348-RA	gi|646781628|gb|KK961514.1|	2585190-2587916	23	9
ACYPI001090-RA	gi|646728609|gb|KK964184.1|	229889-235318	34	14
ACYPI001359-RA	gi|646780441|gb|KK961549.1|	3102433-3103591	21	9.5
ACYPI003246-RA	gi|646780010|gb|KK961559.1|	3021741-3022227	22	10
ACYPI001498-RA	gi|646535382|gb|KK987821.1|	3291-3751	15	6.8
ACYPI002405-RA	gi|646778903|gb|KK961588.1|	3353930-3360933	23	10
ACYPI003389-RA	gi|646748490|gb|KK962146.1|	231238-232079	19	7.7
ACYPI005324-RA	gi|646743772|gb|KK962481.1|	668762-669682	22	8.9
ACYPI082990-RA	gi|646751103|gb|KK962006.1|	261931-262497	20	8.3
ACYPI001496-RA	gi|646782043|gb|KK961502.1|	1234905-1235390	20	9.2
ACYPI009713-RA	gi|646744140|gb|KK962454.1|	469016-469672	21	9.3
ACYPI001596-RA	gi|646763164|gb|KK961904.1|	1034080-1035768	22	10
ACYPI003480-RA	gi|646763164|gb|KK961904.1|	1065661-1066084	22	10

ACYPI006207-RA	gi|646746706|gb|KK962270.1|	402736-408503	23	11
ACYPI007925-RA	gi|646766985|gb|KK961844.1|	375301-375925	21	9.7
ACYPI064230-RA	gi|646775702|gb|KK961713.1|	749847-753223	17	7.4
ACYPI087019-RA	gi|646775702|gb|KK961713.1|	934823-935088	17	7.4
ACYPI004564-RA	gi|646718932|gb|KK965097.1|	37328-42450	21	9.2
ACYPI009494-RA	gi|646718932|gb|KK965097.1|	68041-68631	21	9.2
ACYPI000487-RA	gi|646767120|gb|KK961840.1|	817964-819474	20	8.6
ACYPI002732-RA	gi|646734527|gb|KK963409.1|	29706-30189	21	9.3
ACYPI003255-RA	gi|646776024|gb|KK961697.1|	2236328-2236662	22	9.2
ACYPI007125-RA	gi|646781421|gb|KK961521.1|	2386543-2391647	21	8.7
ACYPI009003-RA	gi|646781421|gb|KK961521.1|	2416081-2417780	21	8.7
ACYPI006395-RA	gi|646769001|gb|KK961801.1|	387237-389203	20	8.5
ACYPI42350-RA	gi|646639086|gb|KK969293.1|	2156-2597	29	12
ACYPI45166-RA	gi|646781183|gb|KK961528.1|	637675-639809	20	9.5
ACYPI005894-RA	gi|646776351|gb|KK961680.1|	177597-178342	22	10
ACYPI009654-RA	gi|646776351|gb|KK961680.1|	340762-341404	22	10
ACYPI008439-RA	gi|646776389|gb|KK961678.1|	754686-754947	22	9.5
ACYPI003377-RA	gi|646516981|gb|KK991223.1|	1825-2393	22	8.6
ACYPI003469-RA	gi|646776091|gb|KK961693.1|	144684-146038	23	10
ACYPI005314-RA	gi|646731892|gb|KK963756.1|	99098-101603	22	9.8
ACYPI009151-RA	gi|646771092|gb|KK961771.1|	1029019-1029588	23	9.7
ACYPI009704-RA	gi|646781772|gb|KK961510.1|	2890517-2899635	20	9.2
ACYPI089560-RA	gi|646781690|gb|KK961512.1|	3781815-3782452	21	8.8
ACYPI001521-RA	gi|646776429|gb|KK961676.1|	2025704-2030229	20	9.9
ACYPI007802-RA	gi|646776368|gb|KK961679.1|	1037861-1038343	21	9.7
ACYPI004766-RA	gi|646765857|gb|KK961886.1|	196224-196561	17	7.7
ACYPI008535-RA	gi|646765880|gb|KK961885.1|	586677-587872	23	10
ACYPI084448-RA	gi|646765902|gb|KK961884.1|	231743-233154	22	9.2
ACYPI005517-RA	gi|646782334|gb|KK961495.1|	7245001-7245990	21	9
ACYPI008053-RA	gi|646776184|gb|KK961688.1|	1715588-1716353	21	9.6

ACYPI009263-RA	gi|646780925|gb|KK961536.1|	1949771-1957014	22	9.9
ACYPI009339-RA	gi|646780105|gb|KK961557.1|	3586780-3587698	21	9.7
ACYPI001042-RA	gi|646776024|gb|KK961697.1|	2453043-2453929	22	9.2
ACYPI002287-RA	gi|646775723|gb|KK961712.1|	2017770-2027262	21	9.5
ACYPI003347-RA	gi|646776024|gb|KK961697.1|	385271-387477	22	9.2
ACYPI004209-RA	gi|646766357|gb|KK961865.1|	168058-168389	16	6.5
ACYPI52571-RA	gi|646767230|gb|KK961837.1|	831232-831878	19	5.4
ACYPI001922-RA	gi|646781601|gb|KK961515.1|	3529589-3529937	19	8.6
ACYPI002538-RA	gi|646771448|gb|KK961767.1|	1433164-1434556	21	9.8
ACYPI004435-RA	gi|646747032|gb|KK962248.1|	326198-328710	20	8.7
ACYPI006352-RA	gi|646744833|gb|KK962403.1|	241068-244042	22	9.4
ACYPI006535-RA	gi|646759277|gb|KK961919.1|	901458-902307	16	7.2
ACYPI009511-RA	gi|646782357|gb|KK961494.1|	9107729-9111597	21	9.2
ACYPI010117-RA	gi|646746298|gb|KK962298.1|	1577-1996	18	8.3
ACYPI50768-RA	gi|646779262|gb|KK961578.1|	2305247-2306495	20	8.1
ACYPI001382-RA	gi|646735640|gb|KK963276.1|	51221-51556	18	6.9
ACYPI006407-RA	gi|646781893|gb|KK961506.1|	2143952-2144738	19	8.5
ACYPI008286-RA	gi|646747841|gb|KK962189.1|	709534-710266	23	10
ACYPI000390-RA	gi|646751628|gb|KK961983.1|	560180-563457	18	7.6
ACYPI56570-RA	gi|646757051|gb|KK961928.1|	368290-371212	18	8.9
ACYPI000971-RA	gi|646776024|gb|KK961697.1|	2426175-2428788	22	9.2
ACYPI002870-RA	gi|646781421|gb|KK961521.1|	1408603-1410559	21	8.7
ACYPI004366-RA	gi|646746706|gb|KK962270.1|	816516-817695	23	11
ACYPI004790-RA	gi|646781421|gb|KK961521.1|	4381153-4381475	21	8.7
ACYPI006693-RA	gi|646781421|gb|KK961521.1|	1424533-1425477	21	8.7
ACYPI000018-RA	gi|646740098|gb|KK962812.1|	577984-580281	20	10
ACYPI001128-RA	gi|646776113|gb|KK961692.1|	147032-149028	21	9.6
ACYPI005158-RA	gi|646779898|gb|KK961562.1|	2940979-2943761	19	9.3
ACYPI007100-RA	gi|646737122|gb|KK963108.1|	49598-50074	17	9
ACYPI008357-RA	gi|646781183|gb|KK961528.1|	653951-659326	20	9.5

ACYPI073873-RA	gi|646737528|gb|KK963067.1|	115818-116099	20	6.1
ACYPI37088-RA	gi|646750021|gb|KK962061.1|	396937-397184	16	7.5
ACYPI007666-RA	gi|646767948|gb|KK961821.1|	607554-607823	17	7.1
ACYPI009612-RA	gi|646775471|gb|KK961725.1|	2005985-2006288	20	10
ACYPI43861-RA	gi|646779298|gb|KK961577.1|	3523616-3524495	20	9.1
ACYPI001360-RA	gi|646782168|gb|KK961499.1|	5435547-5436761	21	9.4
ACYPI001392-RA	gi|646781243|gb|KK961526.1|	2012596-2013384	21	8.9
ACYPI003247-RA	gi|646781243|gb|KK961526.1|	2755649-2756304	21	8.9
ACYPI005163-RA	gi|646781243|gb|KK961526.1|	2770162-2774068	21	8.9
ACYPI005208-RA	gi|646753093|gb|KK961958.1|	1121400-1124006	21	9.3
ACYPI001043-RA	gi|646775765|gb|KK961710.1|	1866358-1867038	21	6
ACYPI003742-RA	gi|646731007|gb|KK963869.1|	181941-183265	21	9.8
ACYPI005687-RA	gi|646742319|gb|KK962590.1|	92513-95142	18	8.5
ACYPI002678-RA	gi|646741342|gb|KK962675.1|	216047-219286	18	7.3
ACYPI004278-RA	gi|646743576|gb|KK962494.1|	320925-321152	19	8.2
ACYPI005854-RA	gi|646781243|gb|KK961526.1|	3797242-3797700	21	8.9
ACYPI008383-RA	gi|646606023|gb|KK975235.1|	784-1026	17	4.6
ACYPI000423-RA	gi|646777017|gb|KK961649.1|	993344-994208	18	8
ACYPI003596-RA	gi|646780794|gb|KK961540.1|	1405293-1407435	21	9.5
ACYPI004199-RA	gi|646781344|gb|KK961523.1|	3879833-3880324	21	8.9
ACYPI006737-RA	gi|646781013|gb|KK961533.1|	938435-943564	18	7.6
ACYPI008596-RA	gi|646780358|gb|KK961551.1|	2223879-2225027	21	8.8
ACYPI009384-RA	gi|646780827|gb|KK961539.1|	32281-33043	20	9.4
ACYPI070989-RA	gi|646742319|gb|KK962590.1|	271518-277398	18	8.5
ACYPI071357-RA	gi|646781013|gb|KK961533.1|	961124-961845	18	7.6
ACYPI21591-RA	gi|646780827|gb|KK961539.1|	335-1932	20	9.4
ACYPI29233-RA	gi|646751735|gb|KK961980.1|	1029272-1032140	21	5.5
ACYPI004567-RA	gi|646780858|gb|KK961538.1|	3911494-3911763	21	10
ACYPI25057-RA	gi|646778149|gb|KK961610.1|	1282568-1284511	19	8.9
ACYPI002122-RA	gi|646752143|gb|KK961972.1|	558684-559089	20	5.6

ACYPI008000-RA	gi|646752431|gb|KK961967.1|	1691052-1691379	20	5.6
ACYPI39411-RA	gi|646729796|gb|KK964027.1|	63485-65217	20	7
ACYPI002966-RA	gi|646743944|gb|KK962469.1|	138015-139156	20	9.1
ACYPI006792-RA	gi|646750135|gb|KK962055.1|	55033-56094	16	7.6
ACYPI001461-RA	gi|646780858|gb|KK961538.1|	3326329-3330800	21	10
ACYPI070582-RA	gi|646780270|gb|KK961553.1|	4476758-4478899	22	8.6
ACYPI071747-RA	gi|646736112|gb|KK963221.1|	75728-76216	14	4.7
ACYPI001819-RA	gi|646778903|gb|KK961588.1|	2665240-2665844	23	10
ACYPI009409-RA	gi|646751350|gb|KK961993.1|	578971-585671	20	7.6
ACYPI001796-RA	gi|646763164|gb|KK961904.1|	551273-553813	22	10
ACYPI003679-RA	gi|646775765|gb|KK961710.1|	1769984-1770500	21	6
ACYPI005047-RA	gi|646778112|gb|KK961611.1|	1970725-1976096	22	10
ACYPI006948-RA	gi|646762191|gb|KK961910.1|	902349-903559	18	7.1
ACYPI009467-RA	gi|646778660|gb|KK961595.1|	210197-211004	16	8.2
ACYPI010014-RA	gi|646769774|gb|KK961788.1|	747875-748228	20	9.2
ACYPI002210-RA	gi|646743492|gb|KK962499.1|	216677-216921	24	11
ACYPI004082-RA	gi|646775558|gb|KK961720.1|	1851019-1853017	20	9.4
ACYPI005528-RA	gi|646781212|gb|KK961527.1|	1172672-1178289	21	10
ACYPI009950-RA	gi|646782168|gb|KK961499.1|	3566455-3571274	21	9.4
ACYPI064347-RA	gi|646693619|gb|KK966519.1|	6017-6292	6.5	5.9
ACYPI065342-RA	gi|646709180|gb|KK965509.1|	49780-52888	16	6.2
ACYPI007870-RA	gi|646779262|gb|KK961578.1|	1925811-1926105	20	8.1
ACYPI000278-RA	gi|646739841|gb|KK962838.1|	399284-400000	21	5.7
ACYPI006257-RA	gi|646777991|gb|KK961614.1|	1111952-1112393	18	7.8
ACYPI006656-RA	gi|646778767|gb|KK961592.1|	150800-156186	21	5.4
ACYPI007187-RA	gi|646746942|gb|KK962254.1|	690757-691360	23	9.8
ACYPI001321-RA	gi|646753527|gb|KK961953.1|	1474708-1477209	24	9.2
ACYPI003203-RA	gi|646741034|gb|KK962704.1|	2934-3967	24	10
ACYPI083426-RA	gi|646777955|gb|KK961615.1|	421058-421442	21	10
ACYPI55930-RA	gi|646739875|gb|KK962835.1|	142120-142380	20	9.5

ACYPI004391-RA	gi|646780696|gb|KK961543.1|	3079576-3080501	15	8.8
ACYPI008187-RA	gi|646762191|gb|KK961910.1|	811593-811972	18	7.1
ACYPI009398-RA	gi|646769275|gb|KK961796.1|	1604205-1604932	22	5.6
ACYPI010073-RA	gi|646747738|gb|KK962196.1|	386359-389194	20	8.3
ACYPI073731-RA	gi|646782168|gb|KK961499.1|	7218426-7223713	21	9.4
ACYPI29600-RA	gi|646734440|gb|KK963419.1|	331882-332330	19	6.9
ACYPI000994-RA	gi|646769275|gb|KK961796.1|	726234-733161	22	5.6
ACYPI071217-RA	gi|646780889|gb|KK961537.1|	1683498-1684049	22	10
ACYPI22575-RA	gi|646779702|gb|KK961567.1|	506333-510400	23	10
ACYPI004768-RA	gi|646747424|gb|KK962220.1|	138850-139592	22	5.7
ACYPI006674-RA	gi|646775884|gb|KK961704.1|	950525-950771	20	7.6
ACYPI000819-RA	gi|646777416|gb|KK961632.1|	779364-781313	22	8.8
ACYPI001468-RA	gi|646782357|gb|KK961494.1|	2067763-2068606	21	9.2
ACYPI002528-RA	gi|646769971|gb|KK961785.1|	42700-47094	13	7
ACYPI002701-RA	gi|646782357|gb|KK961494.1|	2045966-2046339	21	9.2
ACYPI004238-RA	gi|646743859|gb|KK962475.1|	229741-229993	20	7.9
ACYPI004619-RA	gi|646782357|gb|KK961494.1|	2057160-2057482	21	9.2
ACYPI007170-RA	gi|646778736|gb|KK961593.1|	1212305-1213551	20	7.9
ACYPI007836-RA	gi|646778736|gb|KK961593.1|	269267-270772	20	7.9
ACYPI005155-RA	gi|646775523|gb|KK961722.1|	1176464-1181597	21	9.7
ACYPI008850-RA	gi|646768717|gb|KK961805.1|	1261868-1262852	20	8
ACYPI072156-RA	gi|646749036|gb|KK962114.1|	191533-194277	20	7.5
ACYPI004416-RA	gi|646782168|gb|KK961499.1|	1991983-1992993	21	9.4
ACYPI005175-RA	gi|646732968|gb|KK963602.1|	367061-367781	20	9.9
ACYPI24155-RA	gi|646746040|gb|KK962316.1|	515964-516623	19	6.9
ACYPI000824-RA	gi|646782357|gb|KK961494.1|	6268910-6282826	21	9.2
ACYPI003362-RA	gi|646747046|gb|KK962247.1|	7187-8834	22	9
ACYPI004626-RA	gi|646757520|gb|KK961926.1|	1321254-1321595	20	7.4
ACYPI004781-RA	gi|646726111|gb|KK964544.1|	72503-73742	12	5.4
ACYPI006544-RA	gi|646753527|gb|KK961953.1|	1188121-1188545	24	9.2

ACYPI001137-RA	gi|646768582|gb|KK961808.1|	332352-332796	21	6
ACYPI001776-RA	gi|646639169|gb|KK969278.1|	3857-4665	20	7.8
ACYPI003031-RA	gi|646763535|gb|KK961902.1|	417537-418658	23	10
ACYPI006842-RA	gi|646776219|gb|KK961687.1|	2020931-2025293	21	9.9
ACYPI001303-RA	gi|646782168|gb|KK961499.1|	2213831-2220725	21	9.4
ACYPI000857-RA	gi|646762334|gb|KK961909.1|	435741-437406	19	8.9
ACYPI002129-RA	gi|646779826|gb|KK961564.1|	3352189-3353287	22	9.6
ACYPI003391-RA	gi|646753799|gb|KK961950.1|	1473574-1477874	23	9.2
ACYPI007207-RA	gi|646758527|gb|KK961922.1|	48295-49503	24	10
ACYPI009087-RA	gi|646772786|gb|KK961754.1|	777354-778793	22	9.6
ACYPI009502-RA	gi|646780270|gb|KK961553.1|	2192992-2194499	22	8.6
ACYPI53881-RA	gi|646768582|gb|KK961808.1|	968368-970814	21	6
ACYPI001672-RA	gi|646770477|gb|KK961778.1|	921163-921707	21	9.1
ACYPI003875-RA	gi|646775352|gb|KK961732.1|	70781-71306	18	8.7
ACYPI007348-RA	gi|646781118|gb|KK961530.1|	1145476-1145951	21	10
ACYPI000381-RA	gi|646770998|gb|KK961772.1|	979320-979601	22	9.8
ACYPI000610-RA	gi|646777416|gb|KK961632.1|	1856574-1857088	22	8.8
ACYPI002493-RA	gi|646776514|gb|KK961670.1|	1271926-1272903	20	9.6
ACYPI003020-RA	gi|646767439|gb|KK961832.1|	2087751-2088810	23	10
ACYPI008191-RA	gi|646771845|gb|KK961763.1|	1459448-1460178	23	10
ACYPI008619-RA	gi|646767439|gb|KK961832.1|	2210424-2212112	23	10
ACYPI010077-RA	gi|646778410|gb|KK961602.1|	2641257-2641900	20	9
ACYPI010087-RA	gi|646776855|gb|KK961656.1|	295588-296045	19	8.8
ACYPI086281-RA	gi|646524315|gb|KK989884.1|	11252-11569	17	7
ACYPI000173-RA	gi|646776514|gb|KK961670.1|	733975-734278	20	9.6
ACYPI001113-RA	gi|646781732|gb|KK961511.1|	3881635-3887993	22	9.8
ACYPI008128-RA	gi|646780105|gb|KK961557.1|	94185-96805	21	9.7
ACYPI009043-RA	gi|646776219|gb|KK961687.1|	2393402-2395408	21	9.9
ACYPI34776-RA	gi|646782276|gb|KK961497.1|	1616739-1620637	21	9.7
ACYPI061154-RA	gi|646750820|gb|KK962020.1|	339833-340233	18	7

ACYPI008313-RA	gi|646751569|gb|KK961985.1|	386725-387268	21	9.2
ACYPI000886-RA	gi|646751289|gb|KK961996.1|	338417-338891	24	9.1
ACYPI009101-RA	gi|646781659|gb|KK961513.1|	5021805-5022700	22	9.8
ACYPI000386-RA	gi|646782357|gb|KK961494.1|	8629374-8629605	21	9.2
ACYPI001560-RA	gi|646777182|gb|KK961642.1|	1452881-1456106	20	9
ACYPI007326-RA	gi|646777182|gb|KK961642.1|	1672267-1672533	20	9
ACYPI51789-RA	gi|646771448|gb|KK961767.1|	156537-160052	21	9.8
ACYPI004586-RA	gi|646770563|gb|KK961777.1|	759292-759586	19	8.7
ACYPI005655-RA	gi|646781690|gb|KK961512.1|	1960516-1961106	21	8.8
ACYPI005663-RA	gi|646779826|gb|KK961564.1|	2866763-2868827	22	9.6
ACYPI008874-RA	gi|646779741|gb|KK961566.1|	3296980-3299808	20	8.5
ACYPI010059-RA	gi|646776389|gb|KK961678.1|	361099-364764	22	9.5
ACYPI000821-RA	gi|646782334|gb|KK961495.1|	2642576-2642820	21	9
ACYPI001024-RA	gi|646767813|gb|KK961824.1|	309405-309629	17	6.3
ACYPI002275-RA	gi|646778186|gb|KK961609.1|	2486068-2492649	19	9.4
ACYPI004621-RA	gi|646775658|gb|KK961715.1|	934674-942063	19	8.6
ACYPI005588-RA	gi|646781143|gb|KK961529.1|	4199926-4202060	20	10
ACYPI006089-RA	gi|646768447|gb|KK961811.1|	986296-986508	22	9.7
ACYPI006841-RA	gi|646777207|gb|KK961641.1|	896960-899077	20	8.5
ACYPI007972-RA	gi|646768447|gb|KK961811.1|	996320-996925	22	9.7
ACYPI008384-RA	gi|646745941|gb|KK962322.1|	489178-490054	21	10
ACYPI087089-RA	gi|646780978|gb|KK961534.1|	1220525-1225982	20	8
ACYPI062641-RA	gi|646776241|gb|KK961686.1|	1914874-1927795	18	5.9
ACYPI35510-RA	gi|646753180|gb|KK961957.1|	463664-464690	25	11
ACYPI000893-RA	gi|646781968|gb|KK961504.1|	2772260-2774106	20	9.6
ACYPI002471-RA	gi|646767521|gb|KK961830.1|	1183265-1183570	20	9.3
ACYPI003778-RA	gi|646766137|gb|KK961874.1|	741464-743205	20	9
ACYPI009686-RA	gi|646776184|gb|KK961688.1|	1893735-1894802	21	9.6
ACYPI082503-RA	gi|646767521|gb|KK961830.1|	1203022-1203847	20	9.3
ACYPI005116-RA	gi|646781043|gb|KK961532.1|	1663719-1663990	26	11

ACYPI063378-RA	gi|646750889|gb|KK962017.1|	1904889-1907605	22	6.1
ACYPI000592-RA	gi|646781732|gb|KK961511.1|	2776373-2780343	22	9.8
ACYPI002162-RA	gi|646777474|gb|KK961630.1|	1317614-1318058	21	9.3
ACYPI001704-RA	gi|646741719|gb|KK962642.1|	94145-94505	21	8.9
ACYPI000626-RA	gi|646780858|gb|KK961538.1|	2870715-2871107	21	10
ACYPI001870-RA	gi|646780574|gb|KK961546.1|	4148912-4153074	20	9.9
ACYPI007594-RA	gi|646749727|gb|KK962077.1|	233865-237470	21	8.9
ACYPI008203-RA	gi|646780311|gb|KK961552.1|	4022890-4023698	21	9.7
ACYPI004216-RA	gi|646780858|gb|KK961538.1|	2080313-2081531	21	10
ACYPI004830-RA	gi|646779741|gb|KK961566.1|	3016090-3016898	20	8.5
ACYPI29381-RA	gi|646777233|gb|KK961640.1|	1882377-1882634	21	8.7
ACYPI002140-RA	gi|646751515|gb|KK961987.1|	747600-750552	20	9.2
ACYPI004015-RA	gi|646751515|gb|KK961987.1|	743559-744334	20	9.2
ACYPI004314-RA	gi|646776409|gb|KK961677.1|	607724-608833	17	8.4
ACYPI010179-RA	gi|646767624|gb|KK961828.1|	96674-97620	22	8.9
ACYPI000588-RA	gi|646745197|gb|KK962376.1|	153958-154882	21	8.9
ACYPI003214-RA	gi|646776447|gb|KK961675.1|	830176-830874	22	9.4
ACYPI003413-RA	gi|646781282|gb|KK961525.1|	1746216-1747551	22	9.6
ACYPI004121-RA	gi|646775507|gb|KK961723.1|	1747589-1747887	20	9.8
ACYPI004346-RA	gi|646751458|gb|KK961989.1|	433479-434118	21	5.1
ACYPI009810-RA	gi|646745372|gb|KK962364.1|	841817-846225	23	10
ACYPI49659-RA	gi|646780406|gb|KK961550.1|	648031-648629	22	5.9
ACYPI56610-RA	gi|646781282|gb|KK961525.1|	1448875-1450546	22	9.6
ACYPI001274-RA	gi|646777416|gb|KK961632.1|	373995-374330	22	8.8
ACYPI003160-RA	gi|646754231|gb|KK961946.1|	1094687-1095156	18	8.7
ACYPI000395-RA	gi|646591538|gb|KK978123.1|	8236-8364	11	4.8
ACYPI002289-RA	gi|646781243|gb|KK961526.1|	3286934-3287361	21	8.9
ACYPI009639-RA	gi|646754962|gb|KK961940.1|	1013642-1014660	21	10
ACYPI082098-RA	gi|646744874|gb|KK962400.1|	160037-160797	19	8.3
ACYPI31510-RA	gi|646772677|gb|KK961755.1|	1084110-1085677	18	7.9

ACYPI002952-RA	gi|646551326|gb|KK985982.1|	7703-8114	13	5.2
ACYPI068994-RA	gi|646728148|gb|KK964249.1|	28375-30279	20	6.1
ACYPI073693-RA	gi|646775968|gb|KK961700.1|	227205-228445	20	8.2
ACYPI001530-RA	gi|646767196|gb|KK961838.1|	996400-997450	21	9.7
ACYPI002045-RA	gi|646748044|gb|KK962175.1|	489317-490870	19	7.2
ACYPI005976-RA	gi|646781143|gb|KK961529.1|	3916762-3921647	20	10
ACYPI007840-RA	gi|646782288|gb|KK961496.1|	4505613-4508715	21	9.7
ACYPI006615-RA	gi|646552978|gb|KK985631.1|	1244-2626	18	9.7
ACYPI065331-RA	gi|646775867|gb|KK961705.1|	1909502-1910685	23	10
ACYPI41828-RA	gi|646748142|gb|KK962168.1|	220095-222849	20	9.3
ACYPI001552-RA	gi|646782168|gb|KK961499.1|	920959-921579	21	9.4
ACYPI001832-RA	gi|646749512|gb|KK962088.1|	113199-115185	18	7.2
ACYPI003715-RA	gi|646766868|gb|KK961847.1|	1077556-1077950	21	9.9
ACYPI010098-RA	gi|646769038|gb|KK961800.1|	758021-758718	21	8.9
ACYPI001923-RA	gi|646771092|gb|KK961771.1|	1134784-1138749	23	9.7
ACYPI002483-RA	gi|646620535|gb|KK972589.1|	760-1021	12	6.3
ACYPI010153-RA	gi|646747046|gb|KK962247.1|	622118-627799	22	9
ACYPI001129-RA	gi|646776855|gb|KK961656.1|	377398-378630	19	8.8
ACYPI004145-RA	gi|646750643|gb|KK962028.1|	706083-714538	21	7.5
ACYPI006740-RA	gi|646768447|gb|KK961811.1|	821773-822664	22	9.7
ACYPI007358-RA	gi|646768447|gb|KK961811.1|	691661-691934	22	9.7
ACYPI007658-RA	gi|646741630|gb|KK962650.1|	41081-43331	12	8.2
ACYPI008225-RA	gi|646750354|gb|KK962043.1|	580196-580920	20	9.2
ACYPI009061-RA	gi|646780827|gb|KK961539.1|	3310808-3312546	20	9.4
ACYPI066919-RA	gi|646779298|gb|KK961577.1|	252910-254870	20	9.1
ACYPI46593-RA	gi|646745929|gb|KK962323.1|	335883-336369	20	8.8
ACYPI002517-RA	gi|646732914|gb|KK963609.1|	82774-83849	21	5.6
ACYPI004419-RA	gi|646779375|gb|KK961575.1|	2215035-2215926	22	9.3
ACYPI005497-RA	gi|646781183|gb|KK961528.1|	500931-505230	20	9.5
ACYPI51833-RA	gi|646771092|gb|KK961771.1|	715846-719799	23	9.7

ACYPI27416-RA	gi|646754962|gb|KK961940.1|	222457-222961	21	10
ACYPI005271-RA	gi|646778149|gb|KK961610.1|	1424943-1425127	19	8.9
ACYPI006518-RA	gi|646752849|gb|KK961961.1|	1214235-1215294	21	9.4
ACYPI067116-RA	gi|646766158|gb|KK961873.1|	1609472-1609979	22	5.8
ACYPI072184-RA	gi|646776322|gb|KK961682.1|	1173124-1173688	22	8.9
ACYPI45089-RA	gi|646781212|gb|KK961527.1|	309150-310175	21	10
ACYPI001742-RA	gi|646770901|gb|KK961773.1|	1029991-1031304	22	9.7
ACYPI002579-RA	gi|646782168|gb|KK961499.1|	1184464-1185191	21	9.4
ACYPI006852-RA	gi|646781628|gb|KK961514.1|	4586500-4592451	23	9
ACYPI009960-RA	gi|646751289|gb|KK961996.1|	787784-789366	24	9.1
ACYPI000320-RA	gi|646767754|gb|KK961825.1|	83201-83427	20	8.5
ACYPI001138-RA	gi|646781344|gb|KK961523.1|	100908-104016	21	8.9
ACYPI008359-RA	gi|646775685|gb|KK961714.1|	378488-380385	21	8.5
ACYPI000252-RA	gi|646777474|gb|KK961630.1|	3104034-3109213	21	9.3
ACYPI003751-RA	gi|646767477|gb|KK961831.1|	128473-129125	20	8.4
ACYPI004712-RA	gi|646748103|gb|KK962171.1|	43919-46908	23	10
ACYPI001601-RA	gi|646777182|gb|KK961642.1|	1112524-1112733	20	9
ACYPI001835-RA	gi|646780925|gb|KK961536.1|	1580277-1581489	22	9.9
ACYPI007526-RA	gi|646737766|gb|KK963045.1|	134192-136697	21	9.2
ACYPI007932-RA	gi|646748599|gb|KK962140.1|	250445-253005	21	9.8
ACYPI009423-RA	gi|646780794|gb|KK961540.1|	508230-509368	21	9.5
ACYPI009806-RA	gi|646777125|gb|KK961644.1|	3017208-3017568	20	10
ACYPI065245-RA	gi|646759701|gb|KK961917.1|	1291041-1292134	18	6.1
ACYPI33566-RA	gi|646782211|gb|KK961498.1|	159947-165854	20	9
ACYPI39148-RA	gi|646781873|gb|KK961507.1|	1778081-1779611	20	9.1
ACYPI000843-RA	gi|646776280|gb|KK961684.1|	188247-188457	22	9.3
ACYPI001416-RA	gi|646780574|gb|KK961546.1|	3529070-3538977	20	9.9
ACYPI005899-RA	gi|646772045|gb|KK961761.1|	74250-74772	21	9.6
ACYPI083997-RA	gi|646772045|gb|KK961761.1|	83110-83278	21	9.6
ACYPI001850-RA	gi|646776184|gb|KK961688.1|	1192707-1192950	21	9.6

ACYPI006170-RA	gi|646780827|gb|KK961539.1|	3598551-3599003	20	9.4
ACYPI008124-RA	gi|646766868|gb|KK961847.1|	1276263-1278767	21	9.9
ACYPI009976-RA	gi|646782276|gb|KK961497.1|	5215528-5219235	21	9.7
ACYPI001760-RA	gi|646745543|gb|KK962350.1|	745218-745444	21	10
ACYPI002754-RA	gi|646766598|gb|KK961856.1|	687987-692659	23	10
ACYPI004929-RA	gi|646782334|gb|KK961495.1|	2686152-2687634	21	9
ACYPI005581-RA	gi|646769453|gb|KK961793.1|	1508617-1509246	21	8.8
ACYPI009334-RA	gi|646766598|gb|KK961856.1|	1189105-1189408	23	10
ACYPI000368-RA	gi|646750643|gb|KK962028.1|	26114-27850	21	7.5
ACYPI003885-RA	gi|646782168|gb|KK961499.1|	3626686-3627283	21	9.4
ACYPI005646-RA	gi|646770323|gb|KK961780.1|	235758-237077	19	7.1
ACYPI007521-RA	gi|646758845|gb|KK961921.1|	1895830-1896097	22	9.5
ACYPI008166-RA	gi|646778840|gb|KK961590.1|	1725634-1729354	20	9.9
ACYPI002662-RA	gi|646767230|gb|KK961837.1|	325845-326129	19	5.4
ACYPI001667-RA	gi|646781628|gb|KK961514.1|	2292044-2293417	23	9
ACYPI002426-RA	gi|646782334|gb|KK961495.1|	2903013-2903326	21	9
ACYPI002841-RA	gi|646781628|gb|KK961514.1|	230220-232285	23	9
ACYPI004759-RA	gi|646773018|gb|KK961752.1|	632571-635804	18	7.8
ACYPI006008-RA	gi|646767439|gb|KK961832.1|	1952538-1953162	23	10
ACYPI006262-RA	gi|646748142|gb|KK962168.1|	376038-376379	20	9.3
ACYPI009758-RA	gi|646779662|gb|KK961568.1|	2051425-2052235	20	9.3
ACYPI080270-RA	gi|646781628|gb|KK961514.1|	3367012-3370815	23	9
ACYPI005219-RA	gi|646747841|gb|KK962189.1|	317968-318410	23	10
ACYPI006453-RA	gi|646730953|gb|KK963877.1|	58577-61076	23	11
ACYPI006823-RA	gi|646747658|gb|KK962202.1|	102707-103263	15	7.1
ACYPI009001-RA	gi|646775293|gb|KK961735.1|	787843-788426	19	7.7
ACYPI068502-RA	gi|646742450|gb|KK962579.1|	88405-88905	19	7.6
ACYPI005607-RA	gi|646770477|gb|KK961778.1|	1131394-1131659	21	9.1
ACYPI008781-RA	gi|646781628|gb|KK961514.1|	3632767-3633635	23	9
ACYPI008926-RA	gi|646780147|gb|KK961556.1|	1074436-1075601	18	7.5

ACYPI064034-RA	gi|646775944|gb|KK961701.1|	1890191-1892623	22	9.3
ACYPI000739-RA	gi|646776647|gb|KK961663.1|	1490253-1490755	23	9.2
ACYPI002399-RA	gi|646727841|gb|KK964291.1|	34876-36588	23	9.6
ACYPI002629-RA	gi|646782127|gb|KK961500.1|	3949958-3952082	22	9.6
ACYPI003341-RA	gi|646751103|gb|KK962006.1|	97319-99532	20	8.3
ACYPI003960-RA	gi|646780183|gb|KK961555.1|	1993664-1995548	19	8.6
ACYPI005207-RA	gi|646770998|gb|KK961772.1|	1781680-1783927	22	9.8
ACYPI006229-RA	gi|646747201|gb|KK962236.1|	179273-180805	18	7.3
ACYPI000329-RA	gi|646779498|gb|KK961572.1|	2496952-2503099	22	5.6
ACYPI001057-RA	gi|646776219|gb|KK961687.1|	2596331-2597878	21	9.9
ACYPI002306-RA	gi|646768717|gb|KK961805.1|	531833-532379	20	8
ACYPI004185-RA	gi|646750354|gb|KK962043.1|	497683-499153	20	9.2
ACYPI005535-RA	gi|646748407|gb|KK962151.1|	606025-606543	22	9.1
ACYPI002835-RA	gi|646778983|gb|KK961586.1|	1354364-1354928	20	9.9
ACYPI072241-RA	gi|646775765|gb|KK961710.1|	1984639-1985783	21	6
ACYPI085401-RA	gi|646778983|gb|KK961586.1|	1226591-1233225	20	9.9
ACYPI006434-RA	gi|646752919|gb|KK961960.1|	644155-645038	20	9
ACYPI008307-RA	gi|646752919|gb|KK961960.1|	642282-643993	20	9
ACYPI063189-RA	gi|646740756|gb|KK962735.1|	238133-238498	23	10
ACYPI40809-RA	gi|646777474|gb|KK961630.1|	51693-53919	21	9.3
ACYPI001803-RA	gi|646780406|gb|KK961550.1|	784075-784362	22	5.9
ACYPI000149-RA	gi|646776305|gb|KK961683.1|	2525223-2526435	20	8.8
ACYPI001423-RA	gi|646747841|gb|KK962189.1|	663689-665663	23	10
ACYPI002663-RA	gi|646747161|gb|KK962239.1|	274778-275479	24	11
ACYPI003349-RA	gi|646782127|gb|KK961500.1|	3536955-3542945	22	9.6
ACYPI003937-RA	gi|646750409|gb|KK962040.1|	1084764-1085163	21	8.4
ACYPI003961-RA	gi|646775901|gb|KK961703.1|	825369-831326	20	9.6
ACYPI005090-RA	gi|646781421|gb|KK961521.1|	4249798-4251110	21	8.7
ACYPI005246-RA	gi|646775258|gb|KK961737.1|	596924-598829	19	9.8
ACYPI005282-RA	gi|646778336|gb|KK961604.1|	2771944-2772824	21	10

ACYPI007166-RA	gi|646782357|gb|KK961494.1|	9243960-9246647	21	9.2
ACYPI080661-RA	gi|646782276|gb|KK961497.1|	4267793-4268313	21	9.7
ACYPI004696-RA	gi|646762191|gb|KK961910.1|	205249-205581	18	7.1
ACYPI006602-RA	gi|646487701|gb|KK996683.1|	3249-3437	11	5.6
ACYPI24234-RA	gi|646748474|gb|KK962147.1|	588160-589835	20	9
ACYPI005478-RA	gi|646743261|gb|KK962517.1|	34238-35511	19	8.9
ACYPI002088-RA	gi|646780827|gb|KK961539.1|	3053298-3053810	20	9.4
ACYPI002656-RA	gi|646779337|gb|KK961576.1|	1223498-1223747	18	9
ACYPI005300-RA	gi|646747547|gb|KK962210.1|	393459-395163	19	7.5
ACYPI008930-RA	gi|646780574|gb|KK961546.1|	3155925-3157522	20	9.9
ACYPI39418-RA	gi|646743457|gb|KK962502.1|	347586-348083	15	8.8
ACYPI52843-RA	gi|646747500|gb|KK962213.1|	667429-673762	21	9.4
ACYPI000304-RA	gi|646775595|gb|KK961718.1|	677552-678067	21	8.9
ACYPI001241-RA	gi|646781379|gb|KK961522.1|	3775244-3775774	22	7.6
ACYPI002470-RA	gi|646765880|gb|KK961885.1|	1016510-1017864	23	10
ACYPI003749-RA	gi|646673348|gb|KK967633.1|	2934-3243	12	5.6
ACYPI060717-RA	gi|646775595|gb|KK961718.1|	562273-563685	21	8.9
ACYPI38406-RA	gi|646602815|gb|KK975927.1|	1611-2050	13	3.6
ACYPI006577-RA	gi|646776998|gb|KK961650.1|	2592015-2592210	20	9.6
ACYPI008205-RA	gi|646776322|gb|KK961682.1|	1831607-1832055	22	8.9
ACYPI008877-RA	gi|646776998|gb|KK961650.1|	2677370-2679852	20	9.6
ACYPI009769-RA	gi|646776998|gb|KK961650.1|	2705809-2716115	20	9.6
ACYPI000408-RA	gi|646776368|gb|KK961679.1|	1335718-1335988	21	9.7
ACYPI002950-RA	gi|646743492|gb|KK962499.1|	378863-379166	24	11
ACYPI004183-RA	gi|646779006|gb|KK961585.1|	1259395-1259672	20	9.9
ACYPI004328-RA	gi|646781659|gb|KK961513.1|	701750-706295	22	9.8
ACYPI004979-RA	gi|646766904|gb|KK961846.1|	829517-829819	17	6.9
ACYPI006109-RA	gi|646746475|gb|KK962286.1|	12643-13057	19	8.8
ACYPI007706-RA	gi|646777065|gb|KK961647.1|	353273-353507	20	7.9
ACYPI007769-RA	gi|646766904|gb|KK961846.1|	850023-850437	17	6.9

ACYPI009606-RA	gi|646779006|gb|KK961585.1|	820406-826083	20	9.9
ACYPI009635-RA	gi|646755095|gb|KK961939.1|	157015-158888	15	8.9
ACYPI083537-RA	gi|646776647|gb|KK961663.1|	1748757-1749432	23	9.2
ACYPI20880-RA	gi|646748245|gb|KK962161.1|	597756-598900	18	8.6
ACYPI010131-RA	gi|646741815|gb|KK962634.1|	102187-102988	22	10
ACYPI48847-RA	gi|646776069|gb|KK961694.1|	373602-374294	18	7.8
ACYPI004139-RA	gi|646781659|gb|KK961513.1|	793958-795380	22	9.8
ACYPI009768-RA	gi|646782043|gb|KK961502.1|	1702552-1705209	20	9.2
ACYPI001228-RA	gi|646777313|gb|KK961637.1|	303712-305402	20	8.2
ACYPI002741-RA	gi|646748185|gb|KK962165.1|	936983-937496	22	9.9
ACYPI006270-RA	gi|646771265|gb|KK961769.1|	175276-179149	17	7
ACYPI008837-RA	gi|646746663|gb|KK962273.1|	89374-90746	21	10
ACYPI009787-RA	gi|646775821|gb|KK961707.1|	799151-809580	23	9.1
ACYPI063443-RA	gi|646780925|gb|KK961536.1|	3239464-3241194	22	9.9
ACYPI071107-RA	gi|646765790|gb|KK961889.1|	1008609-1010280	22	8.8
ACYPI39340-RA	gi|646744086|gb|KK962458.1|	91013-96206	20	8.2
ACYPI067086-RA	gi|646642363|gb|KK968637.1|	13902-14498	20	12
ACYPI002147-RA	gi|646751160|gb|KK962003.1|	28515-36721	22	10
ACYPI005266-RA	gi|646780010|gb|KK961559.1|	3167362-3168236	22	10
ACYPI001763-RA	gi|646767521|gb|KK961830.1|	641863-643440	20	9.3
ACYPI000521-RA	gi|646768669|gb|KK961806.1|	417270-417813	18	9.1
ACYPI002256-RA	gi|646754962|gb|KK961940.1|	622144-622441	21	10
ACYPI002404-RA	gi|646776351|gb|KK961680.1|	1844763-1849085	22	10
ACYPI002985-RA	gi|646748785|gb|KK962129.1|	324112-329823	20	8.2
ACYPI004377-RA	gi|646749209|gb|KK962104.1|	783632-784278	21	9.2
ACYPI005144-RA	gi|646779741|gb|KK961566.1|	2939635-2940394	20	8.5
ACYPI006949-RA	gi|646781536|gb|KK961517.1|	2431223-2432212	19	7.7
ACYPI007422-RA	gi|646776351|gb|KK961680.1|	1891809-1892088	22	10
ACYPI061529-RA	gi|646742343|gb|KK962588.1|	370281-370601	20	9.4
ACYPI081493-RA	gi|646779741|gb|KK961566.1|	2582201-2583106	20	8.5

ACYPI27417-RA	gi|646754962|gb|KK961940.1|	186182-186458	21	10
ACYPI000104-RA	gi|646743394|gb|KK962507.1|	357348-358187	20	8.8
ACYPI005951-RA	gi|646779621|gb|KK961569.1|	306651-308950	16	7.3
ACYPI008382-RA	gi|646743394|gb|KK962507.1|	368061-369219	20	8.8
ACYPI22867-RA	gi|646777665|gb|KK961623.1|	1099688-1100283	21	9.7
ACYPI53200-RA	gi|646748728|gb|KK962132.1|	226897-229215	19	8.1
ACYPI009117-RA	gi|646780311|gb|KK961552.1|	4116967-4119489	21	9.7
ACYPI009298-RA	gi|646780441|gb|KK961549.1|	1022086-1025958	21	9.5
ACYPI000430-RA	gi|646770323|gb|KK961780.1|	267737-268155	19	7.1
ACYPI001193-RA	gi|646750165|gb|KK962053.1|	198910-199652	25	10
ACYPI002976-RA	gi|646781379|gb|KK961522.1|	777033-777645	22	7.6
ACYPI006797-RA	gi|646781690|gb|KK961512.1|	2360794-2363706	21	8.8
ACYPI003444-RA	gi|646732968|gb|KK963602.1|	255880-256646	20	9.9
ACYPI004368-RA	gi|646747841|gb|KK962189.1|	749628-752947	23	10
ACYPI005378-RA	gi|646781968|gb|KK961504.1|	954509-958304	20	9.6
ACYPI007250-RA	gi|646752567|gb|KK961965.1|	873778-874571	19	8.4
ACYPI007433-RA	gi|646777729|gb|KK961621.1|	1476257-1478960	21	9.8
ACYPI009536-RA	gi|646736283|gb|KK963203.1|	46215-46751	24	9.7
ACYPI56660-RA	gi|646781968|gb|KK961504.1|	905477-905953	20	9.6
ACYPI000035-RA	gi|646766426|gb|KK961862.1|	956662-956908	20	7.1
ACYPI001186-RA	gi|646744453|gb|KK962431.1|	65744-66168	19	8.5
ACYPI001539-RA	gi|646748580|gb|KK962141.1|	217486-217815	20	5.6
ACYPI004701-RA	gi|646741410|gb|KK962669.1|	35505-37144	21	9.3
ACYPI005987-RA	gi|646781536|gb|KK961517.1|	2281321-2281549	19	7.7
ACYPI006231-RA	gi|646739411|gb|KK962885.1|	237402-239049	18	7.7
ACYPI007232-RA	gi|646776762|gb|KK961659.1|	712414-714184	21	8.9
ACYPI000298-RA	gi|646782043|gb|KK961502.1|	1953837-1954110	20	9.2
ACYPI004774-RA	gi|646781732|gb|KK961511.1|	1228535-1229706	22	9.8
ACYPI008539-RA	gi|646749464|gb|KK962091.1|	361958-362486	18	8.8
ACYPI001593-RA	gi|646728609|gb|KK964184.1|	241138-241372	34	14

ACYPI003962-RA	gi|646748002|gb|KK962178.1|	1530220-1531488	22	6.3
ACYPI008213-RA	gi|646742726|gb|KK962557.1|	26389-29144	20	9.1
ACYPI009257-RA	gi|646630362|gb|KK970809.1|	4347-5074	12	7.2
ACYPI010103-RA	gi|646770477|gb|KK961778.1|	439289-439541	21	9.1
ACYPI072994-RA	gi|646773721|gb|KK961747.1|	1828280-1832506	21	9.2
ACYPI000467-RA	gi|646735649|gb|KK963275.1|	10568-10830	16	7.9
ACYPI001605-RA	gi|646742078|gb|KK962611.1|	45317-45880	18	8.1
ACYPI005434-RA	gi|646782043|gb|KK961502.1|	4113098-4113408	20	9.2
ACYPI008701-RA	gi|646780270|gb|KK961553.1|	4311807-4312104	22	8.6
ACYPI009090-RA	gi|646776477|gb|KK961673.1|	2097340-2101788	22	9
ACYPI071353-RA	gi|646782276|gb|KK961497.1|	517660-520202	21	9.7
ACYPI088146-RA	gi|646746556|gb|KK962281.1|	936945-940109	20	9.1
ACYPI43841-RA	gi|646777416|gb|KK961632.1|	3183863-3194314	22	8.8
ACYPI50219-RA	gi|646769275|gb|KK961796.1|	2415913-2433085	22	5.6
ACYPI001652-RA	gi|646750389|gb|KK962041.1|	598521-602195	21	9.5
ACYPI005675-RA	gi|646752431|gb|KK961967.1|	1416341-1416950	20	5.6
ACYPI005798-RA	gi|646754003|gb|KK961948.1|	1040276-1043620	20	8.8
ACYPI008638-RA	gi|646742706|gb|KK962559.1|	153172-154002	22	5.8
ACYPI009439-RA	gi|646747990|gb|KK962179.1|	110156-112105	21	9.3
ACYPI003442-RA	gi|646782276|gb|KK961497.1|	3204691-3205043	21	9.7
ACYPI004634-RA	gi|646746706|gb|KK962270.1|	916994-918232	23	11
ACYPI004669-RA	gi|646779337|gb|KK961576.1|	1096619-1099299	18	9
ACYPI009065-RA	gi|646745434|gb|KK962359.1|	392426-392979	22	10
ACYPI009098-RA	gi|646746663|gb|KK962273.1|	217142-217761	21	10
ACYPI070418-RA	gi|646750889|gb|KK962017.1|	1659281-1660111	22	6.1
ACYPI084297-RA	gi|646777955|gb|KK961615.1|	735783-739461	21	10
ACYPI000085-RA	gi|646782087|gb|KK961501.1|	1505741-1506608	20	8.7
ACYPI000500-RA	gi|646765966|gb|KK961881.1|	1445629-1446753	19	9.2
ACYPI000532-RA	gi|646782127|gb|KK961500.1|	3721499-3726858	22	9.6
ACYPI002389-RA	gi|646772045|gb|KK961761.1|	784801-787815	21	9.6

ACYPI005534-RA	gi|646775451|gb|KK961726.1|	1856723-1858800	20	5.6
ACYPI083213-RA	gi|646741621|gb|KK962651.1|	203888-207516	18	7.2
ACYPI43820-RA	gi|646746663|gb|KK962273.1|	76102-77262	21	10
ACYPI000876-RA	gi|646751458|gb|KK961989.1|	839991-840232	21	5.1
ACYPI007373-RA	gi|646770641|gb|KK961776.1|	655456-656655	20	9.1
ACYPI070323-RA	gi|646770641|gb|KK961776.1|	406066-406290	20	9.1
ACYPI000257-RA	gi|646742343|gb|KK962588.1|	266900-267672	20	9.4
ACYPI001490-RA	gi|646749209|gb|KK962104.1|	631361-632166	21	9.2
ACYPI002160-RA	gi|646769275|gb|KK961796.1|	127480-128055	22	5.6
ACYPI003333-RA	gi|646758845|gb|KK961921.1|	1054646-1054852	22	9.5
ACYPI004031-RA	gi|646746216|gb|KK962304.1|	782239-783216	########	33
ACYPI004733-RA	gi|646749209|gb|KK962104.1|	704254-706401	21	9.2
ACYPI005313-RA	gi|646751664|gb|KK961982.1|	1163913-1164442	21	9.5
ACYPI007194-RA	gi|646753799|gb|KK961950.1|	1991586-1996265	23	9.2
ACYPI008493-RA	gi|646751664|gb|KK961982.1|	1201899-1208065	21	9.5
ACYPI009150-RA	gi|646746828|gb|KK962262.1|	210587-212808	20	9.2
ACYPI000944-RA	gi|646780147|gb|KK961556.1|	1969066-1969740	18	7.5
ACYPI002533-RA	gi|646772358|gb|KK961758.1|	1874503-1876562	20	9.3
ACYPI002840-RA	gi|646768361|gb|KK961813.1|	448676-449914	15	7.4
ACYPI071406-RA	gi|646780147|gb|KK961556.1|	1958281-1958545	18	7.5
ACYPI001189-RA	gi|646780978|gb|KK961534.1|	604778-605595	20	8
ACYPI001658-RA	gi|646781344|gb|KK961523.1|	1315539-1315998	21	8.9
ACYPI001816-RA	gi|646780978|gb|KK961534.1|	574167-575220	20	8
ACYPI003232-RA	gi|646781772|gb|KK961510.1|	3086344-3089300	20	9.2
ACYPI003702-RA	gi|646780978|gb|KK961534.1|	594995-597805	20	8
ACYPI008002-RA	gi|646765991|gb|KK961880.1|	248045-251425	16	7
ACYPI009407-RA	gi|646775944|gb|KK961701.1|	941997-944835	22	9.3
ACYPI009554-RA	gi|646782211|gb|KK961498.1|	4647878-4648429	20	9
ACYPI000087-RA	gi|646771845|gb|KK961763.1|	299895-301396	23	10
ACYPI000938-RA	gi|646732491|gb|KK963666.1|	43033-43470	21	5.7

ACYPI004755-RA	gi|646772786|gb|KK961754.1|	505086-505610	22	9.6
ACYPI005419-RA	gi|646781690|gb|KK961512.1|	1817046-1818934	21	8.8
ACYPI007288-RA	gi|646780270|gb|KK961553.1|	4128835-4130990	22	8.6
ACYPI009174-RA	gi|646768717|gb|KK961805.1|	142739-146618	20	8
ACYPI084854-RA	gi|646781659|gb|KK961513.1|	2834578-2835918	22	9.8
ACYPI001106-RA	gi|646742413|gb|KK962582.1|	112826-113426	23	10
ACYPI004245-RA	gi|646736790|gb|KK963145.1|	356626-357369	21	9.6
ACYPI005955-RA	gi|646776647|gb|KK961663.1|	2215035-2218548	23	9.2
ACYPI067270-RA	gi|646746974|gb|KK962252.1|	1172276-1175426	23	6.1
ACYPI005418-RA	gi|646776128|gb|KK961691.1|	1702175-1710812	21	9.2
ACYPI007287-RA	gi|646767307|gb|KK961835.1|	568643-568929	20	9
ACYPI086727-RA	gi|646755888|gb|KK961934.1|	1116740-1119206	21	9.8
ACYPI52551-RA	gi|646748116|gb|KK962170.1|	416634-416980	20	8.3
ACYPI001483-RA	gi|646747614|gb|KK962205.1|	738370-742198	21	8.9
ACYPI003568-RA	gi|646780147|gb|KK961556.1|	803865-804403	18	7.5
ACYPI004460-RA	gi|646776113|gb|KK961692.1|	323914-324976	21	9.6
ACYPI006375-RA	gi|646776647|gb|KK961663.1|	349899-352026	23	9.2
ACYPI009264-RA	gi|646781143|gb|KK961529.1|	4242899-4243317	20	10
ACYPI55712-RA	gi|646770998|gb|KK961772.1|	1802369-1803254	22	9.8
ACYPI56745-RA	gi|646740395|gb|KK962776.1|	180371-182212	16	6.3
ACYPI007692-RA	gi|646767850|gb|KK961823.1|	1262634-1263112	21	9.8
ACYPI50560-RA	gi|646752849|gb|KK961961.1|	629199-631502	21	9.4
ACYPI000295-RA	gi|646771568|gb|KK961766.1|	989260-989524	18	7.4
ACYPI000335-RA	gi|646764075|gb|KK961900.1|	964327-964605	18	6.5
ACYPI000985-RA	gi|646759701|gb|KK961917.1|	896485-897848	18	6.1
ACYPI001684-RA	gi|646776219|gb|KK961687.1|	2352867-2353169	21	9.9
ACYPI003456-RA	gi|646777416|gb|KK961632.1|	975707-976600	22	8.8
ACYPI005389-RA	gi|646749302|gb|KK962099.1|	476936-478271	17	7
ACYPI006714-RA	gi|646779936|gb|KK961561.1|	3065230-3065613	19	9.6
ACYPI009902-RA	gi|646778336|gb|KK961604.1|	2110262-2111516	21	10

ACYPI067416-RA	gi|646768717|gb|KK961805.1|	567170-574967	20	8
ACYPI002664-RA	gi|646775702|gb|KK961713.1|	255280-258104	17	7.4
ACYPI005908-RA	gi|646748474|gb|KK962147.1|	366701-367496	20	9
ACYPI010244-RA	gi|646781601|gb|KK961515.1|	2058769-2059045	19	8.6
ACYPI069408-RA	gi|646767120|gb|KK961840.1|	1253222-1253404	20	8.6
ACYPI084497-RA	gi|646732188|gb|KK963710.1|	115514-116411	16	7
ACYPI084620-RA	gi|646745941|gb|KK962322.1|	309896-318346	21	10
ACYPI002812-RA	gi|646743886|gb|KK962473.1|	438766-439070	21	9.3
ACYPI005364-RA	gi|646769774|gb|KK961788.1|	548323-549374	20	9.2
ACYPI007200-RA	gi|646776148|gb|KK961690.1|	530609-534320	21	9.4
ACYPI009082-RA	gi|646781282|gb|KK961525.1|	2650538-2650843	22	9.6
ACYPI23394-RA	gi|646777288|gb|KK961638.1|	2847997-2850240	22	6.1
ACYPI001085-RA	gi|646780311|gb|KK961552.1|	2264830-2265753	21	9.7
ACYPI001928-RA	gi|646778983|gb|KK961586.1|	2716963-2717749	20	9.9
ACYPI002911-RA	gi|646754003|gb|KK961948.1|	555807-556686	20	8.8
ACYPI003577-RA	gi|646778565|gb|KK961598.1|	1510852-1511105	19	7.8
ACYPI004617-RA	gi|646767265|gb|KK961836.1|	1198385-1201978	22	9.9
ACYPI008598-RA	gi|646779262|gb|KK961578.1|	207309-209615	20	8.1
ACYPI000227-RA	gi|646779066|gb|KK961583.1|	1212691-1214372	19	7.9
ACYPI001125-RA	gi|646781564|gb|KK961516.1|	285738-290292	23	9.4
ACYPI001932-RA	gi|646779066|gb|KK961583.1|	1089073-1093749	19	7.9
ACYPI005359-RA	gi|646775540|gb|KK961721.1|	948316-948798	17	8.1
ACYPI006095-RA	gi|646775471|gb|KK961725.1|	1322550-1323354	20	10
ACYPI006584-RA	gi|646778983|gb|KK961586.1|	9773-9973	20	9.9
ACYPI073834-RA	gi|646747482|gb|KK962215.1|	364845-365155	21	9.2
ACYPI001050-RA	gi|646749283|gb|KK962100.1|	555599-563050	12	8.6
ACYPI001756-RA	gi|646778274|gb|KK961606.1|	879451-880017	18	7.5
ACYPI007388-RA	gi|646781659|gb|KK961513.1|	1511399-1512537	22	9.8
ACYPI008275-RA	gi|646770477|gb|KK961778.1|	1198416-1205118	21	9.1
ACYPI010151-RA	gi|646747586|gb|KK962207.1|	14940-15327	20	8.8

ACYPI001813-RA	gi|646782357|gb|KK961494.1|	2093145-2094261	21	9.2
ACYPI002118-RA	gi|646757279|gb|KK961927.1|	173582-176071	21	8.6
ACYPI007139-RA	gi|646739555|gb|KK962870.1|	111678-112791	27	18
ACYPI001764-RA	gi|646748002|gb|KK962178.1|	1601285-1602167	22	6.3
ACYPI001777-RA	gi|646747459|gb|KK962217.1|	874270-880389	21	5.9
ACYPI002918-RA	gi|646747149|gb|KK962240.1|	251630-271281	21	10
ACYPI003651-RA	gi|646775944|gb|KK961701.1|	622841-625467	22	9.3
ACYPI004930-RA	gi|646748002|gb|KK962178.1|	1684267-1685192	22	6.3
ACYPI006221-RA	gi|646747459|gb|KK962217.1|	130776-137818	21	5.9
ACYPI006830-RA	gi|646766482|gb|KK961860.1|	262605-264917	17	6.7
ACYPI007362-RA	gi|646747738|gb|KK962196.1|	367493-369842	20	8.3
ACYPI008606-RA	gi|646780889|gb|KK961537.1|	2378429-2381394	22	10
ACYPI009993-RA	gi|646765198|gb|KK961897.1|	383246-386764	22	5.9
ACYPI39184-RA	gi|646777765|gb|KK961620.1|	1927556-1927849	21	8.7
ACYPI001635-RA	gi|646780270|gb|KK961553.1|	1835154-1836223	22	8.6
ACYPI008409-RA	gi|646780270|gb|KK961553.1|	1853799-1854192	22	8.6
ACYPI081555-RA	gi|646749887|gb|KK962068.1|	754932-756160	20	9.5
ACYPI34207-RA	gi|646768163|gb|KK961817.1|	371055-371336	20	7.6
ACYPI003210-RA	gi|646781809|gb|KK961509.1|	3226289-3230714	19	8.6
ACYPI003804-RA	gi|646778903|gb|KK961588.1|	570610-572163	23	10
ACYPI005735-RA	gi|646781809|gb|KK961509.1|	3324099-3325509	19	8.6
ACYPI007453-RA	gi|646782357|gb|KK961494.1|	3614850-3622344	21	9.2
ACYPI007960-RA	gi|646739598|gb|KK962865.1|	254229-255766	21	10
ACYPI008715-RA	gi|646768534|gb|KK961809.1|	106563-111734	21	9.8
ACYPI061526-RA	gi|646739598|gb|KK962865.1|	240041-240266	21	10
ACYPI38602-RA	gi|646777089|gb|KK961646.1|	479877-480198	20	9.4
ACYPI52381-RA	gi|646750484|gb|KK962036.1|	158007-160816	20	5.5
ACYPI003257-RA	gi|646752294|gb|KK961969.1|	871288-871770	23	9.8
ACYPI004037-RA	gi|646769221|gb|KK961797.1|	737439-737674	19	9.6
ACYPI006164-RA	gi|646781443|gb|KK961520.1|	1794021-1795864	20	8.3

ACYPI073036-RA	gi|646781443|gb|KK961520.1|	2119588-2123878	20	8.3
ACYPI50514-RA	gi|646775312|gb|KK961734.1|	116824-117309	17	7.9
ACYPI002475-RA	gi|646772786|gb|KK961754.1|	1082923-1084009	22	9.6
ACYPI006520-RA	gi|646776351|gb|KK961680.1|	719811-726418	22	10
ACYPI007301-RA	gi|646780147|gb|KK961556.1|	1375140-1376175	18	7.5
ACYPI009695-RA	gi|646749832|gb|KK962071.1|	1476318-1477500	22	6
ACYPI35784-RA	gi|646748129|gb|KK962169.1|	268947-270140	21	9.8
ACYPI001171-RA	gi|646737782|gb|KK963043.1|	49469-50210	23	9.2
ACYPI003059-RA	gi|646747482|gb|KK962215.1|	428732-434848	21	9.2
ACYPI006498-RA	gi|646778699|gb|KK961594.1|	1330627-1331300	21	9.5
ACYPI009387-RA	gi|646769163|gb|KK961798.1|	1558984-1576226	20	9.2
ACYPI067721-RA	gi|646742471|gb|KK962577.1|	426315-427144	22	5.9
ACYPI073612-RA	gi|646780953|gb|KK961535.1|	4376882-4377409	20	9.8
ACYPI001730-RA	gi|646781968|gb|KK961504.1|	2300175-2306420	20	9.6
ACYPI005084-RA	gi|646742078|gb|KK962611.1|	48553-49398	18	8.1
ACYPI009568-RA	gi|646746911|gb|KK962256.1|	12697-13330	17	7
ACYPI002231-RA	gi|646746040|gb|KK962316.1|	185966-191202	19	6.9
ACYPI002255-RA	gi|646747574|gb|KK962208.1|	342225-343615	27	10
ACYPI007949-RA	gi|646758845|gb|KK961921.1|	140389-140629	22	9.5
ACYPI001044-RA	gi|646778865|gb|KK961589.1|	1075002-1076615	20	9.2
ACYPI003793-RA	gi|646780794|gb|KK961540.1|	3210011-3210269	21	9.5
ACYPI006871-RA	gi|646778983|gb|KK961586.1|	342591-342856	20	9.9
ACYPI009224-RA	gi|646777363|gb|KK961635.1|	1927326-1929724	18	9.2
ACYPI009633-RA	gi|646781510|gb|KK961518.1|	3510683-3511105	17	7.8
ACYPI31672-RA	gi|646580918|gb|KK980223.1|	30-302	92	56
ACYPI000262-RA	gi|646752294|gb|KK961969.1|	850302-851220	23	9.8
ACYPI004151-RA	gi|646779936|gb|KK961561.1|	2875180-2875456	19	9.6
ACYPI006281-RA	gi|646747124|gb|KK962242.1|	284248-286225	17	8.7
ACYPI006639-RA	gi|646779936|gb|KK961561.1|	3023369-3038129	19	9.6
ACYPI008547-RA	gi|646781344|gb|KK961523.1|	1146528-1151387	21	8.9

ACYPI009077-RA	gi|646780889|gb|KK961537.1|	498098-498740	22	10
ACYPI010144-RA	gi|646780311|gb|KK961552.1|	1700492-1701028	21	9.7
ACYPI003318-RA	gi|646638312|gb|KK969438.1|	12382-13593	13	6.6
ACYPI000071-RA	gi|646747175|gb|KK962238.1|	313611-314731	20	8.4
ACYPI000674-RA	gi|646752431|gb|KK961967.1|	878384-878812	20	5.6
ACYPI003760-RA	gi|646749672|gb|KK962080.1|	302141-302538	20	7.6
ACYPI009460-RA	gi|646780530|gb|KK961547.1|	1044603-1046004	21	9.2
ACYPI068778-RA	gi|646770323|gb|KK961780.1|	681506-683383	19	7.1
ACYPI003625-RA	gi|646776024|gb|KK961697.1|	1912164-1916312	22	9.2
ACYPI003756-RA	gi|646766426|gb|KK961862.1|	914809-915095	20	7.1
ACYPI005614-RA	gi|646773018|gb|KK961752.1|	407033-411250	18	7.8
ACYPI006383-RA	gi|646781043|gb|KK961532.1|	3267845-3269889	26	11
ACYPI007032-RA	gi|646781772|gb|KK961510.1|	3438186-3448567	20	9.2
ACYPI007495-RA	gi|646775635|gb|KK961716.1|	877273-877818	22	10
ACYPI065995-RA	gi|646775635|gb|KK961716.1|	874345-875280	22	10
ACYPI002168-RA	gi|646749482|gb|KK962090.1|	71506-74816	19	7.1
ACYPI002733-RA	gi|646780752|gb|KK961541.1|	1283544-1285555	21	8
ACYPI003385-RA	gi|646746694|gb|KK962271.1|	307644-307939	19	7.6
ACYPI004740-RA	gi|646781043|gb|KK961532.1|	2081786-2082670	26	11
ACYPI006457-RA	gi|646780953|gb|KK961535.1|	521596-521963	20	9.8
ACYPI073759-RA	gi|646766382|gb|KK961864.1|	458031-467067	22	9.7
ACYPI088483-RA	gi|646775173|gb|KK961741.1|	369731-371454	19	8.8
ACYPI000992-RA	gi|646780270|gb|KK961553.1|	2211583-2212046	22	8.6
ACYPI001724-RA	gi|646732968|gb|KK963602.1|	82536-83641	20	9.9
ACYPI003708-RA	gi|646781732|gb|KK961511.1|	4145078-4146127	22	9.8
ACYPI004818-RA	gi|646775867|gb|KK961705.1|	2300785-2307326	23	10
ACYPI005391-RA	gi|646782334|gb|KK961495.1|	3403864-3405361	21	9
ACYPI005557-RA	gi|646748086|gb|KK962172.1|	851816-852182	20	10
ACYPI062430-RA	gi|646780925|gb|KK961536.1|	3302222-3302570	22	9.9
ACYPI003171-RA	gi|646765747|gb|KK961891.1|	904534-908562	18	7.4

ACYPI004433-RA	gi|646605552|gb|KK975337.1|	4842-5026	15	2.4
ACYPI008211-RA	gi|646771092|gb|KK961771.1|	597519-599804	23	9.7
ACYPI30362-RA	gi|646765219|gb|KK961896.1|	664323-664773	20	8.1
ACYPI001788-RA	gi|646744904|gb|KK962398.1|	140567-141310	18	7.8
ACYPI068223-RA	gi|646780889|gb|KK961537.1|	2005093-2007898	22	10
ACYPI000402-RA	gi|646780105|gb|KK961557.1|	3099164-3099558	21	9.7
ACYPI001833-RA	gi|646767754|gb|KK961825.1|	791015-797238	20	8.5
ACYPI002296-RA	gi|646776184|gb|KK961688.1|	1862925-1863193	21	9.6
ACYPI004658-RA	gi|646765966|gb|KK961881.1|	601970-603132	19	9.2
ACYPI010056-RA	gi|646776998|gb|KK961650.1|	1768073-1769230	20	9.6
ACYPI36103-RA	gi|646743352|gb|KK962510.1|	243669-244955	20	5.4
ACYPI002151-RA	gi|646750043|gb|KK962060.1|	110912-111244	21	8.3
ACYPI006622-RA	gi|646749727|gb|KK962077.1|	430323-435086	21	8.9
ACYPI085402-RA	gi|646629468|gb|KK970983.1|	13427-14322	9.1	6.7
ACYPI001975-RA	gi|646749832|gb|KK962071.1|	571544-571896	22	6
ACYPI003866-RA	gi|646776389|gb|KK961678.1|	588239-589099	22	9.5
ACYPI008067-RA	gi|646782276|gb|KK961497.1|	4973771-4976178	21	9.7
ACYPI008277-RA	gi|646778481|gb|KK961600.1|	528042-530643	20	9.5
ACYPI009755-RA	gi|646753799|gb|KK961950.1|	1860673-1862503	23	9.2
ACYPI009818-RA	gi|646776735|gb|KK961660.1|	1273463-1273681	21	9.6
ACYPI010201-RA	gi|646769275|gb|KK961796.1|	263513-263991	22	5.6
ACYPI072205-RA	gi|646776735|gb|KK961660.1|	1011630-1012852	21	9.6
ACYPI002575-RA	gi|646773423|gb|KK961749.1|	931332-931815	19	8.1
ACYPI002649-RA	gi|646777991|gb|KK961614.1|	1500465-1502545	18	7.8
ACYPI005140-RA	gi|646765689|gb|KK961894.1|	399754-400731	18	7.7
ACYPI005173-RA	gi|646685025|gb|KK967178.1|	20901-22353	17	5.2
ACYPI006051-RA	gi|646743394|gb|KK962507.1|	22051-22367	20	8.8
ACYPI006476-RA	gi|646766451|gb|KK961861.1|	11784-12982	19	8.9
ACYPI006821-RA	gi|646777233|gb|KK961640.1|	2279271-2279635	21	8.7
ACYPI008362-RA	gi|646782334|gb|KK961495.1|	8990436-8991485	21	9

ACYPI008996-RA	gi|646777233|gb|KK961640.1|	2315840-2316150	21	8.7
ACYPI004188-RA	gi|646781968|gb|KK961504.1|	3176056-3176657	20	9.6
ACYPI004580-RA	gi|646782288|gb|KK961496.1|	6421686-6421929	21	9.7
ACYPI004887-RA	gi|646604507|gb|KK975569.1|	3897-4127	12	4.4
ACYPI005014-RA	gi|646767670|gb|KK961827.1|	1617703-1618076	21	5.9
ACYPI005706-RA	gi|646749114|gb|KK962109.1|	552627-555443	20	8.8
ACYPI072081-RA	gi|646747817|gb|KK962191.1|	114546-115382	22	5.3
ACYPI35291-RA	gi|646780147|gb|KK961556.1|	1615751-1616118	18	7.5
ACYPI004920-RA	gi|646746489|gb|KK962285.1|	265641-266357	17	6.8
ACYPI22529-RA	gi|646768294|gb|KK961814.1|	143809-144326	23	10
ACYPI001820-RA	gi|646742450|gb|KK962579.1|	210949-214175	19	7.6
ACYPI003705-RA	gi|646751289|gb|KK961996.1|	1196601-1200875	24	9.1
ACYPI005032-RA	gi|646724600|gb|KK964790.1|	63962-66174	20	5.2
ACYPI005634-RA	gi|646749512|gb|KK962088.1|	524647-527487	18	7.2
ACYPI001585-RA	gi|646778840|gb|KK961590.1|	2503479-2503992	20	9.9
ACYPI004834-RA	gi|646747175|gb|KK962238.1|	551723-555205	20	8.4
ACYPI009262-RA	gi|646780010|gb|KK961559.1|	459098-459345	22	10
ACYPI063239-RA	gi|646746868|gb|KK962259.1|	152215-158740	21	10
ACYPI067185-RA	gi|646780723|gb|KK961542.1|	3698178-3699388	21	9.4
ACYPI002401-RA	gi|646781043|gb|KK961532.1|	3022212-3022812	26	11
ACYPI39748-RA	gi|646778865|gb|KK961589.1|	2595109-2598678	20	9.2
ACYPI001243-RA	gi|646748695|gb|KK962134.1|	215558-215862	20	5.8
ACYPI006492-RA	gi|646754962|gb|KK961940.1|	955576-958371	21	10
ACYPI009620-RA	gi|646744930|gb|KK962396.1|	71652-71965	18	8.5
ACYPI081739-RA	gi|646775723|gb|KK961712.1|	1588623-1591241	21	9.5
ACYPI001804-RA	gi|646563700|gb|KK983468.1|	416-594	7	5.6
ACYPI002046-RA	gi|646776351|gb|KK961680.1|	1012093-1016938	22	10
ACYPI005571-RA	gi|646744453|gb|KK962431.1|	20134-21025	19	8.5
ACYPI006140-RA	gi|646751856|gb|KK961977.1|	161954-163343	18	8.5
ACYPI007445-RA	gi|646758845|gb|KK961921.1|	1857357-1860465	22	9.5

ACYPI008089-RA	gi|646778213|gb|KK961608.1|	2532123-2535071	19	8.8
ACYPI008168-RA	gi|646778699|gb|KK961594.1|	1224258-1227543	21	9.5
ACYPI008776-RA	gi|646777108|gb|KK961645.1|	911087-918267	17	6.5
ACYPI009891-RA	gi|646782127|gb|KK961500.1|	764918-768229	22	9.6
ACYPI22631-RA	gi|646781628|gb|KK961514.1|	569515-571093	23	9
ACYPI31274-RA	gi|646581458|gb|KK980124.1|	1172-1563	39	17
ACYPI000765-RA	gi|646776510|gb|KK961671.1|	896194-899453	20	7.8
ACYPI002367-RA	gi|646739361|gb|KK962890.1|	380529-382111	22	9.6
ACYPI005243-RA	gi|646747884|gb|KK962186.1|	506967-513329	22	11
ACYPI006124-RA	gi|646740004|gb|KK962821.1|	379844-388128	22	9.6
ACYPI006420-RA	gi|646769163|gb|KK961798.1|	1478032-1478282	20	9.2
ACYPI007736-RA	gi|646750889|gb|KK962017.1|	990681-991189	22	6.1
ACYPI008662-RA	gi|646776280|gb|KK961684.1|	1893982-1894302	22	9.3
ACYPI009004-RA	gi|646778983|gb|KK961586.1|	269447-270522	20	9.9
ACYPI000060-RA	gi|646739411|gb|KK962885.1|	225159-225481	18	7.7
ACYPI004192-RA	gi|646739411|gb|KK962885.1|	272786-278319	18	7.7
ACYPI005677-RA	gi|646778699|gb|KK961594.1|	663077-665563	21	9.5
ACYPI010079-RA	gi|646771448|gb|KK961767.1|	1119012-1124505	21	9.8
ACYPI000855-RA	gi|646750600|gb|KK962030.1|	678233-679195	23	8.6
ACYPI001003-RA	gi|646781212|gb|KK961527.1|	2191133-2192019	21	10
ACYPI006503-RA	gi|646775451|gb|KK961726.1|	2455969-2465125	20	5.6
ACYPI008327-RA	gi|646752014|gb|KK961974.1|	144836-150271	18	5.8
ACYPI009134-RA	gi|646750643|gb|KK962028.1|	832510-833975	21	7.5
ACYPI009382-RA	gi|646779898|gb|KK961562.1|	2378809-2380683	19	9.3
ACYPI38630-RA	gi|646781212|gb|KK961527.1|	1279243-1281825	21	10
ACYPI000433-RA	gi|646781732|gb|KK961511.1|	905498-907168	22	9.8
ACYPI001277-RA	gi|646772897|gb|KK961753.1|	1531223-1531918	21	8.7
ACYPI001901-RA	gi|646747682|gb|KK962200.1|	439756-440162	19	7.1
ACYPI002880-RA	gi|646782168|gb|KK961499.1|	2729477-2729980	21	9.4
ACYPI005720-RA	gi|646780889|gb|KK961537.1|	5179999-5180478	22	10

ACYPI007110-RA	gi|646775987|gb|KK961699.1|	857149-857656	19	7.5
ACYPI007997-RA	gi|646776322|gb|KK961682.1|	1950010-1951270	22	8.9
ACYPI008848-RA	gi|646746651|gb|KK962274.1|	77672-78544	16	6.6
ACYPI009870-RA	gi|646768261|gb|KK961815.1|	251426-255133	22	6.6
ACYPI081692-RA	gi|646746651|gb|KK962274.1|	35663-36004	16	6.6
ACYPI001285-RA	gi|646774530|gb|KK961743.1|	466194-467574	20	9.8
ACYPI002557-RA	gi|646779826|gb|KK961564.1|	2711260-2712177	22	9.6
ACYPI003170-RA	gi|646706386|gb|KK965650.1|	32734-34754	20	11
ACYPI005724-RA	gi|646780858|gb|KK961538.1|	2802691-2804992	21	10
ACYPI066154-RA	gi|646740955|gb|KK962713.1|	206838-208297	28	13
ACYPI069316-RA	gi|646750341|gb|KK962044.1|	879043-883189	19	5.6
ACYPI23471-RA	gi|646744833|gb|KK962403.1|	846712-847795	22	9.4
ACYPI005897-RA	gi|646768717|gb|KK961805.1|	1241920-1242802	20	8
ACYPI009644-RA	gi|646751569|gb|KK961985.1|	284442-285635	21	9.2
ACYPI009661-RA	gi|646744833|gb|KK962403.1|	746777-747505	22	9.4
ACYPI001856-RA	gi|646776542|gb|KK961668.1|	2151705-2152568	20	5.6
ACYPI009147-RA	gi|646776542|gb|KK961668.1|	1370838-1375160	20	5.6
ACYPI009325-RA	gi|646752567|gb|KK961965.1|	777613-777911	19	8.4
ACYPI009431-RA	gi|646753273|gb|KK961956.1|	684998-689731	22	9
ACYPI001752-RA	gi|646776647|gb|KK961663.1|	2654761-2658004	23	9.2
ACYPI002361-RA	gi|646747884|gb|KK962186.1|	1107153-1108272	22	11
ACYPI002482-RA	gi|646768294|gb|KK961814.1|	1081772-1083971	23	10
ACYPI003002-RA	gi|646781379|gb|KK961522.1|	572075-572243	22	7.6
ACYPI007409-RA	gi|646776368|gb|KK961679.1|	674816-680837	21	9.7
ACYPI085768-RA	gi|646750643|gb|KK962028.1|	517452-518890	21	7.5
ACYPI087848-RA	gi|646768294|gb|KK961814.1|	1050458-1058358	23	10
ACYPI009237-RA	gi|646767850|gb|KK961823.1|	1681050-1681592	21	9.8
ACYPI48166-RA	gi|646782168|gb|KK961499.1|	6087727-6089978	21	9.4
ACYPI001614-RA	gi|646745186|gb|KK962377.1|	937377-938085	21	9.8
ACYPI003083-RA	gi|646618553|gb|KK972958.1|	39-301	20	8.2

ACYPI004129-RA	gi|646776389|gb|KK961678.1|	1664819-1666527	22	9.5
ACYPI005442-RA	gi|646781659|gb|KK961513.1|	2891118-2891548	22	9.8
ACYPI007070-RA	gi|646780574|gb|KK961546.1|	233294-235124	20	9.9
ACYPI50527-RA	gi|646750372|gb|KK962042.1|	277673-278491	18	9.5
ACYPI002157-RA	gi|646750600|gb|KK962030.1|	952838-960782	23	8.6
ACYPI004029-RA	gi|646732431|gb|KK963675.1|	306302-308057	24	9.6
ACYPI004684-RA	gi|646753339|gb|KK961955.1|	163370-163637	22	11
ACYPI085818-RA	gi|646770817|gb|KK961774.1|	1170611-1171713	18	7.5
ACYPI005200-RA	gi|646781043|gb|KK961532.1|	4197254-4198590	26	11
ACYPI005353-RA	gi|646776389|gb|KK961678.1|	728182-731482	22	9.5
ACYPI007627-RA	gi|646766540|gb|KK961858.1|	1129475-1130256	21	8.5
ACYPI007946-RA	gi|646776904|gb|KK961654.1|	1509855-1511279	21	9.3
ACYPI008725-RA	gi|646780794|gb|KK961540.1|	1578307-1578762	21	9.5
ACYPI38600-RA	gi|646780270|gb|KK961553.1|	963912-967265	22	8.6
ACYPI43746-RA	gi|646777665|gb|KK961623.1|	327429-328354	21	9.7
ACYPI52168-RA	gi|646779826|gb|KK961564.1|	1923560-1929115	22	9.6
ACYPI000767-RA	gi|646782127|gb|KK961500.1|	2268280-2271808	22	9.6
ACYPI008437-RA	gi|646780978|gb|KK961534.1|	1021674-1022500	20	8
ACYPI010036-RA	gi|646778767|gb|KK961592.1|	2181239-2181419	21	5.4
ACYPI006713-RA	gi|646776351|gb|KK961680.1|	1475381-1478565	22	10
ACYPI064433-RA	gi|646782087|gb|KK961501.1|	199567-200874	20	8.7
ACYPI003090-RA	gi|646776929|gb|KK961653.1|	607095-607370	17	8.5
ACYPI003826-RA	gi|646776524|gb|KK961669.1|	987357-988125	17	6.4
ACYPI25351-RA	gi|646776998|gb|KK961650.1|	336669-337083	20	9.6
ACYPI000814-RA	gi|646751569|gb|KK961985.1|	937271-938229	21	9.2
ACYPI002695-RA	gi|646736855|gb|KK963138.1|	62509-63107	19	9.1
ACYPI007245-RA	gi|646778565|gb|KK961598.1|	414641-415120	19	7.8
ACYPI000759-RA	gi|646776929|gb|KK961653.1|	883996-885812	17	8.5
ACYPI004562-RA	gi|646777955|gb|KK961615.1|	753562-757393	21	10
ACYPI007054-RA	gi|646777416|gb|KK961632.1|	2152979-2153324	22	8.8

ACYPI082267-RA	gi|646782357|gb|KK961494.1|	5307330-5309462	21	9.2
ACYPI000654-RA	gi|646752919|gb|KK961960.1|	1667440-1669253	20	9
ACYPI001424-RA	gi|646749051|gb|KK962113.1|	444541-445261	19	8.4
ACYPI008222-RA	gi|646775842|gb|KK961706.1|	651342-651921	22	9
ACYPI46077-RA	gi|646743886|gb|KK962473.1|	530155-530972	21	9.3
ACYPI001511-RA	gi|646766985|gb|KK961844.1|	296011-309627	21	9.7
ACYPI002756-RA	gi|646750061|gb|KK962059.1|	505248-505774	15	7.1
ACYPI003401-RA	gi|646777017|gb|KK961649.1|	627201-627455	18	8
ACYPI004349-RA	gi|646746310|gb|KK962297.1|	209661-211844	23	11
ACYPI005038-RA	gi|646750043|gb|KK962060.1|	757679-757990	21	8.3
ACYPI008129-RA	gi|646781690|gb|KK961512.1|	2519417-2519949	21	8.8
ACYPI010007-RA	gi|646750252|gb|KK962049.1|	356632-359093	22	10
ACYPI000714-RA	gi|646768582|gb|KK961808.1|	282713-283331	21	6
ACYPI001898-RA	gi|646769163|gb|KK961798.1|	1340069-1340325	20	9.2
ACYPI002963-RA	gi|646756446|gb|KK961931.1|	620092-621178	14	6.1
ACYPI004154-RA	gi|646767670|gb|KK961827.1|	2087025-2087580	21	5.9
ACYPI006790-RA	gi|646767156|gb|KK961839.1|	147231-147560	15	6.4
ACYPI007404-RA	gi|646777288|gb|KK961638.1|	98943-106268	22	6.1
ACYPI008244-RA	gi|646779498|gb|KK961572.1|	1075894-1076208	22	5.6
ACYPI008884-RA	gi|646747424|gb|KK962220.1|	1163095-1164089	22	5.7
ACYPI36131-RA	gi|646776904|gb|KK961654.1|	596981-598512	21	9.3
ACYPI000050-RA	gi|646768406|gb|KK961812.1|	207006-207229	20	9.6
ACYPI000870-RA	gi|646754003|gb|KK961948.1|	320783-321399	20	8.8
ACYPI002907-RA	gi|646740619|gb|KK962751.1|	73767-74102	21	9.4
ACYPI007276-RA	gi|646781143|gb|KK961529.1|	3160131-3160728	20	10
ACYPI009727-RA	gi|646775702|gb|KK961713.1|	668235-669152	17	7.4
ACYPI37793-RA	gi|646776542|gb|KK961668.1|	3089558-3092060	20	5.6
ACYPI005718-RA	gi|646777765|gb|KK961620.1|	2539674-2543715	21	8.7
ACYPI087652-RA	gi|646751120|gb|KK962005.1|	591682-591818	21	5.8
ACYPI000453-RA	gi|646776701|gb|KK961661.1|	612430-613848	18	6.7

ACYPI002346-RA	gi|646776647|gb|KK961663.1|	558292-562116	23	9.2
ACYPI008552-RA	gi|646746355|gb|KK962294.1|	790028-790559	22	10
ACYPI084988-RA	gi|646781421|gb|KK961521.1|	4496356-4498668	21	8.7
ACYPI36829-RA	gi|646772786|gb|KK961754.1|	1364473-1365189	22	9.6
ACYPI003839-RA	gi|646763535|gb|KK961902.1|	976968-983363	23	10
ACYPI004640-RA	gi|646771352|gb|KK961768.1|	244012-244308	21	9.6
ACYPI001622-RA	gi|646781143|gb|KK961529.1|	4291110-4292074	20	10
ACYPI003895-RA	gi|646778186|gb|KK961609.1|	1783271-1789757	19	9.4
ACYPI004805-RA	gi|646752294|gb|KK961969.1|	613294-615121	23	9.8
ACYPI007971-RA	gi|646744270|gb|KK962445.1|	230442-230881	21	7.9
ACYPI008564-RA	gi|646780441|gb|KK961549.1|	2921561-2923334	21	9.5
ACYPI009308-RA	gi|646781421|gb|KK961521.1|	12560-13018	21	8.7
ACYPI009596-RA	gi|646780105|gb|KK961557.1|	1629025-1629284	21	9.7
ACYPI000798-RA	gi|646778699|gb|KK961594.1|	2244905-2245200	21	9.5
ACYPI001567-RA	gi|646780858|gb|KK961538.1|	3187395-3189248	21	10
ACYPI001746-RA	gi|646766451|gb|KK961861.1|	109275-110647	19	8.9
ACYPI002386-RA	gi|646767697|gb|KK961826.1|	1371153-1377119	21	9.8
ACYPI002680-RA	gi|646780858|gb|KK961538.1|	3841595-3842102	21	10
ACYPI003001-RA	gi|646770477|gb|KK961778.1|	1290658-1293472	21	9.1
ACYPI22813-RA	gi|646768494|gb|KK961810.1|	1389872-1390467	21	10
ACYPI001584-RA	gi|646776389|gb|KK961678.1|	683114-688070	22	9.5
ACYPI002013-RA	gi|646742343|gb|KK962588.1|	497860-499373	20	9.4
ACYPI006725-RA	gi|646778274|gb|KK961606.1|	785398-785619	18	7.5
ACYPI009821-RA	gi|646741719|gb|KK962642.1|	417418-423149	21	8.9
ACYPI081262-RA	gi|646750702|gb|KK962025.1|	610373-611298	12	6.5
ACYPI064464-RA	gi|646781510|gb|KK961518.1|	934256-935380	17	7.8
ACYPI064813-RA	gi|646701057|gb|KK965959.1|	58376-60016	15	5.9
ACYPI001591-RA	gi|646779262|gb|KK961578.1|	729002-729390	20	8.1
ACYPI002372-RA	gi|646745631|gb|KK962343.1|	6096-6581	18	8.6
ACYPI002624-RA	gi|646769275|gb|KK961796.1|	1970018-1975802	22	5.6

ACYPI003082-RA	gi|646781083|gb|KK961531.1|	2765061-2771749	19	8.5
ACYPI003661-RA	gi|646745941|gb|KK962322.1|	795337-795490	21	10
ACYPI008758-RA	gi|646763164|gb|KK961904.1|	629740-630650	22	10
ACYPI083436-RA	gi|646745372|gb|KK962364.1|	763185-764496	23	10
ACYPI49347-RA	gi|646750600|gb|KK962030.1|	1613889-1614820	23	8.6
ACYPI000045-RA	gi|646767196|gb|KK961838.1|	694862-695862	21	9.7
ACYPI001194-RA	gi|646777665|gb|KK961623.1|	1899629-1900013	21	9.7
ACYPI001554-RA	gi|646766868|gb|KK961847.1|	1328139-1328814	21	9.9
ACYPI005122-RA	gi|646780311|gb|KK961552.1|	3719208-3725697	21	9.7
ACYPI007009-RA	gi|646764075|gb|KK961900.1|	384473-387026	18	6.5
ACYPI007022-RA	gi|646766735|gb|KK961851.1|	877803-880419	20	10
ACYPI002137-RA	gi|646762191|gb|KK961910.1|	707040-707591	18	7.1
ACYPI002484-RA	gi|646775807|gb|KK961708.1|	1741426-1742458	21	8.6
ACYPI004206-RA	gi|646777416|gb|KK961632.1|	666876-667489	22	8.8
ACYPI004809-RA	gi|646781628|gb|KK961514.1|	4768649-4771127	23	9
ACYPI006711-RA	gi|646768294|gb|KK961814.1|	607675-612282	23	10
ACYPI008022-RA	gi|646782288|gb|KK961496.1|	7021789-7023913	21	9.7
ACYPI009893-RA	gi|646777416|gb|KK961632.1|	620981-622626	22	8.8
ACYPI069860-RA	gi|646578414|gb|KK980699.1|	9588-10104	25	9.7
ACYPI000956-RA	gi|646778903|gb|KK961588.1|	3713052-3713946	23	10
ACYPI002523-RA	gi|646775789|gb|KK961709.1|	1212365-1212849	20	9.6
ACYPI004423-RA	gi|646749173|gb|KK962106.1|	245626-245938	21	5.7
ACYPI006340-RA	gi|646780270|gb|KK961553.1|	2142737-2144308	22	8.6
ACYPI008218-RA	gi|646765902|gb|KK961884.1|	1185385-1185612	22	9.2
ACYPI008248-RA	gi|646614736|gb|KK973610.1|	15241-15543	14	6.6
ACYPI087011-RA	gi|646776588|gb|KK961666.1|	2308549-2309008	20	5.8
ACYPI010018-RA	gi|646749755|gb|KK962075.1|	704621-704883	22	9.2
ACYPI071475-RA	gi|646781118|gb|KK961530.1|	5166124-5166912	21	10
ACYPI086253-RA	gi|646780530|gb|KK961547.1|	958159-960064	21	9.2
ACYPI001400-RA	gi|646767381|gb|KK961833.1|	460353-461331	18	6.8

ACYPI005401-RA	gi|646768261|gb|KK961815.1|	1265106-1267726	22	6.6
ACYPI006354-RA	gi|646738248|gb|KK962996.1|	96287-99727	17	6.6
ACYPI006873-RA	gi|646779826|gb|KK961564.1|	949765-950723	22	9.6
ACYPI007272-RA	gi|646779580|gb|KK961570.1|	2071591-2081440	17	8.5
ACYPI008918-RA	gi|646740768|gb|KK962733.1|	159834-162128	13	6.8
ACYPI009163-RA	gi|646729280|gb|KK964096.1|	67066-67276	22	9.3
ACYPI009300-RA	gi|646747614|gb|KK962205.1|	481231-484283	21	8.9
ACYPI080769-RA	gi|646779826|gb|KK961564.1|	1100198-1100451	22	9.6
ACYPI002018-RA	gi|646779662|gb|KK961568.1|	1325710-1325937	20	9.3
ACYPI005416-RA	gi|646780105|gb|KK961557.1|	203172-203790	21	9.7
ACYPI008368-RA	gi|646747956|gb|KK962181.1|	233344-234089	22	9.7
ACYPI008833-RA	gi|646766137|gb|KK961874.1|	468576-469321	20	9
ACYPI24411-RA	gi|646769163|gb|KK961798.1|	1407567-1411721	20	9.2
ACYPI007319-RA	gi|646749155|gb|KK962107.1|	51847-56344	22	9.2
ACYPI007615-RA	gi|646749155|gb|KK962107.1|	794982-795554	22	9.2
ACYPI063020-RA	gi|646734882|gb|KK963367.1|	125590-127143	13	6.8
ACYPI067100-RA	gi|646484557|gb|KK997231.1|	14-1172	6.5	3.4
ACYPI000055-RA	gi|646779262|gb|KK961578.1|	2195924-2197069	20	8.1
ACYPI000431-RA	gi|646779826|gb|KK961564.1|	1056290-1056945	22	9.6
ACYPI001692-RA	gi|646781282|gb|KK961525.1|	3671061-3676017	22	9.6
ACYPI001971-RA	gi|646768534|gb|KK961809.1|	80081-80507	21	9.8
ACYPI002220-RA	gi|646776929|gb|KK961653.1|	559623-564080	17	8.5
ACYPI002792-RA	gi|646782288|gb|KK961496.1|	506752-507969	21	9.7
ACYPI003562-RA	gi|646781118|gb|KK961530.1|	5755346-5756974	21	10
ACYPI004859-RA	gi|646739361|gb|KK962890.1|	484931-487641	22	9.6
ACYPI005512-RA	gi|646673021|gb|KK967651.1|	768-1149	10	6.1
ACYPI006399-RA	gi|646738455|gb|KK962975.1|	457007-457851	23	9.5
ACYPI008279-RA	gi|646775821|gb|KK961707.1|	285309-286134	23	9.1
ACYPI009136-RA	gi|646780858|gb|KK961538.1|	3132481-3132748	21	10
ACYPI009523-RA	gi|646780752|gb|KK961541.1|	1543690-1544207	21	8

ACYPI010196-RA	gi|646768534|gb|KK961809.1|	32013-33951	21	9.8
ACYPI32444-RA	gi|646778865|gb|KK961589.1|	2594019-2595005	20	9.2
ACYPI000076-RA	gi|646738524|gb|KK962968.1|	52854-54376	24	11
ACYPI002684-RA	gi|646778736|gb|KK961593.1|	1747586-1748173	20	7.9
ACYPI003008-RA	gi|646781536|gb|KK961517.1|	254137-262390	19	7.7
ACYPI003204-RA	gi|646752143|gb|KK961972.1|	935633-945789	20	5.6
ACYPI003233-RA	gi|646778660|gb|KK961595.1|	125370-125646	16	8.2
ACYPI005152-RA	gi|646677811|gb|KK967447.1|	6679-7767	13	8.3
ACYPI007640-RA	gi|646782334|gb|KK961495.1|	7333029-7334905	21	9
ACYPI52785-RA	gi|646738466|gb|KK962974.1|	310594-314162	30	14
ACYPI002288-RA	gi|646732787|gb|KK963624.1|	132301-133762	12	6.1
ACYPI003481-RA	gi|646735722|gb|KK963266.1|	172392-173278	17	7.6
ACYPI004169-RA	gi|646781772|gb|KK961510.1|	3202699-3202960	20	9.2
ACYPI006105-RA	gi|646731210|gb|KK963842.1|	80326-81284	19	7.5
ACYPI007012-RA	gi|646748615|gb|KK962139.1|	663980-665717	19	7.8
ACYPI007988-RA	gi|646768631|gb|KK961807.1|	397155-398127	19	9.2
ACYPI009378-RA	gi|646767850|gb|KK961823.1|	1756117-1756626	21	9.8
ACYPI009741-RA	gi|646767265|gb|KK961836.1|	561579-562221	22	9.9
ACYPI080621-RA	gi|646776004|gb|KK961698.1|	962864-968458	19	8.4
ACYPI001427-RA	gi|646777065|gb|KK961647.1|	412661-414713	20	7.9
ACYPI003786-RA	gi|646742685|gb|KK962560.1|	7341-8264	21	9.5
ACYPI004248-RA	gi|646766703|gb|KK961852.1|	799261-799915	20	7.1
ACYPI004370-RA	gi|646747884|gb|KK962186.1|	1186452-1191871	22	11
ACYPI004749-RA	gi|646772574|gb|KK961756.1|	1014560-1015013	21	8
ACYPI009615-RA	gi|646775924|gb|KK961702.1|	392342-392845	14	8.8
ACYPI000890-RA	gi|646748888|gb|KK962123.1|	868885-870145	20	8.1
ACYPI002781-RA	gi|646748888|gb|KK962123.1|	823008-825513	20	8.1
ACYPI007858-RA	gi|646748888|gb|KK962123.1|	764180-766647	20	8.1
ACYPI004106-RA	gi|646751458|gb|KK961989.1|	795942-796584	21	5.1
ACYPI009988-RA	gi|646726809|gb|KK964433.1|	15005-16922	19	12

ACYPI29867-RA	gi|646746475|gb|KK962286.1|	66128-67102	19	8.8
ACYPI000203-RA	gi|646768717|gb|KK961805.1|	720790-721668	20	8
ACYPI000235-RA	gi|646748407|gb|KK962151.1|	147350-164335	22	9.1
ACYPI000904-RA	gi|646780311|gb|KK961552.1|	3538603-3539747	21	9.7
ACYPI001518-RA	gi|646781849|gb|KK961508.1|	2585322-2586509	17	8
ACYPI002154-RA	gi|646781443|gb|KK961520.1|	2193431-2194215	20	8.3
ACYPI002393-RA	gi|646779898|gb|KK961562.1|	1093463-1094224	19	9.3
ACYPI002951-RA	gi|646737822|gb|KK963039.1|	15832-16397	20	9
ACYPI003409-RA	gi|646773018|gb|KK961752.1|	565788-566183	18	7.8
ACYPI003886-RA	gi|646758527|gb|KK961922.1|	565562-565804	24	10
ACYPI004880-RA	gi|646740073|gb|KK962814.1|	10677-11241	18	8.3
ACYPI005668-RA	gi|646682319|gb|KK967352.1|	30010-30570	13	6.2
ACYPI005896-RA	gi|646747046|gb|KK962247.1|	752023-754104	22	9
ACYPI005956-RA	gi|646779662|gb|KK961568.1|	1053753-1054155	20	9.3
ACYPI006624-RA	gi|646771352|gb|KK961768.1|	409073-412458	21	9.6
ACYPI007257-RA	gi|646774530|gb|KK961743.1|	334044-334630	20	9.8
ACYPI007584-RA	gi|646752849|gb|KK961961.1|	613031-613318	21	9.4
ACYPI008033-RA	gi|646777729|gb|KK961621.1|	740303-742314	21	9.8
ACYPI008591-RA	gi|646778061|gb|KK961612.1|	1611854-1614132	20	8.7
ACYPI009732-RA	gi|646766598|gb|KK961856.1|	190697-191273	23	10
ACYPI067763-RA	gi|646781043|gb|KK961532.1|	4250812-4251387	26	11
ACYPI52393-RA	gi|646758527|gb|KK961922.1|	579840-586079	24	10
ACYPI001600-RA	gi|646749632|gb|KK962082.1|	267316-268320	20	8.6
ACYPI002236-RA	gi|646747494|gb|KK962214.1|	406124-408614	18	8.5
ACYPI003488-RA	gi|646775507|gb|KK961723.1|	1824044-1825093	20	9.8
ACYPI004115-RA	gi|646780627|gb|KK961545.1|	341927-342413	20	7.7
ACYPI006047-RA	gi|646780627|gb|KK961545.1|	362646-363780	20	7.7
ACYPI34301-RA	gi|646771448|gb|KK961767.1|	864360-865658	21	9.8
ACYPI001217-RA	gi|646613436|gb|KK973865.1|	6907-7589	21	7.7
ACYPI002491-RA	gi|646779006|gb|KK961585.1|	155446-157105	20	9.9

ACYPI003116-RA	gi|646750910|gb|KK962016.1|	52180-53293	13	8.5
ACYPI004395-RA	gi|646771845|gb|KK961763.1|	1182788-1187814	23	10
ACYPI005673-RA	gi|646766943|gb|KK961845.1|	636703-637583	18	8.9
ACYPI008827-RA	gi|646777125|gb|KK961644.1|	2793360-2794142	20	10
ACYPI009438-RA	gi|646753273|gb|KK961956.1|	927205-927424	22	9
ACYPI010075-RA	gi|646782043|gb|KK961502.1|	1757397-1757570	20	9.2
ACYPI23954-RA	gi|646750745|gb|KK962023.1|	925903-937638	18	9.4
ACYPI001352-RA	gi|646750600|gb|KK962030.1|	1468306-1469781	23	8.6
ACYPI004711-RA	gi|646744270|gb|KK962445.1|	221771-223748	21	7.9
ACYPI006613-RA	gi|646746216|gb|KK962304.1|	860189-870564	########	33
ACYPI080140-RA	gi|646776184|gb|KK961688.1|	620976-622156	21	9.6
ACYPI086980-RA	gi|646737936|gb|KK963027.1|	77246-78538	15	6.9
ACYPI40688-RA	gi|646744652|gb|KK962417.1|	659142-662562	25	11
ACYPI000150-RA	gi|646776368|gb|KK961679.1|	1738757-1741452	21	9.7
ACYPI001458-RA	gi|646777207|gb|KK961641.1|	1368135-1368890	20	8.5
ACYPI004109-RA	gi|646769901|gb|KK961786.1|	1032827-1033797	18	6.7
ACYPI006043-RA	gi|646770998|gb|KK961772.1|	779379-783836	22	9.8
ACYPI006824-RA	gi|646767670|gb|KK961827.1|	2187400-2191753	21	5.9
ACYPI008418-RA	gi|646772574|gb|KK961756.1|	1190864-1191414	21	8
ACYPI000003-RA	gi|646738819|gb|KK962938.1|	60350-61387	19	8.4
ACYPI000479-RA	gi|646747990|gb|KK962179.1|	803511-807000	21	9.3
ACYPI002383-RA	gi|646747046|gb|KK962247.1|	156046-156236	22	9
ACYPI002595-RA	gi|646778865|gb|KK961589.1|	2945518-2946005	20	9.2
ACYPI004570-RA	gi|646781013|gb|KK961533.1|	453122-459133	18	7.6
ACYPI004938-RA	gi|646749616|gb|KK962083.1|	516438-526335	22	10
ACYPI006482-RA	gi|646751160|gb|KK962003.1|	1138606-1138983	22	10
ACYPI007710-RA	gi|646735017|gb|KK963352.1|	254176-255405	22	5.9
ACYPI007807-RA	gi|646752143|gb|KK961972.1|	609184-610441	20	5.6
ACYPI21475-RA	gi|646752076|gb|KK961973.1|	422826-436759	22	9.1
ACYPI000841-RA	gi|646776024|gb|KK961697.1|	2350004-2350735	22	9.2

ACYPI002068-RA	gi|646748785|gb|KK962129.1|	480261-480456	20	8.2
ACYPI003771-RA	gi|646780406|gb|KK961550.1|	483864-484318	22	5.9
ACYPI004259-RA	gi|646729487|gb|KK964068.1|	74132-74875	14	10
ACYPI005496-RA	gi|646750600|gb|KK962030.1|	1570058-1572154	23	8.6
ACYPI008006-RA	gi|646758022|gb|KK961924.1|	195362-195699	16	6.2
ACYPI008431-RA	gi|646773721|gb|KK961747.1|	2000809-2001071	21	9.2
ACYPI009716-RA	gi|646740015|gb|KK962820.1|	48723-49978	20	8.4
ACYPI089540-RA	gi|646781893|gb|KK961506.1|	3656507-3656815	19	8.5
ACYPI000646-RA	gi|646770251|gb|KK961781.1|	594123-594844	20	9.6
ACYPI001292-RA	gi|646782168|gb|KK961499.1|	3534783-3535379	21	9.4
ACYPI002350-RA	gi|646776456|gb|KK961674.1|	1366641-1367070	18	7.9
ACYPI002431-RA	gi|646779141|gb|KK961581.1|	2762906-2763427	21	9.1
ACYPI003185-RA	gi|646759701|gb|KK961917.1|	129480-133948	18	6.1
ACYPI004334-RA	gi|646761166|gb|KK961913.1|	348944-350837	17	7.6
ACYPI008555-RA	gi|646775595|gb|KK961718.1|	1422622-1424548	21	8.9
ACYPI008767-RA	gi|646775595|gb|KK961718.1|	1064491-1066804	21	8.9
ACYPI008864-RA	gi|646777729|gb|KK961621.1|	1144176-1145842	21	9.8
ACYPI009525-RA	gi|646747547|gb|KK962210.1|	499463-501253	19	7.5
ACYPI48246-RA	gi|646776305|gb|KK961683.1|	2478680-2479325	20	8.8
ACYPI009537-RA	gi|646780978|gb|KK961534.1|	401500-401706	20	8
ACYPI004261-RA	gi|646779298|gb|KK961577.1|	413754-415156	20	9.1
ACYPI006196-RA	gi|646747574|gb|KK962208.1|	606216-607517	27	10
ACYPI006680-RA	gi|646746475|gb|KK962286.1|	193534-209576	19	8.8
ACYPI007827-RA	gi|646773423|gb|KK961749.1|	282388-283435	19	8.1
ACYPI008069-RA	gi|646769453|gb|KK961793.1|	1331704-1334519	21	8.8
ACYPI010180-RA	gi|646777393|gb|KK961634.1|	1175302-1175554	18	9.1
ACYPI29397-RA	gi|646780889|gb|KK961537.1|	2666187-2668299	22	10
ACYPI48217-RA	gi|646749616|gb|KK962083.1|	104341-107181	22	10
ACYPI000600-RA	gi|646779262|gb|KK961578.1|	907356-907915	20	8.1
ACYPI002480-RA	gi|646781628|gb|KK961514.1|	4812629-4814190	23	9

ACYPI002650-RA	gi|646776490|gb|KK961672.1|	58431-63884	20	10
ACYPI002961-RA	gi|646745372|gb|KK962364.1|	413868-415003	23	10
ACYPI003224-RA	gi|646781183|gb|KK961528.1|	526413-526760	20	9.5
ACYPI004891-RA	gi|646778307|gb|KK961605.1|	664805-665509	17	8.7
ACYPI006789-RA	gi|646745327|gb|KK962367.1|	20662-21267	18	8.3
ACYPI006958-RA	gi|646777474|gb|KK961630.1|	984506-988456	21	9.3
ACYPI007402-RA	gi|646780827|gb|KK961539.1|	3971722-3972364	20	9.4
ACYPI008272-RA	gi|646781043|gb|KK961532.1|	2250870-2252431	26	11
ACYPI008820-RA	gi|646562296|gb|KK983760.1|	137-341	15	7.2
ACYPI010149-RA	gi|646779826|gb|KK961564.1|	1298888-1299663	22	9.6
ACYPI55567-RA	gi|646781043|gb|KK961532.1|	2241209-2245366	26	11
ACYPI000587-RA	gi|646781183|gb|KK961528.1|	2869693-2871527	20	9.5
ACYPI001665-RA	gi|646777802|gb|KK961619.1|	1451621-1453226	24	9
ACYPI003550-RA	gi|646781690|gb|KK961512.1|	3241721-3242537	21	8.8
ACYPI004846-RA	gi|646750043|gb|KK962060.1|	153122-159262	21	8.3
ACYPI005499-RA	gi|646781690|gb|KK961512.1|	3337509-3338589	21	8.8
ACYPI006750-RA	gi|646750043|gb|KK962060.1|	312487-313594	21	8.3
ACYPI007366-RA	gi|646762191|gb|KK961910.1|	568889-569273	18	7.1
ACYPI000182-RA	gi|646740955|gb|KK962713.1|	168948-171713	28	13
ACYPI001145-RA	gi|646754371|gb|KK961945.1|	560961-561596	20	9.7
ACYPI003626-RA	gi|646779006|gb|KK961585.1|	845085-847674	20	9.9
ACYPI004521-RA	gi|646769001|gb|KK961801.1|	490643-490916	20	8.5
ACYPI006438-RA	gi|646778029|gb|KK961613.1|	1830063-1832107	21	8.6
ACYPI50578-RA	gi|646780105|gb|KK961557.1|	699508-705013	21	9.7
ACYPI005237-RA	gi|646769774|gb|KK961788.1|	844641-855761	20	9.2
ACYPI007190-RA	gi|646778767|gb|KK961592.1|	1253504-1253878	21	5.4
ACYPI005746-RA	gi|646779702|gb|KK961567.1|	2026348-2029252	23	10
ACYPI51632-RA	gi|646747597|gb|KK962206.1|	305040-306231	20	8.1
ACYPI000502-RA	gi|646781809|gb|KK961509.1|	2194102-2194572	19	8.6
ACYPI001088-RA	gi|646780658|gb|KK961544.1|	1571160-1572651	19	8.6

ACYPI003519-RA	gi|646776148|gb|KK961690.1|	1863927-1865939	21	9.4
ACYPI006827-RA	gi|646782276|gb|KK961497.1|	3131877-3132082	21	9.7
ACYPI49982-RA	gi|646776113|gb|KK961692.1|	213711-215428	21	9.6
ACYPI000264-RA	gi|646747494|gb|KK962214.1|	302162-302400	18	8.5
ACYPI000882-RA	gi|646776524|gb|KK961669.1|	1195418-1197855	17	6.4
ACYPI001775-RA	gi|646775576|gb|KK961719.1|	123539-124463	20	8.6
ACYPI004822-RA	gi|646742237|gb|KK962597.1|	483568-487448	21	6
ACYPI006133-RA	gi|646772786|gb|KK961754.1|	551230-551961	22	9.6
ACYPI006318-RA	gi|646779621|gb|KK961569.1|	1459543-1459775	16	7.3
ACYPI007240-RA	gi|646769102|gb|KK961799.1|	231139-234110	20	8.3
ACYPI007299-RA	gi|646776904|gb|KK961654.1|	1825030-1825908	21	9.3
ACYPI007869-RA	gi|646767948|gb|KK961821.1|	464254-466589	17	7.1
ACYPI008113-RA	gi|646769038|gb|KK961800.1|	1672749-1675074	21	8.9
ACYPI008501-RA	gi|646781379|gb|KK961522.1|	348766-349932	22	7.6
ACYPI008046-RA	gi|646781083|gb|KK961531.1|	2500698-2501605	19	8.5
ACYPI080058-RA	gi|646749036|gb|KK962114.1|	658529-658678	20	7.5
ACYPI081400-RA	gi|646710354|gb|KK965458.1|	15650-16905	########	92
ACYPI001755-RA	gi|646769577|gb|KK961791.1|	904027-904515	18	8.3
ACYPI067996-RA	gi|646756839|gb|KK961929.1|	176661-177593	17	9.3
ACYPI40226-RA	gi|646766598|gb|KK961856.1|	258732-259021	23	10
ACYPI000407-RA	gi|646775312|gb|KK961734.1|	987612-988879	17	7.9
ACYPI005448-RA	gi|646767850|gb|KK961823.1|	987586-988003	21	9.8
ACYPI007394-RA	gi|646766382|gb|KK961864.1|	909388-910687	22	9.7
ACYPI008010-RA	gi|646770901|gb|KK961773.1|	964375-967240	22	9.7
ACYPI082601-RA	gi|646766382|gb|KK961864.1|	1007759-1009661	22	9.7
ACYPI001877-RA	gi|646775807|gb|KK961708.1|	1421485-1422075	21	8.6
ACYPI004421-RA	gi|646778699|gb|KK961594.1|	2093315-2098363	21	9.5
ACYPI005270-RA	gi|646778213|gb|KK961608.1|	1397324-1398549	19	8.8
ACYPI006978-RA	gi|646747032|gb|KK962248.1|	408105-408610	20	8.7
ACYPI060391-RA	gi|646776588|gb|KK961666.1|	862739-863308	20	5.8

ACYPI061546-RA	gi|646749868|gb|KK962069.1|	165355-165823	19	7.1
ACYPI46702-RA	gi|646776305|gb|KK961683.1|	1820678-1832946	20	8.8
ACYPI48967-RA	gi|646778517|gb|KK961599.1|	599374-600740	20	9.6
ACYPI009959-RA	gi|646776184|gb|KK961688.1|	1238885-1240849	21	9.6
ACYPI060978-RA	gi|646745135|gb|KK962381.1|	196998-197687	20	8.7
ACYPI068389-RA	gi|646768222|gb|KK961816.1|	627824-628925	25	12
ACYPI55789-RA	gi|646781968|gb|KK961504.1|	3653280-3654657	20	9.6
ACYPI003151-RA	gi|646737822|gb|KK963039.1|	93343-93932	20	9
ACYPI005774-RA	gi|646778767|gb|KK961592.1|	88103-88358	21	5.4
ACYPI006969-RA	gi|646765705|gb|KK961893.1|	658505-659931	22	9.7
ACYPI007587-RA	gi|646751544|gb|KK961986.1|	631750-634155	22	6
ACYPI008238-RA	gi|646780270|gb|KK961553.1|	1892303-1893364	22	8.6
ACYPI067736-RA	gi|646778336|gb|KK961604.1|	2247141-2253266	21	10
ACYPI000413-RA	gi|646747574|gb|KK962208.1|	384898-386581	27	10
ACYPI000753-RA	gi|646779936|gb|KK961561.1|	2061110-2061797	19	9.6
ACYPI000896-RA	gi|646750354|gb|KK962043.1|	326752-327026	20	9.2
ACYPI002791-RA	gi|646738812|gb|KK962939.1|	260051-260397	20	8.1
ACYPI004886-RA	gi|646751176|gb|KK962002.1|	807434-808612	21	9.6
ACYPI007034-RA	gi|646746911|gb|KK962256.1|	537607-538680	17	7
ACYPI007249-RA	gi|646781564|gb|KK961516.1|	2473793-2474564	23	9.4
ACYPI008165-RA	gi|646748615|gb|KK962139.1|	468964-469463	19	7.8
ACYPI070244-RA	gi|646777590|gb|KK961625.1|	578910-582202	19	9.7
ACYPI005622-RA	gi|646765948|gb|KK961882.1|	282593-283083	18	7.3
ACYPI069554-RA	gi|646777877|gb|KK961617.1|	1226040-1227744	19	8.1
ACYPI000044-RA	gi|646781510|gb|KK961518.1|	571481-571850	17	7.8
ACYPI000694-RA	gi|646779038|gb|KK961584.1|	500491-500957	21	6.9
ACYPI001296-RA	gi|646779337|gb|KK961576.1|	1112189-1113450	18	9
ACYPI002441-RA	gi|646781344|gb|KK961523.1|	875002-876772	21	8.9
ACYPI003722-RA	gi|646776069|gb|KK961694.1|	1000502-1002275	18	7.8
ACYPI004343-RA	gi|646775702|gb|KK961713.1|	487250-487567	17	7.4

ACYPI006312-RA	gi|646781344|gb|KK961523.1|	910016-910934	21	8.9
ACYPI061215-RA	gi|646782211|gb|KK961498.1|	5643569-5643792	20	9
ACYPI56627-RA	gi|646782211|gb|KK961498.1|	5556234-5557380	20	9
ACYPI000618-RA	gi|646746257|gb|KK962301.1|	713367-715000	13	7.3
ACYPI001675-RA	gi|646744579|gb|KK962422.1|	353613-363276	22	9.9
ACYPI003039-RA	gi|646748200|gb|KK962164.1|	262832-267114	19	7.7
ACYPI004420-RA	gi|646781243|gb|KK961526.1|	3404757-3405218	21	8.9
ACYPI006338-RA	gi|646745631|gb|KK962343.1|	254418-254676	18	8.6
ACYPI007776-RA	gi|646777207|gb|KK961641.1|	1520778-1520979	20	8.5
ACYPI065816-RA	gi|646748558|gb|KK962142.1|	787217-790543	21	9.9
ACYPI065923-RA	gi|646766426|gb|KK961862.1|	751158-752507	20	7.1
ACYPI069326-RA	gi|646745631|gb|KK962343.1|	280472-280749	18	8.6
ACYPI082039-RA	gi|646778865|gb|KK961589.1|	1604142-1606423	20	9.2
ACYPI35199-RA	gi|646775807|gb|KK961708.1|	1049620-1051151	21	8.6
ACYPI000091-RA	gi|646760564|gb|KK961914.1|	518723-518969	21	8.8
ACYPI000454-RA	gi|646776351|gb|KK961680.1|	424875-425208	22	10
ACYPI001153-RA	gi|646780827|gb|KK961539.1|	3273867-3274228	20	9.4
ACYPI003617-RA	gi|646780270|gb|KK961553.1|	4235772-4237638	22	8.6
ACYPI005360-RA	gi|646734874|gb|KK963368.1|	29893-33208	21	8
ACYPI005565-RA	gi|646780270|gb|KK961553.1|	4208555-4209129	22	8.6
ACYPI005600-RA	gi|646748474|gb|KK962147.1|	469276-470105	20	9
ACYPI006254-RA	gi|646776429|gb|KK961676.1|	1344369-1350059	20	9.9
ACYPI007227-RA	gi|646780794|gb|KK961540.1|	401654-402242	21	9.5
ACYPI007435-RA	gi|646747046|gb|KK962247.1|	1132778-1137329	22	9
ACYPI007477-RA	gi|646778213|gb|KK961608.1|	2569748-2576951	19	8.8
ACYPI009315-RA	gi|646747046|gb|KK962247.1|	68124-68337	22	9
ACYPI009370-RA	gi|646781344|gb|KK961523.1|	1250403-1252070	21	8.9
ACYPI27641-RA	gi|646733041|gb|KK963591.1|	109979-111333	24	8.7
ACYPI008425-RA	gi|646767521|gb|KK961830.1|	455622-464370	20	9.3
ACYPI066212-RA	gi|646781212|gb|KK961527.1|	3862373-3863984	21	10

ACYPI000783-RA	gi|646776128|gb|KK961691.1|	1329857-1332987	21	9.2
ACYPI001430-RA	gi|646732855|gb|KK963616.1|	148516-149839	24	7.1
ACYPI004643-RA	gi|646766765|gb|KK961850.1|	668613-675697	18	8.9
ACYPI006703-RA	gi|646781564|gb|KK961516.1|	2900118-2900526	23	9.4
ACYPI007219-RA	gi|646755714|gb|KK961935.1|	565979-567592	18	7.4
ACYPI009104-RA	gi|646622289|gb|KK972279.1|	598-841	19	8.6
ACYPI26612-RA	gi|646754962|gb|KK961940.1|	1299916-1301154	21	10
ACYPI28156-RA	gi|646752625|gb|KK961964.1|	558690-559592	13	7
ACYPI48897-RA	gi|646776855|gb|KK961656.1|	356795-360257	19	8.8
ACYPI004451-RA	gi|646777955|gb|KK961615.1|	2520089-2522450	21	10
ACYPI008886-RA	gi|646781968|gb|KK961504.1|	3297544-3298546	20	9.6
ACYPI061124-RA	gi|646734508|gb|KK963411.1|	179876-180778	19	8.8
ACYPI064146-RA	gi|646763535|gb|KK961902.1|	864982-865254	23	10
ACYPI065710-RA	gi|646776735|gb|KK961660.1|	1615092-1616543	21	9.6
ACYPI080212-RA	gi|646776447|gb|KK961675.1|	771509-774419	22	9.4
ACYPI34057-RA	gi|646780010|gb|KK961559.1|	2056498-2057680	22	10
ACYPI000740-RA	gi|646738615|gb|KK962959.1|	99329-101936	21	9.5
ACYPI001124-RA	gi|646765705|gb|KK961893.1|	1666052-1674499	22	9.7
ACYPI002364-RA	gi|646776389|gb|KK961678.1|	1535315-1535897	22	9.5
ACYPI002711-RA	gi|646766056|gb|KK961877.1|	361125-362060	18	7.8
ACYPI003062-RA	gi|646776128|gb|KK961691.1|	1317613-1318062	21	9.2
ACYPI004254-RA	gi|646781183|gb|KK961528.1|	1129123-1131677	20	9.5
ACYPI004629-RA	gi|646752567|gb|KK961965.1|	316242-317239	19	8.4
ACYPI004936-RA	gi|646688645|gb|KK967064.1|	31875-32418	14	5
ACYPI006545-RA	gi|646748245|gb|KK962161.1|	572602-576028	18	8.6
ACYPI010209-RA	gi|646775867|gb|KK961705.1|	1359592-1363103	23	10
ACYPI000443-RA	gi|646782168|gb|KK961499.1|	1790146-1791926	21	9.4
ACYPI000513-RA	gi|646745644|gb|KK962342.1|	463448-465229	31	14
ACYPI001002-RA	gi|646740756|gb|KK962735.1|	177514-178189	23	10
ACYPI001047-RA	gi|646782168|gb|KK961499.1|	7346412-7347822	21	9.4

ACYPI002904-RA	gi|646767265|gb|KK961836.1|	537573-538196	22	9.9
ACYPI003571-RA	gi|646529355|gb|KK988894.1|	502-1094	13	2.9
ACYPI004779-RA	gi|646739581|gb|KK962867.1|	354734-357834	18	5.4
ACYPI004832-RA	gi|646779006|gb|KK961585.1|	905670-906187	20	9.9
ACYPI004869-RA	gi|646780794|gb|KK961540.1|	2485636-2487800	21	9.5
ACYPI005485-RA	gi|646776128|gb|KK961691.1|	1357846-1363098	21	9.2
ACYPI005521-RA	gi|646775595|gb|KK961718.1|	1326427-1327601	21	8.9
ACYPI005540-RA	gi|646749616|gb|KK962083.1|	228658-228935	22	10
ACYPI006658-RA	gi|646627554|gb|KK971378.1|	3894-4350	12	5.6
ACYPI006682-RA	gi|646753715|gb|KK961951.1|	814999-815500	19	5.4
ACYPI006728-RA	gi|646738156|gb|KK963004.1|	54603-55300	12	6.9
ACYPI007303-RA	gi|646776447|gb|KK961675.1|	1896365-1898673	22	9.4
ACYPI007945-RA	gi|646776447|gb|KK961675.1|	1917798-1920290	22	9.4
ACYPI007967-RA	gi|646779580|gb|KK961570.1|	1991257-1991513	17	8.5
ACYPI008518-RA	gi|646773721|gb|KK961747.1|	2376125-2377311	21	9.2
ACYPI009202-RA	gi|646765198|gb|KK961897.1|	614027-616647	22	5.9
ACYPI009906-RA	gi|646780311|gb|KK961552.1|	2450301-2451452	21	9.7
ACYPI56634-RA	gi|646775540|gb|KK961721.1|	1257474-1259351	17	8.1
ACYPI062092-RA	gi|646779298|gb|KK961577.1|	308108-309062	20	9.1
ACYPI002842-RA	gi|646776514|gb|KK961670.1|	810483-811318	20	9.6
ACYPI005883-RA	gi|646744916|gb|KK962397.1|	559562-560331	18	8.3
ACYPI006954-RA	gi|646748014|gb|KK962177.1|	294669-295462	15	6.4
ACYPI009066-RA	gi|646745533|gb|KK962351.1|	620817-621330	22	9.7
ACYPI38656-RA	gi|646772358|gb|KK961758.1|	1632057-1632809	20	9.3
ACYPI006684-RA	gi|646776368|gb|KK961679.1|	1057471-1058357	21	9.7
ACYPI065234-RA	gi|646750542|gb|KK962033.1|	853989-855253	15	8.2
ACYPI28444-RA	gi|646778660|gb|KK961595.1|	2085867-2086552	16	8.2
ACYPI36183-RA	gi|646749444|gb|KK962092.1|	170125-172791	15	9
ACYPI37446-RA	gi|646738307|gb|KK962990.1|	179527-180465	37	17
ACYPI000112-RA	gi|646767156|gb|KK961839.1|	121818-122206	15	6.4

ACYPI009436-RA	gi|646779826|gb|KK961564.1|	985419-988515	22	9.6
ACYPI080576-RA	gi|646766137|gb|KK961874.1|	858169-858416	20	9
ACYPI006968-RA	gi|646767697|gb|KK961826.1|	1393124-1393662	21	9.8
ACYPI008923-RA	gi|646772358|gb|KK961758.1|	1492987-1494410	20	9.3
ACYPI063645-RA	gi|646781628|gb|KK961514.1|	367170-369080	23	9
ACYPI001779-RA	gi|646773903|gb|KK961746.1|	1519978-1520924	20	8.9
ACYPI002410-RA	gi|646773903|gb|KK961746.1|	1432289-1438946	20	8.9
ACYPI005594-RA	gi|646765669|gb|KK961895.1|	80366-80653	18	7.2
ACYPI006242-RA	gi|646780723|gb|KK961542.1|	1573125-1579403	21	9.4
ACYPI001025-RA	gi|646736283|gb|KK963203.1|	331952-336414	24	9.7
ACYPI003593-RA	gi|646782127|gb|KK961500.1|	6948696-6949852	22	9.6
ACYPI004392-RA	gi|646773018|gb|KK961752.1|	735405-737062	18	7.8
ACYPI005543-RA	gi|646782357|gb|KK961494.1|	5931366-5934131	21	9.2
ACYPI005578-RA	gi|646736260|gb|KK963205.1|	78048-82136	16	8.4
ACYPI29951-RA	gi|646749755|gb|KK962075.1|	688730-689795	22	9.2
ACYPI45731-RA	gi|646747111|gb|KK962243.1|	641751-648684	21	9.4
ACYPI53217-RA	gi|646740712|gb|KK962740.1|	209435-210015	14	8.5
ACYPI001166-RA	gi|646750043|gb|KK962060.1|	585234-585667	21	8.3
ACYPI006530-RA	gi|646737447|gb|KK963075.1|	347083-354281	22	9.2
ACYPI009457-RA	gi|646768631|gb|KK961807.1|	1346293-1346603	19	9.2
ACYPI087849-RA	gi|646740098|gb|KK962812.1|	168807-183678	20	10
ACYPI089164-RA	gi|646776280|gb|KK961684.1|	880141-880830	22	9.3
ACYPI002949-RA	gi|646750869|gb|KK962018.1|	100741-103753	20	10
ACYPI004239-RA	gi|646768717|gb|KK961805.1|	1357392-1359205	20	8
ACYPI005172-RA	gi|646779066|gb|KK961583.1|	216199-220279	19	7.9
ACYPI005839-RA	gi|646750185|gb|KK962052.1|	465161-465976	18	7.1
ACYPI006777-RA	gi|646775842|gb|KK961706.1|	334285-334895	22	9
ACYPI007072-RA	gi|646747482|gb|KK962215.1|	448128-451396	21	9.2
ACYPI008095-RA	gi|646745186|gb|KK962377.1|	683467-688157	21	9.8
ACYPI008640-RA	gi|646750869|gb|KK962018.1|	47570-58292	20	10

ACYPI009258-RA	gi|646777802|gb|KK961619.1|	1271170-1271967	24	9
ACYPI009973-RA	gi|646777842|gb|KK961618.1|	2074067-2076152	19	8.9
ACYPI067645-RA	gi|646778865|gb|KK961589.1|	1496073-1498523	20	9.2
ACYPI086553-RA	gi|646778803|gb|KK961591.1|	1046979-1049414	15	7.8
ACYPI46554-RA	gi|646776128|gb|KK961691.1|	877478-880898	21	9.2
ACYPI003006-RA	gi|646751515|gb|KK961987.1|	890582-892922	20	9.2
ACYPI008560-RA	gi|646751515|gb|KK961987.1|	1006506-1007171	20	9.2
ACYPI085907-RA	gi|646749402|gb|KK962094.1|	483185-483772	14	7.4
ACYPI51231-RA	gi|646777288|gb|KK961638.1|	652541-653675	22	6.1
ACYPI000159-RA	gi|646736790|gb|KK963145.1|	276003-278956	21	9.6
ACYPI003045-RA	gi|646781143|gb|KK961529.1|	2800464-2802968	20	10
ACYPI004195-RA	gi|646781143|gb|KK961529.1|	2157370-2158054	20	10
ACYPI004365-RA	gi|646752143|gb|KK961972.1|	1122090-1122340	20	5.6
ACYPI004964-RA	gi|646781143|gb|KK961529.1|	2817345-2819427	20	10
ACYPI005458-RA	gi|646782357|gb|KK961494.1|	4268127-4284704	21	9.2
ACYPI005919-RA	gi|646768406|gb|KK961812.1|	1292825-1299079	20	9.6
ACYPI007682-RA	gi|646780794|gb|KK961540.1|	2226219-2227143	21	9.5
ACYPI008164-RA	gi|646752143|gb|KK961972.1|	998990-999453	20	5.6
ACYPI008653-RA	gi|646781143|gb|KK961529.1|	2077904-2078118	20	10
ACYPI009871-RA	gi|646780889|gb|KK961537.1|	1403028-1404343	22	10
ACYPI000582-RA	gi|646743576|gb|KK962494.1|	140136-140844	19	8.2
ACYPI003455-RA	gi|646772045|gb|KK961761.1|	735288-736798	21	9.6
ACYPI003942-RA	gi|646775987|gb|KK961699.1|	302590-302863	19	7.5
ACYPI004076-RA	gi|646780953|gb|KK961535.1|	4503899-4504258	20	9.8
ACYPI062923-RA	gi|646771092|gb|KK961771.1|	737400-739749	23	9.7
ACYPI38307-RA	gi|646777089|gb|KK961646.1|	1531342-1532365	20	9.4
ACYPI47632-RA	gi|646780858|gb|KK961538.1|	3921483-3922506	21	10
ACYPI000063-RA	gi|646765790|gb|KK961889.1|	1012080-1014309	22	8.8
ACYPI002516-RA	gi|646767697|gb|KK961826.1|	1185703-1187629	21	9.8
ACYPI007418-RA	gi|646776998|gb|KK961650.1|	64988-66932	20	9.6

ACYPI007642-RA	gi|646730558|gb|KK963928.1|	112251-112538	19	5.4
ACYPI009677-RA	gi|646782276|gb|KK961497.1|	885836-893063	21	9.7
ACYPI009944-RA	gi|646746231|gb|KK962303.1|	305727-305893	21	6
ACYPI082722-RA	gi|646780794|gb|KK961540.1|	284003-285654	21	9.5
ACYPI088758-RA	gi|646770477|gb|KK961778.1|	1784431-1785451	21	9.1
ACYPI45423-RA	gi|646763164|gb|KK961904.1|	1415240-1417064	22	10
ACYPI000209-RA	gi|646740405|gb|KK962775.1|	353974-354546	20	5.8
ACYPI003754-RA	gi|646779298|gb|KK961577.1|	1448548-1450769	20	9.1
ACYPI003986-RA	gi|646776038|gb|KK961696.1|	1278970-1283843	21	10
ACYPI004024-RA	gi|646757520|gb|KK961926.1|	1744453-1746987	20	7.4
ACYPI005967-RA	gi|646757520|gb|KK961926.1|	1586356-1589015	20	7.4
ACYPI006066-RA	gi|646778029|gb|KK961613.1|	2105827-2109018	21	8.6
ACYPI068671-RA	gi|646752348|gb|KK961968.1|	247742-248223	18	8
ACYPI084147-RA	gi|646778029|gb|KK961613.1|	2084912-2091906	21	8.6
ACYPI20693-RA	gi|646777207|gb|KK961641.1|	1028818-1030646	20	8.5
ACYPI31758-RA	gi|646738028|gb|KK963018.1|	79585-80177	22	7.1
ACYPI34938-RA	gi|646766765|gb|KK961850.1|	973845-978317	18	8.9
ACYPI000575-RA	gi|646749632|gb|KK962082.1|	359901-362282	20	8.6
ACYPI005400-RA	gi|646768631|gb|KK961807.1|	430831-432383	19	9.2
ACYPI005676-RA	gi|646780978|gb|KK961534.1|	393472-394280	20	8
ACYPI006929-RA	gi|646750354|gb|KK962043.1|	297550-299205	20	9.2
ACYPI008551-RA	gi|646777233|gb|KK961640.1|	807520-808071	21	8.7
ACYPI081565-RA	gi|646781628|gb|KK961514.1|	832070-842052	23	9
ACYPI32084-RA	gi|646731162|gb|KK963848.1|	94379-95503	13	6.2
ACYPI002245-RA	gi|646747263|gb|KK962232.1|	581179-583917	18	8.6
ACYPI002529-RA	gi|646781628|gb|KK961514.1|	5029207-5029661	23	9
ACYPI006364-RA	gi|646776447|gb|KK961675.1|	783010-784763	22	9.4
ACYPI008243-RA	gi|646763164|gb|KK961904.1|	1344160-1351566	22	10
ACYPI31343-RA	gi|646782334|gb|KK961495.1|	3533923-3536377	21	9
ACYPI38704-RA	gi|646750341|gb|KK962044.1|	347141-348115	19	5.6

ACYPI000090-RA	gi|646770998|gb|KK961772.1|	1214361-1215446	22	9.8
ACYPI002356-RA	gi|646777089|gb|KK961646.1|	1122186-1122723	20	9.4
ACYPI084542-RA	gi|646750889|gb|KK962017.1|	585521-587322	22	6.1
ACYPI003186-RA	gi|646776447|gb|KK961675.1|	1326802-1327313	22	9.4
ACYPI005308-RA	gi|646775842|gb|KK961706.1|	1240797-1243932	22	9
ACYPI001631-RA	gi|646761166|gb|KK961913.1|	309037-309992	17	7.6
ACYPI005253-RA	gi|646755393|gb|KK961937.1|	203176-204012	19	6.8
ACYPI061017-RA	gi|646732604|gb|KK963650.1|	216977-217618	15	7.1
ACYPI001021-RA	gi|646779869|gb|KK961563.1|	2494782-2495034	17	8.8
ACYPI002805-RA	gi|646779498|gb|KK961572.1|	515432-516503	22	5.6
ACYPI003463-RA	gi|646778186|gb|KK961609.1|	2364964-2366717	19	9.4
ACYPI004854-RA	gi|646746158|gb|KK962308.1|	170150-172710	19	6.6
ACYPI005394-RA	gi|646738229|gb|KK962998.1|	340936-346928	22	8.9
ACYPI006268-RA	gi|646777729|gb|KK961621.1|	1260431-1261805	21	9.8
ACYPI006751-RA	gi|646747884|gb|KK962186.1|	24064-24961	22	11
ACYPI007266-RA	gi|646782357|gb|KK961494.1|	4312275-4314107	21	9.2
ACYPI007901-RA	gi|646747424|gb|KK962220.1|	249592-249932	22	5.7
ACYPI008709-RA	gi|646757520|gb|KK961926.1|	1119234-1125099	20	7.4
ACYPI009158-RA	gi|646766624|gb|KK961855.1|	412484-413071	21	10
ACYPI009771-RA	gi|646782334|gb|KK961495.1|	2711667-2712689	21	9
ACYPI080138-RA	gi|646747424|gb|KK962220.1|	244924-246701	22	5.7
ACYPI000249-RA	gi|646779662|gb|KK961568.1|	1262501-1264477	20	9.3
ACYPI000662-RA	gi|646750185|gb|KK962052.1|	259994-261137	18	7.1
ACYPI000913-RA	gi|646781659|gb|KK961513.1|	5019068-5019421	22	9.8
ACYPI001061-RA	gi|646745327|gb|KK962367.1|	51411-51650	18	8.3
ACYPI001698-RA	gi|646781536|gb|KK961517.1|	1890071-1895298	19	7.7
ACYPI002549-RA	gi|646769774|gb|KK961788.1|	651574-654039	20	9.2
ACYPI002798-RA	gi|646776514|gb|KK961670.1|	1699883-1700329	20	9.6
ACYPI002806-RA	gi|646781659|gb|KK961513.1|	6033433-6036104	22	9.8
ACYPI003581-RA	gi|646766735|gb|KK961851.1|	121456-121694	20	10

ACYPI004142-RA	gi|646781443|gb|KK961520.1|	465271-472935	20	8.3
ACYPI005946-RA	gi|646781043|gb|KK961532.1|	1882690-1883463	26	11
ACYPI006166-RA	gi|646781443|gb|KK961520.1|	2250499-2250746	20	8.3
ACYPI006625-RA	gi|646753527|gb|KK961953.1|	665250-672717	24	9.2
ACYPI007197-RA	gi|646683836|gb|KK967228.1|	14621-15584	13	5.4
ACYPI007757-RA	gi|646725863|gb|KK964584.1|	17933-29154	23	11
ACYPI008039-RA	gi|646724260|gb|KK964850.1|	88830-89418	20	8.3
ACYPI009528-RA	gi|646762191|gb|KK961910.1|	907806-912858	18	7.1
ACYPI009915-RA	gi|646782288|gb|KK961496.1|	7663194-7668733	21	9.7
ACYPI010231-RA	gi|646781510|gb|KK961518.1|	3548539-3549027	17	7.8
ACYPI000259-RA	gi|646780858|gb|KK961538.1|	1131776-1132105	21	10
ACYPI001527-RA	gi|646782288|gb|KK961496.1|	5994411-5994695	21	9.7
ACYPI004878-RA	gi|646779412|gb|KK961574.1|	2165323-2168377	21	5.4
ACYPI005975-RA	gi|646782288|gb|KK961496.1|	6234527-6240225	21	9.7
ACYPI006635-RA	gi|646782288|gb|KK961496.1|	5821016-5826189	21	9.7
ACYPI006817-RA	gi|646746897|gb|KK962257.1|	853714-854487	21	9.2
ACYPI008495-RA	gi|646780858|gb|KK961538.1|	1317995-1318583	21	10
ACYPI009739-RA	gi|646747528|gb|KK962211.1|	244449-246216	18	8.1
ACYPI060244-RA	gi|646484028|gb|KK997324.1|	2547-3275	27	10
ACYPI087955-RA	gi|646741540|gb|KK962657.1|	83873-85557	19	7
ACYPI50224-RA	gi|646659616|gb|KK968060.1|	39506-40115	26	8.6
ACYPI56843-RA	gi|646740158|gb|KK962805.1|	688849-689809	24	6.5
ACYPI000505-RA	gi|646766451|gb|KK961861.1|	566084-566355	19	8.9
ACYPI001718-RA	gi|646749832|gb|KK962071.1|	350826-352043	22	6
ACYPI002126-RA	gi|646748580|gb|KK962141.1|	389025-389658	20	5.6
ACYPI004001-RA	gi|646748474|gb|KK962147.1|	568297-568823	20	9
ACYPI004294-RA	gi|646781243|gb|KK961526.1|	3320544-3320879	21	8.9
ACYPI004372-RA	gi|646781243|gb|KK961526.1|	3668890-3669778	21	8.9
ACYPI004697-RA	gi|646782211|gb|KK961498.1|	988580-990820	20	9
ACYPI005940-RA	gi|646782276|gb|KK961497.1|	7209954-7210503	21	9.7

ACYPI006828-RA	gi|646732863|gb|KK963615.1|	29322-33319	########	39
ACYPI007260-RA	gi|646780406|gb|KK961550.1|	1573678-1574009	22	5.9
ACYPI007809-RA	gi|646782276|gb|KK961497.1|	7066018-7068540	21	9.7
ACYPI008241-RA	gi|646738635|gb|KK962957.1|	192172-193016	20	8.6
ACYPI008734-RA	gi|646775901|gb|KK961703.1|	1238559-1238947	20	9.6
ACYPI008811-RA	gi|646781243|gb|KK961526.1|	3535409-3535857	21	8.9
ACYPI009386-RA	gi|646777955|gb|KK961615.1|	1344620-1347449	21	10
ACYPI010127-RA	gi|646726742|gb|KK964444.1|	38589-39289	20	10
ACYPI071272-RA	gi|646780723|gb|KK961542.1|	603115-603484	21	9.4
ACYPI085561-RA	gi|646766253|gb|KK961869.1|	662945-664917	19	10
ACYPI086258-RA	gi|646780530|gb|KK961547.1|	3306309-3306651	21	9.2
ACYPI44580-RA	gi|646782211|gb|KK961498.1|	808344-811287	20	9
ACYPI001686-RA	gi|646746706|gb|KK962270.1|	254576-255499	23	11
ACYPI002240-RA	gi|646738314|gb|KK962989.1|	177763-178067	20	5.5
ACYPI003770-RA	gi|646749727|gb|KK962077.1|	481504-484258	21	8.9
ACYPI004120-RA	gi|646780827|gb|KK961539.1|	193935-195449	20	9.4
ACYPI006029-RA	gi|646781212|gb|KK961527.1|	4323087-4323480	21	10
ACYPI006052-RA	gi|646725650|gb|KK964619.1|	34352-35542	17	7.7
ACYPI006151-RA	gi|646782127|gb|KK961500.1|	1217271-1217484	22	9.6
ACYPI007491-RA	gi|646780925|gb|KK961536.1|	1395149-1396180	22	9.9
ACYPI008028-RA	gi|646773423|gb|KK961749.1|	651527-654008	19	8.1
ACYPI008231-RA	gi|646771092|gb|KK961771.1|	962290-963609	23	9.7
ACYPI009782-RA	gi|646781212|gb|KK961527.1|	4102236-4103280	21	10
ACYPI009904-RA	gi|646746706|gb|KK962270.1|	279307-280916	23	11
ACYPI068591-RA	gi|646778186|gb|KK961609.1|	1660599-1660983	19	9.4
ACYPI23235-RA	gi|646782127|gb|KK961500.1|	1165373-1169747	22	9.6
ACYPI36566-RA	gi|646752014|gb|KK961974.1|	784041-785190	18	5.8
ACYPI40836-RA	gi|646736615|gb|KK963164.1|	164943-173046	20	10
ACYPI006619-RA	gi|646779066|gb|KK961583.1|	1563970-1564637	19	7.9
ACYPI008866-RA	gi|646778632|gb|KK961596.1|	239659-243015	21	9.2

ACYPI010212-RA	gi|646766137|gb|KK961874.1|	893875-896987	20	9
ACYPI38425-RA	gi|646775821|gb|KK961707.1|	39957-42703	23	9.1
ACYPI005799-RA	gi|646573623|gb|KK981629.1|	1182-1469	13	6.9
ACYPI006851-RA	gi|646779785|gb|KK961565.1|	4577703-4577905	18	8.9
ACYPI50007-RA	gi|646768294|gb|KK961814.1|	270235-274866	23	10
ACYPI000065-RA	gi|646776514|gb|KK961670.1|	230372-233696	20	9.6
ACYPI000222-RA	gi|646751350|gb|KK961993.1|	255525-256015	20	7.6
ACYPI000885-RA	gi|646777363|gb|KK961635.1|	1760438-1760718	18	9.2
ACYPI002123-RA	gi|646778903|gb|KK961588.1|	3490396-3493151	23	10
ACYPI003998-RA	gi|646749464|gb|KK962091.1|	437728-438264	18	8.8
ACYPI004750-RA	gi|646782288|gb|KK961496.1|	3414244-3419062	21	9.7
ACYPI005363-RA	gi|646775789|gb|KK961709.1|	117377-119088	20	9.6
ACYPI006178-RA	gi|646740420|gb|KK962773.1|	256629-257094	20	7.5
ACYPI006875-RA	gi|646748474|gb|KK962147.1|	177934-179046	20	9
ACYPI007058-RA	gi|646754106|gb|KK961947.1|	412407-412747	16	7.6
ACYPI008050-RA	gi|646780658|gb|KK961544.1|	1414887-1422236	19	8.6
ACYPI064487-RA	gi|646576310|gb|KK981127.1|	8295-9048	10	5.7
ACYPI084991-RA	gi|646779898|gb|KK961562.1|	598165-599334	19	9.3
ACYPI23999-RA	gi|646782288|gb|KK961496.1|	1884041-1885841	21	9.7
ACYPI47651-RA	gi|646749098|gb|KK962110.1|	86178-86526	20	7.7
ACYPI000028-RA	gi|646742726|gb|KK962557.1|	172816-175816	20	9.1
ACYPI000349-RA	gi|646779337|gb|KK961576.1|	2276897-2277283	18	9
ACYPI000499-RA	gi|646767265|gb|KK961836.1|	1341643-1349857	22	9.9
ACYPI002010-RA	gi|646780222|gb|KK961554.1|	3556937-3561404	20	9.7
ACYPI002397-RA	gi|646762476|gb|KK961908.1|	202237-202666	19	7.4
ACYPI002657-RA	gi|646775842|gb|KK961706.1|	1190141-1201107	22	9
ACYPI004431-RA	gi|646753339|gb|KK961955.1|	340989-342589	22	11
ACYPI005832-RA	gi|646781536|gb|KK961517.1|	118133-120894	19	7.7
ACYPI006227-RA	gi|646766650|gb|KK961854.1|	261444-268747	22	10
ACYPI006348-RA	gi|646766650|gb|KK961854.1|	1584396-1584957	22	10

ACYPI006486-RA	gi|646776514|gb|KK961670.1|	2108102-2108762	20	9.6
ACYPI008107-RA	gi|646738455|gb|KK962975.1|	332440-333227	23	9.5
ACYPI008366-RA	gi|646780222|gb|KK961554.1|	3767171-3767564	20	9.7
ACYPI080567-RA	gi|646777665|gb|KK961623.1|	2276168-2282448	21	9.7
ACYPI30696-RA	gi|646777416|gb|KK961632.1|	264016-265095	22	8.8
ACYPI37471-RA	gi|646777288|gb|KK961638.1|	2529637-2530863	22	6.1
ACYPI004542-RA	gi|646777474|gb|KK961630.1|	1490570-1490818	21	9.3
ACYPI006271-RA	gi|646779976|gb|KK961560.1|	1237054-1237533	16	7.9
ACYPI010025-RA	gi|646780010|gb|KK961559.1|	1997806-1998585	22	10
ACYPI067648-RA	gi|646725187|gb|KK964688.1|	48949-49496	15	9.6
ACYPI004258-RA	gi|646744168|gb|KK962452.1|	731117-731771	22	8.7
ACYPI008065-RA	gi|646744168|gb|KK962452.1|	726520-727612	22	8.7
ACYPI38812-RA	gi|646769275|gb|KK961796.1|	994325-995120	22	5.6
ACYPI40717-RA	gi|646766650|gb|KK961854.1|	1578055-1579158	22	10
ACYPI001310-RA	gi|646772045|gb|KK961761.1|	628094-628677	21	9.6
ACYPI003159-RA	gi|646781421|gb|KK961521.1|	5034415-5038881	21	8.7
ACYPI004635-RA	gi|646781772|gb|KK961510.1|	3802818-3805096	20	9.2
ACYPI004687-RA	gi|646782357|gb|KK961494.1|	5259186-5260009	21	9.2
ACYPI010163-RA	gi|646782357|gb|KK961494.1|	1115466-1115935	21	9.2
ACYPI31682-RA	gi|646776128|gb|KK961691.1|	504007-504798	21	9.2
ACYPI000508-RA	gi|646778865|gb|KK961589.1|	1722136-1723064	20	9.2
ACYPI001052-RA	gi|646743772|gb|KK962481.1|	773352-774243	22	8.9
ACYPI003579-RA	gi|646744874|gb|KK962400.1|	321101-321398	19	8.3
ACYPI004896-RA	gi|646779101|gb|KK961582.1|	21440-21690	18	7.3
ACYPI005464-RA	gi|646746663|gb|KK962273.1|	134543-134783	21	10
ACYPI006864-RA	gi|646778865|gb|KK961589.1|	2124861-2125059	20	9.2
ACYPI007128-RA	gi|646776069|gb|KK961694.1|	973440-975149	18	7.8
ACYPI008037-RA	gi|646781143|gb|KK961529.1|	3771615-3772187	20	10
ACYPI061188-RA	gi|646740955|gb|KK962713.1|	331588-332886	28	13
ACYPI081260-RA	gi|646775821|gb|KK961707.1|	1755772-1758319	23	9.1

ACYPI001683-RA	gi|646778983|gb|KK961586.1|	242281-243091	20	9.9
ACYPI001929-RA	gi|646779006|gb|KK961585.1|	335176-337504	20	9.9
ACYPI002132-RA	gi|646754371|gb|KK961945.1|	163236-163760	20	9.7
ACYPI003296-RA	gi|646776514|gb|KK961670.1|	1415107-1416474	20	9.6
ACYPI004006-RA	gi|646754371|gb|KK961945.1|	115997-116723	20	9.7
ACYPI004937-RA	gi|646779936|gb|KK961561.1|	1938272-1938798	19	9.6
ACYPI005593-RA	gi|646742471|gb|KK962577.1|	250665-251404	22	5.9
ACYPI006607-RA	gi|646763164|gb|KK961904.1|	2093691-2094059	22	10
ACYPI006951-RA	gi|646732362|gb|KK963685.1|	11321-13126	19	7.5
ACYPI007438-RA	gi|646746525|gb|KK962283.1|	46623-48273	21	6.8
ACYPI008332-RA	gi|646779375|gb|KK961575.1|	1181881-1185780	22	9.3
ACYPI008675-RA	gi|646765219|gb|KK961896.1|	231312-231552	20	8.1
ACYPI56219-RA	gi|646778903|gb|KK961588.1|	3834119-3836957	23	10
ACYPI000665-RA	gi|646747559|gb|KK962209.1|	450102-452433	19	9.5
ACYPI005371-RA	gi|646751160|gb|KK962003.1|	597142-599197	22	10
ACYPI006603-RA	gi|646771092|gb|KK961771.1|	471373-471705	23	9.7
ACYPI007238-RA	gi|646621903|gb|KK972348.1|	14187-15360	21	7.3
ACYPI063236-RA	gi|646776822|gb|KK961657.1|	1715571-1716560	19	7.9
ACYPI085301-RA	gi|646751160|gb|KK962003.1|	605771-607199	22	10
ACYPI33155-RA	gi|646744453|gb|KK962431.1|	351120-352372	19	8.5
ACYPI49250-RA	gi|646752076|gb|KK961973.1|	131593-133977	22	9.1
ACYPI007773-RA	gi|646778245|gb|KK961607.1|	472139-474943	18	6.8
ACYPI36831-RA	gi|646774530|gb|KK961743.1|	434342-435930	20	9.8
ACYPI004948-RA	gi|646778632|gb|KK961596.1|	903106-908833	21	9.2
ACYPI088195-RA	gi|646776128|gb|KK961691.1|	1821160-1824428	21	9.2
ACYPI24140-RA	gi|646782357|gb|KK961494.1|	1994094-1997430	21	9.2
ACYPI41926-RA	gi|646741207|gb|KK962687.1|	173650-175208	17	7.6
ACYPI52372-RA	gi|646767196|gb|KK961838.1|	284198-285199	21	9.7
ACYPI52374-RA	gi|646766279|gb|KK961868.1|	191589-192876	16	7.1
ACYPI000720-RA	gi|646770251|gb|KK961781.1|	1363462-1364195	20	9.6

ACYPI000776-RA	gi|646776608|gb|KK961665.1|	1606664-1612257	23	9.3
ACYPI000929-RA	gi|646781659|gb|KK961513.1|	3379491-3383496	22	9.8
ACYPI001378-RA	gi|646776219|gb|KK961687.1|	457983-458314	21	9.9
ACYPI002609-RA	gi|646781118|gb|KK961530.1|	4577873-4595754	21	10
ACYPI003268-RA	gi|646782043|gb|KK961502.1|	5168929-5169787	20	9.2
ACYPI003303-RA	gi|646748474|gb|KK962147.1|	291027-291463	20	9
ACYPI005532-RA	gi|646777912|gb|KK961616.1|	347394-348595	20	8.6
ACYPI007084-RA	gi|646782043|gb|KK961502.1|	5182636-5183583	20	9.2
ACYPI008665-RA	gi|646775523|gb|KK961722.1|	856667-857798	21	9.7
ACYPI008834-RA	gi|646749632|gb|KK962082.1|	218017-218294	20	8.6
ACYPI009793-RA	gi|646780658|gb|KK961544.1|	1372014-1372863	19	8.6
ACYPI080438-RA	gi|646768494|gb|KK961810.1|	1012524-1014149	21	10
ACYPI001220-RA	gi|646768631|gb|KK961807.1|	835255-836784	19	9.2
ACYPI007771-RA	gi|646778061|gb|KK961612.1|	419236-425807	20	8.7
ACYPI56678-RA	gi|646767850|gb|KK961823.1|	1760912-1761222	21	9.8
ACYPI000005-RA	gi|646737680|gb|KK963053.1|	110613-110837	21	5.9
ACYPI000038-RA	gi|646747841|gb|KK962189.1|	106063-106397	23	10
ACYPI000051-RA	gi|646752143|gb|KK961972.1|	776198-776437	20	5.6
ACYPI002536-RA	gi|646552905|gb|KK985647.1|	3557-3784	13	5.5
ACYPI003033-RA	gi|646746706|gb|KK962270.1|	290887-291265	23	11
ACYPI004378-RA	gi|646776822|gb|KK961657.1|	520840-521825	19	7.9
ACYPI004950-RA	gi|646781690|gb|KK961512.1|	2130605-2131223	21	8.8
ACYPI005068-RA	gi|646767624|gb|KK961828.1|	910521-911041	22	8.9
ACYPI005592-RA	gi|646746706|gb|KK962270.1|	386857-388465	23	11
ACYPI006025-RA	gi|646782357|gb|KK961494.1|	3253890-3257480	21	9.2
ACYPI006844-RA	gi|646766034|gb|KK961878.1|	583539-584139	20	9
ACYPI007184-RA	gi|646775614|gb|KK961717.1|	75748-76482	21	9.7
ACYPI007468-RA	gi|646770477|gb|KK961778.1|	1352230-1352931	21	9.1
ACYPI007860-RA	gi|646782357|gb|KK961494.1|	3285498-3285749	21	9.2
ACYPI008713-RA	gi|646776322|gb|KK961682.1|	957003-957206	22	8.9

ACYPI009867-RA	gi|646776091|gb|KK961693.1|	1676123-1676679	23	10
ACYPI088273-RA	gi|646738455|gb|KK962975.1|	260878-263352	23	9.5
ACYPI001536-RA	gi|646768447|gb|KK961811.1|	1209169-1210629	22	9.7
ACYPI002282-RA	gi|646748185|gb|KK962165.1|	903399-903668	22	9.9
ACYPI003154-RA	gi|646743944|gb|KK962469.1|	338994-339395	20	9.1
ACYPI003322-RA	gi|646765669|gb|KK961895.1|	34683-35153	18	7.2
ACYPI003323-RA	gi|646749832|gb|KK962071.1|	608327-614914	22	6
ACYPI003863-RA	gi|646781732|gb|KK961511.1|	2453921-2454500	22	9.8
ACYPI005150-RA	gi|646780270|gb|KK961553.1|	3815950-3816911	22	8.6
ACYPI005247-RA	gi|646781732|gb|KK961511.1|	2219028-2220589	22	9.8
ACYPI005331-RA	gi|646767265|gb|KK961836.1|	845425-846311	22	9.9
ACYPI005368-RA	gi|646745612|gb|KK962344.1|	170763-171937	20	8.2
ACYPI005747-RA	gi|646775821|gb|KK961707.1|	836522-838985	23	9.1
ACYPI007135-RA	gi|646773721|gb|KK961747.1|	2167222-2170130	21	9.2
ACYPI007623-RA	gi|646775576|gb|KK961719.1|	1807402-1807630	20	8.6
ACYPI007672-RA	gi|646746991|gb|KK962251.1|	445192-445538	22	9.2
ACYPI007878-RA	gi|646740879|gb|KK962721.1|	196770-198571	24	6.4
ACYPI009011-RA	gi|646734451|gb|KK963418.1|	131294-132564	19	9.4
ACYPI009513-RA	gi|646778517|gb|KK961599.1|	621343-621678	20	9.6
ACYPI009832-RA	gi|646780270|gb|KK961553.1|	3584776-3585032	22	8.6
ACYPI071784-RA	gi|646750557|gb|KK962032.1|	716548-719512	20	6.1
ACYPI003590-RA	gi|646779826|gb|KK961564.1|	1464953-1467843	22	9.6
ACYPI069414-RA	gi|646672380|gb|KK967689.1|	29293-29817	15	11
ACYPI000192-RA	gi|646775507|gb|KK961723.1|	1642102-1642297	20	9.8
ACYPI001354-RA	gi|646775595|gb|KK961718.1|	1768330-1768610	21	8.9
ACYPI001487-RA	gi|646770323|gb|KK961780.1|	555078-555719	19	7.1
ACYPI002115-RA	gi|646781772|gb|KK961510.1|	3755720-3755980	20	9.2
ACYPI003235-RA	gi|646743956|gb|KK962468.1|	101635-103667	15	5.5
ACYPI004576-RA	gi|646768534|gb|KK961809.1|	181185-182253	21	9.8
ACYPI004689-RA	gi|646781772|gb|KK961510.1|	3693678-3698707	20	9.2

ACYPI004961-RA	gi|646781873|gb|KK961507.1|	1642806-1649767	20	9.1
ACYPI006488-RA	gi|646782276|gb|KK961497.1|	2956913-2957193	21	9.7
ACYPI007401-RA	gi|646745135|gb|KK962381.1|	223350-225638	20	8.7
ACYPI007695-RA	gi|646780858|gb|KK961538.1|	1185449-1189061	21	10
ACYPI008463-RA	gi|646731000|gb|KK963870.1|	122963-126331	20	8.1
ACYPI009510-RA	gi|646777363|gb|KK961635.1|	1488423-1488869	18	9.2
ACYPI002460-RA	gi|646781379|gb|KK961522.1|	3095925-3097440	22	7.6
ACYPI002758-RA	gi|646767948|gb|KK961821.1|	494674-495316	17	7.1
ACYPI004615-RA	gi|646765689|gb|KK961894.1|	727266-730258	18	7.7
ACYPI006251-RA	gi|646779006|gb|KK961585.1|	186630-194175	20	9.9
ACYPI008122-RA	gi|646746202|gb|KK962305.1|	397075-399965	21	6.4
ACYPI008481-RA	gi|646614268|gb|KK973701.1|	3346-3726	24	7.5
ACYPI061780-RA	gi|646780794|gb|KK961540.1|	395472-397182	21	9.5
ACYPI071169-RA	gi|646767670|gb|KK961827.1|	977551-977832	21	5.9
ACYPI085199-RA	gi|646779785|gb|KK961565.1|	3232106-3243489	18	8.9
ACYPI39295-RA	gi|646780858|gb|KK961538.1|	1505476-1506542	21	10
ACYPI000496-RA	gi|646782357|gb|KK961494.1|	3432145-3433422	21	9.2
ACYPI003043-RA	gi|646749741|gb|KK962076.1|	207401-207772	17	7.6
ACYPI003470-RA	gi|646769774|gb|KK961788.1|	766037-766431	20	9.2
ACYPI004268-RA	gi|646781282|gb|KK961525.1|	1037708-1039666	22	9.6
ACYPI006708-RA	gi|646777313|gb|KK961637.1|	1574829-1575707	20	8.2
ACYPI007482-RA	gi|646766034|gb|KK961878.1|	918629-919914	20	9
ACYPI007779-RA	gi|646767120|gb|KK961840.1|	785244-785949	20	8.6
ACYPI007952-RA	gi|646749317|gb|KK962098.1|	323239-323637	20	5.6
ACYPI008044-RA	gi|646768717|gb|KK961805.1|	788429-788723	20	8
ACYPI008181-RA	gi|646494666|gb|KK995492.1|	7936-8625	20	6.8
ACYPI008721-RA	gi|646782357|gb|KK961494.1|	3502069-3503505	21	9.2
ACYPI066025-RA	gi|646751031|gb|KK962010.1|	877411-878452	23	7.5
ACYPI087807-RA	gi|646750002|gb|KK962062.1|	376229-378433	24	12
ACYPI34476-RA	gi|646775685|gb|KK961714.1|	1492632-1493658	21	8.5

ACYPI005033-RA	gi|646743859|gb|KK962475.1|	471212-472353	20	7.9
ACYPI006266-RA	gi|646765902|gb|KK961884.1|	1130987-1132145	22	9.2
ACYPI006932-RA	gi|646747385|gb|KK962223.1|	236443-236742	21	5.4
ACYPI005058-RA	gi|646778112|gb|KK961611.1|	1398136-1398438	22	10
ACYPI007379-RA	gi|646747559|gb|KK962209.1|	749912-753213	19	9.5
ACYPI25873-RA	gi|646699768|gb|KK966040.1|	36584-41073	18	7.2
ACYPI002986-RA	gi|646782334|gb|KK961495.1|	1818705-1819833	21	9
ACYPI004307-RA	gi|646738812|gb|KK962939.1|	205655-205849	20	8.1
ACYPI006238-RA	gi|646777991|gb|KK961614.1|	1215561-1215949	18	7.8
ACYPI072244-RA	gi|646752431|gb|KK961967.1|	1557184-1557663	20	5.6
ACYPI48834-RA	gi|646777570|gb|KK961626.1|	857242-857864	18	7.5
ACYPI001931-RA	gi|646737056|gb|KK963115.1|	328206-330142	24	11
ACYPI008324-RA	gi|646748295|gb|KK962158.1|	83816-84152	18	8.2
ACYPI008529-RA	gi|646748663|gb|KK962136.1|	88965-90512	18	6.5
ACYPI068309-RA	gi|646714902|gb|KK965265.1|	12298-13095	39	18
ACYPI001453-RA	gi|646744699|gb|KK962413.1|	16934-17480	14	6.9
ACYPI001759-RA	gi|646782357|gb|KK961494.1|	9010681-9011324	21	9.2
ACYPI005826-RA	gi|646782357|gb|KK961494.1|	9003940-9009906	21	9.2
ACYPI006885-RA	gi|646777955|gb|KK961615.1|	947487-947974	21	10
ACYPI008055-RA	gi|646782087|gb|KK961501.1|	2518957-2519331	20	8.7
ACYPI009667-RA	gi|646750745|gb|KK962023.1|	981018-982098	18	9.4
ACYPI010135-RA	gi|646777955|gb|KK961615.1|	815691-819979	21	10
ACYPI060796-RA	gi|646777955|gb|KK961615.1|	961819-962037	21	10
ACYPI082595-RA	gi|646775238|gb|KK961738.1|	614773-616163	17	8.7
ACYPI21464-RA	gi|646775807|gb|KK961708.1|	201257-201530	21	8.6
ACYPI001509-RA	gi|646767156|gb|KK961839.1|	323018-325650	15	6.4
ACYPI003301-RA	gi|646775968|gb|KK961700.1|	112114-112366	20	8.2
ACYPI003398-RA	gi|646747586|gb|KK962207.1|	100173-101079	20	8.8
ACYPI003508-RA	gi|646779826|gb|KK961564.1|	1263755-1264075	22	9.6
ACYPI004014-RA	gi|646738635|gb|KK962957.1|	269002-269203	20	8.6

ACYPI005457-RA	gi|646781313|gb|KK961524.1|	3533812-3535266	19	8.5
ACYPI007767-RA	gi|646782043|gb|KK961502.1|	1802309-1803205	20	9.2
ACYPI009012-RA	gi|646754962|gb|KK961940.1|	920780-922206	21	10
ACYPI009722-RA	gi|646741815|gb|KK962634.1|	53109-55116	22	10
ACYPI006283-RA	gi|646781849|gb|KK961508.1|	1864496-1865138	17	8
ACYPI008151-RA	gi|646775312|gb|KK961734.1|	683458-684813	17	7.9
ACYPI008396-RA	gi|646782168|gb|KK961499.1|	3693910-3695458	21	9.4
ACYPI002031-RA	gi|646766703|gb|KK961852.1|	426083-433431	20	7.1
ACYPI004098-RA	gi|646767850|gb|KK961823.1|	1166449-1168953	21	9.8
ACYPI006036-RA	gi|646781379|gb|KK961522.1|	2615013-2615947	22	7.6
ACYPI006225-RA	gi|646741656|gb|KK962648.1|	281233-283877	24	9.7
ACYPI006423-RA	gi|646782276|gb|KK961497.1|	1293139-1293418	21	9.7
ACYPI006514-RA	gi|646778736|gb|KK961593.1|	1335930-1336693	20	7.9
ACYPI008380-RA	gi|646749727|gb|KK962077.1|	218013-221345	21	8.9
ACYPI008390-RA	gi|646766703|gb|KK961852.1|	379404-379585	20	7.1
ACYPI009868-RA	gi|646750204|gb|KK962051.1|	591516-596055	19	7.5
ACYPI062495-RA	gi|646781421|gb|KK961521.1|	6691533-6693984	21	8.7
ACYPI065217-RA	gi|646779936|gb|KK961561.1|	1946952-1947750	19	9.6
ACYPI088675-RA	gi|646747076|gb|KK962245.1|	556857-559588	23	9.6
ACYPI27182-RA	gi|646656105|gb|KK968158.1|	7929-10242	12	5.2
ACYPI49690-RA	gi|646782276|gb|KK961497.1|	1976400-1978884	21	9.7
ACYPI004271-RA	gi|646749632|gb|KK962082.1|	477596-480226	20	8.6
ACYPI006206-RA	gi|646767196|gb|KK961838.1|	623759-625406	21	9.7
ACYPI008080-RA	gi|646776069|gb|KK961694.1|	982581-987632	18	7.8
ACYPI009955-RA	gi|646775987|gb|KK961699.1|	1008430-1008872	19	7.5
ACYPI003302-RA	gi|646782043|gb|KK961502.1|	1201280-1201540	20	9.2
ACYPI003418-RA	gi|646776024|gb|KK961697.1|	918063-918862	22	9.2
ACYPI003915-RA	gi|646743179|gb|KK962523.1|	184601-187499	18	8.3
ACYPI006509-RA	gi|646775842|gb|KK961706.1|	1487839-1492134	22	9
ACYPI065189-RA	gi|646777089|gb|KK961646.1|	339580-341147	20	9.4

ACYPI002263-RA	gi|646767754|gb|KK961825.1|	63041-63921	20	8.5
ACYPI003831-RA	gi|646777288|gb|KK961638.1|	1484450-1484802	22	6.1
ACYPI005865-RA	gi|646751569|gb|KK961985.1|	217504-218010	21	9.2
ACYPI006698-RA	gi|646776608|gb|KK961665.1|	375296-381750	23	9.3
ACYPI007137-RA	gi|646732578|gb|KK963654.1|	40511-41678	33	13
ACYPI007733-RA	gi|646776647|gb|KK961663.1|	2537767-2538242	23	9.2
ACYPI008202-RA	gi|646744957|gb|KK962394.1|	131978-137604	21	9.8
ACYPI008599-RA	gi|646780105|gb|KK961557.1|	1736546-1747679	21	9.7
ACYPI009628-RA	gi|646766382|gb|KK961864.1|	1098642-1100547	22	9.7
ACYPI066811-RA	gi|646748888|gb|KK962123.1|	144602-145108	20	8.1
ACYPI070389-RA	gi|646777288|gb|KK961638.1|	1471328-1471598	22	6.1
ACYPI26472-RA	gi|646759701|gb|KK961917.1|	211364-211746	18	6.1
ACYPI001885-RA	gi|646781421|gb|KK961521.1|	4090387-4096150	21	8.7
ACYPI010114-RA	gi|646781421|gb|KK961521.1|	4134303-4135268	21	8.7
ACYPI34821-RA	gi|646740405|gb|KK962775.1|	132209-134171	20	5.8
ACYPI000066-RA	gi|646781344|gb|KK961523.1|	3687902-3700144	21	8.9
ACYPI000667-RA	gi|646781118|gb|KK961530.1|	197897-203737	21	10
ACYPI001012-RA	gi|646742343|gb|KK962588.1|	275106-283598	20	9.4
ACYPI006444-RA	gi|646631467|gb|KK970607.1|	3317-4261	13	5
ACYPI066102-RA	gi|646778903|gb|KK961588.1|	1342188-1344475	23	10
ACYPI072123-RA	gi|646781421|gb|KK961521.1|	4300123-4301101	21	8.7
ACYPI087117-RA	gi|646776184|gb|KK961688.1|	1531794-1532957	21	9.6
ACYPI40720-RA	gi|646730558|gb|KK963928.1|	17328-22403	19	5.4
ACYPI52138-RA	gi|646782288|gb|KK961496.1|	4134698-4135983	21	9.7
ACYPI55107-RA	gi|646731491|gb|KK963809.1|	81029-82381	24	6.6
ACYPI000828-RA	gi|646779375|gb|KK961575.1|	1080382-1083185	22	9.3
ACYPI001011-RA	gi|646768163|gb|KK961817.1|	154107-154988	20	7.6
ACYPI001079-RA	gi|646781893|gb|KK961506.1|	397198-398622	19	8.5
ACYPI002023-RA	gi|646778736|gb|KK961593.1|	246752-248137	20	7.9
ACYPI003518-RA	gi|646767850|gb|KK961823.1|	458210-462099	21	9.8

ACYPI006505-RA	gi|646776429|gb|KK961676.1|	1271017-1272467	20	9.9
ACYPI007179-RA	gi|646750389|gb|KK962041.1|	395132-396749	21	9.5
ACYPI009500-RA	gi|646749945|gb|KK962065.1|	43724-44075	22	6.1
ACYPI067814-RA	gi|646758935|gb|KK961920.1|	687113-688281	20	8.5
ACYPI071658-RA	gi|646734014|gb|KK963469.1|	50881-53455	11	5.5
ACYPI073870-RA	gi|646768222|gb|KK961816.1|	994397-997338	25	12
ACYPI088059-RA	gi|646751569|gb|KK961985.1|	193207-193839	21	9.2
ACYPI36232-RA	gi|646732955|gb|KK963604.1|	98370-98848	21	8.1
ACYPI000165-RA	gi|646780485|gb|KK961548.1|	999810-1000053	15	6.1
ACYPI006075-RA	gi|646780794|gb|KK961540.1|	619355-621491	21	9.5
ACYPI008618-RA	gi|646774067|gb|KK961745.1|	1188544-1189697	17	9.3
ACYPI55194-RA	gi|646775558|gb|KK961720.1|	744166-744513	20	9.4
ACYPI000340-RA	gi|646779412|gb|KK961574.1|	1929400-1931351	21	5.4
ACYPI000691-RA	gi|646775523|gb|KK961722.1|	297318-300671	21	9.7
ACYPI000804-RA	gi|646775821|gb|KK961707.1|	241943-248190	23	9.1
ACYPI000955-RA	gi|646780978|gb|KK961534.1|	1853405-1853690	20	8
ACYPI001266-RA	gi|646777991|gb|KK961614.1|	1647059-1647546	18	7.8
ACYPI002179-RA	gi|646748785|gb|KK962129.1|	563086-564614	20	8.2
ACYPI002584-RA	gi|646777991|gb|KK961614.1|	1689943-1690781	18	7.8
ACYPI002799-RA	gi|646780827|gb|KK961539.1|	3536479-3537212	20	9.4
ACYPI003718-RA	gi|646743302|gb|KK962514.1|	136789-137050	23	8.4
ACYPI003981-RA	gi|646776024|gb|KK961697.1|	1209935-1217046	22	9.2
ACYPI004727-RA	gi|646766624|gb|KK961855.1|	181816-186224	21	10
ACYPI006185-RA	gi|646757520|gb|KK961926.1|	1199270-1200193	20	7.4
ACYPI006403-RA	gi|646776429|gb|KK961676.1|	1706638-1711057	20	9.9
ACYPI006626-RA	gi|646753180|gb|KK961957.1|	82882-83624	25	11
ACYPI006716-RA	gi|646782127|gb|KK961500.1|	5747443-5748353	22	9.6
ACYPI007006-RA	gi|646782043|gb|KK961502.1|	5359487-5360211	20	9.2
ACYPI007994-RA	gi|646776184|gb|KK961688.1|	2446723-2448506	21	9.6
ACYPI008056-RA	gi|646749672|gb|KK962080.1|	101374-101587	20	7.6

ACYPI008217-RA	gi|646737917|gb|KK963028.1|	258360-259661	22	7.8
ACYPI008457-RA	gi|646775658|gb|KK961715.1|	1105650-1106279	19	8.6
ACYPI008489-RA	gi|646734749|gb|KK963383.1|	106405-106675	15	5.8
ACYPI008578-RA	gi|646765705|gb|KK961893.1|	595584-596484	22	9.7
ACYPI008757-RA	gi|646763535|gb|KK961902.1|	1167780-1169586	23	10
ACYPI008831-RA	gi|646776038|gb|KK961696.1|	1264429-1275408	21	10
ACYPI009478-RA	gi|646782127|gb|KK961500.1|	5611368-5612015	22	9.6
ACYPI009932-RA	gi|646757520|gb|KK961926.1|	1235780-1237025	20	7.4
ACYPI081020-RA	gi|646725428|gb|KK964653.1|	23002-23505	11	6.3
ACYPI33681-RA	gi|646747076|gb|KK962245.1|	486552-490730	23	9.6
ACYPI56814-RA	gi|646731730|gb|KK963777.1|	12209-12870	15	9.8
ACYPI001806-RA	gi|646750043|gb|KK962060.1|	846602-848206	21	8.3
ACYPI001957-RA	gi|646782127|gb|KK961500.1|	1513451-1514131	22	9.6
ACYPI002580-RA	gi|646778448|gb|KK961601.1|	1850032-1850320	19	8.3
ACYPI003691-RA	gi|646777017|gb|KK961649.1|	681444-682750	18	8
ACYPI004482-RA	gi|646780574|gb|KK961546.1|	62797-63328	20	9.9
ACYPI004584-RA	gi|646777955|gb|KK961615.1|	1553237-1557430	21	10
ACYPI006277-RA	gi|646746828|gb|KK962262.1|	614048-614920	20	9.2
ACYPI006499-RA	gi|646776904|gb|KK961654.1|	855176-855654	21	9.3
ACYPI007533-RA	gi|646737822|gb|KK963039.1|	210631-212837	20	9
ACYPI008922-RA	gi|646779337|gb|KK961576.1|	1186733-1187843	18	9
ACYPI010154-RA	gi|646771092|gb|KK961771.1|	1010457-1011987	23	9.7
ACYPI000157-RA	gi|646778903|gb|KK961588.1|	979585-980246	23	10
ACYPI009032-RA	gi|646776184|gb|KK961688.1|	807830-808041	21	9.6
ACYPI20534-RA	gi|646776974|gb|KK961651.1|	1018748-1020123	17	8.1
ACYPI003834-RA	gi|646738414|gb|KK962979.1|	124918-125844	23	10
ACYPI003840-RA	gi|646773903|gb|KK961746.1|	1365521-1367774	20	8.9
ACYPI003941-RA	gi|646776322|gb|KK961682.1|	2114788-2115084	22	8.9
ACYPI009411-RA	gi|646616290|gb|KK973340.1|	11307-11652	4.5	2.7
ACYPI010068-RA	gi|646750204|gb|KK962051.1|	418708-420561	19	7.5

ACYPI068377-RA	gi|646781893|gb|KK961506.1|	1867101-1867656	19	8.5
ACYPI42415-RA	gi|646773721|gb|KK961747.1|	2612013-2612445	21	9.2
ACYPI000177-RA	gi|646781344|gb|KK961523.1|	3497426-3500166	21	8.9
ACYPI000614-RA	gi|646752014|gb|KK961974.1|	115759-117165	18	5.8
ACYPI000768-RA	gi|646725696|gb|KK964612.1|	179764-182430	22	9.2
ACYPI001561-RA	gi|646747586|gb|KK962207.1|	154837-155416	20	8.8
ACYPI001933-RA	gi|646781628|gb|KK961514.1|	2070931-2071637	23	9
ACYPI002080-RA	gi|646690827|gb|KK966813.1|	27648-28760	19	11
ACYPI002659-RA	gi|646725696|gb|KK964612.1|	144364-154658	22	9.2
ACYPI003451-RA	gi|646775658|gb|KK961715.1|	685337-685578	19	8.6
ACYPI003780-RA	gi|646751289|gb|KK961996.1|	466659-467055	24	9.1
ACYPI003820-RA	gi|646776280|gb|KK961684.1|	1730061-1730307	22	9.3
ACYPI004039-RA	gi|646732202|gb|KK963708.1|	15961-17122	22	9.9
ACYPI004071-RA	gi|646775807|gb|KK961708.1|	2797154-2799162	21	8.6
ACYPI005079-RA	gi|646775842|gb|KK961706.1|	2238916-2240068	22	9
ACYPI005385-RA	gi|646768717|gb|KK961805.1|	821120-822533	20	8
ACYPI005467-RA	gi|646745091|gb|KK962384.1|	331785-333112	19	7.9
ACYPI006313-RA	gi|646738615|gb|KK962959.1|	308763-309253	21	9.5
ACYPI006405-RA	gi|646598593|gb|KK976753.1|	869-1508	15	8.4
ACYPI006608-RA	gi|646780147|gb|KK961556.1|	1264346-1264680	18	7.5
ACYPI006618-RA	gi|646743873|gb|KK962474.1|	164468-164753	20	9.4
ACYPI006957-RA	gi|646781379|gb|KK961522.1|	3378502-3382978	22	7.6
ACYPI007327-RA	gi|646761388|gb|KK961911.1|	386814-388120	19	7.4
ACYPI007428-RA	gi|646746310|gb|KK962297.1|	477979-478914	23	11
ACYPI007628-RA	gi|646728609|gb|KK964184.1|	331029-333014	34	14
ACYPI008178-RA	gi|646766056|gb|KK961877.1|	593774-602301	18	7.8
ACYPI010015-RA	gi|646776184|gb|KK961688.1|	1615807-1622084	21	9.6
ACYPI010064-RA	gi|646744833|gb|KK962403.1|	254686-259971	22	9.4
ACYPI066292-RA	gi|646776588|gb|KK961666.1|	854510-855688	20	5.8
ACYPI068681-RA	gi|646781183|gb|KK961528.1|	2689252-2689977	20	9.5

ACYPI56734-RA	gi|646744833|gb|KK962403.1|	357641-360437	22	9.4
ACYPI000119-RA	gi|646746752|gb|KK962267.1|	493701-496561	20	7.9
ACYPI000887-RA	gi|646755567|gb|KK961936.1|	673563-674476	20	8.6
ACYPI002036-RA	gi|646765790|gb|KK961889.1|	1139133-1139597	22	8.8
ACYPI002136-RA	gi|646747263|gb|KK962232.1|	106956-107147	18	8.6
ACYPI002292-RA	gi|646781536|gb|KK961517.1|	1691228-1691490	19	7.7
ACYPI003921-RA	gi|646766357|gb|KK961865.1|	892596-894509	16	6.5
ACYPI004870-RA	gi|646781536|gb|KK961517.1|	1279193-1279894	19	7.7
ACYPI005367-RA	gi|646759358|gb|KK961918.1|	197880-207517	16	5.4
ACYPI005597-RA	gi|646782357|gb|KK961494.1|	1275957-1277319	21	9.2
ACYPI005988-RA	gi|646779662|gb|KK961568.1|	1205074-1205373	20	9.3
ACYPI065216-RA	gi|646744029|gb|KK962462.1|	464117-464966	19	7.9
ACYPI082110-RA	gi|646744029|gb|KK962462.1|	438562-438870	19	7.9
ACYPI086519-RA	gi|646774797|gb|KK961742.1|	70379-70570	20	8.5
ACYPI002170-RA	gi|646770477|gb|KK961778.1|	416606-419512	21	9.1
ACYPI003298-RA	gi|646745612|gb|KK962344.1|	198103-200309	20	8.2
ACYPI009534-RA	gi|646750770|gb|KK962022.1|	405213-405446	14	7.7
ACYPI009542-RA	gi|646740395|gb|KK962776.1|	232789-233176	16	6.3
ACYPI001575-RA	gi|646738812|gb|KK962939.1|	70326-80013	20	8.1
ACYPI003964-RA	gi|646781659|gb|KK961513.1|	123489-124135	22	9.8
ACYPI004663-RA	gi|646780270|gb|KK961553.1|	2450145-2451340	22	8.6
ACYPI073176-RA	gi|646748002|gb|KK962178.1|	901203-902731	22	6.3
ACYPI089545-RA	gi|646749832|gb|KK962071.1|	972015-972795	22	6
ACYPI37433-RA	gi|646779262|gb|KK961578.1|	935428-938179	20	8.1
ACYPI45188-RA	gi|646781421|gb|KK961521.1|	4620399-4622012	21	8.7
ACYPI53399-RA	gi|646742270|gb|KK962594.1|	174283-180345	21	10
ACYPI002072-RA	gi|646775471|gb|KK961725.1|	1366350-1369116	20	10
ACYPI004075-RA	gi|646781732|gb|KK961511.1|	3134559-3134833	22	9.8
ACYPI004270-RA	gi|646746581|gb|KK962279.1|	42123-43158	21	9.1
ACYPI084112-RA	gi|646775614|gb|KK961717.1|	217202-217475	21	9.7

ACYPI088646-RA	gi|646780222|gb|KK961554.1|	1075743-1076937	20	9.7
ACYPI26223-RA	gi|646775614|gb|KK961717.1|	329528-337109	21	9.7
ACYPI26228-RA	gi|646778840|gb|KK961590.1|	1566624-1567396	20	9.9
ACYPI000617-RA	gi|646780222|gb|KK961554.1|	1227144-1234809	20	9.7
ACYPI004627-RA	gi|646755567|gb|KK961936.1|	867751-868024	20	8.6
ACYPI005041-RA	gi|646776024|gb|KK961697.1|	2409088-2409374	22	9.2
ACYPI007630-RA	gi|646777125|gb|KK961644.1|	399233-399629	20	10
ACYPI008516-RA	gi|646770901|gb|KK961773.1|	1649200-1650400	22	9.7
ACYPI008736-RA	gi|646782043|gb|KK961502.1|	2403086-2407648	20	9.2
ACYPI008771-RA	gi|646776024|gb|KK961697.1|	2544948-2546294	22	9.2
ACYPI008810-RA	gi|646523229|gb|KK990101.1|	6520-6763	19	1.9
ACYPI068762-RA	gi|646733656|gb|KK963509.1|	21345-21766	17	13
ACYPI52419-RA	gi|646738229|gb|KK962998.1|	391917-392613	22	8.9
ACYPI002371-RA	gi|646753093|gb|KK961958.1|	989781-990542	21	9.3
ACYPI003025-RA	gi|646781536|gb|KK961517.1|	1964504-1964791	19	7.7
ACYPI004077-RA	gi|646730815|gb|KK963895.1|	181690-184047	20	9.5
ACYPI008681-RA	gi|646767265|gb|KK961836.1|	146543-151198	22	9.9
ACYPI072603-RA	gi|646781628|gb|KK961514.1|	2460965-2464029	23	9
ACYPI22858-RA	gi|646775523|gb|KK961722.1|	226550-227613	21	9.7
ACYPI000089-RA	gi|646780978|gb|KK961534.1|	511824-512059	20	8
ACYPI000219-RA	gi|646773721|gb|KK961747.1|	2447510-2449323	21	9.2
ACYPI000441-RA	gi|646777665|gb|KK961623.1|	1843214-1843723	21	9.7
ACYPI001031-RA	gi|646780222|gb|KK961554.1|	200954-203281	20	9.7
ACYPI001879-RA	gi|646745008|gb|KK962390.1|	603171-605150	20	7.2
ACYPI003044-RA	gi|646782357|gb|KK961494.1|	9093136-9094973	21	9.2
ACYPI003554-RA	gi|646780889|gb|KK961537.1|	2979795-2980118	22	10
ACYPI004693-RA	gi|646780574|gb|KK961546.1|	3309878-3311574	20	9.9
ACYPI004827-RA	gi|646774067|gb|KK961745.1|	1541836-1542070	17	9.3
ACYPI004851-RA	gi|646776113|gb|KK961692.1|	295601-296267	21	9.6
ACYPI006857-RA	gi|646771951|gb|KK961762.1|	1827439-1829636	21	8.3

ACYPI008015-RA	gi|646766382|gb|KK961864.1|	208968-212515	22	9.7
ACYPI008468-RA	gi|646782276|gb|KK961497.1|	6822031-6826216	21	9.7
ACYPI009253-RA	gi|646732863|gb|KK963615.1|	60691-62792	########	39
ACYPI082181-RA	gi|646782276|gb|KK961497.1|	6819979-6821809	21	9.7
ACYPI082950-RA	gi|646774067|gb|KK961745.1|	1504989-1508632	17	9.3
ACYPI31659-RA	gi|646782357|gb|KK961494.1|	9034622-9035409	21	9.2
ACYPI49420-RA	gi|646732176|gb|KK963712.1|	248018-249319	24	10
ACYPI000785-RA	gi|646768717|gb|KK961805.1|	263920-264646	20	8
ACYPI003316-RA	gi|646744699|gb|KK962413.1|	133486-134144	14	6.9
ACYPI003699-RA	gi|646751031|gb|KK962010.1|	578329-578959	23	7.5
ACYPI003732-RA	gi|646738524|gb|KK962968.1|	311187-315438	24	11
ACYPI004308-RA	gi|646751544|gb|KK961986.1|	775046-780913	22	6
ACYPI004355-RA	gi|646749832|gb|KK962071.1|	879513-880077	22	6
ACYPI005606-RA	gi|646782288|gb|KK961496.1|	7553165-7553397	21	9.7
ACYPI007716-RA	gi|646779498|gb|KK961572.1|	1538910-1539067	22	5.6
ACYPI009312-RA	gi|646777495|gb|KK961629.1|	1785431-1786785	19	8.7
ACYPI072962-RA	gi|646758845|gb|KK961921.1|	711777-712464	22	9.5
ACYPI085603-RA	gi|646769163|gb|KK961798.1|	1336514-1336853	20	9.2
ACYPI22584-RA	gi|646780311|gb|KK961552.1|	2237480-2247841	21	9.7
ACYPI29303-RA	gi|646779298|gb|KK961577.1|	4308594-4309686	20	9.1
ACYPI45266-RA	gi|646776264|gb|KK961685.1|	756027-758505	20	9.1
ACYPI56611-RA	gi|646778336|gb|KK961604.1|	1319970-1320386	21	10
ACYPI000941-RA	gi|646780530|gb|KK961547.1|	2199904-2200192	21	9.2
ACYPI009480-RA	gi|646778186|gb|KK961609.1|	1829477-1829777	19	9.4
ACYPI062389-RA	gi|646781601|gb|KK961515.1|	400366-405260	19	8.6
ACYPI068744-RA	gi|646565955|gb|KK983064.1|	1-664	9.6	7.6
ACYPI083109-RA	gi|646779451|gb|KK961573.1|	1885240-1891964	20	8.9
ACYPI43876-RA	gi|646770401|gb|KK961779.1|	436571-439449	21	8.6
ACYPI43878-RA	gi|646751569|gb|KK961985.1|	35249-36570	21	9.2
ACYPI000208-RA	gi|646778840|gb|KK961590.1|	1377250-1379380	20	9.9

ACYPI000636-RA	gi|646750600|gb|KK962030.1|	381346-381772	23	8.6
ACYPI000981-RA	gi|646736316|gb|KK963199.1|	92016-93540	12	7.5
ACYPI001233-RA	gi|646736855|gb|KK963138.1|	140252-142290	19	9.1
ACYPI001969-RA	gi|646775821|gb|KK961707.1|	480823-481671	23	9.1
ACYPI003744-RA	gi|646782334|gb|KK961495.1|	3498564-3498982	21	9
ACYPI006038-RA	gi|646746556|gb|KK962281.1|	530512-532014	20	9.1
ACYPI006940-RA	gi|646777440|gb|KK961631.1|	1453969-1454277	22	9.7
ACYPI007498-RA	gi|646780147|gb|KK961556.1|	1016761-1016938	18	7.5
ACYPI008556-RA	gi|646746556|gb|KK962281.1|	593040-594447	20	9.1
ACYPI008853-RA	gi|646766034|gb|KK961878.1|	273626-273888	20	9
ACYPI065855-RA	gi|646780147|gb|KK961556.1|	912725-916745	18	7.5
ACYPI066334-RA	gi|646747424|gb|KK962220.1|	464224-465336	22	5.7
ACYPI086043-RA	gi|646769275|gb|KK961796.1|	938361-938978	22	5.6
ACYPI30922-RA	gi|646742298|gb|KK962592.1|	227015-228254	10	7
ACYPI35006-RA	gi|646772786|gb|KK961754.1|	928441-929546	22	9.6
ACYPI41722-RA	gi|646782334|gb|KK961495.1|	3500327-3500819	21	9
ACYPI002522-RA	gi|646775312|gb|KK961734.1|	1168349-1168544	17	7.9
ACYPI005093-RA	gi|646778767|gb|KK961592.1|	1308647-1314006	21	5.4
ACYPI006993-RA	gi|646746974|gb|KK962252.1|	141680-142583	23	6.1
ACYPI070768-RA	gi|646775451|gb|KK961726.1|	2179828-2183485	20	5.6
ACYPI086900-RA	gi|646738359|gb|KK962984.1|	82512-83645	16	11
ACYPI28461-RA	gi|646739806|gb|KK962843.1|	33498-34164	17	12
ACYPI34001-RA	gi|646780953|gb|KK961535.1|	1124455-1127576	20	9.8
ACYPI001053-RA	gi|646782127|gb|KK961500.1|	769481-772794	22	9.6
ACYPI005018-RA	gi|646780222|gb|KK961554.1|	3301874-3303251	20	9.7
ACYPI005885-RA	gi|646556697|gb|KK984938.1|	7423-9162	14	5.9
ACYPI006660-RA	gi|646778903|gb|KK961588.1|	4191659-4191959	23	10
ACYPI006915-RA	gi|646780222|gb|KK961554.1|	3277232-3285712	20	9.7
ACYPI007340-RA	gi|646780270|gb|KK961553.1|	4348328-4349053	22	8.6
ACYPI009274-RA	gi|646778632|gb|KK961596.1|	2376348-2376644	21	9.2

ACYPI068713-RA	gi|646775821|gb|KK961707.1|	1160980-1165516	23	9.1
ACYPI069407-RA	gi|646709960|gb|KK965470.1|	14758-18101	21	11
ACYPI088544-RA	gi|646755264|gb|KK961938.1|	473507-475222	20	5.3
ACYPI34470-RA	gi|646730603|gb|KK963922.1|	140915-143006	18	8.3
ACYPI34471-RA	gi|646753527|gb|KK961953.1|	579780-583108	24	9.2
ACYPI52359-RA	gi|646760466|gb|KK961915.1|	47453-48040	22	8.9
ACYPI002919-RA	gi|646743302|gb|KK962514.1|	36000-37996	23	8.4
ACYPI006595-RA	gi|646777125|gb|KK961644.1|	1698956-1700256	20	10
ACYPI009394-RA	gi|646779826|gb|KK961564.1|	1098957-1099868	22	9.6
ACYPI069017-RA	gi|646780978|gb|KK961534.1|	1758435-1759906	20	8
ACYPI34351-RA	gi|646778903|gb|KK961588.1|	3607186-3607802	23	10
ACYPI000946-RA	gi|646782357|gb|KK961494.1|	4401905-4402253	21	9.2
ACYPI002501-RA	gi|646749499|gb|KK962089.1|	197193-197844	20	8.7
ACYPI002571-RA	gi|646776588|gb|KK961666.1|	2429769-2431628	20	5.8
ACYPI003529-RA	gi|646730017|gb|KK963998.1|	94405-94765	20	8.3
ACYPI004471-RA	gi|646776447|gb|KK961675.1|	2004338-2019098	22	9.4
ACYPI004710-RA	gi|646778186|gb|KK961609.1|	1960272-1962359	19	9.4
ACYPI008106-RA	gi|646767196|gb|KK961838.1|	539468-548134	21	9.7
ACYPI081704-RA	gi|646780311|gb|KK961552.1|	2164047-2166567	21	9.7
ACYPI48566-RA	gi|646707383|gb|KK965596.1|	46853-47281	13	5.8
ACYPI002526-RA	gi|646771951|gb|KK961762.1|	675959-678991	21	8.3
ACYPI006443-RA	gi|646743944|gb|KK962469.1|	298409-298882	20	9.1
ACYPI009367-RA	gi|646781564|gb|KK961516.1|	1145497-1147546	23	9.4
ACYPI002513-RA	gi|646748580|gb|KK962141.1|	674933-675285	20	5.6
ACYPI003141-RA	gi|646767670|gb|KK961827.1|	653931-654235	21	5.9
ACYPI004925-RA	gi|646778517|gb|KK961599.1|	617479-617694	20	9.6
ACYPI005613-RA	gi|646778767|gb|KK961592.1|	208015-211193	21	5.4
ACYPI007953-RA	gi|646775368|gb|KK961731.1|	758781-759874	17	8.4
ACYPI008430-RA	gi|646775807|gb|KK961708.1|	2364544-2365182	21	8.6
ACYPI062104-RA	gi|646779337|gb|KK961576.1|	809322-810479	18	9

ACYPI064886-RA	gi|646745974|gb|KK962320.1|	611307-612949	15	8.4
ACYPI072468-RA	gi|646771352|gb|KK961768.1|	357201-357988	21	9.6
ACYPI072792-RA	gi|646776322|gb|KK961682.1|	1164950-1165502	22	8.9
ACYPI26971-RA	gi|646766511|gb|KK961859.1|	873445-874801	17	8.5
ACYPI000102-RA	gi|646779451|gb|KK961573.1|	833738-834143	20	8.9
ACYPI001323-RA	gi|646782009|gb|KK961503.1|	1767752-1770875	15	7.5
ACYPI001504-RA	gi|646732909|gb|KK963610.1|	172018-172978	13	6.7
ACYPI003788-RA	gi|646763164|gb|KK961904.1|	1005243-1005551	22	10
ACYPI003914-RA	gi|646747884|gb|KK962186.1|	479971-482520	22	11
ACYPI004515-RA	gi|646749188|gb|KK962105.1|	110767-112558	18	7.9
ACYPI004605-RA	gi|646775312|gb|KK961734.1|	912141-913216	17	7.9
ACYPI008308-RA	gi|646779412|gb|KK961574.1|	2285204-2286864	21	5.4
ACYPI35076-RA	gi|646771448|gb|KK961767.1|	865889-867406	21	9.8
ACYPI004988-RA	gi|646781809|gb|KK961509.1|	3408564-3412982	19	8.6
ACYPI006320-RA	gi|646777665|gb|KK961623.1|	741853-742360	21	9.7
ACYPI073588-RA	gi|646744179|gb|KK962451.1|	346365-346826	24	11
ACYPI23430-RA	gi|646746556|gb|KK962281.1|	714815-717195	20	9.1
ACYPI36902-RA	gi|646765725|gb|KK961892.1|	142790-143514	20	11
ACYPI44428-RA	gi|646781772|gb|KK961510.1|	4267141-4268933	20	9.2
ACYPI000082-RA	gi|646752567|gb|KK961965.1|	934807-936511	19	8.4
ACYPI000747-RA	gi|646777159|gb|KK961643.1|	1120471-1120775	14	6.5
ACYPI000792-RA	gi|646778865|gb|KK961589.1|	1907516-1908773	20	9.2
ACYPI002637-RA	gi|646776490|gb|KK961672.1|	975484-976258	20	10
ACYPI008814-RA	gi|646777089|gb|KK961646.1|	880826-881216	20	9.4
ACYPI060627-RA	gi|646776588|gb|KK961666.1|	962159-965131	20	5.8
ACYPI067238-RA	gi|646745289|gb|KK962370.1|	412132-412892	14	8.1
ACYPI069416-RA	gi|646776490|gb|KK961672.1|	1036873-1037562	20	10
ACYPI29505-RA	gi|646703040|gb|KK965835.1|	65854-69064	18	9.2
ACYPI001130-RA	gi|646766326|gb|KK961866.1|	253328-254614	19	7.9
ACYPI004055-RA	gi|646778660|gb|KK961595.1|	547232-548731	16	8.2

ACYPI009859-RA	gi|646752431|gb|KK961967.1|	1457508-1459424	20	5.6
ACYPI066960-RA	gi|646776280|gb|KK961684.1|	868790-869825	22	9.3
ACYPI085886-RA	gi|646715043|gb|KK965260.1|	25678-26383	11	5.5
ACYPI37115-RA	gi|646514870|gb|KK991607.1|	5714-7121	1.5	3.1
ACYPI50981-RA	gi|646733146|gb|KK963575.1|	156968-157706	48	23
ACYPI003157-RA	gi|646782043|gb|KK961502.1|	5249003-5250266	20	9.2
ACYPI004165-RA	gi|646769453|gb|KK961793.1|	1280817-1282788	21	8.8
ACYPI005479-RA	gi|646776368|gb|KK961679.1|	1787788-1788552	21	9.7
ACYPI007342-RA	gi|646781212|gb|KK961527.1|	63294-64773	21	10
ACYPI009229-RA	gi|646774067|gb|KK961745.1|	1235647-1236598	17	9.3
ACYPI010047-RA	gi|646780105|gb|KK961557.1|	2816591-2818303	21	9.7
ACYPI064108-RA	gi|646752076|gb|KK961973.1|	114387-117398	22	9.1
ACYPI080219-RA	gi|646766382|gb|KK961864.1|	489155-490248	22	9.7
ACYPI34516-RA	gi|646754710|gb|KK961942.1|	418778-421323	11	7.1
ACYPI36505-RA	gi|646763535|gb|KK961902.1|	1683481-1686247	23	10
ACYPI40167-RA	gi|646769453|gb|KK961793.1|	1036476-1037469	21	8.8
ACYPI44265-RA	gi|646776447|gb|KK961675.1|	1837668-1839708	22	9.4
ACYPI001415-RA	gi|646751160|gb|KK962003.1|	442667-443644	22	10
ACYPI002180-RA	gi|646757520|gb|KK961926.1|	1451150-1454207	20	7.4
ACYPI002627-RA	gi|646618628|gb|KK972945.1|	14620-14909	18	11
ACYPI002839-RA	gi|646737853|gb|KK963036.1|	137035-137669	42	18
ACYPI004049-RA	gi|646745197|gb|KK962376.1|	568807-573328	21	8.9
ACYPI004536-RA	gi|646741553|gb|KK962656.1|	453225-454115	17	7.9
ACYPI005411-RA	gi|646777288|gb|KK961638.1|	3935866-3937635	22	6.1
ACYPI006432-RA	gi|646780105|gb|KK961557.1|	1927883-1935768	21	9.7
ACYPI009177-RA	gi|646760564|gb|KK961914.1|	131658-132028	21	8.8
ACYPI009664-RA	gi|646751160|gb|KK962003.1|	419817-420047	22	10
ACYPI073264-RA	gi|646749499|gb|KK962089.1|	515581-516202	20	8.7
ACYPI073815-RA	gi|646776879|gb|KK961655.1|	1196781-1199967	21	9.3
ACYPI27762-RA	gi|646736505|gb|KK963177.1|	233470-234321	23	7.5

ACYPI42263-RA	gi|646709877|gb|KK965473.1|	9541-11041	18	8.7
ACYPI001909-RA	gi|646780978|gb|KK961534.1|	537860-538428	20	8
ACYPI003798-RA	gi|646777474|gb|KK961630.1|	613574-614529	21	9.3
ACYPI005126-RA	gi|646781421|gb|KK961521.1|	4256955-4257584	21	8.7
ACYPI005705-RA	gi|646779498|gb|KK961572.1|	828920-829271	22	5.6
ACYPI005727-RA	gi|646775867|gb|KK961705.1|	998516-1007354	23	10
ACYPI006377-RA	gi|646782357|gb|KK961494.1|	9236897-9237991	21	9.2
ACYPI007603-RA	gi|646781421|gb|KK961521.1|	4518233-4519596	21	8.7
ACYPI008157-RA	gi|646779621|gb|KK961569.1|	1204604-1206755	16	7.3
ACYPI008262-RA	gi|646782357|gb|KK961494.1|	9345629-9346103	21	9.2
ACYPI009470-RA	gi|646747385|gb|KK962223.1|	404567-411588	21	5.4
ACYPI009497-RA	gi|646740004|gb|KK962821.1|	601095-604200	22	9.6
ACYPI010042-RA	gi|646768534|gb|KK961809.1|	78697-79160	21	9.8
ACYPI010138-RA	gi|646782357|gb|KK961494.1|	4324816-4328175	21	9.2
ACYPI065942-RA	gi|646777474|gb|KK961630.1|	1155021-1156165	21	9.3
ACYPI069761-RA	gi|646765991|gb|KK961880.1|	294533-297208	16	7
ACYPI081460-RA	gi|646769163|gb|KK961798.1|	2102113-2105099	20	9.2
ACYPI083293-RA	gi|646722233|gb|KK964943.1|	63670-66634	34	14
ACYPI54122-RA	gi|646714902|gb|KK965265.1|	82159-83186	39	18
ACYPI003973-RA	gi|646745372|gb|KK962364.1|	655142-656984	23	10
ACYPI005802-RA	gi|646775884|gb|KK961704.1|	646546-647233	20	7.6
ACYPI008051-RA	gi|646749632|gb|KK962082.1|	171874-172186	20	8.6
ACYPI008134-RA	gi|646781893|gb|KK961506.1|	3696860-3698932	19	8.5
ACYPI009795-RA	gi|646781628|gb|KK961514.1|	2293571-2295459	23	9
ACYPI010011-RA	gi|646769511|gb|KK961792.1|	58754-64899	20	9.9
ACYPI073110-RA	gi|646782357|gb|KK961494.1|	10200669-10202145	21	9.2
ACYPI086562-RA	gi|646775523|gb|KK961722.1|	405241-407394	21	9.7
ACYPI29271-RA	gi|646745053|gb|KK962387.1|	294113-295405	19	8
ACYPI36199-RA	gi|646775821|gb|KK961707.1|	9268-11079	23	9.1
ACYPI41915-RA	gi|646741699|gb|KK962644.1|	524702-529223	20	9.2

ACYPI56854-RA	gi|646745579|gb|KK962347.1|	203922-206237	15	7.6
ACYPI000336-RA	gi|646738330|gb|KK962987.1|	191864-196023	19	7.9
ACYPI001007-RA	gi|646740756|gb|KK962735.1|	93490-104906	23	10
ACYPI001597-RA	gi|646781659|gb|KK961513.1|	2788555-2790950	22	9.8
ACYPI001706-RA	gi|646780311|gb|KK961552.1|	3890551-3890711	21	9.7
ACYPI002909-RA	gi|646778213|gb|KK961608.1|	1363971-1371045	19	8.8
ACYPI005488-RA	gi|646747449|gb|KK962218.1|	759603-776530	22	10
ACYPI006500-RA	gi|646773721|gb|KK961747.1|	2115732-2116004	21	9.2
ACYPI006735-RA	gi|646755888|gb|KK961934.1|	873620-874113	21	9.8
ACYPI007353-RA	gi|646767196|gb|KK961838.1|	453935-454172	21	9.7
ACYPI009863-RA	gi|646780311|gb|KK961552.1|	2869573-2870566	21	9.7
ACYPI010142-RA	gi|646777416|gb|KK961632.1|	1913854-1914405	22	8.8
ACYPI062999-RA	gi|646569474|gb|KK982420.1|	11968-13080	16	8.3
ACYPI066483-RA	gi|646776024|gb|KK961697.1|	2103847-2105467	22	9.2
ACYPI083753-RA	gi|646782127|gb|KK961500.1|	2349304-2350723	22	9.6
ACYPI25951-RA	gi|646776024|gb|KK961697.1|	2328465-2328781	22	9.2
ACYPI29477-RA	gi|646780858|gb|KK961538.1|	468679-473230	21	10
ACYPI31897-RA	gi|646745171|gb|KK962378.1|	255397-255590	19	8.1
ACYPI52244-RA	gi|646749945|gb|KK962065.1|	1959548-1961254	22	6.1
ACYPI001940-RA	gi|646734653|gb|KK963394.1|	162477-164187	20	9.1
ACYPI003404-RA	gi|646781628|gb|KK961514.1|	3448418-3448774	23	9
ACYPI003908-RA	gi|646775968|gb|KK961700.1|	879475-879873	20	8.2
ACYPI005629-RA	gi|646777313|gb|KK961637.1|	405010-405708	20	8.2
ACYPI006442-RA	gi|646766985|gb|KK961844.1|	177535-181226	21	9.7
ACYPI060626-RA	gi|646729674|gb|KK964044.1|	155174-155932	24	9.4
ACYPI087337-RA	gi|646639516|gb|KK969207.1|	8922-10190	14	8.4
ACYPI23751-RA	gi|646746841|gb|KK962261.1|	454735-459480	20	9.6
ACYPI29762-RA	gi|646727570|gb|KK964327.1|	12316-13101	29	11
ACYPI005030-RA	gi|646747046|gb|KK962247.1|	66047-66259	22	9
ACYPI005805-RA	gi|646778061|gb|KK961612.1|	1059995-1061909	20	8.7

ACYPI063616-RA	gi|646737344|gb|KK963086.1|	51513-52281	16		9.9
ACYPI38459-RA	gi|646780978|gb|KK961534.1|	2688628-2691087	20		8
ACYPI55006-RA	gi|646775807|gb|KK961708.1|	2112293-2113377	21		8.6
ACYPI000903-RA	gi|646781849|gb|KK961508.1|	2181341-2183151	17		8
ACYPI005167-RA	gi|646781143|gb|KK961529.1|	2195844-2196738	20		10
ACYPI006699-RA	gi|646782211|gb|KK961498.1|	2483034-2483357	20		9
ACYPI006741-RA	gi|646778336|gb|KK961604.1|	1373678-1374197	21		10
ACYPI082482-RA	gi|646518179|gb|KK991009.1|	122-897	1.2	nan	
ACYPI45646-RA	gi|646751289|gb|KK961996.1|	110477-114188	24		9.1
ACYPI49185-RA	gi|646771568|gb|KK961766.1|	164168-165329	18		7.4
ACYPI000178-RA	gi|646754371|gb|KK961945.1|	288169-289147	20		9.7
ACYPI000563-RA	gi|646748295|gb|KK962158.1|	90371-90660	18		8.2
ACYPI002953-RA	gi|646779826|gb|KK961564.1|	3221840-3223314	22		9.6
ACYPI003639-RA	gi|646727554|gb|KK964329.1|	137375-141745	24		10
ACYPI003907-RA	gi|646729280|gb|KK964096.1|	180522-182494	22		9.3
ACYPI004568-RA	gi|646781772|gb|KK961510.1|	2521949-2522820	20		9.2
ACYPI005585-RA	gi|646743261|gb|KK962517.1|	321893-333156	19		8.9
ACYPI005806-RA	gi|646742726|gb|KK962557.1|	202633-203701	20		9.1
ACYPI005852-RA	gi|646753799|gb|KK961950.1|	2018910-2019690	23		9.2
ACYPI006222-RA	gi|646778245|gb|KK961607.1|	1137461-1139114	18		6.8
ACYPI006931-RA	gi|646748558|gb|KK962142.1|	325191-325747	21		9.9
ACYPI006982-RA	gi|646776647|gb|KK961663.1|	215325-216065	23		9.2
ACYPI007315-RA	gi|646766056|gb|KK961877.1|	293889-294202	18		7.8
ACYPI007671-RA	gi|646745186|gb|KK962377.1|	188086-190382	21		9.8
ACYPI009424-RA	gi|646768669|gb|KK961806.1|	331550-333339	18		9.1
ACYPI009979-RA	gi|646781379|gb|KK961522.1|	2150046-2150622	22		7.6
ACYPI010058-RA	gi|646768669|gb|KK961806.1|	320200-321485	18		9.1
ACYPI066741-RA	gi|646765948|gb|KK961882.1|	503314-503920	18		7.3
ACYPI069332-RA	gi|646782276|gb|KK961497.1|	4338475-4339853	21		9.7
ACYPI085620-RA	gi|646782276|gb|KK961497.1|	4374026-4374458	21		9.7

ACYPI086730-RA	gi|646772045|gb|KK961761.1|	979446-979871	21	9.6
ACYPI007596-RA	gi|646749649|gb|KK962081.1|	75645-77432	19	7.6
ACYPI008432-RA	gi|646776456|gb|KK961674.1|	835443-835673	18	7.9
ACYPI009491-RA	gi|646780441|gb|KK961549.1|	3396317-3402171	21	9.5
ACYPI062254-RA	gi|646775414|gb|KK961728.1|	1394574-1395056	17	9.5
ACYPI53305-RA	gi|646737790|gb|KK963042.1|	92178-92437	14	9.1
ACYPI56625-RA	gi|646780441|gb|KK961549.1|	3529786-3533418	21	9.5
ACYPI001054-RA	gi|646775312|gb|KK961734.1|	670482-670714	17	7.9
ACYPI001459-RA	gi|646782288|gb|KK961496.1|	3892920-3893260	21	9.7
ACYPI001465-RA	gi|646781043|gb|KK961532.1|	1767270-1769326	26	11
ACYPI001769-RA	gi|646777570|gb|KK961626.1|	913822-914047	18	7.5
ACYPI002078-RA	gi|646782288|gb|KK961496.1|	7401554-7401985	21	9.7
ACYPI003282-RA	gi|646775821|gb|KK961707.1|	2268585-2271598	23	9.1
ACYPI003641-RA	gi|646778840|gb|KK961590.1|	1872677-1872923	20	9.9
ACYPI003821-RA	gi|646766137|gb|KK961874.1|	611082-612102	20	9
ACYPI004613-RA	gi|646782288|gb|KK961496.1|	6183624-6187655	21	9.7
ACYPI004940-RA	gi|646751569|gb|KK961985.1|	170070-172197	21	9.2
ACYPI005202-RA	gi|646741767|gb|KK962638.1|	36281-37350	21	10
ACYPI007774-RA	gi|646776735|gb|KK961660.1|	1691483-1692826	21	9.6
ACYPI008403-RA	gi|646779741|gb|KK961566.1|	2353739-2355066	20	8.5
ACYPI008438-RA	gi|646776794|gb|KK961658.1|	627666-628786	20	8.1
ACYPI008607-RA	gi|646725628|gb|KK964623.1|	66223-66493	16	5.7
ACYPI21790-RA	gi|646777665|gb|KK961623.1|	899192-905911	21	9.7
ACYPI24008-RA	gi|646767265|gb|KK961836.1|	1069246-1070619	22	9.9
ACYPI41765-RA	gi|646781212|gb|KK961527.1|	3856435-3857553	21	10
ACYPI47447-RA	gi|646722233|gb|KK964943.1|	61438-61968	34	14
ACYPI000033-RA	gi|646758845|gb|KK961921.1|	621449-621952	22	9.5
ACYPI002959-RA	gi|646771845|gb|KK961763.1|	2098996-2107545	23	10
ACYPI003465-RA	gi|646775789|gb|KK961709.1|	1204281-1205390	20	9.6
ACYPI003701-RA	gi|646771845|gb|KK961763.1|	2275019-2275891	23	10

ACYPI004625-RA	gi|646776368|gb|KK961679.1|	938507-942518	21	9.7
ACYPI004646-RA	gi|646747032|gb|KK962248.1|	441267-441496	20	8.7
ACYPI005113-RA	gi|646776456|gb|KK961674.1|	1311316-1313786	18	7.9
ACYPI006992-RA	gi|646756446|gb|KK961931.1|	350865-351153	14	6.1
ACYPI008452-RA	gi|646750795|gb|KK962021.1|	426982-427776	19	8.9
ACYPI33110-RA	gi|646781536|gb|KK961517.1|	530105-533465	19	7.7
ACYPI000111-RA	gi|646781013|gb|KK961533.1|	1858064-1860911	18	7.6
ACYPI000519-RA	gi|646775867|gb|KK961705.1|	301713-315278	23	10
ACYPI000791-RA	gi|646767697|gb|KK961826.1|	766499-767120	21	9.8
ACYPI000799-RA	gi|646777313|gb|KK961637.1|	750381-750852	20	8.2
ACYPI001188-RA	gi|646777416|gb|KK961632.1|	1719456-1722610	22	8.8
ACYPI001815-RA	gi|646782276|gb|KK961497.1|	6214131-6214943	21	9.7
ACYPI003061-RA	gi|646740879|gb|KK962721.1|	700900-703086	24	6.4
ACYPI004588-RA	gi|646782288|gb|KK961496.1|	473419-480039	21	9.7
ACYPI004977-RA	gi|646735008|gb|KK963353.1|	216593-217591	21	9.4
ACYPI005551-RA	gi|646775789|gb|KK961709.1|	982925-987236	20	9.6
ACYPI005997-RA	gi|646747046|gb|KK962247.1|	612278-614172	22	9
ACYPI007514-RA	gi|646780441|gb|KK961549.1|	3224905-3225689	21	9.5
ACYPI007764-RA	gi|646766703|gb|KK961852.1|	806917-807406	20	7.1
ACYPI008958-RA	gi|646781344|gb|KK961523.1|	4533209-4534364	21	8.9
ACYPI009406-RA	gi|646780441|gb|KK961549.1|	3090287-3095238	21	9.5
ACYPI060355-RA	gi|646779262|gb|KK961578.1|	1073849-1075014	20	8.1
ACYPI071956-RA	gi|646774797|gb|KK961742.1|	961887-970351	20	8.5
ACYPI084509-RA	gi|646745522|gb|KK962352.1|	84396-85742	18	9.3
ACYPI086358-RA	gi|646745579|gb|KK962347.1|	498853-499608	15	7.6
ACYPI29936-RA	gi|646746355|gb|KK962294.1|	522380-523639	22	10
ACYPI000468-RA	gi|646776490|gb|KK961672.1|	515054-520064	20	10
ACYPI000550-RA	gi|646778699|gb|KK961594.1|	2426055-2431226	21	9.5
ACYPI000684-RA	gi|646751289|gb|KK961996.1|	594511-595122	24	9.1
ACYPI001312-RA	gi|646758935|gb|KK961920.1|	123408-123652	20	8.5

ACYPI008063-RA	gi|646768294|gb|KK961814.1|	445194-445815	23	10
ACYPI009643-RA	gi|646781379|gb|KK961522.1|	2599570-2600762	22	7.6
ACYPI009972-RA	gi|646771448|gb|KK961767.1|	770233-771698	21	9.8
ACYPI061797-RA	gi|646780441|gb|KK961549.1|	488116-488769	21	9.5
ACYPI068599-RA	gi|646778112|gb|KK961611.1|	507045-521491	22	10
ACYPI069597-RA	gi|646765725|gb|KK961892.1|	396836-398408	20	11
ACYPI36079-RA	gi|646782043|gb|KK961502.1|	5097797-5102526	20	9.2
ACYPI38677-RA	gi|646758845|gb|KK961921.1|	2104837-2107500	22	9.5
ACYPI48985-RA	gi|646772574|gb|KK961756.1|	1107964-1109722	21	8
ACYPI53939-RA	gi|646733041|gb|KK963591.1|	80324-81139	24	8.7
ACYPI000728-RA	gi|646743341|gb|KK962511.1|	175289-175601	19	5
ACYPI001279-RA	gi|646775867|gb|KK961705.1|	2227489-2229686	23	10
ACYPI003736-RA	gi|646781536|gb|KK961517.1|	2024598-2029654	19	7.7
ACYPI004008-RA	gi|646776952|gb|KK961652.1|	1309825-1310609	19	7.7
ACYPI005949-RA	gi|646772897|gb|KK961753.1|	1491284-1495508	21	8.7
ACYPI007598-RA	gi|646777416|gb|KK961632.1|	2307244-2307905	22	8.8
ACYPI008830-RA	gi|646775451|gb|KK961726.1|	1710281-1710965	20	5.6
ACYPI009250-RA	gi|646746216|gb|KK962304.1|	447052-448861	########	33
ACYPI009403-RA	gi|646751160|gb|KK962003.1|	786743-792980	22	10
ACYPI009444-RA	gi|646778803|gb|KK961591.1|	849316-854104	15	7.8
ACYPI009762-RA	gi|646778983|gb|KK961586.1|	1082028-1083502	20	9.9
ACYPI010082-RA	gi|646781510|gb|KK961518.1|	2834312-2835188	17	7.8
ACYPI084955-RA	gi|646781243|gb|KK961526.1|	3723491-3725070	21	8.9
ACYPI27979-RA	gi|646729796|gb|KK964027.1|	13081-14521	20	7
ACYPI39080-RA	gi|646766451|gb|KK961861.1|	41368-42634	19	8.9
ACYPI42061-RA	gi|646780574|gb|KK961546.1|	1200901-1202177	20	9.9
ACYPI47485-RA	gi|646767670|gb|KK961827.1|	807110-812483	21	5.9
ACYPI51093-RA	gi|646767477|gb|KK961831.1|	482477-490293	20	8.4
ACYPI53252-RA	gi|646780978|gb|KK961534.1|	1805403-1806442	20	8
ACYPI000027-RA	gi|646745959|gb|KK962321.1|	195848-196157	20	6.9

ACYPI001757-RA	gi|646740085|gb|KK962813.1|	288924-289198	20	5.5
ACYPI004267-RA	gi|646729189|gb|KK964108.1|	141240-142007	25	7
ACYPI004983-RA	gi|646741778|gb|KK962637.1|	27686-28358	19	6.1
ACYPI39881-RA	gi|646776447|gb|KK961675.1|	1482272-1483526	22	9.4
ACYPI42871-RA	gi|646773903|gb|KK961746.1|	335905-337726	20	8.9
ACYPI48499-RA	gi|646782288|gb|KK961496.1|	4786204-4787545	21	9.7
ACYPI49169-RA	gi|646780889|gb|KK961537.1|	1835271-1837045	22	10
ACYPI56181-RA	gi|646734398|gb|KK963425.1|	288208-290403	22	5.9
ACYPI000534-RA	gi|646778336|gb|KK961604.1|	2203948-2204913	21	10
ACYPI000538-RA	gi|646775635|gb|KK961716.1|	1847031-1850055	22	10
ACYPI002188-RA	gi|646717826|gb|KK965143.1|	59739-60556	13	7
ACYPI002286-RA	gi|646782334|gb|KK961495.1|	4606095-4606597	21	9
ACYPI003560-RA	gi|646779375|gb|KK961575.1|	1835278-1835852	22	9.3
ACYPI004058-RA	gi|646767439|gb|KK961832.1|	1178391-1179361	23	10
ACYPI005000-RA	gi|646778336|gb|KK961604.1|	2454941-2457328	21	10
ACYPI006521-RA	gi|646772574|gb|KK961756.1|	1138358-1141988	21	8
ACYPI006896-RA	gi|646776514|gb|KK961670.1|	6299-6541	20	9.6
ACYPI008198-RA	gi|646778336|gb|KK961604.1|	2218866-2219711	21	10
ACYPI008847-RA	gi|646747076|gb|KK962245.1|	561257-570758	23	9.6
ACYPI009259-RA	gi|646747841|gb|KK962189.1|	462210-463390	23	10
ACYPI010034-RA	gi|646745372|gb|KK962364.1|	484917-490925	23	10
ACYPI062209-RA	gi|646750969|gb|KK962013.1|	643875-644992	16	7.4
ACYPI068208-RA	gi|646752252|gb|KK961970.1|	655390-656353	18	9
ACYPI072192-RA	gi|646751160|gb|KK962003.1|	120488-123622	22	10
ACYPI085245-RA	gi|646782276|gb|KK961497.1|	5807880-5808912	21	9.7
ACYPI25737-RA	gi|646774530|gb|KK961743.1|	311798-312848	20	9.8
ACYPI30186-RA	gi|646780270|gb|KK961553.1|	4494851-4496200	22	8.6
ACYPI48764-RA	gi|646774530|gb|KK961743.1|	52122-53962	20	9.8
ACYPI49710-RA	gi|646743886|gb|KK962473.1|	194403-196266	21	9.3
ACYPI53679-RA	gi|646725525|gb|KK964639.1|	19735-20803	27	9.8

ACYPI56077-RA	gi|646745372|gb|KK962364.1|	471123-471656	23	10
ACYPI006718-RA	gi|646782288|gb|KK961496.1|	4367497-4367889	21	9.7
ACYPI008756-RA	gi|646750643|gb|KK962028.1|	125528-125860	21	7.5
ACYPI082516-RA	gi|646734551|gb|KK963406.1|	246396-250050	23	6.4
ACYPI085634-RA	gi|646781118|gb|KK961530.1|	4105617-4105751	21	10
ACYPI46858-RA	gi|646781083|gb|KK961531.1|	2559230-2565416	19	8.5
ACYPI53238-RA	gi|646745053|gb|KK962387.1|	374729-375959	19	8
ACYPI000387-RA	gi|646770901|gb|KK961773.1|	1607687-1609334	22	9.7
ACYPI000707-RA	gi|646759701|gb|KK961917.1|	19540-24068	18	6.1
ACYPI000947-RA	gi|646775635|gb|KK961716.1|	400858-402792	22	10
ACYPI001643-RA	gi|646779826|gb|KK961564.1|	2794028-2794970	22	9.6
ACYPI002284-RA	gi|646767265|gb|KK961836.1|	1758940-1759768	22	9.9
ACYPI002801-RA	gi|646748888|gb|KK962123.1|	518666-520320	20	8.1
ACYPI006013-RA	gi|646765880|gb|KK961885.1|	1071354-1073042	23	10
ACYPI006630-RA	gi|646753180|gb|KK961957.1|	508121-516575	25	11
ACYPI006668-RA	gi|646775635|gb|KK961716.1|	410805-411074	22	10
ACYPI007384-RA	gi|646746216|gb|KK962304.1|	254136-257345	########	33
ACYPI068701-RA	gi|646551026|gb|KK986044.1|	6123-6289	5.8	3.8
ACYPI21911-RA	gi|646763164|gb|KK961904.1|	1271775-1273166	22	10
ACYPI39685-RA	gi|646739758|gb|KK962847.1|	276987-278912	17	8.2
ACYPI40034-RA	gi|646780406|gb|KK961550.1|	903343-904034	22	5.9
ACYPI41300-RA	gi|646724905|gb|KK964735.1|	79302-80496	43	20
ACYPI47009-RA	gi|646778632|gb|KK961596.1|	1148104-1148905	21	9.2
ACYPI55208-RA	gi|646782288|gb|KK961496.1|	799138-799364	21	9.7
ACYPI001761-RA	gi|646690976|gb|KK966792.1|	23533-24231	15	6.2
ACYPI002064-RA	gi|646778112|gb|KK961611.1|	1023408-1027764	22	10
ACYPI002794-RA	gi|646775558|gb|KK961720.1|	1695082-1695359	20	9.4
ACYPI004211-RA	gi|646780978|gb|KK961534.1|	1054515-1056896	20	8
ACYPI004680-RA	gi|646752348|gb|KK961968.1|	486633-486947	18	8
ACYPI005221-RA	gi|646771092|gb|KK961771.1|	528931-530193	23	9.7

ACYPI005241-RA	gi|646765790|gb|KK961889.1|	934732-935449	22	8.8
ACYPI006305-RA	gi|646778112|gb|KK961611.1|	852582-853337	22	10
ACYPI007800-RA	gi|646775723|gb|KK961712.1|	1603781-1605377	21	9.5
ACYPI008195-RA	gi|646782334|gb|KK961495.1|	2068955-2069679	21	9
ACYPI008980-RA	gi|646780574|gb|KK961546.1|	1149829-1156303	20	9.9
ACYPI063394-RA	gi|646775558|gb|KK961720.1|	1669273-1675149	20	9.4
ACYPI067369-RA	gi|646781932|gb|KK961505.1|	2659181-2660740	17	8.9
ACYPI081140-RA	gi|646778112|gb|KK961611.1|	791258-791494	22	10
ACYPI38188-RA	gi|646780978|gb|KK961534.1|	5642931-5643835	20	8
ACYPI42579-RA	gi|646770401|gb|KK961779.1|	1719667-1724087	21	8.6
ACYPI000612-RA	gi|646779936|gb|KK961561.1|	1881669-1884221	19	9.6
ACYPI001976-RA	gi|646780010|gb|KK961559.1|	1362996-1363780	22	10
ACYPI002852-RA	gi|646741057|gb|KK962701.1|	169594-170386	21	8.8
ACYPI004040-RA	gi|646776280|gb|KK961684.1|	154960-156884	22	9.3
ACYPI005188-RA	gi|646781344|gb|KK961523.1|	1700270-1703527	21	8.9
ACYPI005770-RA	gi|646779976|gb|KK961560.1|	684147-685126	16	7.9
ACYPI006449-RA	gi|646777416|gb|KK961632.1|	1193042-1197981	22	8.8
ACYPI006676-RA	gi|646781690|gb|KK961512.1|	3342729-3343889	21	8.8
ACYPI007150-RA	gi|646741057|gb|KK962701.1|	227672-228212	21	8.8
ACYPI007324-RA	gi|646769275|gb|KK961796.1|	2310078-2310278	22	5.6
ACYPI007730-RA	gi|646777877|gb|KK961617.1|	816802-818452	19	8.1
ACYPI009034-RA	gi|646776113|gb|KK961692.1|	1668056-1668680	21	9.6
ACYPI009193-RA	gi|646781690|gb|KK961512.1|	3419150-3420771	21	8.8
ACYPI009217-RA	gi|646767196|gb|KK961838.1|	114924-124579	21	9.7
ACYPI009555-RA	gi|646772897|gb|KK961753.1|	1648605-1648967	21	8.7
ACYPI069662-RA	gi|646752143|gb|KK961972.1|	2092077-2093317	20	5.6
ACYPI081137-RA	gi|646734653|gb|KK963394.1|	323246-323858	20	9.1
ACYPI50071-RA	gi|646749832|gb|KK962071.1|	310661-311979	22	6
ACYPI000079-RA	gi|646780953|gb|KK961535.1|	3216707-3217447	20	9.8
ACYPI002279-RA	gi|646737056|gb|KK963115.1|	143574-143943	24	11

ACYPI004146-RA	gi|646734213|gb|KK963446.1|	26113-26702	14	8.6
ACYPI005010-RA	gi|646766086|gb|KK961876.1|	30095-31006	23	9.5
ACYPI006616-RA	gi|646777207|gb|KK961641.1|	1186273-1186463	20	8.5
ACYPI007494-RA	gi|646781443|gb|KK961520.1|	617210-622254	20	8.3
ACYPI008142-RA	gi|646781443|gb|KK961520.1|	3108402-3108976	20	8.3
ACYPI009430-RA	gi|646779186|gb|KK961580.1|	510430-510980	20	8.6
ACYPI009856-RA	gi|646751176|gb|KK962002.1|	771697-774718	21	9.6
ACYPI31012-RA	gi|646775842|gb|KK961706.1|	814033-819183	22	9
ACYPI42631-RA	gi|646781118|gb|KK961530.1|	3832826-3835298	21	10
ACYPI55041-RA	gi|646777631|gb|KK961624.1|	1557436-1558441	20	9.3
ACYPI000787-RA	gi|646775312|gb|KK961734.1|	798851-799244	17	7.9
ACYPI000849-RA	gi|646781118|gb|KK961530.1|	3616282-3617476	21	10
ACYPI002674-RA	gi|646747754|gb|KK962195.1|	713736-714702	21	5.3
ACYPI002787-RA	gi|646777698|gb|KK961622.1|	893775-894467	16	7.6
ACYPI004152-RA	gi|646777991|gb|KK961614.1|	1627252-1627683	18	7.8
ACYPI004966-RA	gi|646780441|gb|KK961549.1|	1007895-1009624	21	9.5
ACYPI005060-RA	gi|646782288|gb|KK961496.1|	7810738-7812362	21	9.7
ACYPI006664-RA	gi|646570180|gb|KK982274.1|	3319-3526	5.4	2.9
ACYPI007199-RA	gi|646779785|gb|KK961565.1|	710503-712060	18	8.9
ACYPI008467-RA	gi|646781659|gb|KK961513.1|	5065619-5066012	22	9.8
ACYPI008499-RA	gi|646773903|gb|KK961746.1|	1539477-1540715	20	8.9
ACYPI060019-RA	gi|646772786|gb|KK961754.1|	758192-759096	22	9.6
ACYPI080017-RA	gi|646778448|gb|KK961601.1|	1793756-1798914	19	8.3
ACYPI25540-RA	gi|646781690|gb|KK961512.1|	2273116-2273984	21	8.8
ACYPI25700-RA	gi|646777288|gb|KK961638.1|	13878-16766	22	6.1
ACYPI000058-RA	gi|646744699|gb|KK962413.1|	70330-71410	14	6.9
ACYPI001872-RA	gi|646762191|gb|KK961910.1|	388628-389029	18	7.1
ACYPI002041-RA	gi|646780723|gb|KK961542.1|	2087042-2087325	21	9.4
ACYPI003924-RA	gi|646780752|gb|KK961541.1|	2225303-2229061	21	8
ACYPI005437-RA	gi|646765669|gb|KK961895.1|	253380-254371	18	7.2

ACYPI006409-RA	gi|646779262|gb|KK961578.1|	849442-849639	20	8.1
ACYPI007425-RA	gi|646781628|gb|KK961514.1|	3544858-3545128	23	9
ACYPI007734-RA	gi|646780752|gb|KK961541.1|	2263420-2265091	21	8
ACYPI082963-RA	gi|646782276|gb|KK961497.1|	4615304-4618952	21	9.7
ACYPI27734-RA	gi|646781421|gb|KK961521.1|	4128887-4133087	21	8.7
ACYPI48702-RA	gi|646781772|gb|KK961510.1|	3093387-3095013	20	9.2
ACYPI52139-RA	gi|646778983|gb|KK961586.1|	1974564-1980666	20	9.9
ACYPI53549-RA	gi|646776368|gb|KK961679.1|	429607-430560	21	9.7
ACYPI000008-RA	gi|646740098|gb|KK962812.1|	541234-543927	20	10
ACYPI000013-RA	gi|646740098|gb|KK962812.1|	612042-612505	20	10
ACYPI000061-RA	gi|646781243|gb|KK961526.1|	2284347-2285039	21	8.9
ACYPI004610-RA	gi|646747586|gb|KK962207.1|	223522-228046	20	8.8
ACYPI005722-RA	gi|646750505|gb|KK962035.1|	248950-249273	19	9.9
ACYPI005867-RA	gi|646782334|gb|KK961495.1|	2446244-2449854	21	9
ACYPI007664-RA	gi|646776241|gb|KK961686.1|	337251-338119	18	5.9
ACYPI063237-RA	gi|646781690|gb|KK961512.1|	1738929-1741938	21	8.8
ACYPI082616-RA	gi|646776608|gb|KK961665.1|	519461-521687	23	9.3
ACYPI086531-RA	gi|646749051|gb|KK962113.1|	193261-195828	19	8.4
ACYPI088840-RA	gi|646738524|gb|KK962968.1|	200385-212651	24	11
ACYPI31744-RA	gi|646745135|gb|KK962381.1|	325035-326319	20	8.7
ACYPI50707-RA	gi|646743492|gb|KK962499.1|	474031-476045	24	11
ACYPI002141-RA	gi|646605298|gb|KK975395.1|	885-1531	48	23
ACYPI002218-RA	gi|646777955|gb|KK961615.1|	2515443-2517007	21	10
ACYPI002433-RA	gi|646740304|gb|KK962788.1|	91622-92181	24	9.6
ACYPI006909-RA	gi|646779006|gb|KK961585.1|	911013-911385	20	9.9
ACYPI007005-RA	gi|646778112|gb|KK961611.1|	1163234-1164019	22	10
ACYPI008769-RA	gi|646782288|gb|KK961496.1|	227922-228617	21	9.7
ACYPI009420-RA	gi|646743377|gb|KK962508.1|	68993-70281	19	8
ACYPI41067-RA	gi|646779621|gb|KK961569.1|	1066113-1068382	16	7.3
ACYPI53334-RA	gi|646734398|gb|KK963425.1|	25535-27788	22	5.9

ACYPI000031-RA	gi|646763164|gb|KK961904.1|	1562058-1566225	22	10
ACYPI000348-RA	gi|646778865|gb|KK961589.1|	2907073-2908107	20	9.2
ACYPI000789-RA	gi|646780270|gb|KK961553.1|	834094-835387	22	8.6
ACYPI000979-RA	gi|646782357|gb|KK961494.1|	5102553-5104237	21	9.2
ACYPI001434-RA	gi|646780978|gb|KK961534.1|	2409142-2409645	20	8
ACYPI001613-RA	gi|646770401|gb|KK961779.1|	741506-742931	21	8.6
ACYPI001671-RA	gi|646775968|gb|KK961700.1|	1210354-1212093	20	8.2
ACYPI002278-RA	gi|646782127|gb|KK961500.1|	4393383-4395678	22	9.6
ACYPI003557-RA	gi|646781344|gb|KK961523.1|	1435331-1445399	21	8.9
ACYPI004127-RA	gi|646778865|gb|KK961589.1|	2087689-2087972	20	9.2
ACYPI004804-RA	gi|646758845|gb|KK961921.1|	326855-327121	22	9.5
ACYPI005103-RA	gi|646744324|gb|KK962441.1|	588-903	20	7.2
ACYPI007027-RA	gi|646770323|gb|KK961780.1|	39011-41165	19	7.1
ACYPI007908-RA	gi|646746407|gb|KK962291.1|	294705-294942	21	9.9
ACYPI007943-RA	gi|646777495|gb|KK961629.1|	1122882-1125010	19	8.7
ACYPI008895-RA	gi|646779375|gb|KK961575.1|	3023645-3024163	22	9.3
ACYPI009045-RA	gi|646751160|gb|KK962003.1|	409690-415606	22	10
ACYPI064056-RA	gi|646634301|gb|KK970114.1|	7444-7874	12	4.9
ACYPI069386-RA	gi|646779006|gb|KK961585.1|	1005467-1010542	20	9.9
ACYPI080395-RA	gi|646751176|gb|KK962002.1|	435680-436816	21	9.6
ACYPI26209-RA	gi|646777288|gb|KK961638.1|	1095950-1096565	22	6.1
ACYPI55966-RA	gi|646778374|gb|KK961603.1|	1164819-1165648	18	5.7
ACYPI000987-RA	gi|646748116|gb|KK962170.1|	568550-568835	20	8.3
ACYPI001871-RA	gi|646767120|gb|KK961840.1|	1179020-1179365	20	8.6
ACYPI002525-RA	gi|646745434|gb|KK962359.1|	90096-90372	22	10
ACYPI004414-RA	gi|646768631|gb|KK961807.1|	1458835-1459078	19	9.2
ACYPI006343-RA	gi|646745434|gb|KK962359.1|	42553-44492	22	10
ACYPI006586-RA	gi|646745434|gb|KK962359.1|	10452-10802	22	10
ACYPI006943-RA	gi|646760564|gb|KK961914.1|	561462-562902	21	8.8
ACYPI008353-RA	gi|646756057|gb|KK961933.1|	462961-463204	16	6.7

ACYPI068284-RA	gi|646740686|gb|KK962743.1|	36546-37906	12	5.5
ACYPI087756-RA	gi|646737098|gb|KK963111.1|	90617-91642	20	8
ACYPI20436-RA	gi|646728017|gb|KK964268.1|	64121-65870	19	9.5
ACYPI000579-RA	gi|646777991|gb|KK961614.1|	1129553-1130515	18	7.8
ACYPI001849-RA	gi|646746942|gb|KK962254.1|	1029902-1030605	23	9.8
ACYPI002227-RA	gi|646734508|gb|KK963411.1|	192010-193748	19	8.8
ACYPI003493-RA	gi|646781873|gb|KK961507.1|	1334500-1335191	20	9.1
ACYPI003697-RA	gi|646782334|gb|KK961495.1|	1054782-1056279	21	9
ACYPI004957-RA	gi|646748871|gb|KK962124.1|	280203-281159	21	8.1
ACYPI005035-RA	gi|646780627|gb|KK961545.1|	1387413-1387624	20	7.7
ACYPI005475-RA	gi|646545108|gb|KK987133.1|	265-513	22	5.8
ACYPI006679-RA	gi|646780752|gb|KK961541.1|	851051-851470	21	8
ACYPI008186-RA	gi|646779337|gb|KK961576.1|	784415-784904	18	9
ACYPI008671-RA	gi|646766624|gb|KK961855.1|	44782-46871	21	10
ACYPI008804-RA	gi|646781043|gb|KK961532.1|	1570080-1570885	26	11
ACYPI008825-RA	gi|646765790|gb|KK961889.1|	561358-567429	22	8.8
ACYPI009808-RA	gi|646782087|gb|KK961501.1|	5245312-5245582	20	8.7
ACYPI010072-RA	gi|646746074|gb|KK962314.1|	519848-529549	17	7
ACYPI081909-RA	gi|646780752|gb|KK961541.1|	926699-927109	21	8
ACYPI082808-RA	gi|646737354|gb|KK963085.1|	267977-269450	20	11
ACYPI22692-RA	gi|646749573|gb|KK962085.1|	640375-641345	23	10
ACYPI26764-RA	gi|646740098|gb|KK962812.1|	630134-630555	20	10
ACYPI38608-RA	gi|646781118|gb|KK961530.1|	2284275-2285334	21	10
ACYPI000731-RA	gi|646767075|gb|KK961841.1|	611346-619730	18	10
ACYPI001069-RA	gi|646737822|gb|KK963039.1|	161589-174539	20	9
ACYPI001272-RA	gi|646775702|gb|KK961713.1|	701833-705008	17	7.4
ACYPI001648-RA	gi|646748200|gb|KK962164.1|	157609-158634	19	7.7
ACYPI002150-RA	gi|646756446|gb|KK961931.1|	566450-568116	14	6.1
ACYPI002620-RA	gi|646771845|gb|KK961763.1|	1420337-1420635	23	10
ACYPI002748-RA	gi|646766426|gb|KK961862.1|	977494-977745	20	7.1

ACYPI004168-RA	gi|646776514|gb|KK961670.1|	1177830-1190106	20	9.6
ACYPI004488-RA	gi|646750643|gb|KK962028.1|	695252-695933	21	7.5
ACYPI004665-RA	gi|646775968|gb|KK961700.1|	693276-693463	20	8.2
ACYPI005847-RA	gi|646743772|gb|KK962481.1|	62948-63865	22	8.9
ACYPI006288-RA	gi|646781809|gb|KK961509.1|	3570858-3571078	19	8.6
ACYPI006701-RA	gi|646738812|gb|KK962939.1|	144737-144991	20	8.1
ACYPI006709-RA	gi|646778307|gb|KK961605.1|	1835067-1836838	17	8.7
ACYPI008566-RA	gi|646778565|gb|KK961598.1|	1782564-1783070	19	7.8
ACYPI008963-RA	gi|646781013|gb|KK961533.1|	1450509-1456424	18	7.6
ACYPI009215-RA	gi|646779375|gb|KK961575.1|	3057633-3059346	22	9.3
ACYPI009485-RA	gi|646768717|gb|KK961805.1|	907602-912094	20	8
ACYPI065069-RA	gi|646775842|gb|KK961706.1|	2000255-2000509	22	9
ACYPI070165-RA	gi|646767670|gb|KK961827.1|	1345466-1346556	21	5.9
ACYPI082349-RA	gi|646779375|gb|KK961575.1|	3300236-3301211	22	9.3
ACYPI085466-RA	gi|646781013|gb|KK961533.1|	1935447-1935993	18	7.6
ACYPI30062-RA	gi|646747032|gb|KK962248.1|	586226-587899	20	8.7
ACYPI30384-RA	gi|646776184|gb|KK961688.1|	1410490-1412307	21	9.6
ACYPI34794-RA	gi|646780222|gb|KK961554.1|	3535165-3537207	20	9.7
ACYPI45212-RA	gi|646766985|gb|KK961844.1|	272915-276205	21	9.7
ACYPI56663-RA	gi|646779186|gb|KK961580.1|	1576022-1576693	20	8.6
ACYPI000032-RA	gi|646777416|gb|KK961632.1|	3397743-3397979	22	8.8
ACYPI000077-RA	gi|646782288|gb|KK961496.1|	6774498-6774864	21	9.7
ACYPI004286-RA	gi|646777842|gb|KK961618.1|	2244936-2245562	19	8.9
ACYPI004467-RA	gi|646777802|gb|KK961619.1|	1408031-1408225	24	9
ACYPI006216-RA	gi|646771352|gb|KK961768.1|	2086806-2087568	21	9.6
ACYPI007410-RA	gi|646781118|gb|KK961530.1|	3877970-3879272	21	10
ACYPI008728-RA	gi|646746897|gb|KK962257.1|	1016643-1021994	21	9.2
ACYPI009054-RA	gi|646781772|gb|KK961510.1|	3141405-3144573	20	9.2
ACYPI33427-RA	gi|646780311|gb|KK961552.1|	1209166-1210284	21	9.7
ACYPI36267-RA	gi|646767439|gb|KK961832.1|	1756994-1758341	23	10

ACYPI001107-RA	gi|646744686|gb|KK962414.1|	471483-472831	12	7.3
ACYPI001168-RA	gi|646747627|gb|KK962204.1|	333129-334935	18	7.5
ACYPI002448-RA	gi|646777108|gb|KK961645.1|	288723-288916	17	6.5
ACYPI002830-RA	gi|646745959|gb|KK962321.1|	319481-325472	20	6.9
ACYPI006688-RA	gi|646750389|gb|KK962041.1|	149189-149917	21	9.5
ACYPI006818-RA	gi|646746991|gb|KK962251.1|	338254-350930	22	9.2
ACYPI008276-RA	gi|646780441|gb|KK961549.1|	2399930-2401003	21	9.5
ACYPI008732-RA	gi|646615001|gb|KK973558.1|	2766-4377	11	5.5
ACYPI009383-RA	gi|646747627|gb|KK962204.1|	340280-341914	18	7.5
ACYPI064212-RA	gi|646745186|gb|KK962377.1|	379820-385376	21	9.8
ACYPI085389-RA	gi|646758845|gb|KK961921.1|	1865546-1867208	22	9.5
ACYPI088323-RA	gi|646561926|gb|KK983838.1|	1126-2214	5.4	4
ACYPI48553-RA	gi|646740116|gb|KK962810.1|	35253-38488	12	9.4
ACYPI49124-RA	gi|646748185|gb|KK962165.1|	658433-660314	22	9.9
ACYPI55202-RA	gi|646777729|gb|KK961621.1|	639315-639761	21	9.8
ACYPI000070-RA	gi|646746355|gb|KK962294.1|	502821-503397	22	10
ACYPI001765-RA	gi|646740158|gb|KK962805.1|	312127-312829	24	6.5
ACYPI005094-RA	gi|646769901|gb|KK961786.1|	1012758-1015258	18	6.7
ACYPI007216-RA	gi|646771951|gb|KK961762.1|	1530351-1534902	21	8.3
ACYPI007455-RA	gi|646748057|gb|KK962174.1|	180595-183157	14	7.3
ACYPI008812-RA	gi|646779006|gb|KK961585.1|	523466-523710	20	9.9
ACYPI009280-RA	gi|646779141|gb|KK961581.1|	629196-632506	21	9.1
ACYPI009455-RA	gi|646553387|gb|KK985551.1|	7023-7449	21	7.5
ACYPI062725-RA	gi|646737740|gb|KK963047.1|	65990-66528	15	7.7
ACYPI065062-RA	gi|646748002|gb|KK962178.1|	1636443-1636985	22	6.3
ACYPI068524-RA	gi|646708391|gb|KK965545.1|	26244-27841	17	8.4
ACYPI080990-RA	gi|646779662|gb|KK961568.1|	3123310-3124661	20	9.3
ACYPI082566-RA	gi|646781313|gb|KK961524.1|	3289110-3290164	19	8.5
ACYPI21772-RA	gi|646748728|gb|KK962132.1|	430380-432366	19	8.1
ACYPI23206-RA	gi|646779785|gb|KK961565.1|	3090874-3094975	18	8.9

ACYPI23210-RA	gi|646776389|gb|KK961678.1|	5400-6729	22	9.5
ACYPI000029-RA	gi|646779741|gb|KK961566.1|	2338953-2344250	20	8.5
ACYPI000162-RA	gi|646662646|gb|KK967934.1|	12970-13110	15	5.2
ACYPI000530-RA	gi|646746581|gb|KK962279.1|	274319-276250	21	9.1
ACYPI003545-RA	gi|646773423|gb|KK961749.1|	383372-387975	19	8.1
ACYPI007961-RA	gi|646776929|gb|KK961653.1|	385138-387270	17	8.5
ACYPI008003-RA	gi|646768406|gb|KK961812.1|	159592-167802	20	9.6
ACYPI072921-RA	gi|646744057|gb|KK962460.1|	209770-210606	14	6.5
ACYPI087735-RA	gi|646747076|gb|KK962245.1|	163656-164023	23	9.6
ACYPI34996-RA	gi|646767230|gb|KK961837.1|	572856-578093	19	5.4
ACYPI35828-RA	gi|646604300|gb|KK975617.1|	8068-9285	13	4.9
ACYPI51708-RA	gi|646751544|gb|KK961986.1|	658225-659944	22	6
ACYPI52973-RA	gi|646781732|gb|KK961511.1|	4356009-4357560	22	9.8
ACYPI000734-RA	gi|646782211|gb|KK961498.1|	1230895-1231218	20	9
ACYPI001374-RA	gi|646781443|gb|KK961520.1|	562095-567388	20	8.3
ACYPI001547-RA	gi|646782357|gb|KK961494.1|	9767936-9768929	21	9.2
ACYPI001612-RA	gi|646744957|gb|KK962394.1|	752332-754206	21	9.8
ACYPI001780-RA	gi|646733583|gb|KK963518.1|	57806-60561	20	9.2
ACYPI002098-RA	gi|646743690|gb|KK962487.1|	21568-23753	20	10
ACYPI002622-RA	gi|646740073|gb|KK962814.1|	61569-62315	18	8.3
ACYPI002989-RA	gi|646780827|gb|KK961539.1|	408655-410609	20	9.4
ACYPI004089-RA	gi|646753527|gb|KK961953.1|	1850986-1851761	24	9.2
ACYPI004312-RA	gi|646776647|gb|KK961663.1|	2163546-2164976	23	9.2
ACYPI004530-RA	gi|646775507|gb|KK961723.1|	1563690-1565762	20	9.8
ACYPI004573-RA	gi|646781601|gb|KK961515.1|	3544024-3547790	19	8.6
ACYPI005067-RA	gi|646737959|gb|KK963025.1|	387330-389046	19	7.9
ACYPI005769-RA	gi|646755095|gb|KK961939.1|	396175-397628	15	8.9
ACYPI006028-RA	gi|646780978|gb|KK961534.1|	2496029-2498313	20	8
ACYPI006243-RA	gi|646778840|gb|KK961590.1|	736759-748890	20	9.9
ACYPI006485-RA	gi|646777288|gb|KK961638.1|	133233-135926	22	6.1

ACYPI007079-RA	gi|646762191|gb|KK961910.1|	627589-628025	18	7.1
ACYPI007246-RA	gi|646777159|gb|KK961643.1|	36304-36882	14	6.5
ACYPI007905-RA	gi|646774530|gb|KK961743.1|	1972317-1972705	20	9.8
ACYPI008117-RA	gi|646779375|gb|KK961575.1|	1908575-1911292	22	9.3
ACYPI008679-RA	gi|646740073|gb|KK962814.1|	98929-99906	18	8.3
ACYPI008967-RA	gi|646782211|gb|KK961498.1|	1248851-1250437	20	9
ACYPI080074-RA	gi|646733743|gb|KK963499.1|	22195-23083	18	9.3
ACYPI23840-RA	gi|646782043|gb|KK961502.1|	5398473-5399100	20	9.2
ACYPI38684-RA	gi|646724743|gb|KK964765.1|	63604-65382	16	8.3
ACYPI46812-RA	gi|646727370|gb|KK964352.1|	77171-77878	11	6.7
ACYPI47548-RA	gi|646781421|gb|KK961521.1|	4213757-4218648	21	8.7
ACYPI54344-RA	gi|646756634|gb|KK961930.1|	827075-827878	17	9.4
ACYPI000250-RA	gi|646747586|gb|KK962207.1|	186339-189708	20	8.8
ACYPI000666-RA	gi|646780574|gb|KK961546.1|	958171-961542	20	9.9
ACYPI002524-RA	gi|646757520|gb|KK961926.1|	1355277-1356642	20	7.4
ACYPI003541-RA	gi|646780889|gb|KK961537.1|	2915802-2917548	22	10
ACYPI003975-RA	gi|646747884|gb|KK962186.1|	1230599-1235599	22	11
ACYPI004424-RA	gi|646746216|gb|KK962304.1|	72892-74095	########	33
ACYPI006388-RA	gi|646753339|gb|KK961955.1|	784027-785440	22	11
ACYPI006956-RA	gi|646775867|gb|KK961705.1|	755090-755962	23	10
ACYPI007268-RA	gi|646763164|gb|KK961904.1|	1395764-1410244	22	10
ACYPI008271-RA	gi|646782168|gb|KK961499.1|	1742558-1748485	21	9.4
ACYPI008947-RA	gi|646764801|gb|KK961898.1|	543144-545357	20	9.7
ACYPI009777-RA	gi|646763164|gb|KK961904.1|	10574-15117	22	10
ACYPI010168-RA	gi|646732252|gb|KK963701.1|	154787-155046	19	8
ACYPI066376-RA	gi|646780441|gb|KK961549.1|	595062-597026	21	9.5
ACYPI066519-RA	gi|646775221|gb|KK961739.1|	1469141-1470327	20	10
ACYPI25564-RA	gi|646766868|gb|KK961847.1|	1258944-1263539	21	9.9
ACYPI26413-RA	gi|646747424|gb|KK962220.1|	232191-232687	22	5.7
ACYPI34286-RA	gi|646758845|gb|KK961921.1|	1940102-1941140	22	9.5

ACYPI50440-RA	gi|646770251|gb|KK961781.1|	344525-345495	20	9.6
ACYPI51884-RA	gi|646635790|gb|KK969861.1|	15244-15708	14	4.4
