# Supplementary material for: The X Chromosome of Hemipteran Insects: Conservation, Dosage Compensation and Sex-Biased Expression
Source: Genome Biol Evol. 2015 Nov 10;7(12):3259–68. doi: 10.1093/gbe/evv215 (PMC4700948; doi:10.1093/gbe/evv215)
Supplement: Supplementary Data [file supp_evv215_suppl_data.zip › S2 Data (rev) AP-HV (X).pdf]

AP	HV	gene	covF	covM
ACYPI003888-RA	gi|646751458|gb|KK961989.1|	565234-565510	21	5.1
ACYPI007680-RA	gi|646762797|gb|KK961906.1|	636444-637871	19	5.6
ACYPI49993-RA	gi|646778517|gb|KK961599.1|	2462907-2463559	20	9.6
ACYPI006784-RA	gi|646767307|gb|KK961835.1|	322881-327857	20	9
ACYPI005743-RA	gi|646729045|gb|KK964126.1|	147961-149410	20	11
ACYPI007405-RA	gi|646782334|gb|KK961495.1|	2825622-2826889	21	9
ACYPI001091-RA	gi|646749945|gb|KK962065.1|	1300714-1302205	22	6.1
ACYPI002987-RA	gi|646743352|gb|KK962510.1|	516250-516623	20	5.4
ACYPI004908-RA	gi|646766540|gb|KK961858.1|	1265743-1266136	21	8.5
ACYPI006808-RA	gi|646747802|gb|KK962192.1|	207843-209426	19	7.8
ACYPI006239-RA	gi|646780858|gb|KK961538.1|	3464449-3465340	21	10
ACYPI001170-RA	gi|646745197|gb|KK962376.1|	392665-396975	21	8.9
ACYPI003057-RA	gi|646775884|gb|KK961704.1|	1001963-1002652	20	7.6
ACYPI003598-RA	gi|646776429|gb|KK961676.1|	1452461-1453296	20	9.9
ACYPI001110-RA	gi|646781849|gb|KK961508.1|	2570548-2571249	17	8
ACYPI001535-RA	gi|646778767|gb|KK961592.1|	778804-786113	21	5.4
ACYPI005365-RA	gi|646748310|gb|KK962157.1|	61750-65290	23	6.3
ACYPI000018-RA	gi|646740098|gb|KK962812.1|	577984-580281	20	10
ACYPI001128-RA	gi|646776113|gb|KK961692.1|	147032-149028	21	9.6
ACYPI007100-RA	gi|646737122|gb|KK963108.1|	49598-50074	17	9
ACYPI008357-RA	gi|646781183|gb|KK961528.1|	653951-659326	20	9.5
ACYPI073873-RA	gi|646737528|gb|KK963067.1|	115818-116099	20	6.1
ACYPI37088-RA	gi|646750021|gb|KK962061.1|	396937-397184	16	7.5
ACYPI007673-RA	gi|646767850|gb|KK961823.1|	1162141-1163092	21	9.8
ACYPI001023-RA	gi|646575796|gb|KK981232.1|	19329-20239	21	10
ACYPI001182-RA	gi|646777698|gb|KK961622.1|	392713-393351	16	7.6
ACYPI002925-RA	gi|646769275|gb|KK961796.1|	2835489-2835752	22	5.6
ACYPI004986-RA	gi|646775968|gb|KK961700.1|	1333259-1333531	20	8.2
ACYPI005626-RA	gi|646749945|gb|KK962065.1|	1749651-1756478	22	6.1

ACYPI005729-RA	gi|646743084|gb|KK962530.1|	163288-164055	24	6.2
ACYPI006964-RA	gi|646745091|gb|KK962384.1|	163254-164426	19	7.9
ACYPI009396-RA	gi|646742343|gb|KK962588.1|	315792-316641	20	9.4
ACYPI003290-RA	gi|646747226|gb|KK962234.1|	391655-391992	12	7.8
ACYPI008213-RA	gi|646742726|gb|KK962557.1|	26389-29144	20	9.1
ACYPI009257-RA	gi|646630362|gb|KK970809.1|	4347-5074	12	7.2
ACYPI010103-RA	gi|646770477|gb|KK961778.1|	439289-439541	21	9.1
ACYPI072994-RA	gi|646773721|gb|KK961747.1|	1828280-1832506	21	9.2
ACYPI008719-RA	gi|646767439|gb|KK961832.1|	843293-852153	23	10
ACYPI009068-RA	gi|646780574|gb|KK961546.1|	272205-273668	20	9.9
ACYPI56606-RA	gi|646731712|gb|KK963779.1|	95457-96872	18	7.2
ACYPI000227-RA	gi|646779066|gb|KK961583.1|	1212691-1214372	19	7.9
ACYPI001125-RA	gi|646781564|gb|KK961516.1|	285738-290292	23	9.4
ACYPI001932-RA	gi|646779066|gb|KK961583.1|	1089073-1093749	19	7.9
ACYPI005359-RA	gi|646775540|gb|KK961721.1|	948316-948798	17	8.1
ACYPI006095-RA	gi|646775471|gb|KK961725.1|	1322550-1323354	20	10
ACYPI006584-RA	gi|646778983|gb|KK961586.1|	9773-9973	20	9.9
ACYPI073834-RA	gi|646747482|gb|KK962215.1|	364845-365155	21	9.2
ACYPI003257-RA	gi|646752294|gb|KK961969.1|	871288-871770	23	9.8
ACYPI004037-RA	gi|646769221|gb|KK961797.1|	737439-737674	19	9.6
ACYPI006164-RA	gi|646781443|gb|KK961520.1|	1794021-1795864	20	8.3
ACYPI073036-RA	gi|646781443|gb|KK961520.1|	2119588-2123878	20	8.3
ACYPI50514-RA	gi|646775312|gb|KK961734.1|	116824-117309	17	7.9
ACYPI000704-RA	gi|646777570|gb|KK961626.1|	1443587-1444127	18	7.5
ACYPI004501-RA	gi|646777495|gb|KK961629.1|	1568247-1568438	19	8.7
ACYPI006418-RA	gi|646745197|gb|KK962376.1|	167157-167651	21	8.9
ACYPI001585-RA	gi|646778840|gb|KK961590.1|	2503479-2503992	20	9.9
ACYPI004834-RA	gi|646747175|gb|KK962238.1|	551723-555205	20	8.4
ACYPI009262-RA	gi|646780010|gb|KK961559.1|	459098-459345	22	10
ACYPI063239-RA	gi|646746868|gb|KK962259.1|	152215-158740	21	10

ACYPI067185-RA	gi|646780723|gb|KK961542.1|	3698178-3699388	21	9.4
ACYPI001752-RA	gi|646776647|gb|KK961663.1|	2654761-2658004	23	9.2
ACYPI002361-RA	gi|646747884|gb|KK962186.1|	1107153-1108272	22	11
ACYPI002482-RA	gi|646768294|gb|KK961814.1|	1081772-1083971	23	10
ACYPI003002-RA	gi|646781379|gb|KK961522.1|	572075-572243	22	7.6
ACYPI007409-RA	gi|646776368|gb|KK961679.1|	674816-680837	21	9.7
ACYPI085768-RA	gi|646750643|gb|KK962028.1|	517452-518890	21	7.5
ACYPI087848-RA	gi|646768294|gb|KK961814.1|	1050458-1058358	23	10
ACYPI007048-RA	gi|646777108|gb|KK961645.1|	1400759-1401465	17	6.5
ACYPI008482-RA	gi|646748142|gb|KK962168.1|	492640-493026	20	9.3
ACYPI066762-RA	gi|646751325|gb|KK961994.1|	843976-844912	12	7.8
ACYPI51540-RA	gi|646779141|gb|KK961581.1|	1018864-1019995	21	9.1
ACYPI000654-RA	gi|646752919|gb|KK961960.1|	1667440-1669253	20	9
ACYPI001424-RA	gi|646749051|gb|KK962113.1|	444541-445261	19	8.4
ACYPI008222-RA	gi|646775842|gb|KK961706.1|	651342-651921	22	9
ACYPI46077-RA	gi|646743886|gb|KK962473.1|	530155-530972	21	9.3
ACYPI005622-RA	gi|646765948|gb|KK961882.1|	282593-283083	18	7.3
ACYPI069554-RA	gi|646777877|gb|KK961617.1|	1226040-1227744	19	8.1
ACYPI086553-RA	gi|646778803|gb|KK961591.1|	1046979-1049414	15	7.8
ACYPI46554-RA	gi|646776128|gb|KK961691.1|	877478-880898	21	9.2
ACYPI003186-RA	gi|646776447|gb|KK961675.1|	1326802-1327313	22	9.4
ACYPI005308-RA	gi|646775842|gb|KK961706.1|	1240797-1243932	22	9
ACYPI000065-RA	gi|646776514|gb|KK961670.1|	230372-233696	20	9.6
ACYPI000222-RA	gi|646751350|gb|KK961993.1|	255525-256015	20	7.6
ACYPI000885-RA	gi|646777363|gb|KK961635.1|	1760438-1760718	18	9.2
ACYPI002123-RA	gi|646778903|gb|KK961588.1|	3490396-3493151	23	10
ACYPI003998-RA	gi|646749464|gb|KK962091.1|	437728-438264	18	8.8
ACYPI004750-RA	gi|646782288|gb|KK961496.1|	3414244-3419062	21	9.7
ACYPI005363-RA	gi|646775789|gb|KK961709.1|	117377-119088	20	9.6
ACYPI006178-RA	gi|646740420|gb|KK962773.1|	256629-257094	20	7.5

ACYPI006875-RA	gi|646748474|gb|KK962147.1|	177934-179046	20	9
ACYPI007058-RA	gi|646754106|gb|KK961947.1|	412407-412747	16	7.6
ACYPI008050-RA	gi|646780658|gb|KK961544.1|	1414887-1422236	19	8.6
ACYPI064487-RA	gi|646576310|gb|KK981127.1|	8295-9048	10	5.7
ACYPI084991-RA	gi|646779898|gb|KK961562.1|	598165-599334	19	9.3
ACYPI23999-RA	gi|646782288|gb|KK961496.1|	1884041-1885841	21	9.7
ACYPI47651-RA	gi|646749098|gb|KK962110.1|	86178-86526	20	7.7
ACYPI002850-RA	gi|646781873|gb|KK961507.1|	1603623-1604415	20	9.1
ACYPI004524-RA	gi|646774067|gb|KK961745.1|	1351317-1352506	17	9.3
ACYPI007248-RA	gi|646776447|gb|KK961675.1|	1850801-1851961	22	9.4
ACYPI008429-RA	gi|646779141|gb|KK961581.1|	807247-807874	21	9.1
ACYPI009072-RA	gi|646742541|gb|KK962572.1|	570529-572677	19	10
ACYPI062481-RA	gi|646744140|gb|KK962454.1|	471887-472291	21	9.3
ACYPI081939-RA	gi|646777416|gb|KK961632.1|	3445646-3447549	22	8.8
ACYPI27655-RA	gi|646775807|gb|KK961708.1|	1508426-1510162	21	8.6
ACYPI007773-RA	gi|646778245|gb|KK961607.1|	472139-474943	18	6.8
ACYPI36831-RA	gi|646774530|gb|KK961743.1|	434342-435930	20	9.8
ACYPI000837-RA	gi|646747424|gb|KK962220.1|	1360305-1360572	22	5.7
ACYPI001120-RA	gi|646747884|gb|KK962186.1|	744594-745699	22	11
ACYPI001316-RA	gi|646750889|gb|KK962017.1|	820863-825686	22	6.1
ACYPI003198-RA	gi|646766833|gb|KK961848.1|	318625-322634	17	7
ACYPI003565-RA	gi|646780889|gb|KK961537.1|	4077216-4077409	22	10
ACYPI005123-RA	gi|646766833|gb|KK961848.1|	382584-385833	17	7
ACYPI005583-RA	gi|646768447|gb|KK961811.1|	272092-272353	22	9.7
ACYPI007021-RA	gi|646781313|gb|KK961524.1|	1798838-1800677	19	8.5
ACYPI009070-RA	gi|646739555|gb|KK962870.1|	121424-124548	27	18
ACYPI009978-RA	gi|646742685|gb|KK962560.1|	402147-405466	21	9.5
ACYPI001219-RA	gi|646777545|gb|KK961627.1|	1492777-1493022	21	9.9
ACYPI005028-RA	gi|646774530|gb|KK961743.1|	379038-381487	20	9.8
ACYPI065028-RA	gi|646769275|gb|KK961796.1|	198303-202792	22	5.6

ACYPI081557-RA	gi|646781628|gb|KK961514.1|	2618330-2628198	23	9
ACYPI087266-RA	gi|646781344|gb|KK961523.1|	2282143-2282888	21	8.9
ACYPI38268-RA	gi|646766650|gb|KK961854.1|	1179249-1192510	22	10
ACYPI006283-RA	gi|646781849|gb|KK961508.1|	1864496-1865138	17	8
ACYPI008151-RA	gi|646775312|gb|KK961734.1|	683458-684813	17	7.9
ACYPI008396-RA	gi|646782168|gb|KK961499.1|	3693910-3695458	21	9.4
ACYPI002170-RA	gi|646770477|gb|KK961778.1|	416606-419512	21	9.1
ACYPI003298-RA	gi|646745612|gb|KK962344.1|	198103-200309	20	8.2
ACYPI009534-RA	gi|646750770|gb|KK962022.1|	405213-405446	14	7.7
ACYPI009542-RA	gi|646740395|gb|KK962776.1|	232789-233176	16	6.3
ACYPI002522-RA	gi|646775312|gb|KK961734.1|	1168349-1168544	17	7.9
ACYPI005093-RA	gi|646778767|gb|KK961592.1|	1308647-1314006	21	5.4
ACYPI006993-RA	gi|646746974|gb|KK962252.1|	141680-142583	23	6.1
ACYPI070768-RA	gi|646775451|gb|KK961726.1|	2179828-2183485	20	5.6
ACYPI086900-RA	gi|646738359|gb|KK962984.1|	82512-83645	16	11
ACYPI28461-RA	gi|646739806|gb|KK962843.1|	33498-34164	17	12
ACYPI34001-RA	gi|646780953|gb|KK961535.1|	1124455-1127576	20	9.8
ACYPI000102-RA	gi|646779451|gb|KK961573.1|	833738-834143	20	8.9
ACYPI001323-RA	gi|646782009|gb|KK961503.1|	1767752-1770875	15	7.5
ACYPI001504-RA	gi|646732909|gb|KK963610.1|	172018-172978	13	6.7
ACYPI003788-RA	gi|646763164|gb|KK961904.1|	1005243-1005551	22	10
ACYPI003914-RA	gi|646747884|gb|KK962186.1|	479971-482520	22	11
ACYPI004515-RA	gi|646749188|gb|KK962105.1|	110767-112558	18	7.9
ACYPI004605-RA	gi|646775312|gb|KK961734.1|	912141-913216	17	7.9
ACYPI008308-RA	gi|646779412|gb|KK961574.1|	2285204-2286864	21	5.4
ACYPI35076-RA	gi|646771448|gb|KK961767.1|	865889-867406	21	9.8
ACYPI001030-RA	gi|646766482|gb|KK961860.1|	547849-549145	17	6.7
ACYPI001668-RA	gi|646778632|gb|KK961596.1|	1957857-1958420	21	9.2
ACYPI001797-RA	gi|646735017|gb|KK963352.1|	323473-324256	22	5.9
ACYPI002929-RA	gi|646775558|gb|KK961720.1|	1592801-1594423	20	9.4

ACYPI006758-RA	gi|646765991|gb|KK961880.1|	303369-311399	16	7
ACYPI007368-RA	gi|646775383|gb|KK961730.1|	73349-77881	19	8.7
ACYPI009418-RA	gi|646741719|gb|KK962642.1|	246857-247577	21	8.9
ACYPI061765-RA	gi|646778903|gb|KK961588.1|	4304692-4305494	23	10
ACYPI072215-RA	gi|646752431|gb|KK961967.1|	754979-763797	20	5.6
ACYPI080735-RA	gi|646776477|gb|KK961673.1|	930903-932782	22	9
ACYPI088812-RA	gi|646758845|gb|KK961921.1|	1952809-1956834	22	9.5
ACYPI000534-RA	gi|646778336|gb|KK961604.1|	2203948-2204913	21	10
ACYPI000538-RA	gi|646775635|gb|KK961716.1|	1847031-1850055	22	10
ACYPI002188-RA	gi|646717826|gb|KK965143.1|	59739-60556	13	7
ACYPI002286-RA	gi|646782334|gb|KK961495.1|	4606095-4606597	21	9
ACYPI003560-RA	gi|646779375|gb|KK961575.1|	1835278-1835852	22	9.3
ACYPI004058-RA	gi|646767439|gb|KK961832.1|	1178391-1179361	23	10
ACYPI005000-RA	gi|646778336|gb|KK961604.1|	2454941-2457328	21	10
ACYPI006521-RA	gi|646772574|gb|KK961756.1|	1138358-1141988	21	8
ACYPI006896-RA	gi|646776514|gb|KK961670.1|	6299-6541	20	9.6
ACYPI008198-RA	gi|646778336|gb|KK961604.1|	2218866-2219711	21	10
ACYPI008847-RA	gi|646747076|gb|KK962245.1|	561257-570758	23	9.6
ACYPI009259-RA	gi|646747841|gb|KK962189.1|	462210-463390	23	10
ACYPI010034-RA	gi|646745372|gb|KK962364.1|	484917-490925	23	10
ACYPI062209-RA	gi|646750969|gb|KK962013.1|	643875-644992	16	7.4
ACYPI068208-RA	gi|646752252|gb|KK961970.1|	655390-656353	18	9
ACYPI072192-RA	gi|646751160|gb|KK962003.1|	120488-123622	22	10
ACYPI085245-RA	gi|646782276|gb|KK961497.1|	5807880-5808912	21	9.7
ACYPI25737-RA	gi|646774530|gb|KK961743.1|	311798-312848	20	9.8
ACYPI30186-RA	gi|646780270|gb|KK961553.1|	4494851-4496200	22	8.6
ACYPI48764-RA	gi|646774530|gb|KK961743.1|	52122-53962	20	9.8
ACYPI49710-RA	gi|646743886|gb|KK962473.1|	194403-196266	21	9.3
ACYPI53679-RA	gi|646725525|gb|KK964639.1|	19735-20803	27	9.8
ACYPI56077-RA	gi|646745372|gb|KK962364.1|	471123-471656	23	10

ACYPI000079-RA	gi|646780953|gb|KK961535.1|	3216707-3217447	20	9.8
ACYPI002279-RA	gi|646737056|gb|KK963115.1|	143574-143943	24	11
ACYPI004146-RA	gi|646734213|gb|KK963446.1|	26113-26702	14	8.6
ACYPI005010-RA	gi|646766086|gb|KK961876.1|	30095-31006	23	9.5
ACYPI006616-RA	gi|646777207|gb|KK961641.1|	1186273-1186463	20	8.5
ACYPI007494-RA	gi|646781443|gb|KK961520.1|	617210-622254	20	8.3
ACYPI008142-RA	gi|646781443|gb|KK961520.1|	3108402-3108976	20	8.3
ACYPI009430-RA	gi|646779186|gb|KK961580.1|	510430-510980	20	8.6
ACYPI009856-RA	gi|646751176|gb|KK962002.1|	771697-774718	21	9.6
ACYPI31012-RA	gi|646775842|gb|KK961706.1|	814033-819183	22	9
ACYPI42631-RA	gi|646781118|gb|KK961530.1|	3832826-3835298	21	10
ACYPI55041-RA	gi|646777631|gb|KK961624.1|	1557436-1558441	20	9.3
ACYPI000787-RA	gi|646775312|gb|KK961734.1|	798851-799244	17	7.9
ACYPI000849-RA	gi|646781118|gb|KK961530.1|	3616282-3617476	21	10
ACYPI002674-RA	gi|646747754|gb|KK962195.1|	713736-714702	21	5.3
ACYPI002787-RA	gi|646777698|gb|KK961622.1|	893775-894467	16	7.6
ACYPI004152-RA	gi|646777991|gb|KK961614.1|	1627252-1627683	18	7.8
ACYPI004966-RA	gi|646780441|gb|KK961549.1|	1007895-1009624	21	9.5
ACYPI005060-RA	gi|646782288|gb|KK961496.1|	7810738-7812362	21	9.7
ACYPI006664-RA	gi|646570180|gb|KK982274.1|	3319-3526	5.4	2.9
ACYPI007199-RA	gi|646779785|gb|KK961565.1|	710503-712060	18	8.9
ACYPI008467-RA	gi|646781659|gb|KK961513.1|	5065619-5066012	22	9.8
ACYPI008499-RA	gi|646773903|gb|KK961746.1|	1539477-1540715	20	8.9
ACYPI060019-RA	gi|646772786|gb|KK961754.1|	758192-759096	22	9.6
ACYPI080017-RA	gi|646778448|gb|KK961601.1|	1793756-1798914	19	8.3
ACYPI25540-RA	gi|646781690|gb|KK961512.1|	2273116-2273984	21	8.8
ACYPI25700-RA	gi|646777288|gb|KK961638.1|	13878-16766	22	6.1
ACYPI002141-RA	gi|646605298|gb|KK975395.1|	885-1531	48	23
ACYPI002218-RA	gi|646777955|gb|KK961615.1|	2515443-2517007	21	10
ACYPI002433-RA	gi|646740304|gb|KK962788.1|	91622-92181	24	9.6

ACYPI006909-RA	gi|646779006|gb|KK961585.1|	911013-911385	20	9.9
ACYPI007005-RA	gi|646778112|gb|KK961611.1|	1163234-1164019	22	10
ACYPI008769-RA	gi|646782288|gb|KK961496.1|	227922-228617	21	9.7
ACYPI009420-RA	gi|646743377|gb|KK962508.1|	68993-70281	19	8
ACYPI41067-RA	gi|646779621|gb|KK961569.1|	1066113-1068382	16	7.3
ACYPI53334-RA	gi|646734398|gb|KK963425.1|	25535-27788	22	5.9
