# Supplementary material for: The X Chromosome of Hemipteran Insects: Conservation, Dosage Compensation and Sex-Biased Expression
Source: Genome Biol Evol. 2015 Nov 10;7(12):3259–68. doi: 10.1093/gbe/evv215 (PMC4700948; doi:10.1093/gbe/evv215)
Supplement: Supplementary Data [file supp_evv215_suppl_data.zip › S2 Data (rev) AP-OF (A).pdf]

AP	OF	gene	covF	covM
ACYPI000427-RA	gi|641588267|gb|KK854008.1|	109932-117676	10	40
ACYPI001670-RA	gi|641570727|gb|KK856270.1|	89280-90273	10	35
ACYPI009967-RA	gi|641576570|gb|KK854895.1|	216499-218455	9.9	40
ACYPI065154-RA	gi|641576570|gb|KK854895.1|	269385-270012	9.9	40
ACYPI006033-RA	gi|641586335|gb|KK854256.1|	422890-424570	10	38
ACYPI53104-RA	gi|641576074|gb|KK854988.1|	129922-131166	12	46
ACYPI006974-RA	gi|641587783|gb|KK854060.1|	634998-640549	12	40
ACYPI007586-RA	gi|641557821|gb|KK861408.1|	3789-3985	8.2	34
ACYPI007765-RA	gi|641577837|gb|KK854685.1|	344389-351456	11	42
ACYPI37346-RA	gi|641570930|gb|KK856211.1|	29670-30823	11	39
ACYPI003220-RA	gi|641585423|gb|KK854400.1|	356755-357395	11	36
ACYPI007117-RA	gi|641585446|gb|KK854396.1|	278808-282137	11	39
ACYPI000716-RA	gi|641587757|gb|KK854063.1|	405410-406408	11	38
ACYPI004513-RA	gi|641587757|gb|KK854063.1|	367709-371256	11	38
ACYPI006979-RA	gi|641571612|gb|KK856022.1|	49694-51333	12	43
ACYPI007210-RA	gi|641576315|gb|KK854940.1|	210808-211432	9.9	40
ACYPI008842-RA	gi|641571612|gb|KK856022.1|	43710-46059	12	43
ACYPI005053-RA	gi|641576920|gb|KK854836.1|	127407-127725	11	37
ACYPI003726-RA	gi|641570404|gb|KK856365.1|	43240-44478	10	19
ACYPI003815-RA	gi|641586726|gb|KK854199.1|	378189-379306	11	41
ACYPI56628-RA	gi|641577815|gb|KK854689.1|	144827-147314	11	43
ACYPI003304-RA	gi|641577071|gb|KK854812.1|	293606-294202	13	38
ACYPI000201-RA	gi|641570573|gb|KK856315.1|	134460-142538	10	45
ACYPI004675-RA	gi|641571612|gb|KK856022.1|	152835-153180	12	43
ACYPI28534-RA	gi|641587652|gb|KK854077.1|	60003-61827	10	38
ACYPI001807-RA	gi|641587765|gb|KK854062.1|	759427-760835	10	41
ACYPI006279-RA	gi|641582763|gb|KK854531.1|	409708-413386	10	39
ACYPI010028-RA	gi|641587424|gb|KK854104.1|	61508-61745	11	41
ACYPI31612-RA	gi|641585510|gb|KK854386.1|	214551-216007	11	20

ACYPI006611-RA	gi|641578056|gb|KK854648.1|	66169-67920	11	41
ACYPI006649-RA	gi|641573007|gb|KK855666.1|	49902-52377	11	44
ACYPI087820-RA	gi|641584728|gb|KK854460.1|	135492-136680	9.5	37
ACYPI009254-RA	gi|641574339|gb|KK855345.1|	63733-65232	10	44
ACYPI003976-RA	gi|641574433|gb|KK855322.1|	220311-230259	11	44
ACYPI006101-RA	gi|641576472|gb|KK854913.1|	91163-95029	11	38
ACYPI002250-RA	gi|641586535|gb|KK854227.1|	710833-711880	10	39
ACYPI065159-RA	gi|641580504|gb|KK854565.1|	86244-86583	11	43
ACYPI001710-RA	gi|641577913|gb|KK854671.1|	196722-196969	9.2	40
ACYPI085022-RA	gi|641574668|gb|KK855267.1|	22993-24442	10	38
ACYPI000030-RA	gi|641575946|gb|KK855016.1|	210994-220495	9.5	37
ACYPI001328-RA	gi|641574022|gb|KK855414.1|	148193-148454	11	33
ACYPI004428-RA	gi|641575104|gb|KK855179.1|	38720-43696	10	35
ACYPI009462-RA	gi|641570523|gb|KK856328.1|	178143-181150	12	47
ACYPI007861-RA	gi|641572098|gb|KK855900.1|	133279-136173	9.5	43
ACYPI000265-RA	gi|641582439|gb|KK854536.1|	33343-35639	11	36
ACYPI001324-RA	gi|641564334|gb|KK858483.1|	3716-4056	5.2	18
ACYPI003206-RA	gi|641584835|gb|KK854453.1|	421708-422987	8.3	36
ACYPI002547-RA	gi|641570271|gb|KK856405.1|	24050-24944	9.8	38
ACYPI085714-RA	gi|641587235|gb|KK854127.1|	305451-305676	10	29
ACYPI002351-RA	gi|641575549|gb|KK855090.1|	168413-169535	10	38
ACYPI010003-RA	gi|641586040|gb|KK854300.1|	285943-286616	10	38
ACYPI081484-RA	gi|641587287|gb|KK854120.1|	403433-405091	9.9	37
ACYPI069803-RA	gi|641585871|gb|KK854327.1|	418861-423621	11	38
ACYPI007278-RA	gi|641587937|gb|KK854042.1|	179829-181404	11	42
ACYPI008351-RA	gi|641566809|gb|KK857526.1|	41927-42113	9.8	22
ACYPI000446-RA	gi|641577772|gb|KK854697.1|	258940-261083	10	39
ACYPI001096-RA	gi|641587652|gb|KK854077.1|	500085-502402	10	38
ACYPI001146-RA	gi|641573143|gb|KK855633.1|	115511-115831	9.9	36
ACYPI002411-RA	gi|641572569|gb|KK855781.1|	89141-89669	11	39

ACYPI003035-RA	gi|641586066|gb|KK854296.1|	266109-267407	9.4	37
ACYPI007471-RA	gi|641578330|gb|KK854601.1|	88172-92177	11	38
ACYPI066985-RA	gi|641587256|gb|KK854124.1|	411199-416738	9.7	40
ACYPI068332-RA	gi|641588258|gb|KK854009.1|	307680-309062	11	36
ACYPI21107-RA	gi|641571712|gb|KK856000.1|	161716-164269	10	39
ACYPI003858-RA	gi|641585350|gb|KK854411.1|	254172-254562	11	38
ACYPI005793-RA	gi|641571308|gb|KK856106.1|	33544-36369	8.9	36
ACYPI006102-RA	gi|641575893|gb|KK855025.1|	256060-256351	12	21
ACYPI002300-RA	gi|641574000|gb|KK855418.1|	122043-122918	10	36
ACYPI007522-RA	gi|641569705|gb|KK856587.1|	64456-65046	9.4	37
ACYPI009872-RA	gi|641577905|gb|KK854672.1|	294047-295950	10	37
ACYPI003422-RA	gi|641573394|gb|KK855572.1|	59363-61680	11	40
ACYPI007136-RA	gi|641586817|gb|KK854187.1|	649102-651792	9.3	39
ACYPI004157-RA	gi|641587066|gb|KK854149.1|	646487-647454	11	40
ACYPI006090-RA	gi|641576906|gb|KK854838.1|	266061-266293	9.4	37
ACYPI007973-RA	gi|641576906|gb|KK854838.1|	228206-229309	9.4	37
ACYPI009841-RA	gi|641568414|gb|KK856984.1|	15299-15516	10	39
ACYPI006484-RA	gi|641582757|gb|KK854532.1|	304361-305082	9.6	30
ACYPI002040-RA	gi|641577704|gb|KK854707.1|	309973-312437	10	38
ACYPI002689-RA	gi|641568719|gb|KK856888.1|	91364-91954	11	44
ACYPI005864-RA	gi|641565060|gb|KK858177.1|	6660-7589	9.3	40
ACYPI080213-RA	gi|641574809|gb|KK855240.1|	155778-156485	11	39
ACYPI085715-RA	gi|641574809|gb|KK855240.1|	177906-180855	11	39
ACYPI002739-RA	gi|641572987|gb|KK855671.1|	145732-146241	10	39
ACYPI002789-RA	gi|641576677|gb|KK854879.1|	55802-57216	11	39
ACYPI004656-RA	gi|641572468|gb|KK855806.1|	59116-61102	11	39
ACYPI004722-RA	gi|641585552|gb|KK854379.1|	612583-616401	14	62
ACYPI005456-RA	gi|641578069|gb|KK854646.1|	96534-97246	9.9	20
ACYPI001978-RA	gi|641585971|gb|KK854310.1|	487537-492226	10	40
ACYPI003867-RA	gi|641588136|gb|KK854020.1|	946149-946432	11	36

ACYPI007707-RA	gi|641576084|gb|KK854986.1|	166132-166697	10	38
ACYPI060585-RA	gi|641574990|gb|KK855200.1|	147776-148010	12	37
ACYPI002301-RA	gi|641577837|gb|KK854685.1|	140266-141222	11	42
ACYPI005682-RA	gi|641585946|gb|KK854315.1|	187114-188288	9.8	43
ACYPI27242-RA	gi|641587193|gb|KK854132.1|	304603-305270	11	39
ACYPI003266-RA	gi|641577188|gb|KK854792.1|	129157-130975	9.5	38
ACYPI001059-RA	gi|641586139|gb|KK854284.1|	524856-530442	10	21
ACYPI001660-RA	gi|641578144|gb|KK854633.1|	119870-120090	10	43
ACYPI008110-RA	gi|641578399|gb|KK854595.1|	238868-239570	9.3	36
ACYPI002612-RA	gi|641587660|gb|KK854076.1|	251715-252351	9.9	41
ACYPI004520-RA	gi|641587660|gb|KK854076.1|	255763-257939	9.9	41
ACYPI005299-RA	gi|641574298|gb|KK855354.1|	63805-65387	9.2	38
ACYPI001889-RA	gi|641570617|gb|KK856302.1|	119218-122343	10	33
ACYPI004437-RA	gi|641576414|gb|KK854924.1|	357999-359352	10	40
ACYPI006356-RA	gi|641578246|gb|KK854614.1|	265309-266388	9.5	39
ACYPI007547-RA	gi|641568890|gb|KK856834.1|	32880-33468	11	40
ACYPI005118-RA	gi|641576951|gb|KK854831.1|	322978-323347	8.6	40
ACYPI29847-RA	gi|641586990|gb|KK854161.1|	75938-76218	11	39
ACYPI009613-RA	gi|641572912|gb|KK855690.1|	65983-66823	9.9	38
ACYPI003473-RA	gi|641565788|gb|KK857899.1|	17376-17794	8.5	35
ACYPI004240-RA	gi|641576032|gb|KK854997.1|	156533-156960	9.9	38
ACYPI005422-RA	gi|641568399|gb|KK856989.1|	29740-30547	12	40
ACYPI35323-RA	gi|641573617|gb|KK855515.1|	25572-29338	10	38
ACYPI27507-RA	gi|641575730|gb|KK855054.1|	71259-75663	10	38
ACYPI003236-RA	gi|641572364|gb|KK855831.1|	93981-95142	11	38
ACYPI004947-RA	gi|641569836|gb|KK856546.1|	2777-2979	12	19
ACYPI009357-RA	gi|641575698|gb|KK855061.1|	287769-289345	10	39
ACYPI000271-RA	gi|641572601|gb|KK855773.1|	194693-194928	10	35
ACYPI009848-RA	gi|641586144|gb|KK854283.1|	114601-116017	10	38
ACYPI000181-RA	gi|641585905|gb|KK854321.1|	495675-496013	11	39

ACYPI008558-RA	gi|641586171|gb|KK854278.1|	475981-480606	9	39
ACYPI066772-RA	gi|641546684|gb|KK866707.1|	4-902	8.8	28
ACYPI003234-RA	gi|641558017|gb|KK861320.1|	4684-6386	13	46
ACYPI006272-RA	gi|641571786|gb|KK855980.1|	147943-150971	9.9	19
ACYPI006910-RA	gi|641575826|gb|KK855037.1|	90242-91461	10	39
ACYPI088980-RA	gi|641575826|gb|KK855037.1|	119241-120648	10	39
ACYPI089266-RA	gi|641573351|gb|KK855583.1|	78165-81875	11	41
ACYPI001379-RA	gi|641573714|gb|KK855492.1|	116037-119904	10	37
ACYPI005627-RA	gi|641576074|gb|KK854988.1|	255584-258255	12	46
ACYPI007507-RA	gi|641569999|gb|KK856492.1|	53414-54841	10	38
ACYPI003483-RA	gi|641574800|gb|KK855241.1|	150305-153185	10	37
ACYPI007926-RA	gi|641574742|gb|KK855254.1|	26008-26470	11	41
ACYPI001508-RA	gi|641573457|gb|KK855555.1|	173169-174250	9.7	38
ACYPI005340-RA	gi|641587946|gb|KK854041.1|	1099010-1100150	11	41
ACYPI007821-RA	gi|641574416|gb|KK855326.1|	137812-138323	11	38
ACYPI009721-RA	gi|641586793|gb|KK854190.1|	604512-606687	9.3	41
ACYPI53118-RA	gi|641588343|gb|KK854003.1|	935588-936085	11	40
ACYPI53120-RA	gi|641577511|gb|KK854741.1|	124217-129069	11	38
ACYPI005509-RA	gi|641585642|gb|KK854364.1|	135183-135547	9.9	38
ACYPI000698-RA	gi|641577043|gb|KK854816.1|	311150-313770	11	43
ACYPI002591-RA	gi|641576679|gb|KK854878.1|	157410-158047	9.9	38
ACYPI003241-RA	gi|641552935|gb|KK863732.1|	454-1196	13	21
ACYPI005801-RA	gi|641569979|gb|KK856498.1|	92661-94959	9.2	33
ACYPI008933-RA	gi|641577698|gb|KK854708.1|	57734-58309	9.7	40
ACYPI080332-RA	gi|641587047|gb|KK854151.1|	314586-316489	10	39
ACYPI001791-RA	gi|641573617|gb|KK855515.1|	131876-133163	10	38
ACYPI003522-RA	gi|641576414|gb|KK854924.1|	137776-143145	10	40
ACYPI003669-RA	gi|641575730|gb|KK855054.1|	346708-347099	10	38
ACYPI008417-RA	gi|641581389|gb|KK854557.1|	116105-118480	11	41
ACYPI002653-RA	gi|641571270|gb|KK856115.1|	162152-162527	11	38

ACYPI003687-RA	gi|641585475|gb|KK854392.1|	428029-430880	10	41
ACYPI003777-RA	gi|641586496|gb|KK854233.1|	18232-18563	11	38
ACYPI004569-RA	gi|641571270|gb|KK856115.1|	170208-170410	11	38
ACYPI006362-RA	gi|641587937|gb|KK854042.1|	366511-366715	11	42
ACYPI008364-RA	gi|641585475|gb|KK854392.1|	495251-495637	10	41
ACYPI008927-RA	gi|641577152|gb|KK854799.1|	276270-278017	10	44
ACYPI010124-RA	gi|641584260|gb|KK854495.1|	163032-163385	12	43
ACYPI010192-RA	gi|641584320|gb|KK854490.1|	39097-39488	10	41
ACYPI44294-RA	gi|641572017|gb|KK855921.1|	99496-100520	9.3	35
ACYPI007694-RA	gi|641570952|gb|KK856204.1|	114149-116660	10	21
ACYPI002108-RA	gi|641579960|gb|KK854572.1|	217862-218262	11	40
ACYPI54601-RA	gi|641571435|gb|KK856072.1|	14953-15436	9.1	36
ACYPI002846-RA	gi|641572304|gb|KK855847.1|	137732-137911	11	40
ACYPI003485-RA	gi|641578330|gb|KK854601.1|	94342-97476	11	38
ACYPI007291-RA	gi|641576741|gb|KK854868.1|	172365-172555	9.5	40
ACYPI005044-RA	gi|641574836|gb|KK855234.1|	105075-105240	10	40
ACYPI008188-RA	gi|641571667|gb|KK856008.1|	4820-5515	11	39
ACYPI008325-RA	gi|641576188|gb|KK854966.1|	83930-89497	11	39
ACYPI009957-RA	gi|641576983|gb|KK854825.1|	163430-164126	9.7	37
ACYPI001792-RA	gi|641576766|gb|KK854863.1|	14466-15060	10	38
ACYPI007413-RA	gi|641584305|gb|KK854491.1|	308400-312369	11	39
ACYPI003759-RA	gi|641576442|gb|KK854919.1|	242945-243251	11	36
ACYPI001071-RA	gi|641584205|gb|KK854500.1|	96025-96514	11	41
ACYPI002134-RA	gi|641576266|gb|KK854949.1|	198830-199097	11	45
ACYPI005660-RA	gi|641576266|gb|KK854949.1|	276746-282927	11	45
ACYPI53153-RA	gi|641575372|gb|KK855124.1|	178603-178878	9.5	36
ACYPI003077-RA	gi|641584694|gb|KK854463.1|	6804-7156	9.3	36
ACYPI007561-RA	gi|641588033|gb|KK854031.1|	1129822-1132527	11	38
ACYPI008256-RA	gi|641586620|gb|KK854215.1|	319717-321987	9.1	40
ACYPI009408-RA	gi|641577704|gb|KK854707.1|	65610-66002	10	38

ACYPI005533-RA	gi|641569377|gb|KK856689.1|	37030-37296	9.9	21
ACYPI007392-RA	gi|641569377|gb|KK856689.1|	40663-41409	9.9	21
ACYPI083041-RA	gi|641576172|gb|KK854969.1|	7185-8131	9.9	20
ACYPI002730-RA	gi|641583921|gb|KK854515.1|	196713-197385	11	38
ACYPI005062-RA	gi|641570227|gb|KK856420.1|	107439-107703	10	37
ACYPI000651-RA	gi|641588236|gb|KK854011.1|	780500-780755	9.7	38
ACYPI003764-RA	gi|641571928|gb|KK855946.1|	88091-88532	11	38
ACYPI001409-RA	gi|641580980|gb|KK854561.1|	130339-132214	11	37
ACYPI003371-RA	gi|641564718|gb|KK858315.1|	10865-12195	11	42
ACYPI005306-RA	gi|641574971|gb|KK855204.1|	33793-34257	11	21
ACYPI005858-RA	gi|641574275|gb|KK855359.1|	188970-199118	10	38
ACYPI005292-RA	gi|641578305|gb|KK854605.1|	149932-150581	11	42
ACYPI003276-RA	gi|641587256|gb|KK854124.1|	538695-539111	9.7	40
ACYPI002075-RA	gi|641588073|gb|KK854027.1|	541585-542238	11	41
ACYPI000310-RA	gi|641587272|gb|KK854122.1|	912870-917442	9.3	42
ACYPI002865-RA	gi|641575698|gb|KK855061.1|	347810-348045	10	39
ACYPI39790-RA	gi|641586726|gb|KK854199.1|	145569-150068	11	41
ACYPI000930-RA	gi|641577778|gb|KK854696.1|	282046-284706	9.9	42
ACYPI003888-RA	gi|641575970|gb|KK855012.1|	152130-153208	11	38
ACYPI007680-RA	gi|641573533|gb|KK855535.1|	74742-84171	10	41
ACYPI007176-RA	gi|641571632|gb|KK856018.1|	93777-96711	10	36
ACYPI000702-RA	gi|641575668|gb|KK855066.1|	269826-270087	11	40
ACYPI004498-RA	gi|641573629|gb|KK855512.1|	126969-127222	10	40
ACYPI008937-RA	gi|641574173|gb|KK855380.1|	171169-171604	9.5	39
ACYPI003757-RA	gi|641585521|gb|KK854384.1|	341764-342049	9.7	38
ACYPI005128-RA	gi|641577879|gb|KK854677.1|	287722-291584	18	36
ACYPI069375-RA	gi|641586496|gb|KK854233.1|	472839-473094	11	38
ACYPI085816-RA	gi|641572162|gb|KK855882.1|	22919-23235	12	38
ACYPI006609-RA	gi|641586393|gb|KK854248.1|	33913-37798	9	39
ACYPI002636-RA	gi|641586114|gb|KK854288.1|	365430-366260	10	41

ACYPI004549-RA	gi|641587047|gb|KK854151.1|	407861-409085	10	39
ACYPI005777-RA	gi|641579930|gb|KK854578.1|	7770-8431	9.1	35
ACYPI062391-RA	gi|641587444|gb|KK854102.1|	273244-273469	11	42
ACYPI001396-RA	gi|641568713|gb|KK856890.1|	77631-78976	12	42
ACYPI004883-RA	gi|641586874|gb|KK854178.1|	392524-394880	9.4	38
ACYPI006122-RA	gi|641577047|gb|KK854815.1|	338905-339843	10	41
ACYPI008005-RA	gi|641576123|gb|KK854979.1|	207770-208714	11	22
ACYPI008647-RA	gi|641588194|gb|KK854015.1|	991032-993033	9.4	40
ACYPI008935-RA	gi|641570743|gb|KK856265.1|	18854-22222	9.6	34
ACYPI002247-RA	gi|641587066|gb|KK854149.1|	557889-558485	11	40
ACYPI004126-RA	gi|641585910|gb|KK854320.1|	247453-248579	10	38
ACYPI004824-RA	gi|641543669|gb|KK868158.1|	4001-4205	10	37
ACYPI006059-RA	gi|641575725|gb|KK855055.1|	168327-168540	10	37
ACYPI007941-RA	gi|641576362|gb|KK854933.1|	72375-73268	10	37
ACYPI000937-RA	gi|641587287|gb|KK854120.1|	228753-229046	9.9	37
ACYPI002837-RA	gi|641571749|gb|KK855991.1|	43843-48288	9.4	35
ACYPI000455-RA	gi|641570399|gb|KK856367.1|	51263-56295	11	41
ACYPI009317-RA	gi|641575978|gb|KK855010.1|	173994-174720	10	36
ACYPI000476-RA	gi|641567816|gb|KK857179.1|	76314-76518	12	37
ACYPI005751-RA	gi|641587735|gb|KK854066.1|	737657-737866	11	42
ACYPI006748-RA	gi|641570795|gb|KK856251.1|	76988-77769	9.8	38
ACYPI23907-RA	gi|641566247|gb|KK857731.1|	37794-38931	9.4	35
ACYPI007934-RA	gi|641585340|gb|KK854414.1|	411187-411724	11	42
ACYPI000889-RA	gi|641572227|gb|KK855866.1|	92593-93285	7	32
ACYPI000961-RA	gi|641569727|gb|KK856579.1|	46367-46981	8.3	34
ACYPI23843-RA	gi|641577086|gb|KK854809.1|	312466-312848	9.8	38
ACYPI082935-RA	gi|641577688|gb|KK854709.1|	138696-139468	11	40
ACYPI000806-RA	gi|641585684|gb|KK854357.1|	234578-234779	11	39
ACYPI004608-RA	gi|641569429|gb|KK856673.1|	47806-49532	8.4	36
ACYPI006386-RA	gi|641577725|gb|KK854704.1|	201342-201558	11	42

ACYPI006524-RA	gi|641586470|gb|KK854237.1|	35752-35974	11	42
ACYPI008270-RA	gi|641575368|gb|KK855125.1|	248630-248864	10	36
ACYPI072560-RA	gi|641588033|gb|KK854031.1|	175191-176427	11	38
ACYPI38302-RA	gi|641577725|gb|KK854704.1|	319269-319486	11	42
ACYPI007400-RA	gi|641587287|gb|KK854120.1|	323508-324813	9.9	37
ACYPI000918-RA	gi|641584606|gb|KK854469.1|	390115-393391	13	57
ACYPI001339-RA	gi|641580980|gb|KK854561.1|	256415-256586	11	37
ACYPI005787-RA	gi|641579925|gb|KK854579.1|	223946-224697	9.7	40
ACYPI007646-RA	gi|641586639|gb|KK854212.1|	571378-573404	8.3	41
ACYPI010190-RA	gi|641573587|gb|KK855522.1|	227062-227967	9.3	38
ACYPI000541-RA	gi|641587912|gb|KK854045.1|	361098-361359	11	39
ACYPI001827-RA	gi|641569469|gb|KK856662.1|	60834-63671	9.1	34
ACYPI005644-RA	gi|641587171|gb|KK854135.1|	488624-491178	10	39
ACYPI003991-RA	gi|641587881|gb|KK854049.1|	426345-427066	10	40
ACYPI005480-RA	gi|641570089|gb|KK856463.1|	107132-110839	11	36
ACYPI000572-RA	gi|641587711|gb|KK854069.1|	638096-639559	11	42
ACYPI005672-RA	gi|641575218|gb|KK855155.1|	162305-163819	11	21
ACYPI006928-RA	gi|641587452|gb|KK854101.1|	943490-945993	11	40
ACYPI008793-RA	gi|641584913|gb|KK854447.1|	426340-426884	10	40
ACYPI009437-RA	gi|641571645|gb|KK856014.1|	129770-130070	10	21
ACYPI060526-RA	gi|641576456|gb|KK854916.1|	298771-299598	10	21
ACYPI46839-RA	gi|641576456|gb|KK854916.1|	291933-292461	10	21
ACYPI001736-RA	gi|641573595|gb|KK855520.1|	30042-32216	11	40
ACYPI003573-RA	gi|641587735|gb|KK854066.1|	776187-776395	11	42
ACYPI004227-RA	gi|641571428|gb|KK856074.1|	5390-6292	11	39
ACYPI004872-RA	gi|641573082|gb|KK855648.1|	7162-15362	11	42
ACYPI005524-RA	gi|641556681|gb|KK861957.1|	7030-12038	9.6	38
ACYPI009267-RA	gi|641570730|gb|KK856269.1|	169547-171286	9.1	39
ACYPI071995-RA	gi|641586654|gb|KK854210.1|	254937-255258	11	40
ACYPI000690-RA	gi|641586824|gb|KK854186.1|	497259-497693	10	41

ACYPI004485-RA	gi|641576666|gb|KK854881.1|	17587-18243	10	37
ACYPI004054-RA	gi|641587896|gb|KK854047.1|	852540-852824	9.9	39
ACYPI008778-RA	gi|641586258|gb|KK854267.1|	282632-284535	11	37
ACYPI001995-RA	gi|641586476|gb|KK854236.1|	551513-552631	10	37
ACYPI007675-RA	gi|641575396|gb|KK855118.1|	80665-81779	9	35
ACYPI010223-RA	gi|641569808|gb|KK856554.1|	118724-119284	11	21
ACYPI000068-RA	gi|641569988|gb|KK856495.1|	87740-88795	11	43
ACYPI007981-RA	gi|641582763|gb|KK854531.1|	88592-90905	10	39
ACYPI068525-RA	gi|641582763|gb|KK854531.1|	82803-83820	10	39
ACYPI004579-RA	gi|641576677|gb|KK854879.1|	272723-273220	11	39
ACYPI007803-RA	gi|641574119|gb|KK855392.1|	150662-150924	9.6	37
ACYPI001408-RA	gi|641577161|gb|KK854797.1|	342023-346942	10	38
ACYPI002592-RA	gi|641588082|gb|KK854026.1|	1158045-1162963	11	41
ACYPI004303-RA	gi|641578305|gb|KK854605.1|	29255-29611	11	42
ACYPI000303-RA	gi|641565112|gb|KK858157.1|	7833-12055	9.7	34
ACYPI006026-RA	gi|641588294|gb|KK854006.1|	783720-784688	8.7	40
ACYPI007298-RA	gi|641585953|gb|KK854314.1|	393980-395790	11	43
ACYPI007924-RA	gi|641586528|gb|KK854228.1|	517226-524097	10	42
ACYPI005934-RA	gi|641588194|gb|KK854015.1|	654638-658076	9.4	40
ACYPI067829-RA	gi|641572230|gb|KK855865.1|	3240-3696	9.8	38
ACYPI000800-RA	gi|641587104|gb|KK854144.1|	245827-251242	11	37
ACYPI002810-RA	gi|641578122|gb|KK854636.1|	406306-410622	12	42
ACYPI003918-RA	gi|641576109|gb|KK854982.1|	144500-144781	10	19
ACYPI004218-RA	gi|641576656|gb|KK854883.1|	173777-174036	11	40
ACYPI004594-RA	gi|641586817|gb|KK854187.1|	719359-720789	9.3	39
ACYPI004868-RA	gi|641588090|gb|KK854025.1|	99216-99820	11	43
ACYPI005317-RA	gi|641575593|gb|KK855082.1|	163841-164336	10	38
ACYPI005982-RA	gi|641587527|gb|KK854092.1|	590726-592107	10	38
ACYPI007845-RA	gi|641586943|gb|KK854167.1|	31859-32225	11	38
ACYPI007989-RA	gi|641584818|gb|KK854454.1|	50315-52209	11	42

ACYPI008388-RA	gi|641572615|gb|KK855769.1|	172413-176442	12	43
ACYPI007697-RA	gi|641571667|gb|KK856008.1|	72217-72938	11	39
ACYPI000081-RA	gi|641576091|gb|KK854985.1|	75761-76501	12	24
ACYPI002737-RA	gi|641575047|gb|KK855190.1|	207623-208765	8.3	43
ACYPI005326-RA	gi|641588325|gb|KK854004.1|	1071087-1072532	11	40
ACYPI007206-RA	gi|641576656|gb|KK854883.1|	200051-201305	11	40
ACYPI009717-RA	gi|641577623|gb|KK854722.1|	74912-75117	11	38
ACYPI001211-RA	gi|641587082|gb|KK854147.1|	433025-436082	11	44
ACYPI003105-RA	gi|641587504|gb|KK854095.1|	508921-513839	11	42
ACYPI003832-RA	gi|641588343|gb|KK854003.1|	998331-999157	11	40
ACYPI000754-RA	gi|641588099|gb|KK854024.1|	592114-594104	11	39
ACYPI004632-RA	gi|641587444|gb|KK854102.1|	748732-748911	11	42
ACYPI001984-RA	gi|641585628|gb|KK854366.1|	113589-114063	8.4	39
ACYPI003872-RA	gi|641577228|gb|KK854785.1|	264967-265264	9.7	39
ACYPI004559-RA	gi|641587580|gb|KK854085.1|	469086-469314	10	39
ACYPI005824-RA	gi|641585395|gb|KK854405.1|	453291-455413	10	38
ACYPI007683-RA	gi|641587912|gb|KK854045.1|	288397-288614	11	39
ACYPI071352-RA	gi|641587365|gb|KK854113.1|	119229-120037	12	45
ACYPI081577-RA	gi|641584606|gb|KK854469.1|	498817-499047	13	57
ACYPI001440-RA	gi|641587580|gb|KK854085.1|	646702-650598	10	39
ACYPI003689-RA	gi|641573351|gb|KK855583.1|	50978-51621	11	41
ACYPI005615-RA	gi|641587171|gb|KK854135.1|	346196-346642	10	39
ACYPI006738-RA	gi|641575568|gb|KK855086.1|	309892-310131	11	40
ACYPI007182-RA	gi|641572974|gb|KK855674.1|	198739-199020	9.9	38
ACYPI009063-RA	gi|641575081|gb|KK855183.1|	215715-216462	9.3	37
ACYPI006784-RA	gi|641577595|gb|KK854727.1|	58572-59762	10	20
ACYPI003549-RA	gi|641560616|gb|KK860113.1|	734-987	12	44
ACYPI004190-RA	gi|641576679|gb|KK854878.1|	317889-322000	9.9	38
ACYPI005514-RA	gi|641579937|gb|KK854576.1|	460084-460348	9.4	41
ACYPI003646-RA	gi|641586405|gb|KK854246.1|	369727-370490	10	40

ACYPI009822-RA	gi|641586688|gb|KK854205.1|	60101-63114	11	42
ACYPI061611-RA	gi|641586405|gb|KK854246.1|	366515-366987	10	40
ACYPI066618-RA	gi|641588294|gb|KK854006.1|	859184-860124	8.7	40
ACYPI000605-RA	gi|641575910|gb|KK855021.1|	394918-399195	10	35
ACYPI001390-RA	gi|641587954|gb|KK854040.1|	1015970-1016497	8.9	39
ACYPI003283-RA	gi|641588000|gb|KK854035.1|	368716-370070	11	39
ACYPI004533-RA	gi|641576315|gb|KK854940.1|	174904-175114	9.9	40
ACYPI005339-RA	gi|641576315|gb|KK854940.1|	171178-173268	9.9	40
ACYPI008826-RA	gi|641588109|gb|KK854023.1|	1238560-1240635	10	40
ACYPI001336-RA	gi|641587024|gb|KK854155.1|	311680-312467	10	21
ACYPI003222-RA	gi|641587024|gb|KK854155.1|	459569-461108	10	21
ACYPI005141-RA	gi|641585905|gb|KK854321.1|	423309-424538	11	39
ACYPI005785-RA	gi|641566247|gb|KK857731.1|	39054-43768	9.4	35
ACYPI010216-RA	gi|641586380|gb|KK854250.1|	355490-355972	9.3	38
ACYPI072718-RA	gi|641586072|gb|KK854295.1|	511197-514743	10	20
ACYPI082338-RA	gi|641586301|gb|KK854260.1|	483876-485161	9.8	39
ACYPI088588-RA	gi|641571670|gb|KK856007.1|	69457-70551	11	38
ACYPI003993-RA	gi|641576347|gb|KK854934.1|	313422-314861	11	39
ACYPI007804-RA	gi|641578122|gb|KK854636.1|	420727-421156	12	42
ACYPI000007-RA	gi|641572390|gb|KK855826.1|	56516-58114	10	35
ACYPI000664-RA	gi|641571119|gb|KK856156.1|	70070-70332	11	38
ACYPI004453-RA	gi|641575297|gb|KK855137.1|	195774-197903	9.2	35
ACYPI006370-RA	gi|641588000|gb|KK854035.1|	424968-426705	11	39
ACYPI008255-RA	gi|641568716|gb|KK856889.1|	28408-28817	10	35
ACYPI008888-RA	gi|641588164|gb|KK854017.1|	761934-763437	9.2	40
ACYPI48107-RA	gi|641568716|gb|KK856889.1|	34477-34722	10	35
ACYPI002882-RA	gi|641577879|gb|KK854677.1|	244326-244779	18	36
ACYPI008185-RA	gi|641578138|gb|KK854634.1|	71573-71848	8.2	39
ACYPI000848-RA	gi|641586203|gb|KK854273.1|	324960-325864	9.5	40
ACYPI001218-RA	gi|641588294|gb|KK854006.1|	620000-626234	8.7	40

ACYPI002808-RA	gi|641585562|gb|KK854377.1|	403729-405181	12	47
ACYPI006640-RA	gi|641577843|gb|KK854684.1|	417397-418438	10	40
ACYPI008498-RA	gi|641577896|gb|KK854674.1|	165456-167048	11	40
ACYPI082655-RA	gi|641587652|gb|KK854077.1|	653439-653953	10	38
ACYPI000474-RA	gi|641571315|gb|KK856104.1|	133674-139009	12	46
ACYPI001119-RA	gi|641565021|gb|KK858191.1|	4-3849	10	36
ACYPI005078-RA	gi|641587937|gb|KK854042.1|	175558-175793	11	42
ACYPI008707-RA	gi|641580504|gb|KK854565.1|	345098-345508	11	43
ACYPI009338-RA	gi|641579225|gb|KK854583.1|	17978-22117	10	39
ACYPI068232-RA	gi|641575140|gb|KK855171.1|	289877-290980	9.7	40
ACYPI000023-RA	gi|641573621|gb|KK855514.1|	51915-53233	10	41
ACYPI001212-RA	gi|641577289|gb|KK854776.1|	303573-304486	10	36
ACYPI004351-RA	gi|641586661|gb|KK854209.1|	584743-586316	11	43
ACYPI005023-RA	gi|641586489|gb|KK854234.1|	305108-305856	9.5	39
ACYPI007158-RA	gi|641566395|gb|KK857678.1|	51-519	10	19
ACYPI007537-RA	gi|641568642|gb|KK856914.1|	65616-66744	11	40
ACYPI008785-RA	gi|641580970|gb|KK854563.1|	322943-323194	11	42
ACYPI000527-RA	gi|641587276|gb|KK854121.1|	431239-432417	11	39
ACYPI32327-RA	gi|641570111|gb|KK856458.1|	135853-136796	11	40
ACYPI002352-RA	gi|641570717|gb|KK856273.1|	82511-84071	10	36
ACYPI003042-RA	gi|641566429|gb|KK857664.1|	17082-18381	12	37
ACYPI004235-RA	gi|641574275|gb|KK855359.1|	267082-267664	10	38
ACYPI002456-RA	gi|641575939|gb|KK855017.1|	189691-190597	9.9	41
ACYPI006934-RA	gi|641584445|gb|KK854481.1|	92178-92589	9.9	39
ACYPI000598-RA	gi|641574699|gb|KK855261.1|	81511-84990	9	35
ACYPI000966-RA	gi|641576906|gb|KK854838.1|	169369-169685	9.4	37
ACYPI001579-RA	gi|641586203|gb|KK854273.1|	145471-145719	9.5	40
ACYPI001617-RA	gi|641584806|gb|KK854455.1|	452916-453656	11	43
ACYPI002866-RA	gi|641577535|gb|KK854737.1|	164637-168925	11	42
ACYPI004093-RA	gi|641572955|gb|KK855679.1|	35380-36113	10	40

ACYPI007307-RA	gi|641577772|gb|KK854697.1|	199513-200179	10	39
ACYPI001091-RA	gi|641586936|gb|KK854168.1|	258813-259248	12	42
ACYPI002987-RA	gi|641586139|gb|KK854284.1|	193375-194388	10	21
ACYPI004908-RA	gi|641584419|gb|KK854483.1|	336349-336580	10	42
ACYPI006808-RA	gi|641584728|gb|KK854460.1|	218813-219634	9.5	37
ACYPI000052-RA	gi|641566429|gb|KK857664.1|	66414-67063	12	37
ACYPI001864-RA	gi|641588267|gb|KK854008.1|	56074-59831	10	40
ACYPI005784-RA	gi|641577501|gb|KK854743.1|	109963-110291	9.1	37
ACYPI005917-RA	gi|641574699|gb|KK855261.1|	199522-200784	9	35
ACYPI007791-RA	gi|641576942|gb|KK854833.1|	25471-27111	11	42
ACYPI009225-RA	gi|641577710|gb|KK854706.1|	199148-199612	11	43
ACYPI008861-RA	gi|641587671|gb|KK854074.1|	477951-478521	11	40
ACYPI002938-RA	gi|641572177|gb|KK855878.1|	124730-125775	10	35
ACYPI20476-RA	gi|641570399|gb|KK856367.1|	73110-74909	11	41
ACYPI002802-RA	gi|641587823|gb|KK854055.1|	569834-570486	11	22
ACYPI008491-RA	gi|641587095|gb|KK854145.1|	133940-137608	9.9	40
ACYPI009733-RA	gi|641575516|gb|KK855095.1|	27498-29508	9.6	36
ACYPI31575-RA	gi|641570811|gb|KK856247.1|	12573-13140	9.1	35
ACYPI000585-RA	gi|641585801|gb|KK854337.1|	437800-438557	10	38
ACYPI004250-RA	gi|641587971|gb|KK854038.1|	804060-805157	11	39
ACYPI000953-RA	gi|641586915|gb|KK854171.1|	324778-325722	10	38
ACYPI47094-RA	gi|641585761|gb|KK854344.1|	399444-399849	10	39
ACYPI000888-RA	gi|641587276|gb|KK854121.1|	641090-641535	11	39
ACYPI001502-RA	gi|641573452|gb|KK855556.1|	138398-138644	10	22
ACYPI003428-RA	gi|641584305|gb|KK854491.1|	175587-181117	11	39
ACYPI007853-RA	gi|641571692|gb|KK856004.1|	106826-107582	9.8	37
ACYPI009718-RA	gi|641585562|gb|KK854377.1|	397408-401257	12	47
ACYPI001480-RA	gi|641572891|gb|KK855695.1|	255552-256017	10	36
ACYPI002304-RA	gi|641574370|gb|KK855338.1|	45406-46646	11	38
ACYPI004182-RA	gi|641585446|gb|KK854396.1|	299805-300203	11	39

ACYPI005674-RA	gi|641570807|gb|KK856248.1|	29073-29837	11	38
ACYPI006120-RA	gi|641574416|gb|KK855326.1|	165833-168071	11	38
ACYPI000872-RA	gi|641587937|gb|KK854042.1|	332852-334123	11	42
ACYPI002110-RA	gi|641586344|gb|KK854254.1|	531825-532943	10	40
ACYPI007090-RA	gi|641586033|gb|KK854301.1|	333585-333874	11	21
ACYPI003864-RA	gi|641549627|gb|KK865285.1|	23-408	9	15
ACYPI007529-RA	gi|641587145|gb|KK854138.1|	127743-130100	10	42
ACYPI007655-RA	gi|641573725|gb|KK855489.1|	48969-50625	11	21
ACYPI002900-RA	gi|641570355|gb|KK856378.1|	62186-63841	9.7	35
ACYPI008024-RA	gi|641574833|gb|KK855235.1|	137294-138215	9.4	33
ACYPI000080-RA	gi|641570274|gb|KK856404.1|	107160-108834	9.1	35
ACYPI001206-RA	gi|641588358|gb|KK854002.1|	2226595-2231646	11	43
ACYPI001843-RA	gi|641587992|gb|KK854036.1|	117176-122724	11	41
ACYPI005019-RA	gi|641576010|gb|KK855002.1|	162047-162699	12	43
ACYPI005619-RA	gi|641585423|gb|KK854400.1|	71641-73816	11	36
ACYPI005661-RA	gi|641584945|gb|KK854445.1|	279555-280043	9.9	20
ACYPI007534-RA	gi|641570274|gb|KK856404.1|	121901-122634	9.1	35
ACYPI010029-RA	gi|641587104|gb|KK854144.1|	355010-358995	11	37
ACYPI002086-RA	gi|641568142|gb|KK857071.1|	35652-36129	11	42
ACYPI001745-RA	gi|641588202|gb|KK854014.1|	718695-720197	10	21
ACYPI008698-RA	gi|641587765|gb|KK854062.1|	36184-42410	10	41
ACYPI010049-RA	gi|641564440|gb|KK858439.1|	24294-25273	11	36
ACYPI000047-RA	gi|641573214|gb|KK855616.1|	14320-14563	9.7	34
ACYPI000489-RA	gi|641576161|gb|KK854972.1|	214160-217514	10	36
ACYPI001732-RA	gi|641577837|gb|KK854685.1|	1-3389	11	42
ACYPI004266-RA	gi|641569328|gb|KK856702.1|	25764-28522	9.6	17
ACYPI007431-RA	gi|641575901|gb|KK855023.1|	153314-153826	11	38
ACYPI008075-RA	gi|641588213|gb|KK854013.1|	624716-626027	9.6	39
ACYPI009949-RA	gi|641576469|gb|KK854914.1|	291756-292221	11	21
ACYPI45385-RA	gi|641576469|gb|KK854914.1|	305099-305354	11	21

ACYPI003311-RA	gi|641578138|gb|KK854634.1|	45162-45408	8.2	39
ACYPI009707-RA	gi|641576069|gb|KK854989.1|	275096-276482	11	40
ACYPI004897-RA	gi|641572601|gb|KK855773.1|	122434-122797	10	35
ACYPI006314-RA	gi|641572037|gb|KK855916.1|	115036-115662	13	41
ACYPI003657-RA	gi|641588343|gb|KK854003.1|	2120674-2121062	11	40
ACYPI003904-RA	gi|641587954|gb|KK854040.1|	710604-711778	8.9	39
ACYPI008512-RA	gi|641574876|gb|KK855226.1|	41189-41563	11	39
ACYPI003822-RA	gi|641576414|gb|KK854924.1|	311123-312321	10	40
ACYPI004910-RA	gi|641588258|gb|KK854009.1|	376568-377488	11	36
ACYPI005580-RA	gi|641576608|gb|KK854891.1|	218827-226381	11	37
ACYPI006247-RA	gi|641577184|gb|KK854793.1|	134873-135907	11	40
ACYPI008162-RA	gi|641569519|gb|KK856646.1|	11470-12019	10	39
ACYPI008800-RA	gi|641568762|gb|KK856874.1|	46280-46617	11	41
ACYPI002506-RA	gi|641587186|gb|KK854133.1|	441470-442956	8.1	39
ACYPI003343-RA	gi|641574303|gb|KK855353.1|	227178-230384	10	34
ACYPI005264-RA	gi|641587153|gb|KK854137.1|	869701-871131	10	42
ACYPI007148-RA	gi|641583587|gb|KK854518.1|	192736-193733	10	37
ACYPI009028-RA	gi|641576333|gb|KK854937.1|	152038-152902	9.9	35
ACYPI082729-RA	gi|641575130|gb|KK855173.1|	224050-225095	12	50
ACYPI001646-RA	gi|641577688|gb|KK854709.1|	184207-184535	11	40
ACYPI002777-RA	gi|641576741|gb|KK854868.1|	42692-42911	9.5	40
ACYPI008302-RA	gi|641588090|gb|KK854025.1|	996250-997232	11	43
ACYPI001110-RA	gi|641572364|gb|KK855831.1|	22282-22895	11	38
ACYPI003425-RA	gi|641588073|gb|KK854027.1|	294772-298222	11	41
ACYPI087327-RA	gi|641585293|gb|KK854422.1|	24669-25392	9.1	38
ACYPI000735-RA	gi|641573555|gb|KK855529.1|	97300-97582	8.9	38
ACYPI002246-RA	gi|641586482|gb|KK854235.1|	606376-608934	11	41
ACYPI004125-RA	gi|641585900|gb|KK854322.1|	82028-83778	12	43
ACYPI006058-RA	gi|641568319|gb|KK857014.1|	14952-15195	9.5	33
ACYPI006904-RA	gi|641576994|gb|KK854823.1|	280973-281224	7.7	38

ACYPI007679-RA	gi|641587840|gb|KK854053.1|	354674-355260	11	43
ACYPI008763-RA	gi|641579941|gb|KK854575.1|	112465-112832	10	39
ACYPI000839-RA	gi|641570271|gb|KK856405.1|	108325-109422	9.8	38
ACYPI006557-RA	gi|641578106|gb|KK854639.1|	146076-146525	11	39
ACYPI065145-RA	gi|641585761|gb|KK854344.1|	49699-50498	10	39
ACYPI008242-RA	gi|641574114|gb|KK855393.1|	159342-162609	9.9	40
ACYPI084157-RA	gi|641571841|gb|KK855968.1|	103877-104284	10	36
ACYPI20417-RA	gi|641587962|gb|KK854039.1|	746415-749748	11	40
ACYPI002445-RA	gi|641572891|gb|KK855695.1|	187955-188980	10	36
ACYPI001203-RA	gi|641577851|gb|KK854682.1|	65169-65391	11	34
ACYPI009548-RA	gi|641574876|gb|KK855226.1|	139064-139912	11	39
ACYPI071951-RA	gi|641577851|gb|KK854682.1|	63564-63959	11	34
ACYPI083523-RA	gi|641575603|gb|KK855080.1|	43705-46594	9.4	38
ACYPI000686-RA	gi|641571116|gb|KK856157.1|	239816-241311	11	40
ACYPI001081-RA	gi|641586313|gb|KK854259.1|	459530-459750	12	42
ACYPI002326-RA	gi|641573457|gb|KK855555.1|	183841-184202	9.7	38
ACYPI002342-RA	gi|641543394|gb|KK868292.1|	46-1963	14	56
ACYPI002979-RA	gi|641574085|gb|KK855399.1|	126284-127324	10	38
ACYPI005064-RA	gi|641574173|gb|KK855380.1|	248906-249062	9.5	39
ACYPI006142-RA	gi|641576863|gb|KK854845.1|	272005-274883	11	41
ACYPI007050-RA	gi|641577905|gb|KK854672.1|	416838-417194	10	37
ACYPI008592-RA	gi|641570621|gb|KK856301.1|	93826-95341	10	36
ACYPI23338-RA	gi|641580479|gb|KK854569.1|	303473-306094	10	38
ACYPI000542-RA	gi|641577778|gb|KK854696.1|	217051-219749	9.9	42
ACYPI002382-RA	gi|641586975|gb|KK854163.1|	193402-195937	11	40
ACYPI007374-RA	gi|641578084|gb|KK854643.1|	79905-83124	11	40
ACYPI083936-RA	gi|641577511|gb|KK854741.1|	258777-262347	11	38
ACYPI001090-RA	gi|641571716|gb|KK855999.1|	59771-60818	11	39
ACYPI073321-RA	gi|641583587|gb|KK854518.1|	58578-60318	10	37
ACYPI53110-RA	gi|641571979|gb|KK855932.1|	14369-16411	9.6	38

ACYPI001359-RA	gi|641586793|gb|KK854190.1|	642794-644506	9.3	41
ACYPI003246-RA	gi|641571216|gb|KK856129.1|	36831-37548	12	41
ACYPI088026-RA	gi|641573045|gb|KK855657.1|	3813-4551	14	51
ACYPI001498-RA	gi|641585761|gb|KK854344.1|	93487-94161	10	39
ACYPI002405-RA	gi|641570598|gb|KK856307.1|	53749-55137	11	21
ACYPI005324-RA	gi|641574976|gb|KK855203.1|	212518-212889	11	21
ACYPI008746-RA	gi|641584606|gb|KK854469.1|	165691-165960	13	57
ACYPI001496-RA	gi|641577322|gb|KK854771.1|	99350-100376	10	21
ACYPI009713-RA	gi|641586769|gb|KK854193.1|	274895-275692	12	23
ACYPI001469-RA	gi|641566955|gb|KK857475.1|	40704-41048	10	37
ACYPI001596-RA	gi|641572114|gb|KK855896.1|	145460-146074	10	36
ACYPI002974-RA	gi|641587209|gb|KK854130.1|	393388-393768	11	42
ACYPI003480-RA	gi|641574312|gb|KK855351.1|	100968-101231	8.9	36
ACYPI006207-RA	gi|641575108|gb|KK855178.1|	180173-185543	34	81
ACYPI007925-RA	gi|641587519|gb|KK854093.1|	863002-863426	11	39
ACYPI064230-RA	gi|641571652|gb|KK856012.1|	46063-47748	9.6	35
ACYPI087019-RA	gi|641571652|gb|KK856012.1|	88727-91487	9.6	35
ACYPI53390-RA	gi|641566955|gb|KK857475.1|	18565-19741	10	37
ACYPI004564-RA	gi|641573858|gb|KK855455.1|	44955-46453	9.8	37
ACYPI009494-RA	gi|641573858|gb|KK855455.1|	75809-76439	9.8	37
ACYPI000487-RA	gi|641585971|gb|KK854310.1|	175212-175825	10	40
ACYPI001981-RA	gi|641568869|gb|KK856841.1|	128361-129764	12	50
ACYPI002373-RA	gi|641585971|gb|KK854310.1|	165545-166065	10	40
ACYPI002732-RA	gi|641587896|gb|KK854047.1|	1016138-1016437	9.9	39
ACYPI003255-RA	gi|641587962|gb|KK854039.1|	1113617-1114733	11	40
ACYPI007125-RA	gi|641587671|gb|KK854074.1|	223276-225164	11	40
ACYPI009003-RA	gi|641587671|gb|KK854074.1|	276228-279464	11	40
ACYPI010211-RA	gi|641567905|gb|KK857149.1|	45592-47557	10	38
ACYPI006395-RA	gi|641585521|gb|KK854384.1|	373107-375874	9.7	38
ACYPI42350-RA	gi|641576741|gb|KK854868.1|	114191-115242	9.5	40

ACYPI007153-RA	gi|641584582|gb|KK854471.1|	502092-502336	10	38
ACYPI007763-RA	gi|641588182|gb|KK854016.1|	626961-627365	11	41
ACYPI008439-RA	gi|641584351|gb|KK854488.1|	384637-385073	11	41
ACYPI001492-RA	gi|641572293|gb|KK855850.1|	161818-162165	11	38
ACYPI003377-RA	gi|641586541|gb|KK854226.1|	268842-269769	11	41
ACYPI003469-RA	gi|641569612|gb|KK856617.1|	2544-3197	11	21
ACYPI005314-RA	gi|641576044|gb|KK854994.1|	254456-257819	8.9	37
ACYPI007195-RA	gi|641586372|gb|KK854251.1|	610574-611349	10	38
ACYPI009151-RA	gi|641572022|gb|KK855920.1|	74461-75767	12	42
ACYPI009704-RA	gi|641585936|gb|KK854317.1|	230660-239963	9.9	41
ACYPI001521-RA	gi|641587550|gb|KK854090.1|	137891-139619	9.7	45
ACYPI007802-RA	gi|641573841|gb|KK855459.1|	270792-271383	11	40
ACYPI084662-RA	gi|641570182|gb|KK856435.1|	73094-73602	10	36
ACYPI004766-RA	gi|641572959|gb|KK855678.1|	134025-134442	9.9	36
ACYPI008535-RA	gi|641570121|gb|KK856455.1|	39618-40567	10	40
ACYPI005517-RA	gi|641584681|gb|KK854464.1|	40862-44650	11	42
ACYPI008053-RA	gi|641573202|gb|KK855619.1|	210574-214420	9.6	42
ACYPI009928-RA	gi|641571953|gb|KK855939.1|	59925-60163	10	20
ACYPI000393-RA	gi|641584504|gb|KK854477.1|	523587-524563	9.5	38
ACYPI002287-RA	gi|641581789|gb|KK854549.1|	164122-165413	9.9	37
ACYPI003347-RA	gi|641577341|gb|KK854768.1|	313295-313655	14	58
ACYPI004209-RA	gi|641586817|gb|KK854187.1|	827518-828136	9.3	39
ACYPI006769-RA	gi|641586694|gb|KK854204.1|	421457-424628	12	50
ACYPI007778-RA	gi|641577341|gb|KK854768.1|	275634-276055	14	58
ACYPI008023-RA	gi|641574620|gb|KK855279.1|	90403-90641	10	39
ACYPI52571-RA	gi|641588283|gb|KK854007.1|	1597502-1604467	11	40
ACYPI002538-RA	gi|641584694|gb|KK854463.1|	130845-133454	9.3	36
ACYPI003189-RA	gi|641587276|gb|KK854121.1|	104136-105956	11	39
ACYPI004435-RA	gi|641574876|gb|KK855226.1|	159233-160009	11	39
ACYPI006352-RA	gi|641569883|gb|KK856530.1|	33210-33384	11	45

ACYPI007619-RA	gi|641574468|gb|KK855314.1|	158620-161845	9.9	37
ACYPI009511-RA	gi|641586210|gb|KK854272.1|	616776-617200	10	39
ACYPI001382-RA	gi|641588258|gb|KK854009.1|	333986-335333	11	36
ACYPI006407-RA	gi|641584248|gb|KK854496.1|	304615-304870	9.9	39
ACYPI008286-RA	gi|641572719|gb|KK855742.1|	158226-159383	12	23
ACYPI23268-RA	gi|641588294|gb|KK854006.1|	605409-606956	8.7	40
ACYPI56570-RA	gi|641577805|gb|KK854691.1|	239740-240590	9.4	38
ACYPI000971-RA	gi|641576423|gb|KK854922.1|	195918-196586	18	35
ACYPI002463-RA	gi|641576672|gb|KK854880.1|	179748-180099	9.4	40
ACYPI002870-RA	gi|641588073|gb|KK854027.1|	1075964-1076257	11	41
ACYPI004366-RA	gi|641587954|gb|KK854040.1|	176280-176649	8.9	39
ACYPI004790-RA	gi|641573181|gb|KK855624.1|	199267-199669	11	40
ACYPI006693-RA	gi|641588073|gb|KK854027.1|	1026916-1027178	11	41
ACYPI005158-RA	gi|641570952|gb|KK856204.1|	141929-143031	10	21
ACYPI007100-RA	gi|641577043|gb|KK854816.1|	297515-298663	11	43
ACYPI008357-RA	gi|641571465|gb|KK856064.1|	95177-95459	9.2	35
ACYPI008823-RA	gi|641565511|gb|KK858002.1|	16306-16587	6.4	31
ACYPI073873-RA	gi|641577453|gb|KK854750.1|	294482-295424	10	21
ACYPI007666-RA	gi|641575951|gb|KK855015.1|	243325-244558	9.9	37
ACYPI007713-RA	gi|641587033|gb|KK854153.1|	315975-316243	10	38
ACYPI009612-RA	gi|641582447|gb|KK854535.1|	223710-223869	9.4	44
ACYPI42236-RA	gi|641570579|gb|KK856313.1|	59176-59720	11	38
ACYPI007252-RA	gi|641573548|gb|KK855531.1|	59566-60502	10	20
ACYPI001360-RA	gi|641587263|gb|KK854123.1|	273627-277642	11	42
ACYPI001392-RA	gi|641571123|gb|KK856155.1|	98055-99345	10	42
ACYPI003247-RA	gi|641575324|gb|KK855134.1|	164032-164869	11	38
ACYPI005163-RA	gi|641586496|gb|KK854233.1|	92712-93712	11	38
ACYPI005208-RA	gi|641585957|gb|KK854313.1|	214206-223814	10	37
ACYPI006413-RA	gi|641588309|gb|KK854005.1|	540573-543634	11	40
ACYPI001043-RA	gi|641588246|gb|KK854010.1|	4429-4733	9.2	50

ACYPI003742-RA	gi|641576983|gb|KK854825.1|	297924-298168	9.7	37
ACYPI005687-RA	gi|641585755|gb|KK854345.1|	56916-60099	9.8	39
ACYPI008632-RA	gi|641569223|gb|KK856733.1|	86467-89983	12	42
ACYPI002678-RA	gi|641584913|gb|KK854447.1|	63574-68626	10	40
ACYPI004278-RA	gi|641566897|gb|KK857495.1|	13387-17929	11	41
ACYPI005854-RA	gi|641586528|gb|KK854228.1|	263950-265034	10	42
ACYPI006506-RA	gi|641576328|gb|KK854938.1|	119647-120223	11	36
ACYPI008383-RA	gi|641568815|gb|KK856858.1|	91006-91440	10	34
ACYPI000423-RA	gi|641568716|gb|KK856889.1|	35485-37188	10	35
ACYPI003054-RA	gi|641586521|gb|KK854229.1|	636275-636535	11	41
ACYPI003596-RA	gi|641587992|gb|KK854036.1|	286970-287196	11	41
ACYPI004199-RA	gi|641585755|gb|KK854345.1|	205593-205995	9.8	39
ACYPI006737-RA	gi|641560560|gb|KK860138.1|	8238-8655	8.4	26
ACYPI008596-RA	gi|641573583|gb|KK855523.1|	56055-58144	10	41
ACYPI071357-RA	gi|641587881|gb|KK854049.1|	815859-819795	10	40
ACYPI21591-RA	gi|641586521|gb|KK854229.1|	656304-657364	11	41
ACYPI006480-RA	gi|641584743|gb|KK854459.1|	393321-395763	11	36
ACYPI002122-RA	gi|641578325|gb|KK854602.1|	224959-228435	9.7	38
ACYPI008000-RA	gi|641572955|gb|KK855679.1|	38150-39594	10	40
ACYPI27690-RA	gi|641587814|gb|KK854056.1|	547899-549301	9.7	40
ACYPI002966-RA	gi|641573177|gb|KK855625.1|	76394-77382	9.4	34
ACYPI006792-RA	gi|641574320|gb|KK855349.1|	188081-190196	10	38
ACYPI008656-RA	gi|641576423|gb|KK854922.1|	260466-261318	18	35
ACYPI009286-RA	gi|641574320|gb|KK855349.1|	221118-221288	10	38
ACYPI001461-RA	gi|641566611|gb|KK857597.1|	46972-47584	11	40
ACYPI003384-RA	gi|641570039|gb|KK856479.1|	98522-99757	10	40
ACYPI083652-RA	gi|641571632|gb|KK856018.1|	125138-125640	10	36
ACYPI001420-RA	gi|641574220|gb|KK855370.1|	114202-115607	11	21
ACYPI082432-RA	gi|641587671|gb|KK854074.1|	297029-300486	11	40
ACYPI001819-RA	gi|641560854|gb|KK860006.1|	5-413	9.6	17

ACYPI009409-RA	gi|641572199|gb|KK855873.1|	85171-89569	8.1	36
ACYPI001796-RA	gi|641586335|gb|KK854256.1|	322243-324860	10	38
ACYPI003679-RA	gi|641588073|gb|KK854027.1|	414468-415831	11	41
ACYPI005047-RA	gi|641587519|gb|KK854093.1|	353250-353738	11	39
ACYPI006948-RA	gi|641587519|gb|KK854093.1|	383866-385490	11	39
ACYPI009467-RA	gi|641586599|gb|KK854218.1|	230685-230951	11	42
ACYPI002210-RA	gi|641577843|gb|KK854684.1|	157252-158319	10	40
ACYPI004082-RA	gi|641588164|gb|KK854017.1|	791875-794179	9.2	40
ACYPI005528-RA	gi|641587074|gb|KK854148.1|	585817-586526	11	41
ACYPI009950-RA	gi|641575847|gb|KK855033.1|	316234-316628	10	40
ACYPI007870-RA	gi|641567908|gb|KK857148.1|	29602-29818	9.5	32
ACYPI000278-RA	gi|641587365|gb|KK854113.1|	698368-699804	12	45
ACYPI005309-RA	gi|641588309|gb|KK854005.1|	746950-747350	11	40
ACYPI006257-RA	gi|641587047|gb|KK854151.1|	247234-247433	10	39
ACYPI006656-RA	gi|641573533|gb|KK855535.1|	28624-35255	10	41
ACYPI001321-RA	gi|641572813|gb|KK855716.1|	31805-34765	13	51
ACYPI003203-RA	gi|641576286|gb|KK854946.1|	330565-331118	12	38
ACYPI006860-RA	gi|641577541|gb|KK854736.1|	30346-31251	10	38
ACYPI083426-RA	gi|641573861|gb|KK855454.1|	153278-153610	10	37
ACYPI004391-RA	gi|641575436|gb|KK855110.1|	47777-51009	12	37
ACYPI005628-RA	gi|641585469|gb|KK854393.1|	192759-193488	10	37
ACYPI008187-RA	gi|641586837|gb|KK854184.1|	509466-509801	11	40
ACYPI009398-RA	gi|641577228|gb|KK854785.1|	288420-288825	9.7	39
ACYPI010073-RA	gi|641577623|gb|KK854722.1|	21088-26821	11	38
ACYPI073731-RA	gi|641587562|gb|KK854088.1|	103279-103729	11	42
ACYPI000994-RA	gi|641569808|gb|KK856554.1|	81474-85696	11	21
ACYPI071217-RA	gi|641577890|gb|KK854675.1|	86502-88400	10	40
ACYPI22575-RA	gi|641568580|gb|KK856933.1|	52410-58127	12	25
ACYPI004768-RA	gi|641574699|gb|KK855261.1|	139569-144132	9	35
ACYPI006674-RA	gi|641587365|gb|KK854113.1|	484651-485915	12	45

ACYPI000462-RA	gi|641574066|gb|KK855404.1|	59595-62212	11	36
ACYPI000819-RA	gi|641574951|gb|KK855209.1|	76378-78376	10	38
ACYPI001468-RA	gi|641574676|gb|KK855265.1|	65609-70197	9.4	35
ACYPI002528-RA	gi|641573514|gb|KK855540.1|	168024-168615	9.6	37
ACYPI004238-RA	gi|641574642|gb|KK855274.1|	277930-278352	11	38
ACYPI004619-RA	gi|641586258|gb|KK854267.1|	338208-339084	11	37
ACYPI006176-RA	gi|641573514|gb|KK855540.1|	58977-60024	9.6	37
ACYPI007170-RA	gi|641574036|gb|KK855411.1|	176080-179918	11	37
ACYPI007836-RA	gi|641573514|gb|KK855540.1|	72714-73184	9.6	37
ACYPI000627-RA	gi|641573181|gb|KK855624.1|	31523-31870	11	40
ACYPI002553-RA	gi|641587606|gb|KK854082.1|	438327-439224	11	40
ACYPI005155-RA	gi|641564265|gb|KK858512.1|	4459-5466	11	39
ACYPI008850-RA	gi|641575549|gb|KK855090.1|	71202-71687	10	38
ACYPI072156-RA	gi|641587580|gb|KK854085.1|	699769-700637	10	39
ACYPI005175-RA	gi|641572900|gb|KK855693.1|	244365-246416	11	37
ACYPI24155-RA	gi|641576312|gb|KK854941.1|	237497-241550	9.9	38
ACYPI004626-RA	gi|641584582|gb|KK854471.1|	382671-383339	10	38
ACYPI006544-RA	gi|641583980|gb|KK854512.1|	323395-326097	11	39
ACYPI003031-RA	gi|641572022|gb|KK855920.1|	148707-149204	12	42
ACYPI006842-RA	gi|641585833|gb|KK854332.1|	207656-208632	10	37
ACYPI39596-RA	gi|641585734|gb|KK854348.1|	39156-39808	8.4	33
ACYPI001303-RA	gi|641565608|gb|KK857969.1|	16589-17626	13	42
ACYPI000857-RA	gi|641546561|gb|KK866766.1|	555-1179	8.9	15
ACYPI002129-RA	gi|641573673|gb|KK855501.1|	127225-131973	14	43
ACYPI003391-RA	gi|641566302|gb|KK857711.1|	41442-43496	9.3	36
ACYPI004003-RA	gi|641577133|gb|KK854802.1|	208826-214675	9.9	40
ACYPI005942-RA	gi|641579952|gb|KK854573.1|	205929-206105	11	21
ACYPI007207-RA	gi|641587003|gb|KK854159.1|	66719-68229	10	21
ACYPI009502-RA	gi|641578046|gb|KK854650.1|	356110-357864	10	38
ACYPI068497-RA	gi|641574100|gb|KK855396.1|	119383-120282	11	38

ACYPI073116-RA	gi|641571048|gb|KK856174.1|	53719-53950	11	39
ACYPI34878-RA	gi|641575163|gb|KK855166.1|	24491-25461	10	42
ACYPI001672-RA	gi|641578193|gb|KK854625.1|	286028-286363	11	41
ACYPI003875-RA	gi|641576221|gb|KK854958.1|	205645-205892	9.3	35
ACYPI007348-RA	gi|641586905|gb|KK854172.1|	112497-112869	9.9	38
ACYPI009547-RA	gi|641586843|gb|KK854183.1|	386827-387320	11	41
ACYPI000381-RA	gi|641588283|gb|KK854007.1|	1195488-1197009	11	40
ACYPI000610-RA	gi|641570019|gb|KK856485.1|	48852-51596	12	43
ACYPI003020-RA	gi|641579930|gb|KK854578.1|	122293-122608	9.1	35
ACYPI004159-RA	gi|641588283|gb|KK854007.1|	1245655-1247967	11	40
ACYPI006094-RA	gi|641588283|gb|KK854007.1|	1311821-1312368	11	40
ACYPI008191-RA	gi|641579930|gb|KK854578.1|	52763-53391	9.1	35
ACYPI010077-RA	gi|641572484|gb|KK855802.1|	98614-99493	9.2	36
ACYPI086281-RA	gi|641585967|gb|KK854311.1|	434127-435132	11	40
ACYPI064382-RA	gi|641586452|gb|KK854239.1|	244914-246733	10	19
ACYPI000173-RA	gi|641587555|gb|KK854089.1|	672336-673084	11	39
ACYPI001113-RA	gi|641576842|gb|KK854849.1|	143610-144454	12	38
ACYPI007161-RA	gi|641576248|gb|KK854953.1|	12698-13263	11	41
ACYPI009043-RA	gi|641576238|gb|KK854955.1|	83530-84515	11	40
ACYPI003729-RA	gi|641586258|gb|KK854267.1|	108382-109286	11	37
ACYPI008313-RA	gi|641572734|gb|KK855738.1|	227080-227528	11	40
ACYPI061631-RA	gi|641586328|gb|KK854257.1|	369963-370905	8.4	35
ACYPI000886-RA	gi|641565701|gb|KK857932.1|	2599-2857	11	42
ACYPI001538-RA	gi|641568710|gb|KK856891.1|	15841-16729	10	38
ACYPI001812-RA	gi|641580479|gb|KK854569.1|	136686-137928	10	38
ACYPI001560-RA	gi|641574190|gb|KK855376.1|	177804-180071	11	40
ACYPI004586-RA	gi|641588246|gb|KK854010.1|	728496-729755	9.2	50
ACYPI005655-RA	gi|641580486|gb|KK854568.1|	117604-118579	11	36
ACYPI006400-RA	gi|641584128|gb|KK854505.1|	266383-267562	9.6	37
ACYPI008874-RA	gi|641572637|gb|KK855763.1|	138165-142940	9.5	36

ACYPI010059-RA	gi|641586555|gb|KK854224.1|	45442-46112	11	39
ACYPI000821-RA	gi|641587066|gb|KK854149.1|	220873-221337	11	40
ACYPI001024-RA	gi|641587562|gb|KK854088.1|	542807-544057	11	42
ACYPI002275-RA	gi|641586040|gb|KK854300.1|	316250-316547	10	38
ACYPI003357-RA	gi|641587066|gb|KK854149.1|	262550-262960	11	40
ACYPI004621-RA	gi|641567040|gb|KK857445.1|	28366-32770	11	43
ACYPI005588-RA	gi|641587555|gb|KK854089.1|	302025-302829	11	39
ACYPI006089-RA	gi|641586509|gb|KK854231.1|	511944-513064	9.8	37
ACYPI006841-RA	gi|641577407|gb|KK854756.1|	150713-152613	9.4	39
ACYPI007972-RA	gi|641572963|gb|KK855677.1|	195371-198152	11	43
ACYPI008384-RA	gi|641583958|gb|KK854513.1|	280088-280234	10	22
ACYPI009840-RA	gi|641586898|gb|KK854174.1|	71401-71661	11	39
ACYPI062011-RA	gi|641570807|gb|KK856248.1|	13194-14713	11	38
ACYPI062641-RA	gi|641587390|gb|KK854109.1|	469519-470797	11	44
ACYPI003778-RA	gi|641569564|gb|KK856632.1|	53109-56151	9.8	34
ACYPI009686-RA	gi|641587823|gb|KK854055.1|	477452-480920	11	22
ACYPI005116-RA	gi|641572177|gb|KK855878.1|	128479-129591	10	35
ACYPI063378-RA	gi|641588325|gb|KK854004.1|	262486-263425	11	40
ACYPI081575-RA	gi|641588325|gb|KK854004.1|	288983-290483	11	40
ACYPI000592-RA	gi|641586915|gb|KK854171.1|	304011-304195	10	38
ACYPI002162-RA	gi|641577810|gb|KK854690.1|	445973-446254	9.8	39
ACYPI002617-RA	gi|641586171|gb|KK854278.1|	550701-551299	9	39
ACYPI001704-RA	gi|641587577|gb|KK854086.1|	646483-646726	8.2	41
ACYPI003588-RA	gi|641578106|gb|KK854639.1|	387898-388315	11	39
ACYPI009921-RA	gi|641576741|gb|KK854868.1|	145783-146299	9.5	40
ACYPI000626-RA	gi|641587468|gb|KK854099.1|	604780-605044	10	42
ACYPI006763-RA	gi|641577529|gb|KK854738.1|	221540-221860	9.8	35
ACYPI008203-RA	gi|641587866|gb|KK854050.1|	243237-243456	10	38
ACYPI009489-RA	gi|641588033|gb|KK854031.1|	142559-142931	11	38
ACYPI010132-RA	gi|641584404|gb|KK854484.1|	476614-477037	11	30

ACYPI002333-RA	gi|641564728|gb|KK858311.1|	15637-16597	11	43
ACYPI002477-RA	gi|641578167|gb|KK854629.1|	252113-252572	11	41
ACYPI004216-RA	gi|641576608|gb|KK854891.1|	239405-240511	11	37
ACYPI006955-RA	gi|641573673|gb|KK855501.1|	79796-81170	14	43
ACYPI009231-RA	gi|641570961|gb|KK856201.1|	39795-40511	8.9	38
ACYPI29381-RA	gi|641587606|gb|KK854082.1|	823057-826822	11	40
ACYPI001956-RA	gi|641568223|gb|KK857044.1|	60366-62639	11	39
ACYPI002140-RA	gi|641566693|gb|KK857568.1|	43804-44746	8.5	33
ACYPI002413-RA	gi|641569432|gb|KK856672.1|	40095-40488	13	47
ACYPI004015-RA	gi|641576161|gb|KK854972.1|	225340-226431	10	36
ACYPI004314-RA	gi|641569432|gb|KK856672.1|	45445-46089	13	47
ACYPI005189-RA	gi|641577688|gb|KK854709.1|	117906-118819	11	40
ACYPI006245-RA	gi|641576022|gb|KK854999.1|	328627-330458	10	36
ACYPI010179-RA	gi|641585967|gb|KK854311.1|	463200-463409	11	40
ACYPI000588-RA	gi|641588213|gb|KK854013.1|	1324598-1326630	9.6	39
ACYPI003214-RA	gi|641588021|gb|KK854033.1|	1153237-1156014	12	40
ACYPI003413-RA	gi|641573202|gb|KK855619.1|	194482-194897	9.6	42
ACYPI004121-RA	gi|641577905|gb|KK854672.1|	273652-274627	10	37
ACYPI004683-RA	gi|641586950|gb|KK854166.1|	592407-594372	10	38
ACYPI009810-RA	gi|641587126|gb|KK854141.1|	734179-734795	10	39
ACYPI010030-RA	gi|641586387|gb|KK854249.1|	45336-47577	11	24
ACYPI067965-RA	gi|641577271|gb|KK854779.1|	138903-140040	11	21
ACYPI56610-RA	gi|641575559|gb|KK855088.1|	194613-195059	9.9	37
ACYPI001274-RA	gi|641586990|gb|KK854161.1|	281920-282717	11	39
ACYPI003160-RA	gi|641570589|gb|KK856310.1|	31546-32004	11	36
ACYPI002289-RA	gi|641572501|gb|KK855798.1|	19130-19898	9.8	39
ACYPI007346-RA	gi|641574512|gb|KK855303.1|	134839-135225	9.3	37
ACYPI007748-RA	gi|641587757|gb|KK854063.1|	262374-272220	11	38
ACYPI009639-RA	gi|641574600|gb|KK855283.1|	217763-218798	44	35
ACYPI082098-RA	gi|641574512|gb|KK855303.1|	131685-132026	9.3	37

ACYPI001886-RA	gi|641584862|gb|KK854451.1|	239457-239709	10	38
ACYPI002952-RA	gi|641586817|gb|KK854187.1|	607698-610264	9.3	39
ACYPI073693-RA	gi|641571642|gb|KK856015.1|	166257-166715	11	37
ACYPI001530-RA	gi|641576807|gb|KK854855.1|	128321-129571	9.8	37
ACYPI002045-RA	gi|641573171|gb|KK855626.1|	172175-173913	9.9	40
ACYPI004034-RA	gi|641587981|gb|KK854037.1|	31937-32608	10	40
ACYPI005976-RA	gi|641576437|gb|KK854920.1|	198790-199072	10	39
ACYPI007840-RA	gi|641584108|gb|KK854506.1|	294658-295320	11	40
ACYPI47578-RA	gi|641572114|gb|KK855896.1|	206254-207705	10	36
ACYPI006615-RA	gi|641576946|gb|KK854832.1|	84306-88272	9.4	43
ACYPI065331-RA	gi|641578039|gb|KK854651.1|	469292-470401	10	38
ACYPI080586-RA	gi|641576362|gb|KK854933.1|	298613-302490	10	37
ACYPI41828-RA	gi|641572916|gb|KK855689.1|	93660-97575	9.2	33
ACYPI073199-RA	gi|641577516|gb|KK854740.1|	141079-142003	10	21
ACYPI086047-RA	gi|641577826|gb|KK854687.1|	315702-316254	9.4	37
ACYPI001552-RA	gi|641571067|gb|KK856169.1|	41356-42011	11	39
ACYPI009137-RA	gi|641585521|gb|KK854384.1|	392989-393350	9.7	38
ACYPI063157-RA	gi|641588309|gb|KK854005.1|	1011727-1013218	11	40
ACYPI001198-RA	gi|641586857|gb|KK854181.1|	335115-335566	9.8	39
ACYPI001832-RA	gi|641571950|gb|KK855940.1|	12920-13177	10	37
ACYPI003715-RA	gi|641576882|gb|KK854843.1|	255411-256105	10	39
ACYPI006894-RA	gi|641586880|gb|KK854177.1|	200912-202733	10	20
ACYPI010098-RA	gi|641575804|gb|KK855041.1|	28570-28895	11	40
ACYPI002483-RA	gi|641576371|gb|KK854931.1|	107823-109660	10	38
ACYPI010153-RA	gi|641587912|gb|KK854045.1|	718233-719185	11	39
ACYPI50889-RA	gi|641572247|gb|KK855861.1|	41521-43952	10	37
ACYPI000365-RA	gi|641576442|gb|KK854919.1|	238025-238534	11	36
ACYPI001129-RA	gi|641576392|gb|KK854928.1|	227290-228776	9.7	41
ACYPI004145-RA	gi|641577379|gb|KK854761.1|	252353-258055	11	41
ACYPI006740-RA	gi|641586372|gb|KK854251.1|	543974-548728	10	38

ACYPI007358-RA	gi|641586372|gb|KK854251.1|	592904-593520	10	38
ACYPI009061-RA	gi|641576962|gb|KK854829.1|	342812-343477	10	39
ACYPI46593-RA	gi|641585574|gb|KK854375.1|	158933-160755	11	39
ACYPI002517-RA	gi|641586452|gb|KK854239.1|	426678-427305	10	19
ACYPI004419-RA	gi|641586809|gb|KK854188.1|	179013-181622	10	43
ACYPI000099-RA	gi|641577522|gb|KK854739.1|	88318-95019	11	41
ACYPI005271-RA	gi|641567973|gb|KK857126.1|	55336-55499	10	39
ACYPI006518-RA	gi|641580970|gb|KK854563.1|	305168-307888	11	42
ACYPI062510-RA	gi|641587840|gb|KK854053.1|	675415-675775	11	43
ACYPI067116-RA	gi|641587840|gb|KK854053.1|	785744-786290	11	43
ACYPI072184-RA	gi|641571052|gb|KK856173.1|	14896-15551	8.9	41
ACYPI45089-RA	gi|641586096|gb|KK854291.1|	83256-87483	11	46
ACYPI001742-RA	gi|641576347|gb|KK854934.1|	85516-86354	11	39
ACYPI002579-RA	gi|641577511|gb|KK854741.1|	221813-223353	11	38
ACYPI008278-RA	gi|641575010|gb|KK855196.1|	268989-269206	12	41
ACYPI000320-RA	gi|641570570|gb|KK856316.1|	89342-89634	10	35
ACYPI001138-RA	gi|641568953|gb|KK856815.1|	58826-59321	9.1	41
ACYPI008359-RA	gi|641576887|gb|KK854842.1|	325774-330450	15	41
ACYPI000252-RA	gi|641586843|gb|KK854183.1|	524314-536066	11	41
ACYPI003751-RA	gi|641586943|gb|KK854167.1|	163442-163698	11	38
ACYPI001601-RA	gi|641586661|gb|KK854209.1|	369308-369889	11	43
ACYPI007932-RA	gi|641577760|gb|KK854699.1|	227257-228128	9.8	41
ACYPI008991-RA	gi|641577104|gb|KK854807.1|	123967-127570	10	36
ACYPI009423-RA	gi|641574549|gb|KK855294.1|	122615-123593	8.9	38
ACYPI009806-RA	gi|641587921|gb|KK854044.1|	528720-529611	9.4	41
ACYPI000843-RA	gi|641585940|gb|KK854316.1|	350205-352225	11	42
ACYPI001416-RA	gi|641577644|gb|KK854718.1|	211459-211772	10	40
ACYPI005899-RA	gi|641575377|gb|KK855123.1|	25715-27356	10	38
ACYPI000096-RA	gi|641569976|gb|KK856499.1|	123205-123471	10	43
ACYPI001850-RA	gi|641585622|gb|KK854367.1|	355084-355299	9.9	21

ACYPI006170-RA	gi|641571667|gb|KK856008.1|	105760-107467	11	39
ACYPI008124-RA	gi|641576437|gb|KK854920.1|	414250-414600	10	39
ACYPI009976-RA	gi|641587937|gb|KK854042.1|	138074-139341	11	42
ACYPI081707-RA	gi|641587937|gb|KK854042.1|	201609-206224	11	42
ACYPI001760-RA	gi|641570727|gb|KK856270.1|	141147-141732	10	35
ACYPI002754-RA	gi|641585434|gb|KK854398.1|	151564-155764	13	51
ACYPI003632-RA	gi|641586091|gb|KK854292.1|	336721-337703	9.7	37
ACYPI004929-RA	gi|641587624|gb|KK854080.1|	453905-454210	10	38
ACYPI009334-RA	gi|641587937|gb|KK854042.1|	796973-797800	11	42
ACYPI002577-RA	gi|641588182|gb|KK854016.1|	342557-342911	11	41
ACYPI000368-RA	gi|641575096|gb|KK855181.1|	128586-128796	11	21
ACYPI003885-RA	gi|641584894|gb|KK854448.1|	488267-489072	10	44
ACYPI008166-RA	gi|641577234|gb|KK854784.1|	179623-184486	11	39
ACYPI008605-RA	gi|641575236|gb|KK855151.1|	76033-76816	11	38
ACYPI081683-RA	gi|641586240|gb|KK854269.1|	430999-433684	11	21
ACYPI002662-RA	gi|641587840|gb|KK854053.1|	178836-179852	11	43
ACYPI001667-RA	gi|641570579|gb|KK856313.1|	129453-131170	11	38
ACYPI002841-RA	gi|641570579|gb|KK856313.1|	43225-43878	11	38
ACYPI004063-RA	gi|641588136|gb|KK854020.1|	535260-536802	11	36
ACYPI004759-RA	gi|641573457|gb|KK855555.1|	200513-201000	9.7	38
ACYPI006008-RA	gi|641558930|gb|KK860898.1|	7198-8430	8.8	34
ACYPI006262-RA	gi|641586850|gb|KK854182.1|	150369-150970	10	37
ACYPI006662-RA	gi|641573486|gb|KK855548.1|	182515-182959	9.4	38
ACYPI005219-RA	gi|641567206|gb|KK857389.1|	49552-53372	10	37
ACYPI006823-RA	gi|641575168|gb|KK855165.1|	112036-112546	9.6	36
ACYPI007124-RA	gi|641575337|gb|KK855131.1|	205781-206179	10	37
ACYPI009001-RA	gi|641586903|gb|KK854173.1|	424884-426629	11	45
ACYPI068502-RA	gi|641576104|gb|KK854983.1|	208186-208529	10	20
ACYPI21437-RA	gi|641588202|gb|KK854014.1|	1203755-1204365	10	21
ACYPI000564-RA	gi|641587624|gb|KK854080.1|	467253-467710	10	38

ACYPI001210-RA	gi|641573881|gb|KK855448.1|	101484-101661	9	33
ACYPI005607-RA	gi|641573015|gb|KK855664.1|	83545-84307	11	42
ACYPI008781-RA	gi|641587580|gb|KK854085.1|	321884-323127	10	39
ACYPI008926-RA	gi|641574549|gb|KK855294.1|	92150-95534	8.9	38
ACYPI064034-RA	gi|641587800|gb|KK854058.1|	538978-539906	10	42
ACYPI000739-RA	gi|641577574|gb|KK854731.1|	295485-296034	11	38
ACYPI002084-RA	gi|641571045|gb|KK856175.1|	20192-20509	9.3	35
ACYPI002399-RA	gi|641576260|gb|KK854950.1|	131708-131943	10	39
ACYPI003960-RA	gi|641572177|gb|KK855878.1|	22173-22436	10	35
ACYPI005207-RA	gi|641586857|gb|KK854181.1|	442949-449629	9.8	39
ACYPI006229-RA	gi|641587095|gb|KK854145.1|	132292-132741	9.9	40
ACYPI007132-RA	gi|641588119|gb|KK854022.1|	315655-316106	10	40
ACYPI007703-RA	gi|641576677|gb|KK854879.1|	161762-162186	11	39
ACYPI40062-RA	gi|641565471|gb|KK858017.1|	12751-12941	9.8	40
ACYPI50391-RA	gi|641577574|gb|KK854731.1|	222756-223349	11	38
ACYPI000329-RA	gi|641549615|gb|KK865290.1|	58-1415	12	24
ACYPI001057-RA	gi|641578144|gb|KK854633.1|	112495-112721	10	43
ACYPI002306-RA	gi|641588236|gb|KK854011.1|	290015-292668	9.7	38
ACYPI002895-RA	gi|641586033|gb|KK854301.1|	448471-449376	11	21
ACYPI004185-RA	gi|641585653|gb|KK854362.1|	32202-34531	10	39
ACYPI005535-RA	gi|641570280|gb|KK856402.1|	107703-108266	11	40
ACYPI002835-RA	gi|641588024|gb|KK854032.1|	480266-482661	9.7	41
ACYPI072241-RA	gi|641588325|gb|KK854004.1|	1389588-1390459	11	40
ACYPI085401-RA	gi|641588024|gb|KK854032.1|	343553-343787	9.7	41
ACYPI005683-RA	gi|641576842|gb|KK854849.1|	297327-298693	12	38
ACYPI004514-RA	gi|641578173|gb|KK854628.1|	66871-67451	9.9	21
ACYPI006434-RA	gi|641575763|gb|KK855049.1|	162467-163590	10	40
ACYPI063189-RA	gi|641575568|gb|KK855086.1|	173860-179107	11	40
ACYPI000149-RA	gi|641580970|gb|KK854563.1|	170493-175598	11	42
ACYPI001462-RA	gi|641572022|gb|KK855920.1|	124118-125042	12	42

ACYPI003349-RA	gi|641567905|gb|KK857149.1|	93693-94495	10	38
ACYPI003937-RA	gi|641587249|gb|KK854125.1|	137537-140394	11	37
ACYPI003961-RA	gi|641564341|gb|KK858480.1|	17488-17807	11	19
ACYPI004578-RA	gi|641587171|gb|KK854135.1|	751428-751713	10	39
ACYPI005090-RA	gi|641585350|gb|KK854411.1|	302832-303986	11	38
ACYPI005246-RA	gi|641585412|gb|KK854402.1|	98053-99375	9.8	39
ACYPI005282-RA	gi|641573036|gb|KK855659.1|	185266-190129	13	49
ACYPI007166-RA	gi|641588000|gb|KK854035.1|	1040026-1046739	11	39
ACYPI009008-RA	gi|641577138|gb|KK854801.1|	291381-297215	10	38
ACYPI009634-RA	gi|641573742|gb|KK855485.1|	63956-66942	9.9	36
ACYPI009679-RA	gi|641567358|gb|KK857335.1|	46990-47339	10	36
ACYPI080661-RA	gi|641585690|gb|KK854356.1|	454002-459218	9.6	41
ACYPI007014-RA	gi|641571315|gb|KK856104.1|	171600-174636	12	46
ACYPI008637-RA	gi|641576852|gb|KK854847.1|	250741-252923	13	48
ACYPI34754-RA	gi|641576852|gb|KK854847.1|	249350-250414	13	48
ACYPI004696-RA	gi|641586114|gb|KK854288.1|	198254-202401	10	41
ACYPI006602-RA	gi|641578406|gb|KK854594.1|	258805-258995	10	37
ACYPI24234-RA	gi|641578179|gb|KK854627.1|	92700-94577	10	39
ACYPI003530-RA	gi|641588060|gb|KK854028.1|	698606-699784	11	42
ACYPI005478-RA	gi|641588060|gb|KK854028.1|	715514-715774	11	42
ACYPI002088-RA	gi|641587555|gb|KK854089.1|	904242-904931	11	39
ACYPI002656-RA	gi|641573950|gb|KK855430.1|	123007-123322	10	19
ACYPI003967-RA	gi|641588358|gb|KK854002.1|	1076939-1080307	11	43
ACYPI005300-RA	gi|641566900|gb|KK857494.1|	38695-39377	9.9	40
ACYPI008930-RA	gi|641587511|gb|KK854094.1|	191946-195123	10	41
ACYPI52843-RA	gi|641570707|gb|KK856276.1|	26880-30000	9.4	20
ACYPI000304-RA	gi|641568637|gb|KK856916.1|	75126-76167	10	38
ACYPI000589-RA	gi|641586203|gb|KK854273.1|	579183-580802	9.5	40
ACYPI001241-RA	gi|641588033|gb|KK854031.1|	179262-181521	11	38
ACYPI001460-RA	gi|641577574|gb|KK854731.1|	341242-341687	11	38

ACYPI002470-RA	gi|641548005|gb|KK866068.1|	4-704	9.8	29
ACYPI003749-RA	gi|641569036|gb|KK856790.1|	105472-105984	10	36
ACYPI060717-RA	gi|641570630|gb|KK856298.1|	2-1431	12	47
ACYPI088137-RA	gi|641588033|gb|KK854031.1|	155888-157153	11	38
ACYPI27999-RA	gi|641571380|gb|KK856086.1|	27646-27993	9.5	38
ACYPI38406-RA	gi|641588309|gb|KK854005.1|	662199-662369	11	40
ACYPI007899-RA	gi|641573504|gb|KK855543.1|	261852-262314	13	39
ACYPI008205-RA	gi|641585533|gb|KK854382.1|	419979-420361	10	40
ACYPI008877-RA	gi|641559039|gb|KK860845.1|	10160-10958	8.4	33
ACYPI009769-RA	gi|641575730|gb|KK855054.1|	256154-257944	10	38
ACYPI010095-RA	gi|641586521|gb|KK854229.1|	544257-545384	11	41
ACYPI000408-RA	gi|641583088|gb|KK854527.1|	248264-249291	9.9	39
ACYPI002857-RA	gi|641584606|gb|KK854469.1|	312984-313557	13	57
ACYPI002950-RA	gi|641575837|gb|KK855035.1|	18536-19049	11	46
ACYPI004183-RA	gi|641583088|gb|KK854527.1|	401534-403976	9.9	39
ACYPI004328-RA	gi|641587118|gb|KK854142.1|	369296-370713	11	40
ACYPI004979-RA	gi|641587074|gb|KK854148.1|	626072-627565	11	41
ACYPI006109-RA	gi|641570124|gb|KK856453.1|	117498-118459	8.7	40
ACYPI007706-RA	gi|641577322|gb|KK854771.1|	94424-94943	10	21
ACYPI007769-RA	gi|641577623|gb|KK854722.1|	263042-263482	11	38
ACYPI009606-RA	gi|641583088|gb|KK854527.1|	245670-247152	9.9	39
ACYPI083537-RA	gi|641585871|gb|KK854327.1|	563720-564332	11	38
ACYPI010131-RA	gi|641588182|gb|KK854016.1|	1244468-1245244	11	41
ACYPI009768-RA	gi|641572402|gb|KK855823.1|	95767-96000	11	40
ACYPI001073-RA	gi|641585871|gb|KK854327.1|	251810-252687	11	38
ACYPI001228-RA	gi|641581389|gb|KK854557.1|	338500-342765	11	41
ACYPI002741-RA	gi|641577905|gb|KK854672.1|	31310-31567	10	37
ACYPI003468-RA	gi|641576243|gb|KK854954.1|	316301-316601	10	41
ACYPI009294-RA	gi|641586246|gb|KK854268.1|	330052-331641	10	38
ACYPI063443-RA	gi|641575197|gb|KK855159.1|	106160-107475	11	20

ACYPI51295-RA	gi|641566664|gb|KK857578.1|	62041-63366	9.8	38
ACYPI009851-RA	gi|641567657|gb|KK857233.1|	31443-31659	9.9	35
ACYPI002147-RA	gi|641588136|gb|KK854020.1|	159940-160750	11	36
ACYPI002982-RA	gi|641577634|gb|KK854720.1|	79849-80286	9.5	41
ACYPI005204-RA	gi|641577347|gb|KK854767.1|	411240-411688	9.9	43
ACYPI060618-RA	gi|641586033|gb|KK854301.1|	343208-344770	11	21
ACYPI001763-RA	gi|641573858|gb|KK855455.1|	167987-169455	9.8	37
ACYPI004773-RA	gi|641566230|gb|KK857737.1|	17031-17423	6.3	20
ACYPI000521-RA	gi|641587848|gb|KK854052.1|	631208-633367	11	39
ACYPI002256-RA	gi|641587905|gb|KK854046.1|	131767-132011	10	43
ACYPI002404-RA	gi|641575236|gb|KK855151.1|	365826-371792	11	38
ACYPI003133-RA	gi|641587742|gb|KK854065.1|	796616-797670	11	41
ACYPI005144-RA	gi|641587905|gb|KK854046.1|	533605-533982	10	43
ACYPI006949-RA	gi|641587452|gb|KK854101.1|	225773-229455	11	40
ACYPI007422-RA	gi|641586019|gb|KK854303.1|	356684-356939	11	43
ACYPI061529-RA	gi|641586535|gb|KK854227.1|	42388-43166	10	39
ACYPI070106-RA	gi|641584205|gb|KK854500.1|	62916-63810	11	41
ACYPI000104-RA	gi|641576414|gb|KK854924.1|	81163-82561	10	40
ACYPI008382-RA	gi|641576414|gb|KK854924.1|	86650-87522	10	40
ACYPI22867-RA	gi|641586344|gb|KK854254.1|	451395-451843	10	40
ACYPI008019-RA	gi|641567869|gb|KK857161.1|	66585-68533	11	38
ACYPI008497-RA	gi|641565378|gb|KK858053.1|	25-222	11	35
ACYPI009117-RA	gi|641586626|gb|KK854214.1|	366486-367240	11	44
ACYPI000430-RA	gi|641572219|gb|KK855868.1|	62624-68729	9.8	35
ACYPI001193-RA	gi|641588109|gb|KK854023.1|	588844-589503	10	40
ACYPI002320-RA	gi|641588309|gb|KK854005.1|	460033-461608	11	40
ACYPI006797-RA	gi|641587921|gb|KK854044.1|	327919-328598	9.4	41
ACYPI007330-RA	gi|641577754|gb|KK854700.1|	65600-65793	12	43
ACYPI008664-RA	gi|641571530|gb|KK856044.1|	84770-88688	10	38
ACYPI21190-RA	gi|641571508|gb|KK856050.1|	96814-101943	9.7	33

ACYPI004368-RA	gi|641586399|gb|KK854247.1|	542392-545421	15	68
ACYPI005195-RA	gi|641570587|gb|KK856311.1|	73300-76214	9.9	38
ACYPI006301-RA	gi|641586843|gb|KK854183.1|	359156-361428	11	41
ACYPI006767-RA	gi|641586785|gb|KK854191.1|	231154-232262	11	39
ACYPI007250-RA	gi|641583958|gb|KK854513.1|	442876-444265	10	22
ACYPI007433-RA	gi|641586046|gb|KK854299.1|	271781-273287	10	20
ACYPI009536-RA	gi|641567126|gb|KK857416.1|	49745-52578	11	41
ACYPI56660-RA	gi|641587719|gb|KK854068.1|	186084-186425	10	21
ACYPI000035-RA	gi|641574476|gb|KK855312.1|	158895-159679	10	43
ACYPI001186-RA	gi|641577309|gb|KK854773.1|	204496-205576	9.9	39
ACYPI001539-RA	gi|641588128|gb|KK854021.1|	4084-4769	9.5	42
ACYPI004701-RA	gi|641586496|gb|KK854233.1|	83529-86482	11	38
ACYPI005987-RA	gi|641585967|gb|KK854311.1|	143701-144312	11	40
ACYPI006231-RA	gi|641586528|gb|KK854228.1|	280805-281016	10	42
ACYPI007232-RA	gi|641554387|gb|KK863045.1|	1808-3470	8.9	34
ACYPI49907-RA	gi|641587276|gb|KK854121.1|	653084-654893	11	39
ACYPI004774-RA	gi|641585773|gb|KK854342.1|	97482-97841	12	45
ACYPI008539-RA	gi|641570169|gb|KK856439.1|	24540-24771	10	36
ACYPI001593-RA	gi|641571716|gb|KK855999.1|	78612-79014	11	39
ACYPI003962-RA	gi|641586569|gb|KK854222.1|	93751-96591	12	40
ACYPI006129-RA	gi|641575946|gb|KK855016.1|	126107-127271	9.5	37
ACYPI006138-RA	gi|641585957|gb|KK854313.1|	295909-296414	10	37
ACYPI009257-RA	gi|641587937|gb|KK854042.1|	6600-6973	11	42
ACYPI010103-RA	gi|641588033|gb|KK854031.1|	232678-233225	11	38
ACYPI072994-RA	gi|641567672|gb|KK857228.1|	25216-26937	10	36
ACYPI000467-RA	gi|641570703|gb|KK856277.1|	109132-109483	9.9	36
ACYPI001501-RA	gi|641573752|gb|KK855482.1|	106493-106988	11	37
ACYPI002057-RA	gi|641570703|gb|KK856277.1|	80609-81848	9.9	36
ACYPI005332-RA	gi|641576290|gb|KK854945.1|	259801-260593	9.3	20
ACYPI005434-RA	gi|641574508|gb|KK855304.1|	201492-202297	11	22

ACYPI008701-RA	gi|641585423|gb|KK854400.1|	206389-206732	11	36
ACYPI009090-RA	gi|641570697|gb|KK856279.1|	166834-167257	8.9	37
ACYPI071353-RA	gi|641555084|gb|KK862709.1|	261-639	8.3	27
ACYPI088146-RA	gi|641587219|gb|KK854129.1|	10808-11868	9.9	41
ACYPI005426-RA	gi|641584540|gb|KK854474.1|	62206-63602	9.9	39
ACYPI062442-RA	gi|641568780|gb|KK856870.1|	20971-21332	8.8	42
ACYPI50219-RA	gi|641572124|gb|KK855893.1|	103554-104387	10	40
ACYPI001652-RA	gi|641574320|gb|KK855349.1|	40474-47592	10	38
ACYPI009439-RA	gi|641588325|gb|KK854004.1|	1161609-1166969	11	40
ACYPI003225-RA	gi|641587711|gb|KK854069.1|	666062-666370	11	42
ACYPI003442-RA	gi|641587866|gb|KK854050.1|	589093-589297	10	38
ACYPI004634-RA	gi|641576684|gb|KK854877.1|	6788-7352	10	20
ACYPI004669-RA	gi|641584728|gb|KK854460.1|	395111-402829	9.5	37
ACYPI009065-RA	gi|641574504|gb|KK855305.1|	88090-93539	9.9	39
ACYPI009098-RA	gi|641585778|gb|KK854341.1|	165433-168433	9.7	41
ACYPI084297-RA	gi|641573729|gb|KK855488.1|	194069-196581	10	43
ACYPI000085-RA	gi|641585940|gb|KK854316.1|	469449-469792	11	42
ACYPI000500-RA	gi|641579205|gb|KK854586.1|	154410-154702	10	39
ACYPI000532-RA	gi|641584504|gb|KK854477.1|	134770-136269	9.5	38
ACYPI002389-RA	gi|641569574|gb|KK856629.1|	64067-64549	24	########
ACYPI003672-RA	gi|641586509|gb|KK854231.1|	517194-517938	9.8	37
ACYPI004282-RA	gi|641586850|gb|KK854182.1|	581652-582098	10	37
ACYPI004316-RA	gi|641578046|gb|KK854650.1|	422149-424094	10	38
ACYPI083213-RA	gi|641588128|gb|KK854021.1|	1099394-1099900	9.5	42
ACYPI000876-RA	gi|641578305|gb|KK854605.1|	64482-66730	11	42
ACYPI005350-RA	gi|641588033|gb|KK854031.1|	842532-845820	11	38
ACYPI007373-RA	gi|641567822|gb|KK857177.1|	61341-62200	10	42
ACYPI070323-RA	gi|641542070|gb|KK868923.1|	142-506	6.9	27
ACYPI45536-RA	gi|641566907|gb|KK857492.1|	57-221	11	47
ACYPI000257-RA	gi|641586150|gb|KK854282.1|	456801-457130	11	40

ACYPI003333-RA	gi|641587272|gb|KK854122.1|	306292-306522	9.3	42
ACYPI004031-RA	gi|641587696|gb|KK854071.1|	550344-551738	8.9	39
ACYPI004733-RA	gi|641584517|gb|KK854476.1|	324639-327725	10	39
ACYPI005313-RA	gi|641576328|gb|KK854938.1|	182188-182646	11	36
ACYPI006202-RA	gi|641569815|gb|KK856552.1|	102841-103416	9.5	37
ACYPI007194-RA	gi|641572142|gb|KK855887.1|	175077-178644	10	36
ACYPI008493-RA	gi|641576801|gb|KK854856.1|	342247-342594	10	39
ACYPI009150-RA	gi|641582439|gb|KK854536.1|	36014-37731	11	36
ACYPI000944-RA	gi|641577487|gb|KK854746.1|	128558-131647	11	38
ACYPI002533-RA	gi|641572971|gb|KK855675.1|	185833-186008	9.7	20
ACYPI002840-RA	gi|641567062|gb|KK857438.1|	35453-36137	9.8	36
ACYPI000406-RA	gi|641586161|gb|KK854280.1|	234528-234768	11	39
ACYPI001189-RA	gi|641587800|gb|KK854058.1|	980034-980295	10	42
ACYPI001658-RA	gi|641567435|gb|KK857308.1|	24692-26512	10	38
ACYPI002302-RA	gi|641586161|gb|KK854280.1|	203459-203706	11	39
ACYPI003232-RA	gi|641582439|gb|KK854536.1|	442397-447574	11	36
ACYPI003702-RA	gi|641586893|gb|KK854175.1|	619974-620598	11	41
ACYPI007653-RA	gi|641587417|gb|KK854105.1|	195-644	11	42
ACYPI008002-RA	gi|641575436|gb|KK855110.1|	35050-35312	12	37
ACYPI009554-RA	gi|641571760|gb|KK855988.1|	88155-89507	9.9	37
ACYPI000087-RA	gi|641582439|gb|KK854536.1|	393006-396207	11	36
ACYPI000938-RA	gi|641570086|gb|KK856464.1|	107527-107930	10	35
ACYPI004755-RA	gi|641588090|gb|KK854025.1|	993386-994124	11	43
ACYPI005419-RA	gi|641562852|gb|KK859115.1|	4933-6125	10	35
ACYPI007288-RA	gi|641569833|gb|KK856547.1|	31921-33075	8.9	37
ACYPI009174-RA	gi|641584862|gb|KK854451.1|	383447-384643	10	38
ACYPI084854-RA	gi|641588325|gb|KK854004.1|	1147664-1150408	11	40
ACYPI004245-RA	gi|641586673|gb|KK854207.1|	621323-621637	12	40
ACYPI005925-RA	gi|641575218|gb|KK855155.1|	5278-5596	11	21
ACYPI005955-RA	gi|641556681|gb|KK861957.1|	16026-16331	9.6	38

ACYPI060375-RA	gi|641576298|gb|KK854943.1|	244250-245028	12	36
ACYPI067270-RA	gi|641576055|gb|KK854992.1|	323781-325117	9.7	37
ACYPI003478-RA	gi|641568786|gb|KK856868.1|	75902-76146	11	39
ACYPI007287-RA	gi|641571311|gb|KK856105.1|	41529-41876	9.2	37
ACYPI007454-RA	gi|641587921|gb|KK854044.1|	520837-521133	9.4	41
ACYPI086727-RA	gi|641588283|gb|KK854007.1|	983053-985086	11	40
ACYPI001483-RA	gi|641587624|gb|KK854080.1|	733099-733374	10	38
ACYPI002558-RA	gi|641587047|gb|KK854151.1|	335150-336728	10	39
ACYPI003568-RA	gi|641554782|gb|KK862854.1|	102-305	7.8	21
ACYPI004460-RA	gi|641567657|gb|KK857233.1|	33767-34058	9.9	35
ACYPI006375-RA	gi|641577203|gb|KK854789.1|	288609-290702	11	43
ACYPI007801-RA	gi|641587047|gb|KK854151.1|	396198-398027	10	39
ACYPI55712-RA	gi|641577805|gb|KK854691.1|	412030-412904	9.4	38
ACYPI56745-RA	gi|641587905|gb|KK854046.1|	54465-56561	10	43
ACYPI007692-RA	gi|641572139|gb|KK855888.1|	106079-106632	9.9	36
ACYPI27227-RA	gi|641586639|gb|KK854212.1|	371609-374681	8.3	41
ACYPI38606-RA	gi|641587226|gb|KK854128.1|	369365-370150	9.9	39
ACYPI50560-RA	gi|641586072|gb|KK854295.1|	195371-196458	10	20
ACYPI000295-RA	gi|641587962|gb|KK854039.1|	841203-841667	11	40
ACYPI000335-RA	gi|641584248|gb|KK854496.1|	404807-405231	9.9	39
ACYPI000985-RA	gi|641577574|gb|KK854731.1|	95648-97592	11	38
ACYPI002204-RA	gi|641587962|gb|KK854039.1|	799337-799719	11	40
ACYPI002237-RA	gi|641575454|gb|KK855107.1|	65826-66043	10	38
ACYPI003456-RA	gi|641574990|gb|KK855200.1|	201530-202634	12	37
ACYPI005389-RA	gi|641575759|gb|KK855050.1|	389881-390358	11	38
ACYPI006714-RA	gi|641587365|gb|KK854113.1|	545658-548159	12	45
ACYPI009902-RA	gi|641587981|gb|KK854037.1|	467814-468226	10	40
ACYPI061808-RA	gi|641573361|gb|KK855581.1|	62156-62666	11	24
ACYPI063800-RA	gi|641569377|gb|KK856689.1|	29490-31948	9.9	21
ACYPI067416-RA	gi|641587569|gb|KK854087.1|	691518-691930	11	41

ACYPI56208-RA	gi|641578236|gb|KK854616.1|	269244-269676	8.3	35
ACYPI005908-RA	gi|641573733|gb|KK855487.1|	23403-24610	13	45
ACYPI010244-RA	gi|641586121|gb|KK854287.1|	33800-34240	11	43
ACYPI084620-RA	gi|641576203|gb|KK854962.1|	146165-147967	9.8	36
ACYPI000851-RA	gi|641578128|gb|KK854635.1|	427441-429665	11	41
ACYPI002812-RA	gi|641588033|gb|KK854031.1|	574025-574677	11	38
ACYPI005364-RA	gi|641576791|gb|KK854858.1|	126215-126547	9.2	42
ACYPI009082-RA	gi|641578128|gb|KK854635.1|	382574-382916	11	41
ACYPI23394-RA	gi|641578109|gb|KK854638.1|	117534-120444	14	46
ACYPI001085-RA	gi|641577121|gb|KK854804.1|	18496-18745	16	82
ACYPI002260-RA	gi|641586335|gb|KK854256.1|	408482-408980	10	38
ACYPI003577-RA	gi|641574402|gb|KK855330.1|	239335-240400	11	23
ACYPI003816-RA	gi|641566358|gb|KK857690.1|	20027-25161	12	33
ACYPI004617-RA	gi|641575389|gb|KK855120.1|	82974-83397	10	35
ACYPI073829-RA	gi|641586726|gb|KK854199.1|	453544-453853	11	41
ACYPI000227-RA	gi|641587577|gb|KK854086.1|	48732-49579	8.2	41
ACYPI001125-RA	gi|641574416|gb|KK855326.1|	212242-213675	11	38
ACYPI001177-RA	gi|641572360|gb|KK855832.1|	53637-55443	11	22
ACYPI001932-RA	gi|641587577|gb|KK854086.1|	177759-179416	8.2	41
ACYPI005359-RA	gi|641577704|gb|KK854707.1|	413030-419324	10	38
ACYPI006095-RA	gi|641577905|gb|KK854672.1|	169886-171616	10	37
ACYPI006584-RA	gi|641575794|gb|KK855043.1|	172043-177386	10	36
ACYPI073834-RA	gi|641584351|gb|KK854488.1|	399312-399610	11	41
ACYPI001717-RA	gi|641571956|gb|KK855938.1|	97132-97371	11	42
ACYPI001756-RA	gi|641575112|gb|KK855177.1|	107235-107511	11	39
ACYPI007388-RA	gi|641575332|gb|KK855132.1|	169326-170171	10	39
ACYPI008275-RA	gi|641573765|gb|KK855478.1|	187453-188638	11	42
ACYPI010151-RA	gi|641574173|gb|KK855380.1|	270250-270661	9.5	39
ACYPI001813-RA	gi|641571279|gb|KK856112.1|	185563-185872	9.8	36
ACYPI001852-RA	gi|641585581|gb|KK854374.1|	36494-37351	10	38

ACYPI002118-RA	gi|641587118|gb|KK854142.1|	526922-527972	11	40
ACYPI002673-RA	gi|641575743|gb|KK855052.1|	165629-168226	9.7	38
ACYPI007139-RA	gi|641588060|gb|KK854028.1|	667237-668091	11	42
ACYPI009014-RA	gi|641585446|gb|KK854396.1|	401981-402273	11	39
ACYPI001764-RA	gi|641577121|gb|KK854804.1|	143708-143902	16	82
ACYPI001777-RA	gi|641571315|gb|KK856104.1|	178506-179629	12	46
ACYPI003651-RA	gi|641588073|gb|KK854027.1|	392892-393642	11	41
ACYPI006221-RA	gi|641587527|gb|KK854092.1|	455512-457743	10	38
ACYPI006830-RA	gi|641574266|gb|KK855361.1|	159421-160825	11	38
ACYPI007362-RA	gi|641578300|gb|KK854606.1|	48845-49599	11	40
ACYPI008100-RA	gi|641577879|gb|KK854677.1|	118515-120448	18	36
ACYPI008606-RA	gi|641588051|gb|KK854029.1|	1052570-1054051	9.6	40
ACYPI009993-RA	gi|641576452|gb|KK854917.1|	245256-246911	10	41
ACYPI001635-RA	gi|641585642|gb|KK854364.1|	85844-88073	9.9	38
ACYPI34207-RA	gi|641576635|gb|KK854887.1|	420588-421114	11	40
ACYPI001915-RA	gi|641588309|gb|KK854005.1|	1139075-1139459	11	40
ACYPI003210-RA	gi|641587807|gb|KK854057.1|	170211-171117	10	41
ACYPI005735-RA	gi|641587660|gb|KK854076.1|	548013-553435	9.9	41
ACYPI007453-RA	gi|641584806|gb|KK854455.1|	424746-425229	11	43
ACYPI007611-RA	gi|641585395|gb|KK854405.1|	365289-366904	10	38
ACYPI31718-RA	gi|641571533|gb|KK856043.1|	103109-103304	11	40
ACYPI006807-RA	gi|641569257|gb|KK856724.1|	80621-81226	10	36
ACYPI007960-RA	gi|641585953|gb|KK854314.1|	33271-33818	11	43
ACYPI080807-RA	gi|641575970|gb|KK855012.1|	23208-23490	11	38
ACYPI38602-RA	gi|641571612|gb|KK856022.1|	162706-163018	12	43
ACYPI53596-RA	gi|641571667|gb|KK856008.1|	43474-44069	11	39
ACYPI003257-RA	gi|641586535|gb|KK854227.1|	775053-775943	10	39
ACYPI004037-RA	gi|641587930|gb|KK854043.1|	961692-962191	10	41
ACYPI006164-RA	gi|641587703|gb|KK854070.1|	210947-215659	9.8	42
ACYPI073036-RA	gi|641587703|gb|KK854070.1|	99826-103281	9.8	42

ACYPI50514-RA	gi|641585233|gb|KK854427.1|	423402-423650	10	38
ACYPI006520-RA	gi|641550114|gb|KK865048.1|	39-1938	12	37
ACYPI007301-RA	gi|641577826|gb|KK854687.1|	38828-39649	9.4	37
ACYPI007798-RA	gi|641585574|gb|KK854375.1|	18227-18583	11	39
ACYPI47125-RA	gi|641587688|gb|KK854072.1|	763979-767237	10	38
ACYPI001171-RA	gi|641587614|gb|KK854081.1|	777841-778021	11	40
ACYPI001846-RA	gi|641574654|gb|KK855271.1|	46877-47337	9.5	36
ACYPI002092-RA	gi|641578406|gb|KK854594.1|	334714-336517	10	37
ACYPI003059-RA	gi|641588325|gb|KK854004.1|	1016076-1016602	11	40
ACYPI006498-RA	gi|641576036|gb|KK854996.1|	46467-46990	11	40
ACYPI008376-RA	gi|641587390|gb|KK854109.1|	460890-461128	11	44
ACYPI009387-RA	gi|641588033|gb|KK854031.1|	670089-678906	11	38
ACYPI067721-RA	gi|641588213|gb|KK854013.1|	1082969-1083402	9.6	39
ACYPI073612-RA	gi|641574127|gb|KK855390.1|	14958-16314	10	40
ACYPI083009-RA	gi|641573397|gb|KK855571.1|	35487-36547	10	38
ACYPI001730-RA	gi|641569612|gb|KK856617.1|	65037-65691	11	21
ACYPI004407-RA	gi|641570501|gb|KK856336.1|	44466-45627	10	37
ACYPI005972-RA	gi|641573334|gb|KK855587.1|	171258-171893	10	39
ACYPI009568-RA	gi|641577047|gb|KK854815.1|	365352-366430	10	41
ACYPI000331-RA	gi|641576766|gb|KK854863.1|	197844-198682	10	38
ACYPI000928-RA	gi|641575794|gb|KK855043.1|	214321-214891	10	36
ACYPI002231-RA	gi|641587606|gb|KK854082.1|	770529-770871	11	40
ACYPI002255-RA	gi|641566509|gb|KK857636.1|	55555-60703	11	40
ACYPI007949-RA	gi|641574783|gb|KK855245.1|	221156-221798	10	21
ACYPI009162-RA	gi|641586785|gb|KK854191.1|	205815-206482	11	39
ACYPI001044-RA	gi|641582401|gb|KK854541.1|	146554-147241	11	43
ACYPI001084-RA	gi|641572106|gb|KK855898.1|	20011-21386	7.3	40
ACYPI001902-RA	gi|641588325|gb|KK854004.1|	1422982-1428310	11	40
ACYPI002050-RA	gi|641572341|gb|KK855837.1|	152358-152556	10	36
ACYPI003060-RA	gi|641575666|gb|KK855067.1|	251755-252322	11	39

ACYPI003793-RA	gi|641587800|gb|KK854058.1|	762549-762812	10	42
ACYPI003931-RA	gi|641576414|gb|KK854924.1|	67631-68209	10	40
ACYPI006871-RA	gi|641578046|gb|KK854650.1|	35043-35866	10	38
ACYPI009224-RA	gi|641568119|gb|KK857079.1|	60654-61504	9.8	19
ACYPI009302-RA	gi|641585510|gb|KK854386.1|	70863-71557	11	20
ACYPI009492-RA	gi|641587881|gb|KK854049.1|	688880-694902	10	40
ACYPI009633-RA	gi|641573218|gb|KK855615.1|	149579-149889	9.7	39
ACYPI073394-RA	gi|641570399|gb|KK856367.1|	61812-65289	11	41
ACYPI000262-RA	gi|641587104|gb|KK854144.1|	815457-815780	11	37
ACYPI002867-RA	gi|641574312|gb|KK855351.1|	134736-135473	8.9	36
ACYPI006281-RA	gi|641578411|gb|KK854593.1|	179068-180177	11	43
ACYPI006639-RA	gi|641573737|gb|KK855486.1|	62037-63568	9.1	33
ACYPI008547-RA	gi|641588343|gb|KK854003.1|	1314942-1316573	11	40
ACYPI008782-RA	gi|641578411|gb|KK854593.1|	165711-166311	11	43
ACYPI009077-RA	gi|641585204|gb|KK854429.1|	110559-111892	9.2	36
ACYPI29327-RA	gi|641574923|gb|KK855216.1|	180246-181641	11	38
ACYPI003318-RA	gi|641570593|gb|KK856309.1|	87011-87869	9.6	33
ACYPI004654-RA	gi|641568808|gb|KK856861.1|	65928-68138	10	45
ACYPI51366-RA	gi|641573742|gb|KK855485.1|	120061-127898	9.9	36
ACYPI000071-RA	gi|641571760|gb|KK855988.1|	96541-97388	9.9	37
ACYPI000674-RA	gi|641585022|gb|KK854440.1|	387856-389079	10	39
ACYPI003760-RA	gi|641576055|gb|KK854992.1|	39075-39765	9.7	37
ACYPI005131-RA	gi|641585446|gb|KK854396.1|	415279-415867	11	39
ACYPI009460-RA	gi|641575505|gb|KK855097.1|	174552-176519	11	38
ACYPI068778-RA	gi|641587133|gb|KK854140.1|	461653-464301	9.9	37
ACYPI002565-RA	gi|641578056|gb|KK854648.1|	464859-465312	11	41
ACYPI003625-RA	gi|641573638|gb|KK855510.1|	181524-182031	11	41
ACYPI003756-RA	gi|641567854|gb|KK857166.1|	101175-102372	11	37
ACYPI005614-RA	gi|641550113|gb|KK865049.1|	2201-3140	11	48
ACYPI006383-RA	gi|641587848|gb|KK854052.1|	350484-351920	11	39

ACYPI007032-RA	gi|641577316|gb|KK854772.1|	141220-144073	9.4	36
ACYPI007495-RA	gi|641574249|gb|KK855364.1|	167719-172295	9.2	37
ACYPI060544-RA	gi|641586278|gb|KK854264.1|	631246-631701	11	41
ACYPI081660-RA	gi|641573638|gb|KK855510.1|	170982-172175	11	41
ACYPI002168-RA	gi|641586898|gb|KK854174.1|	3238-3802	11	39
ACYPI002733-RA	gi|641585801|gb|KK854337.1|	540417-540788	10	38
ACYPI004740-RA	gi|641585446|gb|KK854396.1|	379553-379975	11	39
ACYPI006457-RA	gi|641587186|gb|KK854133.1|	289465-289821	8.1	39
ACYPI073759-RA	gi|641587688|gb|KK854072.1|	486555-487281	10	38
ACYPI088483-RA	gi|641588136|gb|KK854020.1|	1110339-1116530	11	36
ACYPI000992-RA	gi|641578046|gb|KK854650.1|	262357-262925	10	38
ACYPI001724-RA	gi|641586185|gb|KK854275.1|	190285-190754	12	40
ACYPI003708-RA	gi|641586777|gb|KK854192.1|	544171-545956	11	30
ACYPI005391-RA	gi|641586203|gb|KK854273.1|	487310-490046	9.5	40
ACYPI005557-RA	gi|641586928|gb|KK854169.1|	130737-132561	11	39
ACYPI008919-RA	gi|641569630|gb|KK856611.1|	40199-40808	11	39
ACYPI065151-RA	gi|641577390|gb|KK854759.1|	35135-35901	9.8	37
ACYPI003171-RA	gi|641565500|gb|KK858006.1|	12012-13302	9.6	19
ACYPI004433-RA	gi|641574220|gb|KK855370.1|	1738-2152	11	21
ACYPI008211-RA	gi|641584419|gb|KK854483.1|	435875-436144	10	42
ACYPI009196-RA	gi|641573218|gb|KK855615.1|	60901-62449	9.7	39
ACYPI21388-RA	gi|641584913|gb|KK854447.1|	447279-451935	10	40
ACYPI000402-RA	gi|641571515|gb|KK856048.1|	37318-41280	9.9	38
ACYPI001833-RA	gi|641585936|gb|KK854317.1|	405920-406669	9.9	41
ACYPI002296-RA	gi|641586809|gb|KK854188.1|	719109-720930	10	43
ACYPI004658-RA	gi|641572166|gb|KK855881.1|	193520-194714	11	41
ACYPI006306-RA	gi|641586476|gb|KK854236.1|	634912-636319	10	37
ACYPI008768-RA	gi|641571213|gb|KK856130.1|	136594-136930	10	43
ACYPI010056-RA	gi|641575959|gb|KK855014.1|	12034-13040	11	41
ACYPI002151-RA	gi|641587111|gb|KK854143.1|	614875-615148	11	40

ACYPI002796-RA	gi|641587111|gb|KK854143.1|	617708-619277	11	40
ACYPI001975-RA	gi|641577430|gb|KK854752.1|	66785-67237	9.7	21
ACYPI003866-RA	gi|641576377|gb|KK854930.1|	181736-188565	10	42
ACYPI009755-RA	gi|641587263|gb|KK854123.1|	252936-253603	11	42
ACYPI009818-RA	gi|641587133|gb|KK854140.1|	62097-62289	9.9	37
ACYPI010201-RA	gi|641586599|gb|KK854218.1|	102587-103126	11	42
ACYPI072205-RA	gi|641573813|gb|KK855466.1|	123643-127729	9.6	37
ACYPI080109-RA	gi|641584474|gb|KK854479.1|	340018-340987	9.5	40
ACYPI000758-RA	gi|641585315|gb|KK854418.1|	464855-466629	10	39
ACYPI002575-RA	gi|641585778|gb|KK854341.1|	154907-155205	9.7	41
ACYPI005140-RA	gi|641588144|gb|KK854019.1|	780050-780337	11	49
ACYPI006821-RA	gi|641574729|gb|KK855257.1|	208642-212290	8.6	33
ACYPI008362-RA	gi|641587832|gb|KK854054.1|	598824-598970	9.8	20
ACYPI008575-RA	gi|641574303|gb|KK855353.1|	115784-118774	10	34
ACYPI008996-RA	gi|641585383|gb|KK854406.1|	216845-217686	11	37
ACYPI004580-RA	gi|641573097|gb|KK855644.1|	32125-32422	10	36
ACYPI004887-RA	gi|641584504|gb|KK854477.1|	217497-220660	9.5	38
ACYPI005706-RA	gi|641578084|gb|KK854643.1|	250661-251047	11	40
ACYPI006125-RA	gi|641586777|gb|KK854192.1|	239239-239427	11	30
ACYPI006494-RA	gi|641587379|gb|KK854111.1|	363091-363738	10	41
ACYPI010118-RA	gi|641572891|gb|KK855695.1|	17863-18418	10	36
ACYPI072081-RA	gi|641584504|gb|KK854477.1|	235075-236774	9.5	38
ACYPI000717-RA	gi|641586372|gb|KK854251.1|	453617-454523	10	38
ACYPI003705-RA	gi|641588325|gb|KK854004.1|	1940069-1941515	11	40
ACYPI004381-RA	gi|641585510|gb|KK854386.1|	370826-371761	11	20
ACYPI005634-RA	gi|641585469|gb|KK854393.1|	54249-56727	10	37
ACYPI006953-RA	gi|641584404|gb|KK854484.1|	391267-391480	11	30
ACYPI008816-RA	gi|641584404|gb|KK854484.1|	503556-503788	11	30
ACYPI001585-RA	gi|641574100|gb|KK855396.1|	75220-76082	11	38
ACYPI063239-RA	gi|641581402|gb|KK854555.1|	296881-298175	12	20

ACYPI067185-RA	gi|641587688|gb|KK854072.1|	170742-172728	10	38
ACYPI001243-RA	gi|641586489|gb|KK854234.1|	263847-266178	9.5	39
ACYPI007241-RA	gi|641571465|gb|KK856064.1|	88449-88725	9.2	35
ACYPI009620-RA	gi|641558787|gb|KK860963.1|	10199-10830	16	########
ACYPI010147-RA	gi|641572501|gb|KK855798.1|	132773-134157	9.8	39
ACYPI55644-RA	gi|641587680|gb|KK854073.1|	1003308-1004153	10	40
ACYPI001108-RA	gi|641586734|gb|KK854198.1|	528200-529074	11	40
ACYPI001804-RA	gi|641579225|gb|KK854583.1|	393013-395261	10	39
ACYPI002999-RA	gi|641578428|gb|KK854590.1|	254133-254427	11	40
ACYPI006140-RA	gi|641585395|gb|KK854405.1|	97838-98842	10	38
ACYPI006274-RA	gi|641573903|gb|KK855443.1|	129188-129656	10	24
ACYPI007445-RA	gi|641570462|gb|KK856348.1|	13712-14325	10	37
ACYPI008020-RA	gi|641588236|gb|KK854011.1|	648610-650162	9.7	38
ACYPI008089-RA	gi|641565378|gb|KK858053.1|	37341-37636	11	35
ACYPI008168-RA	gi|641578310|gb|KK854604.1|	454378-455390	11	39
ACYPI008776-RA	gi|641575209|gb|KK855157.1|	98319-103615	11	38
ACYPI072830-RA	gi|641588021|gb|KK854033.1|	1209832-1210745	12	40
ACYPI000765-RA	gi|641575104|gb|KK855179.1|	94075-95977	10	35
ACYPI002367-RA	gi|641586606|gb|KK854217.1|	373368-377954	11	42
ACYPI004256-RA	gi|641587171|gb|KK854135.1|	174869-176262	10	39
ACYPI005243-RA	gi|641574078|gb|KK855401.1|	215069-221662	11	41
ACYPI006124-RA	gi|641576834|gb|KK854851.1|	329557-329956	10	41
ACYPI007736-RA	gi|641587519|gb|KK854093.1|	158901-159517	11	39
ACYPI008662-RA	gi|641572017|gb|KK855921.1|	15791-16626	9.3	35
ACYPI000060-RA	gi|641572130|gb|KK855891.1|	147850-150539	10	37
ACYPI002958-RA	gi|641587757|gb|KK854063.1|	556255-556783	11	38
ACYPI004192-RA	gi|641572130|gb|KK855891.1|	151168-151534	10	37
ACYPI005677-RA	gi|641586734|gb|KK854198.1|	802019-806121	11	40
ACYPI010079-RA	gi|641577498|gb|KK854744.1|	10555-14276	11	42
ACYPI000855-RA	gi|641588109|gb|KK854023.1|	222111-223328	10	40

ACYPI001003-RA	gi|641570045|gb|KK856477.1|	101916-102138	9.6	43
ACYPI002621-RA	gi|641585953|gb|KK854314.1|	475676-478210	11	43
ACYPI008327-RA	gi|641586886|gb|KK854176.1|	664096-672454	10	41
ACYPI008966-RA	gi|641575888|gb|KK855026.1|	176875-179173	10	41
ACYPI009134-RA	gi|641577905|gb|KK854672.1|	239105-243326	10	37
ACYPI009233-RA	gi|641570897|gb|KK856221.1|	49085-50999	9.1	35
ACYPI009382-RA	gi|641568353|gb|KK857004.1|	67351-68368	10	38
ACYPI010053-RA	gi|641585940|gb|KK854316.1|	278481-278932	11	42
ACYPI38630-RA	gi|641572740|gb|KK855736.1|	154370-158572	11	40
ACYPI001277-RA	gi|641579212|gb|KK854585.1|	203196-203844	10	35
ACYPI001901-RA	gi|641585219|gb|KK854428.1|	234339-234736	11	41
ACYPI003790-RA	gi|641587962|gb|KK854039.1|	771478-772855	11	40
ACYPI005720-RA	gi|641566812|gb|KK857525.1|	66294-66835	11	42
ACYPI006984-RA	gi|641585521|gb|KK854384.1|	395995-398288	9.7	38
ACYPI007110-RA	gi|641587365|gb|KK854113.1|	138327-138565	12	45
ACYPI007997-RA	gi|641553949|gb|KK863251.1|	2779-3523	9.2	32
ACYPI008479-RA	gi|641574048|gb|KK855408.1|	157772-158514	9.9	41
ACYPI008848-RA	gi|641576447|gb|KK854918.1|	35562-35815	12	45
ACYPI009870-RA	gi|641576221|gb|KK854958.1|	164812-165673	9.3	35
ACYPI001285-RA	gi|641572431|gb|KK855815.1|	95127-96397	9.1	35
ACYPI002557-RA	gi|641564032|gb|KK858612.1|	30385-31160	10	35
ACYPI003170-RA	gi|641576766|gb|KK854863.1|	228053-228750	10	38
ACYPI072316-RA	gi|641584351|gb|KK854488.1|	440667-442281	11	41
ACYPI005082-RA	gi|641584876|gb|KK854450.1|	356482-357489	11	25
ACYPI005897-RA	gi|641575549|gb|KK855090.1|	71895-74070	10	38
ACYPI009644-RA	gi|641574031|gb|KK855412.1|	178647-180696	12	39
ACYPI001856-RA	gi|641567104|gb|KK857424.1|	34328-35248	8.5	38
ACYPI003711-RA	gi|641586079|gb|KK854294.1|	182118-184048	8.5	34
ACYPI009147-RA	gi|641574240|gb|KK855366.1|	171428-171917	9.4	38
ACYPI009325-RA	gi|641575218|gb|KK855155.1|	118337-118638	11	21

ACYPI009431-RA	gi|641573722|gb|KK855490.1|	195719-197489	9.7	19
ACYPI001752-RA	gi|641575901|gb|KK855023.1|	245775-246568	11	38
ACYPI002361-RA	gi|641586661|gb|KK854209.1|	542012-542355	11	43
ACYPI002482-RA	gi|641568172|gb|KK857061.1|	74471-75829	11	40
ACYPI003002-RA	gi|641564796|gb|KK858283.1|	4757-4996	10	39
ACYPI005545-RA	gi|641588246|gb|KK854010.1|	1157286-1158645	9.2	50
ACYPI007409-RA	gi|641582763|gb|KK854531.1|	280873-285173	10	39
ACYPI008092-RA	gi|641585865|gb|KK854328.1|	549972-550444	12	42
ACYPI008652-RA	gi|641574668|gb|KK855267.1|	155533-156563	10	38
ACYPI009324-RA	gi|641586817|gb|KK854187.1|	751761-753118	9.3	39
ACYPI009337-RA	gi|641577277|gb|KK854778.1|	255599-259411	11	42
ACYPI085768-RA	gi|641587126|gb|KK854141.1|	334022-336370	10	39
ACYPI087848-RA	gi|641546507|gb|KK866793.1|	6051-7153	10	40
ACYPI006310-RA	gi|641568257|gb|KK857034.1|	23161-23469	10	40
ACYPI008174-RA	gi|641577203|gb|KK854789.1|	287448-287717	11	43
ACYPI000703-RA	gi|641569257|gb|KK856724.1|	41070-42117	10	36
ACYPI001614-RA	gi|641575693|gb|KK855062.1|	141566-143534	11	40
ACYPI003083-RA	gi|641585594|gb|KK854372.1|	331517-332480	10	40
ACYPI004129-RA	gi|641573759|gb|KK855480.1|	127678-128494	9.9	37
ACYPI005442-RA	gi|641569519|gb|KK856646.1|	34454-38585	10	39
ACYPI007070-RA	gi|641576983|gb|KK854825.1|	267001-268730	9.7	37
ACYPI008939-RA	gi|641571870|gb|KK855959.1|	143402-145848	9.7	20
ACYPI088345-RA	gi|641575693|gb|KK855062.1|	143792-145681	11	40
ACYPI088673-RA	gi|641573177|gb|KK855625.1|	100914-102136	9.4	34
ACYPI001522-RA	gi|641577299|gb|KK854775.1|	415118-415670	11	37
ACYPI004029-RA	gi|641567739|gb|KK857205.1|	109817-110020	13	38
ACYPI004684-RA	gi|641577511|gb|KK854741.1|	331705-333443	11	38
ACYPI007834-RA	gi|641585204|gb|KK854429.1|	56225-59671	9.2	36
ACYPI001164-RA	gi|641572824|gb|KK855713.1|	68390-69648	9.8	19
ACYPI005200-RA	gi|641577097|gb|KK854808.1|	130800-136520	11	41

ACYPI007627-RA	gi|641587671|gb|KK854074.1|	901464-902007	11	40
ACYPI007946-RA	gi|641577553|gb|KK854734.1|	82772-85561	9.7	41
ACYPI008725-RA	gi|641571270|gb|KK856115.1|	127170-128037	11	38
ACYPI087213-RA	gi|641585035|gb|KK854439.1|	176580-177578	9.1	40
ACYPI41200-RA	gi|641587671|gb|KK854074.1|	909081-909557	11	40
ACYPI000767-RA	gi|641587652|gb|KK854077.1|	274273-274591	10	38
ACYPI005851-RA	gi|641573641|gb|KK855509.1|	56018-57689	9.8	36
ACYPI008437-RA	gi|641570596|gb|KK856308.1|	116212-117588	9.9	39
ACYPI009835-RA	gi|641571354|gb|KK856093.1|	54582-56942	11	42
ACYPI061734-RA	gi|641588136|gb|KK854020.1|	339168-340768	11	36
ACYPI45421-RA	gi|641578056|gb|KK854648.1|	152774-153281	11	41
ACYPI006713-RA	gi|641574324|gb|KK855348.1|	249663-250823	12	40
ACYPI003090-RA	gi|641584931|gb|KK854446.1|	258608-258816	10	39
ACYPI003826-RA	gi|641585383|gb|KK854406.1|	432441-432827	11	37
ACYPI000814-RA	gi|641585315|gb|KK854418.1|	334099-336057	10	39
ACYPI002695-RA	gi|641587930|gb|KK854043.1|	319793-320387	10	41
ACYPI007245-RA	gi|641585622|gb|KK854367.1|	341501-344100	9.9	21
ACYPI000759-RA	gi|641585376|gb|KK854407.1|	134864-136282	9.8	36
ACYPI007054-RA	gi|641585376|gb|KK854407.1|	130499-131211	9.8	36
ACYPI000014-RA	gi|641578060|gb|KK854647.1|	64753-67419	8.9	35
ACYPI000654-RA	gi|641587462|gb|KK854100.1|	82039-83183	10	21
ACYPI001424-RA	gi|641568831|gb|KK856853.1|	42898-43841	10	30
ACYPI008222-RA	gi|641588283|gb|KK854007.1|	658475-660840	11	40
ACYPI46077-RA	gi|641576064|gb|KK854990.1|	93209-93418	10	39
ACYPI001511-RA	gi|641586399|gb|KK854247.1|	263675-267785	15	68
ACYPI002756-RA	gi|641577075|gb|KK854811.1|	206259-207414	11	36
ACYPI003401-RA	gi|641578060|gb|KK854647.1|	175603-175828	8.9	35
ACYPI004349-RA	gi|641574504|gb|KK855305.1|	68177-68366	9.9	39
ACYPI005038-RA	gi|641588090|gb|KK854025.1|	1013571-1014274	11	43
ACYPI008129-RA	gi|641578315|gb|KK854603.1|	128651-129219	9.8	38

ACYPI010007-RA	gi|641571270|gb|KK856115.1|	75682-79141	11	38
ACYPI21395-RA	gi|641587823|gb|KK854055.1|	467596-468450	11	22
ACYPI000714-RA	gi|641572251|gb|KK855860.1|	174943-176685	10	19
ACYPI001898-RA	gi|641576962|gb|KK854829.1|	258359-258833	10	39
ACYPI002963-RA	gi|641576371|gb|KK854931.1|	89365-90459	10	38
ACYPI004154-RA	gi|641577595|gb|KK854727.1|	168171-168434	10	20
ACYPI004450-RA	gi|641574459|gb|KK855317.1|	122392-123583	10	21
ACYPI006790-RA	gi|641586874|gb|KK854178.1|	623830-624157	9.4	38
ACYPI007404-RA	gi|641557847|gb|KK861397.1|	5674-6196	9.7	30
ACYPI007969-RA	gi|641574220|gb|KK855370.1|	181616-183363	11	21
ACYPI008244-RA	gi|641570627|gb|KK856299.1|	58851-59767	11	20
ACYPI008884-RA	gi|641572297|gb|KK855849.1|	137845-138659	11	21
ACYPI000388-RA	gi|641586620|gb|KK854215.1|	33266-34164	9.1	40
ACYPI000409-RA	gi|641586246|gb|KK854268.1|	125181-125775	10	38
ACYPI000870-RA	gi|641576766|gb|KK854863.1|	157080-157377	10	38
ACYPI002907-RA	gi|641575635|gb|KK855074.1|	296164-299267	9.6	38
ACYPI005406-RA	gi|641582096|gb|KK854546.1|	398563-398852	11	38
ACYPI007276-RA	gi|641570601|gb|KK856306.1|	146612-147336	10	40
ACYPI008807-RA	gi|641588343|gb|KK854003.1|	2036778-2037283	11	40
ACYPI009727-RA	gi|641586606|gb|KK854217.1|	309628-311069	11	42
ACYPI37793-RA	gi|641567493|gb|KK857288.1|	29190-30404	8.6	41
ACYPI29489-RA	gi|641586975|gb|KK854163.1|	411120-413094	11	40
ACYPI000453-RA	gi|641573695|gb|KK855497.1|	21675-23242	9.2	41
ACYPI002346-RA	gi|641569577|gb|KK856628.1|	54946-58205	9.7	36
ACYPI007073-RA	gi|641569964|gb|KK856503.1|	164676-164936	10	36
ACYPI008552-RA	gi|641588294|gb|KK854006.1|	941948-943427	8.7	40
ACYPI001945-RA	gi|641575372|gb|KK855124.1|	133743-133907	9.5	36
ACYPI003839-RA	gi|641575372|gb|KK855124.1|	66926-75766	9.5	36
ACYPI004640-RA	gi|641588258|gb|KK854009.1|	341771-343656	11	36
ACYPI005773-RA	gi|641575372|gb|KK855124.1|	107319-107561	9.5	36

ACYPI007193-RA	gi|641575826|gb|KK855037.1|	215718-216678	10	39
ACYPI001622-RA	gi|641570589|gb|KK856310.1|	30308-31328	11	36
ACYPI003895-RA	gi|641584128|gb|KK854505.1|	236345-237223	9.6	37
ACYPI004805-RA	gi|641572912|gb|KK855690.1|	135988-144279	9.9	38
ACYPI006706-RA	gi|641577166|gb|KK854796.1|	139471-142837	10	38
ACYPI007688-RA	gi|641586837|gb|KK854184.1|	516581-520202	11	40
ACYPI007971-RA	gi|641574303|gb|KK855353.1|	102637-103703	10	34
ACYPI009308-RA	gi|641587946|gb|KK854041.1|	986756-988181	11	41
ACYPI009596-RA	gi|641573858|gb|KK855455.1|	193558-195185	9.8	37
ACYPI000798-RA	gi|641576807|gb|KK854855.1|	59750-62211	9.8	37
ACYPI001567-RA	gi|641587047|gb|KK854151.1|	555059-558798	10	39
ACYPI001746-RA	gi|641586535|gb|KK854227.1|	781024-783240	10	39
ACYPI002386-RA	gi|641587636|gb|KK854079.1|	649757-651319	11	21
ACYPI002680-RA	gi|641566502|gb|KK857638.1|	33820-37686	11	36
ACYPI007130-RA	gi|641578428|gb|KK854590.1|	320857-325405	11	40
ACYPI067549-RA	gi|641587047|gb|KK854151.1|	553005-554459	10	39
ACYPI001584-RA	gi|641586405|gb|KK854246.1|	305967-310196	10	40
ACYPI002013-RA	gi|641577493|gb|KK854745.1|	230451-235737	9.6	41
ACYPI006725-RA	gi|641585515|gb|KK854385.1|	153205-153495	11	40
ACYPI008584-RA	gi|641588060|gb|KK854028.1|	32522-33083	11	42
ACYPI009821-RA	gi|641584305|gb|KK854491.1|	193867-196726	11	39
ACYPI000793-RA	gi|641575358|gb|KK855127.1|	272002-273879	9	41
ACYPI001591-RA	gi|641586096|gb|KK854291.1|	498061-498270	11	46
ACYPI002372-RA	gi|641586025|gb|KK854302.1|	108939-110759	8.5	33
ACYPI002624-RA	gi|641586121|gb|KK854287.1|	11548-12191	11	43
ACYPI003082-RA	gi|641584567|gb|KK854472.1|	67707-71232	9.5	39
ACYPI003661-RA	gi|641586121|gb|KK854287.1|	12793-14494	11	43
ACYPI006445-RA	gi|641587580|gb|KK854085.1|	551097-555408	10	39
ACYPI008758-RA	gi|641586936|gb|KK854168.1|	549874-550917	12	42
ACYPI083436-RA	gi|641576731|gb|KK854870.1|	271103-271790	10	41

ACYPI35175-RA	gi|641564127|gb|KK858571.1|	19593-20250	10	40
ACYPI000045-RA	gi|641587118|gb|KK854142.1|	304062-305430	11	40
ACYPI001194-RA	gi|641585587|gb|KK854373.1|	351503-352162	9.8	41
ACYPI003338-RA	gi|641573164|gb|KK855628.1|	108218-112225	9.9	38
ACYPI005122-RA	gi|641584445|gb|KK854481.1|	213008-213363	9.9	39
ACYPI007009-RA	gi|641573155|gb|KK855630.1|	10643-11451	14	50
ACYPI007022-RA	gi|641587735|gb|KK854066.1|	81087-82509	11	42
ACYPI009714-RA	gi|641574000|gb|KK855418.1|	224032-224533	10	36
ACYPI002137-RA	gi|641578188|gb|KK854626.1|	233875-234121	9.9	39
ACYPI002484-RA	gi|641572695|gb|KK855748.1|	134737-135842	9.7	32
ACYPI004206-RA	gi|641577837|gb|KK854685.1|	386593-387225	11	42
ACYPI006711-RA	gi|641576347|gb|KK854934.1|	234087-235474	11	39
ACYPI008022-RA	gi|641588073|gb|KK854027.1|	106547-107216	11	41
ACYPI009055-RA	gi|641578114|gb|KK854637.1|	315089-317459	9.7	38
ACYPI009893-RA	gi|641577837|gb|KK854685.1|	413563-416052	11	42
ACYPI069860-RA	gi|641575358|gb|KK855127.1|	88426-89155	9	41
ACYPI084171-RA	gi|641586850|gb|KK854182.1|	544098-544711	10	37
ACYPI43057-RA	gi|641587614|gb|KK854081.1|	302005-302649	11	40
ACYPI44907-RA	gi|641586535|gb|KK854227.1|	483605-484223	10	39
ACYPI000956-RA	gi|641572887|gb|KK855696.1|	107162-108046	9.5	26
ACYPI002523-RA	gi|641570299|gb|KK856396.1|	67159-70547	10	33
ACYPI004423-RA	gi|641588000|gb|KK854035.1|	333057-333562	11	39
ACYPI006340-RA	gi|641583980|gb|KK854512.1|	302793-304927	11	39
ACYPI008218-RA	gi|641569596|gb|KK856622.1|	68405-68588	12	43
ACYPI008248-RA	gi|641567299|gb|KK857356.1|	39235-39895	11	38
ACYPI56865-RA	gi|641571315|gb|KK856104.1|	76796-77556	12	46
ACYPI010018-RA	gi|641588309|gb|KK854005.1|	1524617-1524862	11	40
ACYPI37207-RA	gi|641565912|gb|KK857853.1|	27096-27745	12	40
ACYPI005401-RA	gi|641586240|gb|KK854269.1|	362618-364815	11	21
ACYPI006354-RA	gi|641572854|gb|KK855705.1|	177654-178323	11	42

ACYPI007272-RA	gi|641586769|gb|KK854193.1|	354438-357836	12	23
ACYPI009163-RA	gi|641585480|gb|KK854391.1|	179824-180376	11	21
ACYPI060811-RA	gi|641587171|gb|KK854135.1|	551872-553322	10	39
ACYPI067461-RA	gi|641555420|gb|KK862558.1|	4-1228	6.7	25
ACYPI070340-RA	gi|641575666|gb|KK855067.1|	325322-329991	11	39
ACYPI002018-RA	gi|641577309|gb|KK854773.1|	258141-259272	9.9	39
ACYPI006012-RA	gi|641574135|gb|KK855388.1|	116018-117295	9.6	36
ACYPI000373-RA	gi|641587235|gb|KK854127.1|	254724-255225	10	29
ACYPI002497-RA	gi|641577482|gb|KK854747.1|	149442-150650	10	39
ACYPI005416-RA	gi|641577516|gb|KK854740.1|	132328-132623	10	21
ACYPI008368-RA	gi|641586185|gb|KK854275.1|	729440-730289	12	40
ACYPI008833-RA	gi|641579937|gb|KK854576.1|	546261-547493	9.4	41
ACYPI009591-RA	gi|641588128|gb|KK854021.1|	1258244-1258602	9.5	42
ACYPI002992-RA	gi|641556673|gb|KK861961.1|	6121-9537	8.9	35
ACYPI007319-RA	gi|641588358|gb|KK854002.1|	709652-714457	11	43
ACYPI066371-RA	gi|641577851|gb|KK854682.1|	277428-278554	11	34
ACYPI067100-RA	gi|641577677|gb|KK854711.1|	216355-217073	12	39
ACYPI23453-RA	gi|641588073|gb|KK854027.1|	1111560-1112651	11	41
ACYPI000055-RA	gi|641585366|gb|KK854408.1|	368516-371955	12	41
ACYPI000431-RA	gi|641586085|gb|KK854293.1|	98218-99178	11	40
ACYPI001692-RA	gi|641570589|gb|KK856310.1|	98336-100355	11	36
ACYPI001971-RA	gi|641584336|gb|KK854489.1|	160220-162495	10	40
ACYPI002220-RA	gi|641573291|gb|KK855599.1|	83359-88306	9	36
ACYPI002324-RA	gi|641584168|gb|KK854503.1|	289552-289862	10	39
ACYPI002792-RA	gi|641588294|gb|KK854006.1|	877789-879358	8.7	40
ACYPI003562-RA	gi|641577913|gb|KK854671.1|	89841-90140	9.2	40
ACYPI004715-RA	gi|641588294|gb|KK854006.1|	925566-926940	8.7	40
ACYPI004859-RA	gi|641572134|gb|KK855890.1|	80710-84017	10	36
ACYPI005512-RA	gi|641570042|gb|KK856478.1|	90750-91459	10	36
ACYPI006399-RA	gi|641568423|gb|KK856981.1|	66311-66903	11	37

ACYPI008279-RA	gi|641581779|gb|KK854550.1|	251632-251976	11	39
ACYPI009523-RA	gi|641586144|gb|KK854283.1|	545620-546334	10	38
ACYPI010196-RA	gi|641585971|gb|KK854310.1|	38582-39937	10	40
ACYPI064828-RA	gi|641576570|gb|KK854895.1|	248569-253458	9.9	40
ACYPI000076-RA	gi|641572916|gb|KK855689.1|	123851-127276	9.2	33
ACYPI002684-RA	gi|641588213|gb|KK854013.1|	1386491-1387144	9.6	39
ACYPI003204-RA	gi|641577086|gb|KK854809.1|	240058-240779	9.8	38
ACYPI003233-RA	gi|641572782|gb|KK855725.1|	103312-103618	10	36
ACYPI005152-RA	gi|641572782|gb|KK855725.1|	106839-107105	10	36
ACYPI007640-RA	gi|641586967|gb|KK854164.1|	346157-346955	10	27
ACYPI010224-RA	gi|641576846|gb|KK854848.1|	223648-224712	10	38
ACYPI065370-RA	gi|641575288|gb|KK855139.1|	180786-182224	11	38
ACYPI41167-RA	gi|641579947|gb|KK854574.1|	273486-273681	11	39
ACYPI000261-RA	gi|641573837|gb|KK855460.1|	113888-116364	13	24
ACYPI001532-RA	gi|641575759|gb|KK855050.1|	232587-235558	11	38
ACYPI003481-RA	gi|641586700|gb|KK854203.1|	63452-63756	8.4	39
ACYPI004036-RA	gi|641572860|gb|KK855703.1|	43908-44164	9.8	36
ACYPI007012-RA	gi|641587832|gb|KK854054.1|	648239-648803	9.8	20
ACYPI007485-RA	gi|641571680|gb|KK856006.1|	95348-96319	12	42
ACYPI007988-RA	gi|641578154|gb|KK854631.1|	98310-101822	8.2	40
ACYPI009378-RA	gi|641571119|gb|KK856156.1|	184814-185459	11	38
ACYPI009741-RA	gi|641577627|gb|KK854721.1|	361095-362720	11	42
ACYPI080621-RA	gi|641578435|gb|KK854589.1|	375669-377064	9.7	43
ACYPI083200-RA	gi|641570343|gb|KK856382.1|	54438-56922	8.6	35
ACYPI089243-RA	gi|641586278|gb|KK854264.1|	147444-148999	11	41
ACYPI23741-RA	gi|641569874|gb|KK856533.1|	59925-60346	11	39
ACYPI003786-RA	gi|641585594|gb|KK854372.1|	459063-459722	10	40
ACYPI004248-RA	gi|641573919|gb|KK855439.1|	75898-76718	10	37
ACYPI004749-RA	gi|641588021|gb|KK854033.1|	255298-255699	12	40
ACYPI071638-RA	gi|641577289|gb|KK854776.1|	152913-156232	10	36

ACYPI26661-RA	gi|641575734|gb|KK855053.1|	95562-97086	11	39
ACYPI40956-RA	gi|641586990|gb|KK854161.1|	163957-164466	11	39
ACYPI000890-RA	gi|641584205|gb|KK854500.1|	475644-476484	11	41
ACYPI004659-RA	gi|641569478|gb|KK856659.1|	77533-79757	12	42
ACYPI007858-RA	gi|641584205|gb|KK854500.1|	290392-292531	11	41
ACYPI004106-RA	gi|641586521|gb|KK854229.1|	182911-183670	11	41
ACYPI006039-RA	gi|641554246|gb|KK863113.1|	48-804	9.3	13
ACYPI009419-RA	gi|641577896|gb|KK854674.1|	309866-310444	11	40
ACYPI009988-RA	gi|641573796|gb|KK855470.1|	91242-96214	11	46
ACYPI000203-RA	gi|641588236|gb|KK854011.1|	1120983-1121433	9.7	38
ACYPI000235-RA	gi|641588082|gb|KK854026.1|	1083168-1083300	11	41
ACYPI000904-RA	gi|641586785|gb|KK854191.1|	228643-229802	11	39
ACYPI001518-RA	gi|641586606|gb|KK854217.1|	165757-168874	11	42
ACYPI001700-RA	gi|641586843|gb|KK854183.1|	897355-897594	11	41
ACYPI002154-RA	gi|641574127|gb|KK855390.1|	145750-147385	10	40
ACYPI002340-RA	gi|641573933|gb|KK855435.1|	26919-32044	10	40
ACYPI002393-RA	gi|641586599|gb|KK854218.1|	652002-654779	11	42
ACYPI002951-RA	gi|641575140|gb|KK855171.1|	304579-306334	9.7	40
ACYPI003409-RA	gi|641587111|gb|KK854143.1|	358479-359292	11	40
ACYPI003886-RA	gi|641576650|gb|KK854884.1|	242313-247304	9.6	40
ACYPI004025-RA	gi|641574836|gb|KK855234.1|	23660-24996	10	40
ACYPI004880-RA	gi|641552289|gb|KK864038.1|	2878-3552	7	22
ACYPI005346-RA	gi|641577826|gb|KK854687.1|	4848-5882	9.4	37
ACYPI005668-RA	gi|641567546|gb|KK857269.1|	31845-32322	8.5	40
ACYPI005820-RA	gi|641586114|gb|KK854288.1|	356512-357153	10	41
ACYPI005896-RA	gi|641578330|gb|KK854601.1|	311508-313591	11	38
ACYPI006158-RA	gi|641587562|gb|KK854088.1|	912951-914999	11	42
ACYPI006624-RA	gi|641587452|gb|KK854101.1|	1025558-1027416	11	40
ACYPI007220-RA	gi|641586191|gb|KK854274.1|	17048-20294	11	42
ACYPI007257-RA	gi|641587946|gb|KK854041.1|	737899-738721	11	41

ACYPI008033-RA	gi|641575730|gb|KK855054.1|	76562-78366	10	38
ACYPI008591-RA	gi|641575209|gb|KK855157.1|	135587-136067	11	38
ACYPI009105-RA	gi|641587021|gb|KK854156.1|	827265-828863	11	42
ACYPI009276-RA	gi|641588267|gb|KK854008.1|	275960-276394	10	40
ACYPI009908-RA	gi|641573397|gb|KK855571.1|	62423-64589	10	38
ACYPI067763-RA	gi|641588082|gb|KK854026.1|	620604-621771	11	41
ACYPI52393-RA	gi|641575626|gb|KK855076.1|	77400-80548	11	24
ACYPI001600-RA	gi|641586734|gb|KK854198.1|	594106-594894	11	40
ACYPI003488-RA	gi|641575698|gb|KK855061.1|	105484-105727	10	39
ACYPI004115-RA	gi|641585366|gb|KK854408.1|	362929-365996	12	41
ACYPI064172-RA	gi|641575698|gb|KK855061.1|	98481-104039	10	39
ACYPI001217-RA	gi|641585801|gb|KK854337.1|	449458-451053	10	38
ACYPI002491-RA	gi|641586928|gb|KK854169.1|	315058-320740	11	39
ACYPI004395-RA	gi|641576286|gb|KK854946.1|	293017-294829	12	38
ACYPI005673-RA	gi|641586033|gb|KK854301.1|	123736-124572	11	21
ACYPI006936-RA	gi|641587671|gb|KK854074.1|	624201-624856	11	40
ACYPI008827-RA	gi|641576022|gb|KK854999.1|	75869-76399	10	36
ACYPI009438-RA	gi|641578330|gb|KK854601.1|	70568-74566	11	38
ACYPI010075-RA	gi|641587793|gb|KK854059.1|	761869-762049	11	38
ACYPI49333-RA	gi|641586867|gb|KK854179.1|	79487-80128	11	39
ACYPI001352-RA	gi|641586344|gb|KK854254.1|	156976-157525	10	40
ACYPI006613-RA	gi|641561307|gb|KK859801.1|	13587-13777	13	38
ACYPI060229-RA	gi|641571290|gb|KK856111.1|	16790-17045	11	41
ACYPI000150-RA	gi|641572824|gb|KK855713.1|	165584-167439	9.8	19
ACYPI001458-RA	gi|641571548|gb|KK856039.1|	20292-20656	9.9	18
ACYPI006043-RA	gi|641572860|gb|KK855703.1|	5840-9040	9.8	36
ACYPI006154-RA	gi|641570836|gb|KK856240.1|	10842-11809	12	44
ACYPI008418-RA	gi|641587562|gb|KK854088.1|	41262-41915	11	42
ACYPI32163-RA	gi|641574661|gb|KK855269.1|	157372-160791	9.5	36
ACYPI46815-RA	gi|641570377|gb|KK856373.1|	48352-49215	11	39

ACYPI000003-RA	gi|641569982|gb|KK856497.1|	27549-33684	9.9	36
ACYPI000479-RA	gi|641570614|gb|KK856303.1|	19248-19597	9.6	34
ACYPI002383-RA	gi|641577837|gb|KK854685.1|	435056-435918	11	42
ACYPI002595-RA	gi|641588099|gb|KK854024.1|	270490-272616	11	39
ACYPI004570-RA	gi|641585930|gb|KK854318.1|	212445-218613	12	44
ACYPI004938-RA	gi|641576414|gb|KK854924.1|	379718-380804	10	40
ACYPI004956-RA	gi|641586893|gb|KK854175.1|	544230-547849	11	41
ACYPI006482-RA	gi|641574892|gb|KK855223.1|	210168-211751	9.8	39
ACYPI007060-RA	gi|641585905|gb|KK854321.1|	258083-258264	11	39
ACYPI007710-RA	gi|641574085|gb|KK855399.1|	40119-40344	10	38
ACYPI007807-RA	gi|641577081|gb|KK854810.1|	195666-196860	11	20
ACYPI008611-RA	gi|641584238|gb|KK854497.1|	76622-78360	9.4	38
ACYPI21475-RA	gi|641576199|gb|KK854963.1|	268334-268922	12	43
ACYPI002068-RA	gi|641576176|gb|KK854968.1|	162735-163305	11	45
ACYPI002726-RA	gi|641578122|gb|KK854636.1|	462840-463203	12	42
ACYPI003771-RA	gi|641566077|gb|KK857794.1|	11815-12597	9.9	19
ACYPI005194-RA	gi|641586445|gb|KK854240.1|	653024-654973	9.2	39
ACYPI005325-RA	gi|641586874|gb|KK854178.1|	532628-532841	9.4	38
ACYPI005496-RA	gi|641587527|gb|KK854092.1|	149009-150831	10	38
ACYPI008431-RA	gi|641584818|gb|KK854454.1|	208658-209150	11	42
ACYPI009659-RA	gi|641576203|gb|KK854962.1|	101220-101927	9.8	36
ACYPI089540-RA	gi|641586928|gb|KK854169.1|	174835-175511	11	39
ACYPI000548-RA	gi|641588109|gb|KK854023.1|	213584-214396	10	40
ACYPI000646-RA	gi|641578179|gb|KK854627.1|	517236-517471	10	39
ACYPI001292-RA	gi|641587881|gb|KK854049.1|	27088-31073	10	40
ACYPI001295-RA	gi|641573394|gb|KK855572.1|	5926-6682	11	40
ACYPI002350-RA	gi|641585953|gb|KK854314.1|	337257-337903	11	43
ACYPI002534-RA	gi|641588109|gb|KK854023.1|	465188-469209	10	40
ACYPI003185-RA	gi|641585440|gb|KK854397.1|	356440-362755	9	38
ACYPI004334-RA	gi|641587404|gb|KK854107.1|	702164-703401	11	42

ACYPI008555-RA	gi|641575068|gb|KK855186.1|	20290-20716	12	44
ACYPI48246-RA	gi|641588267|gb|KK854008.1|	1118910-1119447	10	40
ACYPI009537-RA	gi|641577138|gb|KK854801.1|	147799-148486	10	38
ACYPI004261-RA	gi|641587133|gb|KK854140.1|	73325-80336	9.9	37
ACYPI005502-RA	gi|641577341|gb|KK854768.1|	207706-207887	14	58
ACYPI006680-RA	gi|641569707|gb|KK856586.1|	54956-55796	8.9	17
ACYPI007789-RA	gi|641576746|gb|KK854867.1|	146108-147710	11	20
ACYPI007827-RA	gi|641586521|gb|KK854229.1|	194198-197247	11	41
ACYPI008069-RA	gi|641569577|gb|KK856628.1|	99941-100288	9.7	36
ACYPI009676-RA	gi|641571632|gb|KK856018.1|	147106-147475	10	36
ACYPI010180-RA	gi|641570138|gb|KK856448.1|	72479-73236	10	21
ACYPI086093-RA	gi|641576409|gb|KK854925.1|	99407-101304	11	44
ACYPI48217-RA	gi|641575255|gb|KK855147.1|	240790-241451	9.4	40
ACYPI000600-RA	gi|641571660|gb|KK856010.1|	173694-174468	11	22
ACYPI000760-RA	gi|641587287|gb|KK854120.1|	419123-421287	9.9	37
ACYPI002480-RA	gi|641575396|gb|KK855118.1|	163763-164723	9	35
ACYPI002650-RA	gi|641570459|gb|KK856349.1|	25114-26078	9.7	40
ACYPI003224-RA	gi|641577104|gb|KK854807.1|	65947-67629	10	36
ACYPI006789-RA	gi|641587003|gb|KK854159.1|	435219-435452	10	21
ACYPI006958-RA	gi|641578046|gb|KK854650.1|	95657-98866	10	38
ACYPI007402-RA	gi|641576238|gb|KK854955.1|	162567-163108	11	40
ACYPI008272-RA	gi|641587937|gb|KK854042.1|	756487-758107	11	42
ACYPI008820-RA	gi|641587171|gb|KK854135.1|	188043-189900	10	39
ACYPI010149-RA	gi|641577498|gb|KK854744.1|	366994-367309	11	42
ACYPI062965-RA	gi|641573464|gb|KK855553.1|	221409-222443	11	39
ACYPI065107-RA	gi|641587074|gb|KK854148.1|	710493-714442	11	41
ACYPI55567-RA	gi|641568389|gb|KK856993.1|	8040-12164	10	41
ACYPI001604-RA	gi|641584931|gb|KK854446.1|	416029-418726	10	39
ACYPI001665-RA	gi|641586700|gb|KK854203.1|	122837-123566	8.4	39
ACYPI003550-RA	gi|641579212|gb|KK854585.1|	44440-46499	10	35

ACYPI004846-RA	gi|641587111|gb|KK854143.1|	599618-602011	11	40
ACYPI005499-RA	gi|641571428|gb|KK856074.1|	123365-124427	11	39
ACYPI006750-RA	gi|641587111|gb|KK854143.1|	528602-529161	11	40
ACYPI007366-RA	gi|641567075|gb|KK857433.1|	55023-58199	10	39
ACYPI009248-RA	gi|641571428|gb|KK856074.1|	77206-78428	11	39
ACYPI060328-RA	gi|641583980|gb|KK854512.1|	453232-453642	11	39
ACYPI063497-RA	gi|641569033|gb|KK856791.1|	102325-103242	11	42
ACYPI070945-RA	gi|641574545|gb|KK855295.1|	227252-230284	11	45
ACYPI001145-RA	gi|641570861|gb|KK856232.1|	38470-42415	11	39
ACYPI003626-RA	gi|641550656|gb|KK864801.1|	3877-4586	7.4	27
ACYPI004521-RA	gi|641576579|gb|KK854894.1|	97177-97423	10	42
ACYPI006438-RA	gi|641577104|gb|KK854807.1|	250704-255588	10	36
ACYPI008315-RA	gi|641574642|gb|KK855274.1|	223733-224421	11	38
ACYPI061132-RA	gi|641573007|gb|KK855666.1|	9748-10611	11	44
ACYPI067901-RA	gi|641587145|gb|KK854138.1|	181888-185686	10	42
ACYPI50578-RA	gi|641586419|gb|KK854244.1|	314168-315126	11	22
ACYPI003075-RA	gi|641587814|gb|KK854056.1|	244222-244751	9.7	40
ACYPI004845-RA	gi|641585847|gb|KK854330.1|	523172-523449	11	41
ACYPI37546-RA	gi|641570660|gb|KK856290.1|	121422-131351	16	69
ACYPI51632-RA	gi|641585383|gb|KK854406.1|	136514-137276	11	37
ACYPI000502-RA	gi|641576957|gb|KK854830.1|	177746-178005	10	43
ACYPI003519-RA	gi|641587921|gb|KK854044.1|	358092-360211	9.4	41
ACYPI089403-RA	gi|641573861|gb|KK855454.1|	49814-50669	10	37
ACYPI56637-RA	gi|641566778|gb|KK857537.1|	26912-27315	12	39
ACYPI000264-RA	gi|641587569|gb|KK854087.1|	340286-342508	11	41
ACYPI000882-RA	gi|641574960|gb|KK855207.1|	278907-280778	10	48
ACYPI001610-RA	gi|641571026|gb|KK856181.1|	112665-113848	7.8	32
ACYPI004201-RA	gi|641584632|gb|KK854467.1|	159757-161953	9.8	37
ACYPI004822-RA	gi|641578014|gb|KK854654.1|	374933-376023	12	44
ACYPI006133-RA	gi|641584632|gb|KK854467.1|	354059-355944	9.8	37

ACYPI006318-RA	gi|641567806|gb|KK857182.1|	5014-5282	9.6	38
ACYPI007240-RA	gi|641576906|gb|KK854838.1|	377655-378307	9.4	37
ACYPI007299-RA	gi|641587562|gb|KK854088.1|	472858-475472	11	42
ACYPI007869-RA	gi|641574031|gb|KK855412.1|	214750-215798	12	39
ACYPI008113-RA	gi|641569718|gb|KK856582.1|	47703-48923	8.4	36
ACYPI008501-RA	gi|641545800|gb|KK867127.1|	2796-4729	9.6	34
ACYPI009989-RA	gi|641588033|gb|KK854031.1|	453266-453475	11	38
ACYPI066082-RA	gi|641574930|gb|KK855214.1|	87148-87435	11	39
ACYPI005100-RA	gi|641585690|gb|KK854356.1|	531274-535887	9.6	41
ACYPI008046-RA	gi|641585350|gb|KK854411.1|	224971-226800	11	38
ACYPI081400-RA	gi|641588283|gb|KK854007.1|	1046085-1046469	11	40
ACYPI44772-RA	gi|641571216|gb|KK856129.1|	124907-125391	12	41
ACYPI060767-RA	gi|641586613|gb|KK854216.1|	402908-404436	11	41
ACYPI56773-RA	gi|641574960|gb|KK855207.1|	10029-11944	10	48
ACYPI001755-RA	gi|641588051|gb|KK854029.1|	539859-541775	9.6	40
ACYPI005417-RA	gi|641586921|gb|KK854170.1|	613915-617331	12	48
ACYPI40226-RA	gi|641587937|gb|KK854042.1|	876448-876800	11	42
ACYPI000407-RA	gi|641575222|gb|KK855154.1|	221423-222184	11	37
ACYPI001663-RA	gi|641586040|gb|KK854300.1|	559476-562612	10	38
ACYPI002303-RA	gi|641586296|gb|KK854261.1|	23163-26624	11	42
ACYPI005448-RA	gi|641574876|gb|KK855226.1|	153072-156281	11	39
ACYPI007308-RA	gi|641570754|gb|KK856261.1|	23075-23502	9.6	38
ACYPI009208-RA	gi|641580470|gb|KK854571.1|	98520-103854	10	41
ACYPI082601-RA	gi|641572963|gb|KK855677.1|	188326-189736	11	43
ACYPI086601-RA	gi|641588136|gb|KK854020.1|	889932-892418	11	36
ACYPI001877-RA	gi|641568433|gb|KK856978.1|	13215-13417	9.8	35
ACYPI004421-RA	gi|641586734|gb|KK854198.1|	698458-700902	11	40
ACYPI005270-RA	gi|641584504|gb|KK854477.1|	176959-177696	9.5	38
ACYPI006978-RA	gi|641573829|gb|KK855462.1|	188384-193291	11	41
ACYPI061546-RA	gi|641544009|gb|KK867995.1|	135-559	11	32

ACYPI48967-RA	gi|641570207|gb|KK856427.1|	111830-115509	11	40
ACYPI007020-RA	gi|641577487|gb|KK854746.1|	17046-19073	11	38
ACYPI008084-RA	gi|641575454|gb|KK855107.1|	290721-291876	10	38
ACYPI009998-RA	gi|641585192|gb|KK854430.1|	416887-418221	10	23
ACYPI003151-RA	gi|641573992|gb|KK855420.1|	138200-138663	10	37
ACYPI005774-RA	gi|641568044|gb|KK857103.1|	26679-28078	10	36
ACYPI007587-RA	gi|641571841|gb|KK855968.1|	29072-30367	10	36
ACYPI067736-RA	gi|641576328|gb|KK854938.1|	237775-238003	11	36
ACYPI20204-RA	gi|641578193|gb|KK854625.1|	348971-349679	11	41
ACYPI000413-RA	gi|641564920|gb|KK858233.1|	27114-27569	11	23
ACYPI000753-RA	gi|641572637|gb|KK855763.1|	18018-21093	9.5	36
ACYPI000896-RA	gi|641585910|gb|KK854320.1|	129892-132882	10	38
ACYPI002791-RA	gi|641572687|gb|KK855750.1|	42845-43279	9.9	39
ACYPI004886-RA	gi|641584977|gb|KK854443.1|	180222-181073	11	24
ACYPI007249-RA	gi|641572687|gb|KK855750.1|	52091-52908	9.9	39
ACYPI008165-RA	gi|641585773|gb|KK854342.1|	608787-609104	12	45
ACYPI070244-RA	gi|641578173|gb|KK854628.1|	50746-52487	9.9	21
ACYPI083931-RA	gi|641572711|gb|KK855744.1|	102371-102651	9.4	31
ACYPI089246-RA	gi|641585773|gb|KK854342.1|	612360-612696	12	45
ACYPI005622-RA	gi|641569260|gb|KK856723.1|	24941-25724	9.7	34
ACYPI005929-RA	gi|641584474|gb|KK854479.1|	249740-250668	9.5	40
ACYPI060353-RA	gi|641573587|gb|KK855522.1|	77385-77877	9.3	38
ACYPI061000-RA	gi|641584145|gb|KK854504.1|	84787-88740	8.9	38
ACYPI069554-RA	gi|641574402|gb|KK855330.1|	85533-85822	11	23
ACYPI085203-RA	gi|641587832|gb|KK854054.1|	664221-664721	9.8	20
ACYPI000044-RA	gi|641578305|gb|KK854605.1|	77673-79547	11	42
ACYPI000694-RA	gi|641587003|gb|KK854159.1|	277619-278020	10	21
ACYPI001296-RA	gi|641575888|gb|KK855026.1|	188622-193373	10	41
ACYPI001437-RA	gi|641583980|gb|KK854512.1|	375360-376158	11	39
ACYPI002441-RA	gi|641575709|gb|KK855059.1|	111571-113002	11	41

ACYPI003722-RA	gi|641570861|gb|KK856232.1|	2196-3235	11	39
ACYPI004343-RA	gi|641579212|gb|KK854585.1|	243762-245519	10	35
ACYPI005659-RA	gi|641571425|gb|KK856075.1|	43573-44914	9.3	36
ACYPI006312-RA	gi|641575709|gb|KK855059.1|	157596-158050	11	41
ACYPI061215-RA	gi|641575709|gb|KK855059.1|	341625-342954	11	41
ACYPI56627-RA	gi|641575709|gb|KK855059.1|	346032-347627	11	41
ACYPI001675-RA	gi|641577341|gb|KK854768.1|	114486-116177	14	58
ACYPI002171-RA	gi|641572875|gb|KK855699.1|	168420-168702	14	37
ACYPI004420-RA	gi|641584632|gb|KK854467.1|	426120-427001	9.8	37
ACYPI005602-RA	gi|641577772|gb|KK854697.1|	416981-417960	10	39
ACYPI006338-RA	gi|641587249|gb|KK854125.1|	245879-248462	11	37
ACYPI007376-RA	gi|641587396|gb|KK854108.1|	688185-688939	11	22
ACYPI007776-RA	gi|641575847|gb|KK855033.1|	191297-198173	10	40
ACYPI008123-RA	gi|641586372|gb|KK854251.1|	76200-76494	10	38
ACYPI065923-RA	gi|641566784|gb|KK857535.1|	77927-78544	9.2	33
ACYPI51196-RA	gi|641578193|gb|KK854625.1|	240316-241675	11	41
ACYPI000091-RA	gi|641577277|gb|KK854778.1|	217865-219704	11	42
ACYPI000454-RA	gi|641542389|gb|KK868779.1|	439-997	11	45
ACYPI001153-RA	gi|641587614|gb|KK854081.1|	704557-704930	11	40
ACYPI005360-RA	gi|641586235|gb|KK854270.1|	99823-101676	11	39
ACYPI005600-RA	gi|641586893|gb|KK854175.1|	507570-510208	11	41
ACYPI006254-RA	gi|641587577|gb|KK854086.1|	398684-398890	8.2	41
ACYPI007435-RA	gi|641574308|gb|KK855352.1|	157446-158790	10	36
ACYPI007477-RA	gi|641586850|gb|KK854182.1|	526445-529572	10	37
ACYPI009315-RA	gi|641576161|gb|KK854972.1|	367196-367866	10	36
ACYPI009370-RA	gi|641584205|gb|KK854500.1|	237854-241065	11	41
ACYPI000783-RA	gi|641586040|gb|KK854300.1|	267082-268549	10	38
ACYPI001430-RA	gi|641585819|gb|KK854334.1|	407671-408118	9.3	38
ACYPI004643-RA	gi|641582740|gb|KK854534.1|	292814-294849	8.1	43
ACYPI006703-RA	gi|641574944|gb|KK855211.1|	214840-216827	10	39

ACYPI007219-RA	gi|641571928|gb|KK855946.1|	110187-111080	11	38
ACYPI004451-RA	gi|641587118|gb|KK854142.1|	223629-224761	11	40
ACYPI008200-RA	gi|641584128|gb|KK854505.1|	442495-443273	9.6	37
ACYPI008886-RA	gi|641569155|gb|KK856752.1|	72581-72807	11	21
ACYPI009529-RA	gi|641578046|gb|KK854650.1|	248081-248562	10	38
ACYPI060066-RA	gi|641585910|gb|KK854320.1|	405520-409222	10	38
ACYPI001124-RA	gi|641587074|gb|KK854148.1|	185784-191231	11	41
ACYPI002711-RA	gi|641587652|gb|KK854077.1|	510488-512305	10	38
ACYPI003062-RA	gi|641586620|gb|KK854215.1|	92126-92460	9.1	40
ACYPI004254-RA	gi|641565780|gb|KK857902.1|	1121-2080	9.9	40
ACYPI004629-RA	gi|641575804|gb|KK855041.1|	277531-279194	11	40
ACYPI004936-RA	gi|641569630|gb|KK856611.1|	8727-9126	11	39
ACYPI004982-RA	gi|641569948|gb|KK856508.1|	39410-42525	10	37
ACYPI006545-RA	gi|641584876|gb|KK854450.1|	288338-289625	11	25
ACYPI008708-RA	gi|641588136|gb|KK854020.1|	281040-281341	11	36
ACYPI010209-RA	gi|641568704|gb|KK856893.1|	67349-67785	10	40
ACYPI000443-RA	gi|641584320|gb|KK854490.1|	426743-429006	10	41
ACYPI000513-RA	gi|641584945|gb|KK854445.1|	394329-397567	9.9	20
ACYPI001047-RA	gi|641580504|gb|KK854565.1|	394640-398081	11	43
ACYPI001687-RA	gi|641586688|gb|KK854205.1|	106586-107579	11	42
ACYPI002904-RA	gi|641575001|gb|KK855197.1|	323997-326642	11	38
ACYPI003499-RA	gi|641588099|gb|KK854024.1|	477462-478707	11	39
ACYPI003537-RA	gi|641585801|gb|KK854337.1|	205854-206612	10	38
ACYPI003571-RA	gi|641587021|gb|KK854156.1|	589985-590553	11	42
ACYPI004832-RA	gi|641583088|gb|KK854527.1|	190333-190637	9.9	39
ACYPI004869-RA	gi|641569291|gb|KK856713.1|	135403-137545	10	21
ACYPI005444-RA	gi|641587452|gb|KK854101.1|	869007-870951	11	40
ACYPI005485-RA	gi|641568762|gb|KK856874.1|	32002-40109	11	41
ACYPI005521-RA	gi|641587866|gb|KK854050.1|	453414-455214	10	38
ACYPI005540-RA	gi|641585383|gb|KK854406.1|	361132-361340	11	37

ACYPI006658-RA	gi|641584460|gb|KK854480.1|	108727-109104	11	40
ACYPI006682-RA	gi|641574036|gb|KK855411.1|	207028-207271	11	37
ACYPI006728-RA	gi|641565803|gb|KK857893.1|	18862-21243	10	35
ACYPI007303-RA	gi|641576188|gb|KK854966.1|	39448-40430	11	39
ACYPI007945-RA	gi|641576188|gb|KK854966.1|	25935-26779	11	39
ACYPI007967-RA	gi|641577677|gb|KK854711.1|	49212-49644	12	39
ACYPI009202-RA	gi|641587452|gb|KK854101.1|	591490-592758	11	40
ACYPI009266-RA	gi|641572364|gb|KK855831.1|	13546-16692	11	38
ACYPI009906-RA	gi|641588258|gb|KK854009.1|	786815-787682	11	36
ACYPI089070-RA	gi|641579930|gb|KK854578.1|	71836-73288	9.1	35
ACYPI56634-RA	gi|641587452|gb|KK854101.1|	752803-753405	11	40
ACYPI002842-RA	gi|641588144|gb|KK854019.1|	1014450-1014679	11	49
ACYPI005883-RA	gi|641575377|gb|KK855123.1|	64068-65287	10	38
ACYPI006954-RA	gi|641576876|gb|KK854844.1|	370973-372217	17	42
ACYPI009066-RA	gi|641574040|gb|KK855410.1|	273304-273985	9.9	44
ACYPI006684-RA	gi|641575368|gb|KK855125.1|	41060-42271	10	36
ACYPI000112-RA	gi|641571048|gb|KK856174.1|	36498-36798	11	39
ACYPI005171-RA	gi|641585946|gb|KK854315.1|	139906-141568	9.8	43
ACYPI009436-RA	gi|641570830|gb|KK856242.1|	23731-27101	9	31
ACYPI086826-RA	gi|641570830|gb|KK856242.1|	27408-28877	9	31
ACYPI006968-RA	gi|641587424|gb|KK854104.1|	787709-788367	11	41
ACYPI008923-RA	gi|641570589|gb|KK856310.1|	88815-91944	11	36
ACYPI001779-RA	gi|641588082|gb|KK854026.1|	333601-335242	11	41
ACYPI005594-RA	gi|641569929|gb|KK856514.1|	83410-83629	10	18
ACYPI006242-RA	gi|641573769|gb|KK855477.1|	124169-128061	9.2	38
ACYPI064692-RA	gi|641577161|gb|KK854797.1|	34103-38895	10	38
ACYPI001025-RA	gi|641575140|gb|KK855171.1|	325434-328817	9.7	40
ACYPI002276-RA	gi|641567358|gb|KK857335.1|	43905-44187	10	36
ACYPI003593-RA	gi|641574990|gb|KK855200.1|	265963-267568	12	37
ACYPI005543-RA	gi|641586955|gb|KK854165.1|	578742-579556	11	40

ACYPI005578-RA	gi|641585790|gb|KK854339.1|	255438-256991	9.7	38
ACYPI060889-RA	gi|641585778|gb|KK854341.1|	387731-388385	9.7	41
ACYPI001166-RA	gi|641586380|gb|KK854250.1|	144189-144519	9.3	38
ACYPI001246-RA	gi|641587569|gb|KK854087.1|	675505-675882	11	41
ACYPI001654-RA	gi|641587840|gb|KK854053.1|	614816-618696	11	43
ACYPI001798-RA	gi|641574061|gb|KK855405.1|	63200-63437	11	21
ACYPI003948-RA	gi|641569782|gb|KK856562.1|	21691-21922	6.8	37
ACYPI006530-RA	gi|641568339|gb|KK857008.1|	5904-9599	11	38
ACYPI009457-RA	gi|641586975|gb|KK854163.1|	225721-226430	11	40
ACYPI086075-RA	gi|641570210|gb|KK856426.1|	21694-23931	11	40
ACYPI087849-RA	gi|641583980|gb|KK854512.1|	356351-360954	11	39
ACYPI47759-RA	gi|641574303|gb|KK855353.1|	169460-170013	10	34
ACYPI001036-RA	gi|641573053|gb|KK855655.1|	187212-187858	11	41
ACYPI002015-RA	gi|641573641|gb|KK855509.1|	60604-60840	9.8	36
ACYPI002949-RA	gi|641577772|gb|KK854697.1|	198093-198437	10	39
ACYPI004239-RA	gi|641587031|gb|KK854154.1|	643098-644799	11	42
ACYPI005172-RA	gi|641584743|gb|KK854459.1|	134000-140372	11	36
ACYPI005839-RA	gi|641577316|gb|KK854772.1|	206720-207362	9.4	36
ACYPI006777-RA	gi|641587866|gb|KK854050.1|	1008888-1009229	10	38
ACYPI008640-RA	gi|641577215|gb|KK854787.1|	159906-167921	11	40
ACYPI009258-RA	gi|641573053|gb|KK855655.1|	251172-252128	11	41
ACYPI009674-RA	gi|641555784|gb|KK862383.1|	5713-5994	########	########
ACYPI009973-RA	gi|641574015|gb|KK855416.1|	96702-97211	10	42
ACYPI066761-RA	gi|641577244|gb|KK854783.1|	128701-130257	9.7	19
ACYPI067645-RA	gi|641575218|gb|KK855155.1|	65554-66497	11	21
ACYPI083672-RA	gi|641587390|gb|KK854109.1|	467880-468138	11	44
ACYPI085314-RA	gi|641586114|gb|KK854288.1|	326956-329678	10	41
ACYPI45971-RA	gi|641586328|gb|KK854257.1|	183175-187668	8.4	35
ACYPI003006-RA	gi|641585778|gb|KK854341.1|	257012-257385	9.7	41
ACYPI008560-RA	gi|641585778|gb|KK854341.1|	299438-299721	9.7	41

ACYPI009292-RA	gi|641586328|gb|KK854257.1|	492974-493200	8.4	35
ACYPI51231-RA	gi|641577623|gb|KK854722.1|	320009-322921	11	38
ACYPI003045-RA	gi|641587614|gb|KK854081.1|	561821-563216	11	40
ACYPI004195-RA	gi|641571667|gb|KK856008.1|	142338-148056	11	39
ACYPI004365-RA	gi|641587111|gb|KK854143.1|	749231-749611	11	40
ACYPI004964-RA	gi|641586359|gb|KK854252.1|	210118-211459	10	39
ACYPI008164-RA	gi|641587111|gb|KK854143.1|	801287-803679	11	40
ACYPI008841-RA	gi|641571813|gb|KK855975.1|	129757-131583	9.5	18
ACYPI068685-RA	gi|641573392|gb|KK855573.1|	91501-92709	8.5	38
ACYPI000582-RA	gi|641586452|gb|KK854239.1|	505286-505784	10	19
ACYPI003455-RA	gi|641577075|gb|KK854811.1|	161155-164885	11	36
ACYPI003942-RA	gi|641575559|gb|KK855088.1|	260800-262258	9.9	37
ACYPI004076-RA	gi|641584271|gb|KK854494.1|	13110-14250	13	45
ACYPI000063-RA	gi|641577920|gb|KK854670.1|	271638-272507	8.5	40
ACYPI007642-RA	gi|641586258|gb|KK854267.1|	696141-696963	11	37
ACYPI009680-RA	gi|641573334|gb|KK855587.1|	154799-158293	10	39
ACYPI009944-RA	gi|641572239|gb|KK855863.1|	84158-86266	11	43
ACYPI070297-RA	gi|641585293|gb|KK854422.1|	162658-163624	9.1	38
ACYPI002153-RA	gi|641587555|gb|KK854089.1|	571977-573234	11	39
ACYPI003754-RA	gi|641586975|gb|KK854163.1|	363994-364405	11	40
ACYPI003986-RA	gi|641572130|gb|KK855891.1|	174453-174824	10	37
ACYPI004024-RA	gi|641585340|gb|KK854414.1|	242560-244760	11	42
ACYPI005967-RA	gi|641588309|gb|KK854005.1|	294109-294307	11	40
ACYPI006066-RA	gi|641586975|gb|KK854163.1|	532106-535528	11	40
ACYPI066036-RA	gi|641587263|gb|KK854123.1|	113345-114656	11	42
ACYPI068671-RA	gi|641575687|gb|KK855063.1|	235261-235921	11	34
ACYPI084147-RA	gi|641586975|gb|KK854163.1|	492895-493557	11	40
ACYPI20693-RA	gi|641575414|gb|KK855115.1|	233010-233774	9.8	39
ACYPI31758-RA	gi|641586754|gb|KK854195.1|	638814-639853	11	41
ACYPI34938-RA	gi|641573969|gb|KK855425.1|	60902-61813	8.9	42

ACYPI49886-RA	gi|641575970|gb|KK855012.1|	41447-43710	11	38
ACYPI56791-RA	gi|641577623|gb|KK854722.1|	376972-380785	11	38
ACYPI000575-RA	gi|641585366|gb|KK854408.1|	388426-390905	12	41
ACYPI004747-RA	gi|641570346|gb|KK856381.1|	59433-60620	9.9	20
ACYPI005400-RA	gi|641575222|gb|KK855154.1|	84349-86176	11	37
ACYPI005676-RA	gi|641575656|gb|KK855069.1|	50248-50699	11	42
ACYPI006929-RA	gi|641585773|gb|KK854342.1|	136565-139150	12	45
ACYPI008551-RA	gi|641577209|gb|KK854788.1|	395918-398664	12	41
ACYPI002245-RA	gi|641587452|gb|KK854101.1|	907055-911734	11	40
ACYPI002529-RA	gi|641585004|gb|KK854441.1|	11004-11710	11	43
ACYPI084647-RA	gi|641586754|gb|KK854195.1|	125012-129373	11	41
ACYPI006364-RA	gi|641588021|gb|KK854033.1|	1097684-1098736	12	40
ACYPI008243-RA	gi|641587162|gb|KK854136.1|	590188-591116	10	40
ACYPI000090-RA	gi|641588343|gb|KK854003.1|	1016518-1016738	11	40
ACYPI006822-RA	gi|641572879|gb|KK855698.1|	195123-195355	10	39
ACYPI000681-RA	gi|641586496|gb|KK854233.1|	623852-626325	11	38
ACYPI003186-RA	gi|641570355|gb|KK856378.1|	6188-6426	9.7	35
ACYPI003944-RA	gi|641567171|gb|KK857401.1|	84734-87637	10	36
ACYPI005106-RA	gi|641570861|gb|KK856232.1|	107684-107978	11	39
ACYPI005308-RA	gi|641585801|gb|KK854337.1|	528369-530177	10	38
ACYPI000648-RA	gi|641587193|gb|KK854132.1|	507941-508323	11	39
ACYPI001631-RA	gi|641575381|gb|KK855122.1|	262263-262727	11	43
ACYPI005253-RA	gi|641576409|gb|KK854925.1|	102260-102986	11	44
ACYPI001021-RA	gi|641573136|gb|KK855635.1|	45632-46972	9.8	26
ACYPI004854-RA	gi|641581389|gb|KK854557.1|	267191-270427	11	41
ACYPI005394-RA	gi|641575987|gb|KK855008.1|	40582-41061	9.9	20
ACYPI006268-RA	gi|641568762|gb|KK856874.1|	16451-17841	11	41
ACYPI007266-RA	gi|641587962|gb|KK854039.1|	1047946-1049128	11	40
ACYPI007901-RA	gi|641585510|gb|KK854386.1|	111591-111997	11	20
ACYPI008616-RA	gi|641588258|gb|KK854009.1|	711818-714755	11	36

ACYPI008709-RA	gi|641576863|gb|KK854845.1|	182756-188864	11	41
ACYPI009158-RA	gi|641586734|gb|KK854198.1|	184729-188199	11	40
ACYPI010205-RA	gi|641587104|gb|KK854144.1|	140457-144470	11	37
ACYPI080138-RA	gi|641585510|gb|KK854386.1|	118212-121205	11	20
ACYPI082626-RA	gi|641576608|gb|KK854891.1|	268192-270841	11	37
ACYPI084669-RA	gi|641575987|gb|KK855008.1|	47630-48008	9.9	20
ACYPI000249-RA	gi|641572891|gb|KK855695.1|	31191-32931	10	36
ACYPI000662-RA	gi|641575385|gb|KK855121.1|	141042-142700	9.4	36
ACYPI000913-RA	gi|641571457|gb|KK856066.1|	186244-186720	12	43
ACYPI001061-RA	gi|641567860|gb|KK857164.1|	1418-3170	11	36
ACYPI001698-RA	gi|641587881|gb|KK854049.1|	613046-614244	10	40
ACYPI002549-RA	gi|641569328|gb|KK856702.1|	97741-98120	9.6	17
ACYPI002798-RA	gi|641585833|gb|KK854332.1|	319838-320301	10	37
ACYPI002806-RA	gi|641588325|gb|KK854004.1|	993003-993701	11	40
ACYPI003581-RA	gi|641571841|gb|KK855968.1|	133530-138589	10	36
ACYPI004142-RA	gi|641575112|gb|KK855177.1|	88837-94473	11	39
ACYPI006166-RA	gi|641588343|gb|KK854003.1|	1343757-1345371	11	40
ACYPI006625-RA	gi|641575743|gb|KK855052.1|	133926-141235	9.7	38
ACYPI006785-RA	gi|641573464|gb|KK855553.1|	178611-179112	11	39
ACYPI007197-RA	gi|641585315|gb|KK854418.1|	21687-22721	10	39
ACYPI007757-RA	gi|641579947|gb|KK854574.1|	414317-414539	11	39
ACYPI009528-RA	gi|641584977|gb|KK854443.1|	282333-284312	11	24
ACYPI009915-RA	gi|641587688|gb|KK854072.1|	510415-511962	10	38
ACYPI010231-RA	gi|641578154|gb|KK854631.1|	95855-97713	8.2	40
ACYPI088944-RA	gi|641576930|gb|KK854835.1|	107321-108300	9.6	19
ACYPI000259-RA	gi|641587040|gb|KK854152.1|	364920-365624	9.1	39
ACYPI001527-RA	gi|641573097|gb|KK855644.1|	184014-184562	10	36
ACYPI004878-RA	gi|641575826|gb|KK855037.1|	139529-139765	10	39
ACYPI005975-RA	gi|641585900|gb|KK854322.1|	662664-662934	12	43
ACYPI006635-RA	gi|641573097|gb|KK855644.1|	104411-111687	10	36

ACYPI006817-RA	gi|641587287|gb|KK854120.1|	161230-163841	9.9	37
ACYPI008495-RA	gi|641587660|gb|KK854076.1|	461594-461882	9.9	41
ACYPI009739-RA	gi|641588309|gb|KK854005.1|	530575-532240	11	40
ACYPI002126-RA	gi|641573850|gb|KK855457.1|	27538-27780	9.3	34
ACYPI003058-RA	gi|641585784|gb|KK854340.1|	605820-606030	9.7	44
ACYPI003388-RA	gi|641587452|gb|KK854101.1|	156979-157382	11	40
ACYPI004001-RA	gi|641587186|gb|KK854133.1|	333281-333586	8.1	39
ACYPI004294-RA	gi|641577374|gb|KK854762.1|	179759-180378	11	42
ACYPI004372-RA	gi|641570188|gb|KK856433.1|	93689-95212	10	38
ACYPI004697-RA	gi|641576973|gb|KK854827.1|	125853-126248	11	38
ACYPI005640-RA	gi|641577879|gb|KK854677.1|	314455-315696	18	36
ACYPI005940-RA	gi|641584281|gb|KK854493.1|	50209-50717	12	41
ACYPI006828-RA	gi|641580504|gb|KK854565.1|	348065-351180	11	43
ACYPI007260-RA	gi|641586967|gb|KK854164.1|	88755-89159	10	27
ACYPI007809-RA	gi|641584281|gb|KK854493.1|	151529-152168	12	41
ACYPI008241-RA	gi|641587383|gb|KK854110.1|	362879-365366	10	37
ACYPI008734-RA	gi|641584681|gb|KK854464.1|	287136-287402	11	42
ACYPI008811-RA	gi|641585653|gb|KK854362.1|	23327-23598	10	39
ACYPI009386-RA	gi|641588000|gb|KK854035.1|	871509-874643	11	39
ACYPI010127-RA	gi|641573464|gb|KK855553.1|	136792-138344	11	39
ACYPI071272-RA	gi|641561323|gb|KK859794.1|	2739-3318	8.5	15
ACYPI086258-RA	gi|641586338|gb|KK854255.1|	565588-566320	14	44
ACYPI22226-RA	gi|641585475|gb|KK854392.1|	199304-202704	10	41
ACYPI44580-RA	gi|641576973|gb|KK854827.1|	13758-14852	11	38
ACYPI001686-RA	gi|641587193|gb|KK854132.1|	658260-659028	11	39
ACYPI003770-RA	gi|641570419|gb|KK856360.1|	130308-131082	12	39
ACYPI004120-RA	gi|641585900|gb|KK854322.1|	117828-118056	12	43
ACYPI006151-RA	gi|641587652|gb|KK854077.1|	830187-830707	10	38
ACYPI008028-RA	gi|641585376|gb|KK854407.1|	180424-187277	9.8	36
ACYPI008231-RA	gi|641577341|gb|KK854768.1|	333027-334677	14	58

ACYPI008576-RA	gi|641566659|gb|KK857580.1|	22827-23709	9.3	35
ACYPI008730-RA	gi|641573617|gb|KK855515.1|	108313-109776	10	38
ACYPI009782-RA	gi|641586837|gb|KK854184.1|	102675-103366	11	40
ACYPI009904-RA	gi|641587193|gb|KK854132.1|	659314-660277	11	39
ACYPI010116-RA	gi|641585967|gb|KK854311.1|	145756-149208	11	40
ACYPI23235-RA	gi|641587652|gb|KK854077.1|	816970-819953	10	38
ACYPI40836-RA	gi|641566659|gb|KK857580.1|	1-834	9.3	35
ACYPI48216-RA	gi|641586351|gb|KK854253.1|	8934-12001	10	37
ACYPI001982-RA	gi|641587504|gb|KK854095.1|	710686-711505	11	42
ACYPI006619-RA	gi|641582408|gb|KK854540.1|	383508-385257	10	41
ACYPI008866-RA	gi|641577058|gb|KK854814.1|	319239-322704	9.1	43
ACYPI008953-RA	gi|641586874|gb|KK854178.1|	189366-189705	9.4	38
ACYPI38425-RA	gi|641585801|gb|KK854337.1|	82386-83614	10	38
ACYPI005799-RA	gi|641584658|gb|KK854465.1|	238742-239030	11	37
ACYPI062437-RA	gi|641573885|gb|KK855447.1|	120529-128768	9.6	37
ACYPI087322-RA	gi|641578149|gb|KK854632.1|	188674-190739	11	40
ACYPI087407-RA	gi|641586850|gb|KK854182.1|	276338-279445	10	37
ACYPI000065-RA	gi|641576347|gb|KK854934.1|	20200-21624	11	39
ACYPI000222-RA	gi|641576260|gb|KK854950.1|	45448-45882	10	39
ACYPI000885-RA	gi|641586452|gb|KK854239.1|	192245-192518	10	19
ACYPI002123-RA	gi|641569294|gb|KK856712.1|	32021-32253	12	22
ACYPI003998-RA	gi|641587606|gb|KK854082.1|	810499-810754	11	40
ACYPI004750-RA	gi|641586626|gb|KK854214.1|	611128-614800	11	44
ACYPI004981-RA	gi|641586191|gb|KK854274.1|	40572-41160	11	42
ACYPI005363-RA	gi|641585725|gb|KK854350.1|	579913-581463	12	41
ACYPI006178-RA	gi|641571605|gb|KK856024.1|	7499-7959	10	38
ACYPI006875-RA	gi|641576887|gb|KK854842.1|	255358-255716	15	41
ACYPI007058-RA	gi|641576677|gb|KK854879.1|	92731-94247	11	39
ACYPI008050-RA	gi|641587856|gb|KK854051.1|	783668-784232	10	41
ACYPI008363-RA	gi|641578109|gb|KK854638.1|	476604-478421	14	46

ACYPI064487-RA	gi|641586936|gb|KK854168.1|	624729-625174	12	42
ACYPI071881-RA	gi|641576689|gb|KK854876.1|	382208-383420	10	40
ACYPI089177-RA	gi|641568328|gb|KK857011.1|	41091-41852	9.7	32
ACYPI23999-RA	gi|641561138|gb|KK859879.1|	12437-15522	12	42
ACYPI32079-RA	gi|641578283|gb|KK854609.1|	312246-316303	12	22
ACYPI47651-RA	gi|641586096|gb|KK854291.1|	638772-639013	11	46
ACYPI000028-RA	gi|641577805|gb|KK854691.1|	404966-405696	9.4	38
ACYPI000349-RA	gi|641587084|gb|KK854146.1|	499204-500331	9	40
ACYPI000499-RA	gi|641564440|gb|KK858439.1|	379-2822	11	36
ACYPI002010-RA	gi|641587606|gb|KK854082.1|	723613-727161	11	40
ACYPI002252-RA	gi|641585812|gb|KK854335.1|	296612-298370	11	42
ACYPI002657-RA	gi|641584818|gb|KK854454.1|	146906-149054	11	42
ACYPI003899-RA	gi|641577905|gb|KK854672.1|	108380-109121	10	37
ACYPI004409-RA	gi|641576100|gb|KK854984.1|	45274-45824	10	40
ACYPI004431-RA	gi|641577265|gb|KK854780.1|	186469-187309	10	40
ACYPI005832-RA	gi|641573729|gb|KK855488.1|	191358-193548	10	43
ACYPI006227-RA	gi|641571982|gb|KK855931.1|	53261-53711	9.8	35
ACYPI006348-RA	gi|641572675|gb|KK855753.1|	133980-135192	11	40
ACYPI008107-RA	gi|641588213|gb|KK854013.1|	1020148-1020832	9.6	39
ACYPI008366-RA	gi|641587866|gb|KK854050.1|	988745-992046	10	38
ACYPI080567-RA	gi|641574119|gb|KK855392.1|	87498-88329	9.6	37
ACYPI30696-RA	gi|641578046|gb|KK854650.1|	29592-31166	10	38
ACYPI49859-RA	gi|641574468|gb|KK855314.1|	91266-91976	9.9	37
ACYPI003871-RA	gi|641587162|gb|KK854136.1|	612957-614718	10	40
ACYPI004542-RA	gi|641571144|gb|KK856149.1|	73873-74073	11	20
ACYPI006271-RA	gi|641573752|gb|KK855482.1|	149708-150092	11	37
ACYPI007285-RA	gi|641586599|gb|KK854218.1|	86380-90927	11	42
ACYPI010025-RA	gi|641584068|gb|KK854508.1|	430414-431046	10	45
ACYPI061311-RA	gi|641570819|gb|KK856245.1|	39769-41418	10	45
ACYPI067648-RA	gi|641587365|gb|KK854113.1|	157924-159522	12	45

ACYPI072429-RA	gi|641585599|gb|KK854371.1|	101841-108089	11	39
ACYPI088096-RA	gi|641578056|gb|KK854648.1|	22281-23600	11	41
ACYPI002029-RA	gi|641577704|gb|KK854707.1|	131700-132030	10	38
ACYPI004258-RA	gi|641586585|gb|KK854220.1|	329669-330917	11	44
ACYPI006678-RA	gi|641573645|gb|KK855508.1|	66824-67160	9.4	40
ACYPI008065-RA	gi|641587992|gb|KK854036.1|	686026-686619	11	41
ACYPI064307-RA	gi|641588309|gb|KK854005.1|	1840464-1841844	11	40
ACYPI40717-RA	gi|641574150|gb|KK855385.1|	104474-107265	10	38
ACYPI001310-RA	gi|641570861|gb|KK856232.1|	77985-79476	11	39
ACYPI004635-RA	gi|641569542|gb|KK856639.1|	25371-25595	9.3	34
ACYPI004687-RA	gi|641574031|gb|KK855412.1|	16706-21476	12	39
ACYPI005081-RA	gi|641588343|gb|KK854003.1|	1745577-1745949	11	40
ACYPI005233-RA	gi|641574704|gb|KK855260.1|	57382-60808	9.3	35
ACYPI009527-RA	gi|641588109|gb|KK854023.1|	67554-69150	10	40
ACYPI088277-RA	gi|641570238|gb|KK856417.1|	34256-35997	9.3	38
ACYPI31682-RA	gi|641587365|gb|KK854113.1|	276177-278154	12	45
ACYPI000508-RA	gi|641585587|gb|KK854373.1|	426424-426985	9.8	41
ACYPI001052-RA	gi|641588164|gb|KK854017.1|	280218-290107	9.2	40
ACYPI003579-RA	gi|641586535|gb|KK854227.1|	613950-614919	10	39
ACYPI005464-RA	gi|641575468|gb|KK855105.1|	109734-110182	9.5	20
ACYPI006864-RA	gi|641576286|gb|KK854946.1|	142021-144034	12	38
ACYPI007128-RA	gi|641575914|gb|KK855020.1|	152122-152578	10	37
ACYPI008037-RA	gi|641587256|gb|KK854124.1|	109531-110540	9.7	40
ACYPI061188-RA	gi|641573788|gb|KK855472.1|	31424-32322	10	37
ACYPI081260-RA	gi|641571667|gb|KK856008.1|	48529-49546	11	39
ACYPI001683-RA	gi|641574876|gb|KK855226.1|	121912-122231	11	39
ACYPI001929-RA	gi|641585383|gb|KK854406.1|	398407-398676	11	37
ACYPI001960-RA	gi|641576791|gb|KK854858.1|	221176-223422	9.2	42
ACYPI002132-RA	gi|641574800|gb|KK855241.1|	134771-135213	10	37
ACYPI002370-RA	gi|641576315|gb|KK854940.1|	118566-118867	9.9	40

ACYPI003296-RA	gi|641576962|gb|KK854829.1|	321264-322112	10	39
ACYPI004006-RA	gi|641587047|gb|KK854151.1|	424829-425148	10	39
ACYPI004937-RA	gi|641574676|gb|KK855265.1|	42480-42804	9.4	35
ACYPI005593-RA	gi|641570141|gb|KK856447.1|	91282-91541	11	40
ACYPI007470-RA	gi|641575730|gb|KK855054.1|	300880-301964	10	38
ACYPI008675-RA	gi|641586850|gb|KK854182.1|	613735-614680	10	37
ACYPI072499-RA	gi|641585552|gb|KK854379.1|	470244-473182	14	62
ACYPI087133-RA	gi|641587052|gb|KK854150.1|	59787-62961	11	38
ACYPI000225-RA	gi|641572601|gb|KK855773.1|	54796-56262	10	35
ACYPI000665-RA	gi|641587082|gb|KK854147.1|	829583-833806	11	44
ACYPI004792-RA	gi|641588194|gb|KK854015.1|	540405-542775	9.4	40
ACYPI005685-RA	gi|641572601|gb|KK855773.1|	50532-51849	10	35
ACYPI006603-RA	gi|641584818|gb|KK854454.1|	397690-398012	11	42
ACYPI006694-RA	gi|641572601|gb|KK855773.1|	52132-53749	10	35
ACYPI007238-RA	gi|641586880|gb|KK854177.1|	228623-232098	10	20
ACYPI007669-RA	gi|641562301|gb|KK859354.1|	10691-11263	9.2	39
ACYPI085301-RA	gi|641570045|gb|KK856477.1|	92062-94767	9.6	43
ACYPI26341-RA	gi|641586741|gb|KK854197.1|	642764-643733	11	43
ACYPI007773-RA	gi|641585107|gb|KK854435.1|	388387-391875	15	45
ACYPI27939-RA	gi|641585407|gb|KK854403.1|	418445-418915	9.4	42
ACYPI54101-RA	gi|641581402|gb|KK854555.1|	182622-184037	12	20
ACYPI004948-RA	gi|641575135|gb|KK855172.1|	206196-210461	11	44
ACYPI066399-RA	gi|641586470|gb|KK854237.1|	409784-411716	11	42
ACYPI41926-RA	gi|641577244|gb|KK854783.1|	102391-105222	9.7	19
ACYPI000720-RA	gi|641569788|gb|KK856560.1|	30690-31881	9.5	37
ACYPI000776-RA	gi|641585871|gb|KK854327.1|	138541-140199	11	38
ACYPI000929-RA	gi|641572134|gb|KK855890.1|	56721-57783	10	36
ACYPI002609-RA	gi|641586328|gb|KK854257.1|	194646-198620	8.4	35
ACYPI003303-RA	gi|641584445|gb|KK854481.1|	139543-146223	9.9	39
ACYPI007084-RA	gi|641587488|gb|KK854097.1|	511710-512191	11	21

ACYPI008834-RA	gi|641587660|gb|KK854076.1|	522839-523332	9.9	41
ACYPI41618-RA	gi|641572773|gb|KK855727.1|	123107-123369	9.7	24
ACYPI001220-RA	gi|641571276|gb|KK856113.1|	89299-90175	9.9	34
ACYPI007771-RA	gi|641574193|gb|KK855375.1|	169090-171505	10	41
ACYPI56678-RA	gi|641571119|gb|KK856156.1|	186130-188457	11	38
ACYPI000038-RA	gi|641567126|gb|KK857416.1|	36342-37408	11	41
ACYPI000051-RA	gi|641568389|gb|KK856993.1|	95561-97314	10	41
ACYPI002409-RA	gi|641575862|gb|KK855031.1|	96392-96524	9.6	45
ACYPI002536-RA	gi|641570016|gb|KK856486.1|	44031-44627	12	20
ACYPI003033-RA	gi|641569948|gb|KK856508.1|	78420-79986	10	37
ACYPI004378-RA	gi|641575965|gb|KK855013.1|	186540-187293	9.2	31
ACYPI004950-RA	gi|641572569|gb|KK855781.1|	16346-17594	11	39
ACYPI005993-RA	gi|641574424|gb|KK855324.1|	131781-132637	10	39
ACYPI006844-RA	gi|641586817|gb|KK854187.1|	578099-578406	9.3	39
ACYPI007468-RA	gi|641570419|gb|KK856360.1|	74048-77389	12	39
ACYPI007860-RA	gi|641564627|gb|KK858354.1|	25000-25242	8.9	35
ACYPI008114-RA	gi|641587193|gb|KK854132.1|	749049-752423	11	39
ACYPI008713-RA	gi|641567114|gb|KK857420.1|	67663-71213	10	38
ACYPI009867-RA	gi|641585480|gb|KK854391.1|	252311-252651	11	21
ACYPI088273-RA	gi|641585233|gb|KK854427.1|	480975-483095	10	38
ACYPI088515-RA	gi|641575047|gb|KK855190.1|	111505-113969	8.3	43
ACYPI089106-RA	gi|641570089|gb|KK856463.1|	53520-53799	11	36
ACYPI002282-RA	gi|641577905|gb|KK854672.1|	26687-27132	10	37
ACYPI002879-RA	gi|641579235|gb|KK854581.1|	349764-350853	9.8	38
ACYPI003154-RA	gi|641584755|gb|KK854458.1|	25114-26019	9.7	41
ACYPI003191-RA	gi|641572928|gb|KK855687.1|	83037-83419	11	38
ACYPI003322-RA	gi|641570058|gb|KK856473.1|	85679-88087	11	40
ACYPI003323-RA	gi|641587624|gb|KK854080.1|	479072-479372	10	38
ACYPI003863-RA	gi|641584445|gb|KK854481.1|	268830-271010	9.9	39
ACYPI005150-RA	gi|641584806|gb|KK854455.1|	194250-194740	11	43

ACYPI005247-RA	gi|641584445|gb|KK854481.1|	415356-416885	9.9	39
ACYPI005331-RA	gi|641583574|gb|KK854520.1|	412333-413035	9.2	40
ACYPI005368-RA	gi|641586096|gb|KK854291.1|	187434-189058	11	46
ACYPI005747-RA	gi|641586915|gb|KK854171.1|	500042-504602	10	38
ACYPI007135-RA	gi|641587624|gb|KK854080.1|	458158-459924	10	38
ACYPI007623-RA	gi|641587383|gb|KK854110.1|	439513-440258	10	37
ACYPI007672-RA	gi|641588267|gb|KK854008.1|	318198-318641	10	40
ACYPI007878-RA	gi|641588294|gb|KK854006.1|	954747-957963	8.7	40
ACYPI009011-RA	gi|641588041|gb|KK854030.1|	977862-978401	11	38
ACYPI009239-RA	gi|641577121|gb|KK854804.1|	331285-331683	16	82
ACYPI009515-RA	gi|641587383|gb|KK854110.1|	468065-468362	10	37
ACYPI009832-RA	gi|641575808|gb|KK855040.1|	87407-87867	9.9	37
ACYPI46801-RA	gi|641569574|gb|KK856629.1|	25228-28986	24	########
ACYPI56547-RA	gi|641575794|gb|KK855043.1|	59665-62878	10	36
ACYPI003590-RA	gi|641585946|gb|KK854315.1|	650300-655433	9.8	43
ACYPI004026-RA	gi|641587003|gb|KK854159.1|	644543-649466	10	21
ACYPI000041-RA	gi|641567348|gb|KK857339.1|	64240-65270	8.5	39
ACYPI000192-RA	gi|641586915|gb|KK854171.1|	329413-330184	10	38
ACYPI001487-RA	gi|641586235|gb|KK854270.1|	295118-295288	11	39
ACYPI002115-RA	gi|641571581|gb|KK856030.1|	199916-200333	10	38
ACYPI004576-RA	gi|641585971|gb|KK854310.1|	413515-415267	10	40
ACYPI006488-RA	gi|641577580|gb|KK854730.1|	12242-12443	11	41
ACYPI007401-RA	gi|641584305|gb|KK854491.1|	330222-339291	11	39
ACYPI007695-RA	gi|641576951|gb|KK854831.1|	274772-278543	8.6	40
ACYPI008463-RA	gi|641584305|gb|KK854491.1|	280359-280752	11	39
ACYPI009510-RA	gi|641586496|gb|KK854233.1|	441890-442501	11	38
ACYPI010237-RA	gi|641587126|gb|KK854141.1|	718352-725883	10	39
ACYPI001800-RA	gi|641568184|gb|KK857057.1|	41747-43913	9.5	39
ACYPI002145-RA	gi|641575730|gb|KK855054.1|	415058-415764	10	38
ACYPI002460-RA	gi|641588267|gb|KK854008.1|	610513-612092	10	40

ACYPI002758-RA	gi|641585647|gb|KK854363.1|	258208-258851	8.8	35
ACYPI004615-RA	gi|641585616|gb|KK854368.1|	285987-289150	8.9	39
ACYPI006251-RA	gi|641575130|gb|KK855173.1|	88810-90602	12	50
ACYPI008122-RA	gi|641575104|gb|KK855179.1|	151037-154790	10	35
ACYPI008481-RA	gi|641586053|gb|KK854298.1|	609578-609753	11	41
ACYPI061780-RA	gi|641584248|gb|KK854496.1|	49894-51558	9.9	39
ACYPI071169-RA	gi|641586452|gb|KK854239.1|	392479-392887	10	19
ACYPI080920-RA	gi|641585833|gb|KK854332.1|	8753-9750	10	37
ACYPI082628-RA	gi|641574646|gb|KK855273.1|	31781-32380	10	38
ACYPI085199-RA	gi|641587074|gb|KK854148.1|	79887-80937	11	41
ACYPI085378-RA	gi|641559061|gb|KK860835.1|	13378-13650	8	35
ACYPI000496-RA	gi|641585423|gb|KK854400.1|	83475-85161	11	36
ACYPI003043-RA	gi|641568815|gb|KK856858.1|	18640-19270	10	34
ACYPI003470-RA	gi|641574532|gb|KK855298.1|	70081-70286	12	20
ACYPI003667-RA	gi|641571523|gb|KK856046.1|	84214-84458	10	43
ACYPI004268-RA	gi|641587404|gb|KK854107.1|	843810-845462	11	42
ACYPI006069-RA	gi|641578198|gb|KK854624.1|	102708-110926	12	40
ACYPI006173-RA	gi|641573595|gb|KK855520.1|	30-1655	11	40
ACYPI006708-RA	gi|641570124|gb|KK856453.1|	155068-155760	8.7	40
ACYPI007779-RA	gi|641574960|gb|KK855207.1|	245839-246039	10	48
ACYPI007952-RA	gi|641588213|gb|KK854013.1|	919377-920385	9.6	39
ACYPI008044-RA	gi|641588236|gb|KK854011.1|	1178455-1186056	9.7	38
ACYPI008181-RA	gi|641586817|gb|KK854187.1|	511230-512619	9.3	39
ACYPI008721-RA	gi|641585423|gb|KK854400.1|	40952-41166	11	36
ACYPI087807-RA	gi|641578330|gb|KK854601.1|	62009-63888	11	38
ACYPI42465-RA	gi|641586489|gb|KK854234.1|	114358-114815	9.5	39
ACYPI001771-RA	gi|641577487|gb|KK854746.1|	174085-174929	11	38
ACYPI008237-RA	gi|641566938|gb|KK857481.1|	21094-21453	11	39
ACYPI34500-RA	gi|641582763|gb|KK854531.1|	171736-174688	10	39
ACYPI004333-RA	gi|641576362|gb|KK854933.1|	226001-226467	10	37

ACYPI006266-RA	gi|641587104|gb|KK854144.1|	720378-721288	11	37
ACYPI006932-RA	gi|641578325|gb|KK854602.1|	269983-277242	9.7	38
ACYPI005058-RA	gi|641587033|gb|KK854153.1|	310248-310804	10	38
ACYPI007379-RA	gi|641576672|gb|KK854880.1|	188212-189661	9.4	40
ACYPI25873-RA	gi|641588099|gb|KK854024.1|	551068-552326	11	39
ACYPI42284-RA	gi|641575693|gb|KK855062.1|	98684-102573	11	40
ACYPI002986-RA	gi|641586793|gb|KK854190.1|	528114-528792	9.3	41
ACYPI004307-RA	gi|641585616|gb|KK854368.1|	170596-171284	8.9	39
ACYPI006238-RA	gi|641568278|gb|KK857027.1|	50838-51986	9.6	38
ACYPI072244-RA	gi|641588343|gb|KK854003.1|	143875-144296	11	40
ACYPI083962-RA	gi|641574783|gb|KK855245.1|	45095-46202	10	21
ACYPI48834-RA	gi|641586874|gb|KK854178.1|	661387-662589	9.4	38
ACYPI001931-RA	gi|641574266|gb|KK855361.1|	220391-221769	11	38
ACYPI004222-RA	gi|641587569|gb|KK854087.1|	136543-136797	11	41
ACYPI005529-RA	gi|641569985|gb|KK856496.1|	102883-103638	11	39
ACYPI008529-RA	gi|641586694|gb|KK854204.1|	75761-78446	12	50
ACYPI001453-RA	gi|641575222|gb|KK855154.1|	36899-39260	11	37
ACYPI001759-RA	gi|641585340|gb|KK854414.1|	13-298	11	42
ACYPI005826-RA	gi|641566738|gb|KK857551.1|	5239-5401	11	39
ACYPI006885-RA	gi|641585961|gb|KK854312.1|	436446-436787	10	40
ACYPI009667-RA	gi|641587126|gb|KK854141.1|	596723-600496	10	39
ACYPI010135-RA	gi|641572022|gb|KK855920.1|	48528-50430	12	42
ACYPI060796-RA	gi|641585961|gb|KK854312.1|	416392-416690	10	40
ACYPI082595-RA	gi|641572405|gb|KK855822.1|	126394-126624	8.7	44
ACYPI001204-RA	gi|641586880|gb|KK854177.1|	248010-248868	10	20
ACYPI003301-RA	gi|641571642|gb|KK856015.1|	101720-102268	11	37
ACYPI003398-RA	gi|641576546|gb|KK854899.1|	67715-69421	11	40
ACYPI003508-RA	gi|641577498|gb|KK854744.1|	339973-340269	11	42
ACYPI004013-RA	gi|641588182|gb|KK854016.1|	289567-292167	11	41
ACYPI004014-RA	gi|641570404|gb|KK856365.1|	71726-73948	10	19

ACYPI005457-RA	gi|641570134|gb|KK856450.1|	101957-102630	10	38
ACYPI005953-RA	gi|641587912|gb|KK854045.1|	678926-680939	11	39
ACYPI009012-RA	gi|641572185|gb|KK855876.1|	98528-99624	8.2	36
ACYPI009722-RA	gi|641577390|gb|KK854759.1|	152256-153570	9.8	37
ACYPI081603-RA	gi|641577890|gb|KK854675.1|	88505-89307	10	40
ACYPI006010-RA	gi|641568098|gb|KK857086.1|	81822-82624	9.9	18
ACYPI006283-RA	gi|641572372|gb|KK855829.1|	122861-123252	12	45
ACYPI008151-RA	gi|641574986|gb|KK855201.1|	123973-124405	9.3	35
ACYPI008396-RA	gi|641571207|gb|KK856132.1|	124825-125119	10	40
ACYPI001655-RA	gi|641577299|gb|KK854775.1|	103023-104151	11	37
ACYPI002031-RA	gi|641584248|gb|KK854496.1|	387530-392950	9.9	39
ACYPI004098-RA	gi|641585847|gb|KK854330.1|	31182-34584	11	41
ACYPI004913-RA	gi|641572402|gb|KK855823.1|	97404-97680	11	40
ACYPI005738-RA	gi|641586372|gb|KK854251.1|	654135-654431	10	38
ACYPI006225-RA	gi|641576243|gb|KK854954.1|	296903-297529	10	41
ACYPI006423-RA	gi|641572581|gb|KK855778.1|	16867-17174	9.9	37
ACYPI006514-RA	gi|641577299|gb|KK854775.1|	284741-287511	11	37
ACYPI007915-RA	gi|641588246|gb|KK854010.1|	685529-686510	9.2	50
ACYPI008380-RA	gi|641577623|gb|KK854722.1|	112486-117087	11	38
ACYPI008390-RA	gi|641569664|gb|KK856600.1|	38142-38368	11	43
ACYPI062495-RA	gi|641571479|gb|KK856060.1|	24072-36768	11	40
ACYPI065217-RA	gi|641574676|gb|KK855265.1|	51955-55920	9.4	35
ACYPI27183-RA	gi|641588090|gb|KK854025.1|	987993-989108	11	43
ACYPI49690-RA	gi|641584474|gb|KK854479.1|	152050-152615	9.5	40
ACYPI004271-RA	gi|641580479|gb|KK854569.1|	152352-153676	10	38
ACYPI009955-RA	gi|641578300|gb|KK854606.1|	447842-448496	11	40
ACYPI000110-RA	gi|641587118|gb|KK854142.1|	326279-333158	11	40
ACYPI001438-RA	gi|641586850|gb|KK854182.1|	32283-32999	10	37
ACYPI003302-RA	gi|641574882|gb|KK855225.1|	56513-56773	9.3	36
ACYPI003418-RA	gi|641569146|gb|KK856755.1|	114080-115703	10	34

ACYPI004393-RA	gi|641587519|gb|KK854093.1|	964936-965989	11	39
ACYPI009380-RA	gi|641587606|gb|KK854082.1|	721035-723396	11	40
ACYPI001512-RA	gi|641567299|gb|KK857356.1|	65474-65940	11	38
ACYPI001987-RA	gi|641577299|gb|KK854775.1|	212071-213472	11	37
ACYPI003831-RA	gi|641587688|gb|KK854072.1|	919401-919687	10	38
ACYPI003874-RA	gi|641577299|gb|KK854775.1|	169045-169591	11	37
ACYPI005865-RA	gi|641570198|gb|KK856430.1|	40541-48319	10	36
ACYPI006428-RA	gi|641588325|gb|KK854004.1|	350970-353220	11	40
ACYPI006698-RA	gi|641585407|gb|KK854403.1|	258696-259400	9.4	42
ACYPI007137-RA	gi|641564369|gb|KK858469.1|	5674-6515	8.4	23
ACYPI007733-RA	gi|641572846|gb|KK855707.1|	280000-280467	13	52
ACYPI008202-RA	gi|641572549|gb|KK855786.1|	52333-52559	9.5	35
ACYPI008599-RA	gi|641587624|gb|KK854080.1|	253427-253931	10	38
ACYPI009531-RA	gi|641577330|gb|KK854770.1|	89090-89303	11	39
ACYPI009628-RA	gi|641575337|gb|KK855131.1|	208473-208965	10	37
ACYPI062879-RA	gi|641588082|gb|KK854026.1|	401503-402610	11	41
ACYPI070389-RA	gi|641587688|gb|KK854072.1|	915498-915781	10	38
ACYPI26472-RA	gi|641587688|gb|KK854072.1|	917497-917741	10	38
ACYPI001885-RA	gi|641570453|gb|KK856351.1|	89554-91937	9.6	36
ACYPI002847-RA	gi|641577574|gb|KK854731.1|	309636-311687	11	38
ACYPI010114-RA	gi|641570453|gb|KK856351.1|	56900-57820	9.6	36
ACYPI000066-RA	gi|641584205|gb|KK854500.1|	65445-66310	11	41
ACYPI000667-RA	gi|641587652|gb|KK854077.1|	887729-889622	10	38
ACYPI001012-RA	gi|641572860|gb|KK855703.1|	118167-121546	9.8	36
ACYPI001163-RA	gi|641586741|gb|KK854197.1|	409709-410214	11	43
ACYPI069209-RA	gi|641578060|gb|KK854647.1|	220371-223120	8.9	35
ACYPI085099-RA	gi|641584336|gb|KK854489.1|	185514-186506	10	40
ACYPI39395-RA	gi|641577546|gb|KK854735.1|	54064-54601	12	46
ACYPI000353-RA	gi|641587145|gb|KK854138.1|	229383-229990	10	42
ACYPI000828-RA	gi|641566255|gb|KK857728.1|	11965-15850	8.3	32

ACYPI002023-RA	gi|641578109|gb|KK854638.1|	129917-131678	14	46
ACYPI002715-RA	gi|641587193|gb|KK854132.1|	773824-775107	11	39
ACYPI003518-RA	gi|641579225|gb|KK854583.1|	329923-330601	10	39
ACYPI006505-RA	gi|641587606|gb|KK854082.1|	203331-204836	11	40
ACYPI007179-RA	gi|641587452|gb|KK854101.1|	219553-220622	11	40
ACYPI007717-RA	gi|641577843|gb|KK854684.1|	152325-156078	10	40
ACYPI009500-RA	gi|641576238|gb|KK854955.1|	326585-327900	11	40
ACYPI067814-RA	gi|641577784|gb|KK854695.1|	444291-445776	9.2	46
ACYPI073870-RA	gi|641545206|gb|KK867413.1|	768-4217	17	31
ACYPI38788-RA	gi|641571730|gb|KK855996.1|	36257-38079	10	20
ACYPI000165-RA	gi|641573181|gb|KK855624.1|	240745-241212	11	40
ACYPI003644-RA	gi|641571402|gb|KK856081.1|	171013-171276	11	39
ACYPI006075-RA	gi|641574549|gb|KK855294.1|	151813-152385	8.9	38
ACYPI006756-RA	gi|641577234|gb|KK854784.1|	188114-188894	11	39
ACYPI000955-RA	gi|641570514|gb|KK856331.1|	101197-107313	9.8	39
ACYPI001266-RA	gi|641588060|gb|KK854028.1|	137691-139650	11	42
ACYPI002179-RA	gi|641586824|gb|KK854186.1|	78363-81899	10	41
ACYPI002584-RA	gi|641588060|gb|KK854028.1|	119261-121195	11	42
ACYPI002799-RA	gi|641586777|gb|KK854192.1|	486323-491448	11	30
ACYPI002947-RA	gi|641586405|gb|KK854246.1|	51911-53094	10	40
ACYPI003718-RA	gi|641588182|gb|KK854016.1|	403241-405057	11	41
ACYPI003981-RA	gi|641587660|gb|KK854076.1|	561282-562910	9.9	41
ACYPI004727-RA	gi|641587652|gb|KK854077.1|	714027-719137	10	38
ACYPI005001-RA	gi|641577299|gb|KK854775.1|	385992-388855	11	37
ACYPI006185-RA	gi|641578263|gb|KK854612.1|	297150-297769	10	39
ACYPI006403-RA	gi|641567672|gb|KK857228.1|	20715-21633	10	36
ACYPI006626-RA	gi|641573007|gb|KK855666.1|	187020-189637	11	44
ACYPI006897-RA	gi|641587569|gb|KK854087.1|	911104-911514	11	41
ACYPI007006-RA	gi|641588073|gb|KK854027.1|	1234954-1237193	11	41
ACYPI007994-RA	gi|641586452|gb|KK854239.1|	123453-125552	10	19

ACYPI008056-RA	gi|641570514|gb|KK856331.1|	85898-86463	9.8	39
ACYPI008217-RA	gi|641571479|gb|KK856060.1|	83596-84806	11	40
ACYPI008281-RA	gi|641579205|gb|KK854586.1|	401592-402101	10	39
ACYPI008457-RA	gi|641576423|gb|KK854922.1|	326523-327253	18	35
ACYPI008489-RA	gi|641586380|gb|KK854250.1|	276069-276602	9.3	38
ACYPI008578-RA	gi|641575236|gb|KK855151.1|	342532-343239	11	38
ACYPI008831-RA	gi|641576377|gb|KK854930.1|	203610-205683	10	42
ACYPI009478-RA	gi|641585363|gb|KK854409.1|	365271-366237	9.3	38
ACYPI009932-RA	gi|641585812|gb|KK854335.1|	204946-206256	11	42
ACYPI073570-RA	gi|641561863|gb|KK859547.1|	10992-11890	9.2	39
ACYPI001806-RA	gi|641587276|gb|KK854121.1|	669761-671524	11	39
ACYPI001842-RA	gi|641578406|gb|KK854594.1|	223216-224600	10	37
ACYPI001927-RA	gi|641574448|gb|KK855319.1|	235813-236952	10	37
ACYPI001957-RA	gi|641587652|gb|KK854077.1|	29390-30777	10	38
ACYPI002580-RA	gi|641588224|gb|KK854012.1|	349291-350387	10	41
ACYPI003691-RA	gi|641577612|gb|KK854724.1|	352469-353133	9.7	37
ACYPI004482-RA	gi|641575516|gb|KK855095.1|	153104-154179	9.6	36
ACYPI004584-RA	gi|641575813|gb|KK855039.1|	95621-95890	11	21
ACYPI006277-RA	gi|641574944|gb|KK855211.1|	239663-241023	10	39
ACYPI006499-RA	gi|641575808|gb|KK855040.1|	258827-259237	9.9	37
ACYPI007533-RA	gi|641576266|gb|KK854949.1|	256940-258236	11	45
ACYPI008146-RA	gi|641586185|gb|KK854275.1|	300307-302227	12	40
ACYPI008922-RA	gi|641587256|gb|KK854124.1|	567313-567531	9.7	40
ACYPI010154-RA	gi|641574448|gb|KK855319.1|	229138-231113	10	37
ACYPI060891-RA	gi|641585107|gb|KK854435.1|	202256-203277	15	45
ACYPI000157-RA	gi|641565939|gb|KK857844.1|	4054-5290	8.8	16
ACYPI009032-RA	gi|641586515|gb|KK854230.1|	704643-705606	11	39
ACYPI20534-RA	gi|641566818|gb|KK857523.1|	38059-39304	10	37
ACYPI003199-RA	gi|641586476|gb|KK854236.1|	140152-140359	10	37
ACYPI003834-RA	gi|641577379|gb|KK854761.1|	170296-171486	11	41

ACYPI40522-RA	gi|641584517|gb|KK854476.1|	369613-370480	10	39
ACYPI003118-RA	gi|641587193|gb|KK854132.1|	100912-103779	11	39
ACYPI003941-RA	gi|641586496|gb|KK854233.1|	367126-370383	11	38
ACYPI000193-RA	gi|641588164|gb|KK854017.1|	753705-755931	9.2	40
ACYPI001254-RA	gi|641555492|gb|KK862524.1|	5719-8268	8.6	36
ACYPI001561-RA	gi|641576328|gb|KK854938.1|	272967-278410	11	36
ACYPI001933-RA	gi|641586144|gb|KK854283.1|	436872-437232	10	38
ACYPI003451-RA	gi|641570089|gb|KK856463.1|	53939-54254	11	36
ACYPI003820-RA	gi|641587671|gb|KK854074.1|	182475-182683	11	40
ACYPI004039-RA	gi|641586809|gb|KK854188.1|	122712-123024	10	43
ACYPI004071-RA	gi|641586515|gb|KK854230.1|	561673-564419	11	39
ACYPI005055-RA	gi|641586127|gb|KK854286.1|	482318-484226	10	43
ACYPI005079-RA	gi|641577193|gb|KK854791.1|	236944-237786	9.5	40
ACYPI005467-RA	gi|641586053|gb|KK854298.1|	179305-185094	11	41
ACYPI005989-RA	gi|641586053|gb|KK854298.1|	116168-116848	11	41
ACYPI006313-RA	gi|641574308|gb|KK855352.1|	130896-132077	10	36
ACYPI006405-RA	gi|641567739|gb|KK857205.1|	81589-82829	13	38
ACYPI006608-RA	gi|641575666|gb|KK855067.1|	229280-229584	11	39
ACYPI006957-RA	gi|641586246|gb|KK854268.1|	515533-516942	10	38
ACYPI007327-RA	gi|641576423|gb|KK854922.1|	337066-337898	18	35
ACYPI007428-RA	gi|641588090|gb|KK854025.1|	27183-28677	11	43
ACYPI007628-RA	gi|641576608|gb|KK854891.1|	364452-365585	11	37
ACYPI007886-RA	gi|641574871|gb|KK855227.1|	158621-159095	10	40
ACYPI008178-RA	gi|641586246|gb|KK854268.1|	26414-35701	10	38
ACYPI010064-RA	gi|641585865|gb|KK854328.1|	527255-527735	12	42
ACYPI068681-RA	gi|641575490|gb|KK855100.1|	91026-91556	11	41
ACYPI082770-RA	gi|641575666|gb|KK855067.1|	214500-215138	11	39
ACYPI21119-RA	gi|641572247|gb|KK855861.1|	71102-76494	10	37
ACYPI25228-RA	gi|641586726|gb|KK854199.1|	542928-544055	11	41
ACYPI34560-RA	gi|641585812|gb|KK854335.1|	302197-304838	11	42

ACYPI38061-RA	gi|641576858|gb|KK854846.1|	343216-343917	10	33
ACYPI42407-RA	gi|641571805|gb|KK855976.1|	15796-19067	11	20
ACYPI56734-RA	gi|641568059|gb|KK857098.1|	46755-47316	12	38
ACYPI000119-RA	gi|641587133|gb|KK854140.1|	612043-612276	9.9	37
ACYPI000887-RA	gi|641579941|gb|KK854575.1|	297254-298997	10	39
ACYPI002036-RA	gi|641588109|gb|KK854023.1|	46746-47033	10	40
ACYPI002136-RA	gi|641551804|gb|KK864267.1|	21-577	9.4	19
ACYPI002292-RA	gi|641572663|gb|KK855756.1|	101017-101788	8.8	38
ACYPI003921-RA	gi|641570720|gb|KK856272.1|	68944-70264	9.1	36
ACYPI004503-RA	gi|641588033|gb|KK854031.1|	1203076-1206284	11	38
ACYPI004870-RA	gi|641573394|gb|KK855572.1|	164240-165459	11	40
ACYPI005367-RA	gi|641587848|gb|KK854052.1|	682585-684933	11	39
ACYPI005597-RA	gi|641585340|gb|KK854414.1|	459480-460016	11	42
ACYPI005988-RA	gi|641569948|gb|KK856508.1|	192939-193218	10	37
ACYPI007282-RA	gi|641587052|gb|KK854150.1|	381847-382391	11	38
ACYPI007473-RA	gi|641585342|gb|KK854413.1|	240834-241731	11	39
ACYPI064387-RA	gi|641578188|gb|KK854626.1|	36026-40934	9.9	39
ACYPI082110-RA	gi|641577104|gb|KK854807.1|	8293-13506	10	36
ACYPI003298-RA	gi|641580470|gb|KK854571.1|	150677-154984	10	41
ACYPI004820-RA	gi|641585515|gb|KK854385.1|	542515-542872	11	40
ACYPI005778-RA	gi|641585871|gb|KK854327.1|	58229-58673	11	38
ACYPI007979-RA	gi|641565734|gb|KK857919.1|	4003-4528	12	45
ACYPI009542-RA	gi|641584845|gb|KK854452.1|	428394-429145	9	36
ACYPI064196-RA	gi|641576333|gb|KK854937.1|	193975-194870	9.9	35
ACYPI066987-RA	gi|641587832|gb|KK854054.1|	373247-377076	9.8	20
ACYPI070927-RA	gi|641573198|gb|KK855620.1|	154693-155385	10	40
ACYPI001575-RA	gi|641573699|gb|KK855496.1|	124324-124856	12	39
ACYPI003964-RA	gi|641577265|gb|KK854780.1|	114139-116705	10	40
ACYPI004663-RA	gi|641574923|gb|KK855216.1|	91698-93305	11	38
ACYPI073176-RA	gi|641585510|gb|KK854386.1|	194300-196529	11	20

ACYPI37433-RA	gi|641575568|gb|KK855086.1|	356079-361383	11	40
ACYPI085162-RA	gi|641586496|gb|KK854233.1|	456021-457347	11	38
ACYPI002072-RA	gi|641567321|gb|KK857348.1|	83512-86810	19	43
ACYPI004075-RA	gi|641582100|gb|KK854545.1|	91749-92304	13	41
ACYPI004270-RA	gi|641587052|gb|KK854150.1|	495914-497788	11	38
ACYPI004373-RA	gi|641574596|gb|KK855284.1|	132494-133335	10	37
ACYPI064361-RA	gi|641585309|gb|KK854419.1|	414547-415492	11	23
ACYPI000514-RA	gi|641575572|gb|KK855085.1|	127087-128569	9.4	35
ACYPI000617-RA	gi|641580479|gb|KK854569.1|	306615-309434	10	38
ACYPI004100-RA	gi|641584336|gb|KK854489.1|	37220-41609	10	40
ACYPI004627-RA	gi|641588109|gb|KK854023.1|	198061-198476	10	40
ACYPI005041-RA	gi|641587066|gb|KK854149.1|	523781-524317	11	40
ACYPI006976-RA	gi|641580479|gb|KK854569.1|	334068-334331	10	38
ACYPI007630-RA	gi|641577968|gb|KK854660.1|	458315-460243	10	42
ACYPI008516-RA	gi|641577109|gb|KK854806.1|	202541-202743	9	32
ACYPI008736-RA	gi|641588202|gb|KK854014.1|	808994-813455	10	21
ACYPI008771-RA	gi|641587946|gb|KK854041.1|	749359-754522	11	41
ACYPI008810-RA	gi|641571760|gb|KK855988.1|	94615-96317	9.9	37
ACYPI064594-RA	gi|641569964|gb|KK856503.1|	149514-151705	10	36
ACYPI067107-RA	gi|641569362|gb|KK856692.1|	82654-86582	10	39
ACYPI068762-RA	gi|641587921|gb|KK854044.1|	726112-728192	9.4	41
ACYPI52419-RA	gi|641577109|gb|KK854806.1|	206711-207086	9	32
ACYPI002371-RA	gi|641585953|gb|KK854314.1|	466140-466639	11	43
ACYPI003025-RA	gi|641578020|gb|KK854653.1|	231920-232190	10	38
ACYPI004077-RA	gi|641586632|gb|KK854213.1|	91623-92094	11	41
ACYPI008681-RA	gi|641581789|gb|KK854549.1|	5868-6514	9.9	37
ACYPI072603-RA	gi|641569664|gb|KK856600.1|	84135-85190	11	43
ACYPI085126-RA	gi|641573533|gb|KK855535.1|	39773-40424	10	41
ACYPI000074-RA	gi|641575100|gb|KK855180.1|	59940-61333	11	41
ACYPI000089-RA	gi|641588099|gb|KK854024.1|	212160-213211	11	39

ACYPI000219-RA	gi|641585569|gb|KK854376.1|	56185-56699	11	47
ACYPI000441-RA	gi|641576437|gb|KK854920.1|	326398-328514	10	39
ACYPI001031-RA	gi|641586915|gb|KK854171.1|	497438-498263	10	38
ACYPI003044-RA	gi|641581779|gb|KK854550.1|	372220-378203	11	39
ACYPI003554-RA	gi|641582091|gb|KK854547.1|	383677-384387	11	42
ACYPI004693-RA	gi|641577644|gb|KK854718.1|	365295-365573	10	40
ACYPI004827-RA	gi|641572191|gb|KK855875.1|	57701-59561	9.7	43
ACYPI006683-RA	gi|641585778|gb|KK854341.1|	404513-405114	9.7	41
ACYPI006857-RA	gi|641575396|gb|KK855118.1|	103150-103770	9	35
ACYPI008015-RA	gi|641588343|gb|KK854003.1|	33801-34479	11	40
ACYPI008468-RA	gi|641587577|gb|KK854086.1|	631076-631480	8.2	41
ACYPI009253-RA	gi|641576333|gb|KK854937.1|	62704-63778	9.9	35
ACYPI009376-RA	gi|641576064|gb|KK854990.1|	104053-105747	10	39
ACYPI066981-RA	gi|641573850|gb|KK855457.1|	94369-95163	9.3	34
ACYPI082950-RA	gi|641570757|gb|KK856260.1|	196439-199568	10	42
ACYPI086401-RA	gi|641577805|gb|KK854691.1|	273205-273998	9.4	38
ACYPI31659-RA	gi|641581779|gb|KK854550.1|	422245-422562	11	39
ACYPI003316-RA	gi|641570198|gb|KK856430.1|	84303-84665	10	36
ACYPI003670-RA	gi|641574689|gb|KK855262.1|	297397-299330	10	38
ACYPI003732-RA	gi|641588090|gb|KK854025.1|	93574-97525	11	43
ACYPI004308-RA	gi|641572239|gb|KK855863.1|	148303-148644	11	43
ACYPI004355-RA	gi|641586059|gb|KK854297.1|	127906-128888	11	22
ACYPI005606-RA	gi|641583313|gb|KK854525.1|	340100-341711	9.5	40
ACYPI007716-RA	gi|641577244|gb|KK854783.1|	156967-158908	9.7	19
ACYPI007795-RA	gi|641588325|gb|KK854004.1|	1150523-1151845	11	40
ACYPI008978-RA	gi|641587209|gb|KK854130.1|	11338-12331	11	42
ACYPI009312-RA	gi|641576666|gb|KK854881.1|	36837-39273	10	37
ACYPI085603-RA	gi|641576962|gb|KK854829.1|	263789-266810	10	39
ACYPI22584-RA	gi|641584658|gb|KK854465.1|	312800-313913	11	37
ACYPI29303-RA	gi|641569801|gb|KK856556.1|	81538-84512	9.6	36

ACYPI45266-RA	gi|641576191|gb|KK854965.1|	191042-194362	11	44
ACYPI56611-RA	gi|641583313|gb|KK854525.1|	437091-437657	9.5	40
ACYPI000941-RA	gi|641570036|gb|KK856480.1|	65780-66033	8.3	33
ACYPI009480-RA	gi|641571380|gb|KK856086.1|	79913-84859	9.5	38
ACYPI062389-RA	gi|641585660|gb|KK854361.1|	239482-242825	11	43
ACYPI083717-RA	gi|641571016|gb|KK856184.1|	144971-149536	10	40
ACYPI43876-RA	gi|641576519|gb|KK854904.1|	85415-87781	10	40
ACYPI001233-RA	gi|641585303|gb|KK854420.1|	215501-217983	10	36
ACYPI002113-RA	gi|641584205|gb|KK854500.1|	227879-229598	11	41
ACYPI003744-RA	gi|641586203|gb|KK854273.1|	244402-244585	9.5	40
ACYPI006038-RA	gi|641585515|gb|KK854385.1|	184366-184707	11	40
ACYPI006940-RA	gi|641587052|gb|KK854150.1|	48659-48972	11	38
ACYPI007498-RA	gi|641585709|gb|KK854353.1|	135731-135954	11	43
ACYPI007920-RA	gi|641579230|gb|KK854582.1|	24666-25668	12	43
ACYPI008556-RA	gi|641585515|gb|KK854385.1|	451785-453086	11	40
ACYPI008853-RA	gi|641577890|gb|KK854675.1|	54969-55589	10	40
ACYPI065855-RA	gi|641585709|gb|KK854353.1|	219731-220046	11	43
ACYPI25751-RA	gi|641577805|gb|KK854691.1|	435330-441238	9.4	38
ACYPI001326-RA	gi|641567496|gb|KK857287.1|	79327-80221	8.3	28
ACYPI002318-RA	gi|641575730|gb|KK855054.1|	251780-253909	10	38
ACYPI002522-RA	gi|641586476|gb|KK854236.1|	499691-501894	10	37
ACYPI005093-RA	gi|641587793|gb|KK854059.1|	827215-829913	11	38
ACYPI006993-RA	gi|641565451|gb|KK858025.1|	30340-32487	9.1	17
ACYPI007164-RA	gi|641586928|gb|KK854169.1|	276877-277367	11	39
ACYPI065096-RA	gi|641586476|gb|KK854236.1|	510402-512180	10	37
ACYPI070768-RA	gi|641568731|gb|KK856884.1|	17513-18613	11	22
ACYPI086900-RA	gi|641588033|gb|KK854031.1|	1149495-1159276	11	38
ACYPI001105-RA	gi|641587711|gb|KK854069.1|	914112-915524	11	42
ACYPI005018-RA	gi|641587249|gb|KK854125.1|	498925-500105	11	37
ACYPI005885-RA	gi|641576362|gb|KK854933.1|	369784-374390	10	37

ACYPI006660-RA	gi|641588099|gb|KK854024.1|	938549-938853	11	39
ACYPI007340-RA	gi|641569670|gb|KK856598.1|	79688-80179	9.8	45
ACYPI008522-RA	gi|641587555|gb|KK854089.1|	178497-182295	11	39
ACYPI009274-RA	gi|641574131|gb|KK855389.1|	26144-26411	10	38
ACYPI068713-RA	gi|641577071|gb|KK854812.1|	139182-143972	13	38
ACYPI069407-RA	gi|641585581|gb|KK854374.1|	310291-313960	10	38
ACYPI071469-RA	gi|641588325|gb|KK854004.1|	246955-249210	11	40
ACYPI088544-RA	gi|641584281|gb|KK854493.1|	102993-103701	12	41
ACYPI34471-RA	gi|641585865|gb|KK854328.1|	488993-491486	12	42
ACYPI071824-RA	gi|641574266|gb|KK855361.1|	227880-230444	11	38
ACYPI089532-RA	gi|641571290|gb|KK856111.1|	161896-162750	11	41
ACYPI002919-RA	gi|641577161|gb|KK854797.1|	27805-30295	10	38
ACYPI008852-RA	gi|641588060|gb|KK854028.1|	247278-249757	11	42
ACYPI009394-RA	gi|641587171|gb|KK854135.1|	554110-555105	10	39
ACYPI080252-RA	gi|641586928|gb|KK854169.1|	624700-626968	11	39
ACYPI000946-RA	gi|641567069|gb|KK857435.1|	25387-25599	6.8	28
ACYPI001255-RA	gi|641577667|gb|KK854713.1|	102310-102984	8.8	36
ACYPI002571-RA	gi|641578198|gb|KK854624.1|	210957-212575	12	40
ACYPI003313-RA	gi|641587468|gb|KK854099.1|	92264-92558	10	42
ACYPI007341-RA	gi|641586713|gb|KK854201.1|	642899-643534	9.1	40
ACYPI008106-RA	gi|641588258|gb|KK854009.1|	935792-938830	11	36
ACYPI009981-RA	gi|641585773|gb|KK854342.1|	590497-591201	12	45
ACYPI089575-RA	gi|641576635|gb|KK854887.1|	98071-99646	11	40
ACYPI002526-RA	gi|641571092|gb|KK856162.1|	64503-67034	11	47
ACYPI006443-RA	gi|641586535|gb|KK854227.1|	350013-351020	10	39
ACYPI007982-RA	gi|641587383|gb|KK854110.1|	371920-376527	10	37
ACYPI009849-RA	gi|641584884|gb|KK854449.1|	392296-393278	11	39
ACYPI010128-RA	gi|641577657|gb|KK854715.1|	418269-419025	11	39
ACYPI48974-RA	gi|641573011|gb|KK855665.1|	84580-85736	9.8	37
ACYPI52004-RA	gi|641577688|gb|KK854709.1|	15219-20553	11	40

ACYPI000475-RA	gi|641585303|gb|KK854420.1|	57616-57928	10	36
ACYPI003141-RA	gi|641569851|gb|KK856541.1|	19721-21216	11	21
ACYPI004374-RA	gi|641587287|gb|KK854120.1|	281853-282397	9.9	37
ACYPI005613-RA	gi|641577271|gb|KK854779.1|	200827-204746	11	21
ACYPI007313-RA	gi|641571334|gb|KK856098.1|	72493-78406	9.3	37
ACYPI009282-RA	gi|641588182|gb|KK854016.1|	589106-589375	11	41
ACYPI061196-RA	gi|641575077|gb|KK855184.1|	58255-63370	12	22
ACYPI071544-RA	gi|641576962|gb|KK854829.1|	225291-228266	10	39
ACYPI072468-RA	gi|641576557|gb|KK854897.1|	124816-128524	8.9	33
ACYPI072602-RA	gi|641588343|gb|KK854003.1|	1725486-1726163	11	40
ACYPI072792-RA	gi|641571052|gb|KK856173.1|	14404-14766	8.9	41
ACYPI26971-RA	gi|641585673|gb|KK854359.1|	572917-575887	10	41
ACYPI39797-RA	gi|641586452|gb|KK854239.1|	139836-146341	10	19
ACYPI000102-RA	gi|641576951|gb|KK854831.1|	209531-210063	8.6	40
ACYPI001323-RA	gi|641588194|gb|KK854015.1|	295639-296775	9.4	40
ACYPI003903-RA	gi|641585684|gb|KK854357.1|	122687-123217	11	39
ACYPI004515-RA	gi|641588082|gb|KK854026.1|	592713-594405	11	41
ACYPI006435-RA	gi|641583980|gb|KK854512.1|	193409-194072	11	39
ACYPI008308-RA	gi|641582439|gb|KK854536.1|	378954-382080	11	36
ACYPI068801-RA	gi|641575987|gb|KK855008.1|	113962-115318	9.9	20
ACYPI38899-RA	gi|641586613|gb|KK854216.1|	194953-197767	11	41
ACYPI004988-RA	gi|641575337|gb|KK855131.1|	167138-172340	10	37
ACYPI062333-RA	gi|641578198|gb|KK854624.1|	434992-438067	12	40
ACYPI44428-RA	gi|641585552|gb|KK854379.1|	150733-155458	14	62
ACYPI000082-RA	gi|641586741|gb|KK854197.1|	680135-682002	11	43
ACYPI000792-RA	gi|641569432|gb|KK856672.1|	56199-57094	13	47
ACYPI002637-RA	gi|641575337|gb|KK855131.1|	94757-95831	10	37
ACYPI003682-RA	gi|641585246|gb|KK854426.1|	133957-134936	10	39
ACYPI005048-RA	gi|641586639|gb|KK854212.1|	523018-523365	8.3	41
ACYPI008814-RA	gi|641575096|gb|KK855181.1|	242206-242521	11	21

ACYPI063806-RA	gi|641573914|gb|KK855440.1|	214307-215158	9.3	36
ACYPI085878-RA	gi|641571652|gb|KK856012.1|	62329-62655	9.6	35
ACYPI31558-RA	gi|641585383|gb|KK854406.1|	221588-221826	11	37
ACYPI33217-RA	gi|641586515|gb|KK854230.1|	295332-296276	11	39
ACYPI001130-RA	gi|641575227|gb|KK855153.1|	39102-39366	9	40
ACYPI003534-RA	gi|641572637|gb|KK855763.1|	77766-78017	9.5	36
ACYPI009859-RA	gi|641587793|gb|KK854059.1|	933574-934973	11	38
ACYPI066960-RA	gi|641576766|gb|KK854863.1|	301126-302710	10	38
ACYPI083025-RA	gi|641571439|gb|KK856071.1|	13502-14689	23	43
ACYPI003157-RA	gi|641587696|gb|KK854071.1|	653508-662646	8.9	39
ACYPI005479-RA	gi|641585342|gb|KK854413.1|	58972-61381	11	39
ACYPI007585-RA	gi|641585395|gb|KK854405.1|	186789-187575	10	38
ACYPI009229-RA	gi|641584977|gb|KK854443.1|	271182-272394	11	24
ACYPI010047-RA	gi|641587462|gb|KK854100.1|	248721-250311	10	21
ACYPI064108-RA	gi|641558438|gb|KK861121.1|	3482-3900	11	39
ACYPI36505-RA	gi|641572663|gb|KK855756.1|	25679-28013	8.8	38
ACYPI44265-RA	gi|641588182|gb|KK854016.1|	567125-568414	11	41
ACYPI49270-RA	gi|641561045|gb|KK859922.1|	6845-7080	11	17
ACYPI50698-RA	gi|641577754|gb|KK854700.1|	247604-247798	12	43
ACYPI000156-RA	gi|641581402|gb|KK854555.1|	46541-46740	12	20
ACYPI001415-RA	gi|641584419|gb|KK854483.1|	402477-402844	10	42
ACYPI002180-RA	gi|641586085|gb|KK854293.1|	110059-112331	11	40
ACYPI002839-RA	gi|641574123|gb|KK855391.1|	127477-128566	9.9	39
ACYPI004049-RA	gi|641584931|gb|KK854446.1|	97699-102186	10	39
ACYPI004536-RA	gi|641572207|gb|KK855871.1|	25467-26603	10	39
ACYPI005411-RA	gi|641576151|gb|KK854974.1|	138735-139998	11	44
ACYPI006432-RA	gi|641570404|gb|KK856365.1|	110417-113754	10	19
ACYPI008876-RA	gi|641586620|gb|KK854215.1|	393342-401318	9.1	40
ACYPI009664-RA	gi|641587896|gb|KK854047.1|	844546-846625	9.9	39
ACYPI066299-RA	gi|641587555|gb|KK854089.1|	913137-914172	11	39

ACYPI069512-RA	gi|641554382|gb|KK863047.1|	6034-6956	6.4	25
ACYPI085777-RA	gi|641587263|gb|KK854123.1|	191533-193847	11	42
ACYPI088724-RA	gi|641578428|gb|KK854590.1|	313581-314970	11	40
ACYPI44545-RA	gi|641575693|gb|KK855062.1|	82995-85309	11	40
ACYPI001909-RA	gi|641584931|gb|KK854446.1|	419315-423333	10	39
ACYPI003146-RA	gi|641574270|gb|KK855360.1|	42299-43401	9.9	37
ACYPI003551-RA	gi|641575808|gb|KK855040.1|	296630-296831	9.9	37
ACYPI003798-RA	gi|641587303|gb|KK854118.1|	366967-369802	11	20
ACYPI005126-RA	gi|641579925|gb|KK854579.1|	238653-239134	9.7	40
ACYPI005234-RA	gi|641586344|gb|KK854254.1|	615553-617814	10	40
ACYPI005705-RA	gi|641584876|gb|KK854450.1|	219293-219645	11	25
ACYPI005727-RA	gi|641574951|gb|KK855209.1|	102379-106981	10	38
ACYPI006179-RA	gi|641580479|gb|KK854569.1|	209154-216032	10	38
ACYPI006377-RA	gi|641588000|gb|KK854035.1|	979782-981150	11	39
ACYPI007603-RA	gi|641543453|gb|KK868263.1|	2189-4064	11	34
ACYPI008157-RA	gi|641575500|gb|KK855098.1|	31559-31871	11	39
ACYPI008262-RA	gi|641588000|gb|KK854035.1|	1026997-1027372	11	39
ACYPI009470-RA	gi|641584351|gb|KK854488.1|	372332-375000	11	41
ACYPI009497-RA	gi|641576842|gb|KK854849.1|	287587-290035	12	38
ACYPI010138-RA	gi|641584931|gb|KK854446.1|	403246-406414	10	39
ACYPI065942-RA	gi|641583931|gb|KK854514.1|	204506-205967	9	38
ACYPI068579-RA	gi|641575808|gb|KK855040.1|	298088-299425	9.9	37
ACYPI081460-RA	gi|641587905|gb|KK854046.1|	175590-177979	10	43
ACYPI56642-RA	gi|641579952|gb|KK854573.1|	384950-386320	11	21
ACYPI001286-RA	gi|641583921|gb|KK854515.1|	284803-286368	11	38
ACYPI001794-RA	gi|641587111|gb|KK854143.1|	415288-417573	11	40
ACYPI003973-RA	gi|641570754|gb|KK856261.1|	104972-106240	9.6	38
ACYPI005802-RA	gi|641578109|gb|KK854638.1|	280794-288242	14	46
ACYPI008134-RA	gi|641586850|gb|KK854182.1|	396237-397769	10	37
ACYPI009795-RA	gi|641574100|gb|KK855396.1|	167451-168438	11	38

ACYPI010011-RA	gi|641572300|gb|KK855848.1|	194267-195000	12	38
ACYPI22227-RA	gi|641588119|gb|KK854022.1|	988678-989196	10	40
ACYPI32351-RA	gi|641586496|gb|KK854233.1|	383373-384114	11	38
ACYPI41915-RA	gi|641575970|gb|KK855012.1|	35652-39016	11	38
ACYPI000336-RA	gi|641577574|gb|KK854731.1|	58409-62851	11	38
ACYPI001007-RA	gi|641569779|gb|KK856563.1|	57403-57893	11	40
ACYPI001597-RA	gi|641586133|gb|KK854285.1|	607011-607796	11	41
ACYPI001706-RA	gi|641577037|gb|KK854817.1|	162792-163124	9.9	37
ACYPI002909-RA	gi|641584128|gb|KK854505.1|	344074-345012	9.6	37
ACYPI005898-RA	gi|641575368|gb|KK855125.1|	76129-76701	10	36
ACYPI006500-RA	gi|641564420|gb|KK858447.1|	3017-5840	13	42
ACYPI006735-RA	gi|641571130|gb|KK856153.1|	164041-164830	10	36
ACYPI007353-RA	gi|641587866|gb|KK854050.1|	373024-373769	10	38
ACYPI008573-RA	gi|641574476|gb|KK855312.1|	67453-68329	10	43
ACYPI010142-RA	gi|641573429|gb|KK855562.1|	125188-125818	9.3	34
ACYPI066275-RA	gi|641570286|gb|KK856400.1|	41278-42842	11	41
ACYPI25951-RA	gi|641573649|gb|KK855507.1|	110967-116928	11	41
ACYPI29477-RA	gi|641578160|gb|KK854630.1|	191766-193492	11	43
ACYPI35143-RA	gi|641586335|gb|KK854256.1|	299496-302428	10	38
ACYPI001132-RA	gi|641573933|gb|KK855435.1|	280566-281641	10	40
ACYPI001940-RA	gi|641573833|gb|KK855461.1|	156434-156799	9.5	35
ACYPI003404-RA	gi|641587021|gb|KK854156.1|	600404-602112	11	42
ACYPI003908-RA	gi|641569574|gb|KK856629.1|	111713-112242	24	########
ACYPI004941-RA	gi|641587774|gb|KK854061.1|	925430-926042	9.1	40
ACYPI008454-RA	gi|641587881|gb|KK854049.1|	4654-5000	10	40
ACYPI009399-RA	gi|641587519|gb|KK854093.1|	513803-514311	11	39
ACYPI060626-RA	gi|641587624|gb|KK854080.1|	265938-267487	10	38
ACYPI066477-RA	gi|641573933|gb|KK855435.1|	221586-223968	10	40
ACYPI080370-RA	gi|641577121|gb|KK854804.1|	276271-276734	16	82
ACYPI55186-RA	gi|641587840|gb|KK854053.1|	214259-218103	11	43

ACYPI005515-RA	gi|641568571|gb|KK856936.1|	62006-64374	8.6	33
ACYPI080648-RA	gi|641588283|gb|KK854007.1|	1199257-1202154	11	40
ACYPI000092-RA	gi|641574746|gb|KK855253.1|	150871-151272	8.9	35
ACYPI000903-RA	gi|641586133|gb|KK854285.1|	606150-606924	11	41
ACYPI004799-RA	gi|641586185|gb|KK854275.1|	29121-29322	12	40
ACYPI005167-RA	gi|641586351|gb|KK854253.1|	79758-82802	10	37
ACYPI006699-RA	gi|641587417|gb|KK854105.1|	84826-85357	11	42
ACYPI006741-RA	gi|641587981|gb|KK854037.1|	100841-101313	10	40
ACYPI007882-RA	gi|641586741|gb|KK854197.1|	613155-614620	11	43
ACYPI008557-RA	gi|641570940|gb|KK856208.1|	73744-74876	10	35
ACYPI25229-RA	gi|641585953|gb|KK854314.1|	468376-468513	11	43
ACYPI34864-RA	gi|641587671|gb|KK854074.1|	673245-674968	11	40
ACYPI40278-RA	gi|641573699|gb|KK855496.1|	65182-66069	12	39
ACYPI50013-RA	gi|641574960|gb|KK855207.1|	13058-14056	10	48
ACYPI000103-RA	gi|641569252|gb|KK856726.1|	23033-23604	11	20
ACYPI000563-RA	gi|641572916|gb|KK855689.1|	101035-107029	9.2	33
ACYPI001767-RA	gi|641570819|gb|KK856245.1|	140618-141193	10	45
ACYPI002596-RA	gi|641576028|gb|KK854998.1|	235243-235654	11	21
ACYPI002953-RA	gi|641588051|gb|KK854029.1|	801660-805013	9.6	40
ACYPI003639-RA	gi|641577778|gb|KK854696.1|	179338-185583	9.9	42
ACYPI003907-RA	gi|641560826|gb|KK860019.1|	4079-4561	9.3	13
ACYPI003912-RA	gi|641567985|gb|KK857122.1|	27158-28826	8.9	39
ACYPI004568-RA	gi|641588073|gb|KK854027.1|	907751-908102	11	41
ACYPI005585-RA	gi|641570819|gb|KK856245.1|	50230-52543	10	45
ACYPI005806-RA	gi|641587527|gb|KK854092.1|	427324-428502	10	38
ACYPI005852-RA	gi|641542181|gb|KK868875.1|	94-437	9.8	32
ACYPI006222-RA	gi|641588060|gb|KK854028.1|	797797-800372	11	42
ACYPI006977-RA	gi|641586006|gb|KK854305.1|	303276-306924	9.7	38
ACYPI007315-RA	gi|641585871|gb|KK854327.1|	511677-511931	11	38
ACYPI007671-RA	gi|641576281|gb|KK854947.1|	53355-53494	12	51

ACYPI008845-RA	gi|641569907|gb|KK856522.1|	42238-42795	9.9	36
ACYPI009424-RA	gi|641575804|gb|KK855041.1|	221451-225733	11	40
ACYPI009706-RA	gi|641586535|gb|KK854227.1|	23484-24208	10	39
ACYPI009979-RA	gi|641585552|gb|KK854379.1|	174470-175741	14	62
ACYPI062225-RA	gi|641588164|gb|KK854017.1|	81921-82255	9.2	40
ACYPI066741-RA	gi|641587742|gb|KK854065.1|	1174254-1177518	11	41
ACYPI069332-RA	gi|641574689|gb|KK855262.1|	17635-18626	10	38
ACYPI082116-RA	gi|641574994|gb|KK855199.1|	129401-129969	13	58
ACYPI083484-RA	gi|641585552|gb|KK854379.1|	48162-51483	14	62
ACYPI20897-RA	gi|641587040|gb|KK854152.1|	228003-229150	9.1	39
ACYPI49263-RA	gi|641587133|gb|KK854140.1|	340528-342737	9.9	37
ACYPI54769-RA	gi|641572106|gb|KK855898.1|	90705-90976	7.3	40
ACYPI003164-RA	gi|641574487|gb|KK855309.1|	253117-253297	11	41
ACYPI005087-RA	gi|641572663|gb|KK855756.1|	38105-40668	8.8	38
ACYPI006204-RA	gi|641585812|gb|KK854335.1|	646350-650806	11	42
ACYPI007596-RA	gi|641572734|gb|KK855738.1|	15662-16071	11	40
ACYPI060124-RA	gi|641588343|gb|KK854003.1|	418189-418650	11	40
ACYPI32906-RA	gi|641577121|gb|KK854804.1|	426294-427466	16	82
ACYPI001054-RA	gi|641574986|gb|KK855201.1|	130802-131216	9.3	35
ACYPI001465-RA	gi|641585303|gb|KK854420.1|	321490-324590	10	36
ACYPI001769-RA	gi|641558279|gb|KK861194.1|	2820-3124	9.5	35
ACYPI001934-RA	gi|641588099|gb|KK854024.1|	709090-709814	11	39
ACYPI002078-RA	gi|641573906|gb|KK855442.1|	121065-121374	11	39
ACYPI003282-RA	gi|641569698|gb|KK856589.1|	93111-93422	11	41
ACYPI003350-RA	gi|641586271|gb|KK854265.1|	551551-555112	10	40
ACYPI003641-RA	gi|641576188|gb|KK854966.1|	158888-159647	11	39
ACYPI003821-RA	gi|641572345|gb|KK855836.1|	53421-54745	9.7	38
ACYPI004171-RA	gi|641569564|gb|KK856632.1|	93335-94551	9.8	34
ACYPI004613-RA	gi|641586983|gb|KK854162.1|	317027-319410	10	41
ACYPI004940-RA	gi|641575867|gb|KK855030.1|	192580-193004	11	39

ACYPI005202-RA	gi|641586033|gb|KK854301.1|	413764-414045	11	21
ACYPI005586-RA	gi|641576188|gb|KK854966.1|	145493-145875	11	39
ACYPI007094-RA	gi|641575140|gb|KK855171.1|	160341-162249	9.7	40
ACYPI008115-RA	gi|641575867|gb|KK855030.1|	126489-127265	11	39
ACYPI008403-RA	gi|641586754|gb|KK854195.1|	517310-517841	11	41
ACYPI008438-RA	gi|641580473|gb|KK854570.1|	97916-104069	11	44
ACYPI008607-RA	gi|641587390|gb|KK854109.1|	658678-659290	11	44
ACYPI21790-RA	gi|641569788|gb|KK856560.1|	40605-41250	9.5	37
ACYPI38780-RA	gi|641586515|gb|KK854230.1|	260860-261075	11	39
ACYPI47447-RA	gi|641586785|gb|KK854191.1|	498820-500956	11	39
ACYPI000033-RA	gi|641586509|gb|KK854231.1|	561560-564107	9.8	37
ACYPI000168-RA	gi|641588164|gb|KK854017.1|	1301922-1302359	9.2	40
ACYPI000844-RA	gi|641577846|gb|KK854683.1|	183293-183988	10	39
ACYPI002729-RA	gi|641559373|gb|KK860689.1|	1710-2273	9.5	18
ACYPI002959-RA	gi|641577946|gb|KK854664.1|	359871-362258	10	38
ACYPI003701-RA	gi|641567788|gb|KK857188.1|	31381-31775	13	43
ACYPI004357-RA	gi|641569510|gb|KK856649.1|	36737-39621	10	37
ACYPI004646-RA	gi|641574110|gb|KK855394.1|	46224-46468	10	21
ACYPI005113-RA	gi|641569673|gb|KK856597.1|	83050-84993	9.2	38
ACYPI006562-RA	gi|641587696|gb|KK854071.1|	410815-411889	8.9	39
ACYPI008261-RA	gi|641550296|gb|KK864961.1|	4429-5224	10	33
ACYPI010137-RA	gi|641574759|gb|KK855250.1|	81418-81768	9.7	37
ACYPI060911-RA	gi|641572916|gb|KK855689.1|	82502-82819	9.2	33
ACYPI069305-RA	gi|641588073|gb|KK854027.1|	1119238-1121101	11	41
ACYPI54351-RA	gi|641585801|gb|KK854337.1|	228956-234007	10	38
ACYPI000111-RA	gi|641576442|gb|KK854919.1|	10082-11256	11	36
ACYPI000519-RA	gi|641586898|gb|KK854174.1|	327042-329558	11	39
ACYPI000799-RA	gi|641584281|gb|KK854493.1|	370854-371128	12	41
ACYPI001188-RA	gi|641574135|gb|KK855388.1|	177826-179223	9.6	36
ACYPI001815-RA	gi|641586850|gb|KK854182.1|	379816-388369	10	37

ACYPI003061-RA	gi|641573417|gb|KK855565.1|	64650-66100	9.5	19
ACYPI004588-RA	gi|641587614|gb|KK854081.1|	604630-606614	11	40
ACYPI004977-RA	gi|641569948|gb|KK856508.1|	31548-32007	10	37
ACYPI005259-RA	gi|641585604|gb|KK854370.1|	528129-528603	9.8	37
ACYPI005997-RA	gi|641587912|gb|KK854045.1|	713341-713667	11	39
ACYPI007514-RA	gi|641562419|gb|KK859303.1|	25785-26250	8.5	36
ACYPI007764-RA	gi|641573919|gb|KK855439.1|	68131-68729	10	37
ACYPI009029-RA	gi|641577215|gb|KK854787.1|	24570-25282	11	40
ACYPI070374-RA	gi|641587606|gb|KK854082.1|	414098-414574	11	40
ACYPI071956-RA	gi|641580486|gb|KK854568.1|	49388-52399	11	36
ACYPI52964-RA	gi|641584474|gb|KK854479.1|	300140-301400	9.5	40
ACYPI000684-RA	gi|641585587|gb|KK854373.1|	364618-365332	9.8	41
ACYPI001312-RA	gi|641585773|gb|KK854342.1|	395091-396466	12	45
ACYPI003177-RA	gi|641578122|gb|KK854636.1|	158313-160172	12	42
ACYPI007077-RA	gi|641576118|gb|KK854980.1|	206277-208022	9.7	37
ACYPI008063-RA	gi|641583094|gb|KK854526.1|	114769-115188	11	41
ACYPI008179-RA	gi|641576118|gb|KK854980.1|	195161-196610	9.7	37
ACYPI009643-RA	gi|641587219|gb|KK854129.1|	238633-239260	9.9	41
ACYPI061797-RA	gi|641567869|gb|KK857161.1|	44511-46672	11	38
ACYPI068599-RA	gi|641572934|gb|KK855685.1|	92370-92954	10	36
ACYPI087358-RA	gi|641574466|gb|KK855315.1|	67162-67809	10	41
ACYPI22564-RA	gi|641575013|gb|KK855195.1|	63635-70791	10	39
ACYPI34358-RA	gi|641585219|gb|KK854428.1|	68035-69699	11	41
ACYPI38677-RA	gi|641578060|gb|KK854647.1|	293483-297300	8.9	35
ACYPI50290-RA	gi|641574352|gb|KK855342.1|	205356-206484	9.4	18
ACYPI000728-RA	gi|641569454|gb|KK856667.1|	48524-50261	9.8	34
ACYPI001279-RA	gi|641574923|gb|KK855216.1|	86554-86801	11	38
ACYPI003736-RA	gi|641588236|gb|KK854011.1|	1247592-1248833	9.7	38
ACYPI004008-RA	gi|641572637|gb|KK855763.1|	251203-251892	9.5	36
ACYPI005949-RA	gi|641569851|gb|KK856541.1|	65317-70785	11	21

ACYPI007598-RA	gi|641587066|gb|KK854149.1|	279457-279811	11	40
ACYPI008815-RA	gi|641573583|gb|KK855523.1|	4330-6632	10	41
ACYPI008830-RA	gi|641585510|gb|KK854386.1|	300394-301654	11	20
ACYPI009250-RA	gi|641566949|gb|KK857477.1|	37704-40088	11	39
ACYPI009444-RA	gi|641572782|gb|KK855725.1|	140030-140844	10	36
ACYPI084955-RA	gi|641587226|gb|KK854128.1|	574708-575972	9.9	39
ACYPI39080-RA	gi|641587519|gb|KK854093.1|	797017-801235	11	39
ACYPI47485-RA	gi|641572364|gb|KK855831.1|	224879-227493	11	38
ACYPI48818-RA	gi|641584305|gb|KK854491.1|	172569-174083	11	39
ACYPI53252-RA	gi|641584945|gb|KK854445.1|	426062-427089	9.9	20
ACYPI000027-RA	gi|641574955|gb|KK855208.1|	187350-187595	8.5	33
ACYPI000813-RA	gi|641578411|gb|KK854593.1|	75847-77036	11	43
ACYPI001757-RA	gi|641570930|gb|KK856211.1|	28516-28730	11	39
ACYPI003629-RA	gi|641570462|gb|KK856348.1|	46408-47002	10	37
ACYPI004267-RA	gi|641574294|gb|KK855355.1|	87139-88083	14	20
ACYPI004983-RA	gi|641572297|gb|KK855849.1|	66004-67023	11	21
ACYPI008514-RA	gi|641575808|gb|KK855040.1|	75012-76452	9.9	37
ACYPI063732-RA	gi|641587153|gb|KK854137.1|	249165-251164	10	42
ACYPI073457-RA	gi|641575288|gb|KK855139.1|	182321-183355	11	38
ACYPI080011-RA	gi|641586975|gb|KK854163.1|	320264-321183	11	40
ACYPI081425-RA	gi|641578411|gb|KK854593.1|	78304-78858	11	43
ACYPI000534-RA	gi|641586344|gb|KK854254.1|	475181-476013	10	40
ACYPI000538-RA	gi|641585847|gb|KK854330.1|	187303-189644	11	41
ACYPI001583-RA	gi|641587962|gb|KK854039.1|	938760-943835	11	40
ACYPI002286-RA	gi|641575730|gb|KK855054.1|	33871-36374	10	38
ACYPI003560-RA	gi|641574110|gb|KK855394.1|	122773-123817	10	21
ACYPI005000-RA	gi|641586515|gb|KK854230.1|	101273-101505	11	39
ACYPI006384-RA	gi|641572565|gb|KK855782.1|	15997-16999	8.7	43
ACYPI006521-RA	gi|641579219|gb|KK854584.1|	184872-188323	12	39
ACYPI006896-RA	gi|641576557|gb|KK854897.1|	178281-180603	8.9	33

ACYPI009259-RA	gi|641571737|gb|KK855994.1|	65417-65950	11	23
ACYPI010034-RA	gi|641580479|gb|KK854569.1|	139738-146127	10	38
ACYPI067449-RA	gi|641578114|gb|KK854637.1|	328960-333632	9.7	38
ACYPI072192-RA	gi|641569463|gb|KK856664.1|	55470-56190	11	46
ACYPI000183-RA	gi|641586535|gb|KK854227.1|	511330-512088	10	39
ACYPI002690-RA	gi|641578056|gb|KK854648.1|	344429-344780	11	41
ACYPI008756-RA	gi|641578084|gb|KK854643.1|	152272-152566	11	40
ACYPI085634-RA	gi|641588033|gb|KK854031.1|	362074-362779	11	38
ACYPI000387-RA	gi|641582768|gb|KK854530.1|	410190-410868	11	41
ACYPI000470-RA	gi|641573617|gb|KK855515.1|	79371-79794	10	38
ACYPI001046-RA	gi|641585720|gb|KK854351.1|	243217-243447	10	39
ACYPI001643-RA	gi|641571138|gb|KK856151.1|	182010-183579	10	45
ACYPI002284-RA	gi|641585796|gb|KK854338.1|	374616-375110	11	43
ACYPI002599-RA	gi|641577341|gb|KK854768.1|	428760-431403	14	58
ACYPI002801-RA	gi|641575730|gb|KK855054.1|	104007-104683	10	38
ACYPI003819-RA	gi|641577442|gb|KK854751.1|	289558-290436	11	22
ACYPI006013-RA	gi|641568533|gb|KK856949.1|	78400-81429	9.7	38
ACYPI006551-RA	gi|641568278|gb|KK857027.1|	56973-58793	9.6	38
ACYPI006630-RA	gi|641585709|gb|KK854353.1|	467547-469362	11	43
ACYPI006668-RA	gi|641575768|gb|KK855048.1|	156177-156512	8.1	41
ACYPI007384-RA	gi|641569577|gb|KK856628.1|	17584-18150	9.7	36
ACYPI009020-RA	gi|641580980|gb|KK854561.1|	498238-498928	11	37
ACYPI066542-RA	gi|641577147|gb|KK854800.1|	303940-304311	10	43
ACYPI068701-RA	gi|641585888|gb|KK854324.1|	522364-523256	10	41
ACYPI087204-RA	gi|641568533|gb|KK856949.1|	70802-72487	9.7	38
ACYPI24406-RA	gi|641572719|gb|KK855742.1|	130409-132128	12	23
ACYPI39685-RA	gi|641576756|gb|KK854865.1|	87016-87809	10	40
ACYPI40034-RA	gi|641561999|gb|KK859490.1|	13149-16393	8.8	34
ACYPI000164-RA	gi|641575730|gb|KK855054.1|	68082-68303	10	38
ACYPI002794-RA	gi|641577890|gb|KK854675.1|	176777-177280	10	40

ACYPI003947-RA	gi|641588343|gb|KK854003.1|	123120-123521	11	40
ACYPI004211-RA	gi|641558402|gb|KK861138.1|	2365-3607	7.6	30
ACYPI005221-RA	gi|641585440|gb|KK854397.1|	400089-406486	9	38
ACYPI005241-RA	gi|641575214|gb|KK855156.1|	191420-191817	8.8	34
ACYPI006305-RA	gi|641571790|gb|KK855979.1|	80019-80411	11	41
ACYPI006324-RA	gi|641586903|gb|KK854173.1|	289245-289406	11	45
ACYPI006593-RA	gi|641574689|gb|KK855262.1|	722-1028	10	38
ACYPI007192-RA	gi|641574230|gb|KK855368.1|	91876-92093	11	20
ACYPI007800-RA	gi|641586059|gb|KK854297.1|	383773-384352	11	22
ACYPI008195-RA	gi|641569495|gb|KK856654.1|	18878-21500	9.5	35
ACYPI008435-RA	gi|641577075|gb|KK854811.1|	143185-144604	11	36
ACYPI008980-RA	gi|641580980|gb|KK854561.1|	355900-359948	11	37
ACYPI009326-RA	gi|641586393|gb|KK854248.1|	470344-472499	9	39
ACYPI063394-RA	gi|641577890|gb|KK854675.1|	138989-139805	10	40
ACYPI066904-RA	gi|641573093|gb|KK855645.1|	195614-195923	10	44
ACYPI081140-RA	gi|641576010|gb|KK855002.1|	373608-374919	12	43
ACYPI084470-RA	gi|641577890|gb|KK854675.1|	159724-163577	10	40
ACYPI25475-RA	gi|641572895|gb|KK855694.1|	33429-33865	8.7	39
ACYPI38188-RA	gi|641586127|gb|KK854286.1|	451793-452056	10	43
ACYPI42579-RA	gi|641577987|gb|KK854657.1|	470598-472914	13	49
ACYPI001976-RA	gi|641575077|gb|KK855184.1|	6836-8863	12	22
ACYPI005188-RA	gi|641586943|gb|KK854167.1|	543069-545195	11	38
ACYPI005770-RA	gi|641587084|gb|KK854146.1|	539067-539335	9	40
ACYPI006676-RA	gi|641577928|gb|KK854668.1|	159761-160032	9.2	37
ACYPI007150-RA	gi|641584755|gb|KK854458.1|	436988-441079	9.7	41
ACYPI007180-RA	gi|641585819|gb|KK854334.1|	506825-507052	9.3	38
ACYPI007324-RA	gi|641586893|gb|KK854175.1|	354291-354660	11	41
ACYPI007730-RA	gi|641584351|gb|KK854488.1|	445574-446134	11	41
ACYPI008335-RA	gi|641587550|gb|KK854090.1|	157286-159554	9.7	45
ACYPI009034-RA	gi|641572293|gb|KK855850.1|	98912-100729	11	38

ACYPI009193-RA	gi|641588033|gb|KK854031.1|	1066939-1067391	11	38
ACYPI009555-RA	gi|641577127|gb|KK854803.1|	268196-268479	10	22
ACYPI067654-RA	gi|641576766|gb|KK854863.1|	330548-330769	10	38
ACYPI081137-RA	gi|641587742|gb|KK854065.1|	263354-264353	11	41
ACYPI000079-RA	gi|641575368|gb|KK855125.1|	91464-93177	10	36
ACYPI000109-RA	gi|641575730|gb|KK855054.1|	312883-318335	10	38
ACYPI000140-RA	gi|641572022|gb|KK855920.1|	70889-71805	12	42
ACYPI001033-RA	gi|641586547|gb|KK854225.1|	57413-57636	10	40
ACYPI006508-RA	gi|641577754|gb|KK854700.1|	285516-286175	12	43
ACYPI006616-RA	gi|641576656|gb|KK854883.1|	19849-25051	11	40
ACYPI006935-RA	gi|641586555|gb|KK854224.1|	440313-441127	11	39
ACYPI007040-RA	gi|641574352|gb|KK855342.1|	225141-225573	9.4	18
ACYPI008142-RA	gi|641568112|gb|KK857081.1|	43747-44432	11	38
ACYPI009430-RA	gi|641566437|gb|KK857661.1|	31830-32219	9.2	34
ACYPI009856-RA	gi|641566296|gb|KK857713.1|	27500-28132	9.7	34
ACYPI000218-RA	gi|641576343|gb|KK854935.1|	42989-44371	9.5	36
ACYPI000787-RA	gi|641587511|gb|KK854094.1|	242785-243982	10	41
ACYPI002674-RA	gi|641575218|gb|KK855155.1|	85984-86668	11	21
ACYPI002787-RA	gi|641573571|gb|KK855526.1|	157932-158244	9.8	36
ACYPI004152-RA	gi|641588099|gb|KK854024.1|	310272-310556	11	39
ACYPI004385-RA	gi|641566380|gb|KK857684.1|	35837-36343	10	38
ACYPI004738-RA	gi|641577453|gb|KK854750.1|	65937-66587	10	21
ACYPI004966-RA	gi|641586713|gb|KK854201.1|	602441-603153	9.1	40
ACYPI005060-RA	gi|641573737|gb|KK855486.1|	20928-21418	9.1	33
ACYPI006316-RA	gi|641587660|gb|KK854076.1|	262255-263089	9.9	41
ACYPI006664-RA	gi|641574249|gb|KK855364.1|	70843-72107	9.2	37
ACYPI007199-RA	gi|641585366|gb|KK854408.1|	198540-199844	12	41
ACYPI008467-RA	gi|641585163|gb|KK854432.1|	15000-15287	11	43
ACYPI009377-RA	gi|641567449|gb|KK857303.1|	46382-47431	8.5	39
ACYPI010096-RA	gi|641568618|gb|KK856922.1|	101334-102114	12	42

ACYPI063955-RA	gi|641575978|gb|KK855010.1|	83403-84629	10	36
ACYPI066875-RA	gi|641575218|gb|KK855155.1|	68443-68759	11	21
ACYPI25540-RA	gi|641587921|gb|KK854044.1|	123855-124138	9.4	41
ACYPI49671-RA	gi|641586831|gb|KK854185.1|	81925-82566	9.5	44
ACYPI000058-RA	gi|641578149|gb|KK854632.1|	229773-230600	11	40
ACYPI001471-RA	gi|641578160|gb|KK854630.1|	226717-229205	11	43
ACYPI001740-RA	gi|641587703|gb|KK854070.1|	782433-783299	9.8	42
ACYPI001872-RA	gi|641579947|gb|KK854574.1|	141043-145053	11	39
ACYPI002041-RA	gi|641575606|gb|KK855079.1|	166553-168521	9.7	40
ACYPI006409-RA	gi|641578350|gb|KK854597.1|	389024-391100	11	21
ACYPI007425-RA	gi|641578114|gb|KK854637.1|	82807-83179	9.7	38
ACYPI007734-RA	gi|641575847|gb|KK855033.1|	290069-291884	10	40
ACYPI086175-RA	gi|641572655|gb|KK855758.1|	86737-90932	19	94
ACYPI49381-RA	gi|641587750|gb|KK854064.1|	12546-13435	11	23
ACYPI52139-RA	gi|641585428|gb|KK854399.1|	383790-385025	9.8	41
ACYPI000013-RA	gi|641578060|gb|KK854647.1|	32081-34839	8.9	35
ACYPI000061-RA	gi|641587742|gb|KK854065.1|	528211-529888	11	41
ACYPI001762-RA	gi|641587444|gb|KK854102.1|	487794-492941	11	42
ACYPI004610-RA	gi|641586599|gb|KK854218.1|	188775-191086	11	42
ACYPI005722-RA	gi|641577022|gb|KK854820.1|	264209-265826	10	21
ACYPI005867-RA	gi|641587066|gb|KK854149.1|	574712-574970	11	40
ACYPI008285-RA	gi|641571950|gb|KK855940.1|	62503-66067	10	37
ACYPI063237-RA	gi|641588153|gb|KK854018.1|	79308-82817	9.2	41
ACYPI070099-RA	gi|641580479|gb|KK854569.1|	447374-449623	10	38
ACYPI081902-RA	gi|641576557|gb|KK854897.1|	64449-64701	8.9	33
ACYPI082616-RA	gi|641588309|gb|KK854005.1|	1579637-1580410	11	40
ACYPI088840-RA	gi|641585581|gb|KK854374.1|	606512-608942	10	38
ACYPI24114-RA	gi|641572569|gb|KK855781.1|	46014-49656	11	39
ACYPI31744-RA	gi|641586824|gb|KK854186.1|	539398-539638	10	41
ACYPI49021-RA	gi|641587287|gb|KK854120.1|	757579-760589	9.9	37

ACYPI50707-RA	gi|641575837|gb|KK855035.1|	188040-188674	11	46
ACYPI000549-RA	gi|641586096|gb|KK854291.1|	135043-135554	11	46
ACYPI002433-RA	gi|641577215|gb|KK854787.1|	126190-127217	11	40
ACYPI006909-RA	gi|641583088|gb|KK854527.1|	192271-193563	9.9	39
ACYPI007005-RA	gi|641585395|gb|KK854405.1|	429043-429785	10	38
ACYPI008769-RA	gi|641585469|gb|KK854393.1|	404812-405277	10	37
ACYPI009420-RA	gi|641567358|gb|KK857335.1|	55886-57599	10	36
ACYPI063903-RA	gi|641575140|gb|KK855171.1|	74146-75610	9.7	40
ACYPI084976-RA	gi|641587303|gb|KK854118.1|	195421-198626	11	20
ACYPI41067-RA	gi|641570488|gb|KK856340.1|	156373-158735	12	45
ACYPI000348-RA	gi|641585905|gb|KK854321.1|	153604-153957	11	39
ACYPI000979-RA	gi|641586066|gb|KK854296.1|	239720-241473	9.4	37
ACYPI001613-RA	gi|641578106|gb|KK854639.1|	236679-244025	11	39
ACYPI001671-RA	gi|641576876|gb|KK854844.1|	388446-391081	17	42
ACYPI003557-RA	gi|641588164|gb|KK854017.1|	267209-267527	9.2	40
ACYPI004127-RA	gi|641579933|gb|KK854577.1|	68139-68369	11	37
ACYPI005103-RA	gi|641577890|gb|KK854675.1|	30485-30932	10	40
ACYPI006093-RA	gi|641586301|gb|KK854260.1|	502639-503489	9.8	39
ACYPI006903-RA	gi|641577104|gb|KK854807.1|	29362-32006	10	36
ACYPI007027-RA	gi|641569133|gb|KK856759.1|	86737-93265	11	41
ACYPI007908-RA	gi|641554069|gb|KK863196.1|	3057-3538	8.5	27
ACYPI007943-RA	gi|641586387|gb|KK854249.1|	101476-102999	11	24
ACYPI008464-RA	gi|641561537|gb|KK859697.1|	13933-15228	9.7	16
ACYPI008869-RA	gi|641575813|gb|KK855039.1|	10485-11963	11	21
ACYPI008895-RA	gi|641586285|gb|KK854263.1|	33879-38036	10	38
ACYPI009816-RA	gi|641581769|gb|KK854552.1|	215417-216492	9.3	38
ACYPI064056-RA	gi|641570423|gb|KK856359.1|	18078-21025	11	43
ACYPI068995-RA	gi|641576059|gb|KK854991.1|	196708-198117	9.3	37
ACYPI069386-RA	gi|641586509|gb|KK854231.1|	499400-504137	9.8	37
ACYPI080583-RA	gi|641575096|gb|KK855181.1|	80790-82269	11	21

ACYPI26209-RA	gi|641587921|gb|KK854044.1|	170211-173293	9.4	41
ACYPI45053-RA	gi|641587888|gb|KK854048.1|	238725-240752	10	43
ACYPI46334-RA	gi|641575910|gb|KK855021.1|	404667-406036	10	35
ACYPI000316-RA	gi|641573536|gb|KK855534.1|	70340-70712	10	38
ACYPI000987-RA	gi|641577837|gb|KK854685.1|	297621-298006	11	42
ACYPI001871-RA	gi|641575372|gb|KK855124.1|	141122-141949	9.5	36
ACYPI002525-RA	gi|641588267|gb|KK854008.1|	270855-271704	10	40
ACYPI004414-RA	gi|641572637|gb|KK855763.1|	189152-189601	9.5	36
ACYPI004876-RA	gi|641585356|gb|KK854410.1|	248494-251584	11	41
ACYPI006018-RA	gi|641557619|gb|KK861506.1|	2460-3138	7.4	27
ACYPI006343-RA	gi|641587424|gb|KK854104.1|	280413-286392	11	41
ACYPI006464-RA	gi|641576983|gb|KK854825.1|	191478-196604	9.7	37
ACYPI006586-RA	gi|641587424|gb|KK854104.1|	331041-331648	11	41
ACYPI006943-RA	gi|641585994|gb|KK854307.1|	451326-451636	11	41
ACYPI008220-RA	gi|641574951|gb|KK855209.1|	68539-70022	10	38
ACYPI008722-RA	gi|641545587|gb|KK867229.1|	775-1201	11	22
ACYPI010094-RA	gi|641569766|gb|KK856567.1|	59736-62290	8.4	38
ACYPI068284-RA	gi|641586372|gb|KK854251.1|	454736-461783	10	38
ACYPI083883-RA	gi|641554468|gb|KK863006.1|	5329-7937	11	36
ACYPI43494-RA	gi|641577810|gb|KK854690.1|	487185-487678	9.8	39
ACYPI45687-RA	gi|641575436|gb|KK855110.1|	240450-243008	12	37
ACYPI000579-RA	gi|641578330|gb|KK854601.1|	65900-67199	11	38
ACYPI001849-RA	gi|641575482|gb|KK855102.1|	226615-227569	11	39
ACYPI002485-RA	gi|641570587|gb|KK856311.1|	45567-46026	9.9	38
ACYPI003493-RA	gi|641583587|gb|KK854518.1|	122334-122954	10	37
ACYPI003697-RA	gi|641576791|gb|KK854858.1|	105870-107321	9.2	42
ACYPI005475-RA	gi|641584474|gb|KK854479.1|	243018-244158	9.5	40
ACYPI006679-RA	gi|641587021|gb|KK854156.1|	586047-587771	11	42
ACYPI008186-RA	gi|641586285|gb|KK854263.1|	24460-27072	10	38
ACYPI008671-RA	gi|641576689|gb|KK854876.1|	354903-355844	10	40

ACYPI008804-RA	gi|641586285|gb|KK854263.1|	126775-128074	10	38
ACYPI008825-RA	gi|641572471|gb|KK855805.1|	43470-45112	13	54
ACYPI009808-RA	gi|641576165|gb|KK854971.1|	35020-35287	11	41
ACYPI010072-RA	gi|641565745|gb|KK857915.1|	48532-48869	11	40
ACYPI061271-RA	gi|641562337|gb|KK859338.1|	12636-14584	5.7	22
ACYPI068718-RA	gi|641574708|gb|KK855259.1|	241993-242958	11	41
ACYPI081417-RA	gi|641572782|gb|KK855725.1|	183587-183833	10	36
ACYPI081909-RA	gi|641585967|gb|KK854311.1|	398923-399263	11	40
ACYPI000862-RA	gi|641586496|gb|KK854233.1|	597034-597888	11	38
ACYPI001069-RA	gi|641586777|gb|KK854192.1|	261403-264682	11	30
ACYPI001272-RA	gi|641571652|gb|KK856012.1|	99182-101204	9.6	35
ACYPI001386-RA	gi|641584884|gb|KK854449.1|	400044-401496	11	39
ACYPI002620-RA	gi|641585690|gb|KK854356.1|	352539-352939	9.6	41
ACYPI002748-RA	gi|641572912|gb|KK855690.1|	37389-37955	9.9	38
ACYPI004168-RA	gi|641588294|gb|KK854006.1|	677955-679275	8.7	40
ACYPI004488-RA	gi|641577244|gb|KK854783.1|	259389-260013	9.7	19
ACYPI004665-RA	gi|641586150|gb|KK854282.1|	191052-191233	11	40
ACYPI004801-RA	gi|641576290|gb|KK854945.1|	76437-77000	9.3	20
ACYPI006097-RA	gi|641585703|gb|KK854354.1|	588957-589615	10	42
ACYPI006288-RA	gi|641585761|gb|KK854344.1|	549648-550233	10	39
ACYPI006701-RA	gi|641586886|gb|KK854176.1|	91548-91917	10	41
ACYPI007992-RA	gi|641577413|gb|KK854755.1|	23466-24812	11	41
ACYPI008963-RA	gi|641577599|gb|KK854726.1|	223084-225329	9.2	40
ACYPI009215-RA	gi|641586000|gb|KK854306.1|	161038-165093	11	22
ACYPI009485-RA	gi|641571652|gb|KK856012.1|	156834-159056	9.6	35
ACYPI065069-RA	gi|641572142|gb|KK855887.1|	48788-49073	10	36
ACYPI067939-RA	gi|641576243|gb|KK854954.1|	149043-149559	10	41
ACYPI070165-RA	gi|641578435|gb|KK854589.1|	239374-241570	9.7	43
ACYPI081937-RA	gi|641585552|gb|KK854379.1|	159412-162015	14	62
ACYPI089040-RA	gi|641566512|gb|KK857635.1|	34702-38719	11	43

ACYPI34041-RA	gi|641570261|gb|KK856409.1|	65009-65282	11	21
ACYPI56663-RA	gi|641563636|gb|KK858785.1|	9951-10651	8.3	33
ACYPI000032-RA	gi|641586734|gb|KK854198.1|	163022-165105	11	40
ACYPI000077-RA	gi|641587735|gb|KK854066.1|	66823-68779	11	42
ACYPI004286-RA	gi|641586983|gb|KK854162.1|	570331-570523	10	41
ACYPI004467-RA	gi|641573053|gb|KK855655.1|	155779-156948	11	41
ACYPI006216-RA	gi|641586983|gb|KK854162.1|	357428-357963	10	41
ACYPI006861-RA	gi|641584567|gb|KK854472.1|	90352-93066	9.5	39
ACYPI007141-RA	gi|641585900|gb|KK854322.1|	291495-292051	12	43
ACYPI008728-RA	gi|641586983|gb|KK854162.1|	578638-582747	10	41
ACYPI009054-RA	gi|641587390|gb|KK854109.1|	568674-572991	11	44
ACYPI069747-RA	gi|641577341|gb|KK854768.1|	88329-90589	14	58
ACYPI28536-RA	gi|641575598|gb|KK855081.1|	28319-29096	10	39
ACYPI34504-RA	gi|641588000|gb|KK854035.1|	422650-424602	11	39
ACYPI51480-RA	gi|641586915|gb|KK854171.1|	279189-281046	10	38
ACYPI51775-RA	gi|641574971|gb|KK855204.1|	62439-65569	11	21
ACYPI000509-RA	gi|641573733|gb|KK855487.1|	64537-64882	13	45
ACYPI000629-RA	gi|641588136|gb|KK854020.1|	682816-684722	11	36
ACYPI001107-RA	gi|641585957|gb|KK854313.1|	365494-371807	10	37
ACYPI001168-RA	gi|641586535|gb|KK854227.1|	356025-357839	10	39
ACYPI002448-RA	gi|641578336|gb|KK854600.1|	139042-139994	10	21
ACYPI002830-RA	gi|641586654|gb|KK854210.1|	143155-144913	11	40
ACYPI002930-RA	gi|641586654|gb|KK854210.1|	150611-150947	11	40
ACYPI003320-RA	gi|641586040|gb|KK854300.1|	568308-572156	10	38
ACYPI003896-RA	gi|641586880|gb|KK854177.1|	594036-594383	10	20
ACYPI004440-RA	gi|641586509|gb|KK854231.1|	456230-456786	9.8	37
ACYPI005570-RA	gi|641585469|gb|KK854393.1|	250779-251231	10	37
ACYPI006335-RA	gi|641572514|gb|KK855795.1|	111173-111432	9.5	39
ACYPI006688-RA	gi|641587688|gb|KK854072.1|	450111-456133	10	38
ACYPI006818-RA	gi|641575910|gb|KK855021.1|	216435-217820	10	35

ACYPI006989-RA	gi|641587562|gb|KK854088.1|	365001-368896	11	42
ACYPI007382-RA	gi|641587179|gb|KK854134.1|	275370-277519	11	40
ACYPI008732-RA	gi|641586191|gb|KK854274.1|	635196-635676	11	42
ACYPI064212-RA	gi|641586452|gb|KK854239.1|	188386-189992	10	19
ACYPI073113-RA	gi|641581389|gb|KK854557.1|	36903-37364	11	41
ACYPI48553-RA	gi|641571472|gb|KK856062.1|	98063-99218	9.9	21
ACYPI55202-RA	gi|641575284|gb|KK855140.1|	172693-172844	8.2	35
ACYPI000070-RA	gi|641577805|gb|KK854691.1|	488167-489736	9.4	38
ACYPI001244-RA	gi|641587962|gb|KK854039.1|	899263-900278	11	40
ACYPI001765-RA	gi|641575389|gb|KK855120.1|	227264-227650	10	35
ACYPI006369-RA	gi|641574324|gb|KK855348.1|	261073-264716	12	40
ACYPI007396-RA	gi|641581789|gb|KK854549.1|	19951-20255	9.9	37
ACYPI007455-RA	gi|641585703|gb|KK854354.1|	517228-518991	10	42
ACYPI008812-RA	gi|641572740|gb|KK855736.1|	70304-72507	11	40
ACYPI009293-RA	gi|641556975|gb|KK861815.1|	2028-2278	7.5	27
ACYPI009455-RA	gi|641575898|gb|KK855024.1|	175947-176529	10	38
ACYPI065062-RA	gi|641577121|gb|KK854804.1|	120175-120860	16	82
ACYPI080880-RA	gi|641576951|gb|KK854831.1|	97815-98587	8.6	40
ACYPI084961-RA	gi|641573722|gb|KK855490.1|	30543-32665	9.7	19
ACYPI088815-RA	gi|641579242|gb|KK854580.1|	62481-63595	11	43
ACYPI45201-RA	gi|641574123|gb|KK855391.1|	186619-187609	9.9	39
ACYPI45498-RA	gi|641570717|gb|KK856273.1|	116550-117844	10	36
ACYPI000162-RA	gi|641586006|gb|KK854305.1|	114816-117276	9.7	38
ACYPI000530-RA	gi|641570396|gb|KK856368.1|	63749-64102	9.5	38
ACYPI003545-RA	gi|641577289|gb|KK854776.1|	192523-193107	10	36
ACYPI003674-RA	gi|641576203|gb|KK854962.1|	131539-131742	9.8	36
ACYPI003966-RA	gi|641573861|gb|KK855454.1|	55644-60665	10	37
ACYPI072921-RA	gi|641588041|gb|KK854030.1|	98871-99350	11	38
ACYPI086231-RA	gi|641576608|gb|KK854891.1|	255090-255760	11	37
ACYPI087735-RA	gi|641577322|gb|KK854771.1|	196261-196896	10	21

ACYPI30823-RA	gi|641588213|gb|KK854013.1|	1239489-1244848	9.6	39
ACYPI34996-RA	gi|641578325|gb|KK854602.1|	301301-303049	9.7	38
ACYPI40392-RA	gi|641585729|gb|KK854349.1|	277297-281215	10	37
ACYPI48898-RA	gi|641585177|gb|KK854431.1|	38380-41910	11	41
ACYPI52973-RA	gi|641577968|gb|KK854660.1|	456150-457691	10	42
ACYPI54877-RA	gi|641570141|gb|KK856447.1|	105819-106195	11	40
ACYPI000734-RA	gi|641576973|gb|KK854827.1|	174448-174739	11	38
ACYPI000901-RA	gi|641571396|gb|KK856083.1|	91272-91899	8.4	35
ACYPI001374-RA	gi|641587111|gb|KK854143.1|	326842-327312	11	40
ACYPI001547-RA	gi|641571315|gb|KK856104.1|	475-639	12	46
ACYPI001612-RA	gi|641573850|gb|KK855457.1|	65140-65548	9.3	34
ACYPI001780-RA	gi|641587912|gb|KK854045.1|	372319-378128	11	39
ACYPI002098-RA	gi|641587783|gb|KK854060.1|	755591-757203	12	40
ACYPI002622-RA	gi|641573906|gb|KK855442.1|	34478-35829	11	39
ACYPI002989-RA	gi|641587249|gb|KK854125.1|	527730-530900	11	37
ACYPI004089-RA	gi|641585860|gb|KK854329.1|	114568-116246	11	43
ACYPI004312-RA	gi|641569596|gb|KK856622.1|	17152-17368	12	43
ACYPI004530-RA	gi|641587066|gb|KK854149.1|	657495-657970	11	40
ACYPI004573-RA	gi|641584336|gb|KK854489.1|	106778-107875	10	40
ACYPI004698-RA	gi|641586626|gb|KK854214.1|	363126-363411	11	44
ACYPI005067-RA	gi|641576333|gb|KK854937.1|	39444-40389	9.9	35
ACYPI005769-RA	gi|641587383|gb|KK854110.1|	124758-125027	10	37
ACYPI006028-RA	gi|641575635|gb|KK855074.1|	268682-271378	9.6	38
ACYPI006243-RA	gi|641579941|gb|KK854575.1|	377799-380928	10	39
ACYPI006485-RA	gi|641573504|gb|KK855543.1|	156999-157226	13	39
ACYPI006604-RA	gi|641588283|gb|KK854007.1|	314748-323065	11	40
ACYPI007079-RA	gi|641559375|gb|KK860688.1|	8068-8479	8.3	26
ACYPI007246-RA	gi|641571956|gb|KK855938.1|	54013-54728	11	42
ACYPI007300-RA	gi|641588099|gb|KK854024.1|	363158-363431	11	39
ACYPI007577-RA	gi|641587962|gb|KK854039.1|	1167352-1167890	11	40

ACYPI007905-RA	gi|641574809|gb|KK855240.1|	189933-190937	11	39
ACYPI008117-RA	gi|641587074|gb|KK854148.1|	599502-601114	11	41
ACYPI008967-RA	gi|641576973|gb|KK854827.1|	177799-178107	11	38
ACYPI009473-RA	gi|641585350|gb|KK854411.1|	352887-353963	11	38
ACYPI009565-RA	gi|641575970|gb|KK855012.1|	252522-253176	11	38
ACYPI009997-RA	gi|641587680|gb|KK854073.1|	579487-579897	10	40
ACYPI072101-RA	gi|641586785|gb|KK854191.1|	377331-377587	11	39
ACYPI080074-RA	gi|641577121|gb|KK854804.1|	285142-289902	16	82
ACYPI088363-RA	gi|641586967|gb|KK854164.1|	585070-586146	10	27
ACYPI25503-RA	gi|641587074|gb|KK854148.1|	550309-552944	11	41
ACYPI25978-RA	gi|641587263|gb|KK854123.1|	205895-210391	11	42
ACYPI28455-RA	gi|641574504|gb|KK855305.1|	74865-79904	9.9	39
ACYPI38686-RA	gi|641574280|gb|KK855358.1|	240947-242014	11	22
ACYPI47548-RA	gi|641588073|gb|KK854027.1|	1130466-1132144	11	41
ACYPI000001-RA	gi|641587660|gb|KK854076.1|	59667-60336	9.9	41
ACYPI000250-RA	gi|641574896|gb|KK855222.1|	292683-295549	11	43
ACYPI000666-RA	gi|641561423|gb|KK859751.1|	5747-6205	8.8	17
ACYPI002524-RA	gi|641570745|gb|KK856264.1|	12187-12463	9.3	18
ACYPI003541-RA	gi|641584305|gb|KK854491.1|	551762-553148	11	39
ACYPI003975-RA	gi|641587971|gb|KK854038.1|	106101-107003	11	39
ACYPI004178-RA	gi|641588343|gb|KK854003.1|	1792205-1793363	11	40
ACYPI005438-RA	gi|641567591|gb|KK857253.1|	7781-8069	10	19
ACYPI005809-RA	gi|641577265|gb|KK854780.1|	306766-308794	10	40
ACYPI006388-RA	gi|641584460|gb|KK854480.1|	14882-16045	11	40
ACYPI006417-RA	gi|641577075|gb|KK854811.1|	390240-394262	11	36
ACYPI006956-RA	gi|641587896|gb|KK854047.1|	55643-57423	9.9	39
ACYPI007075-RA	gi|641577704|gb|KK854707.1|	178464-179560	10	38
ACYPI007268-RA	gi|641587162|gb|KK854136.1|	505438-506974	10	40
ACYPI007996-RA	gi|641586817|gb|KK854187.1|	762328-763361	9.3	39
ACYPI008947-RA	gi|641587896|gb|KK854047.1|	951637-952421	9.9	39

ACYPI009777-RA	gi|641586955|gb|KK854165.1|	789636-790218	11	40
ACYPI084221-RA	gi|641556501|gb|KK862041.1|	2709-3188	8.1	31
ACYPI26967-RA	gi|641587390|gb|KK854109.1|	772637-774392	11	44
ACYPI56670-RA	gi|641575419|gb|KK855114.1|	10678-12938	8.8	35
