# Supplementary material for: The X Chromosome of Hemipteran Insects: Conservation, Dosage Compensation and Sex-Biased Expression
Source: Genome Biol Evol. 2015 Nov 10;7(12):3259–68. doi: 10.1093/gbe/evv215 (PMC4700948; doi:10.1093/gbe/evv215)
Supplement: Supplementary Data [file supp_evv215_suppl_data.zip › S2 Data (rev) AP-OF (X).pdf]

AP	OF	gene	covF	covM
ACYPI003600-RA	gi|641573414|gb|KK855566.1|	102646-103051	9.1	36
ACYPI003104-RA	gi|641587082|gb|KK854147.1|	482312-482712	11	44
ACYPI003888-RA	gi|641575970|gb|KK855012.1|	152130-153208	11	38
ACYPI007680-RA	gi|641573533|gb|KK855535.1|	74742-84171	10	41
ACYPI006784-RA	gi|641577595|gb|KK854727.1|	58572-59762	10	20
ACYPI003611-RA	gi|641585761|gb|KK854344.1|	524816-525129	10	39
ACYPI005743-RA	gi|641587383|gb|KK854110.1|	505574-510334	10	37
ACYPI007405-RA	gi|641577837|gb|KK854685.1|	187613-192307	11	42
ACYPI001091-RA	gi|641586936|gb|KK854168.1|	258813-259248	12	42
ACYPI002987-RA	gi|641586139|gb|KK854284.1|	193375-194388	10	21
ACYPI004908-RA	gi|641584419|gb|KK854483.1|	336349-336580	10	42
ACYPI006808-RA	gi|641584728|gb|KK854460.1|	218813-219634	9.5	37
ACYPI006239-RA	gi|641566611|gb|KK857597.1|	37912-38716	11	40
ACYPI007677-RA	gi|641573906|gb|KK855442.1|	33594-33905	11	39
ACYPI001170-RA	gi|641577749|gb|KK854701.1|	161432-161971	11	20
ACYPI003057-RA	gi|641577493|gb|KK854745.1|	184498-185250	9.6	41
ACYPI003598-RA	gi|641587562|gb|KK854088.1|	497550-505220	11	42
ACYPI061245-RA	gi|641578122|gb|KK854636.1|	68434-69932	12	42
ACYPI001110-RA	gi|641572364|gb|KK855831.1|	22282-22895	11	38
ACYPI003425-RA	gi|641588073|gb|KK854027.1|	294772-298222	11	41
ACYPI087327-RA	gi|641585293|gb|KK854422.1|	24669-25392	9.1	38
ACYPI007100-RA	gi|641577043|gb|KK854816.1|	297515-298663	11	43
ACYPI008357-RA	gi|641571465|gb|KK856064.1|	95177-95459	9.2	35
ACYPI008823-RA	gi|641565511|gb|KK858002.1|	16306-16587	6.4	31
ACYPI073873-RA	gi|641577453|gb|KK854750.1|	294482-295424	10	21
ACYPI005183-RA	gi|641573841|gb|KK855459.1|	124990-125492	11	40
ACYPI001611-RA	gi|641586445|gb|KK854240.1|	551295-552056	9.2	39
ACYPI006233-RA	gi|641572185|gb|KK855876.1|	95882-96377	8.2	36
ACYPI006778-RA	gi|641587383|gb|KK854110.1|	303906-304117	10	37

ACYPI007099-RA	gi|641573210|gb|KK855617.1|	19469-19706	9.2	37
ACYPI41578-RA	gi|641572760|gb|KK855731.1|	25472-25794	9.3	33
ACYPI001023-RA	gi|641586606|gb|KK854217.1|	161019-161487	11	42
ACYPI001182-RA	gi|641583980|gb|KK854512.1|	409592-409864	11	39
ACYPI002925-RA	gi|641586688|gb|KK854205.1|	241435-243061	11	42
ACYPI004848-RA	gi|641587383|gb|KK854110.1|	483715-486430	10	37
ACYPI004986-RA	gi|641587511|gb|KK854094.1|	463978-466404	10	41
ACYPI005626-RA	gi|641587462|gb|KK854100.1|	280175-280563	10	21
ACYPI005729-RA	gi|641573571|gb|KK855526.1|	73764-74598	9.8	36
ACYPI006964-RA	gi|641577623|gb|KK854722.1|	166372-166615	11	38
ACYPI009396-RA	gi|641576666|gb|KK854881.1|	100601-101106	10	37
ACYPI003290-RA	gi|641587856|gb|KK854051.1|	886363-886652	10	41
ACYPI009861-RA	gi|641587671|gb|KK854074.1|	696788-697175	11	40
ACYPI006129-RA	gi|641575946|gb|KK855016.1|	126107-127271	9.5	37
ACYPI006138-RA	gi|641585957|gb|KK854313.1|	295909-296414	10	37
ACYPI009257-RA	gi|641587937|gb|KK854042.1|	6600-6973	11	42
ACYPI010103-RA	gi|641588033|gb|KK854031.1|	232678-233225	11	38
ACYPI072994-RA	gi|641567672|gb|KK857228.1|	25216-26937	10	36
ACYPI007681-RA	gi|641566998|gb|KK857460.1|	44616-45103	11	41
ACYPI009068-RA	gi|641573837|gb|KK855460.1|	80488-81846	13	24
ACYPI009619-RA	gi|641571176|gb|KK856140.1|	69419-71545	9.9	38
ACYPI073714-RA	gi|641586085|gb|KK854293.1|	7079-8599	11	40
ACYPI000227-RA	gi|641587577|gb|KK854086.1|	48732-49579	8.2	41
ACYPI001125-RA	gi|641574416|gb|KK855326.1|	212242-213675	11	38
ACYPI001177-RA	gi|641572360|gb|KK855832.1|	53637-55443	11	22
ACYPI001932-RA	gi|641587577|gb|KK854086.1|	177759-179416	8.2	41
ACYPI005359-RA	gi|641577704|gb|KK854707.1|	413030-419324	10	38
ACYPI006095-RA	gi|641577905|gb|KK854672.1|	169886-171616	10	37
ACYPI006584-RA	gi|641575794|gb|KK855043.1|	172043-177386	10	36
ACYPI073834-RA	gi|641584351|gb|KK854488.1|	399312-399610	11	41

ACYPI003257-RA	gi|641586535|gb|KK854227.1|	775053-775943	10	39
ACYPI004037-RA	gi|641587930|gb|KK854043.1|	961692-962191	10	41
ACYPI006164-RA	gi|641587703|gb|KK854070.1|	210947-215659	9.8	42
ACYPI073036-RA	gi|641587703|gb|KK854070.1|	99826-103281	9.8	42
ACYPI50514-RA	gi|641585233|gb|KK854427.1|	423402-423650	10	38
ACYPI001364-RA	gi|641576679|gb|KK854878.1|	306878-309455	9.9	38
ACYPI003251-RA	gi|641576397|gb|KK854927.1|	64416-65023	9.5	41
ACYPI004501-RA	gi|641577075|gb|KK854811.1|	400970-406165	11	36
ACYPI56763-RA	gi|641571045|gb|KK856175.1|	50074-52572	9.3	35
ACYPI001585-RA	gi|641574100|gb|KK855396.1|	75220-76082	11	38
ACYPI063239-RA	gi|641581402|gb|KK854555.1|	296881-298175	12	20
ACYPI067185-RA	gi|641587688|gb|KK854072.1|	170742-172728	10	38
ACYPI001752-RA	gi|641575901|gb|KK855023.1|	245775-246568	11	38
ACYPI002361-RA	gi|641586661|gb|KK854209.1|	542012-542355	11	43
ACYPI002482-RA	gi|641568172|gb|KK857061.1|	74471-75829	11	40
ACYPI003002-RA	gi|641564796|gb|KK858283.1|	4757-4996	10	39
ACYPI005545-RA	gi|641588246|gb|KK854010.1|	1157286-1158645	9.2	50
ACYPI007409-RA	gi|641582763|gb|KK854531.1|	280873-285173	10	39
ACYPI008092-RA	gi|641585865|gb|KK854328.1|	549972-550444	12	42
ACYPI008652-RA	gi|641574668|gb|KK855267.1|	155533-156563	10	38
ACYPI009324-RA	gi|641586817|gb|KK854187.1|	751761-753118	9.3	39
ACYPI009337-RA	gi|641577277|gb|KK854778.1|	255599-259411	11	42
ACYPI085768-RA	gi|641587126|gb|KK854141.1|	334022-336370	10	39
ACYPI087848-RA	gi|641546507|gb|KK866793.1|	6051-7153	10	40
ACYPI004718-RA	gi|641585767|gb|KK854343.1|	457538-458274	11	22
ACYPI007048-RA	gi|641585383|gb|KK854406.1|	392868-398214	11	37
ACYPI008482-RA	gi|641577922|gb|KK854669.1|	35683-36106	12	21
ACYPI000014-RA	gi|641578060|gb|KK854647.1|	64753-67419	8.9	35
ACYPI000654-RA	gi|641587462|gb|KK854100.1|	82039-83183	10	21
ACYPI001424-RA	gi|641568831|gb|KK856853.1|	42898-43841	10	30

ACYPI008222-RA	gi|641588283|gb|KK854007.1|	658475-660840	11	40
ACYPI46077-RA	gi|641576064|gb|KK854990.1|	93209-93418	10	39
ACYPI005622-RA	gi|641569260|gb|KK856723.1|	24941-25724	9.7	34
ACYPI005929-RA	gi|641584474|gb|KK854479.1|	249740-250668	9.5	40
ACYPI060353-RA	gi|641573587|gb|KK855522.1|	77385-77877	9.3	38
ACYPI061000-RA	gi|641584145|gb|KK854504.1|	84787-88740	8.9	38
ACYPI069554-RA	gi|641574402|gb|KK855330.1|	85533-85822	11	23
ACYPI085203-RA	gi|641587832|gb|KK854054.1|	664221-664721	9.8	20
ACYPI000681-RA	gi|641586496|gb|KK854233.1|	623852-626325	11	38
ACYPI003186-RA	gi|641570355|gb|KK856378.1|	6188-6426	9.7	35
ACYPI003944-RA	gi|641567171|gb|KK857401.1|	84734-87637	10	36
ACYPI005106-RA	gi|641570861|gb|KK856232.1|	107684-107978	11	39
ACYPI005308-RA	gi|641585801|gb|KK854337.1|	528369-530177	10	38
ACYPI000065-RA	gi|641576347|gb|KK854934.1|	20200-21624	11	39
ACYPI000222-RA	gi|641576260|gb|KK854950.1|	45448-45882	10	39
ACYPI000885-RA	gi|641586452|gb|KK854239.1|	192245-192518	10	19
ACYPI002123-RA	gi|641569294|gb|KK856712.1|	32021-32253	12	22
ACYPI003998-RA	gi|641587606|gb|KK854082.1|	810499-810754	11	40
ACYPI004750-RA	gi|641586626|gb|KK854214.1|	611128-614800	11	44
ACYPI004981-RA	gi|641586191|gb|KK854274.1|	40572-41160	11	42
ACYPI005363-RA	gi|641585725|gb|KK854350.1|	579913-581463	12	41
ACYPI006178-RA	gi|641571605|gb|KK856024.1|	7499-7959	10	38
ACYPI006875-RA	gi|641576887|gb|KK854842.1|	255358-255716	15	41
ACYPI007058-RA	gi|641576677|gb|KK854879.1|	92731-94247	11	39
ACYPI008050-RA	gi|641587856|gb|KK854051.1|	783668-784232	10	41
ACYPI008363-RA	gi|641578109|gb|KK854638.1|	476604-478421	14	46
ACYPI064487-RA	gi|641586936|gb|KK854168.1|	624729-625174	12	42
ACYPI071881-RA	gi|641576689|gb|KK854876.1|	382208-383420	10	40
ACYPI089177-RA	gi|641568328|gb|KK857011.1|	41091-41852	9.7	32
ACYPI23999-RA	gi|641561138|gb|KK859879.1|	12437-15522	12	42

ACYPI32079-RA	gi|641578283|gb|KK854609.1|	312246-316303	12	22
ACYPI47651-RA	gi|641586096|gb|KK854291.1|	638772-639013	11	46
ACYPI000161-RA	gi|641586915|gb|KK854171.1|	573794-579809	10	38
ACYPI002850-RA	gi|641586809|gb|KK854188.1|	610368-610773	10	43
ACYPI004524-RA	gi|641577166|gb|KK854796.1|	106151-110994	10	38
ACYPI007248-RA	gi|641575794|gb|KK855043.1|	88920-89619	10	36
ACYPI008429-RA	gi|641587126|gb|KK854141.1|	744065-744613	10	39
ACYPI009072-RA	gi|641585847|gb|KK854330.1|	131481-132194	11	41
ACYPI062481-RA	gi|641586769|gb|KK854193.1|	265879-266459	12	23
ACYPI27655-RA	gi|641584743|gb|KK854459.1|	400551-401716	11	36
ACYPI007773-RA	gi|641585107|gb|KK854435.1|	388387-391875	15	45
ACYPI27939-RA	gi|641585407|gb|KK854403.1|	418445-418915	9.4	42
ACYPI54101-RA	gi|641581402|gb|KK854555.1|	182622-184037	12	20
ACYPI000837-RA	gi|641582096|gb|KK854546.1|	29961-33872	11	38
ACYPI001316-RA	gi|641585246|gb|KK854426.1|	230696-231694	10	39
ACYPI002722-RA	gi|641588109|gb|KK854023.1|	334628-336683	10	40
ACYPI003565-RA	gi|641574809|gb|KK855240.1|	40390-41700	11	39
ACYPI005123-RA	gi|641570135|gb|KK856449.1|	40054-41448	9.7	34
ACYPI005492-RA	gi|641587452|gb|KK854101.1|	302070-302311	11	40
ACYPI005516-RA	gi|641586535|gb|KK854227.1|	498165-499045	10	39
ACYPI005583-RA	gi|641572719|gb|KK855742.1|	196892-199969	12	23
ACYPI006803-RA	gi|641574998|gb|KK855198.1|	150284-151623	10	39
ACYPI007021-RA	gi|641587930|gb|KK854043.1|	482472-483559	10	41
ACYPI008427-RA	gi|641587896|gb|KK854047.1|	1059425-1063863	9.9	39
ACYPI009070-RA	gi|641571169|gb|KK856142.1|	162525-168397	9.6	42
ACYPI009978-RA	gi|641573425|gb|KK855563.1|	59499-61885	9.4	42
ACYPI060771-RA	gi|641575396|gb|KK855118.1|	212692-214092	9	35
ACYPI072126-RA	gi|641575516|gb|KK855095.1|	205871-206287	9.6	36
ACYPI001219-RA	gi|641588060|gb|KK854028.1|	692693-695133	11	42
ACYPI001715-RA	gi|641572740|gb|KK855736.1|	57412-58946	11	40

ACYPI005028-RA	gi|641587688|gb|KK854072.1|	464976-466457	10	38
ACYPI065028-RA	gi|641586694|gb|KK854204.1|	522868-525690	12	50
ACYPI087266-RA	gi|641586694|gb|KK854204.1|	518341-519362	12	50
ACYPI38268-RA	gi|641569798|gb|KK856557.1|	97264-105963	12	46
ACYPI006010-RA	gi|641568098|gb|KK857086.1|	81822-82624	9.9	18
ACYPI006283-RA	gi|641572372|gb|KK855829.1|	122861-123252	12	45
ACYPI008151-RA	gi|641574986|gb|KK855201.1|	123973-124405	9.3	35
ACYPI008396-RA	gi|641571207|gb|KK856132.1|	124825-125119	10	40
ACYPI003298-RA	gi|641580470|gb|KK854571.1|	150677-154984	10	41
ACYPI004820-RA	gi|641585515|gb|KK854385.1|	542515-542872	11	40
ACYPI005778-RA	gi|641585871|gb|KK854327.1|	58229-58673	11	38
ACYPI007979-RA	gi|641565734|gb|KK857919.1|	4003-4528	12	45
ACYPI009542-RA	gi|641584845|gb|KK854452.1|	428394-429145	9	36
ACYPI064196-RA	gi|641576333|gb|KK854937.1|	193975-194870	9.9	35
ACYPI066987-RA	gi|641587832|gb|KK854054.1|	373247-377076	9.8	20
ACYPI070927-RA	gi|641573198|gb|KK855620.1|	154693-155385	10	40
ACYPI001326-RA	gi|641567496|gb|KK857287.1|	79327-80221	8.3	28
ACYPI002318-RA	gi|641575730|gb|KK855054.1|	251780-253909	10	38
ACYPI002522-RA	gi|641586476|gb|KK854236.1|	499691-501894	10	37
ACYPI005093-RA	gi|641587793|gb|KK854059.1|	827215-829913	11	38
ACYPI006993-RA	gi|641565451|gb|KK858025.1|	30340-32487	9.1	17
ACYPI007164-RA	gi|641586928|gb|KK854169.1|	276877-277367	11	39
ACYPI065096-RA	gi|641586476|gb|KK854236.1|	510402-512180	10	37
ACYPI070768-RA	gi|641568731|gb|KK856884.1|	17513-18613	11	22
ACYPI086900-RA	gi|641588033|gb|KK854031.1|	1149495-1159276	11	38
ACYPI000102-RA	gi|641576951|gb|KK854831.1|	209531-210063	8.6	40
ACYPI001323-RA	gi|641588194|gb|KK854015.1|	295639-296775	9.4	40
ACYPI003903-RA	gi|641585684|gb|KK854357.1|	122687-123217	11	39
ACYPI004515-RA	gi|641588082|gb|KK854026.1|	592713-594405	11	41
ACYPI006435-RA	gi|641583980|gb|KK854512.1|	193409-194072	11	39

ACYPI008308-RA	gi|641582439|gb|KK854536.1|	378954-382080	11	36
ACYPI068801-RA	gi|641575987|gb|KK855008.1|	113962-115318	9.9	20
ACYPI38899-RA	gi|641586613|gb|KK854216.1|	194953-197767	11	41
ACYPI001030-RA	gi|641572615|gb|KK855769.1|	190159-190813	12	43
ACYPI001668-RA	gi|641588358|gb|KK854002.1|	1190216-1190790	11	43
ACYPI002929-RA	gi|641585395|gb|KK854405.1|	504492-505497	10	38
ACYPI006758-RA	gi|641585204|gb|KK854429.1|	142132-145872	9.2	36
ACYPI007368-RA	gi|641576161|gb|KK854972.1|	162905-168036	10	36
ACYPI009885-RA	gi|641579192|gb|KK854588.1|	153217-154256	11	41
ACYPI072215-RA	gi|641573950|gb|KK855430.1|	224906-229100	10	19
ACYPI087592-RA	gi|641576161|gb|KK854972.1|	126043-126431	10	36
ACYPI29851-RA	gi|641573368|gb|KK855579.1|	48743-48991	10	20
ACYPI56793-RA	gi|641588246|gb|KK854010.1|	1246276-1246516	9.2	50
ACYPI000534-RA	gi|641586344|gb|KK854254.1|	475181-476013	10	40
ACYPI000538-RA	gi|641585847|gb|KK854330.1|	187303-189644	11	41
ACYPI001583-RA	gi|641587962|gb|KK854039.1|	938760-943835	11	40
ACYPI002286-RA	gi|641575730|gb|KK855054.1|	33871-36374	10	38
ACYPI003560-RA	gi|641574110|gb|KK855394.1|	122773-123817	10	21
ACYPI005000-RA	gi|641586515|gb|KK854230.1|	101273-101505	11	39
ACYPI006384-RA	gi|641572565|gb|KK855782.1|	15997-16999	8.7	43
ACYPI006521-RA	gi|641579219|gb|KK854584.1|	184872-188323	12	39
ACYPI006896-RA	gi|641576557|gb|KK854897.1|	178281-180603	8.9	33
ACYPI009259-RA	gi|641571737|gb|KK855994.1|	65417-65950	11	23
ACYPI010034-RA	gi|641580479|gb|KK854569.1|	139738-146127	10	38
ACYPI067449-RA	gi|641578114|gb|KK854637.1|	328960-333632	9.7	38
ACYPI072192-RA	gi|641569463|gb|KK856664.1|	55470-56190	11	46
ACYPI000079-RA	gi|641575368|gb|KK855125.1|	91464-93177	10	36
ACYPI000109-RA	gi|641575730|gb|KK855054.1|	312883-318335	10	38
ACYPI000140-RA	gi|641572022|gb|KK855920.1|	70889-71805	12	42
ACYPI001033-RA	gi|641586547|gb|KK854225.1|	57413-57636	10	40

ACYPI006508-RA	gi|641577754|gb|KK854700.1|	285516-286175	12	43
ACYPI006616-RA	gi|641576656|gb|KK854883.1|	19849-25051	11	40
ACYPI006935-RA	gi|641586555|gb|KK854224.1|	440313-441127	11	39
ACYPI007040-RA	gi|641574352|gb|KK855342.1|	225141-225573	9.4	18
ACYPI008142-RA	gi|641568112|gb|KK857081.1|	43747-44432	11	38
ACYPI009430-RA	gi|641566437|gb|KK857661.1|	31830-32219	9.2	34
ACYPI009856-RA	gi|641566296|gb|KK857713.1|	27500-28132	9.7	34
ACYPI000218-RA	gi|641576343|gb|KK854935.1|	42989-44371	9.5	36
ACYPI000787-RA	gi|641587511|gb|KK854094.1|	242785-243982	10	41
ACYPI002674-RA	gi|641575218|gb|KK855155.1|	85984-86668	11	21
ACYPI002787-RA	gi|641573571|gb|KK855526.1|	157932-158244	9.8	36
ACYPI004152-RA	gi|641588099|gb|KK854024.1|	310272-310556	11	39
ACYPI004385-RA	gi|641566380|gb|KK857684.1|	35837-36343	10	38
ACYPI004738-RA	gi|641577453|gb|KK854750.1|	65937-66587	10	21
ACYPI004966-RA	gi|641586713|gb|KK854201.1|	602441-603153	9.1	40
ACYPI005060-RA	gi|641573737|gb|KK855486.1|	20928-21418	9.1	33
ACYPI006316-RA	gi|641587660|gb|KK854076.1|	262255-263089	9.9	41
ACYPI006664-RA	gi|641574249|gb|KK855364.1|	70843-72107	9.2	37
ACYPI007199-RA	gi|641585366|gb|KK854408.1|	198540-199844	12	41
ACYPI008467-RA	gi|641585163|gb|KK854432.1|	15000-15287	11	43
ACYPI009377-RA	gi|641567449|gb|KK857303.1|	46382-47431	8.5	39
ACYPI010096-RA	gi|641568618|gb|KK856922.1|	101334-102114	12	42
ACYPI063955-RA	gi|641575978|gb|KK855010.1|	83403-84629	10	36
ACYPI066875-RA	gi|641575218|gb|KK855155.1|	68443-68759	11	21
ACYPI25540-RA	gi|641587921|gb|KK854044.1|	123855-124138	9.4	41
ACYPI49671-RA	gi|641586831|gb|KK854185.1|	81925-82566	9.5	44
ACYPI000549-RA	gi|641586096|gb|KK854291.1|	135043-135554	11	46
ACYPI002433-RA	gi|641577215|gb|KK854787.1|	126190-127217	11	40
ACYPI006909-RA	gi|641583088|gb|KK854527.1|	192271-193563	9.9	39
ACYPI007005-RA	gi|641585395|gb|KK854405.1|	429043-429785	10	38

ACYPI008769-RA	gi|641585469|gb|KK854393.1|	404812-405277	10	37
ACYPI009420-RA	gi|641567358|gb|KK857335.1|	55886-57599	10	36
ACYPI063903-RA	gi|641575140|gb|KK855171.1|	74146-75610	9.7	40
ACYPI084976-RA	gi|641587303|gb|KK854118.1|	195421-198626	11	20
ACYPI41067-RA	gi|641570488|gb|KK856340.1|	156373-158735	12	45
