# Supplementary material for: The X Chromosome of Hemipteran Insects: Conservation, Dosage Compensation and Sex-Biased Expression
Source: Genome Biol Evol. 2015 Nov 10;7(12):3259–68. doi: 10.1093/gbe/evv215 (PMC4700948; doi:10.1093/gbe/evv215)
Supplement: Supplementary Data [file supp_evv215_suppl_data.zip › S2 Data (rev) HH-HV.pdf]

AP	HH	gene	HHcovF	HHcovM	HV	gene	HVcovF	HVcovM
ACYPI000011-RA	ni|645904246|nb|KK920233.1|	638772-640086	16	17	gi|646777495|gb|KK961629.1|	1233265-1237008	19	8.7
ACYPI000013-RA	ni|645904195|nb|KK920254.1|	110171-111932	18	17	gi|646740098|gb|KK962812.1|	612042-612505	20	10
ACYPI000016-RA	ni|645899869|nb|KK924560.1|	1233-1542	nan	nan	gi|646771265|gb|KK961769.1|	157601-159488	17	7
ACYPI000023-RA	ni|645904072|nb|KK920359.1|	947375-948150	17	17	gi|646779785|gb|KK961565.1|	3459246-3460123	18	8.9
ACYPI000027-RA	ni|645902384|nb|KK922045.1|	184957-185581	nan	nan	gi|646745959|gb|KK962321.1|	195848-196157	20	6.9
ACYPI000030-RA	ni|645903923|nb|KK920508.1|	126453-126713	18	18	gi|646776588|gb|KK961666.1|	2584306-2591169	20	5.8
ACYPI000031-RA	ni|645903634|nb|KK920796.1|	572293-572511	nan	nan	gi|646763164|gb|KK961904.1|	1562058-1566225	22	10
ACYPI000032-RA	ni|645903782|nb|KK920648.1|	179359-179729	13	14	gi|646777416|gb|KK961632.1|	3397743-3397979	22	8.8
ACYPI000033-RA	ni|645903879|nb|KK920552.1|	1065460-1065856	18	18	gi|646758845|gb|KK961921.1|	621449-621952	22	9.5
ACYPI000035-RA	ni|645903788|nb|KK920642.1|	730081-730597	16	15	gi|646766426|gb|KK961862.1|	956662-956908	20	7.1
ACYPI000038-RA	ni|645903989|nb|KK920442.1|	731456-731831	17	20	gi|646747841|gb|KK962189.1|	106063-106397	23	10
ACYPI000039-RA	ni|645903755|nb|KK920675.1|	749888-750218	18	16	gi|646742541|gb|KK962572.1|	629400-629683	19	10
ACYPI000051-RA	ni|645903759|nb|KK920671.1|	97725-98872	18	17	gi|646752143|gb|KK961972.1|	776198-776437	20	5.6
ACYPI000052-RA	ni|645902360|nb|KK922069.1|	41628-41906	nan	nan	gi|646775807|gb|KK961708.1|	2100664-2101889	21	8.6
ACYPI000053-RA	ni|645904114|nb|KK920317.1|	2059145-2060225	18	18	gi|646749945|gb|KK962065.1|	1687137-1694597	22	6.1
ACYPI000054-RA	ni|645903534|nb|KK920896.1|	569179-569605	nan	nan	gi|646743302|gb|KK962514.1|	156505-156681	23	8.4
ACYPI000055-RA	ni|645903787|nb|KK920643.1|	1368639-1369809	19	18	gi|646779262|gb|KK961578.1|	2195924-2197069	20	8.1
ACYPI000056-RA	ni|645904133|nb|KK920300.1|	1059081-1059971	18	18	gi|646776024|gb|KK961697.1|	597551-598295	22	9.2
ACYPI000058-RA	ni|645903798|nb|KK920632.1|	767915-768764	18	17	gi|646744699|gb|KK962413.1|	70330-71410	14	6.9
ACYPI000059-RA	ni|645904009|nb|KK920422.1|	608483-608931	17	9.4	gi|646782334|gb|KK961495.1|	4655675-4657262	21	9
ACYPI000061-RA	ni|645903749|nb|KK920681.1|	644306-645583	19	18	gi|646781243|gb|KK961526.1|	2284347-2285039	21	8.9
ACYPI000062-RA	ni|645903923|nb|KK920508.1|	1138568-1139112	18	18	gi|646782334|gb|KK961495.1|	3145231-3146159	21	9
ACYPI000063-RA	ni|645904278|nb|KK920222.1|	543245-543785	15	16	gi|646765790|gb|KK961889.1|	1012080-1014309	22	8.8
ACYPI000065-RA	ni|645903698|nb|KK920732.1|	147048-147807	nan	nan	gi|646776514|gb|KK961670.1|	230372-233696	20	9.6
ACYPI000066-RA	ni|645903749|nb|KK920681.1|	222022-222532	19	18	gi|646781344|gb|KK961523.1|	3687902-3700144	21	8.9
ACYPI000067-RA	ni|645904210|nb|KK920249.1|	1933083-1934378	16	16	gi|646747471|gb|KK962216.1|	172292-176424	24	6.4
ACYPI000068-RA	ni|645903971|nb|KK920460.1|	1942428-1944356	19	18	gi|646775987|gb|KK961699.1|	249584-250665	19	7.5
ACYPI000070-RA	ni|645904160|nb|KK920273.1|	612857-613080	18	18	gi|646746355|gb|KK962294.1|	502821-503397	22	10
ACYPI000071-RA	ni|645903843|nb|KK920587.1|	406702-406961	18	16	gi|646747175|gb|KK962238.1|	313611-314731	20	8.4

ACYPI000072-RA	ni|645904075|nb|KK920356.1|	746654-747204	16	17	gi|646754962|gb|KK961940.1|	752857-753275	21	10
ACYPI000073-RA	ni|645904177|nb|KK920260.1|	2820278-2820690	18	17	gi|646749098|gb|KK962110.1|	745846-746450	20	7.7
ACYPI000076-RA	ni|645902589|nb|KK921840.1|	122803-123041	nan	nan	gi|646738524|gb|KK962968.1|	52854-54376	24	11
ACYPI000077-RA	ni|645903673|nb|KK920757.1|	187211-187723	nan	nan	gi|646782288|gb|KK961496.1|	6774498-6774864	21	9.7
ACYPI000078-RA	ni|645903858|nb|KK920572.1|	709519-709799	16	16	gi|646767265|gb|KK961836.1|	1243106-1247949	22	9.9
ACYPI000079-RA	ni|645904096|nb|KK920335.1|	2233322-2233597	18	17	gi|646780953|gb|KK961535.1|	3216707-3217447	20	9.8
ACYPI000080-RA	ni|645903724|nb|KK920706.1|	262716-263267	nan	nan	gi|646743195|gb|KK962522.1|	250620-251412	14	6.4
ACYPI000082-RA	ni|645903923|nb|KK920508.1|	282216-282451	18	18	gi|646752567|gb|KK961965.1|	934807-936511	19	8.4
ACYPI000083-RA	ni|645904015|nb|KK920416.1|	1558468-1558729	18	16	gi|646776409|gb|KK961677.1|	358171-358575	17	8.4
ACYPI000086-RA	ni|645902589|nb|KK921840.1|	118937-119181	nan	nan	gi|646738524|gb|KK962968.1|	61726-63075	24	11
ACYPI000089-RA	ni|645904130|nb|KK920303.1|	1356279-1356683	18	17	gi|646780978|gb|KK961534.1|	511824-512059	20	8
ACYPI000090-RA	ni|645903841|nb|KK920589.1|	1249445-1250199	17	17	gi|646770998|gb|KK961772.1|	1214361-1215446	22	9.8
ACYPI000091-RA	ni|645904122|nb|KK920309.1|	589900-590358	18	17	gi|646760564|gb|KK961914.1|	518723-518969	21	8.8
ACYPI000100-RA	ni|645902364|nb|KK922065.1|	172748-173317	nan	nan	gi|646778632|gb|KK961596.1|	1194499-1195606	21	9.2
ACYPI000102-RA	ni|645904228|nb|KK920243.1|	803500-803990	17	18	gi|646779451|gb|KK961573.1|	833738-834143	20	8.9
ACYPI000104-RA	ni|645903915|nb|KK920516.1|	207249-208913	17	17	gi|646743394|gb|KK962507.1|	357348-358187	20	8.8
ACYPI000111-RA	ni|645903549|nb|KK920881.1|	19337-19606	nan	nan	gi|646781013|gb|KK961533.1|	1858064-1860911	18	7.6
ACYPI000112-RA	ni|645904174|nb|KK920261.1|	923528-923778	19	17	gi|646767156|gb|KK961839.1|	121818-122206	15	6.4
ACYPI000119-RA	ni|645903743|nb|KK920687.1|	881121-881542	19	18	gi|646746752|gb|KK962267.1|	493701-496561	20	7.9
ACYPI000120-RA	ni|645904129|nb|KK920304.1|	1140307-1140617	16	17	gi|646753799|gb|KK961950.1|	613540-616165	23	9.2
ACYPI000149-RA	ni|645903979|nb|KK920452.1|	532494-536473	15	17	gi|646776305|gb|KK961683.1|	2525223-2526435	20	8.8
ACYPI000150-RA	ni|645903496|nb|KK920934.1|	195331-196100	nan	nan	gi|646776368|gb|KK961679.1|	1738757-1741452	21	9.7
ACYPI000157-RA	ni|645903617|nb|KK920813.1|	290263-290735	nan	nan	gi|646778903|gb|KK961588.1|	979585-980246	23	10
ACYPI000165-RA	ni|645897572|nb|KK926857.1|	730-950	nan	nan	gi|646780485|gb|KK961548.1|	999810-1000053	15	6.1
ACYPI000171-RA	ni|645903705|nb|KK920725.1|	432083-432452	nan	nan	gi|646781043|gb|KK961532.1|	817031-818298	26	11
ACYPI000174-RA	ni|645903749|nb|KK920681.1|	365896-366970	19	18	gi|646782043|gb|KK961502.1|	4966189-4968635	20	9.2
ACYPI000178-RA	ni|645904160|nb|KK920273.1|	2096928-2097166	18	18	gi|646754371|gb|KK961945.1|	288169-289147	20	9.7
ACYPI000181-RA	ni|645903788|nb|KK920642.1|	321728-321979	16	15	gi|646781564|gb|KK961516.1|	637656-637956	23	9.4
ACYPI000191-RA	ni|645904278|nb|KK920222.1|	1295109-1295472	15	16	gi|646748855|gb|KK962125.1|	213474-218196	22	5.8
ACYPI000192-RA	ni|645902694|nb|KK921735.1|	14577-15368	nan	nan	gi|646775507|gb|KK961723.1|	1642102-1642297	20	9.8

ACYPI000201-RA	ni|645903731|nb|KK920699.1|	884810-885019	nan	nan	gi|646778029|gb|KK961613.1|	2338609-2338874	21	8.6
ACYPI000206-RA	ni|645903747|nb|KK920683.1|	74133-74675	19	18	gi|646747385|gb|KK962223.1|	243440-243655	21	5.4
ACYPI000208-RA	ni|645903915|nb|KK920516.1|	1487401-1488626	17	17	gi|646778840|gb|KK961590.1|	1377250-1379380	20	9.9
ACYPI000210-RA	ni|645902026|nb|KK922403.1|	3499-4106	nan	nan	gi|646775987|gb|KK961699.1|	781881-783343	19	7.5
ACYPI000219-RA	ni|645903690|nb|KK920740.1|	1034038-1034219	nan	nan	gi|646773721|gb|KK961747.1|	2447510-2449323	21	9.2
ACYPI000221-RA	ni|645902470|nb|KK921959.1|	127170-128347	nan	nan	gi|646779375|gb|KK961575.1|	2319140-2320297	22	9.3
ACYPI000222-RA	ni|645903607|nb|KK920823.1|	306997-307436	nan	nan	gi|646751350|gb|KK961993.1|	255525-256015	20	7.6
ACYPI000227-RA	ni|645903988|nb|KK920443.1|	96980-98231	14	16	gi|646779066|gb|KK961583.1|	1212691-1214372	19	7.9
ACYPI000235-RA	ni|645904112|nb|KK920319.1|	1717915-1718101	18	17	gi|646748407|gb|KK962151.1|	147350-164335	22	9.1
ACYPI000238-RA	ni|645902469|nb|KK921960.1|	278248-278991	nan	nan	gi|646776219|gb|KK961687.1|	2197359-2198345	21	9.9
ACYPI000243-RA	ni|645903504|nb|KK920926.1|	259682-259871	nan	nan	gi|646766650|gb|KK961854.1|	456289-456754	22	10
ACYPI000249-RA	ni|645904262|nb|KK920227.1|	2592282-2593418	17	17	gi|646779662|gb|KK961568.1|	1262501-1264477	20	9.3
ACYPI000252-RA	ni|645904030|nb|KK920401.1|	406005-408372	14	15	gi|646777474|gb|KK961630.1|	3104034-3109213	21	9.3
ACYPI000257-RA	ni|645904112|nb|KK920319.1|	1677836-1678586	18	17	gi|646742343|gb|KK962588.1|	266900-267672	20	9.4
ACYPI000258-RA	ni|645903597|nb|KK920833.1|	984125-985288	nan	nan	gi|646738615|gb|KK962959.1|	211375-211648	21	9.5
ACYPI000259-RA	ni|645903529|nb|KK920901.1|	101501-103180	nan	nan	gi|646780858|gb|KK961538.1|	1131776-1132105	21	10
ACYPI000262-RA	ni|645903674|nb|KK920756.1|	567814-568257	nan	nan	gi|646752294|gb|KK961969.1|	850302-851220	23	9.8
ACYPI000265-RA	ni|645904106|nb|KK920325.1|	994361-995652	17	17	gi|646595672|gb|KK977392.1|	665-1940	21	8.2
ACYPI000271-RA	ni|645903949|nb|KK920482.1|	2716-3421	17	14	gi|646778448|gb|KK961601.1|	1740135-1741627	19	8.3
ACYPI000278-RA	ni|645904152|nb|KK920281.1|	260381-260684	15	15	gi|646739841|gb|KK962838.1|	399284-400000	21	5.7
ACYPI000286-RA	ni|645903551|nb|KK920879.1|	385576-386048	nan	nan	gi|646750484|gb|KK962036.1|	541864-542093	20	5.5
ACYPI000289-RA	ni|645903634|nb|KK920796.1|	1155220-1155478	nan	nan	gi|646779186|gb|KK961580.1|	2020614-2021080	20	8.6
ACYPI000295-RA	ni|645903717|nb|KK920713.1|	67347-67736	nan	nan	gi|646771568|gb|KK961766.1|	989260-989524	18	7.4
ACYPI000298-RA	ni|645903931|nb|KK920500.1|	567663-571406	16	18	gi|646782043|gb|KK961502.1|	1953837-1954110	20	9.2
ACYPI000303-RA	ni|645902364|nb|KK922065.1|	28028-29134	nan	nan	gi|646781118|gb|KK961530.1|	4711619-4713254	21	10
ACYPI000304-RA	ni|645904106|nb|KK920325.1|	996965-998163	17	17	gi|646775595|gb|KK961718.1|	677552-678067	21	8.9
ACYPI000310-RA	ni|645904171|nb|KK920262.1|	517579-519623	15	16	gi|646775842|gb|KK961706.1|	1806878-1810297	22	9
ACYPI000320-RA	ni|645903768|nb|KK920662.1|	1159763-1159985	18	17	gi|646767754|gb|KK961825.1|	83201-83427	20	8.5
ACYPI000329-RA	ni|645903754|nb|KK920676.1|	99757-100698	14	8.8	gi|646779498|gb|KK961572.1|	2496952-2503099	22	5.6
ACYPI000340-RA	ni|645903957|nb|KK920474.1|	962526-963350	18	17	gi|646779412|gb|KK961574.1|	1929400-1931351	21	5.4

ACYPI000348-RA	ni|645903765|nb|KK920665.1|	929864-930059	17	15	gi|646778865|gb|KK961589.1|	2907073-2908107	20	9.2
ACYPI000362-RA	ni|645904075|nb|KK920356.1|	1037500-1038360	16	17	gi|646768494|gb|KK961810.1|	575133-576685	21	10
ACYPI000383-RA	ni|645903979|nb|KK920452.1|	698803-700771	15	17	gi|646780889|gb|KK961537.1|	4001777-4006160	22	10
ACYPI000387-RA	ni|645903771|nb|KK920659.1|	798050-798518	16	15	gi|646770901|gb|KK961773.1|	1607687-1609334	22	9.7
ACYPI000401-RA	ni|645904058|nb|KK920373.1|	770416-770714	16	15	gi|646776280|gb|KK961684.1|	949639-950510	22	9.3
ACYPI000402-RA	ni|645904262|nb|KK920227.1|	1248071-1251182	17	17	gi|646780105|gb|KK961557.1|	3099164-3099558	21	9.7
ACYPI000405-RA	ni|645904222|nb|KK920245.1|	648405-648723	17	12	gi|646778112|gb|KK961611.1|	1159493-1160574	22	10
ACYPI000413-RA	ni|645903620|nb|KK920810.1|	8735-8932	nan	nan	gi|646747574|gb|KK962208.1|	384898-386581	27	10
ACYPI000423-RA	ni|645904210|nb|KK920249.1|	813969-814148	16	16	gi|646777017|gb|KK961649.1|	993344-994208	18	8
ACYPI000427-RA	ni|645902308|nb|KK922121.1|	113622-115049	nan	nan	gi|646779006|gb|KK961585.1|	202694-213347	20	9.9
ACYPI000430-RA	ni|645903743|nb|KK920687.1|	896515-897013	19	18	gi|646770323|gb|KK961780.1|	267737-268155	19	7.1
ACYPI000431-RA	ni|645904053|nb|KK920378.1|	575073-575639	18	17	gi|646779826|gb|KK961564.1|	1056290-1056945	22	9.6
ACYPI000433-RA	ni|645902311|nb|KK922118.1|	36587-37174	nan	nan	gi|646781732|gb|KK961511.1|	905498-907168	22	9.8
ACYPI000436-RA	ni|645904142|nb|KK920291.1|	1833311-1834094	20	23	gi|646781659|gb|KK961513.1|	3748687-3749643	22	9.8
ACYPI000442-RA	ni|645903804|nb|KK920626.1|	128601-130010	17	17	gi|646777842|gb|KK961618.1|	2148059-2149775	19	8.9
ACYPI000443-RA	ni|645902239|nb|KK922190.1|	60675-62288	nan	nan	gi|646782168|gb|KK961499.1|	1790146-1791926	21	9.4
ACYPI000445-RA	ni|645902642|nb|KK921787.1|	124594-124798	nan	nan	gi|646775807|gb|KK961708.1|	2842833-2843891	21	8.6
ACYPI000446-RA	ni|645902779|nb|KK921650.1|	74199-74708	nan	nan	gi|646781421|gb|KK961521.1|	1473544-1476975	21	8.7
ACYPI000450-RA	ni|645904246|nb|KK920233.1|	991318-992035	16	17	gi|646781732|gb|KK961511.1|	2788372-2792489	22	9.8
ACYPI000453-RA	ni|645903595|nb|KK920835.1|	903022-906579	nan	nan	gi|646776701|gb|KK961661.1|	612430-613848	18	6.7
ACYPI000454-RA	ni|645903688|nb|KK920742.1|	78374-78772	nan	nan	gi|646776351|gb|KK961680.1|	424875-425208	22	10
ACYPI000455-RA	ni|645904234|nb|KK920241.1|	67395-67714	13	15	gi|646776148|gb|KK961690.1|	1761896-1762566	21	9.4
ACYPI000467-RA	ni|645904093|nb|KK920338.1|	23707-24122	13	15	gi|646735649|gb|KK963275.1|	10568-10830	16	7.9
ACYPI000474-RA	ni|645903650|nb|KK920780.1|	143175-143398	nan	nan	gi|646767850|gb|KK961823.1|	1181490-1184713	21	9.8
ACYPI000476-RA	ni|645903837|nb|KK920593.1|	581975-582221	18	9.8	gi|646780222|gb|KK961554.1|	94940-95790	20	9.7
ACYPI000479-RA	ni|645902233|nb|KK922196.1|	159856-160197	nan	nan	gi|646747990|gb|KK962179.1|	803511-807000	21	9.3
ACYPI000484-RA	ni|645904242|nb|KK920237.1|	53205-54736	15	16	gi|646744140|gb|KK962454.1|	286280-288386	21	9.3
ACYPI000487-RA	ni|645904106|nb|KK920325.1|	677994-678427	17	17	gi|646767120|gb|KK961840.1|	817964-819474	20	8.6
ACYPI000496-RA	ni|645904015|nb|KK920416.1|	1300407-1300888	18	16	gi|646782357|gb|KK961494.1|	3432145-3433422	21	9.2
ACYPI000499-RA	ni|645903858|nb|KK920572.1|	822831-823007	16	16	gi|646767265|gb|KK961836.1|	1341643-1349857	22	9.9

ACYPI000500-RA	ni|645903898|nb|KK920533.1|	483988-484252	17	16	gi|646765966|gb|KK961881.1|	1445629-1446753	19	9.2
ACYPI000502-RA	ni|645903759|nb|KK920671.1|	525437-525857	18	17	gi|646781809|gb|KK961509.1|	2194102-2194572	19	8.6
ACYPI000505-RA	ni|645903500|nb|KK920930.1|	190218-190357	nan	nan	gi|646766451|gb|KK961861.1|	566084-566355	19	8.9
ACYPI000507-RA	ni|645904143|nb|KK920290.1|	637388-637886	15	7.9	gi|646766564|gb|KK961857.1|	1258089-1266009	20	9.7
ACYPI000513-RA	ni|645904098|nb|KK920333.1|	21662-24773	16	8.8	gi|646745644|gb|KK962342.1|	463448-465229	31	14
ACYPI000519-RA	ni|645904067|nb|KK920364.1|	1536829-1538669	17	14	gi|646775867|gb|KK961705.1|	301713-315278	23	10
ACYPI000521-RA	ni|645903713|nb|KK920717.1|	446097-447247	nan	nan	gi|646768669|gb|KK961806.1|	417270-417813	18	9.1
ACYPI000523-RA	ni|645903710|nb|KK920720.1|	1038-1528	nan	nan	gi|646771448|gb|KK961767.1|	708643-709058	21	9.8
ACYPI000527-RA	ni|645904130|nb|KK920303.1|	486428-487041	18	17	gi|646751544|gb|KK961986.1|	1295621-1295843	22	6
ACYPI000532-RA	ni|645904077|nb|KK920354.1|	307038-307495	16	17	gi|646782127|gb|KK961500.1|	3721499-3726858	22	9.6
ACYPI000533-RA	ni|645903971|nb|KK920460.1|	1199184-1200576	19	18	gi|646739633|gb|KK962861.1|	266755-267064	17	7.9
ACYPI000534-RA	ni|645902143|nb|KK922286.1|	173814-174760	nan	nan	gi|646778336|gb|KK961604.1|	2203948-2204913	21	10
ACYPI000538-RA	ni|645903755|nb|KK920675.1|	656301-657340	18	16	gi|646775635|gb|KK961716.1|	1847031-1850055	22	10
ACYPI000550-RA	ni|645903812|nb|KK920618.1|	271009-272231	20	18	gi|646778699|gb|KK961594.1|	2426055-2431226	21	9.5
ACYPI000552-RA	ni|645904189|nb|KK920256.1|	1449104-1449831	18	18	gi|646782288|gb|KK961496.1|	791268-791853	21	9.7
ACYPI000563-RA	ni|645902589|nb|KK921840.1|	97255-97493	nan	nan	gi|646748295|gb|KK962158.1|	90371-90660	18	8.2
ACYPI000575-RA	ni|645903787|nb|KK920643.1|	1376064-1377647	19	18	gi|646749632|gb|KK962082.1|	359901-362282	20	8.6
ACYPI000580-RA	ni|645903688|nb|KK920742.1|	1111336-1111644	nan	nan	gi|646737917|gb|KK963028.1|	149263-149945	22	7.8
ACYPI000582-RA	ni|645903541|nb|KK920889.1|	3564-3724	nan	nan	gi|646743576|gb|KK962494.1|	140136-140844	19	8.2
ACYPI000585-RA	ni|645903954|nb|KK920477.1|	197722-197837	35	44	gi|646781772|gb|KK961510.1|	4238842-4240612	20	9.2
ACYPI000592-RA	ni|645904246|nb|KK920233.1|	1091833-1092019	16	17	gi|646781732|gb|KK961511.1|	2776373-2780343	22	9.8
ACYPI000598-RA	ni|645901961|nb|KK922468.1|	109885-111850	nan	nan	gi|646781510|gb|KK961518.1|	184400-187619	17	7.8
ACYPI000600-RA	ni|645903696|nb|KK920734.1|	625575-625788	nan	nan	gi|646779262|gb|KK961578.1|	907356-907915	20	8.1
ACYPI000610-RA	ni|645903970|nb|KK920461.1|	2124908-2125698	17	16	gi|646777416|gb|KK961632.1|	1856574-1857088	22	8.8
ACYPI000613-RA	ni|645903743|nb|KK920687.1|	320413-321814	19	18	gi|646769774|gb|KK961788.1|	490181-499714	20	9.2
ACYPI000617-RA	ni|645903860|nb|KK920570.1|	154050-154973	17	17	gi|646780222|gb|KK961554.1|	1227144-1234809	20	9.7
ACYPI000618-RA	ni|645904174|nb|KK920261.1|	205017-205694	19	17	gi|646746257|gb|KK962301.1|	713367-715000	13	7.3
ACYPI000626-RA	ni|645904155|nb|KK920278.1|	283747-283984	18	9.4	gi|646780858|gb|KK961538.1|	2870715-2871107	21	10
ACYPI000631-RA	ni|645904063|nb|KK920368.1|	503401-504185	16	18	gi|646746339|gb|KK962295.1|	150652-152550	20	8.2
ACYPI000636-RA	ni|645902394|nb|KK922035.1|	65973-66172	nan	nan	gi|646750600|gb|KK962030.1|	381346-381772	23	8.6

ACYPI000662-RA	ni|645903920|nb|KK920511.1|	663830-664865	20	20	gi|646750185|gb|KK962052.1|	259994-261137	18	7.1
ACYPI000663-RA	ni|645903609|nb|KK920821.1|	157522-157654	nan	nan	gi|646748615|gb|KK962139.1|	440751-442875	19	7.8
ACYPI000666-RA	ni|645904043|nb|KK920388.1|	77415-78131	16	9.1	gi|646780574|gb|KK961546.1|	958171-961542	20	9.9
ACYPI000667-RA	ni|645903597|nb|KK920833.1|	566971-569213	nan	nan	gi|646781118|gb|KK961530.1|	197897-203737	21	10
ACYPI000673-RA	ni|645904130|nb|KK920303.1|	2404875-2405391	18	17	gi|646766086|gb|KK961876.1|	207695-208985	23	9.5
ACYPI000686-RA	ni|645903724|nb|KK920706.1|	159437-159822	nan	nan	gi|646780311|gb|KK961552.1|	3429318-3430008	21	9.7
ACYPI000691-RA	ni|645903988|nb|KK920443.1|	125895-127501	14	16	gi|646775523|gb|KK961722.1|	297318-300671	21	9.7
ACYPI000694-RA	ni|645904064|nb|KK920367.1|	761472-761990	17	9.9	gi|646779038|gb|KK961584.1|	500491-500957	21	6.9
ACYPI000695-RA	ni|645903768|nb|KK920662.1|	1193609-1193851	18	17	gi|646781772|gb|KK961510.1|	3879413-3879698	20	9.2
ACYPI000698-RA	ni|645904231|nb|KK920242.1|	630687-631223	16	17	gi|646746243|gb|KK962302.1|	220034-222108	12	7.7
ACYPI000700-RA	ni|645904085|nb|KK920346.1|	617544-618209	15	16	gi|646776050|gb|KK961695.1|	1231942-1233643	19	7.7
ACYPI000702-RA	ni|645904076|nb|KK920355.1|	810963-811167	17	17	gi|646751103|gb|KK962006.1|	587949-591119	20	8.3
ACYPI000704-RA	ni|645903661|nb|KK920769.1|	654640-655669	nan	nan	gi|646777570|gb|KK961626.1|	1443587-1444127	18	7.5
ACYPI000720-RA	ni|645903752|nb|KK920678.1|	652309-652693	18	16	gi|646770251|gb|KK961781.1|	1363462-1364195	20	9.6
ACYPI000724-RA	ni|645903866|nb|KK920564.1|	455494-456067	18	15	gi|646782276|gb|KK961497.1|	7119979-7121066	21	9.7
ACYPI000727-RA	ni|645900910|nb|KK923519.1|	12818-13466	nan	nan	gi|646781083|gb|KK961531.1|	2335878-2336903	19	8.5
ACYPI000735-RA	ni|645903592|nb|KK920838.1|	763281-763503	nan	nan	gi|646744179|gb|KK962451.1|	13535-16722	24	11
ACYPI000737-RA	ni|645903537|nb|KK920893.1|	508337-508812	nan	nan	gi|646748200|gb|KK962164.1|	474820-476214	19	7.7
ACYPI000739-RA	ni|645903969|nb|KK920462.1|	418285-418808	14	16	gi|646776647|gb|KK961663.1|	1490253-1490755	23	9.2
ACYPI000740-RA	ni|645904133|nb|KK920300.1|	936147-936443	18	18	gi|646738615|gb|KK962959.1|	99329-101936	21	9.5
ACYPI000749-RA	ni|645904125|nb|KK920307.1|	2764117-2764389	18	17	gi|646776974|gb|KK961651.1|	486769-489957	17	8.1
ACYPI000753-RA	ni|645904262|nb|KK920227.1|	2473960-2474135	17	17	gi|646779936|gb|KK961561.1|	2061110-2061797	19	9.6
ACYPI000754-RA	ni|645903923|nb|KK920508.1|	1127142-1127692	18	18	gi|646779539|gb|KK961571.1|	1476637-1476921	15	7.5
ACYPI000756-RA	ni|645904071|nb|KK920360.1|	1948746-1949884	17	17	gi|646779702|gb|KK961567.1|	1891062-1891666	23	10
ACYPI000759-RA	ni|645903891|nb|KK920540.1|	214051-214946	15	17	gi|646776929|gb|KK961653.1|	883996-885812	17	8.5
ACYPI000767-RA	ni|645903597|nb|KK920833.1|	1034927-1035235	nan	nan	gi|646782127|gb|KK961500.1|	2268280-2271808	22	9.6
ACYPI000768-RA	ni|645903617|nb|KK920813.1|	105665-106351	nan	nan	gi|646725696|gb|KK964612.1|	179764-182430	22	9.2
ACYPI000776-RA	ni|645904118|nb|KK920313.1|	678422-679063	17	17	gi|646776608|gb|KK961665.1|	1606664-1612257	23	9.3
ACYPI000787-RA	ni|645903716|nb|KK920714.1|	772982-773444	nan	nan	gi|646775312|gb|KK961734.1|	798851-799244	17	7.9
ACYPI000789-RA	ni|645903597|nb|KK920833.1|	695933-696325	nan	nan	gi|646780270|gb|KK961553.1|	834094-835387	22	8.6

ACYPI000814-RA	ni|645903895|nb|KK920536.1|	540572-540824	17	16	gi|646751569|gb|KK961985.1|	937271-938229	21	9.2
ACYPI000816-RA	ni|645903559|nb|KK920871.1|	189904-190635	nan	nan	gi|646771092|gb|KK961771.1|	1087754-1089498	23	9.7
ACYPI000819-RA	ni|645903891|nb|KK920540.1|	90267-90459	15	17	gi|646777416|gb|KK961632.1|	779364-781313	22	8.8
ACYPI000821-RA	ni|645903622|nb|KK920808.1|	572228-572457	nan	nan	gi|646782334|gb|KK961495.1|	2642576-2642820	21	9
ACYPI000824-RA	ni|645903597|nb|KK920833.1|	349204-349726	nan	nan	gi|646782357|gb|KK961494.1|	6268910-6282826	21	9.2
ACYPI000828-RA	ni|645904177|nb|KK920260.1|	782203-785242	18	17	gi|646779375|gb|KK961575.1|	1080382-1083185	22	9.3
ACYPI000837-RA	ni|645903564|nb|KK920866.1|	7905-11322	nan	nan	gi|646747424|gb|KK962220.1|	1360305-1360572	22	5.7
ACYPI000849-RA	ni|645904036|nb|KK920395.1|	1599328-1599778	21	18	gi|646781118|gb|KK961530.1|	3616282-3617476	21	10
ACYPI000854-RA	ni|645903495|nb|KK920935.1|	201271-201682	nan	nan	gi|646776629|gb|KK961664.1|	1076048-1076529	19	8.4
ACYPI000855-RA	ni|645903910|nb|KK920521.1|	544035-544262	19	17	gi|646750600|gb|KK962030.1|	678233-679195	23	8.6
ACYPI000870-RA	ni|645903636|nb|KK920794.1|	239469-240653	nan	nan	gi|646754003|gb|KK961948.1|	320783-321399	20	8.8
ACYPI000873-RA	ni|645903785|nb|KK920645.1|	441178-443405	15	16	gi|646750409|gb|KK962040.1|	816237-821091	21	8.4
ACYPI000876-RA	ni|645904019|nb|KK920412.1|	181000-181322	19	9.4	gi|646751458|gb|KK961989.1|	839991-840232	21	5.1
ACYPI000882-RA	ni|645903827|nb|KK920603.1|	437169-438833	17	18	gi|646776524|gb|KK961669.1|	1195418-1197855	17	6.4
ACYPI000886-RA	ni|645904014|nb|KK920417.1|	794089-794343	19	18	gi|646751289|gb|KK961996.1|	338417-338891	24	9.1
ACYPI000887-RA	ni|645903985|nb|KK920446.1|	1048896-1049470	18	16	gi|646755567|gb|KK961936.1|	673563-674476	20	8.6
ACYPI000890-RA	ni|645902336|nb|KK922093.1|	77307-78304	nan	nan	gi|646748888|gb|KK962123.1|	868885-870145	20	8.1
ACYPI000896-RA	ni|645901860|nb|KK922569.1|	75991-76640	nan	nan	gi|646750354|gb|KK962043.1|	326752-327026	20	9.2
ACYPI000902-RA	ni|645904136|nb|KK920297.1|	1703883-1704234	16	17	gi|646775595|gb|KK961718.1|	873497-873764	21	8.9
ACYPI000904-RA	ni|645903984|nb|KK920447.1|	214903-215208	18	17	gi|646780311|gb|KK961552.1|	3538603-3539747	21	9.7
ACYPI000914-RA	ni|645904081|nb|KK920350.1|	273844-275696	13	18	gi|646776280|gb|KK961684.1|	1742040-1745546	22	9.3
ACYPI000923-RA	ni|645904139|nb|KK920294.1|	273293-273530	15	18	gi|646751373|gb|KK961992.1|	598079-598345	21	8.7
ACYPI000927-RA	ni|645904057|nb|KK920374.1|	176583-176811	16	15	gi|646775944|gb|KK961701.1|	1025966-1026738	22	9.3
ACYPI000929-RA	ni|645903971|nb|KK920460.1|	1606345-1606702	19	18	gi|646781659|gb|KK961513.1|	3379491-3383496	22	9.8
ACYPI000938-RA	ni|645904168|nb|KK920265.1|	1905788-1906395	16	16	gi|646732491|gb|KK963666.1|	43033-43470	21	5.7
ACYPI000941-RA	ni|645904067|nb|KK920364.1|	829093-829550	17	14	gi|646780530|gb|KK961547.1|	2199904-2200192	21	9.2
ACYPI000944-RA	ni|645903919|nb|KK920512.1|	1245222-1245590	17	15	gi|646780147|gb|KK961556.1|	1969066-1969740	18	7.5
ACYPI000946-RA	ni|645904192|nb|KK920255.1|	1153192-1153392	15	16	gi|646782357|gb|KK961494.1|	4401905-4402253	21	9.2
ACYPI000947-RA	ni|645904070|nb|KK920361.1|	558710-558940	17	16	gi|646775635|gb|KK961716.1|	400858-402792	22	10
ACYPI000948-RA	ni|645904054|nb|KK920377.1|	838087-839585	16	16	gi|646779141|gb|KK961581.1|	949954-950678	21	9.1

ACYPI000953-RA	ni|645904246|nb|KK920233.1|	1237740-1237975	16	17	gi|646775507|gb|KK961723.1|	1640561-1641654	20	9.8
ACYPI000955-RA	ni|645904005|nb|KK920426.1|	657821-658181	17	16	gi|646780978|gb|KK961534.1|	1853405-1853690	20	8
ACYPI000961-RA	ni|645903746|nb|KK920684.1|	834812-835345	15	15	gi|646781421|gb|KK961521.1|	918407-918938	21	8.7
ACYPI000965-RA	ni|645902083|nb|KK922346.1|	21095-21346	nan	nan	gi|646776219|gb|KK961687.1|	2698894-2700034	21	9.9
ACYPI000969-RA	ni|645904127|nb|KK920306.1|	343546-343910	15	15	gi|646749755|gb|KK962075.1|	1186925-1203231	22	9.2
ACYPI000979-RA	ni|645903748|nb|KK920682.1|	527271-527526	17	18	gi|646782357|gb|KK961494.1|	5102553-5104237	21	9.2
ACYPI000980-RA	ni|645903597|nb|KK920833.1|	440500-441096	nan	nan	gi|646712812|gb|KK965357.1|	50551-51065	21	9.9
ACYPI000992-RA	ni|645904130|nb|KK920303.1|	737556-738080	18	17	gi|646780270|gb|KK961553.1|	2211583-2212046	22	8.6
ACYPI000994-RA	ni|645903740|nb|KK920690.1|	354203-355126	18	10	gi|646769275|gb|KK961796.1|	726234-733161	22	5.6
ACYPI000998-RA	ni|645904116|nb|KK920315.1|	878343-881037	19	18	gi|646779898|gb|KK961562.1|	488790-491724	19	9.3
ACYPI001001-RA	ni|645903882|nb|KK920549.1|	670516-671469	16	16	gi|646751103|gb|KK962006.1|	353214-355919	20	8.3
ACYPI001003-RA	ni|645904268|nb|KK920225.1|	305124-305324	14	15	gi|646781212|gb|KK961527.1|	2191133-2192019	21	10
ACYPI001007-RA	ni|645904097|nb|KK920334.1|	243359-243893	15	17	gi|646740756|gb|KK962735.1|	93490-104906	23	10
ACYPI001010-RA	ni|645903592|nb|KK920838.1|	928972-929106	nan	nan	gi|646749188|gb|KK962105.1|	306847-311013	18	7.9
ACYPI001011-RA	ni|645903724|nb|KK920706.1|	255608-256096	nan	nan	gi|646768163|gb|KK961817.1|	154107-154988	20	7.6
ACYPI001012-RA	ni|645901733|nb|KK922696.1|	16211-18451	nan	nan	gi|646742343|gb|KK962588.1|	275106-283598	20	9.4
ACYPI001013-RA	ni|645902438|nb|KK921991.1|	30245-30651	nan	nan	gi|646782009|gb|KK961503.1|	2443524-2444005	15	7.5
ACYPI001015-RA	ni|645904004|nb|KK920427.1|	1136898-1137804	16	16	gi|646781443|gb|KK961520.1|	1882162-1882595	20	8.3
ACYPI001018-RA	ni|645904092|nb|KK920339.1|	77546-78078	15	15	gi|646763535|gb|KK961902.1|	841528-843534	23	10
ACYPI001019-RA	ni|645903673|nb|KK920757.1|	304883-309521	nan	nan	gi|646736246|gb|KK963207.1|	338857-341900	19	8.5
ACYPI001024-RA	ni|645903915|nb|KK920516.1|	1437030-1437551	17	17	gi|646767813|gb|KK961824.1|	309405-309629	17	6.3
ACYPI001025-RA	ni|645904051|nb|KK920380.1|	503984-507038	16	17	gi|646736283|gb|KK963203.1|	331952-336414	24	9.7
ACYPI001030-RA	ni|645903934|nb|KK920497.1|	244198-244576	15	16	gi|646766482|gb|KK961860.1|	547849-549145	17	6.7
ACYPI001031-RA	ni|645904246|nb|KK920233.1|	1110042-1110497	16	17	gi|646780222|gb|KK961554.1|	200954-203281	20	9.7
ACYPI001032-RA	ni|645903644|nb|KK920786.1|	737405-737898	nan	nan	gi|646782288|gb|KK961496.1|	6978452-6980594	21	9.7
ACYPI001039-RA	ni|645904281|nb|KK920220.1|	1013060-1013322	15	16	gi|646775723|gb|KK961712.1|	1499360-1499690	21	9.5
ACYPI001042-RA	ni|645902373|nb|KK922056.1|	185613-186176	nan	nan	gi|646776024|gb|KK961697.1|	2453043-2453929	22	9.2
ACYPI001043-RA	ni|645902354|nb|KK922075.1|	45603-45806	nan	nan	gi|646775765|gb|KK961710.1|	1866358-1867038	21	6
ACYPI001044-RA	ni|645903515|nb|KK920915.1|	733824-734944	nan	nan	gi|646778865|gb|KK961589.1|	1075002-1076615	20	9.2
ACYPI001047-RA	ni|645904116|nb|KK920315.1|	466094-466760	19	18	gi|646782168|gb|KK961499.1|	7346412-7347822	21	9.4

ACYPI001051-RA	ni|645904013|nb|KK920418.1|	1161200-1161599	19	18	gi|646770998|gb|KK961772.1|	711159-711623	22	9.8
ACYPI001052-RA	ni|645903511|nb|KK920919.1|	163280-163457	nan	nan	gi|646743772|gb|KK962481.1|	773352-774243	22	8.9
ACYPI001056-RA	ni|645903884|nb|KK920547.1|	400219-400459	16	19	gi|646782357|gb|KK961494.1|	9519081-9523191	21	9.2
ACYPI001057-RA	ni|645903644|nb|KK920786.1|	188924-189137	nan	nan	gi|646776219|gb|KK961687.1|	2596331-2597878	21	9.9
ACYPI001059-RA	ni|645904015|nb|KK920416.1|	187496-193131	18	16	gi|646758845|gb|KK961921.1|	1390254-1396147	22	9.5
ACYPI001061-RA	ni|645904241|nb|KK920238.1|	154535-154849	18	17	gi|646745327|gb|KK962367.1|	51411-51650	18	8.3
ACYPI001071-RA	ni|645903749|nb|KK920681.1|	180990-181603	19	18	gi|646760564|gb|KK961914.1|	16075-16612	21	8.8
ACYPI001079-RA	ni|645903829|nb|KK920601.1|	81478-81647	16	15	gi|646781893|gb|KK961506.1|	397198-398622	19	8.5
ACYPI001081-RA	ni|645903557|nb|KK920873.1|	1071122-1071677	nan	nan	gi|646751289|gb|KK961996.1|	851263-852771	24	9.1
ACYPI001085-RA	ni|645904118|nb|KK920313.1|	733371-734277	17	17	gi|646780311|gb|KK961552.1|	2264830-2265753	21	9.7
ACYPI001088-RA	ni|645903861|nb|KK920569.1|	322258-322457	16	17	gi|646780658|gb|KK961544.1|	1571160-1572651	19	8.6
ACYPI001090-RA	ni|645904153|nb|KK920280.1|	1494710-1495060	17	16	gi|646728609|gb|KK964184.1|	229889-235318	34	14
ACYPI001096-RA	ni|645903597|nb|KK920833.1|	929086-929543	nan	nan	gi|646769102|gb|KK961799.1|	1906841-1907065	20	8.3
ACYPI001106-RA	ni|645903504|nb|KK920926.1|	274425-274907	nan	nan	gi|646742413|gb|KK962582.1|	112826-113426	23	10
ACYPI001107-RA	ni|645903903|nb|KK920528.1|	308925-309243	12	15	gi|646744686|gb|KK962414.1|	471483-472831	12	7.3
ACYPI001110-RA	ni|645904231|nb|KK920242.1|	456319-456444	16	17	gi|646781849|gb|KK961508.1|	2570548-2571249	17	8
ACYPI001111-RA	ni|645903936|nb|KK920495.1|	478645-478975	19	18	gi|646782043|gb|KK961502.1|	1074406-1074590	20	9.2
ACYPI001113-RA	ni|645903530|nb|KK920900.1|	523489-523650	nan	nan	gi|646781732|gb|KK961511.1|	3881635-3887993	22	9.8
ACYPI001119-RA	ni|645902639|nb|KK921790.1|	162983-163480	nan	nan	gi|646745091|gb|KK962384.1|	517591-518107	19	7.9
ACYPI001120-RA	ni|645903503|nb|KK920927.1|	37321-38592	nan	nan	gi|646747884|gb|KK962186.1|	744594-745699	22	11
ACYPI001124-RA	ni|645904133|nb|KK920300.1|	379734-380311	18	18	gi|646765705|gb|KK961893.1|	1666052-1674499	22	9.7
ACYPI001125-RA	ni|645901914|nb|KK922515.1|	54583-55440	nan	nan	gi|646781564|gb|KK961516.1|	285738-290292	23	9.4
ACYPI001129-RA	ni|645904159|nb|KK920274.1|	240197-240382	15	17	gi|646776855|gb|KK961656.1|	377398-378630	19	8.8
ACYPI001145-RA	ni|645903662|nb|KK920768.1|	400678-400881	nan	nan	gi|646754371|gb|KK961945.1|	560961-561596	20	9.7
ACYPI001146-RA	ni|645903800|nb|KK920630.1|	1043669-1044647	18	17	gi|646776024|gb|KK961697.1|	1100000-1100423	22	9.2
ACYPI001153-RA	ni|645902718|nb|KK921711.1|	126947-127213	nan	nan	gi|646780827|gb|KK961539.1|	3273867-3274228	20	9.4
ACYPI001166-RA	ni|645903841|nb|KK920589.1|	1018358-1019195	17	17	gi|646750043|gb|KK962060.1|	585234-585667	21	8.3
ACYPI001167-RA	ni|645904137|nb|KK920296.1|	412930-414139	17	17	gi|646781732|gb|KK961511.1|	646438-646709	22	9.8
ACYPI001168-RA	ni|645903825|nb|KK920605.1|	1008582-1009330	19	17	gi|646747627|gb|KK962204.1|	333129-334935	18	7.5
ACYPI001179-RA	ni|645904155|nb|KK920278.1|	1060648-1061012	18	9.4	gi|646780311|gb|KK961552.1|	2662080-2663106	21	9.7

ACYPI001182-RA	ni|645903716|nb|KK920714.1|	905157-905464	nan	nan	gi|646777698|gb|KK961622.1|	392713-393351	16	7.6
ACYPI001189-RA	ni|645904038|nb|KK920393.1|	489586-489742	17	19	gi|646780978|gb|KK961534.1|	604778-605595	20	8
ACYPI001193-RA	ni|645904028|nb|KK920403.1|	502305-502707	18	17	gi|646750165|gb|KK962053.1|	198910-199652	25	10
ACYPI001194-RA	ni|645904022|nb|KK920409.1|	635284-636006	18	20	gi|646777665|gb|KK961623.1|	1899629-1900013	21	9.7
ACYPI001206-RA	ni|645903680|nb|KK920750.1|	1007743-1008436	nan	nan	gi|646777416|gb|KK961632.1|	1542675-1542970	22	8.8
ACYPI001212-RA	ni|645904281|nb|KK920220.1|	992431-993452	15	16	gi|646775723|gb|KK961712.1|	1678105-1678926	21	9.5
ACYPI001217-RA	ni|645903780|nb|KK920650.1|	756372-757114	19	19	gi|646613436|gb|KK973865.1|	6907-7589	21	7.7
ACYPI001218-RA	ni|645904240|nb|KK920239.1|	1775246-1777473	16	16	gi|646777765|gb|KK961620.1|	1001671-1008255	21	8.7
ACYPI001220-RA	ni|645903635|nb|KK920795.1|	410452-411276	nan	nan	gi|646768631|gb|KK961807.1|	835255-836784	19	9.2
ACYPI001225-RA	ni|645904231|nb|KK920242.1|	90786-93848	16	17	gi|646749964|gb|KK962064.1|	341009-342299	18	8.7
ACYPI001233-RA	ni|645902060|nb|KK922369.1|	9729-11162	nan	nan	gi|646736855|gb|KK963138.1|	140252-142290	19	9.1
ACYPI001243-RA	ni|645903473|nb|KK920957.1|	540287-540462	nan	nan	gi|646748695|gb|KK962134.1|	215558-215862	20	5.8
ACYPI001257-RA	ni|645904153|nb|KK920280.1|	3205368-3205986	17	16	gi|646748129|gb|KK962169.1|	239978-241152	21	9.8
ACYPI001266-RA	ni|645904077|nb|KK920354.1|	1521562-1521763	16	17	gi|646777991|gb|KK961614.1|	1647059-1647546	18	7.8
ACYPI001268-RA	ni|645904156|nb|KK920277.1|	1363820-1364044	17	8.8	gi|646776514|gb|KK961670.1|	161356-162003	20	9.6
ACYPI001270-RA	ni|645903962|nb|KK920469.1|	550079-550587	17	9.8	gi|646776091|gb|KK961693.1|	2171494-2172124	23	10
ACYPI001274-RA	ni|645903824|nb|KK920606.1|	54539-55657	19	17	gi|646777416|gb|KK961632.1|	373995-374330	22	8.8
ACYPI001277-RA	ni|645902313|nb|KK922116.1|	169757-170835	nan	nan	gi|646772897|gb|KK961753.1|	1531223-1531918	21	8.7
ACYPI001279-RA	ni|645904177|nb|KK920260.1|	986799-987029	18	17	gi|646775867|gb|KK961705.1|	2227489-2229686	23	10
ACYPI001285-RA	ni|645903898|nb|KK920533.1|	489653-490437	17	16	gi|646774530|gb|KK961743.1|	466194-467574	20	9.8
ACYPI001292-RA	ni|645903731|nb|KK920699.1|	1221498-1222392	nan	nan	gi|646782168|gb|KK961499.1|	3534783-3535379	21	9.4
ACYPI001296-RA	ni|645903827|nb|KK920603.1|	233378-235221	17	18	gi|646779337|gb|KK961576.1|	1112189-1113450	18	9
ACYPI001299-RA	ni|645904195|nb|KK920254.1|	2280921-2281400	18	17	gi|646782288|gb|KK961496.1|	4418068-4419222	21	9.7
ACYPI001303-RA	ni|645904183|nb|KK920258.1|	873941-877047	25	35	gi|646782168|gb|KK961499.1|	2213831-2220725	21	9.4
ACYPI001310-RA	ni|645901903|nb|KK922526.1|	48739-49992	nan	nan	gi|646772045|gb|KK961761.1|	628094-628677	21	9.6
ACYPI001311-RA	ni|645904279|nb|KK920221.1|	1740436-1740861	16	16	gi|646749945|gb|KK962065.1|	66961-68201	22	6.1
ACYPI001312-RA	ni|645902035|nb|KK922394.1|	208791-209270	nan	nan	gi|646758935|gb|KK961920.1|	123408-123652	20	8.5
ACYPI001316-RA	ni|645904011|nb|KK920420.1|	171865-172077	14	16	gi|646750889|gb|KK962017.1|	820863-825686	22	6.1
ACYPI001329-RA	ni|645903727|nb|KK920703.1|	260071-260482	nan	nan	gi|646733173|gb|KK963571.1|	2768-3167	19	9
ACYPI001336-RA	ni|645901664|nb|KK922765.1|	52325-53615	nan	nan	gi|646769832|gb|KK961787.1|	381980-385374	15	9.8

ACYPI001339-RA	ni|645904118|nb|KK920313.1|	759127-759934	17	17	gi|646775723|gb|KK961712.1|	1181440-1181891	21	9.5
ACYPI001342-RA	ni|645904166|nb|KK920267.1|	3136802-3137034	18	16	gi|646767439|gb|KK961832.1|	1041067-1042130	23	10
ACYPI001352-RA	ni|645902016|nb|KK922413.1|	79165-79360	nan	nan	gi|646750600|gb|KK962030.1|	1468306-1469781	23	8.6
ACYPI001353-RA	ni|645904034|nb|KK920397.1|	361875-363220	15	8.4	gi|646778517|gb|KK961599.1|	840521-842059	20	9.6
ACYPI001354-RA	ni|645903681|nb|KK920749.1|	216850-217555	nan	nan	gi|646775595|gb|KK961718.1|	1768330-1768610	21	8.9
ACYPI001356-RA	ni|645903634|nb|KK920796.1|	1113157-1113379	nan	nan	gi|646776219|gb|KK961687.1|	1973493-1973897	21	9.9
ACYPI001359-RA	ni|645903654|nb|KK920776.1|	1200-2169	nan	nan	gi|646780441|gb|KK961549.1|	3102433-3103591	21	9.5
ACYPI001360-RA	ni|645904071|nb|KK920360.1|	730063-731046	17	17	gi|646782168|gb|KK961499.1|	5435547-5436761	21	9.4
ACYPI001372-RA	ni|645903765|nb|KK920665.1|	559097-560306	17	15	gi|646697572|gb|KK966202.1|	123171-123396	21	5.6
ACYPI001375-RA	ni|645903836|nb|KK920594.1|	270253-270424	18	11	gi|646777340|gb|KK961636.1|	1035157-1035509	18	10
ACYPI001378-RA	ni|645903752|nb|KK920678.1|	620577-620899	18	16	gi|646776219|gb|KK961687.1|	457983-458314	21	9.9
ACYPI001379-RA	ni|645903922|nb|KK920509.1|	463448-463682	15	16	gi|646738604|gb|KK962960.1|	159271-161115	19	8.4
ACYPI001382-RA	ni|645903808|nb|KK920622.1|	617187-617566	17	17	gi|646735640|gb|KK963276.1|	51221-51556	18	6.9
ACYPI001387-RA	ni|645903901|nb|KK920530.1|	683242-684526	17	17	gi|646779898|gb|KK961562.1|	3103134-3105117	19	9.3
ACYPI001392-RA	ni|645904074|nb|KK920357.1|	230139-230496	17	18	gi|646781243|gb|KK961526.1|	2012596-2013384	21	8.9
ACYPI001396-RA	ni|645904258|nb|KK920229.1|	1280567-1281094	16	16	gi|646764075|gb|KK961900.1|	652769-656504	18	6.5
ACYPI001400-RA	ni|645904157|nb|KK920276.1|	42098-42737	15	15	gi|646767381|gb|KK961833.1|	460353-461331	18	6.8
ACYPI001403-RA	ni|645904166|nb|KK920267.1|	3049215-3049420	18	16	gi|646782043|gb|KK961502.1|	4563367-4563783	20	9.2
ACYPI001405-RA	ni|645903518|nb|KK920912.1|	195769-196427	nan	nan	gi|646780627|gb|KK961545.1|	1261614-1262532	20	7.7
ACYPI001408-RA	ni|645903637|nb|KK920793.1|	685066-685738	nan	nan	gi|646749532|gb|KK962087.1|	209132-209467	14	6
ACYPI001415-RA	ni|645904268|nb|KK920225.1|	985511-986014	14	15	gi|646751160|gb|KK962003.1|	442667-443644	22	10
ACYPI001416-RA	ni|645903762|nb|KK920668.1|	130978-131264	17	17	gi|646780574|gb|KK961546.1|	3529070-3538977	20	9.9
ACYPI001423-RA	ni|645903846|nb|KK920584.1|	736311-736504	17	10	gi|646747841|gb|KK962189.1|	663689-665663	23	10
ACYPI001424-RA	ni|645904174|nb|KK920261.1|	1852450-1852684	19	17	gi|646749051|gb|KK962113.1|	444541-445261	19	8.4
ACYPI001430-RA	ni|645904017|nb|KK920414.1|	95643-96303	17	17	gi|646732855|gb|KK963616.1|	148516-149839	24	7.1
ACYPI001434-RA	ni|645904133|nb|KK920300.1|	1034586-1035086	18	18	gi|646780978|gb|KK961534.1|	2409142-2409645	20	8
ACYPI001440-RA	ni|645903607|nb|KK920823.1|	314259-315418	nan	nan	gi|646777313|gb|KK961637.1|	726388-729786	20	8.2
ACYPI001446-RA	ni|645903909|nb|KK920522.1|	247175-247502	16	8.3	gi|646779375|gb|KK961575.1|	1654209-1654884	22	9.3
ACYPI001453-RA	ni|645903787|nb|KK920643.1|	489918-490969	19	18	gi|646744699|gb|KK962413.1|	16934-17480	14	6.9
ACYPI001458-RA	ni|645904150|nb|KK920283.1|	697879-699399	17	17	gi|646777207|gb|KK961641.1|	1368135-1368890	20	8.5

ACYPI001461-RA	ni|645904149|nb|KK920284.1|	216961-217463	16	16	gi|646780858|gb|KK961538.1|	3326329-3330800	21	10
ACYPI001463-RA	ni|645903727|nb|KK920703.1|	1101176-1101700	nan	nan	gi|646611448|gb|KK974227.1|	13621-13915	16	5.4
ACYPI001465-RA	ni|645903899|nb|KK920532.1|	443478-444036	15	16	gi|646781043|gb|KK961532.1|	1767270-1769326	26	11
ACYPI001475-RA	ni|645903834|nb|KK920596.1|	127573-128058	18	17	gi|646775488|gb|KK961724.1|	821706-822264	19	7.8
ACYPI001480-RA	ni|645904262|nb|KK920227.1|	2432336-2433221	17	17	gi|646776280|gb|KK961684.1|	823608-824198	22	9.3
ACYPI001481-RA	ni|645902642|nb|KK921787.1|	194127-194696	nan	nan	gi|646775807|gb|KK961708.1|	56287-56643	21	8.6
ACYPI001483-RA	ni|645904115|nb|KK920316.1|	1772375-1773141	17	18	gi|646747614|gb|KK962205.1|	738370-742198	21	8.9
ACYPI001487-RA	ni|645904254|nb|KK920230.1|	478767-478925	16	16	gi|646770323|gb|KK961780.1|	555078-555719	19	7.1
ACYPI001490-RA	ni|645903822|nb|KK920608.1|	144790-145265	18	20	gi|646749209|gb|KK962104.1|	631361-632166	21	9.2
ACYPI001502-RA	ni|645903863|nb|KK920567.1|	155734-156144	15	9.2	gi|646746651|gb|KK962274.1|	19285-19984	16	6.6
ACYPI001507-RA	ni|645904262|nb|KK920227.1|	667170-667442	17	17	gi|646766985|gb|KK961844.1|	215457-221179	21	9.7
ACYPI001508-RA	ni|645903890|nb|KK920541.1|	58112-58820	17	18	gi|646778948|gb|KK961587.1|	1870106-1870370	20	7.3
ACYPI001509-RA	ni|645904195|nb|KK920254.1|	652880-654017	18	17	gi|646767156|gb|KK961839.1|	323018-325650	15	6.4
ACYPI001511-RA	ni|645902373|nb|KK922056.1|	194351-196978	nan	nan	gi|646766985|gb|KK961844.1|	296011-309627	21	9.7
ACYPI001515-RA	ni|645904114|nb|KK920317.1|	1637996-1639227	18	18	gi|646777665|gb|KK961623.1|	2189050-2190036	21	9.7
ACYPI001527-RA	ni|645901875|nb|KK922554.1|	41241-41460	nan	nan	gi|646782288|gb|KK961496.1|	5994411-5994695	21	9.7
ACYPI001539-RA	ni|645904090|nb|KK920341.1|	254208-255472	15	17	gi|646748580|gb|KK962141.1|	217486-217815	20	5.6
ACYPI001540-RA	ni|645902671|nb|KK921758.1|	70902-71368	nan	nan	gi|646752294|gb|KK961969.1|	654870-657040	23	9.8
ACYPI001542-RA	ni|645903635|nb|KK920795.1|	432848-433167	nan	nan	gi|646770998|gb|KK961772.1|	1126425-1128684	22	9.8
ACYPI001547-RA	ni|645903787|nb|KK920643.1|	476251-476586	19	18	gi|646782357|gb|KK961494.1|	9767936-9768929	21	9.2
ACYPI001560-RA	ni|645904053|nb|KK920378.1|	1114687-1116998	18	17	gi|646777182|gb|KK961642.1|	1452881-1456106	20	9
ACYPI001567-RA	ni|645903973|nb|KK920458.1|	1592954-1594013	20	18	gi|646780858|gb|KK961538.1|	3187395-3189248	21	10
ACYPI001575-RA	ni|645903857|nb|KK920573.1|	251012-251425	16	16	gi|646738812|gb|KK962939.1|	70326-80013	20	8.1
ACYPI001577-RA	ni|645904061|nb|KK920370.1|	34881-35619	17	16	gi|646780530|gb|KK961547.1|	232592-236203	21	9.2
ACYPI001578-RA	ni|645904262|nb|KK920227.1|	337824-338405	17	17	gi|646745372|gb|KK962364.1|	785165-786150	23	10
ACYPI001579-RA	ni|645900333|nb|KK924096.1|	21715-21953	nan	nan	gi|646782357|gb|KK961494.1|	2963857-2964531	21	9.2
ACYPI001584-RA	ni|645903626|nb|KK920804.1|	421339-422422	nan	nan	gi|646776389|gb|KK961678.1|	683114-688070	22	9.5
ACYPI001585-RA	ni|645904225|nb|KK920244.1|	638078-638523	17	17	gi|646778840|gb|KK961590.1|	2503479-2503992	20	9.9
ACYPI001591-RA	ni|645901190|nb|KK923239.1|	15775-15957	nan	nan	gi|646779262|gb|KK961578.1|	729002-729390	20	8.1
ACYPI001593-RA	ni|645904153|nb|KK920280.1|	1495836-1496308	17	16	gi|646728609|gb|KK964184.1|	241138-241372	34	14

ACYPI001596-RA	ni|645903557|nb|KK920873.1|	862467-862681	nan	nan	gi|646763164|gb|KK961904.1|	1034080-1035768	22	10
ACYPI001597-RA	ni|645901319|nb|KK923110.1|	127511-129267	nan	nan	gi|646781659|gb|KK961513.1|	2788555-2790950	22	9.8
ACYPI001600-RA	ni|645902338|nb|KK922091.1|	105712-105924	nan	nan	gi|646749632|gb|KK962082.1|	267316-268320	20	8.6
ACYPI001601-RA	ni|645902659|nb|KK921770.1|	163553-163945	nan	nan	gi|646777182|gb|KK961642.1|	1112524-1112733	20	9
ACYPI001605-RA	ni|645896512|nb|KK927917.1|	992-1347	nan	nan	gi|646742078|gb|KK962611.1|	45317-45880	18	8.1
ACYPI001612-RA	ni|645903551|nb|KK920879.1|	459721-460030	nan	nan	gi|646744957|gb|KK962394.1|	752332-754206	21	9.8
ACYPI001613-RA	ni|645903965|nb|KK920466.1|	1075595-1075976	17	16	gi|646770401|gb|KK961779.1|	741506-742931	21	8.6
ACYPI001614-RA	ni|645904023|nb|KK920408.1|	656810-657813	17	17	gi|646745186|gb|KK962377.1|	937377-938085	21	9.8
ACYPI001617-RA	ni|645902779|nb|KK921650.1|	135035-135804	nan	nan	gi|646768494|gb|KK961810.1|	474833-475486	21	10
ACYPI001622-RA	ni|645903655|nb|KK920775.1|	217072-217635	nan	nan	gi|646781143|gb|KK961529.1|	4291110-4292074	20	10
ACYPI001625-RA	ni|645904042|nb|KK920389.1|	389991-393796	16	16	gi|646746706|gb|KK962270.1|	80951-86147	23	11
ACYPI001631-RA	ni|645904121|nb|KK920310.1|	769101-769364	14	15	gi|646761166|gb|KK961913.1|	309037-309992	17	7.6
ACYPI001633-RA	ni|645904072|nb|KK920359.1|	954035-954609	17	17	gi|646775723|gb|KK961712.1|	1086872-1087662	21	9.5
ACYPI001635-RA	ni|645903970|nb|KK920461.1|	877939-879028	17	16	gi|646780270|gb|KK961553.1|	1835154-1836223	22	8.6
ACYPI001643-RA	ni|645903994|nb|KK920437.1|	232683-233854	15	20	gi|646779826|gb|KK961564.1|	2794028-2794970	22	9.6
ACYPI001646-RA	ni|645903687|nb|KK920743.1|	43586-44176	nan	nan	gi|646781118|gb|KK961530.1|	186752-190040	21	10
ACYPI001649-RA	ni|645904058|nb|KK920373.1|	1047806-1048780	16	15	gi|646753527|gb|KK961953.1|	1457880-1458355	24	9.2
ACYPI001652-RA	ni|645904158|nb|KK920275.1|	1166500-1166931	15	8.3	gi|646750389|gb|KK962041.1|	598521-602195	21	9.5
ACYPI001665-RA	ni|645903466|nb|KK920964.1|	694000-694399	nan	nan	gi|646777802|gb|KK961619.1|	1451621-1453226	24	9
ACYPI001667-RA	ni|645904014|nb|KK920417.1|	1330327-1330589	19	18	gi|646781628|gb|KK961514.1|	2292044-2293417	23	9
ACYPI001668-RA	ni|645903847|nb|KK920583.1|	645752-646613	18	18	gi|646778632|gb|KK961596.1|	1957857-1958420	21	9.2
ACYPI001672-RA	ni|645904245|nb|KK920234.1|	1316654-1317159	16	17	gi|646770477|gb|KK961778.1|	921163-921707	21	9.1
ACYPI001674-RA	ni|645903849|nb|KK920581.1|	317182-317666	17	17	gi|646778699|gb|KK961594.1|	1585114-1585806	21	9.5
ACYPI001675-RA	ni|645903783|nb|KK920647.1|	1524293-1525615	17	17	gi|646744579|gb|KK962422.1|	353613-363276	22	9.9
ACYPI001679-RA	ni|645904048|nb|KK920383.1|	628165-628713	14	14	gi|646779375|gb|KK961575.1|	2294973-2295574	22	9.3
ACYPI001683-RA	ni|645903704|nb|KK920726.1|	404991-405503	nan	nan	gi|646778983|gb|KK961586.1|	242281-243091	20	9.9
ACYPI001686-RA	ni|645904125|nb|KK920307.1|	1973333-1973838	18	17	gi|646746706|gb|KK962270.1|	254576-255499	23	11
ACYPI001692-RA	ni|645903655|nb|KK920775.1|	267027-267260	nan	nan	gi|646781282|gb|KK961525.1|	3671061-3676017	22	9.6
ACYPI001696-RA	ni|645903975|nb|KK920456.1|	1049735-1051325	15	8.2	gi|646766179|gb|KK961872.1|	413458-416916	18	8.2
ACYPI001698-RA	ni|645903727|nb|KK920703.1|	242818-243047	nan	nan	gi|646781536|gb|KK961517.1|	1890071-1895298	19	7.7

ACYPI001704-RA	ni|645904271|nb|KK920224.1|	467005-467470	15	16	gi|646741719|gb|KK962642.1|	94145-94505	21	8.9
ACYPI001706-RA	ni|645904241|nb|KK920238.1|	1387883-1388465	18	17	gi|646780311|gb|KK961552.1|	3890551-3890711	21	9.7
ACYPI001710-RA	ni|645903763|nb|KK920667.1|	69903-70148	26	21	gi|646777182|gb|KK961642.1|	2323435-2330458	20	9
ACYPI001711-RA	ni|645904122|nb|KK920309.1|	1337616-1338574	18	17	gi|646776113|gb|KK961692.1|	589980-591557	21	9.6
ACYPI001724-RA	ni|645903569|nb|KK920861.1|	219433-219752	nan	nan	gi|646732968|gb|KK963602.1|	82536-83641	20	9.9
ACYPI001730-RA	ni|645903885|nb|KK920546.1|	845631-846212	19	12	gi|646781968|gb|KK961504.1|	2300175-2306420	20	9.6
ACYPI001736-RA	ni|645903578|nb|KK920852.1|	203808-205276	nan	nan	gi|646738604|gb|KK962960.1|	28697-32181	19	8.4
ACYPI001742-RA	ni|645903698|nb|KK920732.1|	158467-158767	nan	nan	gi|646770901|gb|KK961773.1|	1029991-1031304	22	9.7
ACYPI001746-RA	ni|645903803|nb|KK920627.1|	887915-889665	17	17	gi|646766451|gb|KK961861.1|	109275-110647	19	8.9
ACYPI001747-RA	ni|645902683|nb|KK921746.1|	70454-70841	nan	nan	gi|646743690|gb|KK962487.1|	697385-697815	20	10
ACYPI001755-RA	ni|645903702|nb|KK920728.1|	421398-422211	nan	nan	gi|646769577|gb|KK961791.1|	904027-904515	18	8.3
ACYPI001756-RA	ni|645902394|nb|KK922035.1|	300175-301057	nan	nan	gi|646778274|gb|KK961606.1|	879451-880017	18	7.5
ACYPI001759-RA	ni|645902468|nb|KK921961.1|	70634-70897	nan	nan	gi|646782357|gb|KK961494.1|	9010681-9011324	21	9.2
ACYPI001760-RA	ni|645904279|nb|KK920221.1|	303514-303727	16	16	gi|646745543|gb|KK962350.1|	745218-745444	21	10
ACYPI001764-RA	ni|645903969|nb|KK920462.1|	266128-266480	14	16	gi|646748002|gb|KK962178.1|	1601285-1602167	22	6.3
ACYPI001765-RA	ni|645903957|nb|KK920474.1|	1047815-1048656	18	17	gi|646740158|gb|KK962805.1|	312127-312829	24	6.5
ACYPI001769-RA	ni|645903627|nb|KK920803.1|	365807-366954	nan	nan	gi|646777570|gb|KK961626.1|	913822-914047	18	7.5
ACYPI001776-RA	ni|645903955|nb|KK920476.1|	514726-514963	18	17	gi|646639169|gb|KK969278.1|	3857-4665	20	7.8
ACYPI001777-RA	ni|645903650|nb|KK920780.1|	174435-174913	nan	nan	gi|646747459|gb|KK962217.1|	874270-880389	21	5.9
ACYPI001780-RA	ni|645904195|nb|KK920254.1|	370779-371520	18	17	gi|646733583|gb|KK963518.1|	57806-60561	20	9.2
ACYPI001782-RA	ni|645904088|nb|KK920343.1|	1280653-1280952	17	18	gi|646778410|gb|KK961602.1|	2739334-2740988	20	9
ACYPI001796-RA	ni|645903557|nb|KK920873.1|	1093184-1094901	nan	nan	gi|646763164|gb|KK961904.1|	551273-553813	22	10
ACYPI001797-RA	ni|645904041|nb|KK920390.1|	136452-138459	20	33	gi|646735017|gb|KK963352.1|	323473-324256	22	5.9
ACYPI001804-RA	ni|645904106|nb|KK920325.1|	774579-776900	17	17	gi|646563700|gb|KK983468.1|	416-594	7	5.6
ACYPI001807-RA	ni|645904053|nb|KK920378.1|	1862598-1863287	18	17	gi|646781659|gb|KK961513.1|	5267786-5268047	22	9.8
ACYPI001810-RA	ni|645903858|nb|KK920572.1|	415604-416129	16	16	gi|646781118|gb|KK961530.1|	5301918-5302631	21	10
ACYPI001813-RA	ni|645902756|nb|KK921673.1|	36146-36726	nan	nan	gi|646782357|gb|KK961494.1|	2093145-2094261	21	9.2
ACYPI001816-RA	ni|645903965|nb|KK920466.1|	263403-264022	17	16	gi|646780978|gb|KK961534.1|	574167-575220	20	8
ACYPI001818-RA	ni|645904045|nb|KK920386.1|	829472-829874	19	21	gi|646778865|gb|KK961589.1|	3062185-3062906	20	9.2
ACYPI001827-RA	ni|645904195|nb|KK920254.1|	673514-673800	18	17	gi|646780441|gb|KK961549.1|	2099232-2099803	21	9.5

ACYPI001832-RA	ni|645903716|nb|KK920714.1|	373150-373390	nan	nan	gi|646749512|gb|KK962088.1|	113199-115185	18	7.2
ACYPI001835-RA	ni|645904064|nb|KK920367.1|	159366-159610	17	9.9	gi|646780925|gb|KK961536.1|	1580277-1581489	22	9.9
ACYPI001838-RA	ni|645903866|nb|KK920564.1|	353378-354905	18	15	gi|646772897|gb|KK961753.1|	1270505-1271903	21	8.7
ACYPI001849-RA	ni|645904149|nb|KK920284.1|	462643-463120	16	16	gi|646746942|gb|KK962254.1|	1029902-1030605	23	9.8
ACYPI001850-RA	ni|645903921|nb|KK920510.1|	579588-579948	16	11	gi|646776184|gb|KK961688.1|	1192707-1192950	21	9.6
ACYPI001856-RA	ni|645904271|nb|KK920224.1|	2044972-2045379	15	16	gi|646776542|gb|KK961668.1|	2151705-2152568	20	5.6
ACYPI001864-RA	ni|645902463|nb|KK921966.1|	60976-61400	nan	nan	gi|646775807|gb|KK961708.1|	1998340-2011589	21	8.6
ACYPI001866-RA	ni|645903900|nb|KK920531.1|	112508-112981	20	30	gi|646776904|gb|KK961654.1|	173568-175042	21	9.3
ACYPI001870-RA	ni|645904064|nb|KK920367.1|	544253-544556	17	9.9	gi|646780574|gb|KK961546.1|	4148912-4153074	20	9.9
ACYPI001871-RA	ni|645903644|nb|KK920786.1|	170154-170877	nan	nan	gi|646767120|gb|KK961840.1|	1179020-1179365	20	8.6
ACYPI001872-RA	ni|645902238|nb|KK922191.1|	50345-50581	nan	nan	gi|646762191|gb|KK961910.1|	388628-389029	18	7.1
ACYPI001877-RA	ni|645904177|nb|KK920260.1|	1634882-1635268	18	17	gi|646775807|gb|KK961708.1|	1421485-1422075	21	8.6
ACYPI001885-RA	ni|645904130|nb|KK920303.1|	1432601-1433229	18	17	gi|646781421|gb|KK961521.1|	4090387-4096150	21	8.7
ACYPI001894-RA	ni|645902052|nb|KK922377.1|	4879-6196	nan	nan	gi|646782043|gb|KK961502.1|	4014346-4016928	20	9.2
ACYPI001895-RA	ni|645904116|nb|KK920315.1|	2344501-2344902	19	18	gi|646770047|gb|KK961784.1|	348407-349322	21	9.1
ACYPI001898-RA	ni|645903954|nb|KK920477.1|	238354-238779	35	44	gi|646769163|gb|KK961798.1|	1340069-1340325	20	9.2
ACYPI001901-RA	ni|645903780|nb|KK920650.1|	472506-473049	19	19	gi|646747682|gb|KK962200.1|	439756-440162	19	7.1
ACYPI001906-RA	ni|645904130|nb|KK920303.1|	364199-364409	18	17	gi|646746390|gb|KK962292.1|	381151-381395	18	6.4
ACYPI001907-RA	ni|645903821|nb|KK920609.1|	759290-762141	17	17	gi|646781421|gb|KK961521.1|	4569597-4570708	21	8.7
ACYPI001909-RA	ni|645904177|nb|KK920260.1|	1151321-1151755	18	17	gi|646780978|gb|KK961534.1|	537860-538428	20	8
ACYPI001917-RA	ni|645904153|nb|KK920280.1|	3194921-3195137	17	16	gi|646748129|gb|KK962169.1|	252266-253520	21	9.8
ACYPI001931-RA	ni|645903938|nb|KK920493.1|	532281-532615	19	18	gi|646737056|gb|KK963115.1|	328206-330142	24	11
ACYPI001932-RA	ni|645903591|nb|KK920839.1|	164055-165291	nan	nan	gi|646779066|gb|KK961583.1|	1089073-1093749	19	7.9
ACYPI001933-RA	ni|645903466|nb|KK920964.1|	261928-262349	nan	nan	gi|646781628|gb|KK961514.1|	2070931-2071637	23	9
ACYPI001939-RA	ni|645904106|nb|KK920325.1|	927454-929067	17	17	gi|646748695|gb|KK962134.1|	130410-130715	20	5.8
ACYPI001943-RA	ni|645903567|nb|KK920863.1|	342222-342440	nan	nan	gi|646666173|gb|KK967831.1|	1079-1592	17	6
ACYPI001946-RA	ni|645904156|nb|KK920277.1|	768602-769384	17	8.8	gi|646776351|gb|KK961680.1|	2732884-2738800	22	10
ACYPI001957-RA	ni|645904067|nb|KK920364.1|	240108-240759	17	14	gi|646782127|gb|KK961500.1|	1513451-1514131	22	9.6
ACYPI001969-RA	ni|645903800|nb|KK920630.1|	201061-201780	18	17	gi|646775821|gb|KK961707.1|	480823-481671	23	9.1
ACYPI001971-RA	ni|645904144|nb|KK920289.1|	915192-916007	16	9	gi|646768534|gb|KK961809.1|	80081-80507	21	9.8

ACYPI001975-RA	ni|645903854|nb|KK920576.1|	378827-379480	16	8.4	gi|646749832|gb|KK962071.1|	571544-571896	22	6
ACYPI001978-RA	ni|645902219|nb|KK922210.1|	189865-191931	nan	nan	gi|646780858|gb|KK961538.1|	2482212-2492121	21	10
ACYPI002006-RA	ni|645903570|nb|KK920860.1|	23627-24169	nan	nan	gi|646777665|gb|KK961623.1|	764968-766748	21	9.7
ACYPI002009-RA	ni|645903747|nb|KK920683.1|	1337315-1338364	19	18	gi|646776024|gb|KK961697.1|	1716286-1718895	22	9.2
ACYPI002010-RA	ni|645903530|nb|KK920900.1|	410134-412502	nan	nan	gi|646780222|gb|KK961554.1|	3556937-3561404	20	9.7
ACYPI002023-RA	ni|645903887|nb|KK920544.1|	95258-96858	15	15	gi|646778736|gb|KK961593.1|	246752-248137	20	7.9
ACYPI002031-RA	ni|645904201|nb|KK920252.1|	384449-385682	17	16	gi|646766703|gb|KK961852.1|	426083-433431	20	7.1
ACYPI002036-RA	ni|645904204|nb|KK920251.1|	590620-590963	15	16	gi|646765790|gb|KK961889.1|	1139133-1139597	22	8.8
ACYPI002040-RA	ni|645904245|nb|KK920234.1|	507990-508183	16	17	gi|646732591|gb|KK963652.1|	829-1456	17	7.3
ACYPI002041-RA	ni|645904107|nb|KK920324.1|	952646-953072	17	9.5	gi|646780723|gb|KK961542.1|	2087042-2087325	21	9.4
ACYPI002045-RA	ni|645904228|nb|KK920243.1|	1269442-1270971	17	18	gi|646748044|gb|KK962175.1|	489317-490870	19	7.2
ACYPI002046-RA	ni|645903644|nb|KK920786.1|	897790-898036	nan	nan	gi|646776351|gb|KK961680.1|	1012093-1016938	22	10
ACYPI002053-RA	ni|645904160|nb|KK920273.1|	2149462-2149864	18	18	gi|646778336|gb|KK961604.1|	135008-135899	21	10
ACYPI002063-RA	ni|645903885|nb|KK920546.1|	104521-104759	19	12	gi|646743341|gb|KK962511.1|	304597-306994	19	5
ACYPI002072-RA	ni|645903617|nb|KK920813.1|	178440-179694	nan	nan	gi|646775471|gb|KK961725.1|	1366350-1369116	20	10
ACYPI002085-RA	ni|645903783|nb|KK920647.1|	1185366-1185682	17	17	gi|646782168|gb|KK961499.1|	4967801-4968081	21	9.4
ACYPI002088-RA	ni|645904219|nb|KK920246.1|	342533-342770	16	17	gi|646780827|gb|KK961539.1|	3053298-3053810	20	9.4
ACYPI002090-RA	ni|645903750|nb|KK920680.1|	486226-486751	14	14	gi|646742553|gb|KK962571.1|	233499-234167	20	8.4
ACYPI002098-RA	ni|645904246|nb|KK920233.1|	911766-912520	16	17	gi|646743690|gb|KK962487.1|	21568-23753	20	10
ACYPI002108-RA	ni|645903637|nb|KK920793.1|	540911-541464	nan	nan	gi|646544887|gb|KK987173.1|	256-865	14	6
ACYPI002110-RA	ni|645903634|nb|KK920796.1|	1217326-1217924	nan	nan	gi|646741767|gb|KK962638.1|	332202-334645	21	10
ACYPI002115-RA	ni|645903768|nb|KK920662.1|	1276766-1277523	18	17	gi|646781772|gb|KK961510.1|	3755720-3755980	20	9.2
ACYPI002118-RA	ni|645904076|nb|KK920355.1|	456636-456861	17	17	gi|646757279|gb|KK961927.1|	173582-176071	21	8.6
ACYPI002122-RA	ni|645896935|nb|KK927494.1|	97-939	nan	nan	gi|646752143|gb|KK961972.1|	558684-559089	20	5.6
ACYPI002123-RA	ni|645904068|nb|KK920363.1|	287474-288119	16	8.6	gi|646778903|gb|KK961588.1|	3490396-3493151	23	10
ACYPI002125-RA	ni|645903977|nb|KK920454.1|	264481-265550	13	16	gi|646694101|gb|KK966473.1|	17275-17620	12	6.2
ACYPI002126-RA	ni|645903551|nb|KK920879.1|	486163-486583	nan	nan	gi|646748580|gb|KK962141.1|	389025-389658	20	5.6
ACYPI002129-RA	ni|645903869|nb|KK920561.1|	444223-445565	17	15	gi|646779826|gb|KK961564.1|	3352189-3353287	22	9.6
ACYPI002132-RA	ni|645904021|nb|KK920410.1|	753525-753751	15	16	gi|646754371|gb|KK961945.1|	163236-163760	20	9.7
ACYPI002133-RA	ni|645903953|nb|KK920478.1|	1611738-1613208	18	17	gi|646776069|gb|KK961694.1|	774424-775731	18	7.8

ACYPI002136-RA	ni|645904098|nb|KK920333.1|	1244288-1244519	16	8.8	gi|646747263|gb|KK962232.1|	106956-107147	18	8.6
ACYPI002137-RA	ni|645903920|nb|KK920511.1|	931872-932434	20	20	gi|646762191|gb|KK961910.1|	707040-707591	18	7.1
ACYPI002140-RA	ni|645902758|nb|KK921671.1|	229334-229536	nan	nan	gi|646751515|gb|KK961987.1|	747600-750552	20	9.2
ACYPI002142-RA	ni|645904158|nb|KK920275.1|	1131655-1131937	15	8.3	gi|646750484|gb|KK962036.1|	762810-765627	20	5.5
ACYPI002147-RA	ni|645903613|nb|KK920817.1|	489615-490018	nan	nan	gi|646751160|gb|KK962003.1|	28515-36721	22	10
ACYPI002150-RA	ni|645904225|nb|KK920244.1|	630516-630984	17	17	gi|646756446|gb|KK961931.1|	566450-568116	14	6.1
ACYPI002154-RA	ni|645903825|nb|KK920605.1|	1057642-1058639	19	17	gi|646781443|gb|KK961520.1|	2193431-2194215	20	8.3
ACYPI002157-RA	ni|645903931|nb|KK920500.1|	498842-499745	16	18	gi|646750600|gb|KK962030.1|	952838-960782	23	8.6
ACYPI002162-RA	ni|645903491|nb|KK920939.1|	597383-597625	nan	nan	gi|646777474|gb|KK961630.1|	1317614-1318058	21	9.3
ACYPI002179-RA	ni|645903466|nb|KK920964.1|	287340-288682	nan	nan	gi|646748785|gb|KK962129.1|	563086-564614	20	8.2
ACYPI002180-RA	ni|645904116|nb|KK920315.1|	677401-679525	19	18	gi|646757520|gb|KK961926.1|	1451150-1454207	20	7.4
ACYPI002193-RA	ni|645904201|nb|KK920252.1|	120041-120896	17	16	gi|646732431|gb|KK963675.1|	221939-222720	24	9.6
ACYPI002199-RA	ni|645903936|nb|KK920495.1|	707231-707756	19	18	gi|646733146|gb|KK963575.1|	157915-158896	48	23
ACYPI002207-RA	ni|645903546|nb|KK920884.1|	617805-619089	nan	nan	gi|646746812|gb|KK962263.1|	426345-426857	16	6.1
ACYPI002210-RA	ni|645903720|nb|KK920710.1|	331001-331671	nan	nan	gi|646743492|gb|KK962499.1|	216677-216921	24	11
ACYPI002214-RA	ni|645903965|nb|KK920466.1|	226244-226569	17	16	gi|646775723|gb|KK961712.1|	2818021-2819224	21	9.5
ACYPI002216-RA	ni|645903787|nb|KK920643.1|	443506-443748	19	18	gi|646775807|gb|KK961708.1|	2991718-2992243	21	8.6
ACYPI002220-RA	ni|645902756|nb|KK921673.1|	274233-274678	nan	nan	gi|646776929|gb|KK961653.1|	559623-564080	17	8.5
ACYPI002221-RA	ni|645904204|nb|KK920251.1|	617206-618300	15	16	gi|646661798|gb|KK967966.1|	14154-14471	12	5.3
ACYPI002228-RA	ni|645903625|nb|KK920805.1|	139707-140389	nan	nan	gi|646781849|gb|KK961508.1|	3597630-3599923	17	8
ACYPI002240-RA	ni|645901974|nb|KK922455.1|	9259-9744	nan	nan	gi|646738314|gb|KK962989.1|	177763-178067	20	5.5
ACYPI002245-RA	ni|645904088|nb|KK920343.1|	1235452-1236597	17	18	gi|646747263|gb|KK962232.1|	581179-583917	18	8.6
ACYPI002247-RA	ni|645902172|nb|KK922257.1|	165026-165677	nan	nan	gi|646782334|gb|KK961495.1|	2443819-2444685	21	9
ACYPI002255-RA	ni|645904015|nb|KK920416.1|	1231196-1233296	18	16	gi|646747574|gb|KK962208.1|	342225-343615	27	10
ACYPI002256-RA	ni|645903837|nb|KK920593.1|	390502-390731	18	9.8	gi|646754962|gb|KK961940.1|	622144-622441	21	10
ACYPI002263-RA	ni|645903768|nb|KK920662.1|	1150166-1150736	18	17	gi|646767754|gb|KK961825.1|	63041-63921	20	8.5
ACYPI002264-RA	ni|645903936|nb|KK920495.1|	378527-380198	19	18	gi|646746974|gb|KK962252.1|	899650-904127	23	6.1
ACYPI002267-RA	ni|645903523|nb|KK920907.1|	209214-209723	nan	nan	gi|646740015|gb|KK962820.1|	299758-300400	20	8.4
ACYPI002277-RA	ni|645904132|nb|KK920301.1|	129061-129423	16	18	gi|646745186|gb|KK962377.1|	310796-311591	21	9.8
ACYPI002279-RA	ni|645904118|nb|KK920313.1|	341388-341918	17	17	gi|646737056|gb|KK963115.1|	143574-143943	24	11

ACYPI002286-RA	ni|645904157|nb|KK920276.1|	992108-994731	15	15	gi|646782334|gb|KK961495.1|	4606095-4606597	21	9
ACYPI002287-RA	ni|645904118|nb|KK920313.1|	897231-898115	17	17	gi|646775723|gb|KK961712.1|	2017770-2027262	21	9.5
ACYPI002289-RA	ni|645903566|nb|KK920864.1|	668231-668439	nan	nan	gi|646781243|gb|KK961526.1|	3286934-3287361	21	8.9
ACYPI002292-RA	ni|645903779|nb|KK920651.1|	604892-605170	18	16	gi|646781536|gb|KK961517.1|	1691228-1691490	19	7.7
ACYPI002296-RA	ni|645903944|nb|KK920487.1|	132787-133451	15	17	gi|646776184|gb|KK961688.1|	1862925-1863193	21	9.6
ACYPI002300-RA	ni|645902463|nb|KK921966.1|	262165-262901	nan	nan	gi|646781732|gb|KK961511.1|	2969307-2971241	22	9.8
ACYPI002301-RA	ni|645903796|nb|KK920634.1|	942702-943875	18	17	gi|646781043|gb|KK961532.1|	2294310-2296497	26	11
ACYPI002304-RA	ni|645903532|nb|KK920898.1|	268766-269396	nan	nan	gi|646732431|gb|KK963675.1|	282660-283322	24	9.6
ACYPI002306-RA	ni|645904058|nb|KK920373.1|	614672-615628	16	15	gi|646768717|gb|KK961805.1|	531833-532379	20	8
ACYPI002331-RA	ni|645901697|nb|KK922732.1|	73272-73756	nan	nan	gi|646775821|gb|KK961707.1|	2916713-2917034	23	9.1
ACYPI002332-RA	ni|645903804|nb|KK920626.1|	143434-143796	17	17	gi|646777842|gb|KK961618.1|	2091230-2091867	19	8.9
ACYPI002342-RA	ni|645903724|nb|KK920706.1|	151621-153446	nan	nan	gi|646778767|gb|KK961592.1|	1756085-1757360	21	5.4
ACYPI002345-RA	ni|645903592|nb|KK920838.1|	341159-341340	nan	nan	gi|646764801|gb|KK961898.1|	322441-322718	20	9.7
ACYPI002346-RA	ni|645903914|nb|KK920517.1|	110775-110977	15	17	gi|646776647|gb|KK961663.1|	558292-562116	23	9.2
ACYPI002350-RA	ni|645903983|nb|KK920448.1|	1024961-1025313	18	17	gi|646776456|gb|KK961674.1|	1366641-1367070	18	7.9
ACYPI002352-RA	ni|645899094|nb|KK925335.1|	7387-8369	nan	nan	gi|646741656|gb|KK962648.1|	158226-160455	24	9.7
ACYPI002361-RA	ni|645903703|nb|KK920727.1|	464445-464826	nan	nan	gi|646747884|gb|KK962186.1|	1107153-1108272	22	11
ACYPI002364-RA	ni|645903564|nb|KK920866.1|	320803-321054	nan	nan	gi|646776389|gb|KK961678.1|	1535315-1535897	22	9.5
ACYPI002367-RA	ni|645903619|nb|KK920811.1|	779382-779518	nan	nan	gi|646739361|gb|KK962890.1|	380529-382111	22	9.6
ACYPI002371-RA	ni|645903780|nb|KK920650.1|	594562-594738	19	19	gi|646753093|gb|KK961958.1|	989781-990542	21	9.3
ACYPI002372-RA	ni|645903779|nb|KK920651.1|	368336-369036	18	16	gi|646745631|gb|KK962343.1|	6096-6581	18	8.6
ACYPI002382-RA	ni|645904049|nb|KK920382.1|	518290-520008	18	18	gi|646768631|gb|KK961807.1|	1304186-1308383	19	9.2
ACYPI002383-RA	ni|645904177|nb|KK920260.1|	2156654-2157402	18	17	gi|646747046|gb|KK962247.1|	156046-156236	22	9
ACYPI002397-RA	ni|645904262|nb|KK920227.1|	2407415-2408168	17	17	gi|646762476|gb|KK961908.1|	202237-202666	19	7.4
ACYPI002401-RA	ni|645903547|nb|KK920883.1|	649534-649957	nan	nan	gi|646781043|gb|KK961532.1|	3022212-3022812	26	11
ACYPI002404-RA	ni|645903597|nb|KK920833.1|	755945-756646	nan	nan	gi|646776351|gb|KK961680.1|	1844763-1849085	22	10
ACYPI002405-RA	ni|645903964|nb|KK920467.1|	637409-637559	15	8	gi|646778903|gb|KK961588.1|	3353930-3360933	23	10
ACYPI002411-RA	ni|645904052|nb|KK920379.1|	512757-513063	18	16	gi|646778948|gb|KK961587.1|	1260121-1261019	20	7.3
ACYPI002414-RA	ni|645903965|nb|KK920466.1|	1684303-1684756	17	16	gi|646776024|gb|KK961697.1|	2255158-2255460	22	9.2
ACYPI002426-RA	ni|645902373|nb|KK922056.1|	156186-156548	nan	nan	gi|646782334|gb|KK961495.1|	2903013-2903326	21	9

ACYPI002433-RA	ni|645903768|nb|KK920662.1|	1317363-1317940	18	17	gi|646740304|gb|KK962788.1|	91622-92181	24	9.6
ACYPI002445-RA	ni|645904262|nb|KK920227.1|	2516351-2516636	17	17	gi|646777065|gb|KK961647.1|	779402-779706	20	7.9
ACYPI002446-RA	ni|645904004|nb|KK920427.1|	1246202-1246407	16	16	gi|646781443|gb|KK961520.1|	1420254-1420972	20	8.3
ACYPI002448-RA	ni|645903524|nb|KK920906.1|	445143-445539	nan	nan	gi|646777108|gb|KK961645.1|	288723-288916	17	6.5
ACYPI002460-RA	ni|645904166|nb|KK920267.1|	965375-966678	18	16	gi|646781379|gb|KK961522.1|	3095925-3097440	22	7.6
ACYPI002469-RA	ni|645902497|nb|KK921932.1|	135733-136152	nan	nan	gi|646781849|gb|KK961508.1|	2909704-2910735	17	8
ACYPI002470-RA	ni|645903879|nb|KK920552.1|	1708908-1709530	18	18	gi|646765880|gb|KK961885.1|	1016510-1017864	23	10
ACYPI002471-RA	ni|645904107|nb|KK920324.1|	685216-685440	17	9.5	gi|646767521|gb|KK961830.1|	1183265-1183570	20	9.3
ACYPI002475-RA	ni|645904136|nb|KK920297.1|	2012897-2013262	16	17	gi|646772786|gb|KK961754.1|	1082923-1084009	22	9.6
ACYPI002478-RA	ni|645903867|nb|KK920563.1|	325156-326335	18	18	gi|646777474|gb|KK961630.1|	2237752-2244425	21	9.3
ACYPI002479-RA	ni|645903920|nb|KK920511.1|	755730-756172	20	20	gi|646742343|gb|KK962588.1|	510380-511704	20	9.4
ACYPI002480-RA	ni|645903780|nb|KK920650.1|	251973-252387	19	19	gi|646781628|gb|KK961514.1|	4812629-4814190	23	9
ACYPI002482-RA	ni|645904102|nb|KK920329.1|	416843-417423	15	16	gi|646768294|gb|KK961814.1|	1081772-1083971	23	10
ACYPI002483-RA	ni|645904225|nb|KK920244.1|	647324-648019	17	17	gi|646620535|gb|KK972589.1|	760-1021	12	6.3
ACYPI002491-RA	ni|645903794|nb|KK920636.1|	416041-416238	25	34	gi|646779006|gb|KK961585.1|	155446-157105	20	9.9
ACYPI002495-RA	ni|645902563|nb|KK921866.1|	165850-166085	nan	nan	gi|646776794|gb|KK961658.1|	1160210-1160937	20	8.1
ACYPI002506-RA	ni|645903858|nb|KK920572.1|	440371-440559	16	16	gi|646776998|gb|KK961650.1|	155617-156732	20	9.6
ACYPI002513-RA	ni|645904128|nb|KK920305.1|	868231-868594	15	8.6	gi|646748580|gb|KK962141.1|	674933-675285	20	5.6
ACYPI002517-RA	ni|645903774|nb|KK920656.1|	460176-460352	16	9.2	gi|646732914|gb|KK963609.1|	82774-83849	21	5.6
ACYPI002524-RA	ni|645902078|nb|KK922351.1|	50868-51074	nan	nan	gi|646757520|gb|KK961926.1|	1355277-1356642	20	7.4
ACYPI002526-RA	ni|645903890|nb|KK920541.1|	1013869-1016550	17	18	gi|646771951|gb|KK961762.1|	675959-678991	21	8.3
ACYPI002527-RA	ni|645903698|nb|KK920732.1|	142370-143258	nan	nan	gi|646780010|gb|KK961559.1|	2231739-2236919	22	10
ACYPI002530-RA	ni|645904057|nb|KK920374.1|	192814-197812	16	15	gi|646746355|gb|KK962294.1|	143486-150254	22	10
ACYPI002533-RA	ni|645903512|nb|KK920918.1|	419909-420112	nan	nan	gi|646772358|gb|KK961758.1|	1874503-1876562	20	9.3
ACYPI002536-RA	ni|645903604|nb|KK920826.1|	121822-122270	nan	nan	gi|646552905|gb|KK985647.1|	3557-3784	13	5.5
ACYPI002537-RA	ni|645903871|nb|KK920559.1|	204887-205386	18	9.4	gi|646778186|gb|KK961609.1|	1713715-1714684	19	9.4
ACYPI002538-RA	ni|645904278|nb|KK920222.1|	481380-482153	15	16	gi|646771448|gb|KK961767.1|	1433164-1434556	21	9.8
ACYPI002544-RA	ni|645904028|nb|KK920403.1|	1831841-1832235	18	17	gi|646782168|gb|KK961499.1|	5396962-5397413	21	9.4
ACYPI002549-RA	ni|645903577|nb|KK920853.1|	594400-594644	nan	nan	gi|646769774|gb|KK961788.1|	651574-654039	20	9.2
ACYPI002557-RA	ni|645904130|nb|KK920303.1|	2292377-2292691	18	17	gi|646779826|gb|KK961564.1|	2711260-2712177	22	9.6

ACYPI002575-RA	ni|645904278|nb|KK920222.1|	1554186-1554452	15	16	gi|646773423|gb|KK961749.1|	931332-931815	19	8.1
ACYPI002578-RA	ni|645904136|nb|KK920297.1|	1679830-1680072	16	17	gi|646775595|gb|KK961718.1|	804248-804475	21	8.9
ACYPI002580-RA	ni|645902673|nb|KK921756.1|	141570-142041	nan	nan	gi|646778448|gb|KK961601.1|	1850032-1850320	19	8.3
ACYPI002584-RA	ni|645904077|nb|KK920354.1|	1490297-1490968	16	17	gi|646777991|gb|KK961614.1|	1689943-1690781	18	7.8
ACYPI002592-RA	ni|645903650|nb|KK920780.1|	794327-794821	nan	nan	gi|646776588|gb|KK961666.1|	2333642-2334573	20	5.8
ACYPI002593-RA	ni|645903751|nb|KK920679.1|	817889-818038	17	16	gi|646741057|gb|KK962701.1|	332940-333300	21	8.8
ACYPI002595-RA	ni|645903923|nb|KK920508.1|	1117897-1118761	18	18	gi|646778865|gb|KK961589.1|	2945518-2946005	20	9.2
ACYPI002598-RA	ni|645904248|nb|KK920232.1|	1113977-1114766	15	16	gi|646782276|gb|KK961497.1|	7381319-7382434	21	9.7
ACYPI002601-RA	ni|645904096|nb|KK920335.1|	910607-910893	18	17	gi|646639844|gb|KK969138.1|	8861-9197	17	6.6
ACYPI002612-RA	ni|645904281|nb|KK920220.1|	827541-827926	15	16	gi|646779298|gb|KK961577.1|	2973102-2974775	20	9.1
ACYPI002620-RA	ni|645901057|nb|KK923372.1|	22671-23217	nan	nan	gi|646771845|gb|KK961763.1|	1420337-1420635	23	10
ACYPI002622-RA	ni|645903866|nb|KK920564.1|	374109-374600	18	15	gi|646740073|gb|KK962814.1|	61569-62315	18	8.3
ACYPI002624-RA	ni|645904035|nb|KK920396.1|	641155-641400	16	16	gi|646769275|gb|KK961796.1|	1970018-1975802	22	5.6
ACYPI002632-RA	ni|645903768|nb|KK920662.1|	1187307-1187692	18	17	gi|646781772|gb|KK961510.1|	3935410-3935647	20	9.2
ACYPI002636-RA	ni|645903650|nb|KK920780.1|	1007873-1008356	nan	nan	gi|646762191|gb|KK961910.1|	768673-769023	18	7.1
ACYPI002650-RA	ni|645904021|nb|KK920410.1|	598172-598882	15	16	gi|646776490|gb|KK961672.1|	58431-63884	20	10
ACYPI002653-RA	ni|645901808|nb|KK922621.1|	145364-146379	nan	nan	gi|646782087|gb|KK961501.1|	1763854-1768289	20	8.7
ACYPI002656-RA	ni|645903538|nb|KK920892.1|	54690-55240	nan	nan	gi|646779337|gb|KK961576.1|	1223498-1223747	18	9
ACYPI002657-RA	ni|645904171|nb|KK920262.1|	732525-733189	15	16	gi|646775842|gb|KK961706.1|	1190141-1201107	22	9
ACYPI002662-RA	ni|645903774|nb|KK920656.1|	526446-526707	16	9.2	gi|646767230|gb|KK961837.1|	325845-326129	19	5.4
ACYPI002674-RA	ni|645904037|nb|KK920394.1|	1132586-1133153	18	17	gi|646747754|gb|KK962195.1|	713736-714702	21	5.3
ACYPI002678-RA	ni|645901760|nb|KK922669.1|	93073-94496	nan	nan	gi|646741342|gb|KK962675.1|	216047-219286	18	7.3
ACYPI002680-RA	ni|645903812|nb|KK920618.1|	550461-551001	20	18	gi|646780858|gb|KK961538.1|	3841595-3842102	21	10
ACYPI002684-RA	ni|645904120|nb|KK920311.1|	282852-283120	18	18	gi|646778736|gb|KK961593.1|	1747586-1748173	20	7.9
ACYPI002689-RA	ni|645904146|nb|KK920287.1|	967584-968143	16	18	gi|646749794|gb|KK962073.1|	646060-646346	20	8.4
ACYPI002692-RA	ni|645903871|nb|KK920559.1|	1075707-1080128	18	9.4	gi|646777288|gb|KK961638.1|	837652-843808	22	6.1
ACYPI002694-RA	ni|645904170|nb|KK920263.1|	322874-323305	15	16	gi|646776608|gb|KK961665.1|	422260-422707	23	9.3
ACYPI002695-RA	ni|645902460|nb|KK921969.1|	14013-14243	nan	nan	gi|646736855|gb|KK963138.1|	62509-63107	19	9.1
ACYPI002698-RA	ni|645904274|nb|KK920223.1|	655074-655655	14	17	gi|646779375|gb|KK961575.1|	575664-577130	22	9.3
ACYPI002711-RA	ni|645903597|nb|KK920833.1|	928664-929004	nan	nan	gi|646766056|gb|KK961877.1|	361125-362060	18	7.8

ACYPI002730-RA	ni|645904014|nb|KK920417.1|	1483679-1484832	19	18	gi|646781873|gb|KK961507.1|	1771011-1773062	20	9.1
ACYPI002732-RA	ni|645903496|nb|KK920934.1|	335367-335584	nan	nan	gi|646734527|gb|KK963409.1|	29706-30189	21	9.3
ACYPI002737-RA	ni|645901565|nb|KK922864.1|	28414-28977	nan	nan	gi|646771352|gb|KK961768.1|	127433-127823	21	9.6
ACYPI002742-RA	ni|645903797|nb|KK920633.1|	412881-413784	17	8.9	gi|646776351|gb|KK961680.1|	2481434-2482398	22	10
ACYPI002749-RA	ni|645903923|nb|KK920508.1|	227849-228304	18	18	gi|646759277|gb|KK961919.1|	522422-523257	16	7.2
ACYPI002752-RA	ni|645904274|nb|KK920223.1|	567917-568252	14	17	gi|646780441|gb|KK961549.1|	2404016-2404718	21	9.5
ACYPI002754-RA	ni|645904119|nb|KK920312.1|	1546830-1550831	19	19	gi|646766598|gb|KK961856.1|	687987-692659	23	10
ACYPI002756-RA	ni|645904125|nb|KK920307.1|	2812631-2812847	18	17	gi|646750061|gb|KK962059.1|	505248-505774	15	7.1
ACYPI002757-RA	ni|645901710|nb|KK922719.1|	44095-44324	nan	nan	gi|646782127|gb|KK961500.1|	3859457-3862296	22	9.6
ACYPI002758-RA	ni|645904265|nb|KK920226.1|	1357564-1358173	14	14	gi|646767948|gb|KK961821.1|	494674-495316	17	7.1
ACYPI002781-RA	ni|645902336|nb|KK922093.1|	9899-10262	nan	nan	gi|646748888|gb|KK962123.1|	823008-825513	20	8.1
ACYPI002787-RA	ni|645903724|nb|KK920706.1|	273927-274285	nan	nan	gi|646777698|gb|KK961622.1|	893775-894467	16	7.6
ACYPI002789-RA	ni|645903495|nb|KK920935.1|	471438-471750	nan	nan	gi|646775488|gb|KK961724.1|	354096-355626	19	7.8
ACYPI002791-RA	ni|645903654|nb|KK920776.1|	646197-646560	nan	nan	gi|646738812|gb|KK962939.1|	260051-260397	20	8.1
ACYPI002792-RA	ni|645901262|nb|KK923167.1|	33437-33676	nan	nan	gi|646782288|gb|KK961496.1|	506752-507969	21	9.7
ACYPI002794-RA	ni|645904281|nb|KK920220.1|	1146197-1146455	15	16	gi|646775558|gb|KK961720.1|	1695082-1695359	20	9.4
ACYPI002798-RA	ni|645902083|nb|KK922346.1|	90008-90229	nan	nan	gi|646776514|gb|KK961670.1|	1699883-1700329	20	9.6
ACYPI002801-RA	ni|645904138|nb|KK920295.1|	871736-872408	15	15	gi|646748888|gb|KK962123.1|	518666-520320	20	8.1
ACYPI002805-RA	ni|645904251|nb|KK920231.1|	1854506-1854709	16	16	gi|646779498|gb|KK961572.1|	515432-516503	22	5.6
ACYPI002806-RA	ni|645903925|nb|KK920506.1|	717164-717635	19	17	gi|646781659|gb|KK961513.1|	6033433-6036104	22	9.8
ACYPI002808-RA	ni|645902404|nb|KK922025.1|	41336-42420	nan	nan	gi|646776647|gb|KK961663.1|	2271830-2274478	23	9.2
ACYPI002820-RA	ni|645904139|nb|KK920294.1|	237606-238219	15	18	gi|646770114|gb|KK961783.1|	1572054-1572635	19	9.7
ACYPI002830-RA	ni|645904159|nb|KK920274.1|	181392-181831	15	17	gi|646745959|gb|KK962321.1|	319481-325472	20	6.9
ACYPI002835-RA	ni|645904265|nb|KK920226.1|	233761-233968	14	14	gi|646778983|gb|KK961586.1|	1354364-1354928	20	9.9
ACYPI002837-RA	ni|645903673|nb|KK920757.1|	83429-86295	nan	nan	gi|646781183|gb|KK961528.1|	3597736-3600845	20	9.5
ACYPI002839-RA	ni|645903476|nb|KK920954.1|	298788-298973	nan	nan	gi|646737853|gb|KK963036.1|	137035-137669	42	18
ACYPI002840-RA	ni|645903661|nb|KK920769.1|	281469-281921	nan	nan	gi|646768361|gb|KK961813.1|	448676-449914	15	7.4
ACYPI002841-RA	ni|645904014|nb|KK920417.1|	1267411-1267664	19	18	gi|646781628|gb|KK961514.1|	230220-232285	23	9
ACYPI002842-RA	ni|645903833|nb|KK920597.1|	34353-34959	17	15	gi|646776514|gb|KK961670.1|	810483-811318	20	9.6
ACYPI002845-RA	ni|645903681|nb|KK920749.1|	229727-230539	nan	nan	gi|646748086|gb|KK962172.1|	551659-551942	20	10

ACYPI002846-RA	ni|645904222|nb|KK920245.1|	946504-946672	17	12	gi|646780010|gb|KK961559.1|	2614772-2615721	22	10
ACYPI002850-RA	ni|645904028|nb|KK920403.1|	1725856-1726547	18	17	gi|646781873|gb|KK961507.1|	1603623-1604415	20	9.1
ACYPI002864-RA	ni|645903821|nb|KK920609.1|	1140419-1140844	17	17	gi|646779141|gb|KK961581.1|	202009-202244	21	9.1
ACYPI002865-RA	ni|645904135|nb|KK920298.1|	549274-549511	14	15	gi|646775258|gb|KK961737.1|	657982-658236	19	9.8
ACYPI002866-RA	ni|645903858|nb|KK920572.1|	639028-643519	16	16	gi|646742413|gb|KK962582.1|	575185-579755	23	10
ACYPI002870-RA	ni|645903999|nb|KK920432.1|	531549-532153	17	15	gi|646781421|gb|KK961521.1|	1408603-1410559	21	8.7
ACYPI002878-RA	ni|645902395|nb|KK922034.1|	13334-13646	nan	nan	gi|646778029|gb|KK961613.1|	453892-455464	21	8.6
ACYPI002880-RA	ni|645904183|nb|KK920258.1|	419934-420233	25	35	gi|646782168|gb|KK961499.1|	2729477-2729980	21	9.4
ACYPI002892-RA	ni|645904137|nb|KK920296.1|	1619679-1619992	17	17	gi|646776514|gb|KK961670.1|	1335911-1337468	20	9.6
ACYPI002900-RA	ni|645903999|nb|KK920432.1|	1120887-1121762	17	15	gi|646778983|gb|KK961586.1|	3162080-3163259	20	9.9
ACYPI002904-RA	ni|645903837|nb|KK920593.1|	949706-949945	18	9.8	gi|646767265|gb|KK961836.1|	537573-538196	22	9.9
ACYPI002907-RA	ni|645903491|nb|KK920939.1|	40510-43295	nan	nan	gi|646740619|gb|KK962751.1|	73767-74102	21	9.4
ACYPI002909-RA	ni|645903543|nb|KK920887.1|	611055-611757	nan	nan	gi|646778213|gb|KK961608.1|	1363971-1371045	19	8.8
ACYPI002929-RA	ni|645904222|nb|KK920245.1|	415542-416016	17	12	gi|646775558|gb|KK961720.1|	1592801-1594423	20	9.4
ACYPI002940-RA	ni|645903820|nb|KK920610.1|	1143394-1144449	18	19	gi|646778865|gb|KK961589.1|	2989579-2990789	20	9.2
ACYPI002948-RA	ni|645904055|nb|KK920376.1|	617728-618095	17	17	gi|646780147|gb|KK961556.1|	1745288-1745969	18	7.5
ACYPI002949-RA	ni|645904098|nb|KK920333.1|	321544-321965	16	8.8	gi|646750869|gb|KK962018.1|	100741-103753	20	10
ACYPI002950-RA	ni|645903773|nb|KK920657.1|	1078881-1079877	18	17	gi|646743492|gb|KK962499.1|	378863-379166	24	11
ACYPI002951-RA	ni|645903594|nb|KK920836.1|	76411-78155	nan	nan	gi|646737822|gb|KK963039.1|	15832-16397	20	9
ACYPI002952-RA	ni|645904008|nb|KK920423.1|	1214544-1215178	16	9.5	gi|646551326|gb|KK985982.1|	7703-8114	13	5.2
ACYPI002953-RA	ni|645903796|nb|KK920634.1|	904946-908161	18	17	gi|646779826|gb|KK961564.1|	3221840-3223314	22	9.6
ACYPI002959-RA	ni|645903824|nb|KK920606.1|	1109982-1111715	19	17	gi|646771845|gb|KK961763.1|	2098996-2107545	23	10
ACYPI002963-RA	ni|645904225|nb|KK920244.1|	629765-630415	17	17	gi|646756446|gb|KK961931.1|	620092-621178	14	6.1
ACYPI002966-RA	ni|645904070|nb|KK920361.1|	384229-385310	17	16	gi|646743944|gb|KK962469.1|	138015-139156	20	9.1
ACYPI002973-RA	ni|645904052|nb|KK920379.1|	726543-727200	18	16	gi|646780978|gb|KK961534.1|	307070-310049	20	8
ACYPI002976-RA	ni|645903879|nb|KK920552.1|	257231-257595	18	18	gi|646781379|gb|KK961522.1|	777033-777645	22	7.6
ACYPI002979-RA	ni|645903743|nb|KK920687.1|	286000-286328	19	18	gi|646750600|gb|KK962030.1|	995287-1000766	23	8.6
ACYPI002985-RA	ni|645904028|nb|KK920403.1|	1868123-1870213	18	17	gi|646748785|gb|KK962129.1|	324112-329823	20	8.2
ACYPI002986-RA	ni|645903725|nb|KK920705.1|	164986-165419	nan	nan	gi|646782334|gb|KK961495.1|	1818705-1819833	21	9
ACYPI002989-RA	ni|645903946|nb|KK920485.1|	49525-49739	16	14	gi|646780827|gb|KK961539.1|	408655-410609	20	9.4

ACYPI002998-RA	ni|645901697|nb|KK922732.1|	104483-105068	nan	nan	gi|646776477|gb|KK961673.1|	991452-991683	22	9
ACYPI003001-RA	ni|645902269|nb|KK922160.1|	73465-73769	nan	nan	gi|646770477|gb|KK961778.1|	1290658-1293472	21	9.1
ACYPI003002-RA	ni|645903879|nb|KK920552.1|	376531-377201	18	18	gi|646781379|gb|KK961522.1|	572075-572243	22	7.6
ACYPI003006-RA	ni|645904278|nb|KK920222.1|	1348709-1349001	15	16	gi|646751515|gb|KK961987.1|	890582-892922	20	9.2
ACYPI003008-RA	ni|645904065|nb|KK920366.1|	757969-759008	18	18	gi|646781536|gb|KK961517.1|	254137-262390	19	7.7
ACYPI003013-RA	ni|645903951|nb|KK920480.1|	252176-252399	16	9	gi|646742450|gb|KK962579.1|	275376-276061	19	7.6
ACYPI003015-RA	ni|645903949|nb|KK920482.1|	625335-625653	17	14	gi|646748955|gb|KK962119.1|	38161-39417	19	9.9
ACYPI003025-RA	ni|645903920|nb|KK920511.1|	1292481-1292778	20	20	gi|646781536|gb|KK961517.1|	1964504-1964791	19	7.7
ACYPI003031-RA	ni|645904100|nb|KK920331.1|	283814-284205	18	22	gi|646763535|gb|KK961902.1|	417537-418658	23	10
ACYPI003033-RA	ni|645903747|nb|KK920683.1|	949998-950947	19	18	gi|646746706|gb|KK962270.1|	290887-291265	23	11
ACYPI003039-RA	ni|645903783|nb|KK920647.1|	1560256-1560671	17	17	gi|646748200|gb|KK962164.1|	262832-267114	19	7.7
ACYPI003042-RA	ni|645904242|nb|KK920237.1|	790552-790887	15	16	gi|646781732|gb|KK961511.1|	3714803-3715407	22	9.8
ACYPI003043-RA	ni|645903578|nb|KK920852.1|	227415-228057	nan	nan	gi|646749741|gb|KK962076.1|	207401-207772	17	7.6
ACYPI003044-RA	ni|645904177|nb|KK920260.1|	900620-900803	18	17	gi|646782357|gb|KK961494.1|	9093136-9094973	21	9.2
ACYPI003045-RA	ni|645904251|nb|KK920231.1|	586083-586627	16	16	gi|646781143|gb|KK961529.1|	2800464-2802968	20	10
ACYPI003049-RA	ni|645904112|nb|KK920319.1|	2577838-2579004	18	17	gi|646778840|gb|KK961590.1|	1158243-1158983	20	9.9
ACYPI003057-RA	ni|645903751|nb|KK920679.1|	773272-773743	17	16	gi|646775884|gb|KK961704.1|	1001963-1002652	20	7.6
ACYPI003059-RA	ni|645903925|nb|KK920506.1|	706742-707595	19	17	gi|646747482|gb|KK962215.1|	428732-434848	21	9.2
ACYPI003061-RA	ni|645904043|nb|KK920388.1|	627384-628441	16	9.1	gi|646740879|gb|KK962721.1|	700900-703086	24	6.4
ACYPI003062-RA	ni|645904162|nb|KK920271.1|	612792-612986	15	18	gi|646776128|gb|KK961691.1|	1317613-1318062	21	9.2
ACYPI003064-RA	ni|645903704|nb|KK920726.1|	471161-472278	nan	nan	gi|646760564|gb|KK961914.1|	951790-952406	21	8.8
ACYPI003073-RA	ni|645902460|nb|KK921969.1|	156280-157168	nan	nan	gi|646740637|gb|KK962749.1|	245123-245698	20	7.3
ACYPI003077-RA	ni|645904130|nb|KK920303.1|	691046-691282	18	17	gi|646751856|gb|KK961977.1|	104921-105121	18	8.5
ACYPI003083-RA	ni|645903566|nb|KK920864.1|	767900-768374	nan	nan	gi|646618553|gb|KK972958.1|	39-301	20	8.2
ACYPI003093-RA	ni|645902471|nb|KK921958.1|	162710-164929	nan	nan	gi|646781893|gb|KK961506.1|	3904830-3914538	19	8.5
ACYPI003099-RA	ni|645903680|nb|KK920750.1|	1003668-1003939	nan	nan	gi|646777416|gb|KK961632.1|	1492866-1494040	22	8.8
ACYPI003113-RA	ni|645903952|nb|KK920479.1|	715098-718755	16	16	gi|646770998|gb|KK961772.1|	1490671-1493006	22	9.8
ACYPI003121-RA	ni|645904122|nb|KK920309.1|	886342-886795	18	17	gi|646781443|gb|KK961520.1|	3344951-3345843	20	8.3
ACYPI003124-RA	ni|645903971|nb|KK920460.1|	1127815-1128499	19	18	gi|646744756|gb|KK962409.1|	206500-208807	18	8.1
ACYPI003141-RA	ni|645901605|nb|KK922824.1|	8520-9309	nan	nan	gi|646767670|gb|KK961827.1|	653931-654235	21	5.9

ACYPI003151-RA	ni|645904014|nb|KK920417.1|	833759-833964	19	18	gi|646737822|gb|KK963039.1|	93343-93932	20	9
ACYPI003157-RA	ni|645903925|nb|KK920506.1|	1557437-1558088	19	17	gi|646782043|gb|KK961502.1|	5249003-5250266	20	9.2
ACYPI003160-RA	ni|645903655|nb|KK920775.1|	221657-222485	nan	nan	gi|646754231|gb|KK961946.1|	1094687-1095156	18	8.7
ACYPI003169-RA	ni|645904135|nb|KK920298.1|	768087-769106	14	15	gi|646743179|gb|KK962523.1|	74132-75289	18	8.3
ACYPI003171-RA	ni|645904169|nb|KK920264.1|	645806-646818	16	9.5	gi|646765747|gb|KK961891.1|	904534-908562	18	7.4
ACYPI003185-RA	ni|645903599|nb|KK920831.1|	216340-217609	nan	nan	gi|646759701|gb|KK961917.1|	129480-133948	18	6.1
ACYPI003186-RA	ni|645903999|nb|KK920432.1|	1196286-1196686	17	15	gi|646776447|gb|KK961675.1|	1326802-1327313	22	9.4
ACYPI003198-RA	ni|645904237|nb|KK920240.1|	1203459-1204047	16	15	gi|646766833|gb|KK961848.1|	318625-322634	17	7
ACYPI003203-RA	ni|645901755|nb|KK922674.1|	8809-9287	nan	nan	gi|646741034|gb|KK962704.1|	2934-3967	24	10
ACYPI003204-RA	ni|645901808|nb|KK922621.1|	50970-52099	nan	nan	gi|646752143|gb|KK961972.1|	935633-945789	20	5.6
ACYPI003208-RA	ni|645903549|nb|KK920881.1|	42243-42713	nan	nan	gi|646781690|gb|KK961512.1|	3492495-3492987	21	8.8
ACYPI003210-RA	ni|645903759|nb|KK920671.1|	82411-82980	18	17	gi|646781809|gb|KK961509.1|	3226289-3230714	19	8.6
ACYPI003214-RA	ni|645903773|nb|KK920657.1|	1120081-1120789	18	17	gi|646776447|gb|KK961675.1|	830176-830874	22	9.4
ACYPI003216-RA	ni|645903831|nb|KK920599.1|	1310947-1311630	18	17	gi|646776904|gb|KK961654.1|	907358-909360	21	9.3
ACYPI003220-RA	ni|645904015|nb|KK920416.1|	916644-917264	18	16	gi|646779038|gb|KK961584.1|	2320231-2320857	21	6.9
ACYPI003221-RA	ni|645904116|nb|KK920315.1|	2042468-2043944	19	18	gi|646777288|gb|KK961638.1|	2042151-2046261	22	6.1
ACYPI003232-RA	ni|645901733|nb|KK922696.1|	67070-67542	nan	nan	gi|646781772|gb|KK961510.1|	3086344-3089300	20	9.2
ACYPI003233-RA	ni|645903678|nb|KK920752.1|	436682-436883	nan	nan	gi|646778660|gb|KK961595.1|	125370-125646	16	8.2
ACYPI003234-RA	ni|645900872|nb|KK923557.1|	33586-35038	nan	nan	gi|646748955|gb|KK962119.1|	669317-676548	19	9.9
ACYPI003235-RA	ni|645903789|nb|KK920641.1|	461715-462182	17	17	gi|646743956|gb|KK962468.1|	101635-103667	15	5.5
ACYPI003241-RA	ni|645903966|nb|KK920465.1|	140514-141562	16	15	gi|646780723|gb|KK961542.1|	1959785-1960954	21	9.4
ACYPI003242-RA	ni|645904157|nb|KK920276.1|	1456385-1457320	15	15	gi|646778274|gb|KK961606.1|	1540844-1542907	18	7.5
ACYPI003244-RA	ni|645903827|nb|KK920603.1|	1139888-1140413	17	18	gi|646776429|gb|KK961676.1|	2429979-2431841	20	9.9
ACYPI003246-RA	ni|645904222|nb|KK920245.1|	1189060-1189599	17	12	gi|646780010|gb|KK961559.1|	3021741-3022227	22	10
ACYPI003252-RA	ni|645904155|nb|KK920278.1|	582125-582337	18	9.4	gi|646763164|gb|KK961904.1|	1513575-1517730	22	10
ACYPI003255-RA	ni|645903965|nb|KK920466.1|	1675427-1676261	17	16	gi|646776024|gb|KK961697.1|	2236328-2236662	22	9.2
ACYPI003261-RA	ni|645903765|nb|KK920665.1|	665224-666551	17	15	gi|646778865|gb|KK961589.1|	2693084-2698239	20	9.2
ACYPI003279-RA	ni|645903701|nb|KK920729.1|	207280-207550	nan	nan	gi|646779298|gb|KK961577.1|	388269-388576	20	9.1
ACYPI003282-RA	ni|645903966|nb|KK920465.1|	336558-337066	16	15	gi|646775821|gb|KK961707.1|	2268585-2271598	23	9.1
ACYPI003283-RA	ni|645903957|nb|KK920474.1|	846525-846814	18	17	gi|646768534|gb|KK961809.1|	339483-342446	21	9.8

ACYPI003284-RA	ni|645903926|nb|KK920505.1|	302194-302363	9.6	12	gi|646749906|gb|KK962067.1|	264901-265421	21	8.2
ACYPI003290-RA	ni|645904186|nb|KK920257.1|	69588-69888	15	16	gi|646747226|gb|KK962234.1|	391655-391992	12	7.8
ACYPI003295-RA	ni|645901747|nb|KK922682.1|	116921-117120	nan	nan	gi|646765705|gb|KK961893.1|	1584153-1584380	22	9.7
ACYPI003296-RA	ni|645903954|nb|KK920477.1|	86066-86415	35	44	gi|646776514|gb|KK961670.1|	1415107-1416474	20	9.6
ACYPI003297-RA	ni|645904005|nb|KK920426.1|	678450-679217	17	16	gi|646775789|gb|KK961709.1|	988971-989423	20	9.6
ACYPI003298-RA	ni|645903679|nb|KK920751.1|	338350-338854	nan	nan	gi|646745612|gb|KK962344.1|	198103-200309	20	8.2
ACYPI003301-RA	ni|645903728|nb|KK920702.1|	999112-999576	nan	nan	gi|646775968|gb|KK961700.1|	112114-112366	20	8.2
ACYPI003303-RA	ni|645903634|nb|KK920796.1|	1078869-1079269	nan	nan	gi|646748474|gb|KK962147.1|	291027-291463	20	9
ACYPI003304-RA	ni|645903965|nb|KK920466.1|	1445001-1445241	17	16	gi|646751569|gb|KK961985.1|	313648-314234	21	9.2
ACYPI003316-RA	ni|645904049|nb|KK920382.1|	141156-141604	18	18	gi|646744699|gb|KK962413.1|	133486-134144	14	6.9
ACYPI003318-RA	ni|645904150|nb|KK920283.1|	967770-968125	17	17	gi|646638312|gb|KK969438.1|	12382-13593	13	6.6
ACYPI003322-RA	ni|645902615|nb|KK921814.1|	32483-34953	nan	nan	gi|646765669|gb|KK961895.1|	34683-35153	18	7.2
ACYPI003327-RA	ni|645904122|nb|KK920309.1|	408932-409406	18	17	gi|646781344|gb|KK961523.1|	356295-357885	21	8.9
ACYPI003333-RA	ni|645904106|nb|KK920325.1|	1022587-1022793	17	17	gi|646758845|gb|KK961921.1|	1054646-1054852	22	9.5
ACYPI003341-RA	ni|645903752|nb|KK920678.1|	1300203-1300430	18	16	gi|646751103|gb|KK962006.1|	97319-99532	20	8.3
ACYPI003343-RA	ni|645903713|nb|KK920717.1|	199518-199736	nan	nan	gi|646778149|gb|KK961610.1|	269958-270647	19	8.9
ACYPI003347-RA	ni|645903891|nb|KK920540.1|	121603-121814	15	17	gi|646776024|gb|KK961697.1|	385271-387477	22	9.2
ACYPI003349-RA	ni|645904141|nb|KK920292.1|	313936-314558	14	14	gi|646782127|gb|KK961500.1|	3536955-3542945	22	9.6
ACYPI003358-RA	ni|645904096|nb|KK920335.1|	1986811-1988455	18	17	gi|646779826|gb|KK961564.1|	1038105-1040174	22	9.6
ACYPI003364-RA	ni|645902263|nb|KK922166.1|	190379-190560	nan	nan	gi|646749852|gb|KK962070.1|	613342-613968	19	8.6
ACYPI003377-RA	ni|645904078|nb|KK920353.1|	563997-564219	17	17	gi|646516981|gb|KK991223.1|	1825-2393	22	8.6
ACYPI003398-RA	ni|645904251|nb|KK920231.1|	369602-369966	16	16	gi|646747586|gb|KK962207.1|	100173-101079	20	8.8
ACYPI003401-RA	ni|645902394|nb|KK922035.1|	344370-344995	nan	nan	gi|646777017|gb|KK961649.1|	627201-627455	18	8
ACYPI003404-RA	ni|645901808|nb|KK922621.1|	116535-116930	nan	nan	gi|646781628|gb|KK961514.1|	3448418-3448774	23	9
ACYPI003409-RA	ni|645903502|nb|KK920928.1|	240818-241179	nan	nan	gi|646773018|gb|KK961752.1|	565788-566183	18	7.8
ACYPI003413-RA	ni|645903817|nb|KK920613.1|	267579-267977	13	16	gi|646781282|gb|KK961525.1|	1746216-1747551	22	9.6
ACYPI003418-RA	ni|645903800|nb|KK920630.1|	1314426-1315385	18	17	gi|646776024|gb|KK961697.1|	918063-918862	22	9.2
ACYPI003428-RA	ni|645903690|nb|KK920740.1|	291212-292032	nan	nan	gi|646781690|gb|KK961512.1|	4507501-4509485	21	8.8
ACYPI003430-RA	ni|645903936|nb|KK920495.1|	805910-806747	19	18	gi|646778112|gb|KK961611.1|	1401896-1403304	22	10
ACYPI003451-RA	ni|645903965|nb|KK920466.1|	1721255-1721440	17	16	gi|646775658|gb|KK961715.1|	685337-685578	19	8.6

ACYPI003455-RA	ni|645903971|nb|KK920460.1|	1173108-1174341	19	18	gi|646772045|gb|KK961761.1|	735288-736798	21	9.6
ACYPI003458-RA	ni|645904189|nb|KK920256.1|	1873228-1873897	18	18	gi|646729261|gb|KK964099.1|	58251-59114	24	10
ACYPI003461-RA	ni|645903561|nb|KK920869.1|	74294-74566	nan	nan	gi|646770401|gb|KK961779.1|	1747695-1748746	21	8.6
ACYPI003469-RA	ni|645903617|nb|KK920813.1|	565660-566135	nan	nan	gi|646776091|gb|KK961693.1|	144684-146038	23	10
ACYPI003470-RA	ni|645903733|nb|KK920697.1|	678373-678510	nan	nan	gi|646769774|gb|KK961788.1|	766037-766431	20	9.2
ACYPI003480-RA	ni|645904258|nb|KK920229.1|	731952-732355	16	16	gi|646763164|gb|KK961904.1|	1065661-1066084	22	10
ACYPI003481-RA	ni|645904210|nb|KK920249.1|	916557-917222	16	16	gi|646735722|gb|KK963266.1|	172392-173278	17	7.6
ACYPI003483-RA	ni|645904021|nb|KK920410.1|	777693-778167	15	16	gi|646782276|gb|KK961497.1|	1548160-1551014	21	9.7
ACYPI003484-RA	ni|645903848|nb|KK920582.1|	576525-578099	18	18	gi|646781344|gb|KK961523.1|	582986-584957	21	8.9
ACYPI003488-RA	ni|645904135|nb|KK920298.1|	310414-311019	14	15	gi|646775507|gb|KK961723.1|	1824044-1825093	20	9.8
ACYPI003489-RA	ni|645904028|nb|KK920403.1|	380822-382773	18	1.70E+01	gi|646732863|gb|KK963615.1|	273712-275036	150	39
ACYPI003491-RA	ni|645904114|nb|KK920317.1|	1373494-1374121	18	18	gi|646780658|gb|KK961544.1|	1904378-1905123	19	8.6
ACYPI003508-RA	ni|645904271|nb|KK920224.1|	1648410-1649755	15	16	gi|646779826|gb|KK961564.1|	1263755-1264075	22	9.6
ACYPI003518-RA	ni|645904106|nb|KK920325.1|	805788-806358	17	17	gi|646767850|gb|KK961823.1|	458210-462099	21	9.8
ACYPI003519-RA	ni|645902511|nb|KK921918.1|	156844-157080	nan	nan	gi|646776148|gb|KK961690.1|	1863927-1865939	21	9.4
ACYPI003522-RA	ni|645903915|nb|KK920516.1|	209340-209601	17	17	gi|646743394|gb|KK962507.1|	418259-418636	20	8.8
ACYPI003532-RA	ni|645904243|nb|KK920236.1|	154812-155009	15	8.5	gi|646779498|gb|KK961572.1|	533389-534148	22	5.6
ACYPI003535-RA	ni|645904088|nb|KK920343.1|	167574-168042	17	18	gi|646771845|gb|KK961763.1|	449987-450193	23	10
ACYPI003541-RA	ni|645903829|nb|KK920601.1|	414787-415281	16	15	gi|646780889|gb|KK961537.1|	2915802-2917548	22	10
ACYPI003545-RA	ni|645903981|nb|KK920450.1|	1135912-1136121	16	8.6	gi|646773423|gb|KK961749.1|	383372-387975	19	8.1
ACYPI003549-RA	ni|645903536|nb|KK920894.1|	858250-858529	nan	nan	gi|646777631|gb|KK961624.1|	1806372-1810062	20	9.3
ACYPI003550-RA	ni|645904065|nb|KK920366.1|	1757004-1757181	18	18	gi|646781690|gb|KK961512.1|	3241721-3242537	21	8.8
ACYPI003552-RA	ni|645903858|nb|KK920572.1|	692794-693015	16	16	gi|646771092|gb|KK961771.1|	1071229-1071679	23	9.7
ACYPI003554-RA	ni|645904047|nb|KK920384.1|	614437-615833	15	16	gi|646780889|gb|KK961537.1|	2979795-2980118	22	10
ACYPI003557-RA	ni|645903992|nb|KK920439.1|	670143-670824	15	15	gi|646781344|gb|KK961523.1|	1435331-1445399	21	8.9
ACYPI003560-RA	ni|645904068|nb|KK920363.1|	362858-363091	16	8.6	gi|646779375|gb|KK961575.1|	1835278-1835852	22	9.3
ACYPI003562-RA	ni|645903763|nb|KK920667.1|	49159-49585	26	21	gi|646781118|gb|KK961530.1|	5755346-5756974	21	10
ACYPI003565-RA	ni|645904057|nb|KK920374.1|	1266431-1267005	16	15	gi|646780889|gb|KK961537.1|	4077216-4077409	22	10
ACYPI003566-RA	ni|645904125|nb|KK920307.1|	2447403-2447636	18	17	gi|646746202|gb|KK962305.1|	84741-87979	21	6.4
ACYPI003567-RA	ni|645903721|nb|KK920709.1|	733562-734860	nan	nan	gi|646776608|gb|KK961665.1|	264136-265583	23	9.3

ACYPI003568-RA	ni|645903727|nb|KK920703.1|	1085041-1085523	nan	nan	gi|646780147|gb|KK961556.1|	803865-804403	18	7.5
ACYPI003571-RA	ni|645901808|nb|KK922621.1|	103745-104204	nan	nan	gi|646529355|gb|KK988894.1|	502-1094	13	2.9
ACYPI003572-RA	ni|645903765|nb|KK920665.1|	1084440-1084695	17	15	gi|646782334|gb|KK961495.1|	3189066-3189559	21	9
ACYPI003574-RA	ni|645904014|nb|KK920417.1|	1273386-1273750	19	18	gi|646748200|gb|KK962164.1|	406362-408061	19	7.7
ACYPI003577-RA	ni|645901994|nb|KK922435.1|	64579-64831	nan	nan	gi|646778565|gb|KK961598.1|	1510852-1511105	19	7.8
ACYPI003579-RA	ni|645904065|nb|KK920366.1|	1116200-1116384	18	18	gi|646744874|gb|KK962400.1|	321101-321398	19	8.3
ACYPI003581-RA	ni|645904192|nb|KK920255.1|	342346-342688	15	16	gi|646766735|gb|KK961851.1|	121456-121694	20	10
ACYPI003590-RA	ni|645904036|nb|KK920395.1|	821842-822574	21	18	gi|646779826|gb|KK961564.1|	1464953-1467843	22	9.6
ACYPI003596-RA	ni|645903831|nb|KK920599.1|	1505400-1505744	18	17	gi|646780794|gb|KK961540.1|	1405293-1407435	21	9.5
ACYPI003598-RA	ni|645903915|nb|KK920516.1|	1444625-1444844	17	17	gi|646776429|gb|KK961676.1|	1452461-1453296	20	9.9
ACYPI003602-RA	ni|645903665|nb|KK920765.1|	154605-154903	nan	nan	gi|646777207|gb|KK961641.1|	1317555-1324263	20	8.5
ACYPI003604-RA	ni|645904107|nb|KK920324.1|	443801-444312	17	9.5	gi|646778903|gb|KK961588.1|	1261298-1261553	23	10
ACYPI003616-RA	ni|645904115|nb|KK920316.1|	1193327-1194219	17	18	gi|646775471|gb|KK961725.1|	325270-325806	20	10
ACYPI003625-RA	ni|645904160|nb|KK920273.1|	979490-979699	18	18	gi|646776024|gb|KK961697.1|	1912164-1916312	22	9.2
ACYPI003631-RA	ni|645904058|nb|KK920373.1|	622297-622935	16	15	gi|646607341|gb|KK974959.1|	6834-6960	7.1	2.6
ACYPI003634-RA	ni|645904081|nb|KK920350.1|	450846-451536	13	18	gi|646779375|gb|KK961575.1|	3605558-3605792	22	9.3
ACYPI003639-RA	ni|645904062|nb|KK920369.1|	141376-148496	14	15	gi|646727554|gb|KK964329.1|	137375-141745	24	10
ACYPI003641-RA	ni|645903681|nb|KK920749.1|	520210-521217	nan	nan	gi|646778840|gb|KK961590.1|	1872677-1872923	20	9.9
ACYPI003646-RA	ni|645903626|nb|KK920804.1|	336789-337409	nan	nan	gi|646776389|gb|KK961678.1|	1305035-1305529	22	9.5
ACYPI003651-RA	ni|645902449|nb|KK921980.1|	100182-100441	nan	nan	gi|646775944|gb|KK961701.1|	622841-625467	22	9.3
ACYPI003657-RA	ni|645901672|nb|KK922757.1|	3755-4399	nan	nan	gi|646776647|gb|KK961663.1|	768432-774690	23	9.2
ACYPI003659-RA	ni|645904174|nb|KK920261.1|	869676-870434	19	17	gi|646738348|gb|KK962985.1|	139845-140214	13	7.1
ACYPI003661-RA	ni|645904035|nb|KK920396.1|	640519-640913	16	16	gi|646745941|gb|KK962322.1|	795337-795490	21	10
ACYPI003669-RA	ni|645903731|nb|KK920699.1|	98773-99067	nan	nan	gi|646773721|gb|KK961747.1|	1007516-1007922	21	9.2
ACYPI003677-RA	ni|645904140|nb|KK920293.1|	361431-361707	22	22	gi|646738524|gb|KK962968.1|	162327-164456	24	11
ACYPI003678-RA	ni|645903847|nb|KK920583.1|	456031-456866	18	18	gi|646777991|gb|KK961614.1|	1475060-1479145	18	7.8
ACYPI003679-RA	ni|645902449|nb|KK921980.1|	82936-83142	nan	nan	gi|646775765|gb|KK961710.1|	1769984-1770500	21	6
ACYPI003691-RA	ni|645904122|nb|KK920309.1|	863712-864135	18	17	gi|646777017|gb|KK961649.1|	681444-682750	18	8
ACYPI003697-RA	ni|645903936|nb|KK920495.1|	566077-567182	19	18	gi|646782334|gb|KK961495.1|	1054782-1056279	21	9
ACYPI003705-RA	ni|645904171|nb|KK920262.1|	864052-864729	15	16	gi|646751289|gb|KK961996.1|	1196601-1200875	24	9.1

ACYPI003710-RA	ni|645903847|nb|KK920583.1|	454760-455680	18	18	gi|646778632|gb|KK961596.1|	2182111-2182325	21	9.2
ACYPI003718-RA	ni|645903639|nb|KK920791.1|	242254-242437	nan	nan	gi|646743302|gb|KK962514.1|	136789-137050	23	8.4
ACYPI003732-RA	ni|645903821|nb|KK920609.1|	1124171-1125294	17	17	gi|646738524|gb|KK962968.1|	311187-315438	24	11
ACYPI003736-RA	ni|645904122|nb|KK920309.1|	1193064-1193353	18	17	gi|646781536|gb|KK961517.1|	2024598-2029654	19	7.7
ACYPI003742-RA	ni|645904116|nb|KK920315.1|	1788704-1788930	19	18	gi|646731007|gb|KK963869.1|	181941-183265	21	9.8
ACYPI003745-RA	ni|645903957|nb|KK920474.1|	546316-547275	18	17	gi|646740917|gb|KK962717.1|	130039-130293	16	7
ACYPI003749-RA	ni|645904241|nb|KK920238.1|	2712552-2712842	18	17	gi|646673348|gb|KK967633.1|	2934-3243	12	5.6
ACYPI003751-RA	ni|645901760|nb|KK922669.1|	114170-114664	nan	nan	gi|646767477|gb|KK961831.1|	128473-129125	20	8.4
ACYPI003754-RA	ni|645904033|nb|KK920398.1|	51728-52526	14	16	gi|646779298|gb|KK961577.1|	1448548-1450769	20	9.1
ACYPI003756-RA	ni|645903788|nb|KK920642.1|	534919-535323	16	15	gi|646766426|gb|KK961862.1|	914809-915095	20	7.1
ACYPI003757-RA	ni|645903720|nb|KK920710.1|	122913-123283	nan	nan	gi|646781893|gb|KK961506.1|	370852-371343	19	8.5
ACYPI003760-RA	ni|645902598|nb|KK921831.1|	93834-94461	nan	nan	gi|646749672|gb|KK962080.1|	302141-302538	20	7.6
ACYPI003764-RA	ni|645903747|nb|KK920683.1|	773739-773928	19	18	gi|646770323|gb|KK961780.1|	218387-218816	19	7.1
ACYPI003771-RA	ni|645902229|nb|KK922200.1|	84208-85489	nan	nan	gi|646780406|gb|KK961550.1|	483864-484318	22	5.9
ACYPI003778-RA	ni|645903899|nb|KK920532.1|	378523-378730	15	16	gi|646766137|gb|KK961874.1|	741464-743205	20	9
ACYPI003779-RA	ni|645903867|nb|KK920563.1|	832377-832967	18	18	gi|646776184|gb|KK961688.1|	358288-359255	21	9.6
ACYPI003780-RA	ni|645901736|nb|KK922693.1|	39046-39418	nan	nan	gi|646751289|gb|KK961996.1|	466659-467055	24	9.1
ACYPI003782-RA	ni|645904123|nb|KK920308.1|	1155604-1155896	16	10	gi|646779702|gb|KK961567.1|	1230883-1231584	23	10
ACYPI003783-RA	ni|645904254|nb|KK920230.1|	98782-99911	16	16	gi|646781379|gb|KK961522.1|	2373417-2377352	22	7.6
ACYPI003786-RA	ni|645903741|nb|KK920689.1|	349057-349477	17	18	gi|646742685|gb|KK962560.1|	7341-8264	21	9.5
ACYPI003795-RA	ni|645903787|nb|KK920643.1|	75263-77034	19	18	gi|646740287|gb|KK962790.1|	90015-99796	20	8.5
ACYPI003798-RA	ni|645903538|nb|KK920892.1|	113270-114186	nan	nan	gi|646777474|gb|KK961630.1|	613574-614529	21	9.3
ACYPI003817-RA	ni|645903496|nb|KK920934.1|	902588-902877	nan	nan	gi|646775702|gb|KK961713.1|	302346-302729	17	7.4
ACYPI003820-RA	ni|645901447|nb|KK922982.1|	162-556	nan	nan	gi|646776280|gb|KK961684.1|	1730061-1730307	22	9.3
ACYPI003821-RA	ni|645903923|nb|KK920508.1|	1141891-1142874	18	18	gi|646766137|gb|KK961874.1|	611082-612102	20	9
ACYPI003822-RA	ni|645902622|nb|KK921807.1|	138278-138478	nan	nan	gi|646739361|gb|KK962890.1|	129238-130324	22	9.6
ACYPI003828-RA	ni|645903655|nb|KK920775.1|	872282-874859	nan	nan	gi|646751120|gb|KK962005.1|	790431-791235	21	5.8
ACYPI003835-RA	ni|645903829|nb|KK920601.1|	602780-603062	16	15	gi|646780889|gb|KK961537.1|	2669593-2671496	22	10
ACYPI003839-RA	ni|645903644|nb|KK920786.1|	134294-134697	nan	nan	gi|646763535|gb|KK961902.1|	976968-983363	23	10
ACYPI003853-RA	ni|645901733|nb|KK922696.1|	24484-25097	nan	nan	gi|646766382|gb|KK961864.1|	34358-34968	22	9.7

ACYPI003863-RA	ni|645903644|nb|KK920786.1|	716927-717168	nan	nan	gi|646781732|gb|KK961511.1|	2453921-2454500	22	9.8
ACYPI003867-RA	ni|645904240|nb|KK920239.1|	955121-955382	16	16	gi|646778948|gb|KK961587.1|	1819307-1819795	20	7.3
ACYPI003876-RA	ni|645903592|nb|KK920838.1|	985370-985888	nan	nan	gi|646729620|gb|KK964051.1|	86615-86858	23	10
ACYPI003885-RA	ni|645903731|nb|KK920699.1|	680621-681065	nan	nan	gi|646782168|gb|KK961499.1|	3626686-3627283	21	9.4
ACYPI003886-RA	ni|645903745|nb|KK920685.1|	316489-319185	16	18	gi|646758527|gb|KK961922.1|	565562-565804	24	10
ACYPI003888-RA	ni|645904130|nb|KK920303.1|	641810-642455	18	17	gi|646751458|gb|KK961989.1|	565234-565510	21	5.1
ACYPI003891-RA	ni|645903473|nb|KK920957.1|	215105-215332	nan	nan	gi|646781344|gb|KK961523.1|	415490-416413	21	8.9
ACYPI003897-RA	ni|645904180|nb|KK920259.1|	1175255-1175500	17	17	gi|646770251|gb|KK961781.1|	76954-79519	20	9.6
ACYPI003904-RA	ni|645904210|nb|KK920249.1|	2063110-2063469	16	16	gi|646750100|gb|KK962057.1|	278314-278701	16	8.2
ACYPI003908-RA	ni|645904228|nb|KK920243.1|	1591174-1591688	17	18	gi|646775968|gb|KK961700.1|	879475-879873	20	8.2
ACYPI003915-RA	ni|645903539|nb|KK920891.1|	57975-58318	nan	nan	gi|646743179|gb|KK962523.1|	184601-187499	18	8.3
ACYPI003918-RA	ni|645903843|nb|KK920587.1|	968274-968543	18	16	gi|646750341|gb|KK962044.1|	885961-886389	19	5.6
ACYPI003922-RA	ni|645903906|nb|KK920525.1|	582924-585620	18	22	gi|646781510|gb|KK961518.1|	1447866-1448481	17	7.8
ACYPI003925-RA	ni|645904122|nb|KK920309.1|	1507549-1507785	18	17	gi|646766650|gb|KK961854.1|	1469365-1476563	22	10
ACYPI003941-RA	ni|645903778|nb|KK920652.1|	211254-211895	12	13	gi|646776322|gb|KK961682.1|	2114788-2115084	22	8.9
ACYPI003942-RA	ni|645904024|nb|KK920407.1|	646272-646516	14	16	gi|646775987|gb|KK961699.1|	302590-302863	19	7.5
ACYPI003943-RA	ni|645903496|nb|KK920934.1|	155025-155361	nan	nan	gi|646781344|gb|KK961523.1|	4364065-4371527	21	8.9
ACYPI003951-RA	ni|645903495|nb|KK920935.1|	314247-314624	nan	nan	gi|646779621|gb|KK961569.1|	1123974-1125732	16	7.3
ACYPI003953-RA	ni|645901697|nb|KK922732.1|	121299-121666	nan	nan	gi|646744874|gb|KK962400.1|	391253-391535	19	8.3
ACYPI003960-RA	ni|645903858|nb|KK920572.1|	446872-447291	16	16	gi|646780183|gb|KK961555.1|	1993664-1995548	19	8.6
ACYPI003961-RA	ni|645904083|nb|KK920348.1|	851600-852307	15	16	gi|646775901|gb|KK961703.1|	825369-831326	20	9.6
ACYPI003971-RA	ni|645904155|nb|KK920278.1|	514449-515369	18	9.4	gi|646747149|gb|KK962240.1|	216691-220176	21	10
ACYPI003972-RA	ni|645904114|nb|KK920317.1|	1954628-1954837	18	18	gi|646780441|gb|KK961549.1|	448971-451404	21	9.5
ACYPI003975-RA	ni|645903911|nb|KK920520.1|	628403-629095	16	17	gi|646747884|gb|KK962186.1|	1230599-1235599	22	11
ACYPI003976-RA	ni|645902649|nb|KK921780.1|	164145-166426	nan	nan	gi|646773721|gb|KK961747.1|	3070756-3087407	21	9.2
ACYPI003981-RA	ni|645903800|nb|KK920630.1|	1002143-1002970	18	17	gi|646776024|gb|KK961697.1|	1209935-1217046	22	9.2
ACYPI003986-RA	ni|645902620|nb|KK921809.1|	209538-209802	nan	nan	gi|646776038|gb|KK961696.1|	1278970-1283843	21	10
ACYPI003991-RA	ni|645904125|nb|KK920307.1|	960143-960680	18	17	gi|646776904|gb|KK961654.1|	1541135-1547468	21	9.3
ACYPI003993-RA	ni|645904139|nb|KK920294.1|	554250-554978	15	18	gi|646775765|gb|KK961710.1|	2033052-2033718	21	6
ACYPI004000-RA	ni|645904268|nb|KK920225.1|	539642-540277	14	15	gi|646748955|gb|KK962119.1|	814945-815243	19	9.9

ACYPI004006-RA	ni|645903973|nb|KK920458.1|	1492006-1492263	20	18	gi|646754371|gb|KK961945.1|	115997-116723	20	9.7
ACYPI004008-RA	ni|645902788|nb|KK921641.1|	253252-254000	nan	nan	gi|646776952|gb|KK961652.1|	1309825-1310609	19	7.7
ACYPI004014-RA	ni|645903871|nb|KK920559.1|	760280-760484	18	9.4	gi|646738635|gb|KK962957.1|	269002-269203	20	8.6
ACYPI004015-RA	ni|645902758|nb|KK921671.1|	201981-202814	nan	nan	gi|646751515|gb|KK961987.1|	743559-744334	20	9.2
ACYPI004018-RA	ni|645903923|nb|KK920508.1|	56345-56485	18	18	gi|646766086|gb|KK961876.1|	331624-332457	23	9.5
ACYPI004019-RA	ni|645902469|nb|KK921960.1|	272464-273018	nan	nan	gi|646776351|gb|KK961680.1|	1800365-1800782	22	10
ACYPI004024-RA	ni|645903743|nb|KK920687.1|	1269681-1270427	19	18	gi|646757520|gb|KK961926.1|	1744453-1746987	20	7.4
ACYPI004029-RA	ni|645903938|nb|KK920493.1|	446255-446423	19	18	gi|646732431|gb|KK963675.1|	306302-308057	24	9.6
ACYPI004030-RA	ni|645903858|nb|KK920572.1|	450258-451156	16	16	gi|646501974|gb|KK994094.1|	200-616	14	7.8
ACYPI004031-RA	ni|645901982|nb|KK922447.1|	193727-194176	nan	nan	gi|646746216|gb|KK962304.1|	782239-783216	130	33
ACYPI004037-RA	ni|645903984|nb|KK920447.1|	764452-764682	18	17	gi|646769221|gb|KK961797.1|	737439-737674	19	9.6
ACYPI004039-RA	ni|645904114|nb|KK920317.1|	1779524-1779867	18	18	gi|646732202|gb|KK963708.1|	15961-17122	22	9.9
ACYPI004047-RA	ni|645902035|nb|KK922394.1|	213785-215047	nan	nan	gi|646758935|gb|KK961920.1|	137592-138206	20	8.5
ACYPI004051-RA	ni|645904177|nb|KK920260.1|	839272-840908	18	17	gi|646753890|gb|KK961949.1|	834535-836511	20	8.9
ACYPI004054-RA	ni|645903496|nb|KK920934.1|	159765-160674	nan	nan	gi|646764801|gb|KK961898.1|	628095-628569	20	9.7
ACYPI004069-RA	ni|645904155|nb|KK920278.1|	969816-970025	18	9.4	gi|646780311|gb|KK961552.1|	4047606-4050625	21	9.7
ACYPI004071-RA	ni|645902642|nb|KK921787.1|	136868-138509	nan	nan	gi|646775807|gb|KK961708.1|	2797154-2799162	21	8.6
ACYPI004075-RA	ni|645903837|nb|KK920593.1|	73821-74327	18	9.8	gi|646781732|gb|KK961511.1|	3134559-3134833	22	9.8
ACYPI004077-RA	ni|645904013|nb|KK920418.1|	1060029-1060248	19	18	gi|646730815|gb|KK963895.1|	181690-184047	20	9.5
ACYPI004078-RA	ni|645904228|nb|KK920243.1|	1660062-1660829	17	18	gi|646781212|gb|KK961527.1|	2239953-2241959	21	10
ACYPI004082-RA	ni|645903511|nb|KK920919.1|	538835-539076	nan	nan	gi|646775558|gb|KK961720.1|	1851019-1853017	20	9.4
ACYPI004089-RA	ni|645903970|nb|KK920461.1|	165284-165710	17	16	gi|646753527|gb|KK961953.1|	1850986-1851761	24	9.2
ACYPI004093-RA	ni|645902226|nb|KK922203.1|	33938-34166	nan	nan	gi|646782357|gb|KK961494.1|	2810374-2810643	21	9.2
ACYPI004098-RA	ni|645904102|nb|KK920329.1|	27343-28738	15	16	gi|646767850|gb|KK961823.1|	1166449-1168953	21	9.8
ACYPI004106-RA	ni|645904168|nb|KK920265.1|	2088813-2089040	16	16	gi|646751458|gb|KK961989.1|	795942-796584	21	5.1
ACYPI004120-RA	ni|645903568|nb|KK920862.1|	266556-267027	nan	nan	gi|646780827|gb|KK961539.1|	193935-195449	20	9.4
ACYPI004126-RA	ni|645904065|nb|KK920366.1|	1614206-1615248	18	18	gi|646781243|gb|KK961526.1|	4103877-4107442	21	8.9
ACYPI004127-RA	ni|645903992|nb|KK920439.1|	683615-684039	15	15	gi|646778865|gb|KK961589.1|	2087689-2087972	20	9.2
ACYPI004128-RA	ni|645903766|nb|KK920664.1|	350567-351118	18	15	gi|646744833|gb|KK962403.1|	130076-131312	22	9.4
ACYPI004129-RA	ni|645897631|nb|KK926798.1|	4349-4507	nan	nan	gi|646776389|gb|KK961678.1|	1664819-1666527	22	9.5

ACYPI004133-RA	ni|645903970|nb|KK920461.1|	345509-346322	17	16	gi|646753527|gb|KK961953.1|	1752479-1753070	24	9.2
ACYPI004142-RA	ni|645902394|nb|KK922035.1|	307797-308242	nan	nan	gi|646781443|gb|KK961520.1|	465271-472935	20	8.3
ACYPI004145-RA	ni|645903751|nb|KK920679.1|	659121-659902	17	16	gi|646750643|gb|KK962028.1|	706083-714538	21	7.5
ACYPI004152-RA	ni|645903923|nb|KK920508.1|	1133407-1133757	18	18	gi|646777991|gb|KK961614.1|	1627252-1627683	18	7.8
ACYPI004154-RA	ni|645904107|nb|KK920324.1|	299475-299869	17	9.5	gi|646767670|gb|KK961827.1|	2087025-2087580	21	5.9
ACYPI004157-RA	ni|645902172|nb|KK922257.1|	212265-212450	nan	nan	gi|646746355|gb|KK962294.1|	1011490-1024685	22	10
ACYPI004165-RA	ni|645903953|nb|KK920478.1|	1851719-1852599	18	17	gi|646769453|gb|KK961793.1|	1280817-1282788	21	8.8
ACYPI004168-RA	ni|645904228|nb|KK920243.1|	1582839-1583310	17	18	gi|646776514|gb|KK961670.1|	1177830-1190106	20	9.6
ACYPI004169-RA	ni|645904120|nb|KK920311.1|	284791-284993	18	18	gi|646781772|gb|KK961510.1|	3202699-3202960	20	9.2
ACYPI004173-RA	ni|645904077|nb|KK920354.1|	602970-603603	16	17	gi|646782357|gb|KK961494.1|	9494725-9495664	21	9.2
ACYPI004185-RA	ni|645904045|nb|KK920386.1|	817728-819194	19	21	gi|646750354|gb|KK962043.1|	497683-499153	20	9.2
ACYPI004195-RA	ni|645904114|nb|KK920317.1|	1648005-1649903	18	18	gi|646781143|gb|KK961529.1|	2157370-2158054	20	10
ACYPI004199-RA	ni|645903651|nb|KK920779.1|	244594-244999	nan	nan	gi|646781344|gb|KK961523.1|	3879833-3880324	21	8.9
ACYPI004203-RA	ni|645903965|nb|KK920466.1|	1599631-1600263	17	16	gi|646779785|gb|KK961565.1|	3434912-3435224	18	8.9
ACYPI004206-RA	ni|645904177|nb|KK920260.1|	2202705-2203229	18	17	gi|646777416|gb|KK961632.1|	666876-667489	22	8.8
ACYPI004209-RA	ni|645903557|nb|KK920873.1|	828896-829330	nan	nan	gi|646766357|gb|KK961865.1|	168058-168389	16	6.5
ACYPI004211-RA	ni|645903971|nb|KK920460.1|	32290-33116	19	18	gi|646780978|gb|KK961534.1|	1054515-1056896	20	8
ACYPI004216-RA	ni|645904153|nb|KK920280.1|	788784-789127	17	16	gi|646780858|gb|KK961538.1|	2080313-2081531	21	10
ACYPI004218-RA	ni|645903827|nb|KK920603.1|	1090808-1091258	17	18	gi|646779337|gb|KK961576.1|	967751-968216	18	9
ACYPI004219-RA	ni|645904075|nb|KK920356.1|	1124869-1125544	16	17	gi|646782357|gb|KK961494.1|	5190612-5191043	21	9.2
ACYPI004223-RA	ni|645903915|nb|KK920516.1|	187974-188368	17	17	gi|646743394|gb|KK962507.1|	337160-340397	20	8.8
ACYPI004224-RA	ni|645903954|nb|KK920477.1|	190165-190969	35	44	gi|646769511|gb|KK961792.1|	14183-16009	20	9.9
ACYPI004227-RA	ni|645899876|nb|KK924553.1|	4626-5541	nan	nan	gi|646781344|gb|KK961523.1|	243344-243909	21	8.9
ACYPI004235-RA	ni|645903848|nb|KK920582.1|	586066-586783	18	18	gi|646781344|gb|KK961523.1|	587351-588847	21	8.9
ACYPI004238-RA	ni|645904094|nb|KK920337.1|	125980-126428	17	16	gi|646743859|gb|KK962475.1|	229741-229993	20	7.9
ACYPI004239-RA	ni|645903808|nb|KK920622.1|	691045-692713	17	17	gi|646768717|gb|KK961805.1|	1357392-1359205	20	8
ACYPI004245-RA	ni|645904120|nb|KK920311.1|	731310-731957	18	18	gi|646736790|gb|KK963145.1|	356626-357369	21	9.6
ACYPI004246-RA	ni|645903982|nb|KK920449.1|	274879-275897	16	14	gi|646778186|gb|KK961609.1|	2601568-2602714	19	9.4
ACYPI004248-RA	ni|645903789|nb|KK920641.1|	487883-488098	17	17	gi|646766703|gb|KK961852.1|	799261-799915	20	7.1
ACYPI004249-RA	ni|645903920|nb|KK920511.1|	1424215-1424642	20	20	gi|646741207|gb|KK962687.1|	169022-169720	17	7.6

ACYPI004258-RA	ni|645902496|nb|KK921933.1|	43143-45569	nan	nan	gi|646744168|gb|KK962452.1|	731117-731771	22	8.7
ACYPI004266-RA	ni|645903960|nb|KK920471.1|	963897-964137	16	8.6	gi|646778565|gb|KK961598.1|	1496804-1497159	19	7.8
ACYPI004268-RA	ni|645904170|nb|KK920263.1|	744155-744693	15	16	gi|646781282|gb|KK961525.1|	1037708-1039666	22	9.6
ACYPI004270-RA	ni|645903770|nb|KK920660.1|	291833-292084	12	15	gi|646746581|gb|KK962279.1|	42123-43158	21	9.1
ACYPI004271-RA	ni|645903860|nb|KK920570.1|	234961-235920	17	17	gi|646749632|gb|KK962082.1|	477596-480226	20	8.6
ACYPI004278-RA	ni|645903650|nb|KK920780.1|	574199-577259	nan	nan	gi|646743576|gb|KK962494.1|	320925-321152	19	8.2
ACYPI004283-RA	ni|645901557|nb|KK922872.1|	37081-37346	nan	nan	gi|646753715|gb|KK961951.1|	974399-974697	19	5.4
ACYPI004286-RA	ni|645903760|nb|KK920670.1|	672622-673008	18	19	gi|646777842|gb|KK961618.1|	2244936-2245562	19	8.9
ACYPI004294-RA	ni|645903566|nb|KK920864.1|	646341-646947	nan	nan	gi|646781243|gb|KK961526.1|	3320544-3320879	21	8.9
ACYPI004297-RA	ni|645903843|nb|KK920587.1|	956077-956240	18	16	gi|646750341|gb|KK962044.1|	887840-892096	19	5.6
ACYPI004307-RA	ni|645904260|nb|KK920228.1|	915794-916419	15	15	gi|646738812|gb|KK962939.1|	205655-205849	20	8.1
ACYPI004308-RA	ni|645904260|nb|KK920228.1|	579285-579507	15	15	gi|646751544|gb|KK961986.1|	775046-780913	22	6
ACYPI004312-RA	ni|645904086|nb|KK920345.1|	395855-397009	14	15	gi|646776647|gb|KK961663.1|	2163546-2164976	23	9.2
ACYPI004328-RA	ni|645904137|nb|KK920296.1|	1649706-1651088	17	17	gi|646781659|gb|KK961513.1|	701750-706295	22	9.8
ACYPI004330-RA	ni|645904251|nb|KK920231.1|	555951-556492	16	16	gi|646782288|gb|KK961496.1|	409653-419079	21	9.7
ACYPI004334-RA	ni|645904258|nb|KK920229.1|	1594016-1594707	16	16	gi|646761166|gb|KK961913.1|	348944-350837	17	7.6
ACYPI004343-RA	ni|645904122|nb|KK920309.1|	276961-277772	18	17	gi|646775702|gb|KK961713.1|	487250-487567	17	7.4
ACYPI004348-RA	ni|645904046|nb|KK920385.1|	535819-536100	17	8.4	gi|646775789|gb|KK961709.1|	1298051-1299294	20	9.6
ACYPI004349-RA	ni|645904116|nb|KK920315.1|	1821169-1823111	19	18	gi|646746310|gb|KK962297.1|	209661-211844	23	11
ACYPI004355-RA	ni|645903721|nb|KK920709.1|	870385-870646	nan	nan	gi|646749832|gb|KK962071.1|	879513-880077	22	6
ACYPI004366-RA	ni|645904088|nb|KK920343.1|	274452-275280	17	18	gi|646746706|gb|KK962270.1|	816516-817695	23	11
ACYPI004368-RA	ni|645903921|nb|KK920510.1|	428521-428770	16	11	gi|646747841|gb|KK962189.1|	749628-752947	23	10
ACYPI004371-RA	ni|645903756|nb|KK920674.1|	670059-671198	16	9	gi|646746407|gb|KK962291.1|	425338-435885	21	9.9
ACYPI004372-RA	ni|645903566|nb|KK920864.1|	455311-455516	nan	nan	gi|646781243|gb|KK961526.1|	3668890-3669778	21	8.9
ACYPI004377-RA	ni|645902706|nb|KK921723.1|	51035-51369	nan	nan	gi|646749209|gb|KK962104.1|	783632-784278	21	9.2
ACYPI004378-RA	ni|645903955|nb|KK920476.1|	173976-174869	18	17	gi|646776822|gb|KK961657.1|	520840-521825	19	7.9
ACYPI004388-RA	ni|645903551|nb|KK920879.1|	321057-321239	nan	nan	gi|646739841|gb|KK962838.1|	152718-152960	21	5.7
ACYPI004395-RA	ni|645901755|nb|KK922674.1|	18471-19736	nan	nan	gi|646771845|gb|KK961763.1|	1182788-1187814	23	10
ACYPI004401-RA	ni|645903971|nb|KK920460.1|	48647-50160	19	18	gi|646775807|gb|KK961708.1|	2766090-2766679	21	8.6
ACYPI004410-RA	ni|645903862|nb|KK920568.1|	172941-173960	17	17	gi|646752431|gb|KK961967.1|	993443-993890	20	5.6

ACYPI004414-RA	ni|645902788|nb|KK921641.1|	205012-205419	nan	nan	gi|646768631|gb|KK961807.1|	1458835-1459078	19	9.2
ACYPI004416-RA	ni|645903944|nb|KK920487.1|	105834-106597	15	17	gi|646782168|gb|KK961499.1|	1991983-1992993	21	9.4
ACYPI004419-RA	ni|645904192|nb|KK920255.1|	410184-410972	15	16	gi|646779375|gb|KK961575.1|	2215035-2215926	22	9.3
ACYPI004420-RA	ni|645903566|nb|KK920864.1|	613351-613600	nan	nan	gi|646781243|gb|KK961526.1|	3404757-3405218	21	8.9
ACYPI004421-RA	ni|645904246|nb|KK920233.1|	933495-935260	16	17	gi|646778699|gb|KK961594.1|	2093315-2098363	21	9.5
ACYPI004423-RA	ni|645903957|nb|KK920474.1|	844498-844636	18	17	gi|646749173|gb|KK962106.1|	245626-245938	21	5.7
ACYPI004424-RA	ni|645903771|nb|KK920659.1|	59045-59718	16	1.50E+01	gi|646746216|gb|KK962304.1|	72892-74095	130	33
ACYPI004428-RA	ni|645903663|nb|KK920767.1|	32890-33099	nan	nan	gi|646751012|gb|KK962011.1|	205541-207542	22	5.6
ACYPI004431-RA	ni|645904116|nb|KK920315.1|	501005-501621	19	18	gi|646753339|gb|KK961955.1|	340989-342589	22	11
ACYPI004435-RA	ni|645903597|nb|KK920833.1|	910585-911169	nan	nan	gi|646747032|gb|KK962248.1|	326198-328710	20	8.7
ACYPI004442-RA	ni|645904028|nb|KK920403.1|	1829708-1830314	18	17	gi|646776510|gb|KK961671.1|	875906-876449	20	7.8
ACYPI004453-RA	ni|645902649|nb|KK921780.1|	204894-206241	nan	nan	gi|646780925|gb|KK961536.1|	1357413-1360757	22	9.9
ACYPI004455-RA	ni|645903996|nb|KK920435.1|	1338693-1338926	17	16	gi|646763535|gb|KK961902.1|	177961-179647	23	10
ACYPI004457-RA	ni|645903511|nb|KK920919.1|	340468-340959	nan	nan	gi|646752348|gb|KK961968.1|	174229-175721	18	8
ACYPI004460-RA	ni|645904231|nb|KK920242.1|	388995-389151	16	17	gi|646776113|gb|KK961692.1|	323914-324976	21	9.6
ACYPI004461-RA	ni|645904177|nb|KK920260.1|	1990201-1991266	18	17	gi|646782357|gb|KK961494.1|	9213771-9214085	21	9.2
ACYPI004463-RA	ni|645902649|nb|KK921780.1|	318731-319224	nan	nan	gi|646743236|gb|KK962519.1|	335978-336617	22	9.1
ACYPI004467-RA	ni|645903697|nb|KK920733.1|	207850-208582	nan	nan	gi|646777802|gb|KK961619.1|	1408031-1408225	24	9
ACYPI004471-RA	ni|645904153|nb|KK920280.1|	2075696-2077098	17	16	gi|646776447|gb|KK961675.1|	2004338-2019098	22	9.4
ACYPI004484-RA	ni|645903895|nb|KK920536.1|	449390-450452	17	16	gi|646752431|gb|KK961967.1|	1539770-1542344	20	5.6
ACYPI004485-RA	ni|645903470|nb|KK920960.1|	206988-207502	nan	nan	gi|646749616|gb|KK962083.1|	1156594-1157883	22	10
ACYPI004488-RA	ni|645903574|nb|KK920856.1|	19612-20166	nan	nan	gi|646750643|gb|KK962028.1|	695252-695933	21	7.5
ACYPI004489-RA	ni|645904065|nb|KK920366.1|	1241140-1241335	18	18	gi|646749464|gb|KK962091.1|	333110-333480	18	8.8
ACYPI004493-RA	ni|645903475|nb|KK920955.1|	261834-262104	nan	nan	gi|646782211|gb|KK961498.1|	5807849-5808235	20	9
ACYPI004496-RA	ni|645904213|nb|KK920248.1|	389585-389802	16	19	gi|646779451|gb|KK961573.1|	117829-118915	20	8.9
ACYPI004501-RA	ni|645903971|nb|KK920460.1|	698189-698696	19	18	gi|646777495|gb|KK961629.1|	1568247-1568438	19	8.7
ACYPI004502-RA	ni|645903773|nb|KK920657.1|	219429-219684	18	17	gi|646750165|gb|KK962053.1|	706955-707220	25	10
ACYPI004505-RA	ni|645904240|nb|KK920239.1|	1798160-1799764	16	16	gi|646780723|gb|KK961542.1|	3554745-3555058	21	9.4
ACYPI004508-RA	ni|645903827|nb|KK920603.1|	710672-711732	17	18	gi|646782357|gb|KK961494.1|	5168018-5169244	21	9.2
ACYPI004513-RA	ni|645904168|nb|KK920265.1|	529291-529720	16	16	gi|646780147|gb|KK961556.1|	1827963-1828393	18	7.5

ACYPI004515-RA	ni|645904112|nb|KK920319.1|	2115216-2116748	18	17	gi|646749188|gb|KK962105.1|	110767-112558	18	7.9
ACYPI004520-RA	ni|645904281|nb|KK920220.1|	831339-831560	15	16	gi|646779298|gb|KK961577.1|	2976283-2980139	20	9.1
ACYPI004521-RA	ni|645903690|nb|KK920740.1|	569499-569954	nan	nan	gi|646769001|gb|KK961801.1|	490643-490916	20	8.5
ACYPI004522-RA	ni|645904111|nb|KK920320.1|	528442-528621	17	17	gi|646738330|gb|KK962987.1|	121609-124441	19	7.9
ACYPI004530-RA	ni|645902172|nb|KK922257.1|	177555-178127	nan	nan	gi|646775507|gb|KK961723.1|	1563690-1565762	20	9.8
ACYPI004534-RA	ni|645903985|nb|KK920446.1|	1013100-1013660	18	16	gi|646749036|gb|KK962114.1|	736188-736508	20	7.5
ACYPI004535-RA	ni|645904054|nb|KK920377.1|	1471087-1471282	16	16	gi|646775884|gb|KK961704.1|	1084525-1085070	20	7.6
ACYPI004544-RA	ni|645903736|nb|KK920694.1|	440807-441515	18	17	gi|646776050|gb|KK961695.1|	124517-124899	19	7.7
ACYPI004549-RA	ni|645903973|nb|KK920458.1|	1471462-1471703	20	18	gi|646781732|gb|KK961511.1|	1664817-1668346	22	9.8
ACYPI004558-RA	ni|645903993|nb|KK920438.1|	298551-298766	19	22	gi|646781344|gb|KK961523.1|	678685-681618	21	8.9
ACYPI004568-RA	ni|645904057|nb|KK920374.1|	166051-166772	16	15	gi|646781772|gb|KK961510.1|	2521949-2522820	20	9.2
ACYPI004570-RA	ni|645904117|nb|KK920314.1|	256015-256460	15	15	gi|646781013|gb|KK961533.1|	453122-459133	18	7.6
ACYPI004575-RA	ni|645904166|nb|KK920267.1|	2702910-2703393	18	16	gi|646737766|gb|KK963045.1|	192969-193275	21	9.2
ACYPI004580-RA	ni|645904228|nb|KK920243.1|	1778676-1779205	17	18	gi|646782288|gb|KK961496.1|	6421686-6421929	21	9.7
ACYPI004588-RA	ni|645904251|nb|KK920231.1|	538202-538573	16	16	gi|646782288|gb|KK961496.1|	473419-480039	21	9.7
ACYPI004613-RA	ni|645903977|nb|KK920454.1|	228959-229861	13	16	gi|646782288|gb|KK961496.1|	6183624-6187655	21	9.7
ACYPI004615-RA	ni|645904260|nb|KK920228.1|	1049634-1050848	15	15	gi|646765689|gb|KK961894.1|	727266-730258	18	7.7
ACYPI004617-RA	ni|645903957|nb|KK920474.1|	976318-976658	18	17	gi|646767265|gb|KK961836.1|	1198385-1201978	22	9.9
ACYPI004619-RA	ni|645903597|nb|KK920833.1|	258266-258724	nan	nan	gi|646782357|gb|KK961494.1|	2057160-2057482	21	9.2
ACYPI004627-RA	ni|645903910|nb|KK920521.1|	561599-562098	19	17	gi|646755567|gb|KK961936.1|	867751-868024	20	8.6
ACYPI004629-RA	ni|645902463|nb|KK921966.1|	231132-232219	nan	nan	gi|646752567|gb|KK961965.1|	316242-317239	19	8.4
ACYPI004634-RA	ni|645903868|nb|KK920562.1|	441720-441967	16	8.7	gi|646746706|gb|KK962270.1|	916994-918232	23	11
ACYPI004635-RA	ni|645903768|nb|KK920662.1|	1306710-1307624	18	17	gi|646781772|gb|KK961510.1|	3802818-3805096	20	9.2
ACYPI004640-RA	ni|645903808|nb|KK920622.1|	988853-989136	17	17	gi|646771352|gb|KK961768.1|	244012-244308	21	9.6
ACYPI004646-RA	ni|645903938|nb|KK920493.1|	1460580-1461744	19	18	gi|646747032|gb|KK962248.1|	441267-441496	20	8.7
ACYPI004656-RA	ni|645902669|nb|KK921760.1|	47359-47563	nan	nan	gi|646775488|gb|KK961724.1|	666126-667962	19	7.8
ACYPI004663-RA	ni|645904177|nb|KK920260.1|	995312-996716	18	17	gi|646780270|gb|KK961553.1|	2450145-2451340	22	8.6
ACYPI004665-RA	ni|645904125|nb|KK920307.1|	36652-37219	18	17	gi|646775968|gb|KK961700.1|	693276-693463	20	8.2
ACYPI004686-RA	ni|645904114|nb|KK920317.1|	1490002-1490945	18	18	gi|646777665|gb|KK961623.1|	1956668-1957068	21	9.7
ACYPI004687-RA	ni|645904077|nb|KK920354.1|	264566-265373	16	17	gi|646782357|gb|KK961494.1|	5259186-5260009	21	9.2

ACYPI004693-RA	ni|645903762|nb|KK920668.1|	344765-345186	17	17	gi|646780574|gb|KK961546.1|	3309878-3311574	20	9.9
ACYPI004696-RA	ni|645903727|nb|KK920703.1|	338427-338812	nan	nan	gi|646762191|gb|KK961910.1|	205249-205581	18	7.1
ACYPI004697-RA	ni|645904112|nb|KK920319.1|	457243-457446	18	17	gi|646782211|gb|KK961498.1|	988580-990820	20	9
ACYPI004699-RA	ni|645904112|nb|KK920319.1|	405784-406071	18	17	gi|646778274|gb|KK961606.1|	907736-908036	18	7.5
ACYPI004701-RA	ni|645904166|nb|KK920267.1|	2675049-2676056	18	16	gi|646741410|gb|KK962669.1|	35505-37144	21	9.3
ACYPI004712-RA	ni|645904142|nb|KK920291.1|	1498568-1500425	20	23	gi|646748103|gb|KK962171.1|	43919-46908	23	10
ACYPI004727-RA	ni|645903597|nb|KK920833.1|	845219-846840	nan	nan	gi|646766624|gb|KK961855.1|	181816-186224	21	10
ACYPI004733-RA	ni|645903822|nb|KK920608.1|	267288-267538	18	20	gi|646749209|gb|KK962104.1|	704254-706401	21	9.2
ACYPI004740-RA	ni|645903957|nb|KK920474.1|	1222746-1223539	18	17	gi|646781043|gb|KK961532.1|	2081786-2082670	26	11
ACYPI004741-RA	ni|645903907|nb|KK920524.1|	228460-229266	16	9	gi|646746974|gb|KK962252.1|	1113532-1116810	23	6.1
ACYPI004748-RA	ni|645903504|nb|KK920926.1|	367299-367742	nan	nan	gi|646771845|gb|KK961763.1|	180243-181887	23	10
ACYPI004749-RA	ni|645902496|nb|KK921933.1|	139905-140729	nan	nan	gi|646772574|gb|KK961756.1|	1014560-1015013	21	8
ACYPI004755-RA	ni|645904112|nb|KK920319.1|	221742-221981	18	17	gi|646772786|gb|KK961754.1|	505086-505610	22	9.6
ACYPI004761-RA	ni|645903855|nb|KK920575.1|	1004450-1004671	16	17	gi|646781143|gb|KK961529.1|	2919162-2919486	20	10
ACYPI004766-RA	ni|645903823|nb|KK920607.1|	360117-360659	16	17	gi|646765857|gb|KK961886.1|	196224-196561	17	7.7
ACYPI004768-RA	ni|645903981|nb|KK920450.1|	124918-125103	16	8.6	gi|646747424|gb|KK962220.1|	138850-139592	22	5.7
ACYPI004770-RA	ni|645901937|nb|KK922492.1|	36931-37140	nan	nan	gi|646744819|gb|KK962404.1|	198161-198386	20	8.6
ACYPI004774-RA	ni|645903752|nb|KK920678.1|	90954-91173	18	16	gi|646781732|gb|KK961511.1|	1228535-1229706	22	9.8
ACYPI004779-RA	ni|645903745|nb|KK920685.1|	24146-24563	16	18	gi|646739581|gb|KK962867.1|	354734-357834	18	5.4
ACYPI004781-RA	ni|645902092|nb|KK922337.1|	12651-13873	nan	nan	gi|646726111|gb|KK964544.1|	72503-73742	12	5.4
ACYPI004783-RA	ni|645903923|nb|KK920508.1|	149019-149721	18	18	gi|646742307|gb|KK962591.1|	130820-131807	23	7.7
ACYPI004804-RA	ni|645903932|nb|KK920499.1|	174152-174303	16	9.1	gi|646758845|gb|KK961921.1|	326855-327121	22	9.5
ACYPI004805-RA	ni|645904166|nb|KK920267.1|	2737685-2737853	18	16	gi|646752294|gb|KK961969.1|	613294-615121	23	9.8
ACYPI004809-RA	ni|645903650|nb|KK920780.1|	141367-142899	nan	nan	gi|646781628|gb|KK961514.1|	4768649-4771127	23	9
ACYPI004814-RA	ni|645903477|nb|KK920953.1|	38571-38849	nan	nan	gi|646780925|gb|KK961536.1|	2022897-2027983	22	9.9
ACYPI004816-RA	ni|645903697|nb|KK920733.1|	104398-105366	nan	nan	gi|646778410|gb|KK961602.1|	1995292-1999872	20	9
ACYPI004822-RA	ni|645902290|nb|KK922139.1|	142812-143238	nan	nan	gi|646742237|gb|KK962597.1|	483568-487448	21	6
ACYPI004824-RA	ni|645904096|nb|KK920335.1|	2416798-2417710	18	17	gi|646743164|gb|KK962524.1|	64291-67104	19	7.1
ACYPI004827-RA	ni|645903983|nb|KK920448.1|	716230-716994	18	17	gi|646774067|gb|KK961745.1|	1541836-1542070	17	9.3
ACYPI004832-RA	ni|645904091|nb|KK920340.1|	657636-657919	15	18	gi|646779006|gb|KK961585.1|	905670-906187	20	9.9

ACYPI004851-RA	ni|645904088|nb|KK920343.1|	1482527-1483333	17	18	gi|646776113|gb|KK961692.1|	295601-296267	21	9.6
ACYPI004870-RA	ni|645903496|nb|KK920934.1|	591520-592676	nan	nan	gi|646781536|gb|KK961517.1|	1279193-1279894	19	7.7
ACYPI004872-RA	ni|645902471|nb|KK921958.1|	210443-211128	nan	nan	gi|646782168|gb|KK961499.1|	7298011-7298940	21	9.4
ACYPI004878-RA	ni|645904132|nb|KK920301.1|	576845-577012	16	18	gi|646779412|gb|KK961574.1|	2165323-2168377	21	5.4
ACYPI004880-RA	ni|645903568|nb|KK920862.1|	224022-224602	nan	nan	gi|646740073|gb|KK962814.1|	10677-11241	18	8.3
ACYPI004883-RA	ni|645903716|nb|KK920714.1|	266928-268964	nan	nan	gi|646780222|gb|KK961554.1|	1181800-1185558	20	9.7
ACYPI004886-RA	ni|645903708|nb|KK920722.1|	27374-27583	nan	nan	gi|646751176|gb|KK962002.1|	807434-808612	21	9.6
ACYPI004896-RA	ni|645903738|nb|KK920692.1|	314062-314314	18	17	gi|646779101|gb|KK961582.1|	21440-21690	18	7.3
ACYPI004908-RA	ni|645903971|nb|KK920460.1|	1573467-1573685	19	18	gi|646766540|gb|KK961858.1|	1265743-1266136	21	8.5
ACYPI004911-RA	ni|645902598|nb|KK921831.1|	63118-64202	nan	nan	gi|646782168|gb|KK961499.1|	6940829-6941078	21	9.4
ACYPI004920-RA	ni|645904004|nb|KK920427.1|	814425-815530	16	16	gi|646746489|gb|KK962285.1|	265641-266357	17	6.8
ACYPI004936-RA	ni|645903785|nb|KK920645.1|	450559-450956	15	16	gi|646688645|gb|KK967064.1|	31875-32418	14	5
ACYPI004938-RA	ni|645902681|nb|KK921748.1|	310537-311067	nan	nan	gi|646749616|gb|KK962083.1|	516438-526335	22	10
ACYPI004940-RA	ni|645904045|nb|KK920386.1|	660891-661449	19	21	gi|646751569|gb|KK961985.1|	170070-172197	21	9.2
ACYPI004948-RA	ni|645901380|nb|KK923049.1|	59135-62089	nan	nan	gi|646778632|gb|KK961596.1|	903106-908833	21	9.2
ACYPI004950-RA	ni|645904090|nb|KK920341.1|	297359-298085	15	17	gi|646781690|gb|KK961512.1|	2130605-2131223	21	8.8
ACYPI004964-RA	ni|645904074|nb|KK920357.1|	1008029-1008284	17	18	gi|646781143|gb|KK961529.1|	2817345-2819427	20	10
ACYPI004966-RA	ni|645903957|nb|KK920474.1|	1149806-1150831	18	17	gi|646780441|gb|KK961549.1|	1007895-1009624	21	9.5
ACYPI004977-RA	ni|645904028|nb|KK920403.1|	377163-379793	18	17	gi|646735008|gb|KK963353.1|	216593-217591	21	9.4
ACYPI004979-RA	ni|645903534|nb|KK920896.1|	618842-619558	nan	nan	gi|646766904|gb|KK961846.1|	829517-829819	17	6.9
ACYPI004983-RA	ni|645903535|nb|KK920895.1|	192452-192719	nan	nan	gi|646741778|gb|KK962637.1|	27686-28358	19	6.1
ACYPI004985-RA	ni|645903781|nb|KK920649.1|	319155-319591	14	15	gi|646770114|gb|KK961783.1|	1455183-1455730	19	9.7
ACYPI004986-RA	ni|645903716|nb|KK920714.1|	622792-623290	nan	nan	gi|646775968|gb|KK961700.1|	1333259-1333531	20	8.2
ACYPI004992-RA	ni|645903612|nb|KK920818.1|	61000-61918	nan	nan	gi|646749188|gb|KK962105.1|	342343-342850	18	7.9
ACYPI004995-RA	ni|645903491|nb|KK920939.1|	426546-427219	nan	nan	gi|646781564|gb|KK961516.1|	1480700-1481894	23	9.4
ACYPI005000-RA	ni|645904113|nb|KK920318.1|	446869-447110	14	15	gi|646778336|gb|KK961604.1|	2454941-2457328	21	10
ACYPI005007-RA	ni|645901858|nb|KK922571.1|	142168-142762	nan	nan	gi|646776351|gb|KK961680.1|	151273-151552	22	10
ACYPI005014-RA	ni|645902350|nb|KK922079.1|	61214-61959	nan	nan	gi|646767670|gb|KK961827.1|	1617703-1618076	21	5.9
ACYPI005018-RA	ni|645903748|nb|KK920682.1|	324107-324781	17	18	gi|646780222|gb|KK961554.1|	3301874-3303251	20	9.7
ACYPI005019-RA	ni|645903603|nb|KK920827.1|	332132-332554	nan	nan	gi|646777065|gb|KK961647.1|	990932-993461	20	7.9

ACYPI005028-RA	ni|645903866|nb|KK920564.1|	507344-508540	18	15	gi|646774530|gb|KK961743.1|	379038-381487	20	9.8
ACYPI005033-RA	ni|645903831|nb|KK920599.1|	1003530-1004000	18	17	gi|646743859|gb|KK962475.1|	471212-472353	20	7.9
ACYPI005035-RA	ni|645903549|nb|KK920881.1|	467638-467842	nan	nan	gi|646780627|gb|KK961545.1|	1387413-1387624	20	7.7
ACYPI005038-RA	ni|645904112|nb|KK920319.1|	232715-233665	18	17	gi|646750043|gb|KK962060.1|	757679-757990	21	8.3
ACYPI005041-RA	ni|645902172|nb|KK922257.1|	110453-111178	nan	nan	gi|646776024|gb|KK961697.1|	2409088-2409374	22	9.2
ACYPI005044-RA	ni|645904115|nb|KK920316.1|	1087675-1088263	17	18	gi|646780147|gb|KK961556.1|	2209925-2210176	18	7.5
ACYPI005047-RA	ni|645903911|nb|KK920520.1|	543859-544272	16	17	gi|646778112|gb|KK961611.1|	1970725-1976096	22	10
ACYPI005053-RA	ni|645900486|nb|KK923943.1|	49912-50218	nan	nan	gi|646747884|gb|KK962186.1|	871535-872155	22	11
ACYPI005060-RA	ni|645902538|nb|KK921891.1|	151786-152139	nan	nan	gi|646782288|gb|KK961496.1|	7810738-7812362	21	9.7
ACYPI005067-RA	ni|645904104|nb|KK920327.1|	313298-313596	15	15	gi|646737959|gb|KK963025.1|	387330-389046	19	7.9
ACYPI005074-RA	ni|645903504|nb|KK920926.1|	597383-597853	nan	nan	gi|646778149|gb|KK961610.1|	2302044-2304193	19	8.9
ACYPI005089-RA	ni|645903983|nb|KK920448.1|	722278-723856	18	17	gi|646722582|gb|KK964929.1|	32148-41758	23	6
ACYPI005091-RA	ni|645904101|nb|KK920330.1|	18591-19093	18	18	gi|646781379|gb|KK961522.1|	3823649-3824187	22	7.6
ACYPI005093-RA	ni|645903975|nb|KK920456.1|	1140547-1141974	15	8.2	gi|646778767|gb|KK961592.1|	1308647-1314006	21	5.4
ACYPI005103-RA	ni|645904012|nb|KK920419.1|	235441-235997	27	32	gi|646744324|gb|KK962441.1|	588-903	20	7.2
ACYPI005113-RA	ni|645903983|nb|KK920448.1|	879016-880592	18	17	gi|646776456|gb|KK961674.1|	1311316-1313786	18	7.9
ACYPI005116-RA	ni|645903787|nb|KK920643.1|	1199864-1200269	19	18	gi|646781043|gb|KK961532.1|	1663719-1663990	26	11
ACYPI005119-RA	ni|645904132|nb|KK920301.1|	173633-173858	16	18	gi|646781183|gb|KK961528.1|	3843931-3844667	20	9.5
ACYPI005122-RA	ni|645903634|nb|KK920796.1|	1096273-1096492	nan	nan	gi|646780311|gb|KK961552.1|	3719208-3725697	21	9.7
ACYPI005123-RA	ni|645904237|nb|KK920240.1|	1198842-1199030	16	15	gi|646766833|gb|KK961848.1|	382584-385833	17	7
ACYPI005124-RA	ni|645903800|nb|KK920630.1|	1556718-1556905	18	17	gi|646775867|gb|KK961705.1|	1959000-1959187	23	10
ACYPI005133-RA	ni|645903965|nb|KK920466.1|	1020492-1020759	17	16	gi|646757279|gb|KK961927.1|	570895-573892	21	8.6
ACYPI005144-RA	ni|645904144|nb|KK920289.1|	673521-674551	16	9	gi|646779741|gb|KK961566.1|	2939635-2940394	20	8.5
ACYPI005152-RA	ni|645903678|nb|KK920752.1|	435236-436098	nan	nan	gi|646677811|gb|KK967447.1|	6679-7767	13	8.3
ACYPI005155-RA	ni|645902293|nb|KK922136.1|	66072-66318	nan	nan	gi|646775523|gb|KK961722.1|	1176464-1181597	21	9.7
ACYPI005167-RA	ni|645904146|nb|KK920287.1|	1377193-1377659	16	18	gi|646781143|gb|KK961529.1|	2195844-2196738	20	10
ACYPI005173-RA	ni|645903811|nb|KK920619.1|	12571-13989	15	15	gi|646685025|gb|KK967178.1|	20901-22353	17	5.2
ACYPI005174-RA	ni|645902129|nb|KK922300.1|	147859-148445	nan	nan	gi|646776490|gb|KK961672.1|	21158-22761	20	10
ACYPI005175-RA	ni|645902690|nb|KK921739.1|	292933-293317	nan	nan	gi|646732968|gb|KK963602.1|	367061-367781	20	9.9
ACYPI005193-RA	ni|645903727|nb|KK920703.1|	1126522-1128064	nan	nan	gi|646781628|gb|KK961514.1|	4639000-4641938	23	9

ACYPI005200-RA	ni|645901741|nb|KK922688.1|	42828-45563	nan	nan	gi|646781043|gb|KK961532.1|	4197254-4198590	26	11
ACYPI005202-RA	ni|645903996|nb|KK920435.1|	1339129-1339390	17	16	gi|646741767|gb|KK962638.1|	36281-37350	21	10
ACYPI005207-RA	ni|645904022|nb|KK920409.1|	209261-209846	18	20	gi|646770998|gb|KK961772.1|	1781680-1783927	22	9.8
ACYPI005208-RA	ni|645901808|nb|KK922621.1|	104436-104610	nan	nan	gi|646753093|gb|KK961958.1|	1121400-1124006	21	9.3
ACYPI005219-RA	ni|645904139|nb|KK920294.1|	385140-388914	15	18	gi|646747841|gb|KK962189.1|	317968-318410	23	10
ACYPI005221-RA	ni|645903953|nb|KK920478.1|	1394055-1394425	18	17	gi|646771092|gb|KK961771.1|	528931-530193	23	9.7
ACYPI005227-RA	ni|645904019|nb|KK920412.1|	152257-152733	19	9.4	gi|646775789|gb|KK961709.1|	1188784-1189471	20	9.6
ACYPI005237-RA	ni|645901808|nb|KK922621.1|	146881-147793	nan	nan	gi|646769774|gb|KK961788.1|	844641-855761	20	9.2
ACYPI005241-RA	ni|645902556|nb|KK921873.1|	49937-50656	nan	nan	gi|646765790|gb|KK961889.1|	934732-935449	22	8.8
ACYPI005243-RA	ni|645903838|nb|KK920592.1|	211824-212061	16	16	gi|646747884|gb|KK962186.1|	506967-513329	22	11
ACYPI005247-RA	ni|645903644|nb|KK920786.1|	397188-398703	nan	nan	gi|646781732|gb|KK961511.1|	2219028-2220589	22	9.8
ACYPI005253-RA	ni|645903495|nb|KK920935.1|	450806-451174	nan	nan	gi|646755393|gb|KK961937.1|	203176-204012	19	6.8
ACYPI005264-RA	ni|645904154|nb|KK920279.1|	710537-711640	14	14	gi|646740304|gb|KK962788.1|	600228-607968	24	9.6
ACYPI005270-RA	ni|645904077|nb|KK920354.1|	705564-705709	16	17	gi|646778213|gb|KK961608.1|	1397324-1398549	19	8.8
ACYPI005271-RA	ni|645903771|nb|KK920659.1|	28378-28555	16	15	gi|646778149|gb|KK961610.1|	1424943-1425127	19	8.9
ACYPI005282-RA	ni|645901710|nb|KK922719.1|	73895-74944	nan	nan	gi|646778336|gb|KK961604.1|	2771944-2772824	21	10
ACYPI005283-RA	ni|645903627|nb|KK920803.1|	506009-507155	nan	nan	gi|646775488|gb|KK961724.1|	721206-722676	19	7.8
ACYPI005295-RA	ni|645903572|nb|KK920858.1|	98432-98634	nan	nan	gi|646781313|gb|KK961524.1|	459701-459957	19	8.5
ACYPI005296-RA	ni|645903513|nb|KK920917.1|	431891-432363	nan	nan	gi|646782211|gb|KK961498.1|	78611-78822	20	9
ACYPI005300-RA	ni|645903855|nb|KK920575.1|	1046028-1046940	16	17	gi|646747547|gb|KK962210.1|	393459-395163	19	7.5
ACYPI005308-RA	ni|645903780|nb|KK920650.1|	689781-690381	19	19	gi|646775842|gb|KK961706.1|	1240797-1243932	22	9
ACYPI005313-RA	ni|645904114|nb|KK920317.1|	1910124-1910376	18	18	gi|646751664|gb|KK961982.1|	1163913-1164442	21	9.5
ACYPI005317-RA	ni|645903643|nb|KK920787.1|	137981-138383	nan	nan	gi|646750600|gb|KK962030.1|	1446627-1447523	23	8.6
ACYPI005320-RA	ni|645903879|nb|KK920552.1|	121750-122797	18	18	gi|646776542|gb|KK961668.1|	3387333-3388591	20	5.6
ACYPI005321-RA	ni|645903825|nb|KK920605.1|	755385-756517	19	17	gi|646769577|gb|KK961791.1|	568856-569535	18	8.3
ACYPI005327-RA	ni|645903702|nb|KK920728.1|	388929-389727	nan	nan	gi|646781118|gb|KK961530.1|	4365006-4365912	21	10
ACYPI005329-RA	ni|645904061|nb|KK920370.1|	1048258-1048497	17	16	gi|646747586|gb|KK962207.1|	405420-405987	20	8.8
ACYPI005331-RA	ni|645904004|nb|KK920427.1|	840532-840922	16	16	gi|646767265|gb|KK961836.1|	845425-846311	22	9.9
ACYPI005335-RA	ni|645904081|nb|KK920350.1|	173227-173470	13	18	gi|646731405|gb|KK963818.1|	9413-10164	24	11
ACYPI005339-RA	ni|645904262|nb|KK920227.1|	656673-657184	17	17	gi|646768294|gb|KK961814.1|	133164-134676	23	10

ACYPI005349-RA	ni|645903918|nb|KK920513.1|	310130-310625	15	18	gi|646767948|gb|KK961821.1|	524252-524526	17	7.1
ACYPI005353-RA	ni|645903592|nb|KK920838.1|	563792-564642	nan	nan	gi|646776389|gb|KK961678.1|	728182-731482	22	9.5
ACYPI005359-RA	ni|645903988|nb|KK920443.1|	505487-506255	14	16	gi|646775540|gb|KK961721.1|	948316-948798	17	8.1
ACYPI005360-RA	ni|645904065|nb|KK920366.1|	963245-963848	18	18	gi|646734874|gb|KK963368.1|	29893-33208	21	8
ACYPI005363-RA	ni|645903958|nb|KK920473.1|	25119-26075	16	16	gi|646775789|gb|KK961709.1|	117377-119088	20	9.6
ACYPI005364-RA	ni|645903574|nb|KK920856.1|	95005-95530	nan	nan	gi|646769774|gb|KK961788.1|	548323-549374	20	9.2
ACYPI005367-RA	ni|645903803|nb|KK920627.1|	819586-820867	17	17	gi|646759358|gb|KK961918.1|	197880-207517	16	5.4
ACYPI005368-RA	ni|645903961|nb|KK920470.1|	866911-867475	17	15	gi|646745612|gb|KK962344.1|	170763-171937	20	8.2
ACYPI005371-RA	ni|645904268|nb|KK920225.1|	324463-324783	14	15	gi|646751160|gb|KK962003.1|	597142-599197	22	10
ACYPI005378-RA	ni|645903800|nb|KK920630.1|	1617453-1618121	18	17	gi|646781968|gb|KK961504.1|	954509-958304	20	9.6
ACYPI005389-RA	ni|645904204|nb|KK920251.1|	782045-786457	15	16	gi|646749302|gb|KK962099.1|	476936-478271	17	7
ACYPI005391-RA	ni|645904177|nb|KK920260.1|	1495333-1496298	18	17	gi|646782334|gb|KK961495.1|	3403864-3405361	21	9
ACYPI005393-RA	ni|645903985|nb|KK920446.1|	453773-454015	18	16	gi|646750252|gb|KK962049.1|	878386-878883	22	10
ACYPI005394-RA	ni|645901763|nb|KK922666.1|	44341-45141	nan	nan	gi|646738229|gb|KK962998.1|	340936-346928	22	8.9
ACYPI005395-RA	ni|645902556|nb|KK921873.1|	55973-56519	nan	nan	gi|646779141|gb|KK961581.1|	2751958-2753981	21	9.1
ACYPI005400-RA	ni|645903787|nb|KK920643.1|	1271270-1272930	19	18	gi|646768631|gb|KK961807.1|	430831-432383	19	9.2
ACYPI005416-RA	ni|645904134|nb|KK920299.1|	441790-442267	17	13	gi|646780105|gb|KK961557.1|	203172-203790	21	9.7
ACYPI005418-RA	ni|645902503|nb|KK921926.1|	132663-133214	nan	nan	gi|646776128|gb|KK961691.1|	1702175-1710812	21	9.2
ACYPI005419-RA	ni|645903873|nb|KK920558.1|	662728-662833	16	15	gi|646781690|gb|KK961512.1|	1817046-1818934	21	8.8
ACYPI005434-RA	ni|645903851|nb|KK920579.1|	246228-247288	14	19	gi|646782043|gb|KK961502.1|	4113098-4113408	20	9.2
ACYPI005456-RA	ni|645903981|nb|KK920450.1|	455935-456537	16	8.6	gi|646780925|gb|KK961536.1|	949915-950232	22	9.9
ACYPI005457-RA	ni|645903547|nb|KK920883.1|	627388-627777	nan	nan	gi|646781313|gb|KK961524.1|	3533812-3535266	19	8.5
ACYPI005458-RA	ni|645902779|nb|KK921650.1|	94716-96301	nan	nan	gi|646782357|gb|KK961494.1|	4268127-4284704	21	9.2
ACYPI005464-RA	ni|645900799|nb|KK923630.1|	6023-6272	nan	nan	gi|646746663|gb|KK962273.1|	134543-134783	21	10
ACYPI005468-RA	ni|645903744|nb|KK920686.1|	575153-575302	17	8.6	gi|646776184|gb|KK961688.1|	873655-874692	21	9.6
ACYPI005470-RA	ni|645904077|nb|KK920354.1|	663260-664414	16	17	gi|646782334|gb|KK961495.1|	2952482-2952715	21	9
ACYPI005476-RA	ni|645904137|nb|KK920296.1|	1303808-1304547	17	17	gi|646776514|gb|KK961670.1|	1595146-1596259	20	9.6
ACYPI005478-RA	ni|645904201|nb|KK920252.1|	334053-334539	17	16	gi|646743261|gb|KK962517.1|	34238-35511	19	8.9
ACYPI005479-RA	ni|645903784|nb|KK920646.1|	340916-341776	17	19	gi|646776368|gb|KK961679.1|	1787788-1788552	21	9.7
ACYPI005482-RA	ni|645904024|nb|KK920407.1|	541012-541576	14	16	gi|646776148|gb|KK961690.1|	1920350-1920892	21	9.4

ACYPI005489-RA	ni|645904168|nb|KK920265.1|	1579185-1579530	16	16	gi|646777416|gb|KK961632.1|	556404-557617	22	8.8
ACYPI005496-RA	ni|645903694|nb|KK920736.1|	422731-423446	nan	nan	gi|646750600|gb|KK962030.1|	1570058-1572154	23	8.6
ACYPI005499-RA	ni|645903612|nb|KK920818.1|	95659-96271	nan	nan	gi|646781690|gb|KK961512.1|	3337509-3338589	21	8.8
ACYPI005509-RA	ni|645903970|nb|KK920461.1|	840452-841449	17	16	gi|646781421|gb|KK961521.1|	4436012-4437649	21	8.7
ACYPI005512-RA	ni|645903627|nb|KK920803.1|	238941-239709	nan	nan	gi|646673021|gb|KK967651.1|	768-1149	10	6.1
ACYPI005514-RA	ni|645903547|nb|KK920883.1|	500931-501158	nan	nan	gi|646781043|gb|KK961532.1|	332887-333200	26	11
ACYPI005517-RA	ni|645903592|nb|KK920838.1|	573285-574973	nan	nan	gi|646782334|gb|KK961495.1|	7245001-7245990	21	9
ACYPI005521-RA	ni|645904134|nb|KK920299.1|	849449-850516	17	13	gi|646775595|gb|KK961718.1|	1326427-1327601	21	8.9
ACYPI005524-RA	ni|645904086|nb|KK920345.1|	410385-411669	14	15	gi|646776647|gb|KK961663.1|	2188197-2190437	23	9.2
ACYPI005525-RA	ni|645904152|nb|KK920281.1|	432807-433798	15	15	gi|646749302|gb|KK962099.1|	208435-210978	17	7
ACYPI005528-RA	ni|645903534|nb|KK920896.1|	579054-579287	nan	nan	gi|646781212|gb|KK961527.1|	1172672-1178289	21	10
ACYPI005532-RA	ni|645904278|nb|KK920222.1|	1011353-1012442	15	16	gi|646777912|gb|KK961616.1|	347394-348595	20	8.6
ACYPI005535-RA	ni|645904058|nb|KK920373.1|	336435-337268	16	15	gi|646748407|gb|KK962151.1|	606025-606543	22	9.1
ACYPI005538-RA	ni|645904219|nb|KK920246.1|	423107-423761	16	17	gi|646781732|gb|KK961511.1|	5295838-5306592	22	9.8
ACYPI005543-RA	ni|645903639|nb|KK920791.1|	413526-414440	nan	nan	gi|646782357|gb|KK961494.1|	5931366-5934131	21	9.2
ACYPI005557-RA	ni|645903569|nb|KK920861.1|	255917-256907	nan	nan	gi|646748086|gb|KK962172.1|	851816-852182	20	10
ACYPI005571-RA	ni|645903879|nb|KK920552.1|	204141-204275	18	18	gi|646744453|gb|KK962431.1|	20134-21025	19	8.5
ACYPI005579-RA	ni|645904077|nb|KK920354.1|	683816-684211	16	17	gi|646738615|gb|KK962959.1|	242512-242939	21	9.5
ACYPI005580-RA	ni|645903762|nb|KK920668.1|	884594-885578	17	17	gi|646777363|gb|KK961635.1|	1885460-1885947	18	9.2
ACYPI005583-RA	ni|645903846|nb|KK920584.1|	55085-55966	17	10	gi|646768447|gb|KK961811.1|	272092-272353	22	9.7
ACYPI005585-RA	ni|645904201|nb|KK920252.1|	183108-183483	17	16	gi|646743261|gb|KK962517.1|	321893-333156	19	8.9
ACYPI005588-RA	ni|645903849|nb|KK920581.1|	60264-61423	17	17	gi|646781143|gb|KK961529.1|	4199926-4202060	20	10
ACYPI005593-RA	ni|645902354|nb|KK922075.1|	125620-125890	nan	nan	gi|646742471|gb|KK962577.1|	250665-251404	22	5.9
ACYPI005594-RA	ni|645904123|nb|KK920308.1|	1606239-1606800	16	10	gi|646765669|gb|KK961895.1|	80366-80653	18	7.2
ACYPI005597-RA	ni|645903464|nb|KK920966.1|	444766-445195	nan	nan	gi|646782357|gb|KK961494.1|	1275957-1277319	21	9.2
ACYPI005600-RA	ni|645903545|nb|KK920885.1|	58888-59114	nan	nan	gi|646748474|gb|KK962147.1|	469276-470105	20	9
ACYPI005606-RA	ni|645904251|nb|KK920231.1|	392565-392735	16	16	gi|646782288|gb|KK961496.1|	7553165-7553397	21	9.7
ACYPI005607-RA	ni|645904027|nb|KK920404.1|	598107-599723	17	17	gi|646770477|gb|KK961778.1|	1131394-1131659	21	9.1
ACYPI005613-RA	ni|645903836|nb|KK920594.1|	169031-170158	18	11	gi|646778767|gb|KK961592.1|	208015-211193	21	5.4
ACYPI005614-RA	ni|645903491|nb|KK920939.1|	648313-649104	nan	nan	gi|646773018|gb|KK961752.1|	407033-411250	18	7.8

ACYPI005619-RA	ni|645904015|nb|KK920416.1|	1253180-1255265	18	16	gi|646782357|gb|KK961494.1|	3452828-3456929	21	9.2
ACYPI005622-RA	ni|645902471|nb|KK921958.1|	183292-184343	nan	nan	gi|646765948|gb|KK961882.1|	282593-283083	18	7.3
ACYPI005626-RA	ni|645903539|nb|KK920891.1|	144225-144461	nan	nan	gi|646749945|gb|KK962065.1|	1749651-1756478	22	6.1
ACYPI005627-RA	ni|645903709|nb|KK920721.1|	260922-262443	nan	nan	gi|646777089|gb|KK961646.1|	757626-761528	20	9.4
ACYPI005638-RA	ni|645901989|nb|KK922440.1|	8684-8899	nan	nan	gi|646767697|gb|KK961826.1|	867427-870484	21	9.8
ACYPI005644-RA	ni|645902662|nb|KK921767.1|	422594-422809	nan	nan	gi|646753890|gb|KK961949.1|	744826-745365	20	8.9
ACYPI005647-RA	ni|645903796|nb|KK920634.1|	913461-913990	18	17	gi|646781043|gb|KK961532.1|	2426872-2430438	26	11
ACYPI005655-RA	ni|645903873|nb|KK920558.1|	469213-470024	16	15	gi|646781690|gb|KK961512.1|	1960516-1961106	21	8.8
ACYPI005660-RA	ni|645904138|nb|KK920295.1|	471760-473136	15	15	gi|646777729|gb|KK961621.1|	1881764-1888857	21	9.8
ACYPI005668-RA	ni|645903648|nb|KK920782.1|	256402-256734	nan	nan	gi|646682319|gb|KK967352.1|	30010-30570	13	6.2
ACYPI005672-RA	ni|645904009|nb|KK920422.1|	839049-840138	17	9.4	gi|646747471|gb|KK962216.1|	712268-712957	24	6.4
ACYPI005673-RA	ni|645903696|nb|KK920734.1|	559883-560274	nan	nan	gi|646766943|gb|KK961845.1|	636703-637583	18	8.9
ACYPI005674-RA	ni|645904051|nb|KK920380.1|	480022-480316	16	17	gi|646736283|gb|KK963203.1|	80665-81078	24	9.7
ACYPI005676-RA	ni|645904052|nb|KK920379.1|	576008-576381	18	16	gi|646780978|gb|KK961534.1|	393472-394280	20	8
ACYPI005677-RA	ni|645904007|nb|KK920424.1|	420823-421043	18	17	gi|646778699|gb|KK961594.1|	663077-665563	21	9.5
ACYPI005687-RA	ni|645902371|nb|KK922058.1|	113237-113981	nan	nan	gi|646742319|gb|KK962590.1|	92513-95142	18	8.5
ACYPI005689-RA	ni|645904093|nb|KK920338.1|	33075-33731	13	15	gi|646781564|gb|KK961516.1|	1295961-1296625	23	9.4
ACYPI005692-RA	ni|645902698|nb|KK921731.1|	94775-96390	nan	nan	gi|646782276|gb|KK961497.1|	4909580-4909813	21	9.7
ACYPI005705-RA	ni|645903642|nb|KK920788.1|	384543-384874	nan	nan	gi|646779498|gb|KK961572.1|	828920-829271	22	5.6
ACYPI005706-RA	ni|645904177|nb|KK920260.1|	2568717-2568910	18	17	gi|646749114|gb|KK962109.1|	552627-555443	20	8.8
ACYPI005710-RA	ni|645903919|nb|KK920512.1|	1246602-1247063	17	15	gi|646780147|gb|KK961556.1|	1971348-1974505	18	7.5
ACYPI005711-RA	ni|645903869|nb|KK920561.1|	957481-959306	17	15	gi|646765705|gb|KK961893.1|	1434813-1435318	22	9.7
ACYPI005720-RA	ni|645903798|nb|KK920632.1|	63777-64298	18	17	gi|646780889|gb|KK961537.1|	5179999-5180478	22	10
ACYPI005722-RA	ni|645901356|nb|KK923073.1|	6022-6956	nan	nan	gi|646750505|gb|KK962035.1|	248950-249273	19	9.9
ACYPI005729-RA	ni|645903906|nb|KK920525.1|	370642-371179	18	22	gi|646743084|gb|KK962530.1|	163288-164055	24	6.2
ACYPI005735-RA	ni|645903973|nb|KK920458.1|	1312921-1313506	20	18	gi|646781809|gb|KK961509.1|	3324099-3325509	19	8.6
ACYPI005743-RA	ni|645904023|nb|KK920408.1|	727289-731538	17	17	gi|646729045|gb|KK964126.1|	147961-149410	20	11
ACYPI005747-RA	ni|645904007|nb|KK920424.1|	332050-334718	18	17	gi|646775821|gb|KK961707.1|	836522-838985	23	9.1
ACYPI005749-RA	ni|645904268|nb|KK920225.1|	246941-247097	14	15	gi|646779141|gb|KK961581.1|	2820620-2820920	21	9.1
ACYPI005761-RA	ni|645902662|nb|KK921767.1|	296505-298449	nan	nan	gi|646751120|gb|KK962005.1|	1469458-1469835	21	5.8

ACYPI005769-RA	ni|645903804|nb|KK920626.1|	618759-619033	17	17	gi|646755095|gb|KK961939.1|	396175-397628	15	8.9
ACYPI005770-RA	ni|645902084|nb|KK922345.1|	118102-118554	nan	nan	gi|646779976|gb|KK961560.1|	684147-685126	16	7.9
ACYPI005774-RA	ni|645903957|nb|KK920474.1|	1424751-1425165	18	17	gi|646778767|gb|KK961592.1|	88103-88358	21	5.4
ACYPI005787-RA	ni|645904115|nb|KK920316.1|	1802020-1802247	17	18	gi|646776219|gb|KK961687.1|	1625147-1625384	21	9.9
ACYPI005789-RA	ni|645902471|nb|KK921958.1|	191580-191700	nan	nan	gi|646738455|gb|KK962975.1|	284769-291267	23	9.5
ACYPI005793-RA	ni|645904015|nb|KK920416.1|	1315364-1316340	18	16	gi|646769102|gb|KK961799.1|	180565-181419	20	8.3
ACYPI005802-RA	ni|645904062|nb|KK920369.1|	82197-82446	14	15	gi|646775884|gb|KK961704.1|	646546-647233	20	7.6
ACYPI005805-RA	ni|645903592|nb|KK920838.1|	739318-739591	nan	nan	gi|646778061|gb|KK961612.1|	1059995-1061909	20	8.7
ACYPI005817-RA	ni|645903688|nb|KK920742.1|	222429-222764	nan	nan	gi|646781421|gb|KK961521.1|	2303331-2303680	21	8.7
ACYPI005824-RA	ni|645904222|nb|KK920245.1|	692309-692579	17	12	gi|646777125|gb|KK961644.1|	2232728-2233437	20	10
ACYPI005826-RA	ni|645902468|nb|KK921961.1|	82852-83463	nan	nan	gi|646782357|gb|KK961494.1|	9003940-9009906	21	9.2
ACYPI005830-RA	ni|645903650|nb|KK920780.1|	820836-820987	nan	nan	gi|646751735|gb|KK961980.1|	638775-641943	21	5.5
ACYPI005832-RA	ni|645903592|nb|KK920838.1|	875477-875682	nan	nan	gi|646781536|gb|KK961517.1|	118133-120894	19	7.7
ACYPI005844-RA	ni|645903971|nb|KK920460.1|	1228698-1229331	19	18	gi|646779337|gb|KK961576.1|	728964-729703	18	9
ACYPI005847-RA	ni|645903959|nb|KK920472.1|	917138-917370	15	15	gi|646743772|gb|KK962481.1|	62948-63865	22	8.9
ACYPI005852-RA	ni|645904116|nb|KK920315.1|	655723-656372	19	18	gi|646753799|gb|KK961950.1|	2018910-2019690	23	9.2
ACYPI005853-RA	ni|645904231|nb|KK920242.1|	586392-588632	16	17	gi|646700461|gb|KK965992.1|	18068-19051	70	24
ACYPI005858-RA	ni|645903848|nb|KK920582.1|	604272-604444	18	18	gi|646781344|gb|KK961523.1|	661397-672200	21	8.9
ACYPI005859-RA	ni|645904122|nb|KK920309.1|	194050-194558	18	17	gi|646746828|gb|KK962262.1|	534864-535128	20	9.2
ACYPI005865-RA	ni|645904052|nb|KK920379.1|	540363-541135	18	16	gi|646751569|gb|KK961985.1|	217504-218010	21	9.2
ACYPI005867-RA	ni|645902172|nb|KK922257.1|	159028-159242	nan	nan	gi|646782334|gb|KK961495.1|	2446244-2449854	21	9
ACYPI005880-RA	ni|645904116|nb|KK920315.1|	2189215-2189699	19	18	gi|646778029|gb|KK961613.1|	2884287-2885428	21	8.6
ACYPI005883-RA	ni|645903635|nb|KK920795.1|	326326-327821	nan	nan	gi|646744916|gb|KK962397.1|	559562-560331	18	8.3
ACYPI005886-RA	ni|645902742|nb|KK921687.1|	140037-144926	nan	nan	gi|646749173|gb|KK962106.1|	1174233-1184062	21	5.7
ACYPI005894-RA	ni|645903783|nb|KK920647.1|	1589483-1589714	17	17	gi|646776351|gb|KK961680.1|	177597-178342	22	10
ACYPI005896-RA	ni|645903734|nb|KK920696.1|	278859-279218	nan	nan	gi|646747046|gb|KK962247.1|	752023-754104	22	9
ACYPI005897-RA	ni|645903738|nb|KK920692.1|	911121-912293	18	17	gi|646768717|gb|KK961805.1|	1241920-1242802	20	8
ACYPI005899-RA	ni|645903635|nb|KK920795.1|	289480-289973	nan	nan	gi|646772045|gb|KK961761.1|	74250-74772	21	9.6
ACYPI005908-RA	ni|645903662|nb|KK920768.1|	210882-211773	nan	nan	gi|646748474|gb|KK962147.1|	366701-367496	20	9
ACYPI005913-RA	ni|645903498|nb|KK920932.1|	509784-510193	nan	nan	gi|646734527|gb|KK963409.1|	161820-162449	21	9.3

ACYPI005914-RA	ni|645904058|nb|KK920373.1|	695194-695784	16	15	gi|646738466|gb|KK962974.1|	130781-135207	30	14
ACYPI005919-RA	ni|645903574|nb|KK920856.1|	164115-169273	nan	nan	gi|646768406|gb|KK961812.1|	1292825-1299079	20	9.6
ACYPI005934-RA	ni|645904245|nb|KK920234.1|	497794-498277	16	17	gi|646779337|gb|KK961576.1|	419527-419980	18	9
ACYPI005940-RA	ni|645903499|nb|KK920931.1|	143518-144160	nan	nan	gi|646782276|gb|KK961497.1|	7209954-7210503	21	9.7
ACYPI005949-RA	ni|645903931|nb|KK920500.1|	564017-564471	16	18	gi|646772897|gb|KK961753.1|	1491284-1495508	21	8.7
ACYPI005955-RA	ni|645904086|nb|KK920345.1|	418009-418377	14	15	gi|646776647|gb|KK961663.1|	2215035-2218548	23	9.2
ACYPI005962-RA	ni|645903847|nb|KK920583.1|	1355387-1356503	18	18	gi|646775807|gb|KK961708.1|	1845211-1845625	21	8.6
ACYPI005967-RA	ni|645902497|nb|KK921932.1|	182742-183088	nan	nan	gi|646757520|gb|KK961926.1|	1586356-1589015	20	7.4
ACYPI005987-RA	ni|645904111|nb|KK920320.1|	1519708-1520820	17	17	gi|646781536|gb|KK961517.1|	2281321-2281549	19	7.7
ACYPI005988-RA	ni|645904088|nb|KK920343.1|	197068-197525	17	18	gi|646779662|gb|KK961568.1|	1205074-1205373	20	9.3
ACYPI005992-RA	ni|645903949|nb|KK920482.1|	351631-351853	17	14	gi|646775221|gb|KK961739.1|	110857-114099	20	10
ACYPI005996-RA	ni|645903773|nb|KK920657.1|	251290-251481	18	17	gi|646743492|gb|KK962499.1|	192142-192945	24	11
ACYPI005997-RA	ni|645903806|nb|KK920624.1|	64708-65009	16	9.3	gi|646747046|gb|KK962247.1|	612278-614172	22	9
ACYPI006008-RA	ni|645903655|nb|KK920775.1|	22059-22780	nan	nan	gi|646767439|gb|KK961832.1|	1952538-1953162	23	10
ACYPI006009-RA	ni|645904152|nb|KK920281.1|	490715-494142	15	15	gi|646775595|gb|KK961718.1|	762310-769966	21	8.9
ACYPI006013-RA	ni|645903665|nb|KK920765.1|	258979-259719	nan	nan	gi|646765880|gb|KK961885.1|	1071354-1073042	23	10
ACYPI006023-RA	ni|645904077|nb|KK920354.1|	749545-749986	16	17	gi|646747046|gb|KK962247.1|	770758-771457	22	9
ACYPI006028-RA	ni|645903622|nb|KK920808.1|	688236-689374	nan	nan	gi|646780978|gb|KK961534.1|	2496029-2498313	20	8
ACYPI006033-RA	ni|645903557|nb|KK920873.1|	1172871-1173128	nan	nan	gi|646778983|gb|KK961586.1|	297548-299398	20	9.9
ACYPI006036-RA	ni|645903938|nb|KK920493.1|	1191107-1192840	19	18	gi|646781379|gb|KK961522.1|	2615013-2615947	22	7.6
ACYPI006038-RA	ni|645903858|nb|KK920572.1|	908277-908695	16	16	gi|646746556|gb|KK962281.1|	530512-532014	20	9.1
ACYPI006043-RA	ni|645903898|nb|KK920533.1|	453573-453923	17	16	gi|646770998|gb|KK961772.1|	779379-783836	22	9.8
ACYPI006052-RA	ni|645902619|nb|KK921810.1|	251936-254055	nan	nan	gi|646725650|gb|KK964619.1|	34352-35542	17	7.7
ACYPI006058-RA	ni|645903568|nb|KK920862.1|	230542-230789	nan	nan	gi|646777313|gb|KK961637.1|	1737743-1738177	20	8.2
ACYPI006059-RA	ni|645904044|nb|KK920387.1|	1355635-1356063	15	16	gi|646781243|gb|KK961526.1|	4078636-4082723	21	8.9
ACYPI006066-RA	ni|645903811|nb|KK920619.1|	546584-546951	15	15	gi|646778029|gb|KK961613.1|	2105827-2109018	21	8.6
ACYPI006067-RA	ni|645904004|nb|KK920427.1|	1268061-1270141	16	16	gi|646780358|gb|KK961551.1|	732792-733481	21	8.8
ACYPI006101-RA	ni|645903832|nb|KK920598.1|	923349-923848	18	8.8	gi|646779869|gb|KK961563.1|	327235-331078	17	8.8
ACYPI006103-RA	ni|645903885|nb|KK920546.1|	278793-279675	19	12	gi|646767521|gb|KK961830.1|	613409-617484	20	9.3
ACYPI006104-RA	ni|645902354|nb|KK922075.1|	147937-150465	nan	nan	gi|646780406|gb|KK961550.1|	2155848-2156104	22	5.9

ACYPI006106-RA	ni|645903511|nb|KK920919.1|	487647-489074	nan	nan	gi|646732905|gb|KK963611.1|	85358-85798	17	7.5
ACYPI006122-RA	ni|645904231|nb|KK920242.1|	622179-623197	16	17	gi|646781313|gb|KK961524.1|	1713596-1714426	19	8.5
ACYPI006124-RA	ni|645904121|nb|KK920310.1|	285995-286702	14	15	gi|646740004|gb|KK962821.1|	379844-388128	22	9.6
ACYPI006131-RA	ni|645904100|nb|KK920331.1|	181368-184208	18	22	gi|646778517|gb|KK961599.1|	700522-703862	20	9.6
ACYPI006140-RA	ni|645904130|nb|KK920303.1|	684546-685003	18	17	gi|646751856|gb|KK961977.1|	161954-163343	18	8.5
ACYPI006141-RA	ni|645903820|nb|KK920610.1|	1103266-1103571	18	19	gi|646775383|gb|KK961730.1|	1124290-1124812	19	8.7
ACYPI006142-RA	ni|645903879|nb|KK920552.1|	951007-951994	18	18	gi|646755567|gb|KK961936.1|	598856-599852	20	8.6
ACYPI006151-RA	ni|645903597|nb|KK920833.1|	558940-559343	nan	nan	gi|646782127|gb|KK961500.1|	1217271-1217484	22	9.6
ACYPI006156-RA	ni|645904155|nb|KK920278.1|	1003957-1004076	18	9.4	gi|646747574|gb|KK962208.1|	112690-112965	27	10
ACYPI006157-RA	ni|645903720|nb|KK920710.1|	93578-94193	nan	nan	gi|646755888|gb|KK961934.1|	941532-941980	21	9.8
ACYPI006163-RA	ni|645904027|nb|KK920404.1|	698723-699189	17	17	gi|646769511|gb|KK961792.1|	895184-895615	20	9.9
ACYPI006164-RA	ni|645903768|nb|KK920662.1|	829449-831659	18	17	gi|646781443|gb|KK961520.1|	1794021-1795864	20	8.3
ACYPI006170-RA	ni|645904114|nb|KK920317.1|	1707595-1708268	18	18	gi|646780827|gb|KK961539.1|	3598551-3599003	20	9.4
ACYPI006177-RA	ni|645903908|nb|KK920523.1|	24030-24541	15	15	gi|646775968|gb|KK961700.1|	776916-777593	20	8.2
ACYPI006178-RA	ni|645903752|nb|KK920678.1|	1088651-1089251	18	16	gi|646740420|gb|KK962773.1|	256629-257094	20	7.5
ACYPI006184-RA	ni|645904262|nb|KK920227.1|	2074409-2074543	17	17	gi|646778307|gb|KK961605.1|	1929034-1933224	17	8.7
ACYPI006186-RA	ni|645901982|nb|KK922447.1|	201285-201594	nan	nan	gi|646781443|gb|KK961520.1|	334450-337082	20	8.3
ACYPI006194-RA	ni|645904156|nb|KK920277.1|	1201301-1201551	17	8.8	gi|646776351|gb|KK961680.1|	2159759-2160207	22	10
ACYPI006205-RA	ni|645904096|nb|KK920335.1|	1532128-1532351	18	17	gi|646775842|gb|KK961706.1|	2110055-2110295	22	9
ACYPI006207-RA	ni|645903674|nb|KK920756.1|	146009-146180	nan	nan	gi|646746706|gb|KK962270.1|	402736-408503	23	11
ACYPI006216-RA	ni|645903876|nb|KK920555.1|	133590-133815	14	15	gi|646771352|gb|KK961768.1|	2086806-2087568	21	9.6
ACYPI006219-RA	ni|645903735|nb|KK920695.1|	127556-127915	16	16	gi|646740287|gb|KK962790.1|	296587-297456	20	8.5
ACYPI006221-RA	ni|645903820|nb|KK920610.1|	838691-841384	18	19	gi|646747459|gb|KK962217.1|	130776-137818	21	5.9
ACYPI006222-RA	ni|645904201|nb|KK920252.1|	250127-252110	17	16	gi|646778245|gb|KK961607.1|	1137461-1139114	18	6.8
ACYPI006225-RA	ni|645904025|nb|KK920406.1|	471446-472143	15	19	gi|646741656|gb|KK962648.1|	281233-283877	24	9.7
ACYPI006227-RA	ni|645904213|nb|KK920248.1|	1725719-1726188	16	19	gi|646766650|gb|KK961854.1|	261444-268747	22	10
ACYPI006229-RA	ni|645904279|nb|KK920221.1|	2631897-2632633	16	16	gi|646747201|gb|KK962236.1|	179273-180805	18	7.3
ACYPI006238-RA	ni|645903710|nb|KK920720.1|	722916-723469	nan	nan	gi|646777991|gb|KK961614.1|	1215561-1215949	18	7.8
ACYPI006240-RA	ni|645903916|nb|KK920515.1|	387444-390438	16	23	gi|646756057|gb|KK961933.1|	382903-383418	16	6.7
ACYPI006242-RA	ni|645903609|nb|KK920821.1|	384374-386063	nan	nan	gi|646780723|gb|KK961542.1|	1573125-1579403	21	9.4

ACYPI006243-RA	ni|645904133|nb|KK920300.1|	2772885-2773556	18	18	gi|646778840|gb|KK961590.1|	736759-748890	20	9.9
ACYPI006248-RA	ni|645904246|nb|KK920233.1|	1095819-1096522	16	17	gi|646781732|gb|KK961511.1|	2762674-2765091	22	9.8
ACYPI006251-RA	ni|645903821|nb|KK920609.1|	1085978-1086994	17	17	gi|646779006|gb|KK961585.1|	186630-194175	20	9.9
ACYPI006254-RA	ni|645904160|nb|KK920273.1|	1906377-1906572	18	18	gi|646776429|gb|KK961676.1|	1344369-1350059	20	9.9
ACYPI006257-RA	ni|645902745|nb|KK921684.1|	116758-117211	nan	nan	gi|646777991|gb|KK961614.1|	1111952-1112393	18	7.8
ACYPI006260-RA	ni|645903654|nb|KK920776.1|	42233-42638	nan	nan	gi|646780441|gb|KK961549.1|	2996151-2997056	21	9.5
ACYPI006262-RA	ni|645903728|nb|KK920702.1|	633118-633588	nan	nan	gi|646748142|gb|KK962168.1|	376038-376379	20	9.3
ACYPI006266-RA	ni|645903674|nb|KK920756.1|	609789-610695	nan	nan	gi|646765902|gb|KK961884.1|	1130987-1132145	22	9.2
ACYPI006268-RA	ni|645902395|nb|KK922034.1|	93398-94604	nan	nan	gi|646777729|gb|KK961621.1|	1260431-1261805	21	9.8
ACYPI006270-RA	ni|645904153|nb|KK920280.1|	1738822-1740379	17	16	gi|646771265|gb|KK961769.1|	175276-179149	17	7
ACYPI006271-RA	ni|645903562|nb|KK920868.1|	428334-428704	nan	nan	gi|646779976|gb|KK961560.1|	1237054-1237533	16	7.9
ACYPI006272-RA	ni|645903566|nb|KK920864.1|	172135-173272	nan	nan	gi|646750354|gb|KK962043.1|	198445-198952	20	9.2
ACYPI006281-RA	ni|645904033|nb|KK920398.1|	154114-154355	14	16	gi|646747124|gb|KK962242.1|	284248-286225	17	8.7
ACYPI006283-RA	ni|645903637|nb|KK920793.1|	189871-190363	nan	nan	gi|646781849|gb|KK961508.1|	1864496-1865138	17	8
ACYPI006305-RA	ni|645903603|nb|KK920827.1|	22406-23147	nan	nan	gi|646778112|gb|KK961611.1|	852582-853337	22	10
ACYPI006313-RA	ni|645903618|nb|KK920812.1|	303349-303582	nan	nan	gi|646738615|gb|KK962959.1|	308763-309253	21	9.5
ACYPI006314-RA	ni|645904216|nb|KK920247.1|	329926-330200	15	16	gi|646776952|gb|KK961652.1|	1685567-1686316	19	7.7
ACYPI006318-RA	ni|645903678|nb|KK920752.1|	366223-366478	nan	nan	gi|646779621|gb|KK961569.1|	1459543-1459775	16	7.3
ACYPI006340-RA	ni|645904119|nb|KK920312.1|	500249-501529	19	19	gi|646780270|gb|KK961553.1|	2142737-2144308	22	8.6
ACYPI006341-RA	ni|645902617|nb|KK921812.1|	83089-83750	nan	nan	gi|646781968|gb|KK961504.1|	2361781-2364161	20	9.6
ACYPI006348-RA	ni|645903606|nb|KK920824.1|	135270-136147	nan	nan	gi|646766650|gb|KK961854.1|	1584396-1584957	22	10
ACYPI006350-RA	ni|645902637|nb|KK921792.1|	272437-272721	nan	nan	gi|646746339|gb|KK962295.1|	551426-552200	20	8.2
ACYPI006351-RA	ni|645904127|nb|KK920306.1|	510173-510408	15	15	gi|646749945|gb|KK962065.1|	1946402-1947000	22	6.1
ACYPI006352-RA	ni|645902662|nb|KK921767.1|	340787-341003	nan	nan	gi|646744833|gb|KK962403.1|	241068-244042	22	9.4
ACYPI006354-RA	ni|645903536|nb|KK920894.1|	320223-320658	nan	nan	gi|646738248|gb|KK962996.1|	96287-99727	17	6.6
ACYPI006364-RA	ni|645903773|nb|KK920657.1|	1109229-1109505	18	17	gi|646776447|gb|KK961675.1|	783010-784763	22	9.4
ACYPI006367-RA	ni|645903846|nb|KK920584.1|	227453-227729	17	10	gi|646768361|gb|KK961813.1|	689097-689295	15	7.4
ACYPI006375-RA	ni|645903929|nb|KK920502.1|	1431575-1432721	17	18	gi|646776647|gb|KK961663.1|	349899-352026	23	9.2
ACYPI006377-RA	ni|645903680|nb|KK920750.1|	633144-633267	nan	nan	gi|646782357|gb|KK961494.1|	9236897-9237991	21	9.2
ACYPI006388-RA	ni|645903902|nb|KK920529.1|	1243762-1244926	19	17	gi|646753339|gb|KK961955.1|	784027-785440	22	11

ACYPI006399-RA	ni|645903563|nb|KK920867.1|	634869-635472	nan	nan	gi|646738455|gb|KK962975.1|	457007-457851	23	9.5
ACYPI006403-RA	ni|645904142|nb|KK920291.1|	69328-71035	20	23	gi|646776429|gb|KK961676.1|	1706638-1711057	20	9.9
ACYPI006405-RA	ni|645903938|nb|KK920493.1|	426643-427800	19	18	gi|646598593|gb|KK976753.1|	869-1508	15	8.4
ACYPI006409-RA	ni|645903684|nb|KK920746.1|	238386-238931	nan	nan	gi|646779262|gb|KK961578.1|	849442-849639	20	8.1
ACYPI006412-RA	ni|645903475|nb|KK920955.1|	246958-247407	nan	nan	gi|646782211|gb|KK961498.1|	5781899-5782435	20	9
ACYPI006418-RA	ni|645902600|nb|KK921829.1|	36785-38549	nan	nan	gi|646745197|gb|KK962376.1|	167157-167651	21	8.9
ACYPI006432-RA	ni|645904107|nb|KK920324.1|	26186-26600	17	9.5	gi|646780105|gb|KK961557.1|	1927883-1935768	21	9.7
ACYPI006436-RA	ni|645904246|nb|KK920233.1|	1689466-1690647	16	17	gi|646777665|gb|KK961623.1|	1577532-1578795	21	9.7
ACYPI006437-RA	ni|645903815|nb|KK920615.1|	406301-406846	19	17	gi|646781809|gb|KK961509.1|	1068569-1070344	19	8.6
ACYPI006441-RA	ni|645903965|nb|KK920466.1|	1714864-1715250	17	16	gi|646744833|gb|KK962403.1|	453282-454519	22	9.4
ACYPI006443-RA	ni|645903825|nb|KK920605.1|	1018135-1018727	19	17	gi|646743944|gb|KK962469.1|	298409-298882	20	9.1
ACYPI006451-RA	ni|645904136|nb|KK920297.1|	2035877-2036321	16	17	gi|646781344|gb|KK961523.1|	3703799-3706804	21	8.9
ACYPI006453-RA	ni|645904133|nb|KK920300.1|	97695-98050	18	18	gi|646730953|gb|KK963877.1|	58577-61076	23	11
ACYPI006456-RA	ni|645904131|nb|KK920302.1|	575078-576144	15	16	gi|646747263|gb|KK962232.1|	380395-382834	18	8.6
ACYPI006482-RA	ni|645904133|nb|KK920300.1|	2963047-2963435	18	18	gi|646751160|gb|KK962003.1|	1138606-1138983	22	10
ACYPI006488-RA	ni|645903753|nb|KK920677.1|	439386-440035	17	9.8	gi|646782276|gb|KK961497.1|	2956913-2957193	21	9.7
ACYPI006497-RA	ni|645902442|nb|KK921987.1|	181041-182619	nan	nan	gi|646777416|gb|KK961632.1|	1236794-1240235	22	8.8
ACYPI006498-RA	ni|645897725|nb|KK926704.1|	3473-3733	nan	nan	gi|646778699|gb|KK961594.1|	1330627-1331300	21	9.5
ACYPI006499-RA	ni|645903796|nb|KK920634.1|	710949-711345	18	17	gi|646776904|gb|KK961654.1|	855176-855654	21	9.3
ACYPI006500-RA	ni|645904171|nb|KK920262.1|	796596-797212	15	16	gi|646773721|gb|KK961747.1|	2115732-2116004	21	9.2
ACYPI006505-RA	ni|645904242|nb|KK920237.1|	789028-790134	15	16	gi|646776429|gb|KK961676.1|	1271017-1272467	20	9.9
ACYPI006509-RA	ni|645904116|nb|KK920315.1|	2319988-2320692	19	18	gi|646775842|gb|KK961706.1|	1487839-1492134	22	9
ACYPI006514-RA	ni|645903703|nb|KK920727.1|	257231-257612	nan	nan	gi|646778736|gb|KK961593.1|	1335930-1336693	20	7.9
ACYPI006518-RA	ni|645903979|nb|KK920452.1|	693623-694953	15	17	gi|646752849|gb|KK961961.1|	1214235-1215294	21	9.4
ACYPI006520-RA	ni|645903545|nb|KK920885.1|	239477-239988	nan	nan	gi|646776351|gb|KK961680.1|	719811-726418	22	10
ACYPI006521-RA	ni|645903780|nb|KK920650.1|	865015-865652	19	19	gi|646772574|gb|KK961756.1|	1138358-1141988	21	8
ACYPI006535-RA	ni|645904076|nb|KK920355.1|	198158-198356	17	17	gi|646759277|gb|KK961919.1|	901458-902307	16	7.2
ACYPI006541-RA	ni|645904258|nb|KK920229.1|	742386-742651	16	16	gi|646746841|gb|KK962261.1|	418139-419060	20	9.6
ACYPI006542-RA	ni|645903806|nb|KK920624.1|	806798-806961	16	9.3	gi|646782043|gb|KK961502.1|	4038506-4038731	20	9.2
ACYPI006544-RA	ni|645904119|nb|KK920312.1|	514158-514550	19	19	gi|646753527|gb|KK961953.1|	1188121-1188545	24	9.2

ACYPI006545-RA	ni|645903774|nb|KK920656.1|	654437-655141	16	9.2	gi|646748245|gb|KK962161.1|	572602-576028	18	8.6
ACYPI006557-RA	ni|645903965|nb|KK920466.1|	1174266-1174946	17	16	gi|646738524|gb|KK962968.1|	127285-127568	24	11
ACYPI006572-RA	ni|645903901|nb|KK920530.1|	580610-581184	17	17	gi|646741719|gb|KK962642.1|	303527-304437	21	8.9
ACYPI006576-RA	ni|645903910|nb|KK920521.1|	353567-354045	19	17	gi|646770563|gb|KK961777.1|	669926-670400	19	8.7
ACYPI006577-RA	ni|645903869|nb|KK920561.1|	669371-669557	17	15	gi|646776998|gb|KK961650.1|	2592015-2592210	20	9.6
ACYPI006581-RA	ni|645903758|nb|KK920672.1|	37398-37615	17	15	gi|646781421|gb|KK961521.1|	5116906-5117972	21	8.7
ACYPI006584-RA	ni|645903773|nb|KK920657.1|	1196725-1197140	18	17	gi|646778983|gb|KK961586.1|	9773-9973	20	9.9
ACYPI006588-RA	ni|645904106|nb|KK920325.1|	1057605-1058232	17	17	gi|646766650|gb|KK961854.1|	714036-714421	22	10
ACYPI006589-RA	ni|645904165|nb|KK920268.1|	766444-766887	16	16	gi|646781849|gb|KK961508.1|	175700-176482	17	8
ACYPI006602-RA	ni|645904192|nb|KK920255.1|	45063-46373	15	16	gi|646487701|gb|KK996683.1|	3249-3437	11	5.6
ACYPI006603-RA	ni|645904131|nb|KK920302.1|	492245-492558	15	16	gi|646771092|gb|KK961771.1|	471373-471705	23	9.7
ACYPI006608-RA	ni|645902648|nb|KK921781.1|	4233-4966	nan	nan	gi|646780147|gb|KK961556.1|	1264346-1264680	18	7.5
ACYPI006611-RA	ni|645903549|nb|KK920881.1|	5588-6823	nan	nan	gi|646776184|gb|KK961688.1|	914142-920365	21	9.6
ACYPI006612-RA	ni|645903925|nb|KK920506.1|	603140-605156	19	17	gi|646762797|gb|KK961906.1|	729578-729895	19	5.6
ACYPI006615-RA	ni|645904160|nb|KK920273.1|	1791608-1791910	18	18	gi|646552978|gb|KK985631.1|	1244-2626	18	9.7
ACYPI006616-RA	ni|645903846|nb|KK920584.1|	256698-256934	17	10	gi|646777207|gb|KK961641.1|	1186273-1186463	20	8.5
ACYPI006617-RA	ni|645904122|nb|KK920309.1|	241291-241586	18	17	gi|646780183|gb|KK961555.1|	1386418-1386695	19	8.6
ACYPI006619-RA	ni|645903695|nb|KK920735.1|	498936-499815	nan	nan	gi|646779066|gb|KK961583.1|	1563970-1564637	19	7.9
ACYPI006624-RA	ni|645904139|nb|KK920294.1|	204208-205253	15	18	gi|646771352|gb|KK961768.1|	409073-412458	21	9.6
ACYPI006625-RA	ni|645904075|nb|KK920356.1|	535648-536240	16	17	gi|646753527|gb|KK961953.1|	665250-672717	24	9.2
ACYPI006626-RA	ni|645904039|nb|KK920392.1|	666983-667491	15	16	gi|646753180|gb|KK961957.1|	82882-83624	25	11
ACYPI006635-RA	ni|645902105|nb|KK922324.1|	61899-65886	nan	nan	gi|646782288|gb|KK961496.1|	5821016-5826189	21	9.7
ACYPI006639-RA	ni|645902538|nb|KK921891.1|	104472-105673	nan	nan	gi|646779936|gb|KK961561.1|	3023369-3038129	19	9.6
ACYPI006649-RA	ni|645904039|nb|KK920392.1|	417356-417592	15	16	gi|646777802|gb|KK961619.1|	91410-96661	24	9
ACYPI006656-RA	ni|645903957|nb|KK920474.1|	1448344-1449893	18	17	gi|646778767|gb|KK961592.1|	150800-156186	21	5.4
ACYPI006658-RA	ni|645903902|nb|KK920529.1|	1120723-1121108	19	17	gi|646627554|gb|KK971378.1|	3894-4350	12	5.6
ACYPI006660-RA	ni|645904222|nb|KK920245.1|	784017-784294	17	12	gi|646778903|gb|KK961588.1|	4191659-4191959	23	10
ACYPI006664-RA	ni|645904070|nb|KK920361.1|	310719-311376	17	16	gi|646570180|gb|KK982274.1|	3319-3526	5.4	2.9
ACYPI006668-RA	ni|645904070|nb|KK920361.1|	564335-564551	17	16	gi|646775635|gb|KK961716.1|	410805-411074	22	10
ACYPI006674-RA	ni|645904152|nb|KK920281.1|	386092-387170	15	15	gi|646775884|gb|KK961704.1|	950525-950771	20	7.6

ACYPI006676-RA	ni|645903938|nb|KK920493.1|	737733-738151	19	18	gi|646781690|gb|KK961512.1|	3342729-3343889	21	8.8
ACYPI006680-RA	ni|645901967|nb|KK922462.1|	6398-6822	nan	nan	gi|646746475|gb|KK962286.1|	193534-209576	19	8.8
ACYPI006682-RA	ni|645904278|nb|KK920222.1|	561997-563106	15	16	gi|646753715|gb|KK961951.1|	814999-815500	19	5.4
ACYPI006688-RA	ni|645903604|nb|KK920826.1|	25905-26096	nan	nan	gi|646750389|gb|KK962041.1|	149189-149917	21	9.5
ACYPI006692-RA	ni|645904180|nb|KK920259.1|	2094267-2095975	17	17	gi|646780953|gb|KK961535.1|	3002168-3003182	20	9.8
ACYPI006693-RA	ni|645903999|nb|KK920432.1|	549019-549429	17	15	gi|646781421|gb|KK961521.1|	1424533-1425477	21	8.7
ACYPI006698-RA	ni|645903523|nb|KK920907.1|	177425-177891	nan	nan	gi|646776608|gb|KK961665.1|	375296-381750	23	9.3
ACYPI006699-RA	ni|645903936|nb|KK920495.1|	1054006-1054287	19	18	gi|646782211|gb|KK961498.1|	2483034-2483357	20	9
ACYPI006701-RA	ni|645903669|nb|KK920761.1|	60069-60796	nan	nan	gi|646738812|gb|KK962939.1|	144737-144991	20	8.1
ACYPI006703-RA	ni|645903699|nb|KK920731.1|	166653-168033	nan	nan	gi|646781564|gb|KK961516.1|	2900118-2900526	23	9.4
ACYPI006708-RA	ni|645902715|nb|KK921714.1|	34127-34575	nan	nan	gi|646777313|gb|KK961637.1|	1574829-1575707	20	8.2
ACYPI006711-RA	ni|645904139|nb|KK920294.1|	500525-501582	15	18	gi|646768294|gb|KK961814.1|	607675-612282	23	10
ACYPI006712-RA	ni|645904248|nb|KK920232.1|	9345-9952	15	16	gi|646781421|gb|KK961521.1|	451694-452266	21	8.7
ACYPI006714-RA	ni|645904152|nb|KK920281.1|	338801-339291	15	15	gi|646779936|gb|KK961561.1|	3065230-3065613	19	9.6
ACYPI006716-RA	ni|645904075|nb|KK920356.1|	1017782-1018551	16	17	gi|646782127|gb|KK961500.1|	5747443-5748353	22	9.6
ACYPI006718-RA	ni|645902469|nb|KK921960.1|	158898-159124	nan	nan	gi|646782288|gb|KK961496.1|	4367497-4367889	21	9.7
ACYPI006725-RA	ni|645903858|nb|KK920572.1|	894147-894397	16	16	gi|646778274|gb|KK961606.1|	785398-785619	18	7.5
ACYPI006727-RA	ni|645904070|nb|KK920361.1|	961045-961589	17	16	gi|646782357|gb|KK961494.1|	5134692-5139197	21	9.2
ACYPI006728-RA	ni|645903984|nb|KK920447.1|	24882-26004	18	17	gi|646738156|gb|KK963004.1|	54603-55300	12	6.9
ACYPI006735-RA	ni|645903968|nb|KK920463.1|	608505-608646	16	16	gi|646755888|gb|KK961934.1|	873620-874113	21	9.8
ACYPI006736-RA	ni|645902034|nb|KK922395.1|	19186-20669	nan	nan	gi|646739932|gb|KK962828.1|	170996-172819	15	8.9
ACYPI006737-RA	ni|645903727|nb|KK920703.1|	958477-958937	nan	nan	gi|646781013|gb|KK961533.1|	938435-943564	18	7.6
ACYPI006740-RA	ni|645903751|nb|KK920679.1|	759317-760058	17	16	gi|646768447|gb|KK961811.1|	821773-822664	22	9.7
ACYPI006741-RA	ni|645903544|nb|KK920886.1|	344250-344705	nan	nan	gi|646778336|gb|KK961604.1|	1373678-1374197	21	10
ACYPI006748-RA	ni|645904130|nb|KK920303.1|	1467726-1469517	18	17	gi|646781601|gb|KK961515.1|	3502615-3503549	19	8.6
ACYPI006758-RA	ni|645903633|nb|KK920797.1|	573722-574669	nan	nan	gi|646765991|gb|KK961880.1|	303369-311399	16	7
ACYPI006761-RA	ni|645903648|nb|KK920782.1|	272636-273115	nan	nan	gi|646749632|gb|KK962082.1|	582288-582940	20	8.6
ACYPI006777-RA	ni|645904156|nb|KK920277.1|	1517327-1517575	17	8.8	gi|646775842|gb|KK961706.1|	334285-334895	22	9
ACYPI006784-RA	ni|645903854|nb|KK920576.1|	31398-31593	16	8.4	gi|646767307|gb|KK961835.1|	322881-327857	20	9
ACYPI006790-RA	ni|645903627|nb|KK920803.1|	529579-530412	nan	nan	gi|646767156|gb|KK961839.1|	147231-147560	15	6.4

ACYPI006792-RA	ni|645904028|nb|KK920403.1|	413374-413757	18	17	gi|646750135|gb|KK962055.1|	55033-56094	16	7.6
ACYPI006797-RA	ni|645901733|nb|KK922696.1|	223835-227076	nan	nan	gi|646781690|gb|KK961512.1|	2360794-2363706	21	8.8
ACYPI006808-RA	ni|645903627|nb|KK920803.1|	520496-520725	nan	nan	gi|646747802|gb|KK962192.1|	207843-209426	19	7.8
ACYPI006817-RA	ni|645901537|nb|KK922892.1|	78395-78995	nan	nan	gi|646746897|gb|KK962257.1|	853714-854487	21	9.2
ACYPI006818-RA	ni|645904132|nb|KK920301.1|	249661-250513	16	18	gi|646746991|gb|KK962251.1|	338254-350930	22	9.2
ACYPI006821-RA	ni|645903899|nb|KK920532.1|	279543-279817	15	16	gi|646777233|gb|KK961640.1|	2279271-2279635	21	8.7
ACYPI006823-RA	ni|645904010|nb|KK920421.1|	1030657-1031841	17	17	gi|646747658|gb|KK962202.1|	102707-103263	15	7.1
ACYPI006827-RA	ni|645903797|nb|KK920633.1|	82754-83399	17	8.9	gi|646782276|gb|KK961497.1|	3131877-3132082	21	9.7
ACYPI006828-RA	ni|645904116|nb|KK920315.1|	424251-426274	19	1.80E+01	gi|646732863|gb|KK963615.1|	29322-33319	150	39
ACYPI006833-RA	ni|645903680|nb|KK920750.1|	68912-69726	nan	nan	gi|646727841|gb|KK964291.1|	121520-128141	23	9.6
ACYPI006841-RA	ni|645904106|nb|KK920325.1|	669062-669656	17	17	gi|646777207|gb|KK961641.1|	896960-899077	20	8.5
ACYPI006852-RA	ni|645903466|nb|KK920964.1|	533802-533989	nan	nan	gi|646781628|gb|KK961514.1|	4586500-4592451	23	9
ACYPI006857-RA	ni|645903780|nb|KK920650.1|	276650-277152	19	19	gi|646771951|gb|KK961762.1|	1827439-1829636	21	8.3
ACYPI006871-RA	ni|645903858|nb|KK920572.1|	844912-845156	16	16	gi|646778983|gb|KK961586.1|	342591-342856	20	9.9
ACYPI006875-RA	ni|645902316|nb|KK922113.1|	5977-6230	nan	nan	gi|646748474|gb|KK962147.1|	177934-179046	20	9
ACYPI006879-RA	ni|645904045|nb|KK920386.1|	210443-210801	19	21	gi|646781118|gb|KK961530.1|	2413756-2426001	21	10
ACYPI006884-RA	ni|645903867|nb|KK920563.1|	769072-770352	18	18	gi|646747494|gb|KK962214.1|	293340-294596	18	8.5
ACYPI006885-RA	ni|645903953|nb|KK920478.1|	1168857-1169879	18	17	gi|646777955|gb|KK961615.1|	947487-947974	21	10
ACYPI006896-RA	ni|645903946|nb|KK920485.1|	33603-33801	16	14	gi|646776514|gb|KK961670.1|	6299-6541	20	9.6
ACYPI006902-RA	ni|645904023|nb|KK920408.1|	768877-769900	17	17	gi|646775523|gb|KK961722.1|	870542-872042	21	9.7
ACYPI006909-RA	ni|645904091|nb|KK920340.1|	663578-663801	15	18	gi|646779006|gb|KK961585.1|	911013-911385	20	9.9
ACYPI006910-RA	ni|645904159|nb|KK920274.1|	1079126-1079292	15	17	gi|646776389|gb|KK961678.1|	1059735-1059932	22	9.5
ACYPI006915-RA	ni|645903941|nb|KK920490.1|	933727-934420	18	18	gi|646780222|gb|KK961554.1|	3277232-3285712	20	9.7
ACYPI006924-RA	ni|645903895|nb|KK920536.1|	422433-422902	17	16	gi|646781043|gb|KK961532.1|	3068738-3069642	26	11
ACYPI006929-RA	ni|645903752|nb|KK920678.1|	73879-75440	18	16	gi|646750354|gb|KK962043.1|	297550-299205	20	9.2
ACYPI006932-RA	ni|645903747|nb|KK920683.1|	98138-99138	19	18	gi|646747385|gb|KK962223.1|	236443-236742	21	5.4
ACYPI006934-RA	ni|645903634|nb|KK920796.1|	1045632-1045976	nan	nan	gi|646767196|gb|KK961838.1|	1107352-1107584	21	9.7
ACYPI006940-RA	ni|645903679|nb|KK920751.1|	395581-396385	nan	nan	gi|646777440|gb|KK961631.1|	1453969-1454277	22	9.7
ACYPI006942-RA	ni|645904165|nb|KK920268.1|	820112-820637	16	16	gi|646782334|gb|KK961495.1|	7010704-7011744	21	9
ACYPI006948-RA	ni|645903911|nb|KK920520.1|	527512-527958	16	17	gi|646762191|gb|KK961910.1|	902349-903559	18	7.1

ACYPI006949-RA	ni|645903742|nb|KK920688.1|	522985-524102	17	16	gi|646781536|gb|KK961517.1|	2431223-2432212	19	7.7
ACYPI006954-RA	ni|645903834|nb|KK920596.1|	42082-43166	18	17	gi|646748014|gb|KK962177.1|	294669-295462	15	6.4
ACYPI006956-RA	ni|645904122|nb|KK920309.1|	1250331-1251362	18	17	gi|646775867|gb|KK961705.1|	755090-755962	23	10
ACYPI006957-RA	ni|645904171|nb|KK920262.1|	1446852-1447285	15	16	gi|646781379|gb|KK961522.1|	3378502-3382978	22	7.6
ACYPI006958-RA	ni|645903950|nb|KK920481.1|	689445-690534	15	15	gi|646777474|gb|KK961630.1|	984506-988456	21	9.3
ACYPI006964-RA	ni|645903831|nb|KK920599.1|	1563083-1563812	18	17	gi|646745091|gb|KK962384.1|	163254-164426	19	7.9
ACYPI006971-RA	ni|645904262|nb|KK920227.1|	2483131-2483362	17	17	gi|646779936|gb|KK961561.1|	2122876-2124034	19	9.6
ACYPI006974-RA	ni|645904045|nb|KK920386.1|	69653-71370	19	21	gi|646694989|gb|KK966396.1|	9127-12831	12	9.8
ACYPI006978-RA	ni|645903938|nb|KK920493.1|	1462006-1465201	19	18	gi|646747032|gb|KK962248.1|	408105-408610	20	8.7
ACYPI006979-RA	ni|645904033|nb|KK920398.1|	96265-96779	14	16	gi|646777089|gb|KK961646.1|	425760-428811	20	9.4
ACYPI006990-RA	ni|645904063|nb|KK920368.1|	468705-469368	16	18	gi|646755567|gb|KK961936.1|	798958-803087	20	8.6
ACYPI006991-RA	ni|645903868|nb|KK920562.1|	474675-475104	16	8.7	gi|646780406|gb|KK961550.1|	2005819-2007292	22	5.9
ACYPI006993-RA	ni|645903521|nb|KK920909.1|	22287-23846	nan	nan	gi|646746974|gb|KK962252.1|	141680-142583	23	6.1
ACYPI007002-RA	ni|645904174|nb|KK920261.1|	653985-654230	19	17	gi|646780858|gb|KK961538.1|	3541720-3543057	21	10
ACYPI007005-RA	ni|645904222|nb|KK920245.1|	631377-631689	17	12	gi|646778112|gb|KK961611.1|	1163234-1164019	22	10
ACYPI007006-RA	ni|645902768|nb|KK921661.1|	246442-247007	nan	nan	gi|646782043|gb|KK961502.1|	5359487-5360211	20	9.2
ACYPI007007-RA	ni|645904251|nb|KK920231.1|	402750-403002	16	16	gi|646500670|gb|KK994326.1|	4284-4906	17	8.9
ACYPI007009-RA	ni|645902760|nb|KK921669.1|	17306-18078	nan	nan	gi|646764075|gb|KK961900.1|	384473-387026	18	6.5
ACYPI007012-RA	ni|645904043|nb|KK920388.1|	547055-547301	16	9.1	gi|646748615|gb|KK962139.1|	663980-665717	19	7.8
ACYPI007021-RA	ni|645904246|nb|KK920233.1|	231517-231963	16	17	gi|646781313|gb|KK961524.1|	1798838-1800677	19	8.5
ACYPI007025-RA	ni|645903800|nb|KK920630.1|	1379640-1379990	18	17	gi|646775867|gb|KK961705.1|	2033466-2034005	23	10
ACYPI007038-RA	ni|645902083|nb|KK922346.1|	145759-146082	nan	nan	gi|646776490|gb|KK961672.1|	138713-142032	20	10
ACYPI007039-RA	ni|645904077|nb|KK920354.1|	990184-990636	16	17	gi|646782357|gb|KK961494.1|	9738478-9742259	21	9.2
ACYPI007054-RA	ni|645903891|nb|KK920540.1|	218032-218752	15	17	gi|646777416|gb|KK961632.1|	2152979-2153324	22	8.8
ACYPI007058-RA	ni|645903627|nb|KK920803.1|	131399-132040	nan	nan	gi|646754106|gb|KK961947.1|	412407-412747	16	7.6
ACYPI007065-RA	ni|645903832|nb|KK920598.1|	1042906-1043137	18	8.8	gi|646782334|gb|KK961495.1|	7711720-7712181	21	9
ACYPI007068-RA	ni|645904234|nb|KK920241.1|	1118185-1118392	13	15	gi|646745372|gb|KK962364.1|	169035-170654	23	10
ACYPI007070-RA	ni|645904094|nb|KK920337.1|	66379-69115	17	16	gi|646780574|gb|KK961546.1|	233294-235124	20	9.9
ACYPI007076-RA	ni|645903973|nb|KK920458.1|	688002-688202	20	18	gi|646782288|gb|KK961496.1|	6512493-6516888	21	9.7
ACYPI007079-RA	ni|645904166|nb|KK920267.1|	2277830-2278076	18	16	gi|646762191|gb|KK961910.1|	627589-628025	18	7.1

ACYPI007084-RA	ni|645904008|nb|KK920423.1|	1363306-1363960	16	9.5	gi|646782043|gb|KK961502.1|	5182636-5183583	20	9.2
ACYPI007086-RA	ni|645903595|nb|KK920835.1|	144259-145854	nan	nan	gi|646776929|gb|KK961653.1|	926074-927190	17	8.5
ACYPI007090-RA	ni|645903835|nb|KK920595.1|	610427-610893	16	8.8	gi|646778903|gb|KK961588.1|	3763475-3766883	23	10
ACYPI007100-RA	ni|645904231|nb|KK920242.1|	651668-652542	16	17	gi|646737122|gb|KK963108.1|	49598-50074	17	9
ACYPI007104-RA	ni|645904163|nb|KK920270.1|	285087-286597	16	16	gi|646751160|gb|KK962003.1|	690377-692271	22	10
ACYPI007109-RA	ni|645903749|nb|KK920681.1|	618151-618956	19	18	gi|646781243|gb|KK961526.1|	2172112-2172611	21	8.9
ACYPI007110-RA	ni|645903713|nb|KK920717.1|	599877-600405	nan	nan	gi|646775987|gb|KK961699.1|	857149-857656	19	7.5
ACYPI007113-RA	ni|645903650|nb|KK920780.1|	1063665-1064210	nan	nan	gi|646775312|gb|KK961734.1|	858306-859009	17	7.9
ACYPI007117-RA	ni|645904015|nb|KK920416.1|	444779-447912	18	16	gi|646748129|gb|KK962169.1|	1016679-1021280	21	9.8
ACYPI007136-RA	ni|645903557|nb|KK920873.1|	724006-725057	nan	nan	gi|646765880|gb|KK961885.1|	914787-922715	23	10
ACYPI007139-RA	ni|645903971|nb|KK920460.1|	61534-62359	19	18	gi|646739555|gb|KK962870.1|	111678-112791	27	18
ACYPI007156-RA	ni|645903470|nb|KK920960.1|	92962-93404	nan	nan	gi|646776456|gb|KK961674.1|	775063-775636	18	7.9
ACYPI007158-RA	ni|645903494|nb|KK920936.1|	334594-334733	nan	nan	gi|646778517|gb|KK961599.1|	540293-540893	20	9.6
ACYPI007166-RA	ni|645903680|nb|KK920750.1|	656416-657225	nan	nan	gi|646782357|gb|KK961494.1|	9243960-9246647	21	9.2
ACYPI007167-RA	ni|645903627|nb|KK920803.1|	496973-497337	nan	nan	gi|646739268|gb|KK962897.1|	179837-180336	18	7.4
ACYPI007171-RA	ni|645903680|nb|KK920750.1|	67470-68135	nan	nan	gi|646776647|gb|KK961663.1|	391420-398477	23	9.2
ACYPI007177-RA	ni|645903572|nb|KK920858.1|	87726-88050	nan	nan	gi|646766158|gb|KK961873.1|	1955302-1957130	22	5.8
ACYPI007179-RA	ni|645903496|nb|KK920934.1|	858330-858799	nan	nan	gi|646750389|gb|KK962041.1|	395132-396749	21	9.5
ACYPI007182-RA	ni|645903697|nb|KK920733.1|	392830-393328	nan	nan	gi|646781443|gb|KK961520.1|	2942491-2943215	20	8.3
ACYPI007184-RA	ni|645904087|nb|KK920344.1|	810877-812424	16	16	gi|646775614|gb|KK961717.1|	75748-76482	21	9.7
ACYPI007190-RA	ni|645904262|nb|KK920227.1|	1223112-1223535	17	17	gi|646778767|gb|KK961592.1|	1253504-1253878	21	5.4
ACYPI007197-RA	ni|645903920|nb|KK920511.1|	306694-307562	20	20	gi|646683836|gb|KK967228.1|	14621-15584	13	5.4
ACYPI007210-RA	ni|645904262|nb|KK920227.1|	492439-492736	17	17	gi|646766985|gb|KK961844.1|	185288-192663	21	9.7
ACYPI007219-RA	ni|645903747|nb|KK920683.1|	752430-753512	19	18	gi|646755714|gb|KK961935.1|	565979-567592	18	7.4
ACYPI007232-RA	ni|645904125|nb|KK920307.1|	618395-618993	18	17	gi|646776762|gb|KK961659.1|	712414-714184	21	8.9
ACYPI007238-RA	ni|645904009|nb|KK920422.1|	706924-707051	17	9.4	gi|646621903|gb|KK972348.1|	14187-15360	21	7.3
ACYPI007240-RA	ni|645899821|nb|KK924608.1|	636-864	nan	nan	gi|646769102|gb|KK961799.1|	231139-234110	20	8.3
ACYPI007245-RA	ni|645901030|nb|KK923399.1|	499-971	nan	nan	gi|646778565|gb|KK961598.1|	414641-415120	19	7.8
ACYPI007246-RA	ni|645904228|nb|KK920243.1|	715934-716163	17	18	gi|646777159|gb|KK961643.1|	36304-36882	14	6.5
ACYPI007248-RA	ni|645903773|nb|KK920657.1|	1227279-1227687	18	17	gi|646776447|gb|KK961675.1|	1850801-1851961	22	9.4

ACYPI007249-RA	ni|645903654|nb|KK920776.1|	676053-676866	nan	nan	gi|646781564|gb|KK961516.1|	2473793-2474564	23	9.4
ACYPI007250-RA	ni|645904157|nb|KK920276.1|	630398-631174	15	15	gi|646752567|gb|KK961965.1|	873778-874571	19	8.4
ACYPI007258-RA	ni|645903572|nb|KK920858.1|	170673-171608	nan	nan	gi|646778865|gb|KK961589.1|	867673-868660	20	9.2
ACYPI007260-RA	ni|645903832|nb|KK920598.1|	931194-931414	18	8.8	gi|646780406|gb|KK961550.1|	1573678-1574009	22	5.9
ACYPI007262-RA	ni|645903981|nb|KK920450.1|	831113-832723	16	8.6	gi|646776091|gb|KK961693.1|	2072834-2073448	23	10
ACYPI007266-RA	ni|645903965|nb|KK920466.1|	1635166-1636273	17	16	gi|646782357|gb|KK961494.1|	4312275-4314107	21	9.2
ACYPI007268-RA	ni|645903518|nb|KK920912.1|	160605-161040	nan	nan	gi|646763164|gb|KK961904.1|	1395764-1410244	22	10
ACYPI007270-RA	ni|645904057|nb|KK920374.1|	1278598-1279087	16	15	gi|646780889|gb|KK961537.1|	4234003-4235158	22	10
ACYPI007272-RA	ni|645903488|nb|KK920942.1|	89331-90743	nan	nan	gi|646779580|gb|KK961570.1|	2071591-2081440	17	8.5
ACYPI007276-RA	ni|645903829|nb|KK920601.1|	387120-387358	16	15	gi|646781143|gb|KK961529.1|	3160131-3160728	20	10
ACYPI007287-RA	ni|645901591|nb|KK922838.1|	43492-43825	nan	nan	gi|646767307|gb|KK961835.1|	568643-568929	20	9
ACYPI007298-RA	ni|645903740|nb|KK920690.1|	398952-399831	18	10	gi|646777288|gb|KK961638.1|	1509819-1512984	22	6.1
ACYPI007299-RA	ni|645904125|nb|KK920307.1|	240239-240645	18	17	gi|646776904|gb|KK961654.1|	1825030-1825908	21	9.3
ACYPI007301-RA	ni|645904065|nb|KK920366.1|	10037-10282	18	18	gi|646780147|gb|KK961556.1|	1375140-1376175	18	7.5
ACYPI007303-RA	ni|645903773|nb|KK920657.1|	1419473-1419637	18	17	gi|646776447|gb|KK961675.1|	1896365-1898673	22	9.4
ACYPI007307-RA	ni|645904098|nb|KK920333.1|	296655-296910	16	8.8	gi|646782357|gb|KK961494.1|	2769530-2773845	21	9.2
ACYPI007315-RA	ni|645903666|nb|KK920764.1|	77098-77333	nan	nan	gi|646766056|gb|KK961877.1|	293889-294202	18	7.8
ACYPI007324-RA	ni|645903984|nb|KK920447.1|	57630-58023	18	17	gi|646769275|gb|KK961796.1|	2310078-2310278	22	5.6
ACYPI007326-RA	ni|645904053|nb|KK920378.1|	1386856-1387233	18	17	gi|646777182|gb|KK961642.1|	1672267-1672533	20	9
ACYPI007327-RA	ni|645904097|nb|KK920334.1|	241706-242276	15	17	gi|646761388|gb|KK961911.1|	386814-388120	19	7.4
ACYPI007331-RA	ni|645903540|nb|KK920890.1|	343551-344231	nan	nan	gi|646748490|gb|KK962146.1|	198398-198880	19	7.7
ACYPI007340-RA	ni|645902779|nb|KK921650.1|	252092-252496	nan	nan	gi|646780270|gb|KK961553.1|	4348328-4349053	22	8.6
ACYPI007342-RA	ni|645903784|nb|KK920646.1|	322383-322874	17	19	gi|646781212|gb|KK961527.1|	63294-64773	21	10
ACYPI007344-RA	ni|645904078|nb|KK920353.1|	595345-595823	17	17	gi|646780441|gb|KK961549.1|	1134567-1135304	21	9.5
ACYPI007348-RA	ni|645902473|nb|KK921956.1|	219352-219785	nan	nan	gi|646781118|gb|KK961530.1|	1145476-1145951	21	10
ACYPI007351-RA	ni|645903788|nb|KK920642.1|	480222-480467	16	15	gi|646775807|gb|KK961708.1|	2888612-2889447	21	8.6
ACYPI007352-RA	ni|645904281|nb|KK920220.1|	1026791-1027937	15	16	gi|646775723|gb|KK961712.1|	1568517-1568847	21	9.5
ACYPI007353-RA	ni|645903949|nb|KK920482.1|	265249-265524	17	14	gi|646767196|gb|KK961838.1|	453935-454172	21	9.7
ACYPI007358-RA	ni|645903751|nb|KK920679.1|	742570-743052	17	16	gi|646768447|gb|KK961811.1|	691661-691934	22	9.7
ACYPI007364-RA	ni|645902463|nb|KK921966.1|	133294-135240	nan	nan	gi|646782043|gb|KK961502.1|	4395897-4402116	20	9.2

ACYPI007366-RA	ni|645904210|nb|KK920249.1|	1910079-1910246	16	16	gi|646762191|gb|KK961910.1|	568889-569273	18	7.1
ACYPI007368-RA	ni|645903795|nb|KK920635.1|	147135-147517	14	16	gi|646775383|gb|KK961730.1|	73349-77881	19	8.7
ACYPI007369-RA	ni|645903931|nb|KK920500.1|	455112-455730	16	18	gi|646741233|gb|KK962685.1|	286322-287952	18	8.2
ACYPI007373-RA	ni|645903901|nb|KK920530.1|	1076969-1077983	17	17	gi|646770641|gb|KK961776.1|	655456-656655	20	9.1
ACYPI007374-RA	ni|645904177|nb|KK920260.1|	2420978-2421580	18	17	gi|646738455|gb|KK962975.1|	420513-425076	23	9.5
ACYPI007375-RA	ni|645904065|nb|KK920366.1|	952992-953220	18	18	gi|646753180|gb|KK961957.1|	444739-448684	25	11
ACYPI007379-RA	ni|645903831|nb|KK920599.1|	356965-358299	18	17	gi|646747559|gb|KK962209.1|	749912-753213	19	9.5
ACYPI007381-RA	ni|645904009|nb|KK920422.1|	844244-844619	17	9.4	gi|646781212|gb|KK961527.1|	4400181-4400594	21	10
ACYPI007388-RA	ni|645903874|nb|KK920557.1|	512753-513426	18	18	gi|646781659|gb|KK961513.1|	1511399-1512537	22	9.8
ACYPI007397-RA	ni|645904053|nb|KK920378.1|	1756810-1757098	18	17	gi|646781659|gb|KK961513.1|	5275561-5276399	22	9.8
ACYPI007401-RA	ni|645903829|nb|KK920601.1|	679214-679590	16	15	gi|646745135|gb|KK962381.1|	223350-225638	20	8.7
ACYPI007402-RA	ni|645903812|nb|KK920618.1|	842794-843046	20	18	gi|646780827|gb|KK961539.1|	3971722-3972364	20	9.4
ACYPI007404-RA	ni|645904234|nb|KK920241.1|	187521-187724	13	15	gi|646777288|gb|KK961638.1|	98943-106268	22	6.1
ACYPI007405-RA	ni|645903622|nb|KK920808.1|	608084-608331	nan	nan	gi|646782334|gb|KK961495.1|	2825622-2826889	21	9
ACYPI007413-RA	ni|645904004|nb|KK920427.1|	957413-959059	16	16	gi|646782334|gb|KK961495.1|	6585760-6587408	21	9
ACYPI007418-RA	ni|645904160|nb|KK920273.1|	759761-760100	18	18	gi|646776998|gb|KK961650.1|	64988-66932	20	9.6
ACYPI007422-RA	ni|645904155|nb|KK920278.1|	919335-919641	18	9.4	gi|646776351|gb|KK961680.1|	1891809-1892088	22	10
ACYPI007426-RA	ni|645903805|nb|KK920625.1|	256521-256690	12	13	gi|646741594|gb|KK962653.1|	259680-264345	17	6.6
ACYPI007433-RA	ni|645904068|nb|KK920363.1|	1109941-1110970	16	8.6	gi|646777729|gb|KK961621.1|	1476257-1478960	21	9.8
ACYPI007436-RA	ni|645904023|nb|KK920408.1|	643817-644780	17	17	gi|646771352|gb|KK961768.1|	1383645-1383986	21	9.6
ACYPI007437-RA	ni|645903746|nb|KK920684.1|	220471-220662	15	15	gi|646749098|gb|KK962110.1|	171616-174188	20	7.7
ACYPI007442-RA	ni|645903509|nb|KK920921.1|	801341-801516	nan	nan	gi|646776128|gb|KK961691.1|	1417400-1419683	21	9.2
ACYPI007445-RA	ni|645903650|nb|KK920780.1|	796964-797450	nan	nan	gi|646758845|gb|KK961921.1|	1857357-1860465	22	9.5
ACYPI007451-RA	ni|645903910|nb|KK920521.1|	618035-618562	19	17	gi|646750135|gb|KK962055.1|	195182-197638	16	7.6
ACYPI007453-RA	ni|645902779|nb|KK921650.1|	36038-37050	nan	nan	gi|646782357|gb|KK961494.1|	3614850-3622344	21	9.2
ACYPI007457-RA	ni|645903513|nb|KK920917.1|	768230-769428	nan	nan	gi|646765669|gb|KK961895.1|	440266-445525	18	7.2
ACYPI007468-RA	ni|645903978|nb|KK920453.1|	528780-528974	16	17	gi|646770477|gb|KK961778.1|	1352230-1352931	21	9.1
ACYPI007477-RA	ni|645903728|nb|KK920702.1|	519649-522439	nan	nan	gi|646778213|gb|KK961608.1|	2569748-2576951	19	8.8
ACYPI007495-RA	ni|645904070|nb|KK920361.1|	183874-184096	17	16	gi|646775635|gb|KK961716.1|	877273-877818	22	10
ACYPI007505-RA	ni|645903985|nb|KK920446.1|	818354-818538	18	16	gi|646769901|gb|KK961786.1|	954081-954580	18	6.7

ACYPI007507-RA	ni|645904153|nb|KK920280.1|	3183413-3183965	17	16	gi|646775635|gb|KK961716.1|	2367226-2367574	22	10
ACYPI007519-RA	ni|645904153|nb|KK920280.1|	780147-785460	17	16	gi|646776822|gb|KK961657.1|	950695-951151	19	7.9
ACYPI007522-RA	ni|645904260|nb|KK920228.1|	1347963-1348396	15	15	gi|646775723|gb|KK961712.1|	1905555-1905868	21	9.5
ACYPI007524-RA	ni|645904219|nb|KK920246.1|	385131-385899	16	17	gi|646730603|gb|KK963922.1|	9891-10380	18	8.3
ACYPI007525-RA	ni|645903634|nb|KK920796.1|	982076-982346	nan	nan	gi|646776929|gb|KK961653.1|	307586-319613	17	8.5
ACYPI007533-RA	ni|645903569|nb|KK920861.1|	167593-168964	nan	nan	gi|646737822|gb|KK963039.1|	210631-212837	20	9
ACYPI007534-RA	ni|645904008|nb|KK920423.1|	276725-276966	16	9.5	gi|646750081|gb|KK962058.1|	417054-421332	19	8.6
ACYPI007545-RA	ni|645904180|nb|KK920259.1|	213282-213565	17	17	gi|646735916|gb|KK963243.1|	60019-60292	20	6.7
ACYPI007561-RA	ni|645903496|nb|KK920934.1|	941503-941833	nan	nan	gi|646780358|gb|KK961551.1|	2280632-2284883	21	8.8
ACYPI007566-RA	ni|645903702|nb|KK920728.1|	650891-651037	nan	nan	gi|646780311|gb|KK961552.1|	3686717-3687399	21	9.7
ACYPI007567-RA	ni|645903633|nb|KK920797.1|	383322-383968	nan	nan	gi|646767381|gb|KK961833.1|	722629-723425	18	6.8
ACYPI007584-RA	ni|645904106|nb|KK920325.1|	701620-701886	17	17	gi|646752849|gb|KK961961.1|	613031-613318	21	9.4
ACYPI007586-RA	ni|645903970|nb|KK920461.1|	2342158-2342363	17	16	gi|646782357|gb|KK961494.1|	6680439-6681649	21	9.2
ACYPI007594-RA	ni|645904112|nb|KK920319.1|	2274504-2275518	18	17	gi|646749727|gb|KK962077.1|	233865-237470	21	8.9
ACYPI007598-RA	ni|645903923|nb|KK920508.1|	43228-43568	18	18	gi|646777416|gb|KK961632.1|	2307244-2307905	22	8.8
ACYPI007627-RA	ni|645904245|nb|KK920234.1|	57388-57661	16	17	gi|646766540|gb|KK961858.1|	1129475-1130256	21	8.5
ACYPI007628-RA	ni|645904153|nb|KK920280.1|	1404610-1404953	17	16	gi|646728609|gb|KK964184.1|	331029-333014	34	14
ACYPI007630-RA	ni|645904088|nb|KK920343.1|	99647-100036	17	18	gi|646777125|gb|KK961644.1|	399233-399629	20	10
ACYPI007635-RA	ni|645904096|nb|KK920335.1|	2031129-2033444	18	17	gi|646777313|gb|KK961637.1|	406427-408194	20	8.2
ACYPI007640-RA	ni|645903780|nb|KK920650.1|	506717-509085	19	19	gi|646782334|gb|KK961495.1|	7333029-7334905	21	9
ACYPI007642-RA	ni|645901974|nb|KK922455.1|	40252-40780	nan	nan	gi|646730558|gb|KK963928.1|	112251-112538	19	5.4
ACYPI007666-RA	ni|645902589|nb|KK921840.1|	254022-254912	nan	nan	gi|646767948|gb|KK961821.1|	607554-607823	17	7.1
ACYPI007679-RA	ni|645904241|nb|KK920238.1|	116765-117564	18	17	gi|646749512|gb|KK962088.1|	483441-483797	18	7.2
ACYPI007689-RA	ni|645903744|nb|KK920686.1|	66442-67759	17	8.6	gi|646780105|gb|KK961557.1|	4107596-4111062	21	9.7
ACYPI007692-RA	ni|645903773|nb|KK920657.1|	417116-417575	18	17	gi|646767850|gb|KK961823.1|	1262634-1263112	21	9.8
ACYPI007695-RA	ni|645903989|nb|KK920442.1|	295902-296254	17	20	gi|646780858|gb|KK961538.1|	1185449-1189061	21	10
ACYPI007697-RA	ni|645904114|nb|KK920317.1|	1729093-1729808	18	18	gi|646780827|gb|KK961539.1|	3648534-3649122	20	9.4
ACYPI007705-RA	ni|645903580|nb|KK920850.1|	184001-184447	nan	nan	gi|646745941|gb|KK962322.1|	295709-296172	21	10
ACYPI007706-RA	ni|645903599|nb|KK920831.1|	114256-114454	nan	nan	gi|646777065|gb|KK961647.1|	353273-353507	20	7.9
ACYPI007710-RA	ni|645903743|nb|KK920687.1|	423212-423721	19	18	gi|646735017|gb|KK963352.1|	254176-255405	22	5.9

ACYPI007716-RA	ni|645902470|nb|KK921959.1|	46818-46963	nan	nan	gi|646779498|gb|KK961572.1|	1538910-1539067	22	5.6
ACYPI007731-RA	ni|645903981|nb|KK920450.1|	522865-523075	16	8.6	gi|646769275|gb|KK961796.1|	1067103-1067331	22	5.6
ACYPI007733-RA	ni|645902285|nb|KK922144.1|	22629-23257	nan	nan	gi|646776647|gb|KK961663.1|	2537767-2538242	23	9.2
ACYPI007734-RA	ni|645903783|nb|KK920647.1|	123344-123764	17	17	gi|646780752|gb|KK961541.1|	2263420-2265091	21	8
ACYPI007736-RA	ni|645904258|nb|KK920229.1|	1021535-1021889	16	16	gi|646750889|gb|KK962017.1|	990681-991189	22	6.1
ACYPI007744-RA	ni|645904132|nb|KK920301.1|	890931-892730	16	18	gi|646777065|gb|KK961647.1|	336603-336825	20	7.9
ACYPI007759-RA	ni|645902471|nb|KK921958.1|	117750-118390	nan	nan	gi|646781536|gb|KK961517.1|	1138040-1138842	19	7.7
ACYPI007760-RA	ni|645903549|nb|KK920881.1|	49841-50532	nan	nan	gi|646781690|gb|KK961512.1|	3515422-3517792	21	8.8
ACYPI007764-RA	ni|645903789|nb|KK920641.1|	477941-478376	17	17	gi|646766703|gb|KK961852.1|	806917-807406	20	7.1
ACYPI007767-RA	ni|645904145|nb|KK920288.1|	903758-904512	16	9.3	gi|646782043|gb|KK961502.1|	1802309-1803205	20	9.2
ACYPI007769-RA	ni|645903534|nb|KK920896.1|	623885-624405	nan	nan	gi|646766904|gb|KK961846.1|	850023-850437	17	6.9
ACYPI007771-RA	ni|645903902|nb|KK920529.1|	249637-251076	19	17	gi|646778061|gb|KK961612.1|	419236-425807	20	8.7
ACYPI007773-RA	ni|645903917|nb|KK920514.1|	1338436-1340705	19	17	gi|646778245|gb|KK961607.1|	472139-474943	18	6.8
ACYPI007776-RA	ni|645903879|nb|KK920552.1|	523748-525691	18	18	gi|646777207|gb|KK961641.1|	1520778-1520979	20	8.5
ACYPI007791-RA	ni|645903902|nb|KK920529.1|	684141-685269	19	17	gi|646781282|gb|KK961525.1|	3620595-3621917	22	9.6
ACYPI007793-RA	ni|645904077|nb|KK920354.1|	1105828-1110256	16	17	gi|646747046|gb|KK962247.1|	276101-280534	22	9
ACYPI007800-RA	ni|645904242|nb|KK920237.1|	1338734-1338925	15	16	gi|646775723|gb|KK961712.1|	1603781-1605377	21	9.5
ACYPI007802-RA	ni|645903734|nb|KK920696.1|	910962-911422	nan	nan	gi|646776368|gb|KK961679.1|	1037861-1038343	21	9.7
ACYPI007807-RA	ni|645904222|nb|KK920245.1|	1639262-1640460	17	12	gi|646752143|gb|KK961972.1|	609184-610441	20	5.6
ACYPI007820-RA	ni|645903858|nb|KK920572.1|	676412-677032	16	16	gi|646779662|gb|KK961568.1|	1533485-1533736	20	9.3
ACYPI007832-RA	ni|645904258|nb|KK920229.1|	757947-758410	16	16	gi|646782276|gb|KK961497.1|	3791136-3793081	21	9.7
ACYPI007858-RA	ni|645903749|nb|KK920681.1|	132994-134621	19	18	gi|646748888|gb|KK962123.1|	764180-766647	20	8.1
ACYPI007878-RA	ni|645904240|nb|KK920239.1|	1875285-1876724	16	16	gi|646740879|gb|KK962721.1|	196770-198571	24	6.4
ACYPI007901-RA	ni|645904019|nb|KK920412.1|	141997-142358	19	9.4	gi|646747424|gb|KK962220.1|	249592-249932	22	5.7
ACYPI007905-RA	ni|645904057|nb|KK920374.1|	1370747-1370991	16	15	gi|646774530|gb|KK961743.1|	1972317-1972705	20	9.8
ACYPI007911-RA	ni|645902724|nb|KK921705.1|	80612-80844	nan	nan	gi|646782357|gb|KK961494.1|	2864684-2864942	21	9.2
ACYPI007926-RA	ni|645903955|nb|KK920476.1|	559090-559345	18	17	gi|646730603|gb|KK963922.1|	93526-93748	18	8.3
ACYPI007932-RA	ni|645904137|nb|KK920296.1|	1275581-1276237	17	17	gi|646748599|gb|KK962140.1|	250445-253005	21	9.8
ACYPI007934-RA	ni|645903464|nb|KK920966.1|	477450-478748	nan	nan	gi|646782357|gb|KK961494.1|	8662262-8664381	21	9.2
ACYPI007943-RA	ni|645903721|nb|KK920709.1|	759956-761446	nan	nan	gi|646777495|gb|KK961629.1|	1122882-1125010	19	8.7

ACYPI007945-RA	ni|645903773|nb|KK920657.1|	1414144-1414902	18	17	gi|646776447|gb|KK961675.1|	1917798-1920290	22	9.4
ACYPI007946-RA	ni|645903527|nb|KK920903.1|	515311-517771	nan	nan	gi|646776904|gb|KK961654.1|	1509855-1511279	21	9.3
ACYPI007949-RA	ni|645904133|nb|KK920300.1|	3069084-3069244	18	18	gi|646758845|gb|KK961921.1|	140389-140629	22	9.5
ACYPI007952-RA	ni|645904120|nb|KK920311.1|	329033-329441	18	18	gi|646749317|gb|KK962098.1|	323239-323637	20	5.6
ACYPI007955-RA	ni|645904106|nb|KK920325.1|	1083601-1083849	17	17	gi|646766650|gb|KK961854.1|	675144-675737	22	10
ACYPI007960-RA	ni|645904079|nb|KK920352.1|	313224-313632	15	16	gi|646739598|gb|KK962865.1|	254229-255766	21	10
ACYPI007961-RA	ni|645903671|nb|KK920759.1|	1122835-1125211	nan	nan	gi|646776929|gb|KK961653.1|	385138-387270	17	8.5
ACYPI007967-RA	ni|645902344|nb|KK922085.1|	278849-279094	nan	nan	gi|646779580|gb|KK961570.1|	1991257-1991513	17	8.5
ACYPI007971-RA	ni|645904118|nb|KK920313.1|	730336-730825	17	17	gi|646744270|gb|KK962445.1|	230442-230881	21	7.9
ACYPI007972-RA	ni|645904237|nb|KK920240.1|	824927-827638	16	15	gi|646768447|gb|KK961811.1|	996320-996925	22	9.7
ACYPI007984-RA	ni|645903771|nb|KK920659.1|	84105-84386	16	15	gi|646781564|gb|KK961516.1|	766814-771175	23	9.4
ACYPI007986-RA	ni|645904243|nb|KK920236.1|	149185-150280	15	8.5	gi|646779498|gb|KK961572.1|	536944-537172	22	5.6
ACYPI007988-RA	ni|645902785|nb|KK921644.1|	105108-106086	nan	nan	gi|646768631|gb|KK961807.1|	397155-398127	19	9.2
ACYPI007989-RA	ni|645904171|nb|KK920262.1|	722019-724441	15	16	gi|646720990|gb|KK965002.1|	73604-74083	20	8.4
ACYPI007990-RA	ni|645904118|nb|KK920313.1|	376393-377342	17	17	gi|646781282|gb|KK961525.1|	1387951-1388828	22	9.6
ACYPI007994-RA	ni|645904246|nb|KK920233.1|	2171131-2171357	16	17	gi|646776184|gb|KK961688.1|	2446723-2448506	21	9.6
ACYPI008000-RA	ni|645903466|nb|KK920964.1|	698959-700474	nan	nan	gi|646752431|gb|KK961967.1|	1691052-1691379	20	5.6
ACYPI008002-RA	ni|645903607|nb|KK920823.1|	96658-96897	nan	nan	gi|646765991|gb|KK961880.1|	248045-251425	16	7
ACYPI008005-RA	ni|645904136|nb|KK920297.1|	1673877-1674535	16	17	gi|646738812|gb|KK962939.1|	198961-200077	20	8.1
ACYPI008007-RA	ni|645903495|nb|KK920935.1|	373205-375045	nan	nan	gi|646781212|gb|KK961527.1|	3906190-3908250	21	10
ACYPI008010-RA	ni|645903785|nb|KK920645.1|	255363-256690	15	16	gi|646770901|gb|KK961773.1|	964375-967240	22	9.7
ACYPI008022-RA	ni|645904058|nb|KK920373.1|	1239274-1239605	16	15	gi|646782288|gb|KK961496.1|	7021789-7023913	21	9.7
ACYPI008024-RA	ni|645903644|nb|KK920786.1|	346022-346513	nan	nan	gi|646766382|gb|KK961864.1|	1216679-1217538	22	9.7
ACYPI008028-RA	ni|645903957|nb|KK920474.1|	896646-897817	18	17	gi|646773423|gb|KK961749.1|	651527-654008	19	8.1
ACYPI008033-RA	ni|645904138|nb|KK920295.1|	667482-668200	15	15	gi|646777729|gb|KK961621.1|	740303-742314	21	9.8
ACYPI008034-RA	ni|645904027|nb|KK920404.1|	582679-583498	17	17	gi|646770477|gb|KK961778.1|	1159251-1159672	21	9.1
ACYPI008035-RA	ni|645904274|nb|KK920223.1|	630382-631063	14	17	gi|646782276|gb|KK961497.1|	5777177-5779293	21	9.7
ACYPI008037-RA	ni|645903983|nb|KK920448.1|	208182-208887	18	17	gi|646781143|gb|KK961529.1|	3771615-3772187	20	10
ACYPI008050-RA	ni|645904251|nb|KK920231.1|	519172-519987	16	16	gi|646780658|gb|KK961544.1|	1414887-1422236	19	8.6
ACYPI008053-RA	ni|645904088|nb|KK920343.1|	1517099-1520331	17	18	gi|646776184|gb|KK961688.1|	1715588-1716353	21	9.6

ACYPI008055-RA	ni|645903614|nb|KK920816.1|	827187-827825	nan	nan	gi|646782087|gb|KK961501.1|	2518957-2519331	20	8.7
ACYPI008056-RA	ni|645904005|nb|KK920426.1|	649656-650128	17	16	gi|646749672|gb|KK962080.1|	101374-101587	20	7.6
ACYPI008058-RA	ni|645903662|nb|KK920768.1|	32480-32763	nan	nan	gi|646754962|gb|KK961940.1|	1174428-1181694	21	10
ACYPI008063-RA	ni|645904106|nb|KK920325.1|	1970771-1971261	17	17	gi|646768294|gb|KK961814.1|	445194-445815	23	10
ACYPI008065-RA	ni|645902496|nb|KK921933.1|	72732-75601	nan	nan	gi|646744168|gb|KK962452.1|	726520-727612	22	8.7
ACYPI008075-RA	ni|645904168|nb|KK920265.1|	682992-683261	16	16	gi|646750889|gb|KK962017.1|	1634077-1635940	22	6.1
ACYPI008076-RA	ni|645904271|nb|KK920224.1|	1355126-1355530	15	16	gi|646776542|gb|KK961668.1|	2645998-2649608	20	5.6
ACYPI008080-RA	ni|645904049|nb|KK920382.1|	413680-413891	18	18	gi|646776069|gb|KK961694.1|	982581-987632	18	7.8
ACYPI008098-RA	ni|645904142|nb|KK920291.1|	1645913-1646647	20	23	gi|646751160|gb|KK962003.1|	621515-622789	22	10
ACYPI008107-RA	ni|645904177|nb|KK920260.1|	2522848-2523442	18	17	gi|646738455|gb|KK962975.1|	332440-333227	23	9.5
ACYPI008113-RA	ni|645903813|nb|KK920617.1|	480728-481433	17	18	gi|646769038|gb|KK961800.1|	1672749-1675074	21	8.9
ACYPI008122-RA	ni|645902779|nb|KK921650.1|	60287-60820	nan	nan	gi|646746202|gb|KK962305.1|	397075-399965	21	6.4
ACYPI008128-RA	ni|645903854|nb|KK920576.1|	403864-404055	16	8.4	gi|646780105|gb|KK961557.1|	94185-96805	21	9.7
ACYPI008129-RA	ni|645904166|nb|KK920267.1|	1937975-1938696	18	16	gi|646781690|gb|KK961512.1|	2519417-2519949	21	8.8
ACYPI008131-RA	ni|645903484|nb|KK920946.1|	143916-144472	nan	nan	gi|646770901|gb|KK961773.1|	1248590-1249019	22	9.7
ACYPI008134-RA	ni|645902035|nb|KK922394.1|	198241-199816	nan	nan	gi|646781893|gb|KK961506.1|	3696860-3698932	19	8.5
ACYPI008142-RA	ni|645904122|nb|KK920309.1|	1068006-1068724	18	17	gi|646781443|gb|KK961520.1|	3108402-3108976	20	8.3
ACYPI008149-RA	ni|645904046|nb|KK920385.1|	282472-283276	17	8.4	gi|646775789|gb|KK961709.1|	1266376-1268753	20	9.6
ACYPI008157-RA	ni|645903495|nb|KK920935.1|	254128-254414	nan	nan	gi|646779621|gb|KK961569.1|	1204604-1206755	16	7.3
ACYPI008158-RA	ni|645904177|nb|KK920260.1|	1060377-1060771	18	17	gi|646748129|gb|KK962169.1|	620766-624905	21	9.8
ACYPI008162-RA	ni|645903988|nb|KK920443.1|	248706-249243	14	16	gi|646781083|gb|KK961531.1|	2092356-2092959	19	8.5
ACYPI008165-RA	ni|645903953|nb|KK920478.1|	1425661-1426058	18	17	gi|646748615|gb|KK962139.1|	468964-469463	19	7.8
ACYPI008166-RA	ni|645903821|nb|KK920609.1|	1054354-1054855	17	17	gi|646778840|gb|KK961590.1|	1725634-1729354	20	9.9
ACYPI008168-RA	ni|645902668|nb|KK921761.1|	142400-145561	nan	nan	gi|646778699|gb|KK961594.1|	1224258-1227543	21	9.5
ACYPI008180-RA	ni|645904061|nb|KK920370.1|	923966-924909	17	16	gi|646776735|gb|KK961660.1|	830516-840025	21	9.6
ACYPI008184-RA	ni|645904158|nb|KK920275.1|	1907043-1907595	15	8.3	gi|646770723|gb|KK961775.1|	1380322-1381050	20	8.8
ACYPI008186-RA	ni|645902011|nb|KK922418.1|	89405-91913	nan	nan	gi|646779337|gb|KK961576.1|	784415-784904	18	9
ACYPI008188-RA	ni|645904114|nb|KK920317.1|	1764971-1765254	18	18	gi|646782276|gb|KK961497.1|	4729408-4733508	21	9.7
ACYPI008191-RA	ni|645904088|nb|KK920343.1|	1323348-1323905	17	18	gi|646771845|gb|KK961763.1|	1459448-1460178	23	10
ACYPI008195-RA	ni|645903999|nb|KK920432.1|	1241125-1241995	17	15	gi|646782334|gb|KK961495.1|	2068955-2069679	21	9

ACYPI008202-RA	ni|645904157|nb|KK920276.1|	475768-475998	15	15	gi|646744957|gb|KK962394.1|	131978-137604	21	9.8
ACYPI008203-RA	ni|645904144|nb|KK920289.1|	1052544-1052950	16	9	gi|646780311|gb|KK961552.1|	4022890-4023698	21	9.7
ACYPI008211-RA	ni|645904268|nb|KK920225.1|	1076619-1077067	14	15	gi|646771092|gb|KK961771.1|	597519-599804	23	9.7
ACYPI008218-RA	ni|645904086|nb|KK920345.1|	347455-347600	14	15	gi|646765902|gb|KK961884.1|	1185385-1185612	22	9.2
ACYPI008222-RA	ni|645903674|nb|KK920756.1|	246220-248151	nan	nan	gi|646775842|gb|KK961706.1|	651342-651921	22	9
ACYPI008225-RA	ni|645903617|nb|KK920813.1|	496192-496801	nan	nan	gi|646750354|gb|KK962043.1|	580196-580920	20	9.2
ACYPI008231-RA	ni|645903831|nb|KK920599.1|	610607-611519	18	17	gi|646771092|gb|KK961771.1|	962290-963609	23	9.7
ACYPI008241-RA	ni|645903832|nb|KK920598.1|	1034509-1034878	18	8.8	gi|646738635|gb|KK962957.1|	192172-193016	20	8.6
ACYPI008242-RA	ni|645904222|nb|KK920245.1|	2329941-2330336	17	12	gi|646782357|gb|KK961494.1|	8255281-8258040	21	9.2
ACYPI008243-RA	ni|645903518|nb|KK920912.1|	151403-152288	nan	nan	gi|646763164|gb|KK961904.1|	1344160-1351566	22	10
ACYPI008244-RA	ni|645902229|nb|KK922200.1|	198305-198659	nan	nan	gi|646779498|gb|KK961572.1|	1075894-1076208	22	5.6
ACYPI008248-RA	ni|645904057|nb|KK920374.1|	57181-57562	16	15	gi|646614736|gb|KK973610.1|	15241-15543	14	6.6
ACYPI008255-RA	ni|645904210|nb|KK920249.1|	795621-795857	16	16	gi|646777017|gb|KK961649.1|	902831-903859	18	8
ACYPI008256-RA	ni|645904240|nb|KK920239.1|	388562-390169	16	16	gi|646746339|gb|KK962295.1|	245856-252050	20	8.2
ACYPI008262-RA	ni|645903680|nb|KK920750.1|	644973-645217	nan	nan	gi|646782357|gb|KK961494.1|	9345629-9346103	21	9.2
ACYPI008265-RA	ni|645904114|nb|KK920317.1|	1949883-1950138	18	18	gi|646775414|gb|KK961728.1|	1385684-1386523	17	9.5
ACYPI008271-RA	ni|645903902|nb|KK920529.1|	1140000-1140811	19	17	gi|646782168|gb|KK961499.1|	1742558-1748485	21	9.4
ACYPI008272-RA	ni|645902662|nb|KK921767.1|	276242-276971	nan	nan	gi|646781043|gb|KK961532.1|	2250870-2252431	26	11
ACYPI008275-RA	ni|645904274|nb|KK920223.1|	603859-604286	14	17	gi|646770477|gb|KK961778.1|	1198416-1205118	21	9.1
ACYPI008279-RA	ni|645903923|nb|KK920508.1|	1343612-1344248	18	18	gi|646775821|gb|KK961707.1|	285309-286134	23	9.1
ACYPI008290-RA	ni|645903564|nb|KK920866.1|	311355-312136	nan	nan	gi|646776322|gb|KK961682.1|	128486-131685	22	8.9
ACYPI008299-RA	ni|645903466|nb|KK920964.1|	205451-205812	nan	nan	gi|646748785|gb|KK962129.1|	604909-605420	20	8.2
ACYPI008301-RA	ni|645903631|nb|KK920799.1|	330051-331826	nan	nan	gi|646780925|gb|KK961536.1|	1171445-1172262	22	9.9
ACYPI008302-RA	ni|645904112|nb|KK920319.1|	223064-223885	18	17	gi|646772786|gb|KK961754.1|	520437-521419	22	9.6
ACYPI008308-RA	ni|645904009|nb|KK920422.1|	208078-208633	17	9.4	gi|646779412|gb|KK961574.1|	2285204-2286864	21	5.4
ACYPI008317-RA	ni|645902415|nb|KK922014.1|	18270-18456	nan	nan	gi|646775576|gb|KK961719.1|	1132574-1134047	20	8.6
ACYPI008318-RA	ni|645901319|nb|KK923110.1|	53804-54327	nan	nan	gi|646776929|gb|KK961653.1|	814956-815368	17	8.5
ACYPI008325-RA	ni|645904014|nb|KK920417.1|	822928-823625	19	18	gi|646772786|gb|KK961754.1|	357201-358960	22	9.6
ACYPI008327-RA	ni|645904130|nb|KK920303.1|	218328-220002	18	17	gi|646752014|gb|KK961974.1|	144836-150271	18	5.8
ACYPI008351-RA	ni|645903540|nb|KK920890.1|	182497-182752	nan	nan	gi|646746868|gb|KK962259.1|	620512-623808	21	10

ACYPI008357-RA	ni|645903664|nb|KK920766.1|	200467-200729	nan	nan	gi|646781183|gb|KK961528.1|	653951-659326	20	9.5
ACYPI008362-RA	ni|645896832|nb|KK927597.1|	2087-2490	nan	nan	gi|646782334|gb|KK961495.1|	8990436-8991485	21	9
ACYPI008366-RA	ni|645903952|nb|KK920479.1|	959333-960791	16	16	gi|646780222|gb|KK961554.1|	3767171-3767564	20	9.7
ACYPI008368-RA	ni|645904279|nb|KK920221.1|	2642380-2643820	16	16	gi|646747956|gb|KK962181.1|	233344-234089	22	9.7
ACYPI008371-RA	ni|645903992|nb|KK920439.1|	723641-724224	15	15	gi|646771845|gb|KK961763.1|	1421382-1421674	23	10
ACYPI008384-RA	ni|645904009|nb|KK920422.1|	897228-897413	17	9.4	gi|646745941|gb|KK962322.1|	489178-490054	21	10
ACYPI008388-RA	ni|645903934|nb|KK920497.1|	257626-257958	15	16	gi|646766482|gb|KK961860.1|	398798-399806	17	6.7
ACYPI008390-RA	ni|645904201|nb|KK920252.1|	402088-402335	17	16	gi|646766703|gb|KK961852.1|	379404-379585	20	7.1
ACYPI008392-RA	ni|645903662|nb|KK920768.1|	265946-267858	nan	nan	gi|646781849|gb|KK961508.1|	2731463-2734086	17	8
ACYPI008396-RA	ni|645904116|nb|KK920315.1|	2470307-2470964	19	18	gi|646782168|gb|KK961499.1|	3693910-3695458	21	9.4
ACYPI008403-RA	ni|645903833|nb|KK920597.1|	245191-246411	17	15	gi|646779741|gb|KK961566.1|	2353739-2355066	20	8.5
ACYPI008409-RA	ni|645904177|nb|KK920260.1|	1203793-1204261	18	17	gi|646780270|gb|KK961553.1|	1853799-1854192	22	8.6
ACYPI008415-RA	ni|645903915|nb|KK920516.1|	298896-300382	17	17	gi|646782043|gb|KK961502.1|	4060838-4061174	20	9.2
ACYPI008418-RA	ni|645904079|nb|KK920352.1|	1102974-1104379	15	16	gi|646772574|gb|KK961756.1|	1190864-1191414	21	8
ACYPI008429-RA	ni|645903741|nb|KK920689.1|	233027-233229	17	18	gi|646779141|gb|KK961581.1|	807247-807874	21	9.1
ACYPI008431-RA	ni|645904171|nb|KK920262.1|	605547-605867	15	16	gi|646773721|gb|KK961747.1|	2000809-2001071	21	9.2
ACYPI008437-RA	ni|645903800|nb|KK920630.1|	1377677-1378069	18	17	gi|646780978|gb|KK961534.1|	1021674-1022500	20	8
ACYPI008438-RA	ni|645903980|nb|KK920451.1|	84506-85063	11	14	gi|646776794|gb|KK961658.1|	627666-628786	20	8.1
ACYPI008452-RA	ni|645903965|nb|KK920466.1|	375425-376062	17	16	gi|646750795|gb|KK962021.1|	426982-427776	19	8.9
ACYPI008463-RA	ni|645904004|nb|KK920427.1|	964748-965362	16	16	gi|646731000|gb|KK963870.1|	122963-126331	20	8.1
ACYPI008467-RA	ni|645902445|nb|KK921984.1|	210468-210982	nan	nan	gi|646781659|gb|KK961513.1|	5065619-5066012	22	9.8
ACYPI008468-RA	ni|645903504|nb|KK920926.1|	341109-341299	nan	nan	gi|646782276|gb|KK961497.1|	6822031-6826216	21	9.7
ACYPI008472-RA	ni|645902346|nb|KK922083.1|	26162-28212	nan	nan	gi|646781243|gb|KK961526.1|	1249478-1252833	21	8.9
ACYPI008481-RA	ni|645904016|nb|KK920415.1|	151231-151467	12	14	gi|646614268|gb|KK973701.1|	3346-3726	24	7.5
ACYPI008482-RA	ni|645903629|nb|KK920801.1|	127081-127314	nan	nan	gi|646748142|gb|KK962168.1|	492640-493026	20	9.3
ACYPI008489-RA	ni|645903743|nb|KK920687.1|	1241440-1241806	19	18	gi|646734749|gb|KK963383.1|	106405-106675	15	5.8
ACYPI008491-RA	ni|645904279|nb|KK920221.1|	2628854-2630142	16	16	gi|646747201|gb|KK962236.1|	177019-178705	18	7.3
ACYPI008492-RA	ni|645902426|nb|KK922003.1|	199333-199603	nan	nan	gi|646781893|gb|KK961506.1|	3652002-3652358	19	8.5
ACYPI008493-RA	ni|645904174|nb|KK920261.1|	361095-361770	19	17	gi|646751664|gb|KK961982.1|	1201899-1208065	21	9.5
ACYPI008495-RA	ni|645902463|nb|KK921966.1|	127148-127419	nan	nan	gi|646780858|gb|KK961538.1|	1317995-1318583	21	10

ACYPI008507-RA	ni|645903889|nb|KK920542.1|	508382-508601	16	19	gi|646780222|gb|KK961554.1|	3078919-3079657	20	9.7
ACYPI008512-RA	ni|645903704|nb|KK920726.1|	204369-205036	nan	nan	gi|646749036|gb|KK962114.1|	10465-11041	20	7.5
ACYPI008516-RA	ni|645903594|nb|KK920836.1|	552289-552505	nan	nan	gi|646770901|gb|KK961773.1|	1649200-1650400	22	9.7
ACYPI008521-RA	ni|645903772|nb|KK920658.1|	281065-282562	15	16	gi|646746284|gb|KK962299.1|	242466-243866	16	6.7
ACYPI008535-RA	ni|645903552|nb|KK920878.1|	629324-631169	nan	nan	gi|646765880|gb|KK961885.1|	586677-587872	23	10
ACYPI008536-RA	ni|645904195|nb|KK920254.1|	2135742-2136232	18	17	gi|646782276|gb|KK961497.1|	1179218-1179677	21	9.7
ACYPI008539-RA	ni|645903847|nb|KK920583.1|	825801-825996	18	18	gi|646749464|gb|KK962091.1|	361958-362486	18	8.8
ACYPI008541-RA	ni|645903812|nb|KK920618.1|	811247-811598	20	18	gi|646749632|gb|KK962082.1|	447267-453128	20	8.6
ACYPI008545-RA	ni|645903634|nb|KK920796.1|	1240388-1241503	nan	nan	gi|646776219|gb|KK961687.1|	1919596-1920742	21	9.9
ACYPI008552-RA	ni|645904240|nb|KK920239.1|	1868699-1869848	16	16	gi|646746355|gb|KK962294.1|	790028-790559	22	10
ACYPI008555-RA	ni|645903518|nb|KK920912.1|	96093-96497	nan	nan	gi|646775595|gb|KK961718.1|	1422622-1424548	21	8.9
ACYPI008556-RA	ni|645903829|nb|KK920601.1|	714638-715795	16	15	gi|646746556|gb|KK962281.1|	593040-594447	20	9.1
ACYPI008558-RA	ni|645903984|nb|KK920447.1|	402221-403164	18	17	gi|646781421|gb|KK961521.1|	4773198-4780250	21	8.7
ACYPI008560-RA	ni|645904278|nb|KK920222.1|	1322795-1323147	15	16	gi|646751515|gb|KK961987.1|	1006506-1007171	20	9.2
ACYPI008563-RA	ni|645903617|nb|KK920813.1|	198033-198528	nan	nan	gi|646765880|gb|KK961885.1|	814878-815400	23	10
ACYPI008564-RA	ni|645903654|nb|KK920776.1|	55618-56120	nan	nan	gi|646780441|gb|KK961549.1|	2921561-2923334	21	9.5
ACYPI008566-RA	ni|645904143|nb|KK920290.1|	604666-605675	15	7.9	gi|646778565|gb|KK961598.1|	1782564-1783070	19	7.8
ACYPI008578-RA	ni|645903597|nb|KK920833.1|	497897-498481	nan	nan	gi|646765705|gb|KK961893.1|	595584-596484	22	9.7
ACYPI008586-RA	ni|645903834|nb|KK920596.1|	83243-83424	18	17	gi|646746663|gb|KK962273.1|	235779-236127	21	10
ACYPI008591-RA	ni|645904166|nb|KK920267.1|	2471076-2471954	18	16	gi|646778061|gb|KK961612.1|	1611854-1614132	20	8.7
ACYPI008596-RA	ni|645903496|nb|KK920934.1|	949022-949806	nan	nan	gi|646780358|gb|KK961551.1|	2223879-2225027	21	8.8
ACYPI008606-RA	ni|645903532|nb|KK920898.1|	880246-880776	nan	nan	gi|646780889|gb|KK961537.1|	2378429-2381394	22	10
ACYPI008607-RA	ni|645904251|nb|KK920231.1|	448430-448934	16	16	gi|646725628|gb|KK964623.1|	66223-66493	16	5.7
ACYPI008618-RA	ni|645903830|nb|KK920600.1|	41407-42066	19	24	gi|646774067|gb|KK961745.1|	1188544-1189697	17	9.3
ACYPI008619-RA	ni|645904088|nb|KK920343.1|	1433232-1433497	17	18	gi|646767439|gb|KK961832.1|	2210424-2212112	23	10
ACYPI008621-RA	ni|645904049|nb|KK920382.1|	452132-453098	18	18	gi|646749532|gb|KK962087.1|	327321-328078	14	6
ACYPI008623-RA	ni|645904142|nb|KK920291.1|	1886435-1887084	20	23	gi|646777631|gb|KK961624.1|	312626-314082	20	9.3
ACYPI008630-RA	ni|645904122|nb|KK920309.1|	1189676-1190439	18	17	gi|646770477|gb|KK961778.1|	894302-898464	21	9.1
ACYPI008640-RA	ni|645904098|nb|KK920333.1|	304677-305873	16	8.8	gi|646750869|gb|KK962018.1|	47570-58292	20	10
ACYPI008641-RA	ni|645903548|nb|KK920882.1|	451442-452508	nan	nan	gi|646747990|gb|KK962179.1|	465682-466776	21	9.3

ACYPI008653-RA	ni|645904146|nb|KK920287.1|	1462381-1462532	16	18	gi|646781143|gb|KK961529.1|	2077904-2078118	20	10
ACYPI008668-RA	ni|645904101|nb|KK920330.1|	642971-643569	18	18	gi|646746974|gb|KK962252.1|	341502-343651	23	6.1
ACYPI008671-RA	ni|645904133|nb|KK920300.1|	1015481-1016086	18	18	gi|646766624|gb|KK961855.1|	44782-46871	21	10
ACYPI008675-RA	ni|645903953|nb|KK920478.1|	1661335-1662416	18	17	gi|646765219|gb|KK961896.1|	231312-231552	20	8.1
ACYPI008677-RA	ni|645904118|nb|KK920313.1|	825177-825588	17	17	gi|646777912|gb|KK961616.1|	1701006-1703372	20	8.6
ACYPI008693-RA	ni|645903965|nb|KK920466.1|	1208597-1209041	17	16	gi|646776477|gb|KK961673.1|	1387426-1387748	22	9
ACYPI008698-RA	ni|645904073|nb|KK920358.1|	1104368-1108545	16	18	gi|646780978|gb|KK961534.1|	2915836-2920336	20	8
ACYPI008701-RA	ni|645904015|nb|KK920416.1|	1217734-1218076	18	16	gi|646780270|gb|KK961553.1|	4311807-4312104	22	8.6
ACYPI008707-RA	ni|645904116|nb|KK920315.1|	420920-421158	19	18	gi|646768294|gb|KK961814.1|	419566-421241	23	10
ACYPI008713-RA	ni|645904258|nb|KK920229.1|	241912-242535	16	16	gi|646776322|gb|KK961682.1|	957003-957206	22	8.9
ACYPI008717-RA	ni|645903744|nb|KK920686.1|	793998-794662	17	8.6	gi|646745387|gb|KK962363.1|	370653-370892	20	8.6
ACYPI008720-RA	ni|645904015|nb|KK920416.1|	178062-178286	18	16	gi|646746407|gb|KK962291.1|	225283-228535	21	9.9
ACYPI008721-RA	ni|645904015|nb|KK920416.1|	1249980-1250325	18	16	gi|646782357|gb|KK961494.1|	3502069-3503505	21	9.2
ACYPI008728-RA	ni|645903760|nb|KK920670.1|	705601-707676	18	19	gi|646746897|gb|KK962257.1|	1016643-1021994	21	9.2
ACYPI008736-RA	ni|645904043|nb|KK920388.1|	330047-334814	16	9.1	gi|646782043|gb|KK961502.1|	2403086-2407648	20	9.2
ACYPI008744-RA	ni|645903752|nb|KK920678.1|	325459-325598	18	16	gi|646780311|gb|KK961552.1|	2912345-2912655	21	9.7
ACYPI008756-RA	ni|645904177|nb|KK920260.1|	2525990-2526624	18	17	gi|646750643|gb|KK962028.1|	125528-125860	21	7.5
ACYPI008757-RA	ni|645903491|nb|KK920939.1|	495703-496133	nan	nan	gi|646763535|gb|KK961902.1|	1167780-1169586	23	10
ACYPI008758-RA	ni|645904258|nb|KK920229.1|	1062965-1063748	16	16	gi|646763164|gb|KK961904.1|	629740-630650	22	10
ACYPI008763-RA	ni|645904271|nb|KK920224.1|	458452-459710	15	16	gi|646781659|gb|KK961513.1|	5765849-5768012	22	9.8
ACYPI008765-RA	ni|645903957|nb|KK920474.1|	1454881-1455097	18	17	gi|646741815|gb|KK962634.1|	67974-69553	22	10
ACYPI008769-RA	ni|645903760|nb|KK920670.1|	740770-741114	18	19	gi|646782288|gb|KK961496.1|	227922-228617	21	9.7
ACYPI008771-RA	ni|645903989|nb|KK920442.1|	279222-279509	17	20	gi|646776024|gb|KK961697.1|	2544948-2546294	22	9.2
ACYPI008778-RA	ni|645903597|nb|KK920833.1|	235130-235288	nan	nan	gi|646501544|gb|KK994170.1|	1242-2507	20	8.3
ACYPI008781-RA	ni|645904079|nb|KK920352.1|	110690-111591	15	16	gi|646781628|gb|KK961514.1|	3632767-3633635	23	9
ACYPI008785-RA	ni|645902580|nb|KK921849.1|	57156-57395	nan	nan	gi|646780889|gb|KK961537.1|	4035156-4037119	22	10
ACYPI008789-RA	ni|645904153|nb|KK920280.1|	2054240-2054490	17	16	gi|646777089|gb|KK961646.1|	1064232-1064560	20	9.4
ACYPI008790-RA	ni|645903971|nb|KK920460.1|	1037958-1038214	19	18	gi|646776974|gb|KK961651.1|	718588-719743	17	8.1
ACYPI008793-RA	ni|645904170|nb|KK920263.1|	1927624-1928348	15	16	gi|646776429|gb|KK961676.1|	1359768-1360536	20	9.9
ACYPI008797-RA	ni|645904162|nb|KK920271.1|	719103-719283	15	18	gi|646777440|gb|KK961631.1|	1415480-1415950	22	9.7

ACYPI008800-RA	ni|645903820|nb|KK920610.1|	394818-395314	18	19	gi|646766158|gb|KK961873.1|	1042121-1042559	22	5.8
ACYPI008804-RA	ni|645903702|nb|KK920728.1|	537841-538709	nan	nan	gi|646781043|gb|KK961532.1|	1570080-1570885	26	11
ACYPI008806-RA	ni|645904165|nb|KK920268.1|	779389-779610	16	16	gi|646767697|gb|KK961826.1|	274483-275407	21	9.8
ACYPI008810-RA	ni|645903843|nb|KK920587.1|	403015-403161	18	16	gi|646523229|gb|KK990101.1|	6520-6763	19	1.9
ACYPI008811-RA	ni|645903566|nb|KK920864.1|	599818-600189	nan	nan	gi|646781243|gb|KK961526.1|	3535409-3535857	21	8.9
ACYPI008830-RA	ni|645901593|nb|KK922836.1|	1586-1846	nan	nan	gi|646775451|gb|KK961726.1|	1710281-1710965	20	5.6
ACYPI008831-RA	ni|645902620|nb|KK921809.1|	180239-181284	nan	nan	gi|646776038|gb|KK961696.1|	1264429-1275408	21	10
ACYPI008833-RA	ni|645903547|nb|KK920883.1|	608168-608673	nan	nan	gi|646766137|gb|KK961874.1|	468576-469321	20	9
ACYPI008834-RA	ni|645902463|nb|KK921966.1|	161240-161656	nan	nan	gi|646749632|gb|KK962082.1|	218017-218294	20	8.6
ACYPI008835-RA	ni|645904204|nb|KK920251.1|	589540-590404	15	16	gi|646765790|gb|KK961889.1|	1133475-1134287	22	8.8
ACYPI008847-RA	ni|645904158|nb|KK920275.1|	979395-979785	15	8.3	gi|646747076|gb|KK962245.1|	561257-570758	23	9.6
ACYPI008848-RA	ni|645904050|nb|KK920381.1|	530500-531337	18	20	gi|646746651|gb|KK962274.1|	77672-78544	16	6.6
ACYPI008850-RA	ni|645903738|nb|KK920692.1|	914605-914994	18	17	gi|646768717|gb|KK961805.1|	1261868-1262852	20	8
ACYPI008851-RA	ni|645904052|nb|KK920379.1|	202567-204032	18	16	gi|646722582|gb|KK964929.1|	127492-129551	23	6
ACYPI008853-RA	ni|645904012|nb|KK920419.1|	241925-242178	27	32	gi|646766034|gb|KK961878.1|	273626-273888	20	9
ACYPI008861-RA	ni|645904231|nb|KK920242.1|	48525-49467	16	17	gi|646685323|gb|KK967172.1|	15217-15509	11	6.4
ACYPI008863-RA	ni|645903627|nb|KK920803.1|	486091-487591	nan	nan	gi|646777474|gb|KK961630.1|	559820-561283	21	9.3
ACYPI008866-RA	ni|645903912|nb|KK920519.1|	278636-280118	17	18	gi|646778632|gb|KK961596.1|	239659-243015	21	9.2
ACYPI008874-RA	ni|645903837|nb|KK920593.1|	552939-554801	18	9.8	gi|646779741|gb|KK961566.1|	3296980-3299808	20	8.5
ACYPI008877-RA	ni|645903869|nb|KK920561.1|	462134-462578	17	15	gi|646776998|gb|KK961650.1|	2677370-2679852	20	9.6
ACYPI008884-RA	ni|645903836|nb|KK920594.1|	893375-893865	18	11	gi|646747424|gb|KK962220.1|	1163095-1164089	22	5.7
ACYPI008886-RA	ni|645903539|nb|KK920891.1|	252857-253068	nan	nan	gi|646781968|gb|KK961504.1|	3297544-3298546	20	9.6
ACYPI008888-RA	ni|645903511|nb|KK920919.1|	481925-482124	nan	nan	gi|646750795|gb|KK962021.1|	618720-619779	19	8.9
ACYPI008895-RA	ni|645902011|nb|KK922418.1|	94606-97129	nan	nan	gi|646779375|gb|KK961575.1|	3023645-3024163	22	9.3
ACYPI008902-RA	ni|645904130|nb|KK920303.1|	1421486-1423724	18	17	gi|646781421|gb|KK961521.1|	4076552-4080007	21	8.7
ACYPI008920-RA	ni|645904246|nb|KK920233.1|	676293-677589	16	17	gi|646778865|gb|KK961589.1|	980458-982829	20	9.2
ACYPI008922-RA	ni|645903827|nb|KK920603.1|	205549-205711	17	18	gi|646779337|gb|KK961576.1|	1186733-1187843	18	9
ACYPI008923-RA	ni|645903910|nb|KK920521.1|	634275-636995	19	17	gi|646772358|gb|KK961758.1|	1492987-1494410	20	9.3
ACYPI008930-RA	ni|645903987|nb|KK920444.1|	795163-797652	15	17	gi|646780574|gb|KK961546.1|	3155925-3157522	20	9.9
ACYPI008931-RA	ni|645901860|nb|KK922569.1|	90936-91345	nan	nan	gi|646775821|gb|KK961707.1|	2979644-2980859	23	9.1

ACYPI008933-RA	ni|645901860|nb|KK922569.1|	108241-108487	nan	nan	gi|646780925|gb|KK961536.1|	1887988-1888248	22	9.9
ACYPI008947-RA	ni|645903496|nb|KK920934.1|	277533-277921	nan	nan	gi|646764801|gb|KK961898.1|	543144-545357	20	9.7
ACYPI008955-RA	ni|645903728|nb|KK920702.1|	46399-46982	nan	nan	gi|646776389|gb|KK961678.1|	2209118-2211754	22	9.5
ACYPI008958-RA	ni|645903742|nb|KK920688.1|	499134-499927	17	16	gi|646781344|gb|KK961523.1|	4533209-4534364	21	8.9
ACYPI008963-RA	ni|645900068|nb|KK924361.1|	32900-34808	nan	nan	gi|646781013|gb|KK961533.1|	1450509-1456424	18	7.6
ACYPI008967-RA	ni|645904112|nb|KK920319.1|	434115-434313	18	17	gi|646782211|gb|KK961498.1|	1248851-1250437	20	9
ACYPI008971-RA	ni|645903812|nb|KK920618.1|	458518-458824	20	18	gi|646741656|gb|KK962648.1|	264321-266085	24	9.7
ACYPI008974-RA	ni|645903910|nb|KK920521.1|	323363-323549	19	17	gi|646750204|gb|KK962051.1|	64911-65267	19	7.5
ACYPI008980-RA	ni|645896873|nb|KK927556.1|	2477-4051	nan	nan	gi|646780574|gb|KK961546.1|	1149829-1156303	20	9.9
ACYPI008983-RA	ni|645904225|nb|KK920244.1|	696118-696389	17	17	gi|646504416|gb|KK993594.1|	977-1247	16	7.8
ACYPI008990-RA	ni|645903925|nb|KK920506.1|	1359898-1360837	19	17	gi|646781732|gb|KK961511.1|	1686649-1689533	22	9.8
ACYPI008993-RA	ni|645903594|nb|KK920836.1|	590027-590352	nan	nan	gi|646770901|gb|KK961773.1|	1693454-1693843	22	9.7
ACYPI009003-RA	ni|645904240|nb|KK920239.1|	1829221-1829755	16	16	gi|646781421|gb|KK961521.1|	2416081-2417780	21	8.7
ACYPI009004-RA	ni|645903957|nb|KK920474.1|	952969-953494	18	17	gi|646778983|gb|KK961586.1|	269447-270522	20	9.9
ACYPI009012-RA	ni|645902671|nb|KK921758.1|	61708-61961	nan	nan	gi|646754962|gb|KK961940.1|	920780-922206	21	10
ACYPI009018-RA	ni|645901741|nb|KK922688.1|	72695-73322	nan	nan	gi|646782276|gb|KK961497.1|	1086721-1088181	21	9.7
ACYPI009032-RA	ni|645904015|nb|KK920416.1|	1373377-1374462	18	16	gi|646776184|gb|KK961688.1|	807830-808041	21	9.6
ACYPI009034-RA	ni|645903484|nb|KK920946.1|	179069-180146	nan	nan	gi|646776113|gb|KK961692.1|	1668056-1668680	21	9.6
ACYPI009038-RA	ni|645901697|nb|KK922732.1|	113387-114303	nan	nan	gi|646744874|gb|KK962400.1|	387724-387950	19	8.3
ACYPI009043-RA	ni|645903812|nb|KK920618.1|	794574-794914	20	18	gi|646776219|gb|KK961687.1|	2393402-2395408	21	9.9
ACYPI009045-RA	ni|645903521|nb|KK920909.1|	475818-476511	nan	nan	gi|646751160|gb|KK962003.1|	409690-415606	22	10
ACYPI009048-RA	ni|645904009|nb|KK920422.1|	1288433-1291240	17	9.4	gi|646781772|gb|KK961510.1|	3373322-3373647	20	9.2
ACYPI009052-RA	ni|645903713|nb|KK920717.1|	670860-671804	nan	nan	gi|646746752|gb|KK962267.1|	5014-6815	20	7.9
ACYPI009057-RA	ni|645904076|nb|KK920355.1|	1536129-1536950	17	17	gi|646734440|gb|KK963419.1|	333408-333669	19	6.9
ACYPI009061-RA	ni|645903467|nb|KK920963.1|	102154-103009	nan	nan	gi|646780827|gb|KK961539.1|	3310808-3312546	20	9.4
ACYPI009065-RA	ni|645902755|nb|KK921674.1|	73065-77441	nan	nan	gi|646745434|gb|KK962359.1|	392426-392979	22	10
ACYPI009066-RA	ni|645904054|nb|KK920377.1|	604310-604521	16	16	gi|646745533|gb|KK962351.1|	620817-621330	22	9.7
ACYPI009068-RA	ni|645903740|nb|KK920690.1|	273330-275198	18	10	gi|646780574|gb|KK961546.1|	272205-273668	20	9.9
ACYPI009070-RA	ni|645904106|nb|KK920325.1|	917795-921017	17	17	gi|646739555|gb|KK962870.1|	121424-124548	27	18
ACYPI009071-RA	ni|645901697|nb|KK922732.1|	122779-123331	nan	nan	gi|646744874|gb|KK962400.1|	385557-387582	19	8.3

ACYPI009072-RA	ni|645903755|nb|KK920675.1|	733067-733703	18	16	gi|646742541|gb|KK962572.1|	570529-572677	19	10
ACYPI009089-RA	ni|645904153|nb|KK920280.1|	723012-724345	17	16	gi|646746556|gb|KK962281.1|	301199-303090	20	9.1
ACYPI009090-RA	ni|645903669|nb|KK920761.1|	25222-26286	nan	nan	gi|646776477|gb|KK961673.1|	2097340-2101788	22	9
ACYPI009098-RA	ni|645902673|nb|KK921756.1|	13452-13943	nan	nan	gi|646746663|gb|KK962273.1|	217142-217761	21	10
ACYPI009132-RA	ni|645904241|nb|KK920238.1|	696919-698752	18	17	gi|646763164|gb|KK961904.1|	1703087-1706524	22	10
ACYPI009136-RA	ni|645904151|nb|KK920282.1|	442439-442616	16	20	gi|646780858|gb|KK961538.1|	3132481-3132748	21	10
ACYPI009144-RA	ni|645903572|nb|KK920858.1|	185126-185922	nan	nan	gi|646777182|gb|KK961642.1|	1699808-1701036	20	9
ACYPI009147-RA	ni|645904271|nb|KK920224.1|	2844807-2845651	15	16	gi|646776542|gb|KK961668.1|	1370838-1375160	20	5.6
ACYPI009151-RA	ni|645904159|nb|KK920274.1|	1063712-1064149	15	17	gi|646771092|gb|KK961771.1|	1029019-1029588	23	9.7
ACYPI009157-RA	ni|645903910|nb|KK920521.1|	575489-576148	19	17	gi|646751373|gb|KK961992.1|	540642-540913	21	8.7
ACYPI009158-RA	ni|645903782|nb|KK920648.1|	143193-143403	13	14	gi|646766624|gb|KK961855.1|	412484-413071	21	10
ACYPI009170-RA	ni|645903665|nb|KK920765.1|	10177-11108	nan	nan	gi|646780827|gb|KK961539.1|	3630757-3633266	20	9.4
ACYPI009174-RA	ni|645903919|nb|KK920512.1|	791168-791782	17	15	gi|646768717|gb|KK961805.1|	142739-146618	20	8
ACYPI009187-RA	ni|645903620|nb|KK920810.1|	610878-611032	nan	nan	gi|646775614|gb|KK961717.1|	833748-836436	21	9.7
ACYPI009193-RA	ni|645903938|nb|KK920493.1|	596777-597180	19	18	gi|646781690|gb|KK961512.1|	3419150-3420771	21	8.8
ACYPI009202-RA	ni|645903743|nb|KK920687.1|	1045386-1045619	19	18	gi|646765198|gb|KK961897.1|	614027-616647	22	5.9
ACYPI009223-RA	ni|645904088|nb|KK920343.1|	1246546-1247967	17	18	gi|646766985|gb|KK961844.1|	91030-101463	21	9.7
ACYPI009224-RA	ni|645898360|nb|KK926069.1|	8524-9062	nan	nan	gi|646777363|gb|KK961635.1|	1927326-1929724	18	9.2
ACYPI009232-RA	ni|645904240|nb|KK920239.1|	1879723-1879927	16	16	gi|646781421|gb|KK961521.1|	5008801-5016367	21	8.7
ACYPI009237-RA	ni|645903694|nb|KK920736.1|	442129-442610	nan	nan	gi|646767850|gb|KK961823.1|	1681050-1681592	21	9.8
ACYPI009250-RA	ni|645903968|nb|KK920463.1|	469656-471177	16	1.60E+01	gi|646746216|gb|KK962304.1|	447052-448861	130	33
ACYPI009253-RA	ni|645904061|nb|KK920370.1|	101849-102864	17	1.60E+01	gi|646732863|gb|KK963615.1|	60691-62792	150	39
ACYPI009254-RA	ni|645903816|nb|KK920614.1|	202350-203080	14	15	gi|646781893|gb|KK961506.1|	1553013-1556132	19	8.5
ACYPI009257-RA	ni|645904207|nb|KK920250.1|	545992-547422	18	20	gi|646630362|gb|KK970809.1|	4347-5074	12	7.2
ACYPI009258-RA	ni|645903697|nb|KK920733.1|	167567-168183	nan	nan	gi|646777802|gb|KK961619.1|	1271170-1271967	24	9
ACYPI009259-RA	ni|645904128|nb|KK920305.1|	959376-960551	15	8.6	gi|646747841|gb|KK962189.1|	462210-463390	23	10
ACYPI009262-RA	ni|645904009|nb|KK920422.1|	1096853-1097117	17	9.4	gi|646780010|gb|KK961559.1|	459098-459345	22	10
ACYPI009267-RA	ni|645902193|nb|KK922236.1|	54992-55570	nan	nan	gi|646769102|gb|KK961799.1|	1848323-1855596	20	8.3
ACYPI009274-RA	ni|645904053|nb|KK920378.1|	517838-518305	18	17	gi|646778632|gb|KK961596.1|	2376348-2376644	21	9.2
ACYPI009277-RA	ni|645902598|nb|KK921831.1|	18016-19000	nan	nan	gi|646771845|gb|KK961763.1|	644293-645904	23	10

ACYPI009280-RA	ni|645903796|nb|KK920634.1|	819026-819611	18	17	gi|646779141|gb|KK961581.1|	629196-632506	21	9.1
ACYPI009306-RA	ni|645903768|nb|KK920662.1|	1375833-1376773	18	17	gi|646743236|gb|KK962519.1|	160632-162206	22	9.1
ACYPI009308-RA	ni|645903775|nb|KK920655.1|	144433-144677	16	16	gi|646781421|gb|KK961521.1|	12560-13018	21	8.7
ACYPI009311-RA	ni|645904036|nb|KK920395.1|	1244478-1244710	21	18	gi|646775807|gb|KK961708.1|	2548305-2548555	21	8.6
ACYPI009312-RA	ni|645903470|nb|KK920960.1|	163104-163979	nan	nan	gi|646777495|gb|KK961629.1|	1785431-1786785	19	8.7
ACYPI009316-RA	ni|645904145|nb|KK920288.1|	674070-674818	16	9.3	gi|646752143|gb|KK961972.1|	1942412-1943645	20	5.6
ACYPI009317-RA	ni|645903525|nb|KK920905.1|	82010-82229	nan	nan	gi|646781421|gb|KK961521.1|	1809600-1809864	21	8.7
ACYPI009325-RA	ni|645904014|nb|KK920417.1|	209646-210874	19	18	gi|646752567|gb|KK961965.1|	777613-777911	19	8.4
ACYPI009332-RA	ni|645904020|nb|KK920411.1|	267134-267810	17	19	gi|646742541|gb|KK962572.1|	472378-475879	19	10
ACYPI009334-RA	ni|645904119|nb|KK920312.1|	1987572-1987965	19	19	gi|646766598|gb|KK961856.1|	1189105-1189408	23	10
ACYPI009338-RA	ni|645903472|nb|KK920958.1|	229918-230887	nan	nan	gi|646776588|gb|KK961666.1|	981015-981822	20	5.8
ACYPI009339-RA	ni|645904046|nb|KK920385.1|	1212651-1213385	17	8.4	gi|646780105|gb|KK961557.1|	3586780-3587698	21	9.7
ACYPI009357-RA	ni|645903855|nb|KK920575.1|	75829-76211	16	17	gi|646768406|gb|KK961812.1|	258091-258331	20	9.6
ACYPI009370-RA	ni|645903749|nb|KK920681.1|	173250-173737	19	18	gi|646781344|gb|KK961523.1|	1250403-1252070	21	8.9
ACYPI009374-RA	ni|645904005|nb|KK920426.1|	611880-612393	17	16	gi|646766382|gb|KK961864.1|	83706-85647	22	9.7
ACYPI009378-RA	ni|645903557|nb|KK920873.1|	916386-916644	nan	nan	gi|646767850|gb|KK961823.1|	1756117-1756626	21	9.8
ACYPI009382-RA	ni|645903999|nb|KK920432.1|	1356881-1357073	17	15	gi|646779898|gb|KK961562.1|	2378809-2380683	19	9.3
ACYPI009386-RA	ni|645903680|nb|KK920750.1|	441315-443132	nan	nan	gi|646777955|gb|KK961615.1|	1344620-1347449	21	10
ACYPI009392-RA	ni|645904035|nb|KK920396.1|	584797-584926	16	16	gi|646779141|gb|KK961581.1|	2984927-2985336	21	9.1
ACYPI009394-RA	ni|645904053|nb|KK920378.1|	594785-595281	18	17	gi|646779826|gb|KK961564.1|	1098957-1099868	22	9.6
ACYPI009395-RA	ni|645903860|nb|KK920570.1|	19575-19837	17	17	gi|646781732|gb|KK961511.1|	3690283-3690972	22	9.8
ACYPI009396-RA	ni|645903470|nb|KK920960.1|	111484-112130	nan	nan	gi|646742343|gb|KK962588.1|	315792-316641	20	9.4
ACYPI009407-RA	ni|645903938|nb|KK920493.1|	545332-545510	19	18	gi|646775944|gb|KK961701.1|	941997-944835	22	9.3
ACYPI009409-RA	ni|645904240|nb|KK920239.1|	4415-5106	16	16	gi|646751350|gb|KK961993.1|	578971-585671	20	7.6
ACYPI009413-RA	ni|645903594|nb|KK920836.1|	547041-547257	nan	nan	gi|646780889|gb|KK961537.1|	2358813-2365594	22	10
ACYPI009420-RA	ni|645904053|nb|KK920378.1|	185444-186415	18	17	gi|646743377|gb|KK962508.1|	68993-70281	19	8
ACYPI009424-RA	ni|645904045|nb|KK920386.1|	671713-672080	19	21	gi|646768669|gb|KK961806.1|	331550-333339	18	9.1
ACYPI009428-RA	ni|645903814|nb|KK920616.1|	415992-416758	17	16	gi|646760564|gb|KK961914.1|	1315264-1317272	21	8.8
ACYPI009430-RA	ni|645902129|nb|KK922300.1|	139007-140643	nan	nan	gi|646779186|gb|KK961580.1|	510430-510980	20	8.6
ACYPI009436-RA	ni|645904237|nb|KK920240.1|	901127-901479	16	15	gi|646779826|gb|KK961564.1|	985419-988515	22	9.6

ACYPI009438-RA	ni|645903858|nb|KK920572.1|	374401-376611	16	16	gi|646753273|gb|KK961956.1|	927205-927424	22	9
ACYPI009439-RA	ni|645903925|nb|KK920506.1|	596336-597133	19	17	gi|646747990|gb|KK962179.1|	110156-112105	21	9.3
ACYPI009441-RA	ni|645901953|nb|KK922476.1|	31239-32457	nan	nan	gi|646776113|gb|KK961692.1|	131031-136075	21	9.6
ACYPI009443-RA	ni|645903952|nb|KK920479.1|	684017-685000	16	16	gi|646752849|gb|KK961961.1|	332523-332867	21	9.4
ACYPI009454-RA	ni|645903484|nb|KK920946.1|	130156-130525	nan	nan	gi|646770901|gb|KK961773.1|	1284772-1287128	22	9.7
ACYPI009455-RA	ni|645904112|nb|KK920319.1|	2494607-2495327	18	17	gi|646553387|gb|KK985551.1|	7023-7449	21	7.5
ACYPI009457-RA	ni|645904049|nb|KK920382.1|	477429-477868	18	18	gi|646768631|gb|KK961807.1|	1346293-1346603	19	9.2
ACYPI009460-RA	ni|645903688|nb|KK920742.1|	375787-375945	nan	nan	gi|646780530|gb|KK961547.1|	1044603-1046004	21	9.2
ACYPI009467-RA	ni|645903637|nb|KK920793.1|	780139-780405	nan	nan	gi|646778660|gb|KK961595.1|	210197-211004	16	8.2
ACYPI009470-RA	ni|645903979|nb|KK920452.1|	494737-495943	15	17	gi|646747385|gb|KK962223.1|	404567-411588	21	5.4
ACYPI009478-RA	ni|645903639|nb|KK920791.1|	746128-746832	nan	nan	gi|646782127|gb|KK961500.1|	5611368-5612015	22	9.6
ACYPI009480-RA	ni|645904131|nb|KK920302.1|	558772-559055	15	16	gi|646778186|gb|KK961609.1|	1829477-1829777	19	9.4
ACYPI009487-RA	ni|645899152|nb|KK925277.1|	43101-43681	nan	nan	gi|646780752|gb|KK961541.1|	3351133-3351684	21	8
ACYPI009498-RA	ni|645903696|nb|KK920734.1|	11356-11545	nan	nan	gi|646776091|gb|KK961693.1|	611546-613120	23	10
ACYPI009500-RA	ni|645903812|nb|KK920618.1|	977459-978237	20	18	gi|646749945|gb|KK962065.1|	43724-44075	22	6.1
ACYPI009503-RA	ni|645903936|nb|KK920495.1|	196417-197600	19	18	gi|646776762|gb|KK961659.1|	1278958-1284746	21	8.9
ACYPI009511-RA	ni|645903765|nb|KK920665.1|	1194221-1194721	17	15	gi|646782357|gb|KK961494.1|	9107729-9111597	21	9.2
ACYPI009513-RA	ni|645903494|nb|KK920936.1|	436158-436346	nan	nan	gi|646778517|gb|KK961599.1|	621343-621678	20	9.6
ACYPI009523-RA	ni|645903731|nb|KK920699.1|	142800-144005	nan	nan	gi|646780752|gb|KK961541.1|	1543690-1544207	21	8
ACYPI009525-RA	ni|645903949|nb|KK920482.1|	566371-567845	17	14	gi|646747547|gb|KK962210.1|	499463-501253	19	7.5
ACYPI009526-RA	ni|645903626|nb|KK920804.1|	794037-794306	nan	nan	gi|646781083|gb|KK961531.1|	2566482-2568795	19	8.5
ACYPI009528-RA	ni|645904009|nb|KK920422.1|	657532-658077	17	9.4	gi|646762191|gb|KK961910.1|	907806-912858	18	7.1
ACYPI009536-RA	ni|645904148|nb|KK920285.1|	1274335-1276011	16	16	gi|646736283|gb|KK963203.1|	46215-46751	24	9.7
ACYPI009537-RA	ni|645904052|nb|KK920379.1|	572945-573618	18	16	gi|646780978|gb|KK961534.1|	401500-401706	20	8
ACYPI009538-RA	ni|645904177|nb|KK920260.1|	1987746-1988257	18	17	gi|646782357|gb|KK961494.1|	9220670-9221668	21	9.2
ACYPI009542-RA	ni|645904271|nb|KK920224.1|	4049223-4049732	15	16	gi|646740395|gb|KK962776.1|	232789-233176	16	6.3
ACYPI009548-RA	ni|645903704|nb|KK920726.1|	434282-434826	nan	nan	gi|646742541|gb|KK962572.1|	504419-505534	19	10
ACYPI009550-RA	ni|645904133|nb|KK920300.1|	1002743-1005662	18	18	gi|646781968|gb|KK961504.1|	416712-417117	20	9.6
ACYPI009554-RA	ni|645903843|nb|KK920587.1|	419452-419901	18	16	gi|646782211|gb|KK961498.1|	4647878-4648429	20	9
ACYPI009568-RA	ni|645904231|nb|KK920242.1|	599775-600035	16	17	gi|646746911|gb|KK962256.1|	12697-13330	17	7

ACYPI009576-RA	ni|645904008|nb|KK920423.1|	995692-995859	16	9.5	gi|646778903|gb|KK961588.1|	2729793-2730289	23	10
ACYPI009593-RA	ni|645904028|nb|KK920403.1|	1870443-1871762	18	17	gi|646770047|gb|KK961784.1|	358450-361876	21	9.1
ACYPI009596-RA	ni|645903747|nb|KK920683.1|	14922-16502	19	18	gi|646780105|gb|KK961557.1|	1629025-1629284	21	9.7
ACYPI009612-RA	ni|645902683|nb|KK921746.1|	125609-125751	nan	nan	gi|646775471|gb|KK961725.1|	2005985-2006288	20	10
ACYPI009613-RA	ni|645904122|nb|KK920309.1|	1169824-1170769	18	17	gi|646779741|gb|KK961566.1|	3087859-3088087	20	8.5
ACYPI009618-RA	ni|645903971|nb|KK920460.1|	937027-938031	19	18	gi|646766540|gb|KK961858.1|	58260-58445	21	8.5
ACYPI009620-RA	ni|645903716|nb|KK920714.1|	555445-556307	nan	nan	gi|646744930|gb|KK962396.1|	71652-71965	18	8.5
ACYPI009625-RA	ni|645904081|nb|KK920350.1|	255493-255710	13	18	gi|646749499|gb|KK962089.1|	801908-802385	20	8.7
ACYPI009633-RA	ni|645904010|nb|KK920421.1|	827123-827952	17	17	gi|646781510|gb|KK961518.1|	3510683-3511105	17	7.8
ACYPI009635-RA	ni|645904231|nb|KK920242.1|	280597-282308	16	17	gi|646755095|gb|KK961939.1|	157015-158888	15	8.9
ACYPI009639-RA	ni|645904077|nb|KK920354.1|	158206-159024	16	17	gi|646754962|gb|KK961940.1|	1013642-1014660	21	10
ACYPI009640-RA	ni|645904207|nb|KK920250.1|	763564-763750	18	20	gi|646781282|gb|KK961525.1|	243200-243676	22	9.6
ACYPI009651-RA	ni|645904122|nb|KK920309.1|	888721-890688	18	17	gi|646781443|gb|KK961520.1|	3321427-3321646	20	8.3
ACYPI009662-RA	ni|645903944|nb|KK920487.1|	471288-471627	15	17	gi|646748116|gb|KK962170.1|	95436-95812	20	8.3
ACYPI009684-RA	ni|645903827|nb|KK920603.1|	1101545-1102930	17	18	gi|646781659|gb|KK961513.1|	5995003-5996183	22	9.8
ACYPI009686-RA	ni|645903962|nb|KK920469.1|	68256-68614	17	9.8	gi|646776184|gb|KK961688.1|	1893735-1894802	21	9.6
ACYPI009704-RA	ni|645904210|nb|KK920249.1|	367814-373916	16	16	gi|646781772|gb|KK961510.1|	2890517-2899635	20	9.2
ACYPI009707-RA	ni|645904129|nb|KK920304.1|	1130026-1130759	16	17	gi|646741656|gb|KK962648.1|	661141-669664	24	9.7
ACYPI009711-RA	ni|645903757|nb|KK920673.1|	77025-77407	18	17	gi|646742842|gb|KK962548.1|	276634-282758	22	10
ACYPI009713-RA	ni|645904019|nb|KK920412.1|	726396-726638	19	9.4	gi|646744140|gb|KK962454.1|	469016-469672	21	9.3
ACYPI009718-RA	ni|645903811|nb|KK920619.1|	38980-40053	15	15	gi|646763796|gb|KK961901.1|	485985-486726	14	6.4
ACYPI009719-RA	ni|645903688|nb|KK920742.1|	1053979-1054215	nan	nan	gi|646768494|gb|KK961810.1|	364714-365914	21	10
ACYPI009720-RA	ni|645903747|nb|KK920683.1|	1090810-1093567	19	18	gi|646781690|gb|KK961512.1|	777801-785027	21	8.8
ACYPI009739-RA	ni|645903931|nb|KK920500.1|	530063-530717	16	18	gi|646747528|gb|KK962211.1|	244449-246216	18	8.1
ACYPI009741-RA	ni|645904163|nb|KK920270.1|	337608-337857	16	16	gi|646767265|gb|KK961836.1|	561579-562221	22	9.9
ACYPI009744-RA	ni|645904122|nb|KK920309.1|	1506520-1507419	18	17	gi|646766650|gb|KK961854.1|	1450255-1450922	22	10
ACYPI009745-RA	ni|645903612|nb|KK920818.1|	118958-119202	nan	nan	gi|646781536|gb|KK961517.1|	1923530-1923800	19	7.7
ACYPI009751-RA	ni|645903925|nb|KK920506.1|	547726-547945	19	17	gi|646776368|gb|KK961679.1|	476780-478102	21	9.7
ACYPI009755-RA	ni|645904071|nb|KK920360.1|	709807-710539	17	17	gi|646753799|gb|KK961950.1|	1860673-1862503	23	9.2
ACYPI009758-RA	ni|645904207|nb|KK920250.1|	562802-563314	18	20	gi|646779662|gb|KK961568.1|	2051425-2052235	20	9.3

ACYPI009769-RA	ni|645903690|nb|KK920740.1|	203871-204642	nan	nan	gi|646776998|gb|KK961650.1|	2705809-2716115	20	9.6
ACYPI009771-RA	ni|645903803|nb|KK920627.1|	830837-831067	17	17	gi|646782334|gb|KK961495.1|	2711667-2712689	21	9
ACYPI009777-RA	ni|645903680|nb|KK920750.1|	158914-159126	nan	nan	gi|646763164|gb|KK961904.1|	10574-15117	22	10
ACYPI009782-RA	ni|645903681|nb|KK920749.1|	232986-233688	nan	nan	gi|646781212|gb|KK961527.1|	4102236-4103280	21	10
ACYPI009786-RA	ni|645901288|nb|KK923141.1|	16166-16419	nan	nan	gi|646782357|gb|KK961494.1|	2947856-2948116	21	9.2
ACYPI009787-RA	ni|645903879|nb|KK920552.1|	1495229-1495809	18	18	gi|646775821|gb|KK961707.1|	799151-809580	23	9.1
ACYPI009795-RA	ni|645904014|nb|KK920417.1|	1416692-1417378	19	18	gi|646781628|gb|KK961514.1|	2293571-2295459	23	9
ACYPI009806-RA	ni|645903829|nb|KK920601.1|	629199-629807	16	15	gi|646777125|gb|KK961644.1|	3017208-3017568	20	10
ACYPI009808-RA	ni|645904150|nb|KK920283.1|	310181-310941	17	17	gi|646782087|gb|KK961501.1|	5245312-5245582	20	8.7
ACYPI009821-RA	ni|645904004|nb|KK920427.1|	994362-994583	16	16	gi|646741719|gb|KK962642.1|	417418-423149	21	8.9
ACYPI009841-RA	ni|645903651|nb|KK920779.1|	588907-589975	nan	nan	gi|646775944|gb|KK961701.1|	56031-56296	22	9.3
ACYPI009846-RA	ni|645900712|nb|KK923717.1|	83222-84668	nan	nan	gi|646781421|gb|KK961521.1|	3944449-3952235	21	8.7
ACYPI009848-RA	ni|645903749|nb|KK920681.1|	277623-278061	19	18	gi|646748407|gb|KK962151.1|	233521-235352	22	9.1
ACYPI009856-RA	ni|645904169|nb|KK920264.1|	120999-121484	16	9.5	gi|646751176|gb|KK962002.1|	771697-774718	21	9.6
ACYPI009859-RA	ni|645904123|nb|KK920308.1|	1644219-1645013	16	10	gi|646752431|gb|KK961967.1|	1457508-1459424	20	5.6
ACYPI009860-RA	ni|645903936|nb|KK920495.1|	756515-757394	19	18	gi|646776477|gb|KK961673.1|	897705-898200	22	9
ACYPI009867-RA	ni|645903868|nb|KK920562.1|	393543-393956	16	8.7	gi|646776091|gb|KK961693.1|	1676123-1676679	23	10
ACYPI009872-RA	ni|645903860|nb|KK920570.1|	181468-181760	17	17	gi|646775471|gb|KK961725.1|	1403012-1403250	20	10
ACYPI009884-RA	ni|645903496|nb|KK920934.1|	867760-868029	nan	nan	gi|646753180|gb|KK961957.1|	639579-641038	25	11
ACYPI009886-RA	ni|645903773|nb|KK920657.1|	619950-621286	18	17	gi|646742541|gb|KK962572.1|	69600-70437	19	10
ACYPI009904-RA	ni|645904125|nb|KK920307.1|	1975375-1975562	18	17	gi|646746706|gb|KK962270.1|	279307-280916	23	11
ACYPI009906-RA	ni|645904156|nb|KK920277.1|	1567846-1568297	17	8.8	gi|646780311|gb|KK961552.1|	2450301-2451452	21	9.7
ACYPI009915-RA	ni|645903866|nb|KK920564.1|	529253-529783	18	15	gi|646782288|gb|KK961496.1|	7663194-7668733	21	9.7
ACYPI009943-RA	ni|645903885|nb|KK920546.1|	789093-790243	19	12	gi|646751160|gb|KK962003.1|	944050-945286	22	10
ACYPI009944-RA	ni|645904260|nb|KK920228.1|	665441-665829	15	15	gi|646746231|gb|KK962303.1|	305727-305893	21	6
ACYPI009947-RA	ni|645903639|nb|KK920791.1|	723347-723804	nan	nan	gi|646781421|gb|KK961521.1|	1134670-1136244	21	8.7
ACYPI009948-RA	ni|645903874|nb|KK920557.1|	861682-862258	18	18	gi|646778903|gb|KK961588.1|	1827451-1831899	23	10
ACYPI009950-RA	ni|645903783|nb|KK920647.1|	139010-139195	17	17	gi|646782168|gb|KK961499.1|	3566455-3571274	21	9.4
ACYPI009955-RA	ni|645904078|nb|KK920353.1|	352390-353002	17	17	gi|646775987|gb|KK961699.1|	1008430-1008872	19	7.5
ACYPI009956-RA	ni|645903779|nb|KK920651.1|	761163-761450	18	16	gi|646782357|gb|KK961494.1|	4172728-4177538	21	9.2

ACYPI009960-RA	ni|645903823|nb|KK920607.1|	99711-100073	16	17	gi|646751289|gb|KK961996.1|	787784-789366	24	9.1
ACYPI009968-RA	ni|645904123|nb|KK920308.1|	1119846-1120068	16	10	gi|646782043|gb|KK961502.1|	2289584-2291200	20	9.2
ACYPI009972-RA	ni|645904002|nb|KK920429.1|	392667-393196	14	18	gi|646771448|gb|KK961767.1|	770233-771698	21	9.8
ACYPI009973-RA	ni|645903804|nb|KK920626.1|	148489-149298	17	17	gi|646777842|gb|KK961618.1|	2074067-2076152	19	8.9
ACYPI009988-RA	ni|645904000|nb|KK920431.1|	357066-359305	18	19	gi|646726809|gb|KK964433.1|	15005-16922	19	12
ACYPI009993-RA	ni|645903650|nb|KK920780.1|	341364-342209	nan	nan	gi|646765198|gb|KK961897.1|	383246-386764	22	5.9
ACYPI010009-RA	ni|645903923|nb|KK920508.1|	1033704-1034005	18	18	gi|646780889|gb|KK961537.1|	2762164-2762920	22	10
ACYPI010018-RA	ni|645903952|nb|KK920479.1|	1085468-1085697	16	16	gi|646749755|gb|KK962075.1|	704621-704883	22	9.2
ACYPI010019-RA	ni|645904195|nb|KK920254.1|	2238338-2238818	18	17	gi|646779936|gb|KK961561.1|	1521301-1521777	19	9.6
ACYPI010020-RA	ni|645904231|nb|KK920242.1|	101241-102305	16	17	gi|646749964|gb|KK962064.1|	453075-453294	18	8.7
ACYPI010027-RA	ni|645904078|nb|KK920353.1|	926213-926759	17	17	gi|646768534|gb|KK961809.1|	240825-242417	21	9.8
ACYPI010028-RA	ni|645903548|nb|KK920882.1|	745298-745714	nan	nan	gi|646747482|gb|KK962215.1|	284871-285500	21	9.2
ACYPI010029-RA	ni|645904122|nb|KK920309.1|	541252-543711	18	17	gi|646769275|gb|KK961796.1|	2451712-2452216	22	5.6
ACYPI010034-RA	ni|645903860|nb|KK920570.1|	217249-217482	17	17	gi|646745372|gb|KK962364.1|	484917-490925	23	10
ACYPI010036-RA	ni|645904019|nb|KK920412.1|	280048-280268	19	9.4	gi|646778767|gb|KK961592.1|	2181239-2181419	21	5.4
ACYPI010039-RA	ni|645904159|nb|KK920274.1|	238667-238841	15	17	gi|646776855|gb|KK961656.1|	397839-402178	19	8.8
ACYPI010042-RA	ni|645904144|nb|KK920289.1|	913890-914433	16	9	gi|646768534|gb|KK961809.1|	78697-79160	21	9.8
ACYPI010044-RA	ni|645904113|nb|KK920318.1|	690950-691699	14	15	gi|646751160|gb|KK962003.1|	116679-120414	22	10
ACYPI010047-RA	ni|645902673|nb|KK921756.1|	147732-149229	nan	nan	gi|646780105|gb|KK961557.1|	2816591-2818303	21	9.7
ACYPI010049-RA	ni|645903594|nb|KK920836.1|	68071-68597	nan	nan	gi|646742413|gb|KK962582.1|	127362-127636	23	10
ACYPI010054-RA	ni|645903801|nb|KK920629.1|	729453-729940	15	14	gi|646734653|gb|KK963394.1|	286111-289814	20	9.1
ACYPI010056-RA	ni|645904058|nb|KK920373.1|	313892-314368	16	15	gi|646776998|gb|KK961650.1|	1768073-1769230	20	9.6
ACYPI010058-RA	ni|645904045|nb|KK920386.1|	685895-686433	19	21	gi|646768669|gb|KK961806.1|	320200-321485	18	9.1
ACYPI010059-RA	ni|645904219|nb|KK920246.1|	692992-694048	16	17	gi|646776389|gb|KK961678.1|	361099-364764	22	9.5
ACYPI010060-RA	ni|645902373|nb|KK922056.1|	137381-138440	nan	nan	gi|646777877|gb|KK961617.1|	364945-366069	19	8.1
ACYPI010073-RA	ni|645903496|nb|KK920934.1|	472271-473782	nan	nan	gi|646747738|gb|KK962196.1|	386359-389194	20	8.3
ACYPI010075-RA	ni|645903975|nb|KK920456.1|	1089045-1089210	15	8.2	gi|646782043|gb|KK961502.1|	1757397-1757570	20	9.2
ACYPI010077-RA	ni|645904088|nb|KK920343.1|	1253879-1254536	17	18	gi|646778410|gb|KK961602.1|	2641257-2641900	20	9
ACYPI010079-RA	ni|645904060|nb|KK920371.1|	179255-180872	15	16	gi|646771448|gb|KK961767.1|	1119012-1124505	21	9.8
ACYPI010087-RA	ni|645903788|nb|KK920642.1|	536483-536835	16	15	gi|646776855|gb|KK961656.1|	295588-296045	19	8.8

ACYPI010089-RA	ni|645903545|nb|KK920885.1|	40184-41584	nan	nan	gi|646747884|gb|KK962186.1|	691825-695338	22	11
ACYPI010091-RA	ni|645904258|nb|KK920229.1|	24403-25694	16	16	gi|646770901|gb|KK961773.1|	1322384-1326765	22	9.7
ACYPI010100-RA	ni|645904122|nb|KK920309.1|	509069-509586	18	17	gi|646616457|gb|KK973314.1|	7296-7668	15	5.6
ACYPI010103-RA	ni|645903778|nb|KK920652.1|	238027-238475	12	13	gi|646770477|gb|KK961778.1|	439289-439541	21	9.1
ACYPI010107-RA	ni|645904107|nb|KK920324.1|	773662-773977	17	9.5	gi|646778903|gb|KK961588.1|	1215307-1215524	23	10
ACYPI010112-RA	ni|645904088|nb|KK920343.1|	1327475-1328948	17	18	gi|646779662|gb|KK961568.1|	1095999-1096635	20	9.3
ACYPI010114-RA	ni|645904130|nb|KK920303.1|	1458370-1459773	18	17	gi|646781421|gb|KK961521.1|	4134303-4135268	21	8.7
ACYPI010117-RA	ni|645903563|nb|KK920867.1|	186680-187279	nan	nan	gi|646746298|gb|KK962298.1|	1577-1996	18	8.3
ACYPI010124-RA	ni|645903771|nb|KK920659.1|	84857-85258	16	15	gi|646781564|gb|KK961516.1|	730325-730644	23	9.4
ACYPI010127-RA	ni|645904174|nb|KK920261.1|	377187-377618	19	17	gi|646726742|gb|KK964444.1|	38589-39289	20	10
ACYPI010129-RA	ni|645903822|nb|KK920608.1|	357691-361135	18	20	gi|646782334|gb|KK961495.1|	6403892-6404463	21	9
ACYPI010131-RA	ni|645904072|nb|KK920359.1|	421302-421700	17	17	gi|646741815|gb|KK962634.1|	102187-102988	22	10
ACYPI010134-RA	ni|645901860|nb|KK922569.1|	80337-80822	nan	nan	gi|646747124|gb|KK962242.1|	250936-251295	17	8.7
ACYPI010135-RA	ni|645903953|nb|KK920478.1|	1310910-1311870	18	17	gi|646777955|gb|KK961615.1|	815691-819979	21	10
ACYPI010138-RA	ni|645904177|nb|KK920260.1|	1138601-1139757	18	17	gi|646782357|gb|KK961494.1|	4324816-4328175	21	9.2
ACYPI010142-RA	ni|645903664|nb|KK920766.1|	175448-175612	nan	nan	gi|646777416|gb|KK961632.1|	1913854-1914405	22	8.8
ACYPI010148-RA	ni|645904174|nb|KK920261.1|	402320-403119	19	17	gi|646732202|gb|KK963708.1|	239843-241194	22	9.9
ACYPI010149-RA	ni|645903795|nb|KK920635.1|	47902-48204	14	16	gi|646779826|gb|KK961564.1|	1298888-1299663	22	9.6
ACYPI010151-RA	ni|645903929|nb|KK920502.1|	1319426-1320004	17	18	gi|646747586|gb|KK962207.1|	14940-15327	20	8.8
ACYPI010153-RA	ni|645903491|nb|KK920939.1|	606494-606927	nan	nan	gi|646747046|gb|KK962247.1|	622118-627799	22	9
ACYPI010154-RA	ni|645904094|nb|KK920337.1|	75686-75854	17	16	gi|646771092|gb|KK961771.1|	1010457-1011987	23	9.7
ACYPI010163-RA	ni|645904036|nb|KK920395.1|	1173974-1174601	21	18	gi|646782357|gb|KK961494.1|	1115466-1115935	21	9.2
ACYPI010168-RA	ni|645904004|nb|KK920427.1|	293331-293571	16	16	gi|646732252|gb|KK963701.1|	154787-155046	19	8
ACYPI010174-RA	ni|645903957|nb|KK920474.1|	909871-910352	18	17	gi|646747586|gb|KK962207.1|	456905-459322	20	8.8
ACYPI010179-RA	ni|645904116|nb|KK920315.1|	2067854-2068338	19	18	gi|646767624|gb|KK961828.1|	96674-97620	22	8.9
ACYPI010180-RA	ni|645903854|nb|KK920576.1|	350740-350985	16	8.4	gi|646777393|gb|KK961634.1|	1175302-1175554	18	9.1
ACYPI010188-RA	ni|645903806|nb|KK920624.1|	525233-526587	16	9.3	gi|646746974|gb|KK962252.1|	709420-712207	23	6.1
ACYPI010190-RA	ni|645904174|nb|KK920261.1|	902505-902862	19	17	gi|646750267|gb|KK962048.1|	490400-490683	16	8.2
ACYPI010200-RA	ni|645903549|nb|KK920881.1|	35211-36045	nan	nan	gi|646632634|gb|KK970403.1|	9317-9521	11	6.4
ACYPI010201-RA	ni|645901835|nb|KK922594.1|	26833-27796	nan	nan	gi|646769275|gb|KK961796.1|	263513-263991	22	5.6

ACYPI010209-RA	ni|645903671|nb|KK920759.1|	772245-772536	nan	nan	gi|646775867|gb|KK961705.1|	1359592-1363103	23	10
ACYPI010216-RA	ni|645904117|nb|KK920314.1|	227743-227969	15	15	gi|646745778|gb|KK962333.1|	345179-351942	17	7.7
ACYPI010217-RA	ni|645903938|nb|KK920493.1|	1134781-1135333	19	18	gi|646775635|gb|KK961716.1|	1542420-1542683	22	10
ACYPI010225-RA	ni|645903898|nb|KK920533.1|	464010-465601	17	16	gi|646780311|gb|KK961552.1|	2765239-2765477	21	9.7
ACYPI010226-RA	ni|645904077|nb|KK920354.1|	1403373-1403621	16	17	gi|646778632|gb|KK961596.1|	1629901-1630186	21	9.2
ACYPI010229-RA	ni|645904180|nb|KK920259.1|	658779-659757	17	17	gi|646750889|gb|KK962017.1|	1722045-1723264	22	6.1
ACYPI010231-RA	ni|645902785|nb|KK921644.1|	106287-107581	nan	nan	gi|646781510|gb|KK961518.1|	3548539-3549027	17	7.8
ACYPI010236-RA	ni|645903475|nb|KK920955.1|	296296-296743	nan	nan	gi|646767477|gb|KK961831.1|	172662-173868	20	8.4
ACYPI010244-RA	ni|645904035|nb|KK920396.1|	684433-684719	16	16	gi|646781601|gb|KK961515.1|	2058769-2059045	19	8.6
ACYPI060216-RA	ni|645903578|nb|KK920852.1|	241574-242642	nan	nan	gi|646775867|gb|KK961705.1|	2221951-2222924	23	10
ACYPI060526-RA	ni|645903567|nb|KK920863.1|	210532-210741	nan	nan	gi|646780406|gb|KK961550.1|	589392-597180	22	5.9
ACYPI060550-RA	ni|645904159|nb|KK920274.1|	190468-190721	15	17	gi|646781659|gb|KK961513.1|	5847286-5848312	22	9.8
ACYPI060717-RA	ni|645904088|nb|KK920343.1|	541953-545536	17	18	gi|646775595|gb|KK961718.1|	562273-563685	21	8.9
ACYPI060796-RA	ni|645903953|nb|KK920478.1|	1152914-1153282	18	17	gi|646777955|gb|KK961615.1|	961819-962037	21	10
ACYPI060844-RA	ni|645904136|nb|KK920297.1|	1199723-1199966	16	17	gi|646780889|gb|KK961537.1|	1829595-1830219	22	10
ACYPI061188-RA	ni|645903567|nb|KK920863.1|	143230-144218	nan	nan	gi|646740955|gb|KK962713.1|	331588-332886	28	13
ACYPI061215-RA	ni|645903993|nb|KK920438.1|	124966-126232	19	22	gi|646782211|gb|KK961498.1|	5643569-5643792	20	9
ACYPI061275-RA	ni|645902490|nb|KK921939.1|	20693-20910	nan	nan	gi|646740645|gb|KK962748.1|	170833-171025	11	6.7
ACYPI061477-RA	ni|645904119|nb|KK920312.1|	429069-429643	19	19	gi|646749616|gb|KK962083.1|	64242-64505	22	10
ACYPI061529-RA	ni|645903938|nb|KK920493.1|	720240-720787	19	18	gi|646742343|gb|KK962588.1|	370281-370601	20	9.4
ACYPI061546-RA	ni|645902609|nb|KK921820.1|	121195-121412	nan	nan	gi|646749868|gb|KK962069.1|	165355-165823	19	7.1
ACYPI061797-RA	ni|645904166|nb|KK920267.1|	2784302-2785059	18	16	gi|646780441|gb|KK961549.1|	488116-488769	21	9.5
ACYPI062376-RA	ni|645904189|nb|KK920256.1|	528381-528856	18	18	gi|646782288|gb|KK961496.1|	1471608-1472973	21	9.7
ACYPI062389-RA	ni|645903987|nb|KK920444.1|	328008-331687	15	17	gi|646781601|gb|KK961515.1|	400366-405260	19	8.6
ACYPI062429-RA	ni|645903925|nb|KK920506.1|	711222-711607	19	17	gi|646781659|gb|KK961513.1|	6041263-6043113	22	9.8
ACYPI062495-RA	ni|645903973|nb|KK920458.1|	1614504-1614703	20	18	gi|646781421|gb|KK961521.1|	6691533-6693984	21	8.7
ACYPI062519-RA	ni|645903783|nb|KK920647.1|	1227798-1228343	17	17	gi|646782168|gb|KK961499.1|	5014281-5015201	21	9.4
ACYPI062651-RA	ni|645902497|nb|KK921932.1|	150458-152179	nan	nan	gi|646778948|gb|KK961587.1|	1792658-1797419	20	7.3
ACYPI063189-RA	ni|645904133|nb|KK920300.1|	2162114-2162975	18	18	gi|646740756|gb|KK962735.1|	238133-238498	23	10
ACYPI063239-RA	ni|645903740|nb|KK920690.1|	292545-293973	18	10	gi|646746868|gb|KK962259.1|	152215-158740	21	10

ACYPI063276-RA	ni|645904107|nb|KK920324.1|	928890-931594	17	9.5	gi|646778903|gb|KK961588.1|	3061449-3072335	23	10
ACYPI063394-RA	ni|645904281|nb|KK920220.1|	1193080-1193831	15	16	gi|646775558|gb|KK961720.1|	1669273-1675149	20	9.4
ACYPI063651-RA	ni|645901866|nb|KK922563.1|	7907-8171	nan	nan	gi|646772786|gb|KK961754.1|	146405-146581	22	9.6
ACYPI064034-RA	ni|645903935|nb|KK920496.1|	963918-964772	17	18	gi|646775944|gb|KK961701.1|	1890191-1892623	22	9.3
ACYPI064056-RA	ni|645903985|nb|KK920446.1|	1030509-1033100	18	16	gi|646634301|gb|KK970114.1|	7444-7874	12	4.9
ACYPI064212-RA	ni|645902771|nb|KK921658.1|	44404-46394	nan	nan	gi|646745186|gb|KK962377.1|	379820-385376	21	9.8
ACYPI064239-RA	ni|645904116|nb|KK920315.1|	1613019-1613962	19	18	gi|646778186|gb|KK961609.1|	2034693-2034977	19	9.4
ACYPI064464-RA	ni|645904177|nb|KK920260.1|	1006492-1008125	18	17	gi|646781510|gb|KK961518.1|	934256-935380	17	7.8
ACYPI065062-RA	ni|645903969|nb|KK920462.1|	268977-269416	14	16	gi|646748002|gb|KK962178.1|	1636443-1636985	22	6.3
ACYPI065070-RA	ni|645902511|nb|KK921918.1|	141187-141789	nan	nan	gi|646747161|gb|KK962239.1|	131359-133528	24	11
ACYPI065097-RA	ni|645903625|nb|KK920805.1|	199394-200534	nan	nan	gi|646744731|gb|KK962411.1|	392105-392850	13	6.5
ACYPI065154-RA	ni|645904065|nb|KK920366.1|	63698-64092	18	18	gi|646776762|gb|KK961659.1|	699217-699426	21	8.9
ACYPI065189-RA	ni|645903999|nb|KK920432.1|	1279878-1280184	17	15	gi|646777089|gb|KK961646.1|	339580-341147	20	9.4
ACYPI065331-RA	ni|645904156|nb|KK920277.1|	248842-249374	17	8.8	gi|646775867|gb|KK961705.1|	1909502-1910685	23	10
ACYPI065684-RA	ni|645904133|nb|KK920300.1|	1551065-1551786	18	18	gi|646775312|gb|KK961734.1|	69027-71666	17	7.9
ACYPI065923-RA	ni|645904166|nb|KK920267.1|	2311041-2311466	18	16	gi|646766426|gb|KK961862.1|	751158-752507	20	7.1
ACYPI066741-RA	ni|645904171|nb|KK920262.1|	778712-778900	15	16	gi|646765948|gb|KK961882.1|	503314-503920	18	7.3
ACYPI066776-RA	ni|645904170|nb|KK920263.1|	1742696-1745680	15	16	gi|646762476|gb|KK961908.1|	518210-522592	19	7.4
ACYPI066811-RA	ni|645903552|nb|KK920878.1|	212369-212505	nan	nan	gi|646748888|gb|KK962123.1|	144602-145108	20	8.1
ACYPI066960-RA	ni|645903636|nb|KK920794.1|	196511-196815	nan	nan	gi|646776280|gb|KK961684.1|	868790-869825	22	9.3
ACYPI066985-RA	ni|645903800|nb|KK920630.1|	991301-991451	18	17	gi|646740694|gb|KK962742.1|	92818-93987	20	7.9
ACYPI067416-RA	ni|645903496|nb|KK920934.1|	915259-916095	nan	nan	gi|646768717|gb|KK961805.1|	567170-574967	20	8
ACYPI067466-RA	ni|645903501|nb|KK920929.1|	72804-74726	nan	nan	gi|646732119|gb|KK963722.1|	68693-70735	20	9.4
ACYPI067736-RA	ni|645904114|nb|KK920317.1|	2078039-2078737	18	18	gi|646778336|gb|KK961604.1|	2247141-2253266	21	10
ACYPI067762-RA	ni|645903957|nb|KK920474.1|	1106693-1107177	18	17	gi|646780658|gb|KK961544.1|	1398178-1402006	19	8.6
ACYPI067763-RA	ni|645903564|nb|KK920866.1|	334772-335057	nan	nan	gi|646781043|gb|KK961532.1|	4250812-4251387	26	11
ACYPI068502-RA	ni|645903836|nb|KK920594.1|	139219-139673	18	11	gi|646742450|gb|KK962579.1|	88405-88905	19	7.6
ACYPI068591-RA	ni|645904207|nb|KK920250.1|	1107127-1108889	18	20	gi|646778186|gb|KK961609.1|	1660599-1660983	19	9.4
ACYPI068599-RA	ni|645901290|nb|KK923139.1|	43029-43492	nan	nan	gi|646778112|gb|KK961611.1|	507045-521491	22	10
ACYPI068631-RA	ni|645904115|nb|KK920316.1|	1813884-1814034	17	18	gi|646782288|gb|KK961496.1|	4410633-4411578	21	9.7

ACYPI068671-RA	ni|645903811|nb|KK920619.1|	356216-356819	15	15	gi|646752348|gb|KK961968.1|	247742-248223	18	8
ACYPI068681-RA	ni|645904150|nb|KK920283.1|	1041291-1041787	17	17	gi|646781183|gb|KK961528.1|	2689252-2689977	20	9.5
ACYPI068701-RA	ni|645902788|nb|KK921641.1|	279060-279276	nan	nan	gi|646551026|gb|KK986044.1|	6123-6289	5.8	3.8
ACYPI068713-RA	ni|645903576|nb|KK920854.1|	61640-66745	nan	nan	gi|646775821|gb|KK961707.1|	1160980-1165516	23	9.1
ACYPI069167-RA	ni|645903688|nb|KK920742.1|	1079199-1080017	nan	nan	gi|646782334|gb|KK961495.1|	8884562-8890841	21	9
ACYPI069326-RA	ni|645902644|nb|KK921785.1|	70423-71842	nan	nan	gi|646745631|gb|KK962343.1|	280472-280749	18	8.6
ACYPI069332-RA	ni|645903833|nb|KK920597.1|	122824-123405	17	15	gi|646782276|gb|KK961497.1|	4338475-4339853	21	9.7
ACYPI069386-RA	ni|645903879|nb|KK920552.1|	1086942-1089661	18	18	gi|646779006|gb|KK961585.1|	1005467-1010542	20	9.9
ACYPI069416-RA	ni|645903548|nb|KK920882.1|	256275-256851	nan	nan	gi|646776490|gb|KK961672.1|	1036873-1037562	20	10
ACYPI069453-RA	ni|645904125|nb|KK920307.1|	2676174-2677198	18	17	gi|646782009|gb|KK961503.1|	1531208-1532865	15	7.5
ACYPI069547-RA	ni|645903622|nb|KK920808.1|	604899-605295	nan	nan	gi|646782334|gb|KK961495.1|	2812463-2815880	21	9
ACYPI069554-RA	ni|645904019|nb|KK920412.1|	623072-623241	19	9.4	gi|646777877|gb|KK961617.1|	1226040-1227744	19	8.1
ACYPI069585-RA	ni|645903873|nb|KK920558.1|	607939-608699	16	15	gi|646781772|gb|KK961510.1|	2652763-2653486	20	9.2
ACYPI069860-RA	ni|645903920|nb|KK920511.1|	1112520-1112619	20	20	gi|646578414|gb|KK980699.1|	9588-10104	25	9.7
ACYPI070244-RA	ni|645901817|nb|KK922612.1|	84775-85007	nan	nan	gi|646777590|gb|KK961625.1|	578910-582202	19	9.7
ACYPI070323-RA	ni|645903952|nb|KK920479.1|	45914-46164	16	16	gi|646770641|gb|KK961776.1|	406066-406290	20	9.1
ACYPI070418-RA	ni|645903592|nb|KK920838.1|	877950-878893	nan	nan	gi|646750889|gb|KK962017.1|	1659281-1660111	22	6.1
ACYPI071107-RA	ni|645904153|nb|KK920280.1|	1821357-1822580	17	16	gi|646765790|gb|KK961889.1|	1008609-1010280	22	8.8
ACYPI071133-RA	ni|645903578|nb|KK920852.1|	273377-274389	nan	nan	gi|646775968|gb|KK961700.1|	731504-732689	20	8.2
ACYPI071144-RA	ni|645902633|nb|KK921796.1|	281975-282471	nan	nan	gi|646776050|gb|KK961695.1|	1243458-1247054	19	7.7
ACYPI071157-RA	ni|645904140|nb|KK920293.1|	78333-79005	22	22	gi|646781344|gb|KK961523.1|	4300933-4307094	21	8.9
ACYPI071169-RA	ni|645903774|nb|KK920656.1|	479324-480395	16	9.2	gi|646767670|gb|KK961827.1|	977551-977832	21	5.9
ACYPI071217-RA	ni|645904012|nb|KK920419.1|	305201-305741	27	32	gi|646780889|gb|KK961537.1|	1683498-1684049	22	10
ACYPI071228-RA	ni|645903971|nb|KK920460.1|	1059703-1059992	19	18	gi|646776974|gb|KK961651.1|	738494-738995	17	8.1
ACYPI071231-RA	ni|645903690|nb|KK920740.1|	1030654-1031247	nan	nan	gi|646750165|gb|KK962053.1|	741293-742978	25	10
ACYPI071272-RA	ni|645904145|nb|KK920288.1|	1224093-1224638	16	9.3	gi|646780723|gb|KK961542.1|	603115-603484	21	9.4
ACYPI071352-RA	ni|645904152|nb|KK920281.1|	862607-863077	15	15	gi|646743501|gb|KK962498.1|	353080-353332	19	9.5
ACYPI071357-RA	ni|645903727|nb|KK920703.1|	952093-954572	nan	nan	gi|646781013|gb|KK961533.1|	961124-961845	18	7.6
ACYPI071951-RA	ni|645903743|nb|KK920687.1|	545031-545451	19	18	gi|646768494|gb|KK961810.1|	1387747-1388176	21	10
ACYPI071956-RA	ni|645903873|nb|KK920558.1|	438336-438560	16	15	gi|646774797|gb|KK961742.1|	961887-970351	20	8.5

ACYPI071995-RA	ni|645901786|nb|KK922643.1|	74287-74502	nan	nan	gi|646745171|gb|KK962378.1|	721517-721903	19	8.1
ACYPI072156-RA	ni|645903472|nb|KK920958.1|	367392-367613	nan	nan	gi|646749036|gb|KK962114.1|	191533-194277	20	7.5
ACYPI072184-RA	ni|645903679|nb|KK920751.1|	63150-63420	nan	nan	gi|646776322|gb|KK961682.1|	1173124-1173688	22	8.9
ACYPI072205-RA	ni|645904061|nb|KK920370.1|	789963-792181	17	16	gi|646776735|gb|KK961660.1|	1011630-1012852	21	9.6
ACYPI072215-RA	ni|645903905|nb|KK920526.1|	76972-78488	15	8.3	gi|646752431|gb|KK961967.1|	754979-763797	20	5.6
ACYPI072241-RA	ni|645902354|nb|KK922075.1|	112303-112676	nan	nan	gi|646775765|gb|KK961710.1|	1984639-1985783	21	6
ACYPI072244-RA	ni|645903895|nb|KK920536.1|	451323-452561	17	16	gi|646752431|gb|KK961967.1|	1557184-1557663	20	5.6
ACYPI072746-RA	ni|645903973|nb|KK920458.1|	338196-338499	20	18	gi|646779141|gb|KK961581.1|	29519-29658	21	9.1
ACYPI072792-RA	ni|645903679|nb|KK920751.1|	63523-63749	nan	nan	gi|646776322|gb|KK961682.1|	1164950-1165502	22	8.9
ACYPI072856-RA	ni|645903780|nb|KK920650.1|	269020-269766	19	19	gi|646770047|gb|KK961784.1|	256021-256577	21	9.1
ACYPI072921-RA	ni|645903748|nb|KK920682.1|	801623-802390	17	18	gi|646744057|gb|KK962460.1|	209770-210606	14	6.5
ACYPI072975-RA	ni|645903722|nb|KK920708.1|	599956-600371	nan	nan	gi|646747500|gb|KK962213.1|	532984-533672	21	9.4
ACYPI072994-RA	ni|645903860|nb|KK920570.1|	348064-348497	17	17	gi|646773721|gb|KK961747.1|	1828280-1832506	21	9.2
ACYPI073040-RA	ni|645903773|nb|KK920657.1|	194326-196137	18	17	gi|646781659|gb|KK961513.1|	1294818-1295856	22	9.8
ACYPI073612-RA	ni|645903840|nb|KK920590.1|	224391-225284	18	17	gi|646780953|gb|KK961535.1|	4376882-4377409	20	9.8
ACYPI073693-RA	ni|645903728|nb|KK920702.1|	1089584-1090311	nan	nan	gi|646775968|gb|KK961700.1|	227205-228445	20	8.2
ACYPI073700-RA	ni|645902673|nb|KK921756.1|	49810-50611	nan	nan	gi|646755264|gb|KK961938.1|	616268-617496	20	5.3
ACYPI073759-RA	ni|645903866|nb|KK920564.1|	515014-515439	18	15	gi|646766382|gb|KK961864.1|	458031-467067	22	9.7
ACYPI073870-RA	ni|645904059|nb|KK920372.1|	458491-461892	16	9	gi|646768222|gb|KK961816.1|	994397-997338	25	12
ACYPI073873-RA	ni|645904243|nb|KK920236.1|	1733080-1733496	15	8.5	gi|646737528|gb|KK963067.1|	115818-116099	20	6.1
ACYPI073889-RA	ni|645899152|nb|KK925277.1|	81668-82199	nan	nan	gi|646781849|gb|KK961508.1|	2857569-2858685	17	8
ACYPI080074-RA	ni|645904119|nb|KK920312.1|	604534-605729	19	19	gi|646733743|gb|KK963499.1|	22195-23083	18	9.3
ACYPI080138-RA	ni|645904019|nb|KK920412.1|	144059-144390	19	9.4	gi|646747424|gb|KK962220.1|	244924-246701	22	5.7
ACYPI080140-RA	ni|645903867|nb|KK920563.1|	1044435-1044666	18	18	gi|646776184|gb|KK961688.1|	620976-622156	21	9.6
ACYPI080240-RA	ni|645904177|nb|KK920260.1|	878816-879784	18	17	gi|646775807|gb|KK961708.1|	2167444-2168157	21	8.6
ACYPI080343-RA	ni|645903900|nb|KK920531.1|	241882-242489	20	30	gi|646779741|gb|KK961566.1|	478496-478832	20	8.5
ACYPI080661-RA	ni|645903697|nb|KK920733.1|	56927-57419	nan	nan	gi|646782276|gb|KK961497.1|	4267793-4268313	21	9.7
ACYPI081137-RA	ni|645904156|nb|KK920277.1|	411435-411682	17	8.8	gi|646734653|gb|KK963394.1|	323246-323858	20	9.1
ACYPI081140-RA	ni|645903603|nb|KK920827.1|	66248-66959	nan	nan	gi|646778112|gb|KK961611.1|	791258-791494	22	10
ACYPI081400-RA	ni|645903665|nb|KK920765.1|	456905-457093	nan	nan	gi|646710354|gb|KK965458.1|	15650-16905	130	92

ACYPI081754-RA	ni|645903588|nb|KK920842.1|	521131-521393	nan	nan	gi|646750409|gb|KK962040.1|	172135-172430	21	8.4
ACYPI081909-RA	ni|645904116|nb|KK920315.1|	2115574-2116251	19	18	gi|646780752|gb|KK961541.1|	926699-927109	21	8
ACYPI082110-RA	ni|645903762|nb|KK920668.1|	865990-866960	17	17	gi|646744029|gb|KK962462.1|	438562-438870	19	7.9
ACYPI082181-RA	ni|645904160|nb|KK920273.1|	1628541-1628832	18	18	gi|646782276|gb|KK961497.1|	6819979-6821809	21	9.7
ACYPI082267-RA	ni|645902373|nb|KK922056.1|	77481-77714	nan	nan	gi|646782357|gb|KK961494.1|	5307330-5309462	21	9.2
ACYPI082338-RA	ni|645904058|nb|KK920373.1|	1109523-1110556	16	15	gi|646776952|gb|KK961652.1|	1459298-1470166	19	7.7
ACYPI082349-RA	ni|645903494|nb|KK920936.1|	434699-435178	nan	nan	gi|646779375|gb|KK961575.1|	3300236-3301211	22	9.3
ACYPI082499-RA	ni|645904170|nb|KK920263.1|	321012-321219	15	16	gi|646776608|gb|KK961665.1|	423321-425795	23	9.3
ACYPI082565-RA	ni|645903685|nb|KK920745.1|	386802-387215	nan	nan	gi|646748379|gb|KK962153.1|	12147-13170	14	8.9
ACYPI082595-RA	ni|645904274|nb|KK920223.1|	754622-754880	14	17	gi|646775238|gb|KK961738.1|	614773-616163	17	8.7
ACYPI082601-RA	ni|645903548|nb|KK920882.1|	206915-207236	nan	nan	gi|646766382|gb|KK961864.1|	1007759-1009661	22	9.7
ACYPI082655-RA	ni|645903597|nb|KK920833.1|	788549-788978	nan	nan	gi|646750002|gb|KK962062.1|	353610-354217	24	12
ACYPI082722-RA	ni|645901974|nb|KK922455.1|	35775-36152	nan	nan	gi|646780794|gb|KK961540.1|	284003-285654	21	9.5
ACYPI082856-RA	ni|645903752|nb|KK920678.1|	647046-647661	18	16	gi|646770251|gb|KK961781.1|	1374188-1375503	20	9.6
ACYPI082950-RA	ni|645903983|nb|KK920448.1|	705762-707673	18	17	gi|646774067|gb|KK961745.1|	1504989-1508632	17	9.3
ACYPI083041-RA	ni|645903533|nb|KK920897.1|	188118-188373	nan	nan	gi|646779498|gb|KK961572.1|	1200915-1201372	22	5.6
ACYPI083213-RA	ni|645903736|nb|KK920694.1|	305120-305513	18	17	gi|646741621|gb|KK962651.1|	203888-207516	18	7.2
ACYPI083398-RA	ni|645904074|nb|KK920357.1|	907697-907946	17	18	gi|646749709|gb|KK962078.1|	527224-527491	18	12
ACYPI083423-RA	ni|645903594|nb|KK920836.1|	883003-883909	nan	nan	gi|646770641|gb|KK961776.1|	946840-948042	20	9.1
ACYPI083436-RA	ni|645904262|nb|KK920227.1|	336091-336742	17	17	gi|646745372|gb|KK962364.1|	763185-764496	23	10
ACYPI083523-RA	ni|645904262|nb|KK920227.1|	285265-285757	17	17	gi|646748129|gb|KK962169.1|	533550-536495	21	9.8
ACYPI083537-RA	ni|645900840|nb|KK923589.1|	1025-1494	nan	nan	gi|646776647|gb|KK961663.1|	1748757-1749432	23	9.2
ACYPI084112-RA	ni|645901982|nb|KK922447.1|	95119-96148	nan	nan	gi|646775614|gb|KK961717.1|	217202-217475	21	9.7
ACYPI084147-RA	ni|645904033|nb|KK920398.1|	119946-120331	14	16	gi|646778029|gb|KK961613.1|	2084912-2091906	21	8.6
ACYPI084287-RA	ni|645903959|nb|KK920472.1|	893249-894023	15	15	gi|646778307|gb|KK961605.1|	1642683-1644660	17	8.7
ACYPI084620-RA	ni|645903580|nb|KK920850.1|	180239-180662	nan	nan	gi|646745941|gb|KK962322.1|	309896-318346	21	10
ACYPI084755-RA	ni|645902490|nb|KK921939.1|	10171-10728	nan	nan	gi|646775194|gb|KK961740.1|	562521-562909	18	7.3
ACYPI084854-RA	ni|645903876|nb|KK920555.1|	294777-295109	14	15	gi|646781659|gb|KK961513.1|	2834578-2835918	22	9.8
ACYPI084955-RA	ni|645904157|nb|KK920276.1|	1433404-1434161	15	15	gi|646781243|gb|KK961526.1|	3723491-3725070	21	8.9
ACYPI084988-RA	ni|645903970|nb|KK920461.1|	839323-839555	17	16	gi|646781421|gb|KK961521.1|	4496356-4498668	21	8.7

ACYPI084991-RA	ni|645902630|nb|KK921799.1|	63048-63443	nan	nan	gi|646779898|gb|KK961562.1|	598165-599334	19	9.3
ACYPI085022-RA	ni|645903937|nb|KK920494.1|	288673-290802	11	12	gi|646750600|gb|KK962030.1|	1121578-1123517	23	8.6
ACYPI085301-RA	ni|645904268|nb|KK920225.1|	317448-317732	14	15	gi|646751160|gb|KK962003.1|	605771-607199	22	10
ACYPI085389-RA	ni|645903955|nb|KK920476.1|	531055-531570	18	17	gi|646758845|gb|KK961921.1|	1865546-1867208	22	9.5
ACYPI085401-RA	ni|645901587|nb|KK922842.1|	124-323	nan	nan	gi|646778983|gb|KK961586.1|	1226591-1233225	20	9.9
ACYPI085603-RA	ni|645903954|nb|KK920477.1|	249860-250558	35	44	gi|646769163|gb|KK961798.1|	1336514-1336853	20	9.2
ACYPI085620-RA	ni|645903833|nb|KK920597.1|	204997-205425	17	15	gi|646782276|gb|KK961497.1|	4374026-4374458	21	9.7
ACYPI086079-RA	ni|645903873|nb|KK920558.1|	866644-867041	16	15	gi|646740544|gb|KK962759.1|	4382-6625	20	8
ACYPI086258-RA	ni|645903848|nb|KK920582.1|	95901-96402	18	18	gi|646780530|gb|KK961547.1|	3306309-3306651	21	9.2
ACYPI086281-RA	ni|645904116|nb|KK920315.1|	2079937-2080922	19	18	gi|646524315|gb|KK989884.1|	11252-11569	17	7
ACYPI086445-RA	ni|645903706|nb|KK920724.1|	174693-175446	nan	nan	gi|646775685|gb|KK961714.1|	1523772-1528778	21	8.5
ACYPI087089-RA	ni|645904130|nb|KK920303.1|	778273-778986	18	17	gi|646780978|gb|KK961534.1|	1220525-1225982	20	8
ACYPI087189-RA	ni|645903626|nb|KK920804.1|	433216-433481	nan	nan	gi|646777570|gb|KK961626.1|	459296-460121	18	7.5
ACYPI087467-RA	ni|645904002|nb|KK920429.1|	382792-383230	14	18	gi|646741656|gb|KK962648.1|	863862-865651	24	9.7
ACYPI087566-RA	ni|645903953|nb|KK920478.1|	1375604-1376188	18	17	gi|646747547|gb|KK962210.1|	434999-436588	19	7.5
ACYPI087735-RA	ni|645903695|nb|KK920735.1|	179600-180348	nan	nan	gi|646747076|gb|KK962245.1|	163656-164023	23	9.6
ACYPI087743-RA	ni|645904115|nb|KK920316.1|	134721-135287	17	18	gi|646781421|gb|KK961521.1|	417491-418092	21	8.7
ACYPI087793-RA	ni|645904138|nb|KK920295.1|	500083-500440	15	15	gi|646770114|gb|KK961783.1|	1607442-1608249	19	9.7
ACYPI087848-RA	ni|645904102|nb|KK920329.1|	423218-423763	15	16	gi|646768294|gb|KK961814.1|	1050458-1058358	23	10
ACYPI088059-RA	ni|645904065|nb|KK920366.1|	1259587-1260269	18	18	gi|646751569|gb|KK961985.1|	193207-193839	21	9.2
ACYPI088146-RA	ni|645903814|nb|KK920616.1|	453469-453922	17	16	gi|646746556|gb|KK962281.1|	936945-940109	20	9.1
ACYPI088207-RA	ni|645903736|nb|KK920694.1|	401908-402366	18	17	gi|646776904|gb|KK961654.1|	954945-955385	21	9.3
ACYPI088273-RA	ni|645903941|nb|KK920490.1|	609045-610870	18	18	gi|646738455|gb|KK962975.1|	260878-263352	23	9.5
ACYPI088486-RA	ni|645903836|nb|KK920594.1|	814112-814348	18	11	gi|646769275|gb|KK961796.1|	2007657-2007935	22	5.6
ACYPI088544-RA	ni|645904045|nb|KK920386.1|	561180-562312	19	21	gi|646755264|gb|KK961938.1|	473507-475222	20	5.3
ACYPI088840-RA	ni|645903965|nb|KK920466.1|	1492083-1493317	17	16	gi|646738524|gb|KK962968.1|	200385-212651	24	11
ACYPI089164-RA	ni|645903636|nb|KK920794.1|	205924-206110	nan	nan	gi|646776280|gb|KK961684.1|	880141-880830	22	9.3
ACYPI089315-RA	ni|645903961|nb|KK920470.1|	1388809-1389071	17	15	gi|646745867|gb|KK962327.1|	14154-14446	16	8.4
ACYPI089540-RA	ni|645902426|nb|KK922003.1|	197837-198248	nan	nan	gi|646781893|gb|KK961506.1|	3656507-3656815	19	8.5
ACYPI089560-RA	ni|645903722|nb|KK920708.1|	842682-842941	nan	nan	gi|646781690|gb|KK961512.1|	3781815-3782452	21	8.8

ACYPI20302-RA	ni|645904243|nb|KK920236.1|	1094976-1095165	15	8.5	gi|646776091|gb|KK961693.1|	2724586-2724832	23	10
ACYPI20477-RA	ni|645904168|nb|KK920265.1|	308543-309824	16	16	gi|646769001|gb|KK961801.1|	682974-688284	20	8.5
ACYPI20534-RA	ni|645902196|nb|KK922233.1|	154324-155563	nan	nan	gi|646776974|gb|KK961651.1|	1018748-1020123	17	8.1
ACYPI20738-RA	ni|645903701|nb|KK920729.1|	186380-186622	nan	nan	gi|646738563|gb|KK962964.1|	209531-211097	15	7.3
ACYPI21373-RA	ni|645902497|nb|KK921932.1|	93913-94468	nan	nan	gi|646746525|gb|KK962283.1|	211384-216183	21	6.8
ACYPI21475-RA	ni|645903874|nb|KK920557.1|	919431-919924	18	18	gi|646752076|gb|KK961973.1|	422826-436759	22	9.1
ACYPI21591-RA	ni|645903662|nb|KK920768.1|	384806-385049	nan	nan	gi|646780827|gb|KK961539.1|	335-1932	20	9.4
ACYPI21611-RA	ni|645903742|nb|KK920688.1|	295328-296574	17	16	gi|646781690|gb|KK961512.1|	4005891-4008511	21	8.8
ACYPI21777-RA	ni|645903925|nb|KK920506.1|	1153027-1153937	19	17	gi|646781183|gb|KK961528.1|	1845538-1845829	20	9.5
ACYPI21906-RA	ni|645903738|nb|KK920692.1|	316177-316355	18	17	gi|646775258|gb|KK961737.1|	1664963-1665169	19	9.8
ACYPI22575-RA	ni|645904064|nb|KK920367.1|	634999-635783	17	9.9	gi|646779702|gb|KK961567.1|	506333-510400	23	10
ACYPI22584-RA	ni|645904195|nb|KK920254.1|	2298196-2298496	18	17	gi|646780311|gb|KK961552.1|	2237480-2247841	21	9.7
ACYPI22631-RA	ni|645903956|nb|KK920475.1|	781402-785776	20	20	gi|646781628|gb|KK961514.1|	569515-571093	23	9
ACYPI22867-RA	ni|645903734|nb|KK920696.1|	708677-709201	nan	nan	gi|646777665|gb|KK961623.1|	1099688-1100283	21	9.7
ACYPI23224-RA	ni|645903687|nb|KK920743.1|	381265-383122	nan	nan	gi|646746025|gb|KK962317.1|	240967-242045	19	8.4
ACYPI23235-RA	ni|645903597|nb|KK920833.1|	519791-522166	nan	nan	gi|646782127|gb|KK961500.1|	1165373-1169747	22	9.6
ACYPI23338-RA	ni|645903860|nb|KK920570.1|	155282-157970	17	17	gi|646780222|gb|KK961554.1|	1257230-1261312	20	9.7
ACYPI23394-RA	ni|645903887|nb|KK920544.1|	84001-85004	15	15	gi|646777288|gb|KK961638.1|	2847997-2850240	22	6.1
ACYPI23999-RA	ni|645904189|nb|KK920256.1|	267210-267474	18	18	gi|646782288|gb|KK961496.1|	1884041-1885841	21	9.7
ACYPI24155-RA	ni|645903960|nb|KK920471.1|	910992-911316	16	8.6	gi|646746040|gb|KK962316.1|	515964-516623	19	6.9
ACYPI24234-RA	ni|645903688|nb|KK920742.1|	193095-193674	nan	nan	gi|646748474|gb|KK962147.1|	588160-589835	20	9
ACYPI24622-RA	ni|645903666|nb|KK920764.1|	129499-131700	nan	nan	gi|646746074|gb|KK962314.1|	571415-574093	17	7
ACYPI24841-RA	ni|645902722|nb|KK921707.1|	53698-54878	nan	nan	gi|646749755|gb|KK962075.1|	591262-592392	22	9.2
ACYPI25279-RA	ni|645903739|nb|KK920691.1|	516146-516490	17	16	gi|646781282|gb|KK961525.1|	4672151-4672955	22	9.6
ACYPI25494-RA	ni|645903787|nb|KK920643.1|	27896-28918	19	18	gi|646766868|gb|KK961847.1|	718900-721468	21	9.9
ACYPI25540-RA	ni|645901733|nb|KK922696.1|	246594-247027	nan	nan	gi|646781690|gb|KK961512.1|	2273116-2273984	21	8.8
ACYPI25873-RA	ni|645903923|nb|KK920508.1|	1172449-1173015	18	18	gi|646699768|gb|KK966040.1|	36584-41073	18	7.2
ACYPI26209-RA	ni|645904115|nb|KK920316.1|	1557721-1558398	17	18	gi|646777288|gb|KK961638.1|	1095950-1096565	22	6.1
ACYPI26223-RA	ni|645903868|nb|KK920562.1|	614213-614381	16	8.7	gi|646775614|gb|KK961717.1|	329528-337109	21	9.7
ACYPI26228-RA	ni|645903569|nb|KK920861.1|	43628-44350	nan	nan	gi|646778840|gb|KK961590.1|	1566624-1567396	20	9.9

ACYPI27242-RA	ni|645904153|nb|KK920280.1|	660052-660584	17	16	gi|646738652|gb|KK962955.1|	129394-130166	19	8.8
ACYPI28767-RA	ni|645904168|nb|KK920265.1|	2095100-2095359	16	16	gi|646767477|gb|KK961831.1|	263847-264105	20	8.4
ACYPI28781-RA	ni|645903870|nb|KK920560.1|	170147-170483	14	23	gi|646744534|gb|KK962425.1|	29794-32360	19	8.6
ACYPI29050-RA	ni|645904009|nb|KK920422.1|	1363128-1363328	17	9.4	gi|646770723|gb|KK961775.1|	384002-384548	20	8.8
ACYPI29303-RA	ni|645904112|nb|KK920319.1|	2181838-2182623	18	17	gi|646779298|gb|KK961577.1|	4308594-4309686	20	9.1
ACYPI29397-RA	ni|645903494|nb|KK920936.1|	405062-406234	nan	nan	gi|646780889|gb|KK961537.1|	2666187-2668299	22	10
ACYPI29477-RA	ni|645904137|nb|KK920296.1|	2391514-2391814	17	17	gi|646780858|gb|KK961538.1|	468679-473230	21	10
ACYPI29600-RA	ni|645904112|nb|KK920319.1|	2322643-2323547	18	17	gi|646734440|gb|KK963419.1|	331882-332330	19	6.9
ACYPI29680-RA	ni|645903972|nb|KK920459.1|	568325-569449	15	16	gi|646775807|gb|KK961708.1|	418853-422210	21	8.6
ACYPI30820-RA	ni|645903599|nb|KK920831.1|	222759-223687	nan	nan	gi|646750341|gb|KK962044.1|	758408-759351	19	5.6
ACYPI31143-RA	ni|645903960|nb|KK920471.1|	404171-404634	16	8.6	gi|646780183|gb|KK961555.1|	66752-69382	19	8.6
ACYPI31510-RA	ni|645903612|nb|KK920818.1|	127967-128460	nan	nan	gi|646772677|gb|KK961755.1|	1084110-1085677	18	7.9
ACYPI31659-RA	ni|645904177|nb|KK920260.1|	901342-901899	18	17	gi|646782357|gb|KK961494.1|	9034622-9035409	21	9.2
ACYPI31758-RA	ni|645904262|nb|KK920227.1|	1392042-1392269	17	17	gi|646738028|gb|KK963018.1|	79585-80177	22	7.1
ACYPI34001-RA	ni|645903538|nb|KK920892.1|	234004-236988	nan	nan	gi|646780953|gb|KK961535.1|	1124455-1127576	20	9.8
ACYPI34621-RA	ni|645903787|nb|KK920643.1|	814254-814958	19	18	gi|646781118|gb|KK961530.1|	6295386-6296813	21	10
ACYPI34776-RA	ni|645897392|nb|KK927037.1|	3550-3709	nan	nan	gi|646782276|gb|KK961497.1|	1616739-1620637	21	9.7
ACYPI34873-RA	ni|645903768|nb|KK920662.1|	416036-416593	18	17	gi|646771735|gb|KK961764.1|	1069592-1070440	19	9.5
ACYPI34996-RA	ni|645903747|nb|KK920683.1|	104035-104488	19	18	gi|646767230|gb|KK961837.1|	572856-578093	19	5.4
ACYPI35199-RA	ni|645901638|nb|KK922791.1|	5981-6551	nan	nan	gi|646775807|gb|KK961708.1|	1049620-1051151	21	8.6
ACYPI35291-RA	ni|645903578|nb|KK920852.1|	262639-262841	nan	nan	gi|646780147|gb|KK961556.1|	1615751-1616118	18	7.5
ACYPI35323-RA	ni|645903831|nb|KK920599.1|	724480-725693	18	17	gi|646767813|gb|KK961824.1|	234889-241469	17	6.3
ACYPI36103-RA	ni|645903572|nb|KK920858.1|	999029-999670	nan	nan	gi|646743352|gb|KK962510.1|	243669-244955	20	5.4
ACYPI36199-RA	ni|645904059|nb|KK920372.1|	714530-715541	16	9	gi|646775821|gb|KK961707.1|	9268-11079	23	9.1
ACYPI36232-RA	ni|645903506|nb|KK920924.1|	99654-100452	nan	nan	gi|646732955|gb|KK963604.1|	98370-98848	21	8.1
ACYPI36355-RA	ni|645901996|nb|KK922433.1|	36734-38492	nan	nan	gi|646776024|gb|KK961697.1|	1632771-1634123	22	9.2
ACYPI36505-RA	ni|645903765|nb|KK920665.1|	534599-537011	17	15	gi|646763535|gb|KK961902.1|	1683481-1686247	23	10
ACYPI36797-RA	ni|645904140|nb|KK920293.1|	69457-69940	22	22	gi|646743873|gb|KK962474.1|	193927-208922	20	9.4
ACYPI37088-RA	ni|645903716|nb|KK920714.1|	418040-418587	nan	nan	gi|646750021|gb|KK962061.1|	396937-397184	16	7.5
ACYPI37793-RA	ni|645904271|nb|KK920224.1|	841398-841921	15	16	gi|646776542|gb|KK961668.1|	3089558-3092060	20	5.6

ACYPI38188-RA	ni|645903618|nb|KK920812.1|	322903-323233	nan	nan	gi|646780978|gb|KK961534.1|	5642931-5643835	20	8
ACYPI38268-RA	ni|645897346|nb|KK927083.1|	41626-47887	nan	nan	gi|646766650|gb|KK961854.1|	1179249-1192510	22	10
ACYPI38303-RA	ni|645903690|nb|KK920740.1|	998974-999823	nan	nan	gi|646758845|gb|KK961921.1|	1042669-1042911	22	9.5
ACYPI38602-RA	ni|645904262|nb|KK920227.1|	710264-710776	17	17	gi|646777089|gb|KK961646.1|	479877-480198	20	9.4
ACYPI38874-RA	ni|645904055|nb|KK920376.1|	672586-673737	17	17	gi|646743873|gb|KK962474.1|	364197-365506	20	9.4
ACYPI39080-RA	ni|645904146|nb|KK920287.1|	434569-435645	16	18	gi|646766451|gb|KK961861.1|	41368-42634	19	8.9
ACYPI39445-RA	ni|645904231|nb|KK920242.1|	647051-648391	16	17	gi|646780311|gb|KK961552.1|	1061459-1062146	21	9.7
ACYPI39685-RA	ni|645904097|nb|KK920334.1|	713426-714218	15	17	gi|646739758|gb|KK962847.1|	276987-278912	17	8.2
ACYPI39748-RA	ni|645904177|nb|KK920260.1|	277788-278479	18	17	gi|646778865|gb|KK961589.1|	2595109-2598678	20	9.2
ACYPI40226-RA	ni|645904119|nb|KK920312.1|	1078292-1078905	19	19	gi|646766598|gb|KK961856.1|	258732-259021	23	10
ACYPI40717-RA	ni|645903780|nb|KK920650.1|	847265-848343	19	19	gi|646766650|gb|KK961854.1|	1578055-1579158	22	10
ACYPI40836-RA	ni|645904087|nb|KK920344.1|	794162-794863	16	16	gi|646736615|gb|KK963164.1|	164943-173046	20	10
ACYPI40853-RA	ni|645904174|nb|KK920261.1|	812352-813326	19	17	gi|646769832|gb|KK961787.1|	1162123-1162658	15	9.8
ACYPI41722-RA	ni|645904177|nb|KK920260.1|	1166857-1168338	18	17	gi|646782334|gb|KK961495.1|	3500327-3500819	21	9
ACYPI42165-RA	ni|645902494|nb|KK921935.1|	62323-62451	nan	nan	gi|646742237|gb|KK962597.1|	335386-335946	21	6
ACYPI42170-RA	ni|645904204|nb|KK920251.1|	754515-755254	15	16	gi|646745462|gb|KK962357.1|	55865-56371	23	11
ACYPI42350-RA	ni|645904136|nb|KK920297.1|	912875-913912	16	17	gi|646639086|gb|KK969293.1|	2156-2597	29	12
ACYPI42579-RA	ni|645903561|nb|KK920869.1|	80183-81266	nan	nan	gi|646770401|gb|KK961779.1|	1719667-1724087	21	8.6
ACYPI43876-RA	ni|645904037|nb|KK920394.1|	1477728-1478876	18	17	gi|646770401|gb|KK961779.1|	436571-439449	21	8.6
ACYPI46077-RA	ni|645903857|nb|KK920573.1|	545029-545350	16	16	gi|646743886|gb|KK962473.1|	530155-530972	21	9.3
ACYPI46836-RA	ni|645903567|nb|KK920863.1|	264906-265274	nan	nan	gi|646780406|gb|KK961550.1|	607204-608114	22	5.9
ACYPI47548-RA	ni|645903999|nb|KK920432.1|	572824-574232	17	15	gi|646781421|gb|KK961521.1|	4213757-4218648	21	8.7
ACYPI47651-RA	ni|645903961|nb|KK920470.1|	460205-460444	17	15	gi|646749098|gb|KK962110.1|	86178-86526	20	7.7
ACYPI48166-RA	ni|645904071|nb|KK920360.1|	1222594-1223379	17	17	gi|646782168|gb|KK961499.1|	6087727-6089978	21	9.4
ACYPI48246-RA	ni|645903747|nb|KK920683.1|	876580-876856	19	18	gi|646776305|gb|KK961683.1|	2478680-2479325	20	8.8
ACYPI48834-RA	ni|645903627|nb|KK920803.1|	347973-348960	nan	nan	gi|646777570|gb|KK961626.1|	857242-857864	18	7.5
ACYPI49124-RA	ni|645904134|nb|KK920299.1|	393202-393792	17	13	gi|646748185|gb|KK962165.1|	658433-660314	22	9.9
ACYPI49734-RA	ni|645902238|nb|KK922191.1|	189752-190371	nan	nan	gi|646735026|gb|KK963351.1|	107368-117016	21	8.9
ACYPI49735-RA	ni|645902238|nb|KK922191.1|	178861-180498	nan	nan	gi|646735026|gb|KK963351.1|	95068-102550	21	8.9
ACYPI49959-RA	ni|645902788|nb|KK921641.1|	285490-286084	nan	nan	gi|646780058|gb|KK961558.1|	409669-410952	15	6.7

ACYPI50514-RA	ni|645904133|nb|KK920300.1|	1623860-1624336	18	18	gi|646775312|gb|KK961734.1|	116824-117309	17	7.9
ACYPI50527-RA	ni|645904013|nb|KK920418.1|	1384871-1385151	19	18	gi|646750372|gb|KK962042.1|	277673-278491	18	9.5
ACYPI50578-RA	ni|645902418|nb|KK922011.1|	70358-70727	nan	nan	gi|646780105|gb|KK961557.1|	699508-705013	21	9.7
ACYPI50902-RA	ni|645904013|nb|KK920418.1|	716342-716903	19	18	gi|646781628|gb|KK961514.1|	1417682-1421156	23	9
ACYPI51052-RA	ni|645903685|nb|KK920745.1|	522246-523151	nan	nan	gi|646748379|gb|KK962153.1|	20937-22146	14	8.9
ACYPI51124-RA	ni|645904070|nb|KK920361.1|	344150-344490	17	16	gi|646746355|gb|KK962294.1|	891255-891649	22	10
ACYPI52139-RA	ni|645904153|nb|KK920280.1|	2320493-2321736	17	16	gi|646778983|gb|KK961586.1|	1974564-1980666	20	9.9
ACYPI52393-RA	ni|645903541|nb|KK920889.1|	271090-272483	nan	nan	gi|646758527|gb|KK961922.1|	579840-586079	24	10
ACYPI52551-RA	ni|645902511|nb|KK921918.1|	108350-109032	nan	nan	gi|646748116|gb|KK962170.1|	416634-416980	20	8.3
ACYPI52571-RA	ni|645903743|nb|KK920687.1|	924656-928329	19	18	gi|646767230|gb|KK961837.1|	831232-831878	19	5.4
ACYPI52843-RA	ni|645903896|nb|KK920535.1|	459081-460504	16	9.1	gi|646747500|gb|KK962213.1|	667429-673762	21	9.4
ACYPI53099-RA	ni|645903577|nb|KK920853.1|	622743-623462	nan	nan	gi|646736885|gb|KK963134.1|	188014-189544	18	9.4
ACYPI53104-RA	ni|645903709|nb|KK920721.1|	90870-92494	nan	nan	gi|646767850|gb|KK961823.1|	847900-852526	21	9.8
ACYPI53120-RA	ni|645902600|nb|KK921829.1|	154361-154889	nan	nan	gi|646779976|gb|KK961560.1|	1397731-1398391	16	7.9
ACYPI53200-RA	ni|645904135|nb|KK920298.1|	350799-351591	14	15	gi|646748728|gb|KK962132.1|	226897-229215	19	8.1
ACYPI55194-RA	ni|645903922|nb|KK920509.1|	144063-144158	15	16	gi|646775558|gb|KK961720.1|	744166-744513	20	9.4
ACYPI55202-RA	ni|645904029|nb|KK920402.1|	575031-575226	14	15	gi|646777729|gb|KK961621.1|	639315-639761	21	9.8
ACYPI55208-RA	ni|645904189|nb|KK920256.1|	1442314-1442520	18	18	gi|646782288|gb|KK961496.1|	799138-799364	21	9.7
ACYPI55567-RA	ni|645901786|nb|KK922643.1|	62098-63715	nan	nan	gi|646781043|gb|KK961532.1|	2241209-2245366	26	11
ACYPI55712-RA	ni|645903505|nb|KK920925.1|	98540-99214	nan	nan	gi|646770998|gb|KK961772.1|	1802369-1803254	22	9.8
ACYPI55872-RA	ni|645902561|nb|KK921868.1|	3335-3594	nan	nan	gi|646780953|gb|KK961535.1|	201198-203770	20	9.8
ACYPI56077-RA	ni|645903860|nb|KK920570.1|	209729-210132	17	17	gi|646745372|gb|KK962364.1|	471123-471656	23	10
ACYPI56610-RA	ni|645897009|nb|KK927420.1|	980-1583	nan	nan	gi|646781282|gb|KK961525.1|	1448875-1450546	22	9.6
ACYPI56611-RA	ni|645904251|nb|KK920231.1|	352483-353339	16	16	gi|646778336|gb|KK961604.1|	1319970-1320386	21	10
ACYPI56627-RA	ni|645903993|nb|KK920438.1|	132747-133864	19	22	gi|646782211|gb|KK961498.1|	5556234-5557380	20	9
ACYPI56635-RA	ni|645903809|nb|KK920621.1|	219501-220065	18	16	gi|646781510|gb|KK961518.1|	3426034-3428246	17	7.8
ACYPI56643-RA	ni|645904174|nb|KK920261.1|	274830-275661	19	17	gi|646782288|gb|KK961496.1|	273783-275111	21	9.7
ACYPI56655-RA	ni|645903970|nb|KK920461.1|	371301-371634	17	16	gi|646750002|gb|KK962062.1|	322645-323032	24	12
ACYPI56660-RA	ni|645901937|nb|KK922492.1|	4406-4714	nan	nan	gi|646781968|gb|KK961504.1|	905477-905953	20	9.6
ACYPI56663-RA	ni|645904074|nb|KK920357.1|	1028780-1029133	17	18	gi|646779186|gb|KK961580.1|	1576022-1576693	20	8.6

ACYPI56678-RA	ni|645903557|nb|KK920873.1|	917401-917614	nan	nan	gi|646767850|gb|KK961823.1|	1760912-1761222	21	9.8
ACYPI56745-RA	ni|645902694|nb|KK921735.1|	178952-179243	nan	nan	gi|646740395|gb|KK962776.1|	180371-182212	16	6.3
ACYPI56857-RA	ni|645904222|nb|KK920245.1|	655881-656414	17	12	gi|646740085|gb|KK962813.1|	165388-169538	20	5.5
