# Supplementary material for: The X Chromosome of Hemipteran Insects: Conservation, Dosage Compensation and Sex-Biased Expression
Source: Genome Biol Evol. 2015 Nov 10;7(12):3259–68. doi: 10.1093/gbe/evv215 (PMC4700948; doi:10.1093/gbe/evv215)
Supplement: Supplementary Data [file supp_evv215_suppl_data.zip › S2 Data (rev) HH-OF.pdf]

AP	HH	gene	HHcovF	HHcovM	OF	gene	OFcovF	OFcovM
ACYPI000007-RA	ni|645904141|nb|KK920292.1|	555475-556035	14	14	gi|641572390|gb|KK855826.1|	56516-58114	10	35
ACYPI000011-RA	ni|645904246|nb|KK920233.1|	638772-640086	16	17	gi|641574095|gb|KK855397.1|	175037-177744	11	39
ACYPI000013-RA	ni|645904195|nb|KK920254.1|	110171-111932	18	17	gi|641578060|gb|KK854647.1|	32081-34839	8.9	35
ACYPI000014-RA	ni|645904195|nb|KK920254.1|	121981-122438	18	17	gi|641578060|gb|KK854647.1|	64753-67419	8.9	35
ACYPI000016-RA	ni|645899869|nb|KK924560.1|	1233-1542	nan	nan	gi|641578198|gb|KK854624.1|	511137-511499	12	40
ACYPI000023-RA	ni|645904072|nb|KK920359.1|	947375-948150	17	17	gi|641573621|gb|KK855514.1|	51915-53233	10	41
ACYPI000027-RA	ni|645902384|nb|KK922045.1|	184957-185581	nan	nan	gi|641574955|gb|KK855208.1|	187350-187595	8.5	33
ACYPI000030-RA	ni|645903923|nb|KK920508.1|	126453-126713	18	18	gi|641575946|gb|KK855016.1|	210994-220495	9.5	37
ACYPI000032-RA	ni|645903782|nb|KK920648.1|	179359-179729	13	14	gi|641586734|gb|KK854198.1|	163022-165105	11	40
ACYPI000033-RA	ni|645903879|nb|KK920552.1|	1065460-1065856	18	18	gi|641586509|gb|KK854231.1|	561560-564107	9.8	37
ACYPI000035-RA	ni|645903788|nb|KK920642.1|	730081-730597	16	15	gi|641574476|gb|KK855312.1|	158895-159679	10	43
ACYPI000038-RA	ni|645903989|nb|KK920442.1|	731456-731831	17	20	gi|641567126|gb|KK857416.1|	36342-37408	11	41
ACYPI000039-RA	ni|645903755|nb|KK920675.1|	749888-750218	18	16	gi|641585847|gb|KK854330.1|	170644-172139	11	41
ACYPI000051-RA	ni|645903759|nb|KK920671.1|	97725-98872	18	17	gi|641568389|gb|KK856993.1|	95561-97314	10	41
ACYPI000052-RA	ni|645902360|nb|KK922069.1|	41628-41906	nan	nan	gi|641566429|gb|KK857664.1|	66414-67063	12	37
ACYPI000053-RA	ni|645904114|nb|KK920317.1|	2059145-2060225	18	18	gi|641572247|gb|KK855861.1|	155478-157522	10	37
ACYPI000054-RA	ni|645903534|nb|KK920896.1|	569179-569605	nan	nan	gi|641587074|gb|KK854148.1|	559178-561366	11	41
ACYPI000055-RA	ni|645903787|nb|KK920643.1|	1368639-1369809	19	18	gi|641585366|gb|KK854408.1|	368516-371955	12	41
ACYPI000056-RA	ni|645904133|nb|KK920300.1|	1059081-1059971	18	18	gi|641575583|gb|KK855084.1|	170446-171490	11	37
ACYPI000058-RA	ni|645903798|nb|KK920632.1|	767915-768764	18	17	gi|641578149|gb|KK854632.1|	229773-230600	11	40
ACYPI000059-RA	ni|645904009|nb|KK920422.1|	608483-608931	17	9.4	gi|641588309|gb|KK854005.1|	525454-526730	11	40
ACYPI000061-RA	ni|645903749|nb|KK920681.1|	644306-645583	19	18	gi|641587742|gb|KK854065.1|	528211-529888	11	41
ACYPI000062-RA	ni|645903923|nb|KK920508.1|	1138568-1139112	18	18	gi|641578310|gb|KK854604.1|	93489-93988	11	39
ACYPI000063-RA	ni|645904278|nb|KK920222.1|	543245-543785	15	16	gi|641577920|gb|KK854670.1|	271638-272507	8.5	40
ACYPI000065-RA	ni|645903698|nb|KK920732.1|	147048-147807	nan	nan	gi|641576347|gb|KK854934.1|	20200-21624	11	39
ACYPI000066-RA	ni|645903749|nb|KK920681.1|	222022-222532	19	18	gi|641584205|gb|KK854500.1|	65445-66310	11	41
ACYPI000067-RA	ni|645904210|nb|KK920249.1|	1933083-1934378	16	16	gi|641577121|gb|KK854804.1|	193382-195033	16	82
ACYPI000068-RA	ni|645903971|nb|KK920460.1|	1942428-1944356	19	18	gi|641569988|gb|KK856495.1|	87740-88795	11	43
ACYPI000070-RA	ni|645904160|nb|KK920273.1|	612857-613080	18	18	gi|641577805|gb|KK854691.1|	488167-489736	9.4	38
ACYPI000071-RA	ni|645903843|nb|KK920587.1|	406702-406961	18	16	gi|641571760|gb|KK855988.1|	96541-97388	9.9	37
ACYPI000072-RA	ni|645904075|nb|KK920356.1|	746654-747204	16	17	gi|641584336|gb|KK854489.1|	176419-176832	10	40

ACYPI000073-RA	ni|645904177|nb|KK920260.1|	2820278-2820690	18	17	gi|641573567|gb|KK855527.1|	77166-77635	12	47
ACYPI000076-RA	ni|645902589|nb|KK921840.1|	122803-123041	nan	nan	gi|641572916|gb|KK855689.1|	123851-127276	9.2	33
ACYPI000077-RA	ni|645903673|nb|KK920757.1|	187211-187723	nan	nan	gi|641587735|gb|KK854066.1|	66823-68779	11	42
ACYPI000078-RA	ni|645903858|nb|KK920572.1|	709519-709799	16	16	gi|641577535|gb|KK854737.1|	4514-10672	11	42
ACYPI000079-RA	ni|645904096|nb|KK920335.1|	2233322-2233597	18	17	gi|641575368|gb|KK855125.1|	91464-93177	10	36
ACYPI000080-RA	ni|645903724|nb|KK920706.1|	262716-263267	nan	nan	gi|641570274|gb|KK856404.1|	107160-108834	9.1	35
ACYPI000082-RA	ni|645903923|nb|KK920508.1|	282216-282451	18	18	gi|641586741|gb|KK854197.1|	680135-682002	11	43
ACYPI000083-RA	ni|645904015|nb|KK920416.1|	1558468-1558729	18	16	gi|641586515|gb|KK854230.1|	651769-652480	11	39
ACYPI000089-RA	ni|645904130|nb|KK920303.1|	1356279-1356683	18	17	gi|641588099|gb|KK854024.1|	212160-213211	11	39
ACYPI000090-RA	ni|645903841|nb|KK920589.1|	1249445-1250199	17	17	gi|641588343|gb|KK854003.1|	1016518-1016738	11	40
ACYPI000091-RA	ni|645904122|nb|KK920309.1|	589900-590358	18	17	gi|641577277|gb|KK854778.1|	217865-219704	11	42
ACYPI000096-RA	ni|645904096|nb|KK920335.1|	177494-177915	18	17	gi|641569976|gb|KK856499.1|	123205-123471	10	43
ACYPI000099-RA	ni|645903870|nb|KK920560.1|	235406-236741	14	23	gi|641577522|gb|KK854739.1|	88318-95019	11	41
ACYPI000100-RA	ni|645902364|nb|KK922065.1|	172748-173317	nan	nan	gi|641570526|gb|KK856327.1|	100135-100754	9.1	37
ACYPI000102-RA	ni|645904228|nb|KK920243.1|	803500-803990	17	18	gi|641576951|gb|KK854831.1|	209531-210063	8.6	40
ACYPI000103-RA	ni|645903836|nb|KK920594.1|	719371-719729	18	11	gi|641569252|gb|KK856726.1|	23033-23604	11	20
ACYPI000104-RA	ni|645903915|nb|KK920516.1|	207249-208913	17	17	gi|641576414|gb|KK854924.1|	81163-82561	10	40
ACYPI000109-RA	ni|645901808|nb|KK922621.1|	148239-149469	nan	nan	gi|641575730|gb|KK855054.1|	312883-318335	10	38
ACYPI000111-RA	ni|645903549|nb|KK920881.1|	19337-19606	nan	nan	gi|641576442|gb|KK854919.1|	10082-11256	11	36
ACYPI000112-RA	ni|645904174|nb|KK920261.1|	923528-923778	19	17	gi|641571048|gb|KK856174.1|	36498-36798	11	39
ACYPI000119-RA	ni|645903743|nb|KK920687.1|	881121-881542	19	18	gi|641587133|gb|KK854140.1|	612043-612276	9.9	37
ACYPI000120-RA	ni|645904129|nb|KK920304.1|	1140307-1140617	16	17	gi|641576069|gb|KK854989.1|	264933-266515	11	40
ACYPI000130-RA	ni|645903967|nb|KK920464.1|	155992-156862	13	14	gi|641577896|gb|KK854674.1|	180912-183814	11	40
ACYPI000149-RA	ni|645903979|nb|KK920452.1|	532494-536473	15	17	gi|641580970|gb|KK854563.1|	170493-175598	11	42
ACYPI000150-RA	ni|645903496|nb|KK920934.1|	195331-196100	nan	nan	gi|641572824|gb|KK855713.1|	165584-167439	9.8	19
ACYPI000156-RA	ni|645903476|nb|KK920954.1|	175927-176047	nan	nan	gi|641581402|gb|KK854555.1|	46541-46740	12	20
ACYPI000157-RA	ni|645903617|nb|KK920813.1|	290263-290735	nan	nan	gi|641565939|gb|KK857844.1|	4054-5290	8.8	16
ACYPI000164-RA	ni|645902751|nb|KK921678.1|	149947-150147	nan	nan	gi|641575730|gb|KK855054.1|	68082-68303	10	38
ACYPI000165-RA	ni|645897572|nb|KK926857.1|	730-950	nan	nan	gi|641573181|gb|KK855624.1|	240745-241212	11	40
ACYPI000171-RA	ni|645903705|nb|KK920725.1|	432083-432452	nan	nan	gi|641573673|gb|KK855501.1|	155796-159785	14	43
ACYPI000181-RA	ni|645903788|nb|KK920642.1|	321728-321979	16	15	gi|641585905|gb|KK854321.1|	495675-496013	11	39
ACYPI000183-RA	ni|645904065|nb|KK920366.1|	981607-981746	18	18	gi|641586535|gb|KK854227.1|	511330-512088	10	39

ACYPI000191-RA	ni|645904278|nb|KK920222.1|	1295109-1295472	15	16	gi|641585778|gb|KK854341.1|	337880-338147	9.7	41
ACYPI000192-RA	ni|645902694|nb|KK921735.1|	14577-15368	nan	nan	gi|641586915|gb|KK854171.1|	329413-330184	10	38
ACYPI000193-RA	ni|645903511|nb|KK920919.1|	462549-463071	nan	nan	gi|641588164|gb|KK854017.1|	753705-755931	9.2	40
ACYPI000201-RA	ni|645903731|nb|KK920699.1|	884810-885019	nan	nan	gi|641570573|gb|KK856315.1|	134460-142538	10	45
ACYPI000210-RA	ni|645902026|nb|KK922403.1|	3499-4106	nan	nan	gi|641586837|gb|KK854184.1|	87782-88434	11	40
ACYPI000218-RA	ni|645902229|nb|KK922200.1|	21019-21430	nan	nan	gi|641576343|gb|KK854935.1|	42989-44371	9.5	36
ACYPI000219-RA	ni|645903690|nb|KK920740.1|	1034038-1034219	nan	nan	gi|641585569|gb|KK854376.1|	56185-56699	11	47
ACYPI000222-RA	ni|645903607|nb|KK920823.1|	306997-307436	nan	nan	gi|641576260|gb|KK854950.1|	45448-45882	10	39
ACYPI000227-RA	ni|645903988|nb|KK920443.1|	96980-98231	14	16	gi|641587577|gb|KK854086.1|	48732-49579	8.2	41
ACYPI000235-RA	ni|645904112|nb|KK920319.1|	1717915-1718101	18	17	gi|641588082|gb|KK854026.1|	1083168-1083300	11	41
ACYPI000238-RA	ni|645902469|nb|KK921960.1|	278248-278991	nan	nan	gi|641566606|gb|KK857599.1|	32825-33433	10	40
ACYPI000243-RA	ni|645903504|nb|KK920926.1|	259682-259871	nan	nan	gi|641578084|gb|KK854643.1|	245531-245751	11	40
ACYPI000249-RA	ni|645904262|nb|KK920227.1|	2592282-2593418	17	17	gi|641572891|gb|KK855695.1|	31191-32931	10	36
ACYPI000252-RA	ni|645904030|nb|KK920401.1|	406005-408372	14	15	gi|641586843|gb|KK854183.1|	524314-536066	11	41
ACYPI000257-RA	ni|645904112|nb|KK920319.1|	1677836-1678586	18	17	gi|641586150|gb|KK854282.1|	456801-457130	11	40
ACYPI000258-RA	ni|645903597|nb|KK920833.1|	984125-985288	nan	nan	gi|641587652|gb|KK854077.1|	598010-598705	10	38
ACYPI000259-RA	ni|645903529|nb|KK920901.1|	101501-103180	nan	nan	gi|641587040|gb|KK854152.1|	364920-365624	9.1	39
ACYPI000261-RA	ni|645903773|nb|KK920657.1|	1520215-1523516	18	17	gi|641573837|gb|KK855460.1|	113888-116364	13	24
ACYPI000262-RA	ni|645903674|nb|KK920756.1|	567814-568257	nan	nan	gi|641587104|gb|KK854144.1|	815457-815780	11	37
ACYPI000265-RA	ni|645904106|nb|KK920325.1|	994361-995652	17	17	gi|641582439|gb|KK854536.1|	33343-35639	11	36
ACYPI000271-RA	ni|645903949|nb|KK920482.1|	2716-3421	17	14	gi|641572601|gb|KK855773.1|	194693-194928	10	35
ACYPI000272-RA	ni|645902715|nb|KK921714.1|	203137-203363	nan	nan	gi|641571045|gb|KK856175.1|	85235-86808	9.3	35
ACYPI000278-RA	ni|645904152|nb|KK920281.1|	260381-260684	15	15	gi|641587365|gb|KK854113.1|	698368-699804	12	45
ACYPI000286-RA	ni|645903551|nb|KK920879.1|	385576-386048	nan	nan	gi|641573850|gb|KK855457.1|	214665-215347	9.3	34
ACYPI000289-RA	ni|645903634|nb|KK920796.1|	1155220-1155478	nan	nan	gi|641584445|gb|KK854481.1|	227492-229988	9.9	39
ACYPI000295-RA	ni|645903717|nb|KK920713.1|	67347-67736	nan	nan	gi|641587962|gb|KK854039.1|	841203-841667	11	40
ACYPI000303-RA	ni|645902364|nb|KK922065.1|	28028-29134	nan	nan	gi|641565112|gb|KK858157.1|	7833-12055	9.7	34
ACYPI000304-RA	ni|645904106|nb|KK920325.1|	996965-998163	17	17	gi|641568637|gb|KK856916.1|	75126-76167	10	38
ACYPI000310-RA	ni|645904171|nb|KK920262.1|	517579-519623	15	16	gi|641587272|gb|KK854122.1|	912870-917442	9.3	42
ACYPI000317-RA	ni|645904072|nb|KK920359.1|	1018088-1018705	17	17	gi|641577047|gb|KK854815.1|	383395-384200	10	41
ACYPI000320-RA	ni|645903768|nb|KK920662.1|	1159763-1159985	18	17	gi|641570570|gb|KK856316.1|	89342-89634	10	35
ACYPI000323-RA	ni|645903982|nb|KK920449.1|	430455-430683	16	14	gi|641584945|gb|KK854445.1|	23268-23552	9.9	20

ACYPI000326-RA	ni|645903688|nb|KK920742.1|	282227-282604	nan	nan	gi|641572857|gb|KK855704.1|	31350-32496	11	41
ACYPI000328-RA	ni|645903820|nb|KK920610.1|	290223-290776	18	19	gi|641572185|gb|KK855876.1|	106699-107899	8.2	36
ACYPI000329-RA	ni|645903754|nb|KK920676.1|	99757-100698	14	8.8	gi|641549615|gb|KK865290.1|	58-1415	12	24
ACYPI000348-RA	ni|645903765|nb|KK920665.1|	929864-930059	17	15	gi|641585905|gb|KK854321.1|	153604-153957	11	39
ACYPI000362-RA	ni|645904075|nb|KK920356.1|	1037500-1038360	16	17	gi|641563408|gb|KK858883.1|	3730-4211	9.5	21
ACYPI000365-RA	ni|645904112|nb|KK920319.1|	2214714-2215473	18	17	gi|641576442|gb|KK854919.1|	238025-238534	11	36
ACYPI000383-RA	ni|645903979|nb|KK920452.1|	698803-700771	15	17	gi|641580970|gb|KK854563.1|	309588-312071	11	42
ACYPI000387-RA	ni|645903771|nb|KK920659.1|	798050-798518	16	15	gi|641582768|gb|KK854530.1|	410190-410868	11	41
ACYPI000402-RA	ni|645904262|nb|KK920227.1|	1248071-1251182	17	17	gi|641571515|gb|KK856048.1|	37318-41280	9.9	38
ACYPI000405-RA	ni|645904222|nb|KK920245.1|	648405-648723	17	12	gi|641585395|gb|KK854405.1|	405520-407871	10	38
ACYPI000413-RA	ni|645903620|nb|KK920810.1|	8735-8932	nan	nan	gi|641564920|gb|KK858233.1|	27114-27569	11	23
ACYPI000420-RA	ni|645903688|nb|KK920742.1|	234687-234859	nan	nan	gi|641572857|gb|KK855704.1|	115605-116659	11	41
ACYPI000423-RA	ni|645904210|nb|KK920249.1|	813969-814148	16	16	gi|641568716|gb|KK856889.1|	35485-37188	10	35
ACYPI000427-RA	ni|645902308|nb|KK922121.1|	113622-115049	nan	nan	gi|641588267|gb|KK854008.1|	109932-117676	10	40
ACYPI000430-RA	ni|645903743|nb|KK920687.1|	896515-897013	19	18	gi|641572219|gb|KK855868.1|	62624-68729	9.8	35
ACYPI000431-RA	ni|645904053|nb|KK920378.1|	575073-575639	18	17	gi|641586085|gb|KK854293.1|	98218-99178	11	40
ACYPI000436-RA	ni|645904142|nb|KK920291.1|	1833311-1834094	20	23	gi|641568213|gb|KK857047.1|	45198-47412	9.2	39
ACYPI000442-RA	ni|645903804|nb|KK920626.1|	128601-130010	17	17	gi|641574015|gb|KK855416.1|	17520-20959	10	42
ACYPI000443-RA	ni|645902239|nb|KK922190.1|	60675-62288	nan	nan	gi|641584320|gb|KK854490.1|	426743-429006	10	41
ACYPI000445-RA	ni|645902642|nb|KK921787.1|	124594-124798	nan	nan	gi|641586515|gb|KK854230.1|	583971-588039	11	39
ACYPI000446-RA	ni|645902779|nb|KK921650.1|	74199-74708	nan	nan	gi|641577772|gb|KK854697.1|	258940-261083	10	39
ACYPI000453-RA	ni|645903595|nb|KK920835.1|	903022-906579	nan	nan	gi|641573695|gb|KK855497.1|	21675-23242	9.2	41
ACYPI000454-RA	ni|645903688|nb|KK920742.1|	78374-78772	nan	nan	gi|641542389|gb|KK868779.1|	439-997	11	45
ACYPI000455-RA	ni|645904234|nb|KK920241.1|	67395-67714	13	15	gi|641570399|gb|KK856367.1|	51263-56295	11	41
ACYPI000467-RA	ni|645904093|nb|KK920338.1|	23707-24122	13	15	gi|641570703|gb|KK856277.1|	109132-109483	9.9	36
ACYPI000474-RA	ni|645903650|nb|KK920780.1|	143175-143398	nan	nan	gi|641571315|gb|KK856104.1|	133674-139009	12	46
ACYPI000475-RA	ni|645903587|nb|KK920843.1|	222774-222914	nan	nan	gi|641585303|gb|KK854420.1|	57616-57928	10	36
ACYPI000476-RA	ni|645903837|nb|KK920593.1|	581975-582221	18	9.8	gi|641567816|gb|KK857179.1|	76314-76518	12	37
ACYPI000479-RA	ni|645902233|nb|KK922196.1|	159856-160197	nan	nan	gi|641570614|gb|KK856303.1|	19248-19597	9.6	34
ACYPI000487-RA	ni|645904106|nb|KK920325.1|	677994-678427	17	17	gi|641585971|gb|KK854310.1|	175212-175825	10	40
ACYPI000489-RA	ni|645902758|nb|KK921671.1|	220670-223892	nan	nan	gi|641576161|gb|KK854972.1|	214160-217514	10	36
ACYPI000496-RA	ni|645904015|nb|KK920416.1|	1300407-1300888	18	16	gi|641585423|gb|KK854400.1|	83475-85161	11	36

ACYPI000499-RA	ni|645903858|nb|KK920572.1|	822831-823007	16	16	gi|641564440|gb|KK858439.1|	379-2822	11	36
ACYPI000500-RA	ni|645903898|nb|KK920533.1|	483988-484252	17	16	gi|641579205|gb|KK854586.1|	154410-154702	10	39
ACYPI000502-RA	ni|645903759|nb|KK920671.1|	525437-525857	18	17	gi|641576957|gb|KK854830.1|	177746-178005	10	43
ACYPI000509-RA	ni|645903662|nb|KK920768.1|	240183-240947	nan	nan	gi|641573733|gb|KK855487.1|	64537-64882	13	45
ACYPI000513-RA	ni|645904098|nb|KK920333.1|	21662-24773	16	8.8	gi|641584945|gb|KK854445.1|	394329-397567	9.9	20
ACYPI000514-RA	ni|645902454|nb|KK921975.1|	158491-159962	nan	nan	gi|641575572|gb|KK855085.1|	127087-128569	9.4	35
ACYPI000519-RA	ni|645904067|nb|KK920364.1|	1536829-1538669	17	14	gi|641586898|gb|KK854174.1|	327042-329558	11	39
ACYPI000521-RA	ni|645903713|nb|KK920717.1|	446097-447247	nan	nan	gi|641587848|gb|KK854052.1|	631208-633367	11	39
ACYPI000523-RA	ni|645903710|nb|KK920720.1|	1038-1528	nan	nan	gi|641586285|gb|KK854263.1|	293171-293643	10	38
ACYPI000527-RA	ni|645904130|nb|KK920303.1|	486428-487041	18	17	gi|641587276|gb|KK854121.1|	431239-432417	11	39
ACYPI000532-RA	ni|645904077|nb|KK920354.1|	307038-307495	16	17	gi|641584504|gb|KK854477.1|	134770-136269	9.5	38
ACYPI000533-RA	ni|645903971|nb|KK920460.1|	1199184-1200576	19	18	gi|641577075|gb|KK854811.1|	97974-99437	11	36
ACYPI000534-RA	ni|645902143|nb|KK922286.1|	173814-174760	nan	nan	gi|641586344|gb|KK854254.1|	475181-476013	10	40
ACYPI000538-RA	ni|645903755|nb|KK920675.1|	656301-657340	18	16	gi|641585847|gb|KK854330.1|	187303-189644	11	41
ACYPI000541-RA	ni|645903789|nb|KK920641.1|	752792-753539	17	17	gi|641587912|gb|KK854045.1|	361098-361359	11	39
ACYPI000552-RA	ni|645904189|nb|KK920256.1|	1449104-1449831	18	18	gi|641586171|gb|KK854278.1|	15939-18288	9	39
ACYPI000563-RA	ni|645902589|nb|KK921840.1|	97255-97493	nan	nan	gi|641572916|gb|KK855689.1|	101035-107029	9.2	33
ACYPI000564-RA	ni|645904029|nb|KK920402.1|	244073-245021	14	15	gi|641587624|gb|KK854080.1|	467253-467710	10	38
ACYPI000568-RA	ni|645903760|nb|KK920670.1|	645969-646126	18	19	gi|641587614|gb|KK854081.1|	865942-866112	11	40
ACYPI000572-RA	ni|645902427|nb|KK922002.1|	143925-144072	nan	nan	gi|641587711|gb|KK854069.1|	638096-639559	11	42
ACYPI000575-RA	ni|645903787|nb|KK920643.1|	1376064-1377647	19	18	gi|641585366|gb|KK854408.1|	388426-390905	12	41
ACYPI000580-RA	ni|645903688|nb|KK920742.1|	1111336-1111644	nan	nan	gi|641586171|gb|KK854278.1|	552666-553443	9	39
ACYPI000582-RA	ni|645903541|nb|KK920889.1|	3564-3724	nan	nan	gi|641586452|gb|KK854239.1|	505286-505784	10	19
ACYPI000585-RA	ni|645903954|nb|KK920477.1|	197722-197837	35	44	gi|641585801|gb|KK854337.1|	437800-438557	10	38
ACYPI000589-RA	ni|645904177|nb|KK920260.1|	1584120-1584841	18	17	gi|641586203|gb|KK854273.1|	579183-580802	9.5	40
ACYPI000592-RA	ni|645904246|nb|KK920233.1|	1091833-1092019	16	17	gi|641586915|gb|KK854171.1|	304011-304195	10	38
ACYPI000598-RA	ni|645901961|nb|KK922468.1|	109885-111850	nan	nan	gi|641574699|gb|KK855261.1|	81511-84990	9	35
ACYPI000600-RA	ni|645903696|nb|KK920734.1|	625575-625788	nan	nan	gi|641571660|gb|KK856010.1|	173694-174468	11	22
ACYPI000605-RA	ni|645904132|nb|KK920301.1|	71003-71455	16	18	gi|641575910|gb|KK855021.1|	394918-399195	10	35
ACYPI000610-RA	ni|645903970|nb|KK920461.1|	2124908-2125698	17	16	gi|641570019|gb|KK856485.1|	48852-51596	12	43
ACYPI000613-RA	ni|645903743|nb|KK920687.1|	320413-321814	19	18	gi|641574085|gb|KK855399.1|	124910-125954	10	38
ACYPI000617-RA	ni|645903860|nb|KK920570.1|	154050-154973	17	17	gi|641580479|gb|KK854569.1|	306615-309434	10	38

ACYPI000624-RA	ni|645903769|nb|KK920661.1|	574335-574620	22	24	gi|641568849|gb|KK856847.1|	81111-82065	10	41
ACYPI000626-RA	ni|645904155|nb|KK920278.1|	283747-283984	18	9.4	gi|641587468|gb|KK854099.1|	604780-605044	10	42
ACYPI000627-RA	ni|645903728|nb|KK920702.1|	961199-961505	nan	nan	gi|641573181|gb|KK855624.1|	31523-31870	11	40
ACYPI000629-RA	ni|645904240|nb|KK920239.1|	654026-655526	16	16	gi|641588136|gb|KK854020.1|	682816-684722	11	36
ACYPI000631-RA	ni|645904063|nb|KK920368.1|	503401-504185	16	18	gi|641588343|gb|KK854003.1|	1166852-1168318	11	40
ACYPI000644-RA	ni|645902394|nb|KK922035.1|	94380-94727	nan	nan	gi|641576221|gb|KK854958.1|	141326-142070	9.3	35
ACYPI000648-RA	ni|645904156|nb|KK920277.1|	163195-163420	17	8.8	gi|641587193|gb|KK854132.1|	507941-508323	11	39
ACYPI000662-RA	ni|645903920|nb|KK920511.1|	663830-664865	20	20	gi|641575385|gb|KK855121.1|	141042-142700	9.4	36
ACYPI000663-RA	ni|645903609|nb|KK920821.1|	157522-157654	nan	nan	gi|641586509|gb|KK854231.1|	319185-321408	9.8	37
ACYPI000666-RA	ni|645904043|nb|KK920388.1|	77415-78131	16	9.1	gi|641561423|gb|KK859751.1|	5747-6205	8.8	17
ACYPI000667-RA	ni|645903597|nb|KK920833.1|	566971-569213	nan	nan	gi|641587652|gb|KK854077.1|	887729-889622	10	38
ACYPI000681-RA	ni|645904210|nb|KK920249.1|	882672-883125	16	16	gi|641586496|gb|KK854233.1|	623852-626325	11	38
ACYPI000686-RA	ni|645903724|nb|KK920706.1|	159437-159822	nan	nan	gi|641571116|gb|KK856157.1|	239816-241311	11	40
ACYPI000690-RA	ni|645903549|nb|KK920881.1|	463009-463704	nan	nan	gi|641586824|gb|KK854186.1|	497259-497693	10	41
ACYPI000694-RA	ni|645904064|nb|KK920367.1|	761472-761990	17	9.9	gi|641587003|gb|KK854159.1|	277619-278020	10	21
ACYPI000695-RA	ni|645903768|nb|KK920662.1|	1193609-1193851	18	17	gi|641584835|gb|KK854453.1|	254573-255248	8.3	36
ACYPI000698-RA	ni|645904231|nb|KK920242.1|	630687-631223	16	17	gi|641577043|gb|KK854816.1|	311150-313770	11	43
ACYPI000700-RA	ni|645904085|nb|KK920346.1|	617544-618209	15	16	gi|641573935|gb|KK855434.1|	218264-223101	11	41
ACYPI000702-RA	ni|645904076|nb|KK920355.1|	810963-811167	17	17	gi|641575668|gb|KK855066.1|	269826-270087	11	40
ACYPI000716-RA	ni|645904168|nb|KK920265.1|	509938-510710	16	16	gi|641587757|gb|KK854063.1|	405410-406408	11	38
ACYPI000717-RA	ni|645902035|nb|KK922394.1|	237948-239067	nan	nan	gi|641586372|gb|KK854251.1|	453617-454523	10	38
ACYPI000720-RA	ni|645903752|nb|KK920678.1|	652309-652693	18	16	gi|641569788|gb|KK856560.1|	30690-31881	9.5	37
ACYPI000724-RA	ni|645903866|nb|KK920564.1|	455494-456067	18	15	gi|641570244|gb|KK856415.1|	66239-67248	10	34
ACYPI000727-RA	ni|645900910|nb|KK923519.1|	12818-13466	nan	nan	gi|641573351|gb|KK855583.1|	89102-91875	11	41
ACYPI000735-RA	ni|645903592|nb|KK920838.1|	763281-763503	nan	nan	gi|641573555|gb|KK855529.1|	97300-97582	8.9	38
ACYPI000739-RA	ni|645903969|nb|KK920462.1|	418285-418808	14	16	gi|641577574|gb|KK854731.1|	295485-296034	11	38
ACYPI000749-RA	ni|645904125|nb|KK920307.1|	2764117-2764389	18	17	gi|641585761|gb|KK854344.1|	323336-323522	10	39
ACYPI000753-RA	ni|645904262|nb|KK920227.1|	2473960-2474135	17	17	gi|641572637|gb|KK855763.1|	18018-21093	9.5	36
ACYPI000754-RA	ni|645903923|nb|KK920508.1|	1127142-1127692	18	18	gi|641588099|gb|KK854024.1|	592114-594104	11	39
ACYPI000756-RA	ni|645904071|nb|KK920360.1|	1948746-1949884	17	17	gi|641586975|gb|KK854163.1|	14899-15804	11	40
ACYPI000758-RA	ni|645903895|nb|KK920536.1|	657557-660370	17	16	gi|641585315|gb|KK854418.1|	464855-466629	10	39
ACYPI000759-RA	ni|645903891|nb|KK920540.1|	214051-214946	15	17	gi|641585376|gb|KK854407.1|	134864-136282	9.8	36

ACYPI000767-RA	ni|645903597|nb|KK920833.1|	1034927-1035235	nan	nan	gi|641587652|gb|KK854077.1|	274273-274591	10	38
ACYPI000775-RA	ni|645902469|nb|KK921960.1|	20668-20791	nan	nan	gi|641572797|gb|KK855721.1|	128656-130067	9.3	39
ACYPI000776-RA	ni|645904118|nb|KK920313.1|	678422-679063	17	17	gi|641585871|gb|KK854327.1|	138541-140199	11	38
ACYPI000787-RA	ni|645903716|nb|KK920714.1|	772982-773444	nan	nan	gi|641587511|gb|KK854094.1|	242785-243982	10	41
ACYPI000806-RA	ni|645902503|nb|KK921926.1|	37124-37276	nan	nan	gi|641585684|gb|KK854357.1|	234578-234779	11	39
ACYPI000814-RA	ni|645903895|nb|KK920536.1|	540572-540824	17	16	gi|641585315|gb|KK854418.1|	334099-336057	10	39
ACYPI000816-RA	ni|645903559|nb|KK920871.1|	189904-190635	nan	nan	gi|641572655|gb|KK855758.1|	154318-156033	19	94
ACYPI000819-RA	ni|645903891|nb|KK920540.1|	90267-90459	15	17	gi|641574951|gb|KK855209.1|	76378-78376	10	38
ACYPI000821-RA	ni|645903622|nb|KK920808.1|	572228-572457	nan	nan	gi|641587066|gb|KK854149.1|	220873-221337	11	40
ACYPI000828-RA	ni|645904177|nb|KK920260.1|	782203-785242	18	17	gi|641566255|gb|KK857728.1|	11965-15850	8.3	32
ACYPI000837-RA	ni|645903564|nb|KK920866.1|	7905-11322	nan	nan	gi|641582096|gb|KK854546.1|	29961-33872	11	38
ACYPI000839-RA	ni|645903714|nb|KK920716.1|	204343-204510	nan	nan	gi|641570271|gb|KK856405.1|	108325-109422	9.8	38
ACYPI000844-RA	ni|645903892|nb|KK920539.1|	401026-401886	20	18	gi|641577846|gb|KK854683.1|	183293-183988	10	39
ACYPI000848-RA	ni|645904177|nb|KK920260.1|	1202685-1203630	18	17	gi|641586203|gb|KK854273.1|	324960-325864	9.5	40
ACYPI000854-RA	ni|645903495|nb|KK920935.1|	201271-201682	nan	nan	gi|641585366|gb|KK854408.1|	70695-71817	12	41
ACYPI000855-RA	ni|645903910|nb|KK920521.1|	544035-544262	19	17	gi|641588109|gb|KK854023.1|	222111-223328	10	40
ACYPI000862-RA	ni|645904210|nb|KK920249.1|	887986-888169	16	16	gi|641586496|gb|KK854233.1|	597034-597888	11	38
ACYPI000864-RA	ni|645904130|nb|KK920303.1|	768086-768588	18	17	gi|641578315|gb|KK854603.1|	231634-232129	9.8	38
ACYPI000870-RA	ni|645903636|nb|KK920794.1|	239469-240653	nan	nan	gi|641576766|gb|KK854863.1|	157080-157377	10	38
ACYPI000876-RA	ni|645904019|nb|KK920412.1|	181000-181322	19	9.4	gi|641578305|gb|KK854605.1|	64482-66730	11	42
ACYPI000882-RA	ni|645903827|nb|KK920603.1|	437169-438833	17	18	gi|641574960|gb|KK855207.1|	278907-280778	10	48
ACYPI000886-RA	ni|645904014|nb|KK920417.1|	794089-794343	19	18	gi|641565701|gb|KK857932.1|	2599-2857	11	42
ACYPI000887-RA	ni|645903985|nb|KK920446.1|	1048896-1049470	18	16	gi|641579941|gb|KK854575.1|	297254-298997	10	39
ACYPI000889-RA	ni|645904258|nb|KK920229.1|	591162-591769	16	16	gi|641572227|gb|KK855866.1|	92593-93285	7	32
ACYPI000890-RA	ni|645902336|nb|KK922093.1|	77307-78304	nan	nan	gi|641584205|gb|KK854500.1|	475644-476484	11	41
ACYPI000896-RA	ni|645901860|nb|KK922569.1|	75991-76640	nan	nan	gi|641585910|gb|KK854320.1|	129892-132882	10	38
ACYPI000901-RA	ni|645904067|nb|KK920364.1|	876361-876993	17	14	gi|641571396|gb|KK856083.1|	91272-91899	8.4	35
ACYPI000904-RA	ni|645903984|nb|KK920447.1|	214903-215208	18	17	gi|641586785|gb|KK854191.1|	228643-229802	11	39
ACYPI000914-RA	ni|645904081|nb|KK920350.1|	273844-275696	13	18	gi|641573954|gb|KK855429.1|	161967-167857	10	42
ACYPI000923-RA	ni|645904139|nb|KK920294.1|	273293-273530	15	18	gi|641570630|gb|KK856298.1|	150400-151002	12	47
ACYPI000928-RA	ni|645903879|nb|KK920552.1|	1212873-1213647	18	18	gi|641575794|gb|KK855043.1|	214321-214891	10	36
ACYPI000929-RA	ni|645903971|nb|KK920460.1|	1606345-1606702	19	18	gi|641572134|gb|KK855890.1|	56721-57783	10	36

ACYPI000938-RA	ni|645904168|nb|KK920265.1|	1905788-1906395	16	16	gi|641570086|gb|KK856464.1|	107527-107930	10	35
ACYPI000941-RA	ni|645904067|nb|KK920364.1|	829093-829550	17	14	gi|641570036|gb|KK856480.1|	65780-66033	8.3	33
ACYPI000942-RA	ni|645904065|nb|KK920366.1|	856978-857405	18	18	gi|641573160|gb|KK855629.1|	90920-91311	11	38
ACYPI000944-RA	ni|645903919|nb|KK920512.1|	1245222-1245590	17	15	gi|641577487|gb|KK854746.1|	128558-131647	11	38
ACYPI000946-RA	ni|645904192|nb|KK920255.1|	1153192-1153392	15	16	gi|641567069|gb|KK857435.1|	25387-25599	6.8	28
ACYPI000948-RA	ni|645904054|nb|KK920377.1|	838087-839585	16	16	gi|641586313|gb|KK854259.1|	176119-176709	12	42
ACYPI000953-RA	ni|645904246|nb|KK920233.1|	1237740-1237975	16	17	gi|641586915|gb|KK854171.1|	324778-325722	10	38
ACYPI000955-RA	ni|645904005|nb|KK920426.1|	657821-658181	17	16	gi|641570514|gb|KK856331.1|	101197-107313	9.8	39
ACYPI000961-RA	ni|645903746|nb|KK920684.1|	834812-835345	15	15	gi|641569727|gb|KK856579.1|	46367-46981	8.3	34
ACYPI000969-RA	ni|645904127|nb|KK920306.1|	343546-343910	15	15	gi|641571055|gb|KK856172.1|	109181-114601	10	38
ACYPI000979-RA	ni|645903748|nb|KK920682.1|	527271-527526	17	18	gi|641586066|gb|KK854296.1|	239720-241473	9.4	37
ACYPI000980-RA	ni|645903597|nb|KK920833.1|	440500-441096	nan	nan	gi|641575236|gb|KK855151.1|	247019-247483	11	38
ACYPI000992-RA	ni|645904130|nb|KK920303.1|	737556-738080	18	17	gi|641578046|gb|KK854650.1|	262357-262925	10	38
ACYPI000994-RA	ni|645903740|nb|KK920690.1|	354203-355126	18	10	gi|641569808|gb|KK856554.1|	81474-85696	11	21
ACYPI000998-RA	ni|645904116|nb|KK920315.1|	878343-881037	19	18	gi|641586928|gb|KK854169.1|	660534-667228	11	39
ACYPI001001-RA	ni|645903882|nb|KK920549.1|	670516-671469	16	16	gi|641585900|gb|KK854322.1|	221865-223064	12	43
ACYPI001003-RA	ni|645904268|nb|KK920225.1|	305124-305324	14	15	gi|641570045|gb|KK856477.1|	101916-102138	9.6	43
ACYPI001007-RA	ni|645904097|nb|KK920334.1|	243359-243893	15	17	gi|641569779|gb|KK856563.1|	57403-57893	11	40
ACYPI001010-RA	ni|645903592|nb|KK920838.1|	928972-929106	nan	nan	gi|641586509|gb|KK854231.1|	117779-118102	9.8	37
ACYPI001012-RA	ni|645901733|nb|KK922696.1|	16211-18451	nan	nan	gi|641572860|gb|KK855703.1|	118167-121546	9.8	36
ACYPI001015-RA	ni|645904004|nb|KK920427.1|	1136898-1137804	16	16	gi|641584305|gb|KK854491.1|	155524-155995	11	39
ACYPI001018-RA	ni|645904092|nb|KK920339.1|	77546-78078	15	15	gi|641586613|gb|KK854216.1|	117156-120306	11	41
ACYPI001019-RA	ni|645903673|nb|KK920757.1|	304883-309521	nan	nan	gi|641587735|gb|KK854066.1|	213810-215396	11	42
ACYPI001024-RA	ni|645903915|nb|KK920516.1|	1437030-1437551	17	17	gi|641587562|gb|KK854088.1|	542807-544057	11	42
ACYPI001025-RA	ni|645904051|nb|KK920380.1|	503984-507038	16	17	gi|641575140|gb|KK855171.1|	325434-328817	9.7	40
ACYPI001030-RA	ni|645903934|nb|KK920497.1|	244198-244576	15	16	gi|641572615|gb|KK855769.1|	190159-190813	12	43
ACYPI001031-RA	ni|645904246|nb|KK920233.1|	1110042-1110497	16	17	gi|641586915|gb|KK854171.1|	497438-498263	10	38
ACYPI001032-RA	ni|645903644|nb|KK920786.1|	737405-737898	nan	nan	gi|641576036|gb|KK854996.1|	68613-74077	11	40
ACYPI001039-RA	ni|645904281|nb|KK920220.1|	1013060-1013322	15	16	gi|641577289|gb|KK854776.1|	280040-281931	10	36
ACYPI001043-RA	ni|645902354|nb|KK922075.1|	45603-45806	nan	nan	gi|641588246|gb|KK854010.1|	4429-4733	9.2	50
ACYPI001044-RA	ni|645903515|nb|KK920915.1|	733824-734944	nan	nan	gi|641582401|gb|KK854541.1|	146554-147241	11	43
ACYPI001046-RA	ni|645903837|nb|KK920593.1|	861735-861955	18	9.8	gi|641585720|gb|KK854351.1|	243217-243447	10	39

ACYPI001047-RA	ni|645904116|nb|KK920315.1|	466094-466760	19	18	gi|641580504|gb|KK854565.1|	394640-398081	11	43
ACYPI001051-RA	ni|645904013|nb|KK920418.1|	1161200-1161599	19	18	gi|641572207|gb|KK855871.1|	63082-65006	10	39
ACYPI001052-RA	ni|645903511|nb|KK920919.1|	163280-163457	nan	nan	gi|641588164|gb|KK854017.1|	280218-290107	9.2	40
ACYPI001056-RA	ni|645903884|nb|KK920547.1|	400219-400459	16	19	gi|641587624|gb|KK854080.1|	393388-393753	10	38
ACYPI001057-RA	ni|645903644|nb|KK920786.1|	188924-189137	nan	nan	gi|641578144|gb|KK854633.1|	112495-112721	10	43
ACYPI001059-RA	ni|645904015|nb|KK920416.1|	187496-193131	18	16	gi|641586139|gb|KK854284.1|	524856-530442	10	21
ACYPI001061-RA	ni|645904241|nb|KK920238.1|	154535-154849	18	17	gi|641567860|gb|KK857164.1|	1418-3170	11	36
ACYPI001071-RA	ni|645903749|nb|KK920681.1|	180990-181603	19	18	gi|641584205|gb|KK854500.1|	96025-96514	11	41
ACYPI001081-RA	ni|645903557|nb|KK920873.1|	1071122-1071677	nan	nan	gi|641586313|gb|KK854259.1|	459530-459750	12	42
ACYPI001085-RA	ni|645904118|nb|KK920313.1|	733371-734277	17	17	gi|641577121|gb|KK854804.1|	18496-18745	16	82
ACYPI001090-RA	ni|645904153|nb|KK920280.1|	1494710-1495060	17	16	gi|641571716|gb|KK855999.1|	59771-60818	11	39
ACYPI001096-RA	ni|645903597|nb|KK920833.1|	929086-929543	nan	nan	gi|641587652|gb|KK854077.1|	500085-502402	10	38
ACYPI001100-RA	ni|645904141|nb|KK920292.1|	609955-610514	14	14	gi|641572390|gb|KK855826.1|	75048-75352	10	35
ACYPI001107-RA	ni|645903903|nb|KK920528.1|	308925-309243	12	15	gi|641585957|gb|KK854313.1|	365494-371807	10	37
ACYPI001108-RA	ni|645903812|nb|KK920618.1|	740357-740592	20	18	gi|641586734|gb|KK854198.1|	528200-529074	11	40
ACYPI001110-RA	ni|645904231|nb|KK920242.1|	456319-456444	16	17	gi|641572364|gb|KK855831.1|	22282-22895	11	38
ACYPI001111-RA	ni|645903936|nb|KK920495.1|	478645-478975	19	18	gi|641570921|gb|KK856214.1|	106021-107147	8.7	39
ACYPI001113-RA	ni|645903530|nb|KK920900.1|	523489-523650	nan	nan	gi|641576842|gb|KK854849.1|	143610-144454	12	38
ACYPI001119-RA	ni|645902639|nb|KK921790.1|	162983-163480	nan	nan	gi|641565021|gb|KK858191.1|	4-3849	10	36
ACYPI001124-RA	ni|645904133|nb|KK920300.1|	379734-380311	18	18	gi|641587074|gb|KK854148.1|	185784-191231	11	41
ACYPI001125-RA	ni|645901914|nb|KK922515.1|	54583-55440	nan	nan	gi|641574416|gb|KK855326.1|	212242-213675	11	38
ACYPI001129-RA	ni|645904159|nb|KK920274.1|	240197-240382	15	17	gi|641576392|gb|KK854928.1|	227290-228776	9.7	41
ACYPI001132-RA	ni|645904096|nb|KK920335.1|	1556835-1557239	18	17	gi|641573933|gb|KK855435.1|	280566-281641	10	40
ACYPI001145-RA	ni|645903662|nb|KK920768.1|	400678-400881	nan	nan	gi|641570861|gb|KK856232.1|	38470-42415	11	39
ACYPI001146-RA	ni|645903800|nb|KK920630.1|	1043669-1044647	18	17	gi|641573143|gb|KK855633.1|	115511-115831	9.9	36
ACYPI001153-RA	ni|645902718|nb|KK921711.1|	126947-127213	nan	nan	gi|641587614|gb|KK854081.1|	704557-704930	11	40
ACYPI001163-RA	ni|645904258|nb|KK920229.1|	1479260-1479645	16	16	gi|641586741|gb|KK854197.1|	409709-410214	11	43
ACYPI001164-RA	ni|645903921|nb|KK920510.1|	389127-389769	16	11	gi|641572824|gb|KK855713.1|	68390-69648	9.8	19
ACYPI001166-RA	ni|645903841|nb|KK920589.1|	1018358-1019195	17	17	gi|641586380|gb|KK854250.1|	144189-144519	9.3	38
ACYPI001167-RA	ni|645904137|nb|KK920296.1|	412930-414139	17	17	gi|641576645|gb|KK854885.1|	337967-340574	10	40
ACYPI001168-RA	ni|645903825|nb|KK920605.1|	1008582-1009330	19	17	gi|641586535|gb|KK854227.1|	356025-357839	10	39
ACYPI001182-RA	ni|645903716|nb|KK920714.1|	905157-905464	nan	nan	gi|641583980|gb|KK854512.1|	409592-409864	11	39

ACYPI001189-RA	ni|645904038|nb|KK920393.1|	489586-489742	17	19	gi|641587800|gb|KK854058.1|	980034-980295	10	42
ACYPI001193-RA	ni|645904028|nb|KK920403.1|	502305-502707	18	17	gi|641588109|gb|KK854023.1|	588844-589503	10	40
ACYPI001194-RA	ni|645904022|nb|KK920409.1|	635284-636006	18	20	gi|641585587|gb|KK854373.1|	351503-352162	9.8	41
ACYPI001198-RA	ni|645904022|nb|KK920409.1|	18476-18737	18	20	gi|641586857|gb|KK854181.1|	335115-335566	9.8	39
ACYPI001204-RA	ni|645903885|nb|KK920546.1|	913844-914087	19	12	gi|641586880|gb|KK854177.1|	248010-248868	10	20
ACYPI001206-RA	ni|645903680|nb|KK920750.1|	1007743-1008436	nan	nan	gi|641588358|gb|KK854002.1|	2226595-2231646	11	43
ACYPI001210-RA	ni|645903543|nb|KK920887.1|	553406-553699	nan	nan	gi|641573881|gb|KK855448.1|	101484-101661	9	33
ACYPI001212-RA	ni|645904281|nb|KK920220.1|	992431-993452	15	16	gi|641577289|gb|KK854776.1|	303573-304486	10	36
ACYPI001217-RA	ni|645903780|nb|KK920650.1|	756372-757114	19	19	gi|641585801|gb|KK854337.1|	449458-451053	10	38
ACYPI001218-RA	ni|645904240|nb|KK920239.1|	1775246-1777473	16	16	gi|641588294|gb|KK854006.1|	620000-626234	8.7	40
ACYPI001220-RA	ni|645903635|nb|KK920795.1|	410452-411276	nan	nan	gi|641571276|gb|KK856113.1|	89299-90175	9.9	34
ACYPI001225-RA	ni|645904231|nb|KK920242.1|	90786-93848	16	17	gi|641587671|gb|KK854074.1|	423271-426435	11	40
ACYPI001229-RA	ni|645904068|nb|KK920363.1|	916406-916620	16	8.6	gi|641569291|gb|KK856713.1|	67388-67721	10	21
ACYPI001233-RA	ni|645902060|nb|KK922369.1|	9729-11162	nan	nan	gi|641585303|gb|KK854420.1|	215501-217983	10	36
ACYPI001234-RA	ni|645903688|nb|KK920742.1|	1100679-1102360	nan	nan	gi|641586785|gb|KK854191.1|	369899-372369	11	39
ACYPI001243-RA	ni|645903473|nb|KK920957.1|	540287-540462	nan	nan	gi|641586489|gb|KK854234.1|	263847-266178	9.5	39
ACYPI001244-RA	ni|645903717|nb|KK920713.1|	112334-112905	nan	nan	gi|641587962|gb|KK854039.1|	899263-900278	11	40
ACYPI001246-RA	ni|645903679|nb|KK920751.1|	545156-546016	nan	nan	gi|641587569|gb|KK854087.1|	675505-675882	11	41
ACYPI001254-RA	ni|645904119|nb|KK920312.1|	486359-487030	19	19	gi|641555492|gb|KK862524.1|	5719-8268	8.6	36
ACYPI001255-RA	ni|645901950|nb|KK922479.1|	128801-129479	nan	nan	gi|641577667|gb|KK854713.1|	102310-102984	8.8	36
ACYPI001257-RA	ni|645904153|nb|KK920280.1|	3205368-3205986	17	16	gi|641573102|gb|KK855643.1|	53475-53715	10	37
ACYPI001258-RA	ni|645903713|nb|KK920717.1|	425786-426297	nan	nan	gi|641565722|gb|KK857924.1|	41797-42237	9.8	31
ACYPI001266-RA	ni|645904077|nb|KK920354.1|	1521562-1521763	16	17	gi|641588060|gb|KK854028.1|	137691-139650	11	42
ACYPI001268-RA	ni|645904156|nb|KK920277.1|	1363820-1364044	17	8.8	gi|641575001|gb|KK855197.1|	169599-171873	11	38
ACYPI001270-RA	ni|645903962|nb|KK920469.1|	550079-550587	17	9.8	gi|641587133|gb|KK854140.1|	670792-675164	9.9	37
ACYPI001274-RA	ni|645903824|nb|KK920606.1|	54539-55657	19	17	gi|641586990|gb|KK854161.1|	281920-282717	11	39
ACYPI001277-RA	ni|645902313|nb|KK922116.1|	169757-170835	nan	nan	gi|641579212|gb|KK854585.1|	203196-203844	10	35
ACYPI001279-RA	ni|645904177|nb|KK920260.1|	986799-987029	18	17	gi|641574923|gb|KK855216.1|	86554-86801	11	38
ACYPI001285-RA	ni|645903898|nb|KK920533.1|	489653-490437	17	16	gi|641572431|gb|KK855815.1|	95127-96397	9.1	35
ACYPI001286-RA	ni|645904014|nb|KK920417.1|	1442671-1442982	19	18	gi|641583921|gb|KK854515.1|	284803-286368	11	38
ACYPI001292-RA	ni|645903731|nb|KK920699.1|	1221498-1222392	nan	nan	gi|641587881|gb|KK854049.1|	27088-31073	10	40
ACYPI001295-RA	ni|645903496|nb|KK920934.1|	847339-847712	nan	nan	gi|641573394|gb|KK855572.1|	5926-6682	11	40

ACYPI001296-RA	ni|645903827|nb|KK920603.1|	233378-235221	17	18	gi|641575888|gb|KK855026.1|	188622-193373	10	41
ACYPI001299-RA	ni|645904195|nb|KK920254.1|	2280921-2281400	18	17	gi|641584658|gb|KK854465.1|	355920-357635	11	37
ACYPI001303-RA	ni|645904183|nb|KK920258.1|	873941-877047	25	35	gi|641565608|gb|KK857969.1|	16589-17626	13	42
ACYPI001310-RA	ni|645901903|nb|KK922526.1|	48739-49992	nan	nan	gi|641570861|gb|KK856232.1|	77985-79476	11	39
ACYPI001312-RA	ni|645902035|nb|KK922394.1|	208791-209270	nan	nan	gi|641585773|gb|KK854342.1|	395091-396466	12	45
ACYPI001313-RA	ni|645904274|nb|KK920223.1|	576154-576576	14	17	gi|641566967|gb|KK857471.1|	74329-74758	9	39
ACYPI001316-RA	ni|645904011|nb|KK920420.1|	171865-172077	14	16	gi|641585246|gb|KK854426.1|	230696-231694	10	39
ACYPI001324-RA	ni|645904107|nb|KK920324.1|	763473-764537	17	9.5	gi|641564334|gb|KK858483.1|	3716-4056	5.2	18
ACYPI001326-RA	ni|645903627|nb|KK920803.1|	174437-174606	nan	nan	gi|641567496|gb|KK857287.1|	79327-80221	8.3	28
ACYPI001328-RA	ni|645898594|nb|KK925835.1|	2869-3716	nan	nan	gi|641574022|gb|KK855414.1|	148193-148454	11	33
ACYPI001329-RA	ni|645903727|nb|KK920703.1|	260071-260482	nan	nan	gi|641571380|gb|KK856086.1|	131443-131675	9.5	38
ACYPI001336-RA	ni|645901664|nb|KK922765.1|	52325-53615	nan	nan	gi|641587024|gb|KK854155.1|	311680-312467	10	21
ACYPI001339-RA	ni|645904118|nb|KK920313.1|	759127-759934	17	17	gi|641580980|gb|KK854561.1|	256415-256586	11	37
ACYPI001342-RA	ni|645904166|nb|KK920267.1|	3136802-3137034	18	16	gi|641586569|gb|KK854222.1|	39754-40061	12	40
ACYPI001352-RA	ni|645902016|nb|KK922413.1|	79165-79360	nan	nan	gi|641586344|gb|KK854254.1|	156976-157525	10	40
ACYPI001353-RA	ni|645904034|nb|KK920397.1|	361875-363220	15	8.4	gi|641570788|gb|KK856253.1|	85502-86815	10	20
ACYPI001359-RA	ni|645903654|nb|KK920776.1|	1200-2169	nan	nan	gi|641586793|gb|KK854190.1|	642794-644506	9.3	41
ACYPI001360-RA	ni|645904071|nb|KK920360.1|	730063-731046	17	17	gi|641587263|gb|KK854123.1|	273627-277642	11	42
ACYPI001379-RA	ni|645903922|nb|KK920509.1|	463448-463682	15	16	gi|641573714|gb|KK855492.1|	116037-119904	10	37
ACYPI001382-RA	ni|645903808|nb|KK920622.1|	617187-617566	17	17	gi|641588258|gb|KK854009.1|	333986-335333	11	36
ACYPI001387-RA	ni|645903901|nb|KK920530.1|	683242-684526	17	17	gi|641572671|gb|KK855754.1|	150090-151719	10	38
ACYPI001392-RA	ni|645904074|nb|KK920357.1|	230139-230496	17	18	gi|641571123|gb|KK856155.1|	98055-99345	10	42
ACYPI001396-RA	ni|645904258|nb|KK920229.1|	1280567-1281094	16	16	gi|641568713|gb|KK856890.1|	77631-78976	12	42
ACYPI001403-RA	ni|645904166|nb|KK920267.1|	3049215-3049420	18	16	gi|641585527|gb|KK854383.1|	404681-405615	11	39
ACYPI001408-RA	ni|645903637|nb|KK920793.1|	685066-685738	nan	nan	gi|641577161|gb|KK854797.1|	342023-346942	10	38
ACYPI001410-RA	ni|645904005|nb|KK920426.1|	356339-356776	17	16	gi|641570593|gb|KK856309.1|	109685-109851	9.6	33
ACYPI001415-RA	ni|645904268|nb|KK920225.1|	985511-986014	14	15	gi|641584419|gb|KK854483.1|	402477-402844	10	42
ACYPI001416-RA	ni|645903762|nb|KK920668.1|	130978-131264	17	17	gi|641577644|gb|KK854718.1|	211459-211772	10	40
ACYPI001417-RA	ni|645903788|nb|KK920642.1|	326794-326973	16	15	gi|641585905|gb|KK854321.1|	491332-492325	11	39
ACYPI001421-RA	ni|645904186|nb|KK920257.1|	697929-698216	15	16	gi|641570295|gb|KK856397.1|	126433-126944	10	37
ACYPI001424-RA	ni|645904174|nb|KK920261.1|	1852450-1852684	19	17	gi|641568831|gb|KK856853.1|	42898-43841	10	30
ACYPI001430-RA	ni|645904017|nb|KK920414.1|	95643-96303	17	17	gi|641585819|gb|KK854334.1|	407671-408118	9.3	38

ACYPI001437-RA	ni|645903716|nb|KK920714.1|	894373-894735	nan	nan	gi|641583980|gb|KK854512.1|	375360-376158	11	39
ACYPI001438-RA	ni|645902035|nb|KK922394.1|	47759-47998	nan	nan	gi|641586850|gb|KK854182.1|	32283-32999	10	37
ACYPI001440-RA	ni|645903607|nb|KK920823.1|	314259-315418	nan	nan	gi|641587580|gb|KK854085.1|	646702-650598	10	39
ACYPI001446-RA	ni|645903909|nb|KK920522.1|	247175-247502	16	8.3	gi|641586452|gb|KK854239.1|	260803-261614	10	19
ACYPI001453-RA	ni|645903787|nb|KK920643.1|	489918-490969	19	18	gi|641575222|gb|KK855154.1|	36899-39260	11	37
ACYPI001458-RA	ni|645904150|nb|KK920283.1|	697879-699399	17	17	gi|641571548|gb|KK856039.1|	20292-20656	9.9	18
ACYPI001460-RA	ni|645902696|nb|KK921733.1|	5333-5930	nan	nan	gi|641577574|gb|KK854731.1|	341242-341687	11	38
ACYPI001461-RA	ni|645904149|nb|KK920284.1|	216961-217463	16	16	gi|641566611|gb|KK857597.1|	46972-47584	11	40
ACYPI001462-RA	ni|645904159|nb|KK920274.1|	1051715-1052406	15	17	gi|641572022|gb|KK855920.1|	124118-125042	12	42
ACYPI001463-RA	ni|645903727|nb|KK920703.1|	1101176-1101700	nan	nan	gi|641586928|gb|KK854169.1|	437164-438228	11	39
ACYPI001465-RA	ni|645903899|nb|KK920532.1|	443478-444036	15	16	gi|641585303|gb|KK854420.1|	321490-324590	10	36
ACYPI001475-RA	ni|645903834|nb|KK920596.1|	127573-128058	18	17	gi|641576876|gb|KK854844.1|	402018-402751	17	42
ACYPI001480-RA	ni|645904262|nb|KK920227.1|	2432336-2433221	17	17	gi|641572891|gb|KK855695.1|	255552-256017	10	36
ACYPI001481-RA	ni|645902642|nb|KK921787.1|	194127-194696	nan	nan	gi|641587624|gb|KK854080.1|	584479-585285	10	38
ACYPI001483-RA	ni|645904115|nb|KK920316.1|	1772375-1773141	17	18	gi|641587624|gb|KK854080.1|	733099-733374	10	38
ACYPI001487-RA	ni|645904254|nb|KK920230.1|	478767-478925	16	16	gi|641586235|gb|KK854270.1|	295118-295288	11	39
ACYPI001501-RA	ni|645903562|nb|KK920868.1|	392843-393532	nan	nan	gi|641573752|gb|KK855482.1|	106493-106988	11	37
ACYPI001502-RA	ni|645903863|nb|KK920567.1|	155734-156144	15	9.2	gi|641573452|gb|KK855556.1|	138398-138644	10	22
ACYPI001508-RA	ni|645903890|nb|KK920541.1|	58112-58820	17	18	gi|641573457|gb|KK855555.1|	173169-174250	9.7	38
ACYPI001511-RA	ni|645902373|nb|KK922056.1|	194351-196978	nan	nan	gi|641586399|gb|KK854247.1|	263675-267785	15	68
ACYPI001512-RA	ni|645904057|nb|KK920374.1|	56065-56342	16	15	gi|641567299|gb|KK857356.1|	65474-65940	11	38
ACYPI001515-RA	ni|645904114|nb|KK920317.1|	1637996-1639227	18	18	gi|641571667|gb|KK856008.1|	160769-161522	11	39
ACYPI001522-RA	ni|645903703|nb|KK920727.1|	329219-329707	nan	nan	gi|641577299|gb|KK854775.1|	415118-415670	11	37
ACYPI001527-RA	ni|645901875|nb|KK922554.1|	41241-41460	nan	nan	gi|641573097|gb|KK855644.1|	184014-184562	10	36
ACYPI001532-RA	ni|645903773|nb|KK920657.1|	1509079-1509276	18	17	gi|641575759|gb|KK855050.1|	232587-235558	11	38
ACYPI001538-RA	ni|645904014|nb|KK920417.1|	479278-480177	19	18	gi|641568710|gb|KK856891.1|	15841-16729	10	38
ACYPI001539-RA	ni|645904090|nb|KK920341.1|	254208-255472	15	17	gi|641588128|gb|KK854021.1|	4084-4769	9.5	42
ACYPI001540-RA	ni|645902671|nb|KK921758.1|	70902-71368	nan	nan	gi|641587468|gb|KK854099.1|	253433-254478	10	42
ACYPI001542-RA	ni|645903635|nb|KK920795.1|	432848-433167	nan	nan	gi|641568386|gb|KK856994.1|	12772-14857	8.9	32
ACYPI001547-RA	ni|645903787|nb|KK920643.1|	476251-476586	19	18	gi|641571315|gb|KK856104.1|	475-639	12	46
ACYPI001560-RA	ni|645904053|nb|KK920378.1|	1114687-1116998	18	17	gi|641574190|gb|KK855376.1|	177804-180071	11	40
ACYPI001567-RA	ni|645903973|nb|KK920458.1|	1592954-1594013	20	18	gi|641587047|gb|KK854151.1|	555059-558798	10	39

ACYPI001575-RA	ni|645903857|nb|KK920573.1|	251012-251425	16	16	gi|641573699|gb|KK855496.1|	124324-124856	12	39
ACYPI001578-RA	ni|645904262|nb|KK920227.1|	337824-338405	17	17	gi|641548848|gb|KK865654.1|	1792-3245	7.4	20
ACYPI001579-RA	ni|645900333|nb|KK924096.1|	21715-21953	nan	nan	gi|641586203|gb|KK854273.1|	145471-145719	9.5	40
ACYPI001583-RA	ni|645903836|nb|KK920594.1|	260909-261504	18	11	gi|641587962|gb|KK854039.1|	938760-943835	11	40
ACYPI001584-RA	ni|645903626|nb|KK920804.1|	421339-422422	nan	nan	gi|641586405|gb|KK854246.1|	305967-310196	10	40
ACYPI001585-RA	ni|645904225|nb|KK920244.1|	638078-638523	17	17	gi|641574100|gb|KK855396.1|	75220-76082	11	38
ACYPI001591-RA	ni|645901190|nb|KK923239.1|	15775-15957	nan	nan	gi|641586096|gb|KK854291.1|	498061-498270	11	46
ACYPI001593-RA	ni|645904153|nb|KK920280.1|	1495836-1496308	17	16	gi|641571716|gb|KK855999.1|	78612-79014	11	39
ACYPI001596-RA	ni|645903557|nb|KK920873.1|	862467-862681	nan	nan	gi|641572114|gb|KK855896.1|	145460-146074	10	36
ACYPI001597-RA	ni|645901319|nb|KK923110.1|	127511-129267	nan	nan	gi|641586133|gb|KK854285.1|	607011-607796	11	41
ACYPI001600-RA	ni|645902338|nb|KK922091.1|	105712-105924	nan	nan	gi|641586734|gb|KK854198.1|	594106-594894	11	40
ACYPI001601-RA	ni|645902659|nb|KK921770.1|	163553-163945	nan	nan	gi|641586661|gb|KK854209.1|	369308-369889	11	43
ACYPI001604-RA	ni|645904177|nb|KK920260.1|	1149866-1150800	18	17	gi|641584931|gb|KK854446.1|	416029-418726	10	39
ACYPI001611-RA	ni|645902717|nb|KK921712.1|	10272-10529	nan	nan	gi|641586445|gb|KK854240.1|	551295-552056	9.2	39
ACYPI001612-RA	ni|645903551|nb|KK920879.1|	459721-460030	nan	nan	gi|641573850|gb|KK855457.1|	65140-65548	9.3	34
ACYPI001613-RA	ni|645903965|nb|KK920466.1|	1075595-1075976	17	16	gi|641578106|gb|KK854639.1|	236679-244025	11	39
ACYPI001614-RA	ni|645904023|nb|KK920408.1|	656810-657813	17	17	gi|641575693|gb|KK855062.1|	141566-143534	11	40
ACYPI001617-RA	ni|645902779|nb|KK921650.1|	135035-135804	nan	nan	gi|641584806|gb|KK854455.1|	452916-453656	11	43
ACYPI001621-RA	ni|645903598|nb|KK920832.1|	300080-303128	nan	nan	gi|641585628|gb|KK854366.1|	39131-42825	8.4	39
ACYPI001622-RA	ni|645903655|nb|KK920775.1|	217072-217635	nan	nan	gi|641570589|gb|KK856310.1|	30308-31328	11	36
ACYPI001625-RA	ni|645904042|nb|KK920389.1|	389991-393796	16	16	gi|641567243|gb|KK857377.1|	61436-65043	11	43
ACYPI001631-RA	ni|645904121|nb|KK920310.1|	769101-769364	14	15	gi|641575381|gb|KK855122.1|	262263-262727	11	43
ACYPI001633-RA	ni|645904072|nb|KK920359.1|	954035-954609	17	17	gi|641570419|gb|KK856360.1|	100337-100863	12	39
ACYPI001635-RA	ni|645903970|nb|KK920461.1|	877939-879028	17	16	gi|641585642|gb|KK854364.1|	85844-88073	9.9	38
ACYPI001639-RA	ni|645904242|nb|KK920237.1|	637512-637960	15	16	gi|641587606|gb|KK854082.1|	421322-422096	11	40
ACYPI001643-RA	ni|645903994|nb|KK920437.1|	232683-233854	15	20	gi|641571138|gb|KK856151.1|	182010-183579	10	45
ACYPI001646-RA	ni|645903687|nb|KK920743.1|	43586-44176	nan	nan	gi|641577688|gb|KK854709.1|	184207-184535	11	40
ACYPI001649-RA	ni|645904058|nb|KK920373.1|	1047806-1048780	16	15	gi|641587660|gb|KK854076.1|	84683-86709	9.9	41
ACYPI001652-RA	ni|645904158|nb|KK920275.1|	1166500-1166931	15	8.3	gi|641574320|gb|KK855349.1|	40474-47592	10	38
ACYPI001663-RA	ni|645904246|nb|KK920233.1|	224451-226144	16	17	gi|641586040|gb|KK854300.1|	559476-562612	10	38
ACYPI001665-RA	ni|645903466|nb|KK920964.1|	694000-694399	nan	nan	gi|641586700|gb|KK854203.1|	122837-123566	8.4	39
ACYPI001667-RA	ni|645904014|nb|KK920417.1|	1330327-1330589	19	18	gi|641570579|gb|KK856313.1|	129453-131170	11	38

ACYPI001668-RA	ni|645903847|nb|KK920583.1|	645752-646613	18	18	gi|641588358|gb|KK854002.1|	1190216-1190790	11	43
ACYPI001672-RA	ni|645904245|nb|KK920234.1|	1316654-1317159	16	17	gi|641578193|gb|KK854625.1|	286028-286363	11	41
ACYPI001674-RA	ni|645903849|nb|KK920581.1|	317182-317666	17	17	gi|641585940|gb|KK854316.1|	519805-521340	11	42
ACYPI001675-RA	ni|645903783|nb|KK920647.1|	1524293-1525615	17	17	gi|641577341|gb|KK854768.1|	114486-116177	14	58
ACYPI001679-RA	ni|645904048|nb|KK920383.1|	628165-628713	14	14	gi|641585395|gb|KK854405.1|	462091-463540	10	38
ACYPI001682-RA	ni|645903983|nb|KK920448.1|	806015-807096	18	17	gi|641586809|gb|KK854188.1|	629575-633270	10	43
ACYPI001683-RA	ni|645903704|nb|KK920726.1|	404991-405503	nan	nan	gi|641574876|gb|KK855226.1|	121912-122231	11	39
ACYPI001686-RA	ni|645904125|nb|KK920307.1|	1973333-1973838	18	17	gi|641587193|gb|KK854132.1|	658260-659028	11	39
ACYPI001692-RA	ni|645903655|nb|KK920775.1|	267027-267260	nan	nan	gi|641570589|gb|KK856310.1|	98336-100355	11	36
ACYPI001696-RA	ni|645903975|nb|KK920456.1|	1049735-1051325	15	8.2	gi|641574844|gb|KK855232.1|	230115-232697	10	23
ACYPI001698-RA	ni|645903727|nb|KK920703.1|	242818-243047	nan	nan	gi|641587881|gb|KK854049.1|	613046-614244	10	40
ACYPI001700-RA	ni|645904065|nb|KK920366.1|	940193-941009	18	18	gi|641586843|gb|KK854183.1|	897355-897594	11	41
ACYPI001704-RA	ni|645904271|nb|KK920224.1|	467005-467470	15	16	gi|641587577|gb|KK854086.1|	646483-646726	8.2	41
ACYPI001706-RA	ni|645904241|nb|KK920238.1|	1387883-1388465	18	17	gi|641577037|gb|KK854817.1|	162792-163124	9.9	37
ACYPI001710-RA	ni|645903763|nb|KK920667.1|	69903-70148	26	21	gi|641577913|gb|KK854671.1|	196722-196969	9.2	40
ACYPI001711-RA	ni|645904122|nb|KK920309.1|	1337616-1338574	18	17	gi|641577821|gb|KK854688.1|	75498-76479	7.8	37
ACYPI001717-RA	ni|645904228|nb|KK920243.1|	710294-712388	17	18	gi|641571956|gb|KK855938.1|	97132-97371	11	42
ACYPI001724-RA	ni|645903569|nb|KK920861.1|	219433-219752	nan	nan	gi|641586185|gb|KK854275.1|	190285-190754	12	40
ACYPI001730-RA	ni|645903885|nb|KK920546.1|	845631-846212	19	12	gi|641569612|gb|KK856617.1|	65037-65691	11	21
ACYPI001732-RA	ni|645903688|nb|KK920742.1|	217204-218425	nan	nan	gi|641577837|gb|KK854685.1|	1-3389	11	42
ACYPI001736-RA	ni|645903578|nb|KK920852.1|	203808-205276	nan	nan	gi|641573595|gb|KK855520.1|	30042-32216	11	40
ACYPI001738-RA	ni|645903771|nb|KK920659.1|	756263-756454	16	15	gi|641571508|gb|KK856050.1|	102438-106221	9.7	33
ACYPI001740-RA	ni|645903768|nb|KK920662.1|	188788-189038	18	17	gi|641587703|gb|KK854070.1|	782433-783299	9.8	42
ACYPI001742-RA	ni|645903698|nb|KK920732.1|	158467-158767	nan	nan	gi|641576347|gb|KK854934.1|	85516-86354	11	39
ACYPI001745-RA	ni|645904046|nb|KK920385.1|	1050279-1050433	17	8.4	gi|641588202|gb|KK854014.1|	718695-720197	10	21
ACYPI001746-RA	ni|645903803|nb|KK920627.1|	887915-889665	17	17	gi|641586535|gb|KK854227.1|	781024-783240	10	39
ACYPI001755-RA	ni|645903702|nb|KK920728.1|	421398-422211	nan	nan	gi|641588051|gb|KK854029.1|	539859-541775	9.6	40
ACYPI001756-RA	ni|645902394|nb|KK922035.1|	300175-301057	nan	nan	gi|641575112|gb|KK855177.1|	107235-107511	11	39
ACYPI001759-RA	ni|645902468|nb|KK921961.1|	70634-70897	nan	nan	gi|641585340|gb|KK854414.1|	13-298	11	42
ACYPI001760-RA	ni|645904279|nb|KK920221.1|	303514-303727	16	16	gi|641570727|gb|KK856270.1|	141147-141732	10	35
ACYPI001762-RA	ni|645903780|nb|KK920650.1|	579350-580736	19	19	gi|641587444|gb|KK854102.1|	487794-492941	11	42
ACYPI001764-RA	ni|645903969|nb|KK920462.1|	266128-266480	14	16	gi|641577121|gb|KK854804.1|	143708-143902	16	82

ACYPI001765-RA	ni|645903957|nb|KK920474.1|	1047815-1048656	18	17	gi|641575389|gb|KK855120.1|	227264-227650	10	35
ACYPI001767-RA	ni|645904201|nb|KK920252.1|	141257-142165	17	16	gi|641570819|gb|KK856245.1|	140618-141193	10	45
ACYPI001769-RA	ni|645903627|nb|KK920803.1|	365807-366954	nan	nan	gi|641558279|gb|KK861194.1|	2820-3124	9.5	35
ACYPI001771-RA	ni|645903919|nb|KK920512.1|	1260224-1260382	17	15	gi|641577487|gb|KK854746.1|	174085-174929	11	38
ACYPI001777-RA	ni|645903650|nb|KK920780.1|	174435-174913	nan	nan	gi|641571315|gb|KK856104.1|	178506-179629	12	46
ACYPI001780-RA	ni|645904195|nb|KK920254.1|	370779-371520	18	17	gi|641587912|gb|KK854045.1|	372319-378128	11	39
ACYPI001782-RA	ni|645904088|nb|KK920343.1|	1280653-1280952	17	18	gi|641572484|gb|KK855802.1|	168265-168518	9.2	36
ACYPI001796-RA	ni|645903557|nb|KK920873.1|	1093184-1094901	nan	nan	gi|641586335|gb|KK854256.1|	322243-324860	10	38
ACYPI001798-RA	ni|645904052|nb|KK920379.1|	522385-524539	18	16	gi|641574061|gb|KK855405.1|	63200-63437	11	21
ACYPI001804-RA	ni|645904106|nb|KK920325.1|	774579-776900	17	17	gi|641579225|gb|KK854583.1|	393013-395261	10	39
ACYPI001807-RA	ni|645904053|nb|KK920378.1|	1862598-1863287	18	17	gi|641587765|gb|KK854062.1|	759427-760835	10	41
ACYPI001810-RA	ni|645903858|nb|KK920572.1|	415604-416129	16	16	gi|641578046|gb|KK854650.1|	6593-7555	10	38
ACYPI001812-RA	ni|645903860|nb|KK920570.1|	206810-207053	17	17	gi|641580479|gb|KK854569.1|	136686-137928	10	38
ACYPI001813-RA	ni|645902756|nb|KK921673.1|	36146-36726	nan	nan	gi|641571279|gb|KK856112.1|	185563-185872	9.8	36
ACYPI001818-RA	ni|645904045|nb|KK920386.1|	829472-829874	19	21	gi|641575867|gb|KK855030.1|	45058-48539	11	39
ACYPI001823-RA	ni|645904049|nb|KK920382.1|	509393-510076	18	18	gi|641554601|gb|KK862942.1|	1-1817	10	28
ACYPI001827-RA	ni|645904195|nb|KK920254.1|	673514-673800	18	17	gi|641569469|gb|KK856662.1|	60834-63671	9.1	34
ACYPI001832-RA	ni|645903716|nb|KK920714.1|	373150-373390	nan	nan	gi|641571950|gb|KK855940.1|	12920-13177	10	37
ACYPI001838-RA	ni|645903866|nb|KK920564.1|	353378-354905	18	15	gi|641570648|gb|KK856293.1|	56063-57010	10	21
ACYPI001846-RA	ni|645903853|nb|KK920577.1|	677892-678142	16	16	gi|641574654|gb|KK855271.1|	46877-47337	9.5	36
ACYPI001849-RA	ni|645904149|nb|KK920284.1|	462643-463120	16	16	gi|641575482|gb|KK855102.1|	226615-227569	11	39
ACYPI001850-RA	ni|645903921|nb|KK920510.1|	579588-579948	16	11	gi|641585622|gb|KK854367.1|	355084-355299	9.9	21
ACYPI001852-RA	ni|645904279|nb|KK920221.1|	1518632-1519343	16	16	gi|641585581|gb|KK854374.1|	36494-37351	10	38
ACYPI001856-RA	ni|645904271|nb|KK920224.1|	2044972-2045379	15	16	gi|641567104|gb|KK857424.1|	34328-35248	8.5	38
ACYPI001864-RA	ni|645902463|nb|KK921966.1|	60976-61400	nan	nan	gi|641588267|gb|KK854008.1|	56074-59831	10	40
ACYPI001866-RA	ni|645903900|nb|KK920531.1|	112508-112981	20	30	gi|641586161|gb|KK854280.1|	334992-335313	11	39
ACYPI001871-RA	ni|645903644|nb|KK920786.1|	170154-170877	nan	nan	gi|641575372|gb|KK855124.1|	141122-141949	9.5	36
ACYPI001872-RA	ni|645902238|nb|KK922191.1|	50345-50581	nan	nan	gi|641579947|gb|KK854574.1|	141043-145053	11	39
ACYPI001877-RA	ni|645904177|nb|KK920260.1|	1634882-1635268	18	17	gi|641568433|gb|KK856978.1|	13215-13417	9.8	35
ACYPI001881-RA	ni|645903825|nb|KK920605.1|	1070899-1071156	19	17	gi|641588033|gb|KK854031.1|	354851-355738	11	38
ACYPI001885-RA	ni|645904130|nb|KK920303.1|	1432601-1433229	18	17	gi|641570453|gb|KK856351.1|	89554-91937	9.6	36
ACYPI001886-RA	ni|645904086|nb|KK920345.1|	374776-374980	14	15	gi|641584862|gb|KK854451.1|	239457-239709	10	38

ACYPI001894-RA	ni|645902052|nb|KK922377.1|	4879-6196	nan	nan	gi|641578283|gb|KK854609.1|	101019-103567	12	22
ACYPI001898-RA	ni|645903954|nb|KK920477.1|	238354-238779	35	44	gi|641576962|gb|KK854829.1|	258359-258833	10	39
ACYPI001901-RA	ni|645903780|nb|KK920650.1|	472506-473049	19	19	gi|641585219|gb|KK854428.1|	234339-234736	11	41
ACYPI001902-RA	ni|645903610|nb|KK920820.1|	535344-535755	nan	nan	gi|641588325|gb|KK854004.1|	1422982-1428310	11	40
ACYPI001906-RA	ni|645904130|nb|KK920303.1|	364199-364409	18	17	gi|641574778|gb|KK855246.1|	127737-128035	9.7	32
ACYPI001907-RA	ni|645903821|nb|KK920609.1|	759290-762141	17	17	gi|641587856|gb|KK854051.1|	799821-806413	10	41
ACYPI001909-RA	ni|645904177|nb|KK920260.1|	1151321-1151755	18	17	gi|641584931|gb|KK854446.1|	419315-423333	10	39
ACYPI001917-RA	ni|645904153|nb|KK920280.1|	3194921-3195137	17	16	gi|641573102|gb|KK855643.1|	47756-47991	10	37
ACYPI001931-RA	ni|645903938|nb|KK920493.1|	532281-532615	19	18	gi|641574266|gb|KK855361.1|	220391-221769	11	38
ACYPI001932-RA	ni|645903591|nb|KK920839.1|	164055-165291	nan	nan	gi|641587577|gb|KK854086.1|	177759-179416	8.2	41
ACYPI001933-RA	ni|645903466|nb|KK920964.1|	261928-262349	nan	nan	gi|641586144|gb|KK854283.1|	436872-437232	10	38
ACYPI001934-RA	ni|645903796|nb|KK920634.1|	784931-786308	18	17	gi|641588099|gb|KK854024.1|	709090-709814	11	39
ACYPI001939-RA	ni|645904106|nb|KK920325.1|	927454-929067	17	17	gi|641575112|gb|KK855177.1|	202976-203277	11	39
ACYPI001943-RA	ni|645903567|nb|KK920863.1|	342222-342440	nan	nan	gi|641586046|gb|KK854299.1|	149693-151090	10	20
ACYPI001945-RA	ni|645903644|nb|KK920786.1|	164963-165304	nan	nan	gi|641575372|gb|KK855124.1|	133743-133907	9.5	36
ACYPI001946-RA	ni|645904156|nb|KK920277.1|	768602-769384	17	8.8	gi|641574796|gb|KK855242.1|	122404-122589	10	42
ACYPI001948-RA	ni|645903855|nb|KK920575.1|	1063778-1065702	16	17	gi|641574508|gb|KK855304.1|	58269-62299	11	22
ACYPI001957-RA	ni|645904067|nb|KK920364.1|	240108-240759	17	14	gi|641587652|gb|KK854077.1|	29390-30777	10	38
ACYPI001960-RA	ni|645903713|nb|KK920717.1|	521722-523395	nan	nan	gi|641576791|gb|KK854858.1|	221176-223422	9.2	42
ACYPI001964-RA	ni|645903925|nb|KK920506.1|	1320396-1320670	19	17	gi|641573984|gb|KK855422.1|	197789-199568	9.5	36
ACYPI001971-RA	ni|645904144|nb|KK920289.1|	915192-916007	16	9	gi|641584336|gb|KK854489.1|	160220-162495	10	40
ACYPI001975-RA	ni|645903854|nb|KK920576.1|	378827-379480	16	8.4	gi|641577430|gb|KK854752.1|	66785-67237	9.7	21
ACYPI001978-RA	ni|645902219|nb|KK922210.1|	189865-191931	nan	nan	gi|641585971|gb|KK854310.1|	487537-492226	10	40
ACYPI001982-RA	ni|645903918|nb|KK920513.1|	303681-304599	15	18	gi|641587504|gb|KK854095.1|	710686-711505	11	42
ACYPI001984-RA	ni|645903598|nb|KK920832.1|	341205-341773	nan	nan	gi|641585628|gb|KK854366.1|	113589-114063	8.4	39
ACYPI001995-RA	ni|645904027|nb|KK920404.1|	611613-612299	17	17	gi|641586476|gb|KK854236.1|	551513-552631	10	37
ACYPI002006-RA	ni|645903570|nb|KK920860.1|	23627-24169	nan	nan	gi|641586096|gb|KK854291.1|	48952-59308	11	46
ACYPI002008-RA	ni|645903957|nb|KK920474.1|	1104615-1105173	18	17	gi|641570807|gb|KK856248.1|	126584-128732	11	38
ACYPI002009-RA	ni|645903747|nb|KK920683.1|	1337315-1338364	19	18	gi|641587660|gb|KK854076.1|	582187-582477	9.9	41
ACYPI002010-RA	ni|645903530|nb|KK920900.1|	410134-412502	nan	nan	gi|641587606|gb|KK854082.1|	723613-727161	11	40
ACYPI002023-RA	ni|645903887|nb|KK920544.1|	95258-96858	15	15	gi|641578109|gb|KK854638.1|	129917-131678	14	46
ACYPI002029-RA	ni|645904231|nb|KK920242.1|	154505-155068	16	17	gi|641577704|gb|KK854707.1|	131700-132030	10	38

ACYPI002031-RA	ni|645904201|nb|KK920252.1|	384449-385682	17	16	gi|641584248|gb|KK854496.1|	387530-392950	9.9	39
ACYPI002036-RA	ni|645904204|nb|KK920251.1|	590620-590963	15	16	gi|641588109|gb|KK854023.1|	46746-47033	10	40
ACYPI002040-RA	ni|645904245|nb|KK920234.1|	507990-508183	16	17	gi|641577704|gb|KK854707.1|	309973-312437	10	38
ACYPI002041-RA	ni|645904107|nb|KK920324.1|	952646-953072	17	9.5	gi|641575606|gb|KK855079.1|	166553-168521	9.7	40
ACYPI002045-RA	ni|645904228|nb|KK920243.1|	1269442-1270971	17	18	gi|641573171|gb|KK855626.1|	172175-173913	9.9	40
ACYPI002053-RA	ni|645904160|nb|KK920273.1|	2149462-2149864	18	18	gi|641574746|gb|KK855253.1|	51029-51395	8.9	35
ACYPI002057-RA	ni|645904133|nb|KK920300.1|	2564518-2565376	18	18	gi|641570703|gb|KK856277.1|	80609-81848	9.9	36
ACYPI002063-RA	ni|645903885|nb|KK920546.1|	104521-104759	19	12	gi|641586059|gb|KK854297.1|	8732-9791	11	22
ACYPI002072-RA	ni|645903617|nb|KK920813.1|	178440-179694	nan	nan	gi|641567321|gb|KK857348.1|	83512-86810	19	43
ACYPI002075-RA	ni|645903911|nb|KK920520.1|	143165-143371	16	17	gi|641588073|gb|KK854027.1|	541585-542238	11	41
ACYPI002084-RA	ni|645902715|nb|KK921714.1|	154324-154501	nan	nan	gi|641571045|gb|KK856175.1|	20192-20509	9.3	35
ACYPI002085-RA	ni|645903783|nb|KK920647.1|	1185366-1185682	17	17	gi|641573447|gb|KK855557.1|	130621-130914	10	41
ACYPI002088-RA	ni|645904219|nb|KK920246.1|	342533-342770	16	17	gi|641587555|gb|KK854089.1|	904242-904931	11	39
ACYPI002090-RA	ni|645903750|nb|KK920680.1|	486226-486751	14	14	gi|641585103|gb|KK854436.1|	220753-221263	9.8	39
ACYPI002092-RA	ni|645903780|nb|KK920650.1|	175459-177094	19	19	gi|641578406|gb|KK854594.1|	334714-336517	10	37
ACYPI002098-RA	ni|645904246|nb|KK920233.1|	911766-912520	16	17	gi|641587783|gb|KK854060.1|	755591-757203	12	40
ACYPI002108-RA	ni|645903637|nb|KK920793.1|	540911-541464	nan	nan	gi|641579960|gb|KK854572.1|	217862-218262	11	40
ACYPI002110-RA	ni|645903634|nb|KK920796.1|	1217326-1217924	nan	nan	gi|641586344|gb|KK854254.1|	531825-532943	10	40
ACYPI002114-RA	ni|645903944|nb|KK920487.1|	216854-217378	15	17	gi|641586648|gb|KK854211.1|	289852-290193	11	29
ACYPI002115-RA	ni|645903768|nb|KK920662.1|	1276766-1277523	18	17	gi|641571581|gb|KK856030.1|	199916-200333	10	38
ACYPI002118-RA	ni|645904076|nb|KK920355.1|	456636-456861	17	17	gi|641587118|gb|KK854142.1|	526922-527972	11	40
ACYPI002122-RA	ni|645896935|nb|KK927494.1|	97-939	nan	nan	gi|641578325|gb|KK854602.1|	224959-228435	9.7	38
ACYPI002123-RA	ni|645904068|nb|KK920363.1|	287474-288119	16	8.6	gi|641569294|gb|KK856712.1|	32021-32253	12	22
ACYPI002125-RA	ni|645903977|nb|KK920454.1|	264481-265550	13	16	gi|641571778|gb|KK855982.1|	86940-89717	11	43
ACYPI002126-RA	ni|645903551|nb|KK920879.1|	486163-486583	nan	nan	gi|641573850|gb|KK855457.1|	27538-27780	9.3	34
ACYPI002129-RA	ni|645903869|nb|KK920561.1|	444223-445565	17	15	gi|641573673|gb|KK855501.1|	127225-131973	14	43
ACYPI002132-RA	ni|645904021|nb|KK920410.1|	753525-753751	15	16	gi|641574800|gb|KK855241.1|	134771-135213	10	37
ACYPI002133-RA	ni|645903953|nb|KK920478.1|	1611738-1613208	18	17	gi|641588258|gb|KK854009.1|	573053-577471	11	36
ACYPI002134-RA	ni|645904138|nb|KK920295.1|	653486-653745	15	15	gi|641576266|gb|KK854949.1|	198830-199097	11	45
ACYPI002136-RA	ni|645904098|nb|KK920333.1|	1244288-1244519	16	8.8	gi|641551804|gb|KK864267.1|	21-577	9.4	19
ACYPI002137-RA	ni|645903920|nb|KK920511.1|	931872-932434	20	20	gi|641578188|gb|KK854626.1|	233875-234121	9.9	39
ACYPI002140-RA	ni|645902758|nb|KK921671.1|	229334-229536	nan	nan	gi|641566693|gb|KK857568.1|	43804-44746	8.5	33

ACYPI002143-RA	ni|645902469|nb|KK921960.1|	269878-270236	nan	nan	gi|641566606|gb|KK857599.1|	50100-50497	10	40
ACYPI002147-RA	ni|645903613|nb|KK920817.1|	489615-490018	nan	nan	gi|641588136|gb|KK854020.1|	159940-160750	11	36
ACYPI002154-RA	ni|645903825|nb|KK920605.1|	1057642-1058639	19	17	gi|641574127|gb|KK855390.1|	145750-147385	10	40
ACYPI002162-RA	ni|645903491|nb|KK920939.1|	597383-597625	nan	nan	gi|641577810|gb|KK854690.1|	445973-446254	9.8	39
ACYPI002171-RA	ni|645904043|nb|KK920388.1|	835475-835772	16	9.1	gi|641572875|gb|KK855699.1|	168420-168702	14	37
ACYPI002179-RA	ni|645903466|nb|KK920964.1|	287340-288682	nan	nan	gi|641586824|gb|KK854186.1|	78363-81899	10	41
ACYPI002180-RA	ni|645904116|nb|KK920315.1|	677401-679525	19	18	gi|641586085|gb|KK854293.1|	110059-112331	11	40
ACYPI002199-RA	ni|645903936|nb|KK920495.1|	707231-707756	19	18	gi|641586470|gb|KK854237.1|	65502-66236	11	42
ACYPI002204-RA	ni|645903717|nb|KK920713.1|	42725-43374	nan	nan	gi|641587962|gb|KK854039.1|	799337-799719	11	40
ACYPI002207-RA	ni|645903546|nb|KK920884.1|	617805-619089	nan	nan	gi|641585860|gb|KK854329.1|	450517-451101	11	43
ACYPI002210-RA	ni|645903720|nb|KK920710.1|	331001-331671	nan	nan	gi|641577843|gb|KK854684.1|	157252-158319	10	40
ACYPI002214-RA	ni|645903965|nb|KK920466.1|	226244-226569	17	16	gi|641576661|gb|KK854882.1|	43498-45593	9.7	38
ACYPI002220-RA	ni|645902756|nb|KK921673.1|	274233-274678	nan	nan	gi|641573291|gb|KK855599.1|	83359-88306	9	36
ACYPI002221-RA	ni|645904204|nb|KK920251.1|	617206-618300	15	16	gi|641575759|gb|KK855050.1|	309492-311149	11	38
ACYPI002226-RA	ni|645903984|nb|KK920447.1|	18973-20135	18	17	gi|641587671|gb|KK854074.1|	637482-638819	11	40
ACYPI002228-RA	ni|645903625|nb|KK920805.1|	139707-140389	nan	nan	gi|641576635|gb|KK854887.1|	370804-371232	11	40
ACYPI002229-RA	ni|645902438|nb|KK921991.1|	168681-169039	nan	nan	gi|641577879|gb|KK854677.1|	108117-108675	18	36
ACYPI002237-RA	ni|645901808|nb|KK922621.1|	169276-169783	nan	nan	gi|641575454|gb|KK855107.1|	65826-66043	10	38
ACYPI002245-RA	ni|645904088|nb|KK920343.1|	1235452-1236597	17	18	gi|641587452|gb|KK854101.1|	907055-911734	11	40
ACYPI002246-RA	ni|645903742|nb|KK920688.1|	536200-537907	17	16	gi|641586482|gb|KK854235.1|	606376-608934	11	41
ACYPI002247-RA	ni|645902172|nb|KK922257.1|	165026-165677	nan	nan	gi|641587066|gb|KK854149.1|	557889-558485	11	40
ACYPI002250-RA	ni|645904065|nb|KK920366.1|	1184436-1184704	18	18	gi|641586535|gb|KK854227.1|	710833-711880	10	39
ACYPI002255-RA	ni|645904015|nb|KK920416.1|	1231196-1233296	18	16	gi|641566509|gb|KK857636.1|	55555-60703	11	40
ACYPI002256-RA	ni|645903837|nb|KK920593.1|	390502-390731	18	9.8	gi|641587905|gb|KK854046.1|	131767-132011	10	43
ACYPI002267-RA	ni|645903523|nb|KK920907.1|	209214-209723	nan	nan	gi|641585407|gb|KK854403.1|	362730-363535	9.4	42
ACYPI002276-RA	ni|645904053|nb|KK920378.1|	172377-172852	18	17	gi|641567358|gb|KK857335.1|	43905-44187	10	36
ACYPI002277-RA	ni|645904132|nb|KK920301.1|	129061-129423	16	18	gi|641575910|gb|KK855021.1|	334264-334789	10	35
ACYPI002286-RA	ni|645904157|nb|KK920276.1|	992108-994731	15	15	gi|641575730|gb|KK855054.1|	33871-36374	10	38
ACYPI002287-RA	ni|645904118|nb|KK920313.1|	897231-898115	17	17	gi|641581789|gb|KK854549.1|	164122-165413	9.9	37
ACYPI002289-RA	ni|645903566|nb|KK920864.1|	668231-668439	nan	nan	gi|641572501|gb|KK855798.1|	19130-19898	9.8	39
ACYPI002292-RA	ni|645903779|nb|KK920651.1|	604892-605170	18	16	gi|641572663|gb|KK855756.1|	101017-101788	8.8	38
ACYPI002296-RA	ni|645903944|nb|KK920487.1|	132787-133451	15	17	gi|641586809|gb|KK854188.1|	719109-720930	10	43

ACYPI002300-RA	ni|645902463|nb|KK921966.1|	262165-262901	nan	nan	gi|641574000|gb|KK855418.1|	122043-122918	10	36
ACYPI002301-RA	ni|645903796|nb|KK920634.1|	942702-943875	18	17	gi|641577837|gb|KK854685.1|	140266-141222	11	42
ACYPI002304-RA	ni|645903532|nb|KK920898.1|	268766-269396	nan	nan	gi|641574370|gb|KK855338.1|	45406-46646	11	38
ACYPI002306-RA	ni|645904058|nb|KK920373.1|	614672-615628	16	15	gi|641588236|gb|KK854011.1|	290015-292668	9.7	38
ACYPI002324-RA	ni|645904265|nb|KK920226.1|	1381277-1381567	14	14	gi|641584168|gb|KK854503.1|	289552-289862	10	39
ACYPI002330-RA	ni|645904078|nb|KK920353.1|	393590-393862	17	17	gi|641578300|gb|KK854606.1|	466526-466877	11	40
ACYPI002331-RA	ni|645901697|nb|KK922732.1|	73272-73756	nan	nan	gi|641586066|gb|KK854296.1|	419206-419503	9.4	37
ACYPI002332-RA	ni|645903804|nb|KK920626.1|	143434-143796	17	17	gi|641574015|gb|KK855416.1|	62453-64837	10	42
ACYPI002333-RA	ni|645902420|nb|KK922009.1|	93122-93831	nan	nan	gi|641564728|gb|KK858311.1|	15637-16597	11	43
ACYPI002342-RA	ni|645903724|nb|KK920706.1|	151621-153446	nan	nan	gi|641543394|gb|KK868292.1|	46-1963	14	56
ACYPI002346-RA	ni|645903914|nb|KK920517.1|	110775-110977	15	17	gi|641569577|gb|KK856628.1|	54946-58205	9.7	36
ACYPI002350-RA	ni|645903983|nb|KK920448.1|	1024961-1025313	18	17	gi|641585953|gb|KK854314.1|	337257-337903	11	43
ACYPI002351-RA	ni|645903738|nb|KK920692.1|	1023137-1023627	18	17	gi|641575549|gb|KK855090.1|	168413-169535	10	38
ACYPI002352-RA	ni|645899094|nb|KK925335.1|	7387-8369	nan	nan	gi|641570717|gb|KK856273.1|	82511-84071	10	36
ACYPI002361-RA	ni|645903703|nb|KK920727.1|	464445-464826	nan	nan	gi|641586661|gb|KK854209.1|	542012-542355	11	43
ACYPI002363-RA	ni|645904262|nb|KK920227.1|	731503-731757	17	17	gi|641572471|gb|KK855805.1|	4440-4895	13	54
ACYPI002367-RA	ni|645903619|nb|KK920811.1|	779382-779518	nan	nan	gi|641586606|gb|KK854217.1|	373368-377954	11	42
ACYPI002370-RA	ni|645904262|nb|KK920227.1|	757744-757990	17	17	gi|641576315|gb|KK854940.1|	118566-118867	9.9	40
ACYPI002371-RA	ni|645903780|nb|KK920650.1|	594562-594738	19	19	gi|641585953|gb|KK854314.1|	466140-466639	11	43
ACYPI002372-RA	ni|645903779|nb|KK920651.1|	368336-369036	18	16	gi|641586025|gb|KK854302.1|	108939-110759	8.5	33
ACYPI002373-RA	ni|645904155|nb|KK920278.1|	870870-871470	18	9.4	gi|641585971|gb|KK854310.1|	165545-166065	10	40
ACYPI002382-RA	ni|645904049|nb|KK920382.1|	518290-520008	18	18	gi|641586975|gb|KK854163.1|	193402-195937	11	40
ACYPI002383-RA	ni|645904177|nb|KK920260.1|	2156654-2157402	18	17	gi|641577837|gb|KK854685.1|	435056-435918	11	42
ACYPI002404-RA	ni|645903597|nb|KK920833.1|	755945-756646	nan	nan	gi|641575236|gb|KK855151.1|	365826-371792	11	38
ACYPI002405-RA	ni|645903964|nb|KK920467.1|	637409-637559	15	8	gi|641570598|gb|KK856307.1|	53749-55137	11	21
ACYPI002407-RA	ni|645903768|nb|KK920662.1|	810851-811358	18	17	gi|641587703|gb|KK854070.1|	291221-291558	9.8	42
ACYPI002411-RA	ni|645904052|nb|KK920379.1|	512757-513063	18	16	gi|641572569|gb|KK855781.1|	89141-89669	11	39
ACYPI002414-RA	ni|645903965|nb|KK920466.1|	1684303-1684756	17	16	gi|641587962|gb|KK854039.1|	1140500-1140989	11	40
ACYPI002433-RA	ni|645903768|nb|KK920662.1|	1317363-1317940	18	17	gi|641577215|gb|KK854787.1|	126190-127217	11	40
ACYPI002445-RA	ni|645904262|nb|KK920227.1|	2516351-2516636	17	17	gi|641572891|gb|KK855695.1|	187955-188980	10	36
ACYPI002446-RA	ni|645904004|nb|KK920427.1|	1246202-1246407	16	16	gi|641584305|gb|KK854491.1|	74908-75107	11	39
ACYPI002448-RA	ni|645903524|nb|KK920906.1|	445143-445539	nan	nan	gi|641578336|gb|KK854600.1|	139042-139994	10	21

ACYPI002460-RA	ni|645904166|nb|KK920267.1|	965375-966678	18	16	gi|641588267|gb|KK854008.1|	610513-612092	10	40
ACYPI002463-RA	ni|645903674|nb|KK920756.1|	560964-561467	nan	nan	gi|641576672|gb|KK854880.1|	179748-180099	9.4	40
ACYPI002469-RA	ni|645902497|nb|KK921932.1|	135733-136152	nan	nan	gi|641581789|gb|KK854549.1|	158038-159096	9.9	37
ACYPI002470-RA	ni|645903879|nb|KK920552.1|	1708908-1709530	18	18	gi|641548005|gb|KK866068.1|	4-704	9.8	29
ACYPI002477-RA	ni|645904118|nb|KK920313.1|	1620303-1621083	17	17	gi|641578167|gb|KK854629.1|	252113-252572	11	41
ACYPI002478-RA	ni|645903867|nb|KK920563.1|	325156-326335	18	18	gi|641569892|gb|KK856527.1|	115717-117174	9.5	20
ACYPI002479-RA	ni|645903920|nb|KK920511.1|	755730-756172	20	20	gi|641573447|gb|KK855557.1|	256045-256264	10	41
ACYPI002480-RA	ni|645903780|nb|KK920650.1|	251973-252387	19	19	gi|641575396|gb|KK855118.1|	163763-164723	9	35
ACYPI002482-RA	ni|645904102|nb|KK920329.1|	416843-417423	15	16	gi|641568172|gb|KK857061.1|	74471-75829	11	40
ACYPI002483-RA	ni|645904225|nb|KK920244.1|	647324-648019	17	17	gi|641576371|gb|KK854931.1|	107823-109660	10	38
ACYPI002485-RA	ni|645904201|nb|KK920252.1|	437267-437522	17	16	gi|641570587|gb|KK856311.1|	45567-46026	9.9	38
ACYPI002491-RA	ni|645903794|nb|KK920636.1|	416041-416238	25	34	gi|641586928|gb|KK854169.1|	315058-320740	11	39
ACYPI002497-RA	ni|645904281|nb|KK920220.1|	1628644-1628950	15	16	gi|641577482|gb|KK854747.1|	149442-150650	10	39
ACYPI002506-RA	ni|645903858|nb|KK920572.1|	440371-440559	16	16	gi|641587186|gb|KK854133.1|	441470-442956	8.1	39
ACYPI002517-RA	ni|645903774|nb|KK920656.1|	460176-460352	16	9.2	gi|641586452|gb|KK854239.1|	426678-427305	10	19
ACYPI002524-RA	ni|645902078|nb|KK922351.1|	50868-51074	nan	nan	gi|641570745|gb|KK856264.1|	12187-12463	9.3	18
ACYPI002526-RA	ni|645903890|nb|KK920541.1|	1013869-1016550	17	18	gi|641571092|gb|KK856162.1|	64503-67034	11	47
ACYPI002527-RA	ni|645903698|nb|KK920732.1|	142370-143258	nan	nan	gi|641582439|gb|KK854536.1|	109026-110642	11	36
ACYPI002530-RA	ni|645904057|nb|KK920374.1|	192814-197812	16	15	gi|641588073|gb|KK854027.1|	832591-839359	11	41
ACYPI002533-RA	ni|645903512|nb|KK920918.1|	419909-420112	nan	nan	gi|641572971|gb|KK855675.1|	185833-186008	9.7	20
ACYPI002536-RA	ni|645903604|nb|KK920826.1|	121822-122270	nan	nan	gi|641570016|gb|KK856486.1|	44031-44627	12	20
ACYPI002537-RA	ni|645903871|nb|KK920559.1|	204887-205386	18	9.4	gi|641577766|gb|KK854698.1|	24598-26758	9.6	28
ACYPI002538-RA	ni|645904278|nb|KK920222.1|	481380-482153	15	16	gi|641584694|gb|KK854463.1|	130845-133454	9.3	36
ACYPI002547-RA	ni|645903714|nb|KK920716.1|	271011-271906	nan	nan	gi|641570271|gb|KK856405.1|	24050-24944	9.8	38
ACYPI002549-RA	ni|645903577|nb|KK920853.1|	594400-594644	nan	nan	gi|641569328|gb|KK856702.1|	97741-98120	9.6	17
ACYPI002557-RA	ni|645904130|nb|KK920303.1|	2292377-2292691	18	17	gi|641564032|gb|KK858612.1|	30385-31160	10	35
ACYPI002558-RA	ni|645903973|nb|KK920458.1|	1403221-1403443	20	18	gi|641587047|gb|KK854151.1|	335150-336728	10	39
ACYPI002565-RA	ni|645904072|nb|KK920359.1|	1042977-1043231	17	17	gi|641578056|gb|KK854648.1|	464859-465312	11	41
ACYPI002575-RA	ni|645904278|nb|KK920222.1|	1554186-1554452	15	16	gi|641585778|gb|KK854341.1|	154907-155205	9.7	41
ACYPI002580-RA	ni|645902673|nb|KK921756.1|	141570-142041	nan	nan	gi|641588224|gb|KK854012.1|	349291-350387	10	41
ACYPI002584-RA	ni|645904077|nb|KK920354.1|	1490297-1490968	16	17	gi|641588060|gb|KK854028.1|	119261-121195	11	42
ACYPI002587-RA	ni|645904166|nb|KK920267.1|	2706517-2707286	18	16	gi|641569400|gb|KK856682.1|	110544-112090	9.7	34

ACYPI002588-RA	ni|645903800|nb|KK920630.1|	1324161-1325000	18	17	gi|641570596|gb|KK856308.1|	40425-41451	9.9	39
ACYPI002591-RA	ni|645904168|nb|KK920265.1|	1339397-1339804	16	16	gi|641576679|gb|KK854878.1|	157410-158047	9.9	38
ACYPI002592-RA	ni|645903650|nb|KK920780.1|	794327-794821	nan	nan	gi|641588082|gb|KK854026.1|	1158045-1162963	11	41
ACYPI002593-RA	ni|645903751|nb|KK920679.1|	817889-818038	17	16	gi|641574085|gb|KK855399.1|	212843-213938	10	38
ACYPI002595-RA	ni|645903923|nb|KK920508.1|	1117897-1118761	18	18	gi|641588099|gb|KK854024.1|	270490-272616	11	39
ACYPI002596-RA	ni|645901830|nb|KK922599.1|	36884-37080	nan	nan	gi|641576028|gb|KK854998.1|	235243-235654	11	21
ACYPI002598-RA	ni|645904248|nb|KK920232.1|	1113977-1114766	15	16	gi|641571116|gb|KK856157.1|	45423-45827	11	40
ACYPI002599-RA	ni|645903699|nb|KK920731.1|	823030-825201	nan	nan	gi|641577341|gb|KK854768.1|	428760-431403	14	58
ACYPI002612-RA	ni|645904281|nb|KK920220.1|	827541-827926	15	16	gi|641587660|gb|KK854076.1|	251715-252351	9.9	41
ACYPI002617-RA	ni|645903688|nb|KK920742.1|	1109903-1110303	nan	nan	gi|641586171|gb|KK854278.1|	550701-551299	9	39
ACYPI002618-RA	ni|645904198|nb|KK920253.1|	367334-367933	11	14	gi|641572809|gb|KK855717.1|	148455-150535	8.9	33
ACYPI002620-RA	ni|645901057|nb|KK923372.1|	22671-23217	nan	nan	gi|641585690|gb|KK854356.1|	352539-352939	9.6	41
ACYPI002621-RA	ni|645903780|nb|KK920650.1|	599152-601193	19	19	gi|641585953|gb|KK854314.1|	475676-478210	11	43
ACYPI002622-RA	ni|645903866|nb|KK920564.1|	374109-374600	18	15	gi|641573906|gb|KK855442.1|	34478-35829	11	39
ACYPI002624-RA	ni|645904035|nb|KK920396.1|	641155-641400	16	16	gi|641586121|gb|KK854287.1|	11548-12191	11	43
ACYPI002632-RA	ni|645903768|nb|KK920662.1|	1187307-1187692	18	17	gi|641584835|gb|KK854453.1|	238627-239249	8.3	36
ACYPI002636-RA	ni|645903650|nb|KK920780.1|	1007873-1008356	nan	nan	gi|641586114|gb|KK854288.1|	365430-366260	10	41
ACYPI002650-RA	ni|645904021|nb|KK920410.1|	598172-598882	15	16	gi|641570459|gb|KK856349.1|	25114-26078	9.7	40
ACYPI002653-RA	ni|645901808|nb|KK922621.1|	145364-146379	nan	nan	gi|641571270|gb|KK856115.1|	162152-162527	11	38
ACYPI002656-RA	ni|645903538|nb|KK920892.1|	54690-55240	nan	nan	gi|641573950|gb|KK855430.1|	123007-123322	10	19
ACYPI002657-RA	ni|645904171|nb|KK920262.1|	732525-733189	15	16	gi|641584818|gb|KK854454.1|	146906-149054	11	42
ACYPI002662-RA	ni|645903774|nb|KK920656.1|	526446-526707	16	9.2	gi|641587840|gb|KK854053.1|	178836-179852	11	43
ACYPI002673-RA	ni|645903705|nb|KK920725.1|	449863-450660	nan	nan	gi|641575743|gb|KK855052.1|	165629-168226	9.7	38
ACYPI002674-RA	ni|645904037|nb|KK920394.1|	1132586-1133153	18	17	gi|641575218|gb|KK855155.1|	85984-86668	11	21
ACYPI002676-RA	ni|645904103|nb|KK920328.1|	116161-116455	15	19	gi|641576699|gb|KK854874.1|	298102-298818	8.5	42
ACYPI002678-RA	ni|645901760|nb|KK922669.1|	93073-94496	nan	nan	gi|641584913|gb|KK854447.1|	63574-68626	10	40
ACYPI002680-RA	ni|645903812|nb|KK920618.1|	550461-551001	20	18	gi|641566502|gb|KK857638.1|	33820-37686	11	36
ACYPI002684-RA	ni|645904120|nb|KK920311.1|	282852-283120	18	18	gi|641588213|gb|KK854013.1|	1386491-1387144	9.6	39
ACYPI002689-RA	ni|645904146|nb|KK920287.1|	967584-968143	16	18	gi|641568719|gb|KK856888.1|	91364-91954	11	44
ACYPI002690-RA	ni|645904072|nb|KK920359.1|	1156789-1157004	17	17	gi|641578056|gb|KK854648.1|	344429-344780	11	41
ACYPI002691-RA	ni|645903689|nb|KK920741.1|	39213-41703	nan	nan	gi|641587588|gb|KK854084.1|	596506-599323	9.9	40
ACYPI002692-RA	ni|645903871|nb|KK920559.1|	1075707-1080128	18	9.4	gi|641577922|gb|KK854669.1|	71346-76254	12	21

ACYPI002694-RA	ni|645904170|nb|KK920263.1|	322874-323305	15	16	gi|641584694|gb|KK854463.1|	82732-83252	9.3	36
ACYPI002695-RA	ni|645902460|nb|KK921969.1|	14013-14243	nan	nan	gi|641587930|gb|KK854043.1|	319793-320387	10	41
ACYPI002698-RA	ni|645904274|nb|KK920223.1|	655074-655655	14	17	gi|641573397|gb|KK855571.1|	81962-87577	10	38
ACYPI002711-RA	ni|645903597|nb|KK920833.1|	928664-929004	nan	nan	gi|641587652|gb|KK854077.1|	510488-512305	10	38
ACYPI002715-RA	ni|645903699|nb|KK920731.1|	207825-208680	nan	nan	gi|641587193|gb|KK854132.1|	773824-775107	11	39
ACYPI002722-RA	ni|645903910|nb|KK920521.1|	371370-372329	19	17	gi|641588109|gb|KK854023.1|	334628-336683	10	40
ACYPI002730-RA	ni|645904014|nb|KK920417.1|	1483679-1484832	19	18	gi|641583921|gb|KK854515.1|	196713-197385	11	38
ACYPI002732-RA	ni|645903496|nb|KK920934.1|	335367-335584	nan	nan	gi|641587896|gb|KK854047.1|	1016138-1016437	9.9	39
ACYPI002735-RA	ni|645904022|nb|KK920409.1|	699615-700028	18	20	gi|641585587|gb|KK854373.1|	312360-312591	9.8	41
ACYPI002737-RA	ni|645901565|nb|KK922864.1|	28414-28977	nan	nan	gi|641575047|gb|KK855190.1|	207623-208765	8.3	43
ACYPI002739-RA	ni|645903831|nb|KK920599.1|	1307472-1307731	18	17	gi|641572987|gb|KK855671.1|	145732-146241	10	39
ACYPI002749-RA	ni|645903923|nb|KK920508.1|	227849-228304	18	18	gi|641586741|gb|KK854197.1|	586117-588546	11	43
ACYPI002754-RA	ni|645904119|nb|KK920312.1|	1546830-1550831	19	19	gi|641585434|gb|KK854398.1|	151564-155764	13	51
ACYPI002756-RA	ni|645904125|nb|KK920307.1|	2812631-2812847	18	17	gi|641577075|gb|KK854811.1|	206259-207414	11	36
ACYPI002757-RA	ni|645901710|nb|KK922719.1|	44095-44324	nan	nan	gi|641586399|gb|KK854247.1|	463583-464709	15	68
ACYPI002758-RA	ni|645904265|nb|KK920226.1|	1357564-1358173	14	14	gi|641585647|gb|KK854363.1|	258208-258851	8.8	35
ACYPI002763-RA	ni|645904042|nb|KK920389.1|	104333-104962	16	16	gi|641572390|gb|KK855826.1|	109748-110011	10	35
ACYPI002787-RA	ni|645903724|nb|KK920706.1|	273927-274285	nan	nan	gi|641573571|gb|KK855526.1|	157932-158244	9.8	36
ACYPI002789-RA	ni|645903495|nb|KK920935.1|	471438-471750	nan	nan	gi|641576677|gb|KK854879.1|	55802-57216	11	39
ACYPI002791-RA	ni|645903654|nb|KK920776.1|	646197-646560	nan	nan	gi|641572687|gb|KK855750.1|	42845-43279	9.9	39
ACYPI002792-RA	ni|645901262|nb|KK923167.1|	33437-33676	nan	nan	gi|641588294|gb|KK854006.1|	877789-879358	8.7	40
ACYPI002794-RA	ni|645904281|nb|KK920220.1|	1146197-1146455	15	16	gi|641577890|gb|KK854675.1|	176777-177280	10	40
ACYPI002798-RA	ni|645902083|nb|KK922346.1|	90008-90229	nan	nan	gi|641585833|gb|KK854332.1|	319838-320301	10	37
ACYPI002801-RA	ni|645904138|nb|KK920295.1|	871736-872408	15	15	gi|641575730|gb|KK855054.1|	104007-104683	10	38
ACYPI002806-RA	ni|645903925|nb|KK920506.1|	717164-717635	19	17	gi|641588325|gb|KK854004.1|	993003-993701	11	40
ACYPI002808-RA	ni|645902404|nb|KK922025.1|	41336-42420	nan	nan	gi|641585562|gb|KK854377.1|	403729-405181	12	47
ACYPI002820-RA	ni|645904139|nb|KK920294.1|	237606-238219	15	18	gi|641584433|gb|KK854482.1|	452935-453537	10	40
ACYPI002823-RA	ni|645903504|nb|KK920926.1|	586460-586890	nan	nan	gi|641586393|gb|KK854248.1|	165448-166235	9	39
ACYPI002824-RA	ni|645904166|nb|KK920267.1|	2847874-2848129	18	16	gi|641577075|gb|KK854811.1|	243484-245341	11	36
ACYPI002830-RA	ni|645904159|nb|KK920274.1|	181392-181831	15	17	gi|641586654|gb|KK854210.1|	143155-144913	11	40
ACYPI002835-RA	ni|645904265|nb|KK920226.1|	233761-233968	14	14	gi|641588024|gb|KK854032.1|	480266-482661	9.7	41
ACYPI002837-RA	ni|645903673|nb|KK920757.1|	83429-86295	nan	nan	gi|641571749|gb|KK855991.1|	43843-48288	9.4	35

ACYPI002839-RA	ni|645903476|nb|KK920954.1|	298788-298973	nan	nan	gi|641574123|gb|KK855391.1|	127477-128566	9.9	39
ACYPI002840-RA	ni|645903661|nb|KK920769.1|	281469-281921	nan	nan	gi|641567062|gb|KK857438.1|	35453-36137	9.8	36
ACYPI002841-RA	ni|645904014|nb|KK920417.1|	1267411-1267664	19	18	gi|641570579|gb|KK856313.1|	43225-43878	11	38
ACYPI002842-RA	ni|645903833|nb|KK920597.1|	34353-34959	17	15	gi|641588144|gb|KK854019.1|	1014450-1014679	11	49
ACYPI002846-RA	ni|645904222|nb|KK920245.1|	946504-946672	17	12	gi|641572304|gb|KK855847.1|	137732-137911	11	40
ACYPI002850-RA	ni|645904028|nb|KK920403.1|	1725856-1726547	18	17	gi|641586809|gb|KK854188.1|	610368-610773	10	43
ACYPI002857-RA	ni|645903901|nb|KK920530.1|	479419-480429	17	17	gi|641584606|gb|KK854469.1|	312984-313557	13	57
ACYPI002864-RA	ni|645903821|nb|KK920609.1|	1140419-1140844	17	17	gi|641588090|gb|KK854025.1|	968908-969667	11	43
ACYPI002865-RA	ni|645904135|nb|KK920298.1|	549274-549511	14	15	gi|641575698|gb|KK855061.1|	347810-348045	10	39
ACYPI002866-RA	ni|645903858|nb|KK920572.1|	639028-643519	16	16	gi|641577535|gb|KK854737.1|	164637-168925	11	42
ACYPI002867-RA	ni|645903999|nb|KK920432.1|	506688-507466	17	15	gi|641574312|gb|KK855351.1|	134736-135473	8.9	36
ACYPI002870-RA	ni|645903999|nb|KK920432.1|	531549-532153	17	15	gi|641588073|gb|KK854027.1|	1075964-1076257	11	41
ACYPI002892-RA	ni|645904137|nb|KK920296.1|	1619679-1619992	17	17	gi|641587052|gb|KK854150.1|	336155-341282	11	38
ACYPI002895-RA	ni|645903567|nb|KK920863.1|	14677-14843	nan	nan	gi|641586033|gb|KK854301.1|	448471-449376	11	21
ACYPI002900-RA	ni|645903999|nb|KK920432.1|	1120887-1121762	17	15	gi|641570355|gb|KK856378.1|	62186-63841	9.7	35
ACYPI002902-RA	ni|645903723|nb|KK920707.1|	132203-132412	nan	nan	gi|641578198|gb|KK854624.1|	115877-117586	12	40
ACYPI002904-RA	ni|645903837|nb|KK920593.1|	949706-949945	18	9.8	gi|641575001|gb|KK855197.1|	323997-326642	11	38
ACYPI002907-RA	ni|645903491|nb|KK920939.1|	40510-43295	nan	nan	gi|641575635|gb|KK855074.1|	296164-299267	9.6	38
ACYPI002909-RA	ni|645903543|nb|KK920887.1|	611055-611757	nan	nan	gi|641584128|gb|KK854505.1|	344074-345012	9.6	37
ACYPI002929-RA	ni|645904222|nb|KK920245.1|	415542-416016	17	12	gi|641585395|gb|KK854405.1|	504492-505497	10	38
ACYPI002930-RA	ni|645904159|nb|KK920274.1|	168652-168850	15	17	gi|641586654|gb|KK854210.1|	150611-150947	11	40
ACYPI002938-RA	ni|645903787|nb|KK920643.1|	1196813-1197302	19	18	gi|641572177|gb|KK855878.1|	124730-125775	10	35
ACYPI002940-RA	ni|645903820|nb|KK920610.1|	1143394-1144449	18	19	gi|641567011|gb|KK857455.1|	7380-11731	11	40
ACYPI002948-RA	ni|645904055|nb|KK920376.1|	617728-618095	17	17	gi|641587774|gb|KK854061.1|	763926-764194	9.1	40
ACYPI002949-RA	ni|645904098|nb|KK920333.1|	321544-321965	16	8.8	gi|641577772|gb|KK854697.1|	198093-198437	10	39
ACYPI002950-RA	ni|645903773|nb|KK920657.1|	1078881-1079877	18	17	gi|641575837|gb|KK855035.1|	18536-19049	11	46
ACYPI002951-RA	ni|645903594|nb|KK920836.1|	76411-78155	nan	nan	gi|641575140|gb|KK855171.1|	304579-306334	9.7	40
ACYPI002952-RA	ni|645904008|nb|KK920423.1|	1214544-1215178	16	9.5	gi|641586817|gb|KK854187.1|	607698-610264	9.3	39
ACYPI002953-RA	ni|645903796|nb|KK920634.1|	904946-908161	18	17	gi|641588051|gb|KK854029.1|	801660-805013	9.6	40
ACYPI002958-RA	ni|645904168|nb|KK920265.1|	352625-352925	16	16	gi|641587757|gb|KK854063.1|	556255-556783	11	38
ACYPI002959-RA	ni|645903824|nb|KK920606.1|	1109982-1111715	19	17	gi|641577946|gb|KK854664.1|	359871-362258	10	38
ACYPI002963-RA	ni|645904225|nb|KK920244.1|	629765-630415	17	17	gi|641576371|gb|KK854931.1|	89365-90459	10	38

ACYPI002966-RA	ni|645904070|nb|KK920361.1|	384229-385310	17	16	gi|641573177|gb|KK855625.1|	76394-77382	9.4	34
ACYPI002967-RA	ni|645904036|nb|KK920395.1|	1224530-1224697	21	18	gi|641584884|gb|KK854449.1|	12320-12775	11	39
ACYPI002973-RA	ni|645904052|nb|KK920379.1|	726543-727200	18	16	gi|641587783|gb|KK854060.1|	919533-920086	12	40
ACYPI002975-RA	ni|645904130|nb|KK920303.1|	1384659-1385437	18	17	gi|641588099|gb|KK854024.1|	218443-219563	11	39
ACYPI002979-RA	ni|645903743|nb|KK920687.1|	286000-286328	19	18	gi|641574085|gb|KK855399.1|	126284-127324	10	38
ACYPI002982-RA	ni|645904078|nb|KK920353.1|	1136941-1137323	17	17	gi|641577634|gb|KK854720.1|	79849-80286	9.5	41
ACYPI002986-RA	ni|645903725|nb|KK920705.1|	164986-165419	nan	nan	gi|641586793|gb|KK854190.1|	528114-528792	9.3	41
ACYPI002989-RA	ni|645903946|nb|KK920485.1|	49525-49739	16	14	gi|641587249|gb|KK854125.1|	527730-530900	11	37
ACYPI002998-RA	ni|645901697|nb|KK922732.1|	104483-105068	nan	nan	gi|641586066|gb|KK854296.1|	379544-380222	9.4	37
ACYPI002999-RA	ni|645903812|nb|KK920618.1|	739434-740185	20	18	gi|641578428|gb|KK854590.1|	254133-254427	11	40
ACYPI003002-RA	ni|645903879|nb|KK920552.1|	376531-377201	18	18	gi|641564796|gb|KK858283.1|	4757-4996	10	39
ACYPI003006-RA	ni|645904278|nb|KK920222.1|	1348709-1349001	15	16	gi|641585778|gb|KK854341.1|	257012-257385	9.7	41
ACYPI003015-RA	ni|645903949|nb|KK920482.1|	625335-625653	17	14	gi|641576109|gb|KK854982.1|	251293-251986	10	19
ACYPI003025-RA	ni|645903920|nb|KK920511.1|	1292481-1292778	20	20	gi|641578020|gb|KK854653.1|	231920-232190	10	38
ACYPI003031-RA	ni|645904100|nb|KK920331.1|	283814-284205	18	22	gi|641572022|gb|KK855920.1|	148707-149204	12	42
ACYPI003033-RA	ni|645903747|nb|KK920683.1|	949998-950947	19	18	gi|641569948|gb|KK856508.1|	78420-79986	10	37
ACYPI003035-RA	ni|645903679|nb|KK920751.1|	386971-387601	nan	nan	gi|641586066|gb|KK854296.1|	266109-267407	9.4	37
ACYPI003042-RA	ni|645904242|nb|KK920237.1|	790552-790887	15	16	gi|641566429|gb|KK857664.1|	17082-18381	12	37
ACYPI003043-RA	ni|645903578|nb|KK920852.1|	227415-228057	nan	nan	gi|641568815|gb|KK856858.1|	18640-19270	10	34
ACYPI003044-RA	ni|645904177|nb|KK920260.1|	900620-900803	18	17	gi|641581779|gb|KK854550.1|	372220-378203	11	39
ACYPI003045-RA	ni|645904251|nb|KK920231.1|	586083-586627	16	16	gi|641587614|gb|KK854081.1|	561821-563216	11	40
ACYPI003049-RA	ni|645904112|nb|KK920319.1|	2577838-2579004	18	17	gi|641576188|gb|KK854966.1|	136114-136593	11	39
ACYPI003054-RA	ni|645903662|nb|KK920768.1|	373262-373655	nan	nan	gi|641586521|gb|KK854229.1|	636275-636535	11	41
ACYPI003057-RA	ni|645903751|nb|KK920679.1|	773272-773743	17	16	gi|641577493|gb|KK854745.1|	184498-185250	9.6	41
ACYPI003058-RA	ni|645903516|nb|KK920914.1|	489975-490205	nan	nan	gi|641585784|gb|KK854340.1|	605820-606030	9.7	44
ACYPI003059-RA	ni|645903925|nb|KK920506.1|	706742-707595	19	17	gi|641588325|gb|KK854004.1|	1016076-1016602	11	40
ACYPI003060-RA	ni|645901695|nb|KK922734.1|	10733-10961	nan	nan	gi|641575666|gb|KK855067.1|	251755-252322	11	39
ACYPI003061-RA	ni|645904043|nb|KK920388.1|	627384-628441	16	9.1	gi|641573417|gb|KK855565.1|	64650-66100	9.5	19
ACYPI003062-RA	ni|645904162|nb|KK920271.1|	612792-612986	15	18	gi|641586620|gb|KK854215.1|	92126-92460	9.1	40
ACYPI003064-RA	ni|645903704|nb|KK920726.1|	471161-472278	nan	nan	gi|641574876|gb|KK855226.1|	165904-170477	11	39
ACYPI003073-RA	ni|645902460|nb|KK921969.1|	156280-157168	nan	nan	gi|641587930|gb|KK854043.1|	462698-462970	10	41
ACYPI003075-RA	ni|645904228|nb|KK920243.1|	376485-376722	17	18	gi|641587814|gb|KK854056.1|	244222-244751	9.7	40

ACYPI003077-RA	ni|645904130|nb|KK920303.1|	691046-691282	18	17	gi|641584694|gb|KK854463.1|	6804-7156	9.3	36
ACYPI003083-RA	ni|645903566|nb|KK920864.1|	767900-768374	nan	nan	gi|641585594|gb|KK854372.1|	331517-332480	10	40
ACYPI003093-RA	ni|645902471|nb|KK921958.1|	162710-164929	nan	nan	gi|641585599|gb|KK854371.1|	155229-157542	11	39
ACYPI003121-RA	ni|645904122|nb|KK920309.1|	886342-886795	18	17	gi|641568799|gb|KK856864.1|	81479-82156	9.2	35
ACYPI003124-RA	ni|645903971|nb|KK920460.1|	1127815-1128499	19	18	gi|641577075|gb|KK854811.1|	272889-273348	11	36
ACYPI003133-RA	ni|645903749|nb|KK920681.1|	353834-354568	19	18	gi|641587742|gb|KK854065.1|	796616-797670	11	41
ACYPI003141-RA	ni|645901605|nb|KK922824.1|	8520-9309	nan	nan	gi|641569851|gb|KK856541.1|	19721-21216	11	21
ACYPI003151-RA	ni|645904014|nb|KK920417.1|	833759-833964	19	18	gi|641573992|gb|KK855420.1|	138200-138663	10	37
ACYPI003157-RA	ni|645903925|nb|KK920506.1|	1557437-1558088	19	17	gi|641587696|gb|KK854071.1|	653508-662646	8.9	39
ACYPI003160-RA	ni|645903655|nb|KK920775.1|	221657-222485	nan	nan	gi|641570589|gb|KK856310.1|	31546-32004	11	36
ACYPI003164-RA	ni|645902662|nb|KK921767.1|	286650-286833	nan	nan	gi|641574487|gb|KK855309.1|	253117-253297	11	41
ACYPI003169-RA	ni|645904135|nb|KK920298.1|	768087-769106	14	15	gi|641587930|gb|KK854043.1|	521469-523671	10	41
ACYPI003171-RA	ni|645904169|nb|KK920264.1|	645806-646818	16	9.5	gi|641565500|gb|KK858006.1|	12012-13302	9.6	19
ACYPI003177-RA	ni|645903862|nb|KK920568.1|	174507-175633	17	17	gi|641578122|gb|KK854636.1|	158313-160172	12	42
ACYPI003185-RA	ni|645903599|nb|KK920831.1|	216340-217609	nan	nan	gi|641585440|gb|KK854397.1|	356440-362755	9	38
ACYPI003186-RA	ni|645903999|nb|KK920432.1|	1196286-1196686	17	15	gi|641570355|gb|KK856378.1|	6188-6426	9.7	35
ACYPI003203-RA	ni|645901755|nb|KK922674.1|	8809-9287	nan	nan	gi|641576286|gb|KK854946.1|	330565-331118	12	38
ACYPI003204-RA	ni|645901808|nb|KK922621.1|	50970-52099	nan	nan	gi|641577086|gb|KK854809.1|	240058-240779	9.8	38
ACYPI003208-RA	ni|645903549|nb|KK920881.1|	42243-42713	nan	nan	gi|641570861|gb|KK856232.1|	54279-55633	11	39
ACYPI003210-RA	ni|645903759|nb|KK920671.1|	82411-82980	18	17	gi|641587807|gb|KK854057.1|	170211-171117	10	41
ACYPI003214-RA	ni|645903773|nb|KK920657.1|	1120081-1120789	18	17	gi|641588021|gb|KK854033.1|	1153237-1156014	12	40
ACYPI003216-RA	ni|645903831|nb|KK920599.1|	1310947-1311630	18	17	gi|641572987|gb|KK855671.1|	127137-127936	10	39
ACYPI003220-RA	ni|645904015|nb|KK920416.1|	916644-917264	18	16	gi|641585423|gb|KK854400.1|	356755-357395	11	36
ACYPI003221-RA	ni|645904116|nb|KK920315.1|	2042468-2043944	19	18	gi|641585967|gb|KK854311.1|	541346-542394	11	40
ACYPI003222-RA	ni|645903806|nb|KK920624.1|	586237-587179	16	9.3	gi|641587024|gb|KK854155.1|	459569-461108	10	21
ACYPI003225-RA	ni|645904070|nb|KK920361.1|	165378-165549	17	16	gi|641587711|gb|KK854069.1|	666062-666370	11	42
ACYPI003232-RA	ni|645901733|nb|KK922696.1|	67070-67542	nan	nan	gi|641582439|gb|KK854536.1|	442397-447574	11	36
ACYPI003233-RA	ni|645903678|nb|KK920752.1|	436682-436883	nan	nan	gi|641572782|gb|KK855725.1|	103312-103618	10	36
ACYPI003234-RA	ni|645900872|nb|KK923557.1|	33586-35038	nan	nan	gi|641558017|gb|KK861320.1|	4684-6386	13	46
ACYPI003241-RA	ni|645903966|nb|KK920465.1|	140514-141562	16	15	gi|641552935|gb|KK863732.1|	454-1196	13	21
ACYPI003245-RA	ni|645903825|nb|KK920605.1|	1094581-1095000	19	17	gi|641584433|gb|KK854482.1|	393284-393892	10	40
ACYPI003246-RA	ni|645904222|nb|KK920245.1|	1189060-1189599	17	12	gi|641571216|gb|KK856129.1|	36831-37548	12	41

ACYPI003251-RA	ni|645903661|nb|KK920769.1|	396573-397204	nan	nan	gi|641576397|gb|KK854927.1|	64416-65023	9.5	41
ACYPI003252-RA	ni|645904155|nb|KK920278.1|	582125-582337	18	9.4	gi|641575177|gb|KK855163.1|	24771-25060	11	43
ACYPI003255-RA	ni|645903965|nb|KK920466.1|	1675427-1676261	17	16	gi|641587962|gb|KK854039.1|	1113617-1114733	11	40
ACYPI003261-RA	ni|645903765|nb|KK920665.1|	665224-666551	17	15	gi|641571138|gb|KK856151.1|	171234-172511	10	45
ACYPI003279-RA	ni|645903701|nb|KK920729.1|	207280-207550	nan	nan	gi|641570304|gb|KK856395.1|	12761-13243	8.8	33
ACYPI003282-RA	ni|645903966|nb|KK920465.1|	336558-337066	16	15	gi|641569698|gb|KK856589.1|	93111-93422	11	41
ACYPI003283-RA	ni|645903957|nb|KK920474.1|	846525-846814	18	17	gi|641588000|gb|KK854035.1|	368716-370070	11	39
ACYPI003284-RA	ni|645903926|nb|KK920505.1|	302194-302363	9.6	12	gi|641573733|gb|KK855487.1|	113201-118652	13	45
ACYPI003290-RA	ni|645904186|nb|KK920257.1|	69588-69888	15	16	gi|641587856|gb|KK854051.1|	886363-886652	10	41
ACYPI003295-RA	ni|645901747|nb|KK922682.1|	116921-117120	nan	nan	gi|641580987|gb|KK854560.1|	17261-17443	11	40
ACYPI003296-RA	ni|645903954|nb|KK920477.1|	86066-86415	35	44	gi|641576962|gb|KK854829.1|	321264-322112	10	39
ACYPI003297-RA	ni|645904005|nb|KK920426.1|	678450-679217	17	16	gi|641570514|gb|KK856331.1|	127926-130200	9.8	39
ACYPI003298-RA	ni|645903679|nb|KK920751.1|	338350-338854	nan	nan	gi|641580470|gb|KK854571.1|	150677-154984	10	41
ACYPI003300-RA	ni|645903639|nb|KK920791.1|	326606-326780	nan	nan	gi|641586210|gb|KK854272.1|	144858-145055	10	39
ACYPI003301-RA	ni|645903728|nb|KK920702.1|	999112-999576	nan	nan	gi|641571642|gb|KK856015.1|	101720-102268	11	37
ACYPI003303-RA	ni|645903634|nb|KK920796.1|	1078869-1079269	nan	nan	gi|641584445|gb|KK854481.1|	139543-146223	9.9	39
ACYPI003304-RA	ni|645903965|nb|KK920466.1|	1445001-1445241	17	16	gi|641577071|gb|KK854812.1|	293606-294202	13	38
ACYPI003311-RA	ni|645904129|nb|KK920304.1|	994384-994837	16	17	gi|641578138|gb|KK854634.1|	45162-45408	8.2	39
ACYPI003316-RA	ni|645904049|nb|KK920382.1|	141156-141604	18	18	gi|641570198|gb|KK856430.1|	84303-84665	10	36
ACYPI003318-RA	ni|645904150|nb|KK920283.1|	967770-968125	17	17	gi|641570593|gb|KK856309.1|	87011-87869	9.6	33
ACYPI003322-RA	ni|645902615|nb|KK921814.1|	32483-34953	nan	nan	gi|641570058|gb|KK856473.1|	85679-88087	11	40
ACYPI003327-RA	ni|645904122|nb|KK920309.1|	408932-409406	18	17	gi|641586155|gb|KK854281.1|	562760-563337	9.8	38
ACYPI003333-RA	ni|645904106|nb|KK920325.1|	1022587-1022793	17	17	gi|641587272|gb|KK854122.1|	306292-306522	9.3	42
ACYPI003343-RA	ni|645903713|nb|KK920717.1|	199518-199736	nan	nan	gi|641574303|gb|KK855353.1|	227178-230384	10	34
ACYPI003347-RA	ni|645903891|nb|KK920540.1|	121603-121814	15	17	gi|641577341|gb|KK854768.1|	313295-313655	14	58
ACYPI003349-RA	ni|645904141|nb|KK920292.1|	313936-314558	14	14	gi|641567905|gb|KK857149.1|	93693-94495	10	38
ACYPI003357-RA	ni|645903622|nb|KK920808.1|	593426-593710	nan	nan	gi|641587066|gb|KK854149.1|	262550-262960	11	40
ACYPI003358-RA	ni|645904096|nb|KK920335.1|	1986811-1988455	18	17	gi|641575951|gb|KK855015.1|	220825-224887	9.9	37
ACYPI003364-RA	ni|645902263|nb|KK922166.1|	190379-190560	nan	nan	gi|641587021|gb|KK854156.1|	763994-766245	11	42
ACYPI003367-RA	ni|645904077|nb|KK920354.1|	1133818-1133993	16	17	gi|641584504|gb|KK854477.1|	514716-515364	9.5	38
ACYPI003377-RA	ni|645904078|nb|KK920353.1|	563997-564219	17	17	gi|641586541|gb|KK854226.1|	268842-269769	11	41
ACYPI003398-RA	ni|645904251|nb|KK920231.1|	369602-369966	16	16	gi|641576546|gb|KK854899.1|	67715-69421	11	40

ACYPI003401-RA	ni|645902394|nb|KK922035.1|	344370-344995	nan	nan	gi|641578060|gb|KK854647.1|	175603-175828	8.9	35
ACYPI003404-RA	ni|645901808|nb|KK922621.1|	116535-116930	nan	nan	gi|641587021|gb|KK854156.1|	600404-602112	11	42
ACYPI003409-RA	ni|645903502|nb|KK920928.1|	240818-241179	nan	nan	gi|641587111|gb|KK854143.1|	358479-359292	11	40
ACYPI003413-RA	ni|645903817|nb|KK920613.1|	267579-267977	13	16	gi|641573202|gb|KK855619.1|	194482-194897	9.6	42
ACYPI003418-RA	ni|645903800|nb|KK920630.1|	1314426-1315385	18	17	gi|641569146|gb|KK856755.1|	114080-115703	10	34
ACYPI003425-RA	ni|645903511|nb|KK920919.1|	774604-776708	nan	nan	gi|641588073|gb|KK854027.1|	294772-298222	11	41
ACYPI003428-RA	ni|645903690|nb|KK920740.1|	291212-292032	nan	nan	gi|641584305|gb|KK854491.1|	175587-181117	11	39
ACYPI003451-RA	ni|645903965|nb|KK920466.1|	1721255-1721440	17	16	gi|641570089|gb|KK856463.1|	53939-54254	11	36
ACYPI003455-RA	ni|645903971|nb|KK920460.1|	1173108-1174341	19	18	gi|641577075|gb|KK854811.1|	161155-164885	11	36
ACYPI003458-RA	ni|645904189|nb|KK920256.1|	1873228-1873897	18	18	gi|641588294|gb|KK854006.1|	372471-373803	8.7	40
ACYPI003461-RA	ni|645903561|nb|KK920869.1|	74294-74566	nan	nan	gi|641577987|gb|KK854657.1|	483319-489271	13	49
ACYPI003469-RA	ni|645903617|nb|KK920813.1|	565660-566135	nan	nan	gi|641569612|gb|KK856617.1|	2544-3197	11	21
ACYPI003470-RA	ni|645903733|nb|KK920697.1|	678373-678510	nan	nan	gi|641574532|gb|KK855298.1|	70081-70286	12	20
ACYPI003478-RA	ni|645904013|nb|KK920418.1|	1265675-1265965	19	18	gi|641568786|gb|KK856868.1|	75902-76146	11	39
ACYPI003480-RA	ni|645904258|nb|KK920229.1|	731952-732355	16	16	gi|641574312|gb|KK855351.1|	100968-101231	8.9	36
ACYPI003481-RA	ni|645904210|nb|KK920249.1|	916557-917222	16	16	gi|641586700|gb|KK854203.1|	63452-63756	8.4	39
ACYPI003483-RA	ni|645904021|nb|KK920410.1|	777693-778167	15	16	gi|641574800|gb|KK855241.1|	150305-153185	10	37
ACYPI003484-RA	ni|645903848|nb|KK920582.1|	576525-578099	18	18	gi|641569425|gb|KK856674.1|	111565-112737	11	42
ACYPI003488-RA	ni|645904135|nb|KK920298.1|	310414-311019	14	15	gi|641575698|gb|KK855061.1|	105484-105727	10	39
ACYPI003489-RA	ni|645904028|nb|KK920403.1|	380822-382773	18	17	gi|641586509|gb|KK854231.1|	531347-533813	9.8	37
ACYPI003491-RA	ni|645904114|nb|KK920317.1|	1373494-1374121	18	18	gi|641571769|gb|KK855985.1|	141949-143044	9	35
ACYPI003499-RA	ni|645901914|nb|KK922515.1|	103453-103691	nan	nan	gi|641588099|gb|KK854024.1|	477462-478707	11	39
ACYPI003508-RA	ni|645904271|nb|KK920224.1|	1648410-1649755	15	16	gi|641577498|gb|KK854744.1|	339973-340269	11	42
ACYPI003518-RA	ni|645904106|nb|KK920325.1|	805788-806358	17	17	gi|641579225|gb|KK854583.1|	329923-330601	10	39
ACYPI003519-RA	ni|645902511|nb|KK921918.1|	156844-157080	nan	nan	gi|641587921|gb|KK854044.1|	358092-360211	9.4	41
ACYPI003522-RA	ni|645903915|nb|KK920516.1|	209340-209601	17	17	gi|641576414|gb|KK854924.1|	137776-143145	10	40
ACYPI003530-RA	ni|645904201|nb|KK920252.1|	290873-291099	17	16	gi|641588060|gb|KK854028.1|	698606-699784	11	42
ACYPI003532-RA	ni|645904243|nb|KK920236.1|	154812-155009	15	8.5	gi|641567609|gb|KK857247.1|	29664-31582	9.7	19
ACYPI003535-RA	ni|645904088|nb|KK920343.1|	167574-168042	17	18	gi|641577968|gb|KK854660.1|	336803-337021	10	42
ACYPI003537-RA	ni|645903823|nb|KK920607.1|	34946-35223	16	17	gi|641585801|gb|KK854337.1|	205854-206612	10	38
ACYPI003538-RA	ni|645904251|nb|KK920231.1|	464823-465163	16	16	gi|641587390|gb|KK854109.1|	639125-639584	11	44
ACYPI003541-RA	ni|645903829|nb|KK920601.1|	414787-415281	16	15	gi|641584305|gb|KK854491.1|	551762-553148	11	39

ACYPI003545-RA	ni|645903981|nb|KK920450.1|	1135912-1136121	16	8.6	gi|641577289|gb|KK854776.1|	192523-193107	10	36
ACYPI003549-RA	ni|645903536|nb|KK920894.1|	858250-858529	nan	nan	gi|641560616|gb|KK860113.1|	734-987	12	44
ACYPI003550-RA	ni|645904065|nb|KK920366.1|	1757004-1757181	18	18	gi|641579212|gb|KK854585.1|	44440-46499	10	35
ACYPI003552-RA	ni|645903858|nb|KK920572.1|	692794-693015	16	16	gi|641577535|gb|KK854737.1|	58519-59440	11	42
ACYPI003554-RA	ni|645904047|nb|KK920384.1|	614437-615833	15	16	gi|641582091|gb|KK854547.1|	383677-384387	11	42
ACYPI003557-RA	ni|645903992|nb|KK920439.1|	670143-670824	15	15	gi|641588164|gb|KK854017.1|	267209-267527	9.2	40
ACYPI003560-RA	ni|645904068|nb|KK920363.1|	362858-363091	16	8.6	gi|641574110|gb|KK855394.1|	122773-123817	10	21
ACYPI003562-RA	ni|645903763|nb|KK920667.1|	49159-49585	26	21	gi|641577913|gb|KK854671.1|	89841-90140	9.2	40
ACYPI003565-RA	ni|645904057|nb|KK920374.1|	1266431-1267005	16	15	gi|641574809|gb|KK855240.1|	40390-41700	11	39
ACYPI003566-RA	ni|645904125|nb|KK920307.1|	2447403-2447636	18	17	gi|641575183|gb|KK855162.1|	25466-25948	10	42
ACYPI003567-RA	ni|645903721|nb|KK920709.1|	733562-734860	nan	nan	gi|641575077|gb|KK855184.1|	103870-105114	12	22
ACYPI003568-RA	ni|645903727|nb|KK920703.1|	1085041-1085523	nan	nan	gi|641554782|gb|KK862854.1|	102-305	7.8	21
ACYPI003571-RA	ni|645901808|nb|KK922621.1|	103745-104204	nan	nan	gi|641587021|gb|KK854156.1|	589985-590553	11	42
ACYPI003572-RA	ni|645903765|nb|KK920665.1|	1084440-1084695	17	15	gi|641586577|gb|KK854221.1|	160457-160756	10	40
ACYPI003573-RA	ni|645903786|nb|KK920644.1|	118074-118640	18	19	gi|641587735|gb|KK854066.1|	776187-776395	11	42
ACYPI003574-RA	ni|645904014|nb|KK920417.1|	1273386-1273750	19	18	gi|641570579|gb|KK856313.1|	1714-2565	11	38
ACYPI003577-RA	ni|645901994|nb|KK922435.1|	64579-64831	nan	nan	gi|641574402|gb|KK855330.1|	239335-240400	11	23
ACYPI003579-RA	ni|645904065|nb|KK920366.1|	1116200-1116384	18	18	gi|641586535|gb|KK854227.1|	613950-614919	10	39
ACYPI003581-RA	ni|645904192|nb|KK920255.1|	342346-342688	15	16	gi|641571841|gb|KK855968.1|	133530-138589	10	36
ACYPI003590-RA	ni|645904036|nb|KK920395.1|	821842-822574	21	18	gi|641585946|gb|KK854315.1|	650300-655433	9.8	43
ACYPI003596-RA	ni|645903831|nb|KK920599.1|	1505400-1505744	18	17	gi|641587992|gb|KK854036.1|	286970-287196	11	41
ACYPI003598-RA	ni|645903915|nb|KK920516.1|	1444625-1444844	17	17	gi|641587562|gb|KK854088.1|	497550-505220	11	42
ACYPI003600-RA	ni|645903834|nb|KK920596.1|	292220-293215	18	17	gi|641573414|gb|KK855566.1|	102646-103051	9.1	36
ACYPI003604-RA	ni|645904107|nb|KK920324.1|	443801-444312	17	9.5	gi|641585334|gb|KK854415.1|	289256-292338	10	39
ACYPI003616-RA	ni|645904115|nb|KK920316.1|	1193327-1194219	17	18	gi|641576565|gb|KK854896.1|	216178-217820	11	40
ACYPI003625-RA	ni|645904160|nb|KK920273.1|	979490-979699	18	18	gi|641573638|gb|KK855510.1|	181524-182031	11	41
ACYPI003631-RA	ni|645904058|nb|KK920373.1|	622297-622935	16	15	gi|641588236|gb|KK854011.1|	278418-280096	9.7	38
ACYPI003632-RA	ni|645902693|nb|KK921736.1|	119626-120102	nan	nan	gi|641586091|gb|KK854292.1|	336721-337703	9.7	37
ACYPI003634-RA	ni|645904081|nb|KK920350.1|	450846-451536	13	18	gi|641571730|gb|KK855996.1|	111657-112310	10	20
ACYPI003639-RA	ni|645904062|nb|KK920369.1|	141376-148496	14	15	gi|641577778|gb|KK854696.1|	179338-185583	9.9	42
ACYPI003641-RA	ni|645903681|nb|KK920749.1|	520210-521217	nan	nan	gi|641576188|gb|KK854966.1|	158888-159647	11	39
ACYPI003646-RA	ni|645903626|nb|KK920804.1|	336789-337409	nan	nan	gi|641586405|gb|KK854246.1|	369727-370490	10	40

ACYPI003651-RA	ni|645902449|nb|KK921980.1|	100182-100441	nan	nan	gi|641588073|gb|KK854027.1|	392892-393642	11	41
ACYPI003657-RA	ni|645901672|nb|KK922757.1|	3755-4399	nan	nan	gi|641588343|gb|KK854003.1|	2120674-2121062	11	40
ACYPI003659-RA	ni|645904174|nb|KK920261.1|	869676-870434	19	17	gi|641572300|gb|KK855848.1|	29325-29712	12	38
ACYPI003661-RA	ni|645904035|nb|KK920396.1|	640519-640913	16	16	gi|641586121|gb|KK854287.1|	12793-14494	11	43
ACYPI003667-RA	ni|645903532|nb|KK920898.1|	530982-531254	nan	nan	gi|641571523|gb|KK856046.1|	84214-84458	10	43
ACYPI003669-RA	ni|645903731|nb|KK920699.1|	98773-99067	nan	nan	gi|641575730|gb|KK855054.1|	346708-347099	10	38
ACYPI003670-RA	ni|645903833|nb|KK920597.1|	326172-326681	17	15	gi|641574689|gb|KK855262.1|	297397-299330	10	38
ACYPI003672-RA	ni|645903879|nb|KK920552.1|	1080299-1081130	18	18	gi|641586509|gb|KK854231.1|	517194-517938	9.8	37
ACYPI003674-RA	ni|645904159|nb|KK920274.1|	672353-672558	15	17	gi|641576203|gb|KK854962.1|	131539-131742	9.8	36
ACYPI003677-RA	ni|645904140|nb|KK920293.1|	361431-361707	22	22	gi|641573850|gb|KK855457.1|	90173-90979	9.3	34
ACYPI003679-RA	ni|645902449|nb|KK921980.1|	82936-83142	nan	nan	gi|641588073|gb|KK854027.1|	414468-415831	11	41
ACYPI003682-RA	ni|645901914|nb|KK922515.1|	117307-117808	nan	nan	gi|641585246|gb|KK854426.1|	133957-134936	10	39
ACYPI003689-RA	ni|645900910|nb|KK923519.1|	34359-35064	nan	nan	gi|641573351|gb|KK855583.1|	50978-51621	11	41
ACYPI003691-RA	ni|645904122|nb|KK920309.1|	863712-864135	18	17	gi|641577612|gb|KK854724.1|	352469-353133	9.7	37
ACYPI003694-RA	ni|645903868|nb|KK920562.1|	509947-510112	16	8.7	gi|641572887|gb|KK855696.1|	137344-137790	9.5	26
ACYPI003697-RA	ni|645903936|nb|KK920495.1|	566077-567182	19	18	gi|641576791|gb|KK854858.1|	105870-107321	9.2	42
ACYPI003705-RA	ni|645904171|nb|KK920262.1|	864052-864729	15	16	gi|641588325|gb|KK854004.1|	1940069-1941515	11	40
ACYPI003710-RA	ni|645903847|nb|KK920583.1|	454760-455680	18	18	gi|641580479|gb|KK854569.1|	472888-473215	10	38
ACYPI003711-RA	ni|645904271|nb|KK920224.1|	3629925-3630457	15	16	gi|641586079|gb|KK854294.1|	182118-184048	8.5	34
ACYPI003718-RA	ni|645903639|nb|KK920791.1|	242254-242437	nan	nan	gi|641588182|gb|KK854016.1|	403241-405057	11	41
ACYPI003729-RA	ni|645902468|nb|KK921961.1|	44084-44541	nan	nan	gi|641586258|gb|KK854267.1|	108382-109286	11	37
ACYPI003732-RA	ni|645903821|nb|KK920609.1|	1124171-1125294	17	17	gi|641588090|gb|KK854025.1|	93574-97525	11	43
ACYPI003736-RA	ni|645904122|nb|KK920309.1|	1193064-1193353	18	17	gi|641588236|gb|KK854011.1|	1247592-1248833	9.7	38
ACYPI003742-RA	ni|645904116|nb|KK920315.1|	1788704-1788930	19	18	gi|641576983|gb|KK854825.1|	297924-298168	9.7	37
ACYPI003748-RA	ni|645904163|nb|KK920270.1|	1168170-1169077	16	16	gi|641576962|gb|KK854829.1|	99186-99448	10	39
ACYPI003749-RA	ni|645904241|nb|KK920238.1|	2712552-2712842	18	17	gi|641569036|gb|KK856790.1|	105472-105984	10	36
ACYPI003751-RA	ni|645901760|nb|KK922669.1|	114170-114664	nan	nan	gi|641586943|gb|KK854167.1|	163442-163698	11	38
ACYPI003754-RA	ni|645904033|nb|KK920398.1|	51728-52526	14	16	gi|641586975|gb|KK854163.1|	363994-364405	11	40
ACYPI003756-RA	ni|645903788|nb|KK920642.1|	534919-535323	16	15	gi|641567854|gb|KK857166.1|	101175-102372	11	37
ACYPI003757-RA	ni|645903720|nb|KK920710.1|	122913-123283	nan	nan	gi|641585521|gb|KK854384.1|	341764-342049	9.7	38
ACYPI003759-RA	ni|645904112|nb|KK920319.1|	2208263-2208527	18	17	gi|641576442|gb|KK854919.1|	242945-243251	11	36
ACYPI003760-RA	ni|645902598|nb|KK921831.1|	93834-94461	nan	nan	gi|641576055|gb|KK854992.1|	39075-39765	9.7	37

ACYPI003764-RA	ni|645903747|nb|KK920683.1|	773739-773928	19	18	gi|641571928|gb|KK855946.1|	88091-88532	11	38
ACYPI003771-RA	ni|645902229|nb|KK922200.1|	84208-85489	nan	nan	gi|641566077|gb|KK857794.1|	11815-12597	9.9	19
ACYPI003777-RA	ni|645904166|nb|KK920267.1|	2650791-2651081	18	16	gi|641586496|gb|KK854233.1|	18232-18563	11	38
ACYPI003778-RA	ni|645903899|nb|KK920532.1|	378523-378730	15	16	gi|641569564|gb|KK856632.1|	53109-56151	9.8	34
ACYPI003779-RA	ni|645903867|nb|KK920563.1|	832377-832967	18	18	gi|641570155|gb|KK856443.1|	126919-127878	10	20
ACYPI003783-RA	ni|645904254|nb|KK920230.1|	98782-99911	16	16	gi|641586235|gb|KK854270.1|	525499-527966	11	39
ACYPI003786-RA	ni|645903741|nb|KK920689.1|	349057-349477	17	18	gi|641585594|gb|KK854372.1|	459063-459722	10	40
ACYPI003790-RA	ni|645903717|nb|KK920713.1|	20131-20327	nan	nan	gi|641587962|gb|KK854039.1|	771478-772855	11	40
ACYPI003795-RA	ni|645903787|nb|KK920643.1|	75263-77034	19	18	gi|641577584|gb|KK854729.1|	253777-255577	9.9	37
ACYPI003796-RA	ni|645904090|nb|KK920341.1|	118809-118981	15	17	gi|641571515|gb|KK856048.1|	219277-220121	9.9	38
ACYPI003798-RA	ni|645903538|nb|KK920892.1|	113270-114186	nan	nan	gi|641587303|gb|KK854118.1|	366967-369802	11	20
ACYPI003817-RA	ni|645903496|nb|KK920934.1|	902588-902877	nan	nan	gi|641588033|gb|KK854031.1|	1117347-1119140	11	38
ACYPI003820-RA	ni|645901447|nb|KK922982.1|	162-556	nan	nan	gi|641587671|gb|KK854074.1|	182475-182683	11	40
ACYPI003821-RA	ni|645903923|nb|KK920508.1|	1141891-1142874	18	18	gi|641572345|gb|KK855836.1|	53421-54745	9.7	38
ACYPI003822-RA	ni|645902622|nb|KK921807.1|	138278-138478	nan	nan	gi|641576414|gb|KK854924.1|	311123-312321	10	40
ACYPI003828-RA	ni|645903655|nb|KK920775.1|	872282-874859	nan	nan	gi|641567423|gb|KK857312.1|	19588-25554	11	37
ACYPI003832-RA	ni|645903841|nb|KK920589.1|	1239705-1240393	17	17	gi|641588343|gb|KK854003.1|	998331-999157	11	40
ACYPI003835-RA	ni|645903829|nb|KK920601.1|	602780-603062	16	15	gi|641584305|gb|KK854491.1|	405448-405959	11	39
ACYPI003839-RA	ni|645903644|nb|KK920786.1|	134294-134697	nan	nan	gi|641575372|gb|KK855124.1|	66926-75766	9.5	36
ACYPI003863-RA	ni|645903644|nb|KK920786.1|	716927-717168	nan	nan	gi|641584445|gb|KK854481.1|	268830-271010	9.9	39
ACYPI003864-RA	ni|645903871|nb|KK920559.1|	798954-799442	18	9.4	gi|641549627|gb|KK865285.1|	23-408	9	15
ACYPI003867-RA	ni|645904240|nb|KK920239.1|	955121-955382	16	16	gi|641588136|gb|KK854020.1|	946149-946432	11	36
ACYPI003874-RA	ni|645903703|nb|KK920727.1|	203556-203898	nan	nan	gi|641577299|gb|KK854775.1|	169045-169591	11	37
ACYPI003876-RA	ni|645903592|nb|KK920838.1|	985370-985888	nan	nan	gi|641577347|gb|KK854767.1|	389694-389929	9.9	43
ACYPI003885-RA	ni|645903731|nb|KK920699.1|	680621-681065	nan	nan	gi|641584894|gb|KK854448.1|	488267-489072	10	44
ACYPI003886-RA	ni|645903745|nb|KK920685.1|	316489-319185	16	18	gi|641576650|gb|KK854884.1|	242313-247304	9.6	40
ACYPI003888-RA	ni|645904130|nb|KK920303.1|	641810-642455	18	17	gi|641575970|gb|KK855012.1|	152130-153208	11	38
ACYPI003890-RA	ni|645902639|nb|KK921790.1|	169275-169765	nan	nan	gi|641574119|gb|KK855392.1|	11356-11836	9.6	37
ACYPI003891-RA	ni|645903473|nb|KK920957.1|	215105-215332	nan	nan	gi|641573825|gb|KK855463.1|	113447-113839	10	39
ACYPI003896-RA	ni|645904207|nb|KK920250.1|	1076650-1078867	18	20	gi|641586880|gb|KK854177.1|	594036-594383	10	20
ACYPI003897-RA	ni|645904180|nb|KK920259.1|	1175255-1175500	17	17	gi|641578160|gb|KK854630.1|	189542-190912	11	43
ACYPI003903-RA	ni|645904061|nb|KK920370.1|	252640-252912	17	16	gi|641585684|gb|KK854357.1|	122687-123217	11	39

ACYPI003904-RA	ni|645904210|nb|KK920249.1|	2063110-2063469	16	16	gi|641587954|gb|KK854040.1|	710604-711778	8.9	39
ACYPI003908-RA	ni|645904228|nb|KK920243.1|	1591174-1591688	17	18	gi|641569574|gb|KK856629.1|	111713-112242	24	120
ACYPI003912-RA	ni|645903981|nb|KK920450.1|	25428-25624	16	8.6	gi|641567985|gb|KK857122.1|	27158-28826	8.9	39
ACYPI003918-RA	ni|645903843|nb|KK920587.1|	968274-968543	18	16	gi|641576109|gb|KK854982.1|	144500-144781	10	19
ACYPI003941-RA	ni|645903778|nb|KK920652.1|	211254-211895	12	13	gi|641586496|gb|KK854233.1|	367126-370383	11	38
ACYPI003942-RA	ni|645904024|nb|KK920407.1|	646272-646516	14	16	gi|641575559|gb|KK855088.1|	260800-262258	9.9	37
ACYPI003943-RA	ni|645903496|nb|KK920934.1|	155025-155361	nan	nan	gi|641587896|gb|KK854047.1|	826843-829594	9.9	39
ACYPI003944-RA	ni|645904271|nb|KK920224.1|	844435-846478	15	16	gi|641567171|gb|KK857401.1|	84734-87637	10	36
ACYPI003947-RA	ni|645902571|nb|KK921858.1|	128717-129534	nan	nan	gi|641588343|gb|KK854003.1|	123120-123521	11	40
ACYPI003951-RA	ni|645903495|nb|KK920935.1|	314247-314624	nan	nan	gi|641587074|gb|KK854148.1|	498367-498861	11	41
ACYPI003953-RA	ni|645901697|nb|KK922732.1|	121299-121666	nan	nan	gi|641586066|gb|KK854296.1|	324029-324269	9.4	37
ACYPI003960-RA	ni|645903858|nb|KK920572.1|	446872-447291	16	16	gi|641572177|gb|KK855878.1|	22173-22436	10	35
ACYPI003961-RA	ni|645904083|nb|KK920348.1|	851600-852307	15	16	gi|641564341|gb|KK858480.1|	17488-17807	11	19
ACYPI003966-RA	ni|645903861|nb|KK920569.1|	340700-340917	16	17	gi|641573861|gb|KK855454.1|	55644-60665	10	37
ACYPI003971-RA	ni|645904155|nb|KK920278.1|	514449-515369	18	9.4	gi|641582100|gb|KK854545.1|	295269-297080	13	41
ACYPI003975-RA	ni|645903911|nb|KK920520.1|	628403-629095	16	17	gi|641587971|gb|KK854038.1|	106101-107003	11	39
ACYPI003976-RA	ni|645902649|nb|KK921780.1|	164145-166426	nan	nan	gi|641574433|gb|KK855322.1|	220311-230259	11	44
ACYPI003981-RA	ni|645903800|nb|KK920630.1|	1002143-1002970	18	17	gi|641587660|gb|KK854076.1|	561282-562910	9.9	41
ACYPI003986-RA	ni|645902620|nb|KK921809.1|	209538-209802	nan	nan	gi|641572130|gb|KK855891.1|	174453-174824	10	37
ACYPI003991-RA	ni|645904125|nb|KK920307.1|	960143-960680	18	17	gi|641587881|gb|KK854049.1|	426345-427066	10	40
ACYPI003993-RA	ni|645904139|nb|KK920294.1|	554250-554978	15	18	gi|641576347|gb|KK854934.1|	313422-314861	11	39
ACYPI004000-RA	ni|645904268|nb|KK920225.1|	539642-540277	14	15	gi|641577522|gb|KK854739.1|	276696-279485	11	41
ACYPI004003-RA	ni|645904241|nb|KK920238.1|	1879707-1882732	18	17	gi|641577133|gb|KK854802.1|	208826-214675	9.9	40
ACYPI004006-RA	ni|645903973|nb|KK920458.1|	1492006-1492263	20	18	gi|641587047|gb|KK854151.1|	424829-425148	10	39
ACYPI004008-RA	ni|645902788|nb|KK921641.1|	253252-254000	nan	nan	gi|641572637|gb|KK855763.1|	251203-251892	9.5	36
ACYPI004013-RA	ni|645904009|nb|KK920422.1|	1179061-1179398	17	9.4	gi|641588182|gb|KK854016.1|	289567-292167	11	41
ACYPI004014-RA	ni|645903871|nb|KK920559.1|	760280-760484	18	9.4	gi|641570404|gb|KK856365.1|	71726-73948	10	19
ACYPI004015-RA	ni|645902758|nb|KK921671.1|	201981-202814	nan	nan	gi|641576161|gb|KK854972.1|	225340-226431	10	36
ACYPI004019-RA	ni|645902469|nb|KK921960.1|	272464-273018	nan	nan	gi|641566606|gb|KK857599.1|	44283-45293	10	40
ACYPI004024-RA	ni|645903743|nb|KK920687.1|	1269681-1270427	19	18	gi|641585340|gb|KK854414.1|	242560-244760	11	42
ACYPI004025-RA	ni|645903634|nb|KK920796.1|	954886-956206	nan	nan	gi|641574836|gb|KK855234.1|	23660-24996	10	40
ACYPI004029-RA	ni|645903938|nb|KK920493.1|	446255-446423	19	18	gi|641567739|gb|KK857205.1|	109817-110020	13	38

ACYPI004030-RA	ni|645903858|nb|KK920572.1|	450258-451156	16	16	gi|641577535|gb|KK854737.1|	386114-386834	11	42
ACYPI004031-RA	ni|645901982|nb|KK922447.1|	193727-194176	nan	nan	gi|641587696|gb|KK854071.1|	550344-551738	8.9	39
ACYPI004034-RA	ni|645903544|nb|KK920886.1|	311834-312444	nan	nan	gi|641587981|gb|KK854037.1|	31937-32608	10	40
ACYPI004036-RA	ni|645903898|nb|KK920533.1|	466747-466992	17	16	gi|641572860|gb|KK855703.1|	43908-44164	9.8	36
ACYPI004037-RA	ni|645903984|nb|KK920447.1|	764452-764682	18	17	gi|641587930|gb|KK854043.1|	961692-962191	10	41
ACYPI004039-RA	ni|645904114|nb|KK920317.1|	1779524-1779867	18	18	gi|641586809|gb|KK854188.1|	122712-123024	10	43
ACYPI004047-RA	ni|645902035|nb|KK922394.1|	213785-215047	nan	nan	gi|641585773|gb|KK854342.1|	403149-403624	12	45
ACYPI004051-RA	ni|645904177|nb|KK920260.1|	839272-840908	18	17	gi|641576423|gb|KK854922.1|	275088-276594	18	35
ACYPI004054-RA	ni|645903496|nb|KK920934.1|	159765-160674	nan	nan	gi|641587896|gb|KK854047.1|	852540-852824	9.9	39
ACYPI004063-RA	ni|645903635|nb|KK920795.1|	528552-529148	nan	nan	gi|641588136|gb|KK854020.1|	535260-536802	11	36
ACYPI004069-RA	ni|645904155|nb|KK920278.1|	969816-970025	18	9.4	gi|641587866|gb|KK854050.1|	275332-276289	10	38
ACYPI004071-RA	ni|645902642|nb|KK921787.1|	136868-138509	nan	nan	gi|641586515|gb|KK854230.1|	561673-564419	11	39
ACYPI004075-RA	ni|645903837|nb|KK920593.1|	73821-74327	18	9.8	gi|641582100|gb|KK854545.1|	91749-92304	13	41
ACYPI004077-RA	ni|645904013|nb|KK920418.1|	1060029-1060248	19	18	gi|641586632|gb|KK854213.1|	91623-92094	11	41
ACYPI004078-RA	ni|645904228|nb|KK920243.1|	1660062-1660829	17	18	gi|641573097|gb|KK855644.1|	82077-83948	10	36
ACYPI004079-RA	ni|645904146|nb|KK920287.1|	679733-680554	16	18	gi|641587390|gb|KK854109.1|	828586-828866	11	44
ACYPI004082-RA	ni|645903511|nb|KK920919.1|	538835-539076	nan	nan	gi|641588164|gb|KK854017.1|	791875-794179	9.2	40
ACYPI004089-RA	ni|645903970|nb|KK920461.1|	165284-165710	17	16	gi|641585860|gb|KK854329.1|	114568-116246	11	43
ACYPI004090-RA	ni|645904246|nb|KK920233.1|	1261954-1262094	16	17	gi|641586734|gb|KK854198.1|	708544-709120	11	40
ACYPI004093-RA	ni|645902226|nb|KK922203.1|	33938-34166	nan	nan	gi|641572955|gb|KK855679.1|	35380-36113	10	40
ACYPI004098-RA	ni|645904102|nb|KK920329.1|	27343-28738	15	16	gi|641585847|gb|KK854330.1|	31182-34584	11	41
ACYPI004100-RA	ni|645904156|nb|KK920277.1|	228291-228504	17	8.8	gi|641584336|gb|KK854489.1|	37220-41609	10	40
ACYPI004106-RA	ni|645904168|nb|KK920265.1|	2088813-2089040	16	16	gi|641586521|gb|KK854229.1|	182911-183670	11	41
ACYPI004120-RA	ni|645903568|nb|KK920862.1|	266556-267027	nan	nan	gi|641585900|gb|KK854322.1|	117828-118056	12	43
ACYPI004126-RA	ni|645904065|nb|KK920366.1|	1614206-1615248	18	18	gi|641585910|gb|KK854320.1|	247453-248579	10	38
ACYPI004127-RA	ni|645903992|nb|KK920439.1|	683615-684039	15	15	gi|641579933|gb|KK854577.1|	68139-68369	11	37
ACYPI004128-RA	ni|645903766|nb|KK920664.1|	350567-351118	18	15	gi|641570555|gb|KK856321.1|	109418-109996	11	38
ACYPI004129-RA	ni|645897631|nb|KK926798.1|	4349-4507	nan	nan	gi|641573759|gb|KK855480.1|	127678-128494	9.9	37
ACYPI004133-RA	ni|645903970|nb|KK920461.1|	345509-346322	17	16	gi|641570991|gb|KK856192.1|	64040-65559	11	38
ACYPI004142-RA	ni|645902394|nb|KK922035.1|	307797-308242	nan	nan	gi|641575112|gb|KK855177.1|	88837-94473	11	39
ACYPI004145-RA	ni|645903751|nb|KK920679.1|	659121-659902	17	16	gi|641577379|gb|KK854761.1|	252353-258055	11	41
ACYPI004152-RA	ni|645903923|nb|KK920508.1|	1133407-1133757	18	18	gi|641588099|gb|KK854024.1|	310272-310556	11	39

ACYPI004154-RA	ni|645904107|nb|KK920324.1|	299475-299869	17	9.5	gi|641577595|gb|KK854727.1|	168171-168434	10	20
ACYPI004157-RA	ni|645902172|nb|KK922257.1|	212265-212450	nan	nan	gi|641587066|gb|KK854149.1|	646487-647454	11	40
ACYPI004168-RA	ni|645904228|nb|KK920243.1|	1582839-1583310	17	18	gi|641588294|gb|KK854006.1|	677955-679275	8.7	40
ACYPI004173-RA	ni|645904077|nb|KK920354.1|	602970-603603	16	17	gi|641569246|gb|KK856728.1|	22365-23068	8.7	32
ACYPI004185-RA	ni|645904045|nb|KK920386.1|	817728-819194	19	21	gi|641585653|gb|KK854362.1|	32202-34531	10	39
ACYPI004195-RA	ni|645904114|nb|KK920317.1|	1648005-1649903	18	18	gi|641571667|gb|KK856008.1|	142338-148056	11	39
ACYPI004199-RA	ni|645903651|nb|KK920779.1|	244594-244999	nan	nan	gi|641585755|gb|KK854345.1|	205593-205995	9.8	39
ACYPI004203-RA	ni|645903965|nb|KK920466.1|	1599631-1600263	17	16	gi|641585581|gb|KK854374.1|	463799-464715	10	38
ACYPI004206-RA	ni|645904177|nb|KK920260.1|	2202705-2203229	18	17	gi|641577837|gb|KK854685.1|	386593-387225	11	42
ACYPI004209-RA	ni|645903557|nb|KK920873.1|	828896-829330	nan	nan	gi|641586817|gb|KK854187.1|	827518-828136	9.3	39
ACYPI004211-RA	ni|645903971|nb|KK920460.1|	32290-33116	19	18	gi|641558402|gb|KK861138.1|	2365-3607	7.6	30
ACYPI004216-RA	ni|645904153|nb|KK920280.1|	788784-789127	17	16	gi|641576608|gb|KK854891.1|	239405-240511	11	37
ACYPI004218-RA	ni|645903827|nb|KK920603.1|	1090808-1091258	17	18	gi|641576656|gb|KK854883.1|	173777-174036	11	40
ACYPI004219-RA	ni|645904075|nb|KK920356.1|	1124869-1125544	16	17	gi|641563481|gb|KK858852.1|	648-1152	8.7	35
ACYPI004222-RA	ni|645904163|nb|KK920270.1|	1220871-1222568	16	16	gi|641587569|gb|KK854087.1|	136543-136797	11	41
ACYPI004223-RA	ni|645903915|nb|KK920516.1|	187974-188368	17	17	gi|641576414|gb|KK854924.1|	105220-106089	10	40
ACYPI004224-RA	ni|645903954|nb|KK920477.1|	190165-190969	35	44	gi|641573015|gb|KK855664.1|	29768-31156	11	42
ACYPI004227-RA	ni|645899876|nb|KK924553.1|	4626-5541	nan	nan	gi|641571428|gb|KK856074.1|	5390-6292	11	39
ACYPI004235-RA	ni|645903848|nb|KK920582.1|	586066-586783	18	18	gi|641574275|gb|KK855359.1|	267082-267664	10	38
ACYPI004238-RA	ni|645904094|nb|KK920337.1|	125980-126428	17	16	gi|641574642|gb|KK855274.1|	277930-278352	11	38
ACYPI004239-RA	ni|645903808|nb|KK920622.1|	691045-692713	17	17	gi|641587031|gb|KK854154.1|	643098-644799	11	42
ACYPI004240-RA	ni|645904155|nb|KK920278.1|	691774-691900	18	9.4	gi|641576032|gb|KK854997.1|	156533-156960	9.9	38
ACYPI004245-RA	ni|645904120|nb|KK920311.1|	731310-731957	18	18	gi|641586673|gb|KK854207.1|	621323-621637	12	40
ACYPI004246-RA	ni|645903982|nb|KK920449.1|	274879-275897	16	14	gi|641574343|gb|KK855344.1|	59580-60829	12	21
ACYPI004248-RA	ni|645903789|nb|KK920641.1|	487883-488098	17	17	gi|641573919|gb|KK855439.1|	75898-76718	10	37
ACYPI004249-RA	ni|645903920|nb|KK920511.1|	1424215-1424642	20	20	gi|641587823|gb|KK854055.1|	615130-616832	11	22
ACYPI004253-RA	ni|645903748|nb|KK920682.1|	521859-522048	17	18	gi|641586066|gb|KK854296.1|	244284-244503	9.4	37
ACYPI004258-RA	ni|645902496|nb|KK921933.1|	43143-45569	nan	nan	gi|641586585|gb|KK854220.1|	329669-330917	11	44
ACYPI004266-RA	ni|645903960|nb|KK920471.1|	963897-964137	16	8.6	gi|641569328|gb|KK856702.1|	25764-28522	9.6	17
ACYPI004268-RA	ni|645904170|nb|KK920263.1|	744155-744693	15	16	gi|641587404|gb|KK854107.1|	843810-845462	11	42
ACYPI004270-RA	ni|645903770|nb|KK920660.1|	291833-292084	12	15	gi|641587052|gb|KK854150.1|	495914-497788	11	38
ACYPI004271-RA	ni|645903860|nb|KK920570.1|	234961-235920	17	17	gi|641580479|gb|KK854569.1|	152352-153676	10	38

ACYPI004278-RA	ni|645903650|nb|KK920780.1|	574199-577259	nan	nan	gi|641566897|gb|KK857495.1|	13387-17929	11	41
ACYPI004282-RA	ni|645903953|nb|KK920478.1|	1626516-1626760	18	17	gi|641586850|gb|KK854182.1|	581652-582098	10	37
ACYPI004283-RA	ni|645901557|nb|KK922872.1|	37081-37346	nan	nan	gi|641576746|gb|KK854867.1|	102110-102503	11	20
ACYPI004286-RA	ni|645903760|nb|KK920670.1|	672622-673008	18	19	gi|641586983|gb|KK854162.1|	570331-570523	10	41
ACYPI004294-RA	ni|645903566|nb|KK920864.1|	646341-646947	nan	nan	gi|641577374|gb|KK854762.1|	179759-180378	11	42
ACYPI004297-RA	ni|645903843|nb|KK920587.1|	956077-956240	18	16	gi|641576109|gb|KK854982.1|	147009-147235	10	19
ACYPI004307-RA	ni|645904260|nb|KK920228.1|	915794-916419	15	15	gi|641585616|gb|KK854368.1|	170596-171284	8.9	39
ACYPI004308-RA	ni|645904260|nb|KK920228.1|	579285-579507	15	15	gi|641572239|gb|KK855863.1|	148303-148644	11	43
ACYPI004312-RA	ni|645904086|nb|KK920345.1|	395855-397009	14	15	gi|641569596|gb|KK856622.1|	17152-17368	12	43
ACYPI004328-RA	ni|645904137|nb|KK920296.1|	1649706-1651088	17	17	gi|641587118|gb|KK854142.1|	369296-370713	11	40
ACYPI004330-RA	ni|645904251|nb|KK920231.1|	555951-556492	16	16	gi|641587614|gb|KK854081.1|	579202-579465	11	40
ACYPI004333-RA	ni|645904096|nb|KK920335.1|	2213452-2213887	18	17	gi|641576362|gb|KK854933.1|	226001-226467	10	37
ACYPI004334-RA	ni|645904258|nb|KK920229.1|	1594016-1594707	16	16	gi|641587404|gb|KK854107.1|	702164-703401	11	42
ACYPI004343-RA	ni|645904122|nb|KK920309.1|	276961-277772	18	17	gi|641579212|gb|KK854585.1|	243762-245519	10	35
ACYPI004349-RA	ni|645904116|nb|KK920315.1|	1821169-1823111	19	18	gi|641574504|gb|KK855305.1|	68177-68366	9.9	39
ACYPI004355-RA	ni|645903721|nb|KK920709.1|	870385-870646	nan	nan	gi|641586059|gb|KK854297.1|	127906-128888	11	22
ACYPI004366-RA	ni|645904088|nb|KK920343.1|	274452-275280	17	18	gi|641587954|gb|KK854040.1|	176280-176649	8.9	39
ACYPI004368-RA	ni|645903921|nb|KK920510.1|	428521-428770	16	11	gi|641586399|gb|KK854247.1|	542392-545421	15	68
ACYPI004371-RA	ni|645903756|nb|KK920674.1|	670059-671198	16	9	gi|641575991|gb|KK855007.1|	177440-184903	9.8	19
ACYPI004372-RA	ni|645903566|nb|KK920864.1|	455311-455516	nan	nan	gi|641570188|gb|KK856433.1|	93689-95212	10	38
ACYPI004374-RA	ni|645903491|nb|KK920939.1|	576190-576438	nan	nan	gi|641587287|gb|KK854120.1|	281853-282397	9.9	37
ACYPI004378-RA	ni|645903955|nb|KK920476.1|	173976-174869	18	17	gi|641575965|gb|KK855013.1|	186540-187293	9.2	31
ACYPI004385-RA	ni|645903727|nb|KK920703.1|	279408-280206	nan	nan	gi|641566380|gb|KK857684.1|	35837-36343	10	38
ACYPI004388-RA	ni|645903551|nb|KK920879.1|	321057-321239	nan	nan	gi|641581389|gb|KK854557.1|	57116-58589	11	41
ACYPI004393-RA	ni|645904042|nb|KK920389.1|	138625-138784	16	16	gi|641587519|gb|KK854093.1|	964936-965989	11	39
ACYPI004395-RA	ni|645901755|nb|KK922674.1|	18471-19736	nan	nan	gi|641576286|gb|KK854946.1|	293017-294829	12	38
ACYPI004398-RA	ni|645904013|nb|KK920418.1|	534548-535373	19	18	gi|641586837|gb|KK854184.1|	33652-33874	11	40
ACYPI004401-RA	ni|645903971|nb|KK920460.1|	48647-50160	19	18	gi|641588060|gb|KK854028.1|	627875-628203	11	42
ACYPI004409-RA	ni|645903699|nb|KK920731.1|	378448-378904	nan	nan	gi|641576100|gb|KK854984.1|	45274-45824	10	40
ACYPI004410-RA	ni|645903862|nb|KK920568.1|	172941-173960	17	17	gi|641578122|gb|KK854636.1|	175253-177425	12	42
ACYPI004414-RA	ni|645902788|nb|KK921641.1|	205012-205419	nan	nan	gi|641572637|gb|KK855763.1|	189152-189601	9.5	36
ACYPI004419-RA	ni|645904192|nb|KK920255.1|	410184-410972	15	16	gi|641586809|gb|KK854188.1|	179013-181622	10	43

ACYPI004420-RA	ni|645903566|nb|KK920864.1|	613351-613600	nan	nan	gi|641584632|gb|KK854467.1|	426120-427001	9.8	37
ACYPI004421-RA	ni|645904246|nb|KK920233.1|	933495-935260	16	17	gi|641586734|gb|KK854198.1|	698458-700902	11	40
ACYPI004423-RA	ni|645903957|nb|KK920474.1|	844498-844636	18	17	gi|641588000|gb|KK854035.1|	333057-333562	11	39
ACYPI004428-RA	ni|645903663|nb|KK920767.1|	32890-33099	nan	nan	gi|641575104|gb|KK855179.1|	38720-43696	10	35
ACYPI004431-RA	ni|645904116|nb|KK920315.1|	501005-501621	19	18	gi|641577265|gb|KK854780.1|	186469-187309	10	40
ACYPI004435-RA	ni|645903597|nb|KK920833.1|	910585-911169	nan	nan	gi|641574876|gb|KK855226.1|	159233-160009	11	39
ACYPI004440-RA	ni|645903879|nb|KK920552.1|	1099466-1100015	18	18	gi|641586509|gb|KK854231.1|	456230-456786	9.8	37
ACYPI004449-RA	ni|645902783|nb|KK921646.1|	207128-207914	nan	nan	gi|641571841|gb|KK855968.1|	120538-121368	10	36
ACYPI004450-RA	ni|645903871|nb|KK920559.1|	408493-409714	18	9.4	gi|641574459|gb|KK855317.1|	122392-123583	10	21
ACYPI004453-RA	ni|645902649|nb|KK921780.1|	204894-206241	nan	nan	gi|641575297|gb|KK855137.1|	195774-197903	9.2	35
ACYPI004455-RA	ni|645903996|nb|KK920435.1|	1338693-1338926	17	16	gi|641586033|gb|KK854301.1|	427381-427833	11	21
ACYPI004457-RA	ni|645903511|nb|KK920919.1|	340468-340959	nan	nan	gi|641588164|gb|KK854017.1|	599840-600853	9.2	40
ACYPI004460-RA	ni|645904231|nb|KK920242.1|	388995-389151	16	17	gi|641567657|gb|KK857233.1|	33767-34058	9.9	35
ACYPI004461-RA	ni|645904177|nb|KK920260.1|	1990201-1991266	18	17	gi|641576946|gb|KK854832.1|	66010-71603	9.4	43
ACYPI004467-RA	ni|645903697|nb|KK920733.1|	207850-208582	nan	nan	gi|641573053|gb|KK855655.1|	155779-156948	11	41
ACYPI004478-RA	ni|645904114|nb|KK920317.1|	1963858-1964100	18	18	gi|641587390|gb|KK854109.1|	712104-716821	11	44
ACYPI004484-RA	ni|645903895|nb|KK920536.1|	449390-450452	17	16	gi|641567002|gb|KK857458.1|	14734-15492	8.3	33
ACYPI004485-RA	ni|645903470|nb|KK920960.1|	206988-207502	nan	nan	gi|641576666|gb|KK854881.1|	17587-18243	10	37
ACYPI004488-RA	ni|645903574|nb|KK920856.1|	19612-20166	nan	nan	gi|641577244|gb|KK854783.1|	259389-260013	9.7	19
ACYPI004489-RA	ni|645904065|nb|KK920366.1|	1241140-1241335	18	18	gi|641575324|gb|KK855134.1|	124294-125743	11	38
ACYPI004493-RA	ni|645903475|nb|KK920955.1|	261834-262104	nan	nan	gi|641576546|gb|KK854899.1|	104812-112033	11	40
ACYPI004498-RA	ni|645903768|nb|KK920662.1|	1343180-1343435	18	17	gi|641573629|gb|KK855512.1|	126969-127222	10	40
ACYPI004501-RA	ni|645903971|nb|KK920460.1|	698189-698696	19	18	gi|641577075|gb|KK854811.1|	400970-406165	11	36
ACYPI004502-RA	ni|645903773|nb|KK920657.1|	219429-219684	18	17	gi|641577843|gb|KK854684.1|	129690-130091	10	40
ACYPI004505-RA	ni|645904240|nb|KK920239.1|	1798160-1799764	16	16	gi|641587614|gb|KK854081.1|	735354-735819	11	40
ACYPI004513-RA	ni|645904168|nb|KK920265.1|	529291-529720	16	16	gi|641587757|gb|KK854063.1|	367709-371256	11	38
ACYPI004514-RA	ni|645901817|nb|KK922612.1|	72837-73613	nan	nan	gi|641578173|gb|KK854628.1|	66871-67451	9.9	21
ACYPI004515-RA	ni|645904112|nb|KK920319.1|	2115216-2116748	18	17	gi|641588082|gb|KK854026.1|	592713-594405	11	41
ACYPI004520-RA	ni|645904281|nb|KK920220.1|	831339-831560	15	16	gi|641587660|gb|KK854076.1|	255763-257939	9.9	41
ACYPI004521-RA	ni|645903690|nb|KK920740.1|	569499-569954	nan	nan	gi|641576579|gb|KK854894.1|	97177-97423	10	42
ACYPI004522-RA	ni|645904111|nb|KK920320.1|	528442-528621	17	17	gi|641576533|gb|KK854902.1|	12781-15419	10	39
ACYPI004530-RA	ni|645902172|nb|KK922257.1|	177555-178127	nan	nan	gi|641587066|gb|KK854149.1|	657495-657970	11	40

ACYPI004533-RA	ni|645904262|nb|KK920227.1|	732425-732612	17	17	gi|641576315|gb|KK854940.1|	174904-175114	9.9	40
ACYPI004534-RA	ni|645903985|nb|KK920446.1|	1013100-1013660	18	16	gi|641577553|gb|KK854734.1|	95091-96425	9.7	41
ACYPI004535-RA	ni|645904054|nb|KK920377.1|	1471087-1471282	16	16	gi|641587992|gb|KK854036.1|	544366-545052	11	41
ACYPI004544-RA	ni|645903736|nb|KK920694.1|	440807-441515	18	17	gi|641576229|gb|KK854956.1|	264004-264277	9.8	36
ACYPI004549-RA	ni|645903973|nb|KK920458.1|	1471462-1471703	20	18	gi|641587047|gb|KK854151.1|	407861-409085	10	39
ACYPI004558-RA	ni|645903993|nb|KK920438.1|	298551-298766	19	22	gi|641577648|gb|KK854717.1|	120265-120872	9.2	38
ACYPI004559-RA	ni|645904079|nb|KK920352.1|	217402-217949	15	16	gi|641587580|gb|KK854085.1|	469086-469314	10	39
ACYPI004568-RA	ni|645904057|nb|KK920374.1|	166051-166772	16	15	gi|641588073|gb|KK854027.1|	907751-908102	11	41
ACYPI004570-RA	ni|645904117|nb|KK920314.1|	256015-256460	15	15	gi|641585930|gb|KK854318.1|	212445-218613	12	44
ACYPI004575-RA	ni|645904166|nb|KK920267.1|	2702910-2703393	18	16	gi|641569400|gb|KK856682.1|	103771-105097	9.7	34
ACYPI004580-RA	ni|645904228|nb|KK920243.1|	1778676-1779205	17	18	gi|641573097|gb|KK855644.1|	32125-32422	10	36
ACYPI004588-RA	ni|645904251|nb|KK920231.1|	538202-538573	16	16	gi|641587614|gb|KK854081.1|	604630-606614	11	40
ACYPI004594-RA	ni|645903557|nb|KK920873.1|	777735-779356	nan	nan	gi|641586817|gb|KK854187.1|	719359-720789	9.3	39
ACYPI004597-RA	ni|645903474|nb|KK920956.1|	374142-374867	nan	nan	gi|641581402|gb|KK854555.1|	273388-275537	12	20
ACYPI004608-RA	ni|645904244|nb|KK920235.1|	387642-387829	15	14	gi|641569429|gb|KK856673.1|	47806-49532	8.4	36
ACYPI004613-RA	ni|645903977|nb|KK920454.1|	228959-229861	13	16	gi|641586983|gb|KK854162.1|	317027-319410	10	41
ACYPI004615-RA	ni|645904260|nb|KK920228.1|	1049634-1050848	15	15	gi|641585616|gb|KK854368.1|	285987-289150	8.9	39
ACYPI004617-RA	ni|645903957|nb|KK920474.1|	976318-976658	18	17	gi|641575389|gb|KK855120.1|	82974-83397	10	35
ACYPI004619-RA	ni|645903597|nb|KK920833.1|	258266-258724	nan	nan	gi|641586258|gb|KK854267.1|	338208-339084	11	37
ACYPI004623-RA	ni|645903688|nb|KK920742.1|	210991-212187	nan	nan	gi|641578179|gb|KK854627.1|	75534-77289	10	39
ACYPI004627-RA	ni|645903910|nb|KK920521.1|	561599-562098	19	17	gi|641588109|gb|KK854023.1|	198061-198476	10	40
ACYPI004629-RA	ni|645902463|nb|KK921966.1|	231132-232219	nan	nan	gi|641575804|gb|KK855041.1|	277531-279194	11	40
ACYPI004634-RA	ni|645903868|nb|KK920562.1|	441720-441967	16	8.7	gi|641576684|gb|KK854877.1|	6788-7352	10	20
ACYPI004635-RA	ni|645903768|nb|KK920662.1|	1306710-1307624	18	17	gi|641569542|gb|KK856639.1|	25371-25595	9.3	34
ACYPI004640-RA	ni|645903808|nb|KK920622.1|	988853-989136	17	17	gi|641588258|gb|KK854009.1|	341771-343656	11	36
ACYPI004646-RA	ni|645903938|nb|KK920493.1|	1460580-1461744	19	18	gi|641574110|gb|KK855394.1|	46224-46468	10	21
ACYPI004654-RA	ni|645903956|nb|KK920475.1|	801681-803612	20	20	gi|641568808|gb|KK856861.1|	65928-68138	10	45
ACYPI004656-RA	ni|645902669|nb|KK921760.1|	47359-47563	nan	nan	gi|641572468|gb|KK855806.1|	59116-61102	11	39
ACYPI004663-RA	ni|645904177|nb|KK920260.1|	995312-996716	18	17	gi|641574923|gb|KK855216.1|	91698-93305	11	38
ACYPI004665-RA	ni|645904125|nb|KK920307.1|	36652-37219	18	17	gi|641586150|gb|KK854282.1|	191052-191233	11	40
ACYPI004666-RA	ni|645903923|nb|KK920508.1|	165291-165500	18	18	gi|641586741|gb|KK854197.1|	548921-549560	11	43
ACYPI004674-RA	ni|645903787|nb|KK920643.1|	549706-549961	19	18	gi|641543386|gb|KK868296.1|	3022-3328	12	35

ACYPI004675-RA	ni|645904262|nb|KK920227.1|	732757-732976	17	17	gi|641571612|gb|KK856022.1|	152835-153180	12	43
ACYPI004687-RA	ni|645904077|nb|KK920354.1|	264566-265373	16	17	gi|641574031|gb|KK855412.1|	16706-21476	12	39
ACYPI004693-RA	ni|645903762|nb|KK920668.1|	344765-345186	17	17	gi|641577644|gb|KK854718.1|	365295-365573	10	40
ACYPI004696-RA	ni|645903727|nb|KK920703.1|	338427-338812	nan	nan	gi|641586114|gb|KK854288.1|	198254-202401	10	41
ACYPI004697-RA	ni|645904112|nb|KK920319.1|	457243-457446	18	17	gi|641576973|gb|KK854827.1|	125853-126248	11	38
ACYPI004698-RA	ni|645904271|nb|KK920224.1|	452193-452584	15	16	gi|641586626|gb|KK854214.1|	363126-363411	11	44
ACYPI004699-RA	ni|645904112|nb|KK920319.1|	405784-406071	18	17	gi|641576973|gb|KK854827.1|	272840-273139	11	38
ACYPI004701-RA	ni|645904166|nb|KK920267.1|	2675049-2676056	18	16	gi|641586496|gb|KK854233.1|	83529-86482	11	38
ACYPI004715-RA	ni|645901262|nb|KK923167.1|	56562-57239	nan	nan	gi|641588294|gb|KK854006.1|	925566-926940	8.7	40
ACYPI004718-RA	ni|645903541|nb|KK920889.1|	128640-129120	nan	nan	gi|641585767|gb|KK854343.1|	457538-458274	11	22
ACYPI004727-RA	ni|645903597|nb|KK920833.1|	845219-846840	nan	nan	gi|641587652|gb|KK854077.1|	714027-719137	10	38
ACYPI004729-RA	ni|645902395|nb|KK922034.1|	91774-92293	nan	nan	gi|641568762|gb|KK856874.1|	6124-6396	11	41
ACYPI004733-RA	ni|645903822|nb|KK920608.1|	267288-267538	18	20	gi|641584517|gb|KK854476.1|	324639-327725	10	39
ACYPI004738-RA	ni|645904183|nb|KK920258.1|	969466-969957	25	35	gi|641577453|gb|KK854750.1|	65937-66587	10	21
ACYPI004740-RA	ni|645903957|nb|KK920474.1|	1222746-1223539	18	17	gi|641585446|gb|KK854396.1|	379553-379975	11	39
ACYPI004741-RA	ni|645903907|nb|KK920524.1|	228460-229266	16	9	gi|641577922|gb|KK854669.1|	260458-261103	12	21
ACYPI004747-RA	ni|645903981|nb|KK920450.1|	78644-79726	16	8.6	gi|641570346|gb|KK856381.1|	59433-60620	9.9	20
ACYPI004748-RA	ni|645903504|nb|KK920926.1|	367299-367742	nan	nan	gi|641586393|gb|KK854248.1|	410119-410688	9	39
ACYPI004749-RA	ni|645902496|nb|KK921933.1|	139905-140729	nan	nan	gi|641588021|gb|KK854033.1|	255298-255699	12	40
ACYPI004755-RA	ni|645904112|nb|KK920319.1|	221742-221981	18	17	gi|641588090|gb|KK854025.1|	993386-994124	11	43
ACYPI004761-RA	ni|645903855|nb|KK920575.1|	1004450-1004671	16	17	gi|641586166|gb|KK854279.1|	313648-313882	11	45
ACYPI004766-RA	ni|645903823|nb|KK920607.1|	360117-360659	16	17	gi|641572959|gb|KK855678.1|	134025-134442	9.9	36
ACYPI004768-RA	ni|645903981|nb|KK920450.1|	124918-125103	16	8.6	gi|641574699|gb|KK855261.1|	139569-144132	9	35
ACYPI004770-RA	ni|645901937|nb|KK922492.1|	36931-37140	nan	nan	gi|641581402|gb|KK854555.1|	337499-338001	12	20
ACYPI004774-RA	ni|645903752|nb|KK920678.1|	90954-91173	18	16	gi|641585773|gb|KK854342.1|	97482-97841	12	45
ACYPI004783-RA	ni|645903923|nb|KK920508.1|	149019-149721	18	18	gi|641586741|gb|KK854197.1|	516849-517130	11	43
ACYPI004792-RA	ni|645904268|nb|KK920225.1|	868600-868959	14	15	gi|641588194|gb|KK854015.1|	540405-542775	9.4	40
ACYPI004799-RA	ni|645903936|nb|KK920495.1|	964041-964281	19	18	gi|641586185|gb|KK854275.1|	29121-29322	12	40
ACYPI004805-RA	ni|645904166|nb|KK920267.1|	2737685-2737853	18	16	gi|641572912|gb|KK855690.1|	135988-144279	9.9	38
ACYPI004816-RA	ni|645903697|nb|KK920733.1|	104398-105366	nan	nan	gi|641585690|gb|KK854356.1|	513313-518583	9.6	41
ACYPI004820-RA	ni|645904067|nb|KK920364.1|	260046-260291	17	14	gi|641585515|gb|KK854385.1|	542515-542872	11	40
ACYPI004822-RA	ni|645902290|nb|KK922139.1|	142812-143238	nan	nan	gi|641578014|gb|KK854654.1|	374933-376023	12	44

ACYPI004824-RA	ni|645904096|nb|KK920335.1|	2416798-2417710	18	17	gi|641543669|gb|KK868158.1|	4001-4205	10	37
ACYPI004826-RA	ni|645904195|nb|KK920254.1|	2326212-2326516	18	17	gi|641584658|gb|KK854465.1|	389744-389911	11	37
ACYPI004827-RA	ni|645903983|nb|KK920448.1|	716230-716994	18	17	gi|641572191|gb|KK855875.1|	57701-59561	9.7	43
ACYPI004832-RA	ni|645904091|nb|KK920340.1|	657636-657919	15	18	gi|641583088|gb|KK854527.1|	190333-190637	9.9	39
ACYPI004845-RA	ni|645903755|nb|KK920675.1|	110707-110922	18	16	gi|641585847|gb|KK854330.1|	523172-523449	11	41
ACYPI004849-RA	ni|645902522|nb|KK921907.1|	83644-84113	nan	nan	gi|641571439|gb|KK856071.1|	26987-28542	23	43
ACYPI004868-RA	ni|645903751|nb|KK920679.1|	668456-669410	17	16	gi|641588090|gb|KK854025.1|	99216-99820	11	43
ACYPI004870-RA	ni|645903496|nb|KK920934.1|	591520-592676	nan	nan	gi|641573394|gb|KK855572.1|	164240-165459	11	40
ACYPI004872-RA	ni|645902471|nb|KK921958.1|	210443-211128	nan	nan	gi|641573082|gb|KK855648.1|	7162-15362	11	42
ACYPI004878-RA	ni|645904132|nb|KK920301.1|	576845-577012	16	18	gi|641575826|gb|KK855037.1|	139529-139765	10	39
ACYPI004880-RA	ni|645903568|nb|KK920862.1|	224022-224602	nan	nan	gi|641552289|gb|KK864038.1|	2878-3552	7	22
ACYPI004883-RA	ni|645903716|nb|KK920714.1|	266928-268964	nan	nan	gi|641586874|gb|KK854178.1|	392524-394880	9.4	38
ACYPI004886-RA	ni|645903708|nb|KK920722.1|	27374-27583	nan	nan	gi|641584977|gb|KK854443.1|	180222-181073	11	24
ACYPI004908-RA	ni|645903971|nb|KK920460.1|	1573467-1573685	19	18	gi|641584419|gb|KK854483.1|	336349-336580	10	42
ACYPI004909-RA	ni|645903727|nb|KK920703.1|	1397007-1397585	nan	nan	gi|641565711|gb|KK857928.1|	4647-6760	11	36
ACYPI004910-RA	ni|645903808|nb|KK920622.1|	971282-971634	17	17	gi|641588258|gb|KK854009.1|	376568-377488	11	36
ACYPI004911-RA	ni|645902598|nb|KK921831.1|	63118-64202	nan	nan	gi|641585350|gb|KK854411.1|	364501-364905	11	38
ACYPI004913-RA	ni|645904198|nb|KK920253.1|	288076-288524	11	14	gi|641572402|gb|KK855823.1|	97404-97680	11	40
ACYPI004936-RA	ni|645903785|nb|KK920645.1|	450559-450956	15	16	gi|641569630|gb|KK856611.1|	8727-9126	11	39
ACYPI004938-RA	ni|645902681|nb|KK921748.1|	310537-311067	nan	nan	gi|641576414|gb|KK854924.1|	379718-380804	10	40
ACYPI004940-RA	ni|645904045|nb|KK920386.1|	660891-661449	19	21	gi|641575867|gb|KK855030.1|	192580-193004	11	39
ACYPI004941-RA	ni|645903985|nb|KK920446.1|	848569-849261	18	16	gi|641587774|gb|KK854061.1|	925430-926042	9.1	40
ACYPI004947-RA	ni|645903836|nb|KK920594.1|	686180-687064	18	11	gi|641569836|gb|KK856546.1|	2777-2979	12	19
ACYPI004948-RA	ni|645901380|nb|KK923049.1|	59135-62089	nan	nan	gi|641575135|gb|KK855172.1|	206196-210461	11	44
ACYPI004950-RA	ni|645904090|nb|KK920341.1|	297359-298085	15	17	gi|641572569|gb|KK855781.1|	16346-17594	11	39
ACYPI004964-RA	ni|645904074|nb|KK920357.1|	1008029-1008284	17	18	gi|641586359|gb|KK854252.1|	210118-211459	10	39
ACYPI004966-RA	ni|645903957|nb|KK920474.1|	1149806-1150831	18	17	gi|641586713|gb|KK854201.1|	602441-603153	9.1	40
ACYPI004977-RA	ni|645904028|nb|KK920403.1|	377163-379793	18	17	gi|641569948|gb|KK856508.1|	31548-32007	10	37
ACYPI004979-RA	ni|645903534|nb|KK920896.1|	618842-619558	nan	nan	gi|641587074|gb|KK854148.1|	626072-627565	11	41
ACYPI004981-RA	ni|645903513|nb|KK920917.1|	413628-414737	nan	nan	gi|641586191|gb|KK854274.1|	40572-41160	11	42
ACYPI004983-RA	ni|645903535|nb|KK920895.1|	192452-192719	nan	nan	gi|641572297|gb|KK855849.1|	66004-67023	11	21
ACYPI004985-RA	ni|645903781|nb|KK920649.1|	319155-319591	14	15	gi|641575068|gb|KK855186.1|	96154-96894	12	44

ACYPI004986-RA	ni|645903716|nb|KK920714.1|	622792-623290	nan	nan	gi|641587511|gb|KK854094.1|	463978-466404	10	41
ACYPI004995-RA	ni|645903491|nb|KK920939.1|	426546-427219	nan	nan	gi|641577400|gb|KK854757.1|	266871-267138	11	37
ACYPI005000-RA	ni|645904113|nb|KK920318.1|	446869-447110	14	15	gi|641586515|gb|KK854230.1|	101273-101505	11	39
ACYPI005001-RA	ni|645903703|nb|KK920727.1|	301262-301530	nan	nan	gi|641577299|gb|KK854775.1|	385992-388855	11	37
ACYPI005017-RA	ni|645902313|nb|KK922116.1|	140204-141664	nan	nan	gi|641586843|gb|KK854183.1|	748737-749300	11	41
ACYPI005018-RA	ni|645903748|nb|KK920682.1|	324107-324781	17	18	gi|641587249|gb|KK854125.1|	498925-500105	11	37
ACYPI005019-RA	ni|645903603|nb|KK920827.1|	332132-332554	nan	nan	gi|641576010|gb|KK855002.1|	162047-162699	12	43
ACYPI005028-RA	ni|645903866|nb|KK920564.1|	507344-508540	18	15	gi|641587688|gb|KK854072.1|	464976-466457	10	38
ACYPI005038-RA	ni|645904112|nb|KK920319.1|	232715-233665	18	17	gi|641588090|gb|KK854025.1|	1013571-1014274	11	43
ACYPI005040-RA	ni|645904090|nb|KK920341.1|	312204-312325	15	17	gi|641571569|gb|KK856033.1|	112423-112821	9.9	37
ACYPI005041-RA	ni|645902172|nb|KK922257.1|	110453-111178	nan	nan	gi|641587066|gb|KK854149.1|	523781-524317	11	40
ACYPI005044-RA	ni|645904115|nb|KK920316.1|	1087675-1088263	17	18	gi|641574836|gb|KK855234.1|	105075-105240	10	40
ACYPI005047-RA	ni|645903911|nb|KK920520.1|	543859-544272	16	17	gi|641587519|gb|KK854093.1|	353250-353738	11	39
ACYPI005053-RA	ni|645900486|nb|KK923943.1|	49912-50218	nan	nan	gi|641576920|gb|KK854836.1|	127407-127725	11	37
ACYPI005055-RA	ni|645903803|nb|KK920627.1|	872855-873066	17	17	gi|641586127|gb|KK854286.1|	482318-484226	10	43
ACYPI005060-RA	ni|645902538|nb|KK921891.1|	151786-152139	nan	nan	gi|641573737|gb|KK855486.1|	20928-21418	9.1	33
ACYPI005062-RA	ni|645903492|nb|KK920938.1|	61847-62348	nan	nan	gi|641570227|gb|KK856420.1|	107439-107703	10	37
ACYPI005064-RA	ni|645903929|nb|KK920502.1|	1363339-1363518	17	18	gi|641574173|gb|KK855380.1|	248906-249062	9.5	39
ACYPI005067-RA	ni|645904104|nb|KK920327.1|	313298-313596	15	15	gi|641576333|gb|KK854937.1|	39444-40389	9.9	35
ACYPI005081-RA	ni|645903931|nb|KK920500.1|	593917-594619	16	18	gi|641588343|gb|KK854003.1|	1745577-1745949	11	40
ACYPI005089-RA	ni|645903983|nb|KK920448.1|	722278-723856	18	17	gi|641570757|gb|KK856260.1|	153966-157700	10	42
ACYPI005091-RA	ni|645904101|nb|KK920330.1|	18591-19093	18	18	gi|641562121|gb|KK859434.1|	518-867	8	32
ACYPI005093-RA	ni|645903975|nb|KK920456.1|	1140547-1141974	15	8.2	gi|641587793|gb|KK854059.1|	827215-829913	11	38
ACYPI005100-RA	ni|645903697|nb|KK920733.1|	120724-121715	nan	nan	gi|641585690|gb|KK854356.1|	531274-535887	9.6	41
ACYPI005103-RA	ni|645904012|nb|KK920419.1|	235441-235997	27	32	gi|641577890|gb|KK854675.1|	30485-30932	10	40
ACYPI005113-RA	ni|645903983|nb|KK920448.1|	879016-880592	18	17	gi|641569673|gb|KK856597.1|	83050-84993	9.2	38
ACYPI005116-RA	ni|645903787|nb|KK920643.1|	1199864-1200269	19	18	gi|641572177|gb|KK855878.1|	128479-129591	10	35
ACYPI005119-RA	ni|645904132|nb|KK920301.1|	173633-173858	16	18	gi|641575910|gb|KK855021.1|	283615-283976	10	35
ACYPI005122-RA	ni|645903634|nb|KK920796.1|	1096273-1096492	nan	nan	gi|641584445|gb|KK854481.1|	213008-213363	9.9	39
ACYPI005123-RA	ni|645904237|nb|KK920240.1|	1198842-1199030	16	15	gi|641570135|gb|KK856449.1|	40054-41448	9.7	34
ACYPI005124-RA	ni|645903800|nb|KK920630.1|	1556718-1556905	18	17	gi|641575763|gb|KK855049.1|	78639-78851	10	40
ACYPI005125-RA	ni|645901334|nb|KK923095.1|	18231-18468	nan	nan	gi|641586278|gb|KK854264.1|	405651-405897	11	41

ACYPI005133-RA	ni|645903965|nb|KK920466.1|	1020492-1020759	17	16	gi|641578106|gb|KK854639.1|	273424-273694	11	39
ACYPI005144-RA	ni|645904144|nb|KK920289.1|	673521-674551	16	9	gi|641587905|gb|KK854046.1|	533605-533982	10	43
ACYPI005152-RA	ni|645903678|nb|KK920752.1|	435236-436098	nan	nan	gi|641572782|gb|KK855725.1|	106839-107105	10	36
ACYPI005155-RA	ni|645902293|nb|KK922136.1|	66072-66318	nan	nan	gi|641564265|gb|KK858512.1|	4459-5466	11	39
ACYPI005166-RA	ni|645903687|nb|KK920743.1|	54120-54365	nan	nan	gi|641586857|gb|KK854181.1|	237705-238223	9.8	39
ACYPI005167-RA	ni|645904146|nb|KK920287.1|	1377193-1377659	16	18	gi|641586351|gb|KK854253.1|	79758-82802	10	37
ACYPI005171-RA	ni|645902600|nb|KK921829.1|	76985-77420	nan	nan	gi|641585946|gb|KK854315.1|	139906-141568	9.8	43
ACYPI005174-RA	ni|645902129|nb|KK922300.1|	147859-148445	nan	nan	gi|641566437|gb|KK857661.1|	22421-24579	9.2	34
ACYPI005175-RA	ni|645902690|nb|KK921739.1|	292933-293317	nan	nan	gi|641572900|gb|KK855693.1|	244365-246416	11	37
ACYPI005189-RA	ni|645902758|nb|KK921671.1|	360126-360387	nan	nan	gi|641577688|gb|KK854709.1|	117906-118819	11	40
ACYPI005191-RA	ni|645904213|nb|KK920248.1|	1711212-1711489	16	19	gi|641571982|gb|KK855931.1|	32659-35316	9.8	35
ACYPI005193-RA	ni|645903727|nb|KK920703.1|	1126522-1128064	nan	nan	gi|641584582|gb|KK854471.1|	457316-461642	10	38
ACYPI005195-RA	ni|645904201|nb|KK920252.1|	469930-471385	17	16	gi|641570587|gb|KK856311.1|	73300-76214	9.9	38
ACYPI005197-RA	ni|645903800|nb|KK920630.1|	989915-990324	18	17	gi|641587660|gb|KK854076.1|	570033-570805	9.9	41
ACYPI005200-RA	ni|645901741|nb|KK922688.1|	42828-45563	nan	nan	gi|641577097|gb|KK854808.1|	130800-136520	11	41
ACYPI005202-RA	ni|645903996|nb|KK920435.1|	1339129-1339390	17	16	gi|641586033|gb|KK854301.1|	413764-414045	11	21
ACYPI005204-RA	ni|645903592|nb|KK920838.1|	964818-965024	nan	nan	gi|641577347|gb|KK854767.1|	411240-411688	9.9	43
ACYPI005207-RA	ni|645904022|nb|KK920409.1|	209261-209846	18	20	gi|641586857|gb|KK854181.1|	442949-449629	9.8	39
ACYPI005208-RA	ni|645901808|nb|KK922621.1|	104436-104610	nan	nan	gi|641585957|gb|KK854313.1|	214206-223814	10	37
ACYPI005213-RA	ni|645904088|nb|KK920343.1|	480239-480546	17	18	gi|641574600|gb|KK855283.1|	219037-220746	44	35
ACYPI005219-RA	ni|645904139|nb|KK920294.1|	385140-388914	15	18	gi|641567206|gb|KK857389.1|	49552-53372	10	37
ACYPI005220-RA	ni|645902229|nb|KK922200.1|	171682-171902	nan	nan	gi|641587326|gb|KK854115.1|	37680-37900	10	34
ACYPI005221-RA	ni|645903953|nb|KK920478.1|	1394055-1394425	18	17	gi|641585440|gb|KK854397.1|	400089-406486	9	38
ACYPI005227-RA	ni|645904019|nb|KK920412.1|	152257-152733	19	9.4	gi|641574508|gb|KK855304.1|	156978-160016	11	22
ACYPI005230-RA	ni|645904009|nb|KK920422.1|	310576-310755	17	9.4	gi|641568558|gb|KK856940.1|	58998-59488	11	41
ACYPI005233-RA	ni|645904213|nb|KK920248.1|	991614-991998	16	19	gi|641574704|gb|KK855260.1|	57382-60808	9.3	35
ACYPI005234-RA	ni|645902529|nb|KK921900.1|	52812-53954	nan	nan	gi|641586344|gb|KK854254.1|	615553-617814	10	40
ACYPI005241-RA	ni|645902556|nb|KK921873.1|	49937-50656	nan	nan	gi|641575214|gb|KK855156.1|	191420-191817	8.8	34
ACYPI005243-RA	ni|645903838|nb|KK920592.1|	211824-212061	16	16	gi|641574078|gb|KK855401.1|	215069-221662	11	41
ACYPI005247-RA	ni|645903644|nb|KK920786.1|	397188-398703	nan	nan	gi|641584445|gb|KK854481.1|	415356-416885	9.9	39
ACYPI005253-RA	ni|645903495|nb|KK920935.1|	450806-451174	nan	nan	gi|641576409|gb|KK854925.1|	102260-102986	11	44
ACYPI005259-RA	ni|645903917|nb|KK920514.1|	728107-728820	19	17	gi|641585604|gb|KK854370.1|	528129-528603	9.8	37

ACYPI005264-RA	ni|645904154|nb|KK920279.1|	710537-711640	14	14	gi|641587153|gb|KK854137.1|	869701-871131	10	42
ACYPI005270-RA	ni|645904077|nb|KK920354.1|	705564-705709	16	17	gi|641584504|gb|KK854477.1|	176959-177696	9.5	38
ACYPI005271-RA	ni|645903771|nb|KK920659.1|	28378-28555	16	15	gi|641567973|gb|KK857126.1|	55336-55499	10	39
ACYPI005282-RA	ni|645901710|nb|KK922719.1|	73895-74944	nan	nan	gi|641573036|gb|KK855659.1|	185266-190129	13	49
ACYPI005283-RA	ni|645903627|nb|KK920803.1|	506009-507155	nan	nan	gi|641584728|gb|KK854460.1|	191603-192662	9.5	37
ACYPI005295-RA	ni|645903572|nb|KK920858.1|	98432-98634	nan	nan	gi|641577535|gb|KK854737.1|	469401-470162	11	42
ACYPI005296-RA	ni|645903513|nb|KK920917.1|	431891-432363	nan	nan	gi|641586928|gb|KK854169.1|	579498-585939	11	39
ACYPI005300-RA	ni|645903855|nb|KK920575.1|	1046028-1046940	16	17	gi|641566900|gb|KK857494.1|	38695-39377	9.9	40
ACYPI005308-RA	ni|645903780|nb|KK920650.1|	689781-690381	19	19	gi|641585801|gb|KK854337.1|	528369-530177	10	38
ACYPI005309-RA	ni|645904002|nb|KK920429.1|	357420-357664	14	18	gi|641588309|gb|KK854005.1|	746950-747350	11	40
ACYPI005313-RA	ni|645904114|nb|KK920317.1|	1910124-1910376	18	18	gi|641576328|gb|KK854938.1|	182188-182646	11	36
ACYPI005317-RA	ni|645903643|nb|KK920787.1|	137981-138383	nan	nan	gi|641575593|gb|KK855082.1|	163841-164336	10	38
ACYPI005321-RA	ni|645903825|nb|KK920605.1|	755385-756517	19	17	gi|641574022|gb|KK855414.1|	109481-110349	11	33
ACYPI005325-RA	ni|645903627|nb|KK920803.1|	474225-474436	nan	nan	gi|641586874|gb|KK854178.1|	532628-532841	9.4	38
ACYPI005326-RA	ni|645903925|nb|KK920506.1|	676544-677276	19	17	gi|641588325|gb|KK854004.1|	1071087-1072532	11	40
ACYPI005327-RA	ni|645903702|nb|KK920728.1|	388929-389727	nan	nan	gi|641588294|gb|KK854006.1|	410837-423597	8.7	40
ACYPI005329-RA	ni|645904061|nb|KK920370.1|	1048258-1048497	17	16	gi|641574746|gb|KK855253.1|	100521-101398	8.9	35
ACYPI005331-RA	ni|645904004|nb|KK920427.1|	840532-840922	16	16	gi|641583574|gb|KK854520.1|	412333-413035	9.2	40
ACYPI005339-RA	ni|645904262|nb|KK920227.1|	656673-657184	17	17	gi|641576315|gb|KK854940.1|	171178-173268	9.9	40
ACYPI005346-RA	ni|645904065|nb|KK920366.1|	18734-19163	18	18	gi|641577826|gb|KK854687.1|	4848-5882	9.4	37
ACYPI005349-RA	ni|645903918|nb|KK920513.1|	310130-310625	15	18	gi|641588164|gb|KK854017.1|	936166-936869	9.2	40
ACYPI005350-RA	ni|645902772|nb|KK921657.1|	258437-260296	nan	nan	gi|641588033|gb|KK854031.1|	842532-845820	11	38
ACYPI005357-RA	ni|645903834|nb|KK920596.1|	1428012-1428255	18	17	gi|641573323|gb|KK855590.1|	245640-245861	10	43
ACYPI005359-RA	ni|645903988|nb|KK920443.1|	505487-506255	14	16	gi|641577704|gb|KK854707.1|	413030-419324	10	38
ACYPI005360-RA	ni|645904065|nb|KK920366.1|	963245-963848	18	18	gi|641586235|gb|KK854270.1|	99823-101676	11	39
ACYPI005363-RA	ni|645903958|nb|KK920473.1|	25119-26075	16	16	gi|641585725|gb|KK854350.1|	579913-581463	12	41
ACYPI005364-RA	ni|645903574|nb|KK920856.1|	95005-95530	nan	nan	gi|641576791|gb|KK854858.1|	126215-126547	9.2	42
ACYPI005367-RA	ni|645903803|nb|KK920627.1|	819586-820867	17	17	gi|641587848|gb|KK854052.1|	682585-684933	11	39
ACYPI005368-RA	ni|645903961|nb|KK920470.1|	866911-867475	17	15	gi|641586096|gb|KK854291.1|	187434-189058	11	46
ACYPI005389-RA	ni|645904204|nb|KK920251.1|	782045-786457	15	16	gi|641575759|gb|KK855050.1|	389881-390358	11	38
ACYPI005391-RA	ni|645904177|nb|KK920260.1|	1495333-1496298	18	17	gi|641586203|gb|KK854273.1|	487310-490046	9.5	40
ACYPI005393-RA	ni|645903985|nb|KK920446.1|	453773-454015	18	16	gi|641579952|gb|KK854573.1|	297496-297707	11	21

ACYPI005394-RA	ni|645901763|nb|KK922666.1|	44341-45141	nan	nan	gi|641575987|gb|KK855008.1|	40582-41061	9.9	20
ACYPI005395-RA	ni|645902556|nb|KK921873.1|	55973-56519	nan	nan	gi|641563481|gb|KK858852.1|	20967-21424	8.7	35
ACYPI005400-RA	ni|645903787|nb|KK920643.1|	1271270-1272930	19	18	gi|641575222|gb|KK855154.1|	84349-86176	11	37
ACYPI005416-RA	ni|645904134|nb|KK920299.1|	441790-442267	17	13	gi|641577516|gb|KK854740.1|	132328-132623	10	21
ACYPI005417-RA	ni|645902593|nb|KK921836.1|	52075-55794	nan	nan	gi|641586921|gb|KK854170.1|	613915-617331	12	48
ACYPI005419-RA	ni|645903873|nb|KK920558.1|	662728-662833	16	15	gi|641562852|gb|KK859115.1|	4933-6125	10	35
ACYPI005422-RA	ni|645904164|nb|KK920269.1|	347242-347695	19	20	gi|641568399|gb|KK856989.1|	29740-30547	12	40
ACYPI005423-RA	ni|645904133|nb|KK920300.1|	2068967-2071457	18	18	gi|641588309|gb|KK854005.1|	401534-404042	11	40
ACYPI005434-RA	ni|645903851|nb|KK920579.1|	246228-247288	14	19	gi|641574508|gb|KK855304.1|	201492-202297	11	22
ACYPI005444-RA	ni|645903743|nb|KK920687.1|	1183192-1183357	19	18	gi|641587452|gb|KK854101.1|	869007-870951	11	40
ACYPI005456-RA	ni|645903981|nb|KK920450.1|	455935-456537	16	8.6	gi|641578069|gb|KK854646.1|	96534-97246	9.9	20
ACYPI005457-RA	ni|645903547|nb|KK920883.1|	627388-627777	nan	nan	gi|641570134|gb|KK856450.1|	101957-102630	10	38
ACYPI005464-RA	ni|645900799|nb|KK923630.1|	6023-6272	nan	nan	gi|641575468|gb|KK855105.1|	109734-110182	9.5	20
ACYPI005468-RA	ni|645903744|nb|KK920686.1|	575153-575302	17	8.6	gi|641577179|gb|KK854794.1|	17765-18113	11	22
ACYPI005470-RA	ni|645904077|nb|KK920354.1|	663260-664414	16	17	gi|641567321|gb|KK857348.1|	63741-65314	19	43
ACYPI005476-RA	ni|645904137|nb|KK920296.1|	1303808-1304547	17	17	gi|641577760|gb|KK854699.1|	177971-178961	9.8	41
ACYPI005478-RA	ni|645904201|nb|KK920252.1|	334053-334539	17	16	gi|641588060|gb|KK854028.1|	715514-715774	11	42
ACYPI005479-RA	ni|645903784|nb|KK920646.1|	340916-341776	17	19	gi|641585342|gb|KK854413.1|	58972-61381	11	39
ACYPI005480-RA	ni|645904246|nb|KK920233.1|	1205132-1207608	16	17	gi|641570089|gb|KK856463.1|	107132-110839	11	36
ACYPI005482-RA	ni|645904024|nb|KK920407.1|	541012-541576	14	16	gi|641587671|gb|KK854074.1|	285826-287868	11	40
ACYPI005489-RA	ni|645904168|nb|KK920265.1|	1579185-1579530	16	16	gi|641571849|gb|KK855965.1|	84351-84907	11	40
ACYPI005492-RA	ni|645903742|nb|KK920688.1|	498277-498503	17	16	gi|641587452|gb|KK854101.1|	302070-302311	11	40
ACYPI005496-RA	ni|645903694|nb|KK920736.1|	422731-423446	nan	nan	gi|641587527|gb|KK854092.1|	149009-150831	10	38
ACYPI005499-RA	ni|645903612|nb|KK920818.1|	95659-96271	nan	nan	gi|641571428|gb|KK856074.1|	123365-124427	11	39
ACYPI005502-RA	ni|645904112|nb|KK920319.1|	2851364-2851641	18	17	gi|641577341|gb|KK854768.1|	207706-207887	14	58
ACYPI005509-RA	ni|645903970|nb|KK920461.1|	840452-841449	17	16	gi|641585642|gb|KK854364.1|	135183-135547	9.9	38
ACYPI005512-RA	ni|645903627|nb|KK920803.1|	238941-239709	nan	nan	gi|641570042|gb|KK856478.1|	90750-91459	10	36
ACYPI005514-RA	ni|645903547|nb|KK920883.1|	500931-501158	nan	nan	gi|641579937|gb|KK854576.1|	460084-460348	9.4	41
ACYPI005516-RA	ni|645904065|nb|KK920366.1|	972657-973459	18	18	gi|641586535|gb|KK854227.1|	498165-499045	10	39
ACYPI005517-RA	ni|645903592|nb|KK920838.1|	573285-574973	nan	nan	gi|641584681|gb|KK854464.1|	40862-44650	11	42
ACYPI005521-RA	ni|645904134|nb|KK920299.1|	849449-850516	17	13	gi|641587866|gb|KK854050.1|	453414-455214	10	38
ACYPI005523-RA	ni|645902144|nb|KK922285.1|	38421-39046	nan	nan	gi|641587009|gb|KK854158.1|	448125-450914	11	41

ACYPI005524-RA	ni|645904086|nb|KK920345.1|	410385-411669	14	15	gi|641556681|gb|KK861957.1|	7030-12038	9.6	38
ACYPI005525-RA	ni|645904152|nb|KK920281.1|	432807-433798	15	15	gi|641587365|gb|KK854113.1|	381765-382475	12	45
ACYPI005528-RA	ni|645903534|nb|KK920896.1|	579054-579287	nan	nan	gi|641587074|gb|KK854148.1|	585817-586526	11	41
ACYPI005529-RA	ni|645904163|nb|KK920270.1|	1185945-1186372	16	16	gi|641569985|gb|KK856496.1|	102883-103638	11	39
ACYPI005535-RA	ni|645904058|nb|KK920373.1|	336435-337268	16	15	gi|641570280|gb|KK856402.1|	107703-108266	11	40
ACYPI005543-RA	ni|645903639|nb|KK920791.1|	413526-414440	nan	nan	gi|641586955|gb|KK854165.1|	578742-579556	11	40
ACYPI005545-RA	ni|645903865|nb|KK920565.1|	365577-366039	15	15	gi|641588246|gb|KK854010.1|	1157286-1158645	9.2	50
ACYPI005557-RA	ni|645903569|nb|KK920861.1|	255917-256907	nan	nan	gi|641586928|gb|KK854169.1|	130737-132561	11	39
ACYPI005570-RA	ni|645903977|nb|KK920454.1|	201631-202200	13	16	gi|641585469|gb|KK854393.1|	250779-251231	10	37
ACYPI005579-RA	ni|645904077|nb|KK920354.1|	683816-684211	16	17	gi|641584504|gb|KK854477.1|	196657-197768	9.5	38
ACYPI005580-RA	ni|645903762|nb|KK920668.1|	884594-885578	17	17	gi|641576608|gb|KK854891.1|	218827-226381	11	37
ACYPI005583-RA	ni|645903846|nb|KK920584.1|	55085-55966	17	10	gi|641572719|gb|KK855742.1|	196892-199969	12	23
ACYPI005585-RA	ni|645904201|nb|KK920252.1|	183108-183483	17	16	gi|641570819|gb|KK856245.1|	50230-52543	10	45
ACYPI005586-RA	ni|645904112|nb|KK920319.1|	2574053-2574610	18	17	gi|641576188|gb|KK854966.1|	145493-145875	11	39
ACYPI005588-RA	ni|645903849|nb|KK920581.1|	60264-61423	17	17	gi|641587555|gb|KK854089.1|	302025-302829	11	39
ACYPI005593-RA	ni|645902354|nb|KK922075.1|	125620-125890	nan	nan	gi|641570141|gb|KK856447.1|	91282-91541	11	40
ACYPI005594-RA	ni|645904123|nb|KK920308.1|	1606239-1606800	16	10	gi|641569929|gb|KK856514.1|	83410-83629	10	18
ACYPI005597-RA	ni|645903464|nb|KK920966.1|	444766-445195	nan	nan	gi|641585340|gb|KK854414.1|	459480-460016	11	42
ACYPI005600-RA	ni|645903545|nb|KK920885.1|	58888-59114	nan	nan	gi|641586893|gb|KK854175.1|	507570-510208	11	41
ACYPI005606-RA	ni|645904251|nb|KK920231.1|	392565-392735	16	16	gi|641583313|gb|KK854525.1|	340100-341711	9.5	40
ACYPI005607-RA	ni|645904027|nb|KK920404.1|	598107-599723	17	17	gi|641573015|gb|KK855664.1|	83545-84307	11	42
ACYPI005613-RA	ni|645903836|nb|KK920594.1|	169031-170158	18	11	gi|641577271|gb|KK854779.1|	200827-204746	11	21
ACYPI005614-RA	ni|645903491|nb|KK920939.1|	648313-649104	nan	nan	gi|641550113|gb|KK865049.1|	2201-3140	11	48
ACYPI005619-RA	ni|645904015|nb|KK920416.1|	1253180-1255265	18	16	gi|641585423|gb|KK854400.1|	71641-73816	11	36
ACYPI005621-RA	ni|645903716|nb|KK920714.1|	315558-315789	nan	nan	gi|641586874|gb|KK854178.1|	295555-296148	9.4	38
ACYPI005622-RA	ni|645902471|nb|KK921958.1|	183292-184343	nan	nan	gi|641569260|gb|KK856723.1|	24941-25724	9.7	34
ACYPI005626-RA	ni|645903539|nb|KK920891.1|	144225-144461	nan	nan	gi|641587462|gb|KK854100.1|	280175-280563	10	21
ACYPI005627-RA	ni|645903709|nb|KK920721.1|	260922-262443	nan	nan	gi|641576074|gb|KK854988.1|	255584-258255	12	46
ACYPI005638-RA	ni|645901989|nb|KK922440.1|	8684-8899	nan	nan	gi|641587256|gb|KK854124.1|	319168-322305	9.7	40
ACYPI005644-RA	ni|645902662|nb|KK921767.1|	422594-422809	nan	nan	gi|641587171|gb|KK854135.1|	488624-491178	10	39
ACYPI005647-RA	ni|645903796|nb|KK920634.1|	913461-913990	18	17	gi|641588051|gb|KK854029.1|	813722-817983	9.6	40
ACYPI005655-RA	ni|645903873|nb|KK920558.1|	469213-470024	16	15	gi|641580486|gb|KK854568.1|	117604-118579	11	36

ACYPI005658-RA	ni|645903736|nb|KK920694.1|	300146-300787	18	17	gi|641576229|gb|KK854956.1|	34945-35810	9.8	36
ACYPI005659-RA	ni|645903762|nb|KK920668.1|	528044-528549	17	17	gi|641571425|gb|KK856075.1|	43573-44914	9.3	36
ACYPI005660-RA	ni|645904138|nb|KK920295.1|	471760-473136	15	15	gi|641576266|gb|KK854949.1|	276746-282927	11	45
ACYPI005661-RA	ni|645903982|nb|KK920449.1|	56583-57174	16	14	gi|641584945|gb|KK854445.1|	279555-280043	9.9	20
ACYPI005668-RA	ni|645903648|nb|KK920782.1|	256402-256734	nan	nan	gi|641567546|gb|KK857269.1|	31845-32322	8.5	40
ACYPI005672-RA	ni|645904009|nb|KK920422.1|	839049-840138	17	9.4	gi|641575218|gb|KK855155.1|	162305-163819	11	21
ACYPI005673-RA	ni|645903696|nb|KK920734.1|	559883-560274	nan	nan	gi|641586033|gb|KK854301.1|	123736-124572	11	21
ACYPI005674-RA	ni|645904051|nb|KK920380.1|	480022-480316	16	17	gi|641570807|gb|KK856248.1|	29073-29837	11	38
ACYPI005676-RA	ni|645904052|nb|KK920379.1|	576008-576381	18	16	gi|641575656|gb|KK855069.1|	50248-50699	11	42
ACYPI005677-RA	ni|645904007|nb|KK920424.1|	420823-421043	18	17	gi|641586734|gb|KK854198.1|	802019-806121	11	40
ACYPI005682-RA	ni|645902600|nb|KK921829.1|	137335-137614	nan	nan	gi|641585946|gb|KK854315.1|	187114-188288	9.8	43
ACYPI005687-RA	ni|645902371|nb|KK922058.1|	113237-113981	nan	nan	gi|641585755|gb|KK854345.1|	56916-60099	9.8	39
ACYPI005689-RA	ni|645904093|nb|KK920338.1|	33075-33731	13	15	gi|641574882|gb|KK855225.1|	166390-167975	9.3	36
ACYPI005703-RA	ni|645904228|nb|KK920243.1|	1201831-1202641	17	18	gi|641577541|gb|KK854736.1|	133913-134291	10	38
ACYPI005705-RA	ni|645903642|nb|KK920788.1|	384543-384874	nan	nan	gi|641584876|gb|KK854450.1|	219293-219645	11	25
ACYPI005706-RA	ni|645904177|nb|KK920260.1|	2568717-2568910	18	17	gi|641578084|gb|KK854643.1|	250661-251047	11	40
ACYPI005711-RA	ni|645903869|nb|KK920561.1|	957481-959306	17	15	gi|641570861|gb|KK856232.1|	164144-164695	11	39
ACYPI005720-RA	ni|645903798|nb|KK920632.1|	63777-64298	18	17	gi|641566812|gb|KK857525.1|	66294-66835	11	42
ACYPI005722-RA	ni|645901356|nb|KK923073.1|	6022-6956	nan	nan	gi|641577022|gb|KK854820.1|	264209-265826	10	21
ACYPI005729-RA	ni|645903906|nb|KK920525.1|	370642-371179	18	22	gi|641573571|gb|KK855526.1|	73764-74598	9.8	36
ACYPI005735-RA	ni|645903973|nb|KK920458.1|	1312921-1313506	20	18	gi|641587660|gb|KK854076.1|	548013-553435	9.9	41
ACYPI005743-RA	ni|645904023|nb|KK920408.1|	727289-731538	17	17	gi|641587383|gb|KK854110.1|	505574-510334	10	37
ACYPI005747-RA	ni|645904007|nb|KK920424.1|	332050-334718	18	17	gi|641586915|gb|KK854171.1|	500042-504602	10	38
ACYPI005751-RA	ni|645904240|nb|KK920239.1|	1219655-1220125	16	16	gi|641587735|gb|KK854066.1|	737657-737866	11	42
ACYPI005761-RA	ni|645902662|nb|KK921767.1|	296505-298449	nan	nan	gi|641569155|gb|KK856752.1|	97415-98955	11	21
ACYPI005769-RA	ni|645903804|nb|KK920626.1|	618759-619033	17	17	gi|641587383|gb|KK854110.1|	124758-125027	10	37
ACYPI005770-RA	ni|645902084|nb|KK922345.1|	118102-118554	nan	nan	gi|641587084|gb|KK854146.1|	539067-539335	9	40
ACYPI005773-RA	ni|645903644|nb|KK920786.1|	147865-148815	nan	nan	gi|641575372|gb|KK855124.1|	107319-107561	9.5	36
ACYPI005774-RA	ni|645903957|nb|KK920474.1|	1424751-1425165	18	17	gi|641568044|gb|KK857103.1|	26679-28078	10	36
ACYPI005777-RA	ni|645904088|nb|KK920343.1|	1297357-1297871	17	18	gi|641579930|gb|KK854578.1|	7770-8431	9.1	35
ACYPI005778-RA	ni|645904118|nb|KK920313.1|	707702-708492	17	17	gi|641585871|gb|KK854327.1|	58229-58673	11	38
ACYPI005784-RA	ni|645903985|nb|KK920446.1|	775004-775409	18	16	gi|641577501|gb|KK854743.1|	109963-110291	9.1	37

ACYPI005787-RA	ni|645904115|nb|KK920316.1|	1802020-1802247	17	18	gi|641579925|gb|KK854579.1|	223946-224697	9.7	40
ACYPI005789-RA	ni|645902471|nb|KK921958.1|	191580-191700	nan	nan	gi|641588033|gb|KK854031.1|	1256161-1256507	11	38
ACYPI005791-RA	ni|645903849|nb|KK920581.1|	540033-540226	17	17	gi|641576404|gb|KK854926.1|	45672-45888	11	40
ACYPI005793-RA	ni|645904015|nb|KK920416.1|	1315364-1316340	18	16	gi|641571308|gb|KK856106.1|	33544-36369	8.9	36
ACYPI005802-RA	ni|645904062|nb|KK920369.1|	82197-82446	14	15	gi|641578109|gb|KK854638.1|	280794-288242	14	46
ACYPI005809-RA	ni|645904116|nb|KK920315.1|	650221-651171	19	18	gi|641577265|gb|KK854780.1|	306766-308794	10	40
ACYPI005817-RA	ni|645903688|nb|KK920742.1|	222429-222764	nan	nan	gi|641587614|gb|KK854081.1|	621167-622162	11	40
ACYPI005824-RA	ni|645904222|nb|KK920245.1|	692309-692579	17	12	gi|641585395|gb|KK854405.1|	453291-455413	10	38
ACYPI005826-RA	ni|645902468|nb|KK921961.1|	82852-83463	nan	nan	gi|641566738|gb|KK857551.1|	5239-5401	11	39
ACYPI005830-RA	ni|645903650|nb|KK920780.1|	820836-820987	nan	nan	gi|641588082|gb|KK854026.1|	1173913-1174812	11	41
ACYPI005832-RA	ni|645903592|nb|KK920838.1|	875477-875682	nan	nan	gi|641573729|gb|KK855488.1|	191358-193548	10	43
ACYPI005844-RA	ni|645903971|nb|KK920460.1|	1228698-1229331	19	18	gi|641585833|gb|KK854332.1|	167986-168474	10	37
ACYPI005852-RA	ni|645904116|nb|KK920315.1|	655723-656372	19	18	gi|641542181|gb|KK868875.1|	94-437	9.8	32
ACYPI005853-RA	ni|645904231|nb|KK920242.1|	586392-588632	16	17	gi|641571886|gb|KK855956.1|	70977-73719	9.4	35
ACYPI005858-RA	ni|645903848|nb|KK920582.1|	604272-604444	18	18	gi|641574275|gb|KK855359.1|	188970-199118	10	38
ACYPI005859-RA	ni|645904122|nb|KK920309.1|	194050-194558	18	17	gi|641588033|gb|KK854031.1|	489134-489475	11	38
ACYPI005864-RA	ni|645903588|nb|KK920842.1|	613875-614319	nan	nan	gi|641565060|gb|KK858177.1|	6660-7589	9.3	40
ACYPI005865-RA	ni|645904052|nb|KK920379.1|	540363-541135	18	16	gi|641570198|gb|KK856430.1|	40541-48319	10	36
ACYPI005867-RA	ni|645902172|nb|KK922257.1|	159028-159242	nan	nan	gi|641587066|gb|KK854149.1|	574712-574970	11	40
ACYPI005877-RA	ni|645903958|nb|KK920473.1|	480480-480656	16	16	gi|641585982|gb|KK854309.1|	574028-574209	11	41
ACYPI005880-RA	ni|645904116|nb|KK920315.1|	2189215-2189699	19	18	gi|641554119|gb|KK863172.1|	1850-2091	7	24
ACYPI005883-RA	ni|645903635|nb|KK920795.1|	326326-327821	nan	nan	gi|641575377|gb|KK855123.1|	64068-65287	10	38
ACYPI005886-RA	ni|645902742|nb|KK921687.1|	140037-144926	nan	nan	gi|641587840|gb|KK854053.1|	1022709-1023872	11	43
ACYPI005896-RA	ni|645903734|nb|KK920696.1|	278859-279218	nan	nan	gi|641578330|gb|KK854601.1|	311508-313591	11	38
ACYPI005897-RA	ni|645903738|nb|KK920692.1|	911121-912293	18	17	gi|641575549|gb|KK855090.1|	71895-74070	10	38
ACYPI005899-RA	ni|645903635|nb|KK920795.1|	289480-289973	nan	nan	gi|641575377|gb|KK855123.1|	25715-27356	10	38
ACYPI005900-RA	ni|645903919|nb|KK920512.1|	1191073-1191295	17	15	gi|641566085|gb|KK857791.1|	15560-15773	11	38
ACYPI005903-RA	ni|645903848|nb|KK920582.1|	558886-559416	18	18	gi|641572637|gb|KK855763.1|	114130-118036	9.5	36
ACYPI005908-RA	ni|645903662|nb|KK920768.1|	210882-211773	nan	nan	gi|641573733|gb|KK855487.1|	23403-24610	13	45
ACYPI005913-RA	ni|645903498|nb|KK920932.1|	509784-510193	nan	nan	gi|641588119|gb|KK854022.1|	935624-936217	10	40
ACYPI005914-RA	ni|645904058|nb|KK920373.1|	695194-695784	16	15	gi|641588236|gb|KK854011.1|	384740-385938	9.7	38
ACYPI005925-RA	ni|645903696|nb|KK920734.1|	407629-408730	nan	nan	gi|641575218|gb|KK855155.1|	5278-5596	11	21

ACYPI005928-RA	ni|645904116|nb|KK920315.1|	1839006-1839207	19	18	gi|641574504|gb|KK855305.1|	9130-9511	9.9	39
ACYPI005934-RA	ni|645904245|nb|KK920234.1|	497794-498277	16	17	gi|641588194|gb|KK854015.1|	654638-658076	9.4	40
ACYPI005940-RA	ni|645903499|nb|KK920931.1|	143518-144160	nan	nan	gi|641584281|gb|KK854493.1|	50209-50717	12	41
ACYPI005943-RA	ni|645904050|nb|KK920381.1|	36720-37081	18	20	gi|641570098|gb|KK856462.1|	79896-80143	12	43
ACYPI005949-RA	ni|645903931|nb|KK920500.1|	564017-564471	16	18	gi|641569851|gb|KK856541.1|	65317-70785	11	21
ACYPI005955-RA	ni|645904086|nb|KK920345.1|	418009-418377	14	15	gi|641556681|gb|KK861957.1|	16026-16331	9.6	38
ACYPI005962-RA	ni|645903847|nb|KK920583.1|	1355387-1356503	18	18	gi|641575867|gb|KK855030.1|	339703-344149	11	39
ACYPI005967-RA	ni|645902497|nb|KK921932.1|	182742-183088	nan	nan	gi|641588309|gb|KK854005.1|	294109-294307	11	40
ACYPI005972-RA	ni|645903619|nb|KK920811.1|	600346-600751	nan	nan	gi|641573334|gb|KK855587.1|	171258-171893	10	39
ACYPI005987-RA	ni|645904111|nb|KK920320.1|	1519708-1520820	17	17	gi|641585967|gb|KK854311.1|	143701-144312	11	40
ACYPI005988-RA	ni|645904088|nb|KK920343.1|	197068-197525	17	18	gi|641569948|gb|KK856508.1|	192939-193218	10	37
ACYPI005993-RA	ni|645903884|nb|KK920547.1|	431836-432278	16	19	gi|641574424|gb|KK855324.1|	131781-132637	10	39
ACYPI005997-RA	ni|645903806|nb|KK920624.1|	64708-65009	16	9.3	gi|641587912|gb|KK854045.1|	713341-713667	11	39
ACYPI006008-RA	ni|645903655|nb|KK920775.1|	22059-22780	nan	nan	gi|641558930|gb|KK860898.1|	7198-8430	8.8	34
ACYPI006009-RA	ni|645904152|nb|KK920281.1|	490715-494142	15	15	gi|641587365|gb|KK854113.1|	307805-312010	12	45
ACYPI006010-RA	ni|645903512|nb|KK920918.1|	476158-476593	nan	nan	gi|641568098|gb|KK857086.1|	81822-82624	9.9	18
ACYPI006013-RA	ni|645903665|nb|KK920765.1|	258979-259719	nan	nan	gi|641568533|gb|KK856949.1|	78400-81429	9.7	38
ACYPI006018-RA	ni|645903491|nb|KK920939.1|	537069-537568	nan	nan	gi|641557619|gb|KK861506.1|	2460-3138	7.4	27
ACYPI006021-RA	ni|645903751|nb|KK920679.1|	1164339-1165335	17	16	gi|641587256|gb|KK854124.1|	232268-232538	9.7	40
ACYPI006023-RA	ni|645904077|nb|KK920354.1|	749545-749986	16	17	gi|641575449|gb|KK855108.1|	35986-36476	11	45
ACYPI006026-RA	ni|645904228|nb|KK920243.1|	1273740-1274345	17	18	gi|641588294|gb|KK854006.1|	783720-784688	8.7	40
ACYPI006028-RA	ni|645903622|nb|KK920808.1|	688236-689374	nan	nan	gi|641575635|gb|KK855074.1|	268682-271378	9.6	38
ACYPI006033-RA	ni|645903557|nb|KK920873.1|	1172871-1173128	nan	nan	gi|641586335|gb|KK854256.1|	422890-424570	10	38
ACYPI006038-RA	ni|645903858|nb|KK920572.1|	908277-908695	16	16	gi|641585515|gb|KK854385.1|	184366-184707	11	40
ACYPI006039-RA	ni|645904145|nb|KK920288.1|	429623-430445	16	9.3	gi|641554246|gb|KK863113.1|	48-804	9.3	13
ACYPI006043-RA	ni|645903898|nb|KK920533.1|	453573-453923	17	16	gi|641572860|gb|KK855703.1|	5840-9040	9.8	36
ACYPI006058-RA	ni|645903568|nb|KK920862.1|	230542-230789	nan	nan	gi|641568319|gb|KK857014.1|	14952-15195	9.5	33
ACYPI006059-RA	ni|645904044|nb|KK920387.1|	1355635-1356063	15	16	gi|641575725|gb|KK855055.1|	168327-168540	10	37
ACYPI006064-RA	ni|645903853|nb|KK920577.1|	658866-659624	16	16	gi|641574654|gb|KK855271.1|	79488-80365	9.5	36
ACYPI006066-RA	ni|645903811|nb|KK920619.1|	546584-546951	15	15	gi|641586975|gb|KK854163.1|	532106-535528	11	40
ACYPI006067-RA	ni|645904004|nb|KK920427.1|	1268061-1270141	16	16	gi|641584305|gb|KK854491.1|	31967-34288	11	39
ACYPI006069-RA	ni|645903723|nb|KK920707.1|	81759-82009	nan	nan	gi|641578198|gb|KK854624.1|	102708-110926	12	40

ACYPI006076-RA	ni|645902238|nb|KK922191.1|	197399-197773	nan	nan	gi|641570080|gb|KK856466.1|	84781-85192	11	39
ACYPI006093-RA	ni|645904058|nb|KK920373.1|	1124013-1124171	16	15	gi|641586301|gb|KK854260.1|	502639-503489	9.8	39
ACYPI006101-RA	ni|645903832|nb|KK920598.1|	923349-923848	18	8.8	gi|641576472|gb|KK854913.1|	91163-95029	11	38
ACYPI006102-RA	ni|645903765|nb|KK920665.1|	592158-594403	17	15	gi|641575893|gb|KK855025.1|	256060-256351	12	21
ACYPI006103-RA	ni|645903885|nb|KK920546.1|	278793-279675	19	12	gi|641587881|gb|KK854049.1|	584793-586578	10	40
ACYPI006104-RA	ni|645902354|nb|KK922075.1|	147937-150465	nan	nan	gi|641570141|gb|KK856447.1|	125411-128455	11	40
ACYPI006106-RA	ni|645903511|nb|KK920919.1|	487647-489074	nan	nan	gi|641588164|gb|KK854017.1|	824013-824257	9.2	40
ACYPI006108-RA	ni|645903954|nb|KK920477.1|	266054-266314	35	44	gi|641576962|gb|KK854829.1|	232455-234517	10	39
ACYPI006122-RA	ni|645904231|nb|KK920242.1|	622179-623197	16	17	gi|641577047|gb|KK854815.1|	338905-339843	10	41
ACYPI006124-RA	ni|645904121|nb|KK920310.1|	285995-286702	14	15	gi|641576834|gb|KK854851.1|	329557-329956	10	41
ACYPI006125-RA	ni|645902441|nb|KK921988.1|	81551-81703	nan	nan	gi|641586777|gb|KK854192.1|	239239-239427	11	30
ACYPI006129-RA	ni|645904007|nb|KK920424.1|	214486-214810	18	17	gi|641575946|gb|KK855016.1|	126107-127271	9.5	37
ACYPI006140-RA	ni|645904130|nb|KK920303.1|	684546-685003	18	17	gi|641585395|gb|KK854405.1|	97838-98842	10	38
ACYPI006141-RA	ni|645903820|nb|KK920610.1|	1103266-1103571	18	19	gi|641567011|gb|KK857455.1|	26442-29198	11	40
ACYPI006142-RA	ni|645903879|nb|KK920552.1|	951007-951994	18	18	gi|641576863|gb|KK854845.1|	272005-274883	11	41
ACYPI006151-RA	ni|645903597|nb|KK920833.1|	558940-559343	nan	nan	gi|641587652|gb|KK854077.1|	830187-830707	10	38
ACYPI006154-RA	ni|645904060|nb|KK920371.1|	430259-430449	15	16	gi|641570836|gb|KK856240.1|	10842-11809	12	44
ACYPI006156-RA	ni|645904155|nb|KK920278.1|	1003957-1004076	18	9.4	gi|641587866|gb|KK854050.1|	313752-314484	10	38
ACYPI006157-RA	ni|645903720|nb|KK920710.1|	93578-94193	nan	nan	gi|641572416|gb|KK855819.1|	128516-129050	10	37
ACYPI006163-RA	ni|645904027|nb|KK920404.1|	698723-699189	17	17	gi|641573015|gb|KK855664.1|	57578-58874	11	42
ACYPI006164-RA	ni|645903768|nb|KK920662.1|	829449-831659	18	17	gi|641587703|gb|KK854070.1|	210947-215659	9.8	42
ACYPI006170-RA	ni|645904114|nb|KK920317.1|	1707595-1708268	18	18	gi|641571667|gb|KK856008.1|	105760-107467	11	39
ACYPI006177-RA	ni|645903908|nb|KK920523.1|	24030-24541	15	15	gi|641586793|gb|KK854190.1|	679665-684081	9.3	41
ACYPI006178-RA	ni|645903752|nb|KK920678.1|	1088651-1089251	18	16	gi|641571605|gb|KK856024.1|	7499-7959	10	38
ACYPI006179-RA	ni|645903860|nb|KK920570.1|	143051-143482	17	17	gi|641580479|gb|KK854569.1|	209154-216032	10	38
ACYPI006184-RA	ni|645904262|nb|KK920227.1|	2074409-2074543	17	17	gi|641575808|gb|KK855040.1|	165053-165619	9.9	37
ACYPI006186-RA	ni|645901982|nb|KK922447.1|	201285-201594	nan	nan	gi|641587452|gb|KK854101.1|	209441-210145	11	40
ACYPI006205-RA	ni|645904096|nb|KK920335.1|	1532128-1532351	18	17	gi|641586246|gb|KK854268.1|	642725-643469	10	38
ACYPI006207-RA	ni|645903674|nb|KK920756.1|	146009-146180	nan	nan	gi|641575108|gb|KK855178.1|	180173-185543	34	81
ACYPI006216-RA	ni|645903876|nb|KK920555.1|	133590-133815	14	15	gi|641586983|gb|KK854162.1|	357428-357963	10	41
ACYPI006219-RA	ni|645903735|nb|KK920695.1|	127556-127915	16	16	gi|641587671|gb|KK854074.1|	1058363-1058819	11	40
ACYPI006221-RA	ni|645903820|nb|KK920610.1|	838691-841384	18	19	gi|641587527|gb|KK854092.1|	455512-457743	10	38

ACYPI006222-RA	ni|645904201|nb|KK920252.1|	250127-252110	17	16	gi|641588060|gb|KK854028.1|	797797-800372	11	42
ACYPI006225-RA	ni|645904025|nb|KK920406.1|	471446-472143	15	19	gi|641576243|gb|KK854954.1|	296903-297529	10	41
ACYPI006227-RA	ni|645904213|nb|KK920248.1|	1725719-1726188	16	19	gi|641571982|gb|KK855931.1|	53261-53711	9.8	35
ACYPI006229-RA	ni|645904279|nb|KK920221.1|	2631897-2632633	16	16	gi|641587095|gb|KK854145.1|	132292-132741	9.9	40
ACYPI006233-RA	ni|645903820|nb|KK920610.1|	304701-304902	18	19	gi|641572185|gb|KK855876.1|	95882-96377	8.2	36
ACYPI006238-RA	ni|645903710|nb|KK920720.1|	722916-723469	nan	nan	gi|641568278|gb|KK857027.1|	50838-51986	9.6	38
ACYPI006240-RA	ni|645903916|nb|KK920515.1|	387444-390438	16	23	gi|641573583|gb|KK855523.1|	19844-24851	10	41
ACYPI006242-RA	ni|645903609|nb|KK920821.1|	384374-386063	nan	nan	gi|641573769|gb|KK855477.1|	124169-128061	9.2	38
ACYPI006243-RA	ni|645904133|nb|KK920300.1|	2772885-2773556	18	18	gi|641579941|gb|KK854575.1|	377799-380928	10	39
ACYPI006245-RA	ni|645904125|nb|KK920307.1|	2064819-2065058	18	17	gi|641576022|gb|KK854999.1|	328627-330458	10	36
ACYPI006247-RA	ni|645903988|nb|KK920443.1|	138738-139187	14	16	gi|641577184|gb|KK854793.1|	134873-135907	11	40
ACYPI006248-RA	ni|645904246|nb|KK920233.1|	1095819-1096522	16	17	gi|641587040|gb|KK854152.1|	309571-310403	9.1	39
ACYPI006251-RA	ni|645903821|nb|KK920609.1|	1085978-1086994	17	17	gi|641575130|gb|KK855173.1|	88810-90602	12	50
ACYPI006254-RA	ni|645904160|nb|KK920273.1|	1906377-1906572	18	18	gi|641587577|gb|KK854086.1|	398684-398890	8.2	41
ACYPI006257-RA	ni|645902745|nb|KK921684.1|	116758-117211	nan	nan	gi|641587047|gb|KK854151.1|	247234-247433	10	39
ACYPI006262-RA	ni|645903728|nb|KK920702.1|	633118-633588	nan	nan	gi|641586850|gb|KK854182.1|	150369-150970	10	37
ACYPI006266-RA	ni|645903674|nb|KK920756.1|	609789-610695	nan	nan	gi|641587104|gb|KK854144.1|	720378-721288	11	37
ACYPI006268-RA	ni|645902395|nb|KK922034.1|	93398-94604	nan	nan	gi|641568762|gb|KK856874.1|	16451-17841	11	41
ACYPI006271-RA	ni|645903562|nb|KK920868.1|	428334-428704	nan	nan	gi|641573752|gb|KK855482.1|	149708-150092	11	37
ACYPI006272-RA	ni|645903566|nb|KK920864.1|	172135-173272	nan	nan	gi|641571786|gb|KK855980.1|	147943-150971	9.9	19
ACYPI006274-RA	ni|645903960|nb|KK920471.1|	1068883-1069102	16	8.6	gi|641573903|gb|KK855443.1|	129188-129656	10	24
ACYPI006281-RA	ni|645904033|nb|KK920398.1|	154114-154355	14	16	gi|641578411|gb|KK854593.1|	179068-180177	11	43
ACYPI006283-RA	ni|645903637|nb|KK920793.1|	189871-190363	nan	nan	gi|641572372|gb|KK855829.1|	122861-123252	12	45
ACYPI006304-RA	ni|645903564|nb|KK920866.1|	282647-283161	nan	nan	gi|641582096|gb|KK854546.1|	322103-322585	11	38
ACYPI006305-RA	ni|645903603|nb|KK920827.1|	22406-23147	nan	nan	gi|641571790|gb|KK855979.1|	80019-80411	11	41
ACYPI006306-RA	ni|645904027|nb|KK920404.1|	633549-634046	17	17	gi|641586476|gb|KK854236.1|	634912-636319	10	37
ACYPI006310-RA	ni|645903849|nb|KK920581.1|	138521-138751	17	17	gi|641568257|gb|KK857034.1|	23161-23469	10	40
ACYPI006313-RA	ni|645903618|nb|KK920812.1|	303349-303582	nan	nan	gi|641574308|gb|KK855352.1|	130896-132077	10	36
ACYPI006314-RA	ni|645904216|nb|KK920247.1|	329926-330200	15	16	gi|641572037|gb|KK855916.1|	115036-115662	13	41
ACYPI006316-RA	ni|645904281|nb|KK920220.1|	816769-817407	15	16	gi|641587660|gb|KK854076.1|	262255-263089	9.9	41
ACYPI006318-RA	ni|645903678|nb|KK920752.1|	366223-366478	nan	nan	gi|641567806|gb|KK857182.1|	5014-5282	9.6	38
ACYPI006324-RA	ni|645904153|nb|KK920280.1|	2096918-2097247	17	16	gi|641586903|gb|KK854173.1|	289245-289406	11	45

ACYPI006331-RA	ni|645904139|nb|KK920294.1|	589331-589519	15	18	gi|641576347|gb|KK854934.1|	330660-333804	11	39
ACYPI006335-RA	ni|645902638|nb|KK921791.1|	115764-115998	nan	nan	gi|641572514|gb|KK855795.1|	111173-111432	9.5	39
ACYPI006340-RA	ni|645904119|nb|KK920312.1|	500249-501529	19	19	gi|641583980|gb|KK854512.1|	302793-304927	11	39
ACYPI006341-RA	ni|645902617|nb|KK921812.1|	83089-83750	nan	nan	gi|641577869|gb|KK854679.1|	313480-315964	9.2	39
ACYPI006348-RA	ni|645903606|nb|KK920824.1|	135270-136147	nan	nan	gi|641572675|gb|KK855753.1|	133980-135192	11	40
ACYPI006351-RA	ni|645904127|nb|KK920306.1|	510173-510408	15	15	gi|641570322|gb|KK856389.1|	7902-8582	11	40
ACYPI006352-RA	ni|645902662|nb|KK921767.1|	340787-341003	nan	nan	gi|641569883|gb|KK856530.1|	33210-33384	11	45
ACYPI006354-RA	ni|645903536|nb|KK920894.1|	320223-320658	nan	nan	gi|641572854|gb|KK855705.1|	177654-178323	11	42
ACYPI006364-RA	ni|645903773|nb|KK920657.1|	1109229-1109505	18	17	gi|641588021|gb|KK854033.1|	1097684-1098736	12	40
ACYPI006367-RA	ni|645903846|nb|KK920584.1|	227453-227729	17	10	gi|641587921|gb|KK854044.1|	345558-347805	9.4	41
ACYPI006369-RA	ni|645903724|nb|KK920706.1|	23762-23905	nan	nan	gi|641574324|gb|KK855348.1|	261073-264716	12	40
ACYPI006375-RA	ni|645903929|nb|KK920502.1|	1431575-1432721	17	18	gi|641577203|gb|KK854789.1|	288609-290702	11	43
ACYPI006377-RA	ni|645903680|nb|KK920750.1|	633144-633267	nan	nan	gi|641588000|gb|KK854035.1|	979782-981150	11	39
ACYPI006388-RA	ni|645903902|nb|KK920529.1|	1243762-1244926	19	17	gi|641584460|gb|KK854480.1|	14882-16045	11	40
ACYPI006399-RA	ni|645903563|nb|KK920867.1|	634869-635472	nan	nan	gi|641568423|gb|KK856981.1|	66311-66903	11	37
ACYPI006403-RA	ni|645904142|nb|KK920291.1|	69328-71035	20	23	gi|641567672|gb|KK857228.1|	20715-21633	10	36
ACYPI006405-RA	ni|645903938|nb|KK920493.1|	426643-427800	19	18	gi|641567739|gb|KK857205.1|	81589-82829	13	38
ACYPI006409-RA	ni|645903684|nb|KK920746.1|	238386-238931	nan	nan	gi|641578350|gb|KK854597.1|	389024-391100	11	21
ACYPI006412-RA	ni|645903475|nb|KK920955.1|	246958-247407	nan	nan	gi|641576546|gb|KK854899.1|	122833-123214	11	40
ACYPI006413-RA	ni|645903743|nb|KK920687.1|	126791-129557	19	18	gi|641588309|gb|KK854005.1|	540573-543634	11	40
ACYPI006417-RA	ni|645904125|nb|KK920307.1|	2824394-2824691	18	17	gi|641577075|gb|KK854811.1|	390240-394262	11	36
ACYPI006432-RA	ni|645904107|nb|KK920324.1|	26186-26600	17	9.5	gi|641570404|gb|KK856365.1|	110417-113754	10	19
ACYPI006436-RA	ni|645904246|nb|KK920233.1|	1689466-1690647	16	17	gi|641574484|gb|KK855310.1|	130781-131872	11	41
ACYPI006441-RA	ni|645903965|nb|KK920466.1|	1714864-1715250	17	16	gi|641570089|gb|KK856463.1|	40948-42728	11	36
ACYPI006443-RA	ni|645903825|nb|KK920605.1|	1018135-1018727	19	17	gi|641586535|gb|KK854227.1|	350013-351020	10	39
ACYPI006448-RA	ni|645903985|nb|KK920446.1|	906917-908285	18	16	gi|641577553|gb|KK854734.1|	245063-246684	9.7	41
ACYPI006451-RA	ni|645904136|nb|KK920297.1|	2035877-2036321	16	17	gi|641566026|gb|KK857812.1|	5483-5684	10	36
ACYPI006456-RA	ni|645904131|nb|KK920302.1|	575078-576144	15	16	gi|641577623|gb|KK854722.1|	285116-286207	11	38
ACYPI006460-RA	ni|645903654|nb|KK920776.1|	317568-318145	nan	nan	gi|641574487|gb|KK855309.1|	81851-82180	11	41
ACYPI006464-RA	ni|645904094|nb|KK920337.1|	8220-9045	17	16	gi|641576983|gb|KK854825.1|	191478-196604	9.7	37
ACYPI006482-RA	ni|645904133|nb|KK920300.1|	2963047-2963435	18	18	gi|641574892|gb|KK855223.1|	210168-211751	9.8	39
ACYPI006488-RA	ni|645903753|nb|KK920677.1|	439386-440035	17	9.8	gi|641577580|gb|KK854730.1|	12242-12443	11	41

ACYPI006494-RA	ni|645904022|nb|KK920409.1|	663842-664394	18	20	gi|641587379|gb|KK854111.1|	363091-363738	10	41
ACYPI006497-RA	ni|645902442|nb|KK921987.1|	181041-182619	nan	nan	gi|641588000|gb|KK854035.1|	615452-617116	11	39
ACYPI006498-RA	ni|645897725|nb|KK926704.1|	3473-3733	nan	nan	gi|641576036|gb|KK854996.1|	46467-46990	11	40
ACYPI006499-RA	ni|645903796|nb|KK920634.1|	710949-711345	18	17	gi|641575808|gb|KK855040.1|	258827-259237	9.9	37
ACYPI006500-RA	ni|645904171|nb|KK920262.1|	796596-797212	15	16	gi|641564420|gb|KK858447.1|	3017-5840	13	42
ACYPI006505-RA	ni|645904242|nb|KK920237.1|	789028-790134	15	16	gi|641587606|gb|KK854082.1|	203331-204836	11	40
ACYPI006506-RA	ni|645904149|nb|KK920284.1|	504474-505015	16	16	gi|641576328|gb|KK854938.1|	119647-120223	11	36
ACYPI006514-RA	ni|645903703|nb|KK920727.1|	257231-257612	nan	nan	gi|641577299|gb|KK854775.1|	284741-287511	11	37
ACYPI006518-RA	ni|645903979|nb|KK920452.1|	693623-694953	15	17	gi|641580970|gb|KK854563.1|	305168-307888	11	42
ACYPI006520-RA	ni|645903545|nb|KK920885.1|	239477-239988	nan	nan	gi|641550114|gb|KK865048.1|	39-1938	12	37
ACYPI006521-RA	ni|645903780|nb|KK920650.1|	865015-865652	19	19	gi|641579219|gb|KK854584.1|	184872-188323	12	39
ACYPI006541-RA	ni|645904258|nb|KK920229.1|	742386-742651	16	16	gi|641575288|gb|KK855139.1|	239521-240813	11	38
ACYPI006544-RA	ni|645904119|nb|KK920312.1|	514158-514550	19	19	gi|641583980|gb|KK854512.1|	323395-326097	11	39
ACYPI006545-RA	ni|645903774|nb|KK920656.1|	654437-655141	16	9.2	gi|641584876|gb|KK854450.1|	288338-289625	11	25
ACYPI006551-RA	ni|645904043|nb|KK920388.1|	528913-529749	16	9.1	gi|641568278|gb|KK857027.1|	56973-58793	9.6	38
ACYPI006557-RA	ni|645903965|nb|KK920466.1|	1174266-1174946	17	16	gi|641578106|gb|KK854639.1|	146076-146525	11	39
ACYPI006562-RA	ni|645902552|nb|KK921877.1|	387747-388300	nan	nan	gi|641587696|gb|KK854071.1|	410815-411889	8.9	39
ACYPI006576-RA	ni|645903910|nb|KK920521.1|	353567-354045	19	17	gi|641588109|gb|KK854023.1|	393708-394509	10	40
ACYPI006584-RA	ni|645903773|nb|KK920657.1|	1196725-1197140	18	17	gi|641575794|gb|KK855043.1|	172043-177386	10	36
ACYPI006588-RA	ni|645904106|nb|KK920325.1|	1057605-1058232	17	17	gi|641587410|gb|KK854106.1|	458544-458859	10	39
ACYPI006589-RA	ni|645904165|nb|KK920268.1|	766444-766887	16	16	gi|641576207|gb|KK854961.1|	201124-202848	11	38
ACYPI006593-RA	ni|645897070|nb|KK927359.1|	1338-2331	nan	nan	gi|641574689|gb|KK855262.1|	722-1028	10	38
ACYPI006602-RA	ni|645904192|nb|KK920255.1|	45063-46373	15	16	gi|641578406|gb|KK854594.1|	258805-258995	10	37
ACYPI006603-RA	ni|645904131|nb|KK920302.1|	492245-492558	15	16	gi|641584818|gb|KK854454.1|	397690-398012	11	42
ACYPI006608-RA	ni|645902648|nb|KK921781.1|	4233-4966	nan	nan	gi|641575666|gb|KK855067.1|	229280-229584	11	39
ACYPI006611-RA	ni|645903549|nb|KK920881.1|	5588-6823	nan	nan	gi|641578056|gb|KK854648.1|	66169-67920	11	41
ACYPI006612-RA	ni|645903925|nb|KK920506.1|	603140-605156	19	17	gi|641588325|gb|KK854004.1|	1135590-1138378	11	40
ACYPI006614-RA	ni|645904159|nb|KK920274.1|	241457-241641	15	17	gi|641576392|gb|KK854928.1|	206419-207234	9.7	41
ACYPI006615-RA	ni|645904160|nb|KK920273.1|	1791608-1791910	18	18	gi|641576946|gb|KK854832.1|	84306-88272	9.4	43
ACYPI006616-RA	ni|645903846|nb|KK920584.1|	256698-256934	17	10	gi|641576656|gb|KK854883.1|	19849-25051	11	40
ACYPI006617-RA	ni|645904122|nb|KK920309.1|	241291-241586	18	17	gi|641586950|gb|KK854166.1|	658820-659587	10	38
ACYPI006619-RA	ni|645903695|nb|KK920735.1|	498936-499815	nan	nan	gi|641582408|gb|KK854540.1|	383508-385257	10	41

ACYPI006624-RA	ni|645904139|nb|KK920294.1|	204208-205253	15	18	gi|641587452|gb|KK854101.1|	1025558-1027416	11	40
ACYPI006625-RA	ni|645904075|nb|KK920356.1|	535648-536240	16	17	gi|641575743|gb|KK855052.1|	133926-141235	9.7	38
ACYPI006626-RA	ni|645904039|nb|KK920392.1|	666983-667491	15	16	gi|641573007|gb|KK855666.1|	187020-189637	11	44
ACYPI006635-RA	ni|645902105|nb|KK922324.1|	61899-65886	nan	nan	gi|641573097|gb|KK855644.1|	104411-111687	10	36
ACYPI006639-RA	ni|645902538|nb|KK921891.1|	104472-105673	nan	nan	gi|641573737|gb|KK855486.1|	62037-63568	9.1	33
ACYPI006640-RA	ni|645904096|nb|KK920335.1|	1995033-1995242	18	17	gi|641577843|gb|KK854684.1|	417397-418438	10	40
ACYPI006649-RA	ni|645904039|nb|KK920392.1|	417356-417592	15	16	gi|641573007|gb|KK855666.1|	49902-52377	11	44
ACYPI006656-RA	ni|645903957|nb|KK920474.1|	1448344-1449893	18	17	gi|641573533|gb|KK855535.1|	28624-35255	10	41
ACYPI006658-RA	ni|645903902|nb|KK920529.1|	1120723-1121108	19	17	gi|641584460|gb|KK854480.1|	108727-109104	11	40
ACYPI006660-RA	ni|645904222|nb|KK920245.1|	784017-784294	17	12	gi|641588099|gb|KK854024.1|	938549-938853	11	39
ACYPI006664-RA	ni|645904070|nb|KK920361.1|	310719-311376	17	16	gi|641574249|gb|KK855364.1|	70843-72107	9.2	37
ACYPI006668-RA	ni|645904070|nb|KK920361.1|	564335-564551	17	16	gi|641575768|gb|KK855048.1|	156177-156512	8.1	41
ACYPI006674-RA	ni|645904152|nb|KK920281.1|	386092-387170	15	15	gi|641587365|gb|KK854113.1|	484651-485915	12	45
ACYPI006676-RA	ni|645903938|nb|KK920493.1|	737733-738151	19	18	gi|641577928|gb|KK854668.1|	159761-160032	9.2	37
ACYPI006680-RA	ni|645901967|nb|KK922462.1|	6398-6822	nan	nan	gi|641569707|gb|KK856586.1|	54956-55796	8.9	17
ACYPI006682-RA	ni|645904278|nb|KK920222.1|	561997-563106	15	16	gi|641574036|gb|KK855411.1|	207028-207271	11	37
ACYPI006683-RA	ni|645904278|nb|KK920222.1|	1175767-1176278	15	16	gi|641585778|gb|KK854341.1|	404513-405114	9.7	41
ACYPI006688-RA	ni|645903604|nb|KK920826.1|	25905-26096	nan	nan	gi|641587688|gb|KK854072.1|	450111-456133	10	38
ACYPI006692-RA	ni|645904180|nb|KK920259.1|	2094267-2095975	17	17	gi|641574491|gb|KK855308.1|	8334-8934	11	43
ACYPI006693-RA	ni|645903999|nb|KK920432.1|	549019-549429	17	15	gi|641588073|gb|KK854027.1|	1026916-1027178	11	41
ACYPI006694-RA	ni|645902349|nb|KK922080.1|	33226-33377	nan	nan	gi|641572601|gb|KK855773.1|	52132-53749	10	35
ACYPI006698-RA	ni|645903523|nb|KK920907.1|	177425-177891	nan	nan	gi|641585407|gb|KK854403.1|	258696-259400	9.4	42
ACYPI006699-RA	ni|645903936|nb|KK920495.1|	1054006-1054287	19	18	gi|641587417|gb|KK854105.1|	84826-85357	11	42
ACYPI006701-RA	ni|645903669|nb|KK920761.1|	60069-60796	nan	nan	gi|641586886|gb|KK854176.1|	91548-91917	10	41
ACYPI006703-RA	ni|645903699|nb|KK920731.1|	166653-168033	nan	nan	gi|641574944|gb|KK855211.1|	214840-216827	10	39
ACYPI006708-RA	ni|645902715|nb|KK921714.1|	34127-34575	nan	nan	gi|641570124|gb|KK856453.1|	155068-155760	8.7	40
ACYPI006711-RA	ni|645904139|nb|KK920294.1|	500525-501582	15	18	gi|641576347|gb|KK854934.1|	234087-235474	11	39
ACYPI006714-RA	ni|645904152|nb|KK920281.1|	338801-339291	15	15	gi|641587365|gb|KK854113.1|	545658-548159	12	45
ACYPI006725-RA	ni|645903858|nb|KK920572.1|	894147-894397	16	16	gi|641585515|gb|KK854385.1|	153205-153495	11	40
ACYPI006727-RA	ni|645904070|nb|KK920361.1|	961045-961589	17	16	gi|641587319|gb|KK854116.1|	652479-654994	11	43
ACYPI006728-RA	ni|645903984|nb|KK920447.1|	24882-26004	18	17	gi|641565803|gb|KK857893.1|	18862-21243	10	35
ACYPI006735-RA	ni|645903968|nb|KK920463.1|	608505-608646	16	16	gi|641571130|gb|KK856153.1|	164041-164830	10	36

ACYPI006737-RA	ni|645903727|nb|KK920703.1|	958477-958937	nan	nan	gi|641560560|gb|KK860138.1|	8238-8655	8.4	26
ACYPI006738-RA	ni|645904133|nb|KK920300.1|	2280485-2280994	18	18	gi|641575568|gb|KK855086.1|	309892-310131	11	40
ACYPI006740-RA	ni|645903751|nb|KK920679.1|	759317-760058	17	16	gi|641586372|gb|KK854251.1|	543974-548728	10	38
ACYPI006741-RA	ni|645903544|nb|KK920886.1|	344250-344705	nan	nan	gi|641587981|gb|KK854037.1|	100841-101313	10	40
ACYPI006748-RA	ni|645904130|nb|KK920303.1|	1467726-1469517	18	17	gi|641570795|gb|KK856251.1|	76988-77769	9.8	38
ACYPI006756-RA	ni|645903821|nb|KK920609.1|	1060752-1061343	17	17	gi|641577234|gb|KK854784.1|	188114-188894	11	39
ACYPI006758-RA	ni|645903633|nb|KK920797.1|	573722-574669	nan	nan	gi|641585204|gb|KK854429.1|	142132-145872	9.2	36
ACYPI006761-RA	ni|645903648|nb|KK920782.1|	272636-273115	nan	nan	gi|641587478|gb|KK854098.1|	260617-261002	10	41
ACYPI006763-RA	ni|645903789|nb|KK920641.1|	720826-721147	17	17	gi|641577529|gb|KK854738.1|	221540-221860	9.8	35
ACYPI006767-RA	ni|645903984|nb|KK920447.1|	213395-214666	18	17	gi|641586785|gb|KK854191.1|	231154-232262	11	39
ACYPI006777-RA	ni|645904156|nb|KK920277.1|	1517327-1517575	17	8.8	gi|641587866|gb|KK854050.1|	1008888-1009229	10	38
ACYPI006784-RA	ni|645903854|nb|KK920576.1|	31398-31593	16	8.4	gi|641577595|gb|KK854727.1|	58572-59762	10	20
ACYPI006785-RA	ni|645904216|nb|KK920247.1|	225407-225673	15	16	gi|641573464|gb|KK855553.1|	178611-179112	11	39
ACYPI006790-RA	ni|645903627|nb|KK920803.1|	529579-530412	nan	nan	gi|641586874|gb|KK854178.1|	623830-624157	9.4	38
ACYPI006792-RA	ni|645904028|nb|KK920403.1|	413374-413757	18	17	gi|641574320|gb|KK855349.1|	188081-190196	10	38
ACYPI006797-RA	ni|645901733|nb|KK922696.1|	223835-227076	nan	nan	gi|641587921|gb|KK854044.1|	327919-328598	9.4	41
ACYPI006803-RA	ni|645903959|nb|KK920472.1|	718521-718767	15	15	gi|641574998|gb|KK855198.1|	150284-151623	10	39
ACYPI006807-RA	ni|645903780|nb|KK920650.1|	979338-980184	19	19	gi|641569257|gb|KK856724.1|	80621-81226	10	36
ACYPI006808-RA	ni|645903627|nb|KK920803.1|	520496-520725	nan	nan	gi|641584728|gb|KK854460.1|	218813-219634	9.5	37
ACYPI006817-RA	ni|645901537|nb|KK922892.1|	78395-78995	nan	nan	gi|641587287|gb|KK854120.1|	161230-163841	9.9	37
ACYPI006818-RA	ni|645904132|nb|KK920301.1|	249661-250513	16	18	gi|641575910|gb|KK855021.1|	216435-217820	10	35
ACYPI006821-RA	ni|645903899|nb|KK920532.1|	279543-279817	15	16	gi|641574729|gb|KK855257.1|	208642-212290	8.6	33
ACYPI006822-RA	ni|645903923|nb|KK920508.1|	1014195-1015151	18	18	gi|641572879|gb|KK855698.1|	195123-195355	10	39
ACYPI006823-RA	ni|645904010|nb|KK920421.1|	1030657-1031841	17	17	gi|641575168|gb|KK855165.1|	112036-112546	9.6	36
ACYPI006828-RA	ni|645904116|nb|KK920315.1|	424251-426274	19	18	gi|641580504|gb|KK854565.1|	348065-351180	11	43
ACYPI006833-RA	ni|645903680|nb|KK920750.1|	68912-69726	nan	nan	gi|641576010|gb|KK855002.1|	52176-55478	12	43
ACYPI006841-RA	ni|645904106|nb|KK920325.1|	669062-669656	17	17	gi|641577407|gb|KK854756.1|	150713-152613	9.4	39
ACYPI006857-RA	ni|645903780|nb|KK920650.1|	276650-277152	19	19	gi|641575396|gb|KK855118.1|	103150-103770	9	35
ACYPI006860-RA	ni|645904228|nb|KK920243.1|	1219030-1219556	17	18	gi|641577541|gb|KK854736.1|	30346-31251	10	38
ACYPI006871-RA	ni|645903858|nb|KK920572.1|	844912-845156	16	16	gi|641578046|gb|KK854650.1|	35043-35866	10	38
ACYPI006875-RA	ni|645902316|nb|KK922113.1|	5977-6230	nan	nan	gi|641576887|gb|KK854842.1|	255358-255716	15	41
ACYPI006879-RA	ni|645904045|nb|KK920386.1|	210443-210801	19	21	gi|641577778|gb|KK854696.1|	261553-262644	9.9	42

ACYPI006884-RA	ni|645903867|nb|KK920563.1|	769072-770352	18	18	gi|641570258|gb|KK856410.1|	10007-11245	8.4	41
ACYPI006885-RA	ni|645903953|nb|KK920478.1|	1168857-1169879	18	17	gi|641585961|gb|KK854312.1|	436446-436787	10	40
ACYPI006896-RA	ni|645903946|nb|KK920485.1|	33603-33801	16	14	gi|641576557|gb|KK854897.1|	178281-180603	8.9	33
ACYPI006897-RA	ni|645904231|nb|KK920242.1|	339818-340689	16	17	gi|641587569|gb|KK854087.1|	911104-911514	11	41
ACYPI006902-RA	ni|645904023|nb|KK920408.1|	768877-769900	17	17	gi|641575693|gb|KK855062.1|	49577-50830	11	40
ACYPI006903-RA	ni|645903762|nb|KK920668.1|	875636-876172	17	17	gi|641577104|gb|KK854807.1|	29362-32006	10	36
ACYPI006909-RA	ni|645904091|nb|KK920340.1|	663578-663801	15	18	gi|641583088|gb|KK854527.1|	192271-193563	9.9	39
ACYPI006910-RA	ni|645904159|nb|KK920274.1|	1079126-1079292	15	17	gi|641575826|gb|KK855037.1|	90242-91461	10	39
ACYPI006928-RA	ni|645904088|nb|KK920343.1|	1230362-1231781	17	18	gi|641587452|gb|KK854101.1|	943490-945993	11	40
ACYPI006929-RA	ni|645903752|nb|KK920678.1|	73879-75440	18	16	gi|641585773|gb|KK854342.1|	136565-139150	12	45
ACYPI006932-RA	ni|645903747|nb|KK920683.1|	98138-99138	19	18	gi|641578325|gb|KK854602.1|	269983-277242	9.7	38
ACYPI006934-RA	ni|645903634|nb|KK920796.1|	1045632-1045976	nan	nan	gi|641584445|gb|KK854481.1|	92178-92589	9.9	39
ACYPI006936-RA	ni|645903984|nb|KK920447.1|	9665-10132	18	17	gi|641587671|gb|KK854074.1|	624201-624856	11	40
ACYPI006940-RA	ni|645903679|nb|KK920751.1|	395581-396385	nan	nan	gi|641587052|gb|KK854150.1|	48659-48972	11	38
ACYPI006941-RA	ni|645903871|nb|KK920559.1|	819021-819632	18	9.4	gi|641578350|gb|KK854597.1|	351451-352039	11	21
ACYPI006942-RA	ni|645904165|nb|KK920268.1|	820112-820637	16	16	gi|641565407|gb|KK858043.1|	30235-30584	11	20
ACYPI006948-RA	ni|645903911|nb|KK920520.1|	527512-527958	16	17	gi|641587519|gb|KK854093.1|	383866-385490	11	39
ACYPI006949-RA	ni|645903742|nb|KK920688.1|	522985-524102	17	16	gi|641587452|gb|KK854101.1|	225773-229455	11	40
ACYPI006953-RA	ni|645904145|nb|KK920288.1|	1264246-1264639	16	9.3	gi|641584404|gb|KK854484.1|	391267-391480	11	30
ACYPI006954-RA	ni|645903834|nb|KK920596.1|	42082-43166	18	17	gi|641576876|gb|KK854844.1|	370973-372217	17	42
ACYPI006956-RA	ni|645904122|nb|KK920309.1|	1250331-1251362	18	17	gi|641587896|gb|KK854047.1|	55643-57423	9.9	39
ACYPI006957-RA	ni|645904171|nb|KK920262.1|	1446852-1447285	15	16	gi|641586246|gb|KK854268.1|	515533-516942	10	38
ACYPI006958-RA	ni|645903950|nb|KK920481.1|	689445-690534	15	15	gi|641578046|gb|KK854650.1|	95657-98866	10	38
ACYPI006964-RA	ni|645903831|nb|KK920599.1|	1563083-1563812	18	17	gi|641577623|gb|KK854722.1|	166372-166615	11	38
ACYPI006974-RA	ni|645904045|nb|KK920386.1|	69653-71370	19	21	gi|641587783|gb|KK854060.1|	634998-640549	12	40
ACYPI006977-RA	ni|645903466|nb|KK920964.1|	284805-286825	nan	nan	gi|641586006|gb|KK854305.1|	303276-306924	9.7	38
ACYPI006978-RA	ni|645903938|nb|KK920493.1|	1462006-1465201	19	18	gi|641573829|gb|KK855462.1|	188384-193291	11	41
ACYPI006979-RA	ni|645904033|nb|KK920398.1|	96265-96779	14	16	gi|641571612|gb|KK856022.1|	49694-51333	12	43
ACYPI006990-RA	ni|645904063|nb|KK920368.1|	468705-469368	16	18	gi|641588343|gb|KK854003.1|	1100981-1101215	11	40
ACYPI006993-RA	ni|645903521|nb|KK920909.1|	22287-23846	nan	nan	gi|641565451|gb|KK858025.1|	30340-32487	9.1	17
ACYPI007000-RA	ni|645904112|nb|KK920319.1|	246263-247259	18	17	gi|641576973|gb|KK854827.1|	383506-383696	11	38
ACYPI007002-RA	ni|645904174|nb|KK920261.1|	653985-654230	19	17	gi|641577184|gb|KK854793.1|	373532-374549	11	40

ACYPI007005-RA	ni|645904222|nb|KK920245.1|	631377-631689	17	12	gi|641585395|gb|KK854405.1|	429043-429785	10	38
ACYPI007006-RA	ni|645902768|nb|KK921661.1|	246442-247007	nan	nan	gi|641588073|gb|KK854027.1|	1234954-1237193	11	41
ACYPI007007-RA	ni|645904251|nb|KK920231.1|	402750-403002	16	16	gi|641586785|gb|KK854191.1|	324708-327123	11	39
ACYPI007009-RA	ni|645902760|nb|KK921669.1|	17306-18078	nan	nan	gi|641573155|gb|KK855630.1|	10643-11451	14	50
ACYPI007012-RA	ni|645904043|nb|KK920388.1|	547055-547301	16	9.1	gi|641587832|gb|KK854054.1|	648239-648803	9.8	20
ACYPI007014-RA	ni|645903650|nb|KK920780.1|	162023-163087	nan	nan	gi|641571315|gb|KK856104.1|	171600-174636	12	46
ACYPI007020-RA	ni|645903919|nb|KK920512.1|	805405-805944	17	15	gi|641577487|gb|KK854746.1|	17046-19073	11	38
ACYPI007021-RA	ni|645904246|nb|KK920233.1|	231517-231963	16	17	gi|641587930|gb|KK854043.1|	482472-483559	10	41
ACYPI007025-RA	ni|645903800|nb|KK920630.1|	1379640-1379990	18	17	gi|641570596|gb|KK856308.1|	129098-129578	9.9	39
ACYPI007038-RA	ni|645902083|nb|KK922346.1|	145759-146082	nan	nan	gi|641585833|gb|KK854332.1|	404690-405297	10	37
ACYPI007039-RA	ni|645904077|nb|KK920354.1|	990184-990636	16	17	gi|641584504|gb|KK854477.1|	361012-362241	9.5	38
ACYPI007040-RA	ni|645904009|nb|KK920422.1|	1250919-1251405	17	9.4	gi|641574352|gb|KK855342.1|	225141-225573	9.4	18
ACYPI007045-RA	ni|645903511|nb|KK920919.1|	763174-763367	nan	nan	gi|641588073|gb|KK854027.1|	346716-347049	11	41
ACYPI007054-RA	ni|645903891|nb|KK920540.1|	218032-218752	15	17	gi|641585376|gb|KK854407.1|	130499-131211	9.8	36
ACYPI007058-RA	ni|645903627|nb|KK920803.1|	131399-132040	nan	nan	gi|641576677|gb|KK854879.1|	92731-94247	11	39
ACYPI007060-RA	ni|645903965|nb|KK920466.1|	1741246-1741496	17	16	gi|641585905|gb|KK854321.1|	258083-258264	11	39
ACYPI007070-RA	ni|645904094|nb|KK920337.1|	66379-69115	17	16	gi|641576983|gb|KK854825.1|	267001-268730	9.7	37
ACYPI007076-RA	ni|645903973|nb|KK920458.1|	688002-688202	20	18	gi|641574000|gb|KK855418.1|	54321-54518	10	36
ACYPI007077-RA	ni|645903506|nb|KK920924.1|	234869-235461	nan	nan	gi|641576118|gb|KK854980.1|	206277-208022	9.7	37
ACYPI007079-RA	ni|645904166|nb|KK920267.1|	2277830-2278076	18	16	gi|641559375|gb|KK860688.1|	8068-8479	8.3	26
ACYPI007084-RA	ni|645904008|nb|KK920423.1|	1363306-1363960	16	9.5	gi|641587488|gb|KK854097.1|	511710-512191	11	21
ACYPI007086-RA	ni|645903595|nb|KK920835.1|	144259-145854	nan	nan	gi|641575709|gb|KK855059.1|	23568-23961	11	41
ACYPI007090-RA	ni|645903835|nb|KK920595.1|	610427-610893	16	8.8	gi|641586033|gb|KK854301.1|	333585-333874	11	21
ACYPI007094-RA	ni|645903765|nb|KK920665.1|	519942-520641	17	15	gi|641575140|gb|KK855171.1|	160341-162249	9.7	40
ACYPI007099-RA	ni|645904045|nb|KK920386.1|	197113-197533	19	21	gi|641573210|gb|KK855617.1|	19469-19706	9.2	37
ACYPI007100-RA	ni|645904231|nb|KK920242.1|	651668-652542	16	17	gi|641577043|gb|KK854816.1|	297515-298663	11	43
ACYPI007104-RA	ni|645904163|nb|KK920270.1|	285087-286597	16	16	gi|641579192|gb|KK854588.1|	238582-239926	11	41
ACYPI007109-RA	ni|645903749|nb|KK920681.1|	618151-618956	19	18	gi|641587742|gb|KK854065.1|	586168-586422	11	41
ACYPI007110-RA	ni|645903713|nb|KK920717.1|	599877-600405	nan	nan	gi|641587365|gb|KK854113.1|	138327-138565	12	45
ACYPI007113-RA	ni|645903650|nb|KK920780.1|	1063665-1064210	nan	nan	gi|641587383|gb|KK854110.1|	273707-276075	10	37
ACYPI007117-RA	ni|645904015|nb|KK920416.1|	444779-447912	18	16	gi|641585446|gb|KK854396.1|	278808-282137	11	39
ACYPI007123-RA	ni|645904013|nb|KK920418.1|	519866-520109	19	18	gi|641571928|gb|KK855946.1|	43551-43983	11	38

ACYPI007134-RA	ni|645903792|nb|KK920638.1|	239608-239896	19	21	gi|641569892|gb|KK856527.1|	67837-68271	9.5	20
ACYPI007136-RA	ni|645903557|nb|KK920873.1|	724006-725057	nan	nan	gi|641586817|gb|KK854187.1|	649102-651792	9.3	39
ACYPI007139-RA	ni|645903971|nb|KK920460.1|	61534-62359	19	18	gi|641588060|gb|KK854028.1|	667237-668091	11	42
ACYPI007141-RA	ni|645903882|nb|KK920549.1|	701186-701692	16	16	gi|641585900|gb|KK854322.1|	291495-292051	12	43
ACYPI007156-RA	ni|645903470|nb|KK920960.1|	92962-93404	nan	nan	gi|641576666|gb|KK854881.1|	164477-165021	10	37
ACYPI007158-RA	ni|645903494|nb|KK920936.1|	334594-334733	nan	nan	gi|641566395|gb|KK857678.1|	51-519	10	19
ACYPI007164-RA	ni|645902426|nb|KK922003.1|	152396-152585	nan	nan	gi|641586928|gb|KK854169.1|	276877-277367	11	39
ACYPI007166-RA	ni|645903680|nb|KK920750.1|	656416-657225	nan	nan	gi|641588000|gb|KK854035.1|	1040026-1046739	11	39
ACYPI007167-RA	ni|645903627|nb|KK920803.1|	496973-497337	nan	nan	gi|641584728|gb|KK854460.1|	178884-180247	9.5	37
ACYPI007172-RA	ni|645904096|nb|KK920335.1|	862399-862582	18	17	gi|641568095|gb|KK857087.1|	41821-42841	11	43
ACYPI007174-RA	ni|645903910|nb|KK920521.1|	347245-347655	19	17	gi|641588109|gb|KK854023.1|	405117-405362	10	40
ACYPI007176-RA	ni|645904075|nb|KK920356.1|	739586-741800	16	17	gi|641571632|gb|KK856018.1|	93777-96711	10	36
ACYPI007179-RA	ni|645903496|nb|KK920934.1|	858330-858799	nan	nan	gi|641587452|gb|KK854101.1|	219553-220622	11	40
ACYPI007182-RA	ni|645903697|nb|KK920733.1|	392830-393328	nan	nan	gi|641572974|gb|KK855674.1|	198739-199020	9.9	38
ACYPI007192-RA	ni|645903541|nb|KK920889.1|	326333-326548	nan	nan	gi|641574230|gb|KK855368.1|	91876-92093	11	20
ACYPI007193-RA	ni|645904132|nb|KK920301.1|	481853-482184	16	18	gi|641575826|gb|KK855037.1|	215718-216678	10	39
ACYPI007195-RA	ni|645903754|nb|KK920676.1|	112124-112395	14	8.8	gi|641586372|gb|KK854251.1|	610574-611349	10	38
ACYPI007197-RA	ni|645903920|nb|KK920511.1|	306694-307562	20	20	gi|641585315|gb|KK854418.1|	21687-22721	10	39
ACYPI007204-RA	ni|645903879|nb|KK920552.1|	138491-138698	18	18	gi|641588283|gb|KK854007.1|	789541-793077	11	40
ACYPI007210-RA	ni|645904262|nb|KK920227.1|	492439-492736	17	17	gi|641576315|gb|KK854940.1|	210808-211432	9.9	40
ACYPI007219-RA	ni|645903747|nb|KK920683.1|	752430-753512	19	18	gi|641571928|gb|KK855946.1|	110187-111080	11	38
ACYPI007220-RA	ni|645904038|nb|KK920393.1|	360832-361350	17	19	gi|641586191|gb|KK854274.1|	17048-20294	11	42
ACYPI007226-RA	ni|645901860|nb|KK922569.1|	8707-9276	nan	nan	gi|641585876|gb|KK854326.1|	78537-79408	9.5	39
ACYPI007232-RA	ni|645904125|nb|KK920307.1|	618395-618993	18	17	gi|641554387|gb|KK863045.1|	1808-3470	8.9	34
ACYPI007238-RA	ni|645904009|nb|KK920422.1|	706924-707051	17	9.4	gi|641586880|gb|KK854177.1|	228623-232098	10	20
ACYPI007240-RA	ni|645899821|nb|KK924608.1|	636-864	nan	nan	gi|641576906|gb|KK854838.1|	377655-378307	9.4	37
ACYPI007241-RA	ni|645903664|nb|KK920766.1|	194474-194750	nan	nan	gi|641571465|gb|KK856064.1|	88449-88725	9.2	35
ACYPI007245-RA	ni|645901030|nb|KK923399.1|	499-971	nan	nan	gi|641585622|gb|KK854367.1|	341501-344100	9.9	21
ACYPI007246-RA	ni|645904228|nb|KK920243.1|	715934-716163	17	18	gi|641571956|gb|KK855938.1|	54013-54728	11	42
ACYPI007248-RA	ni|645903773|nb|KK920657.1|	1227279-1227687	18	17	gi|641575794|gb|KK855043.1|	88920-89619	10	36
ACYPI007249-RA	ni|645903654|nb|KK920776.1|	676053-676866	nan	nan	gi|641572687|gb|KK855750.1|	52091-52908	9.9	39
ACYPI007250-RA	ni|645904157|nb|KK920276.1|	630398-631174	15	15	gi|641583958|gb|KK854513.1|	442876-444265	10	22

ACYPI007258-RA	ni|645903572|nb|KK920858.1|	170673-171608	nan	nan	gi|641547881|gb|KK866127.1|	86-1426	12	43
ACYPI007260-RA	ni|645903832|nb|KK920598.1|	931194-931414	18	8.8	gi|641586967|gb|KK854164.1|	88755-89159	10	27
ACYPI007262-RA	ni|645903981|nb|KK920450.1|	831113-832723	16	8.6	gi|641574532|gb|KK855298.1|	199541-201933	12	20
ACYPI007266-RA	ni|645903965|nb|KK920466.1|	1635166-1636273	17	16	gi|641587962|gb|KK854039.1|	1047946-1049128	11	40
ACYPI007268-RA	ni|645903518|nb|KK920912.1|	160605-161040	nan	nan	gi|641587162|gb|KK854136.1|	505438-506974	10	40
ACYPI007270-RA	ni|645904057|nb|KK920374.1|	1278598-1279087	16	15	gi|641574809|gb|KK855240.1|	71065-72636	11	39
ACYPI007272-RA	ni|645903488|nb|KK920942.1|	89331-90743	nan	nan	gi|641586769|gb|KK854193.1|	354438-357836	12	23
ACYPI007276-RA	ni|645903829|nb|KK920601.1|	387120-387358	16	15	gi|641570601|gb|KK856306.1|	146612-147336	10	40
ACYPI007277-RA	ni|645903492|nb|KK920938.1|	41138-42534	nan	nan	gi|641577215|gb|KK854787.1|	220904-225222	11	40
ACYPI007282-RA	ni|645904137|nb|KK920296.1|	1638944-1639499	17	17	gi|641587052|gb|KK854150.1|	381847-382391	11	38
ACYPI007287-RA	ni|645901591|nb|KK922838.1|	43492-43825	nan	nan	gi|641571311|gb|KK856105.1|	41529-41876	9.2	37
ACYPI007291-RA	ni|645903614|nb|KK920816.1|	849350-849641	nan	nan	gi|641576741|gb|KK854868.1|	172365-172555	9.5	40
ACYPI007298-RA	ni|645903740|nb|KK920690.1|	398952-399831	18	10	gi|641585953|gb|KK854314.1|	393980-395790	11	43
ACYPI007299-RA	ni|645904125|nb|KK920307.1|	240239-240645	18	17	gi|641587562|gb|KK854088.1|	472858-475472	11	42
ACYPI007301-RA	ni|645904065|nb|KK920366.1|	10037-10282	18	18	gi|641577826|gb|KK854687.1|	38828-39649	9.4	37
ACYPI007303-RA	ni|645903773|nb|KK920657.1|	1419473-1419637	18	17	gi|641576188|gb|KK854966.1|	39448-40430	11	39
ACYPI007307-RA	ni|645904098|nb|KK920333.1|	296655-296910	16	8.8	gi|641577772|gb|KK854697.1|	199513-200179	10	39
ACYPI007315-RA	ni|645903666|nb|KK920764.1|	77098-77333	nan	nan	gi|641585871|gb|KK854327.1|	511677-511931	11	38
ACYPI007324-RA	ni|645903984|nb|KK920447.1|	57630-58023	18	17	gi|641586893|gb|KK854175.1|	354291-354660	11	41
ACYPI007327-RA	ni|645904097|nb|KK920334.1|	241706-242276	15	17	gi|641576423|gb|KK854922.1|	337066-337898	18	35
ACYPI007330-RA	ni|645902723|nb|KK921706.1|	142630-143014	nan	nan	gi|641577754|gb|KK854700.1|	65600-65793	12	43
ACYPI007331-RA	ni|645903540|nb|KK920890.1|	343551-344231	nan	nan	gi|641569526|gb|KK856644.1|	9345-9671	9.4	18
ACYPI007334-RA	ni|645903533|nb|KK920897.1|	180030-180231	nan	nan	gi|641576172|gb|KK854969.1|	18008-20491	9.9	20
ACYPI007340-RA	ni|645902779|nb|KK921650.1|	252092-252496	nan	nan	gi|641569670|gb|KK856598.1|	79688-80179	9.8	45
ACYPI007341-RA	ni|645903957|nb|KK920474.1|	1168310-1168709	18	17	gi|641586713|gb|KK854201.1|	642899-643534	9.1	40
ACYPI007344-RA	ni|645904078|nb|KK920353.1|	595345-595823	17	17	gi|641586541|gb|KK854226.1|	315982-316243	11	41
ACYPI007346-RA	ni|645903961|nb|KK920470.1|	75335-75999	17	15	gi|641574512|gb|KK855303.1|	134839-135225	9.3	37
ACYPI007348-RA	ni|645902473|nb|KK921956.1|	219352-219785	nan	nan	gi|641586905|gb|KK854172.1|	112497-112869	9.9	38
ACYPI007351-RA	ni|645903788|nb|KK920642.1|	480222-480467	16	15	gi|641568682|gb|KK856900.1|	35655-35887	10	38
ACYPI007352-RA	ni|645904281|nb|KK920220.1|	1026791-1027937	15	16	gi|641577289|gb|KK854776.1|	255128-256313	10	36
ACYPI007353-RA	ni|645903949|nb|KK920482.1|	265249-265524	17	14	gi|641587866|gb|KK854050.1|	373024-373769	10	38
ACYPI007358-RA	ni|645903751|nb|KK920679.1|	742570-743052	17	16	gi|641586372|gb|KK854251.1|	592904-593520	10	38

ACYPI007364-RA	ni|645902463|nb|KK921966.1|	133294-135240	nan	nan	gi|641587660|gb|KK854076.1|	471346-473456	9.9	41
ACYPI007366-RA	ni|645904210|nb|KK920249.1|	1910079-1910246	16	16	gi|641567075|gb|KK857433.1|	55023-58199	10	39
ACYPI007368-RA	ni|645903795|nb|KK920635.1|	147135-147517	14	16	gi|641576161|gb|KK854972.1|	162905-168036	10	36
ACYPI007373-RA	ni|645903901|nb|KK920530.1|	1076969-1077983	17	17	gi|641567822|gb|KK857177.1|	61341-62200	10	42
ACYPI007374-RA	ni|645904177|nb|KK920260.1|	2420978-2421580	18	17	gi|641578084|gb|KK854643.1|	79905-83124	11	40
ACYPI007375-RA	ni|645904065|nb|KK920366.1|	952992-953220	18	18	gi|641586843|gb|KK854183.1|	863143-863974	11	41
ACYPI007379-RA	ni|645903831|nb|KK920599.1|	356965-358299	18	17	gi|641576672|gb|KK854880.1|	188212-189661	9.4	40
ACYPI007381-RA	ni|645904009|nb|KK920422.1|	844244-844619	17	9.4	gi|641580970|gb|KK854563.1|	134065-137500	11	42
ACYPI007382-RA	ni|645903925|nb|KK920506.1|	1189184-1189389	19	17	gi|641587179|gb|KK854134.1|	275370-277519	11	40
ACYPI007387-RA	ni|645904152|nb|KK920281.1|	426820-426963	15	15	gi|641587365|gb|KK854113.1|	395780-398085	12	45
ACYPI007388-RA	ni|645903874|nb|KK920557.1|	512753-513426	18	18	gi|641575332|gb|KK855132.1|	169326-170171	10	39
ACYPI007397-RA	ni|645904053|nb|KK920378.1|	1756810-1757098	18	17	gi|641582096|gb|KK854546.1|	44265-46812	11	38
ACYPI007401-RA	ni|645903829|nb|KK920601.1|	679214-679590	16	15	gi|641584305|gb|KK854491.1|	330222-339291	11	39
ACYPI007402-RA	ni|645903812|nb|KK920618.1|	842794-843046	20	18	gi|641576238|gb|KK854955.1|	162567-163108	11	40
ACYPI007404-RA	ni|645904234|nb|KK920241.1|	187521-187724	13	15	gi|641557847|gb|KK861397.1|	5674-6196	9.7	30
ACYPI007405-RA	ni|645903622|nb|KK920808.1|	608084-608331	nan	nan	gi|641577837|gb|KK854685.1|	187613-192307	11	42
ACYPI007413-RA	ni|645904004|nb|KK920427.1|	957413-959059	16	16	gi|641584305|gb|KK854491.1|	308400-312369	11	39
ACYPI007422-RA	ni|645904155|nb|KK920278.1|	919335-919641	18	9.4	gi|641586019|gb|KK854303.1|	356684-356939	11	43
ACYPI007433-RA	ni|645904068|nb|KK920363.1|	1109941-1110970	16	8.6	gi|641586046|gb|KK854299.1|	271781-273287	10	20
ACYPI007436-RA	ni|645904023|nb|KK920408.1|	643817-644780	17	17	gi|641575693|gb|KK855062.1|	168512-168805	11	40
ACYPI007437-RA	ni|645903746|nb|KK920684.1|	220471-220662	15	15	gi|641584619|gb|KK854468.1|	259738-260306	10	39
ACYPI007442-RA	ni|645903509|nb|KK920921.1|	801341-801516	nan	nan	gi|641575043|gb|KK855191.1|	81543-82476	12	43
ACYPI007445-RA	ni|645903650|nb|KK920780.1|	796964-797450	nan	nan	gi|641570462|gb|KK856348.1|	13712-14325	10	37
ACYPI007451-RA	ni|645903910|nb|KK920521.1|	618035-618562	19	17	gi|641569429|gb|KK856673.1|	33743-34169	8.4	36
ACYPI007453-RA	ni|645902779|nb|KK921650.1|	36038-37050	nan	nan	gi|641584806|gb|KK854455.1|	424746-425229	11	43
ACYPI007468-RA	ni|645903978|nb|KK920453.1|	528780-528974	16	17	gi|641570419|gb|KK856360.1|	74048-77389	12	39
ACYPI007471-RA	ni|645903734|nb|KK920696.1|	425967-426404	nan	nan	gi|641578330|gb|KK854601.1|	88172-92177	11	38
ACYPI007473-RA	ni|645903464|nb|KK920966.1|	224393-224963	nan	nan	gi|641585342|gb|KK854413.1|	240834-241731	11	39
ACYPI007476-RA	ni|645904177|nb|KK920260.1|	4044156-4044396	18	17	gi|641586291|gb|KK854262.1|	427830-428061	8.9	39
ACYPI007477-RA	ni|645903728|nb|KK920702.1|	519649-522439	nan	nan	gi|641586850|gb|KK854182.1|	526445-529572	10	37
ACYPI007480-RA	ni|645904158|nb|KK920275.1|	1096232-1096645	15	8.3	gi|641586880|gb|KK854177.1|	1477-1952	10	20
ACYPI007485-RA	ni|645897959|nb|KK926470.1|	42725-43504	nan	nan	gi|641571680|gb|KK856006.1|	95348-96319	12	42

ACYPI007495-RA	ni|645904070|nb|KK920361.1|	183874-184096	17	16	gi|641574249|gb|KK855364.1|	167719-172295	9.2	37
ACYPI007505-RA	ni|645903985|nb|KK920446.1|	818354-818538	18	16	gi|641577501|gb|KK854743.1|	44236-47103	9.1	37
ACYPI007507-RA	ni|645904153|nb|KK920280.1|	3183413-3183965	17	16	gi|641569999|gb|KK856492.1|	53414-54841	10	38
ACYPI007519-RA	ni|645904153|nb|KK920280.1|	780147-785460	17	16	gi|641576608|gb|KK854891.1|	282113-289839	11	37
ACYPI007522-RA	ni|645904260|nb|KK920228.1|	1347963-1348396	15	15	gi|641569705|gb|KK856587.1|	64456-65046	9.4	37
ACYPI007524-RA	ni|645904219|nb|KK920246.1|	385131-385899	16	17	gi|641586555|gb|KK854224.1|	114953-115346	11	39
ACYPI007533-RA	ni|645903569|nb|KK920861.1|	167593-168964	nan	nan	gi|641576266|gb|KK854949.1|	256940-258236	11	45
ACYPI007534-RA	ni|645904008|nb|KK920423.1|	276725-276966	16	9.5	gi|641570274|gb|KK856404.1|	121901-122634	9.1	35
ACYPI007537-RA	ni|645903563|nb|KK920867.1|	699462-700637	nan	nan	gi|641568642|gb|KK856914.1|	65616-66744	11	40
ACYPI007545-RA	ni|645904180|nb|KK920259.1|	213282-213565	17	17	gi|641585287|gb|KK854423.1|	132012-132285	11	40
ACYPI007546-RA	ni|645903771|nb|KK920659.1|	722910-723259	16	15	gi|641571508|gb|KK856050.1|	67416-68003	9.7	33
ACYPI007561-RA	ni|645903496|nb|KK920934.1|	941503-941833	nan	nan	gi|641588033|gb|KK854031.1|	1129822-1132527	11	38
ACYPI007567-RA	ni|645903633|nb|KK920797.1|	383322-383968	nan	nan	gi|641585527|gb|KK854383.1|	566803-567981	11	39
ACYPI007585-RA	ni|645904222|nb|KK920245.1|	680499-681176	17	12	gi|641585395|gb|KK854405.1|	186789-187575	10	38
ACYPI007586-RA	ni|645903970|nb|KK920461.1|	2342158-2342363	17	16	gi|641557821|gb|KK861408.1|	3789-3985	8.2	34
ACYPI007598-RA	ni|645903923|nb|KK920508.1|	43228-43568	18	18	gi|641587066|gb|KK854149.1|	279457-279811	11	40
ACYPI007602-RA	ni|645901537|nb|KK922892.1|	1087-1274	nan	nan	gi|641587287|gb|KK854120.1|	251080-251494	9.9	37
ACYPI007605-RA	ni|645903965|nb|KK920466.1|	1438213-1438545	17	16	gi|641576055|gb|KK854992.1|	205208-205646	9.7	37
ACYPI007611-RA	ni|645904222|nb|KK920245.1|	621357-622768	17	12	gi|641585395|gb|KK854405.1|	365289-366904	10	38
ACYPI007627-RA	ni|645904245|nb|KK920234.1|	57388-57661	16	17	gi|641587671|gb|KK854074.1|	901464-902007	11	40
ACYPI007628-RA	ni|645904153|nb|KK920280.1|	1404610-1404953	17	16	gi|641576608|gb|KK854891.1|	364452-365585	11	37
ACYPI007630-RA	ni|645904088|nb|KK920343.1|	99647-100036	17	18	gi|641577968|gb|KK854660.1|	458315-460243	10	42
ACYPI007635-RA	ni|645904096|nb|KK920335.1|	2031129-2033444	18	17	gi|641587256|gb|KK854124.1|	254814-259009	9.7	40
ACYPI007640-RA	ni|645903780|nb|KK920650.1|	506717-509085	19	19	gi|641586967|gb|KK854164.1|	346157-346955	10	27
ACYPI007642-RA	ni|645901974|nb|KK922455.1|	40252-40780	nan	nan	gi|641586258|gb|KK854267.1|	696141-696963	11	37
ACYPI007653-RA	ni|645903936|nb|KK920495.1|	972501-972783	19	18	gi|641587417|gb|KK854105.1|	195-644	11	42
ACYPI007666-RA	ni|645902589|nb|KK921840.1|	254022-254912	nan	nan	gi|641575951|gb|KK855015.1|	243325-244558	9.9	37
ACYPI007669-RA	ni|645904268|nb|KK920225.1|	516702-516930	14	15	gi|641562301|gb|KK859354.1|	10691-11263	9.2	39
ACYPI007677-RA	ni|645903866|nb|KK920564.1|	375640-375831	18	15	gi|641573906|gb|KK855442.1|	33594-33905	11	39
ACYPI007679-RA	ni|645904241|nb|KK920238.1|	116765-117564	18	17	gi|641587840|gb|KK854053.1|	354674-355260	11	43
ACYPI007681-RA	ni|645903752|nb|KK920678.1|	405416-405704	18	16	gi|641566998|gb|KK857460.1|	44616-45103	11	41
ACYPI007683-RA	ni|645903789|nb|KK920641.1|	777339-778494	17	17	gi|641587912|gb|KK854045.1|	288397-288614	11	39

ACYPI007689-RA	ni|645903744|nb|KK920686.1|	66442-67759	17	8.6	gi|641587241|gb|KK854126.1|	414754-416218	11	22
ACYPI007692-RA	ni|645903773|nb|KK920657.1|	417116-417575	18	17	gi|641572139|gb|KK855888.1|	106079-106632	9.9	36
ACYPI007694-RA	ni|645903521|nb|KK920909.1|	510215-510402	nan	nan	gi|641570952|gb|KK856204.1|	114149-116660	10	21
ACYPI007695-RA	ni|645903989|nb|KK920442.1|	295902-296254	17	20	gi|641576951|gb|KK854831.1|	274772-278543	8.6	40
ACYPI007697-RA	ni|645904114|nb|KK920317.1|	1729093-1729808	18	18	gi|641571667|gb|KK856008.1|	72217-72938	11	39
ACYPI007703-RA	ni|645903627|nb|KK920803.1|	82985-83485	nan	nan	gi|641576677|gb|KK854879.1|	161762-162186	11	39
ACYPI007705-RA	ni|645903580|nb|KK920850.1|	184001-184447	nan	nan	gi|641553623|gb|KK863408.1|	9812-10294	11	43
ACYPI007706-RA	ni|645903599|nb|KK920831.1|	114256-114454	nan	nan	gi|641577322|gb|KK854771.1|	94424-94943	10	21
ACYPI007707-RA	ni|645903598|nb|KK920832.1|	282275-283028	nan	nan	gi|641576084|gb|KK854986.1|	166132-166697	10	38
ACYPI007710-RA	ni|645903743|nb|KK920687.1|	423212-423721	19	18	gi|641574085|gb|KK855399.1|	40119-40344	10	38
ACYPI007713-RA	ni|645903997|nb|KK920434.1|	203274-203542	15	16	gi|641587033|gb|KK854153.1|	315975-316243	10	38
ACYPI007716-RA	ni|645902470|nb|KK921959.1|	46818-46963	nan	nan	gi|641577244|gb|KK854783.1|	156967-158908	9.7	19
ACYPI007717-RA	ni|645903773|nb|KK920657.1|	223658-225109	18	17	gi|641577843|gb|KK854684.1|	152325-156078	10	40
ACYPI007731-RA	ni|645903981|nb|KK920450.1|	522865-523075	16	8.6	gi|641578069|gb|KK854646.1|	255909-256139	9.9	20
ACYPI007733-RA	ni|645902285|nb|KK922144.1|	22629-23257	nan	nan	gi|641572846|gb|KK855707.1|	280000-280467	13	52
ACYPI007734-RA	ni|645903783|nb|KK920647.1|	123344-123764	17	17	gi|641575847|gb|KK855033.1|	290069-291884	10	40
ACYPI007736-RA	ni|645904258|nb|KK920229.1|	1021535-1021889	16	16	gi|641587519|gb|KK854093.1|	158901-159517	11	39
ACYPI007744-RA	ni|645904132|nb|KK920301.1|	890931-892730	16	18	gi|641577322|gb|KK854771.1|	82953-83154	10	21
ACYPI007748-RA	ni|645904120|nb|KK920311.1|	757724-758236	18	18	gi|641587757|gb|KK854063.1|	262374-272220	11	38
ACYPI007750-RA	ni|645901892|nb|KK922537.1|	45529-45717	nan	nan	gi|641587504|gb|KK854095.1|	528242-528733	11	42
ACYPI007752-RA	ni|645903885|nb|KK920546.1|	333321-335493	19	12	gi|641571476|gb|KK856061.1|	118693-123275	12	21
ACYPI007759-RA	ni|645902471|nb|KK921958.1|	117750-118390	nan	nan	gi|641574537|gb|KK855297.1|	153099-153776	9.9	37
ACYPI007763-RA	ni|645903866|nb|KK920564.1|	355348-355500	18	15	gi|641588182|gb|KK854016.1|	626961-627365	11	41
ACYPI007764-RA	ni|645903789|nb|KK920641.1|	477941-478376	17	17	gi|641573919|gb|KK855439.1|	68131-68729	10	37
ACYPI007769-RA	ni|645903534|nb|KK920896.1|	623885-624405	nan	nan	gi|641577623|gb|KK854722.1|	263042-263482	11	38
ACYPI007771-RA	ni|645903902|nb|KK920529.1|	249637-251076	19	17	gi|641574193|gb|KK855375.1|	169090-171505	10	41
ACYPI007773-RA	ni|645903917|nb|KK920514.1|	1338436-1340705	19	17	gi|641585107|gb|KK854435.1|	388387-391875	15	45
ACYPI007776-RA	ni|645903879|nb|KK920552.1|	523748-525691	18	18	gi|641575847|gb|KK855033.1|	191297-198173	10	40
ACYPI007778-RA	ni|645903891|nb|KK920540.1|	106464-107629	15	17	gi|641577341|gb|KK854768.1|	275634-276055	14	58
ACYPI007790-RA	ni|645903804|nb|KK920626.1|	449145-450026	17	17	gi|641587866|gb|KK854050.1|	145051-148050	10	38
ACYPI007791-RA	ni|645903902|nb|KK920529.1|	684141-685269	19	17	gi|641576942|gb|KK854833.1|	25471-27111	11	42
ACYPI007793-RA	ni|645904077|nb|KK920354.1|	1105828-1110256	16	17	gi|641584504|gb|KK854477.1|	485898-490280	9.5	38

ACYPI007795-RA	ni|645903876|nb|KK920555.1|	292698-292896	14	15	gi|641588325|gb|KK854004.1|	1150523-1151845	11	40
ACYPI007798-RA	ni|645902344|nb|KK922085.1|	187154-187280	nan	nan	gi|641585574|gb|KK854375.1|	18227-18583	11	39
ACYPI007800-RA	ni|645904242|nb|KK920237.1|	1338734-1338925	15	16	gi|641586059|gb|KK854297.1|	383773-384352	11	22
ACYPI007801-RA	ni|645903973|nb|KK920458.1|	1433224-1433756	20	18	gi|641587047|gb|KK854151.1|	396198-398027	10	39
ACYPI007802-RA	ni|645903734|nb|KK920696.1|	910962-911422	nan	nan	gi|641573841|gb|KK855459.1|	270792-271383	11	40
ACYPI007807-RA	ni|645904222|nb|KK920245.1|	1639262-1640460	17	12	gi|641577081|gb|KK854810.1|	195666-196860	11	20
ACYPI007817-RA	ni|645903895|nb|KK920536.1|	483380-483633	17	16	gi|641586955|gb|KK854165.1|	678597-680742	11	40
ACYPI007832-RA	ni|645904258|nb|KK920229.1|	757947-758410	16	16	gi|641575288|gb|KK855139.1|	303086-303898	11	38
ACYPI007834-RA	ni|645903713|nb|KK920717.1|	498638-502396	nan	nan	gi|641585204|gb|KK854429.1|	56225-59671	9.2	36
ACYPI007858-RA	ni|645903749|nb|KK920681.1|	132994-134621	19	18	gi|641584205|gb|KK854500.1|	290392-292531	11	41
ACYPI007878-RA	ni|645904240|nb|KK920239.1|	1875285-1876724	16	16	gi|641588294|gb|KK854006.1|	954747-957963	8.7	40
ACYPI007879-RA	ni|645904152|nb|KK920281.1|	454959-455474	15	15	gi|641587365|gb|KK854113.1|	349868-350256	12	45
ACYPI007882-RA	ni|645903923|nb|KK920508.1|	258417-258686	18	18	gi|641586741|gb|KK854197.1|	613155-614620	11	43
ACYPI007886-RA	ni|645903639|nb|KK920791.1|	282456-282914	nan	nan	gi|641574871|gb|KK855227.1|	158621-159095	10	40
ACYPI007895-RA	ni|645903846|nb|KK920584.1|	542355-542895	17	10	gi|641583958|gb|KK854513.1|	44556-45126	10	22
ACYPI007899-RA	ni|645904039|nb|KK920392.1|	218176-219136	15	16	gi|641573504|gb|KK855543.1|	261852-262314	13	39
ACYPI007901-RA	ni|645904019|nb|KK920412.1|	141997-142358	19	9.4	gi|641585510|gb|KK854386.1|	111591-111997	11	20
ACYPI007905-RA	ni|645904057|nb|KK920374.1|	1370747-1370991	16	15	gi|641574809|gb|KK855240.1|	189933-190937	11	39
ACYPI007920-RA	ni|645903598|nb|KK920832.1|	290445-291398	nan	nan	gi|641579230|gb|KK854582.1|	24666-25668	12	43
ACYPI007924-RA	ni|645904116|nb|KK920315.1|	1098828-1100927	19	18	gi|641586528|gb|KK854228.1|	517226-524097	10	42
ACYPI007926-RA	ni|645903955|nb|KK920476.1|	559090-559345	18	17	gi|641574742|gb|KK855254.1|	26008-26470	11	41
ACYPI007932-RA	ni|645904137|nb|KK920296.1|	1275581-1276237	17	17	gi|641577760|gb|KK854699.1|	227257-228128	9.8	41
ACYPI007934-RA	ni|645903464|nb|KK920966.1|	477450-478748	nan	nan	gi|641585340|gb|KK854414.1|	411187-411724	11	42
ACYPI007941-RA	ni|645904096|nb|KK920335.1|	2432498-2432922	18	17	gi|641576362|gb|KK854933.1|	72375-73268	10	37
ACYPI007943-RA	ni|645903721|nb|KK920709.1|	759956-761446	nan	nan	gi|641586387|gb|KK854249.1|	101476-102999	11	24
ACYPI007945-RA	ni|645903773|nb|KK920657.1|	1414144-1414902	18	17	gi|641576188|gb|KK854966.1|	25935-26779	11	39
ACYPI007946-RA	ni|645903527|nb|KK920903.1|	515311-517771	nan	nan	gi|641577553|gb|KK854734.1|	82772-85561	9.7	41
ACYPI007949-RA	ni|645904133|nb|KK920300.1|	3069084-3069244	18	18	gi|641574783|gb|KK855245.1|	221156-221798	10	21
ACYPI007952-RA	ni|645904120|nb|KK920311.1|	329033-329441	18	18	gi|641588213|gb|KK854013.1|	919377-920385	9.6	39
ACYPI007960-RA	ni|645904079|nb|KK920352.1|	313224-313632	15	16	gi|641585953|gb|KK854314.1|	33271-33818	11	43
ACYPI007967-RA	ni|645902344|nb|KK922085.1|	278849-279094	nan	nan	gi|641577677|gb|KK854711.1|	49212-49644	12	39
ACYPI007971-RA	ni|645904118|nb|KK920313.1|	730336-730825	17	17	gi|641574303|gb|KK855353.1|	102637-103703	10	34

ACYPI007972-RA	ni|645904237|nb|KK920240.1|	824927-827638	16	15	gi|641572963|gb|KK855677.1|	195371-198152	11	43
ACYPI007984-RA	ni|645903771|nb|KK920659.1|	84105-84386	16	15	gi|641579933|gb|KK854577.1|	404609-405084	11	37
ACYPI007986-RA	ni|645904243|nb|KK920236.1|	149185-150280	15	8.5	gi|641567609|gb|KK857247.1|	36618-37642	9.7	19
ACYPI007988-RA	ni|645902785|nb|KK921644.1|	105108-106086	nan	nan	gi|641578154|gb|KK854631.1|	98310-101822	8.2	40
ACYPI007989-RA	ni|645904171|nb|KK920262.1|	722019-724441	15	16	gi|641584818|gb|KK854454.1|	50315-52209	11	42
ACYPI007990-RA	ni|645904118|nb|KK920313.1|	376393-377342	17	17	gi|641587133|gb|KK854140.1|	572865-573708	9.9	37
ACYPI007994-RA	ni|645904246|nb|KK920233.1|	2171131-2171357	16	17	gi|641586452|gb|KK854239.1|	123453-125552	10	19
ACYPI007996-RA	ni|645903557|nb|KK920873.1|	752108-752334	nan	nan	gi|641586817|gb|KK854187.1|	762328-763361	9.3	39
ACYPI008000-RA	ni|645903466|nb|KK920964.1|	698959-700474	nan	nan	gi|641572955|gb|KK855679.1|	38150-39594	10	40
ACYPI008002-RA	ni|645903607|nb|KK920823.1|	96658-96897	nan	nan	gi|641575436|gb|KK855110.1|	35050-35312	12	37
ACYPI008004-RA	ni|645901319|nb|KK923110.1|	55057-55333	nan	nan	gi|641588000|gb|KK854035.1|	520715-523770	11	39
ACYPI008005-RA	ni|645904136|nb|KK920297.1|	1673877-1674535	16	17	gi|641576123|gb|KK854979.1|	207770-208714	11	22
ACYPI008007-RA	ni|645903495|nb|KK920935.1|	373205-375045	nan	nan	gi|641576409|gb|KK854925.1|	16134-18316	11	44
ACYPI008019-RA	ni|645904166|nb|KK920267.1|	2788074-2788701	18	16	gi|641567869|gb|KK857161.1|	66585-68533	11	38
ACYPI008020-RA	ni|645904058|nb|KK920373.1|	1008378-1009294	16	15	gi|641588236|gb|KK854011.1|	648610-650162	9.7	38
ACYPI008022-RA	ni|645904058|nb|KK920373.1|	1239274-1239605	16	15	gi|641588073|gb|KK854027.1|	106547-107216	11	41
ACYPI008024-RA	ni|645903644|nb|KK920786.1|	346022-346513	nan	nan	gi|641574833|gb|KK855235.1|	137294-138215	9.4	33
ACYPI008028-RA	ni|645903957|nb|KK920474.1|	896646-897817	18	17	gi|641585376|gb|KK854407.1|	180424-187277	9.8	36
ACYPI008033-RA	ni|645904138|nb|KK920295.1|	667482-668200	15	15	gi|641575730|gb|KK855054.1|	76562-78366	10	38
ACYPI008034-RA	ni|645904027|nb|KK920404.1|	582679-583498	17	17	gi|641585684|gb|KK854357.1|	281126-281836	11	39
ACYPI008035-RA	ni|645904274|nb|KK920223.1|	630382-631063	14	17	gi|641586726|gb|KK854199.1|	428431-429580	11	41
ACYPI008037-RA	ni|645903983|nb|KK920448.1|	208182-208887	18	17	gi|641587256|gb|KK854124.1|	109531-110540	9.7	40
ACYPI008050-RA	ni|645904251|nb|KK920231.1|	519172-519987	16	16	gi|641587856|gb|KK854051.1|	783668-784232	10	41
ACYPI008053-RA	ni|645904088|nb|KK920343.1|	1517099-1520331	17	18	gi|641573202|gb|KK855619.1|	210574-214420	9.6	42
ACYPI008056-RA	ni|645904005|nb|KK920426.1|	649656-650128	17	16	gi|641570514|gb|KK856331.1|	85898-86463	9.8	39
ACYPI008063-RA	ni|645904106|nb|KK920325.1|	1970771-1971261	17	17	gi|641583094|gb|KK854526.1|	114769-115188	11	41
ACYPI008064-RA	ni|645903639|nb|KK920791.1|	471012-471520	nan	nan	gi|641566198|gb|KK857747.1|	2954-4686	11	20
ACYPI008065-RA	ni|645902496|nb|KK921933.1|	72732-75601	nan	nan	gi|641587992|gb|KK854036.1|	686026-686619	11	41
ACYPI008075-RA	ni|645904168|nb|KK920265.1|	682992-683261	16	16	gi|641588213|gb|KK854013.1|	624716-626027	9.6	39
ACYPI008076-RA	ni|645904271|nb|KK920224.1|	1355126-1355530	15	16	gi|641585989|gb|KK854308.1|	282895-285702	9.3	39
ACYPI008084-RA	ni|645903633|nb|KK920797.1|	373456-373931	nan	nan	gi|641575454|gb|KK855107.1|	290721-291876	10	38
ACYPI008100-RA	ni|645902438|nb|KK921991.1|	157788-158064	nan	nan	gi|641577879|gb|KK854677.1|	118515-120448	18	36

ACYPI008107-RA	ni|645904177|nb|KK920260.1|	2522848-2523442	18	17	gi|641588213|gb|KK854013.1|	1020148-1020832	9.6	39
ACYPI008110-RA	ni|645902638|nb|KK921791.1|	166061-166293	nan	nan	gi|641578399|gb|KK854595.1|	238868-239570	9.3	36
ACYPI008112-RA	ni|645904081|nb|KK920350.1|	403742-404550	13	18	gi|641586983|gb|KK854162.1|	434207-435022	10	41
ACYPI008113-RA	ni|645903813|nb|KK920617.1|	480728-481433	17	18	gi|641569718|gb|KK856582.1|	47703-48923	8.4	36
ACYPI008114-RA	ni|645903699|nb|KK920731.1|	201054-201684	nan	nan	gi|641587193|gb|KK854132.1|	749049-752423	11	39
ACYPI008115-RA	ni|645904115|nb|KK920316.1|	1125426-1126130	17	18	gi|641575867|gb|KK855030.1|	126489-127265	11	39
ACYPI008122-RA	ni|645902779|nb|KK921650.1|	60287-60820	nan	nan	gi|641575104|gb|KK855179.1|	151037-154790	10	35
ACYPI008129-RA	ni|645904166|nb|KK920267.1|	1937975-1938696	18	16	gi|641578315|gb|KK854603.1|	128651-129219	9.8	38
ACYPI008131-RA	ni|645903484|nb|KK920946.1|	143916-144472	nan	nan	gi|641572293|gb|KK855850.1|	187958-188495	11	38
ACYPI008134-RA	ni|645902035|nb|KK922394.1|	198241-199816	nan	nan	gi|641586850|gb|KK854182.1|	396237-397769	10	37
ACYPI008142-RA	ni|645904122|nb|KK920309.1|	1068006-1068724	18	17	gi|641568112|gb|KK857081.1|	43747-44432	11	38
ACYPI008149-RA	ni|645904046|nb|KK920385.1|	282472-283276	17	8.4	gi|641575987|gb|KK855008.1|	90699-91827	9.9	20
ACYPI008157-RA	ni|645903495|nb|KK920935.1|	254128-254414	nan	nan	gi|641575500|gb|KK855098.1|	31559-31871	11	39
ACYPI008158-RA	ni|645904177|nb|KK920260.1|	1060377-1060771	18	17	gi|641586661|gb|KK854209.1|	488936-489616	11	43
ACYPI008162-RA	ni|645903988|nb|KK920443.1|	248706-249243	14	16	gi|641569519|gb|KK856646.1|	11470-12019	10	39
ACYPI008165-RA	ni|645903953|nb|KK920478.1|	1425661-1426058	18	17	gi|641585773|gb|KK854342.1|	608787-609104	12	45
ACYPI008166-RA	ni|645903821|nb|KK920609.1|	1054354-1054855	17	17	gi|641577234|gb|KK854784.1|	179623-184486	11	39
ACYPI008168-RA	ni|645902668|nb|KK921761.1|	142400-145561	nan	nan	gi|641578310|gb|KK854604.1|	454378-455390	11	39
ACYPI008174-RA	ni|645903929|nb|KK920502.1|	1423681-1424121	17	18	gi|641577203|gb|KK854789.1|	287448-287717	11	43
ACYPI008179-RA	ni|645903506|nb|KK920924.1|	206225-206395	nan	nan	gi|641576118|gb|KK854980.1|	195161-196610	9.7	37
ACYPI008180-RA	ni|645904061|nb|KK920370.1|	923966-924909	17	16	gi|641573318|gb|KK855592.1|	125082-125808	10	41
ACYPI008184-RA	ni|645904158|nb|KK920275.1|	1907043-1907595	15	8.3	gi|641586880|gb|KK854177.1|	544407-545683	10	20
ACYPI008186-RA	ni|645902011|nb|KK922418.1|	89405-91913	nan	nan	gi|641586285|gb|KK854263.1|	24460-27072	10	38
ACYPI008188-RA	ni|645904114|nb|KK920317.1|	1764971-1765254	18	18	gi|641571667|gb|KK856008.1|	4820-5515	11	39
ACYPI008191-RA	ni|645904088|nb|KK920343.1|	1323348-1323905	17	18	gi|641579930|gb|KK854578.1|	52763-53391	9.1	35
ACYPI008195-RA	ni|645903999|nb|KK920432.1|	1241125-1241995	17	15	gi|641569495|gb|KK856654.1|	18878-21500	9.5	35
ACYPI008202-RA	ni|645904157|nb|KK920276.1|	475768-475998	15	15	gi|641572549|gb|KK855786.1|	52333-52559	9.5	35
ACYPI008203-RA	ni|645904144|nb|KK920289.1|	1052544-1052950	16	9	gi|641587866|gb|KK854050.1|	243237-243456	10	38
ACYPI008211-RA	ni|645904268|nb|KK920225.1|	1076619-1077067	14	15	gi|641584419|gb|KK854483.1|	435875-436144	10	42
ACYPI008218-RA	ni|645904086|nb|KK920345.1|	347455-347600	14	15	gi|641569596|gb|KK856622.1|	68405-68588	12	43
ACYPI008220-RA	ni|645904077|nb|KK920354.1|	294671-295333	16	17	gi|641574951|gb|KK855209.1|	68539-70022	10	38
ACYPI008222-RA	ni|645903674|nb|KK920756.1|	246220-248151	nan	nan	gi|641588283|gb|KK854007.1|	658475-660840	11	40

ACYPI008231-RA	ni|645903831|nb|KK920599.1|	610607-611519	18	17	gi|641577341|gb|KK854768.1|	333027-334677	14	58
ACYPI008241-RA	ni|645903832|nb|KK920598.1|	1034509-1034878	18	8.8	gi|641587383|gb|KK854110.1|	362879-365366	10	37
ACYPI008242-RA	ni|645904222|nb|KK920245.1|	2329941-2330336	17	12	gi|641574114|gb|KK855393.1|	159342-162609	9.9	40
ACYPI008243-RA	ni|645903518|nb|KK920912.1|	151403-152288	nan	nan	gi|641587162|gb|KK854136.1|	590188-591116	10	40
ACYPI008244-RA	ni|645902229|nb|KK922200.1|	198305-198659	nan	nan	gi|641570627|gb|KK856299.1|	58851-59767	11	20
ACYPI008248-RA	ni|645904057|nb|KK920374.1|	57181-57562	16	15	gi|641567299|gb|KK857356.1|	39235-39895	11	38
ACYPI008253-RA	ni|645903871|nb|KK920559.1|	951960-952230	18	9.4	gi|641576896|gb|KK854840.1|	306103-306880	10	21
ACYPI008255-RA	ni|645904210|nb|KK920249.1|	795621-795857	16	16	gi|641568716|gb|KK856889.1|	28408-28817	10	35
ACYPI008256-RA	ni|645904240|nb|KK920239.1|	388562-390169	16	16	gi|641586620|gb|KK854215.1|	319717-321987	9.1	40
ACYPI008259-RA	ni|645904122|nb|KK920309.1|	807306-807452	18	17	gi|641577612|gb|KK854724.1|	206041-206221	9.7	37
ACYPI008261-RA	ni|645904262|nb|KK920227.1|	1163111-1163678	17	17	gi|641550296|gb|KK864961.1|	4429-5224	10	33
ACYPI008262-RA	ni|645903680|nb|KK920750.1|	644973-645217	nan	nan	gi|641588000|gb|KK854035.1|	1026997-1027372	11	39
ACYPI008265-RA	ni|645904114|nb|KK920317.1|	1949883-1950138	18	18	gi|641564209|gb|KK858536.1|	19907-20161	11	34
ACYPI008270-RA	ni|645901628|nb|KK922801.1|	9728-10461	nan	nan	gi|641575368|gb|KK855125.1|	248630-248864	10	36
ACYPI008272-RA	ni|645902662|nb|KK921767.1|	276242-276971	nan	nan	gi|641587937|gb|KK854042.1|	756487-758107	11	42
ACYPI008275-RA	ni|645904274|nb|KK920223.1|	603859-604286	14	17	gi|641573765|gb|KK855478.1|	187453-188638	11	42
ACYPI008278-RA	ni|645904116|nb|KK920315.1|	1559610-1559981	19	18	gi|641575010|gb|KK855196.1|	268989-269206	12	41
ACYPI008279-RA	ni|645903923|nb|KK920508.1|	1343612-1344248	18	18	gi|641581779|gb|KK854550.1|	251632-251976	11	39
ACYPI008281-RA	ni|645904271|nb|KK920224.1|	4107686-4108670	15	16	gi|641579205|gb|KK854586.1|	401592-402101	10	39
ACYPI008290-RA	ni|645903564|nb|KK920866.1|	311355-312136	nan	nan	gi|641587126|gb|KK854141.1|	340099-343824	10	39
ACYPI008299-RA	ni|645903466|nb|KK920964.1|	205451-205812	nan	nan	gi|641586144|gb|KK854283.1|	481849-483619	10	38
ACYPI008301-RA	ni|645903631|nb|KK920799.1|	330051-331826	nan	nan	gi|641576650|gb|KK854884.1|	118227-120924	9.6	40
ACYPI008302-RA	ni|645904112|nb|KK920319.1|	223064-223885	18	17	gi|641588090|gb|KK854025.1|	996250-997232	11	43
ACYPI008308-RA	ni|645904009|nb|KK920422.1|	208078-208633	17	9.4	gi|641582439|gb|KK854536.1|	378954-382080	11	36
ACYPI008315-RA	ni|645904028|nb|KK920403.1|	1957327-1957653	18	17	gi|641574642|gb|KK855274.1|	223733-224421	11	38
ACYPI008318-RA	ni|645901319|nb|KK923110.1|	53804-54327	nan	nan	gi|641588000|gb|KK854035.1|	516184-517516	11	39
ACYPI008325-RA	ni|645904014|nb|KK920417.1|	822928-823625	19	18	gi|641576188|gb|KK854966.1|	83930-89497	11	39
ACYPI008327-RA	ni|645904130|nb|KK920303.1|	218328-220002	18	17	gi|641586886|gb|KK854176.1|	664096-672454	10	41
ACYPI008333-RA	ni|645901760|nb|KK922669.1|	70086-70938	nan	nan	gi|641570111|gb|KK856458.1|	104412-105407	11	40
ACYPI008335-RA	ni|645902426|nb|KK922003.1|	95772-96580	nan	nan	gi|641587550|gb|KK854090.1|	157286-159554	9.7	45
ACYPI008351-RA	ni|645903540|nb|KK920890.1|	182497-182752	nan	nan	gi|641566809|gb|KK857526.1|	41927-42113	9.8	22
ACYPI008357-RA	ni|645903664|nb|KK920766.1|	200467-200729	nan	nan	gi|641571465|gb|KK856064.1|	95177-95459	9.2	35

ACYPI008362-RA	ni|645896832|nb|KK927597.1|	2087-2490	nan	nan	gi|641587832|gb|KK854054.1|	598824-598970	9.8	20
ACYPI008365-RA	ni|645904133|nb|KK920300.1|	2786070-2787012	18	18	gi|641587390|gb|KK854109.1|	395190-396353	11	44
ACYPI008366-RA	ni|645903952|nb|KK920479.1|	959333-960791	16	16	gi|641587866|gb|KK854050.1|	988745-992046	10	38
ACYPI008368-RA	ni|645904279|nb|KK920221.1|	2642380-2643820	16	16	gi|641586185|gb|KK854275.1|	729440-730289	12	40
ACYPI008371-RA	ni|645903992|nb|KK920439.1|	723641-724224	15	15	gi|641586210|gb|KK854272.1|	398988-399267	10	39
ACYPI008376-RA	ni|645904133|nb|KK920300.1|	2796953-2797695	18	18	gi|641587390|gb|KK854109.1|	460890-461128	11	44
ACYPI008384-RA	ni|645904009|nb|KK920422.1|	897228-897413	17	9.4	gi|641583958|gb|KK854513.1|	280088-280234	10	22
ACYPI008388-RA	ni|645903934|nb|KK920497.1|	257626-257958	15	16	gi|641572615|gb|KK855769.1|	172413-176442	12	43
ACYPI008390-RA	ni|645904201|nb|KK920252.1|	402088-402335	17	16	gi|641569664|gb|KK856600.1|	38142-38368	11	43
ACYPI008392-RA	ni|645903662|nb|KK920768.1|	265946-267858	nan	nan	gi|641566986|gb|KK857464.1|	48304-52837	11	41
ACYPI008396-RA	ni|645904116|nb|KK920315.1|	2470307-2470964	19	18	gi|641571207|gb|KK856132.1|	124825-125119	10	40
ACYPI008403-RA	ni|645903833|nb|KK920597.1|	245191-246411	17	15	gi|641586754|gb|KK854195.1|	517310-517841	11	41
ACYPI008415-RA	ni|645903915|nb|KK920516.1|	298896-300382	17	17	gi|641582439|gb|KK854536.1|	176136-179145	11	36
ACYPI008418-RA	ni|645904079|nb|KK920352.1|	1102974-1104379	15	16	gi|641587562|gb|KK854088.1|	41262-41915	11	42
ACYPI008427-RA	ni|645903496|nb|KK920934.1|	311412-312191	nan	nan	gi|641587896|gb|KK854047.1|	1059425-1063863	9.9	39
ACYPI008429-RA	ni|645903741|nb|KK920689.1|	233027-233229	17	18	gi|641587126|gb|KK854141.1|	744065-744613	10	39
ACYPI008431-RA	ni|645904171|nb|KK920262.1|	605547-605867	15	16	gi|641584818|gb|KK854454.1|	208658-209150	11	42
ACYPI008435-RA	ni|645903971|nb|KK920460.1|	1152948-1153305	19	18	gi|641577075|gb|KK854811.1|	143185-144604	11	36
ACYPI008437-RA	ni|645903800|nb|KK920630.1|	1377677-1378069	18	17	gi|641570596|gb|KK856308.1|	116212-117588	9.9	39
ACYPI008438-RA	ni|645903980|nb|KK920451.1|	84506-85063	11	14	gi|641580473|gb|KK854570.1|	97916-104069	11	44
ACYPI008454-RA	ni|645903731|nb|KK920699.1|	1196390-1196631	nan	nan	gi|641587881|gb|KK854049.1|	4654-5000	10	40
ACYPI008463-RA	ni|645904004|nb|KK920427.1|	964748-965362	16	16	gi|641584305|gb|KK854491.1|	280359-280752	11	39
ACYPI008464-RA	ni|645903539|nb|KK920891.1|	292513-292654	nan	nan	gi|641561537|gb|KK859697.1|	13933-15228	9.7	16
ACYPI008467-RA	ni|645902445|nb|KK921984.1|	210468-210982	nan	nan	gi|641585163|gb|KK854432.1|	15000-15287	11	43
ACYPI008468-RA	ni|645903504|nb|KK920926.1|	341109-341299	nan	nan	gi|641587577|gb|KK854086.1|	631076-631480	8.2	41
ACYPI008475-RA	ni|645901974|nb|KK922455.1|	63414-65581	nan	nan	gi|641578198|gb|KK854624.1|	446389-449285	12	40
ACYPI008479-RA	ni|645904248|nb|KK920232.1|	709544-709774	15	16	gi|641574048|gb|KK855408.1|	157772-158514	9.9	41
ACYPI008481-RA	ni|645904016|nb|KK920415.1|	151231-151467	12	14	gi|641586053|gb|KK854298.1|	609578-609753	11	41
ACYPI008482-RA	ni|645903629|nb|KK920801.1|	127081-127314	nan	nan	gi|641577922|gb|KK854669.1|	35683-36106	12	21
ACYPI008484-RA	ni|645903766|nb|KK920664.1|	120988-122605	18	15	gi|641570349|gb|KK856380.1|	37120-38857	8.8	38
ACYPI008489-RA	ni|645903743|nb|KK920687.1|	1241440-1241806	19	18	gi|641586380|gb|KK854250.1|	276069-276602	9.3	38
ACYPI008491-RA	ni|645904279|nb|KK920221.1|	2628854-2630142	16	16	gi|641587095|gb|KK854145.1|	133940-137608	9.9	40

ACYPI008492-RA	ni|645902426|nb|KK922003.1|	199333-199603	nan	nan	gi|641586928|gb|KK854169.1|	170275-171738	11	39
ACYPI008493-RA	ni|645904174|nb|KK920261.1|	361095-361770	19	17	gi|641576801|gb|KK854856.1|	342247-342594	10	39
ACYPI008495-RA	ni|645902463|nb|KK921966.1|	127148-127419	nan	nan	gi|641587660|gb|KK854076.1|	461594-461882	9.9	41
ACYPI008512-RA	ni|645903704|nb|KK920726.1|	204369-205036	nan	nan	gi|641574876|gb|KK855226.1|	41189-41563	11	39
ACYPI008516-RA	ni|645903594|nb|KK920836.1|	552289-552505	nan	nan	gi|641577109|gb|KK854806.1|	202541-202743	9	32
ACYPI008521-RA	ni|645903772|nb|KK920658.1|	281065-282562	15	16	gi|641586503|gb|KK854232.1|	217244-217740	8.3	40
ACYPI008535-RA	ni|645903552|nb|KK920878.1|	629324-631169	nan	nan	gi|641570121|gb|KK856455.1|	39618-40567	10	40
ACYPI008536-RA	ni|645904195|nb|KK920254.1|	2135742-2136232	18	17	gi|641584728|gb|KK854460.1|	82910-83530	9.5	37
ACYPI008539-RA	ni|645903847|nb|KK920583.1|	825801-825996	18	18	gi|641570169|gb|KK856439.1|	24540-24771	10	36
ACYPI008541-RA	ni|645903812|nb|KK920618.1|	811247-811598	20	18	gi|641576238|gb|KK854955.1|	124493-125020	11	40
ACYPI008552-RA	ni|645904240|nb|KK920239.1|	1868699-1869848	16	16	gi|641588294|gb|KK854006.1|	941948-943427	8.7	40
ACYPI008555-RA	ni|645903518|nb|KK920912.1|	96093-96497	nan	nan	gi|641575068|gb|KK855186.1|	20290-20716	12	44
ACYPI008556-RA	ni|645903829|nb|KK920601.1|	714638-715795	16	15	gi|641585515|gb|KK854385.1|	451785-453086	11	40
ACYPI008557-RA	ni|645903874|nb|KK920557.1|	201467-202088	18	18	gi|641570940|gb|KK856208.1|	73744-74876	10	35
ACYPI008558-RA	ni|645903984|nb|KK920447.1|	402221-403164	18	17	gi|641586171|gb|KK854278.1|	475981-480606	9	39
ACYPI008560-RA	ni|645904278|nb|KK920222.1|	1322795-1323147	15	16	gi|641585778|gb|KK854341.1|	299438-299721	9.7	41
ACYPI008563-RA	ni|645903617|nb|KK920813.1|	198033-198528	nan	nan	gi|641564966|gb|KK858214.1|	14529-15636	9.6	39
ACYPI008573-RA	ni|645904114|nb|KK920317.1|	441494-441665	18	18	gi|641574476|gb|KK855312.1|	67453-68329	10	43
ACYPI008575-RA	ni|645903713|nb|KK920717.1|	399990-401389	nan	nan	gi|641574303|gb|KK855353.1|	115784-118774	10	34
ACYPI008576-RA	ni|645904087|nb|KK920344.1|	779535-780301	16	16	gi|641566659|gb|KK857580.1|	22827-23709	9.3	35
ACYPI008578-RA	ni|645903597|nb|KK920833.1|	497897-498481	nan	nan	gi|641575236|gb|KK855151.1|	342532-343239	11	38
ACYPI008584-RA	ni|645904120|nb|KK920311.1|	719855-720350	18	18	gi|641588060|gb|KK854028.1|	32522-33083	11	42
ACYPI008591-RA	ni|645904166|nb|KK920267.1|	2471076-2471954	18	16	gi|641575209|gb|KK855157.1|	135587-136067	11	38
ACYPI008596-RA	ni|645903496|nb|KK920934.1|	949022-949806	nan	nan	gi|641573583|gb|KK855523.1|	56055-58144	10	41
ACYPI008605-RA	ni|645903597|nb|KK920833.1|	369926-370350	nan	nan	gi|641575236|gb|KK855151.1|	76033-76816	11	38
ACYPI008606-RA	ni|645903532|nb|KK920898.1|	880246-880776	nan	nan	gi|641588051|gb|KK854029.1|	1052570-1054051	9.6	40
ACYPI008607-RA	ni|645904251|nb|KK920231.1|	448430-448934	16	16	gi|641587390|gb|KK854109.1|	658678-659290	11	44
ACYPI008615-RA	ni|645903607|nb|KK920823.1|	46062-46341	nan	nan	gi|641575436|gb|KK855110.1|	56585-57098	12	37
ACYPI008621-RA	ni|645904049|nb|KK920382.1|	452132-453098	18	18	gi|641586975|gb|KK854163.1|	276069-276294	11	40
ACYPI008630-RA	ni|645904122|nb|KK920309.1|	1189676-1190439	18	17	gi|641576294|gb|KK854944.1|	112031-112692	9.9	39
ACYPI008632-RA	ni|645903862|nb|KK920568.1|	337828-338042	17	17	gi|641569223|gb|KK856733.1|	86467-89983	12	42
ACYPI008637-RA	ni|645904231|nb|KK920242.1|	1112045-1113932	16	17	gi|641576852|gb|KK854847.1|	250741-252923	13	48

ACYPI008640-RA	ni|645904098|nb|KK920333.1|	304677-305873	16	8.8	gi|641577215|gb|KK854787.1|	159906-167921	11	40
ACYPI008641-RA	ni|645903548|nb|KK920882.1|	451442-452508	nan	nan	gi|641573923|gb|KK855438.1|	174998-176352	12	42
ACYPI008652-RA	ni|645903807|nb|KK920623.1|	213172-213410	12	14	gi|641574668|gb|KK855267.1|	155533-156563	10	38
ACYPI008668-RA	ni|645904101|nb|KK920330.1|	642971-643569	18	18	gi|641569397|gb|KK856683.1|	43687-45114	9.5	42
ACYPI008671-RA	ni|645904133|nb|KK920300.1|	1015481-1016086	18	18	gi|641576689|gb|KK854876.1|	354903-355844	10	40
ACYPI008675-RA	ni|645903953|nb|KK920478.1|	1661335-1662416	18	17	gi|641586850|gb|KK854182.1|	613735-614680	10	37
ACYPI008677-RA	ni|645904118|nb|KK920313.1|	825177-825588	17	17	gi|641581789|gb|KK854549.1|	279561-280333	9.9	37
ACYPI008693-RA	ni|645903965|nb|KK920466.1|	1208597-1209041	17	16	gi|641578106|gb|KK854639.1|	90116-90385	11	39
ACYPI008698-RA	ni|645904073|nb|KK920358.1|	1104368-1108545	16	18	gi|641587765|gb|KK854062.1|	36184-42410	10	41
ACYPI008701-RA	ni|645904015|nb|KK920416.1|	1217734-1218076	18	16	gi|641585423|gb|KK854400.1|	206389-206732	11	36
ACYPI008707-RA	ni|645904116|nb|KK920315.1|	420920-421158	19	18	gi|641580504|gb|KK854565.1|	345098-345508	11	43
ACYPI008708-RA	ni|645903896|nb|KK920535.1|	387555-388105	16	9.1	gi|641588136|gb|KK854020.1|	281040-281341	11	36
ACYPI008713-RA	ni|645904258|nb|KK920229.1|	241912-242535	16	16	gi|641567114|gb|KK857420.1|	67663-71213	10	38
ACYPI008720-RA	ni|645904015|nb|KK920416.1|	178062-178286	18	16	gi|641575096|gb|KK855181.1|	54636-55463	11	21
ACYPI008721-RA	ni|645904015|nb|KK920416.1|	1249980-1250325	18	16	gi|641585423|gb|KK854400.1|	40952-41166	11	36
ACYPI008722-RA	ni|645903774|nb|KK920656.1|	157199-158161	16	9.2	gi|641545587|gb|KK867229.1|	775-1201	11	22
ACYPI008728-RA	ni|645903760|nb|KK920670.1|	705601-707676	18	19	gi|641586983|gb|KK854162.1|	578638-582747	10	41
ACYPI008730-RA	ni|645903831|nb|KK920599.1|	955318-956461	18	17	gi|641573617|gb|KK855515.1|	108313-109776	10	38
ACYPI008736-RA	ni|645904043|nb|KK920388.1|	330047-334814	16	9.1	gi|641588202|gb|KK854014.1|	808994-813455	10	21
ACYPI008744-RA	ni|645903752|nb|KK920678.1|	325459-325598	18	16	gi|641566998|gb|KK857460.1|	27454-28140	11	41
ACYPI008746-RA	ni|645904056|nb|KK920375.1|	759901-760171	16	16	gi|641584606|gb|KK854469.1|	165691-165960	13	57
ACYPI008756-RA	ni|645904177|nb|KK920260.1|	2525990-2526624	18	17	gi|641578084|gb|KK854643.1|	152272-152566	11	40
ACYPI008758-RA	ni|645904258|nb|KK920229.1|	1062965-1063748	16	16	gi|641586936|gb|KK854168.1|	549874-550917	12	42
ACYPI008763-RA	ni|645904271|nb|KK920224.1|	458452-459710	15	16	gi|641579941|gb|KK854575.1|	112465-112832	10	39
ACYPI008765-RA	ni|645903957|nb|KK920474.1|	1454881-1455097	18	17	gi|641573533|gb|KK855535.1|	22693-23811	10	41
ACYPI008769-RA	ni|645903760|nb|KK920670.1|	740770-741114	18	19	gi|641585469|gb|KK854393.1|	404812-405277	10	37
ACYPI008771-RA	ni|645903989|nb|KK920442.1|	279222-279509	17	20	gi|641587946|gb|KK854041.1|	749359-754522	11	41
ACYPI008778-RA	ni|645903597|nb|KK920833.1|	235130-235288	nan	nan	gi|641586258|gb|KK854267.1|	282632-284535	11	37
ACYPI008781-RA	ni|645904079|nb|KK920352.1|	110690-111591	15	16	gi|641587580|gb|KK854085.1|	321884-323127	10	39
ACYPI008782-RA	ni|645903654|nb|KK920776.1|	631302-631455	nan	nan	gi|641578411|gb|KK854593.1|	165711-166311	11	43
ACYPI008785-RA	ni|645902580|nb|KK921849.1|	57156-57395	nan	nan	gi|641580970|gb|KK854563.1|	322943-323194	11	42
ACYPI008790-RA	ni|645903971|nb|KK920460.1|	1037958-1038214	19	18	gi|641585761|gb|KK854344.1|	154131-154635	10	39

ACYPI008793-RA	ni|645904170|nb|KK920263.1|	1927624-1928348	15	16	gi|641584913|gb|KK854447.1|	426340-426884	10	40
ACYPI008797-RA	ni|645904162|nb|KK920271.1|	719103-719283	15	18	gi|641586620|gb|KK854215.1|	44003-44203	9.1	40
ACYPI008800-RA	ni|645903820|nb|KK920610.1|	394818-395314	18	19	gi|641568762|gb|KK856874.1|	46280-46617	11	41
ACYPI008804-RA	ni|645903702|nb|KK920728.1|	537841-538709	nan	nan	gi|641586285|gb|KK854263.1|	126775-128074	10	38
ACYPI008806-RA	ni|645904165|nb|KK920268.1|	779389-779610	16	16	gi|641576207|gb|KK854961.1|	141266-141958	11	38
ACYPI008807-RA	ni|645901672|nb|KK922757.1|	58782-59091	nan	nan	gi|641588343|gb|KK854003.1|	2036778-2037283	11	40
ACYPI008810-RA	ni|645903843|nb|KK920587.1|	403015-403161	18	16	gi|641571760|gb|KK855988.1|	94615-96317	9.9	37
ACYPI008811-RA	ni|645903566|nb|KK920864.1|	599818-600189	nan	nan	gi|641585653|gb|KK854362.1|	23327-23598	10	39
ACYPI008815-RA	ni|645903496|nb|KK920934.1|	854265-854460	nan	nan	gi|641573583|gb|KK855523.1|	4330-6632	10	41
ACYPI008816-RA	ni|645904145|nb|KK920288.1|	1303592-1303856	16	9.3	gi|641584404|gb|KK854484.1|	503556-503788	11	30
ACYPI008826-RA	ni|645903834|nb|KK920596.1|	166448-167734	18	17	gi|641588109|gb|KK854023.1|	1238560-1240635	10	40
ACYPI008830-RA	ni|645901593|nb|KK922836.1|	1586-1846	nan	nan	gi|641585510|gb|KK854386.1|	300394-301654	11	20
ACYPI008831-RA	ni|645902620|nb|KK921809.1|	180239-181284	nan	nan	gi|641576377|gb|KK854930.1|	203610-205683	10	42
ACYPI008833-RA	ni|645903547|nb|KK920883.1|	608168-608673	nan	nan	gi|641579937|gb|KK854576.1|	546261-547493	9.4	41
ACYPI008834-RA	ni|645902463|nb|KK921966.1|	161240-161656	nan	nan	gi|641587660|gb|KK854076.1|	522839-523332	9.9	41
ACYPI008835-RA	ni|645904204|nb|KK920251.1|	589540-590404	15	16	gi|641588109|gb|KK854023.1|	49521-49756	10	40
ACYPI008845-RA	ni|645901334|nb|KK923095.1|	46647-46940	nan	nan	gi|641569907|gb|KK856522.1|	42238-42795	9.9	36
ACYPI008848-RA	ni|645904050|nb|KK920381.1|	530500-531337	18	20	gi|641576447|gb|KK854918.1|	35562-35815	12	45
ACYPI008850-RA	ni|645903738|nb|KK920692.1|	914605-914994	18	17	gi|641575549|gb|KK855090.1|	71202-71687	10	38
ACYPI008851-RA	ni|645904052|nb|KK920379.1|	202567-204032	18	16	gi|641567362|gb|KK857334.1|	68177-69957	14	24
ACYPI008852-RA	ni|645903916|nb|KK920515.1|	200513-201243	16	23	gi|641588060|gb|KK854028.1|	247278-249757	11	42
ACYPI008853-RA	ni|645904012|nb|KK920419.1|	241925-242178	27	32	gi|641577890|gb|KK854675.1|	54969-55589	10	40
ACYPI008861-RA	ni|645904231|nb|KK920242.1|	48525-49467	16	17	gi|641587671|gb|KK854074.1|	477951-478521	11	40
ACYPI008863-RA	ni|645903627|nb|KK920803.1|	486091-487591	nan	nan	gi|641586874|gb|KK854178.1|	545698-547202	9.4	38
ACYPI008866-RA	ni|645903912|nb|KK920519.1|	278636-280118	17	18	gi|641577058|gb|KK854814.1|	319239-322704	9.1	43
ACYPI008874-RA	ni|645903837|nb|KK920593.1|	552939-554801	18	9.8	gi|641572637|gb|KK855763.1|	138165-142940	9.5	36
ACYPI008876-RA	ni|645904146|nb|KK920287.1|	1227542-1227893	16	18	gi|641586620|gb|KK854215.1|	393342-401318	9.1	40
ACYPI008877-RA	ni|645903869|nb|KK920561.1|	462134-462578	17	15	gi|641559039|gb|KK860845.1|	10160-10958	8.4	33
ACYPI008884-RA	ni|645903836|nb|KK920594.1|	893375-893865	18	11	gi|641572297|gb|KK855849.1|	137845-138659	11	21
ACYPI008886-RA	ni|645903539|nb|KK920891.1|	252857-253068	nan	nan	gi|641569155|gb|KK856752.1|	72581-72807	11	21
ACYPI008887-RA	ni|645904122|nb|KK920309.1|	2057117-2057829	18	17	gi|641588343|gb|KK854003.1|	882958-883185	11	40
ACYPI008888-RA	ni|645903511|nb|KK920919.1|	481925-482124	nan	nan	gi|641588164|gb|KK854017.1|	761934-763437	9.2	40

ACYPI008895-RA	ni|645902011|nb|KK922418.1|	94606-97129	nan	nan	gi|641586285|gb|KK854263.1|	33879-38036	10	38
ACYPI008899-RA	ni|645903804|nb|KK920626.1|	54980-55207	17	17	gi|641566938|gb|KK857481.1|	29825-30287	11	39
ACYPI008902-RA	ni|645904130|nb|KK920303.1|	1421486-1423724	18	17	gi|641570453|gb|KK856351.1|	115987-116582	9.6	36
ACYPI008911-RA	ni|645903509|nb|KK920921.1|	180104-180293	nan	nan	gi|641588283|gb|KK854007.1|	794214-794590	11	40
ACYPI008922-RA	ni|645903827|nb|KK920603.1|	205549-205711	17	18	gi|641587256|gb|KK854124.1|	567313-567531	9.7	40
ACYPI008923-RA	ni|645903910|nb|KK920521.1|	634275-636995	19	17	gi|641570589|gb|KK856310.1|	88815-91944	11	36
ACYPI008927-RA	ni|645903716|nb|KK920714.1|	598067-598438	nan	nan	gi|641577152|gb|KK854799.1|	276270-278017	10	44
ACYPI008930-RA	ni|645903987|nb|KK920444.1|	795163-797652	15	17	gi|641587511|gb|KK854094.1|	191946-195123	10	41
ACYPI008931-RA	ni|645901860|nb|KK922569.1|	90936-91345	nan	nan	gi|641585910|gb|KK854320.1|	114925-116402	10	38
ACYPI008933-RA	ni|645901860|nb|KK922569.1|	108241-108487	nan	nan	gi|641577698|gb|KK854708.1|	57734-58309	9.7	40
ACYPI008937-RA	ni|645904100|nb|KK920331.1|	399596-399999	18	22	gi|641574173|gb|KK855380.1|	171169-171604	9.5	39
ACYPI008946-RA	ni|645897924|nb|KK926505.1|	2575-3210	nan	nan	gi|641580987|gb|KK854560.1|	391644-392049	11	40
ACYPI008947-RA	ni|645903496|nb|KK920934.1|	277533-277921	nan	nan	gi|641587896|gb|KK854047.1|	951637-952421	9.9	39
ACYPI008953-RA	ni|645902075|nb|KK922354.1|	14125-14338	nan	nan	gi|641586874|gb|KK854178.1|	189366-189705	9.4	38
ACYPI008963-RA	ni|645900068|nb|KK924361.1|	32900-34808	nan	nan	gi|641577599|gb|KK854726.1|	223084-225329	9.2	40
ACYPI008967-RA	ni|645904112|nb|KK920319.1|	434115-434313	18	17	gi|641576973|gb|KK854827.1|	177799-178107	11	38
ACYPI008971-RA	ni|645903812|nb|KK920618.1|	458518-458824	20	18	gi|641566502|gb|KK857638.1|	22094-22425	11	36
ACYPI008980-RA	ni|645896873|nb|KK927556.1|	2477-4051	nan	nan	gi|641580980|gb|KK854561.1|	355900-359948	11	37
ACYPI008983-RA	ni|645904225|nb|KK920244.1|	696118-696389	17	17	gi|641587680|gb|KK854073.1|	365500-365836	10	40
ACYPI008993-RA	ni|645903594|nb|KK920836.1|	590027-590352	nan	nan	gi|641577109|gb|KK854806.1|	175075-175396	9	32
ACYPI009003-RA	ni|645904240|nb|KK920239.1|	1829221-1829755	16	16	gi|641587671|gb|KK854074.1|	276228-279464	11	40
ACYPI009012-RA	ni|645902671|nb|KK921758.1|	61708-61961	nan	nan	gi|641572185|gb|KK855876.1|	98528-99624	8.2	36
ACYPI009014-RA	ni|645903734|nb|KK920696.1|	415403-415597	nan	nan	gi|641585446|gb|KK854396.1|	401981-402273	11	39
ACYPI009020-RA	ni|645903768|nb|KK920662.1|	960888-961077	18	17	gi|641580980|gb|KK854561.1|	498238-498928	11	37
ACYPI009028-RA	ni|645904104|nb|KK920327.1|	289588-290609	15	15	gi|641576333|gb|KK854937.1|	152038-152902	9.9	35
ACYPI009032-RA	ni|645904015|nb|KK920416.1|	1373377-1374462	18	16	gi|641586515|gb|KK854230.1|	704643-705606	11	39
ACYPI009034-RA	ni|645903484|nb|KK920946.1|	179069-180146	nan	nan	gi|641572293|gb|KK855850.1|	98912-100729	11	38
ACYPI009038-RA	ni|645901697|nb|KK922732.1|	113387-114303	nan	nan	gi|641586066|gb|KK854296.1|	312930-313223	9.4	37
ACYPI009043-RA	ni|645903812|nb|KK920618.1|	794574-794914	20	18	gi|641576238|gb|KK854955.1|	83530-84515	11	40
ACYPI009048-RA	ni|645904009|nb|KK920422.1|	1288433-1291240	17	9.4	gi|641577725|gb|KK854704.1|	230082-231588	11	42
ACYPI009050-RA	ni|645904281|nb|KK920220.1|	1474435-1475065	15	16	gi|641586515|gb|KK854230.1|	151462-151616	11	39
ACYPI009052-RA	ni|645903713|nb|KK920717.1|	670860-671804	nan	nan	gi|641572136|gb|KK855889.1|	128252-128460	11	41

ACYPI009055-RA	ni|645904111|nb|KK920320.1|	281504-282092	17	17	gi|641578114|gb|KK854637.1|	315089-317459	9.7	38
ACYPI009057-RA	ni|645904076|nb|KK920355.1|	1536129-1536950	17	17	gi|641567546|gb|KK857269.1|	48213-48756	8.5	40
ACYPI009061-RA	ni|645903467|nb|KK920963.1|	102154-103009	nan	nan	gi|641576962|gb|KK854829.1|	342812-343477	10	39
ACYPI009063-RA	ni|645902554|nb|KK921875.1|	21660-22323	nan	nan	gi|641575081|gb|KK855183.1|	215715-216462	9.3	37
ACYPI009065-RA	ni|645902755|nb|KK921674.1|	73065-77441	nan	nan	gi|641574504|gb|KK855305.1|	88090-93539	9.9	39
ACYPI009066-RA	ni|645904054|nb|KK920377.1|	604310-604521	16	16	gi|641574040|gb|KK855410.1|	273304-273985	9.9	44
ACYPI009068-RA	ni|645903740|nb|KK920690.1|	273330-275198	18	10	gi|641573837|gb|KK855460.1|	80488-81846	13	24
ACYPI009070-RA	ni|645904106|nb|KK920325.1|	917795-921017	17	17	gi|641571169|gb|KK856142.1|	162525-168397	9.6	42
ACYPI009071-RA	ni|645901697|nb|KK922732.1|	122779-123331	nan	nan	gi|641586066|gb|KK854296.1|	345875-348325	9.4	37
ACYPI009072-RA	ni|645903755|nb|KK920675.1|	733067-733703	18	16	gi|641585847|gb|KK854330.1|	131481-132194	11	41
ACYPI009079-RA	ni|645904210|nb|KK920249.1|	873954-874716	16	16	gi|641586496|gb|KK854233.1|	604776-605388	11	38
ACYPI009089-RA	ni|645904153|nb|KK920280.1|	723012-724345	17	16	gi|641587193|gb|KK854132.1|	429703-431013	11	39
ACYPI009090-RA	ni|645903669|nb|KK920761.1|	25222-26286	nan	nan	gi|641570697|gb|KK856279.1|	166834-167257	8.9	37
ACYPI009098-RA	ni|645902673|nb|KK921756.1|	13452-13943	nan	nan	gi|641585778|gb|KK854341.1|	165433-168433	9.7	41
ACYPI009105-RA	ni|645904038|nb|KK920393.1|	334054-334324	17	19	gi|641587021|gb|KK854156.1|	827265-828863	11	42
ACYPI009110-RA	ni|645903699|nb|KK920731.1|	192635-193342	nan	nan	gi|641587193|gb|KK854132.1|	711000-713710	11	39
ACYPI009120-RA	ni|645903626|nb|KK920804.1|	412181-412451	nan	nan	gi|641586405|gb|KK854246.1|	313929-314516	10	40
ACYPI009130-RA	ni|645903920|nb|KK920511.1|	688251-688546	20	20	gi|641573653|gb|KK855506.1|	193422-194139	8.1	33
ACYPI009132-RA	ni|645904241|nb|KK920238.1|	696919-698752	18	17	gi|641575672|gb|KK855065.1|	194158-197587	9.8	38
ACYPI009140-RA	ni|645903690|nb|KK920740.1|	762648-762880	nan	nan	gi|641577511|gb|KK854741.1|	382344-383987	11	38
ACYPI009141-RA	ni|645903743|nb|KK920687.1|	1250016-1250233	19	18	gi|641588343|gb|KK854003.1|	1052625-1053170	11	40
ACYPI009144-RA	ni|645903572|nb|KK920858.1|	185126-185922	nan	nan	gi|641585287|gb|KK854423.1|	166870-168829	11	40
ACYPI009147-RA	ni|645904271|nb|KK920224.1|	2844807-2845651	15	16	gi|641574240|gb|KK855366.1|	171428-171917	9.4	38
ACYPI009151-RA	ni|645904159|nb|KK920274.1|	1063712-1064149	15	17	gi|641572022|gb|KK855920.1|	74461-75767	12	42
ACYPI009152-RA	ni|645903800|nb|KK920630.1|	533636-535150	18	17	gi|641584619|gb|KK854468.1|	490488-491244	10	39
ACYPI009157-RA	ni|645903910|nb|KK920521.1|	575489-576148	19	17	gi|641588109|gb|KK854023.1|	134320-135433	10	40
ACYPI009158-RA	ni|645903782|nb|KK920648.1|	143193-143403	13	14	gi|641586734|gb|KK854198.1|	184729-188199	11	40
ACYPI009162-RA	ni|645903984|nb|KK920447.1|	222678-222911	18	17	gi|641586785|gb|KK854191.1|	205815-206482	11	39
ACYPI009170-RA	ni|645903665|nb|KK920765.1|	10177-11108	nan	nan	gi|641587757|gb|KK854063.1|	334210-336544	11	38
ACYPI009174-RA	ni|645903919|nb|KK920512.1|	791168-791782	17	15	gi|641584862|gb|KK854451.1|	383447-384643	10	38
ACYPI009193-RA	ni|645903938|nb|KK920493.1|	596777-597180	19	18	gi|641588033|gb|KK854031.1|	1066939-1067391	11	38
ACYPI009196-RA	ni|645904010|nb|KK920421.1|	926770-928049	17	17	gi|641573218|gb|KK855615.1|	60901-62449	9.7	39

ACYPI009202-RA	ni|645903743|nb|KK920687.1|	1045386-1045619	19	18	gi|641587452|gb|KK854101.1|	591490-592758	11	40
ACYPI009208-RA	ni|645903743|nb|KK920687.1|	176528-176884	19	18	gi|641580470|gb|KK854571.1|	98520-103854	10	41
ACYPI009223-RA	ni|645904088|nb|KK920343.1|	1246546-1247967	17	18	gi|641572484|gb|KK855802.1|	92299-93777	9.2	36
ACYPI009224-RA	ni|645898360|nb|KK926069.1|	8524-9062	nan	nan	gi|641568119|gb|KK857079.1|	60654-61504	9.8	19
ACYPI009239-RA	ni|645904210|nb|KK920249.1|	1964765-1964940	16	16	gi|641577121|gb|KK854804.1|	331285-331683	16	82
ACYPI009250-RA	ni|645903968|nb|KK920463.1|	469656-471177	16	16	gi|641566949|gb|KK857477.1|	37704-40088	11	39
ACYPI009253-RA	ni|645904061|nb|KK920370.1|	101849-102864	17	16	gi|641576333|gb|KK854937.1|	62704-63778	9.9	35
ACYPI009254-RA	ni|645903816|nb|KK920614.1|	202350-203080	14	15	gi|641574339|gb|KK855345.1|	63733-65232	10	44
ACYPI009257-RA	ni|645904207|nb|KK920250.1|	545992-547422	18	20	gi|641587937|gb|KK854042.1|	6600-6973	11	42
ACYPI009258-RA	ni|645903697|nb|KK920733.1|	167567-168183	nan	nan	gi|641573053|gb|KK855655.1|	251172-252128	11	41
ACYPI009259-RA	ni|645904128|nb|KK920305.1|	959376-960551	15	8.6	gi|641571737|gb|KK855994.1|	65417-65950	11	23
ACYPI009267-RA	ni|645902193|nb|KK922236.1|	54992-55570	nan	nan	gi|641570730|gb|KK856269.1|	169547-171286	9.1	39
ACYPI009274-RA	ni|645904053|nb|KK920378.1|	517838-518305	18	17	gi|641574131|gb|KK855389.1|	26144-26411	10	38
ACYPI009277-RA	ni|645902598|nb|KK921831.1|	18016-19000	nan	nan	gi|641565038|gb|KK858185.1|	20843-22387	9.8	35
ACYPI009286-RA	ni|645903879|nb|KK920552.1|	1696389-1697091	18	18	gi|641574320|gb|KK855349.1|	221118-221288	10	38
ACYPI009292-RA	ni|645904258|nb|KK920229.1|	689516-689792	16	16	gi|641586328|gb|KK854257.1|	492974-493200	8.4	35
ACYPI009293-RA	ni|645903944|nb|KK920487.1|	232200-232646	15	17	gi|641556975|gb|KK861815.1|	2028-2278	7.5	27
ACYPI009306-RA	ni|645903768|nb|KK920662.1|	1375833-1376773	18	17	gi|641567435|gb|KK857308.1|	65524-66777	10	38
ACYPI009308-RA	ni|645903775|nb|KK920655.1|	144433-144677	16	16	gi|641587946|gb|KK854041.1|	986756-988181	11	41
ACYPI009311-RA	ni|645904036|nb|KK920395.1|	1244478-1244710	21	18	gi|641584884|gb|KK854449.1|	79992-81072	11	39
ACYPI009312-RA	ni|645903470|nb|KK920960.1|	163104-163979	nan	nan	gi|641576666|gb|KK854881.1|	36837-39273	10	37
ACYPI009316-RA	ni|645904145|nb|KK920288.1|	674070-674818	16	9.3	gi|641586240|gb|KK854269.1|	242644-243854	11	21
ACYPI009317-RA	ni|645903525|nb|KK920905.1|	82010-82229	nan	nan	gi|641575978|gb|KK855010.1|	173994-174720	10	36
ACYPI009320-RA	ni|645902016|nb|KK922413.1|	194730-197968	nan	nan	gi|641584294|gb|KK854492.1|	37234-38389	10	41
ACYPI009324-RA	ni|645903557|nb|KK920873.1|	705219-705432	nan	nan	gi|641586817|gb|KK854187.1|	751761-753118	9.3	39
ACYPI009325-RA	ni|645904014|nb|KK920417.1|	209646-210874	19	18	gi|641575218|gb|KK855155.1|	118337-118638	11	21
ACYPI009332-RA	ni|645904020|nb|KK920411.1|	267134-267810	17	19	gi|641587757|gb|KK854063.1|	658361-666357	11	38
ACYPI009334-RA	ni|645904119|nb|KK920312.1|	1987572-1987965	19	19	gi|641587937|gb|KK854042.1|	796973-797800	11	42
ACYPI009338-RA	ni|645903472|nb|KK920958.1|	229918-230887	nan	nan	gi|641579225|gb|KK854583.1|	17978-22117	10	39
ACYPI009357-RA	ni|645903855|nb|KK920575.1|	75829-76211	16	17	gi|641575698|gb|KK855061.1|	287769-289345	10	39
ACYPI009364-RA	ni|645904251|nb|KK920231.1|	1913887-1914121	16	16	gi|641586240|gb|KK854269.1|	208088-208314	11	21
ACYPI009370-RA	ni|645903749|nb|KK920681.1|	173250-173737	19	18	gi|641584205|gb|KK854500.1|	237854-241065	11	41

ACYPI009376-RA	ni|645903857|nb|KK920573.1|	547497-547693	16	16	gi|641576064|gb|KK854990.1|	104053-105747	10	39
ACYPI009378-RA	ni|645903557|nb|KK920873.1|	916386-916644	nan	nan	gi|641571119|gb|KK856156.1|	184814-185459	11	38
ACYPI009382-RA	ni|645903999|nb|KK920432.1|	1356881-1357073	17	15	gi|641568353|gb|KK857004.1|	67351-68368	10	38
ACYPI009386-RA	ni|645903680|nb|KK920750.1|	441315-443132	nan	nan	gi|641588000|gb|KK854035.1|	871509-874643	11	39
ACYPI009394-RA	ni|645904053|nb|KK920378.1|	594785-595281	18	17	gi|641587171|gb|KK854135.1|	554110-555105	10	39
ACYPI009395-RA	ni|645903860|nb|KK920570.1|	19575-19837	17	17	gi|641580479|gb|KK854569.1|	245745-246227	10	38
ACYPI009396-RA	ni|645903470|nb|KK920960.1|	111484-112130	nan	nan	gi|641576666|gb|KK854881.1|	100601-101106	10	37
ACYPI009399-RA	ni|645904042|nb|KK920389.1|	694925-695154	16	16	gi|641587519|gb|KK854093.1|	513803-514311	11	39
ACYPI009408-RA	ni|645904240|nb|KK920239.1|	1132942-1134040	16	16	gi|641577704|gb|KK854707.1|	65610-66002	10	38
ACYPI009409-RA	ni|645904240|nb|KK920239.1|	4415-5106	16	16	gi|641572199|gb|KK855873.1|	85171-89569	8.1	36
ACYPI009413-RA	ni|645903594|nb|KK920836.1|	547041-547257	nan	nan	gi|641577109|gb|KK854806.1|	223825-228417	9	32
ACYPI009420-RA	ni|645904053|nb|KK920378.1|	185444-186415	18	17	gi|641567358|gb|KK857335.1|	55886-57599	10	36
ACYPI009424-RA	ni|645904045|nb|KK920386.1|	671713-672080	19	21	gi|641575804|gb|KK855041.1|	221451-225733	11	40
ACYPI009428-RA	ni|645903814|nb|KK920616.1|	415992-416758	17	16	gi|641566559|gb|KK857617.1|	47173-50813	10	40
ACYPI009430-RA	ni|645902129|nb|KK922300.1|	139007-140643	nan	nan	gi|641566437|gb|KK857661.1|	31830-32219	9.2	34
ACYPI009436-RA	ni|645904237|nb|KK920240.1|	901127-901479	16	15	gi|641570830|gb|KK856242.1|	23731-27101	9	31
ACYPI009437-RA	ni|645904098|nb|KK920333.1|	119008-119192	16	8.8	gi|641571645|gb|KK856014.1|	129770-130070	10	21
ACYPI009438-RA	ni|645903858|nb|KK920572.1|	374401-376611	16	16	gi|641578330|gb|KK854601.1|	70568-74566	11	38
ACYPI009439-RA	ni|645903925|nb|KK920506.1|	596336-597133	19	17	gi|641588325|gb|KK854004.1|	1161609-1166969	11	40
ACYPI009441-RA	ni|645901953|nb|KK922476.1|	31239-32457	nan	nan	gi|641571726|gb|KK855997.1|	77777-80734	10	39
ACYPI009443-RA	ni|645903952|nb|KK920479.1|	684017-685000	16	16	gi|641588309|gb|KK854005.1|	1256613-1258251	11	40
ACYPI009448-RA	ni|645904207|nb|KK920250.1|	702891-704097	18	20	gi|641573889|gb|KK855446.1|	114943-116045	10	36
ACYPI009454-RA	ni|645903484|nb|KK920946.1|	130156-130525	nan	nan	gi|641572293|gb|KK855850.1|	212689-213935	11	38
ACYPI009455-RA	ni|645904112|nb|KK920319.1|	2494607-2495327	18	17	gi|641575898|gb|KK855024.1|	175947-176529	10	38
ACYPI009457-RA	ni|645904049|nb|KK920382.1|	477429-477868	18	18	gi|641586975|gb|KK854163.1|	225721-226430	11	40
ACYPI009460-RA	ni|645903688|nb|KK920742.1|	375787-375945	nan	nan	gi|641575505|gb|KK855097.1|	174552-176519	11	38
ACYPI009467-RA	ni|645903637|nb|KK920793.1|	780139-780405	nan	nan	gi|641586599|gb|KK854218.1|	230685-230951	11	42
ACYPI009470-RA	ni|645903979|nb|KK920452.1|	494737-495943	15	17	gi|641584351|gb|KK854488.1|	372332-375000	11	41
ACYPI009478-RA	ni|645903639|nb|KK920791.1|	746128-746832	nan	nan	gi|641585363|gb|KK854409.1|	365271-366237	9.3	38
ACYPI009480-RA	ni|645904131|nb|KK920302.1|	558772-559055	15	16	gi|641571380|gb|KK856086.1|	79913-84859	9.5	38
ACYPI009489-RA	ni|645903938|nb|KK920493.1|	1187032-1187400	19	18	gi|641588033|gb|KK854031.1|	142559-142931	11	38
ACYPI009493-RA	ni|645903974|nb|KK920457.1|	497176-497302	15	8.7	gi|641582757|gb|KK854532.1|	64477-67314	9.6	30

ACYPI009500-RA	ni|645903812|nb|KK920618.1|	977459-978237	20	18	gi|641576238|gb|KK854955.1|	326585-327900	11	40
ACYPI009511-RA	ni|645903765|nb|KK920665.1|	1194221-1194721	17	15	gi|641586210|gb|KK854272.1|	616776-617200	10	39
ACYPI009523-RA	ni|645903731|nb|KK920699.1|	142800-144005	nan	nan	gi|641586144|gb|KK854283.1|	545620-546334	10	38
ACYPI009526-RA	ni|645903626|nb|KK920804.1|	794037-794306	nan	nan	gi|641576600|gb|KK854893.1|	181535-185477	9.5	41
ACYPI009528-RA	ni|645904009|nb|KK920422.1|	657532-658077	17	9.4	gi|641584977|gb|KK854443.1|	282333-284312	11	24
ACYPI009536-RA	ni|645904148|nb|KK920285.1|	1274335-1276011	16	16	gi|641567126|gb|KK857416.1|	49745-52578	11	41
ACYPI009537-RA	ni|645904052|nb|KK920379.1|	572945-573618	18	16	gi|641577138|gb|KK854801.1|	147799-148486	10	38
ACYPI009538-RA	ni|645904177|nb|KK920260.1|	1987746-1988257	18	17	gi|641576946|gb|KK854832.1|	71850-80364	9.4	43
ACYPI009542-RA	ni|645904271|nb|KK920224.1|	4049223-4049732	15	16	gi|641584845|gb|KK854452.1|	428394-429145	9	36
ACYPI009548-RA	ni|645903704|nb|KK920726.1|	434282-434826	nan	nan	gi|641574876|gb|KK855226.1|	139064-139912	11	39
ACYPI009550-RA	ni|645904133|nb|KK920300.1|	1002743-1005662	18	18	gi|641567675|gb|KK857227.1|	42582-48047	11	42
ACYPI009554-RA	ni|645903843|nb|KK920587.1|	419452-419901	18	16	gi|641571760|gb|KK855988.1|	88155-89507	9.9	37
ACYPI009565-RA	ni|645904170|nb|KK920263.1|	460380-460647	15	16	gi|641575970|gb|KK855012.1|	252522-253176	11	38
ACYPI009568-RA	ni|645904231|nb|KK920242.1|	599775-600035	16	17	gi|641577047|gb|KK854815.1|	365352-366430	10	41
ACYPI009576-RA	ni|645904008|nb|KK920423.1|	995692-995859	16	9.5	gi|641587719|gb|KK854068.1|	308661-309223	10	21
ACYPI009593-RA	ni|645904028|nb|KK920403.1|	1870443-1871762	18	17	gi|641574504|gb|KK855305.1|	111921-117205	9.9	39
ACYPI009596-RA	ni|645903747|nb|KK920683.1|	14922-16502	19	18	gi|641573858|gb|KK855455.1|	193558-195185	9.8	37
ACYPI009610-RA	ni|645904228|nb|KK920243.1|	787303-788351	17	18	gi|641576951|gb|KK854831.1|	233155-242962	8.6	40
ACYPI009612-RA	ni|645902683|nb|KK921746.1|	125609-125751	nan	nan	gi|641582447|gb|KK854535.1|	223710-223869	9.4	44
ACYPI009613-RA	ni|645904122|nb|KK920309.1|	1169824-1170769	18	17	gi|641572912|gb|KK855690.1|	65983-66823	9.9	38
ACYPI009618-RA	ni|645903971|nb|KK920460.1|	937027-938031	19	18	gi|641577905|gb|KK854672.1|	115195-117571	10	37
ACYPI009619-RA	ni|645904133|nb|KK920300.1|	2854802-2855298	18	18	gi|641571176|gb|KK856140.1|	69419-71545	9.9	38
ACYPI009620-RA	ni|645903716|nb|KK920714.1|	555445-556307	nan	nan	gi|641558787|gb|KK860963.1|	10199-10830	16	140
ACYPI009625-RA	ni|645904081|nb|KK920350.1|	255493-255710	13	18	gi|641572315|gb|KK855844.1|	175181-175744	9.4	41
ACYPI009629-RA	ni|645904174|nb|KK920261.1|	357731-359996	19	17	gi|641574512|gb|KK855303.1|	258165-260096	9.3	37
ACYPI009633-RA	ni|645904010|nb|KK920421.1|	827123-827952	17	17	gi|641573218|gb|KK855615.1|	149579-149889	9.7	39
ACYPI009634-RA	ni|645904118|nb|KK920313.1|	320004-320166	17	17	gi|641573742|gb|KK855485.1|	63956-66942	9.9	36
ACYPI009639-RA	ni|645904077|nb|KK920354.1|	158206-159024	16	17	gi|641574600|gb|KK855283.1|	217763-218798	44	35
ACYPI009640-RA	ni|645904207|nb|KK920250.1|	763564-763750	18	20	gi|641580470|gb|KK854571.1|	219187-219769	10	41
ACYPI009651-RA	ni|645904122|nb|KK920309.1|	888721-890688	18	17	gi|641568799|gb|KK856864.1|	65763-66048	9.2	35
ACYPI009659-RA	ni|645903472|nb|KK920958.1|	210696-211466	nan	nan	gi|641576203|gb|KK854962.1|	101220-101927	9.8	36
ACYPI009662-RA	ni|645903944|nb|KK920487.1|	471288-471627	15	17	gi|641577704|gb|KK854707.1|	25017-29924	10	38

ACYPI009679-RA	ni|645904053|nb|KK920378.1|	174667-174980	18	17	gi|641567358|gb|KK857335.1|	46990-47339	10	36
ACYPI009680-RA	ni|645903788|nb|KK920642.1|	14105-14388	16	15	gi|641573334|gb|KK855587.1|	154799-158293	10	39
ACYPI009686-RA	ni|645903962|nb|KK920469.1|	68256-68614	17	9.8	gi|641587823|gb|KK854055.1|	477452-480920	11	22
ACYPI009692-RA	ni|645902516|nb|KK921913.1|	91970-93550	nan	nan	gi|641577309|gb|KK854773.1|	259513-260055	9.9	39
ACYPI009704-RA	ni|645904210|nb|KK920249.1|	367814-373916	16	16	gi|641585936|gb|KK854317.1|	230660-239963	9.9	41
ACYPI009707-RA	ni|645904129|nb|KK920304.1|	1130026-1130759	16	17	gi|641576069|gb|KK854989.1|	275096-276482	11	40
ACYPI009713-RA	ni|645904019|nb|KK920412.1|	726396-726638	19	9.4	gi|641586769|gb|KK854193.1|	274895-275692	12	23
ACYPI009714-RA	ni|645902463|nb|KK921966.1|	303667-303869	nan	nan	gi|641574000|gb|KK855418.1|	224032-224533	10	36
ACYPI009717-RA	ni|645904116|nb|KK920315.1|	1587396-1587626	19	18	gi|641577623|gb|KK854722.1|	74912-75117	11	38
ACYPI009718-RA	ni|645903811|nb|KK920619.1|	38980-40053	15	15	gi|641585562|gb|KK854377.1|	397408-401257	12	47
ACYPI009719-RA	ni|645903688|nb|KK920742.1|	1053979-1054215	nan	nan	gi|641586785|gb|KK854191.1|	456393-457436	11	39
ACYPI009721-RA	ni|645904120|nb|KK920311.1|	999835-1000370	18	18	gi|641586793|gb|KK854190.1|	604512-606687	9.3	41
ACYPI009728-RA	ni|645903834|nb|KK920596.1|	189912-190306	18	17	gi|641567062|gb|KK857438.1|	30062-31435	9.8	36
ACYPI009739-RA	ni|645903931|nb|KK920500.1|	530063-530717	16	18	gi|641588309|gb|KK854005.1|	530575-532240	11	40
ACYPI009741-RA	ni|645904163|nb|KK920270.1|	337608-337857	16	16	gi|641577627|gb|KK854721.1|	361095-362720	11	42
ACYPI009744-RA	ni|645904122|nb|KK920309.1|	1506520-1507419	18	17	gi|641585755|gb|KK854345.1|	342551-345765	9.8	39
ACYPI009745-RA	ni|645903612|nb|KK920818.1|	118958-119202	nan	nan	gi|641571428|gb|KK856074.1|	139369-140140	11	39
ACYPI009755-RA	ni|645904071|nb|KK920360.1|	709807-710539	17	17	gi|641587263|gb|KK854123.1|	252936-253603	11	42
ACYPI009760-RA	ni|645904177|nb|KK920260.1|	2544427-2545026	18	17	gi|641578084|gb|KK854643.1|	199235-200641	11	40
ACYPI009769-RA	ni|645903690|nb|KK920740.1|	203871-204642	nan	nan	gi|641575730|gb|KK855054.1|	256154-257944	10	38
ACYPI009777-RA	ni|645903680|nb|KK920750.1|	158914-159126	nan	nan	gi|641586955|gb|KK854165.1|	789636-790218	11	40
ACYPI009782-RA	ni|645903681|nb|KK920749.1|	232986-233688	nan	nan	gi|641586837|gb|KK854184.1|	102675-103366	11	40
ACYPI009795-RA	ni|645904014|nb|KK920417.1|	1416692-1417378	19	18	gi|641574100|gb|KK855396.1|	167451-168438	11	38
ACYPI009806-RA	ni|645903829|nb|KK920601.1|	629199-629807	16	15	gi|641587921|gb|KK854044.1|	528720-529611	9.4	41
ACYPI009808-RA	ni|645904150|nb|KK920283.1|	310181-310941	17	17	gi|641576165|gb|KK854971.1|	35020-35287	11	41
ACYPI009821-RA	ni|645904004|nb|KK920427.1|	994362-994583	16	16	gi|641584305|gb|KK854491.1|	193867-196726	11	39
ACYPI009841-RA	ni|645903651|nb|KK920779.1|	588907-589975	nan	nan	gi|641568414|gb|KK856984.1|	15299-15516	10	39
ACYPI009846-RA	ni|645900712|nb|KK923717.1|	83222-84668	nan	nan	gi|641577152|gb|KK854799.1|	265284-268656	10	44
ACYPI009848-RA	ni|645903749|nb|KK920681.1|	277623-278061	19	18	gi|641586144|gb|KK854283.1|	114601-116017	10	38
ACYPI009849-RA	ni|645902011|nb|KK922418.1|	23793-24187	nan	nan	gi|641584884|gb|KK854449.1|	392296-393278	11	39
ACYPI009851-RA	ni|645904231|nb|KK920242.1|	388105-388739	16	17	gi|641567657|gb|KK857233.1|	31443-31659	9.9	35
ACYPI009856-RA	ni|645904169|nb|KK920264.1|	120999-121484	16	9.5	gi|641566296|gb|KK857713.1|	27500-28132	9.7	34

ACYPI009859-RA	ni|645904123|nb|KK920308.1|	1644219-1645013	16	10	gi|641587793|gb|KK854059.1|	933574-934973	11	38
ACYPI009860-RA	ni|645903936|nb|KK920495.1|	756515-757394	19	18	gi|641576248|gb|KK854953.1|	52840-55169	11	41
ACYPI009861-RA	ni|645904245|nb|KK920234.1|	311780-312543	16	17	gi|641587671|gb|KK854074.1|	696788-697175	11	40
ACYPI009867-RA	ni|645903868|nb|KK920562.1|	393543-393956	16	8.7	gi|641585480|gb|KK854391.1|	252311-252651	11	21
ACYPI009872-RA	ni|645903860|nb|KK920570.1|	181468-181760	17	17	gi|641577905|gb|KK854672.1|	294047-295950	10	37
ACYPI009884-RA	ni|645903496|nb|KK920934.1|	867760-868029	nan	nan	gi|641587696|gb|KK854071.1|	535741-539440	8.9	39
ACYPI009885-RA	ni|645903608|nb|KK920822.1|	283435-283713	nan	nan	gi|641579192|gb|KK854588.1|	153217-154256	11	41
ACYPI009886-RA	ni|645903773|nb|KK920657.1|	619950-621286	18	17	gi|641572459|gb|KK855808.1|	163973-167125	11	45
ACYPI009904-RA	ni|645904125|nb|KK920307.1|	1975375-1975562	18	17	gi|641587193|gb|KK854132.1|	659314-660277	11	39
ACYPI009906-RA	ni|645904156|nb|KK920277.1|	1567846-1568297	17	8.8	gi|641588258|gb|KK854009.1|	786815-787682	11	36
ACYPI009908-RA	ni|645903946|nb|KK920485.1|	582119-582401	16	14	gi|641573397|gb|KK855571.1|	62423-64589	10	38
ACYPI009915-RA	ni|645903866|nb|KK920564.1|	529253-529783	18	15	gi|641587688|gb|KK854072.1|	510415-511962	10	38
ACYPI009928-RA	ni|645903609|nb|KK920821.1|	283028-283227	nan	nan	gi|641571953|gb|KK855939.1|	59925-60163	10	20
ACYPI009929-RA	ni|645903596|nb|KK920834.1|	449718-450947	nan	nan	gi|641570176|gb|KK856437.1|	18307-25317	11	40
ACYPI009943-RA	ni|645903885|nb|KK920546.1|	789093-790243	19	12	gi|641586171|gb|KK854278.1|	490303-493216	9	39
ACYPI009944-RA	ni|645904260|nb|KK920228.1|	665441-665829	15	15	gi|641572239|gb|KK855863.1|	84158-86266	11	43
ACYPI009948-RA	ni|645903874|nb|KK920557.1|	861682-862258	18	18	gi|641567788|gb|KK857188.1|	3-1581	13	43
ACYPI009950-RA	ni|645903783|nb|KK920647.1|	139010-139195	17	17	gi|641575847|gb|KK855033.1|	316234-316628	10	40
ACYPI009955-RA	ni|645904078|nb|KK920353.1|	352390-353002	17	17	gi|641578300|gb|KK854606.1|	447842-448496	11	40
ACYPI009957-RA	ni|645904116|nb|KK920315.1|	2552016-2552377	19	18	gi|641576983|gb|KK854825.1|	163430-164126	9.7	37
ACYPI009967-RA	ni|645904065|nb|KK920366.1|	575237-575928	18	18	gi|641576570|gb|KK854895.1|	216499-218455	9.9	40
ACYPI009973-RA	ni|645903804|nb|KK920626.1|	148489-149298	17	17	gi|641574015|gb|KK855416.1|	96702-97211	10	42
ACYPI009981-RA	ni|645903953|nb|KK920478.1|	1411822-1412942	18	17	gi|641585773|gb|KK854342.1|	590497-591201	12	45
ACYPI009982-RA	ni|645904189|nb|KK920256.1|	526296-526475	18	18	gi|641576978|gb|KK854826.1|	7691-7885	7.6	43
ACYPI009988-RA	ni|645904000|nb|KK920431.1|	357066-359305	18	19	gi|641573796|gb|KK855470.1|	91242-96214	11	46
ACYPI009989-RA	ni|645904122|nb|KK920309.1|	167167-167773	18	17	gi|641588033|gb|KK854031.1|	453266-453475	11	38
ACYPI009993-RA	ni|645903650|nb|KK920780.1|	341364-342209	nan	nan	gi|641576452|gb|KK854917.1|	245256-246911	10	41
ACYPI009997-RA	ni|645902615|nb|KK921814.1|	92722-94146	nan	nan	gi|641587680|gb|KK854073.1|	579487-579897	10	40
ACYPI010009-RA	ni|645903923|nb|KK920508.1|	1033704-1034005	18	18	gi|641572879|gb|KK855698.1|	216603-219353	10	39
ACYPI010018-RA	ni|645903952|nb|KK920479.1|	1085468-1085697	16	16	gi|641588309|gb|KK854005.1|	1524617-1524862	11	40
ACYPI010020-RA	ni|645904231|nb|KK920242.1|	101241-102305	16	17	gi|641587671|gb|KK854074.1|	402341-403647	11	40
ACYPI010028-RA	ni|645903548|nb|KK920882.1|	745298-745714	nan	nan	gi|641587424|gb|KK854104.1|	61508-61745	11	41

ACYPI010029-RA	ni|645904122|nb|KK920309.1|	541252-543711	18	17	gi|641587104|gb|KK854144.1|	355010-358995	11	37
ACYPI010034-RA	ni|645903860|nb|KK920570.1|	217249-217482	17	17	gi|641580479|gb|KK854569.1|	139738-146127	10	38
ACYPI010039-RA	ni|645904159|nb|KK920274.1|	238667-238841	15	17	gi|641576392|gb|KK854928.1|	211881-213005	9.7	41
ACYPI010045-RA	ni|645904169|nb|KK920264.1|	502865-503115	16	9.5	gi|641587750|gb|KK854064.1|	830949-831663	11	23
ACYPI010047-RA	ni|645902673|nb|KK921756.1|	147732-149229	nan	nan	gi|641587462|gb|KK854100.1|	248721-250311	10	21
ACYPI010049-RA	ni|645903594|nb|KK920836.1|	68071-68597	nan	nan	gi|641564440|gb|KK858439.1|	24294-25273	11	36
ACYPI010054-RA	ni|645903801|nb|KK920629.1|	729453-729940	15	14	gi|641587742|gb|KK854065.1|	265400-266022	11	41
ACYPI010056-RA	ni|645904058|nb|KK920373.1|	313892-314368	16	15	gi|641575959|gb|KK855014.1|	12034-13040	11	41
ACYPI010059-RA	ni|645904219|nb|KK920246.1|	692992-694048	16	17	gi|641586555|gb|KK854224.1|	45442-46112	11	39
ACYPI010060-RA	ni|645902373|nb|KK922056.1|	137381-138440	nan	nan	gi|641586399|gb|KK854247.1|	223396-225052	15	68
ACYPI010073-RA	ni|645903496|nb|KK920934.1|	472271-473782	nan	nan	gi|641577623|gb|KK854722.1|	21088-26821	11	38
ACYPI010075-RA	ni|645903975|nb|KK920456.1|	1089045-1089210	15	8.2	gi|641587793|gb|KK854059.1|	761869-762049	11	38
ACYPI010077-RA	ni|645904088|nb|KK920343.1|	1253879-1254536	17	18	gi|641572484|gb|KK855802.1|	98614-99493	9.2	36
ACYPI010079-RA	ni|645904060|nb|KK920371.1|	179255-180872	15	16	gi|641577498|gb|KK854744.1|	10555-14276	11	42
ACYPI010091-RA	ni|645904258|nb|KK920229.1|	24403-25694	16	16	gi|641588325|gb|KK854004.1|	334317-335008	11	40
ACYPI010095-RA	ni|645903977|nb|KK920454.1|	32080-32542	13	16	gi|641586521|gb|KK854229.1|	544257-545384	11	41
ACYPI010096-RA	ni|645904105|nb|KK920326.1|	60496-60640	13	15	gi|641568618|gb|KK856922.1|	101334-102114	12	42
ACYPI010100-RA	ni|645904122|nb|KK920309.1|	509069-509586	18	17	gi|641577407|gb|KK854756.1|	206240-206645	9.4	39
ACYPI010103-RA	ni|645903778|nb|KK920652.1|	238027-238475	12	13	gi|641588033|gb|KK854031.1|	232678-233225	11	38
ACYPI010107-RA	ni|645904107|nb|KK920324.1|	773662-773977	17	9.5	gi|641576456|gb|KK854916.1|	232056-233605	10	21
ACYPI010108-RA	ni|645903464|nb|KK920966.1|	186711-187105	nan	nan	gi|641585342|gb|KK854413.1|	309042-309292	11	39
ACYPI010112-RA	ni|645904088|nb|KK920343.1|	1327475-1328948	17	18	gi|641579930|gb|KK854578.1|	81038-81493	9.1	35
ACYPI010114-RA	ni|645904130|nb|KK920303.1|	1458370-1459773	18	17	gi|641570453|gb|KK856351.1|	56900-57820	9.6	36
ACYPI010116-RA	ni|645904111|nb|KK920320.1|	1516205-1519339	17	17	gi|641585967|gb|KK854311.1|	145756-149208	11	40
ACYPI010118-RA	ni|645904262|nb|KK920227.1|	2612837-2613430	17	17	gi|641572891|gb|KK855695.1|	17863-18418	10	36
ACYPI010124-RA	ni|645903771|nb|KK920659.1|	84857-85258	16	15	gi|641584260|gb|KK854495.1|	163032-163385	12	43
ACYPI010127-RA	ni|645904174|nb|KK920261.1|	377187-377618	19	17	gi|641573464|gb|KK855553.1|	136792-138344	11	39
ACYPI010128-RA	ni|645899372|nb|KK925057.1|	487-1146	nan	nan	gi|641577657|gb|KK854715.1|	418269-419025	11	39
ACYPI010129-RA	ni|645903822|nb|KK920608.1|	357691-361135	18	20	gi|641584517|gb|KK854476.1|	401689-408130	10	39
ACYPI010131-RA	ni|645904072|nb|KK920359.1|	421302-421700	17	17	gi|641588182|gb|KK854016.1|	1244468-1245244	11	41
ACYPI010132-RA	ni|645903863|nb|KK920567.1|	8773-9010	15	9.2	gi|641584404|gb|KK854484.1|	476614-477037	11	30
ACYPI010134-RA	ni|645901860|nb|KK922569.1|	80337-80822	nan	nan	gi|641585910|gb|KK854320.1|	124632-125809	10	38

ACYPI010135-RA	ni|645903953|nb|KK920478.1|	1310910-1311870	18	17	gi|641572022|gb|KK855920.1|	48528-50430	12	42
ACYPI010136-RA	ni|645904050|nb|KK920381.1|	540544-540889	18	20	gi|641575056|gb|KK855188.1|	102917-103132	8.8	35
ACYPI010137-RA	ni|645903738|nb|KK920692.1|	312955-313469	18	17	gi|641574759|gb|KK855250.1|	81418-81768	9.7	37
ACYPI010138-RA	ni|645904177|nb|KK920260.1|	1138601-1139757	18	17	gi|641584931|gb|KK854446.1|	403246-406414	10	39
ACYPI010142-RA	ni|645903664|nb|KK920766.1|	175448-175612	nan	nan	gi|641573429|gb|KK855562.1|	125188-125818	9.3	34
ACYPI010148-RA	ni|645904174|nb|KK920261.1|	402320-403119	19	17	gi|641577184|gb|KK854793.1|	217262-222427	11	40
ACYPI010149-RA	ni|645903795|nb|KK920635.1|	47902-48204	14	16	gi|641577498|gb|KK854744.1|	366994-367309	11	42
ACYPI010151-RA	ni|645903929|nb|KK920502.1|	1319426-1320004	17	18	gi|641574173|gb|KK855380.1|	270250-270661	9.5	39
ACYPI010153-RA	ni|645903491|nb|KK920939.1|	606494-606927	nan	nan	gi|641587912|gb|KK854045.1|	718233-719185	11	39
ACYPI010154-RA	ni|645904094|nb|KK920337.1|	75686-75854	17	16	gi|641574448|gb|KK855319.1|	229138-231113	10	37
ACYPI010171-RA	ni|645902785|nb|KK921644.1|	44900-46018	nan	nan	gi|641576766|gb|KK854863.1|	320760-321218	10	38
ACYPI010174-RA	ni|645903957|nb|KK920474.1|	909871-910352	18	17	gi|641585376|gb|KK854407.1|	223863-224523	9.8	36
ACYPI010179-RA	ni|645904116|nb|KK920315.1|	2067854-2068338	19	18	gi|641585967|gb|KK854311.1|	463200-463409	11	40
ACYPI010180-RA	ni|645903854|nb|KK920576.1|	350740-350985	16	8.4	gi|641570138|gb|KK856448.1|	72479-73236	10	21
ACYPI010190-RA	ni|645904174|nb|KK920261.1|	902505-902862	19	17	gi|641573587|gb|KK855522.1|	227062-227967	9.3	38
ACYPI010192-RA	ni|645903935|nb|KK920496.1|	1067206-1067448	17	18	gi|641584320|gb|KK854490.1|	39097-39488	10	41
ACYPI010200-RA	ni|645903549|nb|KK920881.1|	35211-36045	nan	nan	gi|641572928|gb|KK855687.1|	121749-123342	11	38
ACYPI010201-RA	ni|645901835|nb|KK922594.1|	26833-27796	nan	nan	gi|641586599|gb|KK854218.1|	102587-103126	11	42
ACYPI010205-RA	ni|645903633|nb|KK920797.1|	501209-502117	nan	nan	gi|641587104|gb|KK854144.1|	140457-144470	11	37
ACYPI010209-RA	ni|645903671|nb|KK920759.1|	772245-772536	nan	nan	gi|641568704|gb|KK856893.1|	67349-67785	10	40
ACYPI010211-RA	ni|645903891|nb|KK920540.1|	41989-42456	15	17	gi|641567905|gb|KK857149.1|	45592-47557	10	38
ACYPI010216-RA	ni|645904117|nb|KK920314.1|	227743-227969	15	15	gi|641586380|gb|KK854250.1|	355490-355972	9.3	38
ACYPI010217-RA	ni|645903938|nb|KK920493.1|	1134781-1135333	19	18	gi|641572671|gb|KK855754.1|	66377-66925	10	38
ACYPI010224-RA	ni|645904112|nb|KK920319.1|	2378044-2378321	18	17	gi|641576846|gb|KK854848.1|	223648-224712	10	38
ACYPI010225-RA	ni|645903898|nb|KK920533.1|	464010-465601	17	16	gi|641572860|gb|KK855703.1|	32436-34628	9.8	36
ACYPI010226-RA	ni|645904077|nb|KK920354.1|	1403373-1403621	16	17	gi|641576022|gb|KK854999.1|	259671-263053	10	36
ACYPI010229-RA	ni|645904180|nb|KK920259.1|	658779-659757	17	17	gi|641572501|gb|KK855798.1|	164980-165901	9.8	39
ACYPI010231-RA	ni|645902785|nb|KK921644.1|	106287-107581	nan	nan	gi|641578154|gb|KK854631.1|	95855-97713	8.2	40
ACYPI010236-RA	ni|645903475|nb|KK920955.1|	296296-296743	nan	nan	gi|641576546|gb|KK854899.1|	82699-83464	11	40
ACYPI010237-RA	ni|645904028|nb|KK920403.1|	1050661-1051730	18	17	gi|641587126|gb|KK854141.1|	718352-725883	10	39
ACYPI010244-RA	ni|645904035|nb|KK920396.1|	684433-684719	16	16	gi|641586121|gb|KK854287.1|	33800-34240	11	43
ACYPI010248-RA	ni|645902360|nb|KK922069.1|	67709-68516	nan	nan	gi|641586955|gb|KK854165.1|	555150-558097	11	40

ACYPI060526-RA	ni|645903567|nb|KK920863.1|	210532-210741	nan	nan	gi|641576456|gb|KK854916.1|	298771-299598	10	21
ACYPI060544-RA	ni|645903491|nb|KK920939.1|	720202-720614	nan	nan	gi|641586278|gb|KK854264.1|	631246-631701	11	41
ACYPI060618-RA	ni|645903662|nb|KK920768.1|	259026-260190	nan	nan	gi|641586033|gb|KK854301.1|	343208-344770	11	21
ACYPI060717-RA	ni|645904088|nb|KK920343.1|	541953-545536	17	18	gi|641570630|gb|KK856298.1|	2-1431	12	47
ACYPI060796-RA	ni|645903953|nb|KK920478.1|	1152914-1153282	18	17	gi|641585961|gb|KK854312.1|	416392-416690	10	40
ACYPI060811-RA	ni|645904053|nb|KK920378.1|	595396-596242	18	17	gi|641587171|gb|KK854135.1|	551872-553322	10	39
ACYPI060844-RA	ni|645904136|nb|KK920297.1|	1199723-1199966	16	17	gi|641577965|gb|KK854661.1|	212630-219019	11	40
ACYPI061188-RA	ni|645903567|nb|KK920863.1|	143230-144218	nan	nan	gi|641573788|gb|KK855472.1|	31424-32322	10	37
ACYPI061196-RA	ni|645903874|nb|KK920557.1|	721664-722070	18	18	gi|641575077|gb|KK855184.1|	58255-63370	12	22
ACYPI061215-RA	ni|645903993|nb|KK920438.1|	124966-126232	19	22	gi|641575709|gb|KK855059.1|	341625-342954	11	41
ACYPI061275-RA	ni|645902490|nb|KK921939.1|	20693-20910	nan	nan	gi|641572655|gb|KK855758.1|	40869-43069	19	94
ACYPI061311-RA	ni|645903832|nb|KK920598.1|	225647-226085	18	8.8	gi|641570819|gb|KK856245.1|	39769-41418	10	45
ACYPI061477-RA	ni|645904119|nb|KK920312.1|	429069-429643	19	19	gi|641587179|gb|KK854134.1|	544301-545238	11	40
ACYPI061529-RA	ni|645903938|nb|KK920493.1|	720240-720787	19	18	gi|641586535|gb|KK854227.1|	42388-43166	10	39
ACYPI061546-RA	ni|645902609|nb|KK921820.1|	121195-121412	nan	nan	gi|641544009|gb|KK867995.1|	135-559	11	32
ACYPI061611-RA	ni|645903626|nb|KK920804.1|	338135-338282	nan	nan	gi|641586405|gb|KK854246.1|	366515-366987	10	40
ACYPI061797-RA	ni|645904166|nb|KK920267.1|	2784302-2785059	18	16	gi|641567869|gb|KK857161.1|	44511-46672	11	38
ACYPI062389-RA	ni|645903987|nb|KK920444.1|	328008-331687	15	17	gi|641585660|gb|KK854361.1|	239482-242825	11	43
ACYPI062442-RA	ni|645904160|nb|KK920273.1|	2363982-2364169	18	18	gi|641568780|gb|KK856870.1|	20971-21332	8.8	42
ACYPI062495-RA	ni|645903973|nb|KK920458.1|	1614504-1614703	20	18	gi|641571479|gb|KK856060.1|	24072-36768	11	40
ACYPI062519-RA	ni|645903783|nb|KK920647.1|	1227798-1228343	17	17	gi|641573447|gb|KK855557.1|	193884-194267	10	41
ACYPI062576-RA	ni|645903731|nb|KK920699.1|	125458-125917	nan	nan	gi|641586144|gb|KK854283.1|	522191-522836	10	38
ACYPI062587-RA	ni|645904116|nb|KK920315.1|	844237-844927	19	18	gi|641586928|gb|KK854169.1|	679508-680148	11	39
ACYPI062651-RA	ni|645902497|nb|KK921932.1|	150458-152179	nan	nan	gi|641572912|gb|KK855690.1|	232957-234846	9.9	38
ACYPI063189-RA	ni|645904133|nb|KK920300.1|	2162114-2162975	18	18	gi|641575568|gb|KK855086.1|	173860-179107	11	40
ACYPI063239-RA	ni|645903740|nb|KK920690.1|	292545-293973	18	10	gi|641581402|gb|KK854555.1|	296881-298175	12	20
ACYPI063276-RA	ni|645904107|nb|KK920324.1|	928890-931594	17	9.5	gi|641585452|gb|KK854395.1|	43005-49800	10	21
ACYPI063394-RA	ni|645904281|nb|KK920220.1|	1193080-1193831	15	16	gi|641577890|gb|KK854675.1|	138989-139805	10	40
ACYPI063806-RA	ni|645903832|nb|KK920598.1|	959378-959882	18	8.8	gi|641573914|gb|KK855440.1|	214307-215158	9.3	36
ACYPI064034-RA	ni|645903935|nb|KK920496.1|	963918-964772	17	18	gi|641587800|gb|KK854058.1|	538978-539906	10	42
ACYPI064056-RA	ni|645903985|nb|KK920446.1|	1030509-1033100	18	16	gi|641570423|gb|KK856359.1|	18078-21025	11	43
ACYPI064085-RA	ni|645903588|nb|KK920842.1|	594307-594683	nan	nan	gi|641585946|gb|KK854315.1|	315519-316169	9.8	43

ACYPI064172-RA	ni|645904135|nb|KK920298.1|	308063-308336	14	15	gi|641575698|gb|KK855061.1|	98481-104039	10	39
ACYPI064196-RA	ni|645904104|nb|KK920327.1|	302549-302872	15	15	gi|641576333|gb|KK854937.1|	193975-194870	9.9	35
ACYPI064212-RA	ni|645902771|nb|KK921658.1|	44404-46394	nan	nan	gi|641586452|gb|KK854239.1|	188386-189992	10	19
ACYPI064239-RA	ni|645904116|nb|KK920315.1|	1613019-1613962	19	18	gi|641588082|gb|KK854026.1|	305345-305726	11	41
ACYPI065062-RA	ni|645903969|nb|KK920462.1|	268977-269416	14	16	gi|641577121|gb|KK854804.1|	120175-120860	16	82
ACYPI065154-RA	ni|645904065|nb|KK920366.1|	63698-64092	18	18	gi|641576570|gb|KK854895.1|	269385-270012	9.9	40
ACYPI065159-RA	ni|645904065|nb|KK920366.1|	1181080-1182159	18	18	gi|641580504|gb|KK854565.1|	86244-86583	11	43
ACYPI065331-RA	ni|645904156|nb|KK920277.1|	248842-249374	17	8.8	gi|641578039|gb|KK854651.1|	469292-470401	10	38
ACYPI065684-RA	ni|645904133|nb|KK920300.1|	1551065-1551786	18	18	gi|641585233|gb|KK854427.1|	443557-445444	10	38
ACYPI065830-RA	ni|645904112|nb|KK920319.1|	2842638-2842952	18	17	gi|641577341|gb|KK854768.1|	250987-251227	14	58
ACYPI065923-RA	ni|645904166|nb|KK920267.1|	2311041-2311466	18	16	gi|641566784|gb|KK857535.1|	77927-78544	9.2	33
ACYPI065957-RA	ni|645903901|nb|KK920530.1|	237339-242577	17	17	gi|641584145|gb|KK854504.1|	118489-122374	8.9	38
ACYPI066036-RA	ni|645904071|nb|KK920360.1|	1701562-1702383	17	17	gi|641587263|gb|KK854123.1|	113345-114656	11	42
ACYPI066389-RA	ni|645903716|nb|KK920714.1|	669393-671847	nan	nan	gi|641568226|gb|KK857043.1|	52828-54662	9.5	18
ACYPI066741-RA	ni|645904171|nb|KK920262.1|	778712-778900	15	16	gi|641587742|gb|KK854065.1|	1174254-1177518	11	41
ACYPI066776-RA	ni|645904170|nb|KK920263.1|	1742696-1745680	15	16	gi|641586264|gb|KK854266.1|	106726-110665	11	46
ACYPI066810-RA	ni|645904152|nb|KK920281.1|	545415-545761	15	15	gi|641587365|gb|KK854113.1|	249483-249818	12	45
ACYPI066875-RA	ni|645903620|nb|KK920810.1|	554716-555165	nan	nan	gi|641575218|gb|KK855155.1|	68443-68759	11	21
ACYPI066904-RA	ni|645903774|nb|KK920656.1|	586900-587258	16	9.2	gi|641573093|gb|KK855645.1|	195614-195923	10	44
ACYPI066960-RA	ni|645903636|nb|KK920794.1|	196511-196815	nan	nan	gi|641576766|gb|KK854863.1|	301126-302710	10	38
ACYPI066973-RA	ni|645904140|nb|KK920293.1|	158049-159504	22	22	gi|641569146|gb|KK856755.1|	34-896	10	34
ACYPI066981-RA	ni|645904140|nb|KK920293.1|	357688-358565	22	22	gi|641573850|gb|KK855457.1|	94369-95163	9.3	34
ACYPI066985-RA	ni|645903800|nb|KK920630.1|	991301-991451	18	17	gi|641587256|gb|KK854124.1|	411199-416738	9.7	40
ACYPI066987-RA	ni|645903627|nb|KK920803.1|	501669-505207	nan	nan	gi|641587832|gb|KK854054.1|	373247-377076	9.8	20
ACYPI067359-RA	ni|645902170|nb|KK922259.1|	13204-13896	nan	nan	gi|641574575|gb|KK855289.1|	151642-157897	25	130
ACYPI067416-RA	ni|645903496|nb|KK920934.1|	915259-916095	nan	nan	gi|641587569|gb|KK854087.1|	691518-691930	11	41
ACYPI067549-RA	ni|645904100|nb|KK920331.1|	279904-280673	18	22	gi|641587047|gb|KK854151.1|	553005-554459	10	39
ACYPI067654-RA	ni|645902785|nb|KK921644.1|	25524-26177	nan	nan	gi|641576766|gb|KK854863.1|	330548-330769	10	38
ACYPI067736-RA	ni|645904114|nb|KK920317.1|	2078039-2078737	18	18	gi|641576328|gb|KK854938.1|	237775-238003	11	36
ACYPI067762-RA	ni|645903957|nb|KK920474.1|	1106693-1107177	18	17	gi|641570807|gb|KK856248.1|	119539-120193	11	38
ACYPI067763-RA	ni|645903564|nb|KK920866.1|	334772-335057	nan	nan	gi|641588082|gb|KK854026.1|	620604-621771	11	41
ACYPI067829-RA	ni|645902130|nb|KK922299.1|	29268-30056	nan	nan	gi|641572230|gb|KK855865.1|	3240-3696	9.8	38

ACYPI067901-RA	ni|645901878|nb|KK922551.1|	10788-11135	nan	nan	gi|641587145|gb|KK854138.1|	181888-185686	10	42
ACYPI068502-RA	ni|645903836|nb|KK920594.1|	139219-139673	18	11	gi|641576104|gb|KK854983.1|	208186-208529	10	20
ACYPI068599-RA	ni|645901290|nb|KK923139.1|	43029-43492	nan	nan	gi|641572934|gb|KK855685.1|	92370-92954	10	36
ACYPI068606-RA	ni|645903972|nb|KK920459.1|	532753-533732	15	16	gi|641551029|gb|KK864642.1|	4676-5025	7	27
ACYPI068631-RA	ni|645904115|nb|KK920316.1|	1813884-1814034	17	18	gi|641566429|gb|KK857664.1|	24073-24299	12	37
ACYPI068671-RA	ni|645903811|nb|KK920619.1|	356216-356819	15	15	gi|641575687|gb|KK855063.1|	235261-235921	11	34
ACYPI068681-RA	ni|645904150|nb|KK920283.1|	1041291-1041787	17	17	gi|641575490|gb|KK855100.1|	91026-91556	11	41
ACYPI068685-RA	ni|645904078|nb|KK920353.1|	931978-932360	17	17	gi|641573392|gb|KK855573.1|	91501-92709	8.5	38
ACYPI068701-RA	ni|645902788|nb|KK921641.1|	279060-279276	nan	nan	gi|641585888|gb|KK854324.1|	522364-523256	10	41
ACYPI068713-RA	ni|645903576|nb|KK920854.1|	61640-66745	nan	nan	gi|641577071|gb|KK854812.1|	139182-143972	13	38
ACYPI069332-RA	ni|645903833|nb|KK920597.1|	122824-123405	17	15	gi|641574689|gb|KK855262.1|	17635-18626	10	38
ACYPI069386-RA	ni|645903879|nb|KK920552.1|	1086942-1089661	18	18	gi|641586509|gb|KK854231.1|	499400-504137	9.8	37
ACYPI069453-RA	ni|645904125|nb|KK920307.1|	2676174-2677198	18	17	gi|641577198|gb|KK854790.1|	364612-365246	10	39
ACYPI069533-RA	ni|645904174|nb|KK920261.1|	1579111-1579312	19	17	gi|641576788|gb|KK854859.1|	75687-75846	11	34
ACYPI069547-RA	ni|645903622|nb|KK920808.1|	604899-605295	nan	nan	gi|641576533|gb|KK854902.1|	140271-140535	10	39
ACYPI069554-RA	ni|645904019|nb|KK920412.1|	623072-623241	19	9.4	gi|641574402|gb|KK855330.1|	85533-85822	11	23
ACYPI069585-RA	ni|645903873|nb|KK920558.1|	607939-608699	16	15	gi|641580486|gb|KK854568.1|	242338-242972	11	36
ACYPI069860-RA	ni|645903920|nb|KK920511.1|	1112520-1112619	20	20	gi|641575358|gb|KK855127.1|	88426-89155	9	41
ACYPI070244-RA	ni|645901817|nb|KK922612.1|	84775-85007	nan	nan	gi|641578173|gb|KK854628.1|	50746-52487	9.9	21
ACYPI070297-RA	ni|645904219|nb|KK920246.1|	813898-814077	16	17	gi|641585293|gb|KK854422.1|	162658-163624	9.1	38
ACYPI070323-RA	ni|645903952|nb|KK920479.1|	45914-46164	16	16	gi|641542070|gb|KK868923.1|	142-506	6.9	27
ACYPI070374-RA	ni|645904242|nb|KK920237.1|	639850-640379	15	16	gi|641587606|gb|KK854082.1|	414098-414574	11	40
ACYPI070386-RA	ni|645904136|nb|KK920297.1|	1681987-1682228	16	17	gi|641587896|gb|KK854047.1|	216383-217556	9.9	39
ACYPI070462-RA	ni|645904139|nb|KK920294.1|	501687-502465	15	18	gi|641576347|gb|KK854934.1|	236092-237662	11	39
ACYPI071133-RA	ni|645903578|nb|KK920852.1|	273377-274389	nan	nan	gi|641568815|gb|KK856858.1|	137035-138945	10	34
ACYPI071157-RA	ni|645904140|nb|KK920293.1|	78333-79005	22	22	gi|641568126|gb|KK857076.1|	2004-7180	11	42
ACYPI071169-RA	ni|645903774|nb|KK920656.1|	479324-480395	16	9.2	gi|641586452|gb|KK854239.1|	392479-392887	10	19
ACYPI071217-RA	ni|645904012|nb|KK920419.1|	305201-305741	27	32	gi|641577890|gb|KK854675.1|	86502-88400	10	40
ACYPI071228-RA	ni|645903971|nb|KK920460.1|	1059703-1059992	19	18	gi|641585761|gb|KK854344.1|	122244-126947	10	39
ACYPI071231-RA	ni|645903690|nb|KK920740.1|	1030654-1031247	nan	nan	gi|641585569|gb|KK854376.1|	68057-69219	11	47
ACYPI071272-RA	ni|645904145|nb|KK920288.1|	1224093-1224638	16	9.3	gi|641561323|gb|KK859794.1|	2739-3318	8.5	15
ACYPI071352-RA	ni|645904152|nb|KK920281.1|	862607-863077	15	15	gi|641587365|gb|KK854113.1|	119229-120037	12	45

ACYPI071357-RA	ni|645903727|nb|KK920703.1|	952093-954572	nan	nan	gi|641587881|gb|KK854049.1|	815859-819795	10	40
ACYPI071951-RA	ni|645903743|nb|KK920687.1|	545031-545451	19	18	gi|641577851|gb|KK854682.1|	63564-63959	11	34
ACYPI071956-RA	ni|645903873|nb|KK920558.1|	438336-438560	16	15	gi|641580486|gb|KK854568.1|	49388-52399	11	36
ACYPI071995-RA	ni|645901786|nb|KK922643.1|	74287-74502	nan	nan	gi|641586654|gb|KK854210.1|	254937-255258	11	40
ACYPI072101-RA	ni|645903688|nb|KK920742.1|	1099562-1100097	nan	nan	gi|641586785|gb|KK854191.1|	377331-377587	11	39
ACYPI072156-RA	ni|645903472|nb|KK920958.1|	367392-367613	nan	nan	gi|641587580|gb|KK854085.1|	699769-700637	10	39
ACYPI072184-RA	ni|645903679|nb|KK920751.1|	63150-63420	nan	nan	gi|641571052|gb|KK856173.1|	14896-15551	8.9	41
ACYPI072205-RA	ni|645904061|nb|KK920370.1|	789963-792181	17	16	gi|641573813|gb|KK855466.1|	123643-127729	9.6	37
ACYPI072215-RA	ni|645903905|nb|KK920526.1|	76972-78488	15	8.3	gi|641573950|gb|KK855430.1|	224906-229100	10	19
ACYPI072241-RA	ni|645902354|nb|KK922075.1|	112303-112676	nan	nan	gi|641588325|gb|KK854004.1|	1389588-1390459	11	40
ACYPI072244-RA	ni|645903895|nb|KK920536.1|	451323-452561	17	16	gi|641588343|gb|KK854003.1|	143875-144296	11	40
ACYPI072792-RA	ni|645903679|nb|KK920751.1|	63523-63749	nan	nan	gi|641571052|gb|KK856173.1|	14404-14766	8.9	41
ACYPI072830-RA	ni|645903773|nb|KK920657.1|	1153809-1154029	18	17	gi|641588021|gb|KK854033.1|	1209832-1210745	12	40
ACYPI072856-RA	ni|645903780|nb|KK920650.1|	269020-269766	19	19	gi|641560205|gb|KK860298.1|	2586-3050	6.4	28
ACYPI072921-RA	ni|645903748|nb|KK920682.1|	801623-802390	17	18	gi|641588041|gb|KK854030.1|	98871-99350	11	38
ACYPI072994-RA	ni|645903860|nb|KK920570.1|	348064-348497	17	17	gi|641567672|gb|KK857228.1|	25216-26937	10	36
ACYPI073116-RA	ni|645904174|nb|KK920261.1|	913928-914530	19	17	gi|641571048|gb|KK856174.1|	53719-53950	11	39
ACYPI073321-RA	ni|645902443|nb|KK921986.1|	12019-12412	nan	nan	gi|641583587|gb|KK854518.1|	58578-60318	10	37
ACYPI073612-RA	ni|645903840|nb|KK920590.1|	224391-225284	18	17	gi|641574127|gb|KK855390.1|	14958-16314	10	40
ACYPI073693-RA	ni|645903728|nb|KK920702.1|	1089584-1090311	nan	nan	gi|641571642|gb|KK856015.1|	166257-166715	11	37
ACYPI073700-RA	ni|645902673|nb|KK921756.1|	49810-50611	nan	nan	gi|641581774|gb|KK854551.1|	161918-162762	10	19
ACYPI073714-RA	ni|645904201|nb|KK920252.1|	354533-355894	17	16	gi|641586085|gb|KK854293.1|	7079-8599	11	40
ACYPI073759-RA	ni|645903866|nb|KK920564.1|	515014-515439	18	15	gi|641587688|gb|KK854072.1|	486555-487281	10	38
ACYPI073829-RA	ni|645904274|nb|KK920223.1|	622623-622814	14	17	gi|641586726|gb|KK854199.1|	453544-453853	11	41
ACYPI073870-RA	ni|645904059|nb|KK920372.1|	458491-461892	16	9	gi|641545206|gb|KK867413.1|	768-4217	17	31
ACYPI073873-RA	ni|645904243|nb|KK920236.1|	1733080-1733496	15	8.5	gi|641577453|gb|KK854750.1|	294482-295424	10	21
ACYPI073889-RA	ni|645899152|nb|KK925277.1|	81668-82199	nan	nan	gi|641586837|gb|KK854184.1|	244155-244380	11	40
ACYPI080074-RA	ni|645904119|nb|KK920312.1|	604534-605729	19	19	gi|641577121|gb|KK854804.1|	285142-289902	16	82
ACYPI080138-RA	ni|645904019|nb|KK920412.1|	144059-144390	19	9.4	gi|641585510|gb|KK854386.1|	118212-121205	11	20
ACYPI080181-RA	ni|645904095|nb|KK920336.1|	417054-418206	16	19	gi|641586246|gb|KK854268.1|	580653-582136	10	38
ACYPI080213-RA	ni|645904057|nb|KK920374.1|	1341602-1341761	16	15	gi|641574809|gb|KK855240.1|	155778-156485	11	39
ACYPI080240-RA	ni|645904177|nb|KK920260.1|	878816-879784	18	17	gi|641572955|gb|KK855679.1|	219275-219815	10	40

ACYPI080332-RA	ni|645903973|nb|KK920458.1|	1335332-1335565	20	18	gi|641587047|gb|KK854151.1|	314586-316489	10	39
ACYPI080343-RA	ni|645903900|nb|KK920531.1|	241882-242489	20	30	gi|641574575|gb|KK855289.1|	360098-361542	25	130
ACYPI080648-RA	ni|645904008|nb|KK920423.1|	490787-491306	16	9.5	gi|641588283|gb|KK854007.1|	1199257-1202154	11	40
ACYPI080661-RA	ni|645903697|nb|KK920733.1|	56927-57419	nan	nan	gi|641585690|gb|KK854356.1|	454002-459218	9.6	41
ACYPI080807-RA	ni|645904262|nb|KK920227.1|	503562-504087	17	17	gi|641575970|gb|KK855012.1|	23208-23490	11	38
ACYPI080920-RA	ni|645903847|nb|KK920583.1|	58591-59777	18	18	gi|641585833|gb|KK854332.1|	8753-9750	10	37
ACYPI081137-RA	ni|645904156|nb|KK920277.1|	411435-411682	17	8.8	gi|641587742|gb|KK854065.1|	263354-264353	11	41
ACYPI081140-RA	ni|645903603|nb|KK920827.1|	66248-66959	nan	nan	gi|641576010|gb|KK855002.1|	373608-374919	12	43
ACYPI081400-RA	ni|645903665|nb|KK920765.1|	456905-457093	nan	nan	gi|641588283|gb|KK854007.1|	1046085-1046469	11	40
ACYPI081417-RA	ni|645903678|nb|KK920752.1|	369376-369608	nan	nan	gi|641572782|gb|KK855725.1|	183587-183833	10	36
ACYPI081647-RA	ni|645904107|nb|KK920324.1|	783307-783659	17	9.5	gi|641576456|gb|KK854916.1|	231127-231721	10	21
ACYPI081736-RA	ni|645904274|nb|KK920223.1|	576725-577213	14	17	gi|641566967|gb|KK857471.1|	77507-77766	9	39
ACYPI081754-RA	ni|645903588|nb|KK920842.1|	521131-521393	nan	nan	gi|641585946|gb|KK854315.1|	315148-315384	9.8	43
ACYPI081909-RA	ni|645904116|nb|KK920315.1|	2115574-2116251	19	18	gi|641585967|gb|KK854311.1|	398923-399263	11	40
ACYPI082053-RA	ni|645903854|nb|KK920576.1|	99453-99681	16	8.4	gi|641572824|gb|KK855713.1|	84953-85156	9.8	19
ACYPI082110-RA	ni|645903762|nb|KK920668.1|	865990-866960	17	17	gi|641577104|gb|KK854807.1|	8293-13506	10	36
ACYPI082338-RA	ni|645904058|nb|KK920373.1|	1109523-1110556	16	15	gi|641586301|gb|KK854260.1|	483876-485161	9.8	39
ACYPI082499-RA	ni|645904170|nb|KK920263.1|	321012-321219	15	16	gi|641568014|gb|KK857113.1|	18811-19483	21	81
ACYPI082595-RA	ni|645904274|nb|KK920223.1|	754622-754880	14	17	gi|641572405|gb|KK855822.1|	126394-126624	8.7	44
ACYPI082601-RA	ni|645903548|nb|KK920882.1|	206915-207236	nan	nan	gi|641572963|gb|KK855677.1|	188326-189736	11	43
ACYPI082655-RA	ni|645903597|nb|KK920833.1|	788549-788978	nan	nan	gi|641587652|gb|KK854077.1|	653439-653953	10	38
ACYPI082856-RA	ni|645903752|nb|KK920678.1|	647046-647661	18	16	gi|641569788|gb|KK856560.1|	28740-29917	9.5	37
ACYPI082950-RA	ni|645903983|nb|KK920448.1|	705762-707673	18	17	gi|641570757|gb|KK856260.1|	196439-199568	10	42
ACYPI083009-RA	ni|645903946|nb|KK920485.1|	568168-568417	16	14	gi|641573397|gb|KK855571.1|	35487-36547	10	38
ACYPI083041-RA	ni|645903533|nb|KK920897.1|	188118-188373	nan	nan	gi|641576172|gb|KK854969.1|	7185-8131	9.9	20
ACYPI083213-RA	ni|645903736|nb|KK920694.1|	305120-305513	18	17	gi|641588128|gb|KK854021.1|	1099394-1099900	9.5	42
ACYPI083436-RA	ni|645904262|nb|KK920227.1|	336091-336742	17	17	gi|641576731|gb|KK854870.1|	271103-271790	10	41
ACYPI083523-RA	ni|645904262|nb|KK920227.1|	285265-285757	17	17	gi|641575603|gb|KK855080.1|	43705-46594	9.4	38
ACYPI083537-RA	ni|645900840|nb|KK923589.1|	1025-1494	nan	nan	gi|641585871|gb|KK854327.1|	563720-564332	11	38
ACYPI083652-RA	ni|645904075|nb|KK920356.1|	715621-715925	16	17	gi|641571632|gb|KK856018.1|	125138-125640	10	36
ACYPI083672-RA	ni|645904133|nb|KK920300.1|	2795566-2795810	18	18	gi|641587390|gb|KK854109.1|	467880-468138	11	44
ACYPI083717-RA	ni|645903971|nb|KK920460.1|	2093327-2093575	19	18	gi|641571016|gb|KK856184.1|	144971-149536	10	40

ACYPI083883-RA	ni|645903902|nb|KK920529.1|	1139340-1139682	19	17	gi|641554468|gb|KK863006.1|	5329-7937	11	36
ACYPI084147-RA	ni|645904033|nb|KK920398.1|	119946-120331	14	16	gi|641586975|gb|KK854163.1|	492895-493557	11	40
ACYPI084179-RA	ni|645902170|nb|KK922259.1|	17149-17644	nan	nan	gi|641586399|gb|KK854247.1|	454082-455243	15	68
ACYPI084287-RA	ni|645903959|nb|KK920472.1|	893249-894023	15	15	gi|641585876|gb|KK854326.1|	409020-409565	9.5	39
ACYPI084470-RA	ni|645904281|nb|KK920220.1|	1137111-1137383	15	16	gi|641577890|gb|KK854675.1|	159724-163577	10	40
ACYPI084494-RA	ni|645904058|nb|KK920373.1|	993841-994584	16	15	gi|641588236|gb|KK854011.1|	639884-640763	9.7	38
ACYPI084620-RA	ni|645903580|nb|KK920850.1|	180239-180662	nan	nan	gi|641576203|gb|KK854962.1|	146165-147967	9.8	36
ACYPI084662-RA	ni|645904135|nb|KK920298.1|	607439-607929	14	15	gi|641570182|gb|KK856435.1|	73094-73602	10	36
ACYPI084675-RA	ni|645904009|nb|KK920422.1|	1341191-1342931	17	9.4	gi|641569833|gb|KK856547.1|	61790-62714	8.9	37
ACYPI084854-RA	ni|645903876|nb|KK920555.1|	294777-295109	14	15	gi|641588325|gb|KK854004.1|	1147664-1150408	11	40
ACYPI084955-RA	ni|645904157|nb|KK920276.1|	1433404-1434161	15	15	gi|641587226|gb|KK854128.1|	574708-575972	9.9	39
ACYPI085022-RA	ni|645903937|nb|KK920494.1|	288673-290802	11	12	gi|641574668|gb|KK855267.1|	22993-24442	10	38
ACYPI085126-RA	ni|645903957|nb|KK920474.1|	1445637-1445788	18	17	gi|641573533|gb|KK855535.1|	39773-40424	10	41
ACYPI085203-RA	ni|645904043|nb|KK920388.1|	547753-548066	16	9.1	gi|641587832|gb|KK854054.1|	664221-664721	9.8	20
ACYPI085301-RA	ni|645904268|nb|KK920225.1|	317448-317732	14	15	gi|641570045|gb|KK856477.1|	92062-94767	9.6	43
ACYPI085401-RA	ni|645901587|nb|KK922842.1|	124-323	nan	nan	gi|641588024|gb|KK854032.1|	343553-343787	9.7	41
ACYPI085603-RA	ni|645903954|nb|KK920477.1|	249860-250558	35	44	gi|641576962|gb|KK854829.1|	263789-266810	10	39
ACYPI085715-RA	ni|645904057|nb|KK920374.1|	1352763-1353041	16	15	gi|641574809|gb|KK855240.1|	177906-180855	11	39
ACYPI085777-RA	ni|645904071|nb|KK920360.1|	687116-688095	17	17	gi|641587263|gb|KK854123.1|	191533-193847	11	42
ACYPI085938-RA	ni|645903557|nb|KK920873.1|	717027-718142	nan	nan	gi|641586817|gb|KK854187.1|	629608-633135	9.3	39
ACYPI086093-RA	ni|645903495|nb|KK920935.1|	455897-456189	nan	nan	gi|641576409|gb|KK854925.1|	99407-101304	11	44
ACYPI086258-RA	ni|645903848|nb|KK920582.1|	95901-96402	18	18	gi|641586338|gb|KK854255.1|	565588-566320	14	44
ACYPI086281-RA	ni|645904116|nb|KK920315.1|	2079937-2080922	19	18	gi|641585967|gb|KK854311.1|	434127-435132	11	40
ACYPI086445-RA	ni|645903706|nb|KK920724.1|	174693-175446	nan	nan	gi|641575150|gb|KK855169.1|	57432-64936	11	39
ACYPI087052-RA	ni|645903739|nb|KK920691.1|	798386-798617	17	16	gi|641586990|gb|KK854161.1|	79768-80003	11	39
ACYPI087167-RA	ni|645903773|nb|KK920657.1|	1186315-1187056	18	17	gi|641575794|gb|KK855043.1|	159827-160708	10	36
ACYPI087467-RA	ni|645904002|nb|KK920429.1|	382792-383230	14	18	gi|641577574|gb|KK854731.1|	161111-161593	11	38
ACYPI087487-RA	ni|645904248|nb|KK920232.1|	701508-701763	15	16	gi|641587774|gb|KK854061.1|	892873-893224	9.1	40
ACYPI087566-RA	ni|645903953|nb|KK920478.1|	1375604-1376188	18	17	gi|641578084|gb|KK854643.1|	428277-429245	11	40
ACYPI087592-RA	ni|645903795|nb|KK920635.1|	93841-94075	14	16	gi|641576161|gb|KK854972.1|	126043-126431	10	36
ACYPI087735-RA	ni|645903695|nb|KK920735.1|	179600-180348	nan	nan	gi|641577322|gb|KK854771.1|	196261-196896	10	21
ACYPI087793-RA	ni|645904138|nb|KK920295.1|	500083-500440	15	15	gi|641565175|gb|KK858134.1|	2-1280	12	46

ACYPI087836-RA	ni|645903733|nb|KK920697.1|	69361-69472	nan	nan	gi|641587444|gb|KK854102.1|	560642-561329	11	42
ACYPI087848-RA	ni|645904102|nb|KK920329.1|	423218-423763	15	16	gi|641546507|gb|KK866793.1|	6051-7153	10	40
ACYPI087918-RA	ni|645904088|nb|KK920343.1|	1261899-1263169	17	18	gi|641572484|gb|KK855802.1|	128121-130339	9.2	36
ACYPI088026-RA	ni|645903588|nb|KK920842.1|	622246-623104	nan	nan	gi|641573045|gb|KK855657.1|	3813-4551	14	51
ACYPI088146-RA	ni|645903814|nb|KK920616.1|	453469-453922	17	16	gi|641587219|gb|KK854129.1|	10808-11868	9.9	41
ACYPI088207-RA	ni|645903736|nb|KK920694.1|	401908-402366	18	17	gi|641576229|gb|KK854956.1|	167867-169582	9.8	36
ACYPI088273-RA	ni|645903941|nb|KK920490.1|	609045-610870	18	18	gi|641585233|gb|KK854427.1|	480975-483095	10	38
ACYPI088277-RA	ni|645902312|nb|KK922117.1|	11287-12741	nan	nan	gi|641570238|gb|KK856417.1|	34256-35997	9.3	38
ACYPI088345-RA	ni|645904023|nb|KK920408.1|	666362-666891	17	17	gi|641575693|gb|KK855062.1|	143792-145681	11	40
ACYPI088486-RA	ni|645903836|nb|KK920594.1|	814112-814348	18	11	gi|641576469|gb|KK854914.1|	33253-33507	11	21
ACYPI088512-RA	ni|645902127|nb|KK922302.1|	43396-47089	nan	nan	gi|641582439|gb|KK854536.1|	51318-52172	11	36
ACYPI088544-RA	ni|645904045|nb|KK920386.1|	561180-562312	19	21	gi|641584281|gb|KK854493.1|	102993-103701	12	41
ACYPI088724-RA	ni|645903971|nb|KK920460.1|	762283-763116	19	18	gi|641578428|gb|KK854590.1|	313581-314970	11	40
ACYPI088840-RA	ni|645903965|nb|KK920466.1|	1492083-1493317	17	16	gi|641585581|gb|KK854374.1|	606512-608942	10	38
ACYPI088980-RA	ni|645904132|nb|KK920301.1|	614949-616150	16	18	gi|641575826|gb|KK855037.1|	119241-120648	10	39
ACYPI089177-RA	ni|645903837|nb|KK920593.1|	777960-778412	18	9.8	gi|641568328|gb|KK857011.1|	41091-41852	9.7	32
ACYPI089246-RA	ni|645903953|nb|KK920478.1|	1430144-1430687	18	17	gi|641585773|gb|KK854342.1|	612360-612696	12	45
ACYPI089403-RA	ni|645903861|nb|KK920569.1|	346960-347337	16	17	gi|641573861|gb|KK855454.1|	49814-50669	10	37
ACYPI089540-RA	ni|645902426|nb|KK922003.1|	197837-198248	nan	nan	gi|641586928|gb|KK854169.1|	174835-175511	11	39
ACYPI20302-RA	ni|645904243|nb|KK920236.1|	1094976-1095165	15	8.5	gi|641572011|gb|KK855923.1|	67296-67972	10	21
ACYPI20476-RA	ni|645904168|nb|KK920265.1|	316824-317136	16	16	gi|641570399|gb|KK856367.1|	73110-74909	11	41
ACYPI20534-RA	ni|645902196|nb|KK922233.1|	154324-155563	nan	nan	gi|641566818|gb|KK857523.1|	38059-39304	10	37
ACYPI21475-RA	ni|645903874|nb|KK920557.1|	919431-919924	18	18	gi|641576199|gb|KK854963.1|	268334-268922	12	43
ACYPI21591-RA	ni|645903662|nb|KK920768.1|	384806-385049	nan	nan	gi|641586521|gb|KK854229.1|	656304-657364	11	41
ACYPI21611-RA	ni|645903742|nb|KK920688.1|	295328-296574	17	16	gi|641587896|gb|KK854047.1|	441515-444196	9.9	39
ACYPI21777-RA	ni|645903925|nb|KK920506.1|	1153027-1153937	19	17	gi|641587179|gb|KK854134.1|	164008-171260	11	40
ACYPI22226-RA	ni|645902614|nb|KK921815.1|	50461-51444	nan	nan	gi|641585475|gb|KK854392.1|	199304-202704	10	41
ACYPI22227-RA	ni|645904065|nb|KK920366.1|	743716-743908	18	18	gi|641588119|gb|KK854022.1|	988678-989196	10	40
ACYPI22519-RA	ni|645903566|nb|KK920864.1|	449216-449956	nan	nan	gi|641569291|gb|KK856713.1|	114773-115684	10	21
ACYPI22575-RA	ni|645904064|nb|KK920367.1|	634999-635783	17	9.9	gi|641568580|gb|KK856933.1|	52410-58127	12	25
ACYPI22584-RA	ni|645904195|nb|KK920254.1|	2298196-2298496	18	17	gi|641584658|gb|KK854465.1|	312800-313913	11	37
ACYPI22867-RA	ni|645903734|nb|KK920696.1|	708677-709201	nan	nan	gi|641586344|gb|KK854254.1|	451395-451843	10	40

ACYPI23235-RA	ni|645903597|nb|KK920833.1|	519791-522166	nan	nan	gi|641587652|gb|KK854077.1|	816970-819953	10	38
ACYPI23338-RA	ni|645903860|nb|KK920570.1|	155282-157970	17	17	gi|641580479|gb|KK854569.1|	303473-306094	10	38
ACYPI23394-RA	ni|645903887|nb|KK920544.1|	84001-85004	15	15	gi|641578109|gb|KK854638.1|	117534-120444	14	46
ACYPI23453-RA	ni|645904012|nb|KK920419.1|	434518-435598	27	32	gi|641588073|gb|KK854027.1|	1111560-1112651	11	41
ACYPI23999-RA	ni|645904189|nb|KK920256.1|	267210-267474	18	18	gi|641561138|gb|KK859879.1|	12437-15522	12	42
ACYPI24155-RA	ni|645903960|nb|KK920471.1|	910992-911316	16	8.6	gi|641576312|gb|KK854941.1|	237497-241550	9.9	38
ACYPI24234-RA	ni|645903688|nb|KK920742.1|	193095-193674	nan	nan	gi|641578179|gb|KK854627.1|	92700-94577	10	39
ACYPI24841-RA	ni|645902722|nb|KK921707.1|	53698-54878	nan	nan	gi|641585971|gb|KK854310.1|	385080-388704	10	40
ACYPI25279-RA	ni|645903739|nb|KK920691.1|	516146-516490	17	16	gi|641587696|gb|KK854071.1|	709096-709877	8.9	39
ACYPI25475-RA	ni|645904094|nb|KK920337.1|	799952-800421	17	16	gi|641572895|gb|KK855694.1|	33429-33865	8.7	39
ACYPI25494-RA	ni|645903787|nb|KK920643.1|	27896-28918	19	18	gi|641587606|gb|KK854082.1|	757838-760778	11	40
ACYPI25540-RA	ni|645901733|nb|KK922696.1|	246594-247027	nan	nan	gi|641587921|gb|KK854044.1|	123855-124138	9.4	41
ACYPI25873-RA	ni|645903923|nb|KK920508.1|	1172449-1173015	18	18	gi|641588099|gb|KK854024.1|	551068-552326	11	39
ACYPI26209-RA	ni|645904115|nb|KK920316.1|	1557721-1558398	17	18	gi|641587921|gb|KK854044.1|	170211-173293	9.4	41
ACYPI27126-RA	ni|645904177|nb|KK920260.1|	2538035-2538169	18	17	gi|641578084|gb|KK854643.1|	134650-135260	11	40
ACYPI27183-RA	ni|645903821|nb|KK920609.1|	1125950-1126406	17	17	gi|641588090|gb|KK854025.1|	987993-989108	11	43
ACYPI27242-RA	ni|645904153|nb|KK920280.1|	660052-660584	17	16	gi|641587193|gb|KK854132.1|	304603-305270	11	39
ACYPI27507-RA	ni|645904116|nb|KK920315.1|	1847470-1849096	19	18	gi|641575730|gb|KK855054.1|	71259-75663	10	38
ACYPI27999-RA	ni|645903936|nb|KK920495.1|	674426-674742	19	18	gi|641571380|gb|KK856086.1|	27646-27993	9.5	38
ACYPI28767-RA	ni|645904168|nb|KK920265.1|	2095100-2095359	16	16	gi|641572326|gb|KK855841.1|	72069-72665	12	41
ACYPI28781-RA	ni|645903870|nb|KK920560.1|	170147-170483	14	23	gi|641577522|gb|KK854739.1|	52634-56161	11	41
ACYPI29004-RA	ni|645904098|nb|KK920333.1|	354832-355439	16	8.8	gi|641571123|gb|KK856155.1|	85483-86049	10	42
ACYPI29303-RA	ni|645904112|nb|KK920319.1|	2181838-2182623	18	17	gi|641569801|gb|KK856556.1|	81538-84512	9.6	36
ACYPI29477-RA	ni|645904137|nb|KK920296.1|	2391514-2391814	17	17	gi|641578160|gb|KK854630.1|	191766-193492	11	43
ACYPI29680-RA	ni|645903972|nb|KK920459.1|	568325-569449	15	16	gi|641577390|gb|KK854759.1|	140469-142452	9.8	37
ACYPI29847-RA	ni|645903739|nb|KK920691.1|	819840-820525	17	16	gi|641586990|gb|KK854161.1|	75938-76218	11	39
ACYPI29851-RA	ni|645903985|nb|KK920446.1|	148456-148691	18	16	gi|641573368|gb|KK855579.1|	48743-48991	10	20
ACYPI31143-RA	ni|645903960|nb|KK920471.1|	404171-404634	16	8.6	gi|641571960|gb|KK855937.1|	66724-67167	9.4	17
ACYPI31575-RA	ni|645903626|nb|KK920804.1|	93016-93141	nan	nan	gi|641570811|gb|KK856247.1|	12573-13140	9.1	35
ACYPI31659-RA	ni|645904177|nb|KK920260.1|	901342-901899	18	17	gi|641581779|gb|KK854550.1|	422245-422562	11	39
ACYPI31758-RA	ni|645904262|nb|KK920227.1|	1392042-1392269	17	17	gi|641586754|gb|KK854195.1|	638814-639853	11	41
ACYPI34041-RA	ni|645903500|nb|KK920930.1|	203018-203278	nan	nan	gi|641570261|gb|KK856409.1|	65009-65282	11	21

ACYPI34873-RA	ni|645903768|nb|KK920662.1|	416036-416593	18	17	gi|641587703|gb|KK854070.1|	663771-664739	9.8	42
ACYPI34996-RA	ni|645903747|nb|KK920683.1|	104035-104488	19	18	gi|641578325|gb|KK854602.1|	301301-303049	9.7	38
ACYPI35323-RA	ni|645903831|nb|KK920599.1|	724480-725693	18	17	gi|641573617|gb|KK855515.1|	25572-29338	10	38
ACYPI36505-RA	ni|645903765|nb|KK920665.1|	534599-537011	17	15	gi|641572663|gb|KK855756.1|	25679-28013	8.8	38
ACYPI36710-RA	ni|645904122|nb|KK920309.1|	867575-868100	18	17	gi|641577482|gb|KK854747.1|	27128-27586	10	39
ACYPI36842-RA	ni|645903574|nb|KK920856.1|	154915-155796	nan	nan	gi|641576628|gb|KK854888.1|	209320-211582	11	22
ACYPI37546-RA	ni|645903782|nb|KK920648.1|	154534-155176	13	14	gi|641570660|gb|KK856290.1|	121422-131351	16	69
ACYPI37774-RA	ni|645903953|nb|KK920478.1|	1639705-1639914	18	17	gi|641586850|gb|KK854182.1|	612763-613449	10	37
ACYPI37793-RA	ni|645904271|nb|KK920224.1|	841398-841921	15	16	gi|641567493|gb|KK857288.1|	29190-30404	8.6	41
ACYPI38061-RA	ni|645903704|nb|KK920726.1|	209286-210380	nan	nan	gi|641576858|gb|KK854846.1|	343216-343917	10	33
ACYPI38188-RA	ni|645903618|nb|KK920812.1|	322903-323233	nan	nan	gi|641586127|gb|KK854286.1|	451793-452056	10	43
ACYPI38268-RA	ni|645897346|nb|KK927083.1|	41626-47887	nan	nan	gi|641569798|gb|KK856557.1|	97264-105963	12	46
ACYPI38602-RA	ni|645904262|nb|KK920227.1|	710264-710776	17	17	gi|641571612|gb|KK856022.1|	162706-163018	12	43
ACYPI38993-RA	ni|645903787|nb|KK920643.1|	207331-207537	19	18	gi|641586091|gb|KK854292.1|	9729-9950	9.7	37
ACYPI38997-RA	ni|645903557|nb|KK920873.1|	933841-934025	nan	nan	gi|641571119|gb|KK856156.1|	202411-206229	11	38
ACYPI39080-RA	ni|645904146|nb|KK920287.1|	434569-435645	16	18	gi|641587519|gb|KK854093.1|	797017-801235	11	39
ACYPI39685-RA	ni|645904097|nb|KK920334.1|	713426-714218	15	17	gi|641576756|gb|KK854865.1|	87016-87809	10	40
ACYPI40062-RA	ni|645903762|nb|KK920668.1|	840971-841138	17	17	gi|641565471|gb|KK858017.1|	12751-12941	9.8	40
ACYPI40118-RA	ni|645904251|nb|KK920231.1|	1915224-1916911	16	16	gi|641576290|gb|KK854945.1|	213794-217048	9.3	20
ACYPI40226-RA	ni|645904119|nb|KK920312.1|	1078292-1078905	19	19	gi|641587937|gb|KK854042.1|	876448-876800	11	42
ACYPI40278-RA	ni|645903857|nb|KK920573.1|	316508-316741	16	16	gi|641573699|gb|KK855496.1|	65182-66069	12	39
ACYPI40717-RA	ni|645903780|nb|KK920650.1|	847265-848343	19	19	gi|641574150|gb|KK855385.1|	104474-107265	10	38
ACYPI40836-RA	ni|645904087|nb|KK920344.1|	794162-794863	16	16	gi|641566659|gb|KK857580.1|	1-834	9.3	35
ACYPI40853-RA	ni|645904174|nb|KK920261.1|	812352-813326	19	17	gi|641587783|gb|KK854060.1|	582759-584422	12	40
ACYPI41167-RA	ni|645902238|nb|KK922191.1|	138899-139230	nan	nan	gi|641579947|gb|KK854574.1|	273486-273681	11	39
ACYPI41200-RA	ni|645904245|nb|KK920234.1|	52453-52736	16	17	gi|641587671|gb|KK854074.1|	909081-909557	11	40
ACYPI41578-RA	ni|645904129|nb|KK920304.1|	1294829-1295273	16	17	gi|641572760|gb|KK855731.1|	25472-25794	9.3	33
ACYPI42170-RA	ni|645904204|nb|KK920251.1|	754515-755254	15	16	gi|641575759|gb|KK855050.1|	379736-380381	11	38
ACYPI42284-RA	ni|645903541|nb|KK920889.1|	95139-95315	nan	nan	gi|641575693|gb|KK855062.1|	98684-102573	11	40
ACYPI42350-RA	ni|645904136|nb|KK920297.1|	912875-913912	16	17	gi|641576741|gb|KK854868.1|	114191-115242	9.5	40
ACYPI42579-RA	ni|645903561|nb|KK920869.1|	80183-81266	nan	nan	gi|641577987|gb|KK854657.1|	470598-472914	13	49
ACYPI43494-RA	ni|645903491|nb|KK920939.1|	560529-560693	nan	nan	gi|641577810|gb|KK854690.1|	487185-487678	9.8	39

ACYPI43876-RA	ni|645904037|nb|KK920394.1|	1477728-1478876	18	17	gi|641576519|gb|KK854904.1|	85415-87781	10	40
ACYPI45053-RA	ni|645903760|nb|KK920670.1|	676641-677963	18	19	gi|641587888|gb|KK854048.1|	238725-240752	10	43
ACYPI45421-RA	ni|645904005|nb|KK920426.1|	177812-178022	17	16	gi|641578056|gb|KK854648.1|	152774-153281	11	41
ACYPI45536-RA	ni|645904195|nb|KK920254.1|	2351366-2351531	18	17	gi|641566907|gb|KK857492.1|	57-221	11	47
ACYPI45687-RA	ni|645904164|nb|KK920269.1|	296101-296712	19	20	gi|641575436|gb|KK855110.1|	240450-243008	12	37
ACYPI46077-RA	ni|645903857|nb|KK920573.1|	545029-545350	16	16	gi|641576064|gb|KK854990.1|	93209-93418	10	39
ACYPI46726-RA	ni|645904219|nb|KK920246.1|	734853-735126	16	17	gi|641578325|gb|KK854602.1|	133708-134018	9.7	38
ACYPI46801-RA	ni|645904078|nb|KK920353.1|	522324-524679	17	17	gi|641569574|gb|KK856629.1|	25228-28986	24	120
ACYPI46815-RA	ni|645903661|nb|KK920769.1|	729398-730003	nan	nan	gi|641570377|gb|KK856373.1|	48352-49215	11	39
ACYPI46836-RA	ni|645903567|nb|KK920863.1|	264906-265274	nan	nan	gi|641576456|gb|KK854916.1|	284651-285034	10	21
ACYPI46839-RA	ni|645903567|nb|KK920863.1|	232665-232868	nan	nan	gi|641576456|gb|KK854916.1|	291933-292461	10	21
ACYPI47106-RA	ni|645903575|nb|KK920855.1|	240904-241131	nan	nan	gi|641587303|gb|KK854118.1|	665698-666663	11	20
ACYPI47548-RA	ni|645903999|nb|KK920432.1|	572824-574232	17	15	gi|641588073|gb|KK854027.1|	1130466-1132144	11	41
ACYPI47651-RA	ni|645903961|nb|KK920470.1|	460205-460444	17	15	gi|641586096|gb|KK854291.1|	638772-639013	11	46
ACYPI48107-RA	ni|645904210|nb|KK920249.1|	809385-809692	16	16	gi|641568716|gb|KK856889.1|	34477-34722	10	35
ACYPI48246-RA	ni|645903747|nb|KK920683.1|	876580-876856	19	18	gi|641588267|gb|KK854008.1|	1118910-1119447	10	40
ACYPI48834-RA	ni|645903627|nb|KK920803.1|	347973-348960	nan	nan	gi|641586874|gb|KK854178.1|	661387-662589	9.4	38
ACYPI49270-RA	ni|645903975|nb|KK920456.1|	1234424-1234827	15	8.2	gi|641561045|gb|KK859922.1|	6845-7080	11	17
ACYPI49333-RA	ni|645904244|nb|KK920235.1|	229483-230537	15	14	gi|641586867|gb|KK854179.1|	79487-80128	11	39
ACYPI49734-RA	ni|645902238|nb|KK922191.1|	189752-190371	nan	nan	gi|641561581|gb|KK859676.1|	18619-19341	10	38
ACYPI49735-RA	ni|645902238|nb|KK922191.1|	178861-180498	nan	nan	gi|641561581|gb|KK859676.1|	2-9962	10	38
ACYPI49959-RA	ni|645902788|nb|KK921641.1|	285490-286084	nan	nan	gi|641585888|gb|KK854324.1|	482523-490920	10	41
ACYPI50290-RA	ni|645904174|nb|KK920261.1|	356416-357550	19	17	gi|641574352|gb|KK855342.1|	205356-206484	9.4	18
ACYPI50391-RA	ni|645898156|nb|KK926273.1|	6080-6266	nan	nan	gi|641577574|gb|KK854731.1|	222756-223349	11	38
ACYPI50514-RA	ni|645904133|nb|KK920300.1|	1623860-1624336	18	18	gi|641585233|gb|KK854427.1|	423402-423650	10	38
ACYPI50578-RA	ni|645902418|nb|KK922011.1|	70358-70727	nan	nan	gi|641586419|gb|KK854244.1|	314168-315126	11	22
ACYPI50943-RA	ni|645902313|nb|KK922116.1|	148919-152618	nan	nan	gi|641586950|gb|KK854166.1|	714051-719946	10	38
ACYPI51052-RA	ni|645903685|nb|KK920745.1|	522246-523151	nan	nan	gi|641575363|gb|KK855126.1|	299676-300096	11	42
ACYPI51124-RA	ni|645904070|nb|KK920361.1|	344150-344490	17	16	gi|641574249|gb|KK855364.1|	20161-20352	9.2	37
ACYPI51194-RA	ni|645903697|nb|KK920733.1|	52263-52778	nan	nan	gi|641585690|gb|KK854356.1|	438004-439251	9.6	41
ACYPI51366-RA	ni|645902664|nb|KK921765.1|	248185-248939	nan	nan	gi|641573742|gb|KK855485.1|	120061-127898	9.9	36
ACYPI52009-RA	ni|645902696|nb|KK921733.1|	59508-59717	nan	nan	gi|641577968|gb|KK854660.1|	272460-277391	10	42

ACYPI52139-RA	ni|645904153|nb|KK920280.1|	2320493-2321736	17	16	gi|641585428|gb|KK854399.1|	383790-385025	9.8	41
ACYPI52393-RA	ni|645903541|nb|KK920889.1|	271090-272483	nan	nan	gi|641575626|gb|KK855076.1|	77400-80548	11	24
ACYPI52571-RA	ni|645903743|nb|KK920687.1|	924656-928329	19	18	gi|641588283|gb|KK854007.1|	1597502-1604467	11	40
ACYPI52843-RA	ni|645903896|nb|KK920535.1|	459081-460504	16	9.1	gi|641570707|gb|KK856276.1|	26880-30000	9.4	20
ACYPI52893-RA	ni|645899682|nb|KK924747.1|	13519-13721	nan	nan	gi|641585475|gb|KK854392.1|	90313-91125	10	41
ACYPI53104-RA	ni|645903709|nb|KK920721.1|	90870-92494	nan	nan	gi|641576074|gb|KK854988.1|	129922-131166	12	46
ACYPI53120-RA	ni|645902600|nb|KK921829.1|	154361-154889	nan	nan	gi|641577511|gb|KK854741.1|	124217-129069	11	38
ACYPI53153-RA	ni|645903644|nb|KK920786.1|	214279-214967	nan	nan	gi|641575372|gb|KK855124.1|	178603-178878	9.5	36
ACYPI53596-RA	ni|645904114|nb|KK920317.1|	1751645-1751899	18	18	gi|641571667|gb|KK856008.1|	43474-44069	11	39
ACYPI53903-RA	ni|645904045|nb|KK920386.1|	651027-651718	19	21	gi|641575867|gb|KK855030.1|	203805-204018	11	39
ACYPI54769-RA	ni|645904102|nb|KK920329.1|	200895-201281	15	16	gi|641572106|gb|KK855898.1|	90705-90976	7.3	40
ACYPI54877-RA	ni|645902354|nb|KK922075.1|	138068-138259	nan	nan	gi|641570141|gb|KK856447.1|	105819-106195	11	40
ACYPI55124-RA	ni|645903806|nb|KK920624.1|	385335-385552	16	9.3	gi|641587024|gb|KK854155.1|	310964-311339	10	21
ACYPI55202-RA	ni|645904029|nb|KK920402.1|	575031-575226	14	15	gi|641575284|gb|KK855140.1|	172693-172844	8.2	35
ACYPI55567-RA	ni|645901786|nb|KK922643.1|	62098-63715	nan	nan	gi|641568389|gb|KK856993.1|	8040-12164	10	41
ACYPI55712-RA	ni|645903505|nb|KK920925.1|	98540-99214	nan	nan	gi|641577805|gb|KK854691.1|	412030-412904	9.4	38
ACYPI55872-RA	ni|645902561|nb|KK921868.1|	3335-3594	nan	nan	gi|641587009|gb|KK854158.1|	542147-544768	11	41
ACYPI56610-RA	ni|645897009|nb|KK927420.1|	980-1583	nan	nan	gi|641575559|gb|KK855088.1|	194613-195059	9.9	37
ACYPI56611-RA	ni|645904251|nb|KK920231.1|	352483-353339	16	16	gi|641583313|gb|KK854525.1|	437091-437657	9.5	40
ACYPI56627-RA	ni|645903993|nb|KK920438.1|	132747-133864	19	22	gi|641575709|gb|KK855059.1|	346032-347627	11	41
ACYPI56635-RA	ni|645903809|nb|KK920621.1|	219501-220065	18	16	gi|641576225|gb|KK854957.1|	50263-54712	11	44
ACYPI56637-RA	ni|645903973|nb|KK920458.1|	1225789-1226308	20	18	gi|641566778|gb|KK857537.1|	26912-27315	12	39
ACYPI56642-RA	ni|645903836|nb|KK920594.1|	964993-965471	18	11	gi|641579952|gb|KK854573.1|	384950-386320	11	21
ACYPI56643-RA	ni|645904174|nb|KK920261.1|	274830-275661	19	17	gi|641577184|gb|KK854793.1|	71198-71550	11	40
ACYPI56655-RA	ni|645903970|nb|KK920461.1|	371301-371634	17	16	gi|641588051|gb|KK854029.1|	947744-948917	9.6	40
ACYPI56660-RA	ni|645901937|nb|KK922492.1|	4406-4714	nan	nan	gi|641587719|gb|KK854068.1|	186084-186425	10	21
ACYPI56663-RA	ni|645904074|nb|KK920357.1|	1028780-1029133	17	18	gi|641563636|gb|KK858785.1|	9951-10651	8.3	33
ACYPI56670-RA	ni|645902703|nb|KK921726.1|	89253-89510	nan	nan	gi|641575419|gb|KK855114.1|	10678-12938	8.8	35
ACYPI56678-RA	ni|645903557|nb|KK920873.1|	917401-917614	nan	nan	gi|641571119|gb|KK856156.1|	186130-188457	11	38
ACYPI56745-RA	ni|645902694|nb|KK921735.1|	178952-179243	nan	nan	gi|641587905|gb|KK854046.1|	54465-56561	10	43
ACYPI56773-RA	ni|645904183|nb|KK920258.1|	923160-924975	25	35	gi|641574960|gb|KK855207.1|	10029-11944	10	48
ACYPI56793-RA	ni|645903865|nb|KK920565.1|	236202-236438	15	15	gi|641588246|gb|KK854010.1|	1246276-1246516	9.2	50
