# Supplementary material for: The X Chromosome of Hemipteran Insects: Conservation, Dosage Compensation and Sex-Biased Expression
Source: Genome Biol Evol. 2015 Nov 10;7(12):3259–68. doi: 10.1093/gbe/evv215 (PMC4700948; doi:10.1093/gbe/evv215)
Supplement: Supplementary Data [file supp_evv215_suppl_data.zip › S2 Data (rev) HV-OF.pdf]

AP	HV	gene	HVcovF	HVcovM	OF	gene	OFcovF	OFcovM
ACYPI000003-RA	gi|646738819|gb|KK962938.1|	60350-61387	19	8.4	gi|641569982|gb|KK856497.1|	27549-33684	9.9	36
ACYPI000004-RA	gi|646767697|gb|KK961826.1|	774230-774825	21	9.8	gi|641570570|gb|KK856316.1|	50659-50971	10	35
ACYPI000011-RA	gi|646777495|gb|KK961629.1|	1233265-1237008	19	8.7	gi|641574095|gb|KK855397.1|	175037-177744	11	39
ACYPI000013-RA	gi|646740098|gb|KK962812.1|	612042-612505	20	10	gi|641578060|gb|KK854647.1|	32081-34839	8.9	35
ACYPI000016-RA	gi|646771265|gb|KK961769.1|	157601-159488	17	7	gi|641578198|gb|KK854624.1|	511137-511499	12	40
ACYPI000023-RA	gi|646779785|gb|KK961565.1|	3459246-3460123	18	8.9	gi|641573621|gb|KK855514.1|	51915-53233	10	41
ACYPI000027-RA	gi|646745959|gb|KK962321.1|	195848-196157	20	6.9	gi|641574955|gb|KK855208.1|	187350-187595	8.5	33
ACYPI000028-RA	gi|646742726|gb|KK962557.1|	172816-175816	20	9.1	gi|641577805|gb|KK854691.1|	404966-405696	9.4	38
ACYPI000030-RA	gi|646776588|gb|KK961666.1|	2584306-2591169	20	5.8	gi|641575946|gb|KK855016.1|	210994-220495	9.5	37
ACYPI000032-RA	gi|646777416|gb|KK961632.1|	3397743-3397979	22	8.8	gi|641586734|gb|KK854198.1|	163022-165105	11	40
ACYPI000033-RA	gi|646758845|gb|KK961921.1|	621449-621952	22	9.5	gi|641586509|gb|KK854231.1|	561560-564107	9.8	37
ACYPI000035-RA	gi|646766426|gb|KK961862.1|	956662-956908	20	7.1	gi|641574476|gb|KK855312.1|	158895-159679	10	43
ACYPI000038-RA	gi|646747841|gb|KK962189.1|	106063-106397	23	10	gi|641567126|gb|KK857416.1|	36342-37408	11	41
ACYPI000039-RA	gi|646742541|gb|KK962572.1|	629400-629683	19	10	gi|641585847|gb|KK854330.1|	170644-172139	11	41
ACYPI000044-RA	gi|646781510|gb|KK961518.1|	571481-571850	17	7.8	gi|641578305|gb|KK854605.1|	77673-79547	11	42
ACYPI000045-RA	gi|646767196|gb|KK961838.1|	694862-695862	21	9.7	gi|641587118|gb|KK854142.1|	304062-305430	11	40
ACYPI000046-RA	gi|646767477|gb|KK961831.1|	536027-536740	20	8.4	gi|641586509|gb|KK854231.1|	158422-158646	9.8	37
ACYPI000047-RA	gi|646771951|gb|KK961762.1|	59483-59825	21	8.3	gi|641573214|gb|KK855616.1|	14320-14563	9.7	34
ACYPI000051-RA	gi|646752143|gb|KK961972.1|	776198-776437	20	5.6	gi|641568389|gb|KK856993.1|	95561-97314	10	41
ACYPI000052-RA	gi|646775807|gb|KK961708.1|	2100664-2101889	21	8.6	gi|641566429|gb|KK857664.1|	66414-67063	12	37
ACYPI000053-RA	gi|646749945|gb|KK962065.1|	1687137-1694597	22	6.1	gi|641572247|gb|KK855861.1|	155478-157522	10	37
ACYPI000054-RA	gi|646743302|gb|KK962514.1|	156505-156681	23	8.4	gi|641587074|gb|KK854148.1|	559178-561366	11	41
ACYPI000055-RA	gi|646779262|gb|KK961578.1|	2195924-2197069	20	8.1	gi|641585366|gb|KK854408.1|	368516-371955	12	41
ACYPI000056-RA	gi|646776024|gb|KK961697.1|	597551-598295	22	9.2	gi|641575583|gb|KK855084.1|	170446-171490	11	37
ACYPI000058-RA	gi|646744699|gb|KK962413.1|	70330-71410	14	6.9	gi|641578149|gb|KK854632.1|	229773-230600	11	40
ACYPI000059-RA	gi|646782334|gb|KK961495.1|	4655675-4657262	21	9	gi|641588309|gb|KK854005.1|	525454-526730	11	40
ACYPI000060-RA	gi|646739411|gb|KK962885.1|	225159-225481	18	7.7	gi|641572130|gb|KK855891.1|	147850-150539	10	37
ACYPI000061-RA	gi|646781243|gb|KK961526.1|	2284347-2285039	21	8.9	gi|641587742|gb|KK854065.1|	528211-529888	11	41
ACYPI000062-RA	gi|646782334|gb|KK961495.1|	3145231-3146159	21	9	gi|641578310|gb|KK854604.1|	93489-93988	11	39
ACYPI000063-RA	gi|646765790|gb|KK961889.1|	1012080-1014309	22	8.8	gi|641577920|gb|KK854670.1|	271638-272507	8.5	40
ACYPI000065-RA	gi|646776514|gb|KK961670.1|	230372-233696	20	9.6	gi|641576347|gb|KK854934.1|	20200-21624	11	39

ACYPI000066-RA	gi|646781344|gb|KK961523.1|	3687902-3700144	21	8.9	gi|641584205|gb|KK854500.1|	65445-66310	11	41
ACYPI000067-RA	gi|646747471|gb|KK962216.1|	172292-176424	24	6.4	gi|641577121|gb|KK854804.1|	193382-195033	16	82
ACYPI000068-RA	gi|646775987|gb|KK961699.1|	249584-250665	19	7.5	gi|641569988|gb|KK856495.1|	87740-88795	11	43
ACYPI000069-RA	gi|646751120|gb|KK962005.1|	1057607-1058112	21	5.8	gi|641573950|gb|KK855430.1|	143252-145343	10	19
ACYPI000070-RA	gi|646746355|gb|KK962294.1|	502821-503397	22	10	gi|641577805|gb|KK854691.1|	488167-489736	9.4	38
ACYPI000071-RA	gi|646747175|gb|KK962238.1|	313611-314731	20	8.4	gi|641571760|gb|KK855988.1|	96541-97388	9.9	37
ACYPI000072-RA	gi|646754962|gb|KK961940.1|	752857-753275	21	10	gi|641584336|gb|KK854489.1|	176419-176832	10	40
ACYPI000073-RA	gi|646749098|gb|KK962110.1|	745846-746450	20	7.7	gi|641573567|gb|KK855527.1|	77166-77635	12	47
ACYPI000076-RA	gi|646738524|gb|KK962968.1|	52854-54376	24	11	gi|641572916|gb|KK855689.1|	123851-127276	9.2	33
ACYPI000077-RA	gi|646782288|gb|KK961496.1|	6774498-6774864	21	9.7	gi|641587735|gb|KK854066.1|	66823-68779	11	42
ACYPI000078-RA	gi|646767265|gb|KK961836.1|	1243106-1247949	22	9.9	gi|641577535|gb|KK854737.1|	4514-10672	11	42
ACYPI000079-RA	gi|646780953|gb|KK961535.1|	3216707-3217447	20	9.8	gi|641575368|gb|KK855125.1|	91464-93177	10	36
ACYPI000080-RA	gi|646743195|gb|KK962522.1|	250620-251412	14	6.4	gi|641570274|gb|KK856404.1|	107160-108834	9.1	35
ACYPI000081-RA	gi|646777495|gb|KK961629.1|	1861075-1861463	19	8.7	gi|641576091|gb|KK854985.1|	75761-76501	12	24
ACYPI000082-RA	gi|646752567|gb|KK961965.1|	934807-936511	19	8.4	gi|641586741|gb|KK854197.1|	680135-682002	11	43
ACYPI000083-RA	gi|646776409|gb|KK961677.1|	358171-358575	17	8.4	gi|641586515|gb|KK854230.1|	651769-652480	11	39
ACYPI000084-RA	gi|646746505|gb|KK962284.1|	460428-460607	20	7.6	gi|641572846|gb|KK855707.1|	131310-131940	13	52
ACYPI000085-RA	gi|646782087|gb|KK961501.1|	1505741-1506608	20	8.7	gi|641585940|gb|KK854316.1|	469449-469792	11	42
ACYPI000087-RA	gi|646771845|gb|KK961763.1|	299895-301396	23	10	gi|641582439|gb|KK854536.1|	393006-396207	11	36
ACYPI000089-RA	gi|646780978|gb|KK961534.1|	511824-512059	20	8	gi|641588099|gb|KK854024.1|	212160-213211	11	39
ACYPI000090-RA	gi|646770998|gb|KK961772.1|	1214361-1215446	22	9.8	gi|641588343|gb|KK854003.1|	1016518-1016738	11	40
ACYPI000091-RA	gi|646760564|gb|KK961914.1|	518723-518969	21	8.8	gi|641577277|gb|KK854778.1|	217865-219704	11	42
ACYPI000100-RA	gi|646778632|gb|KK961596.1|	1194499-1195606	21	9.2	gi|641570526|gb|KK856327.1|	100135-100754	9.1	37
ACYPI000102-RA	gi|646779451|gb|KK961573.1|	833738-834143	20	8.9	gi|641576951|gb|KK854831.1|	209531-210063	8.6	40
ACYPI000104-RA	gi|646743394|gb|KK962507.1|	357348-358187	20	8.8	gi|641576414|gb|KK854924.1|	81163-82561	10	40
ACYPI000106-RA	gi|646775746|gb|KK961711.1|	147270-149139	15	8.2	gi|641574334|gb|KK855346.1|	110656-111654	9.8	39
ACYPI000111-RA	gi|646781013|gb|KK961533.1|	1858064-1860911	18	7.6	gi|641576442|gb|KK854919.1|	10082-11256	11	36
ACYPI000112-RA	gi|646767156|gb|KK961839.1|	121818-122206	15	6.4	gi|641571048|gb|KK856174.1|	36498-36798	11	39
ACYPI000119-RA	gi|646746752|gb|KK962267.1|	493701-496561	20	7.9	gi|641587133|gb|KK854140.1|	612043-612276	9.9	37
ACYPI000120-RA	gi|646753799|gb|KK961950.1|	613540-616165	23	9.2	gi|641576069|gb|KK854989.1|	264933-266515	11	40
ACYPI000138-RA	gi|646767120|gb|KK961840.1|	1038645-1038912	20	8.6	gi|641576032|gb|KK854997.1|	269952-271550	9.9	38
ACYPI000149-RA	gi|646776305|gb|KK961683.1|	2525223-2526435	20	8.8	gi|641580970|gb|KK854563.1|	170493-175598	11	42

ACYPI000150-RA	gi|646776368|gb|KK961679.1|	1738757-1741452	21	9.7	gi|641572824|gb|KK855713.1|	165584-167439	9.8	19
ACYPI000157-RA	gi|646778903|gb|KK961588.1|	979585-980246	23	10	gi|641565939|gb|KK857844.1|	4054-5290	8.8	16
ACYPI000162-RA	gi|646662646|gb|KK967934.1|	12970-13110	15	5.2	gi|641586006|gb|KK854305.1|	114816-117276	9.7	38
ACYPI000165-RA	gi|646780485|gb|KK961548.1|	999810-1000053	15	6.1	gi|641573181|gb|KK855624.1|	240745-241212	11	40
ACYPI000171-RA	gi|646781043|gb|KK961532.1|	817031-818298	26	11	gi|641573673|gb|KK855501.1|	155796-159785	14	43
ACYPI000173-RA	gi|646776514|gb|KK961670.1|	733975-734278	20	9.6	gi|641587555|gb|KK854089.1|	672336-673084	11	39
ACYPI000181-RA	gi|646781564|gb|KK961516.1|	637656-637956	23	9.4	gi|641585905|gb|KK854321.1|	495675-496013	11	39
ACYPI000191-RA	gi|646748855|gb|KK962125.1|	213474-218196	22	5.8	gi|641585778|gb|KK854341.1|	337880-338147	9.7	41
ACYPI000192-RA	gi|646775507|gb|KK961723.1|	1642102-1642297	20	9.8	gi|641586915|gb|KK854171.1|	329413-330184	10	38
ACYPI000201-RA	gi|646778029|gb|KK961613.1|	2338609-2338874	21	8.6	gi|641570573|gb|KK856315.1|	134460-142538	10	45
ACYPI000203-RA	gi|646768717|gb|KK961805.1|	720790-721668	20	8	gi|641588236|gb|KK854011.1|	1120983-112143[illegible]	9.7	38
ACYPI000210-RA	gi|646775987|gb|KK961699.1|	781881-783343	19	7.5	gi|641586837|gb|KK854184.1|	87782-88434	11	40
ACYPI000219-RA	gi|646773721|gb|KK961747.1|	2447510-2449323	21	9.2	gi|641585569|gb|KK854376.1|	56185-56699	11	47
ACYPI000222-RA	gi|646751350|gb|KK961993.1|	255525-256015	20	7.6	gi|641576260|gb|KK854950.1|	45448-45882	10	39
ACYPI000224-RA	gi|646748086|gb|KK962172.1|	592013-593208	20	10	gi|641576442|gb|KK854919.1|	338335-344626	11	36
ACYPI000227-RA	gi|646779066|gb|KK961583.1|	1212691-1214372	19	7.9	gi|641587577|gb|KK854086.1|	48732-49579	8.2	41
ACYPI000235-RA	gi|646748407|gb|KK962151.1|	147350-164335	22	9.1	gi|641588082|gb|KK854026.1|	1083168-108330[illegible]	11	41
ACYPI000238-RA	gi|646776219|gb|KK961687.1|	2197359-2198345	21	9.9	gi|641566606|gb|KK857599.1|	32825-33433	10	40
ACYPI000243-RA	gi|646766650|gb|KK961854.1|	456289-456754	22	10	gi|641578084|gb|KK854643.1|	245531-245751	11	40
ACYPI000249-RA	gi|646779662|gb|KK961568.1|	1262501-1264477	20	9.3	gi|641572891|gb|KK855695.1|	31191-32931	10	36
ACYPI000250-RA	gi|646747586|gb|KK962207.1|	186339-189708	20	8.8	gi|641574896|gb|KK855222.1|	292683-295549	11	43
ACYPI000252-RA	gi|646777474|gb|KK961630.1|	3104034-3109213	21	9.3	gi|641586843|gb|KK854183.1|	524314-536066	11	41
ACYPI000257-RA	gi|646742343|gb|KK962588.1|	266900-267672	20	9.4	gi|641586150|gb|KK854282.1|	456801-457130	11	40
ACYPI000258-RA	gi|646738615|gb|KK962959.1|	211375-211648	21	9.5	gi|641587652|gb|KK854077.1|	598010-598705	10	38
ACYPI000259-RA	gi|646780858|gb|KK961538.1|	1131776-1132105	21	10	gi|641587040|gb|KK854152.1|	364920-365624	9.1	39
ACYPI000262-RA	gi|646752294|gb|KK961969.1|	850302-851220	23	9.8	gi|641587104|gb|KK854144.1|	815457-815780	11	37
ACYPI000264-RA	gi|646747494|gb|KK962214.1|	302162-302400	18	8.5	gi|641587569|gb|KK854087.1|	340286-342508	11	41
ACYPI000265-RA	gi|646595672|gb|KK977392.1|	665-1940	21	8.2	gi|641582439|gb|KK854536.1|	33343-35639	11	36
ACYPI000267-RA	gi|646780406|gb|KK961550.1|	2739109-2739642	22	5.9	gi|641585574|gb|KK854375.1|	504621-504906	11	39
ACYPI000271-RA	gi|646778448|gb|KK961601.1|	1740135-1741627	19	8.3	gi|641572601|gb|KK855773.1|	194693-194928	10	35
ACYPI000278-RA	gi|646739841|gb|KK962838.1|	399284-400000	21	5.7	gi|641587365|gb|KK854113.1|	698368-699804	12	45
ACYPI000286-RA	gi|646750484|gb|KK962036.1|	541864-542093	20	5.5	gi|641573850|gb|KK855457.1|	214665-215347	9.3	34

ACYPI000289-RA	gi|646779186|gb|KK961580.1|	2020614-2021080	20	8.6	gi|641584445|gb|KK854481.1|	227492-229988	9.9	39
ACYPI000295-RA	gi|646771568|gb|KK961766.1|	989260-989524	18	7.4	gi|641587962|gb|KK854039.1|	841203-841667	11	40
ACYPI000303-RA	gi|646781118|gb|KK961530.1|	4711619-4713254	21	10	gi|641565112|gb|KK858157.1|	7833-12055	9.7	34
ACYPI000304-RA	gi|646775595|gb|KK961718.1|	677552-678067	21	8.9	gi|641568637|gb|KK856916.1|	75126-76167	10	38
ACYPI000307-RA	gi|646782276|gb|KK961497.1|	3678865-3679391	21	9.7	gi|641577710|gb|KK854706.1|	375992-378221	11	43
ACYPI000310-RA	gi|646775842|gb|KK961706.1|	1806878-1810297	22	9	gi|641587272|gb|KK854122.1|	912870-917442	9.3	42
ACYPI000320-RA	gi|646767754|gb|KK961825.1|	83201-83427	20	8.5	gi|641570570|gb|KK856316.1|	89342-89634	10	35
ACYPI000329-RA	gi|646779498|gb|KK961572.1|	2496952-2503099	22	5.6	gi|641549615|gb|KK865290.1|	58-1415	12	24
ACYPI000335-RA	gi|646764075|gb|KK961900.1|	964327-964605	18	6.5	gi|641584248|gb|KK854496.1|	404807-405231	9.9	39
ACYPI000336-RA	gi|646738330|gb|KK962987.1|	191864-196023	19	7.9	gi|641577574|gb|KK854731.1|	58409-62851	11	38
ACYPI000343-RA	gi|646781659|gb|KK961513.1|	5472275-5472487	22	9.8	gi|641578039|gb|KK854651.1|	354338-354549	10	38
ACYPI000348-RA	gi|646778865|gb|KK961589.1|	2907073-2908107	20	9.2	gi|641585905|gb|KK854321.1|	153604-153957	11	39
ACYPI000349-RA	gi|646779337|gb|KK961576.1|	2276897-2277283	18	9	gi|641587084|gb|KK854146.1|	499204-500331	9	40
ACYPI000362-RA	gi|646768494|gb|KK961810.1|	575133-576685	21	10	gi|641563408|gb|KK858883.1|	3730-4211	9.5	21
ACYPI000368-RA	gi|646750643|gb|KK962028.1|	26114-27850	21	7.5	gi|641575096|gb|KK855181.1|	128586-128796	11	21
ACYPI000381-RA	gi|646770998|gb|KK961772.1|	979320-979601	22	9.8	gi|641588283|gb|KK854007.1|	1195488-1197009	11	40
ACYPI000383-RA	gi|646780889|gb|KK961537.1|	4001777-4006160	22	10	gi|641580970|gb|KK854563.1|	309588-312071	11	42
ACYPI000387-RA	gi|646770901|gb|KK961773.1|	1607687-1609334	22	9.7	gi|641582768|gb|KK854530.1|	410190-410868	11	41
ACYPI000402-RA	gi|646780105|gb|KK961557.1|	3099164-3099558	21	9.7	gi|641571515|gb|KK856048.1|	37318-41280	9.9	38
ACYPI000405-RA	gi|646778112|gb|KK961611.1|	1159493-1160574	22	10	gi|641585395|gb|KK854405.1|	405520-407871	10	38
ACYPI000407-RA	gi|646775312|gb|KK961734.1|	987612-988879	17	7.9	gi|641575222|gb|KK855154.1|	221423-222184	11	37
ACYPI000408-RA	gi|646776368|gb|KK961679.1|	1335718-1335988	21	9.7	gi|641583088|gb|KK854527.1|	248264-249291	9.9	39
ACYPI000413-RA	gi|646747574|gb|KK962208.1|	384898-386581	27	10	gi|641564920|gb|KK858233.1|	27114-27569	11	23
ACYPI000423-RA	gi|646777017|gb|KK961649.1|	993344-994208	18	8	gi|641568716|gb|KK856889.1|	35485-37188	10	35
ACYPI000427-RA	gi|646779006|gb|KK961585.1|	202694-213347	20	9.9	gi|641588267|gb|KK854008.1|	109932-117676	10	40
ACYPI000430-RA	gi|646770323|gb|KK961780.1|	267737-268155	19	7.1	gi|641572219|gb|KK855868.1|	62624-68729	9.8	35
ACYPI000431-RA	gi|646779826|gb|KK961564.1|	1056290-1056945	22	9.6	gi|641586085|gb|KK854293.1|	98218-99178	11	40
ACYPI000436-RA	gi|646781659|gb|KK961513.1|	3748687-3749643	22	9.8	gi|641568213|gb|KK857047.1|	45198-47412	9.2	39
ACYPI000437-RA	gi|646746974|gb|KK962252.1|	566052-567981	23	6.1	gi|641574324|gb|KK855348.1|	109384-110493	12	40
ACYPI000441-RA	gi|646777665|gb|KK961623.1|	1843214-1843723	21	9.7	gi|641576437|gb|KK854920.1|	326398-328514	10	39
ACYPI000442-RA	gi|646777842|gb|KK961618.1|	2148059-2149775	19	8.9	gi|641574015|gb|KK855416.1|	17520-20959	10	42
ACYPI000443-RA	gi|646782168|gb|KK961499.1|	1790146-1791926	21	9.4	gi|641584320|gb|KK854490.1|	426743-429006	10	41

ACYPI000445-RA	gi|646775807|gb|KK961708.1|	2842833-2843891	21	8.6	gi|641586515|gb|KK854230.1|	583971-588039	11	39
ACYPI000446-RA	gi|646781421|gb|KK961521.1|	1473544-1476975	21	8.7	gi|641577772|gb|KK854697.1|	258940-261083	10	39
ACYPI000453-RA	gi|646776701|gb|KK961661.1|	612430-613848	18	6.7	gi|641573695|gb|KK855497.1|	21675-23242	9.2	41
ACYPI000454-RA	gi|646776351|gb|KK961680.1|	424875-425208	22	10	gi|641542389|gb|KK868779.1|	439-997	11	45
ACYPI000455-RA	gi|646776148|gb|KK961690.1|	1761896-1762566	21	9.4	gi|641570399|gb|KK856367.1|	51263-56295	11	41
ACYPI000467-RA	gi|646735649|gb|KK963275.1|	10568-10830	16	7.9	gi|641570703|gb|KK856277.1|	109132-109483	9.9	36
ACYPI000474-RA	gi|646767850|gb|KK961823.1|	1181490-1184713	21	9.8	gi|641571315|gb|KK856104.1|	133674-139009	12	46
ACYPI000476-RA	gi|646780222|gb|KK961554.1|	94940-95790	20	9.7	gi|641567816|gb|KK857179.1|	76314-76518	12	37
ACYPI000479-RA	gi|646747990|gb|KK962179.1|	803511-807000	21	9.3	gi|641570614|gb|KK856303.1|	19248-19597	9.6	34
ACYPI000480-RA	gi|646779066|gb|KK961583.1|	371692-372450	19	7.9	gi|641587179|gb|KK854134.1|	712821-713589	11	40
ACYPI000487-RA	gi|646767120|gb|KK961840.1|	817964-819474	20	8.6	gi|641585971|gb|KK854310.1|	175212-175825	10	40
ACYPI000496-RA	gi|646782357|gb|KK961494.1|	3432145-3433422	21	9.2	gi|641585423|gb|KK854400.1|	83475-85161	11	36
ACYPI000499-RA	gi|646767265|gb|KK961836.1|	1341643-1349857	22	9.9	gi|641564440|gb|KK858439.1|	379-2822	11	36
ACYPI000500-RA	gi|646765966|gb|KK961881.1|	1445629-1446753	19	9.2	gi|641579205|gb|KK854586.1|	154410-154702	10	39
ACYPI000502-RA	gi|646781809|gb|KK961509.1|	2194102-2194572	19	8.6	gi|641576957|gb|KK854830.1|	177746-178005	10	43
ACYPI000508-RA	gi|646778865|gb|KK961589.1|	1722136-1723064	20	9.2	gi|641585587|gb|KK854373.1|	426424-426985	9.8	41
ACYPI000513-RA	gi|646745644|gb|KK962342.1|	463448-465229	31	14	gi|641584945|gb|KK854445.1|	394329-397567	9.9	20
ACYPI000519-RA	gi|646775867|gb|KK961705.1|	301713-315278	23	10	gi|641586898|gb|KK854174.1|	327042-329558	11	39
ACYPI000521-RA	gi|646768669|gb|KK961806.1|	417270-417813	18	9.1	gi|641587848|gb|KK854052.1|	631208-633367	11	39
ACYPI000523-RA	gi|646771448|gb|KK961767.1|	708643-709058	21	9.8	gi|641586285|gb|KK854263.1|	293171-293643	10	38
ACYPI000527-RA	gi|646751544|gb|KK961986.1|	1295621-1295843	22	6	gi|641587276|gb|KK854121.1|	431239-432417	11	39
ACYPI000530-RA	gi|646746581|gb|KK962279.1|	274319-276250	21	9.1	gi|641570396|gb|KK856368.1|	63749-64102	9.5	38
ACYPI000532-RA	gi|646782127|gb|KK961500.1|	3721499-3726858	22	9.6	gi|641584504|gb|KK854477.1|	134770-136269	9.5	38
ACYPI000533-RA	gi|646739633|gb|KK962861.1|	266755-267064	17	7.9	gi|641577075|gb|KK854811.1|	97974-99437	11	36
ACYPI000534-RA	gi|646778336|gb|KK961604.1|	2203948-2204913	21	10	gi|641586344|gb|KK854254.1|	475181-476013	10	40
ACYPI000538-RA	gi|646775635|gb|KK961716.1|	1847031-1850055	22	10	gi|641585847|gb|KK854330.1|	187303-189644	11	41
ACYPI000552-RA	gi|646782288|gb|KK961496.1|	791268-791853	21	9.7	gi|641586171|gb|KK854278.1|	15939-18288	9	39
ACYPI000563-RA	gi|646748295|gb|KK962158.1|	90371-90660	18	8.2	gi|641572916|gb|KK855689.1|	101035-107029	9.2	33
ACYPI000575-RA	gi|646749632|gb|KK962082.1|	359901-362282	20	8.6	gi|641585366|gb|KK854408.1|	388426-390905	12	41
ACYPI000579-RA	gi|646777991|gb|KK961614.1|	1129553-1130515	18	7.8	gi|641578330|gb|KK854601.1|	65900-67199	11	38
ACYPI000580-RA	gi|646737917|gb|KK963028.1|	149263-149945	22	7.8	gi|641586171|gb|KK854278.1|	552666-553443	9	39
ACYPI000582-RA	gi|646743576|gb|KK962494.1|	140136-140844	19	8.2	gi|641586452|gb|KK854239.1|	505286-505784	10	19

ACYPI000585-RA	gi|646781772|gb|KK961510.1|	4238842-4240612	20	9.2	gi|641585801|gb|KK854337.1|	437800-438557	10	38
ACYPI000588-RA	gi|646745197|gb|KK962376.1|	153958-154882	21	8.9	gi|641588213|gb|KK854013.1|	1324598-132663[illegible]	9.6	39
ACYPI000592-RA	gi|646781732|gb|KK961511.1|	2776373-2780343	22	9.8	gi|641586915|gb|KK854171.1|	304011-304195	10	38
ACYPI000598-RA	gi|646781510|gb|KK961518.1|	184400-187619	17	7.8	gi|641574699|gb|KK855261.1|	81511-84990	9	35
ACYPI000600-RA	gi|646779262|gb|KK961578.1|	907356-907915	20	8.1	gi|641571660|gb|KK856010.1|	173694-174468	11	22
ACYPI000602-RA	gi|646781243|gb|KK961526.1|	2098312-2099887	21	8.9	gi|641587021|gb|KK854156.1|	776681-777242	11	42
ACYPI000610-RA	gi|646777416|gb|KK961632.1|	1856574-1857088	22	8.8	gi|641570019|gb|KK856485.1|	48852-51596	12	43
ACYPI000611-RA	gi|646781282|gb|KK961525.1|	3403329-3404135	22	9.6	gi|641587249|gb|KK854125.1|	4172-4960	11	37
ACYPI000613-RA	gi|646769774|gb|KK961788.1|	490181-499714	20	9.2	gi|641574085|gb|KK855399.1|	124910-125954	10	38
ACYPI000617-RA	gi|646780222|gb|KK961554.1|	1227144-1234809	20	9.7	gi|641580479|gb|KK854569.1|	306615-309434	10	38
ACYPI000626-RA	gi|646780858|gb|KK961538.1|	2870715-2871107	21	10	gi|641587468|gb|KK854099.1|	604780-605044	10	42
ACYPI000631-RA	gi|646746339|gb|KK962295.1|	150652-152550	20	8.2	gi|641588343|gb|KK854003.1|	1166852-116831[illegible]	11	40
ACYPI000646-RA	gi|646770251|gb|KK961781.1|	594123-594844	20	9.6	gi|641578179|gb|KK854627.1|	517236-517471	10	39
ACYPI000654-RA	gi|646752919|gb|KK961960.1|	1667440-1669253	20	9	gi|641587462|gb|KK854100.1|	82039-83183	10	21
ACYPI000662-RA	gi|646750185|gb|KK962052.1|	259994-261137	18	7.1	gi|641575385|gb|KK855121.1|	141042-142700	9.4	36
ACYPI000663-RA	gi|646748615|gb|KK962139.1|	440751-442875	19	7.8	gi|641586509|gb|KK854231.1|	319185-321408	9.8	37
ACYPI000664-RA	gi|646750185|gb|KK962052.1|	340664-341625	18	7.1	gi|641571119|gb|KK856156.1|	70070-70332	11	38
ACYPI000665-RA	gi|646747559|gb|KK962209.1|	450102-452433	19	9.5	gi|641587082|gb|KK854147.1|	829583-833806	11	44
ACYPI000666-RA	gi|646780574|gb|KK961546.1|	958171-961542	20	9.9	gi|641561423|gb|KK859751.1|	5747-6205	8.8	17
ACYPI000667-RA	gi|646781118|gb|KK961530.1|	197897-203737	21	10	gi|641587652|gb|KK854077.1|	887729-889622	10	38
ACYPI000669-RA	gi|646776524|gb|KK961669.1|	409949-410657	17	6.4	gi|641588000|gb|KK854035.1|	850506-853466	11	39
ACYPI000674-RA	gi|646752431|gb|KK961967.1|	878384-878812	20	5.6	gi|641585022|gb|KK854440.1|	387856-389079	10	39
ACYPI000684-RA	gi|646751289|gb|KK961996.1|	594511-595122	24	9.1	gi|641585587|gb|KK854373.1|	364618-365332	9.8	41
ACYPI000686-RA	gi|646780311|gb|KK961552.1|	3429318-3430008	21	9.7	gi|641571116|gb|KK856157.1|	239816-241311	11	40
ACYPI000694-RA	gi|646779038|gb|KK961584.1|	500491-500957	21	6.9	gi|641587003|gb|KK854159.1|	277619-278020	10	21
ACYPI000695-RA	gi|646781772|gb|KK961510.1|	3879413-3879698	20	9.2	gi|641584835|gb|KK854453.1|	254573-255248	8.3	36
ACYPI000698-RA	gi|646746243|gb|KK962302.1|	220034-222108	12	7.7	gi|641577043|gb|KK854816.1|	311150-313770	11	43
ACYPI000700-RA	gi|646776050|gb|KK961695.1|	1231942-1233643	19	7.7	gi|641573935|gb|KK855434.1|	218264-223101	11	41
ACYPI000702-RA	gi|646751103|gb|KK962006.1|	587949-591119	20	8.3	gi|641575668|gb|KK855066.1|	269826-270087	11	40
ACYPI000714-RA	gi|646768582|gb|KK961808.1|	282713-283331	21	6	gi|641572251|gb|KK855860.1|	174943-176685	10	19
ACYPI000720-RA	gi|646770251|gb|KK961781.1|	1363462-1364195	20	9.6	gi|641569788|gb|KK856560.1|	30690-31881	9.5	37
ACYPI000724-RA	gi|646782276|gb|KK961497.1|	7119979-7121066	21	9.7	gi|641570244|gb|KK856415.1|	66239-67248	10	34

ACYPI000725-RA	gi|646750484|gb|KK962036.1|	343126-343779	20	5.5	gi|641584108|gb|KK854506.1|	251641-252337	11	40
ACYPI000727-RA	gi|646781083|gb|KK961531.1|	2335878-2336903	19	8.5	gi|641573351|gb|KK855583.1|	89102-91875	11	41
ACYPI000728-RA	gi|646743341|gb|KK962511.1|	175289-175601	19	5	gi|641569454|gb|KK856667.1|	48524-50261	9.8	34
ACYPI000734-RA	gi|646782211|gb|KK961498.1|	1230895-1231218	20	9	gi|641576973|gb|KK854827.1|	174448-174739	11	38
ACYPI000735-RA	gi|646744179|gb|KK962451.1|	13535-16722	24	11	gi|641573555|gb|KK855529.1|	97300-97582	8.9	38
ACYPI000739-RA	gi|646776647|gb|KK961663.1|	1490253-1490755	23	9.2	gi|641577574|gb|KK854731.1|	295485-296034	11	38
ACYPI000749-RA	gi|646776974|gb|KK961651.1|	486769-489957	17	8.1	gi|641585761|gb|KK854344.1|	323336-323522	10	39
ACYPI000750-RA	gi|646777416|gb|KK961632.1|	3716548-3718473	22	8.8	gi|641570459|gb|KK856349.1|	48604-49266	9.7	40
ACYPI000752-RA	gi|646779498|gb|KK961572.1|	2018552-2018828	22	5.6	gi|641574792|gb|KK855243.1|	136945-138648	8.7	36
ACYPI000753-RA	gi|646779936|gb|KK961561.1|	2061110-2061797	19	9.6	gi|641572637|gb|KK855763.1|	18018-21093	9.5	36
ACYPI000754-RA	gi|646779539|gb|KK961571.1|	1476637-1476921	15	7.5	gi|641588099|gb|KK854024.1|	592114-594104	11	39
ACYPI000756-RA	gi|646779702|gb|KK961567.1|	1891062-1891666	23	10	gi|641586975|gb|KK854163.1|	14899-15804	11	40
ACYPI000759-RA	gi|646776929|gb|KK961653.1|	883996-885812	17	8.5	gi|641585376|gb|KK854407.1|	134864-136282	9.8	36
ACYPI000765-RA	gi|646776510|gb|KK961671.1|	896194-899453	20	7.8	gi|641575104|gb|KK855179.1|	94075-95977	10	35
ACYPI000767-RA	gi|646782127|gb|KK961500.1|	2268280-2271808	22	9.6	gi|641587652|gb|KK854077.1|	274273-274591	10	38
ACYPI000770-RA	gi|646741422|gb|KK962668.1|	271081-272044	19	11	gi|641586817|gb|KK854187.1|	526012-527144	9.3	39
ACYPI000776-RA	gi|646776608|gb|KK961665.1|	1606664-1612257	23	9.3	gi|641585871|gb|KK854327.1|	138541-140199	11	38
ACYPI000783-RA	gi|646776128|gb|KK961691.1|	1329857-1332987	21	9.2	gi|641586040|gb|KK854300.1|	267082-268549	10	38
ACYPI000787-RA	gi|646775312|gb|KK961734.1|	798851-799244	17	7.9	gi|641587511|gb|KK854094.1|	242785-243982	10	41
ACYPI000792-RA	gi|646778865|gb|KK961589.1|	1907516-1908773	20	9.2	gi|641569432|gb|KK856672.1|	56199-57094	13	47
ACYPI000798-RA	gi|646778699|gb|KK961594.1|	2244905-2245200	21	9.5	gi|641576807|gb|KK854855.1|	59750-62211	9.8	37
ACYPI000799-RA	gi|646777313|gb|KK961637.1|	750381-750852	20	8.2	gi|641584281|gb|KK854493.1|	370854-371128	12	41
ACYPI000814-RA	gi|646751569|gb|KK961985.1|	937271-938229	21	9.2	gi|641585315|gb|KK854418.1|	334099-336057	10	39
ACYPI000816-RA	gi|646771092|gb|KK961771.1|	1087754-1089498	23	9.7	gi|641572655|gb|KK855758.1|	154318-156033	19	94
ACYPI000819-RA	gi|646777416|gb|KK961632.1|	779364-781313	22	8.8	gi|641574951|gb|KK855209.1|	76378-78376	10	38
ACYPI000821-RA	gi|646782334|gb|KK961495.1|	2642576-2642820	21	9	gi|641587066|gb|KK854149.1|	220873-221337	11	40
ACYPI000828-RA	gi|646779375|gb|KK961575.1|	1080382-1083185	22	9.3	gi|641566255|gb|KK857728.1|	11965-15850	8.3	32
ACYPI000837-RA	gi|646747424|gb|KK962220.1|	1360305-1360572	22	5.7	gi|641582096|gb|KK854546.1|	29961-33872	11	38
ACYPI000843-RA	gi|646776280|gb|KK961684.1|	188247-188457	22	9.3	gi|641585940|gb|KK854316.1|	350205-352225	11	42
ACYPI000854-RA	gi|646776629|gb|KK961664.1|	1076048-1076529	19	8.4	gi|641585366|gb|KK854408.1|	70695-71817	12	41
ACYPI000855-RA	gi|646750600|gb|KK962030.1|	678233-679195	23	8.6	gi|641588109|gb|KK854023.1|	222111-223328	10	40
ACYPI000857-RA	gi|646762334|gb|KK961909.1|	435741-437406	19	8.9	gi|641546561|gb|KK866766.1|	555-1179	8.9	15

ACYPI000870-RA	gi|646754003|gb|KK961948.1|	320783-321399	20	8.8	gi|641576766|gb|KK854863.1|	157080-157377	10	38
ACYPI000872-RA	gi|646782276|gb|KK961497.1|	6436723-6437127	21	9.7	gi|641587937|gb|KK854042.1|	332852-334123	11	42
ACYPI000876-RA	gi|646751458|gb|KK961989.1|	839991-840232	21	5.1	gi|641578305|gb|KK854605.1|	64482-66730	11	42
ACYPI000882-RA	gi|646776524|gb|KK961669.1|	1195418-1197855	17	6.4	gi|641574960|gb|KK855207.1|	278907-280778	10	48
ACYPI000885-RA	gi|646777363|gb|KK961635.1|	1760438-1760718	18	9.2	gi|641586452|gb|KK854239.1|	192245-192518	10	19
ACYPI000886-RA	gi|646751289|gb|KK961996.1|	338417-338891	24	9.1	gi|641565701|gb|KK857932.1|	2599-2857	11	42
ACYPI000887-RA	gi|646755567|gb|KK961936.1|	673563-674476	20	8.6	gi|641579941|gb|KK854575.1|	297254-298997	10	39
ACYPI000890-RA	gi|646748888|gb|KK962123.1|	868885-870145	20	8.1	gi|641584205|gb|KK854500.1|	475644-476484	11	41
ACYPI000896-RA	gi|646750354|gb|KK962043.1|	326752-327026	20	9.2	gi|641585910|gb|KK854320.1|	129892-132882	10	38
ACYPI000903-RA	gi|646781849|gb|KK961508.1|	2181341-2183151	17	8	gi|641586133|gb|KK854285.1|	606150-606924	11	41
ACYPI000904-RA	gi|646780311|gb|KK961552.1|	3538603-3539747	21	9.7	gi|641586785|gb|KK854191.1|	228643-229802	11	39
ACYPI000913-RA	gi|646781659|gb|KK961513.1|	5019068-5019421	22	9.8	gi|641571457|gb|KK856066.1|	186244-186720	12	43
ACYPI000914-RA	gi|646776280|gb|KK961684.1|	1742040-1745546	22	9.3	gi|641573954|gb|KK855429.1|	161967-167857	10	42
ACYPI000918-RA	gi|646767439|gb|KK961832.1|	17249-20743	23	10	gi|641584606|gb|KK854469.1|	390115-393391	13	57
ACYPI000923-RA	gi|646751373|gb|KK961992.1|	598079-598345	21	8.7	gi|641570630|gb|KK856298.1|	150400-151002	12	47
ACYPI000929-RA	gi|646781659|gb|KK961513.1|	3379491-3383496	22	9.8	gi|641572134|gb|KK855890.1|	56721-57783	10	36
ACYPI000930-RA	gi|646732431|gb|KK963675.1|	257184-258001	24	9.6	gi|641577778|gb|KK854696.1|	282046-284706	9.9	42
ACYPI000938-RA	gi|646732491|gb|KK963666.1|	43033-43470	21	5.7	gi|641570086|gb|KK856464.1|	107527-107930	10	35
ACYPI000941-RA	gi|646780530|gb|KK961547.1|	2199904-2200192	21	9.2	gi|641570036|gb|KK856480.1|	65780-66033	8.3	33
ACYPI000944-RA	gi|646780147|gb|KK961556.1|	1969066-1969740	18	7.5	gi|641577487|gb|KK854746.1|	128558-131647	11	38
ACYPI000946-RA	gi|646782357|gb|KK961494.1|	4401905-4402253	21	9.2	gi|641567069|gb|KK857435.1|	25387-25599	6.8	28
ACYPI000948-RA	gi|646779141|gb|KK961581.1|	949954-950678	21	9.1	gi|641586313|gb|KK854259.1|	176119-176709	12	42
ACYPI000953-RA	gi|646775507|gb|KK961723.1|	1640561-1641654	20	9.8	gi|641586915|gb|KK854171.1|	324778-325722	10	38
ACYPI000955-RA	gi|646780978|gb|KK961534.1|	1853405-1853690	20	8	gi|641570514|gb|KK856331.1|	101197-107313	9.8	39
ACYPI000956-RA	gi|646778903|gb|KK961588.1|	3713052-3713946	23	10	gi|641572887|gb|KK855696.1|	107162-108046	9.5	26
ACYPI000961-RA	gi|646781421|gb|KK961521.1|	918407-918938	21	8.7	gi|641569727|gb|KK856579.1|	46367-46981	8.3	34
ACYPI000969-RA	gi|646749755|gb|KK962075.1|	1186925-1203231	22	9.2	gi|641571055|gb|KK856172.1|	109181-114601	10	38
ACYPI000971-RA	gi|646776024|gb|KK961697.1|	2426175-2428788	22	9.2	gi|641576423|gb|KK854922.1|	195918-196586	18	35
ACYPI000979-RA	gi|646782357|gb|KK961494.1|	5102553-5104237	21	9.2	gi|641586066|gb|KK854296.1|	239720-241473	9.4	37
ACYPI000980-RA	gi|646712812|gb|KK965357.1|	50551-51065	21	9.9	gi|641575236|gb|KK855151.1|	247019-247483	11	38
ACYPI000985-RA	gi|646759701|gb|KK961917.1|	896485-897848	18	6.1	gi|641577574|gb|KK854731.1|	95648-97592	11	38
ACYPI000987-RA	gi|646748116|gb|KK962170.1|	568550-568835	20	8.3	gi|641577837|gb|KK854685.1|	297621-298006	11	42

ACYPI000992-RA	gi|646780270|gb|KK961553.1|	2211583-2212046	22	8.6	gi|641578046|gb|KK854650.1|	262357-262925	10	38
ACYPI000994-RA	gi|646769275|gb|KK961796.1|	726234-733161	22	5.6	gi|641569808|gb|KK856554.1|	81474-85696	11	21
ACYPI000998-RA	gi|646779898|gb|KK961562.1|	488790-491724	19	9.3	gi|641586928|gb|KK854169.1|	660534-667228	11	39
ACYPI001001-RA	gi|646751103|gb|KK962006.1|	353214-355919	20	8.3	gi|641585900|gb|KK854322.1|	221865-223064	12	43
ACYPI001003-RA	gi|646781212|gb|KK961527.1|	2191133-2192019	21	10	gi|641570045|gb|KK856477.1|	101916-102138	9.6	43
ACYPI001007-RA	gi|646740756|gb|KK962735.1|	93490-104906	23	10	gi|641569779|gb|KK856563.1|	57403-57893	11	40
ACYPI001010-RA	gi|646749188|gb|KK962105.1|	306847-311013	18	7.9	gi|641586509|gb|KK854231.1|	117779-118102	9.8	37
ACYPI001012-RA	gi|646742343|gb|KK962588.1|	275106-283598	20	9.4	gi|641572860|gb|KK855703.1|	118167-121546	9.8	36
ACYPI001015-RA	gi|646781443|gb|KK961520.1|	1882162-1882595	20	8.3	gi|641584305|gb|KK854491.1|	155524-155995	11	39
ACYPI001018-RA	gi|646763535|gb|KK961902.1|	841528-843534	23	10	gi|641586613|gb|KK854216.1|	117156-120306	11	41
ACYPI001019-RA	gi|646736246|gb|KK963207.1|	338857-341900	19	8.5	gi|641587735|gb|KK854066.1|	213810-215396	11	42
ACYPI001021-RA	gi|646779869|gb|KK961563.1|	2494782-2495034	17	8.8	gi|641573136|gb|KK855635.1|	45632-46972	9.8	26
ACYPI001023-RA	gi|646575796|gb|KK981232.1|	19329-20239	21	10	gi|641586606|gb|KK854217.1|	161019-161487	11	42
ACYPI001024-RA	gi|646767813|gb|KK961824.1|	309405-309629	17	6.3	gi|641587562|gb|KK854088.1|	542807-544057	11	42
ACYPI001025-RA	gi|646736283|gb|KK963203.1|	331952-336414	24	9.7	gi|641575140|gb|KK855171.1|	325434-328817	9.7	40
ACYPI001030-RA	gi|646766482|gb|KK961860.1|	547849-549145	17	6.7	gi|641572615|gb|KK855769.1|	190159-190813	12	43
ACYPI001031-RA	gi|646780222|gb|KK961554.1|	200954-203281	20	9.7	gi|641586915|gb|KK854171.1|	497438-498263	10	38
ACYPI001032-RA	gi|646782288|gb|KK961496.1|	6978452-6980594	21	9.7	gi|641576036|gb|KK854996.1|	68613-74077	11	40
ACYPI001039-RA	gi|646775723|gb|KK961712.1|	1499360-1499690	21	9.5	gi|641577289|gb|KK854776.1|	280040-281931	10	36
ACYPI001043-RA	gi|646775765|gb|KK961710.1|	1866358-1867038	21	6	gi|641588246|gb|KK854010.1|	4429-4733	9.2	50
ACYPI001044-RA	gi|646778865|gb|KK961589.1|	1075002-1076615	20	9.2	gi|641582401|gb|KK854541.1|	146554-147241	11	43
ACYPI001047-RA	gi|646782168|gb|KK961499.1|	7346412-7347822	21	9.4	gi|641580504|gb|KK854565.1|	394640-398081	11	43
ACYPI001051-RA	gi|646770998|gb|KK961772.1|	711159-711623	22	9.8	gi|641572207|gb|KK855871.1|	63082-65006	10	39
ACYPI001052-RA	gi|646743772|gb|KK962481.1|	773352-774243	22	8.9	gi|641588164|gb|KK854017.1|	280218-290107	9.2	40
ACYPI001054-RA	gi|646775312|gb|KK961734.1|	670482-670714	17	7.9	gi|641574986|gb|KK855201.1|	130802-131216	9.3	35
ACYPI001056-RA	gi|646782357|gb|KK961494.1|	9519081-9523191	21	9.2	gi|641587624|gb|KK854080.1|	393388-393753	10	38
ACYPI001057-RA	gi|646776219|gb|KK961687.1|	2596331-2597878	21	9.9	gi|641578144|gb|KK854633.1|	112495-112721	10	43
ACYPI001059-RA	gi|646758845|gb|KK961921.1|	1390254-1396147	22	9.5	gi|641586139|gb|KK854284.1|	524856-530442	10	21
ACYPI001061-RA	gi|646745327|gb|KK962367.1|	51411-51650	18	8.3	gi|641567860|gb|KK857164.1|	1418-3170	11	36
ACYPI001069-RA	gi|646737822|gb|KK963039.1|	161589-174539	20	9	gi|641586777|gb|KK854192.1|	261403-264682	11	30
ACYPI001071-RA	gi|646760564|gb|KK961914.1|	16075-16612	21	8.8	gi|641584205|gb|KK854500.1|	96025-96514	11	41
ACYPI001081-RA	gi|646751289|gb|KK961996.1|	851263-852771	24	9.1	gi|641586313|gb|KK854259.1|	459530-459750	12	42

ACYPI001085-RA	gi|646780311|gb|KK961552.1|	2264830-2265753	21	9.7	gi|641577121|gb|KK854804.1|	18496-18745	16	82
ACYPI001090-RA	gi|646728609|gb|KK964184.1|	229889-235318	34	14	gi|641571716|gb|KK855999.1|	59771-60818	11	39
ACYPI001091-RA	gi|646749945|gb|KK962065.1|	1300714-1302205	22	6.1	gi|641586936|gb|KK854168.1|	258813-259248	12	42
ACYPI001093-RA	gi|646775807|gb|KK961708.1|	2530982-2532190	21	8.6	gi|641584884|gb|KK854449.1|	92625-95034	11	39
ACYPI001096-RA	gi|646769102|gb|KK961799.1|	1906841-1907065	20	8.3	gi|641587652|gb|KK854077.1|	500085-502402	10	38
ACYPI001107-RA	gi|646744686|gb|KK962414.1|	471483-472831	12	7.3	gi|641585957|gb|KK854313.1|	365494-371807	10	37
ACYPI001110-RA	gi|646781849|gb|KK961508.1|	2570548-2571249	17	8	gi|641572364|gb|KK855831.1|	22282-22895	11	38
ACYPI001111-RA	gi|646782043|gb|KK961502.1|	1074406-1074590	20	9.2	gi|641570921|gb|KK856214.1|	106021-107147	8.7	39
ACYPI001113-RA	gi|646781732|gb|KK961511.1|	3881635-3887993	22	9.8	gi|641576842|gb|KK854849.1|	143610-144454	12	38
ACYPI001119-RA	gi|646745091|gb|KK962384.1|	517591-518107	19	01/00	gi|641565021|gb|KK858191.1|	4-3849	10	36
ACYPI001124-RA	gi|646765705|gb|KK961893.1|	1666052-1674499	22	9.7	gi|641587074|gb|KK854148.1|	185784-191231	11	41
ACYPI001125-RA	gi|646781564|gb|KK961516.1|	285738-290292	23	9.4	gi|641574416|gb|KK855326.1|	212242-213675	11	38
ACYPI001129-RA	gi|646776855|gb|KK961656.1|	377398-378630	19	8.8	gi|641576392|gb|KK854928.1|	227290-228776	9.7	41
ACYPI001130-RA	gi|646766326|gb|KK961866.1|	253328-254614	19	7.9	gi|641575227|gb|KK855153.1|	39102-39366	9	40
ACYPI001138-RA	gi|646781344|gb|KK961523.1|	100908-104016	21	8.9	gi|641568953|gb|KK856815.1|	58826-59321	9.1	41
ACYPI001145-RA	gi|646754371|gb|KK961945.1|	560961-561596	20	9.7	gi|641570861|gb|KK856232.1|	38470-42415	11	39
ACYPI001146-RA	gi|646776024|gb|KK961697.1|	1100000-1100423	22	9.2	gi|641573143|gb|KK855633.1|	115511-115831	9.9	36
ACYPI001153-RA	gi|646780827|gb|KK961539.1|	3273867-3274228	20	9.4	gi|641587614|gb|KK854081.1|	704557-704930	11	40
ACYPI001157-RA	gi|646776974|gb|KK961651.1|	413212-414600	17	8.1	gi|641566611|gb|KK857597.1|	53771-54362	11	40
ACYPI001166-RA	gi|646750043|gb|KK962060.1|	585234-585667	21	8.3	gi|641586380|gb|KK854250.1|	144189-144519	9.3	38
ACYPI001167-RA	gi|646781732|gb|KK961511.1|	646438-646709	22	9.8	gi|641576645|gb|KK854885.1|	337967-340574	10	40
ACYPI001168-RA	gi|646747627|gb|KK962204.1|	333129-334935	18	7.5	gi|641586535|gb|KK854227.1|	356025-357839	10	39
ACYPI001170-RA	gi|646745197|gb|KK962376.1|	392665-396975	21	8.9	gi|641577749|gb|KK854701.1|	161432-161971	11	20
ACYPI001171-RA	gi|646737782|gb|KK963043.1|	49469-50210	23	9.2	gi|641587614|gb|KK854081.1|	777841-778021	11	40
ACYPI001182-RA	gi|646777698|gb|KK961622.1|	392713-393351	16	7.6	gi|641583980|gb|KK854512.1|	409592-409864	11	39
ACYPI001186-RA	gi|646744453|gb|KK962431.1|	65744-66168	19	8.5	gi|641577309|gb|KK854773.1|	204496-205576	9.9	39
ACYPI001188-RA	gi|646777416|gb|KK961632.1|	1719456-1722610	22	8.8	gi|641574135|gb|KK855388.1|	177826-179223	9.6	36
ACYPI001189-RA	gi|646780978|gb|KK961534.1|	604778-605595	20	8	gi|641587800|gb|KK854058.1|	980034-980295	10	42
ACYPI001193-RA	gi|646750165|gb|KK962053.1|	198910-199652	25	10	gi|641588109|gb|KK854023.1|	588844-589503	10	40
ACYPI001194-RA	gi|646777665|gb|KK961623.1|	1899629-1900013	21	9.7	gi|641585587|gb|KK854373.1|	351503-352162	9.8	41
ACYPI001206-RA	gi|646777416|gb|KK961632.1|	1542675-1542970	22	8.8	gi|641588358|gb|KK854002.1|	226595-223164	11	43
ACYPI001211-RA	gi|646745434|gb|KK962359.1|	138459-139791	22	10	gi|641587082|gb|KK854147.1|	433025-436082	11	44

ACYPI001212-RA	gi|646775723|gb|KK961712.1|	1678105-1678926	21	9.5	gi|641577289|gb|KK854776.1|	303573-304486	10	36
ACYPI001217-RA	gi|646613436|gb|KK973865.1|	6907-7589	21	7.7	gi|641585801|gb|KK854337.1|	449458-451053	10	38
ACYPI001218-RA	gi|646777765|gb|KK961620.1|	1001671-1008255	21	8.7	gi|641588294|gb|KK854006.1|	620000-626234	8.7	40
ACYPI001219-RA	gi|646777545|gb|KK961627.1|	1492777-1493022	21	9.9	gi|641588060|gb|KK854028.1|	692693-695133	11	42
ACYPI001220-RA	gi|646768631|gb|KK961807.1|	835255-836784	19	9.2	gi|641571276|gb|KK856113.1|	89299-90175	9.9	34
ACYPI001225-RA	gi|646749964|gb|KK962064.1|	341009-342299	18	8.7	gi|641587671|gb|KK854074.1|	423271-426435	11	40
ACYPI001228-RA	gi|646777313|gb|KK961637.1|	303712-305402	20	8.2	gi|641581389|gb|KK854557.1|	338500-342765	11	41
ACYPI001233-RA	gi|646736855|gb|KK963138.1|	140252-142290	19	9.1	gi|641585303|gb|KK854420.1|	215501-217983	10	36
ACYPI001238-RA	gi|646780441|gb|KK961549.1|	2474696-2476140	21	9.5	gi|641587111|gb|KK854143.1|	708491-709003	11	40
ACYPI001241-RA	gi|646781379|gb|KK961522.1|	3775244-3775774	22	7.6	gi|641588033|gb|KK854031.1|	179262-181521	11	38
ACYPI001243-RA	gi|646748695|gb|KK962134.1|	215558-215862	20	5.8	gi|641586489|gb|KK854234.1|	263847-266178	9.5	39
ACYPI001257-RA	gi|646748129|gb|KK962169.1|	239978-241152	21	9.8	gi|641573102|gb|KK855643.1|	53475-53715	10	37
ACYPI001266-RA	gi|646777991|gb|KK961614.1|	1647059-1647546	18	7.8	gi|641588060|gb|KK854028.1|	137691-139650	11	42
ACYPI001268-RA	gi|646776514|gb|KK961670.1|	161356-162003	20	9.6	gi|641575001|gb|KK855197.1|	169599-171873	11	38
ACYPI001270-RA	gi|646776091|gb|KK961693.1|	2171494-2172124	23	10	gi|641587133|gb|KK854140.1|	670792-675164	9.9	37
ACYPI001272-RA	gi|646775702|gb|KK961713.1|	701833-705008	17	7.4	gi|641571652|gb|KK856012.1|	99182-101204	9.6	35
ACYPI001274-RA	gi|646777416|gb|KK961632.1|	373995-374330	22	8.8	gi|641586990|gb|KK854161.1|	281920-282717	11	39
ACYPI001277-RA	gi|646772897|gb|KK961753.1|	1531223-1531918	21	8.7	gi|641579212|gb|KK854585.1|	203196-203844	10	35
ACYPI001279-RA	gi|646775867|gb|KK961705.1|	2227489-2229686	23	10	gi|641574923|gb|KK855216.1|	86554-86801	11	38
ACYPI001285-RA	gi|646774530|gb|KK961743.1|	466194-467574	20	9.8	gi|641572431|gb|KK855815.1|	95127-96397	9.1	35
ACYPI001292-RA	gi|646782168|gb|KK961499.1|	3534783-3535379	21	9.4	gi|641587881|gb|KK854049.1|	27088-31073	10	40
ACYPI001296-RA	gi|646779337|gb|KK961576.1|	1112189-1113450	18	9	gi|641575888|gb|KK855026.1|	188622-193373	10	41
ACYPI001299-RA	gi|646782288|gb|KK961496.1|	4418068-4419222	21	9.7	gi|641584658|gb|KK854465.1|	355920-357635	11	37
ACYPI001303-RA	gi|646782168|gb|KK961499.1|	2213831-2220725	21	9.4	gi|641565608|gb|KK857969.1|	16589-17626	13	42
ACYPI001310-RA	gi|646772045|gb|KK961761.1|	628094-628677	21	9.6	gi|641570861|gb|KK856232.1|	77985-79476	11	39
ACYPI001312-RA	gi|646758935|gb|KK961920.1|	123408-123652	20	8.5	gi|641585773|gb|KK854342.1|	395091-396466	12	45
ACYPI001314-RA	gi|646777495|gb|KK961629.1|	1606164-1606864	19	8.7	gi|641577879|gb|KK854677.1|	145229-147318	18	36
ACYPI001316-RA	gi|646750889|gb|KK962017.1|	820863-825686	22	6.1	gi|641585246|gb|KK854426.1|	230696-231694	10	39
ACYPI001321-RA	gi|646753527|gb|KK961953.1|	1474708-1477209	24	9.2	gi|641572813|gb|KK855716.1|	31805-34765	13	51
ACYPI001323-RA	gi|646782009|gb|KK961503.1|	1767752-1770875	15	7.5	gi|641588194|gb|KK854015.1|	295639-296775	9.4	40
ACYPI001329-RA	gi|646733173|gb|KK963571.1|	2768-3167	19	9	gi|641571380|gb|KK856086.1|	131443-131675	9.5	38
ACYPI001335-RA	gi|646782087|gb|KK961501.1|	5629862-5630431	20	8.7	gi|641583795|gb|KK854516.1|	99860-102228	8.5	40

ACYPI001336-RA	gi|646769832|gb|KK961787.1|	381980-385374	15	9.8	gi|641587024|gb|KK854155.1|	311680-312467	10	21
ACYPI001339-RA	gi|646775723|gb|KK961712.1|	1181440-1181891	21	9.5	gi|641580980|gb|KK854561.1|	256415-256586	11	37
ACYPI001342-RA	gi|646767439|gb|KK961832.1|	1041067-1042130	23	10	gi|641586569|gb|KK854222.1|	39754-40061	12	40
ACYPI001352-RA	gi|646750600|gb|KK962030.1|	1468306-1469781	23	8.6	gi|641586344|gb|KK854254.1|	156976-157525	10	40
ACYPI001353-RA	gi|646778517|gb|KK961599.1|	840521-842059	20	9.6	gi|641570788|gb|KK856253.1|	85502-86815	10	20
ACYPI001359-RA	gi|646780441|gb|KK961549.1|	3102433-3103591	21	9.5	gi|641586793|gb|KK854190.1|	642794-644506	9.3	41
ACYPI001360-RA	gi|646782168|gb|KK961499.1|	5435547-5436761	21	9.4	gi|641587263|gb|KK854123.1|	273627-277642	11	42
ACYPI001374-RA	gi|646781443|gb|KK961520.1|	562095-567388	20	8.3	gi|641587111|gb|KK854143.1|	326842-327312	11	40
ACYPI001379-RA	gi|646738604|gb|KK962960.1|	159271-161115	19	8.4	gi|641573714|gb|KK855492.1|	116037-119904	10	37
ACYPI001382-RA	gi|646735640|gb|KK963276.1|	51221-51556	18	6.9	gi|641588258|gb|KK854009.1|	333986-335333	11	36
ACYPI001387-RA	gi|646779898|gb|KK961562.1|	3103134-3105117	19	9.3	gi|641572671|gb|KK855754.1|	150090-151719	10	38
ACYPI001390-RA	gi|646766985|gb|KK961844.1|	212494-214239	21	9.7	gi|641587954|gb|KK854040.1|	1015970-101649[illegible]	8.9	39
ACYPI001392-RA	gi|646781243|gb|KK961526.1|	2012596-2013384	21	8.9	gi|641571123|gb|KK856155.1|	98055-99345	10	42
ACYPI001396-RA	gi|646764075|gb|KK961900.1|	652769-656504	18	6.5	gi|641568713|gb|KK856890.1|	77631-78976	12	42
ACYPI001403-RA	gi|646782043|gb|KK961502.1|	4563367-4563783	20	9.2	gi|641585527|gb|KK854383.1|	404681-405615	11	39
ACYPI001408-RA	gi|646749532|gb|KK962087.1|	209132-209467	14	6	gi|641577161|gb|KK854797.1|	342023-346942	10	38
ACYPI001415-RA	gi|646751160|gb|KK962003.1|	442667-443644	22	10	gi|641584419|gb|KK854483.1|	402477-402844	10	42
ACYPI001416-RA	gi|646780574|gb|KK961546.1|	3529070-3538977	20	9.9	gi|641577644|gb|KK854718.1|	211459-211772	10	40
ACYPI001424-RA	gi|646749051|gb|KK962113.1|	444541-445261	19	8.4	gi|641568831|gb|KK856853.1|	42898-43841	10	30
ACYPI001430-RA	gi|646732855|gb|KK963616.1|	148516-149839	24	7.1	gi|641585819|gb|KK854334.1|	407671-408118	9.3	38
ACYPI001440-RA	gi|646777313|gb|KK961637.1|	726388-729786	20	8.2	gi|641587580|gb|KK854085.1|	646702-650598	10	39
ACYPI001441-RA	gi|646781536|gb|KK961517.1|	2417520-2418159	19	7.7	gi|641577407|gb|KK854756.1|	266558-267142	9.4	39
ACYPI001446-RA	gi|646779375|gb|KK961575.1|	1654209-1654884	22	9.3	gi|641586452|gb|KK854239.1|	260803-261614	10	19
ACYPI001453-RA	gi|646744699|gb|KK962413.1|	16934-17480	14	6.9	gi|641575222|gb|KK855154.1|	36899-39260	11	37
ACYPI001458-RA	gi|646777207|gb|KK961641.1|	1368135-1368890	20	8.5	gi|641571548|gb|KK856039.1|	20292-20656	9.9	18
ACYPI001461-RA	gi|646780858|gb|KK961538.1|	3326329-3330800	21	10	gi|641566611|gb|KK857597.1|	46972-47584	11	40
ACYPI001463-RA	gi|646611448|gb|KK974227.1|	13621-13915	16	5.4	gi|641586928|gb|KK854169.1|	437164-438228	11	39
ACYPI001465-RA	gi|646781043|gb|KK961532.1|	1767270-1769326	26	11	gi|641585303|gb|KK854420.1|	321490-324590	10	36
ACYPI001468-RA	gi|646782357|gb|KK961494.1|	2067763-2068606	21	9.2	gi|641574676|gb|KK855265.1|	65609-70197	9.4	35
ACYPI001475-RA	gi|646775488|gb|KK961724.1|	821706-822264	19	7.8	gi|641576876|gb|KK854844.1|	402018-402751	17	42
ACYPI001480-RA	gi|646776280|gb|KK961684.1|	823608-824198	22	9.3	gi|641572891|gb|KK855695.1|	255552-256017	10	36
ACYPI001481-RA	gi|646775807|gb|KK961708.1|	56287-56643	21	8.6	gi|641587624|gb|KK854080.1|	584479-585285	10	38

ACYPI001483-RA	gi|646747614|gb|KK962205.1|	738370-742198	21	8.9	gi|641587624|gb|KK854080.1|	733099-733374	10	38
ACYPI001487-RA	gi|646770323|gb|KK961780.1|	555078-555719	19	7.1	gi|641586235|gb|KK854270.1|	295118-295288	11	39
ACYPI001496-RA	gi|646782043|gb|KK961502.1|	1234905-1235390	20	9.2	gi|641577322|gb|KK854771.1|	99350-100376	10	21
ACYPI001498-RA	gi|646535382|gb|KK987821.1|	3291-3751	15	6.8	gi|641585761|gb|KK854344.1|	93487-94161	10	39
ACYPI001502-RA	gi|646746651|gb|KK962274.1|	19285-19984	16	6.6	gi|641573452|gb|KK855556.1|	138398-138644	10	22
ACYPI001508-RA	gi|646778948|gb|KK961587.1|	1870106-1870370	20	7.3	gi|641573457|gb|KK855555.1|	173169-174250	9.7	38
ACYPI001511-RA	gi|646766985|gb|KK961844.1|	296011-309627	21	9.7	gi|641586399|gb|KK854247.1|	263675-267785	15	68
ACYPI001515-RA	gi|646777665|gb|KK961623.1|	2189050-2190036	21	9.7	gi|641571667|gb|KK856008.1|	160769-161522	11	39
ACYPI001518-RA	gi|646781849|gb|KK961508.1|	2585322-2586509	17	8	gi|641586606|gb|KK854217.1|	165757-168874	11	42
ACYPI001521-RA	gi|646776429|gb|KK961676.1|	2025704-2030229	20	9.9	gi|641587550|gb|KK854090.1|	137891-139619	9.7	45
ACYPI001527-RA	gi|646782288|gb|KK961496.1|	5994411-5994695	21	9.7	gi|641573097|gb|KK855644.1|	184014-184562	10	36
ACYPI001530-RA	gi|646767196|gb|KK961838.1|	996400-997450	21	9.7	gi|641576807|gb|KK854855.1|	128321-129571	9.8	37
ACYPI001539-RA	gi|646748580|gb|KK962141.1|	217486-217815	20	5.6	gi|641588128|gb|KK854021.1|	4084-4769	9.5	42
ACYPI001540-RA	gi|646752294|gb|KK961969.1|	654870-657040	23	9.8	gi|641587468|gb|KK854099.1|	253433-254478	10	42
ACYPI001542-RA	gi|646770998|gb|KK961772.1|	1126425-1128684	22	9.8	gi|641568386|gb|KK856994.1|	12772-14857	8.9	32
ACYPI001546-RA	gi|646731841|gb|KK963762.1|	36872-38929	20	8.9	gi|641571786|gb|KK855980.1|	81101-81722	9.9	19
ACYPI001547-RA	gi|646782357|gb|KK961494.1|	9767936-9768929	21	9.2	gi|641571315|gb|KK856104.1|	475-639	12	46
ACYPI001552-RA	gi|646782168|gb|KK961499.1|	920959-921579	21	9.4	gi|641571067|gb|KK856169.1|	41356-42011	11	39
ACYPI001560-RA	gi|646777182|gb|KK961642.1|	1452881-1456106	20	9	gi|641574190|gb|KK855376.1|	177804-180071	11	40
ACYPI001561-RA	gi|646747586|gb|KK962207.1|	154837-155416	20	8.8	gi|641576328|gb|KK854938.1|	272967-278410	11	36
ACYPI001567-RA	gi|646780858|gb|KK961538.1|	3187395-3189248	21	10	gi|641587047|gb|KK854151.1|	555059-558798	10	39
ACYPI001575-RA	gi|646738812|gb|KK962939.1|	70326-80013	20	8.1	gi|641573699|gb|KK855496.1|	124324-124856	12	39
ACYPI001578-RA	gi|646745372|gb|KK962364.1|	785165-786150	23	10	gi|641548848|gb|KK865654.1|	1792-3245	7.4	20
ACYPI001579-RA	gi|646782357|gb|KK961494.1|	2963857-2964531	21	9.2	gi|641586203|gb|KK854273.1|	145471-145719	9.5	40
ACYPI001584-RA	gi|646776389|gb|KK961678.1|	683114-688070	22	9.5	gi|641586405|gb|KK854246.1|	305967-310196	10	40
ACYPI001585-RA	gi|646778840|gb|KK961590.1|	2503479-2503992	20	9.9	gi|641574100|gb|KK855396.1|	75220-76082	11	38
ACYPI001591-RA	gi|646779262|gb|KK961578.1|	729002-729390	20	8.1	gi|641586096|gb|KK854291.1|	498061-498270	11	46
ACYPI001593-RA	gi|646728609|gb|KK964184.1|	241138-241372	34	14	gi|641571716|gb|KK855999.1|	78612-79014	11	39
ACYPI001596-RA	gi|646763164|gb|KK961904.1|	1034080-1035768	22	10	gi|641572114|gb|KK855896.1|	145460-146074	10	36
ACYPI001597-RA	gi|646781659|gb|KK961513.1|	2788555-2790950	22	9.8	gi|641586133|gb|KK854285.1|	607011-607796	11	41
ACYPI001600-RA	gi|646749632|gb|KK962082.1|	267316-268320	20	8.6	gi|641586734|gb|KK854198.1|	594106-594894	11	40
ACYPI001601-RA	gi|646777182|gb|KK961642.1|	1112524-1112733	20	9	gi|641586661|gb|KK854209.1|	369308-369889	11	43

ACYPI001612-RA	gi|646744957|gb|KK962394.1|	752332-754206	21	9.8	gi|641573850|gb|KK855457.1|	65140-65548	9.3	34
ACYPI001613-RA	gi|646770401|gb|KK961779.1|	741506-742931	21	8.6	gi|641578106|gb|KK854639.1|	236679-244025	11	39
ACYPI001614-RA	gi|646745186|gb|KK962377.1|	937377-938085	21	9.8	gi|641575693|gb|KK855062.1|	141566-143534	11	40
ACYPI001617-RA	gi|646768494|gb|KK961810.1|	474833-475486	21	10	gi|641584806|gb|KK854455.1|	452916-453656	11	43
ACYPI001622-RA	gi|646781143|gb|KK961529.1|	4291110-4292074	20	10	gi|641570589|gb|KK856310.1|	30308-31328	11	36
ACYPI001625-RA	gi|646746706|gb|KK962270.1|	80951-86147	23	11	gi|641567243|gb|KK857377.1|	61436-65043	11	43
ACYPI001631-RA	gi|646761166|gb|KK961913.1|	309037-309992	17	7.6	gi|641575381|gb|KK855122.1|	262263-262727	11	43
ACYPI001633-RA	gi|646775723|gb|KK961712.1|	1086872-1087662	21	9.5	gi|641570419|gb|KK856360.1|	100337-100863	12	39
ACYPI001635-RA	gi|646780270|gb|KK961553.1|	1835154-1836223	22	8.6	gi|641585642|gb|KK854364.1|	85844-88073	9.9	38
ACYPI001643-RA	gi|646779826|gb|KK961564.1|	2794028-2794970	22	9.6	gi|641571138|gb|KK856151.1|	182010-183579	10	45
ACYPI001646-RA	gi|646781118|gb|KK961530.1|	186752-190040	21	10	gi|641577688|gb|KK854709.1|	184207-184535	11	40
ACYPI001649-RA	gi|646753527|gb|KK961953.1|	1457880-1458355	24	9.2	gi|641587660|gb|KK854076.1|	84683-86709	9.9	41
ACYPI001652-RA	gi|646750389|gb|KK962041.1|	598521-602195	21	9.5	gi|641574320|gb|KK855349.1|	40474-47592	10	38
ACYPI001658-RA	gi|646781344|gb|KK961523.1|	1315539-1315998	21	8.9	gi|641567435|gb|KK857308.1|	24692-26512	10	38
ACYPI001659-RA	gi|646775507|gb|KK961723.1|	561951-565124	20	9.8	gi|641585773|gb|KK854342.1|	203026-207007	12	45
ACYPI001660-RA	gi|646712499|gb|KK965372.1|	33689-34165	14	6.5	gi|641578144|gb|KK854633.1|	119870-120090	10	43
ACYPI001665-RA	gi|646777802|gb|KK961619.1|	1451621-1453226	24	9	gi|641586700|gb|KK854203.1|	122837-123566	8.4	39
ACYPI001667-RA	gi|646781628|gb|KK961514.1|	2292044-2293417	23	9	gi|641570579|gb|KK856313.1|	129453-131170	11	38
ACYPI001668-RA	gi|646778632|gb|KK961596.1|	1957857-1958420	21	9.2	gi|641588358|gb|KK854002.1|	1190216-119079[illegible]	11	43
ACYPI001670-RA	gi|646745543|gb|KK962350.1|	694900-695263	21	10	gi|641570727|gb|KK856270.1|	89280-90273	10	35
ACYPI001671-RA	gi|646775968|gb|KK961700.1|	1210354-1212093	20	8.2	gi|641576876|gb|KK854844.1|	388446-391081	17	42
ACYPI001672-RA	gi|646770477|gb|KK961778.1|	921163-921707	21	9.1	gi|641578193|gb|KK854625.1|	286028-286363	11	41
ACYPI001674-RA	gi|646778699|gb|KK961594.1|	1585114-1585806	21	9.5	gi|641585940|gb|KK854316.1|	519805-521340	11	42
ACYPI001675-RA	gi|646744579|gb|KK962422.1|	353613-363276	22	9.9	gi|641577341|gb|KK854768.1|	114486-116177	14	58
ACYPI001679-RA	gi|646779375|gb|KK961575.1|	2294973-2295574	22	9.3	gi|641585395|gb|KK854405.1|	462091-463540	10	38
ACYPI001683-RA	gi|646778983|gb|KK961586.1|	242281-243091	20	9.9	gi|641574876|gb|KK855226.1|	121912-122231	11	39
ACYPI001686-RA	gi|646746706|gb|KK962270.1|	254576-255499	23	11	gi|641587193|gb|KK854132.1|	658260-659028	11	39
ACYPI001689-RA	gi|646762982|gb|KK961905.1|	1013095-1016866	17	8.2	gi|641588246|gb|KK854010.1|	1089735-109046[illegible]	9.2	50
ACYPI001692-RA	gi|646781282|gb|KK961525.1|	3671061-3676017	22	9.6	gi|641570589|gb|KK856310.1|	98336-100355	11	36
ACYPI001696-RA	gi|646766179|gb|KK961872.1|	413458-416916	18	8.2	gi|641574844|gb|KK855232.1|	230115-232697	10	23
ACYPI001698-RA	gi|646781536|gb|KK961517.1|	1890071-1895298	19	7.7	gi|641587881|gb|KK854049.1|	613046-614244	10	40
ACYPI001704-RA	gi|646741719|gb|KK962642.1|	94145-94505	21	8.9	gi|641587577|gb|KK854086.1|	646483-646726	8.2	41

ACYPI001706-RA	gi|646780311|gb|KK961552.1|	3890551-3890711	21	9.7	gi|641577037|gb|KK854817.1|	162792-163124	9.9	37
ACYPI001710-RA	gi|646777182|gb|KK961642.1|	2323435-2330458	20	9	gi|641577913|gb|KK854671.1|	196722-196969	9.2	40
ACYPI001711-RA	gi|646776113|gb|KK961692.1|	589980-591557	21	9.6	gi|641577821|gb|KK854688.1|	75498-76479	7.8	37
ACYPI001724-RA	gi|646732968|gb|KK963602.1|	82536-83641	20	9.9	gi|641586185|gb|KK854275.1|	190285-190754	12	40
ACYPI001730-RA	gi|646781968|gb|KK961504.1|	2300175-2306420	20	9.6	gi|641569612|gb|KK856617.1|	65037-65691	11	21
ACYPI001736-RA	gi|646738604|gb|KK962960.1|	28697-32181	19	8.4	gi|641573595|gb|KK855520.1|	30042-32216	11	40
ACYPI001742-RA	gi|646770901|gb|KK961773.1|	1029991-1031304	22	9.7	gi|641576347|gb|KK854934.1|	85516-86354	11	39
ACYPI001746-RA	gi|646766451|gb|KK961861.1|	109275-110647	19	8.9	gi|641586535|gb|KK854227.1|	781024-783240	10	39
ACYPI001752-RA	gi|646776647|gb|KK961663.1|	2654761-2658004	23	9.2	gi|641575901|gb|KK855023.1|	245775-246568	11	38
ACYPI001754-RA	gi|646746407|gb|KK962291.1|	64700-65180	21	9.9	gi|641576906|gb|KK854838.1|	129383-130393	9.4	37
ACYPI001755-RA	gi|646769577|gb|KK961791.1|	904027-904515	18	8.3	gi|641588051|gb|KK854029.1|	539859-541775	9.6	40
ACYPI001756-RA	gi|646778274|gb|KK961606.1|	879451-880017	18	7.5	gi|641575112|gb|KK855177.1|	107235-107511	11	39
ACYPI001757-RA	gi|646740085|gb|KK962813.1|	288924-289198	20	5.5	gi|641570930|gb|KK856211.1|	28516-28730	11	39
ACYPI001759-RA	gi|646782357|gb|KK961494.1|	9010681-9011324	21	9.2	gi|641585340|gb|KK854414.1|	13-298	11	42
ACYPI001760-RA	gi|646745543|gb|KK962350.1|	745218-745444	21	10	gi|641570727|gb|KK856270.1|	141147-141732	10	35
ACYPI001763-RA	gi|646767521|gb|KK961830.1|	641863-643440	20	9.3	gi|641573858|gb|KK855455.1|	167987-169455	9.8	37
ACYPI001764-RA	gi|646748002|gb|KK962178.1|	1601285-1602167	22	6.3	gi|641577121|gb|KK854804.1|	143708-143902	16	82
ACYPI001765-RA	gi|646740158|gb|KK962805.1|	312127-312829	24	6.5	gi|641575389|gb|KK855120.1|	227264-227650	10	35
ACYPI001766-RA	gi|646722582|gb|KK964929.1|	64721-67035	23	6	gi|641575821|gb|KK855038.1|	246124-249218	11	21
ACYPI001768-RA	gi|646776322|gb|KK961682.1|	2458364-2460496	22	8.9	gi|641586950|gb|KK854166.1|	570259-570795	10	38
ACYPI001769-RA	gi|646777570|gb|KK961626.1|	913822-914047	18	7.5	gi|641558279|gb|KK861194.1|	2820-3124	9.5	35
ACYPI001777-RA	gi|646747459|gb|KK962217.1|	874270-880389	21	5.9	gi|641571315|gb|KK856104.1|	178506-179629	12	46
ACYPI001779-RA	gi|646773903|gb|KK961746.1|	1519978-1520924	20	8.9	gi|641588082|gb|KK854026.1|	333601-335242	11	41
ACYPI001780-RA	gi|646733583|gb|KK963518.1|	57806-60561	20	9.2	gi|641587912|gb|KK854045.1|	372319-378128	11	39
ACYPI001782-RA	gi|646778410|gb|KK961602.1|	2739334-2740988	20	9	gi|641572484|gb|KK855802.1|	168265-168518	9.2	36
ACYPI001790-RA	gi|646780953|gb|KK961535.1|	472381-473239	20	9.8	gi|641574930|gb|KK855214.1|	171482-171788	11	39
ACYPI001791-RA	gi|646775901|gb|KK961703.1|	294861-295278	20	9.6	gi|641573617|gb|KK855515.1|	131876-133163	10	38
ACYPI001795-RA	gi|646769774|gb|KK961788.1|	513498-519478	20	9.2	gi|641574205|gb|KK855372.1|	247992-249092	11	47
ACYPI001796-RA	gi|646763164|gb|KK961904.1|	551273-553813	22	10	gi|641586335|gb|KK854256.1|	322243-324860	10	38
ACYPI001799-RA	gi|646777877|gb|KK961617.1|	28231-28572	19	8.1	gi|641585383|gb|KK854406.1|	455509-456106	11	37
ACYPI001802-RA	gi|646740158|gb|KK962805.1|	383988-384224	24	6.5	gi|641578341|gb|KK854599.1|	366674-366966	11	21
ACYPI001804-RA	gi|646563700|gb|KK983468.1|	416-594	7	5.6	gi|641579225|gb|KK854583.1|	393013-395261	10	39

ACYPI001806-RA	gi|646750043|gb|KK962060.1|	846602-848206	21	8.3	gi|641587276|gb|KK854121.1|	669761-671524	11	39
ACYPI001807-RA	gi|646781659|gb|KK961513.1|	5267786-5268047	22	9.8	gi|641587765|gb|KK854062.1|	759427-760835	10	41
ACYPI001810-RA	gi|646781118|gb|KK961530.1|	5301918-5302631	21	10	gi|641578046|gb|KK854650.1|	6593-7555	10	38
ACYPI001813-RA	gi|646782357|gb|KK961494.1|	2093145-2094261	21	9.2	gi|641571279|gb|KK856112.1|	185563-185872	9.8	36
ACYPI001815-RA	gi|646782276|gb|KK961497.1|	6214131-6214943	21	9.7	gi|641586850|gb|KK854182.1|	379816-388369	10	37
ACYPI001818-RA	gi|646778865|gb|KK961589.1|	3062185-3062906	20	9.2	gi|641575867|gb|KK855030.1|	45058-48539	11	39
ACYPI001819-RA	gi|646778903|gb|KK961588.1|	2665240-2665844	23	10	gi|641560854|gb|KK860006.1|	5-413	9.6	17
ACYPI001827-RA	gi|646780441|gb|KK961549.1|	2099232-2099803	21	9.5	gi|641569469|gb|KK856662.1|	60834-63671	9.1	34
ACYPI001832-RA	gi|646749512|gb|KK962088.1|	113199-115185	18	7.2	gi|641571950|gb|KK855940.1|	12920-13177	10	37
ACYPI001833-RA	gi|646767754|gb|KK961825.1|	791015-797238	20	8.5	gi|641585936|gb|KK854317.1|	405920-406669	9.9	41
ACYPI001838-RA	gi|646772897|gb|KK961753.1|	1270505-1271903	21	8.7	gi|641570648|gb|KK856293.1|	56063-57010	10	21
ACYPI001847-RA	gi|646776904|gb|KK961654.1|	2032228-2033135	21	9.3	gi|641577316|gb|KK854772.1|	132429-134748	9.4	36
ACYPI001849-RA	gi|646746942|gb|KK962254.1|	1029902-1030605	23	9.8	gi|641575482|gb|KK855102.1|	226615-227569	11	39
ACYPI001850-RA	gi|646776184|gb|KK961688.1|	1192707-1192950	21	9.6	gi|641585622|gb|KK854367.1|	355084-355299	9.9	21
ACYPI001856-RA	gi|646776542|gb|KK961668.1|	2151705-2152568	20	5.6	gi|641567104|gb|KK857424.1|	34328-35248	8.5	38
ACYPI001864-RA	gi|646775807|gb|KK961708.1|	1998340-2011589	21	8.6	gi|641588267|gb|KK854008.1|	56074-59831	10	40
ACYPI001866-RA	gi|646776904|gb|KK961654.1|	173568-175042	21	9.3	gi|641586161|gb|KK854280.1|	334992-335313	11	39
ACYPI001871-RA	gi|646767120|gb|KK961840.1|	1179020-1179365	20	8.6	gi|641575372|gb|KK855124.1|	141122-141949	9.5	36
ACYPI001872-RA	gi|646762191|gb|KK961910.1|	388628-389029	18	7.1	gi|641579947|gb|KK854574.1|	141043-145053	11	39
ACYPI001877-RA	gi|646775807|gb|KK961708.1|	1421485-1422075	21	8.6	gi|641568433|gb|KK856978.1|	13215-13417	9.8	35
ACYPI001882-RA	gi|646777495|gb|KK961629.1|	1624028-1624527	19	8.7	gi|641586606|gb|KK854217.1|	197868-198226	11	42
ACYPI001885-RA	gi|646781421|gb|KK961521.1|	4090387-4096150	21	8.7	gi|641570453|gb|KK856351.1|	89554-91937	9.6	36
ACYPI001894-RA	gi|646782043|gb|KK961502.1|	4014346-4016928	20	9.2	gi|641578283|gb|KK854609.1|	101019-103567	12	22
ACYPI001898-RA	gi|646769163|gb|KK961798.1|	1340069-1340325	20	9.2	gi|641576962|gb|KK854829.1|	258359-258833	10	39
ACYPI001901-RA	gi|646747682|gb|KK962200.1|	439756-440162	19	7.1	gi|641585219|gb|KK854428.1|	234339-234736	11	41
ACYPI001906-RA	gi|646746390|gb|KK962292.1|	381151-381395	18	6.4	gi|641574778|gb|KK855246.1|	127737-128035	9.7	32
ACYPI001907-RA	gi|646781421|gb|KK961521.1|	4569597-4570708	21	8.7	gi|641587856|gb|KK854051.1|	799821-806413	10	41
ACYPI001909-RA	gi|646780978|gb|KK961534.1|	537860-538428	20	8	gi|641584931|gb|KK854446.1|	419315-423333	10	39
ACYPI001917-RA	gi|646748129|gb|KK962169.1|	252266-253520	21	9.8	gi|641573102|gb|KK855643.1|	47756-47991	10	37
ACYPI001921-RA	gi|646753606|gb|KK961952.1|	165577-166554	17	6.2	gi|641575568|gb|KK855086.1|	132685-137926	11	40
ACYPI001929-RA	gi|646779006|gb|KK961585.1|	335176-337504	20	9.9	gi|641585383|gb|KK854406.1|	398407-398676	11	37
ACYPI001931-RA	gi|646737056|gb|KK963115.1|	328206-330142	24	11	gi|641574266|gb|KK855361.1|	220391-221769	11	38

ACYPI001932-RA	gi|646779066|gb|KK961583.1|	1089073-1093749	19	7.9	gi|641587577|gb|KK854086.1|	177759-179416	8.2	41
ACYPI001933-RA	gi|646781628|gb|KK961514.1|	2070931-2071637	23	9	gi|641586144|gb|KK854283.1|	436872-437232	10	38
ACYPI001939-RA	gi|646748695|gb|KK962134.1|	130410-130715	20	5.8	gi|641575112|gb|KK855177.1|	202976-203277	11	39
ACYPI001940-RA	gi|646734653|gb|KK963394.1|	162477-164187	20	9.1	gi|641573833|gb|KK855461.1|	156434-156799	9.5	35
ACYPI001943-RA	gi|646666173|gb|KK967831.1|	1079-1592	17	6	gi|641586046|gb|KK854299.1|	149693-151090	10	20
ACYPI001946-RA	gi|646776351|gb|KK961680.1|	2732884-2738800	22	10	gi|641574796|gb|KK855242.1|	122404-122589	10	42
ACYPI001957-RA	gi|646782127|gb|KK961500.1|	1513451-1514131	22	9.6	gi|641587652|gb|KK854077.1|	29390-30777	10	38
ACYPI001971-RA	gi|646768534|gb|KK961809.1|	80081-80507	21	9.8	gi|641584336|gb|KK854489.1|	160220-162495	10	40
ACYPI001975-RA	gi|646749832|gb|KK962071.1|	571544-571896	22	6	gi|641577430|gb|KK854752.1|	66785-67237	9.7	21
ACYPI001976-RA	gi|646780010|gb|KK961559.1|	1362996-1363780	22	10	gi|641575077|gb|KK855184.1|	6836-8863	12	22
ACYPI001978-RA	gi|646780858|gb|KK961538.1|	2482212-2492121	21	10	gi|641585971|gb|KK854310.1|	487537-492226	10	40
ACYPI001980-RA	gi|646777416|gb|KK961632.1|	3855271-3856924	22	8.8	gi|641586246|gb|KK854268.1|	609982-611001	10	38
ACYPI002006-RA	gi|646777665|gb|KK961623.1|	764968-766748	21	9.7	gi|641586096|gb|KK854291.1|	48952-59308	11	46
ACYPI002009-RA	gi|646776024|gb|KK961697.1|	1716286-1718895	22	9.2	gi|641587660|gb|KK854076.1|	582187-582477	9.9	41
ACYPI002010-RA	gi|646780222|gb|KK961554.1|	3556937-3561404	20	9.7	gi|641587606|gb|KK854082.1|	723613-727161	11	40
ACYPI002013-RA	gi|646742343|gb|KK962588.1|	497860-499373	20	9.4	gi|641577493|gb|KK854745.1|	230451-235737	9.6	41
ACYPI002018-RA	gi|646779662|gb|KK961568.1|	1325710-1325937	20	9.3	gi|641577309|gb|KK854773.1|	258141-259272	9.9	39
ACYPI002023-RA	gi|646778736|gb|KK961593.1|	246752-248137	20	7.9	gi|641578109|gb|KK854638.1|	129917-131678	14	46
ACYPI002031-RA	gi|646766703|gb|KK961852.1|	426083-433431	20	7.1	gi|641584248|gb|KK854496.1|	387530-392950	9.9	39
ACYPI002036-RA	gi|646765790|gb|KK961889.1|	1139133-1139597	22	8.8	gi|641588109|gb|KK854023.1|	46746-47033	10	40
ACYPI002040-RA	gi|646732591|gb|KK963652.1|	829-1456	17	7.3	gi|641577704|gb|KK854707.1|	309973-312437	10	38
ACYPI002041-RA	gi|646780723|gb|KK961542.1|	2087042-2087325	21	9.4	gi|641575606|gb|KK855079.1|	166553-168521	9.7	40
ACYPI002045-RA	gi|646748044|gb|KK962175.1|	489317-490870	19	7.2	gi|641573171|gb|KK855626.1|	172175-173913	9.9	40
ACYPI002053-RA	gi|646778336|gb|KK961604.1|	135008-135899	21	10	gi|641574746|gb|KK855253.1|	51029-51395	8.9	35
ACYPI002063-RA	gi|646743341|gb|KK962511.1|	304597-306994	19	5	gi|641586059|gb|KK854297.1|	8732-9791	11	22
ACYPI002068-RA	gi|646748785|gb|KK962129.1|	480261-480456	20	8.2	gi|641576176|gb|KK854968.1|	162735-163305	11	45
ACYPI002072-RA	gi|646775471|gb|KK961725.1|	1366350-1369116	20	10	gi|641567321|gb|KK857348.1|	83512-86810	19	43
ACYPI002078-RA	gi|646782288|gb|KK961496.1|	7401554-7401985	21	9.7	gi|641573906|gb|KK855442.1|	121065-121374	11	39
ACYPI002085-RA	gi|646782168|gb|KK961499.1|	4967801-4968081	21	9.4	gi|641573447|gb|KK855557.1|	130621-130914	10	41
ACYPI002088-RA	gi|646780827|gb|KK961539.1|	3053298-3053810	20	9.4	gi|641587555|gb|KK854089.1|	904242-904931	11	39
ACYPI002090-RA	gi|646742553|gb|KK962571.1|	233499-234167	20	8.4	gi|641585103|gb|KK854436.1|	220753-221263	9.8	39
ACYPI002098-RA	gi|646743690|gb|KK962487.1|	21568-23753	20	10	gi|641587783|gb|KK854060.1|	755591-757203	12	40

ACYPI002108-RA	gi|646544887|gb|KK987173.1|	256-865	14	6	gi|641579960|gb|KK854572.1|	217862-218262	11	40
ACYPI002110-RA	gi|646741767|gb|KK962638.1|	332202-334645	21	10	gi|641586344|gb|KK854254.1|	531825-532943	10	40
ACYPI002115-RA	gi|646781772|gb|KK961510.1|	3755720-3755980	20	9.2	gi|641571581|gb|KK856030.1|	199916-200333	10	38
ACYPI002118-RA	gi|646757279|gb|KK961927.1|	173582-176071	21	8.6	gi|641587118|gb|KK854142.1|	526922-527972	11	40
ACYPI002122-RA	gi|646752143|gb|KK961972.1|	558684-559089	20	5.6	gi|641578325|gb|KK854602.1|	224959-228435	9.7	38
ACYPI002123-RA	gi|646778903|gb|KK961588.1|	3490396-3493151	23	10	gi|641569294|gb|KK856712.1|	32021-32253	12	22
ACYPI002125-RA	gi|646694101|gb|KK966473.1|	17275-17620	12	6.2	gi|641571778|gb|KK855982.1|	86940-89717	11	43
ACYPI002126-RA	gi|646748580|gb|KK962141.1|	389025-389658	20	5.6	gi|641573850|gb|KK855457.1|	27538-27780	9.3	34
ACYPI002129-RA	gi|646779826|gb|KK961564.1|	3352189-3353287	22	9.6	gi|641573673|gb|KK855501.1|	127225-131973	14	43
ACYPI002132-RA	gi|646754371|gb|KK961945.1|	163236-163760	20	9.7	gi|641574800|gb|KK855241.1|	134771-135213	10	37
ACYPI002133-RA	gi|646776069|gb|KK961694.1|	774424-775731	18	7.8	gi|641588258|gb|KK854009.1|	573053-577471	11	36
ACYPI002136-RA	gi|646747263|gb|KK962232.1|	106956-107147	18	8.6	gi|641551804|gb|KK864267.1|	21-577	9.4	19
ACYPI002137-RA	gi|646762191|gb|KK961910.1|	707040-707591	18	7.1	gi|641578188|gb|KK854626.1|	233875-234121	9.9	39
ACYPI002140-RA	gi|646751515|gb|KK961987.1|	747600-750552	20	9.2	gi|641566693|gb|KK857568.1|	43804-44746	8.5	33
ACYPI002147-RA	gi|646751160|gb|KK962003.1|	28515-36721	22	10	gi|641588136|gb|KK854020.1|	159940-160750	11	36
ACYPI002151-RA	gi|646750043|gb|KK962060.1|	110912-111244	21	8.3	gi|641587111|gb|KK854143.1|	614875-615148	11	40
ACYPI002154-RA	gi|646781443|gb|KK961520.1|	2193431-2194215	20	8.3	gi|641574127|gb|KK855390.1|	145750-147385	10	40
ACYPI002162-RA	gi|646777474|gb|KK961630.1|	1317614-1318058	21	9.3	gi|641577810|gb|KK854690.1|	445973-446254	9.8	39
ACYPI002168-RA	gi|646749482|gb|KK962090.1|	71506-74816	19	7.1	gi|641586898|gb|KK854174.1|	3238-3802	11	39
ACYPI002176-RA	gi|646782276|gb|KK961497.1|	3306429-3309782	21	9.7	gi|641584743|gb|KK854459.1|	293612-293844	11	36
ACYPI002179-RA	gi|646748785|gb|KK962129.1|	563086-564614	20	8.2	gi|641586824|gb|KK854186.1|	78363-81899	10	41
ACYPI002180-RA	gi|646757520|gb|KK961926.1|	1451150-1454207	20	7.4	gi|641586085|gb|KK854293.1|	110059-112331	11	40
ACYPI002195-RA	gi|646780889|gb|KK961537.1|	3524624-3525874	22	10	gi|641557275|gb|KK861674.1|	7336-7773	11	31
ACYPI002199-RA	gi|646733146|gb|KK963575.1|	157915-158896	48	23	gi|641586470|gb|KK854237.1|	65502-66236	11	42
ACYPI002207-RA	gi|646746812|gb|KK962263.1|	426345-426857	16	6.1	gi|641585860|gb|KK854329.1|	450517-451101	11	43
ACYPI002210-RA	gi|646743492|gb|KK962499.1|	216677-216921	24	11	gi|641577843|gb|KK854684.1|	157252-158319	10	40
ACYPI002214-RA	gi|646775723|gb|KK961712.1|	2818021-2819224	21	9.5	gi|641576661|gb|KK854882.1|	43498-45593	9.7	38
ACYPI002220-RA	gi|646776929|gb|KK961653.1|	559623-564080	17	8.5	gi|641573291|gb|KK855599.1|	83359-88306	9	36
ACYPI002221-RA	gi|646661798|gb|KK967966.1|	14154-14471	12	5.3	gi|641575759|gb|KK855050.1|	309492-311149	11	38
ACYPI002228-RA	gi|646781849|gb|KK961508.1|	3597630-3599923	17	8	gi|641576635|gb|KK854887.1|	370804-371232	11	40
ACYPI002231-RA	gi|646746040|gb|KK962316.1|	185966-191202	19	6.9	gi|641587606|gb|KK854082.1|	770529-770871	11	40
ACYPI002245-RA	gi|646747263|gb|KK962232.1|	581179-583917	18	8.6	gi|641587452|gb|KK854101.1|	907055-911734	11	40

ACYPI002247-RA	gi|646782334|gb|KK961495.1|	2443819-2444685	21	9	gi|641587066|gb|KK854149.1|	557889-558485	11	40
ACYPI002255-RA	gi|646747574|gb|KK962208.1|	342225-343615	27	10	gi|641566509|gb|KK857636.1|	55555-60703	11	40
ACYPI002256-RA	gi|646754962|gb|KK961940.1|	622144-622441	21	10	gi|641587905|gb|KK854046.1|	131767-132011	10	43
ACYPI002267-RA	gi|646740015|gb|KK962820.1|	299758-300400	20	8.4	gi|641585407|gb|KK854403.1|	362730-363535	9.4	42
ACYPI002275-RA	gi|646778186|gb|KK961609.1|	2486068-2492649	19	9.4	gi|641586040|gb|KK854300.1|	316250-316547	10	38
ACYPI002277-RA	gi|646745186|gb|KK962377.1|	310796-311591	21	9.8	gi|641575910|gb|KK855021.1|	334264-334789	10	35
ACYPI002282-RA	gi|646748185|gb|KK962165.1|	903399-903668	22	9.9	gi|641577905|gb|KK854672.1|	26687-27132	10	37
ACYPI002284-RA	gi|646767265|gb|KK961836.1|	1758940-1759768	22	9.9	gi|641585796|gb|KK854338.1|	374616-375110	11	43
ACYPI002286-RA	gi|646782334|gb|KK961495.1|	4606095-4606597	21	9	gi|641575730|gb|KK855054.1|	33871-36374	10	38
ACYPI002287-RA	gi|646775723|gb|KK961712.1|	2017770-2027262	21	9.5	gi|641581789|gb|KK854549.1|	164122-165413	9.9	37
ACYPI002289-RA	gi|646781243|gb|KK961526.1|	3286934-3287361	21	8.9	gi|641572501|gb|KK855798.1|	19130-19898	9.8	39
ACYPI002292-RA	gi|646781536|gb|KK961517.1|	1691228-1691490	19	7.7	gi|641572663|gb|KK855756.1|	101017-101788	8.8	38
ACYPI002296-RA	gi|646776184|gb|KK961688.1|	1862925-1863193	21	9.6	gi|641586809|gb|KK854188.1|	719109-720930	10	43
ACYPI002300-RA	gi|646781732|gb|KK961511.1|	2969307-2971241	22	9.8	gi|641574000|gb|KK855418.1|	122043-122918	10	36
ACYPI002301-RA	gi|646781043|gb|KK961532.1|	2294310-2296497	26	11	gi|641577837|gb|KK854685.1|	140266-141222	11	42
ACYPI002304-RA	gi|646732431|gb|KK963675.1|	282660-283322	24	9.6	gi|641574370|gb|KK855338.1|	45406-46646	11	38
ACYPI002306-RA	gi|646768717|gb|KK961805.1|	531833-532379	20	8	gi|641588236|gb|KK854011.1|	290015-292668	9.7	38
ACYPI002312-RA	gi|646744453|gb|KK962431.1|	85913-86210	19	8.5	gi|641577309|gb|KK854773.1|	220905-224083	9.9	39
ACYPI002331-RA	gi|646775821|gb|KK961707.1|	2916713-2917034	23	9.1	gi|641586066|gb|KK854296.1|	419206-419503	9.4	37
ACYPI002332-RA	gi|646777842|gb|KK961618.1|	2091230-2091867	19	8.9	gi|641574015|gb|KK855416.1|	62453-64837	10	42
ACYPI002342-RA	gi|646778767|gb|KK961592.1|	1756085-1757360	21	5.4	gi|641543394|gb|KK868292.1|	46-1963	14	56
ACYPI002346-RA	gi|646776647|gb|KK961663.1|	558292-562116	23	9.2	gi|641569577|gb|KK856628.1|	54946-58205	9.7	36
ACYPI002350-RA	gi|646776456|gb|KK961674.1|	1366641-1367070	18	7.9	gi|641585953|gb|KK854314.1|	337257-337903	11	43
ACYPI002352-RA	gi|646741656|gb|KK962648.1|	158226-160455	24	9.7	gi|641570717|gb|KK856273.1|	82511-84071	10	36
ACYPI002361-RA	gi|646747884|gb|KK962186.1|	1107153-1108272	22	11	gi|641586661|gb|KK854209.1|	542012-542355	11	43
ACYPI002366-RA	gi|646775867|gb|KK961705.1|	2574033-2575096	23	10	gi|641584517|gb|KK854476.1|	382470-382884	10	39
ACYPI002367-RA	gi|646739361|gb|KK962890.1|	380529-382111	22	9.6	gi|641586606|gb|KK854217.1|	373368-377954	11	42
ACYPI002371-RA	gi|646753093|gb|KK961958.1|	989781-990542	21	9.3	gi|641585953|gb|KK854314.1|	466140-466639	11	43
ACYPI002372-RA	gi|646745631|gb|KK962343.1|	6096-6581	18	8.6	gi|641586025|gb|KK854302.1|	108939-110759	8.5	33
ACYPI002382-RA	gi|646768631|gb|KK961807.1|	1304186-1308383	19	9.2	gi|641586975|gb|KK854163.1|	193402-195937	11	40
ACYPI002383-RA	gi|646747046|gb|KK962247.1|	156046-156236	22	9	gi|641577837|gb|KK854685.1|	435056-435918	11	42
ACYPI002386-RA	gi|646767697|gb|KK961826.1|	1371153-1377119	21	9.8	gi|641587636|gb|KK854079.1|	649757-651319	11	21

ACYPI002389-RA	gi|646772045|gb|KK961761.1|	784801-787815	21	9.6	gi|641569574|gb|KK856629.1|	64067-64549	24	120
ACYPI002393-RA	gi|646779898|gb|KK961562.1|	1093463-1094224	19	9.3	gi|641586599|gb|KK854218.1|	652002-654779	11	42
ACYPI002399-RA	gi|646727841|gb|KK964291.1|	34876-36588	23	9.6	gi|641576260|gb|KK854950.1|	131708-131943	10	39
ACYPI002404-RA	gi|646776351|gb|KK961680.1|	1844763-1849085	22	10	gi|641575236|gb|KK855151.1|	365826-371792	11	38
ACYPI002405-RA	gi|646778903|gb|KK961588.1|	3353930-3360933	23	10	gi|641570598|gb|KK856307.1|	53749-55137	11	21
ACYPI002411-RA	gi|646778948|gb|KK961587.1|	1260121-1261019	20	7.3	gi|641572569|gb|KK855781.1|	89141-89669	11	39
ACYPI002414-RA	gi|646776024|gb|KK961697.1|	2255158-2255460	22	9.2	gi|641587962|gb|KK854039.1|	1140500-1140989	11	40
ACYPI002433-RA	gi|646740304|gb|KK962788.1|	91622-92181	24	9.6	gi|641577215|gb|KK854787.1|	126190-127217	11	40
ACYPI002438-RA	gi|646747046|gb|KK962247.1|	52390-52694	22	9	gi|641576161|gb|KK854972.1|	358809-359708	10	36
ACYPI002441-RA	gi|646781344|gb|KK961523.1|	875002-876772	21	8.9	gi|641575709|gb|KK855059.1|	111571-113002	11	41
ACYPI002445-RA	gi|646777065|gb|KK961647.1|	779402-779706	20	7.9	gi|641572891|gb|KK855695.1|	187955-188980	10	36
ACYPI002446-RA	gi|646781443|gb|KK961520.1|	1420254-1420972	20	8.3	gi|641584305|gb|KK854491.1|	74908-75107	11	39
ACYPI002447-RA	gi|646735753|gb|KK963262.1|	170013-170528	19	7.6	gi|641571224|gb|KK856127.1|	70376-70851	10	21
ACYPI002448-RA	gi|646777108|gb|KK961645.1|	288723-288916	17	6.5	gi|641578336|gb|KK854600.1|	139042-139994	10	21
ACYPI002456-RA	gi|646782276|gb|KK961497.1|	6277666-6282973	21	9.7	gi|641575939|gb|KK855017.1|	189691-190597	9.9	41
ACYPI002460-RA	gi|646781379|gb|KK961522.1|	3095925-3097440	22	7.6	gi|641588267|gb|KK854008.1|	610513-612092	10	40
ACYPI002469-RA	gi|646781849|gb|KK961508.1|	2909704-2910735	17	8	gi|641581789|gb|KK854549.1|	158038-159096	9.9	37
ACYPI002470-RA	gi|646765880|gb|KK961885.1|	1016510-1017864	23	10	gi|641548005|gb|KK866068.1|	4-704	9.8	29
ACYPI002478-RA	gi|646777474|gb|KK961630.1|	2237752-2244425	21	9.3	gi|641569892|gb|KK856527.1|	115717-117174	9.5	20
ACYPI002479-RA	gi|646742343|gb|KK962588.1|	510380-511704	20	9.4	gi|641573447|gb|KK855557.1|	256045-256264	10	41
ACYPI002480-RA	gi|646781628|gb|KK961514.1|	4812629-4814190	23	9	gi|641575396|gb|KK855118.1|	163763-164723	9	35
ACYPI002482-RA	gi|646768294|gb|KK961814.1|	1081772-1083971	23	10	gi|641568172|gb|KK857061.1|	74471-75829	11	40
ACYPI002483-RA	gi|646620535|gb|KK972589.1|	760-1021	12	6.3	gi|641576371|gb|KK854931.1|	107823-109660	10	38
ACYPI002484-RA	gi|646775807|gb|KK961708.1|	1741426-1742458	21	8.6	gi|641572695|gb|KK855748.1|	134737-135842	9.7	32
ACYPI002491-RA	gi|646779006|gb|KK961585.1|	155446-157105	20	9.9	gi|641586928|gb|KK854169.1|	315058-320740	11	39
ACYPI002506-RA	gi|646776998|gb|KK961650.1|	155617-156732	20	9.6	gi|641587186|gb|KK854133.1|	441470-442956	8.1	39
ACYPI002517-RA	gi|646732914|gb|KK963609.1|	82774-83849	21	5.6	gi|641586452|gb|KK854239.1|	426678-427305	10	19
ACYPI002522-RA	gi|646775312|gb|KK961734.1|	1168349-1168544	17	7.9	gi|641586476|gb|KK854236.1|	499691-501894	10	37
ACYPI002523-RA	gi|646775789|gb|KK961709.1|	1212365-1212849	20	9.6	gi|641570299|gb|KK856396.1|	67159-70547	10	33
ACYPI002524-RA	gi|646757520|gb|KK961926.1|	1355277-1356642	20	7.4	gi|641570745|gb|KK856264.1|	12187-12463	9.3	18
ACYPI002525-RA	gi|646745434|gb|KK962359.1|	90096-90372	22	10	gi|641588267|gb|KK854008.1|	270855-271704	10	40
ACYPI002526-RA	gi|646771951|gb|KK961762.1|	675959-678991	21	8.3	gi|641571092|gb|KK856162.1|	64503-67034	11	47

ACYPI002527-RA	gi|646780010|gb|KK961559.1|	2231739-2236919	22	10	gi|641582439|gb|KK854536.1|	109026-110642	11	36
ACYPI002528-RA	gi|646769971|gb|KK961785.1|	42700-47094	13	7	gi|641573514|gb|KK855540.1|	168024-168615	9.6	37
ACYPI002529-RA	gi|646781628|gb|KK961514.1|	5029207-5029661	23	9	gi|641585004|gb|KK854441.1|	11004-11710	11	43
ACYPI002530-RA	gi|646746355|gb|KK962294.1|	143486-150254	22	10	gi|641588073|gb|KK854027.1|	832591-839359	11	41
ACYPI002533-RA	gi|646772358|gb|KK961758.1|	1874503-1876562	20	9.3	gi|641572971|gb|KK855675.1|	185833-186008	9.7	20
ACYPI002536-RA	gi|646552905|gb|KK985647.1|	3557-3784	13	5.5	gi|641570016|gb|KK856486.1|	44031-44627	12	20
ACYPI002537-RA	gi|646778186|gb|KK961609.1|	1713715-1714684	19	9.4	gi|641577766|gb|KK854698.1|	24598-26758	9.6	28
ACYPI002538-RA	gi|646771448|gb|KK961767.1|	1433164-1434556	21	9.8	gi|641584694|gb|KK854463.1|	130845-133454	9.3	36
ACYPI002549-RA	gi|646769774|gb|KK961788.1|	651574-654039	20	9.2	gi|641569328|gb|KK856702.1|	97741-98120	9.6	17
ACYPI002557-RA	gi|646779826|gb|KK961564.1|	2711260-2712177	22	9.6	gi|641564032|gb|KK858612.1|	30385-31160	10	35
ACYPI002571-RA	gi|646776588|gb|KK961666.1|	2429769-2431628	20	5.8	gi|641578198|gb|KK854624.1|	210957-212575	12	40
ACYPI002575-RA	gi|646773423|gb|KK961749.1|	931332-931815	19	8.1	gi|641585778|gb|KK854341.1|	154907-155205	9.7	41
ACYPI002579-RA	gi|646782168|gb|KK961499.1|	1184464-1185191	21	9.4	gi|641577511|gb|KK854741.1|	221813-223353	11	38
ACYPI002580-RA	gi|646778448|gb|KK961601.1|	1850032-1850320	19	8.3	gi|641588224|gb|KK854012.1|	349291-350387	10	41
ACYPI002584-RA	gi|646777991|gb|KK961614.1|	1689943-1690781	18	7.8	gi|641588060|gb|KK854028.1|	119261-121195	11	42
ACYPI002592-RA	gi|646776588|gb|KK961666.1|	2333642-2334573	20	5.8	gi|641588082|gb|KK854026.1|	1158045-1162963	11	41
ACYPI002593-RA	gi|646741057|gb|KK962701.1|	332940-333300	21	8.8	gi|641574085|gb|KK855399.1|	212843-213938	10	38
ACYPI002595-RA	gi|646778865|gb|KK961589.1|	2945518-2946005	20	9.2	gi|641588099|gb|KK854024.1|	270490-272616	11	39
ACYPI002598-RA	gi|646782276|gb|KK961497.1|	7381319-7382434	21	9.7	gi|641571116|gb|KK856157.1|	45423-45827	11	40
ACYPI002607-RA	gi|646748185|gb|KK962165.1|	508948-509492	22	9.9	gi|641558453|gb|KK861114.1|	90-814	9.8	35
ACYPI002609-RA	gi|646781118|gb|KK961530.1|	4577873-4595754	21	10	gi|641586328|gb|KK854257.1|	194646-198620	8.4	35
ACYPI002612-RA	gi|646779298|gb|KK961577.1|	2973102-2974775	20	9.1	gi|641587660|gb|KK854076.1|	251715-252351	9.9	41
ACYPI002620-RA	gi|646771845|gb|KK961763.1|	1420337-1420635	23	10	gi|641585690|gb|KK854356.1|	352539-352939	9.6	41
ACYPI002622-RA	gi|646740073|gb|KK962814.1|	61569-62315	18	8.3	gi|641573906|gb|KK855442.1|	34478-35829	11	39
ACYPI002624-RA	gi|646769275|gb|KK961796.1|	1970018-1975802	22	5.6	gi|641586121|gb|KK854287.1|	11548-12191	11	43
ACYPI002632-RA	gi|646781772|gb|KK961510.1|	3935410-3935647	20	9.2	gi|641584835|gb|KK854453.1|	238627-239249	8.3	36
ACYPI002636-RA	gi|646762191|gb|KK961910.1|	768673-769023	18	7.1	gi|641586114|gb|KK854288.1|	365430-366260	10	41
ACYPI002637-RA	gi|646776490|gb|KK961672.1|	975484-976258	20	10	gi|641575337|gb|KK855131.1|	94757-95831	10	37
ACYPI002648-RA	gi|646780723|gb|KK961542.1|	1663828-1664213	21	9.4	gi|641587396|gb|KK854108.1|	442344-442716	11	22
ACYPI002650-RA	gi|646776490|gb|KK961672.1|	58431-63884	20	10	gi|641570459|gb|KK856349.1|	25114-26078	9.7	40
ACYPI002653-RA	gi|646782087|gb|KK961501.1|	1763854-1768289	20	8.7	gi|641571270|gb|KK856115.1|	162152-162527	11	38
ACYPI002656-RA	gi|646779337|gb|KK961576.1|	1223498-1223747	18	9	gi|641573950|gb|KK855430.1|	123007-123322	10	19

ACYPI002657-RA	gi|646775842|gb|KK961706.1|	1190141-1201107	22	9	gi|641584818|gb|KK854454.1|	146906-149054	11	42
ACYPI002661-RA	gi|646753339|gb|KK961955.1|	164779-165150	22	11	gi|641577511|gb|KK854741.1|	345833-346642	11	38
ACYPI002662-RA	gi|646767230|gb|KK961837.1|	325845-326129	19	5.4	gi|641587840|gb|KK854053.1|	178836-179852	11	43
ACYPI002674-RA	gi|646747754|gb|KK962195.1|	713736-714702	21	5.3	gi|641575218|gb|KK855155.1|	85984-86668	11	21
ACYPI002678-RA	gi|646741342|gb|KK962675.1|	216047-219286	18	7.3	gi|641584913|gb|KK854447.1|	63574-68626	10	40
ACYPI002680-RA	gi|646780858|gb|KK961538.1|	3841595-3842102	21	10	gi|641566502|gb|KK857638.1|	33820-37686	11	36
ACYPI002684-RA	gi|646778736|gb|KK961593.1|	1747586-1748173	20	7.9	gi|641588213|gb|KK854013.1|	1386491-1387144	9.6	39
ACYPI002689-RA	gi|646749794|gb|KK962073.1|	646060-646346	20	8.4	gi|641568719|gb|KK856888.1|	91364-91954	11	44
ACYPI002692-RA	gi|646777288|gb|KK961638.1|	837652-843808	22	6.1	gi|641577922|gb|KK854669.1|	71346-76254	12	21
ACYPI002694-RA	gi|646776608|gb|KK961665.1|	422260-422707	23	9.3	gi|641584694|gb|KK854463.1|	82732-83252	9.3	36
ACYPI002695-RA	gi|646736855|gb|KK963138.1|	62509-63107	19	9.1	gi|641587930|gb|KK854043.1|	319793-320387	10	41
ACYPI002698-RA	gi|646779375|gb|KK961575.1|	575664-577130	22	9.3	gi|641573397|gb|KK855571.1|	81962-87577	10	38
ACYPI002711-RA	gi|646766056|gb|KK961877.1|	361125-362060	18	7.8	gi|641587652|gb|KK854077.1|	510488-512305	10	38
ACYPI002730-RA	gi|646781873|gb|KK961507.1|	1771011-1773062	20	9.1	gi|641583921|gb|KK854515.1|	196713-197385	11	38
ACYPI002732-RA	gi|646734527|gb|KK963409.1|	29706-30189	21	9.3	gi|641587896|gb|KK854047.1|	1016138-1016437	9.9	39
ACYPI002733-RA	gi|646780752|gb|KK961541.1|	1283544-1285555	21	8	gi|641585801|gb|KK854337.1|	540417-540788	10	38
ACYPI002737-RA	gi|646771352|gb|KK961768.1|	127433-127823	21	9.6	gi|641575047|gb|KK855190.1|	207623-208765	8.3	43
ACYPI002741-RA	gi|646748185|gb|KK962165.1|	936983-937496	22	9.9	gi|641577905|gb|KK854672.1|	31310-31567	10	37
ACYPI002748-RA	gi|646766426|gb|KK961862.1|	977494-977745	20	7.1	gi|641572912|gb|KK855690.1|	37389-37955	9.9	38
ACYPI002749-RA	gi|646759277|gb|KK961919.1|	522422-523257	16	7.2	gi|641586741|gb|KK854197.1|	586117-588546	11	43
ACYPI002754-RA	gi|646766598|gb|KK961856.1|	687987-692659	23	10	gi|641585434|gb|KK854398.1|	151564-155764	13	51
ACYPI002756-RA	gi|646750061|gb|KK962059.1|	505248-505774	15	7.1	gi|641577075|gb|KK854811.1|	206259-207414	11	36
ACYPI002757-RA	gi|646782127|gb|KK961500.1|	3859457-3862296	22	9.6	gi|641586399|gb|KK854247.1|	463583-464709	15	68
ACYPI002758-RA	gi|646767948|gb|KK961821.1|	494674-495316	17	7.1	gi|641585647|gb|KK854363.1|	258208-258851	8.8	35
ACYPI002759-RA	gi|646777570|gb|KK961626.1|	439823-443677	18	7.5	gi|641559751|gb|KK860512.1|	105-665	9.8	31
ACYPI002787-RA	gi|646777698|gb|KK961622.1|	893775-894467	16	7.6	gi|641573571|gb|KK855526.1|	157932-158244	9.8	36
ACYPI002789-RA	gi|646775488|gb|KK961724.1|	354096-355626	19	7.8	gi|641576677|gb|KK854879.1|	55802-57216	11	39
ACYPI002791-RA	gi|646738812|gb|KK962939.1|	260051-260397	20	8.1	gi|641572687|gb|KK855750.1|	42845-43279	9.9	39
ACYPI002792-RA	gi|646782288|gb|KK961496.1|	506752-507969	21	9.7	gi|641588294|gb|KK854006.1|	877789-879358	8.7	40
ACYPI002794-RA	gi|646775558|gb|KK961720.1|	1695082-1695359	20	9.4	gi|641577890|gb|KK854675.1|	176777-177280	10	40
ACYPI002798-RA	gi|646776514|gb|KK961670.1|	1699883-1700329	20	9.6	gi|641585833|gb|KK854332.1|	319838-320301	10	37
ACYPI002799-RA	gi|646780827|gb|KK961539.1|	3536479-3537212	20	9.4	gi|641586777|gb|KK854192.1|	486323-491448	11	30

ACYPI002801-RA	gi|646748888|gb|KK962123.1|	518666-520320	20	8.1	gi|641575730|gb|KK855054.1|	104007-104683	10	38
ACYPI002806-RA	gi|646781659|gb|KK961513.1|	6033433-6036104	22	9.8	gi|641588325|gb|KK854004.1|	993003-993701	11	40
ACYPI002808-RA	gi|646776647|gb|KK961663.1|	2271830-2274478	23	9.2	gi|641585562|gb|KK854377.1|	403729-405181	12	47
ACYPI002810-RA	gi|646745372|gb|KK962364.1|	894991-895620	23	10	gi|641578122|gb|KK854636.1|	406306-410622	12	42
ACYPI002812-RA	gi|646743886|gb|KK962473.1|	438766-439070	21	9.3	gi|641588033|gb|KK854031.1|	574025-574677	11	38
ACYPI002820-RA	gi|646770114|gb|KK961783.1|	1572054-1572635	19	9.7	gi|641584433|gb|KK854482.1|	452935-453537	10	40
ACYPI002830-RA	gi|646745959|gb|KK962321.1|	319481-325472	20	6.9	gi|641586654|gb|KK854210.1|	143155-144913	11	40
ACYPI002835-RA	gi|646778983|gb|KK961586.1|	1354364-1354928	20	9.9	gi|641588024|gb|KK854032.1|	480266-482661	9.7	41
ACYPI002837-RA	gi|646781183|gb|KK961528.1|	3597736-3600845	20	9.5	gi|641571749|gb|KK855991.1|	43843-48288	9.4	35
ACYPI002839-RA	gi|646737853|gb|KK963036.1|	137035-137669	42	18	gi|641574123|gb|KK855391.1|	127477-128566	9.9	39
ACYPI002840-RA	gi|646768361|gb|KK961813.1|	448676-449914	15	7.4	gi|641567062|gb|KK857438.1|	35453-36137	9.8	36
ACYPI002841-RA	gi|646781628|gb|KK961514.1|	230220-232285	23	9	gi|641570579|gb|KK856313.1|	43225-43878	11	38
ACYPI002842-RA	gi|646776514|gb|KK961670.1|	810483-811318	20	9.6	gi|641588144|gb|KK854019.1|	1014450-101467[illegible]	11	49
ACYPI002846-RA	gi|646780010|gb|KK961559.1|	2614772-2615721	22	10	gi|641572304|gb|KK855847.1|	137732-137911	11	40
ACYPI002850-RA	gi|646781873|gb|KK961507.1|	1603623-1604415	20	9.1	gi|641586809|gb|KK854188.1|	610368-610773	10	43
ACYPI002864-RA	gi|646779141|gb|KK961581.1|	202009-202244	21	9.1	gi|641588090|gb|KK854025.1|	968908-969667	11	43
ACYPI002865-RA	gi|646775258|gb|KK961737.1|	657982-658236	19	9.8	gi|641575698|gb|KK855061.1|	347810-348045	10	39
ACYPI002866-RA	gi|646742413|gb|KK962582.1|	575185-579755	23	10	gi|641577535|gb|KK854737.1|	164637-168925	11	42
ACYPI002870-RA	gi|646781421|gb|KK961521.1|	1408603-1410559	21	8.7	gi|641588073|gb|KK854027.1|	1075964-107625[illegible]	11	41
ACYPI002882-RA	gi|646768582|gb|KK961808.1|	235534-235766	21	6	gi|641577879|gb|KK854677.1|	244326-244779	18	36
ACYPI002892-RA	gi|646776514|gb|KK961670.1|	1335911-1337468	20	9.6	gi|641587052|gb|KK854150.1|	336155-341282	11	38
ACYPI002900-RA	gi|646778983|gb|KK961586.1|	3162080-3163259	20	9.9	gi|641570355|gb|KK856378.1|	62186-63841	9.7	35
ACYPI002904-RA	gi|646767265|gb|KK961836.1|	537573-538196	22	9.9	gi|641575001|gb|KK855197.1|	323997-326642	11	38
ACYPI002907-RA	gi|646740619|gb|KK962751.1|	73767-74102	21	9.4	gi|641575635|gb|KK855074.1|	296164-299267	9.6	38
ACYPI002909-RA	gi|646778213|gb|KK961608.1|	1363971-1371045	19	8.8	gi|641584128|gb|KK854505.1|	344074-345012	9.6	37
ACYPI002917-RA	gi|646765725|gb|KK961892.1|	1523199-1523978	20	11	gi|641579192|gb|KK854588.1|	415176-415718	11	41
ACYPI002919-RA	gi|646743302|gb|KK962514.1|	36000-37996	23	8.4	gi|641577161|gb|KK854797.1|	27805-30295	10	38
ACYPI002925-RA	gi|646769275|gb|KK961796.1|	2835489-2835752	22	5.6	gi|641586688|gb|KK854205.1|	241435-243061	11	42
ACYPI002926-RA	gi|646782357|gb|KK961494.1|	2221556-2228635	21	9.2	gi|641576906|gb|KK854838.1|	62330-62535	9.4	37
ACYPI002929-RA	gi|646775558|gb|KK961720.1|	1592801-1594423	20	9.4	gi|641585395|gb|KK854405.1|	504492-505497	10	38
ACYPI002940-RA	gi|646778865|gb|KK961589.1|	2989579-2990789	20	9.2	gi|641567011|gb|KK857455.1|	7380-11731	11	40
ACYPI002948-RA	gi|646780147|gb|KK961556.1|	1745288-1745969	18	7.5	gi|641587774|gb|KK854061.1|	763926-764194	9.1	40

ACYPI002949-RA	gi|646750869|gb|KK962018.1|	100741-103753	20	10	gi|641577772|gb|KK854697.1|	198093-198437	10	39
ACYPI002950-RA	gi|646743492|gb|KK962499.1|	378863-379166	24	11	gi|641575837|gb|KK855035.1|	18536-19049	11	46
ACYPI002951-RA	gi|646737822|gb|KK963039.1|	15832-16397	20	9	gi|641575140|gb|KK855171.1|	304579-306334	9.7	40
ACYPI002952-RA	gi|646551326|gb|KK985982.1|	7703-8114	13	5.2	gi|641586817|gb|KK854187.1|	607698-610264	9.3	39
ACYPI002953-RA	gi|646779826|gb|KK961564.1|	3221840-3223314	22	9.6	gi|641588051|gb|KK854029.1|	801660-805013	9.6	40
ACYPI002959-RA	gi|646771845|gb|KK961763.1|	2098996-2107545	23	10	gi|641577946|gb|KK854664.1|	359871-362258	10	38
ACYPI002963-RA	gi|646756446|gb|KK961931.1|	620092-621178	14	6.1	gi|641576371|gb|KK854931.1|	89365-90459	10	38
ACYPI002966-RA	gi|646743944|gb|KK962469.1|	138015-139156	20	9.1	gi|641573177|gb|KK855625.1|	76394-77382	9.4	34
ACYPI002973-RA	gi|646780978|gb|KK961534.1|	307070-310049	20	8	gi|641587783|gb|KK854060.1|	919533-920086	12	40
ACYPI002979-RA	gi|646750600|gb|KK962030.1|	995287-1000766	23	8.6	gi|641574085|gb|KK855399.1|	126284-127324	10	38
ACYPI002986-RA	gi|646782334|gb|KK961495.1|	1818705-1819833	21	9	gi|641586793|gb|KK854190.1|	528114-528792	9.3	41
ACYPI002987-RA	gi|646743352|gb|KK962510.1|	516250-516623	20	5.4	gi|641586139|gb|KK854284.1|	193375-194388	10	21
ACYPI002988-RA	gi|646781873|gb|KK961507.1|	1104412-1104703	20	9.1	gi|641587580|gb|KK854085.1|	94835-95557	10	39
ACYPI002989-RA	gi|646780827|gb|KK961539.1|	408655-410609	20	9.4	gi|641587249|gb|KK854125.1|	527730-530900	11	37
ACYPI002998-RA	gi|646776477|gb|KK961673.1|	991452-991683	22	9	gi|641586066|gb|KK854296.1|	379544-380222	9.4	37
ACYPI003002-RA	gi|646781379|gb|KK961522.1|	572075-572243	22	7.6	gi|641564796|gb|KK858283.1|	4757-4996	10	39
ACYPI003006-RA	gi|646751515|gb|KK961987.1|	890582-892922	20	9.2	gi|641585778|gb|KK854341.1|	257012-257385	9.7	41
ACYPI003015-RA	gi|646748955|gb|KK962119.1|	38161-39417	19	9.9	gi|641576109|gb|KK854982.1|	251293-251986	10	19
ACYPI003020-RA	gi|646767439|gb|KK961832.1|	2087751-2088810	23	10	gi|641579930|gb|KK854578.1|	122293-122608	9.1	35
ACYPI003025-RA	gi|646781536|gb|KK961517.1|	1964504-1964791	19	7.7	gi|641578020|gb|KK854653.1|	231920-232190	10	38
ACYPI003027-RA	gi|646778112|gb|KK961611.1|	1992412-1998546	22	10	gi|641585767|gb|KK854343.1|	274160-278277	11	22
ACYPI003031-RA	gi|646763535|gb|KK961902.1|	417537-418658	23	10	gi|641572022|gb|KK855920.1|	148707-149204	12	42
ACYPI003033-RA	gi|646746706|gb|KK962270.1|	290887-291265	23	11	gi|641569948|gb|KK856508.1|	78420-79986	10	37
ACYPI003042-RA	gi|646781732|gb|KK961511.1|	3714803-3715407	22	9.8	gi|641566429|gb|KK857664.1|	17082-18381	12	37
ACYPI003043-RA	gi|646749741|gb|KK962076.1|	207401-207772	17	7.6	gi|641568815|gb|KK856858.1|	18640-19270	10	34
ACYPI003044-RA	gi|646782357|gb|KK961494.1|	9093136-9094973	21	9.2	gi|641581779|gb|KK854550.1|	372220-378203	11	39
ACYPI003045-RA	gi|646781143|gb|KK961529.1|	2800464-2802968	20	10	gi|641587614|gb|KK854081.1|	561821-563216	11	40
ACYPI003048-RA	gi|646746663|gb|KK962273.1|	336355-336870	21	10	gi|641585342|gb|KK854413.1|	257277-261388	11	39
ACYPI003049-RA	gi|646778840|gb|KK961590.1|	1158243-1158983	20	9.9	gi|641576188|gb|KK854966.1|	136114-136593	11	39
ACYPI003053-RA	gi|646776389|gb|KK961678.1|	956457-957763	22	9.5	gi|641576846|gb|KK854848.1|	195265-196699	10	38
ACYPI003057-RA	gi|646775884|gb|KK961704.1|	1001963-1002652	20	7.6	gi|641577493|gb|KK854745.1|	184498-185250	9.6	41
ACYPI003059-RA	gi|646747482|gb|KK962215.1|	428732-434848	21	9.2	gi|641588325|gb|KK854004.1|	1016076-1016602	11	40

ACYPI003061-RA	gi|646740879|gb|KK962721.1|	700900-703086	24	6.4	gi|641573417|gb|KK855565.1|	64650-66100	9.5	19
ACYPI003062-RA	gi|646776128|gb|KK961691.1|	1317613-1318062	21	9.2	gi|641586620|gb|KK854215.1|	92126-92460	9.1	40
ACYPI003064-RA	gi|646760564|gb|KK961914.1|	951790-952406	21	8.8	gi|641574876|gb|KK855226.1|	165904-170477	11	39
ACYPI003073-RA	gi|646740637|gb|KK962749.1|	245123-245698	20	7.3	gi|641587930|gb|KK854043.1|	462698-462970	10	41
ACYPI003077-RA	gi|646751856|gb|KK961977.1|	104921-105121	18	8.5	gi|641584694|gb|KK854463.1|	6804-7156	9.3	36
ACYPI003082-RA	gi|646781083|gb|KK961531.1|	2765061-2771749	19	8.5	gi|641584567|gb|KK854472.1|	67707-71232	9.5	39
ACYPI003083-RA	gi|646618553|gb|KK972958.1|	39-301	20	8.2	gi|641585594|gb|KK854372.1|	331517-332480	10	40
ACYPI003090-RA	gi|646776929|gb|KK961653.1|	607095-607370	17	8.5	gi|641584931|gb|KK854446.1|	258608-258816	10	39
ACYPI003093-RA	gi|646781893|gb|KK961506.1|	3904830-3914538	19	8.5	gi|641585599|gb|KK854371.1|	155229-157542	11	39
ACYPI003121-RA	gi|646781443|gb|KK961520.1|	3344951-3345843	20	8.3	gi|641568799|gb|KK856864.1|	81479-82156	9.2	35
ACYPI003124-RA	gi|646744756|gb|KK962409.1|	206500-208807	18	8.1	gi|641577075|gb|KK854811.1|	272889-273348	11	36
ACYPI003141-RA	gi|646767670|gb|KK961827.1|	653931-654235	21	5.9	gi|641569851|gb|KK856541.1|	19721-21216	11	21
ACYPI003151-RA	gi|646737822|gb|KK963039.1|	93343-93932	20	9	gi|641573992|gb|KK855420.1|	138200-138663	10	37
ACYPI003154-RA	gi|646743944|gb|KK962469.1|	338994-339395	20	9.1	gi|641584755|gb|KK854458.1|	25114-26019	9.7	41
ACYPI003157-RA	gi|646782043|gb|KK961502.1|	5249003-5250266	20	9.2	gi|641587696|gb|KK854071.1|	653508-662646	8.9	39
ACYPI003160-RA	gi|646754231|gb|KK961946.1|	1094687-1095156	18	8.7	gi|641570589|gb|KK856310.1|	31546-32004	11	36
ACYPI003169-RA	gi|646743179|gb|KK962523.1|	74132-75289	18	8.3	gi|641587930|gb|KK854043.1|	521469-523671	10	41
ACYPI003170-RA	gi|646706386|gb|KK965650.1|	32734-34754	20	11	gi|641576766|gb|KK854863.1|	228053-228750	10	38
ACYPI003171-RA	gi|646765747|gb|KK961891.1|	904534-908562	18	7.4	gi|641565500|gb|KK858006.1|	12012-13302	9.6	19
ACYPI003176-RA	gi|646781282|gb|KK961525.1|	4584306-4585877	22	9.6	gi|641587680|gb|KK854073.1|	738845-739297	10	40
ACYPI003185-RA	gi|646759701|gb|KK961917.1|	129480-133948	18	6.1	gi|641585440|gb|KK854397.1|	356440-362755	9	38
ACYPI003186-RA	gi|646776447|gb|KK961675.1|	1326802-1327313	22	9.4	gi|641570355|gb|KK856378.1|	6188-6426	9.7	35
ACYPI003203-RA	gi|646741034|gb|KK962704.1|	2934-3967	24	10	gi|641576286|gb|KK854946.1|	330565-331118	12	38
ACYPI003204-RA	gi|646752143|gb|KK961972.1|	935633-945789	20	5.6	gi|641577086|gb|KK854809.1|	240058-240779	9.8	38
ACYPI003206-RA	gi|646766426|gb|KK961862.1|	316962-317482	20	7.1	gi|641584835|gb|KK854453.1|	421708-422987	8.3	36
ACYPI003208-RA	gi|646781690|gb|KK961512.1|	3492495-3492987	21	8.8	gi|641570861|gb|KK856232.1|	54279-55633	11	39
ACYPI003210-RA	gi|646781809|gb|KK961509.1|	3226289-3230714	19	8.6	gi|641587807|gb|KK854057.1|	170211-171117	10	41
ACYPI003214-RA	gi|646776447|gb|KK961675.1|	830176-830874	22	9.4	gi|641588021|gb|KK854033.1|	1153237-1156014	12	40
ACYPI003216-RA	gi|646776904|gb|KK961654.1|	907358-909360	21	9.3	gi|641572987|gb|KK855671.1|	127137-127936	10	39
ACYPI003220-RA	gi|646779038|gb|KK961584.1|	2320231-2320857	21	6.9	gi|641585423|gb|KK854400.1|	356755-357395	11	36
ACYPI003221-RA	gi|646777288|gb|KK961638.1|	2042151-2046261	22	6.1	gi|641585967|gb|KK854311.1|	541346-542394	11	40
ACYPI003224-RA	gi|646781183|gb|KK961528.1|	526413-526760	20	9.5	gi|641577104|gb|KK854807.1|	65947-67629	10	36

ACYPI003230-RA	gi|646744534|gb|KK962425.1|	148438-149555	19	8.6	gi|641579235|gb|KK854581.1|	347058-348811	9.8	38
ACYPI003232-RA	gi|646781772|gb|KK961510.1|	3086344-3089300	20	9.2	gi|641582439|gb|KK854536.1|	442397-447574	11	36
ACYPI003233-RA	gi|646778660|gb|KK961595.1|	125370-125646	16	8.2	gi|641572782|gb|KK855725.1|	103312-103618	10	36
ACYPI003234-RA	gi|646748955|gb|KK962119.1|	669317-676548	19	9.9	gi|641558017|gb|KK861320.1|	4684-6386	13	46
ACYPI003236-RA	gi|646780311|gb|KK961552.1|	1346051-1346654	21	9.7	gi|641572364|gb|KK855831.1|	93981-95142	11	38
ACYPI003241-RA	gi|646780723|gb|KK961542.1|	1959785-1960954	21	9.4	gi|641552935|gb|KK863732.1|	454-1196	13	21
ACYPI003246-RA	gi|646780010|gb|KK961559.1|	3021741-3022227	#######	10	gi|641571216|gb|KK856129.1|	36831-37548	12	41
ACYPI003247-RA	gi|646781243|gb|KK961526.1|	2755649-2756304	21	8.9	gi|641575324|gb|KK855134.1|	164032-164869	11	38
ACYPI003252-RA	gi|646763164|gb|KK961904.1|	1513575-1517730	22	10	gi|641575177|gb|KK855163.1|	24771-25060	11	43
ACYPI003255-RA	gi|646776024|gb|KK961697.1|	2236328-2236662	22	9.2	gi|641587962|gb|KK854039.1|	1113617-111473[illegible]	11	40
ACYPI003257-RA	gi|646752294|gb|KK961969.1|	871288-871770	23	9.8	gi|641586535|gb|KK854227.1|	775053-775943	10	39
ACYPI003261-RA	gi|646778865|gb|KK961589.1|	2693084-2698239	20	9.2	gi|641571138|gb|KK856151.1|	171234-172511	10	45
ACYPI003266-RA	gi|646777955|gb|KK961615.1|	638449-638730	21	10	gi|641577188|gb|KK854792.1|	129157-130975	9.5	38
ACYPI003279-RA	gi|646779298|gb|KK961577.1|	388269-388576	20	9.1	gi|641570304|gb|KK856395.1|	12761-13243	8.8	33
ACYPI003282-RA	gi|646775821|gb|KK961707.1|	2268585-2271598	23	9.1	gi|641569698|gb|KK856589.1|	93111-93422	11	41
ACYPI003283-RA	gi|646768534|gb|KK961809.1|	339483-342446	21	9.8	gi|641588000|gb|KK854035.1|	368716-370070	11	39
ACYPI003284-RA	gi|646749906|gb|KK962067.1|	264901-265421	21	8.2	gi|641573733|gb|KK855487.1|	113201-118652	13	45
ACYPI003290-RA	gi|646747226|gb|KK962234.1|	391655-391992	12	7.8	gi|641587856|gb|KK854051.1|	886363-886652	10	41
ACYPI003295-RA	gi|646765705|gb|KK961893.1|	1584153-1584380	22	9.7	gi|641580987|gb|KK854560.1|	17261-17443	11	40
ACYPI003296-RA	gi|646776514|gb|KK961670.1|	1415107-1416474	20	9.6	gi|641576962|gb|KK854829.1|	321264-322112	10	39
ACYPI003297-RA	gi|646775789|gb|KK961709.1|	988971-989423	20	9.6	gi|641570514|gb|KK856331.1|	127926-130200	9.8	39
ACYPI003298-RA	gi|646745612|gb|KK962344.1|	198103-200309	20	8.2	gi|641580470|gb|KK854571.1|	150677-154984	10	41
ACYPI003301-RA	gi|646775968|gb|KK961700.1|	112114-112366	20	8.2	gi|641571642|gb|KK856015.1|	101720-102268	11	37
ACYPI003302-RA	gi|646782043|gb|KK961502.1|	1201280-1201540	20	9.2	gi|641574882|gb|KK855225.1|	56513-56773	9.3	36
ACYPI003303-RA	gi|646748474|gb|KK962147.1|	291027-291463	20	9	gi|641584445|gb|KK854481.1|	139543-146223	9.9	39
ACYPI003304-RA	gi|646751569|gb|KK961985.1|	313648-314234	21	9.2	gi|641577071|gb|KK854812.1|	293606-294202	13	38
ACYPI003316-RA	gi|646744699|gb|KK962413.1|	133486-134144	14	6.9	gi|641570198|gb|KK856430.1|	84303-84665	10	36
ACYPI003318-RA	gi|646638312|gb|KK969438.1|	12382-13593	13	6.6	gi|641570593|gb|KK856309.1|	87011-87869	9.6	33
ACYPI003322-RA	gi|646765669|gb|KK961895.1|	34683-35153	18	7.2	gi|641570058|gb|KK856473.1|	85679-88087	11	40
ACYPI003323-RA	gi|646749832|gb|KK962071.1|	608327-614914	22	6	gi|641587624|gb|KK854080.1|	479072-479372	10	38
ACYPI003327-RA	gi|646781344|gb|KK961523.1|	356295-357885	21	8.9	gi|641586155|gb|KK854281.1|	562760-563337	9.8	38
ACYPI003332-RA	gi|646765790|gb|KK961889.1|	1246915-1248291	22	8.8	gi|641573210|gb|KK855617.1|	29173-34305	9.2	37

ACYPI003333-RA	gi|646758845|gb|KK961921.1|	1054646-1054852	22	9.5	gi|641587272|gb|KK854122.1|	306292-306522	9.3	42
ACYPI003343-RA	gi|646778149|gb|KK961610.1|	269958-270647	19	8.9	gi|641574303|gb|KK855353.1|	227178-230384	10	34
ACYPI003347-RA	gi|646776024|gb|KK961697.1|	385271-387477	22	9.2	gi|641577341|gb|KK854768.1|	313295-313655	14	58
ACYPI003349-RA	gi|646782127|gb|KK961500.1|	3536955-3542945	22	9.6	gi|641567905|gb|KK857149.1|	93693-94495	10	38
ACYPI003358-RA	gi|646779826|gb|KK961564.1|	1038105-1040174	22	9.6	gi|641575951|gb|KK855015.1|	220825-224887	9.9	37
ACYPI003364-RA	gi|646749852|gb|KK962070.1|	613342-613968	19	8.6	gi|641587021|gb|KK854156.1|	763994-766245	11	42
ACYPI003377-RA	gi|646516981|gb|KK991223.1|	1825-2393	22	8.6	gi|641586541|gb|KK854226.1|	268842-269769	11	41
ACYPI003391-RA	gi|646753799|gb|KK961950.1|	1473574-1477874	23	9.2	gi|641566302|gb|KK857711.1|	41442-43496	9.3	36
ACYPI003398-RA	gi|646747586|gb|KK962207.1|	100173-101079	20	8.8	gi|641576546|gb|KK854899.1|	67715-69421	11	40
ACYPI003401-RA	gi|646777017|gb|KK961649.1|	627201-627455	18	8	gi|641578060|gb|KK854647.1|	175603-175828	8.9	35
ACYPI003404-RA	gi|646781628|gb|KK961514.1|	3448418-3448774	23	9	gi|641587021|gb|KK854156.1|	600404-602112	11	42
ACYPI003409-RA	gi|646773018|gb|KK961752.1|	565788-566183	18	7.8	gi|641587111|gb|KK854143.1|	358479-359292	11	40
ACYPI003412-RA	gi|646777264|gb|KK961639.1|	1614125-1614420	15	7.2	gi|641585494|gb|KK854389.1|	255535-255719	10	37
ACYPI003413-RA	gi|646781282|gb|KK961525.1|	1746216-1747551	22	9.6	gi|641573202|gb|KK855619.1|	194482-194897	9.6	42
ACYPI003418-RA	gi|646776024|gb|KK961697.1|	918063-918862	22	9.2	gi|641569146|gb|KK856755.1|	114080-115703	10	34
ACYPI003422-RA	gi|646698808|gb|KK966113.1|	31629-34291	23	6.5	gi|641573394|gb|KK855572.1|	59363-61680	11	40
ACYPI003426-RA	gi|646749065|gb|KK962112.1|	330030-334325	20	7.9	gi|641573111|gb|KK855641.1|	94339-94737	11	160
ACYPI003428-RA	gi|646781690|gb|KK961512.1|	4507501-4509485	21	8.8	gi|641584305|gb|KK854491.1|	175587-181117	11	39
ACYPI003442-RA	gi|646782276|gb|KK961497.1|	3204691-3205043	21	9.7	gi|641587866|gb|KK854050.1|	589093-589297	10	38
ACYPI003451-RA	gi|646775658|gb|KK961715.1|	685337-685578	19	8.6	gi|641570089|gb|KK856463.1|	53939-54254	11	36
ACYPI003455-RA	gi|646772045|gb|KK961761.1|	735288-736798	21	9.6	gi|641577075|gb|KK854811.1|	161155-164885	11	36
ACYPI003456-RA	gi|646777416|gb|KK961632.1|	975707-976600	22	8.8	gi|641574990|gb|KK855200.1|	201530-202634	12	37
ACYPI003458-RA	gi|646729261|gb|KK964099.1|	58251-59114	24	10	gi|641588294|gb|KK854006.1|	372471-373803	8.7	40
ACYPI003461-RA	gi|646770401|gb|KK961779.1|	1747695-1748746	21	8.6	gi|641577987|gb|KK854657.1|	483319-489271	13	49
ACYPI003469-RA	gi|646776091|gb|KK961693.1|	144684-146038	23	10	gi|641569612|gb|KK856617.1|	2544-3197	11	21
ACYPI003470-RA	gi|646769774|gb|KK961788.1|	766037-766431	20	9.2	gi|641574532|gb|KK855298.1|	70081-70286	12	20
ACYPI003480-RA	gi|646763164|gb|KK961904.1|	1065661-1066084	22	10	gi|641574312|gb|KK855351.1|	100968-101231	8.9	36
ACYPI003481-RA	gi|646735722|gb|KK963266.1|	172392-173278	17	7.6	gi|641586700|gb|KK854203.1|	63452-63756	8.4	39
ACYPI003483-RA	gi|646782276|gb|KK961497.1|	1548160-1551014	21	9.7	gi|641574800|gb|KK855241.1|	150305-153185	10	37
ACYPI003484-RA	gi|646781344|gb|KK961523.1|	582986-584957	21	8.9	gi|641569425|gb|KK856674.1|	111565-112737	11	42
ACYPI003488-RA	gi|646775507|gb|KK961723.1|	1824044-1825093	20	9.8	gi|641575698|gb|KK855061.1|	105484-105727	10	39
ACYPI003489-RA	gi|646732863|gb|KK963615.1|	273712-275036	150	39	gi|641586509|gb|KK854231.1|	531347-533813	9.8	37

ACYPI003491-RA	gi|646780658|gb|KK961544.1|	1904378-1905123	19	8.6	gi|641571769|gb|KK855985.1|	141949-143044	9	35
ACYPI003493-RA	gi|646781873|gb|KK961507.1|	1334500-1335191	20	9.1	gi|641583587|gb|KK854518.1|	122334-122954	10	37
ACYPI003508-RA	gi|646779826|gb|KK961564.1|	1263755-1264075	22	9.6	gi|641577498|gb|KK854744.1|	339973-340269	11	42
ACYPI003518-RA	gi|646767850|gb|KK961823.1|	458210-462099	21	9.8	gi|641579225|gb|KK854583.1|	329923-330601	10	39
ACYPI003519-RA	gi|646776148|gb|KK961690.1|	1863927-1865939	21	9.4	gi|641587921|gb|KK854044.1|	358092-360211	9.4	41
ACYPI003522-RA	gi|646743394|gb|KK962507.1|	418259-418636	20	8.8	gi|641576414|gb|KK854924.1|	137776-143145	10	40
ACYPI003532-RA	gi|646779498|gb|KK961572.1|	533389-534148	22	5.6	gi|641567609|gb|KK857247.1|	29664-31582	9.7	19
ACYPI003535-RA	gi|646771845|gb|KK961763.1|	449987-450193	23	10	gi|641577968|gb|KK854660.1|	336803-337021	10	42
ACYPI003541-RA	gi|646780889|gb|KK961537.1|	2915802-2917548	22	10	gi|641584305|gb|KK854491.1|	551762-553148	11	39
ACYPI003545-RA	gi|646773423|gb|KK961749.1|	383372-387975	19	8.1	gi|641577289|gb|KK854776.1|	192523-193107	10	36
ACYPI003549-RA	gi|646777631|gb|KK961624.1|	1806372-1810062	20	9.3	gi|641560616|gb|KK860113.1|	734-987	12	44
ACYPI003550-RA	gi|646781690|gb|KK961512.1|	3241721-3242537	21	8.8	gi|641579212|gb|KK854585.1|	44440-46499	10	35
ACYPI003552-RA	gi|646771092|gb|KK961771.1|	1071229-1071679	23	9.7	gi|641577535|gb|KK854737.1|	58519-59440	11	42
ACYPI003554-RA	gi|646780889|gb|KK961537.1|	2979795-2980118	22	10	gi|641582091|gb|KK854547.1|	383677-384387	11	42
ACYPI003557-RA	gi|646781344|gb|KK961523.1|	1435331-1445399	21	8.9	gi|641588164|gb|KK854017.1|	267209-267527	9.2	40
ACYPI003560-RA	gi|646779375|gb|KK961575.1|	1835278-1835852	22	9.3	gi|641574110|gb|KK855394.1|	122773-123817	10	21
ACYPI003562-RA	gi|646781118|gb|KK961530.1|	5755346-5756974	21	10	gi|641577913|gb|KK854671.1|	89841-90140	9.2	40
ACYPI003565-RA	gi|646780889|gb|KK961537.1|	4077216-4077409	22	10	gi|641574809|gb|KK855240.1|	40390-41700	11	39
ACYPI003566-RA	gi|646746202|gb|KK962305.1|	84741-87979	21	6.4	gi|641575183|gb|KK855162.1|	25466-25948	10	42
ACYPI003567-RA	gi|646776608|gb|KK961665.1|	264136-265583	23	9.3	gi|641575077|gb|KK855184.1|	103870-105114	12	22
ACYPI003568-RA	gi|646780147|gb|KK961556.1|	803865-804403	18	7.5	gi|641554782|gb|KK862854.1|	102-305	7.8	21
ACYPI003571-RA	gi|646529355|gb|KK988894.1|	502-1094	13	2.9	gi|641587021|gb|KK854156.1|	589985-590553	11	42
ACYPI003572-RA	gi|646782334|gb|KK961495.1|	3189066-3189559	21	9	gi|641586577|gb|KK854221.1|	160457-160756	10	40
ACYPI003574-RA	gi|646748200|gb|KK962164.1|	406362-408061	19	7.7	gi|641570579|gb|KK856313.1|	1714-2565	11	38
ACYPI003577-RA	gi|646778565|gb|KK961598.1|	1510852-1511105	19	7.8	gi|641574402|gb|KK855330.1|	239335-240400	11	23
ACYPI003579-RA	gi|646744874|gb|KK962400.1|	321101-321398	19	8.3	gi|641586535|gb|KK854227.1|	613950-614919	10	39
ACYPI003581-RA	gi|646766735|gb|KK961851.1|	121456-121694	20	10	gi|641571841|gb|KK855968.1|	133530-138589	10	36
ACYPI003589-RA	gi|646775658|gb|KK961715.1|	859693-859990	19	8.6	gi|641570245|gb|KK856414.1|	31362-31741	11	41
ACYPI003590-RA	gi|646779826|gb|KK961564.1|	1464953-1467843	22	9.6	gi|641585946|gb|KK854315.1|	650300-655433	9.8	43
ACYPI003593-RA	gi|646782127|gb|KK961500.1|	6948696-6949852	22	9.6	gi|641574990|gb|KK855200.1|	265963-267568	12	37
ACYPI003596-RA	gi|646780794|gb|KK961540.1|	1405293-1407435	21	9.5	gi|641587992|gb|KK854036.1|	286970-287196	11	41
ACYPI003598-RA	gi|646776429|gb|KK961676.1|	1452461-1453296	20	9.9	gi|641587562|gb|KK854088.1|	497550-505220	11	42

ACYPI003604-RA	gi|646778903|gb|KK961588.1|	1261298-1261553	23	10	gi|641585334|gb|KK854415.1|	289256-292338	10	39
ACYPI003616-RA	gi|646775471|gb|KK961725.1|	325270-325806	20	10	gi|641576565|gb|KK854896.1|	216178-217820	11	40
ACYPI003625-RA	gi|646776024|gb|KK961697.1|	1912164-1916312	22	9.2	gi|641573638|gb|KK855510.1|	181524-182031	11	41
ACYPI003626-RA	gi|646779006|gb|KK961585.1|	845085-847674	20	9.9	gi|641550656|gb|KK864801.1|	3877-4586	7.4	27
ACYPI003631-RA	gi|646607341|gb|KK974959.1|	6834-6960	7.1	2.6	gi|641588236|gb|KK854011.1|	278418-280096	9.7	38
ACYPI003634-RA	gi|646779375|gb|KK961575.1|	3605558-3605792	22	9.3	gi|641571730|gb|KK855996.1|	111657-112310	10	20
ACYPI003638-RA	gi|646751084|gb|KK962007.1|	576204-577850	20	5.4	gi|641585423|gb|KK854400.1|	462165-462899	11	36
ACYPI003639-RA	gi|646727554|gb|KK964329.1|	137375-141745	24	10	gi|641577778|gb|KK854696.1|	179338-185583	9.9	42
ACYPI003641-RA	gi|646778840|gb|KK961590.1|	1872677-1872923	20	9.9	gi|641576188|gb|KK854966.1|	158888-159647	11	39
ACYPI003645-RA	gi|646749616|gb|KK962083.1|	1370198-1370442	22	10	gi|641570714|gb|KK856274.1|	74517-75120	10	38
ACYPI003646-RA	gi|646776389|gb|KK961678.1|	1305035-1305529	22	9.5	gi|641586405|gb|KK854246.1|	369727-370490	10	40
ACYPI003651-RA	gi|646775944|gb|KK961701.1|	622841-625467	22	9.3	gi|641588073|gb|KK854027.1|	392892-393642	11	41
ACYPI003657-RA	gi|646776647|gb|KK961663.1|	768432-774690	23	9.2	gi|641588343|gb|KK854003.1|	2120674-2121062	11	40
ACYPI003659-RA	gi|646738348|gb|KK962985.1|	139845-140214	13	7.1	gi|641572300|gb|KK855848.1|	29325-29712	12	38
ACYPI003661-RA	gi|646745941|gb|KK962322.1|	795337-795490	21	10	gi|641586121|gb|KK854287.1|	12793-14494	11	43
ACYPI003668-RA	gi|646780953|gb|KK961535.1|	478682-479178	20	9.8	gi|641574930|gb|KK855214.1|	180616-181220	11	39
ACYPI003669-RA	gi|646773721|gb|KK961747.1|	1007516-1007922	21	9.2	gi|641575730|gb|KK855054.1|	346708-347099	10	38
ACYPI003677-RA	gi|646738524|gb|KK962968.1|	162327-164456	24	11	gi|641573850|gb|KK855457.1|	90173-90979	9.3	34
ACYPI003679-RA	gi|646775765|gb|KK961710.1|	1769984-1770500	21	6	gi|641588073|gb|KK854027.1|	414468-415831	11	41
ACYPI003681-RA	gi|646734312|gb|KK963435.1|	303986-304909	22	11	gi|641574169|gb|KK855381.1|	157663-158755	9.9	38
ACYPI003687-RA	gi|646776409|gb|KK961677.1|	40185-54017	17	8.4	gi|641585475|gb|KK854392.1|	428029-430880	10	41
ACYPI003691-RA	gi|646777017|gb|KK961649.1|	681444-682750	18	8	gi|641577612|gb|KK854724.1|	352469-353133	9.7	37
ACYPI003697-RA	gi|646782334|gb|KK961495.1|	1054782-1056279	21	9	gi|641576791|gb|KK854858.1|	105870-107321	9.2	42
ACYPI003701-RA	gi|646771845|gb|KK961763.1|	2275019-2275891	23	10	gi|641567788|gb|KK857188.1|	31381-31775	13	43
ACYPI003702-RA	gi|646780978|gb|KK961534.1|	594995-597805	20	8	gi|641586893|gb|KK854175.1|	619974-620598	11	41
ACYPI003705-RA	gi|646751289|gb|KK961996.1|	1196601-1200875	24	9.1	gi|641588325|gb|KK854004.1|	1940069-1941515	11	40
ACYPI003708-RA	gi|646781732|gb|KK961511.1|	4145078-4146127	22	9.8	gi|641586777|gb|KK854192.1|	544171-545956	11	30
ACYPI003710-RA	gi|646778632|gb|KK961596.1|	2182111-2182325	21	9.2	gi|641580479|gb|KK854569.1|	472888-473215	10	38
ACYPI003715-RA	gi|646766868|gb|KK961847.1|	1077556-1077950	21	9.9	gi|641576882|gb|KK854843.1|	255411-256105	10	39
ACYPI003718-RA	gi|646743302|gb|KK962514.1|	136789-137050	23	8.4	gi|641588182|gb|KK854016.1|	403241-405057	11	41
ACYPI003722-RA	gi|646776069|gb|KK961694.1|	1000502-1002275	18	7.8	gi|641570861|gb|KK856232.1|	2196-3235	11	39
ACYPI003732-RA	gi|646738524|gb|KK962968.1|	311187-315438	24	11	gi|641588090|gb|KK854025.1|	93574-97525	11	43

ACYPI003736-RA	gi|646781536|gb|KK961517.1|	2024598-2029654	19	7.7	gi|641588236|gb|KK854011.1|	1247592-124883[illegible]	9.7	38
ACYPI003742-RA	gi|646731007|gb|KK963869.1|	181941-183265	21	9.8	gi|641576983|gb|KK854825.1|	297924-298168	9.7	37
ACYPI003744-RA	gi|646782334|gb|KK961495.1|	3498564-3498982	21	9	gi|641586203|gb|KK854273.1|	244402-244585	9.5	40
ACYPI003749-RA	gi|646673348|gb|KK967633.1|	2934-3243	12	5.6	gi|641569036|gb|KK856790.1|	105472-105984	10	36
ACYPI003750-RA	gi|646778699|gb|KK961594.1|	2236170-2237671	21	9.5	gi|641576807|gb|KK854855.1|	54456-57031	9.8	37
ACYPI003751-RA	gi|646767477|gb|KK961831.1|	128473-129125	20	8.4	gi|641586943|gb|KK854167.1|	163442-163698	11	38
ACYPI003754-RA	gi|646779298|gb|KK961577.1|	1448548-1450769	20	9.1	gi|641586975|gb|KK854163.1|	363994-364405	11	40
ACYPI003756-RA	gi|646766426|gb|KK961862.1|	914809-915095	20	7.1	gi|641567854|gb|KK857166.1|	101175-102372	11	37
ACYPI003757-RA	gi|646781893|gb|KK961506.1|	370852-371343	19	8.5	gi|641585521|gb|KK854384.1|	341764-342049	9.7	38
ACYPI003760-RA	gi|646749672|gb|KK962080.1|	302141-302538	20	7.6	gi|641576055|gb|KK854992.1|	39075-39765	9.7	37
ACYPI003764-RA	gi|646770323|gb|KK961780.1|	218387-218816	19	7.1	gi|641571928|gb|KK855946.1|	88091-88532	11	38
ACYPI003770-RA	gi|646749727|gb|KK962077.1|	481504-484258	21	8.9	gi|641570419|gb|KK856360.1|	130308-131082	12	39
ACYPI003771-RA	gi|646780406|gb|KK961550.1|	483864-484318	22	5.9	gi|641566077|gb|KK857794.1|	11815-12597	9.9	19
ACYPI003778-RA	gi|646766137|gb|KK961874.1|	741464-743205	20	9	gi|641569564|gb|KK856632.1|	53109-56151	9.8	34
ACYPI003779-RA	gi|646776184|gb|KK961688.1|	358288-359255	21	9.6	gi|641570155|gb|KK856443.1|	126919-127878	10	20
ACYPI003783-RA	gi|646781379|gb|KK961522.1|	2373417-2377352	22	7.6	gi|641586235|gb|KK854270.1|	525499-527966	11	39
ACYPI003786-RA	gi|646742685|gb|KK962560.1|	7341-8264	21	9.5	gi|641585594|gb|KK854372.1|	459063-459722	10	40
ACYPI003793-RA	gi|646780794|gb|KK961540.1|	3210011-3210269	21	9.5	gi|641587800|gb|KK854058.1|	762549-762812	10	42
ACYPI003795-RA	gi|646740287|gb|KK962790.1|	90015-99796	20	8.5	gi|641577584|gb|KK854729.1|	253777-255577	9.9	37
ACYPI003798-RA	gi|646777474|gb|KK961630.1|	613574-614529	21	9.3	gi|641587303|gb|KK854118.1|	366967-369802	11	20
ACYPI003807-RA	gi|646780010|gb|KK961559.1|	52996-58722	22	10	gi|641577161|gb|KK854797.1|	150972-156719	10	38
ACYPI003815-RA	gi|646745997|gb|KK962319.1|	522269-522522	21	8.5	gi|641586726|gb|KK854199.1|	378189-379306	11	41
ACYPI003817-RA	gi|646775702|gb|KK961713.1|	302346-302729	17	7.4	gi|641588033|gb|KK854031.1|	1117347-111914[illegible]	11	38
ACYPI003820-RA	gi|646776280|gb|KK961684.1|	1730061-1730307	22	9.3	gi|641587671|gb|KK854074.1|	182475-182683	11	40
ACYPI003821-RA	gi|646766137|gb|KK961874.1|	611082-612102	20	9	gi|641572345|gb|KK855836.1|	53421-54745	9.7	38
ACYPI003822-RA	gi|646739361|gb|KK962890.1|	129238-130324	22	9.6	gi|641576414|gb|KK854924.1|	311123-312321	10	40
ACYPI003826-RA	gi|646776524|gb|KK961669.1|	987357-988125	17	6.4	gi|641585383|gb|KK854406.1|	432441-432827	11	37
ACYPI003828-RA	gi|646751120|gb|KK962005.1|	790431-791235	21	5.8	gi|641567423|gb|KK857312.1|	19588-25554	11	37
ACYPI003831-RA	gi|646777288|gb|KK961638.1|	1484450-1484802	22	6.1	gi|641587688|gb|KK854072.1|	919401-919687	10	38
ACYPI003834-RA	gi|646738414|gb|KK962979.1|	124918-125844	23	10	gi|641577379|gb|KK854761.1|	170296-171486	11	41
ACYPI003835-RA	gi|646780889|gb|KK961537.1|	2669593-2671496	22	10	gi|641584305|gb|KK854491.1|	405448-405959	11	39
ACYPI003839-RA	gi|646763535|gb|KK961902.1|	976968-983363	23	10	gi|641575372|gb|KK855124.1|	66926-75766	9.5	36

ACYPI003856-RA	gi|646782127|gb|KK961500.1|	3587979-3588222	22	9.6	gi|641583958|gb|KK854513.1|	294997-295236	10	22
ACYPI003863-RA	gi|646781732|gb|KK961511.1|	2453921-2454500	22	9.8	gi|641584445|gb|KK854481.1|	268830-271010	9.9	39
ACYPI003866-RA	gi|646776389|gb|KK961678.1|	588239-589099	22	9.5	gi|641576377|gb|KK854930.1|	181736-188565	10	42
ACYPI003867-RA	gi|646778948|gb|KK961587.1|	1819307-1819795	20	7.3	gi|641588136|gb|KK854020.1|	946149-946432	11	36
ACYPI003872-RA	gi|646738028|gb|KK963018.1|	516618-517119	22	7.1	gi|641577228|gb|KK854785.1|	264967-265264	9.7	39
ACYPI003875-RA	gi|646775352|gb|KK961732.1|	70781-71306	18	8.7	gi|641576221|gb|KK854958.1|	205645-205892	9.3	35
ACYPI003876-RA	gi|646729620|gb|KK964051.1|	86615-86858	23	10	gi|641577347|gb|KK854767.1|	389694-389929	9.9	43
ACYPI003885-RA	gi|646782168|gb|KK961499.1|	3626686-3627283	21	9.4	gi|641584894|gb|KK854448.1|	488267-489072	10	44
ACYPI003886-RA	gi|646758527|gb|KK961922.1|	565562-565804	24	10	gi|641576650|gb|KK854884.1|	242313-247304	9.6	40
ACYPI003888-RA	gi|646751458|gb|KK961989.1|	565234-565510	21	5.1	gi|641575970|gb|KK855012.1|	152130-153208	11	38
ACYPI003891-RA	gi|646781344|gb|KK961523.1|	415490-416413	21	8.9	gi|641573825|gb|KK855463.1|	113447-113839	10	39
ACYPI003895-RA	gi|646778186|gb|KK961609.1|	1783271-1789757	19	9.4	gi|641584128|gb|KK854505.1|	236345-237223	9.6	37
ACYPI003897-RA	gi|646770251|gb|KK961781.1|	76954-79519	20	9.6	gi|641578160|gb|KK854630.1|	189542-190912	11	43
ACYPI003904-RA	gi|646750100|gb|KK962057.1|	278314-278701	16	8.2	gi|641587954|gb|KK854040.1|	710604-711778	8.9	39
ACYPI003907-RA	gi|646729280|gb|KK964096.1|	180522-182494	22	9.3	gi|641560826|gb|KK860019.1|	4079-4561	9.3	13
ACYPI003908-RA	gi|646775968|gb|KK961700.1|	879475-879873	20	8.2	gi|641569574|gb|KK856629.1|	111713-112242	24	120
ACYPI003909-RA	gi|646738819|gb|KK962938.1|	151625-154415	19	8.4	gi|641556774|gb|KK861912.1|	3087-3967	7	36
ACYPI003918-RA	gi|646750341|gb|KK962044.1|	885961-886389	19	5.6	gi|641576109|gb|KK854982.1|	144500-144781	10	19
ACYPI003921-RA	gi|646766357|gb|KK961865.1|	892596-894509	16	6.5	gi|641570720|gb|KK856272.1|	68944-70264	9.1	36
ACYPI003934-RA	gi|646750043|gb|KK962060.1|	799471-800688	21	8.3	gi|641573171|gb|KK855626.1|	78315-82298	9.9	40
ACYPI003937-RA	gi|646750409|gb|KK962040.1|	1084764-1085163	21	8.4	gi|641587249|gb|KK854125.1|	137537-140394	11	37
ACYPI003941-RA	gi|646776322|gb|KK961682.1|	2114788-2115084	22	8.9	gi|641586496|gb|KK854233.1|	367126-370383	11	38
ACYPI003942-RA	gi|646775987|gb|KK961699.1|	302590-302863	19	7.5	gi|641575559|gb|KK855088.1|	260800-262258	9.9	37
ACYPI003943-RA	gi|646781344|gb|KK961523.1|	4364065-4371527	21	8.9	gi|641587896|gb|KK854047.1|	826843-829594	9.9	39
ACYPI003951-RA	gi|646779621|gb|KK961569.1|	1123974-1125732	16	7.3	gi|641587074|gb|KK854148.1|	498367-498861	11	41
ACYPI003953-RA	gi|646744874|gb|KK962400.1|	391253-391535	19	8.3	gi|641586066|gb|KK854296.1|	324029-324269	9.4	37
ACYPI003960-RA	gi|646780183|gb|KK961555.1|	1993664-1995548	19	8.6	gi|641572177|gb|KK855878.1|	22173-22436	10	35
ACYPI003961-RA	gi|646775901|gb|KK961703.1|	825369-831326	20	9.6	gi|641564341|gb|KK858480.1|	17488-17807	11	19
ACYPI003962-RA	gi|646748002|gb|KK962178.1|	1530220-1531488	22	6.3	gi|641586569|gb|KK854222.1|	93751-96591	12	40
ACYPI003964-RA	gi|646781659|gb|KK961513.1|	123489-124135	22	9.8	gi|641577265|gb|KK854780.1|	114139-116705	10	40
ACYPI003971-RA	gi|646747149|gb|KK962240.1|	216691-220176	21	10	gi|641582100|gb|KK854545.1|	295269-297080	13	41
ACYPI003973-RA	gi|646745372|gb|KK962364.1|	655142-656984	23	10	gi|641570754|gb|KK856261.1|	104972-106240	9.6	38

ACYPI003975-RA	gi|646747884|gb|KK962186.1|	1230599-1235599	22	11	gi|641587971|gb|KK854038.1|	106101-107003	11	39
ACYPI003976-RA	gi|646773721|gb|KK961747.1|	3070756-3087407	21	9.2	gi|641574433|gb|KK855322.1|	220311-230259	11	44
ACYPI003981-RA	gi|646776024|gb|KK961697.1|	1209935-1217046	22	9.2	gi|641587660|gb|KK854076.1|	561282-562910	9.9	41
ACYPI003986-RA	gi|646776038|gb|KK961696.1|	1278970-1283843	21	10	gi|641572130|gb|KK855891.1|	174453-174824	10	37
ACYPI003991-RA	gi|646776904|gb|KK961654.1|	1541135-1547468	21	9.3	gi|641587881|gb|KK854049.1|	426345-427066	10	40
ACYPI003993-RA	gi|646775765|gb|KK961710.1|	2033052-2033718	21	6	gi|641576347|gb|KK854934.1|	313422-314861	11	39
ACYPI003998-RA	gi|646749464|gb|KK962091.1|	437728-438264	18	8.8	gi|641587606|gb|KK854082.1|	810499-810754	11	40
ACYPI004000-RA	gi|646748955|gb|KK962119.1|	814945-815243	19	9.9	gi|641577522|gb|KK854739.1|	276696-279485	11	41
ACYPI004001-RA	gi|646748474|gb|KK962147.1|	568297-568823	20	9	gi|641587186|gb|KK854133.1|	333281-333586	8.1	39
ACYPI004004-RA	gi|646778865|gb|KK961589.1|	1819267-1821004	20	9.2	gi|641586328|gb|KK854257.1|	450153-450724	8.4	35
ACYPI004006-RA	gi|646754371|gb|KK961945.1|	115997-116723	20	9.7	gi|641587047|gb|KK854151.1|	424829-425148	10	39
ACYPI004008-RA	gi|646776952|gb|KK961652.1|	1309825-1310609	19	7.7	gi|641572637|gb|KK855763.1|	251203-251892	9.5	36
ACYPI004014-RA	gi|646738635|gb|KK962957.1|	269002-269203	20	8.6	gi|641570404|gb|KK856365.1|	71726-73948	10	19
ACYPI004015-RA	gi|646751515|gb|KK961987.1|	743559-744334	20	9.2	gi|641576161|gb|KK854972.1|	225340-226431	10	36
ACYPI004019-RA	gi|646776351|gb|KK961680.1|	1800365-1800782	22	10	gi|641566606|gb|KK857599.1|	44283-45293	10	40
ACYPI004024-RA	gi|646757520|gb|KK961926.1|	1744453-1746987	20	7.4	gi|641585340|gb|KK854414.1|	242560-244760	11	42
ACYPI004029-RA	gi|646732431|gb|KK963675.1|	306302-308057	24	9.6	gi|641567739|gb|KK857205.1|	109817-110020	13	38
ACYPI004030-RA	gi|646501974|gb|KK994094.1|	200-616	14	7.8	gi|641577535|gb|KK854737.1|	386114-386834	11	42
ACYPI004031-RA	gi|646746216|gb|KK962304.1|	782239-783216	130	33	gi|641587696|gb|KK854071.1|	550344-551738	8.9	39
ACYPI004033-RA	gi|646776514|gb|KK961670.1|	95511-96976	20	9.6	gi|641575672|gb|KK855065.1|	223324-223847	9.8	38
ACYPI004037-RA	gi|646769221|gb|KK961797.1|	737439-737674	19	9.6	gi|641587930|gb|KK854043.1|	961692-962191	10	41
ACYPI004038-RA	gi|646780723|gb|KK961542.1|	2235132-2235835	21	9.4	gi|641587971|gb|KK854038.1|	788774-789024	11	39
ACYPI004039-RA	gi|646732202|gb|KK963708.1|	15961-17122	22	9.9	gi|641586809|gb|KK854188.1|	122712-123024	10	43
ACYPI004047-RA	gi|646758935|gb|KK961920.1|	137592-138206	20	8.5	gi|641585773|gb|KK854342.1|	403149-403624	12	45
ACYPI004049-RA	gi|646745197|gb|KK962376.1|	568807-573328	21	8.9	gi|641584931|gb|KK854446.1|	97699-102186	10	39
ACYPI004051-RA	gi|646753890|gb|KK961949.1|	834535-836511	20	8.9	gi|641576423|gb|KK854922.1|	275088-276594	18	35
ACYPI004054-RA	gi|646764801|gb|KK961898.1|	628095-628569	20	9.7	gi|641587896|gb|KK854047.1|	852540-852824	9.9	39
ACYPI004065-RA	gi|646777416|gb|KK961632.1|	3350199-3352673	22	8.8	gi|641588000|gb|KK854035.1|	533725-535010	11	39
ACYPI004069-RA	gi|646780311|gb|KK961552.1|	4047606-4050625	21	9.7	gi|641587866|gb|KK854050.1|	275332-276289	10	38
ACYPI004071-RA	gi|646775807|gb|KK961708.1|	2797154-2799162	21	8.6	gi|641586515|gb|KK854230.1|	561673-564419	11	39
ACYPI004075-RA	gi|646781732|gb|KK961511.1|	3134559-3134833	22	9.8	gi|641582100|gb|KK854545.1|	91749-92304	13	41
ACYPI004076-RA	gi|646780953|gb|KK961535.1|	4503899-4504258	20	9.8	gi|641584271|gb|KK854494.1|	13110-14250	13	45

ACYPI004077-RA	gi|646730815|gb|KK963895.1|	181690-184047	20	9.5	gi|641586632|gb|KK854213.1|	91623-92094	11	41
ACYPI004078-RA	gi|646781212|gb|KK961527.1|	2239953-2241959	21	10	gi|641573097|gb|KK855644.1|	82077-83948	10	36
ACYPI004082-RA	gi|646775558|gb|KK961720.1|	1851019-1853017	20	9.4	gi|641588164|gb|KK854017.1|	791875-794179	9.2	40
ACYPI004089-RA	gi|646753527|gb|KK961953.1|	1850986-1851761	24	9.2	gi|641585860|gb|KK854329.1|	114568-116246	11	43
ACYPI004093-RA	gi|646782357|gb|KK961494.1|	2810374-2810643	21	9.2	gi|641572955|gb|KK855679.1|	35380-36113	10	40
ACYPI004098-RA	gi|646767850|gb|KK961823.1|	1166449-1168953	21	9.8	gi|641585847|gb|KK854330.1|	31182-34584	11	41
ACYPI004106-RA	gi|646751458|gb|KK961989.1|	795942-796584	21	5.1	gi|641586521|gb|KK854229.1|	182911-183670	11	41
ACYPI004115-RA	gi|646780627|gb|KK961545.1|	341927-342413	20	7.7	gi|641585366|gb|KK854408.1|	362929-365996	12	41
ACYPI004117-RA	gi|646750165|gb|KK962053.1|	782148-784467	25	10	gi|641587219|gb|KK854129.1|	161652-167657	9.9	41
ACYPI004120-RA	gi|646780827|gb|KK961539.1|	193935-195449	20	9.4	gi|641585900|gb|KK854322.1|	117828-118056	12	43
ACYPI004121-RA	gi|646775507|gb|KK961723.1|	1747589-1747887	20	9.8	gi|641577905|gb|KK854672.1|	273652-274627	10	37
ACYPI004126-RA	gi|646781243|gb|KK961526.1|	4103877-4107442	21	8.9	gi|641585910|gb|KK854320.1|	247453-248579	10	38
ACYPI004127-RA	gi|646778865|gb|KK961589.1|	2087689-2087972	20	9.2	gi|641579933|gb|KK854577.1|	68139-68369	11	37
ACYPI004128-RA	gi|646744833|gb|KK962403.1|	130076-131312	22	9.4	gi|641570555|gb|KK856321.1|	109418-109996	11	38
ACYPI004129-RA	gi|646776389|gb|KK961678.1|	1664819-1666527	22	9.5	gi|641573759|gb|KK855480.1|	127678-128494	9.9	37
ACYPI004133-RA	gi|646753527|gb|KK961953.1|	1752479-1753070	24	9.2	gi|641570991|gb|KK856192.1|	64040-65559	11	38
ACYPI004142-RA	gi|646781443|gb|KK961520.1|	465271-472935	20	8.3	gi|641575112|gb|KK855177.1|	88837-94473	11	39
ACYPI004145-RA	gi|646750643|gb|KK962028.1|	706083-714538	21	7.5	gi|641577379|gb|KK854761.1|	252353-258055	11	41
ACYPI004152-RA	gi|646777991|gb|KK961614.1|	1627252-1627683	18	7.8	gi|641588099|gb|KK854024.1|	310272-310556	11	39
ACYPI004154-RA	gi|646767670|gb|KK961827.1|	2087025-2087580	21	5.9	gi|641577595|gb|KK854727.1|	168171-168434	10	20
ACYPI004157-RA	gi|646746355|gb|KK962294.1|	1011490-1024685	22	10	gi|641587066|gb|KK854149.1|	646487-647454	11	40
ACYPI004168-RA	gi|646776514|gb|KK961670.1|	1177830-1190106	20	9.6	gi|641588294|gb|KK854006.1|	677955-679275	8.7	40
ACYPI004173-RA	gi|646782357|gb|KK961494.1|	9494725-9495664	21	9.2	gi|641569246|gb|KK856728.1|	22365-23068	8.7	32
ACYPI004182-RA	gi|646776024|gb|KK961697.1|	2703197-2703698	22	9.2	gi|641585446|gb|KK854396.1|	299805-300203	11	39
ACYPI004183-RA	gi|646779006|gb|KK961585.1|	1259395-1259672	20	9.9	gi|641583088|gb|KK854527.1|	401534-403976	9.9	39
ACYPI004185-RA	gi|646750354|gb|KK962043.1|	497683-499153	20	9.2	gi|641585653|gb|KK854362.1|	32202-34531	10	39
ACYPI004190-RA	gi|646781659|gb|KK961513.1|	3464204-3476907	22	9.8	gi|641576679|gb|KK854878.1|	317889-322000	9.9	38
ACYPI004192-RA	gi|646739411|gb|KK962885.1|	272786-278319	18	7.7	gi|641572130|gb|KK855891.1|	151168-151534	10	37
ACYPI004195-RA	gi|646781143|gb|KK961529.1|	2157370-2158054	20	10	gi|641571667|gb|KK856008.1|	142338-148056	11	39
ACYPI004199-RA	gi|646781344|gb|KK961523.1|	3879833-3880324	21	8.9	gi|641585755|gb|KK854345.1|	205593-205995	9.8	39
ACYPI004203-RA	gi|646779785|gb|KK961565.1|	3434912-3435224	18	8.9	gi|641585581|gb|KK854374.1|	463799-464715	10	38
ACYPI004206-RA	gi|646777416|gb|KK961632.1|	666876-667489	22	8.8	gi|641577837|gb|KK854685.1|	386593-387225	11	42

ACYPI004209-RA	gi|646766357|gb|KK961865.1|	168058-168389	16	6.5	gi|641586817|gb|KK854187.1|	827518-828136	9.3	39
ACYPI004211-RA	gi|646780978|gb|KK961534.1|	1054515-1056896	20	8	gi|641558402|gb|KK861138.1|	2365-3607	7.6	30
ACYPI004216-RA	gi|646780858|gb|KK961538.1|	2080313-2081531	21	10	gi|641576608|gb|KK854891.1|	239405-240511	11	37
ACYPI004218-RA	gi|646779337|gb|KK961576.1|	967751-968216	18	9	gi|641576656|gb|KK854883.1|	173777-174036	11	40
ACYPI004219-RA	gi|646782357|gb|KK961494.1|	5190612-5191043	21	9.2	gi|641563481|gb|KK858852.1|	648-1152	8.7	35
ACYPI004223-RA	gi|646743394|gb|KK962507.1|	337160-340397	20	8.8	gi|641576414|gb|KK854924.1|	105220-106089	10	40
ACYPI004224-RA	gi|646769511|gb|KK961792.1|	14183-16009	20	9.9	gi|641573015|gb|KK855664.1|	29768-31156	11	42
ACYPI004227-RA	gi|646781344|gb|KK961523.1|	243344-243909	21	8.9	gi|641571428|gb|KK856074.1|	5390-6292	11	39
ACYPI004235-RA	gi|646781344|gb|KK961523.1|	587351-588847	21	8.9	gi|641574275|gb|KK855359.1|	267082-267664	10	38
ACYPI004238-RA	gi|646743859|gb|KK962475.1|	229741-229993	20	7.9	gi|641574642|gb|KK855274.1|	277930-278352	11	38
ACYPI004239-RA	gi|646768717|gb|KK961805.1|	1357392-1359205	20	8	gi|641587031|gb|KK854154.1|	643098-644799	11	42
ACYPI004245-RA	gi|646736790|gb|KK963145.1|	356626-357369	21	9.6	gi|641586673|gb|KK854207.1|	621323-621637	12	40
ACYPI004246-RA	gi|646778186|gb|KK961609.1|	2601568-2602714	19	9.4	gi|641574343|gb|KK855344.1|	59580-60829	12	21
ACYPI004248-RA	gi|646766703|gb|KK961852.1|	799261-799915	20	7.1	gi|641573919|gb|KK855439.1|	75898-76718	10	37
ACYPI004249-RA	gi|646741207|gb|KK962687.1|	169022-169720	17	7.6	gi|641587823|gb|KK854055.1|	615130-616832	11	22
ACYPI004250-RA	gi|646780222|gb|KK961554.1|	269962-274301	20	9.7	gi|641587971|gb|KK854038.1|	804060-805157	11	39
ACYPI004254-RA	gi|646781183|gb|KK961528.1|	1129123-1131677	20	9.5	gi|641565780|gb|KK857902.1|	1121-2080	9.9	40
ACYPI004258-RA	gi|646744168|gb|KK962452.1|	731117-731771	22	8.7	gi|641586585|gb|KK854220.1|	329669-330917	11	44
ACYPI004261-RA	gi|646779298|gb|KK961577.1|	413754-415156	20	9.1	gi|641587133|gb|KK854140.1|	73325-80336	9.9	37
ACYPI004266-RA	gi|646778565|gb|KK961598.1|	1496804-1497159	19	7.8	gi|641569328|gb|KK856702.1|	25764-28522	9.6	17
ACYPI004267-RA	gi|646729189|gb|KK964108.1|	141240-142007	25	7	gi|641574294|gb|KK855355.1|	87139-88083	14	20
ACYPI004268-RA	gi|646781282|gb|KK961525.1|	1037708-1039666	22	9.6	gi|641587404|gb|KK854107.1|	843810-845462	11	42
ACYPI004270-RA	gi|646746581|gb|KK962279.1|	42123-43158	21	9.1	gi|641587052|gb|KK854150.1|	495914-497788	11	38
ACYPI004271-RA	gi|646749632|gb|KK962082.1|	477596-480226	20	8.6	gi|641580479|gb|KK854569.1|	152352-153676	10	38
ACYPI004277-RA	gi|646781421|gb|KK961521.1|	3378338-3382537	21	8.7	gi|641587588|gb|KK854084.1|	371873-374077	9.9	40
ACYPI004278-RA	gi|646743576|gb|KK962494.1|	320925-321152	19	8.2	gi|641566897|gb|KK857495.1|	13387-17929	11	41
ACYPI004283-RA	gi|646753715|gb|KK961951.1|	974399-974697	19	5.4	gi|641576746|gb|KK854867.1|	102110-102503	11	20
ACYPI004286-RA	gi|646777842|gb|KK961618.1|	2244936-2245562	19	8.9	gi|641586983|gb|KK854162.1|	570331-570523	10	41
ACYPI004289-RA	gi|646776091|gb|KK961693.1|	2853627-2853886	23	10	gi|641571946|gb|KK855941.1|	5892-6153	11	23
ACYPI004294-RA	gi|646781243|gb|KK961526.1|	3320544-3320879	21	8.9	gi|641577374|gb|KK854762.1|	179759-180378	11	42
ACYPI004297-RA	gi|646750341|gb|KK962044.1|	887840-892096	19	5.6	gi|641576109|gb|KK854982.1|	147009-147235	10	19
ACYPI004299-RA	gi|646779621|gb|KK961569.1|	907240-909903	16	7.3	gi|641577874|gb|KK854678.1|	298291-301048	8.9	38

ACYPI004303-RA	gi|646776514|gb|KK961670.1|	903227-909723	20	9.6	gi|641578305|gb|KK854605.1|	29255-29611	11	42
ACYPI004307-RA	gi|646738812|gb|KK962939.1|	205655-205849	20	8.1	gi|641585616|gb|KK854368.1|	170596-171284	8.9	39
ACYPI004308-RA	gi|646751544|gb|KK961986.1|	775046-780913	22	6	gi|641572239|gb|KK855863.1|	148303-148644	11	43
ACYPI004312-RA	gi|646776647|gb|KK961663.1|	2163546-2164976	23	9.2	gi|641569596|gb|KK856622.1|	17152-17368	12	43
ACYPI004314-RA	gi|646776409|gb|KK961677.1|	607724-608833	17	8.4	gi|641569432|gb|KK856672.1|	45445-46089	13	47
ACYPI004328-RA	gi|646781659|gb|KK961513.1|	701750-706295	22	9.8	gi|641587118|gb|KK854142.1|	369296-370713	11	40
ACYPI004330-RA	gi|646782288|gb|KK961496.1|	409653-419079	21	9.7	gi|641587614|gb|KK854081.1|	579202-579465	11	40
ACYPI004334-RA	gi|646761166|gb|KK961913.1|	348944-350837	17	7.6	gi|641587404|gb|KK854107.1|	702164-703401	11	42
ACYPI004343-RA	gi|646775702|gb|KK961713.1|	487250-487567	17	7.4	gi|641579212|gb|KK854585.1|	243762-245519	10	35
ACYPI004349-RA	gi|646746310|gb|KK962297.1|	209661-211844	23	11	gi|641574504|gb|KK855305.1|	68177-68366	9.9	39
ACYPI004351-RA	gi|646738229|gb|KK962998.1|	162168-164516	22	8.9	gi|641586661|gb|KK854209.1|	584743-586316	11	43
ACYPI004355-RA	gi|646749832|gb|KK962071.1|	879513-880077	22	6	gi|641586059|gb|KK854297.1|	127906-128888	11	22
ACYPI004360-RA	gi|646778374|gb|KK961603.1|	297961-298450	18	5.7	gi|641586271|gb|KK854265.1|	162281-164967	10	40
ACYPI004365-RA	gi|646752143|gb|KK961972.1|	1122090-1122340	20	5.6	gi|641587111|gb|KK854143.1|	749231-749611	11	40
ACYPI004366-RA	gi|646746706|gb|KK962270.1|	816516-817695	23	11	gi|641587954|gb|KK854040.1|	176280-176649	8.9	39
ACYPI004368-RA	gi|646747841|gb|KK962189.1|	749628-752947	23	10	gi|641586399|gb|KK854247.1|	542392-545421	15	68
ACYPI004371-RA	gi|646746407|gb|KK962291.1|	425338-435885	21	9.9	gi|641575991|gb|KK855007.1|	177440-184903	9.8	19
ACYPI004372-RA	gi|646781243|gb|KK961526.1|	3668890-3669778	21	8.9	gi|641570188|gb|KK856433.1|	93689-95212	10	38
ACYPI004378-RA	gi|646776822|gb|KK961657.1|	520840-521825	19	7.9	gi|641575965|gb|KK855013.1|	186540-187293	9.2	31
ACYPI004388-RA	gi|646739841|gb|KK962838.1|	152718-152960	21	5.7	gi|641581389|gb|KK854557.1|	57116-58589	11	41
ACYPI004391-RA	gi|646780696|gb|KK961543.1|	3079576-3080501	15	8.8	gi|641575436|gb|KK855110.1|	47777-51009	12	37
ACYPI004395-RA	gi|646771845|gb|KK961763.1|	1182788-1187814	23	10	gi|641576286|gb|KK854946.1|	293017-294829	12	38
ACYPI004401-RA	gi|646775807|gb|KK961708.1|	2766090-2766679	21	8.6	gi|641588060|gb|KK854028.1|	627875-628203	11	42
ACYPI004410-RA	gi|646752431|gb|KK961967.1|	993443-993890	20	5.6	gi|641578122|gb|KK854636.1|	175253-177425	12	42
ACYPI004414-RA	gi|646768631|gb|KK961807.1|	1458835-1459078	19	9.2	gi|641572637|gb|KK855763.1|	189152-189601	9.5	36
ACYPI004419-RA	gi|646779375|gb|KK961575.1|	2215035-2215926	22	9.3	gi|641586809|gb|KK854188.1|	179013-181622	10	43
ACYPI004420-RA	gi|646781243|gb|KK961526.1|	3404757-3405218	21	8.9	gi|641584632|gb|KK854467.1|	426120-427001	9.8	37
ACYPI004421-RA	gi|646778699|gb|KK961594.1|	2093315-2098363	21	9.5	gi|641586734|gb|KK854198.1|	698458-700902	11	40
ACYPI004423-RA	gi|646749173|gb|KK962106.1|	245626-245938	21	5.7	gi|641588000|gb|KK854035.1|	333057-333562	11	39
ACYPI004428-RA	gi|646751012|gb|KK962011.1|	205541-207542	22	5.6	gi|641575104|gb|KK855179.1|	38720-43696	10	35
ACYPI004431-RA	gi|646753339|gb|KK961955.1|	340989-342589	22	11	gi|641577265|gb|KK854780.1|	186469-187309	10	40
ACYPI004433-RA	gi|646605552|gb|KK975337.1|	4842-5026	15	2.4	gi|641574220|gb|KK855370.1|	1738-2152	11	21

ACYPI004435-RA	gi|646747032|gb|KK962248.1|	326198-328710	20	8.7	gi|641574876|gb|KK855226.1|	159233-160009	11	39
ACYPI004437-RA	gi|646776389|gb|KK961678.1|	2082231-2082627	22	9.5	gi|641576414|gb|KK854924.1|	357999-359352	10	40
ACYPI004451-RA	gi|646777955|gb|KK961615.1|	2520089-2522450	21	10	gi|641587118|gb|KK854142.1|	223629-224761	11	40
ACYPI004453-RA	gi|646780925|gb|KK961536.1|	1357413-1360757	22	9.9	gi|641575297|gb|KK855137.1|	195774-197903	9.2	35
ACYPI004455-RA	gi|646763535|gb|KK961902.1|	177961-179647	23	10	gi|641586033|gb|KK854301.1|	427381-427833	11	21
ACYPI004457-RA	gi|646752348|gb|KK961968.1|	174229-175721	18	8	gi|641588164|gb|KK854017.1|	599840-600853	9.2	40
ACYPI004460-RA	gi|646776113|gb|KK961692.1|	323914-324976	21	9.6	gi|641567657|gb|KK857233.1|	33767-34058	9.9	35
ACYPI004461-RA	gi|646782357|gb|KK961494.1|	9213771-9214085	21	9.2	gi|641576946|gb|KK854832.1|	66010-71603	9.4	43
ACYPI004467-RA	gi|646777802|gb|KK961619.1|	1408031-1408225	24	9	gi|641573053|gb|KK855655.1|	155779-156948	11	41
ACYPI004482-RA	gi|646780574|gb|KK961546.1|	62797-63328	20	9.9	gi|641575516|gb|KK855095.1|	153104-154179	9.6	36
ACYPI004484-RA	gi|646752431|gb|KK961967.1|	1539770-1542344	20	5.6	gi|641567002|gb|KK857458.1|	14734-15492	8.3	33
ACYPI004485-RA	gi|646749616|gb|KK962083.1|	1156594-1157883	22	10	gi|641576666|gb|KK854881.1|	17587-18243	10	37
ACYPI004488-RA	gi|646750643|gb|KK962028.1|	695252-695933	21	7.5	gi|641577244|gb|KK854783.1|	259389-260013	9.7	19
ACYPI004489-RA	gi|646749464|gb|KK962091.1|	333110-333480	18	8.8	gi|641575324|gb|KK855134.1|	124294-125743	11	38
ACYPI004493-RA	gi|646782211|gb|KK961498.1|	5807849-5808235	20	9	gi|641576546|gb|KK854899.1|	104812-112033	11	40
ACYPI004501-RA	gi|646777495|gb|KK961629.1|	1568247-1568438	19	8.7	gi|641577075|gb|KK854811.1|	400970-406165	11	36
ACYPI004502-RA	gi|646750165|gb|KK962053.1|	706955-707220	25	10	gi|641577843|gb|KK854684.1|	129690-130091	10	40
ACYPI004505-RA	gi|646780723|gb|KK961542.1|	3554745-3555058	21	9.4	gi|641587614|gb|KK854081.1|	735354-735819	11	40
ACYPI004513-RA	gi|646780147|gb|KK961556.1|	1827963-1828393	18	7.5	gi|641587757|gb|KK854063.1|	367709-371256	11	38
ACYPI004515-RA	gi|646749188|gb|KK962105.1|	110767-112558	18	7.9	gi|641588082|gb|KK854026.1|	592713-594405	11	41
ACYPI004520-RA	gi|646779298|gb|KK961577.1|	2976283-2980139	20	9.1	gi|641587660|gb|KK854076.1|	255763-257939	9.9	41
ACYPI004521-RA	gi|646769001|gb|KK961801.1|	490643-490916	20	8.5	gi|641576579|gb|KK854894.1|	97177-97423	10	42
ACYPI004522-RA	gi|646738330|gb|KK962987.1|	121609-124441	19	7.9	gi|641576533|gb|KK854902.1|	12781-15419	10	39
ACYPI004524-RA	gi|646774067|gb|KK961745.1|	1351317-1352506	17	9.3	gi|641577166|gb|KK854796.1|	106151-110994	10	38
ACYPI004530-RA	gi|646775507|gb|KK961723.1|	1563690-1565762	20	9.8	gi|641587066|gb|KK854149.1|	657495-657970	11	40
ACYPI004534-RA	gi|646749036|gb|KK962114.1|	736188-736508	20	7.5	gi|641577553|gb|KK854734.1|	95091-96425	9.7	41
ACYPI004535-RA	gi|646775884|gb|KK961704.1|	1084525-1085070	20	7.6	gi|641587992|gb|KK854036.1|	544366-545052	11	41
ACYPI004536-RA	gi|646741553|gb|KK962656.1|	453225-454115	17	7.9	gi|641572207|gb|KK855871.1|	25467-26603	10	39
ACYPI004542-RA	gi|646777474|gb|KK961630.1|	1490570-1490818	21	9.3	gi|641571144|gb|KK856149.1|	73873-74073	11	20
ACYPI004544-RA	gi|646776050|gb|KK961695.1|	124517-124899	19	7.7	gi|641576229|gb|KK854956.1|	264004-264277	9.8	36
ACYPI004549-RA	gi|646781732|gb|KK961511.1|	1664817-1668346	22	9.8	gi|641587047|gb|KK854151.1|	407861-409085	10	39
ACYPI004558-RA	gi|646781344|gb|KK961523.1|	678685-681618	21	8.9	gi|641577648|gb|KK854717.1|	120265-120872	9.2	38

ACYPI004564-RA	gi|646718932|gb|KK965097.1|	37328-42450	21	9.2	gi|641573858|gb|KK855455.1|	44955-46453	9.8	37
ACYPI004568-RA	gi|646781772|gb|KK961510.1|	2521949-2522820	20	9.2	gi|641588073|gb|KK854027.1|	907751-908102	11	41
ACYPI004570-RA	gi|646781013|gb|KK961533.1|	453122-459133	18	7.6	gi|641585930|gb|KK854318.1|	212445-218613	12	44
ACYPI004573-RA	gi|646781601|gb|KK961515.1|	3544024-3547790	19	8.6	gi|641584336|gb|KK854489.1|	106778-107875	10	40
ACYPI004575-RA	gi|646737766|gb|KK963045.1|	192969-193275	21	9.2	gi|641569400|gb|KK856682.1|	103771-105097	9.7	34
ACYPI004576-RA	gi|646768534|gb|KK961809.1|	181185-182253	21	9.8	gi|641585971|gb|KK854310.1|	413515-415267	10	40
ACYPI004580-RA	gi|646782288|gb|KK961496.1|	6421686-6421929	21	9.7	gi|641573097|gb|KK855644.1|	32125-32422	10	36
ACYPI004584-RA	gi|646777955|gb|KK961615.1|	1553237-1557430	21	10	gi|641575813|gb|KK855039.1|	95621-95890	11	21
ACYPI004586-RA	gi|646770563|gb|KK961777.1|	759292-759586	19	8.7	gi|641588246|gb|KK854010.1|	728496-729755	9.2	50
ACYPI004588-RA	gi|646782288|gb|KK961496.1|	473419-480039	21	9.7	gi|641587614|gb|KK854081.1|	604630-606614	11	40
ACYPI004610-RA	gi|646747586|gb|KK962207.1|	223522-228046	20	8.8	gi|641586599|gb|KK854218.1|	188775-191086	11	42
ACYPI004611-RA	gi|646747547|gb|KK962210.1|	349406-350134	19	7.5	gi|641575436|gb|KK855110.1|	282042-282225	12	37
ACYPI004613-RA	gi|646782288|gb|KK961496.1|	6183624-6187655	21	9.7	gi|641586983|gb|KK854162.1|	317027-319410	10	41
ACYPI004615-RA	gi|646765689|gb|KK961894.1|	727266-730258	18	7.7	gi|641585616|gb|KK854368.1|	285987-289150	8.9	39
ACYPI004617-RA	gi|646767265|gb|KK961836.1|	1198385-1201978	22	9.9	gi|641575389|gb|KK855120.1|	82974-83397	10	35
ACYPI004619-RA	gi|646782357|gb|KK961494.1|	2057160-2057482	21	9.2	gi|641586258|gb|KK854267.1|	338208-339084	11	37
ACYPI004621-RA	gi|646775658|gb|KK961715.1|	934674-942063	19	8.6	gi|641567040|gb|KK857445.1|	28366-32770	11	43
ACYPI004626-RA	gi|646757520|gb|KK961926.1|	1321254-1321595	20	7.4	gi|641584582|gb|KK854471.1|	382671-383339	10	38
ACYPI004627-RA	gi|646755567|gb|KK961936.1|	867751-868024	20	8.6	gi|641588109|gb|KK854023.1|	198061-198476	10	40
ACYPI004629-RA	gi|646752567|gb|KK961965.1|	316242-317239	19	8.4	gi|641575804|gb|KK855041.1|	277531-279194	11	40
ACYPI004632-RA	gi|646775451|gb|KK961726.1|	2326009-2326304	20	5.6	gi|641587444|gb|KK854102.1|	748732-748911	11	42
ACYPI004634-RA	gi|646746706|gb|KK962270.1|	916994-918232	23	11	gi|641576684|gb|KK854877.1|	6788-7352	10	20
ACYPI004635-RA	gi|646781772|gb|KK961510.1|	3802818-3805096	20	9.2	gi|641569542|gb|KK856639.1|	25371-25595	9.3	34
ACYPI004640-RA	gi|646771352|gb|KK961768.1|	244012-244308	21	9.6	gi|641588258|gb|KK854009.1|	341771-343656	11	36
ACYPI004643-RA	gi|646766765|gb|KK961850.1|	668613-675697	18	8.9	gi|641582740|gb|KK854534.1|	292814-294849	8.1	43
ACYPI004646-RA	gi|646747032|gb|KK962248.1|	441267-441496	20	8.7	gi|641574110|gb|KK855394.1|	46224-46468	10	21
ACYPI004656-RA	gi|646775488|gb|KK961724.1|	666126-667962	19	7.8	gi|641572468|gb|KK855806.1|	59116-61102	11	39
ACYPI004658-RA	gi|646765966|gb|KK961881.1|	601970-603132	19	9.2	gi|641572166|gb|KK855881.1|	193520-194714	11	41
ACYPI004663-RA	gi|646780270|gb|KK961553.1|	2450145-2451340	22	8.6	gi|641574923|gb|KK855216.1|	91698-93305	11	38
ACYPI004665-RA	gi|646775968|gb|KK961700.1|	693276-693463	20	8.2	gi|641586150|gb|KK854282.1|	191052-191233	11	40
ACYPI004669-RA	gi|646779337|gb|KK961576.1|	1096619-1099299	18	9	gi|641584728|gb|KK854460.1|	395111-402829	9.5	37
ACYPI004684-RA	gi|646753339|gb|KK961955.1|	163370-163637	22	11	gi|641577511|gb|KK854741.1|	331705-333443	11	38

ACYPI004687-RA	gi|646782357|gb|KK961494.1|	5259186-5260009	21	9.2	gi|641574031|gb|KK855412.1|	16706-21476	12	39
ACYPI004693-RA	gi|646780574|gb|KK961546.1|	3309878-3311574	20	9.9	gi|641577644|gb|KK854718.1|	365295-365573	10	40
ACYPI004695-RA	gi|646747471|gb|KK962216.1|	745041-746935	24	6.4	gi|641575799|gb|KK855042.1|	154561-155023	10	20
ACYPI004696-RA	gi|646762191|gb|KK961910.1|	205249-205581	18	7.1	gi|641586114|gb|KK854288.1|	198254-202401	10	41
ACYPI004697-RA	gi|646782211|gb|KK961498.1|	988580-990820	20	9	gi|641576973|gb|KK854827.1|	125853-126248	11	38
ACYPI004699-RA	gi|646778274|gb|KK961606.1|	907736-908036	18	7.5	gi|641576973|gb|KK854827.1|	272840-273139	11	38
ACYPI004701-RA	gi|646741410|gb|KK962669.1|	35505-37144	21	9.3	gi|641586496|gb|KK854233.1|	83529-86482	11	38
ACYPI004727-RA	gi|646766624|gb|KK961855.1|	181816-186224	21	10	gi|641587652|gb|KK854077.1|	714027-719137	10	38
ACYPI004733-RA	gi|646749209|gb|KK962104.1|	704254-706401	21	9.2	gi|641584517|gb|KK854476.1|	324639-327725	10	39
ACYPI004740-RA	gi|646781043|gb|KK961532.1|	2081786-2082670	26	11	gi|641585446|gb|KK854396.1|	379553-379975	11	39
ACYPI004741-RA	gi|646746974|gb|KK962252.1|	1113532-1116810	23	6.1	gi|641577922|gb|KK854669.1|	260458-261103	12	21
ACYPI004748-RA	gi|646771845|gb|KK961763.1|	180243-181887	23	10	gi|641586393|gb|KK854248.1|	410119-410688	9	39
ACYPI004749-RA	gi|646772574|gb|KK961756.1|	1014560-1015013	21	8	gi|641588021|gb|KK854033.1|	255298-255699	12	40
ACYPI004750-RA	gi|646782288|gb|KK961496.1|	3414244-3419062	21	9.7	gi|641586626|gb|KK854214.1|	611128-614800	11	44
ACYPI004755-RA	gi|646772786|gb|KK961754.1|	505086-505610	22	9.6	gi|641588090|gb|KK854025.1|	993386-994124	11	43
ACYPI004759-RA	gi|646773018|gb|KK961752.1|	632571-635804	18	7.8	gi|641573457|gb|KK855555.1|	200513-201000	9.7	38
ACYPI004761-RA	gi|646781143|gb|KK961529.1|	2919162-2919486	20	10	gi|641586166|gb|KK854279.1|	313648-313882	11	45
ACYPI004766-RA	gi|646765857|gb|KK961886.1|	196224-196561	17	7.7	gi|641572959|gb|KK855678.1|	134025-134442	9.9	36
ACYPI004768-RA	gi|646747424|gb|KK962220.1|	138850-139592	22	5.7	gi|641574699|gb|KK855261.1|	139569-144132	9	35
ACYPI004770-RA	gi|646744819|gb|KK962404.1|	198161-198386	20	8.6	gi|641581402|gb|KK854555.1|	337499-338001	12	20
ACYPI004772-RA	gi|646766624|gb|KK961855.1|	1117000-1117679	21	10	gi|641573649|gb|KK855507.1|	178986-180336	11	41
ACYPI004774-RA	gi|646781732|gb|KK961511.1|	1228535-1229706	22	9.8	gi|641585773|gb|KK854342.1|	97482-97841	12	45
ACYPI004783-RA	gi|646742307|gb|KK962591.1|	130820-131807	23	7.7	gi|641586741|gb|KK854197.1|	516849-517130	11	43
ACYPI004790-RA	gi|646781421|gb|KK961521.1|	4381153-4381475	21	8.7	gi|641573181|gb|KK855624.1|	199267-199669	11	40
ACYPI004791-RA	gi|646778699|gb|KK961594.1|	2143770-2144028	21	9.5	gi|641575337|gb|KK855131.1|	70144-71266	10	37
ACYPI004803-RA	gi|646587481|gb|KK978994.1|	1270-1805	19	4.2	gi|641586335|gb|KK854256.1|	223632-224980	10	38
ACYPI004805-RA	gi|646752294|gb|KK961969.1|	613294-615121	23	9.8	gi|641572912|gb|KK855690.1|	135988-144279	9.9	38
ACYPI004816-RA	gi|646778410|gb|KK961602.1|	1995292-1999872	20	9	gi|641585690|gb|KK854356.1|	513313-518583	9.6	41
ACYPI004822-RA	gi|646742237|gb|KK962597.1|	483568-487448	21	6	gi|641578014|gb|KK854654.1|	374933-376023	12	44
ACYPI004824-RA	gi|646743164|gb|KK962524.1|	64291-67104	19	7.1	gi|641543669|gb|KK868158.1|	4001-4205	10	37
ACYPI004827-RA	gi|646774067|gb|KK961745.1|	1541836-1542070	17	9.3	gi|641572191|gb|KK855875.1|	57701-59561	9.7	43
ACYPI004832-RA	gi|646779006|gb|KK961585.1|	905670-906187	20	9.9	gi|641583088|gb|KK854527.1|	190333-190637	9.9	39

ACYPI004846-RA	gi|646750043|gb|KK962060.1|	153122-159262	21	8.3	gi|641587111|gb|KK854143.1|	599618-602011	11	40
ACYPI004854-RA	gi|646746158|gb|KK962308.1|	170150-172710	19	6.6	gi|641581389|gb|KK854557.1|	267191-270427	11	41
ACYPI004859-RA	gi|646739361|gb|KK962890.1|	484931-487641	22	9.6	gi|641572134|gb|KK855890.1|	80710-84017	10	36
ACYPI004869-RA	gi|646780794|gb|KK961540.1|	2485636-2487800	21	9.5	gi|641569291|gb|KK856713.1|	135403-137545	10	21
ACYPI004870-RA	gi|646781536|gb|KK961517.1|	1279193-1279894	19	7.7	gi|641573394|gb|KK855572.1|	164240-165459	11	40
ACYPI004872-RA	gi|646782168|gb|KK961499.1|	7298011-7298940	21	9.4	gi|641573082|gb|KK855648.1|	7162-15362	11	42
ACYPI004878-RA	gi|646779412|gb|KK961574.1|	2165323-2168377	21	5.4	gi|641575826|gb|KK855037.1|	139529-139765	10	39
ACYPI004880-RA	gi|646740073|gb|KK962814.1|	10677-11241	18	8.3	gi|641552289|gb|KK864038.1|	2878-3552	7	22
ACYPI004883-RA	gi|646780222|gb|KK961554.1|	1181800-1185558	20	9.7	gi|641586874|gb|KK854178.1|	392524-394880	9.4	38
ACYPI004886-RA	gi|646751176|gb|KK962002.1|	807434-808612	21	9.6	gi|641584977|gb|KK854443.1|	180222-181073	11	24
ACYPI004887-RA	gi|646604507|gb|KK975569.1|	3897-4127	12	4.4	gi|641584504|gb|KK854477.1|	217497-220660	9.5	38
ACYPI004908-RA	gi|646766540|gb|KK961858.1|	1265743-1266136	21	8.5	gi|641584419|gb|KK854483.1|	336349-336580	10	42
ACYPI004911-RA	gi|646782168|gb|KK961499.1|	6940829-6941078	21	9.4	gi|641585350|gb|KK854411.1|	364501-364905	11	38
ACYPI004929-RA	gi|646782334|gb|KK961495.1|	2686152-2687634	21	9	gi|641587624|gb|KK854080.1|	453905-454210	10	38
ACYPI004936-RA	gi|646688645|gb|KK967064.1|	31875-32418	14	5	gi|641569630|gb|KK856611.1|	8727-9126	11	39
ACYPI004937-RA	gi|646779936|gb|KK961561.1|	1938272-1938798	19	9.6	gi|641574676|gb|KK855265.1|	42480-42804	9.4	35
ACYPI004938-RA	gi|646749616|gb|KK962083.1|	516438-526335	22	10	gi|641576414|gb|KK854924.1|	379718-380804	10	40
ACYPI004940-RA	gi|646751569|gb|KK961985.1|	170070-172197	21	9.2	gi|641575867|gb|KK855030.1|	192580-193004	11	39
ACYPI004948-RA	gi|646778632|gb|KK961596.1|	903106-908833	21	9.2	gi|641575135|gb|KK855172.1|	206196-210461	11	44
ACYPI004950-RA	gi|646781690|gb|KK961512.1|	2130605-2131223	21	8.8	gi|641572569|gb|KK855781.1|	16346-17594	11	39
ACYPI004964-RA	gi|646781143|gb|KK961529.1|	2817345-2819427	20	10	gi|641586359|gb|KK854252.1|	210118-211459	10	39
ACYPI004966-RA	gi|646780441|gb|KK961549.1|	1007895-1009624	21	9.5	gi|641586713|gb|KK854201.1|	602441-603153	9.1	40
ACYPI004977-RA	gi|646735008|gb|KK963353.1|	216593-217591	21	9.4	gi|641569948|gb|KK856508.1|	31548-32007	10	37
ACYPI004979-RA	gi|646766904|gb|KK961846.1|	829517-829819	17	6.9	gi|641587074|gb|KK854148.1|	626072-627565	11	41
ACYPI004983-RA	gi|646741778|gb|KK962637.1|	27686-28358	19	6.1	gi|641572297|gb|KK855849.1|	66004-67023	11	21
ACYPI004985-RA	gi|646770114|gb|KK961783.1|	1455183-1455730	19	9.7	gi|641575068|gb|KK855186.1|	96154-96894	12	44
ACYPI004986-RA	gi|646775968|gb|KK961700.1|	1333259-1333531	20	8.2	gi|641587511|gb|KK854094.1|	463978-466404	10	41
ACYPI004988-RA	gi|646781809|gb|KK961509.1|	3408564-3412982	19	8.6	gi|641575337|gb|KK855131.1|	167138-172340	10	37
ACYPI004995-RA	gi|646781564|gb|KK961516.1|	1480700-1481894	23	9.4	gi|641577400|gb|KK854757.1|	266871-267138	11	37
ACYPI005000-RA	gi|646778336|gb|KK961604.1|	2454941-2457328	21	10	gi|641586515|gb|KK854230.1|	101273-101505	11	39
ACYPI005005-RA	gi|646775944|gb|KK961701.1|	1101703-1102351	22	9.3	gi|641577900|gb|KK854673.1|	10221-11002	10	41
ACYPI005018-RA	gi|646780222|gb|KK961554.1|	3301874-3303251	20	9.7	gi|641587249|gb|KK854125.1|	498925-500105	11	37

ACYPI005019-RA	gi|646777065|gb|KK961647.1|	990932-993461	20	7.9	gi|641576010|gb|KK855002.1|	162047-162699	12	43
ACYPI005028-RA	gi|646774530|gb|KK961743.1|	379038-381487	20	9.8	gi|641587688|gb|KK854072.1|	464976-466457	10	38
ACYPI005038-RA	gi|646750043|gb|KK962060.1|	757679-757990	21	8.3	gi|641588090|gb|KK854025.1|	1013571-1014274	11	43
ACYPI005041-RA	gi|646776024|gb|KK961697.1|	2409088-2409374	22	9.2	gi|641587066|gb|KK854149.1|	523781-524317	11	40
ACYPI005044-RA	gi|646780147|gb|KK961556.1|	2209925-2210176	18	7.5	gi|641574836|gb|KK855234.1|	105075-105240	10	40
ACYPI005047-RA	gi|646778112|gb|KK961611.1|	1970725-1976096	22	10	gi|641587519|gb|KK854093.1|	353250-353738	11	39
ACYPI005053-RA	gi|646747884|gb|KK962186.1|	871535-872155	22	11	gi|641576920|gb|KK854836.1|	127407-127725	11	37
ACYPI005058-RA	gi|646778112|gb|KK961611.1|	1398136-1398438	22	10	gi|641587033|gb|KK854153.1|	310248-310804	10	38
ACYPI005060-RA	gi|646782288|gb|KK961496.1|	7810738-7812362	21	9.7	gi|641573737|gb|KK855486.1|	20928-21418	9.1	33
ACYPI005067-RA	gi|646737959|gb|KK963025.1|	387330-389046	19	7.9	gi|641576333|gb|KK854937.1|	39444-40389	9.9	35
ACYPI005077-RA	gi|646749945|gb|KK962065.1|	1334214-1334546	22	6.1	gi|641571990|gb|KK855929.1|	134302-134860	9.7	19
ACYPI005079-RA	gi|646775842|gb|KK961706.1|	2238916-2240068	22	9	gi|641577193|gb|KK854791.1|	236944-237786	9.5	40
ACYPI005089-RA	gi|646722582|gb|KK964929.1|	32148-41758	23	6	gi|641570757|gb|KK856260.1|	153966-157700	10	42
ACYPI005090-RA	gi|646781421|gb|KK961521.1|	4249798-4251110	21	8.7	gi|641585350|gb|KK854411.1|	302832-303986	11	38
ACYPI005091-RA	gi|646781379|gb|KK961522.1|	3823649-3824187	22	7.6	gi|641562121|gb|KK859434.1|	518-867	8	32
ACYPI005093-RA	gi|646778767|gb|KK961592.1|	1308647-1314006	21	5.4	gi|641587793|gb|KK854059.1|	827215-829913	11	38
ACYPI005103-RA	gi|646744324|gb|KK962441.1|	588-903	20	7.2	gi|641577890|gb|KK854675.1|	30485-30932	10	40
ACYPI005113-RA	gi|646776456|gb|KK961674.1|	1311316-1313786	18	7.9	gi|641569673|gb|KK856597.1|	83050-84993	9.2	38
ACYPI005116-RA	gi|646781043|gb|KK961532.1|	1663719-1663990	26	11	gi|641572177|gb|KK855878.1|	128479-129591	10	35
ACYPI005119-RA	gi|646781183|gb|KK961528.1|	3843931-3844667	20	9.5	gi|641575910|gb|KK855021.1|	283615-283976	10	35
ACYPI005122-RA	gi|646780311|gb|KK961552.1|	3719208-3725697	21	9.7	gi|641584445|gb|KK854481.1|	213008-213363	9.9	39
ACYPI005123-RA	gi|646766833|gb|KK961848.1|	382584-385833	17	7	gi|641570135|gb|KK856449.1|	40054-41448	9.7	34
ACYPI005124-RA	gi|646775867|gb|KK961705.1|	1959000-1959187	23	10	gi|641575763|gb|KK855049.1|	78639-78851	10	40
ACYPI005126-RA	gi|646781421|gb|KK961521.1|	4256955-4257584	21	8.7	gi|641579925|gb|KK854579.1|	238653-239134	9.7	40
ACYPI005133-RA	gi|646757279|gb|KK961927.1|	570895-573892	21	8.6	gi|641578106|gb|KK854639.1|	273424-273694	11	39
ACYPI005140-RA	gi|646765689|gb|KK961894.1|	399754-400731	18	7.7	gi|641588144|gb|KK854019.1|	780050-780337	11	49
ACYPI005141-RA	gi|646746974|gb|KK962252.1|	536137-540254	23	6.1	gi|641585905|gb|KK854321.1|	423309-424538	11	39
ACYPI005144-RA	gi|646779741|gb|KK961566.1|	2939635-2940394	20	8.5	gi|641587905|gb|KK854046.1|	533605-533982	10	43
ACYPI005150-RA	gi|646780270|gb|KK961553.1|	3815950-3816911	22	8.6	gi|641584806|gb|KK854455.1|	194250-194740	11	43
ACYPI005152-RA	gi|646677811|gb|KK967447.1|	6679-7767	13	8.3	gi|641572782|gb|KK855725.1|	106839-107105	10	36
ACYPI005155-RA	gi|646775523|gb|KK961722.1|	1176464-1181597	21	9.7	gi|641564265|gb|KK858512.1|	4459-5466	11	39
ACYPI005157-RA	gi|646776608|gb|KK961665.1|	1585651-1587020	23	9.3	gi|641585905|gb|KK854321.1|	454183-454988	11	39

ACYPI005158-RA	gi|646779898|gb|KK961562.1|	2940979-2943761	19	9.3	gi|641570952|gb|KK856204.1|	141929-143031	10	21
ACYPI005163-RA	gi|646781243|gb|KK961526.1|	2770162-2774068	21	8.9	gi|641586496|gb|KK854233.1|	92712-93712	11	38
ACYPI005167-RA	gi|646781143|gb|KK961529.1|	2195844-2196738	20	10	gi|641586351|gb|KK854253.1|	79758-82802	10	37
ACYPI005172-RA	gi|646779066|gb|KK961583.1|	216199-220279	19	7.9	gi|641584743|gb|KK854459.1|	134000-140372	11	36
ACYPI005174-RA	gi|646776490|gb|KK961672.1|	21158-22761	20	10	gi|641566437|gb|KK857661.1|	22421-24579	9.2	34
ACYPI005175-RA	gi|646732968|gb|KK963602.1|	367061-367781	20	9.9	gi|641572900|gb|KK855693.1|	244365-246416	11	37
ACYPI005188-RA	gi|646781344|gb|KK961523.1|	1700270-1703527	21	8.9	gi|641586943|gb|KK854167.1|	543069-545195	11	38
ACYPI005193-RA	gi|646781628|gb|KK961514.1|	4639000-4641938	23	9	gi|641584582|gb|KK854471.1|	457316-461642	10	38
ACYPI005200-RA	gi|646781043|gb|KK961532.1|	4197254-4198590	26	11	gi|641577097|gb|KK854808.1|	130800-136520	11	41
ACYPI005202-RA	gi|646741767|gb|KK962638.1|	36281-37350	21	10	gi|641586033|gb|KK854301.1|	413764-414045	11	21
ACYPI005207-RA	gi|646770998|gb|KK961772.1|	1781680-1783927	22	9.8	gi|641586857|gb|KK854181.1|	442949-449629	9.8	39
ACYPI005208-RA	gi|646753093|gb|KK961958.1|	1121400-1124006	21	9.3	gi|641585957|gb|KK854313.1|	214206-223814	10	37
ACYPI005219-RA	gi|646747841|gb|KK962189.1|	317968-318410	23	10	gi|641567206|gb|KK857389.1|	49552-53372	10	37
ACYPI005221-RA	gi|646771092|gb|KK961771.1|	528931-530193	23	9.7	gi|641585440|gb|KK854397.1|	400089-406486	9	38
ACYPI005227-RA	gi|646775789|gb|KK961709.1|	1188784-1189471	20	9.6	gi|641574508|gb|KK855304.1|	156978-160016	11	22
ACYPI005241-RA	gi|646765790|gb|KK961889.1|	934732-935449	22	8.8	gi|641575214|gb|KK855156.1|	191420-191817	8.8	34
ACYPI005243-RA	gi|646747884|gb|KK962186.1|	506967-513329	22	11	gi|641574078|gb|KK855401.1|	215069-221662	11	41
ACYPI005246-RA	gi|646775258|gb|KK961737.1|	596924-598829	19	9.8	gi|641585412|gb|KK854402.1|	98053-99375	9.8	39
ACYPI005247-RA	gi|646781732|gb|KK961511.1|	2219028-2220589	22	9.8	gi|641584445|gb|KK854481.1|	415356-416885	9.9	39
ACYPI005253-RA	gi|646755393|gb|KK961937.1|	203176-204012	19	6.8	gi|641576409|gb|KK854925.1|	102260-102986	11	44
ACYPI005264-RA	gi|646740304|gb|KK962788.1|	600228-607968	24	9.6	gi|641587153|gb|KK854137.1|	869701-871131	10	42
ACYPI005270-RA	gi|646778213|gb|KK961608.1|	1397324-1398549	19	8.8	gi|641584504|gb|KK854477.1|	176959-177696	9.5	38
ACYPI005271-RA	gi|646778149|gb|KK961610.1|	1424943-1425127	19	8.9	gi|641567973|gb|KK857126.1|	55336-55499	10	39
ACYPI005282-RA	gi|646778336|gb|KK961604.1|	2771944-2772824	21	10	gi|641573036|gb|KK855659.1|	185266-190129	13	49
ACYPI005283-RA	gi|646775488|gb|KK961724.1|	721206-722676	19	7.8	gi|641584728|gb|KK854460.1|	191603-192662	9.5	37
ACYPI005292-RA	gi|646576893|gb|KK981008.1|	13725-13998	8.2	3.8	gi|641578305|gb|KK854605.1|	149932-150581	11	42
ACYPI005295-RA	gi|646781313|gb|KK961524.1|	459701-459957	19	8.5	gi|641577535|gb|KK854737.1|	469401-470162	11	42
ACYPI005296-RA	gi|646782211|gb|KK961498.1|	78611-78822	20	9	gi|641586928|gb|KK854169.1|	579498-585939	11	39
ACYPI005299-RA	gi|646742413|gb|KK962582.1|	832617-841435	23	10	gi|641574298|gb|KK855354.1|	63805-65387	9.2	38
ACYPI005300-RA	gi|646747547|gb|KK962210.1|	393459-395163	19	7.5	gi|641566900|gb|KK857494.1|	38695-39377	9.9	40
ACYPI005306-RA	gi|646775451|gb|KK961726.1|	1211785-1213304	20	5.6	gi|641574971|gb|KK855204.1|	33793-34257	11	21
ACYPI005308-RA	gi|646775842|gb|KK961706.1|	1240797-1243932	22	9	gi|641585801|gb|KK854337.1|	528369-530177	10	38

ACYPI005313-RA	gi|646751664|gb|KK961982.1|	1163913-1164442	21	9.5	gi|641576328|gb|KK854938.1|	182188-182646	11	36
ACYPI005314-RA	gi|646731892|gb|KK963756.1|	99098-101603	22	9.8	gi|641576044|gb|KK854994.1|	254456-257819	8.9	37
ACYPI005317-RA	gi|646750600|gb|KK962030.1|	1446627-1447523	23	8.6	gi|641575593|gb|KK855082.1|	163841-164336	10	38
ACYPI005321-RA	gi|646769577|gb|KK961791.1|	568856-569535	18	8.3	gi|641574022|gb|KK855414.1|	109481-110349	11	33
ACYPI005324-RA	gi|646743772|gb|KK962481.1|	668762-669682	22	8.9	gi|641574976|gb|KK855203.1|	212518-212889	11	21
ACYPI005327-RA	gi|646781118|gb|KK961530.1|	4365006-4365912	21	10	gi|641588294|gb|KK854006.1|	410837-423597	8.7	40
ACYPI005329-RA	gi|646747586|gb|KK962207.1|	405420-405987	20	8.8	gi|641574746|gb|KK855253.1|	100521-101398	8.9	35
ACYPI005331-RA	gi|646767265|gb|KK961836.1|	845425-846311	22	9.9	gi|641583574|gb|KK854520.1|	412333-413035	9.2	40
ACYPI005339-RA	gi|646768294|gb|KK961814.1|	133164-134676	23	10	gi|641576315|gb|KK854940.1|	171178-173268	9.9	40
ACYPI005349-RA	gi|646767948|gb|KK961821.1|	524252-524526	17	7.1	gi|641588164|gb|KK854017.1|	936166-936869	9.2	40
ACYPI005359-RA	gi|646775540|gb|KK961721.1|	948316-948798	17	8.1	gi|641577704|gb|KK854707.1|	413030-419324	10	38
ACYPI005360-RA	gi|646734874|gb|KK963368.1|	29893-33208	21	8	gi|641586235|gb|KK854270.1|	99823-101676	11	39
ACYPI005363-RA	gi|646775789|gb|KK961709.1|	117377-119088	20	9.6	gi|641585725|gb|KK854350.1|	579913-581463	12	41
ACYPI005364-RA	gi|646769774|gb|KK961788.1|	548323-549374	20	9.2	gi|641576791|gb|KK854858.1|	126215-126547	9.2	42
ACYPI005367-RA	gi|646759358|gb|KK961918.1|	197880-207517	16	5.4	gi|641587848|gb|KK854052.1|	682585-684933	11	39
ACYPI005368-RA	gi|646745612|gb|KK962344.1|	170763-171937	20	8.2	gi|641586096|gb|KK854291.1|	187434-189058	11	46
ACYPI005380-RA	gi|646779498|gb|KK961572.1|	2042685-2050360	22	5.6	gi|641587832|gb|KK854054.1|	417565-420747	9.8	20
ACYPI005389-RA	gi|646749302|gb|KK962099.1|	476936-478271	17	7	gi|641575759|gb|KK855050.1|	389881-390358	11	38
ACYPI005391-RA	gi|646782334|gb|KK961495.1|	3403864-3405361	21	9	gi|641586203|gb|KK854273.1|	487310-490046	9.5	40
ACYPI005393-RA	gi|646750252|gb|KK962049.1|	878386-878883	22	10	gi|641579952|gb|KK854573.1|	297496-297707	11	21
ACYPI005394-RA	gi|646738229|gb|KK962998.1|	340936-346928	22	8.9	gi|641575987|gb|KK855008.1|	40582-41061	9.9	20
ACYPI005395-RA	gi|646779141|gb|KK961581.1|	2751958-2753981	21	9.1	gi|641563481|gb|KK858852.1|	20967-21424	8.7	35
ACYPI005400-RA	gi|646768631|gb|KK961807.1|	430831-432383	19	9.2	gi|641575222|gb|KK855154.1|	84349-86176	11	37
ACYPI005401-RA	gi|646768261|gb|KK961815.1|	1265106-1267726	22	6.6	gi|641586240|gb|KK854269.1|	362618-364815	11	21
ACYPI005411-RA	gi|646777288|gb|KK961638.1|	3935866-3937635	22	6.1	gi|641576151|gb|KK854974.1|	138735-139998	11	44
ACYPI005416-RA	gi|646780105|gb|KK961557.1|	203172-203790	21	9.7	gi|641577516|gb|KK854740.1|	132328-132623	10	21
ACYPI005419-RA	gi|646781690|gb|KK961512.1|	1817046-1818934	21	8.8	gi|641562852|gb|KK859115.1|	4933-6125	10	35
ACYPI005429-RA	gi|646766382|gb|KK961864.1|	1020344-1020675	22	9.7	gi|641587555|gb|KK854089.1|	545846-546238	11	39
ACYPI005434-RA	gi|646782043|gb|KK961502.1|	4113098-4113408	20	9.2	gi|641574508|gb|KK855304.1|	201492-202297	11	22
ACYPI005442-RA	gi|646781659|gb|KK961513.1|	2891118-2891548	22	9.8	gi|641569519|gb|KK856646.1|	34454-38585	10	39
ACYPI005448-RA	gi|646767850|gb|KK961823.1|	987586-988003	21	9.8	gi|641574876|gb|KK855226.1|	153072-156281	11	39
ACYPI005456-RA	gi|646780925|gb|KK961536.1|	949915-950232	22	9.9	gi|641578069|gb|KK854646.1|	96534-97246	9.9	20

ACYPI005457-RA	gi|646781313|gb|KK961524.1|	3533812-3535266	19	8.5	gi|641570134|gb|KK856450.1|	101957-102630	10	38
ACYPI005464-RA	gi|646746663|gb|KK962273.1|	134543-134783	21	10	gi|641575468|gb|KK855105.1|	109734-110182	9.5	20
ACYPI005465-RA	gi|646778410|gb|KK961602.1|	1903059-1903606	20	9	gi|641588051|gb|KK854029.1|	544386-544951	9.6	40
ACYPI005467-RA	gi|646745091|gb|KK962384.1|	331785-333112	19	7.9	gi|641586053|gb|KK854298.1|	179305-185094	11	41
ACYPI005468-RA	gi|646776184|gb|KK961688.1|	873655-874692	21	9.6	gi|641577179|gb|KK854794.1|	17765-18113	11	22
ACYPI005470-RA	gi|646782334|gb|KK961495.1|	2952482-2952715	21	9	gi|641567321|gb|KK857348.1|	63741-65314	19	43
ACYPI005475-RA	gi|646545108|gb|KK987133.1|	265-513	22	5.8	gi|641584474|gb|KK854479.1|	243018-244158	9.5	40
ACYPI005476-RA	gi|646776514|gb|KK961670.1|	1595146-1596259	20	9.6	gi|641577760|gb|KK854699.1|	177971-178961	9.8	41
ACYPI005478-RA	gi|646743261|gb|KK962517.1|	34238-35511	19	8.9	gi|641588060|gb|KK854028.1|	715514-715774	11	42
ACYPI005479-RA	gi|646776368|gb|KK961679.1|	1787788-1788552	21	9.7	gi|641585342|gb|KK854413.1|	58972-61381	11	39
ACYPI005482-RA	gi|646776148|gb|KK961690.1|	1920350-1920892	21	9.4	gi|641587671|gb|KK854074.1|	285826-287868	11	40
ACYPI005485-RA	gi|646776128|gb|KK961691.1|	1357846-1363098	21	9.2	gi|641568762|gb|KK856874.1|	32002-40109	11	41
ACYPI005489-RA	gi|646777416|gb|KK961632.1|	556404-557617	22	8.8	gi|641571849|gb|KK855965.1|	84351-84907	11	40
ACYPI005495-RA	gi|646775507|gb|KK961723.1|	866117-866347	20	9.8	gi|641587118|gb|KK854142.1|	264655-265555	11	40
ACYPI005496-RA	gi|646750600|gb|KK962030.1|	1570058-1572154	23	8.6	gi|641587527|gb|KK854092.1|	149009-150831	10	38
ACYPI005499-RA	gi|646781690|gb|KK961512.1|	3337509-3338589	21	8.8	gi|641571428|gb|KK856074.1|	123365-124427	11	39
ACYPI005509-RA	gi|646781421|gb|KK961521.1|	4436012-4437649	21	8.7	gi|641585642|gb|KK854364.1|	135183-135547	9.9	38
ACYPI005512-RA	gi|646673021|gb|KK967651.1|	768-1149	10	6.1	gi|641570042|gb|KK856478.1|	90750-91459	10	36
ACYPI005513-RA	gi|646777440|gb|KK961631.1|	510973-513975	22	9.7	gi|641576490|gb|KK854909.1|	221015-223174	12	46
ACYPI005514-RA	gi|646781043|gb|KK961532.1|	332887-333200	26	11	gi|641579937|gb|KK854576.1|	460084-460348	9.4	41
ACYPI005517-RA	gi|646782334|gb|KK961495.1|	7245001-7245990	21	9	gi|641584681|gb|KK854464.1|	40862-44650	11	42
ACYPI005519-RA	gi|646781564|gb|KK961516.1|	1781752-1782253	23	9.4	gi|641585022|gb|KK854440.1|	221326-221787	10	39
ACYPI005521-RA	gi|646775595|gb|KK961718.1|	1326427-1327601	21	8.9	gi|641587866|gb|KK854050.1|	453414-455214	10	38
ACYPI005524-RA	gi|646776647|gb|KK961663.1|	2188197-2190437	23	9.2	gi|641556681|gb|KK861957.1|	7030-12038	9.6	38
ACYPI005525-RA	gi|646749302|gb|KK962099.1|	208435-210978	17	7	gi|641587365|gb|KK854113.1|	381765-382475	12	45
ACYPI005528-RA	gi|646781212|gb|KK961527.1|	1172672-1178289	21	10	gi|641587074|gb|KK854148.1|	585817-586526	11	41
ACYPI005535-RA	gi|646748407|gb|KK962151.1|	606025-606543	22	9.1	gi|641570280|gb|KK856402.1|	107703-108266	11	40
ACYPI005540-RA	gi|646749616|gb|KK962083.1|	228658-228935	22	10	gi|641585383|gb|KK854406.1|	361132-361340	11	37
ACYPI005543-RA	gi|646782357|gb|KK961494.1|	5931366-5934131	21	9.2	gi|641586955|gb|KK854165.1|	578742-579556	11	40
ACYPI005557-RA	gi|646748086|gb|KK962172.1|	851816-852182	20	10	gi|641586928|gb|KK854169.1|	130737-132561	11	39
ACYPI005578-RA	gi|646736260|gb|KK963205.1|	78048-82136	16	8.4	gi|641585790|gb|KK854339.1|	255438-256991	9.7	38
ACYPI005579-RA	gi|646738615|gb|KK962959.1|	242512-242939	21	9.5	gi|641584504|gb|KK854477.1|	196657-197768	9.5	38

ACYPI005580-RA	gi|646777363|gb|KK961635.1|	1885460-1885947	18	9.2	gi|641576608|gb|KK854891.1|	218827-226381	11	37
ACYPI005583-RA	gi|646768447|gb|KK961811.1|	272092-272353	22	9.7	gi|641572719|gb|KK855742.1|	196892-199969	12	23
ACYPI005585-RA	gi|646743261|gb|KK962517.1|	321893-333156	19	8.9	gi|641570819|gb|KK856245.1|	50230-52543	10	45
ACYPI005588-RA	gi|646781143|gb|KK961529.1|	4199926-4202060	20	10	gi|641587555|gb|KK854089.1|	302025-302829	11	39
ACYPI005593-RA	gi|646742471|gb|KK962577.1|	250665-251404	22	5.9	gi|641570141|gb|KK856447.1|	91282-91541	11	40
ACYPI005594-RA	gi|646765669|gb|KK961895.1|	80366-80653	18	7.2	gi|641569929|gb|KK856514.1|	83410-83629	10	18
ACYPI005597-RA	gi|646782357|gb|KK961494.1|	1275957-1277319	21	9.2	gi|641585340|gb|KK854414.1|	459480-460016	11	42
ACYPI005600-RA	gi|646748474|gb|KK962147.1|	469276-470105	20	9	gi|641586893|gb|KK854175.1|	507570-510208	11	41
ACYPI005603-RA	gi|646779621|gb|KK961569.1|	1268973-1272135	16	7.3	gi|641569574|gb|KK856629.1|	142373-144946	24	120
ACYPI005606-RA	gi|646782288|gb|KK961496.1|	7553165-7553397	21	9.7	gi|641583313|gb|KK854525.1|	340100-341711	9.5	40
ACYPI005607-RA	gi|646770477|gb|KK961778.1|	1131394-1131659	21	9.1	gi|641573015|gb|KK855664.1|	83545-84307	11	42
ACYPI005612-RA	gi|646778448|gb|KK961601.1|	1513488-1516818	19	8.3	gi|641587981|gb|KK854037.1|	1047750-1057299	10	40
ACYPI005613-RA	gi|646778767|gb|KK961592.1|	208015-211193	21	5.4	gi|641577271|gb|KK854779.1|	200827-204746	11	21
ACYPI005614-RA	gi|646773018|gb|KK961752.1|	407033-411250	18	7.8	gi|641550113|gb|KK865049.1|	2201-3140	11	48
ACYPI005615-RA	gi|646782127|gb|KK961500.1|	2709931-2710151	22	9.6	gi|641587171|gb|KK854135.1|	346196-346642	10	39
ACYPI005619-RA	gi|646782357|gb|KK961494.1|	3452828-3456929	21	9.2	gi|641585423|gb|KK854400.1|	71641-73816	11	36
ACYPI005622-RA	gi|646765948|gb|KK961882.1|	282593-283083	18	7.3	gi|641569260|gb|KK856723.1|	24941-25724	9.7	34
ACYPI005626-RA	gi|646749945|gb|KK962065.1|	1749651-1756478	22	6.1	gi|641587462|gb|KK854100.1|	280175-280563	10	21
ACYPI005627-RA	gi|646777089|gb|KK961646.1|	757626-761528	20	9.4	gi|641576074|gb|KK854988.1|	255584-258255	12	46
ACYPI005634-RA	gi|646749512|gb|KK962088.1|	524647-527487	18	7.2	gi|641585469|gb|KK854393.1|	54249-56727	10	37
ACYPI005638-RA	gi|646767697|gb|KK961826.1|	867427-870484	21	9.8	gi|641587256|gb|KK854124.1|	319168-322305	9.7	40
ACYPI005644-RA	gi|646753890|gb|KK961949.1|	744826-745365	20	8.9	gi|641587171|gb|KK854135.1|	488624-491178	10	39
ACYPI005647-RA	gi|646781043|gb|KK961532.1|	2426872-2430438	26	11	gi|641588051|gb|KK854029.1|	813722-817983	9.6	40
ACYPI005655-RA	gi|646781690|gb|KK961512.1|	1960516-1961106	21	8.8	gi|641580486|gb|KK854568.1|	117604-118579	11	36
ACYPI005660-RA	gi|646777729|gb|KK961621.1|	1881764-1888857	21	9.8	gi|641576266|gb|KK854949.1|	276746-282927	11	45
ACYPI005668-RA	gi|646682319|gb|KK967352.1|	30010-30570	13	6.2	gi|641567546|gb|KK857269.1|	31845-32322	8.5	40
ACYPI005672-RA	gi|646747471|gb|KK962216.1|	712268-712957	24	6.4	gi|641575218|gb|KK855155.1|	162305-163819	11	21
ACYPI005673-RA	gi|646766943|gb|KK961845.1|	636703-637583	18	8.9	gi|641586033|gb|KK854301.1|	123736-124572	11	21
ACYPI005674-RA	gi|646736283|gb|KK963203.1|	80665-81078	24	9.7	gi|641570807|gb|KK856248.1|	29073-29837	11	38
ACYPI005676-RA	gi|646780978|gb|KK961534.1|	393472-394280	20	8	gi|641575656|gb|KK855069.1|	50248-50699	11	42
ACYPI005677-RA	gi|646778699|gb|KK961594.1|	663077-665563	21	9.5	gi|641586734|gb|KK854198.1|	802019-806121	11	40
ACYPI005684-RA	gi|646780270|gb|KK961553.1|	4065796-4066676	22	8.6	gi|641571562|gb|KK856035.1|	125139-125835	9.8	36

ACYPI005687-RA	gi|646742319|gb|KK962590.1|	92513-95142	18	8.5	gi|641585755|gb|KK854345.1|	56916-60099	9.8	39
ACYPI005689-RA	gi|646781564|gb|KK961516.1|	1295961-1296625	23	9.4	gi|641574882|gb|KK855225.1|	166390-167975	9.3	36
ACYPI005700-RA	gi|646763535|gb|KK961902.1|	630096-630345	23	10	gi|641574454|gb|KK855318.1|	34954-37019	8.5	41
ACYPI005705-RA	gi|646779498|gb|KK961572.1|	828920-829271	22	5.6	gi|641584876|gb|KK854450.1|	219293-219645	11	25
ACYPI005706-RA	gi|646749114|gb|KK962109.1|	552627-555443	20	8.8	gi|641578084|gb|KK854643.1|	250661-251047	11	40
ACYPI005711-RA	gi|646765705|gb|KK961893.1|	1434813-1435318	22	9.7	gi|641570861|gb|KK856232.1|	164144-164695	11	39
ACYPI005720-RA	gi|646780889|gb|KK961537.1|	5179999-5180478	22	10	gi|641566812|gb|KK857525.1|	66294-66835	11	42
ACYPI005722-RA	gi|646750505|gb|KK962035.1|	248950-249273	19	9.9	gi|641577022|gb|KK854820.1|	264209-265826	10	21
ACYPI005727-RA	gi|646775867|gb|KK961705.1|	998516-1007354	23	10	gi|641574951|gb|KK855209.1|	102379-106981	10	38
ACYPI005729-RA	gi|646743084|gb|KK962530.1|	163288-164055	24	6.2	gi|641573571|gb|KK855526.1|	73764-74598	9.8	36
ACYPI005735-RA	gi|646781809|gb|KK961509.1|	3324099-3325509	19	8.6	gi|641587660|gb|KK854076.1|	548013-553435	9.9	41
ACYPI005743-RA	gi|646729045|gb|KK964126.1|	147961-149410	20	11	gi|641587383|gb|KK854110.1|	505574-510334	10	37
ACYPI005747-RA	gi|646775821|gb|KK961707.1|	836522-838985	23	9.1	gi|641586915|gb|KK854171.1|	500042-504602	10	38
ACYPI005761-RA	gi|646751120|gb|KK962005.1|	1469458-1469835	21	5.8	gi|641569155|gb|KK856752.1|	97415-98955	11	21
ACYPI005769-RA	gi|646755095|gb|KK961939.1|	396175-397628	15	8.9	gi|641587383|gb|KK854110.1|	124758-125027	10	37
ACYPI005770-RA	gi|646779976|gb|KK961560.1|	684147-685126	16	7.9	gi|641587084|gb|KK854146.1|	539067-539335	9	40
ACYPI005774-RA	gi|646778767|gb|KK961592.1|	88103-88358	21	5.4	gi|641568044|gb|KK857103.1|	26679-28078	10	36
ACYPI005785-RA	gi|646770047|gb|KK961784.1|	167561-169527	21	9.1	gi|641566247|gb|KK857731.1|	39054-43768	9.4	35
ACYPI005787-RA	gi|646776219|gb|KK961687.1|	1625147-1625384	21	9.9	gi|641579925|gb|KK854579.1|	223946-224697	9.7	40
ACYPI005789-RA	gi|646738455|gb|KK962975.1|	284769-291267	23	9.5	gi|641588033|gb|KK854031.1|	1256161-125650[illegible]	11	38
ACYPI005793-RA	gi|646769102|gb|KK961799.1|	180565-181419	20	8.3	gi|641571308|gb|KK856106.1|	33544-36369	8.9	36
ACYPI005799-RA	gi|646573623|gb|KK981629.1|	1182-1469	13	6.9	gi|641584658|gb|KK854465.1|	238742-239030	11	37
ACYPI005802-RA	gi|646775884|gb|KK961704.1|	646546-647233	20	7.6	gi|641578109|gb|KK854638.1|	280794-288242	14	46
ACYPI005806-RA	gi|646742726|gb|KK962557.1|	202633-203701	20	9.1	gi|641587527|gb|KK854092.1|	427324-428502	10	38
ACYPI005817-RA	gi|646781421|gb|KK961521.1|	2303331-2303680	21	8.7	gi|641587614|gb|KK854081.1|	621167-622162	11	40
ACYPI005821-RA	gi|646780358|gb|KK961551.1|	1980517-1983748	21	8.8	gi|641570307|gb|KK856394.1|	6007-7196	8.9	39
ACYPI005824-RA	gi|646777125|gb|KK961644.1|	2232728-2233437	20	10	gi|641585395|gb|KK854405.1|	453291-455413	10	38
ACYPI005826-RA	gi|646782357|gb|KK961494.1|	9003940-9009906	21	9.2	gi|641566738|gb|KK857551.1|	5239-5401	11	39
ACYPI005830-RA	gi|646751735|gb|KK961980.1|	638775-641943	21	5.5	gi|641588082|gb|KK854026.1|	1173913-117481[illegible]	11	41
ACYPI005832-RA	gi|646781536|gb|KK961517.1|	118133-120894	19	7.7	gi|641573729|gb|KK855488.1|	191358-193548	10	43
ACYPI005839-RA	gi|646750185|gb|KK962052.1|	465161-465976	18	7.1	gi|641577316|gb|KK854772.1|	206720-207362	9.4	36
ACYPI005844-RA	gi|646779337|gb|KK961576.1|	728964-729703	18	9	gi|641585833|gb|KK854332.1|	167986-168474	10	37

ACYPI005848-RA	gi|646780889|gb|KK961537.1|	640572-643363	22	10	gi|641571154|gb|KK856146.1|	77940-78822	9.1	35
ACYPI005852-RA	gi|646753799|gb|KK961950.1|	2018910-2019690	23	9.2	gi|641542181|gb|KK868875.1|	94-437	9.8	32
ACYPI005853-RA	gi|646700461|gb|KK965992.1|	18068-19051	70	24	gi|641571886|gb|KK855956.1|	70977-73719	9.4	35
ACYPI005854-RA	gi|646781243|gb|KK961526.1|	3797242-3797700	21	8.9	gi|641586528|gb|KK854228.1|	263950-265034	10	42
ACYPI005858-RA	gi|646781344|gb|KK961523.1|	661397-672200	21	8.9	gi|641574275|gb|KK855359.1|	188970-199118	10	38
ACYPI005859-RA	gi|646746828|gb|KK962262.1|	534864-535128	20	9.2	gi|641588033|gb|KK854031.1|	489134-489475	11	38
ACYPI005865-RA	gi|646751569|gb|KK961985.1|	217504-218010	21	9.2	gi|641570198|gb|KK856430.1|	40541-48319	10	36
ACYPI005867-RA	gi|646782334|gb|KK961495.1|	2446244-2449854	21	9	gi|641587066|gb|KK854149.1|	574712-574970	11	40
ACYPI005880-RA	gi|646778029|gb|KK961613.1|	2884287-2885428	21	8.6	gi|641554119|gb|KK863172.1|	1850-2091	7	24
ACYPI005883-RA	gi|646744916|gb|KK962397.1|	559562-560331	18	8.3	gi|641575377|gb|KK855123.1|	64068-65287	10	38
ACYPI005885-RA	gi|646556697|gb|KK984938.1|	7423-9162	14	5.9	gi|641576362|gb|KK854933.1|	369784-374390	10	37
ACYPI005886-RA	gi|646749173|gb|KK962106.1|	1174233-1184062	21	5.7	gi|641587840|gb|KK854053.1|	1022709-1023872	11	43
ACYPI005896-RA	gi|646747046|gb|KK962247.1|	752023-754104	22	9	gi|641578330|gb|KK854601.1|	311508-313591	11	38
ACYPI005897-RA	gi|646768717|gb|KK961805.1|	1241920-1242802	20	8	gi|641575549|gb|KK855090.1|	71895-74070	10	38
ACYPI005899-RA	gi|646772045|gb|KK961761.1|	74250-74772	21	9.6	gi|641575377|gb|KK855123.1|	25715-27356	10	38
ACYPI005908-RA	gi|646748474|gb|KK962147.1|	366701-367496	20	9	gi|641573733|gb|KK855487.1|	23403-24610	13	45
ACYPI005913-RA	gi|646734527|gb|KK963409.1|	161820-162449	21	9.3	gi|641588119|gb|KK854022.1|	935624-936217	10	40
ACYPI005914-RA	gi|646738466|gb|KK962974.1|	130781-135207	30	14	gi|641588236|gb|KK854011.1|	384740-385938	9.7	38
ACYPI005917-RA	gi|646741540|gb|KK962657.1|	403975-404317	19	7	gi|641574699|gb|KK855261.1|	199522-200784	9	35
ACYPI005934-RA	gi|646779337|gb|KK961576.1|	419527-419980	18	9	gi|641588194|gb|KK854015.1|	654638-658076	9.4	40
ACYPI005940-RA	gi|646782276|gb|KK961497.1|	7209954-7210503	21	9.7	gi|641584281|gb|KK854493.1|	50209-50717	12	41
ACYPI005949-RA	gi|646772897|gb|KK961753.1|	1491284-1495508	21	8.7	gi|641569851|gb|KK856541.1|	65317-70785	11	21
ACYPI005955-RA	gi|646776647|gb|KK961663.1|	2215035-2218548	23	9.2	gi|641556681|gb|KK861957.1|	16026-16331	9.6	38
ACYPI005962-RA	gi|646775807|gb|KK961708.1|	1845211-1845625	21	8.6	gi|641575867|gb|KK855030.1|	339703-344149	11	39
ACYPI005967-RA	gi|646757520|gb|KK961926.1|	1586356-1589015	20	7.4	gi|641588309|gb|KK854005.1|	294109-294307	11	40
ACYPI005975-RA	gi|646782288|gb|KK961496.1|	6234527-6240225	21	9.7	gi|641585900|gb|KK854322.1|	662664-662934	12	43
ACYPI005976-RA	gi|646781143|gb|KK961529.1|	3916762-3921647	20	10	gi|641576437|gb|KK854920.1|	198790-199072	10	39
ACYPI005982-RA	gi|646781212|gb|KK961527.1|	2286713-2289432	21	10	gi|641587527|gb|KK854092.1|	590726-592107	10	38
ACYPI005987-RA	gi|646781536|gb|KK961517.1|	2281321-2281549	19	7.7	gi|641585967|gb|KK854311.1|	143701-144312	11	40
ACYPI005988-RA	gi|646779662|gb|KK961568.1|	1205074-1205373	20	9.3	gi|641569948|gb|KK856508.1|	192939-193218	10	37
ACYPI005997-RA	gi|646747046|gb|KK962247.1|	612278-614172	22	9	gi|641587912|gb|KK854045.1|	713341-713667	11	39
ACYPI006008-RA	gi|646767439|gb|KK961832.1|	1952538-1953162	23	10	gi|641558930|gb|KK860898.1|	7198-8430	8.8	34

ACYPI006009-RA	gi|646775595|gb|KK961718.1|	762310-769966	21	8.9	gi|641587365|gb|KK854113.1|	307805-312010	12	45
ACYPI006013-RA	gi|646765880|gb|KK961885.1|	1071354-1073042	23	10	gi|641568533|gb|KK856949.1|	78400-81429	9.7	38
ACYPI006023-RA	gi|646747046|gb|KK962247.1|	770758-771457	22	9	gi|641575449|gb|KK855108.1|	35986-36476	11	45
ACYPI006028-RA	gi|646780978|gb|KK961534.1|	2496029-2498313	20	8	gi|641575635|gb|KK855074.1|	268682-271378	9.6	38
ACYPI006033-RA	gi|646778983|gb|KK961586.1|	297548-299398	20	9.9	gi|641586335|gb|KK854256.1|	422890-424570	10	38
ACYPI006038-RA	gi|646746556|gb|KK962281.1|	530512-532014	20	9.1	gi|641585515|gb|KK854385.1|	184366-184707	11	40
ACYPI006043-RA	gi|646770998|gb|KK961772.1|	779379-783836	22	9.8	gi|641572860|gb|KK855703.1|	5840-9040	9.8	36
ACYPI006058-RA	gi|646777313|gb|KK961637.1|	1737743-1738177	20	8.2	gi|641568319|gb|KK857014.1|	14952-15195	9.5	33
ACYPI006059-RA	gi|646781243|gb|KK961526.1|	4078636-4082723	21	8.9	gi|641575725|gb|KK855055.1|	168327-168540	10	37
ACYPI006066-RA	gi|646778029|gb|KK961613.1|	2105827-2109018	21	8.6	gi|641586975|gb|KK854163.1|	532106-535528	11	40
ACYPI006067-RA	gi|646780358|gb|KK961551.1|	732792-733481	21	8.8	gi|641584305|gb|KK854491.1|	31967-34288	11	39
ACYPI006075-RA	gi|646780794|gb|KK961540.1|	619355-621491	21	9.5	gi|641574549|gb|KK855294.1|	151813-152385	8.9	38
ACYPI006089-RA	gi|646768447|gb|KK961811.1|	986296-986508	22	9.7	gi|641586509|gb|KK854231.1|	511944-513064	9.8	37
ACYPI006090-RA	gi|646769102|gb|KK961799.1|	355238-358125	20	8.3	gi|641576906|gb|KK854838.1|	266061-266293	9.4	37
ACYPI006095-RA	gi|646775471|gb|KK961725.1|	1322550-1323354	20	10	gi|641577905|gb|KK854672.1|	169886-171616	10	37
ACYPI006101-RA	gi|646779869|gb|KK961563.1|	327235-331078	17	8.8	gi|641576472|gb|KK854913.1|	91163-95029	11	38
ACYPI006103-RA	gi|646767521|gb|KK961830.1|	613409-617484	20	9.3	gi|641587881|gb|KK854049.1|	584793-586578	10	40
ACYPI006104-RA	gi|646780406|gb|KK961550.1|	2155848-2156104	22	5.9	gi|641570141|gb|KK856447.1|	125411-128455	11	40
ACYPI006106-RA	gi|646732905|gb|KK963611.1|	85358-85798	17	7.5	gi|641588164|gb|KK854017.1|	824013-824257	9.2	40
ACYPI006109-RA	gi|646746475|gb|KK962286.1|	12643-13057	19	8.8	gi|641570124|gb|KK856453.1|	117498-118459	8.7	40
ACYPI006120-RA	gi|646782357|gb|KK961494.1|	6325792-6326798	21	9.2	gi|641574416|gb|KK855326.1|	165833-168071	11	38
ACYPI006122-RA	gi|646781313|gb|KK961524.1|	1713596-1714426	19	8.5	gi|641577047|gb|KK854815.1|	338905-339843	10	41
ACYPI006124-RA	gi|646740004|gb|KK962821.1|	379844-388128	22	9.6	gi|641576834|gb|KK854851.1|	329557-329956	10	41
ACYPI006133-RA	gi|646772786|gb|KK961754.1|	551230-551961	22	9.6	gi|641584632|gb|KK854467.1|	354059-355944	9.8	37
ACYPI006140-RA	gi|646751856|gb|KK961977.1|	161954-163343	18	8.5	gi|641585395|gb|KK854405.1|	97838-98842	10	38
ACYPI006141-RA	gi|646775383|gb|KK961730.1|	1124290-1124812	19	8.7	gi|641567011|gb|KK857455.1|	26442-29198	11	40
ACYPI006142-RA	gi|646755567|gb|KK961936.1|	598856-599852	20	8.6	gi|641576863|gb|KK854845.1|	272005-274883	11	41
ACYPI006151-RA	gi|646782127|gb|KK961500.1|	1217271-1217484	22	9.6	gi|641587652|gb|KK854077.1|	830187-830707	10	38
ACYPI006156-RA	gi|646747574|gb|KK962208.1|	112690-112965	27	10	gi|641587866|gb|KK854050.1|	313752-314484	10	38
ACYPI006157-RA	gi|646755888|gb|KK961934.1|	941532-941980	21	9.8	gi|641572416|gb|KK855819.1|	128516-129050	10	37
ACYPI006162-RA	gi|646781628|gb|KK961514.1|	453044-454059	23	9	gi|641587580|gb|KK854085.1|	502624-508442	10	39
ACYPI006163-RA	gi|646769511|gb|KK961792.1|	895184-895615	20	9.9	gi|641573015|gb|KK855664.1|	57578-58874	11	42

ACYPI006164-RA	gi|646781443|gb|KK961520.1|	1794021-1795864	20	8.3	gi|641587703|gb|KK854070.1|	210947-215659	9.8	42
ACYPI006166-RA	gi|646781443|gb|KK961520.1|	2250499-2250746	20	8.3	gi|641588343|gb|KK854003.1|	1343757-134537[illegible]	11	40
ACYPI006170-RA	gi|646780827|gb|KK961539.1|	3598551-3599003	20	9.4	gi|641571667|gb|KK856008.1|	105760-107467	11	39
ACYPI006177-RA	gi|646775968|gb|KK961700.1|	776916-777593	20	8.2	gi|641586793|gb|KK854190.1|	679665-684081	9.3	41
ACYPI006178-RA	gi|646740420|gb|KK962773.1|	256629-257094	20	7.5	gi|641571605|gb|KK856024.1|	7499-7959	10	38
ACYPI006184-RA	gi|646778307|gb|KK961605.1|	1929034-1933224	17	8.7	gi|641575808|gb|KK855040.1|	165053-165619	9.9	37
ACYPI006185-RA	gi|646757520|gb|KK961926.1|	1199270-1200193	20	7.4	gi|641578263|gb|KK854612.1|	297150-297769	10	39
ACYPI006186-RA	gi|646781443|gb|KK961520.1|	334450-337082	20	8.3	gi|641587452|gb|KK854101.1|	209441-210145	11	40
ACYPI006187-RA	gi|646776389|gb|KK961678.1|	884442-885593	22	9.5	gi|641580479|gb|KK854569.1|	388500-389618	10	38
ACYPI006197-RA	gi|646777955|gb|KK961615.1|	2141990-2142614	21	10	gi|641586240|gb|KK854269.1|	608774-609787	11	21
ACYPI006205-RA	gi|646775842|gb|KK961706.1|	2110055-2110295	22	9	gi|641586246|gb|KK854268.1|	642725-643469	10	38
ACYPI006207-RA	gi|646746706|gb|KK962270.1|	402736-408503	23	11	gi|641575108|gb|KK855178.1|	180173-185543	34	81
ACYPI006216-RA	gi|646771352|gb|KK961768.1|	2086806-2087568	21	9.6	gi|641586983|gb|KK854162.1|	357428-357963	10	41
ACYPI006219-RA	gi|646740287|gb|KK962790.1|	296587-297456	20	8.5	gi|641587671|gb|KK854074.1|	1058363-105881[illegible]	11	40
ACYPI006221-RA	gi|646747459|gb|KK962217.1|	130776-137818	21	5.9	gi|641587527|gb|KK854092.1|	455512-457743	10	38
ACYPI006222-RA	gi|646778245|gb|KK961607.1|	1137461-1139114	18	6.8	gi|641588060|gb|KK854028.1|	797797-800372	11	42
ACYPI006225-RA	gi|646741656|gb|KK962648.1|	281233-283877	24	9.7	gi|641576243|gb|KK854954.1|	296903-297529	10	41
ACYPI006226-RA	gi|646776608|gb|KK961665.1|	1723495-1723827	23	9.3	gi|641578300|gb|KK854606.1|	560798-561347	11	40
ACYPI006227-RA	gi|646766650|gb|KK961854.1|	261444-268747	22	10	gi|641571982|gb|KK855931.1|	53261-53711	9.8	35
ACYPI006229-RA	gi|646747201|gb|KK962236.1|	179273-180805	18	7.3	gi|641587095|gb|KK854145.1|	132292-132741	9.9	40
ACYPI006231-RA	gi|646739411|gb|KK962885.1|	237402-239049	18	7.7	gi|641586528|gb|KK854228.1|	280805-281016	10	42
ACYPI006238-RA	gi|646777991|gb|KK961614.1|	1215561-1215949	18	7.8	gi|641568278|gb|KK857027.1|	50838-51986	9.6	38
ACYPI006239-RA	gi|646780858|gb|KK961538.1|	3464449-3465340	21	10	gi|641566611|gb|KK857597.1|	37912-38716	11	40
ACYPI006240-RA	gi|646756057|gb|KK961933.1|	382903-383418	16	6.7	gi|641573583|gb|KK855523.1|	19844-24851	10	41
ACYPI006242-RA	gi|646780723|gb|KK961542.1|	1573125-1579403	21	9.4	gi|641573769|gb|KK855477.1|	124169-128061	9.2	38
ACYPI006243-RA	gi|646778840|gb|KK961590.1|	736759-748890	20	9.9	gi|641579941|gb|KK854575.1|	377799-380928	10	39
ACYPI006248-RA	gi|646781732|gb|KK961511.1|	2762674-2765091	22	9.8	gi|641587040|gb|KK854152.1|	309571-310403	9.1	39
ACYPI006251-RA	gi|646779006|gb|KK961585.1|	186630-194175	20	9.9	gi|641575130|gb|KK855173.1|	88810-90602	12	50
ACYPI006254-RA	gi|646776429|gb|KK961676.1|	1344369-1350059	20	9.9	gi|641587577|gb|KK854086.1|	398684-398890	8.2	41
ACYPI006257-RA	gi|646777991|gb|KK961614.1|	1111952-1112393	18	7.8	gi|641587047|gb|KK854151.1|	247234-247433	10	39
ACYPI006262-RA	gi|646748142|gb|KK962168.1|	376038-376379	20	9.3	gi|641586850|gb|KK854182.1|	150369-150970	10	37
ACYPI006266-RA	gi|646765902|gb|KK961884.1|	1130987-1132145	22	9.2	gi|641587104|gb|KK854144.1|	720378-721288	11	37

ACYPI006268-RA	gi|646777729|gb|KK961621.1|	1260431-1261805	21	9.8	gi|641568762|gb|KK856874.1|	16451-17841	11	41
ACYPI006271-RA	gi|646779976|gb|KK961560.1|	1237054-1237533	16	7.9	gi|641573752|gb|KK855482.1|	149708-150092	11	37
ACYPI006272-RA	gi|646750354|gb|KK962043.1|	198445-198952	20	9.2	gi|641571786|gb|KK855980.1|	147943-150971	9.9	19
ACYPI006277-RA	gi|646746828|gb|KK962262.1|	614048-614920	20	9.2	gi|641574944|gb|KK855211.1|	239663-241023	10	39
ACYPI006279-RA	gi|646640511|gb|KK968988.1|	35927-36632	21	10	gi|641582763|gb|KK854531.1|	409708-413386	10	39
ACYPI006281-RA	gi|646747124|gb|KK962242.1|	284248-286225	17	8.7	gi|641578411|gb|KK854593.1|	179068-180177	11	43
ACYPI006283-RA	gi|646781849|gb|KK961508.1|	1864496-1865138	17	8	gi|641572372|gb|KK855829.1|	122861-123252	12	45
ACYPI006288-RA	gi|646781809|gb|KK961509.1|	3570858-3571078	19	8.6	gi|641585761|gb|KK854344.1|	549648-550233	10	39
ACYPI006305-RA	gi|646778112|gb|KK961611.1|	852582-853337	22	10	gi|641571790|gb|KK855979.1|	80019-80411	11	41
ACYPI006312-RA	gi|646781344|gb|KK961523.1|	910016-910934	21	8.9	gi|641575709|gb|KK855059.1|	157596-158050	11	41
ACYPI006313-RA	gi|646738615|gb|KK962959.1|	308763-309253	21	9.5	gi|641574308|gb|KK855352.1|	130896-132077	10	36
ACYPI006314-RA	gi|646776952|gb|KK961652.1|	1685567-1686316	19	7.7	gi|641572037|gb|KK855916.1|	115036-115662	13	41
ACYPI006318-RA	gi|646779621|gb|KK961569.1|	1459543-1459775	16	7.3	gi|641567806|gb|KK857182.1|	5014-5282	9.6	38
ACYPI006338-RA	gi|646745631|gb|KK962343.1|	254418-254676	18	8.6	gi|641587249|gb|KK854125.1|	245879-248462	11	37
ACYPI006340-RA	gi|646780270|gb|KK961553.1|	2142737-2144308	22	8.6	gi|641583980|gb|KK854512.1|	302793-304927	11	39
ACYPI006341-RA	gi|646781968|gb|KK961504.1|	2361781-2364161	20	9.6	gi|641577869|gb|KK854679.1|	313480-315964	9.2	39
ACYPI006343-RA	gi|646745434|gb|KK962359.1|	42553-44492	22	10	gi|641587424|gb|KK854104.1|	280413-286392	11	41
ACYPI006348-RA	gi|646766650|gb|KK961854.1|	1584396-1584957	22	10	gi|641572675|gb|KK855753.1|	133980-135192	11	40
ACYPI006351-RA	gi|646749945|gb|KK962065.1|	1946402-1947000	22	6.1	gi|641570322|gb|KK856389.1|	7902-8582	11	40
ACYPI006352-RA	gi|646744833|gb|KK962403.1|	241068-244042	22	9.4	gi|641569883|gb|KK856530.1|	33210-33384	11	45
ACYPI006354-RA	gi|646738248|gb|KK962996.1|	96287-99727	17	6.6	gi|641572854|gb|KK855705.1|	177654-178323	11	42
ACYPI006356-RA	gi|646781421|gb|KK961521.1|	1630913-1631321	21	8.7	gi|641578246|gb|KK854614.1|	265309-266388	9.5	39
ACYPI006364-RA	gi|646776447|gb|KK961675.1|	783010-784763	22	9.4	gi|641588021|gb|KK854033.1|	1097684-1098736	12	40
ACYPI006367-RA	gi|646768361|gb|KK961813.1|	689097-689295	15	7.4	gi|641587921|gb|KK854044.1|	345558-347805	9.4	41
ACYPI006370-RA	gi|646781243|gb|KK961526.1|	4125546-4127408	21	8.9	gi|641588000|gb|KK854035.1|	424968-426705	11	39
ACYPI006371-RA	gi|646776429|gb|KK961676.1|	2517858-2521114	20	9.9	gi|641569257|gb|KK856724.1|	8570-14510	10	36
ACYPI006375-RA	gi|646776647|gb|KK961663.1|	349899-352026	23	9.2	gi|641577203|gb|KK854789.1|	288609-290702	11	43
ACYPI006377-RA	gi|646782357|gb|KK961494.1|	9236897-9237991	21	9.2	gi|641588000|gb|KK854035.1|	979782-981150	11	39
ACYPI006383-RA	gi|646781043|gb|KK961532.1|	3267845-3269889	26	11	gi|641587848|gb|KK854052.1|	350484-351920	11	39
ACYPI006386-RA	gi|646776762|gb|KK961659.1|	1286762-1289278	21	8.9	gi|641577725|gb|KK854704.1|	201342-201558	11	42
ACYPI006388-RA	gi|646753339|gb|KK961955.1|	784027-785440	22	11	gi|641584460|gb|KK854480.1|	14882-16045	11	40
ACYPI006395-RA	gi|646769001|gb|KK961801.1|	387237-389203	20	8.5	gi|641585521|gb|KK854384.1|	373107-375874	9.7	38

ACYPI006399-RA	gi|646738455|gb|KK962975.1|	457007-457851	23	9.5	gi|641568423|gb|KK856981.1|	66311-66903	11	37
ACYPI006403-RA	gi|646776429|gb|KK961676.1|	1706638-1711057	20	9.9	gi|641567672|gb|KK857228.1|	20715-21633	10	36
ACYPI006405-RA	gi|646598593|gb|KK976753.1|	869-1508	15	8.4	gi|641567739|gb|KK857205.1|	81589-82829	13	38
ACYPI006407-RA	gi|646781893|gb|KK961506.1|	2143952-2144738	19	8.5	gi|641584248|gb|KK854496.1|	304615-304870	9.9	39
ACYPI006409-RA	gi|646779262|gb|KK961578.1|	849442-849639	20	8.1	gi|641578350|gb|KK854597.1|	389024-391100	11	21
ACYPI006412-RA	gi|646782211|gb|KK961498.1|	5781899-5782435	20	9	gi|641576546|gb|KK854899.1|	122833-123214	11	40
ACYPI006423-RA	gi|646782276|gb|KK961497.1|	1293139-1293418	21	9.7	gi|641572581|gb|KK855778.1|	16867-17174	9.9	37
ACYPI006430-RA	gi|646741926|gb|KK962624.1|	264099-268209	17	9.1	gi|641574990|gb|KK855200.1|	257092-261138	12	37
ACYPI006432-RA	gi|646780105|gb|KK961557.1|	1927883-1935768	21	9.7	gi|641570404|gb|KK856365.1|	110417-113754	10	19
ACYPI006434-RA	gi|646752919|gb|KK961960.1|	644155-645038	20	9	gi|641575763|gb|KK855049.1|	162467-163590	10	40
ACYPI006436-RA	gi|646777665|gb|KK961623.1|	1577532-1578795	21	9.7	gi|641574484|gb|KK855310.1|	130781-131872	11	41
ACYPI006438-RA	gi|646778029|gb|KK961613.1|	1830063-1832107	21	8.6	gi|641577104|gb|KK854807.1|	250704-255588	10	36
ACYPI006441-RA	gi|646744833|gb|KK962403.1|	453282-454519	22	9.4	gi|641570089|gb|KK856463.1|	40948-42728	11	36
ACYPI006443-RA	gi|646743944|gb|KK962469.1|	298409-298882	20	9.1	gi|641586535|gb|KK854227.1|	350013-351020	10	39
ACYPI006451-RA	gi|646781344|gb|KK961523.1|	3703799-3706804	21	8.9	gi|641566026|gb|KK857812.1|	5483-5684	10	36
ACYPI006456-RA	gi|646747263|gb|KK962232.1|	380395-382834	18	8.6	gi|641577623|gb|KK854722.1|	285116-286207	11	38
ACYPI006457-RA	gi|646780953|gb|KK961535.1|	521596-521963	20	9.8	gi|641587186|gb|KK854133.1|	289465-289821	8.1	39
ACYPI006463-RA	gi|646781564|gb|KK961516.1|	2342261-2345599	23	9.4	gi|641572435|gb|KK855814.1|	99006-100218	10	39
ACYPI006482-RA	gi|646751160|gb|KK962003.1|	1138606-1138983	22	10	gi|641574892|gb|KK855223.1|	210168-211751	9.8	39
ACYPI006483-RA	gi|646724827|gb|KK964750.1|	31470-33949	20	8.3	gi|641588236|gb|KK854011.1|	848699-851165	9.7	38
ACYPI006485-RA	gi|646777288|gb|KK961638.1|	133233-135926	22	6.1	gi|641573504|gb|KK855543.1|	156999-157226	13	39
ACYPI006488-RA	gi|646782276|gb|KK961497.1|	2956913-2957193	21	9.7	gi|641577580|gb|KK854730.1|	12242-12443	11	41
ACYPI006491-RA	gi|646781628|gb|KK961514.1|	1026795-1027637	23	9	gi|641574100|gb|KK855396.1|	169645-170000	11	38
ACYPI006497-RA	gi|646777416|gb|KK961632.1|	1236794-1240235	22	8.8	gi|641588000|gb|KK854035.1|	615452-617116	11	39
ACYPI006498-RA	gi|646778699|gb|KK961594.1|	1330627-1331300	21	9.5	gi|641576036|gb|KK854996.1|	46467-46990	11	40
ACYPI006499-RA	gi|646776904|gb|KK961654.1|	855176-855654	21	9.3	gi|641575808|gb|KK855040.1|	258827-259237	9.9	37
ACYPI006500-RA	gi|646773721|gb|KK961747.1|	2115732-2116004	21	9.2	gi|641564420|gb|KK858447.1|	3017-5840	13	42
ACYPI006505-RA	gi|646776429|gb|KK961676.1|	1271017-1272467	20	9.9	gi|641587606|gb|KK854082.1|	203331-204836	11	40
ACYPI006514-RA	gi|646778736|gb|KK961593.1|	1335930-1336693	20	7.9	gi|641577299|gb|KK854775.1|	284741-287511	11	37
ACYPI006518-RA	gi|646752849|gb|KK961961.1|	1214235-1215294	21	9.4	gi|641580970|gb|KK854563.1|	305168-307888	11	42
ACYPI006520-RA	gi|646776351|gb|KK961680.1|	719811-726418	22	10	gi|641550114|gb|KK865048.1|	39-1938	12	37
ACYPI006521-RA	gi|646772574|gb|KK961756.1|	1138358-1141988	21	8	gi|641579219|gb|KK854584.1|	184872-188323	12	39

ACYPI006530-RA	gi|646737447|gb|KK963075.1|	347083-354281	22	9.2	gi|641568339|gb|KK857008.1|	5904-9599	11	38
ACYPI006541-RA	gi|646746841|gb|KK962261.1|	418139-419060	20	9.6	gi|641575288|gb|KK855139.1|	239521-240813	11	38
ACYPI006544-RA	gi|646753527|gb|KK961953.1|	1188121-1188545	24	9.2	gi|641583980|gb|KK854512.1|	323395-326097	11	39
ACYPI006545-RA	gi|646748245|gb|KK962161.1|	572602-576028	18	8.6	gi|641584876|gb|KK854450.1|	288338-289625	11	25
ACYPI006557-RA	gi|646738524|gb|KK962968.1|	127285-127568	24	11	gi|641578106|gb|KK854639.1|	146076-146525	11	39
ACYPI006576-RA	gi|646770563|gb|KK961777.1|	669926-670400	19	8.7	gi|641588109|gb|KK854023.1|	393708-394509	10	40
ACYPI006584-RA	gi|646778983|gb|KK961586.1|	9773-9973	20	9.9	gi|641575794|gb|KK855043.1|	172043-177386	10	36
ACYPI006586-RA	gi|646745434|gb|KK962359.1|	10452-10802	22	10	gi|641587424|gb|KK854104.1|	331041-331648	11	41
ACYPI006588-RA	gi|646766650|gb|KK961854.1|	714036-714421	22	10	gi|641587410|gb|KK854106.1|	458544-458859	10	39
ACYPI006589-RA	gi|646781849|gb|KK961508.1|	175700-176482	17	8	gi|641576207|gb|KK854961.1|	201124-202848	11	38
ACYPI006602-RA	gi|646487701|gb|KK996683.1|	3249-3437	11	5.6	gi|641578406|gb|KK854594.1|	258805-258995	10	37
ACYPI006603-RA	gi|646771092|gb|KK961771.1|	471373-471705	23	9.7	gi|641584818|gb|KK854454.1|	397690-398012	11	42
ACYPI006608-RA	gi|646780147|gb|KK961556.1|	1264346-1264680	18	7.5	gi|641575666|gb|KK855067.1|	229280-229584	11	39
ACYPI006611-RA	gi|646776184|gb|KK961688.1|	914142-920365	21	9.6	gi|641578056|gb|KK854648.1|	66169-67920	11	41
ACYPI006612-RA	gi|646762797|gb|KK961906.1|	729578-729895	19	5.6	gi|641588325|gb|KK854004.1|	1135590-1138378	11	40
ACYPI006613-RA	gi|646746216|gb|KK962304.1|	860189-870564	130	33	gi|641561307|gb|KK859801.1|	13587-13777	13	38
ACYPI006615-RA	gi|646552978|gb|KK985631.1|	1244-2626	18	9.7	gi|641576946|gb|KK854832.1|	84306-88272	9.4	43
ACYPI006616-RA	gi|646777207|gb|KK961641.1|	1186273-1186463	20	8.5	gi|641576656|gb|KK854883.1|	19849-25051	11	40
ACYPI006617-RA	gi|646780183|gb|KK961555.1|	1386418-1386695	19	8.6	gi|641586950|gb|KK854166.1|	658820-659587	10	38
ACYPI006619-RA	gi|646779066|gb|KK961583.1|	1563970-1564637	19	7.9	gi|641582408|gb|KK854540.1|	383508-385257	10	41
ACYPI006624-RA	gi|646771352|gb|KK961768.1|	409073-412458	21	9.6	gi|641587452|gb|KK854101.1|	1025558-1027416	11	40
ACYPI006625-RA	gi|646753527|gb|KK961953.1|	665250-672717	24	9.2	gi|641575743|gb|KK855052.1|	133926-141235	9.7	38
ACYPI006626-RA	gi|646753180|gb|KK961957.1|	82882-83624	25	11	gi|641573007|gb|KK855666.1|	187020-189637	11	44
ACYPI006630-RA	gi|646753180|gb|KK961957.1|	508121-516575	25	11	gi|641585709|gb|KK854353.1|	467547-469362	11	43
ACYPI006635-RA	gi|646782288|gb|KK961496.1|	5821016-5826189	21	9.7	gi|641573097|gb|KK855644.1|	104411-111687	10	36
ACYPI006639-RA	gi|646779936|gb|KK961561.1|	3023369-3038129	19	9.6	gi|641573737|gb|KK855486.1|	62037-63568	9.1	33
ACYPI006649-RA	gi|646777802|gb|KK961619.1|	91410-96661	24	9	gi|641573007|gb|KK855666.1|	49902-52377	11	44
ACYPI006656-RA	gi|646778767|gb|KK961592.1|	150800-156186	21	5.4	gi|641573533|gb|KK855535.1|	28624-35255	10	41
ACYPI006658-RA	gi|646627554|gb|KK971378.1|	3894-4350	12	5.6	gi|641584460|gb|KK854480.1|	108727-109104	11	40
ACYPI006660-RA	gi|646778903|gb|KK961588.1|	4191659-4191959	23	10	gi|641588099|gb|KK854024.1|	938549-938853	11	39
ACYPI006664-RA	gi|646570180|gb|KK982274.1|	3319-3526	5.4	2.9	gi|641574249|gb|KK855364.1|	70843-72107	9.2	37
ACYPI006668-RA	gi|646775635|gb|KK961716.1|	410805-411074	22	10	gi|641575768|gb|KK855048.1|	156177-156512	8.1	41

ACYPI006674-RA	gi|646775884|gb|KK961704.1|	950525-950771	20	7.6	gi|641587365|gb|KK854113.1|	484651-485915	12	45
ACYPI006676-RA	gi|646781690|gb|KK961512.1|	3342729-3343889	21	8.8	gi|641577928|gb|KK854668.1|	159761-160032	9.2	37
ACYPI006679-RA	gi|646780752|gb|KK961541.1|	851051-851470	21	8	gi|641587021|gb|KK854156.1|	586047-587771	11	42
ACYPI006680-RA	gi|646746475|gb|KK962286.1|	193534-209576	19	8.8	gi|641569707|gb|KK856586.1|	54956-55796	8.9	17
ACYPI006682-RA	gi|646753715|gb|KK961951.1|	814999-815500	19	5.4	gi|641574036|gb|KK855411.1|	207028-207271	11	37
ACYPI006684-RA	gi|646776368|gb|KK961679.1|	1057471-1058357	21	9.7	gi|641575368|gb|KK855125.1|	41060-42271	10	36
ACYPI006688-RA	gi|646750389|gb|KK962041.1|	149189-149917	21	9.5	gi|641587688|gb|KK854072.1|	450111-456133	10	38
ACYPI006692-RA	gi|646780953|gb|KK961535.1|	3002168-3003182	20	9.8	gi|641574491|gb|KK855308.1|	8334-8934	11	43
ACYPI006693-RA	gi|646781421|gb|KK961521.1|	1424533-1425477	21	8.7	gi|641588073|gb|KK854027.1|	1026916-1027178	11	41
ACYPI006698-RA	gi|646776608|gb|KK961665.1|	375296-381750	23	9.3	gi|641585407|gb|KK854403.1|	258696-259400	9.4	42
ACYPI006699-RA	gi|646782211|gb|KK961498.1|	2483034-2483357	20	9	gi|641587417|gb|KK854105.1|	84826-85357	11	42
ACYPI006701-RA	gi|646738812|gb|KK962939.1|	144737-144991	20	8.1	gi|641586886|gb|KK854176.1|	91548-91917	10	41
ACYPI006703-RA	gi|646781564|gb|KK961516.1|	2900118-2900526	23	9.4	gi|641574944|gb|KK855211.1|	214840-216827	10	39
ACYPI006705-RA	gi|646776904|gb|KK961654.1|	299241-305206	21	9.3	gi|641587527|gb|KK854092.1|	670707-671336	10	38
ACYPI006708-RA	gi|646777313|gb|KK961637.1|	1574829-1575707	20	8.2	gi|641570124|gb|KK856453.1|	155068-155760	8.7	40
ACYPI006711-RA	gi|646768294|gb|KK961814.1|	607675-612282	23	10	gi|641576347|gb|KK854934.1|	234087-235474	11	39
ACYPI006713-RA	gi|646776351|gb|KK961680.1|	1475381-1478565	22	10	gi|641574324|gb|KK855348.1|	249663-250823	12	40
ACYPI006714-RA	gi|646779936|gb|KK961561.1|	3065230-3065613	19	9.6	gi|641587365|gb|KK854113.1|	545658-548159	12	45
ACYPI006721-RA	gi|646777233|gb|KK961640.1|	118235-120628	21	8.7	gi|641572908|gb|KK855691.1|	14838-19049	12	45
ACYPI006725-RA	gi|646778274|gb|KK961606.1|	785398-785619	18	7.5	gi|641585515|gb|KK854385.1|	153205-153495	11	40
ACYPI006727-RA	gi|646782357|gb|KK961494.1|	5134692-5139197	21	9.2	gi|641587319|gb|KK854116.1|	652479-654994	11	43
ACYPI006728-RA	gi|646738156|gb|KK963004.1|	54603-55300	12	6.9	gi|641565803|gb|KK857893.1|	18862-21243	10	35
ACYPI006735-RA	gi|646755888|gb|KK961934.1|	873620-874113	21	9.8	gi|641571130|gb|KK856153.1|	164041-164830	10	36
ACYPI006737-RA	gi|646781013|gb|KK961533.1|	938435-943564	18	7.6	gi|641560560|gb|KK860138.1|	8238-8655	8.4	26
ACYPI006740-RA	gi|646768447|gb|KK961811.1|	821773-822664	22	9.7	gi|641586372|gb|KK854251.1|	543974-548728	10	38
ACYPI006741-RA	gi|646778336|gb|KK961604.1|	1373678-1374197	21	10	gi|641587981|gb|KK854037.1|	100841-101313	10	40
ACYPI006748-RA	gi|646781601|gb|KK961515.1|	3502615-3503549	19	8.6	gi|641570795|gb|KK856251.1|	76988-77769	9.8	38
ACYPI006750-RA	gi|646750043|gb|KK962060.1|	312487-313594	21	8.3	gi|641587111|gb|KK854143.1|	528602-529161	11	40
ACYPI006758-RA	gi|646765991|gb|KK961880.1|	303369-311399	16	7	gi|641585204|gb|KK854429.1|	142132-145872	9.2	36
ACYPI006761-RA	gi|646749632|gb|KK962082.1|	582288-582940	20	8.6	gi|641587478|gb|KK854098.1|	260617-261002	10	41
ACYPI006777-RA	gi|646775842|gb|KK961706.1|	334285-334895	22	9	gi|641587866|gb|KK854050.1|	1008888-1009229	10	38
ACYPI006784-RA	gi|646767307|gb|KK961835.1|	322881-327857	20	9	gi|641577595|gb|KK854727.1|	58572-59762	10	20

ACYPI006786-RA	gi|646770114|gb|KK961783.1|	1674129-1675661	19	9.7	gi|641581389|gb|KK854557.1|	324875-327532	11	41
ACYPI006789-RA	gi|646745327|gb|KK962367.1|	20662-21267	18	8.3	gi|641587003|gb|KK854159.1|	435219-435452	10	21
ACYPI006790-RA	gi|646767156|gb|KK961839.1|	147231-147560	15	6.4	gi|641586874|gb|KK854178.1|	623830-624157	9.4	38
ACYPI006792-RA	gi|646750135|gb|KK962055.1|	55033-56094	16	7.6	gi|641574320|gb|KK855349.1|	188081-190196	10	38
ACYPI006797-RA	gi|646781690|gb|KK961512.1|	2360794-2363706	21	8.8	gi|641587921|gb|KK854044.1|	327919-328598	9.4	41
ACYPI006808-RA	gi|646747802|gb|KK962192.1|	207843-209426	19	7.8	gi|641584728|gb|KK854460.1|	218813-219634	9.5	37
ACYPI006817-RA	gi|646746897|gb|KK962257.1|	853714-854487	21	9.2	gi|641587287|gb|KK854120.1|	161230-163841	9.9	37
ACYPI006818-RA	gi|646746991|gb|KK962251.1|	338254-350930	22	9.2	gi|641575910|gb|KK855021.1|	216435-217820	10	35
ACYPI006821-RA	gi|646777233|gb|KK961640.1|	2279271-2279635	21	8.7	gi|641574729|gb|KK855257.1|	208642-212290	8.6	33
ACYPI006823-RA	gi|646747658|gb|KK962202.1|	102707-103263	15	7.1	gi|641575168|gb|KK855165.1|	112036-112546	9.6	36
ACYPI006828-RA	gi|646732863|gb|KK963615.1|	29322-33319	150	39	gi|641580504|gb|KK854565.1|	348065-351180	11	43
ACYPI006830-RA	gi|646766482|gb|KK961860.1|	262605-264917	17	6.7	gi|641574266|gb|KK855361.1|	159421-160825	11	38
ACYPI006833-RA	gi|646727841|gb|KK964291.1|	121520-128141	23	9.6	gi|641576010|gb|KK855002.1|	52176-55478	12	43
ACYPI006837-RA	gi|646778660|gb|KK961595.1|	2002245-2002733	16	8.2	gi|641586271|gb|KK854265.1|	36367-37784	10	40
ACYPI006841-RA	gi|646777207|gb|KK961641.1|	896960-899077	20	8.5	gi|641577407|gb|KK854756.1|	150713-152613	9.4	39
ACYPI006842-RA	gi|646776219|gb|KK961687.1|	2020931-2025293	21	9.9	gi|641585833|gb|KK854332.1|	207656-208632	10	37
ACYPI006844-RA	gi|646766034|gb|KK961878.1|	583539-584139	20	9	gi|641586817|gb|KK854187.1|	578099-578406	9.3	39
ACYPI006857-RA	gi|646771951|gb|KK961762.1|	1827439-1829636	21	8.3	gi|641575396|gb|KK855118.1|	103150-103770	9	35
ACYPI006864-RA	gi|646778865|gb|KK961589.1|	2124861-2125059	20	9.2	gi|641576286|gb|KK854946.1|	142021-144034	12	38
ACYPI006871-RA	gi|646778983|gb|KK961586.1|	342591-342856	20	9.9	gi|641578046|gb|KK854650.1|	35043-35866	10	38
ACYPI006875-RA	gi|646748474|gb|KK962147.1|	177934-179046	20	9	gi|641576887|gb|KK854842.1|	255358-255716	15	41
ACYPI006879-RA	gi|646781118|gb|KK961530.1|	2413756-2426001	21	10	gi|641577778|gb|KK854696.1|	261553-262644	9.9	42
ACYPI006884-RA	gi|646747494|gb|KK962214.1|	293340-294596	18	8.5	gi|641570258|gb|KK856410.1|	10007-11245	8.4	41
ACYPI006885-RA	gi|646777955|gb|KK961615.1|	947487-947974	21	10	gi|641585961|gb|KK854312.1|	436446-436787	10	40
ACYPI006888-RA	gi|646748086|gb|KK962172.1|	718310-718683	20	10	gi|641571982|gb|KK855931.1|	89942-95091	9.8	35
ACYPI006896-RA	gi|646776514|gb|KK961670.1|	6299-6541	20	9.6	gi|641576557|gb|KK854897.1|	178281-180603	8.9	33
ACYPI006902-RA	gi|646775523|gb|KK961722.1|	870542-872042	21	9.7	gi|641575693|gb|KK855062.1|	49577-50830	11	40
ACYPI006909-RA	gi|646779006|gb|KK961585.1|	911013-911385	20	9.9	gi|641583088|gb|KK854527.1|	192271-193563	9.9	39
ACYPI006910-RA	gi|646776389|gb|KK961678.1|	1059735-1059932	22	9.5	gi|641575826|gb|KK855037.1|	90242-91461	10	39
ACYPI006929-RA	gi|646750354|gb|KK962043.1|	297550-299205	20	9.2	gi|641585773|gb|KK854342.1|	136565-139150	12	45
ACYPI006932-RA	gi|646747385|gb|KK962223.1|	236443-236742	21	5.4	gi|641578325|gb|KK854602.1|	269983-277242	9.7	38
ACYPI006934-RA	gi|646767196|gb|KK961838.1|	1107352-1107584	21	9.7	gi|641584445|gb|KK854481.1|	92178-92589	9.9	39

ACYPI006940-RA	gi|646777440|gb|KK961631.1|	1453969-1454277	22	9.7	gi|641587052|gb|KK854150.1|	48659-48972	11	38
ACYPI006942-RA	gi|646782334|gb|KK961495.1|	7010704-7011744	21	9	gi|641565407|gb|KK858043.1|	30235-30584	11	20
ACYPI006943-RA	gi|646760564|gb|KK961914.1|	561462-562902	21	8.8	gi|641585994|gb|KK854307.1|	451326-451636	11	41
ACYPI006948-RA	gi|646762191|gb|KK961910.1|	902349-903559	18	7.1	gi|641587519|gb|KK854093.1|	383866-385490	11	39
ACYPI006949-RA	gi|646781536|gb|KK961517.1|	2431223-2432212	19	7.7	gi|641587452|gb|KK854101.1|	225773-229455	11	40
ACYPI006954-RA	gi|646748014|gb|KK962177.1|	294669-295462	15	6.4	gi|641576876|gb|KK854844.1|	370973-372217	17	42
ACYPI006956-RA	gi|646775867|gb|KK961705.1|	755090-755962	23	10	gi|641587896|gb|KK854047.1|	55643-57423	9.9	39
ACYPI006957-RA	gi|646781379|gb|KK961522.1|	3378502-3382978	22	7.6	gi|641586246|gb|KK854268.1|	515533-516942	10	38
ACYPI006958-RA	gi|646777474|gb|KK961630.1|	984506-988456	21	9.3	gi|641578046|gb|KK854650.1|	95657-98866	10	38
ACYPI006964-RA	gi|646745091|gb|KK962384.1|	163254-164426	19	7.9	gi|641577623|gb|KK854722.1|	166372-166615	11	38
ACYPI006968-RA	gi|646767697|gb|KK961826.1|	1393124-1393662	21	9.8	gi|641587424|gb|KK854104.1|	787709-788367	11	41
ACYPI006974-RA	gi|646694989|gb|KK966396.1|	9127-12831	12	9.8	gi|641587783|gb|KK854060.1|	634998-640549	12	40
ACYPI006978-RA	gi|646747032|gb|KK962248.1|	408105-408610	20	8.7	gi|641573829|gb|KK855462.1|	188384-193291	11	41
ACYPI006979-RA	gi|646777089|gb|KK961646.1|	425760-428811	20	9.4	gi|641571612|gb|KK856022.1|	49694-51333	12	43
ACYPI006990-RA	gi|646755567|gb|KK961936.1|	798958-803087	20	8.6	gi|641588343|gb|KK854003.1|	1100981-1101215	11	40
ACYPI006993-RA	gi|646746974|gb|KK962252.1|	141680-142583	23	6.1	gi|641565451|gb|KK858025.1|	30340-32487	9.1	17
ACYPI007002-RA	gi|646780858|gb|KK961538.1|	3541720-3543057	21	10	gi|641577184|gb|KK854793.1|	373532-374549	11	40
ACYPI007005-RA	gi|646778112|gb|KK961611.1|	1163234-1164019	22	10	gi|641585395|gb|KK854405.1|	429043-429785	10	38
ACYPI007006-RA	gi|646782043|gb|KK961502.1|	5359487-5360211	20	9.2	gi|641588073|gb|KK854027.1|	1234954-1237193	11	41
ACYPI007007-RA	gi|646500670|gb|KK994326.1|	4284-4906	17	8.9	gi|641586785|gb|KK854191.1|	324708-327123	11	39
ACYPI007009-RA	gi|646764075|gb|KK961900.1|	384473-387026	18	6.5	gi|641573155|gb|KK855630.1|	10643-11451	14	50
ACYPI007012-RA	gi|646748615|gb|KK962139.1|	663980-665717	19	7.8	gi|641587832|gb|KK854054.1|	648239-648803	9.8	20
ACYPI007021-RA	gi|646781313|gb|KK961524.1|	1798838-1800677	19	8.5	gi|641587930|gb|KK854043.1|	482472-483559	10	41
ACYPI007022-RA	gi|646766735|gb|KK961851.1|	877803-880419	20	10	gi|641587735|gb|KK854066.1|	81087-82509	11	42
ACYPI007025-RA	gi|646775867|gb|KK961705.1|	2033466-2034005	23	10	gi|641570596|gb|KK856308.1|	129098-129578	9.9	39
ACYPI007027-RA	gi|646770323|gb|KK961780.1|	39011-41165	19	7.1	gi|641569133|gb|KK856759.1|	86737-93265	11	41
ACYPI007032-RA	gi|646781772|gb|KK961510.1|	3438186-3448567	20	9.2	gi|641577316|gb|KK854772.1|	141220-144073	9.4	36
ACYPI007038-RA	gi|646776490|gb|KK961672.1|	138713-142032	20	10	gi|641585833|gb|KK854332.1|	404690-405297	10	37
ACYPI007039-RA	gi|646782357|gb|KK961494.1|	9738478-9742259	21	9.2	gi|641584504|gb|KK854477.1|	361012-362241	9.5	38
ACYPI007048-RA	gi|646777108|gb|KK961645.1|	1400759-1401465	17	6.5	gi|641585383|gb|KK854406.1|	392868-398214	11	37
ACYPI007054-RA	gi|646777416|gb|KK961632.1|	2152979-2153324	22	8.8	gi|641585376|gb|KK854407.1|	130499-131211	9.8	36
ACYPI007058-RA	gi|646754106|gb|KK961947.1|	412407-412747	16	7.6	gi|641576677|gb|KK854879.1|	92731-94247	11	39

ACYPI007070-RA	gi|646780574|gb|KK961546.1|	233294-235124	20	9.9	gi|641576983|gb|KK854825.1|	267001-268730	9.7	37
ACYPI007076-RA	gi|646782288|gb|KK961496.1|	6512493-6516888	21	9.7	gi|641574000|gb|KK855418.1|	54321-54518	10	36
ACYPI007079-RA	gi|646762191|gb|KK961910.1|	627589-628025	18	7.1	gi|641559375|gb|KK860688.1|	8068-8479	8.3	26
ACYPI007084-RA	gi|646782043|gb|KK961502.1|	5182636-5183583	20	9.2	gi|641587488|gb|KK854097.1|	511710-512191	11	21
ACYPI007086-RA	gi|646776929|gb|KK961653.1|	926074-927190	17	8.5	gi|641575709|gb|KK855059.1|	23568-23961	11	41
ACYPI007090-RA	gi|646778903|gb|KK961588.1|	3763475-3766883	23	10	gi|641586033|gb|KK854301.1|	333585-333874	11	21
ACYPI007100-RA	gi|646737122|gb|KK963108.1|	49598-50074	17	9	gi|641577043|gb|KK854816.1|	297515-298663	11	43
ACYPI007104-RA	gi|646751160|gb|KK962003.1|	690377-692271	22	10	gi|641579192|gb|KK854588.1|	238582-239926	11	41
ACYPI007109-RA	gi|646781243|gb|KK961526.1|	2172112-2172611	21	8.9	gi|641587742|gb|KK854065.1|	586168-586422	11	41
ACYPI007110-RA	gi|646775987|gb|KK961699.1|	857149-857656	19	7.5	gi|641587365|gb|KK854113.1|	138327-138565	12	45
ACYPI007113-RA	gi|646775312|gb|KK961734.1|	858306-859009	17	7.9	gi|641587383|gb|KK854110.1|	273707-276075	10	37
ACYPI007117-RA	gi|646748129|gb|KK962169.1|	1016679-1021280	21	9.8	gi|641585446|gb|KK854396.1|	278808-282137	11	39
ACYPI007125-RA	gi|646781421|gb|KK961521.1|	2386543-2391647	21	8.7	gi|641587671|gb|KK854074.1|	223276-225164	11	40
ACYPI007128-RA	gi|646776069|gb|KK961694.1|	973440-975149	18	7.8	gi|641575914|gb|KK855020.1|	152122-152578	10	37
ACYPI007135-RA	gi|646773721|gb|KK961747.1|	2167222-2170130	21	9.2	gi|641587624|gb|KK854080.1|	458158-459924	10	38
ACYPI007136-RA	gi|646765880|gb|KK961885.1|	914787-922715	23	10	gi|641586817|gb|KK854187.1|	649102-651792	9.3	39
ACYPI007137-RA	gi|646732578|gb|KK963654.1|	40511-41678	33	13	gi|641564369|gb|KK858469.1|	5674-6515	8.4	23
ACYPI007139-RA	gi|646739555|gb|KK962870.1|	111678-112791	27	18	gi|641588060|gb|KK854028.1|	667237-668091	11	42
ACYPI007148-RA	gi|646781873|gb|KK961507.1|	2026607-2028522	20	9.1	gi|641583587|gb|KK854518.1|	192736-193733	10	37
ACYPI007150-RA	gi|646741057|gb|KK962701.1|	227672-228212	21	8.8	gi|641584755|gb|KK854458.1|	436988-441079	9.7	41
ACYPI007156-RA	gi|646776456|gb|KK961674.1|	775063-775636	18	7.9	gi|641576666|gb|KK854881.1|	164477-165021	10	37
ACYPI007158-RA	gi|646778517|gb|KK961599.1|	540293-540893	20	9.6	gi|641566395|gb|KK857678.1|	51-519	10	19
ACYPI007166-RA	gi|646782357|gb|KK961494.1|	9243960-9246647	21	9.2	gi|641588000|gb|KK854035.1|	1040026-104673[illegible]	11	39
ACYPI007167-RA	gi|646739268|gb|KK962897.1|	179837-180336	18	7.4	gi|641584728|gb|KK854460.1|	178884-180247	9.5	37
ACYPI007170-RA	gi|646778736|gb|KK961593.1|	1212305-1213551	20	7.9	gi|641574036|gb|KK855411.1|	176080-179918	11	37
ACYPI007179-RA	gi|646750389|gb|KK962041.1|	395132-396749	21	9.5	gi|641587452|gb|KK854101.1|	219553-220622	11	40
ACYPI007182-RA	gi|646781443|gb|KK961520.1|	2942491-2943215	20	8.3	gi|641572974|gb|KK855674.1|	198739-199020	9.9	38
ACYPI007194-RA	gi|646753799|gb|KK961950.1|	1991586-1996265	23	9.2	gi|641572142|gb|KK855887.1|	175077-178644	10	36
ACYPI007197-RA	gi|646683836|gb|KK967228.1|	14621-15584	13	5.4	gi|641585315|gb|KK854418.1|	21687-22721	10	39
ACYPI007199-RA	gi|646779785|gb|KK961565.1|	710503-712060	18	8.9	gi|641585366|gb|KK854408.1|	198540-199844	12	41
ACYPI007207-RA	gi|646758527|gb|KK961922.1|	48295-49503	24	10	gi|641587003|gb|KK854159.1|	66719-68229	10	21
ACYPI007210-RA	gi|646766985|gb|KK961844.1|	185288-192663	21	9.7	gi|641576315|gb|KK854940.1|	210808-211432	9.9	40

ACYPI007219-RA	gi|646755714|gb|KK961935.1|	565979-567592	18	7.4	gi|641571928|gb|KK855946.1|	110187-111080	11	38
ACYPI007224-RA	gi|646776069|gb|KK961694.1|	1247455-1248949	18	7.8	gi|641585147|gb|KK854433.1|	353629-356273	9.5	39
ACYPI007232-RA	gi|646776762|gb|KK961659.1|	712414-714184	21	8.9	gi|641554387|gb|KK863045.1|	1808-3470	8.9	34
ACYPI007238-RA	gi|646621903|gb|KK972348.1|	14187-15360	21	7.3	gi|641586880|gb|KK854177.1|	228623-232098	10	20
ACYPI007240-RA	gi|646769102|gb|KK961799.1|	231139-234110	20	8.3	gi|641576906|gb|KK854838.1|	377655-378307	9.4	37
ACYPI007245-RA	gi|646778565|gb|KK961598.1|	414641-415120	19	7.8	gi|641585622|gb|KK854367.1|	341501-344100	9.9	21
ACYPI007246-RA	gi|646777159|gb|KK961643.1|	36304-36882	14	6.5	gi|641571956|gb|KK855938.1|	54013-54728	11	42
ACYPI007247-RA	gi|646778767|gb|KK961592.1|	1072725-1072993	21	5.4	gi|641587235|gb|KK854127.1|	262296-262553	10	29
ACYPI007248-RA	gi|646776447|gb|KK961675.1|	1850801-1851961	22	9.4	gi|641575794|gb|KK855043.1|	88920-89619	10	36
ACYPI007249-RA	gi|646781564|gb|KK961516.1|	2473793-2474564	23	9.4	gi|641572687|gb|KK855750.1|	52091-52908	9.9	39
ACYPI007250-RA	gi|646752567|gb|KK961965.1|	873778-874571	19	8.4	gi|641583958|gb|KK854513.1|	442876-444265	10	22
ACYPI007257-RA	gi|646774530|gb|KK961743.1|	334044-334630	20	9.8	gi|641587946|gb|KK854041.1|	737899-738721	11	41
ACYPI007258-RA	gi|646778865|gb|KK961589.1|	867673-868660	20	9.2	gi|641547881|gb|KK866127.1|	86-1426	12	43
ACYPI007260-RA	gi|646780406|gb|KK961550.1|	1573678-1574009	22	5.9	gi|641586967|gb|KK854164.1|	88755-89159	10	27
ACYPI007262-RA	gi|646776091|gb|KK961693.1|	2072834-2073448	23	10	gi|641574532|gb|KK855298.1|	199541-201933	12	20
ACYPI007266-RA	gi|646782357|gb|KK961494.1|	4312275-4314107	21	9.2	gi|641587962|gb|KK854039.1|	1047946-1049128	11	40
ACYPI007268-RA	gi|646763164|gb|KK961904.1|	1395764-1410244	22	10	gi|641587162|gb|KK854136.1|	505438-506974	10	40
ACYPI007270-RA	gi|646780889|gb|KK961537.1|	4234003-4235158	22	10	gi|641574809|gb|KK855240.1|	71065-72636	11	39
ACYPI007272-RA	gi|646779580|gb|KK961570.1|	2071591-2081440	17	8.5	gi|641586769|gb|KK854193.1|	354438-357836	12	23
ACYPI007276-RA	gi|646781143|gb|KK961529.1|	3160131-3160728	20	10	gi|641570601|gb|KK856306.1|	146612-147336	10	40
ACYPI007278-RA	gi|646780147|gb|KK961556.1|	2320679-2321695	18	7.5	gi|641587937|gb|KK854042.1|	179829-181404	11	42
ACYPI007287-RA	gi|646767307|gb|KK961835.1|	568643-568929	20	9	gi|641571311|gb|KK856105.1|	41529-41876	9.2	37
ACYPI007288-RA	gi|646780270|gb|KK961553.1|	4128835-4130990	22	8.6	gi|641569833|gb|KK856547.1|	31921-33075	8.9	37
ACYPI007298-RA	gi|646777288|gb|KK961638.1|	1509819-1512984	22	6.1	gi|641585953|gb|KK854314.1|	393980-395790	11	43
ACYPI007299-RA	gi|646776904|gb|KK961654.1|	1825030-1825908	21	9.3	gi|641587562|gb|KK854088.1|	472858-475472	11	42
ACYPI007301-RA	gi|646780147|gb|KK961556.1|	1375140-1376175	18	7.5	gi|641577826|gb|KK854687.1|	38828-39649	9.4	37
ACYPI007303-RA	gi|646776447|gb|KK961675.1|	1896365-1898673	22	9.4	gi|641576188|gb|KK854966.1|	39448-40430	11	39
ACYPI007307-RA	gi|646782357|gb|KK961494.1|	2769530-2773845	21	9.2	gi|641577772|gb|KK854697.1|	199513-200179	10	39
ACYPI007309-RA	gi|646744324|gb|KK962441.1|	170413-171110	20	7.2	gi|641570207|gb|KK856427.1|	164922-165443	11	40
ACYPI007315-RA	gi|646766056|gb|KK961877.1|	293889-294202	18	7.8	gi|641585871|gb|KK854327.1|	511677-511931	11	38
ACYPI007319-RA	gi|646749155|gb|KK962107.1|	51847-56344	22	9.2	gi|641588358|gb|KK854002.1|	709652-714457	11	43
ACYPI007324-RA	gi|646769275|gb|KK961796.1|	2310078-2310278	22	5.6	gi|641586893|gb|KK854175.1|	354291-354660	11	41

ACYPI007327-RA	gi|646761388|gb|KK961911.1|	386814-388120	19	7.4	gi|641576423|gb|KK854922.1|	337066-337898	18	35
ACYPI007331-RA	gi|646748490|gb|KK962146.1|	198398-198880	19	7.7	gi|641569526|gb|KK856644.1|	9345-9671	9.4	18
ACYPI007340-RA	gi|646780270|gb|KK961553.1|	4348328-4349053	22	8.6	gi|641569670|gb|KK856598.1|	79688-80179	9.8	45
ACYPI007344-RA	gi|646780441|gb|KK961549.1|	1134567-1135304	21	9.5	gi|641586541|gb|KK854226.1|	315982-316243	11	41
ACYPI007348-RA	gi|646781118|gb|KK961530.1|	1145476-1145951	21	10	gi|641586905|gb|KK854172.1|	112497-112869	9.9	38
ACYPI007351-RA	gi|646775807|gb|KK961708.1|	2888612-2889447	21	8.6	gi|641568682|gb|KK856900.1|	35655-35887	10	38
ACYPI007352-RA	gi|646775723|gb|KK961712.1|	1568517-1568847	21	9.5	gi|641577289|gb|KK854776.1|	255128-256313	10	36
ACYPI007353-RA	gi|646767196|gb|KK961838.1|	453935-454172	21	9.7	gi|641587866|gb|KK854050.1|	373024-373769	10	38
ACYPI007355-RA	gi|646781536|gb|KK961517.1|	747751-748213	19	7.7	gi|641586509|gb|KK854231.1|	89916-90854	9.8	37
ACYPI007358-RA	gi|646768447|gb|KK961811.1|	691661-691934	22	9.7	gi|641586372|gb|KK854251.1|	592904-593520	10	38
ACYPI007362-RA	gi|646747738|gb|KK962196.1|	367493-369842	20	8.3	gi|641578300|gb|KK854606.1|	48845-49599	11	40
ACYPI007364-RA	gi|646782043|gb|KK961502.1|	4395897-4402116	20	9.2	gi|641587660|gb|KK854076.1|	471346-473456	9.9	41
ACYPI007366-RA	gi|646762191|gb|KK961910.1|	568889-569273	18	7.1	gi|641567075|gb|KK857433.1|	55023-58199	10	39
ACYPI007368-RA	gi|646775383|gb|KK961730.1|	73349-77881	19	8.7	gi|641576161|gb|KK854972.1|	162905-168036	10	36
ACYPI007373-RA	gi|646770641|gb|KK961776.1|	655456-656655	20	9.1	gi|641567822|gb|KK857177.1|	61341-62200	10	42
ACYPI007374-RA	gi|646738455|gb|KK962975.1|	420513-425076	23	9.5	gi|641578084|gb|KK854643.1|	79905-83124	11	40
ACYPI007375-RA	gi|646753180|gb|KK961957.1|	444739-448684	25	11	gi|641586843|gb|KK854183.1|	863143-863974	11	41
ACYPI007379-RA	gi|646747559|gb|KK962209.1|	749912-753213	19	9.5	gi|641576672|gb|KK854880.1|	188212-189661	9.4	40
ACYPI007381-RA	gi|646781212|gb|KK961527.1|	4400181-4400594	21	10	gi|641580970|gb|KK854563.1|	134065-137500	11	42
ACYPI007384-RA	gi|646746216|gb|KK962304.1|	254136-257345	130	33	gi|641569577|gb|KK856628.1|	17584-18150	9.7	36
ACYPI007388-RA	gi|646781659|gb|KK961513.1|	1511399-1512537	22	9.8	gi|641575332|gb|KK855132.1|	169326-170171	10	39
ACYPI007392-RA	gi|646747914|gb|KK962184.1|	874016-876573	23	10	gi|641569377|gb|KK856689.1|	40663-41409	9.9	21
ACYPI007397-RA	gi|646781659|gb|KK961513.1|	5275561-5276399	22	9.8	gi|641582096|gb|KK854546.1|	44265-46812	11	38
ACYPI007401-RA	gi|646745135|gb|KK962381.1|	223350-225638	20	8.7	gi|641584305|gb|KK854491.1|	330222-339291	11	39
ACYPI007402-RA	gi|646780827|gb|KK961539.1|	3971722-3972364	20	9.4	gi|641576238|gb|KK854955.1|	162567-163108	11	40
ACYPI007404-RA	gi|646777288|gb|KK961638.1|	98943-106268	22	6.1	gi|641557847|gb|KK861397.1|	5674-6196	9.7	30
ACYPI007405-RA	gi|646782334|gb|KK961495.1|	2825622-2826889	21	9	gi|641577837|gb|KK854685.1|	187613-192307	11	42
ACYPI007409-RA	gi|646776368|gb|KK961679.1|	674816-680837	21	9.7	gi|641582763|gb|KK854531.1|	280873-285173	10	39
ACYPI007411-RA	gi|646778699|gb|KK961594.1|	2078755-2079068	21	9.5	gi|641575337|gb|KK855131.1|	86108-86669	10	37
ACYPI007413-RA	gi|646782334|gb|KK961495.1|	6585760-6587408	21	9	gi|641584305|gb|KK854491.1|	308400-312369	11	39
ACYPI007422-RA	gi|646776351|gb|KK961680.1|	1891809-1892088	22	10	gi|641586019|gb|KK854303.1|	356684-356939	11	43
ACYPI007425-RA	gi|646781628|gb|KK961514.1|	3544858-3545128	23	9	gi|641578114|gb|KK854637.1|	82807-83179	9.7	38

ACYPI007428-RA	gi|646746310|gb|KK962297.1|	477979-478914	23	11	gi|641588090|gb|KK854025.1|	27183-28677	11	43
ACYPI007433-RA	gi|646777729|gb|KK961621.1|	1476257-1478960	21	9.8	gi|641586046|gb|KK854299.1|	271781-273287	10	20
ACYPI007435-RA	gi|646747046|gb|KK962247.1|	1132778-1137329	22	9	gi|641574308|gb|KK855352.1|	157446-158790	10	36
ACYPI007436-RA	gi|646771352|gb|KK961768.1|	1383645-1383986	21	9.6	gi|641575693|gb|KK855062.1|	168512-168805	11	40
ACYPI007437-RA	gi|646749098|gb|KK962110.1|	171616-174188	20	7.7	gi|641584619|gb|KK854468.1|	259738-260306	10	39
ACYPI007442-RA	gi|646776128|gb|KK961691.1|	1417400-1419683	21	9.2	gi|641575043|gb|KK855191.1|	81543-82476	12	43
ACYPI007445-RA	gi|646758845|gb|KK961921.1|	1857357-1860465	22	9.5	gi|641570462|gb|KK856348.1|	13712-14325	10	37
ACYPI007451-RA	gi|646750135|gb|KK962055.1|	195182-197638	16	7.6	gi|641569429|gb|KK856673.1|	33743-34169	8.4	36
ACYPI007453-RA	gi|646782357|gb|KK961494.1|	3614850-3622344	21	9.2	gi|641584806|gb|KK854455.1|	424746-425229	11	43
ACYPI007455-RA	gi|646748057|gb|KK962174.1|	180595-183157	14	7.3	gi|641585703|gb|KK854354.1|	517228-518991	10	42
ACYPI007468-RA	gi|646770477|gb|KK961778.1|	1352230-1352931	21	9.1	gi|641570419|gb|KK856360.1|	74048-77389	12	39
ACYPI007477-RA	gi|646778213|gb|KK961608.1|	2569748-2576951	19	8.8	gi|641586850|gb|KK854182.1|	526445-529572	10	37
ACYPI007495-RA	gi|646775635|gb|KK961716.1|	877273-877818	22	10	gi|641574249|gb|KK855364.1|	167719-172295	9.2	37
ACYPI007498-RA	gi|646780147|gb|KK961556.1|	1016761-1016938	18	7.5	gi|641585709|gb|KK854353.1|	135731-135954	11	43
ACYPI007505-RA	gi|646769901|gb|KK961786.1|	954081-954580	18	6.7	gi|641577501|gb|KK854743.1|	44236-47103	9.1	37
ACYPI007507-RA	gi|646775635|gb|KK961716.1|	2367226-2367574	22	10	gi|641569999|gb|KK856492.1|	53414-54841	10	38
ACYPI007514-RA	gi|646780441|gb|KK961549.1|	3224905-3225689	21	9.5	gi|641562419|gb|KK859303.1|	25785-26250	8.5	36
ACYPI007519-RA	gi|646776822|gb|KK961657.1|	950695-951151	19	7.9	gi|641576608|gb|KK854891.1|	282113-289839	11	37
ACYPI007522-RA	gi|646775723|gb|KK961712.1|	1905555-1905868	21	9.5	gi|641569705|gb|KK856587.1|	64456-65046	9.4	37
ACYPI007524-RA	gi|646730603|gb|KK963922.1|	9891-10380	18	8.3	gi|641586555|gb|KK854224.1|	114953-115346	11	39
ACYPI007529-RA	gi|646779375|gb|KK961575.1|	914355-915245	22	9.3	gi|641587145|gb|KK854138.1|	127743-130100	10	42
ACYPI007533-RA	gi|646737822|gb|KK963039.1|	210631-212837	20	9	gi|641576266|gb|KK854949.1|	256940-258236	11	45
ACYPI007534-RA	gi|646750081|gb|KK962058.1|	417054-421332	19	8.6	gi|641570274|gb|KK856404.1|	121901-122634	9.1	35
ACYPI007545-RA	gi|646735916|gb|KK963243.1|	60019-60292	20	6.7	gi|641585287|gb|KK854423.1|	132012-132285	11	40
ACYPI007547-RA	gi|646780311|gb|KK961552.1|	1447646-1448282	21	9.7	gi|641568890|gb|KK856834.1|	32880-33468	11	40
ACYPI007561-RA	gi|646780358|gb|KK961551.1|	2280632-2284883	21	8.8	gi|641588033|gb|KK854031.1|	1129822-1132527	11	38
ACYPI007567-RA	gi|646767381|gb|KK961833.1|	722629-723425	18	6.8	gi|641585527|gb|KK854383.1|	566803-567981	11	39
ACYPI007586-RA	gi|646782357|gb|KK961494.1|	6680439-6681649	21	9.2	gi|641557821|gb|KK861408.1|	3789-3985	8.2	34
ACYPI007587-RA	gi|646751544|gb|KK961986.1|	631750-634155	22	6	gi|641571841|gb|KK855968.1|	29072-30367	10	36
ACYPI007591-RA	gi|646778213|gb|KK961608.1|	1386104-1391679	19	8.8	gi|641572162|gb|KK855882.1|	36233-36798	12	38
ACYPI007596-RA	gi|646749649|gb|KK962081.1|	75645-77432	19	7.6	gi|641572734|gb|KK855738.1|	15662-16071	11	40
ACYPI007598-RA	gi|646777416|gb|KK961632.1|	2307244-2307905	22	8.8	gi|641587066|gb|KK854149.1|	279457-279811	11	40

ACYPI007603-RA	gi|646781421|gb|KK961521.1|	4518233-4519596	21	8.7	gi|641543453|gb|KK868263.1|	2189-4064	11	34
ACYPI007620-RA	gi|646781809|gb|KK961509.1|	3642232-3644279	19	8.6	gi|641572416|gb|KK855819.1|	177497-180767	10	37
ACYPI007623-RA	gi|646775576|gb|KK961719.1|	1807402-1807630	20	8.6	gi|641587383|gb|KK854110.1|	439513-440258	10	37
ACYPI007627-RA	gi|646766540|gb|KK961858.1|	1129475-1130256	21	8.5	gi|641587671|gb|KK854074.1|	901464-902007	11	40
ACYPI007628-RA	gi|646728609|gb|KK964184.1|	331029-333014	34	14	gi|641576608|gb|KK854891.1|	364452-365585	11	37
ACYPI007630-RA	gi|646777125|gb|KK961644.1|	399233-399629	20	10	gi|641577968|gb|KK854660.1|	458315-460243	10	42
ACYPI007635-RA	gi|646777313|gb|KK961637.1|	406427-408194	20	8.2	gi|641587256|gb|KK854124.1|	254814-259009	9.7	40
ACYPI007640-RA	gi|646782334|gb|KK961495.1|	7333029-7334905	21	9	gi|641586967|gb|KK854164.1|	346157-346955	10	27
ACYPI007642-RA	gi|646730558|gb|KK963928.1|	112251-112538	19	5.4	gi|641586258|gb|KK854267.1|	696141-696963	11	37
ACYPI007646-RA	gi|646747482|gb|KK962215.1|	547666-549340	21	9.2	gi|641586639|gb|KK854212.1|	571378-573404	8.3	41
ACYPI007655-RA	gi|646779539|gb|KK961571.1|	459883-460620	15	7.5	gi|641573725|gb|KK855489.1|	48969-50625	11	21
ACYPI007666-RA	gi|646767948|gb|KK961821.1|	607554-607823	17	7.1	gi|641575951|gb|KK855015.1|	243325-244558	9.9	37
ACYPI007670-RA	gi|646775293|gb|KK961735.1|	632066-635826	19	7.7	gi|641575500|gb|KK855098.1|	162229-170407	11	39
ACYPI007671-RA	gi|646745186|gb|KK962377.1|	188086-190382	21	9.8	gi|641576281|gb|KK854947.1|	53355-53494	12	51
ACYPI007672-RA	gi|646746991|gb|KK962251.1|	445192-445538	22	9.2	gi|641588267|gb|KK854008.1|	318198-318641	10	40
ACYPI007675-RA	gi|646781873|gb|KK961507.1|	4637236-4639260	20	9.1	gi|641575396|gb|KK855118.1|	80665-81779	9	35
ACYPI007679-RA	gi|646749512|gb|KK962088.1|	483441-483797	18	7.2	gi|641587840|gb|KK854053.1|	354674-355260	11	43
ACYPI007680-RA	gi|646762797|gb|KK961906.1|	636444-637871	19	5.6	gi|641573533|gb|KK855535.1|	74742-84171	10	41
ACYPI007689-RA	gi|646780105|gb|KK961557.1|	4107596-4111062	21	9.7	gi|641587241|gb|KK854126.1|	414754-416218	11	22
ACYPI007692-RA	gi|646767850|gb|KK961823.1|	1262634-1263112	21	9.8	gi|641572139|gb|KK855888.1|	106079-106632	9.9	36
ACYPI007693-RA	gi|646782276|gb|KK961497.1|	5053910-5054343	21	9.7	gi|641576762|gb|KK854864.1|	138428-138777	10	40
ACYPI007695-RA	gi|646780858|gb|KK961538.1|	1185449-1189061	21	10	gi|641576951|gb|KK854831.1|	274772-278543	8.6	40
ACYPI007697-RA	gi|646780827|gb|KK961539.1|	3648534-3649122	20	9.4	gi|641571667|gb|KK856008.1|	72217-72938	11	39
ACYPI007705-RA	gi|646745941|gb|KK962322.1|	295709-296172	21	10	gi|641553623|gb|KK863408.1|	9812-10294	11	43
ACYPI007706-RA	gi|646777065|gb|KK961647.1|	353273-353507	20	7.9	gi|641577322|gb|KK854771.1|	94424-94943	10	21
ACYPI007710-RA	gi|646735017|gb|KK963352.1|	254176-255405	22	5.9	gi|641574085|gb|KK855399.1|	40119-40344	10	38
ACYPI007716-RA	gi|646779498|gb|KK961572.1|	1538910-1539067	22	5.6	gi|641577244|gb|KK854783.1|	156967-158908	9.7	19
ACYPI007723-RA	gi|646781243|gb|KK961526.1|	2321847-2322194	21	8.9	gi|641587742|gb|KK854065.1|	551298-551827	11	41
ACYPI007730-RA	gi|646777877|gb|KK961617.1|	816802-818452	19	8.1	gi|641584351|gb|KK854488.1|	445574-446134	11	41
ACYPI007731-RA	gi|646769275|gb|KK961796.1|	1067103-1067331	22	5.6	gi|641578069|gb|KK854646.1|	255909-256139	9.9	20
ACYPI007733-RA	gi|646776647|gb|KK961663.1|	2537767-2538242	23	9.2	gi|641572846|gb|KK855707.1|	280000-280467	13	52
ACYPI007734-RA	gi|646780752|gb|KK961541.1|	2263420-2265091	21	8	gi|641575847|gb|KK855033.1|	290069-291884	10	40

ACYPI007736-RA	gi|646750889|gb|KK962017.1|	990681-991189	22	6.1	gi|641587519|gb|KK854093.1|	158901-159517	11	39
ACYPI007744-RA	gi|646777065|gb|KK961647.1|	336603-336825	20	7.9	gi|641577322|gb|KK854771.1|	82953-83154	10	21
ACYPI007757-RA	gi|646725863|gb|KK964584.1|	17933-29154	23	11	gi|641579947|gb|KK854574.1|	414317-414539	11	39
ACYPI007759-RA	gi|646781536|gb|KK961517.1|	1138040-1138842	19	7.7	gi|641574537|gb|KK855297.1|	153099-153776	9.9	37
ACYPI007764-RA	gi|646766703|gb|KK961852.1|	806917-807406	20	7.1	gi|641573919|gb|KK855439.1|	68131-68729	10	37
ACYPI007765-RA	gi|646777065|gb|KK961647.1|	579470-580885	20	7.9	gi|641577837|gb|KK854685.1|	344389-351456	11	42
ACYPI007768-RA	gi|646768361|gb|KK961813.1|	1036679-1038233	15	7.4	gi|641576725|gb|KK854871.1|	91065-92690	8.9	37
ACYPI007769-RA	gi|646766904|gb|KK961846.1|	850023-850437	17	6.9	gi|641577623|gb|KK854722.1|	263042-263482	11	38
ACYPI007771-RA	gi|646778061|gb|KK961612.1|	419236-425807	20	8.7	gi|641574193|gb|KK855375.1|	169090-171505	10	41
ACYPI007773-RA	gi|646778245|gb|KK961607.1|	472139-474943	18	6.8	gi|641585107|gb|KK854435.1|	388387-391875	15	45
ACYPI007776-RA	gi|646777207|gb|KK961641.1|	1520778-1520979	20	8.5	gi|641575847|gb|KK855033.1|	191297-198173	10	40
ACYPI007779-RA	gi|646767120|gb|KK961840.1|	785244-785949	20	8.6	gi|641574960|gb|KK855207.1|	245839-246039	10	48
ACYPI007781-RA	gi|646747046|gb|KK962247.1|	401113-401548	22	9	gi|641576238|gb|KK854955.1|	288155-289151	11	40
ACYPI007791-RA	gi|646781282|gb|KK961525.1|	3620595-3621917	22	9.6	gi|641576942|gb|KK854833.1|	25471-27111	11	42
ACYPI007793-RA	gi|646747046|gb|KK962247.1|	276101-280534	22	9	gi|641584504|gb|KK854477.1|	485898-490280	9.5	38
ACYPI007800-RA	gi|646775723|gb|KK961712.1|	1603781-1605377	21	9.5	gi|641586059|gb|KK854297.1|	383773-384352	11	22
ACYPI007802-RA	gi|646776368|gb|KK961679.1|	1037861-1038343	21	9.7	gi|641573841|gb|KK855459.1|	270792-271383	11	40
ACYPI007803-RA	gi|646777665|gb|KK961623.1|	2286062-2289263	21	9.7	gi|641574119|gb|KK855392.1|	150662-150924	9.6	37
ACYPI007807-RA	gi|646752143|gb|KK961972.1|	609184-610441	20	5.6	gi|641577081|gb|KK854810.1|	195666-196860	11	20
ACYPI007809-RA	gi|646782276|gb|KK961497.1|	7066018-7068540	21	9.7	gi|641584281|gb|KK854493.1|	151529-152168	12	41
ACYPI007827-RA	gi|646773423|gb|KK961749.1|	282388-283435	19	8.1	gi|641586521|gb|KK854229.1|	194198-197247	11	41
ACYPI007832-RA	gi|646782276|gb|KK961497.1|	3791136-3793081	21	9.7	gi|641575288|gb|KK855139.1|	303086-303898	11	38
ACYPI007836-RA	gi|646778736|gb|KK961593.1|	269267-270772	20	7.9	gi|641573514|gb|KK855540.1|	72714-73184	9.6	37
ACYPI007840-RA	gi|646782288|gb|KK961496.1|	4505613-4508715	21	9.7	gi|641584108|gb|KK854506.1|	294658-295320	11	40
ACYPI007845-RA	gi|646777955|gb|KK961615.1|	705608-706507	21	10	gi|641586943|gb|KK854167.1|	31859-32225	11	38
ACYPI007858-RA	gi|646748888|gb|KK962123.1|	764180-766647	20	8.1	gi|641584205|gb|KK854500.1|	290392-292531	11	41
ACYPI007860-RA	gi|646782357|gb|KK961494.1|	3285498-3285749	21	9.2	gi|641564627|gb|KK858354.1|	25000-25242	8.9	35
ACYPI007869-RA	gi|646767948|gb|KK961821.1|	464254-466589	17	7.1	gi|641574031|gb|KK855412.1|	214750-215798	12	39
ACYPI007870-RA	gi|646779262|gb|KK961578.1|	1925811-1926105	20	8.1	gi|641567908|gb|KK857148.1|	29602-29818	9.5	32
ACYPI007878-RA	gi|646740879|gb|KK962721.1|	196770-198571	24	6.4	gi|641588294|gb|KK854006.1|	954747-957963	8.7	40
ACYPI007897-RA	gi|646775842|gb|KK961706.1|	1993763-1994230	22	9	gi|641572142|gb|KK855887.1|	70363-70921	10	36
ACYPI007901-RA	gi|646747424|gb|KK962220.1|	249592-249932	22	5.7	gi|641585510|gb|KK854386.1|	111591-111997	11	20

ACYPI007905-RA	gi|646774530|gb|KK961743.1|	1972317-1972705	20	9.8	gi|641574809|gb|KK855240.1|	189933-190937	11	39
ACYPI007908-RA	gi|646746407|gb|KK962291.1|	294705-294942	21	9.9	gi|641554069|gb|KK863196.1|	3057-3538	8.5	27
ACYPI007925-RA	gi|646766985|gb|KK961844.1|	375301-375925	21	9.7	gi|641587519|gb|KK854093.1|	863002-863426	11	39
ACYPI007926-RA	gi|646730603|gb|KK963922.1|	93526-93748	18	8.3	gi|641574742|gb|KK855254.1|	26008-26470	11	41
ACYPI007932-RA	gi|646748599|gb|KK962140.1|	250445-253005	21	9.8	gi|641577760|gb|KK854699.1|	227257-228128	9.8	41
ACYPI007934-RA	gi|646782357|gb|KK961494.1|	8662262-8664381	21	9.2	gi|641585340|gb|KK854414.1|	411187-411724	11	42
ACYPI007943-RA	gi|646777495|gb|KK961629.1|	1122882-1125010	19	8.7	gi|641586387|gb|KK854249.1|	101476-102999	11	24
ACYPI007945-RA	gi|646776447|gb|KK961675.1|	1917798-1920290	22	9.4	gi|641576188|gb|KK854966.1|	25935-26779	11	39
ACYPI007946-RA	gi|646776904|gb|KK961654.1|	1509855-1511279	21	9.3	gi|641577553|gb|KK854734.1|	82772-85561	9.7	41
ACYPI007949-RA	gi|646758845|gb|KK961921.1|	140389-140629	22	9.5	gi|641574783|gb|KK855245.1|	221156-221798	10	21
ACYPI007952-RA	gi|646749317|gb|KK962098.1|	323239-323637	20	5.6	gi|641588213|gb|KK854013.1|	919377-920385	9.6	39
ACYPI007960-RA	gi|646739598|gb|KK962865.1|	254229-255766	21	10	gi|641585953|gb|KK854314.1|	33271-33818	11	43
ACYPI007967-RA	gi|646779580|gb|KK961570.1|	1991257-1991513	17	8.5	gi|641577677|gb|KK854711.1|	49212-49644	12	39
ACYPI007971-RA	gi|646744270|gb|KK962445.1|	230442-230881	21	7.9	gi|641574303|gb|KK855353.1|	102637-103703	10	34
ACYPI007972-RA	gi|646768447|gb|KK961811.1|	996320-996925	22	9.7	gi|641572963|gb|KK855677.1|	195371-198152	11	43
ACYPI007973-RA	gi|646769102|gb|KK961799.1|	322512-322982	20	8.3	gi|641576906|gb|KK854838.1|	228206-229309	9.4	37
ACYPI007981-RA	gi|646777207|gb|KK961641.1|	589995-590360	20	8.5	gi|641582763|gb|KK854531.1|	88592-90905	10	39
ACYPI007984-RA	gi|646781564|gb|KK961516.1|	766814-771175	23	9.4	gi|641579933|gb|KK854577.1|	404609-405084	11	37
ACYPI007986-RA	gi|646779498|gb|KK961572.1|	536944-537172	22	5.6	gi|641567609|gb|KK857247.1|	36618-37642	9.7	19
ACYPI007988-RA	gi|646768631|gb|KK961807.1|	397155-398127	19	9.2	gi|641578154|gb|KK854631.1|	98310-101822	8.2	40
ACYPI007989-RA	gi|646720990|gb|KK965002.1|	73604-74083	20	8.4	gi|641584818|gb|KK854454.1|	50315-52209	11	42
ACYPI007990-RA	gi|646781282|gb|KK961525.1|	1387951-1388828	22	9.6	gi|641587133|gb|KK854140.1|	572865-573708	9.9	37
ACYPI007993-RA	gi|646782334|gb|KK961495.1|	7536230-7537111	21	9	gi|641586967|gb|KK854164.1|	309513-309774	10	27
ACYPI007994-RA	gi|646776184|gb|KK961688.1|	2446723-2448506	21	9.6	gi|641586452|gb|KK854239.1|	123453-125552	10	19
ACYPI007997-RA	gi|646776322|gb|KK961682.1|	1950010-1951270	22	8.9	gi|641553949|gb|KK863251.1|	2779-3523	9.2	32
ACYPI008000-RA	gi|646752431|gb|KK961967.1|	1691052-1691379	20	5.6	gi|641572955|gb|KK855679.1|	38150-39594	10	40
ACYPI008002-RA	gi|646765991|gb|KK961880.1|	248045-251425	16	7	gi|641575436|gb|KK855110.1|	35050-35312	12	37
ACYPI008005-RA	gi|646738812|gb|KK962939.1|	198961-200077	20	8.1	gi|641576123|gb|KK854979.1|	207770-208714	11	22
ACYPI008007-RA	gi|646781212|gb|KK961527.1|	3906190-3908250	21	10	gi|641576409|gb|KK854925.1|	16134-18316	11	44
ACYPI008015-RA	gi|646766382|gb|KK961864.1|	208968-212515	22	9.7	gi|641588343|gb|KK854003.1|	33801-34479	11	40
ACYPI008022-RA	gi|646782288|gb|KK961496.1|	7021789-7023913	21	9.7	gi|641588073|gb|KK854027.1|	106547-107216	11	41
ACYPI008024-RA	gi|646766382|gb|KK961864.1|	1216679-1217538	22	9.7	gi|641574833|gb|KK855235.1|	137294-138215	9.4	33

ACYPI008028-RA	gi|646773423|gb|KK961749.1|	651527-654008	19	8.1	gi|641585376|gb|KK854407.1|	180424-187277	9.8	36
ACYPI008033-RA	gi|646777729|gb|KK961621.1|	740303-742314	21	9.8	gi|641575730|gb|KK855054.1|	76562-78366	10	38
ACYPI008034-RA	gi|646770477|gb|KK961778.1|	1159251-1159672	21	9.1	gi|641585684|gb|KK854357.1|	281126-281836	11	39
ACYPI008035-RA	gi|646782276|gb|KK961497.1|	5777177-5779293	21	9.7	gi|641586726|gb|KK854199.1|	428431-429580	11	41
ACYPI008037-RA	gi|646781143|gb|KK961529.1|	3771615-3772187	20	10	gi|641587256|gb|KK854124.1|	109531-110540	9.7	40
ACYPI008044-RA	gi|646768717|gb|KK961805.1|	788429-788723	20	8	gi|641588236|gb|KK854011.1|	1178455-118605[illegible]	9.7	38
ACYPI008046-RA	gi|646781083|gb|KK961531.1|	2500698-2501605	19	8.5	gi|641585350|gb|KK854411.1|	224971-226800	11	38
ACYPI008048-RA	gi|646777108|gb|KK961645.1|	1146353-1147180	17	6.5	gi|641571321|gb|KK856102.1|	32639-34779	15	57
ACYPI008050-RA	gi|646780658|gb|KK961544.1|	1414887-1422236	19	8.6	gi|641587856|gb|KK854051.1|	783668-784232	10	41
ACYPI008053-RA	gi|646776184|gb|KK961688.1|	1715588-1716353	21	9.6	gi|641573202|gb|KK855619.1|	210574-214420	9.6	42
ACYPI008056-RA	gi|646749672|gb|KK962080.1|	101374-101587	20	7.6	gi|641570514|gb|KK856331.1|	85898-86463	9.8	39
ACYPI008063-RA	gi|646768294|gb|KK961814.1|	445194-445815	23	10	gi|641583094|gb|KK854526.1|	114769-115188	11	41
ACYPI008065-RA	gi|646744168|gb|KK962452.1|	726520-727612	22	8.7	gi|641587992|gb|KK854036.1|	686026-686619	11	41
ACYPI008069-RA	gi|646769453|gb|KK961793.1|	1331704-1334519	21	8.8	gi|641569577|gb|KK856628.1|	99941-100288	9.7	36
ACYPI008075-RA	gi|646750889|gb|KK962017.1|	1634077-1635940	22	6.1	gi|641588213|gb|KK854013.1|	624716-626027	9.6	39
ACYPI008076-RA	gi|646776542|gb|KK961668.1|	2645998-2649608	20	5.6	gi|641585989|gb|KK854308.1|	282895-285702	9.3	39
ACYPI008087-RA	gi|646750389|gb|KK962041.1|	333011-333229	21	9.5	gi|641586837|gb|KK854184.1|	93868-94582	11	40
ACYPI008089-RA	gi|646778213|gb|KK961608.1|	2532123-2535071	19	8.8	gi|641565378|gb|KK858053.1|	37341-37636	11	35
ACYPI008106-RA	gi|646767196|gb|KK961838.1|	539468-548134	21	9.7	gi|641588258|gb|KK854009.1|	935792-938830	11	36
ACYPI008107-RA	gi|646738455|gb|KK962975.1|	332440-333227	23	9.5	gi|641588213|gb|KK854013.1|	1020148-102083[illegible]	9.6	39
ACYPI008113-RA	gi|646769038|gb|KK961800.1|	1672749-1675074	21	8.9	gi|641569718|gb|KK856582.1|	47703-48923	8.4	36
ACYPI008117-RA	gi|646779375|gb|KK961575.1|	1908575-1911292	22	9.3	gi|641587074|gb|KK854148.1|	599502-601114	11	41
ACYPI008122-RA	gi|646746202|gb|KK962305.1|	397075-399965	21	6.4	gi|641575104|gb|KK855179.1|	151037-154790	10	35
ACYPI008124-RA	gi|646766868|gb|KK961847.1|	1276263-1278767	21	9.9	gi|641576437|gb|KK854920.1|	414250-414600	10	39
ACYPI008129-RA	gi|646781690|gb|KK961512.1|	2519417-2519949	21	8.8	gi|641578315|gb|KK854603.1|	128651-129219	9.8	38
ACYPI008131-RA	gi|646770901|gb|KK961773.1|	1248590-1249019	22	9.7	gi|641572293|gb|KK855850.1|	187958-188495	11	38
ACYPI008134-RA	gi|646781893|gb|KK961506.1|	3696860-3698932	19	8.5	gi|641586850|gb|KK854182.1|	396237-397769	10	37
ACYPI008142-RA	gi|646781443|gb|KK961520.1|	3108402-3108976	20	8.3	gi|641568112|gb|KK857081.1|	43747-44432	11	38
ACYPI008149-RA	gi|646775789|gb|KK961709.1|	1266376-1268753	20	9.6	gi|641575987|gb|KK855008.1|	90699-91827	9.9	20
ACYPI008151-RA	gi|646775312|gb|KK961734.1|	683458-684813	17	7.9	gi|641574986|gb|KK855201.1|	123973-124405	9.3	35
ACYPI008157-RA	gi|646779621|gb|KK961569.1|	1204604-1206755	16	7.3	gi|641575500|gb|KK855098.1|	31559-31871	11	39
ACYPI008158-RA	gi|646748129|gb|KK962169.1|	620766-624905	21	9.8	gi|641586661|gb|KK854209.1|	488936-489616	11	43

ACYPI008162-RA	gi|646781083|gb|KK961531.1|	2092356-2092959	19	8.5	gi|641569519|gb|KK856646.1|	11470-12019	10	39
ACYPI008164-RA	gi|646752143|gb|KK961972.1|	998990-999453	20	5.6	gi|641587111|gb|KK854143.1|	801287-803679	11	40
ACYPI008165-RA	gi|646748615|gb|KK962139.1|	468964-469463	19	7.8	gi|641585773|gb|KK854342.1|	608787-609104	12	45
ACYPI008166-RA	gi|646778840|gb|KK961590.1|	1725634-1729354	20	9.9	gi|641577234|gb|KK854784.1|	179623-184486	11	39
ACYPI008168-RA	gi|646778699|gb|KK961594.1|	1224258-1227543	21	9.5	gi|641578310|gb|KK854604.1|	454378-455390	11	39
ACYPI008178-RA	gi|646766056|gb|KK961877.1|	593774-602301	18	7.8	gi|641586246|gb|KK854268.1|	26414-35701	10	38
ACYPI008180-RA	gi|646776735|gb|KK961660.1|	830516-840025	21	9.6	gi|641573318|gb|KK855592.1|	125082-125808	10	41
ACYPI008181-RA	gi|646494666|gb|KK995492.1|	7936-8625	20	6.8	gi|641586817|gb|KK854187.1|	511230-512619	9.3	39
ACYPI008184-RA	gi|646770723|gb|KK961775.1|	1380322-1381050	20	8.8	gi|641586880|gb|KK854177.1|	544407-545683	10	20
ACYPI008186-RA	gi|646779337|gb|KK961576.1|	784415-784904	18	9	gi|641586285|gb|KK854263.1|	24460-27072	10	38
ACYPI008187-RA	gi|646762191|gb|KK961910.1|	811593-811972	18	7.1	gi|641586837|gb|KK854184.1|	509466-509801	11	40
ACYPI008188-RA	gi|646782276|gb|KK961497.1|	4729408-4733508	21	9.7	gi|641571667|gb|KK856008.1|	4820-5515	11	39
ACYPI008191-RA	gi|646771845|gb|KK961763.1|	1459448-1460178	23	10	gi|641579930|gb|KK854578.1|	52763-53391	9.1	35
ACYPI008195-RA	gi|646782334|gb|KK961495.1|	2068955-2069679	21	9	gi|641569495|gb|KK856654.1|	18878-21500	9.5	35
ACYPI008202-RA	gi|646744957|gb|KK962394.1|	131978-137604	21	9.8	gi|641572549|gb|KK855786.1|	52333-52559	9.5	35
ACYPI008203-RA	gi|646780311|gb|KK961552.1|	4022890-4023698	21	9.7	gi|641587866|gb|KK854050.1|	243237-243456	10	38
ACYPI008205-RA	gi|646776322|gb|KK961682.1|	1831607-1832055	22	8.9	gi|641585533|gb|KK854382.1|	419979-420361	10	40
ACYPI008211-RA	gi|646771092|gb|KK961771.1|	597519-599804	23	9.7	gi|641584419|gb|KK854483.1|	435875-436144	10	42
ACYPI008217-RA	gi|646737917|gb|KK963028.1|	258360-259661	22	7.8	gi|641571479|gb|KK856060.1|	83596-84806	11	40
ACYPI008218-RA	gi|646765902|gb|KK961884.1|	1185385-1185612	22	9.2	gi|641569596|gb|KK856622.1|	68405-68588	12	43
ACYPI008222-RA	gi|646775842|gb|KK961706.1|	651342-651921	22	9	gi|641588283|gb|KK854007.1|	658475-660840	11	40
ACYPI008226-RA	gi|646747111|gb|KK962243.1|	509834-510517	21	9.4	gi|641587219|gb|KK854129.1|	375941-376563	9.9	41
ACYPI008231-RA	gi|646771092|gb|KK961771.1|	962290-963609	23	9.7	gi|641577341|gb|KK854768.1|	333027-334677	14	58
ACYPI008241-RA	gi|646738635|gb|KK962957.1|	192172-193016	20	8.6	gi|641587383|gb|KK854110.1|	362879-365366	10	37
ACYPI008242-RA	gi|646782357|gb|KK961494.1|	8255281-8258040	21	9.2	gi|641574114|gb|KK855393.1|	159342-162609	9.9	40
ACYPI008243-RA	gi|646763164|gb|KK961904.1|	1344160-1351566	22	10	gi|641587162|gb|KK854136.1|	590188-591116	10	40
ACYPI008244-RA	gi|646779498|gb|KK961572.1|	1075894-1076208	22	5.6	gi|641570627|gb|KK856299.1|	58851-59767	11	20
ACYPI008248-RA	gi|646614736|gb|KK973610.1|	15241-15543	14	6.6	gi|641567299|gb|KK857356.1|	39235-39895	11	38
ACYPI008255-RA	gi|646777017|gb|KK961649.1|	902831-903859	18	8	gi|641568716|gb|KK856889.1|	28408-28817	10	35
ACYPI008256-RA	gi|646746339|gb|KK962295.1|	245856-252050	20	8.2	gi|641586620|gb|KK854215.1|	319717-321987	9.1	40
ACYPI008262-RA	gi|646782357|gb|KK961494.1|	9345629-9346103	21	9.2	gi|641588000|gb|KK854035.1|	1026997-102737[illegible]	11	39
ACYPI008265-RA	gi|646775414|gb|KK961728.1|	1385684-1386523	17	9.5	gi|641564209|gb|KK858536.1|	19907-20161	11	34

ACYPI008272-RA	gi|646781043|gb|KK961532.1|	2250870-2252431	26	11	gi|641587937|gb|KK854042.1|	756487-758107	11	42
ACYPI008275-RA	gi|646770477|gb|KK961778.1|	1198416-1205118	21	9.1	gi|641573765|gb|KK855478.1|	187453-188638	11	42
ACYPI008279-RA	gi|646775821|gb|KK961707.1|	285309-286134	23	9.1	gi|641581779|gb|KK854550.1|	251632-251976	11	39
ACYPI008286-RA	gi|646747841|gb|KK962189.1|	709534-710266	23	10	gi|641572719|gb|KK855742.1|	158226-159383	12	23
ACYPI008290-RA	gi|646776322|gb|KK961682.1|	128486-131685	22	8.9	gi|641587126|gb|KK854141.1|	340099-343824	10	39
ACYPI008293-RA	gi|646778865|gb|KK961589.1|	2209401-2213978	20	9.2	gi|641587624|gb|KK854080.1|	[illegible]020313-102653[illegible]	10	38
ACYPI008299-RA	gi|646748785|gb|KK962129.1|	604909-605420	20	8.2	gi|641586144|gb|KK854283.1|	481849-483619	10	38
ACYPI008301-RA	gi|646780925|gb|KK961536.1|	1171445-1172262	22	9.9	gi|641576650|gb|KK854884.1|	118227-120924	9.6	40
ACYPI008302-RA	gi|646772786|gb|KK961754.1|	520437-521419	22	9.6	gi|641588090|gb|KK854025.1|	996250-997232	11	43
ACYPI008308-RA	gi|646779412|gb|KK961574.1|	2285204-2286864	21	5.4	gi|641582439|gb|KK854536.1|	378954-382080	11	36
ACYPI008313-RA	gi|646751569|gb|KK961985.1|	386725-387268	21	9.2	gi|641572734|gb|KK855738.1|	227080-227528	11	40
ACYPI008318-RA	gi|646776929|gb|KK961653.1|	814956-815368	17	8.5	gi|641588000|gb|KK854035.1|	516184-517516	11	39
ACYPI008325-RA	gi|646772786|gb|KK961754.1|	357201-358960	22	9.6	gi|641576188|gb|KK854966.1|	83930-89497	11	39
ACYPI008327-RA	gi|646752014|gb|KK961974.1|	144836-150271	18	5.8	gi|641586886|gb|KK854176.1|	664096-672454	10	41
ACYPI008351-RA	gi|646746868|gb|KK962259.1|	620512-623808	21	10	gi|641566809|gb|KK857526.1|	41927-42113	9.8	22
ACYPI008357-RA	gi|646781183|gb|KK961528.1|	653951-659326	20	9.5	gi|641571465|gb|KK856064.1|	95177-95459	9.2	35
ACYPI008359-RA	gi|646775685|gb|KK961714.1|	378488-380385	21	8.5	gi|641576887|gb|KK854842.1|	325774-330450	15	41
ACYPI008362-RA	gi|646782334|gb|KK961495.1|	8990436-8991485	21	9	gi|641587832|gb|KK854054.1|	598824-598970	9.8	20
ACYPI008366-RA	gi|646780222|gb|KK961554.1|	3767171-3767564	20	9.7	gi|641587866|gb|KK854050.1|	988745-992046	10	38
ACYPI008368-RA	gi|646747956|gb|KK962181.1|	233344-234089	22	9.7	gi|641586185|gb|KK854275.1|	729440-730289	12	40
ACYPI008371-RA	gi|646771845|gb|KK961763.1|	1421382-1421674	23	10	gi|641586210|gb|KK854272.1|	398988-399267	10	39
ACYPI008380-RA	gi|646749727|gb|KK962077.1|	218013-221345	21	8.9	gi|641577623|gb|KK854722.1|	112486-117087	11	38
ACYPI008382-RA	gi|646743394|gb|KK962507.1|	368061-369219	20	8.8	gi|641576414|gb|KK854924.1|	86650-87522	10	40
ACYPI008383-RA	gi|646606023|gb|KK975235.1|	784-1026	17	4.6	gi|641568815|gb|KK856858.1|	91006-91440	10	34
ACYPI008384-RA	gi|646745941|gb|KK962322.1|	489178-490054	21	10	gi|641583958|gb|KK854513.1|	280088-280234	10	22
ACYPI008388-RA	gi|646766482|gb|KK961860.1|	398798-399806	17	6.7	gi|641572615|gb|KK855769.1|	172413-176442	12	43
ACYPI008390-RA	gi|646766703|gb|KK961852.1|	379404-379585	20	7.1	gi|641569664|gb|KK856600.1|	38142-38368	11	43
ACYPI008391-RA	gi|646781443|gb|KK961520.1|	1315063-1316043	20	8.3	gi|641573695|gb|KK855497.1|	134631-135749	9.2	41
ACYPI008392-RA	gi|646781849|gb|KK961508.1|	2731463-2734086	17	8	gi|641566986|gb|KK857464.1|	48304-52837	11	41
ACYPI008396-RA	gi|646782168|gb|KK961499.1|	3693910-3695458	21	9.4	gi|641571207|gb|KK856132.1|	124825-125119	10	40
ACYPI008400-RA	gi|646731210|gb|KK963842.1|	154965-156113	19	7.5	gi|641570720|gb|KK856272.1|	23334-24207	9.1	36
ACYPI008403-RA	gi|646779741|gb|KK961566.1|	2353739-2355066	20	8.5	gi|641586754|gb|KK854195.1|	517310-517841	11	41

ACYPI008415-RA	gi|646782043|gb|KK961502.1|	4060838-4061174	20	9.2	gi|641582439|gb|KK854536.1|	176136-179145	11	36
ACYPI008418-RA	gi|646772574|gb|KK961756.1|	1190864-1191414	21	8	gi|641587562|gb|KK854088.1|	41262-41915	11	42
ACYPI008429-RA	gi|646779141|gb|KK961581.1|	807247-807874	21	9.1	gi|641587126|gb|KK854141.1|	744065-744613	10	39
ACYPI008431-RA	gi|646773721|gb|KK961747.1|	2000809-2001071	21	9.2	gi|641584818|gb|KK854454.1|	208658-209150	11	42
ACYPI008437-RA	gi|646780978|gb|KK961534.1|	1021674-1022500	20	8	gi|641570596|gb|KK856308.1|	116212-117588	9.9	39
ACYPI008438-RA	gi|646776794|gb|KK961658.1|	627666-628786	20	8.1	gi|641580473|gb|KK854570.1|	97916-104069	11	44
ACYPI008439-RA	gi|646776389|gb|KK961678.1|	754686-754947	22	9.5	gi|641584351|gb|KK854488.1|	384637-385073	11	41
ACYPI008457-RA	gi|646775658|gb|KK961715.1|	1105650-1106279	19	8.6	gi|641576423|gb|KK854922.1|	326523-327253	18	35
ACYPI008463-RA	gi|646731000|gb|KK963870.1|	122963-126331	20	8.1	gi|641584305|gb|KK854491.1|	280359-280752	11	39
ACYPI008467-RA	gi|646781659|gb|KK961513.1|	5065619-5066012	22	9.8	gi|641585163|gb|KK854432.1|	15000-15287	11	43
ACYPI008468-RA	gi|646782276|gb|KK961497.1|	6822031-6826216	21	9.7	gi|641587577|gb|KK854086.1|	631076-631480	8.2	41
ACYPI008481-RA	gi|646614268|gb|KK973701.1|	3346-3726	24	7.5	gi|641586053|gb|KK854298.1|	609578-609753	11	41
ACYPI008482-RA	gi|646748142|gb|KK962168.1|	492640-493026	20	9.3	gi|641577922|gb|KK854669.1|	35683-36106	12	21
ACYPI008489-RA	gi|646734749|gb|KK963383.1|	106405-106675	15	5.8	gi|641586380|gb|KK854250.1|	276069-276602	9.3	38
ACYPI008491-RA	gi|646747201|gb|KK962236.1|	177019-178705	18	7.3	gi|641587095|gb|KK854145.1|	133940-137608	9.9	40
ACYPI008492-RA	gi|646781893|gb|KK961506.1|	3652002-3652358	19	8.5	gi|641586928|gb|KK854169.1|	170275-171738	11	39
ACYPI008493-RA	gi|646751664|gb|KK961982.1|	1201899-1208065	21	9.5	gi|641576801|gb|KK854856.1|	342247-342594	10	39
ACYPI008495-RA	gi|646780858|gb|KK961538.1|	1317995-1318583	21	10	gi|641587660|gb|KK854076.1|	461594-461882	9.9	41
ACYPI008498-RA	gi|646745186|gb|KK962377.1|	703333-703725	21	9.8	gi|641577896|gb|KK854674.1|	165456-167048	11	40
ACYPI008501-RA	gi|646781379|gb|KK961522.1|	348766-349932	22	7.6	gi|641545800|gb|KK867127.1|	2796-4729	9.6	34
ACYPI008512-RA	gi|646749036|gb|KK962114.1|	10465-11041	20	7.5	gi|641574876|gb|KK855226.1|	41189-41563	11	39
ACYPI008516-RA	gi|646770901|gb|KK961773.1|	1649200-1650400	22	9.7	gi|641577109|gb|KK854806.1|	202541-202743	9	32
ACYPI008521-RA	gi|646746284|gb|KK962299.1|	242466-243866	16	6.7	gi|641586503|gb|KK854232.1|	217244-217740	8.3	40
ACYPI008529-RA	gi|646748663|gb|KK962136.1|	88965-90512	18	6.5	gi|641586694|gb|KK854204.1|	75761-78446	12	50
ACYPI008530-RA	gi|646780953|gb|KK961535.1|	842514-844068	20	9.8	gi|641587971|gb|KK854038.1|	641120-642005	11	39
ACYPI008535-RA	gi|646765880|gb|KK961885.1|	586677-587872	23	10	gi|641570121|gb|KK856455.1|	39618-40567	10	40
ACYPI008536-RA	gi|646782276|gb|KK961497.1|	1179218-1179677	21	9.7	gi|641584728|gb|KK854460.1|	82910-83530	9.5	37
ACYPI008539-RA	gi|646749464|gb|KK962091.1|	361958-362486	18	8.8	gi|641570169|gb|KK856439.1|	24540-24771	10	36
ACYPI008541-RA	gi|646749632|gb|KK962082.1|	447267-453128	20	8.6	gi|641576238|gb|KK854955.1|	124493-125020	11	40
ACYPI008547-RA	gi|646781344|gb|KK961523.1|	1146528-1151387	21	8.9	gi|641588343|gb|KK854003.1|	1314942-1316573	11	40
ACYPI008551-RA	gi|646777233|gb|KK961640.1|	807520-808071	21	8.7	gi|641577209|gb|KK854788.1|	395918-398664	12	41
ACYPI008552-RA	gi|646746355|gb|KK962294.1|	790028-790559	22	10	gi|641588294|gb|KK854006.1|	941948-943427	8.7	40

ACYPI008555-RA	gi|646775595|gb|KK961718.1|	1422622-1424548	21	8.9	gi|641575068|gb|KK855186.1|	20290-20716	12	44
ACYPI008556-RA	gi|646746556|gb|KK962281.1|	593040-594447	20	9.1	gi|641585515|gb|KK854385.1|	451785-453086	11	40
ACYPI008558-RA	gi|646781421|gb|KK961521.1|	4773198-4780250	21	8.7	gi|641586171|gb|KK854278.1|	475981-480606	9	39
ACYPI008560-RA	gi|646751515|gb|KK961987.1|	1006506-1007171	20	9.2	gi|641585778|gb|KK854341.1|	299438-299721	9.7	41
ACYPI008563-RA	gi|646765880|gb|KK961885.1|	814878-815400	23	10	gi|641564966|gb|KK858214.1|	14529-15636	9.6	39
ACYPI008578-RA	gi|646765705|gb|KK961893.1|	595584-596484	22	9.7	gi|641575236|gb|KK855151.1|	342532-343239	11	38
ACYPI008591-RA	gi|646778061|gb|KK961612.1|	1611854-1614132	20	8.7	gi|641575209|gb|KK855157.1|	135587-136067	11	38
ACYPI008592-RA	gi|646770251|gb|KK961781.1|	697741-698014	20	9.6	gi|641570621|gb|KK856301.1|	93826-95341	10	36
ACYPI008595-RA	gi|646766985|gb|KK961844.1|	207350-209202	21	9.7	gi|641571612|gb|KK856022.1|	18390-18797	12	43
ACYPI008596-RA	gi|646780358|gb|KK961551.1|	2223879-2225027	21	8.8	gi|641573583|gb|KK855523.1|	56055-58144	10	41
ACYPI008599-RA	gi|646780105|gb|KK961557.1|	1736546-1747679	21	9.7	gi|641587624|gb|KK854080.1|	253427-253931	10	38
ACYPI008601-RA	gi|646746355|gb|KK962294.1|	909486-911980	22	10	gi|641568445|gb|KK856974.1|	71166-74651	11	43
ACYPI008606-RA	gi|646780889|gb|KK961537.1|	2378429-2381394	22	10	gi|641588051|gb|KK854029.1|	1052570-105405[illegible]	9.6	40
ACYPI008607-RA	gi|646725628|gb|KK964623.1|	66223-66493	16	5.7	gi|641587390|gb|KK854109.1|	658678-659290	11	44
ACYPI008621-RA	gi|646749532|gb|KK962087.1|	327321-328078	14	6	gi|641586975|gb|KK854163.1|	276069-276294	11	40
ACYPI008622-RA	gi|646749098|gb|KK962110.1|	474265-475262	20	7.7	gi|641587276|gb|KK854121.1|	541257-542331	11	39
ACYPI008630-RA	gi|646770477|gb|KK961778.1|	894302-898464	21	9.1	gi|641576294|gb|KK854944.1|	112031-112692	9.9	39
ACYPI008640-RA	gi|646750869|gb|KK962018.1|	47570-58292	20	10	gi|641577215|gb|KK854787.1|	159906-167921	11	40
ACYPI008641-RA	gi|646747990|gb|KK962179.1|	465682-466776	21	9.3	gi|641573923|gb|KK855438.1|	174998-176352	12	42
ACYPI008662-RA	gi|646776280|gb|KK961684.1|	1893982-1894302	22	9.3	gi|641572017|gb|KK855921.1|	15791-16626	9.3	35
ACYPI008668-RA	gi|646746974|gb|KK962252.1|	341502-343651	23	6.1	gi|641569397|gb|KK856683.1|	43687-45114	9.5	42
ACYPI008671-RA	gi|646766624|gb|KK961855.1|	44782-46871	21	10	gi|641576689|gb|KK854876.1|	354903-355844	10	40
ACYPI008675-RA	gi|646765219|gb|KK961896.1|	231312-231552	20	8.1	gi|641586850|gb|KK854182.1|	613735-614680	10	37
ACYPI008677-RA	gi|646777912|gb|KK961616.1|	1701006-1703372	20	8.6	gi|641581789|gb|KK854549.1|	279561-280333	9.9	37
ACYPI008681-RA	gi|646767265|gb|KK961836.1|	146543-151198	22	9.9	gi|641581789|gb|KK854549.1|	5868-6514	9.9	37
ACYPI008685-RA	gi|646750820|gb|KK962020.1|	517624-518415	18	7	gi|641586726|gb|KK854199.1|	27492-28547	11	41
ACYPI008693-RA	gi|646776477|gb|KK961673.1|	1387426-1387748	22	9	gi|641578106|gb|KK854639.1|	90116-90385	11	39
ACYPI008698-RA	gi|646780978|gb|KK961534.1|	2915836-2920336	20	8	gi|641587765|gb|KK854062.1|	36184-42410	10	41
ACYPI008701-RA	gi|646780270|gb|KK961553.1|	4311807-4312104	22	8.6	gi|641585423|gb|KK854400.1|	206389-206732	11	36
ACYPI008707-RA	gi|646768294|gb|KK961814.1|	419566-421241	23	10	gi|641580504|gb|KK854565.1|	345098-345508	11	43
ACYPI008709-RA	gi|646757520|gb|KK961926.1|	1119234-1125099	20	7.4	gi|641576863|gb|KK854845.1|	182756-188864	11	41
ACYPI008713-RA	gi|646776322|gb|KK961682.1|	957003-957206	22	8.9	gi|641567114|gb|KK857420.1|	67663-71213	10	38

ACYPI008720-RA	gi|646746407|gb|KK962291.1|	225283-228535	21	9.9	gi|641575096|gb|KK855181.1|	54636-55463	11	21
ACYPI008721-RA	gi|646782357|gb|KK961494.1|	3502069-3503505	21	9.2	gi|641585423|gb|KK854400.1|	40952-41166	11	36
ACYPI008724-RA	gi|646753339|gb|KK961955.1|	179742-180792	22	11	gi|641574100|gb|KK855396.1|	46918-47602	11	38
ACYPI008725-RA	gi|646780794|gb|KK961540.1|	1578307-1578762	21	9.5	gi|641571270|gb|KK856115.1|	127170-128037	11	38
ACYPI008728-RA	gi|646746897|gb|KK962257.1|	1016643-1021994	21	9.2	gi|641586983|gb|KK854162.1|	578638-582747	10	41
ACYPI008732-RA	gi|646615001|gb|KK973558.1|	2766-4377	11	5.5	gi|641586191|gb|KK854274.1|	635196-635676	11	42
ACYPI008734-RA	gi|646775901|gb|KK961703.1|	1238559-1238947	20	9.6	gi|641584681|gb|KK854464.1|	287136-287402	11	42
ACYPI008736-RA	gi|646782043|gb|KK961502.1|	2403086-2407648	20	9.2	gi|641588202|gb|KK854014.1|	808994-813455	10	21
ACYPI008744-RA	gi|646780311|gb|KK961552.1|	2912345-2912655	21	9.7	gi|641566998|gb|KK857460.1|	27454-28140	11	41
ACYPI008756-RA	gi|646750643|gb|KK962028.1|	125528-125860	21	7.5	gi|641578084|gb|KK854643.1|	152272-152566	11	40
ACYPI008758-RA	gi|646763164|gb|KK961904.1|	629740-630650	22	10	gi|641586936|gb|KK854168.1|	549874-550917	12	42
ACYPI008763-RA	gi|646781659|gb|KK961513.1|	5765849-5768012	22	9.8	gi|641579941|gb|KK854575.1|	112465-112832	10	39
ACYPI008765-RA	gi|646741815|gb|KK962634.1|	67974-69553	22	10	gi|641573533|gb|KK855535.1|	22693-23811	10	41
ACYPI008769-RA	gi|646782288|gb|KK961496.1|	227922-228617	21	9.7	gi|641585469|gb|KK854393.1|	404812-405277	10	37
ACYPI008771-RA	gi|646776024|gb|KK961697.1|	2544948-2546294	22	9.2	gi|641587946|gb|KK854041.1|	749359-754522	11	41
ACYPI008776-RA	gi|646777108|gb|KK961645.1|	911087-918267	17	6.5	gi|641575209|gb|KK855157.1|	98319-103615	11	38
ACYPI008778-RA	gi|646501544|gb|KK994170.1|	1242-2507	20	8.3	gi|641586258|gb|KK854267.1|	282632-284535	11	37
ACYPI008781-RA	gi|646781628|gb|KK961514.1|	3632767-3633635	23	9	gi|641587580|gb|KK854085.1|	321884-323127	10	39
ACYPI008785-RA	gi|646780889|gb|KK961537.1|	4035156-4037119	22	10	gi|641580970|gb|KK854563.1|	322943-323194	11	42
ACYPI008790-RA	gi|646776974|gb|KK961651.1|	718588-719743	17	8.1	gi|641585761|gb|KK854344.1|	154131-154635	10	39
ACYPI008793-RA	gi|646776429|gb|KK961676.1|	1359768-1360536	20	9.9	gi|641584913|gb|KK854447.1|	426340-426884	10	40
ACYPI008797-RA	gi|646777440|gb|KK961631.1|	1415480-1415950	22	9.7	gi|641586620|gb|KK854215.1|	44003-44203	9.1	40
ACYPI008800-RA	gi|646766158|gb|KK961873.1|	1042121-1042559	22	5.8	gi|641568762|gb|KK856874.1|	46280-46617	11	41
ACYPI008804-RA	gi|646781043|gb|KK961532.1|	1570080-1570885	26	11	gi|641586285|gb|KK854263.1|	126775-128074	10	38
ACYPI008806-RA	gi|646767697|gb|KK961826.1|	274483-275407	21	9.8	gi|641576207|gb|KK854961.1|	141266-141958	11	38
ACYPI008810-RA	gi|646523229|gb|KK990101.1|	6520-6763	19	1.9	gi|641571760|gb|KK855988.1|	94615-96317	9.9	37
ACYPI008811-RA	gi|646781243|gb|KK961526.1|	3535409-3535857	21	8.9	gi|641585653|gb|KK854362.1|	23327-23598	10	39
ACYPI008812-RA	gi|646779006|gb|KK961585.1|	523466-523710	20	9.9	gi|641572740|gb|KK855736.1|	70304-72507	11	40
ACYPI008814-RA	gi|646777089|gb|KK961646.1|	880826-881216	20	9.4	gi|641575096|gb|KK855181.1|	242206-242521	11	21
ACYPI008820-RA	gi|646562296|gb|KK983760.1|	137-341	15	7.2	gi|641587171|gb|KK854135.1|	188043-189900	10	39
ACYPI008825-RA	gi|646765790|gb|KK961889.1|	561358-567429	22	8.8	gi|641572471|gb|KK855805.1|	43470-45112	13	54
ACYPI008827-RA	gi|646777125|gb|KK961644.1|	2793360-2794142	20	10	gi|641576022|gb|KK854999.1|	75869-76399	10	36

ACYPI008830-RA	gi|646775451|gb|KK961726.1|	1710281-1710965	20	5.6	gi|641585510|gb|KK854386.1|	300394-301654	11	20
ACYPI008831-RA	gi|646776038|gb|KK961696.1|	1264429-1275408	21	10	gi|641576377|gb|KK854930.1|	203610-205683	10	42
ACYPI008833-RA	gi|646766137|gb|KK961874.1|	468576-469321	20	9	gi|641579937|gb|KK854576.1|	546261-547493	9.4	41
ACYPI008834-RA	gi|646749632|gb|KK962082.1|	218017-218294	20	8.6	gi|641587660|gb|KK854076.1|	522839-523332	9.9	41
ACYPI008835-RA	gi|646765790|gb|KK961889.1|	1133475-1134287	22	8.8	gi|641588109|gb|KK854023.1|	49521-49756	10	40
ACYPI008848-RA	gi|646746651|gb|KK962274.1|	77672-78544	16	6.6	gi|641576447|gb|KK854918.1|	35562-35815	12	45
ACYPI008850-RA	gi|646768717|gb|KK961805.1|	1261868-1262852	20	8	gi|641575549|gb|KK855090.1|	71202-71687	10	38
ACYPI008851-RA	gi|646722582|gb|KK964929.1|	127492-129551	23	6	gi|641567362|gb|KK857334.1|	68177-69957	14	24
ACYPI008853-RA	gi|646766034|gb|KK961878.1|	273626-273888	20	9	gi|641577890|gb|KK854675.1|	54969-55589	10	40
ACYPI008861-RA	gi|646685323|gb|KK967172.1|	15217-15509	11	6.4	gi|641587671|gb|KK854074.1|	477951-478521	11	40
ACYPI008863-RA	gi|646777474|gb|KK961630.1|	559820-561283	21	9.3	gi|641586874|gb|KK854178.1|	545698-547202	9.4	38
ACYPI008866-RA	gi|646778632|gb|KK961596.1|	239659-243015	21	9.2	gi|641577058|gb|KK854814.1|	319239-322704	9.1	43
ACYPI008874-RA	gi|646779741|gb|KK961566.1|	3296980-3299808	20	8.5	gi|641572637|gb|KK855763.1|	138165-142940	9.5	36
ACYPI008877-RA	gi|646776998|gb|KK961650.1|	2677370-2679852	20	9.6	gi|641559039|gb|KK860845.1|	10160-10958	8.4	33
ACYPI008884-RA	gi|646747424|gb|KK962220.1|	1163095-1164089	22	5.7	gi|641572297|gb|KK855849.1|	137845-138659	11	21
ACYPI008886-RA	gi|646781968|gb|KK961504.1|	3297544-3298546	20	9.6	gi|641569155|gb|KK856752.1|	72581-72807	11	21
ACYPI008888-RA	gi|646750795|gb|KK962021.1|	618720-619779	19	8.9	gi|641588164|gb|KK854017.1|	761934-763437	9.2	40
ACYPI008895-RA	gi|646779375|gb|KK961575.1|	3023645-3024163	22	9.3	gi|641586285|gb|KK854263.1|	33879-38036	10	38
ACYPI008902-RA	gi|646781421|gb|KK961521.1|	4076552-4080007	21	8.7	gi|641570453|gb|KK856351.1|	115987-116582	9.6	36
ACYPI008922-RA	gi|646779337|gb|KK961576.1|	1186733-1187843	18	9	gi|641587256|gb|KK854124.1|	567313-567531	9.7	40
ACYPI008923-RA	gi|646772358|gb|KK961758.1|	1492987-1494410	20	9.3	gi|641570589|gb|KK856310.1|	88815-91944	11	36
ACYPI008926-RA	gi|646780147|gb|KK961556.1|	1074436-1075601	18	7.5	gi|641574549|gb|KK855294.1|	92150-95534	8.9	38
ACYPI008930-RA	gi|646780574|gb|KK961546.1|	3155925-3157522	20	9.9	gi|641587511|gb|KK854094.1|	191946-195123	10	41
ACYPI008931-RA	gi|646775821|gb|KK961707.1|	2979644-2980859	23	9.1	gi|641585910|gb|KK854320.1|	114925-116402	10	38
ACYPI008933-RA	gi|646780925|gb|KK961536.1|	1887988-1888248	22	9.9	gi|641577698|gb|KK854708.1|	57734-58309	9.7	40
ACYPI008935-RA	gi|646745372|gb|KK962364.1|	231280-233646	23	10	gi|641570743|gb|KK856265.1|	18854-22222	9.6	34
ACYPI008947-RA	gi|646764801|gb|KK961898.1|	543144-545357	20	9.7	gi|641587896|gb|KK854047.1|	951637-952421	9.9	39
ACYPI008949-RA	gi|646744579|gb|KK962422.1|	201714-212829	22	9.9	gi|641584128|gb|KK854505.1|	142610-143266	9.6	37
ACYPI008961-RA	gi|646738882|gb|KK962932.1|	102056-104354	14	7	gi|641584876|gb|KK854450.1|	269225-272498	11	25
ACYPI008963-RA	gi|646781013|gb|KK961533.1|	1450509-1456424	18	7.6	gi|641577599|gb|KK854726.1|	223084-225329	9.2	40
ACYPI008967-RA	gi|646782211|gb|KK961498.1|	1248851-1250437	20	9	gi|641576973|gb|KK854827.1|	177799-178107	11	38
ACYPI008971-RA	gi|646741656|gb|KK962648.1|	264321-266085	24	9.7	gi|641566502|gb|KK857638.1|	22094-22425	11	36

ACYPI008980-RA	gi|646780574|gb|KK961546.1|	1149829-1156303	20	9.9	gi|641580980|gb|KK854561.1|	355900-359948	11	37
ACYPI008983-RA	gi|646504416|gb|KK993594.1|	977-1247	16	7.8	gi|641587680|gb|KK854073.1|	365500-365836	10	40
ACYPI008986-RA	gi|646782276|gb|KK961497.1|	5320877-5324108	21	9.7	gi|641574285|gb|KK855357.1|	158997-161446	9.7	37
ACYPI008988-RA	gi|646750252|gb|KK962049.1|	455680-458310	22	10	gi|641574668|gb|KK855267.1|	251486-253215	10	38
ACYPI008993-RA	gi|646770901|gb|KK961773.1|	1693454-1693843	22	9.7	gi|641577109|gb|KK854806.1|	175075-175396	9	32
ACYPI008996-RA	gi|646777233|gb|KK961640.1|	2315840-2316150	21	8.7	gi|641585383|gb|KK854406.1|	216845-217686	11	37
ACYPI009001-RA	gi|646775293|gb|KK961735.1|	787843-788426	19	7.7	gi|641586903|gb|KK854173.1|	424884-426629	11	45
ACYPI009003-RA	gi|646781421|gb|KK961521.1|	2416081-2417780	21	8.7	gi|641587671|gb|KK854074.1|	276228-279464	11	40
ACYPI009011-RA	gi|646734451|gb|KK963418.1|	131294-132564	19	9.4	gi|641588041|gb|KK854030.1|	977862-978401	11	38
ACYPI009012-RA	gi|646754962|gb|KK961940.1|	920780-922206	21	10	gi|641572185|gb|KK855876.1|	98528-99624	8.2	36
ACYPI009032-RA	gi|646776184|gb|KK961688.1|	807830-808041	21	9.6	gi|641586515|gb|KK854230.1|	704643-705606	11	39
ACYPI009034-RA	gi|646776113|gb|KK961692.1|	1668056-1668680	21	9.6	gi|641572293|gb|KK855850.1|	98912-100729	11	38
ACYPI009038-RA	gi|646744874|gb|KK962400.1|	387724-387950	19	8.3	gi|641586066|gb|KK854296.1|	312930-313223	9.4	37
ACYPI009043-RA	gi|646776219|gb|KK961687.1|	2393402-2395408	21	9.9	gi|641576238|gb|KK854955.1|	83530-84515	11	40
ACYPI009048-RA	gi|646781772|gb|KK961510.1|	3373322-3373647	20	9.2	gi|641577725|gb|KK854704.1|	230082-231588	11	42
ACYPI009052-RA	gi|646746752|gb|KK962267.1|	5014-6815	20	7.9	gi|641572136|gb|KK855889.1|	128252-128460	11	41
ACYPI009054-RA	gi|646781772|gb|KK961510.1|	3141405-3144573	20	9.2	gi|641587390|gb|KK854109.1|	568674-572991	11	44
ACYPI009057-RA	gi|646734440|gb|KK963419.1|	333408-333669	19	6.9	gi|641567546|gb|KK857269.1|	48213-48756	8.5	40
ACYPI009061-RA	gi|646780827|gb|KK961539.1|	3310808-3312546	20	9.4	gi|641576962|gb|KK854829.1|	342812-343477	10	39
ACYPI009065-RA	gi|646745434|gb|KK962359.1|	392426-392979	22	10	gi|641574504|gb|KK855305.1|	88090-93539	9.9	39
ACYPI009066-RA	gi|646745533|gb|KK962351.1|	620817-621330	22	9.7	gi|641574040|gb|KK855410.1|	273304-273985	9.9	44
ACYPI009068-RA	gi|646780574|gb|KK961546.1|	272205-273668	20	9.9	gi|641573837|gb|KK855460.1|	80488-81846	13	24
ACYPI009070-RA	gi|646739555|gb|KK962870.1|	121424-124548	27	18	gi|641571169|gb|KK856142.1|	162525-168397	9.6	42
ACYPI009071-RA	gi|646744874|gb|KK962400.1|	385557-387582	19	8.3	gi|641586066|gb|KK854296.1|	345875-348325	9.4	37
ACYPI009072-RA	gi|646742541|gb|KK962572.1|	570529-572677	19	10	gi|641585847|gb|KK854330.1|	131481-132194	11	41
ACYPI009075-RA	gi|646781421|gb|KK961521.1|	3711164-3711445	21	8.7	gi|641577152|gb|KK854799.1|	232230-232653	10	44
ACYPI009077-RA	gi|646780889|gb|KK961537.1|	498098-498740	22	10	gi|641585204|gb|KK854429.1|	110559-111892	9.2	36
ACYPI009082-RA	gi|646781282|gb|KK961525.1|	2650538-2650843	22	9.6	gi|641578128|gb|KK854635.1|	382574-382916	11	41
ACYPI009089-RA	gi|646746556|gb|KK962281.1|	301199-303090	20	9.1	gi|641587193|gb|KK854132.1|	429703-431013	11	39
ACYPI009090-RA	gi|646776477|gb|KK961673.1|	2097340-2101788	22	9	gi|641570697|gb|KK856279.1|	166834-167257	8.9	37
ACYPI009098-RA	gi|646746663|gb|KK962273.1|	217142-217761	21	10	gi|641585778|gb|KK854341.1|	165433-168433	9.7	41
ACYPI009117-RA	gi|646780311|gb|KK961552.1|	4116967-4119489	21	9.7	gi|641586626|gb|KK854214.1|	366486-367240	11	44

ACYPI009132-RA	gi|646763164|gb|KK961904.1|	1703087-1706524	22	10	gi|641575672|gb|KK855065.1|	194158-197587	9.8	38
ACYPI009134-RA	gi|646750643|gb|KK962028.1|	832510-833975	21	7.5	gi|641577905|gb|KK854672.1|	239105-243326	10	37
ACYPI009144-RA	gi|646777182|gb|KK961642.1|	1699808-1701036	20	9	gi|641585287|gb|KK854423.1|	166870-168829	11	40
ACYPI009147-RA	gi|646776542|gb|KK961668.1|	1370838-1375160	20	5.6	gi|641574240|gb|KK855366.1|	171428-171917	9.4	38
ACYPI009150-RA	gi|646746828|gb|KK962262.1|	210587-212808	20	9.2	gi|641582439|gb|KK854536.1|	36014-37731	11	36
ACYPI009151-RA	gi|646771092|gb|KK961771.1|	1029019-1029588	23	9.7	gi|641572022|gb|KK855920.1|	74461-75767	12	42
ACYPI009157-RA	gi|646751373|gb|KK961992.1|	540642-540913	21	8.7	gi|641588109|gb|KK854023.1|	134320-135433	10	40
ACYPI009158-RA	gi|646766624|gb|KK961855.1|	412484-413071	21	10	gi|641586734|gb|KK854198.1|	184729-188199	11	40
ACYPI009163-RA	gi|646729280|gb|KK964096.1|	67066-67276	22	9.3	gi|641585480|gb|KK854391.1|	179824-180376	11	21
ACYPI009170-RA	gi|646780827|gb|KK961539.1|	3630757-3633266	20	9.4	gi|641587757|gb|KK854063.1|	334210-336544	11	38
ACYPI009174-RA	gi|646768717|gb|KK961805.1|	142739-146618	20	8	gi|641584862|gb|KK854451.1|	383447-384643	10	38
ACYPI009193-RA	gi|646781690|gb|KK961512.1|	3419150-3420771	21	8.8	gi|641588033|gb|KK854031.1|	1066939-106739[illegible]	11	38
ACYPI009202-RA	gi|646765198|gb|KK961897.1|	614027-616647	22	5.9	gi|641587452|gb|KK854101.1|	591490-592758	11	40
ACYPI009215-RA	gi|646779375|gb|KK961575.1|	3057633-3059346	22	9.3	gi|641586000|gb|KK854306.1|	161038-165093	11	22
ACYPI009223-RA	gi|646766985|gb|KK961844.1|	91030-101463	21	9.7	gi|641572484|gb|KK855802.1|	92299-93777	9.2	36
ACYPI009224-RA	gi|646777363|gb|KK961635.1|	1927326-1929724	18	9.2	gi|641568119|gb|KK857079.1|	60654-61504	9.8	19
ACYPI009229-RA	gi|646774067|gb|KK961745.1|	1235647-1236598	17	9.3	gi|641584977|gb|KK854443.1|	271182-272394	11	24
ACYPI009250-RA	gi|646746216|gb|KK962304.1|	447052-448861	130	33	gi|641566949|gb|KK857477.1|	37704-40088	11	39
ACYPI009253-RA	gi|646732863|gb|KK963615.1|	60691-62792	150	39	gi|641576333|gb|KK854937.1|	62704-63778	9.9	35
ACYPI009254-RA	gi|646781893|gb|KK961506.1|	1553013-1556132	19	8.5	gi|641574339|gb|KK855345.1|	63733-65232	10	44
ACYPI009257-RA	gi|646630362|gb|KK970809.1|	4347-5074	12	7.2	gi|641587937|gb|KK854042.1|	6600-6973	11	42
ACYPI009258-RA	gi|646777802|gb|KK961619.1|	1271170-1271967	24	9	gi|641573053|gb|KK855655.1|	251172-252128	11	41
ACYPI009259-RA	gi|646747841|gb|KK962189.1|	462210-463390	23	10	gi|641571737|gb|KK855994.1|	65417-65950	11	23
ACYPI009267-RA	gi|646769102|gb|KK961799.1|	1848323-1855596	20	8.3	gi|641570730|gb|KK856269.1|	169547-171286	9.1	39
ACYPI009274-RA	gi|646778632|gb|KK961596.1|	2376348-2376644	21	9.2	gi|641574131|gb|KK855389.1|	26144-26411	10	38
ACYPI009277-RA	gi|646771845|gb|KK961763.1|	644293-645904	23	10	gi|641565038|gb|KK858185.1|	20843-22387	9.8	35
ACYPI009306-RA	gi|646743236|gb|KK962519.1|	160632-162206	22	9.1	gi|641567435|gb|KK857308.1|	65524-66777	10	38
ACYPI009308-RA	gi|646781421|gb|KK961521.1|	12560-13018	21	8.7	gi|641587946|gb|KK854041.1|	986756-988181	11	41
ACYPI009311-RA	gi|646775807|gb|KK961708.1|	2548305-2548555	21	8.6	gi|641584884|gb|KK854449.1|	79992-81072	11	39
ACYPI009312-RA	gi|646777495|gb|KK961629.1|	1785431-1786785	19	8.7	gi|641576666|gb|KK854881.1|	36837-39273	10	37
ACYPI009315-RA	gi|646747046|gb|KK962247.1|	68124-68337	22	9	gi|641576161|gb|KK854972.1|	367196-367866	10	36
ACYPI009316-RA	gi|646752143|gb|KK961972.1|	1942412-1943645	20	5.6	gi|641586240|gb|KK854269.1|	242644-243854	11	21

ACYPI009317-RA	gi|646781421|gb|KK961521.1|	1809600-1809864	21	8.7	gi|641575978|gb|KK855010.1|	173994-174720	10	36
ACYPI009325-RA	gi|646752567|gb|KK961965.1|	777613-777911	19	8.4	gi|641575218|gb|KK855155.1|	118337-118638	11	21
ACYPI009332-RA	gi|646742541|gb|KK962572.1|	472378-475879	19	10	gi|641587757|gb|KK854063.1|	658361-666357	11	38
ACYPI009334-RA	gi|646766598|gb|KK961856.1|	1189105-1189408	23	10	gi|641587937|gb|KK854042.1|	796973-797800	11	42
ACYPI009338-RA	gi|646776588|gb|KK961666.1|	981015-981822	20	5.8	gi|641579225|gb|KK854583.1|	17978-22117	10	39
ACYPI009357-RA	gi|646768406|gb|KK961812.1|	258091-258331	20	9.6	gi|641575698|gb|KK855061.1|	287769-289345	10	39
ACYPI009370-RA	gi|646781344|gb|KK961523.1|	1250403-1252070	21	8.9	gi|641584205|gb|KK854500.1|	237854-241065	11	41
ACYPI009378-RA	gi|646767850|gb|KK961823.1|	1756117-1756626	21	9.8	gi|641571119|gb|KK856156.1|	184814-185459	11	38
ACYPI009382-RA	gi|646779898|gb|KK961562.1|	2378809-2380683	19	9.3	gi|641568353|gb|KK857004.1|	67351-68368	10	38
ACYPI009386-RA	gi|646777955|gb|KK961615.1|	1344620-1347449	21	10	gi|641588000|gb|KK854035.1|	871509-874643	11	39
ACYPI009387-RA	gi|646769163|gb|KK961798.1|	1558984-1576226	20	9.2	gi|641588033|gb|KK854031.1|	670089-678906	11	38
ACYPI009394-RA	gi|646779826|gb|KK961564.1|	1098957-1099868	22	9.6	gi|641587171|gb|KK854135.1|	554110-555105	10	39
ACYPI009395-RA	gi|646781732|gb|KK961511.1|	3690283-3690972	22	9.8	gi|641580479|gb|KK854569.1|	245745-246227	10	38
ACYPI009396-RA	gi|646742343|gb|KK962588.1|	315792-316641	20	9.4	gi|641576666|gb|KK854881.1|	100601-101106	10	37
ACYPI009398-RA	gi|646769275|gb|KK961796.1|	1604205-1604932	22	5.6	gi|641577228|gb|KK854785.1|	288420-288825	9.7	39
ACYPI009404-RA	gi|646781282|gb|KK961525.1|	3412786-3413356	22	9.6	gi|641576858|gb|KK854846.1|	365764-366239	10	33
ACYPI009409-RA	gi|646751350|gb|KK961993.1|	578971-585671	20	7.6	gi|641572199|gb|KK855873.1|	85171-89569	8.1	36
ACYPI009413-RA	gi|646780889|gb|KK961537.1|	2358813-2365594	22	10	gi|641577109|gb|KK854806.1|	223825-228417	9	32
ACYPI009420-RA	gi|646743377|gb|KK962508.1|	68993-70281	19	8	gi|641567358|gb|KK857335.1|	55886-57599	10	36
ACYPI009423-RA	gi|646780794|gb|KK961540.1|	508230-509368	21	9.5	gi|641574549|gb|KK855294.1|	122615-123593	8.9	38
ACYPI009424-RA	gi|646768669|gb|KK961806.1|	331550-333339	18	9.1	gi|641575804|gb|KK855041.1|	221451-225733	11	40
ACYPI009428-RA	gi|646760564|gb|KK961914.1|	1315264-1317272	21	8.8	gi|641566559|gb|KK857617.1|	47173-50813	10	40
ACYPI009430-RA	gi|646779186|gb|KK961580.1|	510430-510980	20	8.6	gi|641566437|gb|KK857661.1|	31830-32219	9.2	34
ACYPI009431-RA	gi|646753273|gb|KK961956.1|	684998-689731	22	9	gi|641573722|gb|KK855490.1|	195719-197489	9.7	19
ACYPI009436-RA	gi|646779826|gb|KK961564.1|	985419-988515	22	9.6	gi|641570830|gb|KK856242.1|	23731-27101	9	31
ACYPI009438-RA	gi|646753273|gb|KK961956.1|	927205-927424	22	9	gi|641578330|gb|KK854601.1|	70568-74566	11	38
ACYPI009439-RA	gi|646747990|gb|KK962179.1|	110156-112105	21	9.3	gi|641588325|gb|KK854004.1|	1161609-116696[illegible]	11	40
ACYPI009441-RA	gi|646776113|gb|KK961692.1|	131031-136075	21	9.6	gi|641571726|gb|KK855997.1|	77777-80734	10	39
ACYPI009443-RA	gi|646752849|gb|KK961961.1|	332523-332867	21	9.4	gi|641588309|gb|KK854005.1|	1256613-125825[illegible]	11	40
ACYPI009444-RA	gi|646778803|gb|KK961591.1|	849316-854104	15	7.8	gi|641572782|gb|KK855725.1|	140030-140844	10	36
ACYPI009454-RA	gi|646770901|gb|KK961773.1|	1284772-1287128	22	9.7	gi|641572293|gb|KK855850.1|	212689-213935	11	38
ACYPI009455-RA	gi|646553387|gb|KK985551.1|	7023-7449	21	7.5	gi|641575898|gb|KK855024.1|	175947-176529	10	38

ACYPI009457-RA	gi|646768631|gb|KK961807.1|	1346293-1346603	19	9.2	gi|641586975|gb|KK854163.1|	225721-226430	11	40
ACYPI009460-RA	gi|646780530|gb|KK961547.1|	1044603-1046004	21	9.2	gi|641575505|gb|KK855097.1|	174552-176519	11	38
ACYPI009462-RA	gi|646771092|gb|KK961771.1|	1163884-1164640	23	9.7	gi|641570523|gb|KK856328.1|	178143-181150	12	47
ACYPI009463-RA	gi|646780147|gb|KK961556.1|	1782623-1782889	18	7.5	gi|641567123|gb|KK857417.1|	74651-74893	11	41
ACYPI009467-RA	gi|646778660|gb|KK961595.1|	210197-211004	16	8.2	gi|641586599|gb|KK854218.1|	230685-230951	11	42
ACYPI009470-RA	gi|646747385|gb|KK962223.1|	404567-411588	21	5.4	gi|641584351|gb|KK854488.1|	372332-375000	11	41
ACYPI009478-RA	gi|646782127|gb|KK961500.1|	5611368-5612015	22	9.6	gi|641585363|gb|KK854409.1|	365271-366237	9.3	38
ACYPI009480-RA	gi|646778186|gb|KK961609.1|	1829477-1829777	19	9.4	gi|641571380|gb|KK856086.1|	79913-84859	9.5	38
ACYPI009485-RA	gi|646768717|gb|KK961805.1|	907602-912094	20	8	gi|641571652|gb|KK856012.1|	156834-159056	9.6	35
ACYPI009494-RA	gi|646718932|gb|KK965097.1|	68041-68631	21	9.2	gi|641573858|gb|KK855455.1|	75809-76439	9.8	37
ACYPI009497-RA	gi|646740004|gb|KK962821.1|	601095-604200	22	9.6	gi|641576842|gb|KK854849.1|	287587-290035	12	38
ACYPI009500-RA	gi|646749945|gb|KK962065.1|	43724-44075	22	6.1	gi|641576238|gb|KK854955.1|	326585-327900	11	40
ACYPI009502-RA	gi|646780270|gb|KK961553.1|	2192992-2194499	22	8.6	gi|641578046|gb|KK854650.1|	356110-357864	10	38
ACYPI009510-RA	gi|646777363|gb|KK961635.1|	1488423-1488869	18	9.2	gi|641586496|gb|KK854233.1|	441890-442501	11	38
ACYPI009511-RA	gi|646782357|gb|KK961494.1|	9107729-9111597	21	9.2	gi|641586210|gb|KK854272.1|	616776-617200	10	39
ACYPI009523-RA	gi|646780752|gb|KK961541.1|	1543690-1544207	21	8	gi|641586144|gb|KK854283.1|	545620-546334	10	38
ACYPI009526-RA	gi|646781083|gb|KK961531.1|	2566482-2568795	19	8.5	gi|641576600|gb|KK854893.1|	181535-185477	9.5	41
ACYPI009528-RA	gi|646762191|gb|KK961910.1|	907806-912858	18	7.1	gi|641584977|gb|KK854443.1|	282333-284312	11	24
ACYPI009536-RA	gi|646736283|gb|KK963203.1|	46215-46751	24	9.7	gi|641567126|gb|KK857416.1|	49745-52578	11	41
ACYPI009537-RA	gi|646780978|gb|KK961534.1|	401500-401706	20	8	gi|641577138|gb|KK854801.1|	147799-148486	10	38
ACYPI009538-RA	gi|646782357|gb|KK961494.1|	9220670-9221668	21	9.2	gi|641576946|gb|KK854832.1|	71850-80364	9.4	43
ACYPI009542-RA	gi|646740395|gb|KK962776.1|	232789-233176	16	6.3	gi|641584845|gb|KK854452.1|	428394-429145	9	36
ACYPI009548-RA	gi|646742541|gb|KK962572.1|	504419-505534	19	10	gi|641574876|gb|KK855226.1|	139064-139912	11	39
ACYPI009550-RA	gi|646781968|gb|KK961504.1|	416712-417117	20	9.6	gi|641567675|gb|KK857227.1|	42582-48047	11	42
ACYPI009552-RA	gi|646732089|gb|KK963727.1|	172324-173312	24	13	gi|641578109|gb|KK854638.1|	464764-465214	14	46
ACYPI009554-RA	gi|646782211|gb|KK961498.1|	4647878-4648429	20	9	gi|641571760|gb|KK855988.1|	88155-89507	9.9	37
ACYPI009555-RA	gi|646772897|gb|KK961753.1|	1648605-1648967	21	8.7	gi|641577127|gb|KK854803.1|	268196-268479	10	22
ACYPI009568-RA	gi|646746911|gb|KK962256.1|	12697-13330	17	7	gi|641577047|gb|KK854815.1|	365352-366430	10	41
ACYPI009576-RA	gi|646778903|gb|KK961588.1|	2729793-2730289	23	10	gi|641587719|gb|KK854068.1|	308661-309223	10	21
ACYPI009593-RA	gi|646770047|gb|KK961784.1|	358450-361876	21	9.1	gi|641574504|gb|KK855305.1|	111921-117205	9.9	39
ACYPI009596-RA	gi|646780105|gb|KK961557.1|	1629025-1629284	21	9.7	gi|641573858|gb|KK855455.1|	193558-195185	9.8	37
ACYPI009606-RA	gi|646779006|gb|KK961585.1|	820406-826083	20	9.9	gi|641583088|gb|KK854527.1|	245670-247152	9.9	39

ACYPI009612-RA	gi|646775471|gb|KK961725.1|	2005985-2006288	20	10	gi|641582447|gb|KK854535.1|	223710-223869	9.4	44
ACYPI009613-RA	gi|646779741|gb|KK961566.1|	3087859-3088087	20	8.5	gi|641572912|gb|KK855690.1|	65983-66823	9.9	38
ACYPI009618-RA	gi|646766540|gb|KK961858.1|	58260-58445	21	8.5	gi|641577905|gb|KK854672.1|	115195-117571	10	37
ACYPI009620-RA	gi|646744930|gb|KK962396.1|	71652-71965	18	8.5	gi|641558787|gb|KK860963.1|	10199-10830	16	140
ACYPI009622-RA	gi|646768361|gb|KK961813.1|	942176-942355	15	7.4	gi|641584743|gb|KK854459.1|	352242-352681	11	36
ACYPI009625-RA	gi|646749499|gb|KK962089.1|	801908-802385	20	8.7	gi|641572315|gb|KK855844.1|	175181-175744	9.4	41
ACYPI009628-RA	gi|646766382|gb|KK961864.1|	1098642-1100547	22	9.7	gi|641575337|gb|KK855131.1|	208473-208965	10	37
ACYPI009633-RA	gi|646781510|gb|KK961518.1|	3510683-3511105	17	7.8	gi|641573218|gb|KK855615.1|	149579-149889	9.7	39
ACYPI009639-RA	gi|646754962|gb|KK961940.1|	1013642-1014660	21	10	gi|641574600|gb|KK855283.1|	217763-218798	44	35
ACYPI009640-RA	gi|646781282|gb|KK961525.1|	243200-243676	22	9.6	gi|641580470|gb|KK854571.1|	219187-219769	10	41
ACYPI009643-RA	gi|646781379|gb|KK961522.1|	2599570-2600762	22	7.6	gi|641587219|gb|KK854129.1|	238633-239260	9.9	41
ACYPI009644-RA	gi|646751569|gb|KK961985.1|	284442-285635	21	9.2	gi|641574031|gb|KK855412.1|	178647-180696	12	39
ACYPI009651-RA	gi|646781443|gb|KK961520.1|	3321427-3321646	20	8.3	gi|641568799|gb|KK856864.1|	65763-66048	9.2	35
ACYPI009652-RA	gi|646775968|gb|KK961700.1|	338924-342139	20	8.2	gi|641588325|gb|KK854004.1|	1174063-117821[illegible]	11	40
ACYPI009662-RA	gi|646748116|gb|KK962170.1|	95436-95812	20	8.3	gi|641577704|gb|KK854707.1|	25017-29924	10	38
ACYPI009664-RA	gi|646751160|gb|KK962003.1|	419817-420047	22	10	gi|641587896|gb|KK854047.1|	844546-846625	9.9	39
ACYPI009667-RA	gi|646750745|gb|KK962023.1|	981018-982098	18	9.4	gi|641587126|gb|KK854141.1|	596723-600496	10	39
ACYPI009686-RA	gi|646776184|gb|KK961688.1|	1893735-1894802	21	9.6	gi|641587823|gb|KK854055.1|	477452-480920	11	22
ACYPI009704-RA	gi|646781772|gb|KK961510.1|	2890517-2899635	20	9.2	gi|641585936|gb|KK854317.1|	230660-239963	9.9	41
ACYPI009707-RA	gi|646741656|gb|KK962648.1|	661141-669664	24	9.7	gi|641576069|gb|KK854989.1|	275096-276482	11	40
ACYPI009713-RA	gi|646744140|gb|KK962454.1|	469016-469672	21	9.3	gi|641586769|gb|KK854193.1|	274895-275692	12	23
ACYPI009718-RA	gi|646763796|gb|KK961901.1|	485985-486726	14	6.4	gi|641585562|gb|KK854377.1|	397408-401257	12	47
ACYPI009719-RA	gi|646768494|gb|KK961810.1|	364714-365914	21	10	gi|641586785|gb|KK854191.1|	456393-457436	11	39
ACYPI009722-RA	gi|646741815|gb|KK962634.1|	53109-55116	22	10	gi|641577390|gb|KK854759.1|	152256-153570	9.8	37
ACYPI009727-RA	gi|646775702|gb|KK961713.1|	668235-669152	17	7.4	gi|641586606|gb|KK854217.1|	309628-311069	11	42
ACYPI009738-RA	gi|646763535|gb|KK961902.1|	1356348-1357758	23	10	gi|641552354|gb|KK864007.1|	141-1341	9.1	31
ACYPI009739-RA	gi|646747528|gb|KK962211.1|	244449-246216	18	8.1	gi|641588309|gb|KK854005.1|	530575-532240	11	40
ACYPI009741-RA	gi|646767265|gb|KK961836.1|	561579-562221	22	9.9	gi|641577627|gb|KK854721.1|	361095-362720	11	42
ACYPI009744-RA	gi|646766650|gb|KK961854.1|	1450255-1450922	22	10	gi|641585755|gb|KK854345.1|	342551-345765	9.8	39
ACYPI009745-RA	gi|646781536|gb|KK961517.1|	1923530-1923800	19	7.7	gi|641571428|gb|KK856074.1|	139369-140140	11	39
ACYPI009755-RA	gi|646753799|gb|KK961950.1|	1860673-1862503	23	9.2	gi|641587263|gb|KK854123.1|	252936-253603	11	42
ACYPI009768-RA	gi|646782043|gb|KK961502.1|	1702552-1705209	20	9.2	gi|641572402|gb|KK855823.1|	95767-96000	11	40

ACYPI009769-RA	gi|646776998|gb|KK961650.1|	2705809-2716115	20	9.6	gi|641575730|gb|KK855054.1|	256154-257944	10	38
ACYPI009777-RA	gi|646763164|gb|KK961904.1|	10574-15117	22	10	gi|641586955|gb|KK854165.1|	789636-790218	11	40
ACYPI009782-RA	gi|646781212|gb|KK961527.1|	4102236-4103280	21	10	gi|641586837|gb|KK854184.1|	102675-103366	11	40
ACYPI009795-RA	gi|646781628|gb|KK961514.1|	2293571-2295459	23	9	gi|641574100|gb|KK855396.1|	167451-168438	11	38
ACYPI009806-RA	gi|646777125|gb|KK961644.1|	3017208-3017568	20	10	gi|641587921|gb|KK854044.1|	528720-529611	9.4	41
ACYPI009808-RA	gi|646782087|gb|KK961501.1|	5245312-5245582	20	8.7	gi|641576165|gb|KK854971.1|	35020-35287	11	41
ACYPI009810-RA	gi|646745372|gb|KK962364.1|	841817-846225	23	10	gi|641587126|gb|KK854141.1|	734179-734795	10	39
ACYPI009818-RA	gi|646776735|gb|KK961660.1|	1273463-1273681	21	9.6	gi|641587133|gb|KK854140.1|	62097-62289	9.9	37
ACYPI009821-RA	gi|646741719|gb|KK962642.1|	417418-423149	21	8.9	gi|641584305|gb|KK854491.1|	193867-196726	11	39
ACYPI009832-RA	gi|646780270|gb|KK961553.1|	3584776-3585032	22	8.6	gi|641575808|gb|KK855040.1|	87407-87867	9.9	37
ACYPI009841-RA	gi|646775944|gb|KK961701.1|	56031-56296	22	9.3	gi|641568414|gb|KK856984.1|	15299-15516	10	39
ACYPI009846-RA	gi|646781421|gb|KK961521.1|	3944449-3952235	21	8.7	gi|641577152|gb|KK854799.1|	265284-268656	10	44
ACYPI009848-RA	gi|646748407|gb|KK962151.1|	233521-235352	22	9.1	gi|641586144|gb|KK854283.1|	114601-116017	10	38
ACYPI009856-RA	gi|646751176|gb|KK962002.1|	771697-774718	21	9.6	gi|641566296|gb|KK857713.1|	27500-28132	9.7	34
ACYPI009859-RA	gi|646752431|gb|KK961967.1|	1457508-1459424	20	5.6	gi|641587793|gb|KK854059.1|	933574-934973	11	38
ACYPI009860-RA	gi|646776477|gb|KK961673.1|	897705-898200	22	9	gi|641576248|gb|KK854953.1|	52840-55169	11	41
ACYPI009867-RA	gi|646776091|gb|KK961693.1|	1676123-1676679	23	10	gi|641585480|gb|KK854391.1|	252311-252651	11	21
ACYPI009870-RA	gi|646768261|gb|KK961815.1|	251426-255133	22	6.6	gi|641576221|gb|KK854958.1|	164812-165673	9.3	35
ACYPI009872-RA	gi|646775471|gb|KK961725.1|	1403012-1403250	20	10	gi|641577905|gb|KK854672.1|	294047-295950	10	37
ACYPI009884-RA	gi|646753180|gb|KK961957.1|	639579-641038	25	11	gi|641587696|gb|KK854071.1|	535741-539440	8.9	39
ACYPI009886-RA	gi|646742541|gb|KK962572.1|	69600-70437	19	10	gi|641572459|gb|KK855808.1|	163973-167125	11	45
ACYPI009893-RA	gi|646777416|gb|KK961632.1|	620981-622626	22	8.8	gi|641577837|gb|KK854685.1|	413563-416052	11	42
ACYPI009902-RA	gi|646778336|gb|KK961604.1|	2110262-2111516	21	10	gi|641587981|gb|KK854037.1|	467814-468226	10	40
ACYPI009904-RA	gi|646746706|gb|KK962270.1|	279307-280916	23	11	gi|641587193|gb|KK854132.1|	659314-660277	11	39
ACYPI009906-RA	gi|646780311|gb|KK961552.1|	2450301-2451452	21	9.7	gi|641588258|gb|KK854009.1|	786815-787682	11	36
ACYPI009912-RA	gi|646781183|gb|KK961528.1|	1284561-1293392	20	9.5	gi|641577896|gb|KK854674.1|	146235-150106	11	40
ACYPI009915-RA	gi|646782288|gb|KK961496.1|	7663194-7668733	21	9.7	gi|641587688|gb|KK854072.1|	510415-511962	10	38
ACYPI009932-RA	gi|646757520|gb|KK961926.1|	1235780-1237025	20	7.4	gi|641585812|gb|KK854335.1|	204946-206256	11	42
ACYPI009943-RA	gi|646751160|gb|KK962003.1|	944050-945286	22	10	gi|641586171|gb|KK854278.1|	490303-493216	9	39
ACYPI009944-RA	gi|646746231|gb|KK962303.1|	305727-305893	21	6	gi|641572239|gb|KK855863.1|	84158-86266	11	43
ACYPI009945-RA	gi|646733153|gb|KK963574.1|	139013-140650	17	8.8	gi|641580479|gb|KK854569.1|	283371-285198	10	38
ACYPI009948-RA	gi|646778903|gb|KK961588.1|	1827451-1831899	23	10	gi|641567788|gb|KK857188.1|	3-1581	13	43

ACYPI009949-RA	gi|646766511|gb|KK961859.1|	836677-836948	17	8.5	gi|641576469|gb|KK854914.1|	291756-292221	11	21
ACYPI009950-RA	gi|646782168|gb|KK961499.1|	3566455-3571274	21	9.4	gi|641575847|gb|KK855033.1|	316234-316628	10	40
ACYPI009955-RA	gi|646775987|gb|KK961699.1|	1008430-1008872	19	7.5	gi|641578300|gb|KK854606.1|	447842-448496	11	40
ACYPI009966-RA	gi|646778517|gb|KK961599.1|	2228363-2228650	20	9.6	gi|641585475|gb|KK854392.1|	386650-387959	10	41
ACYPI009973-RA	gi|646777842|gb|KK961618.1|	2074067-2076152	19	8.9	gi|641574015|gb|KK855416.1|	96702-97211	10	42
ACYPI009976-RA	gi|646782276|gb|KK961497.1|	5215528-5219235	21	9.7	gi|641587937|gb|KK854042.1|	138074-139341	11	42
ACYPI009978-RA	gi|646742685|gb|KK962560.1|	402147-405466	21	9.5	gi|641573425|gb|KK855563.1|	59499-61885	9.4	42
ACYPI009979-RA	gi|646781379|gb|KK961522.1|	2150046-2150622	22	7.6	gi|641585552|gb|KK854379.1|	174470-175741	14	62
ACYPI009988-RA	gi|646726809|gb|KK964433.1|	15005-16922	19	12	gi|641573796|gb|KK855470.1|	91242-96214	11	46
ACYPI009993-RA	gi|646765198|gb|KK961897.1|	383246-386764	22	5.9	gi|641576452|gb|KK854917.1|	245256-246911	10	41
ACYPI010007-RA	gi|646750252|gb|KK962049.1|	356632-359093	22	10	gi|641571270|gb|KK856115.1|	75682-79141	11	38
ACYPI010009-RA	gi|646780889|gb|KK961537.1|	2762164-2762920	22	10	gi|641572879|gb|KK855698.1|	216603-219353	10	39
ACYPI010011-RA	gi|646769511|gb|KK961792.1|	58754-64899	20	9.9	gi|641572300|gb|KK855848.1|	194267-195000	12	38
ACYPI010013-RA	gi|646748129|gb|KK962169.1|	365515-366962	21	9.8	gi|641567952|gb|KK857133.1|	45761-47314	9.7	40
ACYPI010018-RA	gi|646749755|gb|KK962075.1|	704621-704883	22	9.2	gi|641588309|gb|KK854005.1|	1524617-152486[illegible]	11	40
ACYPI010020-RA	gi|646749964|gb|KK962064.1|	453075-453294	18	8.7	gi|641587671|gb|KK854074.1|	402341-403647	11	40
ACYPI010025-RA	gi|646780010|gb|KK961559.1|	1997806-1998585	22	10	gi|641584068|gb|KK854508.1|	430414-431046	10	45
ACYPI010028-RA	gi|646747482|gb|KK962215.1|	284871-285500	21	9.2	gi|641587424|gb|KK854104.1|	61508-61745	11	41
ACYPI010029-RA	gi|646769275|gb|KK961796.1|	2451712-2452216	22	5.6	gi|641587104|gb|KK854144.1|	355010-358995	11	37
ACYPI010034-RA	gi|646745372|gb|KK962364.1|	484917-490925	23	10	gi|641580479|gb|KK854569.1|	139738-146127	10	38
ACYPI010039-RA	gi|646776855|gb|KK961656.1|	397839-402178	19	8.8	gi|641576392|gb|KK854928.1|	211881-213005	9.7	41
ACYPI010047-RA	gi|646780105|gb|KK961557.1|	2816591-2818303	21	9.7	gi|641587462|gb|KK854100.1|	248721-250311	10	21
ACYPI010049-RA	gi|646742413|gb|KK962582.1|	127362-127636	23	10	gi|641564440|gb|KK858439.1|	24294-25273	11	36
ACYPI010054-RA	gi|646734653|gb|KK963394.1|	286111-289814	20	9.1	gi|641587742|gb|KK854065.1|	265400-266022	11	41
ACYPI010056-RA	gi|646776998|gb|KK961650.1|	1768073-1769230	20	9.6	gi|641575959|gb|KK855014.1|	12034-13040	11	41
ACYPI010059-RA	gi|646776389|gb|KK961678.1|	361099-364764	22	9.5	gi|641586555|gb|KK854224.1|	45442-46112	11	39
ACYPI010060-RA	gi|646777877|gb|KK961617.1|	364945-366069	19	8.1	gi|641586399|gb|KK854247.1|	223396-225052	15	68
ACYPI010064-RA	gi|646744833|gb|KK962403.1|	254686-259971	22	9.4	gi|641585865|gb|KK854328.1|	527255-527735	12	42
ACYPI010072-RA	gi|646746074|gb|KK962314.1|	519848-529549	17	7	gi|641565745|gb|KK857915.1|	48532-48869	11	40
ACYPI010073-RA	gi|646747738|gb|KK962196.1|	386359-389194	20	8.3	gi|641577623|gb|KK854722.1|	21088-26821	11	38
ACYPI010075-RA	gi|646782043|gb|KK961502.1|	1757397-1757570	20	9.2	gi|641587793|gb|KK854059.1|	761869-762049	11	38
ACYPI010077-RA	gi|646778410|gb|KK961602.1|	2641257-2641900	20	9	gi|641572484|gb|KK855802.1|	98614-99493	9.2	36

ACYPI010079-RA	gi|646771448|gb|KK961767.1|	1119012-1124505	21	9.8	gi|641577498|gb|KK854744.1|	10555-14276	11	42
ACYPI010091-RA	gi|646770901|gb|KK961773.1|	1322384-1326765	22	9.7	gi|641588325|gb|KK854004.1|	334317-335008	11	40
ACYPI010098-RA	gi|646769038|gb|KK961800.1|	758021-758718	21	8.9	gi|641575804|gb|KK855041.1|	28570-28895	11	40
ACYPI010100-RA	gi|646616457|gb|KK973314.1|	7296-7668	15	5.6	gi|641577407|gb|KK854756.1|	206240-206645	9.4	39
ACYPI010103-RA	gi|646770477|gb|KK961778.1|	439289-439541	21	9.1	gi|641588033|gb|KK854031.1|	232678-233225	11	38
ACYPI010107-RA	gi|646778903|gb|KK961588.1|	1215307-1215524	23	10	gi|641576456|gb|KK854916.1|	232056-233605	10	21
ACYPI010112-RA	gi|646779662|gb|KK961568.1|	1095999-1096635	20	9.3	gi|641579930|gb|KK854578.1|	81038-81493	9.1	35
ACYPI010114-RA	gi|646781421|gb|KK961521.1|	4134303-4135268	21	8.7	gi|641570453|gb|KK856351.1|	56900-57820	9.6	36
ACYPI010124-RA	gi|646781564|gb|KK961516.1|	730325-730644	23	9.4	gi|641584260|gb|KK854495.1|	163032-163385	12	43
ACYPI010127-RA	gi|646726742|gb|KK964444.1|	38589-39289	20	10	gi|641573464|gb|KK855553.1|	136792-138344	11	39
ACYPI010129-RA	gi|646782334|gb|KK961495.1|	6403892-6404463	21	9	gi|641584517|gb|KK854476.1|	401689-408130	10	39
ACYPI010131-RA	gi|646741815|gb|KK962634.1|	102187-102988	22	10	gi|641588182|gb|KK854016.1|	1244468-1245244	11	41
ACYPI010134-RA	gi|646747124|gb|KK962242.1|	250936-251295	17	8.7	gi|641585910|gb|KK854320.1|	124632-125809	10	38
ACYPI010135-RA	gi|646777955|gb|KK961615.1|	815691-819979	21	10	gi|641572022|gb|KK855920.1|	48528-50430	12	42
ACYPI010138-RA	gi|646782357|gb|KK961494.1|	4324816-4328175	21	9.2	gi|641584931|gb|KK854446.1|	403246-406414	10	39
ACYPI010142-RA	gi|646777416|gb|KK961632.1|	1913854-1914405	22	8.8	gi|641573429|gb|KK855562.1|	125188-125818	9.3	34
ACYPI010148-RA	gi|646732202|gb|KK963708.1|	239843-241194	22	9.9	gi|641577184|gb|KK854793.1|	217262-222427	11	40
ACYPI010149-RA	gi|646779826|gb|KK961564.1|	1298888-1299663	22	9.6	gi|641577498|gb|KK854744.1|	366994-367309	11	42
ACYPI010151-RA	gi|646747586|gb|KK962207.1|	14940-15327	20	8.8	gi|641574173|gb|KK855380.1|	270250-270661	9.5	39
ACYPI010153-RA	gi|646747046|gb|KK962247.1|	622118-627799	22	9	gi|641587912|gb|KK854045.1|	718233-719185	11	39
ACYPI010154-RA	gi|646771092|gb|KK961771.1|	1010457-1011987	23	9.7	gi|641574448|gb|KK855319.1|	229138-231113	10	37
ACYPI010174-RA	gi|646747586|gb|KK962207.1|	456905-459322	20	8.8	gi|641585376|gb|KK854407.1|	223863-224523	9.8	36
ACYPI010179-RA	gi|646767624|gb|KK961828.1|	96674-97620	22	8.9	gi|641585967|gb|KK854311.1|	463200-463409	11	40
ACYPI010180-RA	gi|646777393|gb|KK961634.1|	1175302-1175554	18	9.1	gi|641570138|gb|KK856448.1|	72479-73236	10	21
ACYPI010190-RA	gi|646750267|gb|KK962048.1|	490400-490683	16	8.2	gi|641573587|gb|KK855522.1|	227062-227967	9.3	38
ACYPI010196-RA	gi|646768534|gb|KK961809.1|	32013-33951	21	9.8	gi|641585971|gb|KK854310.1|	38582-39937	10	40
ACYPI010200-RA	gi|646632634|gb|KK970403.1|	9317-9521	11	6.4	gi|641572928|gb|KK855687.1|	121749-123342	11	38
ACYPI010201-RA	gi|646769275|gb|KK961796.1|	263513-263991	22	5.6	gi|641586599|gb|KK854218.1|	102587-103126	11	42
ACYPI010209-RA	gi|646775867|gb|KK961705.1|	1359592-1363103	23	10	gi|641568704|gb|KK856893.1|	67349-67785	10	40
ACYPI010216-RA	gi|646745778|gb|KK962333.1|	345179-351942	17	7.7	gi|641586380|gb|KK854250.1|	355490-355972	9.3	38
ACYPI010217-RA	gi|646775635|gb|KK961716.1|	1542420-1542683	22	10	gi|641572671|gb|KK855754.1|	66377-66925	10	38
ACYPI010223-RA	gi|646748086|gb|KK962172.1|	949892-950239	20	10	gi|641569808|gb|KK856554.1|	118724-119284	11	21

ACYPI010225-RA	gi|646780311|gb|KK961552.1|	2765239-2765477	21	9.7	gi|641572860|gb|KK855703.1|	32436-34628	9.8	36
ACYPI010226-RA	gi|646778632|gb|KK961596.1|	1629901-1630186	21	9.2	gi|641576022|gb|KK854999.1|	259671-263053	10	36
ACYPI010229-RA	gi|646750889|gb|KK962017.1|	1722045-1723264	22	6.1	gi|641572501|gb|KK855798.1|	164980-165901	9.8	39
ACYPI010231-RA	gi|646781510|gb|KK961518.1|	3548539-3549027	17	7.8	gi|641578154|gb|KK854631.1|	95855-97713	8.2	40
ACYPI010236-RA	gi|646767477|gb|KK961831.1|	172662-173868	20	8.4	gi|641576546|gb|KK854899.1|	82699-83464	11	40
ACYPI010244-RA	gi|646781601|gb|KK961515.1|	2058769-2059045	19	8.6	gi|641586121|gb|KK854287.1|	33800-34240	11	43
ACYPI060218-RA	gi|646780010|gb|KK961559.1|	323459-326007	22	10	gi|641574078|gb|KK855401.1|	221779-224216	11	41
ACYPI060219-RA	gi|646779785|gb|KK961565.1|	3220298-3220464	18	8.9	gi|641576418|gb|KK854923.1|	165555-165816	10	45
ACYPI060325-RA	gi|646618102|gb|KK973038.1|	5574-6183	14	9.2	gi|641572162|gb|KK855882.1|	172980-173774	12	38
ACYPI060526-RA	gi|646780406|gb|KK961550.1|	589392-597180	22	5.9	gi|641576456|gb|KK854916.1|	298771-299598	10	21
ACYPI060585-RA	gi|646780270|gb|KK961553.1|	2583802-2585072	22	8.6	gi|641574990|gb|KK855200.1|	147776-148010	12	37
ACYPI060626-RA	gi|646729674|gb|KK964044.1|	155174-155932	24	9.4	gi|641587624|gb|KK854080.1|	265938-267487	10	38
ACYPI060662-RA	gi|646751735|gb|KK961980.1|	536771-539635	21	5.5	gi|641571130|gb|KK856153.1|	38555-41300	10	36
ACYPI060717-RA	gi|646775595|gb|KK961718.1|	562273-563685	21	8.9	gi|641570630|gb|KK856298.1|	2-1431	12	47
ACYPI060796-RA	gi|646777955|gb|KK961615.1|	961819-962037	21	10	gi|641585961|gb|KK854312.1|	416392-416690	10	40
ACYPI060844-RA	gi|646780889|gb|KK961537.1|	1829595-1830219	22	10	gi|641577965|gb|KK854661.1|	212630-219019	11	40
ACYPI061188-RA	gi|646740955|gb|KK962713.1|	331588-332886	28	13	gi|641573788|gb|KK855472.1|	31424-32322	10	37
ACYPI061215-RA	gi|646782211|gb|KK961498.1|	5643569-5643792	20	9	gi|641575709|gb|KK855059.1|	341625-342954	11	41
ACYPI061275-RA	gi|646740645|gb|KK962748.1|	170833-171025	11	6.7	gi|641572655|gb|KK855758.1|	40869-43069	19	94
ACYPI061477-RA	gi|646749616|gb|KK962083.1|	64242-64505	22	10	gi|641587179|gb|KK854134.1|	544301-545238	11	40
ACYPI061529-RA	gi|646742343|gb|KK962588.1|	370281-370601	20	9.4	gi|641586535|gb|KK854227.1|	42388-43166	10	39
ACYPI061546-RA	gi|646749868|gb|KK962069.1|	165355-165823	19	7.1	gi|641544009|gb|KK867995.1|	135-559	11	32
ACYPI061780-RA	gi|646780794|gb|KK961540.1|	395472-397182	21	9.5	gi|641584248|gb|KK854496.1|	49894-51558	9.9	39
ACYPI061797-RA	gi|646780441|gb|KK961549.1|	488116-488769	21	9.5	gi|641567869|gb|KK857161.1|	44511-46672	11	38
ACYPI062389-RA	gi|646781601|gb|KK961515.1|	400366-405260	19	8.6	gi|641585660|gb|KK854361.1|	239482-242825	11	43
ACYPI062481-RA	gi|646744140|gb|KK962454.1|	471887-472291	21	9.3	gi|641586769|gb|KK854193.1|	265879-266459	12	23
ACYPI062495-RA	gi|646781421|gb|KK961521.1|	6691533-6693984	21	8.7	gi|641571479|gb|KK856060.1|	24072-36768	11	40
ACYPI062519-RA	gi|646782168|gb|KK961499.1|	5014281-5015201	21	9.4	gi|641573447|gb|KK855557.1|	193884-194267	10	41
ACYPI062641-RA	gi|646776241|gb|KK961686.1|	1914874-1927795	18	5.9	gi|641587390|gb|KK854109.1|	469519-470797	11	44
ACYPI062651-RA	gi|646778948|gb|KK961587.1|	1792658-1797419	20	7.3	gi|641572912|gb|KK855690.1|	232957-234846	9.9	38
ACYPI062920-RA	gi|646775723|gb|KK961712.1|	373542-374292	21	9.5	gi|641567243|gb|KK857377.1|	52094-52558	11	43
ACYPI063102-RA	gi|646779141|gb|KK961581.1|	4926-5302	21	9.1	gi|641577121|gb|KK854804.1|	421606-426170	16	82

ACYPI063189-RA	gi|646740756|gb|KK962735.1|	238133-238498	23	10	gi|641575568|gb|KK855086.1|	173860-179107	11	40
ACYPI063237-RA	gi|646781690|gb|KK961512.1|	1738929-1741938	21	8.8	gi|641588153|gb|KK854018.1|	79308-82817	9.2	41
ACYPI063239-RA	gi|646746868|gb|KK962259.1|	152215-158740	21	10	gi|641581402|gb|KK854555.1|	296881-298175	12	20
ACYPI063276-RA	gi|646778903|gb|KK961588.1|	3061449-3072335	23	10	gi|641585452|gb|KK854395.1|	43005-49800	10	21
ACYPI063378-RA	gi|646750889|gb|KK962017.1|	1904889-1907605	22	6.1	gi|641588325|gb|KK854004.1|	262486-263425	11	40
ACYPI063394-RA	gi|646775558|gb|KK961720.1|	1669273-1675149	20	9.4	gi|641577890|gb|KK854675.1|	138989-139805	10	40
ACYPI063443-RA	gi|646780925|gb|KK961536.1|	3239464-3241194	22	9.9	gi|641575197|gb|KK855159.1|	106160-107475	11	20
ACYPI063722-RA	gi|646778903|gb|KK961588.1|	760656-761268	23	10	gi|641572162|gb|KK855882.1|	148818-151850	12	38
ACYPI064034-RA	gi|646775944|gb|KK961701.1|	1890191-1892623	22	9.3	gi|641587800|gb|KK854058.1|	538978-539906	10	42
ACYPI064056-RA	gi|646634301|gb|KK970114.1|	7444-7874	12	4.9	gi|641570423|gb|KK856359.1|	18078-21025	11	43
ACYPI064108-RA	gi|646752076|gb|KK961973.1|	114387-117398	22	9.1	gi|641558438|gb|KK861121.1|	3482-3900	11	39
ACYPI064212-RA	gi|646745186|gb|KK962377.1|	379820-385376	21	9.8	gi|641586452|gb|KK854239.1|	188386-189992	10	19
ACYPI064230-RA	gi|646775702|gb|KK961713.1|	749847-753223	17	7.4	gi|641571652|gb|KK856012.1|	46063-47748	9.6	35
ACYPI064239-RA	gi|646778186|gb|KK961609.1|	2034693-2034977	19	9.4	gi|641588082|gb|KK854026.1|	305345-305726	11	41
ACYPI064487-RA	gi|646576310|gb|KK981127.1|	8295-9048	10	5.7	gi|641586936|gb|KK854168.1|	624729-625174	12	42
ACYPI065028-RA	gi|646769275|gb|KK961796.1|	198303-202792	22	5.6	gi|641586694|gb|KK854204.1|	522868-525690	12	50
ACYPI065062-RA	gi|646748002|gb|KK962178.1|	1636443-1636985	22	6.3	gi|641577121|gb|KK854804.1|	120175-120860	16	82
ACYPI065069-RA	gi|646775842|gb|KK961706.1|	2000255-2000509	22	9	gi|641572142|gb|KK855887.1|	48788-49073	10	36
ACYPI065154-RA	gi|646776762|gb|KK961659.1|	699217-699426	21	8.9	gi|641576570|gb|KK854895.1|	269385-270012	9.9	40
ACYPI065217-RA	gi|646779936|gb|KK961561.1|	1946952-1947750	19	9.6	gi|641574676|gb|KK855265.1|	51955-55920	9.4	35
ACYPI065331-RA	gi|646775867|gb|KK961705.1|	1909502-1910685	23	10	gi|641578039|gb|KK854651.1|	469292-470401	10	38
ACYPI065684-RA	gi|646775312|gb|KK961734.1|	69027-71666	17	7.9	gi|641585233|gb|KK854427.1|	443557-445444	10	38
ACYPI065817-RA	gi|646778374|gb|KK961603.1|	153710-156453	18	5.7	gi|641574836|gb|KK855234.1|	214354-217126	10	40
ACYPI065855-RA	gi|646780147|gb|KK961556.1|	912725-916745	18	7.5	gi|641585709|gb|KK854353.1|	219731-220046	11	43
ACYPI065923-RA	gi|646766426|gb|KK961862.1|	751158-752507	20	7.1	gi|641566784|gb|KK857535.1|	77927-78544	9.2	33
ACYPI065942-RA	gi|646777474|gb|KK961630.1|	1155021-1156165	21	9.3	gi|641583931|gb|KK854514.1|	204506-205967	9	38
ACYPI066741-RA	gi|646765948|gb|KK961882.1|	503314-503920	18	7.3	gi|641587742|gb|KK854065.1|	1174254-1177518	11	41
ACYPI066776-RA	gi|646762476|gb|KK961908.1|	518210-522592	19	7.4	gi|641586264|gb|KK854266.1|	106726-110665	11	46
ACYPI066960-RA	gi|646776280|gb|KK961684.1|	868790-869825	22	9.3	gi|641576766|gb|KK854863.1|	301126-302710	10	38
ACYPI066985-RA	gi|646740694|gb|KK962742.1|	92818-93987	20	7.9	gi|641587256|gb|KK854124.1|	411199-416738	9.7	40
ACYPI067064-RA	gi|646748871|gb|KK962124.1|	66155-66513	21	8.1	gi|641572655|gb|KK855758.1|	4083-7819	19	94
ACYPI067100-RA	gi|646484557|gb|KK997231.1|	14-1172	6.5	3.4	gi|641577677|gb|KK854711.1|	216355-217073	12	39

ACYPI067116-RA	gi|646766158|gb|KK961873.1|	1609472-1609979	22	5.8	gi|641587840|gb|KK854053.1|	785744-786290	11	43
ACYPI067185-RA	gi|646780723|gb|KK961542.1|	3698178-3699388	21	9.4	gi|641587688|gb|KK854072.1|	170742-172728	10	38
ACYPI067270-RA	gi|646746974|gb|KK962252.1|	1172276-1175426	23	6.1	gi|641576055|gb|KK854992.1|	323781-325117	9.7	37
ACYPI067416-RA	gi|646768717|gb|KK961805.1|	567170-574967	20	8	gi|641587569|gb|KK854087.1|	691518-691930	11	41
ACYPI067645-RA	gi|646778865|gb|KK961589.1|	1496073-1498523	20	9.2	gi|641575218|gb|KK855155.1|	65554-66497	11	21
ACYPI067648-RA	gi|646725187|gb|KK964688.1|	48949-49496	15	9.6	gi|641587365|gb|KK854113.1|	157924-159522	12	45
ACYPI067721-RA	gi|646742471|gb|KK962577.1|	426315-427144	22	5.9	gi|641588213|gb|KK854013.1|	1082969-108340[illegible]	9.6	39
ACYPI067726-RA	gi|646777665|gb|KK961623.1|	1807220-1807718	21	9.7	gi|641576437|gb|KK854920.1|	384006-385588	10	39
ACYPI067736-RA	gi|646778336|gb|KK961604.1|	2247141-2253266	21	10	gi|641576328|gb|KK854938.1|	237775-238003	11	36
ACYPI067762-RA	gi|646780658|gb|KK961544.1|	1398178-1402006	19	8.6	gi|641570807|gb|KK856248.1|	119539-120193	11	38
ACYPI067763-RA	gi|646781043|gb|KK961532.1|	4250812-4251387	26	11	gi|641588082|gb|KK854026.1|	620604-621771	11	41
ACYPI067814-RA	gi|646758935|gb|KK961920.1|	687113-688281	20	8.5	gi|641577784|gb|KK854695.1|	444291-445776	9.2	46
ACYPI067972-RA	gi|646749852|gb|KK962070.1|	259927-261140	19	8.6	gi|641586351|gb|KK854253.1|	303100-304924	10	37
ACYPI068284-RA	gi|646740686|gb|KK962743.1|	36546-37906	12	5.5	gi|641586372|gb|KK854251.1|	454736-461783	10	38
ACYPI068502-RA	gi|646742450|gb|KK962579.1|	88405-88905	19	7.6	gi|641576104|gb|KK854983.1|	208186-208529	10	20
ACYPI068599-RA	gi|646778112|gb|KK961611.1|	507045-521491	22	10	gi|641572934|gb|KK855685.1|	92370-92954	10	36
ACYPI068631-RA	gi|646782288|gb|KK961496.1|	4410633-4411578	21	9.7	gi|641566429|gb|KK857664.1|	24073-24299	12	37
ACYPI068671-RA	gi|646752348|gb|KK961968.1|	247742-248223	18	8	gi|641575687|gb|KK855063.1|	235261-235921	11	34
ACYPI068681-RA	gi|646781183|gb|KK961528.1|	2689252-2689977	20	9.5	gi|641575490|gb|KK855100.1|	91026-91556	11	41
ACYPI068701-RA	gi|646551026|gb|KK986044.1|	6123-6289	5.8	3.8	gi|641585888|gb|KK854324.1|	522364-523256	10	41
ACYPI068713-RA	gi|646775821|gb|KK961707.1|	1160980-1165516	23	9.1	gi|641577071|gb|KK854812.1|	139182-143972	13	38
ACYPI068762-RA	gi|646733656|gb|KK963509.1|	21345-21766	17	13	gi|641587921|gb|KK854044.1|	726112-728192	9.4	41
ACYPI068778-RA	gi|646770323|gb|KK961780.1|	681506-683383	19	7.1	gi|641587133|gb|KK854140.1|	461653-464301	9.9	37
ACYPI069332-RA	gi|646782276|gb|KK961497.1|	4338475-4339853	21	9.7	gi|641574689|gb|KK855262.1|	17635-18626	10	38
ACYPI069386-RA	gi|646779006|gb|KK961585.1|	1005467-1010542	20	9.9	gi|641586509|gb|KK854231.1|	499400-504137	9.8	37
ACYPI069407-RA	gi|646709960|gb|KK965470.1|	14758-18101	21	11	gi|641585581|gb|KK854374.1|	310291-313960	10	38
ACYPI069428-RA	gi|646775312|gb|KK961734.1|	573385-574538	17	7.9	gi|641574986|gb|KK855201.1|	87510-87743	9.3	35
ACYPI069453-RA	gi|646782009|gb|KK961503.1|	1531208-1532865	15	7.5	gi|641577198|gb|KK854790.1|	364612-365246	10	39
ACYPI069547-RA	gi|646782334|gb|KK961495.1|	2812463-2815880	21	9	gi|641576533|gb|KK854902.1|	140271-140535	10	39
ACYPI069554-RA	gi|646777877|gb|KK961617.1|	1226040-1227744	19	8.1	gi|641574402|gb|KK855330.1|	85533-85822	11	23
ACYPI069585-RA	gi|646781772|gb|KK961510.1|	2652763-2653486	20	9.2	gi|641580486|gb|KK854568.1|	242338-242972	11	36
ACYPI069627-RA	gi|646735722|gb|KK963266.1|	93423-93861	17	7.6	gi|641576362|gb|KK854933.1|	29154-29390	10	37

ACYPI069698-RA	gi|646744890|gb|KK962399.1|	356508-357595	18	8.5	gi|641586592|gb|KK854219.1|	469767-476199	11	41
ACYPI069803-RA	gi|646769275|gb|KK961796.1|	2079979-2087886	22	5.6	gi|641585871|gb|KK854327.1|	418861-423621	11	38
ACYPI069860-RA	gi|646578414|gb|KK980699.1|	9588-10104	25	9.7	gi|641575358|gb|KK855127.1|	88426-89155	9	41
ACYPI070165-RA	gi|646767670|gb|KK961827.1|	1345466-1346556	21	5.9	gi|641578435|gb|KK854589.1|	239374-241570	9.7	43
ACYPI070244-RA	gi|646777590|gb|KK961625.1|	578910-582202	19	9.7	gi|641578173|gb|KK854628.1|	50746-52487	9.9	21
ACYPI070323-RA	gi|646770641|gb|KK961776.1|	406066-406290	20	9.1	gi|641542070|gb|KK868923.1|	142-506	6.9	27
ACYPI070387-RA	gi|646760564|gb|KK961914.1|	164713-165766	21	8.8	gi|641587881|gb|KK854049.1|	450548-451157	10	40
ACYPI070389-RA	gi|646777288|gb|KK961638.1|	1471328-1471598	22	6.1	gi|641587688|gb|KK854072.1|	915498-915781	10	38
ACYPI070508-RA	gi|646745579|gb|KK962347.1|	118092-118599	15	7.6	gi|641585642|gb|KK854364.1|	33642-34608	9.9	38
ACYPI070768-RA	gi|646775451|gb|KK961726.1|	2179828-2183485	20	5.6	gi|641568731|gb|KK856884.1|	17513-18613	11	22
ACYPI071133-RA	gi|646775968|gb|KK961700.1|	731504-732689	20	8.2	gi|641568815|gb|KK856858.1|	137035-138945	10	34
ACYPI071157-RA	gi|646781344|gb|KK961523.1|	4300933-4307094	21	8.9	gi|641568126|gb|KK857076.1|	2004-7180	11	42
ACYPI071169-RA	gi|646767670|gb|KK961827.1|	977551-977832	21	5.9	gi|641586452|gb|KK854239.1|	392479-392887	10	19
ACYPI071217-RA	gi|646780889|gb|KK961537.1|	1683498-1684049	22	10	gi|641577890|gb|KK854675.1|	86502-88400	10	40
ACYPI071228-RA	gi|646776974|gb|KK961651.1|	738494-738995	17	8.1	gi|641585761|gb|KK854344.1|	122244-126947	10	39
ACYPI071231-RA	gi|646750165|gb|KK962053.1|	741293-742978	25	10	gi|641585569|gb|KK854376.1|	68057-69219	11	47
ACYPI071272-RA	gi|646780723|gb|KK961542.1|	603115-603484	21	9.4	gi|641561323|gb|KK859794.1|	2739-3318	8.5	15
ACYPI071352-RA	gi|646743501|gb|KK962498.1|	353080-353332	19	9.5	gi|641587365|gb|KK854113.1|	119229-120037	12	45
ACYPI071353-RA	gi|646782276|gb|KK961497.1|	517660-520202	21	9.7	gi|641555084|gb|KK862709.1|	261-639	8.3	27
ACYPI071357-RA	gi|646781013|gb|KK961533.1|	961124-961845	18	7.6	gi|641587881|gb|KK854049.1|	815859-819795	10	40
ACYPI071669-RA	gi|646781601|gb|KK961515.1|	1552780-1552997	19	8.6	gi|641586496|gb|KK854233.1|	73751-73968	11	38
ACYPI071737-RA	gi|646761166|gb|KK961913.1|	660838-660969	17	7.6	gi|641572136|gb|KK855889.1|	200082-202229	11	41
ACYPI071951-RA	gi|646768494|gb|KK961810.1|	1387747-1388176	21	10	gi|641577851|gb|KK854682.1|	63564-63959	11	34
ACYPI071956-RA	gi|646774797|gb|KK961742.1|	961887-970351	20	8.5	gi|641580486|gb|KK854568.1|	49388-52399	11	36
ACYPI071995-RA	gi|646745171|gb|KK962378.1|	721517-721903	19	8.1	gi|641586654|gb|KK854210.1|	254937-255258	11	40
ACYPI072081-RA	gi|646747817|gb|KK962191.1|	114546-115382	22	5.3	gi|641584504|gb|KK854477.1|	235075-236774	9.5	38
ACYPI072156-RA	gi|646749036|gb|KK962114.1|	191533-194277	20	7.5	gi|641587580|gb|KK854085.1|	699769-700637	10	39
ACYPI072174-RA	gi|646782288|gb|KK961496.1|	6560148-6560353	21	9.7	gi|641570621|gb|KK856301.1|	89761-90507	10	36
ACYPI072184-RA	gi|646776322|gb|KK961682.1|	1173124-1173688	22	8.9	gi|641571052|gb|KK856173.1|	14896-15551	8.9	41
ACYPI072192-RA	gi|646751160|gb|KK962003.1|	120488-123622	22	10	gi|641569463|gb|KK856664.1|	55470-56190	11	46
ACYPI072205-RA	gi|646776735|gb|KK961660.1|	1011630-1012852	21	9.6	gi|641573813|gb|KK855466.1|	123643-127729	9.6	37
ACYPI072215-RA	gi|646752431|gb|KK961967.1|	754979-763797	20	5.6	gi|641573950|gb|KK855430.1|	224906-229100	10	19

ACYPI072241-RA	gi|646775765|gb|KK961710.1|	1984639-1985783	21	6	gi|641588325|gb|KK854004.1|	1389588-1390459	11	40
ACYPI072244-RA	gi|646752431|gb|KK961967.1|	1557184-1557663	20	5.6	gi|641588343|gb|KK854003.1|	143875-144296	11	40
ACYPI072423-RA	gi|646776091|gb|KK961693.1|	1055032-1059924	23	10	gi|641577996|gb|KK854656.1|	52073-53574	11	23
ACYPI072468-RA	gi|646771352|gb|KK961768.1|	357201-357988	21	9.6	gi|641576557|gb|KK854897.1|	124816-128524	8.9	33
ACYPI072488-RA	gi|646758845|gb|KK961921.1|	1083998-1084999	22	9.5	gi|641584351|gb|KK854488.1|	496839-497645	11	41
ACYPI072603-RA	gi|646781628|gb|KK961514.1|	2460965-2464029	23	9	gi|641569664|gb|KK856600.1|	84135-85190	11	43
ACYPI072792-RA	gi|646776322|gb|KK961682.1|	1164950-1165502	22	8.9	gi|641571052|gb|KK856173.1|	14404-14766	8.9	41
ACYPI072856-RA	gi|646770047|gb|KK961784.1|	256021-256577	21	9.1	gi|641560205|gb|KK860298.1|	2586-3050	6.4	28
ACYPI072921-RA	gi|646744057|gb|KK962460.1|	209770-210606	14	6.5	gi|641588041|gb|KK854030.1|	98871-99350	11	38
ACYPI072994-RA	gi|646773721|gb|KK961747.1|	1828280-1832506	21	9.2	gi|641567672|gb|KK857228.1|	25216-26937	10	36
ACYPI073036-RA	gi|646781443|gb|KK961520.1|	2119588-2123878	20	8.3	gi|641587703|gb|KK854070.1|	99826-103281	9.8	42
ACYPI073176-RA	gi|646748002|gb|KK962178.1|	901203-902731	22	6.3	gi|641585510|gb|KK854386.1|	194300-196529	11	20
ACYPI073612-RA	gi|646780953|gb|KK961535.1|	4376882-4377409	20	9.8	gi|641574127|gb|KK855390.1|	14958-16314	10	40
ACYPI073693-RA	gi|646775968|gb|KK961700.1|	227205-228445	20	8.2	gi|641571642|gb|KK856015.1|	166257-166715	11	37
ACYPI073700-RA	gi|646755264|gb|KK961938.1|	616268-617496	20	5.3	gi|641581774|gb|KK854551.1|	161918-162762	10	19
ACYPI073731-RA	gi|646782168|gb|KK961499.1|	7218426-7223713	21	9.4	gi|641587562|gb|KK854088.1|	103279-103729	11	42
ACYPI073759-RA	gi|646766382|gb|KK961864.1|	458031-467067	22	9.7	gi|641587688|gb|KK854072.1|	486555-487281	10	38
ACYPI073803-RA	gi|646781732|gb|KK961511.1|	1971185-1971652	22	9.8	gi|641574882|gb|KK855225.1|	99877-104762	9.3	36
ACYPI073834-RA	gi|646747482|gb|KK962215.1|	364845-365155	21	9.2	gi|641584351|gb|KK854488.1|	399312-399610	11	41
ACYPI073870-RA	gi|646768222|gb|KK961816.1|	994397-997338	25	12	gi|641545206|gb|KK867413.1|	768-4217	17	31
ACYPI073873-RA	gi|646737528|gb|KK963067.1|	115818-116099	20	6.1	gi|641577453|gb|KK854750.1|	294482-295424	10	21
ACYPI073889-RA	gi|646781849|gb|KK961508.1|	2857569-2858685	17	8	gi|641586837|gb|KK854184.1|	244155-244380	11	40
ACYPI080074-RA	gi|646733743|gb|KK963499.1|	22195-23083	18	9.3	gi|641577121|gb|KK854804.1|	285142-289902	16	82
ACYPI080138-RA	gi|646747424|gb|KK962220.1|	244924-246701	22	5.7	gi|641585510|gb|KK854386.1|	118212-121205	11	20
ACYPI080240-RA	gi|646775807|gb|KK961708.1|	2167444-2168157	21	8.6	gi|641572955|gb|KK855679.1|	219275-219815	10	40
ACYPI080343-RA	gi|646779741|gb|KK961566.1|	478496-478832	20	8.5	gi|641574575|gb|KK855289.1|	360098-361542	25	130
ACYPI080567-RA	gi|646777665|gb|KK961623.1|	2276168-2282448	21	9.7	gi|641574119|gb|KK855392.1|	87498-88329	9.6	37
ACYPI080621-RA	gi|646776004|gb|KK961698.1|	962864-968458	19	8.4	gi|641578435|gb|KK854589.1|	375669-377064	9.7	43
ACYPI080661-RA	gi|646782276|gb|KK961497.1|	4267793-4268313	21	9.7	gi|641585690|gb|KK854356.1|	454002-459218	9.6	41
ACYPI081137-RA	gi|646734653|gb|KK963394.1|	323246-323858	20	9.1	gi|641587742|gb|KK854065.1|	263354-264353	11	41
ACYPI081140-RA	gi|646778112|gb|KK961611.1|	791258-791494	22	10	gi|641576010|gb|KK855002.1|	373608-374919	12	43
ACYPI081260-RA	gi|646775821|gb|KK961707.1|	1755772-1758319	23	9.1	gi|641571667|gb|KK856008.1|	48529-49546	11	39

ACYPI081400-RA	gi|646710354|gb|KK965458.1|	15650-16905	130	92	gi|641588283|gb|KK854007.1|	1046085-1046469	11	40
ACYPI081460-RA	gi|646769163|gb|KK961798.1|	2102113-2105099	20	9.2	gi|641587905|gb|KK854046.1|	175590-177979	10	43
ACYPI081754-RA	gi|646750409|gb|KK962040.1|	172135-172430	21	8.4	gi|641585946|gb|KK854315.1|	315148-315384	9.8	43
ACYPI081909-RA	gi|646780752|gb|KK961541.1|	926699-927109	21	8	gi|641585967|gb|KK854311.1|	398923-399263	11	40
ACYPI082098-RA	gi|646744874|gb|KK962400.1|	160037-160797	19	8.3	gi|641574512|gb|KK855303.1|	131685-132026	9.3	37
ACYPI082110-RA	gi|646744029|gb|KK962462.1|	438562-438870	19	7.9	gi|641577104|gb|KK854807.1|	8293-13506	10	36
ACYPI082338-RA	gi|646776952|gb|KK961652.1|	1459298-1470166	19	7.7	gi|641586301|gb|KK854260.1|	483876-485161	9.8	39
ACYPI082499-RA	gi|646776608|gb|KK961665.1|	423321-425795	23	9.3	gi|641568014|gb|KK857113.1|	18811-19483	21	81
ACYPI082595-RA	gi|646775238|gb|KK961738.1|	614773-616163	17	8.7	gi|641572405|gb|KK855822.1|	126394-126624	8.7	44
ACYPI082601-RA	gi|646766382|gb|KK961864.1|	1007759-1009661	22	9.7	gi|641572963|gb|KK855677.1|	188326-189736	11	43
ACYPI082616-RA	gi|646776608|gb|KK961665.1|	519461-521687	23	9.3	gi|641588309|gb|KK854005.1|	1579637-1580410	11	40
ACYPI082655-RA	gi|646750002|gb|KK962062.1|	353610-354217	24	12	gi|641587652|gb|KK854077.1|	653439-653953	10	38
ACYPI082856-RA	gi|646770251|gb|KK961781.1|	1374188-1375503	20	9.6	gi|641569788|gb|KK856560.1|	28740-29917	9.5	37
ACYPI082935-RA	gi|646564073|gb|KK983396.1|	4085-4753	4.5	5.2	gi|641577688|gb|KK854709.1|	138696-139468	11	40
ACYPI082950-RA	gi|646774067|gb|KK961745.1|	1504989-1508632	17	9.3	gi|641570757|gb|KK856260.1|	196439-199568	10	42
ACYPI082960-RA	gi|646727168|gb|KK964382.1|	102871-108947	16	7.1	gi|641570210|gb|KK856426.1|	124202-125012	11	40
ACYPI083041-RA	gi|646779498|gb|KK961572.1|	1200915-1201372	22	5.6	gi|641576172|gb|KK854969.1|	7185-8131	9.9	20
ACYPI083213-RA	gi|646741621|gb|KK962651.1|	203888-207516	18	7.2	gi|641588128|gb|KK854021.1|	1099394-1099900	9.5	42
ACYPI083426-RA	gi|646777955|gb|KK961615.1|	421058-421442	21	10	gi|641573861|gb|KK855454.1|	153278-153610	10	37
ACYPI083436-RA	gi|646745372|gb|KK962364.1|	763185-764496	23	10	gi|641576731|gb|KK854870.1|	271103-271790	10	41
ACYPI083523-RA	gi|646748129|gb|KK962169.1|	533550-536495	21	9.8	gi|641575603|gb|KK855080.1|	43705-46594	9.4	38
ACYPI083537-RA	gi|646776647|gb|KK961663.1|	1748757-1749432	23	9.2	gi|641585871|gb|KK854327.1|	563720-564332	11	38
ACYPI083635-RA	gi|646782357|gb|KK961494.1|	3442788-3445611	21	9.2	gi|641571660|gb|KK856010.1|	160875-162863	11	22
ACYPI084147-RA	gi|646778029|gb|KK961613.1|	2084912-2091906	21	8.6	gi|641586975|gb|KK854163.1|	492895-493557	11	40
ACYPI084157-RA	gi|646782127|gb|KK961500.1|	2673116-2676517	22	9.6	gi|641571841|gb|KK855968.1|	103877-104284	10	36
ACYPI084287-RA	gi|646778307|gb|KK961605.1|	1642683-1644660	17	8.7	gi|641585876|gb|KK854326.1|	409020-409565	9.5	39
ACYPI084297-RA	gi|646777955|gb|KK961615.1|	735783-739461	21	10	gi|641573729|gb|KK855488.1|	194069-196581	10	43
ACYPI084620-RA	gi|646745941|gb|KK962322.1|	309896-318346	21	10	gi|641576203|gb|KK854962.1|	146165-147967	9.8	36
ACYPI084854-RA	gi|646781659|gb|KK961513.1|	2834578-2835918	22	9.8	gi|641588325|gb|KK854004.1|	1147664-1150408	11	40
ACYPI084955-RA	gi|646781243|gb|KK961526.1|	3723491-3725070	21	8.9	gi|641587226|gb|KK854128.1|	574708-575972	9.9	39
ACYPI085022-RA	gi|646750600|gb|KK962030.1|	1121578-1123517	23	8.6	gi|641574668|gb|KK855267.1|	22993-24442	10	38
ACYPI085070-RA	gi|646598427|gb|KK976785.1|	6168-6390	13	6	gi|641577075|gb|KK854811.1|	281679-282296	11	36

ACYPI085199-RA	gi|646779785|gb|KK961565.1|	3232106-3243489	18	8.9	gi|641587074|gb|KK854148.1|	79887-80937	11	41
ACYPI085301-RA	gi|646751160|gb|KK962003.1|	605771-607199	22	10	gi|641570045|gb|KK856477.1|	92062-94767	9.6	43
ACYPI085401-RA	gi|646778983|gb|KK961586.1|	1226591-1233225	20	9.9	gi|641588024|gb|KK854032.1|	343553-343787	9.7	41
ACYPI085603-RA	gi|646769163|gb|KK961798.1|	1336514-1336853	20	9.2	gi|641576962|gb|KK854829.1|	263789-266810	10	39
ACYPI085634-RA	gi|646781118|gb|KK961530.1|	4105617-4105751	21	10	gi|641588033|gb|KK854031.1|	362074-362779	11	38
ACYPI085768-RA	gi|646750643|gb|KK962028.1|	517452-518890	21	7.5	gi|641587126|gb|KK854141.1|	334022-336370	10	39
ACYPI086258-RA	gi|646780530|gb|KK961547.1|	3306309-3306651	21	9.2	gi|641586338|gb|KK854255.1|	565588-566320	14	44
ACYPI086268-RA	gi|646748771|gb|KK962130.1|	838799-849247	21	8.3	gi|641588194|gb|KK854015.1|	606773-607937	9.4	40
ACYPI086281-RA	gi|646524315|gb|KK989884.1|	11252-11569	17	7	gi|641585967|gb|KK854311.1|	434127-435132	11	40
ACYPI086445-RA	gi|646775685|gb|KK961714.1|	1523772-1528778	21	8.5	gi|641575150|gb|KK855169.1|	57432-64936	11	39
ACYPI086727-RA	gi|646755888|gb|KK961934.1|	1116740-1119206	21	9.8	gi|641588283|gb|KK854007.1|	983053-985086	11	40
ACYPI086900-RA	gi|646738359|gb|KK962984.1|	82512-83645	16	11	gi|641588033|gb|KK854031.1|	1149495-115927[illegible]	11	38
ACYPI087019-RA	gi|646775702|gb|KK961713.1|	934823-935088	17	7.4	gi|641571652|gb|KK856012.1|	88727-91487	9.6	35
ACYPI087266-RA	gi|646781344|gb|KK961523.1|	2282143-2282888	21	8.9	gi|641586694|gb|KK854204.1|	518341-519362	12	50
ACYPI087286-RA	gi|646767015|gb|KK961843.1|	681175-681332	15	8.6	gi|641574541|gb|KK855296.1|	116737-117859	11	44
ACYPI087467-RA	gi|646741656|gb|KK962648.1|	863862-865651	24	9.7	gi|641577574|gb|KK854731.1|	161111-161593	11	38
ACYPI087566-RA	gi|646747547|gb|KK962210.1|	434999-436588	19	7.5	gi|641578084|gb|KK854643.1|	428277-429245	11	40
ACYPI087735-RA	gi|646747076|gb|KK962245.1|	163656-164023	23	9.6	gi|641577322|gb|KK854771.1|	196261-196896	10	21
ACYPI087793-RA	gi|646770114|gb|KK961783.1|	1607442-1608249	19	9.7	gi|641565175|gb|KK858134.1|	2-1280	12	46
ACYPI087807-RA	gi|646750002|gb|KK962062.1|	376229-378433	24	12	gi|641578330|gb|KK854601.1|	62009-63888	11	38
ACYPI087848-RA	gi|646768294|gb|KK961814.1|	1050458-1058358	23	10	gi|641546507|gb|KK866793.1|	6051-7153	10	40
ACYPI087849-RA	gi|646740098|gb|KK962812.1|	168807-183678	20	10	gi|641583980|gb|KK854512.1|	356351-360954	11	39
ACYPI088146-RA	gi|646746556|gb|KK962281.1|	936945-940109	20	9.1	gi|641587219|gb|KK854129.1|	10808-11868	9.9	41
ACYPI088207-RA	gi|646776904|gb|KK961654.1|	954945-955385	21	9.3	gi|641576229|gb|KK854956.1|	167867-169582	9.8	36
ACYPI088273-RA	gi|646738455|gb|KK962975.1|	260878-263352	23	9.5	gi|641585233|gb|KK854427.1|	480975-483095	10	38
ACYPI088483-RA	gi|646775173|gb|KK961741.1|	369731-371454	19	8.8	gi|641588136|gb|KK854020.1|	1110339-111653[illegible]	11	36
ACYPI088486-RA	gi|646769275|gb|KK961796.1|	2007657-2007935	22	5.6	gi|641576469|gb|KK854914.1|	33253-33507	11	21
ACYPI088496-RA	gi|646744324|gb|KK962441.1|	365321-366717	20	7.2	gi|641576303|gb|KK854942.1|	46437-49807	9.6	37
ACYPI088544-RA	gi|646755264|gb|KK961938.1|	473507-475222	20	5.3	gi|641584281|gb|KK854493.1|	102993-103701	12	41
ACYPI088840-RA	gi|646738524|gb|KK962968.1|	200385-212651	24	11	gi|641585581|gb|KK854374.1|	606512-608942	10	38
ACYPI089540-RA	gi|646781893|gb|KK961506.1|	3656507-3656815	19	8.5	gi|641586928|gb|KK854169.1|	174835-175511	11	39
ACYPI089582-RA	gi|646747094|gb|KK962244.1|	541814-542857	20	10	gi|641561674|gb|KK859632.1|	14682-19318	38	430

ACYPI20302-RA	gi|646776091|gb|KK961693.1|	2724586-2724832	23	10	gi|641572011|gb|KK855923.1|	67296-67972	10	21
ACYPI20534-RA	gi|646776974|gb|KK961651.1|	1018748-1020123	17	8.1	gi|641566818|gb|KK857523.1|	38059-39304	10	37
ACYPI20693-RA	gi|646777207|gb|KK961641.1|	1028818-1030646	20	8.5	gi|641575414|gb|KK855115.1|	233010-233774	9.8	39
ACYPI21107-RA	gi|646749755|gb|KK962075.1|	489313-490584	22	9.2	gi|641571712|gb|KK856000.1|	161716-164269	10	39
ACYPI21221-RA	gi|646749984|gb|KK962063.1|	230107-231214	15	7.3	gi|641573053|gb|KK855655.1|	209619-210432	11	41
ACYPI21475-RA	gi|646752076|gb|KK961973.1|	422826-436759	22	9.1	gi|641576199|gb|KK854963.1|	268334-268922	12	43
ACYPI21591-RA	gi|646780827|gb|KK961539.1|	335-1932	20	9.4	gi|641586521|gb|KK854229.1|	656304-657364	11	41
ACYPI21611-RA	gi|646781690|gb|KK961512.1|	4005891-4008511	21	8.8	gi|641587896|gb|KK854047.1|	441515-444196	9.9	39
ACYPI21777-RA	gi|646781183|gb|KK961528.1|	1845538-1845829	20	9.5	gi|641587179|gb|KK854134.1|	164008-171260	11	40
ACYPI21790-RA	gi|646777665|gb|KK961623.1|	899192-905911	21	9.7	gi|641569788|gb|KK856560.1|	40605-41250	9.5	37
ACYPI22575-RA	gi|646779702|gb|KK961567.1|	506333-510400	23	10	gi|641568580|gb|KK856933.1|	52410-58127	12	25
ACYPI22584-RA	gi|646780311|gb|KK961552.1|	2237480-2247841	21	9.7	gi|641584658|gb|KK854465.1|	312800-313913	11	37
ACYPI22867-RA	gi|646777665|gb|KK961623.1|	1099688-1100283	21	9.7	gi|641586344|gb|KK854254.1|	451395-451843	10	40
ACYPI23235-RA	gi|646782127|gb|KK961500.1|	1165373-1169747	22	9.6	gi|641587652|gb|KK854077.1|	816970-819953	10	38
ACYPI23338-RA	gi|646780222|gb|KK961554.1|	1257230-1261312	20	9.7	gi|641580479|gb|KK854569.1|	303473-306094	10	38
ACYPI23394-RA	gi|646777288|gb|KK961638.1|	2847997-2850240	22	6.1	gi|641578109|gb|KK854638.1|	117534-120444	14	46
ACYPI23999-RA	gi|646782288|gb|KK961496.1|	1884041-1885841	21	9.7	gi|641561138|gb|KK859879.1|	12437-15522	12	42
ACYPI24155-RA	gi|646746040|gb|KK962316.1|	515964-516623	19	6.9	gi|641576312|gb|KK854941.1|	237497-241550	9.9	38
ACYPI24234-RA	gi|646748474|gb|KK962147.1|	588160-589835	20	9	gi|641578179|gb|KK854627.1|	92700-94577	10	39
ACYPI24841-RA	gi|646749755|gb|KK962075.1|	591262-592392	22	9.2	gi|641585971|gb|KK854310.1|	385080-388704	10	40
ACYPI25279-RA	gi|646781282|gb|KK961525.1|	4672151-4672955	22	9.6	gi|641587696|gb|KK854071.1|	709096-709877	8.9	39
ACYPI25494-RA	gi|646766868|gb|KK961847.1|	718900-721468	21	9.9	gi|641587606|gb|KK854082.1|	757838-760778	11	40
ACYPI25540-RA	gi|646781690|gb|KK961512.1|	2273116-2273984	21	8.8	gi|641587921|gb|KK854044.1|	123855-124138	9.4	41
ACYPI25873-RA	gi|646699768|gb|KK966040.1|	36584-41073	18	7.2	gi|641588099|gb|KK854024.1|	551068-552326	11	39
ACYPI25951-RA	gi|646776024|gb|KK961697.1|	2328465-2328781	22	9.2	gi|641573649|gb|KK855507.1|	110967-116928	11	41
ACYPI26146-RA	gi|646778213|gb|KK961608.1|	1382216-1385959	19	8.8	gi|641586114|gb|KK854288.1|	254154-256130	10	41
ACYPI26209-RA	gi|646777288|gb|KK961638.1|	1095950-1096565	22	6.1	gi|641587921|gb|KK854044.1|	170211-173293	9.4	41
ACYPI26224-RA	gi|646769453|gb|KK961793.1|	1500835-1503441	21	8.8	gi|641572928|gb|KK855687.1|	58298-63733	11	38
ACYPI26472-RA	gi|646759701|gb|KK961917.1|	211364-211746	18	6.1	gi|641587688|gb|KK854072.1|	917497-917741	10	38
ACYPI26971-RA	gi|646766511|gb|KK961859.1|	873445-874801	17	8.5	gi|641585673|gb|KK854359.1|	572917-575887	10	41
ACYPI27242-RA	gi|646738652|gb|KK962955.1|	129394-130166	19	8.8	gi|641587193|gb|KK854132.1|	304603-305270	11	39
ACYPI27655-RA	gi|646775807|gb|KK961708.1|	1508426-1510162	21	8.6	gi|641584743|gb|KK854459.1|	400551-401716	11	36

ACYPI27662-RA	gi|646780441|gb|KK961549.1|	1543914-1546414	21	9.5	gi|641577683|gb|KK854710.1|	206953-208088	10	43
ACYPI28767-RA	gi|646767477|gb|KK961831.1|	263847-264105	20	8.4	gi|641572326|gb|KK855841.1|	72069-72665	12	41
ACYPI28781-RA	gi|646744534|gb|KK962425.1|	29794-32360	19	8.6	gi|641577522|gb|KK854739.1|	52634-56161	11	41
ACYPI29303-RA	gi|646779298|gb|KK961577.1|	4308594-4309686	20	9.1	gi|641569801|gb|KK856556.1|	81538-84512	9.6	36
ACYPI29381-RA	gi|646777233|gb|KK961640.1|	1882377-1882634	21	8.7	gi|641587606|gb|KK854082.1|	823057-826822	11	40
ACYPI29477-RA	gi|646780858|gb|KK961538.1|	468679-473230	21	10	gi|641578160|gb|KK854630.1|	191766-193492	11	43
ACYPI29680-RA	gi|646775807|gb|KK961708.1|	418853-422210	21	8.6	gi|641577390|gb|KK854759.1|	140469-142452	9.8	37
ACYPI30696-RA	gi|646777416|gb|KK961632.1|	264016-265095	22	8.8	gi|641578046|gb|KK854650.1|	29592-31166	10	38
ACYPI31143-RA	gi|646780183|gb|KK961555.1|	66752-69382	19	8.6	gi|641571960|gb|KK855937.1|	66724-67167	9.4	17
ACYPI31180-RA	gi|646726077|gb|KK964550.1|	130978-131727	18	7.9	gi|641575813|gb|KK855039.1|	72860-73574	11	21
ACYPI31612-RA	gi|646746974|gb|KK962252.1|	1269536-1273215	23	6.1	gi|641585510|gb|KK854386.1|	214551-216007	11	20
ACYPI31659-RA	gi|646782357|gb|KK961494.1|	9034622-9035409	21	9.2	gi|641581779|gb|KK854550.1|	422245-422562	11	39
ACYPI31682-RA	gi|646776128|gb|KK961691.1|	504007-504798	21	9.2	gi|641587365|gb|KK854113.1|	276177-278154	12	45
ACYPI31744-RA	gi|646745135|gb|KK962381.1|	325035-326319	20	8.7	gi|641586824|gb|KK854186.1|	539398-539638	10	41
ACYPI31758-RA	gi|646738028|gb|KK963018.1|	79585-80177	22	7.1	gi|641586754|gb|KK854195.1|	638814-639853	11	41
ACYPI34207-RA	gi|646768163|gb|KK961817.1|	371055-371336	20	7.6	gi|641576635|gb|KK854887.1|	420588-421114	11	40
ACYPI34471-RA	gi|646753527|gb|KK961953.1|	579780-583108	24	9.2	gi|641585865|gb|KK854328.1|	488993-491486	12	42
ACYPI34873-RA	gi|646771735|gb|KK961764.1|	1069592-1070440	19	9.5	gi|641587703|gb|KK854070.1|	663771-664739	9.8	42
ACYPI34938-RA	gi|646766765|gb|KK961850.1|	973845-978317	18	8.9	gi|641573969|gb|KK855425.1|	60902-61813	8.9	42
ACYPI34996-RA	gi|646767230|gb|KK961837.1|	572856-578093	19	5.4	gi|641578325|gb|KK854602.1|	301301-303049	9.7	38
ACYPI35323-RA	gi|646767813|gb|KK961824.1|	234889-241469	17	6.3	gi|641573617|gb|KK855515.1|	25572-29338	10	38
ACYPI35928-RA	gi|646777955|gb|KK961615.1|	2637996-2638510	21	10	gi|641587390|gb|KK854109.1|	632535-637948	11	44
ACYPI36505-RA	gi|646763535|gb|KK961902.1|	1683481-1686247	23	10	gi|641572663|gb|KK855756.1|	25679-28013	8.8	38
ACYPI37433-RA	gi|646779262|gb|KK961578.1|	935428-938179	20	8.1	gi|641575568|gb|KK855086.1|	356079-361383	11	40
ACYPI37793-RA	gi|646776542|gb|KK961668.1|	3089558-3092060	20	5.6	gi|641567493|gb|KK857288.1|	29190-30404	8.6	41
ACYPI38188-RA	gi|646780978|gb|KK961534.1|	5642931-5643835	20	8	gi|641586127|gb|KK854286.1|	451793-452056	10	43
ACYPI38268-RA	gi|646766650|gb|KK961854.1|	1179249-1192510	22	10	gi|641569798|gb|KK856557.1|	97264-105963	12	46
ACYPI38406-RA	gi|646602815|gb|KK975927.1|	1611-2050	13	3.6	gi|641588309|gb|KK854005.1|	662199-662369	11	40
ACYPI38425-RA	gi|646775821|gb|KK961707.1|	39957-42703	23	9.1	gi|641585801|gb|KK854337.1|	82386-83614	10	38
ACYPI38602-RA	gi|646777089|gb|KK961646.1|	479877-480198	20	9.4	gi|641571612|gb|KK856022.1|	162706-163018	12	43
ACYPI38630-RA	gi|646781212|gb|KK961527.1|	1279243-1281825	21	10	gi|641572740|gb|KK855736.1|	154370-158572	11	40
ACYPI38677-RA	gi|646758845|gb|KK961921.1|	2104837-2107500	22	9.5	gi|641578060|gb|KK854647.1|	293483-297300	8.9	35

ACYPI39080-RA	gi|646766451|gb|KK961861.1|	41368-42634	19	8.9	gi|641587519|gb|KK854093.1|	797017-801235	11	39
ACYPI39685-RA	gi|646739758|gb|KK962847.1|	276987-278912	17	8.2	gi|641576756|gb|KK854865.1|	87016-87809	10	40
ACYPI39790-RA	gi|646747094|gb|KK962244.1|	222205-225192	20	10	gi|641586726|gb|KK854199.1|	145569-150068	11	41
ACYPI40034-RA	gi|646780406|gb|KK961550.1|	903343-904034	22	5.9	gi|641561999|gb|KK859490.1|	13149-16393	8.8	34
ACYPI40226-RA	gi|646766598|gb|KK961856.1|	258732-259021	23	10	gi|641587937|gb|KK854042.1|	876448-876800	11	42
ACYPI40717-RA	gi|646766650|gb|KK961854.1|	1578055-1579158	22	10	gi|641574150|gb|KK855385.1|	104474-107265	10	38
ACYPI40836-RA	gi|646736615|gb|KK963164.1|	164943-173046	20	10	gi|641566659|gb|KK857580.1|	1-834	9.3	35
ACYPI40853-RA	gi|646769832|gb|KK961787.1|	1162123-1162658	15	9.8	gi|641587783|gb|KK854060.1|	582759-584422	12	40
ACYPI41067-RA	gi|646779621|gb|KK961569.1|	1066113-1068382	16	7.3	gi|641570488|gb|KK856340.1|	156373-158735	12	45
ACYPI41828-RA	gi|646748142|gb|KK962168.1|	220095-222849	20	9.3	gi|641572916|gb|KK855689.1|	93660-97575	9.2	33
ACYPI41915-RA	gi|646741699|gb|KK962644.1|	524702-529223	20	9.2	gi|641575970|gb|KK855012.1|	35652-39016	11	38
ACYPI41926-RA	gi|646741207|gb|KK962687.1|	173650-175208	17	7.6	gi|641577244|gb|KK854783.1|	102391-105222	9.7	19
ACYPI42170-RA	gi|646745462|gb|KK962357.1|	55865-56371	23	11	gi|641575759|gb|KK855050.1|	379736-380381	11	38
ACYPI42350-RA	gi|646639086|gb|KK969293.1|	2156-2597	29	12	gi|641576741|gb|KK854868.1|	114191-115242	9.5	40
ACYPI42579-RA	gi|646770401|gb|KK961779.1|	1719667-1724087	21	8.6	gi|641577987|gb|KK854657.1|	470598-472914	13	49
ACYPI43876-RA	gi|646770401|gb|KK961779.1|	436571-439449	21	8.6	gi|641576519|gb|KK854904.1|	85415-87781	10	40
ACYPI44265-RA	gi|646776447|gb|KK961675.1|	1837668-1839708	22	9.4	gi|641588182|gb|KK854016.1|	567125-568414	11	41
ACYPI44428-RA	gi|646781772|gb|KK961510.1|	4267141-4268933	20	9.2	gi|641585552|gb|KK854379.1|	150733-155458	14	62
ACYPI44580-RA	gi|646782211|gb|KK961498.1|	808344-811287	20	9	gi|641576973|gb|KK854827.1|	13758-14852	11	38
ACYPI44937-RA	gi|646776241|gb|KK961686.1|	1776106-1777444	18	5.9	gi|641586880|gb|KK854177.1|	587532-589533	10	20
ACYPI45089-RA	gi|646781212|gb|KK961527.1|	309150-310175	21	10	gi|641586096|gb|KK854291.1|	83256-87483	11	46
ACYPI45266-RA	gi|646776264|gb|KK961685.1|	756027-758505	20	9.1	gi|641576191|gb|KK854965.1|	191042-194362	11	44
ACYPI46077-RA	gi|646743886|gb|KK962473.1|	530155-530972	21	9.3	gi|641576064|gb|KK854990.1|	93209-93418	10	39
ACYPI46593-RA	gi|646745929|gb|KK962323.1|	335883-336369	20	8.8	gi|641585574|gb|KK854375.1|	158933-160755	11	39
ACYPI46836-RA	gi|646780406|gb|KK961550.1|	607204-608114	22	5.9	gi|641576456|gb|KK854916.1|	284651-285034	10	21
ACYPI47447-RA	gi|646722233|gb|KK964943.1|	61438-61968	34	14	gi|641586785|gb|KK854191.1|	498820-500956	11	39
ACYPI47472-RA	gi|646777545|gb|KK961627.1|	1286081-1289460	21	9.9	gi|641585741|gb|KK854347.1|	54046-56813	10	43
ACYPI47485-RA	gi|646767670|gb|KK961827.1|	807110-812483	21	5.9	gi|641572364|gb|KK855831.1|	224879-227493	11	38
ACYPI47548-RA	gi|646781421|gb|KK961521.1|	4213757-4218648	21	8.7	gi|641588073|gb|KK854027.1|	1130466-113214[illegible]	11	41
ACYPI47651-RA	gi|646749098|gb|KK962110.1|	86178-86526	20	7.7	gi|641586096|gb|KK854291.1|	638772-639013	11	46
ACYPI48217-RA	gi|646749616|gb|KK962083.1|	104341-107181	22	10	gi|641575255|gb|KK855147.1|	240790-241451	9.4	40
ACYPI48246-RA	gi|646776305|gb|KK961683.1|	2478680-2479325	20	8.8	gi|641588267|gb|KK854008.1|	1118910-111944[illegible]	10	40

ACYPI48553-RA	gi|646740116|gb|KK962810.1|	35253-38488	12	9.4	gi|641571472|gb|KK856062.1|	98063-99218	9.9	21
ACYPI48834-RA	gi|646777570|gb|KK961626.1|	857242-857864	18	7.5	gi|641586874|gb|KK854178.1|	661387-662589	9.4	38
ACYPI48967-RA	gi|646778517|gb|KK961599.1|	599374-600740	20	9.6	gi|641570207|gb|KK856427.1|	111830-115509	11	40
ACYPI49690-RA	gi|646782276|gb|KK961497.1|	1976400-1978884	21	9.7	gi|641584474|gb|KK854479.1|	152050-152615	9.5	40
ACYPI49734-RA	gi|646735026|gb|KK963351.1|	107368-117016	21	8.9	gi|641561581|gb|KK859676.1|	18619-19341	10	38
ACYPI49735-RA	gi|646735026|gb|KK963351.1|	95068-102550	21	01/00	gi|641561581|gb|KK859676.1|	2-9962	10	38
ACYPI49959-RA	gi|646780058|gb|KK961558.1|	409669-410952	15	6.7	gi|641585888|gb|KK854324.1|	482523-490920	10	41
ACYPI50219-RA	gi|646769275|gb|KK961796.1|	2415913-2433085	22	5.6	gi|641572124|gb|KK855893.1|	103554-104387	10	40
ACYPI50514-RA	gi|646775312|gb|KK961734.1|	116824-117309	17	7.9	gi|641585233|gb|KK854427.1|	423402-423650	10	38
ACYPI50560-RA	gi|646752849|gb|KK961961.1|	629199-631502	21	9.4	gi|641586072|gb|KK854295.1|	195371-196458	10	20
ACYPI50578-RA	gi|646780105|gb|KK961557.1|	699508-705013	21	9.7	gi|641586419|gb|KK854244.1|	314168-315126	11	22
ACYPI50707-RA	gi|646743492|gb|KK962499.1|	474031-476045	24	11	gi|641575837|gb|KK855035.1|	188040-188674	11	46
ACYPI51052-RA	gi|646748379|gb|KK962153.1|	20937-22146	14	8.9	gi|641575363|gb|KK855126.1|	299676-300096	11	42
ACYPI51124-RA	gi|646746355|gb|KK962294.1|	891255-891649	22	10	gi|641574249|gb|KK855364.1|	20161-20352	9.2	37
ACYPI51231-RA	gi|646777288|gb|KK961638.1|	652541-653675	22	6.1	gi|641577623|gb|KK854722.1|	320009-322921	11	38
ACYPI51632-RA	gi|646747597|gb|KK962206.1|	305040-306231	20	8.1	gi|641585383|gb|KK854406.1|	136514-137276	11	37
ACYPI52139-RA	gi|646778983|gb|KK961586.1|	1974564-1980666	20	9.9	gi|641585428|gb|KK854399.1|	383790-385025	9.8	41
ACYPI52393-RA	gi|646758527|gb|KK961922.1|	579840-586079	24	10	gi|641575626|gb|KK855076.1|	77400-80548	11	24
ACYPI52419-RA	gi|646738229|gb|KK962998.1|	391917-392613	22	8.9	gi|641577109|gb|KK854806.1|	206711-207086	9	32
ACYPI52571-RA	gi|646767230|gb|KK961837.1|	831232-831878	19	5.4	gi|641588283|gb|KK854007.1|	1597502-160446[illegible]	11	40
ACYPI52843-RA	gi|646747500|gb|KK962213.1|	667429-673762	21	9.4	gi|641570707|gb|KK856276.1|	26880-30000	9.4	20
ACYPI52973-RA	gi|646781732|gb|KK961511.1|	4356009-4357560	22	9.8	gi|641577968|gb|KK854660.1|	456150-457691	10	42
ACYPI53104-RA	gi|646767850|gb|KK961823.1|	847900-852526	21	9.8	gi|641576074|gb|KK854988.1|	129922-131166	12	46
ACYPI53120-RA	gi|646779976|gb|KK961560.1|	1397731-1398391	16	7.9	gi|641577511|gb|KK854741.1|	124217-129069	11	38
ACYPI53252-RA	gi|646780978|gb|KK961534.1|	1805403-1806442	20	8	gi|641584945|gb|KK854445.1|	426062-427089	9.9	20
ACYPI53313-RA	gi|646781628|gb|KK961514.1|	4420407-4420942	23	9	gi|641577104|gb|KK854807.1|	16572-18363	10	36
ACYPI53812-RA	gi|646751432|gb|KK961990.1|	74314-77218	15	7.7	gi|641577309|gb|KK854773.1|	186546-187593	9.9	39
ACYPI54017-RA	gi|646742553|gb|KK962571.1|	375678-378150	20	8.4	gi|641581389|gb|KK854557.1|	299231-301683	11	41
ACYPI54601-RA	gi|646749573|gb|KK962085.1|	286841-287824	23	10	gi|641571435|gb|KK856072.1|	14953-15436	9.1	36
ACYPI55202-RA	gi|646777729|gb|KK961621.1|	639315-639761	21	9.8	gi|641575284|gb|KK855140.1|	172693-172844	8.2	35
ACYPI55541-RA	gi|646780827|gb|KK961539.1|	626623-629143	20	9.4	gi|641574768|gb|KK855248.1|	31670-32539	9.8	37
ACYPI55567-RA	gi|646781043|gb|KK961532.1|	2241209-2245366	26	11	gi|641568389|gb|KK856993.1|	8040-12164	10	41

ACYPI55712-RA	gi|646770998|gb|KK961772.1|	1802369-1803254	22	9.8	gi|641577805|gb|KK854691.1|	412030-412904	9.4	38
ACYPI55872-RA	gi|646780953|gb|KK961535.1|	201198-203770	20	9.8	gi|641587009|gb|KK854158.1|	542147-544768	11	41
ACYPI56570-RA	gi|646757051|gb|KK961928.1|	368290-371212	18	8.9	gi|641577805|gb|KK854691.1|	239740-240590	9.4	38
ACYPI56610-RA	gi|646781282|gb|KK961525.1|	1448875-1450546	22	9.6	gi|641575559|gb|KK855088.1|	194613-195059	9.9	37
ACYPI56611-RA	gi|646778336|gb|KK961604.1|	1319970-1320386	21	10	gi|641583313|gb|KK854525.1|	437091-437657	9.5	40
ACYPI56627-RA	gi|646782211|gb|KK961498.1|	5556234-5557380	20	9	gi|641575709|gb|KK855059.1|	346032-347627	11	41
ACYPI56628-RA	gi|646750341|gb|KK962044.1|	489726-490876	19	5.6	gi|641577815|gb|KK854689.1|	144827-147314	11	43
ACYPI56634-RA	gi|646775540|gb|KK961721.1|	1257474-1259351	17	8.1	gi|641587452|gb|KK854101.1|	752803-753405	11	40
ACYPI56635-RA	gi|646781510|gb|KK961518.1|	3426034-3428246	17	7.8	gi|641576225|gb|KK854957.1|	50263-54712	11	44
ACYPI56643-RA	gi|646782288|gb|KK961496.1|	273783-275111	21	9.7	gi|641577184|gb|KK854793.1|	71198-71550	11	40
ACYPI56655-RA	gi|646750002|gb|KK962062.1|	322645-323032	24	12	gi|641588051|gb|KK854029.1|	947744-948917	9.6	40
ACYPI56660-RA	gi|646781968|gb|KK961504.1|	905477-905953	20	9.6	gi|641587719|gb|KK854068.1|	186084-186425	10	21
ACYPI56663-RA	gi|646779186|gb|KK961580.1|	1576022-1576693	20	8.6	gi|641563636|gb|KK858785.1|	9951-10651	8.3	33
ACYPI56678-RA	gi|646767850|gb|KK961823.1|	1760912-1761222	21	9.8	gi|641571119|gb|KK856156.1|	186130-188457	11	38
ACYPI56734-RA	gi|646744833|gb|KK962403.1|	357641-360437	22	9.4	gi|641568059|gb|KK857098.1|	46755-47316	12	38
ACYPI56745-RA	gi|646740395|gb|KK962776.1|	180371-182212	16	6.3	gi|641587905|gb|KK854046.1|	54465-56561	10	43
ACYPI56897-RA	gi|646744324|gb|KK962441.1|	440286-461843	20	7.2	gi|641570474|gb|KK856344.1|	991-1325	7.5	31
