# Supplementary material for: The X Chromosome of Hemipteran Insects: Conservation, Dosage Compensation and Sex-Biased Expression
Source: Genome Biol Evol. 2015 Nov 10;7(12):3259–68. doi: 10.1093/gbe/evv215 (PMC4700948; doi:10.1093/gbe/evv215)
Supplement: Supplementary Data [file supp_evv215_suppl_data.zip › S3 Data (rev).docx]

**R-Script used for analysis**

**Locating genes on the X and autosomes (Figure 1)**

par(mfrow=c(1,3), cex=1, cex.axis=0.8, cex.main=0.9)

OF<-read.table("D:\\Arka\\project austria\\work\\revision analysis\\O.fasciatus_gene.exp_result_new", head=T)

OF.X<-subset(OF, log(covM/covF, base=2)<(median(log(OF$covM/OF$covF, base=2), na.rm=T)-0.5) & expF>1 & expM>1)

OF.A<-subset(OF, log(covM/covF, base=2)>(median(log(OF$covM/OF$covF, base=2), na.rm=T)-0.5) & expF>1 & expM>1)

hist(log(OF.A$covM/OF.A$covF, base=2), breaks=55, main="O.fasciatus", xlim = c(0,3), xlab="Log2(Mcov/Fcov)", col="darkgrey")

hist(log(OF.X$covM/OF.X$covF, base=2), breaks=30, xlab="Log2(Mcov/Fcov)", col="red", add=T)

HH<-read.table("D:\\Arka\\project austria\\work\\revision analysis\\H.halys_gene.exp_result_new", head=T)

HH.X<-subset(HH, log(covM/covF, base=2)< (median(log(HH$covM/HH$covF, base=2), na.rm=T)-0.5) & expF>1 & expM>1)

HH.A<-subset(HH, log(covM/covF, base=2)> (median(log(HH$covM/HH$covF, base=2), na.rm=T)-0.5) & expF>1 & expM>1)

hist(log(HH.A$covM/HH.A$covF, base=2), breaks=15, xlim=c(-1.5,1.2), main="H.halys", xlab="Log2(Mcov/Fcov)", col="darkgrey")

hist(log(HH.X$covM/HH.X$covF, base=2), breaks=5, xlab="Log2(Mcov/Fcov)", col="red", add=T)

HV<-read.table("D:\\Arka\\project austria\\work\\revision analysis\\H.vitripennis_gene.exp_result_new", head=T)

HV.X<-subset(HV, log(covM/covF, base=2)< (median(log(HV$covM/HV$covF, base=2), na.rm=T)-0.5) & expF>1 & expM>1)

HV.A<-subset(HV, log(covM/covF, base=2)> (median(log(HV$covM/HV$covF, base=2), na.rm=T)-0.5) & expF>1 & expM>1)

hist(log(HV.A$covM/HV.A$covF, base=2), breaks=40, main="H.vitripennis", xlab="Log2(Mcov/Fcov)", xlim = c(-2.5,0.5), col="darkgrey")

hist(log(HV.X$covM/HV.X$covF, base=2), breaks=25, xlab="Log2(Mcov/Fcov)", col="red", add=T)
