# Supplementary material for: The X Chromosome of Hemipteran Insects: Conservation, Dosage Compensation and Sex-Biased Expression
Source: Genome Biol Evol. 2015 Nov 10;7(12):3259–68. doi: 10.1093/gbe/evv215 (PMC4700948; doi:10.1093/gbe/evv215)
Supplement: Supplementary Data [file supp_evv215_suppl_data.zip › S3 Table.pdf]

	<0.4 (female-biased)	0.4-0.6 (Uncompensated)	0.6-0.9 (Partially compensated)	0.9-1.1 (Fully compensated)	>1.1 (Male-biased)
O. fasciatus					
Autosomes	6782	6277	9437	5401	19542
X_expected	737	682	1026	587	2124
X_observed	989	792	1070	586	1720
H. halys					
	<0.4 (female-biased)	0.4-0.6 (Uncompensated)	0.6-0.9 (Partially compensated)	0.9-1.1 (Fully compensated)	>1.1 (Male-biased)
Autosomes	1326	3025	10839	8138	18368
X_expected	162	370	1325	995	2246
X_observed	195	477	2229	1151	1046
H.vitripennis					
	<0.4 (female-biased)	0.4-0.6 (Uncompensated)	0.6-0.9 (Partially compensated)	0.9-1.1 (Fully compensated)	>1.1 (Male-biased)
Autosomes	5076	5615	9484	5203	16089
X_expected	439	485	820	450	1391
X_observed	490	529	884	430	1252
A. pisum					
	<0.4 (female-biased)	0.4-0.6 (Uncompensated)	0.6-0.9 (Partially compensated)	0.9-1.1 (Fully compensated)	>1.1 (Male-biased)
Autosomes	4181	2496	2862	1395	7207
X_expected	327	195	224	109	564
X_observed	362	184	185	112	577
