# Supplementary figures and images for: The X Chromosome of Hemipteran Insects: Conservation, Dosage Compensation and Sex-Biased Expression
Source: Genome Biol Evol. 2015 Nov 10;7(12):3259–68. doi: 10.1093/gbe/evv215 (PMC4700948; doi:10.1093/gbe/evv215)

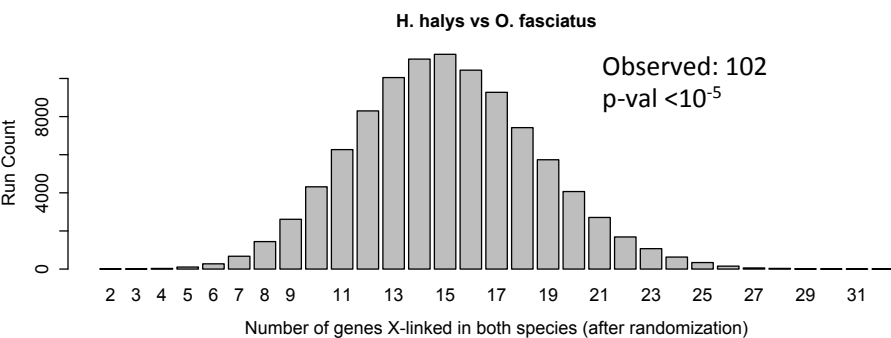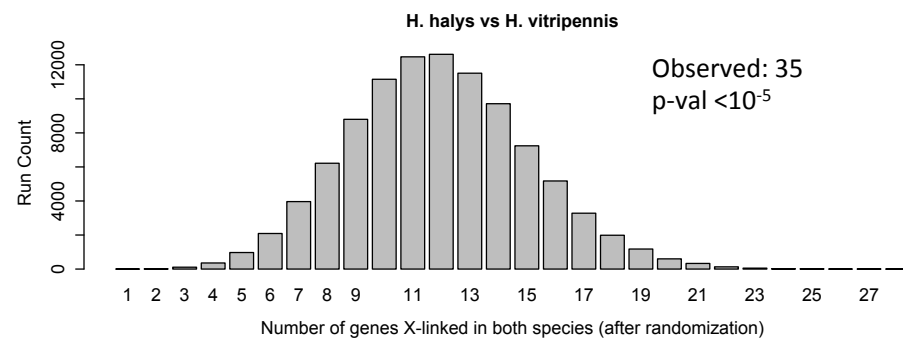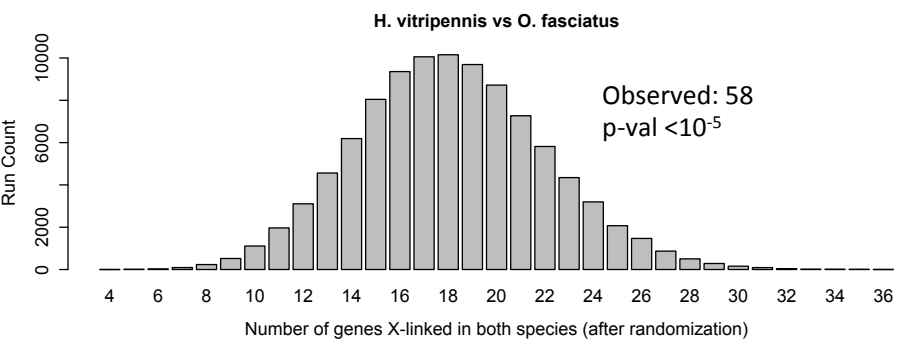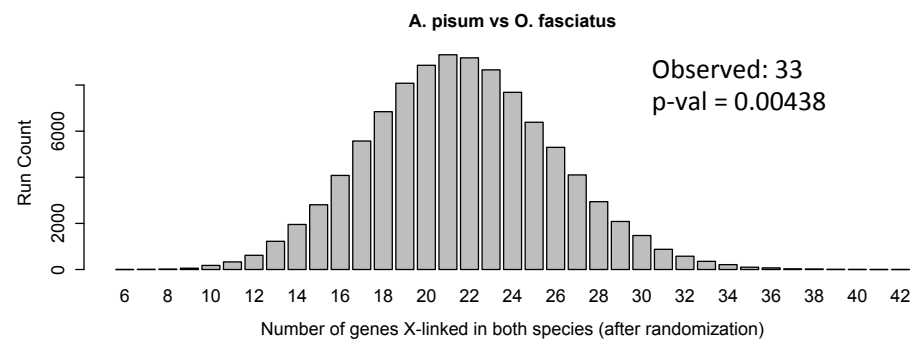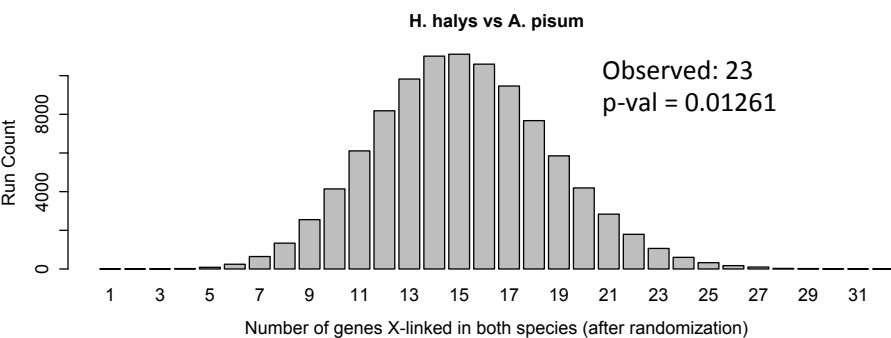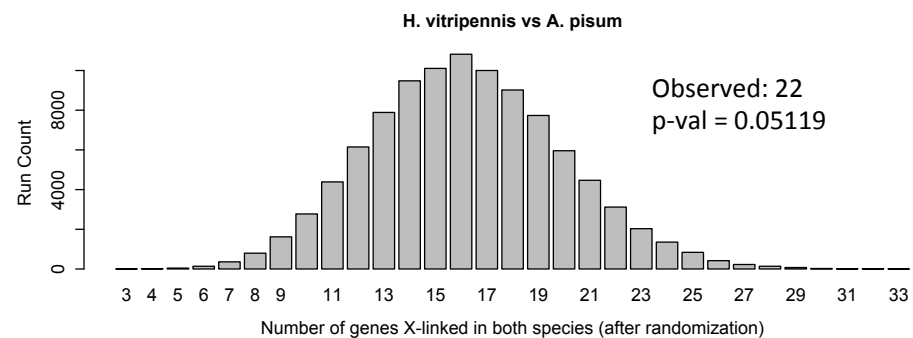

Supplement: Supplementary Data [file supp_evv215_suppl_data.zip › FigS2_Pease.pdf]

*O. fasciatus*

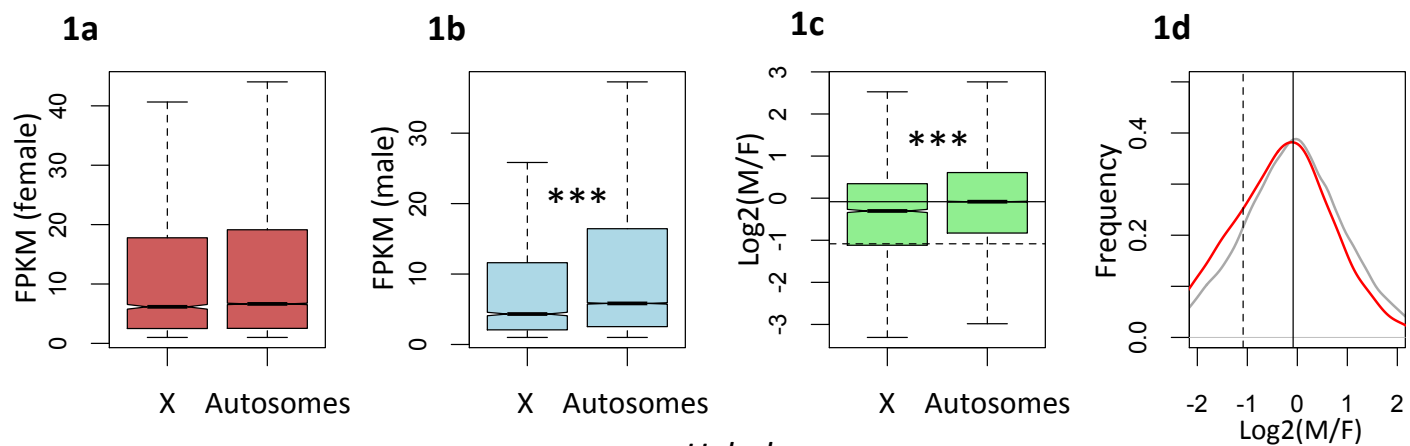

*H. halys*

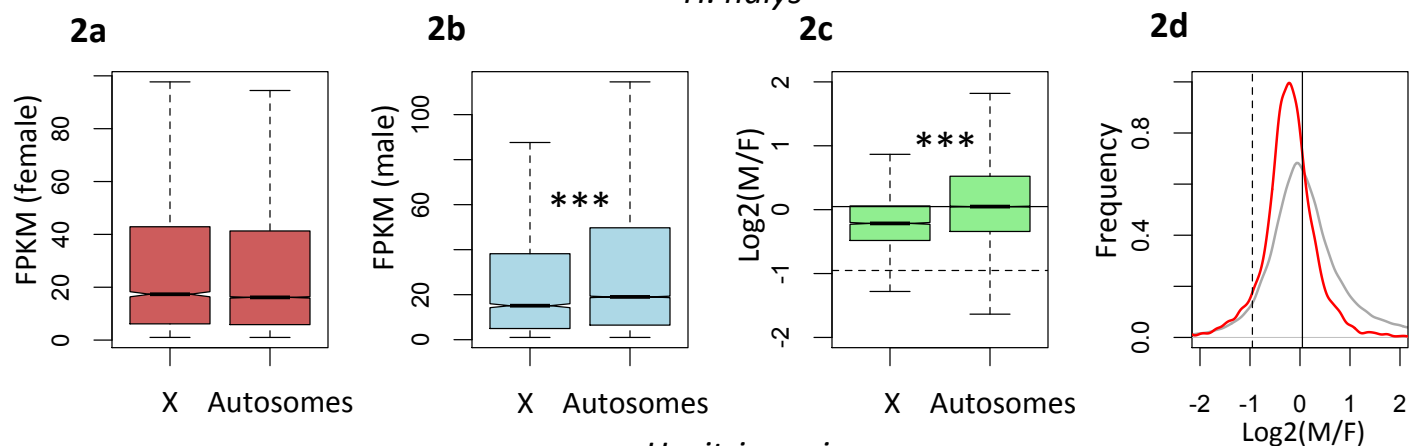

*H. vitripennis*

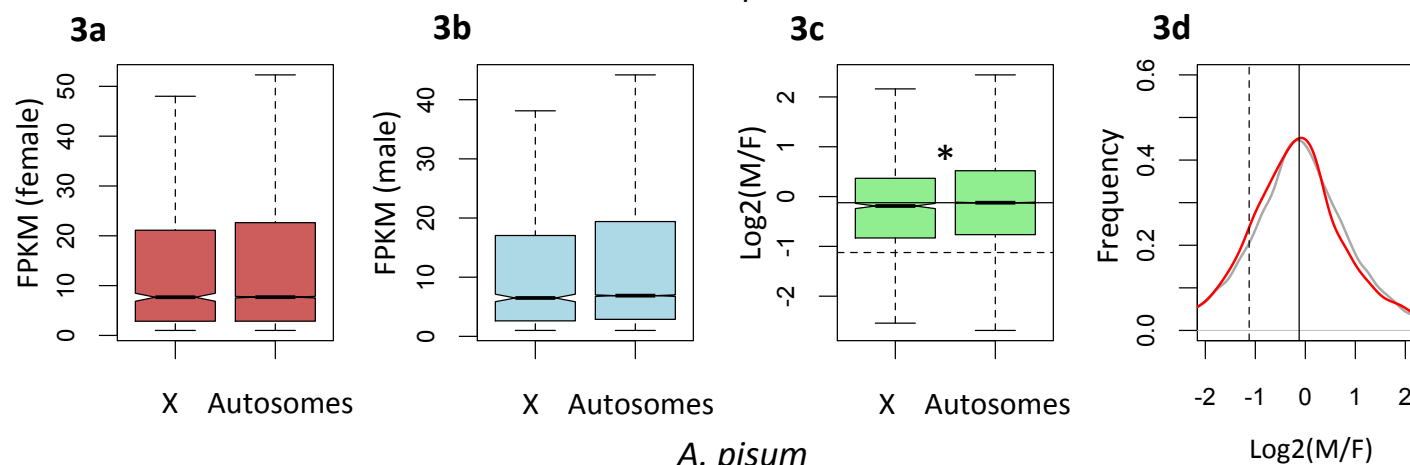

*A. pisum*

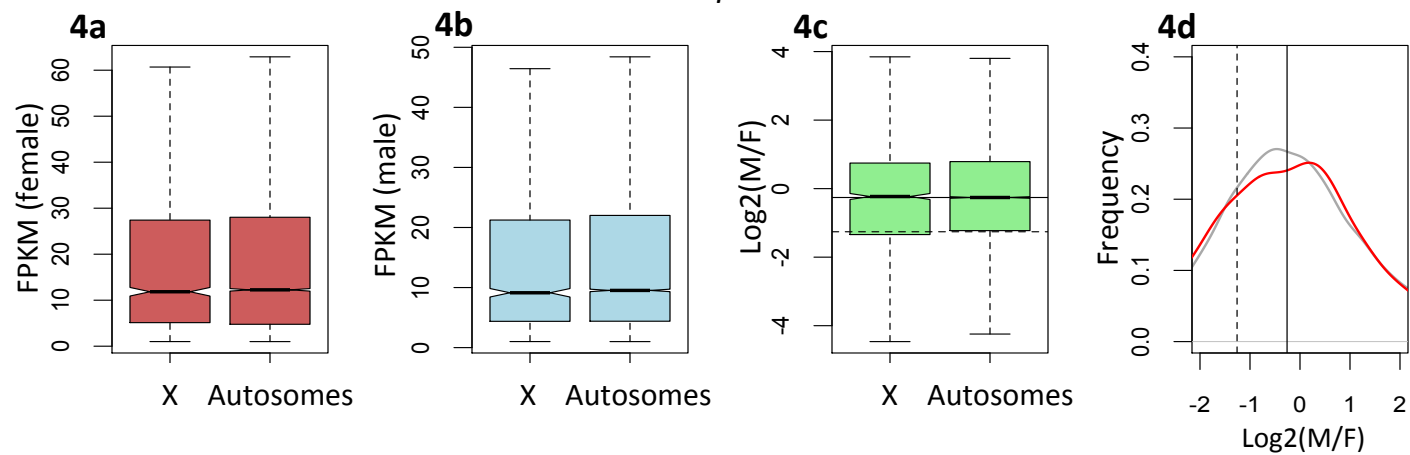

Supplement: Supplementary Data [file supp_evv215_suppl_data.zip › FigureS1_Rev1.pdf]
