# Supplementary material for: The X Chromosome of Hemipteran Insects: Conservation, Dosage Compensation and Sex-Biased Expression
Source: Genome Biol Evol. 2015 Nov 10;7(12):3259–68. doi: 10.1093/gbe/evv215 (PMC4700948; doi:10.1093/gbe/evv215)
Supplement: Supplementary Data [file S1 Data (rev) H.halys.pdf › S1 Data (rev) H.halys.pdf]

scaffold	gene	covF	covM	expF	expM	log2(fold_change)	p-value	significance
ni|645903463|nb|KK920967.1|	11186-11672	nan	nan	14	91	3	0.0621	no
ni|645903463|nb|KK920967.1|	11850-12729	nan	nan	15	121	3	0.0167	no
ni|645903463|nb|KK920967.1|	162482-162588	nan	nan	11	67	3	0.0709	no
ni|645903463|nb|KK920967.1|	229089-229257	nan	nan	27	15	-0.811941	0.4862	no
ni|645903463|nb|KK920967.1|	239532-239712	nan	nan	0	32	inf	5.00E-05	yes
ni|645903463|nb|KK920967.1|	260929-261096	nan	nan	0	6	inf	5.00E-05	yes
ni|645903463|nb|KK920967.1|	30402-30540	nan	nan	19	67	2	0.13635	no
ni|645903463|nb|KK920967.1|	7381-8111	nan	nan	16	8	-1	0.37055	no
ni|645903464|nb|KK920966.1|	121814-122227	nan	nan	0	23	inf	5.00E-05	yes
ni|645903464|nb|KK920966.1|	122468-122646	nan	nan	306	296	-0.0438095	0.97165	no
ni|645903464|nb|KK920966.1|	123122-123257	nan	nan	96	111	0.205175	0.8422	no
ni|645903464|nb|KK920966.1|	124569-124995	nan	nan	11	11	-0.12165	0.91705	no
ni|645903464|nb|KK920966.1|	128246-128516	nan	nan	20	14	-0.526681	0.65	no
ni|645903464|nb|KK920966.1|	129240-129424	nan	nan	2	2	-0.191803	0.88855	no
ni|645903464|nb|KK920966.1|	130097-130268	nan	nan	13	11	-0.154734	0.90135	no
ni|645903464|nb|KK920966.1|	130584-130926	nan	nan	2	2	-0.259647	0.8692	no
ni|645903464|nb|KK920966.1|	132455-132595	nan	nan	39	50	0.361009	0.75345	no
ni|645903464|nb|KK920966.1|	132713-133390	nan	nan	62	83	0.41003	0.7114	no
ni|645903464|nb|KK920966.1|	133595-133803	nan	nan	19	22	0.221237	0.84255	no
ni|645903464|nb|KK920966.1|	135454-135728	nan	nan	19	20	0.0620667	0.95855	no
ni|645903464|nb|KK920966.1|	135838-135966	nan	nan	11	14	0.335751	0.77215	no
ni|645903464|nb|KK920966.1|	185849-186040	nan	nan	0	9	inf	0.00015	yes
ni|645903464|nb|KK920966.1|	186140-186469	nan	nan	113	76	-0.584161	0.6058	no
ni|645903464|nb|KK920966.1|	186710-187105	nan	nan	6	12	0.932767	0.4787	no
ni|645903464|nb|KK920966.1|	188084-188460	nan	nan	1	0.996673	-0.235768	0.8697	no
ni|645903464|nb|KK920966.1|	191623-191809	nan	nan	7	7	-0.107366	0.92905	no
ni|645903464|nb|KK920966.1|	192635-192769	nan	nan	0.451064	303	9	0.04435	no
ni|645903464|nb|KK920966.1|	193392-193579	nan	nan	0	93	inf	5.00E-05	yes
ni|645903464|nb|KK920966.1|	193736-193954	nan	nan	0	93	inf	5.00E-05	yes

ni|645903464|nb|KK920966.1|	195129-195310	nan	nan	68	63	-0.122396	0.9321	no
ni|645903464|nb|KK920966.1|	196223-196415	nan	nan	53	17	-2	0.1946	no
ni|645903464|nb|KK920966.1|	198546-199140	nan	nan	95	55	-0.798575	0.50065	no
ni|645903464|nb|KK920966.1|	201485-201807	nan	nan	15	9	-0.652166	0.5903	no
ni|645903464|nb|KK920966.1|	202483-202679	nan	nan	0.784262	0.999341	0.349642	0.792	no
ni|645903464|nb|KK920966.1|	205012-205318	nan	nan	0.663339	0.845107	0.349387	0.81755	no
ni|645903464|nb|KK920966.1|	205791-207217	nan	nan	22	21	-0.098239	0.93345	no
ni|645903464|nb|KK920966.1|	207420-208069	nan	nan	19	17	-0.195532	0.8663	no
ni|645903464|nb|KK920966.1|	211066-211188	nan	nan	22	19	-0.188446	0.8692	no
ni|645903464|nb|KK920966.1|	211629-211789	nan	nan	1	0.306959	-2	0.14495	no
ni|645903464|nb|KK920966.1|	212791-212962	nan	nan	16	13	-0.356778	0.77485	no
ni|645903464|nb|KK920966.1|	213188-213366	nan	nan	0.275515	8	5	0.12055	no
ni|645903464|nb|KK920966.1|	213552-213781	nan	nan	2	6	2	0.23715	no
ni|645903464|nb|KK920966.1|	215849-216473	nan	nan	5	2	-2	0.2437	no
ni|645903464|nb|KK920966.1|	222226-222575	nan	nan	0.662993	0.253484	-1	0.35155	no
ni|645903464|nb|KK920966.1|	223068-223767	nan	nan	3	2	-0.789443	0.5027	no
ni|645903464|nb|KK920966.1|	223974-224298	nan	nan	5	5	-0.0672061	0.9414	no
ni|645903464|nb|KK920966.1|	224392-224963	nan	nan	13	8	-0.772984	0.51115	no
ni|645903464|nb|KK920966.1|	227269-227990	nan	nan	39	34	-0.205463	0.87975	no
ni|645903464|nb|KK920966.1|	228074-228256	nan	nan	20	14	-0.557341	0.6184	no
ni|645903464|nb|KK920966.1|	310291-310654	nan	nan	20	11	-0.836494	0.4828	no
ni|645903464|nb|KK920966.1|	352593-352733	nan	nan	0.823169	2	1	0.3858	no
ni|645903464|nb|KK920966.1|	429656-430091	nan	nan	105	51	-1	0.356	no
ni|645903464|nb|KK920966.1|	430200-431013	nan	nan	125	75	-0.739196	0.5587	no
ni|645903464|nb|KK920966.1|	431495-431635	nan	nan	1	2	0.888943	0.4698	no
ni|645903464|nb|KK920966.1|	431962-432337	nan	nan	43	55	0.344003	0.7686	no
ni|645903464|nb|KK920966.1|	435280-435476	nan	nan	66	65	-0.0231098	0.98885	no
ni|645903464|nb|KK920966.1|	435675-436132	nan	nan	115	116	0.0156657	0.98925	no
ni|645903464|nb|KK920966.1|	438818-439034	nan	nan	1	0.401024	-2	0.3149	no
ni|645903464|nb|KK920966.1|	439269-439568	nan	nan	1	0.782644	-0.702869	0.6038	no

ni|645903464|nb|KK920966.1|	441016-441299	nan	nan	178	143	-0.313315	0.8339	no
ni|645903464|nb|KK920966.1|	442941-443192	nan	nan	3	2	-0.769716	0.5207	no
ni|645903464|nb|KK920966.1|	444765-445195	nan	nan	0	46	inf	5.00E-05	yes
ni|645903464|nb|KK920966.1|	445766-446280	nan	nan	0.289619	0.593347	1	0.4471	no
ni|645903464|nb|KK920966.1|	447432-447595	nan	nan	23	19	-0.232287	0.8417	no
ni|645903464|nb|KK920966.1|	448359-448477	nan	nan	38	22	-0.78103	0.5207	no
ni|645903464|nb|KK920966.1|	451261-451437	nan	nan	6	3	-0.874567	0.5293	no
ni|645903464|nb|KK920966.1|	452143-452280	nan	nan	15	14	-0.144613	0.9005	no
ni|645903464|nb|KK920966.1|	453158-453774	nan	nan	10	7	-0.436526	0.7067	no
ni|645903464|nb|KK920966.1|	453919-454304	nan	nan	15	13	-0.143083	0.922	no
ni|645903464|nb|KK920966.1|	454850-455103	nan	nan	37	113	2	0.1674	no
ni|645903464|nb|KK920966.1|	455191-455308	nan	nan	47	29	-0.676088	0.565	no
ni|645903464|nb|KK920966.1|	455558-455695	nan	nan	71	10	-3	0.1498	no
ni|645903464|nb|KK920966.1|	456146-456266	nan	nan	61	55	-0.149183	0.8913	no
ni|645903464|nb|KK920966.1|	456370-456694	nan	nan	22	25	0.202857	0.86705	no
ni|645903464|nb|KK920966.1|	462461-463284	nan	nan	54	39	-0.446623	0.74215	no
ni|645903464|nb|KK920966.1|	463347-463646	nan	nan	60	62	0.0522091	0.9699	no
ni|645903464|nb|KK920966.1|	463818-464286	nan	nan	0	57	inf	5.00E-05	yes
ni|645903464|nb|KK920966.1|	464418-464999	nan	nan	12	12	0.0152117	0.9674	no
ni|645903464|nb|KK920966.1|	465665-465952	nan	nan	22	22	-0.0243999	0.9814	no
ni|645903464|nb|KK920966.1|	466801-467144	nan	nan	32	32	-0.0108431	0.992	no
ni|645903464|nb|KK920966.1|	467300-467836	nan	nan	2	0.902292	-1	0.4023	no
ni|645903464|nb|KK920966.1|	468080-468494	nan	nan	0.630002	0.860098	0.449145	0.7543	no
ni|645903464|nb|KK920966.1|	468672-468904	nan	nan	19	34	0.889426	0.4325	no
ni|645903464|nb|KK920966.1|	469749-470688	nan	nan	61	140	1	0.3487	no
ni|645903464|nb|KK920966.1|	470746-470896	nan	nan	2	3	0.196262	0.87955	no
ni|645903464|nb|KK920966.1|	477449-478748	nan	nan	1	0.793578	-0.443864	0.74215	no
ni|645903464|nb|KK920966.1|	482175-482736	nan	nan	4	6	0.397901	0.73745	no
ni|645903464|nb|KK920966.1|	487123-487720	nan	nan	52	45	-0.191529	0.85655	no
ni|645903464|nb|KK920966.1|	488875-489101	nan	nan	87	74	-0.230996	0.83255	no

ni|645903464|nb|KK920966.1|	489163-489281	nan	nan	0	5	inf	0.0003	yes
ni|645903466|nb|KK920964.1|	100423-100541	nan	nan	169	178	0.071108	0.96235	no
ni|645903466|nb|KK920964.1|	125257-125688	nan	nan	1	0.649394	-0.958592	0.51225	no
ni|645903466|nb|KK920964.1|	126208-126376	nan	nan	43	51	0.228174	0.835	no
ni|645903466|nb|KK920964.1|	126480-126982	nan	nan	53	58	0.128655	0.9281	no
ni|645903466|nb|KK920964.1|	127195-127671	nan	nan	18	21	0.277119	0.8099	no
ni|645903466|nb|KK920964.1|	128427-128582	nan	nan	65	71	0.132794	0.93155	no
ni|645903466|nb|KK920964.1|	128723-128895	nan	nan	3	0.680319	-2	0.15435	no
ni|645903466|nb|KK920964.1|	129156-129356	nan	nan	1	0.278543	-2	0.3207	no
ni|645903466|nb|KK920964.1|	129622-129754	nan	nan	1	0.386482	-2	0.2093	no
ni|645903466|nb|KK920964.1|	129928-130649	nan	nan	1	0.288319	-2	0.09395	no
ni|645903466|nb|KK920964.1|	132189-132468	nan	nan	0.794153	0.151855	-2	0.1164	no
ni|645903466|nb|KK920964.1|	15597-16012	nan	nan	0.4304	10	5	0.1211	no
ni|645903466|nb|KK920964.1|	157278-157406	nan	nan	2	0.443243	-2	0.13005	no
ni|645903466|nb|KK920964.1|	157769-157984	nan	nan	7	3	-1	0.31025	no
ni|645903466|nb|KK920964.1|	158406-159057	nan	nan	0	15	inf	5.00E-05	yes
ni|645903466|nb|KK920964.1|	159133-159470	nan	nan	0	20	inf	5.00E-05	yes
ni|645903466|nb|KK920964.1|	160202-161033	nan	nan	1	0.368351	-2	0.2996	no
ni|645903466|nb|KK920964.1|	161166-161868	nan	nan	0.548059	0.419125	-0.386953	0.78725	no
ni|645903466|nb|KK920964.1|	17932-18337	nan	nan	0	6	inf	0.00015	yes
ni|645903466|nb|KK920964.1|	19326-19958	nan	nan	53	74	0.479532	0.73795	no
ni|645903466|nb|KK920964.1|	205450-205812	nan	nan	3	2	-0.581883	0.70815	no
ni|645903466|nb|KK920964.1|	205977-206173	nan	nan	2	2	0.517816	0.7279	no
ni|645903466|nb|KK920964.1|	206319-206473	nan	nan	99	0	-inf	5.00E-05	yes
ni|645903466|nb|KK920964.1|	206640-206898	nan	nan	120	72	-0.743728	0.6113	no
ni|645903466|nb|KK920964.1|	20689-21193	nan	nan	1	0.815351	-0.306303	0.8123	no
ni|645903466|nb|KK920964.1|	207215-207693	nan	nan	54	42	-0.363047	0.7397	no
ni|645903466|nb|KK920964.1|	208192-208726	nan	nan	45	56	0.294338	0.8297	no
ni|645903466|nb|KK920964.1|	208885-209431	nan	nan	0.751288	1	0.754564	0.536	no
ni|645903466|nb|KK920964.1|	210846-211012	nan	nan	0.382646	0.609716	0.672129	0.62105	no

ni|645903466|nb|KK920964.1|	211638-211858	nan	nan	57	70	0.288487	0.8058	no
ni|645903466|nb|KK920964.1|	213366-213528	nan	nan	80	112.87	0.500469	0.6452	no
ni|645903466|nb|KK920964.1|	213651-213983	nan	nan	0.396438	0.26532	-0.579363	0.70815	no
ni|645903466|nb|KK920964.1|	214276-214527	nan	nan	79	101.12	0.356031	0.74485	no
ni|645903466|nb|KK920964.1|	214739-214886	nan	nan	56	76	0.427042	0.71255	no
ni|645903466|nb|KK920964.1|	215059-215280	nan	nan	58	75	0.380247	0.7396	no
ni|645903466|nb|KK920964.1|	23049-23593	nan	nan	7	8	0.0289445	0.98105	no
ni|645903466|nb|KK920964.1|	24020-24608	nan	nan	24	35	0.577705	0.6223	no
ni|645903466|nb|KK920964.1|	24761-24975	nan	nan	4	1	-2	0.3149	no
ni|645903466|nb|KK920964.1|	253859-254004	nan	nan	34	53	0.643305	0.60185	no
ni|645903466|nb|KK920964.1|	255496-255662	nan	nan	40	63	0.645958	0.572	no
ni|645903466|nb|KK920964.1|	258336-258603	nan	nan	38	63	0.723979	0.5178	no
ni|645903466|nb|KK920964.1|	259218-259405	nan	nan	19	32	0.749964	0.5094	no
ni|645903466|nb|KK920964.1|	25989-26183	nan	nan	3	2	-0.597327	0.64595	no
ni|645903466|nb|KK920964.1|	260399-260672	nan	nan	34	52	0.611379	0.62895	no
ni|645903466|nb|KK920964.1|	261927-262349	nan	nan	23	39	0.761978	0.49535	no
ni|645903466|nb|KK920964.1|	265052-265718	nan	nan	0.171793	4	4	0.01595	no
ni|645903466|nb|KK920964.1|	265798-265913	nan	nan	10	14	0.586021	0.6174	no
ni|645903466|nb|KK920964.1|	266075-266253	nan	nan	5	8	0.641508	0.5869	no
ni|645903466|nb|KK920964.1|	266422-266702	nan	nan	7	10	0.464422	0.687	no
ni|645903466|nb|KK920964.1|	266981-267259	nan	nan	24	24	-0.000239775	0.99345	no
ni|645903466|nb|KK920964.1|	267490-267682	nan	nan	28	29	0.0332854	0.9787	no
ni|645903466|nb|KK920964.1|	267772-267910	nan	nan	40	46	0.202432	0.8567	no
ni|645903466|nb|KK920964.1|	268371-268493	nan	nan	16	17	0.139856	0.90445	no
ni|645903466|nb|KK920964.1|	269246-269527	nan	nan	28	26	-0.0673914	0.9528	no
ni|645903466|nb|KK920964.1|	270162-270315	nan	nan	33	29	-0.197606	0.8593	no
ni|645903466|nb|KK920964.1|	270417-270701	nan	nan	17	16	-0.127832	0.9086	no
ni|645903466|nb|KK920964.1|	272056-272187	nan	nan	27	14	-0.915469	0.4969	no
ni|645903466|nb|KK920964.1|	272387-272591	nan	nan	38	25	-0.569706	0.6375	no
ni|645903466|nb|KK920964.1|	272974-273089	nan	nan	57	46	-0.306881	0.79185	no

ni|645903466|nb|KK920964.1|	273991-274120	nan	nan	0.49089	0.794272	0.694234	0.58445	no
ni|645903466|nb|KK920964.1|	277025-277320	nan	nan	0.803566	0.23634	-2	0.22995	no
ni|645903466|nb|KK920964.1|	279056-279941	nan	nan	1	0.905888	-0.650692	0.64935	no
ni|645903466|nb|KK920964.1|	280811-281484	nan	nan	2	2	-0.0655198	0.95835	no
ni|645903466|nb|KK920964.1|	281861-283364	nan	nan	6	8	0.345813	0.80555	no
ni|645903466|nb|KK920964.1|	283681-283867	nan	nan	5	5	0.0983393	0.93645	no
ni|645903466|nb|KK920964.1|	284058-284355	nan	nan	17	14	-0.304692	0.7893	no
ni|645903466|nb|KK920964.1|	284804-286825	nan	nan	14	15	0.0250834	0.98605	no
ni|645903466|nb|KK920964.1|	286976-287183	nan	nan	3	4	0.391332	0.7287	no
ni|645903466|nb|KK920964.1|	287339-288682	nan	nan	4	4	-0.00693396	0.994	no
ni|645903466|nb|KK920964.1|	291280-291478	nan	nan	2	3	0.446512	0.6983	no
ni|645903466|nb|KK920964.1|	29738-29975	nan	nan	52	43	-0.265183	0.81585	no
ni|645903466|nb|KK920964.1|	308451-308907	nan	nan	8	10	0.282918	0.8099	no
ni|645903466|nb|KK920964.1|	30904-32117	nan	nan	17	19	0.105335	0.9189	no
ni|645903466|nb|KK920964.1|	32440-32834	nan	nan	2	2	-0.148017	0.9109	no
ni|645903466|nb|KK920964.1|	336959-337134	nan	nan	2	4	0.846602	0.49255	no
ni|645903466|nb|KK920964.1|	34419-35468	nan	nan	147	162	0.132924	0.9073	no
ni|645903466|nb|KK920964.1|	399168-399329	nan	nan	16	21	0.375313	0.74865	no
ni|645903466|nb|KK920964.1|	424115-424423	nan	nan	10	19	0.919574	0.44285	no
ni|645903466|nb|KK920964.1|	50359-50468	nan	nan	192	165	-0.217815	0.85025	no
ni|645903466|nb|KK920964.1|	50600-52230	nan	nan	121	197	0.70385	0.53045	no
ni|645903466|nb|KK920964.1|	519729-519870	nan	nan	0.05766	77	10	0.03715	no
ni|645903466|nb|KK920964.1|	52429-52597	nan	nan	0.886663	0.896445	0.0158294	0.9894	no
ni|645903466|nb|KK920964.1|	533801-533989	nan	nan	29	28	-0.00110722	0.99045	no
ni|645903466|nb|KK920964.1|	53492-53851	nan	nan	3	13	2	0.10035	no
ni|645903466|nb|KK920964.1|	54331-54713	nan	nan	9	9	-0.0206715	0.98505	no
ni|645903466|nb|KK920964.1|	55855-55984	nan	nan	6	5	-0.205188	0.86005	no
ni|645903466|nb|KK920964.1|	56100-56589	nan	nan	69	63	-0.134957	0.9083	no
ni|645903466|nb|KK920964.1|	57257-57432	nan	nan	37	35	-0.0792641	0.9461	no
ni|645903466|nb|KK920964.1|	57538-57667	nan	nan	7	4	-0.545768	0.643	no

ni|645903466|nb|KK920964.1|	58114-58517	nan	nan	20	23	0.16706	0.89635	no
ni|645903466|nb|KK920964.1|	59339-59482	nan	nan	21	15	-0.526011	0.6432	no
ni|645903466|nb|KK920964.1|	59774-60324	nan	nan	55	27	-1	0.35275	no
ni|645903466|nb|KK920964.1|	599104-599294	nan	nan	3	3	0.441097	0.71395	no
ni|645903466|nb|KK920964.1|	603561-603749	nan	nan	5	4	-0.281758	0.8142	no
ni|645903466|nb|KK920964.1|	60680-61162	nan	nan	10	4	-1	0.26265	no
ni|645903466|nb|KK920964.1|	610862-611220	nan	nan	6	5	-0.0316735	0.97895	no
ni|645903466|nb|KK920964.1|	63405-63890	nan	nan	28	34	0.308097	0.77925	no
ni|645903466|nb|KK920964.1|	642599-642813	nan	nan	2	1	-0.225851	0.85555	no
ni|645903466|nb|KK920964.1|	645678-645840	nan	nan	36	9	-2	0.1631	no
ni|645903466|nb|KK920964.1|	646977-647205	nan	nan	18	11	-0.652267	0.5967	no
ni|645903466|nb|KK920964.1|	647366-647750	nan	nan	9	6	-0.727295	0.53585	no
ni|645903466|nb|KK920964.1|	650007-650137	nan	nan	3	2	-0.78207	0.53215	no
ni|645903466|nb|KK920964.1|	650801-650962	nan	nan	0.316075	0.125934	-1	0.3479	no
ni|645903466|nb|KK920964.1|	651107-651420	nan	nan	1	1	-0.203223	0.87975	no
ni|645903466|nb|KK920964.1|	65175-65395	nan	nan	6	7	0.178268	0.87395	no
ni|645903466|nb|KK920964.1|	652473-652607	nan	nan	8	7	-0.201116	0.8635	no
ni|645903466|nb|KK920964.1|	653158-653496	nan	nan	0.875611	48	6	0.0444	no
ni|645903466|nb|KK920964.1|	658628-658846	nan	nan	0.360931	39	7	0.00325	no
ni|645903466|nb|KK920964.1|	662525-662712	nan	nan	15	32	1	0.346	no
ni|645903466|nb|KK920964.1|	66606-66731	nan	nan	1	35	5	0.04535	no
ni|645903466|nb|KK920964.1|	666336-666759	nan	nan	14	13	-0.0675775	0.9302	no
ni|645903466|nb|KK920964.1|	666895-668155	nan	nan	115	141	0.292999	0.79555	no
ni|645903466|nb|KK920964.1|	669569-669689	nan	nan	96	91	-0.0901586	0.9348	no
ni|645903466|nb|KK920964.1|	673425-673668	nan	nan	98	111	0.181092	0.8726	no
ni|645903466|nb|KK920964.1|	673771-674007	nan	nan	23	22	-0.0816294	0.94385	no
ni|645903466|nb|KK920964.1|	674189-674611	nan	nan	23	24	0.026893	0.9808	no
ni|645903466|nb|KK920964.1|	676688-676848	nan	nan	1	0.760505	-0.460755	0.6964	no
ni|645903466|nb|KK920964.1|	678931-679204	nan	nan	79	76	-0.039493	0.97115	no
ni|645903466|nb|KK920964.1|	679387-680211	nan	nan	138	123	-0.169397	0.8802	no

ni|645903466|nb|KK920964.1|	681582-681844	nan	nan	11	9	-0.295682	0.7931	no
ni|645903466|nb|KK920964.1|	68451-68671	nan	nan	14	8	-0.841105	0.4802	no
ni|645903466|nb|KK920964.1|	686444-686616	nan	nan	8	26	2	0.19855	no
ni|645903466|nb|KK920964.1|	691400-691560	nan	nan	14	39	1	0.2197	no
ni|645903466|nb|KK920964.1|	691970-692180	nan	nan	5	4	-0.507679	0.68875	no
ni|645903466|nb|KK920964.1|	693624-693818	nan	nan	1	0.314261	-2	0.1501	no
ni|645903466|nb|KK920964.1|	693999-694399	nan	nan	0.527243	0.542859	0.0421106	0.9599	no
ni|645903466|nb|KK920964.1|	698958-700474	nan	nan	9	0.25186	-5	0.0185	no
ni|645903466|nb|KK920964.1|	700541-700765	nan	nan	6	0	-inf	5.00E-05	yes
ni|645903466|nb|KK920964.1|	70550-70840	nan	nan	0	14	inf	0.00025	yes
ni|645903466|nb|KK920964.1|	70992-71138	nan	nan	1	0.586458	-1	0.33395	no
ni|645903466|nb|KK920964.1|	72146-72337	nan	nan	151	155	0.0401241	0.97935	no
ni|645903467|nb|KK920963.1|	100492-100689	nan	nan	28	53	0.944143	0.4113	no
ni|645903467|nb|KK920963.1|	102153-103009	nan	nan	12	22	0.808369	0.4897	no
ni|645903467|nb|KK920963.1|	103204-103520	nan	nan	0	26	inf	5.00E-05	yes
ni|645903467|nb|KK920963.1|	104238-104469	nan	nan	2	2	-0.444736	0.74215	no
ni|645903467|nb|KK920963.1|	104591-104746	nan	nan	12	8	-0.526638	0.67145	no
ni|645903467|nb|KK920963.1|	104949-105244	nan	nan	142	157	0.148407	0.8937	no
ni|645903467|nb|KK920963.1|	106256-107124	nan	nan	3	0.634434	-2	0.15765	no
ni|645903467|nb|KK920963.1|	107406-107691	nan	nan	1	0.406198	-2	0.21345	no
ni|645903467|nb|KK920963.1|	108424-108718	nan	nan	0.622614	0.267899	-1	0.37315	no
ni|645903467|nb|KK920963.1|	110036-110288	nan	nan	19	24	0.373137	0.7622	no
ni|645903467|nb|KK920963.1|	111714-112034	nan	nan	4	9	1	0.34485	no
ni|645903467|nb|KK920963.1|	112952-113214	nan	nan	166	123	-0.433336	0.69535	no
ni|645903467|nb|KK920963.1|	114157-114375	nan	nan	60	52	-0.196582	0.8649	no
ni|645903467|nb|KK920963.1|	115645-115960	nan	nan	34	28	-0.283275	0.786	no
ni|645903467|nb|KK920963.1|	78165-78356	nan	nan	2	3	0.826019	0.55535	no
ni|645903467|nb|KK920963.1|	920-990	nan	nan	2	1	-0.065183	0.9574	no
ni|645903467|nb|KK920963.1|	99403-99784	nan	nan	4	4	-0.0671964	0.94635	no
ni|645903469|nb|KK920961.1|	42757-42996	nan	nan	1	1	-0.0656068	0.9467	no

ni|645903469|nb|KK920961.1|	505917-506359	nan	nan	1	1	-0.0665672	0.923	no
ni|645903469|nb|KK920961.1|	52261-52709	nan	nan	1	0.917894	-0.596126	0.64595	no
ni|645903469|nb|KK920961.1|	580915-581420	nan	nan	24	41	0.784037	0.4904	no
ni|645903469|nb|KK920961.1|	588143-588705	nan	nan	9	1	-3	0.1378	no
ni|645903469|nb|KK920961.1|	588934-589408	nan	nan	25	27	0.0628687	0.95675	no
ni|645903469|nb|KK920961.1|	589519-589785	nan	nan	0.999483	1	0.0863473	0.9436	no
ni|645903469|nb|KK920961.1|	589895-590488	nan	nan	22	18	-0.276492	0.81095	no
ni|645903469|nb|KK920961.1|	625365-626295	nan	nan	98	75	-0.391673	0.7111	no
ni|645903469|nb|KK920961.1|	626408-626678	nan	nan	12	3	-2	0.16905	no
ni|645903469|nb|KK920961.1|	627187-627495	nan	nan	20	32	0.663975	0.5344	no
ni|645903469|nb|KK920961.1|	630394-630892	nan	nan	1	1	0.197457	0.87955	no
ni|645903469|nb|KK920961.1|	632300-632439	nan	nan	1	0.864217	-0.775837	0.5539	no
ni|645903469|nb|KK920961.1|	632643-633041	nan	nan	188	119	-0.655635	0.56925	no
ni|645903469|nb|KK920961.1|	633799-633918	nan	nan	3	3	-0.0849915	0.94435	no
ni|645903469|nb|KK920961.1|	642449-642570	nan	nan	4	6	0.717176	0.5449	no
ni|645903469|nb|KK920961.1|	646670-646867	nan	nan	6	4	-0.482235	0.7301	no
ni|645903469|nb|KK920961.1|	649607-649806	nan	nan	265	267	0.0111517	0.99385	no
ni|645903469|nb|KK920961.1|	651831-651984	nan	nan	13	21	0.652614	0.59345	no
ni|645903469|nb|KK920961.1|	657620-657801	nan	nan	2	3	0.0706287	0.94355	no
ni|645903469|nb|KK920961.1|	662785-663284	nan	nan	4	3	-0.355686	0.77565	no
ni|645903469|nb|KK920961.1|	664915-665782	nan	nan	4	4	-0.154275	0.90135	no
ni|645903469|nb|KK920961.1|	668355-668482	nan	nan	24	25	0.0807549	0.9433	no
ni|645903469|nb|KK920961.1|	672501-672649	nan	nan	13	8	-0.59784	0.61085	no
ni|645903469|nb|KK920961.1|	672800-673038	nan	nan	64	96	0.578184	0.68315	no
ni|645903469|nb|KK920961.1|	693561-693704	nan	nan	100	153	0.614933	0.58365	no
ni|645903469|nb|KK920961.1|	701107-701291	nan	nan	65	107	0.711899	0.6291	no
ni|645903469|nb|KK920961.1|	702560-702712	nan	nan	53	90	0.75164	0.5975	no
ni|645903469|nb|KK920961.1|	704226-704612	nan	nan	2	2	0.347714	0.8193	no
ni|645903469|nb|KK920961.1|	704691-704852	nan	nan	14	21	0.599007	0.6124	no
ni|645903469|nb|KK920961.1|	705146-705383	nan	nan	2	2	-0.218703	0.87705	no

ni|645903469|nb|KK920961.1|	709057-709355	nan	nan	2	3	0.256336	0.83575	no
ni|645903469|nb|KK920961.1|	71007-72191	nan	nan	23	34	0.557147	0.63625	no
ni|645903469|nb|KK920961.1|	819531-820053	nan	nan	6	4	-0.499625	0.6758	no
ni|645903469|nb|KK920961.1|	82137-82471	nan	nan	27	30	0.146746	0.90615	no
ni|645903469|nb|KK920961.1|	823873-824118	nan	nan	6	6	0.0967815	0.93755	no
ni|645903469|nb|KK920961.1|	82529-83699	nan	nan	180	182	0.0158678	0.98825	no
ni|645903469|nb|KK920961.1|	829086-829350	nan	nan	17	20	0.224419	0.85195	no
ni|645903469|nb|KK920961.1|	830525-830778	nan	nan	13	19	0.537141	0.6197	no
ni|645903469|nb|KK920961.1|	841977-842266	nan	nan	101.59	2	-6	0.04455	no
ni|645903469|nb|KK920961.1|	844150-845070	nan	nan	0	12	inf	5.00E-05	yes
ni|645903469|nb|KK920961.1|	88402-88716	nan	nan	270	255	-0.0818615	0.95815	no
ni|645903469|nb|KK920961.1|	94209-95536	nan	nan	15	20	0.386974	0.7338	no
ni|645903470|nb|KK920960.1|	100047-100190	nan	nan	6	70	4	0.0229	no
ni|645903470|nb|KK920960.1|	100765-101045	nan	nan	0.863532	0.916896	0.0865083	0.9436	no
ni|645903470|nb|KK920960.1|	102605-102903	nan	nan	2	2	-0.239012	0.83975	no
ni|645903470|nb|KK920960.1|	10397-10549	nan	nan	6	4	-0.778349	0.5088	no
ni|645903470|nb|KK920960.1|	10633-10789	nan	nan	23	31	0.39386	0.73505	no
ni|645903470|nb|KK920960.1|	106806-107194	nan	nan	2	3	0.295247	0.8383	no
ni|645903470|nb|KK920960.1|	107992-108336	nan	nan	2	1	-0.562821	0.6485	no
ni|645903470|nb|KK920960.1|	110355-110547	nan	nan	3	3	0.293295	0.80735	no
ni|645903470|nb|KK920960.1|	111483-112130	nan	nan	0.876015	0.586253	-0.57943	0.6557	no
ni|645903470|nb|KK920960.1|	113653-114030	nan	nan	12	8	-0.574156	0.63205	no
ni|645903470|nb|KK920960.1|	115037-115305	nan	nan	4	5	0.466878	0.6891	no
ni|645903470|nb|KK920960.1|	115801-115939	nan	nan	5	8	0.740122	0.5765	no
ni|645903470|nb|KK920960.1|	116595-116941	nan	nan	19	20	0.0500321	0.9635	no
ni|645903470|nb|KK920960.1|	117366-117624	nan	nan	21	32	0.600217	0.60575	no
ni|645903470|nb|KK920960.1|	118494-118663	nan	nan	18	21	0.229782	0.8428	no
ni|645903470|nb|KK920960.1|	124881-125048	nan	nan	6	8	0.513247	0.66	no
ni|645903470|nb|KK920960.1|	125671-125851	nan	nan	16	18	0.202899	0.86705	no
ni|645903470|nb|KK920960.1|	128232-128554	nan	nan	0.365513	0.271864	-0.427038	0.73235	no

ni|645903470|nb|KK920960.1|	129010-129289	nan	nan	14	16	0.265594	0.81925	no
ni|645903470|nb|KK920960.1|	129837-129987	nan	nan	2	4	0.934467	0.4444	no
ni|645903470|nb|KK920960.1|	130363-130719	nan	nan	18	23	0.389558	0.7404	no
ni|645903470|nb|KK920960.1|	130924-131051	nan	nan	17	19	0.171493	0.8829	no
ni|645903470|nb|KK920960.1|	131910-132371	nan	nan	3	1	-2	0.3149	no
ni|645903470|nb|KK920960.1|	132499-132630	nan	nan	0.76119	0.363757	-1	0.44755	no
ni|645903470|nb|KK920960.1|	133482-133885	nan	nan	2	2	-0.0645006	0.9566	no
ni|645903470|nb|KK920960.1|	134212-134361	nan	nan	0.478236	0.522407	0.127451	0.92555	no
ni|645903470|nb|KK920960.1|	144424-144670	nan	nan	3	3	-0.092082	0.93685	no
ni|645903470|nb|KK920960.1|	148530-148946	nan	nan	2	3	0.481406	0.6999	no
ni|645903470|nb|KK920960.1|	149887-150407	nan	nan	5	5	0.0201714	0.96555	no
ni|645903470|nb|KK920960.1|	151996-152141	nan	nan	3	3	0.104254	0.9282	no
ni|645903470|nb|KK920960.1|	155412-155828	nan	nan	4	5	0.54971	0.6602	no
ni|645903470|nb|KK920960.1|	163103-163979	nan	nan	3	2	-0.206825	0.8572	no
ni|645903470|nb|KK920960.1|	166642-167078	nan	nan	35	27	-0.384948	0.7463	no
ni|645903470|nb|KK920960.1|	187374-187938	nan	nan	29	30	0.0384847	0.97365	no
ni|645903470|nb|KK920960.1|	188388-189112	nan	nan	25	28	0.180023	0.87405	no
ni|645903470|nb|KK920960.1|	189510-189700	nan	nan	10	10	0.0089102	0.9936	no
ni|645903470|nb|KK920960.1|	190430-190617	nan	nan	0	22	inf	5.00E-05	yes
ni|645903470|nb|KK920960.1|	190955-191175	nan	nan	3	2	-0.76704	0.5799	no
ni|645903470|nb|KK920960.1|	191901-192407	nan	nan	2	6	2	0.242	no
ni|645903470|nb|KK920960.1|	192520-192750	nan	nan	1	2	0.313285	0.7992	no
ni|645903470|nb|KK920960.1|	193137-193260	nan	nan	40	23	-0.832751	0.4777	no
ni|645903470|nb|KK920960.1|	193784-194210	nan	nan	22	23	0.0423283	0.972	no
ni|645903470|nb|KK920960.1|	194929-195089	nan	nan	19	16	-0.2865	0.80885	no
ni|645903470|nb|KK920960.1|	195167-195329	nan	nan	0	91	inf	5.00E-05	yes
ni|645903470|nb|KK920960.1|	195822-196402	nan	nan	0	108	inf	5.00E-05	yes
ni|645903470|nb|KK920960.1|	196815-197186	nan	nan	0	402	inf	5.00E-05	yes
ni|645903470|nb|KK920960.1|	200089-200241	nan	nan	28	26	-0.110328	0.93585	no
ni|645903470|nb|KK920960.1|	202571-202684	nan	nan	0.663894	0.996968	0.586594	0.65495	no

ni|645903470|nb|KK920960.1|	204811-204943	nan	nan	107	114	0.0948775	0.9306	no
ni|645903470|nb|KK920960.1|	205437-205658	nan	nan	18	24	0.435229	0.7137	no
ni|645903470|nb|KK920960.1|	206987-207502	nan	nan	1	1	0.0538736	0.9649	no
ni|645903470|nb|KK920960.1|	208981-209150	nan	nan	3	2	-1	0.3864	no
ni|645903470|nb|KK920960.1|	211217-211379	nan	nan	2	2	0.510698	0.6607	no
ni|645903470|nb|KK920960.1|	212640-212854	nan	nan	0.836773	2	0.84949	0.4646	no
ni|645903470|nb|KK920960.1|	222554-222740	nan	nan	0	30	inf	5.00E-05	yes
ni|645903470|nb|KK920960.1|	223643-224481	nan	nan	0.651136	0.233365	-1	0.3864	no
ni|645903470|nb|KK920960.1|	224611-225103	nan	nan	112	88	-0.351875	0.74915	no
ni|645903470|nb|KK920960.1|	225862-226152	nan	nan	0	3	inf	5.00E-05	yes
ni|645903470|nb|KK920960.1|	226209-226450	nan	nan	0	3	inf	0.00055	yes
ni|645903470|nb|KK920960.1|	228417-228963	nan	nan	22	20	-0.105685	0.92745	no
ni|645903470|nb|KK920960.1|	238064-238237	nan	nan	2	0.884648	-0.872251	0.47305	no
ni|645903470|nb|KK920960.1|	242471-243030	nan	nan	14	16	0.230354	0.844	no
ni|645903470|nb|KK920960.1|	244954-245289	nan	nan	8	20	1	0.26665	no
ni|645903470|nb|KK920960.1|	245968-246272	nan	nan	42	68	0.697932	0.53145	no
ni|645903470|nb|KK920960.1|	251644-251863	nan	nan	29	0	-inf	5.00E-05	yes
ni|645903470|nb|KK920960.1|	251965-252108	nan	nan	20	17	-0.261694	0.8166	no
ni|645903470|nb|KK920960.1|	253329-253506	nan	nan	53	47	-0.174119	0.88555	no
ni|645903470|nb|KK920960.1|	253627-253746	nan	nan	2	1	-0.65188	0.6679	no
ni|645903470|nb|KK920960.1|	254655-254954	nan	nan	1	1	-0.0659573	0.9384	no
ni|645903470|nb|KK920960.1|	255082-255392	nan	nan	0	238	inf	5.00E-05	yes
ni|645903470|nb|KK920960.1|	255548-255784	nan	nan	0	284	inf	5.00E-05	yes
ni|645903470|nb|KK920960.1|	256010-256127	nan	nan	29	57	0.96793	0.40355	no
ni|645903470|nb|KK920960.1|	256284-256812	nan	nan	0.547048	0.683936	0.322193	0.7947	no
ni|645903470|nb|KK920960.1|	258281-258669	nan	nan	44	34	-0.356793	0.76225	no
ni|645903470|nb|KK920960.1|	263924-264128	nan	nan	10	10	-0.079021	0.9466	no
ni|645903470|nb|KK920960.1|	264255-264453	nan	nan	22	25	0.180044	0.87155	no
ni|645903470|nb|KK920960.1|	265287-265448	nan	nan	11	13	0.270245	0.81155	no
ni|645903470|nb|KK920960.1|	266096-266384	nan	nan	14	11	-0.366874	0.7706	no

ni|645903470|nb|KK920960.1|	268118-268390	nan	nan	14	10	-0.530205	0.63995	no
ni|645903470|nb|KK920960.1|	38321-38777	nan	nan	20	15	-0.389893	0.73165	no
ni|645903470|nb|KK920960.1|	40499-40896	nan	nan	1	2	0.419477	0.73705	no
ni|645903470|nb|KK920960.1|	48654-49278	nan	nan	8	12	0.579094	0.6331	no
ni|645903470|nb|KK920960.1|	83299-83512	nan	nan	10	49	2	0.0942	no
ni|645903470|nb|KK920960.1|	87957-88161	nan	nan	18	77	2	0.09125	no
ni|645903470|nb|KK920960.1|	90436-90632	nan	nan	8	39	2	0.092	no
ni|645903470|nb|KK920960.1|	92961-93404	nan	nan	22	94	2	0.0852	no
ni|645903470|nb|KK920960.1|	93538-93673	nan	nan	9	45	2	0.0529	no
ni|645903470|nb|KK920960.1|	93845-94238	nan	nan	3	1	-0.906387	0.44855	no
ni|645903470|nb|KK920960.1|	99318-99712	nan	nan	5	90	4	0.0177	no
ni|645903471|nb|KK920959.1|	188886-189218	nan	nan	20	18	-0.185058	0.872	no
ni|645903471|nb|KK920959.1|	190370-190544	nan	nan	0.913065	0.981478	0.104238	0.93335	no
ni|645903471|nb|KK920959.1|	190736-191207	nan	nan	0.842937	0.302051	-1	0.3864	no
ni|645903471|nb|KK920959.1|	296444-296576	nan	nan	7	9	0.239745	0.836	no
ni|645903471|nb|KK920959.1|	318452-318594	nan	nan	16	13	-0.219225	0.8486	no
ni|645903471|nb|KK920959.1|	391151-391311	nan	nan	13	14	0.0608529	0.9584	no
ni|645903472|nb|KK920958.1|	100636-100813	nan	nan	35	41	0.217496	0.84265	no
ni|645903472|nb|KK920958.1|	101449-101613	nan	nan	281	384	0.454639	0.6801	no
ni|645903472|nb|KK920958.1|	102587-102771	nan	nan	0.946429	0.95793	0.0174254	0.9674	no
ni|645903472|nb|KK920958.1|	104963-105366	nan	nan	2	3	0.723245	0.5569	no
ni|645903472|nb|KK920958.1|	106563-107010	nan	nan	3	6	0.82589	0.5554	no
ni|645903472|nb|KK920958.1|	107795-107900	nan	nan	22	27	0.310109	0.77855	no
ni|645903472|nb|KK920958.1|	108146-108465	nan	nan	0.679254	0.998978	0.556501	0.656	no
ni|645903472|nb|KK920958.1|	109153-109612	nan	nan	0.535169	0.4723	-0.180292	0.89165	no
ni|645903472|nb|KK920958.1|	119484-120054	nan	nan	37	34	-0.123774	0.91355	no
ni|645903472|nb|KK920958.1|	120654-121190	nan	nan	2	2	-0.388782	0.78555	no
ni|645903472|nb|KK920958.1|	121303-121511	nan	nan	29	26	-0.12378	0.9374	no
ni|645903472|nb|KK920958.1|	121617-121764	nan	nan	43	48	0.178833	0.8726	no
ni|645903472|nb|KK920958.1|	121841-122217	nan	nan	37	50	0.424414	0.7071	no

ni|645903472|nb|KK920958.1|	122426-122861	nan	nan	22	23	0.0863413	0.9362	no
ni|645903472|nb|KK920958.1|	122931-123437	nan	nan	2	2	0.117434	0.9184	no
ni|645903472|nb|KK920958.1|	123599-125025	nan	nan	3	3	0.370026	0.76295	no
ni|645903472|nb|KK920958.1|	125101-126162	nan	nan	16	9	-0.80446	0.6027	no
ni|645903472|nb|KK920958.1|	126286-126900	nan	nan	4.04	4	-0.0185779	0.98125	no
ni|645903472|nb|KK920958.1|	127570-127948	nan	nan	105	81	-0.373867	0.73755	no
ni|645903472|nb|KK920958.1|	128093-128692	nan	nan	0.968805	0.505017	-0.939876	0.54025	no
ni|645903472|nb|KK920958.1|	130080-130555	nan	nan	26	29	0.137623	0.90565	no
ni|645903472|nb|KK920958.1|	139664-140122	nan	nan	15	12	-0.330262	0.78525	no
ni|645903472|nb|KK920958.1|	140711-141154	nan	nan	2	2	-0.444759	0.74215	no
ni|645903472|nb|KK920958.1|	141516-141758	nan	nan	10	19	0.880262	0.4849	no
ni|645903472|nb|KK920958.1|	144274-144601	nan	nan	12	8	-0.5078	0.68875	no
ni|645903472|nb|KK920958.1|	144947-145283	nan	nan	4	3	-0.445803	0.74215	no
ni|645903472|nb|KK920958.1|	167765-168253	nan	nan	3	3	-0.0657031	0.9568	no
ni|645903472|nb|KK920958.1|	187115-187678	nan	nan	2	3	0.14574	0.90485	no
ni|645903472|nb|KK920958.1|	188444-188903	nan	nan	157	164	0.0553477	0.97045	no
ni|645903472|nb|KK920958.1|	189570-190185	nan	nan	8	4	-0.97409	0.5005	no
ni|645903472|nb|KK920958.1|	191180-191386	nan	nan	3	3	0.125443	0.92845	no
ni|645903472|nb|KK920958.1|	191544-191672	nan	nan	41	33	-0.319758	0.7691	no
ni|645903472|nb|KK920958.1|	192777-192928	nan	nan	46	42	-0.13334	0.90865	no
ni|645903472|nb|KK920958.1|	194847-195186	nan	nan	0.223499	4	4	0.1221	no
ni|645903472|nb|KK920958.1|	195385-196198	nan	nan	110	78	-0.493544	0.6604	no
ni|645903472|nb|KK920958.1|	196317-196924	nan	nan	73	43	-0.754587	0.54495	no
ni|645903472|nb|KK920958.1|	197306-197613	nan	nan	199.98	103	-0.962337	0.41045	no
ni|645903472|nb|KK920958.1|	197721-197890	nan	nan	35	16	-1	0.3424	no
ni|645903472|nb|KK920958.1|	198103-198449	nan	nan	16	10	-0.703761	0.53355	no
ni|645903472|nb|KK920958.1|	198796-199519	nan	nan	11	6	-0.863766	0.4708	no
ni|645903472|nb|KK920958.1|	199678-200052	nan	nan	23	18	-0.363288	0.74255	no
ni|645903472|nb|KK920958.1|	200934-201278	nan	nan	20	19	-0.0889865	0.93915	no
ni|645903472|nb|KK920958.1|	201533-202173	nan	nan	14	9	-0.549037	0.636	no

ni|645903472|nb|KK920958.1|	202408-202749	nan	nan	12	9	-0.434236	0.705	no
ni|645903472|nb|KK920958.1|	202908-203057	nan	nan	2	2	-0.119775	0.91895	no
ni|645903472|nb|KK920958.1|	205544-205995	nan	nan	24	19	-0.347365	0.77315	no
ni|645903472|nb|KK920958.1|	208457-208635	nan	nan	9	10	0.221752	0.85055	no
ni|645903472|nb|KK920958.1|	208730-209119	nan	nan	22	20	-0.158446	0.8823	no
ni|645903472|nb|KK920958.1|	209289-209404	nan	nan	7	7	0.0785548	0.94435	no
ni|645903472|nb|KK920958.1|	209692-210131	nan	nan	21	23	0.110498	0.92395	no
ni|645903472|nb|KK920958.1|	210193-210492	nan	nan	23	26	0.195842	0.8701	no
ni|645903472|nb|KK920958.1|	210695-211466	nan	nan	0.315122	0.150642	-1	0.44965	no
ni|645903472|nb|KK920958.1|	216939-217884	nan	nan	30	30	-0.00303534	0.9954	no
ni|645903472|nb|KK920958.1|	221342-222521	nan	nan	38	35	-0.107844	0.9255	no
ni|645903472|nb|KK920958.1|	222634-222890	nan	nan	42	39	-0.0982233	0.9322	no
ni|645903472|nb|KK920958.1|	225883-226014	nan	nan	21	30	0.472991	0.71005	no
ni|645903472|nb|KK920958.1|	226114-226267	nan	nan	6	8	0.314699	0.78875	no
ni|645903472|nb|KK920958.1|	227631-227785	nan	nan	1	0.506139	-1	0.45115	no
ni|645903472|nb|KK920958.1|	228691-229040	nan	nan	0.633201	0.462961	-0.451773	0.72335	no
ni|645903472|nb|KK920958.1|	229917-230887	nan	nan	41	57	0.453285	0.7043	no
ni|645903472|nb|KK920958.1|	232872-234650	nan	nan	45	57	0.322372	0.78495	no
ni|645903472|nb|KK920958.1|	237814-238131	nan	nan	6	11	0.807265	0.5327	no
ni|645903472|nb|KK920958.1|	242793-242997	nan	nan	0	260	inf	5.00E-05	yes
ni|645903472|nb|KK920958.1|	243833-244201	nan	nan	0	17	inf	5.00E-05	yes
ni|645903472|nb|KK920958.1|	244336-244483	nan	nan	0	258	inf	5.00E-05	yes
ni|645903472|nb|KK920958.1|	245592-245761	nan	nan	3	3	-0.174949	0.88375	no
ni|645903472|nb|KK920958.1|	250908-251594	nan	nan	5	2	-1	0.39585	no
ni|645903472|nb|KK920958.1|	254178-254409	nan	nan	6	25	2	0.0821	no
ni|645903472|nb|KK920958.1|	265978-266134	nan	nan	6	17	2	0.18725	no
ni|645903472|nb|KK920958.1|	275973-276366	nan	nan	2	34	4	0.00175	yes
ni|645903472|nb|KK920958.1|	324237-324484	nan	nan	4	0	-inf	0.0036	no
ni|645903472|nb|KK920958.1|	337200-337599	nan	nan	2	0.468785	-2	0.14175	no
ni|645903472|nb|KK920958.1|	338810-339272	nan	nan	108	116	0.100701	0.9299	no

ni|645903472|nb|KK920958.1|	339639-341171	nan	nan	150.16	143	-0.0719926	0.9613	no
ni|645903472|nb|KK920958.1|	341304-341469	nan	nan	176	192	0.126838	0.92875	no
ni|645903472|nb|KK920958.1|	342136-342745	nan	nan	64	62	-0.0439054	0.9656	no
ni|645903472|nb|KK920958.1|	343482-343947	nan	nan	36	46	0.355412	0.7592	no
ni|645903472|nb|KK920958.1|	346876-347452	nan	nan	61	59	-0.055698	0.96125	no
ni|645903472|nb|KK920958.1|	348725-349040	nan	nan	25	20	-0.311222	0.7861	no
ni|645903472|nb|KK920958.1|	349561-350166	nan	nan	0.78636	0.614943	-0.354737	0.7931	no
ni|645903472|nb|KK920958.1|	350668-350917	nan	nan	1	1	-0.0663116	0.9384	no
ni|645903472|nb|KK920958.1|	351014-351456	nan	nan	2	1	-0.649999	0.59155	no
ni|645903472|nb|KK920958.1|	351589-351750	nan	nan	2	2	-0.0665508	0.9467	no
ni|645903472|nb|KK920958.1|	352534-352737	nan	nan	24	26	0.0939458	0.93265	no
ni|645903472|nb|KK920958.1|	352801-352913	nan	nan	54	59	0.113249	0.92285	no
ni|645903472|nb|KK920958.1|	353024-353277	nan	nan	2	2	0.104497	0.92795	no
ni|645903472|nb|KK920958.1|	353433-353608	nan	nan	3	5	0.418137	0.73705	no
ni|645903472|nb|KK920958.1|	353819-354037	nan	nan	2	2	0.349972	0.762	no
ni|645903472|nb|KK920958.1|	354373-354852	nan	nan	1	1	0.0718528	0.94355	no
ni|645903472|nb|KK920958.1|	354924-355154	nan	nan	25	7	-2	0.2784	no
ni|645903472|nb|KK920958.1|	355346-355481	nan	nan	372	61	-3	0.05465	no
ni|645903472|nb|KK920958.1|	355690-355832	nan	nan	2	1	-1	0.3995	no
ni|645903472|nb|KK920958.1|	355900-356016	nan	nan	0.415176	2	2	0.1256	no
ni|645903472|nb|KK920958.1|	356083-356259	nan	nan	0.513812	1	1	0.4218	no
ni|645903472|nb|KK920958.1|	356505-356682	nan	nan	0.993988	0.356295	-1	0.32095	no
ni|645903472|nb|KK920958.1|	357003-357700	nan	nan	4	3	-0.324358	0.7818	no
ni|645903472|nb|KK920958.1|	357835-358847	nan	nan	6	17	1	0.2305	no
ni|645903472|nb|KK920958.1|	359050-359383	nan	nan	10	21	1	0.34855	no
ni|645903472|nb|KK920958.1|	360122-360411	nan	nan	3	2	-0.551779	0.68265	no
ni|645903472|nb|KK920958.1|	361234-361418	nan	nan	129	226	0.814325	0.4795	no
ni|645903472|nb|KK920958.1|	361591-361725	nan	nan	65	105	0.683202	0.5581	no
ni|645903472|nb|KK920958.1|	363886-364071	nan	nan	90	158	0.803086	0.4706	no
ni|645903472|nb|KK920958.1|	364392-364561	nan	nan	9	23	1	0.2363	no

ni|645903472|nb|KK920958.1|	366759-367014	nan	nan	0	5	inf	5.00E-05	yes
ni|645903472|nb|KK920958.1|	367391-367613	nan	nan	671	519	-0.369007	0.79325	no
ni|645903472|nb|KK920958.1|	369163-369809	nan	nan	367	300	-0.290937	0.8363	no
ni|645903472|nb|KK920958.1|	370223-371453	nan	nan	83	136.07	0.719802	0.5323	no
ni|645903472|nb|KK920958.1|	371927-372381	nan	nan	102	192	0.909217	0.51875	no
ni|645903472|nb|KK920958.1|	373394-373665	nan	nan	74	19	-2	0.2004	no
ni|645903472|nb|KK920958.1|	373767-373989	nan	nan	2	2	-0.267108	0.8253	no
ni|645903472|nb|KK920958.1|	374109-374276	nan	nan	2	2	-0.336503	0.7802	no
ni|645903472|nb|KK920958.1|	384007-384128	nan	nan	13	8	-0.708871	0.557	no
ni|645903472|nb|KK920958.1|	384319-385516	nan	nan	2	0.098582	-4	0.01995	no
ni|645903472|nb|KK920958.1|	387603-387890	nan	nan	2	0.112443	-4	0.11995	no
ni|645903472|nb|KK920958.1|	388053-388703	nan	nan	1	0	-inf	0.00175	yes
ni|645903472|nb|KK920958.1|	390059-391675	nan	nan	1	0	-inf	0.0004	yes
ni|645903472|nb|KK920958.1|	391838-392105	nan	nan	0	1	inf	0.00225	yes
ni|645903472|nb|KK920958.1|	392450-393175	nan	nan	0	131	inf	5.00E-05	yes
ni|645903472|nb|KK920958.1|	394393-394518	nan	nan	96	89	-0.106922	0.94255	no
ni|645903472|nb|KK920958.1|	394746-394881	nan	nan	15	11	-0.372727	0.7461	no
ni|645903472|nb|KK920958.1|	395252-395388	nan	nan	7	7	0.0565987	0.9583	no
ni|645903472|nb|KK920958.1|	395529-395789	nan	nan	7	0.76955	-3	0.244	no
ni|645903472|nb|KK920958.1|	395919-396407	nan	nan	16	17	0.12691	0.91365	no
ni|645903472|nb|KK920958.1|	397204-397540	nan	nan	13	20	0.619522	0.59885	no
ni|645903472|nb|KK920958.1|	397626-397902	nan	nan	39	44	0.170656	0.88855	no
ni|645903472|nb|KK920958.1|	397978-398111	nan	nan	14	6	-1	0.3528	no
ni|645903472|nb|KK920958.1|	398397-398537	nan	nan	15	10	-0.653527	0.54615	no
ni|645903472|nb|KK920958.1|	398821-399143	nan	nan	15	10	-0.589099	0.61755	no
ni|645903472|nb|KK920958.1|	399430-399775	nan	nan	9	8	-0.159462	0.89275	no
ni|645903472|nb|KK920958.1|	399938-400353	nan	nan	2	2	-0.494061	0.6802	no
ni|645903472|nb|KK920958.1|	401395-401593	nan	nan	17	7	-1	0.33495	no
ni|645903472|nb|KK920958.1|	401910-402641	nan	nan	12	6	-1	0.38555	no
ni|645903472|nb|KK920958.1|	403615-403921	nan	nan	5	3	-0.762876	0.51435	no

ni|645903472|nb|KK920958.1|	404153-404572	nan	nan	10	6	-0.678455	0.5607	no
ni|645903472|nb|KK920958.1|	407364-408087	nan	nan	152	208	0.455646	0.7512	no
ni|645903472|nb|KK920958.1|	420860-420974	nan	nan	9	2	-2	0.127	no
ni|645903472|nb|KK920958.1|	421536-421779	nan	nan	0.607835	0.497914	-0.287785	0.8535	no
ni|645903472|nb|KK920958.1|	421858-422113	nan	nan	2	1	-0.487932	0.67735	no
ni|645903472|nb|KK920958.1|	422692-422824	nan	nan	2	1	-0.481129	0.7301	no
ni|645903472|nb|KK920958.1|	422894-423038	nan	nan	16	27	0.746393	0.528	no
ni|645903472|nb|KK920958.1|	423643-424477	nan	nan	4	4	-0.262507	0.82505	no
ni|645903472|nb|KK920958.1|	424762-424925	nan	nan	2	1	-0.649977	0.5826	no
ni|645903472|nb|KK920958.1|	425259-425550	nan	nan	12	8	-0.485566	0.6564	no
ni|645903472|nb|KK920958.1|	426367-426644	nan	nan	116	77	-0.598481	0.5993	no
ni|645903472|nb|KK920958.1|	428623-428999	nan	nan	17	20	0.180724	0.87455	no
ni|645903472|nb|KK920958.1|	430236-430553	nan	nan	15	18	0.254713	0.8243	no
ni|645903472|nb|KK920958.1|	431594-431775	nan	nan	5	4	-0.359495	0.76125	no
ni|645903472|nb|KK920958.1|	432497-432993	nan	nan	31	40	0.379688	0.7694	no
ni|645903472|nb|KK920958.1|	433173-433579	nan	nan	5	3	-0.886554	0.4453	no
ni|645903472|nb|KK920958.1|	433722-434528	nan	nan	2	0.805514	-0.914198	0.53575	no
ni|645903472|nb|KK920958.1|	434864-435363	nan	nan	5	3	-0.941659	0.54025	no
ni|645903472|nb|KK920958.1|	435549-435836	nan	nan	7	7	0.0973845	0.9317	no
ni|645903472|nb|KK920958.1|	436681-436867	nan	nan	4	3	-0.361369	0.7606	no
ni|645903472|nb|KK920958.1|	438684-438840	nan	nan	15	10	-0.576221	0.63215	no
ni|645903472|nb|KK920958.1|	441352-443076	nan	nan	1	1	-0.0657354	0.9467	no
ni|645903472|nb|KK920958.1|	446175-446311	nan	nan	2	2	-0.0657579	0.96005	no
ni|645903472|nb|KK920958.1|	446992-447534	nan	nan	17	13	-0.388384	0.74365	no
ni|645903472|nb|KK920958.1|	449559-449987	nan	nan	18	14	-0.370613	0.7486	no
ni|645903472|nb|KK920958.1|	452098-452484	nan	nan	13	11	-0.305401	0.7946	no
ni|645903472|nb|KK920958.1|	452758-453066	nan	nan	8	4	-0.820884	0.49045	no
ni|645903472|nb|KK920958.1|	453481-453678	nan	nan	7	6	-0.256041	0.8255	no
ni|645903472|nb|KK920958.1|	454396-454551	nan	nan	15	14	-0.0671984	0.95575	no
ni|645903472|nb|KK920958.1|	456331-456641	nan	nan	0.544594	0.520495	-0.0652974	0.9302	no

ni|645903472|nb|KK920958.1|	459046-459212	nan	nan	28	25	-0.158616	0.88555	no
ni|645903472|nb|KK920958.1|	460306-460538	nan	nan	29	36	0.280488	0.81735	no
ni|645903472|nb|KK920958.1|	464610-465108	nan	nan	48	56	0.224024	0.8446	no
ni|645903472|nb|KK920958.1|	468062-468301	nan	nan	85	89	0.0775814	0.94435	no
ni|645903472|nb|KK920958.1|	500353-500627	nan	nan	9	7	-0.209126	0.8594	no
ni|645903472|nb|KK920958.1|	507714-508168	nan	nan	9	7	-0.435247	0.7135	no
ni|645903472|nb|KK920958.1|	511485-511682	nan	nan	6	6	-0.0655284	0.95365	no
ni|645903472|nb|KK920958.1|	512519-512846	nan	nan	4	5	0.282851	0.799	no
ni|645903472|nb|KK920958.1|	514757-514987	nan	nan	6	9	0.498935	0.67355	no
ni|645903472|nb|KK920958.1|	515189-515356	nan	nan	3	2	-0.469533	0.69165	no
ni|645903472|nb|KK920958.1|	516219-516440	nan	nan	10	14	0.456179	0.69095	no
ni|645903472|nb|KK920958.1|	519876-520065	nan	nan	1	1	0.127704	0.9121	no
ni|645903472|nb|KK920958.1|	522924-523124	nan	nan	4	6	0.517769	0.7004	no
ni|645903472|nb|KK920958.1|	525051-525436	nan	nan	6	5	-0.18267	0.889	no
ni|645903472|nb|KK920958.1|	525637-526576	nan	nan	1	2	0.616651	0.59825	no
ni|645903472|nb|KK920958.1|	526627-526741	nan	nan	2	4	0.692395	0.5543	no
ni|645903472|nb|KK920958.1|	533025-533490	nan	nan	7	7	0.0314148	0.97835	no
ni|645903472|nb|KK920958.1|	533874-534255	nan	nan	10	5	-0.984746	0.48245	no
ni|645903472|nb|KK920958.1|	534523-534873	nan	nan	1	2	0.82712	0.55535	no
ni|645903472|nb|KK920958.1|	537242-537386	nan	nan	30	37	0.272518	0.8103	no
ni|645903472|nb|KK920958.1|	538696-539124	nan	nan	0.40422	0.702694	0.797755	0.53465	no
ni|645903472|nb|KK920958.1|	539238-539441	nan	nan	25	28.51	0.169406	0.8762	no
ni|645903472|nb|KK920958.1|	541589-541767	nan	nan	10	18	0.841645	0.45095	no
ni|645903472|nb|KK920958.1|	542230-542359	nan	nan	22	17	-0.365259	0.75255	no
ni|645903472|nb|KK920958.1|	543393-544137	nan	nan	28	60	1	0.36225	no
ni|645903472|nb|KK920958.1|	548763-548937	nan	nan	6	15	1	0.28495	no
ni|645903472|nb|KK920958.1|	556848-557043	nan	nan	3	4	0.566322	0.63235	no
ni|645903472|nb|KK920958.1|	558238-558388	nan	nan	17	87	2	0.05395	no
ni|645903472|nb|KK920958.1|	558853-559211	nan	nan	50	45	-0.142159	0.8992	no
ni|645903472|nb|KK920958.1|	560428-560617	nan	nan	87	106	0.287013	0.7964	no

ni|645903472|nb|KK920958.1|	561553-562026	nan	nan	112.1	80	-0.482239	0.67205	no
ni|645903472|nb|KK920958.1|	563165-563347	nan	nan	95	68	-0.470314	0.684	no
ni|645903472|nb|KK920958.1|	563819-564013	nan	nan	153	112	-0.443407	0.68855	no
ni|645903472|nb|KK920958.1|	564907-565142	nan	nan	0	11	inf	0.0005	yes
ni|645903472|nb|KK920958.1|	570125-570606	nan	nan	0.444375	0.212394	-1	0.44965	no
ni|645903472|nb|KK920958.1|	573553-573668	nan	nan	4	0.982814	-2	0.20695	no
ni|645903472|nb|KK920958.1|	573943-574216	nan	nan	16	42	1	0.23245	no
ni|645903472|nb|KK920958.1|	574629-575094	nan	nan	86	55	-0.646901	0.5841	no
ni|645903472|nb|KK920958.1|	575265-576098	nan	nan	15	13	-0.149992	0.8975	no
ni|645903472|nb|KK920958.1|	576589-576918	nan	nan	40	31	-0.345559	0.7478	no
ni|645903472|nb|KK920958.1|	579810-580141	nan	nan	1	2	0.634367	0.63975	no
ni|645903472|nb|KK920958.1|	584528-585226	nan	nan	44	43	-0.0660639	0.9507	no
ni|645903472|nb|KK920958.1|	585568-586462	nan	nan	5	2	-1	0.44965	no
ni|645903472|nb|KK920958.1|	587259-587540	nan	nan	1	2	0.464459	0.7264	no
ni|645903472|nb|KK920958.1|	587751-587966	nan	nan	4	4	0.162577	0.8907	no
ni|645903472|nb|KK920958.1|	589003-589258	nan	nan	2	3	0.447248	0.7576	no
ni|645903472|nb|KK920958.1|	594206-594392	nan	nan	54	46	-0.21604	0.84855	no
ni|645903472|nb|KK920958.1|	595129-595304	nan	nan	72	56	-0.364501	0.75795	no
ni|645903472|nb|KK920958.1|	595660-595873	nan	nan	12	13	0.128958	0.90655	no
ni|645903472|nb|KK920958.1|	596613-596775	nan	nan	25	25	-0.00284378	0.9978	no
ni|645903472|nb|KK920958.1|	598164-598991	nan	nan	50	49	-0.00724655	0.9887	no
ni|645903472|nb|KK920958.1|	601139-601280	nan	nan	7	10	0.37673	0.7458	no
ni|645903472|nb|KK920958.1|	602619-602944	nan	nan	0	0.92454	inf	0.0003	yes
ni|645903472|nb|KK920958.1|	605315-605470	nan	nan	58	47	-0.290504	0.82015	no
ni|645903472|nb|KK920958.1|	605934-606104	nan	nan	155	116	-0.419086	0.7193	no
ni|645903472|nb|KK920958.1|	606206-606349	nan	nan	139	136	-0.0305855	0.9782	no
ni|645903472|nb|KK920958.1|	610822-611124	nan	nan	0.631209	0.201158	-2	0.24455	no
ni|645903472|nb|KK920958.1|	649047-649204	nan	nan	19	16	-0.190827	0.8622	no
ni|645903472|nb|KK920958.1|	68761-68956	nan	nan	3	2	-0.0333398	0.9778	no
ni|645903472|nb|KK920958.1|	824299-824472	nan	nan	23	20	-0.205117	0.87975	no

ni|645903472|nb|KK920958.1|	93841-93965	nan	nan	3	3	-0.1825	0.889	no
ni|645903472|nb|KK920958.1|	98966-99500	nan	nan	2	0.594691	-2	0.18585	no
ni|645903473|nb|KK920957.1|	107850-108187	nan	nan	0.330337	0.11486	-2	0.3149	no
ni|645903473|nb|KK920957.1|	108643-108868	nan	nan	38	36	-0.0774687	0.9473	no
ni|645903473|nb|KK920957.1|	10927-11398	nan	nan	4	4	-0.0672498	0.94715	no
ni|645903473|nb|KK920957.1|	12514-13215	nan	nan	2	2	-0.335098	0.7774	no
ni|645903473|nb|KK920957.1|	13372-13761	nan	nan	5	5	-0.0671984	0.954	no
ni|645903473|nb|KK920957.1|	13878-14758	nan	nan	3	2	-0.61089	0.6013	no
ni|645903473|nb|KK920957.1|	15239-15400	nan	nan	1	1	-0.152283	0.8951	no
ni|645903473|nb|KK920957.1|	15620-16054	nan	nan	3	3	0.332965	0.77045	no
ni|645903473|nb|KK920957.1|	16195-16755	nan	nan	2	2	0.127991	0.9139	no
ni|645903473|nb|KK920957.1|	16856-16981	nan	nan	9	12	0.463327	0.6832	no
ni|645903473|nb|KK920957.1|	17266-17636	nan	nan	2	2	-0.0661694	0.9467	no
ni|645903473|nb|KK920957.1|	18592-19087	nan	nan	86	179.42	1	0.32875	no
ni|645903473|nb|KK920957.1|	203820-204220	nan	nan	14	13	-0.0649875	0.95425	no
ni|645903473|nb|KK920957.1|	204734-205156	nan	nan	31	26	-0.240193	0.8319	no
ni|645903473|nb|KK920957.1|	206750-207024	nan	nan	30	23	-0.338606	0.77845	no
ni|645903473|nb|KK920957.1|	208807-209066	nan	nan	13	2	-3	0.2671	no
ni|645903473|nb|KK920957.1|	209179-209527	nan	nan	6	10	0.710294	0.61225	no
ni|645903473|nb|KK920957.1|	209717-209840	nan	nan	0.035839	96	11	0.11935	no
ni|645903473|nb|KK920957.1|	210037-210383	nan	nan	0	181.51	inf	5.00E-05	yes
ni|645903473|nb|KK920957.1|	210526-210818	nan	nan	0	4	inf	5.00E-05	yes
ni|645903473|nb|KK920957.1|	213671-213780	nan	nan	0	32	inf	5.00E-05	yes
ni|645903473|nb|KK920957.1|	213918-214036	nan	nan	30	18	-0.723189	0.61175	no
ni|645903473|nb|KK920957.1|	214206-214514	nan	nan	0.702987	0.335991	-1	0.46615	no
ni|645903473|nb|KK920957.1|	215104-215332	nan	nan	0.488906	0.934723	0.934982	0.4817	no
ni|645903473|nb|KK920957.1|	217761-218153	nan	nan	1	2	0.296978	0.8138	no
ni|645903473|nb|KK920957.1|	218313-218867	nan	nan	3	3	-0.271557	0.81055	no
ni|645903473|nb|KK920957.1|	218976-219529	nan	nan	9	5	-0.804289	0.6027	no
ni|645903473|nb|KK920957.1|	22048-22285	nan	nan	70	131	0.908917	0.46035	no

ni|645903473|nb|KK920957.1|	227411-227586	nan	nan	5	0.101988	-6	0.01745	no
ni|645903473|nb|KK920957.1|	23291-23492	nan	nan	68	144	1	0.37905	no
ni|645903473|nb|KK920957.1|	25767-25933	nan	nan	47	117	1	0.2362	no
ni|645903473|nb|KK920957.1|	260919-261170	nan	nan	135	96	-0.491641	0.6584	no
ni|645903473|nb|KK920957.1|	26886-27836	nan	nan	0.409939	0.503978	0.297953	0.8138	no
ni|645903473|nb|KK920957.1|	288417-288879	nan	nan	104	80	-0.37259	0.7339	no
ni|645903473|nb|KK920957.1|	289103-289447	nan	nan	133	83	-0.677445	0.55685	no
ni|645903473|nb|KK920957.1|	291315-291452	nan	nan	95	79	-0.273248	0.8006	no
ni|645903473|nb|KK920957.1|	292771-292946	nan	nan	108	82	-0.396566	0.71015	no
ni|645903473|nb|KK920957.1|	293538-293739	nan	nan	135.8	112.72	-0.268737	0.8011	no
ni|645903473|nb|KK920957.1|	29450-29630	nan	nan	0.349165	0.41727	0.257073	0.86075	no
ni|645903473|nb|KK920957.1|	296922-297262	nan	nan	10	9	-0.253454	0.8222	no
ni|645903473|nb|KK920957.1|	308624-308997	nan	nan	3	317.53	7	0.00145	yes
ni|645903473|nb|KK920957.1|	31305-31501	nan	nan	0.388366	0.464093	0.256996	0.86075	no
ni|645903473|nb|KK920957.1|	313640-313855	nan	nan	2	210	7	0.0005	yes
ni|645903473|nb|KK920957.1|	317208-317323	nan	nan	0.717386	0.514185	-0.480462	0.7594	no
ni|645903473|nb|KK920957.1|	320453-320839	nan	nan	2	0.468673	-2	0.22995	no
ni|645903473|nb|KK920957.1|	32168-32407	nan	nan	0.318264	0.163876	-0.957622	0.51225	no
ni|645903473|nb|KK920957.1|	32682-32794	nan	nan	21	13	-0.727716	0.53795	no
ni|645903473|nb|KK920957.1|	341579-341995	nan	nan	18	14	-0.348017	0.7524	no
ni|645903473|nb|KK920957.1|	34208-34386	nan	nan	15	11	-0.43962	0.70515	no
ni|645903473|nb|KK920957.1|	35376-35547	nan	nan	34	21	-0.682528	0.56015	no
ni|645903473|nb|KK920957.1|	361953-364056	nan	nan	6	4	-0.559264	0.63785	no
ni|645903473|nb|KK920957.1|	367187-368451	nan	nan	5	5	-0.0672163	0.9414	no
ni|645903473|nb|KK920957.1|	369350-369591	nan	nan	2	1	-0.525478	0.7071	no
ni|645903473|nb|KK920957.1|	372137-372294	nan	nan	0.967005	0.654877	-0.5623	0.6485	no
ni|645903473|nb|KK920957.1|	373294-373488	nan	nan	0.765916	0.292812	-1	0.35155	no
ni|645903473|nb|KK920957.1|	375598-376160	nan	nan	1	2	0.25563	0.86075	no
ni|645903473|nb|KK920957.1|	377027-377177	nan	nan	5	5	0.0749998	0.9463	no
ni|645903473|nb|KK920957.1|	386175-386514	nan	nan	3	3	-0.111396	0.92105	no

ni|645903473|nb|KK920957.1|	387213-387386	nan	nan	8	12	0.669546	0.65595	no
ni|645903473|nb|KK920957.1|	387696-388889	nan	nan	8	10	0.46321	0.7264	no
ni|645903473|nb|KK920957.1|	389276-389476	nan	nan	6	8	0.463271	0.7264	no
ni|645903473|nb|KK920957.1|	394247-394483	nan	nan	12	9	-0.366831	0.7706	no
ni|645903473|nb|KK920957.1|	39454-39574	nan	nan	9	5	-1	0.43475	no
ni|645903473|nb|KK920957.1|	438955-439231	nan	nan	8	8	0.0848006	0.949	no
ni|645903473|nb|KK920957.1|	439399-439517	nan	nan	2	2	0.227899	0.8452	no
ni|645903473|nb|KK920957.1|	439659-439994	nan	nan	1	2	0.933841	0.5103	no
ni|645903473|nb|KK920957.1|	440105-440394	nan	nan	2	2	0.103133	0.93335	no
ni|645903473|nb|KK920957.1|	440583-440731	nan	nan	0.370151	53	7	0.0443	no
ni|645903473|nb|KK920957.1|	441108-441484	nan	nan	0	62	inf	5.00E-05	yes
ni|645903473|nb|KK920957.1|	441976-442360	nan	nan	0.102736	27	8	0.11935	no
ni|645903473|nb|KK920957.1|	442522-443063	nan	nan	0.325591	62	8	0.11935	no
ni|645903473|nb|KK920957.1|	443732-443883	nan	nan	0.181323	62	8	0.0443	no
ni|645903473|nb|KK920957.1|	444572-444831	nan	nan	6	17	2	0.20065	no
ni|645903473|nb|KK920957.1|	445992-446170	nan	nan	21	58	1	0.215	no
ni|645903473|nb|KK920957.1|	449283-449496	nan	nan	48	130	1	0.2581	no
ni|645903473|nb|KK920957.1|	46370-46634	nan	nan	38	38	0.00305034	0.99695	no
ni|645903473|nb|KK920957.1|	47061-47383	nan	nan	32	30	-0.090739	0.9373	no
ni|645903473|nb|KK920957.1|	4834-5152	nan	nan	4	4	-0.00474847	0.9752	no
ni|645903473|nb|KK920957.1|	501873-503071	nan	nan	129	347	1	0.25655	no
ni|645903473|nb|KK920957.1|	526756-526947	nan	nan	47	140	2	0.27815	no
ni|645903473|nb|KK920957.1|	532364-532500	nan	nan	14	55	2	0.10075	no
ni|645903473|nb|KK920957.1|	534389-534797	nan	nan	112	337	2	0.22455	no
ni|645903473|nb|KK920957.1|	536848-536994	nan	nan	127	364	2	0.25665	no
ni|645903473|nb|KK920957.1|	540286-540462	nan	nan	64	193	2	0.27905	no
ni|645903473|nb|KK920957.1|	542281-542466	nan	nan	19	78	2	0.1431	no
ni|645903473|nb|KK920957.1|	543502-543783	nan	nan	16	74	2	0.10535	no
ni|645903473|nb|KK920957.1|	545075-545251	nan	nan	11	61	2	0.08215	no
ni|645903473|nb|KK920957.1|	546800-546965	nan	nan	20	123	3	0.03315	no

ni|645903473|nb|KK920957.1|	5528-6092	nan	nan	50	45	-0.164756	0.88485	no
ni|645903473|nb|KK920957.1|	608103-608262	nan	nan	13	69	2	0.0788	no
ni|645903473|nb|KK920957.1|	625078-625187	nan	nan	24	30	0.324804	0.771	no
ni|645903473|nb|KK920957.1|	6952-7539	nan	nan	6	6	0.010568	0.9918	no
ni|645903473|nb|KK920957.1|	7656-8448	nan	nan	7	5	-0.38126	0.7501	no
ni|645903473|nb|KK920957.1|	794115-794349	nan	nan	24	28	0.217046	0.8389	no
ni|645903473|nb|KK920957.1|	795870-796113	nan	nan	11	17	0.602317	0.60735	no
ni|645903473|nb|KK920957.1|	806463-806918	nan	nan	17	25	0.504148	0.64815	no
ni|645903473|nb|KK920957.1|	810231-810410	nan	nan	1	2	0.496815	0.67745	no
ni|645903473|nb|KK920957.1|	811544-811716	nan	nan	6	14	1	0.28335	no
ni|645903473|nb|KK920957.1|	812695-812877	nan	nan	1	4	2	0.1847	no
ni|645903473|nb|KK920957.1|	814018-814245	nan	nan	11	19	0.714081	0.5401	no
ni|645903473|nb|KK920957.1|	816655-816771	nan	nan	1	6	2	0.11815	no
ni|645903473|nb|KK920957.1|	816890-817039	nan	nan	1	5	2	0.1115	no
ni|645903473|nb|KK920957.1|	817553-817753	nan	nan	5	4	-0.0918582	0.93825	no
ni|645903473|nb|KK920957.1|	817942-818225	nan	nan	50	64	0.341044	0.7646	no
ni|645903473|nb|KK920957.1|	818976-819349	nan	nan	0.462098	0.883586	0.935171	0.4672	no
ni|645903473|nb|KK920957.1|	820470-820628	nan	nan	55	72	0.376971	0.73195	no
ni|645903473|nb|KK920957.1|	823789-824211	nan	nan	9	10	0.0365177	0.97375	no
ni|645903473|nb|KK920957.1|	824667-824908	nan	nan	14	17	0.30061	0.83645	no
ni|645903473|nb|KK920957.1|	825660-825830	nan	nan	0	7	inf	5.00E-05	yes
ni|645903473|nb|KK920957.1|	841998-842117	nan	nan	10	12	0.299428	0.80035	no
ni|645903473|nb|KK920957.1|	842563-842771	nan	nan	0	11	inf	5.00E-05	yes
ni|645903473|nb|KK920957.1|	846010-846230	nan	nan	0	2	inf	0.00055	yes
ni|645903473|nb|KK920957.1|	849030-849293	nan	nan	81	30	-1	0.23015	no
ni|645903473|nb|KK920957.1|	854056-854177	nan	nan	236	144	-0.711351	0.53955	no
ni|645903473|nb|KK920957.1|	856302-856454	nan	nan	93	67	-0.470501	0.73265	no
ni|645903473|nb|KK920957.1|	858486-858673	nan	nan	96	80	-0.269064	0.81605	no
ni|645903473|nb|KK920957.1|	863370-863631	nan	nan	0.736186	0.60322	-0.287385	0.8283	no
ni|645903473|nb|KK920957.1|	865818-866628	nan	nan	0.715996	0.760337	0.0866871	0.9436	no

ni|645903473|nb|KK920957.1|	868221-868616	nan	nan	85	62	-0.447143	0.67805	no
ni|645903473|nb|KK920957.1|	868866-869403	nan	nan	160	131	-0.285369	0.84705	no
ni|645903473|nb|KK920957.1|	869921-870118	nan	nan	121	106	-0.198881	0.89025	no
ni|645903473|nb|KK920957.1|	870271-870488	nan	nan	109	94	-0.210007	0.88225	no
ni|645903473|nb|KK920957.1|	870665-871915	nan	nan	99	80	-0.299938	0.7781	no
ni|645903473|nb|KK920957.1|	872077-872807	nan	nan	128	127	-0.0150319	0.9913	no
ni|645903473|nb|KK920957.1|	872916-873237	nan	nan	157	212	0.428973	0.71625	no
ni|645903473|nb|KK920957.1|	8764-8926	nan	nan	40	42	0.0778997	0.94195	no
ni|645903473|nb|KK920957.1|	877453-877941	nan	nan	24	28	0.264894	0.82015	no
ni|645903473|nb|KK920957.1|	878057-878897	nan	nan	8	3	-1	0.43055	no
ni|645903473|nb|KK920957.1|	890842-891218	nan	nan	3	2	-0.176582	0.88435	no
ni|645903473|nb|KK920957.1|	892830-893429	nan	nan	4	3	-0.379603	0.7454	no
ni|645903473|nb|KK920957.1|	896202-897457	nan	nan	2	3	0.329883	0.7767	no
ni|645903473|nb|KK920957.1|	897624-897928	nan	nan	2	1	-0.49585	0.6704	no
ni|645903473|nb|KK920957.1|	898411-898551	nan	nan	2	0.642452	-1	0.3464	no
ni|645903473|nb|KK920957.1|	899570-899773	nan	nan	4	4	0.191412	0.8668	no
ni|645903473|nb|KK920957.1|	904788-905294	nan	nan	0.763545	0.437859	-0.802247	0.6027	no
ni|645903473|nb|KK920957.1|	905389-905583	nan	nan	47	42	-0.139384	0.9046	no
ni|645903473|nb|KK920957.1|	905746-905880	nan	nan	0.503301	0.481141	-0.0649593	0.9467	no
ni|645903473|nb|KK920957.1|	91255-91512	nan	nan	28	26	-0.0579903	0.96155	no
ni|645903473|nb|KK920957.1|	9865-10114	nan	nan	19	20	0.0577541	0.95615	no
ni|645903474|nb|KK920956.1|	155381-155533	nan	nan	15	11	-0.526624	0.66105	no
ni|645903474|nb|KK920956.1|	16038-16492	nan	nan	5	5	0.0270799	0.97955	no
ni|645903474|nb|KK920956.1|	16575-16818	nan	nan	0.826008	0.920639	0.15648	0.9131	no
ni|645903474|nb|KK920956.1|	17051-17165	nan	nan	4	1	-1	0.2898	no
ni|645903474|nb|KK920956.1|	170869-171184	nan	nan	0	4	inf	5.00E-05	yes
ni|645903474|nb|KK920956.1|	171294-171823	nan	nan	0	16	inf	0.00015	yes
ni|645903474|nb|KK920956.1|	17238-17523	nan	nan	3	2	-0.506884	0.68965	no
ni|645903474|nb|KK920956.1|	17617-18014	nan	nan	1	0.826372	-0.436936	0.7272	no
ni|645903474|nb|KK920956.1|	179220-179625	nan	nan	0	14	inf	5.00E-05	yes

ni|645903474|nb|KK920956.1|	179764-181229	nan	nan	1	0.781814	-0.686617	0.58765	no
ni|645903474|nb|KK920956.1|	181331-181963	nan	nan	51	48	-0.0672382	0.9534	no
ni|645903474|nb|KK920956.1|	182070-182358	nan	nan	23	23	0.0470099	0.9672	no
ni|645903474|nb|KK920956.1|	182492-183376	nan	nan	18	21	0.24228	0.82355	no
ni|645903474|nb|KK920956.1|	183641-183919	nan	nan	14	20	0.434219	0.70565	no
ni|645903474|nb|KK920956.1|	18427-18947	nan	nan	4	3	-0.744425	0.54875	no
ni|645903474|nb|KK920956.1|	187145-187282	nan	nan	7	2	-2	0.166	no
ni|645903474|nb|KK920956.1|	188213-188451	nan	nan	27	45	0.739705	0.5033	no
ni|645903474|nb|KK920956.1|	191300-192445	nan	nan	22	8	-2	0.2007	no
ni|645903474|nb|KK920956.1|	195089-195233	nan	nan	73	44	-0.732915	0.5208	no
ni|645903474|nb|KK920956.1|	20241-20502	nan	nan	0.330304	0.526245	0.671938	0.65595	no
ni|645903474|nb|KK920956.1|	20610-20824	nan	nan	0.339621	0.227309	-0.579268	0.70815	no
ni|645903474|nb|KK920956.1|	20946-21106	nan	nan	0.298428	0.28533	-0.0647505	0.9384	no
ni|645903474|nb|KK920956.1|	21840-22098	nan	nan	0.248754	0.185005	-0.42716	0.75965	no
ni|645903474|nb|KK920956.1|	22520-22754	nan	nan	30	26	-0.219476	0.86075	no
ni|645903474|nb|KK920956.1|	229654-229853	nan	nan	59	36	-0.695423	0.5548	no
ni|645903474|nb|KK920956.1|	23358-23761	nan	nan	0.80421	0.56377	-0.512466	0.697	no
ni|645903474|nb|KK920956.1|	24170-24953	nan	nan	1	1	-0.212836	0.853	no
ni|645903474|nb|KK920956.1|	25435-25890	nan	nan	0.685011	0.523908	-0.386815	0.76495	no
ni|645903474|nb|KK920956.1|	271696-271963	nan	nan	27	26	-0.0665027	0.96265	no
ni|645903474|nb|KK920956.1|	272138-272437	nan	nan	53	36	-0.58524	0.60365	no
ni|645903474|nb|KK920956.1|	29646-29934	nan	nan	11	9	-0.376665	0.74405	no
ni|645903474|nb|KK920956.1|	30042-30904	nan	nan	9.29	10	0.0582265	0.9532	no
ni|645903474|nb|KK920956.1|	301872-302074	nan	nan	21	8	-1	0.32095	no
ni|645903474|nb|KK920956.1|	304797-305157	nan	nan	27	26	-0.0936115	0.93175	no
ni|645903474|nb|KK920956.1|	31176-31719	nan	nan	15	14	-0.0672124	0.95425	no
ni|645903474|nb|KK920956.1|	337292-337766	nan	nan	32	37	0.206904	0.8566	no
ni|645903474|nb|KK920956.1|	35645-35939	nan	nan	19	21	0.144214	0.8967	no
ni|645903474|nb|KK920956.1|	368295-368567	nan	nan	30	32	0.0983737	0.9326	no
ni|645903474|nb|KK920956.1|	368829-368970	nan	nan	2	3	0.708597	0.5479	no

ni|645903474|nb|KK920956.1|	370835-371407	nan	nan	21	23	0.133104	0.9093	no
ni|645903474|nb|KK920956.1|	374141-374867	nan	nan	36	25	-0.517036	0.66215	no
ni|645903474|nb|KK920956.1|	376079-376344	nan	nan	33	31	-0.106852	0.92625	no
ni|645903474|nb|KK920956.1|	49375-49503	nan	nan	16	20	0.314383	0.78	no
ni|645903474|nb|KK920956.1|	51685-52130	nan	nan	15	15	-0.00363188	0.99685	no
ni|645903474|nb|KK920956.1|	55471-55917	nan	nan	39	35	-0.155196	0.8911	no
ni|645903474|nb|KK920956.1|	64514-65240	nan	nan	37	38	0.0381445	0.9741	no
ni|645903474|nb|KK920956.1|	65382-66079	nan	nan	55	56	0.0128036	0.99005	no
ni|645903474|nb|KK920956.1|	66849-68014	nan	nan	14	13	-0.0554863	0.962	no
ni|645903474|nb|KK920956.1|	69439-69873	nan	nan	16	17	0.070333	0.95055	no
ni|645903474|nb|KK920956.1|	72666-73263	nan	nan	59	39	-0.61533	0.62875	no
ni|645903474|nb|KK920956.1|	73404-73554	nan	nan	56	50	-0.1444	0.8964	no
ni|645903474|nb|KK920956.1|	73741-74177	nan	nan	5	5	-0.118863	0.91835	no
ni|645903474|nb|KK920956.1|	74403-75172	nan	nan	6	6	-0.067228	0.9525	no
ni|645903474|nb|KK920956.1|	75281-76130	nan	nan	4	3	-0.299718	0.7955	no
ni|645903474|nb|KK920956.1|	76310-76541	nan	nan	0.622001	0.218099	-2	0.24505	no
ni|645903474|nb|KK920956.1|	76643-77482	nan	nan	0	3	inf	0.0003	yes
ni|645903474|nb|KK920956.1|	77632-77993	nan	nan	0	5	inf	0.0009	yes
ni|645903474|nb|KK920956.1|	79115-82796	nan	nan	0	12	inf	5.00E-05	yes
ni|645903474|nb|KK920956.1|	83890-84068	nan	nan	0	19	inf	0.00045	yes
ni|645903474|nb|KK920956.1|	84243-84381	nan	nan	0.861892	0.823824	-0.0651699	0.9554	no
ni|645903475|nb|KK920955.1|	101397-102021	nan	nan	15	10	-0.514803	0.68825	no
ni|645903475|nb|KK920955.1|	106524-107172	nan	nan	5	6	0.456223	0.69885	no
ni|645903475|nb|KK920955.1|	12304-12960	nan	nan	5	5	-0.0959832	0.93585	no
ni|645903475|nb|KK920955.1|	189860-190518	nan	nan	2	0.838704	-0.9134	0.4969	no
ni|645903475|nb|KK920955.1|	192861-193499	nan	nan	0.701321	0.614525	-0.190603	0.88855	no
ni|645903475|nb|KK920955.1|	217419-217504	nan	nan	1	1	-0.0660552	0.9384	no
ni|645903475|nb|KK920955.1|	220482-220693	nan	nan	2	1	-0.718362	0.61925	no
ni|645903475|nb|KK920955.1|	221186-221608	nan	nan	1	1	-0.065741	0.94765	no
ni|645903475|nb|KK920955.1|	222842-223158	nan	nan	2	3	0.65025	0.60985	no

ni|645903475|nb|KK920955.1|	224707-225090	nan	nan	0.974535	2	1	0.39265	no
ni|645903475|nb|KK920955.1|	227638-228108	nan	nan	0.330122	218	9	0.0005	yes
ni|645903475|nb|KK920955.1|	228490-228827	nan	nan	0.216313	168.67	10	0.02725	no
ni|645903475|nb|KK920955.1|	229327-229575	nan	nan	1	3	0.867009	0.5007	no
ni|645903475|nb|KK920955.1|	229963-230277	nan	nan	1	3	1	0.4471	no
ni|645903475|nb|KK920955.1|	230408-230593	nan	nan	2	5	1	0.30495	no
ni|645903475|nb|KK920955.1|	231107-231330	nan	nan	0.349456	0.334072	-0.0649531	0.9302	no
ni|645903475|nb|KK920955.1|	231950-232675	nan	nan	3	2	-0.389238	0.78555	no
ni|645903475|nb|KK920955.1|	243944-244126	nan	nan	309	264	-0.229857	0.83325	no
ni|645903475|nb|KK920955.1|	245223-245360	nan	nan	63	34	-0.887041	0.4756	no
ni|645903475|nb|KK920955.1|	246594-246793	nan	nan	178	171	-0.0570664	0.9659	no
ni|645903475|nb|KK920955.1|	246957-247407	nan	nan	46	47	0.0273304	0.986	no
ni|645903475|nb|KK920955.1|	248526-248668	nan	nan	141	148	0.0757967	0.95915	no
ni|645903475|nb|KK920955.1|	249395-249612	nan	nan	25	26	0.0682109	0.9502	no
ni|645903475|nb|KK920955.1|	250421-250737	nan	nan	2	0.991018	-0.958342	0.44215	no
ni|645903475|nb|KK920955.1|	252612-253033	nan	nan	3	2	-0.729201	0.5717	no
ni|645903475|nb|KK920955.1|	253345-253992	nan	nan	57	52	-0.139567	0.9011	no
ni|645903475|nb|KK920955.1|	255524-255657	nan	nan	12	11	-0.121865	0.91535	no
ni|645903475|nb|KK920955.1|	256450-256622	nan	nan	28	25	-0.166717	0.88045	no
ni|645903475|nb|KK920955.1|	257083-257208	nan	nan	5	1	-2	0.1142	no
ni|645903475|nb|KK920955.1|	258280-258441	nan	nan	16	14	-0.219328	0.85405	no
ni|645903475|nb|KK920955.1|	259586-259754	nan	nan	19	18	-0.0673196	0.95355	no
ni|645903475|nb|KK920955.1|	259883-260088	nan	nan	15	9	-0.804321	0.5588	no
ni|645903475|nb|KK920955.1|	261833-262104	nan	nan	4	3	-0.135785	0.9086	no
ni|645903475|nb|KK920955.1|	262416-262645	nan	nan	2	2	0.17471	0.8916	no
ni|645903475|nb|KK920955.1|	265695-265807	nan	nan	9	14	0.545771	0.65645	no
ni|645903475|nb|KK920955.1|	267971-268661	nan	nan	11	10	-0.171402	0.88315	no
ni|645903475|nb|KK920955.1|	269054-269249	nan	nan	3.8	7	0.906169	0.4469	no
ni|645903475|nb|KK920955.1|	270586-271024	nan	nan	20	29	0.546896	0.63755	no
ni|645903475|nb|KK920955.1|	271930-272293	nan	nan	15.82	26	0.729262	0.5376	no

ni|645903475|nb|KK920955.1|	272410-272868	nan	nan	5	11	1	0.3708	no
ni|645903475|nb|KK920955.1|	277558-277760	nan	nan	16	25	0.66964	0.5682	no
ni|645903475|nb|KK920955.1|	279161-279515	nan	nan	12	26	1	0.3299	no
ni|645903475|nb|KK920955.1|	280011-280477	nan	nan	8	16	1	0.3899	no
ni|645903475|nb|KK920955.1|	280819-281003	nan	nan	8	21	1	0.24285	no
ni|645903475|nb|KK920955.1|	282118-282283	nan	nan	12	31	1	0.28115	no
ni|645903475|nb|KK920955.1|	282827-282957	nan	nan	13	28	1	0.35095	no
ni|645903475|nb|KK920955.1|	283089-283230	nan	nan	7	19	1	0.293	no
ni|645903475|nb|KK920955.1|	285341-285521	nan	nan	11	24.73	1	0.34985	no
ni|645903475|nb|KK920955.1|	287274-287439	nan	nan	14	35	1	0.30485	no
ni|645903475|nb|KK920955.1|	288864-289153	nan	nan	9	11	0.286443	0.81825	no
ni|645903475|nb|KK920955.1|	293054-293443	nan	nan	7	16	1	0.3449	no
ni|645903475|nb|KK920955.1|	293507-294300	nan	nan	7	14	0.989356	0.4064	no
ni|645903475|nb|KK920955.1|	294749-295118	nan	nan	14	21	0.51774	0.66135	no
ni|645903475|nb|KK920955.1|	296295-296743	nan	nan	10	18	0.807261	0.51275	no
ni|645903475|nb|KK920955.1|	296849-297603	nan	nan	13	23	0.807265	0.502	no
ni|645903475|nb|KK920955.1|	297882-298122	nan	nan	8	13	0.710409	0.5589	no
ni|645903475|nb|KK920955.1|	299368-299525	nan	nan	0	127	inf	5.00E-05	yes
ni|645903475|nb|KK920955.1|	303399-303584	nan	nan	0	85	inf	5.00E-05	yes
ni|645903475|nb|KK920955.1|	303807-303947	nan	nan	0	21	inf	5.00E-05	yes
ni|645903475|nb|KK920955.1|	304139-304810	nan	nan	0	37	inf	5.00E-05	yes
ni|645903475|nb|KK920955.1|	306927-307567	nan	nan	0	19	inf	5.00E-05	yes
ni|645903475|nb|KK920955.1|	307958-308107	nan	nan	1	0.975859	-0.534791	0.67295	no
ni|645903475|nb|KK920955.1|	309667-310065	nan	nan	3	2	-0.289642	0.8496	no
ni|645903475|nb|KK920955.1|	310723-311091	nan	nan	2	2	0.0854359	0.9436	no
ni|645903475|nb|KK920955.1|	311332-311664	nan	nan	21	20	-0.0468066	0.9691	no
ni|645903475|nb|KK920955.1|	312045-312349	nan	nan	35	47	0.424102	0.7718	no
ni|645903475|nb|KK920955.1|	318636-319075	nan	nan	130	149	0.187804	0.87265	no
ni|645903475|nb|KK920955.1|	319712-319997	nan	nan	14	15	0.170318	0.87155	no
ni|645903475|nb|KK920955.1|	321783-322097	nan	nan	3	3	0.0703966	0.94355	no

ni|645903475|nb|KK920955.1|	322370-322568	nan	nan	0.826008	1	0.51905	0.7279	no
ni|645903475|nb|KK920955.1|	322751-322892	nan	nan	0.865292	0.578864	-0.579962	0.70815	no
ni|645903475|nb|KK920955.1|	323024-323485	nan	nan	7	7	-0.067279	0.9605	no
ni|645903475|nb|KK920955.1|	331394-331645	nan	nan	5	3	-0.804169	0.6027	no
ni|645903475|nb|KK920955.1|	333969-334252	nan	nan	11	0.711644	-4	0.02485	no
ni|645903475|nb|KK920955.1|	335319-335851	nan	nan	22	20	-0.162674	0.88515	no
ni|645903475|nb|KK920955.1|	337381-337745	nan	nan	17	14	-0.271781	0.81725	no
ni|645903475|nb|KK920955.1|	337870-338014	nan	nan	5	4	-0.199216	0.8686	no
ni|645903475|nb|KK920955.1|	338204-338478	nan	nan	905	680	-0.411684	0.694	no
ni|645903475|nb|KK920955.1|	340102-340221	nan	nan	796	587	-0.439734	0.71005	no
ni|645903475|nb|KK920955.1|	340630-340881	nan	nan	1370.05	1168.7	-0.229323	0.78235	no
ni|645903475|nb|KK920955.1|	340986-341168	nan	nan	792	667	-0.247741	0.82275	no
ni|645903475|nb|KK920955.1|	341914-342080	nan	nan	978	844	-0.212211	0.82465	no
ni|645903475|nb|KK920955.1|	343864-344023	nan	nan	3	3	-0.177712	0.8751	no
ni|645903475|nb|KK920955.1|	345499-345798	nan	nan	2	1	-0.10132	0.9297	no
ni|645903475|nb|KK920955.1|	347610-348001	nan	nan	6	6	-0.116519	0.92245	no
ni|645903475|nb|KK920955.1|	349635-349959	nan	nan	13	6	-1	0.3256	no
ni|645903475|nb|KK920955.1|	350030-350264	nan	nan	7	3.55	-0.944966	0.4255	no
ni|645903475|nb|KK920955.1|	350542-350697	nan	nan	23	11	-1	0.3559	no
ni|645903475|nb|KK920955.1|	350936-351331	nan	nan	10	6	-0.713045	0.53725	no
ni|645903475|nb|KK920955.1|	35152-35808	nan	nan	24	23	-0.0513205	0.9647	no
ni|645903475|nb|KK920955.1|	351742-352121	nan	nan	22	23	0.032041	0.96125	no
ni|645903475|nb|KK920955.1|	352742-353045	nan	nan	17	13	-0.434934	0.71445	no
ni|645903475|nb|KK920955.1|	353179-353508	nan	nan	21	16	-0.354523	0.76125	no
ni|645903475|nb|KK920955.1|	353651-353889	nan	nan	8	10	0.235109	0.8387	no
ni|645903475|nb|KK920955.1|	354314-354536	nan	nan	20	20	0.0116774	0.9915	no
ni|645903475|nb|KK920955.1|	354616-354769	nan	nan	17	14	-0.259541	0.8245	no
ni|645903475|nb|KK920955.1|	354901-355097	nan	nan	8	7	-0.192894	0.8638	no
ni|645903475|nb|KK920955.1|	357237-357342	nan	nan	17	12	-0.545244	0.64545	no
ni|645903475|nb|KK920955.1|	358411-358638	nan	nan	15	18	0.268324	0.81815	no

ni|645903475|nb|KK920955.1|	360060-360436	nan	nan	17	11	-0.606606	0.60915	no
ni|645903475|nb|KK920955.1|	360838-361408	nan	nan	16	16	-0.067271	0.957	no
ni|645903475|nb|KK920955.1|	362016-362386	nan	nan	14	15	0.0438329	0.9707	no
ni|645903475|nb|KK920955.1|	363746-363915	nan	nan	17	17	0.0259437	0.9825	no
ni|645903475|nb|KK920955.1|	368790-369056	nan	nan	4	4	-0.207855	0.85945	no
ni|645903475|nb|KK920955.1|	369174-369398	nan	nan	9	5	-0.929693	0.47045	no
ni|645903475|nb|KK920955.1|	369749-370110	nan	nan	211	126	-0.744493	0.56215	no
ni|645903475|nb|KK920955.1|	372153-372320	nan	nan	1	0.894706	-0.355089	0.7931	no
ni|645903475|nb|KK920955.1|	374834-375062	nan	nan	268	146	-0.876441	0.5328	no
ni|645903475|nb|KK920955.1|	375570-375723	nan	nan	8	8	0.00347571	0.9904	no
ni|645903475|nb|KK920955.1|	380956-381220	nan	nan	57	53	-0.10612	0.9241	no
ni|645903475|nb|KK920955.1|	381879-383834	nan	nan	17	20	0.24872	0.83165	no
ni|645903475|nb|KK920955.1|	480053-480111	nan	nan	55	55	-0.00829864	0.99385	no
ni|645903475|nb|KK920955.1|	569386-569528	nan	nan	53	55	0.0497162	0.9637	no
ni|645903475|nb|KK920955.1|	569994-570471	nan	nan	39	39	-0.00825244	0.99475	no
ni|645903475|nb|KK920955.1|	592451-592660	nan	nan	61	59	-0.0358799	0.9753	no
ni|645903475|nb|KK920955.1|	657238-657351	nan	nan	49	46	-0.0997996	0.94795	no
ni|645903475|nb|KK920955.1|	661170-661382	nan	nan	10	7	-0.412117	0.72175	no
ni|645903475|nb|KK920955.1|	661541-661707	nan	nan	104	80	-0.386557	0.7402	no
ni|645903475|nb|KK920955.1|	665056-665252	nan	nan	63	44	-0.527417	0.6317	no
ni|645903475|nb|KK920955.1|	667421-667584	nan	nan	70	108	0.620818	0.59475	no
ni|645903475|nb|KK920955.1|	669059-669239	nan	nan	8	15	0.817197	0.4959	no
ni|645903475|nb|KK920955.1|	671953-672097	nan	nan	0.141613	12	6	0.11945	no
ni|645903475|nb|KK920955.1|	672213-672363	nan	nan	20	72	2	0.12035	no
ni|645903475|nb|KK920955.1|	672493-672659	nan	nan	6	19	2	0.1684	no
ni|645903475|nb|KK920955.1|	681476-681614	nan	nan	23	68	2	0.1864	no
ni|645903475|nb|KK920955.1|	681828-682474	nan	nan	9	27	2	0.15425	no
ni|645903475|nb|KK920955.1|	686650-686893	nan	nan	40	91	1	0.3169	no
ni|645903475|nb|KK920955.1|	687694-687912	nan	nan	21	50	1	0.3352	no
ni|645903475|nb|KK920955.1|	690409-690553	nan	nan	15	13	-0.173311	0.8792	no

ni|645903475|nb|KK920955.1|	691654-691869	nan	nan	10	7	-0.389122	0.7511	no
ni|645903475|nb|KK920955.1|	693335-693475	nan	nan	29	34	0.25469	0.8268	no
ni|645903475|nb|KK920955.1|	695659-696018	nan	nan	2	2	0.296658	0.8138	no
ni|645903475|nb|KK920955.1|	697310-697682	nan	nan	130	107	-0.278836	0.8446	no
ni|645903475|nb|KK920955.1|	704660-704789	nan	nan	3	4	0.254723	0.86075	no
ni|645903475|nb|KK920955.1|	705285-705933	nan	nan	1	1	-0.364382	0.75765	no
ni|645903475|nb|KK920955.1|	706875-707309	nan	nan	7	6	-0.166728	0.89925	no
ni|645903475|nb|KK920955.1|	707473-707714	nan	nan	3	3	0.255096	0.84735	no
ni|645903475|nb|KK920955.1|	708017-708414	nan	nan	24	21	-0.249136	0.81145	no
ni|645903475|nb|KK920955.1|	709508-710131	nan	nan	6	5	-0.331184	0.77465	no
ni|645903475|nb|KK920955.1|	710786-711696	nan	nan	16	8	-1	0.46615	no
ni|645903475|nb|KK920955.1|	713633-714090	nan	nan	3	2	-0.480268	0.68705	no
ni|645903475|nb|KK920955.1|	714814-714981	nan	nan	29	49	0.757187	0.50635	no
ni|645903475|nb|KK920955.1|	716236-716351	nan	nan	2	4	0.98852	0.44185	no
ni|645903475|nb|KK920955.1|	735-1352	nan	nan	35	29	-0.303648	0.7987	no
ni|645903475|nb|KK920955.1|	89381-90096	nan	nan	6	6	0.0198216	0.9872	no
ni|645903476|nb|KK920954.1|	160230-160613	nan	nan	1	0.292494	-2	0.18825	no
ni|645903476|nb|KK920954.1|	161325-161464	nan	nan	2	0.70373	-1	0.3554	no
ni|645903476|nb|KK920954.1|	161599-161887	nan	nan	1	1	-0.347071	0.77075	no
ni|645903476|nb|KK920954.1|	163656-163827	nan	nan	11	18	0.748575	0.51565	no
ni|645903476|nb|KK920954.1|	164076-164273	nan	nan	17	26	0.618995	0.5915	no
ni|645903476|nb|KK920954.1|	174685-174918	nan	nan	15	24	0.673266	0.5531	no
ni|645903476|nb|KK920954.1|	175926-176047	nan	nan	6	0	-inf	0.0013	yes
ni|645903476|nb|KK920954.1|	179571-180145	nan	nan	52	47	-0.130115	0.9114	no
ni|645903476|nb|KK920954.1|	182005-182401	nan	nan	15	6	-1	0.25725	no
ni|645903476|nb|KK920954.1|	18216-18347	nan	nan	6	4	-0.321287	0.7907	no
ni|645903476|nb|KK920954.1|	185793-186425	nan	nan	3	0.995221	-2	0.2166	no
ni|645903476|nb|KK920954.1|	187986-188486	nan	nan	3	2	-0.246819	0.84435	no
ni|645903476|nb|KK920954.1|	188719-188899	nan	nan	4	0.478397	-3	0.2572	no
ni|645903476|nb|KK920954.1|	189784-190044	nan	nan	4	2	-0.802815	0.51605	no

ni|645903476|nb|KK920954.1|	192480-192971	nan	nan	4	1	-1	0.3464	no
ni|645903476|nb|KK920954.1|	193099-193691	nan	nan	6	0.037781	-7	0.0173	no
ni|645903476|nb|KK920954.1|	196678-197574	nan	nan	5	0	-inf	0.00045	yes
ni|645903476|nb|KK920954.1|	197941-198949	nan	nan	17	0.403978	-5	0.01745	no
ni|645903476|nb|KK920954.1|	201463-201606	nan	nan	5	0.054071	-7	0.2314	no
ni|645903476|nb|KK920954.1|	201722-201840	nan	nan	20	10	-0.941738	0.4199	no
ni|645903476|nb|KK920954.1|	20734-20856	nan	nan	5	5	-0.0663388	0.9578	no
ni|645903476|nb|KK920954.1|	208201-208370	nan	nan	5	3	-0.625364	0.593	no
ni|645903476|nb|KK920954.1|	20920-21171	nan	nan	3	3	-0.246011	0.8347	no
ni|645903476|nb|KK920954.1|	212115-212357	nan	nan	10	8	-0.39134	0.7265	no
ni|645903476|nb|KK920954.1|	215547-215694	nan	nan	3	2	-0.307774	0.7857	no
ni|645903476|nb|KK920954.1|	223937-224575	nan	nan	2	2	-0.307005	0.8123	no
ni|645903476|nb|KK920954.1|	224703-225230	nan	nan	1	0.56373	-1	0.4023	no
ni|645903476|nb|KK920954.1|	22618-22896	nan	nan	3	3	-0.0785622	0.94505	no
ni|645903476|nb|KK920954.1|	227136-227301	nan	nan	0.635081	0.354144	-0.842605	0.5595	no
ni|645903476|nb|KK920954.1|	231170-231318	nan	nan	0.336931	2	2	0.1002	no
ni|645903476|nb|KK920954.1|	231466-231844	nan	nan	0.974535	3	2	0.28535	no
ni|645903476|nb|KK920954.1|	23184-23375	nan	nan	94	94	0.00848242	0.99515	no
ni|645903476|nb|KK920954.1|	233696-233947	nan	nan	1	2	0.809192	0.50525	no
ni|645903476|nb|KK920954.1|	23535-23745	nan	nan	14	32	1	0.303	no
ni|645903476|nb|KK920954.1|	239611-239798	nan	nan	56	49	-0.186787	0.86055	no
ni|645903476|nb|KK920954.1|	298787-298973	nan	nan	61	56	-0.115911	0.91375	no
ni|645903476|nb|KK920954.1|	299165-299488	nan	nan	83	82	-0.0204278	0.98425	no
ni|645903476|nb|KK920954.1|	300448-302305	nan	nan	1	0.702446	-0.765331	0.5354	no
ni|645903476|nb|KK920954.1|	304499-305243	nan	nan	0.906644	1	0.151112	0.89715	no
ni|645903476|nb|KK920954.1|	305470-306275	nan	nan	3	3	0.0142936	0.99065	no
ni|645903476|nb|KK920954.1|	31247-31435	nan	nan	19	26	0.490358	0.6599	no
ni|645903476|nb|KK920954.1|	317335-317602	nan	nan	1	2	0.442405	0.69915	no
ni|645903476|nb|KK920954.1|	320100-320427	nan	nan	28	10	-2	0.2075	no
ni|645903476|nb|KK920954.1|	323622-324019	nan	nan	26	9	-2	0.20965	no

ni|645903476|nb|KK920954.1|	324529-324706	nan	nan	41	31	-0.403689	0.7409	no
ni|645903476|nb|KK920954.1|	325186-325536	nan	nan	4	2	-0.703286	0.56815	no
ni|645903476|nb|KK920954.1|	325703-325947	nan	nan	54	64	0.244006	0.8346	no
ni|645903476|nb|KK920954.1|	326097-326601	nan	nan	0.572088	1	0.852401	0.521	no
ni|645903476|nb|KK920954.1|	327370-327686	nan	nan	6	11	0.933125	0.43015	no
ni|645903476|nb|KK920954.1|	339515-339837	nan	nan	0.690848	0.462224	-0.579777	0.70815	no
ni|645903476|nb|KK920954.1|	340306-340513	nan	nan	0.719621	0.187593	-2	0.24815	no
ni|645903476|nb|KK920954.1|	340739-341078	nan	nan	4	6	0.669733	0.65595	no
ni|645903476|nb|KK920954.1|	341569-341921	nan	nan	12	18	0.584557	0.6992	no
ni|645903476|nb|KK920954.1|	343083-344710	nan	nan	6	4	-0.580052	0.62525	no
ni|645903476|nb|KK920954.1|	344892-345796	nan	nan	42	25	-0.736267	0.49095	no
ni|645903476|nb|KK920954.1|	347325-347510	nan	nan	232	176	-0.394684	0.73445	no
ni|645903476|nb|KK920954.1|	350421-351295	nan	nan	271	279	0.0462683	0.97535	no
ni|645903476|nb|KK920954.1|	361501-361703	nan	nan	1	6	2	0.1054	no
ni|645903476|nb|KK920954.1|	36345-36583	nan	nan	30	44	0.576629	0.62035	no
ni|645903476|nb|KK920954.1|	364947-365380	nan	nan	0	7	inf	5.00E-05	yes
ni|645903476|nb|KK920954.1|	365531-366449	nan	nan	2	3	0.30225	0.7926	no
ni|645903476|nb|KK920954.1|	369063-369174	nan	nan	7	10	0.527944	0.627	no
ni|645903476|nb|KK920954.1|	369835-370117	nan	nan	0.06542	1	4	0.1221	no
ni|645903476|nb|KK920954.1|	370277-370719	nan	nan	3	2	-0.434733	0.7172	no
ni|645903476|nb|KK920954.1|	370914-371115	nan	nan	3	2	-0.960046	0.51205	no
ni|645903476|nb|KK920954.1|	371269-371497	nan	nan	0.961963	0.804219	-0.258392	0.8692	no
ni|645903476|nb|KK920954.1|	377320-377493	nan	nan	3	1	-0.979925	0.4141	no
ni|645903476|nb|KK920954.1|	377957-378254	nan	nan	48	50	0.055847	0.9701	no
ni|645903476|nb|KK920954.1|	378970-379545	nan	nan	11	5	-0.984765	0.48245	no
ni|645903476|nb|KK920954.1|	379649-380509	nan	nan	5	2	-1	0.35155	no
ni|645903476|nb|KK920954.1|	380913-381107	nan	nan	0	6	inf	0.0005	yes
ni|645903476|nb|KK920954.1|	381947-382291	nan	nan	0	5	inf	5.00E-05	yes
ni|645903476|nb|KK920954.1|	382582-382840	nan	nan	1	13	4	0.01885	no
ni|645903476|nb|KK920954.1|	383343-384117	nan	nan	2	6	2	0.1736	no

ni|645903476|nb|KK920954.1|	384232-384339	nan	nan	3	8	1	0.2965	no
ni|645903476|nb|KK920954.1|	385405-386053	nan	nan	0.224123	128	9	0.0443	no
ni|645903476|nb|KK920954.1|	389053-389411	nan	nan	0	2	inf	0.00015	yes
ni|645903476|nb|KK920954.1|	390404-390587	nan	nan	0	261	inf	5.00E-05	yes
ni|645903476|nb|KK920954.1|	390784-391022	nan	nan	0	9	inf	0.00055	yes
ni|645903476|nb|KK920954.1|	391143-391517	nan	nan	0.758194	405	9	0.04435	no
ni|645903476|nb|KK920954.1|	395234-395530	nan	nan	0	107	inf	5.00E-05	yes
ni|645903476|nb|KK920954.1|	395647-395946	nan	nan	0.424374	1	2	0.2479	no
ni|645903476|nb|KK920954.1|	396563-396900	nan	nan	0.168156	1	3	0.08575	no
ni|645903476|nb|KK920954.1|	397428-397875	nan	nan	21	18	-0.159156	0.9118	no
ni|645903476|nb|KK920954.1|	398644-399074	nan	nan	2	1	-0.512174	0.66805	no
ni|645903476|nb|KK920954.1|	400901-402366	nan	nan	9	7	-0.409936	0.71725	no
ni|645903476|nb|KK920954.1|	403202-404256	nan	nan	8	10	0.347618	0.8193	no
ni|645903476|nb|KK920954.1|	410034-410156	nan	nan	2	1	-0.413549	0.76315	no
ni|645903476|nb|KK920954.1|	41443-41634	nan	nan	16	24	0.571427	0.65705	no
ni|645903476|nb|KK920954.1|	416201-416475	nan	nan	27	24	-0.185843	0.894	no
ni|645903476|nb|KK920954.1|	417210-417446	nan	nan	9	12	0.44186	0.70795	no
ni|645903476|nb|KK920954.1|	419052-419170	nan	nan	11	16	0.611498	0.5883	no
ni|645903476|nb|KK920954.1|	42426-42594	nan	nan	30	31	0.0402033	0.97095	no
ni|645903476|nb|KK920954.1|	48165-48349	nan	nan	17	19	0.235731	0.8281	no
ni|645903476|nb|KK920954.1|	52486-52669	nan	nan	23	30	0.379846	0.7694	no
ni|645903476|nb|KK920954.1|	66444-67407	nan	nan	43	49	0.187765	0.8692	no
ni|645903476|nb|KK920954.1|	81253-82042	nan	nan	2	2	-0.0663527	0.9554	no
ni|645903476|nb|KK920954.1|	82577-82763	nan	nan	8	15	0.818261	0.48375	no
ni|645903476|nb|KK920954.1|	88528-88657	nan	nan	8	15	0.91866	0.3992	no
ni|645903477|nb|KK920953.1|	101440-101611	nan	nan	1040.74	812	-0.358911	0.80345	no
ni|645903477|nb|KK920953.1|	11073-11317	nan	nan	2	1	-0.328037	0.7837	no
ni|645903477|nb|KK920953.1|	119989-120461	nan	nan	497	404	-0.298226	0.83695	no
ni|645903477|nb|KK920953.1|	130218-130540	nan	nan	41	35	-0.251261	0.82005	no
ni|645903477|nb|KK920953.1|	130802-130920	nan	nan	2075.93	1838.73	-0.175043	0.8997	no

ni|645903477|nb|KK920953.1|	131164-131415	nan	nan	3	1	-1	0.3131	no
ni|645903477|nb|KK920953.1|	131809-131957	nan	nan	2	0.35617	-3	0.28285	no
ni|645903477|nb|KK920953.1|	132475-134069	nan	nan	1206.78	1137.3	-0.0855513	0.948	no
ni|645903477|nb|KK920953.1|	134184-135504	nan	nan	15	2	-3	0.0785	no
ni|645903477|nb|KK920953.1|	136371-136805	nan	nan	1468.98	1403.16	-0.0661317	0.9625	no
ni|645903477|nb|KK920953.1|	139975-140253	nan	nan	1303.28	1324.55	0.0233557	0.98635	no
ni|645903477|nb|KK920953.1|	140375-140701	nan	nan	15	6	-1	0.3211	no
ni|645903477|nb|KK920953.1|	14156-14318	nan	nan	32.36	46	0.515625	0.64905	no
ni|645903477|nb|KK920953.1|	149204-149404	nan	nan	2	1	-1	0.383	no
ni|645903477|nb|KK920953.1|	15487-15908	nan	nan	0.793459	0.189604	-2	0.20695	no
ni|645903477|nb|KK920953.1|	158953-159265	nan	nan	34	35	0.0767299	0.9441	no
ni|645903477|nb|KK920953.1|	160298-160477	nan	nan	19	21	0.106781	0.9249	no
ni|645903477|nb|KK920953.1|	160668-160832	nan	nan	5	5	-0.0671955	0.94715	no
ni|645903477|nb|KK920953.1|	160902-161010	nan	nan	8	3	-2	0.2821	no
ni|645903477|nb|KK920953.1|	161632-162125	nan	nan	149	85	-0.810802	0.49435	no
ni|645903477|nb|KK920953.1|	162519-162651	nan	nan	38	28	-0.474382	0.6605	no
ni|645903477|nb|KK920953.1|	165986-166163	nan	nan	99	70	-0.49926	0.69105	no
ni|645903477|nb|KK920953.1|	166620-167389	nan	nan	88	66	-0.415035	0.70725	no
ni|645903477|nb|KK920953.1|	167667-167911	nan	nan	37	57	0.630498	0.57445	no
ni|645903477|nb|KK920953.1|	168455-168609	nan	nan	81	176	1	0.38295	no
ni|645903477|nb|KK920953.1|	170027-170284	nan	nan	2	4	0.78813	0.5028	no
ni|645903477|nb|KK920953.1|	170893-172341	nan	nan	8	2	-2	0.22995	no
ni|645903477|nb|KK920953.1|	172486-172988	nan	nan	1	3	1	0.32335	no
ni|645903477|nb|KK920953.1|	174892-175123	nan	nan	64	177.69	1	0.24405	no
ni|645903477|nb|KK920953.1|	17514-17732	nan	nan	1	0.523508	-1	0.3141	no
ni|645903477|nb|KK920953.1|	175832-176272	nan	nan	9	15	0.835512	0.47835	no
ni|645903477|nb|KK920953.1|	179983-181005	nan	nan	22	25	0.168359	0.88585	no
ni|645903477|nb|KK920953.1|	184326-184742	nan	nan	13	17	0.366819	0.7398	no
ni|645903477|nb|KK920953.1|	184887-185123	nan	nan	28	35	0.324548	0.77525	no
ni|645903477|nb|KK920953.1|	201517-202281	nan	nan	8	8	-0.0935273	0.9329	no

ni|645903477|nb|KK920953.1|	205253-205465	nan	nan	25.8	32	0.29034	0.80595	no
ni|645903477|nb|KK920953.1|	207677-208355	nan	nan	20	21	0.0954934	0.93145	no
ni|645903477|nb|KK920953.1|	212476-213233	nan	nan	30	36	0.259032	0.82685	no
ni|645903477|nb|KK920953.1|	216275-216615	nan	nan	46	36	-0.354115	0.7601	no
ni|645903477|nb|KK920953.1|	254866-255164	nan	nan	28	13	-1	0.44755	no
ni|645903477|nb|KK920953.1|	294697-294845	nan	nan	35	39	0.137856	0.9011	no
ni|645903477|nb|KK920953.1|	294968-295156	nan	nan	4	3	-0.650819	0.5904	no
ni|645903477|nb|KK920953.1|	298721-298930	nan	nan	3	7	1	0.37595	no
ni|645903477|nb|KK920953.1|	300681-300884	nan	nan	6	7	0.146998	0.8981	no
ni|645903477|nb|KK920953.1|	302982-303435	nan	nan	6	6	0.114162	0.92325	no
ni|645903477|nb|KK920953.1|	305567-305764	nan	nan	2	0	-inf	0.00455	no
ni|645903477|nb|KK920953.1|	308585-308748	nan	nan	2	0	-inf	0.0008	yes
ni|645903477|nb|KK920953.1|	311291-311626	nan	nan	8	10	0.341016	0.77145	no
ni|645903477|nb|KK920953.1|	313469-313816	nan	nan	36	30	-0.238584	0.84005	no
ni|645903477|nb|KK920953.1|	38570-38849	nan	nan	12	17	0.475751	0.6797	no
ni|645903477|nb|KK920953.1|	39150-39439	nan	nan	32	59	0.895399	0.43765	no
ni|645903477|nb|KK920953.1|	39539-39723	nan	nan	12	15	0.302025	0.79145	no
ni|645903477|nb|KK920953.1|	49484-49748	nan	nan	37	54	0.539071	0.6259	no
ni|645903477|nb|KK920953.1|	50313-50664	nan	nan	30	34	0.212825	0.8556	no
ni|645903477|nb|KK920953.1|	52596-52869	nan	nan	35	46	0.397873	0.716	no
ni|645903477|nb|KK920953.1|	53450-53603	nan	nan	9	12	0.338777	0.7656	no
ni|645903477|nb|KK920953.1|	53753-54263	nan	nan	44	53	0.247154	0.82855	no
ni|645903477|nb|KK920953.1|	54687-55124	nan	nan	40	60	0.589915	0.61385	no
ni|645903477|nb|KK920953.1|	55220-55452	nan	nan	20	20	0.0628107	0.95675	no
ni|645903477|nb|KK920953.1|	55619-56290	nan	nan	36	28	-0.36856	0.74205	no
ni|645903477|nb|KK920953.1|	56626-58124	nan	nan	13	31	1	0.3964	no
ni|645903477|nb|KK920953.1|	58246-58736	nan	nan	39	97	1	0.28245	no
ni|645903477|nb|KK920953.1|	59063-59233	nan	nan	14	11	-0.259949	0.8388	no
ni|645903477|nb|KK920953.1|	59338-59478	nan	nan	15	6	-1	0.37315	no
ni|645903477|nb|KK920953.1|	64046-64278	nan	nan	6	3	-1	0.3203	no

ni|645903477|nb|KK920953.1|	64892-65540	nan	nan	7	5	-0.580947	0.6186	no
ni|645903477|nb|KK920953.1|	67508-67964	nan	nan	14	7	-1	0.3853	no
ni|645903477|nb|KK920953.1|	68402-68693	nan	nan	4	2	-0.759078	0.51715	no
ni|645903477|nb|KK920953.1|	6918-7082	nan	nan	1	0.498073	-1	0.37315	no
ni|645903477|nb|KK920953.1|	74557-75212	nan	nan	0.843188	0.60444	-0.480255	0.7301	no
ni|645903477|nb|KK920953.1|	86446-86570	nan	nan	10	7	-0.482442	0.7594	no
ni|645903477|nb|KK920953.1|	86681-86900	nan	nan	1323.85	846	-0.645495	0.66175	no
ni|645903477|nb|KK920953.1|	92002-92137	nan	nan	6	3	-1	0.3272	no
ni|645903477|nb|KK920953.1|	97711-97970	nan	nan	2108.62	1426.51	-0.563804	0.5932	no
ni|645903478|nb|KK920952.1|	101774-101936	nan	nan	32	32	-0.00313353	0.98685	no
ni|645903478|nb|KK920952.1|	102564-102783	nan	nan	0.040161	12	8	0.11935	no
ni|645903478|nb|KK920952.1|	103984-104369	nan	nan	28	29	0.0541127	0.9619	no
ni|645903478|nb|KK920952.1|	108914-109052	nan	nan	28	28	-0.0224048	0.98295	no
ni|645903478|nb|KK920952.1|	109314-109431	nan	nan	12	12	0.020959	0.9848	no
ni|645903478|nb|KK920952.1|	115290-116278	nan	nan	19	18	-0.0308405	0.9787	no
ni|645903478|nb|KK920952.1|	86819-87082	nan	nan	14	16	0.1957	0.87315	no
ni|645903478|nb|KK920952.1|	91804-92065	nan	nan	30	25	-0.214161	0.85595	no
ni|645903478|nb|KK920952.1|	95548-95718	nan	nan	23	21	-0.166757	0.8828	no
ni|645903478|nb|KK920952.1|	96730-96922	nan	nan	12	14	0.276838	0.814	no
ni|645903479|nb|KK920951.1|	100337-100501	nan	nan	4	5	0.172627	0.87945	no
ni|645903479|nb|KK920951.1|	100599-100897	nan	nan	16	13	-0.338586	0.75845	no
ni|645903479|nb|KK920951.1|	101367-101574	nan	nan	3	2	-0.512051	0.6615	no
ni|645903479|nb|KK920951.1|	101953-102262	nan	nan	45	46	0.0345006	0.9774	no
ni|645903479|nb|KK920951.1|	103480-103685	nan	nan	17	15	-0.154616	0.88965	no
ni|645903479|nb|KK920951.1|	107172-107351	nan	nan	9	3	-2	0.24585	no
ni|645903479|nb|KK920951.1|	107730-109328	nan	nan	27	17	-0.681916	0.5602	no
ni|645903479|nb|KK920951.1|	109430-110427	nan	nan	35	34	-0.0548005	0.9599	no
ni|645903479|nb|KK920951.1|	111604-111899	nan	nan	14	9	-0.609159	0.60505	no
ni|645903479|nb|KK920951.1|	112478-112636	nan	nan	7	2	-1	0.24025	no
ni|645903479|nb|KK920951.1|	120120-120246	nan	nan	10	8	-0.261958	0.8189	no

ni|645903479|nb|KK920951.1|	144869-145057	nan	nan	10	9	-0.20139	0.8635	no
ni|645903479|nb|KK920951.1|	160146-160272	nan	nan	9	8	-0.17937	0.8766	no
ni|645903479|nb|KK920951.1|	166499-166643	nan	nan	2	1	-0.650603	0.60395	no
ni|645903479|nb|KK920951.1|	174341-175059	nan	nan	1	0.743057	-0.768354	0.51165	no
ni|645903479|nb|KK920951.1|	208586-209284	nan	nan	14	10.64	-0.444439	0.70665	no
ni|645903479|nb|KK920951.1|	211859-212528	nan	nan	1	1	-0.0657031	0.9467	no
ni|645903479|nb|KK920951.1|	213291-214141	nan	nan	7	11	0.780759	0.55245	no
ni|645903479|nb|KK920951.1|	214854-215124	nan	nan	9	8	-0.0292979	0.9811	no
ni|645903479|nb|KK920951.1|	216183-216394	nan	nan	14	15	0.0702397	0.9588	no
ni|645903479|nb|KK920951.1|	226095-226582	nan	nan	25	15	-0.767784	0.5318	no
ni|645903479|nb|KK920951.1|	228943-229189	nan	nan	15	8	-0.855782	0.5047	no
ni|645903479|nb|KK920951.1|	230019-230517	nan	nan	8	8	-0.0463065	0.96615	no
ni|645903479|nb|KK920951.1|	231176-231348	nan	nan	11	11	0.0093744	0.9934	no
ni|645903479|nb|KK920951.1|	232364-232538	nan	nan	17	14	-0.218593	0.845	no
ni|645903479|nb|KK920951.1|	232820-232997	nan	nan	8	7	-0.0892401	0.93955	no
ni|645903479|nb|KK920951.1|	233062-233184	nan	nan	20	20	0.0140586	0.99135	no
ni|645903479|nb|KK920951.1|	233683-233924	nan	nan	20	16	-0.316683	0.78495	no
ni|645903479|nb|KK920951.1|	234186-234596	nan	nan	33	18	-0.822292	0.50305	no
ni|645903479|nb|KK920951.1|	234798-235672	nan	nan	3	2	-0.481349	0.7105	no
ni|645903479|nb|KK920951.1|	237104-238009	nan	nan	4	2	-0.832119	0.5313	no
ni|645903479|nb|KK920951.1|	238694-239010	nan	nan	4	2	-0.915199	0.53575	no
ni|645903479|nb|KK920951.1|	239289-239755	nan	nan	4	2	-1	0.44755	no
ni|645903479|nb|KK920951.1|	240846-240982	nan	nan	4	4	-0.0672039	0.9302	no
ni|645903479|nb|KK920951.1|	243209-244684	nan	nan	2	1	-0.429041	0.75965	no
ni|645903479|nb|KK920951.1|	245173-245456	nan	nan	99	56	-0.835321	0.4794	no
ni|645903479|nb|KK920951.1|	248517-248786	nan	nan	19	15	-0.312383	0.78955	no
ni|645903479|nb|KK920951.1|	255237-255349	nan	nan	16	8	-1	0.4093	no
ni|645903479|nb|KK920951.1|	258276-258523	nan	nan	21	14	-0.562339	0.62385	no
ni|645903479|nb|KK920951.1|	262257-262476	nan	nan	20	12	-0.683987	0.55455	no
ni|645903479|nb|KK920951.1|	266706-266825	nan	nan	0.543057	4	3	0.06975	no

ni|645903479|nb|KK920951.1|	280950-281331	nan	nan	24	36	0.583886	0.6782	no
ni|645903479|nb|KK920951.1|	281703-281838	nan	nan	0.349051	14	5	0.0067	no
ni|645903479|nb|KK920951.1|	284950-285220	nan	nan	200	208	0.0548219	0.96855	no
ni|645903479|nb|KK920951.1|	285730-285868	nan	nan	83	79	-0.0673236	0.955	no
ni|645903479|nb|KK920951.1|	301578-302695	nan	nan	93	84	-0.143959	0.91755	no
ni|645903479|nb|KK920951.1|	306926-307374	nan	nan	8	8	-0.0200458	0.98665	no
ni|645903479|nb|KK920951.1|	328621-328962	nan	nan	55	181	2	0.14855	no
ni|645903479|nb|KK920951.1|	6900-7068	nan	nan	12	13	0.176421	0.88285	no
ni|645903479|nb|KK920951.1|	80602-81551	nan	nan	2	0.192578	-4	0.0326	no
ni|645903479|nb|KK920951.1|	87493-87617	nan	nan	2	1	-0.649968	0.58665	no
ni|645903479|nb|KK920951.1|	87940-88930	nan	nan	0.7622	0.624524	-0.287412	0.8283	no
ni|645903479|nb|KK920951.1|	89371-89800	nan	nan	14	15	0.138745	0.90095	no
ni|645903479|nb|KK920951.1|	90375-90498	nan	nan	28	26	-0.122647	0.9139	no
ni|645903479|nb|KK920951.1|	96554-96940	nan	nan	1	2	0.634204	0.63975	no
ni|645903479|nb|KK920951.1|	98252-98660	nan	nan	17	14	-0.302216	0.79605	no
ni|645903479|nb|KK920951.1|	98762-99042	nan	nan	19	28	0.503099	0.66925	no
ni|645903479|nb|KK920951.1|	99118-99345	nan	nan	34	25	-0.426678	0.70735	no
ni|645903480|nb|KK920950.1|	140119-140817	nan	nan	151	104	-0.532858	0.7212	no
ni|645903480|nb|KK920950.1|	159803-159912	nan	nan	4	6	0.720045	0.54195	no
ni|645903480|nb|KK920950.1|	22940-23121	nan	nan	2	2	-0.235003	0.8403	no
ni|645903480|nb|KK920950.1|	247529-247636	nan	nan	0.116283	63	9	0.11935	no
ni|645903480|nb|KK920950.1|	32237-34143	nan	nan	1	2	0.103653	0.93335	no
ni|645903480|nb|KK920950.1|	39023-39281	nan	nan	3	3	-0.327723	0.7793	no
ni|645903480|nb|KK920950.1|	40154-40311	nan	nan	7	8	0.216743	0.8514	no
ni|645903480|nb|KK920950.1|	40450-40784	nan	nan	2	1	-0.56802	0.6631	no
ni|645903480|nb|KK920950.1|	40846-41029	nan	nan	0.471253	6	4	0.04225	no
ni|645903480|nb|KK920950.1|	41462-42106	nan	nan	6	5	-0.04637	0.9663	no
ni|645903480|nb|KK920950.1|	43145-43542	nan	nan	6	21	2	0.20415	no
ni|645903480|nb|KK920950.1|	44153-44506	nan	nan	41	180	2	0.06255	no
ni|645903480|nb|KK920950.1|	44787-45245	nan	nan	30	169	2	0.0788	no

ni|645903480|nb|KK920950.1|	45489-45741	nan	nan	27	164	3	0.0301	no
ni|645903480|nb|KK920950.1|	45850-46203	nan	nan	1	8	2	0.06445	no
ni|645903480|nb|KK920950.1|	47257-47523	nan	nan	0.917304	6	3	0.06305	no
ni|645903480|nb|KK920950.1|	47628-48673	nan	nan	49	92	0.898322	0.53685	no
ni|645903480|nb|KK920950.1|	49586-49913	nan	nan	0	16	inf	0.0003	yes
ni|645903480|nb|KK920950.1|	50554-51033	nan	nan	0.583209	1	1	0.41545	no
ni|645903480|nb|KK920950.1|	55491-55692	nan	nan	3	4	0.377384	0.7467	no
ni|645903480|nb|KK920950.1|	56608-57109	nan	nan	1	0.702746	-0.853672	0.50505	no
ni|645903480|nb|KK920950.1|	57251-57836	nan	nan	27	43	0.657753	0.54135	no
ni|645903480|nb|KK920950.1|	60451-60669	nan	nan	0.860884	0.822979	-0.0649639	0.9615	no
ni|645903480|nb|KK920950.1|	63523-63700	nan	nan	26	25	-0.0652694	0.9523	no
ni|645903480|nb|KK920950.1|	78631-79938	nan	nan	77	54	-0.515094	0.723	no
ni|645903481|nb|KK920949.1|	22392-23238	nan	nan	0.117949	46	9	0.11935	no
ni|645903483|nb|KK920947.1|	389614-391199	nan	nan	0	120	inf	5.00E-05	yes
ni|645903483|nb|KK920947.1|	391273-392074	nan	nan	0	118	inf	5.00E-05	yes
ni|645903483|nb|KK920947.1|	486471-486650	nan	nan	0.048738	2	6	0.11965	no
ni|645903483|nb|KK920947.1|	500526-500696	nan	nan	0.057223	40	9	0.11935	no
ni|645903483|nb|KK920947.1|	503297-503484	nan	nan	0.127705	104	10	0.1194	no
ni|645903483|nb|KK920947.1|	83810-84705	nan	nan	0	56	inf	5.00E-05	yes
ni|645903484|nb|KK920946.1|	114528-114681	nan	nan	0	7	inf	5.00E-05	yes
ni|645903484|nb|KK920946.1|	116497-116906	nan	nan	0.066122	2	5	0.1207	no
ni|645903484|nb|KK920946.1|	119384-119577	nan	nan	0.234044	61	8	0.11935	no
ni|645903484|nb|KK920946.1|	119801-120056	nan	nan	0	54	inf	5.00E-05	yes
ni|645903484|nb|KK920946.1|	122941-123226	nan	nan	0.250566	56	8	0.11935	no
ni|645903484|nb|KK920946.1|	123758-124166	nan	nan	0	3	inf	5.00E-05	yes
ni|645903484|nb|KK920946.1|	124268-124555	nan	nan	0.45903	3	3	0.06975	no
ni|645903484|nb|KK920946.1|	124957-125100	nan	nan	6	4	-0.539229	0.6484	no
ni|645903484|nb|KK920946.1|	125916-126099	nan	nan	2	2	-0.288028	0.8142	no
ni|645903484|nb|KK920946.1|	126409-126910	nan	nan	2	2	-0.0647404	0.9536	no
ni|645903484|nb|KK920946.1|	130155-130525	nan	nan	2	2	-0.185781	0.8779	no

ni|645903484|nb|KK920946.1|	131118-131282	nan	nan	0.885181	2	0.808465	0.56845	no
ni|645903484|nb|KK920946.1|	131388-131790	nan	nan	5	4	-0.552705	0.719	no
ni|645903484|nb|KK920946.1|	132396-132741	nan	nan	3	3	-0.148535	0.91085	no
ni|645903484|nb|KK920946.1|	132876-133339	nan	nan	8	4	-1	0.4103	no
ni|645903484|nb|KK920946.1|	133780-134381	nan	nan	1	2	0.246488	0.83065	no
ni|645903484|nb|KK920946.1|	134519-135125	nan	nan	3	3	-0.328493	0.78125	no
ni|645903484|nb|KK920946.1|	135487-136461	nan	nan	2	3	0.992921	0.4553	no
ni|645903484|nb|KK920946.1|	142762-143103	nan	nan	0	2	inf	0.00015	yes
ni|645903484|nb|KK920946.1|	143915-144472	nan	nan	1	2	0.282247	0.82625	no
ni|645903484|nb|KK920946.1|	146365-146693	nan	nan	2	3	1	0.42705	no
ni|645903484|nb|KK920946.1|	147011-147194	nan	nan	4	9	1	0.43015	no
ni|645903484|nb|KK920946.1|	148094-148241	nan	nan	2	2	-0.218275	0.87705	no
ni|645903484|nb|KK920946.1|	148342-148953	nan	nan	5	6	0.139239	0.90865	no
ni|645903484|nb|KK920946.1|	150717-150899	nan	nan	3	3	0.223549	0.85915	no
ni|645903484|nb|KK920946.1|	151842-151961	nan	nan	2	3	0.933265	0.5103	no
ni|645903484|nb|KK920946.1|	152652-152764	nan	nan	11	4	-1	0.3864	no
ni|645903484|nb|KK920946.1|	155758-155899	nan	nan	23	8	-2	0.19805	no
ni|645903484|nb|KK920946.1|	157661-158170	nan	nan	13	4	-2	0.2996	no
ni|645903484|nb|KK920946.1|	158907-159159	nan	nan	2	0.835324	-1	0.36925	no
ni|645903484|nb|KK920946.1|	159454-160292	nan	nan	30	21	-0.558698	0.63325	no
ni|645903484|nb|KK920946.1|	161384-162174	nan	nan	0.816425	0.156127	-2	0.10835	no
ni|645903484|nb|KK920946.1|	162428-162603	nan	nan	20	19	-0.0276778	0.9819	no
ni|645903484|nb|KK920946.1|	163181-163574	nan	nan	6	5	-0.301785	0.81	no
ni|645903484|nb|KK920946.1|	164644-164965	nan	nan	32	21	-0.615215	0.58585	no
ni|645903484|nb|KK920946.1|	165319-166064	nan	nan	5	3	-0.745369	0.52585	no
ni|645903484|nb|KK920946.1|	174010-174742	nan	nan	2	1	-0.581205	0.70815	no
ni|645903484|nb|KK920946.1|	177902-178652	nan	nan	9	7	-0.370945	0.74775	no
ni|645903484|nb|KK920946.1|	179068-180146	nan	nan	4	2	-0.972781	0.432	no
ni|645903484|nb|KK920946.1|	180367-180790	nan	nan	1	3	1	0.42705	no
ni|645903484|nb|KK920946.1|	181383-181657	nan	nan	21	24	0.182251	0.8773	no

ni|645903484|nb|KK920946.1|	182171-182419	nan	nan	31	29	-0.088258	0.9397	no
ni|645903484|nb|KK920946.1|	182935-183110	nan	nan	28	19	-0.552718	0.62955	no
ni|645903484|nb|KK920946.1|	184129-184275	nan	nan	30	32	0.0982671	0.93125	no
ni|645903484|nb|KK920946.1|	215503-215667	nan	nan	25	15	-0.730163	0.5348	no
ni|645903484|nb|KK920946.1|	527208-527390	nan	nan	19	22	0.202891	0.8667	no
ni|645903484|nb|KK920946.1|	83799-84196	nan	nan	0.070348	10	7	0.0443	no
ni|645903484|nb|KK920946.1|	96516-97059	nan	nan	0.115738	18.91	7	0.11935	no
ni|645903486|nb|KK920944.1|	141512-141850	nan	nan	34	31	-0.132076	0.90725	no
ni|645903486|nb|KK920944.1|	158991-159118	nan	nan	18	6	-1	0.22165	no
ni|645903486|nb|KK920944.1|	472961-473406	nan	nan	32	25	-0.380921	0.7401	no
ni|645903486|nb|KK920944.1|	474382-474881	nan	nan	43	39	-0.140223	0.9042	no
ni|645903488|nb|KK920942.1|	272852-273000	nan	nan	8	15	0.872491	0.4178	no
ni|645903488|nb|KK920942.1|	338622-338940	nan	nan	13	22	0.802858	0.49215	no
ni|645903488|nb|KK920942.1|	427585-427856	nan	nan	12	23	0.975008	0.40975	no
ni|645903488|nb|KK920942.1|	442848-446169	nan	nan	16	24	0.600133	0.60575	no
ni|645903488|nb|KK920942.1|	78086-78377	nan	nan	1	3	0.872875	0.49985	no
ni|645903488|nb|KK920942.1|	81601-81869	nan	nan	4	3	-0.389144	0.7621	no
ni|645903488|nb|KK920942.1|	82128-82365	nan	nan	3	0	-inf	0.002	yes
ni|645903488|nb|KK920942.1|	87354-87493	nan	nan	15	13	-0.195476	0.85885	no
ni|645903488|nb|KK920942.1|	87563-87694	nan	nan	15	16	0.0751319	0.94535	no
ni|645903488|nb|KK920942.1|	88001-88131	nan	nan	17	11	-0.696918	0.5438	no
ni|645903488|nb|KK920942.1|	88591-88715	nan	nan	2	4	1	0.3727	no
ni|645903488|nb|KK920942.1|	89330-90743	nan	nan	8	26	2	0.18765	no
ni|645903488|nb|KK920942.1|	90823-91839	nan	nan	11	16	0.575769	0.612	no
ni|645903489|nb|KK920941.1|	169294-169471	nan	nan	10	9	-0.1151	0.92225	no
ni|645903489|nb|KK920941.1|	242084-242226	nan	nan	0	3	inf	0.00135	yes
ni|645903489|nb|KK920941.1|	255843-256253	nan	nan	2	5	1	0.43015	no
ni|645903489|nb|KK920941.1|	259247-259416	nan	nan	22	11	-1	0.3949	no
ni|645903489|nb|KK920941.1|	269856-269989	nan	nan	42	23	-0.842137	0.4835	no
ni|645903489|nb|KK920941.1|	278345-278470	nan	nan	7	5	-0.416241	0.7156	no

ni|645903490|nb|KK920940.1|	54607-54778	nan	nan	13	10	-0.366351	0.73415	no
ni|645903491|nb|KK920939.1|	106370-106554	nan	nan	4	2	-0.874398	0.53675	no
ni|645903491|nb|KK920939.1|	18275-18389	nan	nan	4	3	-0.228235	0.847	no
ni|645903491|nb|KK920939.1|	20096-20527	nan	nan	3	2	-0.105879	0.93205	no
ni|645903491|nb|KK920939.1|	27632-27904	nan	nan	1	1	-0.112106	0.9225	no
ni|645903491|nb|KK920939.1|	295688-296123	nan	nan	37	26	-0.494771	0.6629	no
ni|645903491|nb|KK920939.1|	297042-297350	nan	nan	0	5	inf	0.0001	yes
ni|645903491|nb|KK920939.1|	297573-297882	nan	nan	0	195	inf	5.00E-05	yes
ni|645903491|nb|KK920939.1|	298197-298421	nan	nan	20	20	-0.0342038	0.97495	no
ni|645903491|nb|KK920939.1|	299856-300079	nan	nan	17	16	-0.14026	0.9063	no
ni|645903491|nb|KK920939.1|	300636-300886	nan	nan	17	17	-0.0427237	0.9678	no
ni|645903491|nb|KK920939.1|	30069-30300	nan	nan	3	2	-0.216986	0.8555	no
ni|645903491|nb|KK920939.1|	301189-301312	nan	nan	41	40	-0.0583772	0.95825	no
ni|645903491|nb|KK920939.1|	302300-302547	nan	nan	8	7	-0.162492	0.89595	no
ni|645903491|nb|KK920939.1|	306119-306230	nan	nan	10	10	0.0316414	0.97995	no
ni|645903491|nb|KK920939.1|	306916-307091	nan	nan	23	19	-0.272808	0.81345	no
ni|645903491|nb|KK920939.1|	307259-307505	nan	nan	4	3	-0.438149	0.72705	no
ni|645903491|nb|KK920939.1|	307620-307912	nan	nan	6	4	-0.48167	0.69415	no
ni|645903491|nb|KK920939.1|	310776-310976	nan	nan	2	2	-0.386549	0.7369	no
ni|645903491|nb|KK920939.1|	311101-311279	nan	nan	23	21	-0.128368	0.91115	no
ni|645903491|nb|KK920939.1|	312178-312461	nan	nan	15	14	-0.0704751	0.94925	no
ni|645903491|nb|KK920939.1|	312852-312977	nan	nan	6	5	-0.3185	0.7941	no
ni|645903491|nb|KK920939.1|	313519-313866	nan	nan	4	4	-0.102177	0.9294	no
ni|645903491|nb|KK920939.1|	314224-314715	nan	nan	2	1	-0.972903	0.5005	no
ni|645903491|nb|KK920939.1|	315637-316880	nan	nan	4	7	0.721274	0.5689	no
ni|645903491|nb|KK920939.1|	320277-320526	nan	nan	67	72	0.0923175	0.9397	no
ni|645903491|nb|KK920939.1|	320717-320989	nan	nan	37	39	0.0930032	0.95055	no
ni|645903491|nb|KK920939.1|	32183-32521	nan	nan	1	1	-0.0654866	0.94765	no
ni|645903491|nb|KK920939.1|	32633-33051	nan	nan	3	0.430669	-3	0.2572	no
ni|645903491|nb|KK920939.1|	394785-395127	nan	nan	52	53	0.0334241	0.9825	no

ni|645903491|nb|KK920939.1|	396335-396500	nan	nan	0.636014	0.607901	-0.0652223	0.94155	no
ni|645903491|nb|KK920939.1|	40509-43295	nan	nan	4	2	-0.669645	0.5837	no
ni|645903491|nb|KK920939.1|	416717-416946	nan	nan	4	3	-0.562959	0.62665	no
ni|645903491|nb|KK920939.1|	426545-427219	nan	nan	2	1	-1	0.44965	no
ni|645903491|nb|KK920939.1|	427705-427949	nan	nan	3	3	-0.419641	0.7284	no
ni|645903491|nb|KK920939.1|	429796-430024	nan	nan	14	11	-0.277679	0.80665	no
ni|645903491|nb|KK920939.1|	431476-431836	nan	nan	63	55	-0.211371	0.85165	no
ni|645903491|nb|KK920939.1|	436899-437042	nan	nan	25	31	0.323129	0.83055	no
ni|645903491|nb|KK920939.1|	437165-438119	nan	nan	49	67	0.454974	0.6921	no
ni|645903491|nb|KK920939.1|	439051-439185	nan	nan	0.458847	0.438615	-0.0650588	0.9384	no
ni|645903491|nb|KK920939.1|	439433-439604	nan	nan	17	167	3	0.02075	no
ni|645903491|nb|KK920939.1|	442218-442403	nan	nan	59	100	0.768219	0.50215	no
ni|645903491|nb|KK920939.1|	443970-444257	nan	nan	4	7	0.838345	0.4557	no
ni|645903491|nb|KK920939.1|	444478-445074	nan	nan	33	61	0.880478	0.4893	no
ni|645903491|nb|KK920939.1|	446306-446415	nan	nan	61	97	0.674548	0.5511	no
ni|645903491|nb|KK920939.1|	446618-446887	nan	nan	41	69	0.747184	0.50055	no
ni|645903491|nb|KK920939.1|	447107-447339	nan	nan	69	124	0.858151	0.45625	no
ni|645903491|nb|KK920939.1|	448579-449207	nan	nan	81	110	0.438982	0.6976	no
ni|645903491|nb|KK920939.1|	449980-450261	nan	nan	30	37	0.274318	0.79685	no
ni|645903491|nb|KK920939.1|	451230-451756	nan	nan	92	85	-0.109871	0.924	no
ni|645903491|nb|KK920939.1|	452158-452321	nan	nan	41	30.42	-0.44119	0.68965	no
ni|645903491|nb|KK920939.1|	454405-454645	nan	nan	5	4	-0.183352	0.87335	no
ni|645903491|nb|KK920939.1|	455525-457230	nan	nan	181.72	151	-0.266327	0.8179	no
ni|645903491|nb|KK920939.1|	463131-463507	nan	nan	0.850383	2	1	0.415	no
ni|645903491|nb|KK920939.1|	463751-464045	nan	nan	1	5	2	0.14055	no
ni|645903491|nb|KK920939.1|	464841-465019	nan	nan	6	14	1	0.3488	no
ni|645903491|nb|KK920939.1|	465456-465643	nan	nan	11	17	0.66217	0.5753	no
ni|645903491|nb|KK920939.1|	471173-471546	nan	nan	3	7	1	0.26625	no
ni|645903491|nb|KK920939.1|	473551-473875	nan	nan	4	7	0.871276	0.49985	no
ni|645903491|nb|KK920939.1|	474032-474221	nan	nan	1	2	0.750666	0.52925	no

ni|645903491|nb|KK920939.1|	474747-474975	nan	nan	0.36259	0.598835	0.723818	0.5686	no
ni|645903491|nb|KK920939.1|	475182-475449	nan	nan	0.393272	0.672992	0.775061	0.52345	no
ni|645903491|nb|KK920939.1|	475559-475705	nan	nan	0.356316	0.340684	-0.0647227	0.9467	no
ni|645903491|nb|KK920939.1|	477016-477164	nan	nan	6	2	-1	0.35155	no
ni|645903491|nb|KK920939.1|	477903-478100	nan	nan	14	19	0.3863	0.7317	no
ni|645903491|nb|KK920939.1|	478307-478487	nan	nan	15	21	0.441528	0.69445	no
ni|645903491|nb|KK920939.1|	478689-479499	nan	nan	0.793736	2	2	0.30335	no
ni|645903491|nb|KK920939.1|	479619-480198	nan	nan	1595.33	1320.67	-0.272589	0.8505	no
ni|645903491|nb|KK920939.1|	480351-480513	nan	nan	0.623184	0.425398	-0.550844	0.71905	no
ni|645903491|nb|KK920939.1|	481053-481266	nan	nan	818	593	-0.463283	0.70365	no
ni|645903491|nb|KK920939.1|	483994-484660	nan	nan	17	12	-0.482651	0.7594	no
ni|645903491|nb|KK920939.1|	485074-485278	nan	nan	33	116	2	0.1302	no
ni|645903491|nb|KK920939.1|	485858-486544	nan	nan	0.536877	0.036665	-4	0.0255	no
ni|645903491|nb|KK920939.1|	487425-487625	nan	nan	23	13	-0.859302	0.43505	no
ni|645903491|nb|KK920939.1|	488604-488797	nan	nan	0.510268	0.650362	0.349987	0.792	no
ni|645903491|nb|KK920939.1|	489128-489745	nan	nan	3	3	-0.0666001	0.9554	no
ni|645903491|nb|KK920939.1|	489941-490239	nan	nan	3	5	1	0.4354	no
ni|645903491|nb|KK920939.1|	491604-491832	nan	nan	0.605002	3	2	0.14335	no
ni|645903491|nb|KK920939.1|	491995-492155	nan	nan	3	6	1	0.35025	no
ni|645903491|nb|KK920939.1|	492304-493030	nan	nan	0	3	inf	5.00E-05	yes
ni|645903491|nb|KK920939.1|	493653-493882	nan	nan	0.020509	4	8	0.11935	no
ni|645903491|nb|KK920939.1|	495414-495595	nan	nan	0	21	inf	0.0001	yes
ni|645903491|nb|KK920939.1|	495702-496133	nan	nan	0.058582	63	10	0.11935	no
ni|645903491|nb|KK920939.1|	497080-497658	nan	nan	0.060417	10	7	0.03715	no
ni|645903491|nb|KK920939.1|	501518-501781	nan	nan	0.066299	1	4	0.12575	no
ni|645903491|nb|KK920939.1|	505266-505487	nan	nan	1	0.260881	-2	0.1906	no
ni|645903491|nb|KK920939.1|	508492-508741	nan	nan	33.24	66	0.992905	0.3781	no
ni|645903491|nb|KK920939.1|	512050-512226	nan	nan	11	18	0.677452	0.56765	no
ni|645903491|nb|KK920939.1|	513061-513275	nan	nan	56	56	-0.00347539	0.9974	no
ni|645903491|nb|KK920939.1|	514129-514280	nan	nan	2	2	0.111954	0.92345	no

ni|645903491|nb|KK920939.1|	516440-516667	nan	nan	2	3	0.711738	0.57115	no
ni|645903491|nb|KK920939.1|	520386-520620	nan	nan	125	132	0.0788129	0.9396	no
ni|645903491|nb|KK920939.1|	520868-521092	nan	nan	3	2	-0.414442	0.76315	no
ni|645903491|nb|KK920939.1|	522433-522729	nan	nan	1964.41	1435.58	-0.452458	0.7577	no
ni|645903491|nb|KK920939.1|	526353-526490	nan	nan	695	476	-0.546073	0.6967	no
ni|645903491|nb|KK920939.1|	528869-529030	nan	nan	10	6	-0.793038	0.4975	no
ni|645903491|nb|KK920939.1|	529463-529695	nan	nan	1834.01	1272.04	-0.527857	0.6686	no
ni|645903491|nb|KK920939.1|	529802-530013	nan	nan	334	400	0.259973	0.8034	no
ni|645903491|nb|KK920939.1|	530233-530495	nan	nan	2264.28	2615.1	0.207819	0.88375	no
ni|645903491|nb|KK920939.1|	530678-530818	nan	nan	1726.86	1907.21	0.143306	0.92275	no
ni|645903491|nb|KK920939.1|	532856-532999	nan	nan	150	211.11	0.495396	0.73145	no
ni|645903491|nb|KK920939.1|	534822-534966	nan	nan	104	129	0.310098	0.7841	no
ni|645903491|nb|KK920939.1|	537068-537568	nan	nan	92	102	0.15487	0.8835	no
ni|645903491|nb|KK920939.1|	541023-541291	nan	nan	86	63	-0.453871	0.7515	no
ni|645903491|nb|KK920939.1|	541847-542030	nan	nan	36	27	-0.3927	0.7355	no
ni|645903491|nb|KK920939.1|	543956-544152	nan	nan	238	60	-2	0.1032	no
ni|645903491|nb|KK920939.1|	548101-548256	nan	nan	2	0.463672	-2	0.16025	no
ni|645903491|nb|KK920939.1|	550465-550598	nan	nan	10	41	2	0.1152	no
ni|645903491|nb|KK920939.1|	552548-552696	nan	nan	24	91	2	0.1057	no
ni|645903491|nb|KK920939.1|	553098-553301	nan	nan	30	15	-0.984852	0.4324	no
ni|645903491|nb|KK920939.1|	555602-555799	nan	nan	0.753601	0.720303	-0.0651967	0.94765	no
ni|645903491|nb|KK920939.1|	557678-557797	nan	nan	71	38	-0.911673	0.4436	no
ni|645903491|nb|KK920939.1|	558644-559076	nan	nan	26	14	-0.859012	0.466	no
ni|645903491|nb|KK920939.1|	560528-560693	nan	nan	15	14	-0.13938	0.90395	no
ni|645903491|nb|KK920939.1|	564650-565073	nan	nan	0.896732	1	0.420003	0.73705	no
ni|645903491|nb|KK920939.1|	568588-569168	nan	nan	15	14	-0.0672441	0.957	no
ni|645903491|nb|KK920939.1|	573909-574136	nan	nan	4	4	-0.0291149	0.98095	no
ni|645903491|nb|KK920939.1|	574306-574454	nan	nan	18	14	-0.405534	0.72625	no
ni|645903491|nb|KK920939.1|	576189-576438	nan	nan	21	14	-0.579065	0.63035	no
ni|645903491|nb|KK920939.1|	577083-577480	nan	nan	7	2	-2	0.20695	no

ni|645903491|nb|KK920939.1|	580067-580279	nan	nan	8	14	0.779439	0.53295	no
ni|645903491|nb|KK920939.1|	580656-580904	nan	nan	19	22	0.163569	0.8902	no
ni|645903491|nb|KK920939.1|	583610-583740	nan	nan	23	30	0.37742	0.735	no
ni|645903491|nb|KK920939.1|	587769-587981	nan	nan	35	25	-0.48902	0.68	no
ni|645903491|nb|KK920939.1|	592481-592698	nan	nan	25	25	0.0412972	0.9705	no
ni|645903491|nb|KK920939.1|	593400-593571	nan	nan	2	1	-0.630468	0.60425	no
ni|645903491|nb|KK920939.1|	594594-594764	nan	nan	44	22	-1	0.39555	no
ni|645903491|nb|KK920939.1|	596097-596270	nan	nan	5	5	-0.0884328	0.93695	no
ni|645903491|nb|KK920939.1|	597382-597625	nan	nan	75	84	0.156328	0.8884	no
ni|645903491|nb|KK920939.1|	599465-599636	nan	nan	31	10	-2	0.1783	no
ni|645903491|nb|KK920939.1|	601325-601504	nan	nan	68	77	0.179585	0.86835	no
ni|645903491|nb|KK920939.1|	603712-603965	nan	nan	0.186108	22	7	0.0443	no
ni|645903491|nb|KK920939.1|	604406-604578	nan	nan	1	79	6	0.00485	no
ni|645903491|nb|KK920939.1|	604866-604985	nan	nan	0.309742	23	6	0.00995	no
ni|645903491|nb|KK920939.1|	606493-606927	nan	nan	0.181571	11	6	0.02725	no
ni|645903491|nb|KK920939.1|	610631-610995	nan	nan	0	5	inf	5.00E-05	yes
ni|645903491|nb|KK920939.1|	611862-612228	nan	nan	3	8	1	0.27225	no
ni|645903491|nb|KK920939.1|	612409-613442	nan	nan	14	19	0.432874	0.7261	no
ni|645903491|nb|KK920939.1|	616704-617201	nan	nan	23	26	0.191376	0.8642	no
ni|645903491|nb|KK920939.1|	617943-618365	nan	nan	6	8	0.463214	0.70795	no
ni|645903491|nb|KK920939.1|	619003-619166	nan	nan	24	31	0.372553	0.75185	no
ni|645903491|nb|KK920939.1|	619340-619541	nan	nan	27	31	0.201936	0.8618	no
ni|645903491|nb|KK920939.1|	619668-619833	nan	nan	33	40	0.288076	0.8042	no
ni|645903491|nb|KK920939.1|	622565-623021	nan	nan	21	19	-0.174186	0.88345	no
ni|645903491|nb|KK920939.1|	623195-623430	nan	nan	0	164	inf	5.00E-05	yes
ni|645903491|nb|KK920939.1|	623865-623984	nan	nan	108	63	-0.778108	0.4848	no
ni|645903491|nb|KK920939.1|	624102-624255	nan	nan	4	3	-0.218384	0.86075	no
ni|645903491|nb|KK920939.1|	624413-624776	nan	nan	2	2	-0.271673	0.8172	no
ni|645903491|nb|KK920939.1|	624924-625185	nan	nan	3	2	-0.627295	0.57515	no
ni|645903491|nb|KK920939.1|	626655-626919	nan	nan	24.46	45	0.871179	0.49675	no

ni|645903491|nb|KK920939.1|	627882-628097	nan	nan	2	1	-0.67088	0.5807	no
ni|645903491|nb|KK920939.1|	628212-628342	nan	nan	130	242	0.897593	0.4785	no
ni|645903491|nb|KK920939.1|	629439-629756	nan	nan	33	65	0.988427	0.37415	no
ni|645903491|nb|KK920939.1|	630016-630347	nan	nan	0.763639	3	2	0.19145	no
ni|645903491|nb|KK920939.1|	632012-632259	nan	nan	0.599701	1	1	0.4359	no
ni|645903491|nb|KK920939.1|	633988-634597	nan	nan	11	76	3	0.02825	no
ni|645903491|nb|KK920939.1|	634721-635047	nan	nan	4	2	-0.777229	0.5539	no
ni|645903491|nb|KK920939.1|	647384-647702	nan	nan	0.884506	1	0.23987	0.8456	no
ni|645903491|nb|KK920939.1|	647876-648148	nan	nan	1	0.503418	-1	0.44755	no
ni|645903491|nb|KK920939.1|	648312-649104	nan	nan	19	33	0.803234	0.5926	no
ni|645903491|nb|KK920939.1|	651210-651535	nan	nan	5	1	-2	0.2784	no
ni|645903491|nb|KK920939.1|	652795-653435	nan	nan	2	2	0.186276	0.87715	no
ni|645903491|nb|KK920939.1|	655835-656242	nan	nan	2	2	0.295674	0.8383	no
ni|645903491|nb|KK920939.1|	659290-659754	nan	nan	4	3	-0.314663	0.8025	no
ni|645903491|nb|KK920939.1|	660901-661065	nan	nan	0.786516	0.805547	0.034492	0.96125	no
ni|645903491|nb|KK920939.1|	670216-670396	nan	nan	5	6	0.193711	0.8689	no
ni|645903491|nb|KK920939.1|	670771-670956	nan	nan	5	8	0.617169	0.599	no
ni|645903491|nb|KK920939.1|	676994-677133	nan	nan	21	38	0.879487	0.4495	no
ni|645903491|nb|KK920939.1|	680170-680346	nan	nan	21	34	0.707706	0.54835	no
ni|645903491|nb|KK920939.1|	684836-685164	nan	nan	3	4	0.556616	0.6361	no
ni|645903491|nb|KK920939.1|	686619-686825	nan	nan	1	2	0.126045	0.92555	no
ni|645903491|nb|KK920939.1|	688381-688930	nan	nan	2	0.765268	-1	0.4219	no
ni|645903491|nb|KK920939.1|	691008-691313	nan	nan	2	2	-0.25808	0.82995	no
ni|645903491|nb|KK920939.1|	697800-698149	nan	nan	11	13	0.314027	0.788	no
ni|645903491|nb|KK920939.1|	703964-704288	nan	nan	1	0.901142	-0.239722	0.83825	no
ni|645903491|nb|KK920939.1|	705546-705697	nan	nan	21	28	0.41423	0.7159	no
ni|645903491|nb|KK920939.1|	710724-710976	nan	nan	11	13	0.264311	0.82005	no
ni|645903491|nb|KK920939.1|	713479-713661	nan	nan	14	45	2	0.22835	no
ni|645903491|nb|KK920939.1|	716254-716423	nan	nan	16	18	0.166795	0.88715	no
ni|645903491|nb|KK920939.1|	720201-720614	nan	nan	0.28154	0.699734	1	0.35675	no

ni|645903491|nb|KK920939.1|	721673-721820	nan	nan	30	34	0.160332	0.915	no
ni|645903491|nb|KK920939.1|	723249-723466	nan	nan	18	19	0.0882277	0.93645	no
ni|645903491|nb|KK920939.1|	725846-726017	nan	nan	0.580198	1	0.934497	0.5295	no
ni|645903491|nb|KK920939.1|	733482-733695	nan	nan	0.685092	0.327411	-1	0.44755	no
ni|645903492|nb|KK920938.1|	18479-18612	nan	nan	14	14	0.0611442	0.9559	no
ni|645903492|nb|KK920938.1|	19694-19869	nan	nan	11	9	-0.344726	0.76915	no
ni|645903492|nb|KK920938.1|	41137-42534	nan	nan	21	18	-0.166786	0.8881	no
ni|645903492|nb|KK920938.1|	57981-58145	nan	nan	1	0.66294	-0.702719	0.6038	no
ni|645903492|nb|KK920938.1|	58249-58407	nan	nan	1	2	0.474971	0.709	no
ni|645903492|nb|KK920938.1|	59124-59227	nan	nan	2	2	0.312116	0.81835	no
ni|645903492|nb|KK920938.1|	59661-59773	nan	nan	1	0.850668	-0.650975	0.6679	no
ni|645903492|nb|KK920938.1|	60751-61076	nan	nan	1	2	0.339664	0.7773	no
ni|645903492|nb|KK920938.1|	61846-62348	nan	nan	2	4	1	0.29595	no
ni|645903492|nb|KK920938.1|	62940-63162	nan	nan	2	2	0.382243	0.75215	no
ni|645903492|nb|KK920938.1|	63718-63943	nan	nan	4	4	0.281754	0.81735	no
ni|645903492|nb|KK920938.1|	68129-68368	nan	nan	1136.74	941	-0.272725	0.82675	no
ni|645903492|nb|KK920938.1|	96859-97087	nan	nan	1244.18	1012.83	-0.2968	0.8068	no
ni|645903494|nb|KK920936.1|	104680-104940	nan	nan	3	1	-1	0.288	no
ni|645903494|nb|KK920936.1|	109164-109316	nan	nan	2	2	-0.169923	0.88545	no
ni|645903494|nb|KK920936.1|	132605-132813	nan	nan	2	2	-0.225276	0.84335	no
ni|645903494|nb|KK920936.1|	145760-145883	nan	nan	5	3	-0.591925	0.6233	no
ni|645903494|nb|KK920936.1|	146272-146468	nan	nan	3	2	-0.363028	0.753	no
ni|645903494|nb|KK920936.1|	173151-173313	nan	nan	2	2	0.04028	0.97145	no
ni|645903494|nb|KK920936.1|	173667-173918	nan	nan	0.908865	1	0.39375	0.77595	no
ni|645903494|nb|KK920936.1|	219821-220446	nan	nan	2	1	-0.479742	0.6835	no
ni|645903494|nb|KK920936.1|	230904-231710	nan	nan	5	3	-0.704672	0.60375	no
ni|645903494|nb|KK920936.1|	232612-235185	nan	nan	3	2	-0.882571	0.47085	no
ni|645903494|nb|KK920936.1|	238792-239443	nan	nan	2	5	1	0.3802	no
ni|645903494|nb|KK920936.1|	239905-240186	nan	nan	39	52	0.418083	0.72235	no
ni|645903494|nb|KK920936.1|	240646-240822	nan	nan	14	19	0.39613	0.7207	no

ni|645903494|nb|KK920936.1|	245250-245402	nan	nan	6	12	0.89421	0.45675	no
ni|645903494|nb|KK920936.1|	246174-246740	nan	nan	21	33	0.687061	0.5414	no
ni|645903494|nb|KK920936.1|	247127-248352	nan	nan	15	19	0.339359	0.7699	no
ni|645903494|nb|KK920936.1|	26069-26258	nan	nan	1	0.973248	-0.288605	0.8535	no
ni|645903494|nb|KK920936.1|	263600-263777	nan	nan	12	25	1	0.3351	no
ni|645903494|nb|KK920936.1|	269276-269396	nan	nan	15	14	-0.0965713	0.92835	no
ni|645903494|nb|KK920936.1|	294370-294652	nan	nan	74	85	0.208584	0.87935	no
ni|645903494|nb|KK920936.1|	297238-298154	nan	nan	3	2	-0.65219	0.64	no
ni|645903494|nb|KK920936.1|	305852-306311	nan	nan	38	40	0.095951	0.9303	no
ni|645903494|nb|KK920936.1|	309719-310000	nan	nan	1	6	2	0.0676	no
ni|645903494|nb|KK920936.1|	312837-312999	nan	nan	51	56	0.128619	0.91	no
ni|645903494|nb|KK920936.1|	332022-332344	nan	nan	67	60.86	-0.144276	0.9032	no
ni|645903494|nb|KK920936.1|	334593-334733	nan	nan	92	99	0.102031	0.9285	no
ni|645903494|nb|KK920936.1|	336761-337148	nan	nan	79	98	0.315855	0.7801	no
ni|645903494|nb|KK920936.1|	353012-353154	nan	nan	0.790944	0.893426	0.175773	0.8916	no
ni|645903494|nb|KK920936.1|	369112-369725	nan	nan	2	2	-0.40914	0.73715	no
ni|645903494|nb|KK920936.1|	37067-37237	nan	nan	14	9	-0.699461	0.5662	no
ni|645903494|nb|KK920936.1|	370730-370847	nan	nan	1	0.2749	-2	0.21345	no
ni|645903494|nb|KK920936.1|	371064-371824	nan	nan	21	22	0.0589729	0.95835	no
ni|645903494|nb|KK920936.1|	372017-372275	nan	nan	43	47	0.116931	0.91425	no
ni|645903494|nb|KK920936.1|	372380-372741	nan	nan	97	158	0.697558	0.54385	no
ni|645903494|nb|KK920936.1|	372855-373123	nan	nan	2	1	-0.246027	0.84435	no
ni|645903494|nb|KK920936.1|	373219-373362	nan	nan	23	31	0.444962	0.6822	no
ni|645903494|nb|KK920936.1|	403799-404300	nan	nan	14	24	0.768676	0.4997	no
ni|645903494|nb|KK920936.1|	405061-406234	nan	nan	24	39	0.670174	0.55345	no
ni|645903494|nb|KK920936.1|	407505-408036	nan	nan	37	31	-0.240053	0.835	no
ni|645903494|nb|KK920936.1|	411929-412849	nan	nan	67	71	0.0889828	0.93395	no
ni|645903494|nb|KK920936.1|	415925-416648	nan	nan	1	0.636344	-0.913866	0.53575	no
ni|645903494|nb|KK920936.1|	432749-432941	nan	nan	32	41	0.379299	0.8006	no
ni|645903494|nb|KK920936.1|	434698-435178	nan	nan	45	62	0.443903	0.74995	no

ni|645903494|nb|KK920936.1|	435288-435981	nan	nan	37	39	0.0687361	0.962	no
ni|645903494|nb|KK920936.1|	436157-436346	nan	nan	33	35	0.100917	0.93005	no
ni|645903494|nb|KK920936.1|	437649-439455	nan	nan	33	31	-0.0997176	0.94585	no
ni|645903494|nb|KK920936.1|	440312-440635	nan	nan	20	18	-0.182778	0.87475	no
ni|645903494|nb|KK920936.1|	440813-440968	nan	nan	11	8	-0.482361	0.7301	no
ni|645903494|nb|KK920936.1|	55303-55508	nan	nan	21	24	0.230412	0.8361	no
ni|645903494|nb|KK920936.1|	55825-55991	nan	nan	0.614424	0.313297	-0.971702	0.5005	no
ni|645903494|nb|KK920936.1|	56106-56317	nan	nan	11	14	0.317782	0.7694	no
ni|645903494|nb|KK920936.1|	7101-7236	nan	nan	185	107	-0.786484	0.46325	no
ni|645903494|nb|KK920936.1|	72674-72822	nan	nan	20	30	0.60247	0.6862	no
ni|645903494|nb|KK920936.1|	86833-87030	nan	nan	21	30	0.515401	0.65135	no
ni|645903495|nb|KK920935.1|	100299-101214	nan	nan	64	100	0.635797	0.6734	no
ni|645903495|nb|KK920935.1|	101349-101767	nan	nan	183	353	0.944087	0.3885	no
ni|645903495|nb|KK920935.1|	101924-102094	nan	nan	0.597172	0.456663	-0.387017	0.78725	no
ni|645903495|nb|KK920935.1|	103710-103949	nan	nan	52	96	0.876109	0.5532	no
ni|645903495|nb|KK920935.1|	104276-104524	nan	nan	164	301	0.880978	0.47725	no
ni|645903495|nb|KK920935.1|	107219-107377	nan	nan	62	105	0.77043	0.53495	no
ni|645903495|nb|KK920935.1|	107872-108136	nan	nan	46	86	0.885146	0.488	no
ni|645903495|nb|KK920935.1|	109670-109936	nan	nan	0.028602	0.984568	5	0.1206	no
ni|645903495|nb|KK920935.1|	113303-113456	nan	nan	211	377	0.835756	0.50405	no
ni|645903495|nb|KK920935.1|	114568-114786	nan	nan	108	81	-0.402146	0.72615	no
ni|645903495|nb|KK920935.1|	117191-117480	nan	nan	0	39	inf	5.00E-05	yes
ni|645903495|nb|KK920935.1|	117721-118636	nan	nan	8	8	-0.0324053	0.9792	no
ni|645903495|nb|KK920935.1|	119351-120070	nan	nan	0.433852	0.241976	-0.842339	0.5595	no
ni|645903495|nb|KK920935.1|	120293-120903	nan	nan	25	36	0.517349	0.68765	no
ni|645903495|nb|KK920935.1|	121099-121260	nan	nan	19	15	-0.389161	0.74325	no
ni|645903495|nb|KK920935.1|	123461-123801	nan	nan	10	9	-0.104757	0.92765	no
ni|645903495|nb|KK920935.1|	124834-125339	nan	nan	5	5	-0.217615	0.85485	no
ni|645903495|nb|KK920935.1|	126079-126763	nan	nan	13	11	-0.33729	0.7641	no
ni|645903495|nb|KK920935.1|	131221-131745	nan	nan	34	22	-0.638056	0.58195	no

ni|645903495|nb|KK920935.1|	135477-135632	nan	nan	13	11	-0.289804	0.79245	no
ni|645903495|nb|KK920935.1|	138445-139226	nan	nan	21	17	-0.323806	0.78595	no
ni|645903495|nb|KK920935.1|	140555-141233	nan	nan	18	8	-1	0.3768	no
ni|645903495|nb|KK920935.1|	142064-142863	nan	nan	17	13	-0.366776	0.74455	no
ni|645903495|nb|KK920935.1|	145775-145923	nan	nan	28	17	-0.671335	0.5829	no
ni|645903495|nb|KK920935.1|	148181-148770	nan	nan	29	21	-0.456102	0.6859	no
ni|645903495|nb|KK920935.1|	149413-149800	nan	nan	12	6.93	-0.741612	0.5176	no
ni|645903495|nb|KK920935.1|	150016-150169	nan	nan	15	14	-0.169235	0.8788	no
ni|645903495|nb|KK920935.1|	150319-151383	nan	nan	3	3	0.119269	0.91685	no
ni|645903495|nb|KK920935.1|	151511-151961	nan	nan	4	3	-0.416488	0.7196	no
ni|645903495|nb|KK920935.1|	160816-160977	nan	nan	16	10	-0.682348	0.5561	no
ni|645903495|nb|KK920935.1|	162103-162721	nan	nan	12	10	-0.367375	0.75275	no
ni|645903495|nb|KK920935.1|	169014-169195	nan	nan	13	8	-0.59927	0.61475	no
ni|645903495|nb|KK920935.1|	169251-169738	nan	nan	11	10	-0.203694	0.8622	no
ni|645903495|nb|KK920935.1|	172732-172876	nan	nan	9	9	-0.00870966	0.98665	no
ni|645903495|nb|KK920935.1|	174590-174766	nan	nan	7	6	-0.204774	0.8706	no
ni|645903495|nb|KK920935.1|	178859-178983	nan	nan	10	7	-0.459508	0.7047	no
ni|645903495|nb|KK920935.1|	181983-182113	nan	nan	6	5	-0.168251	0.88125	no
ni|645903495|nb|KK920935.1|	183757-183925	nan	nan	9	7	-0.330234	0.797	no
ni|645903495|nb|KK920935.1|	187525-187720	nan	nan	4	3	-0.328754	0.7835	no
ni|645903495|nb|KK920935.1|	190152-191174	nan	nan	5	6	0.328822	0.79035	no
ni|645903495|nb|KK920935.1|	191666-191959	nan	nan	1	0.940971	-0.651122	0.6679	no
ni|645903495|nb|KK920935.1|	192702-193080	nan	nan	4	5	0.347835	0.7963	no
ni|645903495|nb|KK920935.1|	195233-195426	nan	nan	1	2	1	0.45555	no
ni|645903495|nb|KK920935.1|	195572-196070	nan	nan	0	9	inf	5.00E-05	yes
ni|645903495|nb|KK920935.1|	196367-196672	nan	nan	0	5	inf	0.00015	yes
ni|645903495|nb|KK920935.1|	196881-197585	nan	nan	2	1	-0.48172	0.7594	no
ni|645903495|nb|KK920935.1|	198260-198392	nan	nan	5	6	0.319764	0.8092	no
ni|645903495|nb|KK920935.1|	198507-198696	nan	nan	8	7	-0.162381	0.89595	no
ni|645903495|nb|KK920935.1|	199808-200130	nan	nan	5	9	0.798645	0.50085	no

ni|645903495|nb|KK920935.1|	200434-200789	nan	nan	13	32	1	0.26655	no
ni|645903495|nb|KK920935.1|	201270-201682	nan	nan	18	40.7	1	0.29895	no
ni|645903495|nb|KK920935.1|	203198-203381	nan	nan	27	50	0.857856	0.5505	no
ni|645903495|nb|KK920935.1|	205695-205968	nan	nan	10	20	1	0.38845	no
ni|645903495|nb|KK920935.1|	206324-206574	nan	nan	9	13	0.57823	0.61905	no
ni|645903495|nb|KK920935.1|	208709-208852	nan	nan	25	25	0.0155382	0.98855	no
ni|645903495|nb|KK920935.1|	210284-210528	nan	nan	33	34	0.0366403	0.97595	no
ni|645903495|nb|KK920935.1|	211642-211980	nan	nan	76	118.85	0.651324	0.58115	no
ni|645903495|nb|KK920935.1|	214059-214466	nan	nan	0	2	inf	0.0001	yes
ni|645903495|nb|KK920935.1|	217857-219103	nan	nan	0	56	inf	5.00E-05	yes
ni|645903495|nb|KK920935.1|	227402-228171	nan	nan	19	32	0.770156	0.51875	no
ni|645903495|nb|KK920935.1|	228460-228992	nan	nan	4	2	-1	0.4064	no
ni|645903495|nb|KK920935.1|	22988-23139	nan	nan	1	0.763097	-0.460757	0.6964	no
ni|645903495|nb|KK920935.1|	230831-231097	nan	nan	59	78	0.394361	0.7491	no
ni|645903495|nb|KK920935.1|	232953-233078	nan	nan	60	77	0.364105	0.7411	no
ni|645903495|nb|KK920935.1|	233762-234244	nan	nan	5	3	-0.850451	0.48515	no
ni|645903495|nb|KK920935.1|	238062-239264	nan	nan	5	4	-0.219498	0.85045	no
ni|645903495|nb|KK920935.1|	239982-240396	nan	nan	17	10	-0.730279	0.5717	no
ni|645903495|nb|KK920935.1|	241846-242120	nan	nan	7	4	-0.652173	0.64	no
ni|645903495|nb|KK920935.1|	242902-243071	nan	nan	9	7	-0.347151	0.77315	no
ni|645903495|nb|KK920935.1|	243263-243996	nan	nan	7	6	-0.0664252	0.95665	no
ni|645903495|nb|KK920935.1|	244532-244977	nan	nan	0.917988	0.233995	-2	0.2004	no
ni|645903495|nb|KK920935.1|	245475-245948	nan	nan	0.616418	0.981716	0.671397	0.65595	no
ni|645903495|nb|KK920935.1|	246110-246401	nan	nan	59	78	0.399812	0.73675	no
ni|645903495|nb|KK920935.1|	247914-248217	nan	nan	41	42	0.0460193	0.9688	no
ni|645903495|nb|KK920935.1|	248546-248856	nan	nan	23	24	0.038781	0.97085	no
ni|645903495|nb|KK920935.1|	249337-249616	nan	nan	36	40	0.136526	0.9012	no
ni|645903495|nb|KK920935.1|	249785-249975	nan	nan	0.438448	0.157172	-1	0.3864	no
ni|645903495|nb|KK920935.1|	253755-253964	nan	nan	6	5	-0.270019	0.81495	no
ni|645903495|nb|KK920935.1|	254127-254414	nan	nan	8	6	-0.425474	0.71575	no

ni|645903495|nb|KK920935.1|	257384-257909	nan	nan	5	6	0.349125	0.7677	no
ni|645903495|nb|KK920935.1|	265256-265961	nan	nan	2	1	-0.365216	0.7706	no
ni|645903495|nb|KK920935.1|	266018-266152	nan	nan	0.573567	1	0.934861	0.4817	no
ni|645903495|nb|KK920935.1|	266235-266373	nan	nan	22	23	0.0869651	0.94065	no
ni|645903495|nb|KK920935.1|	267474-267791	nan	nan	4	3	-0.509841	0.6587	no
ni|645903495|nb|KK920935.1|	267931-268154	nan	nan	1	0.695882	-1	0.45115	no
ni|645903495|nb|KK920935.1|	268726-269245	nan	nan	30	28	-0.127098	0.90975	no
ni|645903495|nb|KK920935.1|	270016-270527	nan	nan	20	21	0.0273665	0.9803	no
ni|645903495|nb|KK920935.1|	277753-278491	nan	nan	375	295	-0.34254	0.81055	no
ni|645903495|nb|KK920935.1|	278789-280073	nan	nan	5	4	-0.0656558	0.95495	no
ni|645903495|nb|KK920935.1|	27949-28894	nan	nan	32	57	0.831167	0.56095	no
ni|645903495|nb|KK920935.1|	280635-280758	nan	nan	3	4	0.312264	0.79995	no
ni|645903495|nb|KK920935.1|	281544-281767	nan	nan	3249.91	2557.59	-0.345616	0.811	no
ni|645903495|nb|KK920935.1|	289110-289483	nan	nan	2814.29	2051.21	-0.456296	0.707	no
ni|645903495|nb|KK920935.1|	289895-290480	nan	nan	280	259.37	-0.111447	0.9154	no
ni|645903495|nb|KK920935.1|	291526-291731	nan	nan	857	810	-0.0810224	0.9523	no
ni|645903495|nb|KK920935.1|	294005-294350	nan	nan	1	1	0.0552557	0.96605	no
ni|645903495|nb|KK920935.1|	295250-295599	nan	nan	2	1	-0.58585	0.62085	no
ni|645903495|nb|KK920935.1|	296769-296983	nan	nan	541	519	-0.0605818	0.9631	no
ni|645903495|nb|KK920935.1|	312424-312611	nan	nan	0.477737	0.913325	0.934911	0.4874	no
ni|645903495|nb|KK920935.1|	314246-314624	nan	nan	3	3	0.133673	0.90805	no
ni|645903495|nb|KK920935.1|	315003-315212	nan	nan	0.226241	0.951685	2	0.14475	no
ni|645903495|nb|KK920935.1|	315314-315494	nan	nan	422	336.35	-0.326727	0.8266	no
ni|645903495|nb|KK920935.1|	317744-317924	nan	nan	11	15	0.386771	0.73465	no
ni|645903495|nb|KK920935.1|	320885-321132	nan	nan	3	6	1	0.39005	no
ni|645903495|nb|KK920935.1|	325847-326367	nan	nan	13	12	-0.0361192	0.9762	no
ni|645903495|nb|KK920935.1|	338956-339076	nan	nan	26	32	0.30823	0.7881	no
ni|645903495|nb|KK920935.1|	339605-339912	nan	nan	0.919577	2	0.998772	0.45905	no
ni|645903495|nb|KK920935.1|	346737-347848	nan	nan	1	1	-0.0655328	0.94765	no
ni|645903495|nb|KK920935.1|	347969-349337	nan	nan	3	5	0.740113	0.5765	no

ni|645903495|nb|KK920935.1|	350891-351073	nan	nan	14	10	-0.482442	0.7301	no
ni|645903495|nb|KK920935.1|	357052-358333	nan	nan	0	128	inf	5.00E-05	yes
ni|645903495|nb|KK920935.1|	358564-358806	nan	nan	0	100	inf	5.00E-05	yes
ni|645903495|nb|KK920935.1|	359089-359558	nan	nan	100	62	-0.691095	0.6396	no
ni|645903495|nb|KK920935.1|	363627-363841	nan	nan	160	101	-0.660095	0.56725	no
ni|645903495|nb|KK920935.1|	3649-4024	nan	nan	0.687674	0.657142	-0.0655198	0.9302	no
ni|645903495|nb|KK920935.1|	365888-366034	nan	nan	69	49	-0.495533	0.74365	no
ni|645903495|nb|KK920935.1|	368270-368502	nan	nan	171	120	-0.515453	0.68185	no
ni|645903495|nb|KK920935.1|	373204-375045	nan	nan	66	47	-0.507761	0.6315	no
ni|645903495|nb|KK920935.1|	378406-378541	nan	nan	0.88051	0.420671	-1	0.44965	no
ni|645903495|nb|KK920935.1|	38184-38524	nan	nan	195	145	-0.427985	0.68495	no
ni|645903495|nb|KK920935.1|	382635-382890	nan	nan	52	38	-0.460429	0.74835	no
ni|645903495|nb|KK920935.1|	383693-384196	nan	nan	0	160	inf	5.00E-05	yes
ni|645903495|nb|KK920935.1|	384490-384698	nan	nan	0	184	inf	5.00E-05	yes
ni|645903495|nb|KK920935.1|	385100-385486	nan	nan	0.518013	105.81	8	0.11935	no
ni|645903495|nb|KK920935.1|	385750-386032	nan	nan	5	8	0.484698	0.68695	no
ni|645903495|nb|KK920935.1|	386186-386348	nan	nan	0	2	inf	5.00E-05	yes
ni|645903495|nb|KK920935.1|	387469-387681	nan	nan	10	13	0.427348	0.708	no
ni|645903495|nb|KK920935.1|	388196-388516	nan	nan	6	8	0.353963	0.7634	no
ni|645903495|nb|KK920935.1|	393480-393630	nan	nan	5	4	-0.214998	0.855	no
ni|645903495|nb|KK920935.1|	394926-395064	nan	nan	3	16	2	0.0893	no
ni|645903495|nb|KK920935.1|	409017-409214	nan	nan	0	2	inf	0.00055	yes
ni|645903495|nb|KK920935.1|	410153-410409	nan	nan	24	21	-0.177316	0.8756	no
ni|645903495|nb|KK920935.1|	410592-411295	nan	nan	27	20	-0.391124	0.73925	no
ni|645903495|nb|KK920935.1|	415104-415494	nan	nan	17	17	-0.0274773	0.98085	no
ni|645903495|nb|KK920935.1|	417821-418749	nan	nan	34	40	0.222274	0.85215	no
ni|645903495|nb|KK920935.1|	419169-419966	nan	nan	19	15	-0.338216	0.749	no
ni|645903495|nb|KK920935.1|	426286-426553	nan	nan	4	3	-0.191794	0.86665	no
ni|645903495|nb|KK920935.1|	428705-429352	nan	nan	4	2	-0.602975	0.61685	no
ni|645903495|nb|KK920935.1|	431002-431301	nan	nan	15	15	0.0201287	0.96555	no

ni|645903495|nb|KK920935.1|	431444-432533	nan	nan	15	21	0.499983	0.67315	no
ni|645903495|nb|KK920935.1|	433-894	nan	nan	0.429847	0.513634	0.256919	0.86075	no
ni|645903495|nb|KK920935.1|	433036-433737	nan	nan	13	16	0.395013	0.73875	no
ni|645903495|nb|KK920935.1|	434240-434503	nan	nan	1	1	-0.318678	0.7938	no
ni|645903495|nb|KK920935.1|	434813-435385	nan	nan	0.825507	0.789151	-0.064978	0.9589	no
ni|645903495|nb|KK920935.1|	437059-437427	nan	nan	2	3	0.324106	0.7731	no
ni|645903495|nb|KK920935.1|	437716-439867	nan	nan	12	17	0.477121	0.69	no
ni|645903495|nb|KK920935.1|	441249-441998	nan	nan	10	18	0.78428	0.50835	no
ni|645903495|nb|KK920935.1|	442115-442519	nan	nan	12	20	0.681268	0.56535	no
ni|645903495|nb|KK920935.1|	442952-443162	nan	nan	7	11	0.754473	0.5144	no
ni|645903495|nb|KK920935.1|	443270-443795	nan	nan	9	14	0.687684	0.57215	no
ni|645903495|nb|KK920935.1|	444467-444777	nan	nan	0	54	inf	5.00E-05	yes
ni|645903495|nb|KK920935.1|	444879-445131	nan	nan	0	21	inf	5.00E-05	yes
ni|645903495|nb|KK920935.1|	445776-446477	nan	nan	0	44	inf	5.00E-05	yes
ni|645903495|nb|KK920935.1|	446602-447754	nan	nan	0	3	inf	5.00E-05	yes
ni|645903495|nb|KK920935.1|	448379-450601	nan	nan	0	44	inf	5.00E-05	yes
ni|645903495|nb|KK920935.1|	450805-451174	nan	nan	131	147	0.169703	0.9059	no
ni|645903495|nb|KK920935.1|	451366-451581	nan	nan	8	6	-0.526695	0.7071	no
ni|645903495|nb|KK920935.1|	451749-451985	nan	nan	2	4	0.895966	0.4567	no
ni|645903495|nb|KK920935.1|	452140-452426	nan	nan	7	6	-0.259949	0.8692	no
ni|645903495|nb|KK920935.1|	455896-456189	nan	nan	4	7	0.767789	0.5252	no
ni|645903495|nb|KK920935.1|	457517-458080	nan	nan	2	8	2	0.19185	no
ni|645903495|nb|KK920935.1|	458961-459154	nan	nan	1	7	2	0.12105	no
ni|645903495|nb|KK920935.1|	459587-459701	nan	nan	2	8	2	0.22835	no
ni|645903495|nb|KK920935.1|	460062-460196	nan	nan	0.304409	2	3	0.06975	no
ni|645903495|nb|KK920935.1|	460384-460945	nan	nan	3	6	1	0.39275	no
ni|645903495|nb|KK920935.1|	464641-464765	nan	nan	4	5	0.362724	0.75955	no
ni|645903495|nb|KK920935.1|	465409-465609	nan	nan	0.712144	2	2	0.205	no
ni|645903495|nb|KK920935.1|	466239-466655	nan	nan	3	4	0.403835	0.7286	no
ni|645903495|nb|KK920935.1|	471437-471750	nan	nan	0.140008	51	9	0.11935	no

ni|645903495|nb|KK920935.1|	476419-476949	nan	nan	6	23	2	0.102	no
ni|645903495|nb|KK920935.1|	478810-478977	nan	nan	28	108	2	0.0995	no
ni|645903495|nb|KK920935.1|	480322-480552	nan	nan	28	95	2	0.13505	no
ni|645903495|nb|KK920935.1|	484576-484781	nan	nan	32	134	2	0.07025	no
ni|645903495|nb|KK920935.1|	486713-486947	nan	nan	42	144	2	0.19275	no
ni|645903495|nb|KK920935.1|	487605-487853	nan	nan	39	139	2	0.1087	no
ni|645903495|nb|KK920935.1|	495278-495432	nan	nan	21	108	2	0.0697	no
ni|645903495|nb|KK920935.1|	497667-497917	nan	nan	36	44	0.310552	0.81835	no
ni|645903495|nb|KK920935.1|	504411-504621	nan	nan	0.929018	0.16143	-3	0.28285	no
ni|645903495|nb|KK920935.1|	508013-508314	nan	nan	13	11	-0.249998	0.83415	no
ni|645903495|nb|KK920935.1|	509656-509904	nan	nan	15	13	-0.246208	0.8319	no
ni|645903495|nb|KK920935.1|	513347-513501	nan	nan	65	41	-0.678685	0.5471	no
ni|645903495|nb|KK920935.1|	516027-516200	nan	nan	2	0.752941	-1	0.312	no
ni|645903495|nb|KK920935.1|	517919-518075	nan	nan	0.575461	0.528283	-0.123407	0.91825	no
ni|645903495|nb|KK920935.1|	519512-519712	nan	nan	1	0.727515	-0.743351	0.56925	no
ni|645903495|nb|KK920935.1|	528485-528669	nan	nan	658.49	557	-0.242264	0.8693	no
ni|645903495|nb|KK920935.1|	532003-532203	nan	nan	0.131793	2	4	0.12505	no
ni|645903495|nb|KK920935.1|	534747-534968	nan	nan	12	13	0.0623965	0.95915	no
ni|645903495|nb|KK920935.1|	542550-542760	nan	nan	13	15	0.150193	0.89225	no
ni|645903495|nb|KK920935.1|	545752-545964	nan	nan	92	101	0.135924	0.9003	no
ni|645903495|nb|KK920935.1|	549357-549485	nan	nan	46	45	-0.039425	0.97075	no
ni|645903495|nb|KK920935.1|	551802-552050	nan	nan	9	8	-0.171297	0.8848	no
ni|645903495|nb|KK920935.1|	553775-553976	nan	nan	16	16	-0.0381785	0.97275	no
ni|645903495|nb|KK920935.1|	555756-555909	nan	nan	5	5	-0.111864	0.92245	no
ni|645903495|nb|KK920935.1|	557242-557454	nan	nan	19	19	0.0343509	0.9739	no
ni|645903495|nb|KK920935.1|	560408-560722	nan	nan	17	22	0.365732	0.7542	no
ni|645903495|nb|KK920935.1|	564634-564772	nan	nan	15	21	0.484344	0.678	no
ni|645903495|nb|KK920935.1|	564954-565368	nan	nan	5	6	0.376466	0.7533	no
ni|645903495|nb|KK920935.1|	574895-575155	nan	nan	4	4	0.20133	0.85755	no
ni|645903495|nb|KK920935.1|	579914-580217	nan	nan	7	8	0.322932	0.7731	no

ni|645903495|nb|KK920935.1|	584966-585081	nan	nan	159	180	0.177108	0.871	no
ni|645903495|nb|KK920935.1|	587127-587592	nan	nan	135	148	0.131866	0.93215	no
ni|645903495|nb|KK920935.1|	587711-588020	nan	nan	58	62	0.0854989	0.9365	no
ni|645903495|nb|KK920935.1|	651372-651774	nan	nan	166	185	0.152694	0.91945	no
ni|645903495|nb|KK920935.1|	653481-653793	nan	nan	178	187	0.0725772	0.96045	no
ni|645903495|nb|KK920935.1|	68848-69152	nan	nan	317	361	0.185502	0.8625	no
ni|645903495|nb|KK920935.1|	78509-78758	nan	nan	148	231	0.643234	0.643	no
ni|645903495|nb|KK920935.1|	88-370	nan	nan	1	0.571876	-0.895247	0.502	no
ni|645903495|nb|KK920935.1|	99662-100016	nan	nan	200	331	0.726736	0.61555	no
ni|645903496|nb|KK920934.1|	1020859-1021023	nan	nan	5	1	-2	0.1791	no
ni|645903496|nb|KK920934.1|	1023421-1023627	nan	nan	2	0.579392	-2	0.2095	no
ni|645903496|nb|KK920934.1|	1025249-1025774	nan	nan	2	0.455126	-2	0.2004	no
ni|645903496|nb|KK920934.1|	1026754-1027008	nan	nan	5	2	-1	0.2739	no
ni|645903496|nb|KK920934.1|	1027395-1027686	nan	nan	0.943588	0.225391	-2	0.3383	no
ni|645903496|nb|KK920934.1|	1028598-1029050	nan	nan	33	26	-0.363583	0.75845	no
ni|645903496|nb|KK920934.1|	1029330-1029466	nan	nan	3	2	-0.501835	0.68865	no
ni|645903496|nb|KK920934.1|	1030640-1030829	nan	nan	4	3	-0.423786	0.7232	no
ni|645903496|nb|KK920934.1|	1031671-1032061	nan	nan	13	10	-0.469421	0.68605	no
ni|645903496|nb|KK920934.1|	1032187-1032312	nan	nan	2	1	-0.444388	0.74215	no
ni|645903496|nb|KK920934.1|	1037680-1038610	nan	nan	3	3	-0.0111254	0.97705	no
ni|645903496|nb|KK920934.1|	1038674-1039225	nan	nan	34	21	-0.682528	0.56015	no
ni|645903496|nb|KK920934.1|	1039410-1039861	nan	nan	36.61	34	-0.115151	0.92285	no
ni|645903496|nb|KK920934.1|	1041082-1041469	nan	nan	10	6	-0.84479	0.48425	no
ni|645903496|nb|KK920934.1|	1045179-1045610	nan	nan	4	4	-0.114997	0.9211	no
ni|645903496|nb|KK920934.1|	1045884-1046137	nan	nan	33	39	0.252552	0.82935	no
ni|645903496|nb|KK920934.1|	126898-127409	nan	nan	243	149	-0.707665	0.5344	no
ni|645903496|nb|KK920934.1|	127789-129210	nan	nan	0.862716	0.706514	-0.288168	0.8535	no
ni|645903496|nb|KK920934.1|	131948-132531	nan	nan	6	7	0.244299	0.8349	no
ni|645903496|nb|KK920934.1|	135511-135635	nan	nan	17	14	-0.252689	0.8207	no
ni|645903496|nb|KK920934.1|	137454-137588	nan	nan	1	23	4	0.0083	no

ni|645903496|nb|KK920934.1|	139160-139379	nan	nan	5	6	0.188262	0.8711	no
ni|645903496|nb|KK920934.1|	141608-141957	nan	nan	10	14	0.496698	0.67765	no
ni|645903496|nb|KK920934.1|	143358-143722	nan	nan	0.244055	7	5	0.0449	no
ni|645903496|nb|KK920934.1|	143830-144029	nan	nan	3	2	-0.652278	0.66125	no
ni|645903496|nb|KK920934.1|	145174-145407	nan	nan	0.736217	4	2	0.0709	no
ni|645903496|nb|KK920934.1|	145521-145942	nan	nan	1700.61	1316.42	-0.36943	0.79625	no
ni|645903496|nb|KK920934.1|	146560-147089	nan	nan	3	8	2	0.16525	no
ni|645903496|nb|KK920934.1|	148293-148437	nan	nan	1	0.845107	-0.387579	0.78555	no
ni|645903496|nb|KK920934.1|	149314-149597	nan	nan	0.843747	1	0.482335	0.6999	no
ni|645903496|nb|KK920934.1|	149948-150207	nan	nan	32	26	-0.306527	0.78	no
ni|645903496|nb|KK920934.1|	151085-151709	nan	nan	41	32	-0.375617	0.7566	no
ni|645903496|nb|KK920934.1|	153111-153505	nan	nan	37	28	-0.408614	0.755	no
ni|645903496|nb|KK920934.1|	154116-154466	nan	nan	26	24	-0.130837	0.90405	no
ni|645903496|nb|KK920934.1|	155024-155361	nan	nan	16	8	-1	0.44755	no
ni|645903496|nb|KK920934.1|	155717-155978	nan	nan	38	33	-0.194036	0.86765	no
ni|645903496|nb|KK920934.1|	156447-157468	nan	nan	25	24.23	-0.0386264	0.9718	no
ni|645903496|nb|KK920934.1|	159764-160674	nan	nan	11	12	0.144197	0.8963	no
ni|645903496|nb|KK920934.1|	160788-160896	nan	nan	4	7	0.726294	0.56145	no
ni|645903496|nb|KK920934.1|	161306-162555	nan	nan	1	0.818596	-0.550482	0.66085	no
ni|645903496|nb|KK920934.1|	163491-163684	nan	nan	0.767602	0.381663	-1	0.4077	no
ni|645903496|nb|KK920934.1|	171099-171451	nan	nan	0.678241	0.353624	-0.939582	0.54025	no
ni|645903496|nb|KK920934.1|	18175-18841	nan	nan	128	103.33	-0.313102	0.78265	no
ni|645903496|nb|KK920934.1|	19260-19439	nan	nan	5	4	-0.244095	0.83805	no
ni|645903496|nb|KK920934.1|	195330-196100	nan	nan	1	0.473369	-1	0.4023	no
ni|645903496|nb|KK920934.1|	19567-20527	nan	nan	78	76	-0.0251867	0.97915	no
ni|645903496|nb|KK920934.1|	20686-20966	nan	nan	90	81	-0.155378	0.88645	no
ni|645903496|nb|KK920934.1|	22055-22222	nan	nan	159	113	-0.487627	0.66705	no
ni|645903496|nb|KK920934.1|	223272-223385	nan	nan	29	33	0.206857	0.8634	no
ni|645903496|nb|KK920934.1|	272328-272511	nan	nan	36	22	-0.665373	0.57145	no
ni|645903496|nb|KK920934.1|	274769-274953	nan	nan	60	34	-0.795455	0.50125	no

ni|645903496|nb|KK920934.1|	275267-275408	nan	nan	0	5	inf	5.00E-05	yes
ni|645903496|nb|KK920934.1|	276344-276515	nan	nan	0	6	inf	5.00E-05	yes
ni|645903496|nb|KK920934.1|	277532-277921	nan	nan	0	4	inf	5.00E-05	yes
ni|645903496|nb|KK920934.1|	27779-29849	nan	nan	96	73	-0.40104	0.7436	no
ni|645903496|nb|KK920934.1|	280215-280358	nan	nan	0.01074	13	10	0.11935	no
ni|645903496|nb|KK920934.1|	280542-280656	nan	nan	18	17	-0.0673551	0.9579	no
ni|645903496|nb|KK920934.1|	280740-280880	nan	nan	8	16	0.934735	0.421	no
ni|645903496|nb|KK920934.1|	281433-281553	nan	nan	0.83001	0.453371	-0.872438	0.53675	no
ni|645903496|nb|KK920934.1|	282128-282638	nan	nan	13	25	0.93405	0.4225	no
ni|645903496|nb|KK920934.1|	283111-283585	nan	nan	39	68	0.802545	0.4874	no
ni|645903496|nb|KK920934.1|	286891-287143	nan	nan	14	3	-2	0.1863	no
ni|645903496|nb|KK920934.1|	288449-288831	nan	nan	4	5	0.402244	0.75355	no
ni|645903496|nb|KK920934.1|	289558-290350	nan	nan	3	4	0.541919	0.64695	no
ni|645903496|nb|KK920934.1|	291184-291417	nan	nan	2	9	2	0.15255	no
ni|645903496|nb|KK920934.1|	293448-293652	nan	nan	2	6	2	0.18445	no
ni|645903496|nb|KK920934.1|	293762-293970	nan	nan	2	4	1	0.2714	no
ni|645903496|nb|KK920934.1|	295096-295324	nan	nan	0.727521	2	1	0.392	no
ni|645903496|nb|KK920934.1|	296049-296332	nan	nan	27	16	-0.730418	0.5717	no
ni|645903496|nb|KK920934.1|	29959-30394	nan	nan	244	171.54	-0.506245	0.73075	no
ni|645903496|nb|KK920934.1|	300295-300527	nan	nan	46	50	0.121239	0.9349	no
ni|645903496|nb|KK920934.1|	301352-301764	nan	nan	87	107	0.297545	0.78455	no
ni|645903496|nb|KK920934.1|	302389-302536	nan	nan	0	6	inf	5.00E-05	yes
ni|645903496|nb|KK920934.1|	302665-303288	nan	nan	70	56	-0.321574	0.78335	no
ni|645903496|nb|KK920934.1|	303399-303920	nan	nan	12	26	1	0.3854	no
ni|645903496|nb|KK920934.1|	305067-305400	nan	nan	1	2	0.862785	0.4667	no
ni|645903496|nb|KK920934.1|	305525-306591	nan	nan	0.4787	9	4	0.00315	no
ni|645903496|nb|KK920934.1|	30565-30935	nan	nan	236	163	-0.533663	0.6248	no
ni|645903496|nb|KK920934.1|	307702-307951	nan	nan	1	2	0.671268	0.62105	no
ni|645903496|nb|KK920934.1|	308165-308330	nan	nan	7	22	2	0.1517	no
ni|645903496|nb|KK920934.1|	309347-309519	nan	nan	30	95	2	0.20965	no

ni|645903496|nb|KK920934.1|	309657-309976	nan	nan	55	187	2	0.18315	no
ni|645903496|nb|KK920934.1|	310135-310369	nan	nan	16	57	2	0.17325	no
ni|645903496|nb|KK920934.1|	311411-312191	nan	nan	1	1	0.282382	0.82625	no
ni|645903496|nb|KK920934.1|	312451-313047	nan	nan	21	24	0.173431	0.8916	no
ni|645903496|nb|KK920934.1|	313164-313586	nan	nan	2	0.260881	-3	0.133	no
ni|645903496|nb|KK920934.1|	313734-315238	nan	nan	3	0.941603	-2	0.2414	no
ni|645903496|nb|KK920934.1|	315475-316379	nan	nan	3	0.877218	-2	0.18585	no
ni|645903496|nb|KK920934.1|	319459-319926	nan	nan	3	0.468961	-3	0.2671	no
ni|645903496|nb|KK920934.1|	322157-322356	nan	nan	0.736205	0	-inf	0.00455	no
ni|645903496|nb|KK920934.1|	322438-322593	nan	nan	5	5	-0.0215943	0.9831	no
ni|645903496|nb|KK920934.1|	322758-322949	nan	nan	59	69	0.210882	0.8561	no
ni|645903496|nb|KK920934.1|	323406-323871	nan	nan	8	8	-0.00955549	0.98865	no
ni|645903496|nb|KK920934.1|	325644-326181	nan	nan	7	6	-0.2267	0.85115	no
ni|645903496|nb|KK920934.1|	327866-328306	nan	nan	0.641588	0.438198	-0.550065	0.661	no
ni|645903496|nb|KK920934.1|	329070-329522	nan	nan	1	1	-0.0105302	0.97705	no
ni|645903496|nb|KK920934.1|	330181-330548	nan	nan	25	22	-0.194279	0.85995	no
ni|645903496|nb|KK920934.1|	331716-332181	nan	nan	103	93	-0.142983	0.8977	no
ni|645903496|nb|KK920934.1|	333739-334164	nan	nan	65	58	-0.152116	0.8932	no
ni|645903496|nb|KK920934.1|	334477-334735	nan	nan	83	83	0.000139592	0.99755	no
ni|645903496|nb|KK920934.1|	335366-335584	nan	nan	74	58	-0.339471	0.7709	no
ni|645903496|nb|KK920934.1|	336325-336555	nan	nan	49	48	-0.0336058	0.9766	no
ni|645903496|nb|KK920934.1|	337128-337443	nan	nan	0	1	inf	0.00055	yes
ni|645903496|nb|KK920934.1|	338999-339245	nan	nan	4	4	0.182494	0.87375	no
ni|645903496|nb|KK920934.1|	339846-340547	nan	nan	10	7	-0.44579	0.74215	no
ni|645903496|nb|KK920934.1|	340918-341756	nan	nan	22	20	-0.13132	0.9097	no
ni|645903496|nb|KK920934.1|	362157-362379	nan	nan	19	17	-0.110275	0.92825	no
ni|645903496|nb|KK920934.1|	363132-363972	nan	nan	18	25	0.463271	0.6936	no
ni|645903496|nb|KK920934.1|	374031-374193	nan	nan	11	19	0.825806	0.51075	no
ni|645903496|nb|KK920934.1|	374765-375006	nan	nan	19	17	-0.116689	0.9224	no
ni|645903496|nb|KK920934.1|	375203-375873	nan	nan	25	17	-0.554967	0.61605	no

ni|645903496|nb|KK920934.1|	376006-376384	nan	nan	19	15	-0.351225	0.75885	no
ni|645903496|nb|KK920934.1|	376474-376940	nan	nan	2	2	-0.104463	0.9266	no
ni|645903496|nb|KK920934.1|	384793-385164	nan	nan	3	3	-0.106821	0.9284	no
ni|645903496|nb|KK920934.1|	386548-387389	nan	nan	25	14	-0.869879	0.4637	no
ni|645903496|nb|KK920934.1|	467635-467825	nan	nan	0	33	inf	5.00E-05	yes
ni|645903496|nb|KK920934.1|	472270-473782	nan	nan	0	101	inf	5.00E-05	yes
ni|645903496|nb|KK920934.1|	473864-474036	nan	nan	0	94	inf	5.00E-05	yes
ni|645903496|nb|KK920934.1|	475141-475286	nan	nan	9	5	-0.974118	0.5005	no
ni|645903496|nb|KK920934.1|	479088-479783	nan	nan	2	3	0.633374	0.63975	no
ni|645903496|nb|KK920934.1|	480960-481084	nan	nan	0.503334	0.541256	0.104796	0.93335	no
ni|645903496|nb|KK920934.1|	519668-520158	nan	nan	5	12	1	0.3144	no
ni|645903496|nb|KK920934.1|	521242-521504	nan	nan	1	1	-0.287556	0.8142	no
ni|645903496|nb|KK920934.1|	544416-544825	nan	nan	0.312987	0.777848	1	0.35675	no
ni|645903496|nb|KK920934.1|	574314-574486	nan	nan	0.41793	0.581213	0.475806	0.709	no
ni|645903496|nb|KK920934.1|	575167-575296	nan	nan	22	14	-0.598034	0.64595	no
ni|645903496|nb|KK920934.1|	575975-576321	nan	nan	4	3	-0.231362	0.8467	no
ni|645903496|nb|KK920934.1|	576384-576527	nan	nan	7	6	-0.177754	0.8763	no
ni|645903496|nb|KK920934.1|	577400-577627	nan	nan	12	9	-0.428413	0.71355	no
ni|645903496|nb|KK920934.1|	578775-578902	nan	nan	230	143	-0.681885	0.5479	no
ni|645903496|nb|KK920934.1|	579376-579705	nan	nan	217	164	-0.399519	0.70995	no
ni|645903496|nb|KK920934.1|	581514-581667	nan	nan	164	123	-0.422032	0.70755	no
ni|645903496|nb|KK920934.1|	581821-582096	nan	nan	0.571562	0.117097	-2	0.1864	no
ni|645903496|nb|KK920934.1|	585088-585320	nan	nan	1	0.170159	-3	0.2671	no
ni|645903496|nb|KK920934.1|	586740-586878	nan	nan	3	4	0.47806	0.6788	no
ni|645903496|nb|KK920934.1|	591519-592676	nan	nan	4	4	0.174859	0.88505	no
ni|645903496|nb|KK920934.1|	6718-7066	nan	nan	44	40	-0.140251	0.8993	no
ni|645903496|nb|KK920934.1|	844750-845210	nan	nan	4	3	-0.329752	0.79705	no
ni|645903496|nb|KK920934.1|	845831-846162	nan	nan	3	4	0.7185	0.5339	no
ni|645903496|nb|KK920934.1|	846437-846545	nan	nan	8	8	-0.0249525	0.9836	no
ni|645903496|nb|KK920934.1|	847338-847712	nan	nan	4	5	0.265293	0.8197	no

ni|645903496|nb|KK920934.1|	848099-848306	nan	nan	6	4	-0.482295	0.7594	no
ni|645903496|nb|KK920934.1|	848523-849431	nan	nan	9	9	-0.0294155	0.97835	no
ni|645903496|nb|KK920934.1|	849935-850387	nan	nan	2	2	0.487976	0.67485	no
ni|645903496|nb|KK920934.1|	851408-851628	nan	nan	2	3	0.555298	0.6571	no
ni|645903496|nb|KK920934.1|	854264-854460	nan	nan	2	2	0.108941	0.9222	no
ni|645903496|nb|KK920934.1|	854742-854927	nan	nan	2	2	0.18223	0.87985	no
ni|645903496|nb|KK920934.1|	858329-858799	nan	nan	9	10	0.0622498	0.95575	no
ni|645903496|nb|KK920934.1|	858940-860240	nan	nan	11	11	-0.111998	0.9193	no
ni|645903496|nb|KK920934.1|	860562-860930	nan	nan	8	7	-0.0645156	0.95355	no
ni|645903496|nb|KK920934.1|	861329-861471	nan	nan	19	12	-0.652457	0.64	no
ni|645903496|nb|KK920934.1|	865098-865448	nan	nan	29	36	0.334283	0.76335	no
ni|645903496|nb|KK920934.1|	866888-867034	nan	nan	19	17	-0.0938541	0.94855	no
ni|645903496|nb|KK920934.1|	867759-868029	nan	nan	3	3	0.255053	0.84735	no
ni|645903496|nb|KK920934.1|	871262-871640	nan	nan	9	6	-0.552345	0.6504	no
ni|645903496|nb|KK920934.1|	871847-872017	nan	nan	11	8	-0.482353	0.68515	no
ni|645903496|nb|KK920934.1|	872279-872652	nan	nan	16	14	-0.227665	0.8507	no
ni|645903496|nb|KK920934.1|	872772-873900	nan	nan	36	25	-0.5236	0.6559	no
ni|645903496|nb|KK920934.1|	874003-874861	nan	nan	23	16	-0.499608	0.66835	no
ni|645903496|nb|KK920934.1|	881350-881634	nan	nan	21	19	-0.134437	0.90875	no
ni|645903496|nb|KK920934.1|	881754-882012	nan	nan	14	13	-0.0471514	0.9639	no
ni|645903496|nb|KK920934.1|	882303-883017	nan	nan	0.758848	0.362639	-1	0.44755	no
ni|645903496|nb|KK920934.1|	884381-884710	nan	nan	39	19	-1	0.4081	no
ni|645903496|nb|KK920934.1|	885024-885461	nan	nan	0.709136	0.52714	-0.427878	0.75965	no
ni|645903496|nb|KK920934.1|	886104-886558	nan	nan	77	41	-0.913888	0.4182	no
ni|645903496|nb|KK920934.1|	888609-889016	nan	nan	13059.6	10779.4	-0.276833	0.70935	no
ni|645903496|nb|KK920934.1|	889486-889595	nan	nan	4	1	-2	0.29725	no
ni|645903496|nb|KK920934.1|	890578-891731	nan	nan	0.891246	0.66241	-0.428099	0.75965	no
ni|645903496|nb|KK920934.1|	892003-892689	nan	nan	11	12	0.19621	0.86035	no
ni|645903496|nb|KK920934.1|	893067-894184	nan	nan	3	2	-0.557086	0.64005	no
ni|645903496|nb|KK920934.1|	896287-896777	nan	nan	22	24	0.0976998	0.93265	no

ni|645903496|nb|KK920934.1|	898478-898909	nan	nan	23	28	0.290728	0.8094	no
ni|645903496|nb|KK920934.1|	899098-899620	nan	nan	32	33	0.0471057	0.96225	no
ni|645903496|nb|KK920934.1|	899742-900275	nan	nan	0.770446	0.736175	-0.0656457	0.9302	no
ni|645903496|nb|KK920934.1|	900423-901077	nan	nan	4	4	-0.0365466	0.9743	no
ni|645903496|nb|KK920934.1|	901296-901519	nan	nan	3	5	0.883832	0.45565	no
ni|645903496|nb|KK920934.1|	902587-902877	nan	nan	2	7	2	0.1683	no
ni|645903496|nb|KK920934.1|	904258-905442	nan	nan	0.519175	2	2	0.234	no
ni|645903496|nb|KK920934.1|	905548-906148	nan	nan	64	56	-0.19908	0.85735	no
ni|645903496|nb|KK920934.1|	910042-910315	nan	nan	0.488757	0.297352	-0.716944	0.61925	no
ni|645903496|nb|KK920934.1|	910443-910822	nan	nan	11	21	0.932505	0.51205	no
ni|645903496|nb|KK920934.1|	914241-914361	nan	nan	70	52	-0.440099	0.7051	no
ni|645903496|nb|KK920934.1|	915258-916095	nan	nan	44	23	-0.936649	0.41555	no
ni|645903496|nb|KK920934.1|	921158-921602	nan	nan	42	40	-0.0674725	0.95525	no
ni|645903496|nb|KK920934.1|	922525-922706	nan	nan	60	52	-0.189531	0.86035	no
ni|645903496|nb|KK920934.1|	923028-923201	nan	nan	0.660609	0.684118	0.0504493	0.95725	no
ni|645903496|nb|KK920934.1|	923281-923520	nan	nan	30	33	0.133066	0.9093	no
ni|645903496|nb|KK920934.1|	923621-923769	nan	nan	43	38	-0.174097	0.8785	no
ni|645903496|nb|KK920934.1|	923899-924184	nan	nan	1	0.416392	-1	0.3479	no
ni|645903496|nb|KK920934.1|	924704-925406	nan	nan	9	9	-0.0577989	0.961	no
ni|645903496|nb|KK920934.1|	925591-927372	nan	nan	25	29	0.195515	0.87315	no
ni|645903496|nb|KK920934.1|	928082-928279	nan	nan	19	15	-0.308726	0.7922	no
ni|645903496|nb|KK920934.1|	929341-929459	nan	nan	3	5	0.476082	0.6773	no
ni|645903496|nb|KK920934.1|	929561-929688	nan	nan	1	0.847291	-0.605895	0.6316	no
ni|645903496|nb|KK920934.1|	929828-929947	nan	nan	0.690521	0.707278	0.0345918	0.96125	no
ni|645903496|nb|KK920934.1|	930450-931183	nan	nan	0.990002	1	0.282495	0.82625	no
ni|645903496|nb|KK920934.1|	931356-931623	nan	nan	0.76028	2	1	0.4471	no
ni|645903496|nb|KK920934.1|	933998-934291	nan	nan	3	1	-1	0.4064	no
ni|645903496|nb|KK920934.1|	935319-936213	nan	nan	2	2	-0.00058063	0.99105	no
ni|645903496|nb|KK920934.1|	936436-936773	nan	nan	8	12	0.643194	0.5963	no
ni|645903496|nb|KK920934.1|	937259-937411	nan	nan	4	5	0.337458	0.77805	no

ni|645903496|nb|KK920934.1|	938026-938226	nan	nan	5	6	0.290701	0.80315	no
ni|645903496|nb|KK920934.1|	941502-941833	nan	nan	4	8	1	0.4054	no
ni|645903496|nb|KK920934.1|	942176-942364	nan	nan	7	9	0.347759	0.7963	no
ni|645903496|nb|KK920934.1|	944588-945428	nan	nan	3	3	-0.116335	0.9214	no
ni|645903496|nb|KK920934.1|	945555-945920	nan	nan	4	2	-0.770043	0.5207	no
ni|645903496|nb|KK920934.1|	946029-946254	nan	nan	7	7	-0.149984	0.894	no
ni|645903496|nb|KK920934.1|	948278-948639	nan	nan	169	133	-0.348134	0.80145	no
ni|645903496|nb|KK920934.1|	949021-949806	nan	nan	0.867906	1	0.256689	0.84735	no
ni|645903496|nb|KK920934.1|	951139-951527	nan	nan	236	197	-0.261443	0.8519	no
ni|645903496|nb|KK920934.1|	952492-952697	nan	nan	2	0.834935	-1	0.37315	no
ni|645903496|nb|KK920934.1|	952911-953071	nan	nan	2	1	-0.264296	0.81915	no
ni|645903496|nb|KK920934.1|	955850-956572	nan	nan	3	2	-0.744623	0.56925	no
ni|645903496|nb|KK920934.1|	957647-957897	nan	nan	2	2	-0.066285	0.94155	no
ni|645903496|nb|KK920934.1|	996485-996936	nan	nan	2	2	0.256545	0.8356	no
ni|645903497|nb|KK920933.1|	106839-107052	nan	nan	33	42	0.322555	0.7894	no
ni|645903497|nb|KK920933.1|	284767-285002	nan	nan	106	90	-0.240225	0.8697	no
ni|645903497|nb|KK920933.1|	423860-424183	nan	nan	0.953055	0.78043	-0.28829	0.8535	no
ni|645903497|nb|KK920933.1|	424403-424719	nan	nan	0.80264	1	0.475322	0.709	no
ni|645903497|nb|KK920933.1|	424771-424891	nan	nan	0.670921	0.769803	0.198346	0.8693	no
ni|645903497|nb|KK920933.1|	441490-441717	nan	nan	2	2	0.071474	0.94355	no
ni|645903497|nb|KK920933.1|	441905-442066	nan	nan	1	0.632735	-0.97224	0.5005	no
ni|645903497|nb|KK920933.1|	442887-443544	nan	nan	15	9	-0.791578	0.51845	no
ni|645903497|nb|KK920933.1|	443692-444013	nan	nan	47	44	-0.0961691	0.9339	no
ni|645903497|nb|KK920933.1|	445130-445430	nan	nan	1	0.639612	-0.842003	0.4717	no
ni|645903497|nb|KK920933.1|	448564-448879	nan	nan	1	0.687201	-0.958653	0.51225	no
ni|645903497|nb|KK920933.1|	453081-453317	nan	nan	3	2	-0.859231	0.49355	no
ni|645903498|nb|KK920932.1|	1004651-100518[illegible]	nan	nan	15	9	-0.804321	0.5588	no
ni|645903498|nb|KK920932.1|	1005487-100568[illegible]	nan	nan	12	9	-0.445704	0.71245	no
ni|645903498|nb|KK920932.1|	1005759-100677[illegible]	nan	nan	6	4	-0.618044	0.60675	no
ni|645903498|nb|KK920932.1|	1007268-100743[illegible]	nan	nan	5	7	0.517676	0.7279	no

ni|645903498|nb|KK920932.1|	1008054-100889[illegible]	nan	nan	2	2	-0.0660727	0.9545	no
ni|645903498|nb|KK920932.1|	1009082-100984[illegible]	nan	nan	4	4	0.13358	0.90795	no
ni|645903498|nb|KK920932.1|	117802-118080	nan	nan	63	61	-0.0388921	0.97075	no
ni|645903498|nb|KK920932.1|	136895-137095	nan	nan	19	18	-0.0287233	0.98165	no
ni|645903498|nb|KK920932.1|	138061-138206	nan	nan	0.167196	9	6	0.04445	no
ni|645903498|nb|KK920932.1|	139374-139793	nan	nan	8	9	0.201333	0.86335	no
ni|645903498|nb|KK920932.1|	139944-140123	nan	nan	11	7	-0.597717	0.62535	no
ni|645903498|nb|KK920932.1|	140764-141473	nan	nan	23	5	-2	0.0535	no
ni|645903498|nb|KK920932.1|	143950-144105	nan	nan	21	5	-2	0.0735	no
ni|645903498|nb|KK920932.1|	145831-146027	nan	nan	66	13	-2	0.053	no
ni|645903498|nb|KK920932.1|	146985-148078	nan	nan	0.607493	0.136669	-2	0.1501	no
ni|645903498|nb|KK920932.1|	148189-148313	nan	nan	34	5	-3	0.02795	no
ni|645903498|nb|KK920932.1|	148628-149554	nan	nan	56	7	-3	0.0194	no
ni|645903498|nb|KK920932.1|	149983-150127	nan	nan	26	4	-3	0.0505	no
ni|645903498|nb|KK920932.1|	152322-152548	nan	nan	33	5	-3	0.02895	no
ni|645903498|nb|KK920932.1|	153289-153739	nan	nan	0	34	inf	5.00E-05	yes
ni|645903498|nb|KK920932.1|	15470-15991	nan	nan	37	35	-0.0651015	0.95015	no
ni|645903498|nb|KK920932.1|	260386-260607	nan	nan	77	149	0.956213	0.511	no
ni|645903498|nb|KK920932.1|	272064-272296	nan	nan	69	105	0.607389	0.67365	no
ni|645903498|nb|KK920932.1|	275729-278965	nan	nan	13	17	0.401851	0.729	no
ni|645903498|nb|KK920932.1|	345882-346320	nan	nan	33	59	0.812792	0.47255	no
ni|645903498|nb|KK920932.1|	415502-416524	nan	nan	70	102	0.556961	0.69805	no
ni|645903498|nb|KK920932.1|	495019-495583	nan	nan	154	211	0.456021	0.6767	no
ni|645903498|nb|KK920932.1|	495756-495914	nan	nan	107	138	0.375679	0.7436	no
ni|645903498|nb|KK920932.1|	502659-502781	nan	nan	40	44	0.154957	0.88605	no
ni|645903498|nb|KK920932.1|	503674-504012	nan	nan	57	66	0.212469	0.88145	no
ni|645903498|nb|KK920932.1|	504248-504522	nan	nan	2	0.496849	-2	0.2784	no
ni|645903498|nb|KK920932.1|	507654-507852	nan	nan	229	258.25	0.173597	0.8753	no
ni|645903498|nb|KK920932.1|	508093-508562	nan	nan	3	3	-0.173466	0.89245	no
ni|645903498|nb|KK920932.1|	509783-510193	nan	nan	24	26	0.139229	0.9042	no

ni|645903498|nb|KK920932.1|	514295-514779	nan	nan	21	20	-0.0673438	0.957	no
ni|645903498|nb|KK920932.1|	573712-574159	nan	nan	18	22	0.304767	0.8009	no
ni|645903498|nb|KK920932.1|	575206-575450	nan	nan	16	19	0.263413	0.82105	no
ni|645903498|nb|KK920932.1|	575751-575927	nan	nan	0.193801	2	3	0.05325	no
ni|645903498|nb|KK920932.1|	576841-576977	nan	nan	2	4	1	0.3106	no
ni|645903498|nb|KK920932.1|	61386-61638	nan	nan	0.738408	0.705753	-0.0652553	0.9467	no
ni|645903498|nb|KK920932.1|	63017-63269	nan	nan	1	0.675078	-0.88814	0.4823	no
ni|645903498|nb|KK920932.1|	663133-663443	nan	nan	2	2	0.00818951	0.9732	no
ni|645903498|nb|KK920932.1|	69401-69758	nan	nan	73	63	-0.229095	0.84365	no
ni|645903498|nb|KK920932.1|	70282-71217	nan	nan	82	68	-0.272933	0.80115	no
ni|645903498|nb|KK920932.1|	71430-71576	nan	nan	66	52	-0.343049	0.7612	no
ni|645903498|nb|KK920932.1|	73279-73439	nan	nan	21.75	19	-0.217755	0.8479	no
ni|645903498|nb|KK920932.1|	77649-78195	nan	nan	0.60399	0.407642	-0.567221	0.6631	no
ni|645903498|nb|KK920932.1|	82031-82281	nan	nan	0	2	inf	0.0003	yes
ni|645903498|nb|KK920932.1|	88609-88790	nan	nan	62	54	-0.199798	0.8935	no
ni|645903498|nb|KK920932.1|	946481-947862	nan	nan	9	200	4	0.05165	no
ni|645903498|nb|KK920932.1|	955039-955322	nan	nan	3	202	6	0.0052	no
ni|645903498|nb|KK920932.1|	962523-962658	nan	nan	2	184.17	7	0.01465	no
ni|645903498|nb|KK920932.1|	965277-965501	nan	nan	0	99	inf	5.00E-05	yes
ni|645903498|nb|KK920932.1|	974924-975076	nan	nan	0	7	inf	0.00015	yes
ni|645903498|nb|KK920932.1|	978703-978840	nan	nan	3	1	-2	0.2996	no
ni|645903498|nb|KK920932.1|	981766-982337	nan	nan	67	67	-0.000516271	0.9974	no
ni|645903498|nb|KK920932.1|	982920-983633	nan	nan	95	82	-0.208558	0.8567	no
ni|645903498|nb|KK920932.1|	984122-984244	nan	nan	65	68	0.0665321	0.95305	no
ni|645903498|nb|KK920932.1|	987211-988130	nan	nan	3	2	-0.409249	0.73715	no
ni|645903498|nb|KK920932.1|	988253-988412	nan	nan	98	91	-0.105183	0.92725	no
ni|645903498|nb|KK920932.1|	988549-988723	nan	nan	63	63	-0.00241273	0.99365	no
ni|645903498|nb|KK920932.1|	990197-990345	nan	nan	34	38	0.150675	0.8898	no
ni|645903498|nb|KK920932.1|	990628-991401	nan	nan	0.105394	13	7	0.0443	no
ni|645903498|nb|KK920932.1|	996010-996908	nan	nan	9	8	-0.160316	0.89795	no

ni|645903499|nb|KK920931.1|	105885-106363	nan	nan	26	36	0.478602	0.682	no
ni|645903499|nb|KK920931.1|	106988-107618	nan	nan	28	39	0.508207	0.6594	no
ni|645903499|nb|KK920931.1|	107982-108253	nan	nan	18	23	0.37357	0.7527	no
ni|645903499|nb|KK920931.1|	108354-108510	nan	nan	7	12	0.865594	0.47505	no
ni|645903499|nb|KK920931.1|	109111-109655	nan	nan	0	10	inf	5.00E-05	yes
ni|645903499|nb|KK920931.1|	114846-115268	nan	nan	0	58	inf	0.00045	yes
ni|645903499|nb|KK920931.1|	116149-116329	nan	nan	0	47	inf	5.00E-05	yes
ni|645903499|nb|KK920931.1|	117978-118186	nan	nan	0	48	inf	5.00E-05	yes
ni|645903499|nb|KK920931.1|	119982-120146	nan	nan	0	33	inf	5.00E-05	yes
ni|645903499|nb|KK920931.1|	122003-122290	nan	nan	0.055547	33	9	0.11935	no
ni|645903499|nb|KK920931.1|	123722-123861	nan	nan	6	3	-0.960287	0.51205	no
ni|645903499|nb|KK920931.1|	125158-125399	nan	nan	2	2	-0.272386	0.8304	no
ni|645903499|nb|KK920931.1|	126749-126885	nan	nan	5	2	-1	0.4105	no
ni|645903499|nb|KK920931.1|	128514-129010	nan	nan	3	6	0.833832	0.50395	no
ni|645903499|nb|KK920931.1|	130398-130588	nan	nan	2	2	-0.0668324	0.94155	no
ni|645903499|nb|KK920931.1|	131023-131223	nan	nan	19	13	-0.558423	0.6183	no
ni|645903499|nb|KK920931.1|	133997-139455	nan	nan	0.43487	579	10	0.0205	no
ni|645903499|nb|KK920931.1|	142458-142703	nan	nan	4	3	-0.34732	0.76755	no
ni|645903499|nb|KK920931.1|	143517-144160	nan	nan	30	7	-2	0.1906	no
ni|645903499|nb|KK920931.1|	144805-145132	nan	nan	0.510655	0.313873	-0.702167	0.6038	no
ni|645903499|nb|KK920931.1|	147368-147525	nan	nan	0.358693	0.17146	-1	0.44965	no
ni|645903499|nb|KK920931.1|	148838-149257	nan	nan	0	17	inf	5.00E-05	yes
ni|645903499|nb|KK920931.1|	149704-150058	nan	nan	3	2	-0.246734	0.8314	no
ni|645903499|nb|KK920931.1|	150192-150718	nan	nan	7	5	-0.444355	0.7012	no
ni|645903499|nb|KK920931.1|	151832-152177	nan	nan	0.140495	0.537329	2	0.1847	no
ni|645903499|nb|KK920931.1|	152333-155326	nan	nan	0.656751	3	2	0.13305	no
ni|645903499|nb|KK920931.1|	156121-156485	nan	nan	2	4	0.854153	0.46585	no
ni|645903499|nb|KK920931.1|	156637-157723	nan	nan	3	1	-0.831617	0.5313	no
ni|645903499|nb|KK920931.1|	158249-158363	nan	nan	2	2	-0.526149	0.7071	no
ni|645903499|nb|KK920931.1|	160927-161154	nan	nan	2	2	-0.289718	0.8496	no

ni|645903499|nb|KK920931.1|	161299-161443	nan	nan	0	41	inf	5.00E-05	yes
ni|645903499|nb|KK920931.1|	161536-162829	nan	nan	0	47	inf	5.00E-05	yes
ni|645903499|nb|KK920931.1|	163444-164056	nan	nan	0	165	inf	5.00E-05	yes
ni|645903499|nb|KK920931.1|	164214-164943	nan	nan	0	15	inf	5.00E-05	yes
ni|645903499|nb|KK920931.1|	165151-165522	nan	nan	102.4	160	0.639948	0.56215	no
ni|645903499|nb|KK920931.1|	165774-165969	nan	nan	100	128.81	0.362186	0.81075	no
ni|645903499|nb|KK920931.1|	166233-166873	nan	nan	0.829018	0.924448	0.157189	0.90275	no
ni|645903499|nb|KK920931.1|	170138-170731	nan	nan	57	72	0.337268	0.7813	no
ni|645903499|nb|KK920931.1|	171013-171398	nan	nan	77	84	0.139215	0.9224	no
ni|645903499|nb|KK920931.1|	227071-227299	nan	nan	0	5	inf	0.0001	yes
ni|645903499|nb|KK920931.1|	269911-270429	nan	nan	0.604491	0.057782	-3	0.03815	no
ni|645903499|nb|KK920931.1|	303963-304105	nan	nan	2	2	-0.228851	0.84895	no
ni|645903499|nb|KK920931.1|	358836-358970	nan	nan	11	12	0.118923	0.9197	no
ni|645903499|nb|KK920931.1|	362403-362630	nan	nan	16	14	-0.237472	0.8697	no
ni|645903499|nb|KK920931.1|	370841-371143	nan	nan	9	5	-0.933949	0.4515	no
ni|645903499|nb|KK920931.1|	71540-71700	nan	nan	4	4	0.264585	0.8186	no
ni|645903499|nb|KK920931.1|	72306-72420	nan	nan	24	21	-0.160366	0.8895	no
ni|645903499|nb|KK920931.1|	72614-72888	nan	nan	19	15	-0.31586	0.7824	no
ni|645903499|nb|KK920931.1|	75367-75513	nan	nan	26	33	0.337999	0.77535	no
ni|645903499|nb|KK920931.1|	86648-86788	nan	nan	37	52	0.479288	0.67135	no
ni|645903499|nb|KK920931.1|	88136-88380	nan	nan	36	36	-0.00161413	0.9937	no
ni|645903499|nb|KK920931.1|	89916-90076	nan	nan	19	24	0.327204	0.7683	no
ni|645903499|nb|KK920931.1|	90912-91034	nan	nan	36	51	0.47115	0.67885	no
ni|645903499|nb|KK920931.1|	91699-91902	nan	nan	27	42	0.636686	0.58545	no
ni|645903500|nb|KK920930.1|	102074-102404	nan	nan	2	0.74993	-1	0.296	no
ni|645903500|nb|KK920930.1|	102597-102988	nan	nan	2	0.835734	-1	0.369	no
ni|645903500|nb|KK920930.1|	104512-104821	nan	nan	4	3	-0.481912	0.7105	no
ni|645903500|nb|KK920930.1|	106452-107050	nan	nan	3	3	0.168167	0.89635	no
ni|645903500|nb|KK920930.1|	112193-112384	nan	nan	6	4	-0.308211	0.81205	no
ni|645903500|nb|KK920930.1|	112568-112877	nan	nan	3	3	-0.237167	0.8697	no

ni|645903500|nb|KK920930.1|	113573-113836	nan	nan	5	3	-0.652164	0.66125	no
ni|645903500|nb|KK920930.1|	114305-115033	nan	nan	6	0	-inf	0.00455	no
ni|645903500|nb|KK920930.1|	115236-115369	nan	nan	8	10	0.19573	0.87955	no
ni|645903500|nb|KK920930.1|	115854-116192	nan	nan	5	4	-0.429812	0.73195	no
ni|645903500|nb|KK920930.1|	118732-119028	nan	nan	6	4	-0.733133	0.5374	no
ni|645903500|nb|KK920930.1|	143522-143699	nan	nan	5	4	-0.0671902	0.94635	no
ni|645903500|nb|KK920930.1|	143999-144150	nan	nan	7	5	-0.408287	0.755	no
ni|645903500|nb|KK920930.1|	144680-144945	nan	nan	10	4	-1	0.24025	no
ni|645903500|nb|KK920930.1|	145366-145506	nan	nan	6	5	-0.378242	0.75755	no
ni|645903500|nb|KK920930.1|	146209-146785	nan	nan	0.929334	0.68327	-0.443742	0.74215	no
ni|645903500|nb|KK920930.1|	190217-190357	nan	nan	10	8	-0.304295	0.7988	no
ni|645903500|nb|KK920930.1|	203017-203278	nan	nan	4	5	0.311295	0.81835	no
ni|645903500|nb|KK920930.1|	206581-206712	nan	nan	5	6	0.0475599	0.96665	no
ni|645903500|nb|KK920930.1|	209255-209554	nan	nan	4	3	-0.599948	0.6143	no
ni|645903500|nb|KK920930.1|	211095-211339	nan	nan	20	13	-0.611529	0.6098	no
ni|645903500|nb|KK920930.1|	212139-212360	nan	nan	13	3	-2	0.1083	no
ni|645903500|nb|KK920930.1|	231296-231531	nan	nan	9	6	-0.726255	0.54605	no
ni|645903500|nb|KK920930.1|	234270-235131	nan	nan	9	5	-0.783336	0.5048	no
ni|645903500|nb|KK920930.1|	235652-235951	nan	nan	8	7	-0.204783	0.8706	no
ni|645903500|nb|KK920930.1|	236868-237322	nan	nan	3	2	-0.506435	0.68965	no
ni|645903500|nb|KK920930.1|	237820-238007	nan	nan	13	8	-0.783416	0.53215	no
ni|645903500|nb|KK920930.1|	238249-238850	nan	nan	7	4	-0.719304	0.61925	no
ni|645903500|nb|KK920930.1|	239244-239512	nan	nan	4	6	0.574711	0.6304	no
ni|645903500|nb|KK920930.1|	239585-240109	nan	nan	6	3	-1	0.40265	no
ni|645903500|nb|KK920930.1|	240226-240621	nan	nan	6	4	-0.564826	0.6291	no
ni|645903500|nb|KK920930.1|	241430-241826	nan	nan	3	4	0.229459	0.849	no
ni|645903500|nb|KK920930.1|	241907-242620	nan	nan	0.279865	0.024329	-4	0.0326	no
ni|645903500|nb|KK920930.1|	94024-94175	nan	nan	6	1	-3	0.15215	no
ni|645903501|nb|KK920929.1|	109705-110010	nan	nan	9	5	-0.65217	0.5967	no
ni|645903501|nb|KK920929.1|	113367-113593	nan	nan	0.87372	0.649394	-0.428077	0.75965	no

ni|645903501|nb|KK920929.1|	11828-12068	nan	nan	7	9	0.209148	0.85925	no
ni|645903501|nb|KK920929.1|	149103-149403	nan	nan	31	40	0.347424	0.7834	no
ni|645903501|nb|KK920929.1|	197193-197507	nan	nan	4	2	-1	0.4023	no
ni|645903501|nb|KK920929.1|	243496-244423	nan	nan	10	4	-1	0.3479	no
ni|645903501|nb|KK920929.1|	246178-247105	nan	nan	5	2	-1	0.3864	no
ni|645903501|nb|KK920929.1|	248230-248388	nan	nan	61	50	-0.273806	0.8131	no
ni|645903501|nb|KK920929.1|	248489-248767	nan	nan	64	62	-0.0519027	0.9629	no
ni|645903501|nb|KK920929.1|	249055-249165	nan	nan	12	5	-1	0.25255	no
ni|645903501|nb|KK920929.1|	249383-249586	nan	nan	17	13	-0.410038	0.717	no
ni|645903501|nb|KK920929.1|	249865-250112	nan	nan	46	30	-0.604255	0.59945	no
ni|645903501|nb|KK920929.1|	250250-250508	nan	nan	93	57	-0.72067	0.53565	no
ni|645903501|nb|KK920929.1|	258913-260131	nan	nan	44	28	-0.662996	0.61155	no
ni|645903501|nb|KK920929.1|	260207-260421	nan	nan	0.684526	2	2	0.22835	no
ni|645903501|nb|KK920929.1|	260960-261437	nan	nan	4	3	-0.370294	0.74965	no
ni|645903501|nb|KK920929.1|	262427-262685	nan	nan	5	4	-0.218381	0.8469	no
ni|645903501|nb|KK920929.1|	262967-263279	nan	nan	7	5	-0.546772	0.65065	no
ni|645903501|nb|KK920929.1|	263380-263539	nan	nan	12	0.881251	-4	0.23575	no
ni|645903501|nb|KK920929.1|	264778-264930	nan	nan	5	0.525964	-3	0.244	no
ni|645903501|nb|KK920929.1|	268481-268600	nan	nan	13	3	-2	0.2949	no
ni|645903501|nb|KK920929.1|	269629-269822	nan	nan	3	6	0.980689	0.43735	no
ni|645903501|nb|KK920929.1|	275214-275431	nan	nan	1	4	2	0.15895	no
ni|645903501|nb|KK920929.1|	296179-296485	nan	nan	5	21	2	0.1738	no
ni|645903501|nb|KK920929.1|	297772-298506	nan	nan	2	15	3	0.0596	no
ni|645903501|nb|KK920929.1|	305812-306093	nan	nan	15	15	0.0294177	0.9803	no
ni|645903501|nb|KK920929.1|	309492-309734	nan	nan	18	21	0.241152	0.83475	no
ni|645903501|nb|KK920929.1|	64831-65014	nan	nan	4	5	0.270262	0.8244	no
ni|645903501|nb|KK920929.1|	70963-71230	nan	nan	1	2	0.234592	0.83745	no
ni|645903501|nb|KK920929.1|	72803-74726	nan	nan	0.272072	0.260141	-0.0646957	0.9384	no
ni|645903502|nb|KK920928.1|	115043-115517	nan	nan	10	11	0.118062	0.91575	no
ni|645903502|nb|KK920928.1|	145128-146197	nan	nan	1	1	-0.316624	0.79415	no

ni|645903502|nb|KK920928.1|	146315-146517	nan	nan	2	1	-0.827813	0.47925	no
ni|645903502|nb|KK920928.1|	187482-187814	nan	nan	2	2	-0.182281	0.87605	no
ni|645903502|nb|KK920928.1|	190059-190223	nan	nan	6	4	-0.432336	0.71155	no
ni|645903502|nb|KK920928.1|	191351-191832	nan	nan	6	0.751597	-3	0.0396	no
ni|645903502|nb|KK920928.1|	193675-193823	nan	nan	1	0.161984	-3	0.2517	no
ni|645903502|nb|KK920928.1|	206253-206380	nan	nan	24	5	-2	0.1106	no
ni|645903502|nb|KK920928.1|	207708-208038	nan	nan	13	5	-1	0.28585	no
ni|645903502|nb|KK920928.1|	210404-210609	nan	nan	1	0.457761	-1	0.3143	no
ni|645903502|nb|KK920928.1|	216540-216808	nan	nan	1	0.48086	-1	0.4064	no
ni|645903502|nb|KK920928.1|	219092-219473	nan	nan	2	0.499161	-2	0.14495	no
ni|645903502|nb|KK920928.1|	232472-232752	nan	nan	2	0.707803	-1	0.33495	no
ni|645903502|nb|KK920928.1|	237763-237960	nan	nan	2	0.668352	-2	0.1901	no
ni|645903502|nb|KK920928.1|	239504-239752	nan	nan	2	0.728391	-1	0.4105	no
ni|645903502|nb|KK920928.1|	240077-240436	nan	nan	0.858141	0.51815	-0.727844	0.5717	no
ni|645903502|nb|KK920928.1|	240817-241179	nan	nan	10	4	-1	0.30195	no
ni|645903502|nb|KK920928.1|	242202-242391	nan	nan	0.446111	0.284379	-0.649587	0.63225	no
ni|645903502|nb|KK920928.1|	243209-243453	nan	nan	0	19	inf	5.00E-05	yes
ni|645903502|nb|KK920928.1|	246094-246333	nan	nan	0	5	inf	5.00E-05	yes
ni|645903502|nb|KK920928.1|	249393-249904	nan	nan	0	68	inf	5.00E-05	yes
ni|645903502|nb|KK920928.1|	251494-251758	nan	nan	11	9	-0.324382	0.78125	no
ni|645903502|nb|KK920928.1|	251955-252411	nan	nan	19	21	0.1343	0.91355	no
ni|645903502|nb|KK920928.1|	253767-254019	nan	nan	12	6	-1	0.4077	no
ni|645903502|nb|KK920928.1|	254503-254853	nan	nan	11	12	0.0736346	0.94765	no
ni|645903502|nb|KK920928.1|	255243-255428	nan	nan	10	18	0.801561	0.50325	no
ni|645903502|nb|KK920928.1|	257426-258201	nan	nan	15	11	-0.42096	0.7156	no
ni|645903502|nb|KK920928.1|	263053-263800	nan	nan	0.464529	0.415594	-0.160595	0.8943	no
ni|645903502|nb|KK920928.1|	265113-265229	nan	nan	11	12	0.169805	0.8892	no
ni|645903502|nb|KK920928.1|	266136-266538	nan	nan	16	15	-0.0420747	0.9719	no
ni|645903502|nb|KK920928.1|	271632-271983	nan	nan	2	2	-0.419201	0.7284	no
ni|645903502|nb|KK920928.1|	275309-275810	nan	nan	6	4	-0.510349	0.66485	no

ni|645903502|nb|KK920928.1|	276381-276518	nan	nan	15	13	-0.209211	0.8594	no
ni|645903502|nb|KK920928.1|	277666-278218	nan	nan	22	12	-0.915224	0.44755	no
ni|645903502|nb|KK920928.1|	278335-278894	nan	nan	25	19	-0.364192	0.75795	no
ni|645903502|nb|KK920928.1|	279756-280077	nan	nan	5	4	-0.255163	0.8278	no
ni|645903502|nb|KK920928.1|	281641-281860	nan	nan	8	6	-0.469449	0.6838	no
ni|645903502|nb|KK920928.1|	283521-283680	nan	nan	21	17	-0.299859	0.793	no
ni|645903502|nb|KK920928.1|	284969-285155	nan	nan	0.509097	0.162218	-2	0.2989	no
ni|645903502|nb|KK920928.1|	287562-287671	nan	nan	28	7	-2	0.15655	no
ni|645903502|nb|KK920928.1|	289958-290125	nan	nan	13	8	-0.650513	0.5742	no
ni|645903502|nb|KK920928.1|	379713-379848	nan	nan	21	15	-0.484097	0.6657	no
ni|645903502|nb|KK920928.1|	433949-434010	nan	nan	13	10	-0.355215	0.76065	no
ni|645903503|nb|KK920927.1|	10716-10877	nan	nan	23	22	-0.0374774	0.97485	no
ni|645903503|nb|KK920927.1|	11147-11283	nan	nan	37	27	-0.428164	0.7154	no
ni|645903503|nb|KK920927.1|	11901-12121	nan	nan	1	1	0.518761	0.7279	no
ni|645903503|nb|KK920927.1|	13937-14135	nan	nan	0.573459	0.822013	0.519472	0.7279	no
ni|645903503|nb|KK920927.1|	16053-16209	nan	nan	0.512848	0.408591	-0.327875	0.80325	no
ni|645903503|nb|KK920927.1|	18039-18210	nan	nan	1	1	-0.147138	0.90605	no
ni|645903503|nb|KK920927.1|	18730-18950	nan	nan	895	798.66	-0.164089	0.8869	no
ni|645903503|nb|KK920927.1|	21829-21970	nan	nan	155	88	-0.817149	0.51945	no
ni|645903503|nb|KK920927.1|	22555-22793	nan	nan	12	7	-0.723117	0.5353	no
ni|645903503|nb|KK920927.1|	25118-25330	nan	nan	1	0.386262	-1	0.35155	no
ni|645903503|nb|KK920927.1|	256538-256854	nan	nan	2	0.532002	-2	0.1501	no
ni|645903503|nb|KK920927.1|	27308-28484	nan	nan	1	2	0.586047	0.65495	no
ni|645903503|nb|KK920927.1|	29645-30073	nan	nan	9	7	-0.526632	0.67145	no
ni|645903503|nb|KK920927.1|	36283-36681	nan	nan	9	6	-0.607675	0.6138	no
ni|645903503|nb|KK920927.1|	37320-38592	nan	nan	17	16	-0.0930664	0.93565	no
ni|645903503|nb|KK920927.1|	43172-44207	nan	nan	51	42	-0.291373	0.7999	no
ni|645903503|nb|KK920927.1|	45747-46183	nan	nan	87	69	-0.337341	0.7582	no
ni|645903503|nb|KK920927.1|	4654-4809	nan	nan	34	24	-0.521833	0.6528	no
ni|645903503|nb|KK920927.1|	49088-49315	nan	nan	26	20	-0.359026	0.7605	no

ni|645903503|nb|KK920927.1|	49442-49890	nan	nan	3	4	0.0575897	0.9602	no
ni|645903503|nb|KK920927.1|	50811-51034	nan	nan	0.873013	0.208635	-2	0.1613	no
ni|645903503|nb|KK920927.1|	51087-51405	nan	nan	14	9	-0.590778	0.62565	no
ni|645903503|nb|KK920927.1|	52731-52841	nan	nan	8	7	-0.139525	0.9059	no
ni|645903503|nb|KK920927.1|	52947-53099	nan	nan	10	7	-0.415174	0.7377	no
ni|645903503|nb|KK920927.1|	53332-53558	nan	nan	13	7	-0.829166	0.49445	no
ni|645903503|nb|KK920927.1|	5421-5766	nan	nan	27	24	-0.16673	0.8828	no
ni|645903503|nb|KK920927.1|	69712-70614	nan	nan	224	234	0.0648386	0.96295	no
ni|645903503|nb|KK920927.1|	70815-71211	nan	nan	45	35	-0.343029	0.76315	no
ni|645903503|nb|KK920927.1|	72535-72979	nan	nan	216	237	0.130285	0.90545	no
ni|645903503|nb|KK920927.1|	73081-73300	nan	nan	200	135	-0.56373	0.617	no
ni|645903503|nb|KK920927.1|	73431-73823	nan	nan	0	206	inf	5.00E-05	yes
ni|645903503|nb|KK920927.1|	74058-74488	nan	nan	0.316101	96	8	0.05065	no
ni|645903503|nb|KK920927.1|	7554-7735	nan	nan	19	15	-0.3526	0.7358	no
ni|645903503|nb|KK920927.1|	77702-78062	nan	nan	0	6	inf	5.00E-05	yes
ni|645903503|nb|KK920927.1|	78662-78808	nan	nan	11	3	-2	0.1613	no
ni|645903503|nb|KK920927.1|	8459-8579	nan	nan	37	26	-0.489468	0.65745	no
ni|645903504|nb|KK920926.1|	193485-193944	nan	nan	5	7	0.259808	0.82295	no
ni|645903504|nb|KK920926.1|	198848-199199	nan	nan	33	30	-0.121739	0.9198	no
ni|645903504|nb|KK920926.1|	199575-199857	nan	nan	12	12	-0.00812992	0.99305	no
ni|645903504|nb|KK920926.1|	202102-202546	nan	nan	16	16	-0.0272231	0.97995	no
ni|645903504|nb|KK920926.1|	202687-203045	nan	nan	8	6	-0.443844	0.70715	no
ni|645903504|nb|KK920926.1|	203411-203816	nan	nan	1	2	0.586004	0.65495	no
ni|645903504|nb|KK920926.1|	205155-205406	nan	nan	5	3	-0.843646	0.4844	no
ni|645903504|nb|KK920926.1|	206917-207334	nan	nan	5	5	-0.0671918	0.94635	no
ni|645903504|nb|KK920926.1|	221130-221890	nan	nan	2	2	0.248101	0.83485	no
ni|645903504|nb|KK920926.1|	226396-226679	nan	nan	14	13.33	-0.0533582	0.97065	no
ni|645903504|nb|KK920926.1|	251870-252081	nan	nan	10	8	-0.226722	0.8429	no
ni|645903504|nb|KK920926.1|	259024-259245	nan	nan	3	2	-1	0.3866	no
ni|645903504|nb|KK920926.1|	259681-259871	nan	nan	0.106734	3	5	0.12045	no

ni|645903504|nb|KK920926.1|	260571-260734	nan	nan	10	17	0.710135	0.61225	no
ni|645903504|nb|KK920926.1|	261289-261729	nan	nan	46	29	-0.681336	0.56	no
ni|645903504|nb|KK920926.1|	262213-262449	nan	nan	30	19	-0.646563	0.58265	no
ni|645903504|nb|KK920926.1|	262955-263456	nan	nan	7	8	0.129335	0.9052	no
ni|645903504|nb|KK920926.1|	263593-263830	nan	nan	1	0.962934	-0.160923	0.8943	no
ni|645903504|nb|KK920926.1|	264006-264250	nan	nan	40	60	0.563763	0.62645	no
ni|645903504|nb|KK920926.1|	264361-266704	nan	nan	6	5	-0.154788	0.90135	no
ni|645903504|nb|KK920926.1|	267459-267615	nan	nan	3	30	3	0.0541	no
ni|645903504|nb|KK920926.1|	267812-268258	nan	nan	0.87352	28	5	0.12045	no
ni|645903504|nb|KK920926.1|	268519-268860	nan	nan	0.878164	23	5	0.0374	no
ni|645903504|nb|KK920926.1|	269293-269558	nan	nan	0.088071	5	6	0.0443	no
ni|645903504|nb|KK920926.1|	274424-274907	nan	nan	0	41.16	inf	5.00E-05	yes
ni|645903504|nb|KK920926.1|	276136-276317	nan	nan	0.650372	21	5	0.12045	no
ni|645903504|nb|KK920926.1|	278676-279744	nan	nan	0.55103	17	5	0.0452	no
ni|645903504|nb|KK920926.1|	280108-280237	nan	nan	0	5	inf	0.00015	yes
ni|645903504|nb|KK920926.1|	280655-281338	nan	nan	6	16	1	0.262	no
ni|645903504|nb|KK920926.1|	282470-282620	nan	nan	7	17	1	0.2429	no
ni|645903504|nb|KK920926.1|	285482-285599	nan	nan	13	27	1	0.3336	no
ni|645903504|nb|KK920926.1|	285894-286350	nan	nan	32	78	1	0.27455	no
ni|645903504|nb|KK920926.1|	286752-286993	nan	nan	6	7	0.254645	0.83635	no
ni|645903504|nb|KK920926.1|	288355-288496	nan	nan	12	19	0.647278	0.5768	no
ni|645903504|nb|KK920926.1|	297779-298305	nan	nan	0.46973	0.314345	-0.579481	0.70815	no
ni|645903504|nb|KK920926.1|	299022-299205	nan	nan	3	2	-0.526244	0.7071	no
ni|645903504|nb|KK920926.1|	299968-300109	nan	nan	0.273997	2	3	0.08575	no
ni|645903504|nb|KK920926.1|	317733-318203	nan	nan	2	1	-0.465628	0.70405	no
ni|645903504|nb|KK920926.1|	318662-319042	nan	nan	0.299334	0.357745	0.257176	0.86075	no
ni|645903504|nb|KK920926.1|	320462-320620	nan	nan	0.706879	0.300264	-1	0.4023	no
ni|645903504|nb|KK920926.1|	321757-321984	nan	nan	11	8	-0.454267	0.722	no
ni|645903504|nb|KK920926.1|	338336-338560	nan	nan	7	8	0.0850733	0.94345	no
ni|645903504|nb|KK920926.1|	338696-338938	nan	nan	1	0.601017	-0.982751	0.48245	no

ni|645903504|nb|KK920926.1|	339942-340191	nan	nan	3	1	-1	0.45115	no
ni|645903504|nb|KK920926.1|	341108-341299	nan	nan	7	8	0.186541	0.87465	no
ni|645903504|nb|KK920926.1|	342279-342424	nan	nan	13	9	-0.518323	0.6593	no
ni|645903504|nb|KK920926.1|	344704-344931	nan	nan	12	12	0.0208298	0.9858	no
ni|645903504|nb|KK920926.1|	349367-349699	nan	nan	923.97	460	-1	0.37165	no
ni|645903504|nb|KK920926.1|	349994-350861	nan	nan	529	523	-0.0175561	0.9895	no
ni|645903504|nb|KK920926.1|	354145-354279	nan	nan	2459.1	2650.4	0.108083	0.94015	no
ni|645903504|nb|KK920926.1|	356743-356926	nan	nan	1190.92	1219.44	0.0341485	0.9804	no
ni|645903504|nb|KK920926.1|	358694-358884	nan	nan	1158.3	1249.5	0.109348	0.9431	no
ni|645903504|nb|KK920926.1|	359043-359849	nan	nan	298	346	0.214872	0.8752	no
ni|645903504|nb|KK920926.1|	360076-360557	nan	nan	19	18	-0.131435	0.9116	no
ni|645903504|nb|KK920926.1|	363104-363270	nan	nan	95	107	0.16982	0.90845	no
ni|645903504|nb|KK920926.1|	366596-366921	nan	nan	114	126	0.141019	0.9264	no
ni|645903504|nb|KK920926.1|	367298-367742	nan	nan	46	39	-0.251552	0.84125	no
ni|645903504|nb|KK920926.1|	368132-368687	nan	nan	4	3	-0.380104	0.7492	no
ni|645903504|nb|KK920926.1|	368855-369254	nan	nan	1875.02	2369.31	0.337561	0.8069	no
ni|645903504|nb|KK920926.1|	369464-369583	nan	nan	11	8	-0.465791	0.70195	no
ni|645903504|nb|KK920926.1|	369768-369939	nan	nan	3	2	-0.407956	0.72535	no
ni|645903504|nb|KK920926.1|	370255-370434	nan	nan	10	7	-0.590762	0.62565	no
ni|645903504|nb|KK920926.1|	370616-370779	nan	nan	3969.06	3614.92	-0.134833	0.89575	no
ni|645903504|nb|KK920926.1|	370900-371023	nan	nan	4369.97	4286.16	-0.0279386	0.9848	no
ni|645903504|nb|KK920926.1|	371495-371707	nan	nan	6	11	0.932773	0.44875	no
ni|645903504|nb|KK920926.1|	372011-372129	nan	nan	2	3	0.518001	0.7004	no
ni|645903504|nb|KK920926.1|	372187-372338	nan	nan	14	22	0.714157	0.5401	no
ni|645903504|nb|KK920926.1|	375038-375166	nan	nan	17	14	-0.231851	0.84165	no
ni|645903504|nb|KK920926.1|	375773-375893	nan	nan	11	10	-0.0964053	0.9308	no
ni|645903504|nb|KK920926.1|	376183-377009	nan	nan	8	6	-0.406658	0.72355	no
ni|645903504|nb|KK920926.1|	378063-378951	nan	nan	7	5	-0.376001	0.7444	no
ni|645903504|nb|KK920926.1|	382463-382698	nan	nan	20	19	-0.0672039	0.95425	no
ni|645903504|nb|KK920926.1|	42163-42419	nan	nan	15	7	-0.965703	0.40675	no

ni|645903504|nb|KK920926.1|	562432-562755	nan	nan	16	14	-0.139377	0.90625	no
ni|645903504|nb|KK920926.1|	571486-571618	nan	nan	3	4	0.117058	0.9185	no
ni|645903504|nb|KK920926.1|	574355-574935	nan	nan	36	27	-0.424094	0.71345	no
ni|645903504|nb|KK920926.1|	575214-575644	nan	nan	19	16	-0.198889	0.8644	no
ni|645903504|nb|KK920926.1|	576458-576612	nan	nan	49	48	-0.0245002	0.98225	no
ni|645903504|nb|KK920926.1|	577108-577418	nan	nan	60	53	-0.172867	0.8788	no
ni|645903504|nb|KK920926.1|	578430-578722	nan	nan	10	10	0.0853034	0.94175	no
ni|645903504|nb|KK920926.1|	578872-579468	nan	nan	15	15	-0.00442294	0.99445	no
ni|645903504|nb|KK920926.1|	580556-580719	nan	nan	3	5	0.460884	0.7011	no
ni|645903504|nb|KK920926.1|	581901-583008	nan	nan	164	170	0.0497641	0.96315	no
ni|645903504|nb|KK920926.1|	583422-583723	nan	nan	101	103	0.0185956	0.991	no
ni|645903504|nb|KK920926.1|	586459-586890	nan	nan	327	374	0.189888	0.89455	no
ni|645903504|nb|KK920926.1|	588287-588876	nan	nan	119	123.73	0.0571349	0.9661	no
ni|645903504|nb|KK920926.1|	589000-589126	nan	nan	255	255	-0.000244817	0.9985	no
ni|645903504|nb|KK920926.1|	589649-589879	nan	nan	6	6	-0.0672504	0.9414	no
ni|645903504|nb|KK920926.1|	597382-597853	nan	nan	1	0.892277	-0.480335	0.7105	no
ni|645903505|nb|KK920925.1|	100449-100707	nan	nan	35	33	-0.0672106	0.95195	no
ni|645903505|nb|KK920925.1|	101201-101862	nan	nan	2	2	0.0788828	0.9453	no
ni|645903505|nb|KK920925.1|	116307-116736	nan	nan	3	33	4	0.0127	no
ni|645903505|nb|KK920925.1|	129519-129679	nan	nan	4	100	5	0.00345	no
ni|645903505|nb|KK920925.1|	132895-133123	nan	nan	0.123245	6	6	0.11985	no
ni|645903505|nb|KK920925.1|	59406-59773	nan	nan	12	24	1	0.3743	no
ni|645903505|nb|KK920925.1|	60724-60863	nan	nan	20	106	2	0.07395	no
ni|645903505|nb|KK920925.1|	64951-65554	nan	nan	30	150	2	0.05805	no
ni|645903505|nb|KK920925.1|	67401-67685	nan	nan	1	1	0.321723	0.8092	no
ni|645903505|nb|KK920925.1|	68749-68887	nan	nan	4	4	0.0582967	0.9532	no
ni|645903505|nb|KK920925.1|	69657-69865	nan	nan	5	7	0.496518	0.66915	no
ni|645903505|nb|KK920925.1|	78428-78573	nan	nan	3	4	0.504899	0.6688	no
ni|645903505|nb|KK920925.1|	80580-80721	nan	nan	11	64	3	0.04485	no
ni|645903505|nb|KK920925.1|	81967-82165	nan	nan	14	87	3	0.06885	no

ni|645903505|nb|KK920925.1|	84488-84788	nan	nan	36	178	2	0.1271	no
ni|645903505|nb|KK920925.1|	86376-86621	nan	nan	34	176	2	0.0505	no
ni|645903505|nb|KK920925.1|	89884-90281	nan	nan	0.954167	4	2	0.15655	no
ni|645903505|nb|KK920925.1|	91826-92033	nan	nan	24	7	-2	0.20995	no
ni|645903505|nb|KK920925.1|	95662-95848	nan	nan	6	9	0.669731	0.5973	no
ni|645903505|nb|KK920925.1|	95949-96099	nan	nan	26	16	-0.67831	0.56025	no
ni|645903505|nb|KK920925.1|	98539-99214	nan	nan	16	17	0.0569992	0.9596	no
ni|645903506|nb|KK920924.1|	116681-116855	nan	nan	17	25	0.54682	0.63755	no
ni|645903506|nb|KK920924.1|	117695-117878	nan	nan	179	138	-0.371188	0.79535	no
ni|645903506|nb|KK920924.1|	118278-118464	nan	nan	21	11	-0.922816	0.4521	no
ni|645903506|nb|KK920924.1|	118587-118816	nan	nan	13	22	0.74368	0.524	no
ni|645903506|nb|KK920924.1|	119121-119312	nan	nan	25	38	0.59649	0.59775	no
ni|645903506|nb|KK920924.1|	121931-122059	nan	nan	57	66	0.215755	0.8464	no
ni|645903506|nb|KK920924.1|	125574-126201	nan	nan	10	13	0.317919	0.7846	no
ni|645903506|nb|KK920924.1|	130107-130398	nan	nan	31	44	0.506503	0.64345	no
ni|645903506|nb|KK920924.1|	134768-135694	nan	nan	40	29	-0.482745	0.7105	no
ni|645903506|nb|KK920924.1|	135843-136055	nan	nan	8	13	0.669631	0.6211	no
ni|645903506|nb|KK920924.1|	141465-141649	nan	nan	38	40	0.0562345	0.9688	no
ni|645903506|nb|KK920924.1|	144988-145161	nan	nan	0.320861	0.35057	0.127755	0.92555	no
ni|645903506|nb|KK920924.1|	147572-147744	nan	nan	0.531335	11	4	0.0028	yes
ni|645903506|nb|KK920924.1|	149368-150267	nan	nan	0.401655	9	4	0.0052	no
ni|645903506|nb|KK920924.1|	199036-199182	nan	nan	0	4	inf	0.00015	yes
ni|645903506|nb|KK920924.1|	206224-206395	nan	nan	41	20	-1	0.37675	no
ni|645903506|nb|KK920924.1|	209284-209521	nan	nan	74	54	-0.459573	0.6861	no
ni|645903506|nb|KK920924.1|	212768-212910	nan	nan	63	50	-0.326068	0.7798	no
ni|645903506|nb|KK920924.1|	221851-222212	nan	nan	1	0.745123	-0.693256	0.58855	no
ni|645903506|nb|KK920924.1|	226666-226797	nan	nan	61	49	-0.332471	0.7835	no
ni|645903506|nb|KK920924.1|	229285-229444	nan	nan	0.607079	0.507695	-0.257922	0.8692	no
ni|645903506|nb|KK920924.1|	234868-235461	nan	nan	28	25	-0.157192	0.8928	no
ni|645903506|nb|KK920924.1|	238745-238880	nan	nan	1	0.320666	-2	0.25615	no

ni|645903506|nb|KK920924.1|	243556-244196	nan	nan	29	22	-0.406449	0.7438	no
ni|645903506|nb|KK920924.1|	244871-245278	nan	nan	23	24	0.0481334	0.9698	no
ni|645903506|nb|KK920924.1|	245426-245810	nan	nan	0	4	inf	0.00015	yes
ni|645903506|nb|KK920924.1|	246324-246473	nan	nan	0	53	inf	5.00E-05	yes
ni|645903506|nb|KK920924.1|	246926-248418	nan	nan	0	105	inf	5.00E-05	yes
ni|645903506|nb|KK920924.1|	248611-248938	nan	nan	0.091845	53	9	0.04435	no
ni|645903506|nb|KK920924.1|	253515-253633	nan	nan	0.250566	101	9	0.11935	no
ni|645903506|nb|KK920924.1|	256457-257196	nan	nan	0	128	inf	5.00E-05	yes
ni|645903506|nb|KK920924.1|	258336-259261	nan	nan	0	111	inf	5.00E-05	yes
ni|645903506|nb|KK920924.1|	259755-260574	nan	nan	0	114	inf	5.00E-05	yes
ni|645903506|nb|KK920924.1|	261897-262378	nan	nan	0	84	inf	5.00E-05	yes
ni|645903506|nb|KK920924.1|	265041-265563	nan	nan	0.089349	61	9	0.11935	no
ni|645903506|nb|KK920924.1|	265836-266373	nan	nan	0	78	inf	5.00E-05	yes
ni|645903506|nb|KK920924.1|	266458-266626	nan	nan	0.221503	89	9	0.11935	no
ni|645903506|nb|KK920924.1|	271317-271427	nan	nan	0	82	inf	5.00E-05	yes
ni|645903506|nb|KK920924.1|	271808-271964	nan	nan	0	72	inf	5.00E-05	yes
ni|645903506|nb|KK920924.1|	272373-272636	nan	nan	346.91	244.04	-0.507442	0.72295	no
ni|645903506|nb|KK920924.1|	273286-273521	nan	nan	390	287	-0.445598	0.7542	no
ni|645903506|nb|KK920924.1|	274334-274582	nan	nan	88	65	-0.442907	0.7578	no
ni|645903506|nb|KK920924.1|	277752-278194	nan	nan	103	82	-0.33817	0.8083	no
ni|645903506|nb|KK920924.1|	279377-279533	nan	nan	626	467	-0.422089	0.77035	no
ni|645903506|nb|KK920924.1|	279807-280057	nan	nan	340	276	-0.301407	0.82645	no
ni|645903506|nb|KK920924.1|	295969-296475	nan	nan	484	401.77	-0.267477	0.8538	no
ni|645903506|nb|KK920924.1|	296874-297180	nan	nan	374	323.97	-0.207299	0.88395	no
ni|645903506|nb|KK920924.1|	299807-300419	nan	nan	573	528	-0.118068	0.9354	no
ni|645903506|nb|KK920924.1|	301964-302340	nan	nan	259	216	-0.260858	0.84995	no
ni|645903506|nb|KK920924.1|	304598-304813	nan	nan	451.27	427	-0.0794045	0.95475	no
ni|645903506|nb|KK920924.1|	304926-305082	nan	nan	224	206.27	-0.115923	0.93325	no
ni|645903506|nb|KK920924.1|	305601-305728	nan	nan	0.973223	2	0.851912	0.521	no
ni|645903506|nb|KK920924.1|	309261-309785	nan	nan	306	315	0.0394055	0.97715	no

ni|645903506|nb|KK920924.1|	312382-312563	nan	nan	1	1	-0.249364	0.84025	no
ni|645903506|nb|KK920924.1|	317059-317636	nan	nan	844.89	806	-0.0673498	0.96465	no
ni|645903506|nb|KK920924.1|	318757-318980	nan	nan	5	4	-0.137143	0.91325	no
ni|645903506|nb|KK920924.1|	320790-320906	nan	nan	8	3	-2	0.3149	no
ni|645903506|nb|KK920924.1|	322115-322245	nan	nan	959	874	-0.133551	0.9215	no
ni|645903506|nb|KK920924.1|	322342-322832	nan	nan	2	8	2	0.1258	no
ni|645903506|nb|KK920924.1|	323025-323861	nan	nan	2	0.898489	-1	0.34	no
ni|645903506|nb|KK920924.1|	325062-325176	nan	nan	1659.9	1464.89	-0.180307	0.8979	no
ni|645903506|nb|KK920924.1|	326072-326262	nan	nan	812	681	-0.254009	0.8572	no
ni|645903506|nb|KK920924.1|	329179-329688	nan	nan	1461.3	1260.01	-0.213809	0.87845	no
ni|645903506|nb|KK920924.1|	363113-363390	nan	nan	4936.09	4518.75	-0.127446	0.90135	no
ni|645903506|nb|KK920924.1|	363963-364228	nan	nan	1409.65	1209.38	-0.221069	0.87745	no
ni|645903506|nb|KK920924.1|	364997-365323	nan	nan	3234.42	2833.71	-0.190818	0.8962	no
ni|645903506|nb|KK920924.1|	365518-365723	nan	nan	3312.33	2553.94	-0.375123	0.7586	no
ni|645903506|nb|KK920924.1|	365841-366321	nan	nan	2	0.993121	-0.75077	0.53775	no
ni|645903506|nb|KK920924.1|	388370-388709	nan	nan	4410.31	3269.69	-0.431729	0.76435	no
ni|645903506|nb|KK920924.1|	391080-391630	nan	nan	2	2	0.0335783	0.96125	no
ni|645903506|nb|KK920924.1|	392550-392699	nan	nan	5035.58	3693.81	-0.447048	0.71825	no
ni|645903506|nb|KK920924.1|	395165-395857	nan	nan	8325.92	5581.83	-0.576871	0.64615	no
ni|645903506|nb|KK920924.1|	396878-397287	nan	nan	4212.29	2995.76	-0.491684	0.73615	no
ni|645903506|nb|KK920924.1|	411097-411591	nan	nan	6667.76	4001.21	-0.736764	0.4088	no
ni|645903506|nb|KK920924.1|	474614-474776	nan	nan	1	0.699566	-0.830273	0.49575	no
ni|645903506|nb|KK920924.1|	99653-100452	nan	nan	3	2	-0.719259	0.61925	no
ni|645903507|nb|KK920923.1|	397436-398497	nan	nan	0.603554	0.72139	0.257296	0.83555	no
ni|645903507|nb|KK920923.1|	402023-402492	nan	nan	1	1	-0.0653269	0.95795	no
ni|645903507|nb|KK920923.1|	412960-413138	nan	nan	2	2	-0.0665344	0.9384	no
ni|645903507|nb|KK920923.1|	422237-423111	nan	nan	0.729293	0.823824	0.175838	0.8916	no
ni|645903507|nb|KK920923.1|	423181-424160	nan	nan	0.330069	0.49586	0.587166	0.65495	no
ni|645903507|nb|KK920923.1|	424420-424856	nan	nan	1	1	-0.16109	0.8943	no
ni|645903507|nb|KK920923.1|	424941-425424	nan	nan	3	2	-0.324054	0.7818	no

ni|645903507|nb|KK920923.1|	425475-425859	nan	nan	2	0.532002	-2	0.3149	no
ni|645903507|nb|KK920923.1|	426167-426382	nan	nan	4	6	0.592429	0.62225	no
ni|645903507|nb|KK920923.1|	426615-426787	nan	nan	3	3	-0.13637	0.91325	no
ni|645903507|nb|KK920923.1|	427361-427720	nan	nan	27	23	-0.259861	0.8277	no
ni|645903507|nb|KK920923.1|	429587-429898	nan	nan	23	22	-0.0816294	0.94385	no
ni|645903507|nb|KK920923.1|	430011-430589	nan	nan	0.418766	0.266897	-0.649863	0.6679	no
ni|645903507|nb|KK920923.1|	430679-431118	nan	nan	2	1	-0.494206	0.6754	no
ni|645903507|nb|KK920923.1|	431451-431615	nan	nan	8	6	-0.405324	0.72585	no
ni|645903507|nb|KK920923.1|	431727-431892	nan	nan	1	0.529113	-1	0.429	no
ni|645903507|nb|KK920923.1|	438425-439140	nan	nan	21	16	-0.3217	0.79495	no
ni|645903507|nb|KK920923.1|	439243-439438	nan	nan	4	2	-1	0.4219	no
ni|645903507|nb|KK920923.1|	439544-440902	nan	nan	2	2	-0.15795	0.89345	no
ni|645903507|nb|KK920923.1|	449204-449769	nan	nan	0.868892	0.934031	0.104294	0.93335	no
ni|645903509|nb|KK920921.1|	114333-114452	nan	nan	2	2	0.0271444	0.9668	no
ni|645903509|nb|KK920921.1|	114992-115349	nan	nan	11	6	-0.915209	0.4631	no
ni|645903509|nb|KK920921.1|	115518-115696	nan	nan	4	0.117752	-5	0.0179	no
ni|645903509|nb|KK920921.1|	115801-115911	nan	nan	0.779846	0.401521	-0.957714	0.4422	no
ni|645903509|nb|KK920921.1|	116046-117785	nan	nan	0.898454	1	0.464978	0.7264	no
ni|645903509|nb|KK920921.1|	133668-133917	nan	nan	149	121	-0.302568	0.83985	no
ni|645903509|nb|KK920921.1|	154031-154197	nan	nan	114	93	-0.288234	0.7912	no
ni|645903509|nb|KK920921.1|	154295-154638	nan	nan	19	16	-0.271758	0.80285	no
ni|645903509|nb|KK920921.1|	156529-156891	nan	nan	37	17	-1	0.3178	no
ni|645903509|nb|KK920921.1|	158076-158429	nan	nan	1	0.123042	-3	0.05725	no
ni|645903509|nb|KK920921.1|	158990-159190	nan	nan	2	2	-0.0661591	0.94765	no
ni|645903509|nb|KK920921.1|	159509-159652	nan	nan	4	4	-0.0666485	0.95835	no
ni|645903509|nb|KK920921.1|	159786-160427	nan	nan	3	3	-0.357224	0.75835	no
ni|645903509|nb|KK920921.1|	161486-161663	nan	nan	4	4	0.190167	0.87185	no
ni|645903509|nb|KK920921.1|	161765-161928	nan	nan	6	4	-0.356488	0.77485	no
ni|645903509|nb|KK920921.1|	162043-162470	nan	nan	3	3	-0.00355249	0.9972	no
ni|645903509|nb|KK920921.1|	162864-163376	nan	nan	0.785041	0.687842	-0.19069	0.88855	no

ni|645903509|nb|KK920921.1|	1641-2912	nan	nan	2	1	-0.908537	0.45135	no
ni|645903509|nb|KK920921.1|	166384-166669	nan	nan	3	2	-0.343824	0.7695	no
ni|645903509|nb|KK920921.1|	167473-167669	nan	nan	1	0.327437	-2	0.162	no
ni|645903509|nb|KK920921.1|	168212-168586	nan	nan	3	1	-1	0.4064	no
ni|645903509|nb|KK920921.1|	169159-170045	nan	nan	0.643952	0.30776	-1	0.44755	no
ni|645903509|nb|KK920921.1|	171397-171758	nan	nan	2	0.671875	-1	0.4023	no
ni|645903509|nb|KK920921.1|	172554-172719	nan	nan	21	10	-1	0.4289	no
ni|645903509|nb|KK920921.1|	174690-174885	nan	nan	10	12	0.281111	0.81175	no
ni|645903509|nb|KK920921.1|	175280-175585	nan	nan	10	9	-0.203842	0.8594	no
ni|645903509|nb|KK920921.1|	176870-176998	nan	nan	13	11	-0.224734	0.8507	no
ni|645903509|nb|KK920921.1|	177220-177400	nan	nan	0	1	inf	0.0003	yes
ni|645903509|nb|KK920921.1|	177774-177928	nan	nan	0	3	inf	5.00E-05	yes
ni|645903509|nb|KK920921.1|	179346-179491	nan	nan	0	6	inf	5.00E-05	yes
ni|645903509|nb|KK920921.1|	180103-180293	nan	nan	0	14	inf	5.00E-05	yes
ni|645903509|nb|KK920921.1|	180500-180633	nan	nan	0	5	inf	5.00E-05	yes
ni|645903509|nb|KK920921.1|	181119-181247	nan	nan	153	202	0.40533	0.77995	no
ni|645903509|nb|KK920921.1|	181453-181561	nan	nan	149	187	0.326636	0.79275	no
ni|645903509|nb|KK920921.1|	182483-182651	nan	nan	138	161.08	0.224338	0.87005	no
ni|645903509|nb|KK920921.1|	183344-183813	nan	nan	197	226	0.197755	0.8533	no
ni|645903509|nb|KK920921.1|	184823-185075	nan	nan	97	106	0.129834	0.9258	no
ni|645903509|nb|KK920921.1|	200955-201123	nan	nan	1	1	-0.0203708	0.9832	no
ni|645903509|nb|KK920921.1|	207315-207780	nan	nan	1	0.80252	-0.8029	0.6027	no
ni|645903509|nb|KK920921.1|	211240-211558	nan	nan	1	0.712731	-0.978877	0.4167	no
ni|645903509|nb|KK920921.1|	251942-252135	nan	nan	296	304	0.0392464	0.9785	no
ni|645903509|nb|KK920921.1|	253553-254053	nan	nan	328	308	-0.0883301	0.9485	no
ni|645903509|nb|KK920921.1|	254350-254590	nan	nan	288	258	-0.154945	0.91235	no
ni|645903509|nb|KK920921.1|	266929-267487	nan	nan	323	236	-0.452114	0.76005	no
ni|645903509|nb|KK920921.1|	269878-270160	nan	nan	1	0.783665	-0.609019	0.61085	no
ni|645903509|nb|KK920921.1|	271179-271421	nan	nan	4	3	-0.621886	0.5679	no
ni|645903509|nb|KK920921.1|	271521-271664	nan	nan	2	6	2	0.234	no

ni|645903509|nb|KK920921.1|	273753-275034	nan	nan	4	4	-0.0672124	0.94715	no
ni|645903509|nb|KK920921.1|	277201-277471	nan	nan	11	10	-0.0784632	0.94615	no
ni|645903509|nb|KK920921.1|	285887-286230	nan	nan	8	0	-inf	0.0004	yes
ni|645903509|nb|KK920921.1|	287068-287346	nan	nan	7	0.908791	-3	0.1378	no
ni|645903509|nb|KK920921.1|	379431-379690	nan	nan	8	0.804399	-3	0.0263	no
ni|645903509|nb|KK920921.1|	426547-426743	nan	nan	4	0.464325	-3	0.1378	no
ni|645903509|nb|KK920921.1|	633638-633879	nan	nan	6	1	-2	0.0877	no
ni|645903509|nb|KK920921.1|	670425-670724	nan	nan	7	0.435791	-4	0.12145	no
ni|645903509|nb|KK920921.1|	699512-699583	nan	nan	6	2	-2	0.20985	no
ni|645903509|nb|KK920921.1|	767543-767788	nan	nan	4	0.251824	-4	0.2337	no
ni|645903509|nb|KK920921.1|	788226-789204	nan	nan	2	0	-inf	0.0036	no
ni|645903509|nb|KK920921.1|	789750-790256	nan	nan	0.391399	24	6	0.03735	no
ni|645903509|nb|KK920921.1|	790821-791019	nan	nan	0	34	inf	5.00E-05	yes
ni|645903509|nb|KK920921.1|	792895-793060	nan	nan	0.560032	8	4	0.04145	no
ni|645903509|nb|KK920921.1|	798931-799168	nan	nan	0.640401	28	5	0.0332	no
ni|645903509|nb|KK920921.1|	799386-799567	nan	nan	0.178401	32	8	0.11935	no
ni|645903509|nb|KK920921.1|	801340-801516	nan	nan	0.4304	25	6	0.11965	no
ni|645903509|nb|KK920921.1|	802432-802657	nan	nan	0.6269	30	6	0.04455	no
ni|645903509|nb|KK920921.1|	804482-804630	nan	nan	0	28	inf	5.00E-05	yes
ni|645903509|nb|KK920921.1|	805947-806187	nan	nan	0.072538	12	7	0.11935	no
ni|645903509|nb|KK920921.1|	806271-806590	nan	nan	15	6	-1	0.279	no
ni|645903509|nb|KK920921.1|	806681-806828	nan	nan	4	2	-1	0.29235	no
ni|645903509|nb|KK920921.1|	806960-807133	nan	nan	8	9	0.306614	0.83935	no
ni|645903509|nb|KK920921.1|	807203-807324	nan	nan	4	3	-0.709233	0.54985	no
ni|645903509|nb|KK920921.1|	807469-807580	nan	nan	17	20	0.180602	0.90345	no
ni|645903509|nb|KK920921.1|	807770-807893	nan	nan	2	2	-0.0665508	0.94765	no
ni|645903509|nb|KK920921.1|	808236-808508	nan	nan	15	10	-0.652318	0.6241	no
ni|645903509|nb|KK920921.1|	808605-808990	nan	nan	7	5	-0.629107	0.5909	no
ni|645903509|nb|KK920921.1|	809045-809198	nan	nan	17	10	-0.726169	0.54605	no
ni|645903509|nb|KK920921.1|	809495-809704	nan	nan	0.752629	0.774781	0.0418496	0.9599	no

ni|645903509|nb|KK920921.1|	809941-810190	nan	nan	13	8	-0.581993	0.70815	no
ni|645903509|nb|KK920921.1|	810536-810672	nan	nan	3	4	0.418339	0.73705	no
ni|645903509|nb|KK920921.1|	810748-810870	nan	nan	16	21	0.324451	0.78535	no
ni|645903509|nb|KK920921.1|	810990-811266	nan	nan	19	22	0.195452	0.8646	no
ni|645903509|nb|KK920921.1|	811516-811688	nan	nan	4	6	0.721227	0.5689	no
ni|645903509|nb|KK920921.1|	811984-812311	nan	nan	3	4	0.435539	0.7373	no
ni|645903509|nb|KK920921.1|	812438-812544	nan	nan	3	2	-0.77902	0.5209	no
ni|645903510|nb|KK920920.1|	116055-116355	nan	nan	4	5	0.412067	0.73235	no
ni|645903510|nb|KK920920.1|	120533-120843	nan	nan	7	11	0.74533	0.51295	no
ni|645903510|nb|KK920920.1|	128790-128941	nan	nan	39	52	0.404649	0.7322	no
ni|645903510|nb|KK920920.1|	141739-141919	nan	nan	18	30	0.676463	0.5588	no
ni|645903510|nb|KK920920.1|	86118-87226	nan	nan	1	2	0.41989	0.7302	no
ni|645903511|nb|KK920919.1|	146296-146496	nan	nan	0	17	inf	5.00E-05	yes
ni|645903511|nb|KK920919.1|	147803-148071	nan	nan	0	141	inf	5.00E-05	yes
ni|645903511|nb|KK920919.1|	148847-149338	nan	nan	0	14	inf	5.00E-05	yes
ni|645903511|nb|KK920919.1|	149508-149723	nan	nan	0	153	inf	5.00E-05	yes
ni|645903511|nb|KK920919.1|	150686-150864	nan	nan	3	2	-0.812452	0.4931	no
ni|645903511|nb|KK920919.1|	150956-151154	nan	nan	1	1	-0.355318	0.7931	no
ni|645903511|nb|KK920919.1|	151449-151618	nan	nan	1	3	1	0.23795	no
ni|645903511|nb|KK920919.1|	157776-159032	nan	nan	2	2	0.182282	0.87985	no
ni|645903511|nb|KK920919.1|	159188-159380	nan	nan	1	2	0.312592	0.81835	no
ni|645903511|nb|KK920919.1|	159487-160006	nan	nan	2	1	-0.717508	0.57	no
ni|645903511|nb|KK920919.1|	160358-160924	nan	nan	2	2	0.125664	0.92555	no
ni|645903511|nb|KK920919.1|	163279-163457	nan	nan	0.927499	0.632934	-0.55129	0.71905	no
ni|645903511|nb|KK920919.1|	164099-164246	nan	nan	1	0.999464	-0.00897855	0.9934	no
ni|645903511|nb|KK920919.1|	167177-167349	nan	nan	0.868336	2	1	0.3444	no
ni|645903511|nb|KK920919.1|	181072-181215	nan	nan	7	4	-0.915275	0.53575	no
ni|645903511|nb|KK920919.1|	189889-190467	nan	nan	18	4	-2	0.0752	no
ni|645903511|nb|KK920919.1|	196773-196998	nan	nan	54	29	-0.903673	0.4235	no
ni|645903511|nb|KK920919.1|	21308-21496	nan	nan	0.727024	0.855354	0.23452	0.85095	no

ni|645903511|nb|KK920919.1|	24421-24616	nan	nan	21	22	0.0796072	0.9458	no
ni|645903511|nb|KK920919.1|	24900-25114	nan	nan	26	27	0.0619879	0.95855	no
ni|645903511|nb|KK920919.1|	25493-26029	nan	nan	2	7	2	0.1637	no
ni|645903511|nb|KK920919.1|	27702-27848	nan	nan	1.26	0.860098	-0.550855	0.68265	no
ni|645903511|nb|KK920919.1|	29544-29865	nan	nan	6	9	0.413221	0.7207	no
ni|645903511|nb|KK920919.1|	304687-304949	nan	nan	20	12	-0.792679	0.50225	no
ni|645903511|nb|KK920919.1|	31035-31338	nan	nan	0	2	inf	0.00055	yes
ni|645903511|nb|KK920919.1|	31553-31848	nan	nan	120	180	0.581893	0.611	no
ni|645903511|nb|KK920919.1|	321364-321588	nan	nan	0.821716	0.448642	-0.873074	0.5788	no
ni|645903511|nb|KK920919.1|	322219-322661	nan	nan	2	5	2	0.1868	no
ni|645903511|nb|KK920919.1|	32247-32408	nan	nan	53	64	0.281557	0.80085	no
ni|645903511|nb|KK920919.1|	322915-323275	nan	nan	5	13	1	0.3244	no
ni|645903511|nb|KK920919.1|	324525-325404	nan	nan	2	2	0.295609	0.8383	no
ni|645903511|nb|KK920919.1|	325685-326493	nan	nan	2	1	-0.693718	0.58855	no
ni|645903511|nb|KK920919.1|	327638-328615	nan	nan	96	38	-1	0.32165	no
ni|645903511|nb|KK920919.1|	32894-33214	nan	nan	8	4	-0.974088	0.5005	no
ni|645903511|nb|KK920919.1|	331075-331415	nan	nan	12	6	-0.970329	0.4008	no
ni|645903511|nb|KK920919.1|	332079-332232	nan	nan	116	72	-0.699276	0.615	no
ni|645903511|nb|KK920919.1|	334172-334359	nan	nan	3	1	-1	0.2563	no
ni|645903511|nb|KK920919.1|	335390-335744	nan	nan	2	0.868415	-1	0.2898	no
ni|645903511|nb|KK920919.1|	33865-33976	nan	nan	2378.84	1669.22	-0.511078	0.7269	no
ni|645903511|nb|KK920919.1|	340083-340267	nan	nan	2	1	-0.649955	0.5826	no
ni|645903511|nb|KK920919.1|	340467-340959	nan	nan	69	50	-0.44581	0.7662	no
ni|645903511|nb|KK920919.1|	34077-34258	nan	nan	24	7	-2	0.16915	no
ni|645903511|nb|KK920919.1|	347174-347308	nan	nan	0	24	inf	5.00E-05	yes
ni|645903511|nb|KK920919.1|	348371-348751	nan	nan	0	129.79	inf	5.00E-05	yes
ni|645903511|nb|KK920919.1|	348882-349180	nan	nan	63	39	-0.712766	0.53625	no
ni|645903511|nb|KK920919.1|	349370-349965	nan	nan	9	6	-0.604385	0.6134	no
ni|645903511|nb|KK920919.1|	350834-351000	nan	nan	2	0.861337	-1	0.35155	no
ni|645903511|nb|KK920919.1|	351221-351412	nan	nan	0.818733	1	0.478101	0.68065	no

ni|645903511|nb|KK920919.1|	35161-35797	nan	nan	71	7	-3	0.02685	no
ni|645903511|nb|KK920919.1|	352450-352645	nan	nan	2	3	0.330092	0.7903	no
ni|645903511|nb|KK920919.1|	353472-353661	nan	nan	3	3	0.186068	0.87345	no
ni|645903511|nb|KK920919.1|	353897-354062	nan	nan	1	2	0.840809	0.5399	no
ni|645903511|nb|KK920919.1|	357799-358036	nan	nan	2	3	0.754389	0.529	no
ni|645903511|nb|KK920919.1|	358698-359031	nan	nan	0.931236	2	1	0.2629	no
ni|645903511|nb|KK920919.1|	359672-359912	nan	nan	2	19	3	0.0447	no
ni|645903511|nb|KK920919.1|	360226-360373	nan	nan	0.698822	0.908783	0.379012	0.7476	no
ni|645903511|nb|KK920919.1|	361230-361993	nan	nan	0.800714	0.459161	-0.802287	0.6027	no
ni|645903511|nb|KK920919.1|	36261-36479	nan	nan	130	12	-3	0.01745	no
ni|645903511|nb|KK920919.1|	363426-363574	nan	nan	0.834342	0.265777	-2	0.2989	no
ni|645903511|nb|KK920919.1|	365326-365606	nan	nan	9	6	-0.62917	0.6001	no
ni|645903511|nb|KK920919.1|	36605-36871	nan	nan	35	25	-0.462907	0.68515	no
ni|645903511|nb|KK920919.1|	366355-366979	nan	nan	6	8	0.371005	0.7476	no
ni|645903511|nb|KK920919.1|	367220-367629	nan	nan	6	4	-0.590087	0.604	no
ni|645903511|nb|KK920919.1|	368447-369512	nan	nan	334.42	303	-0.141902	0.90085	no
ni|645903511|nb|KK920919.1|	37029-37891	nan	nan	0	472	inf	5.00E-05	yes
ni|645903511|nb|KK920919.1|	371767-372268	nan	nan	341	366	0.102348	0.92895	no
ni|645903511|nb|KK920919.1|	373213-373635	nan	nan	9	10	0.188059	0.8705	no
ni|645903511|nb|KK920919.1|	374906-378167	nan	nan	8	8	-0.0220347	0.98455	no
ni|645903511|nb|KK920919.1|	38541-39284	nan	nan	0.075444	455	13	0.03715	no
ni|645903511|nb|KK920919.1|	400305-400547	nan	nan	1	2	0.173448	0.8809	no
ni|645903511|nb|KK920919.1|	42635-43296	nan	nan	0	248	inf	5.00E-05	yes
ni|645903511|nb|KK920919.1|	43916-44322	nan	nan	0	365	inf	5.00E-05	yes
ni|645903511|nb|KK920919.1|	439428-439678	nan	nan	1	2	0.480756	0.68735	no
ni|645903511|nb|KK920919.1|	45020-45644	nan	nan	0	382	inf	5.00E-05	yes
ni|645903511|nb|KK920919.1|	45756-45882	nan	nan	0	16	inf	5.00E-05	yes
ni|645903511|nb|KK920919.1|	459063-459329	nan	nan	0.734952	2	2	0.2119	no
ni|645903511|nb|KK920919.1|	462548-463071	nan	nan	3	3	-0.0672498	0.93835	no
ni|645903511|nb|KK920919.1|	463149-464111	nan	nan	2	3	0.474389	0.709	no

ni|645903511|nb|KK920919.1|	46463-46845	nan	nan	0	17	inf	5.00E-05	yes
ni|645903511|nb|KK920919.1|	46949-47197	nan	nan	0.034056	18	9	0.11935	no
ni|645903511|nb|KK920919.1|	470714-471631	nan	nan	28	27	-0.00207229	0.9985	no
ni|645903511|nb|KK920919.1|	471903-472653	nan	nan	2	1	-0.218104	0.87705	no
ni|645903511|nb|KK920919.1|	473429-473680	nan	nan	0.295895	4	4	0.0541	no
ni|645903511|nb|KK920919.1|	473780-474687	nan	nan	0.946204	0.90479	-0.0645673	0.95495	no
ni|645903511|nb|KK920919.1|	474892-475009	nan	nan	0	24	inf	0.00065	yes
ni|645903511|nb|KK920919.1|	475238-475870	nan	nan	0.314758	27	6	0.05055	no
ni|645903511|nb|KK920919.1|	476498-477070	nan	nan	2	67	5	0.0373	no
ni|645903511|nb|KK920919.1|	480739-480959	nan	nan	0.927427	51	6	0.0373	no
ni|645903511|nb|KK920919.1|	481924-482124	nan	nan	3	67	5	0.0038	no
ni|645903511|nb|KK920919.1|	483064-483568	nan	nan	0	91	inf	5.00E-05	yes
ni|645903511|nb|KK920919.1|	483630-484055	nan	nan	0	55	inf	5.00E-05	yes
ni|645903511|nb|KK920919.1|	484123-484287	nan	nan	3	36	4	0.04035	no
ni|645903511|nb|KK920919.1|	485227-485368	nan	nan	3	61	4	0.02145	no
ni|645903511|nb|KK920919.1|	48532-48704	nan	nan	0	25	inf	5.00E-05	yes
ni|645903511|nb|KK920919.1|	485520-485645	nan	nan	3	52	4	0.02845	no
ni|645903511|nb|KK920919.1|	485952-486078	nan	nan	5	66	4	0.00565	no
ni|645903511|nb|KK920919.1|	486379-487236	nan	nan	3	4	0.447346	0.7576	no
ni|645903511|nb|KK920919.1|	487646-489074	nan	nan	0.917656	1	0.586227	0.65495	no
ni|645903511|nb|KK920919.1|	489177-489796	nan	nan	1	5	2	0.18925	no
ni|645903511|nb|KK920919.1|	495413-495594	nan	nan	1	0.741228	-0.913292	0.4969	no
ni|645903511|nb|KK920919.1|	50718-50965	nan	nan	0.272504	21	6	0.1194	no
ni|645903511|nb|KK920919.1|	51569-52134	nan	nan	0	8	inf	5.00E-05	yes
ni|645903511|nb|KK920919.1|	52189-52490	nan	nan	0	10	inf	5.00E-05	yes
ni|645903511|nb|KK920919.1|	530130-531066	nan	nan	5	3	-0.620389	0.5918	no
ni|645903511|nb|KK920919.1|	53415-54217	nan	nan	96	79	-0.279659	0.81145	no
ni|645903511|nb|KK920919.1|	534973-535610	nan	nan	4	0	-inf	0.0015	yes
ni|645903511|nb|KK920919.1|	538834-539076	nan	nan	6	6	0.001103	0.99265	no
ni|645903511|nb|KK920919.1|	539223-539838	nan	nan	48	56	0.206782	0.8879	no

ni|645903511|nb|KK920919.1|	541022-541304	nan	nan	35	36	0.0174534	0.9899	no
ni|645903511|nb|KK920919.1|	542133-542423	nan	nan	0.679972	0.303366	-1	0.41555	no
ni|645903511|nb|KK920919.1|	542530-542644	nan	nan	0.604823	0.642339	0.0868224	0.9436	no
ni|645903511|nb|KK920919.1|	544425-545944	nan	nan	81	76	-0.089033	0.9354	no
ni|645903511|nb|KK920919.1|	546825-546996	nan	nan	117	118	0.0090818	0.99295	no
ni|645903511|nb|KK920919.1|	548194-548325	nan	nan	23	27	0.200351	0.8954	no
ni|645903511|nb|KK920919.1|	549631-549898	nan	nan	2	3	0.596764	0.63005	no
ni|645903511|nb|KK920919.1|	552357-552728	nan	nan	39	32	-0.246757	0.8315	no
ni|645903511|nb|KK920919.1|	554189-554468	nan	nan	29	31	0.0967066	0.93345	no
ni|645903511|nb|KK920919.1|	556796-556962	nan	nan	30	29	-0.0540761	0.95805	no
ni|645903511|nb|KK920919.1|	557843-558239	nan	nan	33	26	-0.341369	0.82585	no
ni|645903511|nb|KK920919.1|	558347-558607	nan	nan	29	19	-0.600634	0.6136	no
ni|645903511|nb|KK920919.1|	559897-560054	nan	nan	16	7	-1	0.3548	no
ni|645903511|nb|KK920919.1|	560183-560345	nan	nan	23	15	-0.634842	0.5644	no
ni|645903511|nb|KK920919.1|	561411-561709	nan	nan	30	13	-1	0.31655	no
ni|645903511|nb|KK920919.1|	563480-563664	nan	nan	33	18	-0.922924	0.45205	no
ni|645903511|nb|KK920919.1|	565261-565400	nan	nan	12	5	-1	0.3117	no
ni|645903511|nb|KK920919.1|	566625-566902	nan	nan	0.739849	0.235696	-2	0.2989	no
ni|645903511|nb|KK920919.1|	568949-569075	nan	nan	4	9	1	0.38985	no
ni|645903511|nb|KK920919.1|	569673-569844	nan	nan	17	14	-0.319558	0.7653	no
ni|645903511|nb|KK920919.1|	572646-572799	nan	nan	0.658483	0.343328	-0.93956	0.54025	no
ni|645903511|nb|KK920919.1|	574778-575032	nan	nan	2	1	-0.626896	0.60265	no
ni|645903511|nb|KK920919.1|	576568-576776	nan	nan	9	6	-0.610746	0.5727	no
ni|645903511|nb|KK920919.1|	578921-579123	nan	nan	0.651224	0.24904	-1	0.3467	no
ni|645903511|nb|KK920919.1|	581490-582149	nan	nan	29	19	-0.635087	0.66595	no
ni|645903511|nb|KK920919.1|	583693-584063	nan	nan	0.052177	93	11	0.0443	no
ni|645903511|nb|KK920919.1|	60276-60383	nan	nan	172	165	-0.0570979	0.9593	no
ni|645903511|nb|KK920919.1|	612980-613498	nan	nan	0	18	inf	5.00E-05	yes
ni|645903511|nb|KK920919.1|	614965-615112	nan	nan	0.552944	0.884787	0.678197	0.56665	no
ni|645903511|nb|KK920919.1|	615530-615713	nan	nan	0.846415	1	0.388135	0.73725	no

ni|645903511|nb|KK920919.1|	616052-616286	nan	nan	0.158911	10	6	0.11935	no
ni|645903511|nb|KK920919.1|	616723-616970	nan	nan	0.381392	0.607593	0.671829	0.65595	no
ni|645903511|nb|KK920919.1|	620466-620620	nan	nan	0.681262	1	0.995251	0.3868	no
ni|645903511|nb|KK920919.1|	638763-638959	nan	nan	2	2	0.140202	0.90025	no
ni|645903511|nb|KK920919.1|	639917-640104	nan	nan	212	183	-0.216893	0.85235	no
ni|645903511|nb|KK920919.1|	641740-641896	nan	nan	292	219.23	-0.412649	0.715	no
ni|645903511|nb|KK920919.1|	643925-644091	nan	nan	0.339327	0.18024	-0.912754	0.53575	no
ni|645903511|nb|KK920919.1|	644867-645289	nan	nan	264	209	-0.337561	0.8199	no
ni|645903511|nb|KK920919.1|	646705-646862	nan	nan	0.392781	0.331415	-0.245087	0.84465	no
ni|645903511|nb|KK920919.1|	647103-647367	nan	nan	0.383307	0.244308	-0.6498	0.6679	no
ni|645903511|nb|KK920919.1|	668156-668577	nan	nan	0.857576	0.819445	-0.0656163	0.9384	no
ni|645903511|nb|KK920919.1|	67123-67357	nan	nan	1	0.959328	-0.355168	0.7931	no
ni|645903511|nb|KK920919.1|	671668-671880	nan	nan	0.714065	0.735102	0.041889	0.9599	no
ni|645903511|nb|KK920919.1|	674931-675467	nan	nan	43	40	-0.0863417	0.9538	no
ni|645903511|nb|KK920919.1|	676287-676501	nan	nan	11	7	-0.699518	0.5662	no
ni|645903511|nb|KK920919.1|	67767-67915	nan	nan	166	163	-0.0276084	0.9799	no
ni|645903511|nb|KK920919.1|	679684-679815	nan	nan	61	45	-0.445099	0.66905	no
ni|645903511|nb|KK920919.1|	680626-680796	nan	nan	64	49	-0.377596	0.73975	no
ni|645903511|nb|KK920919.1|	681138-681301	nan	nan	19	16	-0.2666	0.8048	no
ni|645903511|nb|KK920919.1|	682475-682630	nan	nan	5	10	1	0.43015	no
ni|645903511|nb|KK920919.1|	683662-683806	nan	nan	57	52	-0.128617	0.91295	no
ni|645903511|nb|KK920919.1|	69968-70739	nan	nan	258	288	0.154136	0.89365	no
ni|645903511|nb|KK920919.1|	705818-706188	nan	nan	178	155	-0.196505	0.8568	no
ni|645903511|nb|KK920919.1|	706683-706972	nan	nan	8	5	-0.56823	0.6386	no
ni|645903511|nb|KK920919.1|	708983-709628	nan	nan	9	5	-0.670863	0.5829	no
ni|645903511|nb|KK920919.1|	71105-71599	nan	nan	1	0.552925	-1	0.4064	no
ni|645903511|nb|KK920919.1|	712421-712612	nan	nan	8	7	-0.172062	0.88105	no
ni|645903511|nb|KK920919.1|	713881-714025	nan	nan	6	5	-0.339894	0.77105	no
ni|645903511|nb|KK920919.1|	715304-715712	nan	nan	11	13	0.280579	0.82625	no
ni|645903511|nb|KK920919.1|	716642-716832	nan	nan	8	15	0.985334	0.39675	no

ni|645903511|nb|KK920919.1|	71705-72261	nan	nan	157	166	0.0856588	0.9324	no
ni|645903511|nb|KK920919.1|	719656-719826	nan	nan	6	18	2	0.1646	no
ni|645903511|nb|KK920919.1|	723597-723975	nan	nan	2	8	2	0.14335	no
ni|645903511|nb|KK920919.1|	725824-726073	nan	nan	7	4	-0.893352	0.44705	no
ni|645903511|nb|KK920919.1|	730941-731123	nan	nan	66	50	-0.397516	0.77625	no
ni|645903511|nb|KK920919.1|	740629-741004	nan	nan	31	51	0.703028	0.5349	no
ni|645903511|nb|KK920919.1|	741291-741694	nan	nan	57	86	0.60897	0.58695	no
ni|645903511|nb|KK920919.1|	741842-742396	nan	nan	36	49	0.43245	0.73145	no
ni|645903511|nb|KK920919.1|	74189-74710	nan	nan	18	23	0.357671	0.74935	no
ni|645903511|nb|KK920919.1|	743460-744103	nan	nan	76	105	0.461594	0.7073	no
ni|645903511|nb|KK920919.1|	744948-745680	nan	nan	24	30	0.333368	0.81735	no
ni|645903511|nb|KK920919.1|	745820-746279	nan	nan	43	52	0.260047	0.8118	no
ni|645903511|nb|KK920919.1|	746386-746943	nan	nan	23	24	0.0690695	0.94495	no
ni|645903511|nb|KK920919.1|	747120-747811	nan	nan	30	32	0.119523	0.9133	no
ni|645903511|nb|KK920919.1|	748789-748933	nan	nan	14	15	0.0791195	0.94525	no
ni|645903511|nb|KK920919.1|	751398-751567	nan	nan	87	94	0.107156	0.93095	no
ni|645903511|nb|KK920919.1|	753761-753885	nan	nan	1	1	-0.287859	0.8283	no
ni|645903511|nb|KK920919.1|	754377-754506	nan	nan	12	10	-0.288572	0.8089	no
ni|645903511|nb|KK920919.1|	755015-755158	nan	nan	13	13	0.0151911	0.9674	no
ni|645903511|nb|KK920919.1|	755611-755746	nan	nan	4	8	1	0.4354	no
ni|645903511|nb|KK920919.1|	756243-756376	nan	nan	3	4	0.141804	0.90275	no
ni|645903511|nb|KK920919.1|	758425-758796	nan	nan	5	7	0.418229	0.73705	no
ni|645903511|nb|KK920919.1|	75876-76283	nan	nan	0	146	inf	5.00E-05	yes
ni|645903511|nb|KK920919.1|	759713-759825	nan	nan	40	34	-0.240158	0.82865	no
ni|645903511|nb|KK920919.1|	760249-760579	nan	nan	20	15	-0.468013	0.68445	no
ni|645903511|nb|KK920919.1|	761213-761513	nan	nan	8	5	-0.580655	0.62925	no
ni|645903511|nb|KK920919.1|	762075-762261	nan	nan	22	17	-0.389307	0.7511	no
ni|645903511|nb|KK920919.1|	763173-763367	nan	nan	16	14	-0.194027	0.8672	no
ni|645903511|nb|KK920919.1|	764027-764235	nan	nan	26	29	0.126633	0.9306	no
ni|645903511|nb|KK920919.1|	764981-765662	nan	nan	45	55	0.302909	0.79485	no

ni|645903511|nb|KK920919.1|	76694-76896	nan	nan	0	240	inf	5.00E-05	yes
ni|645903511|nb|KK920919.1|	769166-769431	nan	nan	13	14	0.118462	0.91645	no
ni|645903511|nb|KK920919.1|	772963-773595	nan	nan	3	1	-1	0.43485	no
ni|645903511|nb|KK920919.1|	77376-77871	nan	nan	0	31	inf	5.00E-05	yes
ni|645903511|nb|KK920919.1|	774603-776708	nan	nan	2	2	-0.32835	0.7854	no
ni|645903511|nb|KK920919.1|	776913-777295	nan	nan	1	0.584171	-0.940029	0.54025	no
ni|645903511|nb|KK920919.1|	78533-78654	nan	nan	0.185075	119	9	0.11935	no
ni|645903511|nb|KK920919.1|	80428-80759	nan	nan	0.088071	159	11	0.0443	no
ni|645903511|nb|KK920919.1|	80994-81146	nan	nan	0	8	inf	5.00E-05	yes
ni|645903511|nb|KK920919.1|	81285-81399	nan	nan	0	1	inf	0.00015	yes
ni|645903511|nb|KK920919.1|	818438-818986	nan	nan	0.865087	0.72329	-0.258271	0.8692	no
ni|645903511|nb|KK920919.1|	81910-82320	nan	nan	0	53	inf	5.00E-05	yes
ni|645903511|nb|KK920919.1|	82542-82742	nan	nan	0	117	inf	5.00E-05	yes
ni|645903511|nb|KK920919.1|	826804-827010	nan	nan	2	2	-0.104558	0.9266	no
ni|645903511|nb|KK920919.1|	829519-829762	nan	nan	5	9	0.850327	0.521	no
ni|645903511|nb|KK920919.1|	831487-831945	nan	nan	3	3	-0.0235553	0.98455	no
ni|645903511|nb|KK920919.1|	832046-832372	nan	nan	5	5	-0.0256918	0.9823	no
ni|645903511|nb|KK920919.1|	83441-83580	nan	nan	0	169	inf	5.00E-05	yes
ni|645903512|nb|KK920918.1|	1064-1264	nan	nan	6	7	0.222268	0.8747	no
ni|645903512|nb|KK920918.1|	393656-393802	nan	nan	10	11	0.0732546	0.9471	no
ni|645903512|nb|KK920918.1|	400927-401080	nan	nan	0	3	inf	0.0001	yes
ni|645903512|nb|KK920918.1|	412523-412638	nan	nan	1	6	2	0.1458	no
ni|645903512|nb|KK920918.1|	415189-415338	nan	nan	5	3	-0.446611	0.70335	no
ni|645903512|nb|KK920918.1|	419908-420112	nan	nan	0.782097	0.398746	-0.971876	0.5005	no
ni|645903512|nb|KK920918.1|	421839-421985	nan	nan	0.238706	0.182628	-0.386323	1	no
ni|645903512|nb|KK920918.1|	422447-422805	nan	nan	0.662584	0.422189	-0.650215	0.6679	no
ni|645903512|nb|KK920918.1|	422903-423248	nan	nan	1	0.803632	-0.567716	0.6631	no
ni|645903512|nb|KK920918.1|	423480-423907	nan	nan	0.414438	0.435852	0.0726842	0.94355	no
ni|645903512|nb|KK920918.1|	424043-424240	nan	nan	1	0.899248	-0.199366	0.86235	no
ni|645903512|nb|KK920918.1|	426044-426170	nan	nan	0.943597	0.5821	-0.696905	0.56625	no

ni|645903512|nb|KK920918.1|	426385-426620	nan	nan	10	7	-0.4168	0.7136	no
ni|645903512|nb|KK920918.1|	426937-427159	nan	nan	5	4	-0.573462	0.6321	no
ni|645903512|nb|KK920918.1|	427305-427488	nan	nan	3	2	-0.310587	0.7885	no
ni|645903512|nb|KK920918.1|	427783-427925	nan	nan	6	3	-0.717888	0.5509	no
ni|645903512|nb|KK920918.1|	432070-432207	nan	nan	33	22	-0.601491	0.5934	no
ni|645903512|nb|KK920918.1|	432322-432603	nan	nan	2	0.947475	-1	0.4048	no
ni|645903512|nb|KK920918.1|	432860-433342	nan	nan	43	29	-0.559845	0.6239	no
ni|645903512|nb|KK920918.1|	433605-433774	nan	nan	3	2	-0.486618	0.6806	no
ni|645903512|nb|KK920918.1|	435561-435723	nan	nan	112	83	-0.437181	0.69045	no
ni|645903512|nb|KK920918.1|	436345-437014	nan	nan	0.959948	1	0.0990492	0.9332	no
ni|645903512|nb|KK920918.1|	437361-437810	nan	nan	2	0.525463	-2	0.2414	no
ni|645903512|nb|KK920918.1|	438020-438410	nan	nan	0.936855	0.667953	-0.48808	0.6796	no
ni|645903512|nb|KK920918.1|	438485-439222	nan	nan	0.8776	0.167741	-2	0.2949	no
ni|645903512|nb|KK920918.1|	439962-440156	nan	nan	1	0.890425	-0.744453	0.59615	no
ni|645903512|nb|KK920918.1|	442611-443002	nan	nan	12	12	-0.105294	0.9251	no
ni|645903512|nb|KK920918.1|	447002-447195	nan	nan	1	2	0.419603	0.73705	no
ni|645903512|nb|KK920918.1|	447865-448463	nan	nan	3	2	-0.513323	0.6589	no
ni|645903512|nb|KK920918.1|	449514-449933	nan	nan	229	247	0.10794	0.93915	no
ni|645903512|nb|KK920918.1|	450212-451050	nan	nan	186	168	-0.146146	0.9226	no
ni|645903512|nb|KK920918.1|	451542-452045	nan	nan	1	0.478161	-1	0.44965	no
ni|645903512|nb|KK920918.1|	453266-453549	nan	nan	0.160981	128	10	0.11935	no
ni|645903512|nb|KK920918.1|	455202-455348	nan	nan	0.229064	75	8	0.0506	no
ni|645903512|nb|KK920918.1|	466762-466893	nan	nan	0.272504	5	4	0.1221	no
ni|645903512|nb|KK920918.1|	470791-471027	nan	nan	12	6	-0.908773	0.4338	no
ni|645903512|nb|KK920918.1|	471240-471382	nan	nan	3	3	0.0719794	0.9525	no
ni|645903512|nb|KK920918.1|	476157-476593	nan	nan	6	2	-1	0.28735	no
ni|645903512|nb|KK920918.1|	480718-480933	nan	nan	17	10	-0.749519	0.52075	no
ni|645903512|nb|KK920918.1|	481765-482137	nan	nan	37	17	-1	0.33435	no
ni|645903512|nb|KK920918.1|	482286-482510	nan	nan	74	25	-2	0.2006	no
ni|645903512|nb|KK920918.1|	483535-483678	nan	nan	0	5	inf	0.0001	yes

ni|645903512|nb|KK920918.1|	488792-489083	nan	nan	0	7	inf	0.0003	yes
ni|645903512|nb|KK920918.1|	490647-490804	nan	nan	0	8	inf	0.00065	yes
ni|645903512|nb|KK920918.1|	496004-496189	nan	nan	0	7	inf	5.00E-05	yes
ni|645903512|nb|KK920918.1|	499913-500255	nan	nan	0	4	inf	5.00E-05	yes
ni|645903512|nb|KK920918.1|	509570-509748	nan	nan	0	12	inf	5.00E-05	yes
ni|645903512|nb|KK920918.1|	510288-510436	nan	nan	0	20	inf	0.00015	yes
ni|645903512|nb|KK920918.1|	510512-511453	nan	nan	0	18	inf	5.00E-05	yes
ni|645903512|nb|KK920918.1|	511895-513930	nan	nan	26	15	-0.825255	0.47085	no
ni|645903512|nb|KK920918.1|	514035-514238	nan	nan	20	9	-1	0.3354	no
ni|645903513|nb|KK920917.1|	100606-100840	nan	nan	31	48	0.623628	0.5648	no
ni|645903513|nb|KK920917.1|	106753-106919	nan	nan	0.911525	0.580926	-0.649926	0.6173	no
ni|645903513|nb|KK920917.1|	132949-133250	nan	nan	7	12	0.681326	0.551	no
ni|645903513|nb|KK920917.1|	133578-133729	nan	nan	6	8	0.536026	0.62715	no
ni|645903513|nb|KK920917.1|	13677-13938	nan	nan	14	9	-0.591456	0.60095	no
ni|645903513|nb|KK920917.1|	139268-139430	nan	nan	2	56	5	0.003	no
ni|645903513|nb|KK920917.1|	148952-149320	nan	nan	0.142429	24	7	0.11935	no
ni|645903513|nb|KK920917.1|	151086-151381	nan	nan	0.441943	8	4	0.029	no
ni|645903513|nb|KK920917.1|	153742-154189	nan	nan	13	15	0.151716	0.89505	no
ni|645903513|nb|KK920917.1|	154731-154853	nan	nan	14	19	0.526107	0.65555	no
ni|645903513|nb|KK920917.1|	156130-156252	nan	nan	1	0.985432	-0.567883	0.6631	no
ni|645903513|nb|KK920917.1|	159820-160098	nan	nan	18	21	0.270952	0.8211	no
ni|645903513|nb|KK920917.1|	161458-161664	nan	nan	21	20	-0.119263	0.91525	no
ni|645903513|nb|KK920917.1|	164089-164208	nan	nan	4	4	-0.0899244	0.9383	no
ni|645903513|nb|KK920917.1|	164318-164448	nan	nan	17	19	0.142252	0.89935	no
ni|645903513|nb|KK920917.1|	177419-177611	nan	nan	15	13	-0.180143	0.8698	no
ni|645903513|nb|KK920917.1|	181076-181329	nan	nan	8	6	-0.243177	0.8345	no
ni|645903513|nb|KK920917.1|	183280-183454	nan	nan	22	24	0.144113	0.90755	no
ni|645903513|nb|KK920917.1|	184414-184918	nan	nan	23	17	-0.464536	0.698	no
ni|645903513|nb|KK920917.1|	192476-192824	nan	nan	13	5	-1	0.35155	no
ni|645903513|nb|KK920917.1|	196541-196821	nan	nan	11	10	-0.231109	0.83705	no

ni|645903513|nb|KK920917.1|	199113-199574	nan	nan	22	16	-0.41681	0.7743	no
ni|645903513|nb|KK920917.1|	200610-201041	nan	nan	4	0	-inf	0.00175	yes
ni|645903513|nb|KK920917.1|	219267-219784	nan	nan	1	137	7	0.01095	no
ni|645903513|nb|KK920917.1|	248514-248787	nan	nan	1	109	6	0.0205	no
ni|645903513|nb|KK920917.1|	255777-255933	nan	nan	0.542535	0.432305	-0.327666	0.79705	no
ni|645903513|nb|KK920917.1|	257832-258225	nan	nan	0.437804	0.557926	0.349791	0.81755	no
ni|645903513|nb|KK920917.1|	259766-260239	nan	nan	0.709136	0.602445	-0.235233	0.8697	no
ni|645903513|nb|KK920917.1|	31598-31983	nan	nan	24	17	-0.455906	0.6956	no
ni|645903513|nb|KK920917.1|	32172-33854	nan	nan	30	17	-0.779192	0.5983	no
ni|645903513|nb|KK920917.1|	352106-352286	nan	nan	2	150	6	0.11935	no
ni|645903513|nb|KK920917.1|	352353-352679	nan	nan	2	134	6	0.00055	yes
ni|645903513|nb|KK920917.1|	353313-353436	nan	nan	0	3	inf	0.00055	yes
ni|645903513|nb|KK920917.1|	353542-354340	nan	nan	3	189	6	0.02725	no
ni|645903513|nb|KK920917.1|	358658-358897	nan	nan	0	12	inf	0.00045	yes
ni|645903513|nb|KK920917.1|	360360-360619	nan	nan	0	18	inf	5.00E-05	yes
ni|645903513|nb|KK920917.1|	36381-36915	nan	nan	0.669035	0.548009	-0.287883	0.8535	no
ni|645903513|nb|KK920917.1|	363823-364016	nan	nan	0	4	inf	0.0003	yes
ni|645903513|nb|KK920917.1|	365054-365223	nan	nan	0	92	inf	5.00E-05	yes
ni|645903513|nb|KK920917.1|	368740-369124	nan	nan	111	130	0.222549	0.88015	no
ni|645903513|nb|KK920917.1|	370294-370477	nan	nan	1	2	0.349412	0.7835	no
ni|645903513|nb|KK920917.1|	372235-372413	nan	nan	0.375773	3	3	0.071	no
ni|645903513|nb|KK920917.1|	374604-374753	nan	nan	25	17	-0.568151	0.6253	no
ni|645903513|nb|KK920917.1|	37610-38126	nan	nan	4	3	-0.219272	0.87705	no
ni|645903513|nb|KK920917.1|	377591-377734	nan	nan	11	10	-0.0656764	0.95495	no
ni|645903513|nb|KK920917.1|	38329-39000	nan	nan	4	2	-1	0.45115	no
ni|645903513|nb|KK920917.1|	386409-387010	nan	nan	0.722645	1	0.742476	0.54275	no
ni|645903513|nb|KK920917.1|	389106-389479	nan	nan	0	79	inf	5.00E-05	yes
ni|645903513|nb|KK920917.1|	389786-390293	nan	nan	4	4	0.153142	0.89415	no
ni|645903513|nb|KK920917.1|	392050-392394	nan	nan	1	1	-0.550262	0.6505	no
ni|645903513|nb|KK920917.1|	396218-396534	nan	nan	3	4	0.0295538	0.97945	no

ni|645903513|nb|KK920917.1|	39869-39998	nan	nan	3	2	-0.399808	0.744	no
ni|645903513|nb|KK920917.1|	398992-399366	nan	nan	8	7	-0.2416	0.8363	no
ni|645903513|nb|KK920917.1|	40109-40388	nan	nan	6	4	-0.651716	0.5959	no
ni|645903513|nb|KK920917.1|	406897-407074	nan	nan	21	49	1	0.3448	no
ni|645903513|nb|KK920917.1|	407392-407583	nan	nan	2	3	0.181846	0.87985	no
ni|645903513|nb|KK920917.1|	407918-408032	nan	nan	1	1	0.175367	0.8916	no
ni|645903513|nb|KK920917.1|	408356-408592	nan	nan	3	3	0.037187	0.97295	no
ni|645903513|nb|KK920917.1|	409413-410362	nan	nan	126	257	1	0.46805	no
ni|645903513|nb|KK920917.1|	410429-410788	nan	nan	6	2	-1	0.3535	no
ni|645903513|nb|KK920917.1|	411000-411183	nan	nan	54	14	-2	0.1001	no
ni|645903513|nb|KK920917.1|	412031-412255	nan	nan	3	1	-1	0.272	no
ni|645903513|nb|KK920917.1|	412838-413571	nan	nan	0.739228	0.192702	-2	0.24815	no
ni|645903513|nb|KK920917.1|	413627-414737	nan	nan	9	6	-0.480907	0.6802	no
ni|645903513|nb|KK920917.1|	41381-41504	nan	nan	4	2	-0.860348	0.4649	no
ni|645903513|nb|KK920917.1|	415171-415423	nan	nan	28	23	-0.2802	0.8115	no
ni|645903513|nb|KK920917.1|	41736-42192	nan	nan	7	2	-2	0.2821	no
ni|645903513|nb|KK920917.1|	419322-419433	nan	nan	61	24	-1	0.31765	no
ni|645903513|nb|KK920917.1|	419829-421000	nan	nan	30	34	0.191096	0.86805	no
ni|645903513|nb|KK920917.1|	42426-42658	nan	nan	0	2	inf	0.00065	yes
ni|645903513|nb|KK920917.1|	426426-426725	nan	nan	14	2	-2	0.17	no
ni|645903513|nb|KK920917.1|	426874-427001	nan	nan	22	18	-0.265566	0.8335	no
ni|645903513|nb|KK920917.1|	427764-428251	nan	nan	13	11	-0.219423	0.87705	no
ni|645903513|nb|KK920917.1|	428354-428519	nan	nan	6	5	-0.178822	0.87685	no
ni|645903513|nb|KK920917.1|	429055-429411	nan	nan	0.409327	0.440214	0.104953	0.93335	no
ni|645903513|nb|KK920917.1|	429605-429768	nan	nan	12	9	-0.366352	0.7578	no
ni|645903513|nb|KK920917.1|	430755-431121	nan	nan	0.674663	0.573175	-0.235192	0.8697	no
ni|645903513|nb|KK920917.1|	431890-432363	nan	nan	14	14	-0.00256939	0.99105	no
ni|645903513|nb|KK920917.1|	435328-435831	nan	nan	2	2	-0.236525	0.8697	no
ni|645903513|nb|KK920917.1|	437683-438196	nan	nan	3	0.996297	-2	0.2784	no
ni|645903513|nb|KK920917.1|	484023-484695	nan	nan	6	6	-0.000858758	0.9954	no

ni|645903513|nb|KK920917.1|	493791-494263	nan	nan	2	0.592185	-2	0.24815	no
ni|645903513|nb|KK920917.1|	495645-495778	nan	nan	5	5	0.162377	0.8845	no
ni|645903513|nb|KK920917.1|	496551-496967	nan	nan	12	13	0.184357	0.87155	no
ni|645903513|nb|KK920917.1|	49730-50075	nan	nan	8	2	-2	0.24815	no
ni|645903513|nb|KK920917.1|	497652-498406	nan	nan	9	17	0.892162	0.47205	no
ni|645903513|nb|KK920917.1|	499329-499694	nan	nan	3	5	0.660095	0.57885	no
ni|645903513|nb|KK920917.1|	499817-499975	nan	nan	3	3	0.210637	0.85605	no
ni|645903513|nb|KK920917.1|	500402-500524	nan	nan	7	11	0.580503	0.6139	no
ni|645903513|nb|KK920917.1|	501088-501231	nan	nan	5	9	0.932676	0.5287	no
ni|645903513|nb|KK920917.1|	501721-502427	nan	nan	8	9	0.138157	0.90545	no
ni|645903513|nb|KK920917.1|	503091-503322	nan	nan	8	15	0.84696	0.4762	no
ni|645903513|nb|KK920917.1|	503834-504001	nan	nan	4	1	-1	0.3864	no
ni|645903513|nb|KK920917.1|	505673-505880	nan	nan	11	2	-3	0.04215	no
ni|645903513|nb|KK920917.1|	506684-506914	nan	nan	29	5	-3	0.03235	no
ni|645903513|nb|KK920917.1|	507189-507336	nan	nan	41	8	-2	0.0631	no
ni|645903513|nb|KK920917.1|	508016-508215	nan	nan	42	7	-3	0.0328	no
ni|645903513|nb|KK920917.1|	508931-509146	nan	nan	52	13	-2	0.0977	no
ni|645903513|nb|KK920917.1|	511744-512141	nan	nan	41	9	-2	0.0792	no
ni|645903513|nb|KK920917.1|	513696-514154	nan	nan	54	13	-2	0.11605	no
ni|645903513|nb|KK920917.1|	514347-514560	nan	nan	37	3	-4	0.03005	no
ni|645903513|nb|KK920917.1|	514880-515129	nan	nan	8	1	-3	0.2671	no
ni|645903513|nb|KK920917.1|	516012-516141	nan	nan	0.971421	1	0.104167	0.93335	no
ni|645903513|nb|KK920917.1|	519635-519921	nan	nan	5	8	0.633212	0.63975	no
ni|645903513|nb|KK920917.1|	522639-523192	nan	nan	13	22	0.740111	0.54335	no
ni|645903513|nb|KK920917.1|	5272-5692	nan	nan	34	19	-0.879565	0.4571	no
ni|645903513|nb|KK920917.1|	533510-533739	nan	nan	17	12	-0.514693	0.6647	no
ni|645903513|nb|KK920917.1|	538690-539055	nan	nan	5	5	-0.00501692	0.9947	no
ni|645903513|nb|KK920917.1|	540515-540796	nan	nan	2	2	-0.105857	0.92825	no
ni|645903513|nb|KK920917.1|	541924-542167	nan	nan	1	0.902094	-0.406166	0.755	no
ni|645903513|nb|KK920917.1|	542433-542622	nan	nan	7	8	0.0397067	0.95995	no

ni|645903513|nb|KK920917.1|	545361-545774	nan	nan	0.123914	0.829294	3	0.081	no
ni|645903513|nb|KK920917.1|	546644-546960	nan	nan	34	40	0.24421	0.82065	no
ni|645903513|nb|KK920917.1|	547093-547550	nan	nan	11	16	0.547658	0.6454	no
ni|645903513|nb|KK920917.1|	547715-547918	nan	nan	65	76	0.230965	0.83865	no
ni|645903513|nb|KK920917.1|	548035-548369	nan	nan	46	81	0.806892	0.502	no
ni|645903513|nb|KK920917.1|	551813-551962	nan	nan	71	71	-0.000503694	0.9974	no
ni|645903513|nb|KK920917.1|	552066-552739	nan	nan	82	78	-0.0674947	0.95295	no
ni|645903513|nb|KK920917.1|	553205-553605	nan	nan	35	40	0.199394	0.85415	no
ni|645903513|nb|KK920917.1|	553977-555258	nan	nan	66	72	0.128359	0.90585	no
ni|645903513|nb|KK920917.1|	559033-559146	nan	nan	60	67	0.146005	0.896	no
ni|645903513|nb|KK920917.1|	559711-560051	nan	nan	15	19	0.299892	0.7969	no
ni|645903513|nb|KK920917.1|	563445-563840	nan	nan	0.379368	1	2	0.2028	no
ni|645903513|nb|KK920917.1|	564402-564545	nan	nan	0.813693	0.937317	0.204051	0.8605	no
ni|645903513|nb|KK920917.1|	565458-565957	nan	nan	3	3	0.102636	0.93335	no
ni|645903513|nb|KK920917.1|	568364-568910	nan	nan	3	2	-0.449787	0.696	no
ni|645903513|nb|KK920917.1|	568970-569685	nan	nan	4	7	0.698201	0.5444	no
ni|645903513|nb|KK920917.1|	569801-570147	nan	nan	2	2	0.1189	0.92325	no
ni|645903513|nb|KK920917.1|	572508-573627	nan	nan	4	10	1	0.3111	no
ni|645903513|nb|KK920917.1|	57276-57795	nan	nan	4	4	-0.0672382	0.923	no
ni|645903513|nb|KK920917.1|	578298-578497	nan	nan	1	4	2	0.24795	no
ni|645903513|nb|KK920917.1|	59462-60063	nan	nan	7	5	-0.482184	0.69675	no
ni|645903513|nb|KK920917.1|	598204-598338	nan	nan	5	9	0.953602	0.4249	no
ni|645903513|nb|KK920917.1|	600044-600973	nan	nan	6	7	0.164323	0.89015	no
ni|645903513|nb|KK920917.1|	606894-607748	nan	nan	10	11	0.236825	0.8377	no
ni|645903513|nb|KK920917.1|	610591-610843	nan	nan	8	12	0.669546	0.65595	no
ni|645903513|nb|KK920917.1|	612622-612786	nan	nan	14	15	0.119223	0.92045	no
ni|645903513|nb|KK920917.1|	613326-613748	nan	nan	6	7	0.329873	0.77515	no
ni|645903513|nb|KK920917.1|	614085-614248	nan	nan	1	2	0.5874	0.6103	no
ni|645903513|nb|KK920917.1|	616968-617155	nan	nan	223.76	171.19	-0.386353	0.7292	no
ni|645903513|nb|KK920917.1|	61713-61842	nan	nan	15	19	0.301775	0.81025	no

ni|645903513|nb|KK920917.1|	618151-618327	nan	nan	91	54	-0.755551	0.51955	no
ni|645903513|nb|KK920917.1|	626754-626832	nan	nan	289	235	-0.302083	0.8277	no
ni|645903513|nb|KK920917.1|	65211-65479	nan	nan	8	6	-0.30062	0.7939	no
ni|645903513|nb|KK920917.1|	681379-681500	nan	nan	4	3	-0.560397	0.63335	no
ni|645903513|nb|KK920917.1|	69404-69576	nan	nan	30	34	0.147042	0.9207	no
ni|645903513|nb|KK920917.1|	70515-70677	nan	nan	8	7	-0.260835	0.81375	no
ni|645903513|nb|KK920917.1|	756253-756392	nan	nan	4	3	-0.474779	0.6835	no
ni|645903513|nb|KK920917.1|	765360-766107	nan	nan	46	39	-0.241649	0.82595	no
ni|645903513|nb|KK920917.1|	766261-766424	nan	nan	10	13	0.448786	0.7066	no
ni|645903513|nb|KK920917.1|	766744-767093	nan	nan	52	33	-0.6776	0.576	no
ni|645903513|nb|KK920917.1|	767480-767603	nan	nan	13	12	-0.0675467	0.9302	no
ni|645903513|nb|KK920917.1|	767868-768063	nan	nan	86	67	-0.354334	0.76085	no
ni|645903513|nb|KK920917.1|	768229-769428	nan	nan	117	80	-0.545892	0.62605	no
ni|645903513|nb|KK920917.1|	771791-772312	nan	nan	0.440406	0.578869	0.394404	0.77595	no
ni|645903513|nb|KK920917.1|	775572-775708	nan	nan	1	0.622483	-0.84307	0.5595	no
ni|645903513|nb|KK920917.1|	777896-778106	nan	nan	199	164	-0.279917	0.81145	no
ni|645903513|nb|KK920917.1|	88048-88790	nan	nan	2	0.537815	-2	0.2261	no
ni|645903513|nb|KK920917.1|	90238-90525	nan	nan	6	4	-0.502841	0.6673	no
ni|645903513|nb|KK920917.1|	92099-92403	nan	nan	10	8	-0.246297	0.83165	no
ni|645903513|nb|KK920917.1|	93463-93589	nan	nan	29	18	-0.692462	0.55275	no
ni|645903513|nb|KK920917.1|	94998-95583	nan	nan	2	2	0.223294	0.8598	no
ni|645903513|nb|KK920917.1|	99605-99847	nan	nan	29	35	0.280575	0.80045	no
ni|645903514|nb|KK920916.1|	276757-276916	nan	nan	1	0.236483	-2	0.1906	no
ni|645903514|nb|KK920916.1|	281367-281868	nan	nan	0.964773	0.921876	-0.0656163	0.94155	no
ni|645903514|nb|KK920916.1|	312256-312575	nan	nan	23	23	-0.000261192	0.9957	no
ni|645903515|nb|KK920915.1|	425557-425700	nan	nan	22	21	-0.0813975	0.93715	no
ni|645903515|nb|KK920915.1|	438841-439159	nan	nan	67	61	-0.129618	0.9116	no
ni|645903515|nb|KK920915.1|	453664-453830	nan	nan	46	64	0.467135	0.6909	no
ni|645903515|nb|KK920915.1|	454580-455048	nan	nan	124	126	0.0199015	0.9864	no
ni|645903515|nb|KK920915.1|	455185-455318	nan	nan	130.51	156	0.256693	0.8378	no

ni|645903515|nb|KK920915.1|	455630-456061	nan	nan	126	151	0.267948	0.82735	no
ni|645903515|nb|KK920915.1|	456393-456548	nan	nan	142	152	0.0979883	0.9346	no
ni|645903515|nb|KK920915.1|	456801-458319	nan	nan	87	110.73	0.350586	0.76045	no
ni|645903515|nb|KK920915.1|	458406-458557	nan	nan	89	137	0.630537	0.55865	no
ni|645903515|nb|KK920915.1|	458938-459116	nan	nan	166	223	0.423688	0.7081	no
ni|645903515|nb|KK920915.1|	459235-459362	nan	nan	91	123	0.446728	0.68895	no
ni|645903515|nb|KK920915.1|	460471-460997	nan	nan	106	113	0.0848047	0.9387	no
ni|645903515|nb|KK920915.1|	461123-461370	nan	nan	29	25	-0.209904	0.85715	no
ni|645903515|nb|KK920915.1|	461500-461692	nan	nan	40	51	0.349016	0.7537	no
ni|645903515|nb|KK920915.1|	461834-465136	nan	nan	37	44	0.268902	0.809	no
ni|645903515|nb|KK920915.1|	465384-465501	nan	nan	15.84	15	-0.0654279	0.9533	no
ni|645903515|nb|KK920915.1|	466231-466377	nan	nan	0	4	inf	5.00E-05	yes
ni|645903515|nb|KK920915.1|	502035-502267	nan	nan	0.033546	4	7	0.11935	no
ni|645903515|nb|KK920915.1|	733823-734944	nan	nan	0	19	inf	5.00E-05	yes
ni|645903516|nb|KK920914.1|	126396-126768	nan	nan	39	44	0.168754	0.88615	no
ni|645903516|nb|KK920914.1|	127149-127825	nan	nan	7	7	-0.0377324	0.9739	no
ni|645903516|nb|KK920914.1|	128034-128540	nan	nan	31	44	0.486401	0.67935	no
ni|645903516|nb|KK920914.1|	137631-137825	nan	nan	43	46	0.1023	0.92575	no
ni|645903516|nb|KK920914.1|	138304-138474	nan	nan	40	46	0.199968	0.8609	no
ni|645903516|nb|KK920914.1|	166295-166994	nan	nan	38	39	0.0276174	0.98175	no
ni|645903516|nb|KK920914.1|	18003-18859	nan	nan	62	39	-0.689331	0.58685	no
ni|645903516|nb|KK920914.1|	181239-181929	nan	nan	77	74	-0.0674529	0.95325	no
ni|645903516|nb|KK920914.1|	184087-184329	nan	nan	54	73	0.438867	0.70295	no
ni|645903516|nb|KK920914.1|	186453-186812	nan	nan	84	73	-0.199422	0.85805	no
ni|645903516|nb|KK920914.1|	187153-187564	nan	nan	55	56	0.0334888	0.9765	no
ni|645903516|nb|KK920914.1|	188271-188620	nan	nan	15	15	0.00316257	0.99725	no
ni|645903516|nb|KK920914.1|	188817-189093	nan	nan	0.5767	9	4	0.12575	no
ni|645903516|nb|KK920914.1|	190272-190547	nan	nan	43	58	0.445175	0.68885	no
ni|645903516|nb|KK920914.1|	191057-191283	nan	nan	15	19	0.318222	0.7754	no
ni|645903516|nb|KK920914.1|	191981-192091	nan	nan	66	78	0.230051	0.82885	no

ni|645903516|nb|KK920914.1|	192242-192372	nan	nan	8	5	-0.593258	0.61395	no
ni|645903516|nb|KK920914.1|	198031-198476	nan	nan	20	14	-0.569717	0.63715	no
ni|645903516|nb|KK920914.1|	198886-200150	nan	nan	10	4	-1	0.27715	no
ni|645903516|nb|KK920914.1|	201286-202271	nan	nan	54	47	-0.181884	0.87825	no
ni|645903516|nb|KK920914.1|	202442-202790	nan	nan	11	10	-0.166788	0.8846	no
ni|645903516|nb|KK920914.1|	205307-206261	nan	nan	68	64	-0.0742635	0.93855	no
ni|645903516|nb|KK920914.1|	206846-207622	nan	nan	28	31	0.148349	0.8887	no
ni|645903516|nb|KK920914.1|	225263-225472	nan	nan	57	71	0.321735	0.7807	no
ni|645903516|nb|KK920914.1|	226906-227042	nan	nan	2	1	-0.898924	0.46065	no
ni|645903516|nb|KK920914.1|	229150-229582	nan	nan	6	7	0.347718	0.7814	no
ni|645903516|nb|KK920914.1|	241944-242346	nan	nan	8	11	0.443762	0.7026	no
ni|645903516|nb|KK920914.1|	242428-242592	nan	nan	4	3	-0.540051	0.6621	no
ni|645903516|nb|KK920914.1|	24780-25546	nan	nan	2	0.515965	-2	0.147	no
ni|645903516|nb|KK920914.1|	251676-251874	nan	nan	15	20	0.403724	0.79455	no
ni|645903516|nb|KK920914.1|	295424-295617	nan	nan	44	74	0.749523	0.52825	no
ni|645903516|nb|KK920914.1|	31217-31926	nan	nan	2	1	-0.650132	0.5978	no
ni|645903516|nb|KK920914.1|	323408-323670	nan	nan	26	52	0.99741	0.5043	no
ni|645903516|nb|KK920914.1|	326162-326415	nan	nan	5	11	1	0.39675	no
ni|645903516|nb|KK920914.1|	357343-357869	nan	nan	2	4	1	0.3727	no
ni|645903516|nb|KK920914.1|	358051-358294	nan	nan	18	39	1	0.32195	no
ni|645903516|nb|KK920914.1|	360962-361200	nan	nan	30	58	0.951722	0.3903	no
ni|645903516|nb|KK920914.1|	361331-361491	nan	nan	15	33	1	0.37525	no
ni|645903516|nb|KK920914.1|	361606-361749	nan	nan	27	56	1	0.3719	no
ni|645903516|nb|KK920914.1|	362778-363344	nan	nan	11	25	1	0.29885	no
ni|645903516|nb|KK920914.1|	364872-364996	nan	nan	4	12	2	0.19825	no
ni|645903516|nb|KK920914.1|	365541-365668	nan	nan	37	110	2	0.19645	no
ni|645903516|nb|KK920914.1|	365885-366032	nan	nan	43279.3	56841.6	0.39327	0.73945	no
ni|645903516|nb|KK920914.1|	373318-373497	nan	nan	0	3	inf	0.00055	yes
ni|645903516|nb|KK920914.1|	377893-378370	nan	nan	0	85	inf	5.00E-05	yes
ni|645903516|nb|KK920914.1|	38219-39277	nan	nan	2	0.691517	-1	0.35155	no

ni|645903516|nb|KK920914.1|	413678-413837	nan	nan	0	8	inf	5.00E-05	yes
ni|645903516|nb|KK920914.1|	4288-4668	nan	nan	35	44	0.313216	0.7911	no
ni|645903516|nb|KK920914.1|	4814-5063	nan	nan	6	7	0.194175	0.8542	no
ni|645903516|nb|KK920914.1|	489974-490205	nan	nan	0	4	inf	0.0001	yes
ni|645903516|nb|KK920914.1|	65673-66480	nan	nan	8	2	-2	0.1999	no
ni|645903516|nb|KK920914.1|	72108-72779	nan	nan	3	3	-0.23625	0.85345	no
ni|645903516|nb|KK920914.1|	728551-728739	nan	nan	0.932883	8	3	0.02255	no
ni|645903516|nb|KK920914.1|	75635-76608	nan	nan	58	55	-0.0723362	0.9569	no
ni|645903516|nb|KK920914.1|	77116-77673	nan	nan	69	62	-0.157078	0.887	no
ni|645903516|nb|KK920914.1|	89776-90507	nan	nan	69	66	-0.0598131	0.9603	no
ni|645903516|nb|KK920914.1|	9232-9613	nan	nan	19	18	-0.0445683	0.9681	no
ni|645903516|nb|KK920914.1|	9784-10116	nan	nan	30	30	-0.00533494	0.99695	no
ni|645903517|nb|KK920913.1|	147876-148060	nan	nan	30	68	1	0.31945	no
ni|645903518|nb|KK920912.1|	102638-102849	nan	nan	19	24	0.381235	0.7426	no
ni|645903518|nb|KK920912.1|	105585-105734	nan	nan	5	3	-0.704756	0.60375	no
ni|645903518|nb|KK920912.1|	108376-108697	nan	nan	1	2	0.256317	0.84735	no
ni|645903518|nb|KK920912.1|	108998-109319	nan	nan	1	1	-0.0660295	0.94155	no
ni|645903518|nb|KK920912.1|	111833-112087	nan	nan	2	2	-0.354489	0.76075	no
ni|645903518|nb|KK920912.1|	112783-114533	nan	nan	3	2	-0.574213	0.63245	no
ni|645903518|nb|KK920912.1|	114788-114905	nan	nan	0.980481	1	0.572696	0.62845	no
ni|645903518|nb|KK920912.1|	117142-117366	nan	nan	5	5	-0.120935	0.91705	no
ni|645903518|nb|KK920912.1|	117505-117728	nan	nan	22	29	0.393723	0.7251	no
ni|645903518|nb|KK920912.1|	118449-118800	nan	nan	25	14	-0.822222	0.50305	no
ni|645903518|nb|KK920912.1|	118921-119097	nan	nan	19	23	0.318371	0.77915	no
ni|645903518|nb|KK920912.1|	120629-120921	nan	nan	9	10	0.125716	0.91285	no
ni|645903518|nb|KK920912.1|	121505-123333	nan	nan	13	16	0.265988	0.81375	no
ni|645903518|nb|KK920912.1|	123475-123688	nan	nan	9	13	0.554209	0.6351	no
ni|645903518|nb|KK920912.1|	124228-124450	nan	nan	8	11	0.388825	0.7443	no
ni|645903518|nb|KK920912.1|	127222-127472	nan	nan	0	11	inf	5.00E-05	yes
ni|645903518|nb|KK920912.1|	135585-135755	nan	nan	163	133	-0.291928	0.7924	no

ni|645903518|nb|KK920912.1|	135816-136035	nan	nan	88	74	-0.250285	0.86175	no
ni|645903518|nb|KK920912.1|	147976-148404	nan	nan	3	3	-0.0131585	0.98035	no
ni|645903518|nb|KK920912.1|	149199-149400	nan	nan	225	170	-0.403602	0.72315	no
ni|645903518|nb|KK920912.1|	149737-150125	nan	nan	668	514	-0.378883	0.7926	no
ni|645903518|nb|KK920912.1|	150325-150699	nan	nan	0	66	inf	5.00E-05	yes
ni|645903518|nb|KK920912.1|	151402-152288	nan	nan	13	18	0.464173	0.686	no
ni|645903518|nb|KK920912.1|	156450-156769	nan	nan	2	2	-0.0673009	0.9302	no
ni|645903518|nb|KK920912.1|	157069-157323	nan	nan	8	9	0.141454	0.9013	no
ni|645903518|nb|KK920912.1|	158899-159458	nan	nan	3	2	-0.597001	0.64595	no
ni|645903518|nb|KK920912.1|	159593-160146	nan	nan	19	21	0.115616	0.9168	no
ni|645903518|nb|KK920912.1|	160604-161040	nan	nan	2	1	-0.45197	0.71035	no
ni|645903518|nb|KK920912.1|	161166-161350	nan	nan	40	37	-0.0877078	0.93715	no
ni|645903518|nb|KK920912.1|	162443-162558	nan	nan	24	19	-0.32289	0.77405	no
ni|645903518|nb|KK920912.1|	163288-163439	nan	nan	41	32	-0.358039	0.7437	no
ni|645903518|nb|KK920912.1|	163529-163643	nan	nan	0.760106	0.403575	-0.913364	0.53575	no
ni|645903518|nb|KK920912.1|	163900-164287	nan	nan	171	82	-1	0.4658	no
ni|645903518|nb|KK920912.1|	164421-164535	nan	nan	312	157	-0.988998	0.4913	no
ni|645903518|nb|KK920912.1|	164794-164921	nan	nan	0.201667	3	4	0.1295	no
ni|645903518|nb|KK920912.1|	165124-165254	nan	nan	0.195302	49	8	0.11935	no
ni|645903518|nb|KK920912.1|	167688-167892	nan	nan	0	35	inf	5.00E-05	yes
ni|645903518|nb|KK920912.1|	170129-170318	nan	nan	0	42	inf	5.00E-05	yes
ni|645903518|nb|KK920912.1|	172895-173339	nan	nan	0	66	inf	5.00E-05	yes
ni|645903518|nb|KK920912.1|	173813-174122	nan	nan	0	2	inf	0.00135	yes
ni|645903518|nb|KK920912.1|	175511-175924	nan	nan	0	4	inf	5.00E-05	yes
ni|645903518|nb|KK920912.1|	176031-176226	nan	nan	0	40	inf	5.00E-05	yes
ni|645903518|nb|KK920912.1|	178738-179130	nan	nan	0	25	inf	0.00015	yes
ni|645903518|nb|KK920912.1|	180894-181238	nan	nan	0	0.786806	inf	0.0009	yes
ni|645903518|nb|KK920912.1|	191960-192148	nan	nan	0	30	inf	5.00E-05	yes
ni|645903518|nb|KK920912.1|	193618-193842	nan	nan	0	70	inf	5.00E-05	yes
ni|645903518|nb|KK920912.1|	194956-195265	nan	nan	1	0	-inf	0.002	yes

ni|645903518|nb|KK920912.1|	195768-196427	nan	nan	5	0	-inf	5.00E-05	yes
ni|645903518|nb|KK920912.1|	200536-200743	nan	nan	2	4	0.913251	0.4326	no
ni|645903518|nb|KK920912.1|	202736-202972	nan	nan	7	10	0.517571	0.7279	no
ni|645903518|nb|KK920912.1|	204736-204891	nan	nan	1	2	0.175825	0.88445	no
ni|645903518|nb|KK920912.1|	205070-205414	nan	nan	2	2	-0.0658111	0.95835	no
ni|645903518|nb|KK920912.1|	206070-206264	nan	nan	82	89.96	0.141261	0.8968	no
ni|645903518|nb|KK920912.1|	207340-207749	nan	nan	30	32	0.0781341	0.94225	no
ni|645903518|nb|KK920912.1|	208269-208427	nan	nan	80	102	0.35546	0.74745	no
ni|645903518|nb|KK920912.1|	209813-209965	nan	nan	93	117	0.334733	0.75855	no
ni|645903518|nb|KK920912.1|	210105-210251	nan	nan	82	98	0.262363	0.833	no
ni|645903518|nb|KK920912.1|	79608-79810	nan	nan	17	17	-0.0527087	0.96495	no
ni|645903518|nb|KK920912.1|	93374-93562	nan	nan	9	9	-0.00962238	0.9923	no
ni|645903518|nb|KK920912.1|	94039-94501	nan	nan	12	15	0.319689	0.8092	no
ni|645903518|nb|KK920912.1|	96092-96497	nan	nan	17	23	0.383451	0.74515	no
ni|645903518|nb|KK920912.1|	97726-97896	nan	nan	18	17	-0.0673551	0.9579	no
ni|645903519|nb|KK920911.1|	440100-440405	nan	nan	66	107	0.701688	0.51465	no
ni|645903519|nb|KK920911.1|	524327-524476	nan	nan	212	231	0.123886	0.9344	no
ni|645903519|nb|KK920911.1|	525813-525966	nan	nan	6	4	-0.507889	0.68875	no
ni|645903519|nb|KK920911.1|	527354-527533	nan	nan	59	76	0.364935	0.79925	no
ni|645903519|nb|KK920911.1|	528750-528935	nan	nan	34	58	0.753639	0.5267	no
ni|645903519|nb|KK920911.1|	531643-531969	nan	nan	43	48	0.163264	0.8806	no
ni|645903519|nb|KK920911.1|	535995-536148	nan	nan	14	13	-0.0673438	0.9582	no
ni|645903519|nb|KK920911.1|	537720-537907	nan	nan	19	27	0.494555	0.6793	no
ni|645903519|nb|KK920911.1|	539585-539783	nan	nan	3	2	-0.319388	0.78835	no
ni|645903519|nb|KK920911.1|	540828-541029	nan	nan	4	3	-0.497942	0.67005	no
ni|645903520|nb|KK920910.1|	186157-186648	nan	nan	5	4	-0.300136	0.80075	no
ni|645903520|nb|KK920910.1|	192266-192529	nan	nan	543	373	-0.543364	0.6946	no
ni|645903520|nb|KK920910.1|	193584-193856	nan	nan	229	182	-0.329324	0.81255	no
ni|645903520|nb|KK920910.1|	205321-205452	nan	nan	9	7	-0.534476	0.636	no
ni|645903520|nb|KK920910.1|	205847-206213	nan	nan	1	0.208606	-2	0.17	no

ni|645903520|nb|KK920910.1|	208688-208825	nan	nan	13	11	-0.200494	0.86755	no
ni|645903520|nb|KK920910.1|	279140-280575	nan	nan	21	13	-0.65219	0.58395	no
ni|645903520|nb|KK920910.1|	281306-281466	nan	nan	9	7	-0.379206	0.74525	no
ni|645903520|nb|KK920910.1|	282354-285649	nan	nan	18	10	-0.941989	0.54025	no
ni|645903520|nb|KK920910.1|	286626-286779	nan	nan	7	7	0.013594	0.99035	no
ni|645903520|nb|KK920910.1|	288084-288448	nan	nan	6	3	-0.804174	0.6027	no
ni|645903520|nb|KK920910.1|	289919-290060	nan	nan	17	11	-0.664456	0.5735	no
ni|645903520|nb|KK920910.1|	293527-293958	nan	nan	4	4	0.0582967	0.9532	no
ni|645903520|nb|KK920910.1|	298824-298964	nan	nan	4	5	0.296872	0.8006	no
ni|645903521|nb|KK920909.1|	11712-11933	nan	nan	12	8	-0.652173	0.5967	no
ni|645903521|nb|KK920909.1|	12089-12594	nan	nan	1	0.288339	-2	0.1613	no
ni|645903521|nb|KK920909.1|	13351-13709	nan	nan	8	9	0.254603	0.8271	no
ni|645903521|nb|KK920909.1|	13879-14434	nan	nan	0.906815	6	3	0.0824	no
ni|645903521|nb|KK920909.1|	144938-145450	nan	nan	15	13	-0.20156	0.85565	no
ni|645903521|nb|KK920909.1|	145669-146032	nan	nan	2	2	0.091086	0.9373	no
ni|645903521|nb|KK920909.1|	154280-154505	nan	nan	23	23	0.00865743	0.9918	no
ni|645903521|nb|KK920909.1|	154591-155162	nan	nan	2	1	-0.904643	0.4582	no
ni|645903521|nb|KK920909.1|	166654-167028	nan	nan	1	1	-0.43821	0.7128	no
ni|645903521|nb|KK920909.1|	168104-168293	nan	nan	12	9	-0.372466	0.73935	no
ni|645903521|nb|KK920909.1|	170269-170407	nan	nan	2	0.965654	-0.698581	0.56445	no
ni|645903521|nb|KK920909.1|	173291-173505	nan	nan	7	7	0.0951648	0.93185	no
ni|645903521|nb|KK920909.1|	173680-173948	nan	nan	0.596255	0.162872	-2	0.21345	no
ni|645903521|nb|KK920909.1|	174795-174903	nan	nan	16	13	-0.256011	0.8222	no
ni|645903521|nb|KK920909.1|	178438-178604	nan	nan	0.818809	0.144988	-2	0.0965	no
ni|645903521|nb|KK920909.1|	181064-181493	nan	nan	25	22	-0.145394	0.89895	no
ni|645903521|nb|KK920909.1|	183475-183680	nan	nan	16	13	-0.254889	0.8244	no
ni|645903521|nb|KK920909.1|	183915-184163	nan	nan	8	6	-0.310026	0.78675	no
ni|645903521|nb|KK920909.1|	184838-184998	nan	nan	18	15	-0.229894	0.83665	no
ni|645903521|nb|KK920909.1|	188566-188688	nan	nan	2	2	-0.0657245	0.95795	no
ni|645903521|nb|KK920909.1|	191777-191988	nan	nan	5	4	-0.231565	0.8467	no

ni|645903521|nb|KK920909.1|	194814-195984	nan	nan	4	4	-0.0126961	0.98885	no
ni|645903521|nb|KK920909.1|	198767-199158	nan	nan	3	2	-0.0661197	0.9589	no
ni|645903521|nb|KK920909.1|	200130-200291	nan	nan	18	17	-0.110176	0.92605	no
ni|645903521|nb|KK920909.1|	200422-200547	nan	nan	8	7	-0.192248	0.8749	no
ni|645903521|nb|KK920909.1|	200671-201024	nan	nan	16	18	0.170517	0.9002	no
ni|645903521|nb|KK920909.1|	201889-202608	nan	nan	16	19	0.259412	0.8081	no
ni|645903521|nb|KK920909.1|	203776-204002	nan	nan	12	14	0.242995	0.832	no
ni|645903521|nb|KK920909.1|	204211-204598	nan	nan	15	17	0.178582	0.86705	no
ni|645903521|nb|KK920909.1|	22286-23846	nan	nan	0	5	inf	0.0007	yes
ni|645903521|nb|KK920909.1|	24056-24537	nan	nan	0	5	inf	5.00E-05	yes
ni|645903521|nb|KK920909.1|	246779-246881	nan	nan	12	17	0.519005	0.65825	no
ni|645903521|nb|KK920909.1|	24945-25501	nan	nan	0	178	inf	5.00E-05	yes
ni|645903521|nb|KK920909.1|	30196-33635	nan	nan	0	103	inf	5.00E-05	yes
ni|645903521|nb|KK920909.1|	33936-34762	nan	nan	88	104	0.240259	0.83625	no
ni|645903521|nb|KK920909.1|	376355-376483	nan	nan	11	13	0.299584	0.7972	no
ni|645903521|nb|KK920909.1|	472633-472843	nan	nan	11	15	0.434974	0.71405	no
ni|645903521|nb|KK920909.1|	473410-473608	nan	nan	12	14	0.235341	0.8369	no
ni|645903521|nb|KK920909.1|	474582-474814	nan	nan	14	25	0.881455	0.4525	no
ni|645903521|nb|KK920909.1|	474959-475151	nan	nan	5	7	0.640942	0.5836	no
ni|645903521|nb|KK920909.1|	475461-475714	nan	nan	10	11	0.125453	0.91735	no
ni|645903521|nb|KK920909.1|	475817-476511	nan	nan	17	15	-0.237502	0.8697	no
ni|645903521|nb|KK920909.1|	478070-478301	nan	nan	0.904531	0.902412	-0.0033836	0.9752	no
ni|645903521|nb|KK920909.1|	478651-479034	nan	nan	67	68	0.0212749	0.985	no
ni|645903521|nb|KK920909.1|	479762-479916	nan	nan	25	28	0.140578	0.8949	no
ni|645903521|nb|KK920909.1|	480862-481077	nan	nan	3	2	-1	0.3576	no
ni|645903521|nb|KK920909.1|	481252-481561	nan	nan	2	0.745687	-1	0.32805	no
ni|645903521|nb|KK920909.1|	482649-482864	nan	nan	17	17	0.00423697	0.9959	no
ni|645903521|nb|KK920909.1|	483424-483899	nan	nan	85	68	-0.319755	0.7819	no
ni|645903521|nb|KK920909.1|	484010-484463	nan	nan	2	2	0.173083	0.88	no
ni|645903521|nb|KK920909.1|	485209-485409	nan	nan	4	3	-0.356806	0.7931	no

ni|645903521|nb|KK920909.1|	486159-486328	nan	nan	0.917505	3	2	0.12065	no
ni|645903521|nb|KK920909.1|	491178-491340	nan	nan	2	1	-0.749411	0.53255	no
ni|645903521|nb|KK920909.1|	49184-49484	nan	nan	0	67	inf	5.00E-05	yes
ni|645903521|nb|KK920909.1|	492338-492619	nan	nan	2	0.51462	-2	0.25615	no
ni|645903521|nb|KK920909.1|	494129-494481	nan	nan	0	31	inf	0.00015	yes
ni|645903521|nb|KK920909.1|	495119-495297	nan	nan	0	17	inf	0.0001	yes
ni|645903521|nb|KK920909.1|	497107-497416	nan	nan	0	9	inf	0.00065	yes
ni|645903521|nb|KK920909.1|	497524-497753	nan	nan	0	6	inf	0.00015	yes
ni|645903521|nb|KK920909.1|	499277-499894	nan	nan	0	9	inf	0.0009	yes
ni|645903521|nb|KK920909.1|	500654-500867	nan	nan	0	6	inf	5.00E-05	yes
ni|645903521|nb|KK920909.1|	504598-504752	nan	nan	5	6	0.237639	0.8456	no
ni|645903521|nb|KK920909.1|	506226-506506	nan	nan	5	3	-0.897376	0.502	no
ni|645903521|nb|KK920909.1|	507045-507217	nan	nan	1	2	0.770923	0.54645	no
ni|645903521|nb|KK920909.1|	50744-51059	nan	nan	0	34	inf	5.00E-05	yes
ni|645903521|nb|KK920909.1|	510214-510402	nan	nan	0.162036	4	5	0.12065	no
ni|645903521|nb|KK920909.1|	511408-511642	nan	nan	9	0.385907	-5	0.00945	no
ni|645903521|nb|KK920909.1|	512785-513041	nan	nan	1	3	1	0.415	no
ni|645903521|nb|KK920909.1|	513522-513872	nan	nan	1	1	-0.066272	0.9384	no
ni|645903521|nb|KK920909.1|	514418-514677	nan	nan	22	162	3	0.01615	no
ni|645903521|nb|KK920909.1|	514969-515270	nan	nan	1	0.879886	-0.355071	0.7931	no
ni|645903521|nb|KK920909.1|	515669-515805	nan	nan	9	147	4	0.00825	no
ni|645903521|nb|KK920909.1|	515894-516045	nan	nan	4	58	4	0.00305	no
ni|645903521|nb|KK920909.1|	516540-516696	nan	nan	11	131	4	0.0167	no
ni|645903521|nb|KK920909.1|	517039-517526	nan	nan	2	32	4	0.0071	no
ni|645903521|nb|KK920909.1|	51856-51960	nan	nan	0.326245	58	7	0.00255	yes
ni|645903521|nb|KK920909.1|	519023-519911	nan	nan	1	55	5	0.00115	yes
ni|645903521|nb|KK920909.1|	521159-521568	nan	nan	5	6	0.127019	0.91245	no
ni|645903521|nb|KK920909.1|	529152-529559	nan	nan	0.769625	0.711414	-0.113466	0.92415	no
ni|645903521|nb|KK920909.1|	529846-530202	nan	nan	2	3	0.282684	0.8153	no
ni|645903521|nb|KK920909.1|	54572-55012	nan	nan	0.069849	90	10	0.0443	no

ni|645903521|nb|KK920909.1|	555625-555941	nan	nan	5	7	0.61704	0.5999	no
ni|645903521|nb|KK920909.1|	557850-558158	nan	nan	15	6	-1	0.3885	no
ni|645903521|nb|KK920909.1|	558608-559003	nan	nan	1	1	0.0600483	0.95295	no
ni|645903521|nb|KK920909.1|	86024-86625	nan	nan	0	26	inf	5.00E-05	yes
ni|645903521|nb|KK920909.1|	90719-91380	nan	nan	3	3	-0.191458	0.87685	no
ni|645903521|nb|KK920909.1|	95789-96954	nan	nan	23	18	-0.389207	0.74325	no
ni|645903521|nb|KK920909.1|	97090-97925	nan	nan	11	10	-0.0573022	0.96035	no
ni|645903522|nb|KK920908.1|	56595-56834	nan	nan	0.561306	0.83735	0.577042	0.63065	no
ni|645903523|nb|KK920907.1|	104203-104360	nan	nan	2	2	0.414201	0.73085	no
ni|645903523|nb|KK920907.1|	105652-105813	nan	nan	10	11	0.0801304	0.94565	no
ni|645903523|nb|KK920907.1|	106773-107246	nan	nan	5	3	-0.605156	0.59235	no
ni|645903523|nb|KK920907.1|	108612-109261	nan	nan	7	8	0.133432	0.9104	no
ni|645903523|nb|KK920907.1|	111777-111921	nan	nan	3	2	-0.694598	0.58855	no
ni|645903523|nb|KK920907.1|	113774-114136	nan	nan	0	147	inf	5.00E-05	yes
ni|645903523|nb|KK920907.1|	115665-115871	nan	nan	0.109369	1	3	0.0515	no
ni|645903523|nb|KK920907.1|	117418-117571	nan	nan	0.4304	150	8	0.11935	no
ni|645903523|nb|KK920907.1|	122505-122662	nan	nan	17	15	-0.192713	0.8646	no
ni|645903523|nb|KK920907.1|	122861-123300	nan	nan	3	2	-0.729085	0.5717	no
ni|645903523|nb|KK920907.1|	123449-123746	nan	nan	17	16	-0.079647	0.94625	no
ni|645903523|nb|KK920907.1|	125821-126249	nan	nan	20	18	-0.211734	0.8562	no
ni|645903523|nb|KK920907.1|	126493-126676	nan	nan	10	13	0.318734	0.7809	no
ni|645903523|nb|KK920907.1|	132223-132405	nan	nan	16	19	0.249255	0.83155	no
ni|645903523|nb|KK920907.1|	145849-146646	nan	nan	6	7	0.221445	0.84765	no
ni|645903523|nb|KK920907.1|	164078-164258	nan	nan	27.87	22	-0.356811	0.76045	no
ni|645903523|nb|KK920907.1|	164733-165245	nan	nan	22	14	-0.643761	0.57845	no
ni|645903523|nb|KK920907.1|	167240-167661	nan	nan	45	34	-0.414222	0.7233	no
ni|645903523|nb|KK920907.1|	168025-168469	nan	nan	0.399632	0.818596	1	0.4471	no
ni|645903523|nb|KK920907.1|	169270-169810	nan	nan	20	16	-0.335965	0.7655	no
ni|645903523|nb|KK920907.1|	170376-170820	nan	nan	3	2	-0.321483	0.7848	no
ni|645903523|nb|KK920907.1|	171013-171221	nan	nan	4	4	-0.258998	0.82995	no

ni|645903523|nb|KK920907.1|	172322-172479	nan	nan	17	13	-0.293746	0.8453	no
ni|645903523|nb|KK920907.1|	173840-174578	nan	nan	52	56	0.102687	0.92965	no
ni|645903523|nb|KK920907.1|	176063-176232	nan	nan	0.345673	0.18361	-0.912766	0.53575	no
ni|645903523|nb|KK920907.1|	177424-177891	nan	nan	4	3	-0.533212	0.64945	no
ni|645903523|nb|KK920907.1|	179632-179758	nan	nan	4	3	-0.514502	0.697	no
ni|645903523|nb|KK920907.1|	180159-180348	nan	nan	52	23	-1	0.35675	no
ni|645903523|nb|KK920907.1|	181793-182057	nan	nan	97	57	-0.781335	0.49695	no
ni|645903523|nb|KK920907.1|	183890-184413	nan	nan	154.1	78	-0.979942	0.39845	no
ni|645903523|nb|KK920907.1|	189266-189476	nan	nan	0	509	inf	5.00E-05	yes
ni|645903523|nb|KK920907.1|	199186-199581	nan	nan	0	1	inf	0.0003	yes
ni|645903523|nb|KK920907.1|	200523-200659	nan	nan	0	188	inf	5.00E-05	yes
ni|645903523|nb|KK920907.1|	204963-205083	nan	nan	0	325	inf	5.00E-05	yes
ni|645903523|nb|KK920907.1|	205971-206119	nan	nan	0	15	inf	5.00E-05	yes
ni|645903523|nb|KK920907.1|	206278-206462	nan	nan	0.333281	188	9	0.0332	no
ni|645903523|nb|KK920907.1|	209213-209723	nan	nan	0.104919	36	8	0.11935	no
ni|645903523|nb|KK920907.1|	211501-211926	nan	nan	0.507679	418	10	0.1194	no
ni|645903523|nb|KK920907.1|	216490-216963	nan	nan	0	285	inf	5.00E-05	yes
ni|645903523|nb|KK920907.1|	217054-217333	nan	nan	0.661353	266	9	0.0205	no
ni|645903523|nb|KK920907.1|	220691-220841	nan	nan	0	41	inf	5.00E-05	yes
ni|645903523|nb|KK920907.1|	220901-221393	nan	nan	0	98	inf	5.00E-05	yes
ni|645903523|nb|KK920907.1|	221964-222124	nan	nan	0	348	inf	5.00E-05	yes
ni|645903523|nb|KK920907.1|	74199-74301	nan	nan	5	3	-0.481791	0.71	no
ni|645903523|nb|KK920907.1|	74642-75135	nan	nan	0.72216	2	1	0.3229	no
ni|645903523|nb|KK920907.1|	75544-75743	nan	nan	5	7	0.380265	0.7694	no
ni|645903523|nb|KK920907.1|	76703-77531	nan	nan	12	14	0.180656	0.87985	no
ni|645903524|nb|KK920906.1|	241768-242038	nan	nan	0	84	inf	5.00E-05	yes
ni|645903524|nb|KK920906.1|	242382-242560	nan	nan	0	11	inf	5.00E-05	yes
ni|645903524|nb|KK920906.1|	244944-245087	nan	nan	0	15	inf	5.00E-05	yes
ni|645903524|nb|KK920906.1|	251186-251310	nan	nan	0	33	inf	5.00E-05	yes
ni|645903524|nb|KK920906.1|	259955-260095	nan	nan	3	3	-0.0652373	0.95265	no

ni|645903524|nb|KK920906.1|	262076-262558	nan	nan	4	2	-0.777077	0.5539	no
ni|645903524|nb|KK920906.1|	262779-262979	nan	nan	7	6	-0.102008	0.92595	no
ni|645903524|nb|KK920906.1|	263423-264047	nan	nan	7	5	-0.608105	0.6058	no
ni|645903524|nb|KK920906.1|	264237-264428	nan	nan	2	2	0.256246	0.83715	no
ni|645903524|nb|KK920906.1|	265247-265658	nan	nan	0.76173	1	0.635522	0.60495	no
ni|645903524|nb|KK920906.1|	269602-270150	nan	nan	2	2	-0.279262	0.81975	no
ni|645903524|nb|KK920906.1|	270372-270668	nan	nan	4	2	-1	0.3948	no
ni|645903524|nb|KK920906.1|	272868-273580	nan	nan	2	1	-0.732763	0.55455	no
ni|645903524|nb|KK920906.1|	274691-274833	nan	nan	7	3	-1	0.29805	no
ni|645903524|nb|KK920906.1|	274996-275124	nan	nan	5	3	-0.941663	0.54025	no
ni|645903524|nb|KK920906.1|	280738-280953	nan	nan	5	2	-1	0.3302	no
ni|645903524|nb|KK920906.1|	281968-282130	nan	nan	3	2	-0.3014	0.78245	no
ni|645903524|nb|KK920906.1|	283735-283922	nan	nan	5	4	-0.413653	0.72545	no
ni|645903524|nb|KK920906.1|	285657-286000	nan	nan	17	13	-0.361491	0.75695	no
ni|645903524|nb|KK920906.1|	286594-288450	nan	nan	3	3	0.0906607	0.9373	no
ni|645903524|nb|KK920906.1|	288819-289326	nan	nan	10	11	0.0685782	0.9515	no
ni|645903524|nb|KK920906.1|	301437-301610	nan	nan	13	15	0.273168	0.8154	no
ni|645903524|nb|KK920906.1|	307960-308067	nan	nan	4	6	0.764219	0.5247	no
ni|645903524|nb|KK920906.1|	40773-41198	nan	nan	0	287	inf	5.00E-05	yes
ni|645903524|nb|KK920906.1|	425238-425419	nan	nan	7	8	0.0710965	0.9486	no
ni|645903524|nb|KK920906.1|	432094-432292	nan	nan	124	121	-0.0354877	0.981	no
ni|645903524|nb|KK920906.1|	432889-433595	nan	nan	8	6	-0.36675	0.7706	no
ni|645903524|nb|KK920906.1|	434698-435181	nan	nan	59	74	0.307776	0.83665	no
ni|645903524|nb|KK920906.1|	435430-435698	nan	nan	46	54	0.254687	0.8528	no
ni|645903524|nb|KK920906.1|	441518-441681	nan	nan	38	42	0.143567	0.90165	no
ni|645903524|nb|KK920906.1|	442081-442248	nan	nan	24	40	0.7218	0.52425	no
ni|645903524|nb|KK920906.1|	445142-445539	nan	nan	15	16	0.088773	0.9527	no
ni|645903524|nb|KK920906.1|	446437-446637	nan	nan	15	18	0.199258	0.8651	no
ni|645903524|nb|KK920906.1|	449160-449421	nan	nan	12	12	-0.00182116	0.99855	no
ni|645903524|nb|KK920906.1|	453655-453810	nan	nan	10	11	0.0760159	0.9485	no

ni|645903524|nb|KK920906.1|	454589-454742	nan	nan	14	14	0.00352979	0.9968	no
ni|645903524|nb|KK920906.1|	461385-461620	nan	nan	12	13	0.119098	0.91655	no
ni|645903524|nb|KK920906.1|	465334-465564	nan	nan	14	15	0.0422092	0.96865	no
ni|645903524|nb|KK920906.1|	472184-472418	nan	nan	14	19	0.450101	0.6912	no
ni|645903524|nb|KK920906.1|	473040-474078	nan	nan	8	8	-0.0170994	0.9887	no
ni|645903524|nb|KK920906.1|	475773-475998	nan	nan	10	10	0.0946702	0.9279	no
ni|645903524|nb|KK920906.1|	477305-478100	nan	nan	0.523795	0.308177	-0.76524	0.5835	no
ni|645903524|nb|KK920906.1|	479719-480542	nan	nan	0.167764	2	4	0.0476	no
ni|645903524|nb|KK920906.1|	481324-481630	nan	nan	10	10	0.124381	0.91265	no
ni|645903524|nb|KK920906.1|	490712-491441	nan	nan	18	21	0.226515	0.8499	no
ni|645903524|nb|KK920906.1|	492689-493007	nan	nan	38	57	0.583781	0.61075	no
ni|645903524|nb|KK920906.1|	493288-493405	nan	nan	52	76	0.541353	0.61455	no
ni|645903524|nb|KK920906.1|	493572-493842	nan	nan	48	61	0.352201	0.76015	no
ni|645903524|nb|KK920906.1|	495545-495718	nan	nan	32	39	0.27636	0.795	no
ni|645903524|nb|KK920906.1|	501853-502651	nan	nan	31	40	0.347424	0.7834	no
ni|645903524|nb|KK920906.1|	502769-503585	nan	nan	35	46	0.419225	0.73715	no
ni|645903524|nb|KK920906.1|	504170-504543	nan	nan	34	38	0.193893	0.856	no
ni|645903524|nb|KK920906.1|	504654-505078	nan	nan	23	21	-0.0970712	0.9465	no
ni|645903524|nb|KK920906.1|	505319-505543	nan	nan	54	43	-0.348365	0.78225	no
ni|645903524|nb|KK920906.1|	506817-507179	nan	nan	113	83	-0.439	0.6772	no
ni|645903524|nb|KK920906.1|	507854-508568	nan	nan	53	39	-0.441341	0.6944	no
ni|645903524|nb|KK920906.1|	517231-517388	nan	nan	3	2	-0.963458	0.4279	no
ni|645903524|nb|KK920906.1|	527063-527234	nan	nan	5	1	-2	0.2949	no
ni|645903524|nb|KK920906.1|	528320-528671	nan	nan	4	1	-2	0.1999	no
ni|645903525|nb|KK920905.1|	101588-101728	nan	nan	64	94	0.557056	0.6323	no
ni|645903525|nb|KK920905.1|	1021-1280	nan	nan	1	0.340271	-2	0.15765	no
ni|645903525|nb|KK920905.1|	106550-106693	nan	nan	31	42	0.414742	0.71625	no
ni|645903525|nb|KK920905.1|	10715-11179	nan	nan	2	0.784751	-1	0.32095	no
ni|645903525|nb|KK920905.1|	109490-109689	nan	nan	86	90	0.0701839	0.95025	no
ni|645903525|nb|KK920905.1|	111133-111338	nan	nan	38	39.55	0.0647479	0.95655	no

ni|645903525|nb|KK920905.1|	11383-11547	nan	nan	0.8776	0.419352	-1	0.44755	no
ni|645903525|nb|KK920905.1|	116419-116600	nan	nan	128	127	-0.00344714	0.99105	no
ni|645903525|nb|KK920905.1|	116806-116954	nan	nan	67	91	0.453436	0.68715	no
ni|645903525|nb|KK920905.1|	11854-12037	nan	nan	0.620437	0.592982	-0.0652948	0.9384	no
ni|645903525|nb|KK920905.1|	119824-119991	nan	nan	52	64	0.282291	0.81835	no
ni|645903525|nb|KK920905.1|	12969-13336	nan	nan	0.616512	0.420847	-0.550833	0.71905	no
ni|645903525|nb|KK920905.1|	13795-14119	nan	nan	1	0.205902	-2	0.1101	no
ni|645903525|nb|KK920905.1|	16906-17060	nan	nan	4	0	-inf	0.0009	yes
ni|645903525|nb|KK920905.1|	21023-21132	nan	nan	1	0.420847	-1	0.3479	no
ni|645903525|nb|KK920905.1|	226706-226909	nan	nan	24	38	0.688119	0.5273	no
ni|645903525|nb|KK920905.1|	24073-24259	nan	nan	10	5	-0.915376	0.53575	no
ni|645903525|nb|KK920905.1|	241438-242278	nan	nan	63	92	0.546695	0.7117	no
ni|645903525|nb|KK920905.1|	25318-25515	nan	nan	17	26	0.624337	0.58995	no
ni|645903525|nb|KK920905.1|	26853-27002	nan	nan	2	3	0.273093	0.813	no
ni|645903525|nb|KK920905.1|	27924-28099	nan	nan	3	3	-0.0167901	0.9887	no
ni|645903525|nb|KK920905.1|	28810-28999	nan	nan	17	7	-1	0.4023	no
ni|645903525|nb|KK920905.1|	299177-299248	nan	nan	32	45	0.494014	0.689	no
ni|645903525|nb|KK920905.1|	31309-31418	nan	nan	4	5	0.348918	0.7605	no
ni|645903525|nb|KK920905.1|	31597-32023	nan	nan	36	31	-0.228957	0.8458	no
ni|645903525|nb|KK920905.1|	32601-32790	nan	nan	74	56	-0.40095	0.73245	no
ni|645903525|nb|KK920905.1|	3272-3531	nan	nan	3	2	-0.482081	0.7301	no
ni|645903525|nb|KK920905.1|	335542-335681	nan	nan	46	70	0.623709	0.5881	no
ni|645903525|nb|KK920905.1|	33896-34113	nan	nan	133.58	140	0.0718256	0.9503	no
ni|645903525|nb|KK920905.1|	341080-341363	nan	nan	21	31	0.555858	0.623	no
ni|645903525|nb|KK920905.1|	343222-343642	nan	nan	0.047425	2	5	0.12	no
ni|645903525|nb|KK920905.1|	344193-344374	nan	nan	13	11	-0.236045	0.84425	no
ni|645903525|nb|KK920905.1|	351417-351598	nan	nan	40	40	-0.00606613	0.99615	no
ni|645903525|nb|KK920905.1|	35191-35329	nan	nan	0	7	inf	5.00E-05	yes
ni|645903525|nb|KK920905.1|	35571-35726	nan	nan	0.539115	6	3	0.05165	no
ni|645903525|nb|KK920905.1|	355755-355978	nan	nan	107.03	112	0.0675907	0.9535	no

ni|645903525|nb|KK920905.1|	38751-38909	nan	nan	0	28	inf	5.00E-05	yes
ni|645903525|nb|KK920905.1|	3959-4394	nan	nan	0.336751	0.071549	-2	0.3207	no
ni|645903525|nb|KK920905.1|	45277-45505	nan	nan	0.171	8	6	0.11975	no
ni|645903525|nb|KK920905.1|	45978-46132	nan	nan	28	29	0.00794064	0.9947	no
ni|645903525|nb|KK920905.1|	49263-49676	nan	nan	9	11	0.249192	0.8316	no
ni|645903525|nb|KK920905.1|	5070-5592	nan	nan	3	0.468961	-3	0.28285	no
ni|645903525|nb|KK920905.1|	51078-52372	nan	nan	19	29	0.582437	0.5988	no
ni|645903525|nb|KK920905.1|	53506-54201	nan	nan	38	51	0.435006	0.7137	no
ni|645903525|nb|KK920905.1|	55596-56288	nan	nan	29	29	-0.0108078	0.97805	no
ni|645903525|nb|KK920905.1|	57410-58122	nan	nan	11.45	12	0.10305	0.9311	no
ni|645903525|nb|KK920905.1|	58661-58828	nan	nan	37	58	0.6758	0.5542	no
ni|645903525|nb|KK920905.1|	60190-60388	nan	nan	12	18	0.559103	0.62775	no
ni|645903525|nb|KK920905.1|	64278-64608	nan	nan	1	0.758969	-0.744182	0.59615	no
ni|645903525|nb|KK920905.1|	66106-66273	nan	nan	9	11	0.343138	0.7682	no
ni|645903525|nb|KK920905.1|	66757-66969	nan	nan	10	15	0.533441	0.62665	no
ni|645903525|nb|KK920905.1|	667590-667816	nan	nan	62	43	-0.506239	0.67255	no
ni|645903525|nb|KK920905.1|	67550-67740	nan	nan	17	28	0.738168	0.5156	no
ni|645903525|nb|KK920905.1|	68756-69171	nan	nan	70	67	-0.0681116	0.96015	no
ni|645903525|nb|KK920905.1|	70252-70475	nan	nan	18	28	0.653815	0.5656	no
ni|645903525|nb|KK920905.1|	7056-7398	nan	nan	1	0.785538	-0.802409	0.5588	no
ni|645903525|nb|KK920905.1|	71726-71906	nan	nan	46	64	0.499258	0.6524	no
ni|645903525|nb|KK920905.1|	73636-73787	nan	nan	12	20	0.696245	0.5481	no
ni|645903525|nb|KK920905.1|	73912-74091	nan	nan	25	48	0.911982	0.43785	no
ni|645903525|nb|KK920905.1|	74302-74617	nan	nan	0	5	inf	0.0009	yes
ni|645903525|nb|KK920905.1|	76635-76798	nan	nan	8	21	1	0.2575	no
ni|645903525|nb|KK920905.1|	777935-778457	nan	nan	0.847462	0.242976	-2	0.2784	no
ni|645903525|nb|KK920905.1|	782788-782918	nan	nan	0.702182	0.276431	-1	0.33495	no
ni|645903525|nb|KK920905.1|	783168-783622	nan	nan	0.570903	0.382012	-0.579629	0.70815	no
ni|645903525|nb|KK920905.1|	783788-784144	nan	nan	0.757225	0.347543	-1	0.37855	no
ni|645903525|nb|KK920905.1|	78753-78917	nan	nan	0.647158	23	5	0.05085	no

ni|645903525|nb|KK920905.1|	788384-789761	nan	nan	3	0.696568	-2	0.1906	no
ni|645903525|nb|KK920905.1|	789884-790313	nan	nan	0.927499	1	0.44871	0.7543	no
ni|645903525|nb|KK920905.1|	790544-790719	nan	nan	4	4	-0.0672018	0.93835	no
ni|645903525|nb|KK920905.1|	79293-79497	nan	nan	0.114663	2	4	0.12175	no
ni|645903525|nb|KK920905.1|	792960-793184	nan	nan	1	0.519101	-1	0.3995	no
ni|645903525|nb|KK920905.1|	80099-80300	nan	nan	267	570	1	0.3541	no
ni|645903525|nb|KK920905.1|	803694-804180	nan	nan	2	0.568896	-2	0.2821	no
ni|645903525|nb|KK920905.1|	805770-806280	nan	nan	1	0.362288	-2	0.24815	no
ni|645903525|nb|KK920905.1|	807732-808079	nan	nan	1	0.858125	-0.551738	0.71905	no
ni|645903525|nb|KK920905.1|	810881-811306	nan	nan	0.758873	0.241791	-2	0.25615	no
ni|645903525|nb|KK920905.1|	811608-812352	nan	nan	0.848985	0.388218	-1	0.38365	no
ni|645903525|nb|KK920905.1|	81194-81707	nan	nan	36	83	1	0.35095	no
ni|645903525|nb|KK920905.1|	812612-812746	nan	nan	2	1	-0.649412	0.5707	no
ni|645903525|nb|KK920905.1|	815630-816057	nan	nan	1	0.816129	-0.428334	0.75965	no
ni|645903525|nb|KK920905.1|	818759-819406	nan	nan	3	2	-0.572759	0.6321	no
ni|645903525|nb|KK920905.1|	82009-82229	nan	nan	93	189.37	1	0.35725	no
ni|645903525|nb|KK920905.1|	83886-84279	nan	nan	53	106	1	0.4151	no
ni|645903525|nb|KK920905.1|	86674-86849	nan	nan	19	38	1	0.42645	no
ni|645903525|nb|KK920905.1|	87039-87156	nan	nan	32	16	-0.997939	0.41055	no
ni|645903525|nb|KK920905.1|	87864-88027	nan	nan	59	65	0.148529	0.8905	no
ni|645903525|nb|KK920905.1|	8903-9100	nan	nan	1	0.609298	-0.802572	0.6027	no
ni|645903525|nb|KK920905.1|	89766-89929	nan	nan	22	28	0.304941	0.79225	no
ni|645903525|nb|KK920905.1|	91324-91469	nan	nan	1	2	1	0.4218	no
ni|645903525|nb|KK920905.1|	92212-92358	nan	nan	1	3	1	0.35025	no
ni|645903525|nb|KK920905.1|	9389-9576	nan	nan	0.937612	0.448007	-1	0.44755	no
ni|645903525|nb|KK920905.1|	97791-97936	nan	nan	0.227261	0.362142	0.672206	0.65595	no
ni|645903526|nb|KK920904.1|	112120-112314	nan	nan	0.838873	0.427677	-0.971931	0.5005	no
ni|645903526|nb|KK920904.1|	115495-115751	nan	nan	2	1	-0.651837	0.6679	no
ni|645903526|nb|KK920904.1|	128270-128668	nan	nan	5	3	-0.896299	0.46435	no
ni|645903526|nb|KK920904.1|	139183-139442	nan	nan	2	2	-0.462459	0.6932	no

ni|645903526|nb|KK920904.1|	146280-146407	nan	nan	2	0.924317	-1	0.31615	no
ni|645903526|nb|KK920904.1|	149708-149855	nan	nan	5	3	-0.662002	0.57355	no
ni|645903526|nb|KK920904.1|	152072-152256	nan	nan	9	4	-1	0.3885	no
ni|645903526|nb|KK920904.1|	153750-154047	nan	nan	3	0.805514	-2	0.2261	no
ni|645903526|nb|KK920904.1|	161467-161615	nan	nan	2	0.48305	-2	0.2004	no
ni|645903526|nb|KK920904.1|	184645-184978	nan	nan	9	8	-0.320599	0.7737	no
ni|645903526|nb|KK920904.1|	48399-48706	nan	nan	2	2	-0.236698	0.8697	no
ni|645903527|nb|KK920903.1|	13034-13209	nan	nan	1	1	-0.0650849	0.959	no
ni|645903527|nb|KK920903.1|	15535-15662	nan	nan	0.750613	0.386475	-0.957695	0.4422	no
ni|645903527|nb|KK920903.1|	16910-17169	nan	nan	132	107	-0.3009	0.79635	no
ni|645903527|nb|KK920903.1|	200126-200824	nan	nan	14	23	0.726753	0.53265	no
ni|645903527|nb|KK920903.1|	20596-20742	nan	nan	6	6	0.0211969	0.98465	no
ni|645903527|nb|KK920903.1|	206603-206731	nan	nan	8	0	-inf	0.00175	yes
ni|645903527|nb|KK920903.1|	206876-207498	nan	nan	1	0	-inf	0.0013	yes
ni|645903527|nb|KK920903.1|	218529-218698	nan	nan	32	0.929326	-5	0.01835	no
ni|645903527|nb|KK920903.1|	219466-219600	nan	nan	55	1	-5	0.01745	no
ni|645903527|nb|KK920903.1|	220026-220460	nan	nan	47	2	-4	0.0107	no
ni|645903527|nb|KK920903.1|	22865-23000	nan	nan	14	18	0.331311	0.79065	no
ni|645903527|nb|KK920903.1|	231855-232200	nan	nan	14	0.229942	-6	0.01735	no
ni|645903527|nb|KK920903.1|	233166-233864	nan	nan	8	7	-0.301032	0.8007	no
ni|645903527|nb|KK920903.1|	235020-235454	nan	nan	2	2	0.0472123	0.9681	no
ni|645903527|nb|KK920903.1|	235775-236198	nan	nan	6	7	0.123979	0.9149	no
ni|645903527|nb|KK920903.1|	236658-237051	nan	nan	7	6	-0.0328884	0.9772	no
ni|645903527|nb|KK920903.1|	240038-240510	nan	nan	9	8	-0.111731	0.92205	no
ni|645903527|nb|KK920903.1|	240735-241031	nan	nan	0.725891	0.390493	-0.894457	0.4542	no
ni|645903527|nb|KK920903.1|	242756-242907	nan	nan	15	19	0.339981	0.7716	no
ni|645903527|nb|KK920903.1|	243043-243251	nan	nan	57	91	0.685801	0.5469	no
ni|645903527|nb|KK920903.1|	25320-25510	nan	nan	0.408784	0.455844	0.1572	0.9131	no
ni|645903527|nb|KK920903.1|	258111-258397	nan	nan	0.846774	0.961247	0.18293	0.87985	no
ni|645903527|nb|KK920903.1|	26747-26933	nan	nan	0.528426	0.210498	-1	0.3479	no

ni|645903527|nb|KK920903.1|	31668-31885	nan	nan	4	2	-1	0.44755	no
ni|645903527|nb|KK920903.1|	39622-39843	nan	nan	0.469681	0.860693	0.873818	0.49985	no
ni|645903527|nb|KK920903.1|	41355-41583	nan	nan	58	79	0.455843	0.67925	no
ni|645903527|nb|KK920903.1|	4249-4414	nan	nan	5	3	-0.638967	0.58205	no
ni|645903527|nb|KK920903.1|	486031-486351	nan	nan	1	2	0.719223	0.55985	no
ni|645903527|nb|KK920903.1|	500553-500720	nan	nan	4	2	-1	0.35155	no
ni|645903527|nb|KK920903.1|	502628-502816	nan	nan	2	0	-inf	5.00E-05	yes
ni|645903527|nb|KK920903.1|	503127-503705	nan	nan	4	2	-1	0.36935	no
ni|645903527|nb|KK920903.1|	503763-504247	nan	nan	11	10	-0.136857	0.90355	no
ni|645903527|nb|KK920903.1|	50912-51081	nan	nan	59	79	0.415673	0.71775	no
ni|645903527|nb|KK920903.1|	514261-515072	nan	nan	4	3	-0.713153	0.54345	no
ni|645903527|nb|KK920903.1|	515310-517771	nan	nan	32	20	-0.652922	0.66125	no
ni|645903527|nb|KK920903.1|	518442-518809	nan	nan	3	12	2	0.11275	no
ni|645903527|nb|KK920903.1|	519008-520433	nan	nan	19	16	-0.249523	0.82955	no
ni|645903527|nb|KK920903.1|	523787-524589	nan	nan	9	18	1	0.39815	no
ni|645903527|nb|KK920903.1|	525857-526747	nan	nan	16	10	-0.651837	0.5742	no
ni|645903527|nb|KK920903.1|	526886-527625	nan	nan	36	32	-0.169388	0.9082	no
ni|645903527|nb|KK920903.1|	529179-529658	nan	nan	1	1	-0.0649205	0.95735	no
ni|645903527|nb|KK920903.1|	53372-53827	nan	nan	58	106	0.877854	0.4267	no
ni|645903527|nb|KK920903.1|	533945-534456	nan	nan	2	3	0.612331	0.62305	no
ni|645903527|nb|KK920903.1|	538957-539319	nan	nan	1	2	0.465235	0.70755	no
ni|645903527|nb|KK920903.1|	541247-541723	nan	nan	1	3	1	0.2955	no
ni|645903527|nb|KK920903.1|	54221-54610	nan	nan	55	116	1	0.40865	no
ni|645903527|nb|KK920903.1|	552891-553063	nan	nan	3	4	0.611747	0.62305	no
ni|645903527|nb|KK920903.1|	559335-559445	nan	nan	71	84	0.250042	0.86225	no
ni|645903527|nb|KK920903.1|	56387-56772	nan	nan	53	117	1	0.3391	no
ni|645903527|nb|KK920903.1|	575962-576094	nan	nan	30	35	0.260011	0.81455	no
ni|645903527|nb|KK920903.1|	57735-57858	nan	nan	43	104	1	0.27615	no
ni|645903527|nb|KK920903.1|	58185-58859	nan	nan	41	86	1	0.3589	no
ni|645903527|nb|KK920903.1|	59042-59617	nan	nan	29	23	-0.297349	0.7902	no

ni|645903527|nb|KK920903.1|	60836-61292	nan	nan	13	11	-0.232679	0.83315	no
ni|645903527|nb|KK920903.1|	618744-619422	nan	nan	19	24	0.354415	0.75105	no
ni|645903527|nb|KK920903.1|	62247-63078	nan	nan	10	9	-0.217478	0.85365	no
ni|645903527|nb|KK920903.1|	63657-63958	nan	nan	3	2	-0.329667	0.80325	no
ni|645903527|nb|KK920903.1|	655493-655629	nan	nan	1	0.467385	-2	0.2989	no
ni|645903527|nb|KK920903.1|	69145-69516	nan	nan	0.173914	7	5	0.0144	no
ni|645903527|nb|KK920903.1|	70577-70710	nan	nan	21	47	1	0.3544	no
ni|645903527|nb|KK920903.1|	74469-76076	nan	nan	9	23	1	0.3229	no
ni|645903527|nb|KK920903.1|	7670-7928	nan	nan	6	5	-0.318393	0.7941	no
ni|645903527|nb|KK920903.1|	830-1648	nan	nan	4	2	-0.79309	0.4965	no
ni|645903529|nb|KK920901.1|	100732-101042	nan	nan	17	68	2	0.0974	no
ni|645903529|nb|KK920901.1|	101500-103180	nan	nan	22	62	1	0.20925	no
ni|645903529|nb|KK920901.1|	104326-104495	nan	nan	18	83	2	0.0572	no
ni|645903529|nb|KK920901.1|	107329-107575	nan	nan	19	73	2	0.1036	no
ni|645903529|nb|KK920901.1|	107687-108390	nan	nan	27	74	1	0.2015	no
ni|645903529|nb|KK920901.1|	108506-108920	nan	nan	42	34	-0.290293	0.8512	no
ni|645903529|nb|KK920901.1|	109596-110288	nan	nan	25	3	-3	0.24205	no
ni|645903529|nb|KK920901.1|	110356-110580	nan	nan	9	38	2	0.1326	no
ni|645903529|nb|KK920901.1|	110734-110885	nan	nan	7	27	2	0.10025	no
ni|645903529|nb|KK920901.1|	113431-113565	nan	nan	9	32	2	0.1032	no
ni|645903529|nb|KK920901.1|	115003-115304	nan	nan	12	35	2	0.1751	no
ni|645903529|nb|KK920901.1|	115769-115965	nan	nan	25	84	2	0.1265	no
ni|645903529|nb|KK920901.1|	116967-117287	nan	nan	28	74	1	0.2451	no
ni|645903529|nb|KK920901.1|	117649-118063	nan	nan	5	15	2	0.1916	no
ni|645903529|nb|KK920901.1|	118164-118566	nan	nan	12	36	2	0.2001	no
ni|645903529|nb|KK920901.1|	118666-121938	nan	nan	17.93	57	2	0.16085	no
ni|645903529|nb|KK920901.1|	122088-122542	nan	nan	9	25	1	0.1948	no
ni|645903529|nb|KK920901.1|	122784-122917	nan	nan	28	78	1	0.19625	no
ni|645903529|nb|KK920901.1|	123137-125307	nan	nan	43	109	1	0.24095	no
ni|645903529|nb|KK920901.1|	125612-126217	nan	nan	25	80	2	0.1546	no

ni|645903529|nb|KK920901.1|	126358-126716	nan	nan	41	67	0.703113	0.55795	no
ni|645903529|nb|KK920901.1|	126793-127778	nan	nan	13	34	1	0.23685	no
ni|645903529|nb|KK920901.1|	127868-128889	nan	nan	24	71	2	0.1847	no
ni|645903529|nb|KK920901.1|	139400-139910	nan	nan	14	37	1	0.2168	no
ni|645903529|nb|KK920901.1|	140016-140937	nan	nan	34	80	1	0.2755	no
ni|645903529|nb|KK920901.1|	141001-142479	nan	nan	12	28	1	0.31835	no
ni|645903529|nb|KK920901.1|	143568-143764	nan	nan	42	98	1	0.2885	no
ni|645903529|nb|KK920901.1|	144230-145217	nan	nan	42	100	1	0.2976	no
ni|645903529|nb|KK920901.1|	147064-147425	nan	nan	44	108.13	1	0.26265	no
ni|645903529|nb|KK920901.1|	147533-148041	nan	nan	35	51	0.554886	0.6243	no
ni|645903529|nb|KK920901.1|	148909-149750	nan	nan	18	13	-0.482694	0.7594	no
ni|645903529|nb|KK920901.1|	149925-153695	nan	nan	27	21	-0.3394	0.7678	no
ni|645903529|nb|KK920901.1|	154577-154716	nan	nan	28	28	-0.0316332	0.97855	no
ni|645903529|nb|KK920901.1|	156647-156948	nan	nan	7	1	-2	0.1349	no
ni|645903529|nb|KK920901.1|	157621-157847	nan	nan	6	7	0.084746	0.9436	no
ni|645903529|nb|KK920901.1|	184174-184636	nan	nan	9	69	3	0.01635	no
ni|645903529|nb|KK920901.1|	205346-205522	nan	nan	5	53	3	0.0262	no
ni|645903529|nb|KK920901.1|	274748-275638	nan	nan	8	76	3	0.01825	no
ni|645903529|nb|KK920901.1|	290145-290204	nan	nan	0.974514	10	3	0.0178	no
ni|645903529|nb|KK920901.1|	416879-417705	nan	nan	0.298625	15	6	0.04445	no
ni|645903529|nb|KK920901.1|	417941-418147	nan	nan	0.111345	7	6	0.0444	no
ni|645903529|nb|KK920901.1|	418588-418728	nan	nan	0	150	inf	5.00E-05	yes
ni|645903529|nb|KK920901.1|	418903-419039	nan	nan	11	17	0.669642	0.56515	no
ni|645903529|nb|KK920901.1|	433716-434219	nan	nan	0.072967	2	5	0.1203	no
ni|645903529|nb|KK920901.1|	463531-463914	nan	nan	9	15	0.648963	0.60985	no
ni|645903529|nb|KK920901.1|	465101-465301	nan	nan	0.766373	0.199774	-2	0.24815	no
ni|645903529|nb|KK920901.1|	50125-50336	nan	nan	0	10	inf	5.00E-05	yes
ni|645903529|nb|KK920901.1|	50443-50585	nan	nan	0	124	inf	5.00E-05	yes
ni|645903529|nb|KK920901.1|	712395-713013	nan	nan	8	2	-2	0.11305	no
ni|645903529|nb|KK920901.1|	713273-713608	nan	nan	6	0.825321	-3	0.1498	no

ni|645903529|nb|KK920901.1|	713726-715722	nan	nan	0	66	inf	5.00E-05	yes
ni|645903529|nb|KK920901.1|	715927-716599	nan	nan	97	58	-0.734891	0.50285	no
ni|645903529|nb|KK920901.1|	717222-717450	nan	nan	83	37	-1	0.32535	no
ni|645903529|nb|KK920901.1|	717601-718191	nan	nan	115	30	-2	0.11025	no
ni|645903529|nb|KK920901.1|	719550-720415	nan	nan	72	25	-2	0.24765	no
ni|645903529|nb|KK920901.1|	721780-721997	nan	nan	3	11	2	0.15455	no
ni|645903529|nb|KK920901.1|	722387-722649	nan	nan	10	28	2	0.2398	no
ni|645903529|nb|KK920901.1|	722778-723402	nan	nan	14	23	0.761687	0.5177	no
ni|645903529|nb|KK920901.1|	723831-724533	nan	nan	4	6	0.561858	0.6321	no
ni|645903529|nb|KK920901.1|	72942-73129	nan	nan	0.100093	136	10	0.05055	no
ni|645903529|nb|KK920901.1|	735324-735680	nan	nan	4	5	0.233873	0.844	no
ni|645903529|nb|KK920901.1|	735926-736096	nan	nan	0.970916	3	1	0.25205	no
ni|645903529|nb|KK920901.1|	737646-738370	nan	nan	0.433852	0.622223	0.520231	0.6876	no
ni|645903529|nb|KK920901.1|	75413-75680	nan	nan	15	59	2	0.08925	no
ni|645903529|nb|KK920901.1|	76514-76686	nan	nan	14	60	2	0.0716	no
ni|645903529|nb|KK920901.1|	77812-78650	nan	nan	9	33	2	0.119	no
ni|645903529|nb|KK920901.1|	79366-79840	nan	nan	4	31	3	0.0309	no
ni|645903529|nb|KK920901.1|	80204-80496	nan	nan	15	53	2	0.1281	no
ni|645903529|nb|KK920901.1|	80889-81254	nan	nan	23	64	1	0.21565	no
ni|645903529|nb|KK920901.1|	81471-81994	nan	nan	14	46	2	0.1561	no
ni|645903529|nb|KK920901.1|	82466-82753	nan	nan	11	44	2	0.11705	no
ni|645903529|nb|KK920901.1|	828423-828837	nan	nan	10	14	0.532376	0.65245	no
ni|645903529|nb|KK920901.1|	832130-832487	nan	nan	15	31	1	0.3766	no
ni|645903529|nb|KK920901.1|	835022-835354	nan	nan	3	3	0.379548	0.74675	no
ni|645903529|nb|KK920901.1|	83964-84158	nan	nan	16	65	2	0.091	no
ni|645903529|nb|KK920901.1|	840335-840638	nan	nan	3	6	1	0.33205	no
ni|645903529|nb|KK920901.1|	840829-841147	nan	nan	0.337221	0.523904	0.635609	0.63975	no
ni|645903529|nb|KK920901.1|	841391-841569	nan	nan	20	22	0.123309	0.9136	no
ni|645903529|nb|KK920901.1|	85525-85963	nan	nan	15	70	2	0.07315	no
ni|645903529|nb|KK920901.1|	857728-860039	nan	nan	15	18	0.237656	0.8461	no

ni|645903529|nb|KK920901.1|	86606-87381	nan	nan	7	28	2	0.1046	no
ni|645903529|nb|KK920901.1|	871931-872221	nan	nan	8	10	0.262898	0.82475	no
ni|645903529|nb|KK920901.1|	873194-873631	nan	nan	30	20	-0.587157	0.6057	no
ni|645903529|nb|KK920901.1|	874848-875420	nan	nan	15	10	-0.594372	0.6019	no
ni|645903529|nb|KK920901.1|	877796-878195	nan	nan	33	23	-0.54303	0.63365	no
ni|645903529|nb|KK920901.1|	878884-879005	nan	nan	0.787929	0.828362	0.0721955	0.94355	no
ni|645903529|nb|KK920901.1|	879825-879945	nan	nan	13	9	-0.607116	0.59795	no
ni|645903529|nb|KK920901.1|	880488-880749	nan	nan	0.722989	0.460655	-0.650287	0.6679	no
ni|645903529|nb|KK920901.1|	882503-882996	nan	nan	0.178053	3	4	0.0464	no
ni|645903529|nb|KK920901.1|	88376-89108	nan	nan	14	63	2	0.07305	no
ni|645903529|nb|KK920901.1|	889327-889491	nan	nan	28	17	-0.702734	0.54865	no
ni|645903529|nb|KK920901.1|	890056-890203	nan	nan	5	3	-0.573258	0.6321	no
ni|645903529|nb|KK920901.1|	890673-891151	nan	nan	14	8	-0.773975	0.50635	no
ni|645903529|nb|KK920901.1|	891390-891651	nan	nan	2	1	-0.159565	0.8913	no
ni|645903529|nb|KK920901.1|	891793-892016	nan	nan	2	1	-0.923351	0.44975	no
ni|645903529|nb|KK920901.1|	892183-892313	nan	nan	1	1	0.0598901	0.95295	no
ni|645903529|nb|KK920901.1|	89742-90183	nan	nan	16	58	2	0.123	no
ni|645903529|nb|KK920901.1|	92147-92430	nan	nan	15	65	2	0.0822	no
ni|645903529|nb|KK920901.1|	94740-95128	nan	nan	11	33	2	0.1937	no
ni|645903529|nb|KK920901.1|	95561-95941	nan	nan	12	45	2	0.13245	no
ni|645903529|nb|KK920901.1|	97606-97904	nan	nan	17	63	2	0.16935	no
ni|645903530|nb|KK920900.1|	101108-101504	nan	nan	36	42	0.222291	0.84765	no
ni|645903530|nb|KK920900.1|	102094-103138	nan	nan	21	62	2	0.1966	no
ni|645903530|nb|KK920900.1|	106914-107049	nan	nan	0.393011	0.375712	-0.0649409	0.9384	no
ni|645903530|nb|KK920900.1|	109161-109789	nan	nan	22	68	2	0.21385	no
ni|645903530|nb|KK920900.1|	11761-11983	nan	nan	2	1	-0.427646	0.7192	no
ni|645903530|nb|KK920900.1|	12086-12304	nan	nan	6	2	-1	0.3535	no
ni|645903530|nb|KK920900.1|	123720-123978	nan	nan	23	27	0.245838	0.83275	no
ni|645903530|nb|KK920900.1|	124151-124356	nan	nan	25	15	-0.722942	0.5371	no
ni|645903530|nb|KK920900.1|	124821-125634	nan	nan	15	10	-0.480562	0.6767	no

ni|645903530|nb|KK920900.1|	125796-126024	nan	nan	17	20	0.223629	0.8417	no
ni|645903530|nb|KK920900.1|	126415-126558	nan	nan	12	6	-0.960419	0.51205	no
ni|645903530|nb|KK920900.1|	126906-127497	nan	nan	6	4	-0.60397	0.614	no
ni|645903530|nb|KK920900.1|	127605-128010	nan	nan	4	9	1	0.42455	no
ni|645903530|nb|KK920900.1|	128740-129057	nan	nan	3	2	-0.682569	0.58925	no
ni|645903530|nb|KK920900.1|	129893-130471	nan	nan	4	8	1	0.4054	no
ni|645903530|nb|KK920900.1|	132442-132820	nan	nan	0.874737	1	0.670977	0.65595	no
ni|645903530|nb|KK920900.1|	136285-136915	nan	nan	2	2	-0.0660639	0.9545	no
ni|645903530|nb|KK920900.1|	141358-141745	nan	nan	2	3	0.517897	0.7004	no
ni|645903530|nb|KK920900.1|	141890-142142	nan	nan	3	1	-1	0.46615	no
ni|645903530|nb|KK920900.1|	147529-147750	nan	nan	4	0	-inf	5.00E-05	yes
ni|645903530|nb|KK920900.1|	148568-148921	nan	nan	3	3	-0.0670435	0.94765	no
ni|645903530|nb|KK920900.1|	151340-151552	nan	nan	13	15	0.138137	0.9053	no
ni|645903530|nb|KK920900.1|	152525-153497	nan	nan	47	35	-0.426252	0.70725	no
ni|645903530|nb|KK920900.1|	154007-154270	nan	nan	46	45	-0.0360818	0.9718	no
ni|645903530|nb|KK920900.1|	154481-155022	nan	nan	53	41	-0.372057	0.7446	no
ni|645903530|nb|KK920900.1|	155147-155406	nan	nan	54	54	-0.00386604	0.99675	no
ni|645903530|nb|KK920900.1|	15699-16643	nan	nan	6	7	0.175729	0.8856	no
ni|645903530|nb|KK920900.1|	157205-157422	nan	nan	0.525565	0.940378	0.839372	0.4814	no
ni|645903530|nb|KK920900.1|	158600-158790	nan	nan	1	1	0.348656	0.81755	no
ni|645903530|nb|KK920900.1|	160471-160709	nan	nan	2	2	-0.0657811	0.9554	no
ni|645903530|nb|KK920900.1|	161280-161505	nan	nan	1	0.853223	-0.401943	0.73545	no
ni|645903530|nb|KK920900.1|	163494-163630	nan	nan	210	113	-0.892557	0.41035	no
ni|645903530|nb|KK920900.1|	165184-165473	nan	nan	114	60	-0.940611	0.527	no
ni|645903530|nb|KK920900.1|	166263-166438	nan	nan	28	21	-0.413881	0.71935	no
ni|645903530|nb|KK920900.1|	166684-166840	nan	nan	27	14	-0.963507	0.4648	no
ni|645903530|nb|KK920900.1|	167540-167655	nan	nan	1	3	1	0.45555	no
ni|645903530|nb|KK920900.1|	168021-168240	nan	nan	12	5	-1	0.2915	no
ni|645903530|nb|KK920900.1|	169979-170142	nan	nan	37	41	0.17721	0.8778	no
ni|645903530|nb|KK920900.1|	171781-171980	nan	nan	18	24	0.417468	0.75735	no

ni|645903530|nb|KK920900.1|	172897-173068	nan	nan	5	7	0.59887	0.61025	no
ni|645903530|nb|KK920900.1|	173799-174195	nan	nan	3	3	0.165527	0.88955	no
ni|645903530|nb|KK920900.1|	177901-178244	nan	nan	10	5	-1	0.33255	no
ni|645903530|nb|KK920900.1|	179917-180081	nan	nan	51	27	-0.935133	0.3879	no
ni|645903530|nb|KK920900.1|	200476-201195	nan	nan	131	61	-1	0.3145	no
ni|645903530|nb|KK920900.1|	206437-207064	nan	nan	40	17	-1	0.29725	no
ni|645903530|nb|KK920900.1|	209973-210652	nan	nan	39	22	-0.833498	0.471	no
ni|645903530|nb|KK920900.1|	211616-211756	nan	nan	0	5	inf	5.00E-05	yes
ni|645903530|nb|KK920900.1|	21761-23246	nan	nan	34	36	0.0660604	0.9553	no
ni|645903530|nb|KK920900.1|	223598-223784	nan	nan	0	9	inf	0.0001	yes
ni|645903530|nb|KK920900.1|	228404-228567	nan	nan	0	2	inf	0.0001	yes
ni|645903530|nb|KK920900.1|	230778-231790	nan	nan	38	34	-0.15959	0.88155	no
ni|645903530|nb|KK920900.1|	231961-232768	nan	nan	13	21	0.669652	0.5973	no
ni|645903530|nb|KK920900.1|	239991-240167	nan	nan	10	6	-0.71939	0.61925	no
ni|645903530|nb|KK920900.1|	244137-245147	nan	nan	2	0.090442	-5	0.11925	no
ni|645903530|nb|KK920900.1|	246682-246797	nan	nan	550	452	-0.283839	0.8506	no
ni|645903530|nb|KK920900.1|	248836-249178	nan	nan	541	436	-0.310855	0.828	no
ni|645903530|nb|KK920900.1|	249510-249903	nan	nan	1554.98	1285.12	-0.274989	0.79415	no
ni|645903530|nb|KK920900.1|	250609-250927	nan	nan	3	3	-0.387791	0.74515	no
ni|645903530|nb|KK920900.1|	252820-253007	nan	nan	5	3	-0.808315	0.49455	no
ni|645903530|nb|KK920900.1|	27060-27311	nan	nan	3	2	-0.480861	0.6968	no
ni|645903530|nb|KK920900.1|	278832-279592	nan	nan	11	7	-0.773528	0.52115	no
ni|645903530|nb|KK920900.1|	282018-282317	nan	nan	0.097463	9	6	0.1194	no
ni|645903530|nb|KK920900.1|	28881-29242	nan	nan	41	36	-0.174955	0.8801	no
ni|645903530|nb|KK920900.1|	289980-290393	nan	nan	21	55	1	0.30495	no
ni|645903530|nb|KK920900.1|	294640-295269	nan	nan	90	148	0.713999	0.5305	no
ni|645903530|nb|KK920900.1|	295409-296018	nan	nan	74	104	0.494657	0.66715	no
ni|645903530|nb|KK920900.1|	296341-296753	nan	nan	0.424474	0.405838	-0.0647719	0.94765	no
ni|645903530|nb|KK920900.1|	29952-30565	nan	nan	46	41	-0.174081	0.8813	no
ni|645903530|nb|KK920900.1|	304693-304891	nan	nan	20	26	0.343116	0.7666	no

ni|645903530|nb|KK920900.1|	305982-306187	nan	nan	1	2	0.384778	0.7446	no
ni|645903530|nb|KK920900.1|	306582-306802	nan	nan	50	70	0.505259	0.6559	no
ni|645903530|nb|KK920900.1|	307578-307904	nan	nan	1	2	0.374995	0.76185	no
ni|645903530|nb|KK920900.1|	308093-308209	nan	nan	2	0.6927	-1	0.3464	no
ni|645903530|nb|KK920900.1|	308864-309038	nan	nan	3	3	-0.218172	0.86075	no
ni|645903530|nb|KK920900.1|	309394-309876	nan	nan	3	3	-0.0394492	0.9743	no
ni|645903530|nb|KK920900.1|	311406-312273	nan	nan	8	3	-2	0.25505	no
ni|645903530|nb|KK920900.1|	312787-313567	nan	nan	2	3	0.0717626	0.9566	no
ni|645903530|nb|KK920900.1|	313690-315145	nan	nan	3	3	0.117602	0.92125	no
ni|645903530|nb|KK920900.1|	316185-316510	nan	nan	62	74	0.243989	0.82855	no
ni|645903530|nb|KK920900.1|	319119-319444	nan	nan	90	77	-0.218972	0.83595	no
ni|645903530|nb|KK920900.1|	320744-321271	nan	nan	21	17	-0.315307	0.8025	no
ni|645903530|nb|KK920900.1|	32932-33315	nan	nan	78	62	-0.323247	0.78515	no
ni|645903530|nb|KK920900.1|	337569-338773	nan	nan	99	88	-0.181224	0.86395	no
ni|645903530|nb|KK920900.1|	339031-339681	nan	nan	2	2	-0.0672893	0.923	no
ni|645903530|nb|KK920900.1|	340649-341866	nan	nan	2	4	1	0.3034	no
ni|645903530|nb|KK920900.1|	34448-34838	nan	nan	64	71	0.154433	0.8959	no
ni|645903530|nb|KK920900.1|	344645-344961	nan	nan	0	39	inf	5.00E-05	yes
ni|645903530|nb|KK920900.1|	347311-347682	nan	nan	5	5	0.214595	0.85505	no
ni|645903530|nb|KK920900.1|	34984-35138	nan	nan	1	0.706514	-0.940244	0.54025	no
ni|645903530|nb|KK920900.1|	36141-36528	nan	nan	2	2	0.0821117	0.94465	no
ni|645903530|nb|KK920900.1|	366896-368605	nan	nan	12	12	0.0771626	0.9481	no
ni|645903530|nb|KK920900.1|	369140-369389	nan	nan	4	6	0.600064	0.6082	no
ni|645903530|nb|KK920900.1|	37000-37136	nan	nan	5	8	0.682036	0.5653	no
ni|645903530|nb|KK920900.1|	370018-370404	nan	nan	0	327	inf	5.00E-05	yes
ni|645903530|nb|KK920900.1|	375352-375473	nan	nan	0	283	inf	5.00E-05	yes
ni|645903530|nb|KK920900.1|	37782-37943	nan	nan	4	3	-0.135611	0.90605	no
ni|645903530|nb|KK920900.1|	381030-383735	nan	nan	0.219541	177	10	0.11935	no
ni|645903530|nb|KK920900.1|	38289-38488	nan	nan	0.757341	0.633518	-0.257558	0.8388	no
ni|645903530|nb|KK920900.1|	38603-38728	nan	nan	1	0.766185	-0.686601	0.58765	no

ni|645903530|nb|KK920900.1|	387090-387719	nan	nan	0.082757	40	9	0.11935	no
ni|645903530|nb|KK920900.1|	38850-39075	nan	nan	1	1	0.157664	0.89095	no
ni|645903530|nb|KK920900.1|	389282-389615	nan	nan	0.125655	0.480627	2	0.1736	no
ni|645903530|nb|KK920900.1|	390220-390772	nan	nan	15	46	2	0.16835	no
ni|645903530|nb|KK920900.1|	390865-391216	nan	nan	6	24	2	0.138	no
ni|645903530|nb|KK920900.1|	392785-392912	nan	nan	7	17	1	0.24315	no
ni|645903530|nb|KK920900.1|	392965-394152	nan	nan	24	55	1	0.33025	no
ni|645903530|nb|KK920900.1|	394583-394779	nan	nan	7	17	1	0.27625	no
ni|645903530|nb|KK920900.1|	401133-401376	nan	nan	0.070066	14	8	0.11935	no
ni|645903530|nb|KK920900.1|	410133-412502	nan	nan	16	16.38	0.0292664	0.9788	no
ni|645903530|nb|KK920900.1|	411-1423	nan	nan	3	2	-0.526475	0.7071	no
ni|645903530|nb|KK920900.1|	412954-413494	nan	nan	0.539089	0.708502	0.394249	0.77595	no
ni|645903530|nb|KK920900.1|	413765-414381	nan	nan	0.833836	3	2	0.2028	no
ni|645903530|nb|KK920900.1|	414807-415457	nan	nan	52	44	-0.246908	0.82355	no
ni|645903530|nb|KK920900.1|	44775-45285	nan	nan	0.900579	0.860504	-0.0656712	0.9384	no
ni|645903530|nb|KK920900.1|	45464-46291	nan	nan	3	3	-0.182289	0.889	no
ni|645903530|nb|KK920900.1|	48827-49143	nan	nan	2	1	-0.731834	0.52305	no
ni|645903530|nb|KK920900.1|	492291-492631	nan	nan	0.724182	0.593503	-0.287096	0.8142	no
ni|645903530|nb|KK920900.1|	494713-495412	nan	nan	1.71	1	-0.612916	0.6301	no
ni|645903530|nb|KK920900.1|	496446-496572	nan	nan	79	68	-0.219026	0.84105	no
ni|645903530|nb|KK920900.1|	498590-498779	nan	nan	168	143.74	-0.224849	0.88055	no
ni|645903530|nb|KK920900.1|	498893-499032	nan	nan	198	147	-0.4314	0.7678	no
ni|645903530|nb|KK920900.1|	499337-499554	nan	nan	156	95	-0.721867	0.51595	no
ni|645903530|nb|KK920900.1|	50361-50821	nan	nan	21	16	-0.427128	0.71175	no
ni|645903530|nb|KK920900.1|	508626-509105	nan	nan	27	13	-1	0.3645	no
ni|645903530|nb|KK920900.1|	510942-511350	nan	nan	0.88609	2	1	0.29125	no
ni|645903530|nb|KK920900.1|	511601-511920	nan	nan	3	3	-0.169962	0.88065	no
ni|645903530|nb|KK920900.1|	513679-514216	nan	nan	1	0.466359	-1	0.35155	no
ni|645903530|nb|KK920900.1|	516043-516748	nan	nan	4	2	-1	0.3302	no
ni|645903530|nb|KK920900.1|	52124-53051	nan	nan	18	7	-1	0.29265	no

ni|645903530|nb|KK920900.1|	523488-523650	nan	nan	34	19	-0.827595	0.45925	no
ni|645903530|nb|KK920900.1|	525031-525294	nan	nan	201.54	209	0.0529677	0.97055	no
ni|645903530|nb|KK920900.1|	526263-526396	nan	nan	232	265	0.19018	0.89595	no
ni|645903530|nb|KK920900.1|	527054-527223	nan	nan	53	64	0.272117	0.8495	no
ni|645903530|nb|KK920900.1|	529592-529752	nan	nan	233	320	0.455415	0.7547	no
ni|645903530|nb|KK920900.1|	529948-530107	nan	nan	296.7	404	0.445399	0.7717	no
ni|645903530|nb|KK920900.1|	533622-533824	nan	nan	194	268	0.466584	0.6747	no
ni|645903530|nb|KK920900.1|	533953-534403	nan	nan	204	296	0.535468	0.61835	no
ni|645903530|nb|KK920900.1|	535741-536362	nan	nan	210	283	0.42697	0.76105	no
ni|645903530|nb|KK920900.1|	537350-537931	nan	nan	229	325	0.503691	0.68955	no
ni|645903530|nb|KK920900.1|	539434-540099	nan	nan	26	39	0.580461	0.64315	no
ni|645903530|nb|KK920900.1|	546436-546556	nan	nan	4	13	2	0.1175	no
ni|645903530|nb|KK920900.1|	547847-548151	nan	nan	10	23	1	0.3235	no
ni|645903530|nb|KK920900.1|	549582-549919	nan	nan	6	23	2	0.1383	no
ni|645903530|nb|KK920900.1|	550261-550559	nan	nan	2	5	2	0.21775	no
ni|645903530|nb|KK920900.1|	552923-553296	nan	nan	14	44	2	0.16305	no
ni|645903530|nb|KK920900.1|	55874-56009	nan	nan	34	21	-0.684987	0.55955	no
ni|645903530|nb|KK920900.1|	568225-568895	nan	nan	8	13	0.80948	0.4783	no
ni|645903530|nb|KK920900.1|	570360-570751	nan	nan	4	6	0.592246	0.62225	no
ni|645903530|nb|KK920900.1|	570874-571132	nan	nan	0.430531	0.293952	-0.550538	0.71905	no
ni|645903530|nb|KK920900.1|	571961-572105	nan	nan	20	28	0.513753	0.6525	no
ni|645903530|nb|KK920900.1|	59409-60529	nan	nan	34	24	-0.496884	0.6737	no
ni|645903530|nb|KK920900.1|	61109-61247	nan	nan	21	11	-0.915653	0.53575	no
ni|645903530|nb|KK920900.1|	61361-62241	nan	nan	0.962467	0.172501	-2	0.17	no
ni|645903530|nb|KK920900.1|	67890-68060	nan	nan	0.992609	0.072977	-4	0.02555	no
ni|645903530|nb|KK920900.1|	69086-69427	nan	nan	1	0.303344	-2	0.2784	no
ni|645903530|nb|KK920900.1|	69647-71743	nan	nan	3	0.796218	-2	0.25615	no
ni|645903530|nb|KK920900.1|	75423-75613	nan	nan	0.957302	0.571662	-0.743813	0.59615	no
ni|645903530|nb|KK920900.1|	75723-76268	nan	nan	13	18	0.517468	0.7004	no
ni|645903530|nb|KK920900.1|	77397-77805	nan	nan	28	18	-0.652175	0.57185	no

ni|645903530|nb|KK920900.1|	78124-78864	nan	nan	0.353947	0.376005	0.0872189	0.9436	no
ni|645903530|nb|KK920900.1|	79089-79449	nan	nan	65	56	-0.214284	0.85335	no
ni|645903530|nb|KK920900.1|	8295-8430	nan	nan	2	3	0.474296	0.709	no
ni|645903530|nb|KK920900.1|	8532-8731	nan	nan	0.985113	1	0.302221	0.7966	no
ni|645903530|nb|KK920900.1|	89085-89402	nan	nan	10	9	-0.152111	0.8927	no
ni|645903530|nb|KK920900.1|	9198-9487	nan	nan	1	1	-0.0387722	0.97225	no
ni|645903530|nb|KK920900.1|	96236-96353	nan	nan	0.66478	0.635418	-0.0651721	0.9467	no
ni|645903530|nb|KK920900.1|	9672-10060	nan	nan	0.541978	1	1	0.4245	no
ni|645903530|nb|KK920900.1|	98921-99369	nan	nan	0.670724	0.789151	0.234582	0.85095	no
ni|645903532|nb|KK920898.1|	123401-123719	nan	nan	7	9	0.348726	0.76005	no
ni|645903532|nb|KK920898.1|	123874-124226	nan	nan	18	14	-0.344433	0.7628	no
ni|645903532|nb|KK920898.1|	124498-125052	nan	nan	15	11	-0.429865	0.73195	no
ni|645903532|nb|KK920898.1|	125333-125631	nan	nan	5	5	-0.133496	0.907	no
ni|645903532|nb|KK920898.1|	125744-125855	nan	nan	5	7	0.347972	0.76915	no
ni|645903532|nb|KK920898.1|	127130-127554	nan	nan	6	7	0.125354	0.91735	no
ni|645903532|nb|KK920898.1|	129133-129336	nan	nan	8	11	0.418223	0.7302	no
ni|645903532|nb|KK920898.1|	148362-148682	nan	nan	11	5	-1	0.46615	no
ni|645903532|nb|KK920898.1|	149821-149962	nan	nan	2	2	0.0121595	0.9704	no
ni|645903532|nb|KK920898.1|	223021-223168	nan	nan	8	10	0.301934	0.79955	no
ni|645903532|nb|KK920898.1|	23631-23741	nan	nan	21	23	0.144106	0.92015	no
ni|645903532|nb|KK920898.1|	238991-239450	nan	nan	9	12	0.418126	0.7244	no
ni|645903532|nb|KK920898.1|	24364-24583	nan	nan	20	14	-0.47686	0.681	no
ni|645903532|nb|KK920898.1|	24770-24888	nan	nan	4	3	-0.282218	0.8093	no
ni|645903532|nb|KK920898.1|	251589-252024	nan	nan	2	5	2	0.1887	no
ni|645903532|nb|KK920898.1|	25225-25419	nan	nan	43	36	-0.275183	0.8118	no
ni|645903532|nb|KK920898.1|	252381-252920	nan	nan	0	77	inf	5.00E-05	yes
ni|645903532|nb|KK920898.1|	254361-254518	nan	nan	0.632391	0.3358	-0.913215	0.53575	no
ni|645903532|nb|KK920898.1|	255268-255764	nan	nan	2	2	-0.367644	0.7554	no
ni|645903532|nb|KK920898.1|	258341-258680	nan	nan	3	2	-0.694267	0.58855	no
ni|645903532|nb|KK920898.1|	260062-260602	nan	nan	4	3	-0.387642	0.7434	no

ni|645903532|nb|KK920898.1|	260864-261080	nan	nan	51	32	-0.691197	0.63955	no
ni|645903532|nb|KK920898.1|	261688-261835	nan	nan	22	11	-0.996415	0.37955	no
ni|645903532|nb|KK920898.1|	261942-262663	nan	nan	12	3	-2	0.1613	no
ni|645903532|nb|KK920898.1|	262843-262958	nan	nan	18	13	-0.509208	0.65405	no
ni|645903532|nb|KK920898.1|	264340-264526	nan	nan	2	2	0.0592589	0.95295	no
ni|645903532|nb|KK920898.1|	264649-264929	nan	nan	63	42	-0.581767	0.62155	no
ni|645903532|nb|KK920898.1|	264988-265100	nan	nan	13	14	0.142919	0.9028	no
ni|645903532|nb|KK920898.1|	265294-265425	nan	nan	4	6	0.439366	0.7118	no
ni|645903532|nb|KK920898.1|	265816-266019	nan	nan	0.43097	0.503563	0.224585	0.8598	no
ni|645903532|nb|KK920898.1|	267565-267942	nan	nan	5	2	-2	0.20015	no
ni|645903532|nb|KK920898.1|	26819-27168	nan	nan	13	10	-0.334047	0.77195	no
ni|645903532|nb|KK920898.1|	268765-269396	nan	nan	1	0.699539	-0.650726	0.6679	no
ni|645903532|nb|KK920898.1|	269584-269705	nan	nan	2	2	-0.279407	0.81975	no
ni|645903532|nb|KK920898.1|	27250-27383	nan	nan	1	0.71234	-1	0.44965	no
ni|645903532|nb|KK920898.1|	27526-28070	nan	nan	45	36	-0.312406	0.78175	no
ni|645903532|nb|KK920898.1|	282222-286673	nan	nan	6	5	-0.404616	0.72395	no
ni|645903532|nb|KK920898.1|	28291-28568	nan	nan	54	46	-0.222174	0.8455	no
ni|645903532|nb|KK920898.1|	28715-29000	nan	nan	20	16	-0.330057	0.7715	no
ni|645903532|nb|KK920898.1|	287850-288097	nan	nan	3	4	0.28204	0.81735	no
ni|645903532|nb|KK920898.1|	291822-292073	nan	nan	3	1	-1	0.30945	no
ni|645903532|nb|KK920898.1|	29204-29825	nan	nan	28	10	-2	0.2091	no
ni|645903532|nb|KK920898.1|	292932-293093	nan	nan	1	2	0.519193	0.6956	no
ni|645903532|nb|KK920898.1|	294058-294263	nan	nan	1	1	0.0599577	0.95295	no
ni|645903532|nb|KK920898.1|	298040-298392	nan	nan	0.496098	1	2	0.24365	no
ni|645903532|nb|KK920898.1|	301349-301553	nan	nan	0.690663	1	1	0.39815	no
ni|645903532|nb|KK920898.1|	302447-302793	nan	nan	437.85	704	0.685176	0.6189	no
ni|645903532|nb|KK920898.1|	303045-303165	nan	nan	0.731258	2	2	0.2394	no
ni|645903532|nb|KK920898.1|	303285-303415	nan	nan	198	504	1	0.36185	no
ni|645903532|nb|KK920898.1|	305225-305352	nan	nan	123.48	301	1	0.3677	no
ni|645903532|nb|KK920898.1|	305488-305643	nan	nan	110	306	1	0.3034	no

ni|645903532|nb|KK920898.1|	305757-306529	nan	nan	0.926312	1	0.256862	0.83705	no
ni|645903532|nb|KK920898.1|	306656-306999	nan	nan	176	565	2	0.1899	no
ni|645903532|nb|KK920898.1|	307363-307535	nan	nan	96	132	0.463281	0.6746	no
ni|645903532|nb|KK920898.1|	307685-308220	nan	nan	11	6	-0.962968	0.41535	no
ni|645903532|nb|KK920898.1|	308429-309582	nan	nan	3	8	1	0.30495	no
ni|645903532|nb|KK920898.1|	309954-310116	nan	nan	127	68	-0.900917	0.43565	no
ni|645903532|nb|KK920898.1|	310884-311627	nan	nan	91	56	-0.695138	0.53695	no
ni|645903532|nb|KK920898.1|	312645-312790	nan	nan	73	40	-0.846157	0.46145	no
ni|645903532|nb|KK920898.1|	312870-313225	nan	nan	38	25	-0.626656	0.5779	no
ni|645903532|nb|KK920898.1|	313532-313729	nan	nan	30	13	-1	0.31155	no
ni|645903532|nb|KK920898.1|	313927-314061	nan	nan	14	5	-1	0.2915	no
ni|645903532|nb|KK920898.1|	31490-31627	nan	nan	25	14	-0.779521	0.51455	no
ni|645903532|nb|KK920898.1|	315518-316102	nan	nan	3	1	-0.896234	0.502	no
ni|645903532|nb|KK920898.1|	316499-316656	nan	nan	4	0.33834	-4	0.2368	no
ni|645903532|nb|KK920898.1|	317542-317690	nan	nan	24	5	-2	0.1501	no
ni|645903532|nb|KK920898.1|	318096-319606	nan	nan	10	1	-3	0.06495	no
ni|645903532|nb|KK920898.1|	31894-32082	nan	nan	3	2	-0.48225	0.7594	no
ni|645903532|nb|KK920898.1|	319983-320288	nan	nan	11	1	-3	0.06135	no
ni|645903532|nb|KK920898.1|	320761-321849	nan	nan	8	0.909643	-3	0.02675	no
ni|645903532|nb|KK920898.1|	322547-323248	nan	nan	1449.06	566	-1	0.35795	no
ni|645903532|nb|KK920898.1|	324578-325029	nan	nan	2	0.33834	-3	0.2671	no
ni|645903532|nb|KK920898.1|	325216-325373	nan	nan	12	12	-0.0624752	0.96785	no
ni|645903532|nb|KK920898.1|	326184-326370	nan	nan	187	167	-0.161054	0.9068	no
ni|645903532|nb|KK920898.1|	326614-326770	nan	nan	28	29	0.059252	0.95695	no
ni|645903532|nb|KK920898.1|	327046-327270	nan	nan	19	12	-0.663927	0.57225	no
ni|645903532|nb|KK920898.1|	328303-328468	nan	nan	34	48	0.50743	0.66165	no
ni|645903532|nb|KK920898.1|	329782-330158	nan	nan	7	3	-1	0.44755	no
ni|645903532|nb|KK920898.1|	330564-330708	nan	nan	32	40	0.330142	0.7545	no
ni|645903532|nb|KK920898.1|	331389-331566	nan	nan	130	199	0.614766	0.6609	no
ni|645903532|nb|KK920898.1|	331694-332241	nan	nan	423	396	-0.0944883	0.94815	no

ni|645903532|nb|KK920898.1|	332974-333218	nan	nan	59	99	0.739844	0.55925	no
ni|645903532|nb|KK920898.1|	333335-333442	nan	nan	411.55	457	0.151641	0.90335	no
ni|645903532|nb|KK920898.1|	33428-33740	nan	nan	209	331	0.661746	0.566	no
ni|645903532|nb|KK920898.1|	334855-335078	nan	nan	17	7	-1	0.31075	no
ni|645903532|nb|KK920898.1|	335217-335410	nan	nan	2214.97	2389.62	0.109497	0.91575	no
ni|645903532|nb|KK920898.1|	335603-335811	nan	nan	25	4	-2	0.0484	no
ni|645903532|nb|KK920898.1|	336915-337130	nan	nan	410	67	-3	0.05145	no
ni|645903532|nb|KK920898.1|	343499-343614	nan	nan	251	40	-3	0.0538	no
ni|645903532|nb|KK920898.1|	36193-36340	nan	nan	6	3	-0.844641	0.4741	no
ni|645903532|nb|KK920898.1|	43441-43671	nan	nan	8	2	-2	0.24585	no
ni|645903532|nb|KK920898.1|	486902-487115	nan	nan	246	19	-4	0.0126	no
ni|645903532|nb|KK920898.1|	530981-531254	nan	nan	12	4	-2	0.2234	no
ni|645903532|nb|KK920898.1|	573547-573704	nan	nan	24	3	-3	0.0299	no
ni|645903532|nb|KK920898.1|	58026-58362	nan	nan	16	16	-0.0156937	0.9893	no
ni|645903532|nb|KK920898.1|	581155-581348	nan	nan	1591.66	793	-1	0.4875	no
ni|645903532|nb|KK920898.1|	651458-651611	nan	nan	24	2	-4	0.004	no
ni|645903532|nb|KK920898.1|	660151-660764	nan	nan	20	6	-2	0.25505	no
ni|645903532|nb|KK920898.1|	660984-661188	nan	nan	15	0.64287	-5	0.01545	no
ni|645903532|nb|KK920898.1|	661465-661638	nan	nan	38	2	-4	0.00245	yes
ni|645903532|nb|KK920898.1|	661912-662181	nan	nan	92	3	-5	0.00555	no
ni|645903532|nb|KK920898.1|	662532-663153	nan	nan	42	2	-4	0.0022	yes
ni|645903532|nb|KK920898.1|	663810-664086	nan	nan	88	5	-4	0.00805	no
ni|645903532|nb|KK920898.1|	670028-670473	nan	nan	15	10	-0.667716	0.5716	no
ni|645903532|nb|KK920898.1|	673933-674423	nan	nan	10	9	-0.144262	0.90165	no
ni|645903532|nb|KK920898.1|	675992-676458	nan	nan	4	4	-0.134156	0.9101	no
ni|645903532|nb|KK920898.1|	677022-677280	nan	nan	64	23	-1	0.2159	no
ni|645903532|nb|KK920898.1|	677761-678393	nan	nan	362	220	-0.713684	0.6105	no
ni|645903532|nb|KK920898.1|	678548-679098	nan	nan	117	73	-0.685411	0.63105	no
ni|645903532|nb|KK920898.1|	679554-679700	nan	nan	40	23	-0.809521	0.5694	no
ni|645903532|nb|KK920898.1|	684307-684491	nan	nan	1	0.746144	-0.428231	0.75965	no

ni|645903532|nb|KK920898.1|	684729-684915	nan	nan	116	75	-0.63982	0.65805	no
ni|645903532|nb|KK920898.1|	685092-685232	nan	nan	139	88	-0.661169	0.65485	no
ni|645903532|nb|KK920898.1|	686393-686514	nan	nan	406	251	-0.691396	0.556	no
ni|645903532|nb|KK920898.1|	690539-690768	nan	nan	3	3	-0.129948	0.9135	no
ni|645903532|nb|KK920898.1|	690894-691712	nan	nan	7	4	-0.683981	0.5892	no
ni|645903532|nb|KK920898.1|	692871-693206	nan	nan	9	5	-0.791119	0.51885	no
ni|645903532|nb|KK920898.1|	695296-695476	nan	nan	2	1	-0.754583	0.52795	no
ni|645903532|nb|KK920898.1|	696544-696769	nan	nan	85	86	0.0224814	0.9818	no
ni|645903532|nb|KK920898.1|	698537-698699	nan	nan	49	48	-0.0458263	0.9744	no
ni|645903532|nb|KK920898.1|	701320-701603	nan	nan	14	15	0.102038	0.9262	no
ni|645903532|nb|KK920898.1|	702372-702537	nan	nan	160	193	0.265103	0.82125	no
ni|645903532|nb|KK920898.1|	703824-703942	nan	nan	2	1	-0.46357	0.70425	no
ni|645903532|nb|KK920898.1|	704795-704975	nan	nan	2	0.63383	-1	0.3464	no
ni|645903532|nb|KK920898.1|	707189-707313	nan	nan	2	3	0.25617	0.83575	no
ni|645903532|nb|KK920898.1|	709022-709380	nan	nan	3	3	-0.0666823	0.9554	no
ni|645903532|nb|KK920898.1|	709801-710024	nan	nan	119.12	69	-0.788378	0.5806	no
ni|645903532|nb|KK920898.1|	710769-710949	nan	nan	98	53	-0.88966	0.48985	no
ni|645903532|nb|KK920898.1|	724224-724406	nan	nan	1	2	0.610945	0.6619	no
ni|645903532|nb|KK920898.1|	724783-724974	nan	nan	1	0.445763	-2	0.22235	no
ni|645903532|nb|KK920898.1|	725105-725617	nan	nan	1	1	-0.481323	0.7594	no
ni|645903532|nb|KK920898.1|	725776-726021	nan	nan	3	2	-0.832069	0.5313	no
ni|645903532|nb|KK920898.1|	727783-727899	nan	nan	75	819	3	0.03365	no
ni|645903532|nb|KK920898.1|	728059-728409	nan	nan	0.57719	0.236537	-1	0.34	no
ni|645903532|nb|KK920898.1|	729943-730179	nan	nan	4	4	-0.0933939	0.9314	no
ni|645903532|nb|KK920898.1|	730509-730698	nan	nan	2	3	0.840274	0.5399	no
ni|645903532|nb|KK920898.1|	730891-731038	nan	nan	4	2	-1	0.429	no
ni|645903532|nb|KK920898.1|	731209-731345	nan	nan	2	0.930624	-0.895089	0.46445	no
ni|645903532|nb|KK920898.1|	732953-733070	nan	nan	5	2	-1	0.3885	no
ni|645903532|nb|KK920898.1|	733368-733602	nan	nan	2	1	-1	0.3926	no
ni|645903532|nb|KK920898.1|	735385-735579	nan	nan	2	0.692205	-1	0.3299	no

ni|645903532|nb|KK920898.1|	737368-737557	nan	nan	35	49	0.482778	0.68945	no
ni|645903532|nb|KK920898.1|	740066-740282	nan	nan	168	159	-0.0790759	0.94665	no
ni|645903532|nb|KK920898.1|	740531-740680	nan	nan	8	12	0.622932	0.5948	no
ni|645903532|nb|KK920898.1|	742267-742792	nan	nan	20	24	0.224976	0.8487	no
ni|645903532|nb|KK920898.1|	745116-745367	nan	nan	1	1	-0.0659126	0.9384	no
ni|645903532|nb|KK920898.1|	746308-746474	nan	nan	0.672131	1	0.694012	0.58445	no
ni|645903532|nb|KK920898.1|	748680-748784	nan	nan	3	2	-0.873889	0.53675	no
ni|645903532|nb|KK920898.1|	749702-749883	nan	nan	2	1	-0.939968	0.4651	no
ni|645903532|nb|KK920898.1|	751796-751917	nan	nan	5	1	-2	0.24815	no
ni|645903532|nb|KK920898.1|	753342-753525	nan	nan	4	2	-1	0.3373	no
ni|645903532|nb|KK920898.1|	802048-802224	nan	nan	0.049721	40	10	0.11935	no
ni|645903532|nb|KK920898.1|	807404-807847	nan	nan	0	82	inf	5.00E-05	yes
ni|645903532|nb|KK920898.1|	809966-810207	nan	nan	0	70	inf	5.00E-05	yes
ni|645903532|nb|KK920898.1|	813214-813364	nan	nan	24	24	0.0229706	0.98525	no
ni|645903532|nb|KK920898.1|	815408-815596	nan	nan	5	9	1	0.37265	no
ni|645903532|nb|KK920898.1|	816484-817029	nan	nan	7	4	-0.86087	0.46595	no
ni|645903532|nb|KK920898.1|	817990-818327	nan	nan	1	0.394326	-2	0.21345	no
ni|645903532|nb|KK920898.1|	820421-820887	nan	nan	7	5	-0.561389	0.62935	no
ni|645903532|nb|KK920898.1|	821530-821734	nan	nan	2	2	0.349008	0.7835	no
ni|645903532|nb|KK920898.1|	821877-822151	nan	nan	10	10	-0.0674725	0.9302	no
ni|645903532|nb|KK920898.1|	822946-823122	nan	nan	6	7	0.27381	0.823	no
ni|645903532|nb|KK920898.1|	825537-826032	nan	nan	2	3	0.0902296	0.9369	no
ni|645903532|nb|KK920898.1|	826813-826958	nan	nan	2	2	-0.0651764	0.9553	no
ni|645903532|nb|KK920898.1|	829493-829672	nan	nan	2	2	0.223991	0.85915	no
ni|645903532|nb|KK920898.1|	830264-830488	nan	nan	1	2	0.540815	0.6539	no
ni|645903532|nb|KK920898.1|	830627-833220	nan	nan	46	34	-0.4063	0.72435	no
ni|645903532|nb|KK920898.1|	833321-834053	nan	nan	43	32	-0.425938	0.714	no
ni|645903532|nb|KK920898.1|	834160-834730	nan	nan	24	23	-0.0845195	0.9438	no
ni|645903532|nb|KK920898.1|	834919-835524	nan	nan	37	28	-0.384563	0.7428	no
ni|645903532|nb|KK920898.1|	835808-836234	nan	nan	42	43	0.0530721	0.96365	no

ni|645903532|nb|KK920898.1|	836380-836537	nan	nan	2	7	1	0.2841	no
ni|645903532|nb|KK920898.1|	836918-837783	nan	nan	3	8	1	0.3067	no
ni|645903532|nb|KK920898.1|	838746-838960	nan	nan	0.122633	3	5	0.1211	no
ni|645903532|nb|KK920898.1|	846122-846279	nan	nan	2	3	0.431839	0.72635	no
ni|645903532|nb|KK920898.1|	846480-850987	nan	nan	2	2	0.176128	0.8772	no
ni|645903532|nb|KK920898.1|	851046-851626	nan	nan	107	146	0.455809	0.7488	no
ni|645903532|nb|KK920898.1|	852730-852887	nan	nan	6	10	0.752061	0.5275	no
ni|645903532|nb|KK920898.1|	859840-860165	nan	nan	9	19	1	0.35025	no
ni|645903532|nb|KK920898.1|	860852-861086	nan	nan	8	11	0.42001	0.7193	no
ni|645903532|nb|KK920898.1|	861163-861334	nan	nan	14	20	0.485221	0.68565	no
ni|645903532|nb|KK920898.1|	861812-862060	nan	nan	5	6	0.303011	0.7975	no
ni|645903532|nb|KK920898.1|	863676-864152	nan	nan	3	4	0.110881	0.9254	no
ni|645903532|nb|KK920898.1|	868581-868757	nan	nan	0.456649	0.476276	0.0607104	0.95295	no
ni|645903532|nb|KK920898.1|	868970-869102	nan	nan	0.35231	0.240571	-0.550386	0.71905	no
ni|645903532|nb|KK920898.1|	870703-870901	nan	nan	4	6	0.603672	0.62205	no
ni|645903532|nb|KK920898.1|	872165-872507	nan	nan	8	8	-0.00312924	0.97495	no
ni|645903532|nb|KK920898.1|	873143-873339	nan	nan	0	10	inf	5.00E-05	yes
ni|645903532|nb|KK920898.1|	873979-874183	nan	nan	0	12	inf	5.00E-05	yes
ni|645903532|nb|KK920898.1|	874467-874748	nan	nan	0	1	inf	0.0003	yes
ni|645903532|nb|KK920898.1|	876258-876676	nan	nan	0	269	inf	5.00E-05	yes
ni|645903532|nb|KK920898.1|	878093-878494	nan	nan	0	50	inf	5.00E-05	yes
ni|645903532|nb|KK920898.1|	878758-878963	nan	nan	0	30	inf	5.00E-05	yes
ni|645903532|nb|KK920898.1|	880245-880776	nan	nan	2	0.791959	-2	0.2366	no
ni|645903532|nb|KK920898.1|	882193-882623	nan	nan	2	0.754018	-1	0.36935	no
ni|645903532|nb|KK920898.1|	886251-886366	nan	nan	2	1	-0.172561	0.89245	no
ni|645903532|nb|KK920898.1|	887411-887552	nan	nan	7	14	1	0.3291	no
ni|645903532|nb|KK920898.1|	888191-888392	nan	nan	10	2	-2	0.15655	no
ni|645903532|nb|KK920898.1|	889809-890043	nan	nan	22	3	-3	0.2563	no
ni|645903532|nb|KK920898.1|	891371-891494	nan	nan	14	11	-0.314689	0.7775	no
ni|645903532|nb|KK920898.1|	891805-892142	nan	nan	2	6	1	0.30495	no

ni|645903532|nb|KK920898.1|	895950-896065	nan	nan	47	40	-0.243998	0.8458	no
ni|645903532|nb|KK920898.1|	896534-896695	nan	nan	17	14	-0.208868	0.85255	no
ni|645903532|nb|KK920898.1|	927586-928031	nan	nan	6	6	0.210115	0.85645	no
ni|645903533|nb|KK920897.1|	123462-124398	nan	nan	0.909488	0.965654	0.0864527	0.9436	no
ni|645903533|nb|KK920897.1|	128645-128785	nan	nan	0.616583	0.707248	0.197921	0.87955	no
ni|645903533|nb|KK920897.1|	136567-138043	nan	nan	0.898454	0.572369	-0.6505	0.6679	no
ni|645903533|nb|KK920897.1|	138249-138400	nan	nan	6	5	-0.193834	0.8562	no
ni|645903533|nb|KK920897.1|	138844-139131	nan	nan	4	3	-0.0671996	0.9302	no
ni|645903533|nb|KK920897.1|	139224-139474	nan	nan	2	2	-0.066381	0.9554	no
ni|645903533|nb|KK920897.1|	161845-162583	nan	nan	5	3	-0.960319	0.51205	no
ni|645903533|nb|KK920897.1|	165866-166043	nan	nan	1	0.251458	-2	0.1417	no
ni|645903533|nb|KK920897.1|	176852-176986	nan	nan	4	3	-0.525201	0.66185	no
ni|645903533|nb|KK920897.1|	177382-177542	nan	nan	2	2	-0.443838	0.7127	no
ni|645903533|nb|KK920897.1|	177605-177850	nan	nan	1	0.478909	-1	0.3141	no
ni|645903533|nb|KK920897.1|	177996-178392	nan	nan	2	0.2749	-3	0.06135	no
ni|645903533|nb|KK920897.1|	178610-179871	nan	nan	9	5	-0.945274	0.4254	no
ni|645903533|nb|KK920897.1|	180029-180231	nan	nan	3	2	-0.480174	0.67875	no
ni|645903533|nb|KK920897.1|	180796-180969	nan	nan	43	23	-0.903673	0.41145	no
ni|645903533|nb|KK920897.1|	181504-182065	nan	nan	20	11	-0.850411	0.46095	no
ni|645903533|nb|KK920897.1|	182285-182690	nan	nan	70	35	-1	0.3587	no
ni|645903533|nb|KK920897.1|	184134-185330	nan	nan	50	31	-0.670661	0.5373	no
ni|645903533|nb|KK920897.1|	185553-186420	nan	nan	28	15	-0.888444	0.43595	no
ni|645903533|nb|KK920897.1|	186523-187132	nan	nan	35	88	1	0.25965	no
ni|645903533|nb|KK920897.1|	187194-187445	nan	nan	12	65.52	2	0.0537	no
ni|645903533|nb|KK920897.1|	188117-188373	nan	nan	60	42	-0.530717	0.72035	no
ni|645903533|nb|KK920897.1|	188499-188829	nan	nan	0.926312	0.110683	-3	0.2479	no
ni|645903533|nb|KK920897.1|	188967-189209	nan	nan	11	10	-0.221219	0.8481	no
ni|645903533|nb|KK920897.1|	189937-190491	nan	nan	28	23	-0.292357	0.7858	no
ni|645903533|nb|KK920897.1|	191765-192741	nan	nan	0.725393	1	0.475403	0.709	no
ni|645903533|nb|KK920897.1|	192851-193006	nan	nan	3	0.430669	-3	0.2671	no

ni|645903533|nb|KK920897.1|	193603-193748	nan	nan	2	27	4	0.05325	no
ni|645903533|nb|KK920897.1|	194224-194532	nan	nan	4	35	3	0.0443	no
ni|645903533|nb|KK920897.1|	197185-197434	nan	nan	3	41	4	0.0193	no
ni|645903533|nb|KK920897.1|	200282-200410	nan	nan	7	46	3	0.04455	no
ni|645903533|nb|KK920897.1|	211673-211824	nan	nan	0	5	inf	5.00E-05	yes
ni|645903533|nb|KK920897.1|	216619-216964	nan	nan	9	6	-0.49789	0.68195	no
ni|645903533|nb|KK920897.1|	222920-223227	nan	nan	0	11	inf	5.00E-05	yes
ni|645903533|nb|KK920897.1|	223533-223700	nan	nan	0.225532	16	6	0.11965	no
ni|645903533|nb|KK920897.1|	252127-252344	nan	nan	0	4	inf	0.00025	yes
ni|645903533|nb|KK920897.1|	261031-261159	nan	nan	0	13	inf	0.00025	yes
ni|645903533|nb|KK920897.1|	261409-261631	nan	nan	0	2	inf	0.00045	yes
ni|645903533|nb|KK920897.1|	269650-269843	nan	nan	0	132	inf	5.00E-05	yes
ni|645903533|nb|KK920897.1|	277406-277590	nan	nan	0.02672	320	14	0.0443	no
ni|645903533|nb|KK920897.1|	281338-281782	nan	nan	5	2	-0.959344	0.44215	no
ni|645903533|nb|KK920897.1|	285207-285457	nan	nan	24	17	-0.511578	0.64185	no
ni|645903533|nb|KK920897.1|	287416-287575	nan	nan	14	12	-0.322479	0.78335	no
ni|645903533|nb|KK920897.1|	288083-288343	nan	nan	15	6	-1	0.28025	no
ni|645903533|nb|KK920897.1|	290680-290877	nan	nan	14	7	-1	0.3992	no
ni|645903534|nb|KK920896.1|	520465-521027	nan	nan	3	2	-0.0666823	0.9545	no
ni|645903534|nb|KK920896.1|	559218-559432	nan	nan	4	6	0.555467	0.6345	no
ni|645903534|nb|KK920896.1|	559528-559694	nan	nan	20	34	0.739835	0.5577	no
ni|645903534|nb|KK920896.1|	560277-560614	nan	nan	21	11	-0.942047	0.54025	no
ni|645903534|nb|KK920896.1|	561178-561348	nan	nan	0.380023	0.322955	-0.234757	0.8697	no
ni|645903534|nb|KK920896.1|	561403-561937	nan	nan	4	3	-0.305807	0.78345	no
ni|645903534|nb|KK920896.1|	562158-562866	nan	nan	2	0.778936	-1	0.40935	no
ni|645903534|nb|KK920896.1|	562987-563339	nan	nan	2	0.140495	-3	0.04415	no
ni|645903534|nb|KK920896.1|	563744-564129	nan	nan	7	7	-0.0672639	0.94635	no
ni|645903534|nb|KK920896.1|	565307-566032	nan	nan	65	63	-0.0422158	0.97685	no
ni|645903534|nb|KK920896.1|	566373-567108	nan	nan	6	9	0.451074	0.69955	no
ni|645903534|nb|KK920896.1|	567712-567845	nan	nan	11	18	0.712609	0.5132	no

ni|645903534|nb|KK920896.1|	569178-569605	nan	nan	5	7	0.42016	0.72065	no
ni|645903534|nb|KK920896.1|	570602-571482	nan	nan	0.268021	1	2	0.13545	no
ni|645903534|nb|KK920896.1|	571725-572171	nan	nan	6	5	-0.19702	0.8692	no
ni|645903534|nb|KK920896.1|	573139-573273	nan	nan	0.945995	2	1	0.2819	no
ni|645903534|nb|KK920896.1|	573658-573957	nan	nan	1	2	0.367876	0.75825	no
ni|645903534|nb|KK920896.1|	576065-576244	nan	nan	17	22	0.347231	0.76235	no
ni|645903534|nb|KK920896.1|	577897-578050	nan	nan	94	101.43	0.115563	0.91525	no
ni|645903534|nb|KK920896.1|	578236-578465	nan	nan	28	29	0.0626844	0.954	no
ni|645903534|nb|KK920896.1|	579053-579287	nan	nan	64	73	0.194687	0.86	no
ni|645903534|nb|KK920896.1|	587177-587818	nan	nan	59	54	-0.112489	0.9211	no
ni|645903534|nb|KK920896.1|	588855-589018	nan	nan	28	15	-0.929949	0.47045	no
ni|645903534|nb|KK920896.1|	589240-589867	nan	nan	29	17	-0.806449	0.48545	no
ni|645903534|nb|KK920896.1|	590418-591009	nan	nan	3	0.992338	-1	0.2534	no
ni|645903534|nb|KK920896.1|	591171-591483	nan	nan	91	55	-0.712304	0.5252	no
ni|645903534|nb|KK920896.1|	591947-592085	nan	nan	134	91	-0.559023	0.696	no
ni|645903534|nb|KK920896.1|	592412-592580	nan	nan	213	167	-0.347375	0.8102	no
ni|645903534|nb|KK920896.1|	592953-593208	nan	nan	53	46	-0.227918	0.8507	no
ni|645903534|nb|KK920896.1|	593367-593528	nan	nan	0.531617	0.781944	0.556678	0.656	no
ni|645903534|nb|KK920896.1|	593720-594201	nan	nan	1889.11	3553.09	0.911368	0.46335	no
ni|645903534|nb|KK920896.1|	599288-600041	nan	nan	2	2	-0.397747	0.73675	no
ni|645903534|nb|KK920896.1|	600142-600564	nan	nan	2	4	0.669724	0.65595	no
ni|645903534|nb|KK920896.1|	600693-601103	nan	nan	2	2	0.519688	0.6668	no
ni|645903534|nb|KK920896.1|	601378-602205	nan	nan	2664.2	4255.96	0.675783	0.5865	no
ni|645903534|nb|KK920896.1|	602764-603128	nan	nan	9	15	0.6787	0.5669	no
ni|645903534|nb|KK920896.1|	605254-605370	nan	nan	171	212	0.306402	0.83675	no
ni|645903534|nb|KK920896.1|	609848-610102	nan	nan	257	342	0.413045	0.74515	no
ni|645903534|nb|KK920896.1|	610723-610954	nan	nan	277	342	0.302934	0.8398	no
ni|645903534|nb|KK920896.1|	613809-614032	nan	nan	279.4	335	0.262381	0.86365	no
ni|645903534|nb|KK920896.1|	614699-615168	nan	nan	80	94	0.230162	0.87635	no
ni|645903534|nb|KK920896.1|	618524-618715	nan	nan	319.07	346	0.11635	0.93565	no

ni|645903534|nb|KK920896.1|	618841-619558	nan	nan	263	295	0.164895	0.91115	no
ni|645903534|nb|KK920896.1|	623480-623604	nan	nan	85	94	0.143721	0.92145	no
ni|645903534|nb|KK920896.1|	623884-624405	nan	nan	137	135	-0.0143654	0.9921	no
ni|645903534|nb|KK920896.1|	624660-625060	nan	nan	27	34	0.31941	0.8092	no
ni|645903534|nb|KK920896.1|	625866-626250	nan	nan	46	57	0.301656	0.79955	no
ni|645903534|nb|KK920896.1|	629837-630026	nan	nan	220	117	-0.916632	0.44755	no
ni|645903534|nb|KK920896.1|	66162-66465	nan	nan	9	3	-2	0.25505	no
ni|645903534|nb|KK920896.1|	66733-67495	nan	nan	2	3	0.585033	0.65495	no
ni|645903535|nb|KK920895.1|	126810-126992	nan	nan	27	25	-0.0793031	0.9429	no
ni|645903535|nb|KK920895.1|	132515-133186	nan	nan	14	6	-1	0.4103	no
ni|645903535|nb|KK920895.1|	134447-135298	nan	nan	2	2	-0.0652576	0.95705	no
ni|645903535|nb|KK920895.1|	136709-137441	nan	nan	15	10	-0.582046	0.70815	no
ni|645903535|nb|KK920895.1|	137519-137729	nan	nan	9	7	-0.393325	0.7282	no
ni|645903535|nb|KK920895.1|	137855-138061	nan	nan	61	6	-3	0.0217	no
ni|645903535|nb|KK920895.1|	153164-153570	nan	nan	12	12	0.0182364	0.98735	no
ni|645903535|nb|KK920895.1|	178652-178848	nan	nan	1	0.316445	-2	0.2092	no
ni|645903535|nb|KK920895.1|	180501-180827	nan	nan	2	0.627314	-2	0.22995	no
ni|645903535|nb|KK920895.1|	181204-181506	nan	nan	164	48	-2	0.23215	no
ni|645903535|nb|KK920895.1|	182859-183094	nan	nan	9	9	-0.0309251	0.97655	no
ni|645903535|nb|KK920895.1|	186617-186756	nan	nan	0.750472	0.425252	-0.81948	0.50325	no
ni|645903535|nb|KK920895.1|	189665-189905	nan	nan	0.725291	0.606489	-0.25808	0.8692	no
ni|645903535|nb|KK920895.1|	192451-192719	nan	nan	0.570618	0.727238	0.349903	0.792	no
ni|645903535|nb|KK920895.1|	197366-197589	nan	nan	14	16	0.14058	0.8984	no
ni|645903535|nb|KK920895.1|	208465-208864	nan	nan	16	5	-2	0.17365	no
ni|645903535|nb|KK920895.1|	209021-209806	nan	nan	4	2	-1	0.30585	no
ni|645903535|nb|KK920895.1|	222528-223039	nan	nan	62	20	-2	0.27265	no
ni|645903535|nb|KK920895.1|	224030-224136	nan	nan	33	12	-1	0.20835	no
ni|645903535|nb|KK920895.1|	231773-232462	nan	nan	3	1	-1	0.4023	no
ni|645903535|nb|KK920895.1|	233553-234140	nan	nan	23	17	-0.415247	0.71835	no
ni|645903535|nb|KK920895.1|	234350-234671	nan	nan	22	10	-1	0.3495	no

ni|645903535|nb|KK920895.1|	235089-235257	nan	nan	50	19	-1	0.2386	no
ni|645903535|nb|KK920895.1|	236582-236963	nan	nan	1	0.62866	-0.978821	0.4167	no
ni|645903535|nb|KK920895.1|	237454-237630	nan	nan	0.798052	0.610559	-0.386353	0.74155	no
ni|645903535|nb|KK920895.1|	238272-238662	nan	nan	1	0.852942	-0.590792	0.6233	no
ni|645903535|nb|KK920895.1|	238738-238945	nan	nan	11	13	0.222302	0.85195	no
ni|645903535|nb|KK920895.1|	374627-375381	nan	nan	0.599614	0.623324	0.0559472	0.96165	no
ni|645903535|nb|KK920895.1|	375489-375655	nan	nan	4	3	-0.614153	0.6288	no
ni|645903535|nb|KK920895.1|	376343-376542	nan	nan	23	19	-0.278088	0.8135	no
ni|645903535|nb|KK920895.1|	376629-377180	nan	nan	0.950861	0.709679	-0.422068	0.71885	no
ni|645903535|nb|KK920895.1|	395359-395554	nan	nan	3	3	-0.0104827	0.9922	no
ni|645903535|nb|KK920895.1|	395774-396269	nan	nan	21.95	22	0.0242481	0.98145	no
ni|645903535|nb|KK920895.1|	396995-397236	nan	nan	21	16	-0.352458	0.7507	no
ni|645903535|nb|KK920895.1|	397439-397723	nan	nan	21	23	0.128699	0.9117	no
ni|645903535|nb|KK920895.1|	398301-398889	nan	nan	28	23	-0.306197	0.7846	no
ni|645903535|nb|KK920895.1|	399043-399427	nan	nan	6	1	-2	0.24815	no
ni|645903535|nb|KK920895.1|	400081-400397	nan	nan	27	20	-0.398753	0.72255	no
ni|645903535|nb|KK920895.1|	401847-402151	nan	nan	6	4	-0.64998	0.5771	no
ni|645903535|nb|KK920895.1|	404903-405351	nan	nan	3	2	-0.651164	0.63225	no
ni|645903535|nb|KK920895.1|	405686-405831	nan	nan	2	2	-0.413912	0.76315	no
ni|645903535|nb|KK920895.1|	409311-409772	nan	nan	2	1	-0.613218	0.6288	no
ni|645903535|nb|KK920895.1|	410054-410400	nan	nan	13	36	2	0.28465	no
ni|645903535|nb|KK920895.1|	410600-410914	nan	nan	45	40	-0.182201	0.86925	no
ni|645903535|nb|KK920895.1|	421553-421900	nan	nan	38	29	-0.401824	0.7212	no
ni|645903535|nb|KK920895.1|	422498-423102	nan	nan	0.46018	0.476642	0.0507093	0.9572	no
ni|645903535|nb|KK920895.1|	423382-424142	nan	nan	10	14	0.575594	0.62455	no
ni|645903535|nb|KK920895.1|	424245-425218	nan	nan	45	47	0.0783884	0.94595	no
ni|645903535|nb|KK920895.1|	425966-426407	nan	nan	9	16	0.825884	0.49415	no
ni|645903535|nb|KK920895.1|	426664-428089	nan	nan	45	30	-0.576419	0.63215	no
ni|645903535|nb|KK920895.1|	428533-428905	nan	nan	13	16	0.216818	0.85565	no
ni|645903535|nb|KK920895.1|	430258-430672	nan	nan	34	27	-0.300233	0.8011	no

ni|645903535|nb|KK920895.1|	431156-431533	nan	nan	37	35.47	-0.0555058	0.9585	no
ni|645903535|nb|KK920895.1|	431814-432499	nan	nan	28	22	-0.393306	0.7382	no
ni|645903535|nb|KK920895.1|	450154-451321	nan	nan	40	37	-0.125603	0.91285	no
ni|645903535|nb|KK920895.1|	451599-451735	nan	nan	0.423902	0.472857	0.157675	0.9026	no
ni|645903535|nb|KK920895.1|	454253-454813	nan	nan	40	51	0.37336	0.7514	no
ni|645903535|nb|KK920895.1|	457598-457813	nan	nan	13	13	-0.0890997	0.9366	no
ni|645903535|nb|KK920895.1|	458621-458863	nan	nan	87	79	-0.144552	0.9201	no
ni|645903535|nb|KK920895.1|	459197-459526	nan	nan	82	91	0.146919	0.89525	no
ni|645903535|nb|KK920895.1|	464908-465063	nan	nan	345	350	0.0210088	0.9873	no
ni|645903535|nb|KK920895.1|	471829-472047	nan	nan	26	13	-1	0.4167	no
ni|645903535|nb|KK920895.1|	473818-474039	nan	nan	0.761743	0.286957	-1	0.26895	no
ni|645903535|nb|KK920895.1|	474532-475189	nan	nan	7	11	0.560793	0.667	no
ni|645903535|nb|KK920895.1|	475623-476291	nan	nan	2	1	-1	0.3535	no
ni|645903535|nb|KK920895.1|	476451-477350	nan	nan	0.062124	3	5	0.11945	no
ni|645903535|nb|KK920895.1|	477855-478087	nan	nan	0	4	inf	5.00E-05	yes
ni|645903535|nb|KK920895.1|	479659-479855	nan	nan	2	1	-0.686865	0.58765	no
ni|645903535|nb|KK920895.1|	481390-481572	nan	nan	2	1	-0.191367	0.88855	no
ni|645903535|nb|KK920895.1|	481835-482795	nan	nan	2	7	2	0.1528	no
ni|645903535|nb|KK920895.1|	483169-484081	nan	nan	196	119	-0.726861	0.61225	no
ni|645903535|nb|KK920895.1|	485466-485628	nan	nan	2	2	0.1519	0.8979	no
ni|645903535|nb|KK920895.1|	494491-494714	nan	nan	1	2	0.393459	0.77595	no
ni|645903535|nb|KK920895.1|	510875-511250	nan	nan	2	3	0.15518	0.9131	no
ni|645903535|nb|KK920895.1|	511301-511974	nan	nan	1	1	0.191721	0.875	no
ni|645903536|nb|KK920894.1|	1068412-107237[illegible]	nan	nan	12	11	-0.0654785	0.95415	no
ni|645903536|nb|KK920894.1|	1072486-107315[illegible]	nan	nan	2	2	-0.126828	0.91305	no
ni|645903536|nb|KK920894.1|	1073443-107383[illegible]	nan	nan	2	4	1	0.38715	no
ni|645903536|nb|KK920894.1|	275316-275447	nan	nan	1	2	0.705361	0.56825	no
ni|645903536|nb|KK920894.1|	286377-286564	nan	nan	9	18	1	0.40655	no
ni|645903536|nb|KK920894.1|	316601-316721	nan	nan	4	2	-0.591609	0.6117	no
ni|645903536|nb|KK920894.1|	318416-318605	nan	nan	11	7	-0.59772	0.62535	no

ni|645903536|nb|KK920894.1|	318946-319282	nan	nan	6	11	0.825884	0.51075	no
ni|645903536|nb|KK920894.1|	320222-320658	nan	nan	2	5	0.932893	0.4817	no
ni|645903536|nb|KK920894.1|	321599-322014	nan	nan	10	15	0.558399	0.693	no
ni|645903536|nb|KK920894.1|	34212-34546	nan	nan	1	0.821617	-0.82416	0.51705	no
ni|645903536|nb|KK920894.1|	342548-342965	nan	nan	0.025004	68	11	0.11935	no
ni|645903536|nb|KK920894.1|	359170-359386	nan	nan	0	156	inf	5.00E-05	yes
ni|645903536|nb|KK920894.1|	361704-361820	nan	nan	2	2	0.32882	0.7754	no
ni|645903536|nb|KK920894.1|	362236-362760	nan	nan	30	35	0.245964	0.83905	no
ni|645903536|nb|KK920894.1|	362953-363081	nan	nan	34	34	0.00645391	0.9732	no
ni|645903536|nb|KK920894.1|	363150-364970	nan	nan	45	64	0.51123	0.6438	no
ni|645903536|nb|KK920894.1|	365048-365506	nan	nan	43	73	0.760086	0.49505	no
ni|645903536|nb|KK920894.1|	367248-367446	nan	nan	61	84	0.465452	0.6876	no
ni|645903536|nb|KK920894.1|	373304-373472	nan	nan	2	2	-0.345931	0.78305	no
ni|645903536|nb|KK920894.1|	373802-375242	nan	nan	30	22	-0.469205	0.6732	no
ni|645903536|nb|KK920894.1|	375536-375757	nan	nan	2	1	-0.729651	0.53715	no
ni|645903536|nb|KK920894.1|	375979-376240	nan	nan	0	46	inf	5.00E-05	yes
ni|645903536|nb|KK920894.1|	377520-378221	nan	nan	6	2	-2	0.20985	no
ni|645903536|nb|KK920894.1|	379138-379361	nan	nan	0.40166	32	6	0.02725	no
ni|645903536|nb|KK920894.1|	383378-383674	nan	nan	0	49	inf	5.00E-05	yes
ni|645903536|nb|KK920894.1|	384381-384653	nan	nan	0.276339	68	8	0.0373	no
ni|645903536|nb|KK920894.1|	384891-385084	nan	nan	0	149	inf	5.00E-05	yes
ni|645903536|nb|KK920894.1|	386254-386583	nan	nan	0.371809	66	7	0.0333	no
ni|645903536|nb|KK920894.1|	387345-387824	nan	nan	0.417658	32	6	0.05055	no
ni|645903536|nb|KK920894.1|	389100-389311	nan	nan	0.723459	0.377187	-0.939633	0.54025	no
ni|645903536|nb|KK920894.1|	390521-390626	nan	nan	0	3	inf	5.00E-05	yes
ni|645903536|nb|KK920894.1|	391839-392060	nan	nan	9	11	0.222139	0.8747	no
ni|645903536|nb|KK920894.1|	392795-392983	nan	nan	0.420639	0.742524	0.819854	0.51315	no
ni|645903536|nb|KK920894.1|	395318-395972	nan	nan	15	9	-0.755258	0.5367	no
ni|645903536|nb|KK920894.1|	397662-398008	nan	nan	6	2	-2	0.18675	no
ni|645903536|nb|KK920894.1|	408226-408690	nan	nan	7	26	2	0.13505	no

ni|645903536|nb|KK920894.1|	454665-454835	nan	nan	0	35	inf	0.00065	yes
ni|645903536|nb|KK920894.1|	557922-558049	nan	nan	13	14	0.0954463	0.93605	no
ni|645903536|nb|KK920894.1|	565360-565874	nan	nan	2	1	-0.461267	0.6964	no
ni|645903536|nb|KK920894.1|	569868-570048	nan	nan	34	35	0.0168138	0.98785	no
ni|645903536|nb|KK920894.1|	571452-572087	nan	nan	17	18	0.119958	0.918	no
ni|645903536|nb|KK920894.1|	572544-572716	nan	nan	29	23	-0.322544	0.78335	no
ni|645903536|nb|KK920894.1|	573935-574360	nan	nan	21	30	0.494524	0.6793	no
ni|645903536|nb|KK920894.1|	575157-575301	nan	nan	15	20	0.402275	0.72895	no
ni|645903536|nb|KK920894.1|	575815-576847	nan	nan	9	3	-1	0.32095	no
ni|645903536|nb|KK920894.1|	578416-578550	nan	nan	11	6	-0.823535	0.47945	no
ni|645903536|nb|KK920894.1|	579207-579481	nan	nan	19	27	0.51776	0.6626	no
ni|645903536|nb|KK920894.1|	580782-580958	nan	nan	22	33	0.568445	0.602	no
ni|645903536|nb|KK920894.1|	581699-582416	nan	nan	41	64	0.668217	0.5513	no
ni|645903536|nb|KK920894.1|	633068-634351	nan	nan	6	9	0.665934	0.5549	no
ni|645903536|nb|KK920894.1|	642431-643128	nan	nan	23	42	0.863433	0.42365	no
ni|645903536|nb|KK920894.1|	643249-643709	nan	nan	0.604614	0.53675	-0.171763	0.89245	no
ni|645903536|nb|KK920894.1|	644170-645247	nan	nan	64	87	0.438864	0.6867	no
ni|645903536|nb|KK920894.1|	646528-646746	nan	nan	394	642	0.702547	0.6139	no
ni|645903536|nb|KK920894.1|	650130-650408	nan	nan	253	453	0.841155	0.5077	no
ni|645903536|nb|KK920894.1|	651469-651651	nan	nan	476	836	0.813258	0.5735	no
ni|645903536|nb|KK920894.1|	651773-651937	nan	nan	1	0.34155	-2	0.2004	no
ni|645903536|nb|KK920894.1|	652284-652427	nan	nan	28	37	0.408266	0.7	no
ni|645903536|nb|KK920894.1|	652806-652915	nan	nan	29	64	1	0.37985	no
ni|645903536|nb|KK920894.1|	653424-653702	nan	nan	69	110	0.665629	0.5524	no
ni|645903536|nb|KK920894.1|	654176-654639	nan	nan	2	0.862934	-0.880306	0.4725	no
ni|645903536|nb|KK920894.1|	655110-655240	nan	nan	31	33	0.0905951	0.93355	no
ni|645903536|nb|KK920894.1|	655661-655770	nan	nan	12	10	-0.260896	0.8221	no
ni|645903536|nb|KK920894.1|	655932-656062	nan	nan	39	60	0.62224	0.59665	no
ni|645903536|nb|KK920894.1|	656420-656861	nan	nan	56	101	0.839633	0.4743	no
ni|645903536|nb|KK920894.1|	656996-657164	nan	nan	25	19	-0.352635	0.7593	no

ni|645903536|nb|KK920894.1|	657745-658006	nan	nan	0.926018	0.465927	-0.990934	0.47555	no
ni|645903536|nb|KK920894.1|	658143-658489	nan	nan	2	1	-0.606315	0.6316	no
ni|645903536|nb|KK920894.1|	658690-659362	nan	nan	55	61	0.139292	0.89185	no
ni|645903536|nb|KK920894.1|	659862-660203	nan	nan	47	41	-0.207491	0.86085	no
ni|645903536|nb|KK920894.1|	660491-662437	nan	nan	3	1	-1	0.38105	no
ni|645903536|nb|KK920894.1|	662839-664029	nan	nan	307	214	-0.516809	0.72205	no
ni|645903536|nb|KK920894.1|	666329-666485	nan	nan	207	170	-0.284493	0.84675	no
ni|645903536|nb|KK920894.1|	666860-667022	nan	nan	18	15	-0.257022	0.82325	no
ni|645903536|nb|KK920894.1|	667361-668210	nan	nan	0.598302	0.444793	-0.427742	0.75965	no
ni|645903536|nb|KK920894.1|	668758-669218	nan	nan	1	0.925864	-0.306415	0.8123	no
ni|645903536|nb|KK920894.1|	669325-669522	nan	nan	3	3	-0.171458	0.8814	no
ni|645903536|nb|KK920894.1|	670593-670752	nan	nan	0.819608	0.569715	-0.524694	0.7071	no
ni|645903536|nb|KK920894.1|	672203-672672	nan	nan	0.379285	0.435226	0.198484	0.8731	no
ni|645903536|nb|KK920894.1|	673316-673711	nan	nan	25	31	0.306321	0.7873	no
ni|645903536|nb|KK920894.1|	674760-675052	nan	nan	0.55354	0.352742	-0.650074	0.6679	no
ni|645903536|nb|KK920894.1|	675438-675686	nan	nan	20	27	0.47123	0.68155	no
ni|645903536|nb|KK920894.1|	676322-676708	nan	nan	36	38	0.107695	0.92895	no
ni|645903536|nb|KK920894.1|	678065-678353	nan	nan	28	24	-0.219784	0.8865	no
ni|645903536|nb|KK920894.1|	678831-679267	nan	nan	0.679641	0.046409	-4	0.0255	no
ni|645903536|nb|KK920894.1|	679774-680227	nan	nan	3	3	-0.00341532	0.9925	no
ni|645903536|nb|KK920894.1|	680629-681240	nan	nan	10	20	0.915759	0.4372	no
ni|645903536|nb|KK920894.1|	682028-682560	nan	nan	106	139	0.390186	0.7348	no
ni|645903536|nb|KK920894.1|	682873-683308	nan	nan	20	29	0.524687	0.65305	no
ni|645903536|nb|KK920894.1|	684279-685585	nan	nan	124	133	0.107098	0.92185	no
ni|645903536|nb|KK920894.1|	685718-686357	nan	nan	0	11	inf	0.0003	yes
ni|645903536|nb|KK920894.1|	774329-774449	nan	nan	44	35	-0.338721	0.77845	no
ni|645903536|nb|KK920894.1|	836258-836478	nan	nan	9	15	0.760347	0.50235	no
ni|645903536|nb|KK920894.1|	843641-843737	nan	nan	42	58	0.480219	0.67505	no
ni|645903536|nb|KK920894.1|	858249-858529	nan	nan	9	9	0.0640367	0.95955	no
ni|645903536|nb|KK920894.1|	861673-861805	nan	nan	1	0.387848	-2	0.3149	no

ni|645903536|nb|KK920894.1|	862362-862526	nan	nan	0.014823	135	13	0.11935	no
ni|645903536|nb|KK920894.1|	866688-866815	nan	nan	4	5	0.277742	0.8144	no
ni|645903536|nb|KK920894.1|	867528-868750	nan	nan	6	7	0.199702	0.85595	no
ni|645903536|nb|KK920894.1|	869368-869841	nan	nan	2	4	1	0.38715	no
ni|645903536|nb|KK920894.1|	870640-871237	nan	nan	4	6	0.717153	0.5449	no
ni|645903536|nb|KK920894.1|	871319-871432	nan	nan	7	11	0.689917	0.55995	no
ni|645903536|nb|KK920894.1|	872196-872412	nan	nan	1	2	0.796933	0.53465	no
ni|645903537|nb|KK920893.1|	144701-145448	nan	nan	1	3	1	0.271	no
ni|645903537|nb|KK920893.1|	152593-152954	nan	nan	2	4	1	0.34405	no
ni|645903537|nb|KK920893.1|	270338-270939	nan	nan	92	76	-0.273054	0.8113	no
ni|645903537|nb|KK920893.1|	279280-279621	nan	nan	36	25	-0.549177	0.6122	no
ni|645903537|nb|KK920893.1|	330026-330555	nan	nan	2	0.589186	-2	0.18015	no
ni|645903537|nb|KK920893.1|	342274-342418	nan	nan	18	19	0.0811808	0.94545	no
ni|645903537|nb|KK920893.1|	342714-343589	nan	nan	2	0.883391	-1	0.40155	no
ni|645903537|nb|KK920893.1|	344928-345232	nan	nan	2	2	-0.139436	0.90955	no
ni|645903537|nb|KK920893.1|	346501-346696	nan	nan	3	1	-1	0.33495	no
ni|645903537|nb|KK920893.1|	347032-347296	nan	nan	25	15	-0.699785	0.54495	no
ni|645903537|nb|KK920893.1|	348190-348674	nan	nan	83	61	-0.455889	0.6824	no
ni|645903537|nb|KK920893.1|	348909-349034	nan	nan	2	1	-0.803778	0.6027	no
ni|645903537|nb|KK920893.1|	349357-349468	nan	nan	15	5	-2	0.24025	no
ni|645903537|nb|KK920893.1|	349519-349840	nan	nan	326	282	-0.207657	0.88995	no
ni|645903537|nb|KK920893.1|	350502-350942	nan	nan	0	34	inf	0.00055	yes
ni|645903537|nb|KK920893.1|	359625-359904	nan	nan	0.260803	45	7	0.0443	no
ni|645903537|nb|KK920893.1|	361193-361730	nan	nan	24	24	-0.0266731	0.9806	no
ni|645903537|nb|KK920893.1|	362107-362437	nan	nan	43	24	-0.820624	0.48805	no
ni|645903537|nb|KK920893.1|	365377-365538	nan	nan	2	2	-0.276897	0.8205	no
ni|645903537|nb|KK920893.1|	367169-367366	nan	nan	11	11	-0.00381734	0.99555	no
ni|645903537|nb|KK920893.1|	368873-369073	nan	nan	7	8	0.170786	0.8842	no
ni|645903537|nb|KK920893.1|	371320-371779	nan	nan	2	0.893549	-1	0.3464	no
ni|645903537|nb|KK920893.1|	372995-373143	nan	nan	0	209	inf	5.00E-05	yes

ni|645903537|nb|KK920893.1|	373333-373470	nan	nan	0	187	inf	5.00E-05	yes
ni|645903537|nb|KK920893.1|	377957-378115	nan	nan	3	4	0.718242	0.55985	no
ni|645903537|nb|KK920893.1|	419255-419478	nan	nan	14	9	-0.652213	0.5967	no
ni|645903537|nb|KK920893.1|	422544-423009	nan	nan	0.030443	206	13	0.05055	no
ni|645903537|nb|KK920893.1|	426829-427490	nan	nan	9	12	0.409235	0.74485	no
ni|645903537|nb|KK920893.1|	428368-428609	nan	nan	0.268004	0.224221	-0.257332	0.8692	no
ni|645903537|nb|KK920893.1|	428712-429162	nan	nan	9	10	0.0281187	0.98115	no
ni|645903537|nb|KK920893.1|	432519-432714	nan	nan	6	7	0.140378	0.90405	no
ni|645903537|nb|KK920893.1|	438943-439091	nan	nan	3	3	-0.355987	0.76085	no
ni|645903537|nb|KK920893.1|	448616-448914	nan	nan	4	4	-0.143195	0.90185	no
ni|645903537|nb|KK920893.1|	449833-450047	nan	nan	15	19	0.318418	0.77565	no
ni|645903537|nb|KK920893.1|	464408-464525	nan	nan	5	4	-0.0818983	0.94805	no
ni|645903537|nb|KK920893.1|	468506-468679	nan	nan	20	26	0.365605	0.7368	no
ni|645903537|nb|KK920893.1|	469789-470066	nan	nan	2	3	0.93408	0.46215	no
ni|645903537|nb|KK920893.1|	472042-472318	nan	nan	3	0.724575	-2	0.2305	no
ni|645903537|nb|KK920893.1|	472962-473439	nan	nan	45	36	-0.330742	0.797	no
ni|645903537|nb|KK920893.1|	487915-488070	nan	nan	0.52146	0.819085	0.651457	0.60985	no
ni|645903537|nb|KK920893.1|	504879-505416	nan	nan	12	15	0.269615	0.8184	no
ni|645903537|nb|KK920893.1|	506473-506766	nan	nan	43	46	0.0919787	0.9378	no
ni|645903537|nb|KK920893.1|	508336-508812	nan	nan	19	15	-0.313453	0.7811	no
ni|645903537|nb|KK920893.1|	519018-519511	nan	nan	6	3	-0.77265	0.52115	no
ni|645903537|nb|KK920893.1|	533344-533736	nan	nan	6	3	-1	0.31245	no
ni|645903537|nb|KK920893.1|	539197-539479	nan	nan	2	0.601017	-1	0.3177	no
ni|645903537|nb|KK920893.1|	541833-542053	nan	nan	4	4	-0.0653994	0.9515	no
ni|645903537|nb|KK920893.1|	546271-546451	nan	nan	5	7	0.633247	0.5763	no
ni|645903537|nb|KK920893.1|	554191-554409	nan	nan	3	3	0.0997486	0.9309	no
ni|645903538|nb|KK920892.1|	100794-101102	nan	nan	317	313	-0.0156495	0.9916	no
ni|645903538|nb|KK920892.1|	109891-110615	nan	nan	226	260	0.198386	0.89615	no
ni|645903538|nb|KK920892.1|	113269-114186	nan	nan	2	3	0.517713	0.7279	no
ni|645903538|nb|KK920892.1|	114477-114624	nan	nan	115	132	0.196116	0.89215	no

ni|645903538|nb|KK920892.1|	137968-138190	nan	nan	15	44	2	0.19575	no
ni|645903538|nb|KK920892.1|	141705-142096	nan	nan	6	17	1	0.23255	no
ni|645903538|nb|KK920892.1|	142855-143060	nan	nan	0	4	inf	5.00E-05	yes
ni|645903538|nb|KK920892.1|	143214-143459	nan	nan	74	167	1	0.3056	no
ni|645903538|nb|KK920892.1|	145002-145836	nan	nan	1	2	0.175233	0.8916	no
ni|645903538|nb|KK920892.1|	146177-146439	nan	nan	0.316101	3	3	0.05625	no
ni|645903538|nb|KK920892.1|	148652-149781	nan	nan	0	5	inf	5.00E-05	yes
ni|645903538|nb|KK920892.1|	165712-165861	nan	nan	0.7358	0.820178	0.156623	0.9131	no
ni|645903538|nb|KK920892.1|	166616-168037	nan	nan	0.769046	0.837571	0.123141	0.91925	no
ni|645903538|nb|KK920892.1|	168152-168390	nan	nan	63	127	0.997579	0.4303	no
ni|645903538|nb|KK920892.1|	168525-169194	nan	nan	157	222	0.493628	0.7253	no
ni|645903538|nb|KK920892.1|	169294-169614	nan	nan	0.454325	0.744453	0.712454	0.61075	no
ni|645903538|nb|KK920892.1|	186974-190858	nan	nan	38202.1	38278.4	0.00287981	0.9949	no
ni|645903538|nb|KK920892.1|	201641-202247	nan	nan	0	126	inf	5.00E-05	yes
ni|645903538|nb|KK920892.1|	202480-202637	nan	nan	0	2	inf	5.00E-05	yes
ni|645903538|nb|KK920892.1|	202747-202875	nan	nan	0	94	inf	5.00E-05	yes
ni|645903538|nb|KK920892.1|	204103-204242	nan	nan	2	4	0.95175	0.42085	no
ni|645903538|nb|KK920892.1|	204437-205221	nan	nan	0.395955	0.447415	0.176279	0.8916	no
ni|645903538|nb|KK920892.1|	20481-20955	nan	nan	55	118	1	0.3407	no
ni|645903538|nb|KK920892.1|	222563-223195	nan	nan	0.5496	0.262658	-1	0.44965	no
ni|645903538|nb|KK920892.1|	223322-224388	nan	nan	5	3	-0.526637	0.7071	no
ni|645903538|nb|KK920892.1|	22575-22759	nan	nan	38	70	0.876839	0.45465	no
ni|645903538|nb|KK920892.1|	232128-232478	nan	nan	1	0.840763	-0.658778	0.5773	no
ni|645903538|nb|KK920892.1|	232597-232880	nan	nan	45	25	-0.866839	0.4209	no
ni|645903538|nb|KK920892.1|	234003-236988	nan	nan	1	0.491158	-1	0.429	no
ni|645903538|nb|KK920892.1|	237305-237481	nan	nan	1	0.215595	-2	0.1791	no
ni|645903538|nb|KK920892.1|	238243-238923	nan	nan	2	1	-1	0.37855	no
ni|645903538|nb|KK920892.1|	239277-239466	nan	nan	28	17	-0.704785	0.568	no
ni|645903538|nb|KK920892.1|	239581-240166	nan	nan	68	43	-0.637481	0.57845	no
ni|645903538|nb|KK920892.1|	240478-240683	nan	nan	1	1	0.0951239	0.93935	no

ni|645903538|nb|KK920892.1|	241490-243755	nan	nan	1	1	0.0477298	0.96865	no
ni|645903538|nb|KK920892.1|	245930-246379	nan	nan	0	12	inf	0.0005	yes
ni|645903538|nb|KK920892.1|	246915-247142	nan	nan	33	10	-2	0.2179	no
ni|645903538|nb|KK920892.1|	248030-248217	nan	nan	116	66	-0.817484	0.56975	no
ni|645903538|nb|KK920892.1|	249134-251291	nan	nan	147	88	-0.745826	0.61435	no
ni|645903538|nb|KK920892.1|	252687-253345	nan	nan	41	24	-0.783754	0.53215	no
ni|645903538|nb|KK920892.1|	253560-253719	nan	nan	38	25	-0.614859	0.5689	no
ni|645903538|nb|KK920892.1|	285348-285585	nan	nan	1	0.76264	-0.693273	0.58855	no
ni|645903538|nb|KK920892.1|	287039-287342	nan	nan	0	53	inf	5.00E-05	yes
ni|645903538|nb|KK920892.1|	287482-287675	nan	nan	5	35	3	0.0439	no
ni|645903538|nb|KK920892.1|	292041-292232	nan	nan	15	27	0.8169	0.4627	no
ni|645903538|nb|KK920892.1|	321860-323508	nan	nan	2	2	0.0853546	0.9436	no
ni|645903538|nb|KK920892.1|	382046-382253	nan	nan	2	2	0.0336729	0.96125	no
ni|645903538|nb|KK920892.1|	45569-45821	nan	nan	25	46	0.894848	0.4141	no
ni|645903538|nb|KK920892.1|	47296-47589	nan	nan	42	41	-0.016645	0.99	no
ni|645903538|nb|KK920892.1|	47986-48634	nan	nan	25	26	0.0702748	0.9493	no
ni|645903538|nb|KK920892.1|	48897-49005	nan	nan	30	24	-0.304234	0.79655	no
ni|645903538|nb|KK920892.1|	51419-52226	nan	nan	29	35	0.278533	0.8046	no
ni|645903538|nb|KK920892.1|	52861-53601	nan	nan	34	27	-0.350344	0.75965	no
ni|645903538|nb|KK920892.1|	54379-54564	nan	nan	32	32	-0.0147488	0.9885	no
ni|645903538|nb|KK920892.1|	54689-55240	nan	nan	2	0	-inf	0.00455	no
ni|645903538|nb|KK920892.1|	55505-55661	nan	nan	23	35	0.636369	0.59155	no
ni|645903538|nb|KK920892.1|	56435-56861	nan	nan	20	27	0.421422	0.71925	no
ni|645903538|nb|KK920892.1|	65660-65800	nan	nan	17	56	2	0.14325	no
ni|645903538|nb|KK920892.1|	89065-89208	nan	nan	34	37	0.100408	0.9265	no
ni|645903538|nb|KK920892.1|	89410-89579	nan	nan	32	38	0.27566	0.8102	no
ni|645903538|nb|KK920892.1|	89761-90464	nan	nan	12	42	2	0.12595	no
ni|645903538|nb|KK920892.1|	90614-90786	nan	nan	63	66	0.0834119	0.9373	no
ni|645903538|nb|KK920892.1|	90963-91076	nan	nan	25	35	0.486069	0.68135	no
ni|645903538|nb|KK920892.1|	91178-91370	nan	nan	29	30	0.0491096	0.96395	no

ni|645903538|nb|KK920892.1|	91535-91752	nan	nan	31	67	1	0.37255	no
ni|645903538|nb|KK920892.1|	91939-92901	nan	nan	36	35	-0.0409906	0.9712	no
ni|645903539|nb|KK920891.1|	10429-11422	nan	nan	1392.63	2422.29	0.798557	0.58415	no
ni|645903539|nb|KK920891.1|	108074-108229	nan	nan	0.704452	0.642958	-0.131777	0.909	no
ni|645903539|nb|KK920891.1|	11736-11928	nan	nan	3	3	0.102675	0.93335	no
ni|645903539|nb|KK920891.1|	12660-12831	nan	nan	0	4	inf	5.00E-05	yes
ni|645903539|nb|KK920891.1|	130910-131465	nan	nan	59	50	-0.245222	0.8671	no
ni|645903539|nb|KK920891.1|	144224-144461	nan	nan	534	322	-0.731716	0.60505	no
ni|645903539|nb|KK920891.1|	145152-145282	nan	nan	1138.13	649	-0.810852	0.57495	no
ni|645903539|nb|KK920891.1|	15041-15707	nan	nan	4	21	2	0.0972	no
ni|645903539|nb|KK920891.1|	163557-165544	nan	nan	3	2	-0.248361	0.82725	no
ni|645903539|nb|KK920891.1|	16416-16676	nan	nan	0.973223	0.619963	-0.650588	0.6679	no
ni|645903539|nb|KK920891.1|	166605-167737	nan	nan	894	576	-0.634697	0.6624	no
ni|645903539|nb|KK920891.1|	16843-16971	nan	nan	365	595.04	0.705202	0.61295	no
ni|645903539|nb|KK920891.1|	168709-169351	nan	nan	0.58547	0.349738	-0.74332	0.59615	no
ni|645903539|nb|KK920891.1|	169995-170925	nan	nan	890	562	-0.66133	0.53575	no
ni|645903539|nb|KK920891.1|	171032-171620	nan	nan	3	2	-0.579848	0.62945	no
ni|645903539|nb|KK920891.1|	172569-172919	nan	nan	388	243	-0.673451	0.6237	no
ni|645903539|nb|KK920891.1|	175119-176029	nan	nan	3	1	-1	0.312	no
ni|645903539|nb|KK920891.1|	17582-17895	nan	nan	4	10	1	0.3349	no
ni|645903539|nb|KK920891.1|	176698-178775	nan	nan	1380.77	851	-0.697912	0.5776	no
ni|645903539|nb|KK920891.1|	178898-179925	nan	nan	9	7	-0.404285	0.73465	no
ni|645903539|nb|KK920891.1|	18083-18753	nan	nan	9	34	2	0.11555	no
ni|645903539|nb|KK920891.1|	183373-183517	nan	nan	6	4	-0.514661	0.68825	no
ni|645903539|nb|KK920891.1|	19002-19311	nan	nan	0.504799	1	2	0.28525	no
ni|645903539|nb|KK920891.1|	190636-191053	nan	nan	5	3	-0.756482	0.5195	no
ni|645903539|nb|KK920891.1|	197708-197833	nan	nan	631	375	-0.752163	0.5909	no
ni|645903539|nb|KK920891.1|	200802-200971	nan	nan	72	86	0.262362	0.8183	no
ni|645903539|nb|KK920891.1|	21314-21477	nan	nan	2	3	0.851926	0.489	no
ni|645903539|nb|KK920891.1|	228378-228661	nan	nan	16	17	0.0620467	0.9559	no

ni|645903539|nb|KK920891.1|	231935-232047	nan	nan	6	9	0.688549	0.5611	no
ni|645903539|nb|KK920891.1|	241018-241129	nan	nan	33	15	-1	0.3267	no
ni|645903539|nb|KK920891.1|	252856-253068	nan	nan	28	13	-1	0.36465	no
ni|645903539|nb|KK920891.1|	253798-253932	nan	nan	6	4	-0.708542	0.542	no
ni|645903539|nb|KK920891.1|	254047-254630	nan	nan	4	4	-0.237119	0.8697	no
ni|645903539|nb|KK920891.1|	254804-255315	nan	nan	3	2	-0.801757	0.4999	no
ni|645903539|nb|KK920891.1|	255990-256215	nan	nan	3	1	-0.915252	0.53575	no
ni|645903539|nb|KK920891.1|	256927-257089	nan	nan	3	2	-0.34539	0.7733	no
ni|645903539|nb|KK920891.1|	257212-257398	nan	nan	25	12	-1	0.3691	no
ni|645903539|nb|KK920891.1|	257885-258064	nan	nan	14	8	-0.85542	0.46345	no
ni|645903539|nb|KK920891.1|	291878-292315	nan	nan	2	1	-0.313363	0.8025	no
ni|645903539|nb|KK920891.1|	292512-292654	nan	nan	4	2	-0.947859	0.4187	no
ni|645903539|nb|KK920891.1|	301657-302030	nan	nan	7	4	-0.754891	0.52225	no
ni|645903539|nb|KK920891.1|	306384-307010	nan	nan	5	2	-1	0.31205	no
ni|645903539|nb|KK920891.1|	307397-307637	nan	nan	10	6	-0.79841	0.4951	no
ni|645903539|nb|KK920891.1|	307856-308245	nan	nan	2	1	-0.448315	0.708	no
ni|645903539|nb|KK920891.1|	308736-308879	nan	nan	2	0.979008	-0.760465	0.51485	no
ni|645903539|nb|KK920891.1|	309353-310038	nan	nan	46	24	-0.934307	0.4211	no
ni|645903539|nb|KK920891.1|	310252-310392	nan	nan	6	11	0.904555	0.44725	no
ni|645903539|nb|KK920891.1|	310662-310815	nan	nan	41	36	-0.180489	0.8766	no
ni|645903539|nb|KK920891.1|	311488-311848	nan	nan	11	26	1	0.2776	no
ni|645903539|nb|KK920891.1|	311975-312240	nan	nan	3	7	1	0.31765	no
ni|645903539|nb|KK920891.1|	312344-313149	nan	nan	1	0.598993	-1	0.46615	no
ni|645903539|nb|KK920891.1|	313582-313881	nan	nan	8	24	2	0.2078	no
ni|645903539|nb|KK920891.1|	317040-317458	nan	nan	9	24	1	0.22795	no
ni|645903539|nb|KK920891.1|	318808-319006	nan	nan	2	7	2	0.06905	no
ni|645903539|nb|KK920891.1|	321233-321542	nan	nan	5	3	-1	0.40475	no
ni|645903539|nb|KK920891.1|	326889-327090	nan	nan	6	3	-0.992148	0.4254	no
ni|645903539|nb|KK920891.1|	335154-335802	nan	nan	2	0.810407	-1	0.3942	no
ni|645903539|nb|KK920891.1|	340940-341293	nan	nan	6	4	-0.526475	0.67145	no

ni|645903539|nb|KK920891.1|	345781-345895	nan	nan	6	3	-0.767193	0.5318	no
ni|645903539|nb|KK920891.1|	346598-346768	nan	nan	1	2	0.705418	0.56825	no
ni|645903539|nb|KK920891.1|	346985-347537	nan	nan	2	4	0.593828	0.61625	no
ni|645903539|nb|KK920891.1|	347814-348036	nan	nan	0.274381	0.585327	1	0.38625	no
ni|645903539|nb|KK920891.1|	348305-349369	nan	nan	0.230904	0.397346	0.783102	0.58145	no
ni|645903539|nb|KK920891.1|	349784-349963	nan	nan	15	8	-0.864958	0.4622	no
ni|645903539|nb|KK920891.1|	350345-350746	nan	nan	29	24	-0.28148	0.81645	no
ni|645903539|nb|KK920891.1|	354378-354516	nan	nan	17	14	-0.359467	0.74935	no
ni|645903539|nb|KK920891.1|	354624-354913	nan	nan	36	24	-0.568503	0.62245	no
ni|645903539|nb|KK920891.1|	355762-355918	nan	nan	15	19	0.316057	0.7827	no
ni|645903539|nb|KK920891.1|	357219-357355	nan	nan	34	21	-0.695083	0.5471	no
ni|645903539|nb|KK920891.1|	358643-358813	nan	nan	16	11	-0.563885	0.6264	no
ni|645903539|nb|KK920891.1|	370064-370494	nan	nan	9	5	-0.713302	0.55545	no
ni|645903539|nb|KK920891.1|	371610-371788	nan	nan	4	3	-0.445727	0.74215	no
ni|645903539|nb|KK920891.1|	372131-372926	nan	nan	2	2	-0.406125	0.73125	no
ni|645903539|nb|KK920891.1|	373087-374469	nan	nan	3	2	-0.535941	0.67295	no
ni|645903539|nb|KK920891.1|	377102-377901	nan	nan	3	2	-0.831975	0.5313	no
ni|645903539|nb|KK920891.1|	378154-378503	nan	nan	3	2	-0.375791	0.75525	no
ni|645903539|nb|KK920891.1|	379967-381042	nan	nan	4	3	-0.389155	0.78555	no
ni|645903539|nb|KK920891.1|	381271-381664	nan	nan	4	2	-0.923745	0.44965	no
ni|645903539|nb|KK920891.1|	382182-382477	nan	nan	91	75	-0.279616	0.82035	no
ni|645903539|nb|KK920891.1|	383052-384125	nan	nan	1	0.774095	-0.428273	0.75965	no
ni|645903539|nb|KK920891.1|	384839-386457	nan	nan	96	80	-0.262981	0.82095	no
ni|645903539|nb|KK920891.1|	38885-39080	nan	nan	6	10	0.705667	0.55055	no
ni|645903539|nb|KK920891.1|	389219-389423	nan	nan	4	6	0.600613	0.6191	no
ni|645903539|nb|KK920891.1|	4091-4239	nan	nan	2	1	-0.514555	0.66435	no
ni|645903539|nb|KK920891.1|	447255-447683	nan	nan	110	98	-0.160766	0.8913	no
ni|645903539|nb|KK920891.1|	454213-454418	nan	nan	25	23	-0.117756	0.91685	no
ni|645903539|nb|KK920891.1|	456014-456423	nan	nan	73	70	-0.0521336	0.96185	no
ni|645903539|nb|KK920891.1|	457177-458602	nan	nan	36	44	0.26671	0.81685	no

ni|645903539|nb|KK920891.1|	459001-459582	nan	nan	24	26	0.145134	0.89945	no
ni|645903539|nb|KK920891.1|	461343-462457	nan	nan	2	1	-0.355676	0.7931	no
ni|645903539|nb|KK920891.1|	462631-463470	nan	nan	2	22	4	0.0514	no
ni|645903539|nb|KK920891.1|	463727-465204	nan	nan	1	7	3	0.06165	no
ni|645903539|nb|KK920891.1|	466450-466732	nan	nan	0.405622	1	2	0.18925	no
ni|645903539|nb|KK920891.1|	468409-468939	nan	nan	63	64	0.0254961	0.98005	no
ni|645903539|nb|KK920891.1|	471541-472248	nan	nan	12	15	0.329109	0.77305	no
ni|645903539|nb|KK920891.1|	472943-473476	nan	nan	0.498798	0.63575	0.350004	0.792	no
ni|645903539|nb|KK920891.1|	473821-474130	nan	nan	6	6	0.100105	0.934	no
ni|645903539|nb|KK920891.1|	476089-476233	nan	nan	5	5	-0.0672639	0.9302	no
ni|645903539|nb|KK920891.1|	477043-477392	nan	nan	30	27	-0.160344	0.8914	no
ni|645903539|nb|KK920891.1|	477829-478507	nan	nan	15	13	-0.15909	0.88335	no
ni|645903539|nb|KK920891.1|	479129-479288	nan	nan	32	36	0.180683	0.87665	no
ni|645903539|nb|KK920891.1|	480705-480814	nan	nan	1	2	0.85178	0.521	no
ni|645903539|nb|KK920891.1|	480969-482727	nan	nan	2	2	0.0709527	0.94355	no
ni|645903539|nb|KK920891.1|	483327-483703	nan	nan	0.431608	0.412561	-0.065113	0.9302	no
ni|645903539|nb|KK920891.1|	483943-484301	nan	nan	0	2	inf	0.0005	yes
ni|645903539|nb|KK920891.1|	5023-5199	nan	nan	16	19	0.212677	0.83495	no
ni|645903539|nb|KK920891.1|	5444-6243	nan	nan	3	2	-0.543226	0.64605	no
ni|645903539|nb|KK920891.1|	55299-55442	nan	nan	0.968979	1	0.464894	0.7264	no
ni|645903539|nb|KK920891.1|	55634-56233	nan	nan	0.079812	7	6	0.11935	no
ni|645903539|nb|KK920891.1|	56313-56608	nan	nan	4	2	-1	0.3864	no
ni|645903539|nb|KK920891.1|	56662-56785	nan	nan	2	2	0.348355	0.792	no
ni|645903539|nb|KK920891.1|	57040-57280	nan	nan	7	14	1	0.3794	no
ni|645903539|nb|KK920891.1|	57974-58318	nan	nan	0.722566	6	3	0.0683	no
ni|645903539|nb|KK920891.1|	59607-63105	nan	nan	0.946622	1	0.419017	0.75735	no
ni|645903539|nb|KK920891.1|	6361-7143	nan	nan	4	4	0.104545	0.9245	no
ni|645903539|nb|KK920891.1|	63986-64252	nan	nan	5	4	-0.245765	0.8305	no
ni|645903539|nb|KK920891.1|	68066-68473	nan	nan	1	1	-0.217717	0.87705	no
ni|645903539|nb|KK920891.1|	69891-71044	nan	nan	5	3	-1	0.3866	no

ni|645903539|nb|KK920891.1|	71400-71802	nan	nan	2	4	0.750136	0.52925	no
ni|645903539|nb|KK920891.1|	7260-7418	nan	nan	2	2	0.321824	0.7924	no
ni|645903539|nb|KK920891.1|	7589-9203	nan	nan	3	1	-1	0.40265	no
ni|645903539|nb|KK920891.1|	76396-76550	nan	nan	3	9	1	0.2232	no
ni|645903539|nb|KK920891.1|	80025-81482	nan	nan	113	70	-0.695923	0.52925	no
ni|645903539|nb|KK920891.1|	81937-83265	nan	nan	106	82	-0.376989	0.723	no
ni|645903539|nb|KK920891.1|	9423-9565	nan	nan	4	4	-0.0660727	0.95525	no
ni|645903540|nb|KK920890.1|	182496-182752	nan	nan	0	2	inf	0.0017	yes
ni|645903540|nb|KK920890.1|	300178-300368	nan	nan	43	28	-0.617565	0.60395	no
ni|645903540|nb|KK920890.1|	326759-326866	nan	nan	23	19	-0.227735	0.83935	no
ni|645903540|nb|KK920890.1|	327570-328182	nan	nan	25	16	-0.652252	0.58285	no
ni|645903540|nb|KK920890.1|	328293-329232	nan	nan	111	89	-0.306879	0.78785	no
ni|645903540|nb|KK920890.1|	330502-331110	nan	nan	0.632211	0.604492	-0.0646835	0.95835	no
ni|645903540|nb|KK920890.1|	331280-331447	nan	nan	1	0.475753	-2	0.3149	no
ni|645903540|nb|KK920890.1|	331812-332198	nan	nan	0.384744	0.306635	-0.327378	0.7838	no
ni|645903540|nb|KK920890.1|	332294-332552	nan	nan	49	34	-0.533275	0.6237	no
ni|645903540|nb|KK920890.1|	332895-333221	nan	nan	2	2	0.0490062	0.95725	no
ni|645903540|nb|KK920890.1|	337812-338055	nan	nan	64	49	-0.381721	0.73735	no
ni|645903540|nb|KK920890.1|	339645-339860	nan	nan	23	17	-0.46151	0.68055	no
ni|645903540|nb|KK920890.1|	343550-344231	nan	nan	3	2	-0.349449	0.77265	no
ni|645903540|nb|KK920890.1|	345016-345269	nan	nan	2	2	-0.346104	0.78305	no
ni|645903540|nb|KK920890.1|	356025-356960	nan	nan	0.817829	0.558152	-0.551141	0.71905	no
ni|645903540|nb|KK920890.1|	358761-358964	nan	nan	2	1	-0.457642	0.7095	no
ni|645903541|nb|KK920889.1|	101776-101999	nan	nan	236	104	-1	0.4312	no
ni|645903541|nb|KK920889.1|	104455-104661	nan	nan	58	22	-1	0.29085	no
ni|645903541|nb|KK920889.1|	106954-107106	nan	nan	76	19	-2	0.1021	no
ni|645903541|nb|KK920889.1|	113835-113987	nan	nan	11	8	-0.543738	0.64955	no
ni|645903541|nb|KK920889.1|	114992-115189	nan	nan	4	0.322206	-4	0.23575	no
ni|645903541|nb|KK920889.1|	117706-118165	nan	nan	6	0.236816	-5	0.0191	no
ni|645903541|nb|KK920889.1|	119279-119840	nan	nan	1	1	-0.14488	0.89955	no

ni|645903541|nb|KK920889.1|	121094-121280	nan	nan	110	68	-0.701048	0.5281	no
ni|645903541|nb|KK920889.1|	122344-122506	nan	nan	54	45	-0.248928	0.86225	no
ni|645903541|nb|KK920889.1|	122573-122872	nan	nan	15	14	-0.0671944	0.9517	no
ni|645903541|nb|KK920889.1|	123843-124194	nan	nan	4	2	-0.830887	0.48175	no
ni|645903541|nb|KK920889.1|	124270-124537	nan	nan	11	7	-0.652197	0.5856	no
ni|645903541|nb|KK920889.1|	125015-125369	nan	nan	56	33	-0.79577	0.4889	no
ni|645903541|nb|KK920889.1|	125456-125623	nan	nan	30	24	-0.299484	0.78435	no
ni|645903541|nb|KK920889.1|	126997-127163	nan	nan	51	40	-0.356375	0.81315	no
ni|645903541|nb|KK920889.1|	127716-128322	nan	nan	19	17	-0.177232	0.87695	no
ni|645903541|nb|KK920889.1|	128639-129120	nan	nan	0.366286	0.350124	-0.0651053	0.923	no
ni|645903541|nb|KK920889.1|	129245-129487	nan	nan	0.388565	0.350935	-0.146951	0.9109	no
ni|645903541|nb|KK920889.1|	129635-129895	nan	nan	3	11	2	0.12695	no
ni|645903541|nb|KK920889.1|	130148-130418	nan	nan	0.204958	3	4	0.05425	no
ni|645903541|nb|KK920889.1|	130648-131175	nan	nan	31	11	-2	0.3149	no
ni|645903541|nb|KK920889.1|	131629-132045	nan	nan	4	3	-0.133284	0.9085	no
ni|645903541|nb|KK920889.1|	133610-133787	nan	nan	19	16	-0.218312	0.8494	no
ni|645903541|nb|KK920889.1|	140182-140297	nan	nan	33	26	-0.327753	0.78315	no
ni|645903541|nb|KK920889.1|	141171-141851	nan	nan	31	27	-0.221564	0.84695	no
ni|645903541|nb|KK920889.1|	1436-1849	nan	nan	0.785421	6	3	0.0437	no
ni|645903541|nb|KK920889.1|	145664-146013	nan	nan	11	9	-0.275777	0.8104	no
ni|645903541|nb|KK920889.1|	146308-146464	nan	nan	22	25	0.188	0.8705	no
ni|645903541|nb|KK920889.1|	148457-148887	nan	nan	4	5	0.361604	0.7645	no
ni|645903541|nb|KK920889.1|	149091-149554	nan	nan	0.972833	0.612014	-0.668627	0.5839	no
ni|645903541|nb|KK920889.1|	149792-149981	nan	nan	2	0.976371	-0.717323	0.57	no
ni|645903541|nb|KK920889.1|	150139-150291	nan	nan	0.945777	0.417212	-1	0.42295	no
ni|645903541|nb|KK920889.1|	150724-150962	nan	nan	1	1	0.296441	0.8383	no
ni|645903541|nb|KK920889.1|	151252-153666	nan	nan	0.683194	0.587708	-0.217197	0.87705	no
ni|645903541|nb|KK920889.1|	153982-154158	nan	nan	0.426824	0.453383	0.0870884	0.9436	no
ni|645903541|nb|KK920889.1|	154985-155245	nan	nan	0.834342	0.442962	-0.913455	0.53575	no
ni|645903541|nb|KK920889.1|	157537-158219	nan	nan	0.447488	3	3	0.06665	no

ni|645903541|nb|KK920889.1|	159030-159192	nan	nan	3	4	0.167226	0.884	no
ni|645903541|nb|KK920889.1|	159695-159824	nan	nan	0.921326	0.135477	-3	0.2592	no
ni|645903541|nb|KK920889.1|	159891-159997	nan	nan	7	7	-0.0163672	0.98915	no
ni|645903541|nb|KK920889.1|	160136-160259	nan	nan	0.386217	0.263711	-0.550457	0.71905	no
ni|645903541|nb|KK920889.1|	160885-161160	nan	nan	24	18	-0.372063	0.7474	no
ni|645903541|nb|KK920889.1|	161287-161642	nan	nan	22	16	-0.507814	0.6621	no
ni|645903541|nb|KK920889.1|	161858-162037	nan	nan	5	4	-0.327752	0.77975	no
ni|645903541|nb|KK920889.1|	162515-162883	nan	nan	0.818207	1	0.3755	0.76185	no
ni|645903541|nb|KK920889.1|	163432-163797	nan	nan	15	15	0.0409803	0.97115	no
ni|645903541|nb|KK920889.1|	163979-164420	nan	nan	2	2	0.224818	0.8498	no
ni|645903541|nb|KK920889.1|	168070-169414	nan	nan	50.72	49	-0.0610867	0.9552	no
ni|645903541|nb|KK920889.1|	169560-169813	nan	nan	135	124.94	-0.113833	0.91765	no
ni|645903541|nb|KK920889.1|	170074-170601	nan	nan	10	8	-0.329683	0.7757	no
ni|645903541|nb|KK920889.1|	170734-170848	nan	nan	9	10	0.125226	0.92845	no
ni|645903541|nb|KK920889.1|	172253-172471	nan	nan	238	192	-0.311059	0.8288	no
ni|645903541|nb|KK920889.1|	174790-174917	nan	nan	1	0.744502	-0.965447	0.4448	no
ni|645903541|nb|KK920889.1|	175481-175713	nan	nan	0.418891	0.350385	-0.257634	0.8692	no
ni|645903541|nb|KK920889.1|	17671-17914	nan	nan	7	11	0.540445	0.64045	no
ni|645903541|nb|KK920889.1|	18457-19284	nan	nan	8	8	0.0175257	0.9866	no
ni|645903541|nb|KK920889.1|	20420-20799	nan	nan	18	26	0.523332	0.7187	no
ni|645903541|nb|KK920889.1|	2384-2588	nan	nan	2	1	-0.946484	0.43225	no
ni|645903541|nb|KK920889.1|	256408-256576	nan	nan	0.819231	0.117486	-3	0.1498	no
ni|645903541|nb|KK920889.1|	271089-272483	nan	nan	1	0.170775	-3	0.2572	no
ni|645903541|nb|KK920889.1|	272688-273227	nan	nan	250	179.98	-0.475816	0.7546	no
ni|645903541|nb|KK920889.1|	273363-273470	nan	nan	69	48	-0.524053	0.73215	no
ni|645903541|nb|KK920889.1|	273574-274245	nan	nan	78	76	-0.0288934	0.9807	no
ni|645903541|nb|KK920889.1|	274382-274517	nan	nan	1	0.749527	-0.717743	0.61925	no
ni|645903541|nb|KK920889.1|	274616-274855	nan	nan	7	11	0.573295	0.6325	no
ni|645903541|nb|KK920889.1|	275220-275371	nan	nan	22	19	-0.278289	0.80225	no
ni|645903541|nb|KK920889.1|	276904-277311	nan	nan	56	53	-0.0905031	0.9493	no

ni|645903541|nb|KK920889.1|	288171-288424	nan	nan	15	19	0.308248	0.779	no
ni|645903541|nb|KK920889.1|	289933-290108	nan	nan	2	2	-0.181458	0.889	no
ni|645903541|nb|KK920889.1|	294338-294776	nan	nan	2	0.735358	-1	0.3479	no
ni|645903541|nb|KK920889.1|	296816-297012	nan	nan	114.3	156	0.44686	0.6849	no
ni|645903541|nb|KK920889.1|	299051-299211	nan	nan	5	5	-0.11035	0.92465	no
ni|645903541|nb|KK920889.1|	304542-304700	nan	nan	1	0.710054	-0.802747	0.6027	no
ni|645903541|nb|KK920889.1|	308336-308615	nan	nan	2	2	-0.539345	0.6621	no
ni|645903541|nb|KK920889.1|	309797-309915	nan	nan	52	68	0.398789	0.782	no
ni|645903541|nb|KK920889.1|	312178-313915	nan	nan	288	325	0.171874	0.9072	no
ni|645903541|nb|KK920889.1|	318232-319084	nan	nan	176	179	0.0228907	0.9848	no
ni|645903541|nb|KK920889.1|	320085-320787	nan	nan	9	16	0.825665	0.5554	no
ni|645903541|nb|KK920889.1|	321127-321267	nan	nan	1	0.600304	-0.775549	0.5539	no
ni|645903541|nb|KK920889.1|	326332-326548	nan	nan	2	1	-1	0.43485	no
ni|645903541|nb|KK920889.1|	327328-327541	nan	nan	19	17	-0.178059	0.87045	no
ni|645903541|nb|KK920889.1|	331008-331249	nan	nan	72	66	-0.131973	0.9116	no
ni|645903541|nb|KK920889.1|	334427-334613	nan	nan	2	0.619101	-2	0.25615	no
ni|645903541|nb|KK920889.1|	336964-337131	nan	nan	0.955944	1	0.275898	0.821	no
ni|645903541|nb|KK920889.1|	338159-338380	nan	nan	14	17	0.251765	0.8269	no
ni|645903541|nb|KK920889.1|	341566-341712	nan	nan	15	13	-0.198568	0.868	no
ni|645903541|nb|KK920889.1|	346109-346312	nan	nan	2	2	-0.328505	0.7854	no
ni|645903541|nb|KK920889.1|	3563-3724	nan	nan	1	5	2	0.136	no
ni|645903541|nb|KK920889.1|	357021-357164	nan	nan	9	11	0.236259	0.84155	no
ni|645903541|nb|KK920889.1|	364017-364208	nan	nan	0.774236	1	0.93417	0.5295	no
ni|645903541|nb|KK920889.1|	369642-369888	nan	nan	29	33	0.207581	0.85565	no
ni|645903541|nb|KK920889.1|	370039-370447	nan	nan	16	10	-0.657373	0.55975	no
ni|645903541|nb|KK920889.1|	374281-374526	nan	nan	0.665156	0.440214	-0.595486	0.64595	no
ni|645903541|nb|KK920889.1|	375341-375595	nan	nan	6	10	0.685112	0.5635	no
ni|645903541|nb|KK920889.1|	376787-377234	nan	nan	5	0.865548	-2	0.2949	no
ni|645903541|nb|KK920889.1|	37775-38385	nan	nan	2	3	0.161843	0.88545	no
ni|645903541|nb|KK920889.1|	383201-383654	nan	nan	11	8	-0.537194	0.61695	no

ni|645903541|nb|KK920889.1|	387605-387770	nan	nan	9	5	-0.892104	0.44755	no
ni|645903541|nb|KK920889.1|	392051-392739	nan	nan	8	6	-0.478411	0.6597	no
ni|645903541|nb|KK920889.1|	393265-393688	nan	nan	15	5	-2	0.2178	no
ni|645903541|nb|KK920889.1|	393753-393948	nan	nan	16	10	-0.676516	0.5881	no
ni|645903541|nb|KK920889.1|	394219-394348	nan	nan	10	10	-0.00432569	0.99645	no
ni|645903541|nb|KK920889.1|	399766-399973	nan	nan	12	9	-0.371254	0.73395	no
ni|645903541|nb|KK920889.1|	401858-402132	nan	nan	21	22	0.0640811	0.95735	no
ni|645903541|nb|KK920889.1|	40320-40784	nan	nan	6	6	0.00263096	0.98695	no
ni|645903541|nb|KK920889.1|	403869-404677	nan	nan	4	9	1	0.288	no
ni|645903541|nb|KK920889.1|	408660-408945	nan	nan	5	18	2	0.13125	no
ni|645903541|nb|KK920889.1|	410670-411906	nan	nan	1	1	0.223846	0.8598	no
ni|645903541|nb|KK920889.1|	412119-412319	nan	nan	75	56	-0.419402	0.76815	no
ni|645903541|nb|KK920889.1|	413101-413359	nan	nan	60	42	-0.509608	0.6493	no
ni|645903541|nb|KK920889.1|	42403-43420	nan	nan	3	2	-0.744512	0.56925	no
ni|645903541|nb|KK920889.1|	427499-427712	nan	nan	33	22	-0.563203	0.61325	no
ni|645903541|nb|KK920889.1|	4338-4625	nan	nan	1	5	2	0.1106	no
ni|645903541|nb|KK920889.1|	453263-453413	nan	nan	13	8	-0.643408	0.57925	no
ni|645903541|nb|KK920889.1|	53191-53440	nan	nan	0.864538	2	0.934027	0.5295	no
ni|645903541|nb|KK920889.1|	54417-55032	nan	nan	2	1	-0.950091	0.44725	no
ni|645903541|nb|KK920889.1|	56003-57160	nan	nan	3	2	-0.118373	0.9184	no
ni|645903541|nb|KK920889.1|	57403-57577	nan	nan	12	6	-0.922934	0.43445	no
ni|645903541|nb|KK920889.1|	58268-59929	nan	nan	24	14	-0.773547	0.52115	no
ni|645903541|nb|KK920889.1|	60157-60652	nan	nan	17	23	0.398368	0.7419	no
ni|645903541|nb|KK920889.1|	6027-6165	nan	nan	7	12	0.836738	0.4777	no
ni|645903541|nb|KK920889.1|	61092-61359	nan	nan	37	26	-0.513864	0.6801	no
ni|645903541|nb|KK920889.1|	74541-74671	nan	nan	82	93	0.189489	0.8621	no
ni|645903541|nb|KK920889.1|	7714-7950	nan	nan	20	29	0.517761	0.665	no
ni|645903541|nb|KK920889.1|	84909-85034	nan	nan	77	70	-0.145949	0.89675	no
ni|645903541|nb|KK920889.1|	85355-85480	nan	nan	11	18	0.708994	0.5178	no
ni|645903541|nb|KK920889.1|	87450-87610	nan	nan	0	3	inf	0.00015	yes

ni|645903541|nb|KK920889.1|	87834-87950	nan	nan	40	21	-0.926739	0.4056	no
ni|645903541|nb|KK920889.1|	95138-95315	nan	nan	0.720643	0.229581	-2	0.2989	no
ni|645903542|nb|KK920888.1|	127979-128105	nan	nan	12	11	-0.191238	0.86045	no
ni|645903542|nb|KK920888.1|	13547-14762	nan	nan	10	7	-0.465395	0.694	no
ni|645903542|nb|KK920888.1|	150424-150818	nan	nan	13	12	-0.0672952	0.9576	no
ni|645903542|nb|KK920888.1|	15157-15526	nan	nan	15	13	-0.218616	0.84915	no
ni|645903542|nb|KK920888.1|	195552-195712	nan	nan	15	20	0.360219	0.76455	no
ni|645903543|nb|KK920887.1|	101180-101553	nan	nan	64	50	-0.360767	0.75095	no
ni|645903543|nb|KK920887.1|	152697-153275	nan	nan	50	38	-0.38411	0.7229	no
ni|645903543|nb|KK920887.1|	157564-157938	nan	nan	58	51	-0.194525	0.8648	no
ni|645903543|nb|KK920887.1|	158393-158584	nan	nan	71	57	-0.316776	0.77305	no
ni|645903543|nb|KK920887.1|	437263-437558	nan	nan	46	34	-0.450315	0.6688	no
ni|645903543|nb|KK920887.1|	437884-438245	nan	nan	5	4	-0.347356	0.76755	no
ni|645903543|nb|KK920887.1|	440361-440670	nan	nan	55	48	-0.174259	0.88075	no
ni|645903543|nb|KK920887.1|	441176-441499	nan	nan	0.966997	1	0.313009	0.81835	no
ni|645903543|nb|KK920887.1|	442241-442387	nan	nan	79	61	-0.377357	0.73525	no
ni|645903543|nb|KK920887.1|	443843-444020	nan	nan	43	34	-0.355642	0.7412	no
ni|645903543|nb|KK920887.1|	444985-445173	nan	nan	56	53	-0.0672273	0.954	no
ni|645903543|nb|KK920887.1|	448998-449140	nan	nan	93	72	-0.358944	0.7476	no
ni|645903543|nb|KK920887.1|	449547-450563	nan	nan	0	27	inf	5.00E-05	yes
ni|645903543|nb|KK920887.1|	454111-454619	nan	nan	0	12	inf	5.00E-05	yes
ni|645903543|nb|KK920887.1|	454804-454998	nan	nan	0	4	inf	5.00E-05	yes
ni|645903543|nb|KK920887.1|	455199-455688	nan	nan	0	4	inf	0.0003	yes
ni|645903543|nb|KK920887.1|	456659-456806	nan	nan	0.535204	545	10	0.05055	no
ni|645903543|nb|KK920887.1|	456951-457084	nan	nan	0.18647	283	11	0.11935	no
ni|645903543|nb|KK920887.1|	457559-457972	nan	nan	0.191896	477	11	0.0443	no
ni|645903543|nb|KK920887.1|	458336-458461	nan	nan	1	931	9	0.11935	no
ni|645903543|nb|KK920887.1|	458628-458839	nan	nan	0	716	inf	5.00E-05	yes
ni|645903543|nb|KK920887.1|	459366-459553	nan	nan	0	954	inf	5.00E-05	yes
ni|645903543|nb|KK920887.1|	460227-460474	nan	nan	0	829	inf	5.00E-05	yes

ni|645903543|nb|KK920887.1|	460628-460753	nan	nan	0.249755	56	8	0.03735	no
ni|645903543|nb|KK920887.1|	462004-462593	nan	nan	0	831	inf	5.00E-05	yes
ni|645903543|nb|KK920887.1|	464277-464986	nan	nan	0.32984	668	11	0.11935	no
ni|645903543|nb|KK920887.1|	465696-465931	nan	nan	0	1	inf	0.00065	yes
ni|645903543|nb|KK920887.1|	46676-47006	nan	nan	16	10	-0.695221	0.57055	no
ni|645903543|nb|KK920887.1|	468017-468169	nan	nan	0	0.57258	inf	5.00E-05	yes
ni|645903543|nb|KK920887.1|	468276-468414	nan	nan	0	119	inf	5.00E-05	yes
ni|645903543|nb|KK920887.1|	468517-468668	nan	nan	1	929	10	0.0443	no
ni|645903543|nb|KK920887.1|	469547-470115	nan	nan	0.042886	988.54	14	0.11935	no
ni|645903543|nb|KK920887.1|	470641-470981	nan	nan	0	1131.61	inf	5.00E-05	yes
ni|645903543|nb|KK920887.1|	471299-471532	nan	nan	0	366.13	inf	5.00E-05	yes
ni|645903543|nb|KK920887.1|	473064-473256	nan	nan	1	0	-inf	0.0004	yes
ni|645903543|nb|KK920887.1|	474853-475393	nan	nan	3	0	-inf	0.0036	no
ni|645903543|nb|KK920887.1|	475665-476146	nan	nan	2	0	-inf	0.002	yes
ni|645903543|nb|KK920887.1|	47860-48084	nan	nan	1	1	-0.428729	0.75965	no
ni|645903543|nb|KK920887.1|	478781-478955	nan	nan	14	9	-0.652457	0.66125	no
ni|645903543|nb|KK920887.1|	479487-479925	nan	nan	7	6	-0.259946	0.82995	no
ni|645903543|nb|KK920887.1|	480709-480872	nan	nan	6	7	0.409542	0.7319	no
ni|645903543|nb|KK920887.1|	481765-482333	nan	nan	3	3	-0.318722	0.7822	no
ni|645903543|nb|KK920887.1|	482466-482593	nan	nan	1	3	1	0.3525	no
ni|645903543|nb|KK920887.1|	482960-483252	nan	nan	13	5	-1	0.29705	no
ni|645903543|nb|KK920887.1|	483352-483535	nan	nan	2	2	-0.124264	0.91825	no
ni|645903543|nb|KK920887.1|	490700-491182	nan	nan	59	106.03	0.847076	0.45955	no
ni|645903543|nb|KK920887.1|	492207-492703	nan	nan	50	86	0.786032	0.4856	no
ni|645903543|nb|KK920887.1|	501630-501780	nan	nan	48	92	0.940285	0.4035	no
ni|645903543|nb|KK920887.1|	502857-503014	nan	nan	20	42	1	0.41485	no
ni|645903543|nb|KK920887.1|	503505-503909	nan	nan	21	41	0.928025	0.4073	no
ni|645903543|nb|KK920887.1|	505203-505400	nan	nan	2	3	0.436941	0.7114	no
ni|645903543|nb|KK920887.1|	507214-507608	nan	nan	4	11	2	0.177	no
ni|645903543|nb|KK920887.1|	519175-519501	nan	nan	2	1	-0.693596	0.54645	no

ni|645903543|nb|KK920887.1|	522897-523016	nan	nan	3	0.807882	-2	0.1593	no
ni|645903543|nb|KK920887.1|	524181-524376	nan	nan	3	1	-0.913954	0.4969	no
ni|645903543|nb|KK920887.1|	527539-527968	nan	nan	3	0.730811	-2	0.18585	no
ni|645903543|nb|KK920887.1|	529819-529951	nan	nan	5	2	-0.960374	0.51205	no
ni|645903543|nb|KK920887.1|	540056-540445	nan	nan	5	1	-2	0.20695	no
ni|645903543|nb|KK920887.1|	544598-544714	nan	nan	32	6	-2	0.03615	no
ni|645903543|nb|KK920887.1|	545070-545471	nan	nan	0	2	inf	0.0001	yes
ni|645903543|nb|KK920887.1|	548724-548978	nan	nan	0.941366	2	0.933909	0.5295	no
ni|645903543|nb|KK920887.1|	549922-550500	nan	nan	42	33	-0.33074	0.757	no
ni|645903543|nb|KK920887.1|	551761-551938	nan	nan	11	5	-1	0.3993	no
ni|645903543|nb|KK920887.1|	552131-552378	nan	nan	7	19	1	0.22915	no
ni|645903543|nb|KK920887.1|	553405-553699	nan	nan	28	9	-2	0.16945	no
ni|645903543|nb|KK920887.1|	554032-554266	nan	nan	14	28	1	0.39515	no
ni|645903543|nb|KK920887.1|	556913-557296	nan	nan	50	24	-1	0.385	no
ni|645903543|nb|KK920887.1|	557527-558044	nan	nan	2	3	0.84042	0.5399	no
ni|645903543|nb|KK920887.1|	558837-559667	nan	nan	124	94	-0.40102	0.70595	no
ni|645903543|nb|KK920887.1|	560130-560244	nan	nan	12	22	0.817331	0.4959	no
ni|645903543|nb|KK920887.1|	560894-561023	nan	nan	5	13	1	0.2855	no
ni|645903543|nb|KK920887.1|	561326-561501	nan	nan	3	3	0.156282	0.8958	no
ni|645903543|nb|KK920887.1|	561609-561782	nan	nan	87	86	-0.0152073	0.9921	no
ni|645903543|nb|KK920887.1|	562135-562457	nan	nan	7	8	0.304459	0.83825	no
ni|645903543|nb|KK920887.1|	563014-563144	nan	nan	8	7	-0.192769	0.87555	no
ni|645903543|nb|KK920887.1|	563307-563563	nan	nan	1	1	0.0423413	0.9711	no
ni|645903543|nb|KK920887.1|	56485-58126	nan	nan	53	25	-1	0.37495	no
ni|645903543|nb|KK920887.1|	599101-599508	nan	nan	1	3	0.981803	0.43735	no
ni|645903543|nb|KK920887.1|	600116-601321	nan	nan	7	8	0.269434	0.8168	no
ni|645903543|nb|KK920887.1|	601549-602664	nan	nan	17	16	-0.0673342	0.9579	no
ni|645903543|nb|KK920887.1|	602946-603381	nan	nan	19	17	-0.154842	0.90135	no
ni|645903543|nb|KK920887.1|	604214-604614	nan	nan	35	29	-0.254563	0.8644	no
ni|645903543|nb|KK920887.1|	606677-607003	nan	nan	11	12	0.212881	0.85985	no

ni|645903543|nb|KK920887.1|	609383-609512	nan	nan	2	2	-0.185941	0.87595	no
ni|645903543|nb|KK920887.1|	609602-609779	nan	nan	10	19	0.912043	0.4415	no
ni|645903543|nb|KK920887.1|	609989-610733	nan	nan	9	11	0.1957	0.87955	no
ni|645903543|nb|KK920887.1|	611054-611757	nan	nan	10	16	0.66965	0.573	no
ni|645903543|nb|KK920887.1|	614039-614524	nan	nan	8	7	-0.0672156	0.9564	no
ni|645903543|nb|KK920887.1|	615264-615516	nan	nan	4	5	0.485417	0.67525	no
ni|645903543|nb|KK920887.1|	617522-617664	nan	nan	12	13	0.102646	0.9329	no
ni|645903543|nb|KK920887.1|	618893-619263	nan	nan	4	7	0.727276	0.5398	no
ni|645903543|nb|KK920887.1|	619495-619894	nan	nan	11	12	0.102779	0.92905	no
ni|645903543|nb|KK920887.1|	620117-620596	nan	nan	7	15	1	0.3655	no
ni|645903543|nb|KK920887.1|	620914-621224	nan	nan	4	5	0.245097	0.8365	no
ni|645903543|nb|KK920887.1|	621668-622372	nan	nan	0.09351	1	3	0.05605	no
ni|645903543|nb|KK920887.1|	622556-623361	nan	nan	100	144	0.528141	0.72805	no
ni|645903543|nb|KK920887.1|	624100-624701	nan	nan	74	85	0.198741	0.86645	no
ni|645903543|nb|KK920887.1|	625095-625476	nan	nan	0	28	inf	0.0001	yes
ni|645903543|nb|KK920887.1|	625793-626237	nan	nan	4	0.083279	-6	0.0177	no
ni|645903543|nb|KK920887.1|	626649-626949	nan	nan	4	0.170775	-4	0.2327	no
ni|645903543|nb|KK920887.1|	627313-627641	nan	nan	58	18	-2	0.1576	no
ni|645903543|nb|KK920887.1|	627774-628134	nan	nan	104	64	-0.709501	0.57635	no
ni|645903543|nb|KK920887.1|	628241-628739	nan	nan	2	1	-0.6502	0.59155	no
ni|645903543|nb|KK920887.1|	628894-629119	nan	nan	10	7	-0.374883	0.8049	no
ni|645903543|nb|KK920887.1|	629378-630236	nan	nan	5	3	-0.941661	0.54025	no
ni|645903543|nb|KK920887.1|	630765-631073	nan	nan	182	122.14	-0.574603	0.60965	no
ni|645903543|nb|KK920887.1|	631373-631638	nan	nan	304	206	-0.565026	0.6851	no
ni|645903543|nb|KK920887.1|	633483-634597	nan	nan	205	142	-0.535362	0.70975	no
ni|645903543|nb|KK920887.1|	637226-637669	nan	nan	0.437057	0.069647	-3	0.2671	no
ni|645903543|nb|KK920887.1|	637787-637924	nan	nan	126	112	-0.171076	0.9079	no
ni|645903543|nb|KK920887.1|	639106-639513	nan	nan	1	0.951069	-0.164762	0.8993	no
ni|645903543|nb|KK920887.1|	639690-639819	nan	nan	1	0.80463	-0.327864	0.7854	no
ni|645903543|nb|KK920887.1|	640108-640250	nan	nan	33	38	0.18714	0.8672	no

ni|645903543|nb|KK920887.1|	640547-641361	nan	nan	3753.2	2688.35	-0.481398	0.5511	no
ni|645903543|nb|KK920887.1|	641786-642138	nan	nan	23	17	-0.449429	0.7251	no
ni|645903543|nb|KK920887.1|	644019-644200	nan	nan	93	88	-0.0790064	0.95425	no
ni|645903543|nb|KK920887.1|	644985-645673	nan	nan	462	384	-0.26835	0.844	no
ni|645903543|nb|KK920887.1|	646006-646386	nan	nan	2	0.862037	-0.802507	0.5588	no
ni|645903543|nb|KK920887.1|	97446-97748	nan	nan	28	23	-0.265145	0.8161	no
ni|645903544|nb|KK920886.1|	273627-274052	nan	nan	1220.62	944	-0.370791	0.7977	no
ni|645903544|nb|KK920886.1|	287706-288169	nan	nan	24	27	0.162177	0.89525	no
ni|645903544|nb|KK920886.1|	292677-292949	nan	nan	0.729673	0.767149	0.0722574	0.94355	no
ni|645903544|nb|KK920886.1|	293165-293489	nan	nan	1	3	1	0.3777	no
ni|645903544|nb|KK920886.1|	294166-294396	nan	nan	8	0	-inf	0.0009	yes
ni|645903544|nb|KK920886.1|	295352-295769	nan	nan	2	2	0.256852	0.8299	no
ni|645903544|nb|KK920886.1|	298680-299234	nan	nan	0.799589	21	5	0.00565	no
ni|645903544|nb|KK920886.1|	306192-306618	nan	nan	0	7	inf	0.0003	yes
ni|645903544|nb|KK920886.1|	306896-307042	nan	nan	0	4	inf	5.00E-05	yes
ni|645903544|nb|KK920886.1|	308631-308819	nan	nan	0	2	inf	0.0003	yes
ni|645903544|nb|KK920886.1|	309985-310216	nan	nan	1	2	0.136919	0.9072	no
ni|645903544|nb|KK920886.1|	310339-310501	nan	nan	7	7	-0.0673009	0.957	no
ni|645903544|nb|KK920886.1|	311254-311630	nan	nan	1	3	1	0.3865	no
ni|645903544|nb|KK920886.1|	311833-312444	nan	nan	21	14	-0.574239	0.63205	no
ni|645903544|nb|KK920886.1|	313093-313341	nan	nan	0.527946	0.504593	-0.0652719	0.9302	no
ni|645903544|nb|KK920886.1|	316042-316527	nan	nan	0.651779	0.511442	-0.34981	0.761	no
ni|645903544|nb|KK920886.1|	319782-320260	nan	nan	2	1	-0.757274	0.54375	no
ni|645903544|nb|KK920886.1|	322439-323183	nan	nan	0	4	inf	5.00E-05	yes
ni|645903544|nb|KK920886.1|	324206-324345	nan	nan	0	11	inf	5.00E-05	yes
ni|645903544|nb|KK920886.1|	324458-324645	nan	nan	2	1	-0.55247	0.71905	no
ni|645903544|nb|KK920886.1|	337931-338056	nan	nan	0.601528	0.359326	-0.743341	0.59615	no
ni|645903544|nb|KK920886.1|	338221-338348	nan	nan	2	2	-0.387277	0.7434	no
ni|645903544|nb|KK920886.1|	338439-338845	nan	nan	2	1	-0.844003	0.5595	no
ni|645903544|nb|KK920886.1|	339196-339359	nan	nan	1	1	0.127302	0.9211	no

ni|645903544|nb|KK920886.1|	339958-340161	nan	nan	4	1	-1	0.2898	no
ni|645903544|nb|KK920886.1|	341031-341231	nan	nan	3	1	-0.913504	0.46325	no
ni|645903544|nb|KK920886.1|	341441-341644	nan	nan	3	1	-1	0.36935	no
ni|645903544|nb|KK920886.1|	342854-343088	nan	nan	2	0.56278	-1	0.26925	no
ni|645903544|nb|KK920886.1|	343352-343526	nan	nan	3	1	-1	0.29705	no
ni|645903544|nb|KK920886.1|	344249-344705	nan	nan	971	611	-0.667383	0.5319	no
ni|645903544|nb|KK920886.1|	344813-345057	nan	nan	0.540992	0.560303	0.0505995	0.95725	no
ni|645903544|nb|KK920886.1|	346329-346799	nan	nan	0	5	inf	5.00E-05	yes
ni|645903544|nb|KK920886.1|	348254-348475	nan	nan	0	10	inf	5.00E-05	yes
ni|645903544|nb|KK920886.1|	355652-355802	nan	nan	0.981822	0.312718	-2	0.2989	no
ni|645903544|nb|KK920886.1|	356242-356434	nan	nan	44	57	0.374382	0.79595	no
ni|645903544|nb|KK920886.1|	357668-358142	nan	nan	30	42	0.482724	0.7514	no
ni|645903544|nb|KK920886.1|	361237-361419	nan	nan	847	501	-0.756791	0.587	no
ni|645903544|nb|KK920886.1|	364701-365111	nan	nan	2	2	-0.327879	0.7834	no
ni|645903544|nb|KK920886.1|	365316-365444	nan	nan	193	133.16	-0.535477	0.70735	no
ni|645903544|nb|KK920886.1|	366092-366374	nan	nan	0.662584	0.562918	-0.235178	0.8697	no
ni|645903544|nb|KK920886.1|	367918-368097	nan	nan	143	116	-0.308726	0.82865	no
ni|645903544|nb|KK920886.1|	389093-389749	nan	nan	170	143	-0.249152	0.8571	no
ni|645903544|nb|KK920886.1|	406189-406452	nan	nan	1	0.689331	-0.940216	0.54025	no
ni|645903544|nb|KK920886.1|	427214-427409	nan	nan	8	3	-1	0.26885	no
ni|645903544|nb|KK920886.1|	427955-428748	nan	nan	476	342	-0.476432	0.74995	no
ni|645903544|nb|KK920886.1|	488535-488724	nan	nan	3	1	-1	0.3061	no
ni|645903544|nb|KK920886.1|	542679-542867	nan	nan	6	4	-0.503991	0.66475	no
ni|645903545|nb|KK920885.1|	11514-11823	nan	nan	2	2	-0.37051	0.76435	no
ni|645903545|nb|KK920885.1|	12443-12638	nan	nan	3	3	-0.0667921	0.95795	no
ni|645903545|nb|KK920885.1|	1302-1449	nan	nan	2	0.568	-2	0.14015	no
ni|645903545|nb|KK920885.1|	218227-218665	nan	nan	69	109	0.657013	0.66355	no
ni|645903545|nb|KK920885.1|	226327-226706	nan	nan	0.474163	0.226685	-1	0.45115	no
ni|645903545|nb|KK920885.1|	230503-231423	nan	nan	185.1	264	0.51211	0.72	no
ni|645903545|nb|KK920885.1|	231913-232191	nan	nan	29	23	-0.336323	0.7596	no

ni|645903545|nb|KK920885.1|	232347-232709	nan	nan	45	36	-0.332936	0.77625	no
ni|645903545|nb|KK920885.1|	234459-234785	nan	nan	26	15	-0.787409	0.4795	no
ni|645903545|nb|KK920885.1|	235782-236155	nan	nan	0.240855	4	4	0.12575	no
ni|645903545|nb|KK920885.1|	237155-237462	nan	nan	1383.79	626	-1	0.3628	no
ni|645903545|nb|KK920885.1|	237928-238562	nan	nan	2	3	0.239674	0.84515	no
ni|645903545|nb|KK920885.1|	239124-239371	nan	nan	109	171	0.651175	0.64985	no
ni|645903545|nb|KK920885.1|	239476-239988	nan	nan	0.1325	50	9	0.11935	no
ni|645903545|nb|KK920885.1|	240390-240746	nan	nan	0.198434	7	5	0.1202	no
ni|645903545|nb|KK920885.1|	240957-241230	nan	nan	0	10	inf	0.00015	yes
ni|645903545|nb|KK920885.1|	241620-241759	nan	nan	0.244172	50	8	0.0275	no
ni|645903545|nb|KK920885.1|	242460-242906	nan	nan	0.436365	11	5	0.0373	no
ni|645903545|nb|KK920885.1|	243174-243507	nan	nan	0.631992	9	4	0.032	no
ni|645903545|nb|KK920885.1|	243576-244082	nan	nan	1	232.35	7	0.11935	no
ni|645903545|nb|KK920885.1|	247039-247166	nan	nan	0	3	inf	5.00E-05	yes
ni|645903545|nb|KK920885.1|	249191-249445	nan	nan	0	4	inf	0.0003	yes
ni|645903545|nb|KK920885.1|	251229-251473	nan	nan	17	11	-0.653839	0.5406	no
ni|645903545|nb|KK920885.1|	253385-254095	nan	nan	37	17	-1	0.31355	no
ni|645903545|nb|KK920885.1|	258306-258473	nan	nan	58	24	-1	0.27435	no
ni|645903545|nb|KK920885.1|	272178-272345	nan	nan	33	13	-1	0.28145	no
ni|645903545|nb|KK920885.1|	276888-277134	nan	nan	22	18	-0.274642	0.81375	no
ni|645903545|nb|KK920885.1|	278490-278687	nan	nan	54	42	-0.344195	0.7434	no
ni|645903545|nb|KK920885.1|	278828-279644	nan	nan	1	0.66643	-0.689104	0.56565	no
ni|645903545|nb|KK920885.1|	288437-288606	nan	nan	0.568733	0.449268	-0.340176	0.7768	no
ni|645903545|nb|KK920885.1|	288769-289948	nan	nan	9	7	-0.468606	0.6866	no
ni|645903545|nb|KK920885.1|	294584-294733	nan	nan	18	23	0.373043	0.75755	no
ni|645903545|nb|KK920885.1|	296138-296928	nan	nan	59	24	-1	0.2528	no
ni|645903545|nb|KK920885.1|	296986-302404	nan	nan	5	6	0.374288	0.7601	no
ni|645903545|nb|KK920885.1|	303116-304360	nan	nan	0	3	inf	0.0003	yes
ni|645903545|nb|KK920885.1|	304575-304882	nan	nan	48	52	0.122061	0.9098	no
ni|645903545|nb|KK920885.1|	307962-308066	nan	nan	4	5	0.127034	0.9146	no

ni|645903545|nb|KK920885.1|	310260-310592	nan	nan	3	4	0.503842	0.67295	no
ni|645903545|nb|KK920885.1|	311294-311681	nan	nan	6	7	0.0482754	0.95725	no
ni|645903545|nb|KK920885.1|	312226-312390	nan	nan	0.41177	0.61236	0.572542	0.67875	no
ni|645903545|nb|KK920885.1|	314413-314605	nan	nan	26	17	-0.607536	0.6029	no
ni|645903545|nb|KK920885.1|	319530-319861	nan	nan	2	1	-0.651035	0.64935	no
ni|645903545|nb|KK920885.1|	320753-321002	nan	nan	18	13	-0.4651	0.6951	no
ni|645903545|nb|KK920885.1|	321167-321352	nan	nan	25	41	0.739976	0.5207	no
ni|645903545|nb|KK920885.1|	322955-323136	nan	nan	35	52	0.567285	0.6195	no
ni|645903545|nb|KK920885.1|	32351-32850	nan	nan	5	3	-0.445791	0.74215	no
ni|645903545|nb|KK920885.1|	327323-327773	nan	nan	3	2	-0.502088	0.68865	no
ni|645903545|nb|KK920885.1|	327938-328151	nan	nan	16	12	-0.38124	0.71745	no
ni|645903545|nb|KK920885.1|	329657-329942	nan	nan	21	33	0.647806	0.5792	no
ni|645903545|nb|KK920885.1|	330690-331113	nan	nan	160	195	0.289427	0.79575	no
ni|645903545|nb|KK920885.1|	33215-33965	nan	nan	2	1	-0.765643	0.5318	no
ni|645903545|nb|KK920885.1|	333019-333148	nan	nan	274.57	325	0.242996	0.86085	no
ni|645903545|nb|KK920885.1|	333730-333902	nan	nan	7	2	-2	0.24815	no
ni|645903545|nb|KK920885.1|	334726-334894	nan	nan	77	97	0.333513	0.75325	no
ni|645903545|nb|KK920885.1|	336691-336849	nan	nan	3	3	-0.0662241	0.96245	no
ni|645903545|nb|KK920885.1|	337113-337250	nan	nan	3	5	0.807677	0.52225	no
ni|645903545|nb|KK920885.1|	341712-342271	nan	nan	228	301.99	0.403683	0.78125	no
ni|645903545|nb|KK920885.1|	342352-342579	nan	nan	8	11	0.56792	0.62025	no
ni|645903545|nb|KK920885.1|	343000-343757	nan	nan	0.690608	0.81563	0.240049	0.8456	no
ni|645903545|nb|KK920885.1|	34381-35028	nan	nan	2	2	0.140215	0.90865	no
ni|645903545|nb|KK920885.1|	343857-344610	nan	nan	4	5	0.254967	0.8373	no
ni|645903545|nb|KK920885.1|	345055-345374	nan	nan	66	136	1	0.3517	no
ni|645903545|nb|KK920885.1|	345978-346215	nan	nan	24	35	0.533193	0.64015	no
ni|645903545|nb|KK920885.1|	35159-36640	nan	nan	0.678788	38	6	0.0105	no
ni|645903545|nb|KK920885.1|	352342-352498	nan	nan	9	12	0.489196	0.69415	no
ni|645903545|nb|KK920885.1|	357387-357624	nan	nan	21	43	1	0.36725	no
ni|645903545|nb|KK920885.1|	358365-358509	nan	nan	60	51	-0.223976	0.84065	no

ni|645903545|nb|KK920885.1|	359545-359674	nan	nan	7	6	-0.131586	0.90745	no
ni|645903545|nb|KK920885.1|	361973-362232	nan	nan	1	1	-0.0644088	0.95435	no
ni|645903545|nb|KK920885.1|	365096-365291	nan	nan	2	2	-0.242274	0.83985	no
ni|645903545|nb|KK920885.1|	36789-37477	nan	nan	0.74706	83	7	0.0443	no
ni|645903545|nb|KK920885.1|	371854-372487	nan	nan	3	4	0.139363	0.90865	no
ni|645903545|nb|KK920885.1|	377919-378159	nan	nan	47	54	0.195969	0.8642	no
ni|645903545|nb|KK920885.1|	379802-380007	nan	nan	41	51	0.319	0.7775	no
ni|645903545|nb|KK920885.1|	381064-381377	nan	nan	39	54	0.463225	0.70905	no
ni|645903545|nb|KK920885.1|	383154-383593	nan	nan	14	16	0.213308	0.8858	no
ni|645903545|nb|KK920885.1|	38799-39728	nan	nan	0.388366	52	7	0.0146	no
ni|645903545|nb|KK920885.1|	388758-388956	nan	nan	9	8	-0.211301	0.8495	no
ni|645903545|nb|KK920885.1|	389593-389753	nan	nan	7	6	-0.247107	0.83435	no
ni|645903545|nb|KK920885.1|	390324-390818	nan	nan	4	3	-0.467618	0.69685	no
ni|645903545|nb|KK920885.1|	391348-391701	nan	nan	2	3	0.698918	0.59985	no
ni|645903545|nb|KK920885.1|	393059-393284	nan	nan	7	4	-0.804156	0.5588	no
ni|645903545|nb|KK920885.1|	393625-394196	nan	nan	0	4	inf	5.00E-05	yes
ni|645903545|nb|KK920885.1|	394375-394910	nan	nan	13	13	-0.0364586	0.97445	no
ni|645903545|nb|KK920885.1|	395588-396361	nan	nan	10	10	-0.0520402	0.96195	no
ni|645903545|nb|KK920885.1|	398334-398671	nan	nan	20	30	0.574251	0.6308	no
ni|645903545|nb|KK920885.1|	399120-399247	nan	nan	27	44	0.669659	0.5652	no
ni|645903545|nb|KK920885.1|	399894-400099	nan	nan	22	33	0.600352	0.60445	no
ni|645903545|nb|KK920885.1|	401077-401440	nan	nan	2	3	0.229873	0.8483	no
ni|645903545|nb|KK920885.1|	40183-41584	nan	nan	0.146655	55	9	0.11935	no
ni|645903545|nb|KK920885.1|	405283-405481	nan	nan	35	132	2	0.1731	no
ni|645903545|nb|KK920885.1|	407177-407324	nan	nan	13	66	2	0.05365	no
ni|645903545|nb|KK920885.1|	408073-408220	nan	nan	3	10	2	0.10565	no
ni|645903545|nb|KK920885.1|	408853-409084	nan	nan	20	27	0.439592	0.71745	no
ni|645903545|nb|KK920885.1|	410684-410876	nan	nan	25	33	0.399795	0.72915	no
ni|645903545|nb|KK920885.1|	412955-413155	nan	nan	13	16	0.330775	0.76575	no
ni|645903545|nb|KK920885.1|	413380-413545	nan	nan	51	56	0.147941	0.92205	no

ni|645903545|nb|KK920885.1|	413831-414030	nan	nan	0.815836	0.059987	-4	0.02555	no
ni|645903545|nb|KK920885.1|	414249-414537	nan	nan	5	4	-0.335945	0.7771	no
ni|645903545|nb|KK920885.1|	414739-415114	nan	nan	68	28	-1	0.38015	no
ni|645903545|nb|KK920885.1|	415985-416487	nan	nan	1	0.285267	-2	0.2949	no
ni|645903545|nb|KK920885.1|	4170-4344	nan	nan	2	1	-0.85845	0.4671	no
ni|645903545|nb|KK920885.1|	417418-417575	nan	nan	0	37	inf	5.00E-05	yes
ni|645903545|nb|KK920885.1|	41790-42097	nan	nan	0.604283	62	7	0.0443	no
ni|645903545|nb|KK920885.1|	421604-421786	nan	nan	4	3	-0.509252	0.67705	no
ni|645903545|nb|KK920885.1|	422665-422865	nan	nan	0.54039	0.563568	0.06059	0.95295	no
ni|645903545|nb|KK920885.1|	423426-423673	nan	nan	1	1	0.349366	0.792	no
ni|645903545|nb|KK920885.1|	429668-429780	nan	nan	14	46	2	0.13285	no
ni|645903545|nb|KK920885.1|	433356-433480	nan	nan	0.368102	1	2	0.22835	no
ni|645903545|nb|KK920885.1|	434397-434571	nan	nan	0.440842	0.511848	0.215454	0.8678	no
ni|645903545|nb|KK920885.1|	439654-439782	nan	nan	18	58	2	0.2189	no
ni|645903545|nb|KK920885.1|	442563-442777	nan	nan	0.197688	3	4	0.0283	no
ni|645903545|nb|KK920885.1|	446050-446175	nan	nan	18	18	-0.0490517	0.96685	no
ni|645903545|nb|KK920885.1|	446388-446504	nan	nan	0.625377	15	5	0.052	no
ni|645903545|nb|KK920885.1|	446727-446950	nan	nan	0	14	inf	5.00E-05	yes
ni|645903545|nb|KK920885.1|	44869-45288	nan	nan	0	39	inf	5.00E-05	yes
ni|645903545|nb|KK920885.1|	4523-4863	nan	nan	1	0.843581	-0.771168	0.5218	no
ni|645903545|nb|KK920885.1|	454164-454396	nan	nan	2	67	5	0.0338	no
ni|645903545|nb|KK920885.1|	45904-46063	nan	nan	0.642891	58	7	0.0443	no
ni|645903545|nb|KK920885.1|	46258-46645	nan	nan	0.605002	55	7	0.05055	no
ni|645903545|nb|KK920885.1|	46819-47166	nan	nan	0.480428	32	6	0.0332	no
ni|645903545|nb|KK920885.1|	47361-47545	nan	nan	0.249125	25	7	0.03715	no
ni|645903545|nb|KK920885.1|	47870-48359	nan	nan	3	6	0.721176	0.5689	no
ni|645903545|nb|KK920885.1|	48941-49169	nan	nan	0	7	inf	0.00015	yes
ni|645903545|nb|KK920885.1|	49354-49619	nan	nan	4	16	2	0.22355	no
ni|645903545|nb|KK920885.1|	5414-5839	nan	nan	2	0.755375	-1	0.3186	no
ni|645903545|nb|KK920885.1|	54276-54548	nan	nan	10	22	1	0.42455	no

ni|645903545|nb|KK920885.1|	55069-55260	nan	nan	0	28	inf	0.00015	yes
ni|645903545|nb|KK920885.1|	55627-55842	nan	nan	151	320	1	0.44705	no
ni|645903545|nb|KK920885.1|	55995-56948	nan	nan	159	294	0.884485	0.4863	no
ni|645903545|nb|KK920885.1|	57348-57559	nan	nan	241	430	0.835254	0.4947	no
ni|645903545|nb|KK920885.1|	58408-58652	nan	nan	234	427	0.868314	0.54865	no
ni|645903545|nb|KK920885.1|	58887-59114	nan	nan	4	3	-0.381988	0.74565	no
ni|645903545|nb|KK920885.1|	62694-62819	nan	nan	3	3	-0.3092	0.79825	no
ni|645903545|nb|KK920885.1|	63023-63235	nan	nan	163	294	0.848086	0.57225	no
ni|645903545|nb|KK920885.1|	65922-66302	nan	nan	233	367	0.657498	0.5603	no
ni|645903545|nb|KK920885.1|	67368-67701	nan	nan	3	3	0.131802	0.90955	no
ni|645903545|nb|KK920885.1|	68153-68700	nan	nan	261.14	391	0.582534	0.6482	no
ni|645903545|nb|KK920885.1|	68991-69597	nan	nan	2	2	-0.651092	0.63225	no
ni|645903545|nb|KK920885.1|	70241-70454	nan	nan	1	0.582771	-0.853529	0.50505	no
ni|645903545|nb|KK920885.1|	8680-9164	nan	nan	3	2	-0.387698	0.74515	no
ni|645903546|nb|KK920884.1|	121123-121396	nan	nan	5	4	-0.114207	0.92225	no
ni|645903546|nb|KK920884.1|	123807-124003	nan	nan	0	26	inf	5.00E-05	yes
ni|645903546|nb|KK920884.1|	124477-125956	nan	nan	0	19	inf	5.00E-05	yes
ni|645903546|nb|KK920884.1|	127283-128278	nan	nan	8	20	1	0.364	no
ni|645903546|nb|KK920884.1|	135090-135409	nan	nan	0.779525	2	1	0.30495	no
ni|645903546|nb|KK920884.1|	136936-137137	nan	nan	0	520	inf	5.00E-05	yes
ni|645903546|nb|KK920884.1|	138441-138845	nan	nan	0	70	inf	5.00E-05	yes
ni|645903546|nb|KK920884.1|	138954-139143	nan	nan	9	7	-0.446702	0.6754	no
ni|645903546|nb|KK920884.1|	140082-140280	nan	nan	0	54	inf	5.00E-05	yes
ni|645903546|nb|KK920884.1|	140864-141066	nan	nan	0	44	inf	5.00E-05	yes
ni|645903546|nb|KK920884.1|	143893-144142	nan	nan	0	5	inf	0.00065	yes
ni|645903546|nb|KK920884.1|	145694-146242	nan	nan	0	26	inf	5.00E-05	yes
ni|645903546|nb|KK920884.1|	147525-148025	nan	nan	0	4	inf	0.00055	yes
ni|645903546|nb|KK920884.1|	149696-150333	nan	nan	3	10	2	0.1606	no
ni|645903546|nb|KK920884.1|	151043-151460	nan	nan	3	10	2	0.17715	no
ni|645903546|nb|KK920884.1|	151707-151856	nan	nan	8	23	2	0.2002	no

ni|645903546|nb|KK920884.1|	152482-152821	nan	nan	6	20	2	0.18165	no
ni|645903546|nb|KK920884.1|	153478-153617	nan	nan	2	16	3	0.06085	no
ni|645903546|nb|KK920884.1|	153909-154184	nan	nan	5	20	2	0.1101	no
ni|645903546|nb|KK920884.1|	161627-162017	nan	nan	3	15	2	0.0767	no
ni|645903546|nb|KK920884.1|	187984-188222	nan	nan	11	16	0.450475	0.7026	no
ni|645903546|nb|KK920884.1|	189485-189660	nan	nan	3	4	0.380498	0.7694	no
ni|645903546|nb|KK920884.1|	189962-190383	nan	nan	2	1	-0.686821	0.58765	no
ni|645903546|nb|KK920884.1|	201010-201197	nan	nan	23	24	0.03517	0.98005	no
ni|645903546|nb|KK920884.1|	319600-320040	nan	nan	18	16	-0.205646	0.8527	no
ni|645903546|nb|KK920884.1|	328411-328737	nan	nan	26	23	-0.209372	0.8557	no
ni|645903546|nb|KK920884.1|	353890-354135	nan	nan	7	5	-0.304782	0.7968	no
ni|645903546|nb|KK920884.1|	532746-532878	nan	nan	21	19	-0.132387	0.89995	no
ni|645903546|nb|KK920884.1|	533057-535029	nan	nan	6	3	-0.790476	0.5191	no
ni|645903546|nb|KK920884.1|	593463-593614	nan	nan	0.638947	0.122148	-2	0.2949	no
ni|645903546|nb|KK920884.1|	595559-595761	nan	nan	5	18	2	0.14785	no
ni|645903546|nb|KK920884.1|	598891-599115	nan	nan	10	29	2	0.1871	no
ni|645903546|nb|KK920884.1|	599544-599703	nan	nan	25	6	-2	0.20695	no
ni|645903546|nb|KK920884.1|	600184-600339	nan	nan	313	189.68	-0.724412	0.6156	no
ni|645903546|nb|KK920884.1|	600575-600853	nan	nan	5	2	-1	0.34	no
ni|645903546|nb|KK920884.1|	601058-601686	nan	nan	2	2	0.0788026	0.9453	no
ni|645903546|nb|KK920884.1|	602141-603939	nan	nan	4	3	-0.429764	0.75965	no
ni|645903546|nb|KK920884.1|	604580-604943	nan	nan	36	20	-0.816574	0.5672	no
ni|645903546|nb|KK920884.1|	605572-605914	nan	nan	3	2	-0.375856	0.75525	no
ni|645903546|nb|KK920884.1|	606048-606467	nan	nan	4	3	-0.650234	0.58665	no
ni|645903546|nb|KK920884.1|	607856-608073	nan	nan	262	147	-0.837482	0.5582	no
ni|645903546|nb|KK920884.1|	610032-610431	nan	nan	50	42	-0.256942	0.8559	no
ni|645903546|nb|KK920884.1|	611356-611491	nan	nan	0.761939	0.468375	-0.702013	0.5682	no
ni|645903546|nb|KK920884.1|	611647-611854	nan	nan	3	2	-0.572484	0.63215	no
ni|645903546|nb|KK920884.1|	614770-615354	nan	nan	48	47	-0.027668	0.98105	no
ni|645903546|nb|KK920884.1|	615925-616077	nan	nan	10	10	0.0171391	0.9876	no

ni|645903546|nb|KK920884.1|	616828-617176	nan	nan	10	10	0.0161597	0.98965	no
ni|645903546|nb|KK920884.1|	617286-617504	nan	nan	10	9	-0.111055	0.9195	no
ni|645903546|nb|KK920884.1|	617804-619089	nan	nan	19	14	-0.4661	0.69375	no
ni|645903546|nb|KK920884.1|	619777-620027	nan	nan	8	5	-0.504814	0.6682	no
ni|645903546|nb|KK920884.1|	622876-623121	nan	nan	45	32	-0.471323	0.67395	no
ni|645903546|nb|KK920884.1|	70606-70882	nan	nan	3	3	0.380667	0.7694	no
ni|645903546|nb|KK920884.1|	744568-744682	nan	nan	7	5	-0.552515	0.6495	no
ni|645903546|nb|KK920884.1|	896064-896344	nan	nan	6	4	-0.315868	0.7845	no
ni|645903547|nb|KK920883.1|	207702-208019	nan	nan	37	25	-0.528886	0.64685	no
ni|645903547|nb|KK920883.1|	248546-248945	nan	nan	3	4	0.425115	0.71095	no
ni|645903547|nb|KK920883.1|	497779-497982	nan	nan	11	11	-0.0266589	0.98055	no
ni|645903547|nb|KK920883.1|	500930-501158	nan	nan	45	42	-0.098556	0.9334	no
ni|645903547|nb|KK920883.1|	501418-501781	nan	nan	61	38	-0.700658	0.54175	no
ni|645903547|nb|KK920883.1|	501951-502101	nan	nan	22	27	0.328537	0.79035	no
ni|645903547|nb|KK920883.1|	502659-502851	nan	nan	58	47	-0.304906	0.8113	no
ni|645903547|nb|KK920883.1|	504758-504972	nan	nan	104	82	-0.347306	0.76535	no
ni|645903547|nb|KK920883.1|	505986-506478	nan	nan	134	94	-0.515427	0.639	no
ni|645903547|nb|KK920883.1|	506672-506831	nan	nan	112	89	-0.336457	0.76105	no
ni|645903547|nb|KK920883.1|	507118-507266	nan	nan	38	29	-0.391992	0.72295	no
ni|645903547|nb|KK920883.1|	507472-507633	nan	nan	104	90	-0.210801	0.8433	no
ni|645903547|nb|KK920883.1|	507742-508062	nan	nan	12	7	-0.773001	0.4951	no
ni|645903547|nb|KK920883.1|	508806-509081	nan	nan	72	39	-0.859308	0.44375	no
ni|645903547|nb|KK920883.1|	51006-51661	nan	nan	8	4	-0.925197	0.44955	no
ni|645903547|nb|KK920883.1|	512047-512344	nan	nan	2	0.928651	-0.803105	0.6027	no
ni|645903547|nb|KK920883.1|	514030-514132	nan	nan	0.657671	0.576297	-0.190555	0.88855	no
ni|645903547|nb|KK920883.1|	546131-546685	nan	nan	3	2	-0.837814	0.4875	no
ni|645903547|nb|KK920883.1|	564202-564466	nan	nan	0.347956	11	5	0.12045	no
ni|645903547|nb|KK920883.1|	569343-569521	nan	nan	6	4	-0.387917	0.7406	no
ni|645903547|nb|KK920883.1|	581472-581593	nan	nan	0	3	inf	0.0003	yes
ni|645903547|nb|KK920883.1|	582410-582729	nan	nan	141	92	-0.613546	0.60385	no

ni|645903547|nb|KK920883.1|	594118-594355	nan	nan	0.741307	0.37303	-0.990778	0.47555	no
ni|645903547|nb|KK920883.1|	598405-598635	nan	nan	0.734436	1	0.448981	0.7543	no
ni|645903547|nb|KK920883.1|	601108-601842	nan	nan	18	16	-0.205329	0.86085	no
ni|645903547|nb|KK920883.1|	603914-605001	nan	nan	3	3	-0.0257828	0.98335	no
ni|645903547|nb|KK920883.1|	605291-605435	nan	nan	20	17	-0.210295	0.8577	no
ni|645903547|nb|KK920883.1|	605836-606201	nan	nan	48	33	-0.541254	0.63425	no
ni|645903547|nb|KK920883.1|	606405-607005	nan	nan	37	32	-0.22113	0.84055	no
ni|645903547|nb|KK920883.1|	607239-607560	nan	nan	48	37	-0.391038	0.72495	no
ni|645903547|nb|KK920883.1|	608167-608673	nan	nan	5	4	-0.323952	0.78	no
ni|645903547|nb|KK920883.1|	611078-611282	nan	nan	7	11	0.675156	0.56365	no
ni|645903547|nb|KK920883.1|	611874-612480	nan	nan	21	33	0.633238	0.5861	no
ni|645903547|nb|KK920883.1|	612539-613092	nan	nan	21	20	-0.0952268	0.9307	no
ni|645903547|nb|KK920883.1|	616022-616270	nan	nan	1	1	0.0499531	0.95725	no
ni|645903547|nb|KK920883.1|	618916-619285	nan	nan	18	18	-0.0114235	0.9903	no
ni|645903547|nb|KK920883.1|	620825-621564	nan	nan	0.880834	0.661535	-0.413052	0.76315	no
ni|645903547|nb|KK920883.1|	625685-625915	nan	nan	2	2	0.140581	0.90865	no
ni|645903547|nb|KK920883.1|	627387-627777	nan	nan	2	2	-0.552752	0.719	no
ni|645903547|nb|KK920883.1|	629552-629703	nan	nan	2	2	0.155103	0.9131	no
ni|645903547|nb|KK920883.1|	629809-629982	nan	nan	3	3	-0.341664	0.7737	no
ni|645903547|nb|KK920883.1|	630571-631068	nan	nan	2	1	-0.743813	0.56925	no
ni|645903547|nb|KK920883.1|	631908-632538	nan	nan	0.778265	1	0.742421	0.54275	no
ni|645903547|nb|KK920883.1|	633932-634346	nan	nan	1	7	2	0.07265	no
ni|645903547|nb|KK920883.1|	634456-634873	nan	nan	5	5	-0.0544604	0.9613	no
ni|645903547|nb|KK920883.1|	636690-636897	nan	nan	28	19	-0.552782	0.647	no
ni|645903547|nb|KK920883.1|	638465-638628	nan	nan	24	21	-0.165516	0.88275	no
ni|645903547|nb|KK920883.1|	638717-639402	nan	nan	26	28	0.0614543	0.96	no
ni|645903547|nb|KK920883.1|	642566-642829	nan	nan	25	28	0.146818	0.90365	no
ni|645903547|nb|KK920883.1|	644754-645271	nan	nan	0.989762	1	0.349691	0.7835	no
ni|645903547|nb|KK920883.1|	646241-646400	nan	nan	50	38	-0.371808	0.7438	no
ni|645903547|nb|KK920883.1|	646666-648133	nan	nan	161	161	-0.00504098	0.9959	no

ni|645903547|nb|KK920883.1|	648283-648432	nan	nan	40	46	0.19786	0.8498	no
ni|645903547|nb|KK920883.1|	648556-649333	nan	nan	162	149	-0.125745	0.9108	no
ni|645903547|nb|KK920883.1|	649533-649957	nan	nan	6	4	-0.767632	0.5799	no
ni|645903547|nb|KK920883.1|	651213-651982	nan	nan	119	140	0.233205	0.83045	no
ni|645903547|nb|KK920883.1|	652452-652610	nan	nan	10	8	-0.383802	0.74115	no
ni|645903547|nb|KK920883.1|	653578-653738	nan	nan	13	5	-1	0.31305	no
ni|645903547|nb|KK920883.1|	653797-653903	nan	nan	4	3	-0.735627	0.5484	no
ni|645903547|nb|KK920883.1|	654436-654737	nan	nan	14	5	-1	0.20155	no
ni|645903547|nb|KK920883.1|	655005-655170	nan	nan	5	2	-2	0.14545	no
ni|645903547|nb|KK920883.1|	655464-655677	nan	nan	0.87449	0.25072	-2	0.2784	no
ni|645903547|nb|KK920883.1|	655740-655855	nan	nan	7	5	-0.396178	0.74215	no
ni|645903547|nb|KK920883.1|	656431-656651	nan	nan	3	2	-0.70679	0.5573	no
ni|645903547|nb|KK920883.1|	715263-715663	nan	nan	16	11	-0.610393	0.583	no
ni|645903548|nb|KK920882.1|	129252-129565	nan	nan	8	5	-0.674011	0.56185	no
ni|645903548|nb|KK920882.1|	13896-14072	nan	nan	4	2	-1	0.3554	no
ni|645903548|nb|KK920882.1|	198214-198362	nan	nan	14	11	-0.342681	0.7809	no
ni|645903548|nb|KK920882.1|	198905-199219	nan	nan	0.205007	241.18	10	0.11935	no
ni|645903548|nb|KK920882.1|	201103-201359	nan	nan	0.079543	129	11	0.0443	no
ni|645903548|nb|KK920882.1|	202648-202870	nan	nan	0	186	inf	5.00E-05	yes
ni|645903548|nb|KK920882.1|	203357-203502	nan	nan	3	5	0.624939	0.61935	no
ni|645903548|nb|KK920882.1|	204196-204481	nan	nan	4	4	-0.146575	0.8874	no
ni|645903548|nb|KK920882.1|	204930-205682	nan	nan	12	6	-0.998033	0.3998	no
ni|645903548|nb|KK920882.1|	205862-206339	nan	nan	0	25	inf	5.00E-05	yes
ni|645903548|nb|KK920882.1|	206914-207236	nan	nan	0	27	inf	0.00015	yes
ni|645903548|nb|KK920882.1|	207782-207979	nan	nan	0.470162	20	5	0.11945	no
ni|645903548|nb|KK920882.1|	213387-213619	nan	nan	0	14	inf	5.00E-05	yes
ni|645903548|nb|KK920882.1|	215891-216319	nan	nan	0	31	inf	5.00E-05	yes
ni|645903548|nb|KK920882.1|	219169-219318	nan	nan	0	28	inf	5.00E-05	yes
ni|645903548|nb|KK920882.1|	221312-221532	nan	nan	0	46	inf	5.00E-05	yes
ni|645903548|nb|KK920882.1|	223784-224155	nan	nan	0	43	inf	5.00E-05	yes

ni|645903548|nb|KK920882.1|	226806-227074	nan	nan	0	33	inf	5.00E-05	yes
ni|645903548|nb|KK920882.1|	228494-228664	nan	nan	0	5	inf	5.00E-05	yes
ni|645903548|nb|KK920882.1|	238349-238687	nan	nan	0	13	inf	5.00E-05	yes
ni|645903548|nb|KK920882.1|	240626-240847	nan	nan	6	21	2	0.22835	no
ni|645903548|nb|KK920882.1|	246516-246875	nan	nan	9	23	1	0.22925	no
ni|645903548|nb|KK920882.1|	249175-249431	nan	nan	14	33	1	0.2959	no
ni|645903548|nb|KK920882.1|	249559-249832	nan	nan	4	15	2	0.15655	no
ni|645903548|nb|KK920882.1|	251001-251354	nan	nan	6	14	1	0.29895	no
ni|645903548|nb|KK920882.1|	254128-254529	nan	nan	13	0	-inf	5.00E-05	yes
ni|645903548|nb|KK920882.1|	254633-254791	nan	nan	2	0	-inf	0.00035	yes
ni|645903548|nb|KK920882.1|	256274-256851	nan	nan	4	0	-inf	0.0013	yes
ni|645903548|nb|KK920882.1|	257485-257591	nan	nan	8	0	-inf	0.0004	yes
ni|645903548|nb|KK920882.1|	259132-259369	nan	nan	9	24	1	0.2447	no
ni|645903548|nb|KK920882.1|	259659-260518	nan	nan	9	7	-0.294869	0.8041	no
ni|645903548|nb|KK920882.1|	263654-264231	nan	nan	21	72	2	0.1258	no
ni|645903548|nb|KK920882.1|	272923-273243	nan	nan	0	41	inf	5.00E-05	yes
ni|645903548|nb|KK920882.1|	273474-274004	nan	nan	4	0	-inf	0.0036	no
ni|645903548|nb|KK920882.1|	274929-275165	nan	nan	0	166	inf	5.00E-05	yes
ni|645903548|nb|KK920882.1|	276341-276549	nan	nan	12	35	2	0.20855	no
ni|645903548|nb|KK920882.1|	277331-277508	nan	nan	11	22	1	0.36005	no
ni|645903548|nb|KK920882.1|	278344-278483	nan	nan	38.39	76	0.980085	0.44295	no
ni|645903548|nb|KK920882.1|	279496-279711	nan	nan	67	122	0.870828	0.46305	no
ni|645903548|nb|KK920882.1|	280153-280626	nan	nan	30	61	1	0.38825	no
ni|645903548|nb|KK920882.1|	281106-281268	nan	nan	21	74	2	0.12235	no
ni|645903548|nb|KK920882.1|	282636-282903	nan	nan	1	1	-0.258836	0.8692	no
ni|645903548|nb|KK920882.1|	451441-452508	nan	nan	31	33	0.082851	0.9419	no
ni|645903548|nb|KK920882.1|	458764-459088	nan	nan	2	2	-0.108368	0.9247	no
ni|645903548|nb|KK920882.1|	460645-462240	nan	nan	19	31	0.686437	0.55265	no
ni|645903548|nb|KK920882.1|	463287-463570	nan	nan	21	35	0.756843	0.518	no
ni|645903548|nb|KK920882.1|	465324-465513	nan	nan	18	45	1	0.2703	no

ni|645903548|nb|KK920882.1|	469250-469448	nan	nan	8	8	-0.0165396	0.97895	no
ni|645903548|nb|KK920882.1|	470641-471123	nan	nan	38	38.34	0.000745258	0.9989	no
ni|645903548|nb|KK920882.1|	474071-474303	nan	nan	14.52	30	1	0.36205	no
ni|645903548|nb|KK920882.1|	482974-483167	nan	nan	15	35	1	0.3782	no
ni|645903548|nb|KK920882.1|	551293-551996	nan	nan	11	20	0.869515	0.46165	no
ni|645903548|nb|KK920882.1|	560527-560685	nan	nan	8	21	1	0.2619	no
ni|645903548|nb|KK920882.1|	572041-572203	nan	nan	24	43	0.878238	0.45415	no
ni|645903548|nb|KK920882.1|	580763-581190	nan	nan	102	108	0.0870905	0.9355	no
ni|645903548|nb|KK920882.1|	585299-585460	nan	nan	0.684002	8	4	0.04315	no
ni|645903548|nb|KK920882.1|	586799-587066	nan	nan	1	34	5	0.02795	no
ni|645903548|nb|KK920882.1|	601575-601815	nan	nan	4	10	1	0.2429	no
ni|645903548|nb|KK920882.1|	707943-708177	nan	nan	0.375354	2	2	0.1097	no
ni|645903548|nb|KK920882.1|	724918-725768	nan	nan	17	32	0.935255	0.512	no
ni|645903548|nb|KK920882.1|	734846-734974	nan	nan	1	28	5	0.02155	no
ni|645903548|nb|KK920882.1|	741324-741482	nan	nan	16	29	0.827614	0.455	no
ni|645903548|nb|KK920882.1|	741901-742054	nan	nan	0.652957	2	1.52	0.23565	no
ni|645903548|nb|KK920882.1|	742233-742506	nan	nan	137	117.41	-0.227788	0.8451	no
ni|645903548|nb|KK920882.1|	742642-743079	nan	nan	81	82	0.0260597	0.9806	no
ni|645903548|nb|KK920882.1|	744029-745181	nan	nan	17	30	0.817649	0.46135	no
ni|645903548|nb|KK920882.1|	745297-745714	nan	nan	30	40	0.438405	0.73235	no
ni|645903548|nb|KK920882.1|	745859-746138	nan	nan	0.041584	0.993914	5	0.1211	no
ni|645903548|nb|KK920882.1|	747004-747170	nan	nan	12	8	-0.652231	0.5859	no
ni|645903548|nb|KK920882.1|	747374-747807	nan	nan	16	12	-0.467733	0.70245	no
ni|645903548|nb|KK920882.1|	748043-748455	nan	nan	10	7	-0.555826	0.6419	no
ni|645903548|nb|KK920882.1|	754684-754801	nan	nan	6	4	-0.405159	0.72995	no
ni|645903548|nb|KK920882.1|	756083-756362	nan	nan	8	9	0.22677	0.8499	no
ni|645903548|nb|KK920882.1|	756425-756591	nan	nan	10	8	-0.280338	0.808	no
ni|645903548|nb|KK920882.1|	758197-758327	nan	nan	3	3	0.181703	0.87985	no
ni|645903548|nb|KK920882.1|	758765-760562	nan	nan	8	6	-0.312052	0.78785	no
ni|645903548|nb|KK920882.1|	760670-760906	nan	nan	7	9	0.348928	0.76065	no

ni|645903548|nb|KK920882.1|	760957-761268	nan	nan	6	10	0.684747	0.5731	no
ni|645903548|nb|KK920882.1|	763629-764683	nan	nan	5	6	0.308136	0.7927	no
ni|645903548|nb|KK920882.1|	764974-766927	nan	nan	9	8	-0.0672061	0.9576	no
ni|645903548|nb|KK920882.1|	767032-767884	nan	nan	6	9	0.591637	0.62225	no
ni|645903548|nb|KK920882.1|	768091-769548	nan	nan	9	6	-0.609481	0.61075	no
ni|645903548|nb|KK920882.1|	769693-770372	nan	nan	5	4	-0.324716	0.7826	no
ni|645903548|nb|KK920882.1|	772530-772787	nan	nan	4	3	-0.342001	0.7737	no
ni|645903548|nb|KK920882.1|	780152-781604	nan	nan	3	1	-1	0.39005	no
ni|645903548|nb|KK920882.1|	790902-791207	nan	nan	33	46	0.49617	0.6741	no
ni|645903548|nb|KK920882.1|	792698-793392	nan	nan	4	2	-1	0.383	no
ni|645903548|nb|KK920882.1|	793529-794042	nan	nan	2	0.303588	-3	0.2517	no
ni|645903548|nb|KK920882.1|	797801-798309	nan	nan	1	1	0.307323	0.7983	no
ni|645903548|nb|KK920882.1|	798755-799708	nan	nan	3	1	-0.941242	0.54025	no
ni|645903548|nb|KK920882.1|	801443-801622	nan	nan	1	0.972764	-0.534788	0.67295	no
ni|645903548|nb|KK920882.1|	801857-802102	nan	nan	2	0.928137	-0.766198	0.5799	no
ni|645903548|nb|KK920882.1|	802224-802833	nan	nan	7	0.794736	-3	0.24205	no
ni|645903548|nb|KK920882.1|	802986-804024	nan	nan	3	1	-1	0.35155	no
ni|645903548|nb|KK920882.1|	804199-804792	nan	nan	3	0.59096	-3	0.28285	no
ni|645903548|nb|KK920882.1|	805722-806234	nan	nan	2	1	-0.552698	0.719	no
ni|645903548|nb|KK920882.1|	807967-808509	nan	nan	3	1	-0.933936	0.439	no
ni|645903548|nb|KK920882.1|	808829-809091	nan	nan	27	59	1	0.4242	no
ni|645903548|nb|KK920882.1|	811514-812006	nan	nan	0.603367	0.288339	-1	0.44965	no
ni|645903548|nb|KK920882.1|	812561-812796	nan	nan	0.986316	0.565513	-0.80249	0.6027	no
ni|645903548|nb|KK920882.1|	818057-818229	nan	nan	0.414868	0.396695	-0.064623	0.95935	no
ni|645903548|nb|KK920882.1|	818566-819137	nan	nan	0.538365	0.583378	0.115845	0.9286	no
ni|645903548|nb|KK920882.1|	819876-820120	nan	nan	51	65	0.34536	0.7835	no
ni|645903548|nb|KK920882.1|	820466-821497	nan	nan	52	55	0.0785507	0.9458	no
ni|645903548|nb|KK920882.1|	823614-823739	nan	nan	68	73	0.103445	0.9457	no
ni|645903548|nb|KK920882.1|	825287-826612	nan	nan	114	127	0.158558	0.8873	no
ni|645903548|nb|KK920882.1|	827141-827471	nan	nan	106.82	106	-0.0134722	0.98995	no

ni|645903548|nb|KK920882.1|	841658-841823	nan	nan	122	111	-0.136265	0.91215	no
ni|645903548|nb|KK920882.1|	847672-847840	nan	nan	28	25	-0.152499	0.88275	no
ni|645903548|nb|KK920882.1|	852353-852604	nan	nan	87	66	-0.390573	0.78085	no
ni|645903549|nb|KK920881.1|	113113-113287	nan	nan	0.425228	0.638733	0.586975	0.65495	no
ni|645903549|nb|KK920881.1|	11424-11576	nan	nan	26	37	0.530612	0.64665	no
ni|645903549|nb|KK920881.1|	11865-12079	nan	nan	29	42	0.545768	0.6394	no
ni|645903549|nb|KK920881.1|	13552-13765	nan	nan	17	24	0.534404	0.642	no
ni|645903549|nb|KK920881.1|	149952-150790	nan	nan	13	35	1	0.22835	no
ni|645903549|nb|KK920881.1|	1523-1735	nan	nan	1	2	0.370772	0.76295	no
ni|645903549|nb|KK920881.1|	15899-16102	nan	nan	29	37	0.35907	0.75695	no
ni|645903549|nb|KK920881.1|	16429-17159	nan	nan	28	45	0.700556	0.55105	no
ni|645903549|nb|KK920881.1|	176343-176845	nan	nan	3	8	1	0.2657	no
ni|645903549|nb|KK920881.1|	178042-178189	nan	nan	3	2	-0.452832	0.69415	no
ni|645903549|nb|KK920881.1|	19033-19232	nan	nan	10	10	-0.0758331	0.9611	no
ni|645903549|nb|KK920881.1|	19336-19606	nan	nan	2	1	-0.513258	0.697	no
ni|645903549|nb|KK920881.1|	201439-201611	nan	nan	15	16	0.108684	0.92825	no
ni|645903549|nb|KK920881.1|	21815-22083	nan	nan	10	17	0.750627	0.5232	no
ni|645903549|nb|KK920881.1|	23376-23499	nan	nan	3	16	2	0.0429	no
ni|645903549|nb|KK920881.1|	24610-24873	nan	nan	10	53	2	0.05	no
ni|645903549|nb|KK920881.1|	258568-258689	nan	nan	4	6	0.584873	0.6992	no
ni|645903549|nb|KK920881.1|	26381-26518	nan	nan	8	60	3	0.0201	no
ni|645903549|nb|KK920881.1|	27048-27484	nan	nan	2	13	3	0.03275	no
ni|645903549|nb|KK920881.1|	274725-274888	nan	nan	17	34	1	0.3968	no
ni|645903549|nb|KK920881.1|	27650-28106	nan	nan	4	20	2	0.0634	no
ni|645903549|nb|KK920881.1|	28217-28341	nan	nan	1	52	5	0.11955	no
ni|645903549|nb|KK920881.1|	2866-3165	nan	nan	8	5	-0.552669	0.65835	no
ni|645903549|nb|KK920881.1|	28730-28934	nan	nan	4	26	3	0.072	no
ni|645903549|nb|KK920881.1|	29744-29944	nan	nan	0.148416	5	5	0.12055	no
ni|645903549|nb|KK920881.1|	30811-31069	nan	nan	0	9	inf	5.00E-05	yes
ni|645903549|nb|KK920881.1|	32913-33212	nan	nan	0.518013	21	5	0.11985	no

ni|645903549|nb|KK920881.1|	33370-33735	nan	nan	0.238542	11	6	0.1198	no
ni|645903549|nb|KK920881.1|	35210-36045	nan	nan	0.364212	27	6	0.00135	yes
ni|645903549|nb|KK920881.1|	36958-37073	nan	nan	118	77	-0.615617	0.6599	no
ni|645903549|nb|KK920881.1|	40775-41095	nan	nan	0.626573	0.513448	-0.287265	0.8283	no
ni|645903549|nb|KK920881.1|	42242-42713	nan	nan	22	18	-0.311647	0.7969	no
ni|645903549|nb|KK920881.1|	430906-431063	nan	nan	8	11	0.513048	0.6522	no
ni|645903549|nb|KK920881.1|	431972-432848	nan	nan	2	2	-0.443611	0.70515	no
ni|645903549|nb|KK920881.1|	439075-439256	nan	nan	110	247	1	0.32135	no
ni|645903549|nb|KK920881.1|	439437-439584	nan	nan	2	10	2	0.07255	no
ni|645903549|nb|KK920881.1|	439743-439879	nan	nan	48	123	1	0.3584	no
ni|645903549|nb|KK920881.1|	44427-44694	nan	nan	51	58	0.186537	0.86545	no
ni|645903549|nb|KK920881.1|	446382-446521	nan	nan	10	61	3	0.03405	no
ni|645903549|nb|KK920881.1|	456958-457121	nan	nan	140.82	349	1	0.3052	no
ni|645903549|nb|KK920881.1|	45814-46170	nan	nan	26	30	0.222739	0.8441	no
ni|645903549|nb|KK920881.1|	459106-459315	nan	nan	10	44	2	0.07265	no
ni|645903549|nb|KK920881.1|	4604-5227	nan	nan	21	24	0.165949	0.8866	no
ni|645903549|nb|KK920881.1|	461864-462014	nan	nan	1	17	3	0.0436	no
ni|645903549|nb|KK920881.1|	46224-46523	nan	nan	4	4	-0.0673257	0.954	no
ni|645903549|nb|KK920881.1|	463008-463704	nan	nan	8	38	2	0.0767	no
ni|645903549|nb|KK920881.1|	466287-466623	nan	nan	16	68	2	0.1022	no
ni|645903549|nb|KK920881.1|	467637-467842	nan	nan	4	20	2	0.0516	no
ni|645903549|nb|KK920881.1|	468001-468699	nan	nan	74	176	1	0.39255	no
ni|645903549|nb|KK920881.1|	46831-47225	nan	nan	170	142	-0.26059	0.8346	no
ni|645903549|nb|KK920881.1|	470930-471207	nan	nan	6	27	2	0.07215	no
ni|645903549|nb|KK920881.1|	471825-472251	nan	nan	132	282	1	0.4473	no
ni|645903549|nb|KK920881.1|	472611-473307	nan	nan	3	2	-0.356424	0.7931	no
ni|645903549|nb|KK920881.1|	47322-47547	nan	nan	262	197	-0.408569	0.75025	no
ni|645903549|nb|KK920881.1|	473550-473791	nan	nan	3	3	0.0901878	0.9378	no
ni|645903549|nb|KK920881.1|	475841-476043	nan	nan	4	2	-0.803798	0.5588	no
ni|645903549|nb|KK920881.1|	47674-47887	nan	nan	220	166	-0.404887	0.71395	no

ni|645903549|nb|KK920881.1|	476852-477011	nan	nan	2	1	-0.434361	0.71745	no
ni|645903549|nb|KK920881.1|	477144-477338	nan	nan	9	7	-0.273935	0.80935	no
ni|645903549|nb|KK920881.1|	478956-479338	nan	nan	17	15	-0.115872	0.9195	no
ni|645903549|nb|KK920881.1|	479664-479878	nan	nan	23	33	0.535075	0.653	no
ni|645903549|nb|KK920881.1|	480102-480930	nan	nan	0.322059	2	2	0.1026	no
ni|645903549|nb|KK920881.1|	481869-482405	nan	nan	0	10	inf	5.00E-05	yes
ni|645903549|nb|KK920881.1|	485368-485659	nan	nan	22	24	0.157505	0.8873	no
ni|645903549|nb|KK920881.1|	487087-487463	nan	nan	51	54	0.0650129	0.9542	no
ni|645903549|nb|KK920881.1|	487933-488190	nan	nan	22	24	0.151476	0.89605	no
ni|645903549|nb|KK920881.1|	488528-488828	nan	nan	51	52	0.0354629	0.97585	no
ni|645903549|nb|KK920881.1|	488998-489238	nan	nan	19	30	0.610837	0.66965	no
ni|645903549|nb|KK920881.1|	48982-49688	nan	nan	205	192	-0.0915798	0.9307	no
ni|645903549|nb|KK920881.1|	491089-491341	nan	nan	0	4	inf	5.00E-05	yes
ni|645903549|nb|KK920881.1|	493732-493908	nan	nan	0	6	inf	0.00045	yes
ni|645903549|nb|KK920881.1|	49840-50532	nan	nan	3	5	0.57013	0.67875	no
ni|645903549|nb|KK920881.1|	51054-51711	nan	nan	0.196856	35.53	7	0.11935	no
ni|645903549|nb|KK920881.1|	52861-53787	nan	nan	0.070853	10	7	0.0443	no
ni|645903549|nb|KK920881.1|	54388-54860	nan	nan	0	7	inf	0.00015	yes
ni|645903549|nb|KK920881.1|	5587-6823	nan	nan	9	9	0.120773	0.92055	no
ni|645903549|nb|KK920881.1|	56597-56844	nan	nan	0	28	inf	5.00E-05	yes
ni|645903549|nb|KK920881.1|	59406-59883	nan	nan	13	10	-0.482557	0.7594	no
ni|645903549|nb|KK920881.1|	60014-60428	nan	nan	17	32	0.913722	0.4319	no
ni|645903549|nb|KK920881.1|	8978-9123	nan	nan	22	25	0.210209	0.85945	no
ni|645903549|nb|KK920881.1|	9440-9866	nan	nan	47	50	0.114933	0.9173	no
ni|645903550|nb|KK920880.1|	143282-143539	nan	nan	7	8	0.167766	0.88995	no
ni|645903550|nb|KK920880.1|	143715-144012	nan	nan	2	1	-0.158588	0.89795	no
ni|645903550|nb|KK920880.1|	186527-186699	nan	nan	8	5	-0.730161	0.5717	no
ni|645903550|nb|KK920880.1|	273106-273356	nan	nan	12	7	-0.852844	0.46185	no
ni|645903550|nb|KK920880.1|	304790-305095	nan	nan	19	13	-0.517856	0.66225	no
ni|645903550|nb|KK920880.1|	318772-318917	nan	nan	10	7	-0.621254	0.5984	no

ni|645903550|nb|KK920880.1|	328251-328520	nan	nan	11	13	0.222294	0.85195	no
ni|645903550|nb|KK920880.1|	83695-84060	nan	nan	13	14	0.0395601	0.95995	no
ni|645903551|nb|KK920879.1|	118027-118225	nan	nan	10	3	-2	0.25505	no
ni|645903551|nb|KK920879.1|	126828-126975	nan	nan	12	6	-1	0.33675	no
ni|645903551|nb|KK920879.1|	130308-130552	nan	nan	2	2	-0.139516	0.90955	no
ni|645903551|nb|KK920879.1|	163290-163793	nan	nan	3	2	-0.498605	0.6775	no
ni|645903551|nb|KK920879.1|	164310-164647	nan	nan	25	22	-0.156021	0.92	no
ni|645903551|nb|KK920879.1|	166031-166215	nan	nan	8	8	-0.0906043	0.93735	no
ni|645903551|nb|KK920879.1|	26654-27426	nan	nan	19	5	-2	0.18825	no
ni|645903551|nb|KK920879.1|	28259-28459	nan	nan	16	14	-0.195624	0.8661	no
ni|645903551|nb|KK920879.1|	29669-29858	nan	nan	11	7	-0.680182	0.5787	no
ni|645903551|nb|KK920879.1|	302801-302968	nan	nan	6	5	-0.0510664	0.9679	no
ni|645903551|nb|KK920879.1|	304152-304280	nan	nan	9	9	0.015065	0.98865	no
ni|645903551|nb|KK920879.1|	305144-305486	nan	nan	4	6	0.347782	0.7834	no
ni|645903551|nb|KK920879.1|	306723-306919	nan	nan	7	4	-0.897271	0.502	no
ni|645903551|nb|KK920879.1|	307373-307552	nan	nan	5	3	-0.809991	0.48645	no
ni|645903551|nb|KK920879.1|	309418-309640	nan	nan	11	4	-2	0.18505	no
ni|645903551|nb|KK920879.1|	310600-311023	nan	nan	27	10	-1	0.21875	no
ni|645903551|nb|KK920879.1|	312208-312396	nan	nan	8	2	-2	0.16095	no
ni|645903551|nb|KK920879.1|	312777-312951	nan	nan	15	6	-1	0.28755	no
ni|645903551|nb|KK920879.1|	314140-314396	nan	nan	0	7	inf	0.0009	yes
ni|645903551|nb|KK920879.1|	315370-315586	nan	nan	22	25	0.210066	0.85855	no
ni|645903551|nb|KK920879.1|	317157-317436	nan	nan	4959.85	4597.91	-0.10932	0.92665	no
ni|645903551|nb|KK920879.1|	318689-319459	nan	nan	9	13	0.489286	0.6794	no
ni|645903551|nb|KK920879.1|	319565-319712	nan	nan	15	17	0.219605	0.85905	no
ni|645903551|nb|KK920879.1|	319846-320001	nan	nan	38	47	0.300504	0.7966	no
ni|645903551|nb|KK920879.1|	321056-321239	nan	nan	16	16	-0.00703376	0.99485	no
ni|645903551|nb|KK920879.1|	321462-323294	nan	nan	16	15	-0.107091	0.92855	no
ni|645903551|nb|KK920879.1|	323769-323951	nan	nan	1	0.738907	-0.830827	0.5313	no
ni|645903551|nb|KK920879.1|	351388-351661	nan	nan	7	6	-0.297216	0.80035	no

ni|645903551|nb|KK920879.1|	351987-352195	nan	nan	0.413468	0.287505	-0.524187	0.7071	no
ni|645903551|nb|KK920879.1|	354061-354270	nan	nan	36	45	0.310965	0.80035	no
ni|645903551|nb|KK920879.1|	354491-354810	nan	nan	45	36	-0.327054	0.82495	no
ni|645903551|nb|KK920879.1|	354961-355277	nan	nan	7	12	0.740156	0.54275	no
ni|645903551|nb|KK920879.1|	355413-356275	nan	nan	6	7	0.237529	0.8456	no
ni|645903551|nb|KK920879.1|	356462-356851	nan	nan	6	9	0.541627	0.6397	no
ni|645903551|nb|KK920879.1|	357148-357662	nan	nan	7	9	0.517584	0.7279	no
ni|645903551|nb|KK920879.1|	357916-358039	nan	nan	7	9	0.347855	0.76865	no
ni|645903551|nb|KK920879.1|	358319-358544	nan	nan	4	7	1	0.38905	no
ni|645903551|nb|KK920879.1|	358852-359013	nan	nan	3	7	1	0.2687	no
ni|645903551|nb|KK920879.1|	360217-360935	nan	nan	0.277639	0.371676	0.420835	0.73705	no
ni|645903551|nb|KK920879.1|	363394-364319	nan	nan	5	13	1	0.3349	no
ni|645903551|nb|KK920879.1|	369091-369306	nan	nan	5	11	1	0.3484	no
ni|645903551|nb|KK920879.1|	369534-369700	nan	nan	3	8	2	0.22065	no
ni|645903551|nb|KK920879.1|	369909-370078	nan	nan	4	7	0.880489	0.4849	no
ni|645903551|nb|KK920879.1|	371652-371798	nan	nan	4	10	1	0.35855	no
ni|645903551|nb|KK920879.1|	374713-375172	nan	nan	0.039265	3	6	0.11935	no
ni|645903551|nb|KK920879.1|	375300-375518	nan	nan	15	21	0.516283	0.6307	no
ni|645903551|nb|KK920879.1|	376358-376689	nan	nan	36	39	0.102657	0.94495	no
ni|645903551|nb|KK920879.1|	377570-377781	nan	nan	34	15	-1	0.3211	no
ni|645903551|nb|KK920879.1|	378596-379028	nan	nan	32.28	34	0.0790203	0.94955	no
ni|645903551|nb|KK920879.1|	380854-381062	nan	nan	2	3	0.561636	0.667	no
ni|645903551|nb|KK920879.1|	381171-382045	nan	nan	4	3	-0.21923	0.87705	no
ni|645903551|nb|KK920879.1|	382600-383104	nan	nan	5	3	-0.915205	0.53575	no
ni|645903551|nb|KK920879.1|	383308-383430	nan	nan	1	1	0.19802	0.8693	no
ni|645903551|nb|KK920879.1|	385575-386048	nan	nan	3	5	0.595838	0.63005	no
ni|645903551|nb|KK920879.1|	395585-395968	nan	nan	0.06542	54	10	0.1194	no
ni|645903551|nb|KK920879.1|	396109-396208	nan	nan	0	102	inf	5.00E-05	yes
ni|645903551|nb|KK920879.1|	399197-399597	nan	nan	0	3	inf	5.00E-05	yes
ni|645903551|nb|KK920879.1|	406241-406499	nan	nan	28	29	0.0105078	0.9704	no

ni|645903551|nb|KK920879.1|	407728-407889	nan	nan	43	37	-0.226522	0.84485	no
ni|645903551|nb|KK920879.1|	408318-408559	nan	nan	35	43	0.27491	0.8167	no
ni|645903551|nb|KK920879.1|	409541-409810	nan	nan	7	6	-0.211689	0.8562	no
ni|645903551|nb|KK920879.1|	410466-410671	nan	nan	3	1	-1	0.46615	no
ni|645903551|nb|KK920879.1|	410873-411882	nan	nan	5	3	-0.836609	0.4978	no
ni|645903551|nb|KK920879.1|	412144-412332	nan	nan	13	10	-0.482557	0.7594	no
ni|645903551|nb|KK920879.1|	412575-412715	nan	nan	16	24	0.556327	0.6165	no
ni|645903551|nb|KK920879.1|	412880-413170	nan	nan	41	53	0.368694	0.7565	no
ni|645903551|nb|KK920879.1|	413656-414048	nan	nan	18	16	-0.192553	0.8697	no
ni|645903551|nb|KK920879.1|	414540-414683	nan	nan	2	5	1	0.29905	no
ni|645903551|nb|KK920879.1|	414832-415557	nan	nan	7	7	-0.0818095	0.94535	no
ni|645903551|nb|KK920879.1|	417255-417404	nan	nan	0	20	inf	5.00E-05	yes
ni|645903551|nb|KK920879.1|	419542-419694	nan	nan	6	12	1	0.4054	no
ni|645903551|nb|KK920879.1|	420115-420394	nan	nan	0	2	inf	0.0001	yes
ni|645903551|nb|KK920879.1|	420511-421388	nan	nan	0	188	inf	5.00E-05	yes
ni|645903551|nb|KK920879.1|	422571-422858	nan	nan	0	52	inf	5.00E-05	yes
ni|645903551|nb|KK920879.1|	423346-424056	nan	nan	16	22	0.437231	0.7064	no
ni|645903551|nb|KK920879.1|	424360-424896	nan	nan	0	378	inf	5.00E-05	yes
ni|645903551|nb|KK920879.1|	425083-425899	nan	nan	22	29	0.422902	0.71505	no
ni|645903551|nb|KK920879.1|	426431-426846	nan	nan	493	287	-0.780792	0.5884	no
ni|645903551|nb|KK920879.1|	428221-428819	nan	nan	2	10	3	0.03345	no
ni|645903551|nb|KK920879.1|	429673-430273	nan	nan	129.15	89	-0.534511	0.7128	no
ni|645903551|nb|KK920879.1|	431166-431470	nan	nan	8	5	-0.581852	0.70815	no
ni|645903551|nb|KK920879.1|	431607-431786	nan	nan	6	5	-0.300831	0.7957	no
ni|645903551|nb|KK920879.1|	435461-435719	nan	nan	5	3	-0.770131	0.5207	no
ni|645903551|nb|KK920879.1|	440219-440553	nan	nan	3	2	-1	0.39745	no
ni|645903551|nb|KK920879.1|	440798-441091	nan	nan	418	310	-0.429147	0.75805	no
ni|645903551|nb|KK920879.1|	442933-443166	nan	nan	59	41	-0.526676	0.6498	no
ni|645903551|nb|KK920879.1|	444980-445177	nan	nan	112	102.58	-0.124463	0.93155	no
ni|645903551|nb|KK920879.1|	448512-449490	nan	nan	1	0.802048	-0.38752	0.78555	no

ni|645903551|nb|KK920879.1|	450495-450634	nan	nan	22	24	0.132044	0.9011	no
ni|645903551|nb|KK920879.1|	453042-453172	nan	nan	6	10	0.706826	0.5514	no
ni|645903551|nb|KK920879.1|	457879-458339	nan	nan	50	50	-0.015811	0.98985	no
ni|645903551|nb|KK920879.1|	459720-460030	nan	nan	88	86	-0.0438189	0.97645	no
ni|645903551|nb|KK920879.1|	465620-465753	nan	nan	292	264	-0.14369	0.9224	no
ni|645903551|nb|KK920879.1|	466134-466255	nan	nan	187	168.84	-0.143738	0.9239	no
ni|645903551|nb|KK920879.1|	466391-466505	nan	nan	2	2	0.5864	0.63255	no
ni|645903551|nb|KK920879.1|	466779-466909	nan	nan	22	13	-0.752214	0.52175	no
ni|645903551|nb|KK920879.1|	467565-467724	nan	nan	12	10	-0.189068	0.87115	no
ni|645903551|nb|KK920879.1|	468012-468133	nan	nan	5	3	-0.915219	0.53575	no
ni|645903551|nb|KK920879.1|	468267-468388	nan	nan	4	5	0.0703071	0.94415	no
ni|645903551|nb|KK920879.1|	468807-468941	nan	nan	3	5	0.517757	0.7004	no
ni|645903551|nb|KK920879.1|	470547-470681	nan	nan	58	21	-1	0.24285	no
ni|645903551|nb|KK920879.1|	471415-471584	nan	nan	152	138	-0.130969	0.92675	no
ni|645903551|nb|KK920879.1|	471935-472088	nan	nan	136	129	-0.0791811	0.94435	no
ni|645903551|nb|KK920879.1|	480147-480405	nan	nan	137	140	0.0301497	0.9742	no
ni|645903551|nb|KK920879.1|	482269-482970	nan	nan	84	93	0.140084	0.8953	no
ni|645903551|nb|KK920879.1|	484484-484743	nan	nan	105	127	0.26896	0.80355	no
ni|645903551|nb|KK920879.1|	484966-485391	nan	nan	116.66	162	0.477939	0.67595	no
ni|645903551|nb|KK920879.1|	485797-485973	nan	nan	118	138	0.223611	0.83715	no
ni|645903551|nb|KK920879.1|	486162-486583	nan	nan	84	119	0.505045	0.65915	no
ni|645903551|nb|KK920879.1|	488811-488965	nan	nan	142	155	0.128918	0.9164	no
ni|645903551|nb|KK920879.1|	489227-489461	nan	nan	73	95	0.385234	0.7359	no
ni|645903551|nb|KK920879.1|	491215-491761	nan	nan	54	66	0.283692	0.82185	no
ni|645903551|nb|KK920879.1|	491894-492326	nan	nan	100	116	0.211896	0.83865	no
ni|645903551|nb|KK920879.1|	492439-492818	nan	nan	61	66	0.109183	0.9183	no
ni|645903551|nb|KK920879.1|	492879-493481	nan	nan	0.702842	0.366443	-0.93961	0.54025	no
ni|645903551|nb|KK920879.1|	49347-49720	nan	nan	12.9	12	-0.160322	0.8872	no
ni|645903551|nb|KK920879.1|	493949-494784	nan	nan	0.418003	0.299693	-0.480025	0.7594	no
ni|645903551|nb|KK920879.1|	495383-495743	nan	nan	26	33	0.296635	0.7968	no

ni|645903551|nb|KK920879.1|	495988-496133	nan	nan	127.77	152	0.246254	0.823	no
ni|645903551|nb|KK920879.1|	496510-497154	nan	nan	66	71	0.0923996	0.9346	no
ni|645903551|nb|KK920879.1|	500320-500792	nan	nan	106	131	0.305292	0.8114	no
ni|645903551|nb|KK920879.1|	501245-501410	nan	nan	85	98	0.209129	0.84775	no
ni|645903551|nb|KK920879.1|	502001-502212	nan	nan	104	137	0.401658	0.74495	no
ni|645903551|nb|KK920879.1|	504464-504624	nan	nan	57	89	0.650984	0.5578	no
ni|645903551|nb|KK920879.1|	505050-505487	nan	nan	109	127	0.229088	0.82985	no
ni|645903551|nb|KK920879.1|	505604-506415	nan	nan	108	143	0.39809	0.70915	no
ni|645903551|nb|KK920879.1|	507312-507432	nan	nan	73	82	0.165825	0.8832	no
ni|645903551|nb|KK920879.1|	507961-508343	nan	nan	55	66	0.27068	0.82395	no
ni|645903551|nb|KK920879.1|	509821-510018	nan	nan	88	115	0.382109	0.72825	no
ni|645903551|nb|KK920879.1|	511827-512126	nan	nan	37	47	0.353604	0.74945	no
ni|645903551|nb|KK920879.1|	513021-513484	nan	nan	5	36	3	0.0282	no
ni|645903551|nb|KK920879.1|	514162-514327	nan	nan	0.648143	948	11	0.03715	no
ni|645903551|nb|KK920879.1|	515664-516271	nan	nan	0.192267	1013.18	12	0.11935	no
ni|645903551|nb|KK920879.1|	516526-516967	nan	nan	0.135142	923	13	0.0443	no
ni|645903551|nb|KK920879.1|	517414-517689	nan	nan	327	152	-1	0.37395	no
ni|645903551|nb|KK920879.1|	517911-518174	nan	nan	889	463	-0.9416	0.5102	no
ni|645903551|nb|KK920879.1|	519173-519416	nan	nan	18	8	-1	0.33	no
ni|645903551|nb|KK920879.1|	519595-519789	nan	nan	12	9	-0.372063	0.762	no
ni|645903551|nb|KK920879.1|	520093-520507	nan	nan	6	4	-0.652287	0.6092	no
ni|645903551|nb|KK920879.1|	521506-521892	nan	nan	0.689484	0.202803	-2	0.23195	no
ni|645903551|nb|KK920879.1|	522627-523449	nan	nan	38	33	-0.176817	0.87665	no
ni|645903551|nb|KK920879.1|	546486-546723	nan	nan	143	163	0.186625	0.9003	no
ni|645903551|nb|KK920879.1|	547524-547939	nan	nan	11	0.200742	-6	0.0177	no
ni|645903551|nb|KK920879.1|	549381-549565	nan	nan	16	16	0.0448366	0.97	no
ni|645903551|nb|KK920879.1|	550287-550412	nan	nan	2	2	-0.218384	0.87705	no
ni|645903551|nb|KK920879.1|	551224-551355	nan	nan	3	4	0.584808	0.6992	no
ni|645903551|nb|KK920879.1|	552504-552663	nan	nan	11	14	0.3885	0.7342	no
ni|645903551|nb|KK920879.1|	554179-554406	nan	nan	2	1	-1	0.38355	no

ni|645903551|nb|KK920879.1|	557062-557227	nan	nan	1	0.486556	-1	0.28135	no
ni|645903551|nb|KK920879.1|	562278-562444	nan	nan	21	16	-0.333913	0.7766	no
ni|645903551|nb|KK920879.1|	562576-562992	nan	nan	20	20	0.0351203	0.9777	no
ni|645903551|nb|KK920879.1|	564653-564984	nan	nan	20	24	0.246441	0.8264	no
ni|645903551|nb|KK920879.1|	566416-566572	nan	nan	9	9	-0.0224254	0.98395	no
ni|645903551|nb|KK920879.1|	569792-570120	nan	nan	13	13	0.0113653	0.9925	no
ni|645903551|nb|KK920879.1|	571110-571387	nan	nan	15	11	-0.413895	0.72175	no
ni|645903551|nb|KK920879.1|	571675-571915	nan	nan	22	20	-0.167192	0.8885	no
ni|645903551|nb|KK920879.1|	572815-572958	nan	nan	43	19	-1	0.33675	no
ni|645903551|nb|KK920879.1|	573373-573562	nan	nan	12	9	-0.4279	0.7134	no
ni|645903551|nb|KK920879.1|	573642-573816	nan	nan	27	29	0.106328	0.9321	no
ni|645903551|nb|KK920879.1|	578998-579141	nan	nan	39	24	-0.692479	0.5474	no
ni|645903551|nb|KK920879.1|	580973-581114	nan	nan	4	2	-1	0.35325	no
ni|645903551|nb|KK920879.1|	582641-582800	nan	nan	10	10	0.0582171	0.9532	no
ni|645903551|nb|KK920879.1|	583080-583266	nan	nan	6	7	0.28939	0.8058	no
ni|645903551|nb|KK920879.1|	586202-586761	nan	nan	41	36	-0.158551	0.88315	no
ni|645903551|nb|KK920879.1|	587801-588029	nan	nan	7	3	-1	0.38245	no
ni|645903551|nb|KK920879.1|	631030-631176	nan	nan	1	2	0.157079	0.8958	no
ni|645903551|nb|KK920879.1|	636714-637164	nan	nan	2	2	-0.649805	0.58435	no
ni|645903551|nb|KK920879.1|	641607-641781	nan	nan	3	3	0.300048	0.7984	no
ni|645903551|nb|KK920879.1|	644391-644865	nan	nan	0.382294	0.109653	-2	0.2784	no
ni|645903551|nb|KK920879.1|	858558-858990	nan	nan	3	2	-0.374399	0.74915	no
ni|645903552|nb|KK920878.1|	187175-187655	nan	nan	2	2	0.519042	0.68765	no
ni|645903552|nb|KK920878.1|	195496-195703	nan	nan	76	66	-0.185245	0.88	no
ni|645903552|nb|KK920878.1|	204436-204567	nan	nan	0	9	inf	5.00E-05	yes
ni|645903552|nb|KK920878.1|	204710-205080	nan	nan	0.105371	4	5	0.0506	no
ni|645903552|nb|KK920878.1|	206904-207093	nan	nan	2	10	2	0.1063	no
ni|645903552|nb|KK920878.1|	212113-212235	nan	nan	113	119	0.0717724	0.9596	no
ni|645903552|nb|KK920878.1|	212368-212505	nan	nan	14	14	0.022898	0.9823	no
ni|645903552|nb|KK920878.1|	213691-213848	nan	nan	2	0.722294	-1	0.36935	no

ni|645903552|nb|KK920878.1|	214043-214178	nan	nan	0.914808	0.426183	-1	0.3573	no
ni|645903552|nb|KK920878.1|	214713-214846	nan	nan	3	3	-0.243575	0.83855	no
ni|645903552|nb|KK920878.1|	215292-215452	nan	nan	122	91	-0.418418	0.71715	no
ni|645903552|nb|KK920878.1|	215754-215985	nan	nan	52	52	0.0158213	0.99065	no
ni|645903552|nb|KK920878.1|	216658-217157	nan	nan	54	40	-0.450216	0.7139	no
ni|645903552|nb|KK920878.1|	268713-268863	nan	nan	72	59	-0.279085	0.80025	no
ni|645903552|nb|KK920878.1|	30657-30832	nan	nan	97	84	-0.196146	0.85855	no
ni|645903552|nb|KK920878.1|	388610-388757	nan	nan	54	32	-0.738989	0.5279	no
ni|645903552|nb|KK920878.1|	388953-389605	nan	nan	88	75	-0.243266	0.8201	no
ni|645903552|nb|KK920878.1|	390766-390905	nan	nan	0.807416	1	0.382208	0.7694	no
ni|645903552|nb|KK920878.1|	391496-391680	nan	nan	4	3	-0.235241	0.8418	no
ni|645903552|nb|KK920878.1|	391971-392719	nan	nan	11	3	-2	0.2784	no
ni|645903552|nb|KK920878.1|	392962-393237	nan	nan	185	275	0.571763	0.60845	no
ni|645903552|nb|KK920878.1|	393834-394003	nan	nan	163	252	0.627583	0.57075	no
ni|645903552|nb|KK920878.1|	394234-394445	nan	nan	90	136	0.585282	0.68965	no
ni|645903552|nb|KK920878.1|	395566-395762	nan	nan	35	49	0.478251	0.66355	no
ni|645903552|nb|KK920878.1|	396898-397893	nan	nan	50	48	-0.0676564	0.957	no
ni|645903552|nb|KK920878.1|	398527-398738	nan	nan	84	88	0.077005	0.9598	no
ni|645903552|nb|KK920878.1|	400657-400820	nan	nan	176	187	0.0918218	0.937	no
ni|645903552|nb|KK920878.1|	402265-402410	nan	nan	57	67	0.241916	0.85085	no
ni|645903552|nb|KK920878.1|	403154-403655	nan	nan	25	28	0.182207	0.8704	no
ni|645903552|nb|KK920878.1|	404661-404869	nan	nan	40	50	0.328047	0.78895	no
ni|645903552|nb|KK920878.1|	405079-405467	nan	nan	133	136	0.0287124	0.97965	no
ni|645903552|nb|KK920878.1|	507980-508185	nan	nan	168	190	0.183097	0.89775	no
ni|645903552|nb|KK920878.1|	509470-509627	nan	nan	146.77	180	0.290656	0.8345	no
ni|645903552|nb|KK920878.1|	514376-514776	nan	nan	13	17	0.393674	0.7355	no
ni|645903552|nb|KK920878.1|	515131-515295	nan	nan	0.437487	0.689033	0.655334	0.5934	no
ni|645903552|nb|KK920878.1|	515734-515836	nan	nan	26	31	0.265777	0.8064	no
ni|645903552|nb|KK920878.1|	515920-516089	nan	nan	158	165.02	0.0668447	0.95205	no
ni|645903552|nb|KK920878.1|	516848-517151	nan	nan	69	88	0.336624	0.81775	no

ni|645903552|nb|KK920878.1|	517765-517997	nan	nan	83	100	0.261539	0.8611	no
ni|645903552|nb|KK920878.1|	519002-519195	nan	nan	35	31	-0.147027	0.89805	no
ni|645903552|nb|KK920878.1|	532037-532216	nan	nan	2	0.688245	-1	0.4064	no
ni|645903552|nb|KK920878.1|	532332-532678	nan	nan	1	0.446138	-2	0.2459	no
ni|645903552|nb|KK920878.1|	540349-540755	nan	nan	0.884452	0.739468	-0.258296	0.8692	no
ni|645903552|nb|KK920878.1|	552402-552794	nan	nan	9	9	-0.00279528	0.99795	no
ni|645903552|nb|KK920878.1|	55316-55753	nan	nan	124	133	0.101423	0.9448	no
ni|645903552|nb|KK920878.1|	556541-556706	nan	nan	5	5	-0.170456	0.88065	no
ni|645903552|nb|KK920878.1|	557635-558206	nan	nan	13	12	-0.0671964	0.9555	no
ni|645903552|nb|KK920878.1|	559174-559414	nan	nan	7	6	-0.265991	0.81915	no
ni|645903552|nb|KK920878.1|	576714-576925	nan	nan	20	14	-0.50162	0.67195	no
ni|645903552|nb|KK920878.1|	578271-578474	nan	nan	7	5	-0.357269	0.76145	no
ni|645903552|nb|KK920878.1|	585189-585310	nan	nan	8	2	-2	0.22995	no
ni|645903552|nb|KK920878.1|	585367-585531	nan	nan	0	70	inf	5.00E-05	yes
ni|645903552|nb|KK920878.1|	585737-585845	nan	nan	0	7	inf	5.00E-05	yes
ni|645903552|nb|KK920878.1|	586099-586538	nan	nan	25	19	-0.430341	0.75965	no
ni|645903552|nb|KK920878.1|	588718-588956	nan	nan	0.866902	0.552283	-0.650461	0.6679	no
ni|645903552|nb|KK920878.1|	589993-590189	nan	nan	0	298.13	inf	5.00E-05	yes
ni|645903552|nb|KK920878.1|	592004-592307	nan	nan	0	108	inf	5.00E-05	yes
ni|645903552|nb|KK920878.1|	595361-595560	nan	nan	0	145	inf	5.00E-05	yes
ni|645903552|nb|KK920878.1|	595813-596439	nan	nan	5	4	-0.259883	0.8692	no
ni|645903552|nb|KK920878.1|	597348-597552	nan	nan	1	0.714354	-0.693221	0.58855	no
ni|645903552|nb|KK920878.1|	599325-599782	nan	nan	63	42	-0.578341	0.6178	no
ni|645903552|nb|KK920878.1|	601070-601574	nan	nan	57	63	0.141785	0.8899	no
ni|645903552|nb|KK920878.1|	602293-602492	nan	nan	2	1	-0.630708	0.60425	no
ni|645903552|nb|KK920878.1|	605264-605520	nan	nan	3	2	-0.782108	0.49885	no
ni|645903552|nb|KK920878.1|	606959-607419	nan	nan	3	4	0.447346	0.7576	no
ni|645903552|nb|KK920878.1|	607898-608177	nan	nan	3	4	0.156107	0.8959	no
ni|645903552|nb|KK920878.1|	610119-610400	nan	nan	3	2	-0.462417	0.69645	no
ni|645903552|nb|KK920878.1|	610504-610800	nan	nan	11	9	-0.301671	0.81	no

ni|645903552|nb|KK920878.1|	611008-611137	nan	nan	10	13	0.284203	0.811	no
ni|645903552|nb|KK920878.1|	611235-611468	nan	nan	8	11	0.580717	0.6298	no
ni|645903552|nb|KK920878.1|	611673-611948	nan	nan	6	7	0.0590907	0.95735	no
ni|645903552|nb|KK920878.1|	612234-612518	nan	nan	7	9	0.425239	0.71255	no
ni|645903552|nb|KK920878.1|	614729-614926	nan	nan	6	8	0.447373	0.7237	no
ni|645903552|nb|KK920878.1|	615975-616644	nan	nan	3	2	-0.551822	0.68265	no
ni|645903552|nb|KK920878.1|	617553-617897	nan	nan	1	0.246208	-2	0.11635	no
ni|645903552|nb|KK920878.1|	618147-618286	nan	nan	1	0.660986	-0.994288	0.4237	no
ni|645903552|nb|KK920878.1|	618520-618967	nan	nan	0.573567	0.974659	0.764936	0.5642	no
ni|645903552|nb|KK920878.1|	619132-619678	nan	nan	4	6	0.554172	0.6571	no
ni|645903552|nb|KK920878.1|	620627-620897	nan	nan	7	9	0.237848	0.83115	no
ni|645903552|nb|KK920878.1|	621046-621412	nan	nan	32	30	-0.0927629	0.93205	no
ni|645903552|nb|KK920878.1|	621721-621902	nan	nan	21	20	-0.0783975	0.9423	no
ni|645903552|nb|KK920878.1|	624078-624215	nan	nan	76	72	-0.0675775	0.95075	no
ni|645903552|nb|KK920878.1|	624374-624539	nan	nan	6	16	1	0.21895	no
ni|645903552|nb|KK920878.1|	624925-625337	nan	nan	51	35	-0.559069	0.6258	no
ni|645903552|nb|KK920878.1|	626040-626419	nan	nan	57	52	-0.14817	0.8948	no
ni|645903552|nb|KK920878.1|	626724-626973	nan	nan	5	5	0.110246	0.9254	no
ni|645903552|nb|KK920878.1|	627229-627869	nan	nan	2	2	0.0014931	0.97495	no
ni|645903552|nb|KK920878.1|	628126-628257	nan	nan	3	5	0.621601	0.6185	no
ni|645903552|nb|KK920878.1|	628539-628940	nan	nan	3	1	-1	0.35155	no
ni|645903552|nb|KK920878.1|	629323-631169	nan	nan	5	1	-2	0.24815	no
ni|645903552|nb|KK920878.1|	631876-632133	nan	nan	1	0.62292	-1	0.4064	no
ni|645903552|nb|KK920878.1|	632708-632986	nan	nan	11	3	-2	0.13085	no
ni|645903552|nb|KK920878.1|	633340-633524	nan	nan	0.841682	0.563079	-0.579937	0.70815	no
ni|645903552|nb|KK920878.1|	634042-634203	nan	nan	32	22	-0.558968	0.6274	no
ni|645903552|nb|KK920878.1|	634770-634919	nan	nan	23	18	-0.320696	0.78395	no
ni|645903552|nb|KK920878.1|	677702-677839	nan	nan	1	0.351879	-2	0.2252	no
ni|645903552|nb|KK920878.1|	678233-678873	nan	nan	3	0.568484	-2	0.1864	no
ni|645903552|nb|KK920878.1|	702923-703183	nan	nan	61	53	-0.198446	0.86325	no

ni|645903552|nb|KK920878.1|	716675-717067	nan	nan	17	16	-0.0672156	0.95195	no
ni|645903552|nb|KK920878.1|	808670-809355	nan	nan	46	69	0.592911	0.6811	no
ni|645903552|nb|KK920878.1|	810327-810634	nan	nan	0.491595	0.391522	-0.328375	0.8122	no
ni|645903552|nb|KK920878.1|	828898-829116	nan	nan	73	53	-0.4531	0.67505	no
ni|645903552|nb|KK920878.1|	829868-830205	nan	nan	0.087137	5	6	0.0446	no
ni|645903552|nb|KK920878.1|	831002-831232	nan	nan	0	2	inf	5.00E-05	yes
ni|645903552|nb|KK920878.1|	831339-831635	nan	nan	0.289134	0.394902	0.449756	0.7543	no
ni|645903552|nb|KK920878.1|	832482-833115	nan	nan	11	8	-0.455626	0.7028	no
ni|645903552|nb|KK920878.1|	844791-844963	nan	nan	2	1	-0.235072	0.84165	no
ni|645903552|nb|KK920878.1|	848819-849654	nan	nan	0.805594	0.855746	0.0871306	0.9485	no
ni|645903552|nb|KK920878.1|	850129-850309	nan	nan	3	5	0.53583	0.65555	no
ni|645903552|nb|KK920878.1|	850409-851135	nan	nan	106.92	195	0.867125	0.4445	no
ni|645903552|nb|KK920878.1|	852443-852993	nan	nan	71	206	2	0.17745	no
ni|645903552|nb|KK920878.1|	853939-855752	nan	nan	22	67	2	0.15925	no
ni|645903552|nb|KK920878.1|	878623-879162	nan	nan	22	82	2	0.1603	no
ni|645903552|nb|KK920878.1|	879858-880151	nan	nan	8	38	2	0.1356	no
ni|645903552|nb|KK920878.1|	880303-880422	nan	nan	19	77.63	2	0.11665	no
ni|645903553|nb|KK920877.1|	106671-106840	nan	nan	5	5	0.0412767	0.96875	no
ni|645903553|nb|KK920877.1|	107156-107906	nan	nan	8	10	0.361602	0.7643	no
ni|645903553|nb|KK920877.1|	109120-109589	nan	nan	15	17	0.18277	0.8733	no
ni|645903553|nb|KK920877.1|	110143-110619	nan	nan	18	18	0.00339736	0.9946	no
ni|645903553|nb|KK920877.1|	110898-111165	nan	nan	13	11	-0.237181	0.8398	no
ni|645903553|nb|KK920877.1|	111417-111604	nan	nan	0.82354	2	0.97464	0.428	no
ni|645903553|nb|KK920877.1|	112566-112748	nan	nan	0.613863	2	1	0.34405	no
ni|645903553|nb|KK920877.1|	114105-114414	nan	nan	2	2	-0.327927	0.7785	no
ni|645903553|nb|KK920877.1|	114584-114778	nan	nan	0.275024	0.569677	1	0.45555	no
ni|645903553|nb|KK920877.1|	122480-127775	nan	nan	0.634395	1	0.742042	0.5765	no
ni|645903553|nb|KK920877.1|	143464-143675	nan	nan	8	7	-0.225559	0.84355	no
ni|645903553|nb|KK920877.1|	173422-173686	nan	nan	22	36	0.700929	0.51205	no
ni|645903553|nb|KK920877.1|	182421-182598	nan	nan	57	88	0.616543	0.6745	no

ni|645903553|nb|KK920877.1|	190848-191060	nan	nan	1	0.415366	-2	0.2784	no
ni|645903553|nb|KK920877.1|	194016-194242	nan	nan	12	8	-0.514681	0.66705	no
ni|645903553|nb|KK920877.1|	211534-211692	nan	nan	0	20	inf	5.00E-05	yes
ni|645903553|nb|KK920877.1|	21516-21731	nan	nan	24	16	-0.623971	0.57425	no
ni|645903553|nb|KK920877.1|	216936-217168	nan	nan	10	8	-0.338997	0.7756	no
ni|645903553|nb|KK920877.1|	22015-22386	nan	nan	18	16	-0.216085	0.8428	no
ni|645903553|nb|KK920877.1|	243497-243825	nan	nan	9.29	5	-0.941774	0.54025	no
ni|645903553|nb|KK920877.1|	249630-250514	nan	nan	66	46	-0.519314	0.6497	no
ni|645903553|nb|KK920877.1|	30682-30841	nan	nan	23	21	-0.103227	0.9235	no
ni|645903553|nb|KK920877.1|	36314-36531	nan	nan	24	22	-0.114328	0.9217	no
ni|645903553|nb|KK920877.1|	40095-40291	nan	nan	37	32	-0.22518	0.8448	no
ni|645903553|nb|KK920877.1|	53371-53579	nan	nan	30	28	-0.0870364	0.93615	no
ni|645903553|nb|KK920877.1|	55617-56373	nan	nan	37	37	-0.00217437	0.9971	no
ni|645903553|nb|KK920877.1|	57009-57147	nan	nan	41	26	-0.652354	0.58225	no
ni|645903553|nb|KK920877.1|	58088-58295	nan	nan	8	9	0.136877	0.90475	no
ni|645903553|nb|KK920877.1|	77473-78085	nan	nan	24	26	0.10673	0.92725	no
ni|645903553|nb|KK920877.1|	80608-81037	nan	nan	22	26	0.24233	0.825	no
ni|645903553|nb|KK920877.1|	91784-91940	nan	nan	5	3	-0.65219	0.64	no
ni|645903554|nb|KK920876.1|	132504-132645	nan	nan	0.387323	0.534877	0.46567	0.7261	no
ni|645903554|nb|KK920876.1|	184545-184740	nan	nan	0.418662	0.360239	-0.216829	0.87705	no
ni|645903554|nb|KK920876.1|	188495-188636	nan	nan	2	7	2	0.11055	no
ni|645903554|nb|KK920876.1|	189422-189563	nan	nan	2	1	-0.551093	0.68265	no
ni|645903554|nb|KK920876.1|	194599-195115	nan	nan	53	61	0.188867	0.8756	no
ni|645903554|nb|KK920876.1|	197375-198149	nan	nan	48	47	-0.0382307	0.97745	no
ni|645903554|nb|KK920876.1|	291245-291457	nan	nan	76	106	0.48151	0.65275	no
ni|645903554|nb|KK920876.1|	293832-293992	nan	nan	7	20	1	0.24765	no
ni|645903554|nb|KK920876.1|	297648-298181	nan	nan	10	22	1	0.36145	no
ni|645903554|nb|KK920876.1|	433715-434319	nan	nan	11	21	0.90581	0.44095	no
ni|645903554|nb|KK920876.1|	436176-436638	nan	nan	16	43	1	0.2175	no
ni|645903554|nb|KK920876.1|	43873-44194	nan	nan	20	15	-0.389363	0.7621	no

ni|645903554|nb|KK920876.1|	466705-466891	nan	nan	5	23	2	0.08915	no
ni|645903554|nb|KK920876.1|	61430-61756	nan	nan	4	12	2	0.16375	no
ni|645903554|nb|KK920876.1|	74353-74920	nan	nan	27	78	2	0.193	no
ni|645903555|nb|KK920875.1|	101130-101533	nan	nan	80	102	0.346862	0.75	no
ni|645903555|nb|KK920875.1|	155175-155460	nan	nan	23	44	0.969083	0.3894	no
ni|645903555|nb|KK920875.1|	159008-159421	nan	nan	82	91	0.148404	0.8936	no
ni|645903555|nb|KK920875.1|	162691-162994	nan	nan	80	84	0.0839241	0.9416	no
ni|645903555|nb|KK920875.1|	164223-164400	nan	nan	97	98	0.0106829	0.9921	no
ni|645903555|nb|KK920875.1|	165444-165619	nan	nan	16	14	-0.192781	0.87175	no
ni|645903555|nb|KK920875.1|	166373-166963	nan	nan	14	14	-0.0451827	0.9683	no
ni|645903555|nb|KK920875.1|	167327-167962	nan	nan	0.203817	44	8	0.03725	no
ni|645903555|nb|KK920875.1|	168357-168639	nan	nan	0.528796	0.643397	0.282999	0.82625	no
ni|645903555|nb|KK920875.1|	168894-169047	nan	nan	2	3	0.534396	0.6509	no
ni|645903555|nb|KK920875.1|	169189-169561	nan	nan	12	19	0.651724	0.57565	no
ni|645903555|nb|KK920875.1|	170623-170814	nan	nan	21	33	0.667429	0.5602	no
ni|645903555|nb|KK920875.1|	171112-171299	nan	nan	27	43	0.695852	0.5401	no
ni|645903555|nb|KK920875.1|	172299-172682	nan	nan	0	12	inf	5.00E-05	yes
ni|645903555|nb|KK920875.1|	172784-172906	nan	nan	27	40	0.542826	0.63405	no
ni|645903555|nb|KK920875.1|	172988-173478	nan	nan	4	4	0.175326	0.88255	no
ni|645903555|nb|KK920875.1|	174172-174644	nan	nan	7	9	0.416152	0.7148	no
ni|645903555|nb|KK920875.1|	174747-175004	nan	nan	28	41.19	0.555177	0.6248	no
ni|645903555|nb|KK920875.1|	175060-175663	nan	nan	1	1	0.0859565	0.9436	no
ni|645903555|nb|KK920875.1|	176580-176698	nan	nan	7	1	-3	0.16545	no
ni|645903555|nb|KK920875.1|	176766-176983	nan	nan	1	1	0.547899	0.65385	no
ni|645903555|nb|KK920875.1|	203708-204063	nan	nan	13	11	-0.164187	0.88645	no
ni|645903555|nb|KK920875.1|	212440-212759	nan	nan	2	2	0.00173143	0.97495	no
ni|645903555|nb|KK920875.1|	236679-236847	nan	nan	0.898275	6	3	0.04715	no
ni|645903555|nb|KK920875.1|	240777-241111	nan	nan	2	8	2	0.05855	no
ni|645903555|nb|KK920875.1|	283038-284284	nan	nan	448	421	-0.0911799	0.95025	no
ni|645903555|nb|KK920875.1|	293813-294055	nan	nan	4	5	0.207951	0.86235	no

ni|645903555|nb|KK920875.1|	294905-295760	nan	nan	450	583	0.375579	0.75755	no
ni|645903555|nb|KK920875.1|	31403-31689	nan	nan	6	19	2	0.18555	no
ni|645903555|nb|KK920875.1|	73189-73575	nan	nan	17	33	0.96845	0.40255	no
ni|645903555|nb|KK920875.1|	82877-83213	nan	nan	30	35	0.238414	0.83085	no
ni|645903557|nb|KK920873.1|	1037069-1037188	nan	nan	6	6	0.0191392	0.98705	no
ni|645903557|nb|KK920873.1|	1058330-1058644	nan	nan	5	4	-0.171846	0.88745	no
ni|645903557|nb|KK920873.1|	1059262-1060019	nan	nan	36	123	2	0.16755	no
ni|645903557|nb|KK920873.1|	1064139-1064378	nan	nan	51	170	2	0.13505	no
ni|645903557|nb|KK920873.1|	1069449-1069567	nan	nan	53	224	2	0.1242	no
ni|645903557|nb|KK920873.1|	1070136-1070799	nan	nan	0.626852	19	5	0.03355	no
ni|645903557|nb|KK920873.1|	1071121-1071677	nan	nan	37	67	0.849668	0.5497	no
ni|645903557|nb|KK920873.1|	1072903-1073856	nan	nan	0.119086	16	7	0.11935	no
ni|645903557|nb|KK920873.1|	1073976-1075025	nan	nan	55	68	0.322756	0.7769	no
ni|645903557|nb|KK920873.1|	1077187-1077746	nan	nan	54	50	-0.104667	0.9291	no
ni|645903557|nb|KK920873.1|	1078233-1078578	nan	nan	42	44	0.0609803	0.959	no
ni|645903557|nb|KK920873.1|	1079948-1080450	nan	nan	88	102	0.214571	0.8462	no
ni|645903557|nb|KK920873.1|	1080895-1081064	nan	nan	20	25	0.328805	0.77615	no
ni|645903557|nb|KK920873.1|	1082276-1082435	nan	nan	70	89	0.330396	0.7695	no
ni|645903557|nb|KK920873.1|	1083640-1083790	nan	nan	41	50	0.285686	0.80805	no
ni|645903557|nb|KK920873.1|	1085551-1085680	nan	nan	100	113	0.174741	0.8727	no
ni|645903557|nb|KK920873.1|	1086930-1087126	nan	nan	7	2	-2	0.32065	no
ni|645903557|nb|KK920873.1|	1087330-1087457	nan	nan	5	4	-0.466546	0.685	no
ni|645903557|nb|KK920873.1|	1088604-1088784	nan	nan	10	7	-0.543475	0.63955	no
ni|645903557|nb|KK920873.1|	1090191-1090591	nan	nan	2	2	-0.223824	0.8494	no
ni|645903557|nb|KK920873.1|	1090989-1091222	nan	nan	3	3	0.0410039	0.9722	no
ni|645903557|nb|KK920873.1|	1092897-1093064	nan	nan	2	3	0.285505	0.8052	no
ni|645903557|nb|KK920873.1|	1093183-1094901	nan	nan	0.445131	0.680637	0.612655	0.6619	no
ni|645903557|nb|KK920873.1|	1095511-1096695	nan	nan	25	12	-1	0.45115	no
ni|645903557|nb|KK920873.1|	1099444-1099638	nan	nan	6	4	-0.613457	0.58745	no
ni|645903557|nb|KK920873.1|	1099915-1100122	nan	nan	13	13	-0.0673551	0.9547	no

ni|645903557|nb|KK920873.1|	1101139-1101592	nan	nan	0.736217	0.645086	-0.19064	0.88855	no
ni|645903557|nb|KK920873.1|	1101962-1102373	nan	nan	0.993505	0.883773	-0.168851	0.8866	no
ni|645903557|nb|KK920873.1|	1102552-1102830	nan	nan	5	3	-0.652179	0.66125	no
ni|645903557|nb|KK920873.1|	1102957-1103394	nan	nan	2	2	-0.132665	0.909	no
ni|645903557|nb|KK920873.1|	1103616-1104004	nan	nan	16	16	0.0502501	0.96345	no
ni|645903557|nb|KK920873.1|	1106979-1107146	nan	nan	7	7	0.0100416	0.9937	no
ni|645903557|nb|KK920873.1|	1107795-1108024	nan	nan	14	17	0.32194	0.7982	no
ni|645903557|nb|KK920873.1|	1109865-1110006	nan	nan	28	28	-0.00374575	0.9967	no
ni|645903557|nb|KK920873.1|	1112974-1113376	nan	nan	3	0.550348	-2	0.1083	no
ni|645903557|nb|KK920873.1|	1113491-1113893	nan	nan	0.335549	0.855354	1.35	0.34405	no
ni|645903557|nb|KK920873.1|	1113986-1114682	nan	nan	51	40	-0.36958	0.7488	no
ni|645903557|nb|KK920873.1|	1114839-1115452	nan	nan	97	65	-0.577445	0.6243	no
ni|645903557|nb|KK920873.1|	1115582-1115838	nan	nan	3	51	4	0.04685	no
ni|645903557|nb|KK920873.1|	1115968-1116262	nan	nan	2	7	2	0.297	no
ni|645903557|nb|KK920873.1|	1116373-1117026	nan	nan	6	29	2	0.11695	no
ni|645903557|nb|KK920873.1|	1117720-1118147	nan	nan	1	6	2	0.089	no
ni|645903557|nb|KK920873.1|	1121800-1121931	nan	nan	9	38	2	0.0812	no
ni|645903557|nb|KK920873.1|	1122152-1122490	nan	nan	5	33	3	0.0767	no
ni|645903557|nb|KK920873.1|	1123658-1124069	nan	nan	7	26	2	0.16985	no
ni|645903557|nb|KK920873.1|	1124323-1124901	nan	nan	10	27	1	0.27955	no
ni|645903557|nb|KK920873.1|	1125875-1126830	nan	nan	19	39	1	0.42705	no
ni|645903557|nb|KK920873.1|	1128963-1129536	nan	nan	3	22	3	0.07865	no
ni|645903557|nb|KK920873.1|	1130331-1130662	nan	nan	4	6	0.824478	0.48	no
ni|645903557|nb|KK920873.1|	1132708-1133114	nan	nan	8	10	0.26274	0.8229	no
ni|645903557|nb|KK920873.1|	1133322-1133745	nan	nan	6	7	0.250794	0.82955	no
ni|645903557|nb|KK920873.1|	1133910-1134194	nan	nan	7	8	0.0614383	0.9554	no
ni|645903557|nb|KK920873.1|	1134297-1134501	nan	nan	1	2	0.828081	0.49315	no
ni|645903557|nb|KK920873.1|	1134675-1134940	nan	nan	9	10	0.126921	0.9158	no
ni|645903557|nb|KK920873.1|	1135008-1135326	nan	nan	1	1	-0.0661591	0.94155	no
ni|645903557|nb|KK920873.1|	1135527-1135901	nan	nan	4	10	1	0.32335	no

<table>
<tr><td>ni|645903557|nb|KK920873.1|</td><td>1136287-1136876</td><td>nan</td><td>nan</td><td>10</td><td>11</td><td>0.212851</td><td>0.86845</td><td>no</td></tr>
<tr><td>ni|645903557|nb|KK920873.1|</td><td>1137201-1137432</td><td>nan</td><td>nan</td><td>17</td><td>11</td><td>-0.652354</td><td>0.6241</td><td>no</td></tr>
<tr><td>ni|645903557|nb|KK920873.1|</td><td>1137648-1137778</td><td>nan</td><td>nan</td><td>11</td><td>9</td><td>-0.389319</td><td>0.78555</td><td>no</td></tr>
<tr><td>ni|645903557|nb|KK920873.1|</td><td>1139210-1139573</td><td>nan</td><td>nan</td><td>7</td><td>6</td><td>-0.141334</td><td>0.90245</td><td>no</td></tr>
<tr><td>ni|645903557|nb|KK920873.1|</td><td>1140383-1140818</td><td>nan</td><td>nan</td><td>11</td><td>9</td><td>-0.278593</td><td>0.8089</td><td>no</td></tr>
<tr><td>ni|645903557|nb|KK920873.1|</td><td>1140991-1141425</td><td>nan</td><td>nan</td><td>12</td><td>15</td><td>0.320147</td><td>0.77495</td><td>no</td></tr>
<tr><td>ni|645903557|nb|KK920873.1|</td><td>1142663-1142811</td><td>nan</td><td>nan</td><td>33</td><td>24</td><td>-0.482808</td><td>0.7301</td><td>no</td></tr>
<tr><td>ni|645903557|nb|KK920873.1|</td><td>1145481-1145647</td><td>nan</td><td>nan</td><td>0.63417</td><td>34</td><td>6</td><td>0.11935</td><td>no</td></tr>
<tr><td>ni|645903557|nb|KK920873.1|</td><td>1145775-1146104</td><td>nan</td><td>nan</td><td>90</td><td>116.01</td><td>0.35895</td><td>0.7669</td><td>no</td></tr>
<tr><td>ni|645903557|nb|KK920873.1|</td><td>1154611-1154823</td><td>nan</td><td>nan</td><td>132</td><td>162</td><td>0.292842</td><td>0.81285</td><td>no</td></tr>
<tr><td>ni|645903557|nb|KK920873.1|</td><td>1156115-1156518</td><td>nan</td><td>nan</td><td>101</td><td>144</td><td>0.520361</td><td>0.67975</td><td>no</td></tr>
<tr><td>ni|645903557|nb|KK920873.1|</td><td>1156819-1156968</td><td>nan</td><td>nan</td><td>31</td><td>40</td><td>0.393914</td><td>0.7813</td><td>no</td></tr>
<tr><td>ni|645903557|nb|KK920873.1|</td><td>1157117-1157620</td><td>nan</td><td>nan</td><td>3</td><td>10</td><td>2</td><td>0.19675</td><td>no</td></tr>
<tr><td>ni|645903557|nb|KK920873.1|</td><td>1158081-1158656</td><td>nan</td><td>nan</td><td>1</td><td>0.457329</td><td>-2</td><td>0.3149</td><td>no</td></tr>
<tr><td>ni|645903557|nb|KK920873.1|</td><td>1159311-1159613</td><td>nan</td><td>nan</td><td>0.384081</td><td>0.146888</td><td>-1</td><td>0.35155</td><td>no</td></tr>
<tr><td>ni|645903557|nb|KK920873.1|</td><td>1160816-1161984</td><td>nan</td><td>nan</td><td>0.560308</td><td>0.856602</td><td>0.612406</td><td>0.6619</td><td>no</td></tr>
<tr><td>ni|645903557|nb|KK920873.1|</td><td>1166242-1166481</td><td>nan</td><td>nan</td><td>0.573459</td><td>0.639344</td><td>0.156902</td><td>0.9131</td><td>no</td></tr>
<tr><td>ni|645903557|nb|KK920873.1|</td><td>1166772-1167660</td><td>nan</td><td>nan</td><td>9</td><td>8</td><td>-0.219327</td><td>0.87705</td><td>no</td></tr>
<tr><td>ni|645903557|nb|KK920873.1|</td><td>1167962-1168160</td><td>nan</td><td>nan</td><td>2</td><td>3</td><td>0.440002</td><td>0.7118</td><td>no</td></tr>
<tr><td>ni|645903557|nb|KK920873.1|</td><td>1168585-1169120</td><td>nan</td><td>nan</td><td>0.347932</td><td>1</td><td>2</td><td>0.1752</td><td>no</td></tr>
<tr><td>ni|645903557|nb|KK920873.1|</td><td>1169908-1170077</td><td>nan</td><td>nan</td><td>0</td><td>10</td><td>inf</td><td>0.0001</td><td>yes</td></tr>
<tr><td>ni|645903557|nb|KK920873.1|</td><td>1170189-1170796</td><td>nan</td><td>nan</td><td>0</td><td>3</td><td>inf</td><td>0.0001</td><td>yes</td></tr>
<tr><td>ni|645903557|nb|KK920873.1|</td><td>1171078-1171294</td><td>nan</td><td>nan</td><td>0.231857</td><td>7</td><td>5</td><td>0.12005</td><td>no</td></tr>
<tr><td>ni|645903557|nb|KK920873.1|</td><td>1171785-1172583</td><td>nan</td><td>nan</td><td>1</td><td>7</td><td>3</td><td>0.0727</td><td>no</td></tr>
<tr><td>ni|645903557|nb|KK920873.1|</td><td>1172870-1173128</td><td>nan</td><td>nan</td><td>0.269557</td><td>7</td><td>5</td><td>0.1211</td><td>no</td></tr>
<tr><td>ni|645903557|nb|KK920873.1|</td><td>1173409-1173541</td><td>nan</td><td>nan</td><td>0.963421</td><td>5</td><td>2</td><td>0.0999</td><td>no</td></tr>
<tr><td>ni|645903557|nb|KK920873.1|</td><td>1173742-1174273</td><td>nan</td><td>nan</td><td>37</td><td>23</td><td>-0.728784</td><td>0.5284</td><td>no</td></tr>
<tr><td>ni|645903557|nb|KK920873.1|</td><td>1174734-1175726</td><td>nan</td><td>nan</td><td>42</td><td>38</td><td>-0.142567</td><td>0.90205</td><td>no</td></tr>
<tr><td>ni|645903557|nb|KK920873.1|</td><td>1175851-1176222</td><td>nan</td><td>nan</td><td>17</td><td>107</td><td>3</td><td>0.0517</td><td>no</td></tr>
<tr><td>ni|645903557|nb|KK920873.1|</td><td>1178458-1178658</td><td>nan</td><td>nan</td><td>19</td><td>13</td><td>-0.559054</td><td>0.6396</td><td>no</td></tr>
</table>

ni|645903557|nb|KK920873.1|	1179421-1179632	nan	nan	25	112	2	0.0755	no
ni|645903557|nb|KK920873.1|	1184865-1185006	nan	nan	0.682146	0.300969	-1	0.42305	no
ni|645903557|nb|KK920873.1|	260918-261102	nan	nan	315	419.98	0.413477	0.7713	no
ni|645903557|nb|KK920873.1|	261237-261390	nan	nan	102	157	0.624124	0.6562	no
ni|645903557|nb|KK920873.1|	264769-264887	nan	nan	0.435701	0.416392	-0.065396	0.89835	no
ni|645903557|nb|KK920873.1|	266377-267890	nan	nan	0.182241	1	3	0.081	no
ni|645903557|nb|KK920873.1|	268085-270650	nan	nan	1491.25	1835.35	0.299527	0.79065	no
ni|645903557|nb|KK920873.1|	277391-277706	nan	nan	831.05	1213.05	0.545637	0.6158	no
ni|645903557|nb|KK920873.1|	281461-281717	nan	nan	0.23198	6	5	0.0208	no
ni|645903557|nb|KK920873.1|	383977-384146	nan	nan	673	819	0.282314	0.7915	no
ni|645903557|nb|KK920873.1|	394987-395367	nan	nan	577	768	0.411605	0.6953	no
ni|645903557|nb|KK920873.1|	429423-429634	nan	nan	0	6	inf	0.00015	yes
ni|645903557|nb|KK920873.1|	431071-431197	nan	nan	78	110	0.488727	0.7344	no
ni|645903557|nb|KK920873.1|	432165-432361	nan	nan	0.317401	23	6	0.11945	no
ni|645903557|nb|KK920873.1|	433660-433839	nan	nan	0.976402	17	4	0.04685	no
ni|645903557|nb|KK920873.1|	434185-434322	nan	nan	0.9955	23	5	0.04545	no
ni|645903557|nb|KK920873.1|	434610-435382	nan	nan	0.904552	19	4	0.12175	no
ni|645903557|nb|KK920873.1|	438884-439193	nan	nan	0.172454	10	6	0.0445	no
ni|645903557|nb|KK920873.1|	439848-440071	nan	nan	2	20	3	0.04185	no
ni|645903557|nb|KK920873.1|	441468-442795	nan	nan	0.259498	0.268833	0.0509873	0.9572	no
ni|645903557|nb|KK920873.1|	446915-447783	nan	nan	3	19	3	0.02615	no
ni|645903557|nb|KK920873.1|	448401-448894	nan	nan	0.068018	0.455246	3	0.0782	no
ni|645903557|nb|KK920873.1|	449287-449633	nan	nan	9	62	3	0.0236	no
ni|645903557|nb|KK920873.1|	450235-450560	nan	nan	11	54	2	0.0672	no
ni|645903557|nb|KK920873.1|	451792-451940	nan	nan	0.269469	0.450892	0.742665	0.5765	no
ni|645903557|nb|KK920873.1|	475740-475883	nan	nan	14	41	2	0.2056	no
ni|645903557|nb|KK920873.1|	476022-476285	nan	nan	6	56	3	0.019	no
ni|645903557|nb|KK920873.1|	476445-476981	nan	nan	1	4	1	0.2759	no
ni|645903557|nb|KK920873.1|	477785-477928	nan	nan	14	54	2	0.1202	no
ni|645903557|nb|KK920873.1|	478361-478519	nan	nan	0.539893	0.430201	-0.327663	0.79705	no

ni|645903557|nb|KK920873.1|	479207-479726	nan	nan	2	0.955701	-0.681846	0.5893	no
ni|645903557|nb|KK920873.1|	479963-480364	nan	nan	55	106	0.934417	0.464	no
ni|645903557|nb|KK920873.1|	483269-483540	nan	nan	7	42	3	0.0432	no
ni|645903557|nb|KK920873.1|	485477-485687	nan	nan	8	58	3	0.053	no
ni|645903557|nb|KK920873.1|	486989-487195	nan	nan	4	21	2	0.0564	no
ni|645903557|nb|KK920873.1|	487910-488050	nan	nan	6	7	0.0482733	0.95725	no
ni|645903557|nb|KK920873.1|	488236-488444	nan	nan	1	1	0.00746625	0.98485	no
ni|645903557|nb|KK920873.1|	488673-488836	nan	nan	6	6	-0.0130885	0.991	no
ni|645903557|nb|KK920873.1|	489536-489698	nan	nan	120	176	0.55708	0.6025	no
ni|645903557|nb|KK920873.1|	490956-491146	nan	nan	12	13	0.135163	0.90585	no
ni|645903557|nb|KK920873.1|	491615-492137	nan	nan	5	6	0.216359	0.84815	no
ni|645903557|nb|KK920873.1|	493856-494186	nan	nan	15	14	-0.166891	0.89925	no
ni|645903557|nb|KK920873.1|	603168-603289	nan	nan	0.864538	0.825894	-0.0659727	0.923	no
ni|645903557|nb|KK920873.1|	683668-683773	nan	nan	30	32	0.0775827	0.9597	no
ni|645903557|nb|KK920873.1|	704111-705061	nan	nan	173	143	-0.28132	0.7991	no
ni|645903557|nb|KK920873.1|	705218-705432	nan	nan	104	74	-0.500967	0.66615	no
ni|645903557|nb|KK920873.1|	707955-708080	nan	nan	93	71	-0.387186	0.73725	no
ni|645903557|nb|KK920873.1|	708996-709321	nan	nan	65	41	-0.687644	0.5566	no
ni|645903557|nb|KK920873.1|	713036-713470	nan	nan	75	43	-0.813925	0.47055	no
ni|645903557|nb|KK920873.1|	717026-718142	nan	nan	12	24	0.996853	0.39115	no
ni|645903557|nb|KK920873.1|	718297-719183	nan	nan	2	1	-0.444388	0.74215	no
ni|645903557|nb|KK920873.1|	719845-720312	nan	nan	6	0.633149	-3	0.01785	no
ni|645903557|nb|KK920873.1|	723345-723841	nan	nan	17	0.321852	-6	0.0176	no
ni|645903557|nb|KK920873.1|	724005-725057	nan	nan	7	0.177846	-5	0.01375	no
ni|645903557|nb|KK920873.1|	726083-727430	nan	nan	3	0	-inf	0.0004	yes
ni|645903557|nb|KK920873.1|	728265-728557	nan	nan	3	0.03701	-6	0.0173	no
ni|645903557|nb|KK920873.1|	729369-729490	nan	nan	0.442593	0.021162	-4	0.01995	no
ni|645903557|nb|KK920873.1|	729747-730908	nan	nan	1	0	-inf	0.0004	yes
ni|645903557|nb|KK920873.1|	731538-731666	nan	nan	0.563402	0.041434	-4	0.02555	no
ni|645903557|nb|KK920873.1|	732185-732338	nan	nan	3	0.53728	-3	0.07365	no

ni|645903557|nb|KK920873.1|	733300-733505	nan	nan	5	0.288514	-4	0.0277	no
ni|645903557|nb|KK920873.1|	744236-744508	nan	nan	30	0.370947	-6	0.0137	no
ni|645903557|nb|KK920873.1|	745576-745711	nan	nan	5	4	-0.173139	0.8837	no
ni|645903557|nb|KK920873.1|	746425-746722	nan	nan	7	15	1	0.3928	no
ni|645903557|nb|KK920873.1|	747267-747450	nan	nan	4	5	0.113344	0.9325	no
ni|645903557|nb|KK920873.1|	752107-752334	nan	nan	23	22	-0.0420393	0.97615	no
ni|645903557|nb|KK920873.1|	753453-753751	nan	nan	15	21	0.430826	0.7086	no
ni|645903557|nb|KK920873.1|	756271-756508	nan	nan	25	27	0.0941128	0.93485	no
ni|645903557|nb|KK920873.1|	756615-757138	nan	nan	23	20	-0.212605	0.8558	no
ni|645903557|nb|KK920873.1|	757475-758233	nan	nan	19	23	0.295254	0.80705	no
ni|645903557|nb|KK920873.1|	759539-759970	nan	nan	27	51	0.906661	0.4475	no
ni|645903557|nb|KK920873.1|	760190-760501	nan	nan	49	78	0.678749	0.5605	no
ni|645903557|nb|KK920873.1|	761358-761506	nan	nan	11	7	-0.68869	0.58685	no
ni|645903557|nb|KK920873.1|	761758-762237	nan	nan	1	0.765717	-0.702847	0.6038	no
ni|645903557|nb|KK920873.1|	763170-763298	nan	nan	11	9	-0.319301	0.78755	no
ni|645903557|nb|KK920873.1|	763440-763971	nan	nan	24	18	-0.389215	0.74325	no
ni|645903557|nb|KK920873.1|	764486-765054	nan	nan	12	10	-0.196582	0.8637	no
ni|645903557|nb|KK920873.1|	765928-766299	nan	nan	4	6	0.585526	0.613	no
ni|645903557|nb|KK920873.1|	767881-768353	nan	nan	16	20	0.360217	0.76455	no
ni|645903557|nb|KK920873.1|	768718-769046	nan	nan	20	21	0.0437361	0.9719	no
ni|645903557|nb|KK920873.1|	769188-769727	nan	nan	17	14	-0.301065	0.7924	no
ni|645903557|nb|KK920873.1|	769952-770622	nan	nan	3	2	-0.373629	0.7575	no
ni|645903557|nb|KK920873.1|	771152-771427	nan	nan	32	21	-0.615007	0.62875	no
ni|645903557|nb|KK920873.1|	771561-772082	nan	nan	14	2	-3	0.0572	no
ni|645903557|nb|KK920873.1|	772469-772612	nan	nan	12	8	-0.576058	0.6249	no
ni|645903557|nb|KK920873.1|	772715-772933	nan	nan	101	55	-0.861127	0.46595	no
ni|645903557|nb|KK920873.1|	773097-773262	nan	nan	15	11	-0.358739	0.7516	no
ni|645903557|nb|KK920873.1|	773571-773791	nan	nan	71	54	-0.382924	0.79965	no
ni|645903557|nb|KK920873.1|	774480-775231	nan	nan	58	46	-0.347505	0.74075	no
ni|645903557|nb|KK920873.1|	775408-775702	nan	nan	26	30	0.220687	0.8509	no

ni|645903557|nb|KK920873.1|	775889-776559	nan	nan	30	35	0.238091	0.83075	no
ni|645903557|nb|KK920873.1|	777734-779356	nan	nan	0.75191	0.071865	-3	0.03815	no
ni|645903557|nb|KK920873.1|	780058-780227	nan	nan	35	40	0.180761	0.87095	no
ni|645903557|nb|KK920873.1|	783136-783430	nan	nan	15	17	0.26132	0.81525	no
ni|645903557|nb|KK920873.1|	788796-789139	nan	nan	31	39	0.307519	0.7843	no
ni|645903557|nb|KK920873.1|	790138-790866	nan	nan	5	9	1	0.42705	no
ni|645903557|nb|KK920873.1|	793488-793670	nan	nan	55	83	0.604022	0.61155	no
ni|645903557|nb|KK920873.1|	796120-796251	nan	nan	55	80	0.522257	0.63765	no
ni|645903557|nb|KK920873.1|	796469-797106	nan	nan	9	8	-0.220318	0.8546	no
ni|645903557|nb|KK920873.1|	801069-802260	nan	nan	11	8	-0.426583	0.7122	no
ni|645903557|nb|KK920873.1|	802388-803559	nan	nan	2	1	-0.983394	0.48245	no
ni|645903557|nb|KK920873.1|	804003-804192	nan	nan	2	0.913325	-0.765528	0.5354	no
ni|645903557|nb|KK920873.1|	807230-807492	nan	nan	12	6	-1	0.45115	no
ni|645903557|nb|KK920873.1|	808281-808408	nan	nan	0.139219	21	7	0.11935	no
ni|645903557|nb|KK920873.1|	809199-809454	nan	nan	0.06574	16	8	0.05065	no
ni|645903557|nb|KK920873.1|	811183-811363	nan	nan	19	15	-0.322364	0.7654	no
ni|645903557|nb|KK920873.1|	812648-812853	nan	nan	36	33	-0.139464	0.9061	no
ni|645903557|nb|KK920873.1|	812962-813227	nan	nan	2	4	0.584798	0.6992	no
ni|645903557|nb|KK920873.1|	815055-815709	nan	nan	58	49	-0.245736	0.8717	no
ni|645903557|nb|KK920873.1|	816267-816471	nan	nan	18	15	-0.273552	0.81635	no
ni|645903557|nb|KK920873.1|	817113-817249	nan	nan	0	7	inf	5.00E-05	yes
ni|645903557|nb|KK920873.1|	817726-817942	nan	nan	0	6	inf	5.00E-05	yes
ni|645903557|nb|KK920873.1|	819613-819873	nan	nan	0	13	inf	5.00E-05	yes
ni|645903557|nb|KK920873.1|	821057-821263	nan	nan	3	2	-0.570914	0.62255	no
ni|645903557|nb|KK920873.1|	821365-821598	nan	nan	0.213858	7	5	0.1206	no
ni|645903557|nb|KK920873.1|	822485-822740	nan	nan	2	2	-0.145766	0.89925	no
ni|645903557|nb|KK920873.1|	822887-823087	nan	nan	18	15	-0.222167	0.83125	no
ni|645903557|nb|KK920873.1|	823188-823497	nan	nan	7	5	-0.44682	0.70695	no
ni|645903557|nb|KK920873.1|	824854-825077	nan	nan	51	42	-0.281072	0.8466	no
ni|645903557|nb|KK920873.1|	825272-825425	nan	nan	8	7	-0.234713	0.8388	no

ni|645903557|nb|KK920873.1|	825558-825722	nan	nan	27	24	-0.171354	0.89285	no
ni|645903557|nb|KK920873.1|	828895-829330	nan	nan	10	9	-0.0887581	0.93855	no
ni|645903557|nb|KK920873.1|	829584-829829	nan	nan	18	16	-0.229185	0.8276	no
ni|645903557|nb|KK920873.1|	830104-830602	nan	nan	57	44	-0.368938	0.79835	no
ni|645903557|nb|KK920873.1|	834366-834644	nan	nan	31	23	-0.384344	0.74125	no
ni|645903557|nb|KK920873.1|	835254-835396	nan	nan	6	4	-0.545624	0.643	no
ni|645903557|nb|KK920873.1|	836213-836465	nan	nan	37	33	-0.143782	0.898	no
ni|645903557|nb|KK920873.1|	837336-837547	nan	nan	2	1	-0.539965	0.64755	no
ni|645903557|nb|KK920873.1|	838699-838928	nan	nan	1.06	1	0.256065	0.86075	no
ni|645903557|nb|KK920873.1|	840385-840789	nan	nan	8	8	0.0152703	0.9674	no
ni|645903557|nb|KK920873.1|	841862-841990	nan	nan	5	4	-0.454315	0.722	no
ni|645903557|nb|KK920873.1|	842172-842426	nan	nan	5	6	0.341766	0.7546	no
ni|645903557|nb|KK920873.1|	843444-843720	nan	nan	0.375784	0.314345	-0.257553	0.8692	no
ni|645903557|nb|KK920873.1|	844507-844694	nan	nan	5	6	0.23564	0.8381	no
ni|645903557|nb|KK920873.1|	849545-849716	nan	nan	5	2	-1	0.3141	no
ni|645903557|nb|KK920873.1|	854825-855047	nan	nan	3	4	0.593107	0.62225	no
ni|645903557|nb|KK920873.1|	856078-856314	nan	nan	0	1	inf	0.00055	yes
ni|645903557|nb|KK920873.1|	857857-858301	nan	nan	15	17	0.18053	0.8726	no
ni|645903557|nb|KK920873.1|	862200-862354	nan	nan	1	0.697408	-1	0.4219	no
ni|645903557|nb|KK920873.1|	862466-862681	nan	nan	19	17	-0.160514	0.89585	no
ni|645903557|nb|KK920873.1|	862828-862950	nan	nan	15	9	-0.753099	0.536	no
ni|645903557|nb|KK920873.1|	865638-865753	nan	nan	3	2	-0.54769	0.6365	no
ni|645903557|nb|KK920873.1|	866008-866527	nan	nan	2	2	-0.672233	0.5667	no
ni|645903557|nb|KK920873.1|	867756-867892	nan	nan	5	5	0.0069128	0.9732	no
ni|645903557|nb|KK920873.1|	869122-869263	nan	nan	2	2	0.197229	0.8731	no
ni|645903557|nb|KK920873.1|	869622-869752	nan	nan	2	1	-0.950001	0.44725	no
ni|645903557|nb|KK920873.1|	870772-870936	nan	nan	4	3	-0.142752	0.8982	no
ni|645903557|nb|KK920873.1|	873018-873216	nan	nan	63	42	-0.581734	0.5853	no
ni|645903557|nb|KK920873.1|	877286-878027	nan	nan	0.742322	0.867104	0.22416	0.8598	no
ni|645903557|nb|KK920873.1|	878845-879029	nan	nan	176	103	-0.777687	0.4935	no

ni|645903557|nb|KK920873.1|	884076-884361	nan	nan	11	11	0.0428697	0.96785	no
ni|645903557|nb|KK920873.1|	887691-887812	nan	nan	13	11	-0.27616	0.80385	no
ni|645903557|nb|KK920873.1|	888071-888277	nan	nan	43	30	-0.531341	0.66715	no
ni|645903557|nb|KK920873.1|	893262-894226	nan	nan	45	108	1	0.2481	no
ni|645903557|nb|KK920873.1|	894939-896271	nan	nan	1	0.696357	-1	0.44755	no
ni|645903557|nb|KK920873.1|	896421-896595	nan	nan	34.95	29	-0.27689	0.79775	no
ni|645903557|nb|KK920873.1|	897017-897317	nan	nan	71	55	-0.378246	0.7948	no
ni|645903557|nb|KK920873.1|	898560-899041	nan	nan	1	5	2	0.0804	no
ni|645903557|nb|KK920873.1|	900595-900767	nan	nan	41	42	0.0520789	0.9617	no
ni|645903557|nb|KK920873.1|	904195-904357	nan	nan	106.66	90	-0.252367	0.86525	no
ni|645903557|nb|KK920873.1|	906401-906691	nan	nan	4	2	-0.694539	0.5559	no
ni|645903557|nb|KK920873.1|	908658-908799	nan	nan	55	54	-0.0248599	0.9838	no
ni|645903557|nb|KK920873.1|	908920-909190	nan	nan	2	2	-0.460782	0.70135	no
ni|645903557|nb|KK920873.1|	909431-909729	nan	nan	10	9	-0.167442	0.88445	no
ni|645903557|nb|KK920873.1|	910695-910914	nan	nan	74	71.26	-0.0507031	0.962	no
ni|645903557|nb|KK920873.1|	915747-916285	nan	nan	113	94	-0.269676	0.81705	no
ni|645903557|nb|KK920873.1|	916385-916644	nan	nan	6	9	0.714438	0.54715	no
ni|645903557|nb|KK920873.1|	917400-917614	nan	nan	3	2	-0.496105	0.683	no
ni|645903557|nb|KK920873.1|	920586-920696	nan	nan	12	3	-2	0.20985	no
ni|645903557|nb|KK920873.1|	921669-921881	nan	nan	26	22	-0.199717	0.85175	no
ni|645903557|nb|KK920873.1|	922174-922543	nan	nan	194	165	-0.235073	0.8702	no
ni|645903557|nb|KK920873.1|	922692-922876	nan	nan	269	226	-0.25227	0.86185	no
ni|645903557|nb|KK920873.1|	923213-923649	nan	nan	2	1	-0.574998	0.62125	no
ni|645903557|nb|KK920873.1|	924199-924498	nan	nan	3	2	-0.71742	0.55395	no
ni|645903557|nb|KK920873.1|	924976-925186	nan	nan	6	2	-1	0.28745	no
ni|645903557|nb|KK920873.1|	930284-930508	nan	nan	6	3	-1	0.30195	no
ni|645903557|nb|KK920873.1|	930638-931830	nan	nan	7	4	-0.737459	0.531	no
ni|645903557|nb|KK920873.1|	931946-932205	nan	nan	0.513791	0.147349	-2	0.2784	no
ni|645903557|nb|KK920873.1|	933134-933373	nan	nan	4	3	-0.389055	0.7621	no
ni|645903557|nb|KK920873.1|	933840-934025	nan	nan	267	203	-0.400462	0.7735	no

ni|645903557|nb|KK920873.1|	935018-935453	nan	nan	33	45	0.44844	0.6831	no
ni|645903557|nb|KK920873.1|	936049-936463	nan	nan	50	60	0.28561	0.80495	no
ni|645903557|nb|KK920873.1|	936919-937095	nan	nan	15	16	0.152114	0.89445	no
ni|645903557|nb|KK920873.1|	940082-940220	nan	nan	5	6	0.133785	0.9104	no
ni|645903557|nb|KK920873.1|	940462-940575	nan	nan	53	52	-0.0255102	0.9809	no
ni|645903557|nb|KK920873.1|	941212-941388	nan	nan	25	9	-2	0.2084	no
ni|645903557|nb|KK920873.1|	941925-942056	nan	nan	0.985961	0.942019	-0.0657753	0.9384	no
ni|645903557|nb|KK920873.1|	945469-945622	nan	nan	1809.3	2132.97	0.237432	0.8316	no
ni|645903557|nb|KK920873.1|	946355-946562	nan	nan	52	40	-0.389541	0.7451	no
ni|645903557|nb|KK920873.1|	947741-948112	nan	nan	39	33	-0.256284	0.82225	no
ni|645903557|nb|KK920873.1|	950854-951024	nan	nan	2877.57	1821.42	-0.659789	0.54155	no
ni|645903557|nb|KK920873.1|	951969-952132	nan	nan	4	0.520223	-3	0.2479	no
ni|645903557|nb|KK920873.1|	953484-953676	nan	nan	0.825325	0.887234	0.104352	0.93335	no
ni|645903557|nb|KK920873.1|	955172-955300	nan	nan	4	2	-0.987648	0.4137	no
ni|645903557|nb|KK920873.1|	956765-956900	nan	nan	9	4	-1	0.3479	no
ni|645903557|nb|KK920873.1|	957111-957548	nan	nan	24	17	-0.477884	0.68375	no
ni|645903557|nb|KK920873.1|	957790-957949	nan	nan	30	20	-0.582102	0.67535	no
ni|645903557|nb|KK920873.1|	958630-958792	nan	nan	6	5	-0.236025	0.8403	no
ni|645903557|nb|KK920873.1|	961749-961910	nan	nan	29	4	-3	0.25165	no
ni|645903557|nb|KK920873.1|	962106-962282	nan	nan	1	0.42824	-1	0.252	no
ni|645903557|nb|KK920873.1|	963753-964080	nan	nan	1	0.401024	-2	0.25615	no
ni|645903557|nb|KK920873.1|	966275-967110	nan	nan	3	2	-0.307743	0.8123	no
ni|645903557|nb|KK920873.1|	967887-968038	nan	nan	19	19	0.00197124	0.9904	no
ni|645903557|nb|KK920873.1|	968108-968588	nan	nan	9	12	0.411075	0.73195	no
ni|645903557|nb|KK920873.1|	969488-971281	nan	nan	43	24	-0.822089	0.48595	no
ni|645903557|nb|KK920873.1|	974214-974347	nan	nan	13	20	0.627197	0.5701	no
ni|645903557|nb|KK920873.1|	975855-976206	nan	nan	37	56	0.607321	0.6036	no
ni|645903557|nb|KK920873.1|	976393-976674	nan	nan	25	46	0.868491	0.47445	no
ni|645903557|nb|KK920873.1|	977082-977234	nan	nan	10	21	1	0.3464	no
ni|645903557|nb|KK920873.1|	977844-978083	nan	nan	18	33	0.860534	0.45675	no

ni|645903557|nb|KK920873.1|	983341-983796	nan	nan	5	16	2	0.1437	no
ni|645903557|nb|KK920873.1|	983903-984140	nan	nan	0	5	inf	5.00E-05	yes
ni|645903557|nb|KK920873.1|	984336-984764	nan	nan	9	11	0.288902	0.8058	no
ni|645903557|nb|KK920873.1|	984870-984980	nan	nan	6	7	0.193609	0.8689	no
ni|645903557|nb|KK920873.1|	986693-988182	nan	nan	14	26	0.925707	0.42975	no
ni|645903557|nb|KK920873.1|	988579-989327	nan	nan	16	32	0.943323	0.40375	no
ni|645903557|nb|KK920873.1|	997666-997850	nan	nan	14	32	1	0.32175	no
ni|645903559|nb|KK920871.1|	122873-123019	nan	nan	0.868898	0.127772	-3	0.2592	no
ni|645903559|nb|KK920871.1|	126569-126759	nan	nan	0.098239	16	7	0.11935	no
ni|645903559|nb|KK920871.1|	128110-128626	nan	nan	3	2	-0.414696	0.76315	no
ni|645903559|nb|KK920871.1|	130411-130607	nan	nan	0	20	inf	5.00E-05	yes
ni|645903559|nb|KK920871.1|	135911-136072	nan	nan	1	0.855802	-0.802987	0.6027	no
ni|645903559|nb|KK920871.1|	140133-140435	nan	nan	3	1	-2	0.162	no
ni|645903559|nb|KK920871.1|	146543-146756	nan	nan	3	1	-2	0.1876	no
ni|645903559|nb|KK920871.1|	147886-148227	nan	nan	2	3	0.603764	0.60725	no
ni|645903559|nb|KK920871.1|	154986-155171	nan	nan	2	2	0.0982244	0.93725	no
ni|645903559|nb|KK920871.1|	164746-164997	nan	nan	1	0.460236	-1	0.4064	no
ni|645903559|nb|KK920871.1|	168423-168760	nan	nan	6	4	-0.719278	0.61925	no
ni|645903559|nb|KK920871.1|	171091-171450	nan	nan	8	6	-0.381419	0.73815	no
ni|645903559|nb|KK920871.1|	176997-177179	nan	nan	69	36	-0.938895	0.40805	no
ni|645903559|nb|KK920871.1|	183242-183523	nan	nan	4	2	-0.723015	0.53525	no
ni|645903559|nb|KK920871.1|	188850-189146	nan	nan	2	2	-0.0653133	0.9574	no
ni|645903559|nb|KK920871.1|	189544-189683	nan	nan	7	4	-0.854194	0.4656	no
ni|645903559|nb|KK920871.1|	189903-190635	nan	nan	2	1	-0.428221	0.73225	no
ni|645903559|nb|KK920871.1|	29818-30010	nan	nan	36	82	1	0.31525	no
ni|645903559|nb|KK920871.1|	45108-47902	nan	nan	35	26	-0.442773	0.70365	no
ni|645903559|nb|KK920871.1|	48044-48249	nan	nan	18	7	-1	0.2776	no
ni|645903559|nb|KK920871.1|	48472-49296	nan	nan	105	26	-2	0.1274	no
ni|645903559|nb|KK920871.1|	76166-77457	nan	nan	0.488382	0.58354	0.256823	0.86075	no
ni|645903559|nb|KK920871.1|	77568-78829	nan	nan	0.6183	0.984966	0.671767	0.62105	no

ni|645903560|nb|KK920870.1|	203988-204230	nan	nan	2	2	-0.191072	0.87685	no
ni|645903560|nb|KK920870.1|	218132-218447	nan	nan	7	3	-1	0.25185	no
ni|645903560|nb|KK920870.1|	221716-221884	nan	nan	272	176	-0.63055	0.5878	no
ni|645903560|nb|KK920870.1|	222529-222722	nan	nan	1	0.07628	-4	0.02125	no
ni|645903560|nb|KK920870.1|	225667-225852	nan	nan	8	4	-1	0.3747	no
ni|645903560|nb|KK920870.1|	225993-226393	nan	nan	31	24	-0.381888	0.7228	no
ni|645903560|nb|KK920870.1|	228431-228677	nan	nan	13	11	-0.187737	0.8684	no
ni|645903560|nb|KK920870.1|	230393-230675	nan	nan	1	0.988349	-0.299101	0.8033	no
ni|645903560|nb|KK920870.1|	233071-233232	nan	nan	0.84655	0.707803	-0.258247	0.8692	no
ni|645903560|nb|KK920870.1|	271103-271629	nan	nan	1	0.910997	-0.551852	0.71905	no
ni|645903560|nb|KK920870.1|	336026-336793	nan	nan	0.964377	0.526835	-0.872246	0.49155	no
ni|645903560|nb|KK920870.1|	336864-337166	nan	nan	28	21	-0.419003	0.71525	no
ni|645903560|nb|KK920870.1|	337299-337698	nan	nan	20	17	-0.24376	0.82975	no
ni|645903560|nb|KK920870.1|	341893-342315	nan	nan	0.464348	0.345254	-0.427548	0.75965	no
ni|645903560|nb|KK920870.1|	342634-343289	nan	nan	28	247	3	0.0298	no
ni|645903560|nb|KK920870.1|	343967-344210	nan	nan	22	273	4	0.01195	no
ni|645903560|nb|KK920870.1|	347360-347708	nan	nan	7	7	0.0702464	0.94415	no
ni|645903560|nb|KK920870.1|	348384-348657	nan	nan	0.215033	0.308398	0.520236	0.7279	no
ni|645903560|nb|KK920870.1|	349231-349631	nan	nan	14	12	-0.249776	0.82635	no
ni|645903560|nb|KK920870.1|	353689-354078	nan	nan	2	1	-0.943432	0.4276	no
ni|645903560|nb|KK920870.1|	355528-355685	nan	nan	0.771856	1	0.641463	0.5998	no
ni|645903560|nb|KK920870.1|	363895-364465	nan	nan	11	6	-0.867258	0.46805	no
ni|645903560|nb|KK920870.1|	365831-366151	nan	nan	2	1	-0.321154	0.7848	no
ni|645903560|nb|KK920870.1|	366262-366816	nan	nan	1	0.300768	-2	0.2784	no
ni|645903560|nb|KK920870.1|	366919-367169	nan	nan	12	18	0.643214	0.58785	no
ni|645903560|nb|KK920870.1|	367343-367675	nan	nan	14	16	0.242286	0.8295	no
ni|645903560|nb|KK920870.1|	367904-368052	nan	nan	220	201	-0.136555	0.92765	no
ni|645903560|nb|KK920870.1|	368944-369264	nan	nan	0.795551	0.456202	-0.802282	0.6027	no
ni|645903560|nb|KK920870.1|	369363-369779	nan	nan	0.824176	0.262677	-2	0.2252	no
ni|645903560|nb|KK920870.1|	371978-372676	nan	nan	15	11	-0.522773	0.62585	no

ni|645903560|nb|KK920870.1|	372775-373008	nan	nan	0.015883	178	13	0.11935	no
ni|645903560|nb|KK920870.1|	376825-377277	nan	nan	57	85	0.581578	0.6133	no
ni|645903560|nb|KK920870.1|	378342-379369	nan	nan	0.019503	48	11	0.11935	no
ni|645903560|nb|KK920870.1|	383601-384976	nan	nan	39	48	0.290233	0.8026	no
ni|645903560|nb|KK920870.1|	385383-385907	nan	nan	27	25	-0.0834023	0.95525	no
ni|645903560|nb|KK920870.1|	388592-388749	nan	nan	14	12	-0.244084	0.8395	no
ni|645903560|nb|KK920870.1|	394713-394916	nan	nan	2	445	8	0.0002	yes
ni|645903560|nb|KK920870.1|	397827-399022	nan	nan	0	124	inf	5.00E-05	yes
ni|645903560|nb|KK920870.1|	401582-401802	nan	nan	2	290	8	0.00925	no
ni|645903560|nb|KK920870.1|	401913-402355	nan	nan	0.189324	23	7	0.11955	no
ni|645903560|nb|KK920870.1|	430301-430459	nan	nan	2	209	7	0.007	no
ni|645903560|nb|KK920870.1|	451152-451444	nan	nan	36	67	0.893876	0.40655	no
ni|645903560|nb|KK920870.1|	606022-606637	nan	nan	4	4	0.0481942	0.95725	no
ni|645903560|nb|KK920870.1|	625376-625594	nan	nan	2	0.805514	-0.914198	0.53575	no
ni|645903561|nb|KK920869.1|	13861-14030	nan	nan	51	52	0.021152	0.9841	no
ni|645903561|nb|KK920869.1|	14198-14441	nan	nan	3	1	-1	0.37315	no
ni|645903561|nb|KK920869.1|	15130-15677	nan	nan	16	11	-0.510431	0.65435	no
ni|645903561|nb|KK920869.1|	151926-152333	nan	nan	37	26	-0.510421	0.65465	no
ni|645903561|nb|KK920869.1|	16392-17269	nan	nan	40	40	0.0134694	0.99205	no
ni|645903561|nb|KK920869.1|	17559-17962	nan	nan	75	75	0.015249	0.99135	no
ni|645903561|nb|KK920869.1|	18072-18387	nan	nan	1	0.380044	-2	0.25615	no
ni|645903561|nb|KK920869.1|	18995-19439	nan	nan	28	8	-2	0.21515	no
ni|645903561|nb|KK920869.1|	20844-21020	nan	nan	21	13	-0.652619	0.66125	no
ni|645903561|nb|KK920869.1|	22323-22579	nan	nan	12	10	-0.183704	0.87205	no
ni|645903561|nb|KK920869.1|	23166-23718	nan	nan	10	9	-0.118836	0.91915	no
ni|645903561|nb|KK920869.1|	23936-24221	nan	nan	0.180659	6	5	0.04485	no
ni|645903561|nb|KK920869.1|	27406-27741	nan	nan	8	17.77	1	0.2776	no
ni|645903561|nb|KK920869.1|	27884-28353	nan	nan	0.302277	3	3	0.0541	no
ni|645903561|nb|KK920869.1|	31756-31992	nan	nan	6	14	1	0.29895	no
ni|645903561|nb|KK920869.1|	32777-32958	nan	nan	6	7	0.183569	0.87365	no

ni|645903561|nb|KK920869.1|	33617-34644	nan	nan	0.870458	0.693309	-0.328276	0.80325	no
ni|645903561|nb|KK920869.1|	34860-35181	nan	nan	13	8	-0.719468	0.61925	no
ni|645903561|nb|KK920869.1|	35837-35974	nan	nan	5	5	-0.0340319	0.9764	no
ni|645903561|nb|KK920869.1|	38205-38936	nan	nan	8	7	-0.168743	0.89145	no
ni|645903561|nb|KK920869.1|	39314-39498	nan	nan	75	98	0.380504	0.7914	no
ni|645903561|nb|KK920869.1|	43817-45218	nan	nan	19	34	0.81705	0.5144	no
ni|645903561|nb|KK920869.1|	45324-45677	nan	nan	123	154	0.316873	0.7697	no
ni|645903561|nb|KK920869.1|	46095-46370	nan	nan	47	58	0.310606	0.8074	no
ni|645903561|nb|KK920869.1|	46760-47427	nan	nan	157	172	0.124094	0.9092	no
ni|645903561|nb|KK920869.1|	47631-47797	nan	nan	50	46	-0.120014	0.91835	no
ni|645903561|nb|KK920869.1|	49138-49323	nan	nan	120	77	-0.631196	0.674	no
ni|645903561|nb|KK920869.1|	49441-49571	nan	nan	1	2	0.400476	0.7408	no
ni|645903561|nb|KK920869.1|	50339-50736	nan	nan	2	2	-0.0665344	0.9384	no
ni|645903561|nb|KK920869.1|	50873-51632	nan	nan	2	2	-0.387457	0.7511	no
ni|645903561|nb|KK920869.1|	516100-516410	nan	nan	82	71	-0.199204	0.8669	no
ni|645903561|nb|KK920869.1|	51836-52110	nan	nan	5	5	-0.0648068	0.95455	no
ni|645903561|nb|KK920869.1|	59523-59724	nan	nan	42	26	-0.686254	0.5653	no
ni|645903561|nb|KK920869.1|	59977-60512	nan	nan	25	19	-0.425651	0.72175	no
ni|645903561|nb|KK920869.1|	60768-61767	nan	nan	47	35	-0.456243	0.6931	no
ni|645903561|nb|KK920869.1|	61907-62458	nan	nan	27	24	-0.163542	0.87955	no
ni|645903561|nb|KK920869.1|	63171-63393	nan	nan	9	9	-0.00173921	0.99105	no
ni|645903561|nb|KK920869.1|	64066-64806	nan	nan	32	26	-0.295435	0.8021	no
ni|645903561|nb|KK920869.1|	65936-66085	nan	nan	26	14	-0.912426	0.429	no
ni|645903561|nb|KK920869.1|	67812-68423	nan	nan	35	39	0.146672	0.90615	no
ni|645903561|nb|KK920869.1|	68784-69176	nan	nan	0.114663	5	5	0.11945	no
ni|645903561|nb|KK920869.1|	69344-69459	nan	nan	28	66	1	0.30005	no
ni|645903561|nb|KK920869.1|	71561-72466	nan	nan	40	65	0.69958	0.5397	no
ni|645903561|nb|KK920869.1|	72709-74008	nan	nan	68	119	0.814158	0.47855	no
ni|645903561|nb|KK920869.1|	74293-74566	nan	nan	33	55	0.731174	0.52895	no
ni|645903561|nb|KK920869.1|	75201-75451	nan	nan	43	70	0.722992	0.5259	no

ni|645903561|nb|KK920869.1|	78325-78435	nan	nan	28	52	0.866355	0.55125	no
ni|645903561|nb|KK920869.1|	78618-78809	nan	nan	67	125	0.899913	0.4378	no
ni|645903561|nb|KK920869.1|	79064-79199	nan	nan	16	21	0.38868	0.7286	no
ni|645903561|nb|KK920869.1|	79411-79533	nan	nan	46	68	0.569362	0.62145	no
ni|645903561|nb|KK920869.1|	80182-81266	nan	nan	39	64	0.709495	0.53925	no
ni|645903561|nb|KK920869.1|	823137-823326	nan	nan	19	15	-0.342939	0.7737	no
ni|645903561|nb|KK920869.1|	82632-83025	nan	nan	6	14	1	0.39005	no
ni|645903561|nb|KK920869.1|	8600-8773	nan	nan	10	15	0.615506	0.60245	no
ni|645903561|nb|KK920869.1|	86170-86556	nan	nan	4	7	0.697084	0.5544	no
ni|645903561|nb|KK920869.1|	86986-87503	nan	nan	0.996521	0.891368	-0.16088	0.8943	no
ni|645903561|nb|KK920869.1|	91879-92019	nan	nan	1	1	-0.135538	0.91325	no
ni|645903561|nb|KK920869.1|	92123-92258	nan	nan	50	52	0.0576431	0.96425	no
ni|645903562|nb|KK920868.1|	146346-146777	nan	nan	7	5	-0.685551	0.56575	no
ni|645903562|nb|KK920868.1|	150931-151262	nan	nan	16	17	0.149093	0.8971	no
ni|645903562|nb|KK920868.1|	151851-152164	nan	nan	20	21	0.0506361	0.96335	no
ni|645903562|nb|KK920868.1|	223586-224336	nan	nan	0.870298	0.554445	-0.650466	0.6679	no
ni|645903562|nb|KK920868.1|	227241-227367	nan	nan	18	22	0.324841	0.7773	no
ni|645903562|nb|KK920868.1|	228719-229119	nan	nan	66	56	-0.237382	0.87385	no
ni|645903562|nb|KK920868.1|	231419-231619	nan	nan	116	85	-0.454674	0.68775	no
ni|645903562|nb|KK920868.1|	232536-232990	nan	nan	129.9	98	-0.411837	0.7223	no
ni|645903562|nb|KK920868.1|	235391-235790	nan	nan	18	14	-0.391575	0.72885	no
ni|645903562|nb|KK920868.1|	238571-238702	nan	nan	99	79	-0.320908	0.77095	no
ni|645903562|nb|KK920868.1|	240261-240757	nan	nan	2	0.910252	-0.972626	0.5005	no
ni|645903562|nb|KK920868.1|	240959-241490	nan	nan	118	104	-0.179265	0.8729	no
ni|645903562|nb|KK920868.1|	241897-242088	nan	nan	104.72	88	-0.245039	0.8257	no
ni|645903562|nb|KK920868.1|	242278-242627	nan	nan	112	85	-0.402339	0.7264	no
ni|645903562|nb|KK920868.1|	242907-243200	nan	nan	14	10	-0.444242	0.70225	no
ni|645903562|nb|KK920868.1|	243345-243556	nan	nan	41	33	-0.324516	0.7684	no
ni|645903562|nb|KK920868.1|	243690-244038	nan	nan	4	3	-0.424336	0.7168	no
ni|645903562|nb|KK920868.1|	244913-245493	nan	nan	62	53	-0.219263	0.8482	no

ni|645903562|nb|KK920868.1|	245593-245744	nan	nan	32	31	-0.0891852	0.93575	no
ni|645903562|nb|KK920868.1|	246345-246534	nan	nan	12	10	-0.258062	0.824	no
ni|645903562|nb|KK920868.1|	247921-248536	nan	nan	34	30.39	-0.176498	0.87845	no
ni|645903562|nb|KK920868.1|	250068-250272	nan	nan	15	14	-0.057852	0.96175	no
ni|645903562|nb|KK920868.1|	250524-250757	nan	nan	37	36	-0.0673071	0.9548	no
ni|645903562|nb|KK920868.1|	251104-251480	nan	nan	8	7	-0.154755	0.8916	no
ni|645903562|nb|KK920868.1|	252102-252840	nan	nan	44	33	-0.395074	0.7337	no
ni|645903562|nb|KK920868.1|	253790-253939	nan	nan	29	30	0.0823122	0.94305	no
ni|645903562|nb|KK920868.1|	254351-254493	nan	nan	46.45	47	0.00931687	0.99005	no
ni|645903562|nb|KK920868.1|	255043-255272	nan	nan	29	32	0.141267	0.9041	no
ni|645903562|nb|KK920868.1|	255541-255723	nan	nan	30	28	-0.0998599	0.9302	no
ni|645903562|nb|KK920868.1|	255958-256186	nan	nan	7	6	-0.263472	0.82045	no
ni|645903562|nb|KK920868.1|	256339-256509	nan	nan	7	8	0.311273	0.81835	no
ni|645903562|nb|KK920868.1|	256775-257119	nan	nan	10	8	-0.366788	0.7706	no
ni|645903562|nb|KK920868.1|	258537-258681	nan	nan	1	0.316915	-2	0.20695	no
ni|645903562|nb|KK920868.1|	258967-259163	nan	nan	1	0.628691	-0.957992	0.4422	no
ni|645903562|nb|KK920868.1|	259428-259839	nan	nan	9	14	0.624627	0.61935	no
ni|645903562|nb|KK920868.1|	260559-260975	nan	nan	6	61	3	0.0161	no
ni|645903562|nb|KK920868.1|	378423-379099	nan	nan	0.534482	1	1	0.3525	no
ni|645903562|nb|KK920868.1|	388324-388841	nan	nan	4	3	-0.065687	0.953	no
ni|645903562|nb|KK920868.1|	390313-390552	nan	nan	10	12	0.29538	0.80305	no
ni|645903562|nb|KK920868.1|	392842-393532	nan	nan	4	3	-0.299554	0.7946	no
ni|645903562|nb|KK920868.1|	396079-396310	nan	nan	8	6	-0.477029	0.6772	no
ni|645903562|nb|KK920868.1|	396968-397129	nan	nan	5	7	0.26089	0.82575	no
ni|645903562|nb|KK920868.1|	400580-400761	nan	nan	3	2	-0.455228	0.7059	no
ni|645903562|nb|KK920868.1|	402308-402580	nan	nan	5	4	-0.605765	0.60075	no
ni|645903562|nb|KK920868.1|	403329-403569	nan	nan	0.201284	6	5	0.0449	no
ni|645903562|nb|KK920868.1|	405972-406234	nan	nan	9	14	0.691741	0.5869	no
ni|645903562|nb|KK920868.1|	408894-409440	nan	nan	0	2	inf	0.0003	yes
ni|645903562|nb|KK920868.1|	410871-411025	nan	nan	3	5	0.844481	0.47295	no

ni|645903562|nb|KK920868.1|	411427-412284	nan	nan	10	14	0.460463	0.69105	no
ni|645903562|nb|KK920868.1|	412985-413366	nan	nan	3	3	-0.218205	0.86075	no
ni|645903562|nb|KK920868.1|	416711-416951	nan	nan	0	4	inf	5.00E-05	yes
ni|645903562|nb|KK920868.1|	419993-420147	nan	nan	0	5	inf	0.00015	yes
ni|645903562|nb|KK920868.1|	420221-420416	nan	nan	12	10	-0.245544	0.82905	no
ni|645903562|nb|KK920868.1|	420732-421082	nan	nan	5	8	0.741574	0.52805	no
ni|645903562|nb|KK920868.1|	421232-421390	nan	nan	15	16	0.129106	0.9084	no
ni|645903562|nb|KK920868.1|	421521-421992	nan	nan	78	83	0.103861	0.92415	no
ni|645903562|nb|KK920868.1|	423199-423536	nan	nan	8	10	0.208672	0.86245	no
ni|645903562|nb|KK920868.1|	423807-424375	nan	nan	15	22	0.599744	0.6103	no
ni|645903562|nb|KK920868.1|	428333-428704	nan	nan	70	86	0.293159	0.84415	no
ni|645903562|nb|KK920868.1|	429755-430319	nan	nan	85	93	0.121084	0.91455	no
ni|645903562|nb|KK920868.1|	430708-431043	nan	nan	81	104	0.361273	0.80725	no
ni|645903562|nb|KK920868.1|	431628-431983	nan	nan	97	104	0.0987066	0.9272	no
ni|645903562|nb|KK920868.1|	442243-442660	nan	nan	8	11	0.452652	0.6913	no
ni|645903562|nb|KK920868.1|	503174-503562	nan	nan	50	54	0.122603	0.91785	no
ni|645903562|nb|KK920868.1|	530504-531126	nan	nan	2	1	-0.569977	0.6347	no
ni|645903562|nb|KK920868.1|	67612-67919	nan	nan	9	8	-0.163867	0.8856	no
ni|645903563|nb|KK920867.1|	157571-157860	nan	nan	1	1	0.208157	0.8624	no
ni|645903563|nb|KK920867.1|	182986-183333	nan	nan	0.578828	0.664098	0.198263	0.8731	no
ni|645903563|nb|KK920867.1|	183447-183587	nan	nan	22	32	0.509203	0.6495	no
ni|645903563|nb|KK920867.1|	183742-183894	nan	nan	6	9	0.595769	0.63005	no
ni|645903563|nb|KK920867.1|	184375-184613	nan	nan	17	20	0.232316	0.83965	no
ni|645903563|nb|KK920867.1|	184736-184876	nan	nan	12	15	0.336121	0.7794	no
ni|645903563|nb|KK920867.1|	185082-185216	nan	nan	2	2	0.309158	0.7927	no
ni|645903563|nb|KK920867.1|	185551-185789	nan	nan	109	130.26	0.260899	0.81475	no
ni|645903563|nb|KK920867.1|	186679-187279	nan	nan	54.42	64	0.241689	0.8442	no
ni|645903563|nb|KK920867.1|	187832-188018	nan	nan	128	134	0.0610823	0.95275	no
ni|645903563|nb|KK920867.1|	188148-188314	nan	nan	52	52.04	-0.00593209	0.9968	no
ni|645903563|nb|KK920867.1|	191197-191364	nan	nan	1	2	0.56222	0.66675	no

ni|645903563|nb|KK920867.1|	193306-193668	nan	nan	55	57	0.0335181	0.97515	no
ni|645903563|nb|KK920867.1|	194788-194953	nan	nan	63	71	0.160183	0.8906	no
ni|645903563|nb|KK920867.1|	197236-197401	nan	nan	47	50	0.0811463	0.94695	no
ni|645903563|nb|KK920867.1|	262944-263449	nan	nan	59	58	-0.00349971	0.99695	no
ni|645903563|nb|KK920867.1|	290449-290519	nan	nan	83	70	-0.233557	0.84585	no
ni|645903563|nb|KK920867.1|	330206-330382	nan	nan	2	2	-0.0648712	0.95495	no
ni|645903563|nb|KK920867.1|	362770-362871	nan	nan	252	361	0.515839	0.6421	no
ni|645903563|nb|KK920867.1|	366548-368228	nan	nan	24	19	-0.31577	0.7863	no
ni|645903563|nb|KK920867.1|	376894-377396	nan	nan	61	83	0.439083	0.7268	no
ni|645903563|nb|KK920867.1|	377976-378149	nan	nan	2	1	-0.345611	0.78305	no
ni|645903563|nb|KK920867.1|	378333-378625	nan	nan	2	2	-0.066139	0.9545	no
ni|645903563|nb|KK920867.1|	380056-380690	nan	nan	6	5	-0.0149024	0.9904	no
ni|645903563|nb|KK920867.1|	380901-381301	nan	nan	7	10	0.391414	0.7306	no
ni|645903563|nb|KK920867.1|	382443-383238	nan	nan	13	11	-0.201683	0.8604	no
ni|645903563|nb|KK920867.1|	383713-383885	nan	nan	52	6	-3	0.012	no
ni|645903563|nb|KK920867.1|	384153-384339	nan	nan	47	2	-5	0.1196	no
ni|645903563|nb|KK920867.1|	384529-384709	nan	nan	4	0.372954	-4	0.0326	no
ni|645903563|nb|KK920867.1|	385123-385834	nan	nan	249.34	351	0.493878	0.732	no
ni|645903563|nb|KK920867.1|	397713-398083	nan	nan	215	293.84	0.451718	0.7526	no
ni|645903563|nb|KK920867.1|	398310-398799	nan	nan	2	119	6	0.00925	no
ni|645903563|nb|KK920867.1|	400197-400441	nan	nan	0.934063	1	0.197568	0.87955	no
ni|645903563|nb|KK920867.1|	403325-403660	nan	nan	0.725736	9	4	0.0498	no
ni|645903563|nb|KK920867.1|	403813-404107	nan	nan	0	9	inf	0.0003	yes
ni|645903563|nb|KK920867.1|	404948-405393	nan	nan	68	59	-0.206886	0.88245	no
ni|645903563|nb|KK920867.1|	407843-408232	nan	nan	48230	53230.7	0.142327	0.9104	no
ni|645903563|nb|KK920867.1|	408514-409901	nan	nan	104.75	123	0.230407	0.85305	no
ni|645903563|nb|KK920867.1|	480503-480695	nan	nan	0	3	inf	0.00175	yes
ni|645903563|nb|KK920867.1|	506345-506555	nan	nan	0	0.959786	inf	0.00175	yes
ni|645903563|nb|KK920867.1|	612868-613000	nan	nan	105	68	-0.611516	0.59985	no
ni|645903563|nb|KK920867.1|	613447-613721	nan	nan	3	2	-0.687331	0.58765	no

ni|645903563|nb|KK920867.1|	618563-618899	nan	nan	7	3	-1	0.4023	no
ni|645903563|nb|KK920867.1|	619335-621658	nan	nan	3	2	-0.4605	0.69615	no
ni|645903563|nb|KK920867.1|	621718-622386	nan	nan	4	2	-0.695866	0.5414	no
ni|645903563|nb|KK920867.1|	622810-623236	nan	nan	122	86	-0.510849	0.6823	no
ni|645903563|nb|KK920867.1|	624312-624806	nan	nan	0.099242	0.80642	3	0.06205	no
ni|645903563|nb|KK920867.1|	625825-626011	nan	nan	0.925377	6	3	0.07235	no
ni|645903563|nb|KK920867.1|	626568-626800	nan	nan	285.07	148	-0.942327	0.4495	no
ni|645903563|nb|KK920867.1|	627869-628032	nan	nan	5	4	-0.342153	0.7737	no
ni|645903563|nb|KK920867.1|	630457-630712	nan	nan	285	167	-0.771294	0.5481	no
ni|645903563|nb|KK920867.1|	631782-632137	nan	nan	170	108	-0.652576	0.5785	no
ni|645903563|nb|KK920867.1|	633447-633729	nan	nan	268	174	-0.625371	0.66525	no
ni|645903563|nb|KK920867.1|	634509-634671	nan	nan	130	97	-0.419672	0.77145	no
ni|645903563|nb|KK920867.1|	634868-635472	nan	nan	0.148416	6	5	0.11975	no
ni|645903563|nb|KK920867.1|	636558-636758	nan	nan	17	48	2	0.297	no
ni|645903563|nb|KK920867.1|	637630-637809	nan	nan	0.799892	0.573421	-0.480212	0.7301	no
ni|645903563|nb|KK920867.1|	638337-638462	nan	nan	6	20	2	0.14905	no
ni|645903563|nb|KK920867.1|	648376-648953	nan	nan	0.531824	0.69896	0.39426	0.77595	no
ni|645903563|nb|KK920867.1|	667466-667643	nan	nan	20	72	2	0.13015	no
ni|645903563|nb|KK920867.1|	668080-668687	nan	nan	11	36	2	0.13475	no
ni|645903563|nb|KK920867.1|	668914-669304	nan	nan	0.475888	0.413609	-0.202352	0.87975	no
ni|645903563|nb|KK920867.1|	669428-669551	nan	nan	35	113	2	0.13715	no
ni|645903563|nb|KK920867.1|	683381-686098	nan	nan	21	68	2	0.20415	no
ni|645903563|nb|KK920867.1|	699461-700637	nan	nan	12	59	2	0.0678	no
ni|645903564|nb|KK920866.1|	22254-22581	nan	nan	15	22	0.542841	0.6474	no
ni|645903564|nb|KK920866.1|	24070-24261	nan	nan	21	17	-0.334375	0.7759	no
ni|645903564|nb|KK920866.1|	250362-250591	nan	nan	21	17	-0.27483	0.8116	no
ni|645903564|nb|KK920866.1|	251709-252557	nan	nan	19	20	0.111471	0.9242	no
ni|645903564|nb|KK920866.1|	259783-259964	nan	nan	10	9	-0.23844	0.8395	no
ni|645903564|nb|KK920866.1|	260089-260275	nan	nan	3	0.11163	-5	0.0191	no
ni|645903564|nb|KK920866.1|	261373-261696	nan	nan	24	13	-0.867791	0.44955	no

ni|645903564|nb|KK920866.1|	265998-266162	nan	nan	37	26	-0.496225	0.6643	no
ni|645903564|nb|KK920866.1|	270466-270619	nan	nan	24	12	-0.963295	0.38405	no
ni|645903564|nb|KK920866.1|	27427-27653	nan	nan	12	7	-0.840779	0.48025	no
ni|645903564|nb|KK920866.1|	277800-278507	nan	nan	32	24	-0.415749	0.71925	no
ni|645903564|nb|KK920866.1|	280121-280398	nan	nan	12	6	-0.889112	0.4489	no
ni|645903564|nb|KK920866.1|	280650-280866	nan	nan	38	23	-0.683519	0.59255	no
ni|645903564|nb|KK920866.1|	281411-281627	nan	nan	52	26	-0.982398	0.4132	no
ni|645903564|nb|KK920866.1|	282646-283161	nan	nan	3	2	-0.854551	0.50505	no
ni|645903564|nb|KK920866.1|	285431-285842	nan	nan	0	43	inf	5.00E-05	yes
ni|645903564|nb|KK920866.1|	290119-290289	nan	nan	0.861315	2	0.934379	0.4874	no
ni|645903564|nb|KK920866.1|	290424-290603	nan	nan	0	47	inf	5.00E-05	yes
ni|645903564|nb|KK920866.1|	290978-291151	nan	nan	0.512848	25	6	0.0045	no
ni|645903564|nb|KK920866.1|	29127-29392	nan	nan	54	45	-0.272998	0.80155	no
ni|645903564|nb|KK920866.1|	293310-293488	nan	nan	41	25	-0.715012	0.5426	no
ni|645903564|nb|KK920866.1|	293579-293720	nan	nan	59	52	-0.185228	0.87185	no
ni|645903564|nb|KK920866.1|	294480-294705	nan	nan	75	64	-0.226926	0.84505	no
ni|645903564|nb|KK920866.1|	294822-295005	nan	nan	25	20	-0.322304	0.7759	no
ni|645903564|nb|KK920866.1|	296786-296893	nan	nan	5	4	-0.456211	0.6981	no
ni|645903564|nb|KK920866.1|	297059-297286	nan	nan	81	60	-0.413188	0.70665	no
ni|645903564|nb|KK920866.1|	300215-300379	nan	nan	56	54	-0.0561452	0.96025	no
ni|645903564|nb|KK920866.1|	302740-303269	nan	nan	5	22	2	0.11685	no
ni|645903564|nb|KK920866.1|	307817-308022	nan	nan	88	81	-0.114722	0.9178	no
ni|645903564|nb|KK920866.1|	310134-310780	nan	nan	54	53	-0.0207584	0.98505	no
ni|645903564|nb|KK920866.1|	311354-312136	nan	nan	39	40	0.0214993	0.9843	no
ni|645903564|nb|KK920866.1|	313074-313321	nan	nan	1	1	0.134362	0.908	no
ni|645903564|nb|KK920866.1|	313432-314205	nan	nan	2	3	0.518088	0.7004	no
ni|645903564|nb|KK920866.1|	314432-314683	nan	nan	0.920766	0.817237	-0.172079	0.89245	no
ni|645903564|nb|KK920866.1|	315026-315215	nan	nan	7	3	-2	0.2563	no
ni|645903564|nb|KK920866.1|	315533-315838	nan	nan	475	220	-1	0.3981	no
ni|645903564|nb|KK920866.1|	317953-318358	nan	nan	371	220	-0.755348	0.60705	no

ni|645903564|nb|KK920866.1|	319036-319187	nan	nan	7	4	-0.818826	0.49455	no
ni|645903564|nb|KK920866.1|	319298-320049	nan	nan	78	40	-0.945817	0.46455	no
ni|645903564|nb|KK920866.1|	320134-320280	nan	nan	128	76	-0.761589	0.54775	no
ni|645903564|nb|KK920866.1|	320802-321054	nan	nan	173.83	78	-1	0.32105	no
ni|645903564|nb|KK920866.1|	321155-321462	nan	nan	222.03	153	-0.533422	0.7226	no
ni|645903564|nb|KK920866.1|	322694-323248	nan	nan	0.493158	0.754018	0.612548	0.6619	no
ni|645903564|nb|KK920866.1|	323400-323603	nan	nan	157	127.23	-0.301959	0.8354	no
ni|645903564|nb|KK920866.1|	324409-325073	nan	nan	151	123.99	-0.28849	0.8154	no
ni|645903564|nb|KK920866.1|	32538-32906	nan	nan	125	97	-0.373066	0.74565	no
ni|645903564|nb|KK920866.1|	325458-325956	nan	nan	70	62	-0.170316	0.87685	no
ni|645903564|nb|KK920866.1|	331124-331397	nan	nan	7	7	-0.0126795	0.99155	no
ni|645903564|nb|KK920866.1|	332878-333016	nan	nan	4	2	-1	0.39475	no
ni|645903564|nb|KK920866.1|	334771-335057	nan	nan	3	3	0.256489	0.8299	no
ni|645903564|nb|KK920866.1|	33490-33674	nan	nan	22	7	-2	0.20615	no
ni|645903564|nb|KK920866.1|	337552-337718	nan	nan	13	9	-0.519535	0.66405	no
ni|645903564|nb|KK920866.1|	338764-338945	nan	nan	10	11	0.1205	0.92055	no
ni|645903564|nb|KK920866.1|	339693-339854	nan	nan	13	12	-0.0316861	0.97855	no
ni|645903564|nb|KK920866.1|	339972-340099	nan	nan	11	13	0.202764	0.8671	no
ni|645903564|nb|KK920866.1|	340628-340797	nan	nan	6	5	-0.19233	0.86875	no
ni|645903564|nb|KK920866.1|	341929-342109	nan	nan	7	7	0.0482666	0.95725	no
ni|645903564|nb|KK920866.1|	343353-343629	nan	nan	4	6	0.436189	0.7114	no
ni|645903564|nb|KK920866.1|	345539-345815	nan	nan	11.88	17	0.485255	0.70275	no
ni|645903564|nb|KK920866.1|	35574-35981	nan	nan	165	142	-0.216067	0.8469	no
ni|645903564|nb|KK920866.1|	38002-38394	nan	nan	116	181	0.648296	0.6471	no
ni|645903564|nb|KK920866.1|	380884-381072	nan	nan	4	5	0.268708	0.8181	no
ni|645903564|nb|KK920866.1|	387314-387461	nan	nan	18	7	-1	0.2643	no
ni|645903564|nb|KK920866.1|	38824-39386	nan	nan	2	3	0.419948	0.7259	no
ni|645903564|nb|KK920866.1|	389593-389982	nan	nan	9	3	-2	0.16375	no
ni|645903564|nb|KK920866.1|	40259-40473	nan	nan	2	1	-0.650159	0.59155	no
ni|645903564|nb|KK920866.1|	41037-41258	nan	nan	2	0.684021	-2	0.1782	no

ni|645903564|nb|KK920866.1|	43626-43845	nan	nan	2	0.774781	-0.95815	0.4422	no
ni|645903564|nb|KK920866.1|	45121-45394	nan	nan	75	123	0.724973	0.62265	no
ni|645903564|nb|KK920866.1|	46561-46733	nan	nan	36	64	0.818059	0.5885	no
ni|645903564|nb|KK920866.1|	47975-48133	nan	nan	1	0.468673	-1	0.35155	no
ni|645903564|nb|KK920866.1|	5934-6061	nan	nan	5	29	3	0.0367	no
ni|645903564|nb|KK920866.1|	630661-631047	nan	nan	4	2	-1	0.27155	no
ni|645903564|nb|KK920866.1|	71511-71899	nan	nan	38	81	1	0.3947	no
ni|645903564|nb|KK920866.1|	7306-7843	nan	nan	12	12	-0.10447	0.92785	no
ni|645903564|nb|KK920866.1|	752424-752717	nan	nan	20	5	-2	0.1092	no
ni|645903564|nb|KK920866.1|	755622-755840	nan	nan	2	0.299693	-3	0.09205	no
ni|645903564|nb|KK920866.1|	756875-756986	nan	nan	0.503249	0.160379	-2	0.25615	no
ni|645903564|nb|KK920866.1|	7904-11322	nan	nan	69	56	-0.289845	0.80925	no
ni|645903564|nb|KK920866.1|	83692-84007	nan	nan	7	5	-0.434843	0.70475	no
ni|645903564|nb|KK920866.1|	84788-84953	nan	nan	13	14	0.126563	0.91585	no
ni|645903566|nb|KK920864.1|	0-1311	nan	nan	21	8	-1	0.22305	no
ni|645903566|nb|KK920864.1|	10242-11232	nan	nan	97	56	-0.80179	0.4855	no
ni|645903566|nb|KK920864.1|	11638-11963	nan	nan	95	56	-0.774009	0.4852	no
ni|645903566|nb|KK920864.1|	12324-12442	nan	nan	39	24	-0.709752	0.5182	no
ni|645903566|nb|KK920864.1|	12514-12664	nan	nan	63	46	-0.449537	0.68265	no
ni|645903566|nb|KK920864.1|	168118-168356	nan	nan	0.879928	1	0.518966	0.7279	no
ni|645903566|nb|KK920864.1|	1691-2715	nan	nan	23	10	-1	0.33905	no
ni|645903566|nb|KK920864.1|	169685-169816	nan	nan	0.131094	4	5	0.12005	no
ni|645903566|nb|KK920864.1|	172134-173272	nan	nan	8	5	-0.58179	0.6557	no
ni|645903566|nb|KK920864.1|	175903-176199	nan	nan	15	13	-0.152805	0.8912	no
ni|645903566|nb|KK920864.1|	176619-177927	nan	nan	25	21	-0.260488	0.8692	no
ni|645903566|nb|KK920864.1|	177994-178345	nan	nan	0.504799	0.82711	0.71237	0.61075	no
ni|645903566|nb|KK920864.1|	24675-25060	nan	nan	12	9	-0.401821	0.7222	no
ni|645903566|nb|KK920864.1|	28044-28398	nan	nan	135	89	-0.613142	0.60365	no
ni|645903566|nb|KK920864.1|	30032-30470	nan	nan	68	51	-0.406633	0.70435	no
ni|645903566|nb|KK920864.1|	30924-31246	nan	nan	41	34.4	-0.252048	0.82035	no

ni|645903566|nb|KK920864.1|	31605-31832	nan	nan	77	66	-0.227356	0.84205	no
ni|645903566|nb|KK920864.1|	3186-3309	nan	nan	27	7	-2	0.1324	no
ni|645903566|nb|KK920864.1|	32521-32835	nan	nan	74	52	-0.494548	0.6564	no
ni|645903566|nb|KK920864.1|	3860-4153	nan	nan	31	10	-2	0.24765	no
ni|645903566|nb|KK920864.1|	412149-412415	nan	nan	4	2	-0.745268	0.59615	no
ni|645903566|nb|KK920864.1|	413913-414338	nan	nan	1	2	0.998589	0.45905	no
ni|645903566|nb|KK920864.1|	414944-415154	nan	nan	1	1	-0.012469	0.98035	no
ni|645903566|nb|KK920864.1|	415348-415477	nan	nan	0.787514	0.347543	-1	0.3561	no
ni|645903566|nb|KK920864.1|	415853-415982	nan	nan	48	37	-0.400702	0.72545	no
ni|645903566|nb|KK920864.1|	416205-416337	nan	nan	13	14	0.0530836	0.96745	no
ni|645903566|nb|KK920864.1|	416513-416823	nan	nan	6	6	0.036931	0.9737	no
ni|645903566|nb|KK920864.1|	420104-420289	nan	nan	64	53	-0.267163	0.8561	no
ni|645903566|nb|KK920864.1|	420918-421043	nan	nan	3	1	-1	0.429	no
ni|645903566|nb|KK920864.1|	421312-421497	nan	nan	0.894319	0.347465	-1	0.25795	no
ni|645903566|nb|KK920864.1|	422602-423252	nan	nan	57	44	-0.3774	0.7354	no
ni|645903566|nb|KK920864.1|	426916-427236	nan	nan	45	38	-0.233555	0.84135	no
ni|645903566|nb|KK920864.1|	427632-428148	nan	nan	68	53	-0.365612	0.7523	no
ni|645903566|nb|KK920864.1|	428536-428986	nan	nan	29	26	-0.157019	0.88535	no
ni|645903566|nb|KK920864.1|	432040-432247	nan	nan	3	1	-1	0.35155	no
ni|645903566|nb|KK920864.1|	432506-432894	nan	nan	2	1	-0.492576	0.6667	no
ni|645903566|nb|KK920864.1|	433110-433318	nan	nan	5	3	-0.704737	0.60375	no
ni|645903566|nb|KK920864.1|	433419-433741	nan	nan	0	7	inf	5.00E-05	yes
ni|645903566|nb|KK920864.1|	433958-434500	nan	nan	0.245616	6	5	0.121	no
ni|645903566|nb|KK920864.1|	434641-434788	nan	nan	0	10	inf	0.0003	yes
ni|645903566|nb|KK920864.1|	4400-4551	nan	nan	19	7	-1	0.2746	no
ni|645903566|nb|KK920864.1|	443397-443590	nan	nan	0.513001	7	4	0.05325	no
ni|645903566|nb|KK920864.1|	444191-444332	nan	nan	0.107988	5	6	0.05065	no
ni|645903566|nb|KK920864.1|	444993-445277	nan	nan	0	11	inf	0.0005	yes
ni|645903566|nb|KK920864.1|	445428-445566	nan	nan	64	72	0.168374	0.8766	no
ni|645903566|nb|KK920864.1|	445751-445870	nan	nan	87	119	0.446731	0.70505	no

ni|645903566|nb|KK920864.1|	446068-446421	nan	nan	11	11	0.104006	0.92725	no
ni|645903566|nb|KK920864.1|	446673-446804	nan	nan	22	24	0.140238	0.90075	no
ni|645903566|nb|KK920864.1|	447071-447609	nan	nan	35	35	-0.00926823	0.9936	no
ni|645903566|nb|KK920864.1|	449215-449956	nan	nan	30	35	0.221398	0.84035	no
ni|645903566|nb|KK920864.1|	450246-450415	nan	nan	45	38	-0.245025	0.83245	no
ni|645903566|nb|KK920864.1|	450951-451121	nan	nan	56	62	0.147817	0.8978	no
ni|645903566|nb|KK920864.1|	452432-452587	nan	nan	42	50	0.257401	0.8264	no
ni|645903566|nb|KK920864.1|	452771-452979	nan	nan	3	3	-0.0660818	0.96245	no
ni|645903566|nb|KK920864.1|	453455-454241	nan	nan	2	2	0.121235	0.91855	no
ni|645903566|nb|KK920864.1|	455310-455516	nan	nan	16	17	0.114761	0.921	no
ni|645903566|nb|KK920864.1|	457258-457365	nan	nan	5	8	0.879637	0.4255	no
ni|645903566|nb|KK920864.1|	459465-459633	nan	nan	1	2	0.562941	0.6385	no
ni|645903566|nb|KK920864.1|	4631-5247	nan	nan	30	7	-2	0.1083	no
ni|645903566|nb|KK920864.1|	5419-5556	nan	nan	3	0	-inf	0.002	yes
ni|645903566|nb|KK920864.1|	5717-5872	nan	nan	0.069185	6	6	0.05055	no
ni|645903566|nb|KK920864.1|	584243-584508	nan	nan	1	2	0.464477	0.7264	no
ni|645903566|nb|KK920864.1|	585706-585861	nan	nan	3	2	-1	0.39285	no
ni|645903566|nb|KK920864.1|	586867-587011	nan	nan	30	33	0.138794	0.90865	no
ni|645903566|nb|KK920864.1|	588550-589278	nan	nan	2	2	0.241516	0.8351	no
ni|645903566|nb|KK920864.1|	589493-590144	nan	nan	1	0.951638	-0.364845	0.7706	no
ni|645903566|nb|KK920864.1|	592481-592730	nan	nan	53	75	0.501871	0.66135	no
ni|645903566|nb|KK920864.1|	593195-593789	nan	nan	53	61	0.214818	0.86425	no
ni|645903566|nb|KK920864.1|	597432-597555	nan	nan	14	17	0.30581	0.7927	no
ni|645903566|nb|KK920864.1|	597666-597850	nan	nan	177	325	0.88079	0.48155	no
ni|645903566|nb|KK920864.1|	598384-598625	nan	nan	27	30	0.162148	0.89525	no
ni|645903566|nb|KK920864.1|	599060-599521	nan	nan	2	2	-0.181482	0.889	no
ni|645903566|nb|KK920864.1|	599817-600189	nan	nan	8	7	-0.175153	0.8774	no
ni|645903566|nb|KK920864.1|	600390-600805	nan	nan	681.43	546	-0.320922	0.8187	no
ni|645903566|nb|KK920864.1|	601188-601785	nan	nan	301	272	-0.143429	0.9171	no
ni|645903566|nb|KK920864.1|	602350-602860	nan	nan	114	63	-0.844896	0.54365	no

ni|645903566|nb|KK920864.1|	603011-603887	nan	nan	7	7	0.123183	0.9094	no
ni|645903566|nb|KK920864.1|	604226-604367	nan	nan	13	29	1	0.3411	no
ni|645903566|nb|KK920864.1|	606984-607140	nan	nan	17	19	0.12535	0.91735	no
ni|645903566|nb|KK920864.1|	611663-611797	nan	nan	0.964773	0.307292	-2	0.2989	no
ni|645903566|nb|KK920864.1|	613350-613600	nan	nan	43	61	0.507916	0.6624	no
ni|645903566|nb|KK920864.1|	613842-614300	nan	nan	3	2	-0.581719	0.70815	no
ni|645903566|nb|KK920864.1|	6143-6267	nan	nan	41	36	-0.187589	0.8754	no
ni|645903566|nb|KK920864.1|	614573-614873	nan	nan	8	5	-0.680278	0.5787	no
ni|645903566|nb|KK920864.1|	615148-615532	nan	nan	0.770656	0.736511	-0.0653796	0.94155	no
ni|645903566|nb|KK920864.1|	618210-618404	nan	nan	21	31	0.519922	0.62995	no
ni|645903566|nb|KK920864.1|	619590-619814	nan	nan	0	50	inf	5.00E-05	yes
ni|645903566|nb|KK920864.1|	620611-620873	nan	nan	6	4	-0.445714	0.74215	no
ni|645903566|nb|KK920864.1|	623573-623895	nan	nan	0.240996	4	4	0.05225	no
ni|645903566|nb|KK920864.1|	625766-626109	nan	nan	1	0.639344	-1	0.45115	no
ni|645903566|nb|KK920864.1|	631755-632032	nan	nan	2	2	-0.0664867	0.9467	no
ni|645903566|nb|KK920864.1|	632325-632559	nan	nan	2	1	-0.718794	0.61925	no
ni|645903566|nb|KK920864.1|	632738-633041	nan	nan	6	8	0.463287	0.7264	no
ni|645903566|nb|KK920864.1|	637068-637459	nan	nan	32	55	0.806887	0.5898	no
ni|645903566|nb|KK920864.1|	638522-638696	nan	nan	63	88	0.47406	0.667	no
ni|645903566|nb|KK920864.1|	640690-641676	nan	nan	2	3	0.519504	0.6682	no
ni|645903566|nb|KK920864.1|	642833-642972	nan	nan	159	102	-0.644225	0.5529	no
ni|645903566|nb|KK920864.1|	644238-644409	nan	nan	0.73757	0.604101	-0.28799	0.8535	no
ni|645903566|nb|KK920864.1|	646340-646947	nan	nan	225	219.02	-0.0419398	0.96885	no
ni|645903566|nb|KK920864.1|	648619-648818	nan	nan	83	75	-0.158557	0.91115	no
ni|645903566|nb|KK920864.1|	650493-650623	nan	nan	5	3	-0.614384	0.6288	no
ni|645903566|nb|KK920864.1|	654189-654431	nan	nan	151	155	0.0413461	0.9783	no
ni|645903566|nb|KK920864.1|	654941-655138	nan	nan	63	56	-0.170734	0.90815	no
ni|645903566|nb|KK920864.1|	661019-661401	nan	nan	2	2	-0.0655971	0.9589	no
ni|645903566|nb|KK920864.1|	661815-662142	nan	nan	5	4	-0.441401	0.7003	no
ni|645903566|nb|KK920864.1|	664481-664693	nan	nan	75	78	0.0752899	0.9511	no

ni|645903566|nb|KK920864.1|	666591-666809	nan	nan	93	104	0.159032	0.91045	no
ni|645903566|nb|KK920864.1|	668230-668439	nan	nan	2	0.622941	-2	0.2095	no
ni|645903566|nb|KK920864.1|	669608-669807	nan	nan	93	83	-0.170728	0.9098	no
ni|645903566|nb|KK920864.1|	670362-670583	nan	nan	115	122	0.0908427	0.9349	no
ni|645903566|nb|KK920864.1|	670652-671074	nan	nan	130	158	0.283373	0.80565	no
ni|645903566|nb|KK920864.1|	6731-7021	nan	nan	1	1	0.206976	0.85905	no
ni|645903566|nb|KK920864.1|	679219-679587	nan	nan	130	140	0.108322	0.94465	no
ni|645903566|nb|KK920864.1|	680518-680694	nan	nan	64	67	0.087765	0.94835	no
ni|645903566|nb|KK920864.1|	687623-687926	nan	nan	123	140	0.18278	0.8561	no
ni|645903566|nb|KK920864.1|	688211-688562	nan	nan	15190	22006.7	0.534826	0.6671	no
ni|645903566|nb|KK920864.1|	688778-688919	nan	nan	8	6	-0.289596	0.82015	no
ni|645903566|nb|KK920864.1|	693795-694161	nan	nan	12447.1	18166.9	0.545507	0.6607	no
ni|645903566|nb|KK920864.1|	694707-694948	nan	nan	35	31	-0.157565	0.8927	no
ni|645903566|nb|KK920864.1|	7318-7438	nan	nan	17	16	-0.104676	0.93055	no
ni|645903566|nb|KK920864.1|	732512-732847	nan	nan	0.075885	785	13	0.11935	no
ni|645903566|nb|KK920864.1|	736794-737260	nan	nan	0	719	inf	5.00E-05	yes
ni|645903566|nb|KK920864.1|	739813-739956	nan	nan	0	475	inf	5.00E-05	yes
ni|645903566|nb|KK920864.1|	741126-741308	nan	nan	3	5	0.840565	0.48945	no
ni|645903566|nb|KK920864.1|	741573-741912	nan	nan	3	6	0.80821	0.50525	no
ni|645903566|nb|KK920864.1|	743501-744083	nan	nan	4	11	2	0.2583	no
ni|645903566|nb|KK920864.1|	747274-747532	nan	nan	4	6	0.726506	0.56145	no
ni|645903566|nb|KK920864.1|	748877-749502	nan	nan	3	3	-0.0652308	0.95265	no
ni|645903566|nb|KK920864.1|	751945-752188	nan	nan	15	7	-1	0.42295	no
ni|645903566|nb|KK920864.1|	752934-753610	nan	nan	0.741248	1	0.998947	0.45905	no
ni|645903566|nb|KK920864.1|	755317-755586	nan	nan	2	7	2	0.12465	no
ni|645903566|nb|KK920864.1|	756616-756880	nan	nan	3	4	0.304018	0.793	no
ni|645903566|nb|KK920864.1|	757746-758002	nan	nan	3	4	0.30823	0.79525	no
ni|645903566|nb|KK920864.1|	759869-760042	nan	nan	0.794999	2	1	0.45555	no
ni|645903566|nb|KK920864.1|	760157-760377	nan	nan	3	6	0.780759	0.58145	no
ni|645903566|nb|KK920864.1|	761946-762136	nan	nan	2	4	0.615867	0.6033	no

ni|645903566|nb|KK920864.1|	763082-763289	nan	nan	5	13	1	0.38715	no
ni|645903566|nb|KK920864.1|	763991-764146	nan	nan	3	6	0.795255	0.5353	no
ni|645903566|nb|KK920864.1|	767899-768374	nan	nan	3	3	0.0106567	0.9852	no
ni|645903566|nb|KK920864.1|	770283-770889	nan	nan	5	6	0.301593	0.79705	no
ni|645903566|nb|KK920864.1|	772079-772543	nan	nan	7	7	-0.0112185	0.9925	no
ni|645903566|nb|KK920864.1|	772726-773559	nan	nan	8	6	-0.514661	0.68825	no
ni|645903566|nb|KK920864.1|	773668-773946	nan	nan	5	2	-1	0.40265	no
ni|645903566|nb|KK920864.1|	778885-779086	nan	nan	0.957302	0.800326	-0.258386	0.8692	no
ni|645903566|nb|KK920864.1|	784365-784492	nan	nan	131	75	-0.799642	0.48305	no
ni|645903566|nb|KK920864.1|	8118-8231	nan	nan	41	38	-0.116853	0.9188	no
ni|645903566|nb|KK920864.1|	8575-8895	nan	nan	44	46	0.0734863	0.95965	no
ni|645903566|nb|KK920864.1|	9228-9408	nan	nan	16	9	-0.74527	0.5313	no
ni|645903566|nb|KK920864.1|	9600-10045	nan	nan	26	17	-0.564151	0.6258	no
ni|645903567|nb|KK920863.1|	11370-11531	nan	nan	20	11	-0.908668	0.4087	no
ni|645903567|nb|KK920863.1|	116320-116730	nan	nan	119	98	-0.278216	0.8051	no
ni|645903567|nb|KK920863.1|	126722-126850	nan	nan	123	99	-0.308224	0.7883	no
ni|645903567|nb|KK920863.1|	127143-127561	nan	nan	34	27	-0.303972	0.78275	no
ni|645903567|nb|KK920863.1|	128232-128338	nan	nan	9	5	-0.974161	0.43165	no
ni|645903567|nb|KK920863.1|	131945-132142	nan	nan	100	69	-0.544414	0.64085	no
ni|645903567|nb|KK920863.1|	1352-1536	nan	nan	34	11	-2	0.1763	no
ni|645903567|nb|KK920863.1|	138916-139243	nan	nan	54	50	-0.102822	0.92925	no
ni|645903567|nb|KK920863.1|	139406-140130	nan	nan	51	40	-0.36974	0.74735	no
ni|645903567|nb|KK920863.1|	141030-142412	nan	nan	13	16	0.296927	0.7892	no
ni|645903567|nb|KK920863.1|	142635-143074	nan	nan	17	19	0.169812	0.8892	no
ni|645903567|nb|KK920863.1|	143229-144218	nan	nan	5	6	0.286076	0.80725	no
ni|645903567|nb|KK920863.1|	145769-146027	nan	nan	22	27	0.280568	0.81735	no
ni|645903567|nb|KK920863.1|	14676-14843	nan	nan	143.85	95	-0.59107	0.601	no
ni|645903567|nb|KK920863.1|	148133-148311	nan	nan	14	17	0.254523	0.84735	no
ni|645903567|nb|KK920863.1|	151256-152027	nan	nan	14	21	0.545774	0.6414	no
ni|645903567|nb|KK920863.1|	152353-152552	nan	nan	19	23	0.259425	0.81565	no

ni|645903567|nb|KK920863.1|	152696-152972	nan	nan	21	24	0.220612	0.8458	no
ni|645903567|nb|KK920863.1|	154611-155104	nan	nan	8	10	0.362113	0.75635	no
ni|645903567|nb|KK920863.1|	161864-162333	nan	nan	22	22	0.0213905	0.98535	no
ni|645903567|nb|KK920863.1|	162474-162757	nan	nan	17	24	0.565684	0.6267	no
ni|645903567|nb|KK920863.1|	1643-1866	nan	nan	0.73368	0.48554	-0.595562	0.64595	no
ni|645903567|nb|KK920863.1|	164310-165047	nan	nan	36	32	-0.14784	0.9011	no
ni|645903567|nb|KK920863.1|	165177-165729	nan	nan	29	22	-0.400618	0.72545	no
ni|645903567|nb|KK920863.1|	166827-167302	nan	nan	30	23	-0.422301	0.7222	no
ni|645903567|nb|KK920863.1|	167514-167659	nan	nan	2	0.345297	-2	0.1417	no
ni|645903567|nb|KK920863.1|	169585-170381	nan	nan	0.495303	102	8	0.001	yes
ni|645903567|nb|KK920863.1|	189100-189409	nan	nan	0.677327	19	5	0.04555	no
ni|645903567|nb|KK920863.1|	198764-199082	nan	nan	0.704356	34	6	0.1198	no
ni|645903567|nb|KK920863.1|	199292-199981	nan	nan	0.936175	10	3	0.0445	no
ni|645903567|nb|KK920863.1|	200313-200646	nan	nan	0.626542	6	3	0.0267	no
ni|645903567|nb|KK920863.1|	203900-204506	nan	nan	0.424009	10	5	0.0345	no
ni|645903567|nb|KK920863.1|	206385-206539	nan	nan	0	25	inf	5.00E-05	yes
ni|645903567|nb|KK920863.1|	207006-207302	nan	nan	0.744201	12	4	0.0532	no
ni|645903567|nb|KK920863.1|	207807-208338	nan	nan	0	24	inf	5.00E-05	yes
ni|645903567|nb|KK920863.1|	210034-210205	nan	nan	0.507348	25	6	0.02735	no
ni|645903567|nb|KK920863.1|	210531-210741	nan	nan	0.245866	4	4	0.0249	no
ni|645903567|nb|KK920863.1|	210876-211566	nan	nan	0.463489	13	5	0.0052	no
ni|645903567|nb|KK920863.1|	213037-213197	nan	nan	0.948589	0.841919	-0.172103	0.89245	no
ni|645903567|nb|KK920863.1|	213448-214060	nan	nan	3	3	-0.240486	0.83825	no
ni|645903567|nb|KK920863.1|	219400-219575	nan	nan	2	1	-0.288203	0.8283	no
ni|645903567|nb|KK920863.1|	220246-221016	nan	nan	33	25	-0.425535	0.70755	no
ni|645903567|nb|KK920863.1|	221202-221955	nan	nan	16	14	-0.181989	0.8688	no
ni|645903567|nb|KK920863.1|	228707-229618	nan	nan	45	60	0.416607	0.7159	no
ni|645903567|nb|KK920863.1|	230023-230323	nan	nan	1	2	0.256214	0.84735	no
ni|645903567|nb|KK920863.1|	231306-231697	nan	nan	2	2	0.140646	0.90865	no
ni|645903567|nb|KK920863.1|	232664-232868	nan	nan	2	1	-0.939904	0.4651	no

ni|645903567|nb|KK920863.1|	256978-257239	nan	nan	4	0.794736	-2	0.2949	no
ni|645903567|nb|KK920863.1|	264905-265274	nan	nan	3	2	-0.33229	0.77515	no
ni|645903567|nb|KK920863.1|	294894-295773	nan	nan	53	72	0.439189	0.71745	no
ni|645903567|nb|KK920863.1|	296381-297010	nan	nan	53	33	-0.671524	0.582	no
ni|645903567|nb|KK920863.1|	298998-299531	nan	nan	5	3	-0.607851	0.63135	no
ni|645903567|nb|KK920863.1|	300379-300961	nan	nan	16	29	0.838633	0.4627	no
ni|645903567|nb|KK920863.1|	301616-302028	nan	nan	0.153183	9	6	0.04435	no
ni|645903567|nb|KK920863.1|	303310-303825	nan	nan	15	17	0.195818	0.8681	no
ni|645903567|nb|KK920863.1|	315545-316639	nan	nan	0.240963	3	4	0.0402	no
ni|645903567|nb|KK920863.1|	322772-323960	nan	nan	15	18	0.242003	0.8377	no
ni|645903567|nb|KK920863.1|	334117-335328	nan	nan	0.445188	0.851328	0.9353	0.4565	no
ni|645903567|nb|KK920863.1|	33490-33770	nan	nan	121	71	-0.774636	0.47995	no
ni|645903567|nb|KK920863.1|	341044-341670	nan	nan	3	5	0.934784	0.4344	no
ni|645903567|nb|KK920863.1|	341945-342130	nan	nan	0.685083	2	2	0.23795	no
ni|645903567|nb|KK920863.1|	342221-342440	nan	nan	3	4	0.583938	0.62725	no
ni|645903567|nb|KK920863.1|	343207-343426	nan	nan	2	3	0.577468	0.62375	no
ni|645903567|nb|KK920863.1|	343650-343935	nan	nan	6	9	0.564949	0.6425	no
ni|645903567|nb|KK920863.1|	34448-35412	nan	nan	37	21	-0.805964	0.46565	no
ni|645903567|nb|KK920863.1|	345591-345734	nan	nan	7	9	0.222923	0.851	no
ni|645903567|nb|KK920863.1|	347122-347355	nan	nan	16	14	-0.174307	0.8924	no
ni|645903567|nb|KK920863.1|	347851-348062	nan	nan	0	1	inf	0.00055	yes
ni|645903567|nb|KK920863.1|	348526-349217	nan	nan	8	16	0.961253	0.41795	no
ni|645903567|nb|KK920863.1|	355129-355257	nan	nan	10	21	1	0.373	no
ni|645903567|nb|KK920863.1|	357685-357975	nan	nan	6	15	1	0.28415	no
ni|645903567|nb|KK920863.1|	358411-358938	nan	nan	3	7	1	0.3398	no
ni|645903567|nb|KK920863.1|	36560-38162	nan	nan	48	30	-0.653828	0.545	no
ni|645903567|nb|KK920863.1|	378169-378933	nan	nan	3	6	1	0.3798	no
ni|645903567|nb|KK920863.1|	379235-379398	nan	nan	0.49795	58	7	0.03715	no
ni|645903567|nb|KK920863.1|	380538-380850	nan	nan	0	11	inf	0.00175	yes
ni|645903567|nb|KK920863.1|	383140-383539	nan	nan	0	1	inf	0.0005	yes

ni|645903567|nb|KK920863.1|	384676-385011	nan	nan	8	9	0.13016	0.9039	no
ni|645903567|nb|KK920863.1|	38691-39243	nan	nan	47	40	-0.221452	0.87335	no
ni|645903567|nb|KK920863.1|	387260-387672	nan	nan	10	7	-0.457516	0.6863	no
ni|645903567|nb|KK920863.1|	40183-40516	nan	nan	0.974514	0.501528	-0.958352	0.51225	no
ni|645903567|nb|KK920863.1|	406135-406328	nan	nan	45	31	-0.528201	0.64885	no
ni|645903567|nb|KK920863.1|	409471-409684	nan	nan	11	5	-1	0.377	no
ni|645903567|nb|KK920863.1|	414827-414997	nan	nan	11	5	-0.963963	0.418	no
ni|645903567|nb|KK920863.1|	419213-419448	nan	nan	56	22	-1	0.2941	no
ni|645903567|nb|KK920863.1|	420763-420977	nan	nan	12	7	-0.778253	0.5147	no
ni|645903567|nb|KK920863.1|	42136-43713	nan	nan	44	43	-0.020829	0.98415	no
ni|645903567|nb|KK920863.1|	425189-425519	nan	nan	30	9	-2	0.17625	no
ni|645903567|nb|KK920863.1|	425674-426176	nan	nan	36	12	-2	0.1941	no
ni|645903567|nb|KK920863.1|	426311-426697	nan	nan	25	12	-1	0.3458	no
ni|645903567|nb|KK920863.1|	427476-427851	nan	nan	13	3	-2	0.11605	no
ni|645903567|nb|KK920863.1|	428549-428750	nan	nan	0.808625	0.552119	-0.550491	0.68265	no
ni|645903567|nb|KK920863.1|	436491-436627	nan	nan	0.734952	0.156091	-2	0.3207	no
ni|645903567|nb|KK920863.1|	438996-439541	nan	nan	4	4	-0.0134233	0.98035	no
ni|645903567|nb|KK920863.1|	44074-45307	nan	nan	25	27	0.134596	0.9235	no
ni|645903567|nb|KK920863.1|	442779-442976	nan	nan	6	6	0.0931857	0.93935	no
ni|645903567|nb|KK920863.1|	445018-445408	nan	nan	4	4	-0.140649	0.90955	no
ni|645903567|nb|KK920863.1|	449340-449700	nan	nan	11	4	-1	0.2873	no
ni|645903567|nb|KK920863.1|	451072-451351	nan	nan	5	4	-0.335773	0.7701	no
ni|645903567|nb|KK920863.1|	452279-452778	nan	nan	7	4	-1	0.39265	no
ni|645903567|nb|KK920863.1|	452985-453879	nan	nan	7	5	-0.540545	0.63595	no
ni|645903567|nb|KK920863.1|	455020-455280	nan	nan	8	5	-0.749658	0.5443	no
ni|645903567|nb|KK920863.1|	45590-45979	nan	nan	20	26	0.402432	0.77385	no
ni|645903567|nb|KK920863.1|	456070-456197	nan	nan	0.385811	0.201212	-0.939183	0.54025	no
ni|645903567|nb|KK920863.1|	48105-48321	nan	nan	0.127401	1	3	0.0499	no
ni|645903567|nb|KK920863.1|	48584-48699	nan	nan	63	123	0.956256	0.50295	no
ni|645903567|nb|KK920863.1|	487322-487528	nan	nan	13	10.29	-0.301698	0.81	no

ni|645903567|nb|KK920863.1|	48836-49015	nan	nan	0.638996	0.57501	-0.152218	0.90135	no
ni|645903567|nb|KK920863.1|	58000-58113	nan	nan	0.444665	0.579727	0.382657	0.7694	no
ni|645903567|nb|KK920863.1|	58778-59591	nan	nan	2	0.704785	-1	0.312	no
ni|645903567|nb|KK920863.1|	63331-63628	nan	nan	258	342	0.404658	0.77735	no
ni|645903567|nb|KK920863.1|	65454-65871	nan	nan	239	209	-0.193784	0.8894	no
ni|645903567|nb|KK920863.1|	75938-76108	nan	nan	146	112	-0.376658	0.73205	no
ni|645903567|nb|KK920863.1|	77681-77872	nan	nan	132.76	109	-0.283838	0.80435	no
ni|645903567|nb|KK920863.1|	81244-82119	nan	nan	41	34	-0.298966	0.7832	no
ni|645903567|nb|KK920863.1|	99632-99757	nan	nan	0.478856	0.503563	0.0725816	0.94355	no
ni|645903567|nb|KK920863.1|	99822-100119	nan	nan	109	86	-0.351237	0.7612	no
ni|645903568|nb|KK920862.1|	159198-160389	nan	nan	0.203324	26	7	0.11935	no
ni|645903568|nb|KK920862.1|	160641-161089	nan	nan	0.15911	23	7	0.0443	no
ni|645903568|nb|KK920862.1|	187308-187653	nan	nan	0.944274	8	3	0.0396	no
ni|645903568|nb|KK920862.1|	189019-189387	nan	nan	0.666577	7	3	0.0258	no
ni|645903568|nb|KK920862.1|	190643-191152	nan	nan	32	38	0.244358	0.8312	no
ni|645903568|nb|KK920862.1|	193407-193621	nan	nan	37	47	0.357727	0.75795	no
ni|645903568|nb|KK920862.1|	195735-196466	nan	nan	8	9	0.0925375	0.93675	no
ni|645903568|nb|KK920862.1|	196638-196889	nan	nan	1	13	3	0.04165	no
ni|645903568|nb|KK920862.1|	201412-201676	nan	nan	3	24	3	0.04375	no
ni|645903568|nb|KK920862.1|	203068-203211	nan	nan	2	22	4	0.04105	no
ni|645903568|nb|KK920862.1|	205474-205633	nan	nan	2	14	3	0.0514	no
ni|645903568|nb|KK920862.1|	208907-209293	nan	nan	8	9	0.100381	0.9366	no
ni|645903568|nb|KK920862.1|	209462-209720	nan	nan	4	19	2	0.08025	no
ni|645903568|nb|KK920862.1|	214121-215258	nan	nan	2	19	4	0.04265	no
ni|645903568|nb|KK920862.1|	215422-215664	nan	nan	2	15	3	0.0477	no
ni|645903568|nb|KK920862.1|	217218-217703	nan	nan	2	17	3	0.04595	no
ni|645903568|nb|KK920862.1|	219967-220575	nan	nan	1	13	3	0.03475	no
ni|645903568|nb|KK920862.1|	221025-221609	nan	nan	1	0.353257	-2	0.24815	no
ni|645903568|nb|KK920862.1|	222106-222519	nan	nan	0	68	inf	5.00E-05	yes
ni|645903568|nb|KK920862.1|	222663-222843	nan	nan	0	59	inf	5.00E-05	yes

ni|645903568|nb|KK920862.1|	224021-224602	nan	nan	0	52	inf	5.00E-05	yes
ni|645903568|nb|KK920862.1|	225842-226004	nan	nan	0	50	inf	5.00E-05	yes
ni|645903568|nb|KK920862.1|	226108-226258	nan	nan	2	1	-0.330037	0.8122	no
ni|645903568|nb|KK920862.1|	227010-228051	nan	nan	1	1	-0.0666485	0.9302	no
ni|645903568|nb|KK920862.1|	228708-228879	nan	nan	1	1	-0.0661591	0.94155	no
ni|645903568|nb|KK920862.1|	229538-229657	nan	nan	0.083316	41	9	0.11935	no
ni|645903568|nb|KK920862.1|	230541-230789	nan	nan	0	105.88	inf	5.00E-05	yes
ni|645903568|nb|KK920862.1|	231209-231325	nan	nan	0	186	inf	5.00E-05	yes
ni|645903568|nb|KK920862.1|	235223-235596	nan	nan	0	14	inf	5.00E-05	yes
ni|645903568|nb|KK920862.1|	238362-238553	nan	nan	3	95	5	0.0036	no
ni|645903568|nb|KK920862.1|	240723-240861	nan	nan	5	63	4	0.03335	no
ni|645903568|nb|KK920862.1|	244978-245466	nan	nan	1	42	5	0.0335	no
ni|645903568|nb|KK920862.1|	246431-246812	nan	nan	0	3	inf	0.00015	yes
ni|645903568|nb|KK920862.1|	247152-248055	nan	nan	0	4	inf	5.00E-05	yes
ni|645903568|nb|KK920862.1|	249149-249404	nan	nan	0.14997	0.372841	1	0.35675	no
ni|645903568|nb|KK920862.1|	249567-250009	nan	nan	2	4	0.612325	0.6154	no
ni|645903568|nb|KK920862.1|	252465-252670	nan	nan	0.512074	0.790872	0.627093	0.61935	no
ni|645903568|nb|KK920862.1|	253092-253321	nan	nan	0.79559	0.526489	-0.595622	0.64595	no
ni|645903568|nb|KK920862.1|	253897-254088	nan	nan	0.297101	0.7304	1	0.35245	no
ni|645903568|nb|KK920862.1|	258894-259115	nan	nan	2	2	-0.00416803	0.9752	no
ni|645903568|nb|KK920862.1|	259384-260544	nan	nan	16	8	-1	0.46615	no
ni|645903568|nb|KK920862.1|	260876-261114	nan	nan	4	3	-0.237147	0.8697	no
ni|645903568|nb|KK920862.1|	261591-261759	nan	nan	8	10	0.428517	0.71635	no
ni|645903568|nb|KK920862.1|	262485-262901	nan	nan	9	13	0.492234	0.68785	no
ni|645903568|nb|KK920862.1|	264247-264392	nan	nan	6	15	1	0.2768	no
ni|645903568|nb|KK920862.1|	266555-267027	nan	nan	9	10	0.139172	0.9021	no
ni|645903568|nb|KK920862.1|	267190-267470	nan	nan	7	8	0.213147	0.85965	no
ni|645903568|nb|KK920862.1|	267660-267816	nan	nan	4	5	0.330894	0.7748	no
ni|645903568|nb|KK920862.1|	269603-269728	nan	nan	2	4	1	0.3943	no
ni|645903568|nb|KK920862.1|	269875-270104	nan	nan	6	8	0.545325	0.64115	no

ni|645903568|nb|KK920862.1|	270980-271228	nan	nan	2	2	0.349455	0.77735	no
ni|645903568|nb|KK920862.1|	275955-276573	nan	nan	4	11	1	0.2461	no
ni|645903568|nb|KK920862.1|	276771-276935	nan	nan	2	2	-0.065741	0.95935	no
ni|645903568|nb|KK920862.1|	277282-277500	nan	nan	8	9	0.147756	0.9196	no
ni|645903568|nb|KK920862.1|	277711-278428	nan	nan	8	8	0.0481524	0.9698	no
ni|645903568|nb|KK920862.1|	283587-284333	nan	nan	4	6	0.53723	0.65175	no
ni|645903568|nb|KK920862.1|	284477-285411	nan	nan	7	9	0.29118	0.7894	no
ni|645903568|nb|KK920862.1|	299836-299949	nan	nan	1	0.592982	-0.87265	0.53675	no
ni|645903568|nb|KK920862.1|	300098-300261	nan	nan	2	2	0.000710606	0.98555	no
ni|645903568|nb|KK920862.1|	300711-302088	nan	nan	5	5	0.102692	0.93335	no
ni|645903568|nb|KK920862.1|	302367-302740	nan	nan	8	15	0.850214	0.521	no
ni|645903568|nb|KK920862.1|	302851-303099	nan	nan	14	9	-0.672929	0.57965	no
ni|645903568|nb|KK920862.1|	303182-303934	nan	nan	4	3	-0.348719	0.7659	no
ni|645903569|nb|KK920861.1|	115286-115492	nan	nan	1	1	-0.0652974	0.95935	no
ni|645903569|nb|KK920861.1|	115712-116103	nan	nan	4	7	0.823034	0.48805	no
ni|645903569|nb|KK920861.1|	116770-117841	nan	nan	8	11	0.430625	0.72125	no
ni|645903569|nb|KK920861.1|	118176-118358	nan	nan	10	14	0.389482	0.73785	no
ni|645903569|nb|KK920861.1|	120481-120593	nan	nan	7	11	0.740522	0.53185	no
ni|645903569|nb|KK920861.1|	120933-121384	nan	nan	8	16	0.99165	0.4553	no
ni|645903569|nb|KK920861.1|	123369-124075	nan	nan	12	11	-0.0674725	0.93835	no
ni|645903569|nb|KK920861.1|	124186-124878	nan	nan	5	6	0.19758	0.8658	no
ni|645903569|nb|KK920861.1|	126169-127088	nan	nan	3	4	0.519686	0.6635	no
ni|645903569|nb|KK920861.1|	128304-128812	nan	nan	7	9	0.31436	0.78545	no
ni|645903569|nb|KK920861.1|	130771-131346	nan	nan	5	5	0.143023	0.9017	no
ni|645903569|nb|KK920861.1|	133122-133292	nan	nan	3	4	0.503806	0.67295	no
ni|645903569|nb|KK920861.1|	141407-141805	nan	nan	2	3	0.634641	0.60495	no
ni|645903569|nb|KK920861.1|	142686-142860	nan	nan	13	14	0.0986866	0.9354	no
ni|645903569|nb|KK920861.1|	142987-143675	nan	nan	17	16	-0.0897636	0.93805	no
ni|645903569|nb|KK920861.1|	147769-148301	nan	nan	14	15	0.060697	0.95995	no
ni|645903569|nb|KK920861.1|	149548-150015	nan	nan	11	13	0.225239	0.8515	no

ni|645903569|nb|KK920861.1|	150919-151144	nan	nan	13	13	-0.0246508	0.98385	no
ni|645903569|nb|KK920861.1|	151969-152199	nan	nan	14	12	-0.196915	0.86275	no
ni|645903569|nb|KK920861.1|	154309-154507	nan	nan	11	11	0.107787	0.92895	no
ni|645903569|nb|KK920861.1|	155803-156070	nan	nan	17	19	0.185506	0.8711	no
ni|645903569|nb|KK920861.1|	157292-157840	nan	nan	14	11	-0.289574	0.80925	no
ni|645903569|nb|KK920861.1|	161587-161764	nan	nan	7	7	0.0457984	0.96875	no
ni|645903569|nb|KK920861.1|	162006-162164	nan	nan	15	15	0.0377712	0.974	no
ni|645903569|nb|KK920861.1|	162612-162767	nan	nan	9	9	-0.0416087	0.96965	no
ni|645903569|nb|KK920861.1|	163422-163576	nan	nan	16	15	-0.108035	0.9267	no
ni|645903569|nb|KK920861.1|	167086-167228	nan	nan	18	14	-0.349012	0.75915	no
ni|645903569|nb|KK920861.1|	167592-168964	nan	nan	20	21	0.0361121	0.9755	no
ni|645903569|nb|KK920861.1|	171549-172606	nan	nan	21	23	0.102729	0.9311	no
ni|645903569|nb|KK920861.1|	172787-173306	nan	nan	5	19	2	0.13495	no
ni|645903569|nb|KK920861.1|	174195-175246	nan	nan	17	14	-0.323197	0.7788	no
ni|645903569|nb|KK920861.1|	175357-175835	nan	nan	55	49	-0.149976	0.8911	no
ni|645903569|nb|KK920861.1|	176429-176830	nan	nan	35	20	-0.824625	0.48645	no
ni|645903569|nb|KK920861.1|	179444-179566	nan	nan	19	19	-0.0386581	0.9718	no
ni|645903569|nb|KK920861.1|	181417-181915	nan	nan	10	7	-0.541436	0.64045	no
ni|645903569|nb|KK920861.1|	182905-183600	nan	nan	27	14	-0.976514	0.41615	no
ni|645903569|nb|KK920861.1|	185665-185973	nan	nan	14	15	0.0480724	0.95725	no
ni|645903569|nb|KK920861.1|	188315-188676	nan	nan	30	17	-0.804438	0.53265	no
ni|645903569|nb|KK920861.1|	188756-189265	nan	nan	22	11	-0.960389	0.44135	no
ni|645903569|nb|KK920861.1|	189582-189790	nan	nan	24	13	-0.940988	0.4143	no
ni|645903569|nb|KK920861.1|	190126-190619	nan	nan	29	15	-0.938494	0.4315	no
ni|645903569|nb|KK920861.1|	191332-191583	nan	nan	15	9	-0.832858	0.5313	no
ni|645903569|nb|KK920861.1|	192054-192236	nan	nan	28	23	-0.28405	0.80805	no
ni|645903569|nb|KK920861.1|	192597-192754	nan	nan	9	5	-0.983429	0.40925	no
ni|645903569|nb|KK920861.1|	193220-193440	nan	nan	23	12	-0.874593	0.4688	no
ni|645903569|nb|KK920861.1|	193570-193801	nan	nan	19	16	-0.277543	0.81015	no
ni|645903569|nb|KK920861.1|	194238-195108	nan	nan	20	16	-0.293494	0.7967	no

ni|645903569|nb|KK920861.1|	198753-198912	nan	nan	5	4	-0.247713	0.82395	no
ni|645903569|nb|KK920861.1|	199958-200127	nan	nan	4	4	-0.153254	0.88795	no
ni|645903569|nb|KK920861.1|	200767-201222	nan	nan	2	2	-0.27689	0.8205	no
ni|645903569|nb|KK920861.1|	207582-207717	nan	nan	4	2	-0.652184	0.66125	no
ni|645903569|nb|KK920861.1|	208360-209039	nan	nan	3	2	-0.574311	0.6323	no
ni|645903569|nb|KK920861.1|	210405-211348	nan	nan	4	3	-0.423824	0.723	no
ni|645903569|nb|KK920861.1|	213707-213913	nan	nan	16	7	-1.26	0.3768	no
ni|645903569|nb|KK920861.1|	214279-214422	nan	nan	97	23	-2	0.20695	no
ni|645903569|nb|KK920861.1|	214563-214687	nan	nan	1	3	1	0.39265	no
ni|645903569|nb|KK920861.1|	214814-215068	nan	nan	41	34	-0.264572	0.81915	no
ni|645903569|nb|KK920861.1|	215238-215363	nan	nan	13	12	-0.106423	0.92815	no
ni|645903569|nb|KK920861.1|	216008-216262	nan	nan	42	29.85	-0.509221	0.6649	no
ni|645903569|nb|KK920861.1|	216539-216797	nan	nan	41	28	-0.545415	0.64545	no
ni|645903569|nb|KK920861.1|	217138-217246	nan	nan	14	11	-0.278071	0.81595	no
ni|645903569|nb|KK920861.1|	217626-217742	nan	nan	1	1	-0.235802	0.8697	no
ni|645903569|nb|KK920861.1|	217857-218594	nan	nan	14	14	0.0397657	0.9708	no
ni|645903569|nb|KK920861.1|	219432-219752	nan	nan	50	47	-0.0681116	0.954	no
ni|645903569|nb|KK920861.1|	221015-221224	nan	nan	13	12	-0.0672272	0.95575	no
ni|645903569|nb|KK920861.1|	222165-222492	nan	nan	16	22	0.456246	0.6969	no
ni|645903569|nb|KK920861.1|	223417-223750	nan	nan	3	2	-0.481977	0.7301	no
ni|645903569|nb|KK920861.1|	224129-224839	nan	nan	18	21	0.278905	0.8118	no
ni|645903569|nb|KK920861.1|	225260-226653	nan	nan	18	23	0.337341	0.7655	no
ni|645903569|nb|KK920861.1|	227308-227526	nan	nan	9	10	0.11335	0.9325	no
ni|645903569|nb|KK920861.1|	227830-227942	nan	nan	23	19	-0.301857	0.81	no
ni|645903569|nb|KK920861.1|	229465-229625	nan	nan	16	26	0.684768	0.5731	no
ni|645903569|nb|KK920861.1|	229749-229956	nan	nan	6	5	-0.307147	0.7919	no
ni|645903569|nb|KK920861.1|	230114-230402	nan	nan	21	29	0.480168	0.68245	no
ni|645903569|nb|KK920861.1|	231318-232101	nan	nan	3	4	0.477484	0.6811	no
ni|645903569|nb|KK920861.1|	232238-232541	nan	nan	10	9	-0.237136	0.8532	no
ni|645903569|nb|KK920861.1|	235438-235784	nan	nan	21	24	0.189123	0.8753	no

ni|645903569|nb|KK920861.1|	240484-240829	nan	nan	23	25	0.150504	0.9016	no
ni|645903569|nb|KK920861.1|	246450-246596	nan	nan	6	7	0.280921	0.81735	no
ni|645903569|nb|KK920861.1|	246671-246951	nan	nan	18	23	0.363348	0.7612	no
ni|645903569|nb|KK920861.1|	249364-249549	nan	nan	8	15	1	0.4359	no
ni|645903569|nb|KK920861.1|	250147-250796	nan	nan	16	23	0.490801	0.6806	no
ni|645903569|nb|KK920861.1|	250923-251515	nan	nan	11	14	0.339603	0.77215	no
ni|645903569|nb|KK920861.1|	251912-252550	nan	nan	3	5	0.73408	0.53775	no
ni|645903569|nb|KK920861.1|	253537-253736	nan	nan	16	16	-0.0165824	0.97895	no
ni|645903569|nb|KK920861.1|	254154-254388	nan	nan	11	13	0.337748	0.769	no
ni|645903569|nb|KK920861.1|	255916-256907	nan	nan	4	3	-0.710154	0.563	no
ni|645903569|nb|KK920861.1|	256976-257434	nan	nan	16	13	-0.223853	0.84705	no
ni|645903569|nb|KK920861.1|	29074-29699	nan	nan	4	0.92037	-2	0.20695	no
ni|645903569|nb|KK920861.1|	29892-30365	nan	nan	3	2	-0.802416	0.51615	no
ni|645903569|nb|KK920861.1|	30689-30930	nan	nan	0.613863	0.879886	0.519399	0.7279	no
ni|645903569|nb|KK920861.1|	31102-31678	nan	nan	2	1	-0.505976	0.68965	no
ni|645903569|nb|KK920861.1|	31941-33128	nan	nan	1	2	0.275616	0.823	no
ni|645903569|nb|KK920861.1|	43627-44350	nan	nan	0.814468	2	1	0.287	no
ni|645903569|nb|KK920861.1|	49171-49416	nan	nan	3	2	-0.42988	0.75965	no
ni|645903569|nb|KK920861.1|	50396-50608	nan	nan	2	4	1	0.41315	no
ni|645903569|nb|KK920861.1|	51580-52004	nan	nan	1	2	0.723519	0.5568	no
ni|645903569|nb|KK920861.1|	61616-62126	nan	nan	0.932352	3	2	0.2306	no
ni|645903569|nb|KK920861.1|	78280-78925	nan	nan	3	2	-0.181964	0.889	no
ni|645903569|nb|KK920861.1|	81041-81216	nan	nan	3	2	-0.767272	0.5799	no
ni|645903569|nb|KK920861.1|	83801-84251	nan	nan	3	2	-0.526328	0.7071	no
ni|645903570|nb|KK920860.1|	23014-23414	nan	nan	21	15	-0.42469	0.697	no
ni|645903570|nb|KK920860.1|	23626-24169	nan	nan	22	16	-0.491698	0.6818	no
ni|645903570|nb|KK920860.1|	24400-24659	nan	nan	23	20	-0.255479	0.8254	no
ni|645903570|nb|KK920860.1|	60648-61024	nan	nan	8	9	0.0624502	0.9556	no
ni|645903571|nb|KK920859.1|	474673-475154	nan	nan	18	13	-0.464408	0.69525	no
ni|645903571|nb|KK920859.1|	504689-504856	nan	nan	10	7	-0.55262	0.65835	no

ni|645903571|nb|KK920859.1|	505930-506119	nan	nan	3	2	-0.419448	0.7284	no
ni|645903571|nb|KK920859.1|	506803-507864	nan	nan	2	2	0.347796	0.8193	no
ni|645903572|nb|KK920858.1|	101824-101989	nan	nan	681	490	-0.473999	0.74615	no
ni|645903572|nb|KK920858.1|	102851-103349	nan	nan	1	0.937145	-0.152102	0.8951	no
ni|645903572|nb|KK920858.1|	110808-111242	nan	nan	3	2	-0.803614	0.5588	no
ni|645903572|nb|KK920858.1|	161518-161621	nan	nan	3	2	-0.859274	0.49355	no
ni|645903572|nb|KK920858.1|	169152-169317	nan	nan	131	97	-0.425574	0.7576	no
ni|645903572|nb|KK920858.1|	169420-169685	nan	nan	346	280.58	-0.302492	0.83375	no
ni|645903572|nb|KK920858.1|	169868-170391	nan	nan	0	4	inf	5.00E-05	yes
ni|645903572|nb|KK920858.1|	170672-171608	nan	nan	2	0.922611	-1	0.3464	no
ni|645903572|nb|KK920858.1|	171720-172433	nan	nan	3	2	-0.803437	0.5588	no
ni|645903572|nb|KK920858.1|	173100-173895	nan	nan	3	2	-0.812369	0.49315	no
ni|645903572|nb|KK920858.1|	174378-174694	nan	nan	3	4	0.15612	0.8958	no
ni|645903572|nb|KK920858.1|	175858-175992	nan	nan	8	6	-0.436341	0.71475	no
ni|645903572|nb|KK920858.1|	183453-184006	nan	nan	190	116	-0.713973	0.5124	no
ni|645903572|nb|KK920858.1|	185125-185922	nan	nan	65	52	-0.315363	0.78115	no
ni|645903572|nb|KK920858.1|	186015-186298	nan	nan	194	179	-0.115657	0.9345	no
ni|645903572|nb|KK920858.1|	189169-189378	nan	nan	116	108	-0.103478	0.925	no
ni|645903572|nb|KK920858.1|	189939-190784	nan	nan	83	81	-0.032446	0.9818	no
ni|645903572|nb|KK920858.1|	191615-191791	nan	nan	0.703458	3	2	0.15655	no
ni|645903572|nb|KK920858.1|	195001-195567	nan	nan	12	13	0.117145	0.915	no
ni|645903572|nb|KK920858.1|	196505-196717	nan	nan	426	581	0.448147	0.6907	no
ni|645903572|nb|KK920858.1|	200536-201130	nan	nan	2	2	0.39376	0.75185	no
ni|645903572|nb|KK920858.1|	688261-688679	nan	nan	3	4	0.463225	0.7264	no
ni|645903572|nb|KK920858.1|	847724-847944	nan	nan	618	977	0.661115	0.643	no
ni|645903572|nb|KK920858.1|	848731-848890	nan	nan	86	145	0.755366	0.61295	no
ni|645903572|nb|KK920858.1|	849173-849303	nan	nan	4	7	0.675403	0.5613	no
ni|645903572|nb|KK920858.1|	854573-854842	nan	nan	2	3	0.296939	0.8014	no
ni|645903572|nb|KK920858.1|	857242-857442	nan	nan	13.9	24	0.795077	0.53535	no
ni|645903572|nb|KK920858.1|	86406-86628	nan	nan	2	1	-0.267181	0.81595	no

ni|645903572|nb|KK920858.1|	87150-87452	nan	nan	21	21	-0.01908	0.9868	no
ni|645903572|nb|KK920858.1|	87725-88050	nan	nan	23	13	-0.794173	0.5063	no
ni|645903572|nb|KK920858.1|	904974-905354	nan	nan	90	197	1	0.35435	no
ni|645903572|nb|KK920858.1|	91687-91855	nan	nan	15	15	0.00478723	0.99585	no
ni|645903572|nb|KK920858.1|	92244-92417	nan	nan	19	14	-0.396091	0.72875	no
ni|645903572|nb|KK920858.1|	93485-94241	nan	nan	173	138	-0.3218	0.7595	no
ni|645903572|nb|KK920858.1|	94615-95539	nan	nan	0.240362	1	3	0.07435	no
ni|645903572|nb|KK920858.1|	95839-96006	nan	nan	0.260149	1	2	0.1002	no
ni|645903572|nb|KK920858.1|	97156-97340	nan	nan	716	560	-0.354083	0.80215	no
ni|645903572|nb|KK920858.1|	975958-976178	nan	nan	5	30	3	0.076	no
ni|645903572|nb|KK920858.1|	98431-98634	nan	nan	471	355	-0.405566	0.77095	no
ni|645903572|nb|KK920858.1|	98947-99620	nan	nan	178.53	152	-0.233374	0.873	no
ni|645903572|nb|KK920858.1|	999028-999670	nan	nan	23	22	-0.0700746	0.9461	no
ni|645903574|nb|KK920856.1|	103724-104333	nan	nan	1	0.383127	-1	0.35155	no
ni|645903574|nb|KK920856.1|	104439-104647	nan	nan	31	46	0.575482	0.60835	no
ni|645903574|nb|KK920856.1|	105060-105318	nan	nan	33	50	0.627845	0.58865	no
ni|645903574|nb|KK920856.1|	105551-106722	nan	nan	11	20	0.891509	0.4396	no
ni|645903574|nb|KK920856.1|	107309-107523	nan	nan	18	27	0.633204	0.57535	no
ni|645903574|nb|KK920856.1|	108058-108388	nan	nan	9	9	0.0202567	0.96555	no
ni|645903574|nb|KK920856.1|	108658-109280	nan	nan	3	2	-0.831975	0.5313	no
ni|645903574|nb|KK920856.1|	110080-110239	nan	nan	3	3	0.282087	0.81735	no
ni|645903574|nb|KK920856.1|	111351-111480	nan	nan	5	11	1	0.3238	no
ni|645903574|nb|KK920856.1|	111736-112401	nan	nan	0.867906	4	2	0.1014	no
ni|645903574|nb|KK920856.1|	114091-114481	nan	nan	2	15	3	0.0594	no
ni|645903574|nb|KK920856.1|	126985-127370	nan	nan	0.456729	3	3	0.0767	no
ni|645903574|nb|KK920856.1|	127450-127787	nan	nan	2	15	3	0.0604	no
ni|645903574|nb|KK920856.1|	136737-137013	nan	nan	5	15	2	0.242	no
ni|645903574|nb|KK920856.1|	13692-14018	nan	nan	17	21	0.307601	0.77645	no
ni|645903574|nb|KK920856.1|	139927-140140	nan	nan	1	10	3	0.06925	no
ni|645903574|nb|KK920856.1|	140251-140432	nan	nan	1	8	3	0.06145	no

ni|645903574|nb|KK920856.1|	140688-141077	nan	nan	3	1	-1	0.4023	no
ni|645903574|nb|KK920856.1|	141179-141891	nan	nan	5	2	-1	0.4064	no
ni|645903574|nb|KK920856.1|	143916-144107	nan	nan	4	3	-0.729757	0.5717	no
ni|645903574|nb|KK920856.1|	146391-146667	nan	nan	2	2	0.00853018	0.9732	no
ni|645903574|nb|KK920856.1|	14686-14878	nan	nan	0.620613	0.158226	-2	0.2004	no
ni|645903574|nb|KK920856.1|	147212-147432	nan	nan	2	2	0.385573	0.74455	no
ni|645903574|nb|KK920856.1|	150899-151014	nan	nan	3	2	-0.320008	0.7811	no
ni|645903574|nb|KK920856.1|	154914-155796	nan	nan	5	5	-0.154754	0.90135	no
ni|645903574|nb|KK920856.1|	156094-156212	nan	nan	33	29	-0.184099	0.90045	no
ni|645903574|nb|KK920856.1|	15619-15754	nan	nan	11	14	0.344742	0.76465	no
ni|645903574|nb|KK920856.1|	156719-156994	nan	nan	43	40	-0.109713	0.9165	no
ni|645903574|nb|KK920856.1|	158644-158810	nan	nan	178	150	-0.246149	0.87205	no
ni|645903574|nb|KK920856.1|	158957-159084	nan	nan	51	42	-0.27833	0.7937	no
ni|645903574|nb|KK920856.1|	159227-160143	nan	nan	181	173.01	-0.0674947	0.95065	no
ni|645903574|nb|KK920856.1|	16018-16303	nan	nan	35	61	0.824762	0.5554	no
ni|645903574|nb|KK920856.1|	160255-160393	nan	nan	102	98	-0.0501676	0.96555	no
ni|645903574|nb|KK920856.1|	160869-161240	nan	nan	69	70	0.020879	0.9839	no
ni|645903574|nb|KK920856.1|	161323-161614	nan	nan	277	277	-5.58E-06	0.9966	no
ni|645903574|nb|KK920856.1|	161872-162287	nan	nan	143	153	0.0936132	0.9465	no
ni|645903574|nb|KK920856.1|	162454-163480	nan	nan	179	186	0.0535367	0.969	no
ni|645903574|nb|KK920856.1|	164114-169273	nan	nan	1	2	0.933633	0.5103	no
ni|645903574|nb|KK920856.1|	16823-17205	nan	nan	0.19918	0.723644	2	0.18925	no
ni|645903574|nb|KK920856.1|	17782-17920	nan	nan	0.56697	0.677385	0.256704	0.86075	no
ni|645903574|nb|KK920856.1|	18068-18259	nan	nan	0.248975	3	4	0.04785	no
ni|645903574|nb|KK920856.1|	18600-18845	nan	nan	7	7	0.18203	0.87635	no
ni|645903574|nb|KK920856.1|	19611-20166	nan	nan	0.618128	0.177255	-2	0.2784	no
ni|645903574|nb|KK920856.1|	21076-21503	nan	nan	0.755832	0.812581	0.104446	0.93335	no
ni|645903574|nb|KK920856.1|	21964-22227	nan	nan	9	10	0.264038	0.8133	no
ni|645903574|nb|KK920856.1|	222921-222982	nan	nan	29929.8	41377.8	0.467275	0.68795	no
ni|645903574|nb|KK920856.1|	22497-22847	nan	nan	29	38	0.413537	0.71135	no

ni|645903574|nb|KK920856.1|	23181-23410	nan	nan	42	48	0.184295	0.8698	no
ni|645903574|nb|KK920856.1|	31656-31997	nan	nan	0.730659	0.43641	-0.743515	0.59615	no
ni|645903574|nb|KK920856.1|	32614-32886	nan	nan	0.557713	0.592333	0.0868861	0.9436	no
ni|645903574|nb|KK920856.1|	33604-33810	nan	nan	6	4	-0.474546	0.65515	no
ni|645903574|nb|KK920856.1|	34095-34524	nan	nan	11	12	0.172122	0.8836	no
ni|645903574|nb|KK920856.1|	35987-36481	nan	nan	28	36	0.385319	0.7387	no
ni|645903574|nb|KK920856.1|	41933-42081	nan	nan	33	34	0.0361756	0.97295	no
ni|645903574|nb|KK920856.1|	43361-44311	nan	nan	51	81	0.674181	0.56685	no
ni|645903574|nb|KK920856.1|	45036-45406	nan	nan	0	18	inf	5.00E-05	yes
ni|645903574|nb|KK920856.1|	45743-45918	nan	nan	0.240855	12	6	0.11975	no
ni|645903574|nb|KK920856.1|	47555-47803	nan	nan	56	38	-0.559662	0.61955	no
ni|645903574|nb|KK920856.1|	49223-49400	nan	nan	0.212028	20	7	0.1194	no
ni|645903574|nb|KK920856.1|	50751-50949	nan	nan	2	0.602445	-2	0.27225	no
ni|645903574|nb|KK920856.1|	63865-63990	nan	nan	0.860188	0.730678	-0.235416	0.8697	no
ni|645903574|nb|KK920856.1|	91859-92007	nan	nan	1	0.497072	-2	0.2234	no
ni|645903574|nb|KK920856.1|	93641-93839	nan	nan	1	0.34279	-2	0.25615	no
ni|645903574|nb|KK920856.1|	95004-95530	nan	nan	1	0.550798	-1	0.27285	no
ni|645903574|nb|KK920856.1|	98432-98850	nan	nan	35	24	-0.521943	0.6746	no
ni|645903575|nb|KK920855.1|	193241-194212	nan	nan	99	301	2	0.2762	no
ni|645903575|nb|KK920855.1|	201877-202678	nan	nan	46	55	0.266959	0.81395	no
ni|645903575|nb|KK920855.1|	211642-211872	nan	nan	77	50	-0.610818	0.60145	no
ni|645903575|nb|KK920855.1|	212258-212554	nan	nan	1	4	1	0.3265	no
ni|645903575|nb|KK920855.1|	213686-213939	nan	nan	3	2	-0.328517	0.78365	no
ni|645903575|nb|KK920855.1|	214830-215011	nan	nan	0.956472	1	0.214914	0.8678	no
ni|645903575|nb|KK920855.1|	215285-215414	nan	nan	2	0.503906	-2	0.2989	no
ni|645903575|nb|KK920855.1|	215549-215681	nan	nan	1	0.840582	-0.481034	0.7594	no
ni|645903575|nb|KK920855.1|	215921-216058	nan	nan	0.314837	22	6	0.0332	no
ni|645903575|nb|KK920855.1|	218298-218585	nan	nan	6	6	-0.163181	0.8921	no
ni|645903575|nb|KK920855.1|	222568-223116	nan	nan	0.713218	2	2	0.2147	no
ni|645903575|nb|KK920855.1|	225806-226079	nan	nan	0.106303	60	9	0.0443	no

ni|645903575|nb|KK920855.1|	226844-226994	nan	nan	12	12	0.0903449	0.9391	no
ni|645903575|nb|KK920855.1|	227226-227952	nan	nan	26	28	0.136514	0.8994	no
ni|645903575|nb|KK920855.1|	229846-230621	nan	nan	2	1	-0.550719	0.66085	no
ni|645903575|nb|KK920855.1|	231062-231962	nan	nan	23	41	0.846497	0.47705	no
ni|645903575|nb|KK920855.1|	232398-232832	nan	nan	0.571786	0.37974	-0.590463	0.6233	no
ni|645903575|nb|KK920855.1|	234942-235605	nan	nan	0.597761	0.464364	-0.364312	0.7706	no
ni|645903575|nb|KK920855.1|	240903-241131	nan	nan	7	11	0.643249	0.6202	no
ni|645903575|nb|KK920855.1|	242278-242425	nan	nan	0.501254	1	1	0.3235	no
ni|645903575|nb|KK920855.1|	246525-246700	nan	nan	6	6	-0.0672639	0.93835	no
ni|645903575|nb|KK920855.1|	304049-304274	nan	nan	4	2	-1	0.27265	no
ni|645903575|nb|KK920855.1|	306894-307267	nan	nan	2	1	-0.858363	0.4671	no
ni|645903575|nb|KK920855.1|	307493-307869	nan	nan	31	48	0.605641	0.603	no
ni|645903575|nb|KK920855.1|	310220-310794	nan	nan	5	9	0.745061	0.51395	no
ni|645903575|nb|KK920855.1|	314354-314477	nan	nan	0.416267	0.489873	0.234898	0.85095	no
ni|645903575|nb|KK920855.1|	314634-315784	nan	nan	0.617712	1	0.819607	0.51315	no
ni|645903575|nb|KK920855.1|	316074-316213	nan	nan	22	37	0.728439	0.53235	no
ni|645903575|nb|KK920855.1|	333853-333992	nan	nan	9	20	1	0.3245	no
ni|645903575|nb|KK920855.1|	357903-358083	nan	nan	5	5	0.0583407	0.9532	no
ni|645903575|nb|KK920855.1|	379703-379881	nan	nan	2	2	-0.193252	0.86635	no
ni|645903575|nb|KK920855.1|	387662-387822	nan	nan	3	8	1	0.30495	no
ni|645903575|nb|KK920855.1|	432755-432902	nan	nan	0.591875	1	0.873656	0.49985	no
ni|645903575|nb|KK920855.1|	433558-434304	nan	nan	0.490526	1	1	0.38645	no
ni|645903575|nb|KK920855.1|	436238-436450	nan	nan	5	6	0.264898	0.821	no
ni|645903575|nb|KK920855.1|	441397-441849	nan	nan	0.732528	0.622306	-0.23526	0.8697	no
ni|645903575|nb|KK920855.1|	493477-493723	nan	nan	0.333125	0.672543	1	0.4082	no
ni|645903575|nb|KK920855.1|	503412-503516	nan	nan	2	3	0.226624	0.84375	no
ni|645903575|nb|KK920855.1|	590712-592314	nan	nan	1	1	0.104878	0.92795	no
ni|645903575|nb|KK920855.1|	88671-88870	nan	nan	34599.4	41736.9	0.270578	0.8154	no
ni|645903576|nb|KK920854.1|	10595-11137	nan	nan	5	4	-0.32869	0.776	no
ni|645903576|nb|KK920854.1|	11252-11544	nan	nan	2	3	0.606176	0.60775	no

ni|645903576|nb|KK920854.1|	114106-114635	nan	nan	1	2	0.518245	0.7279	no
ni|645903576|nb|KK920854.1|	118638-118819	nan	nan	76	82	0.11105	0.94	no
ni|645903576|nb|KK920854.1|	11988-12121	nan	nan	2	0	-inf	0.00175	yes
ni|645903576|nb|KK920854.1|	13218-13508	nan	nan	25	8	-2	0.23205	no
ni|645903576|nb|KK920854.1|	13722-13942	nan	nan	47	18	-1	0.2609	no
ni|645903576|nb|KK920854.1|	146616-146843	nan	nan	2	5	2	0.30335	no
ni|645903576|nb|KK920854.1|	147583-147882	nan	nan	0.105367	4	5	0.11945	no
ni|645903576|nb|KK920854.1|	147982-148296	nan	nan	3	3	0.0997405	0.9309	no
ni|645903576|nb|KK920854.1|	149566-149765	nan	nan	18	13	-0.449367	0.6998	no
ni|645903576|nb|KK920854.1|	15092-15559	nan	nan	18	5	-2	0.1343	no
ni|645903576|nb|KK920854.1|	154192-154968	nan	nan	75	65	-0.201083	0.8604	no
ni|645903576|nb|KK920854.1|	157850-158059	nan	nan	88	68	-0.372468	0.7474	no
ni|645903576|nb|KK920854.1|	161205-161517	nan	nan	0.608817	1	1	0.38715	no
ni|645903576|nb|KK920854.1|	169306-169457	nan	nan	7	14	0.932693	0.43665	no
ni|645903576|nb|KK920854.1|	173682-173839	nan	nan	2	1	-0.802863	0.5588	no
ni|645903576|nb|KK920854.1|	177179-177299	nan	nan	2	1	-0.745033	0.59615	no
ni|645903576|nb|KK920854.1|	18693-19313	nan	nan	34	9	-2	0.12905	no
ni|645903576|nb|KK920854.1|	202003-202200	nan	nan	257.52	486	0.916059	0.4181	no
ni|645903576|nb|KK920854.1|	206455-206728	nan	nan	0.913388	2	1	0.29975	no
ni|645903576|nb|KK920854.1|	208122-208592	nan	nan	1	1	-0.0141932	0.97895	no
ni|645903576|nb|KK920854.1|	208781-208915	nan	nan	2	2	0.223235	0.8598	no
ni|645903576|nb|KK920854.1|	213350-213487	nan	nan	23	40	0.794812	0.4594	no
ni|645903576|nb|KK920854.1|	214126-214756	nan	nan	6	13	1	0.35855	no
ni|645903576|nb|KK920854.1|	215990-216186	nan	nan	6	11	0.910929	0.40215	no
ni|645903576|nb|KK920854.1|	216329-216600	nan	nan	9986.65	33397.5	2	0.18205	no
ni|645903576|nb|KK920854.1|	217333-217598	nan	nan	0.057356	152.09	11	0.11935	no
ni|645903576|nb|KK920854.1|	217993-218318	nan	nan	0	193	inf	5.00E-05	yes
ni|645903576|nb|KK920854.1|	218683-218843	nan	nan	30	4	-3	0.0666	no
ni|645903576|nb|KK920854.1|	221230-221427	nan	nan	22	9	-1	0.2642	no
ni|645903576|nb|KK920854.1|	230943-231131	nan	nan	18	4	-2	0.1191	no

ni|645903576|nb|KK920854.1|	23373-23708	nan	nan	25.4	6	-2	0.11735	no
ni|645903576|nb|KK920854.1|	25163-25342	nan	nan	23	8	-1	0.23405	no
ni|645903576|nb|KK920854.1|	25753-26019	nan	nan	28	6	-2	0.0497	no
ni|645903576|nb|KK920854.1|	277155-277314	nan	nan	9	0	-inf	0.0004	yes
ni|645903576|nb|KK920854.1|	36893-37107	nan	nan	13	1	-3	0.0653	no
ni|645903576|nb|KK920854.1|	37305-37860	nan	nan	36	8	-2	0.0821	no
ni|645903576|nb|KK920854.1|	37937-38130	nan	nan	7	2	-2	0.09175	no
ni|645903576|nb|KK920854.1|	38285-39064	nan	nan	22	5	-2	0.07005	no
ni|645903576|nb|KK920854.1|	39216-39359	nan	nan	22	4	-2	0.0655	no
ni|645903576|nb|KK920854.1|	39840-39968	nan	nan	15	6	-1	0.2133	no
ni|645903576|nb|KK920854.1|	40036-40157	nan	nan	0	8	inf	0.00015	yes
ni|645903576|nb|KK920854.1|	40259-40497	nan	nan	33	33	-0.00651486	0.99245	no
ni|645903576|nb|KK920854.1|	41547-42424	nan	nan	26	35	0.384175	0.7421	no
ni|645903576|nb|KK920854.1|	43168-43537	nan	nan	0	52	inf	5.00E-05	yes
ni|645903576|nb|KK920854.1|	47237-47571	nan	nan	0.075885	1	4	0.12425	no
ni|645903576|nb|KK920854.1|	51647-51820	nan	nan	0	91	inf	5.00E-05	yes
ni|645903576|nb|KK920854.1|	56164-56345	nan	nan	0	3	inf	0.0003	yes
ni|645903576|nb|KK920854.1|	57117-57247	nan	nan	0	2	inf	0.00015	yes
ni|645903576|nb|KK920854.1|	57387-57753	nan	nan	49	47	-0.0683227	0.94635	no
ni|645903576|nb|KK920854.1|	58135-58392	nan	nan	16188.3	13395.4	-0.273218	0.83115	no
ni|645903576|nb|KK920854.1|	60332-60781	nan	nan	2210.04	1739.93	-0.345043	0.8217	no
ni|645903576|nb|KK920854.1|	61639-66745	nan	nan	0.171853	2	4	0.0498	no
ni|645903577|nb|KK920853.1|	197079-197242	nan	nan	2	2	-0.375514	0.75525	no
ni|645903577|nb|KK920853.1|	27385-27572	nan	nan	3	3	-0.287448	0.8112	no
ni|645903577|nb|KK920853.1|	28520-28633	nan	nan	0	18	inf	5.00E-05	yes
ni|645903577|nb|KK920853.1|	29008-29136	nan	nan	56	116	1	0.35125	no
ni|645903577|nb|KK920853.1|	29726-29852	nan	nan	4	9	1	0.3166	no
ni|645903577|nb|KK920853.1|	30622-30841	nan	nan	2	1	-0.803719	0.6027	no
ni|645903577|nb|KK920853.1|	315161-315389	nan	nan	17	22	0.373698	0.75265	no
ni|645903577|nb|KK920853.1|	32765-33116	nan	nan	131.93	239	0.855993	0.504	no

ni|645903577|nb|KK920853.1|	34392-34517	nan	nan	26	34	0.361102	0.805	no
ni|645903577|nb|KK920853.1|	366163-366369	nan	nan	38	62	0.705596	0.54135	no
ni|645903577|nb|KK920853.1|	39978-40361	nan	nan	80	81	0.0262412	0.986	no
ni|645903577|nb|KK920853.1|	418680-419038	nan	nan	25	62	1	0.25975	no
ni|645903577|nb|KK920853.1|	42320-44402	nan	nan	20	18	-0.181951	0.87385	no
ni|645903577|nb|KK920853.1|	435481-435996	nan	nan	21	44	1	0.3398	no
ni|645903577|nb|KK920853.1|	45959-46208	nan	nan	37	37	0.00030665	0.99755	no
ni|645903577|nb|KK920853.1|	542212-542361	nan	nan	29	58	1	0.3868	no
ni|645903577|nb|KK920853.1|	54269-54418	nan	nan	74	71	-0.0737959	0.9456	no
ni|645903577|nb|KK920853.1|	542734-542895	nan	nan	30	70	1	0.30615	no
ni|645903577|nb|KK920853.1|	543222-543360	nan	nan	10	23	1	0.2775	no
ni|645903577|nb|KK920853.1|	543640-543741	nan	nan	0.835803	0.598993	-0.480625	0.7594	no
ni|645903577|nb|KK920853.1|	543896-544008	nan	nan	9	18	0.996345	0.37835	no
ni|645903577|nb|KK920853.1|	544416-544944	nan	nan	2	2	0.447873	0.7543	no
ni|645903577|nb|KK920853.1|	545455-545697	nan	nan	9	18	0.901728	0.42255	no
ni|645903577|nb|KK920853.1|	545868-546015	nan	nan	16	35	1	0.30165	no
ni|645903577|nb|KK920853.1|	546280-546391	nan	nan	8	19	1	0.3053	no
ni|645903577|nb|KK920853.1|	546795-546930	nan	nan	22	55	1	0.2499	no
ni|645903577|nb|KK920853.1|	551615-551808	nan	nan	12	25	1	0.3244	no
ni|645903577|nb|KK920853.1|	552469-552629	nan	nan	33	83	1	0.2495	no
ni|645903577|nb|KK920853.1|	553565-554966	nan	nan	274	231	-0.244459	0.8567	no
ni|645903577|nb|KK920853.1|	55412-55667	nan	nan	34	36	0.0671123	0.95095	no
ni|645903577|nb|KK920853.1|	556758-556923	nan	nan	119	93	-0.346924	0.81135	no
ni|645903577|nb|KK920853.1|	557066-557309	nan	nan	126	102	-0.30196	0.7917	no
ni|645903577|nb|KK920853.1|	559520-559755	nan	nan	65	60	-0.109487	0.94275	no
ni|645903577|nb|KK920853.1|	57943-58154	nan	nan	78	72.03	-0.114929	0.92075	no
ni|645903577|nb|KK920853.1|	591130-591409	nan	nan	2	0.537815	-2	0.24455	no
ni|645903577|nb|KK920853.1|	591927-592175	nan	nan	14	13	-0.0834072	0.9451	no
ni|645903577|nb|KK920853.1|	594399-594644	nan	nan	14	17	0.256771	0.82305	no
ni|645903577|nb|KK920853.1|	59483-59682	nan	nan	55	43	-0.351339	0.81595	no

ni|645903577|nb|KK920853.1|	603756-604628	nan	nan	8	7	-0.219282	0.87705	no
ni|645903577|nb|KK920853.1|	604739-605123	nan	nan	6	5	-0.259905	0.8388	no
ni|645903577|nb|KK920853.1|	611132-612279	nan	nan	16	13	-0.32527	0.77635	no
ni|645903577|nb|KK920853.1|	612678-612805	nan	nan	52	80	0.622646	0.6726	no
ni|645903577|nb|KK920853.1|	613195-613419	nan	nan	2	1	-0.461021	0.6964	no
ni|645903577|nb|KK920853.1|	613691-613818	nan	nan	0.980043	0.857825	-0.192162	0.8709	no
ni|645903577|nb|KK920853.1|	614193-614439	nan	nan	3	2	-0.596245	0.62535	no
ni|645903577|nb|KK920853.1|	614679-614853	nan	nan	134	219	0.707702	0.6381	no
ni|645903577|nb|KK920853.1|	615439-615593	nan	nan	0.478897	0.572213	0.256838	0.86075	no
ni|645903577|nb|KK920853.1|	622742-623462	nan	nan	2	2	-0.482236	0.7594	no
ni|645903577|nb|KK920853.1|	646707-647042	nan	nan	43	84	0.969421	0.44845	no
ni|645903577|nb|KK920853.1|	647160-647411	nan	nan	30	67	1	0.43475	no
ni|645903577|nb|KK920853.1|	647484-648132	nan	nan	33	74	1	0.3287	no
ni|645903577|nb|KK920853.1|	649811-650030	nan	nan	27	93	2	0.1133	no
ni|645903577|nb|KK920853.1|	650119-650259	nan	nan	54	165	2	0.1552	no
ni|645903577|nb|KK920853.1|	652398-653023	nan	nan	26	131	2	0.04885	no
ni|645903577|nb|KK920853.1|	654020-655393	nan	nan	51	164	2	0.20405	no
ni|645903577|nb|KK920853.1|	657842-658882	nan	nan	33	106	2	0.15545	no
ni|645903577|nb|KK920853.1|	659084-660873	nan	nan	47	160	2	0.12595	no
ni|645903577|nb|KK920853.1|	665153-665446	nan	nan	23	75	2	0.19335	no
ni|645903577|nb|KK920853.1|	669907-671591	nan	nan	24	87	2	0.15845	no
ni|645903577|nb|KK920853.1|	671719-672186	nan	nan	44	143	2	0.196	no
ni|645903577|nb|KK920853.1|	672868-673027	nan	nan	29	81	1	0.25265	no
ni|645903577|nb|KK920853.1|	673258-675050	nan	nan	34	117	2	0.12335	no
ni|645903577|nb|KK920853.1|	675211-675469	nan	nan	20	63	2	0.14825	no
ni|645903577|nb|KK920853.1|	675709-676317	nan	nan	20	69	2	0.16275	no
ni|645903577|nb|KK920853.1|	676802-677007	nan	nan	31	92	2	0.2222	no
ni|645903577|nb|KK920853.1|	677847-678002	nan	nan	53	163	2	0.1504	no
ni|645903577|nb|KK920853.1|	689108-689230	nan	nan	30	100	2	0.14155	no
ni|645903577|nb|KK920853.1|	689355-689461	nan	nan	72	169	1	0.29655	no

ni|645903577|nb|KK920853.1|	703091-703197	nan	nan	51	149	2	0.16795	no
ni|645903577|nb|KK920853.1|	72499-72630	nan	nan	6	7	0.289153	0.8043	no
ni|645903577|nb|KK920853.1|	94586-94836	nan	nan	25	24	-0.0517637	0.96	no
ni|645903578|nb|KK920852.1|	164734-164884	nan	nan	24	90	2	0.09105	no
ni|645903578|nb|KK920852.1|	165583-165793	nan	nan	54	184	2	0.11805	no
ni|645903578|nb|KK920852.1|	170900-171450	nan	nan	55	146	1	0.2066	no
ni|645903578|nb|KK920852.1|	179302-179598	nan	nan	50	142	2	0.2528	no
ni|645903578|nb|KK920852.1|	180717-181076	nan	nan	49	162	2	0.1977	no
ni|645903578|nb|KK920852.1|	200150-200605	nan	nan	36	105	2	0.21815	no
ni|645903578|nb|KK920852.1|	201628-202073	nan	nan	23	67	2	0.2264	no
ni|645903578|nb|KK920852.1|	202396-203464	nan	nan	47	136	2	0.18695	no
ni|645903578|nb|KK920852.1|	203807-205276	nan	nan	65	176	1	0.2546	no
ni|645903578|nb|KK920852.1|	205688-206100	nan	nan	59	161	1	0.2722	no
ni|645903578|nb|KK920852.1|	211767-211912	nan	nan	43	114	1	0.235	no
ni|645903578|nb|KK920852.1|	212036-212310	nan	nan	67	180	1	0.19955	no
ni|645903578|nb|KK920852.1|	212546-212674	nan	nan	30	79	1	0.34705	no
ni|645903578|nb|KK920852.1|	213315-213468	nan	nan	51	130	1	0.254	no
ni|645903578|nb|KK920852.1|	213627-213769	nan	nan	71	171	1	0.31565	no
ni|645903578|nb|KK920852.1|	213836-213994	nan	nan	69	189	1	0.20995	no
ni|645903578|nb|KK920852.1|	214091-214742	nan	nan	58	152	1	0.2839	no
ni|645903578|nb|KK920852.1|	214958-215452	nan	nan	66	163	1	0.3069	no
ni|645903578|nb|KK920852.1|	215806-216437	nan	nan	37	108	2	0.16585	no
ni|645903578|nb|KK920852.1|	216575-216824	nan	nan	77	212	1	0.2666	no
ni|645903578|nb|KK920852.1|	218501-218660	nan	nan	35	90	1	0.24275	no
ni|645903578|nb|KK920852.1|	226334-226638	nan	nan	61	141	1	0.3994	no
ni|645903578|nb|KK920852.1|	227414-228057	nan	nan	67	162	1	0.3271	no
ni|645903578|nb|KK920852.1|	231891-232036	nan	nan	87	194	1	0.44435	no
ni|645903578|nb|KK920852.1|	232304-232820	nan	nan	106	251	1	0.33685	no
ni|645903578|nb|KK920852.1|	233544-233828	nan	nan	51	122	1	0.39905	no
ni|645903578|nb|KK920852.1|	234000-234359	nan	nan	28	61	1	0.4507	no

ni|645903578|nb|KK920852.1|	234619-235003	nan	nan	61	138	1	0.41775	no
ni|645903578|nb|KK920852.1|	235476-237188	nan	nan	123	258	1	0.3939	no
ni|645903578|nb|KK920852.1|	237637-238254	nan	nan	110	213	0.959165	0.38005	no
ni|645903578|nb|KK920852.1|	238585-239346	nan	nan	65	143	1	0.3772	no
ni|645903578|nb|KK920852.1|	240046-240353	nan	nan	65	120	0.883969	0.5313	no
ni|645903578|nb|KK920852.1|	240732-241471	nan	nan	46	94	1	0.3452	no
ni|645903578|nb|KK920852.1|	241573-242642	nan	nan	121	183	0.600859	0.57545	no
ni|645903578|nb|KK920852.1|	243903-244178	nan	nan	110	177	0.691846	0.51705	no
ni|645903578|nb|KK920852.1|	244311-244646	nan	nan	103	168	0.708536	0.6212	no
ni|645903578|nb|KK920852.1|	245418-245661	nan	nan	29	47	0.68935	0.5281	no
ni|645903578|nb|KK920852.1|	246601-248883	nan	nan	3	2	-0.513784	0.697	no
ni|645903578|nb|KK920852.1|	249102-249841	nan	nan	43	53	0.28319	0.78415	no
ni|645903578|nb|KK920852.1|	251185-252563	nan	nan	92	57	-0.701691	0.54685	no
ni|645903578|nb|KK920852.1|	254909-255452	nan	nan	71	49	-0.522972	0.68095	no
ni|645903578|nb|KK920852.1|	256145-256299	nan	nan	34	33	-0.0483374	0.96625	no
ni|645903578|nb|KK920852.1|	257380-257528	nan	nan	0	20	inf	0.0001	yes
ni|645903578|nb|KK920852.1|	258271-258489	nan	nan	0	4	inf	5.00E-05	yes
ni|645903578|nb|KK920852.1|	258672-258830	nan	nan	1	50	5	0.0274	no
ni|645903578|nb|KK920852.1|	259654-259892	nan	nan	0.729639	9	4	0.05505	no
ni|645903578|nb|KK920852.1|	261392-261509	nan	nan	0.28823	7	5	0.1211	no
ni|645903578|nb|KK920852.1|	262638-262841	nan	nan	0.87352	46	6	0.1195	no
ni|645903578|nb|KK920852.1|	263941-264073	nan	nan	1	10	3	0.0238	no
ni|645903578|nb|KK920852.1|	264394-264836	nan	nan	0.835769	14	4	0.05195	no
ni|645903578|nb|KK920852.1|	266891-267049	nan	nan	3	1	-1	0.44965	no
ni|645903578|nb|KK920852.1|	267702-269060	nan	nan	0.890496	2	1	0.3264	no
ni|645903578|nb|KK920852.1|	269120-269445	nan	nan	1	3	2	0.2583	no
ni|645903578|nb|KK920852.1|	270144-270305	nan	nan	0.249081	0.555655	1	0.4241	no
ni|645903578|nb|KK920852.1|	270539-271441	nan	nan	0.260299	0.93302	2	0.2028	no
ni|645903578|nb|KK920852.1|	273376-274389	nan	nan	0.219541	10	6	0.11975	no
ni|645903578|nb|KK920852.1|	274567-274715	nan	nan	0	34	inf	5.00E-05	yes

ni|645903578|nb|KK920852.1|	276362-276548	nan	nan	186	199	0.102675	0.92495	no
ni|645903578|nb|KK920852.1|	277391-277653	nan	nan	2	2	-0.210046	0.8588	no
ni|645903578|nb|KK920852.1|	278016-278764	nan	nan	165	179	0.114058	0.939	no
ni|645903578|nb|KK920852.1|	280608-282144	nan	nan	162	180	0.147942	0.91575	no
ni|645903578|nb|KK920852.1|	282648-283103	nan	nan	185	205	0.147815	0.91865	no
ni|645903578|nb|KK920852.1|	285010-285155	nan	nan	0.957066	0.281459	-2	0.22995	no
ni|645903578|nb|KK920852.1|	290417-291038	nan	nan	102	121	0.246797	0.8601	no
ni|645903578|nb|KK920852.1|	301259-301395	nan	nan	1	1	-0.224959	0.84335	no
ni|645903578|nb|KK920852.1|	344466-344582	nan	nan	3	1	-1	0.43485	no
ni|645903578|nb|KK920852.1|	454443-454583	nan	nan	29	33	0.193675	0.85675	no
ni|645903578|nb|KK920852.1|	475856-476075	nan	nan	3	4	0.448917	0.70255	no
ni|645903578|nb|KK920852.1|	497002-497302	nan	nan	48	55	0.189814	0.9015	no
ni|645903578|nb|KK920852.1|	532552-532965	nan	nan	219	273	0.317517	0.7725	no
ni|645903578|nb|KK920852.1|	547607-548004	nan	nan	0.030552	33	10	0.11935	no
ni|645903578|nb|KK920852.1|	583594-583661	nan	nan	6	5	-0.198141	0.8669	no
ni|645903580|nb|KK920850.1|	1581-1780	nan	nan	8	11	0.338464	0.77515	no
ni|645903580|nb|KK920850.1|	175698-175807	nan	nan	3	0	-inf	5.00E-05	yes
ni|645903580|nb|KK920850.1|	175900-176081	nan	nan	14	0	-inf	5.00E-05	yes
ni|645903580|nb|KK920850.1|	176192-176908	nan	nan	0.050849	1	4.52	0.1215	no
ni|645903580|nb|KK920850.1|	178092-178572	nan	nan	0.107228	0.922532	3	0.0589	no
ni|645903580|nb|KK920850.1|	178789-179255	nan	nan	0.297725	0.284619	-0.0649485	0.923	no
ni|645903580|nb|KK920850.1|	180238-180662	nan	nan	84	72	-0.214121	0.8613	no
ni|645903580|nb|KK920850.1|	182857-183871	nan	nan	93	77	-0.272211	0.793	no
ni|645903580|nb|KK920850.1|	184000-184447	nan	nan	21	20	-0.0425975	0.96775	no
ni|645903580|nb|KK920850.1|	189990-190140	nan	nan	1	0.919233	-0.437017	0.7272	no
ni|645903580|nb|KK920850.1|	191878-192236	nan	nan	47	33	-0.490873	0.69725	no
ni|645903580|nb|KK920850.1|	198396-198669	nan	nan	32	31	-0.0123687	0.9921	no
ni|645903580|nb|KK920850.1|	269384-269524	nan	nan	31	16	-0.948626	0.42015	no
ni|645903580|nb|KK920850.1|	290182-290272	nan	nan	3	0.724888	-2	0.1906	no
ni|645903580|nb|KK920850.1|	375206-375393	nan	nan	18	26	0.567321	0.6256	no

ni|645903580|nb|KK920850.1|	398498-398638	nan	nan	22	34	0.627346	0.58725	no
ni|645903580|nb|KK920850.1|	77050-77195	nan	nan	16	0.164864	-7	0.1188	no
ni|645903580|nb|KK920850.1|	8841-9020	nan	nan	62	50	-0.306154	0.791	no
ni|645903581|nb|KK920849.1|	157021-157347	nan	nan	27	34	0.324819	0.77495	no
ni|645903581|nb|KK920849.1|	75460-75854	nan	nan	28	37	0.424176	0.71815	no
ni|645903581|nb|KK920849.1|	85817-86048	nan	nan	25	30	0.223915	0.84175	no
ni|645903581|nb|KK920849.1|	88748-89045	nan	nan	21.52	25	0.24314	0.8392	no
ni|645903583|nb|KK920847.1|	37587-37736	nan	nan	32	37	0.21634	0.85615	no
ni|645903584|nb|KK920846.1|	200262-200772	nan	nan	15	19	0.340871	0.76355	no
ni|645903584|nb|KK920846.1|	277734-277893	nan	nan	26	26	0.0107869	0.99285	no
ni|645903584|nb|KK920846.1|	283697-283838	nan	nan	29	28	-0.0396849	0.97285	no
ni|645903584|nb|KK920846.1|	291827-292028	nan	nan	31	34	0.152819	0.89405	no
ni|645903584|nb|KK920846.1|	292125-292411	nan	nan	11	9	-0.185911	0.8737	no
ni|645903584|nb|KK920846.1|	292761-293302	nan	nan	3	14	2	0.0874	no
ni|645903584|nb|KK920846.1|	293793-294047	nan	nan	18	73	2	0.0946	no
ni|645903584|nb|KK920846.1|	294116-294287	nan	nan	16	43	1	0.23525	no
ni|645903584|nb|KK920846.1|	304794-305453	nan	nan	12	45	2	0.11265	no
ni|645903584|nb|KK920846.1|	358999-359543	nan	nan	21	68	2	0.1954	no
ni|645903584|nb|KK920846.1|	359628-361078	nan	nan	4	12	2	0.17585	no
ni|645903584|nb|KK920846.1|	361136-361677	nan	nan	8	20	1	0.2572	no
ni|645903584|nb|KK920846.1|	373014-373291	nan	nan	26	59	1	0.30955	no
ni|645903584|nb|KK920846.1|	373689-373811	nan	nan	7	30	2	0.11595	no
ni|645903584|nb|KK920846.1|	373981-374468	nan	nan	20	52	1	0.23295	no
ni|645903584|nb|KK920846.1|	375049-375382	nan	nan	3	10	2	0.1386	no
ni|645903584|nb|KK920846.1|	375905-376130	nan	nan	16	55	2	0.1359	no
ni|645903584|nb|KK920846.1|	376326-376514	nan	nan	13	51	2	0.1138	no
ni|645903584|nb|KK920846.1|	376659-377202	nan	nan	4	18	2	0.09925	no
ni|645903584|nb|KK920846.1|	379087-379389	nan	nan	4	12	2	0.16835	no
ni|645903584|nb|KK920846.1|	379780-379917	nan	nan	17	75	2	0.07905	no
ni|645903584|nb|KK920846.1|	384291-384464	nan	nan	19	74	2	0.103	no

ni|645903584|nb|KK920846.1|	384793-384959	nan	nan	26	57	1	0.3336	no
ni|645903584|nb|KK920846.1|	60393-60574	nan	nan	15	16	0.0909923	0.93655	no
ni|645903585|nb|KK920845.1|	416378-416552	nan	nan	28	69	1	0.26855	no
ni|645903585|nb|KK920845.1|	485377-485871	nan	nan	10	20	1	0.3688	no
ni|645903585|nb|KK920845.1|	556193-556360	nan	nan	0.438353	0.59375	0.437763	0.73605	no
ni|645903585|nb|KK920845.1|	625904-626105	nan	nan	4	6	0.690912	0.55985	no
ni|645903587|nb|KK920843.1|	11881-12080	nan	nan	13	20	0.658598	0.56045	no
ni|645903587|nb|KK920843.1|	14486-14643	nan	nan	31	43	0.456218	0.69655	no
ni|645903587|nb|KK920843.1|	201874-202103	nan	nan	25	18	-0.507769	0.6693	no
ni|645903587|nb|KK920843.1|	203950-204339	nan	nan	0.895629	5	2	0.1097	no
ni|645903587|nb|KK920843.1|	209489-209606	nan	nan	5	10	1	0.4218	no
ni|645903587|nb|KK920843.1|	211327-211567	nan	nan	5	8	0.670529	0.5399	no
ni|645903587|nb|KK920843.1|	214432-214893	nan	nan	6	3	-1	0.42295	no
ni|645903587|nb|KK920843.1|	216458-216679	nan	nan	24	20	-0.248356	0.86495	no
ni|645903587|nb|KK920843.1|	220500-220998	nan	nan	25	24	-0.066139	0.95575	no
ni|645903587|nb|KK920843.1|	222773-222914	nan	nan	42	45	0.0937504	0.93645	no
ni|645903587|nb|KK920843.1|	227623-227797	nan	nan	9	6	-0.706743	0.5447	no
ni|645903587|nb|KK920843.1|	233957-234450	nan	nan	11	9	-0.261966	0.81675	no
ni|645903587|nb|KK920843.1|	239609-239728	nan	nan	9	8	-0.174216	0.87735	no
ni|645903587|nb|KK920843.1|	242145-242436	nan	nan	16	142.12	3	0.0257	no
ni|645903587|nb|KK920843.1|	244144-244332	nan	nan	33	34	0.0203636	0.9861	no
ni|645903587|nb|KK920843.1|	244636-245499	nan	nan	4	5	0.113357	0.9325	no
ni|645903587|nb|KK920843.1|	246434-246629	nan	nan	22	27	0.295631	0.79885	no
ni|645903587|nb|KK920843.1|	247062-247538	nan	nan	49	24	-1	0.44755	no
ni|645903587|nb|KK920843.1|	247963-248783	nan	nan	5	7	0.391268	0.72035	no
ni|645903587|nb|KK920843.1|	248887-249739	nan	nan	4	5	0.418185	0.73705	no
ni|645903587|nb|KK920843.1|	249878-250226	nan	nan	8	12	0.589821	0.6164	no
ni|645903587|nb|KK920843.1|	259213-259348	nan	nan	7	6	-0.182676	0.8788	no
ni|645903587|nb|KK920843.1|	264783-264965	nan	nan	4	3	-0.0671657	0.9547	no
ni|645903587|nb|KK920843.1|	265199-265387	nan	nan	3	6	0.893111	0.4682	no

ni|645903587|nb|KK920843.1|	268787-269393	nan	nan	48	52	0.134417	0.90375	no
ni|645903587|nb|KK920843.1|	270265-270815	nan	nan	24	27	0.201347	0.85015	no
ni|645903587|nb|KK920843.1|	270994-271149	nan	nan	0.38575	0.368842	-0.0646648	0.9545	no
ni|645903587|nb|KK920843.1|	272047-272210	nan	nan	10	11	0.113338	0.9177	no
ni|645903587|nb|KK920843.1|	272267-272390	nan	nan	3	2	-0.279595	0.81975	no
ni|645903587|nb|KK920843.1|	272742-273308	nan	nan	35	35	-0.0226567	0.9875	no
ni|645903587|nb|KK920843.1|	274031-274327	nan	nan	12	13	0.199702	0.8556	no
ni|645903587|nb|KK920843.1|	278710-279016	nan	nan	21	21	0.00818914	0.9954	no
ni|645903587|nb|KK920843.1|	386442-387784	nan	nan	25	28	0.161793	0.871	no
ni|645903587|nb|KK920843.1|	394005-394569	nan	nan	16	13	-0.284507	0.8007	no
ni|645903587|nb|KK920843.1|	44231-44371	nan	nan	1	0.751844	-0.427614	0.73225	no
ni|645903587|nb|KK920843.1|	546836-548149	nan	nan	39	34	-0.201049	0.86695	no
ni|645903587|nb|KK920843.1|	572084-572343	nan	nan	29	36	0.320253	0.7826	no
ni|645903587|nb|KK920843.1|	596845-597094	nan	nan	15	15	0.000222635	0.99775	no
ni|645903587|nb|KK920843.1|	76281-76471	nan	nan	25	29	0.243437	0.83735	no
ni|645903587|nb|KK920843.1|	8359-8520	nan	nan	27	34	0.310641	0.783	no
ni|645903588|nb|KK920842.1|	302927-303152	nan	nan	32	29	-0.135963	0.91315	no
ni|645903588|nb|KK920842.1|	377154-377265	nan	nan	19	18	-0.111605	0.9231	no
ni|645903588|nb|KK920842.1|	449645-449872	nan	nan	153	83	-0.885248	0.46855	no
ni|645903588|nb|KK920842.1|	492757-492873	nan	nan	11	9	-0.324349	0.78125	no
ni|645903588|nb|KK920842.1|	502337-502444	nan	nan	239	271	0.18168	0.88735	no
ni|645903588|nb|KK920842.1|	503653-503844	nan	nan	60	81	0.422659	0.7336	no
ni|645903588|nb|KK920842.1|	504950-505123	nan	nan	1	2	0.333464	0.7901	no
ni|645903588|nb|KK920842.1|	506559-506861	nan	nan	2	2	0.329035	0.779	no
ni|645903588|nb|KK920842.1|	507496-508025	nan	nan	70	97	0.458285	0.70845	no
ni|645903588|nb|KK920842.1|	509214-509452	nan	nan	2	1	-0.430156	0.71005	no
ni|645903588|nb|KK920842.1|	510882-511125	nan	nan	13	17	0.349366	0.76425	no
ni|645903588|nb|KK920842.1|	512232-512543	nan	nan	7	4	-0.745376	0.59615	no
ni|645903588|nb|KK920842.1|	513083-513230	nan	nan	89	117	0.400845	0.77045	no
ni|645903588|nb|KK920842.1|	517507-517831	nan	nan	58	70	0.268731	0.84505	no

ni|645903588|nb|KK920842.1|	518441-518584	nan	nan	1	1	0.138124	0.9069	no
ni|645903588|nb|KK920842.1|	519087-519204	nan	nan	1	0.514438	-1	0.40315	no
ni|645903588|nb|KK920842.1|	519933-520061	nan	nan	147.67	142	-0.0553587	0.9576	no
ni|645903588|nb|KK920842.1|	521130-521393	nan	nan	1	0.845338	-0.717882	0.61925	no
ni|645903588|nb|KK920842.1|	522486-522683	nan	nan	3	4	0.442518	0.70325	no
ni|645903588|nb|KK920842.1|	524034-524287	nan	nan	131	128	-0.030909	0.9762	no
ni|645903588|nb|KK920842.1|	555629-556183	nan	nan	0.558577	0.679808	0.283371	0.8168	no
ni|645903588|nb|KK920842.1|	581657-583069	nan	nan	42.99	41	-0.0697374	0.9467	no
ni|645903588|nb|KK920842.1|	583249-583956	nan	nan	0.567276	0.620032	0.12829	0.9142	no
ni|645903588|nb|KK920842.1|	584053-584229	nan	nan	309	273	-0.177209	0.90375	no
ni|645903588|nb|KK920842.1|	586991-587148	nan	nan	215	207	-0.0547124	0.97015	no
ni|645903588|nb|KK920842.1|	590872-591060	nan	nan	26	22	-0.198486	0.8633	no
ni|645903588|nb|KK920842.1|	592051-592179	nan	nan	16	20	0.32274	0.7894	no
ni|645903588|nb|KK920842.1|	592989-593216	nan	nan	15	8	-0.907674	0.43295	no
ni|645903588|nb|KK920842.1|	594306-594683	nan	nan	18	15	-0.320091	0.78755	no
ni|645903588|nb|KK920842.1|	594819-595018	nan	nan	5	11	1	0.2837	no
ni|645903588|nb|KK920842.1|	596115-596329	nan	nan	4	5	0.436941	0.70605	no
ni|645903588|nb|KK920842.1|	601315-601428	nan	nan	9	8	-0.301486	0.7787	no
ni|645903588|nb|KK920842.1|	602426-602563	nan	nan	13	10	-0.318475	0.7862	no
ni|645903588|nb|KK920842.1|	603471-603805	nan	nan	2	2	0.00133204	0.99045	no
ni|645903588|nb|KK920842.1|	604760-604943	nan	nan	4	4	0.113633	0.9325	no
ni|645903588|nb|KK920842.1|	605215-605827	nan	nan	3	2	-0.673114	0.5767	no
ni|645903588|nb|KK920842.1|	607136-607482	nan	nan	4	6	0.414327	0.7309	no
ni|645903588|nb|KK920842.1|	610720-611537	nan	nan	6	10	0.677442	0.5638	no
ni|645903588|nb|KK920842.1|	611718-612237	nan	nan	7	3	-1	0.3993	no
ni|645903588|nb|KK920842.1|	612399-612629	nan	nan	6	6	0.00273614	0.99025	no
ni|645903588|nb|KK920842.1|	612960-613132	nan	nan	7	5	-0.500106	0.6758	no
ni|645903588|nb|KK920842.1|	613304-613743	nan	nan	8	5	-0.652184	0.5967	no
ni|645903588|nb|KK920842.1|	613874-614319	nan	nan	4	3	-0.359413	0.76125	no
ni|645903588|nb|KK920842.1|	617210-617738	nan	nan	0	105	inf	5.00E-05	yes

ni|645903588|nb|KK920842.1|	620227-620481	nan	nan	0.055925	105	11	0.11935	no
ni|645903588|nb|KK920842.1|	620605-620765	nan	nan	0.266673	388	11	0.11935	no
ni|645903588|nb|KK920842.1|	622245-623104	nan	nan	0	2	inf	0.00065	yes
ni|645903588|nb|KK920842.1|	624199-624337	nan	nan	0	1	inf	0.0001	yes
ni|645903588|nb|KK920842.1|	624442-624830	nan	nan	0	2	inf	0.0001	yes
ni|645903588|nb|KK920842.1|	626408-626530	nan	nan	9	6	-0.492881	0.67345	no
ni|645903588|nb|KK920842.1|	626759-626952	nan	nan	16	15	-0.0885912	0.9393	no
ni|645903588|nb|KK920842.1|	627387-627788	nan	nan	4	8	0.744582	0.5237	no
ni|645903588|nb|KK920842.1|	629119-629309	nan	nan	10	11	0.0973795	0.9293	no
ni|645903588|nb|KK920842.1|	629662-629789	nan	nan	7	5	-0.506019	0.66315	no
ni|645903588|nb|KK920842.1|	630322-630812	nan	nan	7	46	3	0.03585	no
ni|645903588|nb|KK920842.1|	638506-638656	nan	nan	0.638526	1	1	0.4292	no
ni|645903588|nb|KK920842.1|	638748-638963	nan	nan	12	77	3	0.03395	no
ni|645903588|nb|KK920842.1|	640387-640611	nan	nan	0.725355	0.742938	0.0345546	0.96125	no
ni|645903589|nb|KK920841.1|	71885-72047	nan	nan	7	65	3	0.012	no
ni|645903590|nb|KK920840.1|	159-412	nan	nan	0.356576	0.624886	0.809384	0.56845	no
ni|645903590|nb|KK920840.1|	167960-168144	nan	nan	18	19	0.147292	0.9031	no
ni|645903590|nb|KK920840.1|	168664-168831	nan	nan	35	20	-0.778071	0.54755	no
ni|645903590|nb|KK920840.1|	171624-171777	nan	nan	32	26	-0.285836	0.80375	no
ni|645903590|nb|KK920840.1|	172929-173108	nan	nan	14	13	-0.147392	0.89825	no
ni|645903590|nb|KK920840.1|	174491-175141	nan	nan	31	28	-0.141999	0.9016	no
ni|645903590|nb|KK920840.1|	175221-175587	nan	nan	28	32	0.206206	0.85985	no
ni|645903590|nb|KK920840.1|	176520-176628	nan	nan	20	19	-0.0883031	0.9397	no
ni|645903590|nb|KK920840.1|	176851-177348	nan	nan	25	23	-0.105294	0.92885	no
ni|645903590|nb|KK920840.1|	177983-178099	nan	nan	45	46	0.0450658	0.9669	no
ni|645903590|nb|KK920840.1|	181591-181743	nan	nan	19	21	0.12661	0.90725	no
ni|645903590|nb|KK920840.1|	181870-181985	nan	nan	3	3	0.17409	0.8916	no
ni|645903590|nb|KK920840.1|	183913-184056	nan	nan	18	17	-0.0984348	0.93075	no
ni|645903590|nb|KK920840.1|	1845-2455	nan	nan	0.661696	6	3	0.0282	no
ni|645903590|nb|KK920840.1|	185742-185888	nan	nan	36	35	-0.0358634	0.9797	no

ni|645903590|nb|KK920840.1|	312606-313384	nan	nan	245	199	-0.29542	0.812	no
ni|645903590|nb|KK920840.1|	316135-316508	nan	nan	1371.66	1184.38	-0.211787	0.88035	no
ni|645903590|nb|KK920840.1|	316640-319237	nan	nan	1330.62	1357.95	0.0293274	0.985	no
ni|645903590|nb|KK920840.1|	36230-36509	nan	nan	0.111854	4	5	0.02755	no
ni|645903590|nb|KK920840.1|	43331-44217	nan	nan	0.678963	0.922513	0.442236	0.71395	no
ni|645903590|nb|KK920840.1|	44787-46119	nan	nan	0.683321	0.653406	-0.0645848	0.95755	no
ni|645903590|nb|KK920840.1|	46226-47215	nan	nan	31	32	0.0786712	0.94545	no
ni|645903590|nb|KK920840.1|	499265-499485	nan	nan	1899.18	1737.26	-0.128563	0.92945	no
ni|645903590|nb|KK920840.1|	500454-500716	nan	nan	410	410	0.000532167	0.9993	no
ni|645903590|nb|KK920840.1|	56568-56690	nan	nan	5	2	-2	0.25675	no
ni|645903590|nb|KK920840.1|	56997-57421	nan	nan	7	6	-0.263238	0.8216	no
ni|645903590|nb|KK920840.1|	575902-576208	nan	nan	5	12	1	0.3217	no
ni|645903590|nb|KK920840.1|	57703-58000	nan	nan	54	56	0.0684209	0.95345	no
ni|645903590|nb|KK920840.1|	58235-58363	nan	nan	28	30	0.101618	0.92895	no
ni|645903590|nb|KK920840.1|	582561-582869	nan	nan	13	26	1	0.3819	no
ni|645903590|nb|KK920840.1|	58840-58990	nan	nan	18	23	0.346529	0.75685	no
ni|645903590|nb|KK920840.1|	59511-60146	nan	nan	45	60	0.427368	0.70425	no
ni|645903590|nb|KK920840.1|	60248-60732	nan	nan	25	25	-0.000388448	0.99625	no
ni|645903590|nb|KK920840.1|	60891-61084	nan	nan	0.551072	0.746349	0.437609	0.73605	no
ni|645903590|nb|KK920840.1|	62017-62456	nan	nan	36	46	0.377266	0.74445	no
ni|645903590|nb|KK920840.1|	62822-62990	nan	nan	19	23	0.306345	0.83175	no
ni|645903590|nb|KK920840.1|	63305-65210	nan	nan	22	25	0.161099	0.88895	no
ni|645903590|nb|KK920840.1|	65330-65584	nan	nan	85	84	-0.0139083	0.9893	no
ni|645903590|nb|KK920840.1|	65761-65877	nan	nan	27	28	0.0631382	0.95495	no
ni|645903590|nb|KK920840.1|	67341-67477	nan	nan	34	45	0.399771	0.73815	no
ni|645903590|nb|KK920840.1|	69896-70055	nan	nan	3	3	0.197894	0.86565	no
ni|645903590|nb|KK920840.1|	70466-71130	nan	nan	9	11	0.376307	0.7506	no
ni|645903590|nb|KK920840.1|	72155-72446	nan	nan	8	10	0.286445	0.81825	no
ni|645903590|nb|KK920840.1|	74235-74443	nan	nan	3	3	-0.243541	0.83855	no
ni|645903590|nb|KK920840.1|	75576-75884	nan	nan	4	5	0.303949	0.79305	no

ni|645903590|nb|KK920840.1|	79077-79459	nan	nan	9	7	-0.3117	0.78305	no
ni|645903590|nb|KK920840.1|	80858-81597	nan	nan	27	26	-0.0870342	0.93735	no
ni|645903590|nb|KK920840.1|	82440-82592	nan	nan	17	19	0.226624	0.84565	no
ni|645903590|nb|KK920840.1|	99054-99544	nan	nan	17	19	0.1741	0.9087	no
ni|645903591|nb|KK920839.1|	10319-10537	nan	nan	14	31	1	0.3514	no
ni|645903591|nb|KK920839.1|	112449-112649	nan	nan	1	0.991017	-0.065021	0.95835	no
ni|645903591|nb|KK920839.1|	126478-126841	nan	nan	20	35	0.77279	0.49375	no
ni|645903591|nb|KK920839.1|	13021-13388	nan	nan	10	17	0.696452	0.54805	no
ni|645903591|nb|KK920839.1|	152300-153017	nan	nan	27	17	-0.668678	0.58015	no
ni|645903591|nb|KK920839.1|	153583-153752	nan	nan	14	10	-0.512049	0.65935	no
ni|645903591|nb|KK920839.1|	155399-155926	nan	nan	17	17	0.0179075	0.98755	no
ni|645903591|nb|KK920839.1|	158579-158758	nan	nan	23	21	-0.0966019	0.93165	no
ni|645903591|nb|KK920839.1|	162215-162440	nan	nan	30	17	-0.817317	0.5025	no
ni|645903591|nb|KK920839.1|	164054-165291	nan	nan	12	10	-0.203256	0.8644	no
ni|645903592|nb|KK920838.1|	1052739-105298	nan	nan	15	13	-0.149607	0.8985	no
ni|645903592|nb|KK920838.1|	116221-116673	nan	nan	22	18	-0.30345	0.7916	no
ni|645903592|nb|KK920838.1|	1166587-116671	nan	nan	47	52	0.158595	0.89295	no
ni|645903592|nb|KK920838.1|	1211100-121131	nan	nan	5	4	-0.251057	0.84	no
ni|645903592|nb|KK920838.1|	1212214-121234	nan	nan	69	98	0.491397	0.67455	no
ni|645903592|nb|KK920838.1|	1212496-121264	nan	nan	39	67	0.758307	0.5165	no
ni|645903592|nb|KK920838.1|	1212952-121338	nan	nan	49	89	0.864114	0.48715	no
ni|645903592|nb|KK920838.1|	1214400-121453	nan	nan	99	173	0.809084	0.4825	no
ni|645903592|nb|KK920838.1|	1216450-1216581	nan	nan	43	67	0.653559	0.58185	no
ni|645903592|nb|KK920838.1|	1217813-121815	nan	nan	3	5	0.712039	0.55715	no
ni|645903592|nb|KK920838.1|	1220947-122111	nan	nan	95	149.8	0.654709	0.55665	no
ni|645903592|nb|KK920838.1|	1221510-122204	nan	nan	44	54	0.298562	0.8438	no
ni|645903592|nb|KK920838.1|	1222322-1222451	nan	nan	63	53	-0.251729	0.83025	no
ni|645903592|nb|KK920838.1|	1223189-122338	nan	nan	0.826544	3	2	0.2452	no
ni|645903592|nb|KK920838.1|	1224109-122448	nan	nan	0.627713	3	2	0.1268	no
ni|645903592|nb|KK920838.1|	1224559-122502	nan	nan	0.406538	0.712393	0.809282	0.56845	no

ni|645903592|nb|KK920838.1|	1225274-122569[illegible]	nan	nan	0.381773	1	1	0.30495	no
ni|645903592|nb|KK920838.1|	135282-135463	nan	nan	12	10	-0.330347	0.76815	no
ni|645903592|nb|KK920838.1|	155080-155367	nan	nan	32	27	-0.23697	0.83465	no
ni|645903592|nb|KK920838.1|	158979-159179	nan	nan	32	30	-0.118861	0.91545	no
ni|645903592|nb|KK920838.1|	183859-184103	nan	nan	9	7	-0.29087	0.80595	no
ni|645903592|nb|KK920838.1|	184586-184894	nan	nan	34	31	-0.145197	0.9025	no
ni|645903592|nb|KK920838.1|	185623-186094	nan	nan	14	10	-0.375391	0.74465	no
ni|645903592|nb|KK920838.1|	186188-186929	nan	nan	36	35	-0.0369049	0.9771	no
ni|645903592|nb|KK920838.1|	187162-187589	nan	nan	31	35	0.16966	0.8892	no
ni|645903592|nb|KK920838.1|	188031-188332	nan	nan	48	33	-0.564034	0.62555	no
ni|645903592|nb|KK920838.1|	189089-189307	nan	nan	0.087602	2	4	0.04515	no
ni|645903592|nb|KK920838.1|	191032-191648	nan	nan	0.095577	2	4	0.1221	no
ni|645903592|nb|KK920838.1|	192439-192698	nan	nan	0	28	inf	5.00E-05	yes
ni|645903592|nb|KK920838.1|	193136-193680	nan	nan	0	3	inf	5.00E-05	yes
ni|645903592|nb|KK920838.1|	194975-195099	nan	nan	0	15	inf	5.00E-05	yes
ni|645903592|nb|KK920838.1|	195315-195469	nan	nan	0	10	inf	5.00E-05	yes
ni|645903592|nb|KK920838.1|	298674-299343	nan	nan	0	81	inf	5.00E-05	yes
ni|645903592|nb|KK920838.1|	314894-315370	nan	nan	0	29	inf	5.00E-05	yes
ni|645903592|nb|KK920838.1|	329843-330234	nan	nan	0	90	inf	5.00E-05	yes
ni|645903592|nb|KK920838.1|	338016-338296	nan	nan	0	100	inf	5.00E-05	yes
ni|645903592|nb|KK920838.1|	339066-339317	nan	nan	0	31	inf	5.00E-05	yes
ni|645903592|nb|KK920838.1|	340490-340929	nan	nan	0	56	inf	5.00E-05	yes
ni|645903592|nb|KK920838.1|	341158-341340	nan	nan	0	100	inf	5.00E-05	yes
ni|645903592|nb|KK920838.1|	341748-342104	nan	nan	2	1	-0.495294	0.6711	no
ni|645903592|nb|KK920838.1|	342372-342651	nan	nan	2	2	-0.0499123	0.96595	no
ni|645903592|nb|KK920838.1|	343066-343203	nan	nan	2	2	0.354745	0.76275	no
ni|645903592|nb|KK920838.1|	343369-343684	nan	nan	0.604283	4	3	0.0782	no
ni|645903592|nb|KK920838.1|	352973-353270	nan	nan	2	2	-0.065805	0.9615	no
ni|645903592|nb|KK920838.1|	354680-355136	nan	nan	0.696702	0.53273	-0.387139	0.78555	no
ni|645903592|nb|KK920838.1|	357477-357891	nan	nan	2	3	0.189946	0.86975	no

ni|645903592|nb|KK920838.1|	371042-371342	nan	nan	15	17	0.141446	0.9016	no
ni|645903592|nb|KK920838.1|	372201-372484	nan	nan	3	4	0.498003	0.6756	no
ni|645903592|nb|KK920838.1|	372584-372846	nan	nan	16	20	0.296506	0.79555	no
ni|645903592|nb|KK920838.1|	373554-373788	nan	nan	6	11	0.864356	0.4626	no
ni|645903592|nb|KK920838.1|	375187-375657	nan	nan	16	19	0.184935	0.86735	no
ni|645903592|nb|KK920838.1|	385104-385340	nan	nan	12	27	1	0.34255	no
ni|645903592|nb|KK920838.1|	389146-389422	nan	nan	4	6	0.844783	0.47905	no
ni|645903592|nb|KK920838.1|	389685-389966	nan	nan	13	22	0.728769	0.53025	no
ni|645903592|nb|KK920838.1|	391377-391759	nan	nan	2	3	0.671559	0.57765	no
ni|645903592|nb|KK920838.1|	392300-392751	nan	nan	13	23	0.818636	0.4699	no
ni|645903592|nb|KK920838.1|	394693-395016	nan	nan	4	6	0.634779	0.5846	no
ni|645903592|nb|KK920838.1|	395287-395471	nan	nan	5	9	0.932729	0.44815	no
ni|645903592|nb|KK920838.1|	403181-403911	nan	nan	0.549707	0.337866	-0.702213	0.6038	no
ni|645903592|nb|KK920838.1|	450565-450928	nan	nan	2	0.812581	-0.895554	0.502	no
ni|645903592|nb|KK920838.1|	451007-451135	nan	nan	6	3	-1	0.43055	no
ni|645903592|nb|KK920838.1|	525929-526147	nan	nan	1	2	0.295938	0.8383	no
ni|645903592|nb|KK920838.1|	532190-532686	nan	nan	0.688571	1	0.867761	0.5007	no
ni|645903592|nb|KK920838.1|	534858-535063	nan	nan	2	1	-0.744947	0.59615	no
ni|645903592|nb|KK920838.1|	550338-550863	nan	nan	1	1	0.40886	0.74735	no
ni|645903592|nb|KK920838.1|	551958-552269	nan	nan	29	35	0.242174	0.825	no
ni|645903592|nb|KK920838.1|	552365-552838	nan	nan	11	11	-0.0299826	0.97845	no
ni|645903592|nb|KK920838.1|	558729-559469	nan	nan	39	60	0.624802	0.6692	no
ni|645903592|nb|KK920838.1|	561719-561872	nan	nan	32	17	-0.906725	0.4506	no
ni|645903592|nb|KK920838.1|	561961-562338	nan	nan	61	34	-0.82458	0.44445	no
ni|645903592|nb|KK920838.1|	562551-563052	nan	nan	14	9	-0.606824	0.5978	no
ni|645903592|nb|KK920838.1|	563263-563419	nan	nan	49	45	-0.107089	0.9275	no
ni|645903592|nb|KK920838.1|	563791-564642	nan	nan	11	8	-0.545007	0.62485	no
ni|645903592|nb|KK920838.1|	564851-565579	nan	nan	2	0.979175	-1	0.39585	no
ni|645903592|nb|KK920838.1|	565707-566413	nan	nan	8	5	-0.605377	0.5922	no
ni|645903592|nb|KK920838.1|	567985-568921	nan	nan	8	7	-0.273436	0.8101	no

ni|645903592|nb|KK920838.1|	569074-569325	nan	nan	7	6	-0.236094	0.84085	no
ni|645903592|nb|KK920838.1|	573284-574973	nan	nan	19	14	-0.380293	0.7968	no
ni|645903592|nb|KK920838.1|	575090-575214	nan	nan	32	21	-0.607923	0.6129	no
ni|645903592|nb|KK920838.1|	575323-575791	nan	nan	24	18	-0.472272	0.66765	no
ni|645903592|nb|KK920838.1|	575897-576331	nan	nan	29	19.88	-0.568972	0.60885	no
ni|645903592|nb|KK920838.1|	576393-576673	nan	nan	23	19	-0.272371	0.8094	no
ni|645903592|nb|KK920838.1|	576876-577073	nan	nan	21	9	-1	0.28715	no
ni|645903592|nb|KK920838.1|	662112-662187	nan	nan	2212.14	8657.01	2	0.19105	no
ni|645903592|nb|KK920838.1|	739317-739591	nan	nan	7	8	0.255963	0.82675	no
ni|645903592|nb|KK920838.1|	763280-763503	nan	nan	7	9	0.412617	0.73085	no
ni|645903592|nb|KK920838.1|	791841-792132	nan	nan	8	7	-0.162058	0.89065	no
ni|645903592|nb|KK920838.1|	830665-830837	nan	nan	0	20	inf	5.00E-05	yes
ni|645903592|nb|KK920838.1|	834998-835362	nan	nan	4	5	0.519488	0.6636	no
ni|645903592|nb|KK920838.1|	836501-836899	nan	nan	0.666525	0.71663	0.10457	0.93335	no
ni|645903592|nb|KK920838.1|	837751-838365	nan	nan	0.192665	19	7	0.03715	no
ni|645903592|nb|KK920838.1|	838786-839354	nan	nan	8	10	0.38713	0.7397	no
ni|645903592|nb|KK920838.1|	839648-840081	nan	nan	5	6	0.111615	0.92295	no
ni|645903592|nb|KK920838.1|	840599-840728	nan	nan	11	6	-0.767744	0.5799	no
ni|645903592|nb|KK920838.1|	840832-841597	nan	nan	5	5	-0.0951223	0.9353	no
ni|645903592|nb|KK920838.1|	842189-842689	nan	nan	0	3	inf	5.00E-05	yes
ni|645903592|nb|KK920838.1|	844296-845261	nan	nan	0	114	inf	5.00E-05	yes
ni|645903592|nb|KK920838.1|	846788-846975	nan	nan	0	50	inf	5.00E-05	yes
ni|645903592|nb|KK920838.1|	847801-848797	nan	nan	0	78	inf	5.00E-05	yes
ni|645903592|nb|KK920838.1|	855361-856484	nan	nan	0	103	inf	5.00E-05	yes
ni|645903592|nb|KK920838.1|	858740-858991	nan	nan	0	26	inf	5.00E-05	yes
ni|645903592|nb|KK920838.1|	859606-860135	nan	nan	0	61	inf	5.00E-05	yes
ni|645903592|nb|KK920838.1|	861010-861363	nan	nan	0	41	inf	5.00E-05	yes
ni|645903592|nb|KK920838.1|	864037-864235	nan	nan	3	66	5	0.01	no
ni|645903592|nb|KK920838.1|	864854-865056	nan	nan	2	61	5	0.0205	no
ni|645903592|nb|KK920838.1|	875111-875415	nan	nan	209	167.19	-0.323927	0.8207	no

ni|645903592|nb|KK920838.1|	875476-875682	nan	nan	206	169	-0.291728	0.8141	no
ni|645903592|nb|KK920838.1|	877949-878893	nan	nan	89	79	-0.173505	0.89845	no
ni|645903592|nb|KK920838.1|	889421-889731	nan	nan	3	6	1	0.3669	no
ni|645903592|nb|KK920838.1|	908917-909111	nan	nan	0	22	inf	5.00E-05	yes
ni|645903592|nb|KK920838.1|	909270-910240	nan	nan	0.028404	65	11	0.11935	no
ni|645903592|nb|KK920838.1|	923677-923924	nan	nan	23	62	1	0.21935	no
ni|645903592|nb|KK920838.1|	925030-925220	nan	nan	26	55	1	0.34405	no
ni|645903592|nb|KK920838.1|	925492-925679	nan	nan	35	66	0.903147	0.4333	no
ni|645903592|nb|KK920838.1|	927432-927664	nan	nan	35	63	0.869101	0.442	no
ni|645903592|nb|KK920838.1|	928971-929106	nan	nan	29	55	0.932743	0.435	no
ni|645903592|nb|KK920838.1|	929353-929574	nan	nan	9	19	1	0.3382	no
ni|645903592|nb|KK920838.1|	933872-934140	nan	nan	13	20	0.694521	0.5516	no
ni|645903592|nb|KK920838.1|	936172-936329	nan	nan	16	33	1	0.36505	no
ni|645903592|nb|KK920838.1|	937342-938042	nan	nan	1	1	0.234744	0.83745	no
ni|645903592|nb|KK920838.1|	938214-938490	nan	nan	25	47	0.9054	0.4283	no
ni|645903592|nb|KK920838.1|	939103-939255	nan	nan	20	27.89	0.505993	0.6658	no
ni|645903592|nb|KK920838.1|	940249-940744	nan	nan	25	29	0.209256	0.84855	no
ni|645903592|nb|KK920838.1|	941000-941120	nan	nan	29	33	0.205614	0.8656	no
ni|645903592|nb|KK920838.1|	941234-941340	nan	nan	46	88	0.932041	0.4586	no
ni|645903592|nb|KK920838.1|	941398-941538	nan	nan	43	40	-0.109663	0.924	no
ni|645903592|nb|KK920838.1|	941759-942114	nan	nan	19	18	-0.0286118	0.97895	no
ni|645903592|nb|KK920838.1|	942538-943144	nan	nan	16	13	-0.326203	0.77425	no
ni|645903592|nb|KK920838.1|	946031-946336	nan	nan	2	3	0.571672	0.64525	no
ni|645903592|nb|KK920838.1|	946910-947189	nan	nan	3	3	0.17508	0.88505	no
ni|645903592|nb|KK920838.1|	955429-955754	nan	nan	0	3	inf	0.00055	yes
ni|645903592|nb|KK920838.1|	956809-957772	nan	nan	0	1	inf	0.0001	yes
ni|645903592|nb|KK920838.1|	960439-960642	nan	nan	0.243213	49	8	0.11935	no
ni|645903592|nb|KK920838.1|	963749-963999	nan	nan	0.166398	43	8	0.11935	no
ni|645903592|nb|KK920838.1|	964118-964284	nan	nan	0	12	inf	0.00025	yes
ni|645903592|nb|KK920838.1|	964817-965024	nan	nan	0	67	inf	5.00E-05	yes

ni|645903592|nb|KK920838.1|	966564-966971	nan	nan	0	49	inf	5.00E-05	yes
ni|645903592|nb|KK920838.1|	969305-969445	nan	nan	0	80	inf	5.00E-05	yes
ni|645903592|nb|KK920838.1|	971045-971179	nan	nan	0	127	inf	5.00E-05	yes
ni|645903592|nb|KK920838.1|	971282-971517	nan	nan	0.185075	143	10	0.11935	no
ni|645903592|nb|KK920838.1|	973293-973431	nan	nan	2	48	5	0.0514	no
ni|645903592|nb|KK920838.1|	983940-984064	nan	nan	140	91	-0.621291	0.5998	no
ni|645903592|nb|KK920838.1|	984189-984857	nan	nan	42	31	-0.420334	0.7353	no
ni|645903592|nb|KK920838.1|	985024-985233	nan	nan	74	93	0.329137	0.7653	no
ni|645903592|nb|KK920838.1|	985369-985888	nan	nan	44	57	0.365304	0.8124	no
ni|645903592|nb|KK920838.1|	986239-986391	nan	nan	66	100.13	0.599071	0.6034	no
ni|645903592|nb|KK920838.1|	988136-988312	nan	nan	118	164	0.475263	0.66435	no
ni|645903592|nb|KK920838.1|	989583-989934	nan	nan	90	128	0.498744	0.73345	no
ni|645903592|nb|KK920838.1|	990669-990850	nan	nan	106	157	0.556283	0.6048	no
ni|645903592|nb|KK920838.1|	994377-994625	nan	nan	0	12	inf	5.00E-05	yes
ni|645903592|nb|KK920838.1|	994819-994971	nan	nan	0	11	inf	0.0003	yes
ni|645903594|nb|KK920836.1|	116008-116227	nan	nan	8	9	0.144888	0.8997	no
ni|645903594|nb|KK920836.1|	117241-117434	nan	nan	4	2	-1	0.41555	no
ni|645903594|nb|KK920836.1|	158088-158214	nan	nan	115	171	0.56758	0.6257	no
ni|645903594|nb|KK920836.1|	158797-158986	nan	nan	216	229.65	0.0896933	0.9495	no
ni|645903594|nb|KK920836.1|	160263-160499	nan	nan	149	190	0.350015	0.81565	no
ni|645903594|nb|KK920836.1|	160629-161060	nan	nan	120.39	151	0.32371	0.8181	no
ni|645903594|nb|KK920836.1|	162338-162482	nan	nan	166	190	0.191745	0.85825	no
ni|645903594|nb|KK920836.1|	163517-163684	nan	nan	252	250.99	-0.0065539	0.99595	no
ni|645903594|nb|KK920836.1|	164508-164860	nan	nan	51	45	-0.175442	0.875	no
ni|645903594|nb|KK920836.1|	165615-165798	nan	nan	129	116	-0.157917	0.88665	no
ni|645903594|nb|KK920836.1|	166831-166977	nan	nan	3	6	0.807265	0.58675	no
ni|645903594|nb|KK920836.1|	214320-214663	nan	nan	4	2	-0.991563	0.4254	no
ni|645903594|nb|KK920836.1|	216897-217187	nan	nan	0.917656	1	0.448724	0.7543	no
ni|645903594|nb|KK920836.1|	218117-218262	nan	nan	129	141	0.129304	0.91115	no
ni|645903594|nb|KK920836.1|	220410-221103	nan	nan	27	24	-0.197737	0.85145	no

ni|645903594|nb|KK920836.1|	221929-222071	nan	nan	107	60	-0.833544	0.47275	no
ni|645903594|nb|KK920836.1|	222353-222739	nan	nan	1	0.414928	-2	0.2261	no
ni|645903594|nb|KK920836.1|	459550-460167	nan	nan	25	34	0.474776	0.7085	no
ni|645903594|nb|KK920836.1|	468331-468571	nan	nan	16	13	-0.272749	0.81835	no
ni|645903594|nb|KK920836.1|	538582-539107	nan	nan	25	33	0.401984	0.7094	no
ni|645903594|nb|KK920836.1|	540679-541046	nan	nan	47	68	0.526124	0.68395	no
ni|645903594|nb|KK920836.1|	543316-543528	nan	nan	126	142	0.168753	0.87565	no
ni|645903594|nb|KK920836.1|	545626-545753	nan	nan	106	130.36	0.300716	0.80105	no
ni|645903594|nb|KK920836.1|	546570-546758	nan	nan	83	108.79	0.386816	0.7226	no
ni|645903594|nb|KK920836.1|	547040-547257	nan	nan	135	164	0.274748	0.8249	no
ni|645903594|nb|KK920836.1|	548470-549121	nan	nan	15	18	0.247547	0.82165	no
ni|645903594|nb|KK920836.1|	550262-550401	nan	nan	140	111	-0.339435	0.7655	no
ni|645903594|nb|KK920836.1|	550706-550884	nan	nan	89	57	-0.634171	0.57175	no
ni|645903594|nb|KK920836.1|	552288-552505	nan	nan	189.05	117	-0.689644	0.58865	no
ni|645903594|nb|KK920836.1|	552684-552838	nan	nan	183	132	-0.46773	0.6707	no
ni|645903594|nb|KK920836.1|	561449-561590	nan	nan	227	201.8	-0.166758	0.8815	no
ni|645903594|nb|KK920836.1|	56806-57158	nan	nan	2	16	3	0.0271	no
ni|645903594|nb|KK920836.1|	57757-58017	nan	nan	89	151	0.755945	0.54435	no
ni|645903594|nb|KK920836.1|	583153-584078	nan	nan	0.509279	0.372388	-0.451652	0.72335	no
ni|645903594|nb|KK920836.1|	586160-586510	nan	nan	136	153	0.17141	0.90655	no
ni|645903594|nb|KK920836.1|	587273-587674	nan	nan	52	48	-0.107506	0.92065	no
ni|645903594|nb|KK920836.1|	588085-588542	nan	nan	1	0.53123	-1	0.43485	no
ni|645903594|nb|KK920836.1|	590026-590352	nan	nan	147	138	-0.0950562	0.9453	no
ni|645903594|nb|KK920836.1|	590475-590779	nan	nan	119	124	0.0545896	0.97085	no
ni|645903594|nb|KK920836.1|	591348-591581	nan	nan	244	209	-0.226076	0.8773	no
ni|645903594|nb|KK920836.1|	618554-618892	nan	nan	2	1	-0.428157	0.73225	no
ni|645903594|nb|KK920836.1|	626797-627667	nan	nan	14	11	-0.274223	0.8017	no
ni|645903594|nb|KK920836.1|	62839-62973	nan	nan	83	156	0.913627	0.43275	no
ni|645903594|nb|KK920836.1|	628485-628623	nan	nan	32	21	-0.594485	0.60855	no
ni|645903594|nb|KK920836.1|	629228-629348	nan	nan	30	28	-0.126299	0.91405	no

ni|645903594|nb|KK920836.1|	630805-630914	nan	nan	28	43	0.642785	0.6202	no
ni|645903594|nb|KK920836.1|	631239-631380	nan	nan	39	38	-0.0451959	0.9683	no
ni|645903594|nb|KK920836.1|	631619-631811	nan	nan	25	29	0.20105	0.86215	no
ni|645903594|nb|KK920836.1|	632718-632874	nan	nan	38	35	-0.119035	0.919	no
ni|645903594|nb|KK920836.1|	633348-633735	nan	nan	14	12	-0.169498	0.88125	no
ni|645903594|nb|KK920836.1|	635268-635410	nan	nan	21	30	0.517746	0.6646	no
ni|645903594|nb|KK920836.1|	635520-635760	nan	nan	47	36	-0.377297	0.73865	no
ni|645903594|nb|KK920836.1|	637147-637471	nan	nan	10	9	-0.147186	0.89825	no
ni|645903594|nb|KK920836.1|	637585-637791	nan	nan	32	30	-0.0992758	0.9257	no
ni|645903594|nb|KK920836.1|	638851-639355	nan	nan	0.490812	0.410509	-0.257754	0.8692	no
ni|645903594|nb|KK920836.1|	641059-641332	nan	nan	1	0.688245	-1	0.44755	no
ni|645903594|nb|KK920836.1|	646801-647139	nan	nan	9	9	-0.0655873	0.9565	no
ni|645903594|nb|KK920836.1|	647738-647909	nan	nan	23	18	-0.312392	0.7895	no
ni|645903594|nb|KK920836.1|	648942-649482	nan	nan	14	13	-0.116678	0.9209	no
ni|645903594|nb|KK920836.1|	650787-650991	nan	nan	20	18	-0.13996	0.90505	no
ni|645903594|nb|KK920836.1|	651993-652140	nan	nan	35	30	-0.204705	0.86165	no
ni|645903594|nb|KK920836.1|	652386-652529	nan	nan	29	31	0.093254	0.93705	no
ni|645903594|nb|KK920836.1|	65299-65572	nan	nan	85	151	0.824115	0.5146	no
ni|645903594|nb|KK920836.1|	653193-653319	nan	nan	34	28	-0.283063	0.8139	no
ni|645903594|nb|KK920836.1|	654217-654359	nan	nan	29	26	-0.158364	0.8935	no
ni|645903594|nb|KK920836.1|	655280-655514	nan	nan	40	37	-0.0800559	0.94455	no
ni|645903594|nb|KK920836.1|	656029-656174	nan	nan	10	14	0.463309	0.70795	no
ni|645903594|nb|KK920836.1|	657153-657327	nan	nan	28	28	-0.016681	0.9891	no
ni|645903594|nb|KK920836.1|	657444-657579	nan	nan	22	29	0.404811	0.7322	no
ni|645903594|nb|KK920836.1|	658945-659142	nan	nan	29	35	0.249814	0.82995	no
ni|645903594|nb|KK920836.1|	659575-659763	nan	nan	40	42	0.0684395	0.95345	no
ni|645903594|nb|KK920836.1|	65971-66836	nan	nan	0.678603	0.432571	-0.64963	0.60395	no
ni|645903594|nb|KK920836.1|	660304-660466	nan	nan	22	18	-0.237137	0.8441	no
ni|645903594|nb|KK920836.1|	661115-661529	nan	nan	13	13	0.0359727	0.97445	no
ni|645903594|nb|KK920836.1|	664689-664944	nan	nan	3	2	-0.481167	0.7105	no

ni|645903594|nb|KK920836.1|	666957-667204	nan	nan	21	25	0.262624	0.82185	no
ni|645903594|nb|KK920836.1|	668008-668272	nan	nan	29	25	-0.217211	0.8503	no
ni|645903594|nb|KK920836.1|	669851-669977	nan	nan	32	24	-0.39991	0.73505	no
ni|645903594|nb|KK920836.1|	67028-67172	nan	nan	136	211	0.634612	0.57875	no
ni|645903594|nb|KK920836.1|	670406-670578	nan	nan	21	20	-0.0915369	0.9365	no
ni|645903594|nb|KK920836.1|	671701-671892	nan	nan	29	30	0.0387678	0.9725	no
ni|645903594|nb|KK920836.1|	68070-68597	nan	nan	50	80	0.667221	0.655	no
ni|645903594|nb|KK920836.1|	68801-70084	nan	nan	15	22	0.558959	0.65515	no
ni|645903594|nb|KK920836.1|	70360-70651	nan	nan	6	12	1	0.3977	no
ni|645903594|nb|KK920836.1|	70902-71090	nan	nan	80	112	0.481353	0.6601	no
ni|645903594|nb|KK920836.1|	71914-72036	nan	nan	85	104	0.295191	0.80515	no
ni|645903594|nb|KK920836.1|	72725-72839	nan	nan	94	66	-0.517323	0.66195	no
ni|645903594|nb|KK920836.1|	75306-75653	nan	nan	1	0.673204	-1	0.45115	no
ni|645903594|nb|KK920836.1|	75957-76257	nan	nan	12	8	-0.542225	0.6418	no
ni|645903594|nb|KK920836.1|	76410-78155	nan	nan	50	45	-0.144034	0.9189	no
ni|645903594|nb|KK920836.1|	825401-825502	nan	nan	306	213	-0.522812	0.66275	no
ni|645903594|nb|KK920836.1|	852404-852837	nan	nan	22	31	0.468959	0.6655	no
ni|645903594|nb|KK920836.1|	856728-857050	nan	nan	57	59	0.043889	0.9671	no
ni|645903594|nb|KK920836.1|	860264-860438	nan	nan	37	42	0.189033	0.87395	no
ni|645903594|nb|KK920836.1|	862055-862257	nan	nan	3	5	0.795557	0.5353	no
ni|645903594|nb|KK920836.1|	862686-862832	nan	nan	73	77	0.0645018	0.95565	no
ni|645903594|nb|KK920836.1|	863302-863443	nan	nan	5	2	-2	0.29845	no
ni|645903594|nb|KK920836.1|	883002-883909	nan	nan	0.612555	0.76143	0.313872	0.7992	no
ni|645903595|nb|KK920835.1|	103005-103479	nan	nan	32	40	0.319748	0.7635	no
ni|645903595|nb|KK920835.1|	110272-110744	nan	nan	22	26	0.27001	0.80765	no
ni|645903595|nb|KK920835.1|	112131-112615	nan	nan	30	36	0.274587	0.8019	no
ni|645903595|nb|KK920835.1|	114602-114810	nan	nan	70	83	0.233046	0.83775	no
ni|645903595|nb|KK920835.1|	119905-120109	nan	nan	50	54	0.116134	0.91595	no
ni|645903595|nb|KK920835.1|	120664-120823	nan	nan	61.37	68	0.145206	0.9008	no
ni|645903595|nb|KK920835.1|	122501-122855	nan	nan	26	31	0.210816	0.85355	no

ni|645903595|nb|KK920835.1|	125820-126169	nan	nan	42	37	-0.208495	0.8492	no
ni|645903595|nb|KK920835.1|	126504-126687	nan	nan	35	41	0.226778	0.84135	no
ni|645903595|nb|KK920835.1|	127165-127509	nan	nan	50	52	0.0419853	0.97075	no
ni|645903595|nb|KK920835.1|	127860-128394	nan	nan	1	0.927742	-0.22552	0.85555	no
ni|645903595|nb|KK920835.1|	129778-129946	nan	nan	5	7	0.554239	0.6571	no
ni|645903595|nb|KK920835.1|	131161-131368	nan	nan	0.936175	4	2	0.1847	no
ni|645903595|nb|KK920835.1|	132326-132954	nan	nan	0.984556	1	0.58717	0.613	no
ni|645903595|nb|KK920835.1|	133411-133588	nan	nan	4	2	-0.652288	0.64	no
ni|645903595|nb|KK920835.1|	134142-134721	nan	nan	1	2	1	0.3294	no
ni|645903595|nb|KK920835.1|	135508-135721	nan	nan	45	54	0.259427	0.81755	no
ni|645903595|nb|KK920835.1|	140229-140634	nan	nan	33	44	0.396328	0.7129	no
ni|645903595|nb|KK920835.1|	142500-142787	nan	nan	26	28	0.143584	0.89635	no
ni|645903595|nb|KK920835.1|	144258-145854	nan	nan	22	29	0.448182	0.75275	no
ni|645903595|nb|KK920835.1|	146380-146547	nan	nan	50	66	0.39805	0.72945	no
ni|645903595|nb|KK920835.1|	147208-147426	nan	nan	41	59	0.515862	0.64905	no
ni|645903595|nb|KK920835.1|	150882-151120	nan	nan	51	72	0.485984	0.6577	no
ni|645903595|nb|KK920835.1|	154561-154967	nan	nan	0.973493	1	0.437168	0.73605	no
ni|645903595|nb|KK920835.1|	167347-168998	nan	nan	3	6	0.956265	0.3974	no
ni|645903595|nb|KK920835.1|	169563-169700	nan	nan	7	7	-0.0672441	0.94715	no
ni|645903595|nb|KK920835.1|	169970-171001	nan	nan	3	2	-0.415069	0.72245	no
ni|645903595|nb|KK920835.1|	173174-173291	nan	nan	20	15	-0.415376	0.7377	no
ni|645903595|nb|KK920835.1|	173469-175060	nan	nan	54	41	-0.397812	0.778	no
ni|645903595|nb|KK920835.1|	175167-175924	nan	nan	5	4	-0.3153	0.7837	no
ni|645903595|nb|KK920835.1|	176575-176798	nan	nan	9	10	0.196484	0.86765	no
ni|645903595|nb|KK920835.1|	177647-177898	nan	nan	3	2	-0.758047	0.54375	no
ni|645903595|nb|KK920835.1|	178428-178541	nan	nan	122	96	-0.347655	0.7699	no
ni|645903595|nb|KK920835.1|	179697-179868	nan	nan	4	3	-0.526711	0.7071	no
ni|645903595|nb|KK920835.1|	180925-181232	nan	nan	45	41	-0.140149	0.89695	no
ni|645903595|nb|KK920835.1|	182303-182809	nan	nan	56	58	0.0357814	0.97875	no
ni|645903595|nb|KK920835.1|	183589-183874	nan	nan	121	103	-0.225969	0.88075	no

ni|645903595|nb|KK920835.1|	184699-185096	nan	nan	75	78	0.042471	0.97795	no
ni|645903595|nb|KK920835.1|	185829-186374	nan	nan	66	63	-0.0760264	0.9566	no
ni|645903595|nb|KK920835.1|	186602-186763	nan	nan	111	97	-0.189827	0.86505	no
ni|645903595|nb|KK920835.1|	187720-188246	nan	nan	60	60	0.00598711	0.9965	no
ni|645903595|nb|KK920835.1|	189093-189689	nan	nan	2	2	0.0911661	0.93675	no
ni|645903595|nb|KK920835.1|	195825-196082	nan	nan	0	4	inf	0.00055	yes
ni|645903595|nb|KK920835.1|	196284-196684	nan	nan	0	3	inf	0.00025	yes
ni|645903595|nb|KK920835.1|	196899-197096	nan	nan	0	7	inf	0.0003	yes
ni|645903595|nb|KK920835.1|	212321-214019	nan	nan	0	62	inf	5.00E-05	yes
ni|645903595|nb|KK920835.1|	272628-272846	nan	nan	2	0.80983	-1	0.4023	no
ni|645903595|nb|KK920835.1|	278464-278705	nan	nan	4	0.50751	-3	0.1498	no
ni|645903595|nb|KK920835.1|	308869-309523	nan	nan	2	1	-0.872226	0.4661	no
ni|645903595|nb|KK920835.1|	311680-312316	nan	nan	0.969798	1	0.243227	0.8361	no
ni|645903595|nb|KK920835.1|	314229-314665	nan	nan	0.738408	0.635178	-0.217258	0.87705	no
ni|645903595|nb|KK920835.1|	392379-392633	nan	nan	7	6	-0.170476	0.88315	no
ni|645903595|nb|KK920835.1|	5041-5224	nan	nan	11	8	-0.379227	0.75395	no
ni|645903595|nb|KK920835.1|	60562-60802	nan	nan	6	5	-0.162514	0.89395	no
ni|645903595|nb|KK920835.1|	60942-62243	nan	nan	0.719614	0.334251	-1	0.3761	no
ni|645903595|nb|KK920835.1|	65851-65960	nan	nan	55	24	-1	0.36895	no
ni|645903595|nb|KK920835.1|	67670-68065	nan	nan	6	4	-0.397939	0.73865	no
ni|645903595|nb|KK920835.1|	68538-69369	nan	nan	2	2	-0.018889	0.9873	no
ni|645903595|nb|KK920835.1|	69526-69941	nan	nan	4	2	-0.725822	0.53905	no
ni|645903595|nb|KK920835.1|	70067-70277	nan	nan	13	11	-0.252328	0.82565	no
ni|645903595|nb|KK920835.1|	72179-72476	nan	nan	8	6	-0.41158	0.7325	no
ni|645903595|nb|KK920835.1|	76881-77304	nan	nan	7	4	-0.642076	0.58205	no
ni|645903595|nb|KK920835.1|	77675-78500	nan	nan	148	160	0.108797	0.93705	no
ni|645903595|nb|KK920835.1|	79043-79463	nan	nan	227	260	0.198398	0.88595	no
ni|645903595|nb|KK920835.1|	82028-82245	nan	nan	415	472	0.185693	0.89395	no
ni|645903595|nb|KK920835.1|	82861-83271	nan	nan	124	136	0.132189	0.92795	no
ni|645903595|nb|KK920835.1|	83650-83963	nan	nan	101	115	0.194245	0.896	no

ni|645903595|nb|KK920835.1|	84198-84709	nan	nan	23	25	0.123738	0.91135	no
ni|645903595|nb|KK920835.1|	85827-85948	nan	nan	315	309	-0.0258721	0.98055	no
ni|645903595|nb|KK920835.1|	86156-86633	nan	nan	189	199	0.078221	0.9554	no
ni|645903595|nb|KK920835.1|	88535-88741	nan	nan	403	397	-0.0218706	0.9871	no
ni|645903595|nb|KK920835.1|	88979-89699	nan	nan	2	1	-0.45474	0.7059	no
ni|645903595|nb|KK920835.1|	901311-901467	nan	nan	4	3	-0.652159	0.66125	no
ni|645903595|nb|KK920835.1|	90151-90392	nan	nan	221	211	-0.0639748	0.9658	no
ni|645903595|nb|KK920835.1|	903021-906579	nan	nan	4	4	0.04286	0.9664	no
ni|645903595|nb|KK920835.1|	907654-907812	nan	nan	3	3	-0.0672004	0.93835	no
ni|645903595|nb|KK920835.1|	907920-908164	nan	nan	6	5	-0.36881	0.7548	no
ni|645903595|nb|KK920835.1|	908350-908514	nan	nan	6	6	-0.0672216	0.9562	no
ni|645903595|nb|KK920835.1|	91265-91641	nan	nan	0.900002	0.430049	-1	0.44755	no
ni|645903595|nb|KK920835.1|	93547-93971	nan	nan	1	1	-0.0652844	0.9589	no
ni|645903595|nb|KK920835.1|	94133-94380	nan	nan	306	296.03	-0.0487156	0.9736	no
ni|645903595|nb|KK920835.1|	95865-96025	nan	nan	508	490	-0.0507438	0.97125	no
ni|645903595|nb|KK920835.1|	96152-96299	nan	nan	312	244	-0.35291	0.74225	no
ni|645903596|nb|KK920834.1|	326334-326630	nan	nan	0	5	inf	0.00015	yes
ni|645903596|nb|KK920834.1|	381483-381978	nan	nan	24	46	0.961555	0.46985	no
ni|645903596|nb|KK920834.1|	382646-383022	nan	nan	0	5	inf	5.00E-05	yes
ni|645903596|nb|KK920834.1|	385382-387571	nan	nan	21	35	0.739886	0.5999	no
ni|645903596|nb|KK920834.1|	391345-391819	nan	nan	26	39	0.579565	0.59165	no
ni|645903596|nb|KK920834.1|	391991-392506	nan	nan	12	20	0.756766	0.50425	no
ni|645903596|nb|KK920834.1|	396015-396526	nan	nan	9	11	0.377614	0.74375	no
ni|645903596|nb|KK920834.1|	396970-397697	nan	nan	4	4	0.134983	0.90545	no
ni|645903596|nb|KK920834.1|	398142-398955	nan	nan	14	13	-0.137402	0.9017	no
ni|645903596|nb|KK920834.1|	399257-399454	nan	nan	13	15	0.191268	0.86805	no
ni|645903596|nb|KK920834.1|	399648-400205	nan	nan	5	4	-0.289889	0.8019	no
ni|645903596|nb|KK920834.1|	400560-400780	nan	nan	22	17	-0.357649	0.7595	no
ni|645903596|nb|KK920834.1|	400969-401462	nan	nan	8	7	-0.281942	0.8079	no
ni|645903596|nb|KK920834.1|	401973-402133	nan	nan	20	17	-0.222483	0.8484	no

ni|645903596|nb|KK920834.1|	408286-408559	nan	nan	2	6	1	0.27715	no
ni|645903596|nb|KK920834.1|	408753-409246	nan	nan	3	5	0.677711	0.5674	no
ni|645903596|nb|KK920834.1|	411306-411692	nan	nan	2	6	1	0.3161	no
ni|645903596|nb|KK920834.1|	411985-412301	nan	nan	9	15.85	0.818825	0.48165	no
ni|645903596|nb|KK920834.1|	414425-414573	nan	nan	9	12	0.406732	0.7474	no
ni|645903596|nb|KK920834.1|	422707-422827	nan	nan	12	21.83	0.858595	0.50475	no
ni|645903596|nb|KK920834.1|	428031-428539	nan	nan	10	16	0.664936	0.55875	no
ni|645903596|nb|KK920834.1|	433983-434180	nan	nan	11	22	1	0.3782	no
ni|645903596|nb|KK920834.1|	434580-434826	nan	nan	1.71	1	-0.580797	0.70815	no
ni|645903596|nb|KK920834.1|	436284-436491	nan	nan	11	15	0.362852	0.7558	no
ni|645903596|nb|KK920834.1|	437150-437479	nan	nan	14	17	0.265286	0.81675	no
ni|645903596|nb|KK920834.1|	437587-437751	nan	nan	7	15	1	0.3398	no
ni|645903596|nb|KK920834.1|	448891-449007	nan	nan	21	1	-4	0.0254	no
ni|645903596|nb|KK920834.1|	449067-449513	nan	nan	20	6	-2	0.12665	no
ni|645903596|nb|KK920834.1|	449717-450947	nan	nan	35	20	-0.771768	0.6042	no
ni|645903596|nb|KK920834.1|	451011-455899	nan	nan	24	17	-0.505479	0.7147	no
ni|645903596|nb|KK920834.1|	456096-456319	nan	nan	13	15	0.283034	0.80465	no
ni|645903596|nb|KK920834.1|	457028-457633	nan	nan	42	48	0.194194	0.9	no
ni|645903596|nb|KK920834.1|	461092-461274	nan	nan	25	61	1	0.28505	no
ni|645903596|nb|KK920834.1|	463935-464130	nan	nan	29	67	1	0.28955	no
ni|645903596|nb|KK920834.1|	466223-466877	nan	nan	0.580763	0.512517	-0.180348	0.89165	no
ni|645903596|nb|KK920834.1|	469817-470145	nan	nan	8	18	1	0.33445	no
ni|645903596|nb|KK920834.1|	472490-472632	nan	nan	21	23	0.161053	0.89125	no
ni|645903596|nb|KK920834.1|	472789-472922	nan	nan	29	31	0.106758	0.92485	no
ni|645903596|nb|KK920834.1|	473997-474180	nan	nan	24	22	-0.104277	0.9294	no
ni|645903596|nb|KK920834.1|	474313-474609	nan	nan	24	20	-0.294512	0.7985	no
ni|645903596|nb|KK920834.1|	474772-474906	nan	nan	19	22	0.232296	0.83965	no
ni|645903596|nb|KK920834.1|	475138-475259	nan	nan	18	8	-1	0.3768	no
ni|645903596|nb|KK920834.1|	475371-475911	nan	nan	32	28	-0.201782	0.8548	no
ni|645903596|nb|KK920834.1|	476796-477126	nan	nan	57	37	-0.627374	0.5653	no

ni|645903596|nb|KK920834.1|	477247-477433	nan	nan	39	33	-0.252159	0.8263	no
ni|645903596|nb|KK920834.1|	477997-478633	nan	nan	0.138543	5	5	0.0506	no
ni|645903596|nb|KK920834.1|	482255-482488	nan	nan	11	15	0.483802	0.68695	no
ni|645903596|nb|KK920834.1|	485263-485382	nan	nan	13.9	27	0.932581	0.46975	no
ni|645903596|nb|KK920834.1|	488205-489260	nan	nan	0.206957	0.692649	2	0.19855	no
ni|645903596|nb|KK920834.1|	489845-489963	nan	nan	16	18	0.154957	0.90275	no
ni|645903596|nb|KK920834.1|	490053-490297	nan	nan	2	5	1	0.29975	no
ni|645903596|nb|KK920834.1|	494944-495166	nan	nan	15	22	0.49903	0.6656	no
ni|645903596|nb|KK920834.1|	495743-496018	nan	nan	10	13	0.373575	0.7525	no
ni|645903596|nb|KK920834.1|	498173-499214	nan	nan	0.446342	0.778295	0.802167	0.51565	no
ni|645903596|nb|KK920834.1|	500307-500871	nan	nan	11	13	0.299993	0.78935	no
ni|645903596|nb|KK920834.1|	501179-501650	nan	nan	0	6	inf	5.00E-05	yes
ni|645903596|nb|KK920834.1|	504552-505234	nan	nan	0	4	inf	5.00E-05	yes
ni|645903596|nb|KK920834.1|	509584-509801	nan	nan	0.213858	21	7	0.1194	no
ni|645903596|nb|KK920834.1|	512818-513298	nan	nan	0.391482	9	5	0.0338	no
ni|645903596|nb|KK920834.1|	518690-518894	nan	nan	0.240855	42	7	0.11935	no
ni|645903596|nb|KK920834.1|	529273-529447	nan	nan	0.338663	44	7	0.11935	no
ni|645903596|nb|KK920834.1|	530781-532127	nan	nan	0.217981	0.511664	1	0.33795	no
ni|645903596|nb|KK920834.1|	532233-533275	nan	nan	0.288502	0.526671	0.868319	0.5007	no
ni|645903596|nb|KK920834.1|	542551-542727	nan	nan	0.32984	45	7	0.11935	no
ni|645903596|nb|KK920834.1|	550039-550198	nan	nan	0	43	inf	5.00E-05	yes
ni|645903596|nb|KK920834.1|	554860-555027	nan	nan	0	26	inf	0.00015	yes
ni|645903596|nb|KK920834.1|	564581-564791	nan	nan	0.227602	53	8	0.11935	no
ni|645903596|nb|KK920834.1|	565772-565900	nan	nan	2	50	5	0.04555	no
ni|645903596|nb|KK920834.1|	565984-566140	nan	nan	2	57	5	0.0277	no
ni|645903596|nb|KK920834.1|	569264-569431	nan	nan	1	72	6	0.03715	no
ni|645903596|nb|KK920834.1|	571133-571302	nan	nan	1	50	5	0.0373	no
ni|645903596|nb|KK920834.1|	579305-579488	nan	nan	1	57	6	0.03725	no
ni|645903596|nb|KK920834.1|	582384-582574	nan	nan	3	61	4	0.011	no
ni|645903596|nb|KK920834.1|	644741-645026	nan	nan	4	1	-1	0.3142	no

ni|645903596|nb|KK920834.1|	645558-645734	nan	nan	5	4	-0.25997	0.8388	no
ni|645903596|nb|KK920834.1|	649865-650235	nan	nan	6	5	-0.291332	0.8	no
ni|645903596|nb|KK920834.1|	651638-651950	nan	nan	1	0	-inf	0.00455	no
ni|645903596|nb|KK920834.1|	653033-653487	nan	nan	0.770726	0	-inf	0.0036	no
ni|645903596|nb|KK920834.1|	656860-657396	nan	nan	0.738753	0.434573	-0.765492	0.5835	no
ni|645903596|nb|KK920834.1|	780607-781034	nan	nan	3	2	-0.524708	0.65835	no
ni|645903596|nb|KK920834.1|	903480-903821	nan	nan	0	6	inf	5.00E-05	yes
ni|645903596|nb|KK920834.1|	903901-904279	nan	nan	0	6	inf	5.00E-05	yes
ni|645903597|nb|KK920833.1|	1000141-1000259	nan	nan	33	32	-0.0674353	0.9553	no
ni|645903597|nb|KK920833.1|	1001591-1001873	nan	nan	21	21	-0.0201015	0.9849	no
ni|645903597|nb|KK920833.1|	1004322-1004451	nan	nan	24	20	-0.281429	0.81645	no
ni|645903597|nb|KK920833.1|	1005885-1006028	nan	nan	40	33	-0.268845	0.81895	no
ni|645903597|nb|KK920833.1|	1007464-1007637	nan	nan	14	16	0.155093	0.89405	no
ni|645903597|nb|KK920833.1|	1008619-1008892	nan	nan	6	5	-0.141922	0.8999	no
ni|645903597|nb|KK920833.1|	1009310-1009684	nan	nan	4	3	-0.287828	0.80025	no
ni|645903597|nb|KK920833.1|	1009983-1010122	nan	nan	8	2	-2	0.25505	no
ni|645903597|nb|KK920833.1|	1011052-1011611	nan	nan	3	4	0.37929	0.7437	no
ni|645903597|nb|KK920833.1|	1012068-1012254	nan	nan	1	3	1	0.43015	no
ni|645903597|nb|KK920833.1|	1012662-1013337	nan	nan	4	3	-0.100469	0.92925	no
ni|645903597|nb|KK920833.1|	1014513-1015094	nan	nan	2	3	0.182951	0.8756	no
ni|645903597|nb|KK920833.1|	1015219-1016120	nan	nan	0.709731	1	0.58172	0.6331	no
ni|645903597|nb|KK920833.1|	1016312-1016738	nan	nan	2	0.873018	-1	0.4167	no
ni|645903597|nb|KK920833.1|	1016912-1017852	nan	nan	1	1	0.0609114	0.9586	no
ni|645903597|nb|KK920833.1|	1018303-1018645	nan	nan	2	1	-0.928065	0.47065	no
ni|645903597|nb|KK920833.1|	1018989-1020308	nan	nan	0.627943	0.484275	-0.374807	0.75535	no
ni|645903597|nb|KK920833.1|	1025436-1025910	nan	nan	10	9	-0.0552544	0.9611	no
ni|645903597|nb|KK920833.1|	1027091-1027267	nan	nan	3	2	-0.652287	0.66125	no
ni|645903597|nb|KK920833.1|	1028325-1028769	nan	nan	0.937484	0.964962	0.0416779	0.95995	no
ni|645903597|nb|KK920833.1|	1030359-1030745	nan	nan	10	11	0.0853874	0.9416	no
ni|645903597|nb|KK920833.1|	1030891-1031032	nan	nan	21	25	0.22976	0.8428	no

ni|645903597|nb|KK920833.1|	1032048-1032174	nan	nan	28	29	0.0257752	0.97995	no
ni|645903597|nb|KK920833.1|	1033369-1033624	nan	nan	15	16	0.0364405	0.97395	no
ni|645903597|nb|KK920833.1|	1034926-1035235	nan	nan	24	26	0.124188	0.9097	no
ni|645903597|nb|KK920833.1|	1036927-1037070	nan	nan	24	21	-0.211601	0.8563	no
ni|645903597|nb|KK920833.1|	1037875-1038112	nan	nan	21	21	0.0323454	0.9776	no
ni|645903597|nb|KK920833.1|	1041245-1041394	nan	nan	24	27	0.1292	0.9084	no
ni|645903597|nb|KK920833.1|	1041767-1041967	nan	nan	24	33	0.452047	0.6963	no
ni|645903597|nb|KK920833.1|	1048101-1048211	nan	nan	37	26	-0.482694	0.7105	no
ni|645903597|nb|KK920833.1|	214575-214806	nan	nan	4	8	0.997705	0.41905	no
ni|645903597|nb|KK920833.1|	217039-217383	nan	nan	0.305665	1	2	0.1537	no
ni|645903597|nb|KK920833.1|	217479-218006	nan	nan	0.116062	0.721131	3	0.0824	no
ni|645903597|nb|KK920833.1|	228301-228437	nan	nan	110	101	-0.113054	0.9171	no
ni|645903597|nb|KK920833.1|	233359-233599	nan	nan	60	46	-0.382853	0.73325	no
ni|645903597|nb|KK920833.1|	235129-235288	nan	nan	110	82	-0.417145	0.71205	no
ni|645903597|nb|KK920833.1|	236179-236339	nan	nan	79	64	-0.298531	0.7975	no
ni|645903597|nb|KK920833.1|	238086-238254	nan	nan	88	73	-0.267677	0.8126	no
ni|645903597|nb|KK920833.1|	239154-239446	nan	nan	24	19	-0.31475	0.78085	no
ni|645903597|nb|KK920833.1|	240467-240608	nan	nan	54	42	-0.353131	0.75835	no
ni|645903597|nb|KK920833.1|	240722-240920	nan	nan	60	54	-0.142639	0.89935	no
ni|645903597|nb|KK920833.1|	242161-242336	nan	nan	52	44	-0.233777	0.83575	no
ni|645903597|nb|KK920833.1|	243158-243354	nan	nan	25	24	-0.0373181	0.97295	no
ni|645903597|nb|KK920833.1|	243987-244125	nan	nan	59	50	-0.219241	0.8476	no
ni|645903597|nb|KK920833.1|	246000-246209	nan	nan	11	10	-0.128193	0.9124	no
ni|645903597|nb|KK920833.1|	248759-249176	nan	nan	5	4	-0.26812	0.82125	no
ni|645903597|nb|KK920833.1|	252598-253205	nan	nan	26	20	-0.354443	0.7469	no
ni|645903597|nb|KK920833.1|	256982-257095	nan	nan	63	24	-1	0.2735	no
ni|645903597|nb|KK920833.1|	257215-257432	nan	nan	271	172	-0.65275	0.6626	no
ni|645903597|nb|KK920833.1|	258018-258145	nan	nan	236	328	0.474626	0.6733	no
ni|645903597|nb|KK920833.1|	258265-258724	nan	nan	107	153.59	0.515733	0.71195	no
ni|645903597|nb|KK920833.1|	259500-259937	nan	nan	53	73	0.48149	0.69515	no

ni|645903597|nb|KK920833.1|	263389-263674	nan	nan	1	0.51462	-2	0.3149	no
ni|645903597|nb|KK920833.1|	265020-265222	nan	nan	85	88	0.0571971	0.95785	no
ni|645903597|nb|KK920833.1|	265336-265484	nan	nan	65	48	-0.444567	0.70325	no
ni|645903597|nb|KK920833.1|	272461-272730	nan	nan	0	14	inf	5.00E-05	yes
ni|645903597|nb|KK920833.1|	275308-275535	nan	nan	2	65	5	0.0144	no
ni|645903597|nb|KK920833.1|	276021-276211	nan	nan	0.835769	31	5	0.0507	no
ni|645903597|nb|KK920833.1|	279081-279451	nan	nan	0.552081	34	6	0.0332	no
ni|645903597|nb|KK920833.1|	279895-280325	nan	nan	0.526337	29	6	0.02055	no
ni|645903597|nb|KK920833.1|	285294-285611	nan	nan	0.687978	20	5	0.0339	no
ni|645903597|nb|KK920833.1|	290401-290772	nan	nan	0.55003	25	6	0.0333	no
ni|645903597|nb|KK920833.1|	291573-292123	nan	nan	1	18	4	0.00555	no
ni|645903597|nb|KK920833.1|	292340-292548	nan	nan	0.927427	53	6	0.03715	no
ni|645903597|nb|KK920833.1|	293654-293872	nan	nan	1	58	6	0.0333	no
ni|645903597|nb|KK920833.1|	295363-295638	nan	nan	0.997001	59	6	0.0205	no
ni|645903597|nb|KK920833.1|	299370-299559	nan	nan	1	62	6	0.0373	no
ni|645903597|nb|KK920833.1|	304988-305507	nan	nan	0.768875	20	5	0.00425	no
ni|645903597|nb|KK920833.1|	305618-305861	nan	nan	0.523868	5	3	0.0586	no
ni|645903597|nb|KK920833.1|	306122-307722	nan	nan	0.773414	11	4	0.0022	yes
ni|645903597|nb|KK920833.1|	308092-308965	nan	nan	0.543643	3	3	0.043	no
ni|645903597|nb|KK920833.1|	309136-309263	nan	nan	5	82	4	0.029	no
ni|645903597|nb|KK920833.1|	311213-311352	nan	nan	4	76	4	0.0351	no
ni|645903597|nb|KK920833.1|	311572-311804	nan	nan	0.567973	15	5	0.05115	no
ni|645903597|nb|KK920833.1|	319098-319366	nan	nan	0.74208	22	5	0.02735	no
ni|645903597|nb|KK920833.1|	319594-319740	nan	nan	1	44	5	0.0445	no
ni|645903597|nb|KK920833.1|	320313-320597	nan	nan	4	84	5	0.00175	yes
ni|645903597|nb|KK920833.1|	322047-322295	nan	nan	1	36	5	0.0146	no
ni|645903597|nb|KK920833.1|	324840-325021	nan	nan	4	67	4	0.0079	no
ni|645903597|nb|KK920833.1|	325709-326201	nan	nan	0.885351	12	4	0.0114	no
ni|645903597|nb|KK920833.1|	326959-327540	nan	nan	0.411891	0.738285	0.841914	0.5399	no
ni|645903597|nb|KK920833.1|	328543-328753	nan	nan	2	1	-0.289165	0.8535	no

ni|645903597|nb|KK920833.1|	330504-331224	nan	nan	0.678308	0.572222	-0.245366	0.84435	no
ni|645903597|nb|KK920833.1|	331921-332339	nan	nan	0.702407	0.596732	-0.235225	0.8697	no
ni|645903597|nb|KK920833.1|	334847-335270	nan	nan	35	32	-0.134234	0.90135	no
ni|645903597|nb|KK920833.1|	335362-335482	nan	nan	46	24	-0.923015	0.45205	no
ni|645903597|nb|KK920833.1|	336822-336974	nan	nan	38.8	32	-0.277759	0.8112	no
ni|645903597|nb|KK920833.1|	337263-338157	nan	nan	14	11	-0.34687	0.7518	no
ni|645903597|nb|KK920833.1|	338514-339191	nan	nan	9	7	-0.355814	0.7518	no
ni|645903597|nb|KK920833.1|	339448-339797	nan	nan	9	6	-0.541271	0.6405	no
ni|645903597|nb|KK920833.1|	339963-340368	nan	nan	10	9	-0.157699	0.89095	no
ni|645903597|nb|KK920833.1|	340576-340836	nan	nan	1	1	-0.0660818	0.9302	no
ni|645903597|nb|KK920833.1|	344293-345261	nan	nan	3	3	0.0148308	0.9891	no
ni|645903597|nb|KK920833.1|	346135-346396	nan	nan	4	2	-0.682744	0.58925	no
ni|645903597|nb|KK920833.1|	347442-348451	nan	nan	0.951374	0.961709	0.0155881	0.9828	no
ni|645903597|nb|KK920833.1|	348653-349034	nan	nan	0.61872	1	0.712198	0.61075	no
ni|645903597|nb|KK920833.1|	349203-349726	nan	nan	5	7	0.329557	0.7768	no
ni|645903597|nb|KK920833.1|	350652-350981	nan	nan	2	3	0.185908	0.87715	no
ni|645903597|nb|KK920833.1|	352286-352603	nan	nan	1	2	0.419735	0.73705	no
ni|645903597|nb|KK920833.1|	352829-353107	nan	nan	8	17	1	0.33725	no
ni|645903597|nb|KK920833.1|	353481-353709	nan	nan	12	29	1	0.2875	no
ni|645903597|nb|KK920833.1|	355063-355202	nan	nan	18	39	1	0.3537	no
ni|645903597|nb|KK920833.1|	357056-357278	nan	nan	11	27	1	0.2973	no
ni|645903597|nb|KK920833.1|	358210-358536	nan	nan	10	21	1	0.36295	no
ni|645903597|nb|KK920833.1|	358954-359286	nan	nan	2	2	0.374956	0.76185	no
ni|645903597|nb|KK920833.1|	359477-360014	nan	nan	9	14	0.70483	0.5319	no
ni|645903597|nb|KK920833.1|	360150-361107	nan	nan	0.519047	0.689326	0.409321	0.74735	no
ni|645903597|nb|KK920833.1|	364220-364442	nan	nan	3	2	-1	0.43485	no
ni|645903597|nb|KK920833.1|	366782-367005	nan	nan	17	24	0.495328	0.67125	no
ni|645903597|nb|KK920833.1|	368084-368295	nan	nan	10	14	0.432052	0.70695	no
ni|645903597|nb|KK920833.1|	369031-369815	nan	nan	14	16	0.188904	0.8616	no
ni|645903597|nb|KK920833.1|	369925-370350	nan	nan	11	14	0.349756	0.76345	no

ni|645903597|nb|KK920833.1|	370713-370966	nan	nan	15	23	0.63551	0.58	no
ni|645903597|nb|KK920833.1|	371265-371700	nan	nan	9	10	0.127387	0.91175	no
ni|645903597|nb|KK920833.1|	372737-372897	nan	nan	20	20	-0.0083114	0.98805	no
ni|645903597|nb|KK920833.1|	375841-376157	nan	nan	7	9	0.360567	0.75585	no
ni|645903597|nb|KK920833.1|	376288-376721	nan	nan	12	16	0.390967	0.72755	no
ni|645903597|nb|KK920833.1|	376927-377153	nan	nan	4	6	0.61157	0.6154	no
ni|645903597|nb|KK920833.1|	377467-377706	nan	nan	15	22	0.587364	0.61235	no
ni|645903597|nb|KK920833.1|	378573-378714	nan	nan	16.31	27	0.701452	0.54975	no
ni|645903597|nb|KK920833.1|	381161-382715	nan	nan	8	12	0.546176	0.61495	no
ni|645903597|nb|KK920833.1|	383050-383429	nan	nan	6	10	0.707172	0.5425	no
ni|645903597|nb|KK920833.1|	383797-384340	nan	nan	11	17	0.650506	0.5634	no
ni|645903597|nb|KK920833.1|	385999-386172	nan	nan	15	36	1	0.2853	no
ni|645903597|nb|KK920833.1|	386759-387023	nan	nan	19	28	0.57967	0.6111	no
ni|645903597|nb|KK920833.1|	388194-388700	nan	nan	9	14	0.723391	0.5254	no
ni|645903597|nb|KK920833.1|	389187-389643	nan	nan	5	7	0.602214	0.605	no
ni|645903597|nb|KK920833.1|	390805-391644	nan	nan	7	12	0.763319	0.50065	no
ni|645903597|nb|KK920833.1|	391867-392072	nan	nan	12	26	1	0.37355	no
ni|645903597|nb|KK920833.1|	393340-393565	nan	nan	15	22	0.543535	0.63935	no
ni|645903597|nb|KK920833.1|	394189-394655	nan	nan	9	16	0.82292	0.4697	no
ni|645903597|nb|KK920833.1|	396062-396617	nan	nan	9	15	0.800231	0.4865	no
ni|645903597|nb|KK920833.1|	397896-398275	nan	nan	6	9	0.761746	0.5113	no
ni|645903597|nb|KK920833.1|	400398-400571	nan	nan	17	24	0.458769	0.7031	no
ni|645903597|nb|KK920833.1|	400692-401080	nan	nan	5	9	0.921439	0.4339	no
ni|645903597|nb|KK920833.1|	401709-401923	nan	nan	11	20	0.832773	0.4866	no
ni|645903597|nb|KK920833.1|	403571-403965	nan	nan	6	10	0.812954	0.4881	no
ni|645903597|nb|KK920833.1|	404197-404356	nan	nan	12	20	0.769299	0.519	no
ni|645903597|nb|KK920833.1|	404716-404943	nan	nan	6	8	0.384175	0.7451	no
ni|645903597|nb|KK920833.1|	405135-405440	nan	nan	4	9	1	0.29525	no
ni|645903597|nb|KK920833.1|	405812-405990	nan	nan	12	17	0.530578	0.6531	no
ni|645903597|nb|KK920833.1|	406479-406636	nan	nan	11	20	0.902428	0.45345	no

ni|645903597|nb|KK920833.1|	408290-408481	nan	nan	10	20	0.9522	0.42565	no
ni|645903597|nb|KK920833.1|	408713-409019	nan	nan	9	13	0.582775	0.6181	no
ni|645903597|nb|KK920833.1|	410464-410747	nan	nan	23	17	-0.413821	0.71955	no
ni|645903597|nb|KK920833.1|	412218-412586	nan	nan	5	9	0.948897	0.4234	no
ni|645903597|nb|KK920833.1|	413276-413471	nan	nan	11	20	0.825997	0.47815	no
ni|645903597|nb|KK920833.1|	414278-414458	nan	nan	13	20	0.627835	0.5884	no
ni|645903597|nb|KK920833.1|	415247-415372	nan	nan	19	35	0.858656	0.48405	no
ni|645903597|nb|KK920833.1|	415649-415775	nan	nan	10	10	-0.0673342	0.94715	no
ni|645903597|nb|KK920833.1|	415913-416157	nan	nan	12	21	0.802508	0.4878	no
ni|645903597|nb|KK920833.1|	417114-417327	nan	nan	12	21	0.810135	0.4934	no
ni|645903597|nb|KK920833.1|	420698-420909	nan	nan	10	19	0.884337	0.4603	no
ni|645903597|nb|KK920833.1|	421361-421610	nan	nan	10	22	1	0.35435	no
ni|645903597|nb|KK920833.1|	423321-423450	nan	nan	12	23	0.983322	0.4461	no
ni|645903597|nb|KK920833.1|	424218-425345	nan	nan	4	7	0.665748	0.5585	no
ni|645903597|nb|KK920833.1|	427482-427896	nan	nan	6	10	0.608189	0.60375	no
ni|645903597|nb|KK920833.1|	428877-429000	nan	nan	3	20	3	0.0821	no
ni|645903597|nb|KK920833.1|	429077-429848	nan	nan	6	11	0.815148	0.47175	no
ni|645903597|nb|KK920833.1|	434375-434565	nan	nan	28	34	0.303461	0.79675	no
ni|645903597|nb|KK920833.1|	434687-435160	nan	nan	0.864215	1	0.69382	0.58445	no
ni|645903597|nb|KK920833.1|	436364-436541	nan	nan	6	7	0.368773	0.7635	no
ni|645903597|nb|KK920833.1|	436789-436905	nan	nan	16	21	0.379986	0.7694	no
ni|645903597|nb|KK920833.1|	437958-438401	nan	nan	12	15	0.304811	0.7886	no
ni|645903597|nb|KK920833.1|	440499-441096	nan	nan	17	20	0.226815	0.83765	no
ni|645903597|nb|KK920833.1|	442249-442537	nan	nan	3	2	-0.341497	0.7737	no
ni|645903597|nb|KK920833.1|	442762-443087	nan	nan	7	10	0.480631	0.6784	no
ni|645903597|nb|KK920833.1|	443876-444013	nan	nan	53	51	-0.0672441	0.9551	no
ni|645903597|nb|KK920833.1|	444733-444925	nan	nan	3	3	-0.0671461	0.94715	no
ni|645903597|nb|KK920833.1|	446530-446696	nan	nan	34	27	-0.365575	0.7523	no
ni|645903597|nb|KK920833.1|	446819-447041	nan	nan	29	33	0.196467	0.86625	no
ni|645903597|nb|KK920833.1|	448617-449181	nan	nan	15	16	0.120639	0.9154	no

ni|645903597|nb|KK920833.1|	449515-450251	nan	nan	10	11	0.0888463	0.93585	no
ni|645903597|nb|KK920833.1|	451772-451893	nan	nan	24	11	-1	0.38555	no
ni|645903597|nb|KK920833.1|	453890-454287	nan	nan	1147.38	1003.08	-0.193902	0.89155	no
ni|645903597|nb|KK920833.1|	455154-455574	nan	nan	1	0.667105	-1	0.39945	no
ni|645903597|nb|KK920833.1|	457173-457486	nan	nan	2	1	-1	0.393	no
ni|645903597|nb|KK920833.1|	457635-457867	nan	nan	2	1	-1	0.46615	no
ni|645903597|nb|KK920833.1|	459173-459539	nan	nan	0	3	inf	0.00045	yes
ni|645903597|nb|KK920833.1|	460991-461183	nan	nan	0	9	inf	0.0001	yes
ni|645903597|nb|KK920833.1|	463078-463348	nan	nan	0.146655	9	6	0.11965	no
ni|645903597|nb|KK920833.1|	463904-464083	nan	nan	0	5	inf	0.00065	yes
ni|645903597|nb|KK920833.1|	465088-465360	nan	nan	0.144935	6	5	0.12	no
ni|645903597|nb|KK920833.1|	466512-466769	nan	nan	0	7	inf	0.00015	yes
ni|645903597|nb|KK920833.1|	477718-478044	nan	nan	0	7	inf	5.00E-05	yes
ni|645903597|nb|KK920833.1|	492096-492501	nan	nan	6	5	-0.252421	0.8253	no
ni|645903597|nb|KK920833.1|	492791-492918	nan	nan	48	35	-0.437692	0.7083	no
ni|645903597|nb|KK920833.1|	493229-493645	nan	nan	6	4	-0.669377	0.56515	no
ni|645903597|nb|KK920833.1|	493730-494317	nan	nan	23	19	-0.251157	0.81955	no
ni|645903597|nb|KK920833.1|	494880-495190	nan	nan	27	32	0.259973	0.81915	no
ni|645903597|nb|KK920833.1|	496933-497481	nan	nan	5	5	-0.173091	0.88305	no
ni|645903597|nb|KK920833.1|	497896-498481	nan	nan	11	14	0.253071	0.8215	no
ni|645903597|nb|KK920833.1|	499338-499517	nan	nan	41	38	-0.112408	0.9218	no
ni|645903597|nb|KK920833.1|	502235-502356	nan	nan	20	20	0.0150708	0.9674	no
ni|645903597|nb|KK920833.1|	502620-503487	nan	nan	0.290069	0.21572	-0.427236	0.75965	no
ni|645903597|nb|KK920833.1|	504342-504684	nan	nan	3	3	-0.109963	0.92475	no
ni|645903597|nb|KK920833.1|	506087-507390	nan	nan	12	14	0.178214	0.86465	no
ni|645903597|nb|KK920833.1|	507527-507766	nan	nan	3	3	0.0332241	0.96125	no
ni|645903597|nb|KK920833.1|	517687-517808	nan	nan	214	97	-1	0.32625	no
ni|645903597|nb|KK920833.1|	518680-518871	nan	nan	3	2	-0.804131	0.6027	no
ni|645903597|nb|KK920833.1|	519018-519726	nan	nan	46	35	-0.411582	0.78365	no
ni|645903597|nb|KK920833.1|	519790-522166	nan	nan	9	11	0.254813	0.8638	no

ni|645903597|nb|KK920833.1|	522398-522610	nan	nan	3	1	-1	0.3479	no
ni|645903597|nb|KK920833.1|	523339-523907	nan	nan	0.688004	0.455328	-0.595512	0.64595	no
ni|645903597|nb|KK920833.1|	530163-530876	nan	nan	0.645726	0.231511	-1	0.32095	no
ni|645903597|nb|KK920833.1|	531212-531490	nan	nan	22	0.936273	-5	0.026	no
ni|645903597|nb|KK920833.1|	534281-534443	nan	nan	100	107	0.0981626	0.93075	no
ni|645903597|nb|KK920833.1|	558939-559343	nan	nan	84	86	0.0319592	0.9833	no
ni|645903597|nb|KK920833.1|	564922-565049	nan	nan	2580.69	1479.71	-0.802436	0.57505	no
ni|645903597|nb|KK920833.1|	565730-565904	nan	nan	5	2	-1	0.45115	no
ni|645903597|nb|KK920833.1|	566006-566294	nan	nan	2083.03	1205.99	-0.788462	0.5913	no
ni|645903597|nb|KK920833.1|	566441-566861	nan	nan	588.95	363	-0.699141	0.6175	no
ni|645903597|nb|KK920833.1|	566970-569213	nan	nan	538	380	-0.503705	0.6563	no
ni|645903597|nb|KK920833.1|	570086-570347	nan	nan	1715.98	1310.33	-0.389108	0.78665	no
ni|645903597|nb|KK920833.1|	570801-571057	nan	nan	2	0.916649	-0.940589	0.54025	no
ni|645903597|nb|KK920833.1|	571169-571299	nan	nan	7	5	-0.482333	0.7594	no
ni|645903597|nb|KK920833.1|	571864-572282	nan	nan	954	776.05	-0.29717	0.84235	no
ni|645903597|nb|KK920833.1|	572999-573161	nan	nan	362	289	-0.32543	0.7859	no
ni|645903597|nb|KK920833.1|	573468-573739	nan	nan	21	18	-0.22586	0.84505	no
ni|645903597|nb|KK920833.1|	573969-574348	nan	nan	14	10	-0.498376	0.6621	no
ni|645903597|nb|KK920833.1|	574726-574838	nan	nan	62	78	0.32474	0.7835	no
ni|645903597|nb|KK920833.1|	575273-575452	nan	nan	1450.2	1191.12	-0.283935	0.8401	no
ni|645903597|nb|KK920833.1|	576438-576643	nan	nan	1194.62	1007.51	-0.245758	0.86075	no
ni|645903597|nb|KK920833.1|	576961-577226	nan	nan	1444.73	1215.79	-0.248906	0.8628	no
ni|645903597|nb|KK920833.1|	586128-586291	nan	nan	0.396837	8	4	0.12175	no
ni|645903597|nb|KK920833.1|	601434-602011	nan	nan	0	1	inf	0.0003	yes
ni|645903597|nb|KK920833.1|	633163-633916	nan	nan	327	322.27	-0.0219811	0.98675	no
ni|645903597|nb|KK920833.1|	634455-634682	nan	nan	31	156	2	0.09895	no
ni|645903597|nb|KK920833.1|	637009-637222	nan	nan	46	204.09	2	0.1206	no
ni|645903597|nb|KK920833.1|	639341-639446	nan	nan	33	138	2	0.1386	no
ni|645903597|nb|KK920833.1|	640780-641034	nan	nan	32	112	2	0.11315	no
ni|645903597|nb|KK920833.1|	641458-641656	nan	nan	3	11	2	0.1177	no

ni|645903597|nb|KK920833.1|	642881-643344	nan	nan	0.683194	0.261203	-1	0.35155	no
ni|645903597|nb|KK920833.1|	643533-643945	nan	nan	2	7	2	0.14325	no
ni|645903597|nb|KK920833.1|	645090-646025	nan	nan	1	0.509424	-1	0.37705	no
ni|645903597|nb|KK920833.1|	646463-646910	nan	nan	2	0.956578	-0.650193	0.60395	no
ni|645903597|nb|KK920833.1|	647540-647779	nan	nan	55	122	1	0.2919	no
ni|645903597|nb|KK920833.1|	651530-652215	nan	nan	0	1	inf	0.0003	yes
ni|645903597|nb|KK920833.1|	652366-653011	nan	nan	0	16	inf	5.00E-05	yes
ni|645903597|nb|KK920833.1|	653121-653238	nan	nan	0	25	inf	0.0005	yes
ni|645903597|nb|KK920833.1|	653360-654522	nan	nan	26	25	-0.0531323	0.97135	no
ni|645903597|nb|KK920833.1|	654828-654956	nan	nan	17	26	0.564954	0.6425	no
ni|645903597|nb|KK920833.1|	655421-655605	nan	nan	14	11	-0.336457	0.7725	no
ni|645903597|nb|KK920833.1|	655785-656493	nan	nan	8	8	-0.0435382	0.9682	no
ni|645903597|nb|KK920833.1|	661370-661575	nan	nan	3	4	0.348163	0.7835	no
ni|645903597|nb|KK920833.1|	661688-661921	nan	nan	17	21	0.368991	0.74555	no
ni|645903597|nb|KK920833.1|	662621-663018	nan	nan	0.668783	0.479371	-0.480395	0.7594	no
ni|645903597|nb|KK920833.1|	665636-665864	nan	nan	18	29	0.736297	0.52625	no
ni|645903597|nb|KK920833.1|	673508-674180	nan	nan	3	5	0.629048	0.5951	no
ni|645903597|nb|KK920833.1|	674666-674919	nan	nan	10	10	0.0226603	0.9866	no
ni|645903597|nb|KK920833.1|	676248-676418	nan	nan	6	11	0.845268	0.49255	no
ni|645903597|nb|KK920833.1|	677292-677456	nan	nan	18	26	0.590672	0.6236	no
ni|645903597|nb|KK920833.1|	680352-680521	nan	nan	16	28	0.780737	0.5028	no
ni|645903597|nb|KK920833.1|	682007-682426	nan	nan	7	5	-0.65026	0.5768	no
ni|645903597|nb|KK920833.1|	683174-683606	nan	nan	1	1	-0.387193	0.7511	no
ni|645903597|nb|KK920833.1|	683804-684441	nan	nan	2	2	0.123551	0.9153	no
ni|645903597|nb|KK920833.1|	684585-685274	nan	nan	2	2	-0.14145	0.89825	no
ni|645903597|nb|KK920833.1|	685538-685911	nan	nan	1	2	1	0.3381	no
ni|645903597|nb|KK920833.1|	686420-687279	nan	nan	3	3	0.017012	0.98905	no
ni|645903597|nb|KK920833.1|	687658-687888	nan	nan	3	3	-0.15955	0.89795	no
ni|645903597|nb|KK920833.1|	691286-691595	nan	nan	0	23	inf	5.00E-05	yes
ni|645903597|nb|KK920833.1|	691989-692404	nan	nan	71	115	0.697768	0.6365	no

ni|645903597|nb|KK920833.1|	693151-693804	nan	nan	0.08951	12	7.12	0.0443	no
ni|645903597|nb|KK920833.1|	694446-695277	nan	nan	90	128	0.516098	0.71125	no
ni|645903597|nb|KK920833.1|	695548-695667	nan	nan	142	198.98	0.491548	0.6708	no
ni|645903597|nb|KK920833.1|	695932-696325	nan	nan	70	102.05	0.549089	0.71535	no
ni|645903597|nb|KK920833.1|	696495-697032	nan	nan	0.623661	1	0.723446	0.5686	no
ni|645903597|nb|KK920833.1|	699655-700135	nan	nan	100	126	0.342065	0.8087	no
ni|645903597|nb|KK920833.1|	708225-708557	nan	nan	0	1174.88	inf	5.00E-05	yes
ni|645903597|nb|KK920833.1|	708961-709253	nan	nan	0	188	inf	5.00E-05	yes
ni|645903597|nb|KK920833.1|	717027-717916	nan	nan	6	6	0.150855	0.89275	no
ni|645903597|nb|KK920833.1|	718044-718567	nan	nan	3	2	-0.777792	0.5088	no
ni|645903597|nb|KK920833.1|	719195-719402	nan	nan	2	2	0.392599	0.77595	no
ni|645903597|nb|KK920833.1|	719866-722238	nan	nan	1	1	-0.0158124	0.98965	no
ni|645903597|nb|KK920833.1|	724096-724379	nan	nan	3	2	-0.28829	0.8142	no
ni|645903597|nb|KK920833.1|	724513-725111	nan	nan	4	6	0.390054	0.73145	no
ni|645903597|nb|KK920833.1|	725458-725659	nan	nan	5	4	-0.619502	0.62755	no
ni|645903597|nb|KK920833.1|	726237-726662	nan	nan	1	0.729765	-0.550709	0.68265	no
ni|645903597|nb|KK920833.1|	726824-727100	nan	nan	3	3	0.407981	0.74735	no
ni|645903597|nb|KK920833.1|	728149-728418	nan	nan	4	3	-0.191535	0.87685	no
ni|645903597|nb|KK920833.1|	730350-730487	nan	nan	16	27	0.740111	0.52995	no
ni|645903597|nb|KK920833.1|	735451-735933	nan	nan	2	3	0.777306	0.508	no
ni|645903597|nb|KK920833.1|	736769-737572	nan	nan	12	18	0.562068	0.60485	no
ni|645903597|nb|KK920833.1|	739053-739641	nan	nan	0.050745	1	4.52	0.1215	no
ni|645903597|nb|KK920833.1|	745152-745474	nan	nan	3	42	4	0.00345	no
ni|645903597|nb|KK920833.1|	745872-746304	nan	nan	0.597884	0.64287	0.10466	0.93335	no
ni|645903597|nb|KK920833.1|	747646-748003	nan	nan	126.29	208	0.717984	0.6181	no
ni|645903597|nb|KK920833.1|	748414-749942	nan	nan	2	2	-0.231315	0.8418	no
ni|645903597|nb|KK920833.1|	750090-750999	nan	nan	2	2	-0.112733	0.92135	no
ni|645903597|nb|KK920833.1|	751149-751831	nan	nan	70	134.3	0.945681	0.50525	no
ni|645903597|nb|KK920833.1|	751934-752367	nan	nan	2	0.854572	-1	0.37855	no
ni|645903597|nb|KK920833.1|	754273-754464	nan	nan	494	1003.01	1	0.4763	no

ni|645903597|nb|KK920833.1|	755944-756646	nan	nan	111	261	1	0.39005	no
ni|645903597|nb|KK920833.1|	756807-757758	nan	nan	1	1	-0.16895	0.8866	no
ni|645903597|nb|KK920833.1|	758167-759372	nan	nan	2	4	0.838822	0.46955	no
ni|645903597|nb|KK920833.1|	759485-759650	nan	nan	4	6	0.517735	0.6956	no
ni|645903597|nb|KK920833.1|	759751-760469	nan	nan	1	1	-0.1106	0.92235	no
ni|645903597|nb|KK920833.1|	760630-760872	nan	nan	91	251	1	0.2535	no
ni|645903597|nb|KK920833.1|	762192-762532	nan	nan	59	149	1	0.297	no
ni|645903597|nb|KK920833.1|	763005-763114	nan	nan	268	634.06	1	0.28595	no
ni|645903597|nb|KK920833.1|	768587-769131	nan	nan	0.446387	0.853419	0.934961	0.4874	no
ni|645903597|nb|KK920833.1|	774284-774555	nan	nan	83	485	3	0.0554	no
ni|645903597|nb|KK920833.1|	778944-779976	nan	nan	68	110	0.690316	0.6207	no
ni|645903597|nb|KK920833.1|	783564-783973	nan	nan	100	167	0.736225	0.60405	no
ni|645903597|nb|KK920833.1|	784160-784469	nan	nan	1	0.682689	-0.940205	0.54025	no
ni|645903597|nb|KK920833.1|	784836-784990	nan	nan	6	4	-0.330227	0.80325	no
ni|645903597|nb|KK920833.1|	786102-786656	nan	nan	1	1	-0.126422	0.91305	no
ni|645903597|nb|KK920833.1|	786891-787038	nan	nan	723	1115.91	0.626484	0.6609	no
ni|645903597|nb|KK920833.1|	788548-788978	nan	nan	377	545.6	0.534976	0.70575	no
ni|645903597|nb|KK920833.1|	791302-791705	nan	nan	213	277	0.378005	0.78435	no
ni|645903597|nb|KK920833.1|	791812-792138	nan	nan	1	0.736175	-0.717722	0.61925	no
ni|645903597|nb|KK920833.1|	794378-794616	nan	nan	79	94	0.252953	0.8164	no
ni|645903597|nb|KK920833.1|	795656-796130	nan	nan	355	360	0.0172324	0.99005	no
ni|645903597|nb|KK920833.1|	796576-796984	nan	nan	5	5	-0.0422433	0.9707	no
ni|645903597|nb|KK920833.1|	797191-797439	nan	nan	3	3	0.114371	0.9325	no
ni|645903597|nb|KK920833.1|	804457-804599	nan	nan	15	14	-0.12611	0.91405	no
ni|645903597|nb|KK920833.1|	819589-819729	nan	nan	8	5	-0.767667	0.5799	no
ni|645903597|nb|KK920833.1|	820001-820422	nan	nan	1	2	0.239562	0.8456	no
ni|645903597|nb|KK920833.1|	821081-821607	nan	nan	3	2	-0.164605	0.891	no
ni|645903597|nb|KK920833.1|	825266-825426	nan	nan	4	4	0.222302	0.8747	no
ni|645903597|nb|KK920833.1|	825603-826133	nan	nan	5	5	-0.0497954	0.96725	no
ni|645903597|nb|KK920833.1|	826244-826627	nan	nan	2	2	-0.0654029	0.958	no

ni|645903597|nb|KK920833.1|	826801-826941	nan	nan	8	3	-1	0.3141	no
ni|645903597|nb|KK920833.1|	829129-829521	nan	nan	0.595268	1	0.934627	0.5103	no
ni|645903597|nb|KK920833.1|	829732-830169	nan	nan	1	3	1	0.3509	no
ni|645903597|nb|KK920833.1|	831456-832380	nan	nan	0.569887	0.717002	0.3313	0.7896	no
ni|645903597|nb|KK920833.1|	833121-833539	nan	nan	0.936543	1	0.256628	0.84735	no
ni|645903597|nb|KK920833.1|	833677-835255	nan	nan	1	1	-0.113877	0.92135	no
ni|645903597|nb|KK920833.1|	836201-836424	nan	nan	14	38	1	0.20865	no
ni|645903597|nb|KK920833.1|	837003-837403	nan	nan	26	20	-0.367005	0.74985	no
ni|645903597|nb|KK920833.1|	837966-838223	nan	nan	43	40	-0.0769167	0.94515	no
ni|645903597|nb|KK920833.1|	838448-839144	nan	nan	15	18	0.321485	0.76445	no
ni|645903597|nb|KK920833.1|	839316-840156	nan	nan	4	4	0.163583	0.88355	no
ni|645903597|nb|KK920833.1|	840685-842186	nan	nan	1	1	0.111734	0.92615	no
ni|645903597|nb|KK920833.1|	844708-845090	nan	nan	2	2	-0.276911	0.8205	no
ni|645903597|nb|KK920833.1|	845218-846840	nan	nan	44	53	0.281733	0.8413	no
ni|645903597|nb|KK920833.1|	846945-847656	nan	nan	56	71.23	0.334637	0.8115	no
ni|645903597|nb|KK920833.1|	847770-848133	nan	nan	29	42	0.521029	0.63505	no
ni|645903597|nb|KK920833.1|	848656-848985	nan	nan	14	17	0.300691	0.7901	no
ni|645903597|nb|KK920833.1|	850796-851083	nan	nan	15	23	0.644624	0.5761	no
ni|645903597|nb|KK920833.1|	852239-852738	nan	nan	16	24	0.602306	0.5856	no
ni|645903597|nb|KK920833.1|	852888-853043	nan	nan	37	56	0.615323	0.5926	no
ni|645903597|nb|KK920833.1|	853533-854121	nan	nan	18	19	0.114628	0.92025	no
ni|645903597|nb|KK920833.1|	854897-855143	nan	nan	40	56	0.464761	0.67985	no
ni|645903597|nb|KK920833.1|	855474-855893	nan	nan	0.544594	0.371782	-0.550724	0.71905	no
ni|645903597|nb|KK920833.1|	856682-856841	nan	nan	52	78	0.58274	0.614	no
ni|645903597|nb|KK920833.1|	859645-859793	nan	nan	61	92	0.597052	0.60045	no
ni|645903597|nb|KK920833.1|	861964-863065	nan	nan	0.345492	0.330377	-0.0645406	0.95935	no
ni|645903597|nb|KK920833.1|	863289-863843	nan	nan	1	3	1	0.3698	no
ni|645903597|nb|KK920833.1|	865285-865807	nan	nan	5	7	0.492614	0.6719	no
ni|645903597|nb|KK920833.1|	866235-867473	nan	nan	5	5	0.0798988	0.94545	no
ni|645903597|nb|KK920833.1|	868549-869169	nan	nan	6	7	0.171783	0.8836	no

ni|645903597|nb|KK920833.1|	869281-869774	nan	nan	16	16	0.0463296	0.9654	no
ni|645903597|nb|KK920833.1|	872683-873202	nan	nan	14	15	0.108948	0.92255	no
ni|645903597|nb|KK920833.1|	873848-874026	nan	nan	12	15	0.347714	0.7749	no
ni|645903597|nb|KK920833.1|	879836-880018	nan	nan	21	25	0.212816	0.8574	no
ni|645903597|nb|KK920833.1|	881700-882053	nan	nan	2	2	-0.0655198	0.959	no
ni|645903597|nb|KK920833.1|	882309-882589	nan	nan	1	1	0.223587	0.8598	no
ni|645903597|nb|KK920833.1|	885685-885824	nan	nan	15	7	-1	0.4167	no
ni|645903597|nb|KK920833.1|	885914-886224	nan	nan	8	6	-0.543052	0.64065	no
ni|645903597|nb|KK920833.1|	886467-886580	nan	nan	22	7	-2	0.25505	no
ni|645903597|nb|KK920833.1|	886913-887091	nan	nan	20	23	0.203324	0.85795	no
ni|645903597|nb|KK920833.1|	887213-887416	nan	nan	19	14	-0.416502	0.71755	no
ni|645903597|nb|KK920833.1|	887769-888291	nan	nan	20	17	-0.24648	0.82655	no
ni|645903597|nb|KK920833.1|	890504-892398	nan	nan	9	6	-0.544352	0.6134	no
ni|645903597|nb|KK920833.1|	893045-893554	nan	nan	0.969562	0.463392	-1	0.43485	no
ni|645903597|nb|KK920833.1|	895208-896131	nan	nan	8	6	-0.535335	0.6352	no
ni|645903597|nb|KK920833.1|	896870-897221	nan	nan	17	11	-0.637891	0.57595	no
ni|645903597|nb|KK920833.1|	902332-902558	nan	nan	3	2	-0.766942	0.5799	no
ni|645903597|nb|KK920833.1|	906298-906439	nan	nan	8	9	0.0323135	0.96125	no
ni|645903597|nb|KK920833.1|	909765-910055	nan	nan	584.94	452	-0.371941	0.7923	no
ni|645903597|nb|KK920833.1|	910584-911169	nan	nan	494	379	-0.380459	0.79305	no
ni|645903597|nb|KK920833.1|	911496-911614	nan	nan	24	8	-2	0.25675	no
ni|645903597|nb|KK920833.1|	911801-911940	nan	nan	30	26	-0.195557	0.8661	no
ni|645903597|nb|KK920833.1|	912049-912446	nan	nan	559	444	-0.332067	0.81255	no
ni|645903597|nb|KK920833.1|	912779-913350	nan	nan	0.210339	0.955099	2	0.133	no
ni|645903597|nb|KK920833.1|	917996-918104	nan	nan	33	45	0.434729	0.7373	no
ni|645903597|nb|KK920833.1|	919335-919541	nan	nan	35	33	-0.0668543	0.95435	no
ni|645903597|nb|KK920833.1|	919798-920300	nan	nan	21	20	-0.0523269	0.96325	no
ni|645903597|nb|KK920833.1|	923568-923974	nan	nan	94	101	0.112889	0.93625	no
ni|645903597|nb|KK920833.1|	925990-926556	nan	nan	98	101	0.0517434	0.971	no
ni|645903597|nb|KK920833.1|	928663-929004	nan	nan	193	194	0.00514653	0.99655	no

ni|645903597|nb|KK920833.1|	929085-929543	nan	nan	14	11	-0.262063	0.81875	no
ni|645903597|nb|KK920833.1|	929906-930601	nan	nan	0.873256	0.556592	-0.649784	0.60395	no
ni|645903597|nb|KK920833.1|	931665-931909	nan	nan	5	5	-0.0189415	0.98125	no
ni|645903597|nb|KK920833.1|	932150-933203	nan	nan	5	4	-0.121464	0.91685	no
ni|645903597|nb|KK920833.1|	933539-933763	nan	nan	7	5	-0.53602	0.6555	no
ni|645903597|nb|KK920833.1|	933986-934877	nan	nan	1	0.687233	-0.89864	0.46065	no
ni|645903597|nb|KK920833.1|	936350-936487	nan	nan	33	29	-0.190101	0.87335	no
ni|645903597|nb|KK920833.1|	940194-940459	nan	nan	41	42	0.0257545	0.9801	no
ni|645903597|nb|KK920833.1|	940802-940926	nan	nan	29	20	-0.533019	0.6606	no
ni|645903597|nb|KK920833.1|	943490-943665	nan	nan	33	29	-0.18761	0.8726	no
ni|645903597|nb|KK920833.1|	944285-944429	nan	nan	33	34	0.0520905	0.9616	no
ni|645903597|nb|KK920833.1|	948814-948962	nan	nan	21	37	0.784702	0.50645	no
ni|645903597|nb|KK920833.1|	949911-950128	nan	nan	42	38	-0.149111	0.89295	no
ni|645903597|nb|KK920833.1|	951863-951989	nan	nan	36	47	0.412659	0.7309	no
ni|645903597|nb|KK920833.1|	953267-953466	nan	nan	30	34	0.200102	0.8621	no
ni|645903597|nb|KK920833.1|	959352-959515	nan	nan	89	116	0.378094	0.7401	no
ni|645903597|nb|KK920833.1|	959627-960347	nan	nan	0.518706	0.495926	-0.0647937	0.9554	no
ni|645903597|nb|KK920833.1|	961185-961434	nan	nan	2	0.640051	-1	0.35155	no
ni|645903597|nb|KK920833.1|	961811-962790	nan	nan	26	46	0.811128	0.57425	no
ni|645903597|nb|KK920833.1|	978592-978981	nan	nan	0.343706	1	2	0.15655	no
ni|645903597|nb|KK920833.1|	984124-985288	nan	nan	377	329	-0.196806	0.89365	no
ni|645903597|nb|KK920833.1|	985796-985949	nan	nan	7	2	-2	0.29715	no
ni|645903597|nb|KK920833.1|	986387-986561	nan	nan	3	2	-0.804273	0.6027	no
ni|645903597|nb|KK920833.1|	986759-987084	nan	nan	1	0.739468	-0.717727	0.61925	no
ni|645903597|nb|KK920833.1|	987213-987438	nan	nan	6	2	-1	0.27985	no
ni|645903597|nb|KK920833.1|	987591-987847	nan	nan	1583.88	1303.47	-0.281101	0.8466	no
ni|645903597|nb|KK920833.1|	987975-988191	nan	nan	2808.02	2163.18	-0.376398	0.7974	no
ni|645903597|nb|KK920833.1|	988348-988511	nan	nan	4	4	-0.0672169	0.94715	no
ni|645903597|nb|KK920833.1|	989235-989467	nan	nan	2201.09	1692.72	-0.378876	0.79085	no
ni|645903597|nb|KK920833.1|	989882-990379	nan	nan	25	14	-0.846369	0.4453	no

ni|645903597|nb|KK920833.1|	990525-990937	nan	nan	39	29	-0.427886	0.69345	no
ni|645903597|nb|KK920833.1|	991188-991577	nan	nan	43	30	-0.537728	0.6209	no
ni|645903597|nb|KK920833.1|	991657-991770	nan	nan	50	45	-0.174462	0.88345	no
ni|645903597|nb|KK920833.1|	991881-992045	nan	nan	49	35	-0.505343	0.65925	no
ni|645903597|nb|KK920833.1|	992151-992384	nan	nan	42	39	-0.098817	0.93275	no
ni|645903597|nb|KK920833.1|	993011-993610	nan	nan	5	4	-0.498602	0.6704	no
ni|645903597|nb|KK920833.1|	995174-995495	nan	nan	3	4	0.171368	0.8844	no
ni|645903597|nb|KK920833.1|	996858-997253	nan	nan	2	0.563079	-2	0.2821	no
ni|645903597|nb|KK920833.1|	997617-997846	nan	nan	2	0.740197	-2	0.3149	no
ni|645903597|nb|KK920833.1|	998786-998988	nan	nan	3	2	-0.704369	0.6038	no
ni|645903597|nb|KK920833.1|	999240-999536	nan	nan	4	2	-1	0.3166	no
ni|645903598|nb|KK920832.1|	148241-148423	nan	nan	10	6	-0.762437	0.53485	no
ni|645903598|nb|KK920832.1|	158855-159013	nan	nan	0.4304	214	9	0.11935	no
ni|645903598|nb|KK920832.1|	159333-159504	nan	nan	0	309	inf	5.00E-05	yes
ni|645903598|nb|KK920832.1|	160864-161347	nan	nan	0.1942	161.37	10	0.05055	no
ni|645903598|nb|KK920832.1|	176886-177109	nan	nan	0	285.8	inf	5.00E-05	yes
ni|645903598|nb|KK920832.1|	178195-178369	nan	nan	0	274	inf	5.00E-05	yes
ni|645903598|nb|KK920832.1|	179936-180152	nan	nan	0	291	inf	5.00E-05	yes
ni|645903598|nb|KK920832.1|	183249-183967	nan	nan	0	49	inf	5.00E-05	yes
ni|645903598|nb|KK920832.1|	200003-200350	nan	nan	0.503211	2	2	0.22835	no
ni|645903598|nb|KK920832.1|	243779-243846	nan	nan	18149.4	0	-inf	0.00175	yes
ni|645903598|nb|KK920832.1|	246379-246657	nan	nan	6	3	-0.864301	0.4756	no
ni|645903598|nb|KK920832.1|	257726-257859	nan	nan	4	7	0.932729	0.5287	no
ni|645903598|nb|KK920832.1|	259788-259930	nan	nan	8	4	-0.960301	0.51205	no
ni|645903598|nb|KK920832.1|	262336-262572	nan	nan	5	3	-0.754409	0.5382	no
ni|645903598|nb|KK920832.1|	263522-263899	nan	nan	3	2	-0.524856	0.66205	no
ni|645903598|nb|KK920832.1|	265093-265579	nan	nan	3	2	-0.459424	0.70065	no
ni|645903598|nb|KK920832.1|	282274-283028	nan	nan	0.151275	2	4	0.03995	no
ni|645903598|nb|KK920832.1|	289860-290028	nan	nan	67	54	-0.29892	0.79155	no
ni|645903598|nb|KK920832.1|	290444-291398	nan	nan	14	11	-0.370878	0.7339	no

ni|645903598|nb|KK920832.1|	298074-298516	nan	nan	0.072538	3	6	0.1198	no
ni|645903598|nb|KK920832.1|	299217-299398	nan	nan	6	6	0.00308844	0.9708	no
ni|645903598|nb|KK920832.1|	300079-303128	nan	nan	20	23	0.240673	0.86475	no
ni|645903598|nb|KK920832.1|	303416-304078	nan	nan	7	10	0.504505	0.65835	no
ni|645903598|nb|KK920832.1|	305798-306325	nan	nan	6	9	0.611318	0.59255	no
ni|645903598|nb|KK920832.1|	309116-309252	nan	nan	34	34	-0.0106669	0.98865	no
ni|645903598|nb|KK920832.1|	310807-311051	nan	nan	27	41	0.596718	0.5971	no
ni|645903598|nb|KK920832.1|	311678-311829	nan	nan	40	51	0.361141	0.7572	no
ni|645903598|nb|KK920832.1|	312648-312775	nan	nan	34	39	0.176602	0.88145	no
ni|645903598|nb|KK920832.1|	312879-313051	nan	nan	16	25	0.637967	0.58625	no
ni|645903598|nb|KK920832.1|	313749-313994	nan	nan	24	33	0.459549	0.68485	no
ni|645903598|nb|KK920832.1|	314898-315036	nan	nan	22	40	0.867667	0.4631	no
ni|645903598|nb|KK920832.1|	315767-316127	nan	nan	9	17	0.889655	0.44235	no
ni|645903598|nb|KK920832.1|	318007-318138	nan	nan	28	44	0.661412	0.57825	no
ni|645903598|nb|KK920832.1|	318298-318467	nan	nan	34	47	0.4397	0.70285	no
ni|645903598|nb|KK920832.1|	319638-319808	nan	nan	6	1	-2	0.1414	no
ni|645903598|nb|KK920832.1|	320233-320431	nan	nan	41	44	0.119941	0.91675	no
ni|645903598|nb|KK920832.1|	321484-321775	nan	nan	39	37	-0.065843	0.95345	no
ni|645903598|nb|KK920832.1|	322748-323042	nan	nan	48	47	-0.0430436	0.96825	no
ni|645903598|nb|KK920832.1|	324188-325383	nan	nan	0.49621	0.388237	-0.354012	0.7757	no
ni|645903598|nb|KK920832.1|	326231-326365	nan	nan	72	80	0.146861	0.9008	no
ni|645903598|nb|KK920832.1|	326581-326748	nan	nan	51	34	-0.580311	0.6169	no
ni|645903598|nb|KK920832.1|	328496-328978	nan	nan	38	38	0.0285769	0.97745	no
ni|645903598|nb|KK920832.1|	329551-329756	nan	nan	54	29	-0.888538	0.4411	no
ni|645903598|nb|KK920832.1|	330183-330488	nan	nan	54	37	-0.542196	0.62805	no
ni|645903598|nb|KK920832.1|	335355-335703	nan	nan	10	9	-0.274128	0.81755	no
ni|645903598|nb|KK920832.1|	336426-336941	nan	nan	30	30	-0.00861578	0.99355	no
ni|645903598|nb|KK920832.1|	337487-337906	nan	nan	18	17	-0.115483	0.91725	no
ni|645903598|nb|KK920832.1|	338594-338782	nan	nan	40	47	0.219498	0.8504	no
ni|645903598|nb|KK920832.1|	341204-341773	nan	nan	7	7	0.0783686	0.94555	no

ni|645903598|nb|KK920832.1|	343063-345130	nan	nan	0.125441	189	11	0.00925	no
ni|645903598|nb|KK920832.1|	352176-352586	nan	nan	0	52.88	inf	5.00E-05	yes
ni|645903598|nb|KK920832.1|	68872-69225	nan	nan	5	9	0.83191	0.48705	no
ni|645903598|nb|KK920832.1|	691943-692599	nan	nan	0	223	inf	5.00E-05	yes
ni|645903598|nb|KK920832.1|	696730-696925	nan	nan	0	5	inf	0.0005	yes
ni|645903598|nb|KK920832.1|	71565-71995	nan	nan	4	5	0.378337	0.7482	no
ni|645903598|nb|KK920832.1|	81065-81583	nan	nan	5	7	0.478741	0.68285	no
ni|645903598|nb|KK920832.1|	82920-83052	nan	nan	15	18	0.25465	0.83635	no
ni|645903598|nb|KK920832.1|	86056-86365	nan	nan	5	6	0.156657	0.89285	no
ni|645903599|nb|KK920831.1|	107076-107203	nan	nan	339	329	-0.0444232	0.96805	no
ni|645903599|nb|KK920831.1|	114255-114454	nan	nan	359	349	-0.0397712	0.97815	no
ni|645903599|nb|KK920831.1|	118639-118918	nan	nan	87	80	-0.119845	0.9094	no
ni|645903599|nb|KK920831.1|	145201-145441	nan	nan	34	21	-0.67037	0.55865	no
ni|645903599|nb|KK920831.1|	160318-160566	nan	nan	38	24	-0.689638	0.54625	no
ni|645903599|nb|KK920831.1|	163652-163821	nan	nan	48	28	-0.76499	0.5176	no
ni|645903599|nb|KK920831.1|	164714-164898	nan	nan	8	3	-1	0.35535	no
ni|645903599|nb|KK920831.1|	182916-183081	nan	nan	10	7	-0.463156	0.6963	no
ni|645903599|nb|KK920831.1|	191202-191362	nan	nan	29	17	-0.804171	0.4983	no
ni|645903599|nb|KK920831.1|	203601-204383	nan	nan	0.217564	0.277357	0.350304	0.81755	no
ni|645903599|nb|KK920831.1|	206114-206276	nan	nan	17	15	-0.208075	0.8597	no
ni|645903599|nb|KK920831.1|	213820-214073	nan	nan	7	6	-0.168029	0.8883	no
ni|645903599|nb|KK920831.1|	216339-217609	nan	nan	2	1	-0.334088	0.77085	no
ni|645903599|nb|KK920831.1|	222758-223687	nan	nan	0.477003	0.59864	0.327691	0.78615	no
ni|645903599|nb|KK920831.1|	226229-226932	nan	nan	0.246179	0.392273	0.672153	0.65595	no
ni|645903599|nb|KK920831.1|	232351-232848	nan	nan	0.686827	0.477464	-0.524555	0.7071	no
ni|645903599|nb|KK920831.1|	233037-233158	nan	nan	24	10	-1	0.33865	no
ni|645903599|nb|KK920831.1|	239878-239998	nan	nan	20	18	-0.154868	0.90135	no
ni|645903599|nb|KK920831.1|	250754-251202	nan	nan	24	18	-0.405144	0.71285	no
ni|645903599|nb|KK920831.1|	257310-257578	nan	nan	10	6	-0.715106	0.54225	no
ni|645903599|nb|KK920831.1|	257910-258071	nan	nan	11	3	-2	0.175	no

ni|645903599|nb|KK920831.1|	258678-258909	nan	nan	12	8	-0.537731	0.65215	no
ni|645903599|nb|KK920831.1|	259043-259506	nan	nan	5	4	-0.56162	0.6325	no
ni|645903599|nb|KK920831.1|	259621-260006	nan	nan	17	8	-1	0.3786	no
ni|645903599|nb|KK920831.1|	260580-262309	nan	nan	0.378766	0.347745	-0.123276	0.91825	no
ni|645903599|nb|KK920831.1|	267643-267809	nan	nan	28	11	-1	0.25025	no
ni|645903599|nb|KK920831.1|	316274-316484	nan	nan	11	7	-0.667165	0.5725	no
ni|645903599|nb|KK920831.1|	339383-339576	nan	nan	10	8	-0.192658	0.8708	no
ni|645903599|nb|KK920831.1|	361986-362268	nan	nan	58	34	-0.788201	0.47905	no
ni|645903599|nb|KK920831.1|	362563-364068	nan	nan	2	2	-0.185175	0.8714	no
ni|645903599|nb|KK920831.1|	364176-364461	nan	nan	2	2	0.0945811	0.93935	no
ni|645903599|nb|KK920831.1|	364588-364762	nan	nan	3	4	0.311205	0.81835	no
ni|645903599|nb|KK920831.1|	364934-365331	nan	nan	2	2	-0.37348	0.7575	no
ni|645903599|nb|KK920831.1|	374885-375755	nan	nan	2	2	-0.204842	0.8627	no
ni|645903599|nb|KK920831.1|	377791-379676	nan	nan	2	1	-0.211202	0.8498	no
ni|645903599|nb|KK920831.1|	381565-385139	nan	nan	2	2	-0.466686	0.67575	no
ni|645903599|nb|KK920831.1|	386286-387617	nan	nan	2	1	-0.305472	0.793	no
ni|645903599|nb|KK920831.1|	404833-405248	nan	nan	1	0.753057	-0.550735	0.68265	no
ni|645903599|nb|KK920831.1|	406548-406769	nan	nan	2	2	0.348454	0.792	no
ni|645903599|nb|KK920831.1|	410180-410720	nan	nan	1	0.807386	-0.802011	0.52275	no
ni|645903599|nb|KK920831.1|	410936-411555	nan	nan	18	11	-0.697752	0.5307	no
ni|645903599|nb|KK920831.1|	416588-416869	nan	nan	49	31	-0.646794	0.56025	no
ni|645903599|nb|KK920831.1|	426631-427204	nan	nan	7	6	-0.330131	0.77275	no
ni|645903599|nb|KK920831.1|	431080-431200	nan	nan	19	21	0.10252	0.9329	no
ni|645903599|nb|KK920831.1|	434543-434710	nan	nan	40	29	-0.464577	0.6976	no
ni|645903599|nb|KK920831.1|	473893-474053	nan	nan	67	43	-0.656655	0.57185	no
ni|645903599|nb|KK920831.1|	477398-477982	nan	nan	47	30	-0.646777	0.60425	no
ni|645903599|nb|KK920831.1|	79917-80502	nan	nan	0.561658	0.34168	-0.717047	0.61925	no
ni|645903599|nb|KK920831.1|	81722-81846	nan	nan	404.98	280	-0.531302	0.6307	no
ni|645903599|nb|KK920831.1|	82104-82249	nan	nan	418	304	-0.458334	0.7155	no
ni|645903599|nb|KK920831.1|	85325-85541	nan	nan	320	269	-0.249897	0.8634	no

ni|645903599|nb|KK920831.1|	86054-86410	nan	nan	1	2	0.671458	0.59635	no
ni|645903599|nb|KK920831.1|	91778-92375	nan	nan	0.398571	0.476281	0.256977	0.86075	no
ni|645903599|nb|KK920831.1|	92478-93383	nan	nan	1	1	0.0348949	0.9763	no
ni|645903599|nb|KK920831.1|	94043-94255	nan	nan	556	455	-0.289514	0.8368	no
ni|645903599|nb|KK920831.1|	96604-97269	nan	nan	181	154.52	-0.224581	0.8718	no
ni|645903599|nb|KK920831.1|	97381-97579	nan	nan	394	332	-0.245409	0.86425	no
ni|645903600|nb|KK920830.1|	395249-395372	nan	nan	33	18	-0.851639	0.4851	no
ni|645903600|nb|KK920830.1|	396171-396423	nan	nan	20	16	-0.35304	0.76045	no
ni|645903600|nb|KK920830.1|	396536-396746	nan	nan	32	16	-1	0.38295	no
ni|645903600|nb|KK920830.1|	399337-399640	nan	nan	19	17	-0.171263	0.881	no
ni|645903600|nb|KK920830.1|	400701-400923	nan	nan	4	5	0.34847	0.77735	no
ni|645903600|nb|KK920830.1|	402323-402676	nan	nan	13	12	-0.0986867	0.9306	no
ni|645903600|nb|KK920830.1|	403803-404402	nan	nan	13	13	0.00389041	0.9971	no
ni|645903600|nb|KK920830.1|	407381-407605	nan	nan	5	5	0.103391	0.9282	no
ni|645903600|nb|KK920830.1|	411493-411610	nan	nan	17	23	0.435047	0.7373	no
ni|645903600|nb|KK920830.1|	485780-485955	nan	nan	13	3	-2	0.11555	no
ni|645903600|nb|KK920830.1|	486394-486595	nan	nan	25	17	-0.581534	0.6193	no
ni|645903600|nb|KK920830.1|	487118-488102	nan	nan	0.950398	0.641494	-0.567095	0.63835	no
ni|645903600|nb|KK920830.1|	488498-488748	nan	nan	4	2	-1	0.369	no
ni|645903600|nb|KK920830.1|	489387-489631	nan	nan	8	4	-1	0.3368	no
ni|645903600|nb|KK920830.1|	490607-490831	nan	nan	8	6	-0.351937	0.75935	no
ni|645903600|nb|KK920830.1|	491048-492321	nan	nan	3	3	-0.291446	0.7984	no
ni|645903600|nb|KK920830.1|	493729-494479	nan	nan	8	6	-0.457908	0.68925	no
ni|645903600|nb|KK920830.1|	494604-494741	nan	nan	7	3	-1	0.4064	no
ni|645903600|nb|KK920830.1|	495360-495699	nan	nan	3	3.38	-0.0225138	0.98165	no
ni|645903600|nb|KK920830.1|	496288-496439	nan	nan	5	3	-0.71927	0.61925	no
ni|645903600|nb|KK920830.1|	496571-496782	nan	nan	6	3	-1	0.37695	no
ni|645903600|nb|KK920830.1|	499908-500851	nan	nan	11	9	-0.283931	0.79125	no
ni|645903600|nb|KK920830.1|	506235-506473	nan	nan	28	17	-0.670396	0.56425	no
ni|645903600|nb|KK920830.1|	508186-508297	nan	nan	19	14	-0.430183	0.75965	no

ni|645903600|nb|KK920830.1|	509177-509373	nan	nan	21	14	-0.556104	0.6354	no
ni|645903600|nb|KK920830.1|	509721-509833	nan	nan	23	13	-0.845185	0.5595	no
ni|645903600|nb|KK920830.1|	510890-511232	nan	nan	1	3	1	0.37025	no
ni|645903600|nb|KK920830.1|	512322-512526	nan	nan	29	27	-0.0913491	0.9385	no
ni|645903601|nb|KK920829.1|	100245-100645	nan	nan	3	4	0.297221	0.79815	no
ni|645903601|nb|KK920829.1|	118801-118936	nan	nan	6	6	0.102668	0.93335	no
ni|645903601|nb|KK920829.1|	137393-137555	nan	nan	8	8	0.00317695	0.9708	no
ni|645903601|nb|KK920829.1|	14453-15125	nan	nan	13	9	-0.475004	0.6685	no
ni|645903601|nb|KK920829.1|	15540-15968	nan	nan	8.7	9	0.0665155	0.9515	no
ni|645903601|nb|KK920829.1|	163033-163148	nan	nan	11	20	0.82559	0.5554	no
ni|645903601|nb|KK920829.1|	21173-21359	nan	nan	21	19	-0.171077	0.88435	no
ni|645903601|nb|KK920829.1|	23984-24642	nan	nan	0.487877	0.424025	-0.20237	0.87975	no
ni|645903601|nb|KK920829.1|	24748-26271	nan	nan	0.48573	0.315209	-0.623846	0.60635	no
ni|645903601|nb|KK920829.1|	31513-31807	nan	nan	17	17	-0.044607	0.9674	no
ni|645903601|nb|KK920829.1|	401239-401636	nan	nan	0.585185	1	0.827727	0.55535	no
ni|645903601|nb|KK920829.1|	49049-49292	nan	nan	8	8	0.0540352	0.9629	no
ni|645903601|nb|KK920829.1|	500463-500759	nan	nan	2	2	-0.246383	0.84435	no
ni|645903601|nb|KK920829.1|	50659-50878	nan	nan	7	6	-0.154079	0.8951	no
ni|645903601|nb|KK920829.1|	528935-529608	nan	nan	0	3	inf	5.00E-05	yes
ni|645903601|nb|KK920829.1|	56257-56423	nan	nan	13	13	0.015228	0.98275	no
ni|645903601|nb|KK920829.1|	56611-56933	nan	nan	9	9	0.0374274	0.97455	no
ni|645903601|nb|KK920829.1|	60737-61064	nan	nan	8	8	0.0286866	0.9785	no
ni|645903601|nb|KK920829.1|	61258-61852	nan	nan	7	7	-0.0143301	0.98955	no
ni|645903601|nb|KK920829.1|	621-1020	nan	nan	0.083035	2	5	0.1203	no
ni|645903601|nb|KK920829.1|	65192-65570	nan	nan	1	0.426937	-1	0.3141	no
ni|645903601|nb|KK920829.1|	65831-66033	nan	nan	2	2	-0.581512	0.70815	no
ni|645903601|nb|KK920829.1|	67705-68029	nan	nan	1	1	-0.181133	0.889	no
ni|645903601|nb|KK920829.1|	69469-70009	nan	nan	0.84462	0.269129	-2	0.24455	no
ni|645903601|nb|KK920829.1|	70458-70601	nan	nan	6	7	0.195824	0.87955	no
ni|645903601|nb|KK920829.1|	79892-80036	nan	nan	6	6	0.0702951	0.94415	no

ni|645903601|nb|KK920829.1|	80339-80816	nan	nan	3	3	-0.435532	0.70925	no
ni|645903601|nb|KK920829.1|	81426-81911	nan	nan	2	2	-0.211988	0.8573	no
ni|645903601|nb|KK920829.1|	82422-82794	nan	nan	2	2	-0.126843	0.91305	no
ni|645903601|nb|KK920829.1|	86067-86221	nan	nan	7	8	0.102733	0.9329	no
ni|645903601|nb|KK920829.1|	91835-92065	nan	nan	6	7	0.188817	0.87005	no
ni|645903601|nb|KK920829.1|	93145-93593	nan	nan	3	5	0.812781	0.4918	no
ni|645903601|nb|KK920829.1|	95168-95521	nan	nan	2	4	1	0.2657	no
ni|645903601|nb|KK920829.1|	97626-98256	nan	nan	0.046706	0.937677	4	0.1221	no
ni|645903602|nb|KK920828.1|	171337-171564	nan	nan	5	7	0.459582	0.69205	no
ni|645903602|nb|KK920828.1|	284971-285368	nan	nan	0	7	inf	5.00E-05	yes
ni|645903602|nb|KK920828.1|	442544-442653	nan	nan	15	36	1	0.38715	no
ni|645903602|nb|KK920828.1|	540036-540152	nan	nan	10	17	0.710135	0.61225	no
ni|645903602|nb|KK920828.1|	640053-640255	nan	nan	6	5	-0.12834	0.91295	no
ni|645903602|nb|KK920828.1|	658937-659077	nan	nan	3	8	1	0.35675	no
ni|645903602|nb|KK920828.1|	663272-663487	nan	nan	2	2	-0.204186	0.87975	no
ni|645903602|nb|KK920828.1|	681401-681646	nan	nan	4	4	-0.130366	0.9135	no
ni|645903602|nb|KK920828.1|	682114-682415	nan	nan	2	3	0.571649	0.64525	no
ni|645903602|nb|KK920828.1|	682577-683058	nan	nan	3	2	-0.59567	0.6113	no
ni|645903602|nb|KK920828.1|	683180-683306	nan	nan	9	8	-0.219337	0.87705	no
ni|645903603|nb|KK920827.1|	109623-110186	nan	nan	0	6	inf	5.00E-05	yes
ni|645903603|nb|KK920827.1|	11733-11858	nan	nan	41	26	-0.652306	0.5797	no
ni|645903603|nb|KK920827.1|	11984-12169	nan	nan	12	21	0.856796	0.46735	no
ni|645903603|nb|KK920827.1|	12995-13332	nan	nan	20	29	0.489313	0.66155	no
ni|645903603|nb|KK920827.1|	13448-13811	nan	nan	28	30	0.128401	0.9057	no
ni|645903603|nb|KK920827.1|	1413-1995	nan	nan	21	31	0.572958	0.5944	no
ni|645903603|nb|KK920827.1|	143807-144024	nan	nan	7	3	-1	0.2814	no
ni|645903603|nb|KK920827.1|	14472-14785	nan	nan	18	25	0.496781	0.6594	no
ni|645903603|nb|KK920827.1|	15587-15783	nan	nan	32	32	0.0134245	0.99035	no
ni|645903603|nb|KK920827.1|	156988-157362	nan	nan	7	4	-0.669507	0.56515	no
ni|645903603|nb|KK920827.1|	175258-175532	nan	nan	0	4	inf	0.00025	yes

ni|645903603|nb|KK920827.1|	18509-18882	nan	nan	12	16	0.443575	0.69875	no
ni|645903603|nb|KK920827.1|	19103-19621	nan	nan	10	13	0.337243	0.7676	no
ni|645903603|nb|KK920827.1|	19954-20161	nan	nan	8	9	0.174176	0.8835	no
ni|645903603|nb|KK920827.1|	20776-20994	nan	nan	22	32	0.532005	0.6425	no
ni|645903603|nb|KK920827.1|	21393-21591	nan	nan	20	25	0.301622	0.795	no
ni|645903603|nb|KK920827.1|	22405-23147	nan	nan	18	19	0.0404114	0.9701	no
ni|645903603|nb|KK920827.1|	23258-23470	nan	nan	7	7	-0.0236665	0.98135	no
ni|645903603|nb|KK920827.1|	23937-24104	nan	nan	19.05	22	0.214529	0.85415	no
ni|645903603|nb|KK920827.1|	245490-245711	nan	nan	6	7	0.155809	0.89315	no
ni|645903603|nb|KK920827.1|	25101-25440	nan	nan	17	21	0.27681	0.8083	no
ni|645903603|nb|KK920827.1|	26008-26179	nan	nan	20	15	-0.433407	0.7142	no
ni|645903603|nb|KK920827.1|	2627-2826	nan	nan	32	41	0.359531	0.75645	no
ni|645903603|nb|KK920827.1|	26809-27250	nan	nan	11	12	0.116467	0.9189	no
ni|645903603|nb|KK920827.1|	27855-28041	nan	nan	20	24	0.27156	0.8192	no
ni|645903603|nb|KK920827.1|	28456-28871	nan	nan	9	13	0.48218	0.674	no
ni|645903603|nb|KK920827.1|	30083-30263	nan	nan	18	30	0.729157	0.53925	no
ni|645903603|nb|KK920827.1|	31198-31371	nan	nan	22	28	0.325018	0.7818	no
ni|645903603|nb|KK920827.1|	32766-33159	nan	nan	6	7	0.159922	0.89075	no
ni|645903603|nb|KK920827.1|	330539-330788	nan	nan	12	9	-0.440586	0.7111	no
ni|645903603|nb|KK920827.1|	330944-331212	nan	nan	27	21	-0.38595	0.7353	no
ni|645903603|nb|KK920827.1|	331307-331465	nan	nan	82	62	-0.406225	0.7317	no
ni|645903603|nb|KK920827.1|	332131-332554	nan	nan	76	76	0.00322183	0.99765	no
ni|645903603|nb|KK920827.1|	333961-334528	nan	nan	0.901633	0.861913	-0.0649989	0.9615	no
ni|645903603|nb|KK920827.1|	334910-335157	nan	nan	82	83	0.0154161	0.9885	no
ni|645903603|nb|KK920827.1|	335407-335881	nan	nan	1	0.697082	-0.605738	0.6316	no
ni|645903603|nb|KK920827.1|	337975-338228	nan	nan	99	109	0.148717	0.8863	no
ni|645903603|nb|KK920827.1|	339680-339865	nan	nan	98	98	0.000449327	0.9977	no
ni|645903603|nb|KK920827.1|	339968-340177	nan	nan	83	82	-0.00805831	0.99275	no
ni|645903603|nb|KK920827.1|	340321-340691	nan	nan	1	2	0.349587	0.7779	no
ni|645903603|nb|KK920827.1|	34066-34179	nan	nan	7	34	2	0.12655	no

ni|645903603|nb|KK920827.1|	341000-341434	nan	nan	1	1	0.115312	0.9325	no
ni|645903603|nb|KK920827.1|	342051-342373	nan	nan	2	1	-0.365226	0.7706	no
ni|645903603|nb|KK920827.1|	343931-344088	nan	nan	100	90	-0.145201	0.9012	no
ni|645903603|nb|KK920827.1|	344965-345167	nan	nan	41	44	0.128981	0.91	no
ni|645903603|nb|KK920827.1|	3459-3807	nan	nan	34	40	0.260687	0.81405	no
ni|645903603|nb|KK920827.1|	34603-34896	nan	nan	19	25	0.390886	0.7302	no
ni|645903603|nb|KK920827.1|	357145-357399	nan	nan	5	5	0.0807024	0.94485	no
ni|645903603|nb|KK920827.1|	36017-36184	nan	nan	19	27	0.517721	0.66145	no
ni|645903603|nb|KK920827.1|	366022-366225	nan	nan	2	4	0.933082	0.4817	no
ni|645903603|nb|KK920827.1|	368409-368681	nan	nan	32	41	0.385004	0.72935	no
ni|645903603|nb|KK920827.1|	39229-39573	nan	nan	11	10	-0.105823	0.92515	no
ni|645903603|nb|KK920827.1|	40777-41041	nan	nan	23	23	-0.00946302	0.99385	no
ni|645903603|nb|KK920827.1|	41191-41405	nan	nan	18	21	0.184839	0.8748	no
ni|645903603|nb|KK920827.1|	414569-414853	nan	nan	2	3	0.349147	0.7779	no
ni|645903603|nb|KK920827.1|	41546-41875	nan	nan	22	24	0.150503	0.89285	no
ni|645903603|nb|KK920827.1|	41979-43035	nan	nan	7	10	0.420867	0.707	no
ni|645903603|nb|KK920827.1|	44014-44681	nan	nan	8	9	0.257073	0.8217	no
ni|645903603|nb|KK920827.1|	45967-47454	nan	nan	14	15	0.128447	0.93115	no
ni|645903603|nb|KK920827.1|	47532-49187	nan	nan	12	14	0.19992	0.89335	no
ni|645903603|nb|KK920827.1|	5024-5274	nan	nan	29	31	0.136912	0.90605	no
ni|645903603|nb|KK920827.1|	50273-50592	nan	nan	13	15	0.220369	0.84905	no
ni|645903603|nb|KK920827.1|	53300-53481	nan	nan	17	18	0.0795405	0.9458	no
ni|645903603|nb|KK920827.1|	53637-53851	nan	nan	18	19	0.101245	0.9315	no
ni|645903603|nb|KK920827.1|	55423-56081	nan	nan	11	13	0.233603	0.83115	no
ni|645903603|nb|KK920827.1|	5618-5835	nan	nan	32	41	0.358039	0.7578	no
ni|645903603|nb|KK920827.1|	56207-56522	nan	nan	15	16	0.146842	0.8979	no
ni|645903603|nb|KK920827.1|	56730-56999	nan	nan	15	18	0.272797	0.81505	no
ni|645903603|nb|KK920827.1|	57317-57607	nan	nan	13	16	0.287166	0.8062	no
ni|645903603|nb|KK920827.1|	58683-59017	nan	nan	9	8	-0.119378	0.9133	no
ni|645903603|nb|KK920827.1|	599-800	nan	nan	32	43	0.418466	0.71645	no

ni|645903603|nb|KK920827.1|	60366-60482	nan	nan	10	14	0.447101	0.7576	no
ni|645903603|nb|KK920827.1|	60707-60942	nan	nan	14	21	0.54949	0.64615	no
ni|645903603|nb|KK920827.1|	61536-61907	nan	nan	6	7	0.22406	0.84505	no
ni|645903603|nb|KK920827.1|	62014-62168	nan	nan	12	15	0.222314	0.85195	no
ni|645903603|nb|KK920827.1|	62272-62466	nan	nan	14	18	0.368992	0.7536	no
ni|645903603|nb|KK920827.1|	62752-63368	nan	nan	6	7	0.137706	0.9028	no
ni|645903603|nb|KK920827.1|	65160-65338	nan	nan	13	12	-0.103849	0.92885	no
ni|645903603|nb|KK920827.1|	66247-66959	nan	nan	5	6	0.186259	0.8702	no
ni|645903603|nb|KK920827.1|	68024-68192	nan	nan	10	20	0.93275	0.43665	no
ni|645903603|nb|KK920827.1|	70267-70489	nan	nan	13	16	0.250915	0.82395	no
ni|645903603|nb|KK920827.1|	71173-71635	nan	nan	5	6	0.222606	0.851	no
ni|645903603|nb|KK920827.1|	7175-7385	nan	nan	31	37	0.248729	0.8274	no
ni|645903603|nb|KK920827.1|	7659-7776	nan	nan	29	32	0.125192	0.91735	no
ni|645903603|nb|KK920827.1|	7866-8082	nan	nan	28	34	0.272645	0.81	no
ni|645903603|nb|KK920827.1|	8327-8484	nan	nan	29	34	0.245959	0.83905	no
ni|645903603|nb|KK920827.1|	9231-9806	nan	nan	20	27	0.396113	0.71445	no
ni|645903603|nb|KK920827.1|	93-246	nan	nan	37	50	0.454763	0.69585	no
ni|645903603|nb|KK920827.1|	9942-10250	nan	nan	38	40	0.0816894	0.9414	no
ni|645903604|nb|KK920826.1|	103851-104308	nan	nan	0.555792	0.53123	-0.0652084	0.9384	no
ni|645903604|nb|KK920826.1|	110936-111516	nan	nan	12	10	-0.304269	0.78725	no
ni|645903604|nb|KK920826.1|	112626-112776	nan	nan	46	31	-0.56196	0.62935	no
ni|645903604|nb|KK920826.1|	112893-113122	nan	nan	14	7	-0.87381	0.461	no
ni|645903604|nb|KK920826.1|	113383-113538	nan	nan	51	33	-0.626622	0.59385	no
ni|645903604|nb|KK920826.1|	113717-114282	nan	nan	34	28	-0.262809	0.8036	no
ni|645903604|nb|KK920826.1|	114943-115403	nan	nan	18	15	-0.332581	0.7684	no
ni|645903604|nb|KK920826.1|	116030-116235	nan	nan	40	31	-0.390939	0.7329	no
ni|645903604|nb|KK920826.1|	117345-117531	nan	nan	52	43	-0.273692	0.8078	no
ni|645903604|nb|KK920826.1|	118093-118217	nan	nan	28	32	0.169684	0.8892	no
ni|645903604|nb|KK920826.1|	118437-118602	nan	nan	52	47	-0.122089	0.91595	no
ni|645903604|nb|KK920826.1|	121049-121390	nan	nan	17	17	-0.0483981	0.9655	no

ni|645903604|nb|KK920826.1|	121543-121703	nan	nan	41	35	-0.206182	0.86055	no
ni|645903604|nb|KK920826.1|	121821-122270	nan	nan	21	20	-0.10462	0.92385	no
ni|645903604|nb|KK920826.1|	123025-123506	nan	nan	29	28	-0.0651552	0.94995	no
ni|645903604|nb|KK920826.1|	125518-125737	nan	nan	35	30	-0.212837	0.85605	no
ni|645903604|nb|KK920826.1|	129045-129213	nan	nan	35	28	-0.312362	0.7838	no
ni|645903604|nb|KK920826.1|	129498-129744	nan	nan	7	8	0.22008	0.8481	no
ni|645903604|nb|KK920826.1|	130589-130727	nan	nan	53	63	0.251123	0.83195	no
ni|645903604|nb|KK920826.1|	22981-23290	nan	nan	3	1	-1	0.33165	no
ni|645903604|nb|KK920826.1|	23680-23829	nan	nan	1014.85	606	-0.744548	0.6106	no
ni|645903604|nb|KK920826.1|	25904-26096	nan	nan	500	359	-0.479044	0.73995	no
ni|645903604|nb|KK920826.1|	29890-30044	nan	nan	1218.25	795.77	-0.614386	0.66905	no
ni|645903604|nb|KK920826.1|	46790-47368	nan	nan	1	0.941055	-0.402016	0.73545	no
ni|645903604|nb|KK920826.1|	50837-50992	nan	nan	10	10	-0.00579384	0.9752	no
ni|645903604|nb|KK920826.1|	52957-53114	nan	nan	10	6	-0.783405	0.53215	no
ni|645903604|nb|KK920826.1|	56117-56866	nan	nan	0.266778	0.291505	0.127881	0.92555	no
ni|645903604|nb|KK920826.1|	57238-57578	nan	nan	11	9	-0.292647	0.79725	no
ni|645903604|nb|KK920826.1|	57703-57970	nan	nan	13	11	-0.208625	0.85805	no
ni|645903604|nb|KK920826.1|	58072-58611	nan	nan	2	2	0.0151234	0.9828	no
ni|645903604|nb|KK920826.1|	61795-62227	nan	nan	2	2	0.0550296	0.96605	no
ni|645903604|nb|KK920826.1|	63690-63845	nan	nan	11	12	0.155198	0.8959	no
ni|645903604|nb|KK920826.1|	64427-64635	nan	nan	6	4	-0.679789	0.5787	no
ni|645903604|nb|KK920826.1|	64830-64959	nan	nan	25	20	-0.330339	0.78285	no
ni|645903604|nb|KK920826.1|	65349-65575	nan	nan	9	8	-0.228774	0.8464	no
ni|645903604|nb|KK920826.1|	70516-70699	nan	nan	12	7	-0.708829	0.557	no
ni|645903604|nb|KK920826.1|	75988-76108	nan	nan	12	11	-0.0674049	0.94635	no
ni|645903604|nb|KK920826.1|	82387-82582	nan	nan	10	9	-0.14509	0.8979	no
ni|645903604|nb|KK920826.1|	95082-95513	nan	nan	0.749626	0.071647	-3	0.03815	no
ni|645903604|nb|KK920826.1|	96911-97026	nan	nan	46	33	-0.466044	0.70195	no
ni|645903604|nb|KK920826.1|	97079-97460	nan	nan	29	17	-0.777886	0.4925	no
ni|645903604|nb|KK920826.1|	97598-97937	nan	nan	28	12	-1	0.2855	no

ni|645903604|nb|KK920826.1|	98092-98232	nan	nan	6	7	0.195807	0.87955	no
ni|645903604|nb|KK920826.1|	98446-98553	nan	nan	59	30	-0.993842	0.47555	no
ni|645903604|nb|KK920826.1|	98767-98875	nan	nan	78	48	-0.705201	0.568	no
ni|645903604|nb|KK920826.1|	98991-99274	nan	nan	25	16	-0.631495	0.5833	no
ni|645903604|nb|KK920826.1|	99560-99787	nan	nan	53	37	-0.515897	0.65015	no
ni|645903604|nb|KK920826.1|	99998-100177	nan	nan	51	38	-0.44404	0.6986	no
ni|645903606|nb|KK920824.1|	100770-101546	nan	nan	0.292669	0.769523	1	0.3034	no
ni|645903606|nb|KK920824.1|	103316-103962	nan	nan	0.498646	2	2	0.0916	no
ni|645903606|nb|KK920824.1|	105733-106056	nan	nan	1	3	2	0.2448	no
ni|645903606|nb|KK920824.1|	107962-108286	nan	nan	0.444215	2	2	0.0999	no
ni|645903606|nb|KK920824.1|	108893-109099	nan	nan	2	5	2	0.28535	no
ni|645903606|nb|KK920824.1|	117698-118206	nan	nan	8	22	1	0.23175	no
ni|645903606|nb|KK920824.1|	120425-120599	nan	nan	27	55	1	0.37825	no
ni|645903606|nb|KK920824.1|	122007-122203	nan	nan	18	57	2	0.1729	no
ni|645903606|nb|KK920824.1|	122792-123196	nan	nan	9	24	1	0.23755	no
ni|645903606|nb|KK920824.1|	128113-128300	nan	nan	30	78	1	0.22915	no
ni|645903606|nb|KK920824.1|	129255-129377	nan	nan	13	36	1	0.26525	no
ni|645903606|nb|KK920824.1|	129711-129910	nan	nan	19	49	1	0.25225	no
ni|645903606|nb|KK920824.1|	132139-132274	nan	nan	13	48	2	0.14235	no
ni|645903606|nb|KK920824.1|	135269-136147	nan	nan	12	37	2	0.20845	no
ni|645903606|nb|KK920824.1|	13996-14171	nan	nan	166	225	0.437919	0.68075	no
ni|645903606|nb|KK920824.1|	189967-191257	nan	nan	2	3	0.29209	0.80295	no
ni|645903606|nb|KK920824.1|	23213-23432	nan	nan	46	64	0.478453	0.668	no
ni|645903606|nb|KK920824.1|	29933-30332	nan	nan	17	25	0.566455	0.62125	no
ni|645903606|nb|KK920824.1|	36376-37140	nan	nan	184	228	0.308187	0.8263	no
ni|645903606|nb|KK920824.1|	41113-41457	nan	nan	0.203777	55	8	0.0443	no
ni|645903606|nb|KK920824.1|	46605-46794	nan	nan	0	491	inf	5.00E-05	yes
ni|645903606|nb|KK920824.1|	54868-55081	nan	nan	0	491	inf	5.00E-05	yes
ni|645903606|nb|KK920824.1|	59350-59682	nan	nan	0	10	inf	5.00E-05	yes
ni|645903606|nb|KK920824.1|	7928-8185	nan	nan	23	11	-1	0.37705	no

ni|645903606|nb|KK920824.1|	95148-95624	nan	nan	8	13	0.683873	0.558	no
ni|645903606|nb|KK920824.1|	95683-96332	nan	nan	10	12	0.260825	0.8186	no
ni|645903606|nb|KK920824.1|	96459-96595	nan	nan	11	4	-1	0.32095	no
ni|645903607|nb|KK920823.1|	100655-101055	nan	nan	0.496541	0.790918	0.671616	0.65595	no
ni|645903607|nb|KK920823.1|	101681-101906	nan	nan	78	61	-0.352732	0.7499	no
ni|645903607|nb|KK920823.1|	103903-104114	nan	nan	141	117	-0.262379	0.80295	no
ni|645903607|nb|KK920823.1|	106790-106997	nan	nan	199	161.33	-0.305687	0.84145	no
ni|645903607|nb|KK920823.1|	108408-108567	nan	nan	154.06	118	-0.390122	0.72305	no
ni|645903607|nb|KK920823.1|	108862-109078	nan	nan	137	111	-0.300462	0.7763	no
ni|645903607|nb|KK920823.1|	109977-110182	nan	nan	219	186	-0.238173	0.87175	no
ni|645903607|nb|KK920823.1|	110457-110872	nan	nan	2	4	0.934692	0.43315	no
ni|645903607|nb|KK920823.1|	111036-111382	nan	nan	33	28	-0.239921	0.8287	no
ni|645903607|nb|KK920823.1|	113527-113738	nan	nan	146	120	-0.285434	0.7853	no
ni|645903607|nb|KK920823.1|	115862-116232	nan	nan	45	34	-0.402952	0.71545	no
ni|645903607|nb|KK920823.1|	119922-120030	nan	nan	116.82	111	-0.067771	0.9541	no
ni|645903607|nb|KK920823.1|	120731-120887	nan	nan	149	102	-0.548323	0.62565	no
ni|645903607|nb|KK920823.1|	123337-123600	nan	nan	3	3	0.223451	0.85915	no
ni|645903607|nb|KK920823.1|	124398-124625	nan	nan	4	3	-0.288879	0.8142	no
ni|645903607|nb|KK920823.1|	126684-126909	nan	nan	5	6	0.25541	0.8267	no
ni|645903607|nb|KK920823.1|	128013-128479	nan	nan	1	2	0.34985	0.76915	no
ni|645903607|nb|KK920823.1|	129968-130160	nan	nan	2	3	0.347891	0.7963	no
ni|645903607|nb|KK920823.1|	131861-132167	nan	nan	0.966668	1	0.393679	0.77595	no
ni|645903607|nb|KK920823.1|	137240-137593	nan	nan	0.785913	3	2	0.19855	no
ni|645903607|nb|KK920823.1|	233704-233934	nan	nan	0	87	inf	5.00E-05	yes
ni|645903607|nb|KK920823.1|	246250-246490	nan	nan	0	16	inf	5.00E-05	yes
ni|645903607|nb|KK920823.1|	246902-247192	nan	nan	0	11	inf	5.00E-05	yes
ni|645903607|nb|KK920823.1|	253895-254145	nan	nan	0	117	inf	5.00E-05	yes
ni|645903607|nb|KK920823.1|	259422-259660	nan	nan	0	8	inf	5.00E-05	yes
ni|645903607|nb|KK920823.1|	265575-265790	nan	nan	0	10	inf	5.00E-05	yes
ni|645903607|nb|KK920823.1|	267799-268206	nan	nan	0.080859	144	11	0.11935	no

ni|645903607|nb|KK920823.1|	270406-270558	nan	nan	0	257	inf	5.00E-05	yes
ni|645903607|nb|KK920823.1|	271420-271867	nan	nan	0.142975	161	10	0.0443	no
ni|645903607|nb|KK920823.1|	274288-274535	nan	nan	0	249	inf	5.00E-05	yes
ni|645903607|nb|KK920823.1|	276389-277091	nan	nan	0	114	inf	5.00E-05	yes
ni|645903607|nb|KK920823.1|	278234-278553	nan	nan	0	217	inf	5.00E-05	yes
ni|645903607|nb|KK920823.1|	282066-282265	nan	nan	0	299	inf	5.00E-05	yes
ni|645903607|nb|KK920823.1|	283599-283845	nan	nan	0	19	inf	5.00E-05	yes
ni|645903607|nb|KK920823.1|	284293-284640	nan	nan	0	99	inf	5.00E-05	yes
ni|645903607|nb|KK920823.1|	287154-287679	nan	nan	0.757967	18	5	0.00495	no
ni|645903607|nb|KK920823.1|	294141-294509	nan	nan	0	24	inf	5.00E-05	yes
ni|645903607|nb|KK920823.1|	294617-294838	nan	nan	0	28	inf	5.00E-05	yes
ni|645903607|nb|KK920823.1|	295833-296382	nan	nan	0.055174	15	8	0.11935	no
ni|645903607|nb|KK920823.1|	296744-297021	nan	nan	3	6	1	0.35335	no
ni|645903607|nb|KK920823.1|	303797-303924	nan	nan	7	37	2	0.09255	no
ni|645903607|nb|KK920823.1|	305959-306099	nan	nan	302	288	-0.0701867	0.94835	no
ni|645903607|nb|KK920823.1|	306996-307436	nan	nan	169	158	-0.101134	0.9464	no
ni|645903607|nb|KK920823.1|	309303-309451	nan	nan	66	84	0.353507	0.7602	no
ni|645903607|nb|KK920823.1|	313010-313289	nan	nan	55	53	-0.0622982	0.95375	no
ni|645903607|nb|KK920823.1|	314258-315418	nan	nan	56	57	0.021351	0.98895	no
ni|645903607|nb|KK920823.1|	315482-316608	nan	nan	39	47	0.263478	0.85335	no
ni|645903607|nb|KK920823.1|	321912-322167	nan	nan	17	25	0.549956	0.6325	no
ni|645903607|nb|KK920823.1|	322977-323252	nan	nan	23	36	0.645538	0.57015	no
ni|645903607|nb|KK920823.1|	323344-323492	nan	nan	31	45	0.533704	0.6519	no
ni|645903607|nb|KK920823.1|	324903-325306	nan	nan	36.46	45	0.294345	0.78365	no
ni|645903607|nb|KK920823.1|	327143-327970	nan	nan	6	4	-0.45105	0.69285	no
ni|645903607|nb|KK920823.1|	328225-328380	nan	nan	7	4	-0.652157	0.6241	no
ni|645903607|nb|KK920823.1|	328490-328654	nan	nan	9	6	-0.619763	0.62755	no
ni|645903607|nb|KK920823.1|	328990-329119	nan	nan	13	10	-0.273755	0.82995	no
ni|645903607|nb|KK920823.1|	34496-34878	nan	nan	39	53	0.434403	0.68535	no
ni|645903607|nb|KK920823.1|	35405-35572	nan	nan	62	80	0.362835	0.74575	no

ni|645903607|nb|KK920823.1|	35857-36265	nan	nan	0.967134	1	0.519636	0.6876	no
ni|645903607|nb|KK920823.1|	38694-38867	nan	nan	52	56	0.112148	0.92255	no
ni|645903607|nb|KK920823.1|	38985-39482	nan	nan	0.749266	0.537147	-0.48016	0.7301	no
ni|645903607|nb|KK920823.1|	40473-40740	nan	nan	121	129	0.0971596	0.949	no
ni|645903607|nb|KK920823.1|	41554-41891	nan	nan	32	36	0.17432	0.8709	no
ni|645903607|nb|KK920823.1|	43110-43253	nan	nan	55	63	0.195824	0.86455	no
ni|645903607|nb|KK920823.1|	44732-44846	nan	nan	129	107	-0.268217	0.82255	no
ni|645903607|nb|KK920823.1|	46061-46341	nan	nan	42	34	-0.297867	0.79165	no
ni|645903607|nb|KK920823.1|	47868-48095	nan	nan	12	15	0.330403	0.77475	no
ni|645903607|nb|KK920823.1|	50095-50331	nan	nan	19	18	-0.0663527	0.9508	no
ni|645903607|nb|KK920823.1|	50423-51018	nan	nan	55	40	-0.433968	0.765	no
ni|645903607|nb|KK920823.1|	81577-82831	nan	nan	5	7	0.394945	0.7197	no
ni|645903607|nb|KK920823.1|	83009-83174	nan	nan	55	62	0.172239	0.88135	no
ni|645903607|nb|KK920823.1|	93991-94229	nan	nan	53	41	-0.376079	0.7364	no
ni|645903607|nb|KK920823.1|	96657-96897	nan	nan	82	66	-0.316787	0.77435	no
ni|645903608|nb|KK920822.1|	11096-11346	nan	nan	3	5	0.753248	0.5361	no
ni|645903608|nb|KK920822.1|	207187-207351	nan	nan	4	3	-0.526653	0.7071	no
ni|645903608|nb|KK920822.1|	210981-211892	nan	nan	0.579049	0.553678	-0.064637	0.95835	no
ni|645903608|nb|KK920822.1|	219666-219967	nan	nan	106	51	-1	0.4079	no
ni|645903608|nb|KK920822.1|	220208-220318	nan	nan	235	168	-0.48737	0.674	no
ni|645903608|nb|KK920822.1|	223791-223969	nan	nan	249	153	-0.70434	0.5789	no
ni|645903608|nb|KK920822.1|	226859-227055	nan	nan	377	240	-0.654528	0.6547	no
ni|645903608|nb|KK920822.1|	229438-229799	nan	nan	76	52	-0.542315	0.66965	no
ni|645903608|nb|KK920822.1|	230166-230378	nan	nan	266	187	-0.502977	0.73625	no
ni|645903608|nb|KK920822.1|	231155-231561	nan	nan	80	61	-0.386897	0.79715	no
ni|645903608|nb|KK920822.1|	234530-234673	nan	nan	12	8	-0.482248	0.71	no
ni|645903608|nb|KK920822.1|	236394-236548	nan	nan	15	15	-0.0241236	0.9813	no
ni|645903608|nb|KK920822.1|	237531-237725	nan	nan	3	4	0.41832	0.73705	no
ni|645903608|nb|KK920822.1|	239593-239710	nan	nan	15	25	0.721043	0.5689	no
ni|645903608|nb|KK920822.1|	240676-240854	nan	nan	21	28	0.396077	0.7425	no

ni|645903608|nb|KK920822.1|	242097-242476	nan	nan	14	15	0.130648	0.90765	no
ni|645903608|nb|KK920822.1|	242598-243127	nan	nan	9	10	0.140797	0.90355	no
ni|645903608|nb|KK920822.1|	245137-245370	nan	nan	16	22	0.421398	0.71415	no
ni|645903608|nb|KK920822.1|	258868-259122	nan	nan	10	4	-1	0.3439	no
ni|645903608|nb|KK920822.1|	260188-260479	nan	nan	4	3	-0.513302	0.6711	no
ni|645903608|nb|KK920822.1|	261319-261880	nan	nan	2	1	-0.615206	0.606	no
ni|645903608|nb|KK920822.1|	261966-262089	nan	nan	9	8	-0.237293	0.8697	no
ni|645903608|nb|KK920822.1|	270221-270546	nan	nan	5	3	-0.94012	0.437	no
ni|645903608|nb|KK920822.1|	271800-272234	nan	nan	9	4	-1	0.36855	no
ni|645903608|nb|KK920822.1|	273234-273481	nan	nan	10	7	-0.556538	0.63515	no
ni|645903608|nb|KK920822.1|	274816-274970	nan	nan	11	4	-2	0.2366	no
ni|645903608|nb|KK920822.1|	275078-275273	nan	nan	12	7	-0.719164	0.55025	no
ni|645903608|nb|KK920822.1|	275699-276281	nan	nan	3	2	-0.927471	0.42845	no
ni|645903608|nb|KK920822.1|	278110-278293	nan	nan	10	2	-2	0.13085	no
ni|645903608|nb|KK920822.1|	278561-278749	nan	nan	13	8	-0.763823	0.52125	no
ni|645903608|nb|KK920822.1|	280333-280673	nan	nan	5	2	-1	0.31	no
ni|645903608|nb|KK920822.1|	283050-283179	nan	nan	23	7	-2	0.2214	no
ni|645903608|nb|KK920822.1|	283434-283713	nan	nan	13	6	-1	0.36795	no
ni|645903608|nb|KK920822.1|	283921-284205	nan	nan	1	0.258719	-2	0.3207	no
ni|645903608|nb|KK920822.1|	284551-284735	nan	nan	15	7	-1	0.39445	no
ni|645903608|nb|KK920822.1|	84376-84776	nan	nan	1	1	-0.257994	0.8388	no
ni|645903609|nb|KK920821.1|	101041-101259	nan	nan	21	21	-0.0244039	0.98385	no
ni|645903609|nb|KK920821.1|	11735-12224	nan	nan	3	2	-0.679849	0.56055	no
ni|645903609|nb|KK920821.1|	128393-128579	nan	nan	22	25	0.157825	0.895	no
ni|645903609|nb|KK920821.1|	144252-144417	nan	nan	22	17	-0.345528	0.7685	no
ni|645903609|nb|KK920821.1|	15054-15312	nan	nan	3	1	-1	0.3131	no
ni|645903609|nb|KK920821.1|	15531-15839	nan	nan	3	2	-1	0.40935	no
ni|645903609|nb|KK920821.1|	157521-157654	nan	nan	28	15	-0.884407	0.4705	no
ni|645903609|nb|KK920821.1|	16403-16719	nan	nan	15	11	-0.521067	0.65635	no
ni|645903609|nb|KK920821.1|	168841-168965	nan	nan	25	21	-0.25178	0.8379	no

ni|645903609|nb|KK920821.1|	171982-172648	nan	nan	0.612122	0.459811	-0.412779	0.76315	no
ni|645903609|nb|KK920821.1|	173536-173942	nan	nan	15	16	0.0513125	0.96235	no
ni|645903609|nb|KK920821.1|	174230-174377	nan	nan	8	3	-2	0.2437	no
ni|645903609|nb|KK920821.1|	174634-174778	nan	nan	6	4	-0.52664	0.7071	no
ni|645903609|nb|KK920821.1|	175392-175797	nan	nan	5	4	-0.115088	0.9211	no
ni|645903609|nb|KK920821.1|	175939-177171	nan	nan	0.719936	0.417249	-0.786959	0.515	no
ni|645903609|nb|KK920821.1|	177754-178325	nan	nan	0.999109	0.854562	-0.225457	0.85555	no
ni|645903609|nb|KK920821.1|	179768-180012	nan	nan	7	6	-0.138397	0.9063	no
ni|645903609|nb|KK920821.1|	181554-182002	nan	nan	38	35	-0.0955443	0.92865	no
ni|645903609|nb|KK920821.1|	182387-182496	nan	nan	20	26	0.391724	0.7763	no
ni|645903609|nb|KK920821.1|	182845-182990	nan	nan	49	41	-0.259851	0.82345	no
ni|645903609|nb|KK920821.1|	183065-183191	nan	nan	50	38	-0.402937	0.73275	no
ni|645903609|nb|KK920821.1|	183321-183808	nan	nan	36	33	-0.140612	0.8962	no
ni|645903609|nb|KK920821.1|	183961-184092	nan	nan	8	6	-0.389215	0.78555	no
ni|645903609|nb|KK920821.1|	184246-184431	nan	nan	66	68	0.0318526	0.97905	no
ni|645903609|nb|KK920821.1|	185356-185524	nan	nan	11	9	-0.281375	0.81645	no
ni|645903609|nb|KK920821.1|	197154-197673	nan	nan	27	23	-0.220931	0.84335	no
ni|645903609|nb|KK920821.1|	197855-197974	nan	nan	121	89	-0.442929	0.70235	no
ni|645903609|nb|KK920821.1|	198448-198633	nan	nan	37	35	-0.0672555	0.95125	no
ni|645903609|nb|KK920821.1|	198740-198939	nan	nan	39	43	0.116305	0.92125	no
ni|645903609|nb|KK920821.1|	199010-199156	nan	nan	22	16	-0.434936	0.71445	no
ni|645903609|nb|KK920821.1|	204978-205878	nan	nan	3	3	-0.0168155	0.98835	no
ni|645903609|nb|KK920821.1|	207399-207897	nan	nan	2	3	0.572309	0.6271	no
ni|645903609|nb|KK920821.1|	207994-208343	nan	nan	2	2	-0.501636	0.68865	no
ni|645903609|nb|KK920821.1|	208756-208995	nan	nan	4	3	-0.682983	0.5892	no
ni|645903609|nb|KK920821.1|	212313-212522	nan	nan	3	3	-0.0667921	0.9545	no
ni|645903609|nb|KK920821.1|	218184-218343	nan	nan	4	4	-0.219206	0.87705	no
ni|645903609|nb|KK920821.1|	218961-219220	nan	nan	3	1	-0.99209	0.47555	no
ni|645903609|nb|KK920821.1|	219403-219637	nan	nan	5	2	-1	0.4077	no
ni|645903609|nb|KK920821.1|	219909-220265	nan	nan	2	2	-0.419144	0.7284	no

ni|645903609|nb|KK920821.1|	220410-220967	nan	nan	3	4	0.0847313	0.94315	no
ni|645903609|nb|KK920821.1|	221026-221145	nan	nan	20	12	-0.745491	0.56925	no
ni|645903609|nb|KK920821.1|	223472-224035	nan	nan	0.588474	0.460266	-0.354512	0.7931	no
ni|645903609|nb|KK920821.1|	231970-233311	nan	nan	0.497411	0.19027	-1	0.297	no
ni|645903609|nb|KK920821.1|	236644-238107	nan	nan	0.760724	0.450355	-0.756309	0.5237	no
ni|645903609|nb|KK920821.1|	239159-239793	nan	nan	0.602609	0.576102	-0.0648979	0.9554	no
ni|645903609|nb|KK920821.1|	242504-242886	nan	nan	0.792659	0.336678	-1	0.4023	no
ni|645903609|nb|KK920821.1|	245961-246430	nan	nan	2	4	0.809288	0.4928	no
ni|645903609|nb|KK920821.1|	257929-259011	nan	nan	0.427662	0.40895	-0.0645486	0.9615	no
ni|645903609|nb|KK920821.1|	26151-26445	nan	nan	7	7	0.0391461	0.9728	no
ni|645903609|nb|KK920821.1|	268310-268813	nan	nan	14	16	0.190322	0.8683	no
ni|645903609|nb|KK920821.1|	283027-283227	nan	nan	35	32	-0.141528	0.8981	no
ni|645903609|nb|KK920821.1|	284034-284175	nan	nan	39	49	0.319801	0.7804	no
ni|645903609|nb|KK920821.1|	284301-284664	nan	nan	2	2	-0.517991	0.6643	no
ni|645903609|nb|KK920821.1|	287162-288054	nan	nan	0.873867	0.507312	-0.784541	0.5276	no
ni|645903609|nb|KK920821.1|	288169-288504	nan	nan	18	20	0.15317	0.8913	no
ni|645903609|nb|KK920821.1|	295020-295508	nan	nan	0.511158	0.488591	-0.0651408	0.9384	no
ni|645903609|nb|KK920821.1|	338612-339540	nan	nan	1	0.713529	-0.508233	0.67775	no
ni|645903609|nb|KK920821.1|	340212-340733	nan	nan	0.706343	0.731454	0.0503995	0.95725	no
ni|645903609|nb|KK920821.1|	340968-341412	nan	nan	1	0.964962	-0.0652373	0.95935	no
ni|645903609|nb|KK920821.1|	346387-350038	nan	nan	1	1	-0.0337114	0.97735	no
ni|645903609|nb|KK920821.1|	350682-350912	nan	nan	16	18	0.16097	0.8855	no
ni|645903609|nb|KK920821.1|	351221-351582	nan	nan	14	18	0.330442	0.77555	no
ni|645903609|nb|KK920821.1|	366989-367179	nan	nan	90	58	-0.645481	0.5697	no
ni|645903609|nb|KK920821.1|	367434-367598	nan	nan	28	13	-1	0.3449	no
ni|645903609|nb|KK920821.1|	367719-367832	nan	nan	50	45	-0.174462	0.88345	no
ni|645903609|nb|KK920821.1|	368345-368481	nan	nan	60	54	-0.152642	0.89725	no
ni|645903609|nb|KK920821.1|	369071-369528	nan	nan	13	12	-0.137965	0.90365	no
ni|645903609|nb|KK920821.1|	371446-372021	nan	nan	0.573538	0.29906	-0.939455	0.54025	no
ni|645903609|nb|KK920821.1|	384373-386063	nan	nan	47	45	-0.0401089	0.97745	no

ni|645903609|nb|KK920821.1|	386138-386560	nan	nan	29	26	-0.15711	0.8864	no
ni|645903609|nb|KK920821.1|	387658-388172	nan	nan	0.419035	0.801099	0.93491	0.5103	no
ni|645903609|nb|KK920821.1|	394446-394555	nan	nan	45	45	0.0102945	0.9704	no
ni|645903609|nb|KK920821.1|	395602-395779	nan	nan	35	34	-0.0403586	0.97085	no
ni|645903609|nb|KK920821.1|	396940-397611	nan	nan	0.693417	0.704372	0.0226129	0.96555	no
ni|645903609|nb|KK920821.1|	397775-398207	nan	nan	1	0.78573	-0.853761	0.50505	no
ni|645903609|nb|KK920821.1|	403611-404209	nan	nan	34	40	0.248105	0.8438	no
ni|645903609|nb|KK920821.1|	420848-421029	nan	nan	37	41	0.143119	0.89905	no
ni|645903609|nb|KK920821.1|	5700-5878	nan	nan	29	21	-0.429893	0.7124	no
ni|645903609|nb|KK920821.1|	70884-71036	nan	nan	19	14	-0.434924	0.71445	no
ni|645903609|nb|KK920821.1|	72618-73387	nan	nan	2	2	-0.00061459	0.98685	no
ni|645903609|nb|KK920821.1|	76489-77064	nan	nan	1	1	-0.000855543	0.97495	no
ni|645903609|nb|KK920821.1|	7781-8614	nan	nan	4	3	-0.521069	0.65185	no
ni|645903609|nb|KK920821.1|	84482-84998	nan	nan	1	0.626395	-0.77558	0.5539	no
ni|645903609|nb|KK920821.1|	86245-89547	nan	nan	0.831854	0.545144	-0.609693	0.60215	no
ni|645903609|nb|KK920821.1|	9502-9801	nan	nan	3	1	-0.940262	0.4651	no
ni|645903609|nb|KK920821.1|	95084-95667	nan	nan	2	1	-0.743045	0.5274	no
ni|645903610|nb|KK920820.1|	115946-116213	nan	nan	3	2	-0.246593	0.84435	no
ni|645903610|nb|KK920820.1|	520870-521513	nan	nan	44	39	-0.172962	0.9059	no
ni|645903610|nb|KK920820.1|	522688-522891	nan	nan	85	78	-0.117396	0.9152	no
ni|645903610|nb|KK920820.1|	524270-524421	nan	nan	83	66	-0.337033	0.76675	no
ni|645903610|nb|KK920820.1|	524752-524895	nan	nan	92	64	-0.531158	0.6469	no
ni|645903610|nb|KK920820.1|	525017-525219	nan	nan	52	38	-0.46611	0.68315	no
ni|645903610|nb|KK920820.1|	525501-525760	nan	nan	59	44	-0.435805	0.6958	no
ni|645903610|nb|KK920820.1|	532609-532894	nan	nan	75	61	-0.295633	0.78345	no
ni|645903610|nb|KK920820.1|	533555-533750	nan	nan	79	59	-0.430343	0.7049	no
ni|645903610|nb|KK920820.1|	535343-535755	nan	nan	14	13	-0.0570955	0.95865	no
ni|645903610|nb|KK920820.1|	536819-537176	nan	nan	25	21	-0.224981	0.838	no
ni|645903610|nb|KK920820.1|	538304-538433	nan	nan	60	54	-0.150962	0.8994	no
ni|645903610|nb|KK920820.1|	538784-539005	nan	nan	25	23.49	-0.0665838	0.95415	no

ni|645903610|nb|KK920820.1|	540254-540441	nan	nan	61	61	-0.000732559	0.99675	no
ni|645903610|nb|KK920820.1|	540586-540811	nan	nan	24	28	0.260292	0.81995	no
ni|645903610|nb|KK920820.1|	543726-543984	nan	nan	53	89	0.766409	0.4888	no
ni|645903610|nb|KK920820.1|	544108-544448	nan	nan	14	22	0.667138	0.5616	no
ni|645903610|nb|KK920820.1|	545309-545893	nan	nan	19	36	0.902478	0.41515	no
ni|645903610|nb|KK920820.1|	546278-546572	nan	nan	0.513475	4	3	0.06165	no
ni|645903610|nb|KK920820.1|	546834-547572	nan	nan	0	4	inf	5.00E-05	yes
ni|645903610|nb|KK920820.1|	549000-550847	nan	nan	0	29	inf	5.00E-05	yes
ni|645903610|nb|KK920820.1|	551394-551898	nan	nan	0.061352	12	8	0.11935	no
ni|645903610|nb|KK920820.1|	557908-558287	nan	nan	12	8	-0.604086	0.6094	no
ni|645903610|nb|KK920820.1|	58723-58960	nan	nan	0	7	inf	5.00E-05	yes
ni|645903610|nb|KK920820.1|	596928-597046	nan	nan	37	32	-0.230934	0.84785	no
ni|645903611|nb|KK920819.1|	167048-167224	nan	nan	152	166.53	0.131176	0.9052	no
ni|645903611|nb|KK920819.1|	169769-170195	nan	nan	80	97	0.2725	0.85235	no
ni|645903611|nb|KK920819.1|	170558-171082	nan	nan	0.584434	0.782122	0.420354	0.73705	no
ni|645903611|nb|KK920819.1|	33304-33445	nan	nan	14	9	-0.683894	0.5892	no
ni|645903611|nb|KK920819.1|	78560-78706	nan	nan	10	9	-0.227669	0.85555	no
ni|645903611|nb|KK920819.1|	79800-80120	nan	nan	1	1	-0.306685	0.8123	no
ni|645903611|nb|KK920819.1|	81205-81339	nan	nan	9	6	-0.597779	0.64595	no
ni|645903611|nb|KK920819.1|	81875-82012	nan	nan	14	15	0.125401	0.91735	no
ni|645903611|nb|KK920819.1|	83390-83609	nan	nan	12	12	-0.0666166	0.9531	no
ni|645903611|nb|KK920819.1|	84254-84660	nan	nan	7	7	-0.129463	0.9128	no
ni|645903611|nb|KK920819.1|	86352-86614	nan	nan	11	5	-1	0.39115	no
ni|645903611|nb|KK920819.1|	87418-87538	nan	nan	11	7	-0.652367	0.66125	no
ni|645903611|nb|KK920819.1|	92285-92415	nan	nan	3	8	1	0.38895	no
ni|645903611|nb|KK920819.1|	95043-95210	nan	nan	9	10	0.164084	0.89015	no
ni|645903612|nb|KK920818.1|	106684-106961	nan	nan	1	9	3	0.0487	no
ni|645903612|nb|KK920818.1|	113025-113196	nan	nan	1	11	3	0.0561	no
ni|645903612|nb|KK920818.1|	117324-117480	nan	nan	26	43	0.722586	0.5331	no
ni|645903612|nb|KK920818.1|	118957-119202	nan	nan	46	91	0.989412	0.3686	no

ni|645903612|nb|KK920818.1|	119603-119843	nan	nan	58	91	0.657247	0.54995	no
ni|645903612|nb|KK920818.1|	121480-121654	nan	nan	65	94	0.537555	0.63045	no
ni|645903612|nb|KK920818.1|	121934-122596	nan	nan	52	75	0.511983	0.7234	no
ni|645903612|nb|KK920818.1|	122927-123354	nan	nan	4	2	-1	0.23135	no
ni|645903612|nb|KK920818.1|	127966-128460	nan	nan	4	4	0.045054	0.96995	no
ni|645903612|nb|KK920818.1|	130427-130652	nan	nan	3	3	0.103407	0.93225	no
ni|645903612|nb|KK920818.1|	132710-132998	nan	nan	5	6	0.248246	0.83245	no
ni|645903612|nb|KK920818.1|	138880-139222	nan	nan	6	8	0.380231	0.74345	no
ni|645903612|nb|KK920818.1|	139721-139840	nan	nan	13	8	-0.581993	0.70815	no
ni|645903612|nb|KK920818.1|	139964-140318	nan	nan	2	2	-0.0655156	0.95755	no
ni|645903612|nb|KK920818.1|	140408-140677	nan	nan	4	3	-0.127405	0.91305	no
ni|645903612|nb|KK920818.1|	141262-141781	nan	nan	3	2	-0.850956	0.4775	no
ni|645903612|nb|KK920818.1|	141983-142189	nan	nan	8	4	-0.896929	0.46425	no
ni|645903612|nb|KK920818.1|	142332-142791	nan	nan	5	6	0.168286	0.8841	no
ni|645903612|nb|KK920818.1|	142859-143064	nan	nan	6	5	-0.2982	0.80195	no
ni|645903612|nb|KK920818.1|	143562-143757	nan	nan	5	8	0.517875	0.66895	no
ni|645903612|nb|KK920818.1|	143828-143996	nan	nan	6	6	0.195785	0.87315	no
ni|645903612|nb|KK920818.1|	144680-144810	nan	nan	8	6	-0.389223	0.78555	no
ni|645903612|nb|KK920818.1|	145513-145701	nan	nan	5	5	0.006785	0.9732	no
ni|645903612|nb|KK920818.1|	201921-202067	nan	nan	11	12	0.0640406	0.95955	no
ni|645903612|nb|KK920818.1|	21378-21827	nan	nan	1	0.815202	-0.567727	0.6631	no
ni|645903612|nb|KK920818.1|	58377-58726	nan	nan	11	10	-0.119476	0.91675	no
ni|645903612|nb|KK920818.1|	59012-59179	nan	nan	11	14	0.360179	0.76455	no
ni|645903612|nb|KK920818.1|	59736-59873	nan	nan	13	18	0.492183	0.68785	no
ni|645903612|nb|KK920818.1|	60353-60743	nan	nan	32	30	-0.0731991	0.9455	no
ni|645903612|nb|KK920818.1|	60999-61918	nan	nan	48	50	0.0455641	0.9745	no
ni|645903612|nb|KK920818.1|	62104-63054	nan	nan	0.988519	0.583814	-0.759759	0.53565	no
ni|645903612|nb|KK920818.1|	63521-63749	nan	nan	2	0.559523	-2	0.20695	no
ni|645903612|nb|KK920818.1|	67916-68077	nan	nan	74	89	0.267488	0.80725	no
ni|645903612|nb|KK920818.1|	68694-69231	nan	nan	0.737054	0.325181	-1	0.42305	no

ni|645903612|nb|KK920818.1|	69497-69660	nan	nan	78	83	0.0928603	0.93215	no
ni|645903612|nb|KK920818.1|	72935-73286	nan	nan	1	0.18926	-3	0.2517	no
ni|645903612|nb|KK920818.1|	73376-73789	nan	nan	34	45	0.406211	0.70105	no
ni|645903612|nb|KK920818.1|	75417-76055	nan	nan	14	10	-0.478333	0.6731	no
ni|645903612|nb|KK920818.1|	78201-78318	nan	nan	283	193	-0.555739	0.6318	no
ni|645903612|nb|KK920818.1|	78477-78660	nan	nan	2	7	2	0.21475	no
ni|645903612|nb|KK920818.1|	79488-79836	nan	nan	1	3	2	0.1919	no
ni|645903612|nb|KK920818.1|	80112-80493	nan	nan	3	8	1	0.31875	no
ni|645903612|nb|KK920818.1|	81226-81350	nan	nan	62	43	-0.506239	0.67255	no
ni|645903612|nb|KK920818.1|	81762-81898	nan	nan	87	71	-0.301048	0.7917	no
ni|645903612|nb|KK920818.1|	82832-82968	nan	nan	64	59.53	-0.0986146	0.93335	no
ni|645903612|nb|KK920818.1|	83268-83509	nan	nan	88	126	0.506094	0.62695	no
ni|645903612|nb|KK920818.1|	84302-84585	nan	nan	61	87	0.505073	0.6373	no
ni|645903612|nb|KK920818.1|	95658-96271	nan	nan	18	37	1	0.4088	no
ni|645903612|nb|KK920818.1|	97075-97844	nan	nan	33	69	1	0.4751	no
ni|645903613|nb|KK920817.1|	288229-288448	nan	nan	9	23	1	0.27485	no
ni|645903613|nb|KK920817.1|	308408-308575	nan	nan	10	16	0.69175	0.5636	no
ni|645903613|nb|KK920817.1|	309465-309623	nan	nan	9	12	0.331349	0.79065	no
ni|645903613|nb|KK920817.1|	319054-319555	nan	nan	4	8	1	0.3694	no
ni|645903613|nb|KK920817.1|	320644-321354	nan	nan	5	7	0.45535	0.69065	no
ni|645903613|nb|KK920817.1|	321490-321797	nan	nan	5	8	0.583346	0.62725	no
ni|645903613|nb|KK920817.1|	322593-322844	nan	nan	10	15	0.572949	0.6318	no
ni|645903613|nb|KK920817.1|	324169-324359	nan	nan	16	20	0.298102	0.79755	no
ni|645903613|nb|KK920817.1|	327190-327448	nan	nan	16	21	0.341984	0.76785	no
ni|645903613|nb|KK920817.1|	400655-400965	nan	nan	2	0.679416	-1	0.3464	no
ni|645903613|nb|KK920817.1|	401293-401518	nan	nan	3	1	-1	0.42295	no
ni|645903613|nb|KK920817.1|	401843-402042	nan	nan	458	348	-0.395124	0.7841	no
ni|645903613|nb|KK920817.1|	403203-404919	nan	nan	1	0.759516	-0.611872	0.5923	no
ni|645903613|nb|KK920817.1|	410124-410899	nan	nan	272	231	-0.233571	0.86745	no
ni|645903613|nb|KK920817.1|	416308-416606	nan	nan	30	36	0.23934	0.82875	no

ni|645903613|nb|KK920817.1|	431686-431845	nan	nan	66	67	0.0403774	0.97175	no
ni|645903613|nb|KK920817.1|	448405-448574	nan	nan	79	82	0.0405964	0.97075	no
ni|645903613|nb|KK920817.1|	451668-451828	nan	nan	96	98	0.0356721	0.974	no
ni|645903613|nb|KK920817.1|	452018-452625	nan	nan	0.488343	0.56022	0.198098	0.87955	no
ni|645903613|nb|KK920817.1|	452752-453392	nan	nan	0.870909	0.438213	-0.990889	0.47555	no
ni|645903613|nb|KK920817.1|	453553-454210	nan	nan	0.488757	0.552226	0.176141	0.8916	no
ni|645903613|nb|KK920817.1|	464550-465943	nan	nan	2	1	-0.43218	0.70745	no
ni|645903613|nb|KK920817.1|	466443-467003	nan	nan	2	2	-0.274464	0.81265	no
ni|645903613|nb|KK920817.1|	467427-467681	nan	nan	53	53	-0.0186919	0.9858	no
ni|645903613|nb|KK920817.1|	467990-468978	nan	nan	23	21	-0.112896	0.942	no
ni|645903613|nb|KK920817.1|	471047-471431	nan	nan	10	8	-0.417701	0.7238	no
ni|645903613|nb|KK920817.1|	471869-472017	nan	nan	9	6	-0.536685	0.67225	no
ni|645903613|nb|KK920817.1|	472408-472543	nan	nan	15	10	-0.552684	0.65835	no
ni|645903613|nb|KK920817.1|	472656-472879	nan	nan	8	8	0.0430737	0.97065	no
ni|645903613|nb|KK920817.1|	473081-473257	nan	nan	9	6	-0.552752	0.647	no
ni|645903613|nb|KK920817.1|	476596-476909	nan	nan	2	2	0.126931	0.9211	no
ni|645903613|nb|KK920817.1|	477195-477817	nan	nan	1	1	0.335623	0.77805	no
ni|645903613|nb|KK920817.1|	478231-479002	nan	nan	1	2	0.579113	0.6231	no
ni|645903613|nb|KK920817.1|	479315-479459	nan	nan	7	4	-0.652171	0.64	no
ni|645903613|nb|KK920817.1|	480327-480476	nan	nan	4	4	0.102728	0.93335	no
ni|645903613|nb|KK920817.1|	480688-481030	nan	nan	1	2	0.403917	0.75355	no
ni|645903613|nb|KK920817.1|	483101-483437	nan	nan	2	2	-0.0655971	0.9573	no
ni|645903613|nb|KK920817.1|	484393-484562	nan	nan	9	7	-0.259905	0.82995	no
ni|645903613|nb|KK920817.1|	485840-486048	nan	nan	3	4	0.447761	0.71915	no
ni|645903613|nb|KK920817.1|	489614-490018	nan	nan	3	3	-0.1478	0.90605	no
ni|645903613|nb|KK920817.1|	492117-492280	nan	nan	10	11	0.205802	0.8656	no
ni|645903613|nb|KK920817.1|	69806-70359	nan	nan	0.382791	0.365921	-0.0650227	0.9302	no
ni|645903613|nb|KK920817.1|	80585-80727	nan	nan	7	19	1	0.26525	no
ni|645903614|nb|KK920816.1|	256900-257225	nan	nan	6	5	-0.185945	0.8763	no
ni|645903614|nb|KK920816.1|	634130-634230	nan	nan	283	207	-0.451824	0.7366	no

ni|645903614|nb|KK920816.1|	793002-793136	nan	nan	7	10	0.517699	0.6956	no
ni|645903614|nb|KK920816.1|	803261-803547	nan	nan	6	9	0.488059	0.6745	no
ni|645903614|nb|KK920816.1|	823585-823783	nan	nan	5	11	1	0.3908	no
ni|645903614|nb|KK920816.1|	824323-824571	nan	nan	9	8	-0.151931	0.897	no
ni|645903614|nb|KK920816.1|	826073-826279	nan	nan	9	13	0.480633	0.6834	no
ni|645903614|nb|KK920816.1|	826414-826567	nan	nan	8	10	0.286447	0.81825	no
ni|645903614|nb|KK920816.1|	827186-827825	nan	nan	3	3	0.284693	0.80385	no
ni|645903614|nb|KK920816.1|	828110-828271	nan	nan	5	8	0.795289	0.5353	no
ni|645903614|nb|KK920816.1|	828964-829091	nan	nan	8	3	-1	0.4023	no
ni|645903614|nb|KK920816.1|	829779-830008	nan	nan	9	7	-0.420092	0.7158	no
ni|645903614|nb|KK920816.1|	830914-831039	nan	nan	10	14	0.517619	0.6956	no
ni|645903614|nb|KK920816.1|	831149-831263	nan	nan	22	6	-2	0.22995	no
ni|645903614|nb|KK920816.1|	831771-831902	nan	nan	8	8	0.0702171	0.94415	no
ni|645903614|nb|KK920816.1|	832286-832670	nan	nan	6	3	-1	0.3661	no
ni|645903614|nb|KK920816.1|	833227-833898	nan	nan	7	2	-2	0.11085	no
ni|645903614|nb|KK920816.1|	834001-834161	nan	nan	8	2	-2	0.1366	no
ni|645903614|nb|KK920816.1|	838810-838924	nan	nan	30	11	-1	0.3115	no
ni|645903614|nb|KK920816.1|	839080-839187	nan	nan	59	48	-0.31577	0.8025	no
ni|645903614|nb|KK920816.1|	839257-839517	nan	nan	14	8	-0.782289	0.50715	no
ni|645903614|nb|KK920816.1|	839732-839890	nan	nan	30	21	-0.531869	0.6491	no
ni|645903614|nb|KK920816.1|	840010-840295	nan	nan	72	133	0.880652	0.48355	no
ni|645903614|nb|KK920816.1|	844920-845064	nan	nan	57	120	1	0.3592	no
ni|645903614|nb|KK920816.1|	846927-847526	nan	nan	0.347346	0.284619	-0.287341	0.8535	no
ni|645903614|nb|KK920816.1|	849349-849641	nan	nan	20	52	1	0.2331	no
ni|645903614|nb|KK920816.1|	851538-851919	nan	nan	19	50	1	0.207	no
ni|645903614|nb|KK920816.1|	854696-854830	nan	nan	51	142	1	0.2163	no
ni|645903614|nb|KK920816.1|	857493-857836	nan	nan	25	99	2	0.1477	no
ni|645903614|nb|KK920816.1|	859595-859706	nan	nan	52	62	0.242727	0.8395	no
ni|645903614|nb|KK920816.1|	860183-860489	nan	nan	12	13	0.0977465	0.93305	no
ni|645903614|nb|KK920816.1|	860562-860863	nan	nan	26	20	-0.334429	0.76875	no

ni|645903614|nb|KK920816.1|	860936-861356	nan	nan	15	12	-0.250406	0.82825	no
ni|645903614|nb|KK920816.1|	867749-867889	nan	nan	13	12	-0.0672273	0.9525	no
ni|645903614|nb|KK920816.1|	867994-868104	nan	nan	21	24	0.22185	0.8747	no
ni|645903614|nb|KK920816.1|	868162-868287	nan	nan	13	17	0.402141	0.75355	no
ni|645903614|nb|KK920816.1|	869023-869355	nan	nan	12	12	-0.0409542	0.9717	no
ni|645903614|nb|KK920816.1|	891320-891648	nan	nan	18	21	0.250972	0.82515	no
ni|645903614|nb|KK920816.1|	930583-930809	nan	nan	22	19	-0.217062	0.85365	no
ni|645903614|nb|KK920816.1|	942405-942591	nan	nan	16	22	0.436801	0.70645	no
ni|645903615|nb|KK920815.1|	96-271	nan	nan	3	3	-0.237241	0.8697	no
ni|645903616|nb|KK920814.1|	471481-471686	nan	nan	4	3	-0.388803	0.7621	no
ni|645903616|nb|KK920814.1|	473909-474128	nan	nan	4	4	-0.289009	0.8142	no
ni|645903616|nb|KK920814.1|	475112-475281	nan	nan	6	2	-1	0.3768	no
ni|645903616|nb|KK920814.1|	478161-478496	nan	nan	2	2	-0.287995	0.8142	no
ni|645903616|nb|KK920814.1|	486747-486969	nan	nan	5	3	-0.718644	0.56995	no
ni|645903616|nb|KK920814.1|	490688-490980	nan	nan	2	1	-0.854332	0.50505	no
ni|645903616|nb|KK920814.1|	491803-491940	nan	nan	4	4	-0.0672441	0.923	no
ni|645903616|nb|KK920814.1|	494342-494722	nan	nan	1	0.678193	-0.765853	0.5799	no
ni|645903616|nb|KK920814.1|	495056-495333	nan	nan	2	0.672574	-2	0.29725	no
ni|645903616|nb|KK920814.1|	495962-496266	nan	nan	1	2	0.562268	0.66675	no
ni|645903616|nb|KK920814.1|	506159-506383	nan	nan	3	2	-0.388463	0.7621	no
ni|645903616|nb|KK920814.1|	506530-506786	nan	nan	2	2	-0.414043	0.76315	no
ni|645903616|nb|KK920814.1|	519958-520138	nan	nan	2	2	0.125335	0.92845	no
ni|645903616|nb|KK920814.1|	521366-521581	nan	nan	3	0.831137	-2	0.1613	no
ni|645903616|nb|KK920814.1|	523280-523489	nan	nan	3	1	-1	0.3464	no
ni|645903616|nb|KK920814.1|	534227-534436	nan	nan	2	1	-0.48183	0.7594	no
ni|645903616|nb|KK920814.1|	541707-541874	nan	nan	4	2	-1	0.44755	no
ni|645903616|nb|KK920814.1|	544178-544367	nan	nan	3	1	-2	0.25505	no
ni|645903617|nb|KK920813.1|	105664-106351	nan	nan	125	33	-2	0.206	no
ni|645903617|nb|KK920813.1|	109794-110288	nan	nan	209	45	-2	0.14775	no
ni|645903617|nb|KK920813.1|	111284-111990	nan	nan	232	47	-2	0.13395	no

ni|645903617|nb|KK920813.1|	112785-113272	nan	nan	208	43	-2	0.08035	no
ni|645903617|nb|KK920813.1|	113381-113516	nan	nan	118	22	-2	0.04905	no
ni|645903617|nb|KK920813.1|	121154-121965	nan	nan	62	29	-1	0.4532	no
ni|645903617|nb|KK920813.1|	124394-124918	nan	nan	77	37	-1	0.39485	no
ni|645903617|nb|KK920813.1|	125274-125995	nan	nan	0.438196	0.152346	-2	0.3149	no
ni|645903617|nb|KK920813.1|	126611-127235	nan	nan	106	47	-1	0.4298	no
ni|645903617|nb|KK920813.1|	127428-127640	nan	nan	213	103	-1	0.42515	no
ni|645903617|nb|KK920813.1|	127751-127928	nan	nan	133	67	-0.998613	0.3721	no
ni|645903617|nb|KK920813.1|	13471-13936	nan	nan	3	2	-0.588752	0.61575	no
ni|645903617|nb|KK920813.1|	137478-138351	nan	nan	386	236	-0.707927	0.6235	no
ni|645903617|nb|KK920813.1|	139936-140428	nan	nan	592	361	-0.711536	0.62165	no
ni|645903617|nb|KK920813.1|	142941-143215	nan	nan	2	0.273704	-3	0.2517	no
ni|645903617|nb|KK920813.1|	144384-145246	nan	nan	123	81	-0.601945	0.6722	no
ni|645903617|nb|KK920813.1|	146044-148230	nan	nan	0.851595	0.791834	-0.104969	0.9275	no
ni|645903617|nb|KK920813.1|	14640-14783	nan	nan	6	4	-0.526642	0.7071	no
ni|645903617|nb|KK920813.1|	148807-149152	nan	nan	502	282	-0.832199	0.5527	no
ni|645903617|nb|KK920813.1|	149275-149495	nan	nan	743	375	-0.985894	0.49135	no
ni|645903617|nb|KK920813.1|	149615-149806	nan	nan	9	6	-0.719242	0.5535	no
ni|645903617|nb|KK920813.1|	150048-150201	nan	nan	7	3	-1	0.45115	no
ni|645903617|nb|KK920813.1|	150422-151922	nan	nan	4	3	-0.740151	0.51365	no
ni|645903617|nb|KK920813.1|	152295-152608	nan	nan	5	6	0.0521226	0.96285	no
ni|645903617|nb|KK920813.1|	152885-153010	nan	nan	690	305	-1	0.2789	no
ni|645903617|nb|KK920813.1|	160076-160244	nan	nan	2	3	0.347788	0.8193	no
ni|645903617|nb|KK920813.1|	160317-160945	nan	nan	1	0.448211	-1	0.2773	no
ni|645903617|nb|KK920813.1|	161949-162430	nan	nan	3	1	-0.899145	0.46045	no
ni|645903617|nb|KK920813.1|	163194-164179	nan	nan	2	0.827658	-1	0.2211	no
ni|645903617|nb|KK920813.1|	164325-164530	nan	nan	5	2	-2	0.27225	no
ni|645903617|nb|KK920813.1|	171750-172264	nan	nan	38	40	0.065861	0.9502	no
ni|645903617|nb|KK920813.1|	172368-172492	nan	nan	81	82	0.0201077	0.9866	no
ni|645903617|nb|KK920813.1|	172940-173230	nan	nan	2	1	-0.19138	0.88855	no

ni|645903617|nb|KK920813.1|	173402-173621	nan	nan	3	3	-0.273067	0.82995	no
ni|645903617|nb|KK920813.1|	173769-174508	nan	nan	69	69	-0.0141478	0.9923	no
ni|645903617|nb|KK920813.1|	17792-18095	nan	nan	1	0.117168	-3	0.04325	no
ni|645903617|nb|KK920813.1|	178439-179694	nan	nan	39	30	-0.387005	0.7877	no
ni|645903617|nb|KK920813.1|	181068-181474	nan	nan	0.162249	1	3	0.0782	no
ni|645903617|nb|KK920813.1|	181835-182151	nan	nan	2	4	0.815658	0.5056	no
ni|645903617|nb|KK920813.1|	182802-182919	nan	nan	11	13	0.254475	0.86075	no
ni|645903617|nb|KK920813.1|	188644-188810	nan	nan	10	19	0.932766	0.4298	no
ni|645903617|nb|KK920813.1|	188915-189098	nan	nan	11	15	0.395689	0.73375	no
ni|645903617|nb|KK920813.1|	189320-189695	nan	nan	6	9	0.443033	0.70795	no
ni|645903617|nb|KK920813.1|	190900-191087	nan	nan	13	18	0.453604	0.6992	no
ni|645903617|nb|KK920813.1|	196362-196566	nan	nan	15	14	-0.0899797	0.93645	no
ni|645903617|nb|KK920813.1|	196673-197036	nan	nan	0.661353	0.812581	0.297092	0.8383	no
ni|645903617|nb|KK920813.1|	198032-198528	nan	nan	4	5	0.398822	0.73415	no
ni|645903617|nb|KK920813.1|	198860-198973	nan	nan	11	24	1	0.42455	no
ni|645903617|nb|KK920813.1|	199918-200543	nan	nan	6	7	0.289754	0.7985	no
ni|645903617|nb|KK920813.1|	206466-206897	nan	nan	7	8	0.0457643	0.96875	no
ni|645903617|nb|KK920813.1|	207006-207118	nan	nan	15	20	0.391854	0.7763	no
ni|645903617|nb|KK920813.1|	208104-208371	nan	nan	1	0.285267	-2	0.3383	no
ni|645903617|nb|KK920813.1|	209173-209566	nan	nan	3	2	-0.787836	0.515	no
ni|645903617|nb|KK920813.1|	209808-209994	nan	nan	6	8	0.418185	0.7259	no
ni|645903617|nb|KK920813.1|	211406-211554	nan	nan	8	6	-0.482237	0.7105	no
ni|645903617|nb|KK920813.1|	212903-213196	nan	nan	1	2	0.741525	0.5765	no
ni|645903617|nb|KK920813.1|	215573-215731	nan	nan	6	7	0.212908	0.86845	no
ni|645903617|nb|KK920813.1|	217571-217751	nan	nan	7	6	-0.278814	0.82035	no
ni|645903617|nb|KK920813.1|	266425-266964	nan	nan	0.620806	1	0.723449	0.5686	no
ni|645903617|nb|KK920813.1|	290262-290735	nan	nan	41	33	-0.31339	0.76835	no
ni|645903617|nb|KK920813.1|	352056-353725	nan	nan	8	5	-0.484375	0.65465	no
ni|645903617|nb|KK920813.1|	355001-355710	nan	nan	21	15	-0.466011	0.67405	no
ni|645903617|nb|KK920813.1|	355800-355984	nan	nan	50	28	-0.838794	0.47585	no

ni|645903617|nb|KK920813.1|	370176-370333	nan	nan	58	51	-0.180855	0.8719	no
ni|645903617|nb|KK920813.1|	375149-375878	nan	nan	0.511257	0.676809	0.404701	0.75355	no
ni|645903617|nb|KK920813.1|	376919-377217	nan	nan	0.880284	1	0.586278	0.65495	no
ni|645903617|nb|KK920813.1|	377433-378718	nan	nan	8	8	0.131567	0.90475	no
ni|645903617|nb|KK920813.1|	378994-379478	nan	nan	16	16	0.0185899	0.98595	no
ni|645903617|nb|KK920813.1|	380218-380563	nan	nan	49	41	-0.269719	0.8095	no
ni|645903617|nb|KK920813.1|	383749-383903	nan	nan	73	50	-0.550794	0.62915	no
ni|645903617|nb|KK920813.1|	386880-387060	nan	nan	50	46	-0.122452	0.9106	no
ni|645903617|nb|KK920813.1|	388344-388778	nan	nan	28	26	-0.119271	0.9123	no
ni|645903617|nb|KK920813.1|	390843-391071	nan	nan	54	45	-0.284545	0.80185	no
ni|645903617|nb|KK920813.1|	392277-393163	nan	nan	0.345934	0.150351	-1	0.4064	no
ni|645903617|nb|KK920813.1|	394325-394637	nan	nan	0.58694	1	0.782278	0.58145	no
ni|645903617|nb|KK920813.1|	394843-395625	nan	nan	0.725214	0.624054	-0.216736	0.8608	no
ni|645903617|nb|KK920813.1|	398244-398396	nan	nan	77	63	-0.300077	0.7948	no
ni|645903617|nb|KK920813.1|	413321-413447	nan	nan	10	5	-0.941803	0.54025	no
ni|645903617|nb|KK920813.1|	413845-413990	nan	nan	25	22	-0.166742	0.88955	no
ni|645903617|nb|KK920813.1|	431632-431819	nan	nan	0.57646	6	3	0.05455	no
ni|645903617|nb|KK920813.1|	432538-432740	nan	nan	0.491233	3	3	0.0824	no
ni|645903617|nb|KK920813.1|	434291-434538	nan	nan	0.339652	6	4	0.04665	no
ni|645903617|nb|KK920813.1|	466801-467144	nan	nan	57	44	-0.364924	0.7355	no
ni|645903617|nb|KK920813.1|	467259-467580	nan	nan	2	1	-0.776165	0.5539	no
ni|645903617|nb|KK920813.1|	467777-469324	nan	nan	11	7	-0.692649	0.5198	no
ni|645903617|nb|KK920813.1|	475852-476037	nan	nan	11	23	1	0.34205	no
ni|645903617|nb|KK920813.1|	477985-478232	nan	nan	38	59	0.657637	0.55945	no
ni|645903617|nb|KK920813.1|	480389-480803	nan	nan	18	27	0.576554	0.6045	no
ni|645903617|nb|KK920813.1|	485891-486799	nan	nan	46	70	0.598977	0.6696	no
ni|645903617|nb|KK920813.1|	487142-487842	nan	nan	51	58	0.18015	0.89935	no
ni|645903617|nb|KK920813.1|	488306-488600	nan	nan	15	10	-0.523674	0.6516	no
ni|645903617|nb|KK920813.1|	489164-489378	nan	nan	60	57	-0.0826413	0.9404	no
ni|645903617|nb|KK920813.1|	490583-491748	nan	nan	0.788519	0.665324	-0.245088	0.836	no

ni|645903617|nb|KK920813.1|	491803-493162	nan	nan	1	0.956481	-0.250012	0.82685	no
ni|645903617|nb|KK920813.1|	496191-496801	nan	nan	0.388366	0.69614	0.841959	0.5399	no
ni|645903617|nb|KK920813.1|	51190-51591	nan	nan	230	131.09	-0.808717	0.5707	no
ni|645903617|nb|KK920813.1|	514967-515559	nan	nan	0.754951	0.625484	-0.27141	0.8304	no
ni|645903617|nb|KK920813.1|	563316-563909	nan	nan	0.301365	0.576194	0.935042	0.5295	no
ni|645903617|nb|KK920813.1|	565659-566135	nan	nan	0.263779	1	2	0.10795	no
ni|645903617|nb|KK920813.1|	56592-56950	nan	nan	0.963224	0.736377	-0.387427	0.78555	no
ni|645903617|nb|KK920813.1|	59066-59777	nan	nan	314	174	-0.852995	0.54065	no
ni|645903617|nb|KK920813.1|	60924-61341	nan	nan	218	107	-1	0.4728	no
ni|645903617|nb|KK920813.1|	62182-62595	nan	nan	601	324	-0.889857	0.5322	no
ni|645903617|nb|KK920813.1|	63366-63876	nan	nan	843	475	-0.829413	0.57695	no
ni|645903617|nb|KK920813.1|	64979-65628	nan	nan	13	7	-0.762734	0.5055	no
ni|645903617|nb|KK920813.1|	66469-66978	nan	nan	564	314	-0.842175	0.556	no
ni|645903617|nb|KK920813.1|	67540-67655	nan	nan	30	11	-2	0.2921	no
ni|645903617|nb|KK920813.1|	84842-85560	nan	nan	0.880663	0.306176	-2	0.27225	no
ni|645903617|nb|KK920813.1|	87728-88248	nan	nan	1	0.789604	-0.579653	0.6557	no
ni|645903617|nb|KK920813.1|	90473-91157	nan	nan	110	82	-0.422905	0.76455	no
ni|645903617|nb|KK920813.1|	94774-95345	nan	nan	97	80	-0.282965	0.84055	no
ni|645903617|nb|KK920813.1|	96334-96564	nan	nan	195	158	-0.301241	0.84085	no
ni|645903617|nb|KK920813.1|	96838-97316	nan	nan	97	74	-0.399326	0.7766	no
ni|645903617|nb|KK920813.1|	97648-97872	nan	nan	188	140	-0.426456	0.73205	no
ni|645903617|nb|KK920813.1|	98855-99055	nan	nan	136	106	-0.361228	0.7431	no
ni|645903618|nb|KK920812.1|	1020172-102038	nan	nan	12	12	-0.0667537	0.9547	no
ni|645903618|nb|KK920812.1|	221462-221617	nan	nan	3	8	2	0.297	no
ni|645903618|nb|KK920812.1|	230611-231029	nan	nan	0.234136	2	3	0.0706	no
ni|645903618|nb|KK920812.1|	301163-301408	nan	nan	6	7	0.281688	0.8159	no
ni|645903618|nb|KK920812.1|	303348-303582	nan	nan	3	4	0.670585	0.59665	no
ni|645903618|nb|KK920812.1|	304646-305055	nan	nan	5	6	0.343481	0.7688	no
ni|645903618|nb|KK920812.1|	307279-307432	nan	nan	24	22	-0.124906	0.91565	no
ni|645903618|nb|KK920812.1|	307918-308082	nan	nan	13	9	-0.54366	0.64955	no

ni|645903618|nb|KK920812.1|	308144-308263	nan	nan	24	14	-0.730384	0.5717	no
ni|645903618|nb|KK920812.1|	308387-308728	nan	nan	5	5	0.161198	0.8859	no
ni|645903618|nb|KK920812.1|	309288-309675	nan	nan	11	10	-0.191922	0.86835	no
ni|645903618|nb|KK920812.1|	309784-309897	nan	nan	13	29	1	0.39265	no
ni|645903618|nb|KK920812.1|	311091-311477	nan	nan	7	12	0.754035	0.51625	no
ni|645903618|nb|KK920812.1|	311681-312288	nan	nan	0.976687	0.980385	0.0054529	0.9708	no
ni|645903618|nb|KK920812.1|	312450-312642	nan	nan	13	31	1	0.31605	no
ni|645903618|nb|KK920812.1|	313148-313295	nan	nan	7	28	2	0.13335	no
ni|645903618|nb|KK920812.1|	314224-314380	nan	nan	14	41	2	0.2114	no
ni|645903618|nb|KK920812.1|	316095-316274	nan	nan	13	32	1	0.27455	no
ni|645903618|nb|KK920812.1|	320684-320896	nan	nan	15	49	2	0.1601	no
ni|645903618|nb|KK920812.1|	321464-321585	nan	nan	16	48	2	0.21845	no
ni|645903618|nb|KK920812.1|	321697-322120	nan	nan	8	24	2	0.17375	no
ni|645903618|nb|KK920812.1|	322902-323233	nan	nan	7	16	1	0.283	no
ni|645903618|nb|KK920812.1|	323870-324029	nan	nan	10	17	0.801553	0.50325	no
ni|645903618|nb|KK920812.1|	325267-325513	nan	nan	0	22	inf	5.00E-05	yes
ni|645903618|nb|KK920812.1|	325697-325907	nan	nan	14	35	1	0.2636	no
ni|645903618|nb|KK920812.1|	326677-327072	nan	nan	4	28	3	0.02495	no
ni|645903618|nb|KK920812.1|	335709-335865	nan	nan	17	20	0.20199	0.8618	no
ni|645903618|nb|KK920812.1|	395578-395743	nan	nan	15	14	-0.0672272	0.9548	no
ni|645903618|nb|KK920812.1|	753408-754205	nan	nan	9	4	-1	0.33735	no
ni|645903618|nb|KK920812.1|	755718-756086	nan	nan	5	6	0.411863	0.7319	no
ni|645903618|nb|KK920812.1|	756413-756615	nan	nan	6	9	0.537132	0.65445	no
ni|645903618|nb|KK920812.1|	758703-758914	nan	nan	13	16	0.290181	0.80145	no
ni|645903618|nb|KK920812.1|	824026-824145	nan	nan	33	31	-0.0674195	0.9532	no
ni|645903618|nb|KK920812.1|	897439-897607	nan	nan	10	13	0.334849	0.77795	no
ni|645903618|nb|KK920812.1|	933493-933724	nan	nan	0	5	inf	0.0003	yes
ni|645903618|nb|KK920812.1|	981489-982004	nan	nan	0.41802	1	2	0.2147	no
ni|645903619|nb|KK920811.1|	161593-162075	nan	nan	0.454325	0.620377	0.44942	0.7543	no
ni|645903619|nb|KK920811.1|	182829-183412	nan	nan	3	5	0.695076	0.55035	no

ni|645903619|nb|KK920811.1|	184499-184638	nan	nan	4	6	0.447341	0.7576	no
ni|645903619|nb|KK920811.1|	186551-186962	nan	nan	0.957749	2	0.80915	0.52225	no
ni|645903619|nb|KK920811.1|	187166-187481	nan	nan	0.925902	1	0.256225	0.86075	no
ni|645903619|nb|KK920811.1|	188526-188892	nan	nan	2	3	0.290677	0.80205	no
ni|645903619|nb|KK920811.1|	189440-189586	nan	nan	3	8	2	0.2306	no
ni|645903619|nb|KK920811.1|	196340-196484	nan	nan	0	144	inf	5.00E-05	yes
ni|645903619|nb|KK920811.1|	196558-196907	nan	nan	0	53	inf	5.00E-05	yes
ni|645903619|nb|KK920811.1|	197059-197344	nan	nan	0.269333	100	9	0.04435	no
ni|645903619|nb|KK920811.1|	197870-198065	nan	nan	0	105	inf	5.00E-05	yes
ni|645903619|nb|KK920811.1|	198193-198573	nan	nan	0.088706	124	10	0.11935	no
ni|645903619|nb|KK920811.1|	204666-204901	nan	nan	16	18	0.193869	0.87435	no
ni|645903619|nb|KK920811.1|	205342-205576	nan	nan	13	13	-0.025739	0.98245	no
ni|645903619|nb|KK920811.1|	206276-206437	nan	nan	19	24	0.339968	0.77155	no
ni|645903619|nb|KK920811.1|	207116-207386	nan	nan	6	7	0.214111	0.84995	no
ni|645903619|nb|KK920811.1|	207500-207623	nan	nan	18	20	0.167098	0.89635	no
ni|645903619|nb|KK920811.1|	207769-207892	nan	nan	15	20	0.447206	0.7237	no
ni|645903619|nb|KK920811.1|	208571-209147	nan	nan	9	10	0.0425001	0.9696	no
ni|645903619|nb|KK920811.1|	209275-209409	nan	nan	16	10	-0.619805	0.62755	no
ni|645903619|nb|KK920811.1|	209605-209759	nan	nan	22	24	0.159579	0.88755	no
ni|645903619|nb|KK920811.1|	209864-210243	nan	nan	13	17	0.368986	0.751	no
ni|645903619|nb|KK920811.1|	210939-211088	nan	nan	31	49	0.674491	0.56675	no
ni|645903619|nb|KK920811.1|	212472-212586	nan	nan	15	29	0.93248	0.4834	no
ni|645903619|nb|KK920811.1|	214498-214708	nan	nan	19	30	0.704958	0.54785	no
ni|645903619|nb|KK920811.1|	216241-216481	nan	nan	23	27	0.185681	0.87265	no
ni|645903619|nb|KK920811.1|	217991-218214	nan	nan	13	15	0.283034	0.80465	no
ni|645903619|nb|KK920811.1|	219260-219506	nan	nan	6	6	-0.188215	0.8746	no
ni|645903619|nb|KK920811.1|	220771-220999	nan	nan	17	19	0.17969	0.8822	no
ni|645903619|nb|KK920811.1|	224313-224516	nan	nan	2	3	0.933082	0.5103	no
ni|645903619|nb|KK920811.1|	224758-225191	nan	nan	6	12	1	0.38695	no
ni|645903619|nb|KK920811.1|	225273-225411	nan	nan	0	52	inf	5.00E-05	yes

ni|645903619|nb|KK920811.1|	227721-228247	nan	nan	0	24	inf	5.00E-05	yes
ni|645903619|nb|KK920811.1|	232549-232691	nan	nan	27	56	1	0.3754	no
ni|645903619|nb|KK920811.1|	233607-233726	nan	nan	44	36	-0.289812	0.80005	no
ni|645903619|nb|KK920811.1|	234694-235448	nan	nan	0.453824	0.216952	-1	0.46615	no
ni|645903619|nb|KK920811.1|	238655-238867	nan	nan	36	49	0.435238	0.70395	no
ni|645903619|nb|KK920811.1|	239578-239889	nan	nan	3	4	0.634715	0.5965	no
ni|645903619|nb|KK920811.1|	241323-241671	nan	nan	8	9	0.191234	0.87145	no
ni|645903619|nb|KK920811.1|	242011-242123	nan	nan	19	24	0.310934	0.81835	no
ni|645903619|nb|KK920811.1|	242288-242398	nan	nan	48	46	-0.0676564	0.9525	no
ni|645903619|nb|KK920811.1|	243386-243726	nan	nan	36	39	0.137514	0.89685	no
ni|645903619|nb|KK920811.1|	262872-263017	nan	nan	33	46	0.468847	0.6886	no
ni|645903619|nb|KK920811.1|	289944-290071	nan	nan	15	24	0.652568	0.59345	no
ni|645903619|nb|KK920811.1|	507528-507633	nan	nan	83	107	0.364848	0.7586	no
ni|645903619|nb|KK920811.1|	530566-530680	nan	nan	91	133	0.552632	0.6371	no
ni|645903619|nb|KK920811.1|	560487-560611	nan	nan	30	56	0.883735	0.4602	no
ni|645903619|nb|KK920811.1|	561614-561873	nan	nan	61	99	0.698789	0.5136	no
ni|645903619|nb|KK920811.1|	562257-562472	nan	nan	64	101	0.658458	0.5514	no
ni|645903619|nb|KK920811.1|	564253-564860	nan	nan	55	96	0.786449	0.59025	no
ni|645903619|nb|KK920811.1|	565822-566013	nan	nan	76	135	0.832738	0.44835	no
ni|645903619|nb|KK920811.1|	569486-570254	nan	nan	37	56	0.623159	0.67435	no
ni|645903619|nb|KK920811.1|	571445-571566	nan	nan	80	135	0.74584	0.5285	no
ni|645903619|nb|KK920811.1|	572203-572432	nan	nan	44	65	0.562323	0.6168	no
ni|645903619|nb|KK920811.1|	572550-573174	nan	nan	0.094486	3	5	0.0446	no
ni|645903619|nb|KK920811.1|	573879-574392	nan	nan	45	93	1	0.4852	no
ni|645903619|nb|KK920811.1|	579835-580115	nan	nan	32	60	0.915055	0.4166	no
ni|645903619|nb|KK920811.1|	581858-582073	nan	nan	9	21	1	0.2961	no
ni|645903619|nb|KK920811.1|	583319-583540	nan	nan	85	168	0.97982	0.45245	no
ni|645903619|nb|KK920811.1|	583727-583925	nan	nan	64	117	0.862588	0.44075	no
ni|645903619|nb|KK920811.1|	585368-585592	nan	nan	92	148	0.680498	0.5974	no
ni|645903619|nb|KK920811.1|	585976-586297	nan	nan	1	2	0.56236	0.66675	no

ni|645903619|nb|KK920811.1|	587063-587181	nan	nan	44	30	-0.526867	0.66105	no
ni|645903619|nb|KK920811.1|	587284-587396	nan	nan	79	81	0.0343021	0.97755	no
ni|645903619|nb|KK920811.1|	587755-587883	nan	nan	12	14	0.212794	0.86845	no
ni|645903619|nb|KK920811.1|	588489-588839	nan	nan	29	26	-0.161917	0.88115	no
ni|645903619|nb|KK920811.1|	589219-589463	nan	nan	7	7	0.0746153	0.9463	no
ni|645903619|nb|KK920811.1|	589832-589937	nan	nan	29	55	0.931888	0.51205	no
ni|645903619|nb|KK920811.1|	590082-590207	nan	nan	12	3	-2	0.20695	no
ni|645903619|nb|KK920811.1|	590285-590394	nan	nan	73	72	-0.0187984	0.97915	no
ni|645903619|nb|KK920811.1|	590485-590592	nan	nan	34	6	-3	0.28285	no
ni|645903619|nb|KK920811.1|	590804-591039	nan	nan	36	30	-0.225064	0.8433	no
ni|645903619|nb|KK920811.1|	591336-591610	nan	nan	68	56	-0.265613	0.8056	no
ni|645903619|nb|KK920811.1|	591898-592063	nan	nan	33	23	-0.532891	0.64655	no
ni|645903619|nb|KK920811.1|	592174-592842	nan	nan	23	16	-0.514056	0.6388	no
ni|645903619|nb|KK920811.1|	593134-593260	nan	nan	11	0.895104	-4	0.02785	no
ni|645903619|nb|KK920811.1|	600345-600751	nan	nan	20	33	0.739497	0.5095	no
ni|645903619|nb|KK920811.1|	601850-602126	nan	nan	19	32	0.747454	0.51375	no
ni|645903619|nb|KK920811.1|	603968-604114	nan	nan	51	86	0.746645	0.5139	no
ni|645903619|nb|KK920811.1|	606883-607041	nan	nan	51	87	0.765902	0.51105	no
ni|645903619|nb|KK920811.1|	607420-607558	nan	nan	21	23	0.0978211	0.9331	no
ni|645903619|nb|KK920811.1|	607931-608053	nan	nan	29	22	-0.375501	0.7568	no
ni|645903619|nb|KK920811.1|	608355-608535	nan	nan	14	8	-0.890432	0.4563	no
ni|645903619|nb|KK920811.1|	609607-609891	nan	nan	22	20	-0.102417	0.92655	no
ni|645903619|nb|KK920811.1|	609991-610156	nan	nan	33	23	-0.499338	0.6678	no
ni|645903619|nb|KK920811.1|	610470-610619	nan	nan	9	4	-1	0.40265	no
ni|645903619|nb|KK920811.1|	615904-616112	nan	nan	0	139	inf	5.00E-05	yes
ni|645903619|nb|KK920811.1|	616298-617095	nan	nan	0	2	inf	5.00E-05	yes
ni|645903619|nb|KK920811.1|	618547-618782	nan	nan	0	224	inf	5.00E-05	yes
ni|645903619|nb|KK920811.1|	621086-621337	nan	nan	0	200	inf	5.00E-05	yes
ni|645903619|nb|KK920811.1|	622903-623345	nan	nan	0	4	inf	5.00E-05	yes
ni|645903619|nb|KK920811.1|	628087-628224	nan	nan	0	39	inf	5.00E-05	yes

ni|645903619|nb|KK920811.1|	629884-630090	nan	nan	0	25	inf	5.00E-05	yes
ni|645903619|nb|KK920811.1|	635919-636180	nan	nan	0	5	inf	0.00015	yes
ni|645903619|nb|KK920811.1|	638520-638686	nan	nan	0	12	inf	0.0001	yes
ni|645903619|nb|KK920811.1|	646852-647399	nan	nan	0	22	inf	5.00E-05	yes
ni|645903619|nb|KK920811.1|	659215-659945	nan	nan	1	19	4	0.0051	no
ni|645903619|nb|KK920811.1|	660109-661076	nan	nan	0.370532	28	6	0.004	no
ni|645903619|nb|KK920811.1|	661458-661680	nan	nan	10	67	3	0.03005	no
ni|645903619|nb|KK920811.1|	66335-66554	nan	nan	2	4	0.79588	0.5353	no
ni|645903619|nb|KK920811.1|	663405-663648	nan	nan	10	95	3	0.0095	no
ni|645903619|nb|KK920811.1|	664521-664732	nan	nan	9	87	3	0.01165	no
ni|645903619|nb|KK920811.1|	671753-671919	nan	nan	7	78	4	0.00955	no
ni|645903619|nb|KK920811.1|	672090-672305	nan	nan	7	91	4	0.00375	no
ni|645903619|nb|KK920811.1|	673430-673734	nan	nan	6	71	4	0.005	no
ni|645903619|nb|KK920811.1|	675906-676340	nan	nan	0.445713	0.781001	0.809209	0.56845	no
ni|645903619|nb|KK920811.1|	676453-677220	nan	nan	2	22.76	3	0.0081	no
ni|645903619|nb|KK920811.1|	677953-678733	nan	nan	6	61	3	0.0198	no
ni|645903619|nb|KK920811.1|	679113-679749	nan	nan	0.877436	1	0.545158	0.66	no
ni|645903619|nb|KK920811.1|	683670-684036	nan	nan	0	7	inf	5.00E-05	yes
ni|645903619|nb|KK920811.1|	686990-687219	nan	nan	0	45	inf	5.00E-05	yes
ni|645903619|nb|KK920811.1|	687338-687701	nan	nan	0	31	inf	5.00E-05	yes
ni|645903619|nb|KK920811.1|	687840-688314	nan	nan	0	36	inf	5.00E-05	yes
ni|645903619|nb|KK920811.1|	688462-688625	nan	nan	0	78	inf	5.00E-05	yes
ni|645903619|nb|KK920811.1|	688727-689423	nan	nan	0	45	inf	5.00E-05	yes
ni|645903619|nb|KK920811.1|	693348-693763	nan	nan	30	28	-0.0807794	0.9417	no
ni|645903619|nb|KK920811.1|	693978-694153	nan	nan	42	48	0.163168	0.8879	no
ni|645903619|nb|KK920811.1|	694335-694509	nan	nan	67	83	0.296068	0.79395	no
ni|645903619|nb|KK920811.1|	695584-696104	nan	nan	0	10	inf	5.00E-05	yes
ni|645903619|nb|KK920811.1|	779381-779518	nan	nan	31	54	0.818585	0.4846	no
ni|645903619|nb|KK920811.1|	818364-818501	nan	nan	14	8	-0.759122	0.54195	no
ni|645903620|nb|KK920810.1|	1018-1167	nan	nan	5	6	0.311314	0.81835	no

ni|645903620|nb|KK920810.1|	11240-11827	nan	nan	1	0.777762	-0.64993	0.5978	no
ni|645903620|nb|KK920810.1|	13191-13709	nan	nan	1	0.453371	-1	0.36935	no
ni|645903620|nb|KK920810.1|	23829-24022	nan	nan	91	89	-0.0375537	0.9718	no
ni|645903620|nb|KK920810.1|	24437-24705	nan	nan	131.2	153.12	0.2229	0.8803	no
ni|645903620|nb|KK920810.1|	27179-27361	nan	nan	127	144	0.174783	0.8755	no
ni|645903620|nb|KK920810.1|	29043-29196	nan	nan	137	132	-0.0573424	0.95755	no
ni|645903620|nb|KK920810.1|	3186-3398	nan	nan	2	2	0.295835	0.8383	no
ni|645903620|nb|KK920810.1|	383592-383793	nan	nan	16	14	-0.180618	0.87635	no
ni|645903620|nb|KK920810.1|	386407-386862	nan	nan	11	13	0.199333	0.8644	no
ni|645903620|nb|KK920810.1|	387638-387772	nan	nan	27	16	-0.753155	0.536	no
ni|645903620|nb|KK920810.1|	388031-388134	nan	nan	37	35	-0.0686347	0.923	no
ni|645903620|nb|KK920810.1|	392255-392361	nan	nan	36	20	-0.804925	0.6027	no
ni|645903620|nb|KK920810.1|	393937-394086	nan	nan	19	18	-0.0671975	0.95425	no
ni|645903620|nb|KK920810.1|	397838-398104	nan	nan	9	6	-0.456819	0.69775	no
ni|645903620|nb|KK920810.1|	398160-398339	nan	nan	20	20	-0.0449518	0.96675	no
ni|645903620|nb|KK920810.1|	403680-403892	nan	nan	5	4	-0.40377	0.73465	no
ni|645903620|nb|KK920810.1|	406125-406282	nan	nan	47	28	-0.764271	0.5111	no
ni|645903620|nb|KK920810.1|	406724-406989	nan	nan	26	26	0.0168984	0.98885	no
ni|645903620|nb|KK920810.1|	407676-407996	nan	nan	30	28	-0.104298	0.9245	no
ni|645903620|nb|KK920810.1|	409366-409505	nan	nan	46	29	-0.672095	0.57915	no
ni|645903620|nb|KK920810.1|	409638-409838	nan	nan	37	30	-0.268121	0.811	no
ni|645903620|nb|KK920810.1|	410352-411149	nan	nan	0.248344	0.271371	0.127925	0.92555	no
ni|645903620|nb|KK920810.1|	411335-411480	nan	nan	29	27	-0.0946869	0.9327	no
ni|645903620|nb|KK920810.1|	413655-413950	nan	nan	8	9	0.17337	0.885	no
ni|645903620|nb|KK920810.1|	417749-417914	nan	nan	36	36	0.0083238	0.99315	no
ni|645903620|nb|KK920810.1|	419369-419662	nan	nan	9	8	-0.172745	0.884	no
ni|645903620|nb|KK920810.1|	423680-423856	nan	nan	34	31	-0.109969	0.9253	no
ni|645903620|nb|KK920810.1|	428401-428677	nan	nan	26	22	-0.230069	0.8402	no
ni|645903620|nb|KK920810.1|	428807-428980	nan	nan	20	16	-0.315227	0.7908	no
ni|645903620|nb|KK920810.1|	437181-438293	nan	nan	8	7	-0.205399	0.8491	no

ni|645903620|nb|KK920810.1|	448337-448780	nan	nan	0.506279	0.276511	-0.872594	0.5788	no
ni|645903620|nb|KK920810.1|	45428-46050	nan	nan	40	22	-0.850321	0.5061	no
ni|645903620|nb|KK920810.1|	454750-454938	nan	nan	18	12	-0.529559	0.6523	no
ni|645903620|nb|KK920810.1|	470001-470287	nan	nan	3	2	-0.406913	0.755	no
ni|645903620|nb|KK920810.1|	543553-543729	nan	nan	226	260.97	0.210051	0.8667	no
ni|645903620|nb|KK920810.1|	552503-552673	nan	nan	523	377	-0.472792	0.741	no
ni|645903620|nb|KK920810.1|	552865-553530	nan	nan	29	23	-0.338059	0.75145	no
ni|645903620|nb|KK920810.1|	553882-554137	nan	nan	150	110	-0.446033	0.7142	no
ni|645903620|nb|KK920810.1|	554715-555165	nan	nan	164	122	-0.424943	0.76435	no
ni|645903620|nb|KK920810.1|	555767-555960	nan	nan	191	133	-0.525541	0.62215	no
ni|645903620|nb|KK920810.1|	556891-557191	nan	nan	34	21	-0.7231	0.52845	no
ni|645903620|nb|KK920810.1|	557334-557889	nan	nan	0.979971	1	0.408912	0.74735	no
ni|645903620|nb|KK920810.1|	558173-558327	nan	nan	455.17	345	-0.401611	0.78905	no
ni|645903620|nb|KK920810.1|	558976-559327	nan	nan	159	117.53	-0.439924	0.76185	no
ni|645903620|nb|KK920810.1|	559441-560143	nan	nan	2	1	-0.412737	0.72545	no
ni|645903620|nb|KK920810.1|	562218-562602	nan	nan	78	59	-0.404488	0.7446	no
ni|645903620|nb|KK920810.1|	562822-563382	nan	nan	0.861572	0.875085	0.0224522	0.96555	no
ni|645903620|nb|KK920810.1|	563845-564082	nan	nan	14	10	-0.544386	0.6423	no
ni|645903620|nb|KK920810.1|	574735-574889	nan	nan	7	4	-0.745264	0.56925	no
ni|645903620|nb|KK920810.1|	575212-575962	nan	nan	5	4	-0.15001	0.8936	no
ni|645903620|nb|KK920810.1|	576122-576755	nan	nan	4	4	-0.162196	0.88885	no
ni|645903620|nb|KK920810.1|	576881-578092	nan	nan	3	2	-0.487999	0.67615	no
ni|645903620|nb|KK920810.1|	579526-579682	nan	nan	6	7	0.125449	0.927	no
ni|645903620|nb|KK920810.1|	579991-580106	nan	nan	14	11	-0.430065	0.75965	no
ni|645903620|nb|KK920810.1|	580561-580801	nan	nan	5	6	0.170741	0.8844	no
ni|645903620|nb|KK920810.1|	580922-581239	nan	nan	8	6	-0.227443	0.84695	no
ni|645903620|nb|KK920810.1|	581417-582155	nan	nan	14	11	-0.296445	0.7867	no
ni|645903620|nb|KK920810.1|	582663-583100	nan	nan	3	2	-0.467351	0.69685	no
ni|645903620|nb|KK920810.1|	584059-584896	nan	nan	3	2	-0.441542	0.70515	no
ni|645903620|nb|KK920810.1|	586887-587067	nan	nan	3	3	-0.06731	0.94715	no

ni|645903620|nb|KK920810.1|	587477-587754	nan	nan	2	0.807088	-0.940411	0.54025	no
ni|645903620|nb|KK920810.1|	589311-589560	nan	nan	2	3	0.320833	0.8092	no
ni|645903620|nb|KK920810.1|	589793-590209	nan	nan	2	2	-0.443817	0.7127	no
ni|645903620|nb|KK920810.1|	590417-590788	nan	nan	1	0.43803	-2	0.24455	no
ni|645903620|nb|KK920810.1|	593489-593885	nan	nan	1	1	-0.152824	0.90135	no
ni|645903620|nb|KK920810.1|	595752-595895	nan	nan	70	53	-0.386161	0.73815	no
ni|645903620|nb|KK920810.1|	610877-611032	nan	nan	98	77	-0.34493	0.7589	no
ni|645903620|nb|KK920810.1|	612254-612481	nan	nan	8	8	-0.0325394	0.9788	no
ni|645903620|nb|KK920810.1|	621780-622022	nan	nan	10	11	0.138548	0.90565	no
ni|645903620|nb|KK920810.1|	8734-8932	nan	nan	175	183	0.0699049	0.95465	no
ni|645903622|nb|KK920808.1|	130485-130941	nan	nan	0	2	inf	0.00045	yes
ni|645903622|nb|KK920808.1|	148895-148958	nan	nan	19971.3	11500.8	-0.796191	0.605	no
ni|645903622|nb|KK920808.1|	200924-200986	nan	nan	22976.1	15266.9	-0.589725	0.66405	no
ni|645903622|nb|KK920808.1|	227765-228179	nan	nan	0	69	inf	5.00E-05	yes
ni|645903622|nb|KK920808.1|	228299-230577	nan	nan	0.011328	135.02	14	0.11935	no
ni|645903622|nb|KK920808.1|	317781-317922	nan	nan	18	13	-0.450551	0.7078	no
ni|645903622|nb|KK920808.1|	322584-322993	nan	nan	8	4	-0.994241	0.3982	no
ni|645903622|nb|KK920808.1|	325241-326191	nan	nan	0.203519	0.639415	2	0.23715	no
ni|645903622|nb|KK920808.1|	328172-328435	nan	nan	6	6	-0.0660552	0.95495	no
ni|645903622|nb|KK920808.1|	336142-336514	nan	nan	21	23	0.126425	0.90935	no
ni|645903622|nb|KK920808.1|	339898-341398	nan	nan	0.440825	1	1	0.27045	no
ni|645903622|nb|KK920808.1|	341550-341788	nan	nan	45	76	0.740208	0.50115	no
ni|645903622|nb|KK920808.1|	342207-342705	nan	nan	0.871936	0.535791	-0.702551	0.6038	no
ni|645903622|nb|KK920808.1|	343649-343841	nan	nan	20	34	0.756824	0.51675	no
ni|645903622|nb|KK920808.1|	343989-344151	nan	nan	36	62	0.787971	0.49155	no
ni|645903622|nb|KK920808.1|	344531-344719	nan	nan	3	6	1	0.39005	no
ni|645903622|nb|KK920808.1|	346343-346575	nan	nan	41	50	0.290155	0.8043	no
ni|645903622|nb|KK920808.1|	347679-348079	nan	nan	1	1	0.234211	0.8527	no
ni|645903622|nb|KK920808.1|	348574-348815	nan	nan	2	0.50751	-2	0.2989	no
ni|645903622|nb|KK920808.1|	349774-349902	nan	nan	9	7	-0.389242	0.78555	no

ni|645903622|nb|KK920808.1|	350094-350242	nan	nan	7	6	-0.182677	0.889	no
ni|645903622|nb|KK920808.1|	350705-351145	nan	nan	7	4	-0.72576	0.5336	no
ni|645903622|nb|KK920808.1|	351264-351527	nan	nan	5	4	-0.509662	0.67705	no
ni|645903622|nb|KK920808.1|	351645-352218	nan	nan	9	7	-0.367018	0.7476	no
ni|645903622|nb|KK920808.1|	353464-353625	nan	nan	23	16	-0.482245	0.6861	no
ni|645903622|nb|KK920808.1|	354100-354583	nan	nan	2	2	-0.574165	0.63245	no
ni|645903622|nb|KK920808.1|	357697-357935	nan	nan	3	1	-0.973216	0.5005	no
ni|645903622|nb|KK920808.1|	363681-363922	nan	nan	11	13	0.162534	0.88715	no
ni|645903622|nb|KK920808.1|	364256-364440	nan	nan	7	6	-0.259916	0.82995	no
ni|645903622|nb|KK920808.1|	364657-365086	nan	nan	0.905051	1	0.671694	0.59755	no
ni|645903622|nb|KK920808.1|	365355-365677	nan	nan	0.784431	1	0.711941	0.61075	no
ni|645903622|nb|KK920808.1|	366135-366628	nan	nan	8	9	0.0802996	0.945	no
ni|645903622|nb|KK920808.1|	367136-367380	nan	nan	38	35	-0.0997219	0.927	no
ni|645903622|nb|KK920808.1|	370045-370257	nan	nan	37	35	-0.0667352	0.95425	no
ni|645903622|nb|KK920808.1|	370873-371069	nan	nan	33	32	-0.0331181	0.97485	no
ni|645903622|nb|KK920808.1|	374472-375488	nan	nan	4	4	-0.073457	0.95135	no
ni|645903622|nb|KK920808.1|	375824-376010	nan	nan	26	26	-0.00311113	0.9932	no
ni|645903622|nb|KK920808.1|	376319-376527	nan	nan	30	30	0.0100035	0.99185	no
ni|645903622|nb|KK920808.1|	377732-378118	nan	nan	9	10	0.0377009	0.97415	no
ni|645903622|nb|KK920808.1|	378544-378707	nan	nan	30	27	-0.166753	0.8845	no
ni|645903622|nb|KK920808.1|	382633-383629	nan	nan	9	9	0.0120324	0.99105	no
ni|645903622|nb|KK920808.1|	384064-384354	nan	nan	8	8	0.0229597	0.9864	no
ni|645903622|nb|KK920808.1|	384476-384897	nan	nan	12	12	0.0324045	0.9759	no
ni|645903622|nb|KK920808.1|	564774-565048	nan	nan	21	12	-0.770141	0.5096	no
ni|645903622|nb|KK920808.1|	565638-565750	nan	nan	15	37	1	0.35025	no
ni|645903622|nb|KK920808.1|	566040-566332	nan	nan	6	7	0.268583	0.81845	no
ni|645903622|nb|KK920808.1|	566712-566829	nan	nan	20	17	-0.174368	0.8924	no
ni|645903622|nb|KK920808.1|	567539-567944	nan	nan	7	8	0.177026	0.87795	no
ni|645903622|nb|KK920808.1|	568019-568149	nan	nan	18	21	0.228161	0.8493	no
ni|645903622|nb|KK920808.1|	568435-568690	nan	nan	12	11	-0.0854945	0.94435	no

ni|645903622|nb|KK920808.1|	569164-569511	nan	nan	14	13	-0.055204	0.95925	no
ni|645903622|nb|KK920808.1|	569967-570196	nan	nan	10	13	0.410866	0.73195	no
ni|645903622|nb|KK920808.1|	570861-571221	nan	nan	13	37	1	0.194	no
ni|645903622|nb|KK920808.1|	571665-571850	nan	nan	14	41	2	0.2073	no
ni|645903622|nb|KK920808.1|	572227-572457	nan	nan	13	53	2	0.0939	no
ni|645903622|nb|KK920808.1|	574264-574383	nan	nan	13	62	2	0.0972	no
ni|645903622|nb|KK920808.1|	576305-576701	nan	nan	7	23	2	0.1412	no
ni|645903622|nb|KK920808.1|	577699-577989	nan	nan	0	2	inf	0.00175	yes
ni|645903622|nb|KK920808.1|	581661-582103	nan	nan	0	3	inf	0.0003	yes
ni|645903622|nb|KK920808.1|	582871-583621	nan	nan	21	18	-0.258519	0.81085	no
ni|645903622|nb|KK920808.1|	583785-584351	nan	nan	5	5	-0.0235807	0.98385	no
ni|645903622|nb|KK920808.1|	584652-585307	nan	nan	0.668899	0.810018	0.276167	0.821	no
ni|645903622|nb|KK920808.1|	586612-586871	nan	nan	95	83	-0.185349	0.86115	no
ni|645903622|nb|KK920808.1|	587051-587371	nan	nan	173.59	147	-0.240308	0.8666	no
ni|645903622|nb|KK920808.1|	587513-587644	nan	nan	197	177	-0.156924	0.88945	no
ni|645903622|nb|KK920808.1|	587762-588349	nan	nan	8	10	0.244202	0.83405	no
ni|645903622|nb|KK920808.1|	589459-589837	nan	nan	26	33	0.317583	0.77015	no
ni|645903622|nb|KK920808.1|	590804-591435	nan	nan	0.466176	0.490236	0.0726015	0.94355	no
ni|645903622|nb|KK920808.1|	591696-591863	nan	nan	41	63	0.605948	0.6028	no
ni|645903622|nb|KK920808.1|	593425-593710	nan	nan	4	6	0.488705	0.67805	no
ni|645903622|nb|KK920808.1|	596281-596982	nan	nan	5	7	0.591701	0.6066	no
ni|645903622|nb|KK920808.1|	598273-598664	nan	nan	0.170665	4	5	0.04565	no
ni|645903622|nb|KK920808.1|	598741-599075	nan	nan	0	11	inf	5.00E-05	yes
ni|645903622|nb|KK920808.1|	600682-600834	nan	nan	353	370	0.0672832	0.9652	no
ni|645903622|nb|KK920808.1|	601315-601918	nan	nan	2	2	-0.15421	0.89595	no
ni|645903622|nb|KK920808.1|	602390-602520	nan	nan	502.72	605	0.268175	0.82575	no
ni|645903622|nb|KK920808.1|	604898-605295	nan	nan	330	400	0.277081	0.8438	no
ni|645903622|nb|KK920808.1|	605813-606013	nan	nan	12	6	-0.981252	0.41625	no
ni|645903622|nb|KK920808.1|	606345-606511	nan	nan	417	546	0.388855	0.7884	no
ni|645903622|nb|KK920808.1|	607126-607278	nan	nan	615	844	0.45769	0.7501	no

ni|645903622|nb|KK920808.1|	607436-607591	nan	nan	11	14	0.392237	0.7426	no
ni|645903622|nb|KK920808.1|	608083-608331	nan	nan	72	112	0.639958	0.55205	no
ni|645903622|nb|KK920808.1|	609299-609607	nan	nan	1	0.800326	-0.428311	0.75965	no
ni|645903622|nb|KK920808.1|	609766-610150	nan	nan	162	228	0.492581	0.72595	no
ni|645903622|nb|KK920808.1|	611722-611980	nan	nan	490	759	0.632119	0.6591	no
ni|645903622|nb|KK920808.1|	628479-628853	nan	nan	38	73	0.931142	0.4714	no
ni|645903622|nb|KK920808.1|	639214-639645	nan	nan	2	2	0.429498	0.72515	no
ni|645903622|nb|KK920808.1|	642663-643969	nan	nan	0.389025	0.293702	-0.405508	0.755	no
ni|645903622|nb|KK920808.1|	646899-648394	nan	nan	181	146	-0.304006	0.8363	no
ni|645903622|nb|KK920808.1|	649334-649835	nan	nan	0	4	inf	5.00E-05	yes
ni|645903622|nb|KK920808.1|	651736-652152	nan	nan	31	31	-0.0187049	0.98655	no
ni|645903622|nb|KK920808.1|	655421-656982	nan	nan	1	0.856366	-0.313771	0.7875	no
ni|645903622|nb|KK920808.1|	658716-659209	nan	nan	0.441544	0.54264	0.297439	0.8383	no
ni|645903622|nb|KK920808.1|	659636-659853	nan	nan	35	48	0.464543	0.68135	no
ni|645903622|nb|KK920808.1|	663837-664533	nan	nan	7	12	0.757593	0.50595	no
ni|645903622|nb|KK920808.1|	669329-669520	nan	nan	13	18	0.465708	0.68925	no
ni|645903622|nb|KK920808.1|	672460-672651	nan	nan	10	21	1	0.35795	no
ni|645903622|nb|KK920808.1|	673018-673291	nan	nan	13	18	0.540685	0.63835	no
ni|645903622|nb|KK920808.1|	674740-674983	nan	nan	14	17	0.262689	0.82245	no
ni|645903622|nb|KK920808.1|	675093-675306	nan	nan	3	3	-0.173632	0.89245	no
ni|645903622|nb|KK920808.1|	676852-676979	nan	nan	17	12	-0.507896	0.68875	no
ni|645903622|nb|KK920808.1|	677385-677535	nan	nan	10	23	1	0.31695	no
ni|645903622|nb|KK920808.1|	678135-678495	nan	nan	3	6	1	0.346	no
ni|645903622|nb|KK920808.1|	678626-678851	nan	nan	16	19	0.248355	0.82615	no
ni|645903622|nb|KK920808.1|	682060-682385	nan	nan	17	21	0.280277	0.81075	no
ni|645903622|nb|KK920808.1|	682471-682654	nan	nan	16	16	0.0107195	0.98925	no
ni|645903622|nb|KK920808.1|	683146-683446	nan	nan	10	6	-0.695418	0.5552	no
ni|645903622|nb|KK920808.1|	683563-683803	nan	nan	9	7	-0.367954	0.7508	no
ni|645903622|nb|KK920808.1|	683908-684069	nan	nan	13	9	-0.436442	0.71475	no
ni|645903622|nb|KK920808.1|	684946-685186	nan	nan	6	4	-0.410253	0.73715	no

ni|645903622|nb|KK920808.1|	688235-689374	nan	nan	18	21	0.20726	0.8909	no
ni|645903622|nb|KK920808.1|	689492-689649	nan	nan	33	33	-0.0113452	0.99055	no
ni|645903622|nb|KK920808.1|	690054-690175	nan	nan	17	20	0.195643	0.87315	no
ni|645903622|nb|KK920808.1|	690614-691137	nan	nan	12	12	0.0423387	0.96855	no
ni|645903622|nb|KK920808.1|	691301-691602	nan	nan	31	34	0.100352	0.928	no
ni|645903622|nb|KK920808.1|	691718-691951	nan	nan	36	34	-0.0739292	0.94885	no
ni|645903622|nb|KK920808.1|	696716-697115	nan	nan	0.664282	2	1	0.32335	no
ni|645903622|nb|KK920808.1|	697210-697570	nan	nan	4	3	-0.387419	0.7393	no
ni|645903622|nb|KK920808.1|	700369-700843	nan	nan	9	8	-0.172085	0.88075	no
ni|645903622|nb|KK920808.1|	702064-702478	nan	nan	7	5	-0.354817	0.76115	no
ni|645903622|nb|KK920808.1|	705473-705629	nan	nan	20	14	-0.471587	0.69135	no
ni|645903622|nb|KK920808.1|	709413-709574	nan	nan	16	12	-0.360939	0.76115	no
ni|645903622|nb|KK920808.1|	709752-709904	nan	nan	17	11	-0.611512	0.6098	no
ni|645903622|nb|KK920808.1|	711668-712228	nan	nan	16	11	-0.500226	0.6589	no
ni|645903622|nb|KK920808.1|	714470-714909	nan	nan	0.878204	0.209843	-2	0.20695	no
ni|645903622|nb|KK920808.1|	716558-716785	nan	nan	41	27	-0.590049	0.61045	no
ni|645903622|nb|KK920808.1|	716900-717830	nan	nan	2	1	-0.767239	0.5162	no
ni|645903622|nb|KK920808.1|	719667-720186	nan	nan	19	13	-0.555081	0.62415	no
ni|645903622|nb|KK920808.1|	720594-720758	nan	nan	26	13	-0.941691	0.42825	no
ni|645903622|nb|KK920808.1|	721144-721358	nan	nan	32	22	-0.528604	0.64695	no
ni|645903622|nb|KK920808.1|	721471-721634	nan	nan	12	4	-2	0.21335	no
ni|645903622|nb|KK920808.1|	726051-726622	nan	nan	1	0.653489	-0.823984	0.51705	no
ni|645903625|nb|KK920805.1|	10463-10592	nan	nan	18	53	2	0.19965	no
ni|645903625|nb|KK920805.1|	119616-119853	nan	nan	3	4	0.518624	0.6723	no
ni|645903625|nb|KK920805.1|	121017-121632	nan	nan	4	3	-0.391735	0.73345	no
ni|645903625|nb|KK920805.1|	122123-122359	nan	nan	2	2	0.0405625	0.95995	no
ni|645903625|nb|KK920805.1|	123146-123359	nan	nan	4	1	-2	0.25885	no
ni|645903625|nb|KK920805.1|	125589-125725	nan	nan	21	18	-0.166786	0.8881	no
ni|645903625|nb|KK920805.1|	126347-126590	nan	nan	22	17	-0.379759	0.74585	no
ni|645903625|nb|KK920805.1|	127011-127223	nan	nan	31	26	-0.253504	0.82735	no

ni|645903625|nb|KK920805.1|	128798-129683	nan	nan	11	11	-0.0567051	0.95635	no
ni|645903625|nb|KK920805.1|	12927-13230	nan	nan	9	20	1	0.3336	no
ni|645903625|nb|KK920805.1|	130511-131431	nan	nan	2.14	2	0.0710237	0.95315	no
ni|645903625|nb|KK920805.1|	131958-132443	nan	nan	14	14	-0.0911421	0.9359	no
ni|645903625|nb|KK920805.1|	133613-134082	nan	nan	18	20	0.207838	0.8511	no
ni|645903625|nb|KK920805.1|	134290-134543	nan	nan	20	17	-0.225929	0.84095	no
ni|645903625|nb|KK920805.1|	134854-135347	nan	nan	14	13	-0.0520154	0.9623	no
ni|645903625|nb|KK920805.1|	135520-135859	nan	nan	6	8.45	0.413071	0.7258	no
ni|645903625|nb|KK920805.1|	136543-136781	nan	nan	18	20	0.216405	0.8559	no
ni|645903625|nb|KK920805.1|	139267-139479	nan	nan	21	22	0.0207277	0.98605	no
ni|645903625|nb|KK920805.1|	139706-140389	nan	nan	8	9	0.139523	0.9002	no
ni|645903625|nb|KK920805.1|	141227-141447	nan	nan	16	19	0.266526	0.8265	no
ni|645903625|nb|KK920805.1|	141699-142152	nan	nan	6	7	0.157175	0.89395	no
ni|645903625|nb|KK920805.1|	143504-143743	nan	nan	13	15	0.219597	0.85145	no
ni|645903625|nb|KK920805.1|	144986-145611	nan	nan	31	27	-0.244044	0.8132	no
ni|645903625|nb|KK920805.1|	14751-15076	nan	nan	15	24	0.675615	0.5586	no
ni|645903625|nb|KK920805.1|	147922-148378	nan	nan	8	11	0.40568	0.73035	no
ni|645903625|nb|KK920805.1|	148830-149064	nan	nan	18	20	0.164399	0.8884	no
ni|645903625|nb|KK920805.1|	149608-149851	nan	nan	12	12	-0.00571752	0.99205	no
ni|645903625|nb|KK920805.1|	150820-151221	nan	nan	5	7	0.355497	0.756	no
ni|645903625|nb|KK920805.1|	153535-154017	nan	nan	9	9	0.0792368	0.9452	no
ni|645903625|nb|KK920805.1|	15499-15694	nan	nan	20	18	-0.185732	0.8672	no
ni|645903625|nb|KK920805.1|	15926-16049	nan	nan	15	21	0.517595	0.6788	no
ni|645903625|nb|KK920805.1|	162402-162631	nan	nan	12	14	0.255473	0.82495	no
ni|645903625|nb|KK920805.1|	165743-165867	nan	nan	16	23	0.517607	0.67245	no
ni|645903625|nb|KK920805.1|	16624-16872	nan	nan	19	19	-0.0283995	0.9796	no
ni|645903625|nb|KK920805.1|	168753-169348	nan	nan	5	6	0.166861	0.88375	no
ni|645903625|nb|KK920805.1|	16990-18003	nan	nan	11	10	-0.115719	0.9176	no
ni|645903625|nb|KK920805.1|	170847-171288	nan	nan	4	5	0.19779	0.8679	no
ni|645903625|nb|KK920805.1|	171462-171750	nan	nan	12	13	0.10226	0.92995	no

ni|645903625|nb|KK920805.1|	174406-174664	nan	nan	14	15	0.134812	0.9065	no
ni|645903625|nb|KK920805.1|	174940-175072	nan	nan	12	10	-0.259924	0.8388	no
ni|645903625|nb|KK920805.1|	178599-178821	nan	nan	12	16	0.458095	0.70175	no
ni|645903625|nb|KK920805.1|	180189-180667	nan	nan	8	8	0.153673	0.8921	no
ni|645903625|nb|KK920805.1|	180826-180988	nan	nan	9	13	0.51775	0.66765	no
ni|645903625|nb|KK920805.1|	181556-181794	nan	nan	3	6	1	0.39145	no
ni|645903625|nb|KK920805.1|	18178-18411	nan	nan	22	21	-0.0540118	0.9634	no
ni|645903625|nb|KK920805.1|	189021-189552	nan	nan	303	134	-1	0.4085	no
ni|645903625|nb|KK920805.1|	189746-190581	nan	nan	26	13	-1	0.42945	no
ni|645903625|nb|KK920805.1|	19081-19436	nan	nan	12	9	-0.499607	0.6699	no
ni|645903625|nb|KK920805.1|	191448-191656	nan	nan	146	89	-0.713672	0.50845	no
ni|645903625|nb|KK920805.1|	191769-191902	nan	nan	25	22	-0.200538	0.86755	no
ni|645903625|nb|KK920805.1|	192663-192796	nan	nan	10	9	-0.067271	0.9547	no
ni|645903625|nb|KK920805.1|	192992-193312	nan	nan	3	2	-0.572636	0.6321	no
ni|645903625|nb|KK920805.1|	193551-193905	nan	nan	10	7	-0.457833	0.6935	no
ni|645903625|nb|KK920805.1|	194049-194407	nan	nan	5	4	-0.489307	0.6807	no
ni|645903625|nb|KK920805.1|	194514-194844	nan	nan	16	10	-0.724589	0.53255	no
ni|645903625|nb|KK920805.1|	195500-196401	nan	nan	35	23	-0.617691	0.6804	no
ni|645903625|nb|KK920805.1|	19556-19746	nan	nan	27	22	-0.312295	0.7838	no
ni|645903625|nb|KK920805.1|	196542-197082	nan	nan	9	4	-1	0.35735	no
ni|645903625|nb|KK920805.1|	197308-197448	nan	nan	22	11	-0.989225	0.4137	no
ni|645903625|nb|KK920805.1|	197685-197814	nan	nan	9.29	2	-2	0.24815	no
ni|645903625|nb|KK920805.1|	198097-199268	nan	nan	84	53	-0.643316	0.64115	no
ni|645903625|nb|KK920805.1|	199393-200534	nan	nan	37	28	-0.38808	0.786	no
ni|645903625|nb|KK920805.1|	200634-201411	nan	nan	58	47	-0.307822	0.8292	no
ni|645903625|nb|KK920805.1|	201755-201988	nan	nan	88	69	-0.346723	0.7537	no
ni|645903625|nb|KK920805.1|	202122-203505	nan	nan	15	14	-0.124728	0.916	no
ni|645903625|nb|KK920805.1|	20227-20359	nan	nan	23	25	0.113294	0.92325	no
ni|645903625|nb|KK920805.1|	203932-204926	nan	nan	41	40	-0.0588306	0.96665	no
ni|645903625|nb|KK920805.1|	207028-207856	nan	nan	0.135848	537	12	0.03715	no

ni|645903625|nb|KK920805.1|	215328-218791	nan	nan	0.036667	3	6	0.02725	no
ni|645903625|nb|KK920805.1|	223632-223785	nan	nan	105	77	-0.441825	0.6992	no
ni|645903625|nb|KK920805.1|	26673-26817	nan	nan	5	4	-0.429779	0.75965	no
ni|645903625|nb|KK920805.1|	305987-306158	nan	nan	59	57	-0.0501039	0.96455	no
ni|645903625|nb|KK920805.1|	3079-3309	nan	nan	13	35	1	0.2362	no
ni|645903625|nb|KK920805.1|	317498-317899	nan	nan	16	83	2	0.0871	no
ni|645903625|nb|KK920805.1|	318251-318390	nan	nan	11	80	3	0.02885	no
ni|645903625|nb|KK920805.1|	323531-323882	nan	nan	4	30	3	0.0236	no
ni|645903625|nb|KK920805.1|	324673-324825	nan	nan	24	119.79	2	0.0573	no
ni|645903625|nb|KK920805.1|	325480-325668	nan	nan	25	119	2	0.0592	no
ni|645903625|nb|KK920805.1|	328926-329049	nan	nan	18	105	3	0.0514	no
ni|645903625|nb|KK920805.1|	330452-330653	nan	nan	10	58	3	0.04565	no
ni|645903625|nb|KK920805.1|	332477-332981	nan	nan	4	18	2	0.07665	no
ni|645903625|nb|KK920805.1|	335043-335164	nan	nan	15	99	3	0.04235	no
ni|645903625|nb|KK920805.1|	336637-336828	nan	nan	29	106	2	0.1131	no
ni|645903625|nb|KK920805.1|	336984-337268	nan	nan	31	142	2	0.10835	no
ni|645903625|nb|KK920805.1|	337975-338089	nan	nan	35	231	3	0.03715	no
ni|645903625|nb|KK920805.1|	343240-344001	nan	nan	0.561252	0.321971	-0.801718	0.5588	no
ni|645903625|nb|KK920805.1|	344545-344700	nan	nan	33	173.11	2	0.0423	no
ni|645903625|nb|KK920805.1|	345671-346024	nan	nan	17	55	2	0.1469	no
ni|645903625|nb|KK920805.1|	349639-349884	nan	nan	0.688765	44	6	0.03715	no
ni|645903625|nb|KK920805.1|	350184-350465	nan	nan	3	4	0.0451187	0.9714	no
ni|645903625|nb|KK920805.1|	350604-350951	nan	nan	2	1	-0.843153	0.49815	no
ni|645903625|nb|KK920805.1|	351063-351328	nan	nan	2	2	-0.172953	0.89245	no
ni|645903625|nb|KK920805.1|	351887-352065	nan	nan	4	1	-2	0.25505	no
ni|645903625|nb|KK920805.1|	352566-352981	nan	nan	8	13	0.709986	0.54735	no
ni|645903625|nb|KK920805.1|	354213-354518	nan	nan	1	1	-0.0657579	0.9467	no
ni|645903625|nb|KK920805.1|	355979-357323	nan	nan	2	2	0.496851	0.6666	no
ni|645903625|nb|KK920805.1|	3588-4223	nan	nan	6	13	1	0.3526	no
ni|645903625|nb|KK920805.1|	358853-359424	nan	nan	0.631017	1	1	0.38815	no

ni|645903625|nb|KK920805.1|	359627-360177	nan	nan	0.220203	12	6	0.03725	no
ni|645903625|nb|KK920805.1|	361321-361523	nan	nan	1	14	3	0.05415	no
ni|645903625|nb|KK920805.1|	362469-362795	nan	nan	1	17	4	0.01245	no
ni|645903625|nb|KK920805.1|	362947-363429	nan	nan	2	27	4	0.00285	yes
ni|645903625|nb|KK920805.1|	364081-364270	nan	nan	3	53	4	0.01045	no
ni|645903625|nb|KK920805.1|	366802-367198	nan	nan	2	21	4	0.00845	no
ni|645903625|nb|KK920805.1|	369500-369841	nan	nan	3	19	3	0.02835	no
ni|645903625|nb|KK920805.1|	372928-373124	nan	nan	5	41	3	0.02525	no
ni|645903625|nb|KK920805.1|	373837-374200	nan	nan	2	17	3	0.0188	no
ni|645903625|nb|KK920805.1|	374510-374648	nan	nan	5	37	3	0.04995	no
ni|645903625|nb|KK920805.1|	384004-385447	nan	nan	7	13	0.851215	0.51605	no
ni|645903625|nb|KK920805.1|	481799-482027	nan	nan	10	9	-0.152201	0.8946	no
ni|645903625|nb|KK920805.1|	514368-514558	nan	nan	8	12	0.598399	0.6133	no
ni|645903625|nb|KK920805.1|	519479-520011	nan	nan	12	10	-0.184111	0.8685	no
ni|645903625|nb|KK920805.1|	533907-534153	nan	nan	6	5	-0.388152	0.74155	no
ni|645903625|nb|KK920805.1|	555725-556094	nan	nan	33	40	0.278743	0.801	no
ni|645903625|nb|KK920805.1|	576527-576689	nan	nan	39	61	0.636656	0.58545	no
ni|645903625|nb|KK920805.1|	580015-580211	nan	nan	20	24	0.232495	0.8387	no
ni|645903625|nb|KK920805.1|	582237-582364	nan	nan	36	40	0.13431	0.90755	no
ni|645903625|nb|KK920805.1|	591634-591826	nan	nan	31	38	0.277188	0.8118	no
ni|645903625|nb|KK920805.1|	601041-601250	nan	nan	23	29	0.305177	0.7913	no
ni|645903625|nb|KK920805.1|	613412-613709	nan	nan	26	32	0.300689	0.7879	no
ni|645903625|nb|KK920805.1|	621917-622043	nan	nan	39	29	-0.459652	0.68725	no
ni|645903625|nb|KK920805.1|	644972-645168	nan	nan	0	10	inf	5.00E-05	yes
ni|645903625|nb|KK920805.1|	655258-655441	nan	nan	31	31	-0.0123165	0.992	no
ni|645903625|nb|KK920805.1|	777936-778099	nan	nan	5	6	0.232343	0.8527	no
ni|645903625|nb|KK920805.1|	801490-801856	nan	nan	0.560438	0.7141	0.349571	0.81755	no
ni|645903625|nb|KK920805.1|	87104-87313	nan	nan	10	13	0.423533	0.7127	no
ni|645903625|nb|KK920805.1|	89258-89603	nan	nan	17	26	0.580337	0.6083	no
ni|645903625|nb|KK920805.1|	90256-90402	nan	nan	17	14	-0.266513	0.81915	no

ni|645903625|nb|KK920805.1|	90528-90699	nan	nan	47	64	0.450569	0.6929	no
ni|645903625|nb|KK920805.1|	90948-91114	nan	nan	52	74	0.496667	0.66895	no
ni|645903625|nb|KK920805.1|	92413-92665	nan	nan	45	62	0.477708	0.6688	no
ni|645903625|nb|KK920805.1|	93463-93994	nan	nan	22	23	0.118902	0.91545	no
ni|645903625|nb|KK920805.1|	94222-94499	nan	nan	62	65	0.0658737	0.9518	no
ni|645903625|nb|KK920805.1|	94627-94749	nan	nan	31	8	-2	0.175	no
ni|645903625|nb|KK920805.1|	94829-94965	nan	nan	45	27	-0.697301	0.5535	no
ni|645903625|nb|KK920805.1|	95361-95500	nan	nan	60	51	-0.229504	0.8461	no
ni|645903626|nb|KK920804.1|	104085-104460	nan	nan	18	19	0.118993	0.91555	no
ni|645903626|nb|KK920804.1|	105634-106567	nan	nan	0.504375	0.226955	-1	0.40265	no
ni|645903626|nb|KK920804.1|	109547-109809	nan	nan	51	48	-0.0880231	0.93905	no
ni|645903626|nb|KK920804.1|	109942-110151	nan	nan	13	4	-2	0.1667	no
ni|645903626|nb|KK920804.1|	110437-110616	nan	nan	41	31	-0.403187	0.73065	no
ni|645903626|nb|KK920804.1|	110727-110863	nan	nan	29	27	-0.101198	0.9325	no
ni|645903626|nb|KK920804.1|	111150-111297	nan	nan	48	33	-0.530602	0.6473	no
ni|645903626|nb|KK920804.1|	111359-111483	nan	nan	51	33	-0.623748	0.5964	no
ni|645903626|nb|KK920804.1|	111706-111843	nan	nan	38	32	-0.227709	0.8415	no
ni|645903626|nb|KK920804.1|	112029-112223	nan	nan	48	37	-0.379051	0.7346	no
ni|645903626|nb|KK920804.1|	113043-113158	nan	nan	46	24	-0.925476	0.44955	no
ni|645903626|nb|KK920804.1|	113503-113787	nan	nan	15	10	-0.564142	0.6276	no
ni|645903626|nb|KK920804.1|	113853-114104	nan	nan	47	25	-0.874804	0.4407	no
ni|645903626|nb|KK920804.1|	114208-114353	nan	nan	24	12	-1	0.40315	no
ni|645903626|nb|KK920804.1|	119621-119754	nan	nan	10	1	-3	0.2563	no
ni|645903626|nb|KK920804.1|	125142-125348	nan	nan	19	22	0.258722	0.82375	no
ni|645903626|nb|KK920804.1|	127026-127314	nan	nan	8	11	0.47019	0.6872	no
ni|645903626|nb|KK920804.1|	128795-128941	nan	nan	11	9	-0.219207	0.86075	no
ni|645903626|nb|KK920804.1|	129337-129961	nan	nan	0	6	inf	5.00E-05	yes
ni|645903626|nb|KK920804.1|	131403-131538	nan	nan	51	44.37	-0.212688	0.857	no
ni|645903626|nb|KK920804.1|	132693-133095	nan	nan	31	21	-0.551542	0.61855	no
ni|645903626|nb|KK920804.1|	133638-133797	nan	nan	44	31	-0.486916	0.67425	no

ni|645903626|nb|KK920804.1|	134102-134358	nan	nan	36	42	0.204278	0.85585	no
ni|645903626|nb|KK920804.1|	134668-135106	nan	nan	26	55	1	0.41225	no
ni|645903626|nb|KK920804.1|	135493-135679	nan	nan	49	105.48	1	0.3308	no
ni|645903626|nb|KK920804.1|	135878-135985	nan	nan	16	60	2	0.1738	no
ni|645903626|nb|KK920804.1|	137771-138199	nan	nan	24	49	1	0.34735	no
ni|645903626|nb|KK920804.1|	138465-139705	nan	nan	5	5	0.0371076	0.97525	no
ni|645903626|nb|KK920804.1|	140078-140206	nan	nan	36	99	1	0.217	no
ni|645903626|nb|KK920804.1|	142135-142295	nan	nan	33	96	2	0.1953	no
ni|645903626|nb|KK920804.1|	143377-143597	nan	nan	34	88	1	0.21975	no
ni|645903626|nb|KK920804.1|	144307-144492	nan	nan	28	76	1	0.2117	no
ni|645903626|nb|KK920804.1|	146674-146971	nan	nan	10	37	2	0.11415	no
ni|645903626|nb|KK920804.1|	151081-151864	nan	nan	22	50	1	0.33505	no
ni|645903626|nb|KK920804.1|	160399-160461	nan	nan	51058.1	50162.8	-0.0255207	0.97445	no
ni|645903626|nb|KK920804.1|	181324-181717	nan	nan	0	5	inf	5.00E-05	yes
ni|645903626|nb|KK920804.1|	181807-182227	nan	nan	0	2	inf	0.00055	yes
ni|645903626|nb|KK920804.1|	212207-212280	nan	nan	26160.5	26559.9	0.0218569	0.9824	no
ni|645903626|nb|KK920804.1|	231722-232063	nan	nan	0	1323.46	inf	5.00E-05	yes
ni|645903626|nb|KK920804.1|	253584-253739	nan	nan	0	252.74	inf	5.00E-05	yes
ni|645903626|nb|KK920804.1|	254676-254857	nan	nan	0.309591	1384.03	12	0.11935	no
ni|645903626|nb|KK920804.1|	260770-261003	nan	nan	0	1618.15	inf	5.00E-05	yes
ni|645903626|nb|KK920804.1|	264155-264542	nan	nan	0.173058	1303.77	13	0.0443	no
ni|645903626|nb|KK920804.1|	264690-264829	nan	nan	0	791	inf	5.00E-05	yes
ni|645903626|nb|KK920804.1|	267867-268055	nan	nan	0.284946	1384.08	12	0.11935	no
ni|645903626|nb|KK920804.1|	268538-268759	nan	nan	0	1408.22	inf	5.00E-05	yes
ni|645903626|nb|KK920804.1|	275715-275853	nan	nan	1	1674.18	10	0.0443	no
ni|645903626|nb|KK920804.1|	282765-282923	nan	nan	0.860801	1523.7	11	0.0443	no
ni|645903626|nb|KK920804.1|	299483-299593	nan	nan	32	33	0.0318793	0.96125	no
ni|645903626|nb|KK920804.1|	30406-30588	nan	nan	31	35	0.179146	0.87715	no
ni|645903626|nb|KK920804.1|	318171-318333	nan	nan	23	25	0.144292	0.90065	no
ni|645903626|nb|KK920804.1|	318896-319209	nan	nan	2	3	0.612358	0.62305	no

ni|645903626|nb|KK920804.1|	319507-320452	nan	nan	0.467945	0.363552	-0.364177	0.7706	no
ni|645903626|nb|KK920804.1|	322005-322184	nan	nan	15	19	0.363315	0.75375	no
ni|645903626|nb|KK920804.1|	322704-322824	nan	nan	25	31	0.295165	0.80305	no
ni|645903626|nb|KK920804.1|	322935-323453	nan	nan	7	8	0.157309	0.8887	no
ni|645903626|nb|KK920804.1|	323809-324057	nan	nan	7	9	0.455751	0.69885	no
ni|645903626|nb|KK920804.1|	324814-325036	nan	nan	24	27	0.159314	0.89005	no
ni|645903626|nb|KK920804.1|	325204-325380	nan	nan	5	0.944405	-2	0.1791	no
ni|645903626|nb|KK920804.1|	326055-326290	nan	nan	8	4	-0.838956	0.4872	no
ni|645903626|nb|KK920804.1|	333549-334047	nan	nan	26	37	0.475023	0.66595	no
ni|645903626|nb|KK920804.1|	336087-336280	nan	nan	168	216	0.359406	0.7798	no
ni|645903626|nb|KK920804.1|	336382-336631	nan	nan	119.11	160	0.43021	0.73065	no
ni|645903626|nb|KK920804.1|	336788-337409	nan	nan	52	66	0.330277	0.82165	no
ni|645903626|nb|KK920804.1|	337514-337766	nan	nan	148.59	179	0.270677	0.8535	no
ni|645903626|nb|KK920804.1|	338134-338282	nan	nan	152	178	0.233899	0.83945	no
ni|645903626|nb|KK920804.1|	339185-339359	nan	nan	179.83	218	0.278869	0.7896	no
ni|645903626|nb|KK920804.1|	343558-343932	nan	nan	42	38	-0.13255	0.90375	no
ni|645903626|nb|KK920804.1|	345027-345163	nan	nan	110	97	-0.179725	0.8737	no
ni|645903626|nb|KK920804.1|	345719-345840	nan	nan	52	22	-1	0.3131	no
ni|645903626|nb|KK920804.1|	364175-364375	nan	nan	62	39	-0.66662	0.5599	no
ni|645903626|nb|KK920804.1|	412180-412451	nan	nan	58	39	-0.594506	0.59485	no
ni|645903626|nb|KK920804.1|	420420-420767	nan	nan	3	3	-0.0110981	0.97705	no
ni|645903626|nb|KK920804.1|	421338-422422	nan	nan	17	10	-0.729524	0.4909	no
ni|645903626|nb|KK920804.1|	422608-422832	nan	nan	4	0.770312	-2	0.1101	no
ni|645903626|nb|KK920804.1|	423159-423517	nan	nan	14	12	-0.317038	0.7865	no
ni|645903626|nb|KK920804.1|	424308-424574	nan	nan	85	52	-0.713389	0.5148	no
ni|645903626|nb|KK920804.1|	425340-425462	nan	nan	59	37	-0.692983	0.56435	no
ni|645903626|nb|KK920804.1|	433215-433481	nan	nan	288	349	0.276945	0.8402	no
ni|645903626|nb|KK920804.1|	437054-437338	nan	nan	3	4	0.399773	0.7408	no
ni|645903626|nb|KK920804.1|	440806-440973	nan	nan	13	11	-0.289634	0.80005	no
ni|645903626|nb|KK920804.1|	444269-444700	nan	nan	0.824589	0.931409	0.175741	0.8916	no

ni|645903626|nb|KK920804.1|	445053-445254	nan	nan	2	1	-0.803927	0.6027	no
ni|645903626|nb|KK920804.1|	445645-445919	nan	nan	1	1	-0.23589	0.8697	no
ni|645903626|nb|KK920804.1|	447047-447364	nan	nan	0.917304	0.657354	-0.48073	0.7594	no
ni|645903626|nb|KK920804.1|	448493-448694	nan	nan	4	8	0.974859	0.43275	no
ni|645903626|nb|KK920804.1|	450011-450172	nan	nan	7	6	-0.237133	0.8532	no
ni|645903626|nb|KK920804.1|	452217-452914	nan	nan	3	4	0.0837032	0.94045	no
ni|645903626|nb|KK920804.1|	455419-455699	nan	nan	3	3	-0.387848	0.74515	no
ni|645903626|nb|KK920804.1|	455995-456352	nan	nan	7	7	0.0300747	0.979	no
ni|645903626|nb|KK920804.1|	456950-457240	nan	nan	3	4	0.156543	0.8953	no
ni|645903626|nb|KK920804.1|	461810-461950	nan	nan	15	11	-0.482265	0.69675	no
ni|645903626|nb|KK920804.1|	464234-464541	nan	nan	5	7	0.36597	0.7547	no
ni|645903626|nb|KK920804.1|	465991-466180	nan	nan	19	19	-0.00306806	0.99105	no
ni|645903626|nb|KK920804.1|	468144-468340	nan	nan	19	11	-0.724959	0.5443	no
ni|645903626|nb|KK920804.1|	468505-468717	nan	nan	27	21	-0.365394	0.75865	no
ni|645903626|nb|KK920804.1|	471919-472993	nan	nan	22	18	-0.28087	0.82	no
ni|645903626|nb|KK920804.1|	47892-48142	nan	nan	4	5	0.303054	0.799	no
ni|645903626|nb|KK920804.1|	482877-483529	nan	nan	0.538039	0.385786	-0.479911	0.7301	no
ni|645903626|nb|KK920804.1|	520190-520332	nan	nan	57	38	-0.58609	0.6151	no
ni|645903626|nb|KK920804.1|	529164-529319	nan	nan	75	61	-0.302682	0.79375	no
ni|645903626|nb|KK920804.1|	531822-532262	nan	nan	0.80264	0.278963	-2	0.3149	no
ni|645903626|nb|KK920804.1|	533307-533606	nan	nan	81	57	-0.501103	0.63545	no
ni|645903626|nb|KK920804.1|	534152-534460	nan	nan	20	13	-0.604767	0.60185	no
ni|645903626|nb|KK920804.1|	534948-535075	nan	nan	137	92	-0.564261	0.62775	no
ni|645903626|nb|KK920804.1|	536641-537432	nan	nan	2	2	-0.0971468	0.93305	no
ni|645903626|nb|KK920804.1|	537637-537812	nan	nan	95	63	-0.580438	0.60545	no
ni|645903626|nb|KK920804.1|	545024-545142	nan	nan	23	8	-2	0.2996	no
ni|645903626|nb|KK920804.1|	545365-545808	nan	nan	17	11	-0.647136	0.56735	no
ni|645903626|nb|KK920804.1|	545910-546621	nan	nan	139	395	2	0.30815	no
ni|645903626|nb|KK920804.1|	546687-547646	nan	nan	81	251	2	0.26195	no
ni|645903626|nb|KK920804.1|	644143-644528	nan	nan	0.609982	2	1	0.3229	no

ni|645903626|nb|KK920804.1|	663583-663857	nan	nan	120	109	-0.14196	0.9267	no
ni|645903626|nb|KK920804.1|	666229-666354	nan	nan	119	125	0.0787344	0.9419	no
ni|645903626|nb|KK920804.1|	667781-668220	nan	nan	36	33	-0.158164	0.8857	no
ni|645903626|nb|KK920804.1|	672566-672773	nan	nan	37	35	-0.0759924	0.94705	no
ni|645903626|nb|KK920804.1|	673067-674721	nan	nan	2	53	5	0.00325	no
ni|645903626|nb|KK920804.1|	676191-676550	nan	nan	2	0.458448	-2	0.1999	no
ni|645903626|nb|KK920804.1|	676918-677581	nan	nan	1	0.588344	-1	0.3967	no
ni|645903626|nb|KK920804.1|	678782-679719	nan	nan	3	0.931706	-2	0.1216	no
ni|645903626|nb|KK920804.1|	680005-680314	nan	nan	3	0.113782	-5	0.01885	no
ni|645903626|nb|KK920804.1|	680966-681169	nan	nan	3	284	6	0.00375	no
ni|645903626|nb|KK920804.1|	682751-682906	nan	nan	0.453407	1778.7	12	0.11935	no
ni|645903626|nb|KK920804.1|	685077-686419	nan	nan	0	0.646397	inf	0.0001	yes
ni|645903626|nb|KK920804.1|	687508-687819	nan	nan	0	307	inf	5.00E-05	yes
ni|645903626|nb|KK920804.1|	689295-689472	nan	nan	0	355	inf	5.00E-05	yes
ni|645903626|nb|KK920804.1|	71248-71391	nan	nan	26	33	0.347827	0.77085	no
ni|645903626|nb|KK920804.1|	732929-733136	nan	nan	10	10	-0.034411	0.97725	no
ni|645903626|nb|KK920804.1|	74814-74980	nan	nan	35	39.44	0.161791	0.8885	no
ni|645903626|nb|KK920804.1|	757139-757356	nan	nan	13	17	0.313624	0.78935	no
ni|645903626|nb|KK920804.1|	757504-757741	nan	nan	0	2	inf	0.003	no
ni|645903626|nb|KK920804.1|	759392-759591	nan	nan	13	33	1	0.2596	no
ni|645903626|nb|KK920804.1|	761266-761446	nan	nan	15	25	0.710298	0.54345	no
ni|645903626|nb|KK920804.1|	765297-765547	nan	nan	18	35	0.979833	0.39895	no
ni|645903626|nb|KK920804.1|	766740-766895	nan	nan	18	38	1	0.36785	no
ni|645903626|nb|KK920804.1|	777897-778209	nan	nan	9	13	0.568996	0.6267	no
ni|645903626|nb|KK920804.1|	778847-779428	nan	nan	14	19	0.374244	0.7334	no
ni|645903626|nb|KK920804.1|	784217-784336	nan	nan	52	22	-1	0.30735	no
ni|645903626|nb|KK920804.1|	784520-784643	nan	nan	20	18	-0.14537	0.90525	no
ni|645903626|nb|KK920804.1|	785031-785212	nan	nan	46	38	-0.307435	0.78805	no
ni|645903626|nb|KK920804.1|	785338-785822	nan	nan	16	14	-0.221594	0.8454	no
ni|645903626|nb|KK920804.1|	785997-786101	nan	nan	99	19	-2	0.11635	no

ni|645903626|nb|KK920804.1|	786608-786841	nan	nan	25	17	-0.576257	0.61725	no
ni|645903626|nb|KK920804.1|	787090-787380	nan	nan	54	42	-0.360033	0.74085	no
ni|645903626|nb|KK920804.1|	787846-788086	nan	nan	25	37	0.57551	0.6188	no
ni|645903626|nb|KK920804.1|	788832-789282	nan	nan	0.779583	0.541908	-0.524656	0.7071	no
ni|645903626|nb|KK920804.1|	794036-794306	nan	nan	19	22	0.167367	0.88295	no
ni|645903626|nb|KK920804.1|	794416-795414	nan	nan	19	30	0.692485	0.56785	no
ni|645903626|nb|KK920804.1|	795557-795942	nan	nan	0.871402	0.499671	-0.802362	0.6027	no
ni|645903626|nb|KK920804.1|	797851-798033	nan	nan	5	1	-2	0.2004	no
ni|645903626|nb|KK920804.1|	798157-798367	nan	nan	5	2	-2	0.2366	no
ni|645903626|nb|KK920804.1|	798644-799506	nan	nan	12	24	0.987308	0.44655	no
ni|645903626|nb|KK920804.1|	799620-799972	nan	nan	4	0.754018	-3	0.0819	no
ni|645903626|nb|KK920804.1|	800281-800484	nan	nan	118	237	1	0.42345	no
ni|645903626|nb|KK920804.1|	801077-801196	nan	nan	27	14	-0.874774	0.48445	no
ni|645903626|nb|KK920804.1|	801382-803245	nan	nan	11	2	-2	0.1217	no
ni|645903626|nb|KK920804.1|	803755-803898	nan	nan	12	4	-2	0.24025	no
ni|645903626|nb|KK920804.1|	805860-806027	nan	nan	24	2	-3	0.04715	no
ni|645903626|nb|KK920804.1|	806162-806294	nan	nan	17	3	-3	0.1043	no
ni|645903626|nb|KK920804.1|	806604-806857	nan	nan	5	0.93477	-2	0.1108	no
ni|645903626|nb|KK920804.1|	807323-807448	nan	nan	24	8	-2	0.2598	no
ni|645903626|nb|KK920804.1|	809126-809653	nan	nan	9	2	-2	0.11085	no
ni|645903626|nb|KK920804.1|	809778-809915	nan	nan	17	3	-3	0.08215	no
ni|645903626|nb|KK920804.1|	810429-810675	nan	nan	769	305	-1	0.3532	no
ni|645903626|nb|KK920804.1|	811363-811479	nan	nan	19	9	-1	0.42295	no
ni|645903626|nb|KK920804.1|	811674-812204	nan	nan	8	4	-0.908943	0.4372	no
ni|645903626|nb|KK920804.1|	812572-813356	nan	nan	239	94	-1	0.34785	no
ni|645903626|nb|KK920804.1|	813644-813995	nan	nan	1	0.37852	-2	0.22995	no
ni|645903626|nb|KK920804.1|	814126-814627	nan	nan	462	192	-1	0.37385	no
ni|645903626|nb|KK920804.1|	815035-815292	nan	nan	1131.61	465	-1	0.3747	no
ni|645903626|nb|KK920804.1|	815492-816144	nan	nan	4	2	-1	0.36815	no
ni|645903626|nb|KK920804.1|	816443-816654	nan	nan	24	12	-1	0.37445	no

ni|645903626|nb|KK920804.1|	816833-817126	nan	nan	635	272.21	-1	0.3838	no
ni|645903626|nb|KK920804.1|	817887-818105	nan	nan	1	2	0.126013	0.92555	no
ni|645903626|nb|KK920804.1|	818347-818534	nan	nan	4	2	-0.767668	0.5799	no
ni|645903626|nb|KK920804.1|	819306-819982	nan	nan	2	0.944672	-0.86321	0.4756	no
ni|645903626|nb|KK920804.1|	820262-821338	nan	nan	128	54	-1	0.3806	no
ni|645903626|nb|KK920804.1|	821671-822212	nan	nan	2	1	-0.328078	0.78365	no
ni|645903626|nb|KK920804.1|	822509-822988	nan	nan	1	0.937967	-0.480197	0.71005	no
ni|645903626|nb|KK920804.1|	840923-841088	nan	nan	2	51	5	0.0375	no
ni|645903626|nb|KK920804.1|	845129-845317	nan	nan	2	43	4	0.0168	no
ni|645903626|nb|KK920804.1|	846794-846943	nan	nan	0.507679	26	6	0.11975	no
ni|645903626|nb|KK920804.1|	865583-865720	nan	nan	0.667423	33	6	0.11965	no
ni|645903626|nb|KK920804.1|	866862-867285	nan	nan	0.07683	25	8	0.11935	no
ni|645903626|nb|KK920804.1|	872824-873024	nan	nan	0.501132	60	7	0.0443	no
ni|645903626|nb|KK920804.1|	878798-879211	nan	nan	0.079299	7	6	0.11935	no
ni|645903626|nb|KK920804.1|	92614-92796	nan	nan	36	43	0.227715	0.8434	no
ni|645903626|nb|KK920804.1|	93015-93141	nan	nan	28	26	-0.116244	0.92085	no
ni|645903626|nb|KK920804.1|	95339-95502	nan	nan	29	33	0.182761	0.8747	no
ni|645903626|nb|KK920804.1|	96185-96312	nan	nan	28	35	0.300408	0.7971	no
ni|645903626|nb|KK920804.1|	97669-97861	nan	nan	41	40	-0.0101902	0.99365	no
ni|645903627|nb|KK920803.1|	119472-119794	nan	nan	0.896493	4	2	0.1112	no
ni|645903627|nb|KK920803.1|	119979-120370	nan	nan	33	49	0.571789	0.5948	no
ni|645903627|nb|KK920803.1|	123700-123821	nan	nan	26	46	0.801364	0.5037	no
ni|645903627|nb|KK920803.1|	125363-125594	nan	nan	61	90	0.567447	0.60395	no
ni|645903627|nb|KK920803.1|	126146-126326	nan	nan	62	94	0.605259	0.59995	no
ni|645903627|nb|KK920803.1|	128030-128165	nan	nan	51	97	0.932743	0.4256	no
ni|645903627|nb|KK920803.1|	131398-132040	nan	nan	15	23	0.590741	0.58665	no
ni|645903627|nb|KK920803.1|	135106-135326	nan	nan	19	36	0.902036	0.4369	no
ni|645903627|nb|KK920803.1|	143411-143713	nan	nan	0.985961	1	0.10415	0.93335	no
ni|645903627|nb|KK920803.1|	145075-145444	nan	nan	0.923577	1	0.419972	0.73705	no
ni|645903627|nb|KK920803.1|	145730-146018	nan	nan	22.26	37.09	0.736577	0.52285	no

ni|645903627|nb|KK920803.1|	148104-148291	nan	nan	22	32	0.49286	0.66745	no
ni|645903627|nb|KK920803.1|	149107-150093	nan	nan	10	15	0.539786	0.61675	no
ni|645903627|nb|KK920803.1|	150532-151683	nan	nan	16	16	-0.00850719	0.99355	no
ni|645903627|nb|KK920803.1|	152291-152523	nan	nan	27	26	-0.0360366	0.97675	no
ni|645903627|nb|KK920803.1|	152989-154153	nan	nan	10	9	-0.131795	0.9032	no
ni|645903627|nb|KK920803.1|	154720-154841	nan	nan	26	15	-0.783598	0.53215	no
ni|645903627|nb|KK920803.1|	156147-156803	nan	nan	0.667691	0.936223	0.487672	0.6923	no
ni|645903627|nb|KK920803.1|	156996-157565	nan	nan	68	57	-0.252685	0.86565	no
ni|645903627|nb|KK920803.1|	157829-157952	nan	nan	16	19	0.273669	0.823	no
ni|645903627|nb|KK920803.1|	158105-158320	nan	nan	13	7	-0.891596	0.44925	no
ni|645903627|nb|KK920803.1|	158706-158911	nan	nan	25	15	-0.693657	0.54815	no
ni|645903627|nb|KK920803.1|	159051-159904	nan	nan	6	6	0.0873269	0.93925	no
ni|645903627|nb|KK920803.1|	160024-160568	nan	nan	13	12	-0.0834954	0.94075	no
ni|645903627|nb|KK920803.1|	160704-161528	nan	nan	57	52	-0.118811	0.9313	no
ni|645903627|nb|KK920803.1|	162040-162430	nan	nan	21	22	0.0509852	0.96245	no
ni|645903627|nb|KK920803.1|	162587-163201	nan	nan	4	4	-0.0130872	0.9918	no
ni|645903627|nb|KK920803.1|	163445-163893	nan	nan	2	2	-0.0652286	0.9574	no
ni|645903627|nb|KK920803.1|	164081-164558	nan	nan	7	7	-0.00103323	0.9954	no
ni|645903627|nb|KK920803.1|	164853-165384	nan	nan	2	1	-0.39948	0.74405	no
ni|645903627|nb|KK920803.1|	165514-166160	nan	nan	50	41	-0.263169	0.8599	no
ni|645903627|nb|KK920803.1|	166334-166689	nan	nan	1	2	0.349526	0.7779	no
ni|645903627|nb|KK920803.1|	166793-167858	nan	nan	2	1	-0.582404	0.62505	no
ni|645903627|nb|KK920803.1|	168032-168463	nan	nan	0.749626	0.143294	-2	0.2949	no
ni|645903627|nb|KK920803.1|	169009-169411	nan	nan	102	80	-0.339627	0.81355	no
ni|645903627|nb|KK920803.1|	173703-174091	nan	nan	2	1	-0.387314	0.7511	no
ni|645903627|nb|KK920803.1|	174206-174346	nan	nan	27	22	-0.245565	0.8369	no
ni|645903627|nb|KK920803.1|	174436-174606	nan	nan	86	92	0.0973029	0.9301	no
ni|645903627|nb|KK920803.1|	174768-174973	nan	nan	82	75	-0.126817	0.903	no
ni|645903627|nb|KK920803.1|	180178-180295	nan	nan	100	103	0.0495865	0.96635	no
ni|645903627|nb|KK920803.1|	201780-202537	nan	nan	0	6	inf	5.00E-05	yes

ni|645903627|nb|KK920803.1|	203711-204470	nan	nan	0	1	inf	0.0001	yes
ni|645903627|nb|KK920803.1|	204656-205436	nan	nan	1	11	3	0.01935	no
ni|645903627|nb|KK920803.1|	205615-206086	nan	nan	0.066838	4	6	0.1195	no
ni|645903627|nb|KK920803.1|	207349-208106	nan	nan	0.150582	19	7	0.03715	no
ni|645903627|nb|KK920803.1|	221133-221424	nan	nan	1	0.872089	-0.580416	0.70815	no
ni|645903627|nb|KK920803.1|	221759-222190	nan	nan	1	0.788116	-0.512726	0.697	no
ni|645903627|nb|KK920803.1|	222897-223177	nan	nan	58	54	-0.0937319	0.92945	no
ni|645903627|nb|KK920803.1|	223439-223770	nan	nan	4	28	3	0.03145	no
ni|645903627|nb|KK920803.1|	223906-224314	nan	nan	60	63	0.0670896	0.9631	no
ni|645903627|nb|KK920803.1|	224489-224979	nan	nan	0.381392	0.78987	1	0.45555	no
ni|645903627|nb|KK920803.1|	225517-225662	nan	nan	165.51	135	-0.2904	0.79415	no
ni|645903627|nb|KK920803.1|	226199-226508	nan	nan	5	4	-0.258381	0.82725	no
ni|645903627|nb|KK920803.1|	227524-228334	nan	nan	0.208956	0.799162	2	0.19135	no
ni|645903627|nb|KK920803.1|	230222-231536	nan	nan	0.02034	108	12	0.11935	no
ni|645903627|nb|KK920803.1|	231597-231897	nan	nan	0.373462	5	4	0.0549	no
ni|645903627|nb|KK920803.1|	238483-238651	nan	nan	45	50	0.173758	0.8773	no
ni|645903627|nb|KK920803.1|	238940-239709	nan	nan	10	12	0.188674	0.867	no
ni|645903627|nb|KK920803.1|	244320-244515	nan	nan	33	48	0.563678	0.62215	no
ni|645903627|nb|KK920803.1|	247434-247728	nan	nan	263	357	0.440355	0.7575	no
ni|645903627|nb|KK920803.1|	248260-248866	nan	nan	2	0.98233	-0.958023	0.4293	no
ni|645903627|nb|KK920803.1|	250212-250524	nan	nan	139	157	0.17772	0.90095	no
ni|645903627|nb|KK920803.1|	251371-251597	nan	nan	226	308	0.449211	0.75315	no
ni|645903627|nb|KK920803.1|	252929-253423	nan	nan	190	215.6	0.180863	0.89605	no
ni|645903627|nb|KK920803.1|	255828-256102	nan	nan	234	247	0.0766602	0.95795	no
ni|645903627|nb|KK920803.1|	256244-256608	nan	nan	2	2	-0.480512	0.69685	no
ni|645903627|nb|KK920803.1|	257140-257432	nan	nan	326	244	-0.415171	0.7671	no
ni|645903627|nb|KK920803.1|	257555-257686	nan	nan	130.68	60	-1	0.33535	no
ni|645903627|nb|KK920803.1|	258465-258945	nan	nan	79	52	-0.598337	0.6847	no
ni|645903627|nb|KK920803.1|	263583-263847	nan	nan	30	28	-0.125548	0.91215	no
ni|645903627|nb|KK920803.1|	265298-265850	nan	nan	0.876895	0.838249	-0.0650244	0.9589	no

ni|645903627|nb|KK920803.1|	340081-340552	nan	nan	0.066838	5	6	0.1194	no
ni|645903627|nb|KK920803.1|	346908-347314	nan	nan	0.081124	1	4	0.12425	no
ni|645903627|nb|KK920803.1|	347972-348960	nan	nan	15	41	1.49	0.24255	no
ni|645903627|nb|KK920803.1|	352607-353710	nan	nan	18	46	1	0.3766	no
ni|645903627|nb|KK920803.1|	356519-356973	nan	nan	27	71	1	0.28705	no
ni|645903627|nb|KK920803.1|	357581-358008	nan	nan	4	11	1	0.21935	no
ni|645903627|nb|KK920803.1|	358894-359137	nan	nan	15	31	1	0.376	no
ni|645903627|nb|KK920803.1|	360287-361845	nan	nan	22	47	1	0.4414	no
ni|645903627|nb|KK920803.1|	361918-362927	nan	nan	25	49	0.974391	0.51015	no
ni|645903627|nb|KK920803.1|	364663-364865	nan	nan	73	87	0.244335	0.82095	no
ni|645903627|nb|KK920803.1|	365004-365643	nan	nan	1	0.482932	-1	0.4105	no
ni|645903627|nb|KK920803.1|	365806-366954	nan	nan	17	20	0.211902	0.86515	no
ni|645903627|nb|KK920803.1|	367205-367694	nan	nan	26	34	0.392372	0.715	no
ni|645903627|nb|KK920803.1|	369208-370598	nan	nan	16	16	-0.00630099	0.9959	no
ni|645903627|nb|KK920803.1|	373337-373750	nan	nan	0.634395	0.454737	-0.480351	0.7594	no
ni|645903627|nb|KK920803.1|	375305-375479	nan	nan	44	32	-0.449178	0.70155	no
ni|645903627|nb|KK920803.1|	377623-378330	nan	nan	58	48	-0.274547	0.8556	no
ni|645903627|nb|KK920803.1|	378437-378631	nan	nan	62	53	-0.223934	0.84155	no
ni|645903627|nb|KK920803.1|	378875-379126	nan	nan	55	51	-0.106062	0.92535	no
ni|645903627|nb|KK920803.1|	381194-381683	nan	nan	0.955944	0.426413	-1	0.41555	no
ni|645903627|nb|KK920803.1|	381943-382523	nan	nan	22	28	0.373061	0.72815	no
ni|645903627|nb|KK920803.1|	384478-386078	nan	nan	32	46	0.531217	0.70515	no
ni|645903627|nb|KK920803.1|	386216-386583	nan	nan	44	59	0.424343	0.68585	no
ni|645903627|nb|KK920803.1|	386779-387900	nan	nan	3	3	-0.105175	0.92775	no
ni|645903627|nb|KK920803.1|	388133-388714	nan	nan	2	3	0.277911	0.81175	no
ni|645903627|nb|KK920803.1|	389176-389370	nan	nan	68	88	0.377332	0.7349	no
ni|645903627|nb|KK920803.1|	394654-394958	nan	nan	13	18	0.47397	0.68085	no
ni|645903627|nb|KK920803.1|	396025-396659	nan	nan	46	68	0.562535	0.69325	no
ni|645903627|nb|KK920803.1|	398619-398781	nan	nan	121	171	0.50163	0.64975	no
ni|645903627|nb|KK920803.1|	399618-399899	nan	nan	16	27	0.762607	0.5032	no

ni|645903627|nb|KK920803.1|	402294-403416	nan	nan	0.435056	0.508474	0.224974	0.85915	no
ni|645903627|nb|KK920803.1|	403550-403835	nan	nan	13	22	0.756578	0.5117	no
ni|645903627|nb|KK920803.1|	405017-405827	nan	nan	0.139304	0.366283	1	0.3311	no
ni|645903627|nb|KK920803.1|	405982-408185	nan	nan	21	40	0.897351	0.5271	no
ni|645903627|nb|KK920803.1|	412127-412355	nan	nan	0.195302	146	10	0.11935	no
ni|645903627|nb|KK920803.1|	420770-420914	nan	nan	0	69	inf	5.00E-05	yes
ni|645903627|nb|KK920803.1|	429261-429441	nan	nan	2	27	4	0.0292	no
ni|645903627|nb|KK920803.1|	430884-432115	nan	nan	0.021836	2	7	0.1194	no
ni|645903627|nb|KK920803.1|	432328-433215	nan	nan	5	8	0.785385	0.4928	no
ni|645903627|nb|KK920803.1|	434451-434920	nan	nan	0.403215	0.770807	0.934819	0.5295	no
ni|645903627|nb|KK920803.1|	435552-435805	nan	nan	112	171	0.612973	0.61575	no
ni|645903627|nb|KK920803.1|	436242-437586	nan	nan	7	6	-0.242903	0.82585	no
ni|645903627|nb|KK920803.1|	437749-437874	nan	nan	15	7	-1	0.41555	no
ni|645903627|nb|KK920803.1|	438613-438745	nan	nan	11	7	-0.552705	0.68145	no
ni|645903627|nb|KK920803.1|	438952-439546	nan	nan	6	4	-0.418593	0.7169	no
ni|645903627|nb|KK920803.1|	439890-441098	nan	nan	15	15	0.0377719	0.96925	no
ni|645903627|nb|KK920803.1|	441257-441906	nan	nan	9	5	-0.672924	0.56235	no
ni|645903627|nb|KK920803.1|	442086-442290	nan	nan	109	135	0.308679	0.7772	no
ni|645903627|nb|KK920803.1|	442553-442838	nan	nan	8	5	-0.639069	0.59255	no
ni|645903627|nb|KK920803.1|	443122-443356	nan	nan	7	9	0.262927	0.8229	no
ni|645903627|nb|KK920803.1|	443661-443916	nan	nan	114	137	0.270396	0.82765	no
ni|645903627|nb|KK920803.1|	446099-446458	nan	nan	124	125	0.012625	0.99285	no
ni|645903627|nb|KK920803.1|	447789-447970	nan	nan	11	8	-0.536786	0.65385	no
ni|645903627|nb|KK920803.1|	448533-449520	nan	nan	8	7	-0.180071	0.87325	no
ni|645903627|nb|KK920803.1|	449579-450104	nan	nan	9	8	-0.133551	0.90815	no
ni|645903627|nb|KK920803.1|	450812-451147	nan	nan	18	23	0.379009	0.73785	no
ni|645903627|nb|KK920803.1|	451605-452028	nan	nan	0.768301	0.587446	-0.387215	0.78555	no
ni|645903627|nb|KK920803.1|	452462-452822	nan	nan	6	10	0.883883	0.45115	no
ni|645903627|nb|KK920803.1|	453405-453867	nan	nan	0.616583	0.523857	-0.235122	0.8697	no
ni|645903627|nb|KK920803.1|	454402-454555	nan	nan	15	20	0.438045	0.71285	no

ni|645903627|nb|KK920803.1|	455706-456099	nan	nan	7	10	0.548976	0.6351	no
ni|645903627|nb|KK920803.1|	456584-456942	nan	nan	1	1	-0.0654989	0.95795	no
ni|645903627|nb|KK920803.1|	457193-457599	nan	nan	8	9	0.200258	0.86315	no
ni|645903627|nb|KK920803.1|	458555-458734	nan	nan	7	13	0.93268	0.4466	no
ni|645903627|nb|KK920803.1|	459474-459681	nan	nan	15	19	0.398831	0.72965	no
ni|645903627|nb|KK920803.1|	462782-463215	nan	nan	0.819608	0.213643	-2	0.24815	no
ni|645903627|nb|KK920803.1|	465211-465567	nan	nan	5	6	0.140944	0.9023	no
ni|645903627|nb|KK920803.1|	465675-466031	nan	nan	4	6	0.455325	0.6979	no
ni|645903627|nb|KK920803.1|	470893-471488	nan	nan	55	172	2	0.26265	no
ni|645903627|nb|KK920803.1|	474224-474436	nan	nan	134.1	311	1	0.335	no
ni|645903627|nb|KK920803.1|	476288-476528	nan	nan	19	41	1	0.35305	no
ni|645903627|nb|KK920803.1|	477483-477615	nan	nan	211.23	401	0.924831	0.4095	no
ni|645903627|nb|KK920803.1|	479540-479668	nan	nan	164	171	0.0575775	0.9586	no
ni|645903627|nb|KK920803.1|	480439-480653	nan	nan	2	0.838488	-0.874055	0.5788	no
ni|645903627|nb|KK920803.1|	486090-487591	nan	nan	37	45	0.281465	0.8437	no
ni|645903627|nb|KK920803.1|	491543-492128	nan	nan	48	37	-0.348292	0.82165	no
ni|645903627|nb|KK920803.1|	493765-494362	nan	nan	7	9	0.452897	0.69095	no
ni|645903627|nb|KK920803.1|	494589-495211	nan	nan	19	24	0.371184	0.7312	no
ni|645903627|nb|KK920803.1|	495356-495959	nan	nan	2	2	0.265206	0.8186	no
ni|645903627|nb|KK920803.1|	496180-496798	nan	nan	1	2	0.413127	0.73235	no
ni|645903627|nb|KK920803.1|	496972-497337	nan	nan	25	29	0.174309	0.87635	no
ni|645903627|nb|KK920803.1|	497888-498032	nan	nan	7	2	-2	0.22995	no
ni|645903627|nb|KK920803.1|	498170-498493	nan	nan	3	2	-0.62754	0.60265	no
ni|645903627|nb|KK920803.1|	498625-499306	nan	nan	5	5	-0.0895	0.93835	no
ni|645903627|nb|KK920803.1|	499524-499679	nan	nan	18	21	0.232366	0.8448	no
ni|645903627|nb|KK920803.1|	499857-500008	nan	nan	241	224	-0.105668	0.92385	no
ni|645903627|nb|KK920803.1|	500353-500604	nan	nan	7	7	0.0713342	0.9485	no
ni|645903627|nb|KK920803.1|	501074-501548	nan	nan	1	2	0.400494	0.7408	no
ni|645903627|nb|KK920803.1|	501668-505207	nan	nan	3	2	-0.541348	0.6299	no
ni|645903627|nb|KK920803.1|	506008-507155	nan	nan	30	33	0.115194	0.93745	no

ni|645903627|nb|KK920803.1|	507564-507848	nan	nan	3	3	0.126754	0.9121	no
ni|645903627|nb|KK920803.1|	508969-509198	nan	nan	79	88	0.156443	0.8834	no
ni|645903627|nb|KK920803.1|	509380-509601	nan	nan	50	57	0.201451	0.8551	no
ni|645903627|nb|KK920803.1|	509826-509987	nan	nan	80	86	0.113364	0.91845	no
ni|645903627|nb|KK920803.1|	510386-511998	nan	nan	0.310172	2	3	0.03415	no
ni|645903627|nb|KK920803.1|	512598-512968	nan	nan	43	50	0.213559	0.84465	no
ni|645903627|nb|KK920803.1|	513710-513955	nan	nan	33	43	0.404945	0.7222	no
ni|645903627|nb|KK920803.1|	514079-515038	nan	nan	7	6	-0.267636	0.809	no
ni|645903627|nb|KK920803.1|	515154-515313	nan	nan	16	16	0.00679811	0.9853	no
ni|645903627|nb|KK920803.1|	515562-515813	nan	nan	1	2	0.63427	0.63975	no
ni|645903627|nb|KK920803.1|	517668-517853	nan	nan	129	146	0.174766	0.86765	no
ni|645903627|nb|KK920803.1|	519182-519312	nan	nan	99	112	0.173713	0.8773	no
ni|645903627|nb|KK920803.1|	520495-520725	nan	nan	101	106	0.0745499	0.9463	no
ni|645903627|nb|KK920803.1|	520904-521192	nan	nan	43	35	-0.263009	0.81445	no
ni|645903627|nb|KK920803.1|	521440-521681	nan	nan	91	78	-0.217351	0.8399	no
ni|645903627|nb|KK920803.1|	522143-522407	nan	nan	5	2	-1	0.33395	no
ni|645903627|nb|KK920803.1|	522747-523424	nan	nan	69	54	-0.348949	0.8098	no
ni|645903627|nb|KK920803.1|	523671-523886	nan	nan	126	84	-0.588058	0.5827	no
ni|645903627|nb|KK920803.1|	525880-526012	nan	nan	21	40	0.959194	0.4175	no
ni|645903627|nb|KK920803.1|	526477-526779	nan	nan	22	39	0.818083	0.47005	no
ni|645903627|nb|KK920803.1|	528435-528941	nan	nan	8	16	0.966146	0.3978	no
ni|645903627|nb|KK920803.1|	529578-530412	nan	nan	7	15	1	0.3703	no
ni|645903627|nb|KK920803.1|	532154-532603	nan	nan	4	8	0.994645	0.3944	no
ni|645903627|nb|KK920803.1|	534786-534979	nan	nan	15	27	0.786484	0.50545	no
ni|645903627|nb|KK920803.1|	541623-542062	nan	nan	9	18	1	0.3507	no
ni|645903627|nb|KK920803.1|	543483-543739	nan	nan	10	20	1	0.37655	no
ni|645903627|nb|KK920803.1|	543884-544561	nan	nan	0.686178	0.943029	0.458719	0.71285	no
ni|645903627|nb|KK920803.1|	545929-546690	nan	nan	1	1	0.082089	0.94455	no
ni|645903627|nb|KK920803.1|	549562-550315	nan	nan	21	31	0.585401	0.6408	no
ni|645903627|nb|KK920803.1|	550420-550660	nan	nan	2	0.681555	-2	0.25615	no

ni|645903627|nb|KK920803.1|	551249-551448	nan	nan	43	45	0.0634999	0.9543	no
ni|645903627|nb|KK920803.1|	551921-552094	nan	nan	38	46	0.270282	0.8151	no
ni|645903627|nb|KK920803.1|	552180-552323	nan	nan	54	58	0.092446	0.9346	no
ni|645903627|nb|KK920803.1|	552475-552820	nan	nan	1	0.872658	-0.59607	0.64595	no
ni|645903627|nb|KK920803.1|	553016-553593	nan	nan	12	14	0.199057	0.86	no
ni|645903627|nb|KK920803.1|	553862-554695	nan	nan	2	1	-0.321849	0.7893	no
ni|645903627|nb|KK920803.1|	555023-555170	nan	nan	22	26	0.247672	0.8297	no
ni|645903627|nb|KK920803.1|	555805-556126	nan	nan	24	29	0.252225	0.82365	no
ni|645903627|nb|KK920803.1|	558119-558492	nan	nan	8	8	-0.081036	0.9443	no
ni|645903627|nb|KK920803.1|	559564-559804	nan	nan	39	42	0.113111	0.9179	no
ni|645903627|nb|KK920803.1|	562275-562694	nan	nan	20	23	0.168891	0.8809	no
ni|645903627|nb|KK920803.1|	564093-564216	nan	nan	73	73	0.0139326	0.9914	no
ni|645903627|nb|KK920803.1|	565228-565375	nan	nan	53	52	-0.0106183	0.993	no
ni|645903627|nb|KK920803.1|	567113-567669	nan	nan	6	3	-0.881571	0.44965	no
ni|645903627|nb|KK920803.1|	573004-573135	nan	nan	0	68	inf	5.00E-05	yes
ni|645903627|nb|KK920803.1|	573237-573373	nan	nan	0	69	inf	5.00E-05	yes
ni|645903627|nb|KK920803.1|	573466-573743	nan	nan	0.140806	55	9	0.11935	no
ni|645903627|nb|KK920803.1|	579755-579991	nan	nan	0	60	inf	5.00E-05	yes
ni|645903627|nb|KK920803.1|	580753-580869	nan	nan	0	54	inf	0.0001	yes
ni|645903627|nb|KK920803.1|	582481-582748	nan	nan	0	6	inf	5.00E-05	yes
ni|645903627|nb|KK920803.1|	588758-589051	nan	nan	0	57	inf	5.00E-05	yes
ni|645903627|nb|KK920803.1|	589210-589386	nan	nan	0	49	inf	5.00E-05	yes
ni|645903627|nb|KK920803.1|	591843-592094	nan	nan	0.495856	53	7	0.05055	no
ni|645903627|nb|KK920803.1|	593930-594145	nan	nan	0	72	inf	5.00E-05	yes
ni|645903627|nb|KK920803.1|	595809-596244	nan	nan	0.074063	24	8	0.11935	no
ni|645903627|nb|KK920803.1|	597389-597614	nan	nan	0	58	inf	5.00E-05	yes
ni|645903627|nb|KK920803.1|	60044-61153	nan	nan	0	58	inf	5.00E-05	yes
ni|645903627|nb|KK920803.1|	614095-614381	nan	nan	0	73	inf	5.00E-05	yes
ni|645903627|nb|KK920803.1|	615271-615751	nan	nan	0	41	inf	5.00E-05	yes
ni|645903627|nb|KK920803.1|	616034-616230	nan	nan	0	83	inf	5.00E-05	yes

ni|645903627|nb|KK920803.1|	617449-617908	nan	nan	0	41	inf	5.00E-05	yes
ni|645903627|nb|KK920803.1|	61893-62642	nan	nan	2	4	1	0.28575	no
ni|645903627|nb|KK920803.1|	621406-621680	nan	nan	0	74	inf	5.00E-05	yes
ni|645903627|nb|KK920803.1|	623240-623375	nan	nan	0	59.16	inf	5.00E-05	yes
ni|645903627|nb|KK920803.1|	624771-624955	nan	nan	0	72	inf	5.00E-05	yes
ni|645903627|nb|KK920803.1|	62821-63868	nan	nan	0.573951	0.698513	0.283359	0.8168	no
ni|645903627|nb|KK920803.1|	628372-628599	nan	nan	0	55	inf	5.00E-05	yes
ni|645903627|nb|KK920803.1|	629646-629879	nan	nan	0	59	inf	5.00E-05	yes
ni|645903627|nb|KK920803.1|	640608-640813	nan	nan	0	46	inf	5.00E-05	yes
ni|645903627|nb|KK920803.1|	64466-65750	nan	nan	5	10	1	0.34585	no
ni|645903627|nb|KK920803.1|	645368-645547	nan	nan	0	75	inf	5.00E-05	yes
ni|645903627|nb|KK920803.1|	646846-647313	nan	nan	0	49	inf	5.00E-05	yes
ni|645903627|nb|KK920803.1|	648013-648321	nan	nan	0	4	inf	0.00015	yes
ni|645903627|nb|KK920803.1|	650210-650444	nan	nan	0	76	inf	5.00E-05	yes
ni|645903627|nb|KK920803.1|	651322-651552	nan	nan	0.384534	78	8	0.0443	no
ni|645903627|nb|KK920803.1|	655224-655470	nan	nan	0.171	89	9	0.11935	no
ni|645903627|nb|KK920803.1|	65883-66953	nan	nan	6	6	-0.0532385	0.9613	no
ni|645903627|nb|KK920803.1|	666615-666857	nan	nan	0.703458	3	2	0.13305	no
ni|645903627|nb|KK920803.1|	668438-668564	nan	nan	11	24	1	0.38845	no
ni|645903627|nb|KK920803.1|	669638-669834	nan	nan	5	14	1	0.25225	no
ni|645903627|nb|KK920803.1|	67138-68411	nan	nan	3	3	-0.0441604	0.9686	no
ni|645903627|nb|KK920803.1|	672361-672926	nan	nan	2	3	0.492993	0.67965	no
ni|645903627|nb|KK920803.1|	673463-673889	nan	nan	2	4	0.884095	0.45565	no
ni|645903627|nb|KK920803.1|	676411-676526	nan	nan	24	15	-0.652457	0.6241	no
ni|645903627|nb|KK920803.1|	676888-677013	nan	nan	20	40	0.966603	0.42405	no
ni|645903627|nb|KK920803.1|	677129-677245	nan	nan	59	51	-0.219476	0.8486	no
ni|645903627|nb|KK920803.1|	677660-677790	nan	nan	21	16	-0.375417	0.7568	no
ni|645903627|nb|KK920803.1|	678763-678887	nan	nan	13	12	-0.182832	0.889	no
ni|645903627|nb|KK920803.1|	679787-680397	nan	nan	1	0.603321	-0.949455	0.44725	no
ni|645903627|nb|KK920803.1|	680854-681066	nan	nan	28	35	0.293308	0.7976	no

ni|645903627|nb|KK920803.1|	681181-681546	nan	nan	15	15	0.0479114	0.96845	no
ni|645903627|nb|KK920803.1|	681923-683204	nan	nan	3	3	0.0618993	0.9562	no
ni|645903627|nb|KK920803.1|	683427-684034	nan	nan	4	7	0.791154	0.50205	no
ni|645903627|nb|KK920803.1|	68530-68694	nan	nan	5	4	-0.289614	0.82015	no
ni|645903627|nb|KK920803.1|	69151-69321	nan	nan	17	31	0.839622	0.47355	no
ni|645903627|nb|KK920803.1|	705472-705820	nan	nan	60	59	-0.0181581	0.9862	no
ni|645903627|nb|KK920803.1|	707148-707402	nan	nan	77	69	-0.153982	0.8907	no
ni|645903627|nb|KK920803.1|	712083-712220	nan	nan	121	122	0.0178725	0.98675	no
ni|645903627|nb|KK920803.1|	712323-712836	nan	nan	23	23	-0.020584	0.9859	no
ni|645903627|nb|KK920803.1|	715154-716051	nan	nan	6	6	-0.153002	0.89165	no
ni|645903627|nb|KK920803.1|	716495-716679	nan	nan	70	54	-0.373932	0.74385	no
ni|645903627|nb|KK920803.1|	726132-726469	nan	nan	1	2	0.474976	0.709	no
ni|645903627|nb|KK920803.1|	727304-727969	nan	nan	1	2	0.539214	0.6538	no
ni|645903627|nb|KK920803.1|	728345-728623	nan	nan	2	1	-0.191466	0.88855	no
ni|645903627|nb|KK920803.1|	728846-729376	nan	nan	2	2	0.465453	0.69295	no
ni|645903627|nb|KK920803.1|	730366-730842	nan	nan	2	2	0.188591	0.87415	no
ni|645903627|nb|KK920803.1|	730972-731520	nan	nan	1	2	0.449542	0.7048	no
ni|645903627|nb|KK920803.1|	733920-734315	nan	nan	2	2	0.157028	0.8958	no
ni|645903627|nb|KK920803.1|	734495-735142	nan	nan	0.542979	0.173034	-2	0.25615	no
ni|645903627|nb|KK920803.1|	742104-742433	nan	nan	3	5	0.877786	0.45965	no
ni|645903627|nb|KK920803.1|	742973-743968	nan	nan	2	3	0.224917	0.8472	no
ni|645903627|nb|KK920803.1|	744081-744238	nan	nan	4	5	0.517764	0.7004	no
ni|645903627|nb|KK920803.1|	744407-744520	nan	nan	50	24	-1	0.39615	no
ni|645903627|nb|KK920803.1|	744896-745781	nan	nan	4	4	-0.0165596	0.98805	no
ni|645903627|nb|KK920803.1|	749809-750092	nan	nan	19	19	0.0137193	0.99155	no
ni|645903627|nb|KK920803.1|	750734-750939	nan	nan	33	31	-0.109	0.922	no
ni|645903627|nb|KK920803.1|	752951-753081	nan	nan	16	14	-0.145298	0.90525	no
ni|645903627|nb|KK920803.1|	753919-754464	nan	nan	5	5	0.0311786	0.97965	no
ni|645903627|nb|KK920803.1|	759958-760219	nan	nan	1	0.887955	-0.651035	0.6679	no
ni|645903627|nb|KK920803.1|	763101-763239	nan	nan	29	28	-0.0672382	0.95405	no

ni|645903627|nb|KK920803.1|	763347-763532	nan	nan	34	18	-0.947957	0.41625	no
ni|645903627|nb|KK920803.1|	765748-765876	nan	nan	0	22	inf	0.00055	yes
ni|645903627|nb|KK920803.1|	80282-80514	nan	nan	59	128	1	0.30465	no
ni|645903627|nb|KK920803.1|	82001-82568	nan	nan	24	54	1	0.3575	no
ni|645903627|nb|KK920803.1|	82984-83485	nan	nan	18	39	1	0.29015	no
ni|645903627|nb|KK920803.1|	85324-85549	nan	nan	88	187	1	0.3884	no
ni|645903627|nb|KK920803.1|	86468-86782	nan	nan	72	155	1	0.3708	no
ni|645903627|nb|KK920803.1|	88658-89141	nan	nan	2	1	-0.480189	0.68545	no
ni|645903627|nb|KK920803.1|	90846-91234	nan	nan	2	1	-0.505959	0.68965	no
ni|645903627|nb|KK920803.1|	91526-91845	nan	nan	1	0.759863	-0.580255	0.70815	no
ni|645903627|nb|KK920803.1|	92528-92881	nan	nan	5	6	0.116684	0.91885	no
ni|645903627|nb|KK920803.1|	93351-93719	nan	nan	14	53	2	0.08825	no
ni|645903627|nb|KK920803.1|	94027-94298	nan	nan	47	58	0.309051	0.77955	no
ni|645903627|nb|KK920803.1|	95314-95749	nan	nan	1	0.991018	-0.427828	0.73225	no
ni|645903627|nb|KK920803.1|	95894-96703	nan	nan	10	12	0.256205	0.81755	no
ni|645903627|nb|KK920803.1|	97006-97417	nan	nan	7	6	-0.201202	0.85845	no
ni|645903627|nb|KK920803.1|	97772-97933	nan	nan	13	9	-0.561972	0.6436	no
ni|645903627|nb|KK920803.1|	98157-98635	nan	nan	15	17	0.196809	0.8579	no
ni|645903627|nb|KK920803.1|	99087-99250	nan	nan	65	56	-0.233867	0.83795	no
ni|645903629|nb|KK920801.1|	104955-105122	nan	nan	19	13	-0.558228	0.63535	no
ni|645903629|nb|KK920801.1|	106373-106504	nan	nan	17	12	-0.459604	0.7047	no
ni|645903629|nb|KK920801.1|	107653-108066	nan	nan	5	2	-1	0.3602	no
ni|645903629|nb|KK920801.1|	124164-124398	nan	nan	27	16	-0.760573	0.51025	no
ni|645903629|nb|KK920801.1|	127080-127314	nan	nan	22	16.74	-0.369773	0.74605	no
ni|645903629|nb|KK920801.1|	129580-129732	nan	nan	18	10	-0.855688	0.48365	no
ni|645903629|nb|KK920801.1|	132824-133100	nan	nan	3	2	-0.458267	0.7095	no
ni|645903629|nb|KK920801.1|	136540-136945	nan	nan	0.569744	0.622306	0.12731	0.92555	no
ni|645903629|nb|KK920801.1|	138088-138936	nan	nan	0.165256	0.474032	2	0.2846	no
ni|645903629|nb|KK920801.1|	156162-156561	nan	nan	0.581247	1	1	0.39265	no
ni|645903629|nb|KK920801.1|	52037-52197	nan	nan	6	10	0.610867	0.6231	no

ni|645903629|nb|KK920801.1|	89533-90069	nan	nan	17	12	-0.553338	0.6276	no
ni|645903629|nb|KK920801.1|	91856-92020	nan	nan	65	48	-0.428558	0.70335	no
ni|645903629|nb|KK920801.1|	92339-92793	nan	nan	25	16	-0.642207	0.572	no
ni|645903629|nb|KK920801.1|	93259-94070	nan	nan	3	2	-0.0840738	0.9452	no
ni|645903629|nb|KK920801.1|	94265-95134	nan	nan	15	11	-0.52182	0.63185	no
ni|645903630|nb|KK920800.1|	11299-11570	nan	nan	6	3	-0.74406	0.5393	no
ni|645903630|nb|KK920800.1|	115047-116631	nan	nan	147	194.4	0.407087	0.77545	no
ni|645903630|nb|KK920800.1|	11645-11934	nan	nan	0.922551	1	0.126789	0.92555	no
ni|645903630|nb|KK920800.1|	12767-12931	nan	nan	5	5	-0.0672216	0.9547	no
ni|645903630|nb|KK920800.1|	13324-14070	nan	nan	5	4	-0.469157	0.68055	no
ni|645903630|nb|KK920800.1|	143732-148395	nan	nan	0.810571	0.573707	-0.498623	0.66355	no
ni|645903630|nb|KK920800.1|	148995-149423	nan	nan	0.680869	0.289222	-1	0.4023	no
ni|645903630|nb|KK920800.1|	183223-183584	nan	nan	0.761662	1	0.519476	0.7004	no
ni|645903630|nb|KK920800.1|	184304-188206	nan	nan	1	2	0.875155	0.43355	no
ni|645903630|nb|KK920800.1|	212016-212192	nan	nan	4	7	0.865561	0.5013	no
ni|645903630|nb|KK920800.1|	215156-220156	nan	nan	8	13	0.668242	0.63335	no
ni|645903630|nb|KK920800.1|	220538-220862	nan	nan	10	12	0.284531	0.802	no
ni|645903630|nb|KK920800.1|	267565-268483	nan	nan	28	58	1	0.4611	no
ni|645903630|nb|KK920800.1|	274954-275336	nan	nan	29	47	0.716277	0.51635	no
ni|645903630|nb|KK920800.1|	302892-303328	nan	nan	21	14	-0.574075	0.61005	no
ni|645903630|nb|KK920800.1|	303621-303798	nan	nan	32	33	0.0312382	0.9787	no
ni|645903630|nb|KK920800.1|	30408-34149	nan	nan	3	4	0.322598	0.7674	no
ni|645903630|nb|KK920800.1|	304721-304870	nan	nan	44	40	-0.118423	0.92275	no
ni|645903630|nb|KK920800.1|	305003-305217	nan	nan	58	57	-0.0342782	0.97415	no
ni|645903630|nb|KK920800.1|	309626-309793	nan	nan	128	168	0.391423	0.7192	no
ni|645903630|nb|KK920800.1|	34709-35970	nan	nan	3	3	0.0585744	0.9585	no
ni|645903630|nb|KK920800.1|	36035-37463	nan	nan	6	6	0.144507	0.8955	no
ni|645903630|nb|KK920800.1|	3700-4902	nan	nan	6	5	-0.309615	0.78215	no
ni|645903630|nb|KK920800.1|	51851-53320	nan	nan	3	0.017245	-7	0.0173	no
ni|645903630|nb|KK920800.1|	6616-7444	nan	nan	2	1	-0.460624	0.70135	no

ni|645903630|nb|KK920800.1|	7545-8066	nan	nan	2	1	-0.524509	0.66205	no
ni|645903630|nb|KK920800.1|	78994-80530	nan	nan	33	22	-0.567733	0.6922	no
ni|645903630|nb|KK920800.1|	80659-80857	nan	nan	3	2	-0.356529	0.7931	no
ni|645903630|nb|KK920800.1|	81424-81826	nan	nan	1	1	-0.364904	0.7706	no
ni|645903630|nb|KK920800.1|	82097-83628	nan	nan	20	13	-0.588684	0.69725	no
ni|645903630|nb|KK920800.1|	84000-84150	nan	nan	8	4	-1	0.40265	no
ni|645903630|nb|KK920800.1|	87561-88003	nan	nan	0.435229	0.485316	0.157151	0.9131	no
ni|645903630|nb|KK920800.1|	90054-91545	nan	nan	81.46	82	0.0177399	0.99035	no
ni|645903631|nb|KK920799.1|	216264-216406	nan	nan	0.590069	185	8	0.11935	no
ni|645903631|nb|KK920799.1|	328028-328214	nan	nan	37	58	0.626245	0.5853	no
ni|645903631|nb|KK920799.1|	330050-331826	nan	nan	50	52	0.0473286	0.97295	no
ni|645903631|nb|KK920799.1|	332731-332856	nan	nan	29	21	-0.450672	0.707	no
ni|645903631|nb|KK920799.1|	333414-334263	nan	nan	17	11	-0.587714	0.5933	no
ni|645903631|nb|KK920799.1|	334817-335202	nan	nan	9	5	-0.811639	0.4908	no
ni|645903631|nb|KK920799.1|	338015-338113	nan	nan	0	8538.97	inf	5.00E-05	yes
ni|645903631|nb|KK920799.1|	339774-339969	nan	nan	0.527697	108	8	0.0443	no
ni|645903631|nb|KK920799.1|	342299-342445	nan	nan	0	104	inf	5.00E-05	yes
ni|645903631|nb|KK920799.1|	344548-344783	nan	nan	0.370151	119	8	0.0443	no
ni|645903631|nb|KK920799.1|	67262-67538	nan	nan	0.991288	1	0.448623	0.7543	no
ni|645903632|nb|KK920798.1|	25885-26404	nan	nan	0.532298	0.452284	-0.235006	0.8697	no
ni|645903632|nb|KK920798.1|	274533-274767	nan	nan	0.932352	1	0.611691	0.6619	no
ni|645903632|nb|KK920798.1|	279737-280108	nan	nan	1	0.43803	-1	0.4064	no
ni|645903632|nb|KK920798.1|	284389-285114	nan	nan	5	3	-0.747931	0.52665	no
ni|645903632|nb|KK920798.1|	285469-285854	nan	nan	17	17	0.0286475	0.97945	no
ni|645903632|nb|KK920798.1|	288754-288946	nan	nan	31	21	-0.542484	0.6489	no
ni|645903632|nb|KK920798.1|	294872-296066	nan	nan	0.428937	0.323827	-0.405543	0.755	no
ni|645903632|nb|KK920798.1|	296736-298486	nan	nan	16	7	-1	0.355	no
ni|645903632|nb|KK920798.1|	299485-299665	nan	nan	11	9	-0.379254	0.75395	no
ni|645903632|nb|KK920798.1|	300359-300644	nan	nan	1	0.643275	-0.743955	0.59615	no
ni|645903632|nb|KK920798.1|	330041-330494	nan	nan	0.140531	1	3	0.0541	no

ni|645903632|nb|KK920798.1|	33219-33465	nan	nan	1	2	0.826861	0.55535	no
ni|645903632|nb|KK920798.1|	357896-358822	nan	nan	0.149606	1	3	0.05925	no
ni|645903633|nb|KK920797.1|	1047233-1047437	nan	nan	11	7	-0.667289	0.5725	no
ni|645903633|nb|KK920797.1|	1138237-1138387	nan	nan	20	19	-0.0671955	0.9557	no
ni|645903633|nb|KK920797.1|	318003-318165	nan	nan	3	4	0.392219	0.7763	no
ni|645903633|nb|KK920797.1|	323645-323838	nan	nan	11	4	-1	0.308	no
ni|645903633|nb|KK920797.1|	326902-328033	nan	nan	4	2	-0.772639	0.51185	no
ni|645903633|nb|KK920797.1|	331930-332202	nan	nan	26	25	-0.0579878	0.9599	no
ni|645903633|nb|KK920797.1|	332358-332715	nan	nan	26	24	-0.12551	0.9113	no
ni|645903633|nb|KK920797.1|	335918-336293	nan	nan	58	169.8	2	0.2131	no
ni|645903633|nb|KK920797.1|	349385-349874	nan	nan	62	171	1	0.321	no
ni|645903633|nb|KK920797.1|	352985-353137	nan	nan	55	139.45	1	0.2457	no
ni|645903633|nb|KK920797.1|	354287-354578	nan	nan	52	118	1	0.36335	no
ni|645903633|nb|KK920797.1|	363008-363512	nan	nan	11	15	0.449464	0.6897	no
ni|645903633|nb|KK920797.1|	364621-365206	nan	nan	14	20	0.523498	0.6376	no
ni|645903633|nb|KK920797.1|	366497-366733	nan	nan	18	23	0.37422	0.75095	no
ni|645903633|nb|KK920797.1|	367662-367893	nan	nan	16	18	0.165522	0.89585	no
ni|645903633|nb|KK920797.1|	372561-372899	nan	nan	2	2	0.118837	0.92325	no
ni|645903633|nb|KK920797.1|	373455-373931	nan	nan	384	525	0.449482	0.75555	no
ni|645903633|nb|KK920797.1|	374046-374166	nan	nan	403	502.09	0.316067	0.77585	no
ni|645903633|nb|KK920797.1|	374512-374926	nan	nan	2	3	0.551361	0.6418	no
ni|645903633|nb|KK920797.1|	381611-382238	nan	nan	7	10	0.411241	0.71625	no
ni|645903633|nb|KK920797.1|	382346-382559	nan	nan	12	22	0.866169	0.45755	no
ni|645903633|nb|KK920797.1|	383321-383968	nan	nan	5	5	-0.101406	0.92935	no
ni|645903633|nb|KK920797.1|	386592-386718	nan	nan	22	29	0.409104	0.7319	no
ni|645903633|nb|KK920797.1|	386782-386893	nan	nan	13	22	0.806856	0.58675	no
ni|645903633|nb|KK920797.1|	387819-389133	nan	nan	24	30	0.285085	0.84435	no
ni|645903633|nb|KK920797.1|	390809-391067	nan	nan	32	49	0.603954	0.59475	no
ni|645903633|nb|KK920797.1|	392350-392630	nan	nan	9	13	0.564846	0.62605	no
ni|645903633|nb|KK920797.1|	393361-393766	nan	nan	5	4	-0.232445	0.84305	no

ni|645903633|nb|KK920797.1|	394101-394350	nan	nan	9	4	-0.959275	0.42265	no
ni|645903633|nb|KK920797.1|	446752-447181	nan	nan	9	5	-0.866368	0.4565	no
ni|645903633|nb|KK920797.1|	448118-448417	nan	nan	9	9	0.0111691	0.99095	no
ni|645903633|nb|KK920797.1|	448960-449413	nan	nan	5	6	0.043035	0.97195	no
ni|645903633|nb|KK920797.1|	449557-449668	nan	nan	40	26	-0.615101	0.62875	no
ni|645903633|nb|KK920797.1|	450141-450260	nan	nan	32	22	-0.541351	0.65845	no
ni|645903633|nb|KK920797.1|	450688-451366	nan	nan	14	13	-0.124866	0.90935	no
ni|645903633|nb|KK920797.1|	451895-452195	nan	nan	15	15	0.0273225	0.9795	no
ni|645903633|nb|KK920797.1|	452700-452905	nan	nan	11	11	-0.00547425	0.9875	no
ni|645903633|nb|KK920797.1|	453149-453695	nan	nan	10	17	0.777424	0.4936	no
ni|645903633|nb|KK920797.1|	453902-454271	nan	nan	16	49	2	0.146	no
ni|645903633|nb|KK920797.1|	455850-456091	nan	nan	18	57	2	0.15525	no
ni|645903633|nb|KK920797.1|	457676-457833	nan	nan	10	44	2	0.08865	no
ni|645903633|nb|KK920797.1|	459698-460359	nan	nan	0.441143	0.253049	-0.801828	0.6027	no
ni|645903633|nb|KK920797.1|	463958-464377	nan	nan	8	32	2	0.09985	no
ni|645903633|nb|KK920797.1|	464589-465083	nan	nan	4	16	2	0.0739	no
ni|645903633|nb|KK920797.1|	465661-466339	nan	nan	1	2	0.520121	0.66315	no
ni|645903633|nb|KK920797.1|	469188-469370	nan	nan	49	38	-0.364785	0.75165	no
ni|645903633|nb|KK920797.1|	471000-471238	nan	nan	45	38	-0.25075	0.82215	no
ni|645903633|nb|KK920797.1|	471791-472459	nan	nan	0.609958	0.333226	-0.872208	0.53675	no
ni|645903633|nb|KK920797.1|	473641-473920	nan	nan	1	0.798003	-0.480965	0.7594	no
ni|645903633|nb|KK920797.1|	474707-475341	nan	nan	1	1	-0.0648979	0.9556	no
ni|645903633|nb|KK920797.1|	475571-476189	nan	nan	88	63	-0.478399	0.7438	no
ni|645903633|nb|KK920797.1|	476504-476716	nan	nan	64	44	-0.53622	0.6324	no
ni|645903633|nb|KK920797.1|	477301-477539	nan	nan	51	31	-0.732256	0.5151	no
ni|645903633|nb|KK920797.1|	478297-478526	nan	nan	11	8	-0.465005	0.6943	no
ni|645903633|nb|KK920797.1|	478660-479461	nan	nan	11	8	-0.525309	0.64165	no
ni|645903633|nb|KK920797.1|	479579-479721	nan	nan	66	41	-0.704646	0.55015	no
ni|645903633|nb|KK920797.1|	479806-479918	nan	nan	14	15	0.125068	0.92845	no
ni|645903633|nb|KK920797.1|	480165-481302	nan	nan	16	13	-0.251241	0.81425	no

ni|645903633|nb|KK920797.1|	482972-483207	nan	nan	38	28	-0.407875	0.7162	no
ni|645903633|nb|KK920797.1|	483518-484767	nan	nan	18	14	-0.33026	0.7895	no
ni|645903633|nb|KK920797.1|	484923-485113	nan	nan	16	14	-0.146617	0.89985	no
ni|645903633|nb|KK920797.1|	485187-485351	nan	nan	5	6	0.232339	0.8527	no
ni|645903633|nb|KK920797.1|	485598-485969	nan	nan	19	16	-0.176194	0.87305	no
ni|645903633|nb|KK920797.1|	486043-486268	nan	nan	13	19	0.467305	0.69595	no
ni|645903633|nb|KK920797.1|	486527-486832	nan	nan	7	7	0.00463144	0.99115	no
ni|645903633|nb|KK920797.1|	487878-488182	nan	nan	3	3	-0.122347	0.9185	no
ni|645903633|nb|KK920797.1|	489717-490145	nan	nan	11	12	0.216664	0.8491	no
ni|645903633|nb|KK920797.1|	492547-492705	nan	nan	45	62	0.470765	0.693	no
ni|645903633|nb|KK920797.1|	494744-494962	nan	nan	32	43	0.417447	0.71625	no
ni|645903633|nb|KK920797.1|	496352-496598	nan	nan	30	37	0.323722	0.7759	no
ni|645903633|nb|KK920797.1|	496768-496907	nan	nan	29	45	0.669733	0.57135	no
ni|645903633|nb|KK920797.1|	498336-498659	nan	nan	12	17	0.487493	0.6743	no
ni|645903633|nb|KK920797.1|	499980-500153	nan	nan	33	42	0.347738	0.76445	no
ni|645903633|nb|KK920797.1|	500257-500508	nan	nan	32	45	0.506149	0.65485	no
ni|645903633|nb|KK920797.1|	501208-502117	nan	nan	12	17	0.441657	0.67805	no
ni|645903633|nb|KK920797.1|	502220-502568	nan	nan	4	10	2	0.1906	no
ni|645903633|nb|KK920797.1|	506434-506767	nan	nan	2	18	3	0.01575	no
ni|645903633|nb|KK920797.1|	536076-536391	nan	nan	0	20	inf	5.00E-05	yes
ni|645903633|nb|KK920797.1|	553386-553632	nan	nan	23	20	-0.190769	0.865	no
ni|645903633|nb|KK920797.1|	553855-554206	nan	nan	1	0.75704	-0.52496	0.7071	no
ni|645903633|nb|KK920797.1|	554340-554999	nan	nan	16	16	0.0319967	0.97615	no
ni|645903633|nb|KK920797.1|	557385-557859	nan	nan	69	58	-0.253679	0.86795	no
ni|645903633|nb|KK920797.1|	558016-558435	nan	nan	10	7	-0.491223	0.6712	no
ni|645903633|nb|KK920797.1|	558761-559190	nan	nan	0.678788	0.216254	-2	0.2989	no
ni|645903633|nb|KK920797.1|	561347-562441	nan	nan	0.994088	0.546592	-0.86291	0.4756	no
ni|645903633|nb|KK920797.1|	567981-568126	nan	nan	194	192	-0.0188431	0.984	no
ni|645903633|nb|KK920797.1|	568281-568885	nan	nan	2	1	-0.434175	0.71745	no
ni|645903633|nb|KK920797.1|	569898-570161	nan	nan	9	7	-0.375383	0.74905	no

ni|645903633|nb|KK920797.1|	570528-570684	nan	nan	199	196	-0.0227045	0.98275	no
ni|645903633|nb|KK920797.1|	570906-571604	nan	nan	78	67	-0.21477	0.88035	no
ni|645903633|nb|KK920797.1|	572798-573024	nan	nan	4	3	-0.300968	0.81	no
ni|645903633|nb|KK920797.1|	573721-574669	nan	nan	93	77	-0.274762	0.8436	no
ni|645903633|nb|KK920797.1|	575377-575615	nan	nan	116.21	66	-0.822899	0.45205	no
ni|645903633|nb|KK920797.1|	575675-576309	nan	nan	141	103	-0.447444	0.7466	no
ni|645903633|nb|KK920797.1|	576885-576993	nan	nan	281	236	-0.250249	0.8282	no
ni|645903633|nb|KK920797.1|	577233-577386	nan	nan	44	14	-2	0.17845	no
ni|645903633|nb|KK920797.1|	579058-579389	nan	nan	8	6	-0.301249	0.79775	no
ni|645903633|nb|KK920797.1|	581589-581783	nan	nan	26	50	0.940235	0.42015	no
ni|645903633|nb|KK920797.1|	586472-586952	nan	nan	7	11	0.736063	0.53195	no
ni|645903633|nb|KK920797.1|	587063-587591	nan	nan	3	7	1	0.36585	no
ni|645903633|nb|KK920797.1|	588787-588956	nan	nan	4	9	1	0.29975	no
ni|645903633|nb|KK920797.1|	589862-590216	nan	nan	2	3	1	0.4039	no
ni|645903633|nb|KK920797.1|	593864-594069	nan	nan	9	10	0.171285	0.88175	no
ni|645903633|nb|KK920797.1|	594176-594391	nan	nan	10	16	0.740673	0.53385	no
ni|645903633|nb|KK920797.1|	594503-594695	nan	nan	6	12	0.963881	0.4265	no
ni|645903633|nb|KK920797.1|	595958-596078	nan	nan	8	6	-0.552832	0.719	no
ni|645903633|nb|KK920797.1|	598601-598999	nan	nan	3	5	0.892825	0.44705	no
ni|645903633|nb|KK920797.1|	599543-600169	nan	nan	2	4	0.746646	0.52545	no
ni|645903633|nb|KK920797.1|	600520-601484	nan	nan	2	3	0.44158	0.7018	no
ni|645903633|nb|KK920797.1|	601680-602048	nan	nan	5	5	0.037635	0.97515	no
ni|645903633|nb|KK920797.1|	602457-602661	nan	nan	2	1	-0.552323	0.71905	no
ni|645903633|nb|KK920797.1|	602767-603208	nan	nan	8	9	0.0828547	0.9432	no
ni|645903633|nb|KK920797.1|	604179-604503	nan	nan	12	11	-0.0656558	0.9544	no
ni|645903633|nb|KK920797.1|	605222-605385	nan	nan	21	17	-0.308225	0.79185	no
ni|645903633|nb|KK920797.1|	605793-605986	nan	nan	19	15	-0.338429	0.77435	no
ni|645903633|nb|KK920797.1|	606096-606531	nan	nan	6	5	-0.38274	0.73675	no
ni|645903633|nb|KK920797.1|	608821-609096	nan	nan	0	167	inf	5.00E-05	yes
ni|645903633|nb|KK920797.1|	609902-610076	nan	nan	0	183	inf	5.00E-05	yes

ni|645903633|nb|KK920797.1|	612180-612414	nan	nan	0	186	inf	5.00E-05	yes
ni|645903633|nb|KK920797.1|	614176-614346	nan	nan	0	66	inf	5.00E-05	yes
ni|645903633|nb|KK920797.1|	616071-616229	nan	nan	0	129	inf	5.00E-05	yes
ni|645903633|nb|KK920797.1|	618451-618979	nan	nan	0	93	inf	5.00E-05	yes
ni|645903633|nb|KK920797.1|	619852-621060	nan	nan	0	90	inf	5.00E-05	yes
ni|645903633|nb|KK920797.1|	621249-621778	nan	nan	0	84	inf	5.00E-05	yes
ni|645903633|nb|KK920797.1|	622182-622359	nan	nan	0	118	inf	5.00E-05	yes
ni|645903633|nb|KK920797.1|	622991-623159	nan	nan	0	99	inf	5.00E-05	yes
ni|645903633|nb|KK920797.1|	623293-623517	nan	nan	0.201667	143	9	0.11935	no
ni|645903633|nb|KK920797.1|	625164-625403	nan	nan	0.179698	118.25	9	0.11935	no
ni|645903633|nb|KK920797.1|	626009-626180	nan	nan	0	95	inf	5.00E-05	yes
ni|645903633|nb|KK920797.1|	632281-632558	nan	nan	0	159	inf	5.00E-05	yes
ni|645903633|nb|KK920797.1|	653522-653821	nan	nan	9	14	0.723787	0.53825	no
ni|645903633|nb|KK920797.1|	808760-809195	nan	nan	0	2	inf	0.0003	yes
ni|645903633|nb|KK920797.1|	814383-814653	nan	nan	0	4	inf	0.0003	yes
ni|645903633|nb|KK920797.1|	910943-911069	nan	nan	20	26	0.398329	0.7419	no
ni|645903633|nb|KK920797.1|	937146-937213	nan	nan	11168.9	13592.6	0.283336	0.8347	no
ni|645903634|nb|KK920796.1|	1000301-1001015	nan	nan	11	12	0.113061	0.91895	no
ni|645903634|nb|KK920796.1|	1008556-1008958	nan	nan	25	24	-0.0701286	0.94605	no
ni|645903634|nb|KK920796.1|	1009321-1009453	nan	nan	25	26	0.0176103	0.98375	no
ni|645903634|nb|KK920796.1|	1009749-1010925	nan	nan	15	15	0.0017797	0.99845	no
ni|645903634|nb|KK920796.1|	1011985-1012667	nan	nan	12	13	0.199958	0.85425	no
ni|645903634|nb|KK920796.1|	1013349-1014116	nan	nan	31	30	-0.0444029	0.97655	no
ni|645903634|nb|KK920796.1|	1015629-1016132	nan	nan	0.615045	0.293952	-1	0.44755	no
ni|645903634|nb|KK920796.1|	1016320-1016609	nan	nan	17	17	-0.00172578	0.9952	no
ni|645903634|nb|KK920796.1|	1016808-1017378	nan	nan	26	29	0.139911	0.89815	no
ni|645903634|nb|KK920796.1|	1021306-1021489	nan	nan	54	42	-0.359464	0.7522	no
ni|645903634|nb|KK920796.1|	1022056-1022491	nan	nan	11	13	0.25667	0.8237	no
ni|645903634|nb|KK920796.1|	1026971-1027494	nan	nan	7	11	0.581617	0.6136	no
ni|645903634|nb|KK920796.1|	1027666-1028039	nan	nan	3	2	-0.775933	0.51985	no

ni|645903634|nb|KK920796.1|	1028261-1028956	nan	nan	0.540587	2	2	0.11225	no
ni|645903634|nb|KK920796.1|	1029329-1030150	nan	nan	135	129.91	-0.0603242	0.9649	no
ni|645903634|nb|KK920796.1|	1031607-1032232	nan	nan	0.612985	0.676173	0.14154	0.90865	no
ni|645903634|nb|KK920796.1|	1032965-1033181	nan	nan	2	5	1	0.2841	no
ni|645903634|nb|KK920796.1|	1033323-1033852	nan	nan	0.808625	1	0.519899	0.67865	no
ni|645903634|nb|KK920796.1|	1033962-1034477	nan	nan	102	94	-0.120121	0.93415	no
ni|645903634|nb|KK920796.1|	1035725-1036840	nan	nan	31	33	0.0642668	0.96295	no
ni|645903634|nb|KK920796.1|	1038722-1039484	nan	nan	24	26	0.0981871	0.9232	no
ni|645903634|nb|KK920796.1|	1039601-1040975	nan	nan	40	32	-0.304242	0.82505	no
ni|645903634|nb|KK920796.1|	1041154-1041530	nan	nan	1.17	0.516059	-1	0.42295	no
ni|645903634|nb|KK920796.1|	1041666-1041926	nan	nan	5	4	-0.162297	0.89395	no
ni|645903634|nb|KK920796.1|	1042565-1043149	nan	nan	14	14	0.0464495	0.964	no
ni|645903634|nb|KK920796.1|	1043325-1043525	nan	nan	38	40	0.0506702	0.9657	no
ni|645903634|nb|KK920796.1|	1044019-1044232	nan	nan	37	39	0.0826604	0.9402	no
ni|645903634|nb|KK920796.1|	1045334-1045512	nan	nan	37	35	-0.0926344	0.93355	no
ni|645903634|nb|KK920796.1|	1045631-1045976	nan	nan	33	39	0.26348	0.81875	no
ni|645903634|nb|KK920796.1|	1047790-1047945	nan	nan	21	30	0.486741	0.67475	no
ni|645903634|nb|KK920796.1|	1050050-1050845	nan	nan	5	7	0.307247	0.7915	no
ni|645903634|nb|KK920796.1|	1050951-1051173	nan	nan	34	43	0.326578	0.7781	no
ni|645903634|nb|KK920796.1|	1056099-1056317	nan	nan	28	40	0.529301	0.6404	no
ni|645903634|nb|KK920796.1|	1064372-1064709	nan	nan	27	34	0.343864	0.7557	no
ni|645903634|nb|KK920796.1|	1064906-1065194	nan	nan	8	13	0.680851	0.56365	no
ni|645903634|nb|KK920796.1|	1065304-1066817	nan	nan	48	59	0.29629	0.82845	no
ni|645903634|nb|KK920796.1|	1072024-1072199	nan	nan	43	46	0.0913818	0.9376	no
ni|645903634|nb|KK920796.1|	1072594-1072717	nan	nan	47	46	-0.0356587	0.9753	no
ni|645903634|nb|KK920796.1|	1073631-1073808	nan	nan	62	71	0.194449	0.8641	no
ni|645903634|nb|KK920796.1|	1074185-1074346	nan	nan	60	61	0.0375884	0.97305	no
ni|645903634|nb|KK920796.1|	1075433-1075916	nan	nan	34	37	0.13165	0.90395	no
ni|645903634|nb|KK920796.1|	1076856-1077035	nan	nan	63	74	0.247266	0.8291	no
ni|645903634|nb|KK920796.1|	1077965-1078142	nan	nan	63	68	0.115004	0.9189	no

ni|645903634|nb|KK920796.1|	1078868-1079269	nan	nan	44	47	0.0994951	0.92415	no
ni|645903634|nb|KK920796.1|	1079662-1079891	nan	nan	73	66	-0.141469	0.8973	no
ni|645903634|nb|KK920796.1|	1080797-1081233	nan	nan	9	9	-0.122741	0.9168	no
ni|645903634|nb|KK920796.1|	1082290-1082705	nan	nan	19	18	-0.0714082	0.94845	no
ni|645903634|nb|KK920796.1|	1086495-1086909	nan	nan	43	40	-0.111114	0.9185	no
ni|645903634|nb|KK920796.1|	1093469-1093788	nan	nan	27	29	0.122391	0.91015	no
ni|645903634|nb|KK920796.1|	1093985-1094149	nan	nan	102	101	-0.023829	0.9809	no
ni|645903634|nb|KK920796.1|	1096272-1096492	nan	nan	68	68	-0.0147612	0.9889	no
ni|645903634|nb|KK920796.1|	1096747-1096874	nan	nan	68	67	-0.0107401	0.9914	no
ni|645903634|nb|KK920796.1|	1097843-1098527	nan	nan	25	25	-0.0314483	0.97545	no
ni|645903634|nb|KK920796.1|	1099740-1099880	nan	nan	93	100.99	0.121807	0.917	no
ni|645903634|nb|KK920796.1|	1100389-1100535	nan	nan	4	5	0.254724	0.86075	no
ni|645903634|nb|KK920796.1|	1101675-1101820	nan	nan	109	110	0.0107964	0.99205	no
ni|645903634|nb|KK920796.1|	1102007-1102157	nan	nan	61	49	-0.32321	0.78515	no
ni|645903634|nb|KK920796.1|	1103728-1105054	nan	nan	0	3	inf	5.00E-05	yes
ni|645903634|nb|KK920796.1|	1106256-1106704	nan	nan	1	10	3	0.02495	no
ni|645903634|nb|KK920796.1|	1107115-1107317	nan	nan	0.736849	11	4	0.05325	no
ni|645903634|nb|KK920796.1|	1108651-1108834	nan	nan	8	87	3	0.0099	no
ni|645903634|nb|KK920796.1|	1110026-1110452	nan	nan	3	43	4	0.0049	no
ni|645903634|nb|KK920796.1|	1113156-1113379	nan	nan	11	103	3	0.01015	no
ni|645903634|nb|KK920796.1|	1116222-1116363	nan	nan	10	165	4	0.0065	no
ni|645903634|nb|KK920796.1|	1118366-1118893	nan	nan	1	17	4	0.0058	no
ni|645903634|nb|KK920796.1|	1119083-1119228	nan	nan	15	217	4	0.0044	no
ni|645903634|nb|KK920796.1|	1120517-1120664	nan	nan	13	198	4	0.0038	no
ni|645903634|nb|KK920796.1|	1122734-1122896	nan	nan	11	204	4	0.00285	yes
ni|645903634|nb|KK920796.1|	1127183-1127413	nan	nan	2	21	4	0.0234	no
ni|645903634|nb|KK920796.1|	1128377-1129637	nan	nan	0.191597	1	2	0.0766	no
ni|645903634|nb|KK920796.1|	1129775-1130035	nan	nan	0.779525	2	1	0.39335	no
ni|645903634|nb|KK920796.1|	1133007-1133404	nan	nan	4	40	3	0.00865	no
ni|645903634|nb|KK920796.1|	1137645-1138311	nan	nan	0	2	inf	5.00E-05	yes

ni|645903634|nb|KK920796.1|	1139210-1139352	nan	nan	26	47	0.86567	0.46695	no
ni|645903634|nb|KK920796.1|	1139593-1139755	nan	nan	33	73	1	0.33625	no
ni|645903634|nb|KK920796.1|	1141487-1141790	nan	nan	47	71	0.591435	0.58485	no
ni|645903634|nb|KK920796.1|	1143491-1143659	nan	nan	53	82	0.63319	0.5813	no
ni|645903634|nb|KK920796.1|	1145154-1145361	nan	nan	55	86	0.649108	0.5628	no
ni|645903634|nb|KK920796.1|	1147304-1147507	nan	nan	59	90	0.598535	0.5924	no
ni|645903634|nb|KK920796.1|	1152079-1152405	nan	nan	24	34	0.52595	0.6394	no
ni|645903634|nb|KK920796.1|	1153225-1153531	nan	nan	1	0.80815	-0.428322	0.75965	no
ni|645903634|nb|KK920796.1|	1154213-1154686	nan	nan	0.731258	0.254167	-2	0.3149	no
ni|645903634|nb|KK920796.1|	1154849-1155044	nan	nan	40	47	0.233666	0.83565	no
ni|645903634|nb|KK920796.1|	1155219-1155478	nan	nan	4	8	1	0.3802	no
ni|645903634|nb|KK920796.1|	1162967-1163117	nan	nan	25	34	0.410126	0.7321	no
ni|645903634|nb|KK920796.1|	1163454-1163582	nan	nan	41	24	-0.763922	0.52125	no
ni|645903634|nb|KK920796.1|	1168046-1168248	nan	nan	26	19	-0.486478	0.6786	no
ni|645903634|nb|KK920796.1|	1168511-1168969	nan	nan	14	11	-0.303003	0.7916	no
ni|645903634|nb|KK920796.1|	1169828-1170211	nan	nan	10	10	0.00859766	0.9936	no
ni|645903634|nb|KK920796.1|	1170797-1171230	nan	nan	37	36	-0.0479498	0.9648	no
ni|645903634|nb|KK920796.1|	1171514-1171702	nan	nan	14	14	-0.00950008	0.98665	no
ni|645903634|nb|KK920796.1|	1171754-1171946	nan	nan	33	30	-0.164993	0.8847	no
ni|645903634|nb|KK920796.1|	1172070-1172255	nan	nan	25	14	-0.787148	0.5017	no
ni|645903634|nb|KK920796.1|	1172309-1172886	nan	nan	28	25	-0.134557	0.89675	no
ni|645903634|nb|KK920796.1|	1172967-1173275	nan	nan	50	43	-0.225801	0.8377	no
ni|645903634|nb|KK920796.1|	1173758-1173964	nan	nan	44	46	0.0664122	0.95625	no
ni|645903634|nb|KK920796.1|	1181926-1182154	nan	nan	46	51	0.141999	0.89695	no
ni|645903634|nb|KK920796.1|	1185336-1185539	nan	nan	17	25	0.552478	0.6391	no
ni|645903634|nb|KK920796.1|	1185791-1185900	nan	nan	30	41	0.434792	0.7373	no
ni|645903634|nb|KK920796.1|	1186617-1186765	nan	nan	40	45	0.17096	0.8821	no
ni|645903634|nb|KK920796.1|	1189354-1189977	nan	nan	0.520672	0.407261	-0.35442	0.7931	no
ni|645903634|nb|KK920796.1|	1195346-1195602	nan	nan	1	0.916649	-0.481157	0.7594	no
ni|645903634|nb|KK920796.1|	1198636-1199246	nan	nan	1	1	0.0419832	0.97395	no

ni|645903634|nb|KK920796.1|	1204516-1204668	nan	nan	23	30	0.362492	0.75795	no
ni|645903634|nb|KK920796.1|	1213293-1213625	nan	nan	17	18	0.133993	0.9076	no
ni|645903634|nb|KK920796.1|	1217325-1217924	nan	nan	8	10	0.251787	0.8234	no
ni|645903634|nb|KK920796.1|	1218104-1218536	nan	nan	0.373678	4	3	0.03845	no
ni|645903634|nb|KK920796.1|	1221222-1221584	nan	nan	6	9	0.612589	0.6008	no
ni|645903634|nb|KK920796.1|	1221784-1222404	nan	nan	3	4	0.278384	0.81085	no
ni|645903634|nb|KK920796.1|	1222836-1223642	nan	nan	3	8	1	0.22225	no
ni|645903634|nb|KK920796.1|	1224630-1225300	nan	nan	6	6	-0.0319365	0.9775	no
ni|645903634|nb|KK920796.1|	1225555-1225973	nan	nan	2	2	-0.360756	0.76745	no
ni|645903634|nb|KK920796.1|	1227305-1227627	nan	nan	1	0.749527	-0.580239	0.70815	no
ni|645903634|nb|KK920796.1|	1228030-1228169	nan	nan	24	25	0.0423919	0.9721	no
ni|645903634|nb|KK920796.1|	1228773-1229094	nan	nan	3	3	-0.00677752	0.9761	no
ni|645903634|nb|KK920796.1|	1230141-1230613	nan	nan	0.599919	0.637134	0.0868289	0.9436	no
ni|645903634|nb|KK920796.1|	1232189-1233127	nan	nan	7	10	0.396891	0.72245	no
ni|645903634|nb|KK920796.1|	1233956-1234100	nan	nan	17	19	0.14852	0.89755	no
ni|645903634|nb|KK920796.1|	1234325-1234719	nan	nan	10	10	0.00389564	0.99515	no
ni|645903634|nb|KK920796.1|	1235182-1235506	nan	nan	7	8	0.318488	0.7842	no
ni|645903634|nb|KK920796.1|	1235720-1235849	nan	nan	23	19	-0.29863	0.80195	no
ni|645903634|nb|KK920796.1|	1236290-1236493	nan	nan	23	15	-0.583709	0.61375	no
ni|645903634|nb|KK920796.1|	1240387-1241503	nan	nan	0.753697	0.999716	0.407534	0.7313	no
ni|645903634|nb|KK920796.1|	1242128-1242568	nan	nan	0.291869	0.697408	1	0.38895	no
ni|645903634|nb|KK920796.1|	1294056-1294688	nan	nan	0.744473	0	-inf	0.0015	yes
ni|645903634|nb|KK920796.1|	1304235-1304695	nan	nan	2	0	-inf	0.00045	yes
ni|645903634|nb|KK920796.1|	1315221-1315826	nan	nan	0	1	inf	5.00E-05	yes
ni|645903634|nb|KK920796.1|	207092-207494	nan	nan	872	520	-0.744667	0.60555	no
ni|645903634|nb|KK920796.1|	207670-207778	nan	nan	19.47	29	0.584306	0.6992	no
ni|645903634|nb|KK920796.1|	208025-208255	nan	nan	1638.31	997	-0.717042	0.6159	no
ni|645903634|nb|KK920796.1|	208361-209378	nan	nan	727	453	-0.681161	0.5845	no
ni|645903634|nb|KK920796.1|	296375-297090	nan	nan	0	76	inf	5.00E-05	yes
ni|645903634|nb|KK920796.1|	394419-395609	nan	nan	220	133	-0.73208	0.6118	no

ni|645903634|nb|KK920796.1|	395998-396693	nan	nan	170	101	-0.74367	0.59275	no
ni|645903634|nb|KK920796.1|	400599-400660	nan	nan	461717	298866	-0.627507	0.58735	no
ni|645903634|nb|KK920796.1|	400762-401443	nan	nan	0.383307	0.570051	0.572592	0.67875	no
ni|645903634|nb|KK920796.1|	401585-402078	nan	nan	0.946165	1	0.275906	0.821	no
ni|645903634|nb|KK920796.1|	405837-406793	nan	nan	33	48	0.527401	0.7167	no
ni|645903634|nb|KK920796.1|	406920-407498	nan	nan	79	88	0.16241	0.9111	no
ni|645903634|nb|KK920796.1|	414617-415123	nan	nan	2	2	0.204984	0.86555	no
ni|645903634|nb|KK920796.1|	425255-425808	nan	nan	36	29	-0.298068	0.7811	no
ni|645903634|nb|KK920796.1|	426079-426547	nan	nan	6	2	-2	0.1823	no
ni|645903634|nb|KK920796.1|	426804-427163	nan	nan	8	3	-1	0.2864	no
ni|645903634|nb|KK920796.1|	439880-440776	nan	nan	547	580	0.084151	0.9463	no
ni|645903634|nb|KK920796.1|	441699-442310	nan	nan	566	587	0.0525113	0.9712	no
ni|645903634|nb|KK920796.1|	442798-443673	nan	nan	1	3	2	0.17985	no
ni|645903634|nb|KK920796.1|	450306-452002	nan	nan	196	142	-0.459064	0.7541	no
ni|645903634|nb|KK920796.1|	462043-462376	nan	nan	28	19	-0.583335	0.6118	no
ni|645903634|nb|KK920796.1|	463057-464398	nan	nan	794	400	-0.987224	0.36805	no
ni|645903634|nb|KK920796.1|	464513-464786	nan	nan	2	2	-0.0659727	0.9589	no
ni|645903634|nb|KK920796.1|	511760-512158	nan	nan	0.083316	2	5	0.1215	no
ni|645903634|nb|KK920796.1|	547461-547783	nan	nan	109.82	139	0.341992	0.81405	no
ni|645903634|nb|KK920796.1|	548150-548577	nan	nan	26	29	0.153558	0.89025	no
ni|645903634|nb|KK920796.1|	549338-549750	nan	nan	163	133	-0.29817	0.83305	no
ni|645903634|nb|KK920796.1|	553296-553432	nan	nan	0	11	inf	0.00135	yes
ni|645903634|nb|KK920796.1|	560034-561023	nan	nan	36	37	0.0380425	0.98015	no
ni|645903634|nb|KK920796.1|	561085-561274	nan	nan	36	41	0.169502	0.88545	no
ni|645903634|nb|KK920796.1|	565487-565918	nan	nan	30	27	-0.139842	0.896	no
ni|645903634|nb|KK920796.1|	566128-566248	nan	nan	18	17	-0.0674049	0.9562	no
ni|645903634|nb|KK920796.1|	566462-566867	nan	nan	30	26	-0.200495	0.8529	no
ni|645903634|nb|KK920796.1|	566926-567174	nan	nan	27	28	0.0285767	0.97875	no
ni|645903634|nb|KK920796.1|	567296-567417	nan	nan	3	16	2	0.13665	no
ni|645903634|nb|KK920796.1|	567480-567814	nan	nan	1	0.812397	-0.766047	0.5799	no

ni|645903634|nb|KK920796.1|	572292-572511	nan	nan	0	117	inf	5.00E-05	yes
ni|645903634|nb|KK920796.1|	573246-573667	nan	nan	0	12	inf	5.00E-05	yes
ni|645903634|nb|KK920796.1|	589915-590116	nan	nan	0	5	inf	0.0007	yes
ni|645903634|nb|KK920796.1|	618022-618626	nan	nan	0.884274	0.140892	-3	0.15235	no
ni|645903634|nb|KK920796.1|	675999-676549	nan	nan	0	3	inf	5.00E-05	yes
ni|645903634|nb|KK920796.1|	805528-806325	nan	nan	0	4	inf	5.00E-05	yes
ni|645903634|nb|KK920796.1|	811207-811712	nan	nan	4	0.292494	-4	0.0392	no
ni|645903634|nb|KK920796.1|	944221-944353	nan	nan	23	34	0.549393	0.6426	no
ni|645903634|nb|KK920796.1|	944562-944802	nan	nan	27	24	-0.173939	0.8804	no
ni|645903634|nb|KK920796.1|	945615-946025	nan	nan	20	19	-0.0596078	0.9568	no
ni|645903634|nb|KK920796.1|	946741-947149	nan	nan	9	11	0.231066	0.8424	no
ni|645903634|nb|KK920796.1|	947954-948328	nan	nan	2	2	-0.267069	0.8253	no
ni|645903634|nb|KK920796.1|	948872-949400	nan	nan	9	10	0.0832571	0.9424	no
ni|645903634|nb|KK920796.1|	950380-950912	nan	nan	16	20	0.316576	0.77835	no
ni|645903634|nb|KK920796.1|	951181-951410	nan	nan	9	9	-0.0373091	0.97325	no
ni|645903634|nb|KK920796.1|	952561-953367	nan	nan	17	16	-0.148544	0.8895	no
ni|645903634|nb|KK920796.1|	953601-953948	nan	nan	8	9	0.181369	0.87655	no
ni|645903634|nb|KK920796.1|	954034-954452	nan	nan	14	11	-0.326741	0.78375	no
ni|645903634|nb|KK920796.1|	954885-956206	nan	nan	7	10	0.517578	0.64175	no
ni|645903634|nb|KK920796.1|	958238-960440	nan	nan	3	4	0.546903	0.6298	no
ni|645903634|nb|KK920796.1|	965174-965292	nan	nan	11	11.42	0.10249	0.93335	no
ni|645903634|nb|KK920796.1|	966026-966398	nan	nan	14	11	-0.340065	0.77005	no
ni|645903634|nb|KK920796.1|	966562-966952	nan	nan	7	6	-0.184024	0.8687	no
ni|645903634|nb|KK920796.1|	967023-967351	nan	nan	4	1	-1	0.2739	no
ni|645903634|nb|KK920796.1|	967441-967610	nan	nan	13	8	-0.777753	0.51535	no
ni|645903634|nb|KK920796.1|	968547-968727	nan	nan	8	9	0.0846931	0.9429	no
ni|645903634|nb|KK920796.1|	968839-968956	nan	nan	11	12	0.102472	0.93335	no
ni|645903634|nb|KK920796.1|	969466-969593	nan	nan	15	6	-1	0.33495	no
ni|645903634|nb|KK920796.1|	969784-970616	nan	nan	3	3	0.391373	0.7378	no
ni|645903634|nb|KK920796.1|	971124-971301	nan	nan	7	8	0.109552	0.9256	no

ni|645903634|nb|KK920796.1|	971650-971957	nan	nan	2	3	0.65046	0.60985	no
ni|645903634|nb|KK920796.1|	972255-973645	nan	nan	0.133975	4	5	0.02095	no
ni|645903634|nb|KK920796.1|	977499-977833	nan	nan	4	6	0.358201	0.757	no
ni|645903634|nb|KK920796.1|	978934-979170	nan	nan	8	8	-0.0980615	0.93415	no
ni|645903634|nb|KK920796.1|	979982-980163	nan	nan	7	8	0.155092	0.8959	no
ni|645903634|nb|KK920796.1|	980559-981011	nan	nan	3	4	0.182707	0.876	no
ni|645903634|nb|KK920796.1|	982075-982346	nan	nan	9	11	0.233572	0.84045	no
ni|645903634|nb|KK920796.1|	985209-985415	nan	nan	10	8	-0.289247	0.80025	no
ni|645903634|nb|KK920796.1|	985518-986146	nan	nan	1	0.80678	-0.354413	0.7757	no
ni|645903634|nb|KK920796.1|	987147-987910	nan	nan	6	9	0.612134	0.58955	no
ni|645903634|nb|KK920796.1|	989773-989901	nan	nan	12	19	0.648893	0.60985	no
ni|645903634|nb|KK920796.1|	990854-991032	nan	nan	14	22	0.66307	0.57745	no
ni|645903634|nb|KK920796.1|	991684-992063	nan	nan	5	7	0.467078	0.6857	no
ni|645903634|nb|KK920796.1|	992936-993194	nan	nan	18	20	0.183878	0.86995	no
ni|645903634|nb|KK920796.1|	996419-996925	nan	nan	5	7	0.489095	0.6759	no
ni|645903634|nb|KK920796.1|	997497-997649	nan	nan	19	18	-0.030666	0.9799	no
ni|645903634|nb|KK920796.1|	997792-997929	nan	nan	15	12	-0.342879	0.7737	no
ni|645903634|nb|KK920796.1|	998182-998386	nan	nan	20	16	-0.336985	0.7663	no
ni|645903634|nb|KK920796.1|	998487-998747	nan	nan	2	4	0.933918	0.45655	no
ni|645903634|nb|KK920796.1|	998932-999977	nan	nan	2	2	0.467656	0.6913	no
ni|645903635|nb|KK920795.1|	115968-116600	nan	nan	0.511825	0.400345	-0.354407	0.7931	no
ni|645903635|nb|KK920795.1|	179762-179897	nan	nan	18	24	0.361586	0.7643	no
ni|645903635|nb|KK920795.1|	207818-207967	nan	nan	43	27	-0.678155	0.55625	no
ni|645903635|nb|KK920795.1|	208415-209195	nan	nan	11	14	0.272682	0.80485	no
ni|645903635|nb|KK920795.1|	210431-210574	nan	nan	22	46	1	0.377	no
ni|645903635|nb|KK920795.1|	214017-214153	nan	nan	12	22	0.93275	0.44845	no
ni|645903635|nb|KK920795.1|	214288-214640	nan	nan	5	3	-0.521204	0.6592	no
ni|645903635|nb|KK920795.1|	215248-215557	nan	nan	0.833601	2	1	0.41545	no
ni|645903635|nb|KK920795.1|	215926-216443	nan	nan	19	34	0.837618	0.4415	no
ni|645903635|nb|KK920795.1|	216870-217219	nan	nan	23	38	0.765336	0.4927	no

ni|645903635|nb|KK920795.1|	217902-218113	nan	nan	37	64	0.793972	0.4855	no
ni|645903635|nb|KK920795.1|	218893-219307	nan	nan	1	0.906561	-0.650273	0.6173	no
ni|645903635|nb|KK920795.1|	228892-229830	nan	nan	5	9	0.91192	0.42385	no
ni|645903635|nb|KK920795.1|	229936-230186	nan	nan	15	12	-0.256511	0.8252	no
ni|645903635|nb|KK920795.1|	230286-230447	nan	nan	13	4	-2	0.1691	no
ni|645903635|nb|KK920795.1|	230772-231098	nan	nan	4	5	0.172514	0.8815	no
ni|645903635|nb|KK920795.1|	234718-234928	nan	nan	9	12	0.46666	0.6898	no
ni|645903635|nb|KK920795.1|	235051-235811	nan	nan	11	14	0.293507	0.79115	no
ni|645903635|nb|KK920795.1|	237361-237715	nan	nan	3	2	-0.650478	0.5959	no
ni|645903635|nb|KK920795.1|	239543-239687	nan	nan	643	411	-0.646487	0.6604	no
ni|645903635|nb|KK920795.1|	240047-240373	nan	nan	305	317	0.0581039	0.96755	no
ni|645903635|nb|KK920795.1|	240726-240848	nan	nan	18	6	-1	0.32095	no
ni|645903635|nb|KK920795.1|	241273-241416	nan	nan	9	7	-0.366771	0.7706	no
ni|645903635|nb|KK920795.1|	241958-242297	nan	nan	129	138	0.100453	0.94665	no
ni|645903635|nb|KK920795.1|	242512-243838	nan	nan	0.543789	0.693367	0.350572	0.76	no
ni|645903635|nb|KK920795.1|	243958-244699	nan	nan	0.656005	0.774781	0.240083	0.8456	no
ni|645903635|nb|KK920795.1|	246297-246401	nan	nan	533	645	0.274822	0.81465	no
ni|645903635|nb|KK920795.1|	246502-246756	nan	nan	97	96	-0.00705234	0.99495	no
ni|645903635|nb|KK920795.1|	247096-247310	nan	nan	10	8	-0.380808	0.74525	no
ni|645903635|nb|KK920795.1|	247521-247718	nan	nan	7	3	-1	0.28135	no
ni|645903635|nb|KK920795.1|	259104-259447	nan	nan	6	6	-0.0898115	0.93795	no
ni|645903635|nb|KK920795.1|	259864-260240	nan	nan	3	6	1	0.3742	no
ni|645903635|nb|KK920795.1|	260348-260574	nan	nan	275	335	0.287588	0.83745	no
ni|645903635|nb|KK920795.1|	272418-272621	nan	nan	440	576	0.388446	0.78055	no
ni|645903635|nb|KK920795.1|	278105-278320	nan	nan	100	67	-0.582603	0.59675	no
ni|645903635|nb|KK920795.1|	278379-278534	nan	nan	282	334	0.244943	0.8488	no
ni|645903635|nb|KK920795.1|	278679-279096	nan	nan	1	0.673452	-0.702733	0.6038	no
ni|645903635|nb|KK920795.1|	279346-279797	nan	nan	1	0.945623	-0.505795	0.68965	no
ni|645903635|nb|KK920795.1|	279953-280404	nan	nan	1	0.607901	-0.802188	0.5588	no
ni|645903635|nb|KK920795.1|	281123-281429	nan	nan	238	300	0.336386	0.81145	no

ni|645903635|nb|KK920795.1|	281872-282427	nan	nan	23	32	0.501417	0.63955	no
ni|645903635|nb|KK920795.1|	283665-284000	nan	nan	77	118	0.617436	0.6825	no
ni|645903635|nb|KK920795.1|	285772-285980	nan	nan	139	200	0.524548	0.6766	no
ni|645903635|nb|KK920795.1|	287995-288259	nan	nan	252.89	390	0.623475	0.65985	no
ni|645903635|nb|KK920795.1|	289479-289973	nan	nan	151	228	0.593333	0.6738	no
ni|645903635|nb|KK920795.1|	293390-293536	nan	nan	98	173	0.820968	0.4733	no
ni|645903635|nb|KK920795.1|	293809-294144	nan	nan	199	324	0.701508	0.61315	no
ni|645903635|nb|KK920795.1|	294396-294574	nan	nan	1	10	3	0.0561	no
ni|645903635|nb|KK920795.1|	296736-296863	nan	nan	25	35	0.44725	0.70505	no
ni|645903635|nb|KK920795.1|	299318-299613	nan	nan	35	55	0.663704	0.5505	no
ni|645903635|nb|KK920795.1|	300510-300695	nan	nan	40	51	0.347782	0.7589	no
ni|645903635|nb|KK920795.1|	31078-31443	nan	nan	0	8	inf	5.00E-05	yes
ni|645903635|nb|KK920795.1|	315148-315577	nan	nan	0.678788	1	0.852266	0.521	no
ni|645903635|nb|KK920795.1|	318352-318537	nan	nan	11	10	-0.106784	0.92625	no
ni|645903635|nb|KK920795.1|	319498-319778	nan	nan	78	96	0.308623	0.7713	no
ni|645903635|nb|KK920795.1|	319987-320190	nan	nan	7	4	-0.78681	0.5273	no
ni|645903635|nb|KK920795.1|	320293-320637	nan	nan	5	3	-0.621953	0.5969	no
ni|645903635|nb|KK920795.1|	321104-321261	nan	nan	5	3	-0.652161	0.64	no
ni|645903635|nb|KK920795.1|	322000-322358	nan	nan	4	4	0.100511	0.934	no
ni|645903635|nb|KK920795.1|	322498-322681	nan	nan	77	116	0.595084	0.59445	no
ni|645903635|nb|KK920795.1|	324049-325765	nan	nan	0.549817	0.540425	-0.0248559	0.98265	no
ni|645903635|nb|KK920795.1|	326325-327821	nan	nan	18	30	0.719801	0.62405	no
ni|645903635|nb|KK920795.1|	328180-328384	nan	nan	98	178	0.86582	0.5016	no
ni|645903635|nb|KK920795.1|	330961-331757	nan	nan	5	5	0.15013	0.89265	no
ni|645903635|nb|KK920795.1|	334545-334774	nan	nan	6	5	-0.208474	0.85945	no
ni|645903635|nb|KK920795.1|	335006-335782	nan	nan	114	173	0.602274	0.6704	no
ni|645903635|nb|KK920795.1|	336102-337260	nan	nan	31	44	0.51499	0.7219	no
ni|645903635|nb|KK920795.1|	337830-337985	nan	nan	264	337	0.350933	0.77925	no
ni|645903635|nb|KK920795.1|	339558-339746	nan	nan	134.78	165	0.292641	0.7879	no
ni|645903635|nb|KK920795.1|	340141-340319	nan	nan	7	4	-0.890446	0.4822	no

ni|645903635|nb|KK920795.1|	345710-345828	nan	nan	77	108	0.483975	0.678	no
ni|645903635|nb|KK920795.1|	345939-346527	nan	nan	0.659678	1	0.989482	0.44185	no
ni|645903635|nb|KK920795.1|	346741-347025	nan	nan	54	87	0.691947	0.5224	no
ni|645903635|nb|KK920795.1|	347573-348023	nan	nan	1	1	-0.0652244	0.96255	no
ni|645903635|nb|KK920795.1|	348293-348492	nan	nan	2	2	0.085001	0.9436	no
ni|645903635|nb|KK920795.1|	349287-349587	nan	nan	20	27	0.409551	0.71755	no
ni|645903635|nb|KK920795.1|	350043-350413	nan	nan	780	1071.98	0.45806	0.7481	no
ni|645903635|nb|KK920795.1|	350568-350735	nan	nan	4	12	2	0.2021	no
ni|645903635|nb|KK920795.1|	350996-351557	nan	nan	2	2	0.319655	0.78905	no
ni|645903635|nb|KK920795.1|	352227-352643	nan	nan	2	4	0.965722	0.4257	no
ni|645903635|nb|KK920795.1|	352809-353123	nan	nan	3	4	0.223798	0.84615	no
ni|645903635|nb|KK920795.1|	358764-358971	nan	nan	10	8	-0.329867	0.77735	no
ni|645903635|nb|KK920795.1|	359406-359730	nan	nan	5	5	-0.160813	0.888	no
ni|645903635|nb|KK920795.1|	359858-360157	nan	nan	5650.58	4475.82	-0.336245	0.7626	no
ni|645903635|nb|KK920795.1|	362637-362824	nan	nan	53	38	-0.463987	0.6819	no
ni|645903635|nb|KK920795.1|	362947-363241	nan	nan	27	24	-0.158017	0.8929	no
ni|645903635|nb|KK920795.1|	363460-363597	nan	nan	26	15	-0.829084	0.49445	no
ni|645903635|nb|KK920795.1|	363952-364282	nan	nan	36	32	-0.178218	0.8718	no
ni|645903635|nb|KK920795.1|	366150-366372	nan	nan	15	11	-0.481605	0.6829	no
ni|645903635|nb|KK920795.1|	367354-367514	nan	nan	12	9	-0.465754	0.70195	no
ni|645903635|nb|KK920795.1|	367629-367814	nan	nan	9	8	-0.117882	0.9212	no
ni|645903635|nb|KK920795.1|	367976-369208	nan	nan	4	3	-0.525824	0.6443	no
ni|645903635|nb|KK920795.1|	369398-369937	nan	nan	5	6	0.155079	0.89205	no
ni|645903635|nb|KK920795.1|	370234-370695	nan	nan	22	19	-0.184498	0.86615	no
ni|645903635|nb|KK920795.1|	375106-375536	nan	nan	43	40	-0.109108	0.92115	no
ni|645903635|nb|KK920795.1|	376217-376665	nan	nan	51	53	0.0557166	0.9633	no
ni|645903635|nb|KK920795.1|	378865-379342	nan	nan	50	49	-0.0014498	0.999	no
ni|645903635|nb|KK920795.1|	380449-380991	nan	nan	52	55	0.0963293	0.947	no
ni|645903635|nb|KK920795.1|	382525-382752	nan	nan	99	100.95	0.0336247	0.9759	no
ni|645903635|nb|KK920795.1|	386637-387038	nan	nan	14	14	-0.00575754	0.99565	no

ni|645903635|nb|KK920795.1|	387314-388165	nan	nan	17	13	-0.317444	0.7724	no
ni|645903635|nb|KK920795.1|	389182-389917	nan	nan	5	4	-0.207222	0.861	no
ni|645903635|nb|KK920795.1|	390428-391215	nan	nan	9	7	-0.263373	0.8183	no
ni|645903635|nb|KK920795.1|	391357-392066	nan	nan	13	9	-0.540255	0.6336	no
ni|645903635|nb|KK920795.1|	392203-392495	nan	nan	4	2	-1	0.2815	no
ni|645903635|nb|KK920795.1|	392903-393174	nan	nan	19	14	-0.382248	0.73775	no
ni|645903635|nb|KK920795.1|	393413-394364	nan	nan	10	8	-0.301942	0.78425	no
ni|645903635|nb|KK920795.1|	394499-394606	nan	nan	37	24	-0.652805	0.64	no
ni|645903635|nb|KK920795.1|	394752-395239	nan	nan	12	9	-0.298202	0.7967	no
ni|645903635|nb|KK920795.1|	395955-396462	nan	nan	14	14	-0.0527197	0.963	no
ni|645903635|nb|KK920795.1|	400288-400655	nan	nan	29	32	0.11511	0.9154	no
ni|645903635|nb|KK920795.1|	401292-402091	nan	nan	25	28	0.187234	0.90485	no
ni|645903635|nb|KK920795.1|	403621-403854	nan	nan	102.21	116	0.177059	0.869	no
ni|645903635|nb|KK920795.1|	405168-405288	nan	nan	77	91	0.236376	0.8357	no
ni|645903635|nb|KK920795.1|	405505-405643	nan	nan	77	95	0.295332	0.7971	no
ni|645903635|nb|KK920795.1|	406980-407343	nan	nan	12	17	0.519484	0.6485	no
ni|645903635|nb|KK920795.1|	408673-408804	nan	nan	92	115	0.32266	0.7769	no
ni|645903635|nb|KK920795.1|	409081-409336	nan	nan	64	64	-0.0164984	0.98715	no
ni|645903635|nb|KK920795.1|	409516-409902	nan	nan	28	35	0.3384	0.7534	no
ni|645903635|nb|KK920795.1|	410451-411276	nan	nan	6	7	0.425778	0.70695	no
ni|645903635|nb|KK920795.1|	419910-420116	nan	nan	49	56	0.188026	0.86425	no
ni|645903635|nb|KK920795.1|	424366-424952	nan	nan	22	27	0.307151	0.77695	no
ni|645903635|nb|KK920795.1|	427349-428232	nan	nan	0.47354	0.905574	0.935345	0.44625	no
ni|645903635|nb|KK920795.1|	428978-429338	nan	nan	1	1	-0.287883	0.8283	no
ni|645903635|nb|KK920795.1|	429760-430346	nan	nan	25	36	0.49953	0.64245	no
ni|645903635|nb|KK920795.1|	432847-433167	nan	nan	43	56	0.384059	0.7266	no
ni|645903635|nb|KK920795.1|	435399-436076	nan	nan	27	36	0.425246	0.7293	no
ni|645903635|nb|KK920795.1|	438509-438665	nan	nan	88	98	0.155196	0.8901	no
ni|645903635|nb|KK920795.1|	439206-439344	nan	nan	69	46	-0.585705	0.61175	no
ni|645903635|nb|KK920795.1|	528551-529148	nan	nan	0.647678	0.381025	-0.765391	0.5835	no

ni|645903636|nb|KK920794.1|	159344-159679	nan	nan	1	1	-0.444114	0.74215	no
ni|645903636|nb|KK920794.1|	169324-171280	nan	nan	1	1	-0.3933	0.7397	no
ni|645903636|nb|KK920794.1|	171830-171952	nan	nan	74	45	-0.707203	0.5517	no
ni|645903636|nb|KK920794.1|	172179-172690	nan	nan	30	28	-0.106573	0.9241	no
ni|645903636|nb|KK920794.1|	174443-174880	nan	nan	13	16	0.321296	0.7789	no
ni|645903636|nb|KK920794.1|	176139-176783	nan	nan	22	25	0.164333	0.8735	no
ni|645903636|nb|KK920794.1|	176914-177405	nan	nan	0.507216	0.484825	-0.0651348	0.9384	no
ni|645903636|nb|KK920794.1|	177683-178042	nan	nan	33	33	-0.00409427	0.99675	no
ni|645903636|nb|KK920794.1|	178598-179149	nan	nan	40	43	0.115897	0.93725	no
ni|645903636|nb|KK920794.1|	192475-193417	nan	nan	10	48	2	0.091	no
ni|645903636|nb|KK920794.1|	196510-196815	nan	nan	11	57	2	0.04875	no
ni|645903636|nb|KK920794.1|	197878-198054	nan	nan	15	67	2	0.07215	no
ni|645903636|nb|KK920794.1|	199548-199725	nan	nan	14	57	2	0.0895	no
ni|645903636|nb|KK920794.1|	203122-203617	nan	nan	0.627566	0.659853	0.0723766	0.94355	no
ni|645903636|nb|KK920794.1|	205923-206110	nan	nan	18	65	2	0.12175	no
ni|645903636|nb|KK920794.1|	207456-207964	nan	nan	3	2	-0.60567	0.6043	no
ni|645903636|nb|KK920794.1|	208253-208454	nan	nan	8	7	-0.204465	0.86145	no
ni|645903636|nb|KK920794.1|	208609-209055	nan	nan	5	6	0.204856	0.8658	no
ni|645903636|nb|KK920794.1|	209201-209350	nan	nan	3	19	3	0.0594	no
ni|645903636|nb|KK920794.1|	210538-211164	nan	nan	3	2	-0.42399	0.7168	no
ni|645903636|nb|KK920794.1|	212527-213174	nan	nan	75	67	-0.15863	0.911	no
ni|645903636|nb|KK920794.1|	213983-214165	nan	nan	164	160	-0.0327183	0.9748	no
ni|645903636|nb|KK920794.1|	215256-215649	nan	nan	127	117	-0.107531	0.9411	no
ni|645903636|nb|KK920794.1|	216838-217019	nan	nan	167	164	-0.0304417	0.97595	no
ni|645903636|nb|KK920794.1|	217133-217822	nan	nan	113	103	-0.132759	0.92515	no
ni|645903636|nb|KK920794.1|	217906-218538	nan	nan	42	59	0.495623	0.73885	no
ni|645903636|nb|KK920794.1|	219631-219867	nan	nan	1	4	2	0.2459	no
ni|645903636|nb|KK920794.1|	220243-220716	nan	nan	0.531824	2	2	0.22595	no
ni|645903636|nb|KK920794.1|	222102-222777	nan	nan	18	33	0.901827	0.4899	no
ni|645903636|nb|KK920794.1|	222862-223150	nan	nan	13	32	1	0.27075	no

ni|645903636|nb|KK920794.1|	223262-223904	nan	nan	2	2	0.0675633	0.95495	no
ni|645903636|nb|KK920794.1|	224465-224736	nan	nan	17	32	0.934012	0.4168	no
ni|645903636|nb|KK920794.1|	224788-225834	nan	nan	18	32	0.796482	0.6022	no
ni|645903636|nb|KK920794.1|	225973-226097	nan	nan	26	33	0.319668	0.7924	no
ni|645903636|nb|KK920794.1|	226724-226870	nan	nan	50	41	-0.268838	0.81975	no
ni|645903636|nb|KK920794.1|	228461-228823	nan	nan	0.853578	0.362534	-1	0.4023	no
ni|645903636|nb|KK920794.1|	229502-229659	nan	nan	74	87	0.22385	0.844	no
ni|645903636|nb|KK920794.1|	229922-230398	nan	nan	25	24	-0.0765704	0.9466	no
ni|645903636|nb|KK920794.1|	231260-231582	nan	nan	31	36	0.237181	0.83175	no
ni|645903636|nb|KK920794.1|	232386-232720	nan	nan	81	95	0.225787	0.87845	no
ni|645903636|nb|KK920794.1|	234518-234905	nan	nan	14	18	0.373785	0.7415	no
ni|645903636|nb|KK920794.1|	239026-239150	nan	nan	69	85	0.304614	0.7956	no
ni|645903636|nb|KK920794.1|	239468-240653	nan	nan	14	19	0.436167	0.72905	no
ni|645903636|nb|KK920794.1|	241543-241869	nan	nan	26	37	0.48248	0.66665	no
ni|645903636|nb|KK920794.1|	248003-248450	nan	nan	1	2	0.782766	0.52795	no
ni|645903636|nb|KK920794.1|	248636-249114	nan	nan	4	5	0.23292	0.8418	no
ni|645903636|nb|KK920794.1|	249697-250232	nan	nan	2	1	-0.765493	0.5216	no
ni|645903636|nb|KK920794.1|	250391-250820	nan	nan	13	18	0.513954	0.65285	no
ni|645903636|nb|KK920794.1|	252553-253096	nan	nan	2	4	0.671925	0.56395	no
ni|645903636|nb|KK920794.1|	254711-254965	nan	nan	22	33	0.591755	0.60205	no
ni|645903636|nb|KK920794.1|	255500-255681	nan	nan	30	42	0.497484	0.66585	no
ni|645903636|nb|KK920794.1|	256694-256959	nan	nan	9	10	0.180602	0.8743	no
ni|645903636|nb|KK920794.1|	258598-258942	nan	nan	6	11	0.798119	0.4972	no
ni|645903636|nb|KK920794.1|	260862-260983	nan	nan	26	32	0.267028	0.8269	no
ni|645903636|nb|KK920794.1|	261344-261967	nan	nan	0.61534	0.135754	-2	0.1906	no
ni|645903636|nb|KK920794.1|	262232-262946	nan	nan	5	7	0.428847	0.71445	no
ni|645903636|nb|KK920794.1|	263060-263447	nan	nan	4	4	-0.0350156	0.97855	no
ni|645903636|nb|KK920794.1|	265039-265173	nan	nan	6	6	0.102661	0.93335	no
ni|645903636|nb|KK920794.1|	265282-265454	nan	nan	7	3	-1	0.37315	no
ni|645903636|nb|KK920794.1|	265871-266315	nan	nan	0.793255	0.82711	0.0602936	0.95295	no

ni|645903636|nb|KK920794.1|	266428-267010	nan	nan	5	6	0.388743	0.7415	no
ni|645903636|nb|KK920794.1|	271044-271187	nan	nan	20	26	0.430289	0.72125	no
ni|645903636|nb|KK920794.1|	277682-277842	nan	nan	15	15	-0.0672051	0.95425	no
ni|645903636|nb|KK920794.1|	278716-278902	nan	nan	31	31	-0.000127267	0.9941	no
ni|645903636|nb|KK920794.1|	279029-279224	nan	nan	29	25	-0.191416	0.8696	no
ni|645903636|nb|KK920794.1|	279897-280074	nan	nan	11	11	0.0151365	0.98275	no
ni|645903636|nb|KK920794.1|	280222-280605	nan	nan	21	18	-0.160981	0.8836	no
ni|645903636|nb|KK920794.1|	280764-281128	nan	nan	42	42	0.0164473	0.98805	no
ni|645903636|nb|KK920794.1|	281456-281638	nan	nan	29	24	-0.262108	0.81825	no
ni|645903636|nb|KK920794.1|	282068-282327	nan	nan	14	9	-0.725054	0.54245	no
ni|645903636|nb|KK920794.1|	287178-287819	nan	nan	0.549026	0.13122	-2	0.20695	no
ni|645903636|nb|KK920794.1|	374196-374334	nan	nan	7	4	-0.719315	0.61925	no
ni|645903637|nb|KK920793.1|	131551-131903	nan	nan	82	73	-0.166302	0.9117	no
ni|645903637|nb|KK920793.1|	178140-178292	nan	nan	6	8	0.435308	0.7373	no
ni|645903637|nb|KK920793.1|	189870-190363	nan	nan	166	168	0.0168746	0.9901	no
ni|645903637|nb|KK920793.1|	472752-473308	nan	nan	0.597552	1	0.867868	0.5007	no
ni|645903637|nb|KK920793.1|	482444-482639	nan	nan	133	127	-0.0699474	0.94825	no
ni|645903637|nb|KK920793.1|	483395-484117	nan	nan	23	21	-0.153484	0.88625	no
ni|645903637|nb|KK920793.1|	484464-484720	nan	nan	8	7	-0.221398	0.85215	no
ni|645903637|nb|KK920793.1|	484846-485124	nan	nan	6	4	-0.616132	0.60575	no
ni|645903637|nb|KK920793.1|	485230-485481	nan	nan	4	5	0.0858337	0.9421	no
ni|645903637|nb|KK920793.1|	485599-485978	nan	nan	6	3	-0.730233	0.5385	no
ni|645903637|nb|KK920793.1|	487404-487741	nan	nan	19	14	-0.429283	0.7113	no
ni|645903637|nb|KK920793.1|	487951-488542	nan	nan	2	2	-0.152424	0.8951	no
ni|645903637|nb|KK920793.1|	497437-497803	nan	nan	82	80	-0.036056	0.9809	no
ni|645903637|nb|KK920793.1|	498013-498442	nan	nan	40	33	-0.314377	0.77355	no
ni|645903637|nb|KK920793.1|	498621-498750	nan	nan	54	32	-0.745376	0.5227	no
ni|645903637|nb|KK920793.1|	499227-499398	nan	nan	38	37	-0.0408068	0.9716	no
ni|645903637|nb|KK920793.1|	499673-500410	nan	nan	7	6	-0.264385	0.81845	no
ni|645903637|nb|KK920793.1|	500535-501653	nan	nan	4	3	-0.205626	0.85825	no

ni|645903637|nb|KK920793.1|	502060-502315	nan	nan	2	1	-0.651092	0.64935	no
ni|645903637|nb|KK920793.1|	502585-502812	nan	nan	8	8	-0.170823	0.88315	no
ni|645903637|nb|KK920793.1|	503383-503720	nan	nan	2	2	-0.158702	0.89795	no
ni|645903637|nb|KK920793.1|	503883-504046	nan	nan	5	3	-0.652179	0.64	no
ni|645903637|nb|KK920793.1|	504260-504381	nan	nan	14	7	-1	0.46615	no
ni|645903637|nb|KK920793.1|	504777-505129	nan	nan	58	52	-0.156353	0.88455	no
ni|645903637|nb|KK920793.1|	505486-505676	nan	nan	88	85	-0.044462	0.9664	no
ni|645903637|nb|KK920793.1|	505973-506096	nan	nan	15	14	-0.0673675	0.9582	no
ni|645903637|nb|KK920793.1|	506323-506484	nan	nan	26	20	-0.372062	0.74175	no
ni|645903637|nb|KK920793.1|	518328-518721	nan	nan	3	10	2	0.1679	no
ni|645903637|nb|KK920793.1|	519024-519326	nan	nan	2	8	2	0.13725	no
ni|645903637|nb|KK920793.1|	520476-520675	nan	nan	4	3	-0.173917	0.89245	no
ni|645903637|nb|KK920793.1|	521058-522142	nan	nan	0.954004	0.840241	-0.183192	0.87685	no
ni|645903637|nb|KK920793.1|	522243-523299	nan	nan	2	1	-0.584567	0.61745	no
ni|645903637|nb|KK920793.1|	523513-524301	nan	nan	1	2	0.41061	0.73135	no
ni|645903637|nb|KK920793.1|	524481-524672	nan	nan	4	3	-0.445677	0.74215	no
ni|645903637|nb|KK920793.1|	525115-527335	nan	nan	6	6	0.169846	0.8782	no
ni|645903637|nb|KK920793.1|	527449-527732	nan	nan	9	12	0.435296	0.70505	no
ni|645903637|nb|KK920793.1|	531825-532248	nan	nan	69	47	-0.552654	0.65915	no
ni|645903637|nb|KK920793.1|	533255-533399	nan	nan	85	79	-0.115789	0.9206	no
ni|645903637|nb|KK920793.1|	533580-533959	nan	nan	52	50	-0.065417	0.95135	no
ni|645903637|nb|KK920793.1|	535025-535238	nan	nan	122	127	0.0612162	0.9531	no
ni|645903637|nb|KK920793.1|	536125-536695	nan	nan	0.73776	0.453382	-0.702424	0.6038	no
ni|645903637|nb|KK920793.1|	537005-537395	nan	nan	25	28	0.183341	0.8692	no
ni|645903637|nb|KK920793.1|	538948-539153	nan	nan	109	124	0.193515	0.85725	no
ni|645903637|nb|KK920793.1|	540003-540221	nan	nan	59	74	0.320999	0.7774	no
ni|645903637|nb|KK920793.1|	540910-541464	nan	nan	57	64	0.162102	0.912	no
ni|645903637|nb|KK920793.1|	543283-543482	nan	nan	97	114	0.238212	0.8303	no
ni|645903637|nb|KK920793.1|	544778-545052	nan	nan	75	101	0.419853	0.6969	no
ni|645903637|nb|KK920793.1|	578353-578450	nan	nan	24636.8	26654.4	0.113558	0.92505	no

ni|645903637|nb|KK920793.1|	673535-674675	nan	nan	20	32	0.684798	0.64075	no
ni|645903637|nb|KK920793.1|	682502-682654	nan	nan	241	150	-0.682528	0.53715	no
ni|645903637|nb|KK920793.1|	683057-683175	nan	nan	158	95	-0.733434	0.5284	no
ni|645903637|nb|KK920793.1|	683490-684132	nan	nan	252	180	-0.487261	0.72375	no
ni|645903637|nb|KK920793.1|	684566-684849	nan	nan	458	341	-0.426938	0.7635	no
ni|645903637|nb|KK920793.1|	685065-685738	nan	nan	152.32	132	-0.203865	0.88545	no
ni|645903637|nb|KK920793.1|	685855-686245	nan	nan	132	120	-0.131557	0.92725	no
ni|645903637|nb|KK920793.1|	686378-686585	nan	nan	280.15	252	-0.155049	0.9158	no
ni|645903637|nb|KK920793.1|	686835-687423	nan	nan	81	84	0.0398937	0.9771	no
ni|645903637|nb|KK920793.1|	687755-688298	nan	nan	166	173	0.0559918	0.96615	no
ni|645903637|nb|KK920793.1|	704511-705023	nan	nan	46	48	0.0768659	0.9572	no
ni|645903637|nb|KK920793.1|	716667-716824	nan	nan	4	6	0.517764	0.6956	no
ni|645903637|nb|KK920793.1|	750633-751614	nan	nan	0.420725	7	4	0.00745	no
ni|645903637|nb|KK920793.1|	753388-753636	nan	nan	9	16	0.905784	0.4434	no
ni|645903637|nb|KK920793.1|	756645-756922	nan	nan	10	11	0.153624	0.8968	no
ni|645903637|nb|KK920793.1|	759797-760088	nan	nan	9	19	1	0.38215	no
ni|645903637|nb|KK920793.1|	761053-761382	nan	nan	12	15	0.253742	0.82585	no
ni|645903637|nb|KK920793.1|	761899-762098	nan	nan	11	15	0.418425	0.7233	no
ni|645903637|nb|KK920793.1|	765373-765907	nan	nan	4	7	0.924297	0.43145	no
ni|645903637|nb|KK920793.1|	766044-766404	nan	nan	9	12	0.455341	0.6934	no
ni|645903637|nb|KK920793.1|	767092-767932	nan	nan	4	5	0.106501	0.9254	no
ni|645903637|nb|KK920793.1|	768139-768264	nan	nan	9	13	0.570086	0.67875	no
ni|645903637|nb|KK920793.1|	768343-768497	nan	nan	14	9	-0.629071	0.6001	no
ni|645903637|nb|KK920793.1|	768681-768807	nan	nan	20	13	-0.652297	0.5967	no
ni|645903637|nb|KK920793.1|	768985-769592	nan	nan	7	7	0.0780215	0.94625	no
ni|645903637|nb|KK920793.1|	769681-770575	nan	nan	9	8	-0.0854065	0.9398	no
ni|645903637|nb|KK920793.1|	771070-771671	nan	nan	7	7	0.00495658	0.9956	no
ni|645903637|nb|KK920793.1|	772191-772379	nan	nan	12	7	-0.66988	0.571	no
ni|645903637|nb|KK920793.1|	773910-774518	nan	nan	4	3	-0.275502	0.8127	no
ni|645903637|nb|KK920793.1|	775509-775685	nan	nan	25	30	0.258445	0.8249	no

ni|645903637|nb|KK920793.1|	776175-776908	nan	nan	10	8	-0.273367	0.8045	no
ni|645903637|nb|KK920793.1|	777667-777903	nan	nan	26	24	-0.138246	0.90785	no
ni|645903637|nb|KK920793.1|	780138-780405	nan	nan	19	19	-0.0331064	0.97675	no
ni|645903637|nb|KK920793.1|	840583-840844	nan	nan	23	21	-0.15473	0.8905	no
ni|645903637|nb|KK920793.1|	845762-845878	nan	nan	45	30	-0.582046	0.63675	no
ni|645903637|nb|KK920793.1|	846783-847122	nan	nan	49	45	-0.134745	0.89895	no
ni|645903637|nb|KK920793.1|	848060-848391	nan	nan	35	30	-0.252639	0.8177	no
ni|645903637|nb|KK920793.1|	848775-849339	nan	nan	41	39	-0.0687806	0.962	no
ni|645903637|nb|KK920793.1|	851524-852568	nan	nan	8	7	-0.162746	0.8823	no
ni|645903637|nb|KK920793.1|	852859-853095	nan	nan	2	0.701774	-2	0.3149	no
ni|645903637|nb|KK920793.1|	854016-854203	nan	nan	46	48	0.0447986	0.96945	no
ni|645903637|nb|KK920793.1|	856154-856794	nan	nan	31	35	0.186649	0.8802	no
ni|645903637|nb|KK920793.1|	856942-857339	nan	nan	8	8	-0.0505609	0.96455	no
ni|645903637|nb|KK920793.1|	862144-862480	nan	nan	12	15	0.32672	0.7805	no
ni|645903637|nb|KK920793.1|	862700-862850	nan	nan	45	58	0.383466	0.7439	no
ni|645903637|nb|KK920793.1|	866128-866264	nan	nan	16	34	1	0.3735	no
ni|645903637|nb|KK920793.1|	867538-867885	nan	nan	14	25	0.797952	0.48365	no
ni|645903637|nb|KK920793.1|	873277-874452	nan	nan	9	10	0.157369	0.8828	no
ni|645903637|nb|KK920793.1|	874738-875096	nan	nan	42	48	0.172234	0.8756	no
ni|645903637|nb|KK920793.1|	877088-877365	nan	nan	0.704029	2	2	0.2306	no
ni|645903637|nb|KK920793.1|	877522-877704	nan	nan	3	7	1	0.392	no
ni|645903637|nb|KK920793.1|	880570-880850	nan	nan	10	7	-0.611888	0.60485	no
ni|645903637|nb|KK920793.1|	880984-881124	nan	nan	22	22	0.0514172	0.96615	no
ni|645903637|nb|KK920793.1|	884631-885003	nan	nan	37	41	0.119687	0.9121	no
ni|645903637|nb|KK920793.1|	885557-885793	nan	nan	91	79	-0.209371	0.84585	no
ni|645903637|nb|KK920793.1|	887102-887546	nan	nan	136	112	-0.285976	0.83775	no
ni|645903637|nb|KK920793.1|	915453-915709	nan	nan	0.319879	3	3	0.0541	no
ni|645903637|nb|KK920793.1|	927720-928134	nan	nan	0.237136	5	4	0.0519	no
ni|645903637|nb|KK920793.1|	949470-949967	nan	nan	0	33	inf	5.00E-05	yes
ni|645903637|nb|KK920793.1|	956687-957194	nan	nan	0.121794	381	12	0.0443	no

ni|645903637|nb|KK920793.1|	959796-960052	nan	nan	0	124	inf	5.00E-05	yes
ni|645903637|nb|KK920793.1|	962515-962816	nan	nan	0	16	inf	5.00E-05	yes
ni|645903637|nb|KK920793.1|	969150-969801	nan	nan	0	1	inf	0.00055	yes
ni|645903638|nb|KK920792.1|	18412-18649	nan	nan	5	5	-0.120787	0.91705	no
ni|645903639|nb|KK920791.1|	1012219-1012408	nan	nan	73	67	-0.118023	0.9129	no
ni|645903639|nb|KK920791.1|	1017938-1018115	nan	nan	82	77	-0.0962383	0.93135	no
ni|645903639|nb|KK920791.1|	1030313-1030556	nan	nan	55	56	0.026565	0.98095	no
ni|645903639|nb|KK920791.1|	1084122-1084247	nan	nan	55	55	0.00794429	0.98955	no
ni|645903639|nb|KK920791.1|	1796-2188	nan	nan	8	6	-0.490679	0.67775	no
ni|645903639|nb|KK920791.1|	211984-212266	nan	nan	107	105	-0.029333	0.98465	no
ni|645903639|nb|KK920791.1|	2359-2738	nan	nan	5	3	-0.786701	0.50465	no
ni|645903639|nb|KK920791.1|	240693-240922	nan	nan	1	2	0.296115	0.8383	no
ni|645903639|nb|KK920791.1|	241323-241729	nan	nan	24	59	1	0.3216	no
ni|645903639|nb|KK920791.1|	242253-242437	nan	nan	152	323	1	0.39365	no
ni|645903639|nb|KK920791.1|	242876-243300	nan	nan	0.919099	0.658826	-0.480322	0.7301	no
ni|645903639|nb|KK920791.1|	243468-243718	nan	nan	107	224	1	0.39105	no
ni|645903639|nb|KK920791.1|	245698-245965	nan	nan	194	350	0.854586	0.54935	no
ni|645903639|nb|KK920791.1|	247867-248070	nan	nan	2	0.928758	-1	0.44965	no
ni|645903639|nb|KK920791.1|	251315-251493	nan	nan	28	21	-0.439292	0.7043	no
ni|645903639|nb|KK920791.1|	252678-253679	nan	nan	12	12	-0.0113211	0.9917	no
ni|645903639|nb|KK920791.1|	253965-254825	nan	nan	152	138	-0.140003	0.92235	no
ni|645903639|nb|KK920791.1|	254944-255458	nan	nan	0.957794	0.915541	-0.0650904	0.9589	no
ni|645903639|nb|KK920791.1|	256619-256759	nan	nan	1247.41	1031.75	-0.273843	0.8468	no
ni|645903639|nb|KK920791.1|	257116-258054	nan	nan	1	0.930592	-0.543788	0.6439	no
ni|645903639|nb|KK920791.1|	259182-259508	nan	nan	2020.88	1578.57	-0.356366	0.7694	no
ni|645903639|nb|KK920791.1|	269635-269893	nan	nan	0.473689	31	6	0.05055	no
ni|645903639|nb|KK920791.1|	271111-271843	nan	nan	0.508821	0.898115	0.819742	0.51315	no
ni|645903639|nb|KK920791.1|	276436-276625	nan	nan	4	9	1	0.33695	no
ni|645903639|nb|KK920791.1|	278825-279351	nan	nan	0.756179	0.722832	-0.0650691	0.9554	no
ni|645903639|nb|KK920791.1|	282455-282914	nan	nan	24	74	2	0.23175	no

ni|645903639|nb|KK920791.1|	283214-283403	nan	nan	81	234	2	0.25595	no
ni|645903639|nb|KK920791.1|	284705-284929	nan	nan	29	70	1	0.2631	no
ni|645903639|nb|KK920791.1|	285280-285400	nan	nan	8	13	0.584672	0.6992	no
ni|645903639|nb|KK920791.1|	285526-286104	nan	nan	10	26	1	0.2229	no
ni|645903639|nb|KK920791.1|	286209-286616	nan	nan	17	51	2	0.1596	no
ni|645903639|nb|KK920791.1|	287611-287857	nan	nan	31	85	1	0.1996	no
ni|645903639|nb|KK920791.1|	2879-3308	nan	nan	9	6	-0.574744	0.6162	no
ni|645903639|nb|KK920791.1|	288866-289465	nan	nan	62	168	1	0.3203	no
ni|645903639|nb|KK920791.1|	289619-289753	nan	nan	75	70	-0.0955529	0.937	no
ni|645903639|nb|KK920791.1|	289952-290109	nan	nan	73	28	-1	0.2466	no
ni|645903639|nb|KK920791.1|	292519-293102	nan	nan	0.153817	3	4	0.05235	no
ni|645903639|nb|KK920791.1|	294915-295606	nan	nan	9	46	2	0.1017	no
ni|645903639|nb|KK920791.1|	299086-299454	nan	nan	130	149	0.194827	0.88895	no
ni|645903639|nb|KK920791.1|	300009-300641	nan	nan	12	20	0.756237	0.49995	no
ni|645903639|nb|KK920791.1|	300781-300918	nan	nan	7	4	-0.581817	0.70815	no
ni|645903639|nb|KK920791.1|	301107-301586	nan	nan	131	150	0.199439	0.88805	no
ni|645903639|nb|KK920791.1|	306703-308756	nan	nan	76	72	-0.0715647	0.95885	no
ni|645903639|nb|KK920791.1|	309202-309456	nan	nan	6	12	1	0.37305	no
ni|645903639|nb|KK920791.1|	311697-311967	nan	nan	12	24	0.959844	0.4154	no
ni|645903639|nb|KK920791.1|	312509-312664	nan	nan	4	10	1	0.364	no
ni|645903639|nb|KK920791.1|	313454-313712	nan	nan	22	32	0.543227	0.63335	no
ni|645903639|nb|KK920791.1|	313835-313958	nan	nan	16	6	-1	0.34635	no
ni|645903639|nb|KK920791.1|	314179-314405	nan	nan	9	9	0.022765	0.9857	no
ni|645903639|nb|KK920791.1|	315467-315599	nan	nan	15	15	0.00672201	0.9732	no
ni|645903639|nb|KK920791.1|	316043-316549	nan	nan	5	5	0.03111	0.97965	no
ni|645903639|nb|KK920791.1|	316995-317142	nan	nan	13	12	-0.126095	0.91405	no
ni|645903639|nb|KK920791.1|	317243-317463	nan	nan	14	6	-1	0.35075	no
ni|645903639|nb|KK920791.1|	318174-318690	nan	nan	15	13	-0.180564	0.87325	no
ni|645903639|nb|KK920791.1|	319175-319318	nan	nan	17	23	0.43236	0.72045	no
ni|645903639|nb|KK920791.1|	319472-319609	nan	nan	25	24	-0.02877	0.98165	no

ni|645903639|nb|KK920791.1|	319776-319936	nan	nan	15	16	0.0808935	0.94585	no
ni|645903639|nb|KK920791.1|	320652-320838	nan	nan	16	8	-1	0.3926	no
ni|645903639|nb|KK920791.1|	322655-323040	nan	nan	39	41	0.0765217	0.9448	no
ni|645903639|nb|KK920791.1|	325437-325988	nan	nan	123	105.12	-0.230942	0.86675	no
ni|645903639|nb|KK920791.1|	326154-326391	nan	nan	113	117.03	0.052183	0.96145	no
ni|645903639|nb|KK920791.1|	326605-326780	nan	nan	100	87	-0.19856	0.8544	no
ni|645903639|nb|KK920791.1|	326952-327237	nan	nan	30	25	-0.266871	0.81535	no
ni|645903639|nb|KK920791.1|	328115-329447	nan	nan	0.561178	0.824148	0.554446	0.6455	no
ni|645903639|nb|KK920791.1|	329618-330138	nan	nan	88	75	-0.241663	0.8672	no
ni|645903639|nb|KK920791.1|	330437-331685	nan	nan	5	5	-0.0886341	0.93525	no
ni|645903639|nb|KK920791.1|	331896-332021	nan	nan	18	15	-0.237269	0.8532	no
ni|645903639|nb|KK920791.1|	332181-332316	nan	nan	23	11	-0.979794	0.43475	no
ni|645903639|nb|KK920791.1|	332626-333206	nan	nan	13	11	-0.191558	0.86355	no
ni|645903639|nb|KK920791.1|	333735-333848	nan	nan	104	77	-0.433675	0.7142	no
ni|645903639|nb|KK920791.1|	334041-334190	nan	nan	36	1	-5	0.1196	no
ni|645903639|nb|KK920791.1|	343406-343575	nan	nan	7	12	0.850278	0.48905	no
ni|645903639|nb|KK920791.1|	344378-344638	nan	nan	5	8	0.61199	0.6031	no
ni|645903639|nb|KK920791.1|	346816-347242	nan	nan	0.532833	1	1	0.4471	no
ni|645903639|nb|KK920791.1|	348224-348706	nan	nan	1	1	-0.147615	0.9109	no
ni|645903639|nb|KK920791.1|	349390-349794	nan	nan	0.898275	0.780455	-0.202842	0.87975	no
ni|645903639|nb|KK920791.1|	350059-351135	nan	nan	2	4	0.623505	0.5913	no
ni|645903639|nb|KK920791.1|	351384-351693	nan	nan	3	12	2	0.15095	no
ni|645903639|nb|KK920791.1|	351900-352411	nan	nan	24	84	2	0.1589	no
ni|645903639|nb|KK920791.1|	352773-352999	nan	nan	8	5	-0.75838	0.5235	no
ni|645903639|nb|KK920791.1|	353835-354203	nan	nan	182	538	2	0.28055	no
ni|645903639|nb|KK920791.1|	354631-354748	nan	nan	146	88	-0.723499	0.53675	no
ni|645903639|nb|KK920791.1|	354876-354983	nan	nan	53	24	-1	0.40265	no
ni|645903639|nb|KK920791.1|	357993-358152	nan	nan	342	312	-0.13117	0.9312	no
ni|645903639|nb|KK920791.1|	358936-359063	nan	nan	422	355	-0.251608	0.82145	no
ni|645903639|nb|KK920791.1|	360357-361243	nan	nan	0.691867	0.631476	-0.131767	0.909	no

ni|645903639|nb|KK920791.1|	362085-362458	nan	nan	91	102	0.168882	0.90865	no
ni|645903639|nb|KK920791.1|	364079-364407	nan	nan	0.87273	1	0.256292	0.86075	no
ni|645903639|nb|KK920791.1|	3642-4178	nan	nan	10	6	-0.633041	0.5794	no
ni|645903639|nb|KK920791.1|	364590-364936	nan	nan	52	53	0.0298467	0.9782	no
ni|645903639|nb|KK920791.1|	367246-367847	nan	nan	11	17	0.721065	0.5313	no
ni|645903639|nb|KK920791.1|	371213-371410	nan	nan	21	15	-0.493858	0.66955	no
ni|645903639|nb|KK920791.1|	371681-371971	nan	nan	14	19	0.408082	0.72475	no
ni|645903639|nb|KK920791.1|	374807-375065	nan	nan	18	19	0.0667938	0.95705	no
ni|645903639|nb|KK920791.1|	377469-377926	nan	nan	8	9	0.25407	0.82555	no
ni|645903639|nb|KK920791.1|	381926-382109	nan	nan	13	16	0.26134	0.82515	no
ni|645903639|nb|KK920791.1|	382285-383111	nan	nan	3	3	0.241965	0.83025	no
ni|645903639|nb|KK920791.1|	385515-385725	nan	nan	12	15	0.33459	0.77005	no
ni|645903639|nb|KK920791.1|	386399-386542	nan	nan	16	16	0.0358829	0.97685	no
ni|645903639|nb|KK920791.1|	389740-389929	nan	nan	7	15	1	0.3815	no
ni|645903639|nb|KK920791.1|	394137-394325	nan	nan	15	13	-0.210174	0.857	no
ni|645903639|nb|KK920791.1|	394472-394634	nan	nan	10	11	0.0903289	0.9391	no
ni|645903639|nb|KK920791.1|	395731-396213	nan	nan	4	4	-0.184452	0.8703	no
ni|645903639|nb|KK920791.1|	396872-397003	nan	nan	15.84	17	0.134347	0.91335	no
ni|645903639|nb|KK920791.1|	403483-403666	nan	nan	10	16	0.721213	0.54385	no
ni|645903639|nb|KK920791.1|	404654-404830	nan	nan	6	13	1	0.3973	no
ni|645903639|nb|KK920791.1|	406225-406336	nan	nan	21	26	0.310898	0.81835	no
ni|645903639|nb|KK920791.1|	406923-407300	nan	nan	5	6	0.0989626	0.9273	no
ni|645903639|nb|KK920791.1|	407572-407722	nan	nan	9	10	0.0771944	0.9481	no
ni|645903639|nb|KK920791.1|	413525-414440	nan	nan	2	2	0.502406	0.67215	no
ni|645903639|nb|KK920791.1|	415183-415325	nan	nan	5	3	-0.915213	0.53575	no
ni|645903639|nb|KK920791.1|	415451-415841	nan	nan	0.770656	0.90018	0.224127	0.8598	no
ni|645903639|nb|KK920791.1|	416204-416462	nan	nan	1	3	1	0.3034	no
ni|645903639|nb|KK920791.1|	421315-421432	nan	nan	41	23	-0.837971	0.4978	no
ni|645903639|nb|KK920791.1|	422352-422852	nan	nan	10	6	-0.746941	0.52205	no
ni|645903639|nb|KK920791.1|	423008-423130	nan	nan	16	17	0.0257304	0.9668	no

ni|645903639|nb|KK920791.1|	423996-424253	nan	nan	10	10	0.0226993	0.9866	no
ni|645903639|nb|KK920791.1|	424514-425207	nan	nan	14	8	-0.860134	0.4469	no
ni|645903639|nb|KK920791.1|	426004-426135	nan	nan	10	2	-3	0.2592	no
ni|645903639|nb|KK920791.1|	426256-426700	nan	nan	28	29	0.0543014	0.9597	no
ni|645903639|nb|KK920791.1|	428224-428703	nan	nan	0.981301	0.437718	-1	0.41555	no
ni|645903639|nb|KK920791.1|	429903-431446	nan	nan	6	10	0.799808	0.46735	no
ni|645903639|nb|KK920791.1|	43370-43939	nan	nan	2	2	0.192162	0.87105	no
ni|645903639|nb|KK920791.1|	434333-434612	nan	nan	3	21	3	0.02975	no
ni|645903639|nb|KK920791.1|	436586-437136	nan	nan	0.605558	0.263122	-1	0.4064	no
ni|645903639|nb|KK920791.1|	438967-439200	nan	nan	50	89	0.821276	0.46035	no
ni|645903639|nb|KK920791.1|	439730-439865	nan	nan	35	69	0.961312	0.4119	no
ni|645903639|nb|KK920791.1|	440187-440451	nan	nan	40	75	0.917339	0.4149	no
ni|645903639|nb|KK920791.1|	441846-442318	nan	nan	6	12	1	0.38685	no
ni|645903639|nb|KK920791.1|	442484-442687	nan	nan	48	78	0.703348	0.53505	no
ni|645903639|nb|KK920791.1|	444086-444247	nan	nan	48	83	0.809521	0.47935	no
ni|645903639|nb|KK920791.1|	444899-445043	nan	nan	79	120	0.604408	0.5977	no
ni|645903639|nb|KK920791.1|	446719-446890	nan	nan	49	79	0.673803	0.55955	no
ni|645903639|nb|KK920791.1|	447367-447516	nan	nan	26	52	1	0.3975	no
ni|645903639|nb|KK920791.1|	448773-448933	nan	nan	29	50	0.792197	0.5017	no
ni|645903639|nb|KK920791.1|	45312-45479	nan	nan	3	2	-0.429812	0.75965	no
ni|645903639|nb|KK920791.1|	46382-47012	nan	nan	1	2	0.454471	0.7007	no
ni|645903639|nb|KK920791.1|	468179-468588	nan	nan	0.963985	1	0.519638	0.6876	no
ni|645903639|nb|KK920791.1|	469253-469957	nan	nan	18	34	0.912052	0.48595	no
ni|645903639|nb|KK920791.1|	470303-470883	nan	nan	2	2	-0.064978	0.9571	no
ni|645903639|nb|KK920791.1|	471011-471520	nan	nan	26	19	-0.501395	0.64505	no
ni|645903639|nb|KK920791.1|	473818-473976	nan	nan	68	52	-0.382296	0.7296	no
ni|645903639|nb|KK920791.1|	475718-475853	nan	nan	80	61	-0.379641	0.7507	no
ni|645903639|nb|KK920791.1|	479247-479424	nan	nan	60	47	-0.35078	0.7619	no
ni|645903639|nb|KK920791.1|	479646-479765	nan	nan	58	41	-0.503519	0.66535	no
ni|645903639|nb|KK920791.1|	480622-480750	nan	nan	17	11	-0.614802	0.62875	no

ni|645903639|nb|KK920791.1|	48103-48271	nan	nan	3	4	0.222257	0.8747	no
ni|645903639|nb|KK920791.1|	481148-481552	nan	nan	14	14	-0.0144141	0.99025	no
ni|645903639|nb|KK920791.1|	481720-481970	nan	nan	30	28	-0.0904273	0.93415	no
ni|645903639|nb|KK920791.1|	482131-483097	nan	nan	6	6	-0.0245273	0.98305	no
ni|645903639|nb|KK920791.1|	483557-483807	nan	nan	2	0.635748	-2	0.21345	no
ni|645903639|nb|KK920791.1|	483991-485196	nan	nan	0.581111	0.299224	-0.957587	0.4422	no
ni|645903639|nb|KK920791.1|	485396-485661	nan	nan	27	22	-0.283067	0.80725	no
ni|645903639|nb|KK920791.1|	51723-52216	nan	nan	0.946165	0.72352	-0.387059	0.7621	no
ni|645903639|nb|KK920791.1|	54672-55044	nan	nan	2	0.87282	-0.830978	0.5313	no
ni|645903639|nb|KK920791.1|	55227-55962	nan	nan	1	0.856614	-0.296103	0.8023	no
ni|645903639|nb|KK920791.1|	560876-561509	nan	nan	22	23	0.0289676	0.979	no
ni|645903639|nb|KK920791.1|	566722-566917	nan	nan	9	5	-0.989085	0.4137	no
ni|645903639|nb|KK920791.1|	56721-57055	nan	nan	1	0.710847	-0.58018	0.70815	no
ni|645903639|nb|KK920791.1|	567395-567861	nan	nan	5	2	-0.961352	0.42095	no
ni|645903639|nb|KK920791.1|	567999-568452	nan	nan	10	8	-0.247809	0.8322	no
ni|645903639|nb|KK920791.1|	569149-569883	nan	nan	0.468194	0.149212	-2	0.25615	no
ni|645903639|nb|KK920791.1|	570162-570399	nan	nan	16	16	-0.000750484	0.9937	no
ni|645903639|nb|KK920791.1|	571118-571540	nan	nan	5	4	-0.214667	0.8523	no
ni|645903639|nb|KK920791.1|	571761-573421	nan	nan	7	6	-0.208607	0.846	no
ni|645903639|nb|KK920791.1|	573480-573615	nan	nan	7	11	0.698278	0.59985	no
ni|645903639|nb|KK920791.1|	588305-588493	nan	nan	3	4	0.173793	0.8916	no
ni|645903639|nb|KK920791.1|	59058-59258	nan	nan	2	2	0.295589	0.8383	no
ni|645903639|nb|KK920791.1|	59656-59922	nan	nan	1	1	-0.0660295	0.9384	no
ni|645903639|nb|KK920791.1|	59987-60263	nan	nan	3	3	0.197085	0.8697	no
ni|645903639|nb|KK920791.1|	60402-60826	nan	nan	3	4	0.519678	0.6577	no
ni|645903639|nb|KK920791.1|	62820-63035	nan	nan	2	5	2	0.262	no
ni|645903639|nb|KK920791.1|	63-1569	nan	nan	15	10	-0.704399	0.57265	no
ni|645903639|nb|KK920791.1|	719472-719670	nan	nan	5	6	0.254906	0.83635	no
ni|645903639|nb|KK920791.1|	719810-720283	nan	nan	0.598302	2	2	0.13945	no
ni|645903639|nb|KK920791.1|	722549-722727	nan	nan	4	6	0.624554	0.61935	no

ni|645903639|nb|KK920791.1|	723346-723804	nan	nan	1	2	0.612866	0.62305	no
ni|645903639|nb|KK920791.1|	724914-725102	nan	nan	11	6	-0.829056	0.49445	no
ni|645903639|nb|KK920791.1|	725697-725856	nan	nan	6	6	-0.0672025	0.9562	no
ni|645903639|nb|KK920791.1|	726034-726174	nan	nan	8	7	-0.182705	0.889	no
ni|645903639|nb|KK920791.1|	726514-726661	nan	nan	5	3	-0.915199	0.53575	no
ni|645903639|nb|KK920791.1|	735396-735833	nan	nan	9	9	-0.145743	0.89945	no
ni|645903639|nb|KK920791.1|	736046-736156	nan	nan	57	37	-0.62405	0.6146	no
ni|645903639|nb|KK920791.1|	739842-740127	nan	nan	10.1	8	-0.387812	0.74	no
ni|645903639|nb|KK920791.1|	740617-740866	nan	nan	97	85	-0.182914	0.86505	no
ni|645903639|nb|KK920791.1|	742028-742235	nan	nan	37	31	-0.242512	0.8299	no
ni|645903639|nb|KK920791.1|	743900-744378	nan	nan	42	36	-0.195865	0.8542	no
ni|645903639|nb|KK920791.1|	744678-744848	nan	nan	63	54	-0.214699	0.85455	no
ni|645903639|nb|KK920791.1|	746127-746832	nan	nan	31	30	-0.034556	0.9811	no
ni|645903639|nb|KK920791.1|	747752-748406	nan	nan	30	28	-0.112604	0.91425	no
ni|645903639|nb|KK920791.1|	749208-749352	nan	nan	52	58	0.14852	0.89465	no
ni|645903639|nb|KK920791.1|	795189-795343	nan	nan	74	67	-0.140721	0.9025	no
ni|645903639|nb|KK920791.1|	799782-799982	nan	nan	16	18	0.207641	0.8583	no
ni|645903639|nb|KK920791.1|	801562-801712	nan	nan	53	46	-0.2237	0.85	no
ni|645903639|nb|KK920791.1|	810562-810760	nan	nan	65	57	-0.179651	0.87505	no
ni|645903639|nb|KK920791.1|	89027-89463	nan	nan	6	165	5	0.00285	yes
ni|645903639|nb|KK920791.1|	961657-962244	nan	nan	1	1	0.313545	0.7992	no
ni|645903640|nb|KK920790.1|	114455-114579	nan	nan	3	11	2	0.22355	no
ni|645903641|nb|KK920789.1|	110028-110257	nan	nan	2	0.555148	-2	0.24815	no
ni|645903641|nb|KK920789.1|	110735-110974	nan	nan	2	2	-0.218315	0.87705	no
ni|645903641|nb|KK920789.1|	118275-118702	nan	nan	2	0.725278	-1	0.2563	no
ni|645903641|nb|KK920789.1|	119436-119647	nan	nan	3	2	-0.597268	0.64595	no
ni|645903641|nb|KK920789.1|	130521-130735	nan	nan	0.439082	7	4	0.04665	no
ni|645903641|nb|KK920789.1|	138093-138314	nan	nan	1	5	2	0.2071	no
ni|645903641|nb|KK920789.1|	147651-147855	nan	nan	0	8	inf	0.00015	yes
ni|645903641|nb|KK920789.1|	217543-217808	nan	nan	2	0.866259	-0.803004	0.6027	no

ni|645903641|nb|KK920789.1|	22620-23006	nan	nan	2	0.497914	-2	0.2095	no
ni|645903641|nb|KK920789.1|	23080-23259	nan	nan	8	10	0.333218	0.7796	no
ni|645903641|nb|KK920789.1|	23693-23837	nan	nan	30	24	-0.362664	0.7615	no
ni|645903641|nb|KK920789.1|	25168-25306	nan	nan	17	6	-1	0.27715	no
ni|645903641|nb|KK920789.1|	25863-26177	nan	nan	2	2	0.374864	0.76185	no
ni|645903641|nb|KK920789.1|	26317-27727	nan	nan	10	9	-0.075759	0.94705	no
ni|645903641|nb|KK920789.1|	29005-29550	nan	nan	10	11	0.136262	0.9019	no
ni|645903641|nb|KK920789.1|	29831-30531	nan	nan	12	12	0.0907767	0.9352	no
ni|645903641|nb|KK920789.1|	30919-31127	nan	nan	7	10	0.405256	0.7322	no
ni|645903641|nb|KK920789.1|	31280-31496	nan	nan	7	9	0.3596	0.7585	no
ni|645903641|nb|KK920789.1|	32769-32891	nan	nan	10	10	0.0846241	0.9436	no
ni|645903641|nb|KK920789.1|	34420-34557	nan	nan	10	10	0.0258653	0.9668	no
ni|645903641|nb|KK920789.1|	34975-35227	nan	nan	5	8	0.589886	0.6097	no
ni|645903641|nb|KK920789.1|	357611-357845	nan	nan	1	2	0.933619	0.5295	no
ni|645903641|nb|KK920789.1|	35860-36775	nan	nan	2	2	0.46588	0.69095	no
ni|645903641|nb|KK920789.1|	37047-37929	nan	nan	3	3	-0.183709	0.87195	no
ni|645903641|nb|KK920789.1|	39262-39581	nan	nan	4	4	0.0993776	0.933	no
ni|645903641|nb|KK920789.1|	42310-44368	nan	nan	3	2	-0.493591	0.66325	no
ni|645903641|nb|KK920789.1|	45361-45912	nan	nan	2	0.787609	-1	0.24205	no
ni|645903641|nb|KK920789.1|	46054-46325	nan	nan	2	2	0.156404	0.90275	no
ni|645903641|nb|KK920789.1|	48526-48745	nan	nan	40	32	-0.338696	0.76265	no
ni|645903641|nb|KK920789.1|	50023-50137	nan	nan	35	24	-0.552946	0.65835	no
ni|645903642|nb|KK920788.1|	361315-361970	nan	nan	103	86	-0.253766	0.8563	no
ni|645903642|nb|KK920788.1|	363108-363377	nan	nan	3	1	-0.831539	0.5313	no
ni|645903642|nb|KK920788.1|	366308-366672	nan	nan	1	0.809485	-0.354981	0.7931	no
ni|645903642|nb|KK920788.1|	367153-367481	nan	nan	0.763639	0.833915	0.127009	0.92555	no
ni|645903642|nb|KK920788.1|	367715-368195	nan	nan	0.782964	0.686024	-0.190688	0.88855	no
ni|645903642|nb|KK920788.1|	369885-370278	nan	nan	39	37	-0.0527701	0.96335	no
ni|645903642|nb|KK920788.1|	371592-371782	nan	nan	89	75	-0.25406	0.82025	no
ni|645903642|nb|KK920788.1|	373093-373663	nan	nan	0.36888	0.35263	-0.064994	0.9302	no

ni|645903642|nb|KK920788.1|	378551-378673	nan	nan	114	101	-0.182856	0.8758	no
ni|645903642|nb|KK920788.1|	379395-379623	nan	nan	65	71	0.132697	0.9058	no
ni|645903642|nb|KK920788.1|	380284-380416	nan	nan	111	92	-0.271419	0.8171	no
ni|645903642|nb|KK920788.1|	382628-382875	nan	nan	67	63	-0.0810097	0.9432	no
ni|645903642|nb|KK920788.1|	383960-384354	nan	nan	60	65	0.102533	0.93345	no
ni|645903642|nb|KK920788.1|	384542-384874	nan	nan	46	50	0.141735	0.89695	no
ni|645903642|nb|KK920788.1|	386004-386300	nan	nan	66	67	0.00957074	0.99205	no
ni|645903642|nb|KK920788.1|	386691-387036	nan	nan	55	74	0.430159	0.7342	no
ni|645903642|nb|KK920788.1|	389532-389933	nan	nan	28	36	0.332768	0.7651	no
ni|645903642|nb|KK920788.1|	391639-391689	nan	nan	1.61E+07	1.05E+07	-0.61581	0.6537	no
ni|645903642|nb|KK920788.1|	481275-481332	nan	nan	97872.7	89149.1	-0.134687	0.91695	no
ni|645903642|nb|KK920788.1|	546504-546694	nan	nan	26	39	0.558138	0.6318	no
ni|645903643|nb|KK920787.1|	109213-109468	nan	nan	2	0.922611	-0.803095	0.6027	no
ni|645903643|nb|KK920787.1|	117209-117410	nan	nan	3	6	1	0.3381	no
ni|645903643|nb|KK920787.1|	118974-119387	nan	nan	2	4	0.882219	0.45985	no
ni|645903643|nb|KK920787.1|	121022-121192	nan	nan	2	5	2	0.28465	no
ni|645903643|nb|KK920787.1|	122194-122396	nan	nan	5	4	-0.459257	0.7095	no
ni|645903643|nb|KK920787.1|	123650-124211	nan	nan	2	5	1	0.3435	no
ni|645903643|nb|KK920787.1|	124341-124562	nan	nan	2	4	0.64391	0.6193	no
ni|645903643|nb|KK920787.1|	126083-126387	nan	nan	1	1	-0.0657637	0.94155	no
ni|645903643|nb|KK920787.1|	133687-134540	nan	nan	0.590958	1	1	0.30915	no
ni|645903643|nb|KK920787.1|	135172-135378	nan	nan	1	1	0.19618	0.8862	no
ni|645903643|nb|KK920787.1|	136062-136656	nan	nan	0.701755	1	0.712651	0.57115	no
ni|645903643|nb|KK920787.1|	137428-137847	nan	nan	0.544594	2	2	0.147	no
ni|645903643|nb|KK920787.1|	137980-138383	nan	nan	80	79	-0.0186098	0.98845	no
ni|645903643|nb|KK920787.1|	182278-183512	nan	nan	4	23	3	0.07335	no
ni|645903643|nb|KK920787.1|	185928-186940	nan	nan	2	14	3	0.0358	no
ni|645903643|nb|KK920787.1|	189167-190463	nan	nan	4	15	2	0.08625	no
ni|645903643|nb|KK920787.1|	206466-208256	nan	nan	11	14	0.248695	0.8722	no
ni|645903643|nb|KK920787.1|	211489-211690	nan	nan	13	16	0.341124	0.7682	no

ni|645903643|nb|KK920787.1|	213154-213944	nan	nan	0	4	inf	5.00E-05	yes
ni|645903643|nb|KK920787.1|	214467-214860	nan	nan	5	6	0.251487	0.8307	no
ni|645903644|nb|KK920786.1|	104717-105359	nan	nan	0.867681	2	1	0.3973	no
ni|645903644|nb|KK920786.1|	1093568-1093764	nan	nan	3	1	-1	0.44755	no
ni|645903644|nb|KK920786.1|	1109096-1109498	nan	nan	0.90426	1	0.475225	0.709	no
ni|645903644|nb|KK920786.1|	131817-131986	nan	nan	28	28	0.006741	0.9922	no
ni|645903644|nb|KK920786.1|	132576-132734	nan	nan	50	45	-0.156997	0.8897	no
ni|645903644|nb|KK920786.1|	133165-133282	nan	nan	15	43	1	0.2531	no
ni|645903644|nb|KK920786.1|	133494-133612	nan	nan	9	9	-0.0674353	0.9302	no
ni|645903644|nb|KK920786.1|	133714-134053	nan	nan	2	1	-0.34569	0.78305	no
ni|645903644|nb|KK920786.1|	134293-134697	nan	nan	43	51	0.239517	0.821	no
ni|645903644|nb|KK920786.1|	137153-137318	nan	nan	2	5	1	0.42455	no
ni|645903644|nb|KK920786.1|	137422-138191	nan	nan	1	0.989672	-0.386673	0.7444	no
ni|645903644|nb|KK920786.1|	138466-139640	nan	nan	4	2	-0.579086	0.6185	no
ni|645903644|nb|KK920786.1|	140289-140483	nan	nan	10	6	-0.855603	0.48415	no
ni|645903644|nb|KK920786.1|	140803-141140	nan	nan	5	3	-0.8432	0.47855	no
ni|645903644|nb|KK920786.1|	141553-141766	nan	nan	8	5	-0.752609	0.536	no
ni|645903644|nb|KK920786.1|	142420-142704	nan	nan	20	31	0.594034	0.60305	no
ni|645903644|nb|KK920786.1|	142973-143147	nan	nan	12	21	0.847804	0.47275	no
ni|645903644|nb|KK920786.1|	144762-145141	nan	nan	11	17	0.661817	0.56465	no
ni|645903644|nb|KK920786.1|	147603-147746	nan	nan	19	57	2	0.1957	no
ni|645903644|nb|KK920786.1|	147864-148815	nan	nan	9	13	0.547616	0.62495	no
ni|645903644|nb|KK920786.1|	151813-152248	nan	nan	21	30	0.552088	0.61575	no
ni|645903644|nb|KK920786.1|	152762-153318	nan	nan	3	15	2	0.06555	no
ni|645903644|nb|KK920786.1|	153550-154221	nan	nan	1	5	2	0.1103	no
ni|645903644|nb|KK920786.1|	163460-163635	nan	nan	42	37	-0.162735	0.89025	no
ni|645903644|nb|KK920786.1|	164050-164829	nan	nan	0.76485	0.835759	0.127909	0.9121	no
ni|645903644|nb|KK920786.1|	164962-165304	nan	nan	21	17.18	-0.286783	0.79935	no
ni|645903644|nb|KK920786.1|	165879-166603	nan	nan	0.872163	1	0.283134	0.8168	no
ni|645903644|nb|KK920786.1|	166727-167408	nan	nan	13	13	0.0126714	0.9907	no

ni|645903644|nb|KK920786.1|	167537-167793	nan	nan	108	104	-0.0469544	0.96035	no
ni|645903644|nb|KK920786.1|	168445-168815	nan	nan	57	55	-0.0631293	0.95295	no
ni|645903644|nb|KK920786.1|	169806-170031	nan	nan	2	2	-0.204022	0.87975	no
ni|645903644|nb|KK920786.1|	170153-170877	nan	nan	96	85	-0.170052	0.8991	no
ni|645903644|nb|KK920786.1|	171530-171754	nan	nan	207	180	-0.197444	0.8931	no
ni|645903644|nb|KK920786.1|	172304-172420	nan	nan	55	45	-0.276926	0.81075	no
ni|645903644|nb|KK920786.1|	173564-174131	nan	nan	149	119	-0.317765	0.8233	no
ni|645903644|nb|KK920786.1|	174297-174473	nan	nan	3	2	-0.482362	0.7594	no
ni|645903644|nb|KK920786.1|	178437-178920	nan	nan	775	643	-0.270555	0.85485	no
ni|645903644|nb|KK920786.1|	181245-181615	nan	nan	1	2	0.723045	0.5686	no
ni|645903644|nb|KK920786.1|	182220-182644	nan	nan	156	166	0.0858686	0.95105	no
ni|645903644|nb|KK920786.1|	18572-18771	nan	nan	46	35	-0.422831	0.70915	no
ni|645903644|nb|KK920786.1|	186636-186933	nan	nan	9	14	0.57726	0.6227	no
ni|645903644|nb|KK920786.1|	187241-187360	nan	nan	32	40	0.333118	0.7796	no
ni|645903644|nb|KK920786.1|	187860-188236	nan	nan	0.720002	0.430049	-0.7435	0.59615	no
ni|645903644|nb|KK920786.1|	188923-189137	nan	nan	54	69	0.33808	0.7634	no
ni|645903644|nb|KK920786.1|	19000-19212	nan	nan	65	52	-0.316848	0.77585	no
ni|645903644|nb|KK920786.1|	190143-190660	nan	nan	8	9	0.212449	0.85205	no
ni|645903644|nb|KK920786.1|	192562-192896	nan	nan	1	1	-0.20311	0.87975	no
ni|645903644|nb|KK920786.1|	193294-193437	nan	nan	42	41	-0.00831695	0.99135	no
ni|645903644|nb|KK920786.1|	194137-194355	nan	nan	2	22	4	0.02485	no
ni|645903644|nb|KK920786.1|	194815-195066	nan	nan	4	16	2	0.08535	no
ni|645903644|nb|KK920786.1|	195461-195653	nan	nan	117	172	0.557709	0.59905	no
ni|645903644|nb|KK920786.1|	197059-197259	nan	nan	2	1	-0.914978	0.53575	no
ni|645903644|nb|KK920786.1|	197457-198002	nan	nan	28	40	0.493173	0.64265	no
ni|645903644|nb|KK920786.1|	19865-20170	nan	nan	9	6	-0.551185	0.63995	no
ni|645903644|nb|KK920786.1|	198714-199981	nan	nan	47	56	0.24334	0.86515	no
ni|645903644|nb|KK920786.1|	200133-200564	nan	nan	1	0.716469	-1	0.4219	no
ni|645903644|nb|KK920786.1|	200863-201108	nan	nan	5	2	-1	0.39245	no
ni|645903644|nb|KK920786.1|	201588-201797	nan	nan	178	211	0.248776	0.87295	no

ni|645903644|nb|KK920786.1|	202252-202369	nan	nan	130	171	0.393388	0.7352	no
ni|645903644|nb|KK920786.1|	208876-209227	nan	nan	0.693191	0.85167	0.297042	0.8383	no
ni|645903644|nb|KK920786.1|	209358-209865	nan	nan	1	3	1	0.39595	no
ni|645903644|nb|KK920786.1|	212566-212950	nan	nan	0.961939	0.417867	-1	0.4064	no
ni|645903644|nb|KK920786.1|	21395-21859	nan	nan	1	0.781447	-0.650155	0.6173	no
ni|645903644|nb|KK920786.1|	214278-214967	nan	nan	2	1	-0.247693	0.82785	no
ni|645903644|nb|KK920786.1|	215739-216287	nan	nan	2	1	-0.44836	0.708	no
ni|645903644|nb|KK920786.1|	218475-219624	nan	nan	1	1	0.308937	0.79515	no
ni|645903644|nb|KK920786.1|	221068-221785	nan	nan	0.601429	0.26834	-1	0.41555	no
ni|645903644|nb|KK920786.1|	221908-222078	nan	nan	4	5	0.610803	0.63765	no
ni|645903644|nb|KK920786.1|	225721-225969	nan	nan	2	4	0.7703	0.5479	no
ni|645903644|nb|KK920786.1|	22617-23574	nan	nan	0.720899	0.661753	-0.123503	0.91825	no
ni|645903644|nb|KK920786.1|	226867-227341	nan	nan	2	2	0.269249	0.82645	no
ni|645903644|nb|KK920786.1|	228453-228686	nan	nan	3	4	0.419031	0.7302	no
ni|645903644|nb|KK920786.1|	230288-230435	nan	nan	3	6	1	0.43015	no
ni|645903644|nb|KK920786.1|	230559-230700	nan	nan	5	3	-0.745294	0.59615	no
ni|645903644|nb|KK920786.1|	230822-231019	nan	nan	5	6	0.125602	0.9121	no
ni|645903644|nb|KK920786.1|	231642-232180	nan	nan	2	3	0.234512	0.84265	no
ni|645903644|nb|KK920786.1|	232675-232829	nan	nan	2	7	2	0.15655	no
ni|645903644|nb|KK920786.1|	233754-234205	nan	nan	2	2	-0.338241	0.78125	no
ni|645903644|nb|KK920786.1|	234674-234865	nan	nan	3	3	-0.204669	0.87975	no
ni|645903644|nb|KK920786.1|	235130-235322	nan	nan	7	10	0.595819	0.6194	no
ni|645903644|nb|KK920786.1|	236691-236893	nan	nan	5	6	0.125706	0.9121	no
ni|645903644|nb|KK920786.1|	238042-238411	nan	nan	2	2	-0.316993	0.79415	no
ni|645903644|nb|KK920786.1|	239531-239893	nan	nan	6	5	-0.421626	0.7166	no
ni|645903644|nb|KK920786.1|	239981-240213	nan	nan	2	2	-0.307418	0.8123	no
ni|645903644|nb|KK920786.1|	240976-241128	nan	nan	10	8	-0.289584	0.8081	no
ni|645903644|nb|KK920786.1|	241756-242037	nan	nan	5	5	-0.106555	0.93205	no
ni|645903644|nb|KK920786.1|	242506-242677	nan	nan	4	4	-0.067279	0.954	no
ni|645903644|nb|KK920786.1|	244934-245367	nan	nan	7.6	7	-0.0938315	0.93585	no

ni|645903644|nb|KK920786.1|	24526-24725	nan	nan	30	19	-0.695033	0.55425	no
ni|645903644|nb|KK920786.1|	245831-246140	nan	nan	4	3	-0.525167	0.66185	no
ni|645903644|nb|KK920786.1|	246480-246736	nan	nan	5	3	-0.606688	0.6138	no
ni|645903644|nb|KK920786.1|	247376-247808	nan	nan	42	33	-0.347387	0.74625	no
ni|645903644|nb|KK920786.1|	251930-252487	nan	nan	13	10	-0.394638	0.7282	no
ni|645903644|nb|KK920786.1|	255077-255337	nan	nan	39	32	-0.285568	0.79425	no
ni|645903644|nb|KK920786.1|	260382-260645	nan	nan	48	35	-0.444567	0.69425	no
ni|645903644|nb|KK920786.1|	27403-27809	nan	nan	0.486746	0.542728	0.15706	0.9131	no
ni|645903644|nb|KK920786.1|	277146-277450	nan	nan	41	29	-0.534267	0.63615	no
ni|645903644|nb|KK920786.1|	27950-28543	nan	nan	0.452047	0.192065	-1	0.4023	no
ni|645903644|nb|KK920786.1|	282293-282696	nan	nan	16	16	-0.0284412	0.97925	no
ni|645903644|nb|KK920786.1|	283917-284399	nan	nan	23	19	-0.319373	0.76525	no
ni|645903644|nb|KK920786.1|	290750-291015	nan	nan	26	20	-0.368885	0.74725	no
ni|645903644|nb|KK920786.1|	291176-291410	nan	nan	12	23	0.944674	0.4241	no
ni|645903644|nb|KK920786.1|	291518-292557	nan	nan	16	24	0.587725	0.6333	no
ni|645903644|nb|KK920786.1|	292956-293241	nan	nan	68	113	0.727994	0.56585	no
ni|645903644|nb|KK920786.1|	293496-293721	nan	nan	3	2	-0.44503	0.74215	no
ni|645903644|nb|KK920786.1|	294490-294956	nan	nan	1	1	0.0172747	0.9674	no
ni|645903644|nb|KK920786.1|	295165-296385	nan	nan	1	1	0.0424182	0.9711	no
ni|645903644|nb|KK920786.1|	298227-299155	nan	nan	60	120	0.991614	0.4837	no
ni|645903644|nb|KK920786.1|	299521-299834	nan	nan	16	30	0.876884	0.44445	no
ni|645903644|nb|KK920786.1|	300576-300723	nan	nan	39	89	1	0.3114	no
ni|645903644|nb|KK920786.1|	302011-302257	nan	nan	115	222	0.952969	0.4405	no
ni|645903644|nb|KK920786.1|	302566-302848	nan	nan	114	222	0.967597	0.5172	no
ni|645903644|nb|KK920786.1|	303376-303537	nan	nan	113	241	1	0.32015	no
ni|645903644|nb|KK920786.1|	305444-305768	nan	nan	0	36	inf	5.00E-05	yes
ni|645903644|nb|KK920786.1|	306127-306259	nan	nan	0	63	inf	5.00E-05	yes
ni|645903644|nb|KK920786.1|	307136-307411	nan	nan	0.142429	54	9	0.11935	no
ni|645903644|nb|KK920786.1|	312164-312325	nan	nan	0	56	inf	5.00E-05	yes
ni|645903644|nb|KK920786.1|	31402-31664	nan	nan	37	24	-0.633324	0.5764	no

ni|645903644|nb|KK920786.1|	317798-317995	nan	nan	141	285	1	0.4201	no
ni|645903644|nb|KK920786.1|	318701-319093	nan	nan	38	90	1	0.3265	no
ni|645903644|nb|KK920786.1|	320280-320703	nan	nan	26	63	1	0.331	no
ni|645903644|nb|KK920786.1|	323876-324163	nan	nan	0.666071	3	2	0.14475	no
ni|645903644|nb|KK920786.1|	32395-32461	nan	nan	18598.6	19129.7	0.0406209	0.95985	no
ni|645903644|nb|KK920786.1|	325384-325489	nan	nan	95	185	0.962915	0.4271	no
ni|645903644|nb|KK920786.1|	325603-325756	nan	nan	85	170	0.995206	0.38905	no
ni|645903644|nb|KK920786.1|	327740-328214	nan	nan	52	116	1	0.43975	no
ni|645903644|nb|KK920786.1|	333613-333751	nan	nan	70	177	1	0.25055	no
ni|645903644|nb|KK920786.1|	334029-335262	nan	nan	0.326954	0.500256	0.613579	0.62145	no
ni|645903644|nb|KK920786.1|	336476-337566	nan	nan	0.748585	2	1	0.3083	no
ni|645903644|nb|KK920786.1|	337844-338982	nan	nan	6	5	-0.191106	0.8639	no
ni|645903644|nb|KK920786.1|	343343-343500	nan	nan	4	5	0.347839	0.7963	no
ni|645903644|nb|KK920786.1|	346021-346513	nan	nan	7	6	-0.143845	0.89965	no
ni|645903644|nb|KK920786.1|	352850-353023	nan	nan	0	30	inf	5.00E-05	yes
ni|645903644|nb|KK920786.1|	353228-353818	nan	nan	21	40.34	0.964713	0.45995	no
ni|645903644|nb|KK920786.1|	353991-354097	nan	nan	60	88	0.545018	0.65645	no
ni|645903644|nb|KK920786.1|	354361-354929	nan	nan	20	34	0.719756	0.4999	no
ni|645903644|nb|KK920786.1|	355694-356192	nan	nan	2	2	-0.493964	0.6802	no
ni|645903644|nb|KK920786.1|	356588-356742	nan	nan	4	2	-0.745264	0.59615	no
ni|645903644|nb|KK920786.1|	356912-357172	nan	nan	5	1	-2	0.2092	no
ni|645903644|nb|KK920786.1|	357568-357918	nan	nan	2	1	-0.802498	0.52275	no
ni|645903644|nb|KK920786.1|	358068-358699	nan	nan	3	2	-0.447372	0.7048	no
ni|645903644|nb|KK920786.1|	359362-359753	nan	nan	4	3	-0.358158	0.75735	no
ni|645903644|nb|KK920786.1|	359827-360096	nan	nan	5	3	-0.606573	0.6138	no
ni|645903644|nb|KK920786.1|	360378-360623	nan	nan	6	3	-0.905771	0.4582	no
ni|645903644|nb|KK920786.1|	361011-361238	nan	nan	36	25	-0.489517	0.6753	no
ni|645903644|nb|KK920786.1|	361807-362027	nan	nan	34	35	0.0352795	0.97495	no
ni|645903644|nb|KK920786.1|	363259-363407	nan	nan	32	22	-0.529543	0.6523	no
ni|645903644|nb|KK920786.1|	363555-363697	nan	nan	29	23	-0.324374	0.7834	no

ni|645903644|nb|KK920786.1|	364099-364416	nan	nan	8	8	-0.0656923	0.95475	no
ni|645903644|nb|KK920786.1|	364498-364751	nan	nan	11	13	0.234717	0.83875	no
ni|645903644|nb|KK920786.1|	364833-365396	nan	nan	4	6	0.314842	0.78565	no
ni|645903644|nb|KK920786.1|	365613-365859	nan	nan	5	4	-0.339243	0.78095	no
ni|645903644|nb|KK920786.1|	366414-367246	nan	nan	6	7	0.165478	0.8866	no
ni|645903644|nb|KK920786.1|	38309-38808	nan	nan	0.621242	0.059383	-3	0.03815	no
ni|645903644|nb|KK920786.1|	392598-392808	nan	nan	3	3	0.380686	0.7694	no
ni|645903644|nb|KK920786.1|	397187-398703	nan	nan	23	34	0.579969	0.68725	no
ni|645903644|nb|KK920786.1|	436654-436965	nan	nan	0	2	inf	0.00065	yes
ni|645903644|nb|KK920786.1|	472028-472196	nan	nan	8	10	0.398414	0.7419	no
ni|645903644|nb|KK920786.1|	499099-499238	nan	nan	63	102	0.681229	0.5622	no
ni|645903644|nb|KK920786.1|	513727-514174	nan	nan	14	15	0.0401222	0.97025	no
ni|645903644|nb|KK920786.1|	607948-608289	nan	nan	0.309497	3	3	0.0631	no
ni|645903644|nb|KK920786.1|	712072-712264	nan	nan	78	102	0.391368	0.7264	no
ni|645903644|nb|KK920786.1|	713537-713732	nan	nan	76	81	0.0799146	0.941	no
ni|645903644|nb|KK920786.1|	714181-714379	nan	nan	65	76	0.230755	0.83975	no
ni|645903644|nb|KK920786.1|	716273-716433	nan	nan	77	79	0.0458576	0.96755	no
ni|645903644|nb|KK920786.1|	716926-717168	nan	nan	78	95	0.275618	0.79775	no
ni|645903644|nb|KK920786.1|	719746-719924	nan	nan	82	81	-0.0229292	0.9836	no
ni|645903644|nb|KK920786.1|	720031-720193	nan	nan	34	33	-0.0672124	0.95215	no
ni|645903644|nb|KK920786.1|	720243-720479	nan	nan	68	54	-0.348124	0.7516	no
ni|645903644|nb|KK920786.1|	720717-720944	nan	nan	5	2	-2	0.2366	no
ni|645903644|nb|KK920786.1|	721262-721437	nan	nan	0	897	inf	5.00E-05	yes
ni|645903644|nb|KK920786.1|	725650-725946	nan	nan	0	538.82	inf	5.00E-05	yes
ni|645903644|nb|KK920786.1|	727861-728177	nan	nan	0	648.75	inf	5.00E-05	yes
ni|645903644|nb|KK920786.1|	734425-734766	nan	nan	419	298	-0.492627	0.7235	no
ni|645903644|nb|KK920786.1|	735221-735748	nan	nan	311	216	-0.525541	0.70355	no
ni|645903644|nb|KK920786.1|	736226-736409	nan	nan	823	590	-0.480733	0.73605	no
ni|645903644|nb|KK920786.1|	737404-737898	nan	nan	364	260	-0.482665	0.73225	no
ni|645903644|nb|KK920786.1|	738996-740372	nan	nan	89	70	-0.348286	0.80575	no

ni|645903644|nb|KK920786.1|	743303-744059	nan	nan	116	94	-0.306872	0.8263	no
ni|645903644|nb|KK920786.1|	744530-744700	nan	nan	53	40	-0.418741	0.71925	no
ni|645903644|nb|KK920786.1|	78167-78657	nan	nan	39	56	0.514377	0.67505	no
ni|645903644|nb|KK920786.1|	79660-79826	nan	nan	547	741	0.43791	0.7543	no
ni|645903644|nb|KK920786.1|	80009-80834	nan	nan	2	2	-0.210548	0.8556	no
ni|645903644|nb|KK920786.1|	81488-82125	nan	nan	1	1	0.164588	0.89345	no
ni|645903644|nb|KK920786.1|	82272-82681	nan	nan	54	69	0.353994	0.7727	no
ni|645903644|nb|KK920786.1|	823637-823882	nan	nan	2	2	-0.32927	0.80325	no
ni|645903644|nb|KK920786.1|	824314-824698	nan	nan	1	1	-0.0653994	0.95935	no
ni|645903644|nb|KK920786.1|	828508-828975	nan	nan	0.945991	0.775028	-0.287578	0.8283	no
ni|645903644|nb|KK920786.1|	829100-829575	nan	nan	1	2	0.127477	0.9121	no
ni|645903644|nb|KK920786.1|	83062-83356	nan	nan	90	145	0.68381	0.5806	no
ni|645903644|nb|KK920786.1|	834642-835099	nan	nan	1	0.730441	-0.605777	0.6316	no
ni|645903644|nb|KK920786.1|	86395-86618	nan	nan	254	393	0.633622	0.6603	no
ni|645903644|nb|KK920786.1|	86844-87361	nan	nan	2	3	0.506072	0.66265	no
ni|645903644|nb|KK920786.1|	87580-88116	nan	nan	2	4	0.531051	0.6466	no
ni|645903644|nb|KK920786.1|	884556-885114	nan	nan	0	92	inf	5.00E-05	yes
ni|645903644|nb|KK920786.1|	886651-887084	nan	nan	0	2	inf	0.00055	yes
ni|645903644|nb|KK920786.1|	887950-888566	nan	nan	0	2	inf	5.00E-05	yes
ni|645903644|nb|KK920786.1|	889001-889358	nan	nan	0	203	inf	5.00E-05	yes
ni|645903644|nb|KK920786.1|	897789-898036	nan	nan	2	2	-0.203716	0.87975	no
ni|645903645|nb|KK920785.1|	126054-128499	nan	nan	0.357682	0.613748	0.778969	0.51085	no
ni|645903645|nb|KK920785.1|	131272-131828	nan	nan	0.434583	0.623149	0.519945	0.7004	no
ni|645903645|nb|KK920785.1|	140513-141055	nan	nan	1	0.910879	-0.621435	0.61715	no
ni|645903645|nb|KK920785.1|	145655-146171	nan	nan	0.774448	0.11389	-3	0.25935	no
ni|645903645|nb|KK920785.1|	146307-146964	nan	nan	15	7	-1	0.339	no
ni|645903645|nb|KK920785.1|	149169-150822	nan	nan	25	12	-1	0.49975	no
ni|645903645|nb|KK920785.1|	151034-151285	nan	nan	29	11	-1	0.25205	no
ni|645903645|nb|KK920785.1|	151655-151847	nan	nan	6	3	-0.941615	0.46495	no
ni|645903645|nb|KK920785.1|	152509-152857	nan	nan	8	5	-0.814733	0.49315	no

ni|645903645|nb|KK920785.1|	153305-153426	nan	nan	10	5	-0.915388	0.53575	no
ni|645903645|nb|KK920785.1|	162535-162933	nan	nan	0.833156	0.796256	-0.0653549	0.9467	no
ni|645903645|nb|KK920785.1|	163445-165535	nan	nan	1	0.865647	-0.300777	0.80065	no
ni|645903645|nb|KK920785.1|	167121-167665	nan	nan	0.781176	0.586726	-0.412962	0.76315	no
ni|645903645|nb|KK920785.1|	169373-169518	nan	nan	4	2	-1	0.44965	no
ni|645903645|nb|KK920785.1|	170027-170329	nan	nan	2	0.353257	-2	0.1906	no
ni|645903645|nb|KK920785.1|	170522-171081	nan	nan	2	2	-0.112318	0.9225	no
ni|645903645|nb|KK920785.1|	171542-172257	nan	nan	0.683844	0.192297	-2	0.1999	no
ni|645903645|nb|KK920785.1|	186671-187082	nan	nan	0.478874	1	2	0.297	no
ni|645903645|nb|KK920785.1|	189601-190095	nan	nan	0.314584	2	3	0.06975	no
ni|645903645|nb|KK920785.1|	205038-205225	nan	nan	2	12	2	0.09255	no
ni|645903645|nb|KK920785.1|	207929-208252	nan	nan	0.669332	5	3	0.07245	no
ni|645903645|nb|KK920785.1|	210113-210398	nan	nan	2	7	2	0.1598	no
ni|645903645|nb|KK920785.1|	219295-219495	nan	nan	1	11	3	0.047	no
ni|645903645|nb|KK920785.1|	220300-220528	nan	nan	2	6	1	0.2754	no
ni|645903645|nb|KK920785.1|	222143-222457	nan	nan	0.813979	1	0.448865	0.7543	no
ni|645903645|nb|KK920785.1|	225116-225297	nan	nan	2	6	2	0.28535	no
ni|645903645|nb|KK920785.1|	232025-232817	nan	nan	0.464554	2	2	0.17655	no
ni|645903645|nb|KK920785.1|	237176-237551	nan	nan	1	0.863243	-0.328466	0.80325	no
ni|645903645|nb|KK920785.1|	237680-238027	nan	nan	2	2	0.182382	0.87985	no
ni|645903645|nb|KK920785.1|	239205-239912	nan	nan	1	2	0.292744	0.80385	no
ni|645903645|nb|KK920785.1|	241991-242149	nan	nan	5	8	0.595765	0.63005	no
ni|645903645|nb|KK920785.1|	262277-262446	nan	nan	3	6	1	0.39265	no
ni|645903645|nb|KK920785.1|	265769-266360	nan	nan	1	5	2	0.17055	no
ni|645903645|nb|KK920785.1|	304322-304475	nan	nan	14	3	-2	0.1184	no
ni|645903645|nb|KK920785.1|	304626-306101	nan	nan	18	4	-2	0.1166	no
ni|645903645|nb|KK920785.1|	31016-31359	nan	nan	23	18	-0.340186	0.77	no
ni|645903645|nb|KK920785.1|	53277-53529	nan	nan	102	69	-0.565168	0.5933	no
ni|645903645|nb|KK920785.1|	55770-55951	nan	nan	89	80	-0.145023	0.8949	no
ni|645903645|nb|KK920785.1|	57301-57443	nan	nan	111	91	-0.290888	0.7991	no

ni|645903645|nb|KK920785.1|	59815-59921	nan	nan	128	105	-0.283688	0.8139	no
ni|645903645|nb|KK920785.1|	6136-6420	nan	nan	32	22	-0.520686	0.6497	no
ni|645903645|nb|KK920785.1|	92916-93703	nan	nan	0.61193	0.378582	-0.69276	0.58855	no
ni|645903645|nb|KK920785.1|	93939-94268	nan	nan	0.543057	3	2	0.08975	no
ni|645903645|nb|KK920785.1|	94823-95132	nan	nan	1	8	2	0.0709	no
ni|645903645|nb|KK920785.1|	95469-95693	nan	nan	2	4	1	0.35025	no
ni|645903645|nb|KK920785.1|	95839-96264	nan	nan	3	12	2	0.15085	no
ni|645903645|nb|KK920785.1|	99271-101076	nan	nan	2	0.055371	-5	0.04475	no
ni|645903646|nb|KK920784.1|	38495-38675	nan	nan	6	6	0.155082	0.8959	no
ni|645903647|nb|KK920783.1|	217986-218248	nan	nan	10	13	0.277238	0.81155	no
ni|645903647|nb|KK920783.1|	219167-219385	nan	nan	5	6	0.348405	0.7655	no
ni|645903647|nb|KK920783.1|	220781-221019	nan	nan	10	12	0.218404	0.85225	no
ni|645903647|nb|KK920783.1|	221710-221950	nan	nan	15	16	0.0839444	0.94045	no
ni|645903647|nb|KK920783.1|	223904-224081	nan	nan	12	16	0.49067	0.6806	no
ni|645903647|nb|KK920783.1|	224190-225217	nan	nan	4	4	-0.0560115	0.9598	no
ni|645903647|nb|KK920783.1|	226138-226566	nan	nan	2	3	1	0.36645	no
ni|645903647|nb|KK920783.1|	228045-228257	nan	nan	2	2	-0.0667352	0.9384	no
ni|645903647|nb|KK920783.1|	228958-229368	nan	nan	2	2	0.0721821	0.9565	no
ni|645903647|nb|KK920783.1|	235392-236022	nan	nan	15	13	-0.217409	0.8457	no
ni|645903647|nb|KK920783.1|	236617-237099	nan	nan	9	7	-0.363915	0.7514	no
ni|645903647|nb|KK920783.1|	237537-238232	nan	nan	0.415836	0.357809	-0.216825	0.87705	no
ni|645903647|nb|KK920783.1|	242176-242421	nan	nan	28	22	-0.348875	0.76095	no
ni|645903647|nb|KK920783.1|	242523-242931	nan	nan	0.72535	1	0.671639	0.62105	no
ni|645903647|nb|KK920783.1|	243066-243700	nan	nan	5	6	0.254562	0.8286	no
ni|645903647|nb|KK920783.1|	245045-245395	nan	nan	11	11	0.0769115	0.94725	no
ni|645903647|nb|KK920783.1|	250584-250843	nan	nan	2	2	0.0493862	0.95725	no
ni|645903647|nb|KK920783.1|	254075-254377	nan	nan	0	31	inf	5.00E-05	yes
ni|645903647|nb|KK920783.1|	255715-256556	nan	nan	0	6	inf	5.00E-05	yes
ni|645903647|nb|KK920783.1|	259040-259609	nan	nan	0	23	inf	5.00E-05	yes
ni|645903647|nb|KK920783.1|	261103-261369	nan	nan	0	17.22	inf	5.00E-05	yes

ni|645903647|nb|KK920783.1|	262116-262418	nan	nan	0	22	inf	5.00E-05	yes
ni|645903647|nb|KK920783.1|	267478-267615	nan	nan	0	75	inf	5.00E-05	yes
ni|645903647|nb|KK920783.1|	270471-270745	nan	nan	0	76	inf	5.00E-05	yes
ni|645903647|nb|KK920783.1|	274492-274665	nan	nan	0	96	inf	5.00E-05	yes
ni|645903647|nb|KK920783.1|	275370-275519	nan	nan	0.507679	50	7	0.11945	no
ni|645903647|nb|KK920783.1|	275680-275799	nan	nan	1	115.77	7	0.1194	no
ni|645903647|nb|KK920783.1|	281986-282638	nan	nan	0.26902	0.814437	2	0.2583	no
ni|645903647|nb|KK920783.1|	282734-283100	nan	nan	0.560438	1	1	0.45555	no
ni|645903647|nb|KK920783.1|	283649-283793	nan	nan	4	4	0.125437	0.92845	no
ni|645903647|nb|KK920783.1|	289962-290382	nan	nan	2	2	0.455537	0.70335	no
ni|645903647|nb|KK920783.1|	293556-293909	nan	nan	1	3	1	0.28525	no
ni|645903647|nb|KK920783.1|	295427-295547	nan	nan	43	54	0.317259	0.7891	no
ni|645903647|nb|KK920783.1|	318759-319286	nan	nan	2	2	0.278887	0.81425	no
ni|645903647|nb|KK920783.1|	358450-361461	nan	nan	40	60.6	0.617123	0.66255	no
ni|645903647|nb|KK920783.1|	361673-361785	nan	nan	27	20	-0.415501	0.7377	no
ni|645903647|nb|KK920783.1|	398129-398412	nan	nan	20	10	-0.963138	0.41135	no
ni|645903647|nb|KK920783.1|	401412-401643	nan	nan	57	29	-1	0.37615	no
ni|645903647|nb|KK920783.1|	457964-458126	nan	nan	19.35	17	-0.192743	0.86665	no
ni|645903647|nb|KK920783.1|	458766-459051	nan	nan	3	2	-0.563383	0.6485	no
ni|645903647|nb|KK920783.1|	462379-462870	nan	nan	6	3	-1	0.3336	no
ni|645903647|nb|KK920783.1|	470171-470488	nan	nan	10	7	-0.435642	0.7032	no
ni|645903647|nb|KK920783.1|	551801-552303	nan	nan	0.616583	2	1	0.2663	no
ni|645903647|nb|KK920783.1|	558391-558668	nan	nan	1	3	1	0.41145	no
ni|645903647|nb|KK920783.1|	559014-559416	nan	nan	4	2	-1	0.35325	no
ni|645903647|nb|KK920783.1|	560693-561100	nan	nan	64	118	0.880714	0.54475	no
ni|645903647|nb|KK920783.1|	562546-562832	nan	nan	94	166	0.827208	0.502	no
ni|645903648|nb|KK920782.1|	122439-122700	nan	nan	0.154928	55	8	0.11935	no
ni|645903648|nb|KK920782.1|	126071-126254	nan	nan	1	36	5	0.0377	no
ni|645903648|nb|KK920782.1|	134573-135839	nan	nan	73	27	-1	0.33215	no
ni|645903648|nb|KK920782.1|	137292-137949	nan	nan	3	0.254873	-4	0.04195	no

ni|645903648|nb|KK920782.1|	139083-139254	nan	nan	64	68	0.098211	0.93025	no
ni|645903648|nb|KK920782.1|	139352-139577	nan	nan	3	0.764096	-2	0.2004	no
ni|645903648|nb|KK920782.1|	139936-140603	nan	nan	0.785621	0.166908	-2	0.1417	no
ni|645903648|nb|KK920782.1|	145623-146003	nan	nan	0.975769	0.339096	-2	0.3149	no
ni|645903648|nb|KK920782.1|	147074-147456	nan	nan	26	31	0.21357	0.84845	no
ni|645903648|nb|KK920782.1|	147587-148096	nan	nan	1	0.405468	-2	0.2821	no
ni|645903648|nb|KK920782.1|	149761-149945	nan	nan	7	36	2	0.05155	no
ni|645903648|nb|KK920782.1|	151555-152043	nan	nan	18	6	-2	0.1799	no
ni|645903648|nb|KK920782.1|	152919-153068	nan	nan	5	0.96915	-2	0.32065	no
ni|645903648|nb|KK920782.1|	153619-153947	nan	nan	3	1	-2	0.24	no
ni|645903648|nb|KK920782.1|	154406-154572	nan	nan	4	3	-0.581807	0.70815	no
ni|645903648|nb|KK920782.1|	244639-244917	nan	nan	0.280016	236	10	0.0443	no
ni|645903648|nb|KK920782.1|	256401-256734	nan	nan	0.106734	335	12	0.11935	no
ni|645903648|nb|KK920782.1|	268275-268457	nan	nan	0.611644	366	9	0.0443	no
ni|645903648|nb|KK920782.1|	270298-270575	nan	nan	0.140806	287	11	0.11935	no
ni|645903648|nb|KK920782.1|	272635-273115	nan	nan	0.65247	218	8	0.00925	no
ni|645903648|nb|KK920782.1|	274100-274212	nan	nan	0	61	inf	0.00015	yes
ni|645903648|nb|KK920782.1|	70062-70348	nan	nan	0.669682	1	0.78212	0.58145	no
ni|645903649|nb|KK920781.1|	118643-119261	nan	nan	2	3	0.596278	0.61415	no
ni|645903649|nb|KK920781.1|	145174-145827	nan	nan	3	3	-0.316411	0.78515	no
ni|645903649|nb|KK920781.1|	146038-146345	nan	nan	3	3	-0.0657465	0.9571	no
ni|645903649|nb|KK920781.1|	146492-146758	nan	nan	6	5	-0.105558	0.9263	no
ni|645903649|nb|KK920781.1|	146914-147378	nan	nan	5	4	-0.486656	0.67725	no
ni|645903649|nb|KK920781.1|	147904-148301	nan	nan	0.668783	1	0.934642	0.4874	no
ni|645903649|nb|KK920781.1|	157504-157724	nan	nan	3	0.796218	-2	0.21345	no
ni|645903649|nb|KK920781.1|	162995-163480	nan	nan	0.450767	0.30776	-0.550574	0.71905	no
ni|645903649|nb|KK920781.1|	163722-164043	nan	nan	0.675434	12	4	0.0363	no
ni|645903649|nb|KK920781.1|	164342-164487	nan	nan	2	37	4	0.039	no
ni|645903649|nb|KK920781.1|	197095-197292	nan	nan	0	7	inf	5.00E-05	yes
ni|645903649|nb|KK920781.1|	201207-201557	nan	nan	2	2	-0.387461	0.7511	no

ni|645903649|nb|KK920781.1|	201877-202007	nan	nan	8	3	-1	0.35155	no
ni|645903649|nb|KK920781.1|	202340-202472	nan	nan	5	4	-0.289671	0.8496	no
ni|645903649|nb|KK920781.1|	202592-202959	nan	nan	11	12	0.170836	0.8831	no
ni|645903649|nb|KK920781.1|	203755-203944	nan	nan	26	24	-0.0671984	0.9548	no
ni|645903649|nb|KK920781.1|	204478-206708	nan	nan	0.891899	0.664681	-0.424219	0.7217	no
ni|645903649|nb|KK920781.1|	207238-207618	nan	nan	0.443532	1	1	0.39335	no
ni|645903649|nb|KK920781.1|	209063-210327	nan	nan	1	0.527485	-0.978753	0.4167	no
ni|645903649|nb|KK920781.1|	216285-217014	nan	nan	0.511257	0.488807	-0.0647841	0.9554	no
ni|645903649|nb|KK920781.1|	218191-218658	nan	nan	0.743278	0.4521	-0.717262	0.61925	no
ni|645903649|nb|KK920781.1|	225108-227355	nan	nan	13	11	-0.213909	0.88645	no
ni|645903649|nb|KK920781.1|	227526-228096	nan	nan	6	5	-0.167174	0.8879	no
ni|645903649|nb|KK920781.1|	228398-229334	nan	nan	5	5	-0.0489402	0.9638	no
ni|645903649|nb|KK920781.1|	229455-229598	nan	nan	6	5	-0.356717	0.7931	no
ni|645903649|nb|KK920781.1|	229799-230113	nan	nan	9	14	0.633289	0.59165	no
ni|645903649|nb|KK920781.1|	230377-230570	nan	nan	23	29	0.339498	0.77465	no
ni|645903649|nb|KK920781.1|	233396-233682	nan	nan	2	1	-0.328911	0.80325	no
ni|645903649|nb|KK920781.1|	233884-234087	nan	nan	3	2	-0.874273	0.53675	no
ni|645903649|nb|KK920781.1|	257086-257536	nan	nan	69	55	-0.316127	0.83395	no
ni|645903649|nb|KK920781.1|	354634-354803	nan	nan	0	279	inf	5.00E-05	yes
ni|645903649|nb|KK920781.1|	377044-377482	nan	nan	0	178	inf	5.00E-05	yes
ni|645903649|nb|KK920781.1|	493602-493764	nan	nan	80	72	-0.164678	0.88105	no
ni|645903649|nb|KK920781.1|	504773-505049	nan	nan	45	38	-0.230731	0.83755	no
ni|645903649|nb|KK920781.1|	507388-507676	nan	nan	1	1	-0.355369	0.7931	no
ni|645903649|nb|KK920781.1|	511882-512318	nan	nan	41	36	-0.159937	0.88305	no
ni|645903649|nb|KK920781.1|	512391-512512	nan	nan	38	15	-1	0.3008	no
ni|645903649|nb|KK920781.1|	516587-517767	nan	nan	0.20582	0.284295	0.466004	0.7261	no
ni|645903649|nb|KK920781.1|	530181-531230	nan	nan	2	2	-0.468953	0.6913	no
ni|645903649|nb|KK920781.1|	532767-533226	nan	nan	0.759933	1	0.723292	0.5686	no
ni|645903649|nb|KK920781.1|	540274-540563	nan	nan	1	1	-0.387784	0.78555	no
ni|645903649|nb|KK920781.1|	548156-548580	nan	nan	2	2	-0.0652844	0.9553	no

ni|645903649|nb|KK920781.1|	68729-69035	nan	nan	0.725001	1	0.671213	0.65595	no
ni|645903649|nb|KK920781.1|	71369-71648	nan	nan	8	9	0.261237	0.8243	no
ni|645903649|nb|KK920781.1|	78660-78930	nan	nan	2	0.980679	-1	0.3768	no
ni|645903649|nb|KK920781.1|	79596-80464	nan	nan	1	0.8002	-0.790491	0.5074	no
ni|645903649|nb|KK920781.1|	80685-81529	nan	nan	10	14	0.592429	0.59155	no
ni|645903649|nb|KK920781.1|	81888-82489	nan	nan	13	17	0.394974	0.7254	no
ni|645903649|nb|KK920781.1|	82850-83196	nan	nan	1	1	-0.287943	0.8283	no
ni|645903649|nb|KK920781.1|	83473-85625	nan	nan	3	3	0.013392	0.9902	no
ni|645903649|nb|KK920781.1|	85825-88085	nan	nan	57	84	0.558808	0.6942	no
ni|645903650|nb|KK920780.1|	1000141-1000257	nan	nan	782	766	-0.0296469	0.9782	no
ni|645903650|nb|KK920780.1|	1002356-1002791	nan	nan	2	1	-0.562757	0.6485	no
ni|645903650|nb|KK920780.1|	1006369-1006477	nan	nan	414	440.53	0.0880999	0.94005	no
ni|645903650|nb|KK920780.1|	1007165-1007397	nan	nan	66	77	0.230281	0.8319	no
ni|645903650|nb|KK920780.1|	1007872-1008356	nan	nan	151	166	0.130879	0.92475	no
ni|645903650|nb|KK920780.1|	1008983-1009399	nan	nan	133.05	178	0.422188	0.7646	no
ni|645903650|nb|KK920780.1|	1009765-1009968	nan	nan	6	3	-1	0.3535	no
ni|645903650|nb|KK920780.1|	1010818-1011412	nan	nan	56	74	0.38455	0.7888	no
ni|645903650|nb|KK920780.1|	1033553-1033979	nan	nan	0	1	inf	0.0005	yes
ni|645903650|nb|KK920780.1|	1063664-1064210	nan	nan	0	34	inf	5.00E-05	yes
ni|645903650|nb|KK920780.1|	139282-139821	nan	nan	1	0.377642	-1	0.312	no
ni|645903650|nb|KK920780.1|	140771-141068	nan	nan	548	411.68	-0.411823	0.76685	no
ni|645903650|nb|KK920780.1|	141366-142899	nan	nan	1915.82	2505.7	0.387252	0.6553	no
ni|645903650|nb|KK920780.1|	143174-143398	nan	nan	2317.96	3329.67	0.522522	0.7239	no
ni|645903650|nb|KK920780.1|	143783-143972	nan	nan	3	3	-0.192729	0.87555	no
ni|645903650|nb|KK920780.1|	144189-144876	nan	nan	1	1	-0.375171	0.75535	no
ni|645903650|nb|KK920780.1|	145432-145990	nan	nan	292	427	0.545352	0.6995	no
ni|645903650|nb|KK920780.1|	146402-146655	nan	nan	1559.93	2356.56	0.595195	0.68675	no
ni|645903650|nb|KK920780.1|	147184-147605	nan	nan	1	216	8	0.0032	no
ni|645903650|nb|KK920780.1|	153293-156200	nan	nan	0.026361	157	13	0.05055	no
ni|645903650|nb|KK920780.1|	156641-157814	nan	nan	0	121	inf	5.00E-05	yes

ni|645903650|nb|KK920780.1|	161250-161408	nan	nan	0	20	inf	0.00015	yes
ni|645903650|nb|KK920780.1|	161569-161897	nan	nan	41	53	0.372485	0.73535	no
ni|645903650|nb|KK920780.1|	162022-163087	nan	nan	28	33	0.229972	0.87515	no
ni|645903650|nb|KK920780.1|	163225-163390	nan	nan	67	84	0.320765	0.7772	no
ni|645903650|nb|KK920780.1|	165567-165798	nan	nan	39	27	-0.541937	0.6399	no
ni|645903650|nb|KK920780.1|	166029-166343	nan	nan	33	23	-0.560244	0.614	no
ni|645903650|nb|KK920780.1|	166852-166972	nan	nan	75	53	-0.498039	0.66925	no
ni|645903650|nb|KK920780.1|	167180-167628	nan	nan	27	19	-0.495533	0.65865	no
ni|645903650|nb|KK920780.1|	167794-168040	nan	nan	21	18	-0.218227	0.84765	no
ni|645903650|nb|KK920780.1|	168185-168326	nan	nan	50	46	-0.120333	0.91805	no
ni|645903650|nb|KK920780.1|	168624-168807	nan	nan	5	4	-0.347391	0.78305	no
ni|645903650|nb|KK920780.1|	169002-169311	nan	nan	52	45	-0.2141	0.84475	no
ni|645903650|nb|KK920780.1|	169594-172893	nan	nan	1	26	4	0.00625	no
ni|645903650|nb|KK920780.1|	173653-174029	nan	nan	0.990002	0.602068	-0.717505	0.61925	no
ni|645903650|nb|KK920780.1|	174434-174913	nan	nan	43	36	-0.258427	0.8043	no
ni|645903650|nb|KK920780.1|	175476-175642	nan	nan	90	63	-0.510841	0.656	no
ni|645903650|nb|KK920780.1|	177931-178221	nan	nan	19	15	-0.294984	0.79465	no
ni|645903650|nb|KK920780.1|	178509-178940	nan	nan	0.899551	0.859763	-0.065267	0.9545	no
ni|645903650|nb|KK920780.1|	179394-181098	nan	nan	5	4	-0.514089	0.64535	no
ni|645903650|nb|KK920780.1|	181705-181877	nan	nan	58	44	-0.387046	0.7405	no
ni|645903650|nb|KK920780.1|	183413-183635	nan	nan	63	58	-0.109493	0.921	no
ni|645903650|nb|KK920780.1|	184231-184338	nan	nan	137.38	116	-0.241872	0.83665	no
ni|645903650|nb|KK920780.1|	19755-20086	nan	nan	12	9	-0.390862	0.73615	no
ni|645903650|nb|KK920780.1|	204139-204207	nan	nan	10132.4	5626.66	-0.848623	0.555	no
ni|645903650|nb|KK920780.1|	24166-24877	nan	nan	0.080981	70	10	0.0443	no
ni|645903650|nb|KK920780.1|	25106-26548	nan	nan	0.055189	71	10	0.05055	no
ni|645903650|nb|KK920780.1|	31632-31821	nan	nan	44	39	-0.171995	0.88085	no
ni|645903650|nb|KK920780.1|	324197-324610	nan	nan	79	61	-0.371784	0.805	no
ni|645903650|nb|KK920780.1|	324716-324867	nan	nan	31	27	-0.161831	0.88785	no
ni|645903650|nb|KK920780.1|	32549-32837	nan	nan	0.927499	2	1	0.4471	no

ni|645903650|nb|KK920780.1|	32911-33658	nan	nan	4	5	0.217634	0.85145	no
ni|645903650|nb|KK920780.1|	337913-338030	nan	nan	70	48	-0.541384	0.64615	no
ni|645903650|nb|KK920780.1|	33889-34216	nan	nan	1	1	-0.181118	0.889	no
ni|645903650|nb|KK920780.1|	338929-339424	nan	nan	13	11	-0.160123	0.88735	no
ni|645903650|nb|KK920780.1|	339616-340026	nan	nan	23	20	-0.225786	0.84185	no
ni|645903650|nb|KK920780.1|	341363-342209	nan	nan	14	14	-0.0442655	0.9676	no
ni|645903650|nb|KK920780.1|	344210-344480	nan	nan	23	23	-0.0299272	0.9788	no
ni|645903650|nb|KK920780.1|	34675-35098	nan	nan	2	2	-0.111091	0.92105	no
ni|645903650|nb|KK920780.1|	35483-36042	nan	nan	11	14	0.353592	0.7521	no
ni|645903650|nb|KK920780.1|	36765-36868	nan	nan	98	169.31	0.789346	0.52185	no
ni|645903650|nb|KK920780.1|	37537-37791	nan	nan	4	6	0.426901	0.72025	no
ni|645903650|nb|KK920780.1|	37948-39601	nan	nan	95	179	0.914354	0.5224	no
ni|645903650|nb|KK920780.1|	39847-40005	nan	nan	19	31	0.702187	0.5522	no
ni|645903650|nb|KK920780.1|	40121-40481	nan	nan	10	13	0.395239	0.7305	no
ni|645903650|nb|KK920780.1|	40783-41026	nan	nan	6	6	0.0942056	0.93725	no
ni|645903650|nb|KK920780.1|	411786-412237	nan	nan	26	23	-0.176412	0.87055	no
ni|645903650|nb|KK920780.1|	41220-41975	nan	nan	4	7	0.76191	0.5073	no
ni|645903650|nb|KK920780.1|	42318-42440	nan	nan	19	17	-0.154842	0.90135	no
ni|645903650|nb|KK920780.1|	42657-43004	nan	nan	8	11	0.323496	0.78125	no
ni|645903650|nb|KK920780.1|	44206-45265	nan	nan	10.15	14	0.429733	0.6935	no
ni|645903650|nb|KK920780.1|	45840-46006	nan	nan	7	14	1	0.39695	no
ni|645903650|nb|KK920780.1|	46164-46305	nan	nan	21	28	0.388457	0.74445	no
ni|645903650|nb|KK920780.1|	46870-47049	nan	nan	12	19	0.700507	0.55105	no
ni|645903650|nb|KK920780.1|	47315-47544	nan	nan	13	18	0.496139	0.66965	no
ni|645903650|nb|KK920780.1|	48069-48317	nan	nan	20	37	0.878657	0.45145	no
ni|645903650|nb|KK920780.1|	48981-49638	nan	nan	16.44	29	0.811019	0.45075	no
ni|645903650|nb|KK920780.1|	49930-51094	nan	nan	15	19	0.331412	0.79265	no
ni|645903650|nb|KK920780.1|	53910-54206	nan	nan	2	4	1	0.32435	no
ni|645903650|nb|KK920780.1|	556683-556800	nan	nan	3	27	3	0.05455	no
ni|645903650|nb|KK920780.1|	562658-563107	nan	nan	0.568603	0.815202	0.519736	0.7004	no

ni|645903650|nb|KK920780.1|	563220-563387	nan	nan	40	75	0.919083	0.42435	no
ni|645903650|nb|KK920780.1|	564125-564295	nan	nan	32	74	1	0.3156	no
ni|645903650|nb|KK920780.1|	564544-564674	nan	nan	33	68	1	0.37675	no
ni|645903650|nb|KK920780.1|	566468-566660	nan	nan	45	87	0.95468	0.3991	no
ni|645903650|nb|KK920780.1|	566897-567288	nan	nan	3	6	0.896149	0.4534	no
ni|645903650|nb|KK920780.1|	568641-569055	nan	nan	6	11	0.887692	0.4497	no
ni|645903650|nb|KK920780.1|	569381-569821	nan	nan	7	4	-0.81248	0.48855	no
ni|645903650|nb|KK920780.1|	570266-570377	nan	nan	31	12	-1	0.34635	no
ni|645903650|nb|KK920780.1|	570620-571630	nan	nan	21	19	-0.12292	0.92125	no
ni|645903650|nb|KK920780.1|	571913-572018	nan	nan	83	59	-0.483149	0.71	no
ni|645903650|nb|KK920780.1|	574198-577259	nan	nan	17	27	0.66088	0.63445	no
ni|645903650|nb|KK920780.1|	577366-578331	nan	nan	11	20	0.914305	0.39615	no
ni|645903650|nb|KK920780.1|	690492-690559	nan	nan	29318.3	30892.2	0.0754427	0.95185	no
ni|645903650|nb|KK920780.1|	69296-69600	nan	nan	0.732165	0.582949	-0.328798	0.8122	no
ni|645903650|nb|KK920780.1|	705598-705663	nan	nan	140700	79239.9	-0.828328	0.474	no
ni|645903650|nb|KK920780.1|	73412-73736	nan	nan	0.777376	0.530565	-0.551083	0.71905	no
ni|645903650|nb|KK920780.1|	779370-779511	nan	nan	204	149	-0.4526	0.6859	no
ni|645903650|nb|KK920780.1|	780655-780863	nan	nan	118	99	-0.260672	0.80925	no
ni|645903650|nb|KK920780.1|	783169-783336	nan	nan	76	70	-0.132337	0.90665	no
ni|645903650|nb|KK920780.1|	783906-784463	nan	nan	7	4	-0.731772	0.531	no
ni|645903650|nb|KK920780.1|	784849-785057	nan	nan	12	11	-0.124528	0.91565	no
ni|645903650|nb|KK920780.1|	787370-787722	nan	nan	15	14	-0.151254	0.89425	no
ni|645903650|nb|KK920780.1|	788370-788908	nan	nan	2	1	-0.397624	0.73675	no
ni|645903650|nb|KK920780.1|	793506-794114	nan	nan	21	16	-0.373688	0.7369	no
ni|645903650|nb|KK920780.1|	794326-794821	nan	nan	91	78	-0.219455	0.87545	no
ni|645903650|nb|KK920780.1|	795771-796086	nan	nan	16	15	-0.128897	0.90865	no
ni|645903650|nb|KK920780.1|	796702-796817	nan	nan	87	57	-0.600927	0.6136	no
ni|645903650|nb|KK920780.1|	796963-797450	nan	nan	91	76	-0.263888	0.8563	no
ni|645903650|nb|KK920780.1|	797681-798250	nan	nan	2	2	-0.202499	0.8631	no
ni|645903650|nb|KK920780.1|	799051-799166	nan	nan	80	71	-0.185331	0.87185	no

ni|645903650|nb|KK920780.1|	800456-800884	nan	nan	78.3	60	-0.387202	0.8006	no
ni|645903650|nb|KK920780.1|	801666-801944	nan	nan	106	75	-0.488626	0.70145	no
ni|645903650|nb|KK920780.1|	802058-802710	nan	nan	1	1	-0.434108	0.71745	no
ni|645903650|nb|KK920780.1|	802952-803786	nan	nan	1	1	-0.436659	0.71285	no
ni|645903650|nb|KK920780.1|	803964-804164	nan	nan	138	111	-0.306557	0.77845	no
ni|645903650|nb|KK920780.1|	805041-805233	nan	nan	5	2	-0.984684	0.48245	no
ni|645903650|nb|KK920780.1|	805349-805663	nan	nan	3	3	-0.0657085	0.9574	no
ni|645903650|nb|KK920780.1|	806013-806182	nan	nan	115	90	-0.354232	0.7507	no
ni|645903650|nb|KK920780.1|	806528-806637	nan	nan	38	2	-4	0.02485	no
ni|645903650|nb|KK920780.1|	806919-807200	nan	nan	137	99	-0.467494	0.76075	no
ni|645903650|nb|KK920780.1|	807507-807666	nan	nan	109	95	-0.195787	0.85945	no
ni|645903650|nb|KK920780.1|	807796-808100	nan	nan	6	6	-0.0943328	0.9337	no
ni|645903650|nb|KK920780.1|	808210-808376	nan	nan	124	81	-0.608607	0.59455	no
ni|645903650|nb|KK920780.1|	808701-809098	nan	nan	2	2	-0.135747	0.91325	no
ni|645903650|nb|KK920780.1|	809205-809566	nan	nan	2	1	-0.606055	0.6316	no
ni|645903650|nb|KK920780.1|	814377-814516	nan	nan	6	2	-1	0.35155	no
ni|645903650|nb|KK920780.1|	814999-815182	nan	nan	3	4	0.311229	0.81835	no
ni|645903650|nb|KK920780.1|	820835-820987	nan	nan	1	8	2	0.1063	no
ni|645903650|nb|KK920780.1|	821187-821660	nan	nan	1	3	1	0.3727	no
ni|645903650|nb|KK920780.1|	825851-826092	nan	nan	2	5	1	0.39275	no
ni|645903650|nb|KK920780.1|	827105-827487	nan	nan	0.528439	1	1	0.38715	no
ni|645903650|nb|KK920780.1|	828457-828817	nan	nan	1	3	1	0.3137	no
ni|645903650|nb|KK920780.1|	830044-830285	nan	nan	3	8	1	0.2442	no
ni|645903650|nb|KK920780.1|	831836-832026	nan	nan	2	6	1	0.2841	no
ni|645903650|nb|KK920780.1|	833920-834235	nan	nan	2	2	0.693289	0.58445	no
ni|645903650|nb|KK920780.1|	890616-891565	nan	nan	0.058217	93	11	0.0443	no
ni|645903650|nb|KK920780.1|	892806-893288	nan	nan	0.194711	56	8	0.0507	no
ni|645903650|nb|KK920780.1|	893357-894034	nan	nan	0	79	inf	5.00E-05	yes
ni|645903650|nb|KK920780.1|	898253-898424	nan	nan	38	21	-0.891429	0.44605	no
ni|645903650|nb|KK920780.1|	898552-898702	nan	nan	24	11	-1	0.37635	no

ni|645903650|nb|KK920780.1|	900673-900857	nan	nan	30	21	-0.521302	0.65865	no
ni|645903650|nb|KK920780.1|	901014-901279	nan	nan	37	19	-0.970011	0.4015	no
ni|645903650|nb|KK920780.1|	902372-902567	nan	nan	28	15	-0.963994	0.415	no
ni|645903650|nb|KK920780.1|	903412-903670	nan	nan	25	16	-0.628414	0.58525	no
ni|645903650|nb|KK920780.1|	905362-905513	nan	nan	19	8	-1	0.3531	no
ni|645903650|nb|KK920780.1|	908766-908949	nan	nan	24	19	-0.344817	0.76615	no
ni|645903650|nb|KK920780.1|	920874-921057	nan	nan	19	10	-0.874638	0.46325	no
ni|645903650|nb|KK920780.1|	992739-992852	nan	nan	2369.55	1995.05	-0.248192	0.86245	no
ni|645903650|nb|KK920780.1|	993007-993462	nan	nan	97	80	-0.269188	0.8558	no
ni|645903650|nb|KK920780.1|	993578-993869	nan	nan	1	2	0.419584	0.73705	no
ni|645903650|nb|KK920780.1|	993984-994172	nan	nan	1629.89	1303.58	-0.322302	0.82205	no
ni|645903650|nb|KK920780.1|	995086-995351	nan	nan	492.41	436	-0.174521	0.90145	no
ni|645903650|nb|KK920780.1|	995509-996097	nan	nan	4	2	-1	0.3101	no
ni|645903650|nb|KK920780.1|	996403-996592	nan	nan	1208.91	1080.48	-0.162035	0.90545	no
ni|645903650|nb|KK920780.1|	996755-997098	nan	nan	270	269	-0.00769841	0.99655	no
ni|645903650|nb|KK920780.1|	997268-997731	nan	nan	2	2	-0.586027	0.62085	no
ni|645903650|nb|KK920780.1|	997864-998470	nan	nan	1	0.98233	-0.427508	0.7192	no
ni|645903650|nb|KK920780.1|	998836-999584	nan	nan	1	0.802879	-0.649727	0.5904	no
ni|645903651|nb|KK920779.1|	101445-101627	nan	nan	39	71.22	0.874761	0.45125	no
ni|645903651|nb|KK920779.1|	104851-104992	nan	nan	36	99	1	0.2215	no
ni|645903651|nb|KK920779.1|	108235-108428	nan	nan	39	60	0.637188	0.57865	no
ni|645903651|nb|KK920779.1|	112315-112429	nan	nan	42	82	0.96105	0.41795	no
ni|645903651|nb|KK920779.1|	113626-113811	nan	nan	37	67	0.867291	0.4484	no
ni|645903651|nb|KK920779.1|	116108-116466	nan	nan	21	37	0.813195	0.4705	no
ni|645903651|nb|KK920779.1|	119213-119375	nan	nan	39	86	1	0.3327	no
ni|645903651|nb|KK920779.1|	120740-120854	nan	nan	49	117	1	0.29095	no
ni|645903651|nb|KK920779.1|	122272-122443	nan	nan	57	105	0.88289	0.4357	no
ni|645903651|nb|KK920779.1|	127224-127442	nan	nan	38	80	1	0.34735	no
ni|645903651|nb|KK920779.1|	132975-133152	nan	nan	25	48	0.932674	0.427	no
ni|645903651|nb|KK920779.1|	135006-135123	nan	nan	51	119	1	0.29505	no

ni|645903651|nb|KK920779.1|	135311-135505	nan	nan	38	75	0.972421	0.39225	no
ni|645903651|nb|KK920779.1|	138201-138374	nan	nan	38	88	1	0.2935	no
ni|645903651|nb|KK920779.1|	141809-142476	nan	nan	18	32	0.860959	0.513	no
ni|645903651|nb|KK920779.1|	143478-143730	nan	nan	50	90	0.848014	0.4339	no
ni|645903651|nb|KK920779.1|	149151-149388	nan	nan	44	75	0.767072	0.49735	no
ni|645903651|nb|KK920779.1|	155141-155366	nan	nan	51	97	0.92221	0.4065	no
ni|645903651|nb|KK920779.1|	159147-159671	nan	nan	17	30	0.765608	0.48805	no
ni|645903651|nb|KK920779.1|	161299-161426	nan	nan	60	108	0.854067	0.46675	no
ni|645903651|nb|KK920779.1|	163770-164026	nan	nan	52	92	0.829711	0.44605	no
ni|645903651|nb|KK920779.1|	167575-167700	nan	nan	44	70	0.669622	0.57135	no
ni|645903651|nb|KK920779.1|	168832-169069	nan	nan	60	99	0.733456	0.4988	no
ni|645903651|nb|KK920779.1|	171548-171732	nan	nan	77	114	0.559777	0.61075	no
ni|645903651|nb|KK920779.1|	173823-173974	nan	nan	50	72	0.527168	0.65075	no
ni|645903651|nb|KK920779.1|	175634-175794	nan	nan	67	113.25	0.765685	0.50105	no
ni|645903651|nb|KK920779.1|	177360-177594	nan	nan	76	110	0.540858	0.61685	no
ni|645903651|nb|KK920779.1|	179484-179617	nan	nan	45	63	0.501572	0.66645	no
ni|645903651|nb|KK920779.1|	181372-181711	nan	nan	12	16	0.39497	0.73135	no
ni|645903651|nb|KK920779.1|	182637-182831	nan	nan	93	126	0.445386	0.68715	no
ni|645903651|nb|KK920779.1|	185978-186107	nan	nan	76	104	0.45207	0.6997	no
ni|645903651|nb|KK920779.1|	187091-187353	nan	nan	33	51	0.605313	0.59565	no
ni|645903651|nb|KK920779.1|	191865-192201	nan	nan	31	49	0.652535	0.5591	no
ni|645903651|nb|KK920779.1|	194750-195141	nan	nan	33	44	0.427564	0.6959	no
ni|645903651|nb|KK920779.1|	201031-201361	nan	nan	0.324408	1	2	0.14335	no
ni|645903651|nb|KK920779.1|	203113-203348	nan	nan	1	24	4	0.0225	no
ni|645903651|nb|KK920779.1|	203835-204717	nan	nan	2	1	-0.699371	0.55645	no
ni|645903651|nb|KK920779.1|	208074-208871	nan	nan	11	9	-0.296046	0.7917	no
ni|645903651|nb|KK920779.1|	213471-213624	nan	nan	20	17	-0.245527	0.8369	no
ni|645903651|nb|KK920779.1|	213822-214409	nan	nan	14	11	-0.249146	0.8253	no
ni|645903651|nb|KK920779.1|	214622-214757	nan	nan	14	9	-0.58183	0.6557	no
ni|645903651|nb|KK920779.1|	215032-215241	nan	nan	7	12	0.661128	0.5751	no

ni|645903651|nb|KK920779.1|	215400-215720	nan	nan	13	15	0.207775	0.85555	no
ni|645903651|nb|KK920779.1|	227591-227730	nan	nan	18	22	0.334866	0.77795	no
ni|645903651|nb|KK920779.1|	229223-229669	nan	nan	10	13	0.430536	0.71155	no
ni|645903651|nb|KK920779.1|	229780-229927	nan	nan	36	34	-0.0672018	0.95675	no
ni|645903651|nb|KK920779.1|	236630-236784	nan	nan	5	2	-1	0.44755	no
ni|645903651|nb|KK920779.1|	236979-237494	nan	nan	6	6	-0.109032	0.92735	no
ni|645903651|nb|KK920779.1|	241552-241717	nan	nan	17	15	-0.169107	0.8883	no
ni|645903651|nb|KK920779.1|	244593-244999	nan	nan	10	12	0.280843	0.80955	no
ni|645903651|nb|KK920779.1|	245571-246288	nan	nan	7	11	0.668867	0.5554	no
ni|645903651|nb|KK920779.1|	247692-247825	nan	nan	23	26	0.148458	0.89755	no
ni|645903651|nb|KK920779.1|	249058-249219	nan	nan	21	22	0.0932569	0.93705	no
ni|645903651|nb|KK920779.1|	253080-253202	nan	nan	29	36	0.319644	0.7924	no
ni|645903651|nb|KK920779.1|	254694-254882	nan	nan	15	18	0.298434	0.7961	no
ni|645903651|nb|KK920779.1|	256886-257165	nan	nan	15	17	0.212608	0.8586	no
ni|645903651|nb|KK920779.1|	272333-272454	nan	nan	30	22	-0.445903	0.71245	no
ni|645903651|nb|KK920779.1|	335513-335601	nan	nan	7084.46	3425.85	-1	0.42925	no
ni|645903651|nb|KK920779.1|	418881-419120	nan	nan	20	19	-0.117842	0.9216	no
ni|645903651|nb|KK920779.1|	437226-437355	nan	nan	18.58	10	-0.941774	0.46495	no
ni|645903651|nb|KK920779.1|	437474-437649	nan	nan	18	13	-0.456358	0.70215	no
ni|645903651|nb|KK920779.1|	437794-437964	nan	nan	15	8	-0.874624	0.46205	no
ni|645903651|nb|KK920779.1|	438105-438253	nan	nan	19	26	0.463315	0.68985	no
ni|645903651|nb|KK920779.1|	438373-438509	nan	nan	18	10	-0.76769	0.5318	no
ni|645903651|nb|KK920779.1|	439070-439199	nan	nan	14	15	0.180623	0.87985	no
ni|645903651|nb|KK920779.1|	439499-439665	nan	nan	14	14	0.048243	0.9687	no
ni|645903651|nb|KK920779.1|	439726-440754	nan	nan	10	10	-0.0454642	0.968	no
ni|645903651|nb|KK920779.1|	440978-441285	nan	nan	5	4	-0.239776	0.83955	no
ni|645903651|nb|KK920779.1|	441487-441606	nan	nan	16	8	-0.960504	0.51205	no
ni|645903651|nb|KK920779.1|	441718-442063	nan	nan	15	15	-0.0371731	0.97385	no
ni|645903651|nb|KK920779.1|	442191-442598	nan	nan	4	6	0.463049	0.69245	no
ni|645903651|nb|KK920779.1|	443808-444095	nan	nan	3.73	4	0.0336661	0.97565	no

ni|645903651|nb|KK920779.1|	444519-444648	nan	nan	12	10	-0.289697	0.82015	no
ni|645903651|nb|KK920779.1|	44471-44729	nan	nan	3	10	1	0.2314	no
ni|645903651|nb|KK920779.1|	444829-444977	nan	nan	10	7	-0.581773	0.6557	no
ni|645903651|nb|KK920779.1|	445200-445325	nan	nan	9	5	-0.915341	0.53575	no
ni|645903651|nb|KK920779.1|	445510-445969	nan	nan	10	24	1	0.2638	no
ni|645903651|nb|KK920779.1|	446408-446666	nan	nan	1	4	2	0.19325	no
ni|645903651|nb|KK920779.1|	44950-45301	nan	nan	12	37	2	0.1601	no
ni|645903651|nb|KK920779.1|	452231-452544	nan	nan	11	35	2	0.1765	no
ni|645903651|nb|KK920779.1|	454800-455209	nan	nan	18	47	1	0.208	no
ni|645903651|nb|KK920779.1|	457664-458235	nan	nan	4	8	0.843085	0.46515	no
ni|645903651|nb|KK920779.1|	461538-462074	nan	nan	0	9	inf	5.00E-05	yes
ni|645903651|nb|KK920779.1|	464189-464758	nan	nan	0.10562	25	8	0.0443	no
ni|645903651|nb|KK920779.1|	465033-466204	nan	nan	0.115308	17	7	0.02745	no
ni|645903651|nb|KK920779.1|	466308-468226	nan	nan	0.040721	8	8	0.05055	no
ni|645903651|nb|KK920779.1|	469681-470221	nan	nan	0.056308	0.861211	4	0.12575	no
ni|645903651|nb|KK920779.1|	472523-473354	nan	nan	25	33	0.420734	0.7831	no
ni|645903651|nb|KK920779.1|	475915-476083	nan	nan	67	74	0.14715	0.8971	no
ni|645903651|nb|KK920779.1|	476492-476932	nan	nan	0.145935	7	6	0.0444	no
ni|645903651|nb|KK920779.1|	484547-484682	nan	nan	70	77	0.148876	0.8978	no
ni|645903651|nb|KK920779.1|	485620-485801	nan	nan	151.08	163	0.113097	0.9181	no
ni|645903651|nb|KK920779.1|	487472-487695	nan	nan	110	104	-0.0853047	0.93595	no
ni|645903651|nb|KK920779.1|	489619-489776	nan	nan	237	207	-0.192487	0.8611	no
ni|645903651|nb|KK920779.1|	492621-492770	nan	nan	123	123	-0.00332527	0.997	no
ni|645903651|nb|KK920779.1|	492957-493119	nan	nan	72	67	-0.108085	0.92725	no
ni|645903651|nb|KK920779.1|	49371-49509	nan	nan	0	16	inf	0.0003	yes
ni|645903651|nb|KK920779.1|	501409-502561	nan	nan	0.962637	2	1	0.35505	no
ni|645903651|nb|KK920779.1|	502761-503846	nan	nan	0.526676	2	2	0.125	no
ni|645903651|nb|KK920779.1|	504820-505758	nan	nan	0.442374	2	2	0.1267	no
ni|645903651|nb|KK920779.1|	507495-507711	nan	nan	1	1	0.418762	0.75735	no
ni|645903651|nb|KK920779.1|	507836-508863	nan	nan	0.399794	0.993966	1	0.2986	no

ni|645903651|nb|KK920779.1|	56476-56610	nan	nan	49	92	0.900556	0.44505	no
ni|645903651|nb|KK920779.1|	566751-566939	nan	nan	0	67	inf	5.00E-05	yes
ni|645903651|nb|KK920779.1|	567011-567196	nan	nan	0	292	inf	5.00E-05	yes
ni|645903651|nb|KK920779.1|	581196-581588	nan	nan	0.170077	111	9	0.0443	no
ni|645903651|nb|KK920779.1|	588906-589975	nan	nan	58	82	0.488788	0.7298	no
ni|645903651|nb|KK920779.1|	590447-590638	nan	nan	156	180	0.210241	0.8397	no
ni|645903651|nb|KK920779.1|	593097-593291	nan	nan	259	304	0.233567	0.87605	no
ni|645903651|nb|KK920779.1|	594635-594828	nan	nan	3	2	-0.356633	0.7931	no
ni|645903651|nb|KK920779.1|	595784-596180	nan	nan	53	63	0.247349	0.8431	no
ni|645903651|nb|KK920779.1|	597469-597993	nan	nan	0.935094	0.726256	-0.364633	0.7706	no
ni|645903651|nb|KK920779.1|	601430-602951	nan	nan	568	394	-0.527572	0.62335	no
ni|645903651|nb|KK920779.1|	603115-603552	nan	nan	6	4	-0.461182	0.69485	no
ni|645903651|nb|KK920779.1|	603920-604155	nan	nan	10	8	-0.340542	0.77105	no
ni|645903651|nb|KK920779.1|	604319-604705	nan	nan	4	3	-0.24373	0.83855	no
ni|645903651|nb|KK920779.1|	605832-606017	nan	nan	3	3	-0.0672555	0.9414	no
ni|645903651|nb|KK920779.1|	60640-60982	nan	nan	17	38	1	0.31815	no
ni|645903651|nb|KK920779.1|	620229-620748	nan	nan	623	192.56	-2	0.25095	no
ni|645903651|nb|KK920779.1|	63849-63962	nan	nan	41	74	0.835156	0.4885	no
ni|645903651|nb|KK920779.1|	65817-65959	nan	nan	24	39	0.71938	0.54075	no
ni|645903651|nb|KK920779.1|	68052-68321	nan	nan	33	69	1	0.3389	no
ni|645903651|nb|KK920779.1|	69524-69649	nan	nan	21	41	0.932656	0.4466	no
ni|645903651|nb|KK920779.1|	73033-73431	nan	nan	13	29	1	0.3146	no
ni|645903651|nb|KK920779.1|	74970-75157	nan	nan	22	54	1	0.2745	no
ni|645903651|nb|KK920779.1|	76115-76310	nan	nan	32	64	1	0.3852	no
ni|645903651|nb|KK920779.1|	88738-88907	nan	nan	41	76	0.88121	0.45225	no
ni|645903651|nb|KK920779.1|	90561-90815	nan	nan	21	47	1	0.3003	no
ni|645903651|nb|KK920779.1|	92943-93110	nan	nan	17	34	0.994159	0.407	no
ni|645903651|nb|KK920779.1|	93972-94128	nan	nan	43	84	0.977198	0.4013	no
ni|645903652|nb|KK920778.1|	109169-109823	nan	nan	0	216	inf	5.00E-05	yes
ni|645903653|nb|KK920777.1|	102233-102662	nan	nan	0.150842	56	9	0.0444	no

ni|645903653|nb|KK920777.1|	108226-108680	nan	nan	1	9	3	0.0307	no
ni|645903653|nb|KK920777.1|	110393-110540	nan	nan	2	15	3	0.0596	no
ni|645903653|nb|KK920777.1|	113457-113839	nan	nan	0.352293	10	5	0.0379	no
ni|645903653|nb|KK920777.1|	119751-121174	nan	nan	13	127	3	0.02055	no
ni|645903653|nb|KK920777.1|	121361-121477	nan	nan	177	160	-0.142111	0.90185	no
ni|645903653|nb|KK920777.1|	124375-124527	nan	nan	746	568	-0.392134	0.78725	no
ni|645903653|nb|KK920777.1|	12801-12951	nan	nan	7	6	-0.289588	0.82015	no
ni|645903653|nb|KK920777.1|	132291-132470	nan	nan	0	423	inf	5.00E-05	yes
ni|645903653|nb|KK920777.1|	134964-135175	nan	nan	0.225532	421.84	11	0.11935	no
ni|645903653|nb|KK920777.1|	135973-136194	nan	nan	0	56	inf	5.00E-05	yes
ni|645903653|nb|KK920777.1|	138578-138701	nan	nan	21	13	-0.688856	0.58685	no
ni|645903653|nb|KK920777.1|	139747-139999	nan	nan	1	2	0.571271	0.67875	no
ni|645903653|nb|KK920777.1|	140467-140712	nan	nan	19	11	-0.812052	0.47985	no
ni|645903653|nb|KK920777.1|	141184-141304	nan	nan	20	16	-0.347513	0.78305	no
ni|645903653|nb|KK920777.1|	141707-141844	nan	nan	9	8	-0.289637	0.82015	no
ni|645903653|nb|KK920777.1|	141922-142080	nan	nan	19	18	-0.0672004	0.9508	no
ni|645903653|nb|KK920777.1|	32418-32549	nan	nan	6	8	0.392145	0.7763	no
ni|645903653|nb|KK920777.1|	39416-39586	nan	nan	98	95	-0.0463599	0.9656	no
ni|645903653|nb|KK920777.1|	41960-42555	nan	nan	57	46	-0.308847	0.83185	no
ni|645903653|nb|KK920777.1|	43539-43881	nan	nan	40	28	-0.504045	0.6475	no
ni|645903653|nb|KK920777.1|	44044-44174	nan	nan	76	66	-0.214137	0.85595	no
ni|645903653|nb|KK920777.1|	47864-48016	nan	nan	0	138	inf	5.00E-05	yes
ni|645903653|nb|KK920777.1|	503610-504315	nan	nan	14	12	-0.226983	0.83795	no
ni|645903653|nb|KK920777.1|	51522-51642	nan	nan	0	52	inf	5.00E-05	yes
ni|645903653|nb|KK920777.1|	53201-53486	nan	nan	1	0.77193	-0.802849	0.6027	no
ni|645903653|nb|KK920777.1|	55063-55553	nan	nan	2	1	-0.257782	0.82995	no
ni|645903653|nb|KK920777.1|	56848-56996	nan	nan	5	3	-0.581773	0.70815	no
ni|645903653|nb|KK920777.1|	57327-57521	nan	nan	4	1	-2	0.1613	no
ni|645903653|nb|KK920777.1|	57858-59032	nan	nan	2	2	0.0809177	0.9453	no
ni|645903653|nb|KK920777.1|	59792-60008	nan	nan	1	9	3	0.0478	no

ni|645903653|nb|KK920777.1|	61513-61768	nan	nan	2	7	2	0.1233	no
ni|645903653|nb|KK920777.1|	62436-62971	nan	nan	0.455667	0.816706	0.841837	0.5399	no
ni|645903653|nb|KK920777.1|	63572-63797	nan	nan	2	9	2	0.1386	no
ni|645903653|nb|KK920777.1|	64978-65214	nan	nan	0.918505	8	3	0.04695	no
ni|645903653|nb|KK920777.1|	66793-66989	nan	nan	1	6	2	0.1258	no
ni|645903653|nb|KK920777.1|	69022-69223	nan	nan	1	7	3	0.08645	no
ni|645903653|nb|KK920777.1|	78407-78682	nan	nan	1	5	2	0.1311	no
ni|645903653|nb|KK920777.1|	83239-83646	nan	nan	0.889444	3	2	0.16315	no
ni|645903653|nb|KK920777.1|	88028-88473	nan	nan	1	4	2	0.1727	no
ni|645903653|nb|KK920777.1|	88763-88955	nan	nan	1	5	2	0.18925	no
ni|645903654|nb|KK920776.1|	101569-101833	nan	nan	2	2	-0.606615	0.6316	no
ni|645903654|nb|KK920776.1|	102565-102926	nan	nan	83	76	-0.123202	0.9346	no
ni|645903654|nb|KK920776.1|	104503-104709	nan	nan	199	210	0.0748438	0.9599	no
ni|645903654|nb|KK920776.1|	109259-109568	nan	nan	100.27	89	-0.172387	0.91	no
ni|645903654|nb|KK920776.1|	111189-111407	nan	nan	80	85	0.0895854	0.93375	no
ni|645903654|nb|KK920776.1|	113060-113203	nan	nan	233	223	-0.063644	0.95065	no
ni|645903654|nb|KK920776.1|	114098-114395	nan	nan	71	76	0.0943895	0.92815	no
ni|645903654|nb|KK920776.1|	117444-117632	nan	nan	11	12	0.0371211	0.9751	no
ni|645903654|nb|KK920776.1|	118491-119078	nan	nan	3	5	0.658823	0.58265	no
ni|645903654|nb|KK920776.1|	119312-119648	nan	nan	6	9	0.640672	0.5837	no
ni|645903654|nb|KK920776.1|	119820-120810	nan	nan	0.721862	0.663708	-0.121175	0.9188	no
ni|645903654|nb|KK920776.1|	1199-2169	nan	nan	253	282	0.156121	0.91215	no
ni|645903654|nb|KK920776.1|	120981-121166	nan	nan	3	3	0.195779	0.87955	no
ni|645903654|nb|KK920776.1|	121269-121799	nan	nan	9	16	0.819461	0.4718	no
ni|645903654|nb|KK920776.1|	13850-14169	nan	nan	0.227216	3	4	0.0514	no
ni|645903654|nb|KK920776.1|	162048-162230	nan	nan	3	4	0.517671	0.6956	no
ni|645903654|nb|KK920776.1|	28339-28600	nan	nan	10	7	-0.519791	0.65605	no
ni|645903654|nb|KK920776.1|	284349-284421	nan	nan	8170.89	10589.5	0.374063	0.77935	no
ni|645903654|nb|KK920776.1|	28885-29020	nan	nan	177	163	-0.113988	0.92135	no
ni|645903654|nb|KK920776.1|	290898-291041	nan	nan	10	19	0.97461	0.43275	no

ni|645903654|nb|KK920776.1|	291239-291372	nan	nan	12	9	-0.366831	0.7706	no
ni|645903654|nb|KK920776.1|	29195-30284	nan	nan	129	200	0.629174	0.65705	no
ni|645903654|nb|KK920776.1|	302216-302429	nan	nan	7	14	0.933283	0.4266	no
ni|645903654|nb|KK920776.1|	302658-302916	nan	nan	16	28	0.770961	0.5066	no
ni|645903654|nb|KK920776.1|	303341-303506	nan	nan	13	19	0.560804	0.63125	no
ni|645903654|nb|KK920776.1|	303954-304158	nan	nan	10	14	0.447677	0.6972	no
ni|645903654|nb|KK920776.1|	308388-308529	nan	nan	7	14	0.991672	0.4553	no
ni|645903654|nb|KK920776.1|	309938-310081	nan	nan	16	21	0.463304	0.6936	no
ni|645903654|nb|KK920776.1|	311668-311936	nan	nan	16	28	0.787855	0.4932	no
ni|645903654|nb|KK920776.1|	312733-312883	nan	nan	13	26	0.959277	0.4175	no
ni|645903654|nb|KK920776.1|	313133-313364	nan	nan	16	29	0.863186	0.45905	no
ni|645903654|nb|KK920776.1|	314322-314789	nan	nan	11	16	0.575441	0.6083	no
ni|645903654|nb|KK920776.1|	315193-315394	nan	nan	16	26	0.714399	0.5419	no
ni|645903654|nb|KK920776.1|	315497-315651	nan	nan	19	25	0.382611	0.74495	no
ni|645903654|nb|KK920776.1|	316808-317060	nan	nan	14	19	0.453619	0.69745	no
ni|645903654|nb|KK920776.1|	317567-318145	nan	nan	6	8	0.47152	0.68365	no
ni|645903654|nb|KK920776.1|	318989-319133	nan	nan	20	22	0.161061	0.89125	no
ni|645903654|nb|KK920776.1|	320249-320418	nan	nan	15	25	0.710348	0.54615	no
ni|645903654|nb|KK920776.1|	320772-320991	nan	nan	22	29	0.375388	0.74765	no
ni|645903654|nb|KK920776.1|	321903-322045	nan	nan	25	26	0.0300809	0.97885	no
ni|645903654|nb|KK920776.1|	322301-322503	nan	nan	26	26	0.0261702	0.98145	no
ni|645903654|nb|KK920776.1|	324976-325130	nan	nan	19	24	0.330143	0.7808	no
ni|645903654|nb|KK920776.1|	325927-326170	nan	nan	19	32	0.701567	0.5452	no
ni|645903654|nb|KK920776.1|	327745-327902	nan	nan	27	38	0.509859	0.6711	no
ni|645903654|nb|KK920776.1|	32795-32956	nan	nan	114	227	0.991065	0.36905	no
ni|645903654|nb|KK920776.1|	329063-329459	nan	nan	11	14	0.355345	0.75915	no
ni|645903654|nb|KK920776.1|	330963-331187	nan	nan	20	28	0.478627	0.67875	no
ni|645903654|nb|KK920776.1|	331868-332022	nan	nan	19	22	0.219112	0.8483	no
ni|645903654|nb|KK920776.1|	332875-333138	nan	nan	22	30	0.420773	0.71525	no
ni|645903654|nb|KK920776.1|	333866-334023	nan	nan	31	37	0.279252	0.813	no

ni|645903654|nb|KK920776.1|	335244-335372	nan	nan	29	34	0.245844	0.83735	no
ni|645903654|nb|KK920776.1|	33541-33704	nan	nan	263	549.22	1	0.39515	no
ni|645903654|nb|KK920776.1|	336351-336534	nan	nan	27	31	0.184904	0.8734	no
ni|645903654|nb|KK920776.1|	337504-337643	nan	nan	23	25	0.120394	0.92055	no
ni|645903654|nb|KK920776.1|	338695-338947	nan	nan	27	32	0.257538	0.82345	no
ni|645903654|nb|KK920776.1|	340389-340759	nan	nan	14	16	0.142241	0.90235	no
ni|645903654|nb|KK920776.1|	340931-341055	nan	nan	43	39	-0.137744	0.90605	no
ni|645903654|nb|KK920776.1|	341907-342092	nan	nan	34	38	0.165944	0.88675	no
ni|645903654|nb|KK920776.1|	342542-342763	nan	nan	25	34	0.400293	0.73315	no
ni|645903654|nb|KK920776.1|	342964-343112	nan	nan	33	36	0.125445	0.9155	no
ni|645903654|nb|KK920776.1|	344308-344574	nan	nan	37	43	0.236853	0.82805	no
ni|645903654|nb|KK920776.1|	34541-34705	nan	nan	131	291	1	0.3831	no
ni|645903654|nb|KK920776.1|	346129-346333	nan	nan	32	37	0.22856	0.8417	no
ni|645903654|nb|KK920776.1|	346614-347060	nan	nan	18	17	-0.0709922	0.94895	no
ni|645903654|nb|KK920776.1|	347245-347963	nan	nan	6	6	-0.0241538	0.98365	no
ni|645903654|nb|KK920776.1|	349280-349630	nan	nan	25	25	0.019523	0.98655	no
ni|645903654|nb|KK920776.1|	350310-350657	nan	nan	0.805138	0.673204	-0.258191	0.8692	no
ni|645903654|nb|KK920776.1|	350939-351418	nan	nan	34	31	-0.147622	0.8918	no
ni|645903654|nb|KK920776.1|	351632-351749	nan	nan	42	27	-0.652415	0.5856	no
ni|645903654|nb|KK920776.1|	352049-352160	nan	nan	29	24	-0.290006	0.82015	no
ni|645903654|nb|KK920776.1|	352527-352745	nan	nan	67	61	-0.141352	0.89575	no
ni|645903654|nb|KK920776.1|	354589-355046	nan	nan	0.555792	1	1	0.4359	no
ni|645903654|nb|KK920776.1|	35685-35845	nan	nan	169	362.4	1	0.3893	no
ni|645903654|nb|KK920776.1|	3586-3942	nan	nan	90	113	0.325177	0.826	no
ni|645903654|nb|KK920776.1|	359123-359305	nan	nan	1	4	2	0.30335	no
ni|645903654|nb|KK920776.1|	36106-36544	nan	nan	35	78	1	0.3658	no
ni|645903654|nb|KK920776.1|	362-545	nan	nan	705	705	0.000983695	0.9991	no
ni|645903654|nb|KK920776.1|	372043-372299	nan	nan	0.799698	3	2	0.14475	no
ni|645903654|nb|KK920776.1|	37740-37850	nan	nan	100	192	0.932344	0.4331	no
ni|645903654|nb|KK920776.1|	382395-383066	nan	nan	7	7	-0.0400465	0.96965	no

ni|645903654|nb|KK920776.1|	383222-383439	nan	nan	17	14	-0.256751	0.82665	no
ni|645903654|nb|KK920776.1|	383901-384832	nan	nan	20	16	-0.351338	0.7371	no
ni|645903654|nb|KK920776.1|	384975-385104	nan	nan	16	12	-0.408341	0.755	no
ni|645903654|nb|KK920776.1|	404596-404942	nan	nan	0	9	inf	5.00E-05	yes
ni|645903654|nb|KK920776.1|	409345-409460	nan	nan	0	266	inf	5.00E-05	yes
ni|645903654|nb|KK920776.1|	412121-412231	nan	nan	0	660	inf	5.00E-05	yes
ni|645903654|nb|KK920776.1|	418425-418598	nan	nan	0	656	inf	5.00E-05	yes
ni|645903654|nb|KK920776.1|	42232-42638	nan	nan	86	206	1	0.3833	no
ni|645903654|nb|KK920776.1|	427443-427986	nan	nan	0.055925	679	14	0.11935	no
ni|645903654|nb|KK920776.1|	44733-44884	nan	nan	173	456	1	0.2929	no
ni|645903654|nb|KK920776.1|	45912-46256	nan	nan	50	188	2	0.1441	no
ni|645903654|nb|KK920776.1|	48025-48343	nan	nan	0	9	inf	5.00E-05	yes
ni|645903654|nb|KK920776.1|	512977-513369	nan	nan	0	2	inf	0.0005	yes
ni|645903654|nb|KK920776.1|	53301-54220	nan	nan	149	147	-0.0148443	0.99175	no
ni|645903654|nb|KK920776.1|	54568-54847	nan	nan	29	26	-0.14393	0.90125	no
ni|645903654|nb|KK920776.1|	54992-55475	nan	nan	84	103	0.304227	0.82965	no
ni|645903654|nb|KK920776.1|	55617-56120	nan	nan	299	299	0.00127476	0.9991	no
ni|645903654|nb|KK920776.1|	56223-56500	nan	nan	314	357	0.186812	0.89245	no
ni|645903654|nb|KK920776.1|	56790-57008	nan	nan	40	38	-0.0512842	0.9632	no
ni|645903654|nb|KK920776.1|	57333-57504	nan	nan	45	39	-0.185599	0.87225	no
ni|645903654|nb|KK920776.1|	57816-58634	nan	nan	244.5	268	0.130201	0.9286	no
ni|645903654|nb|KK920776.1|	59122-59301	nan	nan	3	5	0.473249	0.71005	no
ni|645903654|nb|KK920776.1|	594935-595505	nan	nan	1	0.906764	-0.216997	0.8608	no
ni|645903654|nb|KK920776.1|	59650-60003	nan	nan	3	0.938774	-2	0.17795	no
ni|645903654|nb|KK920776.1|	609884-610323	nan	nan	10	8	-0.297909	0.7981	no
ni|645903654|nb|KK920776.1|	610420-610716	nan	nan	31	24	-0.374497	0.74445	no
ni|645903654|nb|KK920776.1|	611468-611600	nan	nan	60	54	-0.162856	0.889	no
ni|645903654|nb|KK920776.1|	612029-612217	nan	nan	47	41	-0.186363	0.87365	no
ni|645903654|nb|KK920776.1|	612870-613039	nan	nan	32	29	-0.167954	0.8834	no
ni|645903654|nb|KK920776.1|	613270-613394	nan	nan	21	33	0.62779	0.59885	no

ni|645903654|nb|KK920776.1|	613810-614009	nan	nan	31	35	0.158711	0.8879	no
ni|645903654|nb|KK920776.1|	614322-615025	nan	nan	24	32	0.418837	0.7352	no
ni|645903654|nb|KK920776.1|	616913-617170	nan	nan	2	2	-0.06611	0.9467	no
ni|645903654|nb|KK920776.1|	617729-618071	nan	nan	4	4	-0.169905	0.8866	no
ni|645903654|nb|KK920776.1|	618415-619262	nan	nan	3	3	-0.193964	0.864	no
ni|645903654|nb|KK920776.1|	619778-619980	nan	nan	2	2	-0.218942	0.87705	no
ni|645903654|nb|KK920776.1|	620281-620405	nan	nan	9	5	-0.915352	0.53575	no
ni|645903654|nb|KK920776.1|	621223-621813	nan	nan	2	1	-0.4655	0.70405	no
ni|645903654|nb|KK920776.1|	621936-622188	nan	nan	45	80	0.811158	0.46485	no
ni|645903654|nb|KK920776.1|	623792-624242	nan	nan	10	17	0.829504	0.4637	no
ni|645903654|nb|KK920776.1|	627930-628062	nan	nan	22	36	0.740076	0.53185	no
ni|645903654|nb|KK920776.1|	629335-629583	nan	nan	29	41	0.493524	0.6603	no
ni|645903654|nb|KK920776.1|	631301-631455	nan	nan	28	53	0.932808	0.42625	no
ni|645903654|nb|KK920776.1|	633385-634158	nan	nan	16	31	0.986465	0.44265	no
ni|645903654|nb|KK920776.1|	639237-639545	nan	nan	8	25	2	0.161	no
ni|645903654|nb|KK920776.1|	642747-643150	nan	nan	2	2	-0.349134	0.77265	no
ni|645903654|nb|KK920776.1|	644195-645220	nan	nan	3	0.510831	-3	0.0481	no
ni|645903654|nb|KK920776.1|	646196-646560	nan	nan	14	50	2	0.1116	no
ni|645903654|nb|KK920776.1|	656446-656657	nan	nan	2	2	0.0852494	0.9436	no
ni|645903654|nb|KK920776.1|	657778-658461	nan	nan	6	18	2	0.1635	no
ni|645903654|nb|KK920776.1|	660321-660653	nan	nan	0.428788	326	10	0.03715	no
ni|645903654|nb|KK920776.1|	663623-663804	nan	nan	4	2	-0.844908	0.5595	no
ni|645903654|nb|KK920776.1|	676052-676866	nan	nan	3	4	0.180871	0.87455	no
ni|645903654|nb|KK920776.1|	729152-729693	nan	nan	0.05618	1476.57	15	0.11935	no
ni|645903654|nb|KK920776.1|	82925-83216	nan	nan	3	3	0.00127118	0.97495	no
ni|645903654|nb|KK920776.1|	83566-84149	nan	nan	4	4	0.237055	0.84105	no
ni|645903654|nb|KK920776.1|	90548-90657	nan	nan	0	139	inf	5.00E-05	yes
ni|645903654|nb|KK920776.1|	91525-92118	nan	nan	0.40182	10	5	0.0148	no
ni|645903654|nb|KK920776.1|	92556-93513	nan	nan	0.951586	13	4	0.0047	no
ni|645903654|nb|KK920776.1|	94389-95402	nan	nan	0.703631	25	5	0.0027	yes

ni|645903654|nb|KK920776.1|	95697-96106	nan	nan	2	1	-0.617865	0.6277	no
ni|645903654|nb|KK920776.1|	97231-97423	nan	nan	2	75	6	0.0332	no
ni|645903654|nb|KK920776.1|	98235-98733	nan	nan	2	0.892986	-1	0.40935	no
ni|645903654|nb|KK920776.1|	98854-98982	nan	nan	0.87352	75	6	0.11935	no
ni|645903655|nb|KK920775.1|	10103-10222	nan	nan	9	6	-0.552846	0.719	no
ni|645903655|nb|KK920775.1|	1021002-102119	nan	nan	11.11	0	-inf	5.00E-05	yes
ni|645903655|nb|KK920775.1|	1021438-102161	nan	nan	4	0	-inf	0.002	yes
ni|645903655|nb|KK920775.1|	1024224-102438	nan	nan	5	0	-inf	0.002	yes
ni|645903655|nb|KK920775.1|	11368-11503	nan	nan	15	16	0.0582738	0.96205	no
ni|645903655|nb|KK920775.1|	11808-12041	nan	nan	19	16	-0.25082	0.8317	no
ni|645903655|nb|KK920775.1|	12966-13085	nan	nan	13	7	-0.804385	0.6027	no
ni|645903655|nb|KK920775.1|	13235-13511	nan	nan	4	5	0.612122	0.60375	no
ni|645903655|nb|KK920775.1|	16807-17279	nan	nan	4	3	-0.254208	0.8235	no
ni|645903655|nb|KK920775.1|	20384-20532	nan	nan	25	50	1	0.38945	no
ni|645903655|nb|KK920775.1|	205483-205767	nan	nan	1	2	0.0596402	0.95295	no
ni|645903655|nb|KK920775.1|	206482-206928	nan	nan	0.57356	6	3	0.0359	no
ni|645903655|nb|KK920775.1|	207764-208054	nan	nan	67	55	-0.286839	0.7891	no
ni|645903655|nb|KK920775.1|	208681-209191	nan	nan	2	3	0.612974	0.61365	no
ni|645903655|nb|KK920775.1|	209545-209804	nan	nan	149	136	-0.136025	0.9276	no
ni|645903655|nb|KK920775.1|	209942-210087	nan	nan	6	1	-2	0.2949	no
ni|645903655|nb|KK920775.1|	211116-211466	nan	nan	12	12	0.00543376	0.9953	no
ni|645903655|nb|KK920775.1|	211813-212637	nan	nan	41	39	-0.0671046	0.96305	no
ni|645903655|nb|KK920775.1|	21302-21492	nan	nan	17	32	0.920745	0.4297	no
ni|645903655|nb|KK920775.1|	213917-214429	nan	nan	44	44	-0.00557327	0.99625	no
ni|645903655|nb|KK920775.1|	215178-215397	nan	nan	40	38	-0.0437766	0.9704	no
ni|645903655|nb|KK920775.1|	217071-217635	nan	nan	77	86	0.16821	0.90915	no
ni|645903655|nb|KK920775.1|	218195-218368	nan	nan	76	98	0.3688	0.74285	no
ni|645903655|nb|KK920775.1|	219112-219519	nan	nan	39	46	0.233669	0.8265	no
ni|645903655|nb|KK920775.1|	220560-221163	nan	nan	48	56	0.242321	0.86765	no
ni|645903655|nb|KK920775.1|	22058-22780	nan	nan	12	16	0.395475	0.71755	no

ni|645903655|nb|KK920775.1|	221656-222485	nan	nan	6	7	0.143892	0.9002	no
ni|645903655|nb|KK920775.1|	222784-222933	nan	nan	60	109	0.863941	0.4545	no
ni|645903655|nb|KK920775.1|	223273-223403	nan	nan	53	112	1	0.3577	no
ni|645903655|nb|KK920775.1|	223649-223807	nan	nan	39	77	0.979594	0.3975	no
ni|645903655|nb|KK920775.1|	227616-227757	nan	nan	37	67	0.860021	0.46795	no
ni|645903655|nb|KK920775.1|	232115-232352	nan	nan	96	85	-0.177261	0.87045	no
ni|645903655|nb|KK920775.1|	232613-232874	nan	nan	35	24	-0.522302	0.6473	no
ni|645903655|nb|KK920775.1|	233097-233511	nan	nan	30	30	0.00525993	0.9959	no
ni|645903655|nb|KK920775.1|	233585-233812	nan	nan	108	127	0.226348	0.8318	no
ni|645903655|nb|KK920775.1|	235314-235532	nan	nan	90	137	0.597402	0.58385	no
ni|645903655|nb|KK920775.1|	238517-239464	nan	nan	20	23	0.236401	0.84835	no
ni|645903655|nb|KK920775.1|	240512-240657	nan	nan	4	5	0.295364	0.8383	no
ni|645903655|nb|KK920775.1|	246681-249405	nan	nan	0.300816	132	9	0.0001	yes
ni|645903655|nb|KK920775.1|	249916-250713	nan	nan	33	39	0.238381	0.86665	no
ni|645903655|nb|KK920775.1|	250887-251067	nan	nan	8	8	-0.0148426	0.98035	no
ni|645903655|nb|KK920775.1|	251263-251516	nan	nan	5	4	-0.496783	0.683	no
ni|645903655|nb|KK920775.1|	251590-251848	nan	nan	11	6	-0.739872	0.53105	no
ni|645903655|nb|KK920775.1|	251958-252671	nan	nan	5	4	-0.267618	0.8157	no
ni|645903655|nb|KK920775.1|	252902-253077	nan	nan	11	5	-1	0.4045	no
ni|645903655|nb|KK920775.1|	253208-253337	nan	nan	83	73	-0.17422	0.88555	no
ni|645903655|nb|KK920775.1|	261539-261668	nan	nan	49	29	-0.75536	0.51955	no
ni|645903655|nb|KK920775.1|	262083-262483	nan	nan	15	11	-0.393235	0.72605	no
ni|645903655|nb|KK920775.1|	262682-262836	nan	nan	28	25	-0.16504	0.8876	no
ni|645903655|nb|KK920775.1|	262958-263117	nan	nan	30	23	-0.409595	0.72605	no
ni|645903655|nb|KK920775.1|	263198-263371	nan	nan	37	18	-1	0.3742	no
ni|645903655|nb|KK920775.1|	263737-263856	nan	nan	41.7	27	-0.652382	0.5859	no
ni|645903655|nb|KK920775.1|	264129-264672	nan	nan	20	19	-0.0932898	0.9327	no
ni|645903655|nb|KK920775.1|	265193-265688	nan	nan	31	23	-0.402628	0.7155	no
ni|645903655|nb|KK920775.1|	265954-266371	nan	nan	36	31	-0.247746	0.81885	no
ni|645903655|nb|KK920775.1|	267026-267260	nan	nan	33	27	-0.311792	0.7927	no

ni|645903655|nb|KK920775.1|	268856-269011	nan	nan	46	37	-0.313355	0.78755	no
ni|645903655|nb|KK920775.1|	271240-271609	nan	nan	6	8	0.285453	0.80325	no
ni|645903655|nb|KK920775.1|	27224-27444	nan	nan	5	9	0.844133	0.4792	no
ni|645903655|nb|KK920775.1|	272895-273085	nan	nan	24	27	0.181639	0.8789	no
ni|645903655|nb|KK920775.1|	27842-28043	nan	nan	18	33	0.851739	0.46565	no
ni|645903655|nb|KK920775.1|	284988-285137	nan	nan	17	19	0.167268	0.89	no
ni|645903655|nb|KK920775.1|	30580-30931	nan	nan	6	8	0.527846	0.65135	no
ni|645903655|nb|KK920775.1|	313285-313588	nan	nan	0	13.24	inf	5.00E-05	yes
ni|645903655|nb|KK920775.1|	31591-32206	nan	nan	10	26	1	0.20735	no
ni|645903655|nb|KK920775.1|	387292-387486	nan	nan	13	13	-0.00939138	0.98665	no
ni|645903655|nb|KK920775.1|	481516-481652	nan	nan	19	17	-0.121698	0.91705	no
ni|645903655|nb|KK920775.1|	514192-514431	nan	nan	0	115	inf	5.00E-05	yes
ni|645903655|nb|KK920775.1|	685780-686015	nan	nan	0	4	inf	0.00065	yes
ni|645903655|nb|KK920775.1|	6908-7028	nan	nan	20	21	0.0150573	0.9674	no
ni|645903655|nb|KK920775.1|	7584-7817	nan	nan	2	0.538307	-2	0.20695	no
ni|645903655|nb|KK920775.1|	777994-778193	nan	nan	9	13	0.451465	0.70655	no
ni|645903655|nb|KK920775.1|	817969-818356	nan	nan	0	2	inf	0.0003	yes
ni|645903655|nb|KK920775.1|	872281-874859	nan	nan	137	103	-0.414607	0.77385	no
ni|645903655|nb|KK920775.1|	876271-876769	nan	nan	0.871936	1	0.297449	0.8138	no
ni|645903655|nb|KK920775.1|	878063-878363	nan	nan	2	0.475753	-2	0.22995	no
ni|645903655|nb|KK920775.1|	879572-879961	nan	nan	0.945192	0.656955	-0.524814	0.7071	no
ni|645903655|nb|KK920775.1|	883428-884019	nan	nan	0.655629	0.674975	0.0419544	0.9599	no
ni|645903655|nb|KK920775.1|	896316-896877	nan	nan	0.161193	212.85	10	0.05055	no
ni|645903655|nb|KK920775.1|	9286-9702	nan	nan	15	9	-0.665698	0.5653	no
ni|645903655|nb|KK920775.1|	976383-976708	nan	nan	35	31	-0.182627	0.86865	no
ni|645903656|nb|KK920774.1|	13839-14814	nan	nan	36	12	-2	0.2352	no
ni|645903656|nb|KK920774.1|	14945-15464	nan	nan	9	6	-0.453647	0.69855	no
ni|645903656|nb|KK920774.1|	164753-165045	nan	nan	10	5	-0.937103	0.43155	no
ni|645903656|nb|KK920774.1|	172870-173060	nan	nan	3	2	-0.581755	0.70815	no
ni|645903656|nb|KK920774.1|	24747-25533	nan	nan	1079.76	621	-0.797831	0.5312	no

ni|645903656|nb|KK920774.1|	26044-26298	nan	nan	1328.69	638	-1	0.4527	no
ni|645903656|nb|KK920774.1|	34199-35400	nan	nan	168	231	0.456813	0.74855	no
ni|645903656|nb|KK920774.1|	41789-42809	nan	nan	46	77	0.736207	0.6025	no
ni|645903656|nb|KK920774.1|	51306-52265	nan	nan	0.402761	0.330104	-0.287001	0.8283	no
ni|645903656|nb|KK920774.1|	58469-58638	nan	nan	22	30	0.444947	0.70725	no
ni|645903656|nb|KK920774.1|	59066-59572	nan	nan	25	27	0.10206	0.928	no
ni|645903656|nb|KK920774.1|	70252-70757	nan	nan	0.244797	0.701985	2	0.30335	no
ni|645903656|nb|KK920774.1|	80633-81084	nan	nan	2	1	-0.802188	0.51615	no
ni|645903656|nb|KK920774.1|	81326-82161	nan	nan	4	2	-0.833806	0.4739	no
ni|645903656|nb|KK920774.1|	82814-82951	nan	nan	7	6	-0.356751	0.7931	no
ni|645903656|nb|KK920774.1|	83948-84331	nan	nan	2	0.922574	-0.775896	0.5539	no
ni|645903656|nb|KK920774.1|	84775-85022	nan	nan	3	4	0.51875	0.67025	no
ni|645903656|nb|KK920774.1|	91489-91976	nan	nan	2	3	1	0.3802	no
ni|645903657|nb|KK920773.1|	210611-210766	nan	nan	0	12	inf	0.0009	yes
ni|645903658|nb|KK920772.1|	36791-36917	nan	nan	14	34	1	0.3106	no
ni|645903658|nb|KK920772.1|	47146-47296	nan	nan	0	12	inf	0.00055	yes
ni|645903658|nb|KK920772.1|	49693-50060	nan	nan	73	49	-0.57117	0.6491	no
ni|645903658|nb|KK920772.1|	50110-50316	nan	nan	140	112	-0.325023	0.7604	no
ni|645903658|nb|KK920772.1|	51042-51331	nan	nan	3	3	-0.12475	0.91825	no
ni|645903658|nb|KK920772.1|	52387-52586	nan	nan	162	160.2	-0.0183176	0.9848	no
ni|645903658|nb|KK920772.1|	52802-53014	nan	nan	48	29	-0.719762	0.5257	no
ni|645903658|nb|KK920772.1|	53778-54026	nan	nan	28	19	-0.578263	0.6109	no
ni|645903658|nb|KK920772.1|	54259-54539	nan	nan	23	17	-0.475175	0.6811	no
ni|645903658|nb|KK920772.1|	55261-55508	nan	nan	32	17	-0.886988	0.4478	no
ni|645903658|nb|KK920772.1|	55677-55843	nan	nan	19	14	-0.360016	0.75625	no
ni|645903658|nb|KK920772.1|	56583-56725	nan	nan	21	9	-1	0.30995	no
ni|645903658|nb|KK920772.1|	56998-57189	nan	nan	21	16	-0.427062	0.7199	no
ni|645903658|nb|KK920772.1|	58561-58822	nan	nan	17	18	0.0807687	0.9444	no
ni|645903658|nb|KK920772.1|	59729-60146	nan	nan	20	14	-0.568735	0.618	no
ni|645903658|nb|KK920772.1|	60492-60725	nan	nan	3	1	-1	0.40265	no

ni|645903658|nb|KK920772.1|	60875-61060	nan	nan	47	38	-0.323094	0.7768	no
ni|645903658|nb|KK920772.1|	61842-62004	nan	nan	19	17	-0.195536	0.8663	no
ni|645903658|nb|KK920772.1|	62472-62596	nan	nan	30	27	-0.166891	0.8881	no
ni|645903658|nb|KK920772.1|	63491-63648	nan	nan	28	22	-0.316558	0.78495	no
ni|645903658|nb|KK920772.1|	65173-65513	nan	nan	59	55	-0.116925	0.9163	no
ni|645903658|nb|KK920772.1|	66819-67063	nan	nan	128	116	-0.132428	0.91365	no
ni|645903658|nb|KK920772.1|	75157-75473	nan	nan	27	27	-0.0294775	0.97725	no
ni|645903658|nb|KK920772.1|	75711-75873	nan	nan	147	117	-0.3263	0.76435	no
ni|645903658|nb|KK920772.1|	80082-80271	nan	nan	0	19	inf	5.00E-05	yes
ni|645903659|nb|KK920771.1|	219262-220141	nan	nan	0.031732	31	10	0.11935	no
ni|645903661|nb|KK920769.1|	1151531-1151799	nan	nan	0	4	inf	0.00055	yes
ni|645903661|nb|KK920769.1|	1156170-1156512	nan	nan	5	7	0.467305	0.68855	no
ni|645903661|nb|KK920769.1|	1158792-1159050	nan	nan	8	32	2	0.0967	no
ni|645903661|nb|KK920769.1|	1159431-1160140	nan	nan	15	17	0.219983	0.8387	no
ni|645903661|nb|KK920769.1|	1167261-1167505	nan	nan	5	167	5	0.00345	no
ni|645903661|nb|KK920769.1|	1170010-1170759	nan	nan	11	13	0.222036	0.84525	no
ni|645903661|nb|KK920769.1|	1171658-1172013	nan	nan	8	7	-0.258156	0.8232	no
ni|645903661|nb|KK920769.1|	1196720-1196938	nan	nan	1	6	2	0.14705	no
ni|645903661|nb|KK920769.1|	1269341-1269566	nan	nan	28	26	-0.0977694	0.93455	no
ni|645903661|nb|KK920769.1|	1280644-1280872	nan	nan	0	79	inf	5.00E-05	yes
ni|645903661|nb|KK920769.1|	1422148-1422207	nan	nan	35076.4	31938.4	-0.135207	0.92285	no
ni|645903661|nb|KK920769.1|	1497341-1497998	nan	nan	5	4	-0.538119	0.6406	no
ni|645903661|nb|KK920769.1|	1498123-1498475	nan	nan	2	1	-0.843132	0.49815	no
ni|645903661|nb|KK920769.1|	1498579-1498856	nan	nan	2	2	0.197092	0.8731	no
ni|645903661|nb|KK920769.1|	1499965-1500398	nan	nan	2	2	0.127383	0.9121	no
ni|645903661|nb|KK920769.1|	1500546-1501110	nan	nan	0.640582	0.969569	0.597961	0.63	no
ni|645903661|nb|KK920769.1|	1501739-1502516	nan	nan	2	2	-0.226489	0.84745	no
ni|645903661|nb|KK920769.1|	1503114-1503360	nan	nan	18	15	-0.302291	0.796	no
ni|645903661|nb|KK920769.1|	204950-205352	nan	nan	0	5	inf	5.00E-05	yes
ni|645903661|nb|KK920769.1|	248040-248453	nan	nan	42	53	0.329318	0.7581	no

ni|645903661|nb|KK920769.1|	249292-249529	nan	nan	177	223	0.329293	0.8234	no
ni|645903661|nb|KK920769.1|	252886-253052	nan	nan	198	240	0.272786	0.8013	no
ni|645903661|nb|KK920769.1|	253809-253981	nan	nan	35	46	0.418137	0.72015	no
ni|645903661|nb|KK920769.1|	254400-254741	nan	nan	92	95	0.0437742	0.97575	no
ni|645903661|nb|KK920769.1|	259386-259528	nan	nan	310	341	0.137039	0.89565	no
ni|645903661|nb|KK920769.1|	259910-260151	nan	nan	157	194	0.304574	0.83235	no
ni|645903661|nb|KK920769.1|	260347-260897	nan	nan	2	2	-0.158137	0.8877	no
ni|645903661|nb|KK920769.1|	262243-262686	nan	nan	1	0.898661	-0.172155	0.89245	no
ni|645903661|nb|KK920769.1|	263273-263500	nan	nan	4	3	-0.458804	0.7095	no
ni|645903661|nb|KK920769.1|	264433-264748	nan	nan	3	5	0.814003	0.4908	no
ni|645903661|nb|KK920769.1|	265720-266183	nan	nan	0.546555	0.78361	0.519768	0.7004	no
ni|645903661|nb|KK920769.1|	266412-266614	nan	nan	2	1	-0.803905	0.6027	no
ni|645903661|nb|KK920769.1|	267121-267242	nan	nan	326	346	0.0820695	0.94175	no
ni|645903661|nb|KK920769.1|	267789-267944	nan	nan	133	97	-0.453086	0.68455	no
ni|645903661|nb|KK920769.1|	268126-268292	nan	nan	212	165	-0.361049	0.73585	no
ni|645903661|nb|KK920769.1|	268368-268576	nan	nan	208	160	-0.376701	0.80515	no
ni|645903661|nb|KK920769.1|	268718-268823	nan	nan	170	158	-0.103735	0.9312	no
ni|645903661|nb|KK920769.1|	277241-277517	nan	nan	47	66	0.492701	0.6532	no
ni|645903661|nb|KK920769.1|	278627-278844	nan	nan	49	73	0.577963	0.60595	no
ni|645903661|nb|KK920769.1|	279075-279252	nan	nan	43	71	0.710282	0.5353	no
ni|645903661|nb|KK920769.1|	279668-279966	nan	nan	57	74	0.387206	0.7209	no
ni|645903661|nb|KK920769.1|	281468-281921	nan	nan	37	42	0.195503	0.85345	no
ni|645903661|nb|KK920769.1|	282728-283205	nan	nan	23	25	0.0860219	0.93865	no
ni|645903661|nb|KK920769.1|	286082-286281	nan	nan	2	3	0.348035	0.792	no
ni|645903661|nb|KK920769.1|	286518-286633	nan	nan	44	95	1	0.35785	no
ni|645903661|nb|KK920769.1|	287593-288820	nan	nan	0.657397	0.335283	-0.971385	0.43225	no
ni|645903661|nb|KK920769.1|	289440-289682	nan	nan	16	35	1	0.3385	no
ni|645903661|nb|KK920769.1|	291175-291676	nan	nan	9	20	1	0.31065	no
ni|645903661|nb|KK920769.1|	291783-292736	nan	nan	2	2	-0.473077	0.6845	no
ni|645903661|nb|KK920769.1|	294214-295115	nan	nan	4	9	0.995478	0.38855	no

ni|645903661|nb|KK920769.1|	296318-297059	nan	nan	8	21	1	0.21095	no
ni|645903661|nb|KK920769.1|	366880-367173	nan	nan	5	3	-1	0.3971	no
ni|645903661|nb|KK920769.1|	391592-391910	nan	nan	6	2	-2	0.13745	no
ni|645903661|nb|KK920769.1|	396572-397204	nan	nan	5	2	-1	0.27275	no
ni|645903661|nb|KK920769.1|	611419-611831	nan	nan	0.875106	0.760337	-0.20282	0.87975	no
ni|645903661|nb|KK920769.1|	625364-626462	nan	nan	0.470295	0.591735	0.331387	0.7896	no
ni|645903661|nb|KK920769.1|	626571-627078	nan	nan	0.548072	0.11642	-2	0.3207	no
ni|645903661|nb|KK920769.1|	644356-644501	nan	nan	26	27	0.0202567	0.9858	no
ni|645903661|nb|KK920769.1|	645669-645854	nan	nan	29	33	0.173753	0.88275	no
ni|645903661|nb|KK920769.1|	649033-649149	nan	nan	37	34	-0.126366	0.91405	no
ni|645903661|nb|KK920769.1|	652968-653086	nan	nan	52	41	-0.352838	0.7593	no
ni|645903661|nb|KK920769.1|	654639-655669	nan	nan	10	12	0.311259	0.7758	no
ni|645903661|nb|KK920769.1|	656703-656903	nan	nan	29	32	0.128743	0.91085	no
ni|645903661|nb|KK920769.1|	658612-658784	nan	nan	35	44	0.344137	0.7717	no
ni|645903661|nb|KK920769.1|	658900-659222	nan	nan	17	18	0.122194	0.91655	no
ni|645903661|nb|KK920769.1|	660549-660962	nan	nan	16	16	0.0183446	0.98695	no
ni|645903661|nb|KK920769.1|	661074-662009	nan	nan	14	16	0.126174	0.9082	no
ni|645903661|nb|KK920769.1|	662318-662480	nan	nan	25	23	-0.137602	0.9022	no
ni|645903661|nb|KK920769.1|	663732-664110	nan	nan	0.893486	0.768487	-0.217424	0.87705	no
ni|645903661|nb|KK920769.1|	664771-664900	nan	nan	9.29	10	0.0582265	0.9532	no
ni|645903661|nb|KK920769.1|	670012-671368	nan	nan	3	4	0.182555	0.87655	no
ni|645903661|nb|KK920769.1|	671427-671798	nan	nan	2	2	-0.0654468	0.9574	no
ni|645903661|nb|KK920769.1|	671903-672756	nan	nan	3	3	-0.20218	0.8649	no
ni|645903661|nb|KK920769.1|	675082-675243	nan	nan	18	12	-0.604865	0.6156	no
ni|645903661|nb|KK920769.1|	676906-677157	nan	nan	8	5	-0.762777	0.52165	no
ni|645903661|nb|KK920769.1|	67863-68370	nan	nan	0	2	inf	0.00015	yes
ni|645903661|nb|KK920769.1|	682140-682527	nan	nan	24	18	-0.412362	0.717	no
ni|645903661|nb|KK920769.1|	682986-683169	nan	nan	40	32	-0.317261	0.78135	no
ni|645903661|nb|KK920769.1|	684665-685131	nan	nan	11	11	0.0565578	0.9617	no
ni|645903661|nb|KK920769.1|	685244-685453	nan	nan	2	1	-0.803758	0.6027	no

ni|645903661|nb|KK920769.1|	686706-687213	nan	nan	12	12	-0.0794587	0.94345	no
ni|645903661|nb|KK920769.1|	687941-688195	nan	nan	40	38	-0.0544097	0.96495	no
ni|645903661|nb|KK920769.1|	689669-691001	nan	nan	21	24	0.179401	0.9032	no
ni|645903661|nb|KK920769.1|	691157-691895	nan	nan	22	26	0.254069	0.8086	no
ni|645903661|nb|KK920769.1|	692100-693742	nan	nan	15	20	0.43506	0.7657	no
ni|645903661|nb|KK920769.1|	723465-723650	nan	nan	9	14	0.622404	0.59665	no
ni|645903661|nb|KK920769.1|	728027-728423	nan	nan	4	5	0.530249	0.65705	no
ni|645903661|nb|KK920769.1|	728523-729311	nan	nan	45	33	-0.414556	0.77385	no
ni|645903661|nb|KK920769.1|	729397-730003	nan	nan	21	15	-0.463022	0.67185	no
ni|645903661|nb|KK920769.1|	730785-731002	nan	nan	3	0.612608	-2	0.20695	no
ni|645903661|nb|KK920769.1|	731486-731735	nan	nan	2	0.960076	-1	0.46615	no
ni|645903661|nb|KK920769.1|	732160-732341	nan	nan	4	0.29548	-4	0.02785	no
ni|645903661|nb|KK920769.1|	732485-732709	nan	nan	2	2	0.348503	0.792	no
ni|645903661|nb|KK920769.1|	733562-734280	nan	nan	11	8	-0.453838	0.69015	no
ni|645903661|nb|KK920769.1|	738600-738827	nan	nan	55	38	-0.536373	0.63695	no
ni|645903661|nb|KK920769.1|	739084-739751	nan	nan	19	10	-0.942683	0.4018	no
ni|645903661|nb|KK920769.1|	740318-740431	nan	nan	18	10	-0.804512	0.6027	no
ni|645903661|nb|KK920769.1|	741768-741965	nan	nan	4	3	-0.695075	0.58855	no
ni|645903661|nb|KK920769.1|	742748-743209	nan	nan	2	2	-0.0193953	0.97965	no
ni|645903661|nb|KK920769.1|	744883-745016	nan	nan	7	4	-1	0.44755	no
ni|645903661|nb|KK920769.1|	746662-747168	nan	nan	12	13	0.0957487	0.93125	no
ni|645903661|nb|KK920769.1|	747640-747850	nan	nan	38	37	-0.0155473	0.98875	no
ni|645903661|nb|KK920769.1|	748262-748492	nan	nan	38	39	0.0523961	0.96125	no
ni|645903661|nb|KK920769.1|	749226-749660	nan	nan	1	0.497	-1	0.3768	no
ni|645903661|nb|KK920769.1|	750648-750973	nan	nan	2	3	0.934349	0.4458	no
ni|645903661|nb|KK920769.1|	751854-751987	nan	nan	27	22	-0.283	0.8139	no
ni|645903661|nb|KK920769.1|	752910-753111	nan	nan	43	39	-0.12655	0.90925	no
ni|645903661|nb|KK920769.1|	753567-753779	nan	nan	29	31	0.0797509	0.94505	no
ni|645903661|nb|KK920769.1|	754429-754604	nan	nan	28	27	-0.0673159	0.95305	no
ni|645903661|nb|KK920769.1|	755933-756211	nan	nan	13	18	0.476592	0.68005	no

ni|645903661|nb|KK920769.1|	757728-757930	nan	nan	22	34	0.621117	0.5925	no
ni|645903661|nb|KK920769.1|	760041-760186	nan	nan	20	33	0.70062	0.55105	no
ni|645903661|nb|KK920769.1|	760331-760455	nan	nan	24	32	0.392077	0.7426	no
ni|645903661|nb|KK920769.1|	760764-760875	nan	nan	25	24	-0.0676133	0.954	no
ni|645903661|nb|KK920769.1|	761265-761594	nan	nan	2	4	1	0.3446	no
ni|645903661|nb|KK920769.1|	761815-762124	nan	nan	2	3	0.711872	0.57115	no
ni|645903661|nb|KK920769.1|	762381-762704	nan	nan	1	1	0.0598699	0.95295	no
ni|645903661|nb|KK920769.1|	762927-764257	nan	nan	5	9	0.803196	0.47225	no
ni|645903661|nb|KK920769.1|	764472-764820	nan	nan	1	2	0.481946	0.6999	no
ni|645903661|nb|KK920769.1|	896074-896285	nan	nan	0	247	inf	5.00E-05	yes
ni|645903662|nb|KK920768.1|	159816-159920	nan	nan	207.35	188	-0.138712	0.90605	no
ni|645903662|nb|KK920768.1|	172968-173265	nan	nan	4	6	0.652424	0.57805	no
ni|645903662|nb|KK920768.1|	173414-173567	nan	nan	11	12	0.164135	0.89015	no
ni|645903662|nb|KK920768.1|	173623-173754	nan	nan	10	14	0.517676	0.68765	no
ni|645903662|nb|KK920768.1|	173912-175246	nan	nan	8	18	1	0.34965	no
ni|645903662|nb|KK920768.1|	178568-178995	nan	nan	0	2	inf	0.00065	yes
ni|645903662|nb|KK920768.1|	181004-181496	nan	nan	60	47	-0.337386	0.82125	no
ni|645903662|nb|KK920768.1|	181727-183102	nan	nan	1	1	-0.108174	0.92475	no
ni|645903662|nb|KK920768.1|	183562-183783	nan	nan	3	1	-1	0.39005	no
ni|645903662|nb|KK920768.1|	185071-185445	nan	nan	2	1	-0.567936	0.6631	no
ni|645903662|nb|KK920768.1|	185943-186269	nan	nan	91	79	-0.208567	0.8684	no
ni|645903662|nb|KK920768.1|	186576-187590	nan	nan	16	15	-0.0645798	0.9531	no
ni|645903662|nb|KK920768.1|	187769-188107	nan	nan	10	13	0.34945	0.7622	no
ni|645903662|nb|KK920768.1|	188212-188381	nan	nan	36	40	0.146865	0.9008	no
ni|645903662|nb|KK920768.1|	189090-189421	nan	nan	2	2	0.114951	0.9325	no
ni|645903662|nb|KK920768.1|	189527-190492	nan	nan	2	2	-0.165889	0.88585	no
ni|645903662|nb|KK920768.1|	191427-192016	nan	nan	5	5	0.0144705	0.9894	no
ni|645903662|nb|KK920768.1|	192191-192677	nan	nan	2	2	-0.187135	0.8754	no
ni|645903662|nb|KK920768.1|	192797-193004	nan	nan	15	11	-0.452123	0.6966	no
ni|645903662|nb|KK920768.1|	193157-193289	nan	nan	24	16	-0.562043	0.6436	no

ni|645903662|nb|KK920768.1|	194260-194375	nan	nan	49	30	-0.699763	0.5662	no
ni|645903662|nb|KK920768.1|	195267-195397	nan	nan	16	13	-0.30176	0.81	no
ni|645903662|nb|KK920768.1|	196391-196576	nan	nan	3	3	0.222251	0.8747	no
ni|645903662|nb|KK920768.1|	197583-197778	nan	nan	24	18	-0.404957	0.73025	no
ni|645903662|nb|KK920768.1|	198168-198496	nan	nan	16	12	-0.37758	0.74635	no
ni|645903662|nb|KK920768.1|	198832-199054	nan	nan	39	23.1	-0.746167	0.52585	no
ni|645903662|nb|KK920768.1|	199240-199365	nan	nan	42	26	-0.686254	0.5653	no
ni|645903662|nb|KK920768.1|	204826-205028	nan	nan	72	58	-0.3075	0.78595	no
ni|645903662|nb|KK920768.1|	206092-206824	nan	nan	0.665381	0.26195	-1	0.33495	no
ni|645903662|nb|KK920768.1|	207095-207444	nan	nan	26	24	-0.0825435	0.9442	no
ni|645903662|nb|KK920768.1|	209520-209715	nan	nan	2	2	-0.28948	0.8535	no
ni|645903662|nb|KK920768.1|	210881-211773	nan	nan	37	36	-0.0442192	0.97635	no
ni|645903662|nb|KK920768.1|	217547-217867	nan	nan	6	7	0.216095	0.85315	no
ni|645903662|nb|KK920768.1|	220090-220295	nan	nan	4	6	0.570555	0.6454	no
ni|645903662|nb|KK920768.1|	220494-221147	nan	nan	2	3	0.486143	0.6759	no
ni|645903662|nb|KK920768.1|	222192-222317	nan	nan	8	11	0.517619	0.7004	no
ni|645903662|nb|KK920768.1|	222480-222950	nan	nan	6	6	0.066066	0.9547	no
ni|645903662|nb|KK920768.1|	223050-223349	nan	nan	1	1	-0.0657929	0.94155	no
ni|645903662|nb|KK920768.1|	223525-223673	nan	nan	9	7	-0.42977	0.73195	no
ni|645903662|nb|KK920768.1|	224942-225053	nan	nan	19	16	-0.237538	0.8697	no
ni|645903662|nb|KK920768.1|	225203-225334	nan	nan	20	13	-0.62368	0.6146	no
ni|645903662|nb|KK920768.1|	225468-226484	nan	nan	5	5	-0.0573472	0.9596	no
ni|645903662|nb|KK920768.1|	226635-227719	nan	nan	5	4	-0.400483	0.72065	no
ni|645903662|nb|KK920768.1|	227995-228283	nan	nan	2	2	0.126782	0.9211	no
ni|645903662|nb|KK920768.1|	228349-228538	nan	nan	10	7	-0.521764	0.6583	no
ni|645903662|nb|KK920768.1|	228638-230328	nan	nan	10	7	-0.410211	0.70145	no
ni|645903662|nb|KK920768.1|	230680-231248	nan	nan	3	4	0.230459	0.84125	no
ni|645903662|nb|KK920768.1|	231865-232070	nan	nan	6	3	-0.95996	0.44135	no
ni|645903662|nb|KK920768.1|	233085-233249	nan	nan	7	6	-0.315149	0.8025	no
ni|645903662|nb|KK920768.1|	233769-234868	nan	nan	6	6	0.0258448	0.982	no

ni|645903662|nb|KK920768.1|	239310-239757	nan	nan	23	23	0.0177307	0.98665	no
ni|645903662|nb|KK920768.1|	240182-240947	nan	nan	6	7	0.0881029	0.9379	no
ni|645903662|nb|KK920768.1|	244891-245219	nan	nan	6	8	0.336463	0.77465	no
ni|645903662|nb|KK920768.1|	246512-247095	nan	nan	2	1	-0.367536	0.7554	no
ni|645903662|nb|KK920768.1|	247410-247541	nan	nan	10	9	-0.0672865	0.954	no
ni|645903662|nb|KK920768.1|	248363-248578	nan	nan	11	13	0.232878	0.83945	no
ni|645903662|nb|KK920768.1|	248779-249105	nan	nan	4	6	0.532992	0.6531	no
ni|645903662|nb|KK920768.1|	250135-250319	nan	nan	4	13	2	0.143	no
ni|645903662|nb|KK920768.1|	250761-251644	nan	nan	1	0.905574	-0.202159	0.8631	no
ni|645903662|nb|KK920768.1|	251885-252269	nan	nan	4	6	0.529344	0.64815	no
ni|645903662|nb|KK920768.1|	254817-255688	nan	nan	28	18	-0.609741	0.62215	no
ni|645903662|nb|KK920768.1|	256922-257637	nan	nan	5	3	-0.893528	0.4433	no
ni|645903662|nb|KK920768.1|	259025-260190	nan	nan	18.02	21	0.21941	0.88095	no
ni|645903662|nb|KK920768.1|	260572-260988	nan	nan	9	7	-0.427875	0.7185	no
ni|645903662|nb|KK920768.1|	261071-261196	nan	nan	18	4	-2	0.1414	no
ni|645903662|nb|KK920768.1|	264981-265418	nan	nan	12	9	-0.333742	0.77125	no
ni|645903662|nb|KK920768.1|	265578-265881	nan	nan	18	17	-0.105847	0.92705	no
ni|645903662|nb|KK920768.1|	265945-267858	nan	nan	13	14	0.118951	0.93185	no
ni|645903662|nb|KK920768.1|	28213-28335	nan	nan	8	19	1	0.3533	no
ni|645903662|nb|KK920768.1|	30466-30651	nan	nan	7	9	0.453577	0.7043	no
ni|645903662|nb|KK920768.1|	31151-31293	nan	nan	4	12	2	0.28535	no
ni|645903662|nb|KK920768.1|	32479-32763	nan	nan	5	6	0.197144	0.8658	no
ni|645903662|nb|KK920768.1|	32878-33405	nan	nan	8	11	0.379718	0.74735	no
ni|645903662|nb|KK920768.1|	332560-332734	nan	nan	19	23	0.24955	0.83145	no
ni|645903662|nb|KK920768.1|	33537-33689	nan	nan	10	5	-0.929688	0.47045	no
ni|645903662|nb|KK920768.1|	337083-337300	nan	nan	18	18	0.0501651	0.9646	no
ni|645903662|nb|KK920768.1|	33896-34106	nan	nan	6	9	0.500268	0.66545	no
ni|645903662|nb|KK920768.1|	341328-341929	nan	nan	5	5	0.0963355	0.93635	no
ni|645903662|nb|KK920768.1|	347221-347487	nan	nan	18	25	0.523037	0.6549	no
ni|645903662|nb|KK920768.1|	351535-351704	nan	nan	11	11	0.0747594	0.9486	no

ni|645903662|nb|KK920768.1|	35171-35335	nan	nan	9	15	0.795275	0.50935	no
ni|645903662|nb|KK920768.1|	351874-352076	nan	nan	20	20	0.00260175	0.99385	no
ni|645903662|nb|KK920768.1|	355712-355846	nan	nan	22	28	0.300468	0.7971	no
ni|645903662|nb|KK920768.1|	361389-361584	nan	nan	28	31	0.122183	0.9163	no
ni|645903662|nb|KK920768.1|	362357-362862	nan	nan	0.367195	20	6	0.03325	no
ni|645903662|nb|KK920768.1|	365016-365289	nan	nan	43	28	-0.648541	0.5682	no
ni|645903662|nb|KK920768.1|	370360-370884	nan	nan	9	7	-0.446379	0.69705	no
ni|645903662|nb|KK920768.1|	371171-371822	nan	nan	13	12	-0.172169	0.88155	no
ni|645903662|nb|KK920768.1|	37282-38084	nan	nan	0.352242	0.202074	-0.801682	0.6027	no
ni|645903662|nb|KK920768.1|	373261-373655	nan	nan	14	11	-0.278771	0.8055	no
ni|645903662|nb|KK920768.1|	376453-376632	nan	nan	27	25	-0.119159	0.91895	no
ni|645903662|nb|KK920768.1|	377360-377744	nan	nan	7	7	-0.137549	0.90405	no
ni|645903662|nb|KK920768.1|	379415-379675	nan	nan	28	29	0.073643	0.9489	no
ni|645903662|nb|KK920768.1|	380921-381048	nan	nan	55	41	-0.443472	0.71075	no
ni|645903662|nb|KK920768.1|	382026-382324	nan	nan	1	2	0.934201	0.4874	no
ni|645903662|nb|KK920768.1|	38230-39783	nan	nan	4	44	3	0.0215	no
ni|645903662|nb|KK920768.1|	383348-383540	nan	nan	45	45	0.0182454	0.9874	no
ni|645903662|nb|KK920768.1|	384805-385049	nan	nan	49	55	0.183848	0.86685	no
ni|645903662|nb|KK920768.1|	385578-385922	nan	nan	13	15.87	0.238775	0.8338	no
ni|645903662|nb|KK920768.1|	386040-386779	nan	nan	6	5	-0.306592	0.7895	no
ni|645903662|nb|KK920768.1|	387873-388074	nan	nan	34	24	-0.488985	0.67745	no
ni|645903662|nb|KK920768.1|	388222-388360	nan	nan	23	20	-0.192769	0.8708	no
ni|645903662|nb|KK920768.1|	388940-389110	nan	nan	40	18	-1	0.3362	no
ni|645903662|nb|KK920768.1|	392971-393164	nan	nan	18	16	-0.15459	0.89605	no
ni|645903662|nb|KK920768.1|	393287-393934	nan	nan	20	18	-0.111747	0.9195	no
ni|645903662|nb|KK920768.1|	394068-394176	nan	nan	38.94	48	0.294799	0.81435	no
ni|645903662|nb|KK920768.1|	397183-397342	nan	nan	50	43	-0.1961	0.8684	no
ni|645903662|nb|KK920768.1|	397473-398521	nan	nan	7	5	-0.37137	0.74655	no
ni|645903662|nb|KK920768.1|	399262-399875	nan	nan	5	4	-0.349905	0.7652	no
ni|645903662|nb|KK920768.1|	39948-40571	nan	nan	9	10	0.0331717	0.9759	no

ni|645903662|nb|KK920768.1|	400677-400881	nan	nan	57	42	-0.427713	0.7052	no
ni|645903662|nb|KK920768.1|	401606-401798	nan	nan	3	0	-inf	0.00455	no
ni|645903662|nb|KK920768.1|	41445-41668	nan	nan	73	68	-0.102974	0.9268	no
ni|645903662|nb|KK920768.1|	41791-41976	nan	nan	47	56	0.247441	0.82505	no
ni|645903662|nb|KK920768.1|	42143-42398	nan	nan	51	50	-0.0120218	0.99125	no
ni|645903662|nb|KK920768.1|	45061-45202	nan	nan	66	55	-0.265551	0.8197	no
ni|645903662|nb|KK920768.1|	46118-46616	nan	nan	33	32	-0.0384036	0.9691	no
ni|645903662|nb|KK920768.1|	47425-47815	nan	nan	30	34	0.184505	0.86595	no
ni|645903662|nb|KK920768.1|	50413-50607	nan	nan	61	71	0.230466	0.8414	no
ni|645903662|nb|KK920768.1|	64098-64263	nan	nan	66	55	-0.256261	0.8241	no
ni|645903662|nb|KK920768.1|	81912-82062	nan	nan	59.73	67	0.165465	0.88335	no
ni|645903663|nb|KK920767.1|	100690-100821	nan	nan	7	4	-0.915283	0.53575	no
ni|645903663|nb|KK920767.1|	102515-102629	nan	nan	25	6	-2	0.2004	no
ni|645903663|nb|KK920767.1|	103640-104324	nan	nan	0.76266	0.405081	-0.912831	0.4969	no
ni|645903663|nb|KK920767.1|	107314-107525	nan	nan	15	9	-0.761899	0.51825	no
ni|645903663|nb|KK920767.1|	110375-110591	nan	nan	17	9	-0.80989	0.4945	no
ni|645903663|nb|KK920767.1|	113707-114098	nan	nan	9	5	-0.748903	0.51645	no
ni|645903663|nb|KK920767.1|	115609-115807	nan	nan	18	7	-1	0.27585	no
ni|645903663|nb|KK920767.1|	116996-117409	nan	nan	8	6	-0.457631	0.69355	no
ni|645903663|nb|KK920767.1|	136591-136719	nan	nan	6	4	-0.552741	0.719	no
ni|645903663|nb|KK920767.1|	152809-152988	nan	nan	15	9	-0.683991	0.56335	no
ni|645903663|nb|KK920767.1|	1601-1865	nan	nan	24	33	0.429585	0.7012	no
ni|645903663|nb|KK920767.1|	161398-161567	nan	nan	13	9	-0.552686	0.6482	no
ni|645903663|nb|KK920767.1|	163072-164004	nan	nan	0.237639	0.369247	0.635816	0.63975	no
ni|645903663|nb|KK920767.1|	164192-164379	nan	nan	4	3	-0.389156	0.7621	no
ni|645903663|nb|KK920767.1|	165635-165886	nan	nan	18	10	-0.79409	0.4942	no
ni|645903663|nb|KK920767.1|	167588-167865	nan	nan	15	10	-0.657477	0.5734	no
ni|645903663|nb|KK920767.1|	168701-169333	nan	nan	22	17	-0.437748	0.69345	no
ni|645903663|nb|KK920767.1|	170785-170992	nan	nan	20	15	-0.431829	0.71265	no
ni|645903663|nb|KK920767.1|	171783-171939	nan	nan	17	17	0.00680417	0.9853	no

ni|645903663|nb|KK920767.1|	172039-172389	nan	nan	7	7	0.0193561	0.9864	no
ni|645903663|nb|KK920767.1|	172454-172560	nan	nan	39	34	-0.205463	0.87975	no
ni|645903663|nb|KK920767.1|	177275-177834	nan	nan	14	13	-0.127872	0.90855	no
ni|645903663|nb|KK920767.1|	185793-185903	nan	nan	37	30	-0.260301	0.8388	no
ni|645903663|nb|KK920767.1|	19626-19971	nan	nan	15	26	0.835983	0.46405	no
ni|645903663|nb|KK920767.1|	200085-200519	nan	nan	27	28	0.0943584	0.9322	no
ni|645903663|nb|KK920767.1|	211739-211985	nan	nan	21	21	-0.0207811	0.9851	no
ni|645903663|nb|KK920767.1|	22115-22242	nan	nan	5	20	2	0.2071	no
ni|645903663|nb|KK920767.1|	22539-22735	nan	nan	22	24	0.172605	0.87765	no
ni|645903663|nb|KK920767.1|	22843-23023	nan	nan	21	29	0.459937	0.6953	no
ni|645903663|nb|KK920767.1|	23180-23352	nan	nan	18	28	0.624588	0.5935	no
ni|645903663|nb|KK920767.1|	239324-239480	nan	nan	3	5	0.807273	0.58675	no
ni|645903663|nb|KK920767.1|	243021-243257	nan	nan	2	3	0.481135	0.6999	no
ni|645903663|nb|KK920767.1|	244617-244773	nan	nan	2	3	0.195838	0.8862	no
ni|645903663|nb|KK920767.1|	261404-261768	nan	nan	0	2	inf	0.00055	yes
ni|645903663|nb|KK920767.1|	264379-264569	nan	nan	0	9	inf	0.00015	yes
ni|645903663|nb|KK920767.1|	266607-266797	nan	nan	0.27859	7	5	0.12065	no
ni|645903663|nb|KK920767.1|	27839-28023	nan	nan	29	33	0.190797	0.8698	no
ni|645903663|nb|KK920767.1|	30845-31085	nan	nan	21	24	0.205004	0.85435	no
ni|645903663|nb|KK920767.1|	32889-33099	nan	nan	27	37	0.447801	0.69995	no
ni|645903663|nb|KK920767.1|	37114-37282	nan	nan	20	25	0.374755	0.74775	no
ni|645903663|nb|KK920767.1|	37395-37549	nan	nan	27	22	-0.281317	0.80795	no
ni|645903663|nb|KK920767.1|	39602-39764	nan	nan	23	22	-0.0416773	0.97195	no
ni|645903663|nb|KK920767.1|	43255-43512	nan	nan	0.953469	3	2	0.234	no
ni|645903663|nb|KK920767.1|	43640-43875	nan	nan	2	0.530263	-2	0.2784	no
ni|645903663|nb|KK920767.1|	44369-44514	nan	nan	10	9	-0.149668	0.91085	no
ni|645903663|nb|KK920767.1|	45122-45470	nan	nan	12	12	-0.0416947	0.96885	no
ni|645903663|nb|KK920767.1|	45622-45773	nan	nan	13	16	0.36165	0.7643	no
ni|645903663|nb|KK920767.1|	45926-46158	nan	nan	7	6	-0.321667	0.7907	no
ni|645903663|nb|KK920767.1|	46698-46820	nan	nan	9	7	-0.260024	0.8692	no

ni|645903663|nb|KK920767.1|	48936-49273	nan	nan	16	9	-0.755252	0.513	no
ni|645903663|nb|KK920767.1|	49421-49598	nan	nan	12	15	0.299457	0.79895	no
ni|645903663|nb|KK920767.1|	50049-50185	nan	nan	42	20	-1	0.3836	no
ni|645903663|nb|KK920767.1|	50312-50457	nan	nan	16	3	-2	0.1205	no
ni|645903663|nb|KK920767.1|	56321-56487	nan	nan	54	35	-0.602271	0.6021	no
ni|645903663|nb|KK920767.1|	81096-81223	nan	nan	10	13	0.380135	0.7694	no
ni|645903663|nb|KK920767.1|	82265-82412	nan	nan	37	23	-0.672923	0.56785	no
ni|645903663|nb|KK920767.1|	83919-84189	nan	nan	1	0.840582	-0.288389	0.8535	no
ni|645903663|nb|KK920767.1|	84359-84649	nan	nan	54	44	-0.308764	0.78325	no
ni|645903663|nb|KK920767.1|	94966-95150	nan	nan	75	58	-0.386339	0.72995	no
ni|645903663|nb|KK920767.1|	951-1304	nan	nan	12	13	0.127125	0.912	no
ni|645903663|nb|KK920767.1|	95302-95411	nan	nan	103	53	-0.965828	0.42625	no
ni|645903663|nb|KK920767.1|	98236-98733	nan	nan	12	7	-0.933563	0.42525	no
ni|645903664|nb|KK920766.1|	168882-168994	nan	nan	35	52	0.569852	0.64565	no
ni|645903664|nb|KK920766.1|	170205-170353	nan	nan	20	17	-0.227664	0.8507	no
ni|645903664|nb|KK920766.1|	171432-171605	nan	nan	14	9	-0.652262	0.5797	no
ni|645903664|nb|KK920766.1|	172938-173111	nan	nan	4	4	0.0582314	0.9532	no
ni|645903664|nb|KK920766.1|	175447-175612	nan	nan	3	8	2	0.23715	no
ni|645903664|nb|KK920766.1|	175734-175883	nan	nan	20	17	-0.259843	0.82715	no
ni|645903664|nb|KK920766.1|	176865-177066	nan	nan	16	18	0.177458	0.8777	no
ni|645903664|nb|KK920766.1|	177664-178272	nan	nan	8	8	-0.0483521	0.9641	no
ni|645903664|nb|KK920766.1|	178558-178813	nan	nan	27	22	-0.2929	0.7954	no
ni|645903664|nb|KK920766.1|	179119-179337	nan	nan	18	20	0.184907	0.87475	no
ni|645903664|nb|KK920766.1|	181459-181702	nan	nan	20	17	-0.214013	0.8526	no
ni|645903664|nb|KK920766.1|	184112-184237	nan	nan	8	19	1	0.35025	no
ni|645903664|nb|KK920766.1|	18457-19414	nan	nan	2	3	0.623446	0.5926	no
ni|645903664|nb|KK920766.1|	185364-185602	nan	nan	19	19	-0.0529046	0.96485	no
ni|645903664|nb|KK920766.1|	185828-186270	nan	nan	14.29	10	-0.448889	0.70305	no
ni|645903664|nb|KK920766.1|	186500-186682	nan	nan	15	11	-0.442801	0.70365	no
ni|645903664|nb|KK920766.1|	186757-186946	nan	nan	20	16	-0.330233	0.77885	no

ni|645903664|nb|KK920766.1|	191023-191308	nan	nan	99	79	-0.323041	0.79795	no
ni|645903664|nb|KK920766.1|	194473-194750	nan	nan	48	42	-0.189931	0.86195	no
ni|645903664|nb|KK920766.1|	196197-196401	nan	nan	93	85	-0.124229	0.9098	no
ni|645903664|nb|KK920766.1|	197543-197745	nan	nan	105	113	0.10709	0.9237	no
ni|645903664|nb|KK920766.1|	200466-200729	nan	nan	44	43	-0.0315276	0.97595	no
ni|645903664|nb|KK920766.1|	200884-201142	nan	nan	102	101	-0.0135534	0.991	no
ni|645903664|nb|KK920766.1|	203685-203923	nan	nan	110	104	-0.0782978	0.93975	no
ni|645903664|nb|KK920766.1|	205041-205387	nan	nan	43	53	0.292497	0.78725	no
ni|645903664|nb|KK920766.1|	206177-208598	nan	nan	61	86	0.49452	0.7289	no
ni|645903664|nb|KK920766.1|	20750-21044	nan	nan	10	11	0.153958	0.89585	no
ni|645903664|nb|KK920766.1|	209387-210309	nan	nan	0.300673	0.37375	0.313881	0.81835	no
ni|645903664|nb|KK920766.1|	210422-210623	nan	nan	2	2	0.254967	0.86075	no
ni|645903664|nb|KK920766.1|	214279-214590	nan	nan	168	155	-0.121378	0.93085	no
ni|645903664|nb|KK920766.1|	218974-219709	nan	nan	2	1	-0.536846	0.6491	no
ni|645903664|nb|KK920766.1|	222661-223137	nan	nan	1	2	0.349872	0.76915	no
ni|645903664|nb|KK920766.1|	223729-223855	nan	nan	2	50	5	0.0449	no
ni|645903664|nb|KK920766.1|	225649-225879	nan	nan	3	81	5	0.00415	no
ni|645903664|nb|KK920766.1|	228520-228632	nan	nan	0	135	inf	0.00025	yes
ni|645903664|nb|KK920766.1|	231160-231331	nan	nan	2	73	5	0.02115	no
ni|645903664|nb|KK920766.1|	232827-233320	nan	nan	0.315388	13	5	0.02755	no
ni|645903664|nb|KK920766.1|	233423-233631	nan	nan	2	55	5	0.0206	no
ni|645903664|nb|KK920766.1|	233704-233895	nan	nan	0.55103	37	6	0.04445	no
ni|645903664|nb|KK920766.1|	23511-23714	nan	nan	6	7	0.267501	0.8269	no
ni|645903664|nb|KK920766.1|	244251-244389	nan	nan	25	27	0.144266	0.9012	no
ni|645903664|nb|KK920766.1|	244492-244610	nan	nan	40	43	0.113137	0.92335	no
ni|645903664|nb|KK920766.1|	244881-245638	nan	nan	0.527036	0.575888	0.127889	0.9211	no
ni|645903664|nb|KK920766.1|	245753-245862	nan	nan	33	22	-0.598223	0.64595	no
ni|645903664|nb|KK920766.1|	247179-247566	nan	nan	3	2	-0.387317	0.7434	no
ni|645903664|nb|KK920766.1|	252318-252528	nan	nan	38	46	0.301615	0.78705	no
ni|645903664|nb|KK920766.1|	27676-27851	nan	nan	8	9	0.146809	0.90615	no

ni|645903664|nb|KK920766.1|	31987-32103	nan	nan	18	14	-0.330507	0.80325	no
ni|645903664|nb|KK920766.1|	324999-325179	nan	nan	13	15	0.155082	0.89405	no
ni|645903664|nb|KK920766.1|	34033-35287	nan	nan	0	182	inf	5.00E-05	yes
ni|645903664|nb|KK920766.1|	74198-74372	nan	nan	16	13	-0.305467	0.7946	no
ni|645903664|nb|KK920766.1|	74547-74731	nan	nan	13	23	0.777078	0.5159	no
ni|645903664|nb|KK920766.1|	75051-75282	nan	nan	13	17	0.379639	0.74405	no
ni|645903664|nb|KK920766.1|	76969-77200	nan	nan	10	12	0.152215	0.89415	no
ni|645903664|nb|KK920766.1|	78738-78901	nan	nan	12	16	0.404852	0.7322	no
ni|645903664|nb|KK920766.1|	81302-81658	nan	nan	5	7	0.665306	0.56455	no
ni|645903664|nb|KK920766.1|	82518-82700	nan	nan	16	19	0.260283	0.82225	no
ni|645903664|nb|KK920766.1|	91202-94590	nan	nan	50	206	2	0.1145	no
ni|645903664|nb|KK920766.1|	99350-99517	nan	nan	7	9	0.269793	0.82445	no
ni|645903665|nb|KK920765.1|	100889-101157	nan	nan	0.593664	2	2	0.15655	no
ni|645903665|nb|KK920765.1|	10176-11108	nan	nan	21	15	-0.438789	0.67755	no
ni|645903665|nb|KK920765.1|	102093-102742	nan	nan	1	1	0.0683888	0.95495	no
ni|645903665|nb|KK920765.1|	111172-111441	nan	nan	7	2	-2	0.17775	no
ni|645903665|nb|KK920765.1|	111680-111917	nan	nan	9	2	-2	0.0929	no
ni|645903665|nb|KK920765.1|	115616-115787	nan	nan	5	2	-1	0.45115	no
ni|645903665|nb|KK920765.1|	117012-117183	nan	nan	6	3	-1	0.4289	no
ni|645903665|nb|KK920765.1|	12810-12934	nan	nan	5	8	0.610717	0.6619	no
ni|645903665|nb|KK920765.1|	132182-132441	nan	nan	8	9	0.172696	0.8832	no
ni|645903665|nb|KK920765.1|	134303-134523	nan	nan	10	10	-0.0088846	0.98665	no
ni|645903665|nb|KK920765.1|	136203-136380	nan	nan	16	11	-0.541257	0.64615	no
ni|645903665|nb|KK920765.1|	140429-141494	nan	nan	0.358412	0.41617	0.215552	0.8678	no
ni|645903665|nb|KK920765.1|	141679-141831	nan	nan	16	11	-0.510798	0.67685	no
ni|645903665|nb|KK920765.1|	144772-145415	nan	nan	8	7	-0.272577	0.81305	no
ni|645903665|nb|KK920765.1|	145553-145745	nan	nan	3	2	-1	0.46615	no
ni|645903665|nb|KK920765.1|	145878-146050	nan	nan	12	6	-0.906825	0.4582	no
ni|645903665|nb|KK920765.1|	14792-15346	nan	nan	65	42	-0.609798	0.6845	no
ni|645903665|nb|KK920765.1|	149960-150140	nan	nan	25	4	-3	0.0449	no

ni|645903665|nb|KK920765.1|	150406-150539	nan	nan	37	23	-0.637587	0.58345	no
ni|645903665|nb|KK920765.1|	153078-153719	nan	nan	0.366018	0.34992	-0.0648884	0.9384	no
ni|645903665|nb|KK920765.1|	154604-154903	nan	nan	15	13	-0.18127	0.87615	no
ni|645903665|nb|KK920765.1|	155545-155966	nan	nan	1	0.295563	-2	0.2004	no
ni|645903665|nb|KK920765.1|	158642-158900	nan	nan	3	1	-1	0.3131	no
ni|645903665|nb|KK920765.1|	159083-159258	nan	nan	22	25	0.177103	0.8778	no
ni|645903665|nb|KK920765.1|	161293-161425	nan	nan	53	39	-0.447883	0.7043	no
ni|645903665|nb|KK920765.1|	162107-162540	nan	nan	1	0.854572	-0.802228	0.53265	no
ni|645903665|nb|KK920765.1|	163192-163559	nan	nan	2	2	-0.287855	0.8142	no
ni|645903665|nb|KK920765.1|	163683-163974	nan	nan	2	0.872089	-0.958928	0.51225	no
ni|645903665|nb|KK920765.1|	165182-165882	nan	nan	1	0.630786	-0.922797	0.44975	no
ni|645903665|nb|KK920765.1|	166205-166496	nan	nan	1	2	0.571587	0.67875	no
ni|645903665|nb|KK920765.1|	166716-166960	nan	nan	2	0.662465	-2	0.21345	no
ni|645903665|nb|KK920765.1|	16689-16792	nan	nan	122	41	-2	0.2821	no
ni|645903665|nb|KK920765.1|	167172-167714	nan	nan	0.728676	0.642973	-0.180519	0.89165	no
ni|645903665|nb|KK920765.1|	169182-172382	nan	nan	1	0.730499	-0.618851	0.59525	no
ni|645903665|nb|KK920765.1|	174606-175049	nan	nan	1	0.691278	-0.550666	0.68265	no
ni|645903665|nb|KK920765.1|	175353-175617	nan	nan	2	2	0.174962	0.8916	no
ni|645903665|nb|KK920765.1|	179204-179532	nan	nan	0.981822	0.521197	-0.913633	0.53575	no
ni|645903665|nb|KK920765.1|	179722-180311	nan	nan	2	1	-0.264273	0.81915	no
ni|645903665|nb|KK920765.1|	181626-182360	nan	nan	0.780323	0.410333	-0.927275	0.47065	no
ni|645903665|nb|KK920765.1|	186760-187715	nan	nan	0.520265	0.304015	-0.775104	0.5539	no
ni|645903665|nb|KK920765.1|	19079-19398	nan	nan	13	9	-0.578494	0.62615	no
ni|645903665|nb|KK920765.1|	198065-198579	nan	nan	0.83807	0.343328	-1	0.39005	no
ni|645903665|nb|KK920765.1|	213126-213242	nan	nan	24	18	-0.367033	0.7706	no
ni|645903665|nb|KK920765.1|	23841-23979	nan	nan	24	30	0.338018	0.77535	no
ni|645903665|nb|KK920765.1|	241950-242110	nan	nan	3	3	-0.0672051	0.9302	no
ni|645903665|nb|KK920765.1|	255197-255645	nan	nan	31	23	-0.459413	0.67665	no
ni|645903665|nb|KK920765.1|	255751-256333	nan	nan	1	0.83498	-0.835493	0.49955	no
ni|645903665|nb|KK920765.1|	256916-257517	nan	nan	30	23	-0.412869	0.69795	no

ni|645903665|nb|KK920765.1|	258978-259719	nan	nan	28	22	-0.319836	0.80175	no
ni|645903665|nb|KK920765.1|	261874-262559	nan	nan	1	1	0.32219	0.7839	no
ni|645903665|nb|KK920765.1|	262684-263019	nan	nan	0.84655	2	1	0.40865	no
ni|645903665|nb|KK920765.1|	263867-264411	nan	nan	10	7	-0.411489	0.71855	no
ni|645903665|nb|KK920765.1|	264718-265353	nan	nan	0.508943	0.575021	0.176112	0.8916	no
ni|645903665|nb|KK920765.1|	265616-265748	nan	nan	46	37	-0.301744	0.7918	no
ni|645903665|nb|KK920765.1|	268166-268725	nan	nan	1	1	-0.190543	0.87685	no
ni|645903665|nb|KK920765.1|	269319-269729	nan	nan	0.880785	1	0.282602	0.82625	no
ni|645903665|nb|KK920765.1|	270467-270765	nan	nan	8	5	-0.513258	0.66455	no
ni|645903665|nb|KK920765.1|	271219-271407	nan	nan	1	2	0.610856	0.6619	no
ni|645903665|nb|KK920765.1|	272350-272862	nan	nan	2	1	-0.00851055	0.97805	no
ni|645903665|nb|KK920765.1|	276113-276352	nan	nan	15	13	-0.251526	0.82905	no
ni|645903665|nb|KK920765.1|	28367-29193	nan	nan	0.544895	0.521	-0.0646965	0.9589	no
ni|645903665|nb|KK920765.1|	290296-290424	nan	nan	13	7	-0.974204	0.5005	no
ni|645903665|nb|KK920765.1|	299519-299815	nan	nan	11	6	-0.855888	0.4753	no
ni|645903665|nb|KK920765.1|	301532-301895	nan	nan	2	0.812581	-0.983016	0.48245	no
ni|645903665|nb|KK920765.1|	303113-303385	nan	nan	3	2	-0.580554	0.6557	no
ni|645903665|nb|KK920765.1|	309234-309535	nan	nan	16	10	-0.639516	0.58485	no
ni|645903665|nb|KK920765.1|	310563-311230	nan	nan	0.436456	0.166908	-1	0.35155	no
ni|645903665|nb|KK920765.1|	3127-3402	nan	nan	55	47	-0.235882	0.8341	no
ni|645903665|nb|KK920765.1|	314103-314966	nan	nan	2	1	-0.386597	0.74305	no
ni|645903665|nb|KK920765.1|	31505-31729	nan	nan	50	52	0.0784139	0.9424	no
ni|645903665|nb|KK920765.1|	33437-33628	nan	nan	10	8	-0.289558	0.80005	no
ni|645903665|nb|KK920765.1|	3566-3794	nan	nan	2	1	-0.481509	0.7594	no
ni|645903665|nb|KK920765.1|	367442-367862	nan	nan	2	1	-0.858844	0.49355	no
ni|645903665|nb|KK920765.1|	36794-37048	nan	nan	84	63	-0.420394	0.70185	no
ni|645903665|nb|KK920765.1|	368039-368365	nan	nan	3	1	-1	0.37025	no
ni|645903665|nb|KK920765.1|	372089-373318	nan	nan	20	15	-0.479532	0.7031	no
ni|645903665|nb|KK920765.1|	37223-37537	nan	nan	2	3	0.431791	0.72635	no
ni|645903665|nb|KK920765.1|	38136-38251	nan	nan	22	11	-1	0.45115	no

ni|645903665|nb|KK920765.1|	38415-38768	nan	nan	2	1	-0.328554	0.79705	no
ni|645903665|nb|KK920765.1|	39276-39578	nan	nan	1	0.942019	-0.650738	0.64935	no
ni|645903665|nb|KK920765.1|	39979-40450	nan	nan	23	19	-0.274233	0.81005	no
ni|645903665|nb|KK920765.1|	40504-41046	nan	nan	0.952884	0.696554	-0.452065	0.72335	no
ni|645903665|nb|KK920765.1|	41377-42933	nan	nan	0.898546	0.632323	-0.50693	0.664	no
ni|645903665|nb|KK920765.1|	42991-43121	nan	nan	50	24	-1	0.3836	no
ni|645903665|nb|KK920765.1|	456904-457093	nan	nan	1875.49	1406.94	-0.414708	0.77045	no
ni|645903665|nb|KK920765.1|	47414-48395	nan	nan	0.532918	0.241381	-1	0.3995	no
ni|645903665|nb|KK920765.1|	532894-533536	nan	nan	0.959016	0.567567	-0.756765	0.54375	no
ni|645903665|nb|KK920765.1|	534940-535480	nan	nan	0.28154	0.807386	2	0.2846	no
ni|645903665|nb|KK920765.1|	5447-6191	nan	nan	0.460897	0.257053	-0.842376	0.5595	no
ni|645903665|nb|KK920765.1|	549453-549874	nan	nan	38	39	0.0274544	0.9791	no
ni|645903665|nb|KK920765.1|	550155-550324	nan	nan	56	47	-0.226615	0.84795	no
ni|645903665|nb|KK920765.1|	553122-553721	nan	nan	20	18	-0.13189	0.90095	no
ni|645903665|nb|KK920765.1|	557514-557843	nan	nan	25	25	0.0449275	0.96875	no
ni|645903665|nb|KK920765.1|	559914-560896	nan	nan	5	5	-0.0875573	0.93675	no
ni|645903665|nb|KK920765.1|	562152-562507	nan	nan	18	16	-0.130729	0.91075	no
ni|645903665|nb|KK920765.1|	564269-564435	nan	nan	4	3	-0.526666	0.7071	no
ni|645903665|nb|KK920765.1|	564539-565521	nan	nan	2	2	-0.139884	0.9031	no
ni|645903665|nb|KK920765.1|	565654-565998	nan	nan	16	14	-0.247936	0.8261	no
ni|645903665|nb|KK920765.1|	566110-566243	nan	nan	24	5	-2	0.1303	no
ni|645903665|nb|KK920765.1|	579065-579479	nan	nan	20	12	-0.673543	0.54535	no
ni|645903665|nb|KK920765.1|	58086-58419	nan	nan	38	31	-0.275829	0.80265	no
ni|645903665|nb|KK920765.1|	709847-710082	nan	nan	32	24	-0.409053	0.72055	no
ni|645903665|nb|KK920765.1|	90859-91017	nan	nan	12	6	-1	0.39615	no
ni|645903665|nb|KK920765.1|	9572-9936	nan	nan	2	2	-0.387403	0.74515	no
ni|645903665|nb|KK920765.1|	98607-99779	nan	nan	0.737279	2	1	0.24635	no
ni|645903665|nb|KK920765.1|	99919-100244	nan	nan	1	3	1	0.4043	no
ni|645903666|nb|KK920764.1|	100137-100325	nan	nan	40	95	1	0.2832	no
ni|645903666|nb|KK920764.1|	100584-101261	nan	nan	9	20	1	0.3101	no

ni|645903666|nb|KK920764.1|	110126-110277	nan	nan	79	103	0.374316	0.7438	no
ni|645903666|nb|KK920764.1|	112605-112809	nan	nan	59	80	0.44945	0.6887	no
ni|645903666|nb|KK920764.1|	113479-113591	nan	nan	17	39	1	0.39005	no
ni|645903666|nb|KK920764.1|	114350-114537	nan	nan	109	127	0.221586	0.84	no
ni|645903666|nb|KK920764.1|	116133-116405	nan	nan	126	152	0.26718	0.8617	no
ni|645903666|nb|KK920764.1|	117050-117592	nan	nan	18	21	0.239813	0.82945	no
ni|645903666|nb|KK920764.1|	119277-119907	nan	nan	4	5	0.250872	0.8308	no
ni|645903666|nb|KK920764.1|	120520-121323	nan	nan	54	53	-0.0287266	0.9843	no
ni|645903666|nb|KK920764.1|	122034-122147	nan	nan	52	41	-0.340565	0.78085	no
ni|645903666|nb|KK920764.1|	122287-122426	nan	nan	96	58	-0.730198	0.5316	no
ni|645903666|nb|KK920764.1|	123699-123881	nan	nan	100	68	-0.550074	0.6208	no
ni|645903666|nb|KK920764.1|	127163-127643	nan	nan	12	11	-0.192772	0.8688	no
ni|645903666|nb|KK920764.1|	129498-131700	nan	nan	12	13	0.215782	0.8829	no
ni|645903666|nb|KK920764.1|	31442-31702	nan	nan	2	1	-0.355588	0.7931	no
ni|645903666|nb|KK920764.1|	32125-32376	nan	nan	3	2	-0.992169	0.47555	no
ni|645903666|nb|KK920764.1|	35244-35638	nan	nan	2	2	-0.427937	0.7192	no
ni|645903666|nb|KK920764.1|	35916-36335	nan	nan	1	1	-0.0652974	0.95795	no
ni|645903666|nb|KK920764.1|	36557-36865	nan	nan	37	36	-0.047065	0.9656	no
ni|645903666|nb|KK920764.1|	38474-39444	nan	nan	0.369251	0.21728	-0.765042	0.5835	no
ni|645903666|nb|KK920764.1|	39576-39706	nan	nan	31	34	0.111042	0.92485	no
ni|645903666|nb|KK920764.1|	41832-42050	nan	nan	36	30	-0.238693	0.83375	no
ni|645903666|nb|KK920764.1|	46500-46641	nan	nan	30	29	-0.0380758	0.97275	no
ni|645903666|nb|KK920764.1|	46747-47048	nan	nan	25	24	-0.0586566	0.95915	no
ni|645903666|nb|KK920764.1|	47152-47499	nan	nan	12	10	-0.28014	0.81215	no
ni|645903666|nb|KK920764.1|	48192-48310	nan	nan	66	33	-1	0.3958	no
ni|645903666|nb|KK920764.1|	48807-48929	nan	nan	66	46	-0.514838	0.6622	no
ni|645903666|nb|KK920764.1|	49122-49244	nan	nan	42	41	-0.0299043	0.98085	no
ni|645903666|nb|KK920764.1|	55087-55419	nan	nan	3	2	-0.242494	0.83985	no
ni|645903666|nb|KK920764.1|	59705-60044	nan	nan	1	0.894706	-0.48062	0.7301	no
ni|645903666|nb|KK920764.1|	60992-61406	nan	nan	1	0.679921	-0.595825	0.64595	no

ni|645903666|nb|KK920764.1|	62372-62681	nan	nan	3	3	-0.349528	0.77265	no
ni|645903666|nb|KK920764.1|	69263-69751	nan	nan	0.447263	5	3	0.0343	no
ni|645903666|nb|KK920764.1|	70305-70454	nan	nan	2	35	4	0.03875	no
ni|645903666|nb|KK920764.1|	75251-75392	nan	nan	39	57	0.52512	0.6491	no
ni|645903666|nb|KK920764.1|	77097-77333	nan	nan	37	49	0.422147	0.7088	no
ni|645903666|nb|KK920764.1|	80646-80830	nan	nan	18	30	0.740084	0.5276	no
ni|645903667|nb|KK920763.1|	176545-176710	nan	nan	41	38	-0.108255	0.92895	no
ni|645903667|nb|KK920763.1|	179455-179782	nan	nan	0.547877	3	2	0.1002	no
ni|645903667|nb|KK920763.1|	180849-181080	nan	nan	19.46	11	-0.784654	0.50435	no
ni|645903667|nb|KK920763.1|	183379-183535	nan	nan	67	39	-0.782039	0.50125	no
ni|645903667|nb|KK920763.1|	185937-186192	nan	nan	45	28	-0.698397	0.53855	no
ni|645903667|nb|KK920763.1|	18612-19811	nan	nan	0.966327	0.322351	-2	0.21795	no
ni|645903667|nb|KK920763.1|	188796-189242	nan	nan	1	0.411153	-1	0.32095	no
ni|645903667|nb|KK920763.1|	19917-21764	nan	nan	0.579163	0.243172	-1	0.30845	no
ni|645903667|nb|KK920763.1|	212938-213054	nan	nan	28	27	-0.0674725	0.95595	no
ni|645903669|nb|KK920761.1|	101576-101760	nan	nan	5	7	0.517692	0.67245	no
ni|645903669|nb|KK920761.1|	106616-106760	nan	nan	5	10	0.932792	0.4834	no
ni|645903669|nb|KK920761.1|	108586-108782	nan	nan	6	6	0.0484118	0.9698	no
ni|645903669|nb|KK920761.1|	111969-112100	nan	nan	8	8	0.0702171	0.94415	no
ni|645903669|nb|KK920761.1|	112755-113105	nan	nan	2	4	0.895993	0.4567	no
ni|645903669|nb|KK920761.1|	115521-115828	nan	nan	75	64	-0.232208	0.82355	no
ni|645903669|nb|KK920761.1|	116667-116917	nan	nan	128	129	0.00314129	0.99745	no
ni|645903669|nb|KK920761.1|	118474-118726	nan	nan	116	117	0.0152809	0.98705	no
ni|645903669|nb|KK920761.1|	120695-120892	nan	nan	52	56	0.094169	0.9306	no
ni|645903669|nb|KK920761.1|	125717-125977	nan	nan	143	149	0.0602347	0.9659	no
ni|645903669|nb|KK920761.1|	127334-127548	nan	nan	47	56	0.239109	0.8344	no
ni|645903669|nb|KK920761.1|	133168-133330	nan	nan	94	87	-0.105014	0.92245	no
ni|645903669|nb|KK920761.1|	142928-143231	nan	nan	40	36	-0.147197	0.8958	no
ni|645903669|nb|KK920761.1|	16848-17041	nan	nan	15	24	0.706102	0.5495	no
ni|645903669|nb|KK920761.1|	206584-206710	nan	nan	12	11	-0.182811	0.889	no

ni|645903669|nb|KK920761.1|	208296-208557	nan	nan	15	8	-0.923115	0.4324	no
ni|645903669|nb|KK920761.1|	208904-209274	nan	nan	9	5	-0.762058	0.5176	no
ni|645903669|nb|KK920761.1|	209441-209610	nan	nan	4	5	0.155133	0.90275	no
ni|645903669|nb|KK920761.1|	210647-210792	nan	nan	4	2	-1	0.43055	no
ni|645903669|nb|KK920761.1|	211647-211936	nan	nan	4	0.755473	-2	0.1419	no
ni|645903669|nb|KK920761.1|	214417-215303	nan	nan	4	3	-0.675241	0.56295	no
ni|645903669|nb|KK920761.1|	215636-215943	nan	nan	4	2	-1	0.37475	no
ni|645903669|nb|KK920761.1|	216201-216369	nan	nan	14	4	-2	0.19045	no
ni|645903669|nb|KK920761.1|	216812-217093	nan	nan	3	2	-0.84352	0.49805	no
ni|645903669|nb|KK920761.1|	217322-218575	nan	nan	10	7	-0.527985	0.6328	no
ni|645903669|nb|KK920761.1|	230867-231054	nan	nan	8	5	-0.677281	0.576	no
ni|645903669|nb|KK920761.1|	236994-237150	nan	nan	10	6	-0.719273	0.56995	no
ni|645903669|nb|KK920761.1|	239156-239493	nan	nan	4	2	-1	0.3814	no
ni|645903669|nb|KK920761.1|	243008-243253	nan	nan	5	3	-0.499195	0.67645	no
ni|645903669|nb|KK920761.1|	245055-245292	nan	nan	15	9	-0.768953	0.5149	no
ni|645903669|nb|KK920761.1|	246348-246568	nan	nan	9	6	-0.634884	0.59885	no
ni|645903669|nb|KK920761.1|	247445-247602	nan	nan	11	8	-0.482236	0.69675	no
ni|645903669|nb|KK920761.1|	25221-26286	nan	nan	97	107	0.140476	0.91855	no
ni|645903669|nb|KK920761.1|	252345-252550	nan	nan	8	5	-0.526306	0.66105	no
ni|645903669|nb|KK920761.1|	274437-274557	nan	nan	10	6	-0.745477	0.59615	no
ni|645903669|nb|KK920761.1|	286803-287027	nan	nan	10	4	-1	0.3188	no
ni|645903669|nb|KK920761.1|	312223-312481	nan	nan	0	94	inf	5.00E-05	yes
ni|645903669|nb|KK920761.1|	322415-322631	nan	nan	0	126	inf	5.00E-05	yes
ni|645903669|nb|KK920761.1|	325133-325291	nan	nan	0	44	inf	5.00E-05	yes
ni|645903669|nb|KK920761.1|	37169-37488	nan	nan	434	447	0.0452236	0.97105	no
ni|645903669|nb|KK920761.1|	38051-38737	nan	nan	151	155	0.0370657	0.97735	no
ni|645903669|nb|KK920761.1|	42200-42652	nan	nan	0.704664	0.336759	-1	0.44755	no
ni|645903669|nb|KK920761.1|	45357-45547	nan	nan	552	640	0.213226	0.87625	no
ni|645903669|nb|KK920761.1|	46454-46623	nan	nan	379	400	0.0771303	0.9578	no
ni|645903669|nb|KK920761.1|	46768-46875	nan	nan	206.07	206	-0.00371224	0.99105	no

ni|645903669|nb|KK920761.1|	46995-47138	nan	nan	344.29	335	-0.0384993	0.9704	no
ni|645903669|nb|KK920761.1|	47263-47427	nan	nan	152	137	-0.147286	0.8933	no
ni|645903669|nb|KK920761.1|	47841-48018	nan	nan	57	54	-0.092129	0.9364	no
ni|645903669|nb|KK920761.1|	48309-48425	nan	nan	18	7	-1	0.3479	no
ni|645903669|nb|KK920761.1|	54593-54762	nan	nan	4	3	-0.204763	0.87975	no
ni|645903669|nb|KK920761.1|	56419-56786	nan	nan	24	25	0.0512898	0.9658	no
ni|645903669|nb|KK920761.1|	58152-58392	nan	nan	40	56	0.464705	0.6773	no
ni|645903669|nb|KK920761.1|	60068-60796	nan	nan	10	17	0.770777	0.4811	no
ni|645903669|nb|KK920761.1|	65381-65698	nan	nan	11	8	-0.539623	0.6508	no
ni|645903669|nb|KK920761.1|	66252-66526	nan	nan	18	10	-0.791343	0.4942	no
ni|645903669|nb|KK920761.1|	67294-67494	nan	nan	39	28	-0.482019	0.6752	no
ni|645903669|nb|KK920761.1|	67997-68274	nan	nan	34	18	-0.879346	0.4389	no
ni|645903669|nb|KK920761.1|	69013-69261	nan	nan	8	2	-2	0.1728	no
ni|645903669|nb|KK920761.1|	69390-69525	nan	nan	26	24	-0.106785	0.92625	no
ni|645903669|nb|KK920761.1|	70094-70259	nan	nan	7	9	0.328701	0.79035	no
ni|645903669|nb|KK920761.1|	71851-72092	nan	nan	14	16	0.169093	0.88475	no
ni|645903669|nb|KK920761.1|	73005-73198	nan	nan	5	5	-0.145129	0.90525	no
ni|645903669|nb|KK920761.1|	73272-73461	nan	nan	22	16	-0.427094	0.7199	no
ni|645903669|nb|KK920761.1|	73561-74080	nan	nan	15	12	-0.337379	0.7622	no
ni|645903669|nb|KK920761.1|	74642-74872	nan	nan	18	16	-0.146167	0.90055	no
ni|645903669|nb|KK920761.1|	78194-78461	nan	nan	14	10	-0.491327	0.67735	no
ni|645903669|nb|KK920761.1|	91376-91915	nan	nan	7	11	0.683991	0.55785	no
ni|645903669|nb|KK920761.1|	92392-92646	nan	nan	3	5	0.933861	0.44575	no
ni|645903669|nb|KK920761.1|	98681-98869	nan	nan	4	4	-0.0672156	0.9582	no
ni|645903669|nb|KK920761.1|	99000-99128	nan	nan	5	8	0.517649	0.7279	no
ni|645903669|nb|KK920761.1|	99570-99943	nan	nan	2	3	0.612633	0.6154	no
ni|645903670|nb|KK920760.1|	199148-199567	nan	nan	0	5	inf	5.00E-05	yes
ni|645903670|nb|KK920760.1|	202953-203291	nan	nan	0	443	inf	5.00E-05	yes
ni|645903670|nb|KK920760.1|	213802-213944	nan	nan	0	923	inf	5.00E-05	yes
ni|645903670|nb|KK920760.1|	219258-219683	nan	nan	0	920	inf	5.00E-05	yes

ni|645903670|nb|KK920760.1|	224160-224291	nan	nan	0	971	inf	5.00E-05	yes
ni|645903670|nb|KK920760.1|	227360-227731	nan	nan	0.183343	1185.48	13	0.0443	no
ni|645903670|nb|KK920760.1|	232274-232503	nan	nan	0.387545	1438.39	12	0.0443	no
ni|645903670|nb|KK920760.1|	234162-234510	nan	nan	0	550	inf	5.00E-05	yes
ni|645903670|nb|KK920760.1|	239086-239354	nan	nan	0.148416	737	12	0.11935	no
ni|645903670|nb|KK920760.1|	248379-248703	nan	nan	24	20	-0.23051	0.8396	no
ni|645903670|nb|KK920760.1|	248780-249585	nan	nan	33	25	-0.359044	0.8149	no
ni|645903670|nb|KK920760.1|	250465-250848	nan	nan	0.61432	0.670963	0.127242	0.92555	no
ni|645903670|nb|KK920760.1|	299405-299787	nan	nan	11	10	-0.183726	0.87345	no
ni|645903670|nb|KK920760.1|	36730-37211	nan	nan	13	15	0.199087	0.86175	no
ni|645903670|nb|KK920760.1|	39528-39665	nan	nan	55	63	0.187073	0.8713	no
ni|645903670|nb|KK920760.1|	51300-51426	nan	nan	33	22	-0.552761	0.6482	no
ni|645903670|nb|KK920760.1|	51519-51815	nan	nan	45	54	0.274796	0.8027	no
ni|645903670|nb|KK920760.1|	57066-57216	nan	nan	53	71	0.410506	0.72365	no
ni|645903670|nb|KK920760.1|	7293-7530	nan	nan	90	142	0.670039	0.59885	no
ni|645903670|nb|KK920760.1|	7739-8228	nan	nan	336	630.3	0.90902	0.5311	no
ni|645903670|nb|KK920760.1|	9736-10009	nan	nan	0.576358	2	2	0.2229	no
ni|645903671|nb|KK920759.1|	1113233-1113433	nan	nan	56	47	-0.239345	0.83525	no
ni|645903671|nb|KK920759.1|	1115795-1115948	nan	nan	32	28	-0.200457	0.86565	no
ni|645903671|nb|KK920759.1|	1117823-1118053	nan	nan	60	38	-0.656064	0.5631	no
ni|645903671|nb|KK920759.1|	1118655-1119082	nan	nan	50	39	-0.33802	0.74565	no
ni|645903671|nb|KK920759.1|	1119847-1120224	nan	nan	27	18	-0.575909	0.6109	no
ni|645903671|nb|KK920759.1|	1120296-1120424	nan	nan	35	18	-0.92981	0.4425	no
ni|645903671|nb|KK920759.1|	1122834-1125211	nan	nan	15	22	0.610795	0.67015	no
ni|645903671|nb|KK920759.1|	1147970-1148817	nan	nan	0.231669	0.949336	2	0.1457	no
ni|645903671|nb|KK920759.1|	1148918-1150706	nan	nan	0.935702	102.42	7	0.00065	yes
ni|645903671|nb|KK920759.1|	1151497-1151779	nan	nan	1	115	7	0.01485	no
ni|645903671|nb|KK920759.1|	1154019-1154221	nan	nan	3	145	6	0.0067	no
ni|645903671|nb|KK920759.1|	1154490-1154721	nan	nan	0.381569	151.59	9	0.0444	no
ni|645903671|nb|KK920759.1|	1156994-1157873	nan	nan	0.602907	72	7	0.00095	yes

ni|645903671|nb|KK920759.1|	1158818-115920[illegible]	nan	nan	2	3	0.934624	0.4348	no
ni|645903671|nb|KK920759.1|	13639-13753	nan	nan	34	63	0.895955	0.4648	no
ni|645903671|nb|KK920759.1|	14077-14364	nan	nan	13	33	1	0.24345	no
ni|645903671|nb|KK920759.1|	349825-350000	nan	nan	13	12	-0.143265	0.8988	no
ni|645903671|nb|KK920759.1|	395922-396097	nan	nan	8	10	0.301918	0.79955	no
ni|645903671|nb|KK920759.1|	396649-396947	nan	nan	1	2	0.175209	0.8916	no
ni|645903671|nb|KK920759.1|	402051-402258	nan	nan	26	24	-0.159091	0.8898	no
ni|645903671|nb|KK920759.1|	416229-416667	nan	nan	1	41	5	0.00205	yes
ni|645903671|nb|KK920759.1|	417557-417845	nan	nan	1	46	5	0.0068	no
ni|645903671|nb|KK920759.1|	420195-420312	nan	nan	27	531	4	0.0026	yes
ni|645903671|nb|KK920759.1|	420455-420610	nan	nan	0	14	inf	0.00025	yes
ni|645903671|nb|KK920759.1|	421264-421751	nan	nan	3	94	5	0.00285	yes
ni|645903671|nb|KK920759.1|	427813-427955	nan	nan	193	231	0.259117	0.81645	no
ni|645903671|nb|KK920759.1|	433414-433657	nan	nan	6	37	3	0.0312	no
ni|645903671|nb|KK920759.1|	433875-434133	nan	nan	26	109	2	0.06485	no
ni|645903671|nb|KK920759.1|	445506-445777	nan	nan	0.728948	1	0.782008	0.58145	no
ni|645903671|nb|KK920759.1|	446626-446933	nan	nan	7	12	0.738111	0.5301	no
ni|645903671|nb|KK920759.1|	447995-448162	nan	nan	17	50	2	0.18955	no
ni|645903671|nb|KK920759.1|	450806-451005	nan	nan	15	12	-0.310273	0.7916	no
ni|645903671|nb|KK920759.1|	452800-453262	nan	nan	0	3	inf	0.00015	yes
ni|645903671|nb|KK920759.1|	50530-50796	nan	nan	5	11	1	0.3856	no
ni|645903671|nb|KK920759.1|	522003-522118	nan	nan	13	6	-1	0.44965	no
ni|645903671|nb|KK920759.1|	541395-541603	nan	nan	6	1	-2	0.14825	no
ni|645903671|nb|KK920759.1|	643632-644202	nan	nan	4	6	0.410344	0.7228	no
ni|645903671|nb|KK920759.1|	726245-726473	nan	nan	1	3	1	0.4245	no
ni|645903671|nb|KK920759.1|	738405-738669	nan	nan	6	6	-0.0295207	0.98065	no
ni|645903671|nb|KK920759.1|	758919-759052	nan	nan	14	23	0.684801	0.5731	no
ni|645903671|nb|KK920759.1|	764772-765077	nan	nan	23	23	-0.0435051	0.97035	no
ni|645903671|nb|KK920759.1|	766917-767221	nan	nan	10	10	-0.116989	0.9241	no
ni|645903671|nb|KK920759.1|	767754-767861	nan	nan	78	63	-0.319381	0.7941	no

ni|645903671|nb|KK920759.1|	769417-769618	nan	nan	38	47	0.314468	0.78345	no
ni|645903671|nb|KK920759.1|	771296-771493	nan	nan	46	56	0.291899	0.79475	no
ni|645903671|nb|KK920759.1|	771644-771833	nan	nan	45	56	0.343266	0.76035	no
ni|645903671|nb|KK920759.1|	772244-772536	nan	nan	47	47	0.00796487	0.9929	no
ni|645903671|nb|KK920759.1|	772893-773067	nan	nan	53	65	0.291147	0.8007	no
ni|645903671|nb|KK920759.1|	773532-773724	nan	nan	45	49	0.136387	0.90505	no
ni|645903671|nb|KK920759.1|	773999-774326	nan	nan	45	50	0.162801	0.88125	no
ni|645903671|nb|KK920759.1|	774596-774807	nan	nan	49	48	-0.0470354	0.9656	no
ni|645903671|nb|KK920759.1|	775409-775735	nan	nan	3	2	-0.709502	0.563	no
ni|645903671|nb|KK920759.1|	776070-776254	nan	nan	2	4	0.83962	0.5399	no
ni|645903671|nb|KK920759.1|	777006-777235	nan	nan	16	21	0.403995	0.72945	no
ni|645903671|nb|KK920759.1|	777557-778819	nan	nan	24	22.66	-0.0901323	0.94985	no
ni|645903671|nb|KK920759.1|	780004-780538	nan	nan	43	39	-0.138044	0.9107	no
ni|645903671|nb|KK920759.1|	780889-781366	nan	nan	25	26	0.0730337	0.94855	no
ni|645903671|nb|KK920759.1|	781573-781698	nan	nan	34	21	-0.673065	0.57965	no
ni|645903671|nb|KK920759.1|	786304-787042	nan	nan	0.038771	495	14	0.11935	no
ni|645903671|nb|KK920759.1|	793704-793826	nan	nan	0	297	inf	5.00E-05	yes
ni|645903671|nb|KK920759.1|	797824-798430	nan	nan	0.048931	63	10	0.11935	no
ni|645903671|nb|KK920759.1|	799049-799166	nan	nan	0	247.67	inf	5.00E-05	yes
ni|645903671|nb|KK920759.1|	804935-806148	nan	nan	0.066568	20	8	0.0506	no
ni|645903671|nb|KK920759.1|	807275-807522	nan	nan	0	17	inf	5.00E-05	yes
ni|645903671|nb|KK920759.1|	809840-810286	nan	nan	0	185	inf	5.00E-05	yes
ni|645903671|nb|KK920759.1|	818085-818718	nan	nan	0.185768	2	3	0.0445	no
ni|645903671|nb|KK920759.1|	819055-819568	nan	nan	0.120016	93	10	0.0443	no
ni|645903671|nb|KK920759.1|	824771-825028	nan	nan	0	20	inf	5.00E-05	yes
ni|645903671|nb|KK920759.1|	835182-835405	nan	nan	0	41	inf	5.00E-05	yes
ni|645903671|nb|KK920759.1|	841629-842321	nan	nan	0.041795	4	7	0.1194	no
ni|645903671|nb|KK920759.1|	850012-850356	nan	nan	0.509442	2	2	0.18925	no
ni|645903671|nb|KK920759.1|	853661-854114	nan	nan	0	21	inf	5.00E-05	yes
ni|645903671|nb|KK920759.1|	861890-862093	nan	nan	3	0.696568	-2	0.1906	no

ni|645903671|nb|KK920759.1|	863898-864771	nan	nan	0.287811	0.428083	0.572768	0.67875	no
ni|645903671|nb|KK920759.1|	866391-866736	nan	nan	3	3	0.234005	0.83945	no
ni|645903671|nb|KK920759.1|	868081-868254	nan	nan	0	149	inf	5.00E-05	yes
ni|645903671|nb|KK920759.1|	873948-874284	nan	nan	0.105367	74	9	0.11935	no
ni|645903671|nb|KK920759.1|	877107-878157	nan	nan	0.078018	3	5	0.05095	no
ni|645903671|nb|KK920759.1|	886315-886490	nan	nan	0.668391	7	3	0.05215	no
ni|645903671|nb|KK920759.1|	888021-888314	nan	nan	19	16	-0.262631	0.8187	no
ni|645903671|nb|KK920759.1|	888698-888820	nan	nan	23	21	-0.137768	0.9077	no
ni|645903671|nb|KK920759.1|	889075-889251	nan	nan	18	14	-0.42242	0.7222	no
ni|645903671|nb|KK920759.1|	889711-890075	nan	nan	7	6	-0.107295	0.92905	no
ni|645903671|nb|KK920759.1|	894081-894466	nan	nan	9	8	-0.065396	0.953	no
ni|645903671|nb|KK920759.1|	895449-895986	nan	nan	14	13	-0.0955524	0.932	no
ni|645903671|nb|KK920759.1|	899486-899663	nan	nan	21	20	-0.0449578	0.96675	no
ni|645903671|nb|KK920759.1|	901927-902111	nan	nan	23	15	-0.606137	0.61085	no
ni|645903671|nb|KK920759.1|	902959-903122	nan	nan	22	23	0.10562	0.92565	no
ni|645903671|nb|KK920759.1|	904993-905153	nan	nan	12	15	0.311307	0.78895	no
ni|645903671|nb|KK920759.1|	907155-907308	nan	nan	16	13	-0.296672	0.8105	no
ni|645903671|nb|KK920759.1|	907418-908237	nan	nan	3	4	0.297868	0.80095	no
ni|645903671|nb|KK920759.1|	921608-921857	nan	nan	68	52	-0.391676	0.72405	no
ni|645903671|nb|KK920759.1|	937661-938013	nan	nan	0	16	inf	5.00E-05	yes
ni|645903672|nb|KK920758.1|	21178-21625	nan	nan	2	2	-0.152694	0.8951	no
ni|645903672|nb|KK920758.1|	22549-22867	nan	nan	0.570666	0.872425	0.612385	0.6619	no
ni|645903672|nb|KK920758.1|	244616-245011	nan	nan	0	4	inf	5.00E-05	yes
ni|645903672|nb|KK920758.1|	245279-245967	nan	nan	0	2	inf	0.00015	yes
ni|645903672|nb|KK920758.1|	246160-247349	nan	nan	0	2	inf	5.00E-05	yes
ni|645903672|nb|KK920758.1|	247676-247917	nan	nan	0	3	inf	0.0005	yes
ni|645903673|nb|KK920757.1|	101306-101488	nan	nan	4	15	2	0.12	no
ni|645903673|nb|KK920757.1|	101772-102122	nan	nan	1	6	2	0.085	no
ni|645903673|nb|KK920757.1|	104537-104726	nan	nan	2	14	3	0.0626	no
ni|645903673|nb|KK920757.1|	104971-105200	nan	nan	2	9	2	0.1211	no

ni|645903673|nb|KK920757.1|	106092-106310	nan	nan	4	15	2	0.13885	no
ni|645903673|nb|KK920757.1|	106805-106984	nan	nan	3	16	3	0.06385	no
ni|645903673|nb|KK920757.1|	109243-109469	nan	nan	4	10	1	0.31315	no
ni|645903673|nb|KK920757.1|	115095-115259	nan	nan	15	18	0.201965	0.8618	no
ni|645903673|nb|KK920757.1|	158189-159202	nan	nan	0.59538	0.232902	-1	0.3177	no
ni|645903673|nb|KK920757.1|	159631-159916	nan	nan	23	34	0.532911	0.6341	no
ni|645903673|nb|KK920757.1|	163073-163260	nan	nan	11	13	0.299554	0.79895	no
ni|645903673|nb|KK920757.1|	164564-164816	nan	nan	37	34	-0.13782	0.90585	no
ni|645903673|nb|KK920757.1|	164995-165193	nan	nan	29	35	0.285494	0.8042	no
ni|645903673|nb|KK920757.1|	169373-169504	nan	nan	40	51	0.326377	0.78085	no
ni|645903673|nb|KK920757.1|	171123-171718	nan	nan	22	24	0.114455	0.91785	no
ni|645903673|nb|KK920757.1|	174960-175205	nan	nan	32	38	0.244851	0.8276	no
ni|645903673|nb|KK920757.1|	179245-179729	nan	nan	23	20	-0.201032	0.8557	no
ni|645903673|nb|KK920757.1|	180200-180758	nan	nan	6	13	1	0.32705	no
ni|645903673|nb|KK920757.1|	185018-185205	nan	nan	197	396	1	0.40805	no
ni|645903673|nb|KK920757.1|	186281-186636	nan	nan	32	74	1	0.36405	no
ni|645903673|nb|KK920757.1|	187210-187723	nan	nan	92	181	0.966393	0.5006	no
ni|645903673|nb|KK920757.1|	189690-189984	nan	nan	95	142	0.574378	0.7012	no
ni|645903673|nb|KK920757.1|	190981-191135	nan	nan	4	4	0.0848108	0.9436	no
ni|645903673|nb|KK920757.1|	192119-192362	nan	nan	4	4	-0.0662591	0.9573	no
ni|645903673|nb|KK920757.1|	192540-192672	nan	nan	6	7	0.25465	0.86075	no
ni|645903673|nb|KK920757.1|	193118-193293	nan	nan	4	4	0.280607	0.82625	no
ni|645903673|nb|KK920757.1|	195275-195782	nan	nan	0.365382	0.52389	0.519859	0.7279	no
ni|645903673|nb|KK920757.1|	197327-197448	nan	nan	22	15	-0.507964	0.68875	no
ni|645903673|nb|KK920757.1|	197656-197817	nan	nan	483	393	-0.294917	0.84145	no
ni|645903673|nb|KK920757.1|	198176-198807	nan	nan	4	5	0.0980365	0.93355	no
ni|645903673|nb|KK920757.1|	199058-199171	nan	nan	29	12	-1	0.3768	no
ni|645903673|nb|KK920757.1|	199244-199403	nan	nan	303	297	-0.0294178	0.9767	no
ni|645903673|nb|KK920757.1|	199795-200500	nan	nan	7	5.63	-0.386738	0.7342	no
ni|645903673|nb|KK920757.1|	201749-202328	nan	nan	2	4	1	0.3239	no

ni|645903673|nb|KK920757.1|	202655-202822	nan	nan	0.74706	8	3	0.05165	no
ni|645903673|nb|KK920757.1|	227696-228332	nan	nan	0	1	inf	0.0003	yes
ni|645903673|nb|KK920757.1|	263528-263772	nan	nan	5	2	-1	0.3142	no
ni|645903673|nb|KK920757.1|	264756-265124	nan	nan	24	21	-0.180936	0.8667	no
ni|645903673|nb|KK920757.1|	266131-266725	nan	nan	2	0.814615	-1	0.35055	no
ni|645903673|nb|KK920757.1|	266989-267164	nan	nan	68	51	-0.412644	0.71755	no
ni|645903673|nb|KK920757.1|	268440-268810	nan	nan	45	29	-0.631167	0.5677	no
ni|645903673|nb|KK920757.1|	271556-271941	nan	nan	31	20	-0.617937	0.5834	no
ni|645903673|nb|KK920757.1|	272556-273087	nan	nan	34	21	-0.677315	0.53565	no
ni|645903673|nb|KK920757.1|	273880-274444	nan	nan	35	18	-0.952419	0.376	no
ni|645903673|nb|KK920757.1|	277017-277354	nan	nan	1	0.601536	-0.940061	0.54025	no
ni|645903673|nb|KK920757.1|	278617-278967	nan	nan	0.198852	12	6	0.0446	no
ni|645903673|nb|KK920757.1|	280245-280851	nan	nan	0.048931	2	5	0.1198	no
ni|645903673|nb|KK920757.1|	281645-282281	nan	nan	0	33	inf	5.00E-05	yes
ni|645903673|nb|KK920757.1|	286968-287664	nan	nan	4	47	3	0.02445	no
ni|645903673|nb|KK920757.1|	287832-287992	nan	nan	10	81	3	0.01975	no
ni|645903673|nb|KK920757.1|	289877-290367	nan	nan	3	19	3	0.02475	no
ni|645903673|nb|KK920757.1|	291486-292114	nan	nan	0.703254	0.134463	-2	0.1791	no
ni|645903673|nb|KK920757.1|	292335-292706	nan	nan	2	0.788454	-0.982985	0.48245	no
ni|645903673|nb|KK920757.1|	293907-294090	nan	nan	0.604283	47	6	0.0443	no
ni|645903673|nb|KK920757.1|	294609-294723	nan	nan	0	85	inf	0.0001	yes
ni|645903673|nb|KK920757.1|	294849-297110	nan	nan	0.03425	13	9	0.05055	no
ni|645903673|nb|KK920757.1|	298850-299055	nan	nan	0.238542	9	5	0.12	no
ni|645903673|nb|KK920757.1|	300744-300970	nan	nan	7	5	-0.690993	0.56535	no
ni|645903673|nb|KK920757.1|	301098-301335	nan	nan	6	3	-0.788805	0.5129	no
ni|645903673|nb|KK920757.1|	303817-304740	nan	nan	4	5	0.427484	0.71485	no
ni|645903673|nb|KK920757.1|	304882-309521	nan	nan	2	4	0.689139	0.52125	no
ni|645903673|nb|KK920757.1|	66076-66306	nan	nan	17	7	-1	0.24915	no
ni|645903673|nb|KK920757.1|	66379-68799	nan	nan	42	32	-0.411638	0.7722	no
ni|645903673|nb|KK920757.1|	70720-71480	nan	nan	0.974316	0.286631	-2	0.20555	no

ni|645903673|nb|KK920757.1|	71602-72209	nan	nan	0.830184	0.326795	-1	0.33495	no
ni|645903673|nb|KK920757.1|	72468-73084	nan	nan	1	0.36694	-2	0.1861	no
ni|645903673|nb|KK920757.1|	73191-73470	nan	nan	1	0.665003	-1	0.44755	no
ni|645903673|nb|KK920757.1|	74868-75395	nan	nan	1	0.388302	-2	0.22995	no
ni|645903673|nb|KK920757.1|	75574-76047	nan	nan	1	2	0.429593	0.72515	no
ni|645903673|nb|KK920757.1|	77008-77380	nan	nan	2	0.43641	-2	0.1999	no
ni|645903673|nb|KK920757.1|	78566-79270	nan	nan	0.491539	0.313297	-0.649774	0.64935	no
ni|645903673|nb|KK920757.1|	83428-86295	nan	nan	23	22	-0.0162773	0.99175	no
ni|645903673|nb|KK920757.1|	93496-93951	nan	nan	1	11	3	0.02055	no
ni|645903673|nb|KK920757.1|	94962-95170	nan	nan	3	15	2	0.07265	no
ni|645903673|nb|KK920757.1|	99573-99802	nan	nan	2	12	3	0.0609	no
ni|645903674|nb|KK920756.1|	127958-128146	nan	nan	31	24	-0.37916	0.74675	no
ni|645903674|nb|KK920756.1|	128750-129120	nan	nan	5	3	-0.545444	0.643	no
ni|645903674|nb|KK920756.1|	129314-129973	nan	nan	15	21	0.473894	0.66155	no
ni|645903674|nb|KK920756.1|	13195-13617	nan	nan	3	0	-inf	0.0002	yes
ni|645903674|nb|KK920756.1|	133649-133768	nan	nan	9	19	1	0.41635	no
ni|645903674|nb|KK920756.1|	134762-135014	nan	nan	2	6	1	0.30625	no
ni|645903674|nb|KK920756.1|	135169-135309	nan	nan	18	22	0.322719	0.7894	no
ni|645903674|nb|KK920756.1|	13704-14209	nan	nan	0.611992	0	-inf	0.00455	no
ni|645903674|nb|KK920756.1|	137693-137892	nan	nan	15	26	0.805242	0.4882	no
ni|645903674|nb|KK920756.1|	138828-139047	nan	nan	17	30	0.822352	0.4784	no
ni|645903674|nb|KK920756.1|	139234-139839	nan	nan	5	12	1	0.2681	no
ni|645903674|nb|KK920756.1|	140015-140132	nan	nan	22	33	0.576403	0.65045	no
ni|645903674|nb|KK920756.1|	140992-141127	nan	nan	17	34	1	0.3896	no
ni|645903674|nb|KK920756.1|	142869-143052	nan	nan	11	24	1	0.3686	no
ni|645903674|nb|KK920756.1|	146008-146180	nan	nan	13	26	1	0.38435	no
ni|645903674|nb|KK920756.1|	14615-14812	nan	nan	4	0.246639	-4	0.0255	no
ni|645903674|nb|KK920756.1|	148141-148347	nan	nan	13	32	1	0.2702	no
ni|645903674|nb|KK920756.1|	178158-178398	nan	nan	11	15	0.432155	0.7061	no
ni|645903674|nb|KK920756.1|	198967-199026	nan	nan	13153.6	28389.7	1	0.428	no

ni|645903674|nb|KK920756.1|	22730-22943	nan	nan	16	0	-inf	5.00E-05	yes
ni|645903674|nb|KK920756.1|	24603-24854	nan	nan	54	0	-inf	5.00E-05	yes
ni|645903674|nb|KK920756.1|	246219-248151	nan	nan	87	150	0.774573	0.58415	no
ni|645903674|nb|KK920756.1|	248673-249202	nan	nan	133	177.12	0.415604	0.76535	no
ni|645903674|nb|KK920756.1|	249335-249529	nan	nan	129	88	-0.55432	0.61025	no
ni|645903674|nb|KK920756.1|	249804-249933	nan	nan	42	39	-0.126198	0.91725	no
ni|645903674|nb|KK920756.1|	252950-253457	nan	nan	0.060897	5	6	0.11935	no
ni|645903674|nb|KK920756.1|	25797-26031	nan	nan	4	0	-inf	0.00075	yes
ni|645903674|nb|KK920756.1|	3039-3502	nan	nan	1	0.065301	-4	0.02485	no
ni|645903674|nb|KK920756.1|	36436-37547	nan	nan	2	0	-inf	5.00E-05	yes
ni|645903674|nb|KK920756.1|	3644-4585	nan	nan	0.82281	0	-inf	0.0004	yes
ni|645903674|nb|KK920756.1|	40033-40160	nan	nan	9	0	-inf	0.00455	no
ni|645903674|nb|KK920756.1|	402962-403093	nan	nan	25	23	-0.114592	0.9219	no
ni|645903674|nb|KK920756.1|	403299-403407	nan	nan	75	103	0.462744	0.69395	no
ni|645903674|nb|KK920756.1|	403478-403606	nan	nan	59	39	-0.578814	0.62685	no
ni|645903674|nb|KK920756.1|	403926-404170	nan	nan	89	79	-0.176608	0.8727	no
ni|645903674|nb|KK920756.1|	404364-405017	nan	nan	29	32	0.118672	0.91305	no
ni|645903674|nb|KK920756.1|	40440-40656	nan	nan	4	0	-inf	0.0004	yes
ni|645903674|nb|KK920756.1|	405224-406232	nan	nan	12	14	0.316266	0.77025	no
ni|645903674|nb|KK920756.1|	406614-407174	nan	nan	19	24	0.343848	0.75845	no
ni|645903674|nb|KK920756.1|	40722-40940	nan	nan	7	0	-inf	0.0001	yes
ni|645903674|nb|KK920756.1|	408097-408424	nan	nan	29	42	0.508515	0.6508	no
ni|645903674|nb|KK920756.1|	408553-408720	nan	nan	77	106	0.472447	0.67535	no
ni|645903674|nb|KK920756.1|	408930-409344	nan	nan	0.869499	1	0.282613	0.82625	no
ni|645903674|nb|KK920756.1|	409525-409790	nan	nan	79	117	0.554213	0.6636	no
ni|645903674|nb|KK920756.1|	411167-411696	nan	nan	54	79	0.556657	0.704	no
ni|645903674|nb|KK920756.1|	413038-413259	nan	nan	1	2	0.611488	0.6619	no
ni|645903674|nb|KK920756.1|	413366-413568	nan	nan	54	88	0.703579	0.5296	no
ni|645903674|nb|KK920756.1|	413767-415512	nan	nan	6	7	0.273528	0.79895	no
ni|645903674|nb|KK920756.1|	416495-417088	nan	nan	2	0.960323	-0.787424	0.515	no

ni|645903674|nb|KK920756.1|	417873-418555	nan	nan	28	46	0.718441	0.55755	no
ni|645903674|nb|KK920756.1|	41940-42106	nan	nan	6	0	-inf	0.0013	yes
ni|645903674|nb|KK920756.1|	420950-421689	nan	nan	0.309678	0.555153	0.842117	0.5399	no
ni|645903674|nb|KK920756.1|	494807-494986	nan	nan	61	78	0.360876	0.74985	no
ni|645903674|nb|KK920756.1|	499555-499892	nan	nan	3	12	2	0.12155	no
ni|645903674|nb|KK920756.1|	5171-5321	nan	nan	11	0	-inf	0.00045	yes
ni|645903674|nb|KK920756.1|	557856-558757	nan	nan	3	4	0.354186	0.75225	no
ni|645903674|nb|KK920756.1|	559037-559378	nan	nan	6	9	0.609331	0.60695	no
ni|645903674|nb|KK920756.1|	559571-559728	nan	nan	7	13	0.845339	0.49255	no
ni|645903674|nb|KK920756.1|	559854-560051	nan	nan	5	10	0.970431	0.4303	no
ni|645903674|nb|KK920756.1|	560963-561467	nan	nan	2	3	0.319555	0.78905	no
ni|645903674|nb|KK920756.1|	561601-561747	nan	nan	10	18	0.772331	0.5244	no
ni|645903674|nb|KK920756.1|	562155-562510	nan	nan	1	1	-0.0655113	0.9554	no
ni|645903674|nb|KK920756.1|	565331-565781	nan	nan	88	162	0.879815	0.54515	no
ni|645903674|nb|KK920756.1|	566571-567291	nan	nan	1	0.915556	-0.524225	0.66205	no
ni|645903674|nb|KK920756.1|	567813-568257	nan	nan	62	119	0.924732	0.52995	no
ni|645903674|nb|KK920756.1|	569044-569400	nan	nan	10	22	1	0.3333	no
ni|645903674|nb|KK920756.1|	570722-570880	nan	nan	51	104	1	0.37165	no
ni|645903674|nb|KK920756.1|	571271-571456	nan	nan	24	55	1	0.3237	no
ni|645903674|nb|KK920756.1|	573224-573515	nan	nan	34	65	0.92864	0.4095	no
ni|645903674|nb|KK920756.1|	573695-573868	nan	nan	29	56	0.932701	0.43005	no
ni|645903674|nb|KK920756.1|	574669-575100	nan	nan	32	61	0.934733	0.4738	no
ni|645903674|nb|KK920756.1|	575656-575774	nan	nan	21	43	1	0.40415	no
ni|645903674|nb|KK920756.1|	575970-576118	nan	nan	26	40	0.610872	0.60285	no
ni|645903674|nb|KK920756.1|	577615-577776	nan	nan	32	48	0.583208	0.61825	no
ni|645903674|nb|KK920756.1|	578896-579096	nan	nan	8	12	0.503334	0.66925	no
ni|645903674|nb|KK920756.1|	580551-580897	nan	nan	9	16	0.917576	0.4434	no
ni|645903674|nb|KK920756.1|	581644-582004	nan	nan	10	15	0.555998	0.6348	no
ni|645903674|nb|KK920756.1|	584746-584989	nan	nan	0	457.99	inf	5.00E-05	yes
ni|645903674|nb|KK920756.1|	586754-586908	nan	nan	0	294	inf	5.00E-05	yes

ni|645903674|nb|KK920756.1|	588662-588899	nan	nan	0	127	inf	5.00E-05	yes
ni|645903674|nb|KK920756.1|	592065-592406	nan	nan	4	4	-0.217576	0.84925	no
ni|645903674|nb|KK920756.1|	592634-592754	nan	nan	13	21.83	0.721091	0.5689	no
ni|645903674|nb|KK920756.1|	593793-594023	nan	nan	2	2	-0.203944	0.87975	no
ni|645903674|nb|KK920756.1|	594111-594223	nan	nan	23	20	-0.193108	0.87555	no
ni|645903674|nb|KK920756.1|	595188-595609	nan	nan	5	5	-0.023472	0.9844	no
ni|645903674|nb|KK920756.1|	596470-596679	nan	nan	2	4	1	0.35025	no
ni|645903674|nb|KK920756.1|	599566-599794	nan	nan	98	115	0.231112	0.8295	no
ni|645903674|nb|KK920756.1|	599925-600474	nan	nan	48	50	0.0410637	0.97785	no
ni|645903674|nb|KK920756.1|	602075-602249	nan	nan	155	164	0.0853273	0.93885	no
ni|645903674|nb|KK920756.1|	602357-602543	nan	nan	115	125	0.124945	0.9102	no
ni|645903674|nb|KK920756.1|	603848-604272	nan	nan	53	61	0.19775	0.8735	no
ni|645903674|nb|KK920756.1|	608257-608649	nan	nan	62	59	-0.0752345	0.9532	no
ni|645903674|nb|KK920756.1|	609788-610695	nan	nan	85	85	-0.00330373	0.9979	no
ni|645903674|nb|KK920756.1|	613901-614058	nan	nan	250	258	0.0471101	0.9649	no
ni|645903674|nb|KK920756.1|	614242-614663	nan	nan	118	112	-0.0757499	0.9613	no
ni|645903674|nb|KK920756.1|	618733-619010	nan	nan	102	111	0.128453	0.93375	no
ni|645903674|nb|KK920756.1|	619622-620571	nan	nan	101	104	0.0311599	0.98205	no
ni|645903674|nb|KK920756.1|	621720-621836	nan	nan	125	88	-0.505594	0.66715	no
ni|645903674|nb|KK920756.1|	622312-622452	nan	nan	6	2	-1	0.4023	no
ni|645903674|nb|KK920756.1|	623057-623616	nan	nan	2	2	0.0109365	0.9852	no
ni|645903674|nb|KK920756.1|	623749-623852	nan	nan	153	93	-0.712491	0.5592	no
ni|645903674|nb|KK920756.1|	627353-627537	nan	nan	39	28	-0.434642	0.70855	no
ni|645903674|nb|KK920756.1|	627822-628247	nan	nan	31	30	-0.0617328	0.9567	no
ni|645903674|nb|KK920756.1|	628982-630037	nan	nan	18	19	0.0166559	0.9876	no
ni|645903674|nb|KK920756.1|	672652-672949	nan	nan	14	20	0.510544	0.658	no
ni|645903674|nb|KK920756.1|	679899-680106	nan	nan	29	25	-0.20085	0.8579	no
ni|645903674|nb|KK920756.1|	680735-681156	nan	nan	15	13	-0.263232	0.81955	no
ni|645903674|nb|KK920756.1|	681250-681409	nan	nan	18	2	-3	0.04955	no
ni|645903674|nb|KK920756.1|	681622-682048	nan	nan	18	22	0.343526	0.76115	no

ni|645903674|nb|KK920756.1|	682210-682339	nan	nan	7	8	0.254624	0.86075	no
ni|645903674|nb|KK920756.1|	682420-682538	nan	nan	21	13	-0.745507	0.56925	no
ni|645903674|nb|KK920756.1|	682725-683139	nan	nan	19	21	0.192599	0.8662	no
ni|645903674|nb|KK920756.1|	683269-685392	nan	nan	28	36	0.368534	0.798	no
ni|645903674|nb|KK920756.1|	686759-687177	nan	nan	12	18	0.669728	0.55535	no
ni|645903674|nb|KK920756.1|	694159-694370	nan	nan	27	33	0.295816	0.7967	no
ni|645903674|nb|KK920756.1|	694919-695445	nan	nan	11	15	0.453959	0.688	no
ni|645903674|nb|KK920756.1|	696481-696658	nan	nan	24	30	0.342763	0.77315	no
ni|645903674|nb|KK920756.1|	696861-697225	nan	nan	7	10	0.533163	0.6552	no
ni|645903674|nb|KK920756.1|	698163-698401	nan	nan	21	28	0.449513	0.70095	no
ni|645903674|nb|KK920756.1|	700145-700298	nan	nan	19	28	0.565078	0.63375	no
ni|645903674|nb|KK920756.1|	703903-704091	nan	nan	15	21	0.480272	0.68285	no
ni|645903674|nb|KK920756.1|	710386-710532	nan	nan	12	10	-0.204707	0.8706	no
ni|645903674|nb|KK920756.1|	728051-728154	nan	nan	147	204.34	0.475686	0.69195	no
ni|645903674|nb|KK920756.1|	7421-7654	nan	nan	3	0	-inf	0.0009	yes
ni|645903674|nb|KK920756.1|	840479-840584	nan	nan	132	178	0.423742	0.7194	no
ni|645903674|nb|KK920756.1|	853692-853889	nan	nan	24	19	-0.271057	0.81115	no
ni|645903674|nb|KK920756.1|	854286-854511	nan	nan	16	15	-0.0304492	0.97925	no
ni|645903675|nb|KK920755.1|	101242-101905	nan	nan	2	0.084049	-4	0.23295	no
ni|645903675|nb|KK920755.1|	127095-127287	nan	nan	5	0	-inf	0.00055	yes
ni|645903675|nb|KK920755.1|	141019-141340	nan	nan	3	5	0.870198	0.4737	no
ni|645903675|nb|KK920755.1|	141870-142133	nan	nan	3	3	-0.0660552	0.96005	no
ni|645903675|nb|KK920755.1|	146118-146705	nan	nan	7	9	0.286505	0.79755	no
ni|645903675|nb|KK920755.1|	17280-18045	nan	nan	0.520673	0	-inf	0.00195	yes
ni|645903675|nb|KK920755.1|	50686-50905	nan	nan	2	0	-inf	0.00455	no
ni|645903675|nb|KK920755.1|	51836-52298	nan	nan	0.685092	0	-inf	0.00455	no
ni|645903675|nb|KK920755.1|	52964-53357	nan	nan	0.847462	0	-inf	0.00455	no
ni|645903675|nb|KK920755.1|	72782-73065	nan	nan	1	0	-inf	0.0036	no
ni|645903675|nb|KK920755.1|	76314-76716	nan	nan	1	0	-inf	0.0015	yes
ni|645903676|nb|KK920754.1|	109329-109731	nan	nan	5	17	2	0.1163	no

ni|645903676|nb|KK920754.1|	111837-112006	nan	nan	3	15	2	0.0933	no
ni|645903676|nb|KK920754.1|	138971-139235	nan	nan	3	2	-0.506619	0.68965	no
ni|645903676|nb|KK920754.1|	183725-185759	nan	nan	0	54	inf	5.00E-05	yes
ni|645903676|nb|KK920754.1|	202351-202557	nan	nan	18	17	-0.0668543	0.9565	no
ni|645903676|nb|KK920754.1|	205074-205409	nan	nan	46	29	-0.648888	0.5643	no
ni|645903676|nb|KK920754.1|	26469-26591	nan	nan	15	18	0.212729	0.86845	no
ni|645903676|nb|KK920754.1|	33087-33497	nan	nan	2	2	0.0457099	0.9714	no
ni|645903676|nb|KK920754.1|	346215-346842	nan	nan	9	6	-0.690305	0.5506	no
ni|645903676|nb|KK920754.1|	455090-455332	nan	nan	0	4	inf	0.0007	yes
ni|645903676|nb|KK920754.1|	599177-599541	nan	nan	1	0.98937	-0.0654744	0.94765	no
ni|645903676|nb|KK920754.1|	600365-601123	nan	nan	2054.22	1269.62	-0.694191	0.54525	no
ni|645903676|nb|KK920754.1|	610055-610759	nan	nan	476	252	-0.918784	0.52165	no
ni|645903676|nb|KK920754.1|	624827-625092	nan	nan	12	5	-1	0.32985	no
ni|645903676|nb|KK920754.1|	625210-626006	nan	nan	4266.16	2515.9	-0.761863	0.40525	no
ni|645903676|nb|KK920754.1|	627719-628479	nan	nan	2459.02	1679.7	-0.549886	0.5959	no
ni|645903676|nb|KK920754.1|	629536-629934	nan	nan	2	2	0.441605	0.71395	no
ni|645903676|nb|KK920754.1|	79843-80000	nan	nan	21	21	0.0506381	0.96335	no
ni|645903676|nb|KK920754.1|	80096-80248	nan	nan	2	5	0.932808	0.5287	no
ni|645903677|nb|KK920753.1|	24613-25042	nan	nan	0	2	inf	0.00065	yes
ni|645903677|nb|KK920753.1|	490385-490512	nan	nan	22	6	-2	0.1949	no
ni|645903677|nb|KK920753.1|	501181-501362	nan	nan	46	51	0.167655	0.88495	no
ni|645903677|nb|KK920753.1|	502096-502498	nan	nan	9	10	0.111534	0.9209	no
ni|645903677|nb|KK920753.1|	503457-503696	nan	nan	4	2	-1	0.288	no
ni|645903677|nb|KK920753.1|	506787-507079	nan	nan	30	33	0.166422	0.88155	no
ni|645903677|nb|KK920753.1|	507536-507993	nan	nan	9	10	0.121877	0.9138	no
ni|645903677|nb|KK920753.1|	510840-511020	nan	nan	49	57	0.233994	0.8402	no
ni|645903677|nb|KK920753.1|	519818-520159	nan	nan	44	31	-0.503475	0.6519	no
ni|645903677|nb|KK920753.1|	520221-520498	nan	nan	27	31.88	0.222888	0.844	no
ni|645903677|nb|KK920753.1|	521721-521853	nan	nan	10	7	-0.44579	0.74215	no
ni|645903677|nb|KK920753.1|	522401-522548	nan	nan	6	9	0.560829	0.667	no

ni|645903677|nb|KK920753.1|	523726-524033	nan	nan	2	3	0.470306	0.70145	no
ni|645903677|nb|KK920753.1|	524856-525589	nan	nan	5	5	0.0103215	0.9921	no
ni|645903677|nb|KK920753.1|	525922-526531	nan	nan	5	3	-0.425336	0.713	no
ni|645903677|nb|KK920753.1|	527133-527482	nan	nan	19	26	0.463243	0.6788	no
ni|645903677|nb|KK920753.1|	528252-528922	nan	nan	9	10	0.0850895	0.94185	no
ni|645903677|nb|KK920753.1|	529059-529207	nan	nan	7	5	-0.415123	0.7377	no
ni|645903677|nb|KK920753.1|	530319-530654	nan	nan	16	16	0.0476085	0.96565	no
ni|645903677|nb|KK920753.1|	534436-534584	nan	nan	33	30	-0.136462	0.90715	no
ni|645903677|nb|KK920753.1|	537709-538382	nan	nan	7	9	0.405136	0.7181	no
ni|645903677|nb|KK920753.1|	543257-544492	nan	nan	0.28286	0.332915	0.235068	0.85095	no
ni|645903677|nb|KK920753.1|	548083-548201	nan	nan	5	20	2	0.1847	no
ni|645903677|nb|KK920753.1|	548421-548716	nan	nan	13	17	0.34922	0.75915	no
ni|645903677|nb|KK920753.1|	564240-564595	nan	nan	0	5	inf	5.00E-05	yes
ni|645903677|nb|KK920753.1|	569118-569252	nan	nan	8	7	-0.204767	0.87975	no
ni|645903677|nb|KK920753.1|	573359-573651	nan	nan	276	193	-0.512432	0.72415	no
ni|645903677|nb|KK920753.1|	580446-580996	nan	nan	121	67	-0.865191	0.5593	no
ni|645903677|nb|KK920753.1|	582685-582963	nan	nan	1	0.40126	-2	0.2784	no
ni|645903677|nb|KK920753.1|	583198-583528	nan	nan	20	15	-0.39905	0.7315	no
ni|645903677|nb|KK920753.1|	584869-585122	nan	nan	2	0.778975	-1	0.3141	no
ni|645903677|nb|KK920753.1|	586629-587028	nan	nan	171	119.83	-0.514148	0.7136	no
ni|645903677|nb|KK920753.1|	597797-598042	nan	nan	12	0.493393	-5	0.11995	no
ni|645903677|nb|KK920753.1|	609565-609840	nan	nan	20	2	-3	0.01955	no
ni|645903677|nb|KK920753.1|	612585-612731	nan	nan	33	2	-4	0.1196	no
ni|645903677|nb|KK920753.1|	620549-620926	nan	nan	15	0.942672	-4	0.0126	no
ni|645903677|nb|KK920753.1|	626140-626594	nan	nan	6	0.93757	-3	0.0435	no
ni|645903677|nb|KK920753.1|	629405-629755	nan	nan	21	2	-4	0.0103	no
ni|645903677|nb|KK920753.1|	638965-639374	nan	nan	40	20	-0.983507	0.37725	no
ni|645903677|nb|KK920753.1|	646711-647180	nan	nan	68	29	-1	0.3406	no
ni|645903677|nb|KK920753.1|	651092-651251	nan	nan	23	12	-0.915199	0.4387	no
ni|645903677|nb|KK920753.1|	655074-655627	nan	nan	58	32	-0.852647	0.49755	no

ni|645903677|nb|KK920753.1|	661889-662166	nan	nan	98	100	0.0240679	0.9802	no
ni|645903678|nb|KK920752.1|	10233-10400	nan	nan	0	47	inf	5.00E-05	yes
ni|645903678|nb|KK920752.1|	119360-119760	nan	nan	3	1	-1	0.29165	no
ni|645903678|nb|KK920752.1|	151664-151858	nan	nan	5	5	0.0108956	0.9704	no
ni|645903678|nb|KK920752.1|	206142-206255	nan	nan	13	10	-0.289939	0.8496	no
ni|645903678|nb|KK920752.1|	229678-229931	nan	nan	0	23	inf	5.00E-05	yes
ni|645903678|nb|KK920752.1|	346163-346349	nan	nan	4	3	-0.552668	0.68145	no
ni|645903678|nb|KK920752.1|	363818-363954	nan	nan	12	4	-2	0.25675	no
ni|645903678|nb|KK920752.1|	364043-364433	nan	nan	10	19	0.94099	0.41685	no
ni|645903678|nb|KK920752.1|	366222-366478	nan	nan	17	29	0.747002	0.51885	no
ni|645903678|nb|KK920752.1|	366578-366758	nan	nan	20	31	0.605115	0.6039	no
ni|645903678|nb|KK920752.1|	367365-367731	nan	nan	7	15	1	0.31215	no
ni|645903678|nb|KK920752.1|	369375-369608	nan	nan	13	31	1	0.285	no
ni|645903678|nb|KK920752.1|	370663-371056	nan	nan	7	14	0.901077	0.44305	no
ni|645903678|nb|KK920752.1|	371685-371890	nan	nan	19	41	1	0.3363	no
ni|645903678|nb|KK920752.1|	372450-373090	nan	nan	6	12	1	0.3743	no
ni|645903678|nb|KK920752.1|	375310-376006	nan	nan	6	18	2	0.15765	no
ni|645903678|nb|KK920752.1|	377075-377225	nan	nan	15	40	1	0.25765	no
ni|645903678|nb|KK920752.1|	378029-378182	nan	nan	11	35	2	0.17675	no
ni|645903678|nb|KK920752.1|	378725-379253	nan	nan	6	13.78	1	0.2889	no
ni|645903678|nb|KK920752.1|	379647-379807	nan	nan	11	30	1	0.22835	no
ni|645903678|nb|KK920752.1|	379937-380134	nan	nan	14	33	1	0.2931	no
ni|645903678|nb|KK920752.1|	380236-381226	nan	nan	3	7	1	0.3495	no
ni|645903678|nb|KK920752.1|	387941-388504	nan	nan	16	10	-0.649968	0.56455	no
ni|645903678|nb|KK920752.1|	389179-389795	nan	nan	14	14	0.00907728	0.99215	no
ni|645903678|nb|KK920752.1|	390886-391471	nan	nan	10	9	-0.194481	0.85865	no
ni|645903678|nb|KK920752.1|	391941-392098	nan	nan	30	25	-0.244986	0.83245	no
ni|645903678|nb|KK920752.1|	393236-393395	nan	nan	39	41	0.0832144	0.94455	no
ni|645903678|nb|KK920752.1|	434766-435050	nan	nan	21	30	0.494515	0.6594	no
ni|645903678|nb|KK920752.1|	435235-436098	nan	nan	38	43	0.171771	0.90905	no

ni|645903678|nb|KK920752.1|	436681-436883	nan	nan	215	243	0.179772	0.89935	no
ni|645903678|nb|KK920752.1|	438144-438331	nan	nan	240	255	0.0854677	0.95455	no
ni|645903678|nb|KK920752.1|	439372-439564	nan	nan	185	194	0.0664948	0.947	no
ni|645903678|nb|KK920752.1|	441573-441810	nan	nan	149	169	0.184563	0.9	no
ni|645903678|nb|KK920752.1|	444323-444502	nan	nan	216	224	0.0504339	0.9629	no
ni|645903678|nb|KK920752.1|	445950-446092	nan	nan	91	126	0.466897	0.67895	no
ni|645903678|nb|KK920752.1|	446519-446745	nan	nan	209	215	0.039237	0.97815	no
ni|645903678|nb|KK920752.1|	451004-451670	nan	nan	2	1	-1	0.3859	no
ni|645903678|nb|KK920752.1|	458121-458884	nan	nan	22	22	-0.0267201	0.97855	no
ni|645903678|nb|KK920752.1|	459022-459456	nan	nan	32	33	0.0426248	0.97	no
ni|645903678|nb|KK920752.1|	459649-459761	nan	nan	23	26	0.154815	0.90275	no
ni|645903678|nb|KK920752.1|	460092-460554	nan	nan	4	3	-0.333686	0.77455	no
ni|645903678|nb|KK920752.1|	460912-461742	nan	nan	13	14	0.104418	0.92195	no
ni|645903678|nb|KK920752.1|	461962-462222	nan	nan	28	28	0.0127736	0.9911	no
ni|645903678|nb|KK920752.1|	462341-462627	nan	nan	14	9	-0.588555	0.6183	no
ni|645903678|nb|KK920752.1|	462891-463350	nan	nan	24	21	-0.195657	0.8626	no
ni|645903678|nb|KK920752.1|	464163-464296	nan	nan	28	25	-0.147441	0.89825	no
ni|645903678|nb|KK920752.1|	464563-465112	nan	nan	0.993131	1	0.224477	0.85915	no
ni|645903678|nb|KK920752.1|	465522-466018	nan	nan	0.93896	0.718013	-0.387053	0.7621	no
ni|645903678|nb|KK920752.1|	467556-467968	nan	nan	1	1	0.141135	0.90865	no
ni|645903678|nb|KK920752.1|	469303-469483	nan	nan	14	11	-0.311236	0.79705	no
ni|645903678|nb|KK920752.1|	470071-470713	nan	nan	48	53	0.144566	0.92165	no
ni|645903678|nb|KK920752.1|	478253-478565	nan	nan	0.939104	1	0.256209	0.86075	no
ni|645903678|nb|KK920752.1|	479564-479688	nan	nan	100	128	0.347682	0.76415	no
ni|645903678|nb|KK920752.1|	481627-481975	nan	nan	29	43	0.566888	0.6072	no
ni|645903678|nb|KK920752.1|	483060-483613	nan	nan	40	49	0.304404	0.8424	no
ni|645903678|nb|KK920752.1|	483810-484664	nan	nan	13	15	0.17436	0.8748	no
ni|645903678|nb|KK920752.1|	484895-485274	nan	nan	33	38	0.216545	0.83725	no
ni|645903678|nb|KK920752.1|	485854-486440	nan	nan	2	1	-0.112274	0.9225	no
ni|645903678|nb|KK920752.1|	486766-487113	nan	nan	1	0.769376	-0.524978	0.7071	no

ni|645903678|nb|KK920752.1|	487290-487693	nan	nan	1	1	0.0277686	0.9668	no
ni|645903678|nb|KK920752.1|	488395-488624	nan	nan	4	7	0.89507	0.45675	no
ni|645903678|nb|KK920752.1|	489415-489592	nan	nan	42	40	-0.0673257	0.953	no
ni|645903678|nb|KK920752.1|	490301-490622	nan	nan	22	20	-0.149884	0.8964	no
ni|645903678|nb|KK920752.1|	506107-506855	nan	nan	0.419872	0.583912	0.475803	0.709	no
ni|645903678|nb|KK920752.1|	514688-514944	nan	nan	40	45	0.150559	0.89295	no
ni|645903678|nb|KK920752.1|	562068-562670	nan	nan	0	1	inf	0.0003	yes
ni|645903678|nb|KK920752.1|	591134-591287	nan	nan	51	45	-0.177163	0.88225	no
ni|645903678|nb|KK920752.1|	591783-591939	nan	nan	24	29	0.292346	0.7966	no
ni|645903678|nb|KK920752.1|	596424-596616	nan	nan	42	45	0.11841	0.9199	no
ni|645903678|nb|KK920752.1|	598386-598558	nan	nan	1	11	3	0.05625	no
ni|645903678|nb|KK920752.1|	598961-599085	nan	nan	44	40	-0.136068	0.9092	no
ni|645903678|nb|KK920752.1|	599532-599855	nan	nan	20	22	0.187833	0.8685	no
ni|645903678|nb|KK920752.1|	601741-601905	nan	nan	41	49	0.265762	0.8226	no
ni|645903678|nb|KK920752.1|	601977-602107	nan	nan	30	37	0.27784	0.813	no
ni|645903678|nb|KK920752.1|	602929-603055	nan	nan	61	30	-1	0.3946	no
ni|645903678|nb|KK920752.1|	603144-603303	nan	nan	36	29	-0.34372	0.77675	no
ni|645903678|nb|KK920752.1|	603502-603825	nan	nan	15	9	-0.67739	0.56235	no
ni|645903678|nb|KK920752.1|	604404-604517	nan	nan	31	21	-0.570047	0.66295	no
ni|645903678|nb|KK920752.1|	604609-604747	nan	nan	29	28	-0.0672382	0.95405	no
ni|645903678|nb|KK920752.1|	604840-605053	nan	nan	31	50	0.696985	0.5433	no
ni|645903678|nb|KK920752.1|	605473-606314	nan	nan	0.967567	0.733734	-0.399104	0.74405	no
ni|645903678|nb|KK920752.1|	607032-607645	nan	nan	13	18	0.452257	0.6868	no
ni|645903678|nb|KK920752.1|	608179-608416	nan	nan	1	2	0.585738	0.65495	no
ni|645903678|nb|KK920752.1|	608969-609384	nan	nan	1	0.978974	-0.065308	0.9554	no
ni|645903678|nb|KK920752.1|	615127-615249	nan	nan	14	15	0.0395362	0.95995	no
ni|645903678|nb|KK920752.1|	615412-615561	nan	nan	30	50	0.750599	0.52235	no
ni|645903678|nb|KK920752.1|	619468-619702	nan	nan	29	51	0.831739	0.47305	no
ni|645903678|nb|KK920752.1|	622729-622967	nan	nan	2	2	0.085678	0.9436	no
ni|645903678|nb|KK920752.1|	623123-623607	nan	nan	1	1	-0.132263	0.909	no

ni|645903678|nb|KK920752.1|	623719-624191	nan	nan	0.933208	0.318567	-2	0.29725	no
ni|645903678|nb|KK920752.1|	624477-624773	nan	nan	4	3	-0.413734	0.7409	no
ni|645903678|nb|KK920752.1|	624954-625075	nan	nan	40	30	-0.441787	0.7057	no
ni|645903678|nb|KK920752.1|	625519-626066	nan	nan	24	24	-0.000472331	0.9985	no
ni|645903678|nb|KK920752.1|	6812-7165	nan	nan	0	12	inf	5.00E-05	yes
ni|645903678|nb|KK920752.1|	7611-7822	nan	nan	0	14	inf	5.00E-05	yes
ni|645903678|nb|KK920752.1|	8040-8177	nan	nan	0	80	inf	5.00E-05	yes
ni|645903679|nb|KK920751.1|	128460-128690	nan	nan	3	3	0.0330955	0.96125	no
ni|645903679|nb|KK920751.1|	184568-184772	nan	nan	2	3	0.196138	0.87955	no
ni|645903679|nb|KK920751.1|	19176-19356	nan	nan	42	36	-0.191855	0.8643	no
ni|645903679|nb|KK920751.1|	222676-222886	nan	nan	4	5	0.144731	0.90485	no
ni|645903679|nb|KK920751.1|	23445-24196	nan	nan	7	7	0.0757192	0.9473	no
ni|645903679|nb|KK920751.1|	24338-24692	nan	nan	14	10	-0.480553	0.6767	no
ni|645903679|nb|KK920751.1|	25624-25765	nan	nan	33	32	-0.0407499	0.972	no
ni|645903679|nb|KK920751.1|	256517-256582	nan	nan	22581.6	10666.9	-1	0.44525	no
ni|645903679|nb|KK920751.1|	28692-29075	nan	nan	9	9	0.0322079	0.978	no
ni|645903679|nb|KK920751.1|	303172-303489	nan	nan	12	11	-0.175665	0.88275	no
ni|645903679|nb|KK920751.1|	312701-312825	nan	nan	18	14	-0.429925	0.73195	no
ni|645903679|nb|KK920751.1|	31286-31523	nan	nan	37	31	-0.286387	0.8057	no
ni|645903679|nb|KK920751.1|	314987-315240	nan	nan	37	34	-0.123993	0.9125	no
ni|645903679|nb|KK920751.1|	31633-33258	nan	nan	1	0.727505	-0.902128	0.44715	no
ni|645903679|nb|KK920751.1|	316802-317157	nan	nan	12	8	-0.569984	0.62335	no
ni|645903679|nb|KK920751.1|	317390-317842	nan	nan	1	0.471462	-2	0.2821	no
ni|645903679|nb|KK920751.1|	318062-318284	nan	nan	3	0.195763	-4	0.0255	no
ni|645903679|nb|KK920751.1|	318454-319730	nan	nan	1	1	-0.309592	0.7918	no
ni|645903679|nb|KK920751.1|	319837-320308	nan	nan	1	0.830518	-0.271627	0.8304	no
ni|645903679|nb|KK920751.1|	320429-320760	nan	nan	1	1	-0.203125	0.87975	no
ni|645903679|nb|KK920751.1|	321059-321256	nan	nan	3	2	-0.482081	0.7301	no
ni|645903679|nb|KK920751.1|	322280-322583	nan	nan	2	2	0.140681	0.90865	no
ni|645903679|nb|KK920751.1|	323713-324097	nan	nan	1	1	0.141051	0.90865	no

ni|645903679|nb|KK920751.1|	325092-325581	nan	nan	25	24	-0.043224	0.9697	no
ni|645903679|nb|KK920751.1|	326764-327235	nan	nan	0.601545	1	0.764899	0.5642	no
ni|645903679|nb|KK920751.1|	327767-328639	nan	nan	31	28	-0.154377	0.9207	no
ni|645903679|nb|KK920751.1|	328700-328877	nan	nan	64	49	-0.387415	0.73295	no
ni|645903679|nb|KK920751.1|	334757-334971	nan	nan	2444.37	1912.17	-0.35425	0.8038	no
ni|645903679|nb|KK920751.1|	335272-335494	nan	nan	2758.58	2364.82	-0.222196	0.8779	no
ni|645903679|nb|KK920751.1|	336637-337482	nan	nan	125	107.5	-0.216515	0.8783	no
ni|645903679|nb|KK920751.1|	33680-33830	nan	nan	39	40	0.0397197	0.97185	no
ni|645903679|nb|KK920751.1|	338349-338854	nan	nan	766	679	-0.174055	0.9024	no
ni|645903679|nb|KK920751.1|	339366-339498	nan	nan	67	68	0.0289367	0.9813	no
ni|645903679|nb|KK920751.1|	341014-341127	nan	nan	52	34	-0.6036	0.61745	no
ni|645903679|nb|KK920751.1|	345747-345878	nan	nan	38	35	-0.128687	0.9116	no
ni|645903679|nb|KK920751.1|	346559-346962	nan	nan	2	1	-0.678318	0.5787	no
ni|645903679|nb|KK920751.1|	348166-348357	nan	nan	4	3	-0.552593	0.68145	no
ni|645903679|nb|KK920751.1|	348889-351193	nan	nan	31	27	-0.204227	0.8807	no
ni|645903679|nb|KK920751.1|	355151-355435	nan	nan	2	3	0.168575	0.89635	no
ni|645903679|nb|KK920751.1|	357679-358057	nan	nan	6	6	0.160139	0.89055	no
ni|645903679|nb|KK920751.1|	358511-358711	nan	nan	2	0.956795	-0.874336	0.5788	no
ni|645903679|nb|KK920751.1|	359047-359639	nan	nan	0.503301	0.384913	-0.386887	0.78725	no
ni|645903679|nb|KK920751.1|	361228-364137	nan	nan	3	2	-0.467632	0.6717	no
ni|645903679|nb|KK920751.1|	364465-364840	nan	nan	5	3	-0.769039	0.5174	no
ni|645903679|nb|KK920751.1|	365675-365850	nan	nan	4	2	-0.960401	0.51205	no
ni|645903679|nb|KK920751.1|	366127-367016	nan	nan	2	1	-0.33767	0.77685	no
ni|645903679|nb|KK920751.1|	368406-368964	nan	nan	0	0.827221	inf	0.00175	yes
ni|645903679|nb|KK920751.1|	369428-369673	nan	nan	122	138	0.175243	0.886	no
ni|645903679|nb|KK920751.1|	371530-371640	nan	nan	272	333	0.294914	0.7972	no
ni|645903679|nb|KK920751.1|	372871-373054	nan	nan	160	191.48	0.263362	0.80755	no
ni|645903679|nb|KK920751.1|	377928-378031	nan	nan	129	175	0.445938	0.70825	no
ni|645903679|nb|KK920751.1|	379583-379690	nan	nan	91	164	0.855536	0.4677	no
ni|645903679|nb|KK920751.1|	381047-381951	nan	nan	3	3	0.20581	0.8601	no

ni|645903679|nb|KK920751.1|	383024-383365	nan	nan	1	0.887234	-0.35508	0.7931	no
ni|645903679|nb|KK920751.1|	385053-385696	nan	nan	58	61	0.0731361	0.9574	no
ni|645903679|nb|KK920751.1|	386970-387601	nan	nan	51	57	0.155619	0.91485	no
ni|645903679|nb|KK920751.1|	389239-389357	nan	nan	82	30	-1	0.23215	no
ni|645903679|nb|KK920751.1|	389502-391334	nan	nan	62	240	2	0.20025	no
ni|645903679|nb|KK920751.1|	395580-396385	nan	nan	14	15	0.0397044	0.9702	no
ni|645903679|nb|KK920751.1|	39761-40035	nan	nan	13	21	0.683628	0.5512	no
ni|645903679|nb|KK920751.1|	400133-400349	nan	nan	2	3	0.840225	0.5399	no
ni|645903679|nb|KK920751.1|	400920-401211	nan	nan	93	148	0.671528	0.5861	no
ni|645903679|nb|KK920751.1|	403331-403699	nan	nan	95	162	0.772337	0.59565	no
ni|645903679|nb|KK920751.1|	406099-406883	nan	nan	0.325389	0.449384	0.465783	0.7261	no
ni|645903679|nb|KK920751.1|	410320-411012	nan	nan	0.334361	10	5	0.01465	no
ni|645903679|nb|KK920751.1|	443313-443647	nan	nan	6	4	-0.753663	0.5204	no
ni|645903679|nb|KK920751.1|	481456-482623	nan	nan	0.856482	1	0.249593	0.83245	no
ni|645903679|nb|KK920751.1|	48429-48579	nan	nan	34	42	0.300013	0.79435	no
ni|645903679|nb|KK920751.1|	490583-490737	nan	nan	4	15	2	0.1853	no
ni|645903679|nb|KK920751.1|	491271-492923	nan	nan	3	8	2	0.13055	no
ni|645903679|nb|KK920751.1|	493005-493124	nan	nan	9	13	0.584657	0.6992	no
ni|645903679|nb|KK920751.1|	493613-494049	nan	nan	0.443045	0.423452	-0.0652553	0.923	no
ni|645903679|nb|KK920751.1|	495630-495815	nan	nan	76	68	-0.159991	0.88395	no
ni|645903679|nb|KK920751.1|	49595-49823	nan	nan	4	3	-0.43844	0.72705	no
ni|645903679|nb|KK920751.1|	496099-496652	nan	nan	66	70	0.0867397	0.9506	no
ni|645903679|nb|KK920751.1|	498127-498690	nan	nan	3	4	0.413648	0.7258	no
ni|645903679|nb|KK920751.1|	51860-52433	nan	nan	14	18	0.407205	0.7188	no
ni|645903679|nb|KK920751.1|	52786-53150	nan	nan	31	44	0.482925	0.65975	no
ni|645903679|nb|KK920751.1|	528297-528493	nan	nan	3	0.99689	-1	0.35155	no
ni|645903679|nb|KK920751.1|	53766-54241	nan	nan	3	2	-0.524599	0.65835	no
ni|645903679|nb|KK920751.1|	538957-540952	nan	nan	1	0.622665	-1	0.3905	no
ni|645903679|nb|KK920751.1|	541008-541344	nan	nan	1	0.704785	-0.958682	0.51225	no
ni|645903679|nb|KK920751.1|	542351-542602	nan	nan	17	10	-0.779288	0.5088	no

ni|645903679|nb|KK920751.1|	542797-542908	nan	nan	57	34	-0.735038	0.55455	no
ni|645903679|nb|KK920751.1|	543205-543429	nan	nan	33	34	0.0428768	0.96995	no
ni|645903679|nb|KK920751.1|	544623-544725	nan	nan	56	46	-0.291546	0.8496	no
ni|645903679|nb|KK920751.1|	545155-546016	nan	nan	14	12	-0.251549	0.8151	no
ni|645903679|nb|KK920751.1|	54575-55075	nan	nan	20	27	0.402388	0.7151	no
ni|645903679|nb|KK920751.1|	548315-549004	nan	nan	18	18	-0.0382956	0.97225	no
ni|645903679|nb|KK920751.1|	549122-549231	nan	nan	20	29	0.517255	0.7004	no
ni|645903679|nb|KK920751.1|	550065-550361	nan	nan	33	38	0.200027	0.8522	no
ni|645903679|nb|KK920751.1|	551354-551811	nan	nan	23	13	-0.802174	0.4749	no
ni|645903679|nb|KK920751.1|	55674-56119	nan	nan	2	3	0.635205	0.59635	no
ni|645903679|nb|KK920751.1|	56757-57011	nan	nan	3	2	-0.568639	0.66295	no
ni|645903679|nb|KK920751.1|	57185-57762	nan	nan	5	5	-0.019179	0.98605	no
ni|645903679|nb|KK920751.1|	58303-58488	nan	nan	53	43	-0.309714	0.7893	no
ni|645903679|nb|KK920751.1|	600376-600591	nan	nan	45	55	0.292757	0.79765	no
ni|645903679|nb|KK920751.1|	621178-621457	nan	nan	46	55	0.251609	0.8197	no
ni|645903679|nb|KK920751.1|	63149-63420	nan	nan	417	548	0.396102	0.77985	no
ni|645903679|nb|KK920751.1|	63522-63749	nan	nan	231	313	0.440428	0.75765	no
ni|645903679|nb|KK920751.1|	66249-66545	nan	nan	219	283	0.373967	0.79595	no
ni|645903679|nb|KK920751.1|	67318-67647	nan	nan	17	10	-0.716395	0.5392	no
ni|645903679|nb|KK920751.1|	68902-69254	nan	nan	2	0.471261	-2	0.1999	no
ni|645903679|nb|KK920751.1|	69353-69547	nan	nan	2	0.763658	-2	0.2989	no
ni|645903679|nb|KK920751.1|	70348-70628	nan	nan	1	0.132256	-3	0.04325	no
ni|645903680|nb|KK920750.1|	1000837-1001054	nan	nan	14	14	-0.0449535	0.9682	no
ni|645903680|nb|KK920750.1|	1002957-1003457	nan	nan	0.805586	0.3554	-1	0.42295	no
ni|645903680|nb|KK920750.1|	1003667-1003939	nan	nan	39	32	-0.309906	0.78535	no
ni|645903680|nb|KK920750.1|	100418-101727	nan	nan	10	12	0.192747	0.8551	no
ni|645903680|nb|KK920750.1|	1006792-1007064	nan	nan	67	62	-0.100822	0.9264	no
ni|645903680|nb|KK920750.1|	1007742-1008436	nan	nan	36	33	-0.115747	0.93955	no
ni|645903680|nb|KK920750.1|	1010064-1010803	nan	nan	20	15	-0.431702	0.69195	no
ni|645903680|nb|KK920750.1|	1011532-1011748	nan	nan	2	0.411956	-2	0.3383	no

ni|645903680|nb|KK920750.1|	1020910-1021544	nan	nan	0.370836	2	2	0.11095	no
ni|645903680|nb|KK920750.1|	1023442-1025107	nan	nan	15	28	0.900474	0.53515	no
ni|645903680|nb|KK920750.1|	1025382-1026266	nan	nan	23	28	0.288638	0.84755	no
ni|645903680|nb|KK920750.1|	1026691-1026832	nan	nan	33	25	-0.38915	0.74185	no
ni|645903680|nb|KK920750.1|	1026958-1027078	nan	nan	29	14	-1	0.3992	no
ni|645903680|nb|KK920750.1|	1027551-1027704	nan	nan	29	16	-0.828002	0.48695	no
ni|645903680|nb|KK920750.1|	105114-105269	nan	nan	8	8	0.0152678	0.9674	no
ni|645903680|nb|KK920750.1|	122493-122665	nan	nan	25	58	1	0.3007	no
ni|645903680|nb|KK920750.1|	123248-123383	nan	nan	39	57	0.534779	0.6473	no
ni|645903680|nb|KK920750.1|	132504-132679	nan	nan	26	44	0.784839	0.50595	no
ni|645903680|nb|KK920750.1|	137506-137628	nan	nan	120.99	186.85	0.626995	0.59225	no
ni|645903680|nb|KK920750.1|	143977-144102	nan	nan	76	123.6	0.702536	0.5467	no
ni|645903680|nb|KK920750.1|	152424-152628	nan	nan	36	72	0.999338	0.388	no
ni|645903680|nb|KK920750.1|	158913-159126	nan	nan	39	75	0.941457	0.41005	no
ni|645903680|nb|KK920750.1|	161137-161348	nan	nan	46	92	0.99158	0.37875	no
ni|645903680|nb|KK920750.1|	161456-161581	nan	nan	83	171	1	0.37105	no
ni|645903680|nb|KK920750.1|	161997-162140	nan	nan	54	86	0.663603	0.57125	no
ni|645903680|nb|KK920750.1|	163954-164129	nan	nan	48	92	0.942668	0.4101	no
ni|645903680|nb|KK920750.1|	165713-166338	nan	nan	0.565832	0.721251	0.350127	0.7835	no
ni|645903680|nb|KK920750.1|	166502-166755	nan	nan	49	102	1	0.3414	no
ni|645903680|nb|KK920750.1|	169207-169421	nan	nan	52	98	0.914922	0.4084	no
ni|645903680|nb|KK920750.1|	170203-170388	nan	nan	45	85	0.918531	0.42495	no
ni|645903680|nb|KK920750.1|	171174-171475	nan	nan	20	32	0.687425	0.55105	no
ni|645903680|nb|KK920750.1|	172837-173474	nan	nan	9	19	1	0.35605	no
ni|645903680|nb|KK920750.1|	173795-173937	nan	nan	39	64	0.708569	0.5467	no
ni|645903680|nb|KK920750.1|	174800-175449	nan	nan	0.405744	0.172401	-1	0.4023	no
ni|645903680|nb|KK920750.1|	178136-178275	nan	nan	48	95	0.979461	0.39795	no
ni|645903680|nb|KK920750.1|	180593-180763	nan	nan	70	126	0.838156	0.46255	no
ni|645903680|nb|KK920750.1|	180986-181204	nan	nan	53	96	0.862109	0.44155	no
ni|645903680|nb|KK920750.1|	182321-182519	nan	nan	53	101	0.936475	0.4089	no

ni|645903680|nb|KK920750.1|	183026-183199	nan	nan	56	98	0.812791	0.47895	no
ni|645903680|nb|KK920750.1|	186820-187031	nan	nan	54	94	0.795144	0.48025	no
ni|645903680|nb|KK920750.1|	187392-187623	nan	nan	52	89	0.777456	0.488	no
ni|645903680|nb|KK920750.1|	191665-191837	nan	nan	51	100	0.961857	0.3986	no
ni|645903680|nb|KK920750.1|	192721-193167	nan	nan	21	31	0.587293	0.58505	no
ni|645903680|nb|KK920750.1|	193350-193724	nan	nan	0.997276	0.606489	-0.717512	0.61925	no
ni|645903680|nb|KK920750.1|	194222-194797	nan	nan	0.782097	0.797493	0.0281235	0.9668	no
ni|645903680|nb|KK920750.1|	194902-195210	nan	nan	2	1	-0.288133	0.8283	no
ni|645903680|nb|KK920750.1|	196609-196871	nan	nan	89	131	0.55505	0.6592	no
ni|645903680|nb|KK920750.1|	197465-197737	nan	nan	80	123	0.618923	0.62755	no
ni|645903680|nb|KK920750.1|	197892-198074	nan	nan	67	115	0.769734	0.50005	no
ni|645903680|nb|KK920750.1|	198165-198325	nan	nan	89	126	0.501866	0.65175	no
ni|645903680|nb|KK920750.1|	198500-198917	nan	nan	0.704636	0.74828	0.0867004	0.9436	no
ni|645903680|nb|KK920750.1|	199169-199795	nan	nan	10	18	0.78825	0.48795	no
ni|645903680|nb|KK920750.1|	201148-201299	nan	nan	61	83	0.442756	0.7052	no
ni|645903680|nb|KK920750.1|	201666-201924	nan	nan	69	90	0.386412	0.7131	no
ni|645903680|nb|KK920750.1|	204157-204386	nan	nan	61	78	0.371446	0.73625	no
ni|645903680|nb|KK920750.1|	204497-204650	nan	nan	59	39	-0.590031	0.61615	no
ni|645903680|nb|KK920750.1|	204923-205285	nan	nan	35	26	-0.416275	0.71135	no
ni|645903680|nb|KK920750.1|	206904-207126	nan	nan	2	12	3	0.0529	no
ni|645903680|nb|KK920750.1|	24487-25087	nan	nan	1	3	1	0.3272	no
ni|645903680|nb|KK920750.1|	244924-245104	nan	nan	12	5	-1	0.34355	no
ni|645903680|nb|KK920750.1|	245252-245473	nan	nan	9	6	-0.558437	0.6396	no
ni|645903680|nb|KK920750.1|	252381-253295	nan	nan	0.030364	0.464533	4	0.12575	no
ni|645903680|nb|KK920750.1|	26033-26188	nan	nan	3	4	0.295376	0.8383	no
ni|645903680|nb|KK920750.1|	26873-27049	nan	nan	3	5	0.517638	0.6956	no
ni|645903680|nb|KK920750.1|	278184-278622	nan	nan	0.807416	0.420933	-0.93972	0.54025	no
ni|645903680|nb|KK920750.1|	278839-279506	nan	nan	0.654684	0.208635	-2	0.24455	no
ni|645903680|nb|KK920750.1|	279797-280166	nan	nan	19	19	-0.0165449	0.988	no
ni|645903680|nb|KK920750.1|	290297-290824	nan	nan	0.580308	0.499245	-0.21707	0.87705	no

ni|645903680|nb|KK920750.1|	29374-29632	nan	nan	4	6	0.575446	0.63065	no
ni|645903680|nb|KK920750.1|	297732-297845	nan	nan	23	7	-2	0.22995	no
ni|645903680|nb|KK920750.1|	305154-305344	nan	nan	7	5	-0.330216	0.78525	no
ni|645903680|nb|KK920750.1|	33141-33708	nan	nan	2	3	0.533639	0.65285	no
ni|645903680|nb|KK920750.1|	367411-367549	nan	nan	66	84	0.337152	0.77225	no
ni|645903680|nb|KK920750.1|	367768-367894	nan	nan	33	42	0.357972	0.76065	no
ni|645903680|nb|KK920750.1|	369641-370222	nan	nan	9	16	0.809493	0.4713	no
ni|645903680|nb|KK920750.1|	370588-370758	nan	nan	57	86	0.600494	0.58995	no
ni|645903680|nb|KK920750.1|	371025-371215	nan	nan	17	26	0.563584	0.6267	no
ni|645903680|nb|KK920750.1|	371445-372037	nan	nan	0.956271	1	0.330969	0.79	no
ni|645903680|nb|KK920750.1|	372384-372538	nan	nan	34	43	0.323267	0.7803	no
ni|645903680|nb|KK920750.1|	376370-376535	nan	nan	47	76	0.714349	0.53165	no
ni|645903680|nb|KK920750.1|	376896-377066	nan	nan	60	76	0.356382	0.7521	no
ni|645903680|nb|KK920750.1|	381278-381742	nan	nan	16	11	-0.500929	0.66965	no
ni|645903680|nb|KK920750.1|	384730-384878	nan	nan	178	126	-0.50058	0.65885	no
ni|645903680|nb|KK920750.1|	385407-385618	nan	nan	126	91	-0.470611	0.67165	no
ni|645903680|nb|KK920750.1|	387417-387612	nan	nan	146	77	-0.918735	0.40225	no
ni|645903680|nb|KK920750.1|	388066-388215	nan	nan	117	66	-0.814655	0.47715	no
ni|645903680|nb|KK920750.1|	390055-390289	nan	nan	116	59	-0.98083	0.36785	no
ni|645903680|nb|KK920750.1|	392354-392575	nan	nan	53	31	-0.777588	0.49075	no
ni|645903680|nb|KK920750.1|	39312-39512	nan	nan	46	36	-0.363513	0.7498	no
ni|645903680|nb|KK920750.1|	393999-395838	nan	nan	0.113528	0.38001	2	0.19855	no
ni|645903680|nb|KK920750.1|	39710-40243	nan	nan	19	18	-0.126645	0.90905	no
ni|645903680|nb|KK920750.1|	40510-40641	nan	nan	51	56	0.142167	0.9029	no
ni|645903680|nb|KK920750.1|	407066-407646	nan	nan	6	4	-0.656172	0.5715	no
ni|645903680|nb|KK920750.1|	407839-408289	nan	nan	7	6	-0.304895	0.79315	no
ni|645903680|nb|KK920750.1|	40836-41022	nan	nan	40	40	0.0252048	0.982	no
ni|645903680|nb|KK920750.1|	408791-410605	nan	nan	14	11	-0.240248	0.87185	no
ni|645903680|nb|KK920750.1|	410750-411154	nan	nan	2	1	-0.5889	0.62565	no
ni|645903680|nb|KK920750.1|	41435-41634	nan	nan	31	33	0.0790761	0.94175	no

ni|645903680|nb|KK920750.1|	416130-416274	nan	nan	18	13	-0.482246	0.68515	no
ni|645903680|nb|KK920750.1|	41780-41946	nan	nan	33	40	0.271182	0.8182	no
ni|645903680|nb|KK920750.1|	42076-43806	nan	nan	9	9	0.0219178	0.9835	no
ni|645903680|nb|KK920750.1|	437784-437938	nan	nan	132.95	82	-0.690223	0.5391	no
ni|645903680|nb|KK920750.1|	438479-438553	nan	nan	60650.1	32651.4	-0.893363	0.45015	no
ni|645903680|nb|KK920750.1|	441314-443132	nan	nan	0.258564	1	2	0.0836	no
ni|645903680|nb|KK920750.1|	44350-44739	nan	nan	2	2	-0.196627	0.8673	no
ni|645903680|nb|KK920750.1|	44943-46213	nan	nan	4	4	-0.0721348	0.94985	no
ni|645903680|nb|KK920750.1|	46585-46818	nan	nan	30	35	0.186212	0.8688	no
ni|645903680|nb|KK920750.1|	467276-468129	nan	nan	3	4	0.564342	0.6337	no
ni|645903680|nb|KK920750.1|	468277-469180	nan	nan	4	5	0.0832034	0.9399	no
ni|645903680|nb|KK920750.1|	498895-500513	nan	nan	0.422765	0.093302	-2	0.14835	no
ni|645903680|nb|KK920750.1|	515917-516208	nan	nan	2	1	-0.650806	0.6173	no
ni|645903680|nb|KK920750.1|	520863-521141	nan	nan	22	20	-0.13089	0.91215	no
ni|645903680|nb|KK920750.1|	522147-522497	nan	nan	9	7	-0.430297	0.71425	no
ni|645903680|nb|KK920750.1|	524691-524824	nan	nan	16	11	-0.526703	0.67145	no
ni|645903680|nb|KK920750.1|	531457-531695	nan	nan	29	18	-0.669322	0.56605	no
ni|645903680|nb|KK920750.1|	532980-533670	nan	nan	3	3	-0.309246	0.79345	no
ni|645903680|nb|KK920750.1|	534025-534131	nan	nan	43	7	-3	0.2671	no
ni|645903680|nb|KK920750.1|	544451-545887	nan	nan	2	1	-1	0.37815	no
ni|645903680|nb|KK920750.1|	546050-546509	nan	nan	53	37	-0.538301	0.66855	no
ni|645903680|nb|KK920750.1|	547341-548194	nan	nan	85	44	-0.94851	0.51185	no
ni|645903680|nb|KK920750.1|	548792-548974	nan	nan	73	51	-0.531239	0.64525	no
ni|645903680|nb|KK920750.1|	556313-556699	nan	nan	1	0.829856	-0.743464	0.56925	no
ni|645903680|nb|KK920750.1|	556973-559181	nan	nan	182	133	-0.453557	0.75545	no
ni|645903680|nb|KK920750.1|	55876-56032	nan	nan	34	58	0.782908	0.50015	no
ni|645903680|nb|KK920750.1|	559884-563522	nan	nan	269	256	-0.0691179	0.95145	no
ni|645903680|nb|KK920750.1|	564311-566783	nan	nan	97	102	0.0625115	0.9646	no
ni|645903680|nb|KK920750.1|	57475-57726	nan	nan	8	11	0.436331	0.70725	no
ni|645903680|nb|KK920750.1|	579314-579530	nan	nan	44	37	-0.248185	0.82445	no

ni|645903680|nb|KK920750.1|	598893-599507	nan	nan	3	3	-0.126327	0.9131	no
ni|645903680|nb|KK920750.1|	600206-600693	nan	nan	2	1	-0.249567	0.84025	no
ni|645903680|nb|KK920750.1|	601168-601764	nan	nan	1	1	-0.399372	0.74405	no
ni|645903680|nb|KK920750.1|	601958-602166	nan	nan	2	2	-0.38874	0.78555	no
ni|645903680|nb|KK920750.1|	602335-602509	nan	nan	4	2	-0.941776	0.54025	no
ni|645903680|nb|KK920750.1|	602726-602922	nan	nan	3	4	0.643428	0.6202	no
ni|645903680|nb|KK920750.1|	604279-605246	nan	nan	2	3	0.652666	0.57935	no
ni|645903680|nb|KK920750.1|	605306-605470	nan	nan	14	11	-0.379166	0.75395	no
ni|645903680|nb|KK920750.1|	605780-606417	nan	nan	2	2	0.0973776	0.93145	no
ni|645903680|nb|KK920750.1|	606636-606911	nan	nan	9	8	-0.0890409	0.93675	no
ni|645903680|nb|KK920750.1|	607092-607276	nan	nan	8	6	-0.552698	0.647	no
ni|645903680|nb|KK920750.1|	611449-611617	nan	nan	50	37	-0.443626	0.69775	no
ni|645903680|nb|KK920750.1|	613497-613996	nan	nan	7	6	-0.35885	0.75935	no
ni|645903680|nb|KK920750.1|	620973-621713	nan	nan	16	12	-0.364333	0.74445	no
ni|645903680|nb|KK920750.1|	621790-621969	nan	nan	31	31	-0.0384648	0.97445	no
ni|645903680|nb|KK920750.1|	62438-62547	nan	nan	48	98	1	0.3968	no
ni|645903680|nb|KK920750.1|	633143-633267	nan	nan	220	263	0.258557	0.81805	no
ni|645903680|nb|KK920750.1|	634866-634988	nan	nan	21	13	-0.730344	0.5717	no
ni|645903680|nb|KK920750.1|	636413-636815	nan	nan	34	24	-0.526073	0.6318	no
ni|645903680|nb|KK920750.1|	637831-637992	nan	nan	54	43	-0.354306	0.7579	no
ni|645903680|nb|KK920750.1|	638280-638653	nan	nan	45	35	-0.352028	0.74755	no
ni|645903680|nb|KK920750.1|	639126-639557	nan	nan	0.674663	0.859763	0.34977	0.792	no
ni|645903680|nb|KK920750.1|	640367-640661	nan	nan	42	29	-0.533602	0.6323	no
ni|645903680|nb|KK920750.1|	640934-642828	nan	nan	0.687768	0.776157	0.174428	0.88185	no
ni|645903680|nb|KK920750.1|	642988-643506	nan	nan	0.948583	0.566713	-0.743155	0.56925	no
ni|645903680|nb|KK920750.1|	64435-64871	nan	nan	5	7	0.438511	0.71255	no
ni|645903680|nb|KK920750.1|	644972-645217	nan	nan	19	15	-0.362853	0.75445	no
ni|645903680|nb|KK920750.1|	645433-645600	nan	nan	93	84	-0.150383	0.89165	no
ni|645903680|nb|KK920750.1|	646989-647111	nan	nan	21	18	-0.227844	0.85555	no
ni|645903680|nb|KK920750.1|	650037-650208	nan	nan	105	85	-0.310055	0.7814	no

ni|645903680|nb|KK920750.1|	65210-65759	nan	nan	9	11	0.29754	0.79925	no
ni|645903680|nb|KK920750.1|	656415-657225	nan	nan	666	691	0.0538061	0.9588	no
ni|645903680|nb|KK920750.1|	657920-658385	nan	nan	644	615	-0.0659514	0.96015	no
ni|645903680|nb|KK920750.1|	658648-659077	nan	nan	582	538	-0.112434	0.9366	no
ni|645903680|nb|KK920750.1|	65889-66039	nan	nan	14	14	-0.0182859	0.97915	no
ni|645903680|nb|KK920750.1|	659487-659764	nan	nan	1417.07	1247.09	-0.184349	0.90125	no
ni|645903680|nb|KK920750.1|	66280-66607	nan	nan	4	6	0.543168	0.6419	no
ni|645903680|nb|KK920750.1|	664146-665052	nan	nan	3	3	-0.164176	0.88135	no
ni|645903680|nb|KK920750.1|	666105-666303	nan	nan	8	7	-0.0670224	0.95395	no
ni|645903680|nb|KK920750.1|	66878-67112	nan	nan	2	2	-0.066381	0.94155	no
ni|645903680|nb|KK920750.1|	670843-671194	nan	nan	0.792218	1	0.741826	0.5765	no
ni|645903680|nb|KK920750.1|	672753-672976	nan	nan	3	2	-0.551978	0.68265	no
ni|645903680|nb|KK920750.1|	673133-673420	nan	nan	2	2	-0.246442	0.84435	no
ni|645903680|nb|KK920750.1|	674092-674350	nan	nan	18	12	-0.564351	0.62755	no
ni|645903680|nb|KK920750.1|	674517-675202	nan	nan	5	6	0.252507	0.82565	no
ni|645903680|nb|KK920750.1|	67469-68135	nan	nan	10	11	0.241247	0.82915	no
ni|645903680|nb|KK920750.1|	676292-676481	nan	nan	24	21	-0.209803	0.85715	no
ni|645903680|nb|KK920750.1|	677544-677903	nan	nan	11	11	-0.00192969	0.9948	no
ni|645903680|nb|KK920750.1|	678597-679329	nan	nan	15	15	0.074825	0.9466	no
ni|645903680|nb|KK920750.1|	679508-680703	nan	nan	15	17	0.178538	0.88535	no
ni|645903680|nb|KK920750.1|	680853-682161	nan	nan	12	14	0.221737	0.8563	no
ni|645903680|nb|KK920750.1|	68552-68705	nan	nan	37	48	0.370496	0.75125	no
ni|645903680|nb|KK920750.1|	68911-69726	nan	nan	300	252	-0.252443	0.8597	no
ni|645903680|nb|KK920750.1|	70084-70352	nan	nan	436	357	-0.287095	0.84035	no
ni|645903680|nb|KK920750.1|	70551-70716	nan	nan	73	35	-1	0.3766	no
ni|645903680|nb|KK920750.1|	75895-76172	nan	nan	14	14	-0.00935875	0.9934	no
ni|645903680|nb|KK920750.1|	797585-798074	nan	nan	0.191189	1205.84	13	0.05055	no
ni|645903680|nb|KK920750.1|	85943-88116	nan	nan	8	11	0.479804	0.6923	no
ni|645903680|nb|KK920750.1|	88333-88659	nan	nan	0.550319	1	1	0.35675	no
ni|645903680|nb|KK920750.1|	90149-90889	nan	nan	1	30	4	0.0076	no

ni|645903680|nb|KK920750.1|	902534-902662	nan	nan	20	18	-0.131444	0.9116	no
ni|645903680|nb|KK920750.1|	91670-91842	nan	nan	9	9	-0.0148219	0.98035	no
ni|645903680|nb|KK920750.1|	92617-93290	nan	nan	3	3	0.305102	0.794	no
ni|645903680|nb|KK920750.1|	93682-93803	nan	nan	8	8	-0.0673914	0.9302	no
ni|645903680|nb|KK920750.1|	93983-94109	nan	nan	16	17	0.0931305	0.93935	no
ni|645903680|nb|KK920750.1|	95320-95453	nan	nan	12	12	0.0201918	0.96555	no
ni|645903680|nb|KK920750.1|	965689-965794	nan	nan	70	63	-0.155574	0.90135	no
ni|645903680|nb|KK920750.1|	96747-97736	nan	nan	0.917243	0.6113	-0.585425	0.62085	no
ni|645903680|nb|KK920750.1|	997012-997321	nan	nan	2	2	0.0338002	0.96125	no
ni|645903680|nb|KK920750.1|	997507-997731	nan	nan	56	56	0.0048502	0.99715	no
ni|645903680|nb|KK920750.1|	99794-100316	nan	nan	0.704661	2	1	0.29125	no
ni|645903680|nb|KK920750.1|	998435-998606	nan	nan	34	32	-0.112603	0.92285	no
ni|645903680|nb|KK920750.1|	998931-999073	nan	nan	57	65	0.193311	0.8665	no
ni|645903680|nb|KK920750.1|	999218-999351	nan	nan	46	43	-0.09073	0.938	no
ni|645903681|nb|KK920749.1|	141582-142096	nan	nan	9	11	0.202531	0.85885	no
ni|645903681|nb|KK920749.1|	142814-142945	nan	nan	2	16	3	0.081	no
ni|645903681|nb|KK920749.1|	143457-143649	nan	nan	3	2	-0.482184	0.7301	no
ni|645903681|nb|KK920749.1|	146596-147318	nan	nan	1	1	0.072712	0.95225	no
ni|645903681|nb|KK920749.1|	148281-148763	nan	nan	1	0.992604	-0.152616	0.90135	no
ni|645903681|nb|KK920749.1|	149408-149970	nan	nan	1	0.973784	-0.402042	0.73545	no
ni|645903681|nb|KK920749.1|	150108-150349	nan	nan	27	28	0.03263	0.9768	no
ni|645903681|nb|KK920749.1|	150547-150841	nan	nan	2	1	-0.480861	0.7105	no
ni|645903681|nb|KK920749.1|	151046-153056	nan	nan	43	58	0.450447	0.74555	no
ni|645903681|nb|KK920749.1|	157590-157921	nan	nan	0	1510.03	inf	5.00E-05	yes
ni|645903681|nb|KK920749.1|	158961-159326	nan	nan	0	228	inf	5.00E-05	yes
ni|645903681|nb|KK920749.1|	160535-160880	nan	nan	0	364	inf	5.00E-05	yes
ni|645903681|nb|KK920749.1|	163404-163690	nan	nan	0	727.57	inf	5.00E-05	yes
ni|645903681|nb|KK920749.1|	167395-167524	nan	nan	35	11	-2	0.1946	no
ni|645903681|nb|KK920749.1|	167688-167826	nan	nan	85	58	-0.546072	0.6416	no
ni|645903681|nb|KK920749.1|	168624-168814	nan	nan	45	35	-0.35175	0.76245	no

ni|645903681|nb|KK920749.1|	169063-169571	nan	nan	37	34	-0.149777	0.8874	no
ni|645903681|nb|KK920749.1|	170141-170747	nan	nan	0.489313	0.187111	-1	0.35155	no
ni|645903681|nb|KK920749.1|	171140-171309	nan	nan	64	60	-0.0838425	0.9411	no
ni|645903681|nb|KK920749.1|	172410-172739	nan	nan	2	1	-0.888753	0.48225	no
ni|645903681|nb|KK920749.1|	173398-173566	nan	nan	56	50	-0.155907	0.8898	no
ni|645903681|nb|KK920749.1|	173889-174040	nan	nan	42	41	-0.0671936	0.95475	no
ni|645903681|nb|KK920749.1|	174463-174756	nan	nan	30	28	-0.109457	0.92195	no
ni|645903681|nb|KK920749.1|	175165-175385	nan	nan	2	2	-0.204104	0.87975	no
ni|645903681|nb|KK920749.1|	175634-176038	nan	nan	13	13	-0.0125506	0.99005	no
ni|645903681|nb|KK920749.1|	176341-177100	nan	nan	1	0.753552	-0.579327	0.63675	no
ni|645903681|nb|KK920749.1|	177691-178281	nan	nan	7	6	-0.24696	0.82715	no
ni|645903681|nb|KK920749.1|	180217-181207	nan	nan	7	9	0.238985	0.8279	no
ni|645903681|nb|KK920749.1|	181530-181734	nan	nan	20	358.25	4	0.0078	no
ni|645903681|nb|KK920749.1|	191104-191287	nan	nan	17	247	4	0.01515	no
ni|645903681|nb|KK920749.1|	196642-196776	nan	nan	4	54	4	0.04225	no
ni|645903681|nb|KK920749.1|	204436-204573	nan	nan	26	18	-0.545291	0.64545	no
ni|645903681|nb|KK920749.1|	204983-205121	nan	nan	26	42	0.698297	0.5491	no
ni|645903681|nb|KK920749.1|	205477-205682	nan	nan	48	48	-0.0248516	0.98185	no
ni|645903681|nb|KK920749.1|	207070-207309	nan	nan	49	52	0.109538	0.921	no
ni|645903681|nb|KK920749.1|	209462-209707	nan	nan	61.3	58	-0.0825374	0.9418	no
ni|645903681|nb|KK920749.1|	211043-211326	nan	nan	21	20	-0.0564376	0.96285	no
ni|645903681|nb|KK920749.1|	212447-212576	nan	nan	52	54	0.0445885	0.96935	no
ni|645903681|nb|KK920749.1|	212805-212943	nan	nan	56	52	-0.101185	0.9356	no
ni|645903681|nb|KK920749.1|	213959-214091	nan	nan	55	60	0.140526	0.9069	no
ni|645903681|nb|KK920749.1|	215018-215515	nan	nan	13	14	0.147327	0.894	no
ni|645903681|nb|KK920749.1|	216849-217555	nan	nan	9	10	0.242281	0.8302	no
ni|645903681|nb|KK920749.1|	219540-220252	nan	nan	0.121272	0.618376	2	0.1097	no
ni|645903681|nb|KK920749.1|	221230-221606	nan	nan	0.18	1	3	0.06205	no
ni|645903681|nb|KK920749.1|	223549-224312	nan	nan	129	153	0.248316	0.85675	no
ni|645903681|nb|KK920749.1|	224491-225304	nan	nan	528	623	0.236699	0.84705	no

ni|645903681|nb|KK920749.1|	225805-226001	nan	nan	244	188	-0.374378	0.80015	no
ni|645903681|nb|KK920749.1|	226262-226421	nan	nan	23	17	-0.464538	0.698	no
ni|645903681|nb|KK920749.1|	226586-226866	nan	nan	2	2	-0.288312	0.8283	no
ni|645903681|nb|KK920749.1|	227353-227561	nan	nan	218	181	-0.269368	0.8581	no
ni|645903681|nb|KK920749.1|	229726-230539	nan	nan	34	32	-0.0735669	0.9606	no
ni|645903681|nb|KK920749.1|	232985-233688	nan	nan	36	38	0.0644235	0.9661	no
ni|645903681|nb|KK920749.1|	235412-235771	nan	nan	1	0.275069	-2	0.24815	no
ni|645903681|nb|KK920749.1|	236097-236917	nan	nan	6	5	-0.296744	0.7959	no
ni|645903681|nb|KK920749.1|	237029-238845	nan	nan	8	7	-0.213117	0.8446	no
ni|645903681|nb|KK920749.1|	249239-249626	nan	nan	0	3	inf	0.0001	yes
ni|645903681|nb|KK920749.1|	250293-250734	nan	nan	6	8	0.333305	0.77045	no
ni|645903681|nb|KK920749.1|	250988-251519	nan	nan	5	5	0.127585	0.9153	no
ni|645903681|nb|KK920749.1|	251674-252175	nan	nan	0.741754	1	0.671851	0.59755	no
ni|645903681|nb|KK920749.1|	252860-253057	nan	nan	11	9	-0.289436	0.80025	no
ni|645903681|nb|KK920749.1|	254358-254586	nan	nan	34	27	-0.311275	0.78385	no
ni|645903681|nb|KK920749.1|	255137-256101	nan	nan	11	9	-0.225071	0.8363	no
ni|645903681|nb|KK920749.1|	258015-258201	nan	nan	41	47	0.202598	0.85995	no
ni|645903681|nb|KK920749.1|	259254-259360	nan	nan	53	54	0.0251502	0.9668	no
ni|645903681|nb|KK920749.1|	259613-259800	nan	nan	37	38	0.0404858	0.9716	no
ni|645903681|nb|KK920749.1|	259998-260101	nan	nan	171	123	-0.483672	0.69415	no
ni|645903681|nb|KK920749.1|	468919-469101	nan	nan	19	18	-0.0907504	0.938	no
ni|645903681|nb|KK920749.1|	470807-470943	nan	nan	12	13	0.167215	0.89635	no
ni|645903681|nb|KK920749.1|	505796-506157	nan	nan	7	10	0.439306	0.7028	no
ni|645903681|nb|KK920749.1|	506265-506511	nan	nan	27	28	0.0400346	0.9709	no
ni|645903681|nb|KK920749.1|	506622-506973	nan	nan	3	2	-0.785421	0.5276	no
ni|645903681|nb|KK920749.1|	508312-508494	nan	nan	16	18	0.163006	0.88935	no
ni|645903681|nb|KK920749.1|	509299-509453	nan	nan	19	23	0.275695	0.81255	no
ni|645903681|nb|KK920749.1|	509593-509748	nan	nan	22	27	0.272292	0.81505	no
ni|645903681|nb|KK920749.1|	510497-510741	nan	nan	18	21	0.194517	0.86255	no
ni|645903681|nb|KK920749.1|	511059-512282	nan	nan	21	20	-0.035897	0.981	no

ni|645903681|nb|KK920749.1|	513191-513713	nan	nan	30	33	0.102685	0.92615	no
ni|645903681|nb|KK920749.1|	513821-514095	nan	nan	23	18	-0.352488	0.7575	no
ni|645903681|nb|KK920749.1|	514246-514362	nan	nan	43	31	-0.466022	0.70195	no
ni|645903681|nb|KK920749.1|	514647-514808	nan	nan	24	13	-0.949851	0.41825	no
ni|645903681|nb|KK920749.1|	520209-521217	nan	nan	15	16	0.0960238	0.9271	no
ni|645903681|nb|KK920749.1|	522838-523075	nan	nan	89	94	0.0717021	0.9475	no
ni|645903681|nb|KK920749.1|	524952-525313	nan	nan	4	4	-0.0986535	0.9321	no
ni|645903681|nb|KK920749.1|	530297-530418	nan	nan	94	118	0.329944	0.77045	no
ni|645903681|nb|KK920749.1|	531029-531194	nan	nan	97	111	0.188184	0.8701	no
ni|645903681|nb|KK920749.1|	531348-531468	nan	nan	81	97	0.258823	0.82685	no
ni|645903681|nb|KK920749.1|	532647-532997	nan	nan	100	115	0.212794	0.88665	no
ni|645903681|nb|KK920749.1|	607793-608038	nan	nan	7	12	0.81512	0.49655	no
ni|645903682|nb|KK920748.1|	13092-13646	nan	nan	0	18	inf	5.00E-05	yes
ni|645903682|nb|KK920748.1|	137605-137907	nan	nan	1	2	0.474793	0.709	no
ni|645903682|nb|KK920748.1|	145-650	nan	nan	0.305996	22	6	0.02725	no
ni|645903682|nb|KK920748.1|	1512-1805	nan	nan	0.387118	46	7	0.05055	no
ni|645903682|nb|KK920748.1|	24074-24248	nan	nan	10	8	-0.389235	0.74325	no
ni|645903682|nb|KK920748.1|	25158-25469	nan	nan	2	2	0.140726	0.90865	no
ni|645903682|nb|KK920748.1|	25526-25685	nan	nan	2	4	0.932798	0.5287	no
ni|645903682|nb|KK920748.1|	25929-26256	nan	nan	1	0.837613	-0.387569	0.78555	no
ni|645903682|nb|KK920748.1|	28174-28395	nan	nan	11	8	-0.453607	0.7017	no
ni|645903682|nb|KK920748.1|	40815-41262	nan	nan	3	2	-0.26317	0.82235	no
ni|645903682|nb|KK920748.1|	41399-41614	nan	nan	9	6	-0.552109	0.6355	no
ni|645903682|nb|KK920748.1|	419391-419810	nan	nan	6	5	-0.183942	0.8687	no
ni|645903682|nb|KK920748.1|	421338-421696	nan	nan	3	3	-0.0654989	0.95475	no
ni|645903682|nb|KK920748.1|	42283-42490	nan	nan	12	10	-0.368488	0.75055	no
ni|645903682|nb|KK920748.1|	432245-432537	nan	nan	3	3	-0.197081	0.8673	no
ni|645903682|nb|KK920748.1|	44064-44203	nan	nan	13	14	0.0640119	0.95955	no
ni|645903682|nb|KK920748.1|	44943-45082	nan	nan	11	15	0.347805	0.7814	no
ni|645903682|nb|KK920748.1|	450229-450383	nan	nan	4	2	-1	0.44965	no

ni|645903682|nb|KK920748.1|	452665-452869	nan	nan	26	17	-0.61233	0.60085	no
ni|645903682|nb|KK920748.1|	457753-457985	nan	nan	3	1	-1	0.39005	no
ni|645903682|nb|KK920748.1|	460044-460294	nan	nan	30	25	-0.228067	0.84435	no
ni|645903682|nb|KK920748.1|	462612-463104	nan	nan	0.758873	0	-inf	0.002	yes
ni|645903682|nb|KK920748.1|	463477-463686	nan	nan	5	4	-0.278296	0.82035	no
ni|645903682|nb|KK920748.1|	538903-539080	nan	nan	10	4	-1	0.26825	no
ni|645903682|nb|KK920748.1|	546382-546591	nan	nan	6	3	-0.744864	0.54875	no
ni|645903682|nb|KK920748.1|	553069-553224	nan	nan	9	6	-0.688683	0.58685	no
ni|645903682|nb|KK920748.1|	553425-553893	nan	nan	13	10	-0.264492	0.8184	no
ni|645903682|nb|KK920748.1|	554472-555032	nan	nan	0.700027	0.257378	-1	0.3141	no
ni|645903682|nb|KK920748.1|	556269-556810	nan	nan	0.786516	0.698141	-0.171959	0.89245	no
ni|645903682|nb|KK920748.1|	561383-561914	nan	nan	0.804854	1	0.519902	0.67865	no
ni|645903682|nb|KK920748.1|	562267-562766	nan	nan	1	0.415677	-1	0.312	no
ni|645903682|nb|KK920748.1|	565028-565232	nan	nan	4	2	-1	0.3115	no
ni|645903682|nb|KK920748.1|	583381-583542	nan	nan	5	2	-1	0.3479	no
ni|645903682|nb|KK920748.1|	584871-585302	nan	nan	3	1	-0.952792	0.43705	no
ni|645903682|nb|KK920748.1|	585864-586021	nan	nan	5	4	-0.482236	0.7301	no
ni|645903682|nb|KK920748.1|	587482-587629	nan	nan	5	1	-2	0.2949	no
ni|645903682|nb|KK920748.1|	591091-591458	nan	nan	3	1	-1	0.33165	no
ni|645903682|nb|KK920748.1|	615066-615298	nan	nan	18	8	-1	0.31985	no
ni|645903682|nb|KK920748.1|	621939-622351	nan	nan	16	9	-0.851191	0.45755	no
ni|645903683|nb|KK920747.1|	219317-219562	nan	nan	3	5	0.887961	0.46985	no
ni|645903683|nb|KK920747.1|	238043-238403	nan	nan	2	2	-0.0654907	0.96255	no
ni|645903683|nb|KK920747.1|	244848-245019	nan	nan	4	6	0.698256	0.59985	no
ni|645903683|nb|KK920747.1|	247249-247398	nan	nan	6	4	-0.65216	0.64	no
ni|645903683|nb|KK920747.1|	248024-248256	nan	nan	3	6	0.800323	0.51595	no
ni|645903683|nb|KK920747.1|	249239-249448	nan	nan	6	5	-0.318331	0.7941	no
ni|645903683|nb|KK920747.1|	251096-251254	nan	nan	5	7	0.560831	0.667	no
ni|645903683|nb|KK920747.1|	251745-252217	nan	nan	2	3	0.595339	0.6131	no
ni|645903683|nb|KK920747.1|	253498-253732	nan	nan	3.17	3	0.0940837	0.93935	no

ni|645903683|nb|KK920747.1|	390733-390919	nan	nan	32	30	-0.12019	0.91535	no
ni|645903683|nb|KK920747.1|	476222-476543	nan	nan	9	7	-0.480709	0.68025	no
ni|645903683|nb|KK920747.1|	49778-50207	nan	nan	3	3	0.297298	0.8001	no
ni|645903684|nb|KK920746.1|	202602-202772	nan	nan	34	27	-0.366829	0.75	no
ni|645903684|nb|KK920746.1|	202858-203375	nan	nan	25	18	-0.481271	0.66835	no
ni|645903684|nb|KK920746.1|	205239-205470	nan	nan	3	1	-0.87378	0.53675	no
ni|645903684|nb|KK920746.1|	207750-208104	nan	nan	2	1	-0.589078	0.62565	no
ni|645903684|nb|KK920746.1|	211886-212169	nan	nan	1	0.520287	-1	0.44965	no
ni|645903684|nb|KK920746.1|	214439-214753	nan	nan	0.465131	1	2	0.242	no
ni|645903684|nb|KK920746.1|	217987-218151	nan	nan	80	54	-0.563898	0.62805	no
ni|645903684|nb|KK920746.1|	220168-221022	nan	nan	2	2	-0.267492	0.8217	no
ni|645903684|nb|KK920746.1|	221313-221479	nan	nan	74	55	-0.43616	0.71025	no
ni|645903684|nb|KK920746.1|	226326-227562	nan	nan	3	2	-0.127502	0.91395	no
ni|645903684|nb|KK920746.1|	227893-228016	nan	nan	51	24	-1	0.37635	no
ni|645903684|nb|KK920746.1|	228295-228451	nan	nan	236.99	81	-2	0.17625	no
ni|645903684|nb|KK920746.1|	228626-229044	nan	nan	3	3	-0.335389	0.7773	no
ni|645903684|nb|KK920746.1|	229154-229501	nan	nan	2	1	-0.32858	0.79705	no
ni|645903684|nb|KK920746.1|	230557-231586	nan	nan	3	2	-0.402989	0.72905	no
ni|645903684|nb|KK920746.1|	232284-233239	nan	nan	4	4	-0.114536	0.91865	no
ni|645903684|nb|KK920746.1|	233509-233672	nan	nan	4	5	0.463298	0.7264	no
ni|645903684|nb|KK920746.1|	238385-238931	nan	nan	180	130	-0.474606	0.7353	no
ni|645903684|nb|KK920746.1|	267266-267627	nan	nan	12	17	0.470946	0.6791	no
ni|645903684|nb|KK920746.1|	282342-282555	nan	nan	9	0.211492	-5	0.01745	no
ni|645903684|nb|KK920746.1|	282669-282850	nan	nan	6	0	-inf	0.00055	yes
ni|645903684|nb|KK920746.1|	283085-283284	nan	nan	5	0	-inf	0.0004	yes
ni|645903684|nb|KK920746.1|	283531-283800	nan	nan	5	0	-inf	0.0003	yes
ni|645903684|nb|KK920746.1|	285681-285924	nan	nan	3	0	-inf	0.0004	yes
ni|645903684|nb|KK920746.1|	286364-286593	nan	nan	2	0	-inf	0.002	yes
ni|645903684|nb|KK920746.1|	286804-287557	nan	nan	0.643906	0	-inf	0.0009	yes
ni|645903684|nb|KK920746.1|	289399-289739	nan	nan	2	0	-inf	0.0008	yes

ni|645903684|nb|KK920746.1|	289925-290051	nan	nan	11	0	-inf	0.002	yes
ni|645903684|nb|KK920746.1|	290485-291055	nan	nan	2	0	-inf	0.0002	yes
ni|645903684|nb|KK920746.1|	291698-292211	nan	nan	2	0.057361	-5	0.0185	no
ni|645903684|nb|KK920746.1|	293563-293744	nan	nan	3	0	-inf	0.0036	no
ni|645903684|nb|KK920746.1|	293808-294246	nan	nan	3	0	-inf	0.00025	yes
ni|645903684|nb|KK920746.1|	295258-295534	nan	nan	1	0	-inf	0.00455	no
ni|645903684|nb|KK920746.1|	295781-296023	nan	nan	3	0.167969	-4	0.02485	no
ni|645903685|nb|KK920745.1|	34382-34602	nan	nan	0	104	inf	5.00E-05	yes
ni|645903685|nb|KK920745.1|	36315-36608	nan	nan	0	147	inf	5.00E-05	yes
ni|645903685|nb|KK920745.1|	37014-37391	nan	nan	0	8	inf	5.00E-05	yes
ni|645903685|nb|KK920745.1|	386801-387215	nan	nan	8	7	-0.224967	0.8428	no
ni|645903685|nb|KK920745.1|	390679-391144	nan	nan	7	6	-0.207209	0.86065	no
ni|645903685|nb|KK920745.1|	505604-507676	nan	nan	0.012512	14	10	0.11935	no
ni|645903685|nb|KK920745.1|	508399-508725	nan	nan	0	4	inf	0.00015	yes
ni|645903685|nb|KK920745.1|	516698-516865	nan	nan	10098.8	7806.71	-0.37139	0.72425	no
ni|645903685|nb|KK920745.1|	516976-517103	nan	nan	2494.76	1941.59	-0.361662	0.79625	no
ni|645903685|nb|KK920745.1|	517327-517462	nan	nan	139	56	-1	0.26045	no
ni|645903685|nb|KK920745.1|	522245-523151	nan	nan	7	6	-0.397216	0.72715	no
ni|645903685|nb|KK920745.1|	524375-524575	nan	nan	8	6	-0.273432	0.81605	no
ni|645903685|nb|KK920745.1|	524911-525233	nan	nan	4	2	-0.790032	0.5191	no
ni|645903685|nb|KK920745.1|	525334-525567	nan	nan	10	9	-0.0949647	0.93355	no
ni|645903685|nb|KK920745.1|	526179-526482	nan	nan	5	5	0.0101794	0.9852	no
ni|645903685|nb|KK920745.1|	543125-543401	nan	nan	1	0.946994	-0.42852	0.75965	no
ni|645903685|nb|KK920745.1|	601166-601364	nan	nan	52	38	-0.449109	0.6936	no
ni|645903685|nb|KK920745.1|	605947-606062	nan	nan	36	33	-0.131625	0.9116	no
ni|645903685|nb|KK920745.1|	647685-647939	nan	nan	40	27	-0.588241	0.6051	no
ni|645903685|nb|KK920745.1|	650608-650947	nan	nan	20	19	-0.0730771	0.9485	no
ni|645903685|nb|KK920745.1|	651615-651982	nan	nan	0.651379	0.444631	-0.550889	0.71905	no
ni|645903685|nb|KK920745.1|	658131-658559	nan	nan	0.907826	1	0.437226	0.73605	no
ni|645903685|nb|KK920745.1|	663507-663937	nan	nan	0.601528	0.359326	-0.743341	0.59615	no

ni|645903685|nb|KK920745.1|	79281-81151	nan	nan	15	14	-0.141931	0.924	no
ni|645903685|nb|KK920745.1|	81310-81492	nan	nan	12	10	-0.259936	0.8271	no
ni|645903685|nb|KK920745.1|	81608-81896	nan	nan	4	2	-1	0.32805	no
ni|645903687|nb|KK920743.1|	131457-132098	nan	nan	1	1	0.0505888	0.96845	no
ni|645903687|nb|KK920743.1|	132934-133543	nan	nan	0.826907	0.325506	-1	0.33495	no
ni|645903687|nb|KK920743.1|	150799-151253	nan	nan	0.420396	0.468785	0.157177	0.9131	no
ni|645903687|nb|KK920743.1|	151605-152033	nan	nan	0.453913	1	1	0.38715	no
ni|645903687|nb|KK920743.1|	194643-195048	nan	nan	8	4	-1	0.34305	no
ni|645903687|nb|KK920743.1|	357169-357489	nan	nan	3	4	0.388889	0.7395	no
ni|645903687|nb|KK920743.1|	368411-368546	nan	nan	9	7	-0.308265	0.81205	no
ni|645903687|nb|KK920743.1|	368754-368943	nan	nan	11	11	-0.0315745	0.97855	no
ni|645903687|nb|KK920743.1|	371672-371898	nan	nan	5	9	0.903124	0.4529	no
ni|645903687|nb|KK920743.1|	371995-372429	nan	nan	6	8	0.389306	0.7348	no
ni|645903687|nb|KK920743.1|	373379-373827	nan	nan	5	7	0.388803	0.74295	no
ni|645903687|nb|KK920743.1|	374945-376011	nan	nan	0.102297	0.366827	2	0.2028	no
ni|645903687|nb|KK920743.1|	380138-380474	nan	nan	3	4	0.175411	0.88255	no
ni|645903687|nb|KK920743.1|	380648-380829	nan	nan	11	9	-0.200567	0.86755	no
ni|645903687|nb|KK920743.1|	381264-383122	nan	nan	0.364973	0.214799	-0.764803	0.5354	no
ni|645903687|nb|KK920743.1|	386636-388208	nan	nan	0.603689	0.673551	0.157983	0.89725	no
ni|645903687|nb|KK920743.1|	390479-390911	nan	nan	0.971562	0.78573	-0.306273	0.8123	no
ni|645903687|nb|KK920743.1|	392743-393359	nan	nan	2	3	0.101727	0.92925	no
ni|645903687|nb|KK920743.1|	393493-393947	nan	nan	8	7	-0.168309	0.8786	no
ni|645903687|nb|KK920743.1|	395445-395663	nan	nan	9	10	0.0920656	0.9388	no
ni|645903687|nb|KK920743.1|	395973-396132	nan	nan	14	11	-0.454226	0.70885	no
ni|645903687|nb|KK920743.1|	396252-396502	nan	nan	12	12	-0.0261023	0.98185	no
ni|645903687|nb|KK920743.1|	43585-44176	nan	nan	0.958228	1	0.210674	0.8645	no
ni|645903687|nb|KK920743.1|	45501-45946	nan	nan	2	0.618525	-2	0.162	no
ni|645903687|nb|KK920743.1|	47920-48461	nan	nan	0.898876	1	0.256884	0.83705	no
ni|645903687|nb|KK920743.1|	51255-51417	nan	nan	5	3	-0.597727	0.64595	no
ni|645903687|nb|KK920743.1|	52955-53408	nan	nan	0.843188	0.73876	-0.190749	0.88855	no

ni|645903687|nb|KK920743.1|	54119-54365	nan	nan	1	2	0.840666	0.5399	no
ni|645903687|nb|KK920743.1|	56591-56805	nan	nan	3	1	-1	0.3141	no
ni|645903687|nb|KK920743.1|	58808-59268	nan	nan	2	2	0.0417138	0.97455	no
ni|645903687|nb|KK920743.1|	60000-60188	nan	nan	4	3	-0.415139	0.7377	no
ni|645903687|nb|KK920743.1|	70767-70925	nan	nan	3	3	-0.0672004	0.93835	no
ni|645903688|nb|KK920742.1|	1033656-1034033	nan	nan	10	8	-0.217427	0.85365	no
ni|645903688|nb|KK920742.1|	1037714-1038033	nan	nan	1	0.976966	-0.355188	0.7931	no
ni|645903688|nb|KK920742.1|	1040115-1040723	nan	nan	1	0.978448	-0.0649349	0.95505	no
ni|645903688|nb|KK920742.1|	1041317-1041535	nan	nan	23	26	0.180586	0.87475	no
ni|645903688|nb|KK920742.1|	1047069-1047227	nan	nan	124	154	0.312463	0.7734	no
ni|645903688|nb|KK920742.1|	1050634-1050801	nan	nan	98	130.13	0.411088	0.71975	no
ni|645903688|nb|KK920742.1|	1051431-1051608	nan	nan	178	237	0.415456	0.73825	no
ni|645903688|nb|KK920742.1|	1053506-1053705	nan	nan	133	180	0.433238	0.67965	no
ni|645903688|nb|KK920742.1|	1053978-1054215	nan	nan	157	202.19	0.363269	0.80585	no
ni|645903688|nb|KK920742.1|	1055658-1056214	nan	nan	34	39	0.196092	0.85165	no
ni|645903688|nb|KK920742.1|	1058571-1058681	nan	nan	135	109	-0.306443	0.791	no
ni|645903688|nb|KK920742.1|	1058929-1059415	nan	nan	2	2	0.256783	0.82635	no
ni|645903688|nb|KK920742.1|	1059610-1060183	nan	nan	4	2	-0.660147	0.57935	no
ni|645903688|nb|KK920742.1|	1060312-1060628	nan	nan	215	280	0.383773	0.78485	no
ni|645903688|nb|KK920742.1|	1060920-1061173	nan	nan	225.9	169	-0.41834	0.77565	no
ni|645903688|nb|KK920742.1|	1061391-1061504	nan	nan	110	34	-2	0.17185	no
ni|645903688|nb|KK920742.1|	1061737-1061864	nan	nan	32	12	-1	0.2735	no
ni|645903688|nb|KK920742.1|	106417-107560	nan	nan	5	6	0.517918	0.6538	no
ni|645903688|nb|KK920742.1|	1065243-1065546	nan	nan	3	5	0.863841	0.4663	no
ni|645903688|nb|KK920742.1|	1067117-1067253	nan	nan	7	3	-1	0.44755	no
ni|645903688|nb|KK920742.1|	1067602-1068372	nan	nan	0	2	inf	5.00E-05	yes
ni|645903688|nb|KK920742.1|	1069261-1069380	nan	nan	0	29	inf	0.00055	yes
ni|645903688|nb|KK920742.1|	1071355-1071722	nan	nan	86	107	0.318803	0.83235	no
ni|645903688|nb|KK920742.1|	1075930-1076342	nan	nan	0.875106	0.684303	-0.354823	0.7931	no
ni|645903688|nb|KK920742.1|	1076474-1077094	nan	nan	31	39	0.338384	0.7802	no

ni|645903688|nb|KK920742.1|	107668-107848	nan	nan	0	123	inf	5.00E-05	yes
ni|645903688|nb|KK920742.1|	1077159-1077284	nan	nan	44	40	-0.132932	0.9109	no
ni|645903688|nb|KK920742.1|	1077529-1078049	nan	nan	4	3	-0.371183	0.7489	no
ni|645903688|nb|KK920742.1|	1079198-1080017	nan	nan	2	54	5	0.00305	no
ni|645903688|nb|KK920742.1|	1085485-1085615	nan	nan	7	188	5	0.0112	no
ni|645903688|nb|KK920742.1|	1086257-1086368	nan	nan	46	150	2	0.1674	no
ni|645903688|nb|KK920742.1|	1087411-1087631	nan	nan	98	253	1	0.28445	no
ni|645903688|nb|KK920742.1|	1089085-1089282	nan	nan	96	281	2	0.2415	no
ni|645903688|nb|KK920742.1|	108960-109407	nan	nan	1	5	2	0.0964	no
ni|645903688|nb|KK920742.1|	1091750-1091931	nan	nan	64	184	2	0.17275	no
ni|645903688|nb|KK920742.1|	1093015-1093197	nan	nan	111	325	2	0.23945	no
ni|645903688|nb|KK920742.1|	1093414-1094011	nan	nan	9	28	2	0.14295	no
ni|645903688|nb|KK920742.1|	1094425-1094618	nan	nan	60	173	2	0.1734	no
ni|645903688|nb|KK920742.1|	1095208-1095718	nan	nan	25	89	2	0.16015	no
ni|645903688|nb|KK920742.1|	1097033-1097192	nan	nan	42	178	2	0.07635	no
ni|645903688|nb|KK920742.1|	1099561-1100097	nan	nan	55	178	2	0.25825	no
ni|645903688|nb|KK920742.1|	1100678-1102360	nan	nan	14	17	0.300022	0.84515	no
ni|645903688|nb|KK920742.1|	1103070-1103340	nan	nan	20	22	0.148854	0.89415	no
ni|645903688|nb|KK920742.1|	1103787-1103994	nan	nan	18	10	-0.772089	0.51045	no
ni|645903688|nb|KK920742.1|	1104063-1104390	nan	nan	5	3	-0.413564	0.72545	no
ni|645903688|nb|KK920742.1|	1108172-1108455	nan	nan	277	248	-0.158203	0.91205	no
ni|645903688|nb|KK920742.1|	1108789-1109386	nan	nan	1	1	-0.234877	0.84165	no
ni|645903688|nb|KK920742.1|	1109902-1110303	nan	nan	95	99	0.0594532	0.9678	no
ni|645903688|nb|KK920742.1|	1111335-1111644	nan	nan	86	98	0.196294	0.873	no
ni|645903688|nb|KK920742.1|	1112596-1112973	nan	nan	112	128	0.188555	0.8983	no
ni|645903688|nb|KK920742.1|	1113550-1113711	nan	nan	272	352	0.373708	0.75925	no
ni|645903688|nb|KK920742.1|	1113815-1114596	nan	nan	124	156	0.333859	0.80745	no
ni|645903688|nb|KK920742.1|	1117344-1117681	nan	nan	136.71	189	0.464071	0.74205	no
ni|645903688|nb|KK920742.1|	1118332-1118817	nan	nan	156	195	0.320087	0.8244	no
ni|645903688|nb|KK920742.1|	1123734-1123988	nan	nan	1	37	5	0.01465	no

ni|645903688|nb|KK920742.1|	1127529-1128614	nan	nan	38	21	-0.867911	0.55685	no
ni|645903688|nb|KK920742.1|	1129125-1129678	nan	nan	32	19	-0.79709	0.45995	no
ni|645903688|nb|KK920742.1|	1134560-1134729	nan	nan	149	129	-0.207581	0.8448	no
ni|645903688|nb|KK920742.1|	115457-115853	nan	nan	22	35	0.653943	0.5562	no
ni|645903688|nb|KK920742.1|	1155914-1156185	nan	nan	3	1	-1	0.3177	no
ni|645903688|nb|KK920742.1|	119460-120288	nan	nan	14	21	0.590821	0.57915	no
ni|645903688|nb|KK920742.1|	120556-121077	nan	nan	17	37	1	0.30715	no
ni|645903688|nb|KK920742.1|	124264-124525	nan	nan	32	61	0.909445	0.41735	no
ni|645903688|nb|KK920742.1|	124812-124978	nan	nan	39	72	0.889697	0.4435	no
ni|645903688|nb|KK920742.1|	125565-125751	nan	nan	6	11	0.795255	0.50935	no
ni|645903688|nb|KK920742.1|	126122-126259	nan	nan	39	79	1	0.3817	no
ni|645903688|nb|KK920742.1|	126357-126983	nan	nan	12	22	0.866378	0.4387	no
ni|645903688|nb|KK920742.1|	12698-12893	nan	nan	27	187	3	0.02025	no
ni|645903688|nb|KK920742.1|	130695-131352	nan	nan	19	31	0.703659	0.5147	no
ni|645903688|nb|KK920742.1|	131688-131855	nan	nan	10	7	-0.552669	0.647	no
ni|645903688|nb|KK920742.1|	132148-132273	nan	nan	61	49	-0.316703	0.78495	no
ni|645903688|nb|KK920742.1|	132383-132556	nan	nan	30	7	-2	0.0899	no
ni|645903688|nb|KK920742.1|	132702-132902	nan	nan	45	34	-0.419283	0.71195	no
ni|645903688|nb|KK920742.1|	149213-149601	nan	nan	0	2	inf	0.00055	yes
ni|645903688|nb|KK920742.1|	14923-15114	nan	nan	45	204.6	2	0.11915	no
ni|645903688|nb|KK920742.1|	149669-150290	nan	nan	0	3	inf	0.00015	yes
ni|645903688|nb|KK920742.1|	150966-151326	nan	nan	26	32	0.292442	0.7919	no
ni|645903688|nb|KK920742.1|	16545-17077	nan	nan	24	132	2	0.06845	no
ni|645903688|nb|KK920742.1|	182034-182368	nan	nan	6	3	-1	0.3624	no
ni|645903688|nb|KK920742.1|	193094-193674	nan	nan	18	20	0.0878309	0.9365	no
ni|645903688|nb|KK920742.1|	193938-194148	nan	nan	11	9	-0.299433	0.7951	no
ni|645903688|nb|KK920742.1|	19405-19621	nan	nan	39	225	3	0.0723	no
ni|645903688|nb|KK920742.1|	194741-194895	nan	nan	12	12	-0.0147249	0.98035	no
ni|645903688|nb|KK920742.1|	195509-195674	nan	nan	5	6	0.212881	0.86845	no
ni|645903688|nb|KK920742.1|	196004-197181	nan	nan	11.26	8	-0.524311	0.63135	no

ni|645903688|nb|KK920742.1|	197413-197928	nan	nan	13	8	-0.690693	0.5506	no
ni|645903688|nb|KK920742.1|	198096-198927	nan	nan	18	17	-0.0181058	0.98655	no
ni|645903688|nb|KK920742.1|	201495-201644	nan	nan	71	51	-0.482235	0.6785	no
ni|645903688|nb|KK920742.1|	202786-202983	nan	nan	92	89	-0.0469219	0.9648	no
ni|645903688|nb|KK920742.1|	206027-206185	nan	nan	88	68	-0.380358	0.74005	no
ni|645903688|nb|KK920742.1|	207169-207303	nan	nan	131.84	134	0.0248543	0.984	no
ni|645903688|nb|KK920742.1|	207655-208146	nan	nan	62	44	-0.506024	0.68075	no
ni|645903688|nb|KK920742.1|	210990-212187	nan	nan	642	622	-0.0443971	0.96675	no
ni|645903688|nb|KK920742.1|	212345-212568	nan	nan	20	17	-0.22352	0.8487	no
ni|645903688|nb|KK920742.1|	215821-215974	nan	nan	116.13	120	0.0451385	0.96855	no
ni|645903688|nb|KK920742.1|	217203-218425	nan	nan	168	162	-0.0528206	0.97025	no
ni|645903688|nb|KK920742.1|	221234-221633	nan	nan	9	12	0.402634	0.7274	no
ni|645903688|nb|KK920742.1|	222428-222764	nan	nan	45	66	0.565028	0.6001	no
ni|645903688|nb|KK920742.1|	223225-223608	nan	nan	6	8	0.292645	0.80785	no
ni|645903688|nb|KK920742.1|	223737-224020	nan	nan	2	2	-0.0658979	0.9545	no
ni|645903688|nb|KK920742.1|	224177-224856	nan	nan	21	23	0.118778	0.9129	no
ni|645903688|nb|KK920742.1|	22452-22665	nan	nan	43	220	2	0.087	no
ni|645903688|nb|KK920742.1|	224982-225124	nan	nan	23	29	0.347821	0.76205	no
ni|645903688|nb|KK920742.1|	230283-230561	nan	nan	34	28	-0.29726	0.79405	no
ni|645903688|nb|KK920742.1|	230720-230927	nan	nan	16	13	-0.226031	0.84845	no
ni|645903688|nb|KK920742.1|	231269-231638	nan	nan	17	12	-0.409719	0.7191	no
ni|645903688|nb|KK920742.1|	231714-231829	nan	nan	49	44	-0.16371	0.89245	no
ni|645903688|nb|KK920742.1|	232140-232331	nan	nan	21	133	3	0.0311	no
ni|645903688|nb|KK920742.1|	232624-233162	nan	nan	1	0.540725	-0.913046	0.4969	no
ni|645903688|nb|KK920742.1|	233409-233517	nan	nan	25	165	3	0.0586	no
ni|645903688|nb|KK920742.1|	233707-234018	nan	nan	1	0.563479	-1	0.4064	no
ni|645903688|nb|KK920742.1|	234686-234859	nan	nan	37	255	3	0.05825	no
ni|645903688|nb|KK920742.1|	236079-236299	nan	nan	44	302	3	0.0503	no
ni|645903688|nb|KK920742.1|	237525-237720	nan	nan	29	262	3	0.02985	no
ni|645903688|nb|KK920742.1|	238934-239184	nan	nan	14	94	3	0.0215	no

ni|645903688|nb|KK920742.1|	240524-240737	nan	nan	34	222	3	0.0596	no
ni|645903688|nb|KK920742.1|	241532-242244	nan	nan	9	81	3	0.0203	no
ni|645903688|nb|KK920742.1|	243708-244159	nan	nan	19	153	3	0.0297	no
ni|645903688|nb|KK920742.1|	244427-244608	nan	nan	27	221.61	3	0.0124	no
ni|645903688|nb|KK920742.1|	24567-24684	nan	nan	27	115	2	0.09625	no
ni|645903688|nb|KK920742.1|	246950-247095	nan	nan	22	199	3	0.0135	no
ni|645903688|nb|KK920742.1|	247822-248228	nan	nan	10	92	3	0.0299	no
ni|645903688|nb|KK920742.1|	251030-251173	nan	nan	21	195	3	0.01105	no
ni|645903688|nb|KK920742.1|	253498-253649	nan	nan	25	266	3	0.0064	no
ni|645903688|nb|KK920742.1|	256252-257533	nan	nan	7	66	3	0.0202	no
ni|645903688|nb|KK920742.1|	25761-26106	nan	nan	7	6	-0.348489	0.76755	no
ni|645903688|nb|KK920742.1|	258138-258526	nan	nan	0	92	inf	5.00E-05	yes
ni|645903688|nb|KK920742.1|	261230-262312	nan	nan	4	5	0.313963	0.7827	no
ni|645903688|nb|KK920742.1|	262551-263402	nan	nan	2	2	-0.0845772	0.9443	no
ni|645903688|nb|KK920742.1|	263582-264339	nan	nan	5	7	0.285343	0.80235	no
ni|645903688|nb|KK920742.1|	265111-265505	nan	nan	5	6	0.20142	0.86215	no
ni|645903688|nb|KK920742.1|	266493-268239	nan	nan	13	14	0.0106637	0.9946	no
ni|645903688|nb|KK920742.1|	269836-270058	nan	nan	1	2	0.126078	0.92555	no
ni|645903688|nb|KK920742.1|	270417-270564	nan	nan	7	2	-2	0.20985	no
ni|645903688|nb|KK920742.1|	270787-271093	nan	nan	14	10	-0.493614	0.6715	no
ni|645903688|nb|KK920742.1|	275240-275454	nan	nan	59	53	-0.155509	0.8905	no
ni|645903688|nb|KK920742.1|	276451-276619	nan	nan	28	35	0.30982	0.797	no
ni|645903688|nb|KK920742.1|	277032-277224	nan	nan	22	18	-0.301611	0.792	no
ni|645903688|nb|KK920742.1|	2771-3463	nan	nan	0.041795	423	13	0.11935	no
ni|645903688|nb|KK920742.1|	279794-280038	nan	nan	39	35	-0.138561	0.9047	no
ni|645903688|nb|KK920742.1|	280166-280322	nan	nan	33	23	-0.514655	0.6542	no
ni|645903688|nb|KK920742.1|	282226-282604	nan	nan	16	20	0.29805	0.79255	no
ni|645903688|nb|KK920742.1|	284714-284968	nan	nan	36	44	0.30583	0.78645	no
ni|645903688|nb|KK920742.1|	285104-285261	nan	nan	37	36	-0.0671984	0.95215	no
ni|645903688|nb|KK920742.1|	286249-286410	nan	nan	41	51	0.322359	0.78495	no

ni|645903688|nb|KK920742.1|	286791-286970	nan	nan	49	45	-0.124653	0.9135	no
ni|645903688|nb|KK920742.1|	287075-287261	nan	nan	41	36	-0.163815	0.88435	no
ni|645903688|nb|KK920742.1|	296360-296688	nan	nan	18	17	-0.0656358	0.9554	no
ni|645903688|nb|KK920742.1|	297577-297844	nan	nan	36	40	0.144399	0.89735	no
ni|645903688|nb|KK920742.1|	29789-30042	nan	nan	30	197.86	3	0.05395	no
ni|645903688|nb|KK920742.1|	299662-300376	nan	nan	2	0.808966	-1	0.24945	no
ni|645903688|nb|KK920742.1|	300631-301224	nan	nan	3	0.528178	-2	0.0927	no
ni|645903688|nb|KK920742.1|	301303-301569	nan	nan	2	0	-inf	0.0036	no
ni|645903688|nb|KK920742.1|	302503-303302	nan	nan	26	24	-0.107006	0.9312	no
ni|645903688|nb|KK920742.1|	303489-304283	nan	nan	17	17	-0.0499257	0.9636	no
ni|645903688|nb|KK920742.1|	304734-304854	nan	nan	14	21	0.517558	0.68765	no
ni|645903688|nb|KK920742.1|	305187-305417	nan	nan	22	18	-0.336892	0.77625	no
ni|645903688|nb|KK920742.1|	307870-308015	nan	nan	3	3	-0.330241	0.8122	no
ni|645903688|nb|KK920742.1|	308309-308481	nan	nan	30	37	0.317736	0.782	no
ni|645903688|nb|KK920742.1|	309246-309699	nan	nan	4	4	0.188539	0.87035	no
ni|645903688|nb|KK920742.1|	310549-310725	nan	nan	17	21	0.298325	0.7963	no
ni|645903688|nb|KK920742.1|	311988-312424	nan	nan	0.738408	0.494027	-0.579828	0.70815	no
ni|645903688|nb|KK920742.1|	315893-316600	nan	nan	16	12	-0.35759	0.74	no
ni|645903688|nb|KK920742.1|	317014-317276	nan	nan	51	38	-0.414383	0.7139	no
ni|645903688|nb|KK920742.1|	317503-318160	nan	nan	10	9	-0.220285	0.84555	no
ni|645903688|nb|KK920742.1|	321314-322108	nan	nan	2	2	0.117481	0.9184	no
ni|645903688|nb|KK920742.1|	322647-323073	nan	nan	1	0.436509	-1	0.3464	no
ni|645903688|nb|KK920742.1|	324975-325770	nan	nan	3	4	0.211609	0.85695	no
ni|645903688|nb|KK920742.1|	332142-332438	nan	nan	65	50	-0.366023	0.7309	no
ni|645903688|nb|KK920742.1|	332807-333085	nan	nan	62	44	-0.494029	0.6481	no
ni|645903688|nb|KK920742.1|	333403-333700	nan	nan	119	165	0.473899	0.7457	no
ni|645903688|nb|KK920742.1|	334378-334681	nan	nan	3	1	-1	0.383	no
ni|645903688|nb|KK920742.1|	33447-33585	nan	nan	53	228	2	0.0826	no
ni|645903688|nb|KK920742.1|	334845-335002	nan	nan	67	96	0.530364	0.64185	no
ni|645903688|nb|KK920742.1|	335125-335256	nan	nan	159	215	0.431409	0.7023	no

ni|645903688|nb|KK920742.1|	336726-337437	nan	nan	9	15	0.77554	0.494	no
ni|645903688|nb|KK920742.1|	33887-34486	nan	nan	8	4	-0.947297	0.4205	no
ni|645903688|nb|KK920742.1|	340749-341623	nan	nan	144	179	0.309771	0.83025	no
ni|645903688|nb|KK920742.1|	343698-343868	nan	nan	318	356	0.160209	0.913	no
ni|645903688|nb|KK920742.1|	346392-346615	nan	nan	248	275	0.148389	0.918	no
ni|645903688|nb|KK920742.1|	347616-348007	nan	nan	93	99	0.089549	0.9518	no
ni|645903688|nb|KK920742.1|	348490-348987	nan	nan	0.437072	10	5	0.02155	no
ni|645903688|nb|KK920742.1|	34964-35737	nan	nan	0.587957	0.245943	-1	0.3768	no
ni|645903688|nb|KK920742.1|	354664-355231	nan	nan	0.318224	56	7	0.03325	no
ni|645903688|nb|KK920742.1|	355832-356089	nan	nan	0	75	inf	5.00E-05	yes
ni|645903688|nb|KK920742.1|	358981-359141	nan	nan	0.832633	240	8	0.0443	no
ni|645903688|nb|KK920742.1|	35956-36493	nan	nan	0.680357	1	0.597915	0.63	no
ni|645903688|nb|KK920742.1|	361113-361371	nan	nan	0.157896	162	10	0.11935	no
ni|645903688|nb|KK920742.1|	363668-363852	nan	nan	142	148.17	0.0602793	0.95675	no
ni|645903688|nb|KK920742.1|	364914-365136	nan	nan	218	227	0.0582843	0.96885	no
ni|645903688|nb|KK920742.1|	3652-4790	nan	nan	0.095185	607	13	0.03715	no
ni|645903688|nb|KK920742.1|	36546-36914	nan	nan	14	15	0.161134	0.8891	no
ni|645903688|nb|KK920742.1|	366012-366269	nan	nan	251	273	0.11784	0.9339	no
ni|645903688|nb|KK920742.1|	366848-367309	nan	nan	30	28	-0.115356	0.91755	no
ni|645903688|nb|KK920742.1|	367545-368109	nan	nan	1	0.918539	-0.595519	0.62535	no
ni|645903688|nb|KK920742.1|	368263-368488	nan	nan	344	322	-0.093628	0.9477	no
ni|645903688|nb|KK920742.1|	37040-37199	nan	nan	14	15	0.0152597	0.98275	no
ni|645903688|nb|KK920742.1|	37382-37511	nan	nan	8	6	-0.237229	0.8697	no
ni|645903688|nb|KK920742.1|	374398-374792	nan	nan	12	23	1	0.377	no
ni|645903688|nb|KK920742.1|	375437-375634	nan	nan	20	52	1	0.2368	no
ni|645903688|nb|KK920742.1|	375786-375945	nan	nan	11	34	2	0.1772	no
ni|645903688|nb|KK920742.1|	378581-378748	nan	nan	22	44	1	0.38665	no
ni|645903688|nb|KK920742.1|	379044-379154	nan	nan	11	33	2	0.28465	no
ni|645903688|nb|KK920742.1|	379858-380038	nan	nan	18	35	0.957781	0.41595	no
ni|645903688|nb|KK920742.1|	381533-381664	nan	nan	28	57	1	0.3784	no

ni|645903688|nb|KK920742.1|	381819-381945	nan	nan	30	44	0.547376	0.6408	no
ni|645903688|nb|KK920742.1|	382260-382414	nan	nan	18	31	0.834627	0.4848	no
ni|645903688|nb|KK920742.1|	383224-383592	nan	nan	6	10	0.886447	0.45855	no
ni|645903688|nb|KK920742.1|	385454-385817	nan	nan	7	18	1	0.27475	no
ni|645903688|nb|KK920742.1|	386470-386615	nan	nan	28	36	0.368893	0.7536	no
ni|645903688|nb|KK920742.1|	388778-388909	nan	nan	21	44	1	0.36175	no
ni|645903688|nb|KK920742.1|	392417-393155	nan	nan	1	3	0.852763	0.4635	no
ni|645903688|nb|KK920742.1|	396838-397250	nan	nan	7	10	0.357375	0.7633	no
ni|645903688|nb|KK920742.1|	400230-400465	nan	nan	0	63	inf	5.00E-05	yes
ni|645903688|nb|KK920742.1|	400895-401376	nan	nan	0.260299	1	2	0.133	no
ni|645903688|nb|KK920742.1|	406906-407433	nan	nan	5	24	2	0.05825	no
ni|645903688|nb|KK920742.1|	40741-41128	nan	nan	0.173058	2	4	0.0514	no
ni|645903688|nb|KK920742.1|	407942-408950	nan	nan	0.816364	1	0.313929	0.7873	no
ni|645903688|nb|KK920742.1|	410075-410253	nan	nan	17	27	0.653523	0.582	no
ni|645903688|nb|KK920742.1|	411115-411279	nan	nan	25	21	-0.282235	0.80525	no
ni|645903688|nb|KK920742.1|	411706-411950	nan	nan	10	6	-0.778965	0.5088	no
ni|645903688|nb|KK920742.1|	412485-412647	nan	nan	40	28	-0.511997	0.66375	no
ni|645903688|nb|KK920742.1|	412760-412917	nan	nan	30	10	-2	0.2215	no
ni|645903688|nb|KK920742.1|	41612-42242	nan	nan	4	4	0.0945748	0.93575	no
ni|645903688|nb|KK920742.1|	42433-43391	nan	nan	4	2	-0.614806	0.59735	no
ni|645903688|nb|KK920742.1|	43609-44563	nan	nan	13	21.14	0.73803	0.5619	no
ni|645903688|nb|KK920742.1|	444880-445193	nan	nan	2	3	0.457848	0.71285	no
ni|645903688|nb|KK920742.1|	456054-456258	nan	nan	1	5	2	0.2459	no
ni|645903688|nb|KK920742.1|	479323-479648	nan	nan	0	15	inf	5.00E-05	yes
ni|645903688|nb|KK920742.1|	481717-482154	nan	nan	0	3	inf	0.0001	yes
ni|645903688|nb|KK920742.1|	49805-50141	nan	nan	2	2	0.239257	0.8456	no
ni|645903688|nb|KK920742.1|	529308-531638	nan	nan	5122.93	3542.78	-0.53209	0.22715	no
ni|645903688|nb|KK920742.1|	531742-532066	nan	nan	7	3	-1	0.30045	no
ni|645903688|nb|KK920742.1|	541283-541393	nan	nan	71	4	-4	0.2333	no
ni|645903688|nb|KK920742.1|	542759-543027	nan	nan	1	0.567112	-1	0.4023	no

ni|645903688|nb|KK920742.1|	545639-545809	nan	nan	2013.47	1478.86	-0.445191	0.74945	no
ni|645903688|nb|KK920742.1|	546294-546481	nan	nan	2403.84	1867.7	-0.364076	0.80195	no
ni|645903688|nb|KK920742.1|	546610-546765	nan	nan	0	19	inf	5.00E-05	yes
ni|645903688|nb|KK920742.1|	553179-553956	nan	nan	0	2	inf	5.00E-05	yes
ni|645903688|nb|KK920742.1|	571093-571237	nan	nan	31	36	0.195826	0.86685	no
ni|645903688|nb|KK920742.1|	583828-584031	nan	nan	25	32	0.365523	0.7472	no
ni|645903688|nb|KK920742.1|	584451-584851	nan	nan	3	3	-0.0247072	0.9814	no
ni|645903688|nb|KK920742.1|	60944-61325	nan	nan	43	61	0.498837	0.63945	no
ni|645903688|nb|KK920742.1|	616980-617447	nan	nan	11	25	1	0.2801	no
ni|645903688|nb|KK920742.1|	643924-644196	nan	nan	4	1	-2	0.24815	no
ni|645903688|nb|KK920742.1|	64752-65035	nan	nan	1	1	-0.52533	0.7071	no
ni|645903688|nb|KK920742.1|	65161-65325	nan	nan	23	20	-0.170315	0.8817	no
ni|645903688|nb|KK920742.1|	65669-65861	nan	nan	23	142	3	0.02945	no
ni|645903688|nb|KK920742.1|	69976-70149	nan	nan	17	208	4	0.00435	no
ni|645903688|nb|KK920742.1|	702073-702176	nan	nan	227	298	0.394337	0.734	no
ni|645903688|nb|KK920742.1|	71359-71590	nan	nan	21	258	4	0.0153	no
ni|645903688|nb|KK920742.1|	72150-72310	nan	nan	30	366	4	0.0199	no
ni|645903688|nb|KK920742.1|	72426-73057	nan	nan	1	0.757637	-0.621295	0.61715	no
ni|645903688|nb|KK920742.1|	73854-74328	nan	nan	2	10	2	0.0974	no
ni|645903688|nb|KK920742.1|	739525-739798	nan	nan	46	99	1	0.40255	no
ni|645903688|nb|KK920742.1|	743731-743870	nan	nan	71	176	1	0.26435	no
ni|645903688|nb|KK920742.1|	75234-75374	nan	nan	23	262	4	0.00575	no
ni|645903688|nb|KK920742.1|	75889-76077	nan	nan	3	1	-1	0.4064	no
ni|645903688|nb|KK920742.1|	762584-762914	nan	nan	0.973223	0.206654	-2	0.3207	no
ni|645903688|nb|KK920742.1|	76294-76562	nan	nan	1	1	-0.387941	0.78555	no
ni|645903688|nb|KK920742.1|	76676-77734	nan	nan	5	55	4	0.01395	no
ni|645903688|nb|KK920742.1|	773396-774013	nan	nan	0.526741	0.732456	0.475646	0.709	no
ni|645903688|nb|KK920742.1|	779849-780369	nan	nan	0.826038	0.225601	-2	0.21345	no
ni|645903688|nb|KK920742.1|	781497-781955	nan	nan	1	1	0.327111	0.78615	no
ni|645903688|nb|KK920742.1|	78373-78772	nan	nan	8	99	4	0.0151	no

ni|645903688|nb|KK920742.1|	783823-784267	nan	nan	0.721141	0.689258	-0.0652373	0.9467	no
ni|645903688|nb|KK920742.1|	790945-792134	nan	nan	5	4	-0.430635	0.70615	no
ni|645903688|nb|KK920742.1|	79533-79751	nan	nan	3	116	5	0.0032	no
ni|645903688|nb|KK920742.1|	79943-80910	nan	nan	0.912079	0.681357	-0.420748	0.7241	no
ni|645903688|nb|KK920742.1|	81335-81652	nan	nan	0.917304	0.986031	0.104233	0.93335	no
ni|645903688|nb|KK920742.1|	82046-82376	nan	nan	2	2	-0.143628	0.9081	no
ni|645903688|nb|KK920742.1|	83212-83512	nan	nan	3	12	2	0.12875	no
ni|645903688|nb|KK920742.1|	86351-86576	nan	nan	2	2	0.103407	0.93335	no
ni|645903688|nb|KK920742.1|	87184-87348	nan	nan	7	70	3	0.0144	no
ni|645903688|nb|KK920742.1|	88049-88157	nan	nan	11	132.69	4	0.04315	no
ni|645903688|nb|KK920742.1|	928514-928577	nan	nan	53256.8	40252.8	-0.403874	0.739	no
ni|645903688|nb|KK920742.1|	9541-9848	nan	nan	22	121	2	0.07485	no
ni|645903688|nb|KK920742.1|	96005-96112	nan	nan	6	131.07	4	0.04525	no
ni|645903688|nb|KK920742.1|	99431-99540	nan	nan	3	79	5	0.12005	no
ni|645903689|nb|KK920741.1|	1555-1726	nan	nan	181	110	-0.724175	0.5121	no
ni|645903689|nb|KK920741.1|	17707-17835	nan	nan	9	4	-1	0.44755	no
ni|645903689|nb|KK920741.1|	18020-18541	nan	nan	12	13	0.202855	0.85705	no
ni|645903689|nb|KK920741.1|	19134-19382	nan	nan	15	16	0.118223	0.9208	no
ni|645903689|nb|KK920741.1|	20182-20389	nan	nan	18	20	0.158234	0.8949	no
ni|645903689|nb|KK920741.1|	20510-20974	nan	nan	17	19	0.1572	0.88565	no
ni|645903689|nb|KK920741.1|	21345-21514	nan	nan	33	59	0.818569	0.48605	no
ni|645903689|nb|KK920741.1|	22165-22800	nan	nan	0.231338	0.442324	0.935103	0.52835	no
ni|645903689|nb|KK920741.1|	24656-25307	nan	nan	9	22	1	0.25965	no
ni|645903689|nb|KK920741.1|	28229-28387	nan	nan	22	84	2	0.1088	no
ni|645903689|nb|KK920741.1|	29381-29605	nan	nan	21	51	1	0.28385	no
ni|645903689|nb|KK920741.1|	31226-31479	nan	nan	10	25	1	0.25185	no
ni|645903689|nb|KK920741.1|	317163-317564	nan	nan	0	49	inf	5.00E-05	yes
ni|645903689|nb|KK920741.1|	32091-32441	nan	nan	1	1	-0.0655328	0.95795	no
ni|645903689|nb|KK920741.1|	3236-3794	nan	nan	166	99	-0.750436	0.59475	no
ni|645903689|nb|KK920741.1|	33054-33348	nan	nan	1	2	0.634616	0.63975	no

ni|645903689|nb|KK920741.1|	38618-39021	nan	nan	0.98319	1	0.597624	0.63005	no
ni|645903689|nb|KK920741.1|	39212-41703	nan	nan	15	20	0.406467	0.7721	no
ni|645903689|nb|KK920741.1|	45609-46113	nan	nan	0	5	inf	5.00E-05	yes
ni|645903689|nb|KK920741.1|	478796-479240	nan	nan	0	2	inf	0.0003	yes
ni|645903689|nb|KK920741.1|	485338-485725	nan	nan	0	2	inf	5.00E-05	yes
ni|645903689|nb|KK920741.1|	486106-486230	nan	nan	0	349	inf	5.00E-05	yes
ni|645903689|nb|KK920741.1|	486295-486986	nan	nan	0	3	inf	5.00E-05	yes
ni|645903689|nb|KK920741.1|	491674-492011	nan	nan	0	694	inf	5.00E-05	yes
ni|645903689|nb|KK920741.1|	492869-493035	nan	nan	0.379097	242.43	9	0.11935	no
ni|645903689|nb|KK920741.1|	493381-494295	nan	nan	0	63	inf	5.00E-05	yes
ni|645903689|nb|KK920741.1|	499095-499250	nan	nan	0	68	inf	5.00E-05	yes
ni|645903689|nb|KK920741.1|	520906-521188	nan	nan	0	7	inf	5.00E-05	yes
ni|645903689|nb|KK920741.1|	57165-57569	nan	nan	0.163323	27	7	0.0443	no
ni|645903690|nb|KK920740.1|	1000293-1000594	nan	nan	334	166	-1	0.4772	no
ni|645903690|nb|KK920740.1|	1000838-1001033	nan	nan	4	2	-2	0.2996	no
ni|645903690|nb|KK920740.1|	1001648-1001881	nan	nan	89	47	-0.921716	0.40785	no
ni|645903690|nb|KK920740.1|	1002264-1002387	nan	nan	14	6	-1	0.42295	no
ni|645903690|nb|KK920740.1|	1002983-1003131	nan	nan	877	414	-1	0.4694	no
ni|645903690|nb|KK920740.1|	1003340-1003590	nan	nan	9	2	-2	0.0877	no
ni|645903690|nb|KK920740.1|	1003723-1004205	nan	nan	144	71	-1	0.48145	no
ni|645903690|nb|KK920740.1|	1004338-1005234	nan	nan	145	62	-1	0.3847	no
ni|645903690|nb|KK920740.1|	1005355-1005613	nan	nan	386	185	-1	0.46445	no
ni|645903690|nb|KK920740.1|	1006137-1006592	nan	nan	1	0.200339	-3	0.14055	no
ni|645903690|nb|KK920740.1|	1007084-1007324	nan	nan	2	0.170389	-4	0.02555	no
ni|645903690|nb|KK920740.1|	1009673-1010391	nan	nan	79	39	-1	0.48135	no
ni|645903690|nb|KK920740.1|	1010456-1010935	nan	nan	1	0.625311	-1	0.369	no
ni|645903690|nb|KK920740.1|	1016041-1016494	nan	nan	144	62	-1	0.41165	no
ni|645903690|nb|KK920740.1|	1017990-1018169	nan	nan	3	0.908791	-1	0.3864	no
ni|645903690|nb|KK920740.1|	1018432-1018707	nan	nan	163	86	-0.922832	0.46485	no
ni|645903690|nb|KK920740.1|	1019144-1020384	nan	nan	59	30	-0.987356	0.491	no

ni|645903690|nb|KK920740.1|	1020766-1020968	nan	nan	4	0.937922	-2	0.2004	no
ni|645903690|nb|KK920740.1|	1021867-1022152	nan	nan	10	2	-2	0.09055	no
ni|645903690|nb|KK920740.1|	1023158-1023648	nan	nan	5	4	-0.316676	0.7847	no
ni|645903690|nb|KK920740.1|	1024855-1025053	nan	nan	5	4	-0.219026	0.86075	no
ni|645903690|nb|KK920740.1|	1025241-1025416	nan	nan	7	4	-1	0.41285	no
ni|645903690|nb|KK920740.1|	1025816-1026014	nan	nan	4	3	-0.454046	0.722	no
ni|645903690|nb|KK920740.1|	1026994-1027169	nan	nan	5	3	-0.745388	0.56925	no
ni|645903690|nb|KK920740.1|	1027596-1027745	nan	nan	6	4	-0.65216	0.64	no
ni|645903690|nb|KK920740.1|	1028046-1028666	nan	nan	0.333257	0.40962	0.297652	0.8383	no
ni|645903690|nb|KK920740.1|	1029971-1030256	nan	nan	40	20	-1	0.3692	no
ni|645903690|nb|KK920740.1|	1030653-1031247	nan	nan	62	77	0.31626	0.8274	no
ni|645903690|nb|KK920740.1|	1034037-1034219	nan	nan	4	4	-0.0672914	0.9582	no
ni|645903690|nb|KK920740.1|	1036867-1036991	nan	nan	9	5	-0.915352	0.53575	no
ni|645903690|nb|KK920740.1|	1115044-1115226	nan	nan	5	2	-1	0.3768	no
ni|645903690|nb|KK920740.1|	163284-163499	nan	nan	3	3	0.232878	0.8527	no
ni|645903690|nb|KK920740.1|	164013-164163	nan	nan	3	5	0.807274	0.58675	no
ni|645903690|nb|KK920740.1|	164372-164556	nan	nan	1	3	0.780726	0.58145	no
ni|645903690|nb|KK920740.1|	165009-165335	nan	nan	0.550319	4	3	0.069	no
ni|645903690|nb|KK920740.1|	168122-168497	nan	nan	0.993626	1	0.5626	0.66675	no
ni|645903690|nb|KK920740.1|	170702-170925	nan	nan	3	5	0.876866	0.48785	no
ni|645903690|nb|KK920740.1|	174671-175893	nan	nan	0.220111	3	4	0.01805	no
ni|645903690|nb|KK920740.1|	178540-179051	nan	nan	1	2	0.597869	0.61895	no
ni|645903690|nb|KK920740.1|	180213-180412	nan	nan	3	3	-0.0670021	0.94765	no
ni|645903690|nb|KK920740.1|	181084-181275	nan	nan	4	6	0.325152	0.78615	no
ni|645903690|nb|KK920740.1|	181355-181591	nan	nan	3	7	1	0.3973	no
ni|645903690|nb|KK920740.1|	181715-181857	nan	nan	8	4	-1	0.45115	no
ni|645903690|nb|KK920740.1|	200368-201052	nan	nan	2	2	-0.374163	0.75	no
ni|645903690|nb|KK920740.1|	203139-203307	nan	nan	1425.37	1406.83	-0.0188868	0.9881	no
ni|645903690|nb|KK920740.1|	203870-204642	nan	nan	747	759	0.0235947	0.9823	no
ni|645903690|nb|KK920740.1|	205341-205971	nan	nan	4	4	0.276133	0.8146	no

ni|645903690|nb|KK920740.1|	206207-206973	nan	nan	769	716.28	-0.101951	0.92305	no
ni|645903690|nb|KK920740.1|	207228-207385	nan	nan	4	2	-1	0.44755	no
ni|645903690|nb|KK920740.1|	207817-208041	nan	nan	1539.93	1338.99	-0.201716	0.88365	no
ni|645903690|nb|KK920740.1|	208214-208583	nan	nan	2	0.617828	-1	0.33495	no
ni|645903690|nb|KK920740.1|	213025-213157	nan	nan	0	56	inf	5.00E-05	yes
ni|645903690|nb|KK920740.1|	213213-213581	nan	nan	0	39	inf	5.00E-05	yes
ni|645903690|nb|KK920740.1|	214188-214616	nan	nan	0	5	inf	0.00015	yes
ni|645903690|nb|KK920740.1|	214727-214916	nan	nan	0	17	inf	5.00E-05	yes
ni|645903690|nb|KK920740.1|	218614-218859	nan	nan	0	38	inf	5.00E-05	yes
ni|645903690|nb|KK920740.1|	222678-223014	nan	nan	0	65	inf	5.00E-05	yes
ni|645903690|nb|KK920740.1|	226443-226721	nan	nan	0	216	inf	5.00E-05	yes
ni|645903690|nb|KK920740.1|	228648-228821	nan	nan	0	244	inf	5.00E-05	yes
ni|645903690|nb|KK920740.1|	228926-229092	nan	nan	0.758194	281	9	0.04435	no
ni|645903690|nb|KK920740.1|	229890-233078	nan	nan	36	39	0.128622	0.9294	no
ni|645903690|nb|KK920740.1|	240861-241016	nan	nan	8	10	0.286443	0.81825	no
ni|645903690|nb|KK920740.1|	242281-242848	nan	nan	2	4	0.90258	0.44595	no
ni|645903690|nb|KK920740.1|	243999-244571	nan	nan	0.157418	2	4	0.0528	no
ni|645903690|nb|KK920740.1|	244688-244820	nan	nan	8	10	0.17373	0.8916	no
ni|645903690|nb|KK920740.1|	247546-247812	nan	nan	2	1	-0.650992	0.64935	no
ni|645903690|nb|KK920740.1|	248431-248556	nan	nan	8	8	0.102581	0.93335	no
ni|645903690|nb|KK920740.1|	248898-249055	nan	nan	7	10	0.576658	0.65045	no
ni|645903690|nb|KK920740.1|	249755-250026	nan	nan	2	2	-0.0659885	0.95935	no
ni|645903690|nb|KK920740.1|	250654-250986	nan	nan	2	3	0.312895	0.79995	no
ni|645903690|nb|KK920740.1|	252752-252981	nan	nan	3	9	1	0.2657	no
ni|645903690|nb|KK920740.1|	253489-253678	nan	nan	4	10	1	0.3072	no
ni|645903690|nb|KK920740.1|	254143-254614	nan	nan	0.467868	1	1	0.29595	no
ni|645903690|nb|KK920740.1|	256104-256293	nan	nan	6	9	0.610874	0.6155	no
ni|645903690|nb|KK920740.1|	257127-257326	nan	nan	5	6	0.196032	0.8698	no
ni|645903690|nb|KK920740.1|	258545-258897	nan	nan	3	3	0.285948	0.80995	no
ni|645903690|nb|KK920740.1|	260437-260559	nan	nan	4	14	2	0.242	no

ni|645903690|nb|KK920740.1|	263869-264296	nan	nan	2	3	0.626601	0.6021	no
ni|645903690|nb|KK920740.1|	265090-265245	nan	nan	5	6	0.155198	0.90275	no
ni|645903690|nb|KK920740.1|	268426-268593	nan	nan	7	8	0.0702617	0.9588	no
ni|645903690|nb|KK920740.1|	273105-273402	nan	nan	3	3	0.0716986	0.9566	no
ni|645903690|nb|KK920740.1|	274311-274961	nan	nan	1	2	0.520086	0.66315	no
ni|645903690|nb|KK920740.1|	277258-277685	nan	nan	2	3	0.639267	0.5884	no
ni|645903690|nb|KK920740.1|	279678-280169	nan	nan	1	2	0.491259	0.6941	no
ni|645903690|nb|KK920740.1|	282612-282776	nan	nan	4	9	1	0.3067	no
ni|645903690|nb|KK920740.1|	288128-288276	nan	nan	9	7	-0.347308	0.78305	no
ni|645903690|nb|KK920740.1|	291211-292032	nan	nan	156	99	-0.66109	0.636	no
ni|645903690|nb|KK920740.1|	297056-297394	nan	nan	2	3	0.519375	0.6668	no
ni|645903690|nb|KK920740.1|	298595-298741	nan	nan	3	5	0.669762	0.65595	no
ni|645903690|nb|KK920740.1|	304176-304380	nan	nan	4	5	0.392536	0.7519	no
ni|645903690|nb|KK920740.1|	304572-304732	nan	nan	5	4	-0.0672051	0.94715	no
ni|645903690|nb|KK920740.1|	307564-307783	nan	nan	5	5	0.0589143	0.9604	no
ni|645903690|nb|KK920740.1|	312980-313173	nan	nan	4	6	0.517836	0.67245	no
ni|645903690|nb|KK920740.1|	314398-314563	nan	nan	4	5	0.173781	0.8916	no
ni|645903690|nb|KK920740.1|	317643-317992	nan	nan	0.998282	1	0.519425	0.6956	no
ni|645903690|nb|KK920740.1|	320525-320647	nan	nan	25	25	-0.00597844	0.9752	no
ni|645903690|nb|KK920740.1|	320894-321072	nan	nan	21	28	0.396077	0.7425	no
ni|645903690|nb|KK920740.1|	321368-321620	nan	nan	6	5	-0.264099	0.82235	no
ni|645903690|nb|KK920740.1|	322353-323626	nan	nan	7	7	-0.0516563	0.96295	no
ni|645903690|nb|KK920740.1|	323905-324135	nan	nan	53	51	-0.0507301	0.9625	no
ni|645903690|nb|KK920740.1|	325246-325435	nan	nan	3	5	0.435302	0.7373	no
ni|645903690|nb|KK920740.1|	326667-327049	nan	nan	10	8	-0.33334	0.77185	no
ni|645903690|nb|KK920740.1|	327220-327691	nan	nan	3	2	-0.765616	0.5198	no
ni|645903690|nb|KK920740.1|	328074-328337	nan	nan	2	2	-0.481093	0.7105	no
ni|645903690|nb|KK920740.1|	329205-329500	nan	nan	13	20	0.582471	0.6187	no
ni|645903690|nb|KK920740.1|	329705-329967	nan	nan	3	2	-0.235989	0.85345	no
ni|645903690|nb|KK920740.1|	330546-330725	nan	nan	22	26	0.229662	0.8462	no

ni|645903690|nb|KK920740.1|	332199-332340	nan	nan	5	5	-0.0672222	0.93835	no
ni|645903690|nb|KK920740.1|	332629-333549	nan	nan	4	6	0.478511	0.68	no
ni|645903690|nb|KK920740.1|	333787-333890	nan	nan	49	53	0.10129	0.93335	no
ni|645903690|nb|KK920740.1|	336608-337800	nan	nan	2	2	-0.1652	0.88475	no
ni|645903690|nb|KK920740.1|	337917-339381	nan	nan	2	3	0.463306	0.69225	no
ni|645903690|nb|KK920740.1|	339647-339880	nan	nan	19	29	0.583107	0.61875	no
ni|645903690|nb|KK920740.1|	342150-342616	nan	nan	2	4	0.813506	0.4954	no
ni|645903690|nb|KK920740.1|	346470-346584	nan	nan	29	61	1	0.3779	no
ni|645903690|nb|KK920740.1|	349015-349271	nan	nan	0	3	inf	0.0005	yes
ni|645903690|nb|KK920740.1|	349425-350184	nan	nan	16	20	0.336411	0.7522	no
ni|645903690|nb|KK920740.1|	353710-354671	nan	nan	18	4	-2	0.0573	no
ni|645903690|nb|KK920740.1|	560200-560509	nan	nan	11	14	0.326582	0.7793	no
ni|645903690|nb|KK920740.1|	565364-565611	nan	nan	92	93	0.0115096	0.991	no
ni|645903690|nb|KK920740.1|	566549-566819	nan	nan	26	29	0.190152	0.8667	no
ni|645903690|nb|KK920740.1|	567307-567752	nan	nan	1	0.893426	-0.364795	0.7706	no
ni|645903690|nb|KK920740.1|	569498-569954	nan	nan	51	61	0.254737	0.86445	no
ni|645903690|nb|KK920740.1|	570645-570785	nan	nan	108	106	-0.026355	0.9821	no
ni|645903690|nb|KK920740.1|	571783-572332	nan	nan	23	21	-0.0970514	0.9277	no
ni|645903690|nb|KK920740.1|	572602-573088	nan	nan	0.963413	2	0.728404	0.5613	no
ni|645903690|nb|KK920740.1|	574019-574340	nan	nan	85	78	-0.114515	0.92645	no
ni|645903690|nb|KK920740.1|	577111-577319	nan	nan	455.83	532	0.222161	0.87205	no
ni|645903690|nb|KK920740.1|	578178-578328	nan	nan	541	614	0.183175	0.89885	no
ni|645903690|nb|KK920740.1|	578798-579482	nan	nan	85	87	0.0420807	0.97545	no
ni|645903690|nb|KK920740.1|	579862-580340	nan	nan	5	3	-0.392326	0.73045	no
ni|645903690|nb|KK920740.1|	580510-580821	nan	nan	394	486	0.30259	0.8283	no
ni|645903690|nb|KK920740.1|	581097-581343	nan	nan	99	113	0.18601	0.857	no
ni|645903690|nb|KK920740.1|	581467-581653	nan	nan	6	3	-0.855737	0.5047	no
ni|645903690|nb|KK920740.1|	582347-582835	nan	nan	145	183	0.335877	0.81105	no
ni|645903690|nb|KK920740.1|	584070-584288	nan	nan	164	222	0.437626	0.77275	no
ni|645903690|nb|KK920740.1|	587127-587650	nan	nan	2	2	-0.0192706	0.97965	no

ni|645903690|nb|KK920740.1|	588143-588433	nan	nan	90	116	0.363771	0.76795	no
ni|645903690|nb|KK920740.1|	588564-588907	nan	nan	3	2	-0.542002	0.64975	no
ni|645903690|nb|KK920740.1|	589379-589681	nan	nan	2	2	-0.32881	0.79705	no
ni|645903690|nb|KK920740.1|	589851-590155	nan	nan	129	245	0.920574	0.5311	no
ni|645903690|nb|KK920740.1|	597237-597382	nan	nan	0	111.11	inf	5.00E-05	yes
ni|645903690|nb|KK920740.1|	600591-601165	nan	nan	0.05225	1	5	0.1207	no
ni|645903690|nb|KK920740.1|	602206-602415	nan	nan	0	54.61	inf	5.00E-05	yes
ni|645903690|nb|KK920740.1|	608230-609613	nan	nan	0.750513	133.68	7	0.00035	yes
ni|645903690|nb|KK920740.1|	612106-612284	nan	nan	0	102	inf	5.00E-05	yes
ni|645903690|nb|KK920740.1|	612545-612706	nan	nan	0.409615	113	8	0.11935	no
ni|645903690|nb|KK920740.1|	613260-613411	nan	nan	0	115	inf	5.00E-05	yes
ni|645903690|nb|KK920740.1|	616836-617014	nan	nan	0.964337	155	7	0.05055	no
ni|645903690|nb|KK920740.1|	619500-619727	nan	nan	0.393711	113	8	0.0443	no
ni|645903690|nb|KK920740.1|	622712-623074	nan	nan	32	17	-0.939243	0.40945	no
ni|645903690|nb|KK920740.1|	624606-625004	nan	nan	0.749841	0.238877	-2	0.2989	no
ni|645903690|nb|KK920740.1|	625264-626274	nan	nan	2	1	-0.812395	0.48685	no
ni|645903690|nb|KK920740.1|	626904-627651	nan	nan	0.993963	0.365511	-1	0.2773	no
ni|645903690|nb|KK920740.1|	628348-628675	nan	nan	1	0.209403	-3	0.28285	no
ni|645903690|nb|KK920740.1|	630900-631358	nan	nan	20	14	-0.571391	0.61015	no
ni|645903690|nb|KK920740.1|	631463-631674	nan	nan	46	31	-0.572218	0.6195	no
ni|645903690|nb|KK920740.1|	676924-677203	nan	nan	15	3	-2	0.0911	no
ni|645903690|nb|KK920740.1|	749625-749761	nan	nan	36	44	0.270918	0.81325	no
ni|645903690|nb|KK920740.1|	750044-750166	nan	nan	24	42	0.795117	0.5095	no
ni|645903690|nb|KK920740.1|	750601-750874	nan	nan	9	14	0.709596	0.54655	no
ni|645903690|nb|KK920740.1|	751583-752004	nan	nan	20	20	-0.0214045	0.98435	no
ni|645903690|nb|KK920740.1|	753086-753461	nan	nan	2	1	-0.617973	0.6277	no
ni|645903690|nb|KK920740.1|	755653-755880	nan	nan	1	3	0.933513	0.5103	no
ni|645903690|nb|KK920740.1|	756091-756199	nan	nan	53	69	0.384741	0.7548	no
ni|645903690|nb|KK920740.1|	756944-757266	nan	nan	1	0.107075	-4	0.0326	no
ni|645903690|nb|KK920740.1|	761984-762169	nan	nan	7	10	0.435245	0.7137	no

ni|645903690|nb|KK920740.1|	762327-762550	nan	nan	11	13	0.148462	0.89705	no
ni|645903690|nb|KK920740.1|	762647-762880	nan	nan	9	14	0.695445	0.5503	no
ni|645903690|nb|KK920740.1|	772035-772792	nan	nan	1	0.683868	-0.723719	0.5469	no
ni|645903690|nb|KK920740.1|	773094-773789	nan	nan	0.623754	0.477079	-0.38675	0.76495	no
ni|645903690|nb|KK920740.1|	774024-774413	nan	nan	3	3	0.182545	0.87445	no
ni|645903690|nb|KK920740.1|	774830-775424	nan	nan	1	2	0.455876	0.7031	no
ni|645903690|nb|KK920740.1|	776159-776297	nan	nan	4	9	1	0.38715	no
ni|645903690|nb|KK920740.1|	777770-778016	nan	nan	5	4	-0.435458	0.7172	no
ni|645903690|nb|KK920740.1|	794769-794907	nan	nan	5	6	0.102687	0.93335	no
ni|645903690|nb|KK920740.1|	822521-822745	nan	nan	49	78	0.672814	0.54485	no
ni|645903690|nb|KK920740.1|	822901-823125	nan	nan	49	59	0.287363	0.799	no
ni|645903690|nb|KK920740.1|	824884-825575	nan	nan	2	1	-0.299292	0.8033	no
ni|645903690|nb|KK920740.1|	826686-826970	nan	nan	2	2	-0.272342	0.8304	no
ni|645903690|nb|KK920740.1|	827081-827322	nan	nan	41	55	0.434361	0.70085	no
ni|645903690|nb|KK920740.1|	827733-827958	nan	nan	39	77	0.977203	0.3848	no
ni|645903690|nb|KK920740.1|	829184-829638	nan	nan	25	29	0.228304	0.83075	no
ni|645903690|nb|KK920740.1|	831759-832193	nan	nan	81	88	0.115312	0.93795	no
ni|645903690|nb|KK920740.1|	835705-836094	nan	nan	9	13	0.547594	0.63835	no
ni|645903690|nb|KK920740.1|	836617-836841	nan	nan	11	18	0.721961	0.54095	no
ni|645903690|nb|KK920740.1|	837389-837561	nan	nan	11	18	0.706435	0.5522	no
ni|645903690|nb|KK920740.1|	838305-838509	nan	nan	11	20	0.821459	0.48815	no
ni|645903690|nb|KK920740.1|	838793-839012	nan	nan	11	17	0.653276	0.57815	no
ni|645903690|nb|KK920740.1|	839387-839608	nan	nan	10	16	0.723493	0.53535	no
ni|645903690|nb|KK920740.1|	839719-840033	nan	nan	2	5	1	0.28115	no
ni|645903690|nb|KK920740.1|	841404-841551	nan	nan	12	14	0.216591	0.85615	no
ni|645903690|nb|KK920740.1|	842854-843005	nan	nan	9	16	0.814162	0.50645	no
ni|645903690|nb|KK920740.1|	844882-845035	nan	nan	20	21	0.0640544	0.95595	no
ni|645903690|nb|KK920740.1|	845729-846039	nan	nan	4	6	0.533732	0.65165	no
ni|645903690|nb|KK920740.1|	847016-847201	nan	nan	9	17	0.957407	0.4218	no
ni|645903690|nb|KK920740.1|	847618-847740	nan	nan	22	30	0.468674	0.70445	no

ni|645903690|nb|KK920740.1|	847866-847980	nan	nan	24	21	-0.174435	0.8924	no
ni|645903690|nb|KK920740.1|	849472-849685	nan	nan	14	25	0.885189	0.45895	no
ni|645903690|nb|KK920740.1|	850922-851328	nan	nan	6	9	0.61781	0.5976	no
ni|645903690|nb|KK920740.1|	851615-852671	nan	nan	2	3	0.269341	0.8167	no
ni|645903690|nb|KK920740.1|	852982-853230	nan	nan	16	24	0.568005	0.62305	no
ni|645903690|nb|KK920740.1|	853417-854134	nan	nan	5	7	0.479524	0.67895	no
ni|645903690|nb|KK920740.1|	854409-854753	nan	nan	4	4	-0.0655594	0.95475	no
ni|645903690|nb|KK920740.1|	855412-855796	nan	nan	3	3	-0.155597	0.8945	no
ni|645903690|nb|KK920740.1|	855961-856114	nan	nan	20	15	-0.405992	0.7295	no
ni|645903690|nb|KK920740.1|	856301-856504	nan	nan	12	15	0.237937	0.83835	no
ni|645903690|nb|KK920740.1|	856574-857014	nan	nan	8	6	-0.393869	0.7354	no
ni|645903690|nb|KK920740.1|	857946-858517	nan	nan	9	8	-0.18191	0.8706	no
ni|645903690|nb|KK920740.1|	859176-860255	nan	nan	7	5	-0.459306	0.68395	no
ni|645903690|nb|KK920740.1|	860359-861476	nan	nan	2	2	0.0350015	0.9746	no
ni|645903690|nb|KK920740.1|	861661-862100	nan	nan	299	190	-0.653572	0.6427	no
ni|645903690|nb|KK920740.1|	862634-863453	nan	nan	0.378275	0.756249	0.999428	0.45905	no
ni|645903690|nb|KK920740.1|	864233-864528	nan	nan	125	77	-0.70243	0.5826	no
ni|645903690|nb|KK920740.1|	866368-866579	nan	nan	25	40	0.667656	0.564	no
ni|645903690|nb|KK920740.1|	866687-867550	nan	nan	6	7	0.3073	0.78745	no
ni|645903690|nb|KK920740.1|	867971-868396	nan	nan	32	27	-0.239487	0.8277	no
ni|645903690|nb|KK920740.1|	868448-869060	nan	nan	33	28	-0.225642	0.8234	no
ni|645903690|nb|KK920740.1|	870372-870500	nan	nan	4	9	1	0.43015	no
ni|645903690|nb|KK920740.1|	871979-872105	nan	nan	11	31	1	0.2551	no
ni|645903690|nb|KK920740.1|	874795-875125	nan	nan	4	13	2	0.17615	no
ni|645903690|nb|KK920740.1|	877866-878175	nan	nan	0.714515	14	4	0.0351	no
ni|645903690|nb|KK920740.1|	881103-881383	nan	nan	6	3	-1	0.3475	no
ni|645903690|nb|KK920740.1|	881490-881678	nan	nan	18	11	-0.709664	0.54945	no
ni|645903690|nb|KK920740.1|	882552-882721	nan	nan	17	11	-0.590822	0.61575	no
ni|645903690|nb|KK920740.1|	882775-882932	nan	nan	11	15	0.517764	0.66135	no
ni|645903690|nb|KK920740.1|	883360-883984	nan	nan	12	13	0.0755694	0.94385	no

ni|645903690|nb|KK920740.1|	884153-884434	nan	nan	10	11	0.15938	0.89105	no
ni|645903690|nb|KK920740.1|	884943-885183	nan	nan	9	11	0.244042	0.83815	no
ni|645903690|nb|KK920740.1|	885607-885773	nan	nan	11	8	-0.351027	0.7724	no
ni|645903690|nb|KK920740.1|	886756-886945	nan	nan	14	11	-0.259843	0.8278	no
ni|645903690|nb|KK920740.1|	887103-887260	nan	nan	12	18	0.604179	0.60885	no
ni|645903690|nb|KK920740.1|	888253-888450	nan	nan	13	14	0.0138765	0.98935	no
ni|645903690|nb|KK920740.1|	888626-888969	nan	nan	5	5	0.229892	0.8442	no
ni|645903690|nb|KK920740.1|	889963-890322	nan	nan	0.863532	1	0.224012	0.8598	no
ni|645903690|nb|KK920740.1|	891675-892014	nan	nan	2	2	0.197452	0.8697	no
ni|645903690|nb|KK920740.1|	919884-920542	nan	nan	0	19	inf	5.00E-05	yes
ni|645903690|nb|KK920740.1|	966942-967072	nan	nan	12	19	0.610777	0.6231	no
ni|645903690|nb|KK920740.1|	975780-976510	nan	nan	0	65	inf	5.00E-05	yes
ni|645903690|nb|KK920740.1|	978049-978701	nan	nan	0.044837	14	8	0.11935	no
ni|645903690|nb|KK920740.1|	980054-980455	nan	nan	0.16496	128	10	0.0443	no
ni|645903690|nb|KK920740.1|	995902-996513	nan	nan	182	89	-1	0.47075	no
ni|645903690|nb|KK920740.1|	996919-997422	nan	nan	2	0.881856	-0.802077	0.52275	no
ni|645903690|nb|KK920740.1|	998267-998766	nan	nan	427	198	-1	0.43815	no
ni|645903690|nb|KK920740.1|	998973-999823	nan	nan	179	92	-0.96707	0.49525	no
ni|645903691|nb|KK920739.1|	458285-458423	nan	nan	0	108	inf	5.00E-05	yes
ni|645903691|nb|KK920739.1|	464138-464854	nan	nan	6	32	2	0.0888	no
ni|645903691|nb|KK920739.1|	465099-465291	nan	nan	10	0	-inf	5.00E-05	yes
ni|645903691|nb|KK920739.1|	465476-466774	nan	nan	111	0.571623	-8	0.0003	yes
ni|645903691|nb|KK920739.1|	540270-540469	nan	nan	0	8	inf	0.0001	yes
ni|645903691|nb|KK920739.1|	556289-556470	nan	nan	0	9	inf	0.0001	yes
ni|645903691|nb|KK920739.1|	583743-583960	nan	nan	1	1	0.155744	0.9131	no
ni|645903691|nb|KK920739.1|	584680-586040	nan	nan	9	15	0.682135	0.59405	no
ni|645903691|nb|KK920739.1|	587073-587442	nan	nan	0.83122	0.706089	-0.23538	0.8697	no
ni|645903691|nb|KK920739.1|	588269-588464	nan	nan	3	2	-0.219091	0.87705	no
ni|645903691|nb|KK920739.1|	590425-590934	nan	nan	0.424183	0.463392	0.127545	0.92555	no
ni|645903692|nb|KK920738.1|	10576-10753	nan	nan	6	3	-0.695357	0.5883	no

ni|645903692|nb|KK920738.1|	11372-11684	nan	nan	6	6	0.0217437	0.98505	no
ni|645903692|nb|KK920738.1|	121714-121864	nan	nan	21	19	-0.137585	0.90605	no
ni|645903692|nb|KK920738.1|	125209-125428	nan	nan	10	11	0.181311	0.87665	no
ni|645903692|nb|KK920738.1|	125691-126026	nan	nan	3	3	0.0413131	0.97455	no
ni|645903692|nb|KK920738.1|	126182-126373	nan	nan	3	3	-0.0671657	0.94635	no
ni|645903692|nb|KK920738.1|	12754-13448	nan	nan	0.499847	0.477885	-0.0648228	0.9545	no
ni|645903692|nb|KK920738.1|	130433-130870	nan	nan	3	4	0.51971	0.6635	no
ni|645903692|nb|KK920738.1|	131559-131966	nan	nan	1	0.540951	-0.958414	0.51225	no
ni|645903692|nb|KK920738.1|	133148-134102	nan	nan	0.636626	0.525733	-0.276116	0.8205	no
ni|645903692|nb|KK920738.1|	134378-134707	nan	nan	6	6	-0.0391586	0.9733	no
ni|645903692|nb|KK920738.1|	139159-139343	nan	nan	369	308	-0.259415	0.8624	no
ni|645903692|nb|KK920738.1|	139579-140012	nan	nan	4	5	0.349775	0.76415	no
ni|645903692|nb|KK920738.1|	140831-141040	nan	nan	342	256	-0.414892	0.7774	no
ni|645903692|nb|KK920738.1|	142273-143004	nan	nan	0.823249	1	0.297788	0.80095	no
ni|645903692|nb|KK920738.1|	143233-143390	nan	nan	126	92	-0.449221	0.6888	no
ni|645903692|nb|KK920738.1|	143502-144190	nan	nan	0.715371	0.764399	0.0956348	0.93935	no
ni|645903692|nb|KK920738.1|	14377-14526	nan	nan	29	26	-0.1452	0.9004	no
ni|645903692|nb|KK920738.1|	144355-144882	nan	nan	0.580308	0.832075	0.519895	0.6956	no
ni|645903692|nb|KK920738.1|	145170-146126	nan	nan	0.404176	0.634923	0.651597	0.60985	no
ni|645903692|nb|KK920738.1|	147910-149265	nan	nan	1	3	2	0.20395	no
ni|645903692|nb|KK920738.1|	149370-149854	nan	nan	0.322819	0.678844	1	0.4292	no
ni|645903692|nb|KK920738.1|	150392-151152	nan	nan	0.41221	0.788236	0.935247	0.4672	no
ni|645903692|nb|KK920738.1|	151271-151907	nan	nan	0.831255	0.927141	0.157497	0.8958	no
ni|645903692|nb|KK920738.1|	152772-153077	nan	nan	1	0.696103	-0.65072	0.6679	no
ni|645903692|nb|KK920738.1|	154964-157480	nan	nan	0.541162	2	2	0.10125	no
ni|645903692|nb|KK920738.1|	157932-158420	nan	nan	0.638947	0.732887	0.197894	0.87955	no
ni|645903692|nb|KK920738.1|	158722-158953	nan	nan	0.953922	2	0.781572	0.58145	no
ni|645903692|nb|KK920738.1|	160370-160868	nan	nan	204	182	-0.162644	0.90625	no
ni|645903692|nb|KK920738.1|	161772-162310	nan	nan	132	113	-0.22435	0.87945	no
ni|645903692|nb|KK920738.1|	162474-162727	nan	nan	172	143	-0.267988	0.85845	no

ni|645903692|nb|KK920738.1|	165232-165489	nan	nan	440	408	-0.108926	0.9379	no
ni|645903692|nb|KK920738.1|	165589-165845	nan	nan	214	215.26	0.00631896	0.99605	no
ni|645903692|nb|KK920738.1|	16618-16755	nan	nan	18	20	0.132065	0.90925	no
ni|645903692|nb|KK920738.1|	19328-19582	nan	nan	2	2	0.0407762	0.95995	no
ni|645903692|nb|KK920738.1|	196-354	nan	nan	6	2	-1	0.34635	no
ni|645903692|nb|KK920738.1|	211702-212043	nan	nan	155	146	-0.0907315	0.95045	no
ni|645903692|nb|KK920738.1|	264389-264813	nan	nan	0.765916	1	0.70025	0.59985	no
ni|645903692|nb|KK920738.1|	269139-269336	nan	nan	2	2	0.0849596	0.9436	no
ni|645903692|nb|KK920738.1|	284546-285000	nan	nan	0.420396	0.535754	0.349822	0.81755	no
ni|645903692|nb|KK920738.1|	285826-286419	nan	nan	0.40182	0.528178	0.394474	0.77595	no
ni|645903692|nb|KK920738.1|	292293-292968	nan	nan	0.430359	0.617166	0.520117	0.6956	no
ni|645903692|nb|KK920738.1|	293824-294323	nan	nan	0.745491	0.356295	-1	0.46615	no
ni|645903692|nb|KK920738.1|	294952-295266	nan	nan	0.813979	0.888842	0.126937	0.92555	no
ni|645903692|nb|KK920738.1|	311043-311264	nan	nan	2	2	0.0854192	0.9436	no
ni|645903692|nb|KK920738.1|	341605-341827	nan	nan	3	2	-0.28896	0.8283	no
ni|645903692|nb|KK920738.1|	42212-42371	nan	nan	20	17	-0.229474	0.84635	no
ni|645903692|nb|KK920738.1|	4377-4532	nan	nan	10	4	-1	0.31765	no
ni|645903692|nb|KK920738.1|	4957-5274	nan	nan	1	2	0.349345	0.7835	no
ni|645903692|nb|KK920738.1|	6437-6741	nan	nan	3	2	-0.480801	0.6942	no
ni|645903692|nb|KK920738.1|	73115-73244	nan	nan	24	19	-0.401723	0.74285	no
ni|645903692|nb|KK920738.1|	7898-8312	nan	nan	14	10	-0.477571	0.66915	no
ni|645903692|nb|KK920738.1|	8747-9640	nan	nan	0.529891	0.298041	-0.830183	0.5313	no
ni|645903692|nb|KK920738.1|	88512-88701	nan	nan	14	13	-0.156466	0.8942	no
ni|645903692|nb|KK920738.1|	892-1041	nan	nan	5	5	0.0703061	0.94415	no
ni|645903693|nb|KK920737.1|	130184-130318	nan	nan	17	18	0.0482133	0.9698	no
ni|645903694|nb|KK920736.1|	106990-107567	nan	nan	1	0.992669	-0.266617	0.8253	no
ni|645903694|nb|KK920736.1|	222858-223145	nan	nan	2	4	0.93413	0.4458	no
ni|645903694|nb|KK920736.1|	224075-224158	nan	nan	3286.95	6483.87	0.980105	0.5074	no
ni|645903694|nb|KK920736.1|	237064-237340	nan	nan	0.141613	6	5	0.11995	no
ni|645903694|nb|KK920736.1|	33965-34081	nan	nan	13	7	-0.915469	0.53575	no

ni|645903694|nb|KK920736.1|	398908-399304	nan	nan	0.503292	0.961999	0.934639	0.5295	no
ni|645903694|nb|KK920736.1|	417571-418083	nan	nan	0.360925	0.460003	0.349943	0.81755	no
ni|645903694|nb|KK920736.1|	422172-422431	nan	nan	9	8	-0.11953	0.91695	no
ni|645903694|nb|KK920736.1|	422730-423446	nan	nan	60	75	0.329417	0.81155	no
ni|645903694|nb|KK920736.1|	424064-424460	nan	nan	27	28	0.0249715	0.9817	no
ni|645903694|nb|KK920736.1|	425608-425797	nan	nan	67	66	-0.0253782	0.98285	no
ni|645903694|nb|KK920736.1|	431730-432110	nan	nan	5	4	-0.393468	0.7368	no
ni|645903694|nb|KK920736.1|	432380-432989	nan	nan	10	7	-0.656653	0.57525	no
ni|645903694|nb|KK920736.1|	433149-433302	nan	nan	24	14	-0.711046	0.5482	no
ni|645903694|nb|KK920736.1|	433395-433535	nan	nan	21	11	-0.984765	0.4324	no
ni|645903694|nb|KK920736.1|	434969-435096	nan	nan	38	28	-0.459641	0.68725	no
ni|645903694|nb|KK920736.1|	435694-436128	nan	nan	10	8	-0.278504	0.80895	no
ni|645903694|nb|KK920736.1|	437788-438193	nan	nan	15	10	-0.614674	0.5922	no
ni|645903694|nb|KK920736.1|	438443-438614	nan	nan	21	15	-0.458069	0.6973	no
ni|645903694|nb|KK920736.1|	440186-440519	nan	nan	20	20	0.0236558	0.98165	no
ni|645903694|nb|KK920736.1|	441175-441551	nan	nan	2	1	-1	0.4167	no
ni|645903694|nb|KK920736.1|	442128-442610	nan	nan	9	9	-0.105231	0.92755	no
ni|645903694|nb|KK920736.1|	444351-444585	nan	nan	16	10	-0.651344	0.57835	no
ni|645903694|nb|KK920736.1|	444685-444842	nan	nan	9	11	0.365761	0.7586	no
ni|645903694|nb|KK920736.1|	445854-446136	nan	nan	20	20	-0.026911	0.9818	no
ni|645903694|nb|KK920736.1|	448932-449065	nan	nan	13	18	0.489122	0.69415	no
ni|645903694|nb|KK920736.1|	452600-452979	nan	nan	7	8	0.354486	0.7592	no
ni|645903694|nb|KK920736.1|	457863-457995	nan	nan	30	15	-1	0.39265	no
ni|645903694|nb|KK920736.1|	507599-507769	nan	nan	26	137	2	0.0459	no
ni|645903694|nb|KK920736.1|	508699-508853	nan	nan	41	206	2	0.04975	no
ni|645903694|nb|KK920736.1|	510577-510761	nan	nan	41	213	2	0.0393	no
ni|645903694|nb|KK920736.1|	511684-512371	nan	nan	65	369	3	0.10035	no
ni|645903694|nb|KK920736.1|	514349-514752	nan	nan	66	342	2	0.1179	no
ni|645903694|nb|KK920736.1|	516646-516770	nan	nan	61	215	2	0.1254	no
ni|645903694|nb|KK920736.1|	517428-517766	nan	nan	15	11	-0.533574	0.64785	no

ni|645903694|nb|KK920736.1|	528981-529546	nan	nan	107	54	-0.986055	0.50615	no
ni|645903694|nb|KK920736.1|	536863-537109	nan	nan	86	49	-0.818599	0.44965	no
ni|645903694|nb|KK920736.1|	542756-543164	nan	nan	0.080595	77	10	0.11935	no
ni|645903694|nb|KK920736.1|	543967-544842	nan	nan	0	8	inf	5.00E-05	yes
ni|645903694|nb|KK920736.1|	545140-545684	nan	nan	0	33.39	inf	5.00E-05	yes
ni|645903694|nb|KK920736.1|	545798-546021	nan	nan	0.203324	113.97	9	0.11935	no
ni|645903694|nb|KK920736.1|	553107-553305	nan	nan	1	100	7	0.03715	no
ni|645903694|nb|KK920736.1|	556154-556266	nan	nan	0	155	inf	5.00E-05	yes
ni|645903694|nb|KK920736.1|	563468-563639	nan	nan	0	102	inf	5.00E-05	yes
ni|645903694|nb|KK920736.1|	566417-566536	nan	nan	11	8	-0.42999	0.75965	no
ni|645903694|nb|KK920736.1|	566714-566856	nan	nan	6	2	-2	0.3149	no
ni|645903694|nb|KK920736.1|	571059-571390	nan	nan	0.215329	77	8	0.0444	no
ni|645903694|nb|KK920736.1|	619449-619579	nan	nan	0	43	inf	5.00E-05	yes
ni|645903694|nb|KK920736.1|	625244-625470	nan	nan	0	37	inf	5.00E-05	yes
ni|645903694|nb|KK920736.1|	626810-627346	nan	nan	0	27	inf	5.00E-05	yes
ni|645903694|nb|KK920736.1|	628518-629167	nan	nan	0.045083	31	9	0.1194	no
ni|645903694|nb|KK920736.1|	649581-649720	nan	nan	126	128	0.0313312	0.97665	no
ni|645903694|nb|KK920736.1|	671295-671417	nan	nan	480	416.74	-0.202568	0.8546	no
ni|645903694|nb|KK920736.1|	682498-682743	nan	nan	212	176	-0.267459	0.8552	no
ni|645903694|nb|KK920736.1|	683399-683720	nan	nan	1	1	-0.0656712	0.94765	no
ni|645903694|nb|KK920736.1|	684869-685121	nan	nan	219	178	-0.301387	0.83325	no
ni|645903694|nb|KK920736.1|	686099-686364	nan	nan	157	123	-0.35339	0.815	no
ni|645903694|nb|KK920736.1|	686947-687653	nan	nan	0.612388	0.468389	-0.386737	0.76495	no
ni|645903694|nb|KK920736.1|	688802-689053	nan	nan	629	482	-0.382421	0.78815	no
ni|645903694|nb|KK920736.1|	689311-689757	nan	nan	166	108	-0.622797	0.6682	no
ni|645903694|nb|KK920736.1|	98367-98504	nan	nan	21	17	-0.366804	0.7573	no
ni|645903695|nb|KK920735.1|	1006649-100701[illegible]	nan	nan	0	11	inf	5.00E-05	yes
ni|645903695|nb|KK920735.1|	121550-121975	nan	nan	0	3	inf	0.00015	yes
ni|645903695|nb|KK920735.1|	179148-179532	nan	nan	0	116	inf	5.00E-05	yes
ni|645903695|nb|KK920735.1|	179599-180348	nan	nan	0	88	inf	5.00E-05	yes

ni|645903695|nb|KK920735.1|	227859-228143	nan	nan	2	1	-0.650853	0.64935	no
ni|645903695|nb|KK920735.1|	228437-228587	nan	nan	2	2	-0.0671955	0.89835	no
ni|645903695|nb|KK920735.1|	231418-231707	nan	nan	2	1	-0.972747	0.5005	no
ni|645903695|nb|KK920735.1|	279302-279534	nan	nan	3	0.904035	-2	0.18825	no
ni|645903695|nb|KK920735.1|	498935-499815	nan	nan	0.221838	0.878764	2	0.16895	no
ni|645903695|nb|KK920735.1|	51833-52065	nan	nan	10	3	-2	0.21675	no
ni|645903695|nb|KK920735.1|	53423-54303	nan	nan	7	2	-2	0.1855	no
ni|645903695|nb|KK920735.1|	534704-535068	nan	nan	0	6	inf	5.00E-05	yes
ni|645903695|nb|KK920735.1|	582558-582854	nan	nan	4	5	0.22792	0.84935	no
ni|645903695|nb|KK920735.1|	582910-583285	nan	nan	6	4	-0.598306	0.61515	no
ni|645903695|nb|KK920735.1|	584682-585488	nan	nan	8	7	-0.0581099	0.9588	no
ni|645903695|nb|KK920735.1|	586109-586230	nan	nan	16	14	-0.174307	0.8924	no
ni|645903695|nb|KK920735.1|	586629-586897	nan	nan	9	10	0.28546	0.8074	no
ni|645903695|nb|KK920735.1|	587015-587153	nan	nan	14	15	0.125407	0.91735	no
ni|645903695|nb|KK920735.1|	587332-588037	nan	nan	8	11	0.313165	0.78105	no
ni|645903695|nb|KK920735.1|	588137-589185	nan	nan	6	7	0.21297	0.84885	no
ni|645903695|nb|KK920735.1|	589300-589500	nan	nan	16	18	0.222525	0.8451	no
ni|645903695|nb|KK920735.1|	589635-589789	nan	nan	14	11	-0.330227	0.78285	no
ni|645903695|nb|KK920735.1|	594986-595122	nan	nan	25	22	-0.149713	0.9051	no
ni|645903695|nb|KK920735.1|	595370-595530	nan	nan	10	17	0.715203	0.5472	no
ni|645903695|nb|KK920735.1|	596905-597644	nan	nan	1	1	-0.466872	0.6971	no
ni|645903695|nb|KK920735.1|	602443-602709	nan	nan	8	13	0.665774	0.57175	no
ni|645903695|nb|KK920735.1|	602974-603204	nan	nan	9	17	0.93356	0.4323	no
ni|645903695|nb|KK920735.1|	603327-604454	nan	nan	0.432933	0.574993	0.409401	0.74735	no
ni|645903695|nb|KK920735.1|	606271-606747	nan	nan	3	5	0.856832	0.47565	no
ni|645903695|nb|KK920735.1|	606947-607561	nan	nan	1	3	0.762237	0.51905	no
ni|645903695|nb|KK920735.1|	608464-608634	nan	nan	4	8	0.932731	0.46445	no
ni|645903695|nb|KK920735.1|	641049-641238	nan	nan	9	19	1	0.34715	no
ni|645903695|nb|KK920735.1|	793596-793811	nan	nan	0	57	inf	5.00E-05	yes
ni|645903695|nb|KK920735.1|	795143-795300	nan	nan	0.437805	66	7	0.11935	no

ni|645903695|nb|KK920735.1|	797123-797784	nan	nan	0.044114	1	5	0.1215	no
ni|645903695|nb|KK920735.1|	797973-798149	nan	nan	0	34	inf	5.00E-05	yes
ni|645903695|nb|KK920735.1|	834334-834681	nan	nan	2	2	-0.313473	0.8025	no
ni|645903695|nb|KK920735.1|	890781-891020	nan	nan	0	27	inf	5.00E-05	yes
ni|645903695|nb|KK920735.1|	894319-894723	nan	nan	0	134	inf	5.00E-05	yes
ni|645903695|nb|KK920735.1|	896986-897401	nan	nan	0	41	inf	5.00E-05	yes
ni|645903695|nb|KK920735.1|	899893-900285	nan	nan	0	507	inf	5.00E-05	yes
ni|645903695|nb|KK920735.1|	900789-901177	nan	nan	0	2	inf	0.00065	yes
ni|645903695|nb|KK920735.1|	90848-91064	nan	nan	13	6	-1	0.3836	no
ni|645903695|nb|KK920735.1|	969399-969678	nan	nan	2	1	-1	0.40265	no
ni|645903696|nb|KK920734.1|	11355-11545	nan	nan	7	10	0.498415	0.6713	no
ni|645903696|nb|KK920734.1|	262459-262550	nan	nan	3947.53	3021.86	-0.385514	0.7789	no
ni|645903696|nb|KK920734.1|	3075-3534	nan	nan	5	4	-0.127488	0.9133	no
ni|645903696|nb|KK920734.1|	30815-31513	nan	nan	2	2	0.331111	0.7746	no
ni|645903696|nb|KK920734.1|	340436-340760	nan	nan	9	6	-0.493077	0.6734	no
ni|645903696|nb|KK920734.1|	341144-342158	nan	nan	3	2	-0.53926	0.6473	no
ni|645903696|nb|KK920734.1|	343152-343614	nan	nan	5	3	-0.71156	0.5398	no
ni|645903696|nb|KK920734.1|	343753-344149	nan	nan	3	3	-0.0235407	0.98445	no
ni|645903696|nb|KK920734.1|	344405-345240	nan	nan	2	1	-0.588252	0.61575	no
ni|645903696|nb|KK920734.1|	346138-346430	nan	nan	10	7	-0.520402	0.65665	no
ni|645903696|nb|KK920734.1|	347317-348363	nan	nan	2	1	-0.500664	0.66525	no
ni|645903696|nb|KK920734.1|	349434-349642	nan	nan	4	4	0.0206507	0.96555	no
ni|645903696|nb|KK920734.1|	349835-350210	nan	nan	3	2	-0.724395	0.5465	no
ni|645903696|nb|KK920734.1|	350571-350815	nan	nan	3	2	-0.481285	0.7105	no
ni|645903696|nb|KK920734.1|	351055-351574	nan	nan	14	8	-0.744414	0.5217	no
ni|645903696|nb|KK920734.1|	351704-352550	nan	nan	2	2	-0.332615	0.77515	no
ni|645903696|nb|KK920734.1|	358167-358488	nan	nan	8	2	-2	0.16335	no
ni|645903696|nb|KK920734.1|	359675-360057	nan	nan	1	2	0.556082	0.6571	no
ni|645903696|nb|KK920734.1|	360625-361168	nan	nan	1	2	0.496839	0.67745	no
ni|645903696|nb|KK920734.1|	361307-362087	nan	nan	5	4	-0.538106	0.6455	no

ni|645903696|nb|KK920734.1|	371047-371284	nan	nan	15	9	-0.786231	0.502	no
ni|645903696|nb|KK920734.1|	371861-372188	nan	nan	2	0.314105	-3	0.1498	no
ni|645903696|nb|KK920734.1|	373363-374090	nan	nan	5	4	-0.303573	0.7956	no
ni|645903696|nb|KK920734.1|	376076-376389	nan	nan	6	5	-0.211565	0.8542	no
ni|645903696|nb|KK920734.1|	377139-377265	nan	nan	8	14	0.932666	0.48915	no
ni|645903696|nb|KK920734.1|	379194-379376	nan	nan	10	11	0.180636	0.87455	no
ni|645903696|nb|KK920734.1|	381420-381734	nan	nan	2	2	-0.0657085	0.95835	no
ni|645903696|nb|KK920734.1|	387425-387699	nan	nan	3	3	-0.25861	0.82995	no
ni|645903696|nb|KK920734.1|	387928-388134	nan	nan	13	9	-0.516657	0.66215	no
ni|645903696|nb|KK920734.1|	38879-39134	nan	nan	3	2	-0.651092	0.60395	no
ni|645903696|nb|KK920734.1|	389404-389801	nan	nan	0.919577	0.399476	-1	0.4064	no
ni|645903696|nb|KK920734.1|	393852-394167	nan	nan	6	4	-0.365263	0.7553	no
ni|645903696|nb|KK920734.1|	396774-396980	nan	nan	7	4	-0.676908	0.576	no
ni|645903696|nb|KK920734.1|	398077-398252	nan	nan	9	7	-0.308324	0.8005	no
ni|645903696|nb|KK920734.1|	39865-40011	nan	nan	8	6	-0.415127	0.7377	no
ni|645903696|nb|KK920734.1|	399244-399480	nan	nan	10	7	-0.40924	0.7284	no
ni|645903696|nb|KK920734.1|	403793-404320	nan	nan	5	5	-0.000936996	0.9932	no
ni|645903696|nb|KK920734.1|	404503-404645	nan	nan	19	10	-0.979753	0.43475	no
ni|645903696|nb|KK920734.1|	406996-407135	nan	nan	11	12	0.0107699	0.9704	no
ni|645903696|nb|KK920734.1|	407628-408730	nan	nan	4	4	-0.120756	0.9183	no
ni|645903696|nb|KK920734.1|	409563-409805	nan	nan	3	2	-0.803238	0.5588	no
ni|645903696|nb|KK920734.1|	413235-413426	nan	nan	7	6	-0.0671657	0.9528	no
ni|645903696|nb|KK920734.1|	414571-414788	nan	nan	6	6	-0.115558	0.92085	no
ni|645903696|nb|KK920734.1|	417630-417884	nan	nan	8	9	0.014781	0.98935	no
ni|645903696|nb|KK920734.1|	420232-420431	nan	nan	5	10	0.932998	0.4348	no
ni|645903696|nb|KK920734.1|	421832-422007	nan	nan	5	5	-0.0673159	0.9562	no
ni|645903696|nb|KK920734.1|	423010-423220	nan	nan	8	10	0.286864	0.8099	no
ni|645903696|nb|KK920734.1|	432723-433174	nan	nan	4	5	0.0939763	0.93555	no
ni|645903696|nb|KK920734.1|	433505-433745	nan	nan	2	1	-0.873653	0.53675	no
ni|645903696|nb|KK920734.1|	434159-434290	nan	nan	6	11	0.839604	0.5399	no

ni|645903696|nb|KK920734.1|	437923-438104	nan	nan	6	3	-1	0.4219	no
ni|645903696|nb|KK920734.1|	445627-446182	nan	nan	0.653314	0.624524	-0.0650193	0.9545	no
ni|645903696|nb|KK920734.1|	446270-446583	nan	nan	4	6	0.623946	0.59615	no
ni|645903696|nb|KK920734.1|	4497-4982	nan	nan	1	2	0.519816	0.66985	no
ni|645903696|nb|KK920734.1|	458323-458973	nan	nan	0.585005	0.301151	-0.957961	0.51225	no
ni|645903696|nb|KK920734.1|	459942-460510	nan	nan	53	44	-0.257026	0.8681	no
ni|645903696|nb|KK920734.1|	460598-461238	nan	nan	31	23	-0.472203	0.65445	no
ni|645903696|nb|KK920734.1|	5316-5662	nan	nan	3	3	0.104375	0.9244	no
ni|645903696|nb|KK920734.1|	558621-558918	nan	nan	5	3	-0.574819	0.63225	no
ni|645903696|nb|KK920734.1|	559224-559825	nan	nan	12	11	-0.185744	0.86925	no
ni|645903696|nb|KK920734.1|	559882-560274	nan	nan	17	17	-0.0444136	0.9685	no
ni|645903696|nb|KK920734.1|	566115-566333	nan	nan	24	30	0.316696	0.7848	no
ni|645903696|nb|KK920734.1|	567227-567377	nan	nan	18	18	0.010807	0.98435	no
ni|645903696|nb|KK920734.1|	568845-569077	nan	nan	23	28	0.278977	0.80405	no
ni|645903696|nb|KK920734.1|	570459-570773	nan	nan	10	8	-0.285275	0.80425	no
ni|645903696|nb|KK920734.1|	571003-571461	nan	nan	7	6	-0.191963	0.8696	no
ni|645903696|nb|KK920734.1|	577573-577958	nan	nan	6	6	-0.0443344	0.97065	no
ni|645903696|nb|KK920734.1|	580625-580740	nan	nan	22	23	0.032041	0.96125	no
ni|645903696|nb|KK920734.1|	581113-581273	nan	nan	32	27	-0.286518	0.803	no
ni|645903696|nb|KK920734.1|	587689-587933	nan	nan	15	11	-0.436197	0.7026	no
ni|645903696|nb|KK920734.1|	588762-589238	nan	nan	23	22	-0.0237061	0.98315	no
ni|645903696|nb|KK920734.1|	589481-590467	nan	nan	3	2	-0.342579	0.77085	no
ni|645903696|nb|KK920734.1|	591809-592934	nan	nan	1	0.737429	-0.871886	0.45925	no
ni|645903696|nb|KK920734.1|	593402-593979	nan	nan	2	2	-0.234908	0.84185	no
ni|645903696|nb|KK920734.1|	603651-603887	nan	nan	2	2	-0.191884	0.88855	no
ni|645903696|nb|KK920734.1|	606034-606327	nan	nan	2	2	0.275207	0.823	no
ni|645903696|nb|KK920734.1|	606555-608718	nan	nan	0.908939	1	0.495098	0.66775	no
ni|645903696|nb|KK920734.1|	611893-612488	nan	nan	0.250118	0.334749	0.420472	0.75735	no
ni|645903696|nb|KK920734.1|	613648-613976	nan	nan	3	3	-0.42178	0.7241	no
ni|645903696|nb|KK920734.1|	617665-617817	nan	nan	4	3	-0.652154	0.66125	no

ni|645903696|nb|KK920734.1|	618999-619383	nan	nan	1	0.919307	-0.605968	0.6316	no
ni|645903696|nb|KK920734.1|	619468-619815	nan	nan	1	0.769376	-0.650508	0.64935	no
ni|645903696|nb|KK920734.1|	620275-620621	nan	nan	1	0.772523	-0.524982	0.7071	no
ni|645903696|nb|KK920734.1|	620944-621162	nan	nan	8	5	-0.592701	0.62325	no
ni|645903696|nb|KK920734.1|	621864-621974	nan	nan	30	17	-0.768096	0.5799	no
ni|645903696|nb|KK920734.1|	622499-622715	nan	nan	12	9	-0.324463	0.78125	no
ni|645903696|nb|KK920734.1|	623490-623704	nan	nan	13	6	-0.945393	0.42715	no
ni|645903696|nb|KK920734.1|	624330-624438	nan	nan	50	27	-0.915768	0.4969	no
ni|645903696|nb|KK920734.1|	624557-624667	nan	nan	53	52	-0.00625584	0.9752	no
ni|645903696|nb|KK920734.1|	624816-625180	nan	nan	72	47	-0.607756	0.6337	no
ni|645903696|nb|KK920734.1|	625574-625788	nan	nan	144	116.34	-0.3123	0.76695	no
ni|645903696|nb|KK920734.1|	627595-628164	nan	nan	27	22	-0.301935	0.7817	no
ni|645903696|nb|KK920734.1|	630039-630184	nan	nan	116.96	88	-0.402809	0.7273	no
ni|645903696|nb|KK920734.1|	651038-651897	nan	nan	0.325707	0.186857	-0.801637	0.6027	no
ni|645903696|nb|KK920734.1|	6718-6889	nan	nan	6	11	0.850259	0.48905	no
ni|645903696|nb|KK920734.1|	7023-7296	nan	nan	2	4	1	0.3137	no
ni|645903696|nb|KK920734.1|	7520-7693	nan	nan	10	7	-0.603352	0.61745	no
ni|645903696|nb|KK920734.1|	8017-8317	nan	nan	3	3	-0.00920342	0.97805	no
ni|645903697|nb|KK920733.1|	103650-103950	nan	nan	201	216	0.106617	0.9424	no
ni|645903697|nb|KK920733.1|	104397-105366	nan	nan	34	24	-0.529583	0.721	no
ni|645903697|nb|KK920733.1|	10513-10744	nan	nan	34	21	-0.655492	0.5743	no
ni|645903697|nb|KK920733.1|	105494-105838	nan	nan	65	62	-0.0701033	0.9492	no
ni|645903697|nb|KK920733.1|	105971-106127	nan	nan	11	9	-0.192727	0.87175	no
ni|645903697|nb|KK920733.1|	106245-106367	nan	nan	20	14	-0.536864	0.67225	no
ni|645903697|nb|KK920733.1|	106814-106935	nan	nan	14	18	0.347646	0.78955	no
ni|645903697|nb|KK920733.1|	107092-107318	nan	nan	3	0.378989	-3	0.2517	no
ni|645903697|nb|KK920733.1|	107516-108015	nan	nan	6	5	-0.304789	0.79365	no
ni|645903697|nb|KK920733.1|	108232-108850	nan	nan	9	8	-0.164456	0.88565	no
ni|645903697|nb|KK920733.1|	109205-109569	nan	nan	45	42	-0.0898243	0.9327	no
ni|645903697|nb|KK920733.1|	11423-11575	nan	nan	17	13	-0.38912	0.7411	no

ni|645903697|nb|KK920733.1|	11646-11762	nan	nan	18	18	0.0480047	0.95725	no
ni|645903697|nb|KK920733.1|	116983-117192	nan	nan	5	3	-0.874147	0.49155	no
ni|645903697|nb|KK920733.1|	117455-117834	nan	nan	121	131	0.116714	0.9368	no
ni|645903697|nb|KK920733.1|	118275-118794	nan	nan	0.768875	0.282677	-1	0.3141	no
ni|645903697|nb|KK920733.1|	119058-119310	nan	nan	413	411	-0.00717369	0.9959	no
ni|645903697|nb|KK920733.1|	11981-13137	nan	nan	13	14	0.141065	0.89285	no
ni|645903697|nb|KK920733.1|	120723-121715	nan	nan	113	109	-0.0515761	0.97035	no
ni|645903697|nb|KK920733.1|	122150-122351	nan	nan	574	535	-0.101027	0.9417	no
ni|645903697|nb|KK920733.1|	122493-123031	nan	nan	218.12	195	-0.158557	0.9055	no
ni|645903697|nb|KK920733.1|	126836-127291	nan	nan	0	3	inf	5.00E-05	yes
ni|645903697|nb|KK920733.1|	133668-133832	nan	nan	0	66	inf	5.00E-05	yes
ni|645903697|nb|KK920733.1|	13425-13722	nan	nan	0.884789	0.724575	-0.288197	0.8535	no
ni|645903697|nb|KK920733.1|	13908-14323	nan	nan	13	12	-0.0998652	0.9287	no
ni|645903697|nb|KK920733.1|	141917-142117	nan	nan	0	71	inf	5.00E-05	yes
ni|645903697|nb|KK920733.1|	146739-146962	nan	nan	0	62	inf	5.00E-05	yes
ni|645903697|nb|KK920733.1|	14814-15127	nan	nan	16	17	0.0659918	0.95455	no
ni|645903697|nb|KK920733.1|	148804-149049	nan	nan	0	25	inf	5.00E-05	yes
ni|645903697|nb|KK920733.1|	155257-155595	nan	nan	12	8	-0.548529	0.6299	no
ni|645903697|nb|KK920733.1|	155862-156177	nan	nan	69	51	-0.434532	0.68285	no
ni|645903697|nb|KK920733.1|	157150-157439	nan	nan	15	10	-0.520422	0.65245	no
ni|645903697|nb|KK920733.1|	157731-158005	nan	nan	31	21	-0.506054	0.65465	no
ni|645903697|nb|KK920733.1|	158519-158660	nan	nan	7	6	-0.204726	0.87975	no
ni|645903697|nb|KK920733.1|	158777-159159	nan	nan	1	2	0.394025	0.75185	no
ni|645903697|nb|KK920733.1|	159384-159885	nan	nan	6	3	-0.733493	0.5297	no
ni|645903697|nb|KK920733.1|	15969-16099	nan	nan	31	24	-0.361026	0.76115	no
ni|645903697|nb|KK920733.1|	160805-161200	nan	nan	3	3	-0.0278892	0.98205	no
ni|645903697|nb|KK920733.1|	161416-161940	nan	nan	0.818207	0.335195	-1	0.39005	no
ni|645903697|nb|KK920733.1|	162461-163243	nan	nan	0.797735	1	0.761237	0.53415	no
ni|645903697|nb|KK920733.1|	16290-16639	nan	nan	7	18	1	0.2498	no
ni|645903697|nb|KK920733.1|	164030-164318	nan	nan	2	4	0.622193	0.6185	no

ni|645903697|nb|KK920733.1|	165627-166055	nan	nan	22	21	-0.0602563	0.9583	no
ni|645903697|nb|KK920733.1|	167566-168183	nan	nan	9	10	0.260173	0.8171	no
ni|645903697|nb|KK920733.1|	174409-174664	nan	nan	53	53	0.0195158	0.98705	no
ni|645903697|nb|KK920733.1|	17496-17771	nan	nan	25	57	1	0.2935	no
ni|645903697|nb|KK920733.1|	177343-177546	nan	nan	39	35	-0.15884	0.88995	no
ni|645903697|nb|KK920733.1|	177732-177862	nan	nan	10	5	-1	0.46615	no
ni|645903697|nb|KK920733.1|	181273-181921	nan	nan	0.045165	3	6	0.11945	no
ni|645903697|nb|KK920733.1|	182093-183174	nan	nan	20	29	0.533125	0.7213	no
ni|645903697|nb|KK920733.1|	18341-18676	nan	nan	1	0.303344	-2	0.24815	no
ni|645903697|nb|KK920733.1|	184059-185322	nan	nan	9	13	0.553231	0.61035	no
ni|645903697|nb|KK920733.1|	185808-186126	nan	nan	2	0.763372	-1	0.33495	no
ni|645903697|nb|KK920733.1|	186899-187253	nan	nan	8	9	0.238491	0.84055	no
ni|645903697|nb|KK920733.1|	187363-187771	nan	nan	35	48	0.448586	0.67765	no
ni|645903697|nb|KK920733.1|	189564-189800	nan	nan	85	108	0.33541	0.7595	no
ni|645903697|nb|KK920733.1|	190387-190650	nan	nan	27	32	0.245889	0.82925	no
ni|645903697|nb|KK920733.1|	192588-193125	nan	nan	39	37	-0.088067	0.9343	no
ni|645903697|nb|KK920733.1|	196888-197046	nan	nan	32.28	23	-0.514659	0.6542	no
ni|645903697|nb|KK920733.1|	197106-197256	nan	nan	24	22	-0.158343	0.8935	no
ni|645903697|nb|KK920733.1|	19737-20004	nan	nan	21	42	0.998551	0.3849	no
ni|645903697|nb|KK920733.1|	197975-198318	nan	nan	22	42	0.95795	0.39345	no
ni|645903697|nb|KK920733.1|	200153-200525	nan	nan	18	54	2	0.15385	no
ni|645903697|nb|KK920733.1|	200773-201242	nan	nan	0.672026	0.192702	-2	0.2784	no
ni|645903697|nb|KK920733.1|	201824-202024	nan	nan	52	168	2	0.1335	no
ni|645903697|nb|KK920733.1|	203823-203949	nan	nan	60	199	2	0.1454	no
ni|645903697|nb|KK920733.1|	204791-205306	nan	nan	30	95	2	0.19265	no
ni|645903697|nb|KK920733.1|	207849-208582	nan	nan	9	35	2	0.164	no
ni|645903697|nb|KK920733.1|	212759-213227	nan	nan	25	91	2	0.16375	no
ni|645903697|nb|KK920733.1|	213361-213507	nan	nan	33	136	2	0.0905	no
ni|645903697|nb|KK920733.1|	216199-216390	nan	nan	39	144	2	0.10105	no
ni|645903697|nb|KK920733.1|	217364-217675	nan	nan	18.4	51	1	0.1971	no

ni|645903697|nb|KK920733.1|	217971-218424	nan	nan	4	5	0.17397	0.88495	no
ni|645903697|nb|KK920733.1|	218665-218925	nan	nan	4	67	4	0.0041	no
ni|645903697|nb|KK920733.1|	219430-219614	nan	nan	12	20	0.755852	0.51875	no
ni|645903697|nb|KK920733.1|	24079-24653	nan	nan	4	8	1	0.33995	no
ni|645903697|nb|KK920733.1|	24907-25504	nan	nan	6	23	2	0.0987	no
ni|645903697|nb|KK920733.1|	30519-30726	nan	nan	23	64	1	0.2203	no
ni|645903697|nb|KK920733.1|	32965-33124	nan	nan	19	60	2	0.15885	no
ni|645903697|nb|KK920733.1|	37589-38316	nan	nan	0.47344	0.226325	-1	0.46615	no
ni|645903697|nb|KK920733.1|	38719-39098	nan	nan	15	44	2	0.17295	no
ni|645903697|nb|KK920733.1|	389334-389501	nan	nan	48	44	-0.135955	0.90685	no
ni|645903697|nb|KK920733.1|	391258-391498	nan	nan	46	41	-0.159408	0.88845	no
ni|645903697|nb|KK920733.1|	392545-392658	nan	nan	45	21	-1	0.37695	no
ni|645903697|nb|KK920733.1|	392829-393328	nan	nan	15	13	-0.152205	0.8889	no
ni|645903697|nb|KK920733.1|	394569-394690	nan	nan	37	16	-1	0.354	no
ni|645903697|nb|KK920733.1|	395215-395392	nan	nan	48	39	-0.27987	0.80895	no
ni|645903697|nb|KK920733.1|	396530-396775	nan	nan	5	5	-0.165771	0.8886	no
ni|645903697|nb|KK920733.1|	398037-398207	nan	nan	50	38	-0.415192	0.72015	no
ni|645903697|nb|KK920733.1|	398610-398800	nan	nan	46	46	-0.00759324	0.99465	no
ni|645903697|nb|KK920733.1|	399928-400061	nan	nan	60	42	-0.524478	0.65065	no
ni|645903697|nb|KK920733.1|	401772-402039	nan	nan	23	19	-0.237133	0.83865	no
ni|645903697|nb|KK920733.1|	402701-402962	nan	nan	58	42	-0.456948	0.68535	no
ni|645903697|nb|KK920733.1|	404038-404178	nan	nan	3	4	0.4182	0.75735	no
ni|645903697|nb|KK920733.1|	404627-404975	nan	nan	1	0.957819	-0.328576	0.80325	no
ni|645903697|nb|KK920733.1|	405381-405775	nan	nan	2	4	0.993527	0.4086	no
ni|645903697|nb|KK920733.1|	406282-406490	nan	nan	2	3	0.51815	0.6956	no
ni|645903697|nb|KK920733.1|	407476-407680	nan	nan	57	42	-0.447845	0.6914	no
ni|645903697|nb|KK920733.1|	408155-408300	nan	nan	56	43	-0.382079	0.75005	no
ni|645903697|nb|KK920733.1|	408863-409087	nan	nan	59	51	-0.211443	0.85425	no
ni|645903697|nb|KK920733.1|	414025-414184	nan	nan	57	41	-0.47508	0.6852	no
ni|645903697|nb|KK920733.1|	414462-414598	nan	nan	53	31	-0.779448	0.51455	no

ni|645903697|nb|KK920733.1|	415154-415746	nan	nan	18	14	-0.335411	0.7641	no
ni|645903697|nb|KK920733.1|	416283-416431	nan	nan	83	62	-0.420837	0.71795	no
ni|645903697|nb|KK920733.1|	416582-416781	nan	nan	42	41	-0.049827	0.9647	no
ni|645903697|nb|KK920733.1|	417881-418584	nan	nan	38	29	-0.375465	0.76265	no
ni|645903697|nb|KK920733.1|	419159-419557	nan	nan	27	16	-0.773026	0.4933	no
ni|645903697|nb|KK920733.1|	419618-419724	nan	nan	21	31	0.517003	0.7279	no
ni|645903697|nb|KK920733.1|	49888-50153	nan	nan	1	0.577506	-1	0.4023	no
ni|645903697|nb|KK920733.1|	51757-51944	nan	nan	29	73	1	0.25535	no
ni|645903697|nb|KK920733.1|	52262-52778	nan	nan	19	50	1	0.2826	no
ni|645903697|nb|KK920733.1|	54027-54288	nan	nan	20	55	1	0.2155	no
ni|645903697|nb|KK920733.1|	55756-56186	nan	nan	20	54	1	0.2813	no
ni|645903697|nb|KK920733.1|	56576-56684	nan	nan	33	45	0.434729	0.7373	no
ni|645903697|nb|KK920733.1|	56926-57419	nan	nan	18	46	1	0.29495	no
ni|645903697|nb|KK920733.1|	58204-59004	nan	nan	12	31	1	0.29175	no
ni|645903697|nb|KK920733.1|	59173-59387	nan	nan	9	22	1	0.25695	no
ni|645903697|nb|KK920733.1|	60696-60836	nan	nan	35	86	1	0.26815	no
ni|645903697|nb|KK920733.1|	62336-62566	nan	nan	39	80	1	0.36255	no
ni|645903697|nb|KK920733.1|	63444-63902	nan	nan	1	0.794608	-0.387134	0.7621	no
ni|645903697|nb|KK920733.1|	66064-66266	nan	nan	1	3	1	0.30495	no
ni|645903697|nb|KK920733.1|	66388-66938	nan	nan	0.715659	1	0.934972	0.4565	no
ni|645903697|nb|KK920733.1|	67617-67856	nan	nan	1	1	-0.288704	0.8535	no
ni|645903697|nb|KK920733.1|	69608-69749	nan	nan	24	27	0.201964	0.8618	no
ni|645903697|nb|KK920733.1|	71783-72245	nan	nan	0.479564	1	2	0.24365	no
ni|645903697|nb|KK920733.1|	73507-73837	nan	nan	0	5	inf	5.00E-05	yes
ni|645903697|nb|KK920733.1|	76941-77311	nan	nan	0.092013	2	5	0.12065	no
ni|645903697|nb|KK920733.1|	77803-78723	nan	nan	266	336	0.333653	0.815	no
ni|645903697|nb|KK920733.1|	909661-909806	nan	nan	329	176	-0.898357	0.40715	no
ni|645903697|nb|KK920733.1|	910417-910863	nan	nan	0.71695	0.411153	-0.802199	0.6027	no
ni|645903697|nb|KK920733.1|	911077-911853	nan	nan	183	109	-0.746388	0.5973	no
ni|645903697|nb|KK920733.1|	912100-912236	nan	nan	7	5	-0.581824	0.70815	no

ni|645903697|nb|KK920733.1|	913314-913464	nan	nan	4	2	-1	0.44965	no
ni|645903697|nb|KK920733.1|	913590-913881	nan	nan	2	2	0.671123	0.5978	no
ni|645903698|nb|KK920732.1|	142369-143258	nan	nan	383	353	-0.115028	0.93535	no
ni|645903698|nb|KK920732.1|	143396-143517	nan	nan	239	179	-0.419103	0.7103	no
ni|645903698|nb|KK920732.1|	143601-143827	nan	nan	395	320	-0.306984	0.8271	no
ni|645903698|nb|KK920732.1|	144138-144358	nan	nan	2	2	0.0709034	0.94355	no
ni|645903698|nb|KK920732.1|	147047-147807	nan	nan	166	132	-0.335014	0.8063	no
ni|645903698|nb|KK920732.1|	149851-150063	nan	nan	135	121	-0.162704	0.879	no
ni|645903698|nb|KK920732.1|	152331-152948	nan	nan	6	4	-0.528869	0.65425	no
ni|645903698|nb|KK920732.1|	153742-153968	nan	nan	39	31	-0.322232	0.7781	no
ni|645903698|nb|KK920732.1|	155566-155727	nan	nan	102	84	-0.285743	0.79955	no
ni|645903698|nb|KK920732.1|	156132-156490	nan	nan	45	34	-0.370353	0.73475	no
ni|645903698|nb|KK920732.1|	158466-158767	nan	nan	79	57	-0.480069	0.64985	no
ni|645903698|nb|KK920732.1|	160303-160452	nan	nan	15	11	-0.514656	0.66705	no
ni|645903698|nb|KK920732.1|	161974-162180	nan	nan	12	3	-2	0.15355	no
ni|645903698|nb|KK920732.1|	162976-163110	nan	nan	12	8	-0.569764	0.66295	no
ni|645903698|nb|KK920732.1|	164016-164156	nan	nan	19	19	-0.0214236	0.9796	no
ni|645903698|nb|KK920732.1|	165015-165185	nan	nan	16	23	0.539389	0.64975	no
ni|645903698|nb|KK920732.1|	167305-167585	nan	nan	21	21	-0.0201164	0.9849	no
ni|645903698|nb|KK920732.1|	168176-168349	nan	nan	19	21	0.155093	0.8959	no
ni|645903698|nb|KK920732.1|	169254-169422	nan	nan	17	22	0.332358	0.77395	no
ni|645903698|nb|KK920732.1|	172090-172358	nan	nan	27	29	0.134581	0.90755	no
ni|645903698|nb|KK920732.1|	173201-173374	nan	nan	14	25	0.803967	0.4941	no
ni|645903698|nb|KK920732.1|	174102-174231	nan	nan	12	17	0.517658	0.6788	no
ni|645903698|nb|KK920732.1|	176446-176594	nan	nan	22	24	0.121246	0.9171	no
ni|645903698|nb|KK920732.1|	176816-176931	nan	nan	28	39	0.46302	0.70795	no
ni|645903698|nb|KK920732.1|	177038-177155	nan	nan	13	16	0.347585	0.7963	no
ni|645903698|nb|KK920732.1|	180599-180810	nan	nan	143	150	0.0724091	0.94555	no
ni|645903698|nb|KK920732.1|	181059-181192	nan	nan	133	72	-0.892875	0.44135	no
ni|645903698|nb|KK920732.1|	181455-181924	nan	nan	77	49	-0.663997	0.66215	no

ni|645903698|nb|KK920732.1|	182730-182993	nan	nan	30	22	-0.473861	0.68355	no
ni|645903698|nb|KK920732.1|	183472-183632	nan	nan	61	38	-0.672067	0.56015	no
ni|645903698|nb|KK920732.1|	184139-184519	nan	nan	3	3	-0.0654135	0.95355	no
ni|645903698|nb|KK920732.1|	219152-219388	nan	nan	36	95	1	0.21835	no
ni|645903698|nb|KK920732.1|	222186-222348	nan	nan	64	170	1	0.20685	no
ni|645903698|nb|KK920732.1|	224056-224749	nan	nan	31	95	2	0.2795	no
ni|645903698|nb|KK920732.1|	237850-237978	nan	nan	8	28	2	0.1707	no
ni|645903698|nb|KK920732.1|	242998-243523	nan	nan	2	7	2	0.1694	no
ni|645903698|nb|KK920732.1|	248778-248985	nan	nan	0	6	inf	0.0009	yes
ni|645903698|nb|KK920732.1|	94647-94925	nan	nan	4	7	0.986533	0.4076	no
ni|645903699|nb|KK920731.1|	110946-111085	nan	nan	0	91	inf	5.00E-05	yes
ni|645903699|nb|KK920731.1|	126094-126212	nan	nan	0	157	inf	5.00E-05	yes
ni|645903699|nb|KK920731.1|	129981-130091	nan	nan	0	41	inf	0.0005	yes
ni|645903699|nb|KK920731.1|	130227-130784	nan	nan	0	40	inf	5.00E-05	yes
ni|645903699|nb|KK920731.1|	135469-135581	nan	nan	37	7	-2	0.1366	no
ni|645903699|nb|KK920731.1|	135636-135767	nan	nan	113	75	-0.597801	0.60985	no
ni|645903699|nb|KK920731.1|	136058-136193	nan	nan	96	81	-0.247829	0.831	no
ni|645903699|nb|KK920731.1|	137756-138313	nan	nan	4	3	-0.485859	0.6815	no
ni|645903699|nb|KK920731.1|	138597-138907	nan	nan	4	2	-0.717807	0.5539	no
ni|645903699|nb|KK920731.1|	139340-139728	nan	nan	2	1	-0.313313	0.8025	no
ni|645903699|nb|KK920731.1|	139988-140233	nan	nan	6	3	-1	0.3332	no
ni|645903699|nb|KK920731.1|	140609-140933	nan	nan	32	34	0.0718477	0.9448	no
ni|645903699|nb|KK920731.1|	141172-141362	nan	nan	54	85	0.653244	0.56305	no
ni|645903699|nb|KK920731.1|	163246-163752	nan	nan	0.488382	0.700248	0.519857	0.7004	no
ni|645903699|nb|KK920731.1|	166652-168033	nan	nan	71	57	-0.312575	0.82585	no
ni|645903699|nb|KK920731.1|	171040-171293	nan	nan	19	38	1	0.38735	no
ni|645903699|nb|KK920731.1|	172397-172823	nan	nan	16	35	1	0.32595	no
ni|645903699|nb|KK920731.1|	173696-173825	nan	nan	10	35	2	0.1667	no
ni|645903699|nb|KK920731.1|	175941-176142	nan	nan	19	68	2	0.12425	no
ni|645903699|nb|KK920731.1|	178619-179085	nan	nan	15	39	1	0.20135	no

ni|645903699|nb|KK920731.1|	182206-182341	nan	nan	18	32	0.873849	0.466	no
ni|645903699|nb|KK920731.1|	183873-184109	nan	nan	27	59	1	0.33665	no
ni|645903699|nb|KK920731.1|	186429-186785	nan	nan	12	31	1	0.23685	no
ni|645903699|nb|KK920731.1|	186854-187340	nan	nan	8	15	1	0.3686	no
ni|645903699|nb|KK920731.1|	189354-189488	nan	nan	8	9	0.173744	0.8916	no
ni|645903699|nb|KK920731.1|	189902-190034	nan	nan	35	41	0.248223	0.8326	no
ni|645903699|nb|KK920731.1|	191267-191461	nan	nan	37	39	0.0703966	0.95045	no
ni|645903699|nb|KK920731.1|	191801-191976	nan	nan	33	33	0.00447477	0.99385	no
ni|645903699|nb|KK920731.1|	192634-193342	nan	nan	9	9	-0.102775	0.9248	no
ni|645903699|nb|KK920731.1|	193587-193797	nan	nan	26	26	0.00660055	0.99455	no
ni|645903699|nb|KK920731.1|	194060-194500	nan	nan	18	22	0.275178	0.8028	no
ni|645903699|nb|KK920731.1|	195145-195591	nan	nan	8	9	0.157159	0.8969	no
ni|645903699|nb|KK920731.1|	195751-195888	nan	nan	21	32	0.650985	0.58055	no
ni|645903699|nb|KK920731.1|	199032-199251	nan	nan	3	3	-0.0666166	0.95935	no
ni|645903699|nb|KK920731.1|	200504-200983	nan	nan	22	24	0.179543	0.8715	no
ni|645903699|nb|KK920731.1|	201053-201684	nan	nan	114	134.28	0.23234	0.8658	no
ni|645903699|nb|KK920731.1|	203116-203328	nan	nan	6	5	-0.414658	0.7409	no
ni|645903699|nb|KK920731.1|	205688-205803	nan	nan	52	65	0.314376	0.7905	no
ni|645903699|nb|KK920731.1|	207824-208680	nan	nan	37	38	0.0317888	0.98305	no
ni|645903699|nb|KK920731.1|	209165-209629	nan	nan	0.95383	0.651206	-0.550619	0.68265	no
ni|645903699|nb|KK920731.1|	209954-210372	nan	nan	0.312181	1	2	0.14305	no
ni|645903699|nb|KK920731.1|	210785-211303	nan	nan	0.889297	2	0.835381	0.5035	no
ni|645903699|nb|KK920731.1|	212607-212895	nan	nan	51	53	0.0536759	0.96035	no
ni|645903699|nb|KK920731.1|	213010-213783	nan	nan	2	2	0.215367	0.84875	no
ni|645903699|nb|KK920731.1|	214217-214685	nan	nan	2	2	-0.0178772	0.98125	no
ni|645903699|nb|KK920731.1|	214818-215296	nan	nan	2	0.815065	-0.888284	0.4823	no
ni|645903699|nb|KK920731.1|	216142-216692	nan	nan	2	2	0.2569	0.82985	no
ni|645903699|nb|KK920731.1|	219219-219430	nan	nan	2	2	-0.71883	0.61925	no
ni|645903699|nb|KK920731.1|	220066-220296	nan	nan	60	65	0.106545	0.9228	no
ni|645903699|nb|KK920731.1|	221614-222094	nan	nan	5	7	0.4308	0.71075	no

ni|645903699|nb|KK920731.1|	222207-222431	nan	nan	6	5	-0.481572	0.68515	no
ni|645903699|nb|KK920731.1|	233416-233630	nan	nan	45	7	-3	0.035	no
ni|645903699|nb|KK920731.1|	238197-238429	nan	nan	179	21	-3	0.0347	no
ni|645903699|nb|KK920731.1|	301557-301776	nan	nan	3	3	-0.0666166	0.95935	no
ni|645903699|nb|KK920731.1|	304298-304724	nan	nan	2	1	-0.650242	0.5978	no
ni|645903699|nb|KK920731.1|	307027-307348	nan	nan	3	3	-0.0656712	0.954	no
ni|645903699|nb|KK920731.1|	309985-310567	nan	nan	0.976203	0.245582	-2	0.18585	no
ni|645903699|nb|KK920731.1|	311449-311876	nan	nan	3	0.580223	-2	0.1309	no
ni|645903699|nb|KK920731.1|	312037-312378	nan	nan	3	2	-0.480611	0.69425	no
ni|645903699|nb|KK920731.1|	314633-315056	nan	nan	2	0.9546	-0.757165	0.54375	no
ni|645903699|nb|KK920731.1|	323295-323773	nan	nan	2	1	-0.296487	0.8023	no
ni|645903699|nb|KK920731.1|	323903-324065	nan	nan	5	1	-2	0.20695	no
ni|645903699|nb|KK920731.1|	325529-325718	nan	nan	4	1	-1	0.3141	no
ni|645903699|nb|KK920731.1|	329008-329348	nan	nan	1	2	0.519385	0.6876	no
ni|645903699|nb|KK920731.1|	333207-333464	nan	nan	1	0.758969	-0.744182	0.59615	no
ni|645903699|nb|KK920731.1|	334771-335145	nan	nan	0.997276	1	0.175573	0.8916	no
ni|645903699|nb|KK920731.1|	349333-349564	nan	nan	2	0.910997	-1	0.44755	no
ni|645903699|nb|KK920731.1|	355753-355951	nan	nan	2	2	0.669943	0.65595	no
ni|645903699|nb|KK920731.1|	378447-378904	nan	nan	0.764214	0.730441	-0.0652084	0.94765	no
ni|645903699|nb|KK920731.1|	43619-43782	nan	nan	0	152	inf	5.00E-05	yes
ni|645903699|nb|KK920731.1|	473330-474905	nan	nan	0.03347	222	13	0.0443	no
ni|645903699|nb|KK920731.1|	49603-49818	nan	nan	0	8	inf	0.00015	yes
ni|645903699|nb|KK920731.1|	51374-51546	nan	nan	0	6	inf	0.00065	yes
ni|645903699|nb|KK920731.1|	593774-594111	nan	nan	1	1	-0.444104	0.74215	no
ni|645903699|nb|KK920731.1|	629122-629536	nan	nan	0	8	inf	5.00E-05	yes
ni|645903699|nb|KK920731.1|	786664-786832	nan	nan	82	62	-0.400544	0.7231	no
ni|645903699|nb|KK920731.1|	805178-805530	nan	nan	154	124	-0.312352	0.82575	no
ni|645903699|nb|KK920731.1|	823029-825201	nan	nan	185	130	-0.508346	0.7268	no
ni|645903699|nb|KK920731.1|	838267-839980	nan	nan	2	0.248766	-3	0.0395	no
ni|645903699|nb|KK920731.1|	849628-850954	nan	nan	41	10	-2	0.12215	no

ni|645903699|nb|KK920731.1|	89969-90217	nan	nan	0	302.39	inf	5.00E-05	yes
ni|645903699|nb|KK920731.1|	91288-91501	nan	nan	0	415	inf	5.00E-05	yes
ni|645903699|nb|KK920731.1|	93208-93525	nan	nan	0	11	inf	5.00E-05	yes
ni|645903699|nb|KK920731.1|	93801-94035	nan	nan	0	14	inf	5.00E-05	yes
ni|645903700|nb|KK920730.1|	157649-158001	nan	nan	0	96	inf	5.00E-05	yes
ni|645903700|nb|KK920730.1|	160926-161253	nan	nan	0	12	inf	5.00E-05	yes
ni|645903700|nb|KK920730.1|	165713-166082	nan	nan	0	9	inf	5.00E-05	yes
ni|645903700|nb|KK920730.1|	183490-183669	nan	nan	0	188	inf	5.00E-05	yes
ni|645903700|nb|KK920730.1|	193173-193742	nan	nan	0	86	inf	5.00E-05	yes
ni|645903700|nb|KK920730.1|	198561-199093	nan	nan	0	0.932056	inf	0.00135	yes
ni|645903701|nb|KK920729.1|	169831-169951	nan	nan	11	6	-0.915402	0.53575	no
ni|645903701|nb|KK920729.1|	177626-177829	nan	nan	3	3	-0.0669179	0.95935	no
ni|645903701|nb|KK920729.1|	179812-180573	nan	nan	4	6	0.512173	0.6579	no
ni|645903701|nb|KK920729.1|	186379-186622	nan	nan	4	7	0.682202	0.5653	no
ni|645903701|nb|KK920729.1|	188274-188619	nan	nan	1	2	0.860445	0.50465	no
ni|645903701|nb|KK920729.1|	198177-198313	nan	nan	44	58	0.408483	0.7238	no
ni|645903701|nb|KK920729.1|	201323-201457	nan	nan	39	62	0.669702	0.57075	no
ni|645903701|nb|KK920729.1|	202972-203527	nan	nan	29	40	0.457712	0.7243	no
ni|645903701|nb|KK920729.1|	206112-206539	nan	nan	88	143	0.691123	0.63805	no
ni|645903701|nb|KK920729.1|	207279-207550	nan	nan	119.11	230	0.947195	0.5234	no
ni|645903701|nb|KK920729.1|	213793-213904	nan	nan	67	110	0.713746	0.5401	no
ni|645903701|nb|KK920729.1|	214839-215116	nan	nan	149	262	0.811367	0.58045	no
ni|645903701|nb|KK920729.1|	217212-217473	nan	nan	34	58	0.743138	0.51005	no
ni|645903701|nb|KK920729.1|	217823-218078	nan	nan	14	30	1	0.3528	no
ni|645903701|nb|KK920729.1|	222547-222958	nan	nan	17	21	0.259254	0.81505	no
ni|645903701|nb|KK920729.1|	226111-226250	nan	nan	8	8	0.15516	0.90275	no
ni|645903702|nb|KK920728.1|	308548-308693	nan	nan	6	7	0.173802	0.8916	no
ni|645903702|nb|KK920728.1|	308807-308957	nan	nan	33	38	0.206565	0.85555	no
ni|645903702|nb|KK920728.1|	311131-311326	nan	nan	24	24	-0.0360609	0.97505	no
ni|645903702|nb|KK920728.1|	312167-312718	nan	nan	7	9	0.425502	0.712	no

ni|645903702|nb|KK920728.1|	313853-314199	nan	nan	8	11	0.430407	0.7112	no
ni|645903702|nb|KK920728.1|	314423-314599	nan	nan	11	14	0.424528	0.7186	no
ni|645903702|nb|KK920728.1|	314763-316311	nan	nan	6	7	0.187737	0.8657	no
ni|645903702|nb|KK920728.1|	317788-317957	nan	nan	14	18	0.357238	0.7557	no
ni|645903702|nb|KK920728.1|	318613-318973	nan	nan	0.669035	1	0.586586	0.65495	no
ni|645903702|nb|KK920728.1|	319368-319948	nan	nan	2	2	-0.0346043	0.9792	no
ni|645903702|nb|KK920728.1|	320122-320648	nan	nan	2	3	0.519893	0.6577	no
ni|645903702|nb|KK920728.1|	321598-322061	nan	nan	5	7	0.519768	0.65015	no
ni|645903702|nb|KK920728.1|	353098-353246	nan	nan	11	14	0.280724	0.81735	no
ni|645903702|nb|KK920728.1|	371713-371862	nan	nan	3	7	1	0.42455	no
ni|645903702|nb|KK920728.1|	371947-372177	nan	nan	2	3	0.763635	0.5642	no
ni|645903702|nb|KK920728.1|	376917-377889	nan	nan	0.340063	0.46066	0.437899	0.73605	no
ni|645903702|nb|KK920728.1|	388928-389727	nan	nan	0.707517	0.913247	0.368241	0.75825	no
ni|645903702|nb|KK920728.1|	389844-390442	nan	nan	5	3	-0.806882	0.4959	no
ni|645903702|nb|KK920728.1|	391129-391350	nan	nan	2	2	-0.388512	0.78555	no
ni|645903702|nb|KK920728.1|	392333-392562	nan	nan	2	0.740197	-1	0.44965	no
ni|645903702|nb|KK920728.1|	410413-411327	nan	nan	0.455454	0.2613	-0.801601	0.5588	no
ni|645903702|nb|KK920728.1|	411408-412180	nan	nan	0.404824	0.24631	-0.716819	0.61925	no
ni|645903702|nb|KK920728.1|	414623-415522	nan	nan	2	2	0.133901	0.90515	no
ni|645903702|nb|KK920728.1|	418837-419391	nan	nan	0.65476	1	0.742334	0.5577	no
ni|645903702|nb|KK920728.1|	421397-422211	nan	nan	0.831125	2	0.90492	0.45215	no
ni|645903702|nb|KK920728.1|	430991-431554	nan	nan	0.481479	2	2	0.1106	no
ni|645903702|nb|KK920728.1|	484964-485249	nan	nan	0.538666	2	2	0.30335	no
ni|645903702|nb|KK920728.1|	485907-486086	nan	nan	3	4	0.57011	0.67875	no
ni|645903702|nb|KK920728.1|	486184-486395	nan	nan	1	4	2	0.297	no
ni|645903702|nb|KK920728.1|	519647-519839	nan	nan	0	93	inf	5.00E-05	yes
ni|645903702|nb|KK920728.1|	531022-531149	nan	nan	111.26	36	-2	0.1763	no
ni|645903702|nb|KK920728.1|	531378-531836	nan	nan	266	173	-0.621732	0.6508	no
ni|645903702|nb|KK920728.1|	532326-533062	nan	nan	2	2	-0.241655	0.836	no
ni|645903702|nb|KK920728.1|	533718-534222	nan	nan	9	6	-0.679819	0.5613	no

ni|645903702|nb|KK920728.1|	535436-536021	nan	nan	531	408	-0.381224	0.79325	no
ni|645903702|nb|KK920728.1|	536319-537354	nan	nan	1	0.90964	-0.509355	0.661	no
ni|645903702|nb|KK920728.1|	537840-538709	nan	nan	464.99	401	-0.214509	0.88355	no
ni|645903702|nb|KK920728.1|	539023-539373	nan	nan	1	0.190022	-3	0.28285	no
ni|645903702|nb|KK920728.1|	539527-539774	nan	nan	2	1	-1	0.45115	no
ni|645903702|nb|KK920728.1|	540097-540506	nan	nan	715	647	-0.142939	0.9177	no
ni|645903702|nb|KK920728.1|	541589-542175	nan	nan	505	493	-0.034601	0.981	no
ni|645903702|nb|KK920728.1|	542354-542541	nan	nan	1084.61	1068.79	-0.0211967	0.98805	no
ni|645903702|nb|KK920728.1|	542777-543081	nan	nan	311	297	-0.0646321	0.96325	no
ni|645903702|nb|KK920728.1|	544578-544790	nan	nan	1075.48	1135.91	0.0788677	0.95685	no
ni|645903702|nb|KK920728.1|	545424-545647	nan	nan	937	974	0.0555836	0.9688	no
ni|645903702|nb|KK920728.1|	547876-548129	nan	nan	934	1058.63	0.181049	0.8959	no
ni|645903702|nb|KK920728.1|	548503-548989	nan	nan	490.57	581	0.245177	0.86195	no
ni|645903702|nb|KK920728.1|	549657-549877	nan	nan	10	9	-0.189457	0.874	no
ni|645903702|nb|KK920728.1|	550996-551175	nan	nan	4	4	-0.0673196	0.9582	no
ni|645903702|nb|KK920728.1|	551469-551598	nan	nan	8	10	0.46321	0.7264	no
ni|645903702|nb|KK920728.1|	551724-552104	nan	nan	4	4	0.107918	0.92435	no
ni|645903702|nb|KK920728.1|	552333-553088	nan	nan	3	3	-0.0319682	0.9779	no
ni|645903702|nb|KK920728.1|	553655-553817	nan	nan	57	152	1	0.22365	no
ni|645903702|nb|KK920728.1|	554581-554874	nan	nan	2	0.986269	-1	0.43485	no
ni|645903702|nb|KK920728.1|	555165-555542	nan	nan	161	143	-0.170728	0.9037	no
ni|645903702|nb|KK920728.1|	558046-558973	nan	nan	0.35862	0.20003	-0.842235	0.5595	no
ni|645903702|nb|KK920728.1|	597374-597636	nan	nan	606	715	0.239349	0.8643	no
ni|645903702|nb|KK920728.1|	598012-598294	nan	nan	807	962	0.254259	0.86085	no
ni|645903702|nb|KK920728.1|	598759-599482	nan	nan	0.436784	0.303711	-0.524222	0.7071	no
ni|645903702|nb|KK920728.1|	601054-601353	nan	nan	1	3	2	0.19855	no
ni|645903702|nb|KK920728.1|	601544-602179	nan	nan	0.832816	0.575021	-0.534382	0.67295	no
ni|645903702|nb|KK920728.1|	602381-602676	nan	nan	214	279	0.379003	0.7865	no
ni|645903702|nb|KK920728.1|	605220-606537	nan	nan	0	21	inf	5.00E-05	yes
ni|645903702|nb|KK920728.1|	606851-607476	nan	nan	0	9	inf	5.00E-05	yes

ni|645903702|nb|KK920728.1|	610994-611505	nan	nan	6	5	-0.407984	0.7346	no
ni|645903702|nb|KK920728.1|	612326-612513	nan	nan	31	37	0.243974	0.8346	no
ni|645903702|nb|KK920728.1|	613361-613525	nan	nan	41	41	0.0267545	0.98165	no
ni|645903702|nb|KK920728.1|	614500-614878	nan	nan	16	21	0.33583	0.77025	no
ni|645903702|nb|KK920728.1|	615910-617070	nan	nan	6	8	0.277067	0.80125	no
ni|645903702|nb|KK920728.1|	617258-617553	nan	nan	1	3	1	0.35975	no
ni|645903702|nb|KK920728.1|	618918-619159	nan	nan	30	39	0.395501	0.72545	no
ni|645903702|nb|KK920728.1|	619472-619643	nan	nan	24	28	0.237576	0.8455	no
ni|645903702|nb|KK920728.1|	621550-621692	nan	nan	24	28	0.254712	0.8246	no
ni|645903702|nb|KK920728.1|	621897-622104	nan	nan	26	38	0.531069	0.6492	no
ni|645903702|nb|KK920728.1|	622658-622864	nan	nan	23	40	0.771395	0.50995	no
ni|645903702|nb|KK920728.1|	623839-624086	nan	nan	15	29	0.975128	0.4116	no
ni|645903702|nb|KK920728.1|	625297-625576	nan	nan	16	32	1	0.37265	no
ni|645903702|nb|KK920728.1|	628370-628486	nan	nan	19	34	0.81705	0.5144	no
ni|645903702|nb|KK920728.1|	635231-635464	nan	nan	21	32	0.60056	0.59965	no
ni|645903702|nb|KK920728.1|	637033-637303	nan	nan	10	24	1	0.29345	no
ni|645903702|nb|KK920728.1|	648343-648622	nan	nan	11	14	0.34911	0.7644	no
ni|645903702|nb|KK920728.1|	650890-651037	nan	nan	57	69	0.265374	0.82095	no
ni|645903702|nb|KK920728.1|	652139-652485	nan	nan	17	22	0.336776	0.768	no
ni|645903702|nb|KK920728.1|	652976-653318	nan	nan	1	4	1	0.28525	no
ni|645903702|nb|KK920728.1|	695884-696619	nan	nan	0.038955	129.46	12	0.11935	no
ni|645903702|nb|KK920728.1|	696749-697110	nan	nan	0.095208	258	11	0.11935	no
ni|645903702|nb|KK920728.1|	697367-697923	nan	nan	0	109.57	inf	5.00E-05	yes
ni|645903702|nb|KK920728.1|	698040-698370	nan	nan	0	287	inf	5.00E-05	yes
ni|645903702|nb|KK920728.1|	698533-698657	nan	nan	0	220	inf	5.00E-05	yes
ni|645903702|nb|KK920728.1|	698799-699820	nan	nan	0.026827	180	13	0.11935	no
ni|645903702|nb|KK920728.1|	699944-700672	nan	nan	0	306.03	inf	5.00E-05	yes
ni|645903702|nb|KK920728.1|	700776-701666	nan	nan	0	135	inf	5.00E-05	yes
ni|645903702|nb|KK920728.1|	701785-702360	nan	nan	0.05214	257	12	0.11935	no
ni|645903702|nb|KK920728.1|	710773-710994	nan	nan	0.206719	435	11	0.11935	no

ni|645903702|nb|KK920728.1|	711379-711494	nan	nan	0	328	inf	5.00E-05	yes
ni|645903702|nb|KK920728.1|	713554-713861	nan	nan	0	62	inf	5.00E-05	yes
ni|645903702|nb|KK920728.1|	715314-715430	nan	nan	39	11	-2	0.20345	no
ni|645903702|nb|KK920728.1|	716000-716134	nan	nan	28	15	-0.960349	0.428	no
ni|645903702|nb|KK920728.1|	716298-716676	nan	nan	37	32	-0.202219	0.85135	no
ni|645903702|nb|KK920728.1|	716804-717220	nan	nan	30.71	28	-0.137171	0.90185	no
ni|645903702|nb|KK920728.1|	717277-717455	nan	nan	38	33	-0.222046	0.8487	no
ni|645903702|nb|KK920728.1|	718126-718252	nan	nan	21	4	-2	0.1349	no
ni|645903702|nb|KK920728.1|	718532-718672	nan	nan	56	45	-0.311153	0.7908	no
ni|645903702|nb|KK920728.1|	718995-719574	nan	nan	25	20	-0.345087	0.7555	no
ni|645903702|nb|KK920728.1|	719631-719828	nan	nan	20	12	-0.767483	0.5171	no
ni|645903702|nb|KK920728.1|	720270-720439	nan	nan	73	74	0.00923856	0.9938	no
ni|645903702|nb|KK920728.1|	720679-720820	nan	nan	50	59	0.247651	0.8269	no
ni|645903702|nb|KK920728.1|	721836-722324	nan	nan	25	26	0.0808541	0.93995	no
ni|645903702|nb|KK920728.1|	722376-723826	nan	nan	9	8	-0.0826199	0.93955	no
ni|645903702|nb|KK920728.1|	724157-724616	nan	nan	2	1	-0.413127	0.7409	no
ni|645903702|nb|KK920728.1|	724720-725335	nan	nan	17	17.05	0.0315588	0.97685	no
ni|645903702|nb|KK920728.1|	727443-728065	nan	nan	2	0.997442	-0.692946	0.57185	no
ni|645903702|nb|KK920728.1|	728663-728902	nan	nan	1	3	1	0.34405	no
ni|645903702|nb|KK920728.1|	729454-729688	nan	nan	6	8	0.502985	0.67295	no
ni|645903702|nb|KK920728.1|	730648-730810	nan	nan	55	57	0.0449971	0.9695	no
ni|645903702|nb|KK920728.1|	731381-732632	nan	nan	7	12	0.675899	0.53545	no
ni|645903702|nb|KK920728.1|	733245-734963	nan	nan	0.137284	4	5	0.01095	no
ni|645903702|nb|KK920728.1|	759233-759397	nan	nan	0	9	inf	0.0003	yes
ni|645903702|nb|KK920728.1|	767462-767742	nan	nan	0.138439	5	5	0.12	no
ni|645903702|nb|KK920728.1|	836281-836817	nan	nan	0	11	inf	5.00E-05	yes
ni|645903702|nb|KK920728.1|	836873-837208	nan	nan	0	7	inf	5.00E-05	yes
ni|645903702|nb|KK920728.1|	837860-838213	nan	nan	0	2	inf	0.00055	yes
ni|645903702|nb|KK920728.1|	87209-87455	nan	nan	0	3	inf	0.0007	yes
ni|645903702|nb|KK920728.1|	93072-93541	nan	nan	2	2	0.0458506	0.97075	no

ni|645903702|nb|KK920728.1|	95832-97249	nan	nan	0	22	inf	5.00E-05	yes
ni|645903702|nb|KK920728.1|	98606-98951	nan	nan	0	5	inf	5.00E-05	yes
ni|645903703|nb|KK920727.1|	1027832-1028011	nan	nan	97	112	0.206674	0.85175	no
ni|645903703|nb|KK920727.1|	1029020-1029729	nan	nan	108	119	0.136123	0.9234	no
ni|645903703|nb|KK920727.1|	1029792-1031464	nan	nan	84	109	0.388204	0.78325	no
ni|645903703|nb|KK920727.1|	1032127-1032306	nan	nan	10	6	-0.726283	0.54605	no
ni|645903703|nb|KK920727.1|	1033714-1034003	nan	nan	3	2	-0.437825	0.7272	no
ni|645903703|nb|KK920727.1|	116703-116874	nan	nan	22	9	-1	0.29055	no
ni|645903703|nb|KK920727.1|	120289-120577	nan	nan	9	6	-0.538616	0.6479	no
ni|645903703|nb|KK920727.1|	123961-124084	nan	nan	18	5	-2	0.1999	no
ni|645903703|nb|KK920727.1|	126063-126448	nan	nan	0	36	inf	5.00E-05	yes
ni|645903703|nb|KK920727.1|	127692-127974	nan	nan	0	59	inf	5.00E-05	yes
ni|645903703|nb|KK920727.1|	131516-131701	nan	nan	0	52	inf	5.00E-05	yes
ni|645903703|nb|KK920727.1|	132618-132745	nan	nan	0	53	inf	5.00E-05	yes
ni|645903703|nb|KK920727.1|	133882-134092	nan	nan	0	63	inf	5.00E-05	yes
ni|645903703|nb|KK920727.1|	136536-137670	nan	nan	0	24	inf	5.00E-05	yes
ni|645903703|nb|KK920727.1|	144174-144899	nan	nan	0	115	inf	5.00E-05	yes
ni|645903703|nb|KK920727.1|	146204-146455	nan	nan	0.165285	16	7	0.1194	no
ni|645903703|nb|KK920727.1|	147322-147440	nan	nan	0	62	inf	0.00015	yes
ni|645903703|nb|KK920727.1|	150265-150589	nan	nan	0.111054	121	10	0.11935	no
ni|645903703|nb|KK920727.1|	150742-151346	nan	nan	0.098253	228	11	0.0443	no
ni|645903703|nb|KK920727.1|	155726-155861	nan	nan	0	368	inf	5.00E-05	yes
ni|645903703|nb|KK920727.1|	157921-158273	nan	nan	0.098632	324	12	0.11935	no
ni|645903703|nb|KK920727.1|	159763-160020	nan	nan	0	117	inf	5.00E-05	yes
ni|645903703|nb|KK920727.1|	161096-161353	nan	nan	0	424	inf	5.00E-05	yes
ni|645903703|nb|KK920727.1|	163903-164402	nan	nan	0	205	inf	5.00E-05	yes
ni|645903703|nb|KK920727.1|	171389-171622	nan	nan	0	448	inf	5.00E-05	yes
ni|645903703|nb|KK920727.1|	173613-173823	nan	nan	0.227602	459	11	0.11935	no
ni|645903703|nb|KK920727.1|	174385-174679	nan	nan	0.128369	26	8	0.11935	no
ni|645903703|nb|KK920727.1|	175954-176178	nan	nan	0.605002	674	10	0.05055	no

ni|645903703|nb|KK920727.1|	176836-177682	nan	nan	0.09942	233	11	0.05055	no
ni|645903703|nb|KK920727.1|	179925-180146	nan	nan	0.413438	677	11	0.0443	no
ni|645903703|nb|KK920727.1|	183452-183650	nan	nan	458	495	0.112176	0.93535	no
ni|645903703|nb|KK920727.1|	183824-183986	nan	nan	4	2	-0.915209	0.53575	no
ni|645903703|nb|KK920727.1|	185138-185700	nan	nan	58	70	0.268773	0.851	no
ni|645903703|nb|KK920727.1|	185812-186081	nan	nan	389	403	0.0496411	0.97205	no
ni|645903703|nb|KK920727.1|	188108-188366	nan	nan	4	5	0.539621	0.65635	no
ni|645903703|nb|KK920727.1|	188768-189077	nan	nan	3	2	-0.27724	0.8205	no
ni|645903703|nb|KK920727.1|	190024-190376	nan	nan	32	27	-0.28281	0.7993	no
ni|645903703|nb|KK920727.1|	190730-191128	nan	nan	4	3	-0.358137	0.75735	no
ni|645903703|nb|KK920727.1|	191892-192045	nan	nan	17	12	-0.521756	0.6583	no
ni|645903703|nb|KK920727.1|	196342-196644	nan	nan	14	26	0.940768	0.41585	no
ni|645903703|nb|KK920727.1|	201706-201899	nan	nan	18	40	1	0.32995	no
ni|645903703|nb|KK920727.1|	203555-203898	nan	nan	8	17	1	0.3378	no
ni|645903703|nb|KK920727.1|	207188-207566	nan	nan	4	5	0.508315	0.66295	no
ni|645903703|nb|KK920727.1|	207725-207846	nan	nan	25	32	0.331158	0.791	no
ni|645903703|nb|KK920727.1|	209573-209849	nan	nan	11	18	0.671016	0.56445	no
ni|645903703|nb|KK920727.1|	211061-211282	nan	nan	8	16	1	0.38655	no
ni|645903703|nb|KK920727.1|	215445-216009	nan	nan	85	89	0.0578897	0.9682	no
ni|645903703|nb|KK920727.1|	216814-217278	nan	nan	125	124.38	-0.0105409	0.99345	no
ni|645903703|nb|KK920727.1|	220337-220538	nan	nan	2	0.236816	-3	0.03815	no
ni|645903703|nb|KK920727.1|	221450-221891	nan	nan	14	16	0.21398	0.84745	no
ni|645903703|nb|KK920727.1|	222050-222201	nan	nan	97	94	-0.0454986	0.9667	no
ni|645903703|nb|KK920727.1|	223430-223586	nan	nan	86	76	-0.18392	0.87635	no
ni|645903703|nb|KK920727.1|	224400-224548	nan	nan	80.81	98	0.284023	0.8023	no
ni|645903703|nb|KK920727.1|	224768-224902	nan	nan	57	45	-0.348677	0.76085	no
ni|645903703|nb|KK920727.1|	225106-225468	nan	nan	4	3	-0.404284	0.7307	no
ni|645903703|nb|KK920727.1|	225891-226559	nan	nan	5	6	0.359644	0.75825	no
ni|645903703|nb|KK920727.1|	228290-228489	nan	nan	65	74	0.195093	0.86415	no
ni|645903703|nb|KK920727.1|	229936-230103	nan	nan	55	63	0.19091	0.86335	no

ni|645903703|nb|KK920727.1|	230373-230673	nan	nan	75	88	0.22047	0.83445	no
ni|645903703|nb|KK920727.1|	232292-232415	nan	nan	15	26	0.825717	0.51075	no
ni|645903703|nb|KK920727.1|	233217-233372	nan	nan	43	53	0.305465	0.7912	no
ni|645903703|nb|KK920727.1|	235057-235484	nan	nan	27	38	0.497659	0.649	no
ni|645903703|nb|KK920727.1|	236998-237122	nan	nan	50	81	0.710252	0.54345	no
ni|645903703|nb|KK920727.1|	245418-245627	nan	nan	17	14	-0.192323	0.8697	no
ni|645903703|nb|KK920727.1|	245998-246629	nan	nan	38	38	-0.00831854	0.99495	no
ni|645903703|nb|KK920727.1|	252687-252932	nan	nan	64	83	0.361285	0.739	no
ni|645903703|nb|KK920727.1|	253371-253811	nan	nan	9	11	0.383028	0.7378	no
ni|645903703|nb|KK920727.1|	254352-254491	nan	nan	54	94	0.799501	0.49385	no
ni|645903703|nb|KK920727.1|	255563-255776	nan	nan	62	69	0.138706	0.8984	no
ni|645903703|nb|KK920727.1|	257230-257612	nan	nan	75	84	0.162323	0.9145	no
ni|645903703|nb|KK920727.1|	258679-259237	nan	nan	49	63	0.363966	0.80745	no
ni|645903703|nb|KK920727.1|	259951-260111	nan	nan	95	110	0.218846	0.8469	no
ni|645903703|nb|KK920727.1|	260598-260793	nan	nan	63	89	0.493627	0.66165	no
ni|645903703|nb|KK920727.1|	262414-262549	nan	nan	6	9	0.570173	0.67875	no
ni|645903703|nb|KK920727.1|	263574-263699	nan	nan	58	66	0.175513	0.88025	no
ni|645903703|nb|KK920727.1|	263823-264163	nan	nan	27	34	0.321445	0.7691	no
ni|645903703|nb|KK920727.1|	264752-264946	nan	nan	59	84	0.493937	0.6603	no
ni|645903703|nb|KK920727.1|	267060-267207	nan	nan	56	79	0.503955	0.66435	no
ni|645903703|nb|KK920727.1|	267319-267524	nan	nan	71	82	0.209812	0.85155	no
ni|645903703|nb|KK920727.1|	268938-269143	nan	nan	16	29	0.921985	0.4307	no
ni|645903703|nb|KK920727.1|	270895-271082	nan	nan	55	77.03	0.484626	0.6706	no
ni|645903703|nb|KK920727.1|	271288-271452	nan	nan	23	18	-0.359402	0.75655	no
ni|645903703|nb|KK920727.1|	275077-275269	nan	nan	71	104	0.543287	0.62615	no
ni|645903703|nb|KK920727.1|	275854-276007	nan	nan	70	107	0.608459	0.60015	no
ni|645903703|nb|KK920727.1|	277349-277639	nan	nan	37	52	0.510531	0.64325	no
ni|645903703|nb|KK920727.1|	277916-278102	nan	nan	67	80	0.257193	0.8172	no
ni|645903703|nb|KK920727.1|	278826-278994	nan	nan	56	70	0.314179	0.7836	no
ni|645903703|nb|KK920727.1|	281794-281997	nan	nan	69	92	0.412692	0.70655	no

ni|645903703|nb|KK920727.1|	282439-282747	nan	nan	29	33	0.190321	0.86435	no
ni|645903703|nb|KK920727.1|	286718-287288	nan	nan	24	24.13	-0.00346247	0.99765	no
ni|645903703|nb|KK920727.1|	289608-289737	nan	nan	41	56	0.447269	0.7	no
ni|645903703|nb|KK920727.1|	291243-292030	nan	nan	0.323963	5	4	0.01615	no
ni|645903703|nb|KK920727.1|	295291-295998	nan	nan	1	5	2	0.1028	no
ni|645903703|nb|KK920727.1|	296151-296439	nan	nan	0.529999	2	2	0.1847	no
ni|645903703|nb|KK920727.1|	298745-298993	nan	nan	67	43	-0.615095	0.5789	no
ni|645903703|nb|KK920727.1|	301261-301530	nan	nan	22	16	-0.455279	0.6957	no
ni|645903703|nb|KK920727.1|	302258-302718	nan	nan	62	50	-0.295406	0.84095	no
ni|645903703|nb|KK920727.1|	303822-304991	nan	nan	2	2	-0.135773	0.9036	no
ni|645903703|nb|KK920727.1|	307626-307826	nan	nan	4	6	0.545995	0.65645	no
ni|645903703|nb|KK920727.1|	309363-309862	nan	nan	26	29	0.181521	0.86695	no
ni|645903703|nb|KK920727.1|	310557-310782	nan	nan	48	55	0.17449	0.8783	no
ni|645903703|nb|KK920727.1|	312927-313083	nan	nan	57	47	-0.284008	0.8061	no
ni|645903703|nb|KK920727.1|	314684-315129	nan	nan	48	35	-0.46466	0.66115	no
ni|645903703|nb|KK920727.1|	316495-316845	nan	nan	38	29	-0.402768	0.7157	no
ni|645903703|nb|KK920727.1|	317403-317585	nan	nan	106	62	-0.763034	0.50335	no
ni|645903703|nb|KK920727.1|	317933-318219	nan	nan	31	22	-0.480914	0.67745	no
ni|645903703|nb|KK920727.1|	319252-319365	nan	nan	99	60	-0.719623	0.54555	no
ni|645903703|nb|KK920727.1|	328783-329060	nan	nan	0	2	inf	0.00065	yes
ni|645903703|nb|KK920727.1|	329218-329707	nan	nan	12	22	0.852399	0.4534	no
ni|645903703|nb|KK920727.1|	331879-332023	nan	nan	7	25	2	0.1684	no
ni|645903703|nb|KK920727.1|	333317-333426	nan	nan	85	206	1	0.29345	no
ni|645903703|nb|KK920727.1|	334091-334236	nan	nan	51	110	1	0.3409	no
ni|645903703|nb|KK920727.1|	335785-335983	nan	nan	47	83	0.818157	0.4644	no
ni|645903703|nb|KK920727.1|	336316-336454	nan	nan	9	6	-0.704668	0.60375	no
ni|645903703|nb|KK920727.1|	338375-338497	nan	nan	5	15	1	0.30495	no
ni|645903703|nb|KK920727.1|	339423-339852	nan	nan	0.527946	0.648762	0.297298	0.8383	no
ni|645903703|nb|KK920727.1|	340204-340447	nan	nan	2	0.333569	-2	0.3207	no
ni|645903703|nb|KK920727.1|	341744-341870	nan	nan	5	7	0.610738	0.6619	no

ni|645903703|nb|KK920727.1|	342414-342618	nan	nan	5	10	1	0.38095	no
ni|645903703|nb|KK920727.1|	343138-343687	nan	nan	1	2	0.620862	0.60365	no
ni|645903703|nb|KK920727.1|	344882-345392	nan	nan	2	3	0.819425	0.4957	no
ni|645903703|nb|KK920727.1|	347244-347523	nan	nan	1	0.665003	-1	0.44755	no
ni|645903703|nb|KK920727.1|	348512-349076	nan	nan	2	2	-0.0650039	0.957	no
ni|645903703|nb|KK920727.1|	349858-350400	nan	nan	1	2	0.0418732	0.97395	no
ni|645903703|nb|KK920727.1|	350842-351079	nan	nan	1	0.696608	-1	0.44965	no
ni|645903703|nb|KK920727.1|	363553-363764	nan	nan	0	10	inf	0.00015	yes
ni|645903703|nb|KK920727.1|	365465-365665	nan	nan	0	17	inf	5.00E-05	yes
ni|645903703|nb|KK920727.1|	370166-370400	nan	nan	0	9	inf	0.0001	yes
ni|645903703|nb|KK920727.1|	372775-372922	nan	nan	0	16	inf	0.00025	yes
ni|645903703|nb|KK920727.1|	376161-376283	nan	nan	0	22	inf	0.0007	yes
ni|645903703|nb|KK920727.1|	378025-378344	nan	nan	0.113608	6	6	0.11985	no
ni|645903703|nb|KK920727.1|	393291-393590	nan	nan	0	154	inf	5.00E-05	yes
ni|645903703|nb|KK920727.1|	393934-394136	nan	nan	0	21	inf	5.00E-05	yes
ni|645903703|nb|KK920727.1|	395478-395703	nan	nan	0	33	inf	5.00E-05	yes
ni|645903703|nb|KK920727.1|	396533-397045	nan	nan	0	7	inf	5.00E-05	yes
ni|645903703|nb|KK920727.1|	398055-398448	nan	nan	0.084746	51	9	0.11935	no
ni|645903703|nb|KK920727.1|	401528-401715	nan	nan	0	122	inf	5.00E-05	yes
ni|645903703|nb|KK920727.1|	404485-404658	nan	nan	0	63	inf	5.00E-05	yes
ni|645903703|nb|KK920727.1|	406572-406911	nan	nan	0	74	inf	5.00E-05	yes
ni|645903703|nb|KK920727.1|	411112-411719	nan	nan	0.048834	14	8	0.11935	no
ni|645903703|nb|KK920727.1|	413023-413139	nan	nan	0	60	inf	5.00E-05	yes
ni|645903703|nb|KK920727.1|	417910-418286	nan	nan	0	25	inf	5.00E-05	yes
ni|645903703|nb|KK920727.1|	419994-420217	nan	nan	0	156	inf	5.00E-05	yes
ni|645903703|nb|KK920727.1|	423337-423518	nan	nan	0	147	inf	5.00E-05	yes
ni|645903703|nb|KK920727.1|	424335-424518	nan	nan	0.302141	48	7	0.11935	no
ni|645903703|nb|KK920727.1|	426597-426792	nan	nan	30	22	-0.436844	0.70365	no
ni|645903703|nb|KK920727.1|	428806-428999	nan	nan	26	83	2	0.15445	no
ni|645903703|nb|KK920727.1|	430719-430912	nan	nan	32	112	2	0.1231	no

ni|645903703|nb|KK920727.1|	432197-432416	nan	nan	2	1	-0.651579	0.6679	no
ni|645903703|nb|KK920727.1|	432737-433075	nan	nan	2	2	-0.267221	0.8253	no
ni|645903703|nb|KK920727.1|	433643-433883	nan	nan	3	1	-1	0.33495	no
ni|645903703|nb|KK920727.1|	434256-434424	nan	nan	5	2	-2	0.29715	no
ni|645903703|nb|KK920727.1|	434809-435879	nan	nan	2	0.560144	-2	0.0922	no
ni|645903703|nb|KK920727.1|	437577-438938	nan	nan	0.783211	6	3	0.0278	no
ni|645903703|nb|KK920727.1|	439115-439227	nan	nan	27	18	-0.553004	0.68145	no
ni|645903703|nb|KK920727.1|	439792-440451	nan	nan	1	1	-0.110669	0.92235	no
ni|645903703|nb|KK920727.1|	440665-441012	nan	nan	2	1	-0.728511	0.5717	no
ni|645903703|nb|KK920727.1|	441861-442054	nan	nan	3	3	-0.0671268	0.94635	no
ni|645903703|nb|KK920727.1|	442187-442766	nan	nan	21	28	0.404506	0.7074	no
ni|645903703|nb|KK920727.1|	44734-44879	nan	nan	0	238	inf	5.00E-05	yes
ni|645903703|nb|KK920727.1|	450552-450769	nan	nan	86	133	0.63157	0.5616	no
ni|645903703|nb|KK920727.1|	453511-453934	nan	nan	34	67.85	0.99856	0.43355	no
ni|645903703|nb|KK920727.1|	459398-459604	nan	nan	22	21	-0.0668543	0.9555	no
ni|645903703|nb|KK920727.1|	461447-461920	nan	nan	12	11	-0.112478	0.9209	no
ni|645903703|nb|KK920727.1|	462300-462499	nan	nan	24	20	-0.276925	0.81015	no
ni|645903703|nb|KK920727.1|	464444-464826	nan	nan	18	17	-0.129222	0.9072	no
ni|645903703|nb|KK920727.1|	467309-467544	nan	nan	24	24	-0.0339452	0.97405	no
ni|645903703|nb|KK920727.1|	468775-469094	nan	nan	8	8	-0.0656817	0.9569	no
ni|645903703|nb|KK920727.1|	470158-471101	nan	nan	7	7	-0.0417173	0.96875	no
ni|645903703|nb|KK920727.1|	471221-471399	nan	nan	5	5	0.0322124	0.96125	no
ni|645903703|nb|KK920727.1|	471713-471884	nan	nan	177	253	0.511924	0.62165	no
ni|645903703|nb|KK920727.1|	471998-472463	nan	nan	0.67943	0.51953	-0.387118	0.78555	no
ni|645903703|nb|KK920727.1|	473451-473756	nan	nan	37	41	0.163443	0.8821	no
ni|645903703|nb|KK920727.1|	473948-474227	nan	nan	65	43	-0.595076	0.5866	no
ni|645903703|nb|KK920727.1|	474828-474972	nan	nan	5	4	-0.259854	0.8692	no
ni|645903703|nb|KK920727.1|	478242-478389	nan	nan	4	9	1	0.39265	no
ni|645903703|nb|KK920727.1|	478607-478893	nan	nan	26	36	0.456076	0.68555	no
ni|645903703|nb|KK920727.1|	479910-480310	nan	nan	68	104	0.614696	0.68045	no

ni|645903703|nb|KK920727.1|	480713-481499	nan	nan	0.757019	1	0.672236	0.57765	no
ni|645903703|nb|KK920727.1|	481968-482182	nan	nan	147	215	0.547352	0.65665	no
ni|645903703|nb|KK920727.1|	482389-482683	nan	nan	1	0.613218	-1	0.4064	no
ni|645903703|nb|KK920727.1|	483547-483742	nan	nan	35	50	0.510679	0.66065	no
ni|645903703|nb|KK920727.1|	484327-484503	nan	nan	133	207	0.637789	0.55275	no
ni|645903703|nb|KK920727.1|	488528-488755	nan	nan	99	170	0.778953	0.5308	no
ni|645903703|nb|KK920727.1|	490283-490627	nan	nan	1	2	0.80891	0.52225	no
ni|645903703|nb|KK920727.1|	495307-495947	nan	nan	15	12	-0.350432	0.75665	no
ni|645903703|nb|KK920727.1|	496889-497106	nan	nan	4	4	0.167817	0.89635	no
ni|645903703|nb|KK920727.1|	499412-499545	nan	nan	77	88	0.188743	0.87065	no
ni|645903703|nb|KK920727.1|	501331-501739	nan	nan	27	21	-0.374277	0.7401	no
ni|645903703|nb|KK920727.1|	504845-504978	nan	nan	62	47	-0.397916	0.73645	no
ni|645903703|nb|KK920727.1|	51013-51439	nan	nan	0.076119	108	10.47	0.11935	no
ni|645903703|nb|KK920727.1|	511238-511452	nan	nan	75	79	0.0861518	0.9382	no
ni|645903703|nb|KK920727.1|	513100-513229	nan	nan	95	106	0.156911	0.88935	no
ni|645903703|nb|KK920727.1|	516567-516768	nan	nan	96	94	-0.0337942	0.974	no
ni|645903703|nb|KK920727.1|	519092-519204	nan	nan	153	120	-0.34899	0.76085	no
ni|645903703|nb|KK920727.1|	520136-520317	nan	nan	8	7	-0.187595	0.87725	no
ni|645903703|nb|KK920727.1|	522444-522838	nan	nan	58	42	-0.465152	0.66115	no
ni|645903703|nb|KK920727.1|	522972-523382	nan	nan	22	15	-0.536871	0.6381	no
ni|645903703|nb|KK920727.1|	701748-701958	nan	nan	0	11	inf	5.00E-05	yes
ni|645903703|nb|KK920727.1|	703396-703640	nan	nan	0	8	inf	5.00E-05	yes
ni|645903703|nb|KK920727.1|	703762-703899	nan	nan	0	25	inf	0.00015	yes
ni|645903703|nb|KK920727.1|	704337-704521	nan	nan	0	7	inf	0.0003	yes
ni|645903703|nb|KK920727.1|	842953-843567	nan	nan	0	7	inf	5.00E-05	yes
ni|645903703|nb|KK920727.1|	844922-845816	nan	nan	0	38	inf	5.00E-05	yes
ni|645903703|nb|KK920727.1|	846804-847610	nan	nan	0	2	inf	0.0001	yes
ni|645903703|nb|KK920727.1|	951130-951524	nan	nan	0	8	inf	5.00E-05	yes
ni|645903703|nb|KK920727.1|	963579-963864	nan	nan	0	2	inf	0.00225	yes
ni|645903704|nb|KK920726.1|	197124-197445	nan	nan	8	7	-0.222397	0.8508	no

ni|645903704|nb|KK920726.1|	204368-205036	nan	nan	11.11	11	-0.031301	0.9772	no
ni|645903704|nb|KK920726.1|	209285-210380	nan	nan	5	7	0.520419	0.64685	no
ni|645903704|nb|KK920726.1|	210501-212000	nan	nan	9	10	0.0617799	0.9554	no
ni|645903704|nb|KK920726.1|	212115-212263	nan	nan	16	11	-0.450528	0.7078	no
ni|645903704|nb|KK920726.1|	212447-212822	nan	nan	19	17	-0.198915	0.86435	no
ni|645903704|nb|KK920726.1|	221557-221820	nan	nan	37	31	-0.267984	0.81295	no
ni|645903704|nb|KK920726.1|	222354-222492	nan	nan	11	6	-0.984776	0.48245	no
ni|645903704|nb|KK920726.1|	234697-235087	nan	nan	1	2	0.37072	0.76295	no
ni|645903704|nb|KK920726.1|	295023-295821	nan	nan	6	45	3	0.0402	no
ni|645903704|nb|KK920726.1|	296375-296481	nan	nan	395	1062.49	1	0.22105	no
ni|645903704|nb|KK920726.1|	316817-316976	nan	nan	41	44	0.0877722	0.9404	no
ni|645903704|nb|KK920726.1|	38076-38203	nan	nan	7	9	0.254604	0.86075	no
ni|645903704|nb|KK920726.1|	392361-392514	nan	nan	104	92	-0.175106	0.8776	no
ni|645903704|nb|KK920726.1|	402735-402854	nan	nan	420.79	774	0.879632	0.42215	no
ni|645903704|nb|KK920726.1|	403224-403380	nan	nan	127.85	217	0.765076	0.486	no
ni|645903704|nb|KK920726.1|	404990-405503	nan	nan	118	191	0.701741	0.61525	no
ni|645903704|nb|KK920726.1|	406800-407117	nan	nan	0.917304	0.986031	0.104233	0.93335	no
ni|645903704|nb|KK920726.1|	407370-407565	nan	nan	235	414	0.817367	0.58045	no
ni|645903704|nb|KK920726.1|	409808-410102	nan	nan	57	73	0.357333	0.73485	no
ni|645903704|nb|KK920726.1|	413803-414084	nan	nan	60	81	0.427041	0.69085	no
ni|645903704|nb|KK920726.1|	414517-414811	nan	nan	48	39	-0.295972	0.787	no
ni|645903704|nb|KK920726.1|	419378-420269	nan	nan	0.749979	1	0.70794	0.5547	no
ni|645903704|nb|KK920726.1|	422291-422630	nan	nan	1	0.994118	-0.551009	0.68265	no
ni|645903704|nb|KK920726.1|	424771-425295	nan	nan	0.818207	2	0.88246	0.4843	no
ni|645903704|nb|KK920726.1|	426606-426775	nan	nan	83	62	-0.416334	0.71275	no
ni|645903704|nb|KK920726.1|	426894-427055	nan	nan	6	7	0.113364	0.9325	no
ni|645903704|nb|KK920726.1|	427159-427362	nan	nan	82	68	-0.282922	0.79535	no
ni|645903704|nb|KK920726.1|	427535-427673	nan	nan	62	50	-0.330273	0.778	no
ni|645903704|nb|KK920726.1|	427901-428011	nan	nan	27	9	-2	0.25505	no
ni|645903704|nb|KK920726.1|	428666-429158	nan	nan	60	49	-0.275351	0.8518	no

ni|645903704|nb|KK920726.1|	431311-431527	nan	nan	86	85	-0.0131598	0.9901	no
ni|645903704|nb|KK920726.1|	434281-434826	nan	nan	72	65	-0.145335	0.9211	no
ni|645903704|nb|KK920726.1|	438241-438425	nan	nan	100	88	-0.174851	0.8726	no
ni|645903704|nb|KK920726.1|	439455-439759	nan	nan	67	75	0.164879	0.87765	no
ni|645903704|nb|KK920726.1|	443847-444013	nan	nan	65	65	0.00634692	0.99545	no
ni|645903704|nb|KK920726.1|	445895-446281	nan	nan	45	46	0.0334059	0.97455	no
ni|645903704|nb|KK920726.1|	448174-448288	nan	nan	62	48.11	-0.370082	0.75375	no
ni|645903704|nb|KK920726.1|	449858-450010	nan	nan	60	85	0.502423	0.65965	no
ni|645903704|nb|KK920726.1|	454459-454645	nan	nan	96	105	0.129795	0.90465	no
ni|645903704|nb|KK920726.1|	455060-455662	nan	nan	14	19	0.368566	0.7438	no
ni|645903704|nb|KK920726.1|	456779-457568	nan	nan	0.287128	0.480429	0.742628	0.5765	no
ni|645903704|nb|KK920726.1|	458251-458451	nan	nan	97	100	0.0335455	0.97735	no
ni|645903704|nb|KK920726.1|	459555-459886	nan	nan	1	0.720139	-0.580194	0.70815	no
ni|645903704|nb|KK920726.1|	460059-460226	nan	nan	102	105	0.0343984	0.97345	no
ni|645903704|nb|KK920726.1|	463674-463812	nan	nan	30	9	-2	0.1637	no
ni|645903704|nb|KK920726.1|	463909-464034	nan	nan	22	7	-2	0.21515	no
ni|645903704|nb|KK920726.1|	464309-464434	nan	nan	27	13	-1	0.39615	no
ni|645903704|nb|KK920726.1|	464937-465079	nan	nan	6	1	-3	0.28285	no
ni|645903704|nb|KK920726.1|	465215-465338	nan	nan	25	9	-1	0.26925	no
ni|645903704|nb|KK920726.1|	465475-465842	nan	nan	59	24	-1	0.24905	no
ni|645903704|nb|KK920726.1|	467135-467539	nan	nan	16	7	-1	0.32875	no
ni|645903704|nb|KK920726.1|	467715-467958	nan	nan	14	7	-0.946677	0.42235	no
ni|645903704|nb|KK920726.1|	468534-468887	nan	nan	25	11	-1	0.2863	no
ni|645903704|nb|KK920726.1|	469220-469330	nan	nan	50	7	-3	0.1378	no
ni|645903704|nb|KK920726.1|	469676-470148	nan	nan	28	10	-1	0.21625	no
ni|645903704|nb|KK920726.1|	470369-471044	nan	nan	23	9	-1	0.25	no
ni|645903704|nb|KK920726.1|	471160-472278	nan	nan	36	16	-1	0.33615	no
ni|645903704|nb|KK920726.1|	533557-533816	nan	nan	44	20	-1	0.3223	no
ni|645903705|nb|KK920725.1|	113924-114214	nan	nan	0	7	inf	0.00015	yes
ni|645903705|nb|KK920725.1|	116249-116487	nan	nan	0	6	inf	0.00015	yes

ni|645903705|nb|KK920725.1|	19205-19554	nan	nan	0.299485	1	2	0.1537	no
ni|645903705|nb|KK920725.1|	20106-21776	nan	nan	0.990412	3	1	0.2439	no
ni|645903705|nb|KK920725.1|	21969-22642	nan	nan	1	3	0.84769	0.47805	no
ni|645903705|nb|KK920725.1|	35116-35912	nan	nan	0	19	inf	5.00E-05	yes
ni|645903705|nb|KK920725.1|	426551-428579	nan	nan	35	35	0.00790991	0.99525	no
ni|645903705|nb|KK920725.1|	430350-430498	nan	nan	44	45	0.0469714	0.966	no
ni|645903705|nb|KK920725.1|	432082-432452	nan	nan	24	23	-0.0654506	0.95405	no
ni|645903705|nb|KK920725.1|	435045-435251	nan	nan	36	32	-0.144857	0.90025	no
ni|645903705|nb|KK920725.1|	435411-435638	nan	nan	22	21	-0.0149564	0.99055	no
ni|645903705|nb|KK920725.1|	436500-436640	nan	nan	54	37	-0.549379	0.6338	no
ni|645903705|nb|KK920725.1|	437634-437960	nan	nan	51	38	-0.446952	0.6831	no
ni|645903705|nb|KK920725.1|	438148-438263	nan	nan	49	33	-0.562259	0.6436	no
ni|645903705|nb|KK920725.1|	448990-449143	nan	nan	24	25	0.0677394	0.95175	no
ni|645903705|nb|KK920725.1|	449293-449484	nan	nan	187	142	-0.398166	0.7078	no
ni|645903705|nb|KK920725.1|	449862-450660	nan	nan	241	209	-0.202053	0.88775	no
ni|645903705|nb|KK920725.1|	462998-463176	nan	nan	0.642891	5	3	0.0656	no
ni|645903705|nb|KK920725.1|	471988-472846	nan	nan	3	4	0.497747	0.66975	no
ni|645903705|nb|KK920725.1|	479112-479372	nan	nan	18	46	1	0.24435	no
ni|645903705|nb|KK920725.1|	480223-480554	nan	nan	25	57	1	0.27875	no
ni|645903705|nb|KK920725.1|	48163-48337	nan	nan	4	20.04	2	0.0926	no
ni|645903705|nb|KK920725.1|	490440-491263	nan	nan	23	36	0.636235	0.60565	no
ni|645903705|nb|KK920725.1|	500388-500804	nan	nan	2	2	0.382154	0.75215	no
ni|645903705|nb|KK920725.1|	50097-50227	nan	nan	2	16	3	0.0821	no
ni|645903705|nb|KK920725.1|	501253-501762	nan	nan	1	0.637164	-0.853596	0.50505	no
ni|645903705|nb|KK920725.1|	501887-502199	nan	nan	1	1	-0.480757	0.7301	no
ni|645903705|nb|KK920725.1|	511590-511707	nan	nan	13	7	-0.91545	0.53575	no
ni|645903705|nb|KK920725.1|	512013-512411	nan	nan	6	7	0.344521	0.7687	no
ni|645903705|nb|KK920725.1|	513986-514299	nan	nan	2	2	0.403771	0.75355	no
ni|645903705|nb|KK920725.1|	521047-521200	nan	nan	14	13	-0.0182806	0.97915	no
ni|645903705|nb|KK920725.1|	52116-52400	nan	nan	38	94	1	0.2354	no

ni|645903705|nb|KK920725.1|	522444-522618	nan	nan	14	11	-0.380465	0.7493	no
ni|645903705|nb|KK920725.1|	524921-525877	nan	nan	0.519655	0.579712	0.157782	0.89485	no
ni|645903705|nb|KK920725.1|	52533-52873	nan	nan	28	70	1	0.2429	no
ni|645903705|nb|KK920725.1|	526009-526445	nan	nan	1	1	0.12739	0.9211	no
ni|645903705|nb|KK920725.1|	533436-533666	nan	nan	1	2	0.29613	0.8383	no
ni|645903705|nb|KK920725.1|	55785-56125	nan	nan	66	124	0.904769	0.4695	no
ni|645903705|nb|KK920725.1|	562979-567545	nan	nan	0.342317	0.776284	1	0.3163	no
ni|645903705|nb|KK920725.1|	567671-569197	nan	nan	2	6	1	0.25665	no
ni|645903705|nb|KK920725.1|	569572-570309	nan	nan	2	4	0.834697	0.4855	no
ni|645903705|nb|KK920725.1|	573975-574231	nan	nan	2	0.763874	-2	0.24455	no
ni|645903705|nb|KK920725.1|	581463-581674	nan	nan	11	3	-2	0.1363	no
ni|645903705|nb|KK920725.1|	585865-586265	nan	nan	1	0.711827	-1	0.429	no
ni|645903705|nb|KK920725.1|	587604-587940	nan	nan	5	1	-2	0.1135	no
ni|645903705|nb|KK920725.1|	588437-588630	nan	nan	3	1	-2	0.3149	no
ni|645903705|nb|KK920725.1|	593149-593352	nan	nan	9	2	-2	0.121	no
ni|645903705|nb|KK920725.1|	593453-593723	nan	nan	3	1	-1	0.3177	no
ni|645903705|nb|KK920725.1|	594076-594230	nan	nan	9	4	-1	0.31305	no
ni|645903705|nb|KK920725.1|	594369-594665	nan	nan	13	5	-1	0.2503	no
ni|645903705|nb|KK920725.1|	594813-595033	nan	nan	5	0.796218	-3	0.09005	no
ni|645903705|nb|KK920725.1|	595258-595524	nan	nan	3	0.860999	-2	0.17185	no
ni|645903705|nb|KK920725.1|	595663-595958	nan	nan	4	1	-2	0.2366	no
ni|645903705|nb|KK920725.1|	596289-596545	nan	nan	4	1	-1	0.288	no
ni|645903705|nb|KK920725.1|	596668-596798	nan	nan	11	2	-3	0.2592	no
ni|645903705|nb|KK920725.1|	598686-599058	nan	nan	4	2	-1	0.33685	no
ni|645903705|nb|KK920725.1|	599553-600364	nan	nan	2	0.665029	-1	0.26595	no
ni|645903705|nb|KK920725.1|	603378-603909	nan	nan	2	1	-0.775554	0.51985	no
ni|645903705|nb|KK920725.1|	605289-608014	nan	nan	7	6	-0.222663	0.86005	no
ni|645903705|nb|KK920725.1|	608203-608319	nan	nan	18	13	-0.48251	0.7301	no
ni|645903705|nb|KK920725.1|	610145-610295	nan	nan	4	3	-0.652158	0.66125	no
ni|645903705|nb|KK920725.1|	72563-72853	nan	nan	24	26	0.13955	0.9002	no

ni|645903706|nb|KK920724.1|	157008-157569	nan	nan	0.752232	0.513634	-0.550436	0.68265	no
ni|645903706|nb|KK920724.1|	157705-158078	nan	nan	2	2	0.156953	0.8958	no
ni|645903706|nb|KK920724.1|	167422-167918	nan	nan	1	0.718013	-0.72809	0.5717	no
ni|645903706|nb|KK920724.1|	168245-169040	nan	nan	542	598.5	0.143553	0.9246	no
ni|645903706|nb|KK920724.1|	170243-170594	nan	nan	926	863	-0.101607	0.9437	no
ni|645903706|nb|KK920724.1|	171858-172187	nan	nan	2873.64	2615.39	-0.135854	0.8951	no
ni|645903706|nb|KK920724.1|	173618-174093	nan	nan	6	5	-0.189157	0.8712	no
ni|645903706|nb|KK920724.1|	174236-174542	nan	nan	2	2	0.321271	0.8092	no
ni|645903706|nb|KK920724.1|	174692-175446	nan	nan	1458.02	1351.25	-0.109722	0.9208	no
ni|645903706|nb|KK920724.1|	176149-176501	nan	nan	3872.87	3550.48	-0.125389	0.908	no
ni|645903706|nb|KK920724.1|	23158-23961	nan	nan	70	56	-0.324563	0.82355	no
ni|645903706|nb|KK920724.1|	263672-264077	nan	nan	2	1	-0.820223	0.50325	no
ni|645903706|nb|KK920724.1|	8971-9351	nan	nan	0.532238	1163.19	11	0.0332	no
ni|645903706|nb|KK920724.1|	90336-90485	nan	nan	31	47	0.563569	0.6267	no
ni|645903707|nb|KK920723.1|	100921-101297	nan	nan	0.720002	0.516059	-0.480465	0.7594	no
ni|645903707|nb|KK920723.1|	2200-2406	nan	nan	2	3	0.418573	0.73705	no
ni|645903707|nb|KK920723.1|	313486-313599	nan	nan	25	26	0.031989	0.96125	no
ni|645903707|nb|KK920723.1|	34007-34269	nan	nan	2	8	2	0.13545	no
ni|645903707|nb|KK920723.1|	43300-43489	nan	nan	2	9	2	0.11695	no
ni|645903707|nb|KK920723.1|	49708-49873	nan	nan	3	9	2	0.2618	no
ni|645903707|nb|KK920723.1|	65132-65598	nan	nan	0.067756	2	5	0.1211	no
ni|645903707|nb|KK920723.1|	72104-72349	nan	nan	3	2	-0.744308	0.56925	no
ni|645903708|nb|KK920722.1|	105843-105913	nan	nan	20175	27504.3	0.447088	0.7195	no
ni|645903708|nb|KK920722.1|	10871-11364	nan	nan	0.756932	0.120587	-3	0.2671	no
ni|645903708|nb|KK920722.1|	1185-1412	nan	nan	8	7	-0.283298	0.80905	no
ni|645903708|nb|KK920722.1|	13196-14004	nan	nan	0.873117	0.434106	-1	0.4077	no
ni|645903708|nb|KK920722.1|	14820-15238	nan	nan	1	0.745915	-0.443812	0.74215	no
ni|645903708|nb|KK920722.1|	158348-158467	nan	nan	29	37	0.363215	0.7612	no
ni|645903708|nb|KK920722.1|	1645-1794	nan	nan	12	7	-0.683869	0.5892	no
ni|645903708|nb|KK920722.1|	18064-18442	nan	nan	1	0.597712	-0.958505	0.51225	no

ni|645903708|nb|KK920722.1|	1853-2030	nan	nan	15	17	0.159445	0.88765	no
ni|645903708|nb|KK920722.1|	18731-19367	nan	nan	0.969798	0.441496	-1	0.393	no
ni|645903708|nb|KK920722.1|	20853-21044	nan	nan	54	46	-0.215064	0.85375	no
ni|645903708|nb|KK920722.1|	2365-3097	nan	nan	61	41	-0.560596	0.69855	no
ni|645903708|nb|KK920722.1|	246745-246948	nan	nan	12162.4	10447.8	-0.219219	0.8496	no
ni|645903708|nb|KK920722.1|	27373-27583	nan	nan	86	76	-0.165762	0.88325	no
ni|645903708|nb|KK920722.1|	28117-28276	nan	nan	66	65	-0.01241	0.99035	no
ni|645903708|nb|KK920722.1|	301396-301686	nan	nan	30	34	0.15475	0.8888	no
ni|645903708|nb|KK920722.1|	309489-310033	nan	nan	16	15	-0.0448607	0.96785	no
ni|645903708|nb|KK920722.1|	310936-311429	nan	nan	18	17	-0.0599506	0.9576	no
ni|645903708|nb|KK920722.1|	316318-316705	nan	nan	33	28	-0.251541	0.81545	no
ni|645903708|nb|KK920722.1|	316814-316963	nan	nan	22	20	-0.101145	0.9325	no
ni|645903708|nb|KK920722.1|	317980-318247	nan	nan	35	28	-0.343929	0.762	no
ni|645903708|nb|KK920722.1|	318927-319080	nan	nan	17	20	0.182788	0.8733	no
ni|645903708|nb|KK920722.1|	3262-3386	nan	nan	124	94	-0.39818	0.73345	no
ni|645903708|nb|KK920722.1|	329215-329612	nan	nan	0.585185	0.559266	-0.0653579	0.9302	no
ni|645903708|nb|KK920722.1|	36427-36642	nan	nan	91	84	-0.122716	0.90915	no
ni|645903708|nb|KK920722.1|	3902-4130	nan	nan	29	24	-0.316191	0.77725	no
ni|645903708|nb|KK920722.1|	4723-4894	nan	nan	4	1	-1	0.35155	no
ni|645903708|nb|KK920722.1|	490751-491103	nan	nan	1	0	-inf	0.002	yes
ni|645903708|nb|KK920722.1|	491245-492051	nan	nan	0.560383	0	-inf	0.0015	yes
ni|645903708|nb|KK920722.1|	493286-493901	nan	nan	1	0	-inf	0.0004	yes
ni|645903708|nb|KK920722.1|	5168-5733	nan	nan	2	1	-0.495637	0.6831	no
ni|645903708|nb|KK920722.1|	61609-61728	nan	nan	92	59	-0.642534	0.5856	no
ni|645903708|nb|KK920722.1|	6243-6581	nan	nan	2	0.598993	-2	0.1964	no
ni|645903708|nb|KK920722.1|	8204-8379	nan	nan	5	3	-0.897391	0.502	no
ni|645903709|nb|KK920721.1|	260039-260285	nan	nan	31	29	-0.0742614	0.9497	no
ni|645903709|nb|KK920721.1|	260649-260792	nan	nan	50	28	-0.849619	0.4759	no
ni|645903709|nb|KK920721.1|	260921-262443	nan	nan	33	29	-0.18468	0.89415	no
ni|645903709|nb|KK920721.1|	404586-404802	nan	nan	37	33	-0.154128	0.89325	no

ni|645903709|nb|KK920721.1|	90869-92494	nan	nan	4	0.92873	-2	0.0993	no
ni|645903710|nb|KK920720.1|	1037-1528	nan	nan	16	16	-0.0214135	0.9852	no
ni|645903710|nb|KK920720.1|	12897-13104	nan	nan	20	15	-0.401251	0.7335	no
ni|645903710|nb|KK920720.1|	185297-185373	nan	nan	4149.02	6890.89	0.73192	0.6232	no
ni|645903710|nb|KK920720.1|	2471-2906	nan	nan	13	13	-0.0571753	0.9622	no
ni|645903710|nb|KK920720.1|	3265-3511	nan	nan	6	6	-0.0662241	0.95435	no
ni|645903710|nb|KK920720.1|	32886-33078	nan	nan	11	12	0.165515	0.8851	no
ni|645903710|nb|KK920720.1|	4404-4941	nan	nan	12	13	0.135124	0.9062	no
ni|645903710|nb|KK920720.1|	495010-495072	nan	nan	33187.7	17447.9	-0.927594	0.50925	no
ni|645903710|nb|KK920720.1|	5160-5710	nan	nan	18	17	-0.131523	0.90225	no
ni|645903710|nb|KK920720.1|	566425-566541	nan	nan	24	23	-0.0674725	0.9576	no
ni|645903710|nb|KK920720.1|	688617-688774	nan	nan	0	10	inf	0.00055	yes
ni|645903710|nb|KK920720.1|	701189-701580	nan	nan	0	3	inf	0.00015	yes
ni|645903710|nb|KK920720.1|	705420-706791	nan	nan	49	205	2	0.1689	no
ni|645903710|nb|KK920720.1|	709331-709641	nan	nan	3	2	-0.859279	0.49355	no
ni|645903710|nb|KK920720.1|	709758-709892	nan	nan	8	5	-0.719341	0.61925	no
ni|645903710|nb|KK920720.1|	710241-710475	nan	nan	8	4	-1	0.3317	no
ni|645903710|nb|KK920720.1|	710949-711312	nan	nan	2	1	-0.90198	0.46595	no
ni|645903710|nb|KK920720.1|	711740-711882	nan	nan	8	6	-0.41514	0.7377	no
ni|645903710|nb|KK920720.1|	711987-712752	nan	nan	1	0.746732	-0.579322	0.63675	no
ni|645903710|nb|KK920720.1|	713430-713617	nan	nan	5	2	-1	0.33495	no
ni|645903710|nb|KK920720.1|	713753-715028	nan	nan	6	14	1	0.2355	no
ni|645903710|nb|KK920720.1|	715142-715611	nan	nan	36	18	-0.984212	0.38375	no
ni|645903710|nb|KK920720.1|	716300-716556	nan	nan	269	288	0.0983596	0.9432	no
ni|645903710|nb|KK920720.1|	719277-719387	nan	nan	237	222	-0.0956708	0.9324	no
ni|645903710|nb|KK920720.1|	721128-721493	nan	nan	51	59	0.206816	0.84865	no
ni|645903710|nb|KK920720.1|	722915-723469	nan	nan	45	61	0.433999	0.7692	no
ni|645903710|nb|KK920720.1|	764200-764407	nan	nan	0	7	inf	0.00025	yes
ni|645903710|nb|KK920720.1|	764528-764722	nan	nan	0	64	inf	5.00E-05	yes
ni|645903710|nb|KK920720.1|	764923-765440	nan	nan	1	829	10	0.00175	yes

ni|645903710|nb|KK920720.1|	765548-766232	nan	nan	0	1	inf	0.0009	yes
ni|645903710|nb|KK920720.1|	896615-896861	nan	nan	9	4	-1	0.3716	no
ni|645903710|nb|KK920720.1|	897093-897281	nan	nan	23	37	0.66975	0.56515	no
ni|645903710|nb|KK920720.1|	897423-897728	nan	nan	24	28	0.199703	0.8565	no
ni|645903710|nb|KK920720.1|	906458-906609	nan	nan	18	28	0.669772	0.57065	no
ni|645903710|nb|KK920720.1|	912394-912647	nan	nan	24	26	0.0800717	0.94665	no
ni|645903711|nb|KK920719.1|	173615-174845	nan	nan	0.699358	0.39709	-0.816566	0.49545	no
ni|645903711|nb|KK920719.1|	204257-204430	nan	nan	35	27	-0.41395	0.71615	no
ni|645903711|nb|KK920719.1|	204485-206119	nan	nan	36	29	-0.343229	0.81025	no
ni|645903711|nb|KK920719.1|	206243-206619	nan	nan	8	8	0.0915414	0.93845	no
ni|645903711|nb|KK920719.1|	207248-207378	nan	nan	56	37	-0.590857	0.61975	no
ni|645903711|nb|KK920719.1|	216399-216575	nan	nan	37	28	-0.398946	0.73075	no
ni|645903711|nb|KK920719.1|	229598-229893	nan	nan	21	18	-0.226809	0.8473	no
ni|645903711|nb|KK920719.1|	254305-255117	nan	nan	0.625101	0.199228	-2	0.25885	no
ni|645903711|nb|KK920719.1|	256143-256467	nan	nan	20	19	-0.09004	0.9379	no
ni|645903711|nb|KK920719.1|	269801-269957	nan	nan	27	25	-0.115291	0.9208	no
ni|645903711|nb|KK920719.1|	298732-298909	nan	nan	29	25	-0.237251	0.8411	no
ni|645903711|nb|KK920719.1|	417640-417806	nan	nan	34	30	-0.184048	0.87515	no
ni|645903711|nb|KK920719.1|	428701-428996	nan	nan	19	23	0.265892	0.81535	no
ni|645903711|nb|KK920719.1|	576565-577495	nan	nan	0	10	inf	5.00E-05	yes
ni|645903712|nb|KK920718.1|	176982-177132	nan	nan	10	9	-0.141196	0.9095	no
ni|645903712|nb|KK920718.1|	219918-220406	nan	nan	0	13	inf	5.00E-05	yes
ni|645903712|nb|KK920718.1|	247332-247487	nan	nan	4	5	0.347843	0.7963	no
ni|645903712|nb|KK920718.1|	70839-71067	nan	nan	6	12	0.887725	0.453	no
ni|645903713|nb|KK920717.1|	117035-117788	nan	nan	0	3	inf	5.00E-05	yes
ni|645903713|nb|KK920717.1|	117932-118416	nan	nan	0.064564	315	12	0.11935	no
ni|645903713|nb|KK920717.1|	158533-159073	nan	nan	0.84462	0.645909	-0.386973	0.76495	no
ni|645903713|nb|KK920717.1|	164471-164971	nan	nan	0	1	inf	0.0007	yes
ni|645903713|nb|KK920717.1|	199517-199736	nan	nan	69	84	0.279735	0.8004	no
ni|645903713|nb|KK920717.1|	231516-231628	nan	nan	0	103	inf	5.00E-05	yes

ni|645903713|nb|KK920717.1|	234158-234455	nan	nan	0.379195	220	9	0.05055	no
ni|645903713|nb|KK920717.1|	252417-253309	nan	nan	0	144	inf	5.00E-05	yes
ni|645903713|nb|KK920717.1|	268471-268831	nan	nan	0	207	inf	5.00E-05	yes
ni|645903713|nb|KK920717.1|	278696-278860	nan	nan	118	133	0.169385	0.8785	no
ni|645903713|nb|KK920717.1|	317344-317497	nan	nan	54	78	0.521948	0.6549	no
ni|645903713|nb|KK920717.1|	318555-318745	nan	nan	61	77	0.321383	0.77345	no
ni|645903713|nb|KK920717.1|	343666-343842	nan	nan	99	113	0.200527	0.8529	no
ni|645903713|nb|KK920717.1|	346561-346689	nan	nan	66	78	0.239348	0.8319	no
ni|645903713|nb|KK920717.1|	348769-348922	nan	nan	95	136	0.505879	0.64865	no
ni|645903713|nb|KK920717.1|	355026-355461	nan	nan	3	3	0.26405	0.8195	no
ni|645903713|nb|KK920717.1|	356121-356342	nan	nan	4	4	-0.144586	0.90525	no
ni|645903713|nb|KK920717.1|	357964-358816	nan	nan	2	2	-0.386605	0.74145	no
ni|645903713|nb|KK920717.1|	359260-359590	nan	nan	2	2	-0.210016	0.8588	no
ni|645903713|nb|KK920717.1|	361268-361480	nan	nan	4	4	0.0207277	0.96555	no
ni|645903713|nb|KK920717.1|	363069-363285	nan	nan	2	1	-0.914662	0.53575	no
ni|645903713|nb|KK920717.1|	364185-364388	nan	nan	3	3	0.0485593	0.95725	no
ni|645903713|nb|KK920717.1|	365744-365893	nan	nan	5	4	-0.389126	0.78555	no
ni|645903713|nb|KK920717.1|	367273-367417	nan	nan	24	17	-0.493473	0.67245	no
ni|645903713|nb|KK920717.1|	367607-367728	nan	nan	93	109	0.222115	0.8495	no
ni|645903713|nb|KK920717.1|	370376-370735	nan	nan	84	99	0.231296	0.8771	no
ni|645903713|nb|KK920717.1|	370980-371250	nan	nan	141	160	0.175932	0.9049	no
ni|645903713|nb|KK920717.1|	371793-371998	nan	nan	110	112.73	0.035787	0.9763	no
ni|645903713|nb|KK920717.1|	372100-372498	nan	nan	59	64	0.100523	0.9327	no
ni|645903713|nb|KK920717.1|	372811-373062	nan	nan	129	123	-0.0643209	0.9658	no
ni|645903713|nb|KK920717.1|	373554-373777	nan	nan	95	80	-0.23725	0.8268	no
ni|645903713|nb|KK920717.1|	374129-374575	nan	nan	106	76	-0.484821	0.73745	no
ni|645903713|nb|KK920717.1|	377535-378212	nan	nan	8	6	-0.46272	0.6852	no
ni|645903713|nb|KK920717.1|	378462-378850	nan	nan	23	18	-0.358582	0.7502	no
ni|645903713|nb|KK920717.1|	379189-380587	nan	nan	5	4	-0.407989	0.71625	no
ni|645903713|nb|KK920717.1|	385256-385504	nan	nan	146	122	-0.261699	0.86475	no

ni|645903713|nb|KK920717.1|	386512-386695	nan	nan	14	11	-0.273734	0.8173	no
ni|645903713|nb|KK920717.1|	386799-387152	nan	nan	63	60	-0.0520156	0.96125	no
ni|645903713|nb|KK920717.1|	387711-388046	nan	nan	60	53	-0.16605	0.8793	no
ni|645903713|nb|KK920717.1|	388178-388436	nan	nan	21	19	-0.176283	0.8793	no
ni|645903713|nb|KK920717.1|	399989-401389	nan	nan	21	34	0.693002	0.63595	no
ni|645903713|nb|KK920717.1|	401492-401663	nan	nan	1	11	3	0.06085	no
ni|645903713|nb|KK920717.1|	402273-403724	nan	nan	7	9	0.190346	0.86085	no
ni|645903713|nb|KK920717.1|	404952-405364	nan	nan	12	12	0.0308993	0.97795	no
ni|645903713|nb|KK920717.1|	405570-405809	nan	nan	10	10	-0.0147813	0.9889	no
ni|645903713|nb|KK920717.1|	406856-407011	nan	nan	7	2	-2	0.1613	no
ni|645903713|nb|KK920717.1|	408071-408538	nan	nan	1	0.645857	-0.650148	0.63225	no
ni|645903713|nb|KK920717.1|	408638-408835	nan	nan	3	2	-0.844651	0.5595	no
ni|645903713|nb|KK920717.1|	411229-411501	nan	nan	6	5	-0.362962	0.75795	no
ni|645903713|nb|KK920717.1|	411712-411904	nan	nan	22	20	-0.10414	0.9294	no
ni|645903713|nb|KK920717.1|	412085-412340	nan	nan	18	15	-0.316453	0.78825	no
ni|645903713|nb|KK920717.1|	412425-412699	nan	nan	8	4	-0.962871	0.424	no
ni|645903713|nb|KK920717.1|	412800-412919	nan	nan	33	16	-1	0.3945	no
ni|645903713|nb|KK920717.1|	413362-413587	nan	nan	14	11	-0.268152	0.81925	no
ni|645903713|nb|KK920717.1|	413702-414248	nan	nan	5	5	-0.0215333	0.9844	no
ni|645903713|nb|KK920717.1|	414810-415261	nan	nan	14	34	1	0.27345	no
ni|645903713|nb|KK920717.1|	416484-416939	nan	nan	2	1	-0.572173	0.63215	no
ni|645903713|nb|KK920717.1|	417080-417254	nan	nan	15	25	0.758664	0.51995	no
ni|645903713|nb|KK920717.1|	417381-418290	nan	nan	2	2	-0.000507943	0.99105	no
ni|645903713|nb|KK920717.1|	418400-418643	nan	nan	13	31	1	0.28095	no
ni|645903713|nb|KK920717.1|	419263-419435	nan	nan	3	2	-0.482327	0.7594	no
ni|645903713|nb|KK920717.1|	419619-420048	nan	nan	2	3	0.572158	0.6286	no
ni|645903713|nb|KK920717.1|	420535-420844	nan	nan	8	21	1	0.2184	no
ni|645903713|nb|KK920717.1|	421842-422241	nan	nan	5	17	2	0.17775	no
ni|645903713|nb|KK920717.1|	424464-424670	nan	nan	9	30	2	0.1448	no
ni|645903713|nb|KK920717.1|	425785-426297	nan	nan	13	38	2	0.15335	no

ni|645903713|nb|KK920717.1|	426966-427147	nan	nan	15	49	2	0.14305	no
ni|645903713|nb|KK920717.1|	428143-428340	nan	nan	12	37	2	0.17535	no
ni|645903713|nb|KK920717.1|	428821-429000	nan	nan	7	30	2	0.10605	no
ni|645903713|nb|KK920717.1|	430682-430814	nan	nan	17	34	0.996852	0.41905	no
ni|645903713|nb|KK920717.1|	433976-434140	nan	nan	11	32	2	0.18095	no
ni|645903713|nb|KK920717.1|	437664-437866	nan	nan	12	44	2	0.1131	no
ni|645903713|nb|KK920717.1|	438411-438729	nan	nan	2	4	0.741668	0.54335	no
ni|645903713|nb|KK920717.1|	444934-445701	nan	nan	0.556185	1	1	0.31045	no
ni|645903713|nb|KK920717.1|	446096-447247	nan	nan	13	12	-0.0433046	0.96715	no
ni|645903713|nb|KK920717.1|	447545-447774	nan	nan	2	0.925246	-0.914452	0.53575	no
ni|645903713|nb|KK920717.1|	447950-449430	nan	nan	3	12	2	0.05675	no
ni|645903713|nb|KK920717.1|	449649-449951	nan	nan	0.24649	3	3	0.05215	no
ni|645903713|nb|KK920717.1|	457896-458060	nan	nan	70	87	0.32741	0.77415	no
ni|645903713|nb|KK920717.1|	459005-459307	nan	nan	2	2	0.296795	0.8138	no
ni|645903713|nb|KK920717.1|	459821-459991	nan	nan	55	81	0.570161	0.62235	no
ni|645903713|nb|KK920717.1|	460939-461229	nan	nan	21	33	0.680218	0.5433	no
ni|645903713|nb|KK920717.1|	462694-462908	nan	nan	2	1	-0.914697	0.53575	no
ni|645903713|nb|KK920717.1|	465752-465963	nan	nan	2	0.430669	-3	0.28285	no
ni|645903713|nb|KK920717.1|	467804-468155	nan	nan	1	0.56778	-0.939998	0.54025	no
ni|645903713|nb|KK920717.1|	469909-470645	nan	nan	2	1	-0.299242	0.8033	no
ni|645903713|nb|KK920717.1|	471821-472309	nan	nan	13	45	2	0.11535	no
ni|645903713|nb|KK920717.1|	473901-474371	nan	nan	12	109	3	0.0251	no
ni|645903713|nb|KK920717.1|	475762-476004	nan	nan	15	155	3	0.02555	no
ni|645903713|nb|KK920717.1|	477194-477621	nan	nan	12	121	3	0.02345	no
ni|645903713|nb|KK920717.1|	478559-479165	nan	nan	11	116	3	0.0203	no
ni|645903713|nb|KK920717.1|	482187-482501	nan	nan	0	3	inf	0.00065	yes
ni|645903713|nb|KK920717.1|	484039-484515	nan	nan	0.593503	16	5	0.01105	no
ni|645903713|nb|KK920717.1|	491085-491587	nan	nan	4	6	0.689774	0.55245	no
ni|645903713|nb|KK920717.1|	492892-493210	nan	nan	7	16	1	0.29555	no
ni|645903713|nb|KK920717.1|	494716-495000	nan	nan	0.270809	3	4	0.04915	no

ni|645903713|nb|KK920717.1|	496556-497024	nan	nan	4	8	1	0.3513	no
ni|645903713|nb|KK920717.1|	498637-502396	nan	nan	0.26285	19	6	0.00085	yes
ni|645903713|nb|KK920717.1|	508708-509606	nan	nan	2	1	-0.210496	0.8556	no
ni|645903713|nb|KK920717.1|	521721-523395	nan	nan	0.109766	371	12	0.0205	no
ni|645903713|nb|KK920717.1|	523772-524109	nan	nan	0	3	inf	0.0003	yes
ni|645903713|nb|KK920717.1|	525229-526165	nan	nan	0.029562	3	7	0.1194	no
ni|645903713|nb|KK920717.1|	541586-541783	nan	nan	9	12	0.443151	0.71155	no
ni|645903713|nb|KK920717.1|	546553-547093	nan	nan	0	113	inf	5.00E-05	yes
ni|645903713|nb|KK920717.1|	550694-550981	nan	nan	0.532857	244	9	0.03715	no
ni|645903713|nb|KK920717.1|	551674-552139	nan	nan	0.339715	87	8	0.02745	no
ni|645903713|nb|KK920717.1|	557181-558152	nan	nan	0.595795	308	9	0.00095	yes
ni|645903713|nb|KK920717.1|	558258-559349	nan	nan	0.199421	3	4	0.01955	no
ni|645903713|nb|KK920717.1|	560677-560806	nan	nan	20	13	-0.652267	0.5921	no
ni|645903713|nb|KK920717.1|	561202-561529	nan	nan	51.61	41	-0.323172	0.77165	no
ni|645903713|nb|KK920717.1|	562483-562805	nan	nan	34	133.63	2	0.13915	no
ni|645903713|nb|KK920717.1|	565088-565246	nan	nan	110	417	2	0.15485	no
ni|645903713|nb|KK920717.1|	567149-567275	nan	nan	69	259	2	0.10895	no
ni|645903713|nb|KK920717.1|	567536-567732	nan	nan	0.522163	354	9	0.0443	no
ni|645903713|nb|KK920717.1|	569778-570012	nan	nan	20	22	0.171483	0.8833	no
ni|645903713|nb|KK920717.1|	599876-600405	nan	nan	25	29	0.191891	0.86325	no
ni|645903713|nb|KK920717.1|	600726-601390	nan	nan	0.570423	0.293648	-0.957943	0.51225	no
ni|645903713|nb|KK920717.1|	601495-602070	nan	nan	0.521398	0.29906	-0.801951	0.6027	no
ni|645903713|nb|KK920717.1|	602662-602908	nan	nan	34	33	-0.0377957	0.9733	no
ni|645903713|nb|KK920717.1|	603980-604201	nan	nan	51	53	0.0506822	0.9631	no
ni|645903713|nb|KK920717.1|	605319-605687	nan	nan	21	18.25	-0.199137	0.86035	no
ni|645903713|nb|KK920717.1|	606012-606382	nan	nan	22	22	-0.0179502	0.98725	no
ni|645903713|nb|KK920717.1|	607091-607261	nan	nan	35	38	0.112437	0.92325	no
ni|645903713|nb|KK920717.1|	608224-608461	nan	nan	6	5	-0.440734	0.7057	no
ni|645903713|nb|KK920717.1|	610698-610807	nan	nan	35	22	-0.705138	0.60375	no
ni|645903713|nb|KK920717.1|	611219-611408	nan	nan	51	44	-0.192729	0.86215	no

ni|645903713|nb|KK920717.1|	611652-612177	nan	nan	24	23	-0.0721083	0.95225	no
ni|645903713|nb|KK920717.1|	613386-613639	nan	nan	20	18	-0.187455	0.8678	no
ni|645903713|nb|KK920717.1|	616158-616262	nan	nan	69	52	-0.416246	0.7377	no
ni|645903713|nb|KK920717.1|	617462-617955	nan	nan	0.693854	0.18088	-2	0.24815	no
ni|645903713|nb|KK920717.1|	618032-618199	nan	nan	71	62	-0.185886	0.8689	no
ni|645903713|nb|KK920717.1|	619453-619710	nan	nan	85	72	-0.246862	0.8184	no
ni|645903713|nb|KK920717.1|	619959-620398	nan	nan	7	6	-0.217252	0.85635	no
ni|645903713|nb|KK920717.1|	620521-620669	nan	nan	13	7	-0.960284	0.44135	no
ni|645903713|nb|KK920717.1|	620911-621156	nan	nan	12	10	-0.337538	0.77505	no
ni|645903713|nb|KK920717.1|	621269-621428	nan	nan	61	44	-0.472186	0.68025	no
ni|645903713|nb|KK920717.1|	621824-621939	nan	nan	24	5	-2	0.1791	no
ni|645903713|nb|KK920717.1|	622123-622313	nan	nan	62	46	-0.448272	0.69855	no
ni|645903713|nb|KK920717.1|	622633-622824	nan	nan	54	53	-0.0234443	0.98265	no
ni|645903713|nb|KK920717.1|	623065-623209	nan	nan	6	5	-0.0672085	0.94635	no
ni|645903713|nb|KK920717.1|	626370-626571	nan	nan	2	5	1	0.29595	no
ni|645903713|nb|KK920717.1|	628866-629266	nan	nan	1	2	0.835115	0.5035	no
ni|645903713|nb|KK920717.1|	630841-631074	nan	nan	2	4	0.796101	0.5353	no
ni|645903713|nb|KK920717.1|	646772-646971	nan	nan	3	5	1	0.415	no
ni|645903713|nb|KK920717.1|	658255-658455	nan	nan	68	71	0.0603229	0.9566	no
ni|645903713|nb|KK920717.1|	659318-660262	nan	nan	5	5	-0.0020757	0.9964	no
ni|645903713|nb|KK920717.1|	660400-660604	nan	nan	58	75	0.376533	0.73765	no
ni|645903713|nb|KK920717.1|	661093-661505	nan	nan	1	0.532236	-1	0.33495	no
ni|645903713|nb|KK920717.1|	661886-662205	nan	nan	4	4	0.0636013	0.95825	no
ni|645903713|nb|KK920717.1|	664102-664716	nan	nan	0.481663	0.27628	-0.801892	0.6027	no
ni|645903713|nb|KK920717.1|	664892-665230	nan	nan	3	3	0.256341	0.82635	no
ni|645903713|nb|KK920717.1|	665398-665641	nan	nan	2	1	-0.428829	0.75965	no
ni|645903713|nb|KK920717.1|	667120-667290	nan	nan	52	65	0.325049	0.77875	no
ni|645903713|nb|KK920717.1|	667602-668589	nan	nan	0.417871	0.346298	-0.271044	0.8304	no
ni|645903713|nb|KK920717.1|	668725-668917	nan	nan	34	60	0.829543	0.47255	no
ni|645903713|nb|KK920717.1|	669300-669475	nan	nan	49	56	0.184223	0.8707	no

ni|645903713|nb|KK920717.1|	669701-669934	nan	nan	41	64	0.6452	0.5633	no
ni|645903713|nb|KK920717.1|	670859-671804	nan	nan	14	22	0.645371	0.6113	no
ni|645903713|nb|KK920717.1|	672787-673013	nan	nan	37	50	0.427762	0.704	no
ni|645903713|nb|KK920717.1|	716808-716884	nan	nan	10372.5	18793.3	0.857451	0.48495	no
ni|645903713|nb|KK920717.1|	824344-824416	nan	nan	22696.9	30953.8	0.447619	0.7075	no
ni|645903713|nb|KK920717.1|	832923-833657	nan	nan	5	4	-0.425777	0.7092	no
ni|645903713|nb|KK920717.1|	835245-835575	nan	nan	3	2	-0.978163	0.4348	no
ni|645903713|nb|KK920717.1|	888325-888467	nan	nan	11	13	0.208418	0.865	no
ni|645903714|nb|KK920716.1|	201225-201573	nan	nan	6	29	2	0.0703	no
ni|645903714|nb|KK920716.1|	203850-204044	nan	nan	25	93	2	0.1056	no
ni|645903714|nb|KK920716.1|	204342-204510	nan	nan	17	87	2	0.05355	no
ni|645903714|nb|KK920716.1|	204616-204958	nan	nan	7	31	2	0.06995	no
ni|645903714|nb|KK920716.1|	209236-209475	nan	nan	1	4	2	0.22595	no
ni|645903714|nb|KK920716.1|	210203-210342	nan	nan	19	105	2	0.0487	no
ni|645903714|nb|KK920716.1|	211368-212506	nan	nan	13	70	2	0.0741	no
ni|645903714|nb|KK920716.1|	213219-214090	nan	nan	0.57712	1	0.935337	0.44945	no
ni|645903714|nb|KK920716.1|	216893-217069	nan	nan	13	74	2	0.04525	no
ni|645903714|nb|KK920716.1|	218574-218694	nan	nan	0	29	inf	0.0003	yes
ni|645903714|nb|KK920716.1|	219291-219696	nan	nan	4	14	2	0.1857	no
ni|645903714|nb|KK920716.1|	221564-222061	nan	nan	0.749266	0.358098	-1	0.46615	no
ni|645903714|nb|KK920716.1|	222199-222567	nan	nan	0.927046	0.088592	-3	0.03815	no
ni|645903714|nb|KK920716.1|	228860-229065	nan	nan	15	70	2	0.0598	no
ni|645903714|nb|KK920716.1|	237313-238305	nan	nan	3	28	3	0.0271	no
ni|645903714|nb|KK920716.1|	250389-250827	nan	nan	0	7	inf	5.00E-05	yes
ni|645903714|nb|KK920716.1|	261714-261944	nan	nan	32	18	-0.835435	0.46775	no
ni|645903714|nb|KK920716.1|	262009-262384	nan	nan	8	5	-0.650394	0.5786	no
ni|645903714|nb|KK920716.1|	262634-262973	nan	nan	15	11	-0.45727	0.6959	no
ni|645903714|nb|KK920716.1|	263420-263665	nan	nan	31	24	-0.398217	0.7252	no
ni|645903714|nb|KK920716.1|	264946-265277	nan	nan	8	7	-0.203125	0.86465	no
ni|645903714|nb|KK920716.1|	266171-266838	nan	nan	9	8	-0.178065	0.88045	no

ni|645903714|nb|KK920716.1|	267420-267967	nan	nan	14	12	-0.167054	0.8796	no
ni|645903714|nb|KK920716.1|	268687-269520	nan	nan	10	11	0.182224	0.8699	no
ni|645903714|nb|KK920716.1|	270323-270674	nan	nan	8	8	0.0040125	0.99385	no
ni|645903714|nb|KK920716.1|	271010-271906	nan	nan	11	14	0.43093	0.68935	no
ni|645903714|nb|KK920716.1|	283046-283252	nan	nan	16	17	0.0958752	0.9354	no
ni|645903714|nb|KK920716.1|	300889-301166	nan	nan	9	16	0.72595	0.5391	no
ni|645903714|nb|KK920716.1|	312521-312696	nan	nan	2	6	1	0.29655	no
ni|645903714|nb|KK920716.1|	313912-314081	nan	nan	3	9	2	0.19185	no
ni|645903714|nb|KK920716.1|	334367-334555	nan	nan	18	26	0.487373	0.6795	no
ni|645903714|nb|KK920716.1|	56177-56377	nan	nan	2	1	-0.552408	0.71905	no
ni|645903714|nb|KK920716.1|	731406-731563	nan	nan	15	13	-0.200465	0.86755	no
ni|645903715|nb|KK920715.1|	100964-101121	nan	nan	8	23	1	0.22665	no
ni|645903715|nb|KK920715.1|	104764-105484	nan	nan	1	5	2	0.06985	no
ni|645903715|nb|KK920715.1|	113572-113868	nan	nan	0.889339	2	0.827274	0.55535	no
ni|645903715|nb|KK920715.1|	127187-127302	nan	nan	11	9	-0.289887	0.8496	no
ni|645903715|nb|KK920715.1|	146728-146925	nan	nan	8	4	-0.804009	0.516	no
ni|645903715|nb|KK920715.1|	162404-163175	nan	nan	2	2	0.297827	0.7977	no
ni|645903715|nb|KK920715.1|	164508-165134	nan	nan	0.611815	0.539906	-0.180386	0.89165	no
ni|645903715|nb|KK920715.1|	169477-169676	nan	nan	7	7	0.132307	0.90925	no
ni|645903715|nb|KK920715.1|	185785-185993	nan	nan	0.927427	2	1	0.3311	no
ni|645903715|nb|KK920715.1|	197151-197335	nan	nan	3	1	-0.915268	0.53575	no
ni|645903715|nb|KK920715.1|	200968-201414	nan	nan	2	3	0.750342	0.52925	no
ni|645903715|nb|KK920715.1|	202861-203206	nan	nan	2	2	0.27148	0.82415	no
ni|645903715|nb|KK920715.1|	208185-208464	nan	nan	1	1	0.223579	0.8598	no
ni|645903715|nb|KK920715.1|	217239-217447	nan	nan	3	4	0.447761	0.71915	no
ni|645903715|nb|KK920715.1|	226759-226916	nan	nan	10	7	-0.503298	0.68535	no
ni|645903715|nb|KK920715.1|	230688-230880	nan	nan	16	8	-0.94584	0.4268	no
ni|645903715|nb|KK920715.1|	249997-250388	nan	nan	3	1	-0.93211	0.454	no
ni|645903715|nb|KK920715.1|	255787-255942	nan	nan	8	6	-0.330229	0.797	no
ni|645903715|nb|KK920715.1|	274061-274275	nan	nan	3	1	-0.844307	0.5595	no

ni|645903715|nb|KK920715.1|	288188-288380	nan	nan	12	14	0.254782	0.8268	no
ni|645903715|nb|KK920715.1|	293965-294290	nan	nan	4	6	0.767239	0.51555	no
ni|645903715|nb|KK920715.1|	296323-296471	nan	nan	18	18	0.0547909	0.96235	no
ni|645903715|nb|KK920715.1|	309400-309537	nan	nan	13	10	-0.482282	0.71	no
ni|645903715|nb|KK920715.1|	327730-328190	nan	nan	65	37	-0.823839	0.5128	no
ni|645903715|nb|KK920715.1|	333771-333903	nan	nan	255	173	-0.559675	0.62005	no
ni|645903715|nb|KK920715.1|	382311-382939	nan	nan	0.468836	1	2	0.21925	no
ni|645903715|nb|KK920715.1|	407939-409583	nan	nan	4	18	2	0.09855	no
ni|645903715|nb|KK920715.1|	409756-410719	nan	nan	2	17	3	0.0069	no
ni|645903715|nb|KK920715.1|	411035-413394	nan	nan	1	10	3	0.0357	no
ni|645903715|nb|KK920715.1|	73661-73863	nan	nan	3	5	0.933061	0.4672	no
ni|645903715|nb|KK920715.1|	87165-87385	nan	nan	1	5	2	0.16415	no
ni|645903716|nb|KK920714.1|	150412-150825	nan	nan	0	5	inf	5.00E-05	yes
ni|645903716|nb|KK920714.1|	158150-158332	nan	nan	9	5	-0.874646	0.4769	no
ni|645903716|nb|KK920714.1|	163616-163754	nan	nan	20	6	-2	0.21335	no
ni|645903716|nb|KK920714.1|	164781-164974	nan	nan	5	2	-2	0.2921	no
ni|645903716|nb|KK920714.1|	173274-173551	nan	nan	13	6	-1	0.373	no
ni|645903716|nb|KK920714.1|	200218-200375	nan	nan	17	13	-0.398404	0.73505	no
ni|645903716|nb|KK920714.1|	210212-210529	nan	nan	6	3	-0.811936	0.4978	no
ni|645903716|nb|KK920714.1|	210870-211139	nan	nan	17	9	-0.889127	0.444	no
ni|645903716|nb|KK920714.1|	214283-214817	nan	nan	4	2	-1	0.33705	no
ni|645903716|nb|KK920714.1|	215824-216016	nan	nan	16	9	-0.905395	0.4374	no
ni|645903716|nb|KK920714.1|	216827-217225	nan	nan	8	5	-0.773174	0.5103	no
ni|645903716|nb|KK920714.1|	217728-217931	nan	nan	23	11	-1	0.35875	no
ni|645903716|nb|KK920714.1|	218230-218416	nan	nan	15	9	-0.78547	0.51275	no
ni|645903716|nb|KK920714.1|	220007-220748	nan	nan	5	3	-0.835953	0.473	no
ni|645903716|nb|KK920714.1|	223338-223857	nan	nan	0.591442	0.339213	-0.802047	0.6027	no
ni|645903716|nb|KK920714.1|	224717-225250	nan	nan	2	1	-0.421201	0.7241	no
ni|645903716|nb|KK920714.1|	226732-226884	nan	nan	9	8	-0.227657	0.85555	no
ni|645903716|nb|KK920714.1|	227176-227324	nan	nan	19	9	-0.989197	0.4137	no

ni|645903716|nb|KK920714.1|	227992-228315	nan	nan	6	3	-0.90812	0.45145	no
ni|645903716|nb|KK920714.1|	230297-231585	nan	nan	0	6	inf	5.00E-05	yes
ni|645903716|nb|KK920714.1|	231874-232374	nan	nan	19.52	20	0.0365545	0.974	no
ni|645903716|nb|KK920714.1|	232751-233278	nan	nan	13	8	-0.758964	0.50695	no
ni|645903716|nb|KK920714.1|	233523-233691	nan	nan	20	15	-0.335739	0.77345	no
ni|645903716|nb|KK920714.1|	258946-259425	nan	nan	0.915881	9	3	0.01885	no
ni|645903716|nb|KK920714.1|	26062-26236	nan	nan	7	13	0.758664	0.53615	no
ni|645903716|nb|KK920714.1|	264221-264690	nan	nan	0.873633	9	3	0.0194	no
ni|645903716|nb|KK920714.1|	266927-268964	nan	nan	28	27	-0.0623797	0.9643	no
ni|645903716|nb|KK920714.1|	269318-269471	nan	nan	30	41	0.440604	0.70825	no
ni|645903716|nb|KK920714.1|	274748-275224	nan	nan	0.395669	0.756389	0.934834	0.5295	no
ni|645903716|nb|KK920714.1|	275791-276807	nan	nan	0.404608	0.799585	0.982727	0.4373	no
ni|645903716|nb|KK920714.1|	300025-300350	nan	nan	18	17	-0.056662	0.9594	no
ni|645903716|nb|KK920714.1|	300488-300698	nan	nan	20	17	-0.205935	0.8557	no
ni|645903716|nb|KK920714.1|	301249-301817	nan	nan	10	11	0.198037	0.85905	no
ni|645903716|nb|KK920714.1|	302146-302338	nan	nan	29	38	0.398517	0.72425	no
ni|645903716|nb|KK920714.1|	302543-303197	nan	nan	0.714785	0.768776	0.105054	0.93225	no
ni|645903716|nb|KK920714.1|	305185-305387	nan	nan	19	17	-0.163801	0.8952	no
ni|645903716|nb|KK920714.1|	305732-306010	nan	nan	1	0.535013	-1	0.35155	no
ni|645903716|nb|KK920714.1|	306614-307055	nan	nan	9	6	-0.548453	0.6362	no
ni|645903716|nb|KK920714.1|	311078-311287	nan	nan	18	20	0.123539	0.91325	no
ni|645903716|nb|KK920714.1|	311866-312050	nan	nan	16	17	0.111699	0.92075	no
ni|645903716|nb|KK920714.1|	312298-312431	nan	nan	29	30	0.0396442	0.97315	no
ni|645903716|nb|KK920714.1|	314646-314840	nan	nan	14	17	0.265468	0.8193	no
ni|645903716|nb|KK920714.1|	315557-315789	nan	nan	16	15	-0.101176	0.9329	no
ni|645903716|nb|KK920714.1|	316323-316589	nan	nan	2	0.717499	-1	0.3141	no
ni|645903716|nb|KK920714.1|	318802-319319	nan	nan	5	5	-0.0172564	0.98795	no
ni|645903716|nb|KK920714.1|	321024-321326	nan	nan	5	5	0.0466995	0.96945	no
ni|645903716|nb|KK920714.1|	322943-323479	nan	nan	11	11	0.00971576	0.9927	no
ni|645903716|nb|KK920714.1|	333383-333799	nan	nan	147	77	-0.927732	0.5319	no

ni|645903716|nb|KK920714.1|	333985-334326	nan	nan	143.71	78	-0.885671	0.5555	no
ni|645903716|nb|KK920714.1|	334663-334969	nan	nan	10	6	-0.819592	0.49065	no
ni|645903716|nb|KK920714.1|	335055-335238	nan	nan	7	6	-0.330317	0.78525	no
ni|645903716|nb|KK920714.1|	338363-338949	nan	nan	0.968132	0.487106	-0.990968	0.47555	no
ni|645903716|nb|KK920714.1|	339050-339953	nan	nan	0.8003	0.941825	0.234918	0.83745	no
ni|645903716|nb|KK920714.1|	342411-342761	nan	nan	0.894835	0.570065	-0.650495	0.6679	no
ni|645903716|nb|KK920714.1|	346496-346756	nan	nan	2	3	0.670884	0.5978	no
ni|645903716|nb|KK920714.1|	355215-355650	nan	nan	1	0.283148	-2	0.1613	no
ni|645903716|nb|KK920714.1|	360548-361262	nan	nan	10	13	0.387712	0.7283	no
ni|645903716|nb|KK920714.1|	361760-362210	nan	nan	19	35	0.863585	0.44125	no
ni|645903716|nb|KK920714.1|	363453-363667	nan	nan	31	48	0.659849	0.56295	no
ni|645903716|nb|KK920714.1|	364868-365359	nan	nan	13	16	0.335618	0.7648	no
ni|645903716|nb|KK920714.1|	368536-368704	nan	nan	38	51	0.450149	0.70055	no
ni|645903716|nb|KK920714.1|	373149-373390	nan	nan	42	49	0.208822	0.85145	no
ni|645903716|nb|KK920714.1|	373516-373817	nan	nan	37	39	0.0712866	0.94855	no
ni|645903716|nb|KK920714.1|	374600-374868	nan	nan	26	36	0.451836	0.69065	no
ni|645903716|nb|KK920714.1|	376799-377182	nan	nan	2	1	-0.802369	0.53265	no
ni|645903716|nb|KK920714.1|	378233-378336	nan	nan	98	70	-0.483672	0.7105	no
ni|645903716|nb|KK920714.1|	378503-378919	nan	nan	2	2	-0.0653053	0.959	no
ni|645903716|nb|KK920714.1|	379285-379901	nan	nan	3	2	-0.534409	0.6541	no
ni|645903716|nb|KK920714.1|	380238-380644	nan	nan	1	1	-0.257977	0.8388	no
ni|645903716|nb|KK920714.1|	380973-381295	nan	nan	2	0.428301	-2	0.2004	no
ni|645903716|nb|KK920714.1|	382177-382595	nan	nan	0.858498	0.596732	-0.524732	0.7071	no
ni|645903716|nb|KK920714.1|	383408-384287	nan	nan	0.666371	0.485459	-0.456975	0.7095	no
ni|645903716|nb|KK920714.1|	384953-385055	nan	nan	258	215	-0.261798	0.82335	no
ni|645903716|nb|KK920714.1|	390078-390346	nan	nan	134.91	114	-0.244893	0.8721	no
ni|645903716|nb|KK920714.1|	390447-390628	nan	nan	3	2	-0.804266	0.6027	no
ni|645903716|nb|KK920714.1|	392288-392737	nan	nan	0.781829	0.747269	-0.0652265	0.94765	no
ni|645903716|nb|KK920714.1|	393150-393656	nan	nan	1	0.350124	-2	0.2305	no
ni|645903716|nb|KK920714.1|	394293-394602	nan	nan	2	0.455126	-2	0.22995	no

ni|645903716|nb|KK920714.1|	394950-395450	nan	nan	0.743618	0.236933	-2	0.25615	no
ni|645903716|nb|KK920714.1|	400130-400247	nan	nan	170	170	0.0023685	0.9949	no
ni|645903716|nb|KK920714.1|	401576-401879	nan	nan	530	515	-0.0392851	0.97655	no
ni|645903716|nb|KK920714.1|	401983-402235	nan	nan	320	356	0.152527	0.9135	no
ni|645903716|nb|KK920714.1|	406934-407436	nan	nan	3	2	-0.605681	0.6043	no
ni|645903716|nb|KK920714.1|	407974-408125	nan	nan	7	5	-0.514653	0.68825	no
ni|645903716|nb|KK920714.1|	408517-409145	nan	nan	3	2	-0.369761	0.74425	no
ni|645903716|nb|KK920714.1|	410416-411152	nan	nan	3	2	-0.424673	0.72145	no
ni|645903716|nb|KK920714.1|	412088-412601	nan	nan	3	3	0.230364	0.8437	no
ni|645903716|nb|KK920714.1|	413366-414177	nan	nan	4	4	0.00058295	0.9938	no
ni|645903716|nb|KK920714.1|	414824-415334	nan	nan	5	36	3	0.01985	no
ni|645903716|nb|KK920714.1|	415553-415731	nan	nan	5	43	3	0.0233	no
ni|645903716|nb|KK920714.1|	418039-418587	nan	nan	2	16	3	0.01765	no
ni|645903716|nb|KK920714.1|	419028-419544	nan	nan	19	61	2	0.1953	no
ni|645903716|nb|KK920714.1|	424704-424852	nan	nan	111	292	1	0.2169	no
ni|645903716|nb|KK920714.1|	427981-428547	nan	nan	0.478364	0.25405	-0.912997	0.53575	no
ni|645903716|nb|KK920714.1|	429215-429678	nan	nan	0.546555	0.653008	0.256734	0.86075	no
ni|645903716|nb|KK920714.1|	430313-430476	nan	nan	4	6	0.560814	0.667	no
ni|645903716|nb|KK920714.1|	433734-433878	nan	nan	7	4	-0.767648	0.5799	no
ni|645903716|nb|KK920714.1|	448653-448793	nan	nan	79	50	-0.646521	0.5753	no
ni|645903716|nb|KK920714.1|	448868-448980	nan	nan	27	17	-0.705007	0.60375	no
ni|645903716|nb|KK920714.1|	449267-449471	nan	nan	95	63	-0.576757	0.60685	no
ni|645903716|nb|KK920714.1|	449683-449836	nan	nan	3	4	0.669775	0.65595	no
ni|645903716|nb|KK920714.1|	450806-451707	nan	nan	9	9	-0.00345985	0.9975	no
ni|645903716|nb|KK920714.1|	451980-452434	nan	nan	2	17	3	0.0159	no
ni|645903716|nb|KK920714.1|	452837-453169	nan	nan	2	2	-0.0656163	0.95795	no
ni|645903716|nb|KK920714.1|	455173-455989	nan	nan	7	5	-0.346906	0.7651	no
ni|645903716|nb|KK920714.1|	457150-458143	nan	nan	0.276704	12	5.49	0.0093	no
ni|645903716|nb|KK920714.1|	459116-459377	nan	nan	38	30	-0.324385	0.7745	no
ni|645903716|nb|KK920714.1|	460323-460469	nan	nan	38	37	-0.0672039	0.95545	no

ni|645903716|nb|KK920714.1|	460596-461049	nan	nan	11	8	-0.467655	0.68225	no
ni|645903716|nb|KK920714.1|	461194-462548	nan	nan	10	9	-0.162796	0.88065	no
ni|645903716|nb|KK920714.1|	467900-468783	nan	nan	5	6	0.283908	0.8039	no
ni|645903716|nb|KK920714.1|	468906-469141	nan	nan	4	4	0.196668	0.8698	no
ni|645903716|nb|KK920714.1|	469457-470141	nan	nan	1	1	-0.109229	0.9254	no
ni|645903716|nb|KK920714.1|	470543-471390	nan	nan	19	18	-0.143093	0.8946	no
ni|645903716|nb|KK920714.1|	471520-471847	nan	nan	99	81	-0.283232	0.85195	no
ni|645903716|nb|KK920714.1|	472435-472891	nan	nan	0	0.865686	inf	0.003	no
ni|645903716|nb|KK920714.1|	481562-481846	nan	nan	0	4	inf	5.00E-05	yes
ni|645903716|nb|KK920714.1|	504835-505077	nan	nan	18	17	-0.094017	0.93375	no
ni|645903716|nb|KK920714.1|	505643-506069	nan	nan	1	0.50926	-1	0.45115	no
ni|645903716|nb|KK920714.1|	506208-506338	nan	nan	51	40	-0.349066	0.75915	no
ni|645903716|nb|KK920714.1|	508592-508859	nan	nan	19	15	-0.26533	0.81185	no
ni|645903716|nb|KK920714.1|	513777-515824	nan	nan	34	30	-0.202382	0.8838	no
ni|645903716|nb|KK920714.1|	517237-517894	nan	nan	0.666487	0.76462	0.198167	0.8731	no
ni|645903716|nb|KK920714.1|	519040-519648	nan	nan	8	6	-0.369269	0.74655	no
ni|645903716|nb|KK920714.1|	519897-520158	nan	nan	2	0.59197	-1	0.35155	no
ni|645903716|nb|KK920714.1|	521533-522019	nan	nan	16	12	-0.451726	0.69405	no
ni|645903716|nb|KK920714.1|	522308-522983	nan	nan	0.559466	0.370299	-0.59536	0.64595	no
ni|645903716|nb|KK920714.1|	523228-523622	nan	nan	37	25	-0.556708	0.61925	no
ni|645903716|nb|KK920714.1|	524135-524574	nan	nan	72	59	-0.278074	0.85425	no
ni|645903716|nb|KK920714.1|	525083-525902	nan	nan	34	26	-0.397223	0.79415	no
ni|645903716|nb|KK920714.1|	526129-527086	nan	nan	27	18	-0.532057	0.6678	no
ni|645903716|nb|KK920714.1|	527238-528153	nan	nan	13	8	-0.804913	0.4746	no
ni|645903716|nb|KK920714.1|	529308-529660	nan	nan	2	2	-0.0655241	0.95835	no
ni|645903716|nb|KK920714.1|	529767-530397	nan	nan	2	2	0.113434	0.92395	no
ni|645903716|nb|KK920714.1|	530572-531115	nan	nan	16	11	-0.495238	0.66145	no
ni|645903716|nb|KK920714.1|	531772-532529	nan	nan	1	0.791847	-0.605325	0.61485	no
ni|645903716|nb|KK920714.1|	532699-533994	nan	nan	15	9	-0.654448	0.6127	no
ni|645903716|nb|KK920714.1|	534323-534592	nan	nan	20	13	-0.608532	0.60105	no

ni|645903716|nb|KK920714.1|	535296-536400	nan	nan	3	3	-0.438935	0.7026	no
ni|645903716|nb|KK920714.1|	537126-538066	nan	nan	4	4	-0.0761151	0.9452	no
ni|645903716|nb|KK920714.1|	538332-538857	nan	nan	22	15	-0.596423	0.59015	no
ni|645903716|nb|KK920714.1|	539329-539510	nan	nan	3	3	-0.356808	0.7931	no
ni|645903716|nb|KK920714.1|	539663-539830	nan	nan	4	1	-2	0.3149	no
ni|645903716|nb|KK920714.1|	540537-540980	nan	nan	2	1	-0.617781	0.6277	no
ni|645903716|nb|KK920714.1|	541743-541939	nan	nan	74	40	-0.876963	0.44375	no
ni|645903716|nb|KK920714.1|	545170-546277	nan	nan	13	13	-0.0645382	0.9508	no
ni|645903716|nb|KK920714.1|	546415-546879	nan	nan	4	4	-0.065192	0.9532	no
ni|645903716|nb|KK920714.1|	552772-553235	nan	nan	0.956472	0.391805	-1	0.39005	no
ni|645903716|nb|KK920714.1|	554590-554714	nan	nan	44	35	-0.323695	0.777	no
ni|645903716|nb|KK920714.1|	555444-556307	nan	nan	7	7	-0.0906642	0.9362	no
ni|645903716|nb|KK920714.1|	558108-558519	nan	nan	4	4	-0.00391816	0.9875	no
ni|645903716|nb|KK920714.1|	559322-559697	nan	nan	29	30	0.0335102	0.9765	no
ni|645903716|nb|KK920714.1|	561456-561622	nan	nan	58	54	-0.105454	0.9287	no
ni|645903716|nb|KK920714.1|	561909-562072	nan	nan	60	47	-0.34936	0.76255	no
ni|645903716|nb|KK920714.1|	563430-563638	nan	nan	51	62	0.292827	0.7957	no
ni|645903716|nb|KK920714.1|	565203-565356	nan	nan	47	52	0.161434	0.89005	no
ni|645903716|nb|KK920714.1|	565531-565781	nan	nan	40	48	0.261772	0.8151	no
ni|645903716|nb|KK920714.1|	568456-569027	nan	nan	32	36	0.150998	0.88685	no
ni|645903716|nb|KK920714.1|	571578-571747	nan	nan	11	7	-0.532923	0.6606	no
ni|645903716|nb|KK920714.1|	579460-579605	nan	nan	28	11	-1	0.25405	no
ni|645903716|nb|KK920714.1|	581886-582031	nan	nan	14	11	-0.389134	0.7451	no
ni|645903716|nb|KK920714.1|	584145-584347	nan	nan	21	10	-1	0.3652	no
ni|645903716|nb|KK920714.1|	586913-587087	nan	nan	23	11	-1	0.39805	no
ni|645903716|nb|KK920714.1|	588434-588688	nan	nan	10	4	-1	0.3439	no
ni|645903716|nb|KK920714.1|	594127-594279	nan	nan	32	16	-1	0.39155	no
ni|645903716|nb|KK920714.1|	594722-594917	nan	nan	22	11	-0.950274	0.42755	no
ni|645903716|nb|KK920714.1|	598066-598438	nan	nan	14	9	-0.632143	0.58815	no
ni|645903716|nb|KK920714.1|	599490-599623	nan	nan	30	25	-0.259916	0.8271	no

ni|645903716|nb|KK920714.1|	601503-601978	nan	nan	0.727338	0.50561	-0.524599	0.7071	no
ni|645903716|nb|KK920714.1|	603472-603838	nan	nan	0.280219	2	3	0.06975	no
ni|645903716|nb|KK920714.1|	608872-609314	nan	nan	14	14	0.0432827	0.96985	no
ni|645903716|nb|KK920714.1|	610669-610802	nan	nan	461	363	-0.344381	0.7452	no
ni|645903716|nb|KK920714.1|	611356-611682	nan	nan	1	2	0.562385	0.66675	no
ni|645903716|nb|KK920714.1|	612395-612551	nan	nan	6	5	-0.308205	0.81205	no
ni|645903716|nb|KK920714.1|	612758-613000	nan	nan	6	3	-0.978809	0.43475	no
ni|645903716|nb|KK920714.1|	613163-613817	nan	nan	2	2	-0.334058	0.77435	no
ni|645903716|nb|KK920714.1|	615072-615389	nan	nan	1	1	-0.203196	0.87975	no
ni|645903716|nb|KK920714.1|	615890-616918	nan	nan	1	2	0.154067	0.8938	no
ni|645903716|nb|KK920714.1|	618501-618686	nan	nan	310	239.35	-0.374855	0.80205	no
ni|645903716|nb|KK920714.1|	619032-619216	nan	nan	3	0.854826	-2	0.2784	no
ni|645903716|nb|KK920714.1|	619448-619720	nan	nan	1	2	0.419446	0.73705	no
ni|645903716|nb|KK920714.1|	619844-620290	nan	nan	2	1	-0.437202	0.7272	no
ni|645903716|nb|KK920714.1|	620672-621998	nan	nan	1	0.94375	-0.55828	0.6314	no
ni|645903716|nb|KK920714.1|	622151-622418	nan	nan	260	234	-0.151413	0.9147	no
ni|645903716|nb|KK920714.1|	622791-623290	nan	nan	126	125	-0.00979894	0.99455	no
ni|645903716|nb|KK920714.1|	623409-624006	nan	nan	2	7	2	0.14705	no
ni|645903716|nb|KK920714.1|	625088-625352	nan	nan	0	4	inf	0.0003	yes
ni|645903716|nb|KK920714.1|	631673-632876	nan	nan	0	3	inf	5.00E-05	yes
ni|645903716|nb|KK920714.1|	634916-635209	nan	nan	0	16	inf	5.00E-05	yes
ni|645903716|nb|KK920714.1|	636120-636313	nan	nan	0	38	inf	5.00E-05	yes
ni|645903716|nb|KK920714.1|	639783-639946	nan	nan	0	40	inf	5.00E-05	yes
ni|645903716|nb|KK920714.1|	641779-641971	nan	nan	0	41	inf	5.00E-05	yes
ni|645903716|nb|KK920714.1|	642606-642848	nan	nan	0	39	inf	5.00E-05	yes
ni|645903716|nb|KK920714.1|	646659-646884	nan	nan	0	32	inf	5.00E-05	yes
ni|645903716|nb|KK920714.1|	660141-660705	nan	nan	0.533818	0.25515	-1	0.44755	no
ni|645903716|nb|KK920714.1|	662421-662611	nan	nan	0.835769	43	6	0.05055	no
ni|645903716|nb|KK920714.1|	663655-664165	nan	nan	0.725389	1	0.519865	0.6876	no
ni|645903716|nb|KK920714.1|	664818-665078	nan	nan	0.62362	31	6	0.03715	no

ni|645903716|nb|KK920714.1|	665534-665786	nan	nan	0.985127	3	2	0.297	no
ni|645903716|nb|KK920714.1|	666436-666892	nan	nan	0.627032	0.53273	-0.235136	0.8697	no
ni|645903716|nb|KK920714.1|	667850-668189	nan	nan	2	1	-0.717659	0.57	no
ni|645903716|nb|KK920714.1|	668629-669282	nan	nan	0.895104	2	1	0.39815	no
ni|645903716|nb|KK920714.1|	669392-671847	nan	nan	0.722799	2	2	0.13145	no
ni|645903716|nb|KK920714.1|	671953-672813	nan	nan	0.455388	1	2	0.19595	no
ni|645903716|nb|KK920714.1|	673224-673575	nan	nan	0.990273	1	0.419898	0.73705	no
ni|645903716|nb|KK920714.1|	674018-674789	nan	nan	57	61	0.100216	0.9434	no
ni|645903716|nb|KK920714.1|	677227-678538	nan	nan	5	46	3	0.02865	no
ni|645903716|nb|KK920714.1|	679674-679803	nan	nan	10	80	3	0.0346	no
ni|645903716|nb|KK920714.1|	683337-683614	nan	nan	9	91	3	0.0072	no
ni|645903716|nb|KK920714.1|	684679-685628	nan	nan	9	67	3	0.0341	no
ni|645903716|nb|KK920714.1|	686924-687076	nan	nan	12	67	2	0.04915	no
ni|645903716|nb|KK920714.1|	690219-690465	nan	nan	13	87	3	0.0241	no
ni|645903716|nb|KK920714.1|	690820-691122	nan	nan	11	83	3	0.0155	no
ni|645903716|nb|KK920714.1|	692161-692481	nan	nan	0.791615	0.432221	-0.873031	0.5788	no
ni|645903716|nb|KK920714.1|	694956-695200	nan	nan	7	44	3	0.03455	no
ni|645903716|nb|KK920714.1|	695529-696151	nan	nan	4	3	-0.181954	0.87935	no
ni|645903716|nb|KK920714.1|	696789-696990	nan	nan	12	14	0.147164	0.9015	no
ni|645903716|nb|KK920714.1|	697840-698078	nan	nan	7	4	-0.651288	0.5826	no
ni|645903716|nb|KK920714.1|	698415-698591	nan	nan	9	8	-0.119792	0.91835	no
ni|645903716|nb|KK920714.1|	698827-698957	nan	nan	9	11	0.280628	0.82625	no
ni|645903716|nb|KK920714.1|	700843-701051	nan	nan	6	8	0.480676	0.68815	no
ni|645903716|nb|KK920714.1|	701410-701539	nan	nan	12	12	0.0322313	0.96125	no
ni|645903716|nb|KK920714.1|	701764-701885	nan	nan	13	8	-0.719468	0.61925	no
ni|645903716|nb|KK920714.1|	702154-702381	nan	nan	11	11	0.0330489	0.9777	no
ni|645903716|nb|KK920714.1|	702890-703139	nan	nan	11	8	-0.343031	0.7624	no
ni|645903716|nb|KK920714.1|	704021-704294	nan	nan	11	9	-0.275426	0.8097	no
ni|645903716|nb|KK920714.1|	711598-712461	nan	nan	0	2	inf	5.00E-05	yes
ni|645903716|nb|KK920714.1|	713702-714236	nan	nan	0	34	inf	5.00E-05	yes

ni|645903716|nb|KK920714.1|	716966-717138	nan	nan	0	142	inf	5.00E-05	yes
ni|645903716|nb|KK920714.1|	722209-722801	nan	nan	0	95	inf	5.00E-05	yes
ni|645903716|nb|KK920714.1|	722916-723258	nan	nan	0	44	inf	5.00E-05	yes
ni|645903716|nb|KK920714.1|	723469-723877	nan	nan	0	3	inf	0.0003	yes
ni|645903716|nb|KK920714.1|	724514-725862	nan	nan	0	3	inf	5.00E-05	yes
ni|645903716|nb|KK920714.1|	726922-727415	nan	nan	0	12	inf	5.00E-05	yes
ni|645903716|nb|KK920714.1|	729995-731583	nan	nan	0.016589	1	6	0.11965	no
ni|645903716|nb|KK920714.1|	733019-733660	nan	nan	0.045752	76	11	0.11935	no
ni|645903716|nb|KK920714.1|	759680-759991	nan	nan	0	145	inf	5.00E-05	yes
ni|645903716|nb|KK920714.1|	763306-763747	nan	nan	68	63	-0.110948	0.93935	no
ni|645903716|nb|KK920714.1|	766520-766933	nan	nan	50	53	0.0949103	0.9288	no
ni|645903716|nb|KK920714.1|	767124-767363	nan	nan	0	13	inf	5.00E-05	yes
ni|645903716|nb|KK920714.1|	772531-772849	nan	nan	2	2	-0.235612	0.85345	no
ni|645903716|nb|KK920714.1|	772981-773444	nan	nan	45	40	-0.168206	0.87735	no
ni|645903716|nb|KK920714.1|	774305-774682	nan	nan	2	0.68558	-1	0.40265	no
ni|645903716|nb|KK920714.1|	776101-776271	nan	nan	111	88	-0.336812	0.76725	no
ni|645903716|nb|KK920714.1|	778137-779001	nan	nan	0.905992	0.711586	-0.348461	0.77265	no
ni|645903716|nb|KK920714.1|	780267-780799	nan	nan	14	10	-0.383946	0.7318	no
ni|645903716|nb|KK920714.1|	781177-781428	nan	nan	240.16	171	-0.491504	0.74005	no
ni|645903716|nb|KK920714.1|	781708-781841	nan	nan	69	66	-0.0516742	0.9667	no
ni|645903716|nb|KK920714.1|	785588-785828	nan	nan	22	25	0.204663	0.85845	no
ni|645903716|nb|KK920714.1|	788924-789251	nan	nan	8	10	0.380424	0.74345	no
ni|645903716|nb|KK920714.1|	790706-790889	nan	nan	6	7	0.254645	0.83635	no
ni|645903716|nb|KK920714.1|	791074-791633	nan	nan	4	3	-0.487245	0.6795	no
ni|645903716|nb|KK920714.1|	791958-792405	nan	nan	5	4	-0.407623	0.7274	no
ni|645903716|nb|KK920714.1|	793316-793539	nan	nan	6	5	-0.213392	0.8571	no
ni|645903716|nb|KK920714.1|	794862-795489	nan	nan	4	4	-0.25298	0.82785	no
ni|645903716|nb|KK920714.1|	795730-795951	nan	nan	5	7	0.539137	0.65635	no
ni|645903716|nb|KK920714.1|	796054-797332	nan	nan	4	3	-0.424654	0.7083	no
ni|645903716|nb|KK920714.1|	797520-798408	nan	nan	6	6	-0.0493851	0.96425	no

ni|645903716|nb|KK920714.1|	798654-798917	nan	nan	7	9	0.226126	0.8482	no
ni|645903716|nb|KK920714.1|	800743-802045	nan	nan	4	4	-0.0507318	0.96575	no
ni|645903716|nb|KK920714.1|	802199-802658	nan	nan	14	12	-0.171556	0.88205	no
ni|645903716|nb|KK920714.1|	803225-803406	nan	nan	27	35	0.413325	0.7283	no
ni|645903716|nb|KK920714.1|	803628-804632	nan	nan	0.737987	0.940904	0.350453	0.7604	no
ni|645903716|nb|KK920714.1|	807620-808455	nan	nan	0.941853	1	0.485508	0.6835	no
ni|645903716|nb|KK920714.1|	814072-814225	nan	nan	184	130	-0.501991	0.647	no
ni|645903716|nb|KK920714.1|	814479-814612	nan	nan	133	142	0.0927694	0.93605	no
ni|645903716|nb|KK920714.1|	814730-815867	nan	nan	5	4	-0.570806	0.61535	no
ni|645903716|nb|KK920714.1|	816170-816358	nan	nan	967	947	-0.0298499	0.98425	no
ni|645903716|nb|KK920714.1|	818498-819051	nan	nan	161	192	0.254259	0.85065	no
ni|645903716|nb|KK920714.1|	819431-819999	nan	nan	0.635081	0.050592	-4	0.02785	no
ni|645903716|nb|KK920714.1|	820224-820802	nan	nan	590	680	0.204734	0.8884	no
ni|645903716|nb|KK920714.1|	822373-823328	nan	nan	1	0.994957	-0.142613	0.9	no
ni|645903716|nb|KK920714.1|	823589-824204	nan	nan	7	8	0.319086	0.78045	no
ni|645903716|nb|KK920714.1|	827355-827988	nan	nan	36	11	-2	0.2153	no
ni|645903716|nb|KK920714.1|	831269-831391	nan	nan	35	39	0.142074	0.89935	no
ni|645903716|nb|KK920714.1|	832464-833233	nan	nan	0.443613	0.636218	0.520218	0.6876	no
ni|645903716|nb|KK920714.1|	833817-834061	nan	nan	41	53	0.398778	0.7229	no
ni|645903716|nb|KK920714.1|	836058-836343	nan	nan	6	7	0.14711	0.89545	no
ni|645903716|nb|KK920714.1|	845900-846045	nan	nan	50	55	0.125439	0.91425	no
ni|645903716|nb|KK920714.1|	846862-847018	nan	nan	57	61	0.0814477	0.9414	no
ni|645903716|nb|KK920714.1|	850204-850471	nan	nan	11	12	0.101706	0.93275	no
ni|645903716|nb|KK920714.1|	850625-851170	nan	nan	10	10	0.00349462	0.99635	no
ni|645903716|nb|KK920714.1|	861333-861488	nan	nan	38	40	0.079647	0.94665	no
ni|645903716|nb|KK920714.1|	862538-862738	nan	nan	3	4	0.232579	0.8527	no
ni|645903716|nb|KK920714.1|	864706-864814	nan	nan	103	103	0.00817789	0.9852	no
ni|645903716|nb|KK920714.1|	865001-865192	nan	nan	58	57	-0.026332	0.9824	no
ni|645903716|nb|KK920714.1|	867638-868046	nan	nan	26	36	0.4444	0.6846	no
ni|645903716|nb|KK920714.1|	869812-870580	nan	nan	31	36	0.208214	0.88415	no

ni|645903716|nb|KK920714.1|	870690-871204	nan	nan	10	13	0.370646	0.7394	no
ni|645903716|nb|KK920714.1|	876074-876233	nan	nan	170	147	-0.206502	0.85075	no
ni|645903716|nb|KK920714.1|	876348-877580	nan	nan	1	0.980535	-0.440642	0.71185	no
ni|645903716|nb|KK920714.1|	878186-878579	nan	nan	64	67	0.0601608	0.96725	no
ni|645903716|nb|KK920714.1|	878767-878888	nan	nan	18	3	-2	0.17	no
ni|645903716|nb|KK920714.1|	879090-879509	nan	nan	3	3	-0.321637	0.7848	no
ni|645903716|nb|KK920714.1|	880174-880664	nan	nan	3	3	-0.196381	0.8695	no
ni|645903716|nb|KK920714.1|	880878-881380	nan	nan	37	42	0.172943	0.86935	no
ni|645903716|nb|KK920714.1|	883696-884537	nan	nan	88	107	0.279732	0.8429	no
ni|645903716|nb|KK920714.1|	884596-885171	nan	nan	92	176	0.930931	0.5141	no
ni|645903716|nb|KK920714.1|	889474-889757	nan	nan	0	13	inf	5.00E-05	yes
ni|645903716|nb|KK920714.1|	890759-890922	nan	nan	0	8	inf	0.0007	yes
ni|645903716|nb|KK920714.1|	892575-893081	nan	nan	25	38	0.612529	0.56875	no
ni|645903716|nb|KK920714.1|	893729-893910	nan	nan	85	133	0.651651	0.556	no
ni|645903716|nb|KK920714.1|	894372-894735	nan	nan	27	52	0.924537	0.4022	no
ni|645903716|nb|KK920714.1|	895488-895880	nan	nan	7	11	0.664538	0.56635	no
ni|645903716|nb|KK920714.1|	896418-897301	nan	nan	16	27	0.799731	0.5324	no
ni|645903716|nb|KK920714.1|	897744-897878	nan	nan	83	167	1	0.38345	no
ni|645903716|nb|KK920714.1|	898634-899157	nan	nan	77	69	-0.139889	0.92305	no
ni|645903716|nb|KK920714.1|	899477-900346	nan	nan	50	45	-0.144623	0.9192	no
ni|645903716|nb|KK920714.1|	900525-900952	nan	nan	62	40	-0.635325	0.6139	no
ni|645903716|nb|KK920714.1|	901304-901796	nan	nan	77	56	-0.463682	0.74925	no
ni|645903716|nb|KK920714.1|	902145-902513	nan	nan	46	35	-0.41813	0.7045	no
ni|645903716|nb|KK920714.1|	902924-903074	nan	nan	78	76	-0.0489333	0.96465	no
ni|645903716|nb|KK920714.1|	904299-904413	nan	nan	37	56	0.602332	0.6227	no
ni|645903716|nb|KK920714.1|	905156-905464	nan	nan	84	128	0.614131	0.6126	no
ni|645903716|nb|KK920714.1|	907645-908285	nan	nan	12	19	0.742465	0.5034	no
ni|645903716|nb|KK920714.1|	908586-909314	nan	nan	51	86	0.746525	0.6003	no
ni|645903716|nb|KK920714.1|	909544-910454	nan	nan	37	74	0.978362	0.49935	no
ni|645903716|nb|KK920714.1|	915175-915479	nan	nan	4	9	1	0.3722	no

ni|645903716|nb|KK920714.1|	917723-918076	nan	nan	3	8	1	0.30725	no
ni|645903716|nb|KK920714.1|	920537-920727	nan	nan	15	21	0.499859	0.67275	no
ni|645903716|nb|KK920714.1|	921766-922768	nan	nan	0.164362	0.314332	0.935414	0.5295	no
ni|645903716|nb|KK920714.1|	923319-923520	nan	nan	19	28	0.56107	0.63435	no
ni|645903716|nb|KK920714.1|	924517-924914	nan	nan	1	0.719057	-0.802323	0.5588	no
ni|645903716|nb|KK920714.1|	925273-925590	nan	nan	4	5	0.151119	0.8979	no
ni|645903716|nb|KK920714.1|	927937-928136	nan	nan	8	9	0.136531	0.91025	no
ni|645903716|nb|KK920714.1|	929506-929690	nan	nan	10	9	-0.110339	0.92825	no
ni|645903716|nb|KK920714.1|	931791-931966	nan	nan	13	14	0.106713	0.92485	no
ni|645903716|nb|KK920714.1|	935096-935354	nan	nan	9	12	0.355037	0.7603	no
ni|645903716|nb|KK920714.1|	935565-935825	nan	nan	10	13	0.387284	0.73385	no
ni|645903716|nb|KK920714.1|	936576-936682	nan	nan	28	27	-0.0679592	0.93835	no
ni|645903716|nb|KK920714.1|	937896-938110	nan	nan	11	13	0.167766	0.8845	no
ni|645903716|nb|KK920714.1|	939346-939548	nan	nan	14	18	0.399629	0.73345	no
ni|645903716|nb|KK920714.1|	941405-941666	nan	nan	6	8	0.429885	0.70955	no
ni|645903716|nb|KK920714.1|	942946-943113	nan	nan	10	14	0.463273	0.6936	no
ni|645903716|nb|KK920714.1|	944980-945764	nan	nan	3	2	-0.697949	0.5481	no
ni|645903716|nb|KK920714.1|	945976-946201	nan	nan	13	11	-0.183875	0.87285	no
ni|645903716|nb|KK920714.1|	947368-947532	nan	nan	13	12	-0.11029	0.92825	no
ni|645903716|nb|KK920714.1|	948867-949149	nan	nan	1	0.784751	-0.802871	0.6027	no
ni|645903716|nb|KK920714.1|	949756-950041	nan	nan	4	6	0.605494	0.60855	no
ni|645903716|nb|KK920714.1|	953269-953479	nan	nan	9	15	0.735288	0.5287	no
ni|645903716|nb|KK920714.1|	955602-955888	nan	nan	10	12	0.252094	0.8336	no
ni|645903716|nb|KK920714.1|	957232-957402	nan	nan	11	17	0.547441	0.6408	no
ni|645903716|nb|KK920714.1|	958342-958487	nan	nan	13	14	0.164119	0.89015	no
ni|645903716|nb|KK920714.1|	959173-959405	nan	nan	14	14	-0.0098267	0.99165	no
ni|645903716|nb|KK920714.1|	959861-960351	nan	nan	2	2	-0.264446	0.81915	no
ni|645903716|nb|KK920714.1|	965543-965831	nan	nan	13	13	0.0059279	0.99385	no
ni|645903716|nb|KK920714.1|	965964-966391	nan	nan	0.607079	0.580223	-0.0652768	0.9384	no
ni|645903716|nb|KK920714.1|	966720-966878	nan	nan	17	16	-0.104675	0.9274	no

ni|645903716|nb|KK920714.1|	968819-969040	nan	nan	20	19	-0.0960276	0.93765	no
ni|645903716|nb|KK920714.1|	969976-970169	nan	nan	22	17	-0.354703	0.7595	no
ni|645903716|nb|KK920714.1|	970279-970424	nan	nan	13	16	0.316123	0.79115	no
ni|645903716|nb|KK920714.1|	972015-972289	nan	nan	20	18	-0.204485	0.86155	no
ni|645903716|nb|KK920714.1|	972701-973033	nan	nan	10	8	-0.253689	0.82735	no
ni|645903716|nb|KK920714.1|	973308-973695	nan	nan	13	11	-0.230448	0.83695	no
ni|645903716|nb|KK920714.1|	973854-974097	nan	nan	8	2	-2	0.1255	no
ni|645903716|nb|KK920714.1|	974462-974750	nan	nan	12.72	8	-0.650825	0.5764	no
ni|645903716|nb|KK920714.1|	975227-975404	nan	nan	15	11	-0.492631	0.67615	no
ni|645903717|nb|KK920713.1|	100493-101399	nan	nan	5	4	-0.468537	0.6802	no
ni|645903717|nb|KK920713.1|	104084-104222	nan	nan	92	75	-0.298122	0.79545	no
ni|645903717|nb|KK920713.1|	108418-108588	nan	nan	72	74	0.0498637	0.9664	no
ni|645903717|nb|KK920713.1|	108700-108841	nan	nan	89	82	-0.117147	0.9168	no
ni|645903717|nb|KK920713.1|	110044-110211	nan	nan	94	81	-0.217973	0.8431	no
ni|645903717|nb|KK920713.1|	112333-112905	nan	nan	29	27	-0.0968748	0.92855	no
ni|645903717|nb|KK920713.1|	115014-115605	nan	nan	13	15	0.197152	0.86205	no
ni|645903717|nb|KK920713.1|	117362-117730	nan	nan	6	6	0.0582769	0.96105	no
ni|645903717|nb|KK920713.1|	119049-119392	nan	nan	19	20	0.0832994	0.94315	no
ni|645903717|nb|KK920713.1|	11961-12080	nan	nan	54	25	-1	0.36625	no
ni|645903717|nb|KK920713.1|	12183-12419	nan	nan	113	81	-0.468115	0.66185	no
ni|645903717|nb|KK920713.1|	12581-12726	nan	nan	71	49	-0.512617	0.6635	no
ni|645903717|nb|KK920713.1|	129049-129312	nan	nan	10	12	0.273795	0.80865	no
ni|645903717|nb|KK920713.1|	132403-132564	nan	nan	45	48	0.0691137	0.95175	no
ni|645903717|nb|KK920713.1|	13390-13670	nan	nan	31	26	-0.265945	0.81625	no
ni|645903717|nb|KK920713.1|	135397-135534	nan	nan	11	8	-0.366804	0.7706	no
ni|645903717|nb|KK920713.1|	138187-138497	nan	nan	8	6	-0.350459	0.76205	no
ni|645903717|nb|KK920713.1|	138811-138919	nan	nan	28	29	0.0697326	0.94415	no
ni|645903717|nb|KK920713.1|	139167-139426	nan	nan	5	3	-0.62797	0.60265	no
ni|645903717|nb|KK920713.1|	140810-140945	nan	nan	7	10	0.517705	0.6956	no
ni|645903717|nb|KK920713.1|	144533-144901	nan	nan	0.927046	0.797332	-0.217462	0.87705	no

ni|645903717|nb|KK920713.1|	146007-146386	nan	nan	25	25	-0.0109693	0.99245	no
ni|645903717|nb|KK920713.1|	147221-147661	nan	nan	8	8	0.0156738	0.98885	no
ni|645903717|nb|KK920713.1|	14746-15037	nan	nan	38	28	-0.419204	0.7048	no
ni|645903717|nb|KK920713.1|	148069-148797	nan	nan	17	17	-0.0349695	0.97305	no
ni|645903717|nb|KK920713.1|	149022-149326	nan	nan	1	0.816129	-0.843371	0.5595	no
ni|645903717|nb|KK920713.1|	149510-149711	nan	nan	17	13	-0.351691	0.76055	no
ni|645903717|nb|KK920713.1|	149948-150206	nan	nan	2	3	0.518862	0.67875	no
ni|645903717|nb|KK920713.1|	151427-151579	nan	nan	21	21	-0.0016035	0.98555	no
ni|645903717|nb|KK920713.1|	16969-17157	nan	nan	3	3	-0.204719	0.87975	no
ni|645903717|nb|KK920713.1|	17257-17687	nan	nan	12	8	-0.58119	0.62185	no
ni|645903717|nb|KK920713.1|	19077-19284	nan	nan	30	20	-0.618628	0.59635	no
ni|645903717|nb|KK920713.1|	19514-19668	nan	nan	21	18	-0.237117	0.8398	no
ni|645903717|nb|KK920713.1|	20130-20327	nan	nan	31	25	-0.301509	0.79595	no
ni|645903717|nb|KK920713.1|	22024-22180	nan	nan	30	15	-1	0.3928	no
ni|645903717|nb|KK920713.1|	22843-22990	nan	nan	39	20	-0.935098	0.42885	no
ni|645903717|nb|KK920713.1|	24385-24517	nan	nan	45	34	-0.401698	0.7333	no
ni|645903717|nb|KK920713.1|	24642-24757	nan	nan	25	17	-0.608063	0.63135	no
ni|645903717|nb|KK920713.1|	26333-26891	nan	nan	5	4	-0.423468	0.7163	no
ni|645903717|nb|KK920713.1|	27887-28018	nan	nan	17	12	-0.526718	0.67145	no
ni|645903717|nb|KK920713.1|	28155-28594	nan	nan	17	16	-0.121343	0.91065	no
ni|645903717|nb|KK920713.1|	307923-308148	nan	nan	0	4	inf	0.0007	yes
ni|645903717|nb|KK920713.1|	32137-32350	nan	nan	0.664508	6	3	0.058	no
ni|645903717|nb|KK920713.1|	32776-32934	nan	nan	31	130	2	0.08245	no
ni|645903717|nb|KK920713.1|	36138-36405	nan	nan	39	156	2	0.14285	no
ni|645903717|nb|KK920713.1|	37923-38572	nan	nan	12	63	2	0.0849	no
ni|645903717|nb|KK920713.1|	40997-41624	nan	nan	6	8	0.35013	0.76145	no
ni|645903717|nb|KK920713.1|	42724-43374	nan	nan	44	47	0.105874	0.94245	no
ni|645903717|nb|KK920713.1|	43824-44009	nan	nan	117	125	0.0941651	0.9292	no
ni|645903717|nb|KK920713.1|	45316-45597	nan	nan	79	78	-0.0113712	0.99075	no
ni|645903717|nb|KK920713.1|	49199-49395	nan	nan	110	111	0.00608223	0.99465	no

ni|645903717|nb|KK920713.1|	50249-50400	nan	nan	123	107	-0.198984	0.85915	no
ni|645903717|nb|KK920713.1|	51907-52086	nan	nan	159.97	123	-0.382821	0.723	no
ni|645903717|nb|KK920713.1|	54905-55491	nan	nan	0	7	inf	5.00E-05	yes
ni|645903717|nb|KK920713.1|	57926-58129	nan	nan	153	85	-0.844852	0.43935	no
ni|645903717|nb|KK920713.1|	58233-59265	nan	nan	434	396	-0.132436	0.92905	no
ni|645903717|nb|KK920713.1|	59398-59648	nan	nan	3	6	0.971295	0.4302	no
ni|645903717|nb|KK920713.1|	64683-64838	nan	nan	53	90	0.762881	0.50645	no
ni|645903717|nb|KK920713.1|	66478-66603	nan	nan	20	35	0.788266	0.51505	no
ni|645903717|nb|KK920713.1|	67346-67736	nan	nan	16	29	0.849045	0.4535	no
ni|645903717|nb|KK920713.1|	68671-68843	nan	nan	48	94	0.973933	0.4003	no
ni|645903717|nb|KK920713.1|	69888-70095	nan	nan	62	100	0.70482	0.5224	no
ni|645903717|nb|KK920713.1|	71373-71520	nan	nan	35	63	0.8426	0.4731	no
ni|645903717|nb|KK920713.1|	72886-73112	nan	nan	47	82	0.812406	0.4683	no
ni|645903717|nb|KK920713.1|	75401-75615	nan	nan	53	79	0.582653	0.6078	no
ni|645903717|nb|KK920713.1|	75716-75980	nan	nan	0.912411	2	0.933953	0.5295	no
ni|645903717|nb|KK920713.1|	77326-77461	nan	nan	19	45	1	0.29275	no
ni|645903717|nb|KK920713.1|	77598-77756	nan	nan	33	56	0.772335	0.5028	no
ni|645903717|nb|KK920713.1|	83732-83948	nan	nan	74	136	0.890394	0.4225	no
ni|645903717|nb|KK920713.1|	84076-84765	nan	nan	0.924196	1	0.47574	0.6922	no
ni|645903717|nb|KK920713.1|	86023-86336	nan	nan	1	0.781413	-0.580287	0.70815	no
ni|645903717|nb|KK920713.1|	86899-87114	nan	nan	83	143	0.786341	0.46565	no
ni|645903717|nb|KK920713.1|	89148-89255	nan	nan	25	18	-0.48288	0.7594	no
ni|645903717|nb|KK920713.1|	89440-89555	nan	nan	13	12	-0.0674947	0.93835	no
ni|645903717|nb|KK920713.1|	95509-95914	nan	nan	51	40	-0.334403	0.7498	no
ni|645903717|nb|KK920713.1|	97781-98016	nan	nan	116	96	-0.274237	0.8003	no
ni|645903718|nb|KK920712.1|	117156-117308	nan	nan	4	0.914428	-2	0.32065	no
ni|645903719|nb|KK920711.1|	107919-109091	nan	nan	7	7	0.112169	0.92165	no
ni|645903719|nb|KK920711.1|	236003-236202	nan	nan	2	6	2	0.262	no
ni|645903719|nb|KK920711.1|	48586-49971	nan	nan	0	17	inf	5.00E-05	yes
ni|645903720|nb|KK920710.1|	103788-103903	nan	nan	14	9	-0.652457	0.66125	no

ni|645903720|nb|KK920710.1|	105364-105571	nan	nan	3	11	2	0.17255	no
ni|645903720|nb|KK920710.1|	106834-107083	nan	nan	5	13	1	0.2195	no
ni|645903720|nb|KK920710.1|	117635-118131	nan	nan	1	0.359007	-1	0.32095	no
ni|645903720|nb|KK920710.1|	118765-119512	nan	nan	56	40	-0.473879	0.74425	no
ni|645903720|nb|KK920710.1|	119882-120115	nan	nan	424	279.92	-0.597977	0.67565	no
ni|645903720|nb|KK920710.1|	122912-123283	nan	nan	56	37	-0.610945	0.57095	no
ni|645903720|nb|KK920710.1|	124834-125348	nan	nan	20	14	-0.545312	0.62965	no
ni|645903720|nb|KK920710.1|	129161-129712	nan	nan	17	12	-0.517286	0.6508	no
ni|645903720|nb|KK920710.1|	130014-130212	nan	nan	1	3	1	0.39335	no
ni|645903720|nb|KK920710.1|	130496-131054	nan	nan	8	6	-0.382043	0.74265	no
ni|645903720|nb|KK920710.1|	133809-134100	nan	nan	13	9	-0.475931	0.685	no
ni|645903720|nb|KK920710.1|	134824-134962	nan	nan	28	20	-0.449109	0.70165	no
ni|645903720|nb|KK920710.1|	135416-135763	nan	nan	10	8	-0.222515	0.8487	no
ni|645903720|nb|KK920710.1|	136860-137219	nan	nan	3	2	-0.983033	0.43245	no
ni|645903720|nb|KK920710.1|	137850-138287	nan	nan	121	77	-0.64496	0.65775	no
ni|645903720|nb|KK920710.1|	141036-141328	nan	nan	15	7	-1	0.3744	no
ni|645903720|nb|KK920710.1|	143374-143556	nan	nan	139	101	-0.459212	0.6728	no
ni|645903720|nb|KK920710.1|	145585-146009	nan	nan	61	45	-0.428376	0.73275	no
ni|645903720|nb|KK920710.1|	146760-147114	nan	nan	26	16	-0.699947	0.53885	no
ni|645903720|nb|KK920710.1|	331000-331671	nan	nan	0	57	inf	5.00E-05	yes
ni|645903720|nb|KK920710.1|	38534-38649	nan	nan	41	26	-0.680472	0.5787	no
ni|645903720|nb|KK920710.1|	408132-408337	nan	nan	9	5	-0.892845	0.46025	no
ni|645903720|nb|KK920710.1|	446177-446485	nan	nan	0	3	inf	0.0003	yes
ni|645903720|nb|KK920710.1|	60502-60684	nan	nan	176	128	-0.46322	0.6636	no
ni|645903720|nb|KK920710.1|	60809-61068	nan	nan	103.08	90	-0.197022	0.8507	no
ni|645903720|nb|KK920710.1|	61283-61493	nan	nan	253	213	-0.248901	0.8638	no
ni|645903720|nb|KK920710.1|	62800-62940	nan	nan	215	179	-0.266997	0.81085	no
ni|645903720|nb|KK920710.1|	65983-66095	nan	nan	70	52	-0.430148	0.7184	no
ni|645903720|nb|KK920710.1|	66595-66976	nan	nan	86	111	0.375476	0.7965	no
ni|645903720|nb|KK920710.1|	71460-72137	nan	nan	32	33	0.0507839	0.97255	no

ni|645903720|nb|KK920710.1|	74503-75091	nan	nan	164	147	-0.155783	0.91265	no
ni|645903720|nb|KK920710.1|	76696-76983	nan	nan	2	0.381807	-2	0.20695	no
ni|645903720|nb|KK920710.1|	77448-77644	nan	nan	2	3	0.222441	0.8598	no
ni|645903720|nb|KK920710.1|	78158-78803	nan	nan	125	103	-0.27097	0.8425	no
ni|645903720|nb|KK920710.1|	79347-79894	nan	nan	2	1	-0.667698	0.57245	no
ni|645903720|nb|KK920710.1|	81193-81619	nan	nan	174	129	-0.427669	0.762	no
ni|645903720|nb|KK920710.1|	81999-82213	nan	nan	2	0.628866	-1	0.4306	no
ni|645903720|nb|KK920710.1|	85469-85623	nan	nan	1487.38	1115.66	-0.414871	0.76785	no
ni|645903720|nb|KK920710.1|	85746-85963	nan	nan	3	1	-1	0.32095	no
ni|645903720|nb|KK920710.1|	88747-89004	nan	nan	4	4	-0.229609	0.84785	no
ni|645903720|nb|KK920710.1|	91175-91999	nan	nan	2	7	2	0.11565	no
ni|645903720|nb|KK920710.1|	93013-93469	nan	nan	2	6	2	0.17765	no
ni|645903720|nb|KK920710.1|	93577-94193	nan	nan	1	5	2	0.098	no
ni|645903721|nb|KK920709.1|	395476-395686	nan	nan	4	5	0.489621	0.69415	no
ni|645903721|nb|KK920709.1|	433051-433512	nan	nan	33	24	-0.447615	0.68345	no
ni|645903721|nb|KK920709.1|	434038-434302	nan	nan	38	28	-0.449127	0.69645	no
ni|645903721|nb|KK920709.1|	434616-434724	nan	nan	25	29	0.221736	0.8747	no
ni|645903721|nb|KK920709.1|	436043-436159	nan	nan	42	21	-0.967937	0.44425	no
ni|645903721|nb|KK920709.1|	436391-436799	nan	nan	73	66	-0.162034	0.9165	no
ni|645903721|nb|KK920709.1|	437241-437371	nan	nan	72	60	-0.25994	0.8234	no
ni|645903721|nb|KK920709.1|	438180-438524	nan	nan	40	35	-0.176443	0.8686	no
ni|645903721|nb|KK920709.1|	440489-440651	nan	nan	5	0.384775	-4	0.02785	no
ni|645903721|nb|KK920709.1|	440819-441606	nan	nan	1	0.481832	-1	0.37485	no
ni|645903721|nb|KK920709.1|	442174-443996	nan	nan	0.644907	0.466001	-0.468759	0.6933	no
ni|645903721|nb|KK920709.1|	447789-447926	nan	nan	63	66	0.0646676	0.95595	no
ni|645903721|nb|KK920709.1|	451953-452169	nan	nan	121.45	109	-0.159262	0.8823	no
ni|645903721|nb|KK920709.1|	453259-453570	nan	nan	66	63	-0.0554379	0.9595	no
ni|645903721|nb|KK920709.1|	458263-458409	nan	nan	62	59	-0.0798039	0.94205	no
ni|645903721|nb|KK920709.1|	458514-458719	nan	nan	135.73	128	-0.0821685	0.9411	no
ni|645903721|nb|KK920709.1|	460752-460832	nan	nan	46588.4	52752.7	0.179275	0.87755	no

ni|645903721|nb|KK920709.1|	468280-468447	nan	nan	15	0	-inf	5.00E-05	yes
ni|645903721|nb|KK920709.1|	540437-540832	nan	nan	0.757513	0.321759	-1	0.4023	no
ni|645903721|nb|KK920709.1|	577780-577953	nan	nan	111	117	0.0811259	0.94225	no
ni|645903721|nb|KK920709.1|	670179-670254	nan	nan	75950.3	77913.1	0.0368108	0.9759	no
ni|645903721|nb|KK920709.1|	670410-670622	nan	nan	18	24	0.395892	0.73235	no
ni|645903721|nb|KK920709.1|	699821-700302	nan	nan	35	30	-0.204834	0.8476	no
ni|645903721|nb|KK920709.1|	700468-700717	nan	nan	111	91	-0.290039	0.78285	no
ni|645903721|nb|KK920709.1|	700965-701992	nan	nan	0.613018	0.254863	-1	0.34095	no
ni|645903721|nb|KK920709.1|	704330-704954	nan	nan	0.661403	0.316152	-1	0.45115	no
ni|645903721|nb|KK920709.1|	708704-709417	nan	nan	44	41	-0.0835727	0.95485	no
ni|645903721|nb|KK920709.1|	711103-711326	nan	nan	90	91	0.0186166	0.9867	no
ni|645903721|nb|KK920709.1|	720316-720448	nan	nan	108	112	0.0608263	0.9586	no
ni|645903721|nb|KK920709.1|	722820-723222	nan	nan	40	33	-0.246513	0.82185	no
ni|645903721|nb|KK920709.1|	727621-727763	nan	nan	114	110	-0.052343	0.9647	no
ni|645903721|nb|KK920709.1|	733561-734860	nan	nan	18	15	-0.280623	0.85705	no
ni|645903721|nb|KK920709.1|	735704-736152	nan	nan	0.99793	0.204389	-2	0.1864	no
ni|645903721|nb|KK920709.1|	736433-736551	nan	nan	44	39	-0.157633	0.8927	no
ni|645903721|nb|KK920709.1|	737847-738067	nan	nan	1	1	0.155792	0.9131	no
ni|645903721|nb|KK920709.1|	738679-739121	nan	nan	1	0.693309	-0.650204	0.63225	no
ni|645903721|nb|KK920709.1|	739709-739969	nan	nan	1	1	0.103843	0.93335	no
ni|645903721|nb|KK920709.1|	740143-740300	nan	nan	41	38	-0.113993	0.9219	no
ni|645903721|nb|KK920709.1|	742498-742999	nan	nan	2	1	-0.59563	0.62535	no
ni|645903721|nb|KK920709.1|	743523-744198	nan	nan	0.602502	0.534877	-0.17176	0.89245	no
ni|645903721|nb|KK920709.1|	744584-745172	nan	nan	9	6	-0.435948	0.7082	no
ni|645903721|nb|KK920709.1|	746354-746923	nan	nan	3	3	-0.295293	0.80085	no
ni|645903721|nb|KK920709.1|	747223-748159	nan	nan	2	1	-1	0.3657	no
ni|645903721|nb|KK920709.1|	748635-749286	nan	nan	9	6	-0.519714	0.65125	no
ni|645903721|nb|KK920709.1|	759955-761446	nan	nan	124	109	-0.186081	0.89985	no
ni|645903721|nb|KK920709.1|	761821-762033	nan	nan	21	13	-0.636101	0.5887	no
ni|645903721|nb|KK920709.1|	762616-762772	nan	nan	15	13	-0.200463	0.86755	no

ni|645903721|nb|KK920709.1|	764268-764717	nan	nan	2	2	-0.161442	0.8943	no
ni|645903721|nb|KK920709.1|	764800-765171	nan	nan	3	2	-0.448775	0.708	no
ni|645903721|nb|KK920709.1|	765232-765353	nan	nan	9	4	-1	0.44965	no
ni|645903721|nb|KK920709.1|	771155-771464	nan	nan	13	10	-0.45076	0.6989	no
ni|645903721|nb|KK920709.1|	771552-771710	nan	nan	8	2	-2	0.24585	no
ni|645903721|nb|KK920709.1|	773348-773828	nan	nan	4	4	0.00884327	0.99025	no
ni|645903721|nb|KK920709.1|	775505-775655	nan	nan	6	0.950196	-3	0.2671	no
ni|645903721|nb|KK920709.1|	775811-776009	nan	nan	8	7	-0.114328	0.9219	no
ni|645903721|nb|KK920709.1|	776172-776341	nan	nan	8	8	-0.0672596	0.957	no
ni|645903721|nb|KK920709.1|	776626-776777	nan	nan	10	6	-0.804159	0.53265	no
ni|645903721|nb|KK920709.1|	777635-778397	nan	nan	0.934002	0.607246	-0.621145	0.61715	no
ni|645903721|nb|KK920709.1|	779189-780536	nan	nan	6	6	-0.0786269	0.9415	no
ni|645903721|nb|KK920709.1|	780617-781793	nan	nan	8	7	-0.241908	0.82145	no
ni|645903721|nb|KK920709.1|	783003-783248	nan	nan	1	0.822322	-0.744308	0.59615	no
ni|645903721|nb|KK920709.1|	791374-791851	nan	nan	22	25	0.195735	0.858	no
ni|645903721|nb|KK920709.1|	818413-818793	nan	nan	54	53	-0.021234	0.9825	no
ni|645903721|nb|KK920709.1|	819346-819503	nan	nan	4	3	-0.259844	0.8692	no
ni|645903721|nb|KK920709.1|	820313-820502	nan	nan	2	2	0.102727	0.93335	no
ni|645903721|nb|KK920709.1|	820704-821740	nan	nan	0.923862	0.984388	0.09155	0.93675	no
ni|645903721|nb|KK920709.1|	822540-822793	nan	nan	85	77	-0.14015	0.89575	no
ni|645903721|nb|KK920709.1|	847352-847621	nan	nan	137	116	-0.24194	0.8712	no
ni|645903721|nb|KK920709.1|	848263-848566	nan	nan	0.613166	2	2	0.28465	no
ni|645903721|nb|KK920709.1|	849223-849798	nan	nan	0.625678	0.74765	0.256942	0.84735	no
ni|645903721|nb|KK920709.1|	850724-851188	nan	nan	53	48	-0.137058	0.9101	no
ni|645903721|nb|KK920709.1|	852493-853104	nan	nan	29	27	-0.109554	0.917	no
ni|645903721|nb|KK920709.1|	857045-857283	nan	nan	2	2	-0.0663251	0.94155	no
ni|645903721|nb|KK920709.1|	858919-859089	nan	nan	3	3	0.102656	0.93335	no
ni|645903721|nb|KK920709.1|	859596-860436	nan	nan	1	1	-0.0281599	0.9815	no
ni|645903721|nb|KK920709.1|	861573-862153	nan	nan	0.877101	0.641186	-0.452001	0.72335	no
ni|645903721|nb|KK920709.1|	862448-863492	nan	nan	1	0.650685	-0.949088	0.43045	no

ni|645903721|nb|KK920709.1|	863714-864493	nan	nan	2	1	-0.635052	0.58575	no
ni|645903721|nb|KK920709.1|	866166-866370	nan	nan	4	0.459884	-3	0.2479	no
ni|645903721|nb|KK920709.1|	866494-866888	nan	nan	4	6	0.678293	0.56465	no
ni|645903721|nb|KK920709.1|	868969-869136	nan	nan	79	70	-0.180452	0.87855	no
ni|645903721|nb|KK920709.1|	870384-870646	nan	nan	76	61	-0.327931	0.766	no
ni|645903721|nb|KK920709.1|	874967-875171	nan	nan	95	92	-0.0414254	0.96825	no
ni|645903721|nb|KK920709.1|	878052-878429	nan	nan	37	39	0.0635622	0.95255	no
ni|645903721|nb|KK920709.1|	880448-880912	nan	nan	61	51	-0.247271	0.87185	no
ni|645903721|nb|KK920709.1|	881851-882071	nan	nan	133	108	-0.304528	0.7716	no
ni|645903721|nb|KK920709.1|	882327-882508	nan	nan	145	105	-0.457885	0.67305	no
ni|645903721|nb|KK920709.1|	883085-883254	nan	nan	66	27	-1	0.27625	no
ni|645903722|nb|KK920708.1|	196438-196674	nan	nan	0	2	inf	0.0017	yes
ni|645903722|nb|KK920708.1|	359655-360178	nan	nan	1	0.503987	-1	0.429	no
ni|645903722|nb|KK920708.1|	389093-389698	nan	nan	0.539315	1	1	0.4043	no
ni|645903722|nb|KK920708.1|	411133-411325	nan	nan	13	8	-0.794128	0.5063	no
ni|645903722|nb|KK920708.1|	411912-412357	nan	nan	8	5	-0.643771	0.5825	no
ni|645903722|nb|KK920708.1|	413923-414138	nan	nan	5	3	-0.744754	0.54875	no
ni|645903722|nb|KK920708.1|	418048-418462	nan	nan	5	5	-0.224509	0.84935	no
ni|645903722|nb|KK920708.1|	419396-419640	nan	nan	1	0.496849	-1	0.3864	no
ni|645903722|nb|KK920708.1|	419998-420471	nan	nan	1	0.69896	-0.853668	0.50505	no
ni|645903722|nb|KK920708.1|	422750-422868	nan	nan	13	6	-1	0.44755	no
ni|645903722|nb|KK920708.1|	430009-430166	nan	nan	9	8	-0.211588	0.8562	no
ni|645903722|nb|KK920708.1|	440952-441367	nan	nan	2	21	3	0.0156	no
ni|645903722|nb|KK920708.1|	448438-449091	nan	nan	2	20	3	0.0069	no
ni|645903722|nb|KK920708.1|	464203-464328	nan	nan	6	56	3	0.0548	no
ni|645903722|nb|KK920708.1|	472606-472812	nan	nan	5	21	2	0.10415	no
ni|645903722|nb|KK920708.1|	488900-489088	nan	nan	5	5	0.180712	0.87985	no
ni|645903722|nb|KK920708.1|	499453-500065	nan	nan	4	3	-0.372111	0.75075	no
ni|645903722|nb|KK920708.1|	502119-502942	nan	nan	5	4	-0.317241	0.7809	no
ni|645903722|nb|KK920708.1|	503451-503960	nan	nan	0.848367	0.173772	-2	0.1864	no

ni|645903722|nb|KK920708.1|	518539-519040	nan	nan	1	0.47268	-1	0.3487	no
ni|645903722|nb|KK920708.1|	521169-521367	nan	nan	0.511434	2074.44	12	0.0443	no
ni|645903722|nb|KK920708.1|	531597-531671	nan	nan	11815	11357	-0.0570294	0.96445	no
ni|645903722|nb|KK920708.1|	588222-588394	nan	nan	12	13	0.0513552	0.96615	no
ni|645903722|nb|KK920708.1|	589209-589378	nan	nan	9	9	-0.123843	0.918	no
ni|645903722|nb|KK920708.1|	589920-590070	nan	nan	6	8	0.232365	0.8527	no
ni|645903722|nb|KK920708.1|	592407-592686	nan	nan	7	12	0.73316	0.5377	no
ni|645903722|nb|KK920708.1|	598455-599230	nan	nan	5	8	0.726674	0.52905	no
ni|645903722|nb|KK920708.1|	599955-600371	nan	nan	10	17	0.792676	0.49235	no
ni|645903722|nb|KK920708.1|	602013-602179	nan	nan	22	37	0.74721	0.51805	no
ni|645903722|nb|KK920708.1|	602313-603214	nan	nan	7	9	0.347334	0.75755	no
ni|645903722|nb|KK920708.1|	606714-606991	nan	nan	31	36	0.216901	0.8458	no
ni|645903722|nb|KK920708.1|	607224-607347	nan	nan	20	36	0.85463	0.48475	no
ni|645903722|nb|KK920708.1|	607467-607737	nan	nan	4	3	-0.428567	0.7192	no
ni|645903722|nb|KK920708.1|	614319-614694	nan	nan	0.812967	8	3	0.03105	no
ni|645903722|nb|KK920708.1|	616438-616670	nan	nan	13	134	3	0.00845	no
ni|645903722|nb|KK920708.1|	617431-618894	nan	nan	1	4	2	0.1614	no
ni|645903722|nb|KK920708.1|	619116-619618	nan	nan	6	50	3	0.04445	no
ni|645903722|nb|KK920708.1|	619833-620955	nan	nan	11	38	2	0.17535	no
ni|645903722|nb|KK920708.1|	621215-621468	nan	nan	9	8	-0.120597	0.91925	no
ni|645903722|nb|KK920708.1|	621775-622093	nan	nan	2	0.436212	-2	0.2004	no
ni|645903722|nb|KK920708.1|	659366-659526	nan	nan	98	99	0.0162799	0.98725	no
ni|645903722|nb|KK920708.1|	769269-769334	nan	nan	396046	348960	-0.182604	0.8738	no
ni|645903722|nb|KK920708.1|	812891-813256	nan	nan	61	66	0.106656	0.92125	no
ni|645903722|nb|KK920708.1|	821775-822132	nan	nan	3	3	-0.106145	0.93205	no
ni|645903722|nb|KK920708.1|	829822-830154	nan	nan	2	1	-0.650579	0.63225	no
ni|645903722|nb|KK920708.1|	830669-831178	nan	nan	1	0.463392	-1	0.3131	no
ni|645903722|nb|KK920708.1|	831490-832116	nan	nan	4	2	-1	0.3321	no
ni|645903722|nb|KK920708.1|	834691-834964	nan	nan	3	0.688245	-2	0.1349	no
ni|645903722|nb|KK920708.1|	835192-835536	nan	nan	2	0.584171	-2	0.2095	no

ni|645903722|nb|KK920708.1|	835795-836120	nan	nan	0.995009	0.739468	-0.428221	0.75965	no
ni|645903722|nb|KK920708.1|	836272-836741	nan	nan	2	2	0.034355	0.97545	no
ni|645903722|nb|KK920708.1|	837281-839634	nan	nan	954	726	-0.395217	0.6835	no
ni|645903722|nb|KK920708.1|	839713-839922	nan	nan	149	125	-0.25627	0.8077	no
ni|645903722|nb|KK920708.1|	840553-840923	nan	nan	1	0.439662	-1	0.3479	no
ni|645903722|nb|KK920708.1|	842681-842941	nan	nan	147.33	130	-0.185381	0.8972	no
ni|645903722|nb|KK920708.1|	843711-843846	nan	nan	10	6	-0.704687	0.60375	no
ni|645903722|nb|KK920708.1|	844024-844343	nan	nan	4	2	-0.627561	0.60265	no
ni|645903722|nb|KK920708.1|	845141-845674	nan	nan	64.49	63	-0.038422	0.97675	no
ni|645903722|nb|KK920708.1|	846559-847687	nan	nan	26	23	-0.136266	0.9259	no
ni|645903722|nb|KK920708.1|	849258-849714	nan	nan	0.766373	0.998868	0.382248	0.7694	no
ni|645903722|nb|KK920708.1|	850012-850343	nan	nan	4	3	-0.59169	0.6233	no
ni|645903722|nb|KK920708.1|	850443-850821	nan	nan	64	63	-0.0178549	0.98545	no
ni|645903722|nb|KK920708.1|	850921-851243	nan	nan	8	5	-0.650629	0.5846	no
ni|645903722|nb|KK920708.1|	851505-852710	nan	nan	8	11	0.589108	0.58775	no
ni|645903722|nb|KK920708.1|	852887-853697	nan	nan	3	5	0.799636	0.4908	no
ni|645903722|nb|KK920708.1|	857149-857876	nan	nan	5	7	0.471267	0.68485	no
ni|645903722|nb|KK920708.1|	858508-859130	nan	nan	12	14	0.267661	0.81025	no
ni|645903722|nb|KK920708.1|	861585-861791	nan	nan	16	21	0.421645	0.7218	no
ni|645903722|nb|KK920708.1|	863687-863846	nan	nan	15	16	0.0482747	0.9687	no
ni|645903722|nb|KK920708.1|	863958-864343	nan	nan	4	6	0.411926	0.7319	no
ni|645903722|nb|KK920708.1|	869295-869428	nan	nan	19	23	0.232289	0.83965	no
ni|645903722|nb|KK920708.1|	869642-869830	nan	nan	20	26	0.409222	0.7253	no
ni|645903722|nb|KK920708.1|	871227-871458	nan	nan	21	25	0.247683	0.82995	no
ni|645903722|nb|KK920708.1|	872205-872363	nan	nan	17	20	0.225581	0.8461	no
ni|645903722|nb|KK920708.1|	874811-875158	nan	nan	21	21	-0.0254685	0.98235	no
ni|645903722|nb|KK920708.1|	875667-875989	nan	nan	11	11	0.0379565	0.97375	no
ni|645903722|nb|KK920708.1|	876218-876519	nan	nan	2	0.710054	-1	0.39005	no
ni|645903722|nb|KK920708.1|	877076-877209	nan	nan	22	15	-0.581844	0.63675	no
ni|645903722|nb|KK920708.1|	877498-877902	nan	nan	21	19	-0.139339	0.9	no

ni|645903722|nb|KK920708.1|	878044-878176	nan	nan	35	39	0.168789	0.88615	no
ni|645903722|nb|KK920708.1|	878369-878718	nan	nan	11	9	-0.336391	0.7705	no
ni|645903722|nb|KK920708.1|	879587-879993	nan	nan	8	5	-0.702762	0.54455	no
ni|645903722|nb|KK920708.1|	882586-883083	nan	nan	3	10	2	0.18105	no
ni|645903722|nb|KK920708.1|	885875-886351	nan	nan	3	5	0.728383	0.53555	no
ni|645903722|nb|KK920708.1|	886441-886655	nan	nan	10	11	0.196335	0.86775	no
ni|645903722|nb|KK920708.1|	887027-887378	nan	nan	3	5	0.848742	0.4727	no
ni|645903722|nb|KK920708.1|	927706-929618	nan	nan	0	145	inf	5.00E-05	yes
ni|645903722|nb|KK920708.1|	931225-931336	nan	nan	0	198	inf	5.00E-05	yes
ni|645903722|nb|KK920708.1|	940718-940917	nan	nan	0	96	inf	5.00E-05	yes
ni|645903723|nb|KK920707.1|	101755-102157	nan	nan	0.411027	48	7	0.02725	no
ni|645903723|nb|KK920707.1|	102984-103861	nan	nan	0.349953	1	2	0.14845	no
ni|645903723|nb|KK920707.1|	10346-10736	nan	nan	0.685027	0.409173	-0.743451	0.59615	no
ni|645903723|nb|KK920707.1|	105056-105481	nan	nan	0.763545	68	6	0.00955	no
ni|645903723|nb|KK920707.1|	108165-108290	nan	nan	2	156	6	0.0443	no
ni|645903723|nb|KK920707.1|	110212-110403	nan	nan	0.275515	17	6	0.11935	no
ni|645903723|nb|KK920707.1|	111246-111893	nan	nan	2	229	7	0.00015	yes
ni|645903723|nb|KK920707.1|	112358-112735	nan	nan	1	0.942672	-0.512883	0.697	no
ni|645903723|nb|KK920707.1|	112961-113334	nan	nan	3	3	0.0678274	0.95495	no
ni|645903723|nb|KK920707.1|	114090-114338	nan	nan	1	8	3	0.07495	no
ni|645903723|nb|KK920707.1|	115814-117130	nan	nan	0.670107	2	2	0.1502	no
ni|645903723|nb|KK920707.1|	120679-120861	nan	nan	0.305822	35	7	0.1195	no
ni|645903723|nb|KK920707.1|	126634-127042	nan	nan	41	49	0.261674	0.80555	no
ni|645903723|nb|KK920707.1|	129089-129537	nan	nan	11	15	0.409791	0.7201	no
ni|645903723|nb|KK920707.1|	132202-132412	nan	nan	52	66	0.338742	0.7574	no
ni|645903723|nb|KK920707.1|	143743-144489	nan	nan	2	6	2	0.1786	no
ni|645903723|nb|KK920707.1|	155237-155542	nan	nan	0.849993	6	3	0.071	no
ni|645903723|nb|KK920707.1|	156612-156769	nan	nan	3	14	2	0.11395	no
ni|645903723|nb|KK920707.1|	158669-158899	nan	nan	6	20	2	0.1559	no
ni|645903723|nb|KK920707.1|	161422-161580	nan	nan	4	25	3	0.06195	no

ni|645903723|nb|KK920707.1|	162903-163024	nan	nan	8	29	2	0.19185	no
ni|645903723|nb|KK920707.1|	164665-164821	nan	nan	7	22	2	0.20035	no
ni|645903723|nb|KK920707.1|	167315-167550	nan	nan	4	15	2	0.1532	no
ni|645903723|nb|KK920707.1|	167965-168074	nan	nan	23	50	1	0.39005	no
ni|645903723|nb|KK920707.1|	169482-169632	nan	nan	5	18	2	0.16985	no
ni|645903723|nb|KK920707.1|	176913-177053	nan	nan	6	19	2	0.21945	no
ni|645903723|nb|KK920707.1|	185254-185441	nan	nan	3	1	-1	0.3479	no
ni|645903723|nb|KK920707.1|	186229-186442	nan	nan	3	2	-0.552144	0.68265	no
ni|645903723|nb|KK920707.1|	189749-189997	nan	nan	3	1	-1	0.37315	no
ni|645903723|nb|KK920707.1|	193193-194372	nan	nan	4	4	-0.190042	0.86805	no
ni|645903723|nb|KK920707.1|	197721-198018	nan	nan	7	5	-0.47243	0.68975	no
ni|645903723|nb|KK920707.1|	204230-204447	nan	nan	7	5	-0.422792	0.72405	no
ni|645903723|nb|KK920707.1|	218803-221183	nan	nan	2	2	0.157076	0.89125	no
ni|645903723|nb|KK920707.1|	221452-221962	nan	nan	2	2	-0.161313	0.8943	no
ni|645903723|nb|KK920707.1|	222426-223137	nan	nan	1	2	0.44977	0.70075	no
ni|645903723|nb|KK920707.1|	223443-224074	nan	nan	2	2	-0.100526	0.9335	no
ni|645903723|nb|KK920707.1|	225421-226138	nan	nan	0.561333	0.613348	0.127848	0.9211	no
ni|645903723|nb|KK920707.1|	230499-230639	nan	nan	12	6	-0.993227	0.47555	no
ni|645903723|nb|KK920707.1|	232968-233500	nan	nan	2	0.932056	-1	0.34445	no
ni|645903723|nb|KK920707.1|	233732-233867	nan	nan	13	7	-0.915254	0.4969	no
ni|645903723|nb|KK920707.1|	237650-237836	nan	nan	8	3	-2	0.20995	no
ni|645903723|nb|KK920707.1|	239066-239216	nan	nan	8	4	-1	0.40265	no
ni|645903723|nb|KK920707.1|	242691-242855	nan	nan	5	0.372954	-4	0.02555	no
ni|645903723|nb|KK920707.1|	264014-264349	nan	nan	2	1	-0.928099	0.47065	no
ni|645903723|nb|KK920707.1|	265556-265822	nan	nan	3	3	-0.218033	0.86075	no
ni|645903723|nb|KK920707.1|	266244-267365	nan	nan	24	38	0.666262	0.6481	no
ni|645903723|nb|KK920707.1|	267564-268734	nan	nan	30	53	0.814975	0.56725	no
ni|645903723|nb|KK920707.1|	28493-28851	nan	nan	0.674257	2	1	0.3525	no
ni|645903723|nb|KK920707.1|	58706-58865	nan	nan	3	4	0.392229	0.7763	no
ni|645903723|nb|KK920707.1|	62483-62724	nan	nan	2	4	0.877131	0.48785	no

ni|645903723|nb|KK920707.1|	63892-64280	nan	nan	2	3	0.809083	0.50525	no
ni|645903723|nb|KK920707.1|	64709-65012	nan	nan	2	1	-0.743841	0.56925	no
ni|645903723|nb|KK920707.1|	71402-71765	nan	nan	94	53	-0.822805	0.51835	no
ni|645903723|nb|KK920707.1|	72472-72759	nan	nan	184	98	-0.907824	0.5426	no
ni|645903723|nb|KK920707.1|	76519-76687	nan	nan	4	0	-inf	0.002	yes
ni|645903723|nb|KK920707.1|	77287-77500	nan	nan	185	93	-0.995834	0.44165	no
ni|645903723|nb|KK920707.1|	79729-80514	nan	nan	2	1	-0.574925	0.62125	no
ni|645903723|nb|KK920707.1|	81758-82009	nan	nan	200	93	-1	0.3806	no
ni|645903723|nb|KK920707.1|	85227-85407	nan	nan	261	134	-0.960642	0.4545	no
ni|645903723|nb|KK920707.1|	87448-87632	nan	nan	247	108	-1	0.3569	no
ni|645903723|nb|KK920707.1|	97649-99901	nan	nan	0.27515	407	11	0.00105	yes
ni|645903724|nb|KK920706.1|	121552-121689	nan	nan	0	13	inf	0.0005	yes
ni|645903724|nb|KK920706.1|	151620-153446	nan	nan	26	23	-0.154669	0.9149	no
ni|645903724|nb|KK920706.1|	153758-153882	nan	nan	35	18	-0.948711	0.4309	no
ni|645903724|nb|KK920706.1|	153988-154113	nan	nan	17	6	-2	0.2996	no
ni|645903724|nb|KK920706.1|	157229-157375	nan	nan	4	2	-1	0.44965	no
ni|645903724|nb|KK920706.1|	157435-157579	nan	nan	65	57	-0.210944	0.85365	no
ni|645903724|nb|KK920706.1|	159436-159822	nan	nan	50	40	-0.34692	0.74945	no
ni|645903724|nb|KK920706.1|	16228-16423	nan	nan	55	47	-0.212939	0.8503	no
ni|645903724|nb|KK920706.1|	162949-163189	nan	nan	19	15	-0.307306	0.79075	no
ni|645903724|nb|KK920706.1|	164212-164416	nan	nan	105	99	-0.0868415	0.9359	no
ni|645903724|nb|KK920706.1|	165464-165573	nan	nan	33	36	0.138743	0.90865	no
ni|645903724|nb|KK920706.1|	16586-16744	nan	nan	67	67	0.0142272	0.9894	no
ni|645903724|nb|KK920706.1|	16894-17039	nan	nan	38	35	-0.131336	0.9073	no
ni|645903724|nb|KK920706.1|	169033-170155	nan	nan	59	187	2	0.25735	no
ni|645903724|nb|KK920706.1|	170227-170571	nan	nan	37	131	2	0.1669	no
ni|645903724|nb|KK920706.1|	17329-17476	nan	nan	51	50	-0.0232584	0.98375	no
ni|645903724|nb|KK920706.1|	17830-18009	nan	nan	87	72	-0.271334	0.8059	no
ni|645903724|nb|KK920706.1|	200180-200717	nan	nan	5	6	0.157342	0.8942	no
ni|645903724|nb|KK920706.1|	202681-202974	nan	nan	1	0.493135	-2	0.3149	no

ni|645903724|nb|KK920706.1|	203923-204296	nan	nan	1	1	0.115133	0.9325	no
ni|645903724|nb|KK920706.1|	205004-205285	nan	nan	5	4	-0.263852	0.82235	no
ni|645903724|nb|KK920706.1|	206578-207092	nan	nan	0.598621	2	1	0.2759	no
ni|645903724|nb|KK920706.1|	208361-208809	nan	nan	2	3	0.568643	0.63655	no
ni|645903724|nb|KK920706.1|	209277-209524	nan	nan	2	0.648832	-2	0.25615	no
ni|645903724|nb|KK920706.1|	210837-211111	nan	nan	2	2	-0.065965	0.95795	no
ni|645903724|nb|KK920706.1|	211749-211874	nan	nan	11	20	0.932656	0.46975	no
ni|645903724|nb|KK920706.1|	212120-212314	nan	nan	29	36	0.314456	0.78545	no
ni|645903724|nb|KK920706.1|	213729-213910	nan	nan	38	44	0.211415	0.85305	no
ni|645903724|nb|KK920706.1|	216834-217120	nan	nan	1	0.895707	-0.58045	0.70815	no
ni|645903724|nb|KK920706.1|	217355-217933	nan	nan	0.777166	0.941055	0.276056	0.821	no
ni|645903724|nb|KK920706.1|	218723-218960	nan	nan	27	33	0.321265	0.78035	no
ni|645903724|nb|KK920706.1|	219173-219445	nan	nan	36	45	0.350528	0.75985	no
ni|645903724|nb|KK920706.1|	221935-222200	nan	nan	39	44	0.196996	0.8551	no
ni|645903724|nb|KK920706.1|	223355-223606	nan	nan	30	33	0.117628	0.9155	no
ni|645903724|nb|KK920706.1|	223952-224127	nan	nan	28	31	0.108256	0.9251	no
ni|645903724|nb|KK920706.1|	224634-224967	nan	nan	1	0.509941	-1	0.4064	no
ni|645903724|nb|KK920706.1|	225755-225943	nan	nan	53	59	0.16181	0.8865	no
ni|645903724|nb|KK920706.1|	227106-227583	nan	nan	26	27	0.0823936	0.93795	no
ni|645903724|nb|KK920706.1|	227696-227858	nan	nan	41	40	-0.039198	0.9756	no
ni|645903724|nb|KK920706.1|	228144-228249	nan	nan	41	12	-2	0.2784	no
ni|645903724|nb|KK920706.1|	232029-232219	nan	nan	22	29	0.410397	0.72485	no
ni|645903724|nb|KK920706.1|	234448-234904	nan	nan	0.487692	0.53273	0.127434	0.92555	no
ni|645903724|nb|KK920706.1|	235182-235898	nan	nan	0.401606	0.268777	-0.579371	0.70815	no
ni|645903724|nb|KK920706.1|	237069-237178	nan	nan	30	41	0.434792	0.7373	no
ni|645903724|nb|KK920706.1|	23761-23905	nan	nan	59	51	-0.213059	0.8553	no
ni|645903724|nb|KK920706.1|	239332-239527	nan	nan	47	44	-0.124313	0.91515	no
ni|645903724|nb|KK920706.1|	239964-240210	nan	nan	31	35	0.204848	0.86215	no
ni|645903724|nb|KK920706.1|	240857-241997	nan	nan	1	1	-0.160741	0.8911	no
ni|645903724|nb|KK920706.1|	242628-243599	nan	nan	1	0.922379	-0.531728	0.64725	no

ni|645903724|nb|KK920706.1|	243741-244320	nan	nan	9	9	-0.137736	0.9037	no
ni|645903724|nb|KK920706.1|	244728-245159	nan	nan	16	13	-0.365846	0.74735	no
ni|645903724|nb|KK920706.1|	249017-249388	nan	nan	46	29	-0.637431	0.5616	no
ni|645903724|nb|KK920706.1|	24913-25051	nan	nan	47	48	0.00972382	0.99115	no
ni|645903724|nb|KK920706.1|	249884-250196	nan	nan	2	0.448642	-2	0.1501	no
ni|645903724|nb|KK920706.1|	250687-250918	nan	nan	47	30	-0.628024	0.5864	no
ni|645903724|nb|KK920706.1|	251348-251610	nan	nan	7	5	-0.53319	0.6467	no
ni|645903724|nb|KK920706.1|	253724-253910	nan	nan	31	15	-1	0.38655	no
ni|645903724|nb|KK920706.1|	254202-254392	nan	nan	44	31	-0.534575	0.64345	no
ni|645903724|nb|KK920706.1|	255607-256096	nan	nan	32	37	0.20975	0.84015	no
ni|645903724|nb|KK920706.1|	25602-25850	nan	nan	4	2	-1	0.369	no
ni|645903724|nb|KK920706.1|	256376-256906	nan	nan	0.633863	0.991493	0.645431	0.6193	no
ni|645903724|nb|KK920706.1|	259262-259502	nan	nan	29	39	0.431953	0.70055	no
ni|645903724|nb|KK920706.1|	260919-261116	nan	nan	36	43	0.265227	0.8167	no
ni|645903724|nb|KK920706.1|	261402-262094	nan	nan	17	20	0.272654	0.8049	no
ni|645903724|nb|KK920706.1|	26184-26591	nan	nan	2	2	-0.135719	0.91325	no
ni|645903724|nb|KK920706.1|	262715-263267	nan	nan	115	160	0.476745	0.72965	no
ni|645903724|nb|KK920706.1|	263751-263961	nan	nan	89	201	1	0.36235	no
ni|645903724|nb|KK920706.1|	265556-266273	nan	nan	5	9	0.861203	0.45865	no
ni|645903724|nb|KK920706.1|	266544-266992	nan	nan	185.9	271.02	0.543871	0.69915	no
ni|645903724|nb|KK920706.1|	269696-269831	nan	nan	103.54	77	-0.421439	0.71355	no
ni|645903724|nb|KK920706.1|	270171-270403	nan	nan	54	45	-0.267032	0.8092	no
ni|645903724|nb|KK920706.1|	270624-270731	nan	nan	31	30	-0.0678426	0.94635	no
ni|645903724|nb|KK920706.1|	271050-271193	nan	nan	51	53	0.0420184	0.9711	no
ni|645903724|nb|KK920706.1|	271400-271733	nan	nan	14	9	-0.645161	0.57945	no
ni|645903724|nb|KK920706.1|	272176-272341	nan	nan	85	79	-0.113663	0.92	no
ni|645903724|nb|KK920706.1|	272477-273505	nan	nan	22	20	-0.133244	0.928	no
ni|645903724|nb|KK920706.1|	273926-274285	nan	nan	32	32	0.00221004	0.9977	no
ni|645903724|nb|KK920706.1|	275369-275793	nan	nan	1	1	-0.0652844	0.9589	no
ni|645903724|nb|KK920706.1|	276286-276850	nan	nan	22	23	0.0578528	0.9585	no

ni|645903724|nb|KK920706.1|	276998-277188	nan	nan	48	47	-0.0342047	0.9743	no
ni|645903724|nb|KK920706.1|	277491-278121	nan	nan	32	27	-0.230091	0.82855	no
ni|645903724|nb|KK920706.1|	279255-279429	nan	nan	45	47	0.0582238	0.9597	no
ni|645903724|nb|KK920706.1|	28893-29439	nan	nan	3	3	-0.166318	0.8853	no
ni|645903724|nb|KK920706.1|	29970-30308	nan	nan	8	7	-0.242465	0.8334	no
ni|645903724|nb|KK920706.1|	310734-310875	nan	nan	67	74	0.149589	0.8961	no
ni|645903724|nb|KK920706.1|	31410-31634	nan	nan	62	45	-0.464443	0.6787	no
ni|645903724|nb|KK920706.1|	31958-32150	nan	nan	3	5	0.780851	0.55245	no
ni|645903724|nb|KK920706.1|	32763-32984	nan	nan	2	1	-0.259229	0.8692	no
ni|645903724|nb|KK920706.1|	33764-34210	nan	nan	1	0.890831	-0.271684	0.8304	no
ni|645903724|nb|KK920706.1|	34650-35370	nan	nan	1	0.915556	-0.180271	0.8816	no
ni|645903724|nb|KK920706.1|	35581-35970	nan	nan	2	0.903313	-1	0.4105	no
ni|645903724|nb|KK920706.1|	36846-37485	nan	nan	6	6	0.0663535	0.95145	no
ni|645903724|nb|KK920706.1|	377289-377418	nan	nan	83.61	94	0.173704	0.88275	no
ni|645903724|nb|KK920706.1|	38487-38719	nan	nan	12	12	-0.0443839	0.96935	no
ni|645903724|nb|KK920706.1|	38836-38963	nan	nan	6	6	-0.0673236	0.9302	no
ni|645903724|nb|KK920706.1|	39104-39383	nan	nan	3	2	-0.613415	0.6288	no
ni|645903724|nb|KK920706.1|	44763-44923	nan	nan	4	1	-2	0.2784	no
ni|645903724|nb|KK920706.1|	45060-45570	nan	nan	4	8	0.924849	0.4324	no
ni|645903724|nb|KK920706.1|	53805-53947	nan	nan	0	58	inf	5.00E-05	yes
ni|645903724|nb|KK920706.1|	55227-55439	nan	nan	0.446999	37	6	0.04435	no
ni|645903724|nb|KK920706.1|	55998-56345	nan	nan	56	49	-0.182549	0.86675	no
ni|645903724|nb|KK920706.1|	712153-712301	nan	nan	6	5	-0.19273	0.87555	no
ni|645903724|nb|KK920706.1|	783388-783560	nan	nan	3	6	1	0.4359	no
ni|645903724|nb|KK920706.1|	793986-794138	nan	nan	4	5	0.222315	0.8747	no
ni|645903724|nb|KK920706.1|	795507-795663	nan	nan	8	8	0.0152657	0.9674	no
ni|645903724|nb|KK920706.1|	796881-797840	nan	nan	5	4	-0.19014	0.8692	no
ni|645903724|nb|KK920706.1|	797915-798106	nan	nan	7	4	-0.623559	0.6146	no
ni|645903724|nb|KK920706.1|	798816-799304	nan	nan	1	0.671813	-0.998027	0.4532	no
ni|645903725|nb|KK920705.1|	115841-116848	nan	nan	0	91	inf	5.00E-05	yes

ni|645903725|nb|KK920705.1|	121906-122025	nan	nan	35	23	-0.626847	0.6057	no
ni|645903725|nb|KK920705.1|	122133-122276	nan	nan	31	25	-0.330245	0.78165	no
ni|645903725|nb|KK920705.1|	122490-122652	nan	nan	9	5	-0.890335	0.4822	no
ni|645903725|nb|KK920705.1|	122746-123726	nan	nan	55	57	0.0691272	0.9606	no
ni|645903725|nb|KK920705.1|	123933-124299	nan	nan	3	1	-1	0.26885	no
ni|645903725|nb|KK920705.1|	129777-130009	nan	nan	22	22	-0.0298843	0.9805	no
ni|645903725|nb|KK920705.1|	130451-130593	nan	nan	12	8	-0.552643	0.65835	no
ni|645903725|nb|KK920705.1|	130772-131172	nan	nan	8	7	-0.158459	0.8934	no
ni|645903725|nb|KK920705.1|	133081-133209	nan	nan	7	10	0.517649	0.7004	no
ni|645903725|nb|KK920705.1|	133451-133597	nan	nan	11	8	-0.459521	0.7047	no
ni|645903725|nb|KK920705.1|	133804-134128	nan	nan	9	8	-0.122989	0.9178	no
ni|645903725|nb|KK920705.1|	134770-135383	nan	nan	4	3	-0.543581	0.63775	no
ni|645903725|nb|KK920705.1|	136269-136745	nan	nan	1	0.630324	-0.991165	0.47555	no
ni|645903725|nb|KK920705.1|	141542-141876	nan	nan	3	3	0.436894	0.7114	no
ni|645903725|nb|KK920705.1|	142057-142335	nan	nan	15	13	-0.239266	0.83715	no
ni|645903725|nb|KK920705.1|	142669-142840	nan	nan	11	12	0.062004	0.95855	no
ni|645903725|nb|KK920705.1|	142944-143096	nan	nan	11	7	-0.683863	0.5892	no
ni|645903725|nb|KK920705.1|	143721-143859	nan	nan	23	10	-1	0.33285	no
ni|645903725|nb|KK920705.1|	147487-148064	nan	nan	0.674962	0.496335	-0.443494	0.7422	no
ni|645903725|nb|KK920705.1|	151790-151939	nan	nan	24	17	-0.451861	0.69475	no
ni|645903725|nb|KK920705.1|	152177-152449	nan	nan	6	6	0.112357	0.92475	no
ni|645903725|nb|KK920705.1|	152529-153087	nan	nan	19	14	-0.44143	0.68835	no
ni|645903725|nb|KK920705.1|	154347-154455	nan	nan	38.94	8	-2	0.1863	no
ni|645903725|nb|KK920705.1|	154556-154921	nan	nan	6	4	-0.583319	0.62505	no
ni|645903725|nb|KK920705.1|	155565-155974	nan	nan	12	10	-0.197962	0.8611	no
ni|645903725|nb|KK920705.1|	156470-156591	nan	nan	8	11	0.447182	0.7576	no
ni|645903725|nb|KK920705.1|	160203-160356	nan	nan	24	20	-0.308198	0.79185	no
ni|645903725|nb|KK920705.1|	160566-160807	nan	nan	17	15	-0.206766	0.8599	no
ni|645903725|nb|KK920705.1|	160989-161160	nan	nan	20	16	-0.34558	0.7685	no
ni|645903725|nb|KK920705.1|	161406-162024	nan	nan	9	7	-0.208886	0.85955	no

ni|645903725|nb|KK920705.1|	162290-162486	nan	nan	13	13	-0.0390511	0.9715	no
ni|645903725|nb|KK920705.1|	162611-162906	nan	nan	16	17	0.0322149	0.97625	no
ni|645903725|nb|KK920705.1|	164985-165419	nan	nan	13	13.49	0.0207799	0.98535	no
ni|645903725|nb|KK920705.1|	168025-168170	nan	nan	14	12	-0.244084	0.8395	no
ni|645903725|nb|KK920705.1|	229932-230121	nan	nan	13	14	0.0786525	0.9447	no
ni|645903725|nb|KK920705.1|	257021-257537	nan	nan	13	11	-0.294112	0.7956	no
ni|645903725|nb|KK920705.1|	264008-264167	nan	nan	13	9	-0.514661	0.66705	no
ni|645903725|nb|KK920705.1|	63252-63373	nan	nan	32	27	-0.23089	0.84785	no
ni|645903725|nb|KK920705.1|	63556-63994	nan	nan	0.880817	0.561245	-0.650213	0.64935	no
ni|645903725|nb|KK920705.1|	65323-65997	nan	nan	15	17	0.146846	0.89375	no
ni|645903725|nb|KK920705.1|	66823-67307	nan	nan	13	14	0.184327	0.8694	no
ni|645903725|nb|KK920705.1|	67520-67940	nan	nan	12	15	0.290289	0.8003	no
ni|645903725|nb|KK920705.1|	79593-79863	nan	nan	7	11	0.490397	0.6771	no
ni|645903725|nb|KK920705.1|	83888-84047	nan	nan	28	38	0.465019	0.6925	no
ni|645903726|nb|KK920704.1|	161342-161722	nan	nan	23	18	-0.298216	0.78995	no
ni|645903726|nb|KK920704.1|	161870-162224	nan	nan	16	12	-0.34305	0.76255	no
ni|645903726|nb|KK920704.1|	162326-162441	nan	nan	33	21	-0.652457	0.5967	no
ni|645903726|nb|KK920704.1|	164923-165183	nan	nan	10	11	0.0612975	0.95985	no
ni|645903726|nb|KK920704.1|	166573-167005	nan	nan	0.747355	0.78573	0.0722389	0.94355	no
ni|645903726|nb|KK920704.1|	167609-168071	nan	nan	14	18	0.365901	0.74455	no
ni|645903726|nb|KK920704.1|	181549-181780	nan	nan	24	43	0.850434	0.46195	no
ni|645903726|nb|KK920704.1|	182464-182580	nan	nan	36	47	0.391959	0.7426	no
ni|645903726|nb|KK920704.1|	182975-183866	nan	nan	6	10	0.785016	0.47975	no
ni|645903726|nb|KK920704.1|	204937-205687	nan	nan	5	3	-0.98676	0.39745	no
ni|645903726|nb|KK920704.1|	206751-206883	nan	nan	39	15	-1	0.279	no
ni|645903726|nb|KK920704.1|	208693-208837	nan	nan	8	2	-2	0.2004	no
ni|645903726|nb|KK920704.1|	212987-213117	nan	nan	10	4	-1	0.3479	no
ni|645903726|nb|KK920704.1|	218256-218603	nan	nan	7	3	-1	0.31005	no
ni|645903726|nb|KK920704.1|	226539-227512	nan	nan	12	6	-0.903778	0.4171	no
ni|645903726|nb|KK920704.1|	233189-233398	nan	nan	2	1	-0.289185	0.8535	no

ni|645903726|nb|KK920704.1|	234391-234559	nan	nan	5	5	-0.0672498	0.9582	no
ni|645903726|nb|KK920704.1|	236550-237006	nan	nan	1	2	0.578645	0.65045	no
ni|645903726|nb|KK920704.1|	244602-245230	nan	nan	1	3	1	0.3534	no
ni|645903726|nb|KK920704.1|	267435-267898	nan	nan	4	3	-0.0377136	0.97565	no
ni|645903726|nb|KK920704.1|	268139-268434	nan	nan	4	6	0.359489	0.75995	no
ni|645903726|nb|KK920704.1|	268540-268688	nan	nan	17	18	0.142254	0.89935	no
ni|645903726|nb|KK920704.1|	293479-293890	nan	nan	1	2	0.0866844	0.9485	no
ni|645903726|nb|KK920704.1|	297578-298076	nan	nan	0.747374	0.654856	-0.190652	0.88855	no
ni|645903726|nb|KK920704.1|	298853-299105	nan	nan	1	1	-0.236084	0.8697	no
ni|645903726|nb|KK920704.1|	302023-302896	nan	nan	3	2	-0.657238	0.5688	no
ni|645903726|nb|KK920704.1|	303744-303888	nan	nan	5	3	-0.915205	0.53575	no
ni|645903726|nb|KK920704.1|	303998-304953	nan	nan	5	3	-0.597106	0.6049	no
ni|645903726|nb|KK920704.1|	31918-32151	nan	nan	5	10	1	0.3711	no
ni|645903726|nb|KK920704.1|	329133-329407	nan	nan	1	1	-0.065965	0.9384	no
ni|645903726|nb|KK920704.1|	329641-330427	nan	nan	2	1	-0.205592	0.8617	no
ni|645903726|nb|KK920704.1|	330572-330765	nan	nan	9	8	-0.11293	0.92045	no
ni|645903726|nb|KK920704.1|	331116-331534	nan	nan	0.936543	0.074592	-4	0.02785	no
ni|645903726|nb|KK920704.1|	331750-331974	nan	nan	2	2	-0.525966	0.7071	no
ni|645903726|nb|KK920704.1|	332199-332816	nan	nan	0.622513	1	0.69407	0.58445	no
ni|645903726|nb|KK920704.1|	334331-334728	nan	nan	0.919577	0.719057	-0.354864	0.7931	no
ni|645903726|nb|KK920704.1|	335648-336338	nan	nan	3	2	-0.136381	0.90715	no
ni|645903726|nb|KK920704.1|	336828-337209	nan	nan	5	4	-0.238742	0.83445	no
ni|645903726|nb|KK920704.1|	337545-338330	nan	nan	3	3	0.0314848	0.97945	no
ni|645903726|nb|KK920704.1|	9309-9485	nan	nan	41739.3	28867	-0.531986	0.57525	no
ni|645903727|nb|KK920703.1|	1006510-1006618	nan	nan	189	318	0.751657	0.5232	no
ni|645903727|nb|KK920703.1|	1066941-1068499	nan	nan	79	102	0.365015	0.7928	no
ni|645903727|nb|KK920703.1|	1068705-1068927	nan	nan	67	61	-0.139171	0.9021	no
ni|645903727|nb|KK920703.1|	1069056-1069539	nan	nan	66.74	59.4	-0.168089	0.91095	no
ni|645903727|nb|KK920703.1|	1075651-1076031	nan	nan	2	1	-0.345521	0.78305	no
ni|645903727|nb|KK920703.1|	1076204-1076316	nan	nan	33	24	-0.454601	0.722	no

ni|645903727|nb|KK920703.1|	1076420-1076893	nan	nan	94	93	-0.0170443	0.9904	no
ni|645903727|nb|KK920703.1|	1078173-1078284	nan	nan	54	68	0.31941	0.7924	no
ni|645903727|nb|KK920703.1|	1078643-1078835	nan	nan	81	88	0.11514	0.91745	no
ni|645903727|nb|KK920703.1|	1081235-1081572	nan	nan	83	81	-0.0368451	0.9817	no
ni|645903727|nb|KK920703.1|	1082691-1082990	nan	nan	43	43	-0.0163242	0.98745	no
ni|645903727|nb|KK920703.1|	1083217-1083433	nan	nan	97	103	0.0856592	0.9406	no
ni|645903727|nb|KK920703.1|	1085040-1085523	nan	nan	35	39	0.139357	0.896	no
ni|645903727|nb|KK920703.1|	1086684-1087272	nan	nan	9	9	-0.0157779	0.98835	no
ni|645903727|nb|KK920703.1|	1088203-1088451	nan	nan	13	13	0.024564	0.98425	no
ni|645903727|nb|KK920703.1|	1088715-1089026	nan	nan	8	9	0.291827	0.805	no
ni|645903727|nb|KK920703.1|	1089392-1089529	nan	nan	11	1	-3	0.244	no
ni|645903727|nb|KK920703.1|	1089632-1089794	nan	nan	52	52	-0.0123504	0.9915	no
ni|645903727|nb|KK920703.1|	1091348-1091463	nan	nan	30	32	0.0768952	0.9481	no
ni|645903727|nb|KK920703.1|	1091590-1092017	nan	nan	18	21	0.196738	0.8597	no
ni|645903727|nb|KK920703.1|	1092345-1092460	nan	nan	43	45	0.0845084	0.9429	no
ni|645903727|nb|KK920703.1|	1092664-1093046	nan	nan	23	27	0.272094	0.8094	no
ni|645903727|nb|KK920703.1|	1093268-1093392	nan	nan	32	33	0.0201077	0.9822	no
ni|645903727|nb|KK920703.1|	1093501-1093794	nan	nan	8	10	0.286842	0.79975	no
ni|645903727|nb|KK920703.1|	1093978-1094865	nan	nan	8	8	0.000264957	0.99755	no
ni|645903727|nb|KK920703.1|	1095353-1095530	nan	nan	27	30	0.14027	0.90745	no
ni|645903727|nb|KK920703.1|	1101175-1101700	nan	nan	26	25	-0.0650708	0.9503	no
ni|645903727|nb|KK920703.1|	1101857-1102123	nan	nan	21	33	0.64841	0.5736	no
ni|645903727|nb|KK920703.1|	1102530-1102935	nan	nan	0.162784	1	3	0.07095	no
ni|645903727|nb|KK920703.1|	11115-11790	nan	nan	0.043036	21	9	0.11935	no
ni|645903727|nb|KK920703.1|	1125060-1125504	nan	nan	5	5	-0.107057	0.92905	no
ni|645903727|nb|KK920703.1|	1125670-1126059	nan	nan	6	4	-0.661486	0.5725	no
ni|645903727|nb|KK920703.1|	1126297-1126448	nan	nan	9	1	-3	0.14855	no
ni|645903727|nb|KK920703.1|	1126521-1128064	nan	nan	16	25	0.663417	0.647	no
ni|645903727|nb|KK920703.1|	1128368-1128504	nan	nan	6	13	1	0.41145	no
ni|645903727|nb|KK920703.1|	1128640-1128959	nan	nan	7	7	0.0740431	0.94705	no

ni|645903727|nb|KK920703.1|	1129059-1129168	nan	nan	18	53	2	0.27465	no
ni|645903727|nb|KK920703.1|	1129640-1129809	nan	nan	8	24	2	0.20075	no
ni|645903727|nb|KK920703.1|	1130212-1130403	nan	nan	12	24	1	0.39455	no
ni|645903727|nb|KK920703.1|	1131847-1132370	nan	nan	6	13	1	0.2836	no
ni|645903727|nb|KK920703.1|	1133827-1134315	nan	nan	1	2	0.455691	0.7031	no
ni|645903727|nb|KK920703.1|	1135455-1135592	nan	nan	13	29	1	0.35095	no
ni|645903727|nb|KK920703.1|	1136182-1136550	nan	nan	6	11	1	0.37355	no
ni|645903727|nb|KK920703.1|	1137594-1137750	nan	nan	9	18	0.932804	0.43665	no
ni|645903727|nb|KK920703.1|	1147826-1147947	nan	nan	46	100	1	0.35115	no
ni|645903727|nb|KK920703.1|	1273162-1273391	nan	nan	22	23	0.0528435	0.96345	no
ni|645903727|nb|KK920703.1|	1346386-1347100	nan	nan	0.161168	1	3	0.06345	no
ni|645903727|nb|KK920703.1|	1351791-1351920	nan	nan	52	56	0.0870237	0.94065	no
ni|645903727|nb|KK920703.1|	1352066-1352181	nan	nan	11	15	0.447078	0.7576	no
ni|645903727|nb|KK920703.1|	1352850-1353112	nan	nan	2	4	1	0.40635	no
ni|645903727|nb|KK920703.1|	1353877-1354065	nan	nan	9	18	0.977179	0.4091	no
ni|645903727|nb|KK920703.1|	1354606-1354798	nan	nan	20	21	0.084857	0.94405	no
ni|645903727|nb|KK920703.1|	1355090-1355228	nan	nan	23	30	0.418189	0.7187	no
ni|645903727|nb|KK920703.1|	1355352-1355687	nan	nan	16	17	0.086401	0.9423	no
ni|645903727|nb|KK920703.1|	1355872-1355980	nan	nan	42	11	-2	0.2004	no
ni|645903727|nb|KK920703.1|	1356089-1356286	nan	nan	31	25	-0.315723	0.78935	no
ni|645903727|nb|KK920703.1|	1356441-1356666	nan	nan	11	7	-0.584986	0.6078	no
ni|645903727|nb|KK920703.1|	1360538-1360664	nan	nan	22	9	-1	0.34095	no
ni|645903727|nb|KK920703.1|	1361339-1361496	nan	nan	33	36	0.127818	0.91115	no
ni|645903727|nb|KK920703.1|	1361672-1361875	nan	nan	19	12	-0.708464	0.54385	no
ni|645903727|nb|KK920703.1|	1363186-1363601	nan	nan	13	17	0.446591	0.6939	no
ni|645903727|nb|KK920703.1|	1363950-1364069	nan	nan	38	65	0.780577	0.51205	no
ni|645903727|nb|KK920703.1|	1366995-1367227	nan	nan	23	57	1	0.27045	no
ni|645903727|nb|KK920703.1|	1372669-1372917	nan	nan	23	73	2	0.15055	no
ni|645903727|nb|KK920703.1|	1374931-1375126	nan	nan	24	80	2	0.14195	no
ni|645903727|nb|KK920703.1|	1378696-1378804	nan	nan	42	98	1	0.31665	no

ni|645903727|nb|KK920703.1|	1380951-1381153	nan	nan	24	73	2	0.16935	no
ni|645903727|nb|KK920703.1|	1385476-1385737	nan	nan	26	70	1	0.22175	no
ni|645903727|nb|KK920703.1|	1389927-1391160	nan	nan	44	83	0.920363	0.5163	no
ni|645903727|nb|KK920703.1|	1395672-1395845	nan	nan	43	79	0.891394	0.4383	no
ni|645903727|nb|KK920703.1|	1395997-1396448	nan	nan	2	4	1	0.3227	no
ni|645903727|nb|KK920703.1|	1397006-1397585	nan	nan	7	12	0.712628	0.5396	no
ni|645903727|nb|KK920703.1|	1398139-1398562	nan	nan	0.998791	1	0.141164	0.90865	no
ni|645903727|nb|KK920703.1|	1399239-1399495	nan	nan	2	6	1	0.24765	no
ni|645903727|nb|KK920703.1|	1400256-1400412	nan	nan	20	30	0.538525	0.6461	no
ni|645903727|nb|KK920703.1|	1403425-1403554	nan	nan	18.58	28	0.602547	0.6227	no
ni|645903727|nb|KK920703.1|	1404400-1404970	nan	nan	8	9	0.215683	0.84795	no
ni|645903727|nb|KK920703.1|	1408797-1408924	nan	nan	28	28	-0.0215199	0.9796	no
ni|645903727|nb|KK920703.1|	1409297-1409765	nan	nan	9	6	-0.718733	0.53725	no
ni|645903727|nb|KK920703.1|	1412572-1413264	nan	nan	80	64	-0.32786	0.81685	no
ni|645903727|nb|KK920703.1|	1413376-1413785	nan	nan	4	5	0.0893985	0.9364	no
ni|645903727|nb|KK920703.1|	202867-203356	nan	nan	3	1	-1	0.30035	no
ni|645903727|nb|KK920703.1|	214437-214657	nan	nan	3	5	0.550071	0.6602	no
ni|645903727|nb|KK920703.1|	217969-218357	nan	nan	1	0.659249	-0.972277	0.5005	no
ni|645903727|nb|KK920703.1|	221163-221806	nan	nan	0.592578	0.653674	0.141565	0.90865	no
ni|645903727|nb|KK920703.1|	228468-228800	nan	nan	37	29	-0.362952	0.74165	no
ni|645903727|nb|KK920703.1|	229080-229200	nan	nan	7	10	0.517558	0.7279	no
ni|645903727|nb|KK920703.1|	229388-229753	nan	nan	10	6	-0.86955	0.454	no
ni|645903727|nb|KK920703.1|	230011-230158	nan	nan	52	39	-0.429772	0.7141	no
ni|645903727|nb|KK920703.1|	230289-230671	nan	nan	2	2	-0.00127607	0.97495	no
ni|645903727|nb|KK920703.1|	231596-232928	nan	nan	1	1	0.0114853	0.99125	no
ni|645903727|nb|KK920703.1|	234402-234663	nan	nan	3	3	-0.0660727	0.95495	no
ni|645903727|nb|KK920703.1|	235306-235413	nan	nan	87	77	-0.174758	0.88345	no
ni|645903727|nb|KK920703.1|	235681-236069	nan	nan	23	23	0.00370655	0.9968	no
ni|645903727|nb|KK920703.1|	236332-237093	nan	nan	2	1	-0.169089	0.8866	no
ni|645903727|nb|KK920703.1|	237785-237967	nan	nan	4	4	0.155101	0.90275	no

ni|645903727|nb|KK920703.1|	238608-238765	nan	nan	4	3	-0.0671984	0.93835	no
ni|645903727|nb|KK920703.1|	239221-239370	nan	nan	5	5	0.0848056	0.9436	no
ni|645903727|nb|KK920703.1|	239559-240117	nan	nan	6	5	-0.328048	0.77775	no
ni|645903727|nb|KK920703.1|	240404-240597	nan	nan	6	4	-0.590689	0.62565	no
ni|645903727|nb|KK920703.1|	242817-243047	nan	nan	261	247	-0.0792358	0.9559	no
ni|645903727|nb|KK920703.1|	244086-244417	nan	nan	0.861315	0.514385	-0.743693	0.59615	no
ni|645903727|nb|KK920703.1|	247957-248078	nan	nan	282	297	0.0781198	0.94525	no
ni|645903727|nb|KK920703.1|	249806-250355	nan	nan	0.551739	0.210969	-1	0.35155	no
ni|645903727|nb|KK920703.1|	250878-251222	nan	nan	22	22	0.0138751	0.9896	no
ni|645903727|nb|KK920703.1|	253007-253279	nan	nan	2	0.553822	-2	0.22995	no
ni|645903727|nb|KK920703.1|	255046-255571	nan	nan	4	4	0.0953939	0.9374	no
ni|645903727|nb|KK920703.1|	255715-256102	nan	nan	0.692233	0.578864	-0.258034	0.8692	no
ni|645903727|nb|KK920703.1|	257562-257778	nan	nan	4	3	-0.314593	0.8025	no
ni|645903727|nb|KK920703.1|	258615-259321	nan	nan	14	12	-0.145228	0.8976	no
ni|645903727|nb|KK920703.1|	260070-260482	nan	nan	17	16	-0.0987907	0.9276	no
ni|645903727|nb|KK920703.1|	261265-261647	nan	nan	14	11	-0.367676	0.7502	no
ni|645903727|nb|KK920703.1|	265406-265617	nan	nan	3	6	0.726795	0.56145	no
ni|645903727|nb|KK920703.1|	265727-266871	nan	nan	6	12	1	0.34505	no
ni|645903727|nb|KK920703.1|	266972-267115	nan	nan	7	14	0.932789	0.4586	no
ni|645903727|nb|KK920703.1|	273296-273691	nan	nan	15	18	0.295383	0.79685	no
ni|645903727|nb|KK920703.1|	273900-274190	nan	nan	27	31	0.170349	0.88165	no
ni|645903727|nb|KK920703.1|	274539-274743	nan	nan	39	42	0.126876	0.9095	no
ni|645903727|nb|KK920703.1|	275041-276123	nan	nan	6	6	-0.00968666	0.99275	no
ni|645903727|nb|KK920703.1|	276230-276493	nan	nan	2	1	-0.525487	0.7071	no
ni|645903727|nb|KK920703.1|	278032-278229	nan	nan	34	37	0.106493	0.92695	no
ni|645903727|nb|KK920703.1|	279407-280206	nan	nan	7	7	0.169747	0.88345	no
ni|645903727|nb|KK920703.1|	280326-280495	nan	nan	5	8	0.549412	0.6602	no
ni|645903727|nb|KK920703.1|	282452-283242	nan	nan	0	13	inf	5.00E-05	yes
ni|645903727|nb|KK920703.1|	288788-288943	nan	nan	0.453407	104	8	0.11935	no
ni|645903727|nb|KK920703.1|	289641-289810	nan	nan	0	100	inf	5.00E-05	yes

ni|645903727|nb|KK920703.1|	290170-290430	nan	nan	0	32	inf	5.00E-05	yes
ni|645903727|nb|KK920703.1|	296811-296965	nan	nan	33	36	0.120435	0.91925	no
ni|645903727|nb|KK920703.1|	297076-297256	nan	nan	109	81	-0.428104	0.69805	no
ni|645903727|nb|KK920703.1|	297344-297451	nan	nan	125	125	0.00254675	0.98425	no
ni|645903727|nb|KK920703.1|	298550-298718	nan	nan	3	2	-0.259895	0.8692	no
ni|645903727|nb|KK920703.1|	298841-299398	nan	nan	2	3	0.5053	0.6679	no
ni|645903727|nb|KK920703.1|	299708-300762	nan	nan	3	3	0.143547	0.8972	no
ni|645903727|nb|KK920703.1|	301153-301307	nan	nan	5	6	0.418234	0.73705	no
ni|645903727|nb|KK920703.1|	301370-301481	nan	nan	21	14	-0.582187	0.70815	no
ni|645903727|nb|KK920703.1|	302008-303146	nan	nan	9	18	0.931817	0.473	no
ni|645903727|nb|KK920703.1|	303365-303776	nan	nan	6	11	0.913923	0.43715	no
ni|645903727|nb|KK920703.1|	304521-304776	nan	nan	4	3	-0.341764	0.7737	no
ni|645903727|nb|KK920703.1|	306031-306287	nan	nan	2	2	0.0594112	0.95295	no
ni|645903727|nb|KK920703.1|	306513-306716	nan	nan	5	3	-0.929414	0.47065	no
ni|645903727|nb|KK920703.1|	307327-307796	nan	nan	2	1	-0.541619	0.64975	no
ni|645903727|nb|KK920703.1|	307915-308458	nan	nan	2	2	-0.0285145	0.9815	no
ni|645903727|nb|KK920703.1|	309696-309880	nan	nan	73	68	-0.0971413	0.93305	no
ni|645903727|nb|KK920703.1|	311668-311809	nan	nan	54	51	-0.0833418	0.94275	no
ni|645903727|nb|KK920703.1|	312317-312448	nan	nan	97	92	-0.0672865	0.9502	no
ni|645903727|nb|KK920703.1|	312659-312804	nan	nan	109	93	-0.229826	0.8436	no
ni|645903727|nb|KK920703.1|	316546-317007	nan	nan	1	1	-0.065199	0.95505	no
ni|645903727|nb|KK920703.1|	318376-318689	nan	nan	3	2	-0.498673	0.67645	no
ni|645903727|nb|KK920703.1|	320175-320526	nan	nan	0.990273	1	0.197506	0.87955	no
ni|645903727|nb|KK920703.1|	321010-322250	nan	nan	35	27	-0.381082	0.79215	no
ni|645903727|nb|KK920703.1|	322358-322478	nan	nan	51	43.66	-0.211795	0.8563	no
ni|645903727|nb|KK920703.1|	322596-323030	nan	nan	25	18	-0.480297	0.66735	no
ni|645903727|nb|KK920703.1|	335206-335390	nan	nan	48	56	0.214931	0.8497	no
ni|645903727|nb|KK920703.1|	336375-336794	nan	nan	38	36	-0.0888521	0.93615	no
ni|645903727|nb|KK920703.1|	338426-338812	nan	nan	46	44	-0.0735975	0.9473	no
ni|645903727|nb|KK920703.1|	338979-339830	nan	nan	10	10	0.00980059	0.9931	no

ni|645903727|nb|KK920703.1|	342929-343052	nan	nan	54	56	0.067562	0.95175	no
ni|645903727|nb|KK920703.1|	355073-355574	nan	nan	1	1	0.146389	0.9048	no
ni|645903727|nb|KK920703.1|	356010-356788	nan	nan	0.911884	1	0.245603	0.8375	no
ni|645903727|nb|KK920703.1|	358250-358930	nan	nan	17	12	-0.502391	0.64915	no
ni|645903727|nb|KK920703.1|	359677-359893	nan	nan	3	4	0.74069	0.5577	no
ni|645903727|nb|KK920703.1|	361070-361397	nan	nan	62	59	-0.0554088	0.9569	no
ni|645903727|nb|KK920703.1|	361899-362056	nan	nan	63	57	-0.139607	0.9034	no
ni|645903727|nb|KK920703.1|	362214-362540	nan	nan	31	33	0.104854	0.92335	no
ni|645903727|nb|KK920703.1|	363098-363259	nan	nan	61	69	0.190944	0.86335	no
ni|645903727|nb|KK920703.1|	363670-363840	nan	nan	67	62	-0.114063	0.91975	no
ni|645903727|nb|KK920703.1|	365226-365628	nan	nan	25	23	-0.128608	0.90685	no
ni|645903727|nb|KK920703.1|	367357-367553	nan	nan	5	5	0.00332388	0.9708	no
ni|645903727|nb|KK920703.1|	367654-367889	nan	nan	5	5	0.147758	0.9049	no
ni|645903727|nb|KK920703.1|	368339-368923	nan	nan	2	2	0.519991	0.66325	no
ni|645903727|nb|KK920703.1|	370308-370491	nan	nan	41	33	-0.334567	0.77145	no
ni|645903727|nb|KK920703.1|	372509-372629	nan	nan	81	61	-0.405574	0.7321	no
ni|645903727|nb|KK920703.1|	372733-373084	nan	nan	12	13	0.0472395	0.96975	no
ni|645903727|nb|KK920703.1|	374214-374345	nan	nan	53	69	0.374419	0.7546	no
ni|645903727|nb|KK920703.1|	376852-377080	nan	nan	25	21	-0.233581	0.8388	no
ni|645903727|nb|KK920703.1|	380624-380810	nan	nan	54	60	0.162928	0.88785	no
ni|645903727|nb|KK920703.1|	381007-381128	nan	nan	55	71	0.370014	0.74625	no
ni|645903727|nb|KK920703.1|	381765-381923	nan	nan	54	58	0.105267	0.9242	no
ni|645903727|nb|KK920703.1|	383566-383795	nan	nan	54	59	0.131351	0.9055	no
ni|645903727|nb|KK920703.1|	383916-384195	nan	nan	8	6	-0.355434	0.7632	no
ni|645903727|nb|KK920703.1|	553564-553896	nan	nan	48	57	0.243488	0.8282	no
ni|645903727|nb|KK920703.1|	565683-565901	nan	nan	2	1	-1	0.44755	no
ni|645903727|nb|KK920703.1|	566071-566395	nan	nan	0.88843	2	0.841235	0.5399	no
ni|645903727|nb|KK920703.1|	567076-567431	nan	nan	1	2	0.519451	0.67865	no
ni|645903727|nb|KK920703.1|	569039-569532	nan	nan	0.378466	0.422053	0.157262	0.9131	no
ni|645903727|nb|KK920703.1|	570224-570557	nan	nan	1	0.407953	-1	0.35155	no

ni|645903727|nb|KK920703.1|	574596-574804	nan	nan	2	0.664092	-2	0.2784	no
ni|645903727|nb|KK920703.1|	581156-581355	nan	nan	1	2	0.932998	0.54905	no
ni|645903727|nb|KK920703.1|	587219-587422	nan	nan	1	2	0.155475	0.9131	no
ni|645903727|nb|KK920703.1|	759428-759916	nan	nan	0	113	inf	5.00E-05	yes
ni|645903727|nb|KK920703.1|	760061-760855	nan	nan	0	95	inf	5.00E-05	yes
ni|645903727|nb|KK920703.1|	760957-761789	nan	nan	0	54	inf	5.00E-05	yes
ni|645903727|nb|KK920703.1|	761961-762179	nan	nan	0	125	inf	5.00E-05	yes
ni|645903727|nb|KK920703.1|	762240-763385	nan	nan	0	71	inf	5.00E-05	yes
ni|645903727|nb|KK920703.1|	813334-813665	nan	nan	0	2	inf	0.00065	yes
ni|645903727|nb|KK920703.1|	876410-876528	nan	nan	73	100	0.454986	0.69875	no
ni|645903727|nb|KK920703.1|	876721-876873	nan	nan	30	52	0.811502	0.4858	no
ni|645903727|nb|KK920703.1|	878271-878764	nan	nan	0.567699	0.36176	-0.650093	0.6679	no
ni|645903727|nb|KK920703.1|	882317-882476	nan	nan	43	80	0.896997	0.44355	no
ni|645903727|nb|KK920703.1|	883902-884111	nan	nan	5	7	0.497109	0.67765	no
ni|645903727|nb|KK920703.1|	885862-886033	nan	nan	35	47	0.447294	0.69885	no
ni|645903727|nb|KK920703.1|	886152-886291	nan	nan	23	30	0.377552	0.7498	no
ni|645903727|nb|KK920703.1|	886408-886523	nan	nan	25	36	0.517468	0.67245	no
ni|645903727|nb|KK920703.1|	886601-886974	nan	nan	15	24	0.64167	0.5733	no
ni|645903727|nb|KK920703.1|	887067-887216	nan	nan	29	34	0.224569	0.8467	no
ni|645903727|nb|KK920703.1|	887370-887552	nan	nan	47	74	0.645269	0.57735	no
ni|645903727|nb|KK920703.1|	888350-889177	nan	nan	156	135	-0.20768	0.87805	no
ni|645903727|nb|KK920703.1|	890386-890546	nan	nan	0.416317	9	4	0.1223	no
ni|645903727|nb|KK920703.1|	891191-892477	nan	nan	0	2	inf	5.00E-05	yes
ni|645903727|nb|KK920703.1|	915804-916144	nan	nan	0.207197	8	5	0.0447	no
ni|645903727|nb|KK920703.1|	949644-950040	nan	nan	38	33	-0.212771	0.84645	no
ni|645903727|nb|KK920703.1|	950577-951222	nan	nan	12	8	-0.504648	0.657	no
ni|645903727|nb|KK920703.1|	951402-951946	nan	nan	10	9	-0.187029	0.8696	no
ni|645903727|nb|KK920703.1|	952092-954572	nan	nan	20	25	0.307168	0.82685	no
ni|645903727|nb|KK920703.1|	955681-956144	nan	nan	0.204958	1	3	0.098	no
ni|645903727|nb|KK920703.1|	956446-956748	nan	nan	79	409	2	0.07155	no

ni|645903727|nb|KK920703.1|	958476-958937	nan	nan	135	637	2	0.13705	no
ni|645903727|nb|KK920703.1|	959140-959694	nan	nan	20	108	2	0.0695	no
ni|645903727|nb|KK920703.1|	960667-960867	nan	nan	265	1023.53	2	0.1929	no
ni|645903727|nb|KK920703.1|	961113-961394	nan	nan	3	83	5	0.0016	yes
ni|645903727|nb|KK920703.1|	962256-962503	nan	nan	241	1082.74	2	0.14535	no
ni|645903727|nb|KK920703.1|	962604-962704	nan	nan	153	621	2	0.1462	no
ni|645903727|nb|KK920703.1|	963367-963506	nan	nan	96	516	2	0.0894	no
ni|645903727|nb|KK920703.1|	964543-964811	nan	nan	48	418	3	0.02655	no
ni|645903727|nb|KK920703.1|	967013-967421	nan	nan	0.886539	189	8	0.0068	no
ni|645903727|nb|KK920703.1|	973069-973500	nan	nan	0.974514	1	0.556221	0.6571	no
ni|645903727|nb|KK920703.1|	975297-975498	nan	nan	55	63	0.201528	0.8576	no
ni|645903727|nb|KK920703.1|	976561-976767	nan	nan	187	196	0.0688242	0.96395	no
ni|645903727|nb|KK920703.1|	977029-977257	nan	nan	202	185	-0.124842	0.93495	no
ni|645903727|nb|KK920703.1|	977368-977501	nan	nan	90	110	0.28999	0.7965	no
ni|645903727|nb|KK920703.1|	977637-977776	nan	nan	162	155	-0.0672326	0.9527	no
ni|645903727|nb|KK920703.1|	978604-978740	nan	nan	21	35	0.753779	0.5267	no
ni|645903727|nb|KK920703.1|	978816-979051	nan	nan	3	3	-0.329401	0.79705	no
ni|645903727|nb|KK920703.1|	979425-979797	nan	nan	58	50	-0.213419	0.84445	no
ni|645903727|nb|KK920703.1|	987717-988050	nan	nan	34	44	0.350542	0.7516	no
ni|645903727|nb|KK920703.1|	989113-989224	nan	nan	25	42	0.739742	0.5577	no
ni|645903727|nb|KK920703.1|	991705-991863	nan	nan	42	57	0.451828	0.69635	no
ni|645903727|nb|KK920703.1|	992129-992642	nan	nan	0.600078	0.630966	0.0724113	0.94355	no
ni|645903728|nb|KK920702.1|	1000798-100096	nan	nan	47	45	-0.07824	0.94565	no
ni|645903728|nb|KK920702.1|	1001827-100194	nan	nan	51	56	0.145602	0.8963	no
ni|645903728|nb|KK920702.1|	1003211-100339	nan	nan	43	43	0.00407462	0.9952	no
ni|645903728|nb|KK920702.1|	1004692-100496	nan	nan	31	35	0.185074	0.87055	no
ni|645903728|nb|KK920702.1|	10049-10293	nan	nan	8	8	-0.0352203	0.9769	no
ni|645903728|nb|KK920702.1|	1007262-100772	nan	nan	22	20	-0.111897	0.91705	no
ni|645903728|nb|KK920702.1|	1009071-100932	nan	nan	2	2	0.196895	0.87955	no
ni|645903728|nb|KK920702.1|	1012399-101258	nan	nan	45	43	-0.0483965	0.96715	no

ni|645903728|nb|KK920702.1|	1013520-1014054	nan	nan	22	23	0.0535891	0.9603	no
ni|645903728|nb|KK920702.1|	1014644-1014842	nan	nan	30	33	0.125623	0.91315	no
ni|645903728|nb|KK920702.1|	1016051-1016352	nan	nan	38	40	0.0781583	0.94245	no
ni|645903728|nb|KK920702.1|	1016757-1016909	nan	nan	43	37	-0.203075	0.8574	no
ni|645903728|nb|KK920702.1|	1018799-1019255	nan	nan	13	25	0.919761	0.4127	no
ni|645903728|nb|KK920702.1|	1019613-1019979	nan	nan	0	4	inf	0.00015	yes
ni|645903728|nb|KK920702.1|	1023309-1023439	nan	nan	51	67	0.38169	0.74075	no
ni|645903728|nb|KK920702.1|	1024478-1024645	nan	nan	34	40	0.219379	0.84895	no
ni|645903728|nb|KK920702.1|	1024770-1024913	nan	nan	15	19	0.319812	0.7924	no
ni|645903728|nb|KK920702.1|	1026341-1026590	nan	nan	27	34	0.34436	0.76305	no
ni|645903728|nb|KK920702.1|	1031305-1031533	nan	nan	30	48	0.685601	0.5474	no
ni|645903728|nb|KK920702.1|	1032302-1032561	nan	nan	31	46	0.54995	0.6253	no
ni|645903728|nb|KK920702.1|	1032676-1032812	nan	nan	27	37	0.454702	0.69955	no
ni|645903728|nb|KK920702.1|	1032944-1033098	nan	nan	22	37	0.722885	0.5357	no
ni|645903728|nb|KK920702.1|	1035161-1035588	nan	nan	18	26	0.497567	0.6606	no
ni|645903728|nb|KK920702.1|	1045634-1046092	nan	nan	10	16	0.591207	0.6061	no
ni|645903728|nb|KK920702.1|	1047234-1047486	nan	nan	7	9	0.348878	0.76065	no
ni|645903728|nb|KK920702.1|	1048509-1049200	nan	nan	14	18	0.384107	0.72965	no
ni|645903728|nb|KK920702.1|	1049488-1049860	nan	nan	2	1	-0.589005	0.62565	no
ni|645903728|nb|KK920702.1|	1050684-1051090	nan	nan	3	2	-0.433064	0.71505	no
ni|645903728|nb|KK920702.1|	1051840-1052210	nan	nan	0.828121	0.351729	-1	0.4023	no
ni|645903728|nb|KK920702.1|	1052788-1053463	nan	nan	1	7	2	0.0759	no
ni|645903728|nb|KK920702.1|	1053558-1053891	nan	nan	1	3	1	0.3007	no
ni|645903728|nb|KK920702.1|	1057518-1058275	nan	nan	0	1	inf	0.0001	yes
ni|645903728|nb|KK920702.1|	1059125-1059714	nan	nan	0	6	inf	5.00E-05	yes
ni|645903728|nb|KK920702.1|	1077532-1077686	nan	nan	10	8	-0.289585	0.8081	no
ni|645903728|nb|KK920702.1|	1077833-1077966	nan	nan	8	2	-2	0.24815	no
ni|645903728|nb|KK920702.1|	1078381-1078508	nan	nan	370	379	0.0347965	0.9728	no
ni|645903728|nb|KK920702.1|	1078737-1079387	nan	nan	47	64	0.440547	0.7665	no
ni|645903728|nb|KK920702.1|	1080563-1080948	nan	nan	1	0.916063	-0.306404	0.8123	no

ni|645903728|nb|KK920702.1|	1089135-108942[illegible]	nan	nan	184	295	0.68034	0.6323	no
ni|645903728|nb|KK920702.1|	1089583-1090311	nan	nan	79	187.55	1	0.3803	no
ni|645903728|nb|KK920702.1|	1090654-109096[illegible]	nan	nan	3	0.910252	-2	0.20345	no
ni|645903728|nb|KK920702.1|	1091023-109162[illegible]	nan	nan	2	1	-1	0.3054	no
ni|645903728|nb|KK920702.1|	1092024-1092291	nan	nan	2	1	-0.744093	0.56925	no
ni|645903728|nb|KK920702.1|	1092390-109252[illegible]	nan	nan	23	8	-2	0.21685	no
ni|645903728|nb|KK920702.1|	1092802-109310[illegible]	nan	nan	183	261	0.514315	0.71315	no
ni|645903728|nb|KK920702.1|	1096579-109733[illegible]	nan	nan	0.871167	2	0.87111	0.47365	no
ni|645903728|nb|KK920702.1|	1098017-109837[illegible]	nan	nan	201	374	0.900216	0.5261	no
ni|645903728|nb|KK920702.1|	1098659-109882[illegible]	nan	nan	3	5	0.633223	0.63975	no
ni|645903728|nb|KK920702.1|	1099728-110048[illegible]	nan	nan	65	125	0.946017	0.50305	no
ni|645903728|nb|KK920702.1|	1106578-110704[illegible]	nan	nan	0.817569	179	8	0.0045	no
ni|645903728|nb|KK920702.1|	1116417-111666[illegible]	nan	nan	9	8	-0.125118	0.91935	no
ni|645903728|nb|KK920702.1|	1116902-1117021	nan	nan	8	12	0.669546	0.65595	no
ni|645903728|nb|KK920702.1|	1117791-111793[illegible]	nan	nan	8	4	-1	0.45115	no
ni|645903728|nb|KK920702.1|	1118155-111830[illegible]	nan	nan	6	9	0.480292	0.6999	no
ni|645903728|nb|KK920702.1|	1118675-111896[illegible]	nan	nan	1	1	-0.235775	0.8697	no
ni|645903728|nb|KK920702.1|	1119229-111938[illegible]	nan	nan	4	10	1	0.34465	no
ni|645903728|nb|KK920702.1|	1119786-112009[illegible]	nan	nan	2	1	-0.650687	0.63225	no
ni|645903728|nb|KK920702.1|	1120575-112105[illegible]	nan	nan	21	19	-0.177274	0.87255	no
ni|645903728|nb|KK920702.1|	1125558-1126001	nan	nan	17	17	-0.00486687	0.99685	no
ni|645903728|nb|KK920702.1|	1126938-112709[illegible]	nan	nan	13	16	0.311313	0.78895	no
ni|645903728|nb|KK920702.1|	1137924-113810[illegible]	nan	nan	26	20	-0.366884	0.746	no
ni|645903728|nb|KK920702.1|	124559-124738	nan	nan	12	10	-0.270853	0.8181	no
ni|645903728|nb|KK920702.1|	211385-211496	nan	nan	15	22	0.584463	0.6992	no
ni|645903728|nb|KK920702.1|	21213-21364	nan	nan	12	13	0.0438377	0.9719	no
ni|645903728|nb|KK920702.1|	255945-256127	nan	nan	3	5	0.56074	0.667	no
ni|645903728|nb|KK920702.1|	261948-262117	nan	nan	0	6	inf	0.00135	yes
ni|645903728|nb|KK920702.1|	26981-27123	nan	nan	30	25	-0.247789	0.8335	no
ni|645903728|nb|KK920702.1|	27240-27459	nan	nan	5	3	-0.438585	0.72705	no

ni|645903728|nb|KK920702.1|	27850-28273	nan	nan	2	2	-0.185581	0.8779	no
ni|645903728|nb|KK920702.1|	28650-29180	nan	nan	4	4	-0.0650622	0.95565	no
ni|645903728|nb|KK920702.1|	30017-30437	nan	nan	0.697991	0.667105	-0.0652948	0.94155	no
ni|645903728|nb|KK920702.1|	30654-30935	nan	nan	36	29	-0.34328	0.76235	no
ni|645903728|nb|KK920702.1|	31954-32119	nan	nan	42	27	-0.639134	0.5848	no
ni|645903728|nb|KK920702.1|	320-781	nan	nan	8	6	-0.338217	0.76725	no
ni|645903728|nb|KK920702.1|	32221-32378	nan	nan	31	31	-0.00749219	0.99205	no
ni|645903728|nb|KK920702.1|	34001-34115	nan	nan	13	19	0.517443	0.7004	no
ni|645903728|nb|KK920702.1|	34517-34638	nan	nan	13	7	-0.94186	0.54025	no
ni|645903728|nb|KK920702.1|	35034-35196	nan	nan	14	10	-0.552639	0.6482	no
ni|645903728|nb|KK920702.1|	35383-35670	nan	nan	5	4	-0.516531	0.6626	no
ni|645903728|nb|KK920702.1|	35907-36747	nan	nan	7	14	0.998266	0.37475	no
ni|645903728|nb|KK920702.1|	37731-37873	nan	nan	9	25	1	0.26365	no
ni|645903728|nb|KK920702.1|	453588-454733	nan	nan	0	0.768494	inf	0.0001	yes
ni|645903728|nb|KK920702.1|	463833-466032	nan	nan	2	2	-0.194722	0.8642	no
ni|645903728|nb|KK920702.1|	46398-46982	nan	nan	5	14	2	0.17535	no
ni|645903728|nb|KK920702.1|	468037-468207	nan	nan	175	149	-0.22947	0.83575	no
ni|645903728|nb|KK920702.1|	470187-470528	nan	nan	31	29	-0.0510249	0.96105	no
ni|645903728|nb|KK920702.1|	480166-480282	nan	nan	16	28	0.795024	0.53535	no
ni|645903728|nb|KK920702.1|	48175-48601	nan	nan	4	8	1	0.38005	no
ni|645903728|nb|KK920702.1|	483087-483963	nan	nan	0.732665	1	0.456172	0.7031	no
ni|645903728|nb|KK920702.1|	485298-485603	nan	nan	0.607138	2	1	0.35675	no
ni|645903728|nb|KK920702.1|	486461-486608	nan	nan	10	14	0.439758	0.71745	no
ni|645903728|nb|KK920702.1|	487351-487564	nan	nan	1921.53	1378.51	-0.479152	0.7372	no
ni|645903728|nb|KK920702.1|	488053-488546	nan	nan	3	1	-1	0.3505	no
ni|645903728|nb|KK920702.1|	488677-489118	nan	nan	6	3	-1	0.3194	no
ni|645903728|nb|KK920702.1|	489246-489752	nan	nan	194	144.66	-0.422104	0.7646	no
ni|645903728|nb|KK920702.1|	490070-490288	nan	nan	2	1	-0.941101	0.54025	no
ni|645903728|nb|KK920702.1|	496169-496925	nan	nan	8	9	0.301736	0.78715	no
ni|645903728|nb|KK920702.1|	500754-500978	nan	nan	14	24	0.809891	0.4931	no

ni|645903728|nb|KK920702.1|	501851-502042	nan	nan	15	19	0.334933	0.7756	no
ni|645903728|nb|KK920702.1|	503427-503633	nan	nan	24	31	0.372966	0.74735	no
ni|645903728|nb|KK920702.1|	504438-504841	nan	nan	17	21	0.240162	0.8332	no
ni|645903728|nb|KK920702.1|	505648-505817	nan	nan	33	36	0.139191	0.9028	no
ni|645903728|nb|KK920702.1|	508318-508776	nan	nan	20	22	0.127439	0.9055	no
ni|645903728|nb|KK920702.1|	510567-510755	nan	nan	28	33	0.231443	0.8435	no
ni|645903728|nb|KK920702.1|	513140-513720	nan	nan	11	13	0.230731	0.8381	no
ni|645903728|nb|KK920702.1|	513846-514025	nan	nan	10	11	0.187937	0.8705	no
ni|645903728|nb|KK920702.1|	514250-514406	nan	nan	6	9	0.58488	0.6549	no
ni|645903728|nb|KK920702.1|	515030-515147	nan	nan	17	10.71	-0.652415	0.64	no
ni|645903728|nb|KK920702.1|	515901-516141	nan	nan	0.178401	7	5	0.11945	no
ni|645903728|nb|KK920702.1|	519648-522439	nan	nan	22	22	-0.0287021	0.9828	no
ni|645903728|nb|KK920702.1|	525178-525563	nan	nan	0	7	inf	5.00E-05	yes
ni|645903728|nb|KK920702.1|	528537-528935	nan	nan	0	7	inf	5.00E-05	yes
ni|645903728|nb|KK920702.1|	538707-539238	nan	nan	0	3	inf	0.0001	yes
ni|645903728|nb|KK920702.1|	539589-539899	nan	nan	0	5	inf	5.00E-05	yes
ni|645903728|nb|KK920702.1|	540074-540343	nan	nan	0	4	inf	0.0003	yes
ni|645903728|nb|KK920702.1|	544194-544724	nan	nan	23	19	-0.278306	0.80125	no
ni|645903728|nb|KK920702.1|	544851-545021	nan	nan	39	50	0.377516	0.7455	no
ni|645903728|nb|KK920702.1|	554642-554772	nan	nan	102	124	0.279804	0.80655	no
ni|645903728|nb|KK920702.1|	554874-555108	nan	nan	46	63	0.444683	0.68865	no
ni|645903728|nb|KK920702.1|	556399-556875	nan	nan	31	41	0.402092	0.70555	no
ni|645903728|nb|KK920702.1|	557063-557167	nan	nan	59	113	0.931677	0.46445	no
ni|645903728|nb|KK920702.1|	559687-560445	nan	nan	31	41	0.420421	0.77325	no
ni|645903728|nb|KK920702.1|	561658-561815	nan	nan	81	115	0.504708	0.65515	no
ni|645903728|nb|KK920702.1|	566463-566611	nan	nan	80.81	117	0.530041	0.6398	no
ni|645903728|nb|KK920702.1|	569165-569742	nan	nan	0.363441	0.347434	-0.0649827	0.9302	no
ni|645903728|nb|KK920702.1|	570780-570930	nan	nan	52	73	0.499151	0.6682	no
ni|645903728|nb|KK920702.1|	573186-573372	nan	nan	65	85	0.384524	0.7316	no
ni|645903728|nb|KK920702.1|	575917-576135	nan	nan	6	8	0.500408	0.66545	no

ni|645903728|nb|KK920702.1|	576322-576555	nan	nan	79	117.53	0.578149	0.58925	no
ni|645903728|nb|KK920702.1|	587904-588384	nan	nan	39	61	0.650312	0.59735	no
ni|645903728|nb|KK920702.1|	594438-594879	nan	nan	4	8	0.83522	0.4749	no
ni|645903728|nb|KK920702.1|	595801-596174	nan	nan	4	7	0.782558	0.5018	no
ni|645903728|nb|KK920702.1|	596876-597430	nan	nan	0.49107	0.625906	0.350017	0.792	no
ni|645903728|nb|KK920702.1|	597550-597954	nan	nan	0.489968	4	3	0.0734	no
ni|645903728|nb|KK920702.1|	598082-599288	nan	nan	10	12	0.240647	0.82875	no
ni|645903728|nb|KK920702.1|	602114-602311	nan	nan	2	2	-0.259689	0.8692	no
ni|645903728|nb|KK920702.1|	602747-603208	nan	nan	1	2	0.602226	0.61895	no
ni|645903728|nb|KK920702.1|	604272-604779	nan	nan	0.669866	0.5821	-0.202607	0.87975	no
ni|645903728|nb|KK920702.1|	604988-605553	nan	nan	2	3	0.934998	0.4307	no
ni|645903728|nb|KK920702.1|	606205-606343	nan	nan	10	15	0.610834	0.6231	no
ni|645903728|nb|KK920702.1|	607294-607432	nan	nan	7	16	1	0.3865	no
ni|645903728|nb|KK920702.1|	607671-608068	nan	nan	7	13	0.943307	0.42315	no
ni|645903728|nb|KK920702.1|	609891-610489	nan	nan	2	3	0.742405	0.5284	no
ni|645903728|nb|KK920702.1|	610943-611292	nan	nan	8	11	0.513166	0.6611	no
ni|645903728|nb|KK920702.1|	611908-612162	nan	nan	8	4	-0.873494	0.46335	no
ni|645903728|nb|KK920702.1|	612578-612708	nan	nan	15	15	0.0107073	0.9704	no
ni|645903728|nb|KK920702.1|	612774-612897	nan	nan	13	12	-0.0673675	0.954	no
ni|645903728|nb|KK920702.1|	613303-613597	nan	nan	7	5	-0.489631	0.68035	no
ni|645903728|nb|KK920702.1|	613672-613819	nan	nan	10	20	1	0.37295	no
ni|645903728|nb|KK920702.1|	615866-616349	nan	nan	15	11.88	-0.350553	0.7612	no
ni|645903728|nb|KK920702.1|	616886-617237	nan	nan	28	25	-0.144384	0.89835	no
ni|645903728|nb|KK920702.1|	617656-618250	nan	nan	17	26	0.62105	0.56615	no
ni|645903728|nb|KK920702.1|	619857-620203	nan	nan	22	60	1	0.19825	no
ni|645903728|nb|KK920702.1|	620756-621059	nan	nan	0.7358	2	2	0.297	no
ni|645903728|nb|KK920702.1|	621166-621299	nan	nan	29	56	0.932729	0.4278	no
ni|645903728|nb|KK920702.1|	623300-623736	nan	nan	0.664567	1	1	0.41145	no
ni|645903728|nb|KK920702.1|	624059-624254	nan	nan	26	48	0.903165	0.4405	no
ni|645903728|nb|KK920702.1|	625257-625425	nan	nan	19	51	1	0.23545	no

ni|645903728|nb|KK920702.1|	631720-631836	nan	nan	45	52	0.23509	0.8369	no
ni|645903728|nb|KK920702.1|	632038-632653	nan	nan	22	41	0.914408	0.47655	no
ni|645903728|nb|KK920702.1|	632800-632919	nan	nan	76	148	0.968204	0.4082	no
ni|645903728|nb|KK920702.1|	633117-633588	nan	nan	37	76	1	0.41065	no
ni|645903728|nb|KK920702.1|	634131-634800	nan	nan	13	23	0.805637	0.46765	no
ni|645903728|nb|KK920702.1|	635253-635423	nan	nan	15	25	0.710339	0.54615	no
ni|645903728|nb|KK920702.1|	636362-636610	nan	nan	26	37	0.478382	0.67475	no
ni|645903728|nb|KK920702.1|	636796-637050	nan	nan	18	20	0.174869	0.88145	no
ni|645903728|nb|KK920702.1|	637884-638051	nan	nan	27	31	0.205777	0.8608	no
ni|645903728|nb|KK920702.1|	638887-639098	nan	nan	28	28	-0.00926816	0.99515	no
ni|645903728|nb|KK920702.1|	639718-640219	nan	nan	12	16	0.385198	0.7348	no
ni|645903728|nb|KK920702.1|	640359-640541	nan	nan	26	24	-0.102057	0.93235	no
ni|645903728|nb|KK920702.1|	640728-641110	nan	nan	19	40	1	0.34755	no
ni|645903728|nb|KK920702.1|	641964-642192	nan	nan	66	161	1	0.32325	no
ni|645903728|nb|KK920702.1|	646373-646558	nan	nan	64	166	1	0.2234	no
ni|645903728|nb|KK920702.1|	647292-647547	nan	nan	61	146	1	0.3308	no
ni|645903728|nb|KK920702.1|	651494-651892	nan	nan	65	176	1	0.33055	no
ni|645903728|nb|KK920702.1|	652966-653351	nan	nan	38	90	1	0.34575	no
ni|645903728|nb|KK920702.1|	655088-655313	nan	nan	36	90	1	0.24965	no
ni|645903728|nb|KK920702.1|	657761-658108	nan	nan	23	51	1	0.3032	no
ni|645903728|nb|KK920702.1|	658910-659067	nan	nan	28	81	2	0.19875	no
ni|645903728|nb|KK920702.1|	664163-664749	nan	nan	26	44	0.757662	0.54625	no
ni|645903728|nb|KK920702.1|	668032-670416	nan	nan	12	16	0.42675	0.77125	no
ni|645903728|nb|KK920702.1|	677532-677917	nan	nan	0	11	inf	5.00E-05	yes
ni|645903728|nb|KK920702.1|	746642-746851	nan	nan	31	30	-0.0562229	0.9615	no
ni|645903728|nb|KK920702.1|	748655-749140	nan	nan	14	16	0.172293	0.8768	no
ni|645903728|nb|KK920702.1|	750675-750889	nan	nan	29	35	0.289786	0.7986	no
ni|645903728|nb|KK920702.1|	752059-752223	nan	nan	13	14	0.093243	0.93725	no
ni|645903728|nb|KK920702.1|	752803-753115	nan	nan	3	5	0.848551	0.4727	no
ni|645903728|nb|KK920702.1|	753951-754124	nan	nan	4	7	0.932701	0.46975	no

ni|645903728|nb|KK920702.1|	754781-755191	nan	nan	5	6	0.256607	0.8256	no
ni|645903728|nb|KK920702.1|	757759-757922	nan	nan	18	18	0.0258925	0.98455	no
ni|645903728|nb|KK920702.1|	763622-763806	nan	nan	4	0.569884	-3	0.2671	no
ni|645903728|nb|KK920702.1|	764496-764970	nan	nan	0.596694	0.760453	0.349868	0.792	no
ni|645903728|nb|KK920702.1|	765180-765723	nan	nan	5	5	0.0233155	0.98275	no
ni|645903728|nb|KK920702.1|	766000-766248	nan	nan	36	29	-0.322541	0.7752	no
ni|645903728|nb|KK920702.1|	766758-766894	nan	nan	14	9	-0.688739	0.58685	no
ni|645903728|nb|KK920702.1|	770928-771242	nan	nan	24	24	0.00333314	0.9962	no
ni|645903728|nb|KK920702.1|	771677-771970	nan	nan	29	25	-0.23656	0.834	no
ni|645903728|nb|KK920702.1|	777577-777801	nan	nan	8	10	0.29192	0.7992	no
ni|645903728|nb|KK920702.1|	781902-782303	nan	nan	29	35	0.265545	0.8082	no
ni|645903728|nb|KK920702.1|	785914-787255	nan	nan	21	23	0.124889	0.92995	no
ni|645903728|nb|KK920702.1|	790848-790989	nan	nan	373	393	0.0751622	0.9419	no
ni|645903728|nb|KK920702.1|	798328-798552	nan	nan	49	66	0.451029	0.68335	no
ni|645903728|nb|KK920702.1|	862168-863589	nan	nan	0	32	inf	5.00E-05	yes
ni|645903728|nb|KK920702.1|	948349-948440	nan	nan	15461.1	23268.3	0.589718	0.61525	no
ni|645903728|nb|KK920702.1|	952031-952256	nan	nan	86	100	0.212017	0.84535	no
ni|645903728|nb|KK920702.1|	953239-953550	nan	nan	44	49	0.163301	0.8779	no
ni|645903728|nb|KK920702.1|	954730-954919	nan	nan	43	41	-0.0671984	0.95475	no
ni|645903728|nb|KK920702.1|	955678-955839	nan	nan	80	99	0.308455	0.786	no
ni|645903728|nb|KK920702.1|	956021-956571	nan	nan	1	0.631493	-0.872383	0.49155	no
ni|645903728|nb|KK920702.1|	957582-957836	nan	nan	99	93	-0.0828178	0.93765	no
ni|645903728|nb|KK920702.1|	961198-961505	nan	nan	64	51	-0.336394	0.7572	no
ni|645903728|nb|KK920702.1|	963325-963697	nan	nan	1	0.785538	-0.35495	0.7931	no
ni|645903728|nb|KK920702.1|	964306-964764	nan	nan	1	0.529739	-1	0.43485	no
ni|645903728|nb|KK920702.1|	967580-967889	nan	nan	1	0.568908	-0.913732	0.53575	no
ni|645903728|nb|KK920702.1|	969259-970255	nan	nan	7	8	0.134897	0.9053	no
ni|645903728|nb|KK920702.1|	970690-970885	nan	nan	2	2	0.295482	0.8383	no
ni|645903728|nb|KK920702.1|	972797-973156	nan	nan	25	22	-0.222401	0.8456	no
ni|645903728|nb|KK920702.1|	985840-986051	nan	nan	24	16	-0.651716	0.5811	no

ni|645903728|nb|KK920702.1|	986628-986895	nan	nan	3	2	-0.235946	0.85345	no
ni|645903728|nb|KK920702.1|	986984-987141	nan	nan	46	34	-0.423892	0.7187	no
ni|645903728|nb|KK920702.1|	987402-988167	nan	nan	29	26	-0.137763	0.9134	no
ni|645903728|nb|KK920702.1|	990193-990387	nan	nan	3	0.509105	-3	0.28285	no
ni|645903728|nb|KK920702.1|	992681-993086	nan	nan	34	29	-0.224359	0.8403	no
ni|645903728|nb|KK920702.1|	994996-995215	nan	nan	27	31	0.220823	0.84585	no
ni|645903728|nb|KK920702.1|	996808-996997	nan	nan	25	27	0.117226	0.9214	no
ni|645903728|nb|KK920702.1|	997974-998274	nan	nan	18	15	-0.194111	0.8671	no
ni|645903728|nb|KK920702.1|	998699-998931	nan	nan	42	41	-0.0148799	0.9894	no
ni|645903728|nb|KK920702.1|	999111-999576	nan	nan	21	21	0.0206296	0.9849	no
ni|645903730|nb|KK920700.1|	178149-182295	nan	nan	0.646039	0.868567	0.427016	0.7138	no
ni|645903730|nb|KK920700.1|	42944-43135	nan	nan	8	5	-0.626593	0.6057	no
ni|645903730|nb|KK920700.1|	526343-526460	nan	nan	49	50	0.0127174	0.9828	no
ni|645903731|nb|KK920699.1|	104088-105216	nan	nan	98	242	1	0.37165	no
ni|645903731|nb|KK920699.1|	1089342-1089505	nan	nan	22	24	0.128703	0.9117	no
ni|645903731|nb|KK920699.1|	113253-113425	nan	nan	0.347956	30	6	0.1195	no
ni|645903731|nb|KK920699.1|	115688-115927	nan	nan	17	10	-0.77258	0.50895	no
ni|645903731|nb|KK920699.1|	116160-116470	nan	nan	99	69	-0.519149	0.67575	no
ni|645903731|nb|KK920699.1|	116780-117001	nan	nan	3	0.592185	-2	0.1906	no
ni|645903731|nb|KK920699.1|	117836-118241	nan	nan	22.22	14	-0.715149	0.5317	no
ni|645903731|nb|KK920699.1|	118833-118968	nan	nan	82	80	-0.0304203	0.9796	no
ni|645903731|nb|KK920699.1|	1192418-1192479	nan	nan	41703.4	45206.7	0.11637	0.926	no
ni|645903731|nb|KK920699.1|	1196389-1196631	nan	nan	12	10	-0.203775	0.8622	no
ni|645903731|nb|KK920699.1|	119682-120271	nan	nan	39	30	-0.388764	0.75695	no
ni|645903731|nb|KK920699.1|	1198640-1198814	nan	nan	14	17	0.338685	0.7745	no
ni|645903731|nb|KK920699.1|	1198917-1199072	nan	nan	6	10	0.649013	0.60985	no
ni|645903731|nb|KK920699.1|	1199833-1200003	nan	nan	17	16	-0.0982958	0.9325	no
ni|645903731|nb|KK920699.1|	1201443-1201799	nan	nan	15	14	-0.0655071	0.9558	no
ni|645903731|nb|KK920699.1|	1202056-1202172	nan	nan	15	10	-0.582046	0.70815	no
ni|645903731|nb|KK920699.1|	1203917-1204331	nan	nan	4	6	0.548798	0.63655	no

ni|645903731|nb|KK920699.1|	120500-120670	nan	nan	99	77	-0.36723	0.7432	no
ni|645903731|nb|KK920699.1|	1205197-1205538	nan	nan	13	16	0.283155	0.8038	no
ni|645903731|nb|KK920699.1|	1206379-1206855	nan	nan	0.923228	0.189097	-2	0.1864	no
ni|645903731|nb|KK920699.1|	1207213-1207359	nan	nan	36	48	0.405866	0.72855	no
ni|645903731|nb|KK920699.1|	1208340-1208912	nan	nan	1	1	0.367969	0.75825	no
ni|645903731|nb|KK920699.1|	1209233-1209457	nan	nan	26	35	0.449165	0.69525	no
ni|645903731|nb|KK920699.1|	121136-121431	nan	nan	83	69	-0.261332	0.80305	no
ni|645903731|nb|KK920699.1|	1214092-1214802	nan	nan	0.446128	0.193878	-1	0.4064	no
ni|645903731|nb|KK920699.1|	1215239-1215683	nan	nan	72	65	-0.139466	0.926	no
ni|645903731|nb|KK920699.1|	1215880-1216279	nan	nan	311	312	0.0033876	0.9978	no
ni|645903731|nb|KK920699.1|	1216475-1216685	nan	nan	2	1	-0.289165	0.8535	no
ni|645903731|nb|KK920699.1|	1218876-1219545	nan	nan	0.695865	0.623697	-0.157962	0.89795	no
ni|645903731|nb|KK920699.1|	1221497-1222392	nan	nan	219.07	246	0.164865	0.90995	no
ni|645903731|nb|KK920699.1|	122335-122936	nan	nan	31	29	-0.106565	0.9167	no
ni|645903731|nb|KK920699.1|	1232057-1232571	nan	nan	13	5	-2	0.19835	no
ni|645903731|nb|KK920699.1|	1233014-1233218	nan	nan	36.61	14	-1	0.23645	no
ni|645903731|nb|KK920699.1|	1234336-1234591	nan	nan	35	15	-1	0.30975	no
ni|645903731|nb|KK920699.1|	123509-124398	nan	nan	27	26	-0.0244808	0.9871	no
ni|645903731|nb|KK920699.1|	1235860-1236016	nan	nan	36	15	-1	0.31035	no
ni|645903731|nb|KK920699.1|	1237360-1237480	nan	nan	26	17	-0.619946	0.62755	no
ni|645903731|nb|KK920699.1|	1237787-1237956	nan	nan	40	19	-1	0.37455	no
ni|645903731|nb|KK920699.1|	1239445-1239737	nan	nan	4	0.495722	-3	0.07195	no
ni|645903731|nb|KK920699.1|	1241407-1241915	nan	nan	19	9	-1	0.29855	no
ni|645903731|nb|KK920699.1|	1252380-1253027	nan	nan	0.950212	2	1	0.3516	no
ni|645903731|nb|KK920699.1|	125457-125917	nan	nan	28	23	-0.300085	0.78325	no
ni|645903731|nb|KK920699.1|	126098-127340	nan	nan	12	12	-0.0644899	0.95085	no
ni|645903731|nb|KK920699.1|	1261665-1261808	nan	nan	6	2	-2	0.3149	no
ni|645903731|nb|KK920699.1|	1262349-1262970	nan	nan	2	0.681385	-1	0.27175	no
ni|645903731|nb|KK920699.1|	1263875-1264141	nan	nan	12	7	-0.79117	0.4964	no
ni|645903731|nb|KK920699.1|	1267702-1267849	nan	nan	6	4	-0.844809	0.5595	no

ni|645903731|nb|KK920699.1|	1269459-1269758	nan	nan	9	5	-0.714886	0.5423	no
ni|645903731|nb|KK920699.1|	1273728-1274252	nan	nan	7	4	-0.878304	0.45575	no
ni|645903731|nb|KK920699.1|	127464-127838	nan	nan	12	12	-0.00269961	0.9954	no
ni|645903731|nb|KK920699.1|	128065-128645	nan	nan	26	26	-0.00158289	0.99875	no
ni|645903731|nb|KK920699.1|	129344-129669	nan	nan	0.995009	1	0.0863523	0.9436	no
ni|645903731|nb|KK920699.1|	130706-131274	nan	nan	22	22	0.024341	0.98105	no
ni|645903731|nb|KK920699.1|	131898-132510	nan	nan	34	37	0.118517	0.9203	no
ni|645903731|nb|KK920699.1|	133970-134186	nan	nan	38	49	0.366596	0.74825	no
ni|645903731|nb|KK920699.1|	134318-134472	nan	nan	33	58	0.807277	0.4876	no
ni|645903731|nb|KK920699.1|	135305-135551	nan	nan	40	46	0.180416	0.86855	no
ni|645903731|nb|KK920699.1|	136118-136322	nan	nan	40	53	0.388603	0.72955	no
ni|645903731|nb|KK920699.1|	136923-137160	nan	nan	38	44	0.214987	0.851	no
ni|645903731|nb|KK920699.1|	138186-138429	nan	nan	38	41	0.107321	0.92435	no
ni|645903731|nb|KK920699.1|	139111-139355	nan	nan	44.39	51	0.195848	0.86385	no
ni|645903731|nb|KK920699.1|	139885-140349	nan	nan	24	32	0.392412	0.7234	no
ni|645903731|nb|KK920699.1|	141260-141441	nan	nan	29	43	0.548035	0.6421	no
ni|645903731|nb|KK920699.1|	142219-142561	nan	nan	20	30	0.584489	0.604	no
ni|645903731|nb|KK920699.1|	142799-144005	nan	nan	23	31	0.412329	0.77815	no
ni|645903731|nb|KK920699.1|	144523-145045	nan	nan	20	26	0.343675	0.7564	no
ni|645903731|nb|KK920699.1|	145928-146154	nan	nan	29	44	0.599079	0.59815	no
ni|645903731|nb|KK920699.1|	146724-146929	nan	nan	46	56	0.275727	0.80885	no
ni|645903731|nb|KK920699.1|	147308-147504	nan	nan	27	43	0.695137	0.55455	no
ni|645903731|nb|KK920699.1|	148120-148651	nan	nan	22	31	0.508376	0.6413	no
ni|645903731|nb|KK920699.1|	150500-150766	nan	nan	34	47	0.473336	0.68125	no
ni|645903731|nb|KK920699.1|	151616-151941	nan	nan	20	29	0.564776	0.6225	no
ni|645903731|nb|KK920699.1|	153732-154218	nan	nan	16	26	0.720615	0.51065	no
ni|645903731|nb|KK920699.1|	154452-154651	nan	nan	27	38	0.531635	0.64255	no
ni|645903731|nb|KK920699.1|	156019-156443	nan	nan	9	14	0.711277	0.53695	no
ni|645903731|nb|KK920699.1|	158073-158228	nan	nan	18	28	0.669771	0.5636	no
ni|645903731|nb|KK920699.1|	159071-159299	nan	nan	27	39	0.567165	0.62265	no

ni|645903731|nb|KK920699.1|	159803-160187	nan	nan	13	21	0.780354	0.49445	no
ni|645903731|nb|KK920699.1|	161345-161596	nan	nan	21	30	0.466052	0.6863	no
ni|645903731|nb|KK920699.1|	162489-162761	nan	nan	23	34	0.576037	0.61475	no
ni|645903731|nb|KK920699.1|	163474-164022	nan	nan	19	26	0.476604	0.66485	no
ni|645903731|nb|KK920699.1|	165133-165359	nan	nan	21	33	0.648521	0.57635	no
ni|645903731|nb|KK920699.1|	165464-165920	nan	nan	8	11	0.402182	0.72515	no
ni|645903731|nb|KK920699.1|	166052-166269	nan	nan	18	35	0.941715	0.43105	no
ni|645903731|nb|KK920699.1|	166664-166917	nan	nan	20	31	0.649548	0.5765	no
ni|645903731|nb|KK920699.1|	167717-167892	nan	nan	14	21	0.606456	0.6031	no
ni|645903731|nb|KK920699.1|	168029-168159	nan	nan	20	33	0.74006	0.52995	no
ni|645903731|nb|KK920699.1|	168736-169012	nan	nan	18	29	0.67787	0.55535	no
ni|645903731|nb|KK920699.1|	170081-170362	nan	nan	17	27	0.633639	0.5796	no
ni|645903731|nb|KK920699.1|	184172-184325	nan	nan	12	23	0.93281	0.4298	no
ni|645903731|nb|KK920699.1|	194673-194818	nan	nan	28	42	0.610866	0.60285	no
ni|645903731|nb|KK920699.1|	23218-23639	nan	nan	0	9	inf	5.00E-05	yes
ni|645903731|nb|KK920699.1|	50689-51255	nan	nan	19	40	1	0.39795	no
ni|645903731|nb|KK920699.1|	513339-513514	nan	nan	33	37	0.161308	0.89005	no
ni|645903731|nb|KK920699.1|	55918-56322	nan	nan	8	16	1	0.34315	no
ni|645903731|nb|KK920699.1|	58147-58428	nan	nan	6	14	1	0.2444	no
ni|645903731|nb|KK920699.1|	59728-59959	nan	nan	33	70	1	0.33215	no
ni|645903731|nb|KK920699.1|	60574-60839	nan	nan	60	96	0.669484	0.5372	no
ni|645903731|nb|KK920699.1|	62240-62814	nan	nan	28	39	0.455196	0.71445	no
ni|645903731|nb|KK920699.1|	64079-64271	nan	nan	63	75	0.26229	0.8128	no
ni|645903731|nb|KK920699.1|	64328-64631	nan	nan	2	2	0.214338	0.8678	no
ni|645903731|nb|KK920699.1|	65179-65345	nan	nan	3	3	0.102691	0.93335	no
ni|645903731|nb|KK920699.1|	675411-675918	nan	nan	27	23	-0.211713	0.8476	no
ni|645903731|nb|KK920699.1|	676340-677570	nan	nan	4	4	-0.190671	0.86185	no
ni|645903731|nb|KK920699.1|	678685-679636	nan	nan	28	29	0.0281221	0.9825	no
ni|645903731|nb|KK920699.1|	680620-681065	nan	nan	20	20	0.020395	0.98495	no
ni|645903731|nb|KK920699.1|	682218-682391	nan	nan	39	49	0.338215	0.77865	no

ni|645903731|nb|KK920699.1|	685421-685553	nan	nan	36	26	-0.451942	0.69475	no
ni|645903731|nb|KK920699.1|	688277-688423	nan	nan	51	56	0.117828	0.91445	no
ni|645903731|nb|KK920699.1|	689398-689872	nan	nan	18	16	-0.114264	0.91565	no
ni|645903731|nb|KK920699.1|	690546-691121	nan	nan	11	11	-0.0187629	0.98655	no
ni|645903731|nb|KK920699.1|	691647-691872	nan	nan	31	27	-0.192896	0.8674	no
ni|645903731|nb|KK920699.1|	693442-693711	nan	nan	34	33	-0.0348436	0.97565	no
ni|645903731|nb|KK920699.1|	695247-695546	nan	nan	18	17	-0.105594	0.92385	no
ni|645903731|nb|KK920699.1|	699232-699443	nan	nan	34	39	0.204274	0.8576	no
ni|645903731|nb|KK920699.1|	706794-707027	nan	nan	24	24	0.011607	0.99125	no
ni|645903731|nb|KK920699.1|	709120-709288	nan	nan	22	22	0.00430345	0.9895	no
ni|645903731|nb|KK920699.1|	714815-715068	nan	nan	1	2	0.0858541	0.9436	no
ni|645903731|nb|KK920699.1|	715473-716537	nan	nan	18	22	0.222948	0.88335	no
ni|645903731|nb|KK920699.1|	716854-717009	nan	nan	116	249	1	0.3194	no
ni|645903731|nb|KK920699.1|	718175-718329	nan	nan	205	477	1	0.3488	no
ni|645903731|nb|KK920699.1|	718534-719264	nan	nan	0.90309	0.525569	-0.78099	0.53215	no
ni|645903731|nb|KK920699.1|	719653-719782	nan	nan	215	559	1	0.21485	no
ni|645903731|nb|KK920699.1|	819631-819793	nan	nan	144	165	0.205247	0.8496	no
ni|645903731|nb|KK920699.1|	86205-86780	nan	nan	264	351	0.409982	0.7781	no
ni|645903731|nb|KK920699.1|	866909-867350	nan	nan	66	67	0.00709833	0.99595	no
ni|645903731|nb|KK920699.1|	87727-88096	nan	nan	48	83	0.794821	0.5286	no
ni|645903731|nb|KK920699.1|	881385-881955	nan	nan	0.263486	2	3	0.0705	no
ni|645903731|nb|KK920699.1|	883515-883715	nan	nan	265	303	0.195825	0.8917	no
ni|645903731|nb|KK920699.1|	884809-885019	nan	nan	286	279	-0.0372361	0.9796	no
ni|645903731|nb|KK920699.1|	886636-887180	nan	nan	10	9	-0.0816214	0.9429	no
ni|645903731|nb|KK920699.1|	88697-88834	nan	nan	176	390	1	0.2907	no
ni|645903731|nb|KK920699.1|	888078-888621	nan	nan	220	200	-0.132312	0.9242	no
ni|645903731|nb|KK920699.1|	888812-889009	nan	nan	463.26	358	-0.370384	0.79545	no
ni|645903731|nb|KK920699.1|	889163-889656	nan	nan	45	35	-0.35104	0.74045	no
ni|645903731|nb|KK920699.1|	892434-892585	nan	nan	0.488201	21	5	0.11955	no
ni|645903731|nb|KK920699.1|	892707-892839	nan	nan	0	30	inf	0.0003	yes

ni|645903731|nb|KK920699.1|	893806-894013	nan	nan	0	5	inf	0.00055	yes
ni|645903731|nb|KK920699.1|	89401-89675	nan	nan	189	345	0.868014	0.5432	no
ni|645903731|nb|KK920699.1|	896139-896303	nan	nan	0.390743	17	5	0.11955	no
ni|645903731|nb|KK920699.1|	89985-90448	nan	nan	185	349	0.914688	0.51775	no
ni|645903731|nb|KK920699.1|	901337-901509	nan	nan	2	26	4	0.0305	no
ni|645903731|nb|KK920699.1|	903201-903318	nan	nan	0	48	inf	0.00045	yes
ni|645903731|nb|KK920699.1|	90888-91028	nan	nan	332.89	634	0.930089	0.45715	no
ni|645903731|nb|KK920699.1|	918285-918470	nan	nan	0	24	inf	5.00E-05	yes
ni|645903731|nb|KK920699.1|	919858-919994	nan	nan	0.685392	20	5	0.1207	no
ni|645903731|nb|KK920699.1|	93635-94224	nan	nan	81	157	0.952067	0.50505	no
ni|645903731|nb|KK920699.1|	940764-940958	nan	nan	0	6	inf	0.00055	yes
ni|645903731|nb|KK920699.1|	96069-96208	nan	nan	221	405	0.875686	0.4234	no
ni|645903731|nb|KK920699.1|	96281-96445	nan	nan	293	555	0.923115	0.5392	no
ni|645903731|nb|KK920699.1|	97003-97162	nan	nan	172	381	1	0.37405	no
ni|645903731|nb|KK920699.1|	98772-99067	nan	nan	223	480	1	0.4353	no
ni|645903731|nb|KK920699.1|	990795-991047	nan	nan	2	2	-0.19169	0.88855	no
ni|645903732|nb|KK920698.1|	107051-107230	nan	nan	7	5	-0.526751	0.67145	no
ni|645903732|nb|KK920698.1|	139998-140109	nan	nan	84	94	0.165047	0.8857	no
ni|645903732|nb|KK920698.1|	18356-18658	nan	nan	1	24	4	0.01185	no
ni|645903732|nb|KK920698.1|	190990-191143	nan	nan	30	37	0.313082	0.7898	no
ni|645903732|nb|KK920698.1|	195156-195526	nan	nan	7	8	0.0754119	0.94455	no
ni|645903732|nb|KK920698.1|	24254-24570	nan	nan	7	11	0.691031	0.55205	no
ni|645903732|nb|KK920698.1|	28076-28767	nan	nan	0.125599	6	6	0.0506	no
ni|645903732|nb|KK920698.1|	35881-36178	nan	nan	2	0.48305	-2	0.1417	no
ni|645903732|nb|KK920698.1|	40830-41118	nan	nan	0	26	inf	5.00E-05	yes
ni|645903732|nb|KK920698.1|	41920-42267	nan	nan	0.100642	18	7	0.11935	no
ni|645903732|nb|KK920698.1|	46019-46201	nan	nan	0.305822	39	7	0.11935	no
ni|645903732|nb|KK920698.1|	49453-49716	nan	nan	0.30602	3	3	0.05605	no
ni|645903732|nb|KK920698.1|	49858-50046	nan	nan	0.284946	42	7	0.11935	no
ni|645903732|nb|KK920698.1|	52086-52288	nan	nan	0.491233	51	7	0.0443	no

ni|645903732|nb|KK920698.1|	56266-56405	nan	nan	0	45	inf	5.00E-05	yes
ni|645903732|nb|KK920698.1|	63457-63618	nan	nan	0.409615	23	6	0.11955	no
ni|645903732|nb|KK920698.1|	75316-75832	nan	nan	0.119146	49	9	0.0443	no
ni|645903732|nb|KK920698.1|	77817-78157	nan	nan	37	30	-0.300752	0.7859	no
ni|645903732|nb|KK920698.1|	78648-78776	nan	nan	39	39	-0.00457799	0.9871	no
ni|645903732|nb|KK920698.1|	79031-79230	nan	nan	23	36	0.695669	0.55305	no
ni|645903732|nb|KK920698.1|	80187-80966	nan	nan	2	2	0.0581209	0.9598	no
ni|645903732|nb|KK920698.1|	83300-83568	nan	nan	1	2	0.223494	0.8598	no
ni|645903732|nb|KK920698.1|	84318-85128	nan	nan	3	6	0.911043	0.4379	no
ni|645903732|nb|KK920698.1|	86430-86686	nan	nan	40	68	0.774778	0.48585	no
ni|645903732|nb|KK920698.1|	88413-89261	nan	nan	11	14	0.357084	0.7462	no
ni|645903733|nb|KK920697.1|	611459-611643	nan	nan	5	3	-1	0.4289	no
ni|645903733|nb|KK920697.1|	620917-621791	nan	nan	1	0.488604	-1	0.306	no
ni|645903733|nb|KK920697.1|	638715-639007	nan	nan	2	2	-0.328871	0.79705	no
ni|645903733|nb|KK920697.1|	664373-664630	nan	nan	2	0.607175	-2	0.3149	no
ni|645903733|nb|KK920697.1|	665496-665934	nan	nan	23	18.03	-0.321721	0.77095	no
ni|645903733|nb|KK920697.1|	666041-666518	nan	nan	22	17	-0.391392	0.72615	no
ni|645903733|nb|KK920697.1|	667163-667434	nan	nan	39	28	-0.500826	0.6567	no
ni|645903733|nb|KK920697.1|	678372-678510	nan	nan	34	29	-0.24057	0.8337	no
ni|645903733|nb|KK920697.1|	688452-688774	nan	nan	14	14	-0.0882087	0.93605	no
ni|645903733|nb|KK920697.1|	69360-69472	nan	nan	35	35	0.010425	0.9704	no
ni|645903734|nb|KK920696.1|	141214-141374	nan	nan	14	16	0.245953	0.83735	no
ni|645903734|nb|KK920696.1|	240648-241077	nan	nan	9	14	0.684077	0.5507	no
ni|645903734|nb|KK920696.1|	241257-242081	nan	nan	6	9	0.56624	0.6153	no
ni|645903734|nb|KK920696.1|	245372-245939	nan	nan	2	2	-0.0649989	0.95495	no
ni|645903734|nb|KK920696.1|	246619-246749	nan	nan	10	12	0.254633	0.84735	no
ni|645903734|nb|KK920696.1|	247903-248027	nan	nan	5	12	1	0.39335	no
ni|645903734|nb|KK920696.1|	248169-248549	nan	nan	4	5	0.388304	0.74165	no
ni|645903734|nb|KK920696.1|	250085-250462	nan	nan	3	5	0.844378	0.47295	no
ni|645903734|nb|KK920696.1|	251749-251953	nan	nan	3	2	-0.526328	0.7071	no

ni|645903734|nb|KK920696.1|	256733-257017	nan	nan	0.812427	2	0.934109	0.5295	no
ni|645903734|nb|KK920696.1|	257305-257769	nan	nan	2	2	-0.065192	0.9553	no
ni|645903734|nb|KK920696.1|	258659-258959	nan	nan	33	27	-0.323468	0.7763	no
ni|645903734|nb|KK920696.1|	259103-259262	nan	nan	25	18	-0.457992	0.6973	no
ni|645903734|nb|KK920696.1|	259461-260128	nan	nan	12	15	0.344728	0.7594	no
ni|645903734|nb|KK920696.1|	260727-260844	nan	nan	125	159	0.351631	0.75555	no
ni|645903734|nb|KK920696.1|	261303-261538	nan	nan	54	68	0.339998	0.7606	no
ni|645903734|nb|KK920696.1|	262376-262888	nan	nan	0.661696	0.460003	-0.524526	0.7071	no
ni|645903734|nb|KK920696.1|	263597-264569	nan	nan	22	21	-0.0480292	0.9742	no
ni|645903734|nb|KK920696.1|	264837-265167	nan	nan	21	18	-0.256148	0.8248	no
ni|645903734|nb|KK920696.1|	267340-268283	nan	nan	10	14	0.526197	0.6327	no
ni|645903734|nb|KK920696.1|	269117-269513	nan	nan	27	35	0.357472	0.74745	no
ni|645903734|nb|KK920696.1|	270171-270508	nan	nan	2	3	0.118832	0.92325	no
ni|645903734|nb|KK920696.1|	270800-270935	nan	nan	51	66	0.357628	0.7584	no
ni|645903734|nb|KK920696.1|	272909-273631	nan	nan	25	36	0.47985	0.6955	no
ni|645903734|nb|KK920696.1|	275291-275690	nan	nan	31	42	0.43601	0.68965	no
ni|645903734|nb|KK920696.1|	276866-277042	nan	nan	37	49	0.397898	0.7334	no
ni|645903734|nb|KK920696.1|	277125-277316	nan	nan	3	2	-0.719242	0.61925	no
ni|645903734|nb|KK920696.1|	277779-277939	nan	nan	34	22	-0.617402	0.5974	no
ni|645903734|nb|KK920696.1|	278081-278700	nan	nan	52	40	-0.368535	0.8049	no
ni|645903734|nb|KK920696.1|	278858-279218	nan	nan	42	33	-0.325228	0.77105	no
ni|645903734|nb|KK920696.1|	279275-279459	nan	nan	8	8	0.0396445	0.97515	no
ni|645903734|nb|KK920696.1|	279857-280050	nan	nan	4	17	2	0.09325	no
ni|645903734|nb|KK920696.1|	280170-280414	nan	nan	9	27	2	0.1818	no
ni|645903734|nb|KK920696.1|	282614-282896	nan	nan	11	26	1	0.3008	no
ni|645903734|nb|KK920696.1|	287293-287721	nan	nan	6	6	-0.207879	0.85865	no
ni|645903734|nb|KK920696.1|	289017-289315	nan	nan	14	16	0.201485	0.86185	no
ni|645903734|nb|KK920696.1|	292040-292229	nan	nan	14	15	0.102727	0.9311	no
ni|645903734|nb|KK920696.1|	395938-396001	nan	nan	24409.3	32585.6	0.416803	0.73365	no
ni|645903734|nb|KK920696.1|	406204-406346	nan	nan	10	563	6	0.00215	yes

ni|645903734|nb|KK920696.1|	407218-407532	nan	nan	5	263.32	6	0.001	yes
ni|645903734|nb|KK920696.1|	411789-412820	nan	nan	26	91	2	0.22545	no
ni|645903734|nb|KK920696.1|	415402-415597	nan	nan	128	131	0.024725	0.98005	no
ni|645903734|nb|KK920696.1|	418233-419325	nan	nan	2	2	-0.226816	0.84825	no
ni|645903734|nb|KK920696.1|	420166-420320	nan	nan	7	2	-2	0.2437	no
ni|645903734|nb|KK920696.1|	420381-420641	nan	nan	5	2	-1	0.2739	no
ni|645903734|nb|KK920696.1|	424520-425259	nan	nan	41	128	2	0.26565	no
ni|645903734|nb|KK920696.1|	425966-426404	nan	nan	490	721	0.557458	0.69575	no
ni|645903734|nb|KK920696.1|	426478-426615	nan	nan	225	235	0.0636281	0.95655	no
ni|645903734|nb|KK920696.1|	428013-428205	nan	nan	2	2	-0.429716	0.75965	no
ni|645903734|nb|KK920696.1|	434638-434873	nan	nan	2	2	-0.329401	0.80325	no
ni|645903734|nb|KK920696.1|	435902-436096	nan	nan	3	1	-1	0.35155	no
ni|645903734|nb|KK920696.1|	437300-437454	nan	nan	4	3	-0.259837	0.8692	no
ni|645903734|nb|KK920696.1|	441317-441448	nan	nan	2	6	1	0.35215	no
ni|645903734|nb|KK920696.1|	588553-588704	nan	nan	0.488201	17	5	0.1202	no
ni|645903734|nb|KK920696.1|	596761-596996	nan	nan	0	25	inf	5.00E-05	yes
ni|645903734|nb|KK920696.1|	598427-598864	nan	nan	0	7	inf	5.00E-05	yes
ni|645903734|nb|KK920696.1|	600733-600914	nan	nan	0	25	inf	5.00E-05	yes
ni|645903734|nb|KK920696.1|	603254-603817	nan	nan	0	8	inf	5.00E-05	yes
ni|645903734|nb|KK920696.1|	608064-608249	nan	nan	0	22	inf	5.00E-05	yes
ni|645903734|nb|KK920696.1|	608765-609149	nan	nan	0	8	inf	5.00E-05	yes
ni|645903734|nb|KK920696.1|	610802-612354	nan	nan	0.068002	12	7	0.03715	no
ni|645903734|nb|KK920696.1|	615712-615975	nan	nan	0.15301	7	5	0.11995	no
ni|645903734|nb|KK920696.1|	616081-616205	nan	nan	1	32	5	0.12005	no
ni|645903734|nb|KK920696.1|	617327-617570	nan	nan	0.873114	14	4	0.02935	no
ni|645903734|nb|KK920696.1|	619716-619964	nan	nan	0.168668	19	7	0.11935	no
ni|645903734|nb|KK920696.1|	621421-621571	nan	nan	0.49775	27	6	0.11935	no
ni|645903734|nb|KK920696.1|	651228-651839	nan	nan	0.436053	10	5	0.01145	no
ni|645903734|nb|KK920696.1|	658964-659110	nan	nan	0	36	inf	5.00E-05	yes
ni|645903734|nb|KK920696.1|	663716-663866	nan	nan	0.49775	26	6	0.1195	no

ni|645903734|nb|KK920696.1|	674287-675082	nan	nan	0.391381	12	5	0.0068	no
ni|645903734|nb|KK920696.1|	677382-677612	nan	nan	1	25	4	0.0228	no
ni|645903734|nb|KK920696.1|	677806-677936	nan	nan	0.817431	22	5	0.12065	no
ni|645903734|nb|KK920696.1|	687695-687884	nan	nan	0.563465	19	5	0.04455	no
ni|645903734|nb|KK920696.1|	689689-689918	nan	nan	2	36	4	0.01195	no
ni|645903734|nb|KK920696.1|	691315-691486	nan	nan	0.70558	29	5	0.04445	no
ni|645903734|nb|KK920696.1|	700479-700743	nan	nan	0.760343	21	5	0.02785	no
ni|645903734|nb|KK920696.1|	701873-702076	nan	nan	0.243213	26	7	0.11935	no
ni|645903734|nb|KK920696.1|	708432-708567	nan	nan	2	17	3	0.06785	no
ni|645903734|nb|KK920696.1|	708676-709201	nan	nan	0.757967	20	5	0.004	no
ni|645903734|nb|KK920696.1|	709931-710086	nan	nan	0.906815	22	5	0.04505	no
ni|645903734|nb|KK920696.1|	711078-711286	nan	nan	1	25	4	0.03555	no
ni|645903734|nb|KK920696.1|	711493-711684	nan	nan	0	19	inf	5.00E-05	yes
ni|645903734|nb|KK920696.1|	714132-714941	nan	nan	0.558004	12	4	0.00445	no
ni|645903734|nb|KK920696.1|	716728-716950	nan	nan	0.820029	25	5	0.0374	no
ni|645903734|nb|KK920696.1|	724444-724656	nan	nan	2	28	4	0.01775	no
ni|645903734|nb|KK920696.1|	727115-727362	nan	nan	1	27	4	0.02145	no
ni|645903734|nb|KK920696.1|	730193-730460	nan	nan	0.746563	13	4	0.0289	no
ni|645903734|nb|KK920696.1|	733664-733872	nan	nan	2	31	4	0.018	no
ni|645903734|nb|KK920696.1|	734229-734425	nan	nan	0.522163	26	6	0.0447	no
ni|645903734|nb|KK920696.1|	739651-740092	nan	nan	0.945777	11	4	0.01495	no
ni|645903734|nb|KK920696.1|	746089-746667	nan	nan	3	2	-0.261378	0.8282	no
ni|645903734|nb|KK920696.1|	746969-747118	nan	nan	11	12	0.125448	0.91735	no
ni|645903734|nb|KK920696.1|	750736-750941	nan	nan	3	31	4	0.01815	no
ni|645903734|nb|KK920696.1|	753435-753595	nan	nan	2	33	4	0.0383	no
ni|645903734|nb|KK920696.1|	758446-758698	nan	nan	2	27	4	0.0122	no
ni|645903734|nb|KK920696.1|	761168-761431	nan	nan	0.918059	32	5	0.03345	no
ni|645903734|nb|KK920696.1|	762733-763144	nan	nan	0.399062	9	5	0.02785	no
ni|645903734|nb|KK920696.1|	767442-767810	nan	nan	0.556228	8	4	0.039	no
ni|645903734|nb|KK920696.1|	769144-769338	nan	nan	2	23	3	0.03075	no

ni|645903734|nb|KK920696.1|	777794-778426	nan	nan	0.511825	7	4	0.0131	no
ni|645903734|nb|KK920696.1|	785385-785604	nan	nan	3	33	3	0.01735	no
ni|645903734|nb|KK920696.1|	788172-788434	nan	nan	1	22	4	0.0179	no
ni|645903734|nb|KK920696.1|	789174-789382	nan	nan	2	30	4	0.0229	no
ni|645903734|nb|KK920696.1|	791006-791126	nan	nan	0	33	inf	5.00E-05	yes
ni|645903734|nb|KK920696.1|	793895-794350	nan	nan	0.489073	9	4	0.0229	no
ni|645903734|nb|KK920696.1|	796925-797041	nan	nan	4	40	3	0.06165	no
ni|645903734|nb|KK920696.1|	798086-798308	nan	nan	0.615022	17	5	0.0511	no
ni|645903734|nb|KK920696.1|	800112-800251	nan	nan	2	22	4	0.056	no
ni|645903734|nb|KK920696.1|	800666-800926	nan	nan	3	31	4	0.0102	no
ni|645903734|nb|KK920696.1|	910961-911422	nan	nan	0.2061	0.853637	2	0.1537	no
ni|645903734|nb|KK920696.1|	926089-926622	nan	nan	3	2	-0.0326356	0.97875	no
ni|645903734|nb|KK920696.1|	929389-929618	nan	nan	6	9	0.457106	0.69615	no
ni|645903734|nb|KK920696.1|	931130-931677	nan	nan	0.665064	0.900646	0.437467	0.73605	no
ni|645903734|nb|KK920696.1|	934335-934544	nan	nan	5	9	0.967155	0.4234	no
ni|645903734|nb|KK920696.1|	937068-937244	nan	nan	5	8	0.780672	0.52855	no
ni|645903734|nb|KK920696.1|	948132-948271	nan	nan	4	8	0.932767	0.51205	no
ni|645903734|nb|KK920696.1|	954501-954616	nan	nan	14	12	-0.23742	0.8697	no
ni|645903734|nb|KK920696.1|	959897-960040	nan	nan	5	1	-2	0.3383	no
ni|645903735|nb|KK920695.1|	108967-109640	16	16	0.604614	13	4	0.00585	no
ni|645903735|nb|KK920695.1|	109895-110052	16	16	99	80	-0.302415	0.79225	no
ni|645903735|nb|KK920695.1|	110159-110311	16	16	160	127	-0.336268	0.76445	no
ni|645903735|nb|KK920695.1|	110665-111297	16	16	83	63	-0.404272	0.77255	no
ni|645903735|nb|KK920695.1|	111493-111791	16	16	147	118	-0.313804	0.8307	no
ni|645903735|nb|KK920695.1|	114096-114312	16	16	203	190	-0.100722	0.9471	no
ni|645903735|nb|KK920695.1|	115111-115815	16	16	5	4	-0.169781	0.88545	no
ni|645903735|nb|KK920695.1|	117753-118115	16	16	80	79	-0.0202638	0.98805	no
ni|645903735|nb|KK920695.1|	118756-119082	16	16	3	2	-0.19689	0.8673	no
ni|645903735|nb|KK920695.1|	119477-119700	16	16	93	102	0.134394	0.89735	no
ni|645903735|nb|KK920695.1|	123455-124002	16	16	114	114	0.00332135	0.9979	no

ni|645903735|nb|KK920695.1|	126158-126294	16	16	97	90	-0.108473	0.92555	no
ni|645903735|nb|KK920695.1|	127555-127915	16	16	79	89	0.179676	0.90605	no
ni|645903735|nb|KK920695.1|	133692-133888	16	16	67	81	0.272785	0.8115	no
ni|645903735|nb|KK920695.1|	152610-152922	16	16	64	60	-0.0950812	0.92945	no
ni|645903735|nb|KK920695.1|	289430-289653	16	16	31	34	0.101291	0.9307	no
ni|645903735|nb|KK920695.1|	35631-36219	16	16	21	26	0.269335	0.8022	no
ni|645903735|nb|KK920695.1|	406546-406807	16	16	0	2	inf	0.00225	yes
ni|645903735|nb|KK920695.1|	44877-45124	16	16	2	0.486624	-2	0.2989	no
ni|645903736|nb|KK920694.1|	11538-12019	18	17	0.780898	1	0.437345	0.73605	no
ni|645903736|nb|KK920694.1|	12121-12481	18	17	1	0.730678	-0.972381	0.5005	no
ni|645903736|nb|KK920694.1|	12603-13473	18	17	0.449456	0.306968	-0.550091	0.68265	no
ni|645903736|nb|KK920694.1|	135387-135582	18	17	34	22	-0.607656	0.60245	no
ni|645903736|nb|KK920694.1|	136570-137495	18	17	4	3	-0.514154	0.6569	no
ni|645903736|nb|KK920694.1|	13754-15361	18	17	3	3	-0.314181	0.7828	no
ni|645903736|nb|KK920694.1|	138576-139411	18	17	0.908216	0.643253	-0.497649	0.67645	no
ni|645903736|nb|KK920694.1|	141316-141996	18	17	5	4	-0.491849	0.66875	no
ni|645903736|nb|KK920694.1|	142314-142645	18	17	8	6	-0.499518	0.677	no
ni|645903736|nb|KK920694.1|	145509-145863	18	17	9	12	0.369204	0.7503	no
ni|645903736|nb|KK920694.1|	146036-148692	18	17	79	58	-0.46284	0.7414	no
ni|645903736|nb|KK920694.1|	149170-149657	18	17	9	4	-1	0.32655	no
ni|645903736|nb|KK920694.1|	149782-150176	18	17	0.67565	0.32286	-1	0.44965	no
ni|645903736|nb|KK920694.1|	152050-152383	18	17	8	5	-0.747436	0.53595	no
ni|645903736|nb|KK920694.1|	153325-153583	18	17	24	18	-0.416598	0.72205	no
ni|645903736|nb|KK920694.1|	157988-158286	18	17	3	1	-0.802764	0.53265	no
ni|645903736|nb|KK920694.1|	160104-160600	18	17	0.688571	0.658179	-0.065125	0.94765	no
ni|645903736|nb|KK920694.1|	16027-16369	18	17	9	7	-0.321908	0.79305	no
ni|645903736|nb|KK920694.1|	163278-163418	18	17	31	21	-0.5818	0.62925	no
ni|645903736|nb|KK920694.1|	167086-167480	18	17	0.844562	0.242145	-2	0.2784	no
ni|645903736|nb|KK920694.1|	168854-169323	18	17	0.604823	0.642339	0.0868224	0.9436	no
ni|645903736|nb|KK920694.1|	170348-171184	18	17	5	3	-0.604327	0.6082	no

ni|645903736|nb|KK920694.1|	171393-171818	18	17	7	4	-0.864983	0.46015	no
ni|645903736|nb|KK920694.1|	172047-172331	18	17	3	1	-0.998777	0.4532	no
ni|645903736|nb|KK920694.1|	172589-173080	18	17	8	4	-1	0.3532	no
ni|645903736|nb|KK920694.1|	173244-173586	18	17	2	1	-1	0.3995	no
ni|645903736|nb|KK920694.1|	174018-174129	18	17	25	16	-0.652576	0.64	no
ni|645903736|nb|KK920694.1|	174658-174777	18	17	21	12	-0.832954	0.5313	no
ni|645903736|nb|KK920694.1|	175054-175280	18	17	10	6	-0.651465	0.5813	no
ni|645903736|nb|KK920694.1|	175682-176090	18	17	8	7	-0.128063	0.91105	no
ni|645903736|nb|KK920694.1|	176264-176419	18	17	14	8	-0.80416	0.516	no
ni|645903736|nb|KK920694.1|	177779-178243	18	17	3	3	0.0279174	0.9832	no
ni|645903736|nb|KK920694.1|	181565-181836	18	17	2	0.278543	-3	0.2671	no
ni|645903736|nb|KK920694.1|	196729-196951	18	17	2	3	0.311944	0.81835	no
ni|645903736|nb|KK920694.1|	288762-289286	18	17	0	5	inf	5.00E-05	yes
ni|645903736|nb|KK920694.1|	290469-290714	18	17	0	10	inf	5.00E-05	yes
ni|645903736|nb|KK920694.1|	29217-30620	18	17	0.454752	0.199323	-1	0.3535	no
ni|645903736|nb|KK920694.1|	294351-294580	18	17	0	14	inf	5.00E-05	yes
ni|645903736|nb|KK920694.1|	295981-296770	18	17	12	23	0.864988	0.42775	no
ni|645903736|nb|KK920694.1|	297009-297214	18	17	23	46	0.984264	0.3987	no
ni|645903736|nb|KK920694.1|	298644-299131	18	17	11	21	1	0.37765	no
ni|645903736|nb|KK920694.1|	299235-299425	18	17	16	39	1	0.2677	no
ni|645903736|nb|KK920694.1|	300145-300787	18	17	7	16	1	0.3217	no
ni|645903736|nb|KK920694.1|	301539-302308	18	17	10	20	1	0.3606	no
ni|645903736|nb|KK920694.1|	303130-303260	18	17	25	44	0.811399	0.4962	no
ni|645903736|nb|KK920694.1|	304409-305006	18	17	3	7	1	0.2794	no
ni|645903736|nb|KK920694.1|	305119-305513	18	17	181	211	0.220776	0.8752	no
ni|645903736|nb|KK920694.1|	305618-306514	18	17	0.838411	0.861054	0.0384469	0.97585	no
ni|645903736|nb|KK920694.1|	30673-31277	18	17	0.687768	0.23482	-2	0.29725	no
ni|645903736|nb|KK920694.1|	307371-307880	18	17	3	4	0.356727	0.75955	no
ni|645903736|nb|KK920694.1|	313963-314105	18	17	12	73	3	0.04485	no
ni|645903736|nb|KK920694.1|	318060-318336	18	17	11	61	3	0.036	no

ni|645903736|nb|KK920694.1|	386319-387233	18	17	5	20	2	0.0623	no
ni|645903736|nb|KK920694.1|	391148-391265	18	17	32	115	2	0.1359	no
ni|645903736|nb|KK920694.1|	391387-391599	18	17	31	165	2	0.0884	no
ni|645903736|nb|KK920694.1|	396296-396590	18	17	33	45	0.459459	0.67465	no
ni|645903736|nb|KK920694.1|	398243-398565	18	17	3	6	0.680577	0.5605	no
ni|645903736|nb|KK920694.1|	398898-399320	18	17	16	17	0.0614025	0.955	no
ni|645903736|nb|KK920694.1|	399678-399852	18	17	47	50	0.0898763	0.9342	no
ni|645903736|nb|KK920694.1|	400132-400293	18	17	43	57	0.432025	0.7139	no
ni|645903736|nb|KK920694.1|	400665-400846	18	17	19	37	0.944287	0.41795	no
ni|645903736|nb|KK920694.1|	401907-402366	18	17	20.38	24	0.230071	0.8284	no
ni|645903736|nb|KK920694.1|	403036-403234	18	17	41	44	0.101907	0.92905	no
ni|645903736|nb|KK920694.1|	405685-405954	18	17	37	53	0.526632	0.63205	no
ni|645903736|nb|KK920694.1|	407432-407780	18	17	19	22	0.253281	0.82175	no
ni|645903736|nb|KK920694.1|	408452-408588	18	17	32	28	-0.164548	0.8903	no
ni|645903736|nb|KK920694.1|	409584-410104	18	17	0.885041	4	2	0.09835	no
ni|645903736|nb|KK920694.1|	410254-410732	18	17	2	3	0.651046	0.5859	no
ni|645903736|nb|KK920694.1|	410858-411303	18	17	2	2	0.086768	0.9421	no
ni|645903736|nb|KK920694.1|	411484-412674	18	17	2	3	0.367444	0.7511	no
ni|645903736|nb|KK920694.1|	414599-414791	18	17	7	8	0.347891	0.76865	no
ni|645903736|nb|KK920694.1|	414895-415667	18	17	2	2	-0.362423	0.7609	no
ni|645903736|nb|KK920694.1|	416271-416408	18	17	5	4	-0.289637	0.8496	no
ni|645903736|nb|KK920694.1|	417327-417725	18	17	1	1	-0.152818	0.90135	no
ni|645903736|nb|KK920694.1|	418814-419055	18	17	4	4	0.0491922	0.96845	no
ni|645903736|nb|KK920694.1|	419250-419728	18	17	1	2	0.271874	0.82415	no
ni|645903736|nb|KK920694.1|	420036-420275	18	17	21	19	-0.192945	0.8671	no
ni|645903736|nb|KK920694.1|	420433-420715	18	17	2	0.523167	-2	0.22995	no
ni|645903736|nb|KK920694.1|	422391-422497	18	17	46	48	0.038956	0.95995	no
ni|645903736|nb|KK920694.1|	422709-423155	18	17	28	21	-0.394541	0.7197	no
ni|645903736|nb|KK920694.1|	423218-423343	18	17	21	15	-0.526775	0.67145	no
ni|645903736|nb|KK920694.1|	423536-423979	18	17	4	3	-0.373362	0.7539	no

ni|645903736|nb|KK920694.1|	427647-428394	18	17	0	209	inf	5.00E-05	yes
ni|645903736|nb|KK920694.1|	428667-429457	18	17	0	207	inf	5.00E-05	yes
ni|645903736|nb|KK920694.1|	429584-430045	18	17	0	146	inf	5.00E-05	yes
ni|645903736|nb|KK920694.1|	430184-430313	18	17	0	189	inf	5.00E-05	yes
ni|645903736|nb|KK920694.1|	430376-430493	18	17	0	240	inf	5.00E-05	yes
ni|645903736|nb|KK920694.1|	437903-438039	18	17	0.685392	203	8	0.11935	no
ni|645903736|nb|KK920694.1|	438156-438919	18	17	16	74	2	0.09075	no
ni|645903736|nb|KK920694.1|	439268-439533	18	17	69	228	2	0.1847	no
ni|645903736|nb|KK920694.1|	440806-441515	18	17	34	113	2	0.25475	no
ni|645903736|nb|KK920694.1|	442265-442411	18	17	5	4	-0.581777	0.70815	no
ni|645903736|nb|KK920694.1|	442557-442841	18	17	73	241	2	0.1734	no
ni|645903736|nb|KK920694.1|	443364-443655	18	17	87	253	2	0.22155	no
ni|645903736|nb|KK920694.1|	444124-444468	18	17	15	47	2	0.1561	no
ni|645903736|nb|KK920694.1|	4457-4942	18	17	2	2	-0.387075	0.74155	no
ni|645903736|nb|KK920694.1|	446874-447176	18	17	82	223	1	0.2464	no
ni|645903736|nb|KK920694.1|	447930-448197	18	17	2	0	-inf	0.0013	yes
ni|645903736|nb|KK920694.1|	455324-455451	18	17	145	460	2	0.146	no
ni|645903736|nb|KK920694.1|	456503-456949	18	17	33	87	1	0.2869	no
ni|645903736|nb|KK920694.1|	460131-460706	18	17	14	34	1	0.2691	no
ni|645903736|nb|KK920694.1|	461713-462111	18	17	116	265	1	0.40365	no
ni|645903736|nb|KK920694.1|	462448-462570	18	17	89	49	-0.85264	0.4706	no
ni|645903736|nb|KK920694.1|	462681-462786	18	17	37	59	0.668854	0.6211	no
ni|645903736|nb|KK920694.1|	463192-463400	18	17	21	16	-0.38874	0.7434	no
ni|645903736|nb|KK920694.1|	465766-465995	18	17	62	71	0.188316	0.86285	no
ni|645903736|nb|KK920694.1|	466047-466150	18	17	80	257	2	0.1831	no
ni|645903736|nb|KK920694.1|	468112-468376	18	17	13	37	2	0.1884	no
ni|645903736|nb|KK920694.1|	468500-469138	18	17	23	109	2	0.08185	no
ni|645903736|nb|KK920694.1|	475598-476035	18	17	0	4	inf	5.00E-05	yes
ni|645903736|nb|KK920694.1|	5060-5456	18	17	2	2	-0.517873	0.6643	no
ni|645903736|nb|KK920694.1|	539040-539148	18	17	25	56	1	0.39005	no

ni|645903736|nb|KK920694.1|	553307-553609	18	17	0.862716	1	0.448798	0.7543	no
ni|645903736|nb|KK920694.1|	554548-555131	18	17	1	2	0.572457	0.6286	no
ni|645903736|nb|KK920694.1|	555263-558045	18	17	17	33	0.964815	0.4936	no
ni|645903736|nb|KK920694.1|	5566-6180	18	17	2	2	-0.401961	0.7357	no
ni|645903736|nb|KK920694.1|	558225-558736	18	17	7	8	0.0251019	0.9825	no
ni|645903736|nb|KK920694.1|	558950-559294	18	17	12	10	-0.223101	0.8453	no
ni|645903736|nb|KK920694.1|	559433-560525	18	17	2	3	0.227636	0.84625	no
ni|645903736|nb|KK920694.1|	560662-563647	18	17	2	2	0.25619	0.8227	no
ni|645903736|nb|KK920694.1|	564975-565546	18	17	2	3	0.607433	0.60025	no
ni|645903736|nb|KK920694.1|	565699-566049	18	17	0.994262	2	0.612539	0.63765	no
ni|645903736|nb|KK920694.1|	566729-567128	18	17	2	2	-0.065352	0.9556	no
ni|645903736|nb|KK920694.1|	568983-569166	18	17	4	4	0.0322527	0.96125	no
ni|645903736|nb|KK920694.1|	570527-570777	18	17	0.998386	3	2	0.297	no
ni|645903736|nb|KK920694.1|	570958-571104	18	17	6	3	-1	0.4064	no
ni|645903736|nb|KK920694.1|	582298-582655	18	17	34	37	0.122524	0.91135	no
ni|645903736|nb|KK920694.1|	583151-583298	18	17	27	34	0.347836	0.76455	no
ni|645903736|nb|KK920694.1|	583438-584013	18	17	23	25	0.108896	0.9246	no
ni|645903736|nb|KK920694.1|	584227-584552	18	17	13	12	-0.114978	0.91995	no
ni|645903736|nb|KK920694.1|	584691-584984	18	17	33	28	-0.203333	0.86035	no
ni|645903736|nb|KK920694.1|	6579-6978	18	17	27	23	-0.209976	0.84945	no
ni|645903736|nb|KK920694.1|	7341-7685	18	17	146	114	-0.358543	0.804	no
ni|645903736|nb|KK920694.1|	7938-8321	18	17	3	2	-0.830938	0.49575	no
ni|645903736|nb|KK920694.1|	8418-8684	18	17	2	1	-0.355536	0.7931	no
ni|645903736|nb|KK920694.1|	9265-9459	18	17	99	82	-0.270875	0.80675	no
ni|645903736|nb|KK920694.1|	9562-9687	18	17	69	74	0.104837	0.9288	no
ni|645903737|nb|KK920693.1|	122849-122909	16	20	14246.2	61975.2	2	0.14145	no
ni|645903737|nb|KK920693.1|	221577-222571	16	20	0.995018	0.71359	-0.479627	0.68355	no
ni|645903737|nb|KK920693.1|	229899-230077	16	20	12	34	1	0.225	no
ni|645903737|nb|KK920693.1|	232892-233083	16	20	13	27	1	0.3727	no
ni|645903737|nb|KK920693.1|	238378-238881	16	20	6	14	1	0.2697	no

ni|645903737|nb|KK920693.1|	240293-240411	16	20	15	22	0.560596	0.667	no
ni|645903737|nb|KK920693.1|	241423-241950	16	20	8	13	0.774468	0.5025	no
ni|645903737|nb|KK920693.1|	242676-242875	16	20	13	27	1	0.37415	no
ni|645903737|nb|KK920693.1|	244466-245088	16	20	7	11	0.707675	0.5391	no
ni|645903737|nb|KK920693.1|	247954-248336	16	20	11	14	0.395074	0.73415	no
ni|645903737|nb|KK920693.1|	250392-250989	16	20	21	31	0.600402	0.5765	no
ni|645903737|nb|KK920693.1|	251330-251678	16	20	10	14	0.436118	0.70635	no
ni|645903737|nb|KK920693.1|	252587-252950	16	20	8	9	0.0781123	0.95115	no
ni|645903737|nb|KK920693.1|	254027-254134	16	20	37	42	0.15455	0.90275	no
ni|645903737|nb|KK920693.1|	255422-255567	16	20	20	4	-2	0.11305	no
ni|645903737|nb|KK920693.1|	266683-267056	16	20	0.454978	3	3	0.08815	no
ni|645903737|nb|KK920693.1|	268239-268581	16	20	4	4	-0.0308032	0.9803	no
ni|645903737|nb|KK920693.1|	274033-274161	16	20	27	26	-0.0673137	0.95425	no
ni|645903737|nb|KK920693.1|	280688-280911	16	20	0	106	inf	5.00E-05	yes
ni|645903737|nb|KK920693.1|	287422-287593	16	20	0	85	inf	5.00E-05	yes
ni|645903737|nb|KK920693.1|	290181-290650	16	20	0	193	inf	5.00E-05	yes
ni|645903737|nb|KK920693.1|	337648-338387	16	20	0.464517	0.03701	-4	0.02785	no
ni|645903737|nb|KK920693.1|	385533-385718	16	20	8	0	-inf	0.00015	yes
ni|645903737|nb|KK920693.1|	394516-394674	16	20	8	0	-inf	0.0004	yes
ni|645903737|nb|KK920693.1|	397452-397685	16	20	5	0	-inf	0.0004	yes
ni|645903737|nb|KK920693.1|	399014-399379	16	20	6	0	-inf	5.00E-05	yes
ni|645903737|nb|KK920693.1|	412628-413330	16	20	31	56	0.827546	0.5776	no
ni|645903737|nb|KK920693.1|	70659-70719	16	20	10684.7	32463.2	2	0.2412	no
ni|645903738|nb|KK920692.1|	1000610-100260	18	17	9	13	0.425367	0.77515	no
ni|645903738|nb|KK920692.1|	1004253-100440	18	17	13	23	0.82971	0.4847	no
ni|645903738|nb|KK920692.1|	1004512-100478	18	17	12	18	0.625077	0.5942	no
ni|645903738|nb|KK920692.1|	1005079-100561	18	17	25	26	0.0588606	0.95405	no
ni|645903738|nb|KK920692.1|	1010455-101060	18	17	96	78	-0.300696	0.78965	no
ni|645903738|nb|KK920692.1|	1010712-101082	18	17	84	74	-0.189647	0.87405	no
ni|645903738|nb|KK920692.1|	1015642-101580	18	17	97	104	0.0995263	0.9312	no

ni|645903738|nb|KK920692.1|	1016723-1016917	18	17	37	44	0.229875	0.8438	no
ni|645903738|nb|KK920692.1|	1018186-1018382	18	17	96	103	0.110722	0.9188	no
ni|645903738|nb|KK920692.1|	1018707-1018889	18	17	97	114	0.239953	0.82775	no
ni|645903738|nb|KK920692.1|	1023136-1023627	18	17	48	51	0.0838036	0.95335	no
ni|645903738|nb|KK920692.1|	1025911-1026156	18	17	98	99	0.00271175	0.99745	no
ni|645903738|nb|KK920692.1|	1027469-1027661	18	17	101	101	0.00102537	0.998	no
ni|645903738|nb|KK920692.1|	1029635-1029794	18	17	114	108.67	-0.0672025	0.95175	no
ni|645903738|nb|KK920692.1|	1035927-1036104	18	17	112	106	-0.0799995	0.94335	no
ni|645903738|nb|KK920692.1|	1037188-1037572	18	17	29	26	-0.171985	0.88065	no
ni|645903738|nb|KK920692.1|	1037919-1038220	18	17	1	0.946739	-0.387709	0.78555	no
ni|645903738|nb|KK920692.1|	1038439-1039152	18	17	2	2	0.0727022	0.9474	no
ni|645903738|nb|KK920692.1|	1039253-1040024	18	17	1	1	0.0477316	0.96865	no
ni|645903738|nb|KK920692.1|	1040465-1040959	18	17	15	12	-0.237398	0.8353	no
ni|645903738|nb|KK920692.1|	1042053-1042362	18	17	39	37	-0.0968579	0.93205	no
ni|645903738|nb|KK920692.1|	1042484-1043217	18	17	29	26	-0.158547	0.89885	no
ni|645903738|nb|KK920692.1|	1043343-1043569	18	17	58	52	-0.163915	0.88785	no
ni|645903738|nb|KK920692.1|	1044210-1044546	18	17	32	32	0.00388799	0.9963	no
ni|645903738|nb|KK920692.1|	1044606-1044715	18	17	108	112	0.0606161	0.959	no
ni|645903738|nb|KK920692.1|	1044966-1045323	18	17	22	16	-0.45782	0.69605	no
ni|645903738|nb|KK920692.1|	1045536-1045722	18	17	61	53	-0.190252	0.8671	no
ni|645903738|nb|KK920692.1|	1046586-1046716	18	17	16	12	-0.389223	0.7511	no
ni|645903738|nb|KK920692.1|	1046849-1047562	18	17	31	27	-0.209688	0.86295	no
ni|645903738|nb|KK920692.1|	1048180-1048915	18	17	12	8	-0.481403	0.66635	no
ni|645903738|nb|KK920692.1|	1050278-1050409	18	17	69	62	-0.152678	0.89725	no
ni|645903738|nb|KK920692.1|	1050525-1050995	18	17	10	9	-0.132293	0.9094	no
ni|645903738|nb|KK920692.1|	1053456-1053632	18	17	54	41	-0.42242	0.7112	no
ni|645903738|nb|KK920692.1|	1063106-1063492	18	17	2	2	-0.328427	0.7854	no
ni|645903738|nb|KK920692.1|	168196-168354	18	17	4	4	0.0848027	0.9436	no
ni|645903738|nb|KK920692.1|	169850-170028	18	17	5	2	-1	0.40265	no
ni|645903738|nb|KK920692.1|	181318-181448	18	17	6	4	-0.552722	0.719	no

ni|645903738|nb|KK920692.1|	183820-184074	18	17	4	4	0.165187	0.88955	no
ni|645903738|nb|KK920692.1|	195397-195753	18	17	5	5	-0.121002	0.91275	no
ni|645903738|nb|KK920692.1|	227-353	18	17	6	12	1	0.45555	no
ni|645903738|nb|KK920692.1|	254642-255123	18	17	0.325374	1	2	0.1494	no
ni|645903738|nb|KK920692.1|	255967-256105	18	17	3	7	1	0.43015	no
ni|645903738|nb|KK920692.1|	257164-257343	18	17	1	9	3	0.06365	no
ni|645903738|nb|KK920692.1|	260807-261005	18	17	2	6	2	0.262	no
ni|645903738|nb|KK920692.1|	265214-265645	18	17	0.524738	4	3	0.04965	no
ni|645903738|nb|KK920692.1|	265773-266487	18	17	0.926714	4	2	0.0937	no
ni|645903738|nb|KK920692.1|	266836-267224	18	17	0.862268	5	3	0.07015	no
ni|645903738|nb|KK920692.1|	268894-269079	18	17	1	6	2	0.10795	no
ni|645903738|nb|KK920692.1|	270137-270565	18	17	1	4	2	0.1612	no
ni|645903738|nb|KK920692.1|	272210-272391	18	17	2	8	2	0.19185	no
ni|645903738|nb|KK920692.1|	273540-273710	18	17	3	10	2	0.1459	no
ni|645903738|nb|KK920692.1|	276519-277091	18	17	1	5	2	0.12065	no
ni|645903738|nb|KK920692.1|	278266-278795	18	17	1	5	2	0.1046	no
ni|645903738|nb|KK920692.1|	280575-280827	18	17	2	8	2	0.14945	no
ni|645903738|nb|KK920692.1|	283298-283979	18	17	0.809204	2	1	0.3973	no
ni|645903738|nb|KK920692.1|	286740-286923	18	17	3	8	1	0.2759	no
ni|645903738|nb|KK920692.1|	287928-288079	18	17	6	13	0.987254	0.44185	no
ni|645903738|nb|KK920692.1|	292123-292420	18	17	5	5	0.178121	0.88045	no
ni|645903738|nb|KK920692.1|	293278-293468	18	17	4	5	0.554306	0.6571	no
ni|645903738|nb|KK920692.1|	294078-294389	18	17	5	2	-1	0.2675	no
ni|645903738|nb|KK920692.1|	294799-295432	18	17	1	0.488389	-1	0.3302	no
ni|645903738|nb|KK920692.1|	299376-301220	18	17	0.042451	132	12	0.05055	no
ni|645903738|nb|KK920692.1|	301664-302342	18	17	0	65	inf	5.00E-05	yes
ni|645903738|nb|KK920692.1|	302507-302670	18	17	0	73	inf	5.00E-05	yes
ni|645903738|nb|KK920692.1|	306055-306162	18	17	31	18	-0.804808	0.6027	no
ni|645903738|nb|KK920692.1|	306542-306731	18	17	25	15	-0.710042	0.54575	no
ni|645903738|nb|KK920692.1|	307028-307307	18	17	11	9	-0.387856	0.7359	no

ni|645903738|nb|KK920692.1|	307842-308077	18	17	18	46	1	0.25225	no
ni|645903738|nb|KK920692.1|	309126-309434	18	17	10	39	2	0.1017	no
ni|645903738|nb|KK920692.1|	310093-310248	18	17	20	59.29	2	0.18925	no
ni|645903738|nb|KK920692.1|	310998-311544	18	17	8	26	2	0.11375	no
ni|645903738|nb|KK920692.1|	312954-313469	18	17	10	13	0.339487	0.7607	no
ni|645903738|nb|KK920692.1|	314061-314314	18	17	15	20	0.432657	0.706	no
ni|645903738|nb|KK920692.1|	316176-316355	18	17	17	25	0.552832	0.6371	no
ni|645903738|nb|KK920692.1|	318398-318546	18	17	7	13	0.880333	0.4849	no
ni|645903738|nb|KK920692.1|	345794-346018	18	17	0.403335	45	7	0.0443	no
ni|645903738|nb|KK920692.1|	347355-347863	18	17	1	41	5	0.0015	yes
ni|645903738|nb|KK920692.1|	353656-354033	18	17	2	42	4	0.00255	yes
ni|645903738|nb|KK920692.1|	453545-454033	18	17	75	83	0.134365	0.92815	no
ni|645903738|nb|KK920692.1|	454851-455601	18	17	92	86	-0.0936419	0.94705	no
ni|645903738|nb|KK920692.1|	455690-455899	18	17	394	352	-0.16321	0.90925	no
ni|645903738|nb|KK920692.1|	456270-456822	18	17	2	1	-0.421168	0.7241	no
ni|645903738|nb|KK920692.1|	456971-457938	18	17	26	24	-0.0935233	0.95205	no
ni|645903738|nb|KK920692.1|	458143-458432	18	17	188.2	156	-0.271841	0.85005	no
ni|645903738|nb|KK920692.1|	458576-459128	18	17	315	227	-0.47064	0.736	no
ni|645903738|nb|KK920692.1|	473661-474033	18	17	7	7	-0.0472956	0.96835	no
ni|645903738|nb|KK920692.1|	474843-476217	18	17	12	10	-0.271427	0.80265	no
ni|645903738|nb|KK920692.1|	477011-477179	18	17	16	11	-0.526681	0.6575	no
ni|645903738|nb|KK920692.1|	477409-477543	18	17	19	10	-0.860813	0.49335	no
ni|645903738|nb|KK920692.1|	489949-490294	18	17	0	4	inf	5.00E-05	yes
ni|645903738|nb|KK920692.1|	50004-50292	18	17	1	7	2	0.0995	no
ni|645903738|nb|KK920692.1|	502528-503682	18	17	0.492121	0.605053	0.29805	0.80095	no
ni|645903738|nb|KK920692.1|	503818-504142	18	17	0.999483	1	0.464859	0.7264	no
ni|645903738|nb|KK920692.1|	505300-505806	18	17	3	3	-0.0651053	0.95515	no
ni|645903738|nb|KK920692.1|	508319-508593	18	17	32	29	-0.144677	0.8977	no
ni|645903738|nb|KK920692.1|	508939-509227	18	17	24	26	0.0845541	0.9404	no
ni|645903738|nb|KK920692.1|	509982-510181	18	17	22	28	0.348035	0.76425	no

ni|645903738|nb|KK920692.1|	513644-513789	18	17	20	20	0.0107964	0.98435	no
ni|645903738|nb|KK920692.1|	516054-516206	18	17	22	27.89	0.339984	0.77155	no
ni|645903738|nb|KK920692.1|	516863-517095	18	17	24	35	0.582515	0.6168	no
ni|645903738|nb|KK920692.1|	517955-518098	18	17	27	48	0.868659	0.4618	no
ni|645903738|nb|KK920692.1|	520398-520920	18	17	13	22	0.742279	0.51355	no
ni|645903738|nb|KK920692.1|	521316-521509	18	17	25	38	0.583539	0.61665	no
ni|645903738|nb|KK920692.1|	522619-522935	18	17	8	11	0.562334	0.63235	no
ni|645903738|nb|KK920692.1|	523655-523863	18	17	19	27	0.51815	0.6562	no
ni|645903738|nb|KK920692.1|	525266-525509	18	17	21	33	0.625055	0.5855	no
ni|645903738|nb|KK920692.1|	528797-529217	18	17	0.697991	1	0.76478	0.5642	no
ni|645903738|nb|KK920692.1|	529408-529557	18	17	19	41	1	0.35825	no
ni|645903738|nb|KK920692.1|	529707-529903	18	17	10	20	0.914787	0.4399	no
ni|645903738|nb|KK920692.1|	531196-531465	18	17	3	6	0.900829	0.46055	no
ni|645903738|nb|KK920692.1|	531579-532061	18	17	10	22	1	0.32255	no
ni|645903738|nb|KK920692.1|	533398-533820	18	17	6	14	1	0.31295	no
ni|645903738|nb|KK920692.1|	534109-534462	18	17	6	12	1	0.3811	no
ni|645903738|nb|KK920692.1|	55871-56139	18	17	0.890496	8	3	0.05925	no
ni|645903738|nb|KK920692.1|	57066-57315	18	17	0.167525	8	6	0.1198	no
ni|645903738|nb|KK920692.1|	586048-586359	18	17	49	39	-0.314953	0.77645	no
ni|645903738|nb|KK920692.1|	587248-587719	18	17	1	1	-0.235101	0.85345	no
ni|645903738|nb|KK920692.1|	588153-588585	18	17	0.896826	0.42858	-1	0.46615	no
ni|645903738|nb|KK920692.1|	589080-589403	18	17	2	2	0.156731	0.8958	no
ni|645903738|nb|KK920692.1|	589766-590024	18	17	116.37	102.26	-0.18648	0.88405	no
ni|645903738|nb|KK920692.1|	590672-591156	18	17	49	47	-0.063247	0.966	no
ni|645903738|nb|KK920692.1|	593020-593432	18	17	1	0.608269	-1	0.43485	no
ni|645903738|nb|KK920692.1|	594917-595368	18	17	8	6	-0.363746	0.75685	no
ni|645903738|nb|KK920692.1|	596820-597213	18	17	8	11	0.494057	0.6677	no
ni|645903738|nb|KK920692.1|	59792-59938	18	17	3	6	1	0.43015	no
ni|645903738|nb|KK920692.1|	598193-598770	18	17	0.934563	0.397068	-1	0.36935	no
ni|645903738|nb|KK920692.1|	599244-599642	18	17	8	6	-0.361487	0.75665	no

ni|645903738|nb|KK920692.1|	600115-600263	18	17	25	21	-0.289592	0.80925	no
ni|645903738|nb|KK920692.1|	600437-600615	18	17	20	16	-0.32108	0.78215	no
ni|645903738|nb|KK920692.1|	604531-604642	18	17	29	24	-0.290006	0.82015	no
ni|645903738|nb|KK920692.1|	60527-60923	18	17	0.503292	2	2	0.14305	no
ni|645903738|nb|KK920692.1|	605769-606021	18	17	13	11	-0.261415	0.8236	no
ni|645903738|nb|KK920692.1|	613030-613155	18	17	19	20	0.0701597	0.9588	no
ni|645903738|nb|KK920692.1|	615026-615259	18	17	26	18	-0.481433	0.67865	no
ni|645903738|nb|KK920692.1|	62634-62856	18	17	0.410015	2	2	0.14415	no
ni|645903738|nb|KK920692.1|	65145-65366	18	17	7	4	-0.914581	0.44765	no
ni|645903738|nb|KK920692.1|	65808-66254	18	17	4	2	-0.982771	0.416	no
ni|645903738|nb|KK920692.1|	66978-67129	18	17	9	4	-1	0.3487	no
ni|645903738|nb|KK920692.1|	67399-67621	18	17	5	4	-0.278071	0.82035	no
ni|645903738|nb|KK920692.1|	68576-68823	18	17	2	0.486624	-2	0.2989	no
ni|645903738|nb|KK920692.1|	68961-69260	18	17	5	3	-0.690284	0.5654	no
ni|645903738|nb|KK920692.1|	72548-72728	18	17	4	3	-0.330344	0.80325	no
ni|645903738|nb|KK920692.1|	73104-73389	18	17	4	8	0.772365	0.5122	no
ni|645903738|nb|KK920692.1|	74756-74921	18	17	5	1	-2	0.22995	no
ni|645903738|nb|KK920692.1|	78292-78436	18	17	5	4	-0.237133	0.8697	no
ni|645903738|nb|KK920692.1|	82772-83036	18	17	4	4	-0.00946302	0.97805	no
ni|645903738|nb|KK920692.1|	86432-86589	18	17	4	3	-0.429769	0.75965	no
ni|645903738|nb|KK920692.1|	86643-86850	18	17	2	1	-0.803798	0.6027	no
ni|645903738|nb|KK920692.1|	890394-890580	18	17	5	5	0.010761	0.9704	no
ni|645903738|nb|KK920692.1|	909455-909789	18	17	0.743919	5	3	0.07145	no
ni|645903738|nb|KK920692.1|	911120-912293	18	17	241	165	-0.54774	0.70585	no
ni|645903738|nb|KK920692.1|	912646-913080	18	17	3	2	-0.802226	0.5039	no
ni|645903738|nb|KK920692.1|	913888-914197	18	17	1048.19	832	-0.333695	0.8159	no
ni|645903738|nb|KK920692.1|	914604-914994	18	17	153	112.85	-0.436873	0.7572	no
ni|645903738|nb|KK920692.1|	915686-916025	18	17	1	0.695882	-0.958667	0.51225	no
ni|645903738|nb|KK920692.1|	916910-917344	18	17	1	0.497	-1	0.3768	no
ni|645903738|nb|KK920692.1|	918563-919199	18	17	0.461808	0.485645	0.0726084	0.94355	no

ni|645903738|nb|KK920692.1|	919427-919975	18	17	0.442381	46	7	0.01475	no
ni|645903738|nb|KK920692.1|	926327-926462	18	17	43.67	41	-0.0907161	0.938	no
ni|645903738|nb|KK920692.1|	926820-926936	18	17	34	18	-0.890595	0.4822	no
ni|645903738|nb|KK920692.1|	927410-927519	18	17	75	74	-0.0204023	0.9812	no
ni|645903738|nb|KK920692.1|	927572-927679	18	17	59	107	0.854155	0.48475	no
ni|645903738|nb|KK920692.1|	928093-928338	18	17	45	40	-0.146406	0.8976	no
ni|645903738|nb|KK920692.1|	928596-929123	18	17	38	32	-0.270671	0.79475	no
ni|645903738|nb|KK920692.1|	929273-929450	18	17	52	53	0.01961	0.9865	no
ni|645903738|nb|KK920692.1|	929609-929887	18	17	20	7	-1	0.2134	no
ni|645903738|nb|KK920692.1|	930365-930845	18	17	25	21	-0.24873	0.81975	no
ni|645903738|nb|KK920692.1|	933161-933394	18	17	57	49	-0.206768	0.8537	no
ni|645903738|nb|KK920692.1|	994864-995239	18	17	0.541978	0.690595	0.349606	0.81755	no
ni|645903738|nb|KK920692.1|	998723-999057	18	17	0.743919	0.609298	-0.287999	0.8535	no
ni|645903739|nb|KK920691.1|	1009401-1010331	17	16	2	2	-0.202129	0.865	no
ni|645903739|nb|KK920691.1|	1010437-1010604	17	16	6	2	-1	0.32095	no
ni|645903739|nb|KK920691.1|	1011019-1011368	17	16	0.998282	0.19079	-2	0.2949	no
ni|645903739|nb|KK920691.1|	1017465-1017778	17	16	2	0.446522	-2	0.22995	no
ni|645903739|nb|KK920691.1|	1017966-1018732	17	16	9	0.852123	-3	0.01055	no
ni|645903739|nb|KK920691.1|	1021012-1022553	17	16	1	0.049147	-5	0.11935	no
ni|645903739|nb|KK920691.1|	1023218-1024408	17	16	2	0.151675	-4	0.031	no
ni|645903739|nb|KK920691.1|	1025621-1026392	17	16	5	0.563836	-3	0.0178	no
ni|645903739|nb|KK920691.1|	12474-13052	17	16	0.828977	0.445763	-0.895056	0.502	no
ni|645903739|nb|KK920691.1|	133833-133951	17	16	90	110	0.288045	0.80855	no
ni|645903739|nb|KK920691.1|	13430-16141	17	16	5	3	-0.582829	0.5902	no
ni|645903739|nb|KK920691.1|	244094-244216	17	16	31	29	-0.067379	0.95525	no
ni|645903739|nb|KK920691.1|	270187-270484	17	16	0	3	inf	0.00055	yes
ni|645903739|nb|KK920691.1|	46644-46913	17	16	15	38	1	0.2308	no
ni|645903739|nb|KK920691.1|	46974-47192	17	16	13	25	0.921872	0.43815	no
ni|645903739|nb|KK920691.1|	475199-475351	17	16	6	5	-0.330226	0.80325	no
ni|645903739|nb|KK920691.1|	500439-500587	17	16	6	9	0.517763	0.68765	no

ni|645903739|nb|KK920691.1|	501233-501502	17	16	2	3	0.518958	0.68765	no
ni|645903739|nb|KK920691.1|	505767-506389	17	16	2	14	3	0.0346	no
ni|645903739|nb|KK920691.1|	513635-513897	17	16	0.769815	3	2	0.1494	no
ni|645903739|nb|KK920691.1|	513999-514125	17	16	4	24	3	0.0767	no
ni|645903739|nb|KK920691.1|	515259-515474	17	16	4	24	3	0.03895	no
ni|645903739|nb|KK920691.1|	516145-516490	17	16	3	13	2	0.1081	no
ni|645903739|nb|KK920691.1|	5177-6588	17	16	2	1	-0.116911	0.9179	no
ni|645903739|nb|KK920691.1|	519384-519743	17	16	5	23	2	0.07445	no
ni|645903739|nb|KK920691.1|	520243-520452	17	16	4	6	0.463723	0.70795	no
ni|645903739|nb|KK920691.1|	520721-520872	17	16	9	7	-0.429764	0.73195	no
ni|645903739|nb|KK920691.1|	521277-521570	17	16	5	9	0.782167	0.50915	no
ni|645903739|nb|KK920691.1|	528615-529163	17	16	7	11	0.722239	0.52655	no
ni|645903739|nb|KK920691.1|	53032-53181	17	16	13	48	2	0.135	no
ni|645903739|nb|KK920691.1|	564363-564441	17	16	5522.92	5589.42	0.0172671	0.94575	no
ni|645903739|nb|KK920691.1|	621963-622404	17	16	0	5	inf	0.00015	yes
ni|645903739|nb|KK920691.1|	64332-65033	17	16	32	23	-0.49166	0.69235	no
ni|645903739|nb|KK920691.1|	652652-652902	17	16	5	2	-2	0.24815	no
ni|645903739|nb|KK920691.1|	695858-695976	17	16	47	34	-0.441831	0.7057	no
ni|645903739|nb|KK920691.1|	7199-7895	17	16	0.664217	0.476276	-0.479858	0.7105	no
ni|645903739|nb|KK920691.1|	725151-725464	17	16	21	21	-0.0109709	0.99155	no
ni|645903739|nb|KK920691.1|	726485-726606	17	16	18	26	0.517571	0.67245	no
ni|645903739|nb|KK920691.1|	727660-727797	17	16	28	22	-0.372099	0.7474	no
ni|645903739|nb|KK920691.1|	727970-728232	17	16	9	10	0.18448	0.87145	no
ni|645903739|nb|KK920691.1|	728815-729036	17	16	27	28	0.0183051	0.9879	no
ni|645903739|nb|KK920691.1|	729858-730016	17	16	33	37	0.173808	0.8864	no
ni|645903739|nb|KK920691.1|	730704-730831	17	16	39	32	-0.284135	0.80805	no
ni|645903739|nb|KK920691.1|	733414-733623	17	16	14	14	-0.0202495	0.9868	no
ni|645903739|nb|KK920691.1|	736355-736654	17	16	2	2	-0.31372	0.8025	no
ni|645903739|nb|KK920691.1|	743420-743558	17	16	23	25	0.0847649	0.94365	no
ni|645903739|nb|KK920691.1|	744296-744451	17	16	37	39	0.0671067	0.9519	no

ni|645903739|nb|KK920691.1|	748654-748763	17	16	88	69	-0.33901	0.77795	no
ni|645903739|nb|KK920691.1|	781030-781199	17	16	15	13	-0.211649	0.8563	no
ni|645903739|nb|KK920691.1|	792606-793039	17	16	31	26	-0.228621	0.83335	no
ni|645903739|nb|KK920691.1|	795558-795913	17	16	21	18	-0.252389	0.82965	no
ni|645903739|nb|KK920691.1|	796855-797134	17	16	46	40	-0.19304	0.86415	no
ni|645903739|nb|KK920691.1|	798385-798617	17	16	51	46	-0.121482	0.91155	no
ni|645903739|nb|KK920691.1|	801371-802457	17	16	4	2	-0.692578	0.5595	no
ni|645903739|nb|KK920691.1|	802821-803305	17	16	1	0.987409	-0.387077	0.7511	no
ni|645903739|nb|KK920691.1|	804596-805140	17	16	0.892773	0.586726	-0.605607	0.6317	no
ni|645903739|nb|KK920691.1|	805626-805838	17	16	42	32	-0.373397	0.74495	no
ni|645903739|nb|KK920691.1|	807206-807380	17	16	49	36	-0.465856	0.68805	no
ni|645903739|nb|KK920691.1|	816760-817470	17	16	0.8517	0.581635	-0.550231	0.66085	no
ni|645903739|nb|KK920691.1|	817665-818191	17	16	0.814347	0.556024	-0.550496	0.68265	no
ni|645903739|nb|KK920691.1|	818448-818642	17	16	67	55	-0.283763	0.8063	no
ni|645903739|nb|KK920691.1|	819839-820525	17	16	57	47	-0.281701	0.8444	no
ni|645903739|nb|KK920691.1|	820970-821117	17	16	27	15	-0.860751	0.4661	no
ni|645903739|nb|KK920691.1|	829370-829482	17	16	21	24	0.173431	0.8916	no
ni|645903739|nb|KK920691.1|	848234-849016	17	16	7	11	0.693904	0.5426	no
ni|645903739|nb|KK920691.1|	851540-851668	17	16	42	19	-1	0.35525	no
ni|645903739|nb|KK920691.1|	851784-852083	17	16	50	30	-0.756843	0.4903	no
ni|645903739|nb|KK920691.1|	852195-852405	17	16	29	17	-0.830072	0.4833	no
ni|645903739|nb|KK920691.1|	852653-852904	17	16	33	25	-0.411498	0.7185	no
ni|645903739|nb|KK920691.1|	853077-853342	17	16	42	31	-0.421975	0.7104	no
ni|645903739|nb|KK920691.1|	853759-854079	17	16	47	63	0.435593	0.6889	no
ni|645903739|nb|KK920691.1|	858147-858457	17	16	1	0.679416	-1	0.46615	no
ni|645903739|nb|KK920691.1|	862600-863160	17	16	5	8	0.648685	0.57445	no
ni|645903739|nb|KK920691.1|	864263-864468	17	16	75	112	0.575142	0.6034	no
ni|645903739|nb|KK920691.1|	866174-866313	17	16	57	102.9	0.850305	0.47195	no
ni|645903739|nb|KK920691.1|	869442-869720	17	16	0.840048	5	3	0.09155	no
ni|645903739|nb|KK920691.1|	870203-870463	17	16	61	117	0.937594	0.4705	no

ni|645903739|nb|KK920691.1|	870592-871125	17	16	28	58	1	0.42185	no
ni|645903739|nb|KK920691.1|	874693-874824	17	16	46	79	0.775172	0.50885	no
ni|645903739|nb|KK920691.1|	875616-876255	17	16	1	0.834155	-0.276395	0.8205	no
ni|645903739|nb|KK920691.1|	876502-877000	17	16	8	11	0.531716	0.6416	no
ni|645903739|nb|KK920691.1|	878020-878241	17	16	12	17	0.510205	0.65995	no
ni|645903739|nb|KK920691.1|	878371-878742	17	16	2	2	-0.21745	0.8608	no
ni|645903739|nb|KK920691.1|	878958-879668	17	16	5	7	0.392544	0.7345	no
ni|645903739|nb|KK920691.1|	882846-882959	17	16	25	57	1	0.3544	no
ni|645903739|nb|KK920691.1|	883327-883601	17	16	29	42	0.511943	0.6514	no
ni|645903739|nb|KK920691.1|	884214-884640	17	16	7	10	0.562752	0.622	no
ni|645903739|nb|KK920691.1|	884798-885187	17	16	5	9	1	0.38615	no
ni|645903739|nb|KK920691.1|	886869-887094	17	16	15	27	0.806246	0.49475	no
ni|645903739|nb|KK920691.1|	940432-940609	17	16	2	3	0.66964	0.65595	no
ni|645903739|nb|KK920691.1|	945289-945509	17	16	0.416918	4	3	0.0541	no
ni|645903739|nb|KK920691.1|	955630-956119	17	16	0.382378	2	2	0.13595	no
ni|645903739|nb|KK920691.1|	962418-962995	17	16	2	101.6	5	0.0019	yes
ni|645903739|nb|KK920691.1|	969362-969481	17	16	3	48	4	0.0464	no
ni|645903739|nb|KK920691.1|	971112-972540	17	16	5	96	4	0.00455	no
ni|645903739|nb|KK920691.1|	972764-972877	17	16	0	82	inf	5.00E-05	yes
ni|645903739|nb|KK920691.1|	973839-974151	17	16	0.939104	1	0.256209	0.86075	no
ni|645903739|nb|KK920691.1|	974438-974897	17	16	11	9	-0.182243	0.87465	no
ni|645903739|nb|KK920691.1|	975640-975997	17	16	2	2	0.111375	0.9239	no
ni|645903739|nb|KK920691.1|	977738-978009	17	16	22	14	-0.670447	0.56615	no
ni|645903739|nb|KK920691.1|	980991-981396	17	16	103	103	0.00472851	0.99745	no
ni|645903739|nb|KK920691.1|	999796-999949	17	16	5	2	-2	0.3149	no
ni|645903740|nb|KK920690.1|	122473-123809	18	10	0.039954	3	6	0.0443	no
ni|645903740|nb|KK920690.1|	183120-183357	18	10	0.364694	5	4	0.04745	no
ni|645903740|nb|KK920690.1|	221038-221273	18	10	0.370151	3	3	0.0656	no
ni|645903740|nb|KK920690.1|	221953-222266	18	10	0.467331	2	2	0.15655	no
ni|645903740|nb|KK920690.1|	249993-250328	18	10	15	12	-0.336904	0.7735	no

ni|645903740|nb|KK920690.1|	251167-252403	18	10	0.58696	0.478144	-0.295817	0.8023	no
ni|645903740|nb|KK920690.1|	253064-253431	18	10	1	0.978188	-0.606031	0.6316	no
ni|645903740|nb|KK920690.1|	253681-254401	18	10	0.997512	0.839259	-0.249218	0.84025	no
ni|645903740|nb|KK920690.1|	255209-255590	18	10	2	1	-0.152873	0.90135	no
ni|645903740|nb|KK920690.1|	256028-256803	18	10	0.806037	0.945818	0.230716	0.8483	no
ni|645903740|nb|KK920690.1|	258325-258533	18	10	2	2	0.103113	0.93335	no
ni|645903740|nb|KK920690.1|	259757-260597	18	10	1	1	0.414482	0.73085	no
ni|645903740|nb|KK920690.1|	269113-270794	18	10	1	1	-0.366418	0.75525	no
ni|645903740|nb|KK920690.1|	271385-271862	18	10	0.526152	0.377187	-0.480201	0.7594	no
ni|645903740|nb|KK920690.1|	273329-275198	18	10	10	8	-0.235804	0.82705	no
ni|645903740|nb|KK920690.1|	275345-275640	18	10	2	2	-0.148279	0.91085	no
ni|645903740|nb|KK920690.1|	275694-275806	18	10	17	6	-2	0.29845	no
ni|645903740|nb|KK920690.1|	275978-276231	18	10	11	7	-0.596664	0.6153	no
ni|645903740|nb|KK920690.1|	289837-290613	18	10	1	0.944414	-0.264047	0.81915	no
ni|645903740|nb|KK920690.1|	291310-291578	18	10	1	0.70889	-0.744085	0.59615	no
ni|645903740|nb|KK920690.1|	292544-293973	18	10	66	51	-0.382715	0.78605	no
ni|645903740|nb|KK920690.1|	294110-294234	18	10	25	9	-2	0.2598	no
ni|645903740|nb|KK920690.1|	294512-294794	18	10	15	12	-0.28381	0.80855	no
ni|645903740|nb|KK920690.1|	295243-295522	18	10	131	80	-0.703187	0.57335	no
ni|645903740|nb|KK920690.1|	302564-302968	18	10	9	9	-0.0398029	0.97135	no
ni|645903740|nb|KK920690.1|	303327-304656	18	10	18	13	-0.448857	0.7245	no
ni|645903740|nb|KK920690.1|	305183-305456	18	10	2	0.412947	-2	0.20695	no
ni|645903740|nb|KK920690.1|	306173-306535	18	10	36	29	-0.301295	0.786	no
ni|645903740|nb|KK920690.1|	312481-312765	18	10	15	10	-0.494225	0.6648	no
ni|645903740|nb|KK920690.1|	316419-316549	18	10	45	32	-0.491103	0.67885	no
ni|645903740|nb|KK920690.1|	318008-318131	18	10	34	29	-0.209387	0.8594	no
ni|645903740|nb|KK920690.1|	318484-318612	18	10	8	3	-2	0.29845	no
ni|645903740|nb|KK920690.1|	320337-321305	18	10	0.996433	0.353896	-1	0.2415	no
ni|645903740|nb|KK920690.1|	321874-322288	18	10	0.790454	0.679921	-0.217314	0.87705	no
ni|645903740|nb|KK920690.1|	32442-33248	18	10	0.455311	0.401852	-0.18019	0.89165	no

ni|645903740|nb|KK920690.1|	326151-326361	18	10	11	5	-1	0.33675	no
ni|645903740|nb|KK920690.1|	326473-326638	18	10	3	3	-0.0672272	0.9414	no
ni|645903740|nb|KK920690.1|	327042-327370	18	10	0.87273	0.938154	0.104289	0.93335	no
ni|645903740|nb|KK920690.1|	327463-327656	18	10	3	1	-2	0.3149	no
ni|645903740|nb|KK920690.1|	329404-329605	18	10	31	27	-0.200978	0.8578	no
ni|645903740|nb|KK920690.1|	330826-331591	18	10	2	0.960084	-0.667413	0.5746	no
ni|645903740|nb|KK920690.1|	332919-334417	18	10	2	2	-0.214984	0.8552	no
ni|645903740|nb|KK920690.1|	334595-334839	18	10	2	2	-0.0662472	0.9467	no
ni|645903740|nb|KK920690.1|	335140-335743	18	10	8	6	-0.380444	0.74385	no
ni|645903740|nb|KK920690.1|	33533-34052	18	10	0.473154	0.339213	-0.480119	0.7594	no
ni|645903740|nb|KK920690.1|	340819-340963	18	10	8	6	-0.389137	0.7621	no
ni|645903740|nb|KK920690.1|	341087-341497	18	10	14	12	-0.261387	0.8224	no
ni|645903740|nb|KK920690.1|	345729-346008	18	10	14	15	0.0430068	0.97135	no
ni|645903740|nb|KK920690.1|	349450-349710	18	10	31	31	-0.0236465	0.9841	no
ni|645903740|nb|KK920690.1|	352230-352429	18	10	28	22	-0.356509	0.7594	no
ni|645903740|nb|KK920690.1|	352791-353051	18	10	18	13	-0.502181	0.6614	no
ni|645903740|nb|KK920690.1|	353678-354094	18	10	2	0.975859	-1	0.3948	no
ni|645903740|nb|KK920690.1|	354202-355126	18	10	30	26	-0.207862	0.8883	no
ni|645903740|nb|KK920690.1|	366163-366385	18	10	5	30	3	0.036	no
ni|645903740|nb|KK920690.1|	367608-367787	18	10	12	32	1	0.24735	no
ni|645903740|nb|KK920690.1|	371226-371413	18	10	5	21	2	0.12705	no
ni|645903740|nb|KK920690.1|	373508-373744	18	10	2	12	2	0.0709	no
ni|645903740|nb|KK920690.1|	373860-374047	18	10	8	26	2	0.15755	no
ni|645903740|nb|KK920690.1|	376421-377474	18	10	3	14	2	0.07635	no
ni|645903740|nb|KK920690.1|	377591-377776	18	10	6	29	2	0.08535	no
ni|645903740|nb|KK920690.1|	380112-380240	18	10	4	26	3	0.08815	no
ni|645903740|nb|KK920690.1|	382571-383764	18	10	4	13	2	0.11585	no
ni|645903740|nb|KK920690.1|	383875-384071	18	10	7	24	2	0.15265	no
ni|645903740|nb|KK920690.1|	386395-386813	18	10	4	14	2	0.14765	no
ni|645903740|nb|KK920690.1|	38703-38901	18	10	10	7	-0.566593	0.63775	no

ni|645903740|nb|KK920690.1|	387952-388334	18	10	7	19	1	0.2118	no
ni|645903740|nb|KK920690.1|	388435-388646	18	10	7	37	2	0.05585	no
ni|645903740|nb|KK920690.1|	389978-390712	18	10	3	8	2	0.20125	no
ni|645903740|nb|KK920690.1|	39119-39630	18	10	2	0.288204	-2	0.10365	no
ni|645903740|nb|KK920690.1|	393893-394382	18	10	7	20	2	0.2019	no
ni|645903740|nb|KK920690.1|	397564-397759	18	10	9	33	2	0.1142	no
ni|645903740|nb|KK920690.1|	398951-399831	18	10	9	22	1	0.2242	no
ni|645903740|nb|KK920690.1|	401794-401981	18	10	12	36	2	0.2007	no
ni|645903740|nb|KK920690.1|	402369-402595	18	10	16.47	30	0.880386	0.45475	no
ni|645903740|nb|KK920690.1|	404748-405148	18	10	9	15	0.679394	0.5582	no
ni|645903740|nb|KK920690.1|	407491-407646	18	10	14	29	1	0.3776	no
ni|645903740|nb|KK920690.1|	407849-408169	18	10	2	2	0.126969	0.9211	no
ni|645903740|nb|KK920690.1|	408443-409152	18	10	0.60936	1	1	0.40485	no
ni|645903740|nb|KK920690.1|	410605-410852	18	10	19	37	0.978575	0.3971	no
ni|645903740|nb|KK920690.1|	410978-411386	18	10	0.564161	1	0.934673	0.5103	no
ni|645903740|nb|KK920690.1|	411496-411920	18	10	0.765916	1	0.519678	0.6956	no
ni|645903740|nb|KK920690.1|	412192-412391	18	10	14	25	0.798068	0.4945	no
ni|645903740|nb|KK920690.1|	412749-412971	18	10	12	10	-0.252123	0.82565	no
ni|645903740|nb|KK920690.1|	413790-414083	18	10	3	6	0.788319	0.51075	no
ni|645903740|nb|KK920690.1|	414526-414729	18	10	10	15	0.551211	0.63975	no
ni|645903740|nb|KK920690.1|	415305-415498	18	10	6	6	0.117298	0.9234	no
ni|645903740|nb|KK920690.1|	42071-42375	18	10	3	1	-1	0.31025	no
ni|645903740|nb|KK920690.1|	421405-421687	18	10	34	25	-0.420189	0.7083	no
ni|645903740|nb|KK920690.1|	421904-422048	18	10	20	16	-0.33851	0.77845	no
ni|645903740|nb|KK920690.1|	423745-424435	18	10	0.712936	0.160379	-2	0.1501	no
ni|645903740|nb|KK920690.1|	427010-427251	18	10	35	28	-0.329319	0.7696	no
ni|645903740|nb|KK920690.1|	427661-428035	18	10	0.725291	0.606489	-0.25808	0.8692	no
ni|645903740|nb|KK920690.1|	428628-429503	18	10	1	0.457473	-1	0.24245	no
ni|645903740|nb|KK920690.1|	42875-43635	18	10	0.524631	0.179145	-2	0.29735	no
ni|645903740|nb|KK920690.1|	432803-433639	18	10	1	1	-0.350091	0.76095	no

ni|645903740|nb|KK920690.1|	437094-437399	18	10	11	9	-0.341392	0.7679	no
ni|645903740|nb|KK920690.1|	438502-438784	18	10	10	10	-0.0467963	0.9683	no
ni|645903740|nb|KK920690.1|	439458-439582	18	10	15	11	-0.514814	0.68825	no
ni|645903740|nb|KK920690.1|	441808-442357	18	10	1	2	0.455802	0.7031	no
ni|645903740|nb|KK920690.1|	44240-47129	18	10	1	0.490594	-1	0.36195	no
ni|645903740|nb|KK920690.1|	442794-443417	18	10	0.662673	1	0.712694	0.57115	no
ni|645903740|nb|KK920690.1|	444898-445616	18	10	2	1	-0.394944	0.74335	no
ni|645903740|nb|KK920690.1|	446068-446445	18	10	0.627713	1	0.712183	0.61075	no
ni|645903740|nb|KK920690.1|	448063-449050	18	10	2	2	0.185385	0.87225	no
ni|645903740|nb|KK920690.1|	48119-51012	18	10	0.812472	0.278731	-2	0.1993	no
ni|645903740|nb|KK920690.1|	495145-495273	18	10	19	15	-0.35682	0.77485	no
ni|645903740|nb|KK920690.1|	500960-501732	18	10	0.772845	0.562994	-0.45706	0.7095	no
ni|645903740|nb|KK920690.1|	502130-502476	18	10	2	0.386262	-2	0.1417	no
ni|645903740|nb|KK920690.1|	503165-503532	18	10	2	1	-0.872817	0.49155	no
ni|645903740|nb|KK920690.1|	504715-504828	18	10	34	19	-0.856043	0.5047	no
ni|645903740|nb|KK920690.1|	517391-517542	18	10	6	7	0.155199	0.90275	no
ni|645903740|nb|KK920690.1|	51806-53042	18	10	3	1	-1	0.28065	no
ni|645903740|nb|KK920690.1|	520842-520976	18	10	6	4	-0.482301	0.7594	no
ni|645903740|nb|KK920690.1|	521159-521287	18	10	7	14	1	0.4359	no
ni|645903740|nb|KK920690.1|	522139-522406	18	10	3	2	-0.43799	0.7272	no
ni|645903740|nb|KK920690.1|	523510-523700	18	10	8	6	-0.415105	0.7274	no
ni|645903740|nb|KK920690.1|	526655-526790	18	10	6	5	-0.0672571	0.93835	no
ni|645903740|nb|KK920690.1|	533459-533662	18	10	5	3	-0.65188	0.60395	no
ni|645903740|nb|KK920690.1|	534434-534617	18	10	7	7	-0.067283	0.9542	no
ni|645903740|nb|KK920690.1|	537798-537947	18	10	9	7	-0.429768	0.73195	no
ni|645903740|nb|KK920690.1|	538427-538568	18	10	11	7	-0.730187	0.5717	no
ni|645903740|nb|KK920690.1|	539976-540117	18	10	7	8	0.280701	0.82625	no
ni|645903740|nb|KK920690.1|	543273-544583	18	10	0.387736	0.33176	-0.224934	0.85555	no
ni|645903740|nb|KK920690.1|	544717-545056	18	10	1	0.795294	-0.525014	0.7071	no
ni|645903740|nb|KK920690.1|	545382-546238	18	10	0.915609	0.625336	-0.550101	0.6501	no

ni|645903740|nb|KK920690.1|	546724-547764	18	10	0.683369	0.427262	-0.677544	0.58655	no
ni|645903740|nb|KK920690.1|	551334-551849	18	10	0.83604	0.684994	-0.287481	0.8283	no
ni|645903740|nb|KK920690.1|	55414-55686	18	10	5	2	-1	0.3008	no
ni|645903740|nb|KK920690.1|	556277-556475	18	10	12	6	-0.915019	0.45365	no
ni|645903740|nb|KK920690.1|	557605-557846	18	10	2	2	0.174723	0.8916	no
ni|645903740|nb|KK920690.1|	558073-558353	18	10	1	1	-0.217923	0.87705	no
ni|645903740|nb|KK920690.1|	558548-558686	18	10	14	11	-0.356745	0.77485	no
ni|645903740|nb|KK920690.1|	560566-560986	18	10	6	4	-0.332399	0.77745	no
ni|645903740|nb|KK920690.1|	564330-564512	18	10	16	12	-0.382165	0.748	no
ni|645903740|nb|KK920690.1|	564635-564814	18	10	9	10	0.265256	0.82305	no
ni|645903740|nb|KK920690.1|	569930-570351	18	10	2	2	0.290852	0.80205	no
ni|645903740|nb|KK920690.1|	571216-571762	18	10	1	1	0.230421	0.8483	no
ni|645903740|nb|KK920690.1|	578089-578298	18	10	2	2	-0.259437	0.8692	no
ni|645903741|nb|KK920689.1|	10277-10445	17	18	0	24	inf	5.00E-05	yes
ni|645903741|nb|KK920689.1|	103187-103336	17	18	2	9	3	0.08575	no
ni|645903741|nb|KK920689.1|	11294-12076	17	18	0	13	inf	5.00E-05	yes
ni|645903741|nb|KK920689.1|	114730-114953	17	18	0.609971	11	4	0.05235	no
ni|645903741|nb|KK920689.1|	145469-145663	17	18	0.800018	12	4	0.05295	no
ni|645903741|nb|KK920689.1|	149057-149246	17	18	0.281733	18	6	0.11935	no
ni|645903741|nb|KK920689.1|	153683-153827	17	18	0.563923	12	4	0.12175	no
ni|645903741|nb|KK920689.1|	156468-156646	17	18	0.321446	11	5	0.12005	no
ni|645903741|nb|KK920689.1|	159319-159516	17	18	0.258372	9	5	0.12005	no
ni|645903741|nb|KK920689.1|	165283-165504	17	18	0.620157	10	4	0.05235	no
ni|645903741|nb|KK920689.1|	171539-171703	17	18	1	10	3	0.06315	no
ni|645903741|nb|KK920689.1|	177190-177372	17	18	0.305822	10	5	0.12005	no
ni|645903741|nb|KK920689.1|	180854-181012	17	18	0	12	inf	5.00E-05	yes
ni|645903741|nb|KK920689.1|	184460-184881	17	18	0.154623	2	4	0.04785	no
ni|645903741|nb|KK920689.1|	188640-189039	17	18	0.249106	1	3	0.098	no
ni|645903741|nb|KK920689.1|	190625-190859	17	18	0.372941	13	5	0.0451	no
ni|645903741|nb|KK920689.1|	190930-191127	17	18	0.258372	9	5	0.1198	no

ni|645903741|nb|KK920689.1|	196746-197077	17	18	0	3	inf	0.00025	yes
ni|645903741|nb|KK920689.1|	200148-200398	17	18	0.499193	14	5	0.0511	no
ni|645903741|nb|KK920689.1|	204101-204293	17	18	0.545008	14	5	0.045	no
ni|645903741|nb|KK920689.1|	207670-208069	17	18	3	8	1	0.30185	no
ni|645903741|nb|KK920689.1|	209005-209308	17	18	3	1	-1	0.3554	no
ni|645903741|nb|KK920689.1|	212652-212896	17	18	0.173398	8	5	0.11995	no
ni|645903741|nb|KK920689.1|	215326-215609	17	18	0.680754	5	3	0.06975	no
ni|645903741|nb|KK920689.1|	22087-22248	17	18	2	14	3	0.0533	no
ni|645903741|nb|KK920689.1|	221708-221921	17	18	0.664508	10	4	0.05325	no
ni|645903741|nb|KK920689.1|	224363-224812	17	18	0.284301	6	4	0.0379	no
ni|645903741|nb|KK920689.1|	227627-228077	17	18	0.212614	5	5	0.052	no
ni|645903741|nb|KK920689.1|	229091-229313	17	18	58	89	0.615323	0.5794	no
ni|645903741|nb|KK920689.1|	230126-230252	17	18	60	89	0.562022	0.63335	no
ni|645903741|nb|KK920689.1|	233026-233229	17	18	91	140	0.613557	0.57215	no
ni|645903741|nb|KK920689.1|	233570-233719	17	18	77	64	-0.261208	0.81525	no
ni|645903741|nb|KK920689.1|	239631-239747	17	18	16	21	0.379986	0.7694	no
ni|645903741|nb|KK920689.1|	240169-240294	17	18	11	7	-0.719421	0.61925	no
ni|645903741|nb|KK920689.1|	241246-241798	17	18	5	5	0.00676623	0.99385	no
ni|645903741|nb|KK920689.1|	241900-242508	17	18	4	4	-0.0137096	0.9906	no
ni|645903741|nb|KK920689.1|	242598-243110	17	18	3	2	-0.282686	0.81105	no
ni|645903741|nb|KK920689.1|	245269-245471	17	18	9	11	0.254989	0.8315	no
ni|645903741|nb|KK920689.1|	246919-247099	17	18	2	1	-0.330344	0.8122	no
ni|645903741|nb|KK920689.1|	247572-247834	17	18	9	10	0.0494133	0.96425	no
ni|645903741|nb|KK920689.1|	248258-248464	17	18	9	6	-0.651817	0.5826	no
ni|645903741|nb|KK920689.1|	248952-249145	17	18	7	10	0.387439	0.73955	no
ni|645903741|nb|KK920689.1|	251685-251901	17	18	14	12	-0.159775	0.8888	no
ni|645903741|nb|KK920689.1|	254567-255342	17	18	93	73	-0.341426	0.8083	no
ni|645903741|nb|KK920689.1|	25567-25987	17	18	0	2	inf	0.00015	yes
ni|645903741|nb|KK920689.1|	257761-258104	17	18	4	6	0.375009	0.74465	no
ni|645903741|nb|KK920689.1|	262244-262396	17	18	12	9	-0.445703	0.71245	no

ni|645903741|nb|KK920689.1|	265372-265524	17	18	11	15	0.517771	0.66765	no
ni|645903741|nb|KK920689.1|	269132-269294	17	18	9	13	0.51775	0.66765	no
ni|645903741|nb|KK920689.1|	269371-269755	17	18	4	5	0.173387	0.88245	no
ni|645903741|nb|KK920689.1|	273960-274227	17	18	1	2	0.699514	0.59985	no
ni|645903741|nb|KK920689.1|	277840-278372	17	18	6	8	0.356965	0.75205	no
ni|645903741|nb|KK920689.1|	27909-28217	17	18	0.119663	3	5	0.1211	no
ni|645903741|nb|KK920689.1|	279800-280038	17	18	5	4	-0.400744	0.74285	no
ni|645903741|nb|KK920689.1|	286964-287253	17	18	3	4	0.519106	0.6688	no
ni|645903741|nb|KK920689.1|	288559-290201	17	18	4	22	2	0.07755	no
ni|645903741|nb|KK920689.1|	290677-290994	17	18	0.917304	0.876472	-0.0656923	0.9384	no
ni|645903741|nb|KK920689.1|	292847-293202	17	18	1	2	0.728038	0.5613	no
ni|645903741|nb|KK920689.1|	298721-299110	17	18	1	0.903313	-0.190914	0.88855	no
ni|645903741|nb|KK920689.1|	31676-31858	17	18	1	9	3	0.06365	no
ni|645903741|nb|KK920689.1|	317201-317758	17	18	2	0.880854	-0.784908	0.5276	no
ni|645903741|nb|KK920689.1|	320886-320995	17	18	73	60	-0.281833	0.8164	no
ni|645903741|nb|KK920689.1|	321051-321218	17	18	216	172	-0.328782	0.76315	no
ni|645903741|nb|KK920689.1|	321701-322586	17	18	60	51	-0.246187	0.8654	no
ni|645903741|nb|KK920689.1|	323790-323998	17	18	2	1	-1	0.44755	no
ni|645903741|nb|KK920689.1|	324193-324626	17	18	2	1	-0.437231	0.7272	no
ni|645903741|nb|KK920689.1|	326816-327166	17	18	2	2	0.0949319	0.93935	no
ni|645903741|nb|KK920689.1|	327507-327757	17	18	5	6	0.189077	0.86995	no
ni|645903741|nb|KK920689.1|	328662-330166	17	18	5	44	3	0.0225	no
ni|645903741|nb|KK920689.1|	332210-332404	17	18	2	7	2	0.1729	no
ni|645903741|nb|KK920689.1|	334056-334168	17	18	685	696	0.0232377	0.98095	no
ni|645903741|nb|KK920689.1|	336381-336598	17	18	505	529	0.0680736	0.9591	no
ni|645903741|nb|KK920689.1|	336717-336833	17	18	239	244	0.0278754	0.97995	no
ni|645903741|nb|KK920689.1|	336979-337915	17	18	2	1	-0.848893	0.47155	no
ni|645903741|nb|KK920689.1|	338021-338413	17	18	58	60	0.0538254	0.9579	no
ni|645903741|nb|KK920689.1|	340735-341599	17	18	4	4	-0.101194	0.9296	no
ni|645903741|nb|KK920689.1|	341701-342018	17	18	6	4	-0.515495	0.66215	no

ni|645903741|nb|KK920689.1|	342581-342699	17	18	27	16	-0.688924	0.58685	no
ni|645903741|nb|KK920689.1|	343057-343541	17	18	31	23	-0.436405	0.69285	no
ni|645903741|nb|KK920689.1|	343643-343941	17	18	10	8	-0.400983	0.72455	no
ni|645903741|nb|KK920689.1|	344513-344622	17	18	58	38	-0.59127	0.62565	no
ni|645903741|nb|KK920689.1|	345095-345330	17	18	179	179	-0.00658	0.9962	no
ni|645903741|nb|KK920689.1|	345888-346041	17	18	319.24	358	0.163978	0.8958	no
ni|645903741|nb|KK920689.1|	348083-348470	17	18	171	180.44	0.077679	0.95775	no
ni|645903741|nb|KK920689.1|	349056-349477	17	18	205	248	0.274457	0.83975	no
ni|645903741|nb|KK920689.1|	350765-350934	17	18	327.67	341	0.0566728	0.96695	no
ni|645903741|nb|KK920689.1|	352352-352562	17	18	298	362	0.283984	0.84075	no
ni|645903741|nb|KK920689.1|	355759-355887	17	18	300	364	0.282111	0.7992	no
ni|645903741|nb|KK920689.1|	40916-41275	17	18	0.191896	4	4	0.04535	no
ni|645903741|nb|KK920689.1|	41468-41629	17	18	1	18	4	0.05305	no
ni|645903741|nb|KK920689.1|	42-277	17	18	1	3	2	0.297	no
ni|645903741|nb|KK920689.1|	46270-46549	17	18	0.278439	6	4	0.04515	no
ni|645903741|nb|KK920689.1|	483743-483805	17	18	456970	523438	0.19592	0.8626	no
ni|645903741|nb|KK920689.1|	51251-51712	17	18	0.3435	5	4	0.0305	no
ni|645903741|nb|KK920689.1|	54227-54367	17	18	0.618755	12	4	0.1221	no
ni|645903741|nb|KK920689.1|	545170-545317	17	18	228	311	0.450381	0.6765	no
ni|645903741|nb|KK920689.1|	551675-551794	17	18	1	94	6	0.1195	no
ni|645903741|nb|KK920689.1|	553894-554125	17	18	0.190784	10	6	0.11985	no
ni|645903741|nb|KK920689.1|	556683-556905	17	18	0.205007	143	9	0.11935	no
ni|645903741|nb|KK920689.1|	558708-558868	17	18	0.416317	10	5	0.1211	no
ni|645903741|nb|KK920689.1|	563312-563686	17	18	0.090661	73	10	0.11935	no
ni|645903741|nb|KK920689.1|	564599-564774	17	18	0.334195	7	4	0.12175	no
ni|645903741|nb|KK920689.1|	566357-566480	17	18	0	196	inf	5.00E-05	yes
ni|645903741|nb|KK920689.1|	567190-567428	17	18	0	93	inf	5.00E-05	yes
ni|645903741|nb|KK920689.1|	569413-569602	17	18	0	168	inf	5.00E-05	yes
ni|645903741|nb|KK920689.1|	573242-573543	17	18	0	60	inf	5.00E-05	yes
ni|645903741|nb|KK920689.1|	578564-578752	17	18	8	8	-0.0672156	0.9555	no

ni|645903741|nb|KK920689.1|	581838-582008	17	18	6	8	0.286368	0.81825	no
ni|645903741|nb|KK920689.1|	583173-583403	17	18	9	10	0.15174	0.8992	no
ni|645903741|nb|KK920689.1|	584093-584298	17	18	8	8	-0.0224807	0.98295	no
ni|645903741|nb|KK920689.1|	586794-586978	17	18	9	11	0.311241	0.78895	no
ni|645903741|nb|KK920689.1|	58952-59148	17	18	1	12	4	0.04315	no
ni|645903741|nb|KK920689.1|	590993-591409	17	18	4	5	0.261859	0.8243	no
ni|645903741|nb|KK920689.1|	596511-596677	17	18	5	6	0.347803	0.78955	no
ni|645903741|nb|KK920689.1|	597689-597848	17	18	7	11	0.545774	0.65645	no
ni|645903741|nb|KK920689.1|	599144-599293	17	18	8	8	0.025912	0.9668	no
ni|645903741|nb|KK920689.1|	601453-601693	17	18	5	9	0.670667	0.57235	no
ni|645903741|nb|KK920689.1|	602552-602991	17	18	2	6	1	0.27155	no
ni|645903741|nb|KK920689.1|	604210-604420	17	18	5	7	0.698762	0.5659	no
ni|645903741|nb|KK920689.1|	60439-60878	17	18	0.292735	6	4	0.0383	no
ni|645903741|nb|KK920689.1|	605803-605982	17	18	5	7	0.368779	0.7635	no
ni|645903741|nb|KK920689.1|	606119-606288	17	18	5	7	0.554229	0.6571	no
ni|645903741|nb|KK920689.1|	607010-607205	17	18	5	4	-0.14955	0.91085	no
ni|645903741|nb|KK920689.1|	609157-609300	17	18	5	4	-0.259856	0.8692	no
ni|645903741|nb|KK920689.1|	609568-610034	17	18	13	11	-0.302227	0.7905	no
ni|645903741|nb|KK920689.1|	61141-61274	17	18	1	18	4	0.0498	no
ni|645903741|nb|KK920689.1|	66261-66405	17	18	0	12	inf	0.00065	yes
ni|645903741|nb|KK920689.1|	68917-69069	17	18	2	13	3	0.0746	no
ni|645903741|nb|KK920689.1|	74313-74707	17	18	0.337825	12	5	0.0375	no
ni|645903741|nb|KK920689.1|	77184-77629	17	18	0.143808	5	5	0.0446	no
ni|645903741|nb|KK920689.1|	81074-81321	17	18	0.339652	11	5	0.0447	no
ni|645903741|nb|KK920689.1|	87716-87950	17	18	0	9	inf	0.0001	yes
ni|645903741|nb|KK920689.1|	94125-94295	17	18	1	10	3	0.058	no
ni|645903741|nb|KK920689.1|	97253-97566	17	18	0.233665	3	4	0.0514	no
ni|645903741|nb|KK920689.1|	9876-10157	17	18	0.275336	32	7	0.0443	no
ni|645903742|nb|KK920688.1|	100902-101114	17	16	2	4	0.643758	0.6202	no
ni|645903742|nb|KK920688.1|	102087-102250	17	16	2	20	4	0.041	no

ni|645903742|nb|KK920688.1|	102503-102652	17	16	5	16	2	0.1707	no
ni|645903742|nb|KK920688.1|	103736-103945	17	16	2	16	3	0.04165	no
ni|645903742|nb|KK920688.1|	105551-105734	17	16	2	14	3	0.0709	no
ni|645903742|nb|KK920688.1|	107299-107742	17	16	4	6	0.717169	0.5449	no
ni|645903742|nb|KK920688.1|	107892-108025	17	16	80	43	-0.878001	0.4516	no
ni|645903742|nb|KK920688.1|	108504-108912	17	16	19	12	-0.644266	0.5746	no
ni|645903742|nb|KK920688.1|	109147-109267	17	16	67	45	-0.589358	0.61755	no
ni|645903742|nb|KK920688.1|	111163-111551	17	16	15	11	-0.423199	0.7081	no
ni|645903742|nb|KK920688.1|	111957-112829	17	16	39	29	-0.414441	0.7767	no
ni|645903742|nb|KK920688.1|	112963-113093	17	16	45	27	-0.761192	0.51675	no
ni|645903742|nb|KK920688.1|	113213-113329	17	16	21	16	-0.415396	0.7377	no
ni|645903742|nb|KK920688.1|	113648-114013	17	16	12	8	-0.576723	0.6188	no
ni|645903742|nb|KK920688.1|	114728-114838	17	16	39	9	-2	0.1501	no
ni|645903742|nb|KK920688.1|	115042-115329	17	16	7	4	-0.847229	0.4727	no
ni|645903742|nb|KK920688.1|	115435-115648	17	16	22	18	-0.335534	0.7738	no
ni|645903742|nb|KK920688.1|	115822-115977	17	16	23	22	-0.0671944	0.94945	no
ni|645903742|nb|KK920688.1|	116398-116507	17	16	28	31	0.1733	0.8916	no
ni|645903742|nb|KK920688.1|	116771-116968	17	16	25	19	-0.366604	0.7503	no
ni|645903742|nb|KK920688.1|	117139-117492	17	16	42	31	-0.444031	0.6811	no
ni|645903742|nb|KK920688.1|	117674-118168	17	16	19.63	16	-0.268382	0.81035	no
ni|645903742|nb|KK920688.1|	120833-120999	17	16	28	28	-0.0292664	0.9811	no
ni|645903742|nb|KK920688.1|	121320-121481	17	16	6	2	-1	0.34635	no
ni|645903742|nb|KK920688.1|	123303-123893	17	16	2	0.966226	-1	0.2961	no
ni|645903742|nb|KK920688.1|	124003-124287	17	16	2	1	-0.413814	0.76315	no
ni|645903742|nb|KK920688.1|	125595-125941	17	16	2	1	-0.568051	0.6631	no
ni|645903742|nb|KK920688.1|	126092-126313	17	16	12	10	-0.255618	0.8224	no
ni|645903742|nb|KK920688.1|	128479-129022	17	16	21	19	-0.128816	0.90765	no
ni|645903742|nb|KK920688.1|	129583-130346	17	16	17	15	-0.146096	0.89395	no
ni|645903742|nb|KK920688.1|	130507-130888	17	16	8	7	-0.14694	0.89825	no
ni|645903742|nb|KK920688.1|	16857-17051	17	16	2	4	0.932893	0.4874	no

ni|645903742|nb|KK920688.1|	258232-258441	17	16	148	0	-inf	5.00E-05	yes
ni|645903742|nb|KK920688.1|	271139-271868	17	16	129	0.075201	-11	0.2314	no
ni|645903742|nb|KK920688.1|	295327-296574	17	16	2	2	-0.0776637	0.9482	no
ni|645903742|nb|KK920688.1|	406836-407253	17	16	0.54805	0.972764	0.827782	0.55535	no
ni|645903742|nb|KK920688.1|	419440-419794	17	16	13	7	-0.787982	0.50035	no
ni|645903742|nb|KK920688.1|	420192-420561	17	16	29	25	-0.190566	0.8633	no
ni|645903742|nb|KK920688.1|	423517-423791	17	16	21	14	-0.583364	0.60805	no
ni|645903742|nb|KK920688.1|	423900-424044	17	16	24	20	-0.245546	0.8369	no
ni|645903742|nb|KK920688.1|	426247-426618	17	16	0.183343	2	4	0.0489	no
ni|645903742|nb|KK920688.1|	432578-433015	17	16	0.588964	1	1	0.37765	no
ni|645903742|nb|KK920688.1|	437214-437674	17	16	59	50	-0.24127	0.8694	no
ni|645903742|nb|KK920688.1|	437810-437946	17	16	69	75	0.107428	0.9234	no
ni|645903742|nb|KK920688.1|	438193-438383	17	16	106	120	0.180545	0.8673	no
ni|645903742|nb|KK920688.1|	438487-439136	17	16	19.07	24	0.3476	0.74755	no
ni|645903742|nb|KK920688.1|	441788-442194	17	16	3	2	-0.0653324	0.95355	no
ni|645903742|nb|KK920688.1|	442991-443120	17	16	108	134	0.307735	0.78765	no
ni|645903742|nb|KK920688.1|	498276-498503	17	16	92	118	0.362445	0.7336	no
ni|645903742|nb|KK920688.1|	499133-499927	17	16	32	46	0.518115	0.723	no
ni|645903742|nb|KK920688.1|	500182-500593	17	16	28	33	0.248246	0.8204	no
ni|645903742|nb|KK920688.1|	500764-501017	17	16	168	210	0.321367	0.8257	no
ni|645903742|nb|KK920688.1|	501378-501627	17	16	218	289	0.408096	0.7792	no
ni|645903742|nb|KK920688.1|	502927-503394	17	16	1	0.581271	-0.89526	0.502	no
ni|645903742|nb|KK920688.1|	505126-505315	17	16	178.9	207.33	0.212776	0.86335	no
ni|645903742|nb|KK920688.1|	505440-505540	17	16	283	290	0.0336029	0.95995	no
ni|645903742|nb|KK920688.1|	506624-506801	17	16	3	0.310746	-3	0.04325	no
ni|645903742|nb|KK920688.1|	507818-508269	17	16	88	92	0.0660725	0.9637	no
ni|645903742|nb|KK920688.1|	508704-508846	17	16	149	104	-0.513115	0.6505	no
ni|645903742|nb|KK920688.1|	511384-511643	17	16	4	1	-2	0.21515	no
ni|645903742|nb|KK920688.1|	514632-515042	17	16	29	26	-0.106427	0.92275	no
ni|645903742|nb|KK920688.1|	516181-516346	17	16	38	32	-0.270319	0.8172	no

ni|645903742|nb|KK920688.1|	516608-517027	17	16	11	15	0.371655	0.7454	no
ni|645903742|nb|KK920688.1|	520517-521152	17	16	11	12	0.159272	0.8851	no
ni|645903742|nb|KK920688.1|	522984-524102	17	16	13	14	0.110476	0.91665	no
ni|645903742|nb|KK920688.1|	536199-537907	17	16	3	2	-0.668158	0.56195	no
ni|645903742|nb|KK920688.1|	538142-538427	17	16	1	1	-0.203387	0.87975	no
ni|645903742|nb|KK920688.1|	696584-696843	17	16	0	4	inf	0.0003	yes
ni|645903742|nb|KK920688.1|	70158-70426	17	16	1	2	1	0.41545	no
ni|645903742|nb|KK920688.1|	73435-73622	17	16	0.86469	5	2	0.1063	no
ni|645903742|nb|KK920688.1|	778071-778216	17	16	0	181	inf	5.00E-05	yes
ni|645903742|nb|KK920688.1|	779568-779722	17	16	0.461633	19	5	0.11945	no
ni|645903742|nb|KK920688.1|	780214-780374	17	16	1	89	6	0.05055	no
ni|645903742|nb|KK920688.1|	781138-782306	17	16	0.948184	15	4	0.00255	yes
ni|645903742|nb|KK920688.1|	782466-782910	17	16	0.937484	0.896036	-0.0652373	0.9554	no
ni|645903742|nb|KK920688.1|	783168-783711	17	16	0.055925	20	8	0.11935	no
ni|645903742|nb|KK920688.1|	783853-784056	17	16	0	13	inf	5.00E-05	yes
ni|645903742|nb|KK920688.1|	784868-785017	17	16	0	108	inf	5.00E-05	yes
ni|645903742|nb|KK920688.1|	786935-787056	17	16	0	134	inf	5.00E-05	yes
ni|645903742|nb|KK920688.1|	796580-797626	17	16	8	8	-0.124398	0.91055	no
ni|645903742|nb|KK920688.1|	799267-799551	17	16	2	1	-0.743963	0.56925	no
ni|645903742|nb|KK920688.1|	800167-800410	17	16	2	1	-0.580832	0.70815	no
ni|645903742|nb|KK920688.1|	806445-806813	17	16	9	8	-0.24103	0.8355	no
ni|645903742|nb|KK920688.1|	809021-809165	17	16	23	22	-0.102832	0.9324	no
ni|645903742|nb|KK920688.1|	809574-809731	17	16	44	30	-0.575663	0.62555	no
ni|645903742|nb|KK920688.1|	810463-810643	17	16	31	25	-0.339115	0.77695	no
ni|645903742|nb|KK920688.1|	812134-812367	17	16	2	0.897179	-1	0.3141	no
ni|645903742|nb|KK920688.1|	812705-812903	17	16	43	30	-0.505144	0.6656	no
ni|645903742|nb|KK920688.1|	813381-813550	17	16	13	5	-1	0.30285	no
ni|645903742|nb|KK920688.1|	814158-814437	17	16	7	3	-1	0.3162	no
ni|645903742|nb|KK920688.1|	815065-815400	17	16	14	10	-0.46212	0.68815	no
ni|645903742|nb|KK920688.1|	815716-815840	17	16	32	12	-1	0.25325	no

ni|645903742|nb|KK920688.1|	82175-82366	17	16	2	3	0.710442	0.61225	no
ni|645903742|nb|KK920688.1|	97143-97290	17	16	19	13	-0.496045	0.6783	no
ni|645903742|nb|KK920688.1|	97630-97804	17	16	55	33	-0.748946	0.52035	no
ni|645903742|nb|KK920688.1|	98273-99528	17	16	37	138	2	0.197	no
ni|645903743|nb|KK920687.1|	1000388-1000721	19	18	7	6	-0.0421526	0.96855	no
ni|645903743|nb|KK920687.1|	1008208-100850[illegible]	19	18	0.766232	2	2	0.2459	no
ni|645903743|nb|KK920687.1|	1014526-101477[illegible]	19	18	1	5	2	0.11395	no
ni|645903743|nb|KK920687.1|	1021213-102149[illegible]	19	18	0.136151	4	5	0.1211	no
ni|645903743|nb|KK920687.1|	1023121-102367[illegible]	19	18	3	9	2	0.12405	no
ni|645903743|nb|KK920687.1|	1029786-103048[illegible]	19	18	0.287208	0.274591	-0.0648124	0.9302	no
ni|645903743|nb|KK920687.1|	1033388-103352[illegible]	19	18	2	12	2	0.10795	no
ni|645903743|nb|KK920687.1|	1033957-1034281	19	18	2	5	2	0.227	no
ni|645903743|nb|KK920687.1|	1034408-1034531	19	18	7	13	0.825717	0.5554	no
ni|645903743|nb|KK920687.1|	1040850-104139[illegible]	19	18	0.503324	0.855354	0.765035	0.5642	no
ni|645903743|nb|KK920687.1|	1042430-1042661	19	18	13	12	-0.195708	0.86815	no
ni|645903743|nb|KK920687.1|	1042893-104300[illegible]	19	18	140	120	-0.2213	0.8503	no
ni|645903743|nb|KK920687.1|	1043270-104350[illegible]	19	18	453	347	-0.384627	0.79195	no
ni|645903743|nb|KK920687.1|	1043734-104403[illegible]	19	18	119	90	-0.400771	0.78805	no
ni|645903743|nb|KK920687.1|	1044375-104476[illegible]	19	18	132	106	-0.311444	0.83375	no
ni|645903743|nb|KK920687.1|	1044963-104512[illegible]	19	18	329	279	-0.236924	0.84605	no
ni|645903743|nb|KK920687.1|	1045385-104561[illegible]	19	18	406	979	1	0.373	no
ni|645903743|nb|KK920687.1|	1047000-104755[illegible]	19	18	0.927577	61	6	0.00195	yes
ni|645903743|nb|KK920687.1|	1048754-104905[illegible]	19	18	2	2	0.00460238	0.9708	no
ni|645903743|nb|KK920687.1|	1049421-104962[illegible]	19	18	400	608	0.603134	0.6695	no
ni|645903743|nb|KK920687.1|	1050142-105052[illegible]	19	18	106	170	0.680421	0.64035	no
ni|645903743|nb|KK920687.1|	1052723-105304[illegible]	19	18	123	212	0.784801	0.5901	no
ni|645903743|nb|KK920687.1|	105281-106020	19	18	0.464517	22	6	0.0045	no
ni|645903743|nb|KK920687.1|	1055140-1055561	19	18	0.309246	7	4	0.0379	no
ni|645903743|nb|KK920687.1|	1055670-1056351	19	18	0.212948	5	5	0.02795	no
ni|645903743|nb|KK920687.1|	1057408-105775[illegible]	19	18	0.792218	0.66241	-0.258174	0.8692	no

ni|645903743|nb|KK920687.1|	1061431-1061674	19	18	0.349246	3	3	0.05605	no
ni|645903743|nb|KK920687.1|	107126-107322	19	18	4	39	3	0.0172	no
ni|645903743|nb|KK920687.1|	1077129-1077580	19	18	36	28	-0.355043	0.7376	no
ni|645903743|nb|KK920687.1|	1093013-1093164	19	18	39	84	1	0.3487	no
ni|645903743|nb|KK920687.1|	1093736-1094382	19	18	0.407983	5	4	0.02365	no
ni|645903743|nb|KK920687.1|	109428-109961	19	18	2	9	2	0.07415	no
ni|645903743|nb|KK920687.1|	1094565-1095704	19	18	0.237734	6	5	0.01015	no
ni|645903743|nb|KK920687.1|	1095907-1096302	19	18	0.92585	2	1	0.27955	no
ni|645903743|nb|KK920687.1|	1098015-1098211	19	18	72	44	-0.707984	0.5352	no
ni|645903743|nb|KK920687.1|	1098837-1099295	19	18	52	36	-0.527507	0.66545	no
ni|645903743|nb|KK920687.1|	1100649-1101299	19	18	2	0.81741	-1	0.38145	no
ni|645903743|nb|KK920687.1|	1101768-1102374	19	18	1	0.888775	-0.460867	0.6964	no
ni|645903743|nb|KK920687.1|	1102480-1103994	19	18	1	0.784698	-0.464028	0.693	no
ni|645903743|nb|KK920687.1|	1105491-1105857	19	18	1	0.981887	-0.0654664	0.94765	no
ni|645903743|nb|KK920687.1|	1106216-1106476	19	18	1	1	0.296488	0.8383	no
ni|645903743|nb|KK920687.1|	1107027-1107655	19	18	4	3	-0.634762	0.5793	no
ni|645903743|nb|KK920687.1|	1109823-1110055	19	18	75	54	-0.469415	0.66275	no
ni|645903743|nb|KK920687.1|	1113812-1114164	19	18	32	23	-0.488025	0.66175	no
ni|645903743|nb|KK920687.1|	1115765-1115919	19	18	81	69	-0.232003	0.83845	no
ni|645903743|nb|KK920687.1|	1116811-1117110	19	18	13	8	-0.657915	0.5732	no
ni|645903743|nb|KK920687.1|	1120097-1120279	19	18	106	92	-0.215614	0.84555	no
ni|645903743|nb|KK920687.1|	1121770-1122027	19	18	81	70	-0.218428	0.8462	no
ni|645903743|nb|KK920687.1|	1122680-1123183	19	18	92	75	-0.295427	0.8363	no
ni|645903743|nb|KK920687.1|	1123340-1124523	19	18	16	15	-0.154905	0.8775	no
ni|645903743|nb|KK920687.1|	1124874-1125249	19	18	2	0.60427	-1.58	0.2821	no
ni|645903743|nb|KK920687.1|	112533-112679	19	18	7	51	3	0.036	no
ni|645903743|nb|KK920687.1|	1127178-1127481	19	18	2	2	0.321254	0.8092	no
ni|645903743|nb|KK920687.1|	1127584-1128136	19	18	0.931701	0.785859	-0.245597	0.84435	no
ni|645903743|nb|KK920687.1|	1129273-1130038	19	18	0.520673	0.640056	0.297821	0.8138	no
ni|645903743|nb|KK920687.1|	1130359-1130670	19	18	1	1	0.312787	0.81835	no

ni|645903743|nb|KK920687.1|	1130946-1131272	19	18	137.69	35	-2	0.14075	no
ni|645903743|nb|KK920687.1|	1132347-1132481	19	18	42	3	-4	0.0405	no
ni|645903743|nb|KK920687.1|	113375-113661	19	18	9	44	2	0.05235	no
ni|645903743|nb|KK920687.1|	1133752-1134318	19	18	2	0.15243	-4	0.12335	no
ni|645903743|nb|KK920687.1|	1135963-1136150	19	18	5	0	-inf	0.0015	yes
ni|645903743|nb|KK920687.1|	1136880-1137183	19	18	67	225.9	2	0.17005	no
ni|645903743|nb|KK920687.1|	1138595-1138791	19	18	75	154	1	0.34075	no
ni|645903743|nb|KK920687.1|	1138996-1139189	19	18	112	234.66	1	0.4044	no
ni|645903743|nb|KK920687.1|	1139392-1139702	19	18	0.948116	1	0.63471	0.63975	no
ni|645903743|nb|KK920687.1|	113961-114121	19	18	9	58	3	0.03805	no
ni|645903743|nb|KK920687.1|	1139872-1141059	19	18	0.863411	0.890838	0.0451164	0.96965	no
ni|645903743|nb|KK920687.1|	1141404-1141808	19	18	6	11	0.955126	0.42025	no
ni|645903743|nb|KK920687.1|	1142844-1143026	19	18	118	281	1	0.33805	no
ni|645903743|nb|KK920687.1|	1143779-1144035	19	18	95	222	1	0.3251	no
ni|645903743|nb|KK920687.1|	1144445-1144564	19	18	56	181	2	0.1552	no
ni|645903743|nb|KK920687.1|	114525-114688	19	18	7	41	3	0.05245	no
ni|645903743|nb|KK920687.1|	1147374-1147577	19	18	117	277	1	0.327	no
ni|645903743|nb|KK920687.1|	114868-115071	19	18	4	22	3	0.053	no
ni|645903743|nb|KK920687.1|	1149184-1149345	19	18	69	165	1	0.2739	no
ni|645903743|nb|KK920687.1|	1152739-1152871	19	18	44	0.733111	-6	0.0178	no
ni|645903743|nb|KK920687.1|	115725-115907	19	18	13	63	2	0.05755	no
ni|645903743|nb|KK920687.1|	1158658-1159297	19	18	0.50515	0	-inf	0.0036	no
ni|645903743|nb|KK920687.1|	1176805-1177437	19	18	5	43	3	0.03575	no
ni|645903743|nb|KK920687.1|	1178508-1179104	19	18	0.299534	2	3	0.08345	no
ni|645903743|nb|KK920687.1|	1179271-1179563	19	18	25	37	0.560453	0.6143	no
ni|645903743|nb|KK920687.1|	1181220-1181581	19	18	0.571247	2	1	0.3264	no
ni|645903743|nb|KK920687.1|	1181773-1182150	19	18	9	18	0.961797	0.40685	no
ni|645903743|nb|KK920687.1|	1182755-1183050	19	18	10	18	0.839855	0.47145	no
ni|645903743|nb|KK920687.1|	1183191-1183357	19	18	34	59	0.798465	0.48845	no
ni|645903743|nb|KK920687.1|	1183834-1184332	19	18	1	1	0.349917	0.77735	no

ni|645903743|nb|KK920687.1|	1184436-1184931	19	18	1	2	0.4943	0.68725	no
ni|645903743|nb|KK920687.1|	1185097-1185383	19	18	2	4	0.851661	0.48905	no
ni|645903743|nb|KK920687.1|	118856-119048	19	18	7	27	2	0.11345	no
ni|645903743|nb|KK920687.1|	1188876-1189054	19	18	14	35	1	0.2492	no
ni|645903743|nb|KK920687.1|	1191704-1191841	19	18	19	59	2	0.17325	no
ni|645903743|nb|KK920687.1|	1194114-1194586	19	18	17	40	1	0.2737	no
ni|645903743|nb|KK920687.1|	1196665-1197264	19	18	11	25	1	0.30415	no
ni|645903743|nb|KK920687.1|	119679-119829	19	18	6	13	0.987252	0.44185	no
ni|645903743|nb|KK920687.1|	1198729-1199203	19	18	361	287	-0.330698	0.809	no
ni|645903743|nb|KK920687.1|	1199358-1199595	19	18	6	9	0.561692	0.6312	no
ni|645903743|nb|KK920687.1|	1199790-1201306	19	18	28	24	-0.224257	0.87765	no
ni|645903743|nb|KK920687.1|	1202473-1202858	19	18	4	1	-1	0.2613	no
ni|645903743|nb|KK920687.1|	1204513-1204616	19	18	2877.69	2440.41	-0.237792	0.826	no
ni|645903743|nb|KK920687.1|	1204803-1204933	19	18	75	39	-0.947001	0.4242	no
ni|645903743|nb|KK920687.1|	1205023-1205470	19	18	10	7	-0.453047	0.7006	no
ni|645903743|nb|KK920687.1|	1205676-1205859	19	18	30	23	-0.374712	0.74995	no
ni|645903743|nb|KK920687.1|	1208947-1209164	19	18	35	24	-0.545012	0.638	no
ni|645903743|nb|KK920687.1|	1209691-1209842	19	18	32	36	0.155199	0.89555	no
ni|645903743|nb|KK920687.1|	1209977-1210194	19	18	3	0.612608	-2	0.1864	no
ni|645903743|nb|KK920687.1|	1210545-1210773	19	18	44	37	-0.264598	0.8171	no
ni|645903743|nb|KK920687.1|	1212943-1213185	19	18	31	28	-0.142444	0.90135	no
ni|645903743|nb|KK920687.1|	1213338-1213492	19	18	43	36	-0.281931	0.8049	no
ni|645903743|nb|KK920687.1|	1216466-1216657	19	18	46	43	-0.110755	0.9234	no
ni|645903743|nb|KK920687.1|	1217065-1217248	19	18	49	45	-0.121731	0.91525	no
ni|645903743|nb|KK920687.1|	1217375-1218227	19	18	3	2	-0.746501	0.5366	no
ni|645903743|nb|KK920687.1|	1220012-1220538	19	18	6	8	0.294473	0.8002	no
ni|645903743|nb|KK920687.1|	1224754-1224885	19	18	18	4	-2	0.13075	no
ni|645903743|nb|KK920687.1|	1225004-1225159	19	18	18	8	-1	0.34705	no
ni|645903743|nb|KK920687.1|	1225821-1225935	19	18	15	11	-0.43009	0.75965	no
ni|645903743|nb|KK920687.1|	1226379-1226520	19	18	12	10	-0.219225	0.86075	no

ni|645903743|nb|KK920687.1|	1226801-1227718	19	18	13	21	0.683669	0.5204	no
ni|645903743|nb|KK920687.1|	1227776-1228230	19	18	7	8	0.241116	0.8328	no
ni|645903743|nb|KK920687.1|	1236906-1237271	19	18	4	4	0.0688307	0.95395	no
ni|645903743|nb|KK920687.1|	1237636-1237774	19	18	34	25	-0.473231	0.67995	no
ni|645903743|nb|KK920687.1|	1239207-1239444	19	18	39	38	-0.0394972	0.97215	no
ni|645903743|nb|KK920687.1|	1239942-1240153	19	18	29	32	0.120332	0.9149	no
ni|645903743|nb|KK920687.1|	1240525-1241176	19	18	6	5	-0.0648751	0.95265	no
ni|645903743|nb|KK920687.1|	1241439-1241806	19	18	14	18	0.400201	0.72445	no
ni|645903743|nb|KK920687.1|	1245321-1245750	19	18	1	5	2	0.1293	no
ni|645903743|nb|KK920687.1|	1249194-1249411	19	18	75	83	0.15045	0.8913	no
ni|645903743|nb|KK920687.1|	1249565-1249774	19	18	116	138	0.255709	0.8115	no
ni|645903743|nb|KK920687.1|	1250015-1250233	19	18	48	55	0.199576	0.859	no
ni|645903743|nb|KK920687.1|	1251341-1251547	19	18	101	120	0.248285	0.81905	no
ni|645903743|nb|KK920687.1|	1253729-1253946	19	18	132	142	0.105357	0.9192	no
ni|645903743|nb|KK920687.1|	1254064-1254405	19	18	2	2	0.0124293	0.9704	no
ni|645903743|nb|KK920687.1|	1254520-1255056	19	18	1	0.869147	-0.31298	0.80255	no
ni|645903743|nb|KK920687.1|	1255179-1256033	19	18	10	12	0.287706	0.7909	no
ni|645903743|nb|KK920687.1|	1257119-1257804	19	18	0.507567	189	9	0.00455	no
ni|645903743|nb|KK920687.1|	125738-125992	19	18	43	34	-0.346634	0.7591	no
ni|645903743|nb|KK920687.1|	1257976-1258679	19	18	1	413	8	5.00E-05	yes
ni|645903743|nb|KK920687.1|	1258820-1259100	19	18	1	431	8	0.00925	no
ni|645903743|nb|KK920687.1|	1260128-1260476	19	18	1	0.862037	-0.217545	0.87705	no
ni|645903743|nb|KK920687.1|	1260927-1261278	19	18	11	13	0.187014	0.87095	no
ni|645903743|nb|KK920687.1|	1261404-1263017	19	18	5	5	0.0776174	0.9415	no
ni|645903743|nb|KK920687.1|	1263099-1263475	19	18	20	17	-0.271879	0.8141	no
ni|645903743|nb|KK920687.1|	1263546-1263921	19	18	0.903296	0.60427	-0.580005	0.70815	no
ni|645903743|nb|KK920687.1|	1264019-1264887	19	18	0.933447	0.4001	-1	0.3372	no
ni|645903743|nb|KK920687.1|	1265108-1265421	19	18	2	1	-0.983252	0.48245	no
ni|645903743|nb|KK920687.1|	1265929-1266182	19	18	67	60	-0.145916	0.8946	no
ni|645903743|nb|KK920687.1|	1266394-1266726	19	18	310.55	474	0.611148	0.66095	no

ni|645903743|nb|KK920687.1|	126790-129557	19	18	49	87	0.836054	0.5658	no
ni|645903743|nb|KK920687.1|	1269680-1270427	19	18	226	391	0.793914	0.58605	no
ni|645903743|nb|KK920687.1|	1270655-1270787	19	18	0	10	inf	0.003	no
ni|645903743|nb|KK920687.1|	1270917-1271128	19	18	12	37	2	0.16565	no
ni|645903743|nb|KK920687.1|	1271818-1271933	19	18	36	77	1	0.3707	no
ni|645903743|nb|KK920687.1|	147667-147922	19	18	1	0.922611	-0.481167	0.7594	no
ni|645903743|nb|KK920687.1|	148050-149955	19	18	8	4	-0.775788	0.4774	no
ni|645903743|nb|KK920687.1|	150199-150328	19	18	62	48	-0.394115	0.73185	no
ni|645903743|nb|KK920687.1|	150583-150964	19	18	11	11	-0.0184889	0.98625	no
ni|645903743|nb|KK920687.1|	151317-152263	19	18	5	6	0.190198	0.86615	no
ni|645903743|nb|KK920687.1|	162353-162532	19	18	199.01	141	-0.492363	0.6381	no
ni|645903743|nb|KK920687.1|	172218-172424	19	18	84	68	-0.295356	0.78655	no
ni|645903743|nb|KK920687.1|	174112-174275	19	18	152	82	-0.890628	0.4323	no
ni|645903743|nb|KK920687.1|	175170-175461	19	18	79	55	-0.520233	0.62345	no
ni|645903743|nb|KK920687.1|	175980-176317	19	18	32	21	-0.61051	0.5862	no
ni|645903743|nb|KK920687.1|	176527-176884	19	18	103	65	-0.674346	0.58575	no
ni|645903743|nb|KK920687.1|	179284-179866	19	18	11	8	-0.525242	0.6464	no
ni|645903743|nb|KK920687.1|	186680-187175	19	18	0.062757	4	6	0.11945	no
ni|645903743|nb|KK920687.1|	193501-194229	19	18	4	5	0.225892	0.84755	no
ni|645903743|nb|KK920687.1|	194492-195112	19	18	3	3	-0.0230974	0.9847	no
ni|645903743|nb|KK920687.1|	196144-196433	19	18	3	2	-0.288249	0.8142	no
ni|645903743|nb|KK920687.1|	196961-198419	19	18	2	1	-0.452703	0.69875	no
ni|645903743|nb|KK920687.1|	199792-199940	19	18	53	107	1	0.38545	no
ni|645903743|nb|KK920687.1|	204147-204371	19	18	78	145	0.891973	0.4899	no
ni|645903743|nb|KK920687.1|	204479-204624	19	18	56	103	0.867699	0.45705	no
ni|645903743|nb|KK920687.1|	204690-205766	19	18	58	96	0.727211	0.6044	no
ni|645903743|nb|KK920687.1|	206026-206170	19	18	24	14	-0.793034	0.50645	no
ni|645903743|nb|KK920687.1|	206321-206677	19	18	12	4	-2	0.16185	no
ni|645903743|nb|KK920687.1|	206885-207252	19	18	35	14	-1	0.2372	no
ni|645903743|nb|KK920687.1|	207498-207611	19	18	43	7	-3	0.08385	no

ni|645903743|nb|KK920687.1|	229387-229783	19	18	33	10	-2	0.1506	no
ni|645903743|nb|KK920687.1|	233042-233524	19	18	35	12	-2	0.16725	no
ni|645903743|nb|KK920687.1|	233639-234115	19	18	45	17	-1	0.2019	no
ni|645903743|nb|KK920687.1|	235413-235551	19	18	47	16	-2	0.203	no
ni|645903743|nb|KK920687.1|	235750-236912	19	18	10.21	4	-1	0.24745	no
ni|645903743|nb|KK920687.1|	237242-237403	19	18	42	20	-1	0.3556	no
ni|645903743|nb|KK920687.1|	270754-271101	19	18	1	0.288516	-2	0.24815	no
ni|645903743|nb|KK920687.1|	271177-271732	19	18	1	0.312262	-2	0.16025	no
ni|645903743|nb|KK920687.1|	282065-282198	19	18	14	7	-0.99327	0.47555	no
ni|645903743|nb|KK920687.1|	283331-283573	19	18	22	20	-0.136093	0.90605	no
ni|645903743|nb|KK920687.1|	284239-284572	19	18	9	8	-0.172527	0.88185	no
ni|645903743|nb|KK920687.1|	284663-284968	19	18	9	8	-0.162619	0.89595	no
ni|645903743|nb|KK920687.1|	285999-286328	19	18	23	82	2	0.17625	no
ni|645903743|nb|KK920687.1|	288568-288689	19	18	16	59	2	0.14085	no
ni|645903743|nb|KK920687.1|	289996-290225	19	18	25	92	2	0.104	no
ni|645903743|nb|KK920687.1|	295053-295843	19	18	9	31	2	0.2011	no
ni|645903743|nb|KK920687.1|	296415-296613	19	18	3	1	-1	0.46615	no
ni|645903743|nb|KK920687.1|	298391-298555	19	18	3	3	-0.0672216	0.9302	no
ni|645903743|nb|KK920687.1|	298700-298876	19	18	6	6	0.0931398	0.93935	no
ni|645903743|nb|KK920687.1|	299369-299733	19	18	10	36	2	0.1129	no
ni|645903743|nb|KK920687.1|	301785-301999	19	18	26	100	2	0.09175	no
ni|645903743|nb|KK920687.1|	303242-303511	19	18	37	114.86	2	0.2242	no
ni|645903743|nb|KK920687.1|	304751-304943	19	18	35	111	2	0.15185	no
ni|645903743|nb|KK920687.1|	307584-307810	19	18	0	19	inf	5.00E-05	yes
ni|645903743|nb|KK920687.1|	308751-308918	19	18	19.05	88	2	0.07135	no
ni|645903743|nb|KK920687.1|	310800-311073	19	18	24	85	2	0.10715	no
ni|645903743|nb|KK920687.1|	312222-312364	19	18	25	77	2	0.18035	no
ni|645903743|nb|KK920687.1|	315639-315847	19	18	26	112	2	0.07315	no
ni|645903743|nb|KK920687.1|	315966-316161	19	18	27	112	2	0.0844	no
ni|645903743|nb|KK920687.1|	319265-319539	19	18	21	59	1	0.20105	no

ni|645903743|nb|KK920687.1|	320412-321814	19	18	84	83	-0.0247065	0.98605	no
ni|645903743|nb|KK920687.1|	321883-322258	19	18	5	5	-0.14072	0.90255	no
ni|645903743|nb|KK920687.1|	322330-323092	19	18	0.26152	0.571525	1	0.41545	no
ni|645903743|nb|KK920687.1|	336377-336511	19	18	8	10	0.380195	0.7694	no
ni|645903743|nb|KK920687.1|	347629-348096	19	18	0.405425	230	9	0.0332	no
ni|645903743|nb|KK920687.1|	352887-353223	19	18	0.316101	238	10	0.05055	no
ni|645903743|nb|KK920687.1|	357612-358018	19	18	0	247	inf	5.00E-05	yes
ni|645903743|nb|KK920687.1|	358743-358982	19	18	0.179698	332	11	0.11935	no
ni|645903743|nb|KK920687.1|	362383-362719	19	18	0.105367	277	11	0.11935	no
ni|645903743|nb|KK920687.1|	364350-364604	19	18	0.162036	408	11	0.11935	no
ni|645903743|nb|KK920687.1|	373060-373428	19	18	0.185409	161.15	10	0.0443	no
ni|645903743|nb|KK920687.1|	389903-390130	19	18	4	5	0.196548	0.8698	no
ni|645903743|nb|KK920687.1|	411082-411240	19	18	65	115	0.838407	0.45915	no
ni|645903743|nb|KK920687.1|	420863-421051	19	18	58	87	0.575232	0.60955	no
ni|645903743|nb|KK920687.1|	421878-422487	19	18	17	22	0.382526	0.7273	no
ni|645903743|nb|KK920687.1|	423211-423721	19	18	48	63	0.396981	0.78785	no
ni|645903743|nb|KK920687.1|	423824-424038	19	18	77	97	0.33185	0.76	no
ni|645903743|nb|KK920687.1|	424313-424640	19	18	28	31	0.157573	0.8906	no
ni|645903743|nb|KK920687.1|	424891-425015	19	18	39	63	0.66961	0.5636	no
ni|645903743|nb|KK920687.1|	425359-425612	19	18	77	99	0.368621	0.73105	no
ni|645903743|nb|KK920687.1|	426096-426305	19	18	30	40	0.42654	0.7092	no
ni|645903743|nb|KK920687.1|	426390-426505	19	18	24	51	1	0.373	no
ni|645903743|nb|KK920687.1|	427243-427668	19	18	22	29	0.370602	0.7375	no
ni|645903743|nb|KK920687.1|	427990-428310	19	18	14	17	0.334503	0.76565	no
ni|645903743|nb|KK920687.1|	428415-428896	19	18	11	10	-0.169492	0.88315	no
ni|645903743|nb|KK920687.1|	429212-429349	19	18	13	15	0.13439	0.91335	no
ni|645903743|nb|KK920687.1|	540007-540515	19	18	12	39	2	0.13375	no
ni|645903743|nb|KK920687.1|	542491-542822	19	18	50	125	1	0.29685	no
ni|645903743|nb|KK920687.1|	544163-544346	19	18	65	153	1	0.2735	no
ni|645903743|nb|KK920687.1|	545030-545451	19	18	50	115	1	0.3329	no

ni|645903743|nb|KK920687.1|	547480-547680	19	18	77	182	1	0.35385	no
ni|645903743|nb|KK920687.1|	548302-548494	19	18	74	155	1	0.339	no
ni|645903743|nb|KK920687.1|	549603-549900	19	18	68	155	1	0.355	no
ni|645903743|nb|KK920687.1|	551003-551215	19	18	93	201	1	0.385	no
ni|645903743|nb|KK920687.1|	552334-552982	19	18	47	97	1	0.4741	no
ni|645903743|nb|KK920687.1|	553102-553305	19	18	73	156	1	0.3182	no
ni|645903743|nb|KK920687.1|	553450-553621	19	18	67	136	1	0.36745	no
ni|645903743|nb|KK920687.1|	554948-555419	19	18	53	86	0.688705	0.6461	no
ni|645903743|nb|KK920687.1|	556632-556928	19	18	70	49	-0.515422	0.6353	no
ni|645903743|nb|KK920687.1|	557779-558588	19	18	0.976507	0.900329	-0.117178	0.9189	no
ni|645903743|nb|KK920687.1|	558831-558964	19	18	309	230	-0.428848	0.6924	no
ni|645903743|nb|KK920687.1|	560274-560525	19	18	3	2	-0.506742	0.68965	no
ni|645903743|nb|KK920687.1|	560602-560957	19	18	4	3	-0.313439	0.7861	no
ni|645903743|nb|KK920687.1|	608649-608981	19	18	5	5	-0.0656163	0.95525	no
ni|645903743|nb|KK920687.1|	609298-609633	19	18	5	3	-0.781809	0.5117	no
ni|645903743|nb|KK920687.1|	610366-611589	19	18	7	241	5	0.0021	yes
ni|645903743|nb|KK920687.1|	611741-611974	19	18	241	164	-0.55723	0.71205	no
ni|645903743|nb|KK920687.1|	612467-612803	19	18	9	5	-0.824589	0.4778	no
ni|645903743|nb|KK920687.1|	613153-614299	19	18	333	184	-0.857847	0.55585	no
ni|645903743|nb|KK920687.1|	617026-618099	19	18	0.07617	2	5	0.0509	no
ni|645903743|nb|KK920687.1|	642954-643502	19	18	4	2	-0.671689	0.57255	no
ni|645903743|nb|KK920687.1|	646623-647746	19	18	0	4	inf	5.00E-05	yes
ni|645903743|nb|KK920687.1|	672116-672266	19	18	5	5	-0.0671955	0.94635	no
ni|645903743|nb|KK920687.1|	695415-695896	19	18	0.520599	0.559812	0.10477	0.93335	no
ni|645903743|nb|KK920687.1|	752345-753217	19	18	5	4	-0.373991	0.7411	no
ni|645903743|nb|KK920687.1|	753331-753879	19	18	9	7	-0.338792	0.76745	no
ni|645903743|nb|KK920687.1|	754036-754374	19	18	16	14	-0.182627	0.87455	no
ni|645903743|nb|KK920687.1|	757222-757469	19	18	28	25	-0.166377	0.88425	no
ni|645903743|nb|KK920687.1|	758120-758241	19	18	49	39	-0.323731	0.777	no
ni|645903743|nb|KK920687.1|	759282-759617	19	18	8	7	-0.127886	0.91305	no

ni|645903743|nb|KK920687.1|	760731-760956	19	18	26	25	-0.044659	0.97015	no
ni|645903743|nb|KK920687.1|	766399-766707	19	18	1	0.228665	-2	0.2949	no
ni|645903743|nb|KK920687.1|	767566-767757	19	18	36	26	-0.485879	0.6706	no
ni|645903743|nb|KK920687.1|	768287-768841	19	18	1	0.41727	-2	0.27225	no
ni|645903743|nb|KK920687.1|	768956-769684	19	18	14	10	-0.439181	0.691	no
ni|645903743|nb|KK920687.1|	769881-770042	19	18	18	11	-0.804173	0.5034	no
ni|645903743|nb|KK920687.1|	770416-770839	19	18	17	12	-0.537775	0.6405	no
ni|645903743|nb|KK920687.1|	770952-771147	19	18	25	21	-0.213929	0.85635	no
ni|645903743|nb|KK920687.1|	771224-771423	19	18	14	14	0.0110004	0.98925	no
ni|645903743|nb|KK920687.1|	772067-772188	19	18	15	12	-0.308399	0.81205	no
ni|645903743|nb|KK920687.1|	772496-773021	19	18	17	12	-0.585903	0.6051	no
ni|645903743|nb|KK920687.1|	783605-785064	19	18	0.036332	2	6	0.04445	no
ni|645903743|nb|KK920687.1|	785934-786568	19	18	0.046355	2	6	0.11975	no
ni|645903743|nb|KK920687.1|	800062-800901	19	18	0.26764	3	3	0.0277	no
ni|645903743|nb|KK920687.1|	802896-803759	19	18	15	30	1	0.4322	no
ni|645903743|nb|KK920687.1|	804566-805625	19	18	15	21	0.537238	0.66255	no
ni|645903743|nb|KK920687.1|	806101-806368	19	18	33	46	0.457541	0.6893	no
ni|645903743|nb|KK920687.1|	806598-806807	19	18	100	141	0.491453	0.651	no
ni|645903743|nb|KK920687.1|	808265-809164	19	18	16	23	0.545312	0.66685	no
ni|645903743|nb|KK920687.1|	811735-812624	19	18	1	2	0.638956	0.5903	no
ni|645903743|nb|KK920687.1|	812912-813341	19	18	66	101	0.60561	0.67715	no
ni|645903743|nb|KK920687.1|	814069-814429	19	18	3	4	0.568381	0.63655	no
ni|645903743|nb|KK920687.1|	815104-815728	19	18	140	192	0.452821	0.74305	no
ni|645903743|nb|KK920687.1|	816462-816990	19	18	2	2	0.0280438	0.9793	no
ni|645903743|nb|KK920687.1|	817450-817874	19	18	7	9	0.302447	0.79755	no
ni|645903743|nb|KK920687.1|	818457-819018	19	18	0.537309	0.410908	-0.386937	0.78725	no
ni|645903743|nb|KK920687.1|	819261-821115	19	18	0.337667	0.659323	0.965383	0.41525	no
ni|645903743|nb|KK920687.1|	822990-823127	19	18	9	24	1	0.2478	no
ni|645903743|nb|KK920687.1|	825437-825620	19	18	12	17	0.554205	0.63595	no
ni|645903743|nb|KK920687.1|	829515-829673	19	18	15	25	0.757713	0.51965	no

ni|645903743|nb|KK920687.1|	830324-831230	19	18	0.582652	0.293222	-0.99064	0.47555	no
ni|645903743|nb|KK920687.1|	831343-831848	19	18	0.550793	0.93598	0.764968	0.5642	no
ni|645903743|nb|KK920687.1|	832056-832184	19	18	5	11	1	0.45555	no
ni|645903743|nb|KK920687.1|	837159-837684	19	18	17	18	0.081615	0.93955	no
ni|645903743|nb|KK920687.1|	837861-838036	19	18	4	3	-0.526748	0.7071	no
ni|645903743|nb|KK920687.1|	838971-839412	19	18	3	2	-0.280973	0.81425	no
ni|645903743|nb|KK920687.1|	841409-842037	19	18	24	23	-0.0931953	0.9326	no
ni|645903743|nb|KK920687.1|	842218-842328	19	18	78	65	-0.248229	0.83205	no
ni|645903743|nb|KK920687.1|	842476-842658	19	18	6	2	-2	0.2921	no
ni|645903743|nb|KK920687.1|	848071-848650	19	18	37	121.29	2	0.26265	no
ni|645903743|nb|KK920687.1|	848816-848967	19	18	4	5	0.392238	0.7763	no
ni|645903743|nb|KK920687.1|	849214-849728	19	18	3	2	-0.328125	0.77655	no
ni|645903743|nb|KK920687.1|	849921-850193	19	18	46	119	1	0.30645	no
ni|645903743|nb|KK920687.1|	850445-852665	19	18	4	4	0.102495	0.92525	no
ni|645903743|nb|KK920687.1|	852853-852970	19	18	18	12	-0.597968	0.64595	no
ni|645903743|nb|KK920687.1|	853114-854066	19	18	7	6	-0.368481	0.741	no
ni|645903743|nb|KK920687.1|	854488-854954	19	18	7	6	-0.125308	0.91515	no
ni|645903743|nb|KK920687.1|	855066-855208	19	18	9	15	0.726333	0.56145	no
ni|645903743|nb|KK920687.1|	855857-856284	19	18	6	7	0.326148	0.7852	no
ni|645903743|nb|KK920687.1|	856694-857200	19	18	11	24	1	0.3171	no
ni|645903743|nb|KK920687.1|	857392-858021	19	18	2	1	-0.287297	0.80395	no
ni|645903743|nb|KK920687.1|	858268-858588	19	18	4	1	-1	0.30405	no
ni|645903743|nb|KK920687.1|	859634-860038	19	18	32	72	1	0.3671	no
ni|645903743|nb|KK920687.1|	860409-860728	19	18	1	0.868415	-0.525113	0.7071	no
ni|645903743|nb|KK920687.1|	861756-861873	19	18	115	303	1	0.2381	no
ni|645903743|nb|KK920687.1|	862088-862305	19	18	10	6	-0.813882	0.4928	no
ni|645903743|nb|KK920687.1|	865608-865753	19	18	36	31	-0.206931	0.856	no
ni|645903743|nb|KK920687.1|	866120-866408	19	18	85	71	-0.254986	0.8068	no
ni|645903743|nb|KK920687.1|	866701-866823	19	18	120.99	62	-0.966096	0.4113	no
ni|645903743|nb|KK920687.1|	866988-867170	19	18	99	81	-0.303063	0.7878	no

ni|645903743|nb|KK920687.1|	867278-867606	19	18	112.8	105	-0.10955	0.94035	no
ni|645903743|nb|KK920687.1|	871050-871518	19	18	36	44	0.29617	0.7791	no
ni|645903743|nb|KK920687.1|	871968-872213	19	18	88	96	0.123937	0.90815	no
ni|645903743|nb|KK920687.1|	872879-873976	19	18	8	8	0.152487	0.8887	no
ni|645903743|nb|KK920687.1|	876316-876471	19	18	55	57	0.0473654	0.9659	no
ni|645903743|nb|KK920687.1|	877081-877289	19	18	131	163	0.31405	0.7591	no
ni|645903743|nb|KK920687.1|	881120-881542	19	18	37	40	0.112978	0.91805	no
ni|645903743|nb|KK920687.1|	883663-883789	19	18	23	36	0.610738	0.6041	no
ni|645903743|nb|KK920687.1|	884734-884954	19	18	9	8	-0.244937	0.83795	no
ni|645903743|nb|KK920687.1|	887825-888070	19	18	29	29	-0.00734192	0.99435	no
ni|645903743|nb|KK920687.1|	888250-888902	19	18	0.717386	0.943032	0.394558	0.75185	no
ni|645903743|nb|KK920687.1|	889306-889494	19	18	32	26	-0.300193	0.8011	no
ni|645903743|nb|KK920687.1|	890426-890602	19	18	24	26	0.155068	0.8954	no
ni|645903743|nb|KK920687.1|	892199-892451	19	18	14	9	-0.602212	0.60695	no
ni|645903743|nb|KK920687.1|	892571-892758	19	18	25	24	-0.0837161	0.9444	no
ni|645903743|nb|KK920687.1|	896514-897013	19	18	12	11	-0.166814	0.8846	no
ni|645903743|nb|KK920687.1|	898320-898518	19	18	11	11	-0.0670224	0.95195	no
ni|645903743|nb|KK920687.1|	898910-899321	19	18	8	5	-0.821082	0.4803	no
ni|645903743|nb|KK920687.1|	902109-902380	19	18	0	631	inf	5.00E-05	yes
ni|645903743|nb|KK920687.1|	903350-903716	19	18	0.093406	290	12	0.11935	no
ni|645903743|nb|KK920687.1|	908277-908569	19	18	0.129717	262.98	11	0.11935	no
ni|645903743|nb|KK920687.1|	911551-911770	19	18	0	328	inf	5.00E-05	yes
ni|645903743|nb|KK920687.1|	913454-913675	19	18	0	251	inf	5.00E-05	yes
ni|645903743|nb|KK920687.1|	914352-914831	19	18	3	2	-0.427729	0.72205	no
ni|645903743|nb|KK920687.1|	915430-916235	19	18	1596.66	1309.38	-0.286171	0.8032	no
ni|645903743|nb|KK920687.1|	917923-918236	19	18	1	2	0.282209	0.82625	no
ni|645903743|nb|KK920687.1|	918793-919991	19	18	0.76477	0.860387	0.169961	0.8861	no
ni|645903743|nb|KK920687.1|	921374-921656	19	18	2	4	1	0.35855	no
ni|645903743|nb|KK920687.1|	922044-924595	19	18	10	15	0.540307	0.7109	no
ni|645903743|nb|KK920687.1|	924655-928329	19	18	17	18	0.109625	0.9387	no

ni|645903743|nb|KK920687.1|	929276-929404	19	18	10	3	-2	0.20695	no
ni|645903743|nb|KK920687.1|	930020-930285	19	18	1	1	0.126607	0.92555	no
ni|645903743|nb|KK920687.1|	932389-932606	19	18	2	4	0.866237	0.5013	no
ni|645903743|nb|KK920687.1|	934241-934413	19	18	5	33	3	0.0439	no
ni|645903743|nb|KK920687.1|	935896-936067	19	18	6	61	3	0.0145	no
ni|645903743|nb|KK920687.1|	936855-937263	19	18	3	21	3	0.0307	no
ni|645903743|nb|KK920687.1|	938790-939086	19	18	4	40	3	0.0094	no
ni|645903743|nb|KK920687.1|	941056-941199	19	18	6	40	3	0.0543	no
ni|645903743|nb|KK920687.1|	941924-942283	19	18	0.287844	2	3	0.06315	no
ni|645903743|nb|KK920687.1|	943688-943828	19	18	4	54	4	0.03865	no
ni|645903743|nb|KK920687.1|	953487-953711	19	18	5	35	3	0.02685	no
ni|645903743|nb|KK920687.1|	953828-953989	19	18	3	38	4	0.0282	no
ni|645903743|nb|KK920687.1|	954836-955075	19	18	1	1	0.348726	0.81755	no
ni|645903743|nb|KK920687.1|	955285-956497	19	18	0.666283	1	0.886591	0.458	no
ni|645903743|nb|KK920687.1|	957648-958155	19	18	1	18	4	0.00575	no
ni|645903743|nb|KK920687.1|	958291-958854	19	18	0.802465	0.971672	0.276031	0.821	no
ni|645903743|nb|KK920687.1|	959175-959640	19	18	0.543544	0.714354	0.394242	0.77595	no
ni|645903743|nb|KK920687.1|	962106-962305	19	18	6	51	3	0.017	no
ni|645903743|nb|KK920687.1|	962445-962903	19	18	0.762068	3	2	0.1231	no
ni|645903743|nb|KK920687.1|	963956-964180	19	18	1	18	4	0.0305	no
ni|645903743|nb|KK920687.1|	965402-965520	19	18	1	33	5	0.121	no
ni|645903743|nb|KK920687.1|	965624-965815	19	18	0	11	inf	5.00E-05	yes
ni|645903743|nb|KK920687.1|	967560-967898	19	18	2	13	3	0.0303	no
ni|645903743|nb|KK920687.1|	970958-971512	19	18	3	37	4	0.0027	yes
ni|645903743|nb|KK920687.1|	981098-981354	19	18	12	22	0.808349	0.4897	no
ni|645903743|nb|KK920687.1|	986910-987021	19	18	25	44	0.806856	0.5327	no
ni|645903743|nb|KK920687.1|	987214-987429	19	18	13	26	1	0.36655	no
ni|645903743|nb|KK920687.1|	992799-993235	19	18	8	10	0.243376	0.8333	no
ni|645903743|nb|KK920687.1|	996251-996834	19	18	6	3	-0.965438	0.41595	no
ni|645903743|nb|KK920687.1|	997725-998284	19	18	8	7	-0.178021	0.8782	no

ni|645903743|nb|KK920687.1|	998370-998965	19	18	9	8	-0.168666	0.8798	no
ni|645903743|nb|KK920687.1|	999162-999987	19	18	11	10	-0.173721	0.88	no
ni|645903744|nb|KK920686.1|	15845-15975	17	8.6	7	3	-1	0.4023	no
ni|645903744|nb|KK920686.1|	263353-263727	17	8.6	0.997276	0.606489	-0.717512	0.61925	no
ni|645903744|nb|KK920686.1|	422684-423015	17	8.6	14	11	-0.315599	0.7822	no
ni|645903744|nb|KK920686.1|	430211-430355	17	8.6	4	5	0.295362	0.8383	no
ni|645903744|nb|KK920686.1|	431210-431374	17	8.6	7	5	-0.454245	0.722	no
ni|645903744|nb|KK920686.1|	443217-443468	17	8.6	0.991712	2	1	0.45555	no
ni|645903744|nb|KK920686.1|	444704-444906	17	8.6	3	0.937922	-2	0.25615	no
ni|645903744|nb|KK920686.1|	445826-446687	17	8.6	0.552242	0.77652	0.491723	0.6941	no
ni|645903744|nb|KK920686.1|	448513-448739	17	8.6	2	1	-0.651465	0.6679	no
ni|645903744|nb|KK920686.1|	449232-449581	17	8.6	0.598969	1	0.808932	0.56845	no
ni|645903744|nb|KK920686.1|	450294-451227	17	8.6	0.65272	0.48228	-0.436592	0.7272	no
ni|645903744|nb|KK920686.1|	452112-452515	17	8.6	1	2	0.578515	0.65045	no
ni|645903744|nb|KK920686.1|	452614-453417	17	8.6	0.914521	0.70625	-0.372837	0.7575	no
ni|645903744|nb|KK920686.1|	453882-454535	17	8.6	1	2	0.190385	0.86975	no
ni|645903744|nb|KK920686.1|	454658-455014	17	8.6	0.9708	2	0.860492	0.50465	no
ni|645903744|nb|KK920686.1|	457409-457916	17	8.6	1	2	0.782893	0.52115	no
ni|645903744|nb|KK920686.1|	458173-460173	17	8.6	2	3	0.161535	0.88805	no
ni|645903744|nb|KK920686.1|	460495-461590	17	8.6	3	2	-0.40558	0.7295	no
ni|645903744|nb|KK920686.1|	462264-462652	17	8.6	3	3	-0.065386	0.9571	no
ni|645903744|nb|KK920686.1|	462705-463109	17	8.6	2	2	-0.065338	0.9553	no
ni|645903744|nb|KK920686.1|	463558-464088	17	8.6	3	2	-0.406099	0.7314	no
ni|645903744|nb|KK920686.1|	471712-471898	17	8.6	3	1	-1	0.4023	no
ni|645903744|nb|KK920686.1|	472756-473086	17	8.6	0.973223	2	0.934374	0.4817	no
ni|645903744|nb|KK920686.1|	475722-475888	17	8.6	6	4	-0.695265	0.5883	no
ni|645903744|nb|KK920686.1|	477272-477388	17	8.6	13	10	-0.430043	0.75965	no
ni|645903744|nb|KK920686.1|	506045-506265	17	8.6	48	55	0.202721	0.85735	no
ni|645903744|nb|KK920686.1|	507277-507565	17	8.6	39	45	0.194749	0.8608	no
ni|645903744|nb|KK920686.1|	520607-520899	17	8.6	1	0.619652	-0.743908	0.59615	no

ni|645903744|nb|KK920686.1|	536119-536300	17	8.6	5	2	-1	0.33495	no
ni|645903744|nb|KK920686.1|	541617-541786	17	8.6	4	1	-1	0.35155	no
ni|645903744|nb|KK920686.1|	543904-544062	17	8.6	4	2	-0.915197	0.53575	no
ni|645903744|nb|KK920686.1|	555877-556055	17	8.6	14	14	-0.0673233	0.95405	no
ni|645903744|nb|KK920686.1|	556180-556620	17	8.6	0.583738	0.767149	0.394185	0.77595	no
ni|645903744|nb|KK920686.1|	557825-557982	17	8.6	5	2	-2	0.25505	no
ni|645903744|nb|KK920686.1|	559050-559442	17	8.6	1	2	0.693619	0.58445	no
ni|645903744|nb|KK920686.1|	560267-560835	17	8.6	1	0.50592	-1	0.34095	no
ni|645903744|nb|KK920686.1|	562708-562910	17	8.6	26	29	0.170925	0.8836	no
ni|645903744|nb|KK920686.1|	563103-563349	17	8.6	21	16	-0.326876	0.7755	no
ni|645903744|nb|KK920686.1|	565939-566844	17	8.6	0.491265	0.46974	-0.0646408	0.9589	no
ni|645903744|nb|KK920686.1|	567058-568079	17	8.6	4	4	0.064157	0.95655	no
ni|645903744|nb|KK920686.1|	568385-569747	17	8.6	0.626072	0.598716	-0.0644556	0.9536	no
ni|645903744|nb|KK920686.1|	570433-571136	17	8.6	2	0.784546	-1	0.3931	no
ni|645903744|nb|KK920686.1|	571590-572213	17	8.6	6	6	-0.0874559	0.9366	no
ni|645903744|nb|KK920686.1|	572598-572726	17	8.6	36	42	0.21899	0.84835	no
ni|645903744|nb|KK920686.1|	574178-574686	17	8.6	2	0.812933	-0.958186	0.44215	no
ni|645903744|nb|KK920686.1|	575152-575302	17	8.6	45	41	-0.148725	0.897	no
ni|645903744|nb|KK920686.1|	575599-575760	17	8.6	11	13	0.177905	0.87595	no
ni|645903744|nb|KK920686.1|	576015-576151	17	8.6	7	3	-1	0.44755	no
ni|645903744|nb|KK920686.1|	576521-577113	17	8.6	20	16	-0.384205	0.72755	no
ni|645903744|nb|KK920686.1|	577168-577278	17	8.6	34	48	0.484885	0.70275	no
ni|645903744|nb|KK920686.1|	66441-67759	17	8.6	8	10	0.372124	0.73105	no
ni|645903744|nb|KK920686.1|	709746-709948	17	8.6	6	4	-0.54087	0.65845	no
ni|645903744|nb|KK920686.1|	710525-710697	17	8.6	8	4	-0.890412	0.4822	no
ni|645903744|nb|KK920686.1|	711292-711474	17	8.6	4	5	0.347746	0.78955	no
ni|645903744|nb|KK920686.1|	712686-712988	17	8.6	7	7	0.00577799	0.9895	no
ni|645903744|nb|KK920686.1|	716322-716532	17	8.6	5	3	-0.552199	0.66085	no
ni|645903744|nb|KK920686.1|	716675-716837	17	8.6	10	11	0.146912	0.9049	no
ni|645903744|nb|KK920686.1|	718813-719189	17	8.6	5	7	0.434143	0.71695	no

ni|645903744|nb|KK920686.1|	719592-720151	17	8.6	5	7	0.457214	0.69755	no
ni|645903744|nb|KK920686.1|	721034-721225	17	8.6	7	11	0.624712	0.60255	no
ni|645903744|nb|KK920686.1|	728501-728634	17	8.6	5	11	1	0.44715	no
ni|645903744|nb|KK920686.1|	729502-729815	17	8.6	1	3	1	0.3172	no
ni|645903744|nb|KK920686.1|	730412-730724	17	8.6	4	6	0.689168	0.56095	no
ni|645903744|nb|KK920686.1|	756567-756836	17	8.6	18	12	-0.577257	0.61815	no
ni|645903744|nb|KK920686.1|	757204-757352	17	8.6	4	6	0.63324	0.63975	no
ni|645903744|nb|KK920686.1|	757491-757625	17	8.6	8	2	-2	0.24815	no
ni|645903744|nb|KK920686.1|	781901-782197	17	8.6	5	7	0.593152	0.61635	no
ni|645903744|nb|KK920686.1|	789268-789502	17	8.6	30	33	0.116272	0.91935	no
ni|645903744|nb|KK920686.1|	791492-791651	17	8.6	75	84	0.172551	0.8778	no
ni|645903744|nb|KK920686.1|	793997-794662	17	8.6	28	27	-0.0783826	0.94035	no
ni|645903744|nb|KK920686.1|	796572-796713	17	8.6	47	38	-0.30823	0.79065	no
ni|645903744|nb|KK920686.1|	797617-797779	17	8.6	70.95	70	-0.0109442	0.99255	no
ni|645903744|nb|KK920686.1|	800164-800402	17	8.6	114.4	87	-0.398562	0.71095	no
ni|645903744|nb|KK920686.1|	801601-801811	17	8.6	99	66	-0.593239	0.58335	no
ni|645903744|nb|KK920686.1|	802140-802405	17	8.6	109	85	-0.371127	0.76835	no
ni|645903744|nb|KK920686.1|	803331-804017	17	8.6	2	1	-0.634198	0.5939	no
ni|645903744|nb|KK920686.1|	804435-805322	17	8.6	13	9	-0.442093	0.68735	no
ni|645903744|nb|KK920686.1|	809255-809544	17	8.6	81	55	-0.553164	0.60585	no
ni|645903744|nb|KK920686.1|	809929-810444	17	8.6	24	18	-0.442529	0.68715	no
ni|645903744|nb|KK920686.1|	810702-811007	17	8.6	72	62	-0.203994	0.84735	no
ni|645903744|nb|KK920686.1|	811341-811657	17	8.6	95	62	-0.607385	0.63285	no
ni|645903744|nb|KK920686.1|	811896-812054	17	8.6	107	74	-0.521551	0.6391	no
ni|645903744|nb|KK920686.1|	812221-812354	17	8.6	8	2	-2	0.24815	no
ni|645903744|nb|KK920686.1|	812609-813042	17	8.6	54	21	-1	0.3142	no
ni|645903744|nb|KK920686.1|	815573-815710	17	8.6	247	169	-0.548777	0.62325	no
ni|645903744|nb|KK920686.1|	816518-817148	17	8.6	2	0.759072	-1	0.3761	no
ni|645903744|nb|KK920686.1|	818273-818494	17	8.6	154	125	-0.301619	0.81175	no
ni|645903745|nb|KK920685.1|	104644-105067	16	18	0.15366	10	6	0.0443	no

ni|645903745|nb|KK920685.1|	107996-108274	16	18	0.140008	13	7	0.1194	no
ni|645903745|nb|KK920685.1|	114036-114176	16	18	0.618755	22	5	0.1202	no
ni|645903745|nb|KK920685.1|	118546-118727	16	18	0.619182	22	5	0.0448	no
ni|645903745|nb|KK920685.1|	121289-121502	16	18	0.221503	17	6	0.11945	no
ni|645903745|nb|KK920685.1|	137579-137932	16	18	0	492	inf	5.00E-05	yes
ni|645903745|nb|KK920685.1|	15417-15573	16	18	22	16	-0.501599	0.67195	no
ni|645903745|nb|KK920685.1|	15693-15841	16	18	13	15	0.186557	0.87465	no
ni|645903745|nb|KK920685.1|	160781-160926	16	18	4	4	0.125439	0.92845	no
ni|645903745|nb|KK920685.1|	167352-167479	16	18	4	17	2	0.12655	no
ni|645903745|nb|KK920685.1|	17293-17542	16	18	5	6	0.448383	0.70255	no
ni|645903745|nb|KK920685.1|	174515-174637	16	18	21	58	1	0.23255	no
ni|645903745|nb|KK920685.1|	184857-185481	16	18	0	226	inf	5.00E-05	yes
ni|645903745|nb|KK920685.1|	189162-189438	16	18	0	33	inf	5.00E-05	yes
ni|645903745|nb|KK920685.1|	193057-193216	16	18	0	51	inf	5.00E-05	yes
ni|645903745|nb|KK920685.1|	193504-193784	16	18	0	20	inf	5.00E-05	yes
ni|645903745|nb|KK920685.1|	19708-19896	16	18	14	44	2	0.17765	no
ni|645903745|nb|KK920685.1|	24145-24563	16	18	12	28	1	0.28815	no
ni|645903745|nb|KK920685.1|	26199-26543	16	18	8	20	1	0.2748	no
ni|645903745|nb|KK920685.1|	29153-29565	16	18	13	31	1	0.25425	no
ni|645903745|nb|KK920685.1|	30169-30526	16	18	26	49	0.918256	0.40535	no
ni|645903745|nb|KK920685.1|	31477-31893	16	18	24	47	0.979233	0.36325	no
ni|645903745|nb|KK920685.1|	316488-319185	16	18	5	13	1	0.29825	no
ni|645903745|nb|KK920685.1|	32210-32554	16	18	13	11	-0.344095	0.76295	no
ni|645903745|nb|KK920685.1|	32892-33116	16	18	20	18	-0.158653	0.89545	no
ni|645903745|nb|KK920685.1|	33953-34238	16	18	33	19	-0.812697	0.48655	no
ni|645903745|nb|KK920685.1|	342272-342749	16	18	0	2	inf	0.00015	yes
ni|645903745|nb|KK920685.1|	34631-34759	16	18	9	3	-1	0.35155	no
ni|645903745|nb|KK920685.1|	35472-35726	16	18	42	22	-0.929637	0.42475	no
ni|645903745|nb|KK920685.1|	35997-36246	16	18	14	6	-1	0.30655	no
ni|645903745|nb|KK920685.1|	38267-38633	16	18	0	26	inf	5.00E-05	yes

ni|645903745|nb|KK920685.1|	38825-39691	16	18	0	3	inf	5.00E-05	yes
ni|645903745|nb|KK920685.1|	42106-42304	16	18	0	18	inf	5.00E-05	yes
ni|645903745|nb|KK920685.1|	431597-432044	16	18	0.571898	0.341635	-0.743303	0.59615	no
ni|645903745|nb|KK920685.1|	432904-433314	16	18	1	1	-0.158431	0.89795	no
ni|645903745|nb|KK920685.1|	433468-434265	16	18	1	0.644505	-0.904255	0.4582	no
ni|645903745|nb|KK920685.1|	434385-434708	16	18	2	1	-0.272112	0.8304	no
ni|645903745|nb|KK920685.1|	44878-45025	16	18	0	19	inf	0.0001	yes
ni|645903745|nb|KK920685.1|	46753-47008	16	18	0	9	inf	5.00E-05	yes
ni|645903745|nb|KK920685.1|	48510-48982	16	18	0.133315	19	7	0.0443	no
ni|645903745|nb|KK920685.1|	544869-545205	16	18	82	84	0.0351807	0.9809	no
ni|645903745|nb|KK920685.1|	545835-546285	16	18	1	1	-0.0652244	0.9589	no
ni|645903745|nb|KK920685.1|	547469-548460	16	18	138	129	-0.0991873	0.94565	no
ni|645903745|nb|KK920685.1|	59309-60167	16	18	0.163069	12	6	0.02725	no
ni|645903745|nb|KK920685.1|	63423-63576	16	18	0	13	inf	0.0003	yes
ni|645903745|nb|KK920685.1|	65180-65451	16	18	0.437369	19	5	0.05055	no
ni|645903745|nb|KK920685.1|	69695-70049	16	18	0	19	inf	5.00E-05	yes
ni|645903745|nb|KK920685.1|	71193-71352	16	18	0	29	inf	0.00025	yes
ni|645903745|nb|KK920685.1|	75474-75770	16	18	0.127048	4	5	0.1202	no
ni|645903745|nb|KK920685.1|	89196-89341	16	18	0	23	inf	5.00E-05	yes
ni|645903745|nb|KK920685.1|	92600-92774	16	18	0.338663	24	6	0.11965	no
ni|645903746|nb|KK920684.1|	159151-159508	15	15	0.966997	0.184815	-2	0.2949	no
ni|645903746|nb|KK920684.1|	178738-178961	15	15	4	6	0.518412	0.66895	no
ni|645903746|nb|KK920684.1|	187397-187611	15	15	22	22	-0.0386853	0.976	no
ni|645903746|nb|KK920684.1|	189575-189837	15	15	20	25	0.343327	0.76785	no
ni|645903746|nb|KK920684.1|	191090-191363	15	15	8	9	0.15642	0.8945	no
ni|645903746|nb|KK920684.1|	194308-194502	15	15	12	13	0.16896	0.88615	no
ni|645903746|nb|KK920684.1|	195137-195506	15	15	0.923577	1	0.313057	0.81835	no
ni|645903746|nb|KK920684.1|	196724-197197	15	15	10	9	-0.143175	0.90165	no
ni|645903746|nb|KK920684.1|	197304-197744	15	15	0.80264	0.209222	-2	0.24815	no
ni|645903746|nb|KK920684.1|	199741-200302	15	15	0.322385	0.359544	0.157383	0.9131	no

ni|645903746|nb|KK920684.1|	202378-202497	15	15	18	13	-0.415343	0.7377	no
ni|645903746|nb|KK920684.1|	203401-204220	15	15	5	4	-0.0971235	0.9268	no
ni|645903746|nb|KK920684.1|	206787-207090	15	15	3	2	-0.373892	0.7575	no
ni|645903746|nb|KK920684.1|	208290-208422	15	15	12	15	0.325039	0.78615	no
ni|645903746|nb|KK920684.1|	208475-208839	15	15	6	4	-0.411249	0.73255	no
ni|645903746|nb|KK920684.1|	209130-209268	15	15	16	12	-0.463167	0.6963	no
ni|645903746|nb|KK920684.1|	209357-209509	15	15	21	17	-0.31717	0.78875	no
ni|645903746|nb|KK920684.1|	209879-210158	15	15	21	83	2	0.07765	no
ni|645903746|nb|KK920684.1|	210315-210654	15	15	15	70	2	0.05135	no
ni|645903746|nb|KK920684.1|	210836-211145	15	15	8	38	2	0.0618	no
ni|645903746|nb|KK920684.1|	212067-212278	15	15	0.451064	3	3	0.071	no
ni|645903746|nb|KK920684.1|	214217-214390	15	15	20	89	2	0.06695	no
ni|645903746|nb|KK920684.1|	220470-220662	15	15	14	75	2	0.0486	no
ni|645903746|nb|KK920684.1|	221044-221609	15	15	0.639198	0.254599	-1	0.3479	no
ni|645903746|nb|KK920684.1|	221873-222269	15	15	15	45	2	0.1554	no
ni|645903746|nb|KK920684.1|	222429-222632	15	15	198	434	1	0.4481	no
ni|645903746|nb|KK920684.1|	223197-223331	15	15	111	247	1	0.3141	no
ni|645903746|nb|KK920684.1|	228277-228788	15	15	29	65	1	0.36635	no
ni|645903746|nb|KK920684.1|	229421-229584	15	15	152	270	0.823554	0.44835	no
ni|645903746|nb|KK920684.1|	232959-233099	15	15	129.32	306	1	0.26985	no
ni|645903746|nb|KK920684.1|	234380-235418	15	15	1	0.402988	-1	0.24955	no
ni|645903746|nb|KK920684.1|	237269-237729	15	15	32	56	0.779381	0.5397	no
ni|645903746|nb|KK920684.1|	239297-239424	15	15	14	5	-1	0.32095	no
ni|645903746|nb|KK920684.1|	241702-242311	15	15	0.437774	0.279005	-0.649896	0.6679	no
ni|645903746|nb|KK920684.1|	242673-242964	15	15	108	92	-0.22446	0.8529	no
ni|645903746|nb|KK920684.1|	261472-262080	15	15	54	50	-0.103268	0.94205	no
ni|645903746|nb|KK920684.1|	265588-265702	15	15	175	178	0.0264565	0.98175	no
ni|645903746|nb|KK920684.1|	281949-282525	15	15	23	39	0.75574	0.56195	no
ni|645903746|nb|KK920684.1|	282652-283542	15	15	24	43	0.83521	0.57305	no
ni|645903746|nb|KK920684.1|	283622-284343	15	15	18	32	0.854626	0.50365	no

ni|645903746|nb|KK920684.1|	334630-335028	15	15	1	0.079626	-4	0.0255	no
ni|645903746|nb|KK920684.1|	383218-383337	15	15	21	6	-2	0.1999	no
ni|645903746|nb|KK920684.1|	384460-384622	15	15	8	4	-0.993212	0.47555	no
ni|645903746|nb|KK920684.1|	386354-386677	15	15	7	6	-0.355168	0.76785	no
ni|645903746|nb|KK920684.1|	386878-387310	15	15	4	4	0.232416	0.84015	no
ni|645903746|nb|KK920684.1|	431226-431484	15	15	10	10	-0.0661004	0.9528	no
ni|645903746|nb|KK920684.1|	548066-548264	15	15	10	8	-0.470378	0.6922	no
ni|645903746|nb|KK920684.1|	570266-570412	15	15	14	11	-0.375326	0.7568	no
ni|645903746|nb|KK920684.1|	635086-635219	15	15	28	24	-0.189262	0.87405	no
ni|645903746|nb|KK920684.1|	748335-748630	15	15	3	6	0.733884	0.53775	no
ni|645903746|nb|KK920684.1|	834811-835345	15	15	35	22	-0.637026	0.55735	no
ni|645903746|nb|KK920684.1|	864176-864304	15	15	0	53	inf	5.00E-05	yes
ni|645903746|nb|KK920684.1|	866666-867011	15	15	24	24	-0.0111071	0.9915	no
ni|645903746|nb|KK920684.1|	868142-868260	15	15	37	241	3	0.0333	no
ni|645903746|nb|KK920684.1|	871053-872512	15	15	26	189	3	0.068	no
ni|645903746|nb|KK920684.1|	873023-873141	15	15	21	4	-2	0.17	no
ni|645903746|nb|KK920684.1|	873770-873931	15	15	28	22	-0.373311	0.74745	no
ni|645903746|nb|KK920684.1|	874293-874436	15	15	13	14	0.117214	0.9234	no
ni|645903746|nb|KK920684.1|	874792-874955	15	15	12	7	-0.72618	0.54605	no
ni|645903746|nb|KK920684.1|	875086-875412	15	15	49	31	-0.671933	0.5441	no
ni|645903746|nb|KK920684.1|	876150-876561	15	15	18	12	-0.618711	0.5896	no
ni|645903746|nb|KK920684.1|	876672-876800	15	15	50	17	-2	0.19885	no
ni|645903746|nb|KK920684.1|	943737-943891	15	15	3	4	0.125453	0.92845	no
ni|645903746|nb|KK920684.1|	949670-949930	15	15	2	2	0.419345	0.73705	no
ni|645903746|nb|KK920684.1|	952593-952793	15	15	3	4	0.380478	0.7694	no
ni|645903746|nb|KK920684.1|	953586-953768	15	15	3	3	-0.0672914	0.9414	no
ni|645903746|nb|KK920684.1|	956513-956818	15	15	1	1	-0.0657579	0.94155	no
ni|645903746|nb|KK920684.1|	957267-957455	15	15	3	4	0.155177	0.90275	no
ni|645903747|nb|KK920683.1|	100298-100545	19	18	194	243	0.32081	0.82325	no
ni|645903747|nb|KK920683.1|	100772-101126	19	18	107	131	0.296907	0.848	no

ni|645903747|nb|KK920683.1|	101429-101671	19	18	24	19	-0.27438	0.80895	no
ni|645903747|nb|KK920683.1|	102305-102889	19	18	14	11	-0.37285	0.73535	no
ni|645903747|nb|KK920683.1|	1025516-102567[illegible]	19	18	27	25	-0.0906574	0.938	no
ni|645903747|nb|KK920683.1|	1030113-103106[illegible]	19	18	50	47	-0.0747481	0.95795	no
ni|645903747|nb|KK920683.1|	103065-103317	19	18	6	4	-0.544206	0.64605	no
ni|645903747|nb|KK920683.1|	1039129-103932[illegible]	19	18	48	51	0.0981189	0.9293	no
ni|645903747|nb|KK920683.1|	104034-104488	19	18	11	12	0.0557999	0.96055	no
ni|645903747|nb|KK920683.1|	1040491-1040971	19	18	10	12	0.25868	0.8208	no
ni|645903747|nb|KK920683.1|	1041612-104219[illegible]	19	18	0.407634	0.292263	-0.480006	0.7594	no
ni|645903747|nb|KK920683.1|	1042596-104272[illegible]	19	18	24	23	-0.0673236	0.9564	no
ni|645903747|nb|KK920683.1|	1048888-104903[illegible]	19	18	9	11	0.325109	0.78615	no
ni|645903747|nb|KK920683.1|	1056289-105668[illegible]	19	18	0.415176	2	2	0.14475	no
ni|645903747|nb|KK920683.1|	1059422-106042[illegible]	19	18	66	61	-0.11745	0.93465	no
ni|645903747|nb|KK920683.1|	1061303-106151[illegible]	19	18	70	58	-0.28611	0.79915	no
ni|645903747|nb|KK920683.1|	1064296-1065141	19	18	0.597324	0.539405	-0.147144	0.9109	no
ni|645903747|nb|KK920683.1|	1065307-106552[illegible]	19	18	93	81	-0.203118	0.85645	no
ni|645903747|nb|KK920683.1|	1082898-108390[illegible]	19	18	16	22	0.486154	0.6979	no
ni|645903747|nb|KK920683.1|	1084179-108444[illegible]	19	18	7	9	0.348965	0.7687	no
ni|645903747|nb|KK920683.1|	1088413-108947[illegible]	19	18	23	22	-0.0146637	0.9915	no
ni|645903747|nb|KK920683.1|	1089615-108985[illegible]	19	18	5	7	0.571158	0.629	no
ni|645903747|nb|KK920683.1|	1090809-109356[illegible]	19	18	100	110	0.138248	0.9223	no
ni|645903747|nb|KK920683.1|	1093772-1093941	19	18	12	13	0.0932051	0.93725	no
ni|645903747|nb|KK920683.1|	1094406-109460[illegible]	19	18	13	19	0.53638	0.64825	no
ni|645903747|nb|KK920683.1|	1094737-109505[illegible]	19	18	11	10	-0.127759	0.91	no
ni|645903747|nb|KK920683.1|	1095225-109536[illegible]	19	18	10	7	-0.454232	0.722	no
ni|645903747|nb|KK920683.1|	1095490-109598[illegible]	19	18	97	107	0.128214	0.92925	no
ni|645903747|nb|KK920683.1|	1098242-109838[illegible]	19	18	156	193	0.309568	0.77625	no
ni|645903747|nb|KK920683.1|	1135492-113828[illegible]	19	18	69.17	67	-0.0483472	0.975	no
ni|645903747|nb|KK920683.1|	1138719-113888[illegible]	19	18	61	76	0.303127	0.78925	no
ni|645903747|nb|KK920683.1|	1139645-113986[illegible]	19	18	44	58	0.416692	0.71725	no

ni|645903747|nb|KK920683.1|	1140038-1140677	19	18	10	15	0.579233	0.6076	no
ni|645903747|nb|KK920683.1|	1142107-1143215	19	18	0.343087	0.210907	-0.701968	0.6038	no
ni|645903747|nb|KK920683.1|	1156633-1156745	19	18	62	48	-0.367138	0.7573	no
ni|645903747|nb|KK920683.1|	1162176-1162964	19	18	0.503207	0.343664	-0.550155	0.68265	no
ni|645903747|nb|KK920683.1|	1163697-1164090	19	18	0.508477	1	1	0.38715	no
ni|645903747|nb|KK920683.1|	1168153-1168457	19	18	32	18	-0.840204	0.45945	no
ni|645903747|nb|KK920683.1|	1168751-1169047	19	18	3	3	0.00457826	0.9708	no
ni|645903747|nb|KK920683.1|	1169684-1172024	19	18	13	7	-0.874211	0.48485	no
ni|645903747|nb|KK920683.1|	1178986-1179180	19	18	46	55	0.263233	0.8177	no
ni|645903747|nb|KK920683.1|	1181530-1181649	19	18	68	45	-0.612854	0.60485	no
ni|645903747|nb|KK920683.1|	1208443-1208635	19	18	54	57	0.0644136	0.9554	no
ni|645903747|nb|KK920683.1|	1210834-1210963	19	18	83.61	103	0.303339	0.79705	no
ni|645903747|nb|KK920683.1|	1244963-1245127	19	18	49	58	0.231623	0.83815	no
ni|645903747|nb|KK920683.1|	1310194-1310553	19	18	11	6	-0.991494	0.40395	no
ni|645903747|nb|KK920683.1|	1310636-1310745	19	18	28	10	-2	0.3149	no
ni|645903747|nb|KK920683.1|	1310983-1311586	19	18	16	12	-0.481434	0.6643	no
ni|645903747|nb|KK920683.1|	1312471-1312609	19	18	6	2	-1	0.4023	no
ni|645903747|nb|KK920683.1|	1312989-1313411	19	18	14	14	0.0122934	0.99175	no
ni|645903747|nb|KK920683.1|	1314628-1314792	19	18	42	36	-0.208776	0.86325	no
ni|645903747|nb|KK920683.1|	1315972-1316274	19	18	17	14	-0.318317	0.7804	no
ni|645903747|nb|KK920683.1|	1317554-1318041	19	18	0.832789	0.673557	-0.306151	0.8123	no
ni|645903747|nb|KK920683.1|	1318532-1318664	19	18	25	30	0.290273	0.80405	no
ni|645903747|nb|KK920683.1|	1321530-1321962	19	18	0.149471	3	4	0.0468	no
ni|645903747|nb|KK920683.1|	1323599-1323806	19	18	0.468087	10	4	0.04575	no
ni|645903747|nb|KK920683.1|	1324238-1324880	19	18	0.548009	10	4	0.00725	no
ni|645903747|nb|KK920683.1|	13265-13562	19	18	6	8	0.290339	0.8036	no
ni|645903747|nb|KK920683.1|	1326974-1327355	19	18	4	6	0.449163	0.69865	no
ni|645903747|nb|KK920683.1|	1327439-1327674	19	18	2	1	-0.236292	0.8697	no
ni|645903747|nb|KK920683.1|	1332016-1333014	19	18	0	36.52	inf	5.00E-05	yes
ni|645903747|nb|KK920683.1|	1335368-1335792	19	18	0.076592	32	9	0.11935	no

ni|645903747|nb|KK920683.1|	1337314-1338364	19	18	0.052012	164	12	0.0443	no
ni|645903747|nb|KK920683.1|	1346627-1347305	19	18	854	608	-0.489436	0.69185	no
ni|645903747|nb|KK920683.1|	1347501-1347880	19	18	4	1	-2	0.1135	no
ni|645903747|nb|KK920683.1|	1354482-1355693	19	18	2543.47	1747.56	-0.541458	0.55245	no
ni|645903747|nb|KK920683.1|	1359402-1360144	19	18	8	8	-0.146842	0.89695	no
ni|645903747|nb|KK920683.1|	144776-144920	19	18	28	18	-0.623602	0.5964	no
ni|645903747|nb|KK920683.1|	14921-16502	19	18	38	35	-0.129259	0.924	no
ni|645903747|nb|KK920683.1|	149416-149734	19	18	3	2	-0.328721	0.7854	no
ni|645903747|nb|KK920683.1|	16631-16914	19	18	6	3	-1	0.38255	no
ni|645903747|nb|KK920683.1|	17078-17248	19	18	12	9	-0.510876	0.67685	no
ni|645903747|nb|KK920683.1|	17410-17533	19	18	13	9	-0.482405	0.7301	no
ni|645903747|nb|KK920683.1|	17610-18194	19	18	8	5	-0.663355	0.56555	no
ni|645903747|nb|KK920683.1|	18521-18823	19	18	8	8	-0.00258145	0.99195	no
ni|645903747|nb|KK920683.1|	18973-19153	19	18	6	4	-0.482348	0.71	no
ni|645903747|nb|KK920683.1|	19375-19539	19	18	18	14	-0.342856	0.7751	no
ni|645903747|nb|KK920683.1|	19683-19815	19	18	11	7	-0.704708	0.60375	no
ni|645903747|nb|KK920683.1|	20372-20677	19	18	4	3	-0.368321	0.75485	no
ni|645903747|nb|KK920683.1|	20803-20987	19	18	5	7	0.286366	0.81825	no
ni|645903747|nb|KK920683.1|	22153-22627	19	18	12	7	-0.719526	0.5329	no
ni|645903747|nb|KK920683.1|	232507-232621	19	18	37	37	-0.00338926	0.97495	no
ni|645903747|nb|KK920683.1|	23663-23832	19	18	9	7	-0.259905	0.82995	no
ni|645903747|nb|KK920683.1|	24259-24416	19	18	10	3	-2	0.1964	no
ni|645903747|nb|KK920683.1|	24575-24700	19	18	16	15	-0.0673438	0.9576	no
ni|645903747|nb|KK920683.1|	25200-25323	19	18	9	6	-0.65233	0.66125	no
ni|645903747|nb|KK920683.1|	25609-25913	19	18	4	6	0.574694	0.6306	no
ni|645903747|nb|KK920683.1|	26343-27220	19	18	1	1	0.0797306	0.9477	no
ni|645903747|nb|KK920683.1|	28417-28852	19	18	1	0.778657	-0.927754	0.47065	no
ni|645903747|nb|KK920683.1|	29035-30235	19	18	2	2	-0.379014	0.74455	no
ni|645903747|nb|KK920683.1|	31652-31847	19	18	4	4	-0.166623	0.89925	no
ni|645903747|nb|KK920683.1|	319228-319309	19	18	10393.5	9552.49	-0.121735	0.92525	no

ni|645903747|nb|KK920683.1|	32158-32370	19	18	2	2	-0.356242	0.7931	no
ni|645903747|nb|KK920683.1|	33946-34360	19	18	2	3	0.553599	0.6455	no
ni|645903747|nb|KK920683.1|	377217-377279	19	18	28081.9	30533.9	0.120769	0.9276	no
ni|645903747|nb|KK920683.1|	37812-38070	19	18	8	14	0.697832	0.54585	no
ni|645903747|nb|KK920683.1|	38133-38584	19	18	33	39	0.235498	0.8255	no
ni|645903747|nb|KK920683.1|	39088-39282	19	18	69	94	0.454746	0.6848	no
ni|645903747|nb|KK920683.1|	41540-41822	19	18	9	8	-0.110299	0.92435	no
ni|645903747|nb|KK920683.1|	43202-43386	19	18	32	34	0.0604848	0.95715	no
ni|645903747|nb|KK920683.1|	43503-43878	19	18	46	45	-0.0459166	0.9641	no
ni|645903747|nb|KK920683.1|	45104-45559	19	18	16	14	-0.190171	0.86465	no
ni|645903747|nb|KK920683.1|	45676-45844	19	18	5	3	-0.70468	0.60375	no
ni|645903747|nb|KK920683.1|	48778-49021	19	18	9	7	-0.307267	0.79275	no
ni|645903747|nb|KK920683.1|	50148-50627	19	18	41	34	-0.268624	0.799	no
ni|645903747|nb|KK920683.1|	50797-51656	19	18	2	2	0.300978	0.79475	no
ni|645903747|nb|KK920683.1|	52116-53194	19	18	2	2	-0.422598	0.7217	no
ni|645903747|nb|KK920683.1|	53654-53989	19	18	14	35	1	0.2601	no
ni|645903747|nb|KK920683.1|	54116-54419	19	18	2	0.703009	-1	0.32095	no
ni|645903747|nb|KK920683.1|	54639-56215	19	18	10	10	-0.0200143	0.9851	no
ni|645903747|nb|KK920683.1|	57367-57656	19	18	55	53	-0.0487421	0.9636	no
ni|645903747|nb|KK920683.1|	57886-58019	19	18	68	67	-0.0204768	0.9848	no
ni|645903747|nb|KK920683.1|	58138-58320	19	18	54	60	0.159777	0.88545	no
ni|645903747|nb|KK920683.1|	601837-602115	19	18	0.280016	5	4	0.04685	no
ni|645903747|nb|KK920683.1|	602905-603192	19	18	0.799286	38	6	0.0333	no
ni|645903747|nb|KK920683.1|	606341-606589	19	18	0.506003	34	6	0.05055	no
ni|645903747|nb|KK920683.1|	607728-608114	19	18	0.434168	8	4	0.0292	no
ni|645903747|nb|KK920683.1|	60942-61115	19	18	34	38	0.148834	0.89825	no
ni|645903747|nb|KK920683.1|	616136-616426	19	18	2	0.626223	-1	0.3479	no
ni|645903747|nb|KK920683.1|	61669-61820	19	18	42	41	-0.0344037	0.97635	no
ni|645903747|nb|KK920683.1|	616873-617000	19	18	21	26	0.316005	0.79115	no
ni|645903747|nb|KK920683.1|	617875-618300	19	18	2	2	0.214826	0.8585	no

ni|645903747|nb|KK920683.1|	618456-618601	19	18	31	41	0.410841	0.7248	no
ni|645903747|nb|KK920683.1|	620060-620399	19	18	8	6	-0.363664	0.75425	no
ni|645903747|nb|KK920683.1|	62088-62273	19	18	14	10	-0.381364	0.7446	no
ni|645903747|nb|KK920683.1|	621924-622708	19	18	18	16	-0.154929	0.8857	no
ni|645903747|nb|KK920683.1|	628140-628294	19	18	212	87	-1	0.2536	no
ni|645903747|nb|KK920683.1|	628814-629407	19	18	55	26	-1	0.383	no
ni|645903747|nb|KK920683.1|	629696-630033	19	18	35	16	-1	0.2967	no
ni|645903747|nb|KK920683.1|	630738-631117	19	18	1	0.425398	-1	0.3479	no
ni|645903747|nb|KK920683.1|	631539-631714	19	18	10	7	-0.465865	0.70195	no
ni|645903747|nb|KK920683.1|	63659-63960	19	18	5	3	-0.728746	0.5477	no
ni|645903747|nb|KK920683.1|	637621-637747	19	18	13	12	-0.174249	0.8924	no
ni|645903747|nb|KK920683.1|	639509-639638	19	18	55	30	-0.880219	0.45195	no
ni|645903747|nb|KK920683.1|	639768-640377	19	18	0.826907	0.325506	-1	0.33495	no
ni|645903747|nb|KK920683.1|	640834-641600	19	18	34	26	-0.380005	0.7556	no
ni|645903747|nb|KK920683.1|	645555-646421	19	18	3	1	-1	0.26235	no
ni|645903747|nb|KK920683.1|	646568-647269	19	18	0.699836	0.4723	-0.567315	0.6631	no
ni|645903747|nb|KK920683.1|	648921-649599	19	18	0.642174	0.28651	-1	0.41555	no
ni|645903747|nb|KK920683.1|	66894-67075	19	18	50	60	0.259994	0.82005	no
ni|645903747|nb|KK920683.1|	671135-671448	19	18	1	0	-inf	0.0036	no
ni|645903747|nb|KK920683.1|	67391-67569	19	18	4	1	-2	0.25505	no
ni|645903747|nb|KK920683.1|	68910-69634	19	18	0.872163	1	0.475779	0.6922	no
ni|645903747|nb|KK920683.1|	70684-71222	19	18	10	10	0.102989	0.9299	no
ni|645903747|nb|KK920683.1|	71879-72422	19	18	36	78	1	0.36575	no
ni|645903747|nb|KK920683.1|	74132-74675	19	18	44	78	0.82691	0.5791	no
ni|645903747|nb|KK920683.1|	744081-745737	19	18	22	29	0.380328	0.7888	no
ni|645903747|nb|KK920683.1|	747274-747497	19	18	0	6	inf	0.00025	yes
ni|645903747|nb|KK920683.1|	748383-748889	19	18	0.732572	0.116708	-3	0.2671	no
ni|645903747|nb|KK920683.1|	750337-750774	19	18	0.515343	0.492554	-0.065253	0.9302	no
ni|645903747|nb|KK920683.1|	751560-751918	19	18	17	13	-0.448828	0.6921	no
ni|645903747|nb|KK920683.1|	752429-753512	19	18	33	22	-0.589401	0.69045	no

ni|645903747|nb|KK920683.1|	75499-76171	19	18	0.432625	0.330888	-0.386777	0.78725	no
ni|645903747|nb|KK920683.1|	756209-756496	19	18	0.266429	2	3	0.0997	no
ni|645903747|nb|KK920683.1|	764961-765522	19	18	0.859694	1	0.458553	0.71285	no
ni|645903747|nb|KK920683.1|	765635-766276	19	18	1	2	1	0.30455	no
ni|645903747|nb|KK920683.1|	767123-767257	19	18	41	30	-0.448354	0.70515	no
ni|645903747|nb|KK920683.1|	767330-767617	19	18	18	17	-0.0334481	0.9744	no
ni|645903747|nb|KK920683.1|	768278-768400	19	18	41	14	-2	0.2278	no
ni|645903747|nb|KK920683.1|	768781-768941	19	18	25	21	-0.297503	0.79895	no
ni|645903747|nb|KK920683.1|	769242-769381	19	18	12	13	0.144272	0.90755	no
ni|645903747|nb|KK920683.1|	769466-769731	19	18	16	15	-0.0935187	0.9375	no
ni|645903747|nb|KK920683.1|	770348-770692	19	18	2	2	0.275478	0.823	no
ni|645903747|nb|KK920683.1|	772856-772971	19	18	25	29	0.180433	0.8836	no
ni|645903747|nb|KK920683.1|	77324-78365	19	18	0.262554	0.451913	0.78343	0.55245	no
ni|645903747|nb|KK920683.1|	773738-773928	19	18	15	19	0.341283	0.7691	no
ni|645903747|nb|KK920683.1|	774088-774696	19	18	8	10	0.437565	0.69765	no
ni|645903747|nb|KK920683.1|	781108-781772	19	18	456	371	-0.297228	0.83825	no
ni|645903747|nb|KK920683.1|	782849-782963	19	18	100.83	123	0.292376	0.8003	no
ni|645903747|nb|KK920683.1|	783088-783218	19	18	149	138	-0.107484	0.92265	no
ni|645903747|nb|KK920683.1|	784945-785258	19	18	32.83	34	0.0525253	0.96055	no
ni|645903747|nb|KK920683.1|	78564-79137	19	18	0.575975	0.350385	-0.717066	0.61925	no
ni|645903747|nb|KK920683.1|	787713-788296	19	18	1	2	0.626904	0.6021	no
ni|645903747|nb|KK920683.1|	790060-790262	19	18	132	108	-0.296051	0.78255	no
ni|645903747|nb|KK920683.1|	792004-792537	19	18	50	45	-0.157284	0.9207	no
ni|645903747|nb|KK920683.1|	793083-793736	19	18	53	45	-0.248132	0.86735	no
ni|645903747|nb|KK920683.1|	794913-795555	19	18	36	29	-0.29677	0.811	no
ni|645903747|nb|KK920683.1|	797307-798224	19	18	70	50	-0.481342	0.73425	no
ni|645903747|nb|KK920683.1|	798580-798843	19	18	150	95	-0.665784	0.5922	no
ni|645903747|nb|KK920683.1|	800235-800840	19	18	0.637372	0.609318	-0.0649394	0.9554	no
ni|645903747|nb|KK920683.1|	803080-804517	19	18	59	26	-1	0.41945	no
ni|645903747|nb|KK920683.1|	804614-805068	19	18	2	0.93757	-0.901717	0.46595	no

ni|645903747|nb|KK920683.1|	80593-80838	19	18	107	212	0.981518	0.4251	no
ni|645903747|nb|KK920683.1|	806250-806359	19	18	18	41	1	0.39265	no
ni|645903747|nb|KK920683.1|	806413-806750	19	18	35	34	-0.0267729	0.98195	no
ni|645903747|nb|KK920683.1|	808207-808342	19	18	16	22	0.409181	0.7319	no
ni|645903747|nb|KK920683.1|	808815-808941	19	18	15	5	-1	0.32095	no
ni|645903747|nb|KK920683.1|	81020-81429	19	18	2	2	0.542358	0.64695	no
ni|645903747|nb|KK920683.1|	810858-811019	19	18	86	91	0.0758792	0.9473	no
ni|645903747|nb|KK920683.1|	813589-813770	19	18	77	66	-0.238669	0.82625	no
ni|645903747|nb|KK920683.1|	81822-82043	19	18	2	2	-0.192115	0.88855	no
ni|645903747|nb|KK920683.1|	82313-82551	19	18	139	245	0.821251	0.5795	no
ni|645903747|nb|KK920683.1|	826144-826288	19	18	36	65	0.839682	0.47345	no
ni|645903747|nb|KK920683.1|	826490-827034	19	18	52	29	-0.82715	0.51115	no
ni|645903747|nb|KK920683.1|	827914-828426	19	18	24	23	-0.0506309	0.96275	no
ni|645903747|nb|KK920683.1|	830339-830476	19	18	11	69	3	0.04245	no
ni|645903747|nb|KK920683.1|	837422-837741	19	18	32	17	-0.91884	0.41865	no
ni|645903747|nb|KK920683.1|	839428-839548	19	18	24	21	-0.219408	0.86075	no
ni|645903747|nb|KK920683.1|	839736-840197	19	18	1	0.393986	-2	0.2996	no
ni|645903747|nb|KK920683.1|	841445-841742	19	18	1	0.845338	-0.717882	0.61925	no
ni|645903747|nb|KK920683.1|	843993-844345	19	18	0.887685	0.659765	-0.428094	0.75965	no
ni|645903747|nb|KK920683.1|	846374-846534	19	18	70	51	-0.456857	0.68885	no
ni|645903747|nb|KK920683.1|	847003-847314	19	18	4	3	-0.320982	0.7907	no
ni|645903747|nb|KK920683.1|	85559-85694	19	18	188	389	1	0.34065	no
ni|645903747|nb|KK920683.1|	855839-856081	19	18	6	5	-0.481309	0.68305	no
ni|645903747|nb|KK920683.1|	857198-857495	19	18	1	0.845338	-0.580378	0.70815	no
ni|645903747|nb|KK920683.1|	86488-86669	19	18	172	290	0.757403	0.5446	no
ni|645903747|nb|KK920683.1|	872356-872527	19	18	4	2	-0.719356	0.61925	no
ni|645903747|nb|KK920683.1|	876579-876856	19	18	173	120	-0.521555	0.72515	no
ni|645903747|nb|KK920683.1|	879125-879729	19	18	0.491263	0.187856	-1	0.35155	no
ni|645903747|nb|KK920683.1|	883607-883814	19	18	69	56	-0.289225	0.79355	no
ni|645903747|nb|KK920683.1|	888083-888350	19	18	14	16	0.184302	0.87195	no

ni|645903747|nb|KK920683.1|	888760-892015	19	18	17	17	-0.0488027	0.9705	no
ni|645903747|nb|KK920683.1|	901174-901720	19	18	23	23	0.0444162	0.9669	no
ni|645903747|nb|KK920683.1|	901895-902353	19	18	11	10	-0.102681	0.9293	no
ni|645903747|nb|KK920683.1|	902471-903976	19	18	26	24	-0.151461	0.9145	no
ni|645903747|nb|KK920683.1|	907993-908613	19	18	32	28	-0.167533	0.8764	no
ni|645903747|nb|KK920683.1|	908671-910418	19	18	35	30	-0.254482	0.8557	no
ni|645903747|nb|KK920683.1|	90878-91062	19	18	3	3	0.222236	0.8747	no
ni|645903747|nb|KK920683.1|	910559-910822	19	18	42	1	-5	0.02525	no
ni|645903747|nb|KK920683.1|	912924-913908	19	18	0.363387	0.775139	1	0.38625	no
ni|645903747|nb|KK920683.1|	91464-91714	19	18	3	3	-0.0661798	0.9589	no
ni|645903747|nb|KK920683.1|	916095-916647	19	18	0.986507	2	0.852513	0.48895	no
ni|645903747|nb|KK920683.1|	919879-920668	19	18	0.68193	1	0.73174	0.54495	no
ni|645903747|nb|KK920683.1|	920835-921549	19	18	1	1	0.198234	0.8619	no
ni|645903747|nb|KK920683.1|	922089-922256	19	18	17	7	-1	0.2723	no
ni|645903747|nb|KK920683.1|	922374-922883	19	18	21	10	-1	0.3621	no
ni|645903747|nb|KK920683.1|	92239-93223	19	18	2	2	-0.283908	0.80435	no
ni|645903747|nb|KK920683.1|	924292-924458	19	18	0	24	inf	0.00015	yes
ni|645903747|nb|KK920683.1|	924567-925110	19	18	0	0.962274	inf	0.00065	yes
ni|645903747|nb|KK920683.1|	927938-928593	19	18	48	33	-0.548966	0.71075	no
ni|645903747|nb|KK920683.1|	928805-928910	19	18	45	24	-0.942581	0.54025	no
ni|645903747|nb|KK920683.1|	929387-930385	19	18	33	33	0.0108644	0.9947	no
ni|645903747|nb|KK920683.1|	931649-932830	19	18	13	25	0.93549	0.452	no
ni|645903747|nb|KK920683.1|	933311-934127	19	18	0.690663	0.528297	-0.386633	0.7545	no
ni|645903747|nb|KK920683.1|	935187-935615	19	18	15	32	1	0.3274	no
ni|645903747|nb|KK920683.1|	93529-93775	19	18	1.71	0.979971	-0.80319	0.6027	no
ni|645903747|nb|KK920683.1|	939523-940272	19	18	13	23	0.818372	0.4551	no
ni|645903747|nb|KK920683.1|	94022-94441	19	18	2	2	-0.0652974	0.95635	no
ni|645903747|nb|KK920683.1|	94966-95418	19	18	2	1	-0.595735	0.62535	no
ni|645903747|nb|KK920683.1|	949997-950947	19	18	23	45	0.979053	0.5083	no
ni|645903747|nb|KK920683.1|	952673-952913	19	18	55	120	1	0.305	no

ni|645903747|nb|KK920683.1|	953407-953579	19	18	62	122	0.964595	0.38975	no
ni|645903747|nb|KK920683.1|	955570-955820	19	18	26	61	1	0.2802	no
ni|645903747|nb|KK920683.1|	95649-95920	19	18	120	184	0.614926	0.68155	no
ni|645903747|nb|KK920683.1|	957345-957563	19	18	63	115	0.854024	0.4291	no
ni|645903747|nb|KK920683.1|	959091-959256	19	18	54	91	0.751857	0.51415	no
ni|645903747|nb|KK920683.1|	959425-959554	19	18	10	6	-0.652267	0.64	no
ni|645903747|nb|KK920683.1|	961655-962078	19	18	25	19	-0.381677	0.73285	no
ni|645903747|nb|KK920683.1|	962440-962618	19	18	223	183.46	-0.280033	0.78965	no
ni|645903747|nb|KK920683.1|	963124-963287	19	18	152	124	-0.290866	0.7929	no
ni|645903747|nb|KK920683.1|	963474-963593	19	18	215	148	-0.534296	0.64255	no
ni|645903747|nb|KK920683.1|	963940-964959	19	18	24	25	0.0513887	0.97055	no
ni|645903747|nb|KK920683.1|	965625-966923	19	18	9	9	-0.0868409	0.934	no
ni|645903747|nb|KK920683.1|	967523-967844	19	18	70	73	0.0496284	0.96195	no
ni|645903747|nb|KK920683.1|	969355-969507	19	18	122	119	-0.0335087	0.97525	no
ni|645903747|nb|KK920683.1|	969695-970279	19	18	0.460492	0.73369	0.671994	0.62105	no
ni|645903747|nb|KK920683.1|	970815-971343	19	18	0.926312	0.442732	-1	0.43485	no
ni|645903747|nb|KK920683.1|	971480-971880	19	18	1	1	-0.245921	0.84435	no
ni|645903747|nb|KK920683.1|	97252-97815	19	18	18	25	0.468427	0.66995	no
ni|645903747|nb|KK920683.1|	973164-974284	19	18	36	38	0.108651	0.9387	no
ni|645903747|nb|KK920683.1|	98137-99138	19	18	23	30	0.384121	0.79025	no
ni|645903747|nb|KK920683.1|	99549-100179	19	18	1	0.401862	-2	0.22235	no
ni|645903748|nb|KK920682.1|	11669-11861	17	18	74	61	-0.27893	0.8033	no
ni|645903748|nb|KK920682.1|	12652-13123	17	18	1	0.766632	-0.802142	0.53265	no
ni|645903748|nb|KK920682.1|	13470-13776	17	18	44	32	-0.462511	0.67535	no
ni|645903748|nb|KK920682.1|	18654-19699	17	18	3	2	-0.4016	0.734	no
ni|645903748|nb|KK920682.1|	22525-22834	17	18	1	0.910252	-0.387664	0.78555	no
ni|645903748|nb|KK920682.1|	26741-27238	17	18	24	18	-0.402158	0.7179	no
ni|645903748|nb|KK920682.1|	297953-298168	17	18	1	4	2	0.234	no
ni|645903748|nb|KK920682.1|	303420-303684	17	18	1	2	1	0.4471	no
ni|645903748|nb|KK920682.1|	306666-306859	17	18	73	31	-1	0.29	no

ni|645903748|nb|KK920682.1|	307042-307329	17	18	25	10	-1	0.24	no
ni|645903748|nb|KK920682.1|	307723-307839	17	18	39	16	-1	0.3142	no
ni|645903748|nb|KK920682.1|	308540-309063	17	18	32	19	-0.759592	0.48925	no
ni|645903748|nb|KK920682.1|	310122-310256	17	18	40	24	-0.719341	0.54555	no
ni|645903748|nb|KK920682.1|	310482-311157	17	18	15	11	-0.433544	0.6996	no
ni|645903748|nb|KK920682.1|	312118-312294	17	18	26	26	0.00482493	0.99215	no
ni|645903748|nb|KK920682.1|	313803-314171	17	18	1	0.797332	-0.595973	0.64595	no
ni|645903748|nb|KK920682.1|	314334-314541	17	18	11	8	-0.492138	0.67635	no
ni|645903748|nb|KK920682.1|	314668-315345	17	18	0.814836	0.410012	-0.990842	0.47555	no
ni|645903748|nb|KK920682.1|	315744-316252	17	18	0.668216	0.3484	-0.939571	0.54025	no
ni|645903748|nb|KK920682.1|	316520-316695	17	18	36	26	-0.468933	0.6841	no
ni|645903748|nb|KK920682.1|	320332-320790	17	18	0.554231	0.463522	-0.257851	0.8692	no
ni|645903748|nb|KK920682.1|	321510-321679	17	18	24	23	-0.0892859	0.9363	no
ni|645903748|nb|KK920682.1|	324106-324781	17	18	2	3	0.131075	0.9101	no
ni|645903748|nb|KK920682.1|	32649-33213	17	18	0.747345	0.663389	-0.171919	0.89245	no
ni|645903748|nb|KK920682.1|	326876-327019	17	18	22	19	-0.22333	0.8473	no
ni|645903748|nb|KK920682.1|	336139-336779	17	18	0.137512	1	3	0.0631	no
ni|645903748|nb|KK920682.1|	338461-339002	17	18	3	2	-0.71712	0.5464	no
ni|645903748|nb|KK920682.1|	33894-37182	17	18	0.943817	0.680725	-0.471434	0.6823	no
ni|645903748|nb|KK920682.1|	339510-341053	17	18	74	137	0.894285	0.5303	no
ni|645903748|nb|KK920682.1|	39180-39677	17	18	0.686827	0.537147	-0.35463	0.7931	no
ni|645903748|nb|KK920682.1|	39832-40345	17	18	0.900117	0.630966	-0.512551	0.697	no
ni|645903748|nb|KK920682.1|	48496-49444	17	18	0.524571	0.501595	-0.0646149	0.9601	no
ni|645903748|nb|KK920682.1|	492636-492870	17	18	3	0	-inf	0.00195	yes
ni|645903748|nb|KK920682.1|	50074-50513	17	18	2	2	-0.121832	0.9188	no
ni|645903748|nb|KK920682.1|	50626-50813	17	18	80	49	-0.691937	0.54065	no
ni|645903748|nb|KK920682.1|	516553-517019	17	18	20	55	2	0.26075	no
ni|645903748|nb|KK920682.1|	517986-518282	17	18	3	10	2	0.12335	no
ni|645903748|nb|KK920682.1|	518583-518731	17	18	31	87	1	0.211	no
ni|645903748|nb|KK920682.1|	520879-521246	17	18	0.558325	0.711409	0.349575	0.81755	no

ni|645903748|nb|KK920682.1|	521463-521593	17	18	41	76	0.903558	0.44295	no
ni|645903748|nb|KK920682.1|	521858-522048	17	18	34	94	1	0.19975	no
ni|645903748|nb|KK920682.1|	522185-522394	17	18	3	1	-1	0.3479	no
ni|645903748|nb|KK920682.1|	523443-524585	17	18	1	1	0.0930168	0.9367	no
ni|645903748|nb|KK920682.1|	524715-525574	17	18	0.781696	0.560572	-0.479709	0.69685	no
ni|645903748|nb|KK920682.1|	525688-526402	17	18	1	0.808966	-0.479838	0.6944	no
ni|645903748|nb|KK920682.1|	527270-527526	17	18	32	84.79	1	0.2195	no
ni|645903748|nb|KK920682.1|	528715-528842	17	18	31	79	1	0.26645	no
ni|645903748|nb|KK920682.1|	535364-535675	17	18	24	49	1	0.34	no
ni|645903748|nb|KK920682.1|	538087-538585	17	18	25	14	-0.77794	0.48745	no
ni|645903748|nb|KK920682.1|	544101-544232	17	18	11.88	11	-0.0672865	0.9562	no
ni|645903748|nb|KK920682.1|	544283-544504	17	18	2	2	0.392848	0.77595	no
ni|645903748|nb|KK920682.1|	545215-545540	17	18	3	5	0.725763	0.5449	no
ni|645903748|nb|KK920682.1|	547259-547762	17	18	2	2	0.117753	0.92125	no
ni|645903748|nb|KK920682.1|	548019-548197	17	18	6	4	-0.581896	0.6557	no
ni|645903748|nb|KK920682.1|	548285-549264	17	18	3	3	-0.167691	0.87885	no
ni|645903748|nb|KK920682.1|	549351-549505	17	18	5	0.881251	-2	0.2949	no
ni|645903748|nb|KK920682.1|	550150-550640	17	18	4	3	-0.41306	0.73045	no
ni|645903748|nb|KK920682.1|	550788-551472	17	18	1	1	-0.121418	0.9188	no
ni|645903748|nb|KK920682.1|	552178-552812	17	18	0.278127	1	2	0.14305	no
ni|645903748|nb|KK920682.1|	553071-554842	17	18	0.768003	3	2	0.0883	no
ni|645903748|nb|KK920682.1|	555132-555595	17	18	1	2	0.578662	0.65045	no
ni|645903748|nb|KK920682.1|	555721-556170	17	18	1	1	0.0952382	0.93935	no
ni|645903748|nb|KK920682.1|	556903-557125	17	18	1	4	2	0.28535	no
ni|645903748|nb|KK920682.1|	558240-559049	17	18	1	2	0.63573	0.585	no
ni|645903748|nb|KK920682.1|	568679-569216	17	18	9	79	3	0.03325	no
ni|645903748|nb|KK920682.1|	572184-572321	17	18	17	120	3	0.0269	no
ni|645903748|nb|KK920682.1|	575677-576187	17	18	0.060449	9	7	0.11935	no
ni|645903748|nb|KK920682.1|	577459-577949	17	18	0.063565	9	7	0.11935	no
ni|645903748|nb|KK920682.1|	578377-578658	17	18	0	6	inf	5.00E-05	yes

ni|645903748|nb|KK920682.1|	588519-588690	17	18	7	4	-0.804245	0.53265	no
ni|645903748|nb|KK920682.1|	5907-6052	17	18	32	24	-0.433334	0.7142	no
ni|645903748|nb|KK920682.1|	593217-593777	17	18	0.861572	1	0.394421	0.75185	no
ni|645903748|nb|KK920682.1|	600540-600681	17	18	11	8	-0.429792	0.73195	no
ni|645903748|nb|KK920682.1|	603811-603969	17	18	22	13	-0.813444	0.49705	no
ni|645903748|nb|KK920682.1|	606368-606783	17	18	3	4	0.131729	0.90955	no
ni|645903748|nb|KK920682.1|	607418-607609	17	18	11	8	-0.434897	0.71445	no
ni|645903748|nb|KK920682.1|	607728-607894	17	18	8	7	-0.356741	0.77485	no
ni|645903748|nb|KK920682.1|	610378-610541	17	18	8	9	0.328712	0.79035	no
ni|645903748|nb|KK920682.1|	610642-610776	17	18	7	8	0.195771	0.87955	no
ni|645903748|nb|KK920682.1|	617322-617466	17	18	12	9	-0.52664	0.67145	no
ni|645903748|nb|KK920682.1|	619905-620071	17	18	11	15	0.418193	0.7244	no
ni|645903748|nb|KK920682.1|	623324-623793	17	18	0.940836	2	0.712427	0.57115	no
ni|645903748|nb|KK920682.1|	625722-627366	17	18	0.143874	1	3	0.0485	no
ni|645903748|nb|KK920682.1|	629089-630089	17	18	0.164728	0.866337	2	0.1162	no
ni|645903748|nb|KK920682.1|	641467-642522	17	18	0.129348	0.321587	1	0.35675	no
ni|645903748|nb|KK920682.1|	644813-644980	17	18	10	6	-0.822129	0.50305	no
ni|645903748|nb|KK920682.1|	645105-645320	17	18	15	11	-0.432332	0.70845	no
ni|645903748|nb|KK920682.1|	645800-646001	17	18	11	6	-0.771505	0.51925	no
ni|645903748|nb|KK920682.1|	649230-649619	17	18	1	1	0.141068	0.90865	no
ni|645903748|nb|KK920682.1|	650288-650575	17	18	2	0.763613	-2	0.2996	no
ni|645903748|nb|KK920682.1|	651999-652492	17	18	1	0.9044	-0.681802	0.5893	no
ni|645903748|nb|KK920682.1|	654135-654629	17	18	2	1	-0.724092	0.5469	no
ni|645903748|nb|KK920682.1|	657204-657876	17	18	1	0.537692	-1	0.3948	no
ni|645903748|nb|KK920682.1|	658525-659676	17	18	2	0.786571	-1	0.2794	no
ni|645903748|nb|KK920682.1|	6589-6985	17	18	0.671056	0.481	-0.480398	0.7594	no
ni|645903748|nb|KK920682.1|	660474-660915	17	18	0.800273	0.973494	0.282679	0.82625	no
ni|645903748|nb|KK920682.1|	661138-661564	17	18	4	5	0.188477	0.87035	no
ni|645903748|nb|KK920682.1|	663607-663845	17	18	3	6	0.978069	0.42695	no
ni|645903748|nb|KK920682.1|	667388-667853	17	18	0.067943	32	9	0.11935	no

ni|645903748|nb|KK920682.1|	680098-680230	17	18	7	6	-0.237204	0.8697	no
ni|645903748|nb|KK920682.1|	714484-714833	17	18	2	3	0.3495	0.76915	no
ni|645903748|nb|KK920682.1|	775597-776041	17	18	33	29	-0.156868	0.8849	no
ni|645903748|nb|KK920682.1|	776943-777518	17	18	1	0.847336	-0.677963	0.58655	no
ni|645903748|nb|KK920682.1|	778337-778917	17	18	0.464348	0.295932	-0.64994	0.6679	no
ni|645903748|nb|KK920682.1|	780779-780980	17	18	31	38	0.280138	0.8061	no
ni|645903748|nb|KK920682.1|	781180-781387	17	18	25	26	0.0743436	0.94895	no
ni|645903748|nb|KK920682.1|	781664-782110	17	18	6	5	-0.250448	0.82955	no
ni|645903748|nb|KK920682.1|	782220-782730	17	18	5	6	0.250104	0.83535	no
ni|645903748|nb|KK920682.1|	785864-786255	17	18	14	16	0.185808	0.8732	no
ni|645903748|nb|KK920682.1|	787251-787739	17	18	16	15	-0.124276	0.91315	no
ni|645903748|nb|KK920682.1|	788701-788904	17	18	21	22	0.076673	0.95215	no
ni|645903748|nb|KK920682.1|	790955-791136	17	18	20	18	-0.113105	0.9216	no
ni|645903748|nb|KK920682.1|	793293-793511	17	18	3	2	-0.803598	0.5588	no
ni|645903748|nb|KK920682.1|	794267-795241	17	18	0.735113	1	0.726813	0.54425	no
ni|645903748|nb|KK920682.1|	799392-800774	17	18	0.519992	0.202593	-1	0.2905	no
ni|645903748|nb|KK920682.1|	801622-802390	17	18	16	14	-0.233453	0.83405	no
ni|645903748|nb|KK920682.1|	802919-803172	17	18	27	24	-0.155233	0.8893	no
ni|645903748|nb|KK920682.1|	804357-804778	17	18	1	0.443344	-1	0.3464	no
ni|645903748|nb|KK920682.1|	805783-805939	17	18	61	74	0.267223	0.8172	no
ni|645903748|nb|KK920682.1|	806580-806719	17	18	80	85	0.0950388	0.9318	no
ni|645903748|nb|KK920682.1|	807612-807753	17	18	54	62	0.195812	0.86515	no
ni|645903748|nb|KK920682.1|	808011-808346	17	18	41	38	-0.122784	0.90885	no
ni|645903748|nb|KK920682.1|	808647-808940	17	18	37	33	-0.148588	0.89465	no
ni|645903748|nb|KK920682.1|	809154-810105	17	18	74	60	-0.301309	0.8316	no
ni|645903748|nb|KK920682.1|	810244-810475	17	18	77	59	-0.385671	0.7265	no
ni|645903748|nb|KK920682.1|	810591-810846	17	18	66	52	-0.340468	0.7553	no
ni|645903748|nb|KK920682.1|	811199-811310	17	18	13	20	0.669352	0.65595	no
ni|645903748|nb|KK920682.1|	812558-812949	17	18	4	3	-0.480414	0.67875	no
ni|645903748|nb|KK920682.1|	813077-813226	17	18	21	12	-0.874552	0.46205	no

ni|645903748|nb|KK920682.1|	813699-813833	17	18	51	52	0.0118077	0.99145	no
ni|645903748|nb|KK920682.1|	814099-814313	17	18	59	53	-0.165855	0.88345	no
ni|645903748|nb|KK920682.1|	815957-816221	17	18	89	84	-0.0933853	0.9332	no
ni|645903748|nb|KK920682.1|	817313-817710	17	18	35	33	-0.110609	0.9206	no
ni|645903748|nb|KK920682.1|	833358-833941	17	18	9	20	1	0.2807	no
ni|645903748|nb|KK920682.1|	844821-845001	17	18	22	15	-0.602347	0.6083	no
ni|645903748|nb|KK920682.1|	847078-847413	17	18	9	9	-0.0161334	0.99065	no
ni|645903748|nb|KK920682.1|	879669-880200	17	18	0	1	inf	0.00055	yes
ni|645903748|nb|KK920682.1|	899189-899753	17	18	0	1	inf	0.0003	yes
ni|645903748|nb|KK920682.1|	9002-9344	17	18	20	17	-0.238271	0.8394	no
ni|645903749|nb|KK920681.1|	132993-134621	19	18	93	99	0.091643	0.94895	no
ni|645903749|nb|KK920681.1|	134692-134911	19	18	121	102	-0.250359	0.8146	no
ni|645903749|nb|KK920681.1|	156715-157295	19	18	4	5	0.306991	0.79185	no
ni|645903749|nb|KK920681.1|	158704-159155	19	18	33	29	-0.162084	0.88065	no
ni|645903749|nb|KK920681.1|	159642-160101	19	18	3	1	-1	0.3376	no
ni|645903749|nb|KK920681.1|	160229-160513	19	18	47	36.35	-0.361917	0.74865	no
ni|645903749|nb|KK920681.1|	161472-162482	19	18	15	13	-0.216585	0.84065	no
ni|645903749|nb|KK920681.1|	164249-164766	19	18	100	140	0.486831	0.7295	no
ni|645903749|nb|KK920681.1|	165672-166147	19	18	97	151	0.634592	0.65585	no
ni|645903749|nb|KK920681.1|	166953-167497	19	18	0.557983	0.373371	-0.579612	0.70815	no
ni|645903749|nb|KK920681.1|	167618-167937	19	18	1	0.434207	-2	0.22995	no
ni|645903749|nb|KK920681.1|	168676-169008	19	18	111.27	176	0.658786	0.65225	no
ni|645903749|nb|KK920681.1|	169356-170801	19	18	3	3	-0.0465136	0.96825	no
ni|645903749|nb|KK920681.1|	171664-172024	19	18	3	2	-0.51295	0.6711	no
ni|645903749|nb|KK920681.1|	173249-173737	19	18	109	184	0.754372	0.59395	no
ni|645903749|nb|KK920681.1|	176454-176731	19	18	3	11	2	0.11405	no
ni|645903749|nb|KK920681.1|	180989-181603	19	18	0.240832	0.414421	0.783071	0.58145	no
ni|645903749|nb|KK920681.1|	184875-185461	19	18	0.71336	0.048711	-4	0.0255	no
ni|645903749|nb|KK920681.1|	191742-191936	19	18	2	2	-0.0671069	0.923	no
ni|645903749|nb|KK920681.1|	209105-209262	19	18	138	150	0.121434	0.91065	no

ni|645903749|nb|KK920681.1|	211108-211403	19	18	1	0.732061	-0.802783	0.6027	no
ni|645903749|nb|KK920681.1|	212701-213682	19	18	25	27	0.132127	0.9298	no
ni|645903749|nb|KK920681.1|	214897-215154	19	18	120	123	0.0372474	0.97975	no
ni|645903749|nb|KK920681.1|	216326-216648	19	18	144	146	0.0191588	0.98935	no
ni|645903749|nb|KK920681.1|	216718-216833	19	18	20	18	-0.182972	0.889	no
ni|645903749|nb|KK920681.1|	220225-220342	19	18	14	9	-0.582026	0.70815	no
ni|645903749|nb|KK920681.1|	221047-221686	19	18	121	255	1	0.44765	no
ni|645903749|nb|KK920681.1|	222021-222532	19	18	122	298	1	0.36535	no
ni|645903749|nb|KK920681.1|	222693-223121	19	18	77	195	1	0.3666	no
ni|645903749|nb|KK920681.1|	234268-234546	19	18	7	3	-1	0.3594	no
ni|645903749|nb|KK920681.1|	274371-274726	19	18	0.779707	41	6	0.0144	no
ni|645903749|nb|KK920681.1|	275890-276118	19	18	0.585906	33	6	0.0507	no
ni|645903749|nb|KK920681.1|	277622-278061	19	18	0.292735	18	6	0.03715	no
ni|645903749|nb|KK920681.1|	293953-294099	19	18	0.539994	42	6	0.11945	no
ni|645903749|nb|KK920681.1|	29764-30771	19	18	0	37	inf	5.00E-05	yes
ni|645903749|nb|KK920681.1|	315564-315728	19	18	0	35	inf	5.00E-05	yes
ni|645903749|nb|KK920681.1|	319651-319786	19	18	0	59.16	inf	5.00E-05	yes
ni|645903749|nb|KK920681.1|	323999-324147	19	18	0	68	inf	5.00E-05	yes
ni|645903749|nb|KK920681.1|	324269-325725	19	18	0.018206	60	12	0.11935	no
ni|645903749|nb|KK920681.1|	329354-329468	19	18	0	74	inf	5.00E-05	yes
ni|645903749|nb|KK920681.1|	331052-331293	19	18	0	12	inf	5.00E-05	yes
ni|645903749|nb|KK920681.1|	332602-332789	19	18	111	111	-0.00863332	0.9926	no
ni|645903749|nb|KK920681.1|	332927-333508	19	18	33	29	-0.161585	0.8741	no
ni|645903749|nb|KK920681.1|	333828-334308	19	18	158	138	-0.200938	0.88815	no
ni|645903749|nb|KK920681.1|	334563-334872	19	18	7	3	-1	0.3171	no
ni|645903749|nb|KK920681.1|	335635-336462	19	18	23	23	0.0119622	0.98965	no
ni|645903749|nb|KK920681.1|	336577-337003	19	18	3	0.800266	-2	0.14275	no
ni|645903749|nb|KK920681.1|	337304-338038	19	18	71	60	-0.250755	0.8591	no
ni|645903749|nb|KK920681.1|	338390-338512	19	18	474	349	-0.443884	0.6776	no
ni|645903749|nb|KK920681.1|	339094-339513	19	18	1	0.223069	-2	0.1791	no

ni|645903749|nb|KK920681.1|	339924-340364	19	18	28	22	-0.332796	0.7678	no
ni|645903749|nb|KK920681.1|	341389-341832	19	18	23	12	-0.899431	0.43465	no
ni|645903749|nb|KK920681.1|	342005-342130	19	18	460	293	-0.650779	0.54925	no
ni|645903749|nb|KK920681.1|	348040-350055	19	18	25	27	0.098021	0.9452	no
ni|645903749|nb|KK920681.1|	352463-352753	19	18	1	1	-0.217853	0.87705	no
ni|645903749|nb|KK920681.1|	353147-353742	19	18	3	3	-0.0649546	0.9586	no
ni|645903749|nb|KK920681.1|	353833-354568	19	18	5	5	-0.154976	0.89205	no
ni|645903749|nb|KK920681.1|	354674-355855	19	18	22	25	0.168467	0.9099	no
ni|645903749|nb|KK920681.1|	356378-356645	19	18	0.746563	2	2	0.22835	no
ni|645903749|nb|KK920681.1|	357424-358175	19	18	0.721879	1	0.545292	0.66	no
ni|645903749|nb|KK920681.1|	358815-359187	19	18	0.639327	1	0.827642	0.55535	no
ni|645903749|nb|KK920681.1|	365647-365788	19	18	46	58	0.328707	0.77575	no
ni|645903749|nb|KK920681.1|	365895-366970	19	18	40	69	0.813866	0.5656	no
ni|645903749|nb|KK920681.1|	367560-367753	19	18	33	64	0.962377	0.39995	no
ni|645903749|nb|KK920681.1|	368582-368739	19	18	40	70	0.792944	0.4953	no
ni|645903749|nb|KK920681.1|	427310-427864	19	18	0	21	inf	5.00E-05	yes
ni|645903749|nb|KK920681.1|	492315-492483	19	18	8	8	0.125395	0.91735	no
ni|645903749|nb|KK920681.1|	544771-545035	19	18	0	57	inf	5.00E-05	yes
ni|645903749|nb|KK920681.1|	558907-559143	19	18	0.183701	74	9	0.11935	no
ni|645903749|nb|KK920681.1|	562832-562989	19	18	0	36	inf	5.00E-05	yes
ni|645903749|nb|KK920681.1|	566579-566696	19	18	0	76	inf	5.00E-05	yes
ni|645903749|nb|KK920681.1|	569611-570933	19	18	0.404128	49	7	0.00165	yes
ni|645903749|nb|KK920681.1|	571086-571460	19	18	0.181323	46	8	0.0443	no
ni|645903749|nb|KK920681.1|	571685-572546	19	18	0.389818	51	7	0.00455	no
ni|645903749|nb|KK920681.1|	573425-573944	19	18	0.236577	0.565355	1	0.38895	no
ni|645903749|nb|KK920681.1|	577639-577872	19	18	0.375773	93	8	0.0443	no
ni|645903749|nb|KK920681.1|	581063-581387	19	18	0.111054	8	6	0.1194	no
ni|645903749|nb|KK920681.1|	581744-582283	19	18	0	28	inf	5.00E-05	yes
ni|645903749|nb|KK920681.1|	582730-582886	19	18	0	30	inf	5.00E-05	yes
ni|645903749|nb|KK920681.1|	589556-590651	19	18	0.124136	53	9	0.02725	no

ni|645903749|nb|KK920681.1|	595403-595606	19	18	35	21	-0.693103	0.5532	no
ni|645903749|nb|KK920681.1|	595882-596059	19	18	32	18	-0.849145	0.46985	no
ni|645903749|nb|KK920681.1|	596565-596740	19	18	22	20	-0.13443	0.90875	no
ni|645903749|nb|KK920681.1|	597105-597360	19	18	4	2	-1	0.40515	no
ni|645903749|nb|KK920681.1|	597486-597652	19	18	14	8	-0.713597	0.55545	no
ni|645903749|nb|KK920681.1|	597760-598701	19	18	21	19	-0.130063	0.9044	no
ni|645903749|nb|KK920681.1|	599426-600728	19	18	6	8	0.414696	0.7121	no
ni|645903749|nb|KK920681.1|	600887-602014	19	18	3	3	-0.0995772	0.93075	no
ni|645903749|nb|KK920681.1|	602143-602802	19	18	2	3	0.407888	0.7257	no
ni|645903749|nb|KK920681.1|	603191-603571	19	18	13	14	0.0836423	0.94135	no
ni|645903749|nb|KK920681.1|	606378-606636	19	18	39	39	-0.00884311	0.99285	no
ni|645903749|nb|KK920681.1|	607305-607493	19	18	41	41	-0.0279562	0.9804	no
ni|645903749|nb|KK920681.1|	608024-608269	19	18	36	39	0.122068	0.91175	no
ni|645903749|nb|KK920681.1|	611020-611167	19	18	24	26	0.0816616	0.94055	no
ni|645903749|nb|KK920681.1|	611309-611445	19	18	38	42	0.15139	0.896	no
ni|645903749|nb|KK920681.1|	613633-613878	19	18	37	50	0.431542	0.7018	no
ni|645903749|nb|KK920681.1|	614108-615366	19	18	0.746389	0.591403	-0.335787	0.78025	no
ni|645903749|nb|KK920681.1|	618150-618956	19	18	7	10	0.436557	0.70035	no
ni|645903749|nb|KK920681.1|	619171-619923	19	18	16	19	0.256461	0.8121	no
ni|645903749|nb|KK920681.1|	621385-621616	19	18	42	49	0.227821	0.8391	no
ni|645903749|nb|KK920681.1|	622332-623475	19	18	26	25	-0.0552887	0.96895	no
ni|645903749|nb|KK920681.1|	623896-624090	19	18	56	56	-0.00656534	0.99565	no
ni|645903749|nb|KK920681.1|	627233-627978	19	18	2	0.806626	-1	0.3538	no
ni|645903749|nb|KK920681.1|	628652-629176	19	18	2	1	-0.512531	0.6711	no
ni|645903749|nb|KK920681.1|	629877-630012	19	18	14	10	-0.482295	0.71	no
ni|645903749|nb|KK920681.1|	630125-630342	19	18	7	3	-1	0.3041	no
ni|645903749|nb|KK920681.1|	630614-630719	19	18	33	12	-1	0.3864	no
ni|645903749|nb|KK920681.1|	632147-632399	19	18	14	8	-0.786051	0.50205	no
ni|645903749|nb|KK920681.1|	632736-632964	19	18	71	134	0.915582	0.39635	no
ni|645903749|nb|KK920681.1|	634069-634405	19	18	10	29	2	0.1818	no

ni|645903749|nb|KK920681.1|	636372-636526	19	18	92	174	0.911003	0.4168	no
ni|645903749|nb|KK920681.1|	636666-637585	19	18	1	2	0.193166	0.8658	no
ni|645903749|nb|KK920681.1|	637925-638231	19	18	2	2	-0.406789	0.755	no
ni|645903749|nb|KK920681.1|	638345-638712	19	18	15	38	1	0.23445	no
ni|645903749|nb|KK920681.1|	639131-640492	19	18	149.75	140	-0.0957289	0.94865	no
ni|645903749|nb|KK920681.1|	642835-644198	19	18	1593.48	1118.39	-0.51075	0.6346	no
ni|645903749|nb|KK920681.1|	644305-645583	19	18	2996.13	1841.06	-0.702563	0.46915	no
ni|645903749|nb|KK920681.1|	646142-647446	19	18	8	3	-1	0.2439	no
ni|645903749|nb|KK920681.1|	649384-649566	19	18	60	35	-0.787184	0.49435	no
ni|645903749|nb|KK920681.1|	649896-650326	19	18	29	20	-0.539613	0.6258	no
ni|645903749|nb|KK920681.1|	651558-652263	19	18	22	16	-0.491493	0.65235	no
ni|645903749|nb|KK920681.1|	652463-652578	19	18	43	26	-0.734919	0.55455	no
ni|645903749|nb|KK920681.1|	662832-663215	19	18	0.8776	1	0.51956	0.6956	no
ni|645903749|nb|KK920681.1|	670346-670763	19	18	0.078293	478	13	0.11935	no
ni|645903749|nb|KK920681.1|	673034-673347	19	18	0	1092.75	inf	5.00E-05	yes
ni|645903749|nb|KK920681.1|	673686-673834	19	18	0.518013	1134.73	11	0.11935	no
ni|645903749|nb|KK920681.1|	674397-674564	19	18	0.37353	754	11	0.11935	no
ni|645903749|nb|KK920681.1|	675449-675668	19	18	0	727	inf	5.00E-05	yes
ni|645903749|nb|KK920681.1|	675782-675968	19	18	0.291589	269	10	0.11935	no
ni|645903749|nb|KK920681.1|	676719-676831	19	18	0	334.37	inf	5.00E-05	yes
ni|645903749|nb|KK920681.1|	679387-679648	19	18	102	81	-0.32516	0.75995	no
ni|645903749|nb|KK920681.1|	679967-680351	19	18	1	0.835734	-0.830934	0.5313	no
ni|645903749|nb|KK920681.1|	680631-680796	19	18	192	183	-0.0759095	0.9428	no
ni|645903749|nb|KK920681.1|	680927-681356	19	18	5	3	-0.569744	0.63345	no
ni|645903749|nb|KK920681.1|	689061-689611	19	18	0.330304	2	3	0.0734	no
ni|645903749|nb|KK920681.1|	689827-690144	19	18	0.687978	3	2	0.2071	no
ni|645903749|nb|KK920681.1|	69477-69585	19	18	17	40	1	0.38715	no
ni|645903749|nb|KK920681.1|	695704-695839	19	18	18	23	0.37635	0.7506	no
ni|645903749|nb|KK920681.1|	700141-700341	19	18	96	17	-2	0.0422	no
ni|645903749|nb|KK920681.1|	700687-700791	19	18	118	75	-0.653285	0.5921	no

ni|645903749|nb|KK920681.1|	700860-701362	19	18	108	83	-0.379825	0.7951	no
ni|645903749|nb|KK920681.1|	703279-703900	19	18	0.712745	0.31798	-1	0.41555	no
ni|645903749|nb|KK920681.1|	704997-705285	19	18	4	4	0.0761562	0.94775	no
ni|645903749|nb|KK920681.1|	705710-706339	19	18	95	105	0.151774	0.9154	no
ni|645903749|nb|KK920681.1|	708036-708269	19	18	156	192	0.301771	0.8401	no
ni|645903749|nb|KK920681.1|	722827-722960	19	18	6	8	0.392161	0.7763	no
ni|645903749|nb|KK920681.1|	755001-755120	19	18	13	14	0.195615	0.87955	no
ni|645903749|nb|KK920681.1|	814217-814513	19	18	67	55	-0.291413	0.7923	no
ni|645903749|nb|KK920681.1|	816028-817372	19	18	49	29	-0.767247	0.59555	no
ni|645903749|nb|KK920681.1|	819707-820136	19	18	108	69	-0.648225	0.65385	no
ni|645903749|nb|KK920681.1|	821881-822437	19	18	50	26	-0.905135	0.4788	no
ni|645903749|nb|KK920681.1|	832212-832502	19	18	56	55	-0.0195205	0.98555	no
ni|645903749|nb|KK920681.1|	834427-834868	19	18	131	96	-0.446873	0.758	no
ni|645903749|nb|KK920681.1|	835117-835258	19	18	91	44	-1	0.36845	no
ni|645903749|nb|KK920681.1|	835755-836343	19	18	0.811912	0.970203	0.256963	0.83705	no
ni|645903749|nb|KK920681.1|	838521-839289	19	18	133	91	-0.540221	0.6996	no
ni|645903749|nb|KK920681.1|	847539-847720	19	18	4	1	-2	0.25505	no
ni|645903749|nb|KK920681.1|	853495-854031	19	18	2	0.814825	-2	0.21795	no
ni|645903749|nb|KK920681.1|	854419-855281	19	18	2	0.279179	-3	0.06645	no
ni|645903749|nb|KK920681.1|	869948-870629	19	18	2	2	0.520125	0.6633	no
ni|645903749|nb|KK920681.1|	873147-873576	19	18	2	4	0.549438	0.6386	no
ni|645903749|nb|KK920681.1|	874047-874196	19	18	5	8	0.762878	0.5642	no
ni|645903749|nb|KK920681.1|	878238-878616	19	18	12	36	2	0.15965	no
ni|645903749|nb|KK920681.1|	878924-879582	19	18	5	17	2	0.1378	no
ni|645903749|nb|KK920681.1|	881244-881643	19	18	0.913388	0.634863	-0.524784	0.7071	no
ni|645903749|nb|KK920681.1|	883707-884411	19	18	12	32	1	0.2959	no
ni|645903749|nb|KK920681.1|	886511-886813	19	18	7	4	-0.898665	0.44745	no
ni|645903749|nb|KK920681.1|	887581-888033	19	18	17	11	-0.592467	0.6107	no
ni|645903749|nb|KK920681.1|	888315-888777	19	18	17	8	-1	0.3464	no
ni|645903749|nb|KK920681.1|	889115-889243	19	18	40	25	-0.683985	0.56335	no

ni|645903749|nb|KK920681.1|	889391-889535	19	18	25	12	-1	0.3677	no
ni|645903749|nb|KK920681.1|	890195-890338	19	18	7	6	-0.192742	0.87555	no
ni|645903749|nb|KK920681.1|	893276-893884	19	18	5	83	4	0.0074	no
ni|645903749|nb|KK920681.1|	894143-894501	19	18	0.096322	4	5	0.12	no
ni|645903749|nb|KK920681.1|	894612-894982	19	18	0.828121	0.615526	-0.428021	0.75965	no
ni|645903749|nb|KK920681.1|	896817-897153	19	18	4	104	5	0.00445	no
ni|645903749|nb|KK920681.1|	897410-897559	19	18	13	7	-0.804163	0.52275	no
ni|645903749|nb|KK920681.1|	897663-898366	19	18	3	3	-0.161674	0.8967	no
ni|645903749|nb|KK920681.1|	898980-899218	19	18	11	8	-0.458643	0.69925	no
ni|645903749|nb|KK920681.1|	900797-900961	19	18	13	165	4	0.0051	no
ni|645903749|nb|KK920681.1|	901214-902006	19	18	3	2	-0.56996	0.62545	no
ni|645903749|nb|KK920681.1|	903151-903552	19	18	3	43	4	0.00355	no
ni|645903749|nb|KK920681.1|	904952-905099	19	18	6	168	5	0.00735	no
ni|645903749|nb|KK920681.1|	906973-907671	19	18	3	52	4	0.00655	no
ni|645903749|nb|KK920681.1|	912533-912713	19	18	0	58	inf	5.00E-05	yes
ni|645903749|nb|KK920681.1|	913087-913328	19	18	0	37	inf	5.00E-05	yes
ni|645903749|nb|KK920681.1|	916258-916457	19	18	0	53	inf	5.00E-05	yes
ni|645903750|nb|KK920680.1|	412952-413232	14	14	1	1	0.223587	0.8598	no
ni|645903750|nb|KK920680.1|	417066-417358	14	14	2	2	-0.0658364	0.95935	no
ni|645903750|nb|KK920680.1|	421762-422308	14	14	0	7	inf	5.00E-05	yes
ni|645903750|nb|KK920680.1|	424018-425025	14	14	9	24	1	0.2956	no
ni|645903750|nb|KK920680.1|	425322-425523	14	14	21	59	1	0.19665	no
ni|645903750|nb|KK920680.1|	428012-428275	14	14	3	9	2	0.1703	no
ni|645903750|nb|KK920680.1|	428419-428629	14	14	20	59	2	0.18515	no
ni|645903750|nb|KK920680.1|	429540-429796	14	14	6	19	2	0.17635	no
ni|645903750|nb|KK920680.1|	431190-431378	14	14	27	66	1	0.2686	no
ni|645903750|nb|KK920680.1|	432258-432559	14	14	17	49	2	0.1825	no
ni|645903750|nb|KK920680.1|	432668-432998	14	14	21	48	1	0.2854	no
ni|645903750|nb|KK920680.1|	433235-433379	14	14	12	38	2	0.1782	no
ni|645903750|nb|KK920680.1|	434500-434643	14	14	22	83	2	0.11955	no

ni|645903750|nb|KK920680.1|	435950-436125	14	14	19	43	1	0.29765	no
ni|645903750|nb|KK920680.1|	437036-437228	14	14	14	36	1	0.2563	no
ni|645903750|nb|KK920680.1|	439581-440500	14	14	8	23	2	0.24175	no
ni|645903750|nb|KK920680.1|	440594-440730	14	14	25	40	0.693562	0.5533	no
ni|645903750|nb|KK920680.1|	442396-442530	14	14	26	61	1	0.301	no
ni|645903750|nb|KK920680.1|	443502-443656	14	14	18	53	2	0.1857	no
ni|645903750|nb|KK920680.1|	443960-444115	14	14	26	61	1	0.3058	no
ni|645903750|nb|KK920680.1|	444218-444371	14	14	25	58	1	0.29605	no
ni|645903750|nb|KK920680.1|	445597-445997	14	14	14	35	1	0.2439	no
ni|645903750|nb|KK920680.1|	447412-447567	14	14	24	51	1	0.37675	no
ni|645903750|nb|KK920680.1|	448953-449096	14	14	27	48.99	0.884961	0.4506	no
ni|645903750|nb|KK920680.1|	449289-449561	14	14	20	54	1	0.20885	no
ni|645903750|nb|KK920680.1|	449896-450276	14	14	10	21	1	0.34045	no
ni|645903750|nb|KK920680.1|	451663-452377	14	14	8	25	2	0.1659	no
ni|645903750|nb|KK920680.1|	452766-452902	14	14	25	58	1	0.295	no
ni|645903750|nb|KK920680.1|	454531-454715	14	14	24	65	1	0.22385	no
ni|645903750|nb|KK920680.1|	455022-455154	14	14	20	65	2	0.1686	no
ni|645903750|nb|KK920680.1|	455529-456031	14	14	16	38	1	0.2474	no
ni|645903750|nb|KK920680.1|	457728-457974	14	14	24	55	1	0.29535	no
ni|645903750|nb|KK920680.1|	459752-460013	14	14	32	81	1	0.2393	no
ni|645903750|nb|KK920680.1|	460344-460616	14	14	24	60	1	0.2545	no
ni|645903750|nb|KK920680.1|	461738-462138	14	14	8	18	1	0.2776	no
ni|645903750|nb|KK920680.1|	462705-462828	14	14	8	26	2	0.226	no
ni|645903750|nb|KK920680.1|	463380-463577	14	14	4	10	1	0.2503	no
ni|645903750|nb|KK920680.1|	464212-464398	14	14	27	54	1	0.3918	no
ni|645903750|nb|KK920680.1|	464874-465073	14	14	29	66	1	0.3034	no
ni|645903750|nb|KK920680.1|	466475-466669	14	14	6	10	0.705483	0.5556	no
ni|645903750|nb|KK920680.1|	467358-467877	14	14	5	8	0.619057	0.59335	no
ni|645903750|nb|KK920680.1|	473464-473599	14	14	26	71	1	0.23205	no
ni|645903750|nb|KK920680.1|	475924-476075	14	14	26	83	2	0.15805	no

ni|645903750|nb|KK920680.1|	478628-478824	14	14	4	12	1	0.22825	no
ni|645903750|nb|KK920680.1|	479524-480033	14	14	4	11	2	0.1803	no
ni|645903750|nb|KK920680.1|	480591-480992	14	14	18	35	0.93143	0.40825	no
ni|645903750|nb|KK920680.1|	481272-481708	14	14	17	41	1	0.2547	no
ni|645903750|nb|KK920680.1|	484640-485171	14	14	21	40	0.946945	0.3883	no
ni|645903750|nb|KK920680.1|	485638-485816	14	14	46	67	0.547592	0.63135	no
ni|645903750|nb|KK920680.1|	486225-486751	14	14	24	41	0.755172	0.49055	no
ni|645903750|nb|KK920680.1|	487489-487784	14	14	46	65	0.509685	0.6433	no
ni|645903750|nb|KK920680.1|	489574-489740	14	14	26	37	0.496667	0.66835	no
ni|645903750|nb|KK920680.1|	490615-491140	14	14	12	15	0.355261	0.7474	no
ni|645903750|nb|KK920680.1|	491252-491619	14	14	16	26	0.651807	0.5643	no
ni|645903750|nb|KK920680.1|	492520-493637	14	14	16	23	0.504927	0.68265	no
ni|645903750|nb|KK920680.1|	493785-494236	14	14	25	34	0.475088	0.66465	no
ni|645903750|nb|KK920680.1|	495190-495343	14	14	33	54	0.701926	0.55105	no
ni|645903750|nb|KK920680.1|	495468-495633	14	14	38	51	0.447346	0.69885	no
ni|645903750|nb|KK920680.1|	496391-496826	14	14	35	47	0.434016	0.68985	no
ni|645903750|nb|KK920680.1|	498495-498798	14	14	2	0.585841	-2	0.15435	no
ni|645903750|nb|KK920680.1|	498976-499201	14	14	33	6	-3	0.04495	no
ni|645903750|nb|KK920680.1|	501441-501923	14	14	0.584133	54	7	0.01105	no
ni|645903750|nb|KK920680.1|	507344-507547	14	14	0.486426	10	4	0.04515	no
ni|645903750|nb|KK920680.1|	507733-507982	14	14	20	4	-3	0.05005	no
ni|645903750|nb|KK920680.1|	510432-510907	14	14	53	11	-2	0.10295	no
ni|645903750|nb|KK920680.1|	517812-517994	14	14	65	16	-2	0.0887	no
ni|645903751|nb|KK920679.1|	111089-111294	17	16	0	382	inf	5.00E-05	yes
ni|645903751|nb|KK920679.1|	1128605-1129159	17	16	2	0.365112	-2	0.14175	no
ni|645903751|nb|KK920679.1|	1130314-1130514	17	16	3	0	-inf	0.0036	no
ni|645903751|nb|KK920679.1|	1131917-1132231	17	16	1	0.333316	-2	0.20695	no
ni|645903751|nb|KK920679.1|	1132455-1132657	17	16	3	0.468961	-3	0.28285	no
ni|645903751|nb|KK920679.1|	1136833-1137629	17	16	2	0.475583	-2	0.12	no
ni|645903751|nb|KK920679.1|	1145848-1146236	17	16	0.948495	0.082406	-4	0.0326	no

ni|645903751|nb|KK920679.1|	1146790-1147362	17	16	0.787092	0.100323	-3	0.2517	no
ni|645903751|nb|KK920679.1|	1150490-1150704	17	16	4	0.838488	-2	0.1501	no
ni|645903751|nb|KK920679.1|	1152633-1152823	17	16	3	1	-2	0.25505	no
ni|645903751|nb|KK920679.1|	1154288-1154441	17	16	6	1	-2	0.1906	no
ni|645903751|nb|KK920679.1|	1156333-1156724	17	16	2	0.897069	-1	0.383	no
ni|645903751|nb|KK920679.1|	1159172-1159437	17	16	4	1	-1	0.2773	no
ni|645903751|nb|KK920679.1|	1159768-1160416	17	16	2	0.259075	-3	0.0744	no
ni|645903751|nb|KK920679.1|	1160846-1161102	17	16	5	0.458324	-3	0.1251	no
ni|645903751|nb|KK920679.1|	116115-116347	17	16	0	370	inf	5.00E-05	yes
ni|645903751|nb|KK920679.1|	1161268-1161430	17	16	4	0.384775	-3	0.03815	no
ni|645903751|nb|KK920679.1|	1161988-1162128	17	16	29	26	-0.162385	0.88695	no
ni|645903751|nb|KK920679.1|	1163317-1163654	17	16	17	15	-0.206685	0.8577	no
ni|645903751|nb|KK920679.1|	1163885-1164038	17	16	25	32	0.374814	0.74775	no
ni|645903751|nb|KK920679.1|	1164338-1165335	17	16	14	15	0.073676	0.94765	no
ni|645903751|nb|KK920679.1|	1166810-1166953	17	16	24	37	0.606561	0.6031	no
ni|645903751|nb|KK920679.1|	1168396-1168618	17	16	20	37	0.910949	0.43735	no
ni|645903751|nb|KK920679.1|	1168827-1169243	17	16	11	17	0.555685	0.62465	no
ni|645903751|nb|KK920679.1|	1169365-1169633	17	16	3	5	0.656453	0.5865	no
ni|645903751|nb|KK920679.1|	1171380-1171540	17	16	25	43	0.780792	0.50145	no
ni|645903751|nb|KK920679.1|	1172428-1172595	17	16	17	21	0.347796	0.77085	no
ni|645903751|nb|KK920679.1|	1174975-1175143	17	16	13	17	0.38843	0.74445	no
ni|645903751|nb|KK920679.1|	1186382-1186933	17	16	0	1	inf	0.00065	yes
ni|645903751|nb|KK920679.1|	119108-119380	17	16	0	69	inf	5.00E-05	yes
ni|645903751|nb|KK920679.1|	120402-120611	17	16	0	393	inf	5.00E-05	yes
ni|645903751|nb|KK920679.1|	121833-121984	17	16	0	247	inf	5.00E-05	yes
ni|645903751|nb|KK920679.1|	127607-127896	17	16	0	154	inf	5.00E-05	yes
ni|645903751|nb|KK920679.1|	138958-139184	17	16	0	542	inf	5.00E-05	yes
ni|645903751|nb|KK920679.1|	147991-148149	17	16	0.4304	406	10	0.11935	no
ni|645903751|nb|KK920679.1|	150976-151272	17	16	0	292	inf	5.00E-05	yes
ni|645903751|nb|KK920679.1|	433198-434920	17	16	83	27	-2	0.27155	no

ni|645903751|nb|KK920679.1|	621173-622440	17	16	48	74	0.639844	0.64275	no
ni|645903751|nb|KK920679.1|	623695-623852	17	16	284	322	0.179442	0.8851	no
ni|645903751|nb|KK920679.1|	626015-626264	17	16	14	17	0.252339	0.8279	no
ni|645903751|nb|KK920679.1|	627595-627717	17	16	21	34	0.684693	0.5731	no
ni|645903751|nb|KK920679.1|	627865-628028	17	16	22	33	0.551693	0.6371	no
ni|645903751|nb|KK920679.1|	629382-630029	17	16	0.723971	0.648876	-0.15799	0.89795	no
ni|645903751|nb|KK920679.1|	630483-631031	17	16	9	14	0.568646	0.6171	no
ni|645903751|nb|KK920679.1|	631658-632915	17	16	2	2	-0.05041	0.96635	no
ni|645903751|nb|KK920679.1|	633323-633819	17	16	7	11	0.679414	0.5587	no
ni|645903751|nb|KK920679.1|	639550-639776	17	16	17	25	0.607269	0.6001	no
ni|645903751|nb|KK920679.1|	639875-640121	17	16	11.97	16	0.389455	0.74335	no
ni|645903751|nb|KK920679.1|	642270-643040	17	16	27	24	-0.176479	0.8861	no
ni|645903751|nb|KK920679.1|	643408-643953	17	16	37	32.41	-0.200601	0.85145	no
ni|645903751|nb|KK920679.1|	644957-645093	17	16	117	84	-0.476641	0.6783	no
ni|645903751|nb|KK920679.1|	649494-649897	17	16	14	15	0.0637011	0.9553	no
ni|645903751|nb|KK920679.1|	650594-650947	17	16	18	21	0.237043	0.8279	no
ni|645903751|nb|KK920679.1|	651480-651657	17	16	23	26	0.175245	0.8833	no
ni|645903751|nb|KK920679.1|	652511-652997	17	16	7	11	0.696343	0.55095	no
ni|645903751|nb|KK920679.1|	654709-655110	17	16	8	12	0.577332	0.61315	no
ni|645903751|nb|KK920679.1|	658393-658610	17	16	4	4	-0.0666485	0.96245	no
ni|645903751|nb|KK920679.1|	659120-659902	17	16	7	18	1	0.2339	no
ni|645903751|nb|KK920679.1|	660096-660521	17	16	9	22	1	0.2994	no
ni|645903751|nb|KK920679.1|	663450-663619	17	16	20	47	1	0.29765	no
ni|645903751|nb|KK920679.1|	664187-665364	17	16	4	10	1	0.2354	no
ni|645903751|nb|KK920679.1|	665471-665733	17	16	2	3	0.757058	0.55115	no
ni|645903751|nb|KK920679.1|	666004-666992	17	16	6	12	0.948643	0.3966	no
ni|645903751|nb|KK920679.1|	668455-669410	17	16	15	33	1	0.3683	no
ni|645903751|nb|KK920679.1|	670784-670925	17	16	0	30	inf	5.00E-05	yes
ni|645903751|nb|KK920679.1|	671232-671442	17	16	30	81	1	0.21175	no
ni|645903751|nb|KK920679.1|	672777-672917	17	16	35	54	0.623445	0.59345	no

ni|645903751|nb|KK920679.1|	676904-677307	17	16	8	8	0.0361972	0.975	no
ni|645903751|nb|KK920679.1|	677654-677818	17	16	18	20	0.164104	0.89025	no
ni|645903751|nb|KK920679.1|	678077-678528	17	16	11	10	-0.143225	0.9016	no
ni|645903751|nb|KK920679.1|	678677-678936	17	16	6	4	-0.378035	0.75755	no
ni|645903751|nb|KK920679.1|	679196-679328	17	16	30	21	-0.545326	0.64545	no
ni|645903751|nb|KK920679.1|	679489-679666	17	16	14	15	0.121119	0.9171	no
ni|645903751|nb|KK920679.1|	679914-680044	17	16	32	62	0.951083	0.4219	no
ni|645903751|nb|KK920679.1|	680293-680651	17	16	7	12	0.741856	0.52415	no
ni|645903751|nb|KK920679.1|	680821-681077	17	16	18	13	-0.451145	0.6986	no
ni|645903751|nb|KK920679.1|	681438-681617	17	16	14	9	-0.635603	0.5986	no
ni|645903751|nb|KK920679.1|	681992-682115	17	16	39	30	-0.36993	0.75375	no
ni|645903751|nb|KK920679.1|	688301-688569	17	16	7	10	0.603839	0.611	no
ni|645903751|nb|KK920679.1|	689147-689274	17	16	13	18	0.517639	0.6788	no
ni|645903751|nb|KK920679.1|	690056-690225	17	16	7	13	0.820266	0.4962	no
ni|645903751|nb|KK920679.1|	691592-691731	17	16	4	13	2	0.27465	no
ni|645903751|nb|KK920679.1|	693682-693972	17	16	0.131094	10	6	0.1195	no
ni|645903751|nb|KK920679.1|	695901-696087	17	16	6	11	0.830879	0.4964	no
ni|645903751|nb|KK920679.1|	699440-699916	17	16	0.857283	12	4	0.01045	no
ni|645903751|nb|KK920679.1|	709976-710223	17	16	1	4	2	0.24365	no
ni|645903751|nb|KK920679.1|	713953-714396	17	16	0.506279	2	2	0.18075	no
ni|645903751|nb|KK920679.1|	714527-715185	17	16	0.221762	1	2	0.08975	no
ni|645903751|nb|KK920679.1|	717828-718673	17	16	0.099554	20	8	0.05055	no
ni|645903751|nb|KK920679.1|	734192-734432	17	16	4	6	0.518664	0.6612	no
ni|645903751|nb|KK920679.1|	734688-734961	17	16	6	6	-0.136362	0.90855	no
ni|645903751|nb|KK920679.1|	736610-736772	17	16	12	16	0.370909	0.7587	no
ni|645903751|nb|KK920679.1|	737308-737506	17	16	20	24	0.254906	0.82795	no
ni|645903751|nb|KK920679.1|	739287-739737	17	16	7	8	0.19043	0.87145	no
ni|645903751|nb|KK920679.1|	739967-740088	17	16	25	35	0.473177	0.6941	no
ni|645903751|nb|KK920679.1|	740464-740686	17	16	20	29	0.542973	0.63745	no
ni|645903751|nb|KK920679.1|	741667-741788	17	16	11	19	0.698143	0.59985	no

ni|645903751|nb|KK920679.1|	742569-743052	17	16	10	10	0.0539962	0.96285	no
ni|645903751|nb|KK920679.1|	743446-744001	17	16	20	19	-0.0572419	0.95675	no
ni|645903751|nb|KK920679.1|	745631-745788	17	16	24	24	0.0108041	0.98925	no
ni|645903751|nb|KK920679.1|	745896-746044	17	16	19	28	0.556237	0.63935	no
ni|645903751|nb|KK920679.1|	746587-747048	17	16	17	20	0.217579	0.844	no
ni|645903751|nb|KK920679.1|	747125-747267	17	16	40	39	-0.00402244	0.99195	no
ni|645903751|nb|KK920679.1|	747949-748218	17	16	3	3	-0.288397	0.8142	no
ni|645903751|nb|KK920679.1|	748503-749132	17	16	1	29	5	0.0014	yes
ni|645903751|nb|KK920679.1|	749669-749927	17	16	85	84	-0.0213825	0.98355	no
ni|645903751|nb|KK920679.1|	752201-752514	17	16	1	0.781413	-0.717791	0.61925	no
ni|645903751|nb|KK920679.1|	753062-753267	17	16	128	112	-0.201699	0.85375	no
ni|645903751|nb|KK920679.1|	755340-755669	17	16	35	35	-0.00478528	0.9966	no
ni|645903751|nb|KK920679.1|	756830-757671	17	16	3	3	0.164797	0.8857	no
ni|645903751|nb|KK920679.1|	757785-757949	17	16	6	6	0.0258878	0.9668	no
ni|645903751|nb|KK920679.1|	758490-759045	17	16	3	2	-0.817092	0.4927	no
ni|645903751|nb|KK920679.1|	759316-760058	17	16	71	62	-0.202039	0.88445	no
ni|645903751|nb|KK920679.1|	760357-760506	17	16	124	109	-0.190054	0.86835	no
ni|645903751|nb|KK920679.1|	761004-761241	17	16	48	32	-0.560603	0.6271	no
ni|645903751|nb|KK920679.1|	761343-761877	17	16	31	21	-0.573967	0.5961	no
ni|645903751|nb|KK920679.1|	762077-762520	17	16	36	24	-0.578158	0.5994	no
ni|645903751|nb|KK920679.1|	764204-764433	17	16	116	98	-0.240609	0.822	no
ni|645903751|nb|KK920679.1|	765726-766042	17	16	26	20	-0.400393	0.72565	no
ni|645903751|nb|KK920679.1|	769100-769360	17	16	4	2	-1	0.3948	no
ni|645903751|nb|KK920679.1|	769858-770278	17	16	1	1	-0.328329	0.79705	no
ni|645903751|nb|KK920679.1|	770422-771540	17	16	19	16	-0.23898	0.8468	no
ni|645903751|nb|KK920679.1|	772579-772806	17	16	109	133.66	0.288278	0.8213	no
ni|645903751|nb|KK920679.1|	773271-773743	17	16	45	44	-0.0525374	0.9584	no
ni|645903751|nb|KK920679.1|	774809-774985	17	16	87	84	-0.0564775	0.95895	no
ni|645903751|nb|KK920679.1|	776503-776720	17	16	81	85	0.0636079	0.95375	no
ni|645903751|nb|KK920679.1|	777415-777601	17	16	66	73	0.144263	0.89845	no

ni|645903751|nb|KK920679.1|	778519-778630	17	16	111	74	-0.58608	0.6078	no
ni|645903751|nb|KK920679.1|	779334-779534	17	16	23	34	0.574971	0.61765	no
ni|645903751|nb|KK920679.1|	779676-780029	17	16	12	73	3	0.0795	no
ni|645903751|nb|KK920679.1|	780239-781416	17	16	4	24	3	0.0604	no
ni|645903751|nb|KK920679.1|	784620-784788	17	16	17	80	2	0.067	no
ni|645903751|nb|KK920679.1|	786789-787056	17	16	1	0.998436	-0.428591	0.75965	no
ni|645903751|nb|KK920679.1|	787231-787661	17	16	4	13	2	0.1668	no
ni|645903751|nb|KK920679.1|	817888-818038	17	16	459	308	-0.575968	0.6403	no
ni|645903751|nb|KK920679.1|	819019-819183	17	16	401	261	-0.620231	0.6152	no
ni|645903751|nb|KK920679.1|	821858-822468	17	16	0.485458	0.139228	-2	0.2784	no
ni|645903751|nb|KK920679.1|	824089-824219	17	16	317	158	-1	0.3724	no
ni|645903751|nb|KK920679.1|	830829-831190	17	16	2	0.727862	-1	0.3487	no
ni|645903751|nb|KK920679.1|	834474-834693	17	16	1	2	0.447957	0.7543	no
ni|645903751|nb|KK920679.1|	97882-98056	17	16	0	356	inf	5.00E-05	yes
ni|645903752|nb|KK920678.1|	1071504-107174[illegible]	18	16	51	32	-0.675527	0.54945	no
ni|645903752|nb|KK920678.1|	1072075-107221[illegible]	18	16	61	37	-0.712162	0.546	no
ni|645903752|nb|KK920678.1|	1072345-107255[illegible]	18	16	55	37	-0.574621	0.6147	no
ni|645903752|nb|KK920678.1|	1072827-107307[illegible]	18	16	8	9	0.0687602	0.95115	no
ni|645903752|nb|KK920678.1|	1073919-107420[illegible]	18	16	86	68	-0.341637	0.7476	no
ni|645903752|nb|KK920678.1|	1074728-107494[illegible]	18	16	79	69	-0.204136	0.8525	no
ni|645903752|nb|KK920678.1|	1075104-107530[illegible]	18	16	84	70	-0.274933	0.8059	no
ni|645903752|nb|KK920678.1|	1075440-107559[illegible]	18	16	6	6	0.1552	0.90275	no
ni|645903752|nb|KK920678.1|	1077319-107784[illegible]	18	16	4	3	-0.248513	0.83105	no
ni|645903752|nb|KK920678.1|	1078133-107846[illegible]	18	16	38	30	-0.335223	0.7605	no
ni|645903752|nb|KK920678.1|	1078640-107912[illegible]	18	16	3	3	0.323901	0.7731	no
ni|645903752|nb|KK920678.1|	1079557-108044[illegible]	18	16	2	1	-0.228148	0.84515	no
ni|645903752|nb|KK920678.1|	1080686-108094[illegible]	18	16	31	27	-0.158219	0.89025	no
ni|645903752|nb|KK920678.1|	1081462-108225[illegible]	18	16	0.753712	0.960858	0.35031	0.7691	no
ni|645903752|nb|KK920678.1|	1086378-108676[illegible]	18	16	0.945192	1	0.562648	0.66675	no
ni|645903752|nb|KK920678.1|	1087059-108761[illegible]	18	16	7	7	-0.0877411	0.939	no

ni|645903752|nb|KK920678.1|	1088650-1089251	18	16	28	28	0.0200907	0.9858	no
ni|645903752|nb|KK920678.1|	1091674-1091935	18	16	63	62	-0.0310541	0.9772	no
ni|645903752|nb|KK920678.1|	1093886-1094072	18	16	48	46	-0.0672415	0.9541	no
ni|645903752|nb|KK920678.1|	1094682-1094926	18	16	47	42	-0.148709	0.89745	no
ni|645903752|nb|KK920678.1|	1095825-1096269	18	16	22	25	0.172091	0.87245	no
ni|645903752|nb|KK920678.1|	1149258-1149395	18	16	6	10	0.669721	0.6211	no
ni|645903752|nb|KK920678.1|	119204-119352	18	16	8	8	0.0202633	0.96555	no
ni|645903752|nb|KK920678.1|	1270751-1270862	18	16	17	12	-0.482651	0.7594	no
ni|645903752|nb|KK920678.1|	1272403-1272581	18	16	4	4	0.155069	0.90275	no
ni|645903752|nb|KK920678.1|	1298224-1298453	18	16	0	7	inf	0.0001	yes
ni|645903752|nb|KK920678.1|	1300202-1300430	18	16	6	5	-0.166007	0.8886	no
ni|645903752|nb|KK920678.1|	1318858-1318994	18	16	9	5	-0.76769	0.5799	no
ni|645903752|nb|KK920678.1|	1319621-1320110	18	16	5	5	0.0519006	0.96445	no
ni|645903752|nb|KK920678.1|	1320657-1320781	18	16	15	8	-0.974246	0.5005	no
ni|645903752|nb|KK920678.1|	1335860-1337740	18	16	47	59	0.325967	0.80975	no
ni|645903752|nb|KK920678.1|	1337888-1338055	18	16	84	76	-0.153103	0.8938	no
ni|645903752|nb|KK920678.1|	1338135-1338676	18	16	31	22	-0.466954	0.6717	no
ni|645903752|nb|KK920678.1|	1338871-1339186	18	16	17	12	-0.451196	0.69315	no
ni|645903752|nb|KK920678.1|	241846-243129	18	16	0.020874	53	11	0.11935	no
ni|645903752|nb|KK920678.1|	253915-254126	18	16	4	7	0.807715	0.50525	no
ni|645903752|nb|KK920678.1|	321654-322365	18	16	2	3	0.176205	0.8781	no
ni|645903752|nb|KK920678.1|	323181-323302	18	16	45	33	-0.445903	0.70485	no
ni|645903752|nb|KK920678.1|	325458-325598	18	16	38	31	-0.293503	0.80225	no
ni|645903752|nb|KK920678.1|	328703-329076	18	16	4	28	3	0.02545	no
ni|645903752|nb|KK920678.1|	331901-332509	18	16	0.097476	1	3	0.0515	no
ni|645903752|nb|KK920678.1|	333981-334360	18	16	1	13	4	0.01425	no
ni|645903752|nb|KK920678.1|	334528-334722	18	16	5	40	3	0.0204	no
ni|645903752|nb|KK920678.1|	337120-337411	18	16	3	50	4	0.003	no
ni|645903752|nb|KK920678.1|	351779-351950	18	16	6	47	3	0.0245	no
ni|645903752|nb|KK920678.1|	390978-391117	18	16	222	232	0.0665188	0.95335	no

ni|645903752|nb|KK920678.1|	391635-391977	18	16	5	4	-0.499971	0.67235	no
ni|645903752|nb|KK920678.1|	392154-392940	18	16	79	102	0.364377	0.79895	no
ni|645903752|nb|KK920678.1|	401741-401922	18	16	147	236.68	0.686574	0.5935	no
ni|645903752|nb|KK920678.1|	403350-403621	18	16	85	94	0.141454	0.8962	no
ni|645903752|nb|KK920678.1|	405415-405704	18	16	161	256	0.673475	0.63645	no
ni|645903752|nb|KK920678.1|	408576-408683	18	16	53	92	0.798891	0.5162	no
ni|645903752|nb|KK920678.1|	409221-409399	18	16	113	193	0.77197	0.47905	no
ni|645903752|nb|KK920678.1|	410883-411009	18	16	143	280	0.965297	0.4043	no
ni|645903752|nb|KK920678.1|	411729-412038	18	16	2	6	1	0.27095	no
ni|645903752|nb|KK920678.1|	415414-415650	18	16	0.183701	3	4	0.12505	no
ni|645903752|nb|KK920678.1|	416380-416803	18	16	0.07683	19	8	0.11935	no
ni|645903752|nb|KK920678.1|	418376-418644	18	16	0	6	inf	5.00E-05	yes
ni|645903752|nb|KK920678.1|	420289-420479	18	16	0	47	inf	5.00E-05	yes
ni|645903752|nb|KK920678.1|	423778-424775	18	16	0.027547	2	6.49	0.11935	no
ni|645903752|nb|KK920678.1|	426667-426958	18	16	13	131	3	0.02465	no
ni|645903752|nb|KK920678.1|	432587-432755	18	16	3	1	-1	0.44965	no
ni|645903752|nb|KK920678.1|	497072-497351	18	16	2	0.399002	-2	0.24815	no
ni|645903752|nb|KK920678.1|	51934-52557	18	16	1	1	0.365721	0.761	no
ni|645903752|nb|KK920678.1|	57356-57608	18	16	11	24	1	0.3224	no
ni|645903752|nb|KK920678.1|	57733-57918	18	16	34	31	-0.10573	0.92855	no
ni|645903752|nb|KK920678.1|	58298-59327	18	16	2	6	2	0.10395	no
ni|645903752|nb|KK920678.1|	59488-59680	18	16	5	10	1	0.40825	no
ni|645903752|nb|KK920678.1|	594894-595206	18	16	1	2	0.38174	0.7694	no
ni|645903752|nb|KK920678.1|	595859-595998	18	16	10	10.29	0.11334	0.9325	no
ni|645903752|nb|KK920678.1|	597206-597574	18	16	16	14	-0.20895	0.85245	no
ni|645903752|nb|KK920678.1|	598657-599091	18	16	4	4	0.15292	0.8969	no
ni|645903752|nb|KK920678.1|	599360-599657	18	16	1	1	-0.217808	0.87705	no
ni|645903752|nb|KK920678.1|	60004-60218	18	16	2	3	0.196335	0.87955	no
ni|645903752|nb|KK920678.1|	600892-601181	18	16	7	9	0.261309	0.8243	no
ni|645903752|nb|KK920678.1|	603564-604098	18	16	3	5	0.643896	0.5831	no

ni|645903752|nb|KK920678.1|	605020-605134	18	16	24	29	0.29505	0.81435	no
ni|645903752|nb|KK920678.1|	608355-608789	18	16	5	6	0.313252	0.7946	no
ni|645903752|nb|KK920678.1|	609638-609812	18	16	4	1	-2	0.3149	no
ni|645903752|nb|KK920678.1|	610389-610938	18	16	0.772435	1	0.519933	0.67865	no
ni|645903752|nb|KK920678.1|	61116-62188	18	16	2	3	0.214601	0.85105	no
ni|645903752|nb|KK920678.1|	612252-612417	18	16	3	1	-1	0.4023	no
ni|645903752|nb|KK920678.1|	612543-612719	18	16	3	3	-0.389253	0.78555	no
ni|645903752|nb|KK920678.1|	613031-613231	18	16	5	4	-0.408018	0.755	no
ni|645903752|nb|KK920678.1|	613938-614157	18	16	3	2	-0.329651	0.80325	no
ni|645903752|nb|KK920678.1|	614888-615050	18	16	3	3	0.125433	0.92845	no
ni|645903752|nb|KK920678.1|	615957-616144	18	16	3	2	-0.067228	0.9414	no
ni|645903752|nb|KK920678.1|	616696-616928	18	16	2	2	0.049067	0.95725	no
ni|645903752|nb|KK920678.1|	618086-618351	18	16	0.453416	5	4	0.05455	no
ni|645903752|nb|KK920678.1|	618452-618713	18	16	10.69	22	1	0.3884	no
ni|645903752|nb|KK920678.1|	619885-619999	18	16	74	151	1	0.39355	no
ni|645903752|nb|KK920678.1|	620576-620899	18	16	37	74	0.991842	0.3639	no
ni|645903752|nb|KK920678.1|	624225-624341	18	16	40	91	1	0.3226	no
ni|645903752|nb|KK920678.1|	626739-626966	18	16	1	3	1	0.39265	no
ni|645903752|nb|KK920678.1|	627684-628712	18	16	2	1	-0.169543	0.8831	no
ni|645903752|nb|KK920678.1|	629236-630642	18	16	3	5	0.540777	0.6307	no
ni|645903752|nb|KK920678.1|	63043-64041	18	16	1	1	0.102062	0.9286	no
ni|645903752|nb|KK920678.1|	633330-633809	18	16	4	3	-0.299625	0.79275	no
ni|645903752|nb|KK920678.1|	634163-634287	18	16	21	39	0.862256	0.46915	no
ni|645903752|nb|KK920678.1|	636537-636651	18	16	72	119	0.715669	0.54385	no
ni|645903752|nb|KK920678.1|	638632-638796	18	16	43	56	0.380237	0.74345	no
ni|645903752|nb|KK920678.1|	639108-639270	18	16	71	117	0.729504	0.5216	no
ni|645903752|nb|KK920678.1|	639521-639662	18	16	14	14	0.0530721	0.96745	no
ni|645903752|nb|KK920678.1|	640251-640809	18	16	0.649014	0.465312	-0.480052	0.7301	no
ni|645903752|nb|KK920678.1|	647045-647661	18	16	11	10	-0.241001	0.8311	no
ni|645903752|nb|KK920678.1|	64853-65394	18	16	2	2	-0.147506	0.90605	no

ni|645903752|nb|KK920678.1|	650690-651107	18	16	6	6	-0.138366	0.90635	no
ni|645903752|nb|KK920678.1|	652308-652693	18	16	25	20	-0.342624	0.7607	no
ni|645903752|nb|KK920678.1|	65527-65711	18	16	3	4	0.669695	0.6211	no
ni|645903752|nb|KK920678.1|	656407-656512	18	16	66	47	-0.483149	0.7105	no
ni|645903752|nb|KK920678.1|	656703-656860	18	16	37	31	-0.286765	0.8035	no
ni|645903752|nb|KK920678.1|	657075-657261	18	16	36	25	-0.517903	0.6573	no
ni|645903752|nb|KK920678.1|	657412-658184	18	16	19	18	-0.0814209	0.93735	no
ni|645903752|nb|KK920678.1|	658366-658563	18	16	51	47	-0.126239	0.9114	no
ni|645903752|nb|KK920678.1|	660088-660754	18	16	16	16	0.0207197	0.9844	no
ni|645903752|nb|KK920678.1|	660890-662001	18	16	2	2	0.20507	0.8624	no
ni|645903752|nb|KK920678.1|	663132-663388	18	16	33	45	0.442894	0.6926	no
ni|645903752|nb|KK920678.1|	663595-664619	18	16	4	4	-0.0543066	0.9617	no
ni|645903752|nb|KK920678.1|	664994-665348	18	16	16	17	0.113455	0.9223	no
ni|645903752|nb|KK920678.1|	666765-666933	18	16	28	36	0.338389	0.7749	no
ni|645903752|nb|KK920678.1|	667675-667824	18	16	45	51	0.171315	0.88215	no
ni|645903752|nb|KK920678.1|	671323-671458	18	16	45	71	0.646988	0.5829	no
ni|645903752|nb|KK920678.1|	6820-7047	18	16	18	60	2	0.14695	no
ni|645903752|nb|KK920678.1|	73878-75440	18	16	26	32	0.327051	0.81725	no
ni|645903752|nb|KK920678.1|	80679-80815	18	16	11	14	0.325067	0.78615	no
ni|645903752|nb|KK920678.1|	81167-81630	18	16	5	4	-0.531762	0.64225	no
ni|645903752|nb|KK920678.1|	82024-82221	18	16	8	5	-0.726007	0.54605	no
ni|645903752|nb|KK920678.1|	82477-83121	18	16	9	8	-0.22161	0.8458	no
ni|645903752|nb|KK920678.1|	83587-83797	18	16	1	6	2	0.15125	no
ni|645903752|nb|KK920678.1|	88208-88596	18	16	2	2	0.0866171	0.9485	no
ni|645903752|nb|KK920678.1|	89265-89607	18	16	2	3	0.0499086	0.96845	no
ni|645903752|nb|KK920678.1|	894165-894309	18	16	12	19	0.643285	0.5963	no
ni|645903752|nb|KK920678.1|	90341-90541	18	16	8	6	-0.410936	0.73715	no
ni|645903752|nb|KK920678.1|	90953-91173	18	16	6	6	-0.0141327	0.98035	no
ni|645903753|nb|KK920677.1|	131047-131310	17	9.8	3	2	-0.525487	0.67215	no
ni|645903753|nb|KK920677.1|	132775-133074	17	9.8	10	8	-0.297118	0.79855	no

ni|645903753|nb|KK920677.1|	135119-135803	17	9.8	0.63555	0.445589	-0.512293	0.697	no
ni|645903753|nb|KK920677.1|	136862-136991	17	9.8	26	25	-0.0673044	0.95425	no
ni|645903753|nb|KK920677.1|	140140-140324	17	9.8	5	5	-0.0672707	0.9579	no
ni|645903753|nb|KK920677.1|	140940-141916	17	9.8	0.535968	0.29671	-0.853095	0.5051	no
ni|645903753|nb|KK920677.1|	142854-143084	17	9.8	24	19	-0.340879	0.7683	no
ni|645903753|nb|KK920677.1|	144337-144473	17	9.8	37	39	0.0847526	0.9407	no
ni|645903753|nb|KK920677.1|	145700-145849	17	9.8	31	19	-0.712533	0.55245	no
ni|645903753|nb|KK920677.1|	147006-147143	17	9.8	21	8	-1	0.28115	no
ni|645903753|nb|KK920677.1|	148251-148385	17	9.8	15	8	-0.874619	0.48445	no
ni|645903753|nb|KK920677.1|	186047-186275	17	9.8	0.781208	4	2	0.10295	no
ni|645903753|nb|KK920677.1|	190847-191068	17	9.8	2	3	0.570846	0.67875	no
ni|645903753|nb|KK920677.1|	195271-195868	17	9.8	0.298928	0.904935	2	0.2583	no
ni|645903753|nb|KK920677.1|	198503-198711	17	9.8	1	4	2	0.2459	no
ni|645903753|nb|KK920677.1|	220296-221417	17	9.8	0.532257	0.948541	0.833588	0.4959	no
ni|645903753|nb|KK920677.1|	224500-224985	17	9.8	0.708347	1	0.645346	0.6193	no
ni|645903753|nb|KK920677.1|	229364-229643	17	9.8	3	4	0.381531	0.7525	no
ni|645903753|nb|KK920677.1|	229710-229875	17	9.8	3	5	0.825858	0.5554	no
ni|645903753|nb|KK920677.1|	252198-252417	17	9.8	15	16	0.0289611	0.9798	no
ni|645903753|nb|KK920677.1|	253561-253763	17	9.8	2	1	-0.651902	0.6679	no
ni|645903753|nb|KK920677.1|	257304-257459	17	9.8	19	18	-0.032429	0.97865	no
ni|645903753|nb|KK920677.1|	259705-260598	17	9.8	3	4	0.484437	0.67315	no
ni|645903753|nb|KK920677.1|	261031-261564	17	9.8	5	5	0.000531238	0.9937	no
ni|645903753|nb|KK920677.1|	262177-262479	17	9.8	8	7	-0.161991	0.89065	no
ni|645903753|nb|KK920677.1|	266439-267143	17	9.8	0.696347	0.587432	-0.245384	0.84435	no
ni|645903753|nb|KK920677.1|	267361-268298	17	9.8	0.649592	0.988173	0.60523	0.6214	no
ni|645903753|nb|KK920677.1|	270850-271388	17	9.8	0.509097	0.919233	0.852489	0.521	no
ni|645903753|nb|KK920677.1|	272259-272691	17	9.8	7	6	-0.128	0.91145	no
ni|645903753|nb|KK920677.1|	272803-273146	17	9.8	11	8	-0.437533	0.70365	no
ni|645903753|nb|KK920677.1|	273272-273787	17	9.8	10	8	-0.425264	0.70795	no
ni|645903753|nb|KK920677.1|	273929-274067	17	9.8	38	26	-0.532902	0.6529	no

ni|645903753|nb|KK920677.1|	274184-274366	17	9.8	36	19	-0.893262	0.4424	no
ni|645903753|nb|KK920677.1|	274745-275096	17	9.8	0.495137	1	1	0.4292	no
ni|645903753|nb|KK920677.1|	280083-280456	17	9.8	0.63697	0.521763	-0.287832	0.8535	no
ni|645903753|nb|KK920677.1|	282352-283067	17	9.8	0.764296	0.807646	0.0795908	0.9453	no
ni|645903753|nb|KK920677.1|	296415-296647	17	9.8	29	19	-0.632757	0.5864	no
ni|645903753|nb|KK920677.1|	297031-297149	17	9.8	11	13	0.254493	0.86075	no
ni|645903753|nb|KK920677.1|	297394-297792	17	9.8	13	12	-0.12085	0.9171	no
ni|645903753|nb|KK920677.1|	300711-301046	17	9.8	16	19	0.189655	0.8674	no
ni|645903753|nb|KK920677.1|	301209-302657	17	9.8	0.732575	0.560461	-0.386363	0.74215	no
ni|645903753|nb|KK920677.1|	303321-303632	17	9.8	3	2	-0.480762	0.6968	no
ni|645903753|nb|KK920677.1|	304621-305956	17	9.8	2	1	-0.513448	0.6639	no
ni|645903753|nb|KK920677.1|	307255-307584	17	9.8	9	4	-1	0.3067	no
ni|645903753|nb|KK920677.1|	307784-308886	17	9.8	0.567026	0.471495	-0.266174	0.8253	no
ni|645903753|nb|KK920677.1|	310107-310310	17	9.8	2	1	-0.914915	0.53575	no
ni|645903753|nb|KK920677.1|	311847-312315	17	9.8	1	0.96614	-0.158292	0.89795	no
ni|645903753|nb|KK920677.1|	313188-314235	17	9.8	0.443508	0.449044	0.0178981	0.9674	no
ni|645903753|nb|KK920677.1|	314353-314997	17	9.8	0.500487	0.391481	-0.354391	0.7931	no
ni|645903753|nb|KK920677.1|	315306-315705	17	9.8	0.830353	0.952294	0.197682	0.87955	no
ni|645903753|nb|KK920677.1|	317348-317890	17	9.8	3	2	-0.550469	0.63985	no
ni|645903753|nb|KK920677.1|	318640-319713	17	9.8	0.380848	0.63125	0.728997	0.56125	no
ni|645903753|nb|KK920677.1|	319842-320348	17	9.8	1	1	0.16936	0.89635	no
ni|645903753|nb|KK920677.1|	328665-329135	17	9.8	0.73722	0.384357	-0.939648	0.54025	no
ni|645903753|nb|KK920677.1|	330047-330843	17	9.8	0.532933	0.577493	0.115851	0.9286	no
ni|645903753|nb|KK920677.1|	331639-333417	17	9.8	0.352982	0.46417	0.395057	0.74035	no
ni|645903753|nb|KK920677.1|	333849-334444	17	9.8	0.600284	0.71732	0.256973	0.84735	no
ni|645903753|nb|KK920677.1|	334625-335269	17	9.8	0.909976	0.739464	-0.29935	0.8145	no
ni|645903753|nb|KK920677.1|	335715-336219	17	9.8	0.368109	0.469154	0.349928	0.81755	no
ni|645903753|nb|KK920677.1|	336789-337120	17	9.8	7	9	0.311233	0.79025	no
ni|645903753|nb|KK920677.1|	337763-338572	17	9.8	0.313877	0.233419	-0.42728	0.75965	no
ni|645903753|nb|KK920677.1|	338787-339041	17	9.8	14	11	-0.412589	0.7216	no

ni|645903753|nb|KK920677.1|	354057-354403	17	9.8	2	1	-0.246123	0.84435	no
ni|645903753|nb|KK920677.1|	362161-362442	17	9.8	2	1	-0.355419	0.7931	no
ni|645903753|nb|KK920677.1|	363192-364585	17	9.8	2	1	-0.390218	0.73995	no
ni|645903753|nb|KK920677.1|	364893-365069	17	9.8	4	1	-2	0.25505	no
ni|645903753|nb|KK920677.1|	365623-366444	17	9.8	2	2	-0.5008	0.66525	no
ni|645903753|nb|KK920677.1|	366837-367146	17	9.8	1	0.682689	-0.802701	0.6027	no
ni|645903753|nb|KK920677.1|	368222-368662	17	9.8	3	3	-0.10372	0.92855	no
ni|645903753|nb|KK920677.1|	369561-369911	17	9.8	17	9	-0.811216	0.4925	no
ni|645903753|nb|KK920677.1|	370064-370817	17	9.8	0.83329	0.977783	0.230696	0.8483	no
ni|645903753|nb|KK920677.1|	374441-374695	17	9.8	71	43	-0.70504	0.5309	no
ni|645903753|nb|KK920677.1|	375833-376641	17	9.8	0.52387	0.133571	-2	0.2004	no
ni|645903753|nb|KK920677.1|	378276-378442	17	9.8	78	53	-0.56391	0.62785	no
ni|645903753|nb|KK920677.1|	379720-379875	17	9.8	80	47	-0.758442	0.51985	no
ni|645903753|nb|KK920677.1|	391491-391654	17	9.8	85	55	-0.638759	0.5868	no
ni|645903753|nb|KK920677.1|	393383-393580	17	9.8	65	40	-0.692485	0.54105	no
ni|645903753|nb|KK920677.1|	393721-393913	17	9.8	69	48	-0.5458	0.6288	no
ni|645903753|nb|KK920677.1|	415805-416085	17	9.8	30	20	-0.598653	0.59635	no
ni|645903753|nb|KK920677.1|	424675-424818	17	9.8	43	41	-0.0672106	0.9537	no
ni|645903753|nb|KK920677.1|	429427-429551	17	9.8	32	21	-0.607923	0.6129	no
ni|645903753|nb|KK920677.1|	436361-436575	17	9.8	33	38	0.220181	0.851	no
ni|645903753|nb|KK920677.1|	439385-440035	17	9.8	18	16	-0.14649	0.8929	no
ni|645903753|nb|KK920677.1|	440390-440743	17	9.8	8	6	-0.266217	0.82415	no
ni|645903753|nb|KK920677.1|	459083-459251	17	9.8	40	39	-0.0280792	0.981	no
ni|645903753|nb|KK920677.1|	459358-459472	17	9.8	27	21	-0.36708	0.7706	no
ni|645903753|nb|KK920677.1|	461419-461585	17	9.8	51	54	0.07613	0.9473	no
ni|645903753|nb|KK920677.1|	462304-463025	17	9.8	0.557704	0.190433	-2	0.29725	no
ni|645903753|nb|KK920677.1|	464713-466405	17	9.8	0.465098	0.252052	-0.883816	0.4775	no
ni|645903753|nb|KK920677.1|	466922-467581	17	9.8	0.487001	0.46559	-0.0648648	0.94765	no
ni|645903753|nb|KK920677.1|	467868-468552	17	9.8	7	7	0.0862315	0.94165	no
ni|645903753|nb|KK920677.1|	469957-470149	17	9.8	38	32	-0.25535	0.8208	no

ni|645903753|nb|KK920677.1|	474402-474534	17	9.8	39	45	0.214492	0.85415	no
ni|645903753|nb|KK920677.1|	476471-476868	17	9.8	15	14	-0.154292	0.89395	no
ni|645903753|nb|KK920677.1|	479467-479605	17	9.8	40	30	-0.413013	0.7325	no
ni|645903753|nb|KK920677.1|	480245-480579	17	9.8	9	6	-0.558003	0.63725	no
ni|645903753|nb|KK920677.1|	480758-480908	17	9.8	78	61	-0.350589	0.7595	no
ni|645903753|nb|KK920677.1|	482584-482727	17	9.8	46	33	-0.464101	0.69175	no
ni|645903753|nb|KK920677.1|	484061-484399	17	9.8	1	0.399328	-1	0.35155	no
ni|645903753|nb|KK920677.1|	484528-484904	17	9.8	1.71	0.344039	-2	0.1366	no
ni|645903753|nb|KK920677.1|	485080-485566	17	9.8	21	19	-0.132259	0.90725	no
ni|645903753|nb|KK920677.1|	489628-489944	17	9.8	41	38	-0.110594	0.9188	no
ni|645903753|nb|KK920677.1|	490671-490865	17	9.8	59	47	-0.317085	0.77565	no
ni|645903753|nb|KK920677.1|	497135-498109	17	9.8	1	0.729958	-1	0.38275	no
ni|645903753|nb|KK920677.1|	498283-498502	17	9.8	3	2	-0.651579	0.64935	no
ni|645903753|nb|KK920677.1|	502431-502979	17	9.8	0.663572	0.158581	-2	0.20695	no
ni|645903753|nb|KK920677.1|	510420-510989	17	9.8	15	12	-0.24942	0.82875	no
ni|645903753|nb|KK920677.1|	511250-511361	17	9.8	86	48	-0.840203	0.48615	no
ni|645903753|nb|KK920677.1|	511605-511843	17	9.8	55	36	-0.595254	0.59795	no
ni|645903753|nb|KK920677.1|	522511-523585	17	9.8	0.887731	0.897393	0.0156183	0.9828	no
ni|645903753|nb|KK920677.1|	529288-529566	17	9.8	76	63	-0.263167	0.8104	no
ni|645903753|nb|KK920677.1|	529872-530301	17	9.8	1	0.576677	-1	0.40265	no
ni|645903753|nb|KK920677.1|	530552-531053	17	9.8	1	0.827189	-0.505688	0.68965	no
ni|645903753|nb|KK920677.1|	531153-532702	17	9.8	9	7	-0.350066	0.7451	no
ni|645903753|nb|KK920677.1|	534395-534549	17	9.8	264	200	-0.397341	0.71655	no
ni|645903753|nb|KK920677.1|	538714-539315	17	9.8	138	97	-0.503818	0.7252	no
ni|645903753|nb|KK920677.1|	539912-540178	17	9.8	5	4	-0.592098	0.6233	no
ni|645903753|nb|KK920677.1|	540395-540589	17	9.8	5	4	-0.237032	0.85345	no
ni|645903753|nb|KK920677.1|	540935-541153	17	9.8	4	5	0.51833	0.6703	no
ni|645903753|nb|KK920677.1|	542262-542556	17	9.8	2	0.490574	-2	0.25615	no
ni|645903753|nb|KK920677.1|	542717-543427	17	9.8	4	3	-0.372927	0.7542	no
ni|645903753|nb|KK920677.1|	543562-543731	17	9.8	6	3	-0.832794	0.5313	no

ni|645903753|nb|KK920677.1|	543872-544364	17	9.8	44	30	-0.578824	0.5867	no
ni|645903753|nb|KK920677.1|	544521-544724	17	9.8	2	2	-0.388846	0.78555	no
ni|645903753|nb|KK920677.1|	545303-545515	17	9.8	3	2	-0.59725	0.64595	no
ni|645903753|nb|KK920677.1|	547724-548650	17	9.8	3	3	-0.0963367	0.93385	no
ni|645903753|nb|KK920677.1|	548797-549734	17	9.8	1	0.875239	-0.467977	0.6942	no
ni|645903753|nb|KK920677.1|	550240-550554	17	9.8	100	66	-0.593054	0.63865	no
ni|645903753|nb|KK920677.1|	550784-551029	17	9.8	249	165	-0.592105	0.67955	no
ni|645903753|nb|KK920677.1|	555636-555773	17	9.8	29	31	0.0880341	0.93825	no
ni|645903753|nb|KK920677.1|	556365-556561	17	9.8	30	29	-0.0670654	0.953	no
ni|645903753|nb|KK920677.1|	559073-559191	17	9.8	61	57	-0.0991442	0.9341	no
ni|645903753|nb|KK920677.1|	67724-67845	17	9.8	26	4	-3	0.09005	no
ni|645903754|nb|KK920676.1|	100851-100979	14	8.8	9	12	0.418113	0.73705	no
ni|645903754|nb|KK920676.1|	101850-101969	14	8.8	30	7	-2	0.1631	no
ni|645903754|nb|KK920676.1|	102022-102142	14	8.8	29	14	-1	0.3992	no
ni|645903754|nb|KK920676.1|	102288-102499	14	8.8	17	7	-1	0.2945	no
ni|645903754|nb|KK920676.1|	103087-103277	14	8.8	40	26	-0.632381	0.58475	no
ni|645903754|nb|KK920676.1|	104423-104549	14	8.8	31	30	-0.0242655	0.9812	no
ni|645903754|nb|KK920676.1|	110092-110510	14	8.8	80	44	-0.877912	0.48625	no
ni|645903754|nb|KK920676.1|	112123-112395	14	8.8	104	66	-0.662037	0.5289	no
ni|645903754|nb|KK920676.1|	112569-113279	14	8.8	0.608357	0.62041	0.0283048	0.9668	no
ni|645903754|nb|KK920676.1|	114806-115493	14	8.8	1	0.765706	-0.7711	0.5218	no
ni|645903754|nb|KK920676.1|	115603-115940	14	8.8	47	30	-0.62008	0.5765	no
ni|645903754|nb|KK920676.1|	17030-17150	14	8.8	20	17	-0.247977	0.84135	no
ni|645903754|nb|KK920676.1|	20572-20695	14	8.8	22	9	-1	0.33865	no
ni|645903754|nb|KK920676.1|	2365-2588	14	8.8	15	15	-0.0476917	0.9667	no
ni|645903754|nb|KK920676.1|	29489-30796	14	8.8	0.388702	0.273895	-0.505043	0.68965	no
ni|645903754|nb|KK920676.1|	40508-40933	14	8.8	5	5	-0.192661	0.8684	no
ni|645903754|nb|KK920676.1|	441220-441397	14	8.8	20	0	-inf	5.00E-05	yes
ni|645903754|nb|KK920676.1|	50335-50658	14	8.8	19	16	-0.241028	0.8384	no
ni|645903754|nb|KK920676.1|	56498-56630	14	8.8	32	24	-0.415202	0.71685	no

ni|645903754|nb|KK920676.1|	57640-57884	14	8.8	26	18	-0.553599	0.6335	no
ni|645903754|nb|KK920676.1|	58046-58234	14	8.8	25	26	0.106433	0.9321	no
ni|645903754|nb|KK920676.1|	58462-59105	14	8.8	5	3	-0.649848	0.5758	no
ni|645903754|nb|KK920676.1|	59256-59388	14	8.8	9	7	-0.330313	0.80325	no
ni|645903754|nb|KK920676.1|	62300-63006	14	8.8	0.612388	0.858714	0.487732	0.6923	no
ni|645903754|nb|KK920676.1|	70398-70522	14	8.8	31	17	-0.851626	0.4851	no
ni|645903754|nb|KK920676.1|	71859-71988	14	8.8	33	26	-0.352707	0.7593	no
ni|645903754|nb|KK920676.1|	74520-74992	14	8.8	0.933208	0.445994	-1	0.45115	no
ni|645903754|nb|KK920676.1|	76496-76674	14	8.8	35	27	-0.359774	0.75515	no
ni|645903754|nb|KK920676.1|	77066-77270	14	8.8	37	32	-0.226252	0.84815	no
ni|645903754|nb|KK920676.1|	84061-84321	14	8.8	28	25	-0.181559	0.8746	no
ni|645903754|nb|KK920676.1|	86169-86374	14	8.8	31	24	-0.386052	0.73615	no
ni|645903754|nb|KK920676.1|	90541-90783	14	8.8	31	26	-0.261288	0.82015	no
ni|645903754|nb|KK920676.1|	92304-92654	14	8.8	17	15	-0.15517	0.89485	no
ni|645903754|nb|KK920676.1|	92833-92967	14	8.8	35	30	-0.255709	0.82785	no
ni|645903754|nb|KK920676.1|	96314-99124	14	8.8	0.555217	0.348211	-0.673091	0.56585	no
ni|645903754|nb|KK920676.1|	99756-100698	14	8.8	44	37	-0.253421	0.8558	no
ni|645903755|nb|KK920675.1|	101498-101646	18	16	39	110.26	2	0.2052	no
ni|645903755|nb|KK920675.1|	102343-102674	18	16	2	1	-0.687109	0.58765	no
ni|645903755|nb|KK920675.1|	103202-103602	18	16	0.662055	0.395459	-0.743421	0.59615	no
ni|645903755|nb|KK920675.1|	104495-105218	18	16	2	2	0.627087	0.59155	no
ni|645903755|nb|KK920675.1|	105577-105710	18	16	2	30	4	0.05375	no
ni|645903755|nb|KK920675.1|	105931-106202	18	16	0.728948	6	3	0.047	no
ni|645903755|nb|KK920675.1|	106601-106883	18	16	2	5	2	0.2054	no
ni|645903755|nb|KK920675.1|	107721-108111	18	16	3	2	-0.480417	0.684	no
ni|645903755|nb|KK920675.1|	10839-11566	18	16	1	2	0.288851	0.8094	no
ni|645903755|nb|KK920675.1|	110706-110922	18	16	33	93.72	2	0.1791	no
ni|645903755|nb|KK920675.1|	111828-112002	18	16	33	85	1	0.2374	no
ni|645903755|nb|KK920675.1|	112104-112268	18	16	32	97	2	0.1808	no
ni|645903755|nb|KK920675.1|	113453-113605	18	16	28	82	2	0.19335	no

ni|645903755|nb|KK920675.1|	126637-126835	18	16	36	73	1	0.37715	no
ni|645903755|nb|KK920675.1|	127131-127248	18	16	31	42	0.427312	0.7268	no
ni|645903755|nb|KK920675.1|	128197-128497	18	16	22	25	0.187707	0.86865	no
ni|645903755|nb|KK920675.1|	13461-13575	18	16	18	11	-0.719596	0.61925	no
ni|645903755|nb|KK920675.1|	138051-138410	18	16	6	7	0.238286	0.83385	no
ni|645903755|nb|KK920675.1|	138839-138983	18	16	30	45	0.597188	0.60435	no
ni|645903755|nb|KK920675.1|	147051-147181	18	16	47	55	0.204007	0.85805	no
ni|645903755|nb|KK920675.1|	14720-14893	18	16	11	19	0.746287	0.528	no
ni|645903755|nb|KK920675.1|	149551-149658	18	16	131	161	0.294727	0.79955	no
ni|645903755|nb|KK920675.1|	158243-158510	18	16	12	10	-0.240519	0.8361	no
ni|645903755|nb|KK920675.1|	16558-16720	18	16	10	10	0.0482648	0.9698	no
ni|645903755|nb|KK920675.1|	16988-17375	18	16	5	7	0.423358	0.71765	no
ni|645903755|nb|KK920675.1|	17689-18427	18	16	2	3	0.0895533	0.9391	no
ni|645903755|nb|KK920675.1|	2052-2286	18	16	3	3	-0.148843	0.91085	no
ni|645903755|nb|KK920675.1|	226775-226946	18	16	0	122	inf	5.00E-05	yes
ni|645903755|nb|KK920675.1|	24967-25105	18	16	5	4	-0.259883	0.8692	no
ni|645903755|nb|KK920675.1|	269383-269546	18	16	4	3	-0.0672169	0.9414	no
ni|645903755|nb|KK920675.1|	28884-29203	18	16	1	0.651311	-0.650644	0.6679	no
ni|645903755|nb|KK920675.1|	290747-290892	18	16	27	105	2	0.10445	no
ni|645903755|nb|KK920675.1|	296016-296163	18	16	25	103	2	0.09605	no
ni|645903755|nb|KK920675.1|	30008-30369	18	16	17	16	-0.138243	0.9035	no
ni|645903755|nb|KK920675.1|	325170-325373	18	16	81	247	2	0.2221	no
ni|645903755|nb|KK920675.1|	328236-328391	18	16	62	192	2	0.1518	no
ni|645903755|nb|KK920675.1|	32930-33300	18	16	10	6	-0.616143	0.60535	no
ni|645903755|nb|KK920675.1|	332994-333272	18	16	66	195	2	0.22385	no
ni|645903755|nb|KK920675.1|	34410-34780	18	16	19	20	0.066394	0.9533	no
ni|645903755|nb|KK920675.1|	34957-35083	18	16	39	30	-0.372189	0.7474	no
ni|645903755|nb|KK920675.1|	36393-36514	18	16	15	13	-0.182869	0.889	no
ni|645903755|nb|KK920675.1|	37084-37253	18	16	45	37	-0.291577	0.8026	no
ni|645903755|nb|KK920675.1|	37399-37563	18	16	48	44	-0.1266	0.91185	no

ni|645903755|nb|KK920675.1|	38055-38274	18	16	20	23	0.203835	0.86185	no
ni|645903755|nb|KK920675.1|	39148-39256	18	16	72	58	-0.308779	0.8005	no
ni|645903755|nb|KK920675.1|	39698-40436	18	16	8	9	0.168634	0.88	no
ni|645903755|nb|KK920675.1|	40547-40686	18	16	67	64	-0.0809075	0.9424	no
ni|645903755|nb|KK920675.1|	42676-42925	18	16	0	9	inf	5.00E-05	yes
ni|645903755|nb|KK920675.1|	43108-43991	18	16	0.284124	0.211301	-0.427225	0.75965	no
ni|645903755|nb|KK920675.1|	438238-438470	18	16	12	13	0.212306	0.8563	no
ni|645903755|nb|KK920675.1|	44368-44518	18	16	0.49775	108	8	0.11935	no
ni|645903755|nb|KK920675.1|	46793-46904	18	16	0	64	inf	0.00025	yes
ni|645903755|nb|KK920675.1|	49179-49338	18	16	2	117	6	0.0372	no
ni|645903755|nb|KK920675.1|	49469-49580	18	16	0	66	inf	0.00015	yes
ni|645903755|nb|KK920675.1|	52232-52455	18	16	1	107	6	0.0332	no
ni|645903755|nb|KK920675.1|	56850-56996	18	16	1	141.74	7	0.0443	no
ni|645903755|nb|KK920675.1|	62627-62803	18	16	2	89	5	0.0205	no
ni|645903755|nb|KK920675.1|	640816-641076	18	16	53	128	1	0.3192	no
ni|645903755|nb|KK920675.1|	642614-642780	18	16	79	220	1	0.1858	no
ni|645903755|nb|KK920675.1|	644896-645115	18	16	27	83	2	0.1655	no
ni|645903755|nb|KK920675.1|	647193-647518	18	16	18	47	1	0.21075	no
ni|645903755|nb|KK920675.1|	648410-648731	18	16	1	4	2	0.17595	no
ni|645903755|nb|KK920675.1|	648993-649246	18	16	2	0.778975	-1	0.44755	no
ni|645903755|nb|KK920675.1|	65213-66015	18	16	0.84538	17	4	0.0021	yes
ni|645903755|nb|KK920675.1|	652964-653109	18	16	65	171	1	0.22735	no
ni|645903755|nb|KK920675.1|	656300-657340	18	16	52	131	1	0.3507	no
ni|645903755|nb|KK920675.1|	657589-657922	18	16	32	77	1	0.34315	no
ni|645903755|nb|KK920675.1|	658195-658534	18	16	34	73	1	0.31115	no
ni|645903755|nb|KK920675.1|	658932-659307	18	16	81	195	1	0.3875	no
ni|645903755|nb|KK920675.1|	659780-660213	18	16	47	49	0.0465285	0.96535	no
ni|645903755|nb|KK920675.1|	660574-660837	18	16	81	75	-0.110195	0.9163	no
ni|645903755|nb|KK920675.1|	661371-661727	18	16	20	16	-0.351978	0.75905	no
ni|645903755|nb|KK920675.1|	66152-66635	18	16	1	61	5	0.00275	yes

ni|645903755|nb|KK920675.1|	661971-662572	18	16	37	27	-0.444233	0.71995	no
ni|645903755|nb|KK920675.1|	662779-662973	18	16	121	100	-0.2689	0.8091	no
ni|645903755|nb|KK920675.1|	663560-663725	18	16	104	53	-0.984171	0.40185	no
ni|645903755|nb|KK920675.1|	679357-679665	18	16	5	4	-0.428311	0.722	no
ni|645903755|nb|KK920675.1|	680227-680805	18	16	0.259055	2	3	0.0705	no
ni|645903755|nb|KK920675.1|	681798-682031	18	16	18	34	0.88828	0.447	no
ni|645903755|nb|KK920675.1|	68211-68425	18	16	3	113	5	0.0028	yes
ni|645903755|nb|KK920675.1|	686134-686348	18	16	18	47	1	0.25475	no
ni|645903755|nb|KK920675.1|	687810-688008	18	16	10	26	1	0.26005	no
ni|645903755|nb|KK920675.1|	688665-688810	18	16	14	32	1	0.3209	no
ni|645903755|nb|KK920675.1|	690300-690440	18	16	7	11	0.595738	0.63005	no
ni|645903755|nb|KK920675.1|	690839-690955	18	16	10	26	1	0.3533	no
ni|645903755|nb|KK920675.1|	696887-698826	18	16	22	21	-0.0582899	0.96675	no
ni|645903755|nb|KK920675.1|	69750-69892	18	16	2	56	5	0.05095	no
ni|645903755|nb|KK920675.1|	700442-701222	18	16	6	7	0.014554	0.9895	no
ni|645903755|nb|KK920675.1|	701848-702089	18	16	2	1	-0.388213	0.78555	no
ni|645903755|nb|KK920675.1|	702672-702889	18	16	23	98	2	0.07305	no
ni|645903755|nb|KK920675.1|	705072-705608	18	16	8	19	1	0.2998	no
ni|645903755|nb|KK920675.1|	705747-706022	18	16	0.712144	1	0.934043	0.52835	no
ni|645903755|nb|KK920675.1|	708329-708482	18	16	43	125	2	0.1892	no
ni|645903755|nb|KK920675.1|	708819-708973	18	16	38	126	2	0.14875	no
ni|645903755|nb|KK920675.1|	710005-710341	18	16	0.948304	0.704785	-0.428167	0.75965	no
ni|645903755|nb|KK920675.1|	711390-712088	18	16	38	100	1	0.3506	no
ni|645903755|nb|KK920675.1|	714443-714908	18	16	22	31	0.536364	0.62505	no
ni|645903755|nb|KK920675.1|	723163-723438	18	16	0.142429	7	6	0.11975	no
ni|645903755|nb|KK920675.1|	727156-727350	18	16	64	80	0.314625	0.78285	no
ni|645903755|nb|KK920675.1|	729047-729255	18	16	35	54	0.610073	0.59065	no
ni|645903755|nb|KK920675.1|	729789-730018	18	16	33	47	0.48456	0.6693	no
ni|645903755|nb|KK920675.1|	730806-731311	18	16	17	23	0.423931	0.70015	no
ni|645903755|nb|KK920675.1|	732747-732863	18	16	71	71	-0.00857881	0.98805	no

ni|645903755|nb|KK920675.1|	733066-733703	18	16	25	30	0.283801	0.78545	no
ni|645903755|nb|KK920675.1|	734087-734414	18	16	0.986179	2	0.851897	0.521	no
ni|645903755|nb|KK920675.1|	734573-735520	18	16	2	1	-0.73341	0.53455	no
ni|645903755|nb|KK920675.1|	735690-735798	18	16	28	40	0.517192	0.6956	no
ni|645903755|nb|KK920675.1|	735913-736023	18	16	23	26	0.195378	0.87955	no
ni|645903755|nb|KK920675.1|	736267-736432	18	16	18	10	-0.921376	0.43995	no
ni|645903755|nb|KK920675.1|	736894-737032	18	16	98	79	-0.305643	0.7894	no
ni|645903755|nb|KK920675.1|	737678-738115	18	16	2	1	-0.55068	0.66085	no
ni|645903755|nb|KK920675.1|	738238-738511	18	16	3	2	-0.84358	0.49805	no
ni|645903755|nb|KK920675.1|	743608-744723	18	16	0.559747	5	3	0.01625	no
ni|645903755|nb|KK920675.1|	744983-745375	18	16	1	1	-0.258018	0.8388	no
ni|645903755|nb|KK920675.1|	745519-745875	18	16	442	344	-0.362561	0.7918	no
ni|645903755|nb|KK920675.1|	746141-747781	18	16	3	3	-0.0149282	0.9896	no
ni|645903755|nb|KK920675.1|	749887-750218	18	16	612	442	-0.469034	0.74295	no
ni|645903755|nb|KK920675.1|	757123-758179	18	16	0.051685	80	11	0.0443	no
ni|645903755|nb|KK920675.1|	76319-76431	18	16	0	1276.52	inf	5.00E-05	yes
ni|645903755|nb|KK920675.1|	84168-84470	18	16	0.123245	3	5	0.121	no
ni|645903755|nb|KK920675.1|	85112-85439	18	16	0.219151	4	4	0.04655	no
ni|645903755|nb|KK920675.1|	85905-86307	18	16	0.082205	9	7	0.1194	no
ni|645903755|nb|KK920675.1|	86556-86964	18	16	0.080595	353	12	0.11935	no
ni|645903755|nb|KK920675.1|	90139-90258	18	16	0	110	inf	5.00E-05	yes
ni|645903755|nb|KK920675.1|	90839-91094	18	16	0	256	inf	5.00E-05	yes
ni|645903755|nb|KK920675.1|	91768-92069	18	16	0.247726	151	9	0.0443	no
ni|645903755|nb|KK920675.1|	92357-93124	18	16	7	17	1	0.28235	no
ni|645903755|nb|KK920675.1|	9519-9679	18	16	2	4	0.807264	0.58675	no
ni|645903755|nb|KK920675.1|	97639-97910	18	16	0.728948	2	1	0.30495	no
ni|645903755|nb|KK920675.1|	99196-99550	18	16	2	0.187011	-3	0.2403	no
ni|645903756|nb|KK920674.1|	567475-567613	16	9	6	6	-0.0672382	0.9414	no
ni|645903756|nb|KK920674.1|	567676-567895	16	9	2	2	0.0589143	0.95295	no
ni|645903756|nb|KK920674.1|	576896-577108	16	9	7	10	0.44346	0.71155	no

ni|645903756|nb|KK920674.1|	577561-579047	16	9	0.391851	0.238469	-0.716503	0.57	no
ni|645903756|nb|KK920674.1|	581945-582166	16	9	24	21	-0.20776	0.85715	no
ni|645903756|nb|KK920674.1|	584891-585111	16	9	27	22	-0.270472	0.815	no
ni|645903756|nb|KK920674.1|	585666-585836	16	9	8	6	-0.372123	0.762	no
ni|645903756|nb|KK920674.1|	586245-587340	16	9	4	3	-0.249898	0.82745	no
ni|645903756|nb|KK920674.1|	587764-587913	16	9	17	9	-0.941667	0.4392	no
ni|645903756|nb|KK920674.1|	588193-588594	16	9	2	2	-0.172262	0.8837	no
ni|645903756|nb|KK920674.1|	588833-589307	16	9	0.99449	0.697082	-0.512629	0.697	no
ni|645903756|nb|KK920674.1|	589574-590095	16	9	1	0.843986	-0.550505	0.66085	no
ni|645903756|nb|KK920674.1|	590580-590846	16	9	23	18	-0.306164	0.78895	no
ni|645903756|nb|KK920674.1|	593349-593506	16	9	37	23	-0.669234	0.5669	no
ni|645903756|nb|KK920674.1|	594366-594753	16	9	12	11	-0.172304	0.8806	no
ni|645903756|nb|KK920674.1|	595789-595895	16	9	39	34	-0.205463	0.87975	no
ni|645903756|nb|KK920674.1|	595989-596391	16	9	10	7	-0.615541	0.59685	no
ni|645903756|nb|KK920674.1|	596686-596808	16	9	21	4	-2	0.1366	no
ni|645903756|nb|KK920674.1|	597698-598232	16	9	3	2	-0.621449	0.5984	no
ni|645903756|nb|KK920674.1|	651003-651578	16	9	94	42	-1	0.43085	no
ni|645903756|nb|KK920674.1|	654953-655657	16	9	0.655385	0.469946	-0.479849	0.7105	no
ni|645903756|nb|KK920674.1|	655811-656186	16	9	0.993626	0.258973	-2	0.24815	no
ni|645903756|nb|KK920674.1|	658312-658587	16	9	13	13	0.0242405	0.98225	no
ni|645903756|nb|KK920674.1|	658738-658999	16	9	2	1	-0.580646	0.70815	no
ni|645903756|nb|KK920674.1|	659622-660051	16	9	8	8	-0.0387997	0.9731	no
ni|645903756|nb|KK920674.1|	660162-660771	16	9	6	5	-0.383884	0.7395	no
ni|645903756|nb|KK920674.1|	660877-661663	16	9	11	10	-0.234655	0.83125	no
ni|645903756|nb|KK920674.1|	661863-662033	16	9	18	18	0.0151933	0.98835	no
ni|645903756|nb|KK920674.1|	662175-663399	16	9	19	16	-0.238144	0.87755	no
ni|645903756|nb|KK920674.1|	663880-663995	16	9	27	21	-0.347603	0.78305	no
ni|645903756|nb|KK920674.1|	664450-664590	16	9	21	18	-0.200494	0.86755	no
ni|645903756|nb|KK920674.1|	664703-664822	16	9	13	19	0.610652	0.63765	no
ni|645903756|nb|KK920674.1|	665555-665984	16	9	7	7	-0.125393	0.91515	no

ni|645903756|nb|KK920674.1|	666465-667194	16	9	8	5	-0.576124	0.6179	no
ni|645903756|nb|KK920674.1|	667440-667590	16	9	21	10	-1	0.34665	no
ni|645903756|nb|KK920674.1|	667950-668065	16	9	24	5	-2	0.1791	no
ni|645903756|nb|KK920674.1|	668861-669099	16	9	3480.87	2544.44	-0.452102	0.71575	no
ni|645903756|nb|KK920674.1|	670058-671198	16	9	2007.2	1507.69	-0.412846	0.6657	no
ni|645903756|nb|KK920674.1|	672389-672850	16	9	30	20	-0.534684	0.6241	no
ni|645903756|nb|KK920674.1|	673017-673592	16	9	17	11	-0.656643	0.55755	no
ni|645903756|nb|KK920674.1|	674394-674964	16	9	53	37	-0.521372	0.67855	no
ni|645903756|nb|KK920674.1|	675271-675808	16	9	3675.97	2378.54	-0.628052	0.54015	no
ni|645903756|nb|KK920674.1|	676410-676844	16	9	6	5	-0.250475	0.82955	no
ni|645903756|nb|KK920674.1|	679274-679694	16	9	59	38	-0.615424	0.6262	no
ni|645903756|nb|KK920674.1|	680240-680783	16	9	25	16	-0.653248	0.5584	no
ni|645903756|nb|KK920674.1|	681238-681820	16	9	37	26	-0.548419	0.66695	no
ni|645903756|nb|KK920674.1|	682729-683064	16	9	18	13	-0.418274	0.7157	no
ni|645903756|nb|KK920674.1|	684485-685750	16	9	49	38	-0.388275	0.7865	no
ni|645903756|nb|KK920674.1|	688457-688808	16	9	1	0.56778	-0.939998	0.54025	no
ni|645903756|nb|KK920674.1|	689185-689331	16	9	119	88	-0.439173	0.70265	no
ni|645903756|nb|KK920674.1|	691572-691750	16	9	150	123.33	-0.286636	0.7874	no
ni|645903756|nb|KK920674.1|	723991-724368	16	9	46	38	-0.266206	0.80755	no
ni|645903756|nb|KK920674.1|	725722-726088	16	9	5	4	-0.345574	0.7743	no
ni|645903756|nb|KK920674.1|	726199-726550	16	9	3	3	-0.302568	0.799	no
ni|645903756|nb|KK920674.1|	733419-733540	16	9	98	83	-0.228855	0.84215	no
ni|645903756|nb|KK920674.1|	735191-735553	16	9	0.758736	0.453167	-0.743554	0.59615	no
ni|645903756|nb|KK920674.1|	736977-737133	16	9	104	101	-0.0426393	0.9715	no
ni|645903756|nb|KK920674.1|	740559-740964	16	9	73	65	-0.173359	0.9084	no
ni|645903756|nb|KK920674.1|	86854-87234	16	9	1	0	-inf	0.0013	yes
ni|645903757|nb|KK920673.1|	10250-10647	18	17	0.585185	1	1	0.3525	no
ni|645903757|nb|KK920673.1|	143511-143932	18	17	30	24	-0.335052	0.76145	no
ni|645903757|nb|KK920673.1|	145905-146016	18	17	42	40	-0.0676133	0.9605	no
ni|645903757|nb|KK920673.1|	149063-149713	18	17	82	47	-0.796028	0.589	no

ni|645903757|nb|KK920673.1|	16677-16924	18	17	2	1	-0.718289	0.61925	no
ni|645903757|nb|KK920673.1|	183391-183477	18	17	6518.08	5989.89	-0.121917	0.9228	no
ni|645903757|nb|KK920673.1|	2143-2586	18	17	0	22	inf	5.00E-05	yes
ni|645903757|nb|KK920673.1|	22976-23445	18	17	2	2	0.427859	0.71975	no
ni|645903757|nb|KK920673.1|	24958-25191	18	17	6	6	0.0160666	0.98275	no
ni|645903757|nb|KK920673.1|	25540-25696	18	17	7	6	-0.166732	0.89925	no
ni|645903757|nb|KK920673.1|	28379-28533	18	17	5	6	0.311319	0.81835	no
ni|645903757|nb|KK920673.1|	29552-29768	18	17	8	11	0.54477	0.64125	no
ni|645903757|nb|KK920673.1|	30992-31364	18	17	2	3	0.404042	0.7286	no
ni|645903757|nb|KK920673.1|	313405-313567	18	17	16	20	0.311299	0.78925	no
ni|645903757|nb|KK920673.1|	344949-345098	18	17	322	324	0.00805216	0.99355	no
ni|645903757|nb|KK920673.1|	348686-348885	18	17	214	232	0.11367	0.94045	no
ni|645903757|nb|KK920673.1|	34900-35139	18	17	5	7	0.397635	0.7375	no
ni|645903757|nb|KK920673.1|	349476-349733	18	17	3	3	-0.300575	0.81	no
ni|645903757|nb|KK920673.1|	352034-352564	18	17	62	74	0.265971	0.85535	no
ni|645903757|nb|KK920673.1|	352844-353163	18	17	0.681649	2	1	0.34405	no
ni|645903757|nb|KK920673.1|	356936-357067	18	17	285	285	-0.000718839	0.9973	no
ni|645903757|nb|KK920673.1|	360783-361395	18	17	0.628619	0.69341	0.141522	0.90865	no
ni|645903757|nb|KK920673.1|	37172-37713	18	17	0.674157	0.268516	-1	0.3479	no
ni|645903757|nb|KK920673.1|	371891-372306	18	17	1374.39	2115.34	0.6221	0.6159	no
ni|645903757|nb|KK920673.1|	372631-372776	18	17	2	9	2	0.15655	no
ni|645903757|nb|KK920673.1|	373000-373639	18	17	2	3	0.688469	0.55905	no
ni|645903757|nb|KK920673.1|	374203-374737	18	17	77	142	0.884929	0.5374	no
ni|645903757|nb|KK920673.1|	375193-375677	18	17	2	3	0.385512	0.74455	no
ni|645903757|nb|KK920673.1|	378521-378945	18	17	172	291	0.756834	0.5882	no
ni|645903757|nb|KK920673.1|	379393-379722	18	17	1	3	1	0.3865	no
ni|645903757|nb|KK920673.1|	380361-380789	18	17	184	319	0.793422	0.57605	no
ni|645903757|nb|KK920673.1|	381623-381808	18	17	2	4	1	0.45555	no
ni|645903757|nb|KK920673.1|	382483-382973	18	17	194	373	0.942634	0.5097	no
ni|645903757|nb|KK920673.1|	38594-40308	18	17	12	12	-0.0294508	0.9825	no

ni|645903757|nb|KK920673.1|	386567-386882	18	17	71	159	1	0.3485	no
ni|645903757|nb|KK920673.1|	400591-400748	18	17	427	1058.07	1	0.37375	no
ni|645903757|nb|KK920673.1|	40856-41153	18	17	22	20	-0.0913401	0.93795	no
ni|645903757|nb|KK920673.1|	412224-412769	18	17	55	74	0.423509	0.7781	no
ni|645903757|nb|KK920673.1|	414080-415053	18	17	44	83	0.915326	0.52655	no
ni|645903757|nb|KK920673.1|	419019-419172	18	17	126	238	0.911196	0.4113	no
ni|645903757|nb|KK920673.1|	419575-420010	18	17	0.666562	0.566296	-0.235183	0.8697	no
ni|645903757|nb|KK920673.1|	420253-421233	18	17	26	53	1	0.4842	no
ni|645903757|nb|KK920673.1|	423286-423438	18	17	290	741	1	0.283	no
ni|645903757|nb|KK920673.1|	42707-42933	18	17	67	67	-0.00385736	0.99675	no
ni|645903757|nb|KK920673.1|	44137-44452	18	17	0.578689	3	2	0.08975	no
ni|645903757|nb|KK920673.1|	4435-4770	18	17	0.317456	33	7	0.05055	no
ni|645903757|nb|KK920673.1|	48665-48962	18	17	0	8	inf	5.00E-05	yes
ni|645903757|nb|KK920673.1|	49208-49799	18	17	0.252165	31	7	0.02725	no
ni|645903757|nb|KK920673.1|	50563-50817	18	17	0	51	inf	5.00E-05	yes
ni|645903757|nb|KK920673.1|	52813-53067	18	17	0.486108	73	7	0.05055	no
ni|645903757|nb|KK920673.1|	54560-54785	18	17	0.200038	129	9	0.11935	no
ni|645903757|nb|KK920673.1|	55347-55568	18	17	0.413438	97	8	0.0443	no
ni|645903757|nb|KK920673.1|	60972-61123	18	17	0	144	inf	5.00E-05	yes
ni|645903757|nb|KK920673.1|	6409-6743	18	17	0	21	inf	5.00E-05	yes
ni|645903757|nb|KK920673.1|	643-815	18	17	0	134.5	inf	5.00E-05	yes
ni|645903757|nb|KK920673.1|	65740-65855	18	17	24	26	0.113078	0.9325	no
ni|645903757|nb|KK920673.1|	66503-66615	18	17	68	57	-0.242664	0.8355	no
ni|645903757|nb|KK920673.1|	67107-67469	18	17	78	73	-0.0972302	0.95085	no
ni|645903757|nb|KK920673.1|	69514-70236	18	17	42	45	0.115537	0.93595	no
ni|645903757|nb|KK920673.1|	72438-72574	18	17	138	157	0.188588	0.8685	no
ni|645903757|nb|KK920673.1|	74569-74785	18	17	74	77	0.0501019	0.9618	no
ni|645903757|nb|KK920673.1|	75604-75818	18	17	63	88	0.484474	0.6627	no
ni|645903757|nb|KK920673.1|	75963-76081	18	17	105	80	-0.393936	0.7369	no
ni|645903757|nb|KK920673.1|	77024-77407	18	17	33	36	0.134896	0.90085	no

ni|645903757|nb|KK920673.1|	80488-80791	18	17	5	7	0.724777	0.5388	no
ni|645903757|nb|KK920673.1|	81518-81672	18	17	47	52	0.157239	0.8913	no
ni|645903757|nb|KK920673.1|	81774-82413	18	17	2	2	0.0809598	0.9432	no
ni|645903758|nb|KK920672.1|	102836-103138	17	15	10	16	0.714912	0.5347	no
ni|645903758|nb|KK920672.1|	104870-105002	17	15	28.42	34	0.277857	0.813	no
ni|645903758|nb|KK920672.1|	107511-107684	17	15	24	37	0.631531	0.5881	no
ni|645903758|nb|KK920672.1|	110360-110688	17	15	4	6	0.543173	0.6419	no
[truncated: 529,292 more chars]
