# Supplementary material for: The X Chromosome of Hemipteran Insects: Conservation, Dosage Compensation and Sex-Biased Expression
Source: Genome Biol Evol. 2015 Nov 10;7(12):3259–68. doi: 10.1093/gbe/evv215 (PMC4700948; doi:10.1093/gbe/evv215)
Supplement: Supplementary Data [file S1 Data (rev) H.vitripennis.pdf › S1 Data (rev) H.vitripennis.pdf]

scaffold	gene	covF	covM	expF	expM	log2(fold_change)	p-value	significance
gi|646480687|gb|KK997905.1|	324-1137	18	6.6	2	2	-0.410639	0.76285	no
gi|646480849|gb|KK997877.1|	819-1019	24	9.5	0	107	inf	0.00035	yes
gi|646480860|gb|KK997875.1|	5-581	13	13	0	4	inf	0.0014	yes
gi|646480890|gb|KK997870.1|	172-450	12	5.2	20	4	-2	0.20105	no
gi|646480989|gb|KK997853.1|	19-669	13	3.7	0.701862	5	3	0.17585	no
gi|646481080|gb|KK997837.1|	59-625	9.1	12	4	4	0.327786	0.78955	no
gi|646481175|gb|KK997820.1|	8971-9538	32	7.3	3	1	-1	0.4199	no
gi|646481181|gb|KK997819.1|	1-807	6.3	4.6	27	21	-0.332496	0.82965	no
gi|646481214|gb|KK997813.1|	0-604	17	6.1	30	0	-inf	0.00015	yes
gi|646481214|gb|KK997813.1|	1849-2824	17	6.1	36	49	0.450903	0.6813	no
gi|646481418|gb|KK997777.1|	0-437	23	11	18	5	-2	0.20995	no
gi|646481469|gb|KK997768.1|	45-667	4.9	3.8	0	4	inf	0.001	yes
gi|646481753|gb|KK997717.1|	294-553	9	2.7	5	21	2	0.3162	no
gi|646481843|gb|KK997701.1|	582-1080	13	3.6	0.754622	5	3	0.26725	no
gi|646481890|gb|KK997692.1|	2511-3123	15	10	2	1	-0.949675	0.589	no
gi|646481900|gb|KK997690.1|	3785-4194	18	10	6	2	-2	0.21525	no
gi|646482094|gb|KK997656.1|	0-931	13	3.1	31	29	-0.0758133	0.942	no
gi|646482322|gb|KK997616.1|	29-2013	17	5.8	4	4	0.048703	0.97265	no
gi|646482395|gb|KK997603.1|	87-2245	20	4.9	3	4	0.0921967	0.9511	no
gi|646482546|gb|KK997577.1|	1591-1998	13	7.5	12	0	-inf	0.00045	yes
gi|646482626|gb|KK997562.1|	1465-1638	15	4.5	40	164.54	2	0.3137	no
gi|646482626|gb|KK997562.1|	2695-3269	15	4.5	18	52	2	0.3262	no
gi|646483465|gb|KK997422.1|	8-349	13	4.1	8	1	-3	0.20705	no
gi|646483648|gb|KK997390.1|	1891-2210	21	8	44	19	-1	0.39745	no
gi|646483777|gb|KK997366.1|	6047-6936	7.1	3.2	11	8	-0.515976	0.73165	no
gi|646483914|gb|KK997345.1|	25-2413	19	4.5	1	2	0.868805	0.57485	no
gi|646483914|gb|KK997345.1|	4232-5083	19	4.5	2	2	-0.500225	0.70405	no
gi|646484028|gb|KK997324.1|	2546-3275	27	10	3	3	0.370933	0.7632	no
gi|646484356|gb|KK997266.1|	10624-11673	12	6.6	0.116506	8	6	0.0958	no

gi|646484557|gb|KK997231.1|	13-1172	6.5	3.4	0.61399	1	0.739926	1	no
gi|646484587|gb|KK997226.1|	939-1199	42	25	14	10	-0.442712	0.7662	no
gi|646485158|gb|KK997126.1|	2518-2900	17	8.8	1	9	3	0.2646	no
gi|646485295|gb|KK997102.1|	5800-6199	5.3	2.1	33	15	-1	0.4806	no
gi|646485387|gb|KK997086.1|	235-560	16	8.9	40	38	-0.0735282	0.96055	no
gi|646485456|gb|KK997074.1|	1979-2425	18	6.2	7	0.627479	-4	0.17715	no
gi|646485456|gb|KK997074.1|	450-1320	18	6.2	0.601622	2	2	0.2964	no
gi|646485906|gb|KK996993.1|	3285-4756	17	8.4	2	0.876656	-0.997128	0.5218	no
gi|646485906|gb|KK996993.1|	378-1059	17	8.4	5	3	-0.591341	0.6238	no
gi|646485957|gb|KK996983.1|	2-1192	14	6.2	2	0.992216	-0.84389	0.5181	no
gi|646485979|gb|KK996979.1|	1787-2493	13	5.3	11	3	-2	0.1991	no
gi|646486276|gb|KK996926.1|	1724-2666	6.3	2.6	6	14	1	0.4861	no
gi|646486423|gb|KK996900.1|	341-1085	16	9	1	1	0.0029929	0.92345	no
gi|646486502|gb|KK996886.1|	202-840	9.5	3.6	4	11	1	0.34265	no
gi|646486953|gb|KK996805.1|	191-535	10	12	34	36	0.0960616	0.95005	no
gi|646487009|gb|KK996795.1|	3192-3406	11	6.7	74	6	-4	0.17805	no
gi|646487037|gb|KK996790.1|	87-856	19	7.1	5	0	-inf	0.00315	no
gi|646487145|gb|KK996772.1|	5205-5626	2.5	1.9	10	3	-2	0.20625	no
gi|646487255|gb|KK996753.1|	7150-7390	26	5.4	27	18	-0.599293	0.6687	no
gi|646487299|gb|KK996745.1|	30-626	5.3	1	0	4	inf	0.00255	no
gi|646487328|gb|KK996739.1|	2104-2439	40	20	5	18	2	0.29145	no
gi|646487372|gb|KK996731.1|	19-1102	17	8.2	6	4	-0.781093	0.6108	no
gi|646487372|gb|KK996731.1|	5959-6979	17	8.2	5	6	0.153712	0.91895	no
gi|646487433|gb|KK996720.1|	156-1346	15	4.2	31	24	-0.33493	0.7565	no
gi|646487448|gb|KK996717.1|	5146-6090	13	4.4	0.537023	2	2	0.2406	no
gi|646487486|gb|KK996710.1|	301-654	12	9.6	0	16	inf	0.00605	no
gi|646487701|gb|KK996683.1|	3248-3437	11	5.6	148	61	-1	0.357	no
gi|646488213|gb|KK996595.1|	5958-6886	12	7.7	4	2	-0.86352	0.5339	no
gi|646488213|gb|KK996595.1|	897-1746	12	7.7	2	2	-0.278007	0.8333	no
gi|646488585|gb|KK996529.1|	9126-9341	9.1	6.2	22	479	4	0.09995	no

gi|646488603|gb|KK996526.1|	2903-3431	17	6.1	3	1	-1	0.41965	no
gi|646488875|gb|KK996479.1|	5545-6265	15	7.7	3	2	-0.322213	0.79045	no
gi|646488926|gb|KK996470.1|	22-1246	3.60E+00	4.6	21	23	0.140007	0.8915	no
gi|646488969|gb|KK996462.1|	2-736	15	4.9	1	1	-0.0978686	0.9036	no
gi|646488987|gb|KK996459.1|	1291-2311	13	5.5	6	7	0.345254	0.82225	no
gi|646489413|gb|KK996385.1|	2223-2922	110	29	2	1	-0.687937	0.6459	no
gi|646489413|gb|KK996385.1|	5296-5705	110	29	11	5	-1	0.4099	no
gi|646489592|gb|KK996354.1|	571-2181	40	11	1	1	0.114052	0.93675	no
gi|646489909|gb|KK996299.1|	38-1160	19	8.7	8	4	-1	0.46785	no
gi|646490001|gb|KK996279.1|	1929-2579	18	16	4	0.324821	-4	0.17385	no
gi|646490059|gb|KK996269.1|	13-899	15	6.3	3	4	0.130682	0.935	no
gi|646490158|gb|KK996252.1|	11631-11886	11	5.4	64	194	2	0.32215	no
gi|646490279|gb|KK996227.1|	1166-1440	5.8	3.4	16	4	-2	0.28375	no
gi|646490519|gb|KK996178.1|	734-1977	8.2	4.9	4	0.53724	-3	0.1104	no
gi|646490529|gb|KK996176.1|	4790-6548	8.3	4.1	7	4	-0.892426	0.55455	no
gi|646490610|gb|KK996160.1|	7447-8014	20	7.2	1	6	2	0.24775	no
gi|646490871|gb|KK996106.1|	573-1754	17	8.9	2	0.14309	-4	0.18715	no
gi|646490895|gb|KK996101.1|	0-1214	14	6.3	4	3	-0.724408	0.6479	no
gi|646490983|gb|KK996083.1|	367-1492	15	11	2	1	-0.483817	0.7079	no
gi|646490983|gb|KK996083.1|	4116-4883	15	11	1	3	0.813202	0.5504	no
gi|646491073|gb|KK996064.1|	130-797	8.2	3.5	2	3	0.476598	0.72655	no
gi|646491087|gb|KK996061.1|	3775-4899	15	7.8	15	11	-0.393346	0.79565	no
gi|646491546|gb|KK996056.1|	3475-3876	15	8.6	23	9	-1	0.316	no
gi|646491566|gb|KK996052.1|	863-1213	10	6.9	8	6	-0.271521	0.8395	no
gi|646491591|gb|KK996047.1|	6654-7799	17	6.1	2	0.148736	-3	0.1791	no
gi|646491643|gb|KK996037.1|	966-1613	20	6.3	2	7	2	0.2445	no
gi|646492062|gb|KK995950.1|	3274-3438	12	9.5	58	228	2	0.3458	no
gi|646492433|gb|KK995883.1|	2807-3640	9.4	3.8	0.640123	2	1	0.43305	no
gi|646492513|gb|KK995869.1|	5833-6572	11	6	5	3	-0.86016	0.54295	no
gi|646492773|gb|KK995823.1|	6859-7372	17	7.2	9	4	-1	0.47385	no

gi|646492818|gb|KK995815.1|	250-410	21	5.5	526	562	0.0943557	0.949	no
gi|646492943|gb|KK995792.1|	147-359	8.4	4	2163.72	2073.71	-0.0612974	0.9561	no
gi|646493050|gb|KK995773.1|	654-884	8.8	5.4	807.04	743	-0.118515	0.9141	no
gi|646493174|gb|KK995750.1|	3-666	17	5.1	6	6	-0.109091	0.9463	no
gi|646493504|gb|KK995694.1|	463-744	2.4	3.3	14	18	0.365559	0.78965	no
gi|646493683|gb|KK995664.1|	3671-4557	10	5.3	8	2	-2	0.1633	no
gi|646493683|gb|KK995664.1|	475-1227	10	5.3	5	0.522515	-3	0.14335	no
gi|646493801|gb|KK995644.1|	3119-3996	7.8	7.7	2	1	-0.498058	0.7001	no
gi|646494030|gb|KK995605.1|	5264-5875	5.9	2.5	2	1	-0.342225	0.81865	no
gi|646494257|gb|KK995567.1|	0-546	12	5.7	13	5	-1	0.3522	no
gi|646494362|gb|KK995550.1|	443-1091	22	7.7	4	10	1	0.34215	no
gi|646494666|gb|KK995492.1|	2-468	20	6.8	6	2	-2	0.2447	no
gi|646494666|gb|KK995492.1|	7935-8625	20	6.8	12	21	0.797404	0.59555	no
gi|646495235|gb|KK995371.1|	6862-7410	30	12	2	4	1	0.3993	no
gi|646495339|gb|KK995349.1|	930-1586	19	13	31	20	-0.637324	0.66915	no
gi|646495670|gb|KK995288.1|	146-726	8.8	4	1	3	0.942728	0.5627	no
gi|646495730|gb|KK995279.1|	8724-9215	11	4.3	16	20	0.388974	0.79815	no
gi|646495810|gb|KK995262.1|	587-750	12	9.3	3661.17	663	-2	0.13075	no
gi|646495832|gb|KK995258.1|	3467-3652	11	6.7	233	373.98	0.680432	0.6156	no
gi|646495979|gb|KK995229.1|	7921-8273	20	6.9	226	31	-3	0.0485	no
gi|646496004|gb|KK995224.1|	1-888	19	6.3	2	1	-0.497271	0.7125	no
gi|646496171|gb|KK995191.1|	11222-11964	7.7	5.6	0	3	inf	0.00255	no
gi|646496171|gb|KK995191.1|	12158-12444	7.7	5.6	161	63	-1	0.38025	no
gi|646496407|gb|KK995144.1|	3948-4846	2.5	2.2	1	0.815797	-0.666359	0.6592	no
gi|646497301|gb|KK994964.1|	0-509	24	9.2	7	13	0.940327	0.48995	no
gi|646497301|gb|KK994964.1|	1490-2097	24	9.2	1	5	2	0.1961	no
gi|646497518|gb|KK994921.1|	718-1195	10	4.2	11	14.86	0.418727	0.7905	no
gi|646497541|gb|KK994917.1|	4796-5375	16	6.6	3	4	0.60903	0.64685	no
gi|646497611|gb|KK994904.1|	2993-3321	18	7.3	12	9	-0.369537	0.7843	no
gi|646497628|gb|KK994901.1|	1806-3120	19	9.3	35	8	-2	0.12655	no

gi|646497923|gb|KK994844.1|	440-606	13	7	347	359	0.0473651	0.95975	no
gi|646497997|gb|KK994829.1|	511-927	15	7.3	0	47	inf	0.0001	yes
gi|646498142|gb|KK994801.1|	8551-9005	11	8.7	8	13	0.628534	0.65015	no
gi|646498169|gb|KK994796.1|	972-1143	6.3	5.1	130	88	-0.561449	0.73955	no
gi|646498305|gb|KK994770.1|	309-623	19	6.7	21	22	0.0796194	0.95545	no
gi|646498368|gb|KK994758.1|	66-893	35	16	13	8	-0.707511	0.64415	no
gi|646498642|gb|KK994704.1|	140-503	15	5.1	3	9	1	0.3607	no
gi|646498642|gb|KK994704.1|	4771-5020	15	5.1	62	40	-0.649896	0.64445	no
gi|646499022|gb|KK994631.1|	9328-10911	23	11	73	42	-0.813334	0.48175	no
gi|646499404|gb|KK994560.1|	4687-5476	16	5.6	4	5	0.368239	0.81475	no
gi|646499521|gb|KK994538.1|	646-991	4.3	3.4	0	263	inf	5.00E-05	yes
gi|646499527|gb|KK994537.1|	3624-6639	21	8.2	2	2	0.262678	0.86175	no
gi|646499613|gb|KK994520.1|	3153-4249	12	5.7	0.769971	0.785888	0.0295186	1	no
gi|646499718|gb|KK994501.1|	0-408	9.4	4.7	47	31	-0.608393	0.67925	no
gi|646499718|gb|KK994501.1|	1719-2080	9.4	4.7	138	34	-2	0.20245	no
gi|646500143|gb|KK994422.1|	2653-3545	17	10	8	7	-0.304242	0.84075	no
gi|646500231|gb|KK994405.1|	3329-3758	10	5.4	18	26	0.508616	0.7438	no
gi|646500237|gb|KK994404.1|	21-869	13	8	2	2	0.258505	0.83855	no
gi|646500485|gb|KK994358.1|	2775-3400	12	6.4	6	2	-2	0.2829	no
gi|646500485|gb|KK994358.1|	3656-4583	12	6.4	5	2	-2	0.27	no
gi|646500577|gb|KK994342.1|	185-404	10	6.6	61	20	-2	0.2706	no
gi|646500625|gb|KK994334.1|	4853-5460	1.5	1.9	0.786811	3	2	0.29365	no
gi|646500670|gb|KK994326.1|	4283-4906	17	8.9	2	3	0.297921	0.8322	no
gi|646500765|gb|KK994309.1|	1847-2076	14	5.9	4	42	3	0.1087	no
gi|646500833|gb|KK994297.1|	22-807	20	9.7	1	1	0.230298	0.87595	no
gi|646500845|gb|KK994295.1|	125-1088	3.3	2.4	2	0.928995	-0.977434	0.50955	no
gi|646501115|gb|KK994247.1|	6781-7504	11	6.7	3	5	0.926143	0.51295	no
gi|646501150|gb|KK994240.1|	1765-2516	29	10	4	3	-0.318039	0.794	no
gi|646501196|gb|KK994231.1|	0-776	4.6	1.4	0	18	inf	5.00E-05	yes
gi|646501304|gb|KK994213.1|	181-519	18	9.6	102	76	-0.430708	0.7805	no

gi|646501338|gb|KK994207.1|	8722-8898	14	4.4	375	104	-2	0.17255	no
gi|646501544|gb|KK994170.1|	1241-2507	20	8.3	2	4	1	0.45335	no
gi|646501601|gb|KK994160.1|	295-543	14	7.8	35	47	0.427803	0.77015	no
gi|646501703|gb|KK994142.1|	3725-3983	17	9.5	0	437	inf	5.00E-05	yes
gi|646501974|gb|KK994094.1|	199-616	14	7.8	12	17	0.573745	0.67715	no
gi|646501980|gb|KK994093.1|	4170-5054	16	9.3	1	2	0.33257	0.8043	no
gi|646502125|gb|KK994068.1|	3579-4343	26	13	5	5	-0.168047	0.91435	no
gi|646502466|gb|KK994006.1|	7322-7658	19	5.6	4	249	6	0.09345	no
gi|646502519|gb|KK993996.1|	1871-3714	8.2	3.5	1	3	1	0.51705	no
gi|646502634|gb|KK993971.1|	3512-4348	11	6.5	7	9	0.396559	0.79175	no
gi|646502813|gb|KK993933.1|	121-1704	11	4	9	17	0.93225	0.46335	no
gi|646502914|gb|KK993912.1|	159-962	7.8	4	0.506366	2	2	0.25505	no
gi|646502932|gb|KK993908.1|	3208-4082	12	5.2	19	14	-0.464083	0.764	no
gi|646502964|gb|KK993900.1|	1743-3034	12	6	4	1	-2	0.26475	no
gi|646502964|gb|KK993900.1|	3186-3763	12	6	7	0.392993	-4	0.1857	no
gi|646502964|gb|KK993900.1|	4911-6092	12	6	11	2	-3	0.0651	no
gi|646503066|gb|KK993879.1|	737-911	18	9.4	116	64	-0.859714	0.5951	no
gi|646503076|gb|KK993877.1|	728-1423	7.1	7.7	2	3	0.940786	0.4992	no
gi|646503100|gb|KK993872.1|	532-822	19	9.3	2	498	8	0.09445	no
gi|646503158|gb|KK993860.1|	3101-3516	9.1	4	5	4	-0.468454	0.7542	no
gi|646503158|gb|KK993860.1|	4449-5192	9.1	4	3	3	-0.0966729	0.9386	no
gi|646503167|gb|KK993858.1|	36-701	4.5	4.5	13	16	0.343786	0.817	no
gi|646503267|gb|KK993837.1|	10220-10934	13	4.9	20	7	-1	0.34925	no
gi|646503356|gb|KK993817.1|	4643-5669	10	5.9	4	0.684181	-3	0.1561	no
gi|646503356|gb|KK993817.1|	97-908	10	5.9	2	0.938508	-0.963378	0.54505	no
gi|646503468|gb|KK993794.1|	6045-6802	22	9.6	2	1	-0.888447	0.53905	no
gi|646503499|gb|KK993788.1|	9891-10242	1.3	2	7	8	0.122083	0.9266	no
gi|646503630|gb|KK993761.1|	3267-3494	5.2	4	90	96	0.0966534	0.9422	no
gi|646504379|gb|KK993601.1|	2543-3298	10	5.3	6	5	-0.384652	0.8093	no
gi|646504416|gb|KK993594.1|	976-1247	16	7.8	85	59	-0.520232	0.7387	no

gi|646504546|gb|KK993570.1|	9607-11447	12	7.1	1	1	0.169899	0.8949	no
gi|646504683|gb|KK993545.1|	405-996	18	9.8	2	2	0.460226	0.7494	no
gi|646505127|gb|KK993465.1|	9958-10537	8.8	5.1	0	4	inf	0.00255	no
gi|646505153|gb|KK993460.1|	20-752	3.1	4.2	1	2	0.709214	0.63615	no
gi|646505182|gb|KK993455.1|	4128-4901	12	6	3	7	1	0.41535	no
gi|646505328|gb|KK993428.1|	5689-6391	17	5.6	2	2	0.482457	0.7389	no
gi|646505743|gb|KK993351.1|	5823-6071	11	4.8	32	34	0.105875	0.93465	no
gi|646506244|gb|KK993258.1|	2742-3453	5.1	2.2	0	3	inf	0.001	yes
gi|646506790|gb|KK993160.1|	3223-4219	20	7.3	6	6	-0.10303	0.94395	no
gi|646506790|gb|KK993160.1|	338-1629	20	7.3	4	5	0.192639	0.9033	no
gi|646506880|gb|KK993144.1|	5868-6139	26	16	8	27	2	0.26595	no
gi|646507264|gb|KK993075.1|	81-993	18	8.1	6	7	0.311947	0.8345	no
gi|646507277|gb|KK993072.1|	8930-9135	15	4.8	57	27	-1	0.47075	no
gi|646507448|gb|KK993041.1|	1485-2588	12	5.7	143	74	-0.941152	0.42245	no
gi|646507678|gb|KK992997.1|	3216-3816	18	5.9	6	8	0.462496	0.7738	no
gi|646507683|gb|KK992996.1|	2877-3652	10	5	3	0.250194	-4	0.1757	no
gi|646508163|gb|KK992895.1|	68-1444	6.4	2	4	5	0.424707	0.77435	no
gi|646508221|gb|KK992883.1|	4229-4534	19	8.2	1	22	4	0.0975	no
gi|646508289|gb|KK992869.1|	6342-7109	22	5.8	1	1	-0.316081	0.8509	no
gi|646508289|gb|KK992869.1|	764-1675	22	5.8	0.987054	0.600123	-0.717872	1	no
gi|646508625|gb|KK992797.1|	6096-6630	14	6.7	3	0.896512	-2	0.3832	no
gi|646508697|gb|KK992782.1|	7361-8118	19	7.1	1	0.517541	-2	0.38345	no
gi|646508942|gb|KK992730.1|	1783-2460	20	8.2	1	2	0.215328	0.89085	no
gi|646508950|gb|KK992728.1|	2136-2629	19	7.7	2	5	2	0.2822	no
gi|646509032|gb|KK992711.1|	279-980	92	63	3	3	-0.00312567	0.95325	no
gi|646509126|gb|KK992692.1|	469-800	16	6.3	5	14	1	0.3398	no
gi|646509126|gb|KK992692.1|	4874-5390	16	6.3	9	12	0.436196	0.7839	no
gi|646509155|gb|KK992685.1|	1967-2480	13	9.2	3	0.48123	-3	0.2136	no
gi|646509460|gb|KK992620.1|	4526-5356	21	8.3	0	12	inf	0.00045	yes
gi|646509576|gb|KK992596.1|	6727-6953	11	4	88	0	-inf	0.00045	yes

gi|646509595|gb|KK992592.1|	2073-3543	17	9.8	3	3	-0.280943	0.8528	no
gi|646509741|gb|KK992562.1|	1947-2374	18	7.4	58	9	-3	0.0693	no
gi|646509741|gb|KK992562.1|	2997-3775	18	7.4	15	11	-0.407915	0.79015	no
gi|646510118|gb|KK992491.1|	6127-7079	22	7.2	0.796292	2	1	0.34075	no
gi|646510128|gb|KK992489.1|	101-1004	15	5.9	2	3	1	0.4606	no
gi|646510143|gb|KK992486.1|	537-871	7.8	2.5	8	2	-2	0.3038	no
gi|646510210|gb|KK992474.1|	0-783	12	10	3	0.986243	-2	0.25345	no
gi|646510373|gb|KK992444.1|	1731-2945	20	20	1	7	2	0.1062	no
gi|646510411|gb|KK992437.1|	1208-1600	6.2	3.1	4	41	3	0.07665	no
gi|646510411|gb|KK992437.1|	588-782	6.2	3.1	78	71	-0.135291	0.9261	no
gi|646510700|gb|KK992383.1|	0-815	48	21	1	2	0.496449	0.72995	no
gi|646510700|gb|KK992383.1|	8518-9337	48	21	0.164002	4	5	0.10615	no
gi|646510929|gb|KK992340.1|	257-848	11	6.4	3.57	5	0.344749	0.78175	no
gi|646511037|gb|KK992320.1|	242-1984	4.8	4.6	5	2	-2	0.3101	no
gi|646511109|gb|KK992307.1|	37-1421	18	9.4	1	2	0.417526	0.7207	no
gi|646511581|gb|KK992216.1|	5511-5993	7.4	2.5	10	50	2	0.17635	no
gi|646511609|gb|KK992211.1|	199-1201	22	4.9	9	10	0.108186	0.94395	no
gi|646511771|gb|KK992180.1|	597-1914	18	13	3	3	-0.017881	0.98095	no
gi|646511777|gb|KK992179.1|	3949-5599	14	6.5	0.994776	0.765944	-0.377134	1	no
gi|646512508|gb|KK992042.1|	8294-8580	8	2.6	5	21	2	0.1933	no
gi|646512573|gb|KK992030.1|	5331-6719	17	7.5	2	1	-0.263956	0.8181	no
gi|646512806|gb|KK991985.1|	1-205	16	6	22	49	1	0.4526	no
gi|646513168|gb|KK991922.1|	470-925	8.2	2.8	4	5	0.576656	0.69795	no
gi|646513168|gb|KK991922.1|	4838-5598	8.2	2.8	0.915748	3	2	0.2489	no
gi|646513400|gb|KK991876.1|	4396-4800	25	13	7	5	-0.28153	0.8336	no
gi|646513440|gb|KK991869.1|	1889-2179	18	6.7	39	48	0.271863	0.86145	no
gi|646513469|gb|KK991864.1|	7544-8728	14	6.5	2	3	0.518464	0.7069	no
gi|646513469|gb|KK991864.1|	96-925	14	6.5	41	49	0.268704	0.80375	no
gi|646514022|gb|KK991761.1|	178-592	13	8.7	6	3	-0.943172	0.5495	no
gi|646514022|gb|KK991761.1|	4161-4574	13	8.7	5	3	-0.622038	0.6684	no

gi|646514022|gb|KK991761.1|	5634-6006	13	8.7	3	6	0.924563	0.591	no
gi|646514074|gb|KK991752.1|	2-1566	18	8.4	4	2	-0.620591	0.69645	no
gi|646514214|gb|KK991726.1|	8034-8213	15	6.4	1165.81	1056.99	-0.141372	0.9279	no
gi|646514297|gb|KK991710.1|	4986-6211	4.6	4	1	0.68373	-0.617615	1	no
gi|646514325|gb|KK991705.1|	9845-10324	66	30	39	10	-2	0.23635	no
gi|646514362|gb|KK991698.1|	4128-4283	19	6.1	539	730.53	0.438887	0.7612	no
gi|646514431|gb|KK991685.1|	8654-8928	11	7.3	41	20	-1	0.4086	no
gi|646514806|gb|KK991618.1|	63-936	6	6.4	2	0.423822	-2	0.1855	no
gi|646514870|gb|KK991607.1|	5713-7121	1.5	3.1	0.240707	10	5	0.10675	no
gi|646514993|gb|KK991584.1|	4085-4440	9.7	4	6	8	0.512425	0.7261	no
gi|646515005|gb|KK991582.1|	0-456	9.8	3.7	6	3	-1	0.4321	no
gi|646515005|gb|KK991582.1|	5060-5699	9.8	3.7	8	5	-0.751159	0.6442	no
gi|646515347|gb|KK991521.1|	2802-3170	5.2	3.4	11	6	-0.887226	0.50465	no
gi|646515625|gb|KK991471.1|	181-635	5.1	2.8	4	2	-0.593858	0.6685	no
gi|646515697|gb|KK991458.1|	5146-6244	12	5.1	0.768145	2	1	0.44735	no
gi|646515887|gb|KK991422.1|	2894-3169	5.2	3.7	23	28	0.297002	0.81345	no
gi|646516372|gb|KK991334.1|	6066-6595	17	7.4	12	15	0.276093	0.8584	no
gi|646516500|gb|KK991310.1|	837-1536	10	5.2	6	5	-0.185436	0.9106	no
gi|646516533|gb|KK991304.1|	428-1449	19	5.6	1	0.17213	-3	0.19945	no
gi|646516740|gb|KK991267.1|	5252-5613	5.1	7	4	5	0.327076	0.823	no
gi|646516779|gb|KK991260.1|	83-1196	11	5.8	6	2	-1	0.3885	no
gi|646516922|gb|KK991234.1|	53-777	13	7.6	5	2	-1	0.34075	no
gi|646516981|gb|KK991223.1|	1824-2393	22	8.6	2	2	0.191163	0.8995	no
gi|646517017|gb|KK991216.1|	3725-4076	7.1	2.5	0	54	inf	0.00015	yes
gi|646517485|gb|KK991132.1|	360-1097	20	9.2	11	7	-0.751289	0.6257	no
gi|646517646|gb|KK991103.1|	856-1269	15	4.2	18	0.736068	-5	0.17725	no
gi|646517684|gb|KK991096.1|	32-330	17	16	19	24	0.338281	0.78395	no
gi|646517723|gb|KK991089.1|	409-1069	19	5.4	2	5	1	0.4517	no
gi|646517740|gb|KK991086.1|	381-601	6.2	5.7	30	30	-9.38E-05	0.91805	no
gi|646517788|gb|KK991077.1|	603-1252	13	6.7	3	0.325596	-3	0.1994	no

gi|646517825|gb|KK991071.1|	1412-2222	8.7	3.4	11	4	-1	0.40925	no
gi|646517836|gb|KK991069.1|	5422-5714	22	7.7	32	27	-0.266011	0.84415	no
gi|646518109|gb|KK991021.1|	4-637	9	4	9	0	-inf	0.0014	yes
gi|646518179|gb|KK991009.1|	121-897	1.2	nan	0.532593	2	2	0.25505	no
gi|646518305|gb|KK990987.1|	0-532	9.9	4.4	3	5	0.579742	0.67205	no
gi|646518310|gb|KK990986.1|	3885-4304	8.9	6.9	4	4	-0.102792	0.9229	no
gi|646518332|gb|KK990982.1|	1745-2340	22	13	2	2	-0.386748	0.8011	no
gi|646518355|gb|KK990978.1|	4-1265	17	7.2	3	194	6	0.00485	no
gi|646518442|gb|KK990962.1|	0-863	9.5	3.5	0	3	inf	0.00035	yes
gi|646518466|gb|KK990958.1|	4676-5643	9.9	6.9	2	2	-0.119792	0.92185	no
gi|646518558|gb|KK990941.1|	82-1336	20	9.5	0.092602	13	7	0.0946	no
gi|646519029|gb|KK990858.1|	7419-7969	20	7	3	2	-0.873559	0.56495	no
gi|646519096|gb|KK990846.1|	2911-3201	11	8.7	9	15	0.630317	0.6584	no
gi|646519096|gb|KK990846.1|	3341-3768	11	8.7	10	24	1	0.38225	no
gi|646519102|gb|KK990845.1|	3329-4862	19	7.7	11	5	-1	0.49565	no
gi|646519102|gb|KK990845.1|	485-1579	19	7.7	24	13	-0.869058	0.49355	no
gi|646519198|gb|KK990829.1|	1-904	12	4.6	3	1	-2	0.30655	no
gi|646519420|gb|KK990790.1|	5229-5975	9.1	6.3	4	4	0.0963611	0.94295	no
gi|646520066|gb|KK990677.1|	3388-3611	6.6	1.5	56	0	-inf	0.0041	no
gi|646520117|gb|KK990668.1|	846-1124	11	5.4	160	6	-5	0.07895	no
gi|646520157|gb|KK990661.1|	5114-6180	13	4.5	3	7	1	0.39725	no
gi|646520162|gb|KK990660.1|	124-1022	13	7.9	2	0.815797	-1	0.4538	no
gi|646520255|gb|KK990642.1|	1806-2369	10	2.5	3	0.409442	-3	0.2136	no
gi|646520358|gb|KK990623.1|	6-742	6.5	11	0	9	inf	0.0007	yes
gi|646520477|gb|KK990601.1|	1953-2908	12	4.1	0.79288	2	2	0.24925	no
gi|646520477|gb|KK990601.1|	3260-4329	12	4.1	2	4	1	0.34365	no
gi|646520623|gb|KK990576.1|	5-1449	16	8.4	2	1	-1	0.4294	no
gi|646520689|gb|KK990564.1|	47-2606	9.2	3.1	2	1	-1	0.51545	no
gi|646520701|gb|KK990562.1|	1682-2154	9.6	5.3	3	4	0.223509	0.86575	no
gi|646520741|gb|KK990555.1|	24-332	16	9.3	0	47	inf	0.001	yes

gi|646520769|gb|KK990550.1|	34-1258	4.4	2	105	14	-3	0.04735	no
gi|646520785|gb|KK990547.1|	6360-6755	16	5	80	71	-0.183137	0.90285	no
gi|646520813|gb|KK990542.1|	33-920	26	15	1	1	0.140158	0.9186	no
gi|646520892|gb|KK990528.1|	204-874	18	6.8	2	0.620114	-2	0.38325	no
gi|646521347|gb|KK990448.1|	95-742	19	13	0.707196	2	2	0.2936	no
gi|646521387|gb|KK990440.1|	5024-5763	7.9	8.5	2	0.803854	-1	0.47555	no
gi|646521405|gb|KK990436.1|	3282-3725	7.9	2.7	16	57	2	0.25245	no
gi|646521793|gb|KK990365.1|	206-795	5.2	4.9	3	2	-0.803327	0.5828	no
gi|646521793|gb|KK990365.1|	6470-7275	5.2	4.9	1	0.71126	-0.919588	0.5894	no
gi|646521814|gb|KK990361.1|	1075-1599	3.3	1	9	18	1	0.514	no
gi|646522098|gb|KK990314.1|	5116-6137	16	8.2	1	0.51639	-1	0.3882	no
gi|646522110|gb|KK990312.1|	1-2248	15	7.4	1	1	-0.270808	0.8669	no
gi|646522163|gb|KK990303.1|	3635-4324	12	4.5	1	1	-0.104579	0.9035	no
gi|646522168|gb|KK990302.1|	235-664	20	8	0.518388	64	7	0.095	no
gi|646522201|gb|KK990296.1|	1154-1794	11	5.3	2	0.665475	-2	0.29735	no
gi|646522201|gb|KK990296.1|	3-507	11	5.3	17	9	-0.953394	0.55495	no
gi|646522213|gb|KK990294.1|	591-918	16	5.9	70.29	32	-1	0.46995	no
gi|646522220|gb|KK990293.1|	5707-6234	19	6.5	3	0	-inf	0.00655	no
gi|646522289|gb|KK990281.1|	9205-10201	15	7.6	0.37451	2	3	0.134	no
gi|646522438|gb|KK990255.1|	5-362	1.4	1	12	5	-1	0.3812	no
gi|646522577|gb|KK990231.1|	10858-11177	22	11	20	32	0.704545	0.61115	no
gi|646522616|gb|KK990225.1|	10227-10695	7.2	2.9	4	2	-0.755892	0.6076	no
gi|646522727|gb|KK990203.1|	1128-1849	10	4.9	9	8	-0.261948	0.8722	no
gi|646522731|gb|KK990202.1|	313-1012	12	6.6	6	11	0.784551	0.61285	no
gi|646522757|gb|KK990196.1|	5944-6784	21	7.9	0.948698	0.893699	-0.0861612	1	no
gi|646522783|gb|KK990190.1|	565-756	14	5.8	32	106	2	0.2639	no
gi|646522845|gb|KK990178.1|	0-331	140	77	31	6	-2	0.1804	no
gi|646522865|gb|KK990174.1|	5140-5534	20	9.5	8	7	-0.338753	0.7966	no
gi|646522893|gb|KK990168.1|	6201-7224	5.1	4	0	2	inf	0.001	yes
gi|646523143|gb|KK990117.1|	87-1589	15	7.3	5	7	0.527077	0.7232	no

gi|646523229|gb|KK990101.1|	22-1441	19	1.9	2	1	-0.233713	0.86725	no
gi|646523229|gb|KK990101.1|	6519-6763	19	1.9	480	89	-2	0.13275	no
gi|646523297|gb|KK990087.1|	6237-6686	19	6.2	2	6	1	0.4075	no
gi|646523735|gb|KK989998.1|	5-491	14	4.2	19	3	-3	0.12045	no
gi|646523802|gb|KK989984.1|	1016-2143	8.6	2.9	2	0.758648	-2	0.31905	no
gi|646524277|gb|KK989891.1|	2-604	7.9	6.9	4	1	-2	0.2617	no
gi|646524277|gb|KK989891.1|	672-1026	7.9	6.9	10	4	-1	0.39435	no
gi|646524299|gb|KK989887.1|	451-634	9.3	6.2	28	156	2	0.26645	no
gi|646524315|gb|KK989884.1|	11251-1156[illegible]	17	7	16	31	0.918896	0.5129	no
gi|646524463|gb|KK989856.1|	98-843	3.8	1.5	2	0	-inf	0.0041	no
gi|646524542|gb|KK989840.1|	335-878	11	6.9	110	146	0.407925	0.7151	no
gi|646524609|gb|KK989826.1|	47-1127	16	7	2	0	-inf	0.00525	no
gi|646524609|gb|KK989826.1|	6206-7289	16	7	7	0	-inf	0.0002	yes
gi|646524664|gb|KK989815.1|	343-974	16	7.5	0.491366	3	2	0.28375	no
gi|646525289|gb|KK989685.1|	4725-6059	3.3	3.2	2	0.492851	-2	0.2508	no
gi|646525512|gb|KK989639.1|	2033-2442	14	6.7	3	4	0.374729	0.8177	no
gi|646525512|gb|KK989639.1|	265-833	14	6.7	9	3	-2	0.27625	no
gi|646525547|gb|KK989632.1|	1539-3582	14	3.7	3	7	1	0.43445	no
gi|646525572|gb|KK989627.1|	3511-4355	5.3	9.5	1	1	-0.348837	0.81205	no
gi|646526374|gb|KK989460.1|	5986-7529	20	9.5	11	4	-1	0.40085	no
gi|646526641|gb|KK989407.1|	8161-8644	11	5.1	4	3	-0.452763	0.7411	no
gi|646526778|gb|KK989379.1|	0-1023	12	4.3	13	0	-inf	5.00E-05	yes
gi|646526782|gb|KK989378.1|	291-742	17	6.3	19	8	-1	0.34955	no
gi|646526782|gb|KK989378.1|	805-1173	17	6.3	6	8	0.335166	0.80525	no
gi|646526855|gb|KK989362.1|	9737-9861	4.6	3.9	3185.6	1463.66	-1	0.4431	no
gi|646526860|gb|KK989361.1|	340-784	11	10	9	2	-2	0.18055	no
gi|646527411|gb|KK989249.1|	1972-2938	13	6	1	0.370078	-1	1	no
gi|646527411|gb|KK989249.1|	2-583	13	6	4	0.388531	-3	0.179	no
gi|646527456|gb|KK989240.1|	4027-4668	6.2	5	1	4	2	0.25505	no
gi|646527472|gb|KK989237.1|	1043-1258	13	28	78	17	-2	0.18055	no

gi|646528130|gb|KK989116.1|	119-1409	17	4.8	8	6	-0.336064	0.8191	no
gi|646528136|gb|KK989115.1|	2909-3890	18	6.3	1	2	0.772115	0.55405	no
gi|646528285|gb|KK989089.1|	7380-8194	14	9.9	2	1	-0.503649	0.71065	no
gi|646528301|gb|KK989086.1|	2729-2979	8.8	4.3	25	36	0.527319	0.6895	no
gi|646528827|gb|KK988991.1|	2-676	11	4.5	9	14	0.609085	0.6909	no
gi|646528827|gb|KK988991.1|	853-1549	11	4.5	17	12	-0.500339	0.7385	no
gi|646529133|gb|KK988935.1|	289-764	9.1	4.4	23	15	-0.608764	0.68905	no
gi|646529191|gb|KK988924.1|	771-1263	14	5.8	0.773124	36	6	0.23405	no
gi|646529284|gb|KK988907.1|	0-423	12	7.8	62	16.09	-2	0.21835	no
gi|646529284|gb|KK988907.1|	7226-7530	12	7.8	0	16	inf	0.00255	no
gi|646529355|gb|KK988894.1|	501-1094	13	2.9	16	10	-0.617262	0.68755	no
gi|646529528|gb|KK988863.1|	4155-4969	20	6.8	11	16	0.517109	0.72475	no
gi|646529554|gb|KK988858.1|	8-2879	19	6.8	3	0.925825	-2	0.24515	no
gi|646529712|gb|KK988829.1|	972-1487	8.9	5.9	11	12	0.172764	0.9088	no
gi|646529838|gb|KK988807.1|	23-851	7.4	3.9	0.807134	6	3	0.1175	no
gi|646529926|gb|KK988791.1|	2057-2430	2.7	2.6	11	23	1	0.45175	no
gi|646529926|gb|KK988791.1|	364-1093	2.7	2.6	11	14	0.375379	0.79995	no
gi|646530296|gb|KK988725.1|	870-1593	25	11	29	2	-4	0.03015	no
gi|646530320|gb|KK988721.1|	5752-7429	23	9	12	8	-0.579867	0.69765	no
gi|646530531|gb|KK988683.1|	5553-7066	16	10	7	9	0.349759	0.81285	no
gi|646530536|gb|KK988682.1|	92-1719	5.4	nan	21	0	-inf	5.00E-05	yes
gi|646530876|gb|KK988622.1|	6717-7020	9	6.9	75	0	-inf	0.00085	yes
gi|646530925|gb|KK988613.1|	872-1088	15	5.8	49	11	-2	0.2141	no
gi|646530987|gb|KK988602.1|	460-1031	25	4.8	3	2	-0.867153	0.56495	no
gi|646530987|gb|KK988602.1|	5993-7281	25	4.8	0.716439	0.514332	-0.478142	1	no
gi|646531035|gb|KK988594.1|	1497-1700	17	5.4	60	36	-0.761274	0.61065	no
gi|646531276|gb|KK988551.1|	627-863	18	8.4	43	15	-2	0.32295	no
gi|646531387|gb|KK988531.1|	0-398	5.6	5.6	19	14	-0.50142	0.7527	no
gi|646531807|gb|KK988458.1|	3507-5861	21	8.4	0.749313	0.767638	0.0348576	1	no
gi|646531959|gb|KK988430.1|	31-976	18	8.3	2	3	0.407313	0.74615	no

gi|646531994|gb|KK988424.1|	3534-3828	21	12	24	14	-0.776525	0.55685	no
gi|646532091|gb|KK988407.1|	0-580	14	8.4	3	2	-0.542699	0.71625	no
gi|646532362|gb|KK988358.1|	4559-4782	14	6.6	42	90	1	0.4753	no
gi|646532368|gb|KK988357.1|	21-1112	15	4.6	3	6	0.845929	0.5865	no
gi|646532693|gb|KK988300.1|	0-754	17	7.9	2	3	0.779199	0.5545	no
gi|646532881|gb|KK988266.1|	462-980	18	7.2	1	3	1	0.44135	no
gi|646533403|gb|KK988172.1|	10277-10825	8.4	3.1	1	4	2	0.29945	no
gi|646533403|gb|KK988172.1|	11549-12416	8.4	3.1	1	2	1	0.40715	no
gi|646533606|gb|KK988134.1|	288-1011	9.5	5	2	2	0.0261386	0.94695	no
gi|646533617|gb|KK988132.1|	4899-5974	11	6.2	49	26	-0.918964	0.4078	no
gi|646533689|gb|KK988119.1|	0-1190	5.7	3.7	7	6	-0.333221	0.82	no
gi|646533784|gb|KK988102.1|	3290-3508	8.9	5.2	42	16	-1	0.3894	no
gi|646533945|gb|KK988073.1|	6747-8527	14	5	2	0.613895	-2	0.2561	no
gi|646534036|gb|KK988057.1|	6208-6811	9.5	7.3	31	23	-0.465217	0.7578	no
gi|646534194|gb|KK988029.1|	6830-7007	18	8.9	0	202	inf	0.00035	yes
gi|646534355|gb|KK988000.1|	8134-9691	9.3	4.8	0.710548	0.409865	-0.793785	1	no
gi|646534698|gb|KK987939.1|	590-878	15	8.1	91	64	-0.50883	0.7392	no
gi|646534709|gb|KK987937.1|	0-884	11	2.4	7	10	0.593643	0.69935	no
gi|646534709|gb|KK987937.1|	4079-5244	11	2.4	17	21	0.31878	0.8019	no
gi|646534759|gb|KK987929.1|	66-612	4.4	6	0.632815	4	3	0.26725	no
gi|646534930|gb|KK987899.1|	0-362	16	5.3	7	3	-1	0.4325	no
gi|646534930|gb|KK987899.1|	2822-3420	16	5.3	2	3	0.0994311	0.9345	no
gi|646534986|gb|KK987889.1|	8589-9259	4.5	9.6	2	1	-0.982297	0.54505	no
gi|646535119|gb|KK987866.1|	624-1336	23	11	4	2	-1	0.34765	no
gi|646535302|gb|KK987835.1|	508-1243	12	7.6	10	9	-0.111408	0.93805	no
gi|646535343|gb|KK987828.1|	1-296	2.9	5.8	0	49	inf	0.0012	yes
gi|646535382|gb|KK987821.1|	3290-3751	15	6.8	319	145	-1	0.3194	no
gi|646535434|gb|KK987812.1|	5213-5858	20	4.6	2	4	1	0.42595	no
gi|646535570|gb|KK987787.1|	144-644	8.3	4.5	2	3	0.429545	0.75545	no
gi|646535665|gb|KK987770.1|	5912-6320	22	14	4	3	-0.43345	0.76715	no

gi|646535741|gb|KK987757.1|	1915-2155	24	6.1	50	28.12	-0.828112	0.5281	no
gi|646535801|gb|KK987746.1|	93-609	13	12	11	5	-1	0.4605	no
gi|646535812|gb|KK987744.1|	6594-7178	18	6	12	8	-0.508448	0.74125	no
gi|646536326|gb|KK987653.1|	32-792	5.6	7	8	2	-2	0.24215	no
gi|646536600|gb|KK987605.1|	7101-7552	12	8.2	84	100.64	0.254107	0.8395	no
gi|646536707|gb|KK987584.1|	554-1368	8.1	5.9	5	8	0.496351	0.7513	no
gi|646536930|gb|KK987536.1|	0-510	4.9	nan	0	21	inf	0.0002	yes
gi|646536972|gb|KK987529.1|	0-1257	20	9.2	12	4	-1	0.34265	no
gi|646536972|gb|KK987529.1|	4373-6059	20	9.2	19	10	-0.845184	0.50265	no
gi|646537417|gb|KK987437.1|	87-730	5.6	7.7	2	1	-0.335314	0.81865	no
gi|646537528|gb|KK987413.1|	1050-1850	20	11	2	0.956703	-0.964495	0.54505	no
gi|646537528|gb|KK987413.1|	7627-8942	20	11	2	2	0.311128	0.8483	no
gi|646537677|gb|KK987382.1|	131-1784	10	6.5	2	1	-0.872293	0.5291	no
gi|646537718|gb|KK987373.1|	5168-6276	2.5	2.3	0.976039	2	0.804974	0.5518	no
gi|646538212|gb|KK987345.1|	2692-3540	9.5	5.7	1	0.882081	-0.500487	0.73865	no
gi|646538340|gb|KK987319.1|	3635-4067	14	7.8	36	6	-3	0.0864	no
gi|646538404|gb|KK987306.1|	5167-5799	14	6.5	3	1	-2	0.35005	no
gi|646544260|gb|KK987290.1|	1560-2341	16	6.1	64	6	-3	0.03145	no
gi|646544821|gb|KK987186.1|	2259-3272	10	7	3	2	-0.81104	0.57175	no
gi|646544887|gb|KK987173.1|	255-865	14	6	2	3	0.464897	0.74015	no
gi|646544923|gb|KK987166.1|	18-692	21	6.5	3	3	-0.159589	0.90345	no
gi|646544923|gb|KK987166.1|	6518-6958	21	6.5	7	8	0.190992	0.8847	no
gi|646544962|gb|KK987159.1|	5920-6157	20	5.6	21	55	1	0.4103	no
gi|646545108|gb|KK987133.1|	264-513	22	5.8	54	67	0.3206	0.81865	no
gi|646545483|gb|KK987064.1|	4748-4930	21	8.3	431	49	-3	0.09955	no
gi|646545529|gb|KK987056.1|	58-321	22	8.1	5	23	2	0.30035	no
gi|646545595|gb|KK987044.1|	4938-5149	21	7.4	86	78	-0.14884	0.9062	no
gi|646545861|gb|KK986994.1|	1290-2857	12	7	11	11	0.0723823	0.96095	no
gi|646545861|gb|KK986994.1|	13-305	12	7	17	0	-inf	0.00475	no
gi|646546092|gb|KK986951.1|	0-1164	20	7.6	5	3	-0.927061	0.5612	no

gi|646546092|gb|KK986951.1|	11670-12158	20	7.6	5	3	-0.954411	0.5242	no
gi|646546350|gb|KK986902.1|	4962-5886	6.4	5.8	5	3	-0.77467	0.5729	no
gi|646546435|gb|KK986887.1|	0-410	7.8	3.2	5	0.747733	-3	0.21305	no
gi|646546460|gb|KK986882.1|	507-2313	14	7.9	9	6	-0.657792	0.6685	no
gi|646546570|gb|KK986861.1|	6593-7972	9.1	4.9	5	4	-0.2462	0.8724	no
gi|646546708|gb|KK986835.1|	735-1018	7.6	2.5	76	26	-2	0.26345	no
gi|646546725|gb|KK986832.1|	39-659	15	10	4	6	0.549644	0.6917	no
gi|646546808|gb|KK986817.1|	5763-6159	8.6	4.6	66	5	-4	0.0357	no
gi|646547053|gb|KK986772.1|	3743-3990	14	5.6	15	28	0.950615	0.5487	no
gi|646547283|gb|KK986731.1|	8900-10181	21	9.1	0.721326	0.647208	-0.156421	1	no
gi|646547418|gb|KK986707.1|	19-1826	23	4.1	2	0.517172	-2	0.25835	no
gi|646547622|gb|KK986670.1|	493-758	11	5.5	51	133	1	0.3907	no
gi|646548210|gb|KK986563.1|	8-1081	3.4	3.1	7	0.484438	-4	0.0882	no
gi|646548399|gb|KK986530.1|	5359-5594	8	2.5	197	27	-3	0.0755	no
gi|646548462|gb|KK986518.1|	1640-3472	21	6.8	0.586655	0.678784	0.21044	1	no
gi|646548742|gb|KK986468.1|	11361-11788	10	4.8	6	2	-1	0.3618	no
gi|646548851|gb|KK986448.1|	2828-3399	3.5	4	3	0.799759	-2	0.29375	no
gi|646548953|gb|KK986430.1|	1723-1966	17	7.9	3	40	4	0.10165	no
gi|646549089|gb|KK986405.1|	85-1894	21	7.4	13	23	0.794041	0.4629	no
gi|646549095|gb|KK986404.1|	387-903	8.4	4.7	0.351973	55	7	0.09465	no
gi|646549131|gb|KK986398.1|	835-1195	12	5.6	88	78	-0.175513	0.90875	no
gi|646549201|gb|KK986386.1|	4004-4693	10	5.2	2	0.297211	-3	0.2033	no
gi|646549293|gb|KK986369.1|	9573-10834	14	6.4	0.643706	1	0.713689	1	no
gi|646549648|gb|KK986307.1|	1671-1919	2.7	2.7	26	146	2	0.1166	no
gi|646550016|gb|KK986240.1|	7971-8578	19	7.9	26	8	-2	0.256	no
gi|646550149|gb|KK986217.1|	1010-1707	12	5.8	6	9	0.623692	0.69035	no
gi|646550175|gb|KK986213.1|	0-379	14	7.2	2	11	2	0.1606	no
gi|646550246|gb|KK986200.1|	5030-6314	18	5.7	0.449514	1	1	1	no
gi|646550583|gb|KK986137.1|	383-862	14	8.9	4	5	0.130227	0.92715	no
gi|646550623|gb|KK986128.1|	5187-5772	12	7.5	63	58	-0.123122	0.9053	no

gi|646551026|gb|KK986044.1|	6122-6289	5.8	3.8	230	590	1	0.36365	no
gi|646551079|gb|KK986033.1|	5339-6767	19	9	1	0	-inf	1	no
gi|646551131|gb|KK986021.1|	7566-8011	15	3.8	2	3	0.40065	0.81675	no
gi|646551209|gb|KK986006.1|	51-564	5.2	2.6	2	3	0.434998	0.7554	no
gi|646551250|gb|KK985998.1|	41-860	4.9	5.1	1	2	0.496839	0.72995	no
gi|646551326|gb|KK985982.1|	1874-2621	13	5.2	3	0.791377	-2	0.2313	no
gi|646551326|gb|KK985982.1|	49-1359	13	5.2	0.701501	1	0.844419	0.54675	no
gi|646551326|gb|KK985982.1|	7702-8114	13	5.2	9	4	-1	0.36265	no
gi|646551811|gb|KK985882.1|	6268-7044	27	10	3	3	-0.218172	0.8599	no
gi|646551841|gb|KK985875.1|	3556-4573	13	5.2	2	0	-inf	0.0011	yes
gi|646551841|gb|KK985875.1|	872-1339	13	5.2	3	2	-0.393861	0.8098	no
gi|646551869|gb|KK985869.1|	7411-7666	34	11	15	25	0.71266	0.62625	no
gi|646552076|gb|KK985825.1|	0-576	19	6.4	33	64	0.946941	0.45015	no
gi|646552076|gb|KK985825.1|	8468-9893	19	6.4	1	0.341428	-2	0.2263	no
gi|646552131|gb|KK985813.1|	1546-1819	4.1	2.9	22	9	-1	0.4008	no
gi|646552382|gb|KK985759.1|	366-2475	22	8.2	1	0.940191	-0.622178	0.65215	no
gi|646552382|gb|KK985759.1|	4661-5611	22	8.2	3	7	1	0.5073	no
gi|646552416|gb|KK985752.1|	9717-10362	5.4	6.9	0	4	inf	0.0014	yes
gi|646552542|gb|KK985725.1|	2815-3008	19	6.5	0	137	inf	0.00605	no
gi|646552542|gb|KK985725.1|	3134-3364	19	6.5	4	515	7	0.0949	no
gi|646552542|gb|KK985725.1|	3489-3784	19	6.5	1	71	6	0.09735	no
gi|646552822|gb|KK985665.1|	1135-1958	21	7.9	2	0	-inf	0.0035	no
gi|646552826|gb|KK985664.1|	141-1509	19	3.6	2	0.836661	-1	0.3342	no
gi|646552901|gb|KK985648.1|	6-460	18	3.4	7	4	-0.915786	0.4998	no
gi|646552905|gb|KK985647.1|	3556-3784	13	5.5	25	22	-0.229455	0.87595	no
gi|646552955|gb|KK985636.1|	5643-6188	14	6.8	5	5	-0.0557711	0.95765	no
gi|646552973|gb|KK985632.1|	1347-2156	19	7.4	5	0	-inf	0.0033	no
gi|646552973|gb|KK985632.1|	688-1178	19	7.4	4	0	-inf	0.00655	no
gi|646552978|gb|KK985631.1|	1243-2626	18	9.7	4	3	-0.566731	0.7196	no
gi|646552983|gb|KK985630.1|	199-428	23	5.2	78	220	1	0.27805	no

gi|646552983|gb|KK985630.1|	3467-3786	23	5.2	18	66	2	0.1979	no
gi|646552983|gb|KK985630.1|	4303-4526	23	5.2	23	57	1	0.38435	no
gi|646552983|gb|KK985630.1|	949-1604	23	5.2	3	9	2	0.26105	no
gi|646553029|gb|KK985620.1|	2654-3167	17	6.6	0	16	inf	0.0007	yes
gi|646553132|gb|KK985599.1|	0-1138	13	8	6	12	1	0.48065	no
gi|646553298|gb|KK985568.1|	5296-5590	16	7	12	7	-0.776525	0.612	no
gi|646553387|gb|KK985551.1|	7022-7449	21	7.5	3	5	0.873974	0.5771	no
gi|646553417|gb|KK985545.1|	1505-1777	8.5	6	32	9	-2	0.2189	no
gi|646553472|gb|KK985535.1|	0-1201	14	2	2	1	-0.159002	0.9019	no
gi|646553538|gb|KK985521.1|	5281-5720	13	8.5	54	17	-2	0.29705	no
gi|646553552|gb|KK985518.1|	3428-3645	17	9	37	32	-0.235936	0.87175	no
gi|646553682|gb|KK985494.1|	1712-2608	1	3.7	2	2	-0.174656	0.8903	no
gi|646553682|gb|KK985494.1|	8-374	1	3.7	6	5	-0.345174	0.81465	no
gi|646553850|gb|KK985466.1|	3035-3331	18	6.4	34	28	-0.296103	0.85085	no
gi|646554050|gb|KK985430.1|	504-1089	18	11	11	4	-1	0.3221	no
gi|646554055|gb|KK985429.1|	0-984	15	8	4	4	-0.168815	0.92015	no
gi|646554064|gb|KK985427.1|	212-631	2.1	2.3	10	14	0.382635	0.77895	no
gi|646554076|gb|KK985425.1|	29-1015	19	13	9	6	-0.573089	0.71125	no
gi|646554076|gb|KK985425.1|	5967-6212	19	13	12	26	1	0.4712	no
gi|646554109|gb|KK985419.1|	485-1492	4.9	3.1	2	0.87621	-1	0.371	no
gi|646554189|gb|KK985404.1|	5417-5789	20	7.9	30	7	-2	0.18015	no
gi|646554247|gb|KK985393.1|	799-1632	2.4	3.1	4	6	0.55063	0.7317	no
gi|646554310|gb|KK985381.1|	9293-10133	14	4	2	1	-0.375668	0.78485	no
gi|646554512|gb|KK985346.1|	1256-1824	15	6.3	1	2	0.71694	0.64505	no
gi|646554663|gb|KK985319.1|	3442-4672	18	10	17	30	0.851897	0.5076	no
gi|646554721|gb|KK985308.1|	3-557	7.8	5.3	5	7	0.542757	0.7026	no
gi|646554827|gb|KK985288.1|	8101-8806	15	12	4	2	-1	0.34715	no
gi|646555031|gb|KK985251.1|	0-322	10	6.2	11	18	0.651759	0.6178	no
gi|646555085|gb|KK985242.1|	423-1087	15	7.9	1	2	0.476054	0.7721	no
gi|646555485|gb|KK985170.1|	4561-5115	10	7.1	15	3	-2	0.13825	no

gi|646555583|gb|KK985152.1|	208-534	13	4.7	35	46	0.364376	0.8134	no
gi|646555634|gb|KK985143.1|	3424-3836	61	13	0.5697	7	4	0.106	no
gi|646555655|gb|KK985139.1|	121-495	8.6	8.4	7	3	-1	0.43315	no
gi|646556102|gb|KK985059.1|	659-1277	14	5.6	1	2	0.466733	0.7722	no
gi|646556124|gb|KK985055.1|	1675-2054	8.1	7.4	6	0.892243	-3	0.20705	no
gi|646556244|gb|KK985034.1|	3843-4563	21	6	3	1	-0.893369	0.53405	no
gi|646556304|gb|KK985021.1|	98-733	20	11	5	2	-1	0.2989	no
gi|646556331|gb|KK985016.1|	5125-8861	17	10	14	9	-0.643047	0.56175	no
gi|646556335|gb|KK985015.1|	50-581	49	16	4	2	-1	0.46265	no
gi|646556469|gb|KK984986.1|	0-1354	26	7.9	0.084178	18	8	0.09445	no
gi|646556697|gb|KK984938.1|	7422-9162	14	5.9	55	125	1	0.3199	no
gi|646556765|gb|KK984923.1|	1-334	16	11	9	7	-0.294966	0.8326	no
gi|646557225|gb|KK984826.1|	67-384	7	4.1	8	4	-0.957213	0.5991	no
gi|646557262|gb|KK984818.1|	44-886	17	8.8	7	3	-1	0.37895	no
gi|646557262|gb|KK984818.1|	5216-6749	17	8.8	7	5	-0.642236	0.67585	no
gi|646557575|gb|KK984751.1|	0-1432	7	nan	70	7	-3	0.0269	no
gi|646557738|gb|KK984717.1|	592-1704	8.1	4.9	2	0	-inf	0.0011	yes
gi|646557751|gb|KK984714.1|	486-958	9.6	5.9	0	7	inf	0.0008	yes
gi|646557771|gb|KK984710.1|	300-917	12	6	5	2	-1	0.39755	no
gi|646557966|gb|KK984668.1|	1619-4291	18	3.4	0.842127	2	1	0.52165	no
gi|646557966|gb|KK984668.1|	162-1158	18	3.4	2	3	0.772982	0.5915	no
gi|646557992|gb|KK984663.1|	1125-1664	12	6.6	0.324059	45	7	0.0948	no
gi|646558166|gb|KK984627.1|	0-796	16	8.1	16	19	0.292884	0.8418	no
gi|646558612|gb|KK984536.1|	8599-10179	18	11	0.628394	2	1	0.3839	no
gi|646558643|gb|KK984530.1|	2-541	12	7.5	17	13	-0.376331	0.80265	no
gi|646558643|gb|KK984530.1|	9555-10125	12	7.5	1	2	0.717521	0.64505	no
gi|646558862|gb|KK984487.1|	0-808	14	7.9	31	10	-2	0.28395	no
gi|646558984|gb|KK984460.1|	2160-3097	18	6.6	5	4	-0.230644	0.88405	no
gi|646559550|gb|KK984341.1|	5127-5394	11	12	1010.68	1992.36	0.979147	0.39365	no
gi|646559563|gb|KK984338.1|	0-418	17	4.8	18	9	-1	0.44825	no

gi|646559563|gb|KK984338.1|	3520-3796	17	4.8	26	45	0.768896	0.58175	no
gi|646559711|gb|KK984308.1|	45-1837	7	1.8	4	3	-0.490521	0.74795	no
gi|646559889|gb|KK984274.1|	10925-11414	14	6	4	9	1	0.4576	no
gi|646559899|gb|KK984272.1|	4705-5355	13	3.8	8	0.974463	-3	0.12035	no
gi|646559926|gb|KK984267.1|	27-825	12	4.7	1	10	3	0.07735	no
gi|646559976|gb|KK984258.1|	2087-2264	13	6.1	0	202	inf	0.00035	yes
gi|646560019|gb|KK984249.1|	818-1083	9.7	6.9	2	22	3	0.1096	no
gi|646560106|gb|KK984231.1|	5376-5997	19	6.8	2	4	0.756911	0.56745	no
gi|646560111|gb|KK984230.1|	3013-4740	6.10E+01	30	2	2	-0.206026	0.8947	no
gi|646560270|gb|KK984198.1|	2758-3697	18	8.5	4	22	2	0.1428	no
gi|646560270|gb|KK984198.1|	53-1614	18	8.5	4	13	2	0.2467	no
gi|646560380|gb|KK984175.1|	33-467	24	8	8	3	-1	0.3646	no
gi|646560438|gb|KK984164.1|	8228-9660	18	8.6	23	15	-0.567533	0.6525	no
gi|646560453|gb|KK984161.1|	91-411	3.50E+01	41	15	3	-2	0.167	no
gi|646560541|gb|KK984141.1|	112-810	12	13	21	24	0.166958	0.91205	no
gi|646560541|gb|KK984141.1|	5315-5687	12	13	43	0	-inf	0.00025	yes
gi|646560580|gb|KK984133.1|	39-521	19	5.7	8	0	-inf	0.00045	yes
gi|646560619|gb|KK984125.1|	138-491	14	6.5	19	15	-0.334805	0.80785	no
gi|646560867|gb|KK984069.1|	409-957	4.1	3.6	4	5	0.332241	0.78555	no
gi|646560924|gb|KK984055.1|	53-939	12	4.4	0.879557	0.83078	-0.0823115	1	no
gi|646560924|gb|KK984055.1|	6242-8019	12	4.4	10	4	-1	0.451	no
gi|646560940|gb|KK984051.1|	5-494	8.3	4.5	3	1	-2	0.3832	no
gi|646561076|gb|KK984016.1|	444-1702	15	8.3	0.368932	0.926416	1	1	no
gi|646561148|gb|KK984002.1|	11774-12914	19	7.1	6	0.448667	-4	0.0913	no
gi|646561369|gb|KK983956.1|	497-1742	17	7.4	6	4	-0.412345	0.7812	no
gi|646561369|gb|KK983956.1|	5133-6081	17	7.4	11	13	0.272578	0.8541	no
gi|646561374|gb|KK983955.1|	10615-12474	20	9.2	6	6	-0.0953262	0.9529	no
gi|646561374|gb|KK983955.1|	9139-10357	20	9.2	20	16	-0.320238	0.7922	no
gi|646561402|gb|KK983949.1|	3535-3932	6.4	2.4	227	14	-4	0.02335	no
gi|646561438|gb|KK983942.1|	0-1057	8.3	6.1	6	0.329227	-4	0.117	no

gi|646561438|gb|KK983942.1|	2667-2953	8.3	6.1	63	0	-inf	0.0019	no
gi|646561451|gb|KK983939.1|	3591-3767	14	4.3	161	45	-2	0.27165	no
gi|646561451|gb|KK983939.1|	4727-4970	14	4.3	32	20	-0.647744	0.6457	no
gi|646561547|gb|KK983918.1|	192-967	38	14	3	5	0.833244	0.56065	no
gi|646561583|gb|KK983910.1|	18600-18844	10	6.3	31	7	-2	0.2052	no
gi|646561628|gb|KK983901.1|	2479-3111	12	7.8	2	10	3	0.13165	no
gi|646561628|gb|KK983901.1|	418-1205	12	7.8	3	14	2	0.1115	no
gi|646561672|gb|KK983892.1|	10391-10784	16	3.9	6	8	0.360763	0.7912	no
gi|646561722|gb|KK983882.1|	875-1363	5.7	4.9	4	2	-0.897828	0.5544	no
gi|646561735|gb|KK983879.1|	9953-11277	8.2	5.4	16	9	-0.818156	0.58665	no
gi|646561897|gb|KK983845.1|	3598-4312	12	8.3	4	0.845492	-2	0.20295	no
gi|646561926|gb|KK983838.1|	1125-2214	5.4	4	36	8	-2	0.11075	no
gi|646561931|gb|KK983837.1|	29-1167	11	5.9	0.62859	1	0.739104	1	no
gi|646562134|gb|KK983794.1|	8465-8692	5.5	6.3	56	9	-3	0.14725	no
gi|646562148|gb|KK983791.1|	7501-7836	13	6.2	31	11	-1	0.3147	no
gi|646562208|gb|KK983779.1|	69-1489	21	9.2	2	1	-0.0221862	0.9627	no
gi|646562285|gb|KK983762.1|	746-1400	15	9	3	0	-inf	0.0041	no
gi|646562296|gb|KK983760.1|	136-341	15	7.2	50	116	1	0.45685	no
gi|646562447|gb|KK983729.1|	1074-1556	10	2.7	0.402993	24	6	0.09615	no
gi|646562447|gb|KK983729.1|	2286-3167	10	2.7	0.147763	17	7	0.09495	no
gi|646562466|gb|KK983725.1|	7130-7574	5.2	4.9	17	4	-2	0.185	no
gi|646562466|gb|KK983725.1|	7702-8160	5.2	4.9	11	2	-2	0.2086	no
gi|646562529|gb|KK983712.1|	5242-5626	20	7.3	0	9	inf	0.00255	no
gi|646562564|gb|KK983705.1|	9132-9522	12	7	16	22	0.414529	0.79875	no
gi|646562758|gb|KK983662.1|	3304-4756	15	7	16	5	-2	0.21095	no
gi|646562812|gb|KK983651.1|	4952-5384	32	15	5	2	-1	0.38935	no
gi|646562812|gb|KK983651.1|	5491-6356	32	15	2	2	0.348961	0.794	no
gi|646562823|gb|KK983649.1|	10913-11584	15	7.7	4	2	-0.802795	0.5422	no
gi|646562823|gb|KK983649.1|	17622-17858	15	7.7	147	83	-0.833774	0.5959	no
gi|646562920|gb|KK983630.1|	364-644	6.2	2	4	20	3	0.2738	no

gi|646562931|gb|KK983628.1|	611-1414	13	5.2	1	1	0.495245	0.73295	no
gi|646563153|gb|KK983582.1|	326-1436	13	6.2	2	3	0.667556	0.63035	no
gi|646563153|gb|KK983582.1|	6064-6715	13	6.2	2	2	-0.111349	0.9288	no
gi|646563236|gb|KK983565.1|	2851-3697	17	6.9	14	39	1	0.25275	no
gi|646563236|gb|KK983565.1|	43-781	17	6.9	0.765916	3	2	0.2422	no
gi|646563365|gb|KK983537.1|	5905-6148	19	6.6	38	23	-0.688386	0.6086	no
gi|646563370|gb|KK983536.1|	13631-14308	13	3.2	5	5	0.00443141	0.9754	no
gi|646563402|gb|KK983528.1|	5237-6322	19	7.7	9	9	-0.00260468	0.9916	no
gi|646563477|gb|KK983512.1|	6802-7892	8.3	1.5	3	0.949665	-2	0.2909	no
gi|646563522|gb|KK983503.1|	774-1292	14	7.6	29	3	-3	0.0519	no
gi|646563564|gb|KK983494.1|	78-1035	20	9.8	7	8	0.261457	0.8692	no
gi|646563641|gb|KK983480.1|	8907-9777	8.2	4.8	27	23.42	-0.18487	0.9021	no
gi|646563700|gb|KK983468.1|	415-594	7	5.6	79.85	108	0.441123	0.7628	no
gi|646563963|gb|KK983416.1|	2227-2836	15	5.9	3	0.719816	-2	0.20515	no
gi|646563992|gb|KK983411.1|	1865-2191	20	12	12	1	-3	0.191	no
gi|646564008|gb|KK983408.1|	1881-2438	18	7.5	6	10	0.643233	0.64005	no
gi|646564008|gb|KK983408.1|	376-1782	18	7.5	0.723376	0.578262	-0.323023	1	no
gi|646564049|gb|KK983400.1|	7540-7879	8.6	3	140	193	0.466351	0.7071	no
gi|646564073|gb|KK983396.1|	4084-4753	4.5	5.2	0.893125	2	1	0.3715	no
gi|646564217|gb|KK983370.1|	0-659	20	7.5	2	1	-0.332224	0.81865	no
gi|646564269|gb|KK983361.1|	1027-2128	17	10	15	18	0.317863	0.83275	no
gi|646564326|gb|KK983351.1|	2-954	18	5.9	0.663576	3	2	0.2421	no
gi|646564326|gb|KK983351.1|	2705-3626	18	5.9	0.833347	2	0.920267	0.5404	no
gi|646564355|gb|KK983346.1|	0-368	21	5.4	9	22	1	0.3874	no
gi|646564366|gb|KK983344.1|	3926-4444	17	7.4	6	4	-0.39309	0.7602	no
gi|646564537|gb|KK983313.1|	0-530	11	5.9	10	4	-1	0.3414	no
gi|646564548|gb|KK983311.1|	12302-13716	16	10	4	3	-0.260713	0.8642	no
gi|646564548|gb|KK983311.1|	3238-3741	16	10	2	2	0.167808	0.9062	no
gi|646564584|gb|KK983305.1|	5969-6967	15	11	4	4	0.237042	0.8791	no
gi|646564584|gb|KK983305.1|	657-3140	15	11	8	4	-1	0.4988	no

gi|646564584|gb|KK983305.1|	7293-8196	15	11	2	2	-0.4136	0.75455	no
gi|646564681|gb|KK983290.1|	138-595	16	7	31	25	-0.287248	0.84145	no
gi|646564897|gb|KK983252.1|	3511-3992	8.4	7	2	5	1	0.4025	no
gi|646565005|gb|KK983233.1|	4294-4721	26	14	14	12	-0.224429	0.8858	no
gi|646565139|gb|KK983209.1|	12161-12483	16	6.6	9	13	0.595176	0.6475	no
gi|646565139|gb|KK983209.1|	166-985	16	6.6	4	3	-0.446577	0.7468	no
gi|646565223|gb|KK983194.1|	2-575	15	4.9	2	2	0.455348	0.75415	no
gi|646565401|gb|KK983161.1|	5351-5705	22	12	13	2	-3	0.14175	no
gi|646565546|gb|KK983136.1|	2837-4078	12	7.1	4	5	0.308908	0.84185	no
gi|646565955|gb|KK983064.1|	0-664	9.6	7.6	2	0.314345	-3	0.21605	no
gi|646565955|gb|KK983064.1|	3130-4332	9.6	7.6	5	3	-0.895933	0.5758	no
gi|646566086|gb|KK983041.1|	300-901	11	4.7	3	2	-0.800292	0.5828	no
gi|646566203|gb|KK983020.1|	12212-12799	18	9.8	33	29	-0.194642	0.8979	no
gi|646566203|gb|KK983020.1|	6366-7423	18	9.8	2	2	-0.0718202	0.9548	no
gi|646566320|gb|KK982999.1|	253-1884	19	6.8	9	9	0.0485548	0.97365	no
gi|646566320|gb|KK982999.1|	6434-7802	19	6.8	0.997426	1	0.398511	0.75865	no
gi|646566337|gb|KK982996.1|	13369-13796	9.4	5.2	10	6	-0.763456	0.5459	no
gi|646566343|gb|KK982995.1|	7429-8476	11	5.8	5	2	-1	0.3929	no
gi|646566537|gb|KK982959.1|	0-1358	14	4.8	14	10	-0.450829	0.7689	no
gi|646566852|gb|KK982901.1|	4698-6144	17	6.6	2	1	-0.626112	0.62125	no
gi|646566958|gb|KK982882.1|	350-538	10	3.4	129.25	188	0.536818	0.7178	no
gi|646566966|gb|KK982881.1|	4456-4712	8	5.5	30	8	-2	0.25415	no
gi|646567167|gb|KK982844.1|	495-2060	17	5.5	1	0.611106	-0.974209	0.48145	no
gi|646567193|gb|KK982840.1|	12294-12540	19	7.2	3	93	5	0.0997	no
gi|646567728|gb|KK982746.1|	0-1209	11	3.7	9	3	-1	0.34405	no
gi|646567822|gb|KK982730.1|	4168-4363	7.6	5.3	483	0	-inf	0.00045	yes
gi|646567851|gb|KK982725.1|	68-199	7.7	5.6	3400.03	135	-5	0.17075	no
gi|646568259|gb|KK982655.1|	4864-5581	15	8.5	25	11	-1	0.43315	no
gi|646568282|gb|KK982651.1|	201-532	14	5.9	71	76	0.109256	0.9407	no
gi|646568361|gb|KK982638.1|	13074-13371	14	6.1	12	10	-0.185616	0.90165	no

gi|646568367|gb|KK982637.1|	143-579	9.6	3	19	7	-1	0.3147	no
gi|646568385|gb|KK982634.1|	5582-5934	12	5.1	9	4	-1	0.46035	no
gi|646568446|gb|KK982623.1|	0-482	14	5.5	2	3	0.684252	0.6482	no
gi|646568446|gb|KK982623.1|	2585-3692	14	5.5	10	8	-0.248508	0.86405	no
gi|646568446|gb|KK982623.1|	9137-9417	14	5.5	0	20	inf	0.00255	no
gi|646568632|gb|KK982590.1|	6573-7102	4.9	3.5	5	1	-2	0.2597	no
gi|646568809|gb|KK982556.1|	2802-3635	8.7	3.8	2	2	-0.501837	0.7001	no
gi|646568976|gb|KK982521.1|	0-905	8.9	5.2	10	2	-2	0.1359	no
gi|646569039|gb|KK982508.1|	41-1935	13	5.3	8	4	-0.958727	0.5266	no
gi|646569190|gb|KK982478.1|	601-2213	19	8.3	0.545402	1	1	0.41045	no
gi|646569243|gb|KK982467.1|	0-442	7	3.6	7	2	-2	0.2447	no
gi|646569291|gb|KK982457.1|	3964-4701	16	6.3	3	7	1	0.37765	no
gi|646569474|gb|KK982420.1|	11967-13080	16	8.3	2	0.770712	-1	0.37145	no
gi|646569483|gb|KK982418.1|	5666-6419	15	6.7	3	4	0.67014	0.64365	no
gi|646569668|gb|KK982380.1|	2841-3237	8.6	4.1	6	0.807124	-3	0.201	no
gi|646569700|gb|KK982373.1|	18-404	15	3	4	9	1	0.44785	no
gi|646569755|gb|KK982361.1|	2698-3030	5.5	1	8	10	0.288525	0.84215	no
gi|646569755|gb|KK982361.1|	3641-4173	5.5	1	3	2	-0.405758	0.8003	no
gi|646569995|gb|KK982313.1|	6508-7126	15	7.6	17	23	0.401638	0.78645	no
gi|646570003|gb|KK982311.1|	11292-11706	15	4	16	19	0.221214	0.88865	no
gi|646570176|gb|KK982275.1|	9396-9585	16	5.5	80	61	-0.389447	0.79485	no
gi|646570180|gb|KK982274.1|	172-364	5.4	2.9	0	140	inf	0.00605	no
gi|646570180|gb|KK982274.1|	3318-3526	5.4	2.9	205.76	102	-1	0.5274	no
gi|646570180|gb|KK982274.1|	6694-7064	5.4	2.9	10	19	0.851974	0.5441	no
gi|646570180|gb|KK982274.1|	9161-9939	5.4	2.9	11	16	0.49255	0.73845	no
gi|646570218|gb|KK982266.1|	4639-5316	9.9	2.4	3	0	-inf	0.0011	yes
gi|646570259|gb|KK982258.1|	0-639	15	8	20	20	0.00965301	0.9932	no
gi|646570264|gb|KK982257.1|	0-323	20	8.2	105	120	0.196822	0.89545	no
gi|646570264|gb|KK982257.1|	1166-1604	20	8.2	124	115	-0.115983	0.9152	no
gi|646570318|gb|KK982246.1|	351-582	19	3.9	91	24	-2	0.24025	no

gi|646570375|gb|KK982234.1|	1244-1575	15	8.3	18	20	0.117118	0.9317	no
gi|646570375|gb|KK982234.1|	8947-10170	15	8.3	2	2	-0.268674	0.8181	no
gi|646570380|gb|KK982233.1|	2-229	11	4.2	103	53	-0.970461	0.4975	no
gi|646570537|gb|KK982200.1|	2360-2630	17	8.1	29	21	-0.468319	0.7129	no
gi|646570703|gb|KK982166.1|	16-517	10	6.3	15	12	-0.368386	0.8111	no
gi|646570848|gb|KK982137.1|	47-1633	12	6.1	3	3	-0.360298	0.81525	no
gi|646570919|gb|KK982123.1|	69-370	15	4.3	4	20	2	0.18025	no
gi|646571037|gb|KK982099.1|	9179-10921	12	6.2	4	0.269722	-4	0.0869	no
gi|646571067|gb|KK982093.1|	710-1247	6.4	16	11	4	-2	0.28265	no
gi|646571092|gb|KK982088.1|	16022-16938	13	8	0.559766	1	1	0.43305	no
gi|646571133|gb|KK982079.1|	1-406	19	4.2	8	5	-0.744081	0.57515	no
gi|646571158|gb|KK982074.1|	3164-4066	12	7.5	97	31	-2	0.1459	no
gi|646571158|gb|KK982074.1|	75-832	12	7.5	6	3	-1	0.41515	no
gi|646571178|gb|KK982070.1|	7547-7860	3.7	6	13	3	-2	0.2047	no
gi|646571440|gb|KK982017.1|	2972-4135	24	7.6	0.101881	2	5	0.1111	no
gi|646571468|gb|KK982012.1|	2977-3450	10	6.9	3	1	-2	0.3832	no
gi|646571768|gb|KK981956.1|	22-304	3.2	3.1	10	10	-0.0651279	0.91525	no
gi|646571790|gb|KK981952.1|	0-400	23	7.6	106	57	-0.897618	0.5539	no
gi|646571907|gb|KK981932.1|	475-794	25	7.7	8	47	3	0.13365	no
gi|646571925|gb|KK981929.1|	0-405	19	11	4	18	2	0.17005	no
gi|646571925|gb|KK981929.1|	3393-4369	19	11	8	7	-0.304131	0.8409	no
gi|646572069|gb|KK981903.1|	4974-5497	13	4.2	0	10	inf	0.0026	no
gi|646572146|gb|KK981889.1|	7908-8177	6.8	3	19	2	-3	0.2001	no
gi|646572801|gb|KK981772.1|	8009-8702	12	4	2	0.589279	-2	0.29735	no
gi|646573001|gb|KK981736.1|	287-1547	25	5	3	5	0.842941	0.59325	no
gi|646573043|gb|KK981729.1|	36-378	21	6	14	9	-0.6041	0.6341	no
gi|646573146|gb|KK981712.1|	6107-8004	16	7.5	4	3	-0.158717	0.9131	no
gi|646573146|gb|KK981712.1|	8997-9960	16	7.5	5	2	-1	0.4009	no
gi|646573440|gb|KK981661.1|	4884-5404	4.4	5.6	3	0.939439	-2	0.3832	no
gi|646573446|gb|KK981660.1|	4919-5292	13	7.3	4	12	2	0.25165	no

gi|646573623|gb|KK981629.1|	1181-1469	13	6.9	10	7	-0.3739	0.8118	no
gi|646573723|gb|KK981612.1|	827-1113	14	6.5	10	19	0.943731	0.52985	no
gi|646573911|gb|KK981580.1|	4825-6691	18	8.2	1	0.498491	-1	0.3412	no
gi|646573922|gb|KK981578.1|	6231-6871	15	5.3	11	2	-2	0.1265	no
gi|646574053|gb|KK981555.1|	3071-3577	21	5.7	14	3	-2	0.15855	no
gi|646574058|gb|KK981554.1|	248-1083	18	6.2	0	11	inf	0.0004	yes
gi|646574185|gb|KK981532.1|	9515-9922	15	8.4	11	3	-2	0.2007	no
gi|646574484|gb|KK981483.1|	50-473	14	6.7	11	15	0.523138	0.70215	no
gi|646574515|gb|KK981478.1|	0-411	22	9.4	14	1	-3	0.14605	no
gi|646574515|gb|KK981478.1|	6058-8092	22	9.4	2	0.828669	-1	0.3233	no
gi|646574615|gb|KK981461.1|	6312-6697	29	11	3	5	0.616137	0.669	no
gi|646574621|gb|KK981460.1|	3232-3555	41	6.3	209	107	-0.96549	0.52325	no
gi|646574621|gb|KK981460.1|	7448-7865	41	6.3	5	2	-1	0.45395	no
gi|646574712|gb|KK981444.1|	3183-4108	7.8	6.4	7	5	-0.324568	0.83515	no
gi|646574953|gb|KK981401.1|	2-665	20	12	31	11	-2	0.33085	no
gi|646574988|gb|KK981394.1|	1-995	16	10	1	1	0.050402	0.94695	no
gi|646574988|gb|KK981394.1|	3534-4167	16	10	104	101	-0.0387587	0.97095	no
gi|646575005|gb|KK981390.1|	790-1780	5.6	4.2	15	3	-2	0.1878	no
gi|646575038|gb|KK981383.1|	5230-6066	12	8.5	9	15	0.787945	0.59985	no
gi|646575038|gb|KK981383.1|	68-1446	12	8.5	0.741409	2	1	0.3841	no
gi|646575049|gb|KK981381.1|	5252-6160	9.9	4.8	16	14	-0.194527	0.898	no
gi|646575058|gb|KK981379.1|	4799-5570	25	11	0	6	inf	0.00175	no
gi|646575153|gb|KK981360.1|	8629-9326	13	18	0	7	inf	0.00185	no
gi|646575250|gb|KK981340.1|	1282-1609	16	7	6.39	22	2	0.30055	no
gi|646575250|gb|KK981340.1|	2282-3798	16	7	10	14	0.603021	0.62925	no
gi|646575250|gb|KK981340.1|	6085-6648	16	7	6	7	0.0601052	0.9634	no
gi|646575266|gb|KK981337.1|	158-983	8.2	5.2	0	3	inf	0.00035	yes
gi|646575298|gb|KK981330.1|	3933-4338	12	5.2	4	3	-0.213567	0.88655	no
gi|646575347|gb|KK981320.1|	0-1454	15	7.2	2	3	0.632982	0.69265	no
gi|646575347|gb|KK981320.1|	3055-3513	15	7.2	9	14	0.539719	0.69305	no

gi|646575347|gb|KK981320.1|	8016-8488	15	7.2	13	0.561537	-4	0.1804	no
gi|646575699|gb|KK981251.1|	5342-6332	9.5	4.6	26	0.358351	-6	0.10535	no
gi|646575785|gb|KK981234.1|	2394-3280	6	4.1	3	0.41539	-3	0.1295	no
gi|646575796|gb|KK981232.1|	10469-11001	21	10	13	16	0.249594	0.86915	no
gi|646575796|gb|KK981232.1|	19328-20239	21	10	1	3	1	0.3749	no
gi|646575796|gb|KK981232.1|	3688-3974	21	10	10	15	0.621803	0.6595	no
gi|646575796|gb|KK981232.1|	6400-7017	21	10	4	2	-1	0.359	no
gi|646575944|gb|KK981200.1|	1559-2073	25	7.8	13	0	-inf	0.0023	no
gi|646575944|gb|KK981200.1|	2860-4228	25	7.8	38	0	-inf	5.00E-05	yes
gi|646575944|gb|KK981200.1|	9080-9522	25	7.8	25	0	-inf	0.0003	yes
gi|646576310|gb|KK981127.1|	8294-9048	10	5.7	15	17	0.132141	0.9347	no
gi|646576635|gb|KK981061.1|	5922-6328	21	2.9	6	6	0.0503104	0.9428	no
gi|646576819|gb|KK981023.1|	4714-5736	14	9	3	2	-0.363099	0.78995	no
gi|646576893|gb|KK981008.1|	13724-13998	8.2	3.8	10	24	1	0.38465	no
gi|646577012|gb|KK980983.1|	17999-18697	11	3.3	0.417449	3	3	0.2662	no
gi|646577113|gb|KK980962.1|	0-479	14	6.3	4	8	1	0.46065	no
gi|646577176|gb|KK980949.1|	2023-2875	16	7.5	7	6	-0.237104	0.87975	no
gi|646577754|gb|KK980832.1|	0-1896	16	6.4	6	10	0.596159	0.69255	no
gi|646577754|gb|KK980832.1|	9428-10175	16	6.4	0.563947	3	2	0.1544	no
gi|646577783|gb|KK980826.1|	938-1114	11	5.4	124.86	89	-0.481785	0.749	no
gi|646577917|gb|KK980799.1|	755-1469	120	120	11	19	0.738843	0.62135	no
gi|646577922|gb|KK980798.1|	4354-5224	6.8	3.6	3	4	0.349373	0.79785	no
gi|646577937|gb|KK980795.1|	5965-6904	14	8.8	20	11	-0.889474	0.5569	no
gi|646578045|gb|KK980772.1|	1297-2050	20	6.6	14	3	-2	0.12865	no
gi|646578084|gb|KK980764.1|	0-1116	13	4	5	1	-2	0.1803	no
gi|646578084|gb|KK980764.1|	3476-4000	13	4	3	1	-1	0.41965	no
gi|646578184|gb|KK980744.1|	1-994	16	7.4	8	4	-0.777105	0.613	no
gi|646578184|gb|KK980744.1|	2655-3542	16	7.4	16	11	-0.632201	0.6696	no
gi|646578184|gb|KK980744.1|	6479-6837	16	7.4	7	2	-2	0.30735	no
gi|646578384|gb|KK980704.1|	9172-9973	15	6.7	1	3	2	0.2389	no

gi|646578414|gb|KK980699.1|	9587-10104	25	9.7	210	196	-0.0974131	0.93245	no
gi|646578493|gb|KK980684.1|	9385-9833	22	3.6	3	2	-0.819873	0.6134	no
gi|646578750|gb|KK980634.1|	3641-4398	13	8.9	3	4	0.390529	0.75065	no
gi|646578761|gb|KK980632.1|	2-437	11	4.7	12	5	-1	0.42635	no
gi|646578912|gb|KK980601.1|	3156-3797	17	7.8	1	2	0.471639	0.7721	no
gi|646578923|gb|KK980599.1|	8278-8651	15	3.6	16	14	-0.211854	0.89975	no
gi|646578923|gb|KK980599.1|	9050-10076	15	3.6	2	4	1	0.45175	no
gi|646579116|gb|KK980563.1|	259-824	17	6.8	43	70	0.690964	0.58725	no
gi|646579270|gb|KK980534.1|	11-331	23	7.4	0	14	inf	0.00255	no
gi|646579382|gb|KK980511.1|	199-1748	11	7.8	0	9	inf	0.0001	yes
gi|646579445|gb|KK980499.1|	868-1107	11	6.4	7	32	2	0.30225	no
gi|646579484|gb|KK980493.1|	394-1149	12	5.2	2	0	-inf	0.00475	no
gi|646579517|gb|KK980487.1|	5149-6331	26	7.6	48	19	-1	0.3138	no
gi|646579601|gb|KK980471.1|	0-643	6.4	2.1	5	6	0.0359419	0.97375	no
gi|646579601|gb|KK980471.1|	1419-1816	6.4	2.1	5	4	-0.313655	0.84645	no
gi|646579601|gb|KK980471.1|	5582-6132	6.4	2.1	7	5	-0.4261	0.75665	no
gi|646579763|gb|KK980440.1|	9166-9357	13	5.9	96	29	-2	0.2922	no
gi|646579933|gb|KK980407.1|	25-870	19	6.8	2	4	0.861818	0.5452	no
gi|646579933|gb|KK980407.1|	4755-5645	19	6.8	5	3	-0.689685	0.61465	no
gi|646580010|gb|KK980393.1|	436-1567	19	8.3	7	3	-2	0.3488	no
gi|646580010|gb|KK980393.1|	7104-8013	19	8.3	5	2	-2	0.3033	no
gi|646580153|gb|KK980366.1|	1590-1953	17	6.1	9	9	0.0399252	0.9492	no
gi|646580153|gb|KK980366.1|	7399-7921	17	6.1	1	5	2	0.2467	no
gi|646580258|gb|KK980346.1|	2837-4264	12	6.9	7	0	-inf	0.0001	yes
gi|646580275|gb|KK980343.1|	63-602	13	6.7	8	4	-0.970339	0.51755	no
gi|646580344|gb|KK980330.1|	8603-8951	11	4	26	20	-0.377218	0.81325	no
gi|646580344|gb|KK980330.1|	9292-9589	11	4	15	22	0.607933	0.6333	no
gi|646580344|gb|KK980330.1|	9780-10061	11	4	16	8	-0.974291	0.5573	no
gi|646580368|gb|KK980325.1|	1107-1291	11	7.6	295	117	-1	0.3704	no
gi|646580564|gb|KK980289.1|	4-771	16	7	2	2	-0.0936886	0.93195	no

gi|646580564|gb|KK980289.1|	5514-5815	16	7	11	5	-1	0.4638	no
gi|646580725|gb|KK980259.1|	50-1165	6.9	5.8	0.430265	3	3	0.15235	no
gi|646580782|gb|KK980248.1|	9698-10225	5.5	8	2	5	1	0.4188	no
gi|646580918|gb|KK980223.1|	29-302	92	56	89	53	-0.730256	0.64415	no
gi|646581271|gb|KK980156.1|	11357-11874	2.3	3.9	4	6	0.552068	0.66405	no
gi|646581271|gb|KK980156.1|	12006-12625	2.3	3.9	3	2	-0.185119	0.8941	no
gi|646581347|gb|KK980142.1|	7827-9449	23	7.7	8	3	-1	0.35425	no
gi|646581458|gb|KK980124.1|	1171-1563	39	17	16	13	-0.284025	0.8573	no
gi|646581615|gb|KK980094.1|	9257-9932	9.8	5.4	2	2	-0.521985	0.72175	no
gi|646581629|gb|KK980091.1|	2655-4079	23	7.9	14	36	1	0.28545	no
gi|646581708|gb|KK980076.1|	25-252	8.3	4.3	42.91	18	-1	0.40015	no
gi|646581736|gb|KK980071.1|	145-671	22	7	0.67855	4	2	0.2858	no
gi|646581793|gb|KK980060.1|	2977-3265	7.8	5.2	27	0	-inf	0.0012	yes
gi|646581853|gb|KK980049.1|	3141-3839	6	3.8	0	9	inf	0.001	yes
gi|646582278|gb|KK979972.1|	5006-5332	16	5.2	49	7	-3	0.093	no
gi|646582305|gb|KK979967.1|	4342-6389	15	5.6	5	5	0.0995577	0.94975	no
gi|646582379|gb|KK979954.1|	69-1501	9.5	1.8	7	5	-0.707084	0.6442	no
gi|646582393|gb|KK979951.1|	187-607	44	29	3	4	0.646427	0.6668	no
gi|646582532|gb|KK979925.1|	9660-9868	15	7.6	232.31	153	-0.601225	0.70075	no
gi|646582692|gb|KK979897.1|	4615-5324	15	8.5	4	3	-0.516474	0.6801	no
gi|646582711|gb|KK979893.1|	8882-9346	12	4.6	1	4	2	0.29775	no
gi|646582878|gb|KK979862.1|	5422-5927	19	7.6	9	14	0.654087	0.68095	no
gi|646582878|gb|KK979862.1|	6044-7143	19	7.6	0.986445	0.313267	-2	1	no
gi|646582903|gb|KK979857.1|	579-1238	12	6.8	1	3	0.890169	0.5491	no
gi|646582903|gb|KK979857.1|	5925-8017	12	6.8	1	1	-0.0814657	0.9603	no
gi|646582944|gb|KK979850.1|	2-513	4.6	4.5	0	10	inf	0.00315	no
gi|646582961|gb|KK979847.1|	3383-4318	3.4	4.8	2	0	-inf	0.0035	no
gi|646582968|gb|KK979846.1|	8768-8964	6.9	7.4	175	279	0.67329	0.61975	no
gi|646582972|gb|KK979845.1|	0-750	17	7.5	461	202	-1	0.31305	no
gi|646582972|gb|KK979845.1|	2770-3585	17	7.5	6	4	-0.619028	0.69775	no

gi|646583060|gb|KK979829.1|	10401-10809	26	10	16	36	1	0.4465	no
gi|646583060|gb|KK979829.1|	19615-19926	26	10	4	12	2	0.28855	no
gi|646583094|gb|KK979823.1|	5059-5405	10	4.4	22	10	-1	0.45315	no
gi|646583094|gb|KK979823.1|	8516-9805	10	4.4	5	0.89923	-3	0.11085	no
gi|646583161|gb|KK979811.1|	10186-10735	18	6.8	5	4	-0.230027	0.8525	no
gi|646583451|gb|KK979757.1|	2-960	14	4.7	1	0.748319	-0.492319	1	no
gi|646583451|gb|KK979757.1|	8926-9439	14	4.7	1	3	1	0.44135	no
gi|646583597|gb|KK979731.1|	78-460	14	8.2	38	16	-1	0.4299	no
gi|646583610|gb|KK979729.1|	2735-3255	15	4.6	6	4	-0.39231	0.76095	no
gi|646583780|gb|KK979698.1|	10423-10804	14	6.6	28	28	0.0271603	0.9844	no
gi|646583780|gb|KK979698.1|	10911-11615	14	6.6	0.823775	2	1	0.4862	no
gi|646583818|gb|KK979691.1|	25-889	11	4.9	13	15.69	0.281315	0.84835	no
gi|646583880|gb|KK979680.1|	4001-4257	6.3	4	13	42	2	0.3286	no
gi|646584075|gb|KK979645.1|	22-852	6.8	5.8	1	1	-0.180187	0.9042	no
gi|646584081|gb|KK979644.1|	3-400	13	4.9	21	5	-2	0.19085	no
gi|646584081|gb|KK979644.1|	674-2094	13	4.9	4	4	-0.0450141	0.975	no
gi|646584113|gb|KK979638.1|	10906-11703	13	6.4	8	4	-0.920414	0.566	no
gi|646584223|gb|KK979618.1|	11169-11754	9.5	5.9	16	6	-1	0.4043	no
gi|646584238|gb|KK979615.1|	2699-2911	11	3.3	24	41	0.770305	0.62785	no
gi|646584238|gb|KK979615.1|	7867-8782	11	3.3	12	14	0.279514	0.8521	no
gi|646584357|gb|KK979595.1|	1037-1418	13	5	4	4	0.086054	0.91505	no
gi|646584564|gb|KK979557.1|	95-452	13	5.9	0.824084	11	4	0.1007	no
gi|646584662|gb|KK979539.1|	5-1140	7	2.8	0.946098	2	0.806097	0.5518	no
gi|646584662|gb|KK979539.1|	9606-10792	7	2.8	2	2	0.431074	0.75415	no
gi|646584668|gb|KK979538.1|	10470-11336	15	8	7	26	2	0.21645	no
gi|646584668|gb|KK979538.1|	13069-14361	15	8	29	45	0.607367	0.5814	no
gi|646584879|gb|KK979500.1|	4699-6559	19	6.4	1	0.667105	-1	0.43705	no
gi|646584879|gb|KK979500.1|	531-1397	19	6.4	0.60556	2	2	0.36865	no
gi|646584979|gb|KK979484.1|	2606-3241	13	6.8	27	19	-0.568409	0.70535	no
gi|646584979|gb|KK979484.1|	4674-6162	13	6.8	76	46	-0.71578	0.53475	no

gi|646585393|gb|KK979414.1|	10569-10937	12	3.2	27	9	-2	0.27475	no
gi|646585399|gb|KK979413.1|	37-563	8.3	3.8	4	8	0.909531	0.53575	no
gi|646585418|gb|KK979410.1|	8188-8557	9.1	4.9	0	81	inf	5.00E-05	yes
gi|646585451|gb|KK979404.1|	1600-1765	12	4.7	0	241	inf	0.0014	yes
gi|646585532|gb|KK979391.1|	7311-8865	14	9.5	15	13	-0.224695	0.8596	no
gi|646585797|gb|KK979346.1|	6436-7378	7.1	3.6	4	2	-1	0.44875	no
gi|646585871|gb|KK979333.1|	889-1540	16	9.1	98	9	-3	0.02855	no
gi|646585883|gb|KK979331.1|	10339-12956	21	11	3	1	-1	0.43495	no
gi|646585883|gb|KK979331.1|	23125-23447	21	11	56	8	-3	0.0945	no
gi|646585883|gb|KK979331.1|	25-2087	21	11	11	2	-3	0.07475	no
gi|646585908|gb|KK979327.1|	354-1406	13	6.1	9	0	-inf	0.0002	yes
gi|646585920|gb|KK979325.1|	2954-3454	12	5.2	10	11	0.0816212	0.95895	no
gi|646586037|gb|KK979306.1|	187-383	5.2	3.6	607	245	-1	0.39355	no
gi|646586066|gb|KK979301.1|	7428-8106	12	4.2	12	14	0.215501	0.8863	no
gi|646586179|gb|KK979278.1|	7574-7816	21	8.1	157	72	-1	0.4698	no
gi|646586235|gb|KK979267.1|	10693-11945	15	6	31	56	0.862722	0.44305	no
gi|646586286|gb|KK979257.1|	8182-9568	12	4.1	1	1	-0.112939	0.9362	no
gi|646586311|gb|KK979252.1|	1203-3335	7.3	2.7	5	0	-inf	5.00E-05	yes
gi|646586311|gb|KK979252.1|	631-1091	7.3	2.7	22	0	-inf	0.00085	yes
gi|646586906|gb|KK979137.1|	5244-6230	9.5	6.4	0	4	inf	0.00315	no
gi|646587082|gb|KK979093.1|	276-1425	10	4.3	0.931285	0.296174	-2	1	no
gi|646587082|gb|KK979093.1|	5675-6560	10	4.3	1	0.62404	-0.912464	0.58965	no
gi|646587082|gb|KK979093.1|	8157-8806	10	4.3	8	10	0.303301	0.8436	no
gi|646587124|gb|KK979083.1|	10285-10496	24	11	25	60	1.28	0.41865	no
gi|646587182|gb|KK979068.1|	2123-3408	18	6.2	13	7	-0.967615	0.52365	no
gi|646587206|gb|KK979061.1|	1367-2406	19	9.1	3	4	0.204128	0.8979	no
gi|646587255|gb|KK979049.1|	47-289	9.2	7.8	0	75	inf	0.0023	no
gi|646587273|gb|KK979045.1|	165-2039	17	5.9	27	12	-1	0.3022	no
gi|646587273|gb|KK979045.1|	5831-6886	17	5.9	17	15	-0.233506	0.8757	no
gi|646587337|gb|KK979029.1|	5043-5296	9.5	5.7	85	0	-inf	0.0026	no

gi|646587375|gb|KK979020.1|	589-1341	11	7.6	3	5	0.723908	0.60605	no
gi|646587464|gb|KK978998.1|	3330-4151	18	7	19	15	-0.316555	0.8328	no
gi|646587464|gb|KK978998.1|	497-1291	18	7	21	11	-0.889022	0.55565	no
gi|646587476|gb|KK978995.1|	13265-13777	22	8	3	2	-0.0508329	0.92345	no
gi|646587481|gb|KK978994.1|	1269-1805	19	4.2	1	8	3	0.2061	no
gi|646587578|gb|KK978970.1|	2263-3229	10	3.3	0.780616	0.740156	-0.0767848	1	no
gi|646587578|gb|KK978970.1|	4904-6582	10	3.3	2	1	-0.92923	0.50805	no
gi|646587637|gb|KK978954.1|	3834-4027	14	7.5	151	191	0.343953	0.8096	no
gi|646587694|gb|KK978939.1|	10638-11530	5.4	2	5	0.205805	-4	0.17915	no
gi|646587729|gb|KK978930.1|	9044-10020	15	6.9	0.769792	0.730199	-0.0761789	1	no
gi|646587796|gb|KK978913.1|	4893-5390	6.8	4.6	27	32	0.253135	0.8657	no
gi|646587907|gb|KK978884.1|	121-908	12	7	2	2	0.11504	0.93025	no
gi|646587914|gb|KK978882.1|	175-799	19	4.9	145	47	-2	0.1485	no
gi|646587987|gb|KK978863.1|	18375-19301	13	6.5	1	0.391427	-2	0.2991	no
gi|646587991|gb|KK978862.1|	12-794	19	9.9	90	70	-0.360069	0.74665	no
gi|646587991|gb|KK978862.1|	2180-2837	19	9.9	4	3	-0.203315	0.8736	no
gi|646587991|gb|KK978862.1|	8100-9245	19	9.9	0.831529	0.743681	-0.161082	1	no
gi|646588039|gb|KK978850.1|	47-560	54	8.8	2	11	2	0.19815	no
gi|646588039|gb|KK978850.1|	5835-6836	54	8.8	0	3	inf	0.00435	no
gi|646588167|gb|KK978818.1|	51-371	5.8	2.9	7	8	0.270059	0.84425	no
gi|646588195|gb|KK978811.1|	2241-3200	14	7.9	27	20	-0.42387	0.74025	no
gi|646588268|gb|KK978793.1|	6524-6900	6.9	6.5	5	4	-0.463459	0.7629	no
gi|646588353|gb|KK978771.1|	320-947	30	12	0.248302	5	4	0.11325	no
gi|646588353|gb|KK978771.1|	9211-10148	30	12	0.813801	0.770628	-0.0786408	1	no
gi|646588375|gb|KK978765.1|	243-798	7	4.3	2	2	0.449963	0.7747	no
gi|646588413|gb|KK978755.1|	224-662	15	5.2	8	7	-0.369406	0.766	no
gi|646588413|gb|KK978755.1|	5640-6199	15	5.2	2	5	0.910638	0.506	no
gi|646588909|gb|KK978630.1|	3226-3487	17	7.4	18.73	18	-0.0475792	0.93325	no
gi|646589047|gb|KK978595.1|	14-548	16	6.5	0.32975	6	4	0.0968	no
gi|646589047|gb|KK978595.1|	1943-3114	16	6.5	1	5	2	0.1531	no

gi|646589372|gb|KK978519.1|	12043-12402	35	9.4	128	56	-1	0.4376	no
gi|646589538|gb|KK978489.1|	6008-6409	10	9	8	5	-0.747516	0.57515	no
gi|646589591|gb|KK978479.1|	6579-8571	16	8.6	2	1	-0.755293	0.6334	no
gi|646589645|gb|KK978469.1|	201-1899	8.2	3.9	0.577014	0.463034	-0.317488	1	no
gi|646589792|gb|KK978442.1|	826-1066	14	6.2	43	35.15	-0.299733	0.8146	no
gi|646589886|gb|KK978426.1|	10044-10617	9.3	6.2	7	0	-inf	0.00495	no
gi|646589929|gb|KK978419.1|	6714-7301	16	6.2	4	8	1	0.40585	no
gi|646589937|gb|KK978418.1|	0-830	5.1	8.3	2	6	1	0.3707	no
gi|646589937|gb|KK978418.1|	1624-2010	5.1	8.3	6	8	0.354086	0.7976	no
gi|646590281|gb|KK978355.1|	52-595	12	12	4	3	-0.669417	0.62505	no
gi|646590343|gb|KK978344.1|	6352-6609	7.70E+01	97	32	19	-0.759467	0.5736	no
gi|646590348|gb|KK978343.1|	5756-6613	14	7	46	25	-0.903531	0.48425	no
gi|646590357|gb|KK978341.1|	10506-11870	16	8.1	0.667349	1	0.693865	1	no
gi|646590369|gb|KK978339.1|	5530-6143	13	6.6	3	5	0.572509	0.6557	no
gi|646590374|gb|KK978338.1|	731-965	12	6.8	19	31	0.734977	0.6353	no
gi|646590616|gb|KK978293.1|	11010-11527	12	4.6	2	3	0.699625	0.6482	no
gi|646590709|gb|KK978276.1|	5871-6720	20	9.7	13	9	-0.483121	0.7412	no
gi|646590747|gb|KK978269.1|	15566-16024	18	8.3	0.896987	151	7	0.2322	no
gi|646590795|gb|KK978260.1|	5320-5867	13	7.2	2	3	0.447391	0.75535	no
gi|646590920|gb|KK978237.1|	4012-5552	21	9.2	39	12	-2	0.2071	no
gi|646590920|gb|KK978237.1|	5-2183	21	9.2	44	11	-2	0.0773	no
gi|646591114|gb|KK978201.1|	687-1560	4.1	2.5	1	2	0.331695	0.8043	no
gi|646591320|gb|KK978163.1|	1187-1898	14	7.7	0.811165	2	1	0.3715	no
gi|646591495|gb|KK978131.1|	0-268	13	7.1	0	45	inf	0.00315	no
gi|646591533|gb|KK978124.1|	6990-7171	4.4	1	30	115	2	0.3508	no
gi|646591538|gb|KK978123.1|	5637-6080	11	4.8	14	8	-0.873638	0.53665	no
gi|646591538|gb|KK978123.1|	8235-8364	11	4.8	2766.01	1107.26	-1	0.36275	no
gi|646591597|gb|KK978112.1|	149-424	15	6.5	61	46	-0.426158	0.7906	no
gi|646591656|gb|KK978102.1|	525-1489	14	7	0.913287	1	0.285662	1	no
gi|646591887|gb|KK978058.1|	406-1112	10	5.7	7	7	0.0236399	0.9819	no

gi|646591975|gb|KK978041.1|	5315-5894	17	9	0	33	inf	5.00E-05	yes
gi|646592067|gb|KK978025.1|	3093-4322	15	5.5	22	18	-0.316921	0.80125	no
gi|646592067|gb|KK978025.1|	5651-7656	15	5.5	13	9	-0.521027	0.68115	no
gi|646592096|gb|KK978020.1|	1-2817	18	9.1	6	6	-0.0622596	0.96735	no
gi|646592096|gb|KK978020.1|	3227-4203	18	9.1	1	1	0.316139	0.8346	no
gi|646592096|gb|KK978020.1|	4488-7094	18	9.1	0.708354	0.97202	0.456516	1	no
gi|646592152|gb|KK978010.1|	6675-7877	12	8.9	0.293058	14	6	0.1071	no
gi|646592357|gb|KK977975.1|	128-486	4.1	7.7	0	32	inf	0.001	yes
gi|646592466|gb|KK977955.1|	555-1105	29	7.8	2	3	0.933796	0.5629	no
gi|646592506|gb|KK977948.1|	52-1687	15	7.1	0.469274	0.967671	1	1	no
gi|646593031|gb|KK977853.1|	0-603	18	8.7	39	16	-1	0.4217	no
gi|646593057|gb|KK977848.1|	9515-10048	8.6	2.7	2	3	0.705631	0.64555	no
gi|646593094|gb|KK977842.1|	4738-5051	19	6.9	20	7	-1	0.3127	no
gi|646593094|gb|KK977842.1|	7385-8211	19	6.9	48	30	-0.661528	0.60275	no
gi|646593109|gb|KK977839.1|	5233-6511	6.2	1.5	2	2	0.361097	0.79565	no
gi|646593139|gb|KK977834.1|	1571-2316	7.5	4.8	2	2	-0.626928	0.64225	no
gi|646593561|gb|KK977762.1|	14-1136	16	3.4	2	3	0.603521	0.672	no
gi|646593840|gb|KK977712.1|	8836-9558	19	5.5	3	0.83171	-2	0.19355	no
gi|646593902|gb|KK977701.1|	0-496	21	6.1	28	11	-1	0.39495	no
gi|646593941|gb|KK977694.1|	3087-3658	5.5	5	1	2	0.71781	0.64505	no
gi|646593970|gb|KK977689.1|	4273-4509	19	9.5	47	64	0.45137	0.7536	no
gi|646594180|gb|KK977653.1|	4911-5268	4.4	3.4	7	1	-3	0.20705	no
gi|646594263|gb|KK977638.1|	6499-6720	12	3.5	10	39	2	0.32265	no
gi|646594295|gb|KK977632.1|	4006-4651	21	9.3	0.473864	5	3	0.24135	no
gi|646594327|gb|KK977626.1|	170-680	2.4	1.9	6	6	0.0187408	0.9572	no
gi|646594456|gb|KK977603.1|	3666-3848	12	4.2	144	186	0.369875	0.8058	no
gi|646594584|gb|KK977580.1|	1640-2591	16	6.4	0.531624	1	1	0.43305	no
gi|646594646|gb|KK977569.1|	14467-15238	11	3.5	0.537749	7	4	0.1246	no
gi|646594686|gb|KK977562.1|	476-1154	17	6	2	0.91355	-1	0.47355	no
gi|646594847|gb|KK977535.1|	24-449	18	10	6	0	-inf	0.00475	no

gi|646594985|gb|KK977511.1|	259-491	11	3	50	16	-2	0.2641	no
gi|646595002|gb|KK977508.1|	5421-5821	8.6	4.1	18	28	0.63012	0.68275	no
gi|646595038|gb|KK977502.1|	84-1162	2.1	2.8	2	2	-0.418752	0.7201	no
gi|646595118|gb|KK977490.1|	2572-3165	10	6.2	7	10	0.630665	0.6907	no
gi|646595322|gb|KK977453.1|	6-697	1.5	2.8	34	12	-2	0.32245	no
gi|646595334|gb|KK977451.1|	13922-15128	15	5.3	3	8	2	0.3167	no
gi|646595516|gb|KK977419.1|	4733-4995	7.4	11	37	54	0.540169	0.70605	no
gi|646595672|gb|KK977392.1|	664-1940	21	8.2	8	14	0.72381	0.6328	no
gi|646595850|gb|KK977360.1|	8684-9117	70	37	6	3	-0.744746	0.59655	no
gi|646595899|gb|KK977352.1|	3433-3846	12	4.9	11	7	-0.774041	0.5413	no
gi|646596219|gb|KK977296.1|	890-1447	9.7	4.7	4	0	-inf	0.0041	no
gi|646596243|gb|KK977292.1|	0-553	22	6.5	20	9	-1	0.4673	no
gi|646596243|gb|KK977292.1|	1882-2624	22	6.5	10	5	-1	0.50125	no
gi|646596277|gb|KK977286.1|	566-772	23	7.4	42	93	1	0.41255	no
gi|646596277|gb|KK977286.1|	6483-7118	23	7.4	1	6	2	0.21475	no
gi|646596594|gb|KK977229.1|	11628-12473	19	8.2	0.783884	6	3	0.1187	no
gi|646596594|gb|KK977229.1|	4782-5515	19	8.2	7	10	0.408956	0.79265	no
gi|646596594|gb|KK977229.1|	5640-6455	19	8.2	2	2	-0.0885135	0.94285	no
gi|646596847|gb|KK977173.1|	35-842	9.2	4.7	0.670264	1	1	0.48525	no
gi|646596869|gb|KK977168.1|	10427-11251	13	4.9	0.487684	2	2	0.18805	no
gi|646597094|gb|KK977113.1|	14091-14771	15	7.4	4	0.303162	-4	0.17095	no
gi|646597094|gb|KK977113.1|	6729-8145	15	7.4	5	2	-1	0.47195	no
gi|646597111|gb|KK977109.1|	270-495	16	7.3	54	50	-0.10422	0.93455	no
gi|646597227|gb|KK977079.1|	11701-11973	16	7.4	20	11	-0.825847	0.57265	no
gi|646597298|gb|KK977062.1|	129-946	7.4	7.1	0.987512	1	0.496645	0.73295	no
gi|646597326|gb|KK977058.1|	6352-6790	15	16	69	31	-1	0.44315	no
gi|646597355|gb|KK977050.1|	1679-2268	9.3	7.7	4	5	0.459707	0.7088	no
gi|646597355|gb|KK977050.1|	3279-3821	9.3	7.7	1	3	1	0.4404	no
gi|646597429|gb|KK977031.1|	10686-13135	13	7.7	2	0	-inf	0.0018	no
gi|646597429|gb|KK977031.1|	8656-10474	13	7.7	0.828673	0.342389	-1	1	no

gi|646597485|gb|KK977017.1|	62-1063	7.9	3.6	0	6	inf	0.0007	yes
gi|646597583|gb|KK976991.1|	2037-2843	11	5.4	1	2	0.495551	0.72995	no
gi|646597583|gb|KK976991.1|	710-1860	11	5.4	2	1	-0.482814	0.70265	no
gi|646597658|gb|KK976971.1|	8547-9304	46	37	8	7	-0.246901	0.8744	no
gi|646597764|gb|KK976945.1|	20783-21128	7	7.2	3	10	2	0.25215	no
gi|646597885|gb|KK976917.1|	10134-12421	12	8.7	10	4	-1	0.2967	no
gi|646597919|gb|KK976909.1|	4165-4842	12	6.3	2	5	1	0.4432	no
gi|646597919|gb|KK976909.1|	5549-6593	12	6.3	0.93795	2	1	0.3744	no
gi|646598036|gb|KK976878.1|	4369-5465	11	6	7	0.157178	-5	0.16865	no
gi|646598077|gb|KK976868.1|	0-864	16	4.1	13	12	-0.151196	0.9218	no
gi|646598077|gb|KK976868.1|	9756-10104	16	4.1	7	2	-2	0.30625	no
gi|646598115|gb|KK976858.1|	7598-8344	12	6.1	2	3	0.363148	0.76905	no
gi|646598115|gb|KK976858.1|	9902-10575	12	6.1	2	6	1	0.3339	no
gi|646598127|gb|KK976855.1|	1300-2069	18	7.2	9	11	0.282923	0.8532	no
gi|646598176|gb|KK976843.1|	11949-13050	14	8.6	3	3	-0.00839727	0.9741	no
gi|646598202|gb|KK976836.1|	6208-6895	7.7	3.7	3	0	-inf	0.0035	no
gi|646598253|gb|KK976824.1|	3-1016	17	10	3	5	0.65773	0.67975	no
gi|646598253|gb|KK976824.1|	3221-3947	17	10	2	1	-0.683932	0.6459	no
gi|646598336|gb|KK976805.1|	3133-3657	14	7.4	0.683484	63	7	0.2322	no
gi|646598427|gb|KK976785.1|	114-570	13	6	9	3	-2	0.2833	no
gi|646598427|gb|KK976785.1|	6167-6390	13	6	131	85	-0.624533	0.70185	no
gi|646598450|gb|KK976781.1|	1103-2116	4.9	3.6	0.854263	0.521684	-0.711504	1	no
gi|646598486|gb|KK976774.1|	3219-3692	11	4.1	3	3	0.153641	0.9062	no
gi|646598593|gb|KK976753.1|	868-1508	15	8.4	4	1	-2	0.30395	no
gi|646598620|gb|KK976748.1|	238-672	14	5.4	0	7	inf	0.00255	no
gi|646598634|gb|KK976745.1|	7921-8737	11	6.5	1	0.697849	-0.725845	0.66415	no
gi|646598654|gb|KK976741.1|	667-1585	21	8.5	0.697597	0.989989	0.505019	1	no
gi|646598710|gb|KK976730.1|	2691-3182	19	4.7	5	9	0.928001	0.5336	no
gi|646598742|gb|KK976723.1|	111-687	9.9	6.7	3	4	0.166687	0.9081	no
gi|646598780|gb|KK976715.1|	8787-9935	32	9.7	108	83	-0.370834	0.74475	no

gi|646598842|gb|KK976702.1|	20730-21044	22	8.5	10	7	-0.41788	0.76235	no
gi|646598842|gb|KK976702.1|	21717-22212	22	8.5	1	4	2	0.29735	no
gi|646598847|gb|KK976701.1|	14182-15124	14	8.2	1	1	-0.230313	0.86285	no
gi|646598877|gb|KK976695.1|	1551-1840	15	2.8	0	102	inf	0.0002	yes
gi|646598877|gb|KK976695.1|	3901-5507	15	2.8	38	8	-2	0.10045	no
gi|646598922|gb|KK976686.1|	11748-12297	11	4.6	2	7	2	0.2248	no
gi|646599102|gb|KK976650.1|	4782-5040	7.7	3.3	27	22	-0.322904	0.80235	no
gi|646599163|gb|KK976638.1|	6085-6288	11	5	45	29	-0.668165	0.67415	no
gi|646599163|gb|KK976638.1|	7070-7928	11	5	8	5	-0.684048	0.65645	no
gi|646599182|gb|KK976634.1|	5024-5849	8.2	12	2	1	-0.640089	0.65475	no
gi|646599211|gb|KK976628.1|	9090-9713	8.3	4.4	2	2	-0.380151	0.8011	no
gi|646599336|gb|KK976603.1|	5563-6815	25	9.1	0.742303	5	3	0.099	no
gi|646599479|gb|KK976577.1|	6758-7665	23	13	1	0.80491	-0.665694	0.6592	no
gi|646599518|gb|KK976569.1|	0-324	11	3.2	5	8	0.539424	0.73475	no
gi|646599518|gb|KK976569.1|	7109-7333	11	3.2	59	46	-0.361386	0.7842	no
gi|646599567|gb|KK976560.1|	2486-3243	5.8	2.8	2	0.25877	-3	0.1994	no
gi|646599588|gb|KK976556.1|	3233-3490	11	5.8	25	0	-inf	0.00655	no
gi|646599828|gb|KK976509.1|	0-1071	5.8	5.3	43	32	-0.408389	0.70645	no
gi|646599828|gb|KK976509.1|	12347-13007	5.8	5.3	7	9	0.323314	0.83325	no
gi|646599828|gb|KK976509.1|	14288-14689	5.8	5.3	4	8	0.882534	0.53305	no
gi|646599828|gb|KK976509.1|	20721-21410	5.8	5.3	3	1	-1	0.345	no
gi|646599999|gb|KK976477.1|	15-686	12	6.5	8	5	-0.732141	0.6534	no
gi|646599999|gb|KK976477.1|	6899-7778	12	6.5	2	4	1	0.46745	no
gi|646600026|gb|KK976471.1|	6819-7320	8.1	5.8	3	3	-0.418017	0.76145	no
gi|646600031|gb|KK976470.1|	4509-4688	12	6.9	79.85	108	0.441123	0.7628	no
gi|646600041|gb|KK976468.1|	581-794	16	6.5	1331.79	602	-1	0.36925	no
gi|646600046|gb|KK976467.1|	9223-9605	8.1	3.2	7	0.876032	-3	0.201	no
gi|646600103|gb|KK976456.1|	6885-7509	11	4.4	97	37	-1	0.29475	no
gi|646600133|gb|KK976450.1|	2604-4392	13	4.4	5	5	0.00713102	0.9924	no
gi|646600156|gb|KK976446.1|	11779-12517	13	5.7	1	3	1	0.39835	no

gi|646600156|gb|KK976446.1|	9288-10029	13	5.7	2	3	0.109516	0.93025	no
gi|646600190|gb|KK976440.1|	4708-5307	12	5.8	6	4	-0.537751	0.70425	no
gi|646600226|gb|KK976433.1|	481-1162	9.5	7.6	38	9	-2	0.1927	no
gi|646600282|gb|KK976422.1|	11068-11325	12	6.2	12	16	0.396652	0.8017	no
gi|646600282|gb|KK976422.1|	538-754	12	6.2	98	76	-0.380704	0.7512	no
gi|646600435|gb|KK976393.1|	10535-10825	14	7.7	71	31	-1	0.4582	no
gi|646600485|gb|KK976383.1|	5111-5372	11	6.3	224.76	62	-2	0.24265	no
gi|646600543|gb|KK976371.1|	2851-3726	13	6.3	34	45	0.403601	0.7102	no
gi|646600564|gb|KK976367.1|	3959-4715	18	6.5	2	1	-0.96949	0.54505	no
gi|646600564|gb|KK976367.1|	6-2485	18	6.5	11	7	-0.699953	0.5804	no
gi|646600723|gb|KK976337.1|	2063-2838	14	7.1	2	2	-0.507793	0.7106	no
gi|646600787|gb|KK976323.1|	3087-3443	21	19	40	16	-1	0.40965	no
gi|646600787|gb|KK976323.1|	65-1048	21	19	22	11	-1	0.50305	no
gi|646600830|gb|KK976315.1|	4672-5133	13	5.3	48	26	-0.898571	0.55135	no
gi|646600958|gb|KK976290.1|	13703-13902	12	5.3	0	86	inf	0.0014	yes
gi|646600963|gb|KK976289.1|	13006-14434	22	8.2	13	9	-0.50928	0.7359	no
gi|646601057|gb|KK976270.1|	9291-9561	16	7.4	14	30	1	0.42555	no
gi|646601073|gb|KK976267.1|	0-668	16	8.7	32	55	0.758064	0.5493	no
gi|646601073|gb|KK976267.1|	4909-5136	16	8.7	30	35	0.222184	0.8861	no
gi|646601073|gb|KK976267.1|	5907-6183	16	8.7	74	133	0.852727	0.58065	no
gi|646601408|gb|KK976204.1|	1583-2436	18	10	2	0	-inf	0.0035	no
gi|646601457|gb|KK976194.1|	14917-15488	12	7.9	3	3	-0.0597977	0.9366	no
gi|646601457|gb|KK976194.1|	7308-10190	12	7.9	19	16	-0.289087	0.7958	no
gi|646601475|gb|KK976192.1|	1811-2449	14	6.6	2	3	0.319026	0.82475	no
gi|646601566|gb|KK976173.1|	2773-3073	10	7.8	10	20	1	0.4338	no
gi|646601566|gb|KK976173.1|	4703-5960	10	7.8	9	6	-0.494677	0.74395	no
gi|646601566|gb|KK976173.1|	866-1795	10	7.8	0.960701	2	0.698427	0.629	no
gi|646601609|gb|KK976163.1|	9317-10537	13	6	2	2	0.104683	0.94135	no
gi|646601657|gb|KK976153.1|	115-319	17	9	81	223	1	0.32135	no
gi|646601727|gb|KK976139.1|	205-563	13	5.3	13	18	0.493397	0.72855	no

gi|646601789|gb|KK976126.1|	7312-7682	17	5.1	18	32	0.839901	0.59725	no
gi|646601850|gb|KK976114.1|	2643-2932	7.9	6.6	0	20	inf	0.0014	yes
gi|646602041|gb|KK976077.1|	5153-5623	11	3.3	0	48	inf	0.0002	yes
gi|646602041|gb|KK976077.1|	809-1244	11	3.3	0	48	inf	5.00E-05	yes
gi|646602138|gb|KK976060.1|	16-1175	14	5.1	2	3	0.655037	0.632	no
gi|646602138|gb|KK976060.1|	16689-17851	14	5.1	1	2	0.517648	0.66675	no
gi|646602148|gb|KK976058.1|	6661-7291	12	4.2	3	3	0.0543224	0.94925	no
gi|646602196|gb|KK976050.1|	12179-12774	21	12	1	8	3	0.1688	no
gi|646602206|gb|KK976048.1|	254-530	12	4.9	17	17	0.014008	0.9426	no
gi|646602221|gb|KK976045.1|	166-686	9.5	4.2	8	3	-2	0.34095	no
gi|646602266|gb|KK976036.1|	11690-13164	41	8.5	1	5	2	0.1955	no
gi|646602266|gb|KK976036.1|	13444-14451	41	8.5	2	2	-0.0300073	0.95635	no
gi|646602266|gb|KK976036.1|	190-3707	41	8.5	0.397085	1	1	1	no
gi|646602266|gb|KK976036.1|	3915-4766	41	8.5	0.465597	2	2	0.2898	no
gi|646602266|gb|KK976036.1|	4838-6174	41	8.5	0.342316	1	2	1	no
gi|646602286|gb|KK976032.1|	10702-11490	8.6	6.4	2	0.977369	-0.965771	0.54505	no
gi|646602310|gb|KK976027.1|	5375-6463	8	2.10E+01	77	78	0.0216264	0.98535	no
gi|646602385|gb|KK976012.1|	1881-3370	18	8	55	10	-2	0.08525	no
gi|646602385|gb|KK976012.1|	82-812	18	8	0.194698	5	5	0.11045	no
gi|646602484|gb|KK975993.1|	6219-6515	12	6.8	68	98	0.536787	0.72575	no
gi|646602564|gb|KK975976.1|	38-1744	15	6.6	2	2	0.000114767	0.9752	no
gi|646602612|gb|KK975967.1|	10413-10785	10	6.4	4	5	0.3396	0.823	no
gi|646602612|gb|KK975967.1|	1979-3591	10	6.4	20	17	-0.222689	0.8309	no
gi|646602612|gb|KK975967.1|	4420-4918	10	6.4	293	266	-0.137824	0.90215	no
gi|646602624|gb|KK975965.1|	330-1371	18	7.5	24	15	-0.668221	0.5846	no
gi|646602633|gb|KK975963.1|	65-1130	8.4	6.4	2	2	-0.0714364	0.95135	no
gi|646602664|gb|KK975957.1|	6084-6613	8	3.9	4	0	-inf	0.0041	no
gi|646602668|gb|KK975956.1|	9649-9866	14	8.3	37	79	1	0.49595	no
gi|646602815|gb|KK975927.1|	1610-2050	13	3.6	4	9	1	0.3796	no
gi|646602926|gb|KK975907.1|	0-1028	16	3.6	13	17	0.346626	0.8179	no

gi|646603013|gb|KK975890.1|	4285-4556	5.4	2.8	36	66	0.859701	0.52635	no
gi|646603051|gb|KK975883.1|	7145-9400	17	7.3	4	0.805215	-2	0.10795	no
gi|646603163|gb|KK975864.1|	3440-3818	16	6.4	8	6	-0.431611	0.74265	no
gi|646603188|gb|KK975859.1|	2637-3739	11	4.2	7	7	0.0037079	0.99125	no
gi|646603299|gb|KK975838.1|	0-363	15	9.7	58	33	-0.835627	0.5848	no
gi|646603299|gb|KK975838.1|	2911-4156	15	9.7	3	10	2	0.2557	no
gi|646603348|gb|KK975829.1|	3980-4534	15	7.9	2	1	-0.96539	0.589	no
gi|646603533|gb|KK975791.1|	0-335	45	20	9	8	-0.0696617	0.93635	no
gi|646603533|gb|KK975791.1|	6161-6875	45	20	2	1	-0.51573	0.72175	no
gi|646603586|gb|KK975779.1|	2131-2721	22	9.4	2	2	-0.0254594	0.92345	no
gi|646603624|gb|KK975771.1|	0-421	14	5.3	96	0.706589	-7	0.1668	no
gi|646603624|gb|KK975771.1|	1402-2697	14	5.3	33	0.255472	-7	0.10535	no
gi|646603641|gb|KK975767.1|	8733-9327	9.6	3.4	0	6	inf	0.00435	no
gi|646603647|gb|KK975766.1|	9028-9405	11	3.8	5	5	0.122557	0.92165	no
gi|646603651|gb|KK975765.1|	126-1533	11	5.6	1	1	-0.112433	0.9362	no
gi|646603655|gb|KK975764.1|	408-1193	13	7.1	0.174519	3	4	0.09985	no
gi|646603655|gb|KK975764.1|	8572-9184	13	7.1	7	6	-0.25453	0.87725	no
gi|646603736|gb|KK975745.1|	8718-9680	4	3.2	0	26	inf	0.00015	yes
gi|646603765|gb|KK975738.1|	10394-10945	13	5	1	7	2	0.21395	no
gi|646603806|gb|KK975728.1|	18237-18460	12	6.7	70	113	0.690969	0.6277	no
gi|646603806|gb|KK975728.1|	3497-3696	12	6.7	127	110	-0.205112	0.87205	no
gi|646603925|gb|KK975700.1|	5463-5714	16	3	16	12	-0.46953	0.76265	no
gi|646603925|gb|KK975700.1|	5935-6343	16	3	8	2	-2	0.24295	no
gi|646603995|gb|KK975684.1|	1026-1863	14	4.9	1	1	0.0131027	0.92345	no
gi|646603995|gb|KK975684.1|	4761-5527	14	4.9	0.543005	3	2	0.1544	no
gi|646603995|gb|KK975684.1|	8626-9478	14	4.9	2	2	-0.030653	0.9504	no
gi|646604014|gb|KK975680.1|	4039-4470	14	8.1	33	15	-1	0.45525	no
gi|646604084|gb|KK975665.1|	137-905	8.6	4.1	0.901484	1	0.491392	0.7568	no
gi|646604101|gb|KK975661.1|	1234-1426	16	7.3	31	103	2	0.2634	no
gi|646604101|gb|KK975661.1|	369-1018	16	7.3	2	5	2	0.3569	no

gi|646604101|gb|KK975661.1|	7435-8313	16	7.3	1	0.630808	-0.720371	1	no
gi|646604118|gb|KK975657.1|	11397-12414	64	39	2	4	0.800611	0.5641	no
gi|646604242|gb|KK975629.1|	15018-15628	10	8.1	0	5	inf	0.00605	no
gi|646604242|gb|KK975629.1|	9522-10951	10	8.1	10	4	-1	0.35705	no
gi|646604245|gb|KK975628.1|	3847-4092	17	6.7	12	20	0.680958	0.6605	no
gi|646604300|gb|KK975617.1|	8067-9285	13	4.9	3	2	-0.734103	0.59365	no
gi|646604326|gb|KK975611.1|	4551-5789	17	4.3	19	6	-2	0.2092	no
gi|646604399|gb|KK975595.1|	4-1203	9.9	5.5	0.195993	1	3	0.2821	no
gi|646604476|gb|KK975577.1|	1666-3077	11	6.5	7	7	-0.0352088	0.981	no
gi|646604476|gb|KK975577.1|	3376-4070	11	6.5	8	11	0.441666	0.7762	no
gi|646604476|gb|KK975577.1|	6784-8284	11	6.5	0.89169	1	0.642514	0.60905	no
gi|646604476|gb|KK975577.1|	70-1072	11	6.5	6	5	-0.255251	0.87385	no
gi|646604507|gb|KK975569.1|	3896-4127	12	4.4	32	49	0.630395	0.63055	no
gi|646604540|gb|KK975561.1|	221-643	12	4.5	6	6	-0.0745386	0.9459	no
gi|646604540|gb|KK975561.1|	9800-11119	12	4.5	0.347784	1	2	0.26385	no
gi|646604589|gb|KK975548.1|	6907-7136	20	8.1	243	42	-3	0.0984	no
gi|646604601|gb|KK975545.1|	21089-25298	29	13	9	3	-1	0.26825	no
gi|646604635|gb|KK975538.1|	4365-4718	32	11	2	10	2	0.2774	no
gi|646604649|gb|KK975534.1|	15845-16948	11	4.3	2	0	-inf	0.00525	no
gi|646604653|gb|KK975533.1|	5016-5753	18	6.9	2	0.538023	-2	0.2078	no
gi|646604676|gb|KK975528.1|	7848-8020	11	4.5	146	187	0.36165	0.7954	no
gi|646604757|gb|KK975511.1|	11418-13428	17	8.7	9	3	-2	0.3014	no
gi|646604832|gb|KK975495.1|	68-601	14	6.8	3	0.899449	-2	0.3832	no
gi|646604895|gb|KK975485.1|	20-1788	18	8.3	9	8	-0.146551	0.92235	no
gi|646605106|gb|KK975439.1|	3451-4397	17	5.4	0.937072	2	0.869484	0.5462	no
gi|646605106|gb|KK975439.1|	601-1053	17	5.4	29	13	-1	0.4462	no
gi|646605106|gb|KK975439.1|	9207-10475	17	5.4	5	3	-0.919313	0.5506	no
gi|646605129|gb|KK975434.1|	13191-14147	11	4	3	0.375194	-3	0.1238	no
gi|646605129|gb|KK975434.1|	14253-15215	11	4	2	0.930268	-1	0.3995	no
gi|646605133|gb|KK975433.1|	4145-4647	10	4.5	3	2	-0.376942	0.811	no

gi|646605203|gb|KK975417.1|	5782-6264	38	11	3	5	0.783787	0.5828	no
gi|646605286|gb|KK975398.1|	13800-14604	12	5	6	4	-0.665117	0.6803	no
gi|646605298|gb|KK975395.1|	5-793	48	23	2	0.244342	-3	0.2033	no
gi|646605298|gb|KK975395.1|	884-1531	48	23	2	0.654313	-2	0.3832	no
gi|646605318|gb|KK975390.1|	4451-5853	14	7.3	8	7	-0.0370006	0.9788	no
gi|646605415|gb|KK975369.1|	7305-8210	9.8	3.6	1	0.807304	-0.495915	0.73865	no
gi|646605552|gb|KK975337.1|	4841-5026	15	2.4	0	124.66	inf	0.0014	yes
gi|646605593|gb|KK975327.1|	2910-3615	12	5.8	1	1	0.482919	0.7721	no
gi|646605593|gb|KK975327.1|	3703-3993	12	5.8	32	9	-2	0.21585	no
gi|646605598|gb|KK975326.1|	16239-16472	13	6	0	67	inf	0.00435	no
gi|646605610|gb|KK975323.1|	2035-3145	10	3.7	5	0.464009	-3	0.1028	no
gi|646605682|gb|KK975308.1|	7945-8202	14	4.6	22	8	-1	0.37675	no
gi|646605723|gb|KK975299.1|	5893-6675	40	13	2	1	-0.507001	0.7312	no
gi|646605845|gb|KK975272.1|	282-689	7.9	5.5	8	11	0.47999	0.7037	no
gi|646605845|gb|KK975272.1|	776-1210	7.9	5.5	3	3	-0.191521	0.8939	no
gi|646605903|gb|KK975258.1|	2138-2740	15	5.4	5	0.733328	-3	0.1395	no
gi|646605903|gb|KK975258.1|	5719-6333	15	5.4	2	2	0.465824	0.7494	no
gi|646605935|gb|KK975251.1|	1028-1262	62	2.00E+01	37	66	0.82244	0.58655	no
gi|646605951|gb|KK975248.1|	13272-14116	13	6.4	17	30	0.837328	0.57965	no
gi|646605978|gb|KK975243.1|	11560-12425	13	9.4	3	0	-inf	0.0004	yes
gi|646606001|gb|KK975239.1|	1657-2653	15	5.1	2	0.533301	-2	0.2858	no
gi|646606023|gb|KK975235.1|	783-1026	17	4.6	16	20	0.352256	0.81125	no
gi|646606094|gb|KK975223.1|	0-2117	15	8.6	6	6	-0.0246508	0.987	no
gi|646606162|gb|KK975210.1|	4631-5163	13	7.6	5	12	1	0.34745	no
gi|646606195|gb|KK975204.1|	12430-12855	15	7.5	15	0.692678	-4	0.17945	no
gi|646606295|gb|KK975184.1|	1-1000	160	74	2	0.531214	-2	0.2348	no
gi|646606327|gb|KK975176.1|	4203-4363	13	4.6	351	0	-inf	0.00655	no
gi|646606342|gb|KK975173.1|	8310-8896	20	8.3	0	5	inf	0.00035	yes
gi|646606399|gb|KK975160.1|	3707-4316	12	4.2	1	5	2	0.2139	no
gi|646606453|gb|KK975148.1|	4282-4801	30	13	7	3	-1	0.3349	no

gi|646606468|gb|KK975145.1|	91-1060	12	7.5	0.777337	2	1	0.398	no
gi|646606617|gb|KK975113.1|	248-781	7.8	5.2	2	2	0.442596	0.77485	no
gi|646606663|gb|KK975103.1|	5913-6672	15	3.5	3	2	-0.609225	0.6374	no
gi|646606677|gb|KK975100.1|	8462-8793	9.9	7.5	8	8	-0.127994	0.9215	no
gi|646606867|gb|KK975060.1|	2823-3862	10	5.3	0.94393	2	1	0.3744	no
gi|646606935|gb|KK975044.1|	10125-10870	22	7.6	2	2	-0.404535	0.7698	no
gi|646606940|gb|KK975043.1|	458-1806	19	5.9	0.931035	1	0.649056	0.61505	no
gi|646606940|gb|KK975043.1|	6436-7237	19	5.9	77	66	-0.237723	0.8293	no
gi|646606957|gb|KK975039.1|	10883-11402	6.6	1.7	3	0.471331	-3	0.2136	no
gi|646607047|gb|KK975020.1|	3619-4383	14	4.7	3	3	-0.435083	0.7269	no
gi|646607047|gb|KK975020.1|	7876-8052	14	4.7	124.86	89	-0.481785	0.749	no
gi|646607140|gb|KK975002.1|	5027-5779	85	28	2	0.522515	-2	0.2551	no
gi|646607203|gb|KK974988.1|	11777-12177	16	6.8	2	8	2	0.29255	no
gi|646607203|gb|KK974988.1|	5925-7592	16	6.8	73	46	-0.67692	0.5564	no
gi|646607238|gb|KK974980.1|	14-658	19	8.8	3	3	0.209206	0.87205	no
gi|646607281|gb|KK974971.1|	4269-4601	23	10	7	4	-0.933867	0.5991	no
gi|646607341|gb|KK974959.1|	6833-6960	7.1	2.6	3601.09	0	-inf	0.0041	no
gi|646607662|gb|KK974893.1|	4541-6002	10	7.4	2	1	-0.680232	0.61425	no
gi|646607762|gb|KK974875.1|	11865-12348	12	8.3	8	9	0.173778	0.8978	no
gi|646607810|gb|KK974867.1|	24-351	13	7.8	17.04	14	-0.259549	0.8359	no
gi|646607810|gb|KK974867.1|	3158-3435	13	7.8	7	15	0.993673	0.58275	no
gi|646607866|gb|KK974857.1|	1205-1417	15	4.6	48	59	0.284878	0.83565	no
gi|646607938|gb|KK974844.1|	10744-11927	24	6.3	2	2	-0.0290598	0.9627	no
gi|646607938|gb|KK974844.1|	13674-15258	24	6.3	3	4	0.778613	0.6195	no
gi|646608024|gb|KK974829.1|	3261-4437	11	6.6	66	35	-0.901257	0.43015	no
gi|646608041|gb|KK974826.1|	8781-9772	14	5.8	15	3	-3	0.13725	no
gi|646608128|gb|KK974811.1|	1709-2618	14	6.9	2	2	0.388899	0.75195	no
gi|646608128|gb|KK974811.1|	3362-4688	14	6.9	4	12	1	0.36455	no
gi|646608183|gb|KK974800.1|	7931-9364	15	8.7	0.863865	1	0.388096	0.7705	no
gi|646608201|gb|KK974797.1|	1772-2574	14	4.1	13	13	0.0357109	0.98125	no

gi|646608201|gb|KK974797.1|	41-1665	14	4.1	1	0.682725	-0.75078	0.56025	no
gi|646608316|gb|KK974776.1|	3658-3882	13	8.7	46	0	-inf	0.00655	no
gi|646608327|gb|KK974774.1|	17595-17990	12	6	0.631313	8	4	0.106	no
gi|646608394|gb|KK974762.1|	11699-12707	11	6.2	9	3	-2	0.27105	no
gi|646608394|gb|KK974762.1|	8268-8736	11	6.2	9	3	-1	0.2976	no
gi|646608394|gb|KK974762.1|	8802-9478	11	6.2	2	0.91765	-0.744203	0.6333	no
gi|646608544|gb|KK974734.1|	12598-13331	17	7.4	5	11	1	0.43705	no
gi|646608600|gb|KK974724.1|	6844-7147	13	6.3	10	26	1	0.3792	no
gi|646608662|gb|KK974713.1|	9900-10488	14	8.6	2	2	-0.125517	0.9259	no
gi|646608704|gb|KK974705.1|	7552-7930	13	6.8	6	4	-0.332075	0.81475	no
gi|646608704|gb|KK974705.1|	9083-9346	13	6.8	18	23	0.320535	0.83165	no
gi|646608741|gb|KK974698.1|	12169-12624	12	6.2	6	8	0.406731	0.74225	no
gi|646608825|gb|KK974683.1|	3028-3433	6.5	3.6	28	181	3	0.06735	no
gi|646608919|gb|KK974667.1|	3231-3618	7.2	3.4	3	6	0.840489	0.58765	no
gi|646608993|gb|KK974654.1|	11623-13315	14	5.6	3	7	1	0.389	no
gi|646608993|gb|KK974654.1|	6177-7628	14	5.6	10	6	-0.692833	0.6423	no
gi|646609011|gb|KK974651.1|	9063-9797	21	10	2	1	-0.512906	0.7384	no
gi|646609299|gb|KK974603.1|	8960-10587	10	4.5	4	3	-0.333118	0.8288	no
gi|646609333|gb|KK974597.1|	3674-4129	15	6.4	7	2	-2	0.2365	no
gi|646609351|gb|KK974594.1|	174-592	12	5.8	2	6	1	0.39095	no
gi|646609546|gb|KK974559.1|	9665-10498	13	7.1	20	9	-1	0.4431	no
gi|646609610|gb|KK974548.1|	6584-7045	14	5.6	0.442276	7	4	0.09965	no
gi|646609662|gb|KK974539.1|	1561-1773	31	6.1	42	41	-0.0370499	0.92975	no
gi|646609662|gb|KK974539.1|	2859-3358	31	6.1	4	4	-0.0303253	0.94695	no
gi|646609666|gb|KK974538.1|	2204-2610	9	5.3	0	13	inf	0.00435	no
gi|646609729|gb|KK974527.1|	7673-8099	14	5.8	4	13	2	0.2642	no
gi|646609746|gb|KK974524.1|	5259-6263	18	6.9	1	0.175923	-3	0.19945	no
gi|646610238|gb|KK974438.1|	16983-17393	7.4	5.5	1	7	3	0.2756	no
gi|646610238|gb|KK974438.1|	18535-18763	7.4	5.5	38	26	-0.551383	0.70535	no
gi|646610243|gb|KK974437.1|	10727-11381	11	5.9	0.926498	4	2	0.24305	no

gi|646610287|gb|KK974429.1|	0-181	18	7	149	140.21	-0.084791	0.93995	no
gi|646610429|gb|KK974405.1|	23-1326	11	4.2	2	2	0.600295	0.66245	no
gi|646610429|gb|KK974405.1|	2559-5989	11	4.2	5	2	-1	0.41125	no
gi|646610429|gb|KK974405.1|	6999-8153	11	4.2	1	0.294567	-2	0.25535	no
gi|646610429|gb|KK974405.1|	8270-12434	11	4.2	2	3	0.451259	0.76525	no
gi|646610511|gb|KK974390.1|	0-279	12	5	53	31	-0.760068	0.64575	no
gi|646610511|gb|KK974390.1|	17563-17840	12	5	17	8	-0.983607	0.5573	no
gi|646610579|gb|KK974378.1|	651-1514	14	8.2	1	2	0.790304	0.5526	no
gi|646610627|gb|KK974370.1|	783-976	20	5.2	50	46	-0.141474	0.9047	no
gi|646610743|gb|KK974349.1|	12473-14114	10	3.9	0.800988	0.770838	-0.0553524	1	no
gi|646610743|gb|KK974349.1|	14307-15483	10	3.9	0.502216	2	2	0.2703	no
gi|646610780|gb|KK974342.1|	8-502	16	10	12	1	-3	0.1345	no
gi|646610912|gb|KK974319.1|	832-1558	16	5.2	1	1	-0.321362	0.8509	no
gi|646611045|gb|KK974296.1|	14364-15534	19	8.3	2	1	-0.482051	0.7079	no
gi|646611083|gb|KK974290.1|	4054-7073	19	6.7	2	2	-0.0678131	0.9634	no
gi|646611112|gb|KK974285.1|	11550-12626	19	10	0.788724	2	1	0.44735	no
gi|646611265|gb|KK974258.1|	8857-9761	6.2	5.4	3	0.202127	-4	0.17035	no
gi|646611326|gb|KK974248.1|	4790-5344	17	11	0.616174	5	3	0.2509	no
gi|646611448|gb|KK974227.1|	13620-13915	16	5.4	12	12	0.0328319	0.9333	no
gi|646611448|gb|KK974227.1|	3203-3465	16	5.4	18	13	-0.53022	0.7297	no
gi|646611613|gb|KK974199.1|	3335-3938	19	8.6	5	4	-0.46277	0.7078	no
gi|646611730|gb|KK974178.1|	2-352	12	7.5	3	19	2	0.20795	no
gi|646611730|gb|KK974178.1|	4916-5867	12	7.5	23	4	-2	0.1341	no
gi|646611802|gb|KK974166.1|	6268-6870	12	3.5	6	6	0.0035547	0.97425	no
gi|646611802|gb|KK974166.1|	9232-9937	12	3.5	14	13	-0.0696222	0.9608	no
gi|646611881|gb|KK974153.1|	10233-10462	17	6	78	25	-2	0.22395	no
gi|646611881|gb|KK974153.1|	19564-20717	17	6	18	10	-0.834461	0.58075	no
gi|646611986|gb|KK974135.1|	3276-4199	10	2.5	2	3	0.827297	0.5801	no
gi|646612046|gb|KK974124.1|	16-662	12	7.4	4	4	0.173077	0.8889	no
gi|646612131|gb|KK974110.1|	0-3124	21	9.8	4075.53	2	-11	0.0012	yes

gi|646612216|gb|KK974097.1|	5330-5624	8.3	3.7	33	11	-2	0.29885	no
gi|646612544|gb|KK974041.1|	254-749	7.6	5.1	18	4	-2	0.1465	no
gi|646612667|gb|KK974014.1|	5206-6278	9.4	10	0.452906	1	2	0.3671	no
gi|646612679|gb|KK974012.1|	10692-10961	16	7.5	33	16	-1	0.42525	no
gi|646612679|gb|KK974012.1|	11969-12310	16	7.5	5	6	0.301412	0.85295	no
gi|646612715|gb|KK974005.1|	149-338	13	4.6	102	71	-0.529625	0.7078	no
gi|646612715|gb|KK974005.1|	8441-8823	13	4.6	20	0	-inf	0.0033	no
gi|646612744|gb|KK973999.1|	10851-11159	1.00E+01	7.3	26	3	-3	0.11805	no
gi|646612753|gb|KK973997.1|	10628-11068	37	14	5	10	1	0.4782	no
gi|646612948|gb|KK973963.1|	6934-7283	16	8.5	8	5	-0.535852	0.7233	no
gi|646612948|gb|KK973963.1|	89-1973	16	8.5	3	4	0.166879	0.91095	no
gi|646612979|gb|KK973956.1|	5955-6314	18	9.5	8	8	0.00275492	0.9428	no
gi|646613006|gb|KK973950.1|	7701-8001	12	6.8	209	246	0.235199	0.8467	no
gi|646613077|gb|KK973941.1|	11531-11984	13	4	9	16	0.78406	0.5685	no
gi|646613133|gb|KK973930.1|	1213-2777	16	6.4	0.636074	0.611565	-0.0566898	1	no
gi|646613133|gb|KK973930.1|	2845-3991	16	6.4	0.830596	0.594294	-0.482971	1	no
gi|646613213|gb|KK973912.1|	537-1408	8.3	6.8	0.900969	2	1	0.4498	no
gi|646613218|gb|KK973911.1|	4968-5638	11	3.2	2	2	-0.0374383	0.9367	no
gi|646613218|gb|KK973911.1|	6609-7195	11	3.2	4	4	0.0804062	0.94495	no
gi|646613223|gb|KK973910.1|	14110-14515	9.6	6.5	28	9	-2	0.25395	no
gi|646613244|gb|KK973906.1|	5809-6703	14	6.2	4	0	-inf	0.0042	no
gi|646613249|gb|KK973905.1|	6202-6588	7.6	5.1	5	4	-0.13134	0.9193	no
gi|646613292|gb|KK973896.1|	3-753	16	7.2	7	7	-0.0957728	0.9506	no
gi|646613436|gb|KK973865.1|	6906-7589	21	7.7	185	109	-0.766226	0.49565	no
gi|646613606|gb|KK973831.1|	339-881	8.7	4.1	18	27	0.569105	0.7071	no
gi|646613621|gb|KK973828.1|	11442-11864	19	6.9	0	9	inf	0.0008	yes
gi|646613621|gb|KK973828.1|	7658-8455	19	6.9	6	25	2	0.2082	no
gi|646613853|gb|KK973782.1|	3582-4200	4.9	5	2	0.703153	-2	0.3832	no
gi|646613858|gb|KK973781.1|	4984-5495	17	10	6	0.48462	-4	0.17325	no
gi|646613874|gb|KK973778.1|	6649-8236	20	9.8	13	11	-0.311655	0.83725	no

gi|646613900|gb|KK973773.1|	11143-11885	16	6.9	8	2	-2	0.2013	no
gi|646613900|gb|KK973773.1|	9184-9831	16	6.9	2	1	-0.527166	0.73185	no
gi|646613905|gb|KK973772.1|	13954-14759	20	8.1	1	0.474173	-2	0.38345	no
gi|646613905|gb|KK973772.1|	6418-6828	20	8.1	9	13	0.638581	0.65495	no
gi|646613927|gb|KK973768.1|	694-1457	13	8.4	0.728278	4	2	0.2096	no
gi|646613977|gb|KK973758.1|	4077-4351	17	6.8	16	13	-0.235946	0.8676	no
gi|646613993|gb|KK973755.1|	5462-5930	4.8	3	6	0.570792	-3	0.1786	no
gi|646613997|gb|KK973754.1|	6039-6396	14	4.4	17	13	-0.369626	0.75695	no
gi|646614008|gb|KK973752.1|	4334-6352	20	5.4	15	22	0.594813	0.58915	no
gi|646614008|gb|KK973752.1|	8604-8928	20	5.4	3	11	2	0.2864	no
gi|646614016|gb|KK973750.1|	11019-11570	15	2.5	0.311156	44	7	0.09475	no
gi|646614062|gb|KK973741.1|	9855-10153	15	5.6	17	17	-0.0316681	0.9494	no
gi|646614071|gb|KK973739.1|	6806-7120	8.8	4.2	4	29	3	0.1587	no
gi|646614087|gb|KK973736.1|	11226-11847	12	3.9	7	0	-inf	0.0031	no
gi|646614087|gb|KK973736.1|	12338-13101	12	3.9	65	0.255848	-8	0.1665	no
gi|646614087|gb|KK973736.1|	28-236	12	3.9	1088.54	0	-inf	5.00E-05	yes
gi|646614087|gb|KK973736.1|	4358-5025	12	3.9	35	0	-inf	5.00E-05	yes
gi|646614101|gb|KK973733.1|	4092-5353	5.2	3.8	6	0.131959	-6	0.1685	no
gi|646614133|gb|KK973727.1|	3100-3636	10	6.3	13	17	0.369656	0.8095	no
gi|646614163|gb|KK973721.1|	9451-14271	18	8	13	9	-0.627137	0.57235	no
gi|646614268|gb|KK973701.1|	3345-3726	24	7.5	4	10	1	0.38605	no
gi|646614367|gb|KK973682.1|	2739-3359	16	7.1	17	0	-inf	0.00025	yes
gi|646614367|gb|KK973682.1|	3989-4194	16	7.1	344	0	-inf	0.0005	yes
gi|646614395|gb|KK973677.1|	11741-12680	17	5.9	3	6	0.953914	0.55665	no
gi|646614395|gb|KK973677.1|	75-778	17	5.9	1	1	0.219577	0.89085	no
gi|646614395|gb|KK973677.1|	9002-9877	17	5.9	2	4	1	0.4631	no
gi|646614487|gb|KK973659.1|	19759-21005	16	7.8	13	16	0.314122	0.8346	no
gi|646614498|gb|KK973657.1|	6356-6720	16	7.6	12	12	-0.0844416	0.94745	no
gi|646614518|gb|KK973653.1|	10099-10716	17	4.6	2	3	1	0.4534	no
gi|646614518|gb|KK973653.1|	5184-6039	17	4.6	6	10	0.732781	0.63665	no

gi|646614586|gb|KK973640.1|	6336-7139	11	5.4	3	2	-0.6974	0.60075	no
gi|646614627|gb|KK973632.1|	5030-5150	36	15	4223.38	1090.51	-2	0.28355	no
gi|646614627|gb|KK973632.1|	634-801	36	15	43500.5	13504.9	-2	0.1645	no
gi|646614649|gb|KK973628.1|	2057-2955	15	6.7	6	2	-1	0.2898	no
gi|646614649|gb|KK973628.1|	5010-6110	15	6.7	1	2	0.778155	0.5464	no
gi|646614649|gb|KK973628.1|	900-1336	15	6.7	4	4	-0.190168	0.9009	no
gi|646614736|gb|KK973610.1|	15240-15543	14	6.6	11	14	0.410743	0.7823	no
gi|646614901|gb|KK973578.1|	385-829	16	8.5	33	19	-0.801616	0.59375	no
gi|646615001|gb|KK973558.1|	2765-4377	11	5.5	5	5	0.0625563	0.9658	no
gi|646615001|gb|KK973558.1|	377-602	11	5.5	112	86	-0.374618	0.8131	no
gi|646615001|gb|KK973558.1|	8266-8616	11	5.5	8	9	0.143516	0.9206	no
gi|646615001|gb|KK973558.1|	9587-9849	11	5.5	21	3	-3	0.2001	no
gi|646615031|gb|KK973552.1|	0-425	17	7.6	0	8	inf	0.0014	yes
gi|646615067|gb|KK973545.1|	5469-5980	12	6.2	2	3	0.434187	0.7554	no
gi|646615084|gb|KK973542.1|	7031-7942	15	9.3	17	4	-2	0.20755	no
gi|646615147|gb|KK973530.1|	6216-6635	6.3	6.6	3	4	0.645669	0.6668	no
gi|646615213|gb|KK973517.1|	0-3731	12	11	24	17	-0.538733	0.64155	no
gi|646615265|gb|KK973507.1|	13366-14208	28	11	59	13	-2	0.1127	no
gi|646615283|gb|KK973504.1|	1665-1989	11	6.1	4	12	1	0.36975	no
gi|646615288|gb|KK973503.1|	21058-21640	16	5.7	0.846234	4	2	0.16245	no
gi|646615382|gb|KK973490.1|	1396-4306	19	12	41	23	-0.858358	0.4596	no
gi|646615417|gb|KK973484.1|	44082-45533	22	11	0.386757	0.779666	1	1	no
gi|646615417|gb|KK973484.1|	47315-51787	22	11	9	2	-2	0.165	no
gi|646615678|gb|KK973441.1|	10249-11281	17	3.7	1	0.679046	-0.947539	0.54505	no
gi|646615690|gb|KK973439.1|	7740-7971	16	5.3	40	65	0.723505	0.6337	no
gi|646615719|gb|KK973435.1|	8015-9128	9.3	6.1	2	2	-0.0248877	0.95635	no
gi|646615865|gb|KK973412.1|	11754-12746	13	4.9	6	2	-2	0.27775	no
gi|646615865|gb|KK973412.1|	13205-13824	13	4.9	2	3	0.659603	0.6445	no
gi|646615865|gb|KK973412.1|	15964-16353	13	4.9	11	17	0.591457	0.6639	no
gi|646615865|gb|KK973412.1|	5399-6113	13	4.9	17	11	-0.620067	0.67965	no

gi|646615969|gb|KK973394.1|	13060-13438	18	10	9	15	0.73302	0.62605	no
gi|646615969|gb|KK973394.1|	13634-14412	18	10	2	3	1	0.3975	no
gi|646616002|gb|KK973388.1|	10157-10557	9.8	5.8	7	6	-0.410522	0.7745	no
gi|646616147|gb|KK973363.1|	5586-6335	15	1.90E+01	13	18	0.468001	0.74975	no
gi|646616290|gb|KK973340.1|	11306-11652	4.5	2.7	7	23	2	0.28045	no
gi|646616457|gb|KK973314.1|	10346-10720	15	5.6	44	0	-inf	0.00045	yes
gi|646616457|gb|KK973314.1|	2369-2612	15	5.6	38	23	-0.688386	0.6086	no
gi|646616457|gb|KK973314.1|	3228-3470	15	5.6	6	37	3	0.2684	no
gi|646616457|gb|KK973314.1|	5987-6199	15	5.6	36	117	2	0.30885	no
gi|646616457|gb|KK973314.1|	7295-7668	15	5.6	15	48	2	0.2341	no
gi|646616481|gb|KK973310.1|	434-1087	14	3.4	0.696608	3	2	0.25715	no
gi|646616546|gb|KK973299.1|	27-1755	50	22	2	2	0.476506	0.76635	no
gi|646616630|gb|KK973285.1|	2-719	9.3	3.8	4	28	3	0.05965	no
gi|646616740|gb|KK973267.1|	12800-13719	9.9	7.5	9	5	-0.881934	0.5648	no
gi|646616740|gb|KK973267.1|	3613-4403	9.9	7.5	0.691554	2	1	0.4331	no
gi|646616740|gb|KK973267.1|	5-1103	9.9	7.5	0.21947	1	3	0.2655	no
gi|646616744|gb|KK973266.1|	18388-19153	17	7	1	2	0.853606	0.548	no
gi|646616744|gb|KK973266.1|	9449-9768	17	7	7	17	1	0.37435	no
gi|646616849|gb|KK973248.1|	11432-12393	15	7.2	0.917167	2	0.870439	0.5462	no
gi|646616872|gb|KK973244.1|	1292-2909	14	5.2	1	1	-0.0988219	0.93905	no
gi|646616902|gb|KK973239.1|	10863-11291	16	10	26	17	-0.610738	0.6879	no
gi|646616902|gb|KK973239.1|	2914-3741	16	10	13	4	-2	0.30045	no
gi|646616907|gb|KK973238.1|	10259-11043	8.4	5.1	10	11	0.0700101	0.96395	no
gi|646616907|gb|KK973238.1|	8842-9502	8.4	5.1	10	7	-0.557104	0.72075	no
gi|646616925|gb|KK973235.1|	15948-16762	19	6.5	10	0.700249	-4	0.08805	no
gi|646616995|gb|KK973224.1|	10337-11637	11	5.4	2	3	0.974719	0.48475	no
gi|646617074|gb|KK973211.1|	11215-11437	11	5.5	0	53	inf	0.0014	yes
gi|646617121|gb|KK973203.1|	11095-11505	15	7.6	9	7	-0.302526	0.809	no
gi|646617121|gb|KK973203.1|	3029-4379	15	7.6	2	2	0.145066	0.93	no
gi|646617121|gb|KK973203.1|	7330-8396	15	7.6	2	4	0.706219	0.60905	no

gi|646617183|gb|KK973193.1|	18804-19172	13	6.1	5	6	0.112774	0.9237	no
gi|646617367|gb|KK973162.1|	129-598	21	7	0	81	inf	5.00E-05	yes
gi|646617367|gb|KK973162.1|	6722-6954	21	7	0	84	inf	0.0026	no
gi|646617410|gb|KK973155.1|	6071-6821	20	8.7	3	4	0.769298	0.5881	no
gi|646617450|gb|KK973149.1|	16284-17145	15	9.7	5	3	-0.843324	0.5424	no
gi|646617460|gb|KK973147.1|	2524-3587	33	12	4	3	-0.528389	0.7417	no
gi|646617460|gb|KK973147.1|	9223-10299	33	12	2	0.482715	-2	0.19185	no
gi|646617495|gb|KK973141.1|	13-378	14	6.9	6	2	-2	0.30735	no
gi|646617495|gb|KK973141.1|	1350-2725	14	6.9	3	3	-0.0607445	0.97015	no
gi|646617495|gb|KK973141.1|	5325-5691	14	6.9	5	5	-0.152529	0.91095	no
gi|646617573|gb|KK973129.1|	417-880	16	5.1	13	4	-2	0.28675	no
gi|646617608|gb|KK973123.1|	10421-10673	15	10	5	108	4	0.24035	no
gi|646617671|gb|KK973112.1|	125-1521	9.4	4.7	0.729715	2	2	0.3356	no
gi|646617742|gb|KK973100.1|	15004-15693	14	6.6	5	2	-1	0.3754	no
gi|646617742|gb|KK973100.1|	15861-17030	14	6.6	2	1	-0.552478	0.6574	no
gi|646617747|gb|KK973099.1|	8863-9466	9.7	6	3	1	-1	0.39505	no
gi|646617872|gb|KK973078.1|	13476-13737	18	7.5	35	67	0.938615	0.5074	no
gi|646617872|gb|KK973078.1|	15701-16736	18	7.5	1	2	0.386516	0.76215	no
gi|646617900|gb|KK973073.1|	13123-13882	17	7.9	8	8	-0.00154251	0.98635	no
gi|646618039|gb|KK973049.1|	6549-6965	20	5.8	11	15	0.524715	0.70365	no
gi|646618050|gb|KK973047.1|	3482-3907	8.6	6.2	4	8	1	0.43575	no
gi|646618102|gb|KK973038.1|	5573-6183	14	9.2	3	4	0.201862	0.8752	no
gi|646618102|gb|KK973038.1|	6869-7959	14	9.2	2	4	1	0.4299	no
gi|646618210|gb|KK973020.1|	148-1325	18	6	228	257	0.170228	0.88945	no
gi|646618263|gb|KK973011.1|	631-831	22	9.8	57	38	-0.585291	0.67465	no
gi|646618279|gb|KK973008.1|	2087-2584	15	3.6	0.378823	6	4	0.0994	no
gi|646618438|gb|KK972980.1|	13223-13477	14	7.2	0	31	inf	0.0014	yes
gi|646618519|gb|KK972965.1|	15636-16031	24	10	30	24	-0.364378	0.81025	no
gi|646618519|gb|KK972965.1|	3316-3866	24	10	4	3	-0.774024	0.5577	no
gi|646618532|gb|KK972962.1|	10921-11412	15	4.7	16	7	-1	0.35595	no

gi|646618553|gb|KK972958.1|	38-301	20	8.2	5	28	3	0.2613	no
gi|646618553|gb|KK972958.1|	9452-9869	20	8.2	7	17	1	0.3484	no
gi|646618568|gb|KK972955.1|	4555-5734	4.9	4.1	2	2	0.0487968	0.96295	no
gi|646618599|gb|KK972949.1|	16-370	12	5	10	0	-inf	0.0041	no
gi|646618599|gb|KK972949.1|	1651-2721	12	5	32	0	-inf	5.00E-05	yes
gi|646618599|gb|KK972949.1|	4396-5053	12	5	16	0	-inf	5.00E-05	yes
gi|646618628|gb|KK972945.1|	14619-14909	18	11	2	18	4	0.10695	no
gi|646618664|gb|KK972938.1|	13848-15823	16	8.7	40	28	-0.509507	0.65265	no
gi|646618804|gb|KK972910.1|	8693-9231	14	4.7	21	9	-1	0.43085	no
gi|646618853|gb|KK972901.1|	11597-12024	13	5.3	32	10	-2	0.30645	no
gi|646618857|gb|KK972900.1|	7943-9172	29	15	2	0.408591	-2	0.2263	no
gi|646618968|gb|KK972881.1|	3598-4382	14	8	2	0.984455	-0.676703	0.6592	no
gi|646618968|gb|KK972881.1|	97-1315	14	8	1	0.413152	-1	0.35685	no
gi|646618973|gb|KK972880.1|	17439-18682	14	7.7	0.93633	6	3	0.0845	no
gi|646619042|gb|KK972866.1|	9627-10096	17	8.8	5	6	0.289038	0.82155	no
gi|646619074|gb|KK972860.1|	8230-9729	20	8	1	5	2	0.2264	no
gi|646619266|gb|KK972823.1|	15009-15717	13	8.1	6	3	-1	0.43075	no
gi|646619479|gb|KK972788.1|	10069-11281	23	9.4	0.773324	0.554245	-0.48055	1	no
gi|646619479|gb|KK972788.1|	19130-19442	23	9.4	7	13	0.841765	0.5686	no
gi|646619479|gb|KK972788.1|	3366-3701	23	9.4	7	4	-0.929484	0.5991	no
gi|646619479|gb|KK972788.1|	5336-6657	23	9.4	2	2	0.0533129	0.9623	no
gi|646619520|gb|KK972779.1|	9284-9616	13	3.6	5	9	0.773952	0.6126	no
gi|646619546|gb|KK972774.1|	8067-8293	16	7.6	342	71	-2	0.1728	no
gi|646619546|gb|KK972774.1|	8599-9326	16	7.6	2	2	-0.250833	0.8594	no
gi|646619546|gb|KK972774.1|	9451-11878	16	7.6	8	7	-0.357292	0.81535	no
gi|646619551|gb|KK972773.1|	19-967	8.4	6.5	4	9	1	0.44745	no
gi|646619551|gb|KK972773.1|	6038-6317	8.4	6.5	13	10	-0.294405	0.8508	no
gi|646619561|gb|KK972771.1|	14044-14412	19	9.4	11	0.956735	-4	0.17675	no
gi|646619600|gb|KK972763.1|	1472-3291	7	6.4	3	1	-0.965298	0.5511	no
gi|646619697|gb|KK972742.1|	12581-12991	13	4.8	43	22	-0.946382	0.5377	no

gi|646619834|gb|KK972716.1|	9941-10329	16	9.4	13	3	-2	0.1476	no
gi|646619840|gb|KK972715.1|	28257-28552	15	7.2	12	9	-0.452595	0.76135	no
gi|646619870|gb|KK972711.1|	594-1160	5.5	3.3	4	0.405804	-3	0.18585	no
gi|646619912|gb|KK972703.1|	18645-19582	14	11	5	3	-0.779081	0.6261	no
gi|646619934|gb|KK972699.1|	13378-14702	18	4.5	0.605776	0.74605	0.300488	1	no
gi|646619956|gb|KK972695.1|	11743-12130	9.9	6.4	7	9	0.492566	0.70155	no
gi|646619956|gb|KK972695.1|	12521-13217	9.9	6.4	1	4	2	0.24035	no
gi|646620020|gb|KK972684.1|	4051-4449	13	16	14	2	-3	0.19225	no
gi|646620029|gb|KK972682.1|	1633-1825	12	4.3	73	84	0.214828	0.8852	no
gi|646620101|gb|KK972669.1|	1110-1323	8.4	3.3	12	103	3	0.27655	no
gi|646620105|gb|KK972668.1|	13463-14065	6.2	4.2	2	1	-0.537014	0.73	no
gi|646620105|gb|KK972668.1|	14122-15092	6.2	4.2	1	2	0.508422	0.7139	no
gi|646620133|gb|KK972663.1|	10-1263	11	7.6	2	4	1	0.39295	no
gi|646620257|gb|KK972640.1|	1958-4703	7.4	5.3	5	5	-0.00600977	0.99505	no
gi|646620257|gb|KK972640.1|	9572-10124	7.4	5.3	41	53	0.392869	0.79185	no
gi|646620322|gb|KK972628.1|	10500-11099	12	7	1	2	0.462249	0.7722	no
gi|646620338|gb|KK972625.1|	10445-11527	13	7.8	6	22	2	0.25795	no
gi|646620446|gb|KK972606.1|	8131-9162	12	7.1	33	21	-0.683038	0.59295	no
gi|646620535|gb|KK972589.1|	759-1021	12	6.3	35	31	-0.174076	0.8869	no
gi|646620593|gb|KK972579.1|	4117-4425	12	5.9	19	8	-1	0.3435	no
gi|646620628|gb|KK972573.1|	1977-3226	12	5.7	0.372272	1	2	1	no
gi|646620690|gb|KK972562.1|	11076-12523	18	7.3	9	25	1	0.2787	no
gi|646620690|gb|KK972562.1|	12796-13458	18	7.3	5	10	0.996519	0.54095	no
gi|646620690|gb|KK972562.1|	13874-14069	18	7.3	38	69	0.870807	0.5888	no
gi|646620690|gb|KK972562.1|	6537-7312	18	7.3	106	79	-0.4162	0.71645	no
gi|646620776|gb|KK972546.1|	3023-3236	19	6.8	70	40	-0.80984	0.54325	no
gi|646620776|gb|KK972546.1|	5222-5514	19	6.8	14	13	-0.143154	0.9186	no
gi|646620816|gb|KK972540.1|	16483-16837	18	5.9	12	0	-inf	0.00225	no
gi|646620816|gb|KK972540.1|	17104-18451	18	5.9	1	0.121775	-3	1	no
gi|646620816|gb|KK972540.1|	9514-10794	18	5.9	5	0	-inf	0.00055	yes

gi|646620869|gb|KK972530.1|	11678-12764	14	6.2	1	1	0.192571	0.8968	no
gi|646620869|gb|KK972530.1|	2837-3070	14	6.2	30	51	0.753563	0.566	no
gi|646620912|gb|KK972522.1|	2204-2586	5.8	6.1	1	76	6	0.2341	no
gi|646620928|gb|KK972519.1|	10402-10877	17	5.8	103	190	0.875559	0.41925	no
gi|646620969|gb|KK972512.1|	8646-9714	13	6.6	4	2	-1	0.435	no
gi|646621091|gb|KK972493.1|	16-2171	15	9.7	8	3	-2	0.31165	no
gi|646621167|gb|KK972479.1|	19141-19437	7.4	3.9	10	19	0.879535	0.52485	no
gi|646621201|gb|KK972473.1|	16906-17747	14	6	2	2	0.498891	0.7092	no
gi|646621201|gb|KK972473.1|	5462-5785	14	6	3	13	2	0.2031	no
gi|646621258|gb|KK972462.1|	21-1369	15	5.4	4	3	-0.34523	0.8227	no
gi|646621258|gb|KK972462.1|	5523-5961	15	5.4	8	4	-1	0.41045	no
gi|646621362|gb|KK972444.1|	8900-9843	19	8.3	1	0.76412	-0.493282	1	no
gi|646621443|gb|KK972429.1|	7662-8158	11	4.9	3	1	-2	0.3832	no
gi|646621672|gb|KK972389.1|	139-677	11	9.2	7	6	-0.140609	0.9042	no
gi|646621765|gb|KK972372.1|	8345-8787	18	9.4	18	11	-0.761725	0.6374	no
gi|646621793|gb|KK972367.1|	6321-7148	5.3	3.2	10	8	-0.458003	0.7598	no
gi|646621861|gb|KK972355.1|	2339-2585	17	6.4	78	60.85	-0.353186	0.8262	no
gi|646621861|gb|KK972355.1|	7339-7793	17	6.4	4	5	0.236217	0.86565	no
gi|646621890|gb|KK972350.1|	104-1167	9.5	5.3	3	2	-0.541017	0.6937	no
gi|646621890|gb|KK972350.1|	1347-1827	9.5	5.3	26	36	0.487345	0.7411	no
gi|646621890|gb|KK972350.1|	4492-5114	9.5	5.3	3	1	-0.854302	0.56515	no
gi|646621890|gb|KK972350.1|	5616-5972	9.5	5.3	84	35	-1	0.4182	no
gi|646621897|gb|KK972349.1|	12904-13066	17	8.2	64	296	2	0.2938	no
gi|646621897|gb|KK972349.1|	23419-23657	17	8.2	45	18	-1	0.3725	no
gi|646621903|gb|KK972348.1|	14186-15360	21	7.3	3	5	0.721631	0.6483	no
gi|646621920|gb|KK972344.1|	14833-15198	9.5	4.7	0.77537	11.71	4	0.09975	no
gi|646621937|gb|KK972341.1|	10295-11140	23	9.7	32	39	0.295502	0.77945	no
gi|646621986|gb|KK972332.1|	3274-3838	14	6.1	0	28	inf	5.00E-05	yes
gi|646622004|gb|KK972329.1|	7123-7329	15	8.2	0	433	inf	0.0001	yes
gi|646622068|gb|KK972318.1|	12738-13018	14	10	14	18	0.363265	0.78965	no

gi|646622068|gb|KK972318.1|	17374-17812	14	10	3	12	2	0.26365	no
gi|646622068|gb|KK972318.1|	9807-10427	14	10	2	2	0.467182	0.7494	no
gi|646622118|gb|KK972309.1|	20900-21145	18	8.1	30	39	0.35903	0.78195	no
gi|646622289|gb|KK972279.1|	11448-11661	19	8.6	235	63	-2	0.19155	no
gi|646622289|gb|KK972279.1|	597-841	19	8.6	28	59	1	0.47715	no
gi|646622348|gb|KK972268.1|	0-524	9.9	3.9	3	4	0.26937	0.84125	no
gi|646622448|gb|KK972250.1|	1066-1706	4.3	3	3	2	-0.528564	0.6841	no
gi|646622448|gb|KK972250.1|	3023-3334	4.3	3	12	12	-0.066841	0.9425	no
gi|646622448|gb|KK972250.1|	4053-4913	4.3	3	11	11	0.0232239	0.98715	no
gi|646622586|gb|KK972225.1|	8197-8978	12	6.5	15	2	-3	0.0781	no
gi|646622614|gb|KK972220.1|	7589-7991	13	7.1	10	5	-1	0.42885	no
gi|646622635|gb|KK972216.1|	6124-6617	13	5.5	7	7	-0.0141537	0.9646	no
gi|646622672|gb|KK972209.1|	14023-15883	11	7.1	5	3	-0.726669	0.6392	no
gi|646622672|gb|KK972209.1|	5582-5995	11	7.1	39	15	-1	0.37255	no
gi|646622676|gb|KK972208.1|	9518-12105	11	6.1	1	0.63403	-0.955897	0.49895	no
gi|646622718|gb|KK972200.1|	0-505	18	7.5	11	5	-1	0.46045	no
gi|646622718|gb|KK972200.1|	13459-13836	18	7.5	294	145	-1	0.3571	no
gi|646622718|gb|KK972200.1|	3129-3671	18	7.5	8	3	-1	0.3799	no
gi|646622812|gb|KK972183.1|	13502-14569	11	5.7	13	6	-1	0.4529	no
gi|646622812|gb|KK972183.1|	15596-15961	11	5.7	45	64	0.518164	0.7322	no
gi|646622812|gb|KK972183.1|	9684-10677	11	5.7	0.250688	1	3	0.2821	no
gi|646622836|gb|KK972179.1|	17395-18204	18	10	3	7	1	0.3881	no
gi|646622935|gb|KK972162.1|	78-424	170	62	8	18	1	0.4606	no
gi|646622983|gb|KK972155.1|	11157-12905	15	6.7	3	3	0.22458	0.8881	no
gi|646622983|gb|KK972155.1|	19456-20084	15	6.7	0.495284	3	3	0.26695	no
gi|646623017|gb|KK972149.1|	6058-6687	8.2	3.1	1	2	0.469147	0.7494	no
gi|646623041|gb|KK972145.1|	3820-4412	16	3.9	6	3	-1	0.3915	no
gi|646623046|gb|KK972144.1|	8172-8883	14	8.2	0.202791	18	6	0.0953	no
gi|646623063|gb|KK972141.1|	551-807	16	6.1	23	44	0.960749	0.54465	no
gi|646623218|gb|KK972114.1|	14160-15114	16	8.6	4	0.188117	-4	0.1839	no

gi|646623218|gb|KK972114.1|	2996-3159	16	8.6	366	0	-inf	0.0041	no
gi|646623218|gb|KK972114.1|	7062-8452	16	8.6	5	0	-inf	0.00025	yes
gi|646623274|gb|KK972104.1|	15611-15922	3.5	4.3	7	6	-0.329875	0.8178	no
gi|646623282|gb|KK972103.1|	5-1070	8.5	4.1	1	0	-inf	0.00475	no
gi|646623411|gb|KK972080.1|	2658-3094	9.5	2.3	13	7	-1	0.47485	no
gi|646623459|gb|KK972071.1|	12087-13074	12	4.2	13	0	-inf	0.0001	yes
gi|646623459|gb|KK972071.1|	5321-6057	12	4.2	2	0	-inf	0.00655	no
gi|646623736|gb|KK972021.1|	8287-9147	18	9.6	1	0.648907	-0.914492	0.58965	no
gi|646623805|gb|KK972009.1|	1230-1877	46	35	4	3	-0.357241	0.7824	no
gi|646623902|gb|KK971992.1|	11543-12379	16	5	32	20	-0.66076	0.59625	no
gi|646623902|gb|KK971992.1|	14997-15399	16	5	5	2	-2	0.28785	no
gi|646623902|gb|KK971992.1|	16909-17907	16	5	1	0.354605	-2	0.20925	no
gi|646624014|gb|KK971972.1|	11482-11883	9.3	3.9	0	8	inf	0.00255	no
gi|646624035|gb|KK971968.1|	9368-10243	20	9.4	1	5	2	0.22445	no
gi|646624071|gb|KK971962.1|	6205-6610	12	5.1	26	4	-3	0.113	no
gi|646624076|gb|KK971961.1|	5080-5966	23	6.7	0.146593	6	5	0.0982	no
gi|646624168|gb|KK971945.1|	17731-18802	9.2	5.3	3	5	0.813369	0.60115	no
gi|646624305|gb|KK971921.1|	3262-3557	15	5.6	10	10	0.0030846	0.92625	no
gi|646624360|gb|KK971911.1|	12755-13276	8.9	4.1	5	6	0.116223	0.92335	no
gi|646624360|gb|KK971911.1|	18-1151	8.9	4.1	2	2	0.0570782	0.96765	no
gi|646624360|gb|KK971911.1|	4254-4961	8.9	4.1	0.818324	2	1	0.4862	no
gi|646624454|gb|KK971895.1|	12234-13192	15	6.7	1	2	0.659684	0.62755	no
gi|646624495|gb|KK971888.1|	2-798	17	4.5	0.170972	5	5	0.1055	no
gi|646624495|gb|KK971888.1|	5094-6183	17	4.5	0.221838	6	5	0.23775	no
gi|646624512|gb|KK971885.1|	201-1894	12	5	0.836362	0.836315	-8.18E-05	1	no
gi|646624532|gb|KK971881.1|	76-574	13	6	8	2	-2	0.1476	no
gi|646624588|gb|KK971871.1|	8631-10106	12	4.7	13	0	-inf	5.00E-05	yes
gi|646624630|gb|KK971864.1|	7338-7601	10	4.9	32	13	-1	0.3359	no
gi|646624719|gb|KK971848.1|	12561-15486	210	79	41	12	-2	0.12425	no
gi|646624783|gb|KK971837.1|	5293-5531	17	6.7	24	29	0.264278	0.84735	no

gi|646624790|gb|KK971836.1|	0-528	17	7.5	2	2	0.440786	0.77485	no
gi|646624808|gb|KK971833.1|	10355-11328	11	3.5	0.773008	0.916447	0.245569	1	no
gi|646624865|gb|KK971823.1|	1923-2404	8.2	2.2	0	6	inf	0.0014	yes
gi|646624865|gb|KK971823.1|	302-1868	8.2	2.2	0.282268	63	8	0.09345	no
gi|646624865|gb|KK971823.1|	5078-6305	8.2	2.2	0	5	inf	0.00065	yes
gi|646624906|gb|KK971816.1|	3906-5025	20	8.3	8	10	0.343466	0.82055	no
gi|646625075|gb|KK971788.1|	1183-1825	15	6.8	21	17	-0.298678	0.83915	no
gi|646625075|gb|KK971788.1|	11975-12742	15	6.8	1	1	0.22824	0.87595	no
gi|646625075|gb|KK971788.1|	12799-12982	15	6.8	83	108	0.37697	0.7952	no
gi|646625075|gb|KK971788.1|	2608-3595	15	6.8	9	9	-0.0457838	0.97725	no
gi|646625080|gb|KK971787.1|	11258-12496	10	7.3	1	1	-0.117115	0.9313	no
gi|646625080|gb|KK971787.1|	13539-14455	10	7.3	1	2	0.964309	0.49675	no
gi|646625080|gb|KK971787.1|	21565-22114	10	7.3	3	5	0.448045	0.72545	no
gi|646625080|gb|KK971787.1|	29-643	10	7.3	3	3	0.176317	0.89535	no
gi|646625220|gb|KK971763.1|	16594-17404	16	6.4	2	3	0.736962	0.57435	no
gi|646625220|gb|KK971763.1|	9769-10995	16	6.4	0.762176	0.546433	-0.480079	1	no
gi|646625255|gb|KK971757.1|	974-1231	8.1	4.3	112	27	-2	0.16625	no
gi|646625306|gb|KK971749.1|	7288-8999	14	5.4	1	3	1	0.3469	no
gi|646625412|gb|KK971733.1|	3459-4152	15	3.6	3	3	-0.0494812	0.9504	no
gi|646625471|gb|KK971723.1|	2170-3455	11	4.6	0.359262	1	2	0.2937	no
gi|646625500|gb|KK971718.1|	6-498	13	6.9	5	3	-0.574039	0.6912	no
gi|646625516|gb|KK971715.1|	17499-17826	5.6	3	6.39	9	0.503411	0.7318	no
gi|646625590|gb|KK971703.1|	11799-12195	7.9	5.4	14	2	-3	0.1523	no
gi|646625590|gb|KK971703.1|	8527-9537	7.9	5.4	0	2	inf	0.00255	no
gi|646625646|gb|KK971695.1|	2503-3157	11	6.9	0.463249	7	4	0.2526	no
gi|646625766|gb|KK971676.1|	3117-3621	15	12	4	0	-inf	0.00475	no
gi|646625766|gb|KK971676.1|	7366-8287	15	12	2	0	-inf	0.0041	no
gi|646625799|gb|KK971671.1|	3300-4219	24	17	1	2	0.920127	0.48975	no
gi|646625839|gb|KK971664.1|	18893-19256	7	4.3	8	17	1	0.4729	no
gi|646625839|gb|KK971664.1|	3976-4244	7	4.3	13	9	-0.420998	0.7662	no

gi|646625839|gb|KK971664.1|	5150-5369	7	4.3	260	117	-1	0.46755	no
gi|646625972|gb|KK971643.1|	1429-1688	9.9	6.5	22	5	-2	0.22445	no
gi|646625972|gb|KK971643.1|	6808-7570	9.9	6.5	2	0.76899	-1	0.427	no
gi|646626120|gb|KK971619.1|	4791-5081	12	7.2	3	16	2	0.28335	no
gi|646626120|gb|KK971619.1|	589-793	12	7.2	52	56	0.114843	0.92975	no
gi|646626132|gb|KK971617.1|	11520-12189	20	8.9	0.893125	2	1	0.4335	no
gi|646626132|gb|KK971617.1|	12697-14433	20	8.9	3	0.992927	-2	0.24225	no
gi|646626185|gb|KK971609.1|	18361-19334	14	7.7	5	5	0.0579422	0.97025	no
gi|646626297|gb|KK971592.1|	1-752	19	6.5	6	4	-0.610219	0.65355	no
gi|646626319|gb|KK971588.1|	4233-4621	12	4.9	29	12	-1	0.35125	no
gi|646626336|gb|KK971585.1|	11223-11857	14	6.3	0	24	inf	0.0002	yes
gi|646626538|gb|KK971554.1|	4927-5900	38	14	9	2	-2	0.1861	no
gi|646626538|gb|KK971554.1|	6340-6959	38	14	3	1	-1	0.44495	no
gi|646626538|gb|KK971554.1|	8238-9392	38	14	2	0.736417	-1	0.37145	no
gi|646626570|gb|KK971549.1|	8952-9817	8.5	8.6	0	3	inf	0.00605	no
gi|646626625|gb|KK971540.1|	12891-14106	13	5.1	2	2	0.367549	0.7858	no
gi|646626679|gb|KK971532.1|	1207-2170	11	4.4	2	0.743196	-1	0.45505	no
gi|646626679|gb|KK971532.1|	2365-3525	11	4.4	0.919968	0.438992	-1	1	no
gi|646626912|gb|KK971494.1|	21779-22162	4.8	6.3	66	0	-inf	5.00E-05	yes
gi|646627040|gb|KK971475.1|	6303-7438	19	4.6	0.420488	2	2	0.26385	no
gi|646627048|gb|KK971474.1|	14173-15400	14	4.9	8	5	-0.535899	0.7268	no
gi|646627048|gb|KK971474.1|	5640-5930	14	4.9	14	35	1	0.40235	no
gi|646627115|gb|KK971461.1|	941-1914	17	7.9	0	23	inf	0.00015	yes
gi|646627157|gb|KK971453.1|	4107-4925	15	4.2	101	38	-1	0.20385	no
gi|646627275|gb|KK971431.1|	12464-13097	15	6.2	27	13	-1	0.46515	no
gi|646627275|gb|KK971431.1|	6326-7454	15	6.2	2	0.606241	-2	0.2986	no
gi|646627351|gb|KK971416.1|	15749-16427	17	6.3	4	2	-1	0.36105	no
gi|646627432|gb|KK971401.1|	477-993	22	9.7	25	0.952461	-5	0.10955	no
gi|646627437|gb|KK971400.1|	12133-12837	11	5.6	4	3	-0.145266	0.904	no
gi|646627437|gb|KK971400.1|	307-1622	11	5.6	2	0.877629	-1	0.4142	no

gi|646627554|gb|KK971378.1|	2462-2709	12	5.6	138	114	-0.282046	0.85585	no
gi|646627554|gb|KK971378.1|	28800-29641	12	5.6	3	3	0.318319	0.8254	no
gi|646627554|gb|KK971378.1|	3893-4350	12	5.6	12	5	-1	0.42485	no
gi|646627566|gb|KK971376.1|	20414-20845	16	7.1	4	3	-0.608625	0.6685	no
gi|646627640|gb|KK971362.1|	13773-13992	14	6	20	56	1	0.3577	no
gi|646627640|gb|KK971362.1|	16-1019	14	6	12	4	-1	0.3519	no
gi|646627640|gb|KK971362.1|	2669-3205	14	6	9	3	-2	0.2906	no
gi|646627640|gb|KK971362.1|	4033-4336	14	6	5	11	1	0.4821	no
gi|646627795|gb|KK971333.1|	16190-16713	14	7.6	4	2	-1	0.3935	no
gi|646627891|gb|KK971315.1|	107-365	12	4.5	56	19	-2	0.3251	no
gi|646627891|gb|KK971315.1|	21264-22194	12	4.5	0.685199	1	0.991277	0.5626	no
gi|646627971|gb|KK971300.1|	14530-15186	13	7.6	3	0.640496	-2	0.19405	no
gi|646627971|gb|KK971300.1|	5434-6384	13	7.6	10	9	-0.0480426	0.97595	no
gi|646627971|gb|KK971300.1|	9275-9959	13	7.6	3	5	0.649479	0.6548	no
gi|646627976|gb|KK971299.1|	6173-6616	14	7.3	3	9	2	0.25175	no
gi|646628024|gb|KK971290.1|	4417-5107	29	17	1	1	-0.0048799	0.92345	no
gi|646628024|gb|KK971290.1|	7291-8354	29	17	0.572437	1	1	0.36855	no
gi|646628044|gb|KK971286.1|	16564-18436	14	4.9	0.629359	1	0.980322	0.53675	no
gi|646628075|gb|KK971280.1|	19822-20278	12	4.6	54	32	-0.732614	0.62865	no
gi|646628075|gb|KK971280.1|	24576-24865	12	4.6	97	46	-1	0.49045	no
gi|646628199|gb|KK971257.1|	13888-14412	11	5.4	14	43	2	0.31695	no
gi|646628289|gb|KK971241.1|	13736-14306	10	4	3	1	-1	0.4199	no
gi|646628320|gb|KK971235.1|	18229-18678	15	6.3	5	13	1	0.3707	no
gi|646628320|gb|KK971235.1|	22739-23425	15	6.3	2	4	0.894925	0.4983	no
gi|646628400|gb|KK971220.1|	12239-12799	15	5.9	2	10	2	0.1699	no
gi|646628400|gb|KK971220.1|	7667-7997	15	5.9	5	10	0.963624	0.5533	no
gi|646628400|gb|KK971220.1|	8295-8787	15	5.9	2	3	0.425961	0.75545	no
gi|646628400|gb|KK971220.1|	9020-9968	15	5.9	2	6	1	0.37095	no
gi|646628465|gb|KK971208.1|	27766-28979	11	9.4	4	3	-0.368041	0.8197	no
gi|646628543|gb|KK971193.1|	13570-14552	17	8.3	1	14	4	0.0426	no

gi|646628543|gb|KK971193.1|	6460-7679	17	8.3	1	368	8	0.0099	no
gi|646628611|gb|KK971180.1|	10481-11775	41	14	0.534227	0.767137	0.522034	1	no
gi|646628611|gb|KK971180.1|	16245-18010	41	14	37	47	0.353457	0.75765	no
gi|646628611|gb|KK971180.1|	6801-7890	41	14	0.887351	0.633849	-0.485366	1	no
gi|646628674|gb|KK971168.1|	10075-10328	16	8.4	635	2156.14	2	0.1217	no
gi|646628674|gb|KK971168.1|	11681-12577	16	8.4	6	22	2	0.236	no
gi|646628674|gb|KK971168.1|	4650-5085	16	8.4	2643.58	4684.64	0.825445	0.49245	no
gi|646628674|gb|KK971168.1|	9433-9845	16	8.4	27	101	2	0.2355	no
gi|646628708|gb|KK971161.1|	1378-2531	14	7.2	1	0.884661	-0.598175	0.64385	no
gi|646628748|gb|KK971153.1|	13090-13608	14	7.1	4	0	-inf	0.0041	no
gi|646628754|gb|KK971152.1|	15507-16479	13	7.3	2	2	0.383012	0.76225	no
gi|646628754|gb|KK971152.1|	95-1605	13	7.3	1	0.743654	-0.665407	0.6175	no
gi|646628799|gb|KK971142.1|	4834-5807	16	7.8	5	5	-0.239858	0.8769	no
gi|646628848|gb|KK971130.1|	1231-1683	12	9.9	3	2	-0.817441	0.6134	no
gi|646628848|gb|KK971130.1|	71-1032	12	9.9	0.917167	0.558927	-0.714524	1	no
gi|646629005|gb|KK971093.1|	10759-11145	8.4	5.8	221	50	-2	0.11775	no
gi|646629125|gb|KK971064.1|	0-659	15	5.8	2	3	0.934563	0.49995	no
gi|646629125|gb|KK971064.1|	10022-11389	15	5.8	1	2	0.865053	0.54615	no
gi|646629125|gb|KK971064.1|	11846-12920	15	5.8	1	1	-0.263658	0.8336	no
gi|646629125|gb|KK971064.1|	6298-6683	15	5.8	19	3	-2	0.1669	no
gi|646629144|gb|KK971059.1|	1548-1767	8	6.7	36	25	-0.489954	0.74425	no
gi|646629167|gb|KK971054.1|	7943-9011	14	5.8	0.455133	36	6	0.09345	no
gi|646629171|gb|KK971053.1|	18744-19477	15	7	4	4	0.145921	0.9185	no
gi|646629250|gb|KK971035.1|	0-254	13	7.2	26	17	-0.612281	0.6514	no
gi|646629250|gb|KK971035.1|	4558-6015	13	7.2	1	1	-0.101899	0.93395	no
gi|646629250|gb|KK971035.1|	9165-9383	13	7.2	52	83	0.669064	0.65875	no
gi|646629309|gb|KK971020.1|	13233-13621	12	6.5	5	2	-2	0.3081	no
gi|646629368|gb|KK971007.1|	0-673	72	5.2	3	3	0.214631	0.87115	no
gi|646629368|gb|KK971007.1|	776-1118	72	5.2	20	11	-0.767598	0.5421	no
gi|646629368|gb|KK971007.1|	9133-9374	72	5.2	36	7	-2	0.1776	no

gi|646629384|gb|KK971003.1|	15-593	13	5	1	2	0.719785	0.64505	no
gi|646629384|gb|KK971003.1|	2630-3236	13	5	3	1	-0.857977	0.56515	no
gi|646629384|gb|KK971003.1|	26743-27577	13	5	4	2	-1	0.4551	no
gi|646629384|gb|KK971003.1|	808-2368	13	5	2	1	-0.678252	0.61425	no
gi|646629389|gb|KK971002.1|	2-347	10	6.1	12	0	-inf	0.0035	no
gi|646629468|gb|KK970983.1|	13426-14322	9.1	6.7	24	8	-2	0.281	no
gi|646629468|gb|KK970983.1|	14445-15716	9.1	6.7	8	2	-2	0.20355	no
gi|646629511|gb|KK970973.1|	11028-11326	9.9	4.6	14	22	0.609878	0.6333	no
gi|646629529|gb|KK970969.1|	17844-18230	12	6.9	9	0	-inf	0.0035	no
gi|646629581|gb|KK970957.1|	13322-13825	8.2	7.1	8	10	0.293339	0.86005	no
gi|646629581|gb|KK970957.1|	8658-9564	8.2	7.1	3	3	-0.318964	0.8191	no
gi|646629600|gb|KK970953.1|	15994-16965	14	7.2	0.775166	1	0.508483	1	no
gi|646629757|gb|KK970922.1|	1149-2086	9.9	3.7	0	3	inf	0.00605	no
gi|646629811|gb|KK970912.1|	28552-28758	11	7.6	35	93	1	0.3322	no
gi|646629859|gb|KK970903.1|	13204-14385	16	6.5	48	4	-3	0.02805	no
gi|646629859|gb|KK970903.1|	7469-8715	16	6.5	2	3	0.318939	0.8468	no
gi|646629934|gb|KK970889.1|	20107-21765	15	5.4	3	4	0.74139	0.63345	no
gi|646629945|gb|KK970887.1|	18447-18655	12	5	40	26	-0.641867	0.6774	no
gi|646630123|gb|KK970854.1|	13121-13617	21	12	6	7	0.23513	0.84515	no
gi|646630227|gb|KK970834.1|	5210-5783	13	7.6	8	44	2	0.14715	no
gi|646630297|gb|KK970821.1|	4277-4491	9.7	5	378	107	-2	0.25825	no
gi|646630315|gb|KK970818.1|	335-1291	13	5.8	1	0.937984	-0.170517	1	no
gi|646630315|gb|KK970818.1|	8956-9570	13	5.8	2	0.710463	-2	0.3832	no
gi|646630346|gb|KK970812.1|	5017-8885	12	7	1	1	0.0582737	0.9689	no
gi|646630346|gb|KK970812.1|	9599-10887	12	7	0.537329	1	1	0.33975	no
gi|646630362|gb|KK970809.1|	4346-5074	12	7.2	2	10	2	0.14565	no
gi|646630492|gb|KK970785.1|	5761-6687	12	6.3	2	2	-0.494424	0.70515	no
gi|646630508|gb|KK970782.1|	15951-16536	12	5.4	44	37	-0.245216	0.8704	no
gi|646630529|gb|KK970778.1|	9163-9405	14	5.1	6	41	3	0.25855	no
gi|646630554|gb|KK970773.1|	127-873	20	8.7	2	3	0.225644	0.8573	no

gi|646630554|gb|KK970773.1|	2401-3254	20	8.7	2	3	0.307303	0.7997	no
gi|646630564|gb|KK970771.1|	6763-7061	13	6.4	59	218	2	0.23705	no
gi|646630564|gb|KK970771.1|	9043-9438	13	6.4	16	55	2	0.2639	no
gi|646630576|gb|KK970769.1|	15562-16076	16	9.2	58	13	-2	0.1938	no
gi|646630597|gb|KK970765.1|	11459-13032	15	7.3	5	0.607458	-3	0.068	no
gi|646630597|gb|KK970765.1|	18600-19525	15	7.3	2	0.195996	-4	0.17455	no
gi|646630597|gb|KK970765.1|	3182-3943	15	7.3	39	0	-inf	5.00E-05	yes
gi|646630597|gb|KK970765.1|	6255-7128	15	7.3	2	0	-inf	0.0035	no
gi|646630607|gb|KK970763.1|	9267-10534	13	5.2	2	6	2	0.33395	no
gi|646630612|gb|KK970762.1|	14069-14333	17	7.5	63	17	-2	0.23645	no
gi|646630612|gb|KK970762.1|	17740-17952	17	7.5	174.3	0	-inf	0.0033	no
gi|646630623|gb|KK970760.1|	10500-10880	10	5.1	0	9	inf	0.00255	no
gi|646630771|gb|KK970735.1|	0-295	7.6	3.3	24	19	-0.315091	0.8006	no
gi|646630804|gb|KK970729.1|	7879-8268	4.2	2	5	5	-0.0580454	0.9298	no
gi|646630816|gb|KK970727.1|	11819-13097	16	7	0.45215	0.908692	1	1	no
gi|646630825|gb|KK970725.1|	59-1211	22	6.2	1	0.885623	-0.705129	0.5887	no
gi|646630852|gb|KK970720.1|	10739-11128	17	5.1	41	0	-inf	0.0004	yes
gi|646630852|gb|KK970720.1|	12669-12826	17	5.1	284	425	0.582409	0.68675	no
gi|646630852|gb|KK970720.1|	13637-15248	17	5.1	0.682256	2	2	0.27745	no
gi|646630852|gb|KK970720.1|	8609-9683	17	5.1	34	14	-1	0.3211	no
gi|646631050|gb|KK970683.1|	5597-5919	15	6.6	42	38	-0.167325	0.9183	no
gi|646631050|gb|KK970683.1|	56-461	15	6.6	12	5	-1	0.28245	no
gi|646631075|gb|KK970678.1|	20038-20398	13	6.1	7	4	-0.844041	0.58245	no
gi|646631075|gb|KK970678.1|	20485-21141	13	6.1	3	3	-0.162861	0.90235	no
gi|646631075|gb|KK970678.1|	8974-10786	13	6.1	6	4	-0.453092	0.76535	no
gi|646631099|gb|KK970674.1|	807-1078	11	3.6	6	16	1	0.3892	no
gi|646631138|gb|KK970666.1|	2540-3059	8	5.4	2	2	0.437376	0.77485	no
gi|646631177|gb|KK970659.1|	14994-15594	34	6	0.8026	3	2	0.29365	no
gi|646631248|gb|KK970646.1|	0-1100	37	16	22	13	-0.775175	0.5408	no
gi|646631248|gb|KK970646.1|	8508-9698	37	16	0.791516	0.425236	-0.896357	1	no

gi|646631303|gb|KK970636.1|	1086-1515	31	4.6	8	0.67928	-4	0.17465	no
gi|646631337|gb|KK970630.1|	11320-12046	12	6.1	2	0	-inf	0.0041	no
gi|646631337|gb|KK970630.1|	5377-6027	12	6.1	2	0	-inf	0.00655	no
gi|646631467|gb|KK970607.1|	3316-4261	13	5	2	2	-0.0781135	0.94535	no
gi|646631592|gb|KK970586.1|	9942-10674	13	4.5	3	3	0.371345	0.7632	no
gi|646631611|gb|KK970583.1|	357-915	8.9	5.7	119	14	-3	0.03965	no
gi|646631711|gb|KK970566.1|	9514-10702	14	7.3	0.495758	2	2	0.2411	no
gi|646631778|gb|KK970553.1|	34-347	10	3.9	219	117	-0.903384	0.5493	no
gi|646631811|gb|KK970547.1|	1456-1747	11	6.7	156	227	0.539286	0.71965	no
gi|646631866|gb|KK970537.1|	273-550	11	5.6	26	0	-inf	0.00225	no
gi|646632080|gb|KK970498.1|	13968-14247	14	7	11	60	2	0.1513	no
gi|646632080|gb|KK970498.1|	16766-17072	14	7	7	14	1	0.4522	no
gi|646632165|gb|KK970487.1|	11058-11455	12	6.3	4	6	0.58681	0.6634	no
gi|646632198|gb|KK970481.1|	14949-15313	13	6.5	5	4	-0.476759	0.76275	no
gi|646632198|gb|KK970481.1|	7299-7688	13	6.5	7	7	0.0350641	0.9428	no
gi|646632332|gb|KK970458.1|	11521-11873	9.5	4.2	39	7	-2	0.12995	no
gi|646632429|gb|KK970439.1|	6312-7283	14	6.9	3	4	0.569883	0.7228	no
gi|646632429|gb|KK970439.1|	7834-8485	14	6.9	0.933469	3	1	0.3719	no
gi|646632465|gb|KK970433.1|	6665-7744	15	6.5	2	3	0.592183	0.66255	no
gi|646632634|gb|KK970403.1|	9316-9521	11	6.4	1269.29	1500.37	0.241288	0.84885	no
gi|646632634|gb|KK970403.1|	9876-10173	11	6.4	15	44	2	0.28425	no
gi|646632662|gb|KK970398.1|	7635-8053	11	6.3	35	19	-0.91769	0.5475	no
gi|646632734|gb|KK970386.1|	309-1764	13	5.8	0.84803	1	0.767097	0.5448	no
gi|646632826|gb|KK970371.1|	11615-12671	16	8.5	3	0.49444	-3	0.16605	no
gi|646632826|gb|KK970371.1|	5096-6228	16	8.5	6	2	-1	0.38455	no
gi|646632902|gb|KK970358.1|	5538-6045	14	6.5	0	19	inf	0.00055	yes
gi|646632908|gb|KK970357.1|	12058-12275	10	4.9	27	74	1	0.3244	no
gi|646632954|gb|KK970349.1|	41532-43000	24	9.7	1	0.878785	-0.721558	0.57085	no
gi|646632999|gb|KK970342.1|	17117-17940	20	3.8	1	3	1	0.3622	no
gi|646632999|gb|KK970342.1|	9490-9696	20	3.8	98	40	-1	0.3455	no

gi|646633006|gb|KK970341.1|	6759-7325	16	6.2	302	137	-1	0.32125	no
gi|646633200|gb|KK970306.1|	6162-6543	7.4	5	0	39	inf	0.0002	yes
gi|646633249|gb|KK970298.1|	167-2012	12	5	3	3	-0.248828	0.86935	no
gi|646633249|gb|KK970298.1|	27076-27494	12	5	10	16	0.657505	0.63035	no
gi|646633249|gb|KK970298.1|	29927-30346	12	5	15	7	-1	0.45045	no
gi|646633322|gb|KK970285.1|	2494-3745	15	9.5	6	1	-2	0.1696	no
gi|646633353|gb|KK970280.1|	563-1350	14	7.7	2	4	1	0.4787	no
gi|646633353|gb|KK970280.1|	9287-9975	14	7.7	3	0.893583	-2	0.24535	no
gi|646633358|gb|KK970279.1|	3004-5171	11	5.8	2	0.771693	-1	0.3942	no
gi|646633448|gb|KK970264.1|	14188-14909	16	9.6	11	23	1	0.4848	no
gi|646633448|gb|KK970264.1|	1500-2354	16	9.6	2	2	-0.307321	0.81215	no
gi|646633448|gb|KK970264.1|	15873-17058	16	9.6	14	10	-0.565157	0.7052	no
gi|646633476|gb|KK970259.1|	2868-3094	11	5.5	22	27	0.288483	0.84635	no
gi|646633504|gb|KK970254.1|	2263-3056	12	6.4	2	5	1	0.47145	no
gi|646633515|gb|KK970252.1|	49-323	12	10	0	37	inf	0.00435	no
gi|646633543|gb|KK970247.1|	1103-1524	9	4.4	22	8	-1	0.31835	no
gi|646633550|gb|KK970246.1|	2524-5593	15	7.3	2	1	-0.37307	0.8118	no
gi|646633563|gb|KK970244.1|	10788-12474	15	6.2	12	10	-0.263223	0.86485	no
gi|646633563|gb|KK970244.1|	6052-7313	15	6.2	3	9	2	0.3301	no
gi|646633568|gb|KK970243.1|	10859-11309	24	8.1	3	4	0.403744	0.7964	no
gi|646633581|gb|KK970241.1|	7508-7876	13	5.8	3	9	2	0.35885	no
gi|646633632|gb|KK970232.1|	13222-13579	12	7.7	8	2	-2	0.26015	no
gi|646633746|gb|KK970212.1|	17401-17671	20	6.7	178	101	-0.814401	0.5952	no
gi|646633887|gb|KK970188.1|	31972-32870	16	11	0.575431	1	1	0.43305	no
gi|646633903|gb|KK970185.1|	4525-5198	7.3	1.9	0	5	inf	0.00605	no
gi|646633915|gb|KK970183.1|	185-1262	16	5.8	1	4	2	0.2361	no
gi|646634014|gb|KK970166.1|	32757-33215	17	7.2	5	0	-inf	0.0041	no
gi|646634029|gb|KK970163.1|	13960-15507	12	8.1	2	1	-0.57158	0.6774	no
gi|646634029|gb|KK970163.1|	16019-16550	12	8.1	2	2	0.178844	0.89965	no
gi|646634045|gb|KK970160.1|	61-452	12	5.1	6	2	-2	0.2745	no

gi|646634064|gb|KK970156.1|	11025-11412	16	6.8	7	3	-0.966866	0.5492	no
gi|646634064|gb|KK970156.1|	6242-7830	16	6.8	0.694026	0.901103	0.376703	1	no
gi|646634113|gb|KK970147.1|	16544-18856	14	5	2	0.065262	-5	0.17125	no
gi|646634113|gb|KK970147.1|	19942-22984	14	5	5	1	-2	0.2398	no
gi|646634113|gb|KK970147.1|	26173-26559	14	5	7	0	-inf	0.00655	no
gi|646634113|gb|KK970147.1|	2627-3652	14	5	0.720816	1	0.511528	1	no
gi|646634113|gb|KK970147.1|	3779-5501	14	5	2	2	-0.0821102	0.95865	no
gi|646634113|gb|KK970147.1|	7466-11807	14	5	5	2	-1	0.2632	no
gi|646634137|gb|KK970143.1|	11555-12693	15	6.7	3	0.449659	-3	0.1433	no
gi|646634137|gb|KK970143.1|	3062-3934	15	6.7	2	0	-inf	0.00475	no
gi|646634148|gb|KK970141.1|	669-1479	17	8.8	1	3	1	0.3153	no
gi|646634267|gb|KK970120.1|	4130-4445	14	5	11	6	-0.908059	0.559	no
gi|646634267|gb|KK970120.1|	4933-5247	14	5	18	30	0.745618	0.59825	no
gi|646634301|gb|KK970114.1|	7443-7874	12	4.9	12	16	0.452776	0.7743	no
gi|646634307|gb|KK970113.1|	12073-12492	11	5.7	11	1	-3	0.1216	no
gi|646634307|gb|KK970113.1|	5461-5817	11	5.7	26	13	-0.932736	0.49735	no
gi|646634346|gb|KK970106.1|	8449-8633	26	12	80	58	-0.464015	0.7636	no
gi|646634362|gb|KK970104.1|	10388-11458	14	8.6	3	6	0.785064	0.61805	no
gi|646634362|gb|KK970104.1|	21386-22509	14	8.6	3	0.609645	-2	0.2071	no
gi|646634362|gb|KK970104.1|	3047-3412	14	8.6	5	4	-0.475604	0.76275	no
gi|646634362|gb|KK970104.1|	32390-32616	14	8.6	105	49	-1	0.43925	no
gi|646634362|gb|KK970104.1|	4165-5028	14	8.6	2	2	0.375267	0.76805	no
gi|646634362|gb|KK970104.1|	653-2230	14	8.6	17	9	-0.923698	0.4656	no
gi|646634397|gb|KK970098.1|	4548-4834	18	10	0	40	inf	0.0026	no
gi|646634403|gb|KK970097.1|	533-1373	8.7	5.7	12	14	0.172301	0.9057	no
gi|646634445|gb|KK970090.1|	14056-15338	10	4.4	0.630546	0.517273	-0.285674	1	no
gi|646634463|gb|KK970087.1|	14295-15106	9.9	4.1	3	0.234627	-4	0.1757	no
gi|646634595|gb|KK970064.1|	14068-14553	32	12	6	3	-1	0.4352	no
gi|646634595|gb|KK970064.1|	19037-20464	32	12	85	0.113619	-10	0.1665	no
gi|646634604|gb|KK970062.1|	9766-10124	24	11	4	7	0.808899	0.5898	no

gi|646634653|gb|KK970054.1|	5459-5836	17	8.1	9	22	1	0.36025	no
gi|646634772|gb|KK970032.1|	1498-2229	19	8.1	2	0.816728	-0.928352	0.58905	no
gi|646634895|gb|KK970011.1|	10939-12146	18	7	6	8	0.593279	0.6926	no
gi|646634895|gb|KK970011.1|	13217-13711	18	7	19	12	-0.664274	0.6732	no
gi|646634895|gb|KK970011.1|	70-961	18	7	3	4	0.358647	0.79395	no
gi|646635006|gb|KK969992.1|	3413-3996	11	6.8	2	2	-0.126846	0.9025	no
gi|646635006|gb|KK969992.1|	7021-7345	11	6.8	206	591	2	0.26065	no
gi|646635073|gb|KK969980.1|	182-1346	12	5.6	33	16	-1	0.4253	no
gi|646635233|gb|KK969954.1|	4377-4909	10	6.4	7	5	-0.695265	0.63425	no
gi|646635247|gb|KK969952.1|	17872-18977	13	5.8	38	19	-1	0.4448	no
gi|646635247|gb|KK969952.1|	9387-9763	13	5.8	50	15	-2	0.29275	no
gi|646635292|gb|KK969944.1|	21187-21833	15	6.8	0.23633	4	4	0.0994	no
gi|646635297|gb|KK969943.1|	12399-12735	5.1	4	0	39	inf	0.00085	yes
gi|646635385|gb|KK969928.1|	11273-12394	17	8.7	1	1	-0.184423	0.89	no
gi|646635385|gb|KK969928.1|	27893-29682	17	8.7	36	19	-0.93123	0.3962	no
gi|646635385|gb|KK969928.1|	3528-5089	17	8.7	12	9	-0.397782	0.79065	no
gi|646635385|gb|KK969928.1|	800-1599	17	8.7	3	8	1	0.3646	no
gi|646635389|gb|KK969927.1|	2166-6483	200	53	2	0.963532	-0.882746	0.56335	no
gi|646635389|gb|KK969927.1|	360-2026	200	53	1	1	0.249905	0.8327	no
gi|646635389|gb|KK969927.1|	7766-9131	200	53	5	5	-0.228107	0.88135	no
gi|646635481|gb|KK969911.1|	5790-7172	13	6.1	0.985038	0.708403	-0.475608	1	no
gi|646635623|gb|KK969888.1|	15101-16040	11	6.8	1	0.384223	-2	0.26845	no
gi|646635623|gb|KK969888.1|	19042-19326	11	6.8	9	18	1	0.45825	no
gi|646635630|gb|KK969887.1|	7649-8719	9.2	7.2	0.908034	0.648231	-0.486238	1	no
gi|646635648|gb|KK969884.1|	14059-14319	21	9.4	19	5	-2	0.2833	no
gi|646635648|gb|KK969884.1|	17050-17890	21	9.4	0.948698	1	0.498801	0.73295	no
gi|646635648|gb|KK969884.1|	21630-22510	21	9.4	15	4	-2	0.22435	no
gi|646635648|gb|KK969884.1|	27894-29884	21	9.4	3	5	0.906162	0.5585	no
gi|646635648|gb|KK969884.1|	30151-31679	21	9.4	1	4	2	0.18705	no
gi|646635671|gb|KK969880.1|	3893-4384	12	5.7	26	30	0.239087	0.87075	no

gi|646635695|gb|KK969876.1|	11129-11992	10	5.4	2	2	0.152874	0.8971	no
gi|646635695|gb|KK969876.1|	13418-14078	10	5.4	21	20	-0.125075	0.93475	no
gi|646635695|gb|KK969876.1|	6064-7292	10	5.4	4	3	-0.544143	0.727	no
gi|646635695|gb|KK969876.1|	7417-7927	10	5.4	5	2	-1	0.38765	no
gi|646635719|gb|KK969872.1|	12364-12822	21	7.3	36	41	0.177149	0.90565	no
gi|646635790|gb|KK969861.1|	15243-15708	14	4.4	10	6	-0.789229	0.58605	no
gi|646635882|gb|KK969844.1|	12973-13429	11	5.8	7	33	2	0.1258	no
gi|646635886|gb|KK969843.1|	1153-1362	12	4.3	39	37	-0.0518506	0.91805	no
gi|646635886|gb|KK969843.1|	13430-14201	12	4.3	19	4	-2	0.19575	no
gi|646635920|gb|KK969837.1|	1288-1638	17	7.5	39	99	1	0.38715	no
gi|646635920|gb|KK969837.1|	12956-13916	17	7.5	2	5	1	0.42035	no
gi|646635920|gb|KK969837.1|	5040-5668	17	7.5	3	3	-0.231507	0.8577	no
gi|646635920|gb|KK969837.1|	9216-10265	17	7.5	2	4	0.94885	0.4973	no
gi|646635925|gb|KK969836.1|	20694-21404	18	11	0.406471	3	3	0.2662	no
gi|646635954|gb|KK969831.1|	2730-3613	6.2	7.6	5	3	-1	0.4558	no
gi|646635972|gb|KK969828.1|	6678-6996	12	5.9	118	39	-2	0.3092	no
gi|646635999|gb|KK969823.1|	1715-2020	12	6.7	0	19	inf	0.001	yes
gi|646636015|gb|KK969821.1|	7218-7462	14	8.5	19	20	0.0926273	0.9177	no
gi|646636070|gb|KK969811.1|	14343-15637	37	14	8	3	-1	0.41975	no
gi|646636070|gb|KK969811.1|	17679-18761	37	14	3	2	-0.980447	0.4947	no
gi|646636070|gb|KK969811.1|	18844-19492	37	14	2	0.979123	-1	0.4735	no
gi|646636070|gb|KK969811.1|	19931-20164	37	14	76	8	-3	0.11515	no
gi|646636070|gb|KK969811.1|	23205-23686	37	14	3	2	-0.801667	0.6307	no
gi|646636070|gb|KK969811.1|	568-1690	37	14	1	1	0.101021	0.94	no
gi|646636076|gb|KK969810.1|	17148-17391	17	6.7	25	10	-1	0.40655	no
gi|646636076|gb|KK969810.1|	25180-26270	17	6.7	1	2	1	0.41785	no
gi|646636136|gb|KK969800.1|	159-591	10	6.8	12	8	-0.607931	0.65895	no
gi|646636136|gb|KK969800.1|	757-1170	10	6.8	22	31	0.484878	0.746	no
gi|646636154|gb|KK969797.1|	349-719	10	3.8	23	25	0.138092	0.9281	no
gi|646636172|gb|KK969794.1|	10418-10586	12	4.9	172	98	-0.808368	0.5925	no

gi|646636172|gb|KK969794.1|	14021-14226	12	4.9	36	82	1	0.40985	no
gi|646636172|gb|KK969794.1|	20540-20769	12	4.9	111	135.25	0.282687	0.85895	no
gi|646636361|gb|KK969762.1|	24476-24671	16	7	28	130	2	0.24125	no
gi|646636417|gb|KK969752.1|	2610-3526	52	39	0.97959	1	0.504878	0.7286	no
gi|646636455|gb|KK969745.1|	26297-26998	11	5.5	13	6	-1	0.49085	no
gi|646636455|gb|KK969745.1|	27059-27994	11	5.5	6	4	-0.588969	0.70755	no
gi|646636674|gb|KK969707.1|	3869-4101	10	5.6	62	12	-2	0.15325	no
gi|646636776|gb|KK969690.1|	384-968	17	6.5	2	7	2	0.2914	no
gi|646636814|gb|KK969683.1|	5744-6937	9.4	5.7	7	3	-1	0.362	no
gi|646636838|gb|KK969679.1|	6530-7833	13	6.9	0.176546	18	7	0.2322	no
gi|646637092|gb|KK969637.1|	14452-15169	16	6	2	3	0.806637	0.56885	no
gi|646637092|gb|KK969637.1|	27274-27873	16	6	1	2	0.462249	0.7722	no
gi|646637092|gb|KK969637.1|	6123-7549	16	6	0.553018	2	2	0.2429	no
gi|646637092|gb|KK969637.1|	7759-9534	16	6	1	1	0.371152	0.7861	no
gi|646637117|gb|KK969633.1|	10685-11408	10	5.5	2	1	-0.836358	0.57665	no
gi|646637134|gb|KK969630.1|	8995-9557	14	8.8	1053.05	297	-2	0.1328	no
gi|646637207|gb|KK969616.1|	12711-12900	8.9	2.8	91	91	0.00287054	0.94165	no
gi|646637207|gb|KK969616.1|	13639-14294	8.9	2.8	1	3	1	0.45285	no
gi|646637273|gb|KK969605.1|	41-629	9	6	7	6	-0.240994	0.8831	no
gi|646637273|gb|KK969605.1|	5664-5837	9	6	600	378	-0.665875	0.67285	no
gi|646637273|gb|KK969605.1|	6740-6927	9	6	73	268	2	0.2651	no
gi|646637299|gb|KK969601.1|	13153-13622	12	6.4	5	9	0.862028	0.5716	no
gi|646637299|gb|KK969601.1|	358-522	12	6.4	263	91	-2	0.33945	no
gi|646637299|gb|KK969601.1|	3659-4355	12	6.4	3	0.29274	-3	0.1923	no
gi|646637299|gb|KK969601.1|	581-1133	12	6.4	4	0	-inf	0.0035	no
gi|646637365|gb|KK969590.1|	15661-15932	16	5.4	4	23	2	0.27475	no
gi|646637383|gb|KK969587.1|	10367-10735	9.5	9.1	49	46	-0.0798712	0.95455	no
gi|646637389|gb|KK969586.1|	14154-15965	19	8.9	1	9	3	0.05715	no
gi|646637389|gb|KK969586.1|	418-1138	19	8.9	0.596627	3	2	0.1624	no
gi|646637412|gb|KK969582.1|	19438-19961	11	5.2	2	5	1	0.4188	no

gi|646637538|gb|KK969561.1|	13817-14970	14	8.1	283	157.47	-0.844066	0.48615	no
gi|646637538|gb|KK969561.1|	16-841	14	8.1	10	8	-0.309941	0.8296	no
gi|646637538|gb|KK969561.1|	5219-6102	14	8.1	7	8	0.068014	0.96375	no
gi|646637574|gb|KK969555.1|	12175-13016	13	6.1	2	0.89223	-0.823037	0.57835	no
gi|646637586|gb|KK969553.1|	1089-1925	12	7.4	3	1	-0.916599	0.49815	no
gi|646637599|gb|KK969551.1|	20352-21011	16	7.2	2	3	0.837701	0.55995	no
gi|646637599|gb|KK969551.1|	21235-21941	16	7.2	0.6151	2	2	0.25705	no
gi|646637643|gb|KK969544.1|	10128-10443	19	10	207	0	-inf	5.00E-05	yes
gi|646637643|gb|KK969544.1|	105-1277	19	10	26	0	-inf	5.00E-05	yes
gi|646637643|gb|KK969544.1|	8984-9769	19	10	10	1	-3	0.08215	no
gi|646637675|gb|KK969539.1|	35-1196	21	10	0.918953	0.877037	-0.0673528	1	no
gi|646637675|gb|KK969539.1|	7165-7771	21	10	3	2	-0.536049	0.6957	no
gi|646637681|gb|KK969538.1|	13904-14206	11	5	19	3	-3	0.1554	no
gi|646637681|gb|KK969538.1|	14942-15545	11	5	6	7	0.261596	0.8742	no
gi|646637681|gb|KK969538.1|	6881-8586	11	5	1	5	2	0.25945	no
gi|646637681|gb|KK969538.1|	889-1497	11	5	2	1	-0.535574	0.73	no
gi|646637693|gb|KK969536.1|	7368-8158	9.3	4.4	9	4	-1	0.4901	no
gi|646637722|gb|KK969531.1|	16665-17036	9.5	4.8	9	0.938312	-3	0.1889	no
gi|646637722|gb|KK969531.1|	17198-17994	9.5	4.8	5	4	-0.225374	0.88855	no
gi|646637738|gb|KK969528.1|	17948-18208	14	5.1	21	20.99	-0.0276747	0.9426	no
gi|646637745|gb|KK969527.1|	3509-3700	13	6.2	214	154	-0.476021	0.73025	no
gi|646637755|gb|KK969525.1|	85-423	12	8	14	9	-0.609678	0.63405	no
gi|646637784|gb|KK969520.1|	1205-2831	15	8.9	1	6	2	0.1307	no
gi|646637784|gb|KK969520.1|	21818-23812	15	8.9	0.212804	2	3	0.12765	no
gi|646637784|gb|KK969520.1|	24083-25029	15	8.9	2	7	2	0.20295	no
gi|646637784|gb|KK969520.1|	3117-4272	15	8.9	11	19	0.745332	0.62225	no
gi|646637784|gb|KK969520.1|	33644-37431	15	8.9	0.419122	4	3	0.03375	no
gi|646637784|gb|KK969520.1|	40207-42702	15	8.9	0.991271	6	3	0.1265	no
gi|646637784|gb|KK969520.1|	47299-47824	15	8.9	23	30	0.39595	0.7885	no
gi|646637784|gb|KK969520.1|	51235-52801	15	8.9	1	8	3	0.07995	no

gi|646637784|gb|KK969520.1|	9188-9692	15	8.9	3	2	-0.56873	0.72945	no
gi|646637802|gb|KK969517.1|	12393-12751	13	5.8	57	35	-0.718348	0.63465	no
gi|646637802|gb|KK969517.1|	19972-20821	13	5.8	5	22	2	0.19145	no
gi|646637815|gb|KK969515.1|	16796-18557	17	7.5	7	5	-0.377153	0.80055	no
gi|646637815|gb|KK969515.1|	18675-20232	17	7.5	2	1	-0.793785	0.5833	no
gi|646637815|gb|KK969515.1|	25042-26451	17	7.5	0.080166	2	5	0.11005	no
gi|646637815|gb|KK969515.1|	3883-4706	17	7.5	14	7	-0.94572	0.53605	no
gi|646637839|gb|KK969511.1|	1482-1640	8.9	4	0	291	inf	0.00255	no
gi|646637839|gb|KK969511.1|	3647-4284	8.9	4	0	3	inf	0.00255	no
gi|646637839|gb|KK969511.1|	8464-9057	8.9	4	0	12	inf	0.00085	yes
gi|646637858|gb|KK969508.1|	11211-12237	12	6.9	1	2	0.890092	0.4908	no
gi|646637903|gb|KK969500.1|	12887-13896	10	5.3	1	0.349581	-2	0.20925	no
gi|646637998|gb|KK969490.1|	14404-15580	37	13	0.703103	1	1	0.44735	no
gi|646638027|gb|KK969485.1|	20825-22042	9.8	5.1	3	0.689287	-2	0.2101	no
gi|646638077|gb|KK969477.1|	5664-6015	15	7.1	85	31	-1	0.3492	no
gi|646638095|gb|KK969474.1|	12274-13956	22	1.40E+01	0.388989	0.749033	0.945301	1	no
gi|646638095|gb|KK969474.1|	17576-17907	22	1.40E+01	17	10	-0.800419	0.53485	no
gi|646638095|gb|KK969474.1|	19141-20651	22	1.40E+01	12	3	-2	0.17425	no
gi|646638095|gb|KK969474.1|	6904-7425	22	1.40E+01	53	16	-2	0.26905	no
gi|646638095|gb|KK969474.1|	7642-7928	22	1.40E+01	35	44	0.33801	0.80305	no
gi|646638125|gb|KK969469.1|	13207-13995	19	5.4	0	2	inf	0.00255	no
gi|646638138|gb|KK969467.1|	8316-8741	12	6.3	7	0	-inf	0.0035	no
gi|646638166|gb|KK969462.1|	18299-18584	14	7	22	33	0.591608	0.6768	no
gi|646638166|gb|KK969462.1|	2841-3550	14	7	23	13	-0.851458	0.57145	no
gi|646638166|gb|KK969462.1|	9994-10633	14	7	12	9	-0.393837	0.80525	no
gi|646638210|gb|KK969455.1|	15167-15747	14	6.8	9	4	-1	0.3877	no
gi|646638210|gb|KK969455.1|	3154-4717	14	6.8	5	4	-0.2743	0.85285	no
gi|646638210|gb|KK969455.1|	9120-10180	14	6.8	2	3	0.435285	0.7503	no
gi|646638224|gb|KK969453.1|	829-1928	17	7.4	2	2	0.000504102	0.96475	no
gi|646638282|gb|KK969443.1|	0-530	17	5.6	0.334446	5	4	0.0994	no

gi|646638312|gb|KK969438.1|	11294-11939	13	6.6	28	36	0.39649	0.78895	no
gi|646638312|gb|KK969438.1|	12381-13593	13	6.6	13	8	-0.706618	0.64055	no
gi|646638312|gb|KK969438.1|	14686-16003	13	6.6	4	10	1	0.4034	no
gi|646638312|gb|KK969438.1|	20875-21065	13	6.6	55	49	-0.160531	0.90205	no
gi|646638377|gb|KK969427.1|	0-442	16	6.8	62	0	-inf	0.0001	yes
gi|646638417|gb|KK969421.1|	5818-6371	13	6.5	3	0	-inf	0.00475	no
gi|646638436|gb|KK969418.1|	11972-12174	9.1	3.4	70	95	0.441837	0.73385	no
gi|646638436|gb|KK969418.1|	17689-19027	9.1	3.4	3	2	-0.891777	0.5849	no
gi|646638446|gb|KK969416.1|	770-988	9.4	5.4	31	36	0.213384	0.8816	no
gi|646638481|gb|KK969410.1|	9006-10072	20	6.4	2	0.814178	-1	0.43875	no
gi|646638563|gb|KK969396.1|	14535-14759	18	5.9	101	28	-2	0.25015	no
gi|646638573|gb|KK969394.1|	18973-19155	14	8.5	0	198	inf	0.00515	no
gi|646638608|gb|KK969388.1|	5084-5777	9.9	5.5	12	7	-0.741359	0.6331	no
gi|646638623|gb|KK969385.1|	29-387	14	6.2	366	109	-2	0.19915	no
gi|646638716|gb|KK969366.1|	18190-18795	16	7.3	1	2	0.726746	0.64505	no
gi|646638716|gb|KK969366.1|	23240-24244	16	7.3	0.741021	0.879617	0.247361	1	no
gi|646638725|gb|KK969364.1|	11916-12512	17	4.2	0	75	inf	5.00E-05	yes
gi|646638725|gb|KK969364.1|	13009-13560	17	4.2	0	7	inf	0.00435	no
gi|646638749|gb|KK969359.1|	6799-6951	15	6.4	421	153	-1	0.3565	no
gi|646638827|gb|KK969343.1|	10460-11330	9.9	4.9	2	0.425817	-2	0.26845	no
gi|646638827|gb|KK969343.1|	2701-4123	9.9	4.9	3	3	-0.0416927	0.97795	no
gi|646638827|gb|KK969343.1|	789-1739	9.9	4.9	6	3	-1	0.4342	no
gi|646638842|gb|KK969340.1|	5541-6451	11	7.6	2	0.801345	-1	0.41185	no
gi|646638949|gb|KK969320.1|	2463-3053	14	5.8	2	2	-0.0254594	0.92345	no
gi|646638980|gb|KK969315.1|	16364-17684	22	9.3	1	1	-0.307304	0.8119	no
gi|646638980|gb|KK969315.1|	17895-20909	22	9.3	0.60361	0.878225	0.540975	1	no
gi|646638980|gb|KK969315.1|	29542-30105	22	9.3	25	11	-1	0.43575	no
gi|646638980|gb|KK969315.1|	42552-43006	22	9.3	10	7	-0.526744	0.6634	no
gi|646638980|gb|KK969315.1|	5163-6748	22	9.3	0.417355	0.903102	1	1	no
gi|646639008|gb|KK969309.1|	10099-10918	19	7.3	0	3	inf	0.0008	yes

gi|646639008|gb|KK969309.1|	13841-14161	19	7.3	2	15	3	0.25775	no
gi|646639031|gb|KK969304.1|	35-274	9.6	5.5	20	18	-0.187813	0.8995	no
gi|646639079|gb|KK969294.1|	10921-11606	12	8.4	15	9	-0.791581	0.6002	no
gi|646639079|gb|KK969294.1|	15174-16957	12	8.4	2	0.262589	-3	0.13745	no
gi|646639079|gb|KK969294.1|	18176-19219	12	8.4	2	2	-0.639551	0.61155	no
gi|646639086|gb|KK969293.1|	17925-19167	29	12	13	1	-3	0.04015	no
gi|646639086|gb|KK969293.1|	2155-2597	29	12	8	1	-3	0.14	no
gi|646639086|gb|KK969293.1|	40956-42958	29	12	3	2	-0.465682	0.76735	no
gi|646639086|gb|KK969293.1|	44886-45858	29	12	1	1	0.338618	0.80425	no
gi|646639086|gb|KK969293.1|	7540-8724	29	12	0.497892	0.855835	0.781499	1	no
gi|646639098|gb|KK969291.1|	10063-10304	14	7.4	114	0	-inf	0.00185	no
gi|646639098|gb|KK969291.1|	8534-9166	14	7.4	232.05	263	0.178752	0.88005	no
gi|646639169|gb|KK969278.1|	12856-13129	20	7.8	14	33	1	0.43665	no
gi|646639169|gb|KK969278.1|	3856-4665	20	7.8	8	15	0.910891	0.55855	no
gi|646639169|gb|KK969278.1|	6251-6529	20	7.8	33	52	0.662612	0.62805	no
gi|646639169|gb|KK969278.1|	7612-7821	20	7.8	39	25	-0.636813	0.67815	no
gi|646639223|gb|KK969267.1|	18828-21056	17	3.7	0.093784	25	8	0.2322	no
gi|646639223|gb|KK969267.1|	22296-23086	17	3.7	0.172889	56	8	0.09445	no
gi|646639223|gb|KK969267.1|	3199-3689	17	3.7	0	30	inf	0.00035	yes
gi|646639223|gb|KK969267.1|	5875-7070	17	3.7	0.098413	14	7	0.0948	no
gi|646639223|gb|KK969267.1|	8152-11863	17	3.7	0.026771	31.25	10	0.09445	no
gi|646639242|gb|KK969263.1|	13095-13673	10	5.4	2	2	-0.350604	0.81835	no
gi|646639260|gb|KK969259.1|	13829-14163	9.5	4.8	12	5	-1	0.39165	no
gi|646639445|gb|KK969222.1|	6891-7931	14	6.5	0.824887	1	0.512301	0.7286	no
gi|646639445|gb|KK969222.1|	9260-10472	14	6.5	0.86999	2	1	0.3867	no
gi|646639516|gb|KK969207.1|	8921-10190	14	8.4	0.821093	1	0.673293	0.62555	no
gi|646639545|gb|KK969201.1|	13903-14265	20	8.3	16	18	0.176255	0.89445	no
gi|646639585|gb|KK969193.1|	10753-12484	6.7	4.1	5	1	-2	0.1473	no
gi|646639585|gb|KK969193.1|	13751-14300	6.7	4.1	60	46	-0.380344	0.80175	no
gi|646639603|gb|KK969189.1|	72-900	12	7	2	0.683783	-2	0.2858	no

gi|646639603|gb|KK969189.1|	9538-10727	12	7	0.990455	0	-inf	1	no
gi|646639628|gb|KK969184.1|	6627-7919	11	6.3	6	4	-0.64795	0.67275	no
gi|646639647|gb|KK969180.1|	21236-21921	14	5	11	3	-2	0.2308	no
gi|646639658|gb|KK969178.1|	10064-10585	9.8	4.4	8	4	-0.915486	0.534	no
gi|646639658|gb|KK969178.1|	8462-9169	9.8	4.4	8	1	-2	0.14725	no
gi|646639735|gb|KK969162.1|	9725-10026	8.5	3.6	15	7	-1	0.42735	no
gi|646639798|gb|KK969148.1|	11789-12271	11	5.7	7	3	-1	0.41745	no
gi|646639844|gb|KK969138.1|	8860-9197	17	6.6	3	21	3	0.1643	no
gi|646639848|gb|KK969137.1|	26158-26894	9.9	4.9	1	0.808635	-0.735029	0.66415	no
gi|646639892|gb|KK969127.1|	2764-3203	2.20E+01	9.5	7	5	-0.510102	0.69735	no
gi|646639892|gb|KK969127.1|	461-1699	2.20E+01	9.5	2	4	0.868238	0.59475	no
gi|646639892|gb|KK969127.1|	7495-8525	2.20E+01	9.5	4	4	-0.00278395	0.982	no
gi|646639892|gb|KK969127.1|	8634-9384	2.20E+01	9.5	0.934223	3	2	0.2739	no
gi|646639944|gb|KK969115.1|	1-345	16	4.2	5	7	0.568549	0.73385	no
gi|646639944|gb|KK969115.1|	3343-5008	16	4.2	2	8	2	0.2863	no
gi|646639944|gb|KK969115.1|	5828-6471	16	4.2	1	6	2	0.26445	no
gi|646639952|gb|KK969113.1|	12359-13541	18	7.8	3	2	-0.432699	0.79425	no
gi|646640006|gb|KK969101.1|	2703-2886	12	7	69	72	0.0550419	0.91215	no
gi|646640006|gb|KK969101.1|	8789-9390	12	7	27	22	-0.312826	0.8411	no
gi|646640055|gb|KK969089.1|	0-433	14	7.1	4	8	0.97772	0.49265	no
gi|646640055|gb|KK969089.1|	4895-5824	14	7.1	23	13	-0.811833	0.5913	no
gi|646640139|gb|KK969071.1|	26327-26573	16	7.2	39	13	-2	0.2649	no
gi|646640139|gb|KK969071.1|	29696-29906	16	7.2	138.88	98	-0.506291	0.76065	no
gi|646640172|gb|KK969064.1|	161-437	16	5.4	38	21	-0.816067	0.5112	no
gi|646640172|gb|KK969064.1|	8607-9259	16	5.4	4	2	-0.966766	0.46735	no
gi|646640202|gb|KK969057.1|	15671-16429	19	9.6	3	0.516557	-3	0.1518	no
gi|646640202|gb|KK969057.1|	1990-3074	19	9.6	1	0.956357	-0.360061	0.79735	no
gi|646640202|gb|KK969057.1|	3238-4935	19	9.6	0.256629	0.648691	1	1	no
gi|646640202|gb|KK969057.1|	32671-33478	19	9.6	1	1	0.0102253	0.92345	no
gi|646640202|gb|KK969057.1|	33737-35074	19	9.6	0.940498	1	0.38573	0.77315	no

gi|646640202|gb|KK969057.1|	37250-37595	19	9.6	5	12	1	0.3367	no
gi|646640202|gb|KK969057.1|	38352-39104	19	9.6	1	5	2	0.27565	no
gi|646640202|gb|KK969057.1|	41528-42284	19	9.6	14	21	0.562091	0.7067	no
gi|646640224|gb|KK969052.1|	6470-6686	12	5.2	49	188.9	2	0.21425	no
gi|646640224|gb|KK969052.1|	7821-8608	12	5.2	92	187	1	0.3785	no
gi|646640301|gb|KK969034.1|	18426-19207	12	7.1	135	108	-0.317288	0.7821	no
gi|646640353|gb|KK969022.1|	11559-12053	12	4.3	21	0.515452	-5	0.17055	no
gi|646640370|gb|KK969019.1|	14168-14955	7.5	3.5	1	571	9	0.02865	no
gi|646640375|gb|KK969018.1|	34184-36016	16	5.2	29	7	-2	0.1433	no
gi|646640397|gb|KK969013.1|	19799-20158	15	6.8	42	19	-1	0.4799	no
gi|646640448|gb|KK969002.1|	19822-20043	8	5.7	1717.71	563	-2	0.2281	no
gi|646640461|gb|KK968999.1|	28046-28796	19	8.2	0	3	inf	0.00255	no
gi|646640461|gb|KK968999.1|	8670-8900	19	8.2	8	37	2	0.30495	no
gi|646640478|gb|KK968995.1|	17495-17681	9.1	5	100	66	-0.602222	0.6544	no
gi|646640500|gb|KK968990.1|	10584-11710	32	13	5	2	-1	0.49385	no
gi|646640500|gb|KK968990.1|	14226-15257	32	13	3	2	-1	0.4659	no
gi|646640500|gb|KK968990.1|	2608-3065	32	13	9	10	0.15997	0.90875	no
gi|646640500|gb|KK968990.1|	8125-9179	32	13	0	2	inf	0.00605	no
gi|646640511|gb|KK968988.1|	11683-11928	21	10	24	26	0.0959952	0.93635	no
gi|646640511|gb|KK968988.1|	28905-29641	21	10	2	2	0.317438	0.8299	no
gi|646640511|gb|KK968988.1|	358-1267	21	10	0.565756	1	1	0.43305	no
gi|646640511|gb|KK968988.1|	35926-36632	21	10	3	5	0.675717	0.63345	no
gi|646640516|gb|KK968987.1|	6623-7793	7.5	3.6	0.606611	1	1	0.44445	no
gi|646640530|gb|KK968984.1|	10-946	13	7.8	6	4	-0.726406	0.6495	no
gi|646640530|gb|KK968984.1|	1880-2296	13	7.8	19	19	-0.0485186	0.97575	no
gi|646640557|gb|KK968978.1|	676-992	10	6.7	4	11	2	0.2865	no
gi|646640601|gb|KK968968.1|	19445-20099	17	8.3	3	5	0.474189	0.7437	no
gi|646640601|gb|KK968968.1|	25014-25762	17	8.3	4	2	-1	0.418	no
gi|646640601|gb|KK968968.1|	3455-4026	17	8.3	2	3	0.26213	0.863	no
gi|646640621|gb|KK968963.1|	24825-27148	13	4.3	27	22	-0.242559	0.8308	no

gi|646640621|gb|KK968963.1|	28531-29314	13	4.3	64	53	-0.283932	0.79275	no
gi|646640641|gb|KK968958.1|	27494-28056	15	6.5	4	1	-2	0.2829	no
gi|646640641|gb|KK968958.1|	28231-28514	15	6.5	14	28	1	0.4285	no
gi|646640662|gb|KK968952.1|	4094-4478	12	5.6	22	20	-0.168721	0.91545	no
gi|646640693|gb|KK968945.1|	540-872	11	4.2	40	11	-2	0.2206	no
gi|646640734|gb|KK968935.1|	2383-3023	16	7.1	3	0	-inf	0.00475	no
gi|646640777|gb|KK968925.1|	0-281	13	5.5	19	20	0.0581307	0.949	no
gi|646640786|gb|KK968923.1|	13546-14486	28	14	2	2	-0.0239938	0.9504	no
gi|646640817|gb|KK968917.1|	13835-14827	40	110	0.62757	0.893517	0.509719	1	no
gi|646641057|gb|KK968875.1|	17086-17788	14	6.8	2	2	-0.254509	0.8594	no
gi|646641057|gb|KK968875.1|	7319-7621	14	6.8	32	6	-2	0.19915	no
gi|646641106|gb|KK968866.1|	9935-10284	49	19	5	10	0.897107	0.54375	no
gi|646641145|gb|KK968859.1|	24345-26173	16	7.6	0.588147	0.595425	0.0177437	1	no
gi|646641171|gb|KK968854.1|	16132-17346	13	5.8	5	6	0.458117	0.7678	no
gi|646641193|gb|KK968850.1|	119-1501	13	7.4	0	4	inf	0.001	yes
gi|646641299|gb|KK968830.1|	15574-15979	11	7.1	4	3	-0.213567	0.88655	no
gi|646641326|gb|KK968825.1|	7943-9370	23	9.4	25	6	-2	0.1167	no
gi|646641434|gb|KK968806.1|	8803-9165	3.9	4.4	0	22	inf	0.0023	no
gi|646641492|gb|KK968795.1|	22334-23151	18	4.9	2	3	0.371114	0.769	no
gi|646641492|gb|KK968795.1|	9227-10316	18	4.9	2	4	1	0.3678	no
gi|646641564|gb|KK968782.1|	12778-13169	14	5.6	5	7	0.358892	0.80195	no
gi|646641564|gb|KK968782.1|	22046-22953	14	5.6	26	42	0.687095	0.5904	no
gi|646641564|gb|KK968782.1|	7018-7418	14	5.6	6	8	0.367085	0.78015	no
gi|646641591|gb|KK968777.1|	19682-19981	10	7.1	7	10	0.496326	0.74255	no
gi|646641591|gb|KK968777.1|	33-1084	10	7.1	2	0.16582	-3	0.18	no
gi|646641667|gb|KK968763.1|	17876-18152	8.3	4.6	40	0	-inf	0.0004	yes
gi|646641667|gb|KK968763.1|	19598-19803	8.3	4.6	108	61	-0.809431	0.5286	no
gi|646641740|gb|KK968750.1|	15229-15605	18	9.2	80	38	-1	0.47235	no
gi|646641740|gb|KK968750.1|	18066-18330	18	9.2	72	57	-0.3231	0.83455	no
gi|646641750|gb|KK968748.1|	16710-17189	12	6.4	6	3	-0.902195	0.5046	no

gi|646641864|gb|KK968726.1|	8857-9695	12	5.6	2	21	3	0.0396	no
gi|646641869|gb|KK968725.1|	24145-24581	17	7.8	6	5	-0.49829	0.70845	no
gi|646641932|gb|KK968713.1|	15663-16352	12	6	33	0	-inf	5.00E-05	yes
gi|646641932|gb|KK968713.1|	7598-8513	12	6	11	0	-inf	0.00065	yes
gi|646641979|gb|KK968707.1|	4294-4584	16	4.1	11	13	0.215279	0.88515	no
gi|646641993|gb|KK968704.1|	12534-14259	11	7.5	2	1	-1	0.4013	no
gi|646641993|gb|KK968704.1|	15175-15831	11	7.5	3	2	-0.525432	0.6841	no
gi|646641993|gb|KK968704.1|	17924-19785	11	7.5	2	1	-0.715198	0.655	no
gi|646642001|gb|KK968703.1|	5376-5557	11	6.2	59	178	2	0.28215	no
gi|646642018|gb|KK968700.1|	18239-18738	11	5.2	25	15	-0.779002	0.60315	no
gi|646642085|gb|KK968688.1|	7485-8438	9.7	5.4	1	0.753512	-0.662561	0.6593	no
gi|646642130|gb|KK968679.1|	22085-22288	12	6.3	53	578	3	0.0561	no
gi|646642141|gb|KK968677.1|	3841-4434	5.3	3.5	0	16	inf	0.0002	yes
gi|646642164|gb|KK968673.1|	10000-10609	36	19	6	7	0.127627	0.93665	no
gi|646642271|gb|KK968653.1|	10780-11045	98	29	13	22	0.741001	0.5966	no
gi|646642363|gb|KK968637.1|	13901-14498	20	12	5	3	-0.455786	0.72015	no
gi|646642448|gb|KK968622.1|	14698-16347	27	23	1	0.670674	-0.984832	0.4647	no
gi|646642490|gb|KK968614.1|	7439-8222	13	5.7	4	3	-0.506889	0.7216	no
gi|646642495|gb|KK968613.1|	17935-18933	13	6.1	37	59	0.685427	0.52525	no
gi|646642526|gb|KK968608.1|	21648-21992	13	6.5	5	10	0.890477	0.5478	no
gi|646642526|gb|KK968608.1|	6373-6593	13	6.5	30	45	0.584869	0.7117	no
gi|646642546|gb|KK968604.1|	1920-2144	15	6.9	0	51	inf	0.0014	yes
gi|646642546|gb|KK968604.1|	5673-6134	15	6.9	3	6	0.924755	0.52385	no
gi|646642621|gb|KK968590.1|	13488-14398	9.9	4.7	1	2	0.674373	0.6345	no
gi|646642621|gb|KK968590.1|	23142-23609	9.9	4.7	52	24	-1	0.46055	no
gi|646642621|gb|KK968590.1|	26567-27118	9.9	4.7	16	25.48	0.711726	0.6337	no
gi|646642666|gb|KK968582.1|	10258-10673	18	7.2	16	0.728482	-4	0.17945	no
gi|646642666|gb|KK968582.1|	23366-23936	18	7.2	17	2	-3	0.08	no
gi|646642757|gb|KK968565.1|	29144-29362	11	6.6	501	425	-0.236419	0.8801	no
gi|646642796|gb|KK968558.1|	905-1659	21	7.6	3	2	-0.247273	0.8538	no

gi|646642819|gb|KK968554.1|	19006-19343	13	5.9	0.972567	11	3	0.1075	no
gi|646642930|gb|KK968534.1|	18940-19220	13	5.9	653	274	-1	0.33875	no
gi|646642976|gb|KK968526.1|	11834-12686	9.5	8	4	4	0.103933	0.9467	no
gi|646643170|gb|KK968493.1|	18409-18835	11	3	5	6	0.0658992	0.9425	no
gi|646643201|gb|KK968488.1|	7224-7363	15	6.1	1746.08	0	-inf	0.0011	yes
gi|646643201|gb|KK968488.1|	7719-8234	15	6.1	1	5	2	0.2467	no
gi|646643217|gb|KK968485.1|	16761-17701	17	5.7	9	8	-0.135927	0.92535	no
gi|646643283|gb|KK968474.1|	10366-11158	12	8.3	5	4	-0.412798	0.7989	no
gi|646643289|gb|KK968473.1|	1432-2545	27	13	7	0.308285	-4	0.1119	no
gi|646643311|gb|KK968469.1|	21585-21989	10	9.4	7	2	-2	0.2135	no
gi|646643330|gb|KK968466.1|	3232-4082	23	9.1	164	178	0.120765	0.91935	no
gi|646643344|gb|KK968464.1|	5341-6243	12	6.4	1	0.608192	-1	0.4271	no
gi|646643359|gb|KK968461.1|	290-2264	13	3.6	0.484379	0.701362	0.534023	1	no
gi|646643495|gb|KK968437.1|	17418-17605	7.2	4.5	146	21	-3	0.14745	no
gi|646643549|gb|KK968430.1|	7261-8719	19	9.5	0.615211	0.885956	0.526154	1	no
gi|646643564|gb|KK968428.1|	10720-12607	14	6.2	2	2	0.533043	0.73835	no
gi|646643564|gb|KK968428.1|	15090-15947	14	6.2	0.921916	2	1	0.4498	no
gi|646643710|gb|KK968412.1|	18086-18644	12	4.8	5	2	-1	0.35845	no
gi|646643710|gb|KK968412.1|	18829-20446	12	4.8	3	1	-1	0.3948	no
gi|646643875|gb|KK968395.1|	16937-17210	21	8.5	30	9	-2	0.2492	no
gi|646644085|gb|KK968377.1|	15834-16684	11	3.8	2	2	-0.0148853	0.9367	no
gi|646644085|gb|KK968377.1|	20717-21788	11	3.8	2	3	0.513809	0.7171	no
gi|646644862|gb|KK968345.1|	13607-14676	27	6.9	0.681862	0.973509	0.513715	1	no
gi|646645233|gb|KK968334.1|	15992-16169	9.1	5.5	17	996	6	0.0958	no
gi|646645273|gb|KK968333.1|	10507-11766	67	21	0.921411	0.661081	-0.479018	1	no
gi|646645428|gb|KK968330.1|	28813-29270	15	5.3	3	3	-0.0775297	0.92345	no
gi|646645800|gb|KK968315.1|	14709-15338	13	7.7	1	1	-0.115816	0.9032	no
gi|646645800|gb|KK968315.1|	35371-35820	13	7.7	0	6	inf	0.00255	no
gi|646645862|gb|KK968313.1|	11471-12216	9.8	5	2	0.52964	-2	0.2551	no
gi|646645862|gb|KK968313.1|	5222-5758	9.8	5	14	0	-inf	0.00155	yes

gi|646646995|gb|KK968292.1|	10376-10683	12	4.5	3	26	3	0.27165	no
gi|646647433|gb|KK968283.1|	10131-10320	8.6	5.7	91	152	0.739836	0.6291	no
gi|646648130|gb|KK968272.1|	15414-17952	15	7.6	23	16	-0.49465	0.66135	no
gi|646648130|gb|KK968272.1|	202-367	15	7.6	223	88	-1	0.38465	no
gi|646648130|gb|KK968272.1|	3544-4076	15	7.6	65	55	-0.241734	0.87355	no
gi|646649361|gb|KK968258.1|	14166-14858	35	9.9	1	1	-0.00455477	0.92345	no
gi|646649361|gb|KK968258.1|	4179-5313	35	9.9	2	3	0.438548	0.7485	no
gi|646651230|gb|KK968234.1|	10054-11899	28	11	0.116372	0.757475	3	1	no
gi|646651230|gb|KK968234.1|	3973-5665	28	11	5	18	2	0.1306	no
gi|646651230|gb|KK968234.1|	6537-6972	28	11	5	0.660087	-3	0.20135	no
gi|646651829|gb|KK968226.1|	26847-27398	13	4.6	21	21	-0.0159771	0.9902	no
gi|646651829|gb|KK968226.1|	29740-30119	13	4.6	25	27	0.0840008	0.95855	no
gi|646651829|gb|KK968226.1|	32906-33356	13	4.6	27	20	-0.409843	0.78805	no
gi|646652060|gb|KK968219.1|	10493-10806	9.7	4.3	26	0	-inf	0.0004	yes
gi|646656079|gb|KK968161.1|	21383-22153	11	4	3	0.757558	-2	0.268	no
gi|646656105|gb|KK968158.1|	160-868	12	5.2	1	2	0.67602	0.64375	no
gi|646656105|gb|KK968158.1|	7928-10242	12	5.2	0.718935	0.84759	0.237504	1	no
gi|646656304|gb|KK968156.1|	10714-11032	16	7.9	29	4	-3	0.1596	no
gi|646656312|gb|KK968155.1|	14405-15235	11	3.4	10	2	-2	0.153	no
gi|646656851|gb|KK968142.1|	10187-11429	18	7.7	2	4	1	0.3668	no
gi|646657416|gb|KK968135.1|	28629-29851	13	6.5	5	4	-0.245747	0.87785	no
gi|646657416|gb|KK968135.1|	32123-32933	13	6.5	6	5	-0.321182	0.8445	no
gi|646657416|gb|KK968135.1|	37417-39049	13	6.5	7	4	-0.678134	0.64805	no
gi|646658008|gb|KK968126.1|	26361-28405	12	5.6	4	6	0.459781	0.7612	no
gi|646658008|gb|KK968126.1|	31226-31842	12	5.6	7	11	0.613122	0.6993	no
gi|646658008|gb|KK968126.1|	9684-9877	12	5.6	80	91	0.180454	0.8963	no
gi|646658183|gb|KK968124.1|	9666-11317	12	4.4	5	3	-0.920888	0.5511	no
gi|646658190|gb|KK968123.1|	16622-17054	12	6.6	0	7	inf	0.0014	yes
gi|646658190|gb|KK968123.1|	20406-21056	12	6.6	30	33	0.157161	0.91575	no
gi|646658852|gb|KK968112.1|	17460-18740	13	7.8	1	2	0.299229	0.8034	no

gi|646659105|gb|KK968105.1|	10984-11400	15	6.2	30	0	-inf	0.00055	yes
gi|646659105|gb|KK968105.1|	11571-12929	15	6.2	3	8	1	0.36115	no
gi|646659105|gb|KK968105.1|	3565-4203	15	6.2	59	24	-1	0.31685	no
gi|646659105|gb|KK968105.1|	6774-7563	15	6.2	16	8	-0.95369	0.53055	no
gi|646659131|gb|KK968102.1|	2413-3259	15	5.9	3	2	-0.289159	0.812	no
gi|646659131|gb|KK968102.1|	3364-3951	15	5.9	6	7	0.314792	0.8175	no
gi|646659131|gb|KK968102.1|	4904-5382	15	5.9	11	14	0.308201	0.8467	no
gi|646659131|gb|KK968102.1|	8830-9328	15	5.9	10	9	-0.101849	0.9468	no
gi|646659149|gb|KK968100.1|	24053-25695	14	4	19	11	-0.749005	0.55175	no
gi|646659224|gb|KK968093.1|	18476-19004	12	5	2	4	0.803356	0.58155	no
gi|646659224|gb|KK968093.1|	21940-22325	12	5	18	11	-0.701345	0.61155	no
gi|646659255|gb|KK968089.1|	0-1604	14	5.6	14	3	-2	0.13475	no
gi|646659277|gb|KK968087.1|	35810-36238	13	6.2	362	355	-0.0271616	0.9793	no
gi|646659434|gb|KK968073.1|	29745-30227	15	4.5	3	2	-0.99382	0.589	no
gi|646659513|gb|KK968067.1|	14321-15806	14	6.1	2	9	2	0.24445	no
gi|646659513|gb|KK968067.1|	2187-2544	14	6.1	30	13	-1	0.40765	no
gi|646659524|gb|KK968066.1|	24973-25632	15	7.7	5	0	-inf	0.00045	yes
gi|646659616|gb|KK968060.1|	12640-13099	26	8.6	0	6	inf	0.00255	no
gi|646659616|gb|KK968060.1|	39505-40115	26	8.6	12	6	-1	0.52215	no
gi|646659635|gb|KK968058.1|	8286-8498	11	6.7	138	176	0.346279	0.82785	no
gi|646659934|gb|KK968037.1|	18303-19450	13	4.7	2	2	0.324423	0.78225	no
gi|646660102|gb|KK968030.1|	2861-3749	17	5.9	1	0.621183	-1	0.4766	no
gi|646660374|gb|KK968018.1|	30088-30415	7.8	4.9	108	78	-0.470302	0.75715	no
gi|646660430|gb|KK968016.1|	21139-21557	11	7.6	64	1	-5	0.1075	no
gi|646660468|gb|KK968014.1|	6378-9641	14	6.5	5	1	-2	0.24855	no
gi|646660605|gb|KK968005.1|	10291-10904	14	9.5	3	5	0.687986	0.58615	no
gi|646660814|gb|KK967997.1|	20717-21396	18	5.7	0.436081	3	3	0.2662	no
gi|646661067|gb|KK967989.1|	743-2919	12	3.5	16	13	-0.369874	0.73065	no
gi|646661067|gb|KK967989.1|	7854-8592	12	3.5	0.191479	7	5	0.0982	no
gi|646661372|gb|KK967978.1|	11804-12952	15	7.5	5	7	0.452977	0.7696	no

gi|646661372|gb|KK967978.1|	20415-20961	15	7.5	2	2	0.447061	0.77485	no
gi|646661372|gb|KK967978.1|	3549-3950	15	7.5	5	2	-2	0.3081	no
gi|646661660|gb|KK967969.1|	3781-4728	12	7.8	4	6	0.320566	0.84075	no
gi|646661660|gb|KK967969.1|	5866-6981	12	7.8	11	8	-0.430795	0.77585	no
gi|646661752|gb|KK967967.1|	22894-23779	9.6	7.6	1	0.832053	-0.304782	1	no
gi|646661798|gb|KK967966.1|	10252-10522	12	5.3	14	44	2	0.31005	no
gi|646661798|gb|KK967966.1|	14153-14471	12	5.3	15	15	0.0258114	0.9512	no
gi|646661798|gb|KK967966.1|	15038-15453	12	5.3	6	9	0.505074	0.6928	no
gi|646661798|gb|KK967966.1|	5998-6618	12	5.3	3	2	-0.0473914	0.93665	no
gi|646661798|gb|KK967966.1|	6901-7130	12	5.3	41	59	0.523002	0.68475	no
gi|646661798|gb|KK967966.1|	7719-8095	12	5.3	4	11	2	0.26755	no
gi|646661834|gb|KK967964.1|	11095-11514	11	4.8	8	22	2	0.28645	no
gi|646661944|gb|KK967960.1|	25584-26436	22	8.5	0.929635	0.876384	-0.0851008	1	no
gi|646662088|gb|KK967954.1|	11917-13193	9.3	4.8	0.543643	1	1	0.3963	no
gi|646662099|gb|KK967953.1|	12526-12806	13	5.5	34	55	0.7003	0.60805	no
gi|646662099|gb|KK967953.1|	22081-22445	13	5.5	5	7	0.330596	0.8098	no
gi|646662468|gb|KK967943.1|	17021-19375	11	6	6	6	-0.00927641	0.9936	no
gi|646662646|gb|KK967934.1|	12969-13110	15	5.2	16167.3	4748.95	-2	0.26075	no
gi|646662821|gb|KK967928.1|	281-451	17	7.4	0	420	inf	0.00175	no
gi|646662964|gb|KK967923.1|	0-410	11	6.3	54	44	-0.28097	0.85285	no
gi|646663059|gb|KK967920.1|	20749-21921	13	5.4	0.302644	2	2	0.1605	no
gi|646663566|gb|KK967904.1|	17367-17981	12	5.9	2	2	0.0507865	0.9298	no
gi|646663566|gb|KK967904.1|	22724-23634	12	5.9	0.988561	0.801345	-0.302907	1	no
gi|646663650|gb|KK967902.1|	29-1439	14	6.8	2	3	0.736572	0.5979	no
gi|646663703|gb|KK967901.1|	19348-19848	13	5.2	32	11	-2	0.3382	no
gi|646663703|gb|KK967901.1|	33299-35914	13	5.2	3	3	-0.30532	0.84015	no
gi|646663703|gb|KK967901.1|	40194-40910	13	5.2	0.601792	5	3	0.1491	no
gi|646663915|gb|KK967894.1|	51712-52088	10	7.1	23	4	-3	0.1368	no
gi|646663935|gb|KK967893.1|	9811-10554	12	6.3	13	4	-2	0.2943	no
gi|646663968|gb|KK967892.1|	14325-14941	6.2	6	0.255797	5	4	0.0989	no

gi|646663986|gb|KK967891.1|	3334-4752	14	4.1	0.556821	0.801371	0.525255	1	no
gi|646664103|gb|KK967887.1|	11554-12638	11	6.8	4	5	0.0504613	0.9738	no
gi|646664103|gb|KK967887.1|	5106-6500	11	6.8	2	2	0.0885868	0.9538	no
gi|646664228|gb|KK967882.1|	9821-10214	12	6.2	7	2	-2	0.2135	no
gi|646664563|gb|KK967876.1|	17692-19486	75	26	6	3	-0.924191	0.54045	no
gi|646664981|gb|KK967865.1|	14195-16047	17	6.1	1	0.2514	-2	0.2049	no
gi|646664981|gb|KK967865.1|	21302-21485	17	6.1	125	24	-2	0.1837	no
gi|646664981|gb|KK967865.1|	9279-10130	17	6.1	1	0.658351	-0.915263	0.58965	no
gi|646665397|gb|KK967853.1|	11587-11831	17	4.9	3	33	3	0.1082	no
gi|646665787|gb|KK967845.1|	28369-28900	15	8.2	4	9	0.989367	0.5165	no
gi|646665984|gb|KK967841.1|	12104-12577	10	3.5	4	0	-inf	0.00655	no
gi|646666093|gb|KK967839.1|	11995-12364	14	5.6	2	7	2	0.3694	no
gi|646666101|gb|KK967838.1|	17742-18049	16	7.5	12	82	3	0.0931	no
gi|646666119|gb|KK967836.1|	10755-11922	14	7.6	3	1	-1	0.47435	no
gi|646666119|gb|KK967836.1|	16567-18257	14	7.6	0.644701	0.744921	0.208459	1	no
gi|646666119|gb|KK967836.1|	22987-24982	14	7.6	38	12	-2	0.1488	no
gi|646666119|gb|KK967836.1|	26640-27317	14	7.6	35	5	-3	0.1024	no
gi|646666119|gb|KK967836.1|	7557-7890	14	7.6	52	2	-4	0.11315	no
gi|646666152|gb|KK967833.1|	10407-10744	9.2	4.3	19	13	-0.566707	0.6436	no
gi|646666152|gb|KK967833.1|	1294-1483	9.2	4.3	102	20	-2	0.1837	no
gi|646666152|gb|KK967833.1|	30945-32583	9.2	4.3	3	4	0.529561	0.72985	no
gi|646666173|gb|KK967831.1|	1078-1592	17	6	90	31	-2	0.23865	no
gi|646666173|gb|KK967831.1|	11626-13195	17	6	3	3	0.0644172	0.96545	no
gi|646666173|gb|KK967831.1|	23701-24442	17	6	2	6	1	0.3825	no
gi|646666173|gb|KK967831.1|	60-391	17	6	28	9	-2	0.2858	no
gi|646666246|gb|KK967825.1|	19289-20006	11	6.7	13	6	-0.991729	0.52225	no
gi|646666246|gb|KK967825.1|	21377-22109	11	6.7	8	2	-2	0.24215	no
gi|646666310|gb|KK967820.1|	13637-13851	12	6.5	23	39	0.779879	0.62245	no
gi|646666361|gb|KK967816.1|	1-509	210	26	26	32	0.287522	0.8533	no
gi|646667394|gb|KK967785.1|	18806-20209	13	5.5	21	1	-4	0.00725	no

gi|646667394|gb|KK967785.1|	25663-26016	13	5.5	24	0	-inf	0.0031	no
gi|646667717|gb|KK967781.1|	3301-3503	14	6.1	31	73	1	0.4261	no
gi|646667817|gb|KK967779.1|	17852-18399	16	5.4	7	2	-2	0.19355	no
gi|646668247|gb|KK967772.1|	16255-17202	14	5.9	0.267348	6	5	0.23975	no
gi|646668927|gb|KK967759.1|	14148-16298	7.8	4.1	7	2	-2	0.18785	no
gi|646669665|gb|KK967749.1|	6150-6661	27	25	3	2	-0.373168	0.811	no
gi|646670040|gb|KK967744.1|	11449-13827	9.9	4.4	0.740843	0.948813	0.356956	1	no
gi|646670040|gb|KK967744.1|	14453-14642	9.9	4.4	171	51	-2	0.2312	no
gi|646670040|gb|KK967744.1|	6968-7790	9.9	4.4	1	0.920993	-0.502872	0.7384	no
gi|646671486|gb|KK967724.1|	20457-20798	15	6.2	8	1	-3	0.20705	no
gi|646672054|gb|KK967710.1|	0-562	9.3	4.5	107	92	-0.230795	0.82835	no
gi|646672110|gb|KK967706.1|	28162-28409	8.2	3.4	15	22	0.588044	0.66825	no
gi|646672236|gb|KK967698.1|	12762-14124	120	27	0.752123	0.60079	-0.324108	1	no
gi|646672236|gb|KK967698.1|	18132-19031	120	27	2	2	-0.196799	0.87625	no
gi|646672236|gb|KK967698.1|	20487-22316	120	27	1	0.765067	-0.545673	1	no
gi|646672236|gb|KK967698.1|	28467-33109	120	27	2	2	-0.495008	0.73945	no
gi|646672236|gb|KK967698.1|	33162-33880	120	27	2	2	0.0698165	0.92975	no
gi|646672236|gb|KK967698.1|	34668-35470	120	27	1	0.953343	-0.08982	1	no
gi|646672236|gb|KK967698.1|	38473-39269	120	27	2	0.240874	-3	0.2033	no
gi|646672380|gb|KK967689.1|	28850-29191	15	11	8	7	-0.28355	0.8392	no
gi|646672380|gb|KK967689.1|	29292-29817	15	11	3	9	1	0.3676	no
gi|646672466|gb|KK967685.1|	32228-32445	11	4.8	37	69	0.879541	0.5148	no
gi|646672677|gb|KK967672.1|	11050-11686	14	6.2	2	0.672025	-2	0.2673	no
gi|646672677|gb|KK967672.1|	15450-16459	14	6.2	1	0.349581	-2	0.20925	no
gi|646672872|gb|KK967660.1|	23868-24725	11	6.5	6	0.434683	-4	0.1227	no
gi|646672872|gb|KK967660.1|	26487-27418	11	6.5	49	5	-3	0.03505	no
gi|646672891|gb|KK967659.1|	14561-14828	7.3	4.7	51	65	0.331276	0.8341	no
gi|646673021|gb|KK967651.1|	2870-3593	10	6.1	18	8	-1	0.4768	no
gi|646673021|gb|KK967651.1|	767-1149	10	6.1	10	19	0.902645	0.52765	no
gi|646673038|gb|KK967650.1|	27593-28295	8.5	4.1	2	5	1	0.36	no

gi|646673225|gb|KK967640.1|	4167-4581	14	6.9	8	3	-2	0.3114	no
gi|646673348|gb|KK967633.1|	2933-3243	12	5.6	1	18	4	0.1003	no
gi|646673458|gb|KK967628.1|	19465-19877	34	8.9	5	3	-0.622837	0.6684	no
gi|646673458|gb|KK967628.1|	20107-20344	34	8.9	21	18	-0.195027	0.89115	no
gi|646673458|gb|KK967628.1|	22892-23777	34	8.9	2	1	-0.912464	0.49835	no
gi|646673507|gb|KK967625.1|	33812-34611	16	6.9	2	2	0.342829	0.79725	no
gi|646673507|gb|KK967625.1|	34699-35174	16	6.9	2	7	2	0.24175	no
gi|646673520|gb|KK967624.1|	17580-19170	13	7.6	2	4	0.859947	0.57965	no
gi|646673520|gb|KK967624.1|	26682-27201	13	7.6	4	4	-0.0220555	0.94695	no
gi|646673881|gb|KK967606.1|	20526-21179	14	5.2	1	2	0.473998	0.7489	no
gi|646674087|gb|KK967596.1|	16356-16666	11	6.1	6	9	0.516393	0.73725	no
gi|646674087|gb|KK967596.1|	20693-21129	11	6.1	1	5	2	0.2631	no
gi|646674087|gb|KK967596.1|	29455-29912	11	6.1	15	9	-0.772675	0.63505	no
gi|646674103|gb|KK967595.1|	1021-1677	15	7.6	8	2	-2	0.18685	no
gi|646674103|gb|KK967595.1|	18087-19334	15	7.6	7	2	-2	0.2855	no
gi|646674103|gb|KK967595.1|	24793-25102	15	7.6	33	11	-2	0.2916	no
gi|646674215|gb|KK967590.1|	17574-18189	11	5	2	3	0.658698	0.6445	no
gi|646674299|gb|KK967586.1|	48042-48251	16	5.3	52	37	-0.466888	0.7424	no
gi|646674299|gb|KK967586.1|	49086-49363	16	5.3	48	19	-1	0.3639	no
gi|646674614|gb|KK967571.1|	13634-14767	11	8.9	12	5	-1	0.4447	no
gi|646674838|gb|KK967561.1|	18758-19437	9.8	5.1	2	1	-0.843221	0.57665	no
gi|646675268|gb|KK967543.1|	14437-15105	10	4.8	17	4	-2	0.1694	no
gi|646675409|gb|KK967537.1|	17450-18384	13	7.1	9	0	-inf	0.0004	yes
gi|646675882|gb|KK967521.1|	1571-2466	11	7.8	0	4	inf	0.00435	no
gi|646675882|gb|KK967521.1|	7922-8625	11	7.8	0	30	inf	5.00E-05	yes
gi|646676013|gb|KK967516.1|	430-843	12	4.8	1	10	3	0.2422	no
gi|646676197|gb|KK967511.1|	11504-12011	15	7.3	6	6	-0.0699645	0.9538	no
gi|646676197|gb|KK967511.1|	14610-14842	15	7.3	12	28	1	0.42395	no
gi|646676197|gb|KK967511.1|	30976-31421	15	7.3	4	4	-0.014388	0.9298	no
gi|646676197|gb|KK967511.1|	60-822	15	7.3	3	4	0.403213	0.77245	no

gi|646677068|gb|KK967483.1|	17874-19014	12	7.3	4	2	-0.705601	0.6654	no
gi|646677068|gb|KK967483.1|	9986-11076	12	7.3	112	15	-3	0.04655	no
gi|646677110|gb|KK967477.1|	3127-4143	16	6.4	2	1	-1	0.3511	no
gi|646677146|gb|KK967472.1|	18664-18931	12	6.1	30	17	-0.838649	0.5246	no
gi|646677242|gb|KK967467.1|	7002-7264	30	13	18	23	0.317777	0.83165	no
gi|646677541|gb|KK967454.1|	908-1254	20	7.9	8	13	0.723233	0.58905	no
gi|646677811|gb|KK967447.1|	14298-14549	13	8.3	38	36	-0.10696	0.9281	no
gi|646677811|gb|KK967447.1|	20945-21592	13	8.3	99	44	-1	0.36525	no
gi|646677811|gb|KK967447.1|	22522-23802	13	8.3	1	0.518261	-1	0.34475	no
gi|646677811|gb|KK967447.1|	6678-7767	13	8.3	3	2	-0.222332	0.89575	no
gi|646677817|gb|KK967446.1|	6183-6575	14	5.6	7	7	-0.0996003	0.9388	no
gi|646677817|gb|KK967446.1|	9523-10890	14	5.6	20	53	1	0.1993	no
gi|646677880|gb|KK967444.1|	227-575	16	6.2	4	5	0.310838	0.8529	no
gi|646678138|gb|KK967441.1|	46273-46710	10	4.8	7	5	-0.411891	0.75895	no
gi|646678541|gb|KK967432.1|	11047-11387	9.1	8.5	17	17	0.0369884	0.9689	no
gi|646678541|gb|KK967432.1|	19791-20244	9.1	8.5	23	71	2	0.30755	no
gi|646678541|gb|KK967432.1|	2742-3546	9.1	8.5	8	23	2	0.3066	no
gi|646678541|gb|KK967432.1|	4586-5175	9.1	8.5	19	76	2	0.1367	no
gi|646678541|gb|KK967432.1|	6126-7208	9.1	8.5	6	25	2	0.1292	no
gi|646678541|gb|KK967432.1|	9708-10277	9.1	8.5	24	101	2	0.131	no
gi|646678920|gb|KK967424.1|	4474-5391	11	5.4	1	3	2	0.35315	no
gi|646678957|gb|KK967423.1|	31051-32565	15	8.1	7	4	-0.724221	0.62015	no
gi|646679122|gb|KK967419.1|	1412-1855	14	5.4	7	7	-0.0480783	0.95565	no
gi|646679560|gb|KK967415.1|	13916-14335	17	8.5	5	8	0.520138	0.6874	no
gi|646679560|gb|KK967415.1|	191-791	17	8.5	9	4	-1.04	0.45375	no
gi|646680670|gb|KK967395.1|	27433-27665	14	10	78	96	0.312334	0.81975	no
gi|646682091|gb|KK967374.1|	13080-13970	11	6.8	36	19	-0.923666	0.4663	no
gi|646682091|gb|KK967374.1|	24112-24371	11	6.8	89	56	-0.677731	0.6746	no
gi|646682091|gb|KK967374.1|	25447-25727	11	6.8	20	37	0.903833	0.54895	no
gi|646682151|gb|KK967372.1|	797-1093	12	6.7	21	33	0.668031	0.6337	no

gi|646682259|gb|KK967359.1|	5053-6041	16	5.4	0.504795	1	2	0.3671	no
gi|646682319|gb|KK967352.1|	18917-19765	13	6.2	0	54	inf	5.00E-05	yes
gi|646682319|gb|KK967352.1|	23613-24100	13	6.2	0	168	inf	5.00E-05	yes
gi|646682319|gb|KK967352.1|	25216-25559	13	6.2	0.923437	48	6	0.09595	no
gi|646682319|gb|KK967352.1|	30009-30570	13	6.2	0.602304	101	7	0.2322	no
gi|646682521|gb|KK967330.1|	20467-21426	5.8	8.4	3	2	-0.492256	0.7258	no
gi|646682521|gb|KK967330.1|	30195-30597	5.8	8.4	6	4	-0.63117	0.6644	no
gi|646682571|gb|KK967325.1|	13577-14272	9.2	7.6	15	11	-0.423068	0.78465	no
gi|646682642|gb|KK967317.1|	30682-32350	11	5.1	0.130947	9	6	0.23315	no
gi|646682678|gb|KK967313.1|	12365-12602	9.2	5.1	5402.49	3925.54	-0.460733	0.6976	no
gi|646682736|gb|KK967307.1|	7570-8757	17	6.5	2	2	-0.0219957	0.95635	no
gi|646682764|gb|KK967305.1|	13181-14320	27	11	0.209293	3	4	0.2606	no
gi|646682798|gb|KK967302.1|	20400-20944	40	16	5	6	0.146835	0.90795	no
gi|646682847|gb|KK967297.1|	5419-6196	31	16	10	6	-0.618595	0.6806	no
gi|646682958|gb|KK967286.1|	16067-16825	13	6.6	0.919385	1	0.490188	0.7568	no
gi|646682958|gb|KK967286.1|	3312-4216	13	6.6	6	9	0.601308	0.69355	no
gi|646682958|gb|KK967286.1|	6103-7290	13	6.6	15	15	0.0675885	0.96395	no
gi|646682958|gb|KK967286.1|	8121-10530	13	6.6	0.944941	1	0.470629	0.7752	no
gi|646683040|gb|KK967279.1|	19523-19736	14	6.9	53	126	1	0.41105	no
gi|646683062|gb|KK967277.1|	4769-6342	6.3	2.5	1	8	3	0.05585	no
gi|646683144|gb|KK967271.1|	20421-20695	7.3	5.4	17	13	-0.405871	0.78525	no
gi|646683144|gb|KK967271.1|	6416-7298	7.3	5.4	1	1	-0.0826243	0.93265	no
gi|646683362|gb|KK967256.1|	13049-13403	9.5	4.2	10	8	-0.266434	0.8366	no
gi|646683362|gb|KK967256.1|	397-933	9.5	4.2	5	2	-1	0.3416	no
gi|646683362|gb|KK967256.1|	44156-44765	9.5	4.2	4	3	-0.365413	0.77185	no
gi|646683362|gb|KK967256.1|	9991-11964	9.5	4.2	4	3	-0.658632	0.6626	no
gi|646683391|gb|KK967254.1|	33846-35398	17	9.5	1	2	0.659295	0.6315	no
gi|646683391|gb|KK967254.1|	40582-40958	17	9.5	43	29	-0.562994	0.7122	no
gi|646683391|gb|KK967254.1|	41515-42178	17	9.5	77	44	-0.808474	0.4536	no
gi|646683498|gb|KK967247.1|	11416-13366	15	6	3	2	-0.174056	0.9101	no

gi|646683498|gb|KK967247.1|	5948-6638	15	6	9	7	-0.519453	0.7372	no
gi|646683706|gb|KK967236.1|	6860-8312	17	7.2	0.695581	0.667737	-0.0589394	1	no
gi|646683729|gb|KK967234.1|	3326-4378	37	21	5	2	-0.965149	0.48755	no
gi|646683729|gb|KK967234.1|	4601-4963	37	21	7	0.995643	-3	0.20705	no
gi|646683836|gb|KK967228.1|	14620-15584	13	5.4	2	10	3	0.0851	no
gi|646683836|gb|KK967228.1|	8514-10085	13	5.4	10	2	-2	0.20735	no
gi|646684026|gb|KK967219.1|	21117-21879	14	9.8	2	4	1	0.4808	no
gi|646684114|gb|KK967216.1|	33157-34142	18	8.5	4	4	-0.29804	0.85265	no
gi|646684353|gb|KK967205.1|	202-900	13	7.6	2	0.291487	-3	0.2033	no
gi|646684516|gb|KK967198.1|	14440-15237	11	5.6	1	0.961789	-0.090339	1	no
gi|646685000|gb|KK967180.1|	3616-4114	14	3	0.754622	4	2	0.28615	no
gi|646685000|gb|KK967180.1|	8757-10121	14	3	4	2	-0.645985	0.68365	no
gi|646685025|gb|KK967178.1|	20900-22353	17	5.2	2	2	0.263011	0.87665	no
gi|646685273|gb|KK967173.1|	15090-15499	14	6.4	10	2	-3	0.13655	no
gi|646685323|gb|KK967172.1|	15216-15509	11	6.4	5	16	2	0.2677	no
gi|646685337|gb|KK967171.1|	22401-24371	19	11	43	30	-0.525674	0.65065	no
gi|646685579|gb|KK967162.1|	32798-33235	11	5.1	0	8	inf	0.001	yes
gi|646685819|gb|KK967155.1|	10478-11919	94	36	3	3	0.138756	0.9241	no
gi|646686692|gb|KK967128.1|	24081-25261	44	13	0.900081	0.286483	-2	1	no
gi|646686692|gb|KK967128.1|	32354-33365	44	13	0.856522	0.697366	-0.296575	1	no
gi|646686692|gb|KK967128.1|	50239-50386	44	13	1187.65	0	-inf	0.0012	yes
gi|646686711|gb|KK967127.1|	16726-16977	19	5.9	118	375	2	0.2885	no
gi|646686711|gb|KK967127.1|	19821-21005	19	5.9	2	6	1	0.3849	no
gi|646686711|gb|KK967127.1|	268-2115	19	5.9	21	26	0.305442	0.78125	no
gi|646686711|gb|KK967127.1|	7074-7442	19	5.9	5	8	0.750204	0.5987	no
gi|646686723|gb|KK967126.1|	33611-34230	13	6.8	2	4	1	0.4163	no
gi|646687243|gb|KK967115.1|	28477-28728	200	98	25	15	-0.732564	0.63775	no
gi|646687364|gb|KK967113.1|	234-587	11	4.6	133	84	-0.66435	0.65975	no
gi|646687456|gb|KK967109.1|	36270-37285	21	7.7	0.608582	2	2	0.27185	no
gi|646687456|gb|KK967109.1|	3701-4401	21	7.7	7	6	-0.365852	0.8192	no

gi|646687584|gb|KK967106.1|	15338-15881	19	10	6	25	2	0.1669	no
gi|646687612|gb|KK967105.1|	20468-20953	15	11	8	1	-3	0.1213	no
gi|646687812|gb|KK967098.1|	241-1135	15	6.2	0	3	inf	0.00035	yes
gi|646687865|gb|KK967097.1|	13611-14533	13	5.5	22	14	-0.619396	0.68335	no
gi|646688104|gb|KK967089.1|	25400-26062	20	7.5	0	5	inf	0.00605	no
gi|646688316|gb|KK967085.1|	519-1581	6.8	5.9	2	2	0.0728105	0.95495	no
gi|646688501|gb|KK967079.1|	12409-14516	2.80E+01	17	0.899194	2	1	0.3702	no
gi|646688528|gb|KK967076.1|	11385-12078	14	8.8	8	6	-0.344937	0.82345	no
gi|646688528|gb|KK967076.1|	12133-12556	14	8.8	3	4	0.648669	0.6668	no
gi|646688528|gb|KK967076.1|	22246-24184	14	8.8	6	6	0.215268	0.89335	no
gi|646688528|gb|KK967076.1|	8122-9513	14	8.8	5	4	-0.34212	0.81895	no
gi|646688596|gb|KK967070.1|	15582-16594	13	5.9	46	54	0.225551	0.83465	no
gi|646688596|gb|KK967070.1|	17268-18401	13	5.9	4	6	0.714449	0.64175	no
gi|646688596|gb|KK967070.1|	20692-21103	13	5.9	10	26	1	0.33575	no
gi|646688596|gb|KK967070.1|	21741-22910	13	5.9	14	23	0.772013	0.54105	no
gi|646688645|gb|KK967064.1|	25498-26431	14	5	8	13	0.660382	0.66045	no
gi|646688645|gb|KK967064.1|	31874-32418	14	5	4	5	0.446396	0.7252	no
gi|646688645|gb|KK967064.1|	9454-9719	14	5	49	0	-inf	0.00525	no
gi|646688752|gb|KK967052.1|	3873-4221	11	4.6	8	5	-0.537159	0.7233	no
gi|646688929|gb|KK967034.1|	9938-10741	9.2	6.7	1	1	-0.182827	0.90405	no
gi|646688938|gb|KK967033.1|	20087-21049	11	3.8	16	4	-2	0.2184	no
gi|646688967|gb|KK967030.1|	10290-12766	23	5	3	2	-1	0.4987	no
gi|646688967|gb|KK967030.1|	14066-14930	23	5	3	1	-2	0.3017	no
gi|646688967|gb|KK967030.1|	16927-17368	23	5	6	5	-0.302346	0.8209	no
gi|646688967|gb|KK967030.1|	21286-21680	23	5	4	4	-0.12374	0.9214	no
gi|646688967|gb|KK967030.1|	22254-23450	23	5	1	2	0.644425	0.61505	no
gi|646688967|gb|KK967030.1|	31718-33598	23	5	2	1	-0.788968	0.56415	no
gi|646688967|gb|KK967030.1|	5781-6489	23	5	9	0.285377	-5	0.1736	no
gi|646688967|gb|KK967030.1|	8108-8384	23	5	19	9	-1	0.44375	no
gi|646688967|gb|KK967030.1|	8701-10134	23	5	46	1	-5	0.0022	no

gi|646689116|gb|KK967014.1|	7511-9487	19	8.8	6	3	-1	0.4221	no
gi|646689116|gb|KK967014.1|	9556-11967	19	8.8	4	3	-0.734047	0.6284	no
gi|646689286|gb|KK966998.1|	147-1022	7.5	4	2	0	-inf	0.00195	no
gi|646689345|gb|KK966993.1|	846-1614	13	7.3	4	4	0.119423	0.93965	no
gi|646689415|gb|KK966985.1|	24799-26191	7.7	4.3	2	1	-0.263859	0.8181	no
gi|646689488|gb|KK966980.1|	19243-20061	20	7	4	2	-0.766292	0.5942	no
gi|646689488|gb|KK966980.1|	21758-22368	20	7	2	3	0.687289	0.6395	no
gi|646689557|gb|KK966972.1|	10562-11417	5.6	5.7	9	11.12	0.339656	0.8172	no
gi|646689557|gb|KK966972.1|	17783-18182	5.6	5.7	5	2	-1	0.4814	no
gi|646689557|gb|KK966972.1|	8140-10364	5.6	5.7	13	5	-1	0.3135	no
gi|646689694|gb|KK966959.1|	703-1203	6.2	4.2	0	7	inf	0.0008	yes
gi|646689770|gb|KK966949.1|	29863-30787	12	3.2	1	0.588839	-0.909599	1	no
gi|646689770|gb|KK966949.1|	32312-32922	12	3.2	2	1	-0.757496	0.6333	no
gi|646689839|gb|KK966940.1|	11933-12301	6.8	4	249	87	-2	0.24775	no
gi|646689869|gb|KK966936.1|	12524-13088	24	7.6	0	4	inf	0.00255	no
gi|646689916|gb|KK966930.1|	12-1192	9.4	4.7	0.900081	3	2	0.262	no
gi|646689916|gb|KK966930.1|	5806-6508	9.4	4.7	5	3	-0.719177	0.61935	no
gi|646689946|gb|KK966926.1|	2667-3858	9.8	6.4	0.494169	0.707986	0.518716	1	no
gi|646690063|gb|KK966911.1|	12186-12720	12	5	3	7	1	0.48705	no
gi|646690063|gb|KK966911.1|	2562-2760	12	5	69	137	0.976097	0.5559	no
gi|646690063|gb|KK966911.1|	33826-34002	12	5	0	209	inf	0.00035	yes
gi|646690083|gb|KK966908.1|	10979-11809	11	6	1	2	0.497884	0.72155	no
gi|646690090|gb|KK966907.1|	16425-16849	9.8	5	4	6	0.748941	0.59135	no
gi|646690237|gb|KK966887.1|	10228-10616	7.5	4.4	3	21	3	0.14405	no
gi|646690365|gb|KK966869.1|	29000-30090	13	5.5	8	8	-0.0901832	0.9532	no
gi|646690406|gb|KK966864.1|	4656-6021	9.9	4	16	18	0.186524	0.8807	no
gi|646690406|gb|KK966864.1|	8022-9172	9.9	4	8	16	0.936725	0.5418	no
gi|646690427|gb|KK966862.1|	13335-13565	14	7.6	40	21	-0.958474	0.53905	no
gi|646690531|gb|KK966853.1|	2221-2683	11	5.2	4	8	1	0.3784	no
gi|646690537|gb|KK966852.1|	8062-8314	27	11	32	6	-2	0.1666	no

gi|646690563|gb|KK966848.1|	10799-11035	11	7.4	108	45	-1	0.37195	no
gi|646690563|gb|KK966848.1|	5201-5887	11	7.4	2	0.897507	-1	0.4263	no
gi|646690598|gb|KK966843.1|	16274-17349	56	15	3	3	-0.205897	0.8998	no
gi|646690598|gb|KK966843.1|	23030-24750	56	15	5	2	-1	0.411	no
gi|646690598|gb|KK966843.1|	30877-31092	56	15	1208.45	567	-1	0.3873	no
gi|646690598|gb|KK966843.1|	31984-32549	56	15	3	4	0.315517	0.81835	no
gi|646690598|gb|KK966843.1|	33370-33701	56	15	13	15	0.171566	0.8833	no
gi|646690598|gb|KK966843.1|	36384-36820	56	15	11	4	-1	0.3512	no
gi|646690598|gb|KK966843.1|	43503-44391	56	15	2	2	0.180878	0.88335	no
gi|646690667|gb|KK966834.1|	34569-35863	11	7.1	3	2	-0.390504	0.80665	no
gi|646690686|gb|KK966831.1|	19328-19932	12	5.7	0	5	inf	0.00035	yes
gi|646690718|gb|KK966827.1|	77092-77572	16	10	28	21	-0.381829	0.7971	no
gi|646690741|gb|KK966824.1|	20414-21721	14	7.3	7	7	0.0127322	0.9929	no
gi|646690741|gb|KK966824.1|	22029-23731	14	7.3	0.575419	0.461774	-0.317428	1	no
gi|646690741|gb|KK966824.1|	23916-24724	14	7.3	81	93	0.190027	0.86485	no
gi|646690791|gb|KK966817.1|	15251-15710	14	6.5	4	6	0.730975	0.6055	no
gi|646690827|gb|KK966813.1|	18275-19096	1.90E+01	11	1	2	0.666957	0.6347	no
gi|646690827|gb|KK966813.1|	19324-19665	1.90E+01	11	4	7	0.886375	0.5964	no
gi|646690827|gb|KK966813.1|	27647-28760	1.90E+01	11	0.431268	1	2	0.36595	no
gi|646690895|gb|KK966803.1|	21305-23048	27	25	3	4	0.429291	0.77405	no
gi|646690937|gb|KK966797.1|	8118-8538	8.6	4.4	11	0	-inf	0.0004	yes
gi|646690976|gb|KK966792.1|	20812-21651	15	6.2	526	75	-3	0.0224	no
gi|646690976|gb|KK966792.1|	23532-24231	15	6.2	13	3	-2	0.13495	no
gi|646690976|gb|KK966792.1|	26296-27032	15	6.2	2	1	-0.834565	0.57665	no
gi|646691000|gb|KK966789.1|	22275-24172	14	6.8	4	4	0.0477341	0.9741	no
gi|646691063|gb|KK966782.1|	16234-17282	16	6.2	3	0.16643	-4	0.1839	no
gi|646691085|gb|KK966780.1|	11269-13569	17	7.9	2	2	0.0933526	0.9486	no
gi|646691085|gb|KK966780.1|	27686-28615	17	7.9	0.960701	10	3	0.05975	no
gi|646691085|gb|KK966780.1|	29407-30958	17	7.9	14	58	2	0.1474	no
gi|646691085|gb|KK966780.1|	33292-34588	17	7.9	0.710934	0.638079	-0.15598	1	no

gi|646691085|gb|KK966780.1|	35072-35895	17	7.9	0.977084	2	0.719616	0.6352	no
gi|646691085|gb|KK966780.1|	36893-38466	17	7.9	53	155	2	0.199	no
gi|646691085|gb|KK966780.1|	6169-11219	17	7.9	2	3	0.646402	0.66455	no
gi|646691183|gb|KK966768.1|	13684-14162	16	8.5	9	3	-2	0.3016	no
gi|646691259|gb|KK966759.1|	12230-13028	30	11	3	0.480044	-3	0.12945	no
gi|646691365|gb|KK966749.1|	18280-18609	18	7.7	14	13	-0.0944606	0.9386	no
gi|646691365|gb|KK966749.1|	20567-21378	18	7.7	1	4	2	0.26595	no
gi|646691365|gb|KK966749.1|	21765-23318	18	7.7	8	10	0.35339	0.8135	no
gi|646691365|gb|KK966749.1|	25272-26002	18	7.7	2	0.818367	-1	0.4263	no
gi|646691365|gb|KK966749.1|	28731-29393	18	7.7	10	10	-0.0936784	0.95305	no
gi|646691365|gb|KK966749.1|	3341-9638	18	7.7	4	8	1	0.35925	no
gi|646691365|gb|KK966749.1|	9819-11138	18	7.7	0.347784	1	2	0.2405	no
gi|646691432|gb|KK966740.1|	11433-12219	13	5.7	3	4	0.493442	0.7217	no
gi|646691432|gb|KK966740.1|	18937-19273	13	5.7	6	13	1	0.41645	no
gi|646691432|gb|KK966740.1|	20390-21033	13	5.7	22	34	0.633322	0.6698	no
gi|646691432|gb|KK966740.1|	25121-25979	13	5.7	12	17	0.445235	0.76855	no
gi|646691464|gb|KK966736.1|	12835-13485	29	13	2	2	-0.0120067	0.92345	no
gi|646691464|gb|KK966736.1|	15163-15502	29	13	11	4	-2	0.35815	no
gi|646691464|gb|KK966736.1|	20196-21185	29	13	0.378081	1	2	0.255	no
gi|646691474|gb|KK966735.1|	15614-17544	15	6	23	15	-0.654664	0.5493	no
gi|646691474|gb|KK966735.1|	29209-30266	15	6	60	14	-2	0.1284	no
gi|646691489|gb|KK966733.1|	3017-3515	11	6.2	6	0	-inf	0.0011	yes
gi|646691526|gb|KK966730.1|	6669-7031	12	5.5	9	4	-1	0.4507	no
gi|646691712|gb|KK966710.1|	6733-8272	16	6.6	5	0.934902	-2	0.10955	no
gi|646691893|gb|KK966691.1|	19389-20347	15	8.6	0.526331	6	4	0.11565	no
gi|646691982|gb|KK966681.1|	0-925	21	7.7	4	0.195996	-4	0.18315	no
gi|646691997|gb|KK966679.1|	5200-5872	12	9.9	2	2	-0.174587	0.89575	no
gi|646692168|gb|KK966664.1|	5517-6199	12	9.7	6	9	0.526523	0.73525	no
gi|646692192|gb|KK966661.1|	13680-14287	9.9	5.8	221	55	-2	0.08245	no
gi|646692231|gb|KK966655.1|	52031-52395	16	11	23	2	-4	0.1342	no

gi|646692310|gb|KK966647.1|	316-1853	18	5.6	11	2	-3	0.10815	no
gi|646692325|gb|KK966646.1|	21310-21944	21	11	0.975017	7	3	0.155	no
gi|646692325|gb|KK966646.1|	22142-22747	21	11	2	3	0.271067	0.84035	no
gi|646692344|gb|KK966644.1|	16538-16849	29	11	10	9	-0.15995	0.91185	no
gi|646692344|gb|KK966644.1|	42202-43508	29	11	0.968234	0.252854	-2	1	no
gi|646692344|gb|KK966644.1|	60460-61249	29	11	171	105	-0.711223	0.53905	no
gi|646692386|gb|KK966639.1|	20437-21857	15	6.2	2	7	2	0.29135	no
gi|646692527|gb|KK966623.1|	22464-23228	9.9	9	2	3	0.227882	0.8573	no
gi|646692527|gb|KK966623.1|	24295-25071	9.9	9	1	3	1	0.37645	no
gi|646692535|gb|KK966622.1|	12120-13368	17	8.4	9	6	-0.592258	0.69715	no
gi|646692535|gb|KK966622.1|	19310-20556	17	8.4	3	3	-0.157499	0.92425	no
gi|646692535|gb|KK966622.1|	5-1438	17	8.4	0.863865	0.678306	-0.34887	1	no
gi|646692535|gb|KK966622.1|	56394-57283	17	8.4	3	0	-inf	0.00095	yes
gi|646692535|gb|KK966622.1|	64453-65419	17	8.4	0.520411	1	1	1	no
gi|646692535|gb|KK966622.1|	70228-70532	17	8.4	19	5	-2	0.23205	no
gi|646692634|gb|KK966614.1|	20697-21081	9.5	9	0	17	inf	0.00315	no
gi|646692709|gb|KK966605.1|	29168-29517	9.4	11	0	79	inf	0.0002	yes
gi|646692748|gb|KK966601.1|	32054-32630	11	6.1	7	2	-2	0.22675	no
gi|646692898|gb|KK966589.1|	27570-28658	11	5.5	0.666312	0.951885	0.514589	1	no
gi|646692942|gb|KK966585.1|	12514-12842	13	5.3	72	87	0.28254	0.84595	no
gi|646692942|gb|KK966585.1|	13000-13251	13	5.3	16	15	-0.147602	0.9098	no
gi|646692942|gb|KK966585.1|	13447-15276	13	5.3	57	73	0.341163	0.7668	no
gi|646692942|gb|KK966585.1|	17194-17386	13	5.3	62	47	-0.410777	0.81145	no
gi|646692942|gb|KK966585.1|	9449-10699	13	5.3	26	39	0.601431	0.5752	no
gi|646693134|gb|KK966564.1|	19211-19676	10	4.9	8	7	-0.25056	0.83395	no
gi|646693233|gb|KK966554.1|	17164-17359	14	7.6	142	43	-2	0.24205	no
gi|646693258|gb|KK966551.1|	8011-8306	15	7	6	17	2	0.3544	no
gi|646693290|gb|KK966548.1|	45189-45883	13	6.5	3	3	0.0661569	0.9489	no
gi|646693318|gb|KK966545.1|	15912-17082	16	7.7	13	13	0.0798281	0.95965	no
gi|646693318|gb|KK966545.1|	21589-22341	16	7.7	9	9	0.0641365	0.9669	no

gi|646693318|gb|KK966545.1|	23381-26614	16	7.7	10	9	-0.148395	0.889	no
gi|646693351|gb|KK966541.1|	36679-36973	11	5.4	33	70	1	0.49625	no
gi|646693373|gb|KK966539.1|	17973-18893	10	5.6	0.834599	0.987109	0.242125	1	no
gi|646693373|gb|KK966539.1|	20487-20780	10	5.6	113	101	-0.155108	0.91745	no
gi|646693373|gb|KK966539.1|	25960-28124	10	5.6	0.290783	4	4	0.0647	no
gi|646693373|gb|KK966539.1|	36454-37090	10	5.6	0	32	inf	0.00035	yes
gi|646693373|gb|KK966539.1|	37210-37780	10	5.6	0	7	inf	0.00435	no
gi|646693373|gb|KK966539.1|	45650-46709	10	5.6	0	13	inf	0.00025	yes
gi|646693580|gb|KK966523.1|	5871-6427	10	6.4	2	3	1	0.44985	no
gi|646693619|gb|KK966519.1|	6016-6292	6.5	5.9	8	15	0.991288	0.58275	no
gi|646693626|gb|KK966518.1|	28284-29213	20	8.3	4	10	1	0.43255	no
gi|646693746|gb|KK966507.1|	26693-27091	12	5.4	0	9	inf	0.0014	yes
gi|646693796|gb|KK966503.1|	19554-20868	14	6.7	5	7	0.655054	0.6703	no
gi|646693796|gb|KK966503.1|	22125-23001	14	6.7	4	5	0.179934	0.90755	no
gi|646693796|gb|KK966503.1|	24386-26238	14	6.7	6	6	0.0416323	0.9785	no
gi|646693870|gb|KK966495.1|	11321-11876	14	9.6	4	1	-2	0.2829	no
gi|646693870|gb|KK966495.1|	14701-16029	14	9.6	15	6	-1	0.3733	no
gi|646693870|gb|KK966495.1|	9624-10719	14	9.6	38	27	-0.505917	0.6393	no
gi|646693900|gb|KK966493.1|	52063-52324	29	15	16	8	-1	0.48735	no
gi|646693998|gb|KK966484.1|	37703-37996	7.3	7.3	9	11	0.221455	0.885	no
gi|646693998|gb|KK966484.1|	38261-38625	7.3	7.3	101	26	-2	0.2194	no
gi|646693998|gb|KK966484.1|	39907-40490	7.3	7.3	13	8	-0.637041	0.6887	no
gi|646694037|gb|KK966480.1|	26385-27215	17	9.7	16	8	-1	0.51095	no
gi|646694092|gb|KK966474.1|	13055-13606	4.8	3.9	2	3	0.863729	0.5567	no
gi|646694101|gb|KK966473.1|	17274-17620	12	6.2	6	3	-0.914197	0.5995	no
gi|646694101|gb|KK966473.1|	2550-3515	12	6.2	0.130286	2	4	0.1061	no
gi|646694118|gb|KK966471.1|	11-374	17	8.7	6	8	0.329432	0.80525	no
gi|646694183|gb|KK966465.1|	14511-14836	14	5.4	14	4	-2	0.253	no
gi|646694183|gb|KK966465.1|	37121-37912	14	5.4	5	2	-1	0.39895	no
gi|646694259|gb|KK966457.1|	24007-26581	14	8	11	6	-1	0.42205	no

gi|646694259|gb|KK966457.1|	28707-32476	14	8	10	4	-1	0.32835	no
gi|646694259|gb|KK966457.1|	33504-34640	14	8	3	2	-1	0.4743	no
gi|646694371|gb|KK966448.1|	29337-32585	20	7.3	8	10	0.221278	0.83435	no
gi|646694443|gb|KK966441.1|	10765-11291	17	6.5	3	0.920553	-2	0.3832	no
gi|646694626|gb|KK966427.1|	23055-23826	12	5.1	3	0.252051	-4	0.17385	no
gi|646694626|gb|KK966427.1|	3009-3741	12	5.1	4	4	-0.0606658	0.95915	no
gi|646694626|gb|KK966427.1|	47-498	12	5.1	2	4	0.989312	0.5906	no
gi|646694653|gb|KK966425.1|	10465-10609	19	4.3	0	578	inf	0.00255	no
gi|646694653|gb|KK966425.1|	19-1797	19	4.3	0	5	inf	5.00E-05	yes
gi|646694653|gb|KK966425.1|	24511-24820	19	4.3	0	309	inf	5.00E-05	yes
gi|646694653|gb|KK966425.1|	25306-26219	19	4.3	0	4	inf	0.0026	no
gi|646694653|gb|KK966425.1|	28354-28869	19	4.3	0	7	inf	0.00605	no
gi|646694653|gb|KK966425.1|	7190-8494	19	4.3	0	9	inf	5.00E-05	yes
gi|646694720|gb|KK966419.1|	121-739	10	4.7	2	3	0.296808	0.8322	no
gi|646694975|gb|KK966398.1|	46405-46736	16	7.1	4	13	2	0.2988	no
gi|646694989|gb|KK966396.1|	18486-21564	12	9.8	8	36	2	0.0618	no
gi|646694989|gb|KK966396.1|	29513-31137	12	9.8	5	13	1	0.31635	no
gi|646694989|gb|KK966396.1|	6220-8726	12	9.8	33	119	2	0.13035	no
gi|646694989|gb|KK966396.1|	9126-12831	12	9.8	164	490.39	2	0.1972	no
gi|646695153|gb|KK966385.1|	3688-4024	8.8	8.8	0	13	inf	0.0014	yes
gi|646695379|gb|KK966363.1|	35947-37038	21	8.2	0.553268	1	1	0.36645	no
gi|646695379|gb|KK966363.1|	39823-40677	21	8.2	5	10	0.991888	0.5342	no
gi|646695379|gb|KK966363.1|	43348-46787	21	8.2	34	23	-0.595477	0.60525	no
gi|646695379|gb|KK966363.1|	47267-47919	21	8.2	3	2	-0.526194	0.7013	no
gi|646695379|gb|KK966363.1|	53520-53972	21	8.2	8	15	0.902451	0.51685	no
gi|646695379|gb|KK966363.1|	58091-60545	21	8.2	2	0.794171	-0.970969	0.48405	no
gi|646695415|gb|KK966360.1|	5440-5966	24	9.9	8	0.460276	-4	0.1878	no
gi|646695477|gb|KK966356.1|	31549-31892	13	5.4	10	3	-2	0.35815	no
gi|646695528|gb|KK966351.1|	10241-10672	8.8	5	5	3	-0.77855	0.6023	no
gi|646695721|gb|KK966339.1|	3734-3946	11	4.5	222	94	-1	0.44865	no

gi|646695721|gb|KK966339.1|	77-669	11	4.5	16	20	0.281514	0.8511	no
gi|646695893|gb|KK966328.1|	20244-21983	13	6.1	0.311708	2	3	0.17425	no
gi|646695893|gb|KK966328.1|	53362-54946	13	6.1	6	2	-1	0.3851	no
gi|646695948|gb|KK966323.1|	12235-13771	21	11	0.361097	1	2	1	no
gi|646695948|gb|KK966323.1|	28023-28441	21	11	2	8	2	0.2544	no
gi|646695948|gb|KK966323.1|	32837-36120	21	11	12	4	-2	0.189	no
gi|646695948|gb|KK966323.1|	3546-4249	21	11	5	1	-2	0.26135	no
gi|646695948|gb|KK966323.1|	5461-6502	21	11	2	3	0.83428	0.55825	no
gi|646696013|gb|KK966318.1|	0-1522	8.7	5.1	42	20	-1	0.3301	no
gi|646696076|gb|KK966312.1|	49720-50202	11	6.2	2	2	-0.163745	0.8987	no
gi|646696129|gb|KK966307.1|	25123-25294	15	8.9	0	863	inf	0.0004	yes
gi|646696129|gb|KK966307.1|	26542-27204	15	8.9	0	20	inf	0.00025	yes
gi|646696450|gb|KK966284.1|	32119-33193	12	3.4	17	13	-0.347667	0.8138	no
gi|646696450|gb|KK966284.1|	37167-39131	12	3.4	4	5	0.118878	0.93825	no
gi|646696500|gb|KK966279.1|	0-343	13	5.5	110	41	-1	0.36205	no
gi|646696500|gb|KK966279.1|	10695-11469	13	5.5	3	2	-0.922945	0.49515	no
gi|646696500|gb|KK966279.1|	13500-13711	13	5.5	265	42	-3	0.10015	no
gi|646696500|gb|KK966279.1|	21453-21762	13	5.5	10	6	-0.748385	0.6124	no
gi|646696584|gb|KK966272.1|	22133-23584	16	5.2	0.773514	1	0.789036	0.54425	no
gi|646696685|gb|KK966264.1|	870-1433	12	9.5	4	4	0.0373851	0.9493	no
gi|646696691|gb|KK966263.1|	24745-26223	26	17	3	2	-0.717344	0.6518	no
gi|646696719|gb|KK966260.1|	37677-38128	25	9.2	6	3	-0.858685	0.54895	no
gi|646696746|gb|KK966257.1|	1062-1885	9.2	3.6	8	5	-0.564177	0.71705	no
gi|646696746|gb|KK966257.1|	28-1003	9.2	3.6	3	4	0.701369	0.6092	no
gi|646696763|gb|KK966255.1|	11399-12164	17	5.8	3	3	-0.356961	0.77745	no
gi|646696763|gb|KK966255.1|	156-780	17	5.8	3	2	-0.910446	0.53065	no
gi|646696763|gb|KK966255.1|	3746-5658	17	5.8	2	2	-0.228505	0.88345	no
gi|646696763|gb|KK966255.1|	887-2189	17	5.8	4	2	-1	0.3805	no
gi|646696770|gb|KK966254.1|	7984-8894	9.9	7.8	4	2	-0.611029	0.65755	no
gi|646696789|gb|KK966252.1|	26889-27126	13	9.8	21	22	0.068007	0.918	no

gi|646696789|gb|KK966252.1|	4920-5376	13	9.8	3	5	0.599961	0.6563	no
gi|646697061|gb|KK966235.1|	19872-21078	13	4.5	7	0.27883	-5	0.11105	no
gi|646697070|gb|KK966234.1|	14432-16223	13	7.8	6	5	-0.263808	0.8547	no
gi|646697070|gb|KK966234.1|	18582-19600	13	7.8	8	3	-1	0.4154	no
gi|646697070|gb|KK966234.1|	7263-9018	13	7.8	8	4	-1	0.47585	no
gi|646697147|gb|KK966230.1|	56352-56599	13	7.5	32	0	-inf	0.00475	no
gi|646697147|gb|KK966230.1|	62770-64074	13	7.5	1	0	-inf	0.00225	no
gi|646697221|gb|KK966226.1|	17340-18607	21	9	0.274239	150	9	0.1049	no
gi|646697221|gb|KK966226.1|	20662-21940	21	9	0.813871	7	3	0.0655	no
gi|646697221|gb|KK966226.1|	28362-28886	21	9	0.341742	6	4	0.0994	no
gi|646697221|gb|KK966226.1|	30105-31399	21	9	0.445189	3	3	0.13935	no
gi|646697221|gb|KK966226.1|	31858-32838	21	9	2	998	9	0.0067	no
gi|646697221|gb|KK966226.1|	38342-38777	21	9	1	18	4	0.24465	no
gi|646697221|gb|KK966226.1|	42146-43689	21	9	1	0.932048	-0.302195	0.81285	no
gi|646697221|gb|KK966226.1|	7496-9030	21	9	0.216997	89	9	0.1049	no
gi|646697250|gb|KK966223.1|	36352-36908	18	21	2	2	-0.227796	0.87465	no
gi|646697312|gb|KK966219.1|	20935-21461	11	5.3	14	0	-inf	0.00135	yes
gi|646697312|gb|KK966219.1|	40539-41188	11	5.3	17	18	0.130833	0.93495	no
gi|646697572|gb|KK966202.1|	09403-10977	21	5.6	6	4	-0.658235	0.66245	no
gi|646697572|gb|KK966202.1|	23170-12339	21	5.6	39	13	-2	0.3672	no
gi|646697572|gb|KK966202.1|	32537-13563	21	5.6	99	54	-0.872293	0.4658	no
gi|646697572|gb|KK966202.1|	36704-13867	21	5.6	1	0.702573	-0.618012	1	no
gi|646697572|gb|KK966202.1|	39605-14166	21	5.6	8	10	0.284693	0.8503	no
gi|646697572|gb|KK966202.1|	41921-14343	21	5.6	0.586918	1	0.849285	1	no
gi|646697572|gb|KK966202.1|	48753-15042	21	5.6	0.977954	0.470744	-1	1	no
gi|646697572|gb|KK966202.1|	52321-15949	21	5.6	14	11	-0.448068	0.69265	no
gi|646697572|gb|KK966202.1|	59860-16039	21	5.6	7	5	-0.628873	0.61215	no
gi|646697572|gb|KK966202.1|	37141-45560	21	5.6	5	4	-0.372866	0.7357	no
gi|646697572|gb|KK966202.1|	58857-61253	21	5.6	3	1	-2	0.35065	no
gi|646697572|gb|KK966202.1|	75098-75392	21	5.6	9	9	-0.039559	0.91525	no

gi|646697572|gb|KK966202.1|	86966-88497	21	5.6	3	4	0.5998	0.6934	no
gi|646697621|gb|KK966198.1|	1-590	10	4.6	0.82872	3	2	0.2058	no
gi|646697621|gb|KK966198.1|	38792-39575	10	4.6	2	1	-0.885401	0.53905	no
gi|646697621|gb|KK966198.1|	7879-8332	10	4.6	2	7	2	0.32225	no
gi|646697650|gb|KK966195.1|	14639-15237	12	7.5	7	0	-inf	0.00315	no
gi|646697679|gb|KK966193.1|	22562-24161	19	8.5	1	1	-0.0991317	0.93905	no
gi|646697679|gb|KK966193.1|	34925-36233	19	8.5	2	0.883342	-0.918138	0.47575	no
gi|646697679|gb|KK966193.1|	36453-37281	19	8.5	2	0.911711	-1	0.3737	no
gi|646697679|gb|KK966193.1|	39838-40452	19	8.5	0.514416	3	3	0.26695	no
gi|646697679|gb|KK966193.1|	40615-41143	19	8.5	2	3	0.440786	0.75545	no
gi|646697679|gb|KK966193.1|	42343-43295	19	8.5	0.929007	0.9432	0.0218739	1	no
gi|646697679|gb|KK966193.1|	43668-44705	19	8.5	1	2	0.627626	0.622	no
gi|646697679|gb|KK966193.1|	45661-46846	19	8.5	0.994714	2	0.781535	0.5464	no
gi|646697679|gb|KK966193.1|	47568-48871	19	8.5	4	4	-0.285062	0.8578	no
gi|646697679|gb|KK966193.1|	50858-53065	19	8.5	19	16	-0.201972	0.85575	no
gi|646697720|gb|KK966188.1|	10199-10949	13	14	0	3	inf	0.00255	no
gi|646697720|gb|KK966188.1|	21333-24415	13	14	26	1	-4	0.0095	no
gi|646697720|gb|KK966188.1|	24483-25154	13	14	8	0	-inf	0.00185	no
gi|646697836|gb|KK966182.1|	2346-5529	27	7.7	0.853159	1	0.693643	0.6638	no
gi|646697836|gb|KK966182.1|	8888-9935	27	7.7	2	2	0.0651928	0.954	no
gi|646697952|gb|KK966173.1|	14712-16100	42	23	0.89817	0.234908	-2	1	no
gi|646697952|gb|KK966173.1|	31105-32034	42	23	1	1	0.143212	0.9186	no
gi|646697960|gb|KK966172.1|	34536-35494	7.50E+00	4.1	8	4	-0.987084	0.52675	no
gi|646697960|gb|KK966172.1|	36287-36749	7.50E+00	4.1	92	7	-4	0.00725	no
gi|646697989|gb|KK966171.1|	14210-14786	11	5.1	2	2	-0.351161	0.81835	no
gi|646698281|gb|KK966151.1|	28272-29502	18	9.1	2	4	1	0.32945	no
gi|646698360|gb|KK966146.1|	19159-19502	12	5.5	17	39	1	0.3824	no
gi|646698405|gb|KK966143.1|	23067-23598	26	7.9	2	2	-0.365476	0.811	no
gi|646698405|gb|KK966143.1|	70313-70989	26	7.9	10	0	-inf	0.0015	yes
gi|646698581|gb|KK966129.1|	44252-44656	8.4	3.8	13	16	0.303433	0.8545	no

gi|646698682|gb|KK966122.1|	7766-8029	10	5.5	179	161	-0.153171	0.9223	no
gi|646698808|gb|KK966113.1|	11080-12663	23	6.5	2	2	-0.310918	0.8377	no
gi|646698808|gb|KK966113.1|	1714-2274	23	6.5	4	4	-0.185917	0.8883	no
gi|646698808|gb|KK966113.1|	20801-21857	23	6.5	1	1	-0.264514	0.8336	no
gi|646698808|gb|KK966113.1|	23068-23506	23	6.5	5	7	0.258625	0.84835	no
gi|646698808|gb|KK966113.1|	31628-34291	23	6.5	1	1	0.276263	0.8573	no
gi|646698808|gb|KK966113.1|	4513-6624	23	6.5	2	0.650208	-2	0.25955	no
gi|646698845|gb|KK966109.1|	19037-20782	19	7.4	1	1	0.283271	0.835	no
gi|646698845|gb|KK966109.1|	20865-24778	19	7.4	1	1	0.47342	0.7536	no
gi|646698845|gb|KK966109.1|	2744-3906	19	7.4	1	0.438047	-1	1	no
gi|646698951|gb|KK966099.1|	12334-13194	6.5	3.2	0.917346	4	2	0.2375	no
gi|646699089|gb|KK966087.1|	6167-6587	8.3	5.8	21	6	-2	0.2192	no
gi|646699116|gb|KK966084.1|	2865-3885	52	13	0.362763	2	2	0.186	no
gi|646699225|gb|KK966075.1|	35227-35524	11	7.1	12	17	0.55135	0.6943	no
gi|646699225|gb|KK966075.1|	44236-44887	11	7.1	137	151	0.143043	0.90215	no
gi|646699369|gb|KK966063.1|	12556-12725	30	13	259	170	-0.604192	0.6425	no
gi|646699369|gb|KK966063.1|	16323-16973	30	13	3	2	-0.401049	0.7788	no
gi|646699369|gb|KK966063.1|	850-1598	30	13	11	4	-2	0.23805	no
gi|646699467|gb|KK966056.1|	11971-12286	14	6.1	23	22	-0.079171	0.9562	no
gi|646699742|gb|KK966043.1|	43902-45260	20	14	0	3	inf	0.00175	no
gi|646699742|gb|KK966043.1|	54091-54330	20	14	0	68	inf	0.00315	no
gi|646699768|gb|KK966040.1|	36583-41073	18	7.2	151	83	-0.873658	0.46885	no
gi|646699768|gb|KK966040.1|	42003-45684	18	7.2	1	0.708356	-0.901565	0.57195	no
gi|646699768|gb|KK966040.1|	45914-46801	18	7.2	259	151	-0.781454	0.51035	no
gi|646699783|gb|KK966039.1|	24040-24717	7.1	3.7	14	13	-0.141365	0.91935	no
gi|646699848|gb|KK966033.1|	20234-20780	42	15	3	3	0.0320234	0.9298	no
gi|646699848|gb|KK966033.1|	28525-29110	42	15	1	2	0.458652	0.7747	no
gi|646699848|gb|KK966033.1|	31836-32386	42	15	6	3	-0.992204	0.46725	no
gi|646699848|gb|KK966033.1|	32704-33253	42	15	8	1	-3	0.1694	no
gi|646699848|gb|KK966033.1|	46937-49626	42	15	2	1	-0.519456	0.73495	no

gi|646699848|gb|KK966033.1|	59586-59842	42	15	18	30	0.782751	0.5685	no
gi|646699875|gb|KK966031.1|	10560-10983	11	4.8	0	22	inf	0.001	yes
gi|646699875|gb|KK966031.1|	6112-6755	11	4.8	16	6	-2	0.3352	no
gi|646700072|gb|KK966018.1|	16543-17765	11	6.9	0.095666	7	6	0.09545	no
gi|646700072|gb|KK966018.1|	18242-22641	11	6.9	0.044653	13	8	0.2322	no
gi|646700072|gb|KK966018.1|	26802-27595	11	6.9	0	18	inf	5.00E-05	yes
gi|646700072|gb|KK966018.1|	29477-31384	11	6.9	0	16	inf	5.00E-05	yes
gi|646700072|gb|KK966018.1|	31524-32281	11	6.9	0	3	inf	0.0008	yes
gi|646700072|gb|KK966018.1|	6-804	11	6.9	0	5	inf	0.0026	no
gi|646700072|gb|KK966018.1|	7022-7582	11	6.9	0	9	inf	0.0023	no
gi|646700072|gb|KK966018.1|	8909-9524	11	6.9	0	4	inf	0.001	yes
gi|646700095|gb|KK966015.1|	22281-23241	45	16	0.787258	1	0.507806	1	no
gi|646700234|gb|KK966005.1|	18648-18977	9.1	6.8	0	14	inf	0.0014	yes
gi|646700234|gb|KK966005.1|	29860-30037	9.1	6.8	0	3593.32	inf	5.00E-05	yes
gi|646700461|gb|KK965992.1|	18067-19051	70	24	14	14	-0.00170533	0.9943	no
gi|646700461|gb|KK965992.1|	24655-25368	70	24	5	5	0.0316103	0.9778	no
gi|646700461|gb|KK965992.1|	28870-29476	70	24	4	7	0.904524	0.52295	no
gi|646700467|gb|KK965991.1|	9163-9519	13	5.3	12	1	-3	0.1774	no
gi|646700752|gb|KK965974.1|	3441-4408	20	8.7	0.90944	1	0.285847	1	no
gi|646700859|gb|KK965968.1|	15233-16104	32	17	0.600646	2	2	0.36865	no
gi|646700859|gb|KK965968.1|	19681-20161	32	17	2	3	0.683266	0.6482	no
gi|646700964|gb|KK965963.1|	15163-15827	18	6.8	6	0.314345	-4	0.18655	no
gi|646701057|gb|KK965959.1|	32036-33267	15	5.9	1	1	0.060655	1	no
gi|646701057|gb|KK965959.1|	33432-34369	15	5.9	0.542534	1	1	0.43305	no
gi|646701057|gb|KK965959.1|	51452-51780	15	5.9	178	168.12	-0.0849164	0.9533	no
gi|646701057|gb|KK965959.1|	57283-58142	15	5.9	3	5	0.854098	0.535	no
gi|646701057|gb|KK965959.1|	58375-60016	15	5.9	0.800988	1	0.752003	0.5503	no
gi|646701120|gb|KK965957.1|	13549-14531	17	8	2	2	0.0941018	0.93975	no
gi|646701129|gb|KK965956.1|	24601-25379	14	5.4	38	14	-1	0.2709	no
gi|646701129|gb|KK965956.1|	28083-28365	14	5.4	7	12	0.782869	0.62645	no

gi|646701129|gb|KK965956.1|	9533-9988	14	5.4	14	3	-2	0.18995	no
gi|646701164|gb|KK965952.1|	16954-17283	12	5.3	0	55	inf	0.0002	yes
gi|646701164|gb|KK965952.1|	31459-31680	12	5.3	0	83	inf	0.00435	no
gi|646701190|gb|KK965950.1|	35150-35791	6.8	6.7	22	5	-2	0.2172	no
gi|646701413|gb|KK965936.1|	02395-10323	14	5	2	1	-0.237895	0.8627	no
gi|646701413|gb|KK965936.1|	04089-10463	14	5	9	3	-1	0.3511	no
gi|646701413|gb|KK965936.1|	462-870	14	5	3	3	-0.211058	0.88655	no
gi|646701413|gb|KK965936.1|	70049-70673	14	5	2	2	-0.379931	0.8011	no
gi|646701428|gb|KK965934.1|	7212-7511	12	5.9	14	0	-inf	0.00655	no
gi|646701454|gb|KK965933.1|	25298-25751	10	7.4	4	2	-0.764377	0.60755	no
gi|646701454|gb|KK965933.1|	27254-28424	10	7.4	0.606611	1	0.740342	1	no
gi|646701551|gb|KK965927.1|	27104-28186	14	5.3	7	5	-0.597575	0.69195	no
gi|646701643|gb|KK965922.1|	19105-19513	59	16	11	5	-1	0.33755	no
gi|646701643|gb|KK965922.1|	7196-8084	59	16	3	2	-0.345191	0.7847	no
gi|646701671|gb|KK965920.1|	17742-19041	11	4.4	5	1	-2	0.1525	no
gi|646701671|gb|KK965920.1|	23242-24954	11	4.4	3	3	0.313908	0.8341	no
gi|646701683|gb|KK965919.1|	43318-43735	33	8.2	60	0.721042	-6	0.16735	no
gi|646701683|gb|KK965919.1|	49368-50150	33	8.2	2	0	-inf	0.00475	no
gi|646701683|gb|KK965919.1|	50262-51926	33	8.2	2	0	-inf	0.0032	no
gi|646701683|gb|KK965919.1|	60816-61126	33	8.2	21	12	-0.834105	0.51465	no
gi|646702072|gb|KK965897.1|	0-926	13	6.9	7	5	-0.301779	0.8492	no
gi|646702072|gb|KK965897.1|	11521-12697	13	6.9	2	1	-0.581364	0.67605	no
gi|646702072|gb|KK965897.1|	30888-31412	13	6.9	7	6	-0.145667	0.9019	no
gi|646702072|gb|KK965897.1|	40467-41504	13	6.9	3	4	0.319504	0.84575	no
gi|646702072|gb|KK965897.1|	5250-5998	13	6.9	2	4	1	0.37175	no
gi|646702080|gb|KK965896.1|	39374-41844	15	7.2	4	1	-2	0.25225	no
gi|646702080|gb|KK965896.1|	45803-46446	15	7.2	7	2	-2	0.20385	no
gi|646702080|gb|KK965896.1|	47815-48203	15	7.2	101	6	-4	0.0208	no
gi|646702201|gb|KK965890.1|	19628-20131	12	5	10	20	0.98104	0.535	no
gi|646702334|gb|KK965879.1|	38474-39271	12	5.3	5	1	-2	0.24815	no

gi|646702334|gb|KK965879.1|	42181-42808	12	5.3	2	2	-0.37928	0.8011	no
gi|646702482|gb|KK965870.1|	54767-55682	1.70E+01	6.3	4	3	-0.34319	0.83785	no
gi|646702482|gb|KK965870.1|	81659-83167	1.70E+01	6.3	9	3	-1	0.3435	no
gi|646702491|gb|KK965869.1|	15090-15802	9.4	5	3	4	0.203866	0.86565	no
gi|646702509|gb|KK965867.1|	31279-31538	5.4	3.5	22	16	-0.445557	0.74925	no
gi|646702651|gb|KK965860.1|	15863-19049	20	8.6	4	6	0.753155	0.61775	no
gi|646702651|gb|KK965860.1|	4111-5368	20	8.6	17	79	2	0.12315	no
gi|646702651|gb|KK965860.1|	53248-54129	20	8.6	8	1	-3	0.1026	no
gi|646702651|gb|KK965860.1|	7346-7519	20	8.6	180	263	0.547529	0.7229	no
gi|646702651|gb|KK965860.1|	8471-9689	20	8.6	6	17	1	0.3729	no
gi|646702761|gb|KK965852.1|	23059-23932	13	12	2	1	-0.372849	0.78485	no
gi|646702817|gb|KK965849.1|	25506-27041	15	7.3	10	5	-0.869865	0.57145	no
gi|646702817|gb|KK965849.1|	36191-37130	15	7.3	17	12	-0.482405	0.74565	no
gi|646702817|gb|KK965849.1|	39486-40986	15	7.3	6	5	-0.327766	0.8267	no
gi|646702817|gb|KK965849.1|	42954-43228	15	7.3	31	13	-1	0.36145	no
gi|646702817|gb|KK965849.1|	45089-46333	15	7.3	17	8	-1	0.46075	no
gi|646702982|gb|KK965838.1|	46356-47683	17	5.5	3	3	0.179009	0.90815	no
gi|646702982|gb|KK965838.1|	56123-57255	17	5.5	6	7	0.207141	0.89095	no
gi|646703040|gb|KK965835.1|	26994-28898	18	9.2	0.561043	2	2	0.2768	no
gi|646703040|gb|KK965835.1|	34226-35985	18	9.2	0.799665	1	0.415919	1	no
gi|646703040|gb|KK965835.1|	39669-46950	18	9.2	8	5	-0.818669	0.46505	no
gi|646703040|gb|KK965835.1|	49825-50460	18	9.2	2	2	-0.0150164	0.92345	no
gi|646703040|gb|KK965835.1|	5216-5994	18	9.2	65	44	-0.563098	0.6093	no
gi|646703040|gb|KK965835.1|	55181-57868	18	9.2	2	2	-0.323069	0.83245	no
gi|646703040|gb|KK965835.1|	63114-63852	18	9.2	7	0.805442	-3	0.1264	no
gi|646703040|gb|KK965835.1|	65853-69064	18	9.2	2	1	-0.223792	0.888	no
gi|646703040|gb|KK965835.1|	78534-79398	18	9.2	3	1	-1	0.33355	no
gi|646703040|gb|KK965835.1|	80726-82260	18	9.2	1	0.521386	-0.957718	1	no
gi|646703040|gb|KK965835.1|	83016-85967	18	9.2	2	3	0.68957	0.6477	no
gi|646703182|gb|KK965826.1|	15122-15392	12	5.6	14	44	2	0.31005	no

gi|646703182|gb|KK965826.1|	16116-16621	12	5.6	4	10	1	0.3462	no
gi|646703255|gb|KK965822.1|	40783-41009	17	10	2344.16	2604.4	0.151878	0.889	no
gi|646703367|gb|KK965816.1|	41732-42591	36	7.9	5	0.433295	-3	0.13595	no
gi|646703367|gb|KK965816.1|	43847-44395	36	7.9	4	0.428649	-3	0.179	no
gi|646703367|gb|KK965816.1|	46366-46619	36	7.9	16	20	0.344027	0.8089	no
gi|646703367|gb|KK965816.1|	47900-48335	36	7.9	3	3	-0.190842	0.8939	no
gi|646703367|gb|KK965816.1|	48477-49990	36	7.9	1	1	-0.472702	0.70375	no
gi|646703367|gb|KK965816.1|	50966-52571	36	7.9	0.822345	0.791059	-0.0559588	1	no
gi|646703377|gb|KK965815.1|	10-260	13	15	0	81	inf	0.00095	yes
gi|646703417|gb|KK965812.1|	66427-66924	13	5.6	4	3	-0.446247	0.74355	no
gi|646703452|gb|KK965810.1|	20830-22180	12	6.2	0.084485	2	5	0.1055	no
gi|646703452|gb|KK965810.1|	28380-30393	12	6.2	0.052626	9	7	0.09465	no
gi|646703452|gb|KK965810.1|	8814-10019	12	6.2	0.292133	0.97691	2	1	no
gi|646703603|gb|KK965801.1|	23102-24234	12	9.6	2	1	-0.313606	0.8115	no
gi|646703647|gb|KK965797.1|	1589-1977	10	6.6	0	8	inf	0.00255	no
gi|646703647|gb|KK965797.1|	3295-4637	10	6.6	0	2	inf	0.00315	no
gi|646703703|gb|KK965794.1|	51184-52035	15	8	2	2	0.347772	0.79725	no
gi|646703703|gb|KK965794.1|	55717-55950	15	8	27	12	-1	0.4807	no
gi|646703724|gb|KK965792.1|	54422-55213	15	5.9	10	6	-0.663556	0.66975	no
gi|646703758|gb|KK965790.1|	15003-15447	11	5.6	10	4	-1	0.31265	no
gi|646703758|gb|KK965790.1|	57236-57871	11	5.6	1	3	1	0.3433	no
gi|646703758|gb|KK965790.1|	9410-10060	11	5.6	24	7	-2	0.24995	no
gi|646703871|gb|KK965783.1|	24454-26870	23	11	0.856255	0.9323	0.122754	1	no
gi|646704026|gb|KK965775.1|	19176-23185	22	8.5	33	4	-3	0.0461	no
gi|646704026|gb|KK965775.1|	23524-23815	22	8.5	17	4	-2	0.2023	no
gi|646704026|gb|KK965775.1|	2446-2972	22	8.5	28	2	-4	0.05565	no
gi|646704026|gb|KK965775.1|	36370-39796	22	8.5	40	5	-3	0.04065	no
gi|646704026|gb|KK965775.1|	79287-79551	22	8.5	436	324	-0.431624	0.779	no
gi|646704026|gb|KK965775.1|	90106-90279	22	8.5	580	181	-2	0.251	no
gi|646704185|gb|KK965769.1|	13278-13755	11	7.9	0.823449	5	3	0.26725	no

gi|646704317|gb|KK965761.1|	36736-37227	13	13	0	8	inf	0.00605	no
gi|646704375|gb|KK965756.1|	35677-35894	8.7	11	732	0	-inf	0.0002	yes
gi|646704464|gb|KK965750.1|	26318-27289	19	7.7	0.129194	5	5	0.0987	no
gi|646704481|gb|KK965749.1|	4908-5681	10	6.9	3	3	0.0224914	0.9633	no
gi|646704669|gb|KK965739.1|	16607-17276	17	6.8	45	30	-0.574182	0.6464	no
gi|646704669|gb|KK965739.1|	18891-19398	17	6.8	2	2	-0.152427	0.8987	no
gi|646704669|gb|KK965739.1|	27250-28832	17	6.8	46	33	-0.495538	0.6581	no
gi|646704669|gb|KK965739.1|	9345-10776	17	6.8	1	0	-inf	1	no
gi|646704795|gb|KK965732.1|	13709-14271	37	14	0.600372	3	2	0.2858	no
gi|646705060|gb|KK965720.1|	11625-12185	14	6.6	2	2	-0.13345	0.9025	no
gi|646705579|gb|KK965692.1|	10641-11474	44	13	1	2	0.498163	0.72155	no
gi|646705579|gb|KK965692.1|	11553-11941	44	13	5	3	-1	0.48135	no
gi|646705579|gb|KK965692.1|	19976-20788	44	13	3	3	0.0811153	0.94945	no
gi|646705606|gb|KK965690.1|	0-321	120	36	12	7	-0.865844	0.55565	no
gi|646705606|gb|KK965690.1|	15649-16524	120	36	0.596772	3	2	0.1831	no
gi|646705681|gb|KK965684.1|	28212-29618	8.6	6.2	0.964501	1	0.399443	0.75855	no
gi|646705681|gb|KK965684.1|	32608-33578	8.6	6.2	1	0.552105	-0.906615	1	no
gi|646705944|gb|KK965670.1|	15898-16543	13	7.1	4	2	-1	0.3597	no
gi|646705944|gb|KK965670.1|	72670-73143	13	7.1	2	3	0.416676	0.77485	no
gi|646705944|gb|KK965670.1|	88335-89219	13	7.1	3	5	0.917533	0.5072	no
gi|646706225|gb|KK965658.1|	3157-5288	22	5.9	2	0.285937	-3	0.12675	no
gi|646706225|gb|KK965658.1|	41-1037	22	5.9	9	7	-0.430347	0.77475	no
gi|646706225|gb|KK965658.1|	66091-67179	22	5.9	0.444208	1	2	0.29375	no
gi|646706225|gb|KK965658.1|	8585-10093	22	5.9	0.738331	0.425612	-0.79473	1	no
gi|646706386|gb|KK965650.1|	11719-13189	20	11	2	1	-0.473588	0.70375	no
gi|646706386|gb|KK965650.1|	19961-20134	20	11	200	148	-0.43455	0.74305	no
gi|646706386|gb|KK965650.1|	29586-31451	20	11	0.689558	0.831328	0.269744	1	no
gi|646706386|gb|KK965650.1|	32733-34754	20	11	4	7	1	0.5019	no
gi|646706386|gb|KK965650.1|	35622-37851	20	11	135	121.54	-0.151821	0.8983	no
gi|646706386|gb|KK965650.1|	55445-57505	20	11	1	7	2	0.157	no

gi|646706386|gb|KK965650.1|	57861-58546	20	11	9	9	0.0416003	0.9775	no
gi|646706386|gb|KK965650.1|	72206-72829	20	11	5	3	-0.380151	0.76	no
gi|646706386|gb|KK965650.1|	8674-9995	20	11	12	9	-0.367324	0.81105	no
gi|646706424|gb|KK965647.1|	2830-4182	120	31	7	3	-1	0.4727	no
gi|646706424|gb|KK965647.1|	34641-36051	120	31	2	2	-0.0368108	0.98015	no
gi|646706424|gb|KK965647.1|	36407-37269	120	31	2	1	-0.63679	0.65905	no
gi|646706424|gb|KK965647.1|	38259-41695	120	31	3	2	-0.457496	0.7652	no
gi|646706424|gb|KK965647.1|	42093-43347	120	31	0.555613	0.531454	-0.0641369	1	no
gi|646706424|gb|KK965647.1|	45737-46418	120	31	3	2	-0.520952	0.70145	no
gi|646706424|gb|KK965647.1|	61397-62690	120	31	1	1	-0.156067	0.90205	no
gi|646706424|gb|KK965647.1|	70077-73380	120	31	1	0.972354	-0.490354	0.75255	no
gi|646706424|gb|KK965647.1|	75056-76160	120	31	3	2	-0.970132	0.5106	no
gi|646706424|gb|KK965647.1|	86990-91823	120	31	10	5	-0.985964	0.3677	no
gi|646706563|gb|KK965639.1|	16851-17267	15	5	6	13	1	0.41675	no
gi|646706643|gb|KK965632.1|	61003-62053	16	9.3	0.930872	0.498069	-0.902237	1	no
gi|646706830|gb|KK965625.1|	15121-15712	12	5	0	4	inf	0.0014	yes
gi|646706887|gb|KK965621.1|	16831-17812	20	11	1	0.54399	-1	0.4271	no
gi|646706887|gb|KK965621.1|	58751-58983	20	11	50	4	-4	0.17885	no
gi|646706887|gb|KK965621.1|	64792-65569	20	11	3	1	-1	0.43425	no
gi|646706887|gb|KK965621.1|	66591-66903	20	11	9	7	-0.228625	0.87875	no
gi|646706887|gb|KK965621.1|	68513-69022	20	11	3	2	-0.0520602	0.92345	no
gi|646706887|gb|KK965621.1|	69277-71515	20	11	7	4	-0.621066	0.6808	no
gi|646706887|gb|KK965621.1|	75681-76310	20	11	2	1	-0.852781	0.56515	no
gi|646706887|gb|KK965621.1|	81098-82291	20	11	1	2	0.840715	0.5749	no
gi|646707149|gb|KK965607.1|	71329-74151	17	7.4	0.612712	4	3	0.077	no
gi|646707158|gb|KK965606.1|	17894-18442	23	5.7	9	4	-1	0.37915	no
gi|646707158|gb|KK965606.1|	18592-19057	23	5.7	2	3	0.997368	0.5906	no
gi|646707158|gb|KK965606.1|	41163-41748	23	5.7	3	0	-inf	0.00475	no
gi|646707158|gb|KK965606.1|	41810-43121	23	5.7	4	0	-inf	0.0005	yes
gi|646707165|gb|KK965605.1|	54919-55295	6.6	3.2	9	0.909019	-3	0.1825	no

gi|646707290|gb|KK965601.1|	6253-6549	9.1	4.2	4	24	2	0.15155	no
gi|646707370|gb|KK965597.1|	42404-42737	18	8	18	14	-0.420497	0.7214	no
gi|646707383|gb|KK965596.1|	46852-47281	13	5.8	3	3	0.126937	0.91245	no
gi|646707710|gb|KK965578.1|	19126-19561	17	7.1	6	8	0.394121	0.7546	no
gi|646707910|gb|KK965566.1|	05350-10735	22	8.2	0.846349	1	0.44123	0.7595	no
gi|646707910|gb|KK965566.1|	17-1498	22	8.2	2	0.869634	-0.865673	0.4995	no
gi|646707910|gb|KK965566.1|	45028-45316	22	8.2	5	13	1	0.37975	no
gi|646707910|gb|KK965566.1|	78338-79539	22	8.2	0.782314	0.980946	0.326424	1	no
gi|646707910|gb|KK965566.1|	80198-80610	22	8.2	6	10	0.755675	0.55595	no
gi|646708152|gb|KK965555.1|	23538-23835	21	9	13	2	-3	0.20545	no
gi|646708152|gb|KK965555.1|	46983-48530	21	9	1	0.825966	-0.641969	0.6136	no
gi|646708242|gb|KK965551.1|	6152-6614	12	4.6	6	8	0.303828	0.7989	no
gi|646708391|gb|KK965545.1|	13309-16295	1.70E+01	8.4	1	0.739297	-0.722177	0.6581	no
gi|646708391|gb|KK965545.1|	17185-18555	1.70E+01	8.4	1	0.119313	-4	0.17415	no
gi|646708391|gb|KK965545.1|	19325-20443	1.70E+01	8.4	3	3	-0.151533	0.9274	no
gi|646708391|gb|KK965545.1|	20524-21540	1.70E+01	8.4	2	0	-inf	0.0008	yes
gi|646708391|gb|KK965545.1|	22814-23601	1.70E+01	8.4	2	0.489566	-2	0.26845	no
gi|646708391|gb|KK965545.1|	23693-26185	1.70E+01	8.4	2	0.120125	-4	0.1242	no
gi|646708391|gb|KK965545.1|	26243-27841	1.70E+01	8.4	1	0.099389	-3	1	no
gi|646708391|gb|KK965545.1|	33899-36030	1.70E+01	8.4	2	0.214453	-3	0.10255	no
gi|646708391|gb|KK965545.1|	42680-43559	1.70E+01	8.4	3	0	-inf	0.0012	yes
gi|646708391|gb|KK965545.1|	52080-53572	1.70E+01	8.4	3	3	0.041446	0.97815	no
gi|646708391|gb|KK965545.1|	56660-58421	1.70E+01	8.4	2	2	-0.191042	0.9095	no
gi|646708391|gb|KK965545.1|	58625-59642	1.70E+01	8.4	3	5	0.61064	0.70005	no
gi|646708415|gb|KK965544.1|	28096-28688	9.5	4.9	6	8	0.527598	0.69855	no
gi|646708424|gb|KK965543.1|	13603-17275	23	4.9	3	5	0.565915	0.7116	no
gi|646708424|gb|KK965543.1|	30344-31889	23	4.9	2	5	1	0.4185	no
gi|646708534|gb|KK965538.1|	38400-39147	17	7.1	2	0.527585	-2	0.26845	no
gi|646708534|gb|KK965538.1|	49842-51072	17	7.1	0.664169	0.544242	-0.287301	1	no
gi|646708534|gb|KK965538.1|	51238-51655	17	7.1	5	5	0.0185302	0.9365	no

gi|646708558|gb|KK965537.1|	42463-43037	9.5	4	6	1	-2	0.1996	no
gi|646708679|gb|KK965530.1|	16062-16520	26	11	8	2	-2	0.2323	no
gi|646708688|gb|KK965529.1|	12225-13598	32	15	0.744724	3	2	0.2291	no
gi|646708727|gb|KK965528.1|	4929-5728	26	9.4	3	5	0.980259	0.50035	no
gi|646708788|gb|KK965526.1|	21187-21951	9.6	3.9	1	1	-0.316439	0.8509	no
gi|646709159|gb|KK965510.1|	16603-17357	22	9.7	1	1	0.00426601	0.92345	no
gi|646709159|gb|KK965510.1|	22755-23605	22	9.7	3	0.879223	-2	0.2124	no
gi|646709180|gb|KK965509.1|	49779-52888	16	6.2	1	1	-0.00883833	0.9852	no
gi|646709180|gb|KK965509.1|	53115-54253	16	6.2	2	2	0.236603	0.8422	no
gi|646709395|gb|KK965496.1|	54080-54778	17	5.9	2	0.87446	-0.933207	0.589	no
gi|646709526|gb|KK965490.1|	39569-39985	11	6.9	19	25	0.380325	0.8038	no
gi|646709589|gb|KK965487.1|	39320-40526	8.2	4.9	0	3	inf	0.00185	no
gi|646709807|gb|KK965476.1|	13886-14596	15	7.5	32	8	-2	0.2114	no
gi|646709807|gb|KK965476.1|	28721-28978	15	7.5	10	19	0.940973	0.58285	no
gi|646709857|gb|KK965475.1|	3704-4419	11	5.9	4	0.281248	-4	0.171	no
gi|646709877|gb|KK965473.1|	3372-4240	18	8.7	8	10	0.442318	0.76465	no
gi|646709877|gb|KK965473.1|	9540-11041	18	8.7	20	42	1	0.33905	no
gi|646709950|gb|KK965471.1|	0-516	13	7.8	0	79	inf	5.00E-05	yes
gi|646709950|gb|KK965471.1|	4394-4696	13	7.8	0	42	inf	0.0017	no
gi|646709960|gb|KK965470.1|	10884-13850	21	11	27	9	-2	0.15475	no
gi|646709960|gb|KK965470.1|	14757-18101	21	11	10	4	-1	0.34355	no
gi|646709960|gb|KK965470.1|	18336-19124	21	11	3	2	-0.946912	0.4699	no
gi|646709960|gb|KK965470.1|	21144-21997	21	11	26	10	-1	0.38615	no
gi|646709960|gb|KK965470.1|	47897-48458	21	11	2	2	-0.355537	0.81835	no
gi|646710126|gb|KK965466.1|	38637-39350	27	13	3	0.847247	-2	0.28565	no
gi|646710126|gb|KK965466.1|	51047-51864	27	13	0.987512	2	0.719037	0.6352	no
gi|646710126|gb|KK965466.1|	61729-62229	27	13	1	4	1	0.44135	no
gi|646710343|gb|KK965459.1|	867-1402	12	5.2	41	10	-2	0.20125	no
gi|646710354|gb|KK965458.1|	15649-16905	130	92	297	266	-0.159787	0.89195	no
gi|646710623|gb|KK965453.1|	13862-14154	9.7	6.8	22	14	-0.587939	0.62765	no

gi|646710909|gb|KK965445.1|	32294-32907	13	9.4	3	2	-0.912918	0.5306	no
gi|646711086|gb|KK965436.1|	13496-15238	46	16	1	1	0.0905834	0.9429	no
gi|646711086|gb|KK965436.1|	17302-19302	46	16	1	2	0.271263	0.868	no
gi|646711086|gb|KK965436.1|	22776-24906	46	16	1	1	0.139626	0.9288	no
gi|646711086|gb|KK965436.1|	31107-31980	46	16	1	1	-0.0833424	0.93265	no
gi|646711086|gb|KK965436.1|	35228-36960	46	16	4	0.362028	-4	0.0628	no
gi|646711086|gb|KK965436.1|	37713-38703	46	16	2	1	-0.29775	0.8162	no
gi|646711086|gb|KK965436.1|	39381-41015	46	16	0.737966	1	0.655026	0.6075	no
gi|646711086|gb|KK965436.1|	4373-4887	46	16	1	6	2	0.19745	no
gi|646711086|gb|KK965436.1|	5662-6970	46	16	2	1	-0.188058	0.88005	no
gi|646711086|gb|KK965436.1|	56748-57546	46	16	9	0.720066	-4	0.0932	no
gi|646711305|gb|KK965426.1|	31050-33755	14	5.8	4	0.054888	-6	0.1673	no
gi|646711305|gb|KK965426.1|	43384-44833	14	5.8	2	0	-inf	0.0031	no
gi|646711305|gb|KK965426.1|	45234-46441	14	5.8	2	0	-inf	0.00525	no
gi|646711305|gb|KK965426.1|	48614-49170	14	5.8	6	0.418189	-4	0.17325	no
gi|646711305|gb|KK965426.1|	62767-63160	14	5.8	13	6	-1	0.37905	no
gi|646711455|gb|KK965419.1|	44613-45501	17	6.7	3	1	-1	0.4572	no
gi|646711455|gb|KK965419.1|	91918-92424	17	6.7	3	4	0.432117	0.75085	no
gi|646711482|gb|KK965418.1|	36342-37300	9.6	4.9	55	36	-0.59631	0.58535	no
gi|646711521|gb|KK965417.1|	7697-8831	16	12	0.526208	1	1	0.36645	no
gi|646711521|gb|KK965417.1|	795-2580	16	12	0.604675	0.524501	-0.205213	1	no
gi|646711521|gb|KK965417.1|	9993-11915	16	12	0.887977	0.642623	-0.46655	1	no
gi|646711735|gb|KK965410.1|	2887-3203	2.20E+01	15	16.59	10	-0.736472	0.58315	no
gi|646711979|gb|KK965400.1|	67540-67902	8.4	4.2	3	7	1	0.4736	no
gi|646712006|gb|KK965398.1|	19377-19590	11	8.9	35	23	-0.617195	0.67825	no
gi|646712163|gb|KK965389.1|	7009-7477	13	14	0	19	inf	0.0007	yes
gi|646712247|gb|KK965385.1|	211-782	7.4	4.1	15	6	-1	0.42645	no
gi|646712286|gb|KK965382.1|	240-656	14	6.9	3	4	0.380325	0.8169	no
gi|646712286|gb|KK965382.1|	3088-4023	14	6.9	2	2	-0.581275	0.66405	no
gi|646712286|gb|KK965382.1|	9304-9745	14	6.9	34	32	-0.0873332	0.95165	no

gi|646712499|gb|KK965372.1|	33688-34165	14	6.5	16	2	-3	0.11325	no
gi|646712499|gb|KK965372.1|	43090-43252	14	6.5	191.61	296	0.626344	0.64235	no
gi|646712499|gb|KK965372.1|	8808-9376	14	6.5	0	6	inf	0.00515	no
gi|646712723|gb|KK965363.1|	9474-9976	11	9.3	0	8	inf	0.00515	no
gi|646712812|gb|KK965357.1|	25672-27764	21	9.9	2	2	-0.306097	0.8406	no
gi|646712812|gb|KK965357.1|	50550-51065	21	9.9	9	13	0.490246	0.7566	no
gi|646712812|gb|KK965357.1|	79881-80559	21	9.9	6	3	-0.899977	0.5348	no
gi|646712853|gb|KK965353.1|	15166-15685	11	4.5	6	0	-inf	0.0012	yes
gi|646712970|gb|KK965348.1|	33974-34150	27	12	54	119	1	0.4405	no
gi|646712978|gb|KK965347.1|	43269-43694	11	15	4	1	-2	0.3081	no
gi|646713019|gb|KK965346.1|	2917-3371	10	7.1	0	8	inf	0.0008	yes
gi|646713097|gb|KK965344.1|	04475-10951	21	9.6	4	2	-0.599332	0.6947	no
gi|646713097|gb|KK965344.1|	10297-11075	21	9.6	2	4	0.893324	0.56765	no
gi|646713097|gb|KK965344.1|	25516-12855	21	9.6	6	2	-1	0.4239	no
gi|646713097|gb|KK965344.1|	15805-20007	21	9.6	539	808	0.584867	0.6053	no
gi|646713097|gb|KK965344.1|	31289-31444	21	9.6	225	299	0.412415	0.79515	no
gi|646713097|gb|KK965344.1|	37962-38319	21	9.6	4	14	2	0.22895	no
gi|646713097|gb|KK965344.1|	38697-39237	21	9.6	0.322943	4	4	0.1063	no
gi|646713097|gb|KK965344.1|	49424-56349	21	9.6	140	177	0.338417	0.77685	no
gi|646713097|gb|KK965344.1|	56938-58451	21	9.6	20	55	1	0.19595	no
gi|646713097|gb|KK965344.1|	58995-59879	21	9.6	47	137	2	0.18115	no
gi|646713097|gb|KK965344.1|	60259-61026	21	9.6	19	33	0.774581	0.6129	no
gi|646713097|gb|KK965344.1|	62115-66184	21	9.6	5	11	1	0.3296	no
gi|646713097|gb|KK965344.1|	74278-74976	21	9.6	17	16	-0.0587375	0.9686	no
gi|646713097|gb|KK965344.1|	78803-79721	21	9.6	1	0.395996	-2	0.2991	no
gi|646713097|gb|KK965344.1|	8104-8587	21	9.6	6	0	-inf	0.00195	no
gi|646713097|gb|KK965344.1|	81853-82007	21	9.6	189	209	0.140368	0.8941	no
gi|646713109|gb|KK965343.1|	46686-47135	15	9.7	1	4	2	0.29775	no
gi|646713109|gb|KK965343.1|	47314-48142	15	9.7	1	2	0.497697	0.7157	no
gi|646713160|gb|KK965341.1|	112-678	6.1	4.2	4	1	-2	0.3498	no

gi|646713219|gb|KK965338.1|	20869-21348	14	6.8	2	4	1	0.3784	no
gi|646713219|gb|KK965338.1|	37812-38888	14	6.8	3	5	0.514042	0.7419	no
gi|646713219|gb|KK965338.1|	63165-63356	14	6.8	53	48	-0.154093	0.90205	no
gi|646713534|gb|KK965322.1|	15184-15567	13	9.2	6	5	-0.233851	0.867	no
gi|646713629|gb|KK965320.1|	10175-10572	9	5.4	9	13	0.457527	0.74925	no
gi|646713666|gb|KK965318.1|	11161-11399	21	10	7992.5	7	-10	0.10535	no
gi|646713666|gb|KK965318.1|	4921-5463	21	10	24	0	-inf	5.00E-05	yes
gi|646713666|gb|KK965318.1|	61469-62190	21	10	2	1	-0.192786	0.89315	no
gi|646713666|gb|KK965318.1|	7324-8214	21	10	6	0	-inf	0.0008	yes
gi|646713733|gb|KK965315.1|	2535-7306	17	8.3	0	21	inf	5.00E-05	yes
gi|646714037|gb|KK965300.1|	23424-27329	63	34	9	6	-0.772317	0.4781	no
gi|646714037|gb|KK965300.1|	48733-48993	63	34	29	13	-1	0.43705	no
gi|646714168|gb|KK965297.1|	39318-39932	21	11	0	5	inf	0.0008	yes
gi|646714168|gb|KK965297.1|	40046-40489	21	11	0	27	inf	0.00015	yes
gi|646714220|gb|KK965294.1|	21418-22176	19	7.1	3	0	-inf	0.0011	yes
gi|646714269|gb|KK965292.1|	41779-42773	18	12	7	6	-0.0613231	0.96685	no
gi|646714414|gb|KK965285.1|	15937-18328	19	12	1	2	0.878657	0.5755	no
gi|646714414|gb|KK965285.1|	18-377	19	12	8	5	-0.675317	0.65215	no
gi|646714414|gb|KK965285.1|	19601-20881	19	12	2	2	-0.09505	0.95295	no
gi|646714414|gb|KK965285.1|	21736-22544	19	12	3	2	-0.334322	0.79055	no
gi|646714414|gb|KK965285.1|	30814-31839	19	12	5	1	-2	0.2086	no
gi|646714414|gb|KK965285.1|	34350-35508	19	12	2	0.293294	-3	0.1299	no
gi|646714414|gb|KK965285.1|	36204-38021	19	12	2	0.171303	-3	0.1408	no
gi|646714414|gb|KK965285.1|	38881-40192	19	12	2	0.880885	-0.992054	0.4647	no
gi|646714414|gb|KK965285.1|	51827-52550	19	12	4	2	-0.736822	0.5714	no
gi|646714414|gb|KK965285.1|	54495-55145	19	12	1	1	-0.111542	0.9034	no
gi|646714414|gb|KK965285.1|	55422-56754	19	12	1	1	-0.242436	0.84115	no
gi|646714414|gb|KK965285.1|	63939-64951	19	12	0.610993	1	0.996261	0.5626	no
gi|646714414|gb|KK965285.1|	9003-9584	19	12	6	4	-0.612815	0.62245	no
gi|646714463|gb|KK965282.1|	0-426	18	10	9	4	-1	0.36885	no

gi|646714463|gb|KK965282.1|	1924-2216	18	10	20	0	-inf	0.0035	no
gi|646714670|gb|KK965275.1|	51371-52187	11	7.4	1	2	0.496547	0.7218	no
gi|646714857|gb|KK965268.1|	22489-12346	14	7	0	6	inf	0.0006	yes
gi|646714857|gb|KK965268.1|	21448-22231	14	7	4	4	-0.0594303	0.96805	no
gi|646714902|gb|KK965265.1|	12297-13095	39	18	3	4	0.664653	0.6448	no
gi|646714902|gb|KK965265.1|	82158-83186	39	18	7	6	-0.289769	0.8459	no
gi|646714930|gb|KK965264.1|	38164-38824	11	6.9	0	5	inf	0.00515	no
gi|646714985|gb|KK965261.1|	13198-13957	14	3.9	3	0	-inf	0.00225	no
gi|646714985|gb|KK965261.1|	56763-58973	14	3.9	9	9	-0.00576433	0.99615	no
gi|646714985|gb|KK965261.1|	59308-60352	14	3.9	2	0.334501	-2	0.1971	no
gi|646715043|gb|KK965260.1|	25677-26383	11	5.5	0.820133	3	2	0.2977	no
gi|646715494|gb|KK965243.1|	46778-47096	25	13	20	10	-1	0.421	no
gi|646715494|gb|KK965243.1|	47153-48187	25	13	11	6	-0.919344	0.547	no
gi|646715539|gb|KK965241.1|	26491-27582	8.2	6.2	1	0.316186	-2	0.20995	no
gi|646715594|gb|KK965237.1|	76658-77260	11	4.8	20	48	1	0.4021	no
gi|646715594|gb|KK965237.1|	86361-88091	11	4.8	0.439053	0.634394	0.530983	1	no
gi|646715790|gb|KK965229.1|	31257-31578	25	9.5	5	8	0.856622	0.60055	no
gi|646716139|gb|KK965218.1|	10165-11305	22	9.1	21	33	0.659247	0.56505	no
gi|646716139|gb|KK965218.1|	14145-11449	22	9.1	5	30	2	0.1547	no
gi|646716139|gb|KK965218.1|	15513-11750	22	9.1	1	2	0.423214	0.7868	no
gi|646716139|gb|KK965218.1|	22419-22848	22	9.1	8	28	2	0.21915	no
gi|646716180|gb|KK965216.1|	25975-28233	24	7.2	6	8	0.525046	0.733	no
gi|646716621|gb|KK965196.1|	44363-45280	8.8	7.6	4	2	-1	0.42005	no
gi|646716675|gb|KK965193.1|	17090-18818	12	5	0.125614	2	4	0.25965	no
gi|646716723|gb|KK965191.1|	9861-10367	31	19	4	4	0.110188	0.93395	no
gi|646716756|gb|KK965190.1|	8563-9150	11	4.6	2	3	0.459182	0.75425	no
gi|646716990|gb|KK965179.1|	10627-20052	19	9.7	7	7	-0.00130217	0.9988	no
gi|646716990|gb|KK965179.1|	35137-13552	19	9.7	4	5	0.352111	0.80755	no
gi|646716990|gb|KK965179.1|	30278-31515	19	9.7	1	1	0.105245	0.94	no
gi|646716990|gb|KK965179.1|	31-598	19	9.7	1	3	1	0.3774	no

gi|646716990|gb|KK965179.1|	31629-32156	19	9.7	9	10	0.132294	0.93415	no
gi|646716990|gb|KK965179.1|	33612-33883	19	9.7	36	39	0.0891832	0.94985	no
gi|646716990|gb|KK965179.1|	44296-44932	19	9.7	3	0.336012	-3	0.1994	no
gi|646716990|gb|KK965179.1|	45405-46325	19	9.7	3	3	0.133191	0.9336	no
gi|646716990|gb|KK965179.1|	61689-63265	19	9.7	1	2	0.750884	0.58805	no
gi|646716990|gb|KK965179.1|	63461-64738	19	9.7	0.995704	1	0.521532	0.686	no
gi|646716990|gb|KK965179.1|	66783-69028	19	9.7	0.604387	0.741782	0.295523	1	no
gi|646716990|gb|KK965179.1|	69381-71300	19	9.7	0.611585	0.321883	-0.926016	1	no
gi|646716990|gb|KK965179.1|	72516-72855	19	9.7	30	11	-1	0.31485	no
gi|646716990|gb|KK965179.1|	74597-74810	19	9.7	35	23	-0.617195	0.67825	no
gi|646716990|gb|KK965179.1|	77692-78348	19	9.7	2	3	0.474568	0.73205	no
gi|646716990|gb|KK965179.1|	93000-93575	19	9.7	3	2	-0.866015	0.56495	no
gi|646717028|gb|KK965178.1|	45275-46549	14	6.2	13	8	-0.653645	0.66815	no
gi|646717205|gb|KK965171.1|	36536-38292	8.4	4.8	89	65	-0.444799	0.7035	no
gi|646717501|gb|KK965158.1|	33140-34563	41	16	2	3	0.229915	0.88555	no
gi|646717501|gb|KK965158.1|	51008-51494	41	16	5	1	-2	0.19195	no
gi|646717501|gb|KK965158.1|	5256-6491	41	16	1	5	2	0.1903	no
gi|646717749|gb|KK965148.1|	30532-31534	13	7.9	0.743005	0.705523	-0.0746783	1	no
gi|646717826|gb|KK965143.1|	12822-13241	13	7	1	168	7	0.2322	no
gi|646717826|gb|KK965143.1|	16650-17826	13	7	0	14	inf	0.00035	yes
gi|646717826|gb|KK965143.1|	39401-40307	13	7	0	23	inf	5.00E-05	yes
gi|646717826|gb|KK965143.1|	59738-60556	13	7	0.164293	3	4	0.0994	no
gi|646717826|gb|KK965143.1|	75644-76782	13	7	0	42	inf	5.00E-05	yes
gi|646717826|gb|KK965143.1|	89103-89446	13	7	0	11	inf	0.00255	no
gi|646718033|gb|KK965135.1|	19123-19521	36	12	4	6	0.587706	0.6634	no
gi|646718072|gb|KK965133.1|	74168-74779	20	8.9	3	2	-0.409339	0.7788	no
gi|646718072|gb|KK965133.1|	74916-75645	20	8.9	2	1	-0.191662	0.90405	no
gi|646718124|gb|KK965131.1|	33807-34637	12	4.5	75	169	1	0.3237	no
gi|646718386|gb|KK965119.1|	16842-17332	11	6.5	4	4	0.255112	0.8603	no
gi|646718386|gb|KK965119.1|	20192-21556	11	6.5	16	10	-0.638332	0.66945	no

gi|646718386|gb|KK965119.1|	23041-23404	11	6.5	13	8	-0.758031	0.55345	no
gi|646718451|gb|KK965116.1|	21568-22564	16	8.8	1	4	2	0.23555	no
gi|646718465|gb|KK965115.1|	18079-18561	9.8	5.4	11	0	-inf	0.0031	no
gi|646718465|gb|KK965115.1|	18745-19114	9.8	5.4	12	12	0.0367277	0.96045	no
gi|646718465|gb|KK965115.1|	22362-22857	9.8	5.4	45	14	-2	0.3018	no
gi|646718465|gb|KK965115.1|	44846-45167	9.8	5.4	18	4	-2	0.1859	no
gi|646718465|gb|KK965115.1|	5816-6727	9.8	5.4	3	0.200041	-4	0.19055	no
gi|646718465|gb|KK965115.1|	63303-64274	9.8	5.4	4	5	0.0648761	0.96475	no
gi|646718465|gb|KK965115.1|	7423-10350	9.8	5.4	15	0.856217	-4	0.0225	no
gi|646718549|gb|KK965111.1|	9016-9488	17	5.5	3	4	0.638547	0.6597	no
gi|646718695|gb|KK965107.1|	5858-6158	6.4	7.8	24	18	-0.392832	0.74505	no
gi|646718748|gb|KK965105.1|	39010-39632	10	6	0.754962	2	2	0.2936	no
gi|646718932|gb|KK965097.1|	34056-35155	21	9.2	0.65763	0.9398	0.515077	1	no
gi|646718932|gb|KK965097.1|	37327-42450	21	9.2	5	11	1	0.2672	no
gi|646718932|gb|KK965097.1|	44036-44620	21	9.2	3	3	-0.0561884	0.93665	no
gi|646718932|gb|KK965097.1|	47437-49201	21	9.2	5	4	-0.28567	0.84145	no
gi|646718932|gb|KK965097.1|	50617-57645	21	9.2	2	3	0.275565	0.85655	no
gi|646718932|gb|KK965097.1|	68040-68631	21	9.2	16	4	-2	0.17445	no
gi|646718932|gb|KK965097.1|	69927-70503	21	9.2	3	1	-1	0.4702	no
gi|646718967|gb|KK965095.1|	28402-29526	7.7	5.3	0	9	inf	0.00045	yes
gi|646718967|gb|KK965095.1|	39673-43555	7.7	5.3	0	5	inf	0.0001	yes
gi|646718979|gb|KK965094.1|	17444-17991	68	15	3	7	1	0.3691	no
gi|646719049|gb|KK965091.1|	35255-13572	19	12	5	5	-0.0448627	0.94695	no
gi|646719049|gb|KK965091.1|	42784-14317	19	12	11	6	-0.920277	0.4818	no
gi|646719049|gb|KK965091.1|	91006-91680	19	12	3	2	-0.299767	0.82375	no
gi|646719279|gb|KK965081.1|	24873-25607	35	12	2	1	-0.972338	0.54505	no
gi|646719279|gb|KK965081.1|	32336-33212	35	12	1	3	2	0.3063	no
gi|646719279|gb|KK965081.1|	45386-45859	35	12	8	10	0.18221	0.8909	no
gi|646719734|gb|KK965062.1|	21847-22149	12	6	143	133	-0.103926	0.9441	no
gi|646719946|gb|KK965048.1|	16028-17259	17	5.3	2	0	-inf	0.00595	no

gi|646719946|gb|KK965048.1|	18344-20331	17	5.3	1	0	-inf	0.00595	no
gi|646719946|gb|KK965048.1|	28651-29193	17	5.3	16	19	0.261298	0.86475	no
gi|646719946|gb|KK965048.1|	39548-40342	17	5.3	0.343212	3	3	0.2502	no
gi|646719946|gb|KK965048.1|	53738-54698	17	5.3	0.524839	1	1	1	no
gi|646719977|gb|KK965047.1|	92998-19383	13	9.5	0.317862	2	3	0.2655	no
gi|646719977|gb|KK965047.1|	78226-78994	13	9.5	3	3	-0.356605	0.77745	no
gi|646720001|gb|KK965046.1|	16434-11949	15	7.4	0.856667	2	1	0.522	no
gi|646720001|gb|KK965046.1|	19557-12153	15	7.4	2	1	-0.559054	0.73315	no
gi|646720009|gb|KK965045.1|	12559-13171	9.7	6.2	3	2	-0.312245	0.8235	no
gi|646720009|gb|KK965045.1|	13704-14254	9.7	6.2	2	2	-0.229703	0.87465	no
gi|646720009|gb|KK965045.1|	16146-16508	9.7	6.2	22	7	-2	0.27895	no
gi|646720009|gb|KK965045.1|	2032-2506	9.7	6.2	6	0.557018	-3	0.179	no
gi|646720009|gb|KK965045.1|	25398-26197	9.7	6.2	12	3	-2	0.1849	no
gi|646720009|gb|KK965045.1|	2676-3643	9.7	6.2	1	0.369574	-1	1	no
gi|646720009|gb|KK965045.1|	4056-10078	9.7	6.2	11	5	-1	0.32545	no
gi|646720258|gb|KK965036.1|	27733-29094	20	10	0.501869	2	2	0.24775	no
gi|646720258|gb|KK965036.1|	32056-32627	20	10	3	3	0.132847	0.92595	no
gi|646720258|gb|KK965036.1|	34285-35206	20	10	1	1	0.0901921	0.9294	no
gi|646720258|gb|KK965036.1|	36979-37599	20	10	3	4	0.467182	0.7124	no
gi|646720493|gb|KK965022.1|	77056-77874	20	6	1	1	-0.0882204	0.9324	no
gi|646720506|gb|KK965020.1|	22697-22997	11	4	0	20	inf	0.001	yes
gi|646720575|gb|KK965017.1|	17606-18926	10	5.8	0	20	inf	5.00E-05	yes
gi|646720575|gb|KK965017.1|	21749-22180	10	5.8	0	19	inf	0.0012	yes
gi|646720575|gb|KK965017.1|	22666-23162	10	5.8	0	13	inf	0.00155	yes
gi|646720695|gb|KK965012.1|	1164-2017	25	14	0.30936	3	3	0.24735	no
gi|646720695|gb|KK965012.1|	14921-16530	25	14	0.888242	2	0.828633	0.57245	no
gi|646720695|gb|KK965012.1|	16649-17796	25	14	3	1	-1	0.47445	no
gi|646720695|gb|KK965012.1|	22994-24637	25	14	2	2	-0.392355	0.8033	no
gi|646720695|gb|KK965012.1|	30990-31975	25	14	3	0.541098	-2	0.16785	no
gi|646720695|gb|KK965012.1|	32119-33087	25	14	1	0.553607	-1	0.4271	no

gi|646720695|gb|KK965012.1|	6792-8267	25	14	2	0.873833	-1	0.38765	no
gi|646720695|gb|KK965012.1|	8388-12976	25	14	2	1	-1	0.47725	no
gi|646720752|gb|KK965010.1|	9272-9778	8.6	4.3	34	61	0.831724	0.5922	no
gi|646720773|gb|KK965009.1|	33409-34231	16	6.7	1	2	0.497128	0.72995	no
gi|646720990|gb|KK965002.1|	01017-10150	20	8.4	102	91	-0.165443	0.8767	no
gi|646720990|gb|KK965002.1|	03068-10368	20	8.4	1	2	0.951251	0.56265	no
gi|646720990|gb|KK965002.1|	04128-10714	20	8.4	15	10	-0.632943	0.56645	no
gi|646720990|gb|KK965002.1|	14836-11686	20	8.4	2	1	-0.153492	0.92075	no
gi|646720990|gb|KK965002.1|	20215-12103	20	8.4	2	3	0.371693	0.769	no
gi|646720990|gb|KK965002.1|	26181-12701	20	8.4	4	3	-0.260645	0.8561	no
gi|646720990|gb|KK965002.1|	1527-1863	20	8.4	24	10	-1	0.38995	no
gi|646720990|gb|KK965002.1|	45546-45807	20	8.4	26	26	0.00756232	0.9493	no
gi|646720990|gb|KK965002.1|	64154-64385	20	8.4	20	20	0.045433	0.90935	no
gi|646720990|gb|KK965002.1|	73603-74083	20	8.4	2	5	1	0.4025	no
gi|646720990|gb|KK965002.1|	97579-98818	20	8.4	0.658095	0.539375	-0.287007	1	no
gi|646721073|gb|KK964993.1|	23065-23510	11	6.5	0	40	inf	0.0002	yes
gi|646721280|gb|KK964989.1|	19038-19946	16	7.6	0.283311	2	3	0.24675	no
gi|646721345|gb|KK964985.1|	47493-47804	13	5.6	138	42	-2	0.27225	no
gi|646721394|gb|KK964981.1|	10214-11323	14	6.5	0.758253	0.619384	-0.291845	1	no
gi|646721394|gb|KK964981.1|	40780-41007	14	6.5	69	74	0.117002	0.93365	no
gi|646721394|gb|KK964981.1|	48943-49138	14	6.5	19	87	2	0.3022	no
gi|646721414|gb|KK964979.1|	59454-59913	16	6	2	4	0.99401	0.5906	no
gi|646721684|gb|KK964967.1|	14391-14916	15	5.5	1	4	2	0.29945	no
gi|646721684|gb|KK964967.1|	24212-24745	15	5.5	0.661823	6	3	0.24155	no
gi|646721684|gb|KK964967.1|	38617-38773	15	5.5	640	889	0.473898	0.7383	no
gi|646721684|gb|KK964967.1|	46220-47012	15	5.5	0.861225	2	1	0.36955	no
gi|646721927|gb|KK964955.1|	55168-56026	12	12	1	1	-0.177696	0.9042	no
gi|646722070|gb|KK964949.1|	34669-35581	11	9.4	1	0.199746	-3	0.2033	no
gi|646722233|gb|KK964943.1|	22916-24056	34	14	0.940754	0.598223	-0.653134	1	no
gi|646722233|gb|KK964943.1|	59359-60233	34	14	6	6	0.0996025	0.94865	no

gi|646722233|gb|KK964943.1|	61437-61968	34	14	2	1	-0.780514	0.63075	no
gi|646722233|gb|KK964943.1|	63669-66634	34	14	1	0.744979	-0.596737	0.7153	no
gi|646722233|gb|KK964943.1|	80313-80636	34	14	8	5	-0.53253	0.73825	no
gi|646722233|gb|KK964943.1|	86483-86675	34	14	301	253	-0.250836	0.8732	no
gi|646722274|gb|KK964941.1|	24088-24456	20	12	4	5	0.335166	0.823	no
gi|646722470|gb|KK964933.1|	0-249	30	8.6	20	15	-0.376331	0.81185	no
gi|646722470|gb|KK964933.1|	37624-37972	30	8.6	4	23	2	0.182	no
gi|646722470|gb|KK964933.1|	38874-39502	30	8.6	5	32	3	0.07945	no
gi|646722582|gb|KK964929.1|	13904-11608	23	6	66	63	-0.0617423	0.9605	no
gi|646722582|gb|KK964929.1|	27491-12955	23	6	6	22	2	0.17235	no
gi|646722582|gb|KK964929.1|	29752-13134	23	6	0.41455	0.498407	0.265778	1	no
gi|646722582|gb|KK964929.1|	31519-13323	23	6	0.634967	0.733844	0.208791	1	no
gi|646722582|gb|KK964929.1|	42614-14368	23	6	1	1	0.151332	0.9186	no
gi|646722582|gb|KK964929.1|	24101-25496	23	6	2	1	-0.47529	0.70275	no
gi|646722582|gb|KK964929.1|	32147-41758	23	6	9	7	-0.435645	0.7084	no
gi|646722582|gb|KK964929.1|	42370-43442	23	6	83	39	-1	0.3313	no
gi|646722582|gb|KK964929.1|	59637-60442	23	6	3	2	-0.352547	0.77885	no
gi|646722582|gb|KK964929.1|	60664-61996	23	6	0.429484	1	2	0.3354	no
gi|646722582|gb|KK964929.1|	62101-62996	23	6	2	1	-1	0.4371	no
gi|646722582|gb|KK964929.1|	64720-67035	23	6	6	4	-0.635395	0.6691	no
gi|646722582|gb|KK964929.1|	67502-68075	23	6	1	3	1	0.3774	no
gi|646722582|gb|KK964929.1|	82065-82561	23	6	23	14	-0.695607	0.65265	no
gi|646722582|gb|KK964929.1|	90620-90953	23	6	7	7	0.0676039	0.9262	no
gi|646722779|gb|KK964921.1|	45700-46268	12	6.4	2	1	-0.768487	0.633	no
gi|646722807|gb|KK964919.1|	47298-48810	9.2	5.7	37	11	-2	0.17315	no
gi|646722807|gb|KK964919.1|	59029-60204	9.2	5.7	2741.46	1991.68	-0.460957	0.6966	no
gi|646722807|gb|KK964919.1|	65359-65617	9.2	5.7	118	8	-4	0.0897	no
gi|646722992|gb|KK964909.1|	13208-16957	9.3	10	2	0.849026	-1	0.37935	no
gi|646722992|gb|KK964909.1|	1666-2087	9.3	10	8	8	-0.0633132	0.95485	no
gi|646722992|gb|KK964909.1|	25105-27080	9.3	10	1	0	-inf	1	no

gi|646722992|gb|KK964909.1|	28730-31331	9.3	10	2	0.744879	-1	0.37855	no
gi|646722992|gb|KK964909.1|	39243-40617	9.3	10	17	14	-0.224845	0.8558	no
gi|646722992|gb|KK964909.1|	42129-42667	9.3	10	105	15	-3	0.052	no
gi|646722992|gb|KK964909.1|	43723-45130	9.3	10	9	2	-3	0.0836	no
gi|646722992|gb|KK964909.1|	46663-49181	9.3	10	13	2	-3	0.07445	no
gi|646722992|gb|KK964909.1|	56715-57113	9.3	10	42	30	-0.512696	0.7305	no
gi|646722992|gb|KK964909.1|	9221-10586	9.3	10	10	10	0.0661148	0.96375	no
gi|646723115|gb|KK964905.1|	11282-12074	7.5	4.9	1	0.727788	-0.728299	0.66415	no
gi|646723115|gb|KK964905.1|	200-451	7.5	4.9	27	0	-inf	0.00655	no
gi|646723115|gb|KK964905.1|	22839-23124	7.5	4.9	92	2	-6	0.1698	no
gi|646723212|gb|KK964897.1|	3145-4905	12	4.3	0.122944	3	5	0.23805	no
gi|646723239|gb|KK964896.1|	46347-47532	26	10	3	0.569957	-2	0.20715	no
gi|646723239|gb|KK964896.1|	47882-49663	26	10	2	2	0.0552605	0.9712	no
gi|646723239|gb|KK964896.1|	61126-61852	26	10	2	2	-0.0285805	0.9367	no
gi|646723285|gb|KK964894.1|	47682-49247	19	8.3	1	0.916659	-0.389247	0.75645	no
gi|646723285|gb|KK964894.1|	68738-69098	19	8.3	10	1	-3	0.18235	no
gi|646723285|gb|KK964894.1|	76359-77514	19	8.3	12	18	0.577752	0.704	no
gi|646723329|gb|KK964893.1|	15392-16112	16	9.1	0	5	inf	0.00435	no
gi|646723432|gb|KK964889.1|	39247-39972	18	9.7	0	3	inf	0.001	yes
gi|646723432|gb|KK964889.1|	45027-46152	18	9.7	0.21266	67	8	0.2322	no
gi|646723751|gb|KK964877.1|	27395-27962	11	5.4	16	0.404605	-5	0.17035	no
gi|646723751|gb|KK964877.1|	30866-31429	11	5.4	17	2	-3	0.08205	no
gi|646723751|gb|KK964877.1|	32760-34256	11	5.4	3	1	-1	0.3712	no
gi|646723751|gb|KK964877.1|	57032-58385	11	5.4	0.421271	4	3	0.0963	no
gi|646723751|gb|KK964877.1|	68649-69941	11	5.4	2	5	0.924074	0.55455	no
gi|646723769|gb|KK964876.1|	29140-29488	11	5	13	0	-inf	0.0011	yes
gi|646723769|gb|KK964876.1|	35106-35374	11	5	17	19	0.163964	0.91255	no
gi|646723769|gb|KK964876.1|	36833-37315	11	5	9	7	-0.401905	0.76955	no
gi|646723891|gb|KK964871.1|	14352-15120	12	7.4	0.360594	3	3	0.2502	no
gi|646723891|gb|KK964871.1|	77057-17772	12	7.4	2	2	0.476778	0.7479	no

gi|646724047|gb|KK964864.1|	11387-12593	16	7.7	1	0.418246	-1	1	no
gi|646724047|gb|KK964864.1|	17190-18257	16	7.7	0.455694	1	1	0.43305	no
gi|646724047|gb|KK964864.1|	41224-42480	16	7.7	0.739338	0.928226	0.328243	1	no
gi|646724047|gb|KK964864.1|	42895-44453	16	7.7	0.497002	0.819111	0.720807	1	no
gi|646724047|gb|KK964864.1|	48229-48903	16	7.7	1	2	0.740875	0.6148	no
gi|646724090|gb|KK964863.1|	72998-73606	22	9.8	2	3	0.294501	0.83225	no
gi|646724090|gb|KK964863.1|	82774-83241	22	9.8	3	2	-1	0.4814	no
gi|646724161|gb|KK964858.1|	04994-10574	21	9.5	8	7	-0.148751	0.92575	no
gi|646724161|gb|KK964858.1|	08337-10899	21	9.5	18	17	-0.110965	0.94295	no
gi|646724161|gb|KK964858.1|	12919-11439	21	9.5	0.679684	0.979122	0.526624	1	no
gi|646724161|gb|KK964858.1|	16444-11869	21	9.5	1	0.939883	-0.115474	1	no
gi|646724161|gb|KK964858.1|	24792-12793	21	9.5	3	3	-0.303555	0.83665	no
gi|646724161|gb|KK964858.1|	31731-13282	21	9.5	64	49	-0.389151	0.73215	no
gi|646724161|gb|KK964858.1|	32914-13370	21	9.5	9	9	-0.0362082	0.98105	no
gi|646724161|gb|KK964858.1|	34410-13521	21	9.5	0.670264	2	1	0.43305	no
gi|646724161|gb|KK964858.1|	39833-14034	21	9.5	2	6	1	0.38605	no
gi|646724161|gb|KK964858.1|	42357-14293	21	9.5	0.565864	4	3	0.26125	no
gi|646724161|gb|KK964858.1|	44917-14583	21	9.5	2	4	1	0.38395	no
gi|646724161|gb|KK964858.1|	45967-14726	21	9.5	2	1	-0.565342	0.66405	no
gi|646724161|gb|KK964858.1|	53159-53467	21	9.5	21	6	-2	0.2378	no
gi|646724161|gb|KK964858.1|	66966-72767	21	9.5	6	4	-0.58505	0.58725	no
gi|646724161|gb|KK964858.1|	72847-74189	21	9.5	3	3	-0.254241	0.8727	no
gi|646724161|gb|KK964858.1|	75257-75703	21	9.5	5	0.627479	-3	0.20135	no
gi|646724161|gb|KK964858.1|	80282-86322	21	9.5	5	5	-0.205503	0.852	no
gi|646724161|gb|KK964858.1|	95265-95894	21	9.5	21	22	0.048571	0.9738	no
gi|646724203|gb|KK964856.1|	1213-1616	130	27	13	26	1	0.4537	no
gi|646724203|gb|KK964856.1|	35294-36327	130	27	0.713406	0.678197	-0.0730184	1	no
gi|646724203|gb|KK964856.1|	41458-42264	130	27	2	0.236673	-3	0.2033	no
gi|646724203|gb|KK964856.1|	68-635	130	27	2	3	0.260967	0.863	no
gi|646724228|gb|KK964854.1|	21029-21378	19	8.7	0	12	inf	0.0014	yes

gi|646724228|gb|KK964854.1|	68165-69220	19	8.7	1	0.825068	-0.334952	1	no
gi|646724228|gb|KK964854.1|	71701-72115	19	8.7	4	2	-0.843637	0.6128	no
gi|646724260|gb|KK964850.1|	01260-10150	20	8.3	21	88	2	0.2036	no
gi|646724260|gb|KK964850.1|	46374-51168	20	8.3	85	40	-1	0.3627	no
gi|646724260|gb|KK964850.1|	60731-61051	20	8.3	26	25	-0.0835777	0.9556	no
gi|646724260|gb|KK964850.1|	61364-61692	20	8.3	38	40	0.0668113	0.96715	no
gi|646724260|gb|KK964850.1|	64405-65216	20	8.3	12	14	0.125216	0.9302	no
gi|646724260|gb|KK964850.1|	65470-67136	20	8.3	52	28	-0.899111	0.4268	no
gi|646724260|gb|KK964850.1|	83329-83645	20	8.3	18	24	0.4441	0.7534	no
gi|646724260|gb|KK964850.1|	87471-87737	20	8.3	30	22	-0.478722	0.7095	no
gi|646724260|gb|KK964850.1|	88829-89418	20	8.3	31	20	-0.672743	0.65315	no
gi|646724260|gb|KK964850.1|	92426-92784	20	8.3	188	65	-2	0.24495	no
gi|646724265|gb|KK964849.1|	45161-45479	13	6	77	0	-inf	0.00025	yes
gi|646724265|gb|KK964849.1|	46358-47204	13	6	13	0	-inf	0.00025	yes
gi|646724313|gb|KK964840.1|	30265-30499	14	6.2	26	31	0.24955	0.8668	no
gi|646724379|gb|KK964829.1|	6116-6670	10	5.9	4	5	0.449648	0.7215	no
gi|646724379|gb|KK964829.1|	6724-7412	10	5.9	2	1	-0.519781	0.73195	no
gi|646724420|gb|KK964822.1|	19749-20655	21	8.2	0.568362	2	2	0.2964	no
gi|646724420|gb|KK964822.1|	71896-72251	21	8.2	4	10	1	0.39765	no
gi|646724420|gb|KK964822.1|	77591-77887	21	8.2	3	14	2	0.30875	no
gi|646724465|gb|KK964814.1|	39531-40391	19	8.6	1	2	0.500546	0.7299	no
gi|646724488|gb|KK964810.1|	10854-11959	12	7	0	4	inf	0.00095	yes
gi|646724540|gb|KK964801.1|	65122-65322	12	8.7	49	38	-0.362898	0.81265	no
gi|646724572|gb|KK964795.1|	20908-21607	21	7.7	11	2	-2	0.11805	no
gi|646724600|gb|KK964790.1|	13423-13897	20	5.2	40	6	-3	0.07545	no
gi|646724600|gb|KK964790.1|	21502-21826	20	5.2	14	0	-inf	0.0035	no
gi|646724600|gb|KK964790.1|	42153-43666	20	5.2	1	0.741912	-0.665348	0.6175	no
gi|646724600|gb|KK964790.1|	43930-45305	20	5.2	1	1	0.28321	0.8201	no
gi|646724600|gb|KK964790.1|	46680-47601	20	5.2	0.833347	1	0.50523	0.73275	no
gi|646724600|gb|KK964790.1|	48508-49733	20	5.2	0.762962	0.68373	-0.158184	1	no

gi|646724600|gb|KK964790.1|	63961-66174	20	5.2	12	10	-0.217569	0.83745	no
gi|646724600|gb|KK964790.1|	67086-68627	20	5.2	11	8	-0.499638	0.737	no
gi|646724600|gb|KK964790.1|	82930-83529	20	5.2	2	3	0.292324	0.83225	no
gi|646724600|gb|KK964790.1|	85972-87000	20	5.2	0.23934	3	4	0.26465	no
gi|646724633|gb|KK964784.1|	14239-18948	9.5	6.1	0.373911	57	7	0.00165	no
gi|646724633|gb|KK964784.1|	23973-25444	9.5	6.1	0.152159	11	6	0.2329	no
gi|646724633|gb|KK964784.1|	36784-37524	9.5	6.1	0.190691	10	6	0.09675	no
gi|646724633|gb|KK964784.1|	40479-42017	9.5	6.1	0.216321	16	6	0.1049	no
gi|646724633|gb|KK964784.1|	42820-44005	9.5	6.1	0.895243	24	5	0.0169	no
gi|646724633|gb|KK964784.1|	44391-45427	9.5	6.1	0.118444	3	5	0.1111	no
gi|646724633|gb|KK964784.1|	4699-6698	9.5	6.1	0.848776	0.153652	-2	1	no
gi|646724660|gb|KK964779.1|	28213-28882	14	6	5	0	-inf	0.0004	yes
gi|646724660|gb|KK964779.1|	44133-44746	14	6	78	66	-0.258772	0.81435	no
gi|646724720|gb|KK964768.1|	76925-77505	20	5.5	1	2	0.720336	0.64505	no
gi|646724743|gb|KK964765.1|	07326-10865	16	8.3	1	4	2	0.18795	no
gi|646724743|gb|KK964765.1|	19613-20291	16	8.3	15	16	0.0771724	0.95475	no
gi|646724743|gb|KK964765.1|	24109-25498	16	8.3	12	9	-0.51391	0.7306	no
gi|646724743|gb|KK964765.1|	27245-31139	16	8.3	71	47	-0.598681	0.6139	no
gi|646724743|gb|KK964765.1|	35895-36165	16	8.3	18	5	-2	0.22445	no
gi|646724743|gb|KK964765.1|	37690-38295	16	8.3	14	7	-0.9888	0.54115	no
gi|646724743|gb|KK964765.1|	43945-46617	16	8.3	2	0.222523	-3	0.0797	no
gi|646724743|gb|KK964765.1|	53581-53836	16	8.3	51	51	-0.0243057	0.9681	no
gi|646724743|gb|KK964765.1|	54251-55242	16	8.3	9	9	0.129058	0.92615	no
gi|646724743|gb|KK964765.1|	63603-65382	16	8.3	0.424939	1	2	0.31585	no
gi|646724743|gb|KK964765.1|	67248-68318	16	8.3	0.908034	2	1	0.37345	no
gi|646724743|gb|KK964765.1|	68529-69743	16	8.3	4	6	0.623855	0.6886	no
gi|646724743|gb|KK964765.1|	90368-91987	16	8.3	0.74608	0.195768	-2	1	no
gi|646724743|gb|KK964765.1|	92489-93832	16	8.3	10	8	-0.290193	0.84765	no
gi|646724743|gb|KK964765.1|	95713-96185	16	8.3	3	4	0.223509	0.86575	no
gi|646724780|gb|KK964759.1|	1095-1337	28	11	19	14	-0.499184	0.74775	no

gi|646724780|gb|KK964759.1|	26857-27599	28	11	2	3	0.751193	0.56	no
gi|646724780|gb|KK964759.1|	38556-39403	28	11	2	4	0.886448	0.55	no
gi|646724780|gb|KK964759.1|	48483-48904	28	11	4	4	-0.293926	0.847	no
gi|646724780|gb|KK964759.1|	63399-64017	28	11	5	6	0.466733	0.73535	no
gi|646724787|gb|KK964758.1|	12146-12686	49	21	2	8	2	0.1843	no
gi|646724787|gb|KK964758.1|	22882-23405	49	21	8	8	0.00281722	0.97445	no
gi|646724787|gb|KK964758.1|	8317-8803	49	21	3	2	-0.799241	0.6307	no
gi|646724792|gb|KK964757.1|	4748-5257	11	6.5	8	6	-0.441102	0.7502	no
gi|646724792|gb|KK964757.1|	51169-51551	11	6.5	0	17	inf	0.00315	no
gi|646724792|gb|KK964757.1|	5490-6665	11	6.5	3	3	-0.140828	0.9268	no
gi|646724792|gb|KK964757.1|	61813-62352	11	6.5	0	7	inf	0.00605	no
gi|646724827|gb|KK964750.1|	09320-11039	20	8.3	11	13	0.236149	0.8726	no
gi|646724827|gb|KK964750.1|	15963-16240	20	8.3	19	17	-0.13561	0.92595	no
gi|646724827|gb|KK964750.1|	17691-19142	20	8.3	2	2	-0.386536	0.8097	no
gi|646724827|gb|KK964750.1|	28482-30113	20	8.3	0.067234	2	5	0.11045	no
gi|646724827|gb|KK964750.1|	3-2666	20	8.3	20	30	0.582527	0.60555	no
gi|646724827|gb|KK964750.1|	30373-30779	20	8.3	4	20	2	0.15615	no
gi|646724827|gb|KK964750.1|	31469-33949	20	8.3	4	6	0.751207	0.6126	no
gi|646724827|gb|KK964750.1|	34336-35556	20	8.3	22	15	-0.524673	0.68425	no
gi|646724827|gb|KK964750.1|	3914-4279	20	8.3	7	18	1	0.38795	no
gi|646724827|gb|KK964750.1|	46012-46691	20	8.3	13	7	-0.919843	0.55935	no
gi|646724827|gb|KK964750.1|	5042-7171	20	8.3	1	3	0.999493	0.53115	no
gi|646724827|gb|KK964750.1|	59989-61109	20	8.3	0.213889	1	3	0.2655	no
gi|646724827|gb|KK964750.1|	91989-92224	20	8.3	18	22.88	0.32368	0.81255	no
gi|646724843|gb|KK964747.1|	28724-29367	17	11	2	2	-0.206031	0.89315	no
gi|646724843|gb|KK964747.1|	45476-46229	17	11	1	3	1	0.39835	no
gi|646724864|gb|KK964743.1|	15333-15822	14	7.4	0.782708	5	3	0.26725	no
gi|646724905|gb|KK964735.1|	10797-11126	43	20	4	1	-2	0.28945	no
gi|646724905|gb|KK964735.1|	11168-11575	43	20	47	19	-1	0.4086	no
gi|646724905|gb|KK964735.1|	54993-55336	43	20	8	5	-0.865767	0.5704	no

gi|646724905|gb|KK964735.1|	76832-77620	43	20	4	5	0.230626	0.8909	no
gi|646724905|gb|KK964735.1|	77732-78712	43	20	0.893137	1	0.509021	0.7286	no
gi|646724905|gb|KK964735.1|	79301-80496	43	20	3	0.987061	-2	0.2543	no
gi|646724905|gb|KK964735.1|	80589-84142	43	20	5	0.613052	-3	0.09305	no
gi|646724905|gb|KK964735.1|	87260-88010	43	20	3	0	-inf	0.00095	yes
gi|646724915|gb|KK964733.1|	04436-10475	26	9	9	4	-1	0.473	no
gi|646724915|gb|KK964733.1|	08359-10888	26	9	2	2	0.176637	0.89965	no
gi|646724915|gb|KK964733.1|	27154-27754	26	9	1	23	4	0.0679	no
gi|646724915|gb|KK964733.1|	27856-29306	26	9	1	7	2	0.1176	no
gi|646724915|gb|KK964733.1|	29542-30217	26	9	1	2	0.214981	0.89085	no
gi|646724915|gb|KK964733.1|	36532-37129	26	9	2	3	0.876789	0.5493	no
gi|646724915|gb|KK964733.1|	64661-65822	26	9	1	3	1	0.4006	no
gi|646724915|gb|KK964733.1|	6886-7558	26	9	6	5	-0.135487	0.93265	no
gi|646724915|gb|KK964733.1|	83395-84613	26	9	1	0.964022	-0.373431	0.77685	no
gi|646724969|gb|KK964723.1|	08789-10917	20	5.6	14	15	0.0472564	0.96845	no
gi|646724969|gb|KK964723.1|	10212-11047	20	5.6	22	5	-2	0.2127	no
gi|646724969|gb|KK964723.1|	27577-12784	20	5.6	12	26	1	0.4437	no
gi|646724969|gb|KK964723.1|	45924-14618	20	5.6	25	3	-3	0.1901	no
gi|646724969|gb|KK964723.1|	48079-14857	20	5.6	2	3	0.164292	0.9062	no
gi|646724969|gb|KK964723.1|	67892-17634	20	5.6	7	3	-0.963076	0.38865	no
gi|646724969|gb|KK964723.1|	76511-17991	20	5.6	1	1	-0.375142	0.81465	no
gi|646724969|gb|KK964723.1|	80032-18136	20	5.6	0.944864	0.864058	-0.128978	1	no
gi|646724969|gb|KK964723.1|	03901-20432	20	5.6	7	3	-0.992877	0.5242	no
gi|646724969|gb|KK964723.1|	4995-10780	20	5.6	10	7	-0.465499	0.6781	no
gi|646724969|gb|KK964723.1|	77256-84569	20	5.6	1	6	2	0.113	no
gi|646724969|gb|KK964723.1|	86073-86414	20	5.6	12	1	-3	0.18215	no
gi|646724969|gb|KK964723.1|	89082-89315	20	5.6	34	20	-0.794874	0.59895	no
gi|646724969|gb|KK964723.1|	95531-95816	20	5.6	18	6	-2	0.2955	no
gi|646724969|gb|KK964723.1|	97226-97430	20	5.6	74	28	-1	0.36185	no
gi|646724974|gb|KK964722.1|	15838-16469	23	15	2	2	-0.267394	0.83675	no

gi|646724995|gb|KK964718.1|	35274-35648	11	13	6	4	-0.658235	0.66245	no
gi|646725010|gb|KK964715.1|	17244-17713	11	8	0.852952	5	3	0.2756	no
gi|646725010|gb|KK964715.1|	26410-27041	11	8	6	12	1	0.5161	no
gi|646725027|gb|KK964712.1|	5084-5312	8.6	6.9	46	13	-2	0.26155	no
gi|646725040|gb|KK964710.1|	25345-12748	21	9.3	0.049055	5	7	0.09485	no
gi|646725040|gb|KK964710.1|	68264-17252	21	9.3	5	5	-0.0114497	0.9911	no
gi|646725046|gb|KK964709.1|	33181-33784	8.8	10	2	0.731367	-2	0.29375	no
gi|646725062|gb|KK964706.1|	19068-19346	13	6.2	29	36	0.276144	0.8423	no
gi|646725085|gb|KK964702.1|	17095-17554	14	5	4	8	0.787559	0.5417	no
gi|646725085|gb|KK964702.1|	19976-20623	14	5	5	2	-1	0.39765	no
gi|646725113|gb|KK964698.1|	67112-67407	26	13	16	9	-0.912027	0.53875	no
gi|646725113|gb|KK964698.1|	82810-83786	26	13	14	13	-0.0963567	0.949	no
gi|646725179|gb|KK964689.1|	11287-12837	40	25	1	2	1	0.45835	no
gi|646725179|gb|KK964689.1|	9466-9886	40	25	553	541	-0.0302238	0.97965	no
gi|646725179|gb|KK964689.1|	98698-99174	40	25	0.827029	5	3	0.26725	no
gi|646725187|gb|KK964688.1|	48948-49496	15	9.6	4	6	0.547254	0.70265	no
gi|646725216|gb|KK964684.1|	29666-30566	17	8.1	2	0.813352	-1	0.3737	no
gi|646725216|gb|KK964684.1|	44409-44747	17	8.1	6	8	0.519605	0.7234	no
gi|646725216|gb|KK964684.1|	73823-74282	17	8.1	5	2	-1	0.46265	no
gi|646725265|gb|KK964677.1|	28407-28803	12	5.9	0	12	inf	0.00605	no
gi|646725272|gb|KK964676.1|	12374-12764	25	6.6	12	0.835372	-4	0.17255	no
gi|646725294|gb|KK964673.1|	28550-29893	12	8.1	7	58	3	0.04185	no
gi|646725294|gb|KK964673.1|	30258-30832	12	8.1	4	36	3	0.0438	no
gi|646725294|gb|KK964673.1|	32211-32856	12	8.1	11	37	2	0.26295	no
gi|646725294|gb|KK964673.1|	61395-62145	12	8.1	2	0.786797	-1	0.427	no
gi|646725294|gb|KK964673.1|	62245-67013	12	8.1	7	18	1	0.24505	no
gi|646725294|gb|KK964673.1|	71608-73778	12	8.1	1	0.630407	-0.686485	1	no
gi|646725294|gb|KK964673.1|	96173-96990	12	8.1	2	1	-0.640859	0.65475	no
gi|646725294|gb|KK964673.1|	97668-98234	12	8.1	9	7	-0.283648	0.85555	no
gi|646725350|gb|KK964665.1|	20100-21522	16	7.3	2	2	0.11031	0.9375	no

gi|646725350|gb|KK964665.1|	23016-23259	16	7.3	9	37	2	0.2328	no
gi|646725350|gb|KK964665.1|	29028-29343	16	7.3	50	0	-inf	0.00155	yes
gi|646725350|gb|KK964665.1|	81908-82500	16	7.3	4	6	0.460484	0.7473	no
gi|646725370|gb|KK964662.1|	18723-19647	21	11	1	0.588839	-0.909599	1	no
gi|646725370|gb|KK964662.1|	19881-21042	21	11	2	0.877037	-1	0.3648	no
gi|646725370|gb|KK964662.1|	21158-21957	21	11	2	0.239598	-3	0.2033	no
gi|646725370|gb|KK964662.1|	22083-23823	21	11	1	0.990292	-0.533003	0.6991	no
gi|646725370|gb|KK964662.1|	33929-34771	21	11	1	0.890766	-0.501019	0.73865	no
gi|646725370|gb|KK964662.1|	39439-40173	21	11	5	2	-2	0.2711	no
gi|646725370|gb|KK964662.1|	52-6466	21	11	4	2	-1	0.3082	no
gi|646725370|gb|KK964662.1|	54177-54447	21	11	10	42	2	0.24675	no
gi|646725370|gb|KK964662.1|	6577-6932	21	11	10	0	-inf	0.0041	no
gi|646725370|gb|KK964662.1|	7701-15156	21	11	5	2	-1	0.3843	no
gi|646725389|gb|KK964659.1|	35871-36366	18	6.4	23	13	-0.835708	0.59235	no
gi|646725389|gb|KK964659.1|	41653-42248	18	6.4	3	1	-0.998183	0.54505	no
gi|646725389|gb|KK964659.1|	57976-58879	18	6.4	4	0.809712	-2	0.19745	no
gi|646725428|gb|KK964653.1|	23001-23505	11	6.3	3	6	1	0.32645	no
gi|646725465|gb|KK964648.1|	98717-99163	14	9.2	8	5	-0.686186	0.5911	no
gi|646725472|gb|KK964647.1|	16502-17237	28	19	2	1	-0.512771	0.7384	no
gi|646725505|gb|KK964642.1|	12716-13116	11	6.6	8	0	-inf	0.0035	no
gi|646725525|gb|KK964639.1|	19734-20803	27	9.8	3	1	-1	0.4703	no
gi|646725525|gb|KK964639.1|	76224-77827	27	9.8	120	69	-0.806241	0.4908	no
gi|646725570|gb|KK964632.1|	55538-56127	9.5	11	3	6	0.781635	0.59885	no
gi|646725578|gb|KK964631.1|	07022-10825	17	6.7	1	0.813915	-0.70224	0.5887	no
gi|646725578|gb|KK964631.1|	4591-5077	17	6.7	4	2	-0.746773	0.6076	no
gi|646725578|gb|KK964631.1|	63290-64162	17	6.7	1	0.212243	-3	0.2033	no
gi|646725578|gb|KK964631.1|	8531-9015	17	6.7	4	11	1	0.39835	no
gi|646725578|gb|KK964631.1|	95665-96499	17	6.7	4	2	-0.916783	0.53455	no
gi|646725578|gb|KK964631.1|	97891-98435	17	6.7	4	7	0.893855	0.5546	no
gi|646725599|gb|KK964628.1|	64655-65345	12	8.2	6	1	-2	0.2044	no

gi|646725605|gb|KK964627.1|	00867-10222	21	9.5	0.924271	2	1	0.4159	no
gi|646725605|gb|KK964627.1|	55433-56267	21	9.5	7	4	-0.713249	0.64995	no
gi|646725605|gb|KK964627.1|	92571-93023	21	9.5	11	4	-1	0.3991	no
gi|646725605|gb|KK964627.1|	)9365-10049(	21	9.5	7	2	-2	0.20725	no
gi|646725628|gb|KK964623.1|	18643-19868	16	5.7	8	7	-0.0827763	0.95605	no
gi|646725628|gb|KK964623.1|	22315-22627	16	5.7	67	21	-2	0.2246	no
gi|646725628|gb|KK964623.1|	30597-30886	16	5.7	48	74	0.628218	0.6805	no
gi|646725628|gb|KK964623.1|	33824-37880	16	5.7	3	3	0.0159601	0.99175	no
gi|646725628|gb|KK964623.1|	43685-44066	16	5.7	8	6	-0.302988	0.8259	no
gi|646725628|gb|KK964623.1|	45384-47424	16	5.7	23	26	0.229264	0.83605	no
gi|646725628|gb|KK964623.1|	51733-52229	16	5.7	4	2	-1	0.3707	no
gi|646725628|gb|KK964623.1|	52977-53778	16	5.7	24	18	-0.445254	0.7634	no
gi|646725628|gb|KK964623.1|	58335-59001	16	5.7	3	7	1	0.36255	no
gi|646725628|gb|KK964623.1|	66222-66493	16	5.7	47	52	0.171645	0.9109	no
gi|646725628|gb|KK964623.1|	66597-67525	16	5.7	5	8	0.661833	0.6687	no
gi|646725628|gb|KK964623.1|	76295-78892	16	5.7	6	7	0.0319658	0.9838	no
gi|646725644|gb|KK964620.1|	61375-62557	36	15	0.498966	1	1	1	no
gi|646725650|gb|KK964619.1|	2421-3132	17	7.7	0.811165	2	1	0.3715	no
gi|646725650|gb|KK964619.1|	34351-35542	17	7.7	0.197668	2	3	0.24065	no
gi|646725650|gb|KK964619.1|	3935-4833	17	7.7	4	6	0.552476	0.7294	no
gi|646725650|gb|KK964619.1|	42-803	17	7.7	1	2	0.712947	0.6352	no
gi|646725650|gb|KK964619.1|	44532-45609	17	7.7	0.112538	2	4	0.10345	no
gi|646725650|gb|KK964619.1|	51164-52814	17	7.7	0	2	inf	0.00155	yes
gi|646725650|gb|KK964619.1|	5409-6707	17	7.7	11	8	-0.407461	0.78485	no
gi|646725650|gb|KK964619.1|	9605-10363	17	7.7	28	20	-0.471844	0.7545	no
gi|646725663|gb|KK964617.1|	03645-10467	12	7.3	1	2	0.248858	0.8482	no
gi|646725663|gb|KK964617.1|	3089-3901	12	7.3	4	3	-0.558295	0.6876	no
gi|646725663|gb|KK964617.1|	53849-54656	12	7.3	3	2	-0.411238	0.76285	no
gi|646725663|gb|KK964617.1|	87409-89531	12	7.3	5	3	-0.90509	0.54765	no
gi|646725663|gb|KK964617.1|	92423-93118	12	7.3	3	0	-inf	0.00225	no

gi|646725663|gb|KK964617.1|	99496-10020!	12	7.3	4	5	0.313601	0.82375	no
gi|646725696|gb|KK964612.1|	27255-12774	22	9.2	2	4	1	0.44135	no
gi|646725696|gb|KK964612.1|	35469-13566	22	9.2	117	36	-2	0.2607	no
gi|646725696|gb|KK964612.1|	44363-15465	22	9.2	31	6	-2	0.0516	no
gi|646725696|gb|KK964612.1|	15630-17054	22	9.2	0.870515	1	0.38789	0.7705	no
gi|646725696|gb|KK964612.1|	64524-16558	22	9.2	0.918171	0.982911	0.0982977	1	no
gi|646725696|gb|KK964612.1|	66022-16661	22	9.2	21	16	-0.394615	0.78615	no
gi|646725696|gb|KK964612.1|	68250-17050	22	9.2	19	2	-4	0.02605	no
gi|646725696|gb|KK964612.1|	79448-17964	22	9.2	98	100	0.0156132	0.9542	no
gi|646725696|gb|KK964612.1|	79763-18243	22	9.2	7	12	0.646765	0.6062	no
gi|646725696|gb|KK964612.1|	96871-19841	22	9.2	0.93666	4	2	0.15715	no
gi|646725696|gb|KK964612.1|	99853-20063	22	9.2	4	11	1	0.3654	no
gi|646725696|gb|KK964612.1|	00748-20143	22	9.2	6	8	0.42544	0.79025	no
gi|646725696|gb|KK964612.1|	14000-21420	22	9.2	6	69	3	0.10415	no
gi|646725696|gb|KK964612.1|	15812-21610	22	9.2	81	108.13	0.425497	0.78615	no
gi|646725696|gb|KK964612.1|	16365-21693	22	9.2	2	4	0.969349	0.5187	no
gi|646725696|gb|KK964612.1|	17036-21781	22	9.2	3	10	2	0.19735	no
gi|646725696|gb|KK964612.1|	18339-21907	22	9.2	12	52	2	0.12385	no
gi|646725696|gb|KK964612.1|	19862-22035	22	9.2	3	17	3	0.111	no
gi|646725696|gb|KK964612.1|	22351-22277	22	9.2	8	29	2	0.20265	no
gi|646725696|gb|KK964612.1|	23128-22333	22	9.2	68	60	-0.179428	0.89165	no
gi|646725696|gb|KK964612.1|	28003-29195	22	9.2	3	0.424349	-3	0.17015	no
gi|646725696|gb|KK964612.1|	34281-34970	22	9.2	1	1	-0.326972	0.81865	no
gi|646725696|gb|KK964612.1|	38832-39081	22	9.2	23	9	-1	0.43905	no
gi|646725696|gb|KK964612.1|	39839-40426	22	9.2	3	2	-0.862746	0.56495	no
gi|646725696|gb|KK964612.1|	50348-51116	22	9.2	3	2	-0.315963	0.8121	no
gi|646725696|gb|KK964612.1|	8874-10125	22	9.2	26	6	-2	0.1143	no
gi|646725708|gb|KK964610.1|	18123-19470	34	12	0.931887	2	1	0.39205	no
gi|646725708|gb|KK964610.1|	77782-78537	34	12	0.924894	2	0.752852	0.6148	no
gi|646725713|gb|KK964609.1|	62770-63008	27	6.9	14	33	1	0.42655	no

gi|646725713|gb|KK964609.1|	76733-77055	27	6.9	3	9	1	0.37285	no
gi|646725713|gb|KK964609.1|	90886-91850	27	6.9	51	31	-0.72168	0.50335	no
gi|646725713|gb|KK964609.1|	98227-99061	27	6.9	0.479263	2	2	0.2898	no
gi|646725726|gb|KK964607.1|	58517-58791	15	6.5	6	15	1	0.38915	no
gi|646725726|gb|KK964607.1|	93899-95474	15	6.5	2	1	-0.793455	0.5656	no
gi|646725726|gb|KK964607.1|	96695-97737	15	6.5	5	4	-0.350095	0.82	no
gi|646725794|gb|KK964595.1|	56813-57535	10	7.1	2	2	-0.029145	0.9367	no
gi|646725799|gb|KK964594.1|	34648-35541	13	8	0.144985	3	4	0.1128	no
gi|646725799|gb|KK964594.1|	37190-37727	13	8	3	2	-0.234066	0.87465	no
gi|646725863|gb|KK964584.1|	12118-12561	23	11	24	44	0.893195	0.5636	no
gi|646725863|gb|KK964584.1|	16188-17421	23	11	0.472951	1	1	1	no
gi|646725863|gb|KK964584.1|	17932-29154	23	11	3	4	0.227694	0.8353	no
gi|646725863|gb|KK964584.1|	42841-47224	23	11	4	8	0.99567	0.43485	no
gi|646725870|gb|KK964583.1|	10-840	19	9.8	8	12	0.694282	0.6505	no
gi|646725888|gb|KK964580.1|	03509-10414	18	9.4	2	1	-1	0.4735	no
gi|646725888|gb|KK964580.1|	10555-11361	18	9.4	6	2	-2	0.249	no
gi|646725888|gb|KK964580.1|	07877-10860	18	9.4	0.197604	3	4	0.09985	no
gi|646725888|gb|KK964580.1|	14078-11598	18	9.4	0.784034	0.486285	-0.689115	1	no
gi|646725888|gb|KK964580.1|	16113-12303	18	9.4	5	5	0.152262	0.8904	no
gi|646725888|gb|KK964580.1|	24057-12499	18	9.4	13	8	-0.711177	0.63525	no
gi|646725888|gb|KK964580.1|	25915-12710	18	9.4	10	6	-0.795938	0.5978	no
gi|646725888|gb|KK964580.1|	20132-20811	18	9.4	19	9	-1	0.4695	no
gi|646725888|gb|KK964580.1|	42320-42714	18	9.4	12	4	-2	0.2792	no
gi|646725888|gb|KK964580.1|	45683-45998	18	9.4	8	9	0.0394735	0.92625	no
gi|646725931|gb|KK964573.1|	40931-42167	10	5	0.848709	0.270494	-2	1	no
gi|646725931|gb|KK964573.1|	44164-45111	10	5	1	0.949801	-0.493021	0.73195	no
gi|646725931|gb|KK964573.1|	5287-5551	10	5	45	10	-2	0.15665	no
gi|646725951|gb|KK964570.1|	41820-43271	10	5.3	0.309406	3	3	0.143	no
gi|646725969|gb|KK964567.1|	10626-10944	8.9	7.7	0	53	inf	0.00055	yes
gi|646725998|gb|KK964563.1|	23803-25387	13	7.2	0.278446	0.903771	2	1	no

gi|646725998|gb|KK964563.1|	34130-35647	13	7.2	0.43984	1	1	1	no
gi|646726010|gb|KK964561.1|	01093-10152	22	9.3	11	13	0.245582	0.85445	no
gi|646726010|gb|KK964561.1|	11865-11211	22	9.3	65	28	-1	0.4018	no
gi|646726010|gb|KK964561.1|	19886-12032	22	9.3	14	15	0.175051	0.9139	no
gi|646726010|gb|KK964561.1|	24509-12516	22	9.3	7	7	-0.0398162	0.97855	no
gi|646726010|gb|KK964561.1|	26288-12767	22	9.3	30	7	-2	0.12425	no
gi|646726010|gb|KK964561.1|	33102-13436	22	9.3	2	1	-0.106803	0.93385	no
gi|646726010|gb|KK964561.1|	37651-13875	22	9.3	0.875802	0.469358	-0.899916	1	no
gi|646726010|gb|KK964561.1|	40339-14215	22	9.3	0.297095	0.859225	2	1	no
gi|646726010|gb|KK964561.1|	43956-14440	22	9.3	19	12	-0.636197	0.68905	no
gi|646726010|gb|KK964561.1|	45030-14588	22	9.3	1	1	0.277469	0.8456	no
gi|646726010|gb|KK964561.1|	55573-15576	22	9.3	176	115	-0.604776	0.61425	no
gi|646726010|gb|KK964561.1|	59049-16326	22	9.3	38	34	-0.168105	0.88275	no
gi|646726010|gb|KK964561.1|	70938-17133	22	9.3	7	8	0.226914	0.8568	no
gi|646726010|gb|KK964561.1|	73517-17512	22	9.3	1	0	-inf	1	no
gi|646726010|gb|KK964561.1|	76662-18069	22	9.3	20	6	-2	0.11835	no
gi|646726010|gb|KK964561.1|	83609-18618	22	9.3	0.9184	2	0.822573	0.6124	no
gi|646726010|gb|KK964561.1|	91086-19356	22	9.3	4	11	2	0.21615	no
gi|646726010|gb|KK964561.1|	97335-19801	22	9.3	0.872162	2	1	0.3715	no
gi|646726010|gb|KK964561.1|	99803-20040	22	9.3	0.809561	3	2	0.25715	no
gi|646726010|gb|KK964561.1|	07942-20961	22	9.3	1	1	-0.27731	0.81505	no
gi|646726010|gb|KK964561.1|	17490-21978	22	9.3	1	0.985564	-0.36352	0.826	no
gi|646726010|gb|KK964561.1|	21850-23723	22	9.3	1	1	-0.244732	0.85885	no
gi|646726010|gb|KK964561.1|	19932-22083	22	9.3	1	2	0.696435	0.629	no
gi|646726010|gb|KK964561.1|	29777-23201	22	9.3	10	13	0.268254	0.79715	no
gi|646726010|gb|KK964561.1|	39619-24074	22	9.3	6	7	0.0845076	0.95585	no
gi|646726010|gb|KK964561.1|	24008-24755	22	9.3	2	3	0.488807	0.6992	no
gi|646726010|gb|KK964561.1|	40946-24300	22	9.3	0.975483	1	0.286944	0.8344	no
gi|646726010|gb|KK964561.1|	44725-24614	22	9.3	0.316551	1	2	1	no
gi|646726010|gb|KK964561.1|	49873-25224	22	9.3	4	1	-2	0.2521	no

gi|646726010|gb|KK964561.1|	56491-25817	22	9.3	16.65	19	0.154011	0.88615	no
gi|646726041|gb|KK964556.1|	17645-18271	26	12	5	0	-inf	0.0008	yes
gi|646726060|gb|KK964553.1|	79168-79514	18	13	9	4	-1	0.46035	no
gi|646726066|gb|KK964552.1|	24399-26151	16	13	2	3	0.415821	0.7818	no
gi|646726066|gb|KK964552.1|	39384-40180	16	13	2	1	-0.642985	0.65475	no
gi|646726077|gb|KK964550.1|	00533-10084	18	7.9	22	23	0.0692824	0.9613	no
gi|646726077|gb|KK964550.1|	30977-13172	18	7.9	0.373689	3	3	0.2578	no
gi|646726077|gb|KK964550.1|	13987-15202	18	7.9	0.481817	1	2	0.3354	no
gi|646726077|gb|KK964550.1|	44208-14877	18	7.9	16	5	-2	0.12715	no
gi|646726077|gb|KK964550.1|	51627-15294	18	7.9	0	2	inf	0.00605	no
gi|646726077|gb|KK964550.1|	16645-18319	18	7.9	1	2	0.781678	0.57005	no
gi|646726077|gb|KK964550.1|	68432-17281	18	7.9	7	6	-0.040162	0.96995	no
gi|646726077|gb|KK964550.1|	74246-17499	18	7.9	3	0.79446	-2	0.19015	no
gi|646726077|gb|KK964550.1|	18701-19928	18	7.9	0.47587	2	2	0.22555	no
gi|646726077|gb|KK964550.1|	20026-20680	18	7.9	0.926498	2	1	0.4335	no
gi|646726077|gb|KK964550.1|	20778-21529	18	7.9	0.932342	2	1	0.4427	no
gi|646726077|gb|KK964550.1|	34932-37312	18	7.9	12	12	0.0807902	0.93735	no
gi|646726077|gb|KK964550.1|	38108-39856	18	7.9	18	15	-0.233803	0.82975	no
gi|646726077|gb|KK964550.1|	44919-49332	18	7.9	7	5	-0.510639	0.635	no
gi|646726077|gb|KK964550.1|	48-3837	18	7.9	2	3	0.543504	0.7159	no
gi|646726077|gb|KK964550.1|	59645-60640	18	7.9	3	2	-0.111598	0.92575	no
gi|646726077|gb|KK964550.1|	6117-10397	18	7.9	2	3	0.89824	0.56275	no
gi|646726077|gb|KK964550.1|	64584-65338	18	7.9	6	7	0.226658	0.8823	no
gi|646726077|gb|KK964550.1|	65564-70317	18	7.9	9	7	-0.31793	0.77465	no
gi|646726077|gb|KK964550.1|	72122-73777	18	7.9	0.330408	0.667845	1	1	no
gi|646726077|gb|KK964550.1|	74665-79336	18	7.9	15	11	-0.403999	0.7157	no
gi|646726077|gb|KK964550.1|	84453-86324	18	7.9	0.17175	1	3	0.1676	no
gi|646726077|gb|KK964550.1|	89286-90431	18	7.9	1	0.297472	-2	0.20995	no
gi|646726077|gb|KK964550.1|	97161-98231	18	7.9	0.567521	2	2	0.2468	no
gi|646726088|gb|KK964548.1|	6628-7350	12	4.4	5	3	-0.573466	0.68255	no

gi|646726111|gb|KK964544.1|	72502-73742	12	5.4	0.563509	2	2	0.2484	no
gi|646726131|gb|KK964541.1|	28141-28594	9.5	4.8	20	9	-1	0.3821	no
gi|646726165|gb|KK964535.1|	48114-48428	16	16	0	35	inf	0.00185	no
gi|646726191|gb|KK964531.1|	139-712	8.7	10	96	95	-0.00844921	0.9938	no
gi|646726209|gb|KK964528.1|	0-619	27	12	1	2	0.466958	0.7722	no
gi|646726209|gb|KK964528.1|	10615-11114	27	12	3	3	-0.248966	0.87285	no
gi|646726209|gb|KK964528.1|	11423-12082	27	12	2	0.636018	-2	0.29735	no
gi|646726209|gb|KK964528.1|	12136-15652	27	12	4	2	-1	0.49445	no
gi|646726209|gb|KK964528.1|	1927-3273	27	12	1	0.121885	-3	1	no
gi|646726209|gb|KK964528.1|	03417-30391	27	12	5	6	0.187657	0.8818	no
gi|646726209|gb|KK964528.1|	57091-65003	27	12	9	9	-0.0299184	0.9797	no
gi|646726209|gb|KK964528.1|	68352-73272	27	12	4	4	-0.0221184	0.98495	no
gi|646726209|gb|KK964528.1|	8144-8405	27	12	23	10	-1	0.431	no
gi|646726209|gb|KK964528.1|	81623-81946	27	12	11	8	-0.462141	0.73665	no
gi|646726209|gb|KK964528.1|	93667-95587	27	12	0.500087	9	4	0.0254	no
gi|646726233|gb|KK964524.1|	10180-12061	19	7	4	3	-0.162174	0.9108	no
gi|646726233|gb|KK964524.1|	12203-12987	19	7	0.874242	1	0.756256	0.6148	no
gi|646726233|gb|KK964524.1|	13360-14625	19	7	4	3	-0.251422	0.86935	no
gi|646726233|gb|KK964524.1|	18993-20771	19	7	0.91118	1	0.531658	0.7108	no
gi|646726233|gb|KK964524.1|	30960-31607	19	7	6	2	-2	0.2641	no
gi|646726233|gb|KK964524.1|	37362-38399	19	7	2	1	-0.603328	0.64385	no
gi|646726233|gb|KK964524.1|	5142-5944	19	7	0.845486	2	0.980569	0.5626	no
gi|646726233|gb|KK964524.1|	53230-53376	19	7	742	310.85	-1	0.38165	no
gi|646726233|gb|KK964524.1|	55377-57124	19	7	2	1	-0.112629	0.942	no
gi|646726233|gb|KK964524.1|	65662-67326	19	7	1	0.379226	-2	1	no
gi|646726233|gb|KK964524.1|	70733-72454	19	7	1	1	0.189815	0.89595	no
gi|646726233|gb|KK964524.1|	75803-77044	19	7	1	0.269153	-2	1	no
gi|646726233|gb|KK964524.1|	81516-82259	19	7	2	1	-0.681635	0.6459	no
gi|646726264|gb|KK964519.1|	33468-33734	9.1	5.7	65	44	-0.578258	0.72115	no
gi|646726269|gb|KK964518.1|	32209-13256	13	6.8	0	36	inf	0.0003	yes

gi|646726269|gb|KK964518.1|	40366-14082	13	6.8	0	19	inf	0.001	yes
gi|646726269|gb|KK964518.1|	7447-8270	13	6.8	1	3	0.956655	0.4971	no
gi|646726273|gb|KK964517.1|	10-626	25	7.8	2	2	0.688673	0.6395	no
gi|646726273|gb|KK964517.1|	01182-10317	25	7.8	2	2	-0.0507589	0.973	no
gi|646726273|gb|KK964517.1|	04735-10537	25	7.8	2	1	-0.698896	0.64585	no
gi|646726273|gb|KK964517.1|	24624-12665	25	7.8	0.935562	1	0.27162	0.84875	no
gi|646726273|gb|KK964517.1|	33933-13538	25	7.8	1	3	0.962166	0.49355	no
gi|646726273|gb|KK964517.1|	16365-16781	25	7.8	5	9	0.910839	0.4858	no
gi|646726273|gb|KK964517.1|	38994-39298	25	7.8	20	38	0.92073	0.51705	no
gi|646726273|gb|KK964517.1|	40016-40335	25	7.8	8	7	-0.216981	0.8944	no
gi|646726273|gb|KK964517.1|	42026-42598	25	7.8	4	2	-0.866866	0.5197	no
gi|646726273|gb|KK964517.1|	48842-49133	25	7.8	17	5	-2	0.2968	no
gi|646726273|gb|KK964517.1|	50934-52204	25	7.8	0.273428	1	2	0.186	no
gi|646726273|gb|KK964517.1|	56670-58284	25	7.8	0.8169	1	0.751551	0.5503	no
gi|646726273|gb|KK964517.1|	97035-97304	25	7.8	15	26	0.818628	0.54925	no
gi|646726273|gb|KK964517.1|	99878-10101[illegible]	25	7.8	0.735019	2	1	0.31245	no
gi|646726324|gb|KK964508.1|	21976-22861	26	13	7	12	0.811901	0.591	no
gi|646726329|gb|KK964507.1|	12083-12529	9.9	3.6	1	8	3	0.1501	no
gi|646726329|gb|KK964507.1|	17040-18217	9.9	3.6	84	28	-2	0.1682	no
gi|646726371|gb|KK964500.1|	27845-12928	12	6.2	0.545565	1	1	0.39575	no
gi|646726371|gb|KK964500.1|	40710-42393	12	6.2	8	6	-0.437054	0.77075	no
gi|646726405|gb|KK964495.1|	16804-11804	23	5.7	2	3	0.581716	0.72195	no
gi|646726405|gb|KK964495.1|	66044-68922	23	5.7	24	14	-0.838968	0.4523	no
gi|646726405|gb|KK964495.1|	79579-79835	23	5.7	35	14	-1	0.33465	no
gi|646726405|gb|KK964495.1|	81391-82290	23	5.7	2	2	0.181713	0.88335	no
gi|646726405|gb|KK964495.1|	82464-83521	23	5.7	1	0.658453	-0.946289	0.5451	no
gi|646726405|gb|KK964495.1|	97743-98387	23	5.7	1	4	2	0.25505	no
gi|646726456|gb|KK964489.1|	00774-10150	17	7.2	2	4	1	0.41885	no
gi|646726456|gb|KK964489.1|	44731-45295	17	7.2	2	7	2	0.2819	no
gi|646726456|gb|KK964489.1|	47269-48012	17	7.2	0.947603	2	1	0.4427	no

gi|646726456|gb|KK964489.1|	57093-58210	17	7.2	1	5	2	0.1908	no
gi|646726456|gb|KK964489.1|	59211-60850	17	7.2	0.868991	0.578951	-0.5859	1	no
gi|646726456|gb|KK964489.1|	6606-6989	17	7.2	16	18	0.219867	0.89075	no
gi|646726456|gb|KK964489.1|	68521-68933	17	7.2	6	5	-0.13741	0.92275	no
gi|646726456|gb|KK964489.1|	71580-72172	17	7.2	3	2	-0.413985	0.7788	no
gi|646726456|gb|KK964489.1|	82594-83330	17	7.2	362	556.61	0.619706	0.6077	no
gi|646726497|gb|KK964483.1|	35909-36741	14	6.2	2	2	0.346067	0.79725	no
gi|646726497|gb|KK964483.1|	73500-74176	14	6.2	3	16	2	0.1255	no
gi|646726502|gb|KK964482.1|	52336-52851	25	16	0.706587	4	2	0.2858	no
gi|646726518|gb|KK964480.1|	43029-14482	12	12	1	2	0.614335	0.66155	no
gi|646726518|gb|KK964480.1|	55145-15615	12	12	3	4	0.333738	0.83425	no
gi|646726548|gb|KK964475.1|	33091-33646	9.9	4.6	3	0	-inf	0.00475	no
gi|646726560|gb|KK964473.1|	60791-61012	17	9.1	283	49	-3	0.0975	no
gi|646726560|gb|KK964473.1|	61887-62555	17	9.1	16	6	-1	0.373	no
gi|646726560|gb|KK964473.1|	63469-64422	17	9.1	69	41	-0.748516	0.49805	no
gi|646726560|gb|KK964473.1|	72972-73267	17	9.1	160.66	37	-2	0.18595	no
gi|646726560|gb|KK964473.1|	73387-74112	17	9.1	3	2	-0.151577	0.9126	no
gi|646726624|gb|KK964463.1|	73350-75590	12	8	0.512692	1	1	0.3304	no
gi|646726630|gb|KK964462.1|	53059-53560	12	6.4	6	0	-inf	0.0011	yes
gi|646726719|gb|KK964448.1|	48634-50782	16	6.9	0.635354	0.991897	0.642631	1	no
gi|646726719|gb|KK964448.1|	55346-56591	16	6.9	0.093444	2	4	0.11275	no
gi|646726719|gb|KK964448.1|	57069-58278	16	6.9	0	1	inf	0.00255	no
gi|646726719|gb|KK964448.1|	66409-67952	16	6.9	0.071827	3	5	0.09815	no
gi|646726736|gb|KK964445.1|	55851-56504	11	5	29	12	-1	0.38965	no
gi|646726742|gb|KK964444.1|	04615-10533	20	10	9	6	-0.684355	0.66575	no
gi|646726742|gb|KK964444.1|	06800-10804	20	10	2	1	-0.244994	0.84115	no
gi|646726742|gb|KK964444.1|	38588-39289	20	10	3470	1757.74	-0.981209	0.41835	no
gi|646726742|gb|KK964444.1|	90511-91417	20	10	0.852543	2	0.919195	0.5404	no
gi|646726742|gb|KK964444.1|	94803-95710	20	10	3	3	-0.254761	0.85645	no
gi|646726780|gb|KK964438.1|	11321-12132	19	12	17	3	-2	0.0975	no

gi|646726809|gb|KK964433.1|	15004-16922	19	12	1	0.644148	-0.636521	1	no
gi|646726809|gb|KK964433.1|	17182-18644	19	12	0.766466	1	0.789275	0.54425	no
gi|646726809|gb|KK964433.1|	28614-29813	19	12	0.881968	0.5617	-0.650926	1	no
gi|646726819|gb|KK964431.1|	14285-11501	15	7.4	2	0.820011	-1	0.47555	no
gi|646726819|gb|KK964431.1|	17435-18646	15	7.4	3	4	0.519416	0.74455	no
gi|646726819|gb|KK964431.1|	34047-35481	15	7.4	2	1	-0.281733	0.81125	no
gi|646726819|gb|KK964431.1|	44688-45064	15	7.4	8	11	0.469427	0.7149	no
gi|646726819|gb|KK964431.1|	45192-46425	15	7.4	3	0.54261	-2	0.1681	no
gi|646726819|gb|KK964431.1|	46485-47248	15	7.4	4	0	-inf	0.00595	no
gi|646726819|gb|KK964431.1|	48578-51516	15	7.4	3	0.351127	-3	0.0739	no
gi|646726819|gb|KK964431.1|	57922-58772	15	7.4	12	12	-0.0391782	0.9785	no
gi|646726819|gb|KK964431.1|	67200-68525	15	7.4	4	0.62114	-3	0.0974	no
gi|646726819|gb|KK964431.1|	68683-69241	15	7.4	3	0	-inf	0.00655	no
gi|646726819|gb|KK964431.1|	71017-72827	15	7.4	29	5	-3	0.06395	no
gi|646726819|gb|KK964431.1|	74939-76080	15	7.4	31	3	-3	0.03615	no
gi|646726840|gb|KK964428.1|	14370-15791	12	7.3	2	2	-0.268224	0.86925	no
gi|646726840|gb|KK964428.1|	15965-17465	12	7.3	2	0.642447	-2	0.29915	no
gi|646726840|gb|KK964428.1|	25781-27452	12	7.3	2	1	-0.627449	0.6515	no
gi|646726840|gb|KK964428.1|	28471-29568	12	7.3	4	4	-0.0489114	0.9728	no
gi|646726840|gb|KK964428.1|	51544-52090	12	7.3	4	5	0.447061	0.7252	no
gi|646726840|gb|KK964428.1|	56840-57731	12	7.3	1	0.412235	-2	0.2991	no
gi|646726840|gb|KK964428.1|	57872-58736	12	7.3	4	1	-2	0.3296	no
gi|646726840|gb|KK964428.1|	65140-65688	12	7.3	2	3	0.862756	0.5567	no
gi|646726840|gb|KK964428.1|	65739-66650	12	7.3	5	3	-0.839434	0.5434	no
gi|646726840|gb|KK964428.1|	67138-68243	12	7.3	13	9	-0.533571	0.71785	no
gi|646726840|gb|KK964428.1|	68974-69251	12	7.3	11	25	1	0.40125	no
gi|646726840|gb|KK964428.1|	73565-77605	12	7.3	4	7	0.959139	0.44965	no
gi|646726915|gb|KK964418.1|	39-924	15	7.2	6	2	-1	0.344	no
gi|646726926|gb|KK964416.1|	5095-5715	19	13	2	1	-0.702743	0.64185	no
gi|646726926|gb|KK964416.1|	68825-69556	19	13	3	1	-0.891826	0.53905	no

gi|646726947|gb|KK964413.1|	53642-53922	12	5	0	37	inf	0.00435	no
gi|646726947|gb|KK964413.1|	61112-62126	12	5	0.121877	29	8	0.09445	no
gi|646726985|gb|KK964407.1|	27323-27549	13	6.1	0	76	inf	0.00435	no
gi|646726985|gb|KK964407.1|	29368-29908	13	6.1	0.96883	14	4	0.1205	no
gi|646726991|gb|KK964406.1|	82569-83977	12	3.5	2	2	0.203093	0.90035	no
gi|646726991|gb|KK964406.1|	84838-86043	12	3.5	9	3	-1	0.4043	no
gi|646726998|gb|KK964405.1|	06293-10767	24	9.4	5	6	0.214003	0.8873	no
gi|646726998|gb|KK964405.1|	1098-2962	24	9.4	1	0.415919	-2	0.31955	no
gi|646726998|gb|KK964405.1|	16841-19001	24	9.4	9	13	0.54377	0.66625	no
gi|646726998|gb|KK964405.1|	4023-7067	24	9.4	2	0.482697	-2	0.1961	no
gi|646726998|gb|KK964405.1|	46036-52533	24	9.4	13	3	-2	0.08985	no
gi|646726998|gb|KK964405.1|	55476-58759	24	9.4	1	1	0.141461	1	no
gi|646726998|gb|KK964405.1|	58951-60927	24	9.4	1	0.856237	-0.65038	0.644	no
gi|646726998|gb|KK964405.1|	63440-69124	24	9.4	1	1	0.496899	0.74355	no
gi|646726998|gb|KK964405.1|	69334-70667	24	9.4	2	0.616624	-2	0.27885	no
gi|646726998|gb|KK964405.1|	70805-76264	24	9.4	0.942295	1	0.625832	0.6794	no
gi|646726998|gb|KK964405.1|	7126-8425	24	9.4	2	0.636284	-2	0.28995	no
gi|646726998|gb|KK964405.1|	77208-80822	24	9.4	56	127	1	0.33585	no
gi|646727006|gb|KK964404.1|	8702-9022	9.8	5.1	11	18	0.648571	0.6178	no
gi|646727028|gb|KK964401.1|	4629-4927	22	7.7	23	7	-2	0.2346	no
gi|646727028|gb|KK964401.1|	92690-93031	22	7.7	8	3	-1	0.479	no
gi|646727051|gb|KK964398.1|	71023-71599	21	5.4	3	1	-1	0.4702	no
gi|646727108|gb|KK964390.1|	23321-24583	13	5.5	5	0	-inf	0.00085	yes
gi|646727108|gb|KK964390.1|	452-786	13	5.5	8	5	-0.708543	0.64635	no
gi|646727108|gb|KK964390.1|	76934-77758	13	5.5	2	0	-inf	0.0035	no
gi|646727147|gb|KK964385.1|	11685-11260	13	5.7	0.418558	4	3	0.1391	no
gi|646727153|gb|KK964384.1|	94682-95207	14	6.9	4	2	-0.93884	0.5245	no
gi|646727168|gb|KK964382.1|	02870-10894	16	7.1	178	120	-0.568	0.64	no
gi|646727168|gb|KK964382.1|	09098-11005	16	7.1	1	0.56357	-0.907546	1	no
gi|646727168|gb|KK964382.1|	10191-11165	16	7.1	1	0.551472	-1	0.41285	no

gi|646727168|gb|KK964382.1|	20738-12170	16	7.1	2	2	0.160069	0.89675	no
gi|646727168|gb|KK964382.1|	22617-22980	16	7.1	6	8	0.522077	0.71695	no
gi|646727168|gb|KK964382.1|	35771-36478	16	7.1	3	0	-inf	0.00225	no
gi|646727168|gb|KK964382.1|	46850-48004	16	7.1	2	0.44185	-2	0.17375	no
gi|646727168|gb|KK964382.1|	49341-49998	16	7.1	2	1	-0.847171	0.57665	no
gi|646727168|gb|KK964382.1|	61826-63460	16	7.1	12	6	-0.852376	0.57755	no
gi|646727168|gb|KK964382.1|	67364-68195	16	7.1	26	12	-1	0.4774	no
gi|646727168|gb|KK964382.1|	73999-74647	16	7.1	4	2	-0.88954	0.49265	no
gi|646727175|gb|KK964381.1|	12361-14026	12	5.5	0.65613	2	2	0.26155	no
gi|646727175|gb|KK964381.1|	18476-19433	12	5.5	0.395311	1	2	0.255	no
gi|646727175|gb|KK964381.1|	22866-23782	12	5.5	29	36	0.296291	0.77795	no
gi|646727175|gb|KK964381.1|	35710-36906	12	5.5	0.983088	1	0.366891	0.78175	no
gi|646727175|gb|KK964381.1|	9092-12241	12	5.5	2	3	0.562886	0.70225	no
gi|646727205|gb|KK964376.1|	08447-10966	17	7.8	3	2	-0.743347	0.64475	no
gi|646727205|gb|KK964376.1|	10669-11140	17	7.8	2	2	-0.386972	0.7808	no
gi|646727205|gb|KK964376.1|	49423-49831	17	7.8	12	21	0.859332	0.53295	no
gi|646727205|gb|KK964376.1|	75330-75825	17	7.8	3	5	0.579329	0.68905	no
gi|646727205|gb|KK964376.1|	76082-76326	17	7.8	19	26	0.507665	0.7293	no
gi|646727205|gb|KK964376.1|	93885-94147	17	7.8	37	15	-1	0.35185	no
gi|646727205|gb|KK964376.1|	96337-96647	17	7.8	25	23	-0.161679	0.90705	no
gi|646727205|gb|KK964376.1|	9821-10992	17	7.8	19	12	-0.727126	0.63625	no
gi|646727205|gb|KK964376.1|	)9675-10044(	17	7.8	3	2	-1	0.40815	no
gi|646727211|gb|KK964375.1|	44379-45204	18	6.5	9	15	0.760449	0.6132	no
gi|646727216|gb|KK964374.1|	19898-20225	13	7	12.78	14	0.155488	0.9006	no
gi|646727216|gb|KK964374.1|	21443-21886	13	7	6	5	-0.185582	0.893	no
gi|646727216|gb|KK964374.1|	22267-22601	13	7	10	4	-1	0.36945	no
gi|646727222|gb|KK964373.1|	58253-59170	14	5.9	0.978111	0.793149	-0.302406	1	no
gi|646727320|gb|KK964359.1|	19348-19899	15	7.4	2	2	-0.0367356	0.92345	no
gi|646727370|gb|KK964352.1|	12137-13476	11	6.7	35	22	-0.654675	0.54425	no
gi|646727370|gb|KK964352.1|	31426-31957	11	6.7	3	3	-0.143084	0.91785	no

gi|646727370|gb|KK964352.1|	64836-65407	11	6.7	1	2	0.71781	0.64505	no
gi|646727370|gb|KK964352.1|	77170-77878	11	6.7	3	2	-0.217065	0.8729	no
gi|646727394|gb|KK964349.1|	29389-30388	13	5.5	0.373	2	2	0.18785	no
gi|646727408|gb|KK964347.1|	15324-15754	9.4	6.5	0.515639	8	4	0.09965	no
gi|646727408|gb|KK964347.1|	61292-61624	9.4	6.5	0	17	inf	0.00035	yes
gi|646727457|gb|KK964341.1|	09948-11022	13	5.6	14	18	0.363265	0.78965	no
gi|646727457|gb|KK964341.1|	10602-11142	13	5.6	8	7	-0.221293	0.89025	no
gi|646727510|gb|KK964334.1|	95969-96328	12	6.3	12	5	-1	0.3812	no
gi|646727540|gb|KK964331.1|	64648-65463	13	10	0	4	inf	0.00435	no
gi|646727554|gb|KK964329.1|	27906-12829	24	10	4	5	0.358892	0.80755	no
gi|646727554|gb|KK964329.1|	35115-13605	24	10	2	2	-0.171551	0.8903	no
gi|646727554|gb|KK964329.1|	37374-14174	24	10	1	3	1	0.35085	no
gi|646727554|gb|KK964329.1|	43647-14736	24	10	0.641209	3	2	0.2179	no
gi|646727554|gb|KK964329.1|	36324-36560	24	10	32	8	-2	0.2227	no
gi|646727554|gb|KK964329.1|	62480-64601	24	10	1	0.718628	-0.897486	0.5372	no
gi|646727554|gb|KK964329.1|	68212-69731	24	10	3	0.210986	-4	0.12165	no
gi|646727554|gb|KK964329.1|	76931-77912	24	10	2	2	-0.256454	0.83805	no
gi|646727554|gb|KK964329.1|	78019-83421	24	10	0.880826	0.65629	-0.424524	1	no
gi|646727554|gb|KK964329.1|	86812-90292	24	10	9	2	-2	0.1525	no
gi|646727554|gb|KK964329.1|	90868-92026	24	10	3	1	-2	0.3124	no
gi|646727554|gb|KK964329.1|	97813-98098	24	10	25	19	-0.380378	0.76385	no
gi|646727554|gb|KK964329.1|	99419-99630	24	10	86	42	-1	0.4207	no
gi|646727570|gb|KK964327.1|	12315-13101	29	11	2	3	0.90848	0.49355	no
gi|646727570|gb|KK964327.1|	15855-16833	29	11	1	0.9103	-0.491097	0.73195	no
gi|646727570|gb|KK964327.1|	21342-21977	29	11	2	1	-1	0.42175	no
gi|646727570|gb|KK964327.1|	27033-27730	29	11	3	5	0.651598	0.6548	no
gi|646727570|gb|KK964327.1|	8636-8800	29	11	175	137	-0.35656	0.80755	no
gi|646727644|gb|KK964318.1|	56351-16042	15	5.8	9	0	-inf	5.00E-05	yes
gi|646727644|gb|KK964318.1|	58131-58463	15	5.8	25	32	0.345109	0.8279	no
gi|646727652|gb|KK964317.1|	46425-14824	17	8.3	7	0	-inf	0.0003	yes

gi|646727652|gb|KK964317.1|	52416-15383	17	8.3	2	0	-inf	0.0042	no
gi|646727680|gb|KK964313.1|	15842-16446	18	6.7	2	0.729416	-2	0.3832	no
gi|646727680|gb|KK964313.1|	77082-17915	18	6.7	0.203782	2	3	0.12015	no
gi|646727680|gb|KK964313.1|	79986-18101	18	6.7	0.35901	2	3	0.15295	no
gi|646727692|gb|KK964311.1|	94959-19574	16	13	3	2	-0.658452	0.6023	no
gi|646727692|gb|KK964311.1|	7141-8442	16	13	20	19	-0.0498457	0.964	no
gi|646727722|gb|KK964307.1|	69827-70468	20	7.2	6	0.663858	-3	0.1478	no
gi|646727770|gb|KK964301.1|	69304-69781	22	11	16	4	-2	0.2092	no
gi|646727841|gb|KK964291.1|	07878-10875	23	9.6	11	0.210596	-6	0.16865	no
gi|646727841|gb|KK964291.1|	11031-11695	23	9.6	16	12	-0.369479	0.74015	no
gi|646727841|gb|KK964291.1|	21519-12814	23	9.6	78	83	0.0975112	0.93555	no
gi|646727841|gb|KK964291.1|	28969-12947	23	9.6	91	37	-1	0.30795	no
gi|646727841|gb|KK964291.1|	72264-17662	23	9.6	8	4	-1	0.35615	no
gi|646727841|gb|KK964291.1|	77204-17876	23	9.6	4	2	-0.943088	0.5447	no
gi|646727841|gb|KK964291.1|	79526-18092	23	9.6	3	2	-0.944631	0.49015	no
gi|646727841|gb|KK964291.1|	34875-36588	23	9.6	0.507625	0.366674	-0.469266	1	no
gi|646727841|gb|KK964291.1|	79333-81411	23	9.6	6	3	-1	0.4412	no
gi|646727841|gb|KK964291.1|	93179-93461	23	9.6	40	0	-inf	0.00495	no
gi|646727841|gb|KK964291.1|	97944-98408	23	9.6	25	0	-inf	0.0003	yes
gi|646727863|gb|KK964288.1|	01985-10284	12	10	18	4	-2	0.18535	no
gi|646727863|gb|KK964288.1|	30873-13208	12	10	9	10	0.201377	0.89295	no
gi|646727863|gb|KK964288.1|	33790-13446	12	10	18	15	-0.282489	0.8502	no
gi|646727877|gb|KK964286.1|	26363-27087	15	5.7	0	8	inf	0.0012	yes
gi|646727886|gb|KK964285.1|	33313-34071	10	5.9	1	0.774836	-0.732204	0.66415	no
gi|646727899|gb|KK964283.1|	18306-18987	13	13	8	6	-0.484426	0.7618	no
gi|646727907|gb|KK964282.1|	46782-47821	11	6.5	1	0	-inf	0.00655	no
gi|646727962|gb|KK964275.1|	14366-11518	12	7.5	0.818559	1	0.496936	0.7568	no
gi|646728017|gb|KK964268.1|	09070-11016	19	9.5	10	4	-1	0.39395	no
gi|646728017|gb|KK964268.1|	10502-11149	19	9.5	9	4	-1	0.50205	no
gi|646728017|gb|KK964268.1|	43005-14592	19	9.5	1	0.859407	-0.326178	1	no

gi|646728017|gb|KK964268.1|	64120-65870	19	9.5	3	3	0.24887	0.87005	no
gi|646728017|gb|KK964268.1|	66166-66673	19	9.5	7	7	0.0699658	0.95785	no
gi|646728017|gb|KK964268.1|	67612-68540	19	9.5	2	0.975753	-1	0.3995	no
gi|646728032|gb|KK964266.1|	69981-70578	11	2.4	3	0.371646	-3	0.1921	no
gi|646728032|gb|KK964266.1|	72357-72834	11	2.4	20	1	-4	0.1164	no
gi|646728049|gb|KK964263.1|	03152-10392	16	7.9	1	2	0.0763547	0.92975	no
gi|646728049|gb|KK964263.1|	35723-37571	16	7.9	0.987298	0	-inf	1	no
gi|646728049|gb|KK964263.1|	46999-47727	16	7.9	2	0	-inf	0.00655	no
gi|646728049|gb|KK964263.1|	48884-50349	16	7.9	0.917479	0	-inf	1	no
gi|646728049|gb|KK964263.1|	61183-62715	16	7.9	2	0	-inf	0.00495	no
gi|646728053|gb|KK964262.1|	)9553-10077[illegible]	12	6.1	0.76771	2	1	0.3744	no
gi|646728086|gb|KK964257.1|	02715-10309	12	5.1	6	4	-0.654004	0.66245	no
gi|646728086|gb|KK964257.1|	06302-10802	12	5.1	0.693231	4	2	0.11415	no
gi|646728086|gb|KK964257.1|	88170-88488	12	5.1	8	4	-0.955573	0.5991	no
gi|646728127|gb|KK964252.1|	73494-73886	12	11	0	21	inf	0.00155	yes
gi|646728135|gb|KK964251.1|	47554-14831	35	17	2	0.258279	-3	0.21605	no
gi|646728135|gb|KK964251.1|	50754-15187	35	17	2	2	-0.105597	0.93175	no
gi|646728135|gb|KK964251.1|	52701-15335	35	17	4	7	0.619147	0.6537	no
gi|646728148|gb|KK964249.1|	07298-10771	20	6.1	4	13	2	0.34585	no
gi|646728148|gb|KK964249.1|	19696-20059	20	6.1	14	8	-0.840493	0.5105	no
gi|646728148|gb|KK964249.1|	28374-30279	20	6.1	6	6	-0.0646473	0.96625	no
gi|646728148|gb|KK964249.1|	30615-34590	20	6.1	11	8	-0.446039	0.68545	no
gi|646728148|gb|KK964249.1|	37120-38165	20	6.1	11	7	-0.642726	0.6749	no
gi|646728221|gb|KK964239.1|	47390-48455	10	8.9	3	0	-inf	0.00525	no
gi|646728259|gb|KK964234.1|	11680-11903	9.9	5.4	47	47	0.0128974	0.94515	no
gi|646728259|gb|KK964234.1|	22633-24534	9.9	5.4	26	63	1	0.27965	no
gi|646728285|gb|KK964230.1|	12197-12616	9.8	12	6	3	-1	0.46155	no
gi|646728339|gb|KK964223.1|	43876-14479	20	9.4	4	2	-0.817193	0.5715	no
gi|646728339|gb|KK964223.1|	58485-59684	20	9.4	1	3	0.988485	0.52035	no
gi|646728339|gb|KK964223.1|	6979-7707	20	9.4	6	3	-1	0.43205	no

gi|646728370|gb|KK964218.1|	54931-56204	14	8.6	0	6	inf	5.00E-05	yes
gi|646728391|gb|KK964215.1|	25813-12639	16	15	6	0.386338	-4	0.1898	no
gi|646728391|gb|KK964215.1|	29294-12954	16	15	35	41	0.218095	0.86555	no
gi|646728391|gb|KK964215.1|	13719-14389	16	15	2	3	0.614638	0.63615	no
gi|646728391|gb|KK964215.1|	37788-13920	16	15	2	2	-0.211687	0.8909	no
gi|646728391|gb|KK964215.1|	45827-14662	16	15	0.680118	2	2	0.29655	no
gi|646728391|gb|KK964215.1|	47385-15015	16	15	0.478993	0.696365	0.539838	1	no
gi|646728391|gb|KK964215.1|	23887-25939	16	15	6	15	1	0.31595	no
gi|646728391|gb|KK964215.1|	26178-27016	16	15	5	7	0.588818	0.70005	no
gi|646728391|gb|KK964215.1|	29318-29746	16	15	3	11	2	0.2994	no
gi|646728391|gb|KK964215.1|	31806-32023	16	15	21	69	2	0.27235	no
gi|646728391|gb|KK964215.1|	37104-37357	16	15	21	61	2	0.34005	no
gi|646728391|gb|KK964215.1|	37613-41350	16	15	0.637711	2	1	0.3888	no
gi|646728391|gb|KK964215.1|	45343-45527	16	15	54	117	1	0.4601	no
gi|646728391|gb|KK964215.1|	76602-78103	16	15	16	12	-0.512472	0.686	no
gi|646728391|gb|KK964215.1|	78584-79448	16	15	0.911322	1	0.237847	1	no
gi|646728391|gb|KK964215.1|	87456-88433	16	15	17	8	-1	0.44035	no
gi|646728391|gb|KK964215.1|	90222-91186	16	15	1	0.556635	-1	0.3882	no
gi|646728431|gb|KK964209.1|	2774-3416	14	7.7	1	2	0.47184	0.7721	no
gi|646728431|gb|KK964209.1|	60392-60622	14	7.7	0	145	inf	0.0006	yes
gi|646728514|gb|KK964198.1|	565-1523	15	6.5	2	1	-0.492319	0.7125	no
gi|646728514|gb|KK964198.1|	6849-7738	15	6.5	0.583598	2	2	0.20295	no
gi|646728583|gb|KK964188.1|	07683-10877	16	6.7	0.774575	0.474279	-0.707669	1	no
gi|646728609|gb|KK964184.1|	01193-10475	34	14	1	3	1	0.50675	no
gi|646728609|gb|KK964184.1|	08643-10904	34	14	6	2	-2	0.26015	no
gi|646728609|gb|KK964184.1|	10089-11081	34	14	50	69	0.462109	0.66835	no
gi|646728609|gb|KK964184.1|	13374-11415	34	14	5	5	0.0777379	0.96185	no
gi|646728609|gb|KK964184.1|	26980-12966	34	14	0.419502	0.609688	0.539395	1	no
gi|646728609|gb|KK964184.1|	29781-13184	34	14	1	2	1	0.36445	no
gi|646728609|gb|KK964184.1|	32008-13376	34	14	2	0.445405	-2	0.21385	no

gi|646728609|gb|KK964184.1|	50793-15128	34	14	0	14	inf	0.0017	no
gi|646728609|gb|KK964184.1|	67637-16793	34	14	15	9	-0.778545	0.5939	no
gi|646728609|gb|KK964184.1|	82969-18364	34	14	5	5	0.172635	0.89635	no
gi|646728609|gb|KK964184.1|	92013-19674	34	14	23	18	-0.35936	0.754	no
gi|646728609|gb|KK964184.1|	00537-20174	34	14	0.778202	0.418246	-0.895794	1	no
gi|646728609|gb|KK964184.1|	01901-20321	34	14	0.609195	0.875204	0.522715	1	no
gi|646728609|gb|KK964184.1|	07209-20813	34	14	0.692377	1	0.768403	0.6143	no
gi|646728609|gb|KK964184.1|	10404-21148	34	14	0.557939	1	0.999835	1	no
gi|646728609|gb|KK964184.1|	11588-21376	34	14	0.432999	0.558058	0.366051	1	no
gi|646728609|gb|KK964184.1|	17190-22732	34	14	4	3	-0.389299	0.72425	no
gi|646728609|gb|KK964184.1|	29888-23531	34	14	85	51	-0.750688	0.5291	no
gi|646728609|gb|KK964184.1|	35430-23683	34	14	1	0.928383	-0.645047	0.61285	no
gi|646728609|gb|KK964184.1|	38450-24095	34	14	0.656743	0.715369	0.123359	1	no
gi|646728609|gb|KK964184.1|	41137-24137	34	14	135	171.6	0.343046	0.82635	no
gi|646728609|gb|KK964184.1|	43505-24484	34	14	1	1	0.282359	0.8201	no
gi|646728609|gb|KK964184.1|	45411-24755	34	14	0.833931	0.853316	0.0331531	1	no
gi|646728609|gb|KK964184.1|	47680-24799	34	14	7	8	0.268446	0.84425	no
gi|646728609|gb|KK964184.1|	52005-25325	34	14	2	0.802682	-1	0.38595	no
gi|646728609|gb|KK964184.1|	53985-25443	34	14	5	11	1	0.41345	no
gi|646728609|gb|KK964184.1|	62378-26269	34	14	7	14	1	0.4553	no
gi|646728609|gb|KK964184.1|	70832-27148	34	14	9	4	-1	0.35535	no
gi|646728609|gb|KK964184.1|	75790-27602	34	14	36	56	0.649309	0.67465	no
gi|646728609|gb|KK964184.1|	77077-27917	34	14	2	3	0.60567	0.6881	no
gi|646728609|gb|KK964184.1|	21720-32255	34	14	55	40	-0.456629	0.6701	no
gi|646728609|gb|KK964184.1|	28735-33018	34	14	8	4	-1	0.496	no
gi|646728609|gb|KK964184.1|	31028-33301	34	14	7	11	0.625943	0.6235	no
gi|646728609|gb|KK964184.1|	36730-33888	34	14	2	4	0.93313	0.54275	no
gi|646728609|gb|KK964184.1|	39581-34063	34	14	100	131	0.394496	0.7364	no
gi|646728609|gb|KK964184.1|	47144-34764	34	14	3	1	-2	0.28945	no
gi|646728609|gb|KK964184.1|	71866-37225	34	14	6	0.821021	-3	0.20705	no

gi|646728609|gb|KK964184.1|	80621-38127	34	14	2	1	-0.332598	0.81865	no
gi|646728609|gb|KK964184.1|	81634-38244	34	14	2	2	-0.018921	0.9367	no
gi|646728609|gb|KK964184.1|	83196-38345	34	14	29	3	-3	0.18545	no
gi|646728609|gb|KK964184.1|	4497-4935	34	14	4	1	-2	0.3081	no
gi|646728609|gb|KK964184.1|	48609-49141	34	14	14	41	2	0.32125	no
gi|646728609|gb|KK964184.1|	52111-53755	34	14	12	42	2	0.18005	no
gi|646728609|gb|KK964184.1|	56671-56923	34	14	30	32	0.118551	0.92535	no
gi|646728609|gb|KK964184.1|	58574-58882	34	14	15	87	2	0.1076	no
gi|646728609|gb|KK964184.1|	61689-62224	34	14	17	56	2	0.27835	no
gi|646728609|gb|KK964184.1|	95418-98778	34	14	13	10	-0.324474	0.76945	no
gi|646728658|gb|KK964178.1|	85626-86079	7.2	4.7	2	4	0.890975	0.5714	no
gi|646728687|gb|KK964174.1|	00644-10108	18	7	3	3	-0.407338	0.8098	no
gi|646728687|gb|KK964174.1|	12133-11273	18	7	2	0.743292	-2	0.29375	no
gi|646728721|gb|KK964170.1|	12713-14615	35	7.9	2	1	-1	0.39785	no
gi|646728721|gb|KK964170.1|	15332-16648	35	7.9	2	1	-0.555343	0.6596	no
gi|646728721|gb|KK964170.1|	22774-24339	35	7.9	1	0.611106	-1	0.3649	no
gi|646728721|gb|KK964170.1|	24455-28813	35	7.9	2	0.493425	-2	0.30475	no
gi|646728721|gb|KK964170.1|	33532-35134	35	7.9	0.686814	0.099099	-3	1	no
gi|646728721|gb|KK964170.1|	57826-58365	35	7.9	7	2	-2	0.23	no
gi|646728728|gb|KK964169.1|	10867-11344	11	4.5	12	0	-inf	0.0031	no
gi|646728813|gb|KK964157.1|	45893-46388	12	7.3	3	2	-0.742599	0.6076	no
gi|646728843|gb|KK964153.1|	19703-20819	13	5	0.537207	0.768095	0.515807	1	no
gi|646728843|gb|KK964153.1|	85182-86129	13	5	0.66837	2	1	0.43245	no
gi|646728879|gb|KK964148.1|	75224-75638	9.1	4.1	26	6	-2	0.1541	no
gi|646728879|gb|KK964148.1|	75762-76318	9.1	4.1	2	3	0.227884	0.87175	no
gi|646728903|gb|KK964145.1|	14464-11469	18	5.7	8	32	2	0.32265	no
gi|646728903|gb|KK964145.1|	42235-14256	18	5.7	12	19	0.728478	0.6366	no
gi|646728903|gb|KK964145.1|	46005-14674	18	5.7	1	3	1	0.3075	no
gi|646728903|gb|KK964145.1|	53315-15381	18	5.7	6	4	-0.478225	0.71865	no
gi|646728903|gb|KK964145.1|	53973-15726	18	5.7	6	0.931493	-3	0.06605	no

gi|646728903|gb|KK964145.1|	59606-15995	18	5.7	16	2	-3	0.12775	no
gi|646728903|gb|KK964145.1|	09067-20938	18	5.7	12	15	0.258504	0.8511	no
gi|646728903|gb|KK964145.1|	12710-21325	18	5.7	4	3	-0.205681	0.878	no
gi|646728903|gb|KK964145.1|	16993-21826	18	5.7	0.63989	1	0.883799	0.5385	no
gi|646728903|gb|KK964145.1|	19353-22134	18	5.7	0.850727	0.693005	-0.29583	1	no
gi|646728903|gb|KK964145.1|	24765-22525	18	5.7	5	8	0.840999	0.56825	no
gi|646728903|gb|KK964145.1|	25453-22585	18	5.7	5	8	0.689013	0.6147	no
gi|646728903|gb|KK964145.1|	26708-22725	18	5.7	12	20	0.785404	0.6078	no
gi|646728903|gb|KK964145.1|	28760-23206	18	5.7	11	8	-0.387543	0.7226	no
gi|646728903|gb|KK964145.1|	32181-23545	18	5.7	2	1	-0.434559	0.776	no
gi|646728903|gb|KK964145.1|	47527-24786	18	5.7	8	6	-0.385164	0.80435	no
gi|646728903|gb|KK964145.1|	70955-72313	18	5.7	6	0.24117	-5	0.1114	no
gi|646728903|gb|KK964145.1|	74143-76444	18	5.7	0.813941	1	0.826473	0.55095	no
gi|646728903|gb|KK964145.1|	90450-90901	18	5.7	4	5	0.404349	0.7845	no
gi|646728921|gb|KK964142.1|	84639-86475	13	5.4	2	3	0.532418	0.7325	no
gi|646728988|gb|KK964135.1|	16160-17043	23	12	2	1	-0.372052	0.78485	no
gi|646728988|gb|KK964135.1|	77345-17834	23	12	3	0.177303	-4	0.17035	no
gi|646728988|gb|KK964135.1|	17966-18744	23	12	2	0.497638	-2	0.2551	no
gi|646728988|gb|KK964135.1|	80734-18255	23	12	3	0.170442	-4	0.11595	no
gi|646728988|gb|KK964135.1|	39948-40331	23	12	8	0.87075	-3	0.19445	no
gi|646728988|gb|KK964135.1|	93640-94391	23	12	2	4	1	0.42735	no
gi|646728988|gb|KK964135.1|	96189-97293	23	12	2	1	-0.732632	0.5707	no
gi|646729000|gb|KK964133.1|	7217-7573	18	7.8	27	12	-1	0.43485	no
gi|646729013|gb|KK964131.1|	49804-50479	13	8	0	3	inf	0.0014	yes
gi|646729013|gb|KK964131.1|	63746-64985	13	8	0	2	inf	0.00435	no
gi|646729013|gb|KK964131.1|	84661-85189	13	8	0	6	inf	0.00035	yes
gi|646729045|gb|KK964126.1|	00368-10342	20	11	6	0	-inf	0.00015	yes
gi|646729045|gb|KK964126.1|	16974-12459	20	11	8	0.01836	-9	0.1665	no
gi|646729045|gb|KK964126.1|	26550-12805	20	11	1	0	-inf	0.00195	no
gi|646729045|gb|KK964126.1|	31796-13395	20	11	4	0	-inf	0.0002	yes

gi|646729045|gb|KK964126.1|	35133-13559	20	11	5	2	-0.914031	0.5518	no
gi|646729045|gb|KK964126.1|	42995-14517	20	11	33	11	-2	0.1527	no
gi|646729045|gb|KK964126.1|	47960-14941	20	11	2	1	-1	0.42755	no
gi|646729045|gb|KK964126.1|	62493-16391	20	11	0.476865	0.800695	0.747671	1	no
gi|646729045|gb|KK964126.1|	64022-16497	20	11	1	1	0.0921992	0.9294	no
gi|646729045|gb|KK964126.1|	65152-16724	20	11	1	0.583242	-0.924207	1	no
gi|646729045|gb|KK964126.1|	44300-45772	20	11	19	0	-inf	5.00E-05	yes
gi|646729045|gb|KK964126.1|	58827-61056	20	11	3	0	-inf	0.00055	yes
gi|646729045|gb|KK964126.1|	63087-74165	20	11	10	0	-inf	5.00E-05	yes
gi|646729045|gb|KK964126.1|	75747-77899	20	11	3	0	-inf	0.00045	yes
gi|646729045|gb|KK964126.1|	82135-83255	20	11	4	0	-inf	0.0018	no
gi|646729045|gb|KK964126.1|	83317-85572	20	11	3	0	-inf	0.00055	yes
gi|646729045|gb|KK964126.1|	85672-86329	20	11	5	0	-inf	0.0004	yes
gi|646729045|gb|KK964126.1|	86393-88310	20	11	4	0	-inf	5.00E-05	yes
gi|646729077|gb|KK964122.1|	07359-20773	12	7	12	0.909019	-4	0.1728	no
gi|646729084|gb|KK964121.1|	60277-60755	7.7	4.7	4	6	0.556736	0.67275	no
gi|646729084|gb|KK964121.1|	65942-66962	7.7	4.7	2	0	-inf	0.0008	yes
gi|646729084|gb|KK964121.1|	93584-95430	7.7	4.7	0.988539	0.504671	-0.969956	1	no
gi|646729091|gb|KK964120.1|	27539-30011	16	6.4	0.166926	2	4	0.11655	no
gi|646729091|gb|KK964120.1|	30166-31105	16	6.4	0.135237	2	4	0.09905	no
gi|646729091|gb|KK964120.1|	32092-32932	16	6.4	1	5	2	0.2026	no
gi|646729091|gb|KK964120.1|	36096-36307	16	6.4	55	30	-0.889922	0.5566	no
gi|646729091|gb|KK964120.1|	45119-45879	16	6.4	14	7	-0.936989	0.5385	no
gi|646729091|gb|KK964120.1|	46291-48928	16	6.4	33	15	-1	0.33445	no
gi|646729144|gb|KK964114.1|	52366-15314	16	8	0.350358	2	3	0.2655	no
gi|646729150|gb|KK964113.1|	0-757	17	4.6	0	21	inf	0.0001	yes
gi|646729150|gb|KK964113.1|	14955-15894	17	4.6	0	12	inf	0.00045	yes
gi|646729150|gb|KK964113.1|	16260-16621	17	4.6	0	38	inf	0.00055	yes
gi|646729150|gb|KK964113.1|	17102-18900	17	4.6	0.059958	14	8	0.09445	no
gi|646729150|gb|KK964113.1|	19489-20671	17	4.6	0	8	inf	0.00035	yes

gi|646729150|gb|KK964113.1|	28718-30048	17	4.6	0	13	inf	5.00E-05	yes
gi|646729150|gb|KK964113.1|	30302-31772	17	4.6	0.076142	6	6	0.09525	no
gi|646729150|gb|KK964113.1|	3564-3950	17	4.6	0	9	inf	0.00255	no
gi|646729150|gb|KK964113.1|	56293-57277	17	4.6	0	3	inf	0.00315	no
gi|646729150|gb|KK964113.1|	58005-59966	17	4.6	0.05423	7	7	0.09495	no
gi|646729150|gb|KK964113.1|	60168-60862	17	4.6	0	8	inf	0.00095	yes
gi|646729150|gb|KK964113.1|	62819-63508	17	4.6	0.213037	10	6	0.0979	no
gi|646729150|gb|KK964113.1|	64553-66212	17	4.6	0.131787	54	9	0.2322	no
gi|646729175|gb|KK964110.1|	16107-11688	19	8.6	21	12	-0.817763	0.58565	no
gi|646729175|gb|KK964110.1|	17919-11834	19	8.6	6	4	-0.488098	0.71535	no
gi|646729175|gb|KK964110.1|	22903-12381	19	8.6	13	16	0.27186	0.8576	no
gi|646729175|gb|KK964110.1|	58978-15918	19	8.6	91	64	-0.49824	0.7073	no
gi|646729175|gb|KK964110.1|	60258-16089	19	8.6	27	14	-0.956223	0.5239	no
gi|646729175|gb|KK964110.1|	65620-16848	19	8.6	18	11	-0.730795	0.5084	no
gi|646729175|gb|KK964110.1|	91060-19211	19	8.6	1	0.82708	-0.750087	0.59275	no
gi|646729175|gb|KK964110.1|	39728-40301	19	8.6	1	2	0.455348	0.7747	no
gi|646729175|gb|KK964110.1|	50127-50599	19	8.6	5	6	0.0376425	0.9505	no
gi|646729182|gb|KK964109.1|	16795-11820	13	6.3	0.958672	0	-inf	1	no
gi|646729182|gb|KK964109.1|	94313-94901	13	6.3	4	6	0.739553	0.59515	no
gi|646729189|gb|KK964108.1|	12392-12339	25	7	13	23	0.812917	0.495	no
gi|646729189|gb|KK964108.1|	28916-12911	25	7	38	156	2	0.26825	no
gi|646729189|gb|KK964108.1|	41239-14200	25	7	1	1	0.228358	0.87595	no
gi|646729189|gb|KK964108.1|	42173-14352	25	7	0.591935	0.972458	0.716197	1	no
gi|646729189|gb|KK964108.1|	47011-14718	25	7	167	136	-0.290427	0.83605	no
gi|646729189|gb|KK964108.1|	55869-15799	25	7	0.198322	0.862354	2	1	no
gi|646729189|gb|KK964108.1|	16598-17332	25	7	3	0.270618	-3	0.18625	no
gi|646729189|gb|KK964108.1|	42616-24288	25	7	77	25	-2	0.26895	no
gi|646729189|gb|KK964108.1|	46616-24687	25	7	32	5	-3	0.1424	no
gi|646729189|gb|KK964108.1|	86901-87929	25	7	1	0.511845	-1	0.3882	no
gi|646729189|gb|KK964108.1|	93941-95809	25	7	65	105	0.689876	0.5566	no

gi|646729189|gb|KK964108.1|	96019-97294	25	7	0.725568	0.260373	-1	1	no
gi|646729189|gb|KK964108.1|	97409-98107	25	7	2	0.87446	-1	0.38485	no
gi|646729189|gb|KK964108.1|	)8436-10959!	25	7	29	37	0.333502	0.7797	no
gi|646729242|gb|KK964101.1|	52103-52518	12	8.1	1	19	4	0.24715	no
gi|646729242|gb|KK964101.1|	53007-53700	12	8.1	5	1	-2	0.22205	no
gi|646729261|gb|KK964099.1|	00660-10088	24	10	21	39	0.877536	0.56255	no
gi|646729261|gb|KK964099.1|	23343-12365	24	10	49	97	0.977431	0.52945	no
gi|646729261|gb|KK964099.1|	33182-13497	24	10	50	76	0.604677	0.60515	no
gi|646729261|gb|KK964099.1|	80055-18286	24	10	31	11	-1	0.2151	no
gi|646729261|gb|KK964099.1|	88757-19095	24	10	22	15	-0.554448	0.6117	no
gi|646729261|gb|KK964099.1|	92615-19329	24	10	4	1	-2	0.25345	no
gi|646729261|gb|KK964099.1|	58250-59114	24	10	17	61	2	0.167	no
gi|646729261|gb|KK964099.1|	72368-72942	24	10	9	10	0.201875	0.894	no
gi|646729280|gb|KK964096.1|	02972-10419	22	9.3	1	0.274877	-2	0.1855	no
gi|646729280|gb|KK964096.1|	11873-14242	22	9.3	2	2	-0.0100227	0.98395	no
gi|646729280|gb|KK964096.1|	42176-14305	22	9.3	62	204	2	0.1352	no
gi|646729280|gb|KK964096.1|	15971-21737	22	9.3	2	2	-0.00169759	0.9941	no
gi|646729280|gb|KK964096.1|	69449-17103	22	9.3	0.625092	1	1	0.3858	no
gi|646729280|gb|KK964096.1|	71931-17368	22	9.3	9	31	2	0.18585	no
gi|646729280|gb|KK964096.1|	74303-17593	22	9.3	0.737431	1	0.877435	0.4936	no
gi|646729280|gb|KK964096.1|	80521-18249	22	9.3	75	134	0.841336	0.4806	no
gi|646729280|gb|KK964096.1|	22476-23580	22	9.3	0.762717	1	0.877865	0.5385	no
gi|646729280|gb|KK964096.1|	36722-23692	22	9.3	62	73	0.23325	0.86265	no
gi|646729280|gb|KK964096.1|	45231-24610	22	9.3	0.593899	2	2	0.2406	no
gi|646729280|gb|KK964096.1|	24992-26074	22	9.3	0.894861	0.479305	-0.90072	1	no
gi|646729280|gb|KK964096.1|	56485-25672	22	9.3	22	10	-1	0.4847	no
gi|646729280|gb|KK964096.1|	74008-27483	22	9.3	0.814237	1	0.497224	0.7568	no
gi|646729280|gb|KK964096.1|	41238-41816	22	9.3	114	53	-1	0.39525	no
gi|646729280|gb|KK964096.1|	44417-44906	22	9.3	3	5	0.78714	0.5828	no
gi|646729280|gb|KK964096.1|	53411-54429	22	9.3	0.848666	1	0.288765	1	no

gi|646729280|gb|KK964096.1|	56042-56295	22	9.3	29	29	-0.0158688	0.9493	no
gi|646729280|gb|KK964096.1|	61647-62459	22	9.3	0.996374	1	0.496153	0.73295	no
gi|646729280|gb|KK964096.1|	63248-65070	22	9.3	100	41	-1	0.2819	no
gi|646729280|gb|KK964096.1|	67065-67276	22	9.3	142	36	-2	0.2296	no
gi|646729280|gb|KK964096.1|	82778-86913	22	9.3	0.929986	0.660721	-0.493168	1	no
gi|646729280|gb|KK964096.1|	89193-90514	22	9.3	1	0.748107	-0.477202	1	no
gi|646729280|gb|KK964096.1|	90698-95262	22	9.3	0.987704	0.59528	-0.73051	1	no
gi|646729280|gb|KK964096.1|	95395-97354	22	9.3	2	1	-0.658784	0.6302	no
gi|646729280|gb|KK964096.1|	97532-98532	22	9.3	4	3	-0.57229	0.72255	no
gi|646729280|gb|KK964096.1|	99318-10276[illegible]	22	9.3	70	32	-1	0.3468	no
gi|646729332|gb|KK964089.1|	88070-18865	13	6.7	4	1	-2	0.2617	no
gi|646729345|gb|KK964087.1|	45332-47119	25	11	0.42272	0.349218	-0.275575	1	no
gi|646729406|gb|KK964079.1|	13130-21514	21	11	0.946193	0.304546	-2	1	no
gi|646729406|gb|KK964079.1|	15244-21647	21	11	0.473435	0.814729	0.783154	1	no
gi|646729406|gb|KK964079.1|	18581-21954	21	11	0.780616	1	0.508178	1	no
gi|646729406|gb|KK964079.1|	36803-23738	21	11	0.836137	11	4	0.1255	no
gi|646729406|gb|KK964079.1|	39780-24314	21	11	8	21	1	0.2233	no
gi|646729406|gb|KK964079.1|	43658-24471	21	11	0.919311	0.984099	0.0982496	1	no
gi|646729406|gb|KK964079.1|	48813-24936	21	11	14	15	0.108917	0.9397	no
gi|646729406|gb|KK964079.1|	53101-25373	21	11	0.723687	4	2	0.1548	no
gi|646729444|gb|KK964073.1|	33993-34410	13	5.9	0	50	inf	0.0002	yes
gi|646729444|gb|KK964073.1|	34556-35463	13	5.9	0.141873	22	7	0.09455	no
gi|646729444|gb|KK964073.1|	53517-55428	13	5.9	7	6	-0.140175	0.92555	no
gi|646729444|gb|KK964073.1|	87209-87832	13	5.9	150	105	-0.515336	0.6431	no
gi|646729444|gb|KK964073.1|	88379-89551	13	5.9	3	7	1	0.42955	no
gi|646729464|gb|KK964071.1|	02073-10466	37	11	2	1	-0.488578	0.7464	no
gi|646729464|gb|KK964071.1|	05376-10564	37	11	67	35	-0.946197	0.4966	no
gi|646729464|gb|KK964071.1|	7315-7779	37	11	20	13	-0.652275	0.6738	no
gi|646729464|gb|KK964071.1|	88824-90170	37	11	1	1	-0.104558	0.93385	no
gi|646729464|gb|KK964071.1|	90718-94320	37	11	1	2	0.472611	0.7537	no

gi|646729464|gb|KK964071.1|	9296-11262	37	11	4	7	0.652119	0.66805	no
gi|646729487|gb|KK964068.1|	74131-74875	14	10	5	8	0.635261	0.69075	no
gi|646729504|gb|KK964066.1|	14135-11517	17	8.1	1	0.839357	-0.335645	1	no
gi|646729504|gb|KK964066.1|	33528-13378	17	8.1	41	6	-3	0.1377	no
gi|646729504|gb|KK964066.1|	49196-49533	17	8.1	6	10	0.710827	0.62785	no
gi|646729504|gb|KK964066.1|	57246-57647	17	8.1	15	17	0.24243	0.87775	no
gi|646729504|gb|KK964066.1|	66753-67135	17	8.1	218	177	-0.300024	0.77905	no
gi|646729504|gb|KK964066.1|	67383-67673	17	8.1	47	18	-1	0.34835	no
gi|646729531|gb|KK964062.1|	49662-50762	10	5.3	5	15	1	0.35435	no
gi|646729547|gb|KK964060.1|	20012-23236	29	9.7	0.124649	102	10	0.09345	no
gi|646729590|gb|KK964055.1|	32773-33164	11	5.2	8	7	-0.171622	0.89155	no
gi|646729620|gb|KK964051.1|	38702-14829	23	10	5	3	-0.972422	0.3775	no
gi|646729620|gb|KK964051.1|	48501-14964	23	10	2	0.449163	-2	0.19245	no
gi|646729620|gb|KK964051.1|	53472-15391	23	10	14	16	0.136346	0.92955	no
gi|646729620|gb|KK964051.1|	55406-15705	23	10	6	6	0.0312221	0.98215	no
gi|646729620|gb|KK964051.1|	58163-15993	23	10	6	2	-1	0.3508	no
gi|646729620|gb|KK964051.1|	63215-16391	23	10	4	5	0.25928	0.8498	no
gi|646729620|gb|KK964051.1|	64704-16559	23	10	1	1	-0.344732	0.8138	no
gi|646729620|gb|KK964051.1|	65777-16753	23	10	2	0.800153	-1	0.44875	no
gi|646729620|gb|KK964051.1|	69124-16977	23	10	8	1	-3	0.14015	no
gi|646729620|gb|KK964051.1|	78208-18088	23	10	10	4	-2	0.2423	no
gi|646729620|gb|KK964051.1|	80989-18200	23	10	21	7	-1	0.33975	no
gi|646729620|gb|KK964051.1|	92575-19347	23	10	40	2	-4	0.0048	no
gi|646729620|gb|KK964051.1|	95682-19604	23	10	14	4	-2	0.21415	no
gi|646729620|gb|KK964051.1|	96230-19764	23	10	2	0	-inf	0.00525	no
gi|646729620|gb|KK964051.1|	97976-19975	23	10	16	4	-2	0.11335	no
gi|646729620|gb|KK964051.1|	08758-20993	23	10	2	0.863092	-1	0.3648	no
gi|646729620|gb|KK964051.1|	11932-21294	23	10	2	0.523024	-2	0.26895	no
gi|646729620|gb|KK964051.1|	13103-21412	23	10	1	0.343389	-2	1	no
gi|646729620|gb|KK964051.1|	14453-21719	23	10	9	3	-1	0.25145	no

gi|646729620|gb|KK964051.1|	20503-22104	23	10	3	2	-0.555301	0.72945	no
gi|646729620|gb|KK964051.1|	58392-59364	23	10	0	6	inf	0.0006	yes
gi|646729620|gb|KK964051.1|	70509-70802	23	10	17	16	-0.0680511	0.94855	no
gi|646729620|gb|KK964051.1|	72431-72864	23	10	2	5	2	0.2631	no
gi|646729620|gb|KK964051.1|	77439-83309	23	10	2	2	-0.586737	0.69585	no
gi|646729620|gb|KK964051.1|	86614-86858	23	10	124	92	-0.421946	0.78575	no
gi|646729620|gb|KK964051.1|	87843-88913	23	10	49	22	-1	0.371	no
gi|646729674|gb|KK964044.1|	13728-11516	24	9.4	0.471199	0.791357	0.747992	1	no
gi|646729674|gb|KK964044.1|	28592-12989	24	9.4	0.618503	0.507599	-0.285091	1	no
gi|646729674|gb|KK964044.1|	44518-14504	24	9.4	2	3	0.21876	0.88855	no
gi|646729674|gb|KK964044.1|	55173-15593	24	9.4	9	6	-0.479316	0.76055	no
gi|646729674|gb|KK964044.1|	86653-18755	24	9.4	3	0.812135	-2	0.2244	no
gi|646729674|gb|KK964044.1|	94186-19566	24	9.4	4	0.978338	-2	0.1445	no
gi|646729674|gb|KK964044.1|	84775-85260	24	9.4	4	10	1	0.4576	no
gi|646729695|gb|KK964041.1|	44030-44596	13	9.5	2	4	0.815887	0.5637	no
gi|646729718|gb|KK964038.1|	0-489	11	9.9	179.24	241	0.427716	0.70535	no
gi|646729718|gb|KK964038.1|	70923-71233	11	9.9	6	11	0.738785	0.6139	no
gi|646729726|gb|KK964037.1|	01025-10169	8.9	5.3	0	9	inf	0.00105	yes
gi|646729733|gb|KK964036.1|	6435-7205	9.4	7.2	1	2	0.298982	0.83555	no
gi|646729755|gb|KK964033.1|	10983-11232	14	13	22	2	-4	0.03345	no
gi|646729755|gb|KK964033.1|	12938-11371	14	13	16	2	-3	0.0688	no
gi|646729763|gb|KK964032.1|	1-1968	12	7.1	8	1	-3	0.1128	no
gi|646729763|gb|KK964032.1|	10093-11050	12	7.1	11	10	-0.112534	0.94015	no
gi|646729763|gb|KK964032.1|	12396-13394	12	7.1	10	9	-0.142017	0.92325	no
gi|646729763|gb|KK964032.1|	6700-7393	12	7.1	7	6	-0.171043	0.9134	no
gi|646729796|gb|KK964027.1|	13080-14521	20	7	5	6	0.267467	0.8549	no
gi|646729796|gb|KK964027.1|	22526-23045	20	7	6	5	-0.410621	0.75295	no
gi|646729796|gb|KK964027.1|	4927-5671	20	7	4	6	0.625923	0.6494	no
gi|646729796|gb|KK964027.1|	51628-52326	20	7	34	3	-3	0.03525	no
gi|646729796|gb|KK964027.1|	63484-65217	20	7	3	9	2	0.278	no

gi|646729803|gb|KK964026.1|	13244-11396	11	6.7	1	1	-0.000717863	0.92345	no
gi|646729803|gb|KK964026.1|	14067-11518	11	6.7	1	1	-0.014834	0.9504	no
gi|646729811|gb|KK964025.1|	03653-10395	13	9.7	19	0	-inf	0.00225	no
gi|646729811|gb|KK964025.1|	30793-32021	13	9.7	6	1	-2	0.11305	no
gi|646729836|gb|KK964022.1|	33236-33574	17	12	23	21	-0.117825	0.94125	no
gi|646729836|gb|KK964022.1|	53245-54042	17	12	3	3	-0.126865	0.91255	no
gi|646729874|gb|KK964017.1|	46523-47400	24	11	1	0.631787	-1	0.4766	no
gi|646729874|gb|KK964017.1|	48304-50417	24	11	3	0.793854	-2	0.156	no
gi|646729967|gb|KK964005.1|	16436-16967	18	8.5	3	2	-0.728046	0.64125	no
gi|646729988|gb|KK964002.1|	4194-5035	19	7.1	0	3	inf	0.00035	yes
gi|646729988|gb|KK964002.1|	6973-8143	19	7.1	0	4	inf	0.00105	yes
gi|646729988|gb|KK964002.1|	98054-98473	19	7.1	1	6	3	0.2756	no
gi|646730010|gb|KK963999.1|	64810-65161	12	6.4	3	8	2	0.36945	no
gi|646730017|gb|KK963998.1|	05236-10549	20	8.3	10	36	2	0.2471	no
gi|646730017|gb|KK963998.1|	21526-12231	20	8.3	26	17	-0.597926	0.69385	no
gi|646730017|gb|KK963998.1|	25829-12633	20	8.3	31	24	-0.363838	0.81205	no
gi|646730017|gb|KK963998.1|	34914-13668	20	8.3	0.549232	0.529275	-0.0534004	1	no
gi|646730017|gb|KK963998.1|	41123-14297	20	8.3	0.753102	1	0.919642	0.53435	no
gi|646730017|gb|KK963998.1|	44992-14531	20	8.3	19	5	-2	0.21595	no
gi|646730017|gb|KK963998.1|	63196-16463	20	8.3	0.858039	0.224605	-2	1	no
gi|646730017|gb|KK963998.1|	91473-19503	20	8.3	2	2	-0.358934	0.8164	no
gi|646730017|gb|KK963998.1|	95117-20380	20	8.3	2	2	-0.373251	0.75835	no
gi|646730017|gb|KK963998.1|	08044-20973	20	8.3	4	2	-0.868162	0.58325	no
gi|646730017|gb|KK963998.1|	14463-21582	20	8.3	0.585514	0.841852	0.523863	1	no
gi|646730017|gb|KK963998.1|	66839-26745	20	8.3	12	1	-3	0.0909	no
gi|646730017|gb|KK963998.1|	67843-26832	20	8.3	0.409949	5	4	0.1063	no
gi|646730017|gb|KK963998.1|	81028-28161	20	8.3	3	2	-0.711007	0.6413	no
gi|646730017|gb|KK963998.1|	64205-65111	20	8.3	0.568362	1	1	0.43305	no
gi|646730017|gb|KK963998.1|	66682-70073	20	8.3	29	14	-1	0.36625	no
gi|646730017|gb|KK963998.1|	77323-77582	20	8.3	14	37	1	0.3396	no

gi|646730017|gb|KK963998.1|	78426-79177	20	8.3	2	2	-0.288291	0.83195	no
gi|646730017|gb|KK963998.1|	81321-82093	20	8.3	1	1	0.00643389	0.92345	no
gi|646730017|gb|KK963998.1|	82564-83650	20	8.3	3	2	-0.308623	0.8199	no
gi|646730017|gb|KK963998.1|	85270-85690	20	8.3	5	3	-0.786532	0.6023	no
gi|646730017|gb|KK963998.1|	94404-94765	20	8.3	17	16	-0.0652413	0.9611	no
gi|646730026|gb|KK963997.1|	54373-55334	13	7.8	0.655119	356	9	0.05805	no
gi|646730033|gb|KK963996.1|	13699-11417	79	23	3	2	-0.389135	0.80985	no
gi|646730033|gb|KK963996.1|	28979-12937	79	23	3	5	0.627452	0.66895	no
gi|646730033|gb|KK963996.1|	30530-13089	79	23	9	19	1	0.4682	no
gi|646730033|gb|KK963996.1|	15246-16199	79	23	0.795151	0.94189	0.24433	1	no
gi|646730033|gb|KK963996.1|	53811-15425	79	23	5	2	-1	0.3894	no
gi|646730033|gb|KK963996.1|	16266-17356	79	23	0.775503	0.949665	0.292286	1	no
gi|646730033|gb|KK963996.1|	57501-58590	79	23	2	2	-0.148331	0.9014	no
gi|646730033|gb|KK963996.1|	96400-98110	79	23	0.826592	0.551128	-0.584788	1	no
gi|646730049|gb|KK963994.1|	30489-31043	15	8.6	31	1	-5	0.0788	no
gi|646730049|gb|KK963994.1|	51368-51837	15	8.6	5	0	-inf	0.0041	no
gi|646730089|gb|KK963989.1|	50776-51502	11	5.1	2	5	1	0.3721	no
gi|646730120|gb|KK963985.1|	3431-4035	14	4.6	9	0.364708	-5	0.1761	no
gi|646730135|gb|KK963983.1|	13929-14538	23	13	1	2	0.727697	0.64505	no
gi|646730135|gb|KK963983.1|	16466-17497	23	13	1	1	-0.0731215	1	no
gi|646730135|gb|KK963983.1|	18398-20478	23	13	0.96319	1	0.609093	0.6588	no
gi|646730135|gb|KK963983.1|	23477-24732	23	13	1	2	0.520857	0.7175	no
gi|646730135|gb|KK963983.1|	2551-3301	23	13	2	0.524531	-2	0.29735	no
gi|646730135|gb|KK963983.1|	47793-48729	23	13	57	25	-1	0.3774	no
gi|646730135|gb|KK963983.1|	63986-65240	23	13	6	4	-0.655024	0.669	no
gi|646730135|gb|KK963983.1|	9147-9801	23	13	3	1	-1	0.39505	no
gi|646730141|gb|KK963982.1|	20062-12030	12	5.6	269.41	18	-4	0.0421	no
gi|646730141|gb|KK963982.1|	20705-12163	12	5.6	15	0.978567	-4	0.04095	no
gi|646730194|gb|KK963976.1|	04215-10528	16	8.3	0.904704	1	0.683828	0.619	no
gi|646730194|gb|KK963976.1|	34425-35626	16	8.3	1	4	1	0.3028	no

gi|646730194|gb|KK963976.1|	78033-78469	16	8.3	5	0	-inf	0.00655	no
gi|646730200|gb|KK963975.1|	41131-41586	15	11	0.909759	29	5	0.23635	no
gi|646730232|gb|KK963971.1|	09632-11039	28	7.7	3	0.258279	-3	0.18	no
gi|646730232|gb|KK963971.1|	16465-11710	28	7.7	4	3	-0.52816	0.68755	no
gi|646730232|gb|KK963971.1|	22800-12342	28	7.7	7	12	0.811359	0.60755	no
gi|646730232|gb|KK963971.1|	32696-13442	28	7.7	0.940829	0.362511	-1	1	no
gi|646730232|gb|KK963971.1|	38031-14054	28	7.7	4	1	-2	0.34535	no
gi|646730232|gb|KK963971.1|	41040-14224	28	7.7	6	2	-2	0.2159	no
gi|646730232|gb|KK963971.1|	42559-14533	28	7.7	0.991673	0.907764	-0.127546	1	no
gi|646730232|gb|KK963971.1|	57342-16239	28	7.7	2	2	-0.179946	0.90305	no
gi|646730232|gb|KK963971.1|	64468-16680	28	7.7	2	2	-0.304082	0.84185	no
gi|646730232|gb|KK963971.1|	28031-29011	28	7.7	2	1	-0.490979	0.7125	no
gi|646730232|gb|KK963971.1|	30872-31852	28	7.7	3	1	-1	0.38195	no
gi|646730232|gb|KK963971.1|	31962-33866	28	7.7	2	2	-0.466757	0.76115	no
gi|646730232|gb|KK963971.1|	35363-37164	28	7.7	8	0.865251	-3	0.04805	no
gi|646730232|gb|KK963971.1|	43529-44292	28	7.7	2	3	0.365265	0.76905	no
gi|646730239|gb|KK963970.1|	10699-12246	40	19	1	3	1	0.3622	no
gi|646730239|gb|KK963970.1|	13718-14697	40	19	0.766603	0.727264	-0.0760003	1	no
gi|646730239|gb|KK963970.1|	17-2408	40	19	2	2	-0.405797	0.79175	no
gi|646730239|gb|KK963970.1|	25224-27548	40	19	2	2	-0.106717	0.94575	no
gi|646730239|gb|KK963970.1|	31468-31994	40	19	1	3	1	0.48625	no
gi|646730239|gb|KK963970.1|	35119-35861	40	19	1	1	0.00273204	0.92345	no
gi|646730239|gb|KK963970.1|	55754-56942	40	19	3	2	-0.574501	0.7285	no
gi|646730239|gb|KK963970.1|	58301-60542	40	19	2	1	-0.647926	0.6743	no
gi|646730239|gb|KK963970.1|	73504-74817	40	19	3	3	-0.109693	0.94565	no
gi|646730239|gb|KK963970.1|	7714-8249	40	19	2	4	0.805875	0.58155	no
gi|646730239|gb|KK963970.1|	77909-78907	40	19	0.996005	0.70921	-0.48994	1	no
gi|646730239|gb|KK963970.1|	79040-79817	40	19	2	0.498552	-2	0.2078	no
gi|646730239|gb|KK963970.1|	85726-88152	40	19	2	2	-0.269496	0.8588	no
gi|646730239|gb|KK963970.1|	96983-97177	40	19	29	71	1	0.41405	no

gi|646730276|gb|KK963965.1|	6204-6489	10	6.6	5	21	2	0.1933	no
gi|646730276|gb|KK963965.1|	75058-75416	10	6.6	0	20	inf	0.00315	no
gi|646730289|gb|KK963963.1|	11253-11225	20	8.6	0.743005	1	0.925322	0.5404	no
gi|646730289|gb|KK963963.1|	30321-13082	20	8.6	4	0.993732	-2	0.2163	no
gi|646730289|gb|KK963963.1|	41292-14239	20	8.6	0	3	inf	0.00435	no
gi|646730289|gb|KK963963.1|	43933-14550	20	8.6	5	3	-0.842383	0.5785	no
gi|646730289|gb|KK963963.1|	59059-15947	20	8.6	5	4	-0.0386799	0.9298	no
gi|646730289|gb|KK963963.1|	03105-20344	20	8.6	5	6	0.295789	0.8532	no
gi|646730289|gb|KK963963.1|	24394-25037	20	8.6	2	2	0.109471	0.93055	no
gi|646730289|gb|KK963963.1|	3277-4168	20	8.6	2	1	-1	0.37095	no
gi|646730289|gb|KK963963.1|	52313-53441	20	8.6	0.953683	4	2	0.2191	no
gi|646730297|gb|KK963962.1|	46770-47250	8.5	5.7	0	29	inf	0.00025	yes
gi|646730297|gb|KK963962.1|	49071-50513	8.5	5.7	0.077938	23	8	0.09445	no
gi|646730307|gb|KK963961.1|	01148-10212	13	8.4	0	5	inf	0.00155	yes
gi|646730349|gb|KK963955.1|	71604-72294	11	7.3	0	7	inf	0.00185	no
gi|646730381|gb|KK963951.1|	24908-12544	22	5.8	5	0	-inf	0.0011	yes
gi|646730381|gb|KK963951.1|	27164-12941	22	5.8	0.650225	6	3	0.04595	no
gi|646730381|gb|KK963951.1|	33497-13788	22	5.8	12	9	-0.444429	0.6898	no
gi|646730381|gb|KK963951.1|	39148-14069	22	5.8	0.861256	0.724374	-0.249708	1	no
gi|646730381|gb|KK963951.1|	42744-14360	22	5.8	0.306796	2	3	0.2655	no
gi|646730381|gb|KK963951.1|	45040-14670	22	5.8	2	2	0.404419	0.8004	no
gi|646730381|gb|KK963951.1|	49074-15185	22	5.8	63	41	-0.613955	0.60115	no
gi|646730381|gb|KK963951.1|	57291-15790	22	5.8	12	6	-0.978277	0.54335	no
gi|646730381|gb|KK963951.1|	69526-17333	22	5.8	2	2	-0.0301885	0.9832	no
gi|646730381|gb|KK963951.1|	74722-17571	22	5.8	1	0.357407	-1	1	no
gi|646730381|gb|KK963951.1|	77899-19174	22	5.8	6	3	-0.922466	0.4218	no
gi|646730381|gb|KK963951.1|	00764-20245	22	5.8	2	0.465257	-2	0.2662	no
gi|646730381|gb|KK963951.1|	05800-20640	22	5.8	93	92	-0.0207973	0.98515	no
gi|646730381|gb|KK963951.1|	20942-22223	22	5.8	37	29	-0.370641	0.7281	no
gi|646730381|gb|KK963951.1|	25096-23338	22	5.8	6	0.960128	-3	0.069	no

gi|646730381|gb|KK963951.1|	34190-23576	22	5.8	4	0.70976	-2	0.12125	no
gi|646730381|gb|KK963951.1|	86459-28682	22	5.8	2	13	3	0.24655	no
gi|646730381|gb|KK963951.1|	05627-30593	22	5.8	3	15	3	0.27295	no
gi|646730381|gb|KK963951.1|	92500-39532	22	5.8	2	0	-inf	0.00025	yes
gi|646730381|gb|KK963951.1|	97408-39783	22	5.8	9	0	-inf	0.00095	yes
gi|646730381|gb|KK963951.1|	98004-40247	22	5.8	2	0.288434	-3	0.069	no
gi|646730381|gb|KK963951.1|	70694-71276	22	5.8	96	31	-2	0.21475	no
gi|646730381|gb|KK963951.1|	90701-91384	22	5.8	5	2	-1	0.4756	no
gi|646730381|gb|KK963951.1|	910-1093	22	5.8	97	36	-1	0.397	no
gi|646730381|gb|KK963951.1|	92742-92924	22	5.8	72	87	0.270339	0.84515	no
gi|646730381|gb|KK963951.1|	97081-97336	22	5.8	121	51	-1	0.4388	no
gi|646730451|gb|KK963942.1|	22418-22693	14	7	40	80	0.99866	0.47525	no
gi|646730451|gb|KK963942.1|	28527-28895	14	7	5	9	0.697736	0.6266	no
gi|646730451|gb|KK963942.1|	30451-30911	14	7	49	74	0.605537	0.685	no
gi|646730451|gb|KK963942.1|	32689-33825	14	7	0.525014	1	1	0.36645	no
gi|646730451|gb|KK963942.1|	5127-5983	14	7	2	6	1	0.37045	no
gi|646730451|gb|KK963942.1|	7397-9064	14	7	33	32	-0.0419556	0.9689	no
gi|646730451|gb|KK963942.1|	9702-10656	14	7	40	36	-0.146014	0.89015	no
gi|646730465|gb|KK963940.1|	47678-48428	9.1	5.2	2	2	-0.247776	0.85975	no
gi|646730491|gb|KK963937.1|	80216-80790	15	4.2	27	2	-4	0.04605	no
gi|646730558|gb|KK963928.1|	03341-10649	19	5.4	131	73	-0.849528	0.48055	no
gi|646730558|gb|KK963928.1|	07787-10805	19	5.4	34	24	-0.514108	0.69385	no
gi|646730558|gb|KK963928.1|	09408-10962	19	5.4	120	25	-2	0.1915	no
gi|646730558|gb|KK963928.1|	12250-11253	19	5.4	11	13	0.211062	0.8852	no
gi|646730558|gb|KK963928.1|	15515-15827	19	5.4	15	0	-inf	0.0041	no
gi|646730558|gb|KK963928.1|	17327-22403	19	5.4	2	0.056037	-5	0.10735	no
gi|646730558|gb|KK963928.1|	25671-27000	19	5.4	1	0	-inf	0.0012	yes
gi|646730558|gb|KK963928.1|	27-600	19	5.4	8	1	-3	0.14525	no
gi|646730558|gb|KK963928.1|	27278-28242	19	5.4	2	0.185545	-3	0.18	no
gi|646730558|gb|KK963928.1|	6362-8054	19	5.4	2	0.092988	-5	0.1761	no

gi|646730558|gb|KK963928.1|	87358-88414	19	5.4	3	2	-1	0.4598	no
gi|646730558|gb|KK963928.1|	90349-92061	19	5.4	7	4	-0.919084	0.5399	no
gi|646730558|gb|KK963928.1|	9593-11227	19	5.4	5	0.484181	-3	0.0649	no
gi|646730577|gb|KK963925.1|	11266-11189	25	7	3	0.339352	-3	0.1994	no
gi|646730577|gb|KK963925.1|	12158-11347	25	7	18	6	-2	0.20455	no
gi|646730577|gb|KK963925.1|	9754-10064	25	7	39	27	-0.530913	0.73525	no
gi|646730585|gb|KK963924.1|	06092-10716	26	10	79	27	-2	0.17045	no
gi|646730603|gb|KK963922.1|	20237-12057	18	8.3	0.93933	15	4	0.0993	no
gi|646730603|gb|KK963922.1|	26167-12678	18	8.3	1	5	2	0.1836	no
gi|646730603|gb|KK963922.1|	30839-13622	18	8.3	7	5	-0.423948	0.6979	no
gi|646730603|gb|KK963922.1|	40914-14300	18	8.3	5	5	0.0442193	0.97705	no
gi|646730603|gb|KK963922.1|	44597-14583	18	8.3	10	9	-0.163082	0.91395	no
gi|646730603|gb|KK963922.1|	46664-14697	18	8.3	14	7	-0.880701	0.54705	no
gi|646730603|gb|KK963922.1|	63344-16386	18	8.3	4	2	-1	0.3935	no
gi|646730603|gb|KK963922.1|	49543-55320	18	8.3	20	34	0.766899	0.51775	no
gi|646730603|gb|KK963922.1|	63083-65058	18	8.3	0.968195	1	0.0645488	1	no
gi|646730603|gb|KK963922.1|	67444-69749	18	8.3	0.451307	0.785788	0.800031	1	no
gi|646730603|gb|KK963922.1|	76124-77461	18	8.3	0.940498	0.245756	-2	1	no
gi|646730603|gb|KK963922.1|	78044-79280	18	8.3	0.471505	0.946728	1	1	no
gi|646730603|gb|KK963922.1|	8713-8973	18	8.3	33	10	-2	0.25325	no
gi|646730603|gb|KK963922.1|	93525-93748	18	8.3	14	61	2	0.1851	no
gi|646730603|gb|KK963922.1|	97628-97976	18	8.3	4	21	2	0.2133	no
gi|646730603|gb|KK963922.1|	9890-10380	18	8.3	4	4	-0.034395	0.94695	no
gi|646730610|gb|KK963921.1|	02686-10345	17	7.8	0.179597	6	5	0.10345	no
gi|646730610|gb|KK963921.1|	07768-10914	17	7.8	0.576652	0.473918	-0.283062	1	no
gi|646730610|gb|KK963921.1|	32681-32909	17	7.8	0	146	inf	0.0003	yes
gi|646730610|gb|KK963921.1|	46479-46667	17	7.8	0	188	inf	0.00435	no
gi|646730610|gb|KK963921.1|	62-1290	17	7.8	0.665534	0.409002	-0.702405	1	no
gi|646730641|gb|KK963917.1|	98479-99631	16	6.7	18	11	-0.680182	0.65215	no
gi|646730649|gb|KK963916.1|	35524-35841	13	12	4248.07	12345.1	2	0.2089	no

gi|646730684|gb|KK963911.1|	10571-13458	11	7.6	5	9	0.952743	0.4521	no
gi|646730684|gb|KK963911.1|	13711-14240	11	7.6	0.67128	5	3	0.26145	no
gi|646730684|gb|KK963911.1|	14345-15382	11	7.6	0.236586	4	4	0.2485	no
gi|646730699|gb|KK963909.1|	04618-10582	15	7.6	5	3	-0.623611	0.6847	no
gi|646730699|gb|KK963909.1|	07785-10886	15	7.6	3	2	-0.48582	0.7217	no
gi|646730699|gb|KK963909.1|	19437-12111	15	7.6	6	2	-1	0.4007	no
gi|646730699|gb|KK963909.1|	26621-12741	15	7.6	5	6	0.336871	0.8317	no
gi|646730699|gb|KK963909.1|	54111-15449	15	7.6	10	0	-inf	0.00225	no
gi|646730699|gb|KK963909.1|	54962-15547	15	7.6	8	4	-0.919859	0.53045	no
gi|646730699|gb|KK963909.1|	25574-26218	15	7.6	2	4	0.931672	0.50175	no
gi|646730699|gb|KK963909.1|	34312-34547	15	7.6	40	19	-1	0.45625	no
gi|646730699|gb|KK963909.1|	48077-49169	15	7.6	0.663129	1	0.929806	0.53925	no
gi|646730699|gb|KK963909.1|	61708-62216	15	7.6	2	2	-0.15201	0.8987	no
gi|646730699|gb|KK963909.1|	98812-10030	15	7.6	7	5	-0.350229	0.81695	no
gi|646730772|gb|KK963900.1|	16859-11740	14	7.3	2	2	-0.229703	0.87465	no
gi|646730815|gb|KK963895.1|	16319-11756	20	9.5	1	1	-0.0100704	0.9504	no
gi|646730815|gb|KK963895.1|	17709-11860	20	9.5	2	0.409746	-2	0.197	no
gi|646730815|gb|KK963895.1|	26785-12813	20	9.5	30	29	-0.0328942	0.97515	no
gi|646730815|gb|KK963895.1|	28429-12919	20	9.5	1	1	0.0058471	0.92345	no
gi|646730815|gb|KK963895.1|	29669-13321	20	9.5	4	3	-0.391797	0.79455	no
gi|646730815|gb|KK963895.1|	54223-15460	20	9.5	4	6	0.832462	0.58765	no
gi|646730815|gb|KK963895.1|	59635-16111	20	9.5	9	16	0.819321	0.5144	no
gi|646730815|gb|KK963895.1|	61856-16241	20	9.5	1	6	2	0.21915	no
gi|646730815|gb|KK963895.1|	64541-16590	20	9.5	1	2	1	0.4337	no
gi|646730815|gb|KK963895.1|	66689-16697	20	9.5	19	15	-0.369683	0.77905	no
gi|646730815|gb|KK963895.1|	73773-17431	20	9.5	5	7	0.537463	0.70605	no
gi|646730815|gb|KK963895.1|	74711-17622	20	9.5	0.807653	0.211644	-2	1	no
gi|646730815|gb|KK963895.1|	76952-17896	20	9.5	3	0.838914	-2	0.1706	no
gi|646730815|gb|KK963895.1|	79503-18153	20	9.5	0.935562	0.602334	-0.635271	1	no
gi|646730815|gb|KK963895.1|	81689-18404	20	9.5	1	2	0.537387	0.73685	no

gi|646730815|gb|KK963895.1|	85703-18655	20	9.5	2	2	0.210268	0.87615	no
gi|646730815|gb|KK963895.1|	89774-19030	20	9.5	2	2	0.178482	0.89965	no
gi|646730815|gb|KK963895.1|	90893-19138	20	9.5	5	1	-2	0.20435	no
gi|646730815|gb|KK963895.1|	35805-36814	20	9.5	0.981478	2	1	0.3462	no
gi|646730815|gb|KK963895.1|	44935-45452	20	9.5	4	0.474587	-3	0.19565	no
gi|646730815|gb|KK963895.1|	48503-48886	20	9.5	25	0	-inf	0.0014	yes
gi|646730815|gb|KK963895.1|	68797-69768	20	9.5	26	0.551358	-6	0.07395	no
gi|646730815|gb|KK963895.1|	85342-85685	20	9.5	25	0	-inf	0.00315	no
gi|646730815|gb|KK963895.1|	[illegible]	20	9.5	47	0	-inf	5.00E-05	yes
gi|646730953|gb|KK963877.1|	10473-11811	23	11	0.170842	0.982135	3	1	no
gi|646730953|gb|KK963877.1|	12186-11242	23	11	3	41	4	0.10165	no
gi|646730953|gb|KK963877.1|	19302-12012	23	11	3	3	0.0272568	0.9633	no
gi|646730953|gb|KK963877.1|	20313-12092	23	11	3	2	-0.912459	0.53065	no
gi|646730953|gb|KK963877.1|	56611-57597	23	11	12	3	-2	0.2202	no
gi|646730953|gb|KK963877.1|	58576-61076	23	11	11	4	-1	0.27395	no
gi|646730953|gb|KK963877.1|	98918-99717	23	11	4	3	-0.398252	0.77375	no
gi|646730960|gb|KK963876.1|	04578-10494	21	9.5	4	9	1	0.4517	no
gi|646730960|gb|KK963876.1|	75570-17796	21	9.5	1	1	-0.217233	0.8963	no
gi|646730960|gb|KK963876.1|	00749-20246	21	9.5	2	1	-0.710245	0.614	no
gi|646730960|gb|KK963876.1|	10158-21067	21	9.5	8	6	-0.386532	0.778	no
gi|646730960|gb|KK963876.1|	16986-22421	21	9.5	13	10	-0.335006	0.7697	no
gi|646730960|gb|KK963876.1|	48589-49082	21	9.5	0	14	inf	0.0012	yes
gi|646730960|gb|KK963876.1|	53075-53573	21	9.5	0	12	inf	0.00175	no
gi|646730960|gb|KK963876.1|	6590-8916	21	9.5	1	1	-0.121809	0.93635	no
gi|646730993|gb|KK963871.1|	7217-7813	170	84	3	3	-0.316107	0.8235	no
gi|646731000|gb|KK963870.1|	06253-10670	20	8.1	4	4	-0.179414	0.90115	no
gi|646731000|gb|KK963870.1|	08746-10896	20	8.1	49	34	-0.510286	0.7158	no
gi|646731000|gb|KK963870.1|	17428-11837	20	8.1	1	0.56829	-0.907929	1	no
gi|646731000|gb|KK963870.1|	18538-11910	20	8.1	0.300186	4	4	0.10445	no
gi|646731000|gb|KK963870.1|	22962-12633	20	8.1	2	1	-0.844734	0.5829	no

gi|646731000|gb|KK963870.1|	26897-12835	20	8.1	3	3	-0.0376601	0.97825	no
gi|646731000|gb|KK963870.1|	31853-13224	20	8.1	0	9	inf	0.0014	yes
gi|646731000|gb|KK963870.1|	42695-14332	20	8.1	1	3	1	0.4145	no
gi|646731000|gb|KK963870.1|	48223-14866	20	8.1	3	26	3	0.0839	no
gi|646731000|gb|KK963870.1|	55089-15542	20	8.1	8	5	-0.704211	0.64635	no
gi|646731000|gb|KK963870.1|	57113-15795	20	8.1	1	1	0.0131027	0.92345	no
gi|646731000|gb|KK963870.1|	59294-16095	20	8.1	0.262452	2	3	0.18695	no
gi|646731000|gb|KK963870.1|	65923-67451	20	8.1	0.65406	0.628561	-0.0573701	1	no
gi|646731000|gb|KK963870.1|	67656-72811	20	8.1	0.509818	1	1	0.4453	no
gi|646731000|gb|KK963870.1|	81660-81905	20	8.1	0	156	inf	5.00E-05	yes
gi|646731000|gb|KK963870.1|	82395-83044	20	8.1	0	19	inf	0.00025	yes
gi|646731000|gb|KK963870.1|	96008-96259	20	8.1	0	154.67	inf	0.00025	yes
gi|646731007|gb|KK963869.1|	02449-10385	21	9.8	0.482251	0.693914	0.524974	1	no
gi|646731007|gb|KK963869.1|	20370-12227	21	9.8	63	66	0.0668486	0.9527	no
gi|646731007|gb|KK963869.1|	37555-13850	21	9.8	2	0.945829	-1	0.43875	no
gi|646731007|gb|KK963869.1|	56488-15829	21	9.8	18	4	-2	0.12805	no
gi|646731007|gb|KK963869.1|	71701-17469	21	9.8	2	1	-0.976961	0.5281	no
gi|646731007|gb|KK963869.1|	75146-17585	21	9.8	5	7	0.483224	0.7634	no
gi|646731007|gb|KK963869.1|	81940-18326	21	9.8	1	0.745367	-0.79902	0.5562	no
gi|646731007|gb|KK963869.1|	96376-19713	21	9.8	0.554936	2	2	0.25705	no
gi|646731007|gb|KK963869.1|	00585-20110	21	9.8	19	5	-2	0.1775	no
gi|646731007|gb|KK963869.1|	02162-20328	21	9.8	1	0.917559	-0.484025	0.7302	no
gi|646731007|gb|KK963869.1|	63526-63972	21	9.8	2	4	0.886704	0.5714	no
gi|646731035|gb|KK963865.1|	63681-64044	15	8.7	10	7	-0.563653	0.68245	no
gi|646731035|gb|KK963865.1|	66845-67066	15	8.7	59	39	-0.580676	0.6823	no
gi|646731035|gb|KK963865.1|	78711-78907	15	8.7	74	16.89	-2	0.22755	no
gi|646731044|gb|KK963864.1|	09981-11069	25	13	0.802389	3	2	0.2422	no
gi|646731044|gb|KK963864.1|	14572-11521	25	13	2	0.655885	-2	0.3832	no
gi|646731065|gb|KK963861.1|	22471-22799	13	8	0	58	inf	0.00025	yes
gi|646731095|gb|KK963857.1|	34665-13648	16	8.8	1	1	0.297788	0.83125	no

gi|646731138|gb|KK963851.1|	10774-12035	17	8.7	15	5	-1	0.3429	no
gi|646731138|gb|KK963851.1|	14529-15034	17	8.7	8	1	-3	0.19025	no
gi|646731138|gb|KK963851.1|	2-240	17	8.7	41	15	-2	0.3242	no
gi|646731138|gb|KK963851.1|	8181-9006	17	8.7	0.973657	1	0.497414	0.73295	no
gi|646731162|gb|KK963848.1|	25624-26012	13	6.2	4	4	0.0929966	0.91495	no
gi|646731162|gb|KK963848.1|	94378-95503	13	6.2	2	3	0.888151	0.525	no
gi|646731177|gb|KK963846.1|	18279-18718	16	6.4	12	3	-2	0.20835	no
gi|646731177|gb|KK963846.1|	45406-46169	16	6.4	10.56	2	-2	0.1254	no
gi|646731177|gb|KK963846.1|	64005-64513	16	6.4	12	1	-3	0.12745	no
gi|646731177|gb|KK963846.1|	68526-68945	16	6.4	13	9	-0.440488	0.7505	no
gi|646731177|gb|KK963846.1|	96659-97078	16	6.4	11	7	-0.617365	0.6204	no
gi|646731188|gb|KK963845.1|	00926-10308	9.7	5.9	0.097029	134	10	0.2322	no
gi|646731188|gb|KK963845.1|	39463-40085	9.7	5.9	0	4	inf	0.001	yes
gi|646731188|gb|KK963845.1|	88299-88970	9.7	5.9	0	3	inf	0.0014	yes
gi|646731188|gb|KK963845.1|	95205-96312	9.7	5.9	0.217152	7	5	0.2359	no
gi|646731188|gb|KK963845.1|	99142-99942	9.7	5.9	0	47	inf	5.00E-05	yes
gi|646731194|gb|KK963844.1|	03839-10505	15	8.8	2	0.966966	-0.760556	0.5577	no
gi|646731194|gb|KK963844.1|	69689-70423	15	8.8	2	0.811853	-0.927944	0.58905	no
gi|646731194|gb|KK963844.1|	80677-81266	15	8.8	3	2	-0.277258	0.8367	no
gi|646731194|gb|KK963844.1|	97443-98248	15	8.8	2	0.71126	-1	0.47555	no
gi|646731210|gb|KK963842.1|	00329-10270	19	7.5	0.918186	2	0.900049	0.51285	no
gi|646731210|gb|KK963842.1|	07867-10863	19	7.5	0.726848	2	1	0.36865	no
gi|646731210|gb|KK963842.1|	11226-11165	19	7.5	6	14	1	0.40855	no
gi|646731210|gb|KK963842.1|	31046-13609	19	7.5	0.30873	2	3	0.0703	no
gi|646731210|gb|KK963842.1|	36214-13663	19	7.5	69	122	0.813111	0.523	no
gi|646731210|gb|KK963842.1|	37164-13869	19	7.5	0	3	inf	0.00085	yes
gi|646731210|gb|KK963842.1|	49265-15002	19	7.5	7	2	-2	0.26885	no
gi|646731210|gb|KK963842.1|	50808-15340	19	7.5	0.670999	0.114694	-3	1	no
gi|646731210|gb|KK963842.1|	54964-15611	19	7.5	4	2	-0.875171	0.5803	no
gi|646731210|gb|KK963842.1|	56842-16178	19	7.5	26	20	-0.369354	0.75025	no

gi|646731210|gb|KK963842.1|	70730-17178	19	7.5	38	42	0.139639	0.8951	no
gi|646731210|gb|KK963842.1|	81151-18180	19	7.5	2	0.643516	-2	0.3832	no
gi|646731210|gb|KK963842.1|	82131-18547	19	7.5	2	2	-0.139608	0.92095	no
gi|646731210|gb|KK963842.1|	87341-18853	19	7.5	2	0.422149	-2	0.1679	no
gi|646731210|gb|KK963842.1|	80325-81284	19	7.5	17	2	-3	0.0562	no
gi|646731210|gb|KK963842.1|	92089-92967	19	7.5	15	10	-0.601601	0.6925	no
gi|646731239|gb|KK963839.1|	33477-34441	18	5.5	1	0.74218	-0.66187	0.6593	no
gi|646731239|gb|KK963839.1|	35380-36500	18	5.5	1	0.611706	-0.805953	1	no
gi|646731239|gb|KK963839.1|	42537-44648	18	5.5	3	1	-0.84313	0.59645	no
gi|646731239|gb|KK963839.1|	64328-65522	18	5.5	0.886664	0.846933	-0.0661396	1	no
gi|646731239|gb|KK963839.1|	69388-69841	18	5.5	6	4	-0.594452	0.63095	no
gi|646731278|gb|KK963834.1|	54311-54795	9.2	8.4	6	5	-0.162772	0.9014	no
gi|646731293|gb|KK963832.1|	75009-75495	14	6	233	53	-2	0.1203	no
gi|646731301|gb|KK963831.1|	75361-77176	11	6	2	5	0.947187	0.53575	no
gi|646731307|gb|KK963830.1|	9211-9860	18	7.6	11	8	-0.465374	0.7652	no
gi|646731332|gb|KK963827.1|	11570-11786	11	4.7	0	59	inf	0.0014	yes
gi|646731380|gb|KK963821.1|	09224-10975	19	8.1	2	2	-0.144546	0.90125	no
gi|646731380|gb|KK963821.1|	12911-11338	19	8.1	3	2	-0.170394	0.89395	no
gi|646731380|gb|KK963821.1|	21214-12187	19	8.1	1	3	1	0.45285	no
gi|646731380|gb|KK963821.1|	27570-12828	19	8.1	2	2	-0.0298642	0.9367	no
gi|646731380|gb|KK963821.1|	30065-13121	19	8.1	1	1	-0.482775	0.71555	no
gi|646731380|gb|KK963821.1|	34654-13978	19	8.1	24	12	-1	0.35155	no
gi|646731380|gb|KK963821.1|	44205-14458	19	8.1	6	3	-1	0.48125	no
gi|646731380|gb|KK963821.1|	56770-15732	19	8.1	2	3	0.864368	0.5567	no
gi|646731380|gb|KK963821.1|	59752-16073	19	8.1	2	0.725321	-1	0.45505	no
gi|646731380|gb|KK963821.1|	65861-17139	19	8.1	3	7	1	0.30345	no
gi|646731380|gb|KK963821.1|	73400-17594	19	8.1	3	5	1	0.4971	no
gi|646731380|gb|KK963821.1|	76114-17660	19	8.1	4	3	-0.712467	0.62725	no
gi|646731380|gb|KK963821.1|	80266-18165	19	8.1	1	0.351753	-2	0.27245	no
gi|646731380|gb|KK963821.1|	81780-18274	19	8.1	4	5	0.263127	0.86535	no

gi|646731380|gb|KK963821.1|	91475-19340	19	8.1	0.884793	2	1	0.40785	no
gi|646731380|gb|KK963821.1|	93718-19499	19	8.1	0.72628	1	0.980873	0.4966	no
gi|646731380|gb|KK963821.1|	95156-19577	19	8.1	2	2	-0.212248	0.8925	no
gi|646731380|gb|KK963821.1|	95911-19929	19	8.1	26	37	0.507899	0.6646	no
gi|646731380|gb|KK963821.1|	35438-36711	19	8.1	2	2	-0.00215088	0.9741	no
gi|646731380|gb|KK963821.1|	69548-70166	19	8.1	3	6	1	0.4743	no
gi|646731380|gb|KK963821.1|	80644-83356	19	8.1	3	6	0.97926	0.5266	no
gi|646731380|gb|KK963821.1|	88994-93895	19	8.1	6	3	-1	0.4062	no
gi|646731380|gb|KK963821.1|	95594-96640	19	8.1	5	2	-2	0.2676	no
gi|646731387|gb|KK963820.1|	40977-42091	30	12	6	0.153968	-5	0.17045	no
gi|646731387|gb|KK963820.1|	73518-74860	30	12	3	0.489297	-3	0.126	no
gi|646731387|gb|KK963820.1|	77924-78929	30	12	3	0	-inf	0.0004	yes
gi|646731387|gb|KK963820.1|	82420-83635	30	12	30	0	-inf	5.00E-05	yes
gi|646731405|gb|KK963818.1|	1-1602	24	11	18	24	0.414828	0.69825	no
gi|646731405|gb|KK963818.1|	18992-12075	24	11	0	3	inf	0.0006	yes
gi|646731405|gb|KK963818.1|	22222-12572	24	11	0	5	inf	5.00E-05	yes
gi|646731405|gb|KK963818.1|	28296-12927	24	11	6	2	-1	0.29625	no
gi|646731405|gb|KK963818.1|	33526-13561	24	11	1	1	-0.0777994	0.96045	no
gi|646731405|gb|KK963818.1|	37922-13849	24	11	27	6	-2	0.18895	no
gi|646731405|gb|KK963818.1|	14722-15113	24	11	19	4	-2	0.1857	no
gi|646731405|gb|KK963818.1|	65717-17266	24	11	0	6	inf	5.00E-05	yes
gi|646731405|gb|KK963818.1|	16955-17780	24	11	3	3	0.0568416	0.9612	no
gi|646731405|gb|KK963818.1|	93798-19710	24	11	0	3	inf	0.0002	yes
gi|646731405|gb|KK963818.1|	98012-20029	24	11	0	4	inf	0.00035	yes
gi|646731405|gb|KK963818.1|	19564-22056	24	11	0	2	inf	0.00035	yes
gi|646731405|gb|KK963818.1|	22330-22544	24	11	40	22	-0.83483	0.59745	no
gi|646731405|gb|KK963818.1|	2235-2987	24	11	4	4	0.0300107	0.9741	no
gi|646731405|gb|KK963818.1|	23550-24625	24	11	0.902498	0.322192	-1	1	no
gi|646731405|gb|KK963818.1|	34444-35086	24	11	7	4	-0.685701	0.61895	no
gi|646731405|gb|KK963818.1|	3474-4750	24	11	0.63425	0.780372	0.299109	1	no

gi|646731405|gb|KK963818.1|	48799-52433	24	11	21	10	-1	0.31855	no
gi|646731405|gb|KK963818.1|	52589-56313	24	11	5	5	0.231675	0.88215	no
gi|646731405|gb|KK963818.1|	6133-6872	24	11	15	5	-2	0.2996	no
gi|646731405|gb|KK963818.1|	71402-72137	24	11	15	8	-0.862355	0.5799	no
gi|646731405|gb|KK963818.1|	74302-74822	24	11	19	17	-0.134141	0.9295	no
gi|646731405|gb|KK963818.1|	75048-75457	24	11	8	6	-0.325711	0.7969	no
gi|646731405|gb|KK963818.1|	81668-82942	24	11	8	4	-1	0.5064	no
gi|646731405|gb|KK963818.1|	85586-87044	24	11	3	4	0.361095	0.8165	no
gi|646731405|gb|KK963818.1|	9412-10164	24	11	8	8	-0.112008	0.94045	no
gi|646731405|gb|KK963818.1|	94644-95041	24	11	7	4	-0.773086	0.59415	no
gi|646731443|gb|KK963815.1|	29111-29712	17	10	5	9	0.877779	0.52245	no
gi|646731443|gb|KK963815.1|	54339-54850	17	10	2	4	1	0.40035	no
gi|646731491|gb|KK963809.1|	01974-10335	24	6.6	5	0.118067	-5	0.1685	no
gi|646731491|gb|KK963809.1|	07282-10759	24	6.6	26	6	-2	0.1626	no
gi|646731491|gb|KK963809.1|	17105-11766	24	6.6	4	2	-0.926391	0.52635	no
gi|646731491|gb|KK963809.1|	67699-16890	24	6.6	1	0.978924	-0.132937	1	no
gi|646731491|gb|KK963809.1|	84725-18604	24	6.6	1	2	0.522715	0.67535	no
gi|646731491|gb|KK963809.1|	95364-19739	24	6.6	0.313439	0.605356	0.949601	1	no
gi|646731491|gb|KK963809.1|	97651-19838	24	6.6	5	6	0.367203	0.8149	no
gi|646731491|gb|KK963809.1|	99461-20163	24	6.6	18	16	-0.190661	0.8554	no
gi|646731491|gb|KK963809.1|	34354-25281	24	6.6	2	4	0.787259	0.49015	no
gi|646731491|gb|KK963809.1|	70945-27168	24	6.6	8	18	1	0.46425	no
gi|646731491|gb|KK963809.1|	74789-27697	24	6.6	1	4	1	0.35375	no
gi|646731491|gb|KK963809.1|	80017-28028	24	6.6	24	52	1	0.4493	no
gi|646731491|gb|KK963809.1|	86006-28656	24	6.6	0.900558	3	2	0.26115	no
gi|646731491|gb|KK963809.1|	90613-29096	24	6.6	3	29	3	0.12925	no
gi|646731491|gb|KK963809.1|	92022-29323	24	6.6	1	9	3	0.07805	no
gi|646731491|gb|KK963809.1|	11702-31279	24	6.6	7	7	0.0199141	0.9891	no
gi|646731491|gb|KK963809.1|	13704-31398	24	6.6	21	26	0.315632	0.80725	no
gi|646731491|gb|KK963809.1|	15135-31536	24	6.6	13	47	2	0.2371	no

gi|646731491|gb|KK963809.1|	16160-32177	24	6.6	1	1	0.0611491	0.9647	no
gi|646731491|gb|KK963809.1|	22829-32346	24	6.6	22	28	0.370458	0.80145	no
gi|646731491|gb|KK963809.1|	44264-34894	24	6.6	1	2	0.195379	0.8932	no
gi|646731491|gb|KK963809.1|	49080-35037	24	6.6	0.715054	1	0.981349	0.4966	no
gi|646731491|gb|KK963809.1|	66460-36670	24	6.6	65	21	-2	0.22305	no
gi|646731491|gb|KK963809.1|	75719-37672	24	6.6	33	38	0.207228	0.84395	no
gi|646731491|gb|KK963809.1|	79181-37941	24	6.6	27	47	0.830731	0.5418	no
gi|646731491|gb|KK963809.1|	82439-38270	24	6.6	44	36	-0.292721	0.8046	no
gi|646731491|gb|KK963809.1|	18793-42035	24	6.6	13	8	-0.707235	0.64105	no
gi|646731491|gb|KK963809.1|	26165-43032	24	6.6	4	7	0.661706	0.59905	no
gi|646731491|gb|KK963809.1|	41241-44224	24	6.6	45	31	-0.533287	0.6217	no
gi|646731491|gb|KK963809.1|	43252-44401	24	6.6	4	6	0.812482	0.55005	no
gi|646731491|gb|KK963809.1|	47237-45212	24	6.6	107	69	-0.621589	0.60305	no
gi|646731491|gb|KK963809.1|	52249-45313	24	6.6	3	2	-0.497583	0.6935	no
gi|646731491|gb|KK963809.1|	53246-45391	24	6.6	1	2	0.476957	0.7489	no
gi|646731491|gb|KK963809.1|	75850-47601	24	6.6	133.57	338	1	0.42695	no
gi|646731491|gb|KK963809.1|	76576-47761	24	6.6	4	8	0.81881	0.59585	no
gi|646731491|gb|KK963809.1|	78318-47899	24	6.6	3	3	0.062978	0.9491	no
gi|646731491|gb|KK963809.1|	82629-48335	24	6.6	5	0	-inf	0.0042	no
gi|646731491|gb|KK963809.1|	84484-48551	24	6.6	8	0.169549	-6	0.16875	no
gi|646731491|gb|KK963809.1|	91795-49273	24	6.6	4	0.57309	-3	0.1569	no
gi|646731491|gb|KK963809.1|	92850-49983	24	6.6	3	0.080401	-5	0.0479	no
gi|646731491|gb|KK963809.1|	00495-50256	24	6.6	4	0.665805	-3	0.0821	no
gi|646731491|gb|KK963809.1|	02760-50589	24	6.6	3	0.420372	-3	0.0688	no
gi|646731491|gb|KK963809.1|	06056-50655	24	6.6	3	1	-2	0.28945	no
gi|646731491|gb|KK963809.1|	07330-50990	24	6.6	7	0.173729	-5	0.07615	no
gi|646731491|gb|KK963809.1|	61476-65477	24	6.6	29	3	-3	0.03635	no
gi|646731491|gb|KK963809.1|	81028-82381	24	6.6	1	0.605616	-1	0.41145	no
gi|646731491|gb|KK963809.1|	83847-85420	24	6.6	53	15	-2	0.11005	no
gi|646731491|gb|KK963809.1|	93607-93884	24	6.6	91	8	-3	0.07365	no

gi|646731491|gb|KK963809.1|	95516-96007	24	6.6	5	2	-1	0.33665	no
gi|646731491|gb|KK963809.1|	97301-97645	24	6.6	39	7	-3	0.12185	no
gi|646731498|gb|KK963808.1|	18838-19326	9.5	6.2	4	2	-0.897828	0.5544	no
gi|646731545|gb|KK963802.1|	08341-10860	14	4	25	0	-inf	0.00475	no
gi|646731545|gb|KK963802.1|	8938-9742	14	4	0.505444	2	2	0.2898	no
gi|646731552|gb|KK963801.1|	75413-76229	18	11	56	71	0.343595	0.75515	no
gi|646731602|gb|KK963795.1|	31065-13172	13	5.2	4	2	-0.804977	0.5422	no
gi|646731608|gb|KK963794.1|	565-819	93	100	251	123	-1	0.5023	no
gi|646731608|gb|KK963794.1|	85306-86819	93	100	32	12	-1	0.29455	no
gi|646731619|gb|KK963792.1|	37407-38713	11	5.3	7	3	-1	0.34905	no
gi|646731619|gb|KK963792.1|	39922-40215	11	5.3	17	25	0.569379	0.6651	no
gi|646731619|gb|KK963792.1|	79582-80931	11	5.3	0.845622	0.243115	-2	1	no
gi|646731619|gb|KK963792.1|	83453-84750	11	5.3	1	0.509984	-1	1	no
gi|646731619|gb|KK963792.1|	86411-87174	11	5.3	2	2	-0.24617	0.85975	no
gi|646731619|gb|KK963792.1|	87319-88098	11	5.3	5	2	-1	0.3445	no
gi|646731643|gb|KK963789.1|	10286-21198	19	6.4	0.450036	0.928603	1	1	no
gi|646731643|gb|KK963789.1|	12120-21264	19	6.4	0	5	inf	0.0014	yes
gi|646731643|gb|KK963789.1|	20183-22084	19	6.4	3	4	0.253844	0.8366	no
gi|646731643|gb|KK963789.1|	29916-23031	19	6.4	4	5	0.139294	0.9151	no
gi|646731662|gb|KK963786.1|	01092-10318	17	7.2	2	0.291934	-3	0.12365	no
gi|646731662|gb|KK963786.1|	88569-89918	17	7.2	7	3	-1	0.40415	no
gi|646731670|gb|KK963785.1|	00762-10104	23	6	28	10	-1	0.30185	no
gi|646731670|gb|KK963785.1|	11321-11568	23	6	82	19	-2	0.18695	no
gi|646731670|gb|KK963785.1|	12839-13166	23	6	140	93	-0.582479	0.69685	no
gi|646731670|gb|KK963785.1|	28599-12881	23	6	70	17	-2	0.20755	no
gi|646731670|gb|KK963785.1|	62450-16805	23	6	10	7	-0.634328	0.57155	no
gi|646731670|gb|KK963785.1|	17483-18053	23	6	73	14	-2	0.0965	no
gi|646731670|gb|KK963785.1|	23836-24842	23	6	0	3	inf	0.00435	no
gi|646731670|gb|KK963785.1|	54626-25669	23	6	0.967431	2	1	0.44475	no
gi|646731670|gb|KK963785.1|	92576-92849	23	6	22	18	-0.282797	0.8361	no

gi|646731678|gb|KK963784.1|	18346-20366	12	5.2	0	42	inf	5.00E-05	yes
gi|646731706|gb|KK963780.1|	31744-33082	18	8.1	22	18	-0.265752	0.80605	no
gi|646731706|gb|KK963780.1|	43652-44034	18	8.1	11	0.876032	-4	0.1745	no
gi|646731706|gb|KK963780.1|	44231-45446	18	8.1	1	0	-inf	0.00225	no
gi|646731706|gb|KK963780.1|	55032-56289	18	8.1	0.7386	0.397423	-0.894118	1	no
gi|646731706|gb|KK963780.1|	60733-61007	18	8.1	47	44	-0.0839424	0.95955	no
gi|646731712|gb|KK963779.1|	29041-12996	18	7.2	13	8	-0.707214	0.6349	no
gi|646731712|gb|KK963779.1|	34570-13555	18	7.2	4	4	0.174896	0.91015	no
gi|646731712|gb|KK963779.1|	35658-13616	18	7.2	4	1	-1	0.41965	no
gi|646731712|gb|KK963779.1|	38758-13941	18	7.2	2	2	0.281544	0.8397	no
gi|646731712|gb|KK963779.1|	39521-14093	18	7.2	3	3	-0.0597305	0.96835	no
gi|646731712|gb|KK963779.1|	66688-66914	18	7.2	53	27	-0.974551	0.5042	no
gi|646731712|gb|KK963779.1|	69291-69561	18	7.2	16	9	-0.830889	0.58865	no
gi|646731712|gb|KK963779.1|	95456-96872	18	7.2	0.079683	1	4	0.10345	no
gi|646731730|gb|KK963777.1|	12208-12870	15	9.8	1	2	0.738722	0.6148	no
gi|646731751|gb|KK963774.1|	22553-23319	12	13	0	5	inf	0.00435	no
gi|646731751|gb|KK963774.1|	58756-60048	12	13	0	2	inf	0.001	yes
gi|646731781|gb|KK963770.1|	03680-10425	18	8.3	7	7	-0.100199	0.94905	no
gi|646731781|gb|KK963770.1|	32641-13360	18	8.3	1	0.553607	-0.906737	1	no
gi|646731781|gb|KK963770.1|	29471-29791	18	8.3	18	30	0.729491	0.6106	no
gi|646731781|gb|KK963770.1|	33531-34118	18	8.3	4	0.764047	-2	0.194	no
gi|646731781|gb|KK963770.1|	93984-94864	18	8.3	1	1	0.279788	0.8456	no
gi|646731788|gb|KK963769.1|	02805-10324	63	17	4	3	-0.280629	0.85095	no
gi|646731788|gb|KK963769.1|	89977-90557	63	17	8	2	-2	0.2144	no
gi|646731804|gb|KK963767.1|	30466-31541	19	9.3	0.789686	0.644383	-0.293359	1	no
gi|646731804|gb|KK963767.1|	31650-32458	19	9.3	1	0.943402	-0.311602	0.8509	no
gi|646731804|gb|KK963767.1|	9452-9942	19	9.3	2	3	0.425037	0.77485	no
gi|646731841|gb|KK963762.1|	13807-14989	20	8.9	4	2	-1	0.44975	no
gi|646731841|gb|KK963762.1|	59098-15995	20	8.9	39	7	-2	0.0758	no
gi|646731841|gb|KK963762.1|	60482-16074	20	8.9	40	7	-2	0.14995	no

gi|646731841|gb|KK963762.1|	63545-16476	20	8.9	74	8	-3	0.03145	no
gi|646731841|gb|KK963762.1|	87225-18918	20	8.9	2	0.942729	-1	0.33505	no
gi|646731841|gb|KK963762.1|	89293-18994	20	8.9	2	2	0.473806	0.7479	no
gi|646731841|gb|KK963762.1|	91957-19281	20	8.9	43	27	-0.675052	0.6029	no
gi|646731841|gb|KK963762.1|	92918-19321	20	8.9	21	13	-0.669802	0.6107	no
gi|646731841|gb|KK963762.1|	06180-20721	20	8.9	22	16	-0.4878	0.74825	no
gi|646731841|gb|KK963762.1|	16319-21736	20	8.9	0.704355	1	0.734845	0.6352	no
gi|646731841|gb|KK963762.1|	22091-22271	20	8.9	2	0.345357	-3	0.2136	no
gi|646731841|gb|KK963762.1|	22796-22350	20	8.9	2	0.574366	-2	0.29735	no
gi|646731841|gb|KK963762.1|	30317-23054	20	8.9	64	51	-0.330797	0.7807	no
gi|646731841|gb|KK963762.1|	31826-23369	20	8.9	0.458566	2	2	0.27055	no
gi|646731841|gb|KK963762.1|	35135-23676	20	8.9	18	29	0.674603	0.5396	no
gi|646731841|gb|KK963762.1|	41578-24223	20	8.9	5	5	0.0386578	0.9732	no
gi|646731841|gb|KK963762.1|	50756-25182	20	8.9	1	0.484438	-1	0.38955	no
gi|646731841|gb|KK963762.1|	52411-25342	20	8.9	3	0.697366	-2	0.26345	no
gi|646731841|gb|KK963762.1|	53500-25547	20	8.9	1	0.855256	-0.650358	0.644	no
gi|646731841|gb|KK963762.1|	56611-25780	20	8.9	23	6	-2	0.1486	no
gi|646731841|gb|KK963762.1|	66958-26834	20	8.9	25	6	-2	0.1216	no
gi|646731841|gb|KK963762.1|	35630-36513	20	8.9	10	15	0.562958	0.701	no
gi|646731841|gb|KK963762.1|	36871-38929	20	8.9	23	23	-0.00835458	0.99365	no
gi|646731850|gb|KK963761.1|	20999-12133	16	7.3	8	7	-0.126512	0.9215	no
gi|646731868|gb|KK963759.1|	50029-50471	13	6.2	0	40	inf	0.0001	yes
gi|646731875|gb|KK963758.1|	51876-52544	12	6.1	3	0	-inf	0.0011	yes
gi|646731875|gb|KK963758.1|	76258-76570	12	6.1	4	18	2	0.1792	no
gi|646731875|gb|KK963758.1|	83954-84683	12	6.1	3	0.273337	-3	0.18625	no
gi|646731883|gb|KK963757.1|	12091-12855	29	10	2	0.255367	-3	0.21605	no
gi|646731883|gb|KK963757.1|	16950-17559	29	10	4	14	2	0.2536	no
gi|646731883|gb|KK963757.1|	17670-19011	29	10	7	20	2	0.2498	no
gi|646731883|gb|KK963757.1|	20721-22652	29	10	13	28	1	0.29875	no
gi|646731883|gb|KK963757.1|	22752-24586	29	10	3	3	-0.102322	0.94875	no

<table>
<tr><td>gi|646731883|gb|KK963757.1|</td><td>24697-30632</td><td>29</td><td>10</td><td>6</td><td>13</td><td>1</td><td>0.27195</td><td>no</td></tr>
<tr><td>gi|646731883|gb|KK963757.1|</td><td>30735-32042</td><td>29</td><td>10</td><td>3</td><td>9</td><td>2</td><td>0.32955</td><td>no</td></tr>
<tr><td>gi|646731883|gb|KK963757.1|</td><td>34020-35401</td><td>29</td><td>10</td><td>12</td><td>29</td><td>1</td><td>0.31035</td><td>no</td></tr>
<tr><td>gi|646731883|gb|KK963757.1|</td><td>37334-38760</td><td>29</td><td>10</td><td>0.474015</td><td>2</td><td>2</td><td>0.1757</td><td>no</td></tr>
<tr><td>gi|646731883|gb|KK963757.1|</td><td>39173-44280</td><td>29</td><td>10</td><td>44</td><td>61</td><td>0.477547</td><td>0.6881</td><td>no</td></tr>
<tr><td>gi|646731883|gb|KK963757.1|</td><td>44837-52709</td><td>29</td><td>10</td><td>33</td><td>54</td><td>0.702892</td><td>0.56475</td><td>no</td></tr>
<tr><td>gi|646731883|gb|KK963757.1|</td><td>54612-55001</td><td>29</td><td>10</td><td>5</td><td>3</td><td>-0.450363</td><td>0.76665</td><td>no</td></tr>
<tr><td>gi|646731883|gb|KK963757.1|</td><td>55490-56214</td><td>29</td><td>10</td><td>85</td><td>102</td><td>0.2689</td><td>0.81135</td><td>no</td></tr>
<tr><td>gi|646731883|gb|KK963757.1|</td><td>57283-57961</td><td>29</td><td>10</td><td>2</td><td>1</td><td>-0.521465</td><td>0.73195</td><td>no</td></tr>
<tr><td>gi|646731883|gb|KK963757.1|</td><td>59347-60122</td><td>29</td><td>10</td><td>1</td><td>3</td><td>1</td><td>0.4088</td><td>no</td></tr>
<tr><td>gi|646731883|gb|KK963757.1|</td><td>61133-63024</td><td>29</td><td>10</td><td>1</td><td>2</td><td>0.764416</td><td>0.6331</td><td>no</td></tr>
<tr><td>gi|646731883|gb|KK963757.1|</td><td>65106-69434</td><td>29</td><td>10</td><td>200</td><td>167</td><td>-0.262168</td><td>0.82855</td><td>no</td></tr>
<tr><td>gi|646731883|gb|KK963757.1|</td><td>69591-71581</td><td>29</td><td>10</td><td>5</td><td>4</td><td>-0.43478</td><td>0.7713</td><td>no</td></tr>
<tr><td>gi|646731883|gb|KK963757.1|</td><td>71915-72861</td><td>29</td><td>10</td><td>4</td><td>4</td><td>-0.171158</td><td>0.91575</td><td>no</td></tr>
<tr><td>gi|646731892|gb|KK963756.1|</td><td>01846-10321</td><td>22</td><td>9.8</td><td>0.583404</td><td>0.359519</td><td>-0.698427</td><td>1</td><td>no</td></tr>
<tr><td>gi|646731892|gb|KK963756.1|</td><td>10668-17766</td><td>22</td><td>9.8</td><td>14</td><td>16</td><td>0.163203</td><td>0.8892</td><td>no</td></tr>
<tr><td>gi|646731892|gb|KK963756.1|</td><td>15301-11575</td><td>22</td><td>9.8</td><td>2</td><td>6</td><td>1</td><td>0.3611</td><td>no</td></tr>
<tr><td>gi|646731892|gb|KK963756.1|</td><td>28708-12914</td><td>22</td><td>9.8</td><td>4</td><td>8</td><td>0.979757</td><td>0.49265</td><td>no</td></tr>
<tr><td>gi|646731892|gb|KK963756.1|</td><td>29827-13003</td><td>22</td><td>9.8</td><td>43</td><td>68</td><td>0.6645</td><td>0.64075</td><td>no</td></tr>
<tr><td>gi|646731892|gb|KK963756.1|</td><td>42805-14322</td><td>22</td><td>9.8</td><td>13</td><td>14</td><td>0.0440653</td><td>0.97485</td><td>no</td></tr>
<tr><td>gi|646731892|gb|KK963756.1|</td><td>46787-14800</td><td>22</td><td>9.8</td><td>2</td><td>4</td><td>0.696362</td><td>0.6659</td><td>no</td></tr>
<tr><td>gi|646731892|gb|KK963756.1|</td><td>48223-15350</td><td>22</td><td>9.8</td><td>8</td><td>11</td><td>0.369853</td><td>0.7472</td><td>no</td></tr>
<tr><td>gi|646731892|gb|KK963756.1|</td><td>60557-16083</td><td>22</td><td>9.8</td><td>34</td><td>18</td><td>-0.884663</td><td>0.48415</td><td>no</td></tr>
<tr><td>gi|646731892|gb|KK963756.1|</td><td>61726-17003</td><td>22</td><td>9.8</td><td>10</td><td>10</td><td>0.0160753</td><td>0.98865</td><td>no</td></tr>
<tr><td>gi|646731892|gb|KK963756.1|</td><td>32409-23304</td><td>22</td><td>9.8</td><td>0.962423</td><td>2</td><td>1</td><td>0.4355</td><td>no</td></tr>
<tr><td>gi|646731892|gb|KK963756.1|</td><td>51082-25230</td><td>22</td><td>9.8</td><td>0.572812</td><td>0.821304</td><td>0.519855</td><td>1</td><td>no</td></tr>
<tr><td>gi|646731892|gb|KK963756.1|</td><td>37005-37401</td><td>22</td><td>9.8</td><td>19</td><td>8</td><td>-1</td><td>0.40525</td><td>no</td></tr>
<tr><td>gi|646731892|gb|KK963756.1|</td><td>69760-70135</td><td>22</td><td>9.8</td><td>6</td><td>5</td><td>-0.072203</td><td>0.92975</td><td>no</td></tr>
<tr><td>gi|646731892|gb|KK963756.1|</td><td>99097-10160</td><td>22</td><td>9.8</td><td>0.575416</td><td>0.65662</td><td>0.190455</td><td>1</td><td>no</td></tr>
<tr><td>gi|646731899|gb|KK963755.1|</td><td>14033-14424</td><td>7.1</td><td>4.2</td><td>6</td><td>2</td><td>-2</td><td>0.2745</td><td>no</td></tr>
</table>

gi|646731906|gb|KK963754.1|	33584-34250	9.4	5	0	8	inf	0.00095	yes
gi|646731906|gb|KK963754.1|	50711-50951	9.4	5	0	60	inf	0.00435	no
gi|646731924|gb|KK963751.1|	28191-28589	12	6.4	114938	33044.7	-2	0.05315	no
gi|646731939|gb|KK963749.1|	15509-11638	12	4.3	7	8	0.172634	0.9111	no
gi|646731939|gb|KK963749.1|	18817-19151	12	4.3	8	16	0.991897	0.4856	no
gi|646731939|gb|KK963749.1|	34227-34557	12	4.3	25	16	-0.598971	0.66035	no
gi|646731939|gb|KK963749.1|	3535-3883	12	4.3	10	2	-2	0.21025	no
gi|646731939|gb|KK963749.1|	46269-48467	12	4.3	2	1	-0.579331	0.71915	no
gi|646731939|gb|KK963749.1|	49901-50960	12	4.3	1	2	0.272231	0.8231	no
gi|646731939|gb|KK963749.1|	52518-53224	12	4.3	0.820133	2	1	0.4332	no
gi|646731939|gb|KK963749.1|	61420-62364	12	4.3	1	0.953807	-0.63072	0.65905	no
gi|646731939|gb|KK963749.1|	66097-67001	12	4.3	1	0.202127	-3	0.2033	no
gi|646731939|gb|KK963749.1|	95093-96038	12	4.3	1	0.571481	-0.908188	1	no
gi|646731987|gb|KK963743.1|	95548-19602	15	9.6	19	3	-3	0.12175	no
gi|646731987|gb|KK963743.1|	24512-22491	15	9.6	8	12	0.563192	0.6907	no
gi|646731993|gb|KK963742.1|	15065-16555	10	8.8	4	4	-0.113626	0.9367	no
gi|646732004|gb|KK963740.1|	53965-15452	16	8.9	7	6	-0.166083	0.9198	no
gi|646732004|gb|KK963740.1|	82631-83480	16	8.9	1	3	1	0.37615	no
gi|646732017|gb|KK963738.1|	37479-13803	23	9.7	2	7	2	0.2819	no
gi|646732017|gb|KK963738.1|	41511-14260	23	9.7	10	9	-0.0539859	0.97025	no
gi|646732017|gb|KK963738.1|	48531-14882	23	9.7	29	52	0.863634	0.5274	no
gi|646732017|gb|KK963738.1|	49214-14994	23	9.7	1	0.82332	-0.73626	0.66415	no
gi|646732042|gb|KK963734.1|	68387-68791	16	8.7	22	2	-3	0.1133	no
gi|646732048|gb|KK963733.1|	02573-10289	10	5.4	6	10	0.757086	0.61385	no
gi|646732054|gb|KK963732.1|	22226-12294	18	5	3	2	-0.515583	0.68855	no
gi|646732054|gb|KK963732.1|	30036-30555	18	5	3	2	-0.240696	0.8746	no
gi|646732054|gb|KK963732.1|	44840-46442	18	5	2	3	0.166382	0.9174	no
gi|646732054|gb|KK963732.1|	58259-59296	18	5	11	1	-3	0.04455	no
gi|646732078|gb|KK963729.1|	19039-11975	16	7.4	12	2	-3	0.0824	no
gi|646732078|gb|KK963729.1|	21140-23036	16	7.4	3	3	-0.144923	0.924	no

gi|646732078|gb|KK963729.1|	32819-33213	16	7.4	34	38	0.157328	0.91715	no
gi|646732078|gb|KK963729.1|	41426-41694	16	7.4	76	113.5	0.579002	0.7039	no
gi|646732078|gb|KK963729.1|	55466-56158	16	7.4	5	12	1	0.4221	no
gi|646732078|gb|KK963729.1|	58429-61903	16	7.4	3	3	-0.24927	0.86665	no
gi|646732078|gb|KK963729.1|	62017-62427	16	7.4	488	97	-2	0.1023	no
gi|646732078|gb|KK963729.1|	75212-78119	16	7.4	1	1	0.153487	0.9156	no
gi|646732078|gb|KK963729.1|	78259-80165	16	7.4	2	2	0.192229	0.90465	no
gi|646732078|gb|KK963729.1|	80273-83234	16	7.4	4	2	-0.781178	0.60545	no
gi|646732089|gb|KK963727.1|	01300-10227	24	13	9	4	-1	0.50475	no
gi|646732089|gb|KK963727.1|	04027-10994	24	13	6	4	-0.661789	0.5324	no
gi|646732089|gb|KK963727.1|	27441-13009	24	13	6	7	0.109399	0.94505	no
gi|646732089|gb|KK963727.1|	30202-13102	24	13	4	4	-0.0875483	0.9551	no
gi|646732089|gb|KK963727.1|	38378-14192	24	13	7	5	-0.548789	0.6626	no
gi|646732089|gb|KK963727.1|	68263-16904	24	13	356	171	-1	0.3703	no
gi|646732089|gb|KK963727.1|	69585-17151	24	13	0.551348	1	1	0.3699	no
gi|646732089|gb|KK963727.1|	72323-17331	24	13	1	1	0.0501154	0.94695	no
gi|646732089|gb|KK963727.1|	73829-17432	24	13	24	25	0.0643038	0.96425	no
gi|646732089|gb|KK963727.1|	87413-18773	24	13	26	18	-0.549875	0.6894	no
gi|646732101|gb|KK963725.1|	22165-12288	9.7	4.2	0	14	inf	0.00015	yes
gi|646732101|gb|KK963725.1|	27968-12818	9.7	4.2	0	113	inf	0.00175	no
gi|646732101|gb|KK963725.1|	52889-54126	9.7	4.2	2	0.135112	-4	0.17035	no
gi|646732108|gb|KK963724.1|	53869-54270	11	9.1	16	31	0.898476	0.5685	no
gi|646732114|gb|KK963723.1|	00081-10058	12	3.9	2	3	0.43127	0.75545	no
gi|646732119|gb|KK963722.1|	31778-32607	20	9.4	3	6	0.93075	0.4949	no
gi|646732119|gb|KK963722.1|	66170-68118	20	9.4	2	1	-0.858577	0.5886	no
gi|646732119|gb|KK963722.1|	68692-70735	20	9.4	5	2	-1	0.4819	no
gi|646732119|gb|KK963722.1|	71218-72537	20	9.4	10	1	-3	0.05405	no
gi|646732119|gb|KK963722.1|	73208-74206	20	9.4	7	2	-1	0.2872	no
gi|646732130|gb|KK963720.1|	07041-10834	13	6.7	3	2	-0.335717	0.8328	no
gi|646732142|gb|KK963718.1|	13529-13781	13	6	124	0	-inf	0.0015	yes

gi|646732142|gb|KK963718.1|	14240-14700	13	6	11	2	-3	0.1675	no
gi|646732160|gb|KK963715.1|	16746-17923	13	8.2	9	4	-1	0.499	no
gi|646732164|gb|KK963714.1|	97049-19786	22	9.4	3	4	0.496053	0.7207	no
gi|646732164|gb|KK963714.1|	08361-21004	22	9.4	0.453185	0.841501	0.892865	1	no
gi|646732171|gb|KK963713.1|	18930-19441	15	6.4	6	2	-2	0.2499	no
gi|646732171|gb|KK963713.1|	46417-46813	15	6.4	49	10	-2	0.10775	no
gi|646732176|gb|KK963712.1|	38430-14035	24	10	6	3	-1	0.46545	no
gi|646732176|gb|KK963712.1|	40478-14118	24	10	5	5	-0.0401869	0.97245	no
gi|646732176|gb|KK963712.1|	88998-18994	24	10	2	0.378332	-2	0.16115	no
gi|646732176|gb|KK963712.1|	02941-20379	24	10	1	1	0.277728	0.8456	no
gi|646732176|gb|KK963712.1|	22665-22474	24	10	6	1	-2	0.18275	no
gi|646732176|gb|KK963712.1|	45648-24795	24	10	0.450209	1	1	1	no
gi|646732176|gb|KK963712.1|	48017-24931	24	10	1	0.761399	-0.700128	0.595	no
gi|646732176|gb|KK963712.1|	44850-45129	24	10	1012.17	304	-2	0.1221	no
gi|646732176|gb|KK963712.1|	46723-47441	24	10	3	0	-inf	0.00195	no
gi|646732176|gb|KK963712.1|	63118-63671	24	10	2	5	1	0.3482	no
gi|646732176|gb|KK963712.1|	76906-77587	24	10	15	21	0.479048	0.7489	no
gi|646732188|gb|KK963710.1|	15513-11641	16	7	7	18	1	0.36995	no
gi|646732202|gb|KK963708.1|	15960-17122	22	9.9	4	5	0.399003	0.7946	no
gi|646732202|gb|KK963708.1|	09610-21090	22	9.9	1	0.898379	-0.478083	0.71555	no
gi|646732202|gb|KK963708.1|	17544-21982	22	9.9	1	0.729458	-0.463202	1	no
gi|646732202|gb|KK963708.1|	20051-22245	22	9.9	1	1	-0.379822	0.80425	no
gi|646732202|gb|KK963708.1|	23710-22693	22	9.9	39	35	-0.14974	0.89725	no
gi|646732202|gb|KK963708.1|	33913-23586	22	9.9	2	0.868189	-0.96098	0.49785	no
gi|646732202|gb|KK963708.1|	38176-23914	22	9.9	1	1	0.0930787	1	no
gi|646732202|gb|KK963708.1|	39842-24119	22	9.9	3	5	0.679749	0.6586	no
gi|646732202|gb|KK963708.1|	59153-26143	22	9.9	2	1	-0.42011	0.78955	no
gi|646732202|gb|KK963708.1|	4866-5198	22	9.9	1078.04	202	-2	0.03995	no
gi|646732202|gb|KK963708.1|	6803-7402	22	9.9	3	6	0.784177	0.59875	no
gi|646732202|gb|KK963708.1|	74848-75221	22	9.9	0.731537	18	5	0.1072	no

gi|646732202|gb|KK963708.1|	84451-84882	22	9.9	577	649	0.170051	0.88495	no
gi|646732210|gb|KK963707.1|	53894-54557	13	7.4	5	2	-2	0.3356	no
gi|646732210|gb|KK963707.1|	54641-55219	13	7.4	4	0.391868	-3	0.179	no
gi|646732210|gb|KK963707.1|	56051-56561	13	7.4	7	1	-2	0.16995	no
gi|646732252|gb|KK963701.1|	18234-12787	19	8	10	9	-0.138773	0.9035	no
gi|646732252|gb|KK963701.1|	30922-13206	19	8	1	1	0.227324	0.8612	no
gi|646732252|gb|KK963701.1|	44396-14495	19	8	96	21	-2	0.10675	no
gi|646732252|gb|KK963701.1|	54786-15504	19	8	17	8	-1	0.48725	no
gi|646732252|gb|KK963701.1|	57941-15923	19	8	0.978474	0.638679	-0.615441	1	no
gi|646732252|gb|KK963701.1|	59351-15980	19	8	11	11	-0.0677894	0.9643	no
gi|646732252|gb|KK963701.1|	80367-18142	19	8	535	310	-0.788491	0.51235	no
gi|646732252|gb|KK963701.1|	90067-19101	19	8	87	72	-0.276184	0.8014	no
gi|646732252|gb|KK963701.1|	02170-20297	19	8	59	66	0.157228	0.88405	no
gi|646732314|gb|KK963692.1|	24803-25154	22	7.5	41	21	-0.917933	0.56315	no
gi|646732331|gb|KK963690.1|	66499-66868	18	4.9	12	145.43	4	0.00965	no
gi|646732337|gb|KK963689.1|	76390-77237	14	7.1	0.781237	1	0.762459	0.61475	no
gi|646732362|gb|KK963685.1|	11320-13126	19	7.5	31	24.15	-0.355493	0.7487	no
gi|646732362|gb|KK963685.1|	15177-15734	19	7.5	32	0.416917	-6	0.1672	no
gi|646732362|gb|KK963685.1|	23470-25011	19	7.5	14	8	-0.805284	0.59605	no
gi|646732362|gb|KK963685.1|	28442-29327	19	7.5	7	6	-0.0823894	0.95905	no
gi|646732362|gb|KK963685.1|	40520-41166	19	7.5	16	9	-0.764402	0.6237	no
gi|646732362|gb|KK963685.1|	46275-46700	19	7.5	15	17	0.172977	0.9096	no
gi|646732362|gb|KK963685.1|	58002-58895	19	7.5	3	6	1	0.4099	no
gi|646732362|gb|KK963685.1|	89007-89887	19	7.5	0.148	2	4	0.1061	no
gi|646732362|gb|KK963685.1|	95171-98576	19	7.5	2	2	-0.291584	0.8498	no
gi|646732412|gb|KK963678.1|	747-1000	10	14	1848.98	1572.31	-0.233846	0.83475	no
gi|646732426|gb|KK963676.1|	41778-42320	23	15	2	1	-0.77667	0.633	no
gi|646732431|gb|KK963675.1|	14093-11475	24	9.6	2	3	0.612635	0.6366	no
gi|646732431|gb|KK963675.1|	88521-18891	24	9.6	19	7	-1	0.3622	no
gi|646732431|gb|KK963675.1|	05918-21351	24	9.6	13	6	-1	0.3711	no

gi|646732431|gb|KK963675.1|	19671-22097	24	9.6	4	4	0.119051	0.9385	no
gi|646732431|gb|KK963675.1|	21938-22272	24	9.6	12	8	-0.640267	0.67395	no
gi|646732431|gb|KK963675.1|	25121-22612	24	9.6	31	67	1	0.3171	no
gi|646732431|gb|KK963675.1|	30882-23249	24	9.6	7	14	0.944196	0.45775	no
gi|646732431|gb|KK963675.1|	34041-23573	24	9.6	1	0.464619	-1	1	no
gi|646732431|gb|KK963675.1|	37432-24020	24	9.6	8	19	1	0.2523	no
gi|646732431|gb|KK963675.1|	49288-24982	24	9.6	11	31	2	0.3275	no
gi|646732431|gb|KK963675.1|	50412-25079	24	9.6	109	188	0.786718	0.5409	no
gi|646732431|gb|KK963675.1|	53055-25670	24	9.6	5	3	-1	0.4809	no
gi|646732431|gb|KK963675.1|	57183-25800	24	9.6	2	4	1	0.43705	no
gi|646732431|gb|KK963675.1|	70532-27093	24	9.6	4	2	-0.857079	0.6052	no
gi|646732431|gb|KK963675.1|	76166-27676	24	9.6	1	3	1	0.43555	no
gi|646732431|gb|KK963675.1|	82659-28332	24	9.6	8	10	0.220614	0.88765	no
gi|646732431|gb|KK963675.1|	86060-28632	24	9.6	37	12	-2	0.24805	no
gi|646732431|gb|KK963675.1|	94346-29454	24	9.6	53	21	-1	0.44885	no
gi|646732431|gb|KK963675.1|	94715-30285	24	9.6	1	1	-0.113521	0.94015	no
gi|646732431|gb|KK963675.1|	06301-30805	24	9.6	2	1	-0.316643	0.854	no
gi|646732431|gb|KK963675.1|	40510-34092	24	9.6	109	88	-0.312982	0.836	no
gi|646732431|gb|KK963675.1|	48704-34919	24	9.6	4	0.523273	-3	0.21315	no
gi|646732431|gb|KK963675.1|	57468-36968	24	9.6	6	5	-0.148882	0.8948	no
gi|646732431|gb|KK963675.1|	62850-63546	24	9.6	0.209668	3	4	0.1061	no
gi|646732431|gb|KK963675.1|	96866-97097	24	9.6	16	24	0.630395	0.67685	no
gi|646732437|gb|KK963674.1|	61555-62350	10	5.3	2	1	-0.353584	0.81205	no
gi|646732451|gb|KK963672.1|	1840-2050	12	8.6	57	12	-2	0.21405	no
gi|646732451|gb|KK963672.1|	01308-20208	12	8.6	4	2	-1	0.38005	no
gi|646732463|gb|KK963670.1|	17510-18265	12	9.2	25	18	-0.510183	0.72995	no
gi|646732463|gb|KK963670.1|	26805-28440	12	9.2	35	41	0.207583	0.8551	no
gi|646732463|gb|KK963670.1|	29697-30312	12	9.2	41	44	0.107366	0.9448	no
gi|646732463|gb|KK963670.1|	31689-33367	12	9.2	0.845178	5	2	0.11155	no
gi|646732491|gb|KK963666.1|	14006-11698	21	5.7	0.848495	0.543137	-0.643589	1	no

gi|646732491|gb|KK963666.1|	23631-12390	21	5.7	24	9	-1	0.3537	no
gi|646732491|gb|KK963666.1|	65681-16711	21	5.7	0.391006	0.788074	1	1	no
gi|646732491|gb|KK963666.1|	06166-20671	21	5.7	4	2	-1	0.3935	no
gi|646732491|gb|KK963666.1|	26665-22709	21	5.7	3	4	0.65371	0.66225	no
gi|646732491|gb|KK963666.1|	33769-23448	21	5.7	3	2	-0.795691	0.5424	no
gi|646732491|gb|KK963666.1|	38598-24016	21	5.7	2	0.304183	-3	0.18655	no
gi|646732491|gb|KK963666.1|	60767-27711	21	5.7	3	3	0.274156	0.80885	no
gi|646732491|gb|KK963666.1|	77262-27940	21	5.7	2	2	-0.298337	0.84535	no
gi|646732491|gb|KK963666.1|	79528-28056	21	5.7	2	2	0.271343	0.8231	no
gi|646732491|gb|KK963666.1|	01906-30302	21	5.7	1	0.914468	-0.706292	0.5885	no
gi|646732491|gb|KK963666.1|	03185-30916	21	5.7	3	3	-0.0912539	0.94325	no
gi|646732491|gb|KK963666.1|	09476-31379	21	5.7	3	3	0.0896731	0.95315	no
gi|646732491|gb|KK963666.1|	31053-31239	21	5.7	75	44	-0.772147	0.6244	no
gi|646732491|gb|KK963666.1|	22443-32351	21	5.7	0.797464	2	1	0.44735	no
gi|646732491|gb|KK963666.1|	25086-32652	21	5.7	0.467233	1	1	0.3963	no
gi|646732491|gb|KK963666.1|	29212-33169	21	5.7	5	5	-0.128511	0.9307	no
gi|646732491|gb|KK963666.1|	43032-43470	21	5.7	5	8	0.659163	0.61225	no
gi|646732491|gb|KK963666.1|	49771-50240	21	5.7	15	10	-0.502969	0.74635	no
gi|646732491|gb|KK963666.1|	51270-51563	21	5.7	9	20	1	0.43425	no
gi|646732491|gb|KK963666.1|	59042-59521	21	5.7	2	5	1	0.43035	no
gi|646732491|gb|KK963666.1|	65027-69684	21	5.7	12	8	-0.511101	0.6493	no
gi|646732491|gb|KK963666.1|	77860-79790	21	5.7	0.773269	1	0.53354	1	no
gi|646732491|gb|KK963666.1|	79925-83957	21	5.7	1	1	0.305924	0.8465	no
gi|646732491|gb|KK963666.1|	84007-84923	21	5.7	1	1	0.142308	0.9186	no
gi|646732541|gb|KK963660.1|	66202-66407	14	6.5	65	55	-0.242391	0.8608	no
gi|646732546|gb|KK963659.1|	35349-36177	16	8.9	2	3	0.390782	0.74485	no
gi|646732546|gb|KK963659.1|	36383-37087	16	8.9	3	7	1	0.44445	no
gi|646732558|gb|KK963657.1|	37000-13745	15	8.8	0.448493	6	4	0.10465	no
gi|646732558|gb|KK963657.1|	46048-14638	15	8.8	20	32	0.658359	0.629	no
gi|646732558|gb|KK963657.1|	93354-97045	15	8.8	56	46	-0.274255	0.81995	no

gi|646732558|gb|KK963657.1|	)8858-10061ε	15	8.8	0.67619	0.444239	-0.606094	1	no
gi|646732578|gb|KK963654.1|	08259-10878	33	13	2	1	-0.781976	0.63075	no
gi|646732578|gb|KK963654.1|	48852-14977	33	13	2	1	-0.610039	0.64385	no
gi|646732578|gb|KK963654.1|	39072-39552	33	13	2	7	2	0.207	no
gi|646732578|gb|KK963654.1|	40510-41678	33	13	1	0.290158	-2	1	no
gi|646732578|gb|KK963654.1|	73763-74352	33	13	2	2	0.196673	0.89115	no
gi|646732584|gb|KK963653.1|	11682-11280	17	7.1	40	34	-0.198996	0.856	no
gi|646732584|gb|KK963653.1|	14292-11591	17	7.1	36	29	-0.309495	0.7842	no
gi|646732584|gb|KK963653.1|	27075-12813	17	7.1	0.4608	0.986483	1	1	no
gi|646732584|gb|KK963653.1|	36795-13749	17	7.1	1	2	0.25928	0.86925	no
gi|646732584|gb|KK963653.1|	47041-14728	17	7.1	17	60	2	0.2979	no
gi|646732584|gb|KK963653.1|	68370-16860	17	7.1	28	33	0.234171	0.8708	no
gi|646732591|gb|KK963652.1|	27484-28074	17	7.3	21	15	-0.483449	0.74565	no
gi|646732591|gb|KK963652.1|	7395-10527	17	7.3	21	11	-0.966893	0.385	no
gi|646732591|gb|KK963652.1|	828-1456	17	7.3	3	3	-0.11603	0.932	no
gi|646732604|gb|KK963650.1|	04287-10753	15	7.1	3	0.135192	-5	0.0788	no
gi|646732604|gb|KK963650.1|	11782-11230	15	7.1	2	5	0.95349	0.5192	no
gi|646732604|gb|KK963650.1|	12440-11307	15	7.1	2	1	-0.944627	0.589	no
gi|646732604|gb|KK963650.1|	58935-15957	15	7.1	2	1	-0.336944	0.81865	no
gi|646732604|gb|KK963650.1|	78909-17985	15	7.1	10	13	0.308975	0.83535	no
gi|646732604|gb|KK963650.1|	18332-19776	15	7.1	3	9	1	0.3834	no
gi|646732604|gb|KK963650.1|	03563-20392	15	7.1	3	8	1	0.39585	no
gi|646732604|gb|KK963650.1|	16976-21761	15	7.1	5	3	-0.729793	0.61795	no
gi|646732604|gb|KK963650.1|	53404-25421	15	7.1	3	0	-inf	0.0011	yes
gi|646732604|gb|KK963650.1|	59638-26062	15	7.1	1	0.720507	-0.812555	0.57835	no
gi|646732604|gb|KK963650.1|	61013-26187	15	7.1	2	0.869366	-0.95914	0.54505	no
gi|646732604|gb|KK963650.1|	62068-26253	15	7.1	5	1	-2	0.2003	no
gi|646732604|gb|KK963650.1|	41101-42009	15	7.1	7	7	0.0133172	0.9886	no
gi|646732604|gb|KK963650.1|	44112-44636	15	7.1	6	2	-2	0.2403	no
gi|646732615|gb|KK963648.1|	05608-10608	12	6	16	11	-0.620799	0.69625	no

gi|646732615|gb|KK963648.1|	40135-14053	12	6	14	0	-inf	0.00525	no
gi|646732615|gb|KK963648.1|	41778-43538	12	6	2	36	4	0.0166	no
gi|646732615|gb|KK963648.1|	82036-83014	12	6	0.127944	3	5	0.11005	no
gi|646732615|gb|KK963648.1|	89935-90836	12	6	0	10	inf	0.0004	yes
gi|646732623|gb|KK963647.1|	16-684	13	12	2	1	-0.330577	0.81865	no
gi|646732636|gb|KK963645.1|	28967-30249	20	14	0.720623	1	0.691606	0.6189	no
gi|646732658|gb|KK963642.1|	06974-10923	32	13	3	4	0.303467	0.83955	no
gi|646732658|gb|KK963642.1|	14459-11575	32	13	2	0.639884	-2	0.28995	no
gi|646732658|gb|KK963642.1|	16303-11849	32	13	4	1	-2	0.3143	no
gi|646732685|gb|KK963638.1|	16971-17565	16	7.4	2	2	-0.387002	0.8011	no
gi|646732714|gb|KK963634.1|	16810-11736	18	7.9	0	8	inf	0.00315	no
gi|646732714|gb|KK963634.1|	19465-12129	18	7.9	0.058628	14	8	0.09445	no
gi|646732714|gb|KK963634.1|	25893-13058	18	7.9	5	3	-0.561572	0.6499	no
gi|646732720|gb|KK963633.1|	17328-19139	26	12	8	9	0.179082	0.9031	no
gi|646732720|gb|KK963633.1|	20540-21009	26	12	15	13	-0.213462	0.88605	no
gi|646732756|gb|KK963628.1|	24313-12544	13	4.6	0.315725	4	4	0.12695	no
gi|646732756|gb|KK963628.1|	27901-12861	13	4.6	51	15	-2	0.1735	no
gi|646732756|gb|KK963628.1|	32510-13358	13	4.6	30	8	-2	0.13685	no
gi|646732756|gb|KK963628.1|	36729-13758	13	4.6	6	7	0.265741	0.8622	no
gi|646732756|gb|KK963628.1|	13553-21390	13	4.6	8	3	-1	0.4288	no
gi|646732756|gb|KK963628.1|	34906-35143	13	4.6	32	7	-2	0.2227	no
gi|646732756|gb|KK963628.1|	50577-50996	13	4.6	17	4	-2	0.2217	no
gi|646732756|gb|KK963628.1|	51713-55964	13	4.6	1	0.70919	-0.993874	0.5285	no
gi|646732756|gb|KK963628.1|	65244-66962	13	4.6	0.442654	0.913599	1	1	no
gi|646732756|gb|KK963628.1|	77255-78441	13	4.6	0.496823	0.711699	0.518537	1	no
gi|646732770|gb|KK963626.1|	68920-17360	17	6.1	10	0	-inf	5.00E-05	yes
gi|646732770|gb|KK963626.1|	73833-17513	17	6.1	4	0	-inf	0.00025	yes
gi|646732770|gb|KK963626.1|	75422-17755	17	6.1	2	0	-inf	0.0017	no
gi|646732770|gb|KK963626.1|	81968-18312	17	6.1	2	0	-inf	0.00595	no
gi|646732770|gb|KK963626.1|	16413-21756	17	6.1	2	2	0.0696877	0.95335	no

gi|646732770|gb|KK963626.1|	93775-95488	17	6.1	0.824891	0	-inf	1	no
gi|646732770|gb|KK963626.1|	96564-98214	17	6.1	2	0.287229	-3	0.18655	no
gi|646732770|gb|KK963626.1|	)8645-10252(	17	6.1	2	1	-0.804206	0.6021	no
gi|646732778|gb|KK963625.1|	34031-13492	18	8.8	0	10	inf	5.00E-05	yes
gi|646732778|gb|KK963625.1|	74861-75682	18	8.8	0	29	inf	0.0001	yes
gi|646732787|gb|KK963624.1|	32300-13376	12	6.1	10	13	0.377849	0.8012	no
gi|646732793|gb|KK963623.1|	51371-15193	12	9.3	0.300186	4	4	0.1063	no
gi|646732793|gb|KK963623.1|	242-1131	12	9.3	48	50	0.0716765	0.94795	no
gi|646732793|gb|KK963623.1|	6646-6909	12	9.3	45	43	-0.0838551	0.95005	no
gi|646732801|gb|KK963622.1|	23596-12413	13	5.6	4	3	-0.555301	0.69155	no
gi|646732840|gb|KK963618.1|	40833-41596	41	20	4	0	-inf	0.00045	yes
gi|646732840|gb|KK963618.1|	47928-49047	41	20	7	2	-2	0.15335	no
gi|646732840|gb|KK963618.1|	50645-51042	41	20	12	0.80259	-4	0.1711	no
gi|646732840|gb|KK963618.1|	59942-61074	41	20	1	1	-0.0684933	0.9467	no
gi|646732855|gb|KK963616.1|	22671-12404	24	7.1	0.663163	1	0.983549	0.4966	no
gi|646732855|gb|KK963616.1|	33874-13476	24	7.1	2	3	0.695296	0.62065	no
gi|646732855|gb|KK963616.1|	13998-15530	24	7.1	5	2	-1	0.45915	no
gi|646732855|gb|KK963616.1|	41032-14260	24	7.1	1	0.505461	-2	0.27905	no
gi|646732855|gb|KK963616.1|	43759-14672	24	7.1	0.580597	0.496834	-0.224772	1	no
gi|646732855|gb|KK963616.1|	47721-14840	24	7.1	2	2	-0.520952	0.72175	no
gi|646732855|gb|KK963616.1|	48515-14983	24	7.1	0.865394	0.74605	-0.214085	1	no
gi|646732855|gb|KK963616.1|	50001-15081	24	7.1	2	1	-0.641054	0.65475	no
gi|646732855|gb|KK963616.1|	56191-15844	24	7.1	45	31	-0.529972	0.64465	no
gi|646732855|gb|KK963616.1|	58576-15936	24	7.1	1	0.735675	-0.921595	0.5894	no
gi|646732855|gb|KK963616.1|	63803-16548	24	7.1	2	0.2805	-3	0.18655	no
gi|646732855|gb|KK963616.1|	66122-16677	24	7.1	2	2	-0.373618	0.80365	no
gi|646732855|gb|KK963616.1|	66886-16798	24	7.1	1	0.781363	-0.863347	0.54655	no
gi|646732855|gb|KK963616.1|	76736-17810	24	7.1	0.499611	1	1	1	no
gi|646732855|gb|KK963616.1|	53536-55348	24	7.1	1	0.34369	-2	0.20495	no
gi|646732855|gb|KK963616.1|	55752-57365	24	7.1	0.885626	0.393237	-1	1	no

gi|646732855|gb|KK963616.1|	57474-58765	24	7.1	1	0.384656	-2	0.26095	no
gi|646732855|gb|KK963616.1|	59828-60748	24	7.1	0.834599	0.987109	0.242125	1	no
gi|646732855|gb|KK963616.1|	60927-62990	24	7.1	0.818733	1	0.342284	1	no
gi|646732855|gb|KK963616.1|	63106-64766	24	7.1	0.790158	0.380289	-1	1	no
gi|646732855|gb|KK963616.1|	65350-66748	24	7.1	1	0.815124	-0.475218	0.71555	no
gi|646732855|gb|KK963616.1|	69691-70129	24	7.1	4	5	0.203484	0.8848	no
gi|646732855|gb|KK963616.1|	81820-88094	24	7.1	14	15	0.09608	0.9324	no
gi|646732863|gb|KK963615.1|	03441-10453	150	39	5	1	-2	0.22255	no
gi|646732863|gb|KK963615.1|	38826-13922	150	39	11	4	-1	0.29075	no
gi|646732863|gb|KK963615.1|	41196-14285	150	39	4	1	-2	0.19495	no
gi|646732863|gb|KK963615.1|	55856-15733	150	39	3	1	-1	0.43515	no
gi|646732863|gb|KK963615.1|	87854-18824	150	39	5	2	-1	0.4814	no
gi|646732863|gb|KK963615.1|	93132-19387	150	39	3	0	-inf	0.00225	no
gi|646732863|gb|KK963615.1|	94423-19513	150	39	1	2	0.483224	0.7479	no
gi|646732863|gb|KK963615.1|	2083-3444	150	39	3	0.962116	-2	0.3138	no
gi|646732863|gb|KK963615.1|	24855-22617	150	39	2	1	-0.541277	0.6952	no
gi|646732863|gb|KK963615.1|	26911-22780	150	39	4	2	-0.770136	0.58205	no
gi|646732863|gb|KK963615.1|	54921-25688	150	39	4	2	-1	0.4658	no
gi|646732863|gb|KK963615.1|	73711-27503	150	39	3	2	-0.657664	0.68545	no
gi|646732863|gb|KK963615.1|	29321-33319	150	39	3	2	-0.417519	0.7815	no
gi|646732863|gb|KK963615.1|	93378-29452	150	39	2	0.894378	-1	0.4155	no
gi|646732863|gb|KK963615.1|	06179-30658	150	39	6	2	-2	0.21525	no
gi|646732863|gb|KK963615.1|	36676-37344	150	39	2	0.622947	-2	0.2684	no
gi|646732863|gb|KK963615.1|	44981-45632	150	39	4	2	-0.941424	0.49045	no
gi|646732863|gb|KK963615.1|	48920-49585	150	39	2	2	0.283591	0.8397	no
gi|646732863|gb|KK963615.1|	56453-57312	150	39	1	0.433295	-2	0.2991	no
gi|646732863|gb|KK963615.1|	60690-62792	150	39	3	2	-0.853743	0.5798	no
gi|646732869|gb|KK963614.1|	11556-11700	11	6.3	3521.45	5609.93	0.671812	0.6602	no
gi|646732905|gb|KK963611.1|	01535-10182	17	7.5	96	9	-3	0.05995	no
gi|646732905|gb|KK963611.1|	58928-59337	17	7.5	24	2	-4	0.1209	no

gi|646732905|gb|KK963611.1|	85357-85798	17	7.5	66	12	-2	0.1429	no
gi|646732905|gb|KK963611.1|	99709-10000[illegible]	17	7.5	101	9	-4	0.0515	no
gi|646732909|gb|KK963610.1|	72017-17297	13	6.7	0.786143	1	0.922906	0.5404	no
gi|646732914|gb|KK963609.1|	06228-10671	21	5.6	3	4	0.229546	0.86575	no
gi|646732914|gb|KK963609.1|	06887-10730	21	5.6	5	5	0.185317	0.89945	no
gi|646732914|gb|KK963609.1|	11567-11227	21	5.6	2	2	-0.169005	0.90845	no
gi|646732914|gb|KK963609.1|	67291-16877	21	5.6	1	0.648588	-0.97571	0.48145	no
gi|646732914|gb|KK963609.1|	70579-17127	21	5.6	2	2	0.312063	0.8299	no
gi|646732914|gb|KK963609.1|	71454-17338	21	5.6	2	2	-0.108017	0.9447	no
gi|646732914|gb|KK963609.1|	76172-17638	21	5.6	56	0	-inf	0.00655	no
gi|646732914|gb|KK963609.1|	84191-18537	21	5.6	8	0	-inf	0.00025	yes
gi|646732914|gb|KK963609.1|	89140-19191	21	5.6	13	5	-1	0.2951	no
gi|646732914|gb|KK963609.1|	93211-19363	21	5.6	10	8	-0.324122	0.783	no
gi|646732914|gb|KK963609.1|	01438-20167	21	5.6	18	19	0.0643466	0.90935	no
gi|646732914|gb|KK963609.1|	12638-21397	21	5.6	2	2	-0.0243889	0.9627	no
gi|646732914|gb|KK963609.1|	14495-21605	21	5.6	0.710548	1	0.665647	0.6132	no
gi|646732914|gb|KK963609.1|	20137-22151	21	5.6	0.657859	1	0.983774	0.4966	no
gi|646732914|gb|KK963609.1|	27174-22940	21	5.6	0.748752	0.814478	0.121389	1	no
gi|646732914|gb|KK963609.1|	33709-23496	21	5.6	0.739338	0.663019	-0.157184	1	no
gi|646732914|gb|KK963609.1|	35136-23645	21	5.6	0.434322	0.873594	1	1	no
gi|646732914|gb|KK963609.1|	37382-23900	21	5.6	0.406953	0.489419	0.266209	1	no
gi|646732914|gb|KK963609.1|	40164-24055	21	5.6	4	3	-0.225131	0.87385	no
gi|646732914|gb|KK963609.1|	42197-24358	21	5.6	1	0.822179	-0.574996	0.67605	no
gi|646732914|gb|KK963609.1|	47065-24809	21	5.6	1	1	-0.225176	0.86285	no
gi|646732914|gb|KK963609.1|	48439-24934	21	5.6	2	1	-0.981051	0.50955	no
gi|646732914|gb|KK963609.1|	51498-25336	21	5.6	1	1	-0.26071	0.8794	no
gi|646732914|gb|KK963609.1|	53826-25526	21	5.6	0.467233	0.784819	0.748216	1	no
gi|646732914|gb|KK963609.1|	57728-25793	21	5.6	44	49	0.145786	0.9177	no
gi|646732914|gb|KK963609.1|	76846-27712	21	5.6	12	16	0.3928	0.79065	no
gi|646732914|gb|KK963609.1|	80901-28130	21	5.6	7	7	0.0784542	0.947	no

gi|646732914|gb|KK963609.1|	87725-28792	21	5.6	74	23	-2	0.3027	no
gi|646732914|gb|KK963609.1|	82778-38312	21	5.6	18	8	-1	0.3347	no
gi|646732914|gb|KK963609.1|	53465-53650	21	5.6	181	227	0.320536	0.8242	no
gi|646732914|gb|KK963609.1|	73837-75120	21	5.6	6	10	0.71965	0.62945	no
gi|646732914|gb|KK963609.1|	82773-83849	21	5.6	5	5	0.166119	0.9138	no
gi|646732914|gb|KK963609.1|	84530-87678	21	5.6	3	5	0.484365	0.74865	no
gi|646732914|gb|KK963609.1|	87910-90794	21	5.6	426	278	-0.61536	0.61045	no
gi|646732914|gb|KK963609.1|	)9657-10048(	21	5.6	66	0	-inf	5.00E-05	yes
gi|646732955|gb|KK963604.1|	33542-13403	21	8.1	4	1	-2	0.2639	no
gi|646732955|gb|KK963604.1|	61623-16207	21	8.1	7	13	0.890975	0.5323	no
gi|646732955|gb|KK963604.1|	78106-17878	21	8.1	0.218554	3	4	0.1061	no
gi|646732955|gb|KK963604.1|	84478-18564	21	8.1	2	1	-0.73028	0.5707	no
gi|646732955|gb|KK963604.1|	54965-55264	21	8.1	6	13	1	0.4303	no
gi|646732955|gb|KK963604.1|	88395-90079	21	8.1	23	8	-1	0.25785	no
gi|646732955|gb|KK963604.1|	90930-91572	21	8.1	3	3	0.056803	0.94915	no
gi|646732955|gb|KK963604.1|	92906-93709	21	8.1	14	5	-1	0.36335	no
gi|646732955|gb|KK963604.1|	98369-98848	21	8.1	12	5	-1	0.42495	no
gi|646732962|gb|KK963603.1|	45539-14591	18	7.1	42	37	-0.208983	0.8916	no
gi|646732962|gb|KK963603.1|	20602-21632	18	7.1	42	17	-1	0.3045	no
gi|646732962|gb|KK963603.1|	2232-3689	18	7.1	41	15	-1	0.2058	no
gi|646732962|gb|KK963603.1|	32988-35416	18	7.1	23	16	-0.527716	0.63265	no
gi|646732962|gb|KK963603.1|	4274-5561	18	7.1	22	14	-0.619183	0.62645	no
gi|646732962|gb|KK963603.1|	6633-7153	18	7.1	3	2	-0.562235	0.71555	no
gi|646732968|gb|KK963602.1|	07258-10791	20	9.9	2	4	1	0.3212	no
gi|646732968|gb|KK963602.1|	10451-11172	20	9.9	0.36172	1	2	1	no
gi|646732968|gb|KK963602.1|	24420-12551	20	9.9	0.552607	2	2	0.24115	no
gi|646732968|gb|KK963602.1|	28633-12892	20	9.9	34	19	-0.859327	0.4965	no
gi|646732968|gb|KK963602.1|	32388-13326	20	9.9	5	10	0.957781	0.5358	no
gi|646732968|gb|KK963602.1|	33387-13667	20	9.9	3	2	-0.788139	0.5994	no
gi|646732968|gb|KK963602.1|	39432-14060	20	9.9	8	2	-2	0.1357	no

gi|646732968|gb|KK963602.1|	49254-14966	20	9.9	8	11	0.481644	0.7037	no
gi|646732968|gb|KK963602.1|	70666-17149	20	9.9	0.800154	2	1	0.43255	no
gi|646732968|gb|KK963602.1|	18660-19152	20	9.9	48	34	-0.472159	0.75915	no
gi|646732968|gb|KK963602.1|	89797-19070	20	9.9	0.566622	2	2	0.3671	no
gi|646732968|gb|KK963602.1|	99204-20141	20	9.9	6	4	-0.622944	0.67415	no
gi|646732968|gb|KK963602.1|	18725-21898	20	9.9	121	74	-0.697096	0.651	no
gi|646732968|gb|KK963602.1|	24210-22485	20	9.9	2	2	-0.205435	0.89315	no
gi|646732968|gb|KK963602.1|	40351-24180	20	9.9	1	0.999147	-0.210877	0.8761	no
gi|646732968|gb|KK963602.1|	55879-25664	20	9.9	3	3	0.143351	0.906	no
gi|646732968|gb|KK963602.1|	57974-25955	20	9.9	3	2	-0.553827	0.7285	no
gi|646732968|gb|KK963602.1|	61201-26310	20	9.9	1	1	0.0637574	1	no
gi|646732968|gb|KK963602.1|	64043-26534	20	9.9	2	4	0.922773	0.56275	no
gi|646732968|gb|KK963602.1|	65398-26616	20	9.9	0.181002	5	5	0.1046	no
gi|646732968|gb|KK963602.1|	74173-27458	20	9.9	39	27	-0.563731	0.7065	no
gi|646732968|gb|KK963602.1|	27471-28585	20	9.9	0.861532	0.61587	-0.484277	1	no
gi|646732968|gb|KK963602.1|	95486-29579	20	9.9	14	6	-1	0.42745	no
gi|646732968|gb|KK963602.1|	97532-29806	20	9.9	4	4	-0.256783	0.85035	no
gi|646732968|gb|KK963602.1|	30864-31084	20	9.9	30	45	0.584869	0.7117	no
gi|646732968|gb|KK963602.1|	10347-31129	20	9.9	0.802044	1	0.729371	0.6352	no
gi|646732968|gb|KK963602.1|	13871-31563	20	9.9	1	0.6195	-0.748615	1	no
gi|646732968|gb|KK963602.1|	15826-31722	20	9.9	1	0.696883	-0.890183	0.50965	no
gi|646732968|gb|KK963602.1|	18626-32718	20	9.9	4	4	-0.115936	0.91315	no
gi|646732968|gb|KK963602.1|	31920-33414	20	9.9	0.896007	1	0.401383	0.75845	no
gi|646732968|gb|KK963602.1|	33573-35818	20	9.9	1	0.606913	-1	0.40945	no
gi|646732968|gb|KK963602.1|	39769-34154	20	9.9	4	5	0.288747	0.8507	no
gi|646732968|gb|KK963602.1|	51227-35338	20	9.9	1	3	1	0.4145	no
gi|646732968|gb|KK963602.1|	54027-35737	20	9.9	23.16	8	-2	0.15895	no
gi|646732968|gb|KK963602.1|	35927-36538	20	9.9	3	1	-1	0.42175	no
gi|646732968|gb|KK963602.1|	67060-36778	20	9.9	19	7	-1	0.34585	no
gi|646732968|gb|KK963602.1|	41223-43160	20	9.9	2	1	-0.285809	0.85595	no

gi|646732968|gb|KK963602.1|	45240-47633	20	9.9	12	15	0.315241	0.7732	no
gi|646732968|gb|KK963602.1|	56940-61536	20	9.9	14	15	0.0735294	0.94865	no
gi|646732968|gb|KK963602.1|	66615-73478	20	9.9	42	43	0.0323072	0.9791	no
gi|646732968|gb|KK963602.1|	82535-83641	20	9.9	2	3	1	0.4621	no
gi|646732968|gb|KK963602.1|	91276-91521	20	9.9	49	33	-0.582077	0.66445	no
gi|646732982|gb|KK963600.1|	73676-74493	15	8.4	0	8	inf	0.00065	yes
gi|646732999|gb|KK963597.1|	81666-18308	13	8.1	0.397388	0.91508	1	1	no
gi|646732999|gb|KK963597.1|	63068-26331	13	8.1	60	84	0.485151	0.722	no
gi|646733026|gb|KK963593.1|	25249-26307	12	5.3	9	8	-0.118165	0.93395	no
gi|646733041|gb|KK963591.1|	08787-10985	24	8.7	1	1	-0.000999585	0.9367	no
gi|646733041|gb|KK963591.1|	09978-11133	24	8.7	2	2	0.523708	0.70325	no
gi|646733041|gb|KK963591.1|	72725-17321	24	8.7	7	2	-2	0.24985	no
gi|646733041|gb|KK963591.1|	82748-18341	24	8.7	5	0.945213	-2	0.14855	no
gi|646733041|gb|KK963591.1|	18554-19371	24	8.7	9	7	-0.296904	0.84825	no
gi|646733041|gb|KK963591.1|	86720-18769	24	8.7	3	11	2	0.2968	no
gi|646733041|gb|KK963591.1|	24980-25517	24	8.7	5	2	-1	0.4366	no
gi|646733041|gb|KK963591.1|	25743-26331	24	8.7	4	4	-0.081123	0.94965	no
gi|646733041|gb|KK963591.1|	39588-40853	24	8.7	0.732751	0.525791	-0.478833	1	no
gi|646733041|gb|KK963591.1|	80323-81139	24	8.7	1	0.930465	-0.503453	0.7384	no
gi|646733041|gb|KK963591.1|	81672-82487	24	8.7	1	0.699047	-0.725943	0.66415	no
gi|646733041|gb|KK963591.1|	8806-9682	24	8.7	10	7	-0.456318	0.75985	no
gi|646733066|gb|KK963587.1|	13660-13919	23	13	22	8	-1	0.37675	no
gi|646733066|gb|KK963587.1|	14450-16327	23	13	4	2	-0.926508	0.55085	no
gi|646733066|gb|KK963587.1|	42668-44503	23	13	9	2	-2	0.17655	no
gi|646733066|gb|KK963587.1|	51021-53207	23	13	15	12	-0.33174	0.7597	no
gi|646733066|gb|KK963587.1|	65331-66496	23	13	38	27	-0.478397	0.6729	no
gi|646733066|gb|KK963587.1|	87361-88155	23	13	2	0.966928	-1	0.3737	no
gi|646733066|gb|KK963587.1|	88218-89068	23	13	5	4	-0.252385	0.87335	no
gi|646733073|gb|KK963586.1|	87437-89426	14	5	7	12	0.724154	0.56525	no
gi|646733073|gb|KK963586.1|	92461-93385	14	5	14	12	-0.161138	0.91075	no

gi|646733129|gb|KK963578.1|	28384-12871	15	6.6	18	10	-0.878468	0.4862	no
gi|646733133|gb|KK963577.1|	56723-57694	15	6.8	1	0.735144	-0.491517	1	no
gi|646733146|gb|KK963575.1|	06060-10643	48	23	2	7	2	0.2769	no
gi|646733146|gb|KK963575.1|	53665-15447	48	23	2	3	0.148427	0.9046	no
gi|646733146|gb|KK963575.1|	54963-15532	48	23	5	7	0.552988	0.7191	no
gi|646733146|gb|KK963575.1|	56967-15770	48	23	3	4	0.580873	0.6939	no
gi|646733146|gb|KK963575.1|	57914-15889	48	23	1	3	1	0.4297	no
gi|646733146|gb|KK963575.1|	31174-31502	48	23	6	5	-0.302422	0.85055	no
gi|646733146|gb|KK963575.1|	76339-76977	48	23	6	0.668734	-3	0.14575	no
gi|646733146|gb|KK963575.1|	93584-94433	48	23	2	2	-0.500399	0.7001	no
gi|646733153|gb|KK963574.1|	12457-11298	17	8.8	13	5	-1	0.3051	no
gi|646733153|gb|KK963574.1|	39012-14065	17	8.8	18	11	-0.683921	0.58505	no
gi|646733153|gb|KK963574.1|	55573-15592	17	8.8	12	7	-0.804639	0.5631	no
gi|646733153|gb|KK963574.1|	63504-16371	17	8.8	116	20	-3	0.1426	no
gi|646733153|gb|KK963574.1|	66332-16673	17	8.8	31	17	-0.869612	0.58205	no
gi|646733153|gb|KK963574.1|	90570-19139	17	8.8	28	12	-1	0.40545	no
gi|646733153|gb|KK963574.1|	96885-19743	17	8.8	2	2	0.184027	0.8995	no
gi|646733153|gb|KK963574.1|	35228-35606	17	8.8	19	6	-2	0.29335	no
gi|646733153|gb|KK963574.1|	63021-63696	17	8.8	19	3	-3	0.0779	no
gi|646733153|gb|KK963574.1|	69556-69927	17	8.8	11	5	-1	0.38285	no
gi|646733153|gb|KK963574.1|	88684-89231	17	8.8	7	2	-2	0.22595	no
gi|646733153|gb|KK963574.1|	90662-95086	17	8.8	10	12	0.312969	0.7771	no
gi|646733153|gb|KK963574.1|	96274-97766	17	8.8	13	9	-0.531342	0.72685	no
gi|646733173|gb|KK963571.1|	05962-10916	19	9	8	6	-0.387084	0.7593	no
gi|646733173|gb|KK963571.1|	12333-11455	19	9	7	6	-0.328742	0.82415	no
gi|646733173|gb|KK963571.1|	82653-18504	19	9	51	32	-0.646601	0.57625	no
gi|646733173|gb|KK963571.1|	2767-3167	19	9	19	11	-0.779756	0.56845	no
gi|646733173|gb|KK963571.1|	3455-3864	19	9	4	5	0.374729	0.7976	no
gi|646733173|gb|KK963571.1|	72083-76484	19	9	0.647163	0.325557	-0.991219	1	no
gi|646733173|gb|KK963571.1|	95610-96753	19	9	7	6	-0.186108	0.8997	no

gi|646733185|gb|KK963569.1|	86406-87120	15	9	0	6	inf	0.0026	no
gi|646733193|gb|KK963568.1|	28518-23012	24	11	2	2	0.0695548	0.96365	no
gi|646733193|gb|KK963568.1|	30236-23191	24	11	0.648314	0.936291	0.530264	1	no
gi|646733193|gb|KK963568.1|	33076-23493	24	11	0.462579	0.501871	0.117618	1	no
gi|646733193|gb|KK963568.1|	39101-24138	24	11	0.365322	0.463747	0.344169	1	no
gi|646733193|gb|KK963568.1|	42655-24331	24	11	1	2	0.474568	0.7489	no
gi|646733193|gb|KK963568.1|	24308-24807	24	11	0	6	inf	0.001	yes
gi|646733193|gb|KK963568.1|	44229-24638	24	11	0.632348	0.211555	-2	1	no
gi|646733193|gb|KK963568.1|	46575-24903	24	11	3	7	1	0.4561	no
gi|646733193|gb|KK963568.1|	53552-25421	24	11	1	4	1	0.31055	no
gi|646733193|gb|KK963568.1|	58440-25968	24	11	0.279485	2	3	0.13755	no
gi|646733193|gb|KK963568.1|	60463-26394	24	11	20	17	-0.215523	0.8495	no
gi|646733193|gb|KK963568.1|	67659-26907	24	11	16	21	0.347422	0.7472	no
gi|646733193|gb|KK963568.1|	71156-27236	24	11	6	4	-0.480757	0.7542	no
gi|646733193|gb|KK963568.1|	25769-32689	24	11	0.63945	0.762056	0.253066	1	no
gi|646733193|gb|KK963568.1|	28331-32852	24	11	89	8	-3	0.18945	no
gi|646733193|gb|KK963568.1|	57345-59527	24	11	0.240056	5	4	0.06275	no
gi|646733193|gb|KK963568.1|	86855-88769	24	11	0.557662	0.64568	0.21143	1	no
gi|646733193|gb|KK963568.1|	89375-92029	24	11	54	35	-0.634519	0.58865	no
gi|646733193|gb|KK963568.1|	97653-98374	24	11	0.7938	4	2	0.19115	no
gi|646733199|gb|KK963567.1|	11745-11254	23	11	0.336963	3	3	0.25	no
gi|646733199|gb|KK963567.1|	71443-17268	23	11	0.187646	2	3	0.24065	no
gi|646733199|gb|KK963567.1|	32364-33737	23	11	4	1	-2	0.2082	no
gi|646733199|gb|KK963567.1|	44257-46121	23	11	2	6	1	0.3759	no
gi|646733199|gb|KK963567.1|	50243-51548	23	11	0.616736	1	0.88492	0.53825	no
gi|646733207|gb|KK963566.1|	47169-14881	15	8.3	3	4	0.453727	0.7657	no
gi|646733207|gb|KK963566.1|	57081-15780	15	8.3	2	1	-0.651511	0.6547	no
gi|646733213|gb|KK963565.1|	54346-54809	14	6.7	2	6	2	0.322	no
gi|646733264|gb|KK963558.1|	35842-36078	20	14	50	34	-0.573083	0.66445	no
gi|646733264|gb|KK963558.1|	53089-53822	20	14	3	0.542314	-2	0.1599	no

gi|646733271|gb|KK963557.1|	19011-11925	12	7.4	42	19	-1	0.40455	no
gi|646733285|gb|KK963555.1|	06054-10678	18	6.1	3	0.542314	-2	0.1599	no
gi|646733285|gb|KK963555.1|	8843-9605	18	6.1	1	1	-0.187396	0.90405	no
gi|646733292|gb|KK963554.1|	46637-47722	14	6.4	10	0.636824	-4	0.05475	no
gi|646733299|gb|KK963553.1|	43192-14456	17	8	53	40	-0.420753	0.70945	no
gi|646733306|gb|KK963552.1|	01125-10205	50	25	0	4	inf	0.0026	no
gi|646733306|gb|KK963552.1|	47015-47653	50	25	0	4	inf	0.0014	yes
gi|646733306|gb|KK963552.1|	56714-57880	50	25	0.203091	2	3	0.26825	no
gi|646733326|gb|KK963549.1|	20698-12135	16	4.7	3	3	0.211155	0.87205	no
gi|646733326|gb|KK963549.1|	66303-68118	16	4.7	0.415133	0.943355	1	1	no
gi|646733326|gb|KK963549.1|	68797-70820	16	4.7	9	13	0.525971	0.6732	no
gi|646733326|gb|KK963549.1|	82024-84259	16	4.7	2	9	2	0.1966	no
gi|646733326|gb|KK963549.1|	84962-86068	16	4.7	0.543518	0.932267	0.778416	1	no
gi|646733333|gb|KK963548.1|	19860-12049	14	6.7	2	4	0.930255	0.50175	no
gi|646733333|gb|KK963548.1|	20716-12121	14	6.7	2	4	0.912301	0.56505	no
gi|646733342|gb|KK963547.1|	62498-63731	17	6.6	5	4	-0.4254	0.7846	no
gi|646733417|gb|KK963538.1|	06937-10763	19	8.8	10	6	-0.769082	0.61975	no
gi|646733417|gb|KK963538.1|	19540-12012	19	8.8	42	18	-1	0.4309	no
gi|646733417|gb|KK963538.1|	21178-23091	19	8.8	8	7	-0.320433	0.83415	no
gi|646733417|gb|KK963538.1|	25319-25615	19	8.8	21	55	1	0.31005	no
gi|646733417|gb|KK963538.1|	28037-28406	19	8.8	5	6	0.113896	0.9237	no
gi|646733417|gb|KK963538.1|	28981-29497	19	8.8	5	1	-2	0.281	no
gi|646733417|gb|KK963538.1|	33589-34063	19	8.8	5	3	-0.457276	0.74105	no
gi|646733417|gb|KK963538.1|	5507-6218	19	8.8	0	4	inf	0.00035	yes
gi|646733417|gb|KK963538.1|	63156-63704	19	8.8	13	7	-0.822371	0.608	no
gi|646733417|gb|KK963538.1|	76455-76790	19	8.8	11	10	-0.166523	0.9095	no
gi|646733417|gb|KK963538.1|	84694-85760	19	8.8	9	5	-0.919386	0.54795	no
gi|646733423|gb|KK963537.1|	97266-97963	13	7.1	3	3	0.133749	0.9145	no
gi|646733432|gb|KK963536.1|	13401-14399	26	7	1	0.70921	-0.659865	1	no
gi|646733432|gb|KK963536.1|	15364-16604	26	7	2	2	-0.302742	0.82245	no

gi|646733432|gb|KK963536.1|	21220-22140	26	7	0.973699	0.592265	-0.717233	1	no
gi|646733432|gb|KK963536.1|	15358-21642	26	7	1	0.489094	-1	1	no
gi|646733432|gb|KK963536.1|	49219-24995	26	7	0.384548	4	3	0.24125	no
gi|646733432|gb|KK963536.1|	55052-25890	26	7	5	2	-1	0.3399	no
gi|646733432|gb|KK963536.1|	25842-28695	26	7	4	2	-0.959812	0.52915	no
gi|646733432|gb|KK963536.1|	64797-26566	26	7	3	4	0.0654399	0.9649	no
gi|646733432|gb|KK963536.1|	86404-28697	26	7	6	4	-0.623515	0.6203	no
gi|646733432|gb|KK963536.1|	04603-30574	26	7	5	3	-0.772715	0.62485	no
gi|646733432|gb|KK963536.1|	50557-51372	26	7	6	9	0.656914	0.6719	no
gi|646733432|gb|KK963536.1|	52111-52746	26	7	0.729354	3	2	0.25715	no
gi|646733432|gb|KK963536.1|	54006-55052	26	7	1	2	0.512602	0.70715	no
gi|646733432|gb|KK963536.1|	58219-60902	26	7	20	12	-0.686712	0.53785	no
gi|646733432|gb|KK963536.1|	62850-64102	26	7	3	4	0.569672	0.7204	no
gi|646733432|gb|KK963536.1|	65189-66354	26	7	1	2	0.517761	0.6755	no
gi|646733432|gb|KK963536.1|	7329-7578	26	7	48	18	-1	0.3039	no
gi|646733432|gb|KK963536.1|	81202-82011	26	7	1	0.706324	-0.726539	0.66415	no
gi|646733432|gb|KK963536.1|	85332-87270	26	7	3	3	0.282091	0.84775	no
gi|646733432|gb|KK963536.1|	93226-93484	26	7	108	51	-1	0.5006	no
gi|646733456|gb|KK963533.1|	65836-26613	17	9.7	17	13	-0.349763	0.79355	no
gi|646733456|gb|KK963533.1|	79256-27952	17	9.7	13	9	-0.420998	0.7662	no
gi|646733456|gb|KK963533.1|	09479-31065	17	9.7	0.700063	1	1	0.44735	no
gi|646733456|gb|KK963533.1|	15379-31657	17	9.7	0.491544	0.986037	1	1	no
gi|646733456|gb|KK963533.1|	64957-36550	17	9.7	0.936578	3	2	0.29625	no
gi|646733456|gb|KK963533.1|	73663-37396	17	9.7	28	2	-4	0.16905	no
gi|646733456|gb|KK963533.1|	8785-9137	17	9.7	9	7	-0.198568	0.8817	no
gi|646733497|gb|KK963528.1|	28851-29936	12	5.8	0.66872	1	1	0.4476	no
gi|646733524|gb|KK963525.1|	10416-11065	16	5.1	19	20	0.085779	0.91785	no
gi|646733524|gb|KK963525.1|	68754-16956	16	5.1	9	4	-1	0.4424	no
gi|646733524|gb|KK963525.1|	38181-39638	16	5.1	53	50	-0.106448	0.9264	no
gi|646733524|gb|KK963525.1|	41364-42831	16	5.1	0.763304	0.329811	-1	1	no

gi|646733524|gb|KK963525.1|	44468-49235	16	5.1	0.779221	0.658414	-0.243038	1	no
gi|646733524|gb|KK963525.1|	50269-52059	16	5.1	0.421894	0.522817	0.309427	1	no
gi|646733583|gb|KK963518.1|	43964-44260	20	9.2	94	26	-2	0.25165	no
gi|646733583|gb|KK963518.1|	48487-49394	20	9.2	2	1	-0.99827	0.48145	no
gi|646733583|gb|KK963518.1|	49515-50830	20	9.2	1	0.877629	-0.576904	0.67605	no
gi|646733583|gb|KK963518.1|	51795-52597	20	9.2	6	5	-0.312212	0.8456	no
gi|646733583|gb|KK963518.1|	53345-55335	20	9.2	3	1	-1	0.4767	no
gi|646733583|gb|KK963518.1|	55554-57696	20	9.2	1	0.497507	-1	1	no
gi|646733583|gb|KK963518.1|	57805-60561	20	9.2	114	47	-1	0.294	no
gi|646733583|gb|KK963518.1|	68859-72537	20	9.2	6	9	0.583853	0.57845	no
gi|646733583|gb|KK963518.1|	73146-75021	20	9.2	9	9	-0.0427851	0.97705	no
gi|646733583|gb|KK963518.1|	85752-87631	20	9.2	5	5	-0.229553	0.8779	no
gi|646733601|gb|KK963516.1|	53699-55274	12	4.9	14	12	-0.271757	0.8246	no
gi|646733609|gb|KK963515.1|	55004-55386	56	27	10	7	-0.457251	0.72605	no
gi|646733622|gb|KK963513.1|	08057-10997	18	6.9	8	8	0.0840623	0.95525	no
gi|646733656|gb|KK963509.1|	42001-14298	17	13	0	3	inf	0.00515	no
gi|646733656|gb|KK963509.1|	20333-20991	17	13	0.22934	12	6	0.09665	no
gi|646733656|gb|KK963509.1|	21344-21766	17	13	0.53843	8	4	0.10035	no
gi|646733656|gb|KK963509.1|	39567-24102	17	13	18	2	-4	0.00765	no
gi|646733656|gb|KK963509.1|	44253-24550	17	13	8	1	-2	0.09245	no
gi|646733656|gb|KK963509.1|	53589-25422	17	13	0	13	inf	0.00055	yes
gi|646733656|gb|KK963509.1|	38341-39531	17	13	0.593637	0.850471	0.51868	1	no
gi|646733656|gb|KK963509.1|	82340-84078	17	13	2	2	-0.0252946	0.9761	no
gi|646733672|gb|KK963507.1|	62500-16300	18	6.3	6	1	-2	0.19115	no
gi|646733692|gb|KK963505.1|	12115-11305	14	6.3	0.537804	2	2	0.3671	no
gi|646733692|gb|KK963505.1|	48498-48917	14	6.3	2	5	1	0.46025	no
gi|646733692|gb|KK963505.1|	52969-53411	14	6.3	0.484673	6	4	0.10725	no
gi|646733707|gb|KK963503.1|	57716-58326	9	6.5	2	2	0.0498592	0.9298	no
gi|646733707|gb|KK963503.1|	64638-65293	9	6.5	5	2	-2	0.2419	no
gi|646733707|gb|KK963503.1|	74219-75125	9	6.5	2	3	0.504158	0.6864	no

gi|646733725|gb|KK963501.1|	67398-16825	17	11	3	3	0.252449	0.8539	no
gi|646733725|gb|KK963501.1|	81751-18258	17	11	6	3	-0.804033	0.62745	no
gi|646733743|gb|KK963499.1|	20470-21003	18	9.3	4	2	-1	0.46265	no
gi|646733743|gb|KK963499.1|	22194-23083	18	9.3	3	1	-2	0.27815	no
gi|646733743|gb|KK963499.1|	23520-24171	18	9.3	3	2	-0.789421	0.5829	no
gi|646733757|gb|KK963497.1|	3487-3792	12	9.9	12	17	0.533987	0.68555	no
gi|646733757|gb|KK963497.1|	65893-66490	12	9.9	7	7	0.0287922	0.97885	no
gi|646733776|gb|KK963495.1|	10204-11569	16	5.9	6	0.239679	-5	0.11055	no
gi|646733776|gb|KK963495.1|	24045-12442	16	5.9	4	6	0.570458	0.71905	no
gi|646733776|gb|KK963495.1|	25739-12596	16	5.9	9	62	3	0.24935	no
gi|646733776|gb|KK963495.1|	22491-23170	16	5.9	5	1	-2	0.22875	no
gi|646733776|gb|KK963495.1|	30055-32158	16	5.9	5	3	-0.523585	0.7177	no
gi|646733776|gb|KK963495.1|	34619-35478	16	5.9	3	3	0.252534	0.8539	no
gi|646733776|gb|KK963495.1|	47223-48324	16	5.9	35	17	-1	0.4059	no
gi|646733776|gb|KK963495.1|	48469-49479	16	5.9	5	4	-0.523222	0.7383	no
gi|646733776|gb|KK963495.1|	54442-54629	16	5.9	61	54	-0.180395	0.89715	no
gi|646733776|gb|KK963495.1|	95695-98990	16	5.9	123	55	-1	0.33765	no
gi|646733776|gb|KK963495.1|	99332-99894	16	5.9	4	1	-2	0.3498	no
gi|646733785|gb|KK963494.1|	92740-93249	19	5.6	10	25	1	0.39765	no
gi|646733820|gb|KK963492.1|	21906-12336	18	7.6	1	0.221128	-2	1	no
gi|646733820|gb|KK963492.1|	23578-12446	18	7.6	1	0.412862	-1	0.38425	no
gi|646733820|gb|KK963492.1|	24938-12602	18	7.6	1	0.638321	-0.807565	1	no
gi|646733820|gb|KK963492.1|	26123-12738	18	7.6	0.640523	0.656603	0.0357711	1	no
gi|646733820|gb|KK963492.1|	53697-15396	18	7.6	21	2	-3	0.19615	no
gi|646733820|gb|KK963492.1|	84888-18951	18	7.6	4	3	-0.358089	0.7752	no
gi|646733820|gb|KK963492.1|	94925-19685	18	7.6	7	5	-0.344792	0.8155	no
gi|646733820|gb|KK963492.1|	00956-20232	18	7.6	3	3	-0.03991	0.9771	no
gi|646733820|gb|KK963492.1|	02721-20448	18	7.6	9	7	-0.478079	0.75025	no
gi|646733820|gb|KK963492.1|	04605-20501	18	7.6	14	5	-2	0.2983	no
gi|646733820|gb|KK963492.1|	13927-21463	18	7.6	1	2	0.898714	0.545	no

gi|646733820|gb|KK963492.1|	17902-21851	18	7.6	3	2	-0.185569	0.8941	no
gi|646733820|gb|KK963492.1|	69623-27043	18	7.6	1	0.943402	-0.0892095	1	no
gi|646733820|gb|KK963492.1|	54427-54661	18	7.6	41	0	-inf	0.00475	no
gi|646733820|gb|KK963492.1|	68486-68885	18	7.6	7	4	-0.771301	0.59415	no
gi|646733836|gb|KK963490.1|	32311-33188	14	5.6	4	9	1	0.4364	no
gi|646733836|gb|KK963490.1|	56604-57161	14	5.6	2	3	0.450588	0.75415	no
gi|646733836|gb|KK963490.1|	81971-84184	14	5.6	2	1	-0.463729	0.77205	no
gi|646733843|gb|KK963489.1|	06261-10732	18	6.7	1	0.325281	-2	0.26845	no
gi|646733843|gb|KK963489.1|	17899-11843	18	6.7	24	29	0.294681	0.8475	no
gi|646733843|gb|KK963489.1|	19426-12161	18	6.7	0.907502	0.484812	-0.904477	1	no
gi|646733843|gb|KK963489.1|	33916-13531	18	6.7	0.404688	0.815124	1	1	no
gi|646733843|gb|KK963489.1|	44296-14599	18	6.7	0.768819	0.647806	-0.247083	1	no
gi|646733843|gb|KK963489.1|	48757-15060	18	6.7	2	0.168119	-3	0.1495	no
gi|646733843|gb|KK963489.1|	50962-15207	18	6.7	2	2	-0.484362	0.73725	no
gi|646733843|gb|KK963489.1|	54365-15568	18	6.7	2	1	-1	0.46935	no
gi|646733843|gb|KK963489.1|	25066-25261	18	6.7	57	52	-0.129193	0.9119	no
gi|646733843|gb|KK963489.1|	62107-63241	18	6.7	15	5	-2	0.27305	no
gi|646733843|gb|KK963489.1|	64382-66980	18	6.7	2	1	-0.367959	0.81135	no
gi|646733843|gb|KK963489.1|	70831-72763	18	6.7	0.386174	0.558986	0.533563	1	no
gi|646733843|gb|KK963489.1|	95863-96832	18	6.7	0.647781	0.921425	0.508361	1	no
gi|646733897|gb|KK963483.1|	46149-14685	9.5	5.4	0.204131	9	5	0.09795	no
gi|646733922|gb|KK963480.1|	48067-14933	12	6.6	1	0.914818	-0.371734	0.77735	no
gi|646733922|gb|KK963480.1|	20061-20574	12	6.6	3	3	0.0199604	0.9298	no
gi|646733945|gb|KK963477.1|	30635-31404	19	7.6	2	3	0.870022	0.50085	no
gi|646733973|gb|KK963474.1|	00795-10134	19	9.3	67	29	-1	0.3444	no
gi|646733973|gb|KK963474.1|	10859-11370	19	9.3	6	5	-0.276306	0.81935	no
gi|646733973|gb|KK963474.1|	10997-11173	19	9.3	35	34	-0.0622848	0.97045	no
gi|646733973|gb|KK963474.1|	11993-11501	19	9.3	2	2	-0.481347	0.7525	no
gi|646733973|gb|KK963474.1|	19710-12049	19	9.3	0.175511	5	5	0.10345	no
gi|646733973|gb|KK963474.1|	21934-12266	19	9.3	2	2	0.334819	0.7982	no

gi|646733973|gb|KK963474.1|	27844-13103	19	9.3	3	1	-1	0.47535	no
gi|646733973|gb|KK963474.1|	40089-14079	19	9.3	1	2	0.898109	0.545	no
gi|646733973|gb|KK963474.1|	43360-14421	19	9.3	83	30	-1	0.19485	no
gi|646734005|gb|KK963470.1|	51526-15457	17	7.4	46	15	-2	0.17285	no
gi|646734005|gb|KK963470.1|	15968-16828	17	7.4	0	3	inf	0.00605	no
gi|646734005|gb|KK963470.1|	18036-19030	17	7.4	0.250349	5	4	0.2424	no
gi|646734005|gb|KK963470.1|	21167-22399	17	7.4	0	3	inf	0.00155	yes
gi|646734005|gb|KK963470.1|	35816-36555	17	7.4	0	5	inf	0.00435	no
gi|646734005|gb|KK963470.1|	49624-50495	17	7.4	0	16	inf	0.0002	yes
gi|646734005|gb|KK963470.1|	63079-63480	17	7.4	6	3	-0.953967	0.5492	no
gi|646734005|gb|KK963470.1|	65675-66689	17	7.4	23	3	-3	0.09685	no
gi|646734005|gb|KK963470.1|	68225-68994	17	7.4	69	6	-4	0.0263	no
gi|646734005|gb|KK963470.1|	76916-78427	17	7.4	1	0.212306	-3	0.1506	no
gi|646734005|gb|KK963470.1|	81983-82469	17	7.4	9	1	-3	0.15395	no
gi|646734005|gb|KK963470.1|	97137-97752	17	7.4	0	46	inf	5.00E-05	yes
gi|646734014|gb|KK963469.1|	47189-47878	11	5.5	3	4	0.373468	0.75635	no
gi|646734014|gb|KK963469.1|	50880-53455	11	5.5	0.917213	2	1	0.51495	no
gi|646734033|gb|KK963467.1|	43328-44780	11	6.4	1	772	9	0.00135	yes
gi|646734033|gb|KK963467.1|	55377-56268	11	6.4	0.290882	103	8	0.2322	no
gi|646734042|gb|KK963466.1|	12220-14000	14	6.7	0.909986	0.701595	-0.375206	1	no
gi|646734042|gb|KK963466.1|	1677-1891	14	6.7	57	6	-3	0.18995	no
gi|646734042|gb|KK963466.1|	24051-24357	14	6.7	28	19	-0.561067	0.6407	no
gi|646734042|gb|KK963466.1|	5335-5654	14	6.7	10	14	0.420449	0.74595	no
gi|646734068|gb|KK963463.1|	45537-14586	18	7.8	3	9	2	0.3712	no
gi|646734068|gb|KK963463.1|	63232-16364	18	7.8	21	13	-0.746444	0.6464	no
gi|646734068|gb|KK963463.1|	64537-16601	18	7.8	7	7	-0.0583388	0.96795	no
gi|646734068|gb|KK963463.1|	28557-30392	18	7.8	0.878312	1	0.625515	0.66165	no
gi|646734068|gb|KK963463.1|	32375-34232	18	7.8	12	9	-0.326621	0.7905	no
gi|646734068|gb|KK963463.1|	37263-38110	18	7.8	1	2	0.499425	0.72155	no
gi|646734068|gb|KK963463.1|	46856-47253	18	7.8	10	20	1	0.46355	no

gi|646734068|gb|KK963463.1|	63871-65855	18	7.8	30	16	-0.929192	0.40365	no
gi|646734068|gb|KK963463.1|	67092-69212	18	7.8	5	2	-1	0.4055	no
gi|646734076|gb|KK963462.1|	56739-25700	12	6.5	7	17	1	0.38965	no
gi|646734115|gb|KK963457.1|	14577-11482	11	5.5	16	23	0.533589	0.7171	no
gi|646734115|gb|KK963457.1|	98037-98516	11	5.5	18	11	-0.684603	0.6631	no
gi|646734156|gb|KK963453.1|	08004-11201	15	6.8	6	11	0.754539	0.49555	no
gi|646734156|gb|KK963453.1|	16085-11762	15	6.8	0.938121	3	1	0.31705	no
gi|646734156|gb|KK963453.1|	20923-12156	15	6.8	0.241855	3	4	0.10445	no
gi|646734156|gb|KK963453.1|	22705-12667	15	6.8	16	39	1	0.25605	no
gi|646734174|gb|KK963451.1|	03991-10441	17	15	0	7	inf	0.00255	no
gi|646734182|gb|KK963450.1|	42811-43751	12	10	1	0	-inf	0.00475	no
gi|646734182|gb|KK963450.1|	67162-67537	12	10	0.721265	24	5	0.10135	no
gi|646734182|gb|KK963450.1|	67878-68185	12	10	0	25	inf	0.00515	no
gi|646734182|gb|KK963450.1|	78718-80717	12	10	0	11	inf	5.00E-05	yes
gi|646734213|gb|KK963446.1|	37941-13867	14	8.6	0	3	inf	0.0014	yes
gi|646734213|gb|KK963446.1|	40869-14142	14	8.6	0	6	inf	0.00035	yes
gi|646734213|gb|KK963446.1|	26112-26702	14	8.6	3	6	1	0.45415	no
gi|646734213|gb|KK963446.1|	76699-77351	14	8.6	0.465567	5	3	0.24135	no
gi|646734213|gb|KK963446.1|	86384-86644	14	8.6	2	29	4	0.1022	no
gi|646734213|gb|KK963446.1|	96772-97674	14	8.6	0	12	inf	0.00025	yes
gi|646734213|gb|KK963446.1|	98335-98981	14	8.6	4	18	2	0.11365	no
gi|646734268|gb|KK963440.1|	29763-30086	12	4.7	19	24	0.357287	0.7936	no
gi|646734280|gb|KK963439.1|	84283-85214	44	17	2	6	1	0.3084	no
gi|646734289|gb|KK963438.1|	06303-10840	21	5.8	10	0.871585	-4	0.00945	no
gi|646734289|gb|KK963438.1|	72175-17251	21	5.8	7	2	-2	0.30625	no
gi|646734289|gb|KK963438.1|	86147-18654	21	5.8	5	2	-2	0.3081	no
gi|646734289|gb|KK963438.1|	20134-20401	21	5.8	9	17	0.968706	0.58275	no
gi|646734289|gb|KK963438.1|	63362-26391	21	5.8	15	5	-2	0.2803	no
gi|646734289|gb|KK963438.1|	27708-28281	21	5.8	5	1	-2	0.1873	no
gi|646734289|gb|KK963438.1|	77084-27731	21	5.8	128	0	-inf	0.00185	no

gi|646734289|gb|KK963438.1|	77437-27776	21	5.8	23.43	0	-inf	0.00525	no
gi|646734289|gb|KK963438.1|	80552-28119	21	5.8	2	0.668734	-2	0.3832	no
gi|646734289|gb|KK963438.1|	26096-32666	21	5.8	2	1	-0.962017	0.589	no
gi|646734289|gb|KK963438.1|	37594-34361	21	5.8	6	3	-1	0.3675	no
gi|646734289|gb|KK963438.1|	46376-49631	21	5.8	1	0.673498	-0.799136	0.6244	no
gi|646734289|gb|KK963438.1|	55695-56202	21	5.8	24	14	-0.731851	0.6318	no
gi|646734289|gb|KK963438.1|	73286-74793	21	5.8	2	3	0.719823	0.6039	no
gi|646734289|gb|KK963438.1|	76303-78183	21	5.8	7	4	-0.752443	0.61715	no
gi|646734289|gb|KK963438.1|	88287-89727	21	5.8	2	3	0.695682	0.6606	no
gi|646734296|gb|KK963437.1|	63412-64225	20	16	0	5	inf	0.00315	no
gi|646734312|gb|KK963435.1|	11473-11189	22	11	3	14	2	0.2084	no
gi|646734312|gb|KK963435.1|	18357-11859	22	11	31	15	-1	0.465	no
gi|646734312|gb|KK963435.1|	32168-13265	22	11	4	4	0.255112	0.8603	no
gi|646734312|gb|KK963435.1|	61336-16177	22	11	5	2	-1	0.3647	no
gi|646734312|gb|KK963435.1|	63364-16572	22	11	22	38	0.821277	0.46685	no
gi|646734312|gb|KK963435.1|	82082-18294	22	11	2	1	-0.636706	0.65905	no
gi|646734312|gb|KK963435.1|	87475-18847	22	11	0	2	inf	0.0014	yes
gi|646734312|gb|KK963435.1|	44601-24998	22	11	52	33	-0.675258	0.5755	no
gi|646734312|gb|KK963435.1|	51172-25397	22	11	1	1	0.294885	0.8532	no
gi|646734312|gb|KK963435.1|	75905-27675	22	11	0.777299	2	1	0.33895	no
gi|646734312|gb|KK963435.1|	77517-27807	22	11	15	19	0.329353	0.82985	no
gi|646734312|gb|KK963435.1|	97914-29824	22	11	25	25	0.00385315	0.97655	no
gi|646734312|gb|KK963435.1|	03985-30490	22	11	1	3	1	0.47555	no
gi|646734312|gb|KK963435.1|	09338-31353	22	11	0.493387	1	1	1	no
gi|646734312|gb|KK963435.1|	17744-31930	22	11	0.568002	0.614333	0.113124	1	no
gi|646734312|gb|KK963435.1|	21522-32282	22	11	0.79674	0.889963	0.159637	1	no
gi|646734312|gb|KK963435.1|	28251-33045	22	11	1	0.827726	-0.338306	1	no
gi|646734312|gb|KK963435.1|	30520-33533	22	11	9	11	0.314485	0.7737	no
gi|646734312|gb|KK963435.1|	35707-34042	22	11	9	12	0.355052	0.7521	no
gi|646734312|gb|KK963435.1|	50597-35146	22	11	24	17	-0.526023	0.72265	no

gi|646734312|gb|KK963435.1|	41530-42934	22	11	3	3	0.0394992	0.9787	no
gi|646734312|gb|KK963435.1|	84820-85422	22	11	3	3	0.0479488	0.9493	no
gi|646734327|gb|KK963433.1|	)9484-10038(	16	6.9	33	3	-3	0.04415	no
gi|646734344|gb|KK963431.1|	12052-11269	23	11	2	2	0.109671	0.93055	no
gi|646734344|gb|KK963431.1|	44059-14434	23	11	22	25	0.204585	0.8676	no
gi|646734344|gb|KK963431.1|	28774-31182	23	11	6	7	0.167625	0.9117	no
gi|646734344|gb|KK963431.1|	31933-36830	23	11	2	3	0.667217	0.65875	no
gi|646734344|gb|KK963431.1|	36943-41249	23	11	3	3	-0.0602083	0.96915	no
gi|646734344|gb|KK963431.1|	59488-59715	23	11	82	13	-3	0.1206	no
gi|646734344|gb|KK963431.1|	90504-91895	23	11	0.732926	0.58575	-0.323384	1	no
gi|646734352|gb|KK963430.1|	25045-25487	18	9.7	10	14	0.536243	0.69425	no
gi|646734391|gb|KK963426.1|	81216-82442	15	6.5	0.47636	2	2	0.2458	no
gi|646734391|gb|KK963426.1|	82531-84790	15	6.5	0.646332	1	0.729287	1	no
gi|646734391|gb|KK963426.1|	84914-86635	15	6.5	0.883497	0.63823	-0.469148	1	no
gi|646734391|gb|KK963426.1|	87325-89038	15	6.5	0.888344	3	2	0.2564	no
gi|646734398|gb|KK963425.1|	08359-11020	22	5.9	1	0.083956	-4	0.17135	no
gi|646734398|gb|KK963425.1|	11510-12727	22	5.9	1	0.275715	-2	1	no
gi|646734398|gb|KK963425.1|	16448-13572	22	5.9	9	9	0.0869106	0.94105	no
gi|646734398|gb|KK963425.1|	13527-21563	22	5.9	0.749132	0.695619	-0.106923	1	no
gi|646734398|gb|KK963425.1|	42796-14440	22	5.9	17	9	-0.90817	0.4767	no
gi|646734398|gb|KK963425.1|	44482-14782	22	5.9	14	6	-1	0.2979	no
gi|646734398|gb|KK963425.1|	54595-15517	22	5.9	2	5	1	0.42765	no
gi|646734398|gb|KK963425.1|	58302-15893	22	5.9	1	2	0.468933	0.7494	no
gi|646734398|gb|KK963425.1|	95996-19637	22	5.9	29	10	-2	0.25835	no
gi|646734398|gb|KK963425.1|	97735-19830	22	5.9	5	2	-1	0.35845	no
gi|646734398|gb|KK963425.1|	02677-20365	22	5.9	2	1	-0.590812	0.6374	no
gi|646734398|gb|KK963425.1|	05160-20601	22	5.9	0.931194	2	0.722167	0.6352	no
gi|646734398|gb|KK963425.1|	19821-22036	22	5.9	14	12	-0.290236	0.85135	no
gi|646734398|gb|KK963425.1|	30849-23129	22	5.9	9	10	0.135059	0.9279	no
gi|646734398|gb|KK963425.1|	23738-23998	22	5.9	24	31	0.405285	0.75805	no

gi|646734398|gb|KK963425.1|	39067-24011	22	5.9	2	0.835224	-1	0.33035	no
gi|646734398|gb|KK963425.1|	44482-24604	22	5.9	0.425023	0.817261	0.943255	1	no
gi|646734398|gb|KK963425.1|	46170-24743	22	5.9	0.641157	0.657239	0.0357404	1	no
gi|646734398|gb|KK963425.1|	51426-25248	22	5.9	0.576718	1	0.998569	0.5626	no
gi|646734398|gb|KK963425.1|	25534-27788	22	5.9	1	0.469941	-1	1	no
gi|646734398|gb|KK963425.1|	72582-27336	22	5.9	10	9	-0.0659854	0.9685	no
gi|646734398|gb|KK963425.1|	78347-27972	22	5.9	1	3	0.983675	0.49905	no
gi|646734398|gb|KK963425.1|	27959-29331	22	5.9	1	0.595517	-1	0.3735	no
gi|646734398|gb|KK963425.1|	88207-29040	22	5.9	0.476649	0.967648	1	1	no
gi|646734398|gb|KK963425.1|	93562-29383	22	5.9	29	7	-2	0.19715	no
gi|646734398|gb|KK963425.1|	31958-32770	22	5.9	15	8	-0.825775	0.58585	no
gi|646734398|gb|KK963425.1|	08542-50927	22	5.9	42	27	-0.616111	0.62325	no
gi|646734398|gb|KK963425.1|	13655-51510	22	5.9	1	1	-0.304162	0.81285	no
gi|646734398|gb|KK963425.1|	24449-52987	22	5.9	2	2	-0.0531097	0.97175	no
gi|646734398|gb|KK963425.1|	8-803	22	5.9	2	2	0.172485	0.8983	no
gi|646734398|gb|KK963425.1|	956-1869	22	5.9	1	0.598359	-0.910374	0.58965	no
gi|646734406|gb|KK963424.1|	31982-13375	13	6.3	0.852113	0.879836	0.04619	1	no
gi|646734406|gb|KK963424.1|	43378-44294	13	6.3	1	0.397155	-1	1	no
gi|646734432|gb|KK963420.1|	30236-31156	11	6.9	7	3	-1	0.489	no
gi|646734432|gb|KK963420.1|	31865-32273	11	6.9	5	2	-2	0.28785	no
gi|646734440|gb|KK963419.1|	46051-14677	19	6.9	42	17	-1	0.3095	no
gi|646734440|gb|KK963419.1|	48240-14864	19	6.9	6	6	0.0494679	0.9428	no
gi|646734440|gb|KK963419.1|	48843-14961	19	6.9	2	2	0.339271	0.7981	no
gi|646734440|gb|KK963419.1|	49805-15108	19	6.9	2	2	0.521562	0.7048	no
gi|646734440|gb|KK963419.1|	65364-16571	19	6.9	56	97	0.79644	0.60105	no
gi|646734440|gb|KK963419.1|	66855-16737	19	6.9	4	7	1	0.48725	no
gi|646734440|gb|KK963419.1|	68338-16959	19	6.9	3	3	-0.360405	0.82085	no
gi|646734440|gb|KK963419.1|	71028-17201	19	6.9	4	7	0.840637	0.5879	no
gi|646734440|gb|KK963419.1|	72124-17274	19	6.9	3	4	0.468284	0.71795	no
gi|646734440|gb|KK963419.1|	74563-17510	19	6.9	12	12	0.0293159	0.98255	no

gi|646734440|gb|KK963419.1|	82055-18244	19	6.9	57	13	-2	0.18325	no
gi|646734440|gb|KK963419.1|	85625-18611	19	6.9	5	4	-0.16229	0.90765	no
gi|646734440|gb|KK963419.1|	02032-20235	19	6.9	17	1	-4	0.1758	no
gi|646734440|gb|KK963419.1|	04699-20562	19	6.9	1	1	-0.0791805	0.93285	no
gi|646734440|gb|KK963419.1|	19264-22030	19	6.9	28	44	0.662929	0.53985	no
gi|646734440|gb|KK963419.1|	21439-22287	19	6.9	0.784666	1	0.373619	1	no
gi|646734440|gb|KK963419.1|	26435-22660	19	6.9	400.71	464	0.212883	0.88195	no
gi|646734440|gb|KK963419.1|	27014-22726	19	6.9	9	38	2	0.19	no
gi|646734440|gb|KK963419.1|	28863-22950	19	6.9	2	2	0.249649	0.86925	no
gi|646734440|gb|KK963419.1|	47397-24854	19	6.9	0.515651	1	1	1	no
gi|646734440|gb|KK963419.1|	48800-24917	19	6.9	3	7	1	0.46965	no
gi|646734440|gb|KK963419.1|	66419-26786	19	6.9	1	1	0.226375	0.84925	no
gi|646734440|gb|KK963419.1|	00703-30096	19	6.9	20	2	-3	0.2001	no
gi|646734440|gb|KK963419.1|	11674-31206	19	6.9	0.639372	68	7	0.09505	no
gi|646734440|gb|KK963419.1|	27548-32794	19	6.9	0	73	inf	0.00025	yes
gi|646734440|gb|KK963419.1|	30200-33042	19	6.9	0	74	inf	0.00435	no
gi|646734440|gb|KK963419.1|	31881-33233	19	6.9	0	36	inf	0.00035	yes
gi|646734440|gb|KK963419.1|	33407-33366	19	6.9	0	77	inf	0.00105	yes
gi|646734440|gb|KK963419.1|	42528-34267	19	6.9	0	443	inf	0.00255	no
gi|646734440|gb|KK963419.1|	46455-34674	19	6.9	2	149	7	0.095	no
gi|646734451|gb|KK963418.1|	25234-12545	19	9.4	1284.34	309	-2	0.12455	no
gi|646734451|gb|KK963418.1|	31293-13256	19	9.4	439	324.63	-0.434718	0.71205	no
gi|646734451|gb|KK963418.1|	51394-15348	19	9.4	1	1	-0.0497475	1	no
gi|646734451|gb|KK963418.1|	53753-15521	19	9.4	0.766466	0.772689	0.0116676	1	no
gi|646734451|gb|KK963418.1|	66723-16817	19	9.4	14	6	-1	0.3952	no
gi|646734451|gb|KK963418.1|	95684-19591	19	9.4	15	50	2	0.2626	no
gi|646734451|gb|KK963418.1|	97497-19888	19	9.4	6	8	0.419113	0.78275	no
gi|646734451|gb|KK963418.1|	25331-26067	19	9.4	0	9	inf	0.0003	yes
gi|646734451|gb|KK963418.1|	92447-93832	19	9.4	609	1100.82	0.853821	0.47985	no
gi|646734467|gb|KK963416.1|	71316-72502	18	8.5	0.596187	0.56936	-0.0664259	1	no

gi|646734467|gb|KK963416.1|	76162-78455	18	8.5	1	0.856228	-0.40651	0.7688	no
gi|646734467|gb|KK963416.1|	79489-81487	18	8.5	2	1	-0.553186	0.7262	no
gi|646734492|gb|KK963413.1|	48580-15456	19	4.6	28	30	0.0824442	0.94495	no
gi|646734492|gb|KK963413.1|	54764-15715	19	4.6	69	86	0.320502	0.78105	no
gi|646734492|gb|KK963413.1|	57261-16699	19	4.6	145	129	-0.170911	0.8887	no
gi|646734492|gb|KK963413.1|	68012-16981	19	4.6	0.715786	0.862501	0.268998	1	no
gi|646734492|gb|KK963413.1|	70400-17161	19	4.6	1	1	0.140938	0.91015	no
gi|646734492|gb|KK963413.1|	71757-17331	19	4.6	2	2	0.528106	0.7413	no
gi|646734492|gb|KK963413.1|	73445-17406	19	4.6	1	3	1	0.39925	no
gi|646734492|gb|KK963413.1|	74310-17456	19	4.6	34	66	0.975907	0.4947	no
gi|646734492|gb|KK963413.1|	85865-18677	19	4.6	132	23	-3	0.0806	no
gi|646734492|gb|KK963413.1|	2690-3165	19	4.6	11	1	-3	0.1446	no
gi|646734500|gb|KK963412.1|	3336-3938	14	6.3	1	5	2	0.2263	no
gi|646734500|gb|KK963412.1|	483-1048	14	6.3	1	3	0.938447	0.5629	no
gi|646734500|gb|KK963412.1|	5722-6194	14	6.3	0.841654	5	3	0.26725	no
gi|646734508|gb|KK963411.1|	04152-10493	19	8.8	7	2	-1	0.32415	no
gi|646734508|gb|KK963411.1|	15910-11651	19	8.8	2	4	1	0.31165	no
gi|646734508|gb|KK963411.1|	16648-11740	19	8.8	2	6	1	0.35805	no
gi|646734508|gb|KK963411.1|	45157-14546	19	8.8	19	0	-inf	0.00225	no
gi|646734508|gb|KK963411.1|	79875-18077	19	8.8	5	13	1	0.4008	no
gi|646734508|gb|KK963411.1|	82854-18412	19	8.8	2	0.784885	-1	0.4168	no
gi|646734508|gb|KK963411.1|	92009-19374	19	8.8	2	2	0.231553	0.88125	no
gi|646734508|gb|KK963411.1|	95353-19645	19	8.8	2	4	1	0.3021	no
gi|646734508|gb|KK963411.1|	00735-20206	19	8.8	3	7	1	0.497	no
gi|646734508|gb|KK963411.1|	05637-20663	19	8.8	0.748011	2	2	0.2385	no
gi|646734508|gb|KK963411.1|	73169-74070	19	8.8	0.85914	1	0.726182	0.6352	no
gi|646734508|gb|KK963411.1|	97561-97957	19	8.8	6	12	0.948476	0.5431	no
gi|646734527|gb|KK963409.1|	06988-10794	21	9.3	0.394749	1	2	0.2897	no
gi|646734527|gb|KK963409.1|	09752-11000	21	9.3	13	37	2	0.31615	no
gi|646734527|gb|KK963409.1|	60316-16140	21	9.3	2	5	1	0.3297	no

gi|646734527|gb|KK963409.1|	61819-16244	21	9.3	16	6	-1	0.3549	no
gi|646734527|gb|KK963409.1|	63740-16397	21	9.3	28	37	0.404096	0.78825	no
gi|646734527|gb|KK963409.1|	66054-16662	21	9.3	90	71	-0.331155	0.75655	no
gi|646734527|gb|KK963409.1|	14554-21621	21	9.3	1	3	1	0.4012	no
gi|646734527|gb|KK963409.1|	28277-22897	21	9.3	2	4	0.860657	0.5035	no
gi|646734527|gb|KK963409.1|	25990-27484	21	9.3	0.672005	0.215168	-2	1	no
gi|646734527|gb|KK963409.1|	28117-29343	21	9.3	2	2	0.132898	0.91415	no
gi|646734527|gb|KK963409.1|	29705-30189	21	9.3	4	4	-0.0923823	0.9348	no
gi|646734527|gb|KK963409.1|	01909-30261	21	9.3	29	32	0.134688	0.92915	no
gi|646734527|gb|KK963409.1|	02772-30337	21	9.3	3	6	1	0.45415	no
gi|646734527|gb|KK963409.1|	30334-30883	21	9.3	14	16	0.133936	0.9321	no
gi|646734527|gb|KK963409.1|	04220-30460	21	9.3	85	23	-2	0.2278	no
gi|646734527|gb|KK963409.1|	12156-31365	21	9.3	66	29	-1	0.2941	no
gi|646734527|gb|KK963409.1|	17707-31817	21	9.3	77	24	-2	0.278	no
gi|646734527|gb|KK963409.1|	18967-31934	21	9.3	12	15	0.345996	0.8023	no
gi|646734527|gb|KK963409.1|	38220-39138	21	9.3	5	3	-0.632484	0.7019	no
gi|646734527|gb|KK963409.1|	45959-49914	21	9.3	24	20	-0.226701	0.8455	no
gi|646734527|gb|KK963409.1|	50365-50719	21	9.3	39	14	-2	0.2768	no
gi|646734527|gb|KK963409.1|	67170-67436	21	9.3	0	27	inf	0.0014	yes
gi|646734527|gb|KK963409.1|	7119-7944	21	9.3	25	17	-0.530783	0.7267	no
gi|646734527|gb|KK963409.1|	75783-77861	21	9.3	0.253749	5	4	0.06275	no
gi|646734537|gb|KK963408.1|	70248-71198	12	6	0.665486	1	0.770207	0.6143	no
gi|646734551|gb|KK963406.1|	17303-18010	23	6.4	44	29	-0.613452	0.61655	no
gi|646734551|gb|KK963406.1|	76894-17825	23	6.4	1	3	1	0.32315	no
gi|646734551|gb|KK963406.1|	19152-19815	23	6.4	1	2	0.961298	0.5626	no
gi|646734551|gb|KK963406.1|	03899-20431	23	6.4	6	10	0.724281	0.57645	no
gi|646734551|gb|KK963406.1|	21363-21822	23	6.4	26	30	0.19883	0.89065	no
gi|646734551|gb|KK963406.1|	29110-22967	23	6.4	33	16	-1	0.501	no
gi|646734551|gb|KK963406.1|	46395-25005	23	6.4	2	2	0.341515	0.8178	no
gi|646734551|gb|KK963406.1|	54383-25555	23	6.4	0.709262	0.435237	-0.704518	1	no

gi|646734551|gb|KK963406.1|	58461-26044	23	6.4	1	1	0.162119	0.92085	no
gi|646734551|gb|KK963406.1|	26742-27251	23	6.4	6	2	-2	0.3025	no
gi|646734551|gb|KK963406.1|	83525-28619	23	6.4	0.53701	0.390215	-0.460681	1	no
gi|646734551|gb|KK963406.1|	94195-29478	23	6.4	2	3	0.460484	0.73365	no
gi|646734551|gb|KK963406.1|	97043-29833	23	6.4	1	2	0.521975	0.67535	no
gi|646734551|gb|KK963406.1|	98821-29962	23	6.4	9	2	-2	0.14955	no
gi|646734551|gb|KK963406.1|	02281-30419	23	6.4	50	24	-1	0.3346	no
gi|646734551|gb|KK963406.1|	04293-30864	23	6.4	4	1	-2	0.313	no
gi|646734551|gb|KK963406.1|	10788-31351	23	6.4	3	0.816388	-2	0.2782	no
gi|646734551|gb|KK963406.1|	31227-32147	23	6.4	3	3	0.242125	0.86515	no
gi|646734551|gb|KK963406.1|	13638-31539	23	6.4	3	0.445113	-3	0.11905	no
gi|646734551|gb|KK963406.1|	34-508	23	6.4	7	8	0.324084	0.81455	no
gi|646734551|gb|KK963406.1|	44424-44876	23	6.4	2	9	2	0.28235	no
gi|646734551|gb|KK963406.1|	49789-51612	23	6.4	8	10	0.30986	0.83635	no
gi|646734551|gb|KK963406.1|	51759-52622	23	6.4	16	9	-0.787183	0.60925	no
gi|646734551|gb|KK963406.1|	58906-61762	23	6.4	23	15	-0.635738	0.56235	no
gi|646734551|gb|KK963406.1|	72716-74063	23	6.4	38	17	-1	0.2975	no
gi|646734551|gb|KK963406.1|	7964-8526	23	6.4	3	3	0.089551	0.93525	no
gi|646734595|gb|KK963401.1|	37682-13797	16	5.9	12	5	-1	0.4638	no
gi|646734603|gb|KK963400.1|	15333-16122	19	9.6	76	85	0.155353	0.89095	no
gi|646734603|gb|KK963400.1|	30725-31407	19	9.6	6	8	0.379682	0.80705	no
gi|646734603|gb|KK963400.1|	34750-34912	19	9.6	160	222	0.474341	0.7503	no
gi|646734603|gb|KK963400.1|	41960-42899	19	9.6	36	49	0.445291	0.6833	no
gi|646734603|gb|KK963400.1|	48579-54481	19	9.6	36	39	0.0819249	0.94835	no
gi|646734603|gb|KK963400.1|	55326-57385	19	9.6	0.71798	2	2	0.28905	no
gi|646734603|gb|KK963400.1|	61077-65026	19	9.6	120	77	-0.632761	0.5985	no
gi|646734653|gb|KK963394.1|	00348-10480	20	9.1	4	2	-0.695267	0.65055	no
gi|646734653|gb|KK963394.1|	04910-10683	20	9.1	45	22	-0.997252	0.38045	no
gi|646734653|gb|KK963394.1|	27253-13095	20	9.1	3	3	-0.173803	0.90605	no
gi|646734653|gb|KK963394.1|	31803-13571	20	9.1	2	1	-0.620571	0.686	no

gi|646734653|gb|KK963394.1|	36058-13717	20	9.1	4	3	-0.443635	0.7789	no
gi|646734653|gb|KK963394.1|	48000-14833	20	9.1	137	62	-1	0.44785	no
gi|646734653|gb|KK963394.1|	56729-15819	20	9.1	38	41	0.10259	0.925	no
gi|646734653|gb|KK963394.1|	58322-15985	20	9.1	3	10	1	0.34355	no
gi|646734653|gb|KK963394.1|	61232-16141	20	9.1	39	136	2	0.24975	no
gi|646734653|gb|KK963394.1|	62476-16418	20	9.1	2	4	0.816106	0.5997	no
gi|646734653|gb|KK963394.1|	66069-16830	20	9.1	1	2	0.337166	0.8283	no
gi|646734653|gb|KK963394.1|	68615-16994	20	9.1	1	1	-0.0618636	0.9541	no
gi|646734653|gb|KK963394.1|	71039-17253	20	9.1	1	1	0.0414666	1	no
gi|646734653|gb|KK963394.1|	73062-17506	20	9.1	0.954873	2	0.68629	0.62135	no
gi|646734653|gb|KK963394.1|	76518-17688	20	9.1	5	16	2	0.36015	no
gi|646734653|gb|KK963394.1|	78551-18129	20	9.1	2	2	0.347091	0.81495	no
gi|646734653|gb|KK963394.1|	18538-19279	20	9.1	2	3	0.751062	0.56	no
gi|646734653|gb|KK963394.1|	89231-19052	20	9.1	2	3	0.756528	0.58795	no
gi|646734653|gb|KK963394.1|	91320-19207	20	9.1	2	0.780771	-1	0.427	no
gi|646734653|gb|KK963394.1|	93241-19626	20	9.1	2	2	0.297091	0.84785	no
gi|646734653|gb|KK963394.1|	98893-19998	20	9.1	6	9	0.488773	0.7471	no
gi|646734653|gb|KK963394.1|	28555-22887	20	9.1	11	4	-1	0.39645	no
gi|646734653|gb|KK963394.1|	33832-23491	20	9.1	27	20	-0.444995	0.72155	no
gi|646734653|gb|KK963394.1|	39905-24080	20	9.1	3	0.204256	-4	0.17395	no
gi|646734653|gb|KK963394.1|	44589-24971	20	9.1	15	12	-0.269895	0.8202	no
gi|646734653|gb|KK963394.1|	52605-26253	20	9.1	25	14	-0.821338	0.4902	no
gi|646734653|gb|KK963394.1|	74740-27929	20	9.1	3	3	-0.310503	0.8341	no
gi|646734653|gb|KK963394.1|	80826-28348	20	9.1	4	3	-0.342872	0.82145	no
gi|646734653|gb|KK963394.1|	86110-28981	20	9.1	4	4	0.0287084	0.98355	no
gi|646734653|gb|KK963394.1|	92654-29338	20	9.1	2	1	-0.514007	0.7384	no
gi|646734653|gb|KK963394.1|	98249-29874	20	9.1	3	8	2	0.3606	no
gi|646734653|gb|KK963394.1|	99345-30064	20	9.1	2	3	0.827061	0.5526	no
gi|646734653|gb|KK963394.1|	11919-31215	20	9.1	37	19	-0.939354	0.53935	no
gi|646734653|gb|KK963394.1|	23245-32385	20	9.1	4	4	0.0505563	0.95685	no

gi|646734653|gb|KK963394.1|	24242-32444	20	9.1	69	64	-0.111366	0.933	no
gi|646734653|gb|KK963394.1|	38254-34025	20	9.1	0.530485	0.230479	-1	1	no
gi|646734653|gb|KK963394.1|	45578-34635	20	9.1	8	12	0.553493	0.71245	no
gi|646734653|gb|KK963394.1|	73880-74371	20	9.1	0.388147	6	4	0.0994	no
gi|646734653|gb|KK963394.1|	82383-82648	20	9.1	0	27	inf	0.0014	yes
gi|646734653|gb|KK963394.1|	92174-92813	20	9.1	0.481212	10	4	0.24135	no
gi|646734659|gb|KK963393.1|	14031-14524	23	10	9	11	0.233774	0.88675	no
gi|646734659|gb|KK963393.1|	70756-17098	23	10	151	52	-2	0.27715	no
gi|646734659|gb|KK963393.1|	44409-24463	23	10	29	24	-0.258748	0.8749	no
gi|646734659|gb|KK963393.1|	69972-70266	23	10	68	72	0.0891743	0.95275	no
gi|646734659|gb|KK963393.1|	70788-71998	23	10	2	0.277689	-3	0.1506	no
gi|646734659|gb|KK963393.1|	78201-78861	23	10	223	224	0.00458212	0.99665	no
gi|646734668|gb|KK963392.1|	10638-11148	18	10	1	0.444653	-2	0.38425	no
gi|646734668|gb|KK963392.1|	13360-11384	18	10	2	4	1	0.44135	no
gi|646734668|gb|KK963392.1|	86250-18744	18	10	3	0.990148	-1	0.35075	no
gi|646734668|gb|KK963392.1|	02946-20520	18	10	0.692844	0.335009	-1	1	no
gi|646734668|gb|KK963392.1|	05724-20703	18	10	0.349756	0.753646	1	1	no
gi|646734668|gb|KK963392.1|	08286-20932	18	10	0.710666	1	0.512098	1	no
gi|646734668|gb|KK963392.1|	35889-23672	18	10	2	0	-inf	0.0035	no
gi|646734668|gb|KK963392.1|	55908-25679	18	10	0.730649	1	0.502806	1	no
gi|646734668|gb|KK963392.1|	41898-42319	18	10	6	0.706589	-3	0.1891	no
gi|646734668|gb|KK963392.1|	92645-93168	18	10	4	2	-1	0.436	no
gi|646734668|gb|KK963392.1|	94715-95306	18	10	2	2	-0.347129	0.81865	no
gi|646734697|gb|KK963389.1|	29514-12998	16	7.6	8	8	-0.0238971	0.9646	no
gi|646734707|gb|KK963388.1|	15220-11572	14	6	4	4	0.0166571	0.9493	no
gi|646734707|gb|KK963388.1|	41630-14323	14	6	2	2	0.713359	0.6583	no
gi|646734720|gb|KK963386.1|	23600-24329	9.8	9.2	0	5	inf	0.00435	no
gi|646734730|gb|KK963385.1|	62629-63736	11	7.2	3	1	-1	0.3226	no
gi|646734741|gb|KK963384.1|	75627-17599	19	8.5	2	9	2	0.24905	no
gi|646734741|gb|KK963384.1|	)8998-10105[illegible]	19	8.5	0.615411	4	3	0.08155	no

gi|646734749|gb|KK963383.1|	00450-10069	15	5.8	3	40	4	0.10085	no
gi|646734749|gb|KK963383.1|	06404-10667	15	5.8	14	25	0.823722	0.54815	no
gi|646734749|gb|KK963383.1|	78826-80237	15	5.8	6	5	-0.282263	0.84995	no
gi|646734749|gb|KK963383.1|	82368-83656	15	5.8	2	0	-inf	0.00315	no
gi|646734749|gb|KK963383.1|	83873-84876	15	5.8	2	0.176152	-4	0.17105	no
gi|646734749|gb|KK963383.1|	85626-86281	15	5.8	4	2	-1	0.42505	no
gi|646734749|gb|KK963383.1|	94359-95000	15	5.8	5	2	-0.968934	0.46725	no
gi|646734790|gb|KK963379.1|	83039-84514	13	8.6	0	12	inf	5.00E-05	yes
gi|646734798|gb|KK963378.1|	34329-34522	13	8.3	80	36	-1	0.4584	no
gi|646734819|gb|KK963375.1|	23528-24086	11	6	0	6	inf	0.00035	yes
gi|646734827|gb|KK963374.1|	95530-19760	12	5.2	1	1	-0.0213677	0.97615	no
gi|646734827|gb|KK963374.1|	53486-53954	12	5.2	2	5	1	0.45755	no
gi|646734867|gb|KK963369.1|	17551-11912	11	5.8	0	4	inf	0.00045	yes
gi|646734867|gb|KK963369.1|	20848-12160	11	5.8	26	5	-2	0.17115	no
gi|646734867|gb|KK963369.1|	47353-48369	11	5.8	2	1	-0.604427	0.64385	no
gi|646734874|gb|KK963368.1|	1124-2888	21	8	2	2	-0.00458744	0.9797	no
gi|646734874|gb|KK963368.1|	15661-15932	21	8	77	7	-3	0.09985	no
gi|646734874|gb|KK963368.1|	21842-22273	21	8	7	7	0.0434517	0.95385	no
gi|646734874|gb|KK963368.1|	23293-23907	21	8	2	2	0.465824	0.7494	no
gi|646734874|gb|KK963368.1|	25254-28909	21	8	2	2	-0.0179672	0.98985	no
gi|646734874|gb|KK963368.1|	29892-33208	21	8	37	38	0.0552515	0.96245	no
gi|646734874|gb|KK963368.1|	5745-7172	21	8	0.473611	1	1	0.3963	no
gi|646734874|gb|KK963368.1|	7605-8842	21	8	0.56523	1	0.93532	1	no
gi|646734882|gb|KK963367.1|	25589-12714	13	6.8	0.854627	1	0.402557	0.7584	no
gi|646734882|gb|KK963367.1|	28944-12942	13	6.8	9	4	-1	0.4804	no
gi|646734882|gb|KK963367.1|	35657-13633	13	6.8	2	0.915596	-0.74403	0.6333	no
gi|646734882|gb|KK963367.1|	87812-18882	13	6.8	5	4	-0.21375	0.8911	no
gi|646734914|gb|KK963363.1|	75076-17722	16	8.3	224	9	-5	0.00505	no
gi|646734914|gb|KK963363.1|	84290-18516	16	8.3	0.743567	2	1	0.43255	no
gi|646734922|gb|KK963362.1|	78062-78305	14	6.1	44	7	-3	0.13935	no

gi|646734960|gb|KK963358.1|	17032-21749	19	13	6	1	-2	0.15695	no
gi|646734960|gb|KK963358.1|	25065-25255	19	13	6252.08	5781.15	-0.112979	0.9226	no
gi|646734986|gb|KK963355.1|	8748-9299	13	6.4	3	1	-1	0.4702	no
gi|646735008|gb|KK963353.1|	16592-21759	21	9.4	0.994667	0.708285	-0.489884	1	no
gi|646735008|gb|KK963353.1|	42405-24288	21	9.4	21	9	-1	0.46115	no
gi|646735008|gb|KK963353.1|	46462-24674	21	9.4	42	23	-0.873108	0.5413	no
gi|646735008|gb|KK963353.1|	89117-29243	21	9.4	3	3	-0.258112	0.86325	no
gi|646735008|gb|KK963353.1|	04815-30749	21	9.4	1	0.777873	-0.697677	0.61635	no
gi|646735008|gb|KK963353.1|	09216-31685	21	9.4	13	6	-1	0.30325	no
gi|646735008|gb|KK963353.1|	21529-32170	21	9.4	802	282	-2	0.3643	no
gi|646735008|gb|KK963353.1|	24134-32432	21	9.4	80	51	-0.652481	0.66735	no
gi|646735008|gb|KK963353.1|	35546-35956	21	9.4	16	15	-0.0574133	0.97165	no
gi|646735017|gb|KK963352.1|	02013-20223	22	5.9	18	42	1	0.4452	no
gi|646735017|gb|KK963352.1|	14359-21461	22	5.9	18	8	-1	0.487	no
gi|646735017|gb|KK963352.1|	30506-23165	22	5.9	1	0.147765	-3	1	no
gi|646735017|gb|KK963352.1|	33333-23417	22	5.9	2	0.890766	-1	0.3737	no
gi|646735017|gb|KK963352.1|	38128-24321	22	5.9	3	1	-0.770549	0.6039	no
gi|646735017|gb|KK963352.1|	44387-24731	22	5.9	48	17	-2	0.19275	no
gi|646735017|gb|KK963352.1|	49663-25406	22	5.9	10	11	0.154333	0.88995	no
gi|646735017|gb|KK963352.1|	54175-25540	22	5.9	8	8	0.0130938	0.9924	no
gi|646735017|gb|KK963352.1|	23472-32425	22	5.9	2	0.246114	-3	0.2033	no
gi|646735026|gb|KK963351.1|	07367-11701	21	8.9	13	14	0.0897028	0.9352	no
gi|646735026|gb|KK963351.1|	11216-12142	21	8.9	16	25	0.682454	0.655	no
gi|646735026|gb|KK963351.1|	16702-17028	21	8.9	20	40	0.985756	0.47995	no
gi|646735026|gb|KK963351.1|	18413-20210	21	8.9	33	37	0.16257	0.8878	no
gi|646735026|gb|KK963351.1|	31611-32873	21	8.9	0.5512	2	2	0.286	no
gi|646735026|gb|KK963351.1|	36581-36876	21	8.9	3	23	3	0.2488	no
gi|646735026|gb|KK963351.1|	70294-72306	21	8.9	7	7	0.00390666	0.99465	no
gi|646735026|gb|KK963351.1|	87262-90936	21	8.9	10	9	-0.161793	0.8828	no
gi|646735026|gb|KK963351.1|	)5067-10255(	21	8.9	5	5	0.243261	0.82205	no

gi|646735048|gb|KK963348.1|	90705-29106	12	8.3	8	9	0.17268	0.89755	no
gi|646735055|gb|KK963347.1|	02572-10281	21	9	126	48	-1	0.3959	no
gi|646735055|gb|KK963347.1|	09533-11070	21	9	13	3	-2	0.2259	no
gi|646735055|gb|KK963347.1|	10951-11305	21	9	2	2	-0.106256	0.9467	no
gi|646735055|gb|KK963347.1|	14047-11468	21	9	3	6	1	0.43765	no
gi|646735055|gb|KK963347.1|	15311-11646	21	9	27	19	-0.494042	0.69485	no
gi|646735055|gb|KK963347.1|	23162-24266	21	9	1	0.467204	-1	0.38955	no
gi|646735055|gb|KK963347.1|	35136-35979	21	9	2	0.66698	-2	0.2463	no
gi|646735055|gb|KK963347.1|	36116-37404	21	9	1	0.128583	-3	0.1757	no
gi|646735055|gb|KK963347.1|	70190-70431	21	9	42	62	0.551783	0.70385	no
gi|646735055|gb|KK963347.1|	92304-92479	21	9	259	185	-0.489424	0.6824	no
gi|646735055|gb|KK963347.1|	93720-94455	21	9	0.770694	5	3	0.20395	no
gi|646735055|gb|KK963347.1|	94972-96176	21	9	1	2	0.519174	0.6803	no
gi|646735055|gb|KK963347.1|	)8760-10023‹	21	9	1	3	1	0.3235	no
gi|646735119|gb|KK963337.1|	05215-10701	8.3	5	11	8	-0.524302	0.7361	no
gi|646735119|gb|KK963337.1|	10864-11195	8.3	5	35	12	-2	0.2341	no
gi|646735119|gb|KK963337.1|	13662-11596	8.3	5	9	6	-0.53269	0.72075	no
gi|646735119|gb|KK963337.1|	49716-49934	8.3	5	130	41	-2	0.2803	no
gi|646735119|gb|KK963337.1|	63025-63872	8.3	5	11	2	-3	0.08075	no
gi|646735119|gb|KK963337.1|	75973-76222	8.3	5	23	21	-0.0835494	0.93275	no
gi|646735125|gb|KK963336.1|	30413-31000	17	8.1	4	5	0.159622	0.90035	no
gi|646735125|gb|KK963336.1|	33001-33599	17	8.1	23	27	0.242435	0.87045	no
gi|646735125|gb|KK963336.1|	45064-45753	17	8.1	2	3	0.32838	0.7982	no
gi|646735162|gb|KK963332.1|	72975-17377	18	8.3	2	1	-0.767995	0.59035	no
gi|646735162|gb|KK963332.1|	77124-17863	18	8.3	0.367406	0.847237	1	1	no
gi|646735162|gb|KK963332.1|	18041-20731	18	8.3	27	15	-0.855188	0.4471	no
gi|646735162|gb|KK963332.1|	81004-18434	18	8.3	0.480314	0.699422	0.542183	1	no
gi|646735162|gb|KK963332.1|	84485-18648	18	8.3	0.796184	0.691827	-0.202689	1	no
gi|646735162|gb|KK963332.1|	87766-18898	18	8.3	1	0.548643	-1	0.41495	no
gi|646735162|gb|KK963332.1|	89094-18984	18	8.3	0.936111	2	0.97449	0.5626	no

gi|646735162|gb|KK963332.1|	03174-20468	18	8.3	2	2	0.0417307	0.97835	no
gi|646735162|gb|KK963332.1|	14193-21707	18	8.3	3	3	-0.0445613	0.97635	no
gi|646735162|gb|KK963332.1|	17322-22157	18	8.3	2	2	0.388438	0.7962	no
gi|646735162|gb|KK963332.1|	22198-22720	18	8.3	1	2	0.30663	0.84165	no
gi|646735162|gb|KK963332.1|	27455-22954	18	8.3	0.90555	1	0.535224	0.6999	no
gi|646735162|gb|KK963332.1|	29966-23148	18	8.3	1	0.741333	-0.665328	0.6175	no
gi|646735162|gb|KK963332.1|	31706-23417	18	8.3	3	2	-0.461851	0.7592	no
gi|646735162|gb|KK963332.1|	23866-24237	18	8.3	7	4	-0.83142	0.5825	no
gi|646735162|gb|KK963332.1|	29273-30132	18	8.3	2	4	0.587924	0.67255	no
gi|646735162|gb|KK963332.1|	61681-61916	18	8.3	37	65	0.826181	0.58555	no
gi|646735162|gb|KK963332.1|	7332-8780	18	8.3	59	23	-1	0.224	no
gi|646735162|gb|KK963332.1|	74345-75378	18	8.3	1	1	-0.362525	0.79735	no
gi|646735162|gb|KK963332.1|	98309-10000[illegible]	18	8.3	0.450349	0.557543	0.308041	1	no
gi|646735188|gb|KK963329.1|	7152-7540	11	4.9	51	6	-3	0.05745	no
gi|646735213|gb|KK963326.1|	18864-19105	12	6.1	23	14	-0.725057	0.6549	no
gi|646735219|gb|KK963325.1|	45647-46238	56	23	2	1	-0.762166	0.6333	no
gi|646735241|gb|KK963323.1|	49944-15076	14	6.1	0	12	inf	0.00025	yes
gi|646735241|gb|KK963323.1|	55073-15587	14	6.1	0.168175	31	8	0.0947	no
gi|646735241|gb|KK963323.1|	63019-16671	14	6.1	21	7	-2	0.15685	no
gi|646735241|gb|KK963323.1|	66963-16836	14	6.1	2	2	-0.0966101	0.936	no
gi|646735256|gb|KK963321.1|	1180-1742	18	7.7	27	26	-0.0624521	0.96645	no
gi|646735256|gb|KK963321.1|	19439-12047	18	7.7	0	21	inf	0.00015	yes
gi|646735256|gb|KK963321.1|	20226-20954	18	7.7	0.586558	2	2	0.20395	no
gi|646735256|gb|KK963321.1|	27294-28517	18	7.7	0.668971	3	2	0.17505	no
gi|646735256|gb|KK963321.1|	34208-34674	18	7.7	23	21	-0.172011	0.9089	no
gi|646735256|gb|KK963321.1|	40633-41050	18	7.7	0.553653	16	5	0.10415	no
gi|646735256|gb|KK963321.1|	52093-52367	18	7.7	16	180	4	0.0457	no
gi|646735256|gb|KK963321.1|	58850-60527	18	7.7	6	21	2	0.15555	no
gi|646735256|gb|KK963321.1|	87868-88286	18	7.7	0	29	inf	0.00075	yes
gi|646735256|gb|KK963321.1|	89195-89524	18	7.7	0	13	inf	0.00255	no

gi|646735256|gb|KK963321.1|	90573-90758	18	7.7	0	1076.61	inf	0.0002	yes
gi|646735269|gb|KK963320.1|	72236-17252	9.9	7.2	10	10	-0.0562689	0.91525	no
gi|646735297|gb|KK963317.1|	10810-11007	13	8.9	134	99	-0.43916	0.72035	no
gi|646735297|gb|KK963317.1|	92906-93399	13	8.9	5	0	-inf	0.0035	no
gi|646735305|gb|KK963316.1|	50831-51303	18	7.2	0	6	inf	0.0014	yes
gi|646735322|gb|KK963314.1|	16420-11707	13	7.4	2	3	0.625423	0.644	no
gi|646735322|gb|KK963314.1|	19165-11961	13	7.4	3	3	-0.406705	0.8098	no
gi|646735322|gb|KK963314.1|	60272-16097	13	7.4	0.820133	2	1	0.4862	no
gi|646735338|gb|KK963312.1|	09418-10971	21	10	350.69	901	1	0.2267	no
gi|646735338|gb|KK963312.1|	13602-11446	21	10	6	53	3	0.03715	no
gi|646735338|gb|KK963312.1|	80514-81424	21	10	8	24	2	0.3253	no
gi|646735338|gb|KK963312.1|	85053-85718	21	10	3	0.313622	-3	0.179	no
gi|646735338|gb|KK963312.1|	90882-91550	21	10	0.671457	8	4	0.13005	no
gi|646735338|gb|KK963312.1|	92147-94788	21	10	0.504085	9	4	0.009	no
gi|646735338|gb|KK963312.1|	)5235-10154(	21	10	4	13	2	0.14845	no
gi|646735358|gb|KK963310.1|	31774-32313	12	7.7	13	0	-inf	0.0018	no
gi|646735358|gb|KK963310.1|	3288-3787	12	7.7	3	2	-0.985931	0.589	no
gi|646735368|gb|KK963309.1|	22883-32302	14	5.3	671	636	-0.0763514	0.93935	no
gi|646735403|gb|KK963305.1|	155-889	24	18	5	10	0.849664	0.59195	no
gi|646735403|gb|KK963305.1|	7535-7760	24	18	22	23	0.0213104	0.90935	no
gi|646735411|gb|KK963304.1|	00761-10109	19	10	1	67	6	0.096	no
gi|646735411|gb|KK963304.1|	16750-11720	19	10	0.454879	62	7	0.09475	no
gi|646735411|gb|KK963304.1|	28208-13111	19	10	0.174434	16	7	0.05805	no
gi|646735411|gb|KK963304.1|	82841-18356	19	10	0.594078	49	6	0.1049	no
gi|646735411|gb|KK963304.1|	86352-18668	19	10	0	53	inf	0.0002	yes
gi|646735411|gb|KK963304.1|	06326-20827	19	10	1	8	3	0.0569	no
gi|646735411|gb|KK963304.1|	09621-21008	19	10	3	21	3	0.0977	no
gi|646735411|gb|KK963304.1|	18606-21988	19	10	0.816264	8	3	0.05055	no
gi|646735411|gb|KK963304.1|	24118-22448	19	10	5	47	3	0.07105	no
gi|646735411|gb|KK963304.1|	23381-23703	19	10	0	45	inf	0.0007	yes

gi|646735411|gb|KK963304.1|	24518-25332	19	10	0.496405	50	7	0.1049	no
gi|646735411|gb|KK963304.1|	44012-44192	19	10	0	158	inf	0.001	yes
gi|646735411|gb|KK963304.1|	48539-48779	19	10	0	196.84	inf	0.0003	yes
gi|646735411|gb|KK963304.1|	66623-66962	19	10	0	94	inf	0.0001	yes
gi|646735411|gb|KK963304.1|	70058-71846	19	10	0	23	inf	5.00E-05	yes
gi|646735411|gb|KK963304.1|	72677-73222	19	10	0	26	inf	5.00E-05	yes
gi|646735411|gb|KK963304.1|	86848-87580	19	10	0	11	inf	0.00075	yes
gi|646735411|gb|KK963304.1|	96530-96767	19	10	0	723	inf	5.00E-05	yes
gi|646735442|gb|KK963300.1|	07209-10760	13	15	1	8	3	0.26455	no
gi|646735451|gb|KK963299.1|	09524-11000	15	10	1	8	3	0.13615	no
gi|646735451|gb|KK963299.1|	32577-13364	15	10	0	4	inf	0.0017	no
gi|646735451|gb|KK963299.1|	46125-14720	15	10	0	2	inf	0.00255	no
gi|646735459|gb|KK963298.1|	26031-22807	14	9.2	1	1	-0.202262	0.9019	no
gi|646735459|gb|KK963298.1|	28194-22989	14	9.2	3	1	-1	0.3475	no
gi|646735459|gb|KK963298.1|	36609-23732	14	9.2	2	1	-0.515291	0.73195	no
gi|646735494|gb|KK963294.1|	06137-10713	11	6.8	0.744999	1	0.510172	1	no
gi|646735494|gb|KK963294.1|	27823-12868	11	6.8	2	1	-0.63679	0.65905	no
gi|646735494|gb|KK963294.1|	79970-81039	11	6.8	2	1	-0.123715	0.9312	no
gi|646735494|gb|KK963294.1|	84655-85501	11	6.8	5	3	-0.593773	0.71105	no
gi|646735494|gb|KK963294.1|	86300-86779	11	6.8	3	4	0.612378	0.6562	no
gi|646735494|gb|KK963294.1|	97513-97879	11	6.8	15	4	-2	0.19525	no
gi|646735523|gb|KK963290.1|	40828-14184	12	6.5	0	2	inf	0.00255	no
gi|646735551|gb|KK963287.1|	60700-61170	40	17	8	6	-0.432896	0.72985	no
gi|646735567|gb|KK963285.1|	23230-12515	20	8.9	15	13	-0.121754	0.9099	no
gi|646735567|gb|KK963285.1|	12836-13105	20	8.9	10	16	0.651978	0.66495	no
gi|646735567|gb|KK963285.1|	39771-14091	20	8.9	13	8	-0.766556	0.6078	no
gi|646735567|gb|KK963285.1|	44348-14513	20	8.9	2	0.988036	-0.676926	0.6592	no
gi|646735567|gb|KK963285.1|	48839-14952	20	8.9	31	29	-0.120515	0.93865	no
gi|646735567|gb|KK963285.1|	51225-15157	20	8.9	27	18	-0.595265	0.70775	no
gi|646735567|gb|KK963285.1|	56892-15738	20	8.9	4	3	-0.310547	0.8287	no

gi|646735567|gb|KK963285.1|	92942-19328	20	8.9	6	13	1	0.4259	no
gi|646735567|gb|KK963285.1|	95567-19579	20	8.9	16	24	0.634262	0.67685	no
gi|646735567|gb|KK963285.1|	18679-22082	20	8.9	4	3	-0.292536	0.8458	no
gi|646735567|gb|KK963285.1|	33054-23752	20	8.9	14	4	-2	0.1519	no
gi|646735567|gb|KK963285.1|	38052-23899	20	8.9	2	2	-0.203514	0.8674	no
gi|646735567|gb|KK963285.1|	42329-24256	20	8.9	40	8	-2	0.17695	no
gi|646735567|gb|KK963285.1|	268-763	20	8.9	4	4	-0.0321056	0.94695	no
gi|646735567|gb|KK963285.1|	67344-67579	20	8.9	51	19	-1	0.31225	no
gi|646735567|gb|KK963285.1|	69478-70616	20	8.9	0.733355	0.749431	0.0312844	1	no
gi|646735567|gb|KK963285.1|	86765-87137	20	8.9	18	23	0.3396	0.8269	no
gi|646735567|gb|KK963285.1|	89719-90878	20	8.9	13	13	0.0551902	0.9719	no
gi|646735567|gb|KK963285.1|	98272-105357	20	8.9	7	7	-0.095657	0.93365	no
gi|646735598|gb|KK963281.1|	32210-13286	9.9	6.9	5	4	-0.333357	0.7791	no
gi|646735606|gb|KK963280.1|	11254-21175	11	4.9	27	13	-1	0.46485	no
gi|646735615|gb|KK963279.1|	40225-41631	12	8	7	2	-2	0.2155	no
gi|646735615|gb|KK963279.1|	44863-45639	12	8	32	10	-2	0.2904	no
gi|646735615|gb|KK963279.1|	51054-51696	12	8	4	2	-0.850088	0.52155	no
gi|646735640|gb|KK963276.1|	42279-14373	18	6.9	0.772868	0.222579	-2	1	no
gi|646735640|gb|KK963276.1|	14650-15005	18	6.9	525	180	-2	0.17135	no
gi|646735640|gb|KK963276.1|	85538-18834	18	6.9	13	41	2	0.143	no
gi|646735640|gb|KK963276.1|	20181-20407	18	6.9	61	36	-0.781906	0.5411	no
gi|646735640|gb|KK963276.1|	30407-30922	18	6.9	4	0.955771	-2	0.20435	no
gi|646735640|gb|KK963276.1|	48662-50123	18	6.9	0.536971	0.773318	0.526219	1	no
gi|646735640|gb|KK963276.1|	51220-51556	18	6.9	46	37	-0.306039	0.84435	no
gi|646735640|gb|KK963276.1|	57793-60155	18	6.9	76	37	-1	0.38	no
gi|646735649|gb|KK963275.1|	10567-10830	16	7.9	36	18	-1	0.42505	no
gi|646735649|gb|KK963275.1|	11362-12874	16	7.9	11	10	-0.126165	0.93305	no
gi|646735666|gb|KK963273.1|	15316-16460	12	5.5	0.936522	0.297799	-2	1	no
gi|646735715|gb|KK963267.1|	13993-11442	25	10	4	3	-0.607242	0.6685	no
gi|646735715|gb|KK963267.1|	86333-18746	25	10	1	0.451654	-1	1	no

gi|646735715|gb|KK963267.1|	21543-22299	25	10	3	5	0.659866	0.64735	no
gi|646735715|gb|KK963267.1|	35027-23581	25	10	1	1	-0.184086	0.90405	no
gi|646735715|gb|KK963267.1|	44878-24558	25	10	2	2	0.31345	0.8299	no
gi|646735715|gb|KK963267.1|	52835-25312	25	10	10	8	-0.376038	0.8118	no
gi|646735715|gb|KK963267.1|	34688-35387	25	10	3	1	-2	0.30475	no
gi|646735715|gb|KK963267.1|	68956-69420	25	10	10	2	-2	0.19495	no
gi|646735715|gb|KK963267.1|	75432-75641	25	10	104	50	-1	0.41785	no
gi|646735722|gb|KK963266.1|	03744-10497	17	7.6	0.569872	0.680985	0.256986	1	no
gi|646735722|gb|KK963266.1|	10353-11150	17	7.6	0.412981	1	2	0.36595	no
gi|646735722|gb|KK963266.1|	26853-12715	17	7.6	28	30	0.100091	0.9387	no
gi|646735722|gb|KK963266.1|	28968-12940	17	7.6	6	10	0.811166	0.5764	no
gi|646735722|gb|KK963266.1|	29789-13011	17	7.6	7	5	-0.523304	0.7398	no
gi|646735722|gb|KK963266.1|	37067-13903	17	7.6	0.32424	14	5	0.0463	no
gi|646735722|gb|KK963266.1|	41160-14133	17	7.6	45	182	2	0.31375	no
gi|646735722|gb|KK963266.1|	41550-14202	17	7.6	14	6	-1	0.39305	no
gi|646735722|gb|KK963266.1|	53986-15451	17	7.6	9	10	0.0771013	0.96235	no
gi|646735722|gb|KK963266.1|	55286-15602	17	7.6	0.953454	2	1	0.36955	no
gi|646735722|gb|KK963266.1|	61621-16253	17	7.6	9	5	-0.671401	0.6608	no
gi|646735722|gb|KK963266.1|	71391-17159	17	7.6	27	77	2	0.33885	no
gi|646735722|gb|KK963266.1|	72391-17327	17	7.6	5	9	0.759068	0.6245	no
gi|646735722|gb|KK963266.1|	79857-18016	17	7.6	13	50	2	0.2088	no
gi|646735722|gb|KK963266.1|	80423-18071	17	7.6	8	21	1	0.3252	no
gi|646735722|gb|KK963266.1|	91514-19194	17	7.6	3	8	1	0.33355	no
gi|646735722|gb|KK963266.1|	22368-24081	17	7.6	37	10	-2	0.14815	no
gi|646735722|gb|KK963266.1|	55667-58304	17	7.6	6	4	-0.576324	0.7038	no
gi|646735722|gb|KK963266.1|	58990-61250	17	7.6	9	6	-0.425679	0.778	no
gi|646735722|gb|KK963266.1|	62019-63515	17	7.6	15	12	-0.313737	0.84045	no
gi|646735722|gb|KK963266.1|	65661-66046	17	7.6	11	27	1	0.36075	no
gi|646735722|gb|KK963266.1|	74746-75041	17	7.6	115	31	-2	0.2533	no
gi|646735722|gb|KK963266.1|	75803-76323	17	7.6	24	10	-1	0.41675	no

gi|646735722|gb|KK963266.1|	77294-78010	17	7.6	0.601792	3	2	0.1546	no
gi|646735722|gb|KK963266.1|	79109-79352	17	7.6	25	13	-0.910778	0.5688	no
gi|646735722|gb|KK963266.1|	84535-84829	17	7.6	27	47	0.808438	0.5507	no
gi|646735722|gb|KK963266.1|	86205-86777	17	7.6	3	9	2	0.3151	no
gi|646735722|gb|KK963266.1|	87315-87536	17	7.6	39	54	0.463718	0.7266	no
gi|646735722|gb|KK963266.1|	93422-93861	17	7.6	28	34	0.317354	0.8309	no
gi|646735722|gb|KK963266.1|	94783-95080	17	7.6	6	12	1	0.4822	no
gi|646735722|gb|KK963266.1|	96283-97211	17	7.6	0.962128	0.780602	-0.301641	1	no
gi|646735722|gb|KK963266.1|	)9697-10040[illegible]	17	7.6	5	3	-0.779209	0.587	no
gi|646735738|gb|KK963264.1|	73066-17344	26	5.7	0	11	inf	0.0008	yes
gi|646735738|gb|KK963264.1|	92466-29291	26	5.7	0	38	inf	0.0001	yes
gi|646735753|gb|KK963262.1|	16562-11676	19	7.6	74	92	0.315173	0.8153	no
gi|646735753|gb|KK963262.1|	24010-12515	19	7.6	0.312174	1	2	0.25365	no
gi|646735753|gb|KK963262.1|	26283-12729	19	7.6	1	1	-0.225969	0.86285	no
gi|646735753|gb|KK963262.1|	28290-12962	19	7.6	0.597395	1	1	0.39575	no
gi|646735753|gb|KK963262.1|	31194-13208	19	7.6	1	2	0.655116	0.62775	no
gi|646735753|gb|KK963262.1|	34012-13419	19	7.6	50	98	0.978029	0.5813	no
gi|646735753|gb|KK963262.1|	70012-17052	19	7.6	4	9	1	0.46105	no
gi|646735753|gb|KK963262.1|	81780-18420	19	7.6	0.979256	2	0.821665	0.6124	no
gi|646735753|gb|KK963262.1|	88659-18954	19	7.6	15	16	0.0500609	0.97295	no
gi|646735753|gb|KK963262.1|	89873-19151	19	7.6	1	0.965605	-0.318436	0.807	no
gi|646735753|gb|KK963262.1|	96511-19709	19	7.6	3	0.383093	-3	0.19565	no
gi|646735753|gb|KK963262.1|	00113-20400	19	7.6	8	4	-0.963196	0.4573	no
gi|646735753|gb|KK963262.1|	08640-21021	19	7.6	44	3	-4	0.01475	no
gi|646735753|gb|KK963262.1|	12502-21520	19	7.6	40	2	-4	0.01005	no
gi|646735753|gb|KK963262.1|	51509-51722	19	7.6	88	98	0.14834	0.91945	no
gi|646735784|gb|KK963258.1|	28942-12953	17	9	4	3	-0.639052	0.62635	no
gi|646735784|gb|KK963258.1|	59367-16005	17	9	10	13	0.413798	0.78985	no
gi|646735803|gb|KK963256.1|	05526-10971	25	8.8	90	69	-0.386693	0.74465	no
gi|646735803|gb|KK963256.1|	19259-12057	25	8.8	0.435549	0.876011	1	1	no

gi|646735803|gb|KK963256.1|	20737-12166	25	8.8	1	0.785118	-0.81649	0.57835	no
gi|646735803|gb|KK963256.1|	21803-12208	25	8.8	90	317	2	0.16795	no
gi|646735803|gb|KK963256.1|	24366-12697	25	8.8	0.471034	0.570335	0.275978	1	no
gi|646735803|gb|KK963256.1|	27275-12840	25	8.8	6	16	2	0.32535	no
gi|646735803|gb|KK963256.1|	29353-12989	25	8.8	16	53	2	0.2752	no
gi|646735803|gb|KK963256.1|	37158-13763	25	8.8	23	59	1	0.37185	no
gi|646735803|gb|KK963256.1|	40285-14055	25	8.8	127	374	2	0.23025	no
gi|646735803|gb|KK963256.1|	41055-14240	25	8.8	4	9	1	0.3923	no
gi|646735803|gb|KK963256.1|	43622-14457	25	8.8	16	52	2	0.20475	no
gi|646735803|gb|KK963256.1|	45357-14562	25	8.8	34	20	-0.75628	0.5623	no
gi|646735803|gb|KK963256.1|	51629-15336	25	8.8	2	1	-0.375835	0.81225	no
gi|646735803|gb|KK963256.1|	53549-15658	25	8.8	0.665612	1	0.804097	0.56015	no
gi|646735803|gb|KK963256.1|	60354-16279	25	8.8	1	1	0.0404396	1	no
gi|646735803|gb|KK963256.1|	63435-16428	25	8.8	1	2	0.500034	0.7299	no
gi|646735803|gb|KK963256.1|	64535-16635	25	8.8	0.355601	1	2	0.2839	no
gi|646735803|gb|KK963256.1|	68161-17069	25	8.8	0.771666	1	0.934474	0.4951	no
gi|646735803|gb|KK963256.1|	82781-18385	25	8.8	0.904704	0.807396	-0.164169	1	no
gi|646735803|gb|KK963256.1|	85350-18753	25	8.8	0.623821	0.695784	0.157508	1	no
gi|646735803|gb|KK963256.1|	96971-98070	25	8.8	117	120	0.0336973	0.97635	no
gi|646735826|gb|KK963253.1|	79518-18009	22	11	5	3	-0.6324	0.6217	no
gi|646735826|gb|KK963253.1|	15588-21710	22	11	16	16	-0.00683569	0.9946	no
gi|646735826|gb|KK963253.1|	78073-27823	22	11	203	152	-0.417591	0.77485	no
gi|646735826|gb|KK963253.1|	93102-93761	22	11	2	4	0.853643	0.50415	no
gi|646735826|gb|KK963253.1|	93918-95341	22	11	0.475233	0.456003	-0.0595916	1	no
gi|646735862|gb|KK963249.1|	05217-10564	14	5.8	5	15	2	0.3178	no
gi|646735872|gb|KK963248.1|	54828-15566	15	8.3	111	40	-1	0.1919	no
gi|646735872|gb|KK963248.1|	57738-15921	15	8.3	8	3	-2	0.2888	no
gi|646735872|gb|KK963248.1|	66129-16678	15	8.3	2	2	-0.0116206	0.92345	no
gi|646735916|gb|KK963243.1|	60018-60292	20	6.7	33	55	0.735485	0.59795	no
gi|646735916|gb|KK963243.1|	63214-64384	20	6.7	2	4	0.813405	0.6118	no

gi|646735916|gb|KK963243.1|	64489-64975	20	6.7	6	8	0.423152	0.76395	no
gi|646735916|gb|KK963243.1|	65204-66220	20	6.7	2	0.866143	-0.974376	0.50955	no
gi|646735916|gb|KK963243.1|	67903-71431	20	6.7	1	2	0.58026	0.71045	no
gi|646735916|gb|KK963243.1|	87283-88621	20	6.7	33	26	-0.358095	0.7399	no
gi|646735916|gb|KK963243.1|	89642-90642	20	6.7	42	40	-0.0938989	0.93015	no
gi|646735916|gb|KK963243.1|	91059-94691	20	6.7	21	22	0.036223	0.97355	no
gi|646735924|gb|KK963242.1|	47881-48388	12	5.2	16	0	-inf	0.0008	yes
gi|646735970|gb|KK963237.1|	67311-68141	9.4	5.6	3	0	-inf	0.00045	yes
gi|646735970|gb|KK963237.1|	69505-70490	9.4	5.6	3	0	-inf	0.0042	no
gi|646735978|gb|KK963236.1|	34194-13575	9.7	10	0.070892	10	7	0.0952	no
gi|646735978|gb|KK963236.1|	37710-13874	9.7	10	0.119054	2	4	0.0966	no
gi|646735978|gb|KK963236.1|	39167-13986	9.7	10	0	16	inf	0.0003	yes
gi|646735978|gb|KK963236.1|	40314-14054	9.7	10	0	104	inf	0.0017	no
gi|646735978|gb|KK963236.1|	45454-14596	9.7	10	0	9	inf	0.00435	no
gi|646735978|gb|KK963236.1|	54560-15535	9.7	10	0	24	inf	0.0003	yes
gi|646735978|gb|KK963236.1|	67942-16848	9.7	10	0	17	inf	0.00045	yes
gi|646735995|gb|KK963234.1|	55818-57269	15	6.8	4	0.334142	-3	0.1033	no
gi|646735995|gb|KK963234.1|	73494-74810	15	6.8	30	28	-0.117553	0.91375	no
gi|646735995|gb|KK963234.1|	83053-83361	15	6.8	31	21	-0.527753	0.7038	no
gi|646736096|gb|KK963223.1|	53033-15370	24	13	2	2	0.255623	0.86925	no
gi|646736096|gb|KK963223.1|	65801-16649	24	13	5	3	-0.655673	0.6529	no
gi|646736096|gb|KK963223.1|	80532-18109	24	13	5	7	0.281893	0.83825	no
gi|646736112|gb|KK963221.1|	75727-76216	14	4.7	2	3	0.42457	0.77485	no
gi|646736112|gb|KK963221.1|	96839-97353	14	4.7	0	6	inf	0.001	yes
gi|646736135|gb|KK963218.1|	55189-57164	11	5	0.806829	0	-inf	1	no
gi|646736135|gb|KK963218.1|	67013-67853	11	5	2	0	-inf	0.0011	yes
gi|646736143|gb|KK963217.1|	00691-10099	14	6.9	98	69	-0.502842	0.745	no
gi|646736143|gb|KK963217.1|	23315-23673	14	6.9	11	6	-0.89892	0.49785	no
gi|646736143|gb|KK963217.1|	71158-72101	14	6.9	1	2	0.796225	0.55185	no
gi|646736143|gb|KK963217.1|	72203-76517	14	6.9	10	9	-0.280873	0.7968	no

gi|646736163|gb|KK963215.1|	40526-40850	12	6.6	0	78	inf	0.00025	yes
gi|646736196|gb|KK963212.1|	33123-13329	11	4.7	217	88	-1	0.38815	no
gi|646736215|gb|KK963210.1|	57290-15809	13	8.5	1	1	0.232211	0.87595	no
gi|646736215|gb|KK963210.1|	73512-17911	13	8.5	2	5	1	0.3289	no
gi|646736215|gb|KK963210.1|	82028-18299	13	8.5	0.387583	2	2	0.2023	no
gi|646736215|gb|KK963210.1|	45311-34626	13	8.5	1	4	2	0.30965	no
gi|646736215|gb|KK963210.1|	47215-34762	13	8.5	1	7	3	0.26455	no
gi|646736215|gb|KK963210.1|	48758-34902	13	8.5	17	26	0.620782	0.6383	no
gi|646736215|gb|KK963210.1|	49585-34990	13	8.5	9.48	26	1	0.37165	no
gi|646736215|gb|KK963210.1|	56723-57819	13	8.5	0.659975	1	1	0.44445	no
gi|646736215|gb|KK963210.1|	79593-80844	13	8.5	1	0.799529	-0.479269	1	no
gi|646736246|gb|KK963207.1|	46980-14786	19	8.5	1	2	0.639763	0.62475	no
gi|646736246|gb|KK963207.1|	51627-15387	19	8.5	23	23	-0.00743903	0.99495	no
gi|646736246|gb|KK963207.1|	63347-16408	19	8.5	4	5	0.561764	0.6834	no
gi|646736246|gb|KK963207.1|	72260-17286	19	8.5	3	3	0.140814	0.9194	no
gi|646736246|gb|KK963207.1|	75483-17618	19	8.5	4	1	-1	0.3613	no
gi|646736246|gb|KK963207.1|	79767-18029	19	8.5	2	7	2	0.23595	no
gi|646736246|gb|KK963207.1|	82031-18301	19	8.5	0.381196	4	3	0.1391	no
gi|646736246|gb|KK963207.1|	85606-18988	19	8.5	2	3	0.354081	0.81515	no
gi|646736246|gb|KK963207.1|	97389-19764	19	8.5	11	42	2	0.23835	no
gi|646736246|gb|KK963207.1|	36654-33783	19	8.5	0.400469	1	2	0.26385	no
gi|646736246|gb|KK963207.1|	38856-34190	19	8.5	0.331732	1	2	1	no
gi|646736246|gb|KK963207.1|	53729-35473	19	8.5	1	3	2	0.3384	no
gi|646736246|gb|KK963207.1|	63778-36411	19	8.5	25	0	-inf	0.0047	no
gi|646736253|gb|KK963206.1|	33877-13516	13	5.2	1	0.25668	-2	0.15305	no
gi|646736253|gb|KK963206.1|	41311-14408	13	5.2	1	0.480203	-1	1	no
gi|646736253|gb|KK963206.1|	56703-15761	13	5.2	2	0.400672	-2	0.197	no
gi|646736260|gb|KK963205.1|	45680-14641	16	8.4	2	3	0.749582	0.56005	no
gi|646736260|gb|KK963205.1|	17021-31752	16	8.4	4	3	-0.707091	0.628	no
gi|646736260|gb|KK963205.1|	74485-76468	16	8.4	0.803094	1	0.627229	0.66165	no

gi|646736260|gb|KK963205.1|	76583-77955	16	8.4	0.828212	2	0.902654	0.4835	no
gi|646736260|gb|KK963205.1|	78047-82136	16	8.4	0.892879	2	0.826608	0.5987	no
gi|646736260|gb|KK963205.1|	82209-82606	16	8.4	4	4	0.101383	0.9147	no
gi|646736283|gb|KK963203.1|	20902-12328	24	9.7	3	1	-1	0.4889	no
gi|646736283|gb|KK963203.1|	2231-2671	24	9.7	7	10	0.490553	0.7274	no
gi|646736283|gb|KK963203.1|	25068-25633	24	9.7	5	3	-0.634442	0.6217	no
gi|646736283|gb|KK963203.1|	06833-30753	24	9.7	0	3	inf	0.001	yes
gi|646736283|gb|KK963203.1|	09516-30998	24	9.7	7	4	-0.779151	0.5538	no
gi|646736283|gb|KK963203.1|	15512-31730	24	9.7	1	0.693971	-1	0.47525	no
gi|646736283|gb|KK963203.1|	31951-33641	24	9.7	48	51	0.0794725	0.94605	no
gi|646736283|gb|KK963203.1|	52725-35309	24	9.7	6	7	0.139106	0.9183	no
gi|646736283|gb|KK963203.1|	36759-37200	24	9.7	3	3	-0.0873332	0.92325	no
gi|646736283|gb|KK963203.1|	77722-37992	24	9.7	4	2	-0.931944	0.5442	no
gi|646736283|gb|KK963203.1|	46214-46751	24	9.7	7	6	-0.247872	0.8355	no
gi|646736283|gb|KK963203.1|	50146-51847	24	9.7	1	2	0.452552	0.74015	no
gi|646736283|gb|KK963203.1|	55132-55370	24	9.7	14	33	1	0.42655	no
gi|646736283|gb|KK963203.1|	57674-60221	24	9.7	0.686221	0.527718	-0.378908	1	no
gi|646736283|gb|KK963203.1|	61866-62530	24	9.7	0.677987	3	2	0.25715	no
gi|646736283|gb|KK963203.1|	63417-63852	24	9.7	7	14	1	0.4539	no
gi|646736283|gb|KK963203.1|	64970-66685	24	9.7	61	39	-0.650007	0.5761	no
gi|646736283|gb|KK963203.1|	80664-81078	24	9.7	59	59	-0.0135617	0.99205	no
gi|646736283|gb|KK963203.1|	83960-84549	24	9.7	5	5	0.097137	0.939	no
gi|646736283|gb|KK963203.1|	86249-86504	24	9.7	18	37	1	0.4291	no
gi|646736283|gb|KK963203.1|	88762-89723	24	9.7	2	3	0.420406	0.75495	no
gi|646736283|gb|KK963203.1|	89895-90530	24	9.7	5	4	-0.462475	0.701	no
gi|646736307|gb|KK963200.1|	73726-74082	10	8.2	55	17	-2	0.2905	no
gi|646736316|gb|KK963199.1|	70722-71120	12	7.5	6	2	-2	0.28785	no
gi|646736316|gb|KK963199.1|	78651-79375	12	7.5	2	0.828334	-1	0.3853	no
gi|646736316|gb|KK963199.1|	92015-93540	12	7.5	2	2	0.00397205	0.9741	no
gi|646736345|gb|KK963196.1|	18959-11930	16	6.4	0	22	inf	0.00315	no

gi|646736345|gb|KK963196.1|	46387-46757	16	6.4	0	38	inf	0.00075	yes
gi|646736353|gb|KK963195.1|	08611-10895	15	7.1	2	11	3	0.2664	no
gi|646736353|gb|KK963195.1|	37703-38625	15	7.1	132	219	0.728342	0.5351	no
gi|646736353|gb|KK963195.1|	44262-45863	15	7.1	3	5	0.857557	0.577	no
gi|646736353|gb|KK963195.1|	46564-47528	15	7.1	0.521878	3	2	0.21535	no
gi|646736353|gb|KK963195.1|	48346-49197	15	7.1	0.775995	2	2	0.2735	no
gi|646736353|gb|KK963195.1|	49494-50748	15	7.1	5	10	1	0.4599	no
gi|646736353|gb|KK963195.1|	59009-59296	15	7.1	21	110	2	0.11695	no
gi|646736353|gb|KK963195.1|	60732-61194	15	7.1	3	5	0.603388	0.6563	no
gi|646736353|gb|KK963195.1|	61405-62711	15	7.1	2	9	2	0.08925	no
gi|646736353|gb|KK963195.1|	64429-65625	15	7.1	1	3	1	0.35515	no
gi|646736353|gb|KK963195.1|	67036-67630	15	7.1	5	34	3	0.07395	no
gi|646736353|gb|KK963195.1|	76200-76800	15	7.1	1	2	0.725531	0.64505	no
gi|646736353|gb|KK963195.1|	92753-93812	15	7.1	0.690341	1	0.928276	0.53925	no
gi|646736373|gb|KK963193.1|	67184-36753	14	8.6	9	2	-2	0.2103	no
gi|646736373|gb|KK963193.1|	67747-36859	14	8.6	2	0.663718	-1	0.4271	no
gi|646736373|gb|KK963193.1|	49842-55049	14	8.6	43	51	0.227716	0.84875	no
gi|646736381|gb|KK963192.1|	00755-10147	11	6.1	6	0	-inf	0.0017	no
gi|646736381|gb|KK963192.1|	01794-10266	11	6.1	4	0	-inf	0.00595	no
gi|646736381|gb|KK963192.1|	08654-10945	11	6.1	2	0	-inf	0.00225	no
gi|646736381|gb|KK963192.1|	09895-11099	11	6.1	3	0	-inf	0.0047	no
gi|646736381|gb|KK963192.1|	20309-12106	11	6.1	29	0	-inf	5.00E-05	yes
gi|646736389|gb|KK963191.1|	88738-89580	17	6.9	0.472734	2	2	0.2898	no
gi|646736443|gb|KK963185.1|	69172-70165	11	4.6	27	0.178468	-7	0.1665	no
gi|646736443|gb|KK963185.1|	70671-72452	11	4.6	3	0	-inf	0.00045	yes
gi|646736443|gb|KK963185.1|	82130-82555	11	4.6	8	0	-inf	0.00195	no
gi|646736443|gb|KK963185.1|	83385-86227	11	4.6	2	0	-inf	0.00075	yes
gi|646736451|gb|KK963184.1|	11150-11222	15	5.8	47	30	-0.655883	0.55015	no
gi|646736451|gb|KK963184.1|	13137-11364	15	5.8	30	32	0.0960858	0.95115	no
gi|646736451|gb|KK963184.1|	16548-11920	15	5.8	25	17	-0.593341	0.5897	no

gi|646736451|gb|KK963184.1|	20261-12076	15	5.8	22	19	-0.180073	0.90975	no
gi|646736451|gb|KK963184.1|	23902-12412	15	5.8	53	19	-1	0.34585	no
gi|646736451|gb|KK963184.1|	24597-12509	15	5.8	3	5	0.750594	0.5809	no
gi|646736451|gb|KK963184.1|	26043-12683	15	5.8	1	0.977369	-0.506339	0.7384	no
gi|646736451|gb|KK963184.1|	26936-12722	15	5.8	14	15	0.0432555	0.9426	no
gi|646736451|gb|KK963184.1|	32192-13293	15	5.8	2	4	1	0.42735	no
gi|646736451|gb|KK963184.1|	33623-13726	15	5.8	1	0.954718	-0.229471	1	no
gi|646736451|gb|KK963184.1|	37376-13946	15	5.8	1	2	0.294169	0.8522	no
gi|646736451|gb|KK963184.1|	42084-14618	15	5.8	0.79428	0.701972	-0.178236	1	no
gi|646736451|gb|KK963184.1|	46309-14807	15	5.8	0.552146	0.975409	0.820958	1	no
gi|646736451|gb|KK963184.1|	48747-15046	15	5.8	0.505545	0.365194	-0.469177	1	no
gi|646736451|gb|KK963184.1|	51779-15293	15	5.8	0.925078	0.588495	-0.652545	1	no
gi|646736451|gb|KK963184.1|	53316-15489	15	5.8	0.55984	0.908476	0.698434	1	no
gi|646736451|gb|KK963184.1|	62830-16546	15	5.8	0.466482	0.22595	-1	1	no
gi|646736451|gb|KK963184.1|	66329-16903	15	5.8	1	0.934232	-0.180385	1	no
gi|646736451|gb|KK963184.1|	91930-19229	15	5.8	8	5	-0.663712	0.6522	no
gi|646736451|gb|KK963184.1|	33795-36771	15	5.8	10	7	-0.537719	0.61875	no
gi|646736451|gb|KK963184.1|	37308-38682	15	5.8	3	2	-0.363332	0.8207	no
gi|646736451|gb|KK963184.1|	39487-40337	15	5.8	10	7	-0.478938	0.7496	no
gi|646736451|gb|KK963184.1|	58105-59409	15	5.8	0.529135	0.88664	0.744714	1	no
gi|646736451|gb|KK963184.1|	59472-60161	15	5.8	1	1	0.217349	0.89085	no
gi|646736451|gb|KK963184.1|	62734-63223	15	5.8	20	16	-0.26509	0.85985	no
gi|646736451|gb|KK963184.1|	63374-65981	15	5.8	1	1	-0.387016	0.8102	no
gi|646736451|gb|KK963184.1|	66520-67706	15	5.8	0.794917	0.854039	0.103499	1	no
gi|646736451|gb|KK963184.1|	69419-70824	15	5.8	16	14	-0.187563	0.8997	no
gi|646736451|gb|KK963184.1|	71019-74527	15	5.8	1	1	0.377666	0.80825	no
gi|646736451|gb|KK963184.1|	74860-76050	15	5.8	0.989396	2	0.656184	0.6155	no
gi|646736451|gb|KK963184.1|	80948-83244	15	5.8	0.906594	0.789216	-0.200037	1	no
gi|646736451|gb|KK963184.1|	94196-95885	15	5.8	0.709665	0.931791	0.392868	1	no
gi|646736460|gb|KK963183.1|	13564-14553	15	7.2	0.756161	1	0.509547	1	no

gi|646736484|gb|KK963180.1|	68275-16936	16	6.6	2	6	2	0.21065	no
gi|646736484|gb|KK963180.1|	93726-19431	16	6.6	10	14	0.540397	0.72445	no
gi|646736484|gb|KK963180.1|	87225-87862	16	6.6	1	2	0.207789	0.8909	no
gi|646736497|gb|KK963178.1|	3299-3901	11	7.2	21	2	-4	0.05635	no
gi|646736497|gb|KK963178.1|	442-960	11	7.2	27	19	-0.507874	0.73145	no
gi|646736505|gb|KK963177.1|	33469-23432	23	7.5	0.929635	1	0.236827	1	no
gi|646736505|gb|KK963177.1|	28432-29239	23	7.5	3	4	0.665577	0.6448	no
gi|646736505|gb|KK963177.1|	21459-32226	23	7.5	2	0.472522	-2	0.26845	no
gi|646736505|gb|KK963177.1|	46750-47493	23	7.5	4	1	-1	0.30195	no
gi|646736505|gb|KK963177.1|	47596-49970	23	7.5	0.873225	1	0.537501	0.6974	no
gi|646736505|gb|KK963177.1|	86370-86732	23	7.5	6	4	-0.479096	0.76275	no
gi|646736525|gb|KK963175.1|	44502-24498	19	7.7	69	66	-0.0702965	0.9625	no
gi|646736525|gb|KK963175.1|	45729-24691	19	7.7	97	73	-0.417864	0.72095	no
gi|646736525|gb|KK963175.1|	47834-24922	19	7.7	64	41	-0.662933	0.5612	no
gi|646736553|gb|KK963172.1|	8097-8414	13	6.5	12	7	-0.73482	0.6309	no
gi|646736560|gb|KK963171.1|	76466-27701	17	8.6	3	0.423355	-3	0.2136	no
gi|646736560|gb|KK963171.1|	69931-37032	17	8.6	6	3	-0.807321	0.602	no
gi|646736560|gb|KK963171.1|	70599-37125	17	8.6	2	1	-0.332037	0.81865	no
gi|646736560|gb|KK963171.1|	99251-39963	17	8.6	9	5	-0.86733	0.51385	no
gi|646736560|gb|KK963171.1|	75700-76184	17	8.6	16	12	-0.40378	0.79645	no
gi|646736568|gb|KK963170.1|	07385-10791	17	4	4	8	0.911002	0.53575	no
gi|646736575|gb|KK963169.1|	16233-16738	9.4	5.8	9	2	-2	0.21605	no
gi|646736575|gb|KK963169.1|	18063-18573	9.4	5.8	30	8	-2	0.2582	no
gi|646736575|gb|KK963169.1|	25233-25653	9.4	5.8	16	11	-0.523498	0.74285	no
gi|646736606|gb|KK963165.1|	09300-10954	18	9.1	29	10	-1	0.3686	no
gi|646736606|gb|KK963165.1|	10955-11157	18	9.1	2	3	0.829078	0.56315	no
gi|646736606|gb|KK963165.1|	11760-11228	18	9.1	2	2	0.176261	0.89965	no
gi|646736606|gb|KK963165.1|	12706-11323	18	9.1	102	17	-3	0.0728	no
gi|646736606|gb|KK963165.1|	13695-11489	18	9.1	1	1	0.381426	0.7733	no
gi|646736606|gb|KK963165.1|	15547-11596	18	9.1	4	6	0.551795	0.6991	no

gi|646736606|gb|KK963165.1|	93974-95159	18	9.1	5	4	-0.302529	0.84015	no
gi|646736615|gb|KK963164.1|	45608-14663	20	10	3	3	0.309842	0.8482	no
gi|646736615|gb|KK963164.1|	62465-16337	20	10	44	19	-1	0.34285	no
gi|646736615|gb|KK963164.1|	64942-17304	20	10	7	7	-0.0352545	0.97455	no
gi|646736615|gb|KK963164.1|	84287-18524	20	10	5	4	-0.21656	0.8896	no
gi|646736615|gb|KK963164.1|	85731-19006	20	10	2	2	-0.296591	0.8384	no
gi|646736615|gb|KK963164.1|	46514-24720	20	10	0.21158	4	4	0.09875	no
gi|646736615|gb|KK963164.1|	47371-25225	20	10	0.70032	2	1	0.41075	no
gi|646736615|gb|KK963164.1|	52353-25498	20	10	0.505144	2	2	0.20545	no
gi|646736615|gb|KK963164.1|	56000-25661	20	10	6	6	0.0290311	0.97425	no
gi|646736650|gb|KK963160.1|	04353-10568	22	9.9	35	63	0.83233	0.4567	no
gi|646736650|gb|KK963160.1|	1195-1813	22	9.9	3	0.351576	-3	0.1921	no
gi|646736650|gb|KK963160.1|	50201-15191	22	9.9	371	283	-0.390705	0.7427	no
gi|646736650|gb|KK963160.1|	67559-16884	22	9.9	0.447774	0.642916	0.521858	1	no
gi|646736650|gb|KK963160.1|	69847-17130	22	9.9	2	1	-0.232773	0.8673	no
gi|646736650|gb|KK963160.1|	72442-17271	22	9.9	86	76	-0.167319	0.91495	no
gi|646736650|gb|KK963160.1|	79224-18050	22	9.9	64	31	-1	0.3564	no
gi|646736650|gb|KK963160.1|	84930-18804	22	9.9	3	2	-0.201122	0.89065	no
gi|646736650|gb|KK963160.1|	88258-18929	22	9.9	2	1	-0.394844	0.7639	no
gi|646736650|gb|KK963160.1|	91724-19209	22	9.9	5	4	-0.466668	0.7629	no
gi|646736650|gb|KK963160.1|	94003-19578	22	9.9	4	6	0.671477	0.65705	no
gi|646736650|gb|KK963160.1|	27400-28403	22	9.9	4	4	-0.0746226	0.96205	no
gi|646736650|gb|KK963160.1|	78067-27922	22	9.9	3	5	0.961102	0.5478	no
gi|646736650|gb|KK963160.1|	31412-33188	22	9.9	3	2	-0.391201	0.80985	no
gi|646736650|gb|KK963160.1|	7265-22843	22	9.9	3	3	-0.0210151	0.9836	no
gi|646736650|gb|KK963160.1|	81410-84454	22	9.9	0.82933	1	0.356675	1	no
gi|646736650|gb|KK963160.1|	94847-95137	22	9.9	16	35	1	0.44845	no
gi|646736662|gb|KK963159.1|	45894-14642	18	9.4	4	5	0.199409	0.86905	no
gi|646736662|gb|KK963159.1|	60836-16175	18	9.4	4	7	0.549484	0.72385	no
gi|646736662|gb|KK963159.1|	63505-16596	18	9.4	8	8	0.0267118	0.9847	no

gi|646736662|gb|KK963159.1|	75022-17552	18	9.4	8	10	0.285155	0.8325	no
gi|646736662|gb|KK963159.1|	76365-17689	18	9.4	2	2	-0.0431882	0.92345	no
gi|646736662|gb|KK963159.1|	95713-19614	18	9.4	24.62	10	-1	0.433	no
gi|646736662|gb|KK963159.1|	6505-7166	18	9.4	20	6	-2	0.3034	no
gi|646736662|gb|KK963159.1|	65953-66843	18	9.4	2	4	0.807814	0.5567	no
gi|646736687|gb|KK963156.1|	06961-10761	19	8.5	0.698351	3	2	0.2057	no
gi|646736687|gb|KK963156.1|	55326-15664	19	8.5	1	0.499196	-1	0.375	no
gi|646736687|gb|KK963156.1|	59881-16084	19	8.5	2	1	-0.492256	0.70515	no
gi|646736687|gb|KK963156.1|	90276-90744	19	8.5	8	6	-0.29646	0.807	no
gi|646736697|gb|KK963155.1|	35500-13652	8.9	5.5	0	18	inf	0.0001	yes
gi|646736707|gb|KK963154.1|	21533-22139	15	8.9	4	1	-2	0.2617	no
gi|646736724|gb|KK963152.1|	90966-93891	13	4.7	3	2	-0.408183	0.7865	no
gi|646736733|gb|KK963151.1|	15027-11559	18	8.2	6	10	0.645666	0.63855	no
gi|646736733|gb|KK963151.1|	24067-12555	18	8.2	2	3	0.652321	0.68825	no
gi|646736733|gb|KK963151.1|	25969-12704	18	8.2	1	1	0.344255	0.8037	no
gi|646736733|gb|KK963151.1|	58052-15853	18	8.2	2	17	3	0.1205	no
gi|646736733|gb|KK963151.1|	59962-16050	18	8.2	1	8	3	0.1861	no
gi|646736733|gb|KK963151.1|	82074-18296	18	8.2	10	19	0.928112	0.54125	no
gi|646736733|gb|KK963151.1|	84331-18503	18	8.2	14	14	-0.019889	0.98855	no
gi|646736733|gb|KK963151.1|	94235-19454	18	8.2	42	65	0.633485	0.6896	no
gi|646736733|gb|KK963151.1|	25577-26805	18	8.2	1	0.818003	-0.354481	1	no
gi|646736733|gb|KK963151.1|	35780-36856	18	8.2	10	13	0.314733	0.83565	no
gi|646736733|gb|KK963151.1|	41974-43285	18	8.2	12	17	0.458861	0.76455	no
gi|646736733|gb|KK963151.1|	44968-47631	18	8.2	9	13	0.490799	0.6513	no
gi|646736733|gb|KK963151.1|	55526-55907	18	8.2	9	23	1	0.3541	no
gi|646736733|gb|KK963151.1|	99673-10046	18	8.2	0.862831	1	0.75702	0.6148	no
gi|646736746|gb|KK963150.1|	28583-29402	11	13	0.984011	2	0.911877	0.5424	no
gi|646736746|gb|KK963150.1|	73964-74631	11	13	0	7	inf	0.0026	no
gi|646736767|gb|KK963148.1|	38388-13899	14	7.2	2	1	-0.757966	0.6333	no
gi|646736767|gb|KK963148.1|	17594-19009	14	7.2	0.717764	1	0.677188	0.62555	no

gi|646736767|gb|KK963148.1|	20008-22033	14	7.2	8	11	0.46747	0.74735	no
gi|646736775|gb|KK963147.1|	86692-87602	53	22	2	0.400672	-3	0.1506	no
gi|646736775|gb|KK963147.1|	)9955-10066(	53	22	2	0.281831	-3	0.21605	no
gi|646736782|gb|KK963146.1|	60532-16073	14	6.5	84	196	1	0.4206	no
gi|646736790|gb|KK963145.1|	17240-21819	21	9.6	0.797436	2	2	0.30515	no
gi|646736790|gb|KK963145.1|	26141-22638	21	9.6	7	28	2	0.32145	no
gi|646736790|gb|KK963145.1|	27988-22912	21	9.6	0.736691	2	1	0.3574	no
gi|646736790|gb|KK963145.1|	33592-23884	21	9.6	10	17	0.729395	0.5201	no
gi|646736790|gb|KK963145.1|	76002-27895	21	9.6	0.617027	2	1	0.3074	no
gi|646736790|gb|KK963145.1|	80198-28173	21	9.6	3	5	0.835927	0.584	no
gi|646736790|gb|KK963145.1|	93882-29510	21	9.6	6	6	0.143572	0.92255	no
gi|646736790|gb|KK963145.1|	95215-29641	21	9.6	1	0.843423	-0.481036	0.7302	no
gi|646736790|gb|KK963145.1|	96625-29848	21	9.6	1	2	0.684696	0.6219	no
gi|646736790|gb|KK963145.1|	99202-30013	21	9.6	2	2	0.0908806	0.94375	no
gi|646736790|gb|KK963145.1|	02942-30446	21	9.6	1	0.842634	-0.720472	0.57085	no
gi|646736790|gb|KK963145.1|	43117-34336	21	9.6	14	19	0.36891	0.8043	no
gi|646736790|gb|KK963145.1|	52704-35378	21	9.6	1	1	0.362131	0.79375	no
gi|646736790|gb|KK963145.1|	54604-35485	21	9.6	16	21	0.337825	0.80895	no
gi|646736790|gb|KK963145.1|	56625-35736	21	9.6	2	2	0.0733822	0.92975	no
gi|646736790|gb|KK963145.1|	66911-37050	21	9.6	29	21	-0.486361	0.6711	no
gi|646736790|gb|KK963145.1|	70838-37304	21	9.6	0.474449	0.619221	0.384201	1	no
gi|646736790|gb|KK963145.1|	73224-37458	21	9.6	0.420123	1	1	1	no
gi|646736790|gb|KK963145.1|	80933-38586	21	9.6	24	19	-0.383353	0.743	no
gi|646736790|gb|KK963145.1|	38235-39736	21	9.6	0.519735	2	2	0.3161	no
gi|646736790|gb|KK963145.1|	91048-39488	21	9.6	76	44	-0.799278	0.51	no
gi|646736790|gb|KK963145.1|	96762-39777	21	9.6	0.980179	0.523697	-0.904312	1	no
gi|646736790|gb|KK963145.1|	02827-40321	21	9.6	7	26	2	0.21025	no
gi|646736790|gb|KK963145.1|	08899-40932	21	9.6	31	0	-inf	0.0003	yes
gi|646736808|gb|KK963143.1|	27377-12799	13	7.2	4	3	-0.533719	0.68055	no
gi|646736808|gb|KK963143.1|	49187-49684	13	7.2	11	4	-1	0.32075	no

gi|646736808|gb|KK963143.1| 94313-94904	13	7.2	8	2	-2	0.1737	no
gi|646736819|gb|KK963142.1| 53829-54650	19	7.3	0	2	inf	0.00255	no
gi|646736819|gb|KK963142.1| 86302-86546	19	7.3	77	53	-0.551229	0.73305	no
gi|646736829|gb|KK963141.1| 01776-10233	19	8.8	5	4	-0.314328	0.80385	no
gi|646736829|gb|KK963141.1| 03255-10387	19	8.8	9	10	0.115932	0.9374	no
gi|646736829|gb|KK963141.1| 22058-12250	19	8.8	5	15	1	0.3295	no
gi|646736829|gb|KK963141.1| 39420-14021	19	8.8	0.512916	2	2	0.18805	no
gi|646736829|gb|KK963141.1| 41926-14535	19	8.8	28	28	-0.00147012	0.9986	no
gi|646736829|gb|KK963141.1| 50915-15322	19	8.8	7	4	-1	0.5026	no
gi|646736829|gb|KK963141.1| 58999-15934	19	8.8	18	8	-1	0.3347	no
gi|646736829|gb|KK963141.1| 68717-16936	19	8.8	0.931134	4	2	0.2059	no
gi|646736829|gb|KK963141.1| 79247-17954	19	8.8	1	17	4	0.10695	no
gi|646736829|gb|KK963141.1| 38837-40401	19	8.8	2	3	0.528273	0.7351	no
gi|646736829|gb|KK963141.1| 50334-53809	19	8.8	121	143	0.233721	0.84505	no
gi|646736829|gb|KK963141.1| 69193-69457	19	8.8	4	27	3	0.2613	no
gi|646736829|gb|KK963141.1| 72720-73031	19	8.8	14	15	0.117584	0.92845	no
gi|646736829|gb|KK963141.1| 77445-78147	19	8.8	0.82745	3	2	0.205	no
gi|646736829|gb|KK963141.1| 92321-93289	19	8.8	2	5	1	0.38945	no
gi|646736855|gb|KK963138.1| 02817-10400	19	9.1	8	3	-1	0.3897	no
gi|646736855|gb|KK963138.1| 19024-11931	19	9.1	81	7	-3	0.07425	no
gi|646736855|gb|KK963138.1| 26505-12744	19	9.1	2	1	-0.493282	0.7125	no
gi|646736855|gb|KK963138.1| 27735-12887	19	9.1	0.733355	1	0.709356	0.6242	no
gi|646736855|gb|KK963138.1| 29110-12936	19	9.1	42	24	-0.794609	0.545	no
gi|646736855|gb|KK963138.1| 40251-14229	19	9.1	15	7	-1	0.40105	no
gi|646736855|gb|KK963138.1| 60255-16074	19	9.1	7	5	-0.651129	0.6087	no
gi|646736855|gb|KK963138.1| 94180-19584	19	9.1	20	19	-0.0813851	0.93975	no
gi|646736855|gb|KK963138.1| 74550-27477	19	9.1	34	29	-0.218106	0.88855	no
gi|646736855|gb|KK963138.1| 84576-28549	19	9.1	1	3	1	0.3749	no
gi|646736855|gb|KK963138.1| 86469-28691	19	9.1	32	38	0.267167	0.85695	no
gi|646736855|gb|KK963138.1| 53776-55531	19	9.1	38	17	-1	0.3236	no

gi|646736855|gb|KK963138.1|	60796-61583	19	9.1	1	5	2	0.27565	no
gi|646736855|gb|KK963138.1|	62508-63107	19	9.1	3	4	0.599753	0.63685	no
gi|646736855|gb|KK963138.1|	67797-68166	19	9.1	30	16	-0.898177	0.5785	no
gi|646736885|gb|KK963134.1|	47959-14898	18	9.4	2	3	0.189442	0.89005	no
gi|646736885|gb|KK963134.1|	82700-18437	18	9.4	11	11	0.053388	0.97095	no
gi|646736885|gb|KK963134.1|	85549-18649	18	9.4	5	3	-0.933529	0.49795	no
gi|646736885|gb|KK963134.1|	88013-18954	18	9.4	4	5	0.24588	0.8662	no
gi|646736885|gb|KK963134.1|	89651-19058	18	9.4	2	3	0.769155	0.5924	no
gi|646736885|gb|KK963134.1|	90708-19117	18	9.4	7	6	-0.264578	0.8258	no
gi|646736898|gb|KK963132.1|	10674-11174	50	27	3	3	-0.0558823	0.92345	no
gi|646736906|gb|KK963131.1|	89915-90553	10	6.6	4	4	-0.157002	0.89645	no
gi|646736917|gb|KK963130.1|	51288-51818	15	6.7	0	10	inf	0.0023	no
gi|646736917|gb|KK963130.1|	54845-55100	15	6.7	0	42	inf	0.00605	no
gi|646736917|gb|KK963130.1|	68987-69209	15	6.7	0	125	inf	0.0017	no
gi|646736959|gb|KK963125.1|	01078-10251	21	5.3	2	0	-inf	0.00045	yes
gi|646736959|gb|KK963125.1|	10576-11084	21	5.3	35	0	-inf	0.0012	yes
gi|646736959|gb|KK963125.1|	12099-13031	21	5.3	9	2	-2	0.10375	no
gi|646736959|gb|KK963125.1|	13512-13699	21	5.3	1347.17	139	-3	0.0357	no
gi|646736959|gb|KK963125.1|	62981-63475	21	5.3	46	73	0.647562	0.6722	no
gi|646736959|gb|KK963125.1|	66278-67188	21	5.3	3	0	-inf	0.00525	no
gi|646736959|gb|KK963125.1|	71017-71245	21	5.3	122	0	-inf	0.0033	no
gi|646736959|gb|KK963125.1|	90754-92212	21	5.3	2	0.553723	-2	0.2982	no
gi|646736959|gb|KK963125.1|	93046-93394	21	5.3	29	1	-5	0.1751	no
gi|646736985|gb|KK963122.1|	74314-75215	13	4.9	3	3	-0.112882	0.94635	no
gi|646736985|gb|KK963122.1|	83151-83993	13	4.9	1	3	0.914019	0.4934	no
gi|646737012|gb|KK963120.1|	50344-25566	20	9.8	6	5	-0.282439	0.789	no
gi|646737022|gb|KK963119.1|	79060-80031	8.7	5.6	2	3	0.508483	0.67555	no
gi|646737022|gb|KK963119.1|	80642-82210	8.7	5.6	1	1	-0.0566165	0.9618	no
gi|646737056|gb|KK963115.1|	03178-10431	24	11	1	3	1	0.45915	no
gi|646737056|gb|KK963115.1|	04440-10557	24	11	1	2	0.616126	0.6657	no

gi|646737056|gb|KK963115.1|	20386-12180	24	11	2	2	0.110218	0.9375	no
gi|646737056|gb|KK963115.1|	22609-12383	24	11	0.861885	2	1	0.41795	no
gi|646737056|gb|KK963115.1|	1252-2374	24	11	0.960282	0.457748	-1	1	no
gi|646737056|gb|KK963115.1|	32271-13328	24	11	0.609384	1	0.773977	1	no
gi|646737056|gb|KK963115.1|	37285-13838	24	11	0.874767	0.781363	-0.162907	1	no
gi|646737056|gb|KK963115.1|	38535-14176	24	11	3	2	-0.813687	0.59565	no
gi|646737056|gb|KK963115.1|	42359-14266	24	11	17	0	-inf	0.0035	no
gi|646737056|gb|KK963115.1|	43573-14394	24	11	6	5	-0.340671	0.81475	no
gi|646737056|gb|KK963115.1|	47446-14943	24	11	1	1	-0.0824993	0.9581	no
gi|646737056|gb|KK963115.1|	49993-15175	24	11	0.738152	0.177812	-2	1	no
gi|646737056|gb|KK963115.1|	58728-16235	24	11	61	35	-0.827196	0.4864	no
gi|646737056|gb|KK963115.1|	62813-16391	24	11	1	0.4699	-1	0.38955	no
gi|646737056|gb|KK963115.1|	68734-17042	24	11	2	2	-0.0545143	0.9701	no
gi|646737056|gb|KK963115.1|	73338-17495	24	11	0.744988	0.390971	-0.930154	1	no
gi|646737056|gb|KK963115.1|	77260-17979	24	11	1	0.471527	-1	0.38515	no
gi|646737056|gb|KK963115.1|	80013-18270	24	11	0.836246	0.220983	-2	1	no
gi|646737056|gb|KK963115.1|	86504-18992	24	11	18	13	-0.495402	0.65455	no
gi|646737056|gb|KK963115.1|	94800-19779	24	11	11	7	-0.770323	0.5507	no
gi|646737056|gb|KK963115.1|	206-998	24	11	7	2	-2	0.24235	no
gi|646737056|gb|KK963115.1|	08175-20848	24	11	13	5	-1	0.36405	no
gi|646737056|gb|KK963115.1|	36047-23629	24	11	22	27	0.285358	0.83955	no
gi|646737056|gb|KK963115.1|	24028-24387	24	11	470	52	-3	0.0329	no
gi|646737056|gb|KK963115.1|	44800-24560	24	11	1	0.945044	-0.504348	0.7384	no
gi|646737056|gb|KK963115.1|	62414-26290	24	11	18	9	-0.987711	0.54585	no
gi|646737056|gb|KK963115.1|	65071-26537	24	11	11	5	-1	0.4638	no
gi|646737056|gb|KK963115.1|	77307-27835	24	11	2	2	-0.487348	0.6907	no
gi|646737056|gb|KK963115.1|	80927-28120	24	11	16	4	-2	0.2666	no
gi|646737056|gb|KK963115.1|	81456-28269	24	11	1	1	0.257248	0.8481	no
gi|646737056|gb|KK963115.1|	85992-28629	24	11	12	6	-0.927267	0.5576	no
gi|646737056|gb|KK963115.1|	94069-29792	24	11	0.205774	83	9	0.02095	no

gi|646737056|gb|KK963115.1|	01319-30151	24	11	0	132	inf	0.00515	no
gi|646737056|gb|KK963115.1|	09620-30983	24	11	131.17	32	-2	0.207	no
gi|646737056|gb|KK963115.1|	16845-31856	24	11	0.504856	1	0.990284	1	no
gi|646737056|gb|KK963115.1|	3172-3585	24	11	10	4	-1	0.29505	no
gi|646737056|gb|KK963115.1|	23233-32383	24	11	2	1	-0.706213	0.64185	no
gi|646737056|gb|KK963115.1|	28205-33014	24	11	40	22	-0.824682	0.46355	no
gi|646737056|gb|KK963115.1|	35211-33611	24	11	3	2	-0.6213	0.65875	no
gi|646737056|gb|KK963115.1|	65251-36586	24	11	2	2	-0.0191459	0.92345	no
gi|646737056|gb|KK963115.1|	74400-37469	24	11	12	9	-0.456616	0.76135	no
gi|646737056|gb|KK963115.1|	85455-38571	24	11	62	23	-1	0.3334	no
gi|646737056|gb|KK963115.1|	86279-38697	24	11	1	3	1	0.41435	no
gi|646737056|gb|KK963115.1|	88637-39096	24	11	43	32	-0.430135	0.7129	no
gi|646737056|gb|KK963115.1|	98706-40025	24	11	57	23	-1	0.24665	no
gi|646737056|gb|KK963115.1|	07005-40766	24	11	7	0.943035	-3	0.13615	no
gi|646737056|gb|KK963115.1|	10796-41282	24	11	3	2	-0.912905	0.5612	no
gi|646737056|gb|KK963115.1|	70057-75133	24	11	2	2	-0.553625	0.7156	no
gi|646737056|gb|KK963115.1|	79016-84621	24	11	8	4	-0.859911	0.4336	no
gi|646737056|gb|KK963115.1|	8310-9550	24	11	0.845263	0.26942	-2	1	no
gi|646737056|gb|KK963115.1|	85865-90179	24	11	0.341914	2	3	0.0929	no
gi|646737056|gb|KK963115.1|	90280-92641	24	11	0.966476	0.828873	-0.221584	1	no
gi|646737056|gb|KK963115.1|	92805-93830	24	11	1	0.513783	-1	1	no
gi|646737056|gb|KK963115.1|	98004-10072	24	11	0.824726	2	1	0.47525	no
gi|646737056|gb|KK963115.1|	9898-11546	24	11	0.796964	0.287635	-1	1	no
gi|646737089|gb|KK963112.1|	11579-12915	16	6.8	2	2	-0.527419	0.7035	no
gi|646737089|gb|KK963112.1|	70603-71209	16	6.8	5	7	0.389951	0.7755	no
gi|646737098|gb|KK963111.1|	46488-14713	20	8	0.471464	3	2	0.28375	no
gi|646737098|gb|KK963111.1|	71352-17232	20	8	2	0.73714	-1	0.45505	no
gi|646737098|gb|KK963111.1|	72402-17388	20	8	0.831404	0.979908	0.237096	1	no
gi|646737098|gb|KK963111.1|	83084-18422	20	8	0.625051	0.596252	-0.068052	1	no
gi|646737098|gb|KK963111.1|	14654-21503	20	8	7	12	0.83853	0.51835	no

gi|646737098|gb|KK963111.1|	26639-22736	20	8	3	2	-1	0.42505	no
gi|646737098|gb|KK963111.1|	26632-28990	20	8	2	1	-0.365316	0.81035	no
gi|646737098|gb|KK963111.1|	34187-35499	20	8	2	1	-0.936884	0.5333	no
gi|646737098|gb|KK963111.1|	42384-43894	20	8	0.663439	0.318709	-1	1	no
gi|646737098|gb|KK963111.1|	45884-46865	20	8	1	0.54399	-1	0.47685	no
gi|646737098|gb|KK963111.1|	46993-50515	20	8	2	2	-0.594736	0.68955	no
gi|646737098|gb|KK963111.1|	50748-53695	20	8	2	1	-0.485784	0.7469	no
gi|646737098|gb|KK963111.1|	71901-73368	20	8	2	0.879497	-1	0.3405	no
gi|646737098|gb|KK963111.1|	89423-90510	20	8	0.444742	1	2	0.3671	no
gi|646737098|gb|KK963111.1|	90616-91642	20	8	1	0.513136	-1	0.4271	no
gi|646737098|gb|KK963111.1|	92178-94127	20	8	0.546141	0.474385	-0.203214	1	no
gi|646737115|gb|KK963109.1|	05293-30553	13	6.5	38	37	-0.036309	0.9501	no
gi|646737115|gb|KK963109.1|	20742-32091	13	6.5	167	102.14	-0.705464	0.6239	no
gi|646737115|gb|KK963109.1|	21190-32145	13	6.5	10	19	0.844623	0.58005	no
gi|646737115|gb|KK963109.1|	27627-32792	13	6.5	16	22	0.468467	0.7143	no
gi|646737115|gb|KK963109.1|	34320-33463	13	6.5	12	19	0.635314	0.62535	no
gi|646737115|gb|KK963109.1|	38507-34030	13	6.5	23	20	-0.164748	0.879	no
gi|646737115|gb|KK963109.1|	40485-34140	13	6.5	12	4	-2	0.33835	no
gi|646737122|gb|KK963108.1|	06178-10644	17	9	35	7	-2	0.1532	no
gi|646737122|gb|KK963108.1|	10978-11119	17	9	59	21	-1	0.3379	no
gi|646737122|gb|KK963108.1|	11560-14675	17	9	29	23	-0.360267	0.75575	no
gi|646737122|gb|KK963108.1|	21960-12236	17	9	5	8	0.519964	0.70115	no
gi|646737122|gb|KK963108.1|	31695-13284	17	9	14	10	-0.557245	0.7115	no
gi|646737122|gb|KK963108.1|	41326-14191	17	9	2	0.768337	-2	0.3832	no
gi|646737122|gb|KK963108.1|	42299-14285	17	9	2	2	-0.356767	0.81835	no
gi|646737122|gb|KK963108.1|	43149-14347	17	9	20	14	-0.504445	0.6763	no
gi|646737122|gb|KK963108.1|	43974-14622	17	9	8	7	-0.100819	0.94465	no
gi|646737122|gb|KK963108.1|	55395-15814	17	9	34	12	-2	0.1857	no
gi|646737122|gb|KK963108.1|	81906-18212	17	9	38	24	-0.669452	0.6598	no
gi|646737122|gb|KK963108.1|	93812-19448	17	9	3	2	-0.299592	0.82375	no

gi|646737122|gb|KK963108.1| 95322-19590	17	9	1	3	1	0.43555	no
gi|646737122|gb|KK963108.1| 17075-21759	17	9	38	42	0.161045	0.915	no
gi|646737122|gb|KK963108.1| 20770-22169	17	9	4	6	0.554209	0.728	no
gi|646737122|gb|KK963108.1| 22677-27207	17	9	35	19	-0.861914	0.46545	no
gi|646737122|gb|KK963108.1| 49597-50074	17	9	12	6	-1	0.46465	no
gi|646737150|gb|KK963105.1| 28268-12875	15	9	22	13	-0.739871	0.6285	no
gi|646737150|gb|KK963105.1| 50073-15049	15	9	0	14	inf	0.00315	no
gi|646737159|gb|KK963104.1| 70915-17143	15	6.8	4	3	-0.45379	0.735	no
gi|646737180|gb|KK963102.1| 46723-14782	22	9	37	4	-3	0.03515	no
gi|646737180|gb|KK963102.1| 53965-15485	22	9	2	0.205493	-3	0.1994	no
gi|646737180|gb|KK963102.1| 55132-15613	22	9	0.990673	1	0.510284	0.71995	no
gi|646737180|gb|KK963102.1| 59507-16067	22	9	0.812365	0.43617	-0.897238	1	no
gi|646737180|gb|KK963102.1| 60803-16144	22	9	2	1	-0.851104	0.56515	no
gi|646737180|gb|KK963102.1| 84775-85075	22	9	7	21	2	0.26305	no
gi|646737201|gb|KK963100.1| 14138-11538	12	7.3	13	2	-3	0.12085	no
gi|646737201|gb|KK963100.1| 17661-11803	12	7.3	7	7	0.0230217	0.9428	no
gi|646737201|gb|KK963100.1| 96378-19699	12	7.3	2	2	0.20437	0.89115	no
gi|646737201|gb|KK963100.1| 99992-20066	12	7.3	0	5	inf	0.00605	no
gi|646737201|gb|KK963100.1| 02265-20297	12	7.3	0	8	inf	0.0012	yes
gi|646737201|gb|KK963100.1| 09187-21009	12	7.3	0	3	inf	0.00605	no
gi|646737201|gb|KK963100.1| 36120-37268	12	7.3	2	2	0.217547	0.86235	no
gi|646737252|gb|KK963095.1| 00703-20198	16	7.3	2	0	-inf	0.00495	no
gi|646737252|gb|KK963095.1| 29256-22955	16	7.3	378	2	-8	0.1665	no
gi|646737252|gb|KK963095.1| 47097-48864	16	7.3	31	31	0.0136587	0.9898	no
gi|646737252|gb|KK963095.1| 50221-52089	16	7.3	8	15	0.883722	0.4932	no
gi|646737300|gb|KK963090.1| 66312-16805	12	8.4	0	1	inf	0.00515	no
gi|646737300|gb|KK963090.1| 72923-17546	12	8.4	0	1	inf	0.00315	no
gi|646737300|gb|KK963090.1| 76983-17892	12	8.4	0	2	inf	0.0026	no
gi|646737300|gb|KK963090.1| 79341-18034	12	8.4	0	2	inf	0.001	yes
gi|646737300|gb|KK963090.1| 83165-18464	12	8.4	0	1	inf	0.0008	yes

gi|646737300|gb|KK963090.1|	84875-18575	12	8.4	0	3	inf	0.00605	no
gi|646737300|gb|KK963090.1|	89173-19204	12	8.4	0	3	inf	0.0004	yes
gi|646737300|gb|KK963090.1|	92906-19634	12	8.4	0	3	inf	0.00015	yes
gi|646737300|gb|KK963090.1|	96459-19922	12	8.4	0	5	inf	0.0005	yes
gi|646737312|gb|KK963089.1|	48539-49455	18	7.4	0.839649	1	0.72727	0.6352	no
gi|646737312|gb|KK963089.1|	49555-50292	18	7.4	0.191876	2	4	0.1061	no
gi|646737322|gb|KK963088.1|	29665-13003	13	9.2	5	5	-0.146922	0.91555	no
gi|646737322|gb|KK963088.1|	93047-19369	13	9.2	0.950146	2	1	0.4862	no
gi|646737344|gb|KK963086.1|	00535-10162	16	9.9	2	1	-0.392034	0.76465	no
gi|646737344|gb|KK963086.1|	06702-10824	16	9.9	1	2	0.527956	0.7131	no
gi|646737344|gb|KK963086.1|	56193-15678	16	9.9	1	6	2	0.21055	no
gi|646737344|gb|KK963086.1|	57120-15758	16	9.9	3	2	-0.173108	0.89395	no
gi|646737344|gb|KK963086.1|	58735-15923	16	9.9	2	3	1	0.48625	no
gi|646737344|gb|KK963086.1|	73712-17421	16	9.9	20	2	-3	0.0814	no
gi|646737344|gb|KK963086.1|	34871-33583	16	9.9	3	1	-1	0.33475	no
gi|646737344|gb|KK963086.1|	49974-50867	16	9.9	23	33	0.521336	0.6857	no
gi|646737344|gb|KK963086.1|	51512-52281	16	9.9	17	22	0.347919	0.81835	no
gi|646737354|gb|KK963085.1|	16782-11742	20	11	4	3	-0.698896	0.5862	no
gi|646737354|gb|KK963085.1|	33881-13716	20	11	25	24	-0.0880756	0.94315	no
gi|646737354|gb|KK963085.1|	40936-14138	20	11	44	59	0.434117	0.77565	no
gi|646737354|gb|KK963085.1|	49121-14944	20	11	11	33	2	0.31045	no
gi|646737354|gb|KK963085.1|	87747-18885	20	11	1	2	0.75639	0.5508	no
gi|646737354|gb|KK963085.1|	91756-19239	20	11	0.967422	2	1	0.4355	no
gi|646737354|gb|KK963085.1|	01847-20252	20	11	0.439174	3	3	0.2662	no
gi|646737354|gb|KK963085.1|	24844-22567	20	11	4	4	0.22225	0.89	no
gi|646737354|gb|KK963085.1|	41661-24558	20	11	2	2	0.0924439	0.95015	no
gi|646737354|gb|KK963085.1|	59799-26123	20	11	0.312541	1	2	1	no
gi|646737354|gb|KK963085.1|	63587-26420	20	11	1	4	2	0.20625	no
gi|646737354|gb|KK963085.1|	67976-26945	20	11	2	4	0.927035	0.5605	no
gi|646737354|gb|KK963085.1|	75125-27828	20	11	7	14	0.974255	0.3782	no

gi|646737354|gb|KK963085.1|	88513-38923	20	11	0	3	inf	0.0014	yes
gi|646737354|gb|KK963085.1|	61832-63100	20	11	1	0.262134	-2	1	no
gi|646737354|gb|KK963085.1|	67375-71049	20	11	8	19	1	0.25535	no
gi|646737385|gb|KK963082.1|	38922-33929	18	7.7	10	9	-0.148026	0.9084	no
gi|646737394|gb|KK963081.1|	89926-90605	13	7.4	0	7	inf	0.00175	no
gi|646737401|gb|KK963080.1|	16472-21697	15	6.1	4	0	-inf	0.00655	no
gi|646737430|gb|KK963077.1|	52885-53618	15	7.3	4	6	0.631348	0.64665	no
gi|646737447|gb|KK963075.1|	04679-10818	22	9.2	28	25	-0.181573	0.87675	no
gi|646737447|gb|KK963075.1|	14087-11483	22	9.2	2	2	0.126237	0.9235	no
gi|646737447|gb|KK963075.1|	21823-12229	22	9.2	3	3	0.00267068	0.9298	no
gi|646737447|gb|KK963075.1|	32309-13381	22	9.2	0.666093	0.213308	-2	1	no
gi|646737447|gb|KK963075.1|	37087-13838	22	9.2	7	11	0.722144	0.63725	no
gi|646737447|gb|KK963075.1|	45908-14906	22	9.2	2	0.929361	-0.917904	0.555	no
gi|646737447|gb|KK963075.1|	50001-15118	22	9.2	22	14	-0.688942	0.5804	no
gi|646737447|gb|KK963075.1|	56299-15836	22	9.2	1	0.443145	-2	0.2654	no
gi|646737447|gb|KK963075.1|	68216-16975	22	9.2	3	2	-0.320212	0.8453	no
gi|646737447|gb|KK963075.1|	75437-17650	22	9.2	0.793556	0.647458	-0.293546	1	no
gi|646737447|gb|KK963075.1|	86788-18851	22	9.2	0.502457	0.362996	-0.469046	1	no
gi|646737447|gb|KK963075.1|	88666-18957	22	9.2	0.984055	0.797812	-0.302691	1	no
gi|646737447|gb|KK963075.1|	91818-19234	22	9.2	14	15	0.136298	0.9295	no
gi|646737447|gb|KK963075.1|	20877-23608	22	9.2	18	12	-0.602563	0.5814	no
gi|646737447|gb|KK963075.1|	23379-22437	22	9.2	4	5	0.323249	0.83315	no
gi|646737447|gb|KK963075.1|	23722-24540	22	9.2	1	0.695464	-1	0.47555	no
gi|646737447|gb|KK963075.1|	59157-25997	22	9.2	1	0.695464	-1	0.47555	no
gi|646737447|gb|KK963075.1|	66723-26755	22	9.2	20	14	-0.513242	0.7357	no
gi|646737447|gb|KK963075.1|	71473-27290	22	9.2	1	4	2	0.23035	no
gi|646737447|gb|KK963075.1|	77337-27833	22	9.2	0.993332	4	2	0.23635	no
gi|646737447|gb|KK963075.1|	83518-28479	22	9.2	1	6	2	0.1502	no
gi|646737447|gb|KK963075.1|	08153-31101	22	9.2	16	8	-0.92967	0.39855	no
gi|646737447|gb|KK963075.1|	12464-31451	22	9.2	1	0.672773	-1	0.4491	no

gi|646737447|gb|KK963075.1|	14615-31672	22	9.2	0.69674	0.432781	-0.686983	1	no
gi|646737447|gb|KK963075.1|	16841-31884	22	9.2	1	0.383263	-2	1	no
gi|646737447|gb|KK963075.1|	19123-32027	22	9.2	2	1	-0.772243	0.6022	no
gi|646737447|gb|KK963075.1|	32964-35127	22	9.2	0.678848	0.773292	0.187924	1	no
gi|646737447|gb|KK963075.1|	47082-35428	22	9.2	37	35	-0.0755163	0.9475	no
gi|646737447|gb|KK963075.1|	56388-35775	22	9.2	0.998323	2	0.746409	0.5505	no
gi|646737447|gb|KK963075.1|	60187-36136	22	9.2	1	0.573571	-0.803646	1	no
gi|646737447|gb|KK963075.1|	62109-36439	22	9.2	10	17	0.838144	0.43825	no
gi|646737447|gb|KK963075.1|	47023-48277	22	9.2	1	0.664317	-0.742209	1	no
gi|646737447|gb|KK963075.1|	56952-58190	22	9.2	0.564655	0.539911	-0.0646473	1	no
gi|646737486|gb|KK963071.1|	01412-10173	16	10	23	1	-4	0.16905	no
gi|646737486|gb|KK963071.1|	10413-11107	16	10	2	3	0.798345	0.56885	no
gi|646737486|gb|KK963071.1|	40231-41125	16	10	4	5	0.326387	0.83565	no
gi|646737486|gb|KK963071.1|	43395-44106	16	10	1	2	0.74686	0.6148	no
gi|646737486|gb|KK963071.1|	92529-93506	16	10	0.640605	1	0.99427	0.5626	no
gi|646737528|gb|KK963067.1|	10220-10671	20	6.1	48	18	-1	0.3742	no
gi|646737528|gb|KK963067.1|	15817-11609	20	6.1	31	12	-1	0.3039	no
gi|646737528|gb|KK963067.1|	20577-12099	20	6.1	5	6	0.232143	0.86235	no
gi|646737528|gb|KK963067.1|	30661-13090	20	6.1	60	42	-0.522163	0.6646	no
gi|646737528|gb|KK963067.1|	48263-14847	20	6.1	80	45	-0.834512	0.5384	no
gi|646737528|gb|KK963067.1|	61336-16177	20	6.1	21	15	-0.471967	0.7598	no
gi|646737528|gb|KK963067.1|	65518-16594	20	6.1	71	29	-1	0.39365	no
gi|646737528|gb|KK963067.1|	96850-19711	20	6.1	23	3	-3	0.1932	no
gi|646737528|gb|KK963067.1|	04690-20502	20	6.1	8	5	-0.708543	0.64635	no
gi|646737528|gb|KK963067.1|	09303-21015	20	6.1	14	5	-2	0.3195	no
gi|646737528|gb|KK963067.1|	13827-22020	20	6.1	16	10	-0.708199	0.5386	no
gi|646737528|gb|KK963067.1|	41556-24217	20	6.1	2	3	0.882219	0.5493	no
gi|646737528|gb|KK963067.1|	45797-24675	20	6.1	0.131958	2	4	0.1061	no
gi|646737528|gb|KK963067.1|	28203-29988	20	6.1	1	0.611918	-0.748355	1	no
gi|646737528|gb|KK963067.1|	49827-50822	20	6.1	5	2	-1	0.33605	no

gi|646737528|gb|KK963067.1|	81288-84626	20	6.1	34	18	-0.955685	0.4117	no
gi|646737528|gb|KK963067.1|	84801-87408	20	6.1	16	11	-0.551554	0.61095	no
gi|646737549|gb|KK963065.1|	27098-12746	14	9.5	5	5	-0.152529	0.91095	no
gi|646737549|gb|KK963065.1|	14683-41526	14	9.5	104	42	-1	0.32075	no
gi|646737557|gb|KK963064.1|	49801-51187	18	6.1	0.081796	3	5	0.09925	no
gi|646737625|gb|KK963058.1|	90844-91528	17	7.5	5	5	0.257162	0.8498	no
gi|646737644|gb|KK963056.1|	00169-10053	22	10	4	9	1	0.42855	no
gi|646737644|gb|KK963056.1|	08701-10942	22	10	0.99013	3	1	0.33915	no
gi|646737644|gb|KK963056.1|	17715-11873	22	10	2	3	0.330745	0.8105	no
gi|646737644|gb|KK963056.1|	18988-11983	22	10	1	6	2	0.2179	no
gi|646737644|gb|KK963056.1|	12376-12748	22	10	7	2	-2	0.2745	no
gi|646737644|gb|KK963056.1|	39036-13931	22	10	93	39	-1	0.4331	no
gi|646737644|gb|KK963056.1|	15391-15981	22	10	3	2	-0.414502	0.7788	no
gi|646737644|gb|KK963056.1|	92301-19396	22	10	9	2	-2	0.1952	no
gi|646737644|gb|KK963056.1|	94586-19525	22	10	6	0.936559	-3	0.1849	no
gi|646737644|gb|KK963056.1|	2444-4110	22	10	2	2	-0.0318657	0.98135	no
gi|646737644|gb|KK963056.1|	50224-56984	22	10	4	4	-0.0212956	0.98395	no
gi|646737644|gb|KK963056.1|	57101-60961	22	10	3	6	1	0.38505	no
gi|646737644|gb|KK963056.1|	61151-62583	22	10	8	8	0.0289008	0.98385	no
gi|646737644|gb|KK963056.1|	67715-68329	22	10	5	7	0.543826	0.6948	no
gi|646737644|gb|KK963056.1|	68422-69794	22	10	4	8	0.952986	0.53225	no
gi|646737667|gb|KK963054.1|	03410-10377	13	6.9	8	0	-inf	0.00475	no
gi|646737667|gb|KK963054.1|	15377-21572	13	6.9	76	110	0.530388	0.72475	no
gi|646737680|gb|KK963053.1|	06446-10684	21	5.9	24	14	-0.796951	0.62105	no
gi|646737680|gb|KK963053.1|	10612-11083	21	5.9	58	45	-0.357201	0.7842	no
gi|646737680|gb|KK963053.1|	27426-12772	21	5.9	84	47	-0.828992	0.58995	no
gi|646737680|gb|KK963053.1|	40121-14334	21	5.9	16	16	-0.0236949	0.98205	no
gi|646737680|gb|KK963053.1|	49900-15377	21	5.9	14	14	0.0473332	0.96545	no
gi|646737680|gb|KK963053.1|	55034-15621	21	5.9	2	0.716207	-1	0.4405	no
gi|646737680|gb|KK963053.1|	57767-15870	21	5.9	0.817396	3	2	0.26985	no

gi|646737680|gb|KK963053.1|	68495-16944	21	5.9	10	11	0.0795578	0.957	no
gi|646737680|gb|KK963053.1|	73123-17418	21	5.9	1	0.163621	-3	0.2033	no
gi|646737680|gb|KK963053.1|	80905-18127	21	5.9	10	6	-0.881706	0.5051	no
gi|646737680|gb|KK963053.1|	83126-18415	21	5.9	7	11	0.601779	0.6844	no
gi|646737680|gb|KK963053.1|	85997-18624	21	5.9	22	26	0.285272	0.8462	no
gi|646737680|gb|KK963053.1|	89065-18994	21	5.9	2	4	0.781889	0.58135	no
gi|646737680|gb|KK963053.1|	90091-19381	21	5.9	0.879884	0.85486	-0.0416251	1	no
gi|646737680|gb|KK963053.1|	94916-19640	21	5.9	2	1	-0.347576	0.80355	no
gi|646737680|gb|KK963053.1|	2350-2863	21	5.9	13	12	-0.130599	0.9311	no
gi|646737680|gb|KK963053.1|	60122-26032	21	5.9	102	52	-0.968911	0.474	no
gi|646737680|gb|KK963053.1|	78667-28184	21	5.9	5	2	-1	0.47605	no
gi|646737680|gb|KK963053.1|	00406-30068	21	5.9	50	0	-inf	0.0047	no
gi|646737680|gb|KK963053.1|	19650-31991	21	5.9	98	0	-inf	0.00125	yes
gi|646737680|gb|KK963053.1|	35970-36950	21	5.9	0.893137	7	3	0.0929	no
gi|646737680|gb|KK963053.1|	63265-36425	21	5.9	3	2	-0.433985	0.7515	no
gi|646737680|gb|KK963053.1|	69268-37002	21	5.9	1	2	0.659742	0.63585	no
gi|646737680|gb|KK963053.1|	70354-37103	21	5.9	6	2	-2	0.2965	no
gi|646737680|gb|KK963053.1|	38341-43885	21	5.9	5	1	-2	0.23925	no
gi|646737680|gb|KK963053.1|	43223-44441	21	5.9	3	2	-0.574465	0.7285	no
gi|646737680|gb|KK963053.1|	44544-44793	21	5.9	2	1	-0.767992	0.61325	no
gi|646737680|gb|KK963053.1|	48295-45218	21	5.9	3	2	-0.57654	0.6996	no
gi|646737680|gb|KK963053.1|	52530-45393	21	5.9	0.64412	1	0.694851	1	no
gi|646737680|gb|KK963053.1|	45472-46014	21	5.9	3	1	-1	0.41965	no
gi|646737680|gb|KK963053.1|	49772-50900	21	5.9	5	3	-0.483694	0.75515	no
gi|646737680|gb|KK963053.1|	20363-52056	21	5.9	74	28	-1	0.36185	no
gi|646737680|gb|KK963053.1|	26076-52688	21	5.9	2	0.936889	-1	0.3737	no
gi|646737680|gb|KK963053.1|	32821-53341	21	5.9	3	2	-0.539516	0.69565	no
gi|646737680|gb|KK963053.1|	33562-53708	21	5.9	0.989982	1	0.168384	1	no
gi|646737680|gb|KK963053.1|	37257-53874	21	5.9	0.827349	2	0.974187	0.53675	no
gi|646737680|gb|KK963053.1|	38852-53976	21	5.9	4	2	-0.791152	0.575	no

gi|646737680|gb|KK963053.1|	56302-58942	21	5.9	25.37	11	-1	0.30405	no
gi|646737680|gb|KK963053.1|	59167-61534	21	5.9	37	24	-0.612834	0.5971	no
gi|646737680|gb|KK963053.1|	65083-66854	21	5.9	0.793336	0.35285	-1	1	no
gi|646737680|gb|KK963053.1|	66977-68834	21	5.9	0.462001	0.668337	0.53268	1	no
gi|646737680|gb|KK963053.1|	79770-81287	21	5.9	4	1	-2	0.20385	no
gi|646737680|gb|KK963053.1|	81441-84138	21	5.9	4	1	-2	0.23255	no
gi|646737680|gb|KK963053.1|	9055-11111	21	5.9	1	0.744246	-0.465139	1	no
gi|646737680|gb|KK963053.1|	93057-95500	21	5.9	2	2	0.611973	0.69045	no
gi|646737714|gb|KK963050.1|	02266-10772	26	11	11	11	0.0354952	0.9759	no
gi|646737714|gb|KK963050.1|	10635-11250	26	11	12	10	-0.329066	0.7911	no
gi|646737714|gb|KK963050.1|	24872-22620	26	11	0.946622	1	0.385541	0.77315	no
gi|646737714|gb|KK963050.1|	26440-22758	26	11	1	1	0.379288	0.7733	no
gi|646737714|gb|KK963050.1|	47666-24861	26	11	9	10	0.179943	0.9024	no
gi|646737714|gb|KK963050.1|	50290-25099	26	11	2	3	0.482457	0.7265	no
gi|646737714|gb|KK963050.1|	57379-25817	26	11	2	3	0.90945	0.49355	no
gi|646737714|gb|KK963050.1|	77997-27907	26	11	2	4	0.973473	0.4998	no
gi|646737714|gb|KK963050.1|	79341-28039	26	11	1	2	0.891654	0.4908	no
gi|646737714|gb|KK963050.1|	81875-28215	26	11	9	15	0.66936	0.66055	no
gi|646737714|gb|KK963050.1|	86897-28930	26	11	2	1	-0.696742	0.66605	no
gi|646737714|gb|KK963050.1|	89930-29023	26	11	10	26	1	0.38005	no
gi|646737714|gb|KK963050.1|	92007-29511	26	11	6	8	0.260707	0.83595	no
gi|646737714|gb|KK963050.1|	95414-29591	26	11	3	8	1	0.4194	no
gi|646737714|gb|KK963050.1|	96044-29754	26	11	0.521409	1	1	0.35635	no
gi|646737714|gb|KK963050.1|	07515-30790	26	11	3	14	2	0.2368	no
gi|646737714|gb|KK963050.1|	08428-30918	26	11	3	6	0.862279	0.5367	no
gi|646737714|gb|KK963050.1|	10966-31135	26	11	4	4	-0.124664	0.9214	no
gi|646737714|gb|KK963050.1|	12029-31220	26	11	135	111	-0.274752	0.8443	no
gi|646737714|gb|KK963050.1|	13074-31683	26	11	3	6	1	0.36045	no
gi|646737714|gb|KK963050.1|	16987-31789	26	11	0.705039	2	2	0.33695	no
gi|646737714|gb|KK963050.1|	21259-32257	26	11	0.525628	1	1	0.4444	no

gi|646737714|gb|KK963050.1|	26264-32726	26	11	0.248333	2	3	0.2655	no
gi|646737714|gb|KK963050.1|	29770-33026	26	11	2	4	1	0.3784	no
gi|646737714|gb|KK963050.1|	30530-33337	26	11	2	1	-0.301074	0.84275	no
gi|646737714|gb|KK963050.1|	46533-34905	26	11	0.89824	0.177904	-2	1	no
gi|646737714|gb|KK963050.1|	74733-37806	26	11	0.902346	1	0.220235	1	no
gi|646737714|gb|KK963050.1|	78216-37885	26	11	0.727455	3	2	0.25715	no
gi|646737714|gb|KK963050.1|	87898-38855	26	11	6	2	-2	0.2958	no
gi|646737714|gb|KK963050.1|	97592-39916	26	11	0.980462	2	0.808599	0.56595	no
gi|646737714|gb|KK963050.1|	17257-41805	26	11	1	0.721342	-0.920414	0.5894	no
gi|646737714|gb|KK963050.1|	81320-81965	26	11	12	9	-0.334916	0.832	no
gi|646737714|gb|KK963050.1|	99276-99596	26	11	17	10	-0.829476	0.5351	no
gi|646737721|gb|KK963049.1|	39392-14027	13	6.4	0	21	inf	5.00E-05	yes
gi|646737721|gb|KK963049.1|	63447-64805	13	6.4	27	7	-2	0.13715	no
gi|646737740|gb|KK963047.1|	65989-66528	15	7.7	9	12	0.392231	0.809	no
gi|646737766|gb|KK963045.1|	04976-10541	21	9.2	4	3	-0.773137	0.60695	no
gi|646737766|gb|KK963045.1|	19174-12405	21	9.2	11	8	-0.420353	0.70695	no
gi|646737766|gb|KK963045.1|	25415-12765	21	9.2	1	0.337509	-2	1	no
gi|646737766|gb|KK963045.1|	34191-13669	21	9.2	6	6	-0.195547	0.9015	no
gi|646737766|gb|KK963045.1|	38541-14039	21	9.2	12	7	-0.889363	0.47835	no
gi|646737766|gb|KK963045.1|	44092-14437	21	9.2	15	6	-1	0.3917	no
gi|646737766|gb|KK963045.1|	47872-14847	21	9.2	5	3	-1	0.4135	no
gi|646737766|gb|KK963045.1|	61054-16134	21	9.2	19	15	-0.369683	0.77905	no
gi|646737766|gb|KK963045.1|	63145-16399	21	9.2	1	2	0.959032	0.4968	no
gi|646737766|gb|KK963045.1|	64129-16538	21	9.2	1	1	-0.386096	0.76835	no
gi|646737766|gb|KK963045.1|	75120-17569	21	9.2	3	6	0.803837	0.5356	no
gi|646737766|gb|KK963045.1|	77110-17819	21	9.2	3	2	-0.42401	0.75235	no
gi|646737766|gb|KK963045.1|	92968-19327	21	9.2	18	31	0.762652	0.58685	no
gi|646737766|gb|KK963045.1|	94338-19488	21	9.2	11	12	0.114486	0.94035	no
gi|646737766|gb|KK963045.1|	53988-25508	21	9.2	4	5	0.163473	0.9135	no
gi|646737766|gb|KK963045.1|	58107-25840	21	9.2	12	24	0.975927	0.549	no

gi|646737766|gb|KK963045.1|	70530-27162	21	9.2	12	3	-2	0.2402	no
gi|646737766|gb|KK963045.1|	11308-31242	21	9.2	1	1	-0.121455	0.9312	no
gi|646737766|gb|KK963045.1|	88435-88616	21	9.2	89	64	-0.485329	0.76355	no
gi|646737782|gb|KK963043.1|	1669-1986	23	9.2	36	28	-0.367089	0.8164	no
gi|646737782|gb|KK963043.1|	67929-16932	23	9.2	6	5	-0.0392878	0.9802	no
gi|646737782|gb|KK963043.1|	82628-28518	23	9.2	1	1	0.23058	0.88545	no
gi|646737782|gb|KK963043.1|	85300-29098	23	9.2	1	2	0.148892	0.92015	no
gi|646737782|gb|KK963043.1|	99947-30030	23	9.2	0.824084	10	4	0.106	no
gi|646737782|gb|KK963043.1|	04468-31238	23	9.2	9	5	-0.788881	0.4788	no
gi|646737782|gb|KK963043.1|	31525-31756	23	9.2	91	69	-0.390666	0.8072	no
gi|646737782|gb|KK963043.1|	27230-32806	23	9.2	13	5	-1	0.36415	no
gi|646737782|gb|KK963043.1|	36578-37332	23	9.2	0.741396	2	1	0.4859	no
gi|646737782|gb|KK963043.1|	73804-37448	23	9.2	3	0	-inf	0.0035	no
gi|646737782|gb|KK963043.1|	98741-39924	23	9.2	0.372845	10	5	0.1068	no
gi|646737782|gb|KK963043.1|	08700-40893	23	9.2	11	31	1	0.36505	no
gi|646737782|gb|KK963043.1|	46564-44742	23	9.2	0.923449	2	0.915243	0.5424	no
gi|646737782|gb|KK963043.1|	54297-45539	23	9.2	0.329596	1	2	0.25365	no
gi|646737782|gb|KK963043.1|	60513-46071	23	9.2	43	80	0.888634	0.5566	no
gi|646737782|gb|KK963043.1|	62495-46305	23	9.2	6	5	-0.354932	0.76605	no
gi|646737782|gb|KK963043.1|	76967-47745	23	9.2	2	3	0.423152	0.77485	no
gi|646737782|gb|KK963043.1|	49468-50210	23	9.2	15	21	0.451633	0.7633	no
gi|646737782|gb|KK963043.1|	98364-49859	23	9.2	15	42	2	0.34595	no
gi|646737782|gb|KK963043.1|	03051-50405	23	9.2	3	4	0.641751	0.6405	no
gi|646737782|gb|KK963043.1|	04211-50460	23	9.2	3	6	1	0.4686	no
gi|646737782|gb|KK963043.1|	51151-52121	23	9.2	37	14	-1	0.2694	no
gi|646737782|gb|KK963043.1|	21189-52296	23	9.2	97	1	-6	0.0095	no
gi|646737782|gb|KK963043.1|	23457-52452	23	9.2	2	0	-inf	0.00225	no
gi|646737782|gb|KK963043.1|	25948-52695	23	9.2	0.990673	1	0.510284	0.71995	no
gi|646737782|gb|KK963043.1|	29997-53085	23	9.2	111.06	0	-inf	5.00E-05	yes
gi|646737782|gb|KK963043.1|	62617-56354	23	9.2	0.687234	3	2	0.21275	no

gi|646737782|gb|KK963043.1|	56769-57035	23	9.2	78	29	-1	0.3124	no
gi|646737782|gb|KK963043.1|	5910-6071	23	9.2	134	154	0.202621	0.8773	no
gi|646737782|gb|KK963043.1|	93690-59418	23	9.2	5	2	-2	0.2807	no
gi|646737782|gb|KK963043.1|	59436-59685	23	9.2	344	144	-1	0.4137	no
gi|646737782|gb|KK963043.1|	22310-62283	23	9.2	2	3	0.702706	0.64555	no
gi|646737782|gb|KK963043.1|	23590-62417	23	9.2	1	2	1	0.48625	no
gi|646737782|gb|KK963043.1|	25076-62581	23	9.2	3	2	-0.405991	0.7698	no
gi|646737782|gb|KK963043.1|	27071-62850	23	9.2	5	0.226668	-4	0.1139	no
gi|646737782|gb|KK963043.1|	63242-63603	23	9.2	6	2	-2	0.30735	no
gi|646737790|gb|KK963042.1|	68306-68722	14	9.1	154	117	-0.397283	0.70895	no
gi|646737790|gb|KK963042.1|	69743-70152	14	9.1	309	264	-0.227936	0.84095	no
gi|646737790|gb|KK963042.1|	92177-92437	14	9.1	2336.98	2193.46	-0.0914382	0.9354	no
gi|646737815|gb|KK963040.1|	33257-13408	8.8	6.6	3	0	-inf	0.0008	yes
gi|646737815|gb|KK963040.1|	35963-13727	8.8	6.6	23	0	-inf	5.00E-05	yes
gi|646737815|gb|KK963040.1|	39707-14049	8.8	6.6	7	0	-inf	0.0006	yes
gi|646737815|gb|KK963040.1|	42888-14372	8.8	6.6	7	0	-inf	0.00125	yes
gi|646737815|gb|KK963040.1|	43828-14435	8.8	6.6	4	0	-inf	0.0041	no
gi|646737815|gb|KK963040.1|	47101-14766	8.8	6.6	187	0	-inf	5.00E-05	yes
gi|646737815|gb|KK963040.1|	48404-14950	8.8	6.6	1	0	-inf	0.00475	no
gi|646737822|gb|KK963039.1|	44538-14509	20	9	4	8	0.89116	0.5304	no
gi|646737822|gb|KK963039.1|	47555-14863	20	9	13	29.25	1	0.3694	no
gi|646737822|gb|KK963039.1|	55046-16135	20	9	19	20	0.0875339	0.94095	no
gi|646737822|gb|KK963039.1|	15831-16397	20	9	5	5	0.066294	0.9573	no
gi|646737822|gb|KK963039.1|	61588-17453	20	9	19	25	0.400693	0.7354	no
gi|646737822|gb|KK963039.1|	96521-19699	20	9	15	0	-inf	0.0014	yes
gi|646737822|gb|KK963039.1|	10630-21283	20	9	41	9	-2	0.11055	no
gi|646737822|gb|KK963039.1|	40763-24108	20	9	14	4	-2	0.28255	no
gi|646737822|gb|KK963039.1|	42560-24548	20	9	14	8	-0.81099	0.45335	no
gi|646737822|gb|KK963039.1|	45675-24628	20	9	3	2	-0.41028	0.7788	no
gi|646737822|gb|KK963039.1|	63571-26565	20	9	1	0	-inf	0.00495	no

gi|646737822|gb|KK963039.1|	81968-28289	20	9	1	0	-inf	0.00655	no
gi|646737822|gb|KK963039.1|	29343-29698	20	9	112	68	-0.723942	0.62855	no
gi|646737822|gb|KK963039.1|	36653-38882	20	9	3	3	0.088943	0.9549	no
gi|646737822|gb|KK963039.1|	39243-40275	20	9	1	2	0.511893	0.695	no
gi|646737822|gb|KK963039.1|	42130-42768	20	9	10	19	0.852119	0.57875	no
gi|646737822|gb|KK963039.1|	43493-45352	20	9	0.865172	1	0.532705	0.7108	no
gi|646737822|gb|KK963039.1|	45573-46277	20	9	2	3	0.193259	0.8791	no
gi|646737822|gb|KK963039.1|	50506-51004	20	9	117	102	-0.201054	0.85065	no
gi|646737822|gb|KK963039.1|	70561-71785	20	9	0.763749	2	1	0.41115	no
gi|646737822|gb|KK963039.1|	75716-77692	20	9	42	39	-0.127239	0.9103	no
gi|646737822|gb|KK963039.1|	79021-80072	20	9	2	2	0.164926	0.8944	no
gi|646737822|gb|KK963039.1|	85441-85777	20	9	141	19	-3	0.09465	no
gi|646737822|gb|KK963039.1|	85945-87368	20	9	0.712849	0.456003	-0.644554	1	no
gi|646737822|gb|KK963039.1|	93342-93932	20	9	5	3	-0.627495	0.6249	no
gi|646737822|gb|KK963039.1|	96646-97062	20	9	40	28	-0.541673	0.72695	no
gi|646737831|gb|KK963038.1|	13958-11507	10	6.5	0.858534	2	1	0.3239	no
gi|646737853|gb|KK963036.1|	37034-13766	42	18	5	4	-0.404059	0.7728	no
gi|646737853|gb|KK963036.1|	63178-16439	42	18	2	3	0.268182	0.86575	no
gi|646737853|gb|KK963036.1|	64498-16531	42	18	2	1	-0.640761	0.65475	no
gi|646737853|gb|KK963036.1|	76420-17820	42	18	0.54492	0.962826	0.821232	1	no
gi|646737853|gb|KK963036.1|	78387-18009	42	18	1	1	0.143576	0.9072	no
gi|646737853|gb|KK963036.1|	82213-18574	42	18	0.846744	0.740121	-0.194165	1	no
gi|646737853|gb|KK963036.1|	86134-18726	42	18	1	1	0.00173286	1	no
gi|646737853|gb|KK963036.1|	89779-19305	42	18	0.765391	0.802357	0.0680469	1	no
gi|646737853|gb|KK963036.1|	93224-19435	42	18	0.845781	0.453667	-0.89865	1	no
gi|646737853|gb|KK963036.1|	94653-19604	42	18	0.811505	0.817227	0.0101369	1	no
gi|646737853|gb|KK963036.1|	96159-19836	42	18	0.759508	0.757209	-0.00437223	1	no
gi|646737853|gb|KK963036.1|	99673-20163	42	18	0.977285	1	0.270827	0.84875	no
gi|646737853|gb|KK963036.1|	17979-22006	42	18	2	2	0.203905	0.895	no
gi|646737853|gb|KK963036.1|	40475-24190	42	18	0.869028	1	0.387936	0.7705	no

gi|646737853|gb|KK963036.1|	51609-25421	42	18	43	42	-0.0532371	0.9625	no
gi|646737853|gb|KK963036.1|	55278-25677	42	18	2	1	-0.164862	0.89055	no
gi|646737853|gb|KK963036.1|	65100-26809	42	18	55	58	0.0750725	0.94915	no
gi|646737853|gb|KK963036.1|	68466-26878	42	18	9	10	0.0725346	0.9332	no
gi|646737853|gb|KK963036.1|	83489-28498	42	18	2	1	-0.920607	0.5111	no
gi|646737853|gb|KK963036.1|	90404-29211	42	18	1	1	-0.276681	0.81505	no
gi|646737853|gb|KK963036.1|	92617-29334	42	18	3	5	0.568872	0.6862	no
gi|646737853|gb|KK963036.1|	94322-29455	42	18	36	23	-0.672619	0.63615	no
gi|646737853|gb|KK963036.1|	58915-59601	42	18	2	0.299169	-3	0.2033	no
gi|646737853|gb|KK963036.1|	60691-61586	42	18	2	0.204873	-3	0.1757	no
gi|646737853|gb|KK963036.1|	65763-66602	42	18	2	0	-inf	0.00655	no
gi|646737853|gb|KK963036.1|	68722-70085	42	18	1	0.360155	-2	1	no
gi|646737853|gb|KK963036.1|	73181-73942	42	18	2	3	0.112043	0.93025	no
gi|646737853|gb|KK963036.1|	74015-74566	42	18	2	2	-0.358664	0.81835	no
gi|646737853|gb|KK963036.1|	75630-76691	42	18	0.573857	2	2	0.27185	no
gi|646737853|gb|KK963036.1|	76826-77837	42	18	3	3	-0.0741825	0.9612	no
gi|646737853|gb|KK963036.1|	87267-87942	42	18	43	21	-1	0.48445	no
gi|646737902|gb|KK963030.1|	30872-31223	14	11	0	11	inf	0.00255	no
gi|646737902|gb|KK963030.1|	33703-35854	14	11	0	3	inf	0.00055	yes
gi|646737902|gb|KK963030.1|	51359-52811	14	11	0	3	inf	0.00185	no
gi|646737902|gb|KK963030.1|	52909-54268	14	11	0.083796	4	5	0.0988	no
gi|646737911|gb|KK963029.1|	12171-21301	13	9.6	0.158387	2	4	0.1061	no
gi|646737917|gb|KK963028.1|	31267-13167	22	7.8	2	8	2	0.2554	no
gi|646737917|gb|KK963028.1|	38013-13846	22	7.8	1	7	2	0.18125	no
gi|646737917|gb|KK963028.1|	40616-14081	22	7.8	72	202	1	0.32605	no
gi|646737917|gb|KK963028.1|	41440-14187	22	7.8	2	16	3	0.1507	no
gi|646737917|gb|KK963028.1|	42959-14320	22	7.8	30	60.85	1	0.4814	no
gi|646737917|gb|KK963028.1|	43953-14422	22	7.8	80	187	1	0.42935	no
gi|646737917|gb|KK963028.1|	46931-14762	22	7.8	2	4	1	0.3889	no
gi|646737917|gb|KK963028.1|	47896-14834	22	7.8	5	11	1	0.4135	no

gi|646737917|gb|KK963028.1|	49262-14994	22	7.8	3	9	2	0.21525	no
gi|646737917|gb|KK963028.1|	55502-15593	22	7.8	2	5	1	0.4607	no
gi|646737917|gb|KK963028.1|	56548-15691	22	7.8	38	25	-0.635698	0.6902	no
gi|646737917|gb|KK963028.1|	60628-16144	22	7.8	31	18	-0.81767	0.5914	no
gi|646737917|gb|KK963028.1|	17199-21968	22	7.8	2	2	-0.190427	0.8988	no
gi|646737917|gb|KK963028.1|	38290-23877	22	7.8	3	4	0.227586	0.86575	no
gi|646737917|gb|KK963028.1|	42781-24345	22	7.8	16	6	-1	0.36715	no
gi|646737917|gb|KK963028.1|	45686-24617	22	7.8	2	8	2	0.2151	no
gi|646737917|gb|KK963028.1|	54326-25821	22	7.8	11	22	0.996252	0.3823	no
gi|646737917|gb|KK963028.1|	58359-25966	22	7.8	8	10	0.364723	0.8065	no
gi|646737917|gb|KK963028.1|	62582-26285	22	7.8	14	50	2	0.2639	no
gi|646737917|gb|KK963028.1|	64064-26428	22	7.8	5	111	4	0.10795	no
gi|646737917|gb|KK963028.1|	27165-28523	22	7.8	0	2	inf	0.00315	no
gi|646737917|gb|KK963028.1|	95278-29629	22	7.8	0.975014	3	2	0.29805	no
gi|646737917|gb|KK963028.1|	96793-29708	22	7.8	11	7	-0.592076	0.67445	no
gi|646737917|gb|KK963028.1|	03596-40409	22	7.8	8	18	1	0.35795	no
gi|646737917|gb|KK963028.1|	10365-41195	22	7.8	2	5	0.99585	0.52735	no
gi|646737917|gb|KK963028.1|	41279-41515	22	7.8	14	23	0.649309	0.67515	no
gi|646737917|gb|KK963028.1|	34840-43611	22	7.8	0	1	inf	0.00255	no
gi|646737917|gb|KK963028.1|	47706-44836	22	7.8	0	6	inf	0.00315	no
gi|646737917|gb|KK963028.1|	54740-45699	22	7.8	0.046236	3	6	0.0964	no
gi|646737917|gb|KK963028.1|	57696-45848	22	7.8	0	3	inf	0.00035	yes
gi|646737917|gb|KK963028.1|	60345-46161	22	7.8	0	3	inf	0.00315	no
gi|646737917|gb|KK963028.1|	63899-46454	22	7.8	0.238755	15	6	0.09585	no
gi|646737917|gb|KK963028.1|	66196-46680	22	7.8	0	4	inf	0.00255	no
gi|646737936|gb|KK963027.1|	00607-10107	15	6.9	2	5	1	0.4075	no
gi|646737936|gb|KK963027.1|	20113-12045	15	6.9	14	11	-0.279448	0.82965	no
gi|646737936|gb|KK963027.1|	22358-12267	15	6.9	6	19	2	0.2622	no
gi|646737936|gb|KK963027.1|	23580-12385	15	6.9	19	17	-0.137995	0.9194	no
gi|646737936|gb|KK963027.1|	24486-12481	15	6.9	17	19	0.190942	0.90055	no

gi|646737936|gb|KK963027.1|	34075-13439	15	6.9	18	19	0.0774142	0.95375	no
gi|646737936|gb|KK963027.1|	85297-18564	15	6.9	9	8	-0.205051	0.8817	no
gi|646737936|gb|KK963027.1|	94065-19494	15	6.9	1	2	0.332097	0.8043	no
gi|646737936|gb|KK963027.1|	53497-54562	15	6.9	4	14	2	0.24655	no
gi|646737936|gb|KK963027.1|	55424-55799	15	6.9	4	9	1	0.371	no
gi|646737936|gb|KK963027.1|	59499-59883	15	6.9	3	11	2	0.2512	no
gi|646737936|gb|KK963027.1|	61474-64264	15	6.9	20	41	1	0.3805	no
gi|646737936|gb|KK963027.1|	64370-65773	15	6.9	4	8	0.874845	0.57205	no
gi|646737936|gb|KK963027.1|	73825-74336	15	6.9	41	78	0.923225	0.46795	no
gi|646737936|gb|KK963027.1|	77245-78538	15	6.9	30	36	0.288805	0.7888	no
gi|646737936|gb|KK963027.1|	87725-88140	15	6.9	7	7	-0.150972	0.9135	no
gi|646737936|gb|KK963027.1|	89887-90619	15	6.9	6	8	0.583037	0.7193	no
gi|646737936|gb|KK963027.1|	91157-91565	15	6.9	8	11	0.373905	0.7566	no
gi|646737936|gb|KK963027.1|	92112-92568	15	6.9	6	8	0.300401	0.79895	no
gi|646737936|gb|KK963027.1|	92907-93828	15	6.9	48	73	0.622324	0.56285	no
gi|646737936|gb|KK963027.1|	95151-95614	15	6.9	4	5	0.581226	0.69795	no
gi|646737959|gb|KK963025.1|	14949-15127	19	7.9	265	28	-3	0.1187	no
gi|646737959|gb|KK963025.1|	44356-34462	19	7.9	15	21	0.529121	0.71435	no
gi|646737959|gb|KK963025.1|	55240-35545	19	7.9	59	32	-0.888013	0.52625	no
gi|646737959|gb|KK963025.1|	58426-35880	19	7.9	43	34	-0.354603	0.8152	no
gi|646737959|gb|KK963025.1|	66096-36655	19	7.9	11	7	-0.777694	0.5774	no
gi|646737959|gb|KK963025.1|	67858-36815	19	7.9	46	19	-1	0.36015	no
gi|646737959|gb|KK963025.1|	69821-37155	19	7.9	0.876325	0.723572	-0.276328	1	no
gi|646737959|gb|KK963025.1|	71865-37228	19	7.9	6	1	-2	0.20015	no
gi|646737959|gb|KK963025.1|	74566-37642	19	7.9	34	23	-0.545797	0.62625	no
gi|646737959|gb|KK963025.1|	87329-38904	19	7.9	2	2	0.193758	0.9025	no
gi|646737959|gb|KK963025.1|	90412-39165	19	7.9	0.186699	2	3	0.2438	no
gi|646737959|gb|KK963025.1|	93158-39395	19	7.9	0.516735	2	2	0.20395	no
gi|646737959|gb|KK963025.1|	94643-39581	19	7.9	5	5	0.113447	0.93925	no
gi|646737959|gb|KK963025.1|	99121-39965	19	7.9	13.09	13	-0.0368949	0.9801	no

gi|646737959|gb|KK963025.1|	04998-40519	19	7.9	59	36	-0.720253	0.6602	no
gi|646737959|gb|KK963025.1|	20391-42102	19	7.9	14	7	-0.995046	0.5311	no
gi|646737959|gb|KK963025.1|	45657-44760	19	7.9	0	14	inf	5.00E-05	yes
gi|646737959|gb|KK963025.1|	52454-45320	19	7.9	2	2	0.489316	0.7159	no
gi|646737959|gb|KK963025.1|	69113-46978	19	7.9	0.224359	3	4	0.10445	no
gi|646738028|gb|KK963018.1|	29689-13023	22	7.1	8	3	-1	0.4403	no
gi|646738028|gb|KK963018.1|	34296-13570	22	7.1	17	9	-0.883204	0.4866	no
gi|646738028|gb|KK963018.1|	35826-13726	22	7.1	0.546485	0.561978	0.0403302	1	no
gi|646738028|gb|KK963018.1|	37377-13921	22	7.1	17	7	-1	0.309	no
gi|646738028|gb|KK963018.1|	42288-14373	22	7.1	4	1	-2	0.27865	no
gi|646738028|gb|KK963018.1|	56125-15688	22	7.1	3	2	-1	0.42505	no
gi|646738028|gb|KK963018.1|	64056-16432	22	7.1	32	26	-0.286108	0.82	no
gi|646738028|gb|KK963018.1|	66033-16716	22	7.1	3	2	-0.756753	0.5877	no
gi|646738028|gb|KK963018.1|	78077-17919	22	7.1	1	1	-0.0689456	0.9467	no
gi|646738028|gb|KK963018.1|	82476-18465	22	7.1	0.527848	0.695784	0.398516	1	no
gi|646738028|gb|KK963018.1|	87793-18880	22	7.1	0.9763	0.869473	-0.167183	1	no
gi|646738028|gb|KK963018.1|	88971-19156	22	7.1	0.75058	1	0.460923	1	no
gi|646738028|gb|KK963018.1|	91679-19905	22	7.1	7	4	-0.98977	0.372	no
gi|646738028|gb|KK963018.1|	00445-20265	22	7.1	2	5	2	0.26595	no
gi|646738028|gb|KK963018.1|	12599-21342	22	7.1	2	4	0.937701	0.51025	no
gi|646738028|gb|KK963018.1|	16404-21786	22	7.1	1	0.880924	-0.28105	1	no
gi|646738028|gb|KK963018.1|	18439-21928	22	7.1	3	2	-0.50084	0.70405	no
gi|646738028|gb|KK963018.1|	70357-27080	22	7.1	29	28	-0.0505844	0.9729	no
gi|646738028|gb|KK963018.1|	70915-27170	22	7.1	6	9	0.658611	0.6739	no
gi|646738028|gb|KK963018.1|	92671-29389	22	7.1	2	3	0.25682	0.8769	no
gi|646738028|gb|KK963018.1|	01442-30192	22	7.1	5	5	0.00666823	0.9493	no
gi|646738028|gb|KK963018.1|	06215-30682	22	7.1	2	2	0.20279	0.89115	no
gi|646738028|gb|KK963018.1|	71376-37321	22	7.1	2	0	-inf	0.002	no
gi|646738028|gb|KK963018.1|	85681-38663	22	7.1	32	3	-4	0.0066	no
gi|646738028|gb|KK963018.1|	89515-39269	22	7.1	2	1	-0.45836	0.76895	no

gi|646738028|gb|KK963018.1|	92818-39431	22	7.1	2	2	-0.125122	0.9288	no
gi|646738028|gb|KK963018.1|	94608-39780	22	7.1	1	1	-0.136373	0.93065	no
gi|646738028|gb|KK963018.1|	08672-40979	22	7.1	4	1	-2	0.2345	no
gi|646738028|gb|KK963018.1|	10003-41163	22	7.1	1	0.77802	-0.558068	0.66445	no
gi|646738028|gb|KK963018.1|	12393-41385	22	7.1	1	0.885956	-0.643771	0.6136	no
gi|646738028|gb|KK963018.1|	14507-41478	22	7.1	33	9	-2	0.2055	no
gi|646738028|gb|KK963018.1|	20234-42048	22	7.1	323	50	-3	0.11405	no
gi|646738028|gb|KK963018.1|	28726-42898	22	7.1	221	46	-2	0.1709	no
gi|646738028|gb|KK963018.1|	35204-43578	22	7.1	14	23	0.700572	0.6435	no
gi|646738028|gb|KK963018.1|	63260-46353	22	7.1	13	21	0.705595	0.61705	no
gi|646738028|gb|KK963018.1|	77160-47782	22	7.1	0.455312	3	3	0.2662	no
gi|646738028|gb|KK963018.1|	86704-48733	22	7.1	1	9	3	0.1538	no
gi|646738028|gb|KK963018.1|	15720-51600	22	7.1	4	18	2	0.2837	no
gi|646738028|gb|KK963018.1|	16617-51711	22	7.1	1	5	2	0.19575	no
gi|646738028|gb|KK963018.1|	27991-52836	22	7.1	7	25	2	0.2428	no
gi|646738028|gb|KK963018.1|	60021-61638	22	7.1	0.611326	0.392101	-0.640716	1	no
gi|646738028|gb|KK963018.1|	61984-66068	22	7.1	100	29	-2	0.13875	no
gi|646738028|gb|KK963018.1|	67428-67707	22	7.1	34	12	-1	0.2733	no
gi|646738028|gb|KK963018.1|	70105-70311	22	7.1	70	53	-0.389135	0.77585	no
gi|646738028|gb|KK963018.1|	74907-78477	22	7.1	0.697745	0.650591	-0.100948	1	no
gi|646738028|gb|KK963018.1|	78969-79481	22	7.1	3	0.965839	-2	0.29375	no
gi|646738028|gb|KK963018.1|	79584-80177	22	7.1	30	11	-1	0.36235	no
gi|646738046|gb|KK963016.1|	48941-49912	12	10	5	0.918929	-2	0.16265	no
gi|646738066|gb|KK963014.1|	80461-81653	17	8.1	1	2	0.644283	0.61505	no
gi|646738066|gb|KK963014.1|	88917-89545	17	8.1	41	29	-0.479842	0.74775	no
gi|646738083|gb|KK963012.1|	98003-98329	21	9.7	108.58	61	-0.824135	0.5882	no
gi|646738098|gb|KK963011.1|	66483-16693	11	7.1	2	5	1	0.38995	no
gi|646738098|gb|KK963011.1|	75428-17627	11	7.1	12	6	-0.978622	0.5269	no
gi|646738116|gb|KK963009.1|	8103-8330	13	4.4	73	0	-inf	0.0012	yes
gi|646738138|gb|KK963006.1|	76256-76999	15	13	1	3	1	0.39835	no

gi|646738156|gb|KK963004.1|	54602-55300	12	6.9	23	18	-0.355616	0.8114	no
gi|646738156|gb|KK963004.1|	56158-56562	12	6.9	24	29	0.258072	0.86505	no
gi|646738179|gb|KK963001.1|	01695-10235	20	7.7	9	3	-1	0.3067	no
gi|646738179|gb|KK963001.1|	59480-16049	20	7.7	0	3	inf	0.00515	no
gi|646738179|gb|KK963001.1|	61067-16307	20	7.7	0	3	inf	0.00075	yes
gi|646738179|gb|KK963001.1|	63241-16541	20	7.7	0.048111	3	6	0.0954	no
gi|646738179|gb|KK963001.1|	67387-16980	20	7.7	0.128142	7	6	0.10625	no
gi|646738179|gb|KK963001.1|	75921-17730	20	7.7	9	7	-0.307161	0.84095	no
gi|646738179|gb|KK963001.1|	83537-18529	20	7.7	0.061636	3	6	0.0977	no
gi|646738179|gb|KK963001.1|	97046-19730	20	7.7	16	18	0.145066	0.91585	no
gi|646738179|gb|KK963001.1|	05102-20727	20	7.7	0	4	inf	0.0001	yes
gi|646738229|gb|KK962998.1|	09651-11083	22	8.9	1	1	-0.288963	0.82625	no
gi|646738229|gb|KK962998.1|	20335-12143	22	8.9	0.873735	0.312186	-1	1	no
gi|646738229|gb|KK962998.1|	27619-12915	22	8.9	1	0.834858	-0.642236	0.6136	no
gi|646738229|gb|KK962998.1|	38406-13928	22	8.9	1	1	0.139689	0.9186	no
gi|646738229|gb|KK962998.1|	41643-14220	22	8.9	3	1	-1	0.4199	no
gi|646738229|gb|KK962998.1|	51638-15226	22	8.9	16	16	-0.00015317	0.9905	no
gi|646738229|gb|KK962998.1|	52700-15405	22	8.9	87	35	-1	0.25365	no
gi|646738229|gb|KK962998.1|	54347-15474	22	8.9	5	3	-0.636486	0.6683	no
gi|646738229|gb|KK962998.1|	56344-15756	22	8.9	3	2	-0.650305	0.6839	no
gi|646738229|gb|KK962998.1|	60439-16179	22	8.9	1	0.842598	-0.476163	0.71555	no
gi|646738229|gb|KK962998.1|	62167-16451	22	8.9	18	17	-0.0900759	0.93225	no
gi|646738229|gb|KK962998.1|	70480-17140	22	8.9	4	10	1	0.38395	no
gi|646738229|gb|KK962998.1|	74079-17445	22	8.9	19	16	-0.271212	0.8659	no
gi|646738229|gb|KK962998.1|	75455-17855	22	8.9	0.944083	1	0.206893	1	no
gi|646738229|gb|KK962998.1|	79545-18119	22	8.9	0.663184	0.574458	-0.207209	1	no
gi|646738229|gb|KK962998.1|	86824-18880	22	8.9	0.482415	0.465703	-0.0508645	1	no
gi|646738229|gb|KK962998.1|	98021-19948	22	8.9	0.917479	0.550577	-0.736729	1	no
gi|646738229|gb|KK962998.1|	00080-20493	22	8.9	0.783747	0.557322	-0.491876	1	no
gi|646738229|gb|KK962998.1|	05868-20697	22	8.9	0.763617	0.779567	0.0298249	1	no

gi|646738229|gb|KK962998.1|	34580-23492	22	8.9	4	26	3	0.158	no
gi|646738229|gb|KK962998.1|	71886-27247	22	8.9	3	0	-inf	0.0041	no
gi|646738229|gb|KK962998.1|	92615-29322	22	8.9	6	0.363738	-4	0.1895	no
gi|646738229|gb|KK962998.1|	40935-34692	22	8.9	65	125	0.950693	0.4339	no
gi|646738229|gb|KK962998.1|	47200-34812	22	8.9	0.686215	2	1	0.36855	no
gi|646738229|gb|KK962998.1|	48238-34952	22	8.9	38	69	0.86802	0.44285	no
gi|646738229|gb|KK962998.1|	54784-35503	22	8.9	31	6	-2	0.17765	no
gi|646738229|gb|KK962998.1|	55145-35613	22	8.9	0.633586	1	1	0.43245	no
gi|646738229|gb|KK962998.1|	56944-35892	22	8.9	7	20	2	0.2364	no
gi|646738229|gb|KK962998.1|	74680-37668	22	8.9	0.421968	0.840331	0.993822	1	no
gi|646738229|gb|KK962998.1|	91916-39261	22	8.9	1	2	0.674318	0.64375	no
gi|646738229|gb|KK962998.1|	99661-39999	22	8.9	10	11	0.13504	0.9256	no
gi|646738229|gb|KK962998.1|	42664-42900	22	8.9	18	23	0.327381	0.81255	no
gi|646738229|gb|KK962998.1|	58318-59745	22	8.9	0.789351	0.340857	-1	1	no
gi|646738229|gb|KK962998.1|	66890-67451	22	8.9	0.903455	4	2	0.20605	no
gi|646738248|gb|KK962996.1|	03347-10387	17	6.6	29	19	-0.625597	0.67185	no
gi|646738248|gb|KK962996.1|	13528-11417	17	6.6	2	4	1	0.3212	no
gi|646738248|gb|KK962996.1|	96286-99727	17	6.6	8	12	0.527518	0.62775	no
gi|646738248|gb|KK962996.1|	99894-10239	17	6.6	8	12	0.580985	0.58815	no
gi|646738278|gb|KK962993.1|	51508-15173	17	10	17	35	1	0.4759	no
gi|646738278|gb|KK962993.1|	17754-18576	17	10	3	4	0.249201	0.8551	no
gi|646738290|gb|KK962992.1|	91767-92409	30	13	16	2	-3	0.0744	no
gi|646738299|gb|KK962991.1|	02541-20287	13	7.8	7	9	0.489857	0.728	no
gi|646738307|gb|KK962990.1|	65990-16704	37	17	3	1	-1	0.4659	no
gi|646738307|gb|KK962990.1|	79526-18046	37	17	6	7	0.121791	0.9399	no
gi|646738307|gb|KK962990.1|	80730-18335	37	17	3	3	0.254328	0.86405	no
gi|646738307|gb|KK962990.1|	95192-19559	37	17	13	4	-2	0.2269	no
gi|646738307|gb|KK962990.1|	03523-20385	37	17	38	16	-1	0.36305	no
gi|646738307|gb|KK962990.1|	04461-20847	37	17	48	18	-1	0.2546	no
gi|646738307|gb|KK962990.1|	20873-22144	37	17	4	0.79743	-2	0.194	no

gi|646738307|gb|KK962990.1| 63510-26378	37	17	13	10	-0.294405	0.8508	no
gi|646738307|gb|KK962990.1| 68551-26951	37	17	0.908165	0.922678	0.0228734	1	no
gi|646738307|gb|KK962990.1| 82955-28351	37	17	3	6	1	0.4106	no
gi|646738314|gb|KK962989.1| 17546-11902	20	5.5	2	2	0.452581	0.7388	no
gi|646738314|gb|KK962989.1| 20975-12127	20	5.5	39	21	-0.869827	0.5292	no
gi|646738314|gb|KK962989.1| 27087-12816	20	5.5	3	4	0.575627	0.72195	no
gi|646738314|gb|KK962989.1| 64531-16539	20	5.5	4	1	-2	0.299	no
gi|646738314|gb|KK962989.1| 77762-17806	20	5.5	7	16	1	0.4082	no
gi|646738314|gb|KK962989.1| 93032-19329	20	5.5	9	33	2	0.2453	no
gi|646738314|gb|KK962989.1| 27074-23458	20	5.5	11	17	0.55771	0.63515	no
gi|646738314|gb|KK962989.1| 35699-23622	20	5.5	9	9	0.0615336	0.9677	no
gi|646738314|gb|KK962989.1| 46244-46866	20	5.5	4	0	-inf	0.0011	yes
gi|646738330|gb|KK962987.1| 10915-11232	19	7.9	0.559225	0.919732	0.717784	1	no
gi|646738330|gb|KK962987.1| 21608-12444	19	7.9	7	8	0.179577	0.86085	no
gi|646738330|gb|KK962987.1| 28606-13094	19	7.9	8	8	-0.0438682	0.97835	no
gi|646738330|gb|KK962987.1| 35644-13651	19	7.9	3	2	-0.34695	0.7847	no
gi|646738330|gb|KK962987.1| 36763-13705	19	7.9	163	0	-inf	5.00E-05	yes
gi|646738330|gb|KK962987.1| 41934-14287	19	7.9	51	0.385862	-7	0.10535	no
gi|646738330|gb|KK962987.1| 52529-15444	19	7.9	5	1	-2	0.25995	no
gi|646738330|gb|KK962987.1| 54662-15626	19	7.9	2	0.198343	-3	0.14735	no
gi|646738330|gb|KK962987.1| 64200-16561	19	7.9	0.876512	0.68805	-0.34926	1	no
gi|646738330|gb|KK962987.1| 67805-16843	19	7.9	4	4	0.0208249	0.9559	no
gi|646738330|gb|KK962987.1| 78937-18004	19	7.9	1	2	0.75626	0.5508	no
gi|646738330|gb|KK962987.1| 80335-18195	19	7.9	3	2	-0.7186	0.6531	no
gi|646738330|gb|KK962987.1| 91863-19602	19	7.9	14	9	-0.6207	0.58	no
gi|646738330|gb|KK962987.1| 00866-20119	19	7.9	51.12	6	-3	0.093	no
gi|646738330|gb|KK962987.1| 48143-24854	19	7.9	4	2	-0.85619	0.6052	no
gi|646738338|gb|KK962986.1| 14657-11511	15	6.6	3	5	0.820586	0.57265	no
gi|646738338|gb|KK962986.1| 37872-13841	15	6.6	6	0.436841	-4	0.17325	no
gi|646738348|gb|KK962985.1| 06263-10818	13	7.1	1	0.966222	-0.52549	0.70235	no

gi|646738348|gb|KK962985.1|	26778-12724	13	7.1	5	6	0.27653	0.8317	no
gi|646738348|gb|KK962985.1|	32468-13367	13	7.1	0.776569	0.834776	0.104275	1	no
gi|646738348|gb|KK962985.1|	39844-14021	13	7.1	4	4	-0.247562	0.87355	no
gi|646738348|gb|KK962985.1|	77054-17755	13	7.1	5	8	0.623058	0.6567	no
gi|646738348|gb|KK962985.1|	83198-18346	13	7.1	52	42	-0.30738	0.84665	no
gi|646738348|gb|KK962985.1|	19241-19580	13	7.1	0	13	inf	0.0014	yes
gi|646738348|gb|KK962985.1|	33-665	13	7.1	1	2	0.732817	0.64505	no
gi|646738348|gb|KK962985.1|	34521-35045	13	7.1	9	8	-0.0912196	0.95235	no
gi|646738348|gb|KK962985.1|	42103-43606	13	7.1	21	11	-0.898209	0.48	no
gi|646738348|gb|KK962985.1|	44105-45563	13	7.1	3	2	-1	0.39945	no
gi|646738348|gb|KK962985.1|	48586-48963	13	7.1	55	42	-0.398275	0.7917	no
gi|646738348|gb|KK962985.1|	98138-98498	13	7.1	7	2	-2	0.2743	no
gi|646738359|gb|KK962984.1|	42720-43373	16	11	0	4	inf	0.001	yes
gi|646738359|gb|KK962984.1|	82511-83645	16	11	0.947175	3	2	0.3367	no
gi|646738402|gb|KK962980.1|	11650-12705	12	5.5	1	2	0.735438	0.56995	no
gi|646738402|gb|KK962980.1|	25435-12656	12	5.5	3	0.756112	-2	0.28225	no
gi|646738402|gb|KK962980.1|	02466-20330	12	5.5	0.793293	2	2	0.2735	no
gi|646738414|gb|KK962979.1|	02054-10287	23	10	1	0.920993	-0.672797	0.6592	no
gi|646738414|gb|KK962979.1|	03506-10474	23	10	2	0.269956	-3	0.1297	no
gi|646738414|gb|KK962979.1|	12928-11373	23	10	2	2	0.325422	0.8043	no
gi|646738414|gb|KK962979.1|	16081-11642	23	10	5	10	0.890477	0.5478	no
gi|646738414|gb|KK962979.1|	21717-12243	23	10	5	4	-0.309427	0.8523	no
gi|646738414|gb|KK962979.1|	24917-12584	23	10	6	4	-0.879019	0.5835	no
gi|646738414|gb|KK962979.1|	29544-13356	23	10	10	6	-0.846503	0.43675	no
gi|646738414|gb|KK962979.1|	35792-13646	23	10	18	17	-0.0464726	0.97585	no
gi|646738414|gb|KK962979.1|	38083-13874	23	10	2	0.62869	-2	0.2684	no
gi|646738414|gb|KK962979.1|	46161-14813	23	10	4	4	-0.191074	0.8994	no
gi|646738414|gb|KK962979.1|	52043-15318	23	10	7	5	-0.415857	0.7752	no
gi|646738414|gb|KK962979.1|	67191-68119	23	10	4	2	-0.989051	0.4947	no
gi|646738414|gb|KK962979.1|	93000-93467	23	10	6	1	-2	0.16245	no

gi|646738423|gb|KK962978.1|	54132-15441	22	11	18	37	0.993081	0.5272	no
gi|646738423|gb|KK962978.1|	45404-24609	22	11	4	2	-0.881537	0.49395	no
gi|646738455|gb|KK962975.1|	20028-12030	23	9.5	12	9	-0.408328	0.8114	no
gi|646738455|gb|KK962975.1|	45864-14635	23	9.5	6	2	-2	0.22915	no
gi|646738455|gb|KK962975.1|	86290-18974	23	9.5	12	13	0.0375509	0.97115	no
gi|646738455|gb|KK962975.1|	60877-26335	23	9.5	49	31	-0.645244	0.57695	no
gi|646738455|gb|KK962975.1|	63753-26552	23	9.5	1	0.263951	-2	1	no
gi|646738455|gb|KK962975.1|	77001-27758	23	9.5	3	0.383093	-3	0.2136	no
gi|646738455|gb|KK962975.1|	78983-27924	23	9.5	43	45	0.0753359	0.9554	no
gi|646738455|gb|KK962975.1|	84768-29126	23	9.5	2	2	0.110555	0.9404	no
gi|646738455|gb|KK962975.1|	03264-30525	23	9.5	9	7	-0.338863	0.82665	no
gi|646738455|gb|KK962975.1|	12696-31388	23	9.5	1	2	0.625416	0.621	no
gi|646738455|gb|KK962975.1|	32439-33322	23	9.5	2	3	0.872172	0.50085	no
gi|646738455|gb|KK962975.1|	43564-35185	23	9.5	13	9	-0.539366	0.6382	no
gi|646738455|gb|KK962975.1|	79218-38209	23	9.5	2	0.820468	-1	0.43015	no
gi|646738455|gb|KK962975.1|	87903-38888	23	9.5	2	0.726291	-1	0.45505	no
gi|646738455|gb|KK962975.1|	89076-39005	23	9.5	1	0.726291	-0.490979	1	no
gi|646738455|gb|KK962975.1|	93350-39566	23	9.5	0.943144	0.91235	-0.0478904	1	no
gi|646738455|gb|KK962975.1|	97098-39750	23	9.5	6	5	-0.144881	0.9174	no
gi|646738455|gb|KK962975.1|	00886-40133	23	9.5	13	11	-0.235529	0.8845	no
gi|646738455|gb|KK962975.1|	04913-40572	23	9.5	3	2	-0.866616	0.50275	no
gi|646738455|gb|KK962975.1|	05856-40665	23	9.5	2	0.715007	-1	0.427	no
gi|646738455|gb|KK962975.1|	08243-40898	23	9.5	3	5	0.809425	0.566	no
gi|646738455|gb|KK962975.1|	09729-41097	23	9.5	0.651475	0.534068	-0.286687	1	no
gi|646738455|gb|KK962975.1|	13181-41371	23	9.5	10	12	0.237555	0.8757	no
gi|646738455|gb|KK962975.1|	18048-41835	23	9.5	12	10	-0.342305	0.80325	no
gi|646738455|gb|KK962975.1|	20512-42507	23	9.5	11	10	-0.192762	0.86185	no
gi|646738455|gb|KK962975.1|	25309-42674	23	9.5	8	9	0.172809	0.909	no
gi|646738455|gb|KK962975.1|	34239-43586	23	9.5	12	3	-2	0.1717	no
gi|646738455|gb|KK962975.1|	47399-45150	23	9.5	17	14	-0.277334	0.8042	no

gi|646738455|gb|KK962975.1|	52302-45391	23	9.5	0.749928	0.393522	-0.930308	1	no
gi|646738455|gb|KK962975.1|	57006-45785	23	9.5	2	4	1	0.4631	no
gi|646738455|gb|KK962975.1|	62006-46255	23	9.5	3	5	0.585872	0.63685	no
gi|646738455|gb|KK962975.1|	63686-46424	23	9.5	2	3	1	0.44985	no
gi|646738455|gb|KK962975.1|	64978-46584	23	9.5	2	5	2	0.3124	no
gi|646738455|gb|KK962975.1|	71965-47316	23	9.5	0.984134	0.141009	-3	1	no
gi|646738455|gb|KK962975.1|	73263-47591	23	9.5	11	14	0.378789	0.72735	no
gi|646738455|gb|KK962975.1|	77070-47846	23	9.5	7	11	0.624246	0.6776	no
gi|646738455|gb|KK962975.1|	93461-49457	23	9.5	1	0.772467	-0.862916	0.54655	no
gi|646738455|gb|KK962975.1|	08491-50931	23	9.5	2	0.914784	-1	0.4538	no
gi|646738455|gb|KK962975.1|	09432-51045	23	9.5	0.958599	2	0.681558	0.619	no
gi|646738455|gb|KK962975.1|	49498-55014	23	9.5	6	6	-0.139363	0.92885	no
gi|646738455|gb|KK962975.1|	84519-58480	23	9.5	18	6	-2	0.357	no
gi|646738466|gb|KK962974.1|	04115-11053	30	14	2	2	-0.420935	0.7789	no
gi|646738466|gb|KK962974.1|	10705-11312	30	14	65	27	-1	0.2739	no
gi|646738466|gb|KK962974.1|	15183-11615	30	14	3	3	0.356236	0.79495	no
gi|646738466|gb|KK962974.1|	20655-12236	30	14	1	0.829499	-0.621389	0.6236	no
gi|646738466|gb|KK962974.1|	25371-12835	30	14	0.508351	0.887479	0.803887	1	no
gi|646738466|gb|KK962974.1|	12753-13491	30	14	2	0.268481	-3	0.1994	no
gi|646738466|gb|KK962974.1|	29597-13065	30	14	1	1	0.513045	0.7139	no
gi|646738466|gb|KK962974.1|	30780-13520	30	14	3	4	0.156317	0.9183	no
gi|646738466|gb|KK962974.1|	35902-13610	30	14	477	430	-0.146909	0.92145	no
gi|646738466|gb|KK962974.1|	44557-14498	30	14	5	2	-1	0.454	no
gi|646738466|gb|KK962974.1|	17872-18654	30	14	2	0.494018	-2	0.2991	no
gi|646738466|gb|KK962974.1|	18783-19823	30	14	1	0.504238	-1	0.4271	no
gi|646738466|gb|KK962974.1|	20665-22261	30	14	0.758875	0.497676	-0.608656	1	no
gi|646738466|gb|KK962974.1|	19597-22027	30	14	0.866061	2	1	0.4335	no
gi|646738466|gb|KK962974.1|	21506-22228	30	14	224	148	-0.603847	0.6071	no
gi|646738466|gb|KK962974.1|	24174-22545	30	14	0.905186	1	0.199603	1	no
gi|646738466|gb|KK962974.1|	30437-23172	30	14	2	2	0.443767	0.74595	no

gi|646738466|gb|KK962974.1|	31945-23445	30	14	3	4	0.473283	0.74965	no
gi|646738466|gb|KK962974.1|	36065-23757	30	14	0.295097	0.850556	2	1	no
gi|646738466|gb|KK962974.1|	38110-23937	30	14	1	1	-0.0638877	1	no
gi|646738466|gb|KK962974.1|	42821-24376	30	14	9	11	0.41374	0.78165	no
gi|646738466|gb|KK962974.1|	47013-24776	30	14	55	15	-2	0.1585	no
gi|646738466|gb|KK962974.1|	51638-25212	30	14	4	2	-1	0.46265	no
gi|646738466|gb|KK962974.1|	77668-27969	30	14	141	27	-2	0.0534	no
gi|646738466|gb|KK962974.1|	87508-28799	30	14	2	3	0.422673	0.77485	no
gi|646738466|gb|KK962974.1|	29378-37622	30	14	32	23	-0.46773	0.7041	no
gi|646738466|gb|KK962974.1|	98027-29895	30	14	8	11	0.404567	0.78775	no
gi|646738466|gb|KK962974.1|	00017-30082	30	14	2	0.936889	-0.825775	0.5782	no
gi|646738466|gb|KK962974.1|	02214-30238	30	14	140	115	-0.282546	0.84375	no
gi|646738466|gb|KK962974.1|	10593-31416	30	14	2	4	1	0.50165	no
gi|646738466|gb|KK962974.1|	15275-31645	30	14	8	12	0.607919	0.68655	no
gi|646738466|gb|KK962974.1|	31266-33194	30	14	4	10	1	0.3857	no
gi|646738466|gb|KK962974.1|	56174-35652	30	14	64	147	1	0.44155	no
gi|646738466|gb|KK962974.1|	3630-4247	30	14	2	10	3	0.13555	no
gi|646738466|gb|KK962974.1|	76066-37665	30	14	10	7	-0.502608	0.7555	no
gi|646738466|gb|KK962974.1|	78195-38000	30	14	1	1	-0.204855	0.8619	no
gi|646738466|gb|KK962974.1|	80154-38103	30	14	2	3	1	0.48665	no
gi|646738466|gb|KK962974.1|	81163-38288	30	14	0.56835	1	0.820344	1	no
gi|646738466|gb|KK962974.1|	84690-38660	30	14	5	3	-0.517222	0.72655	no
gi|646738466|gb|KK962974.1|	20035-42177	30	14	5	6	0.298968	0.83895	no
gi|646738466|gb|KK962974.1|	46799-49399	30	14	1	0.802516	-0.823627	0.5486	no
gi|646738466|gb|KK962974.1|	57565-58109	30	14	3	2	-0.553604	0.72945	no
gi|646738466|gb|KK962974.1|	64928-65659	30	14	4	5	0.560686	0.68345	no
gi|646738466|gb|KK962974.1|	67540-67743	30	14	121	0	-inf	0.00195	no
gi|646738466|gb|KK962974.1|	70822-72067	30	14	0.560663	0.804267	0.52054	1	no
gi|646738466|gb|KK962974.1|	74561-77353	30	14	342	7	-6	0.0017	no
gi|646738466|gb|KK962974.1|	78567-79774	30	14	1	0.696361	-0.859234	0.5471	no

gi|646738466|gb|KK962974.1|	93175-94019	30	14	0.628172	2	1	0.36865	no
gi|646738466|gb|KK962974.1|	94438-10398	30	14	14	9	-0.532435	0.6479	no
gi|646738476|gb|KK962973.1|	20330-12293	16	6.9	7	5	-0.622916	0.6795	no
gi|646738476|gb|KK962973.1|	43936-14418	16	6.9	25	13	-0.907373	0.5688	no
gi|646738476|gb|KK962973.1|	63829-16477	16	6.9	3	2	-0.69485	0.63085	no
gi|646738476|gb|KK962973.1|	73042-17400	16	6.9	18	12	-0.555388	0.7162	no
gi|646738476|gb|KK962973.1|	06330-21114	16	6.9	4	4	0.316331	0.7932	no
gi|646738476|gb|KK962973.1|	11348-21252	16	6.9	9	7	-0.361534	0.8069	no
gi|646738476|gb|KK962973.1|	23592-22496	16	6.9	3	6	1	0.4647	no
gi|646738476|gb|KK962973.1|	25798-22753	16	6.9	3	5	0.859649	0.57605	no
gi|646738476|gb|KK962973.1|	38560-24070	16	6.9	2	2	0.398159	0.80305	no
gi|646738476|gb|KK962973.1|	46047-24759	16	6.9	0.286641	0.723271	1	1	no
gi|646738476|gb|KK962973.1|	69222-26979	16	6.9	3	7	1	0.39925	no
gi|646738476|gb|KK962973.1|	71660-27191	16	6.9	28	76	1	0.33865	no
gi|646738476|gb|KK962973.1|	93920-29425	16	6.9	7	25	2	0.2482	no
gi|646738476|gb|KK962973.1|	98817-29939	16	6.9	2	6	2	0.35695	no
gi|646738476|gb|KK962973.1|	02730-30309	16	6.9	9	44	2	0.13945	no
gi|646738476|gb|KK962973.1|	18090-32020	16	6.9	23	35	0.630869	0.583	no
gi|646738486|gb|KK962972.1|	80957-28137	17	8.5	4	2	-0.841292	0.6128	no
gi|646738486|gb|KK962972.1|	81549-28332	17	8.5	0.488207	0.882124	0.85349	1	no
gi|646738486|gb|KK962972.1|	13232-41910	17	8.5	11	8	-0.535366	0.63615	no
gi|646738486|gb|KK962972.1|	20104-42209	17	8.5	1	2	0.271169	0.868	no
gi|646738486|gb|KK962972.1|	33300-43393	17	8.5	2	1	-0.529176	0.73185	no
gi|646738486|gb|KK962972.1|	47461-44771	17	8.5	40	15	-1	0.3319	no
gi|646738486|gb|KK962972.1|	49965-45075	17	8.5	6	5	-0.467756	0.7708	no
gi|646738486|gb|KK962972.1|	72028-47772	17	8.5	5	6	0.352538	0.73765	no
gi|646738486|gb|KK962972.1|	06609-50712	17	8.5	4	4	0.145087	0.9183	no
gi|646738512|gb|KK962969.1|	97079-19810	12	7.1	0.120136	14	7	0.0949	no
gi|646738524|gb|KK962968.1|	00037-10063	24	11	7	5	-0.591951	0.66375	no
gi|646738524|gb|KK962968.1|	01446-10324	24	11	0.900538	2	0.872937	0.5449	no

gi|646738524|gb|KK962968.1|	04875-10740	24	11	3	2	-0.631429	0.67185	no
gi|646738524|gb|KK962968.1|	08525-10875	24	11	120	39	-2	0.27495	no
gi|646738524|gb|KK962968.1|	20041-12054	24	11	5	6	0.209724	0.8625	no
gi|646738524|gb|KK962968.1|	24157-12471	24	11	2	3	1	0.44985	no
gi|646738524|gb|KK962968.1|	26109-12662	24	11	5	6	0.319521	0.80575	no
gi|646738524|gb|KK962968.1|	27284-12756	24	11	51	37	-0.456582	0.771	no
gi|646738524|gb|KK962968.1|	28914-12960	24	11	3	3	-0.15672	0.90355	no
gi|646738524|gb|KK962968.1|	38470-13868	24	11	36	41	0.188118	0.8992	no
gi|646738524|gb|KK962968.1|	49213-14979	24	11	3	2	-0.128212	0.9221	no
gi|646738524|gb|KK962968.1|	62326-16445	24	11	57	28	-1	0.3733	no
gi|646738524|gb|KK962968.1|	69484-17143	24	11	1	0.711993	-0.544262	1	no
gi|646738524|gb|KK962968.1|	83744-18403	24	11	43	18	-1	0.3856	no
gi|646738524|gb|KK962968.1|	86129-18724	24	11	0.971484	0.771589	-0.332359	1	no
gi|646738524|gb|KK962968.1|	89761-19005	24	11	14	15	0.0453541	0.9426	no
gi|646738524|gb|KK962968.1|	98376-19997	24	11	0.550268	1	0.988349	1	no
gi|646738524|gb|KK962968.1|	00384-21265	24	11	12	31	1	0.25215	no
gi|646738524|gb|KK962968.1|	26700-22810	24	11	0.888758	0.813727	-0.127246	1	no
gi|646738524|gb|KK962968.1|	33036-23482	24	11	2	3	0.435564	0.7774	no
gi|646738524|gb|KK962968.1|	35007-24256	24	11	1	1	0.456235	0.75875	no
gi|646738524|gb|KK962968.1|	42920-24470	24	11	2	2	0.432271	0.7826	no
gi|646738524|gb|KK962968.1|	46949-24729	24	11	16	7	-1	0.34235	no
gi|646738524|gb|KK962968.1|	48570-24956	24	11	7	3	-1	0.50605	no
gi|646738524|gb|KK962968.1|	60172-26049	24	11	26	26	-0.00718841	0.97655	no
gi|646738524|gb|KK962968.1|	68574-27111	24	11	34	18	-0.933015	0.41435	no
gi|646738524|gb|KK962968.1|	80392-28066	24	11	17	7	-1	0.39135	no
gi|646738524|gb|KK962968.1|	84847-28725	24	11	51	23	-1	0.32755	no
gi|646738524|gb|KK962968.1|	89193-29502	24	11	16	12	-0.340562	0.7671	no
gi|646738524|gb|KK962968.1|	96143-29723	24	11	1	0.788628	-0.863699	0.54655	no
gi|646738524|gb|KK962968.1|	00438-30089	24	11	3	4	0.409616	0.7964	no
gi|646738524|gb|KK962968.1|	11186-31543	24	11	15	8	-0.828606	0.45585	no

gi|646738524|gb|KK962968.1|	55219-35746	24	11	2	2	0.121517	0.9369	no
gi|646738524|gb|KK962968.1|	58280-35893	24	11	2	3	0.473998	0.7389	no
gi|646738524|gb|KK962968.1|	59342-36094	24	11	1	1	0.113932	0.92865	no
gi|646738524|gb|KK962968.1|	61186-36445	24	11	1	1	-0.00235637	1	no
gi|646738524|gb|KK962968.1|	64673-37710	24	11	9	5	-1	0.38625	no
gi|646738524|gb|KK962968.1|	91516-39173	24	11	291	77	-2	0.2411	no
gi|646738524|gb|KK962968.1|	98001-40356	24	11	21	16	-0.434471	0.7096	no
gi|646738524|gb|KK962968.1|	14986-41563	24	11	96	32	-2	0.2291	no
gi|646738524|gb|KK962968.1|	27263-42854	24	11	36	27	-0.408079	0.7092	no
gi|646738524|gb|KK962968.1|	38142-43852	24	11	5	4	-0.133316	0.9193	no
gi|646738524|gb|KK962968.1|	46783-44803	24	11	0.279767	3	3	0.1465	no
gi|646738524|gb|KK962968.1|	50167-45248	24	11	0.314534	0.717191	1	1	no
gi|646738524|gb|KK962968.1|	56216-45651	24	11	22	23	0.0425542	0.96225	no
gi|646738524|gb|KK962968.1|	56896-45816	24	11	2	0.656603	-1	0.37345	no
gi|646738524|gb|KK962968.1|	60678-46130	24	11	3	1	-1	0.3848	no
gi|646738524|gb|KK962968.1|	62962-46747	24	11	0.651411	0.570269	-0.191924	1	no
gi|646738524|gb|KK962968.1|	69558-47160	24	11	0.465428	0.899011	0.949781	1	no
gi|646738524|gb|KK962968.1|	72268-47341	24	11	0.729226	0.596252	-0.290444	1	no
gi|646738524|gb|KK962968.1|	75499-47631	24	11	2	0.466833	-2	0.2551	no
gi|646738524|gb|KK962968.1|	77499-47907	24	11	0.984186	1	0.0429739	1	no
gi|646738524|gb|KK962968.1|	52853-54376	24	11	160	67	-1	0.28945	no
gi|646738524|gb|KK962968.1|	61725-63075	24	11	34	63	0.909321	0.42205	no
gi|646738524|gb|KK962968.1|	63488-68645	24	11	1	2	0.249627	0.8669	no
gi|646738524|gb|KK962968.1|	73755-74112	24	11	16	2	-3	0.12135	no
gi|646738524|gb|KK962968.1|	81955-83609	24	11	1	2	0.529822	0.7073	no
gi|646738524|gb|KK962968.1|	88064-89545	24	11	63	27	-1	0.2745	no
gi|646738524|gb|KK962968.1|	89848-91474	24	11	2	2	-0.14049	0.9268	no
gi|646738524|gb|KK962968.1|	96291-96777	24	11	6	7	0.423152	0.7206	no
gi|646738524|gb|KK962968.1|	98145-98627	24	11	2	3	0.158183	0.9062	no
gi|646738532|gb|KK962967.1|	22894-23555	12	9.3	0	4	inf	0.00035	yes

gi|646738532|gb|KK962967.1|	30638-31153	12	9.3	0	18	inf	0.00055	yes
gi|646738532|gb|KK962967.1|	34346-35231	12	9.3	0	3	inf	0.00035	yes
gi|646738532|gb|KK962967.1|	35876-37853	12	9.3	0	4	inf	0.00025	yes
gi|646738552|gb|KK962965.1|	54704-55228	16	8.2	4	0	-inf	0.00475	no
gi|646738563|gb|KK962964.1|	56866-15889	15	7.3	4	1	-2	0.3419	no
gi|646738563|gb|KK962964.1|	15725-16012	15	7.3	21	4	-2	0.1647	no
gi|646738563|gb|KK962964.1|	59575-16007	15	7.3	3	0.500477	-3	0.21315	no
gi|646738563|gb|KK962964.1|	91774-19239	15	7.3	2	2	0.467626	0.7483	no
gi|646738563|gb|KK962964.1|	09530-21109	15	7.3	0.564107	0.508491	-0.149744	1	no
gi|646738563|gb|KK962964.1|	22181-22665	15	7.3	7	3	-1	0.33385	no
gi|646738563|gb|KK962964.1|	28836-22980	15	7.3	4	4	0.145669	0.92885	no
gi|646738563|gb|KK962964.1|	33516-34582	15	7.3	1	0.814178	-0.62393	0.6602	no
gi|646738563|gb|KK962964.1|	69142-69786	15	7.3	4	3	-0.357834	0.7824	no
gi|646738563|gb|KK962964.1|	71462-71941	15	7.3	5	0.546026	-3	0.1921	no
gi|646738572|gb|KK962963.1|	24160-24363	17	10	53	29	-0.890557	0.58075	no
gi|646738572|gb|KK962963.1|	45564-24618	17	10	6	4	-0.416239	0.75595	no
gi|646738572|gb|KK962963.1|	46875-24716	17	10	20	6	-2	0.2716	no
gi|646738582|gb|KK962962.1|	14576-11527	13	6.5	55	4	-4	0.0062	no
gi|646738604|gb|KK962960.1|	26889-13697	19	8.4	2	2	0.205585	0.84765	no
gi|646738604|gb|KK962960.1|	59270-16111	19	8.4	0.407302	53	7	0.02865	no
gi|646738604|gb|KK962960.1|	63471-16683	19	8.4	8	4	-0.906204	0.4715	no
gi|646738604|gb|KK962960.1|	22782-22306	19	8.4	12	15	0.399411	0.79045	no
gi|646738604|gb|KK962960.1|	24346-25929	19	8.4	1	0	-inf	0.00095	yes
gi|646738604|gb|KK962960.1|	26476-27081	19	8.4	2	11	2	0.1387	no
gi|646738604|gb|KK962960.1|	28696-32181	19	8.4	67	65	-0.0379788	0.97375	no
gi|646738604|gb|KK962960.1|	52978-54085	19	8.4	4	3	-0.484576	0.76355	no
gi|646738604|gb|KK962960.1|	54237-55812	19	8.4	6	4	-0.769868	0.6172	no
gi|646738604|gb|KK962960.1|	58464-61364	19	8.4	52	37	-0.500941	0.6666	no
gi|646738604|gb|KK962960.1|	70341-71239	19	8.4	15	3	-2	0.095	no
gi|646738604|gb|KK962960.1|	71499-72087	19	8.4	43	19	-1	0.4524	no

gi|646738604|gb|KK962960.1|	72212-74760	19	8.4	7	3	-1	0.3858	no
gi|646738604|gb|KK962960.1|	74879-77339	19	8.4	5	2	-2	0.32295	no
gi|646738615|gb|KK962959.1|	54211-15531	21	9.5	4	2	-1	0.42925	no
gi|646738615|gb|KK962959.1|	68919-16957	21	9.5	17	2	-3	0.0731	no
gi|646738615|gb|KK962959.1|	71392-17310	21	9.5	20	14	-0.511101	0.6345	no
gi|646738615|gb|KK962959.1|	96910-19906	21	9.5	1	1	0.313385	0.81795	no
gi|646738615|gb|KK962959.1|	06184-20682	21	9.5	79	63	-0.32503	0.76515	no
gi|646738615|gb|KK962959.1|	11374-21164	21	9.5	151	200	0.401484	0.78725	no
gi|646738615|gb|KK962959.1|	25150-23017	21	9.5	48	55	0.199198	0.86815	no
gi|646738615|gb|KK962959.1|	30334-23274	21	9.5	35	37	0.0689219	0.95155	no
gi|646738615|gb|KK962959.1|	39888-24212	21	9.5	21	29	0.490479	0.6594	no
gi|646738615|gb|KK962959.1|	42511-24293	21	9.5	14	29	1	0.48555	no
gi|646738615|gb|KK962959.1|	47309-24883	21	9.5	0.509513	0.734455	0.527554	1	no
gi|646738615|gb|KK962959.1|	78769-27932	21	9.5	22	29	0.407827	0.78485	no
gi|646738615|gb|KK962959.1|	84254-28459	21	9.5	3	9	2	0.28405	no
gi|646738615|gb|KK962959.1|	89251-28942	21	9.5	232	179	-0.37487	0.7615	no
gi|646738615|gb|KK962959.1|	92420-29321	21	9.5	1	1	0.00877319	0.92345	no
gi|646738615|gb|KK962959.1|	08762-30925	21	9.5	5	14	1	0.36025	no
gi|646738615|gb|KK962959.1|	20358-32081	21	9.5	13	15	0.243232	0.87975	no
gi|646738615|gb|KK962959.1|	9456-14967	21	9.5	5	5	0.0884078	0.93345	no
gi|646738615|gb|KK962959.1|	)9328-10193(	21	9.5	1	3	1	0.49265	no
gi|646738622|gb|KK962958.1|	12257-21314	16	9.3	16	0	-inf	0.0003	yes
gi|646738622|gb|KK962958.1|	14302-21565	16	9.3	3	0	-inf	0.0014	yes
gi|646738622|gb|KK962958.1|	15771-21710	16	9.3	1	0	-inf	1	no
gi|646738622|gb|KK962958.1|	00771-30099	16	9.3	23	28	0.28016	0.84635	no
gi|646738622|gb|KK962958.1|	01543-30180	16	9.3	14	15	0.147852	0.9093	no
gi|646738622|gb|KK962958.1|	03202-30385	16	9.3	4	3	-0.263352	0.8334	no
gi|646738622|gb|KK962958.1|	16590-31688	16	9.3	120	30	-2	0.2317	no
gi|646738635|gb|KK962957.1|	28428-12920	20	8.6	22	19	-0.209597	0.89045	no
gi|646738635|gb|KK962957.1|	29710-13000	20	8.6	68	81	0.251838	0.86745	no

gi|646738635|gb|KK962957.1|	43478-14376	20	8.6	37	53.72	0.5476	0.72445	no
gi|646738635|gb|KK962957.1|	46781-14707	20	8.6	9	29	2	0.32605	no
gi|646738635|gb|KK962957.1|	53188-15338	20	8.6	101	128	0.343953	0.7865	no
gi|646738635|gb|KK962957.1|	56803-15718	20	8.6	14	25	0.834515	0.5439	no
gi|646738635|gb|KK962957.1|	61660-16193	20	8.6	57	48	-0.265935	0.86175	no
gi|646738635|gb|KK962957.1|	62518-16282	20	8.6	32	41	0.359256	0.8182	no
gi|646738635|gb|KK962957.1|	63957-16430	20	8.6	17	38	1	0.41145	no
gi|646738635|gb|KK962957.1|	66854-16712	20	8.6	164	178	0.117198	0.93805	no
gi|646738635|gb|KK962957.1|	69968-17254	20	8.6	1	0.809699	-0.698218	0.61635	no
gi|646738635|gb|KK962957.1|	73433-17527	20	8.6	1	0.337703	-2	1	no
gi|646738635|gb|KK962957.1|	75547-17737	20	8.6	0.588521	0.595799	0.0177309	1	no
gi|646738635|gb|KK962957.1|	77680-17860	20	8.6	1	0.979981	-0.494493	0.73195	no
gi|646738635|gb|KK962957.1|	84138-18483	20	8.6	8	12	0.482145	0.75225	no
gi|646738635|gb|KK962957.1|	84987-18746	20	8.6	115	77	-0.587492	0.6269	no
gi|646738635|gb|KK962957.1|	89780-19157	20	8.6	40	16	-1	0.22915	no
gi|646738635|gb|KK962957.1|	91676-19185	20	8.6	33	154	2	0.29645	no
gi|646738635|gb|KK962957.1|	92171-19301	20	8.6	9	14	0.547343	0.71765	no
gi|646738635|gb|KK962957.1|	05728-20713	20	8.6	98	57	-0.779251	0.50755	no
gi|646738635|gb|KK962957.1|	10393-21187	20	8.6	1	0.978338	-0.110785	1	no
gi|646738635|gb|KK962957.1|	43715-24446	20	8.6	10	4	-1	0.29015	no
gi|646738635|gb|KK962957.1|	44935-24701	20	8.6	21	17	-0.268724	0.80595	no
gi|646738635|gb|KK962957.1|	47199-24795	20	8.6	2	3	0.489693	0.7096	no
gi|646738635|gb|KK962957.1|	55414-25685	20	8.6	8	8	0.0316031	0.9844	no
gi|646738635|gb|KK962957.1|	57058-25937	20	8.6	1	2	0.674583	0.6652	no
gi|646738635|gb|KK962957.1|	59598-26117	20	8.6	2	0.909153	-0.761015	0.6058	no
gi|646738635|gb|KK962957.1|	26726-28266	20	8.6	0.719947	1	1	0.41785	no
gi|646738635|gb|KK962957.1|	67654-26822	20	8.6	1	4	2	0.27405	no
gi|646738635|gb|KK962957.1|	69001-26920	20	8.6	109	44	-1	0.34495	no
gi|646738635|gb|KK962957.1|	72554-27303	20	8.6	45	18	-1	0.398	no
gi|646738635|gb|KK962957.1|	85813-28648	20	8.6	24	23	-0.0750516	0.96075	no

gi|646738635|gb|KK962957.1|	93432-29492	20	8.6	0.07497	7	7	0.0951	no
gi|646738635|gb|KK962957.1|	31513-31894	20	8.6	4	12	2	0.29635	no
gi|646738635|gb|KK962957.1|	37666-40074	20	8.6	6	7	0.0697504	0.96165	no
gi|646738635|gb|KK962957.1|	47844-48223	20	8.6	27	20	-0.441461	0.77905	no
gi|646738635|gb|KK962957.1|	49445-50690	20	8.6	0.840995	0.536178	-0.649385	1	no
gi|646738635|gb|KK962957.1|	55828-57764	20	8.6	1	0.637341	-0.925824	0.5371	no
gi|646738635|gb|KK962957.1|	57967-60016	20	8.6	0.721982	0.672403	-0.102637	1	no
gi|646738635|gb|KK962957.1|	60139-61617	20	8.6	0.680796	0.435864	-0.643343	1	no
gi|646738635|gb|KK962957.1|	74896-75877	20	8.6	20	15	-0.400154	0.786	no
gi|646738635|gb|KK962957.1|	79883-81299	20	8.6	2	0.802725	-1	0.41375	no
gi|646738635|gb|KK962957.1|	93589-94938	20	8.6	0.930184	0.121557	-3	1	no
gi|646738643|gb|KK962956.1|	65656-16711	13	5.5	2	3	0.0497595	0.97395	no
gi|646738643|gb|KK962956.1|	80004-81039	13	5.5	0	4	inf	0.0023	no
gi|646738643|gb|KK962956.1|	83146-85097	13	5.5	0.109099	11	7	0.23265	no
gi|646738643|gb|KK962956.1|	88795-89276	13	5.5	0	5	inf	0.00255	no
gi|646738643|gb|KK962956.1|	89502-90926	13	5.5	0.079138	3	5	0.10045	no
gi|646738643|gb|KK962956.1|	95043-95441	13	5.5	0	9	inf	0.0014	yes
gi|646738652|gb|KK962955.1|	02029-10335	19	8.8	3	4	0.738582	0.6319	no
gi|646738652|gb|KK962955.1|	04675-10666	19	8.8	13	14	0.138594	0.8938	no
gi|646738652|gb|KK962955.1|	11928-11280	19	8.8	8	11	0.445358	0.77015	no
gi|646738652|gb|KK962955.1|	25492-12630	19	8.8	1	1	0.232819	0.87595	no
gi|646738652|gb|KK962955.1|	26548-12762	19	8.8	0.791616	0.968875	0.29151	1	no
gi|646738652|gb|KK962955.1|	27756-12926	19	8.8	0.738921	1	1	0.41785	no
gi|646738652|gb|KK962955.1|	29393-13016	19	8.8	0.892791	2	0.977404	0.5626	no
gi|646738652|gb|KK962955.1|	66208-16710	19	8.8	0.720409	1	0.766526	0.6143	no
gi|646738652|gb|KK962955.1|	77716-78147	19	8.8	22	7	-2	0.25155	no
gi|646738652|gb|KK962955.1|	92058-93566	19	8.8	2	3	0.849126	0.5926	no
gi|646738652|gb|KK962955.1|	94042-95003	19	8.8	6	3	-0.748471	0.63695	no
gi|646738652|gb|KK962955.1|	95258-95988	19	8.8	1	1	-0.0984146	0.9036	no
gi|646738662|gb|KK962954.1|	29129-22935	17	8.8	54	20	-1	0.34585	no

gi|646738662|gb|KK962954.1|	29886-23015	17	8.8	15	7	-1	0.48745	no
gi|646738662|gb|KK962954.1|	32391-23327	17	8.8	2	3	0.409619	0.78245	no
gi|646738662|gb|KK962954.1|	33810-23418	17	8.8	34	11	-2	0.25135	no
gi|646738682|gb|KK962952.1|	32968-13390	12	4.7	1	0.383138	-2	0.2551	no
gi|646738744|gb|KK962946.1|	72378-17315	12	9.9	3	0.751967	-2	0.19015	no
gi|646738753|gb|KK962945.1|	87810-18838	20	7.9	24	15	-0.633724	0.67605	no
gi|646738753|gb|KK962945.1|	04433-20496	20	7.9	4	0	-inf	0.0041	no
gi|646738753|gb|KK962945.1|	05692-20760	20	7.9	3	0.565307	-2	0.1169	no
gi|646738753|gb|KK962945.1|	08071-20831	20	7.9	455	17	-5	0.033	no
gi|646738753|gb|KK962945.1|	11079-21180	20	7.9	21	11	-0.969793	0.52035	no
gi|646738753|gb|KK962945.1|	12263-21432	20	7.9	2	0.814205	-1	0.31205	no
gi|646738753|gb|KK962945.1|	18700-21902	20	7.9	36	32	-0.161067	0.9122	no
gi|646738753|gb|KK962945.1|	45074-24573	20	7.9	6	6	-0.00973957	0.9807	no
gi|646738753|gb|KK962945.1|	53275-25747	20	7.9	2	2	0.277934	0.85455	no
gi|646738753|gb|KK962945.1|	57691-25857	20	7.9	2	0.626923	-1	0.35375	no
gi|646738753|gb|KK962945.1|	58679-25981	20	7.9	10	22	1	0.3848	no
gi|646738753|gb|KK962945.1|	59926-26110	20	7.9	34	30	-0.177047	0.87025	no
gi|646738753|gb|KK962945.1|	62369-26361	20	7.9	10	10	-0.0389189	0.9794	no
gi|646738753|gb|KK962945.1|	78782-28105	20	7.9	0.503312	0.995688	0.984242	1	no
gi|646738753|gb|KK962945.1|	28635-29006	20	7.9	121	0	-inf	0.0001	yes
gi|646738753|gb|KK962945.1|	91464-29255	20	7.9	0.441558	2	2	0.20255	no
gi|646738753|gb|KK962945.1|	92787-29497	20	7.9	0.382897	1	2	0.23155	no
gi|646738753|gb|KK962945.1|	35449-36475	20	7.9	1	4	2	0.25755	no
gi|646738753|gb|KK962945.1|	8947-9192	20	7.9	64	0	-inf	0.0004	yes
gi|646738782|gb|KK962942.1|	95907-19647	22	8.2	4	3	-0.13224	0.9271	no
gi|646738782|gb|KK962942.1|	10941-21197	22	8.2	2	4	0.926775	0.5001	no
gi|646738782|gb|KK962942.1|	31198-23227	22	8.2	0.338025	1	2	0.2023	no
gi|646738803|gb|KK962940.1|	74117-27464	11	8.4	8	7	-0.236919	0.88885	no
gi|646738812|gb|KK962939.1|	05756-10637	20	8.1	0.759061	3	2	0.1897	no
gi|646738812|gb|KK962939.1|	19374-12172	20	8.1	1	0.447578	-1	1	no

gi|646738812|gb|KK962939.1|	23322-12435	20	8.1	23	21	-0.174795	0.9076	no
gi|646738812|gb|KK962939.1|	44736-14499	20	8.1	8	22	2	0.35845	no
gi|646738812|gb|KK962939.1|	48411-14855	20	8.1	357	367	0.0400767	0.9017	no
gi|646738812|gb|KK962939.1|	56043-15658	20	8.1	1	4	2	0.2794	no
gi|646738812|gb|KK962939.1|	76817-17745	20	8.1	6	5	-0.229411	0.88565	no
gi|646738812|gb|KK962939.1|	78053-17939	20	8.1	0.598499	0.860146	0.523234	1	no
gi|646738812|gb|KK962939.1|	79669-18145	20	8.1	4	4	-0.0730556	0.9619	no
gi|646738812|gb|KK962939.1|	84269-18623	20	8.1	6	6	-0.190977	0.904	no
gi|646738812|gb|KK962939.1|	86549-18889	20	8.1	0.398594	0.964092	1	1	no
gi|646738812|gb|KK962939.1|	90108-19069	20	8.1	3	3	-0.00104688	0.94695	no
gi|646738812|gb|KK962939.1|	98960-20007	20	8.1	0.751217	67	6	0.02865	no
gi|646738812|gb|KK962939.1|	05654-20584	20	8.1	85	26	-2	0.29885	no
gi|646738812|gb|KK962939.1|	17239-21750	20	8.1	20	7	-1	0.37725	no
gi|646738812|gb|KK962939.1|	25249-22719	20	8.1	10	12	0.360745	0.77355	no
gi|646738812|gb|KK962939.1|	27933-22844	20	8.1	1	4	2	0.2626	no
gi|646738812|gb|KK962939.1|	29651-23047	20	8.1	1	0.917878	-0.502681	0.7384	no
gi|646738812|gb|KK962939.1|	31224-23166	20	8.1	8	6	-0.364885	0.7661	no
gi|646738812|gb|KK962939.1|	54651-25545	20	8.1	2	2	0.495245	0.7095	no
gi|646738812|gb|KK962939.1|	56533-25706	20	8.1	28	16	-0.819378	0.59095	no
gi|646738812|gb|KK962939.1|	60050-26039	20	8.1	5	27	2	0.19095	no
gi|646738812|gb|KK962939.1|	66958-26755	20	8.1	16	24	0.576153	0.7006	no
gi|646738812|gb|KK962939.1|	71340-27197	20	8.1	1	2	0.734268	0.64505	no
gi|646738812|gb|KK962939.1|	88586-28941	20	8.1	2	0.684934	-2	0.35375	no
gi|646738812|gb|KK962939.1|	91715-29394	20	8.1	0.468681	0.475826	0.0218288	1	no
gi|646738812|gb|KK962939.1|	25003-33005	20	8.1	4	3	-0.696976	0.5677	no
gi|646738812|gb|KK962939.1|	30849-33198	20	8.1	6	3	-1	0.51605	no
gi|646738812|gb|KK962939.1|	41777-34269	20	8.1	10	4	-1	0.41005	no
gi|646738812|gb|KK962939.1|	61975-36560	20	8.1	7	3	-1	0.27945	no
gi|646738812|gb|KK962939.1|	65714-36806	20	8.1	2	2	0.0304052	0.98505	no
gi|646738812|gb|KK962939.1|	69450-37313	20	8.1	5	5	-0.00305971	0.99675	no

gi|646738812|gb|KK962939.1|	77773-37828	20	8.1	2	38	4	0.06435	no
gi|646738812|gb|KK962939.1|	79328-37977	20	8.1	0	7	inf	0.001	yes
gi|646738812|gb|KK962939.1|	53901-54298	20	8.1	9	6	-0.442938	0.73105	no
gi|646738812|gb|KK962939.1|	70325-80013	20	8.1	55	38	-0.53354	0.6535	no
gi|646738812|gb|KK962939.1|	99806-100605	20	8.1	2	0.718794	-2	0.268	no
gi|646738819|gb|KK962938.1|	04646-10827	19	8.4	13	13	-0.0228629	0.9816	no
gi|646738819|gb|KK962938.1|	24738-12883	19	8.4	15	14	-0.137972	0.90285	no
gi|646738819|gb|KK962938.1|	30517-13176	19	8.4	1	2	1	0.453	no
gi|646738819|gb|KK962938.1|	32907-13379	19	8.4	37	22	-0.785516	0.52885	no
gi|646738819|gb|KK962938.1|	36544-14182	19	8.4	28	27	-0.0730631	0.9526	no
gi|646738819|gb|KK962938.1|	42967-14463	19	8.4	0.718685	0.849106	0.240586	1	no
gi|646738819|gb|KK962938.1|	51624-15441	19	8.4	1	3	0.984748	0.53275	no
gi|646738819|gb|KK962938.1|	55491-15730	19	8.4	29	37	0.338276	0.7674	no
gi|646738819|gb|KK962938.1|	21181-22429	19	8.4	0.931616	1	0.520636	0.69625	no
gi|646738819|gb|KK962938.1|	22553-23651	19	8.4	47	63	0.407604	0.7205	no
gi|646738819|gb|KK962938.1|	24924-26260	19	8.4	37	45	0.284019	0.7956	no
gi|646738819|gb|KK962938.1|	30916-32695	19	8.4	2	2	-0.427687	0.78595	no
gi|646738819|gb|KK962938.1|	42256-43777	19	8.4	3	2	-0.642469	0.68485	no
gi|646738819|gb|KK962938.1|	48573-49769	19	8.4	6	4	-0.457647	0.76395	no
gi|646738819|gb|KK962938.1|	50653-52743	19	8.4	9	11	0.306246	0.8066	no
gi|646738819|gb|KK962938.1|	60349-61387	19	8.4	4	8	0.967879	0.53205	no
gi|646738819|gb|KK962938.1|	63425-63697	19	8.4	118	115	-0.0360647	0.9812	no
gi|646738819|gb|KK962938.1|	84065-84400	19	8.4	36	13	-1	0.31865	no
gi|646738819|gb|KK962938.1|	90359-90986	19	8.4	1	11	3	0.11045	no
gi|646738819|gb|KK962938.1|	95391-95773	19	8.4	7	11	0.728615	0.5782	no
gi|646738819|gb|KK962938.1|	99839-100012	19	8.4	620	82	-3	0.10195	no
gi|646738841|gb|KK962936.1|	1-464	13	7.5	11	16	0.465749	0.7683	no
gi|646738872|gb|KK962933.1|	34404-34732	24	10	13	9	-0.495068	0.71245	no
gi|646738882|gb|KK962932.1|	02055-10435	14	7	2	3	0.713829	0.63745	no
gi|646738893|gb|KK962931.1|	83885-18414	18	6.5	17	13	-0.346021	0.82015	no

gi|646738893|gb|KK962931.1|	98131-20202	18	6.5	9	8	-0.268622	0.80285	no
gi|646738893|gb|KK962931.1|	2593-2904	18	6.5	21	13	-0.662451	0.6053	no
gi|646738893|gb|KK962931.1|	39483-40059	18	6.5	3	2	-0.865734	0.56495	no
gi|646738893|gb|KK962931.1|	46-362	18	6.5	6	16	1	0.35005	no
gi|646738904|gb|KK962930.1|	41326-14217	15	6.6	0	53	inf	5.00E-05	yes
gi|646738915|gb|KK962929.1|	22732-12574	21	11	1	0.780084	-0.816568	0.60745	no
gi|646738915|gb|KK962929.1|	44062-15028	21	11	20	10	-1	0.38625	no
gi|646738915|gb|KK962929.1|	50774-15577	21	11	3	2	-0.861012	0.57245	no
gi|646738915|gb|KK962929.1|	57342-15963	21	11	10	9	-0.221144	0.85705	no
gi|646738915|gb|KK962929.1|	93966-94182	21	11	27	32	0.2449	0.8667	no
gi|646738926|gb|KK962928.1|	19550-20000	15	12	0	7	inf	0.0014	yes
gi|646738926|gb|KK962928.1|	87834-88718	15	12	0	3	inf	0.00605	no
gi|646738926|gb|KK962928.1|	90066-91588	15	12	0	2	inf	0.00315	no
gi|646738953|gb|KK962925.1|	34596-13479	14	7.1	74	46	-0.684827	0.62515	no
gi|646738953|gb|KK962925.1|	33002-23320	14	7.1	34	59	0.785976	0.5987	no
gi|646738953|gb|KK962925.1|	82591-28344	14	7.1	8	5	-0.587086	0.70715	no
gi|646738953|gb|KK962925.1|	84308-48618	14	7.1	3	2	-0.214358	0.89405	no
gi|646738953|gb|KK962925.1|	90410-49071	14	7.1	25	15	-0.761039	0.5472	no
gi|646738953|gb|KK962925.1|	94728-49503	14	7.1	21	20	-0.0550795	0.9593	no
gi|646739010|gb|KK962920.1|	40625-14125	12	9.2	8	1	-3	0.1361	no
gi|646739020|gb|KK962919.1|	07760-10820	12	5.7	2	4	1	0.45825	no
gi|646739039|gb|KK962917.1|	47820-14820	13	7.1	3	5	0.617121	0.66895	no
gi|646739056|gb|KK962915.1|	02635-10286	11	5	8	106	4	0.24925	no
gi|646739056|gb|KK962915.1|	08723-10951	11	5	2	42	4	0.00875	no
gi|646739078|gb|KK962913.1|	56662-57058	12	9.4	6	2	-1	0.45395	no
gi|646739078|gb|KK962913.1|	84413-84865	12	9.4	0	6	inf	0.00255	no
gi|646739111|gb|KK962910.1|	31156-13140	9.7	4.6	45	3	-4	0.1732	no
gi|646739121|gb|KK962909.1|	59991-26052	16	9.3	3	2	-0.557048	0.72945	no
gi|646739121|gb|KK962909.1|	63125-27036	16	9.3	9	7	-0.503677	0.659	no
gi|646739121|gb|KK962909.1|	70478-27483	16	9.3	1	1	0.0694117	0.96285	no

gi|646739121|gb|KK962909.1|	75319-27606	16	9.3	2	2	-0.385279	0.7808	no
gi|646739121|gb|KK962909.1|	79899-28025	16	9.3	5	6	0.323472	0.80885	no
gi|646739121|gb|KK962909.1|	86132-28747	16	9.3	0.847947	0.609424	-0.476527	1	no
gi|646739121|gb|KK962909.1|	92070-29276	16	9.3	1	2	0.481831	0.7489	no
gi|646739121|gb|KK962909.1|	93437-29624	16	9.3	18	12	-0.545937	0.6263	no
gi|646739121|gb|KK962909.1|	06491-31268	16	9.3	14	9	-0.696019	0.5476	no
gi|646739121|gb|KK962909.1|	22233-32253	16	9.3	20	16	-0.342305	0.7885	no
gi|646739121|gb|KK962909.1|	25564-32638	16	9.3	18	7	-1	0.40575	no
gi|646739132|gb|KK962908.1|	14765-11528	15	9.4	0	44	inf	0.00025	yes
gi|646739132|gb|KK962908.1|	92786-93556	15	9.4	6	2	-1	0.37955	no
gi|646739132|gb|KK962908.1|	97943-98278	15	9.4	20	17	-0.221665	0.8554	no
gi|646739152|gb|KK962906.1|	51249-15332	18	7.7	0.254307	2	3	0.11705	no
gi|646739152|gb|KK962906.1|	53429-15476	18	7.7	0.25793	4	4	0.12085	no
gi|646739228|gb|KK962900.1|	22788-12324	12	7	7	14	0.930293	0.5109	no
gi|646739228|gb|KK962900.1|	59411-15973	12	7	6	7	0.271659	0.85615	no
gi|646739228|gb|KK962900.1|	62431-16299	12	7	30	15	-1	0.5009	no
gi|646739256|gb|KK962898.1|	00408-10110	11	4.7	3	0.590557	-2	0.2077	no
gi|646739268|gb|KK962897.1|	04995-10560	18	7.4	6	1	-2	0.21885	no
gi|646739268|gb|KK962897.1|	09181-11097	18	7.4	0.661683	0.869687	0.394356	1	no
gi|646739268|gb|KK962897.1|	11140-11145	18	7.4	18	4	-2	0.1984	no
gi|646739268|gb|KK962897.1|	14087-11470	18	7.4	8	2	-2	0.1832	no
gi|646739268|gb|KK962897.1|	15721-11616	18	7.4	8	23	2	0.27505	no
gi|646739268|gb|KK962897.1|	20768-12114	18	7.4	8	40	2	0.13705	no
gi|646739268|gb|KK962897.1|	37965-13933	18	7.4	0.750091	0.11984	-3	1	no
gi|646739268|gb|KK962897.1|	44914-14526	18	7.4	54	22	-1	0.404	no
gi|646739268|gb|KK962897.1|	46026-14725	18	7.4	11	9	-0.325569	0.835	no
gi|646739268|gb|KK962897.1|	47995-14936	18	7.4	1	0.957027	-0.475984	0.71985	no
gi|646739268|gb|KK962897.1|	49505-15004	18	7.4	7	7	-0.0778391	0.96025	no
gi|646739268|gb|KK962897.1|	56604-15704	18	7.4	10	8	-0.293138	0.80355	no
gi|646739268|gb|KK962897.1|	57773-15838	18	7.4	4	4	-0.211791	0.86835	no

gi|646739268|gb|KK962897.1|	63209-16355	18	7.4	10	11	0.168011	0.9034	no
gi|646739268|gb|KK962897.1|	79836-18033	18	7.4	4	5	0.014507	0.9493	no
gi|646739268|gb|KK962897.1|	81696-18266	18	7.4	2	0.917687	-1	0.43875	no
gi|646739268|gb|KK962897.1|	94406-19566	18	7.4	7	3	-1	0.34905	no
gi|646739268|gb|KK962897.1|	17158-22484	18	7.4	6	6	0.117885	0.9152	no
gi|646739268|gb|KK962897.1|	42205-24248	18	7.4	13	39	2	0.30695	no
gi|646739268|gb|KK962897.1|	53282-25371	18	7.4	17	2	-3	0.1264	no
gi|646739268|gb|KK962897.1|	79940-28040	18	7.4	36	19	-0.901857	0.5567	no
gi|646739268|gb|KK962897.1|	73045-74571	18	7.4	0.436726	0.419688	-0.057409	1	no
gi|646739268|gb|KK962897.1|	95983-96720	18	7.4	2	3	0.487497	0.69145	no
gi|646739268|gb|KK962897.1|	98440-10001[illegible]	18	7.4	10	4	-1	0.381	no
gi|646739335|gb|KK962892.1|	17864-18547	12	8.6	0.432023	156.9	9	0.2322	no
gi|646739335|gb|KK962892.1|	48738-25044	12	8.6	4	0.735335	-3	0.10365	no
gi|646739335|gb|KK962892.1|	51391-25297	12	8.6	2	0	-inf	0.0026	no
gi|646739347|gb|KK962891.1|	84262-18516	15	8.8	0	2	inf	0.001	yes
gi|646739347|gb|KK962891.1|	98787-19988	15	8.8	0	2	inf	0.0014	yes
gi|646739347|gb|KK962891.1|	20108-22188	15	8.8	24	24	0.036515	0.974	no
gi|646739347|gb|KK962891.1|	34308-23453	15	8.8	26	27	0.0254487	0.91805	no
gi|646739347|gb|KK962891.1|	54268-25453	15	8.8	23	31	0.410886	0.75805	no
gi|646739347|gb|KK962891.1|	66337-26753	15	8.8	0.591739	2	1	0.33975	no
gi|646739361|gb|KK962890.1|	21769-12200	22	9.6	27	12	-1	0.4807	no
gi|646739361|gb|KK962890.1|	26126-12663	22	9.6	6	7	0.241949	0.8389	no
gi|646739361|gb|KK962890.1|	29237-13032	22	9.6	2	0.317666	-3	0.1387	no
gi|646739361|gb|KK962890.1|	35988-13692	22	9.6	4	1	-2	0.3206	no
gi|646739361|gb|KK962890.1|	43745-14522	22	9.6	24	11	-1	0.3672	no
gi|646739361|gb|KK962890.1|	47209-14782	22	9.6	10	4	-1	0.3136	no
gi|646739361|gb|KK962890.1|	60200-16084	22	9.6	0.705409	3	2	0.2057	no
gi|646739361|gb|KK962890.1|	84600-18494	22	9.6	5	10	0.8958	0.54375	no
gi|646739361|gb|KK962890.1|	92245-19246	22	9.6	37	24	-0.665174	0.6598	no
gi|646739361|gb|KK962890.1|	00429-20104	22	9.6	2	1	-0.340621	0.81865	no

gi|646739361|gb|KK962890.1|	12536-21337	22	9.6	2	1	-1	0.43695	no
gi|646739361|gb|KK962890.1|	17096-21858	22	9.6	6	2	-1	0.4089	no
gi|646739361|gb|KK962890.1|	25249-22636	22	9.6	1	0.923806	-0.599754	0.64385	no
gi|646739361|gb|KK962890.1|	40601-24189	22	9.6	1	0.382129	-2	0.27245	no
gi|646739361|gb|KK962890.1|	50938-25231	22	9.6	59	24	-1	0.24155	no
gi|646739361|gb|KK962890.1|	53969-25483	22	9.6	1	0.647874	-1	0.4766	no
gi|646739361|gb|KK962890.1|	71856-27208	22	9.6	43	36	-0.240207	0.86025	no
gi|646739361|gb|KK962890.1|	76368-27688	22	9.6	4	3	-0.437484	0.74405	no
gi|646739361|gb|KK962890.1|	81654-28305	22	9.6	2	0.815824	-1	0.39895	no
gi|646739361|gb|KK962890.1|	86696-28704	22	9.6	15	3	-2	0.1793	no
gi|646739361|gb|KK962890.1|	90219-29046	22	9.6	45	6	-3	0.1311	no
gi|646739361|gb|KK962890.1|	00219-30346	22	9.6	15	4	-2	0.1604	no
gi|646739361|gb|KK962890.1|	03738-30538	22	9.6	0.732654	1	0.529659	1	no
gi|646739361|gb|KK962890.1|	64079-36492	22	9.6	2	0.440325	-2	0.26845	no
gi|646739361|gb|KK962890.1|	80528-38211	22	9.6	0.696639	1	0.666121	1	no
gi|646739361|gb|KK962890.1|	01062-40367	22	9.6	143	89	-0.689172	0.5637	no
gi|646739361|gb|KK962890.1|	48679-44936	22	9.6	27	16	-0.715537	0.6383	no
gi|646739361|gb|KK962890.1|	57167-45759	22	9.6	0	10	inf	0.00035	yes
gi|646739361|gb|KK962890.1|	63663-46483	22	9.6	5	4	-0.422934	0.7826	no
gi|646739361|gb|KK962890.1|	80175-48135	22	9.6	0.797486	0.571157	-0.481572	1	no
gi|646739361|gb|KK962890.1|	84930-48764	22	9.6	3	3	0.0159929	0.9891	no
gi|646739361|gb|KK962890.1|	89325-49091	22	9.6	2	2	-0.352561	0.82355	no
gi|646739361|gb|KK962890.1|	85138-86167	22	9.6	46	19	-1	0.31475	no
gi|646739361|gb|KK962890.1|	89016-89975	22	9.6	0.394188	3	3	0.134	no
gi|646739402|gb|KK962886.1|	11626-11260	10	5.9	0	3	inf	0.00315	no
gi|646739411|gb|KK962885.1|	35869-13620	18	7.7	4	16	2	0.2342	no
gi|646739411|gb|KK962885.1|	14081-14898	18	7.7	16	12	-0.34529	0.81365	no
gi|646739411|gb|KK962885.1|	44595-14545	18	7.7	5	3	-0.75338	0.57985	no
gi|646739411|gb|KK962885.1|	46862-14718	18	7.7	17	7	-1	0.3249	no
gi|646739411|gb|KK962885.1|	49126-14997	18	7.7	0.777299	2	1	0.33895	no

gi|646739411|gb|KK962885.1| 63986-16592	18	7.7	6	5	-0.374969	0.8018	no
gi|646739411|gb|KK962885.1| 68606-16986	18	7.7	12	9	-0.401645	0.7928	no
gi|646739411|gb|KK962885.1| 71307-17172	18	7.7	20	6	-2	0.27245	no
gi|646739411|gb|KK962885.1| 17579-19364	18	7.7	0.604675	1	1	0.41785	no
gi|646739411|gb|KK962885.1| 85409-18557	18	7.7	732	663	-0.142675	0.9318	no
gi|646739411|gb|KK962885.1| 88127-18847	18	7.7	435	426	-0.0281154	0.9805	no
gi|646739411|gb|KK962885.1| 20443-21475	18	7.7	2	3	1	0.4626	no
gi|646739411|gb|KK962885.1| 21423-22294	18	7.7	226	135	-0.742157	0.53675	no
gi|646739411|gb|KK962885.1| 23862-22411	18	7.7	27	3	-3	0.1932	no
gi|646739411|gb|KK962885.1| 25158-22548	18	7.7	63	70	0.142374	0.92335	no
gi|646739411|gb|KK962885.1| 27737-22892	18	7.7	8	6	-0.322058	0.8282	no
gi|646739411|gb|KK962885.1| 29838-23178	18	7.7	5	5	0.118703	0.9392	no
gi|646739411|gb|KK962885.1| 32576-23284	18	7.7	20.14	45	1	0.45645	no
gi|646739411|gb|KK962885.1| 37401-23904	18	7.7	13	16	0.269662	0.8271	no
gi|646739411|gb|KK962885.1| 39376-24004	18	7.7	3	3	0.0615603	0.9491	no
gi|646739411|gb|KK962885.1| 2442-3451	18	7.7	2	0.174791	-4	0.17415	no
gi|646739411|gb|KK962885.1| 45555-24658	18	7.7	1	0.847747	-0.625559	0.66015	no
gi|646739411|gb|KK962885.1| 55618-25630	18	7.7	1	4	1	0.31035	no
gi|646739411|gb|KK962885.1| 70684-27110	18	7.7	6	3	-0.750402	0.59655	no
gi|646739411|gb|KK962885.1| 72785-27831	18	7.7	8	7	-0.123947	0.9161	no
gi|646739411|gb|KK962885.1| 83543-28448	18	7.7	70	23	-2	0.23925	no
gi|646739411|gb|KK962885.1| 44317-45187	18	7.7	2	3	0.723769	0.5703	no
gi|646739411|gb|KK962885.1| 49626-50093	18	7.7	27	62	1	0.43445	no
gi|646739411|gb|KK962885.1| 50488-50886	18	7.7	14	15	0.153809	0.9228	no
gi|646739411|gb|KK962885.1| 985-2126	18	7.7	1	1	0.00225789	1	no
gi|646739440|gb|KK962882.1| 97379-97879	14	7.7	1	3	1	0.48625	no
gi|646739450|gb|KK962881.1| 04723-10566	16	7.7	0.937072	0.57068	-0.715478	1	no
gi|646739450|gb|KK962881.1| 08614-10952	16	7.7	0.562317	2	2	0.3671	no
gi|646739450|gb|KK962881.1| 48114-24909	16	7.7	16	8	-1	0.4896	no
gi|646739450|gb|KK962881.1| 60195-26060	16	7.7	42	11	-2	0.2559	no

gi|646739458|gb|KK962880.1|	79211-17954	13	6.8	11	4	-2	0.35815	no
gi|646739458|gb|KK962880.1|	85612-18600	13	6.8	8	2	-2	0.169	no
gi|646739458|gb|KK962880.1|	9292-9539	13	6.8	103	60	-0.778738	0.6245	no
gi|646739490|gb|KK962877.1|	51977-54986	13	6.7	2	2	0.511808	0.73675	no
gi|646739490|gb|KK962877.1|	55651-56961	13	6.7	0.526126	2	2	0.24775	no
gi|646739497|gb|KK962876.1|	20007-20840	10	7	0	82	inf	5.00E-05	yes
gi|646739497|gb|KK962876.1|	21471-22933	10	7	0.076647	30	9	0.09445	no
gi|646739497|gb|KK962876.1|	28672-29483	10	7	0.166361	4	4	0.1128	no
gi|646739497|gb|KK962876.1|	9441-9620	10	7	0	163	inf	0.001	yes
gi|646739507|gb|KK962875.1|	68635-16919	22	8.9	0	10	inf	0.00175	no
gi|646739507|gb|KK962875.1|	06308-20668	22	8.9	0	64	inf	0.00015	yes
gi|646739516|gb|KK962874.1|	13044-11488	16	7.4	0.408844	0.760305	0.895026	1	no
gi|646739516|gb|KK962874.1|	34052-13508	16	7.4	2	2	0.511893	0.6858	no
gi|646739516|gb|KK962874.1|	41598-14189	16	7.4	20	7	-1	0.3226	no
gi|646739516|gb|KK962874.1|	44233-14584	16	7.4	4	3	-0.225849	0.8815	no
gi|646739516|gb|KK962874.1|	49736-15089	16	7.4	6	4	-0.405884	0.78685	no
gi|646739516|gb|KK962874.1|	52031-15243	16	7.4	43	18	-1	0.40835	no
gi|646739516|gb|KK962874.1|	60916-16119	16	7.4	0	88	inf	0.0002	yes
gi|646739516|gb|KK962874.1|	84834-18533	16	7.4	0	6	inf	0.001	yes
gi|646739516|gb|KK962874.1|	27126-28200	16	7.4	2	2	-0.251586	0.8381	no
gi|646739516|gb|KK962874.1|	32180-33081	16	7.4	1	0.812135	-0.818138	0.57835	no
gi|646739516|gb|KK962874.1|	42913-43966	16	7.4	2	1	-0.679698	0.60255	no
gi|646739516|gb|KK962874.1|	51397-52259	16	7.4	3	4	0.500714	0.71845	no
gi|646739516|gb|KK962874.1|	54784-56709	16	7.4	0.830983	0.080186	-3	1	no
gi|646739516|gb|KK962874.1|	57233-57732	16	7.4	24	8	-2	0.3277	no
gi|646739516|gb|KK962874.1|	59706-60333	16	7.4	16	18	0.146789	0.92305	no
gi|646739516|gb|KK962874.1|	68544-68783	16	7.4	31	25	-0.287349	0.8374	no
gi|646739516|gb|KK962874.1|	72605-74869	16	7.4	1	3	1	0.4125	no
gi|646739516|gb|KK962874.1|	99628-99846	16	7.4	16	57	2	0.24265	no
gi|646739537|gb|KK962872.1|	16308-16573	14	7.7	44	27	-0.706458	0.5636	no

gi|646739537|gb|KK962872.1|	20039-20921	14	7.7	0.442584	2	2	0.25505	no
gi|646739537|gb|KK962872.1|	21022-21649	14	7.7	2	1	-0.338638	0.81865	no
gi|646739537|gb|KK962872.1|	21795-22302	14	7.7	0.728427	5	3	0.26175	no
gi|646739547|gb|KK962871.1|	16949-11783	16	10	1	2	0.502259	0.7157	no
gi|646739547|gb|KK962871.1|	17983-11887	16	10	0	3	inf	0.00515	no
gi|646739547|gb|KK962871.1|	20529-12098	16	10	0	26	inf	0.0002	yes
gi|646739547|gb|KK962871.1|	33493-13459	16	10	0	7	inf	0.0002	yes
gi|646739547|gb|KK962871.1|	35583-13646	16	10	0	19	inf	0.0001	yes
gi|646739547|gb|KK962871.1|	53626-15510	16	10	0	3	inf	0.00185	no
gi|646739547|gb|KK962871.1|	58263-16111	16	10	0.178471	111	9	0.05805	no
gi|646739547|gb|KK962871.1|	61689-16212	16	10	0	9	inf	0.0008	yes
gi|646739547|gb|KK962871.1|	81044-18285	16	10	0	2	inf	0.00435	no
gi|646739547|gb|KK962871.1|	07444-20806	16	10	0.255097	50	8	0.09445	no
gi|646739547|gb|KK962871.1|	20248-22156	16	10	0.260593	49	8	0.1049	no
gi|646739547|gb|KK962871.1|	32274-23587	16	10	0.110737	42	9	0.09345	no
gi|646739547|gb|KK962871.1|	46432-24820	16	10	0.061066	55	10	0.09445	no
gi|646739547|gb|KK962871.1|	50938-25134	16	10	0.579749	49	6	0.0952	no
gi|646739547|gb|KK962871.1|	55861-25745	16	10	0.138597	1	3	1	no
gi|646739547|gb|KK962871.1|	59287-60111	16	10	0.975367	0.917878	-0.0876433	1	no
gi|646739547|gb|KK962871.1|	60635-61063	16	10	6	9	0.50474	0.6902	no
gi|646739547|gb|KK962871.1|	73417-74361	16	10	0	3	inf	0.00515	no
gi|646739547|gb|KK962871.1|	82019-87672	16	10	0.171402	59	8	0.0126	no
gi|646739555|gb|KK962870.1|	00437-10070	27	18	164	135	-0.279248	0.8502	no
gi|646739555|gb|KK962870.1|	03535-10481	27	18	3	5	0.443708	0.7735	no
gi|646739555|gb|KK962870.1|	05390-10632	27	18	6	7	0.069819	0.96425	no
gi|646739555|gb|KK962870.1|	11677-11279	27	18	8	10	0.374367	0.79705	no
gi|646739555|gb|KK962870.1|	14252-11470	27	18	5	6	0.114843	0.937	no
gi|646739555|gb|KK962870.1|	18832-11951	27	18	0.434042	3	3	0.2662	no
gi|646739555|gb|KK962870.1|	21423-12454	27	18	19	18	-0.0361242	0.97535	no
gi|646739555|gb|KK962870.1|	26712-12855	27	18	1	3	1	0.41685	no

gi|646739555|gb|KK962870.1|	30370-13150	27	18	2	6	1	0.38195	no
gi|646739555|gb|KK962870.1|	33107-13355	27	18	31	48	0.647308	0.66685	no
gi|646739555|gb|KK962870.1|	13459-15834	27	18	1	0.570085	-1	0.37795	no
gi|646739555|gb|KK962870.1|	34798-13524	27	18	10	4	-1	0.3522	no
gi|646739555|gb|KK962870.1|	36582-13743	27	18	34	21	-0.668066	0.59095	no
gi|646739555|gb|KK962870.1|	16696-17233	27	18	15	9	-0.65553	0.67815	no
gi|646739555|gb|KK962870.1|	83759-18418	27	18	9	2	-2	0.19495	no
gi|646739555|gb|KK962870.1|	02411-20326	27	18	2	0.655172	-1	0.35375	no
gi|646739555|gb|KK962870.1|	19958-22240	27	18	2	4	0.924989	0.54835	no
gi|646739555|gb|KK962870.1|	22610-22359	27	18	1	1	0.0944518	1	no
gi|646739555|gb|KK962870.1|	36863-23709	27	18	87	41	-1	0.46235	no
gi|646739555|gb|KK962870.1|	48224-24979	27	18	6	7	0.233017	0.87745	no
gi|646739555|gb|KK962870.1|	34278-35190	27	18	5	3	-0.79797	0.62875	no
gi|646739555|gb|KK962870.1|	37119-38306	27	18	0.893322	0.426572	-1	1	no
gi|646739555|gb|KK962870.1|	43605-44326	27	18	2	1	-0.836643	0.57665	no
gi|646739555|gb|KK962870.1|	57271-57524	27	18	869	199	-2	0.11965	no
gi|646739555|gb|KK962870.1|	68764-69315	27	18	32	12	-1	0.35655	no
gi|646739555|gb|KK962870.1|	70172-73787	27	18	4	1	-2	0.3356	no
gi|646739555|gb|KK962870.1|	7043-8839	27	18	2	0.434009	-2	0.2533	no
gi|646739555|gb|KK962870.1|	78844-79647	27	18	12	5	-1	0.40035	no
gi|646739555|gb|KK962870.1|	94716-95766	27	18	1	1	0.0977627	0.942	no
gi|646739570|gb|KK962868.1|	19993-22179	18	8.1	0.419163	3	3	0.11995	no
gi|646739570|gb|KK962868.1|	22440-22577	18	8.1	3	5	0.787743	0.60445	no
gi|646739570|gb|KK962868.1|	26277-23087	18	8.1	1	3	0.949081	0.53625	no
gi|646739570|gb|KK962868.1|	46513-24743	18	8.1	1	4	2	0.32315	no
gi|646739570|gb|KK962868.1|	40067-40586	18	8.1	12	20	0.734358	0.63955	no
gi|646739570|gb|KK962868.1|	57240-57717	18	8.1	3	3	-0.259345	0.87285	no
gi|646739570|gb|KK962868.1|	64460-64678	18	8.1	16	36	1	0.4424	no
gi|646739570|gb|KK962868.1|	65421-65637	18	8.1	44	65	0.566828	0.6797	no
gi|646739570|gb|KK962868.1|	67018-67558	18	8.1	1	3	1	0.4404	no

gi|646739570|gb|KK962868.1|	76496-77313	18	8.1	0.822927	1	0.759679	0.61475	no
gi|646739570|gb|KK962868.1|	77464-77971	18	8.1	4	4	-0.152427	0.9082	no
gi|646739581|gb|KK962867.1|	3-293	18	5.4	16	11	-0.521687	0.7068	no
gi|646739581|gb|KK962867.1|	04047-30492	18	5.4	4	12	2	0.31995	no
gi|646739581|gb|KK962867.1|	42463-34361	18	5.4	0.412981	1	2	0.29375	no
gi|646739581|gb|KK962867.1|	54733-35783	18	5.4	6	10	0.738865	0.55705	no
gi|646739581|gb|KK962867.1|	98180-39972	18	5.4	0.643446	0.206178	-2	1	no
gi|646739581|gb|KK962867.1|	00789-40239	18	5.4	0.618591	0.595028	-0.0560282	1	no
gi|646739581|gb|KK962867.1|	05153-41077	18	5.4	7	5	-0.624234	0.5634	no
gi|646739581|gb|KK962867.1|	13015-41917	18	5.4	3	2	-0.638744	0.674	no
gi|646739581|gb|KK962867.1|	19288-42741	18	5.4	3	3	-0.168512	0.87715	no
gi|646739581|gb|KK962867.1|	27487-42888	18	5.4	0.64637	2	2	0.2716	no
gi|646739581|gb|KK962867.1|	93657-59599	18	5.4	0.710994	1	0.537235	1	no
gi|646739592|gb|KK962866.1|	44976-14572	14	7	5	7	0.545773	0.73135	no
gi|646739592|gb|KK962866.1|	49094-15030	14	7	0.680927	3	2	0.15655	no
gi|646739592|gb|KK962866.1|	50988-15127	14	7	41	59	0.517106	0.74125	no
gi|646739592|gb|KK962866.1|	52212-15245	14	7	31	75	1	0.39805	no
gi|646739592|gb|KK962866.1|	53994-15421	14	7	83	127	0.617264	0.6552	no
gi|646739592|gb|KK962866.1|	54687-15517	14	7	2	5	1	0.4025	no
gi|646739592|gb|KK962866.1|	55761-15607	14	7	14	29	1	0.4815	no
gi|646739592|gb|KK962866.1|	57389-15931	14	7	7	11	0.645411	0.6738	no
gi|646739592|gb|KK962866.1|	59405-16049	14	7	2	2	0.407853	0.74125	no
gi|646739592|gb|KK962866.1|	01982-20237	14	7	292	0.821021	-8	0.1665	no
gi|646739592|gb|KK962866.1|	04987-20642	14	7	6	0	-inf	0.00065	yes
gi|646739592|gb|KK962866.1|	09012-20974	14	7	50	0.545578	-7	0.10535	no
gi|646739592|gb|KK962866.1|	10143-21095	14	7	10	0	-inf	0.00025	yes
gi|646739592|gb|KK962866.1|	15732-21650	14	7	9	0	-inf	0.00045	yes
gi|646739592|gb|KK962866.1|	18431-21992	14	7	16	0.215338	-6	0.10535	no
gi|646739592|gb|KK962866.1|	24467-22490	14	7	9	7	-0.454555	0.71955	no
gi|646739592|gb|KK962866.1|	25705-22615	14	7	6	0	-inf	0.0035	no

gi|646739592|gb|KK962866.1|	38664-34645	14	7	2	3	0.580902	0.644	no
gi|646739592|gb|KK962866.1|	52892-35326	14	7	0.747445	71	7	0.09515	no
gi|646739592|gb|KK962866.1|	75496-37592	14	7	5	0	-inf	0.00655	no
gi|646739592|gb|KK962866.1|	78017-37827	14	7	50	3	-4	0.16935	no
gi|646739592|gb|KK962866.1|	82973-38356	14	7	3	0	-inf	0.00655	no
gi|646739592|gb|KK962866.1|	85753-38599	14	7	83	22	-2	0.2337	no
gi|646739592|gb|KK962866.1|	87047-38748	14	7	15	0	-inf	0.0033	no
gi|646739592|gb|KK962866.1|	02574-40306	14	7	0	11	inf	0.0026	no
gi|646739592|gb|KK962866.1|	05331-40562	14	7	102	5	-4	0.08335	no
gi|646739592|gb|KK962866.1|	06872-40908	14	7	32	2	-4	0.0187	no
gi|646739592|gb|KK962866.1|	19071-41959	14	7	0	24	inf	0.00025	yes
gi|646739592|gb|KK962866.1|	20632-42121	14	7	2	2	-0.128212	0.9025	no
gi|646739592|gb|KK962866.1|	51571-52495	14	7	3	4	0.168403	0.9199	no
gi|646739592|gb|KK962866.1|	86393-88714	14	7	0.268687	2	3	0.13855	no
gi|646739598|gb|KK962865.1|	12711-13778	21	10	0.227847	2	3	0.25	no
gi|646739598|gb|KK962865.1|	75779-17668	21	10	3	1	-1	0.45725	no
gi|646739598|gb|KK962865.1|	77271-17921	21	10	36	40	0.177111	0.873	no
gi|646739598|gb|KK962865.1|	95992-19648	21	10	3	3	-0.0613265	0.92345	no
gi|646739598|gb|KK962865.1|	98866-19919	21	10	23	5	-2	0.20825	no
gi|646739598|gb|KK962865.1|	03967-20494	21	10	2	0.547649	-2	0.26895	no
gi|646739598|gb|KK962865.1|	11573-21262	21	10	7	7	0.00475166	0.98915	no
gi|646739598|gb|KK962865.1|	19646-22064	21	10	5	6	0.138035	0.92565	no
gi|646739598|gb|KK962865.1|	40040-24026	21	10	18	45	1	0.3917	no
gi|646739598|gb|KK962865.1|	42429-24306	21	10	2	4	0.607497	0.6368	no
gi|646739598|gb|KK962865.1|	54228-25576	21	10	177	168	-0.0725694	0.9507	no
gi|646739598|gb|KK962865.1|	67240-26749	21	10	30	35	0.244082	0.84555	no
gi|646739598|gb|KK962865.1|	70938-27117	21	10	143	158	0.144367	0.92285	no
gi|646739598|gb|KK962865.1|	28528-29087	21	10	2	3	1	0.44985	no
gi|646739598|gb|KK962865.1|	39670-33986	21	10	429	240	-0.839835	0.5891	no
gi|646739598|gb|KK962865.1|	42221-34253	21	10	20	15	-0.419568	0.73405	no

gi|646739598|gb|KK962865.1|	52706-35366	21	10	2	2	0.507492	0.6858	no
gi|646739598|gb|KK962865.1|	55820-35660	21	10	1	5	2	0.23815	no
gi|646739598|gb|KK962865.1|	57939-35857	21	10	1	2	0.470618	0.7721	no
gi|646739598|gb|KK962865.1|	58774-35931	21	10	4	0.443907	-3	0.1921	no
gi|646739598|gb|KK962865.1|	65645-36613	21	10	7	3	-1	0.30505	no
gi|646739598|gb|KK962865.1|	36751-37913	21	10	0.30598	2	2	0.1611	no
gi|646739598|gb|KK962865.1|	41384-42635	21	10	0.464405	2	2	0.22555	no
gi|646739598|gb|KK962865.1|	52021-53750	21	10	0.690409	3	2	0.20145	no
gi|646739607|gb|KK962864.1|	07258-20790	18	7.3	1	2	0.472834	0.7721	no
gi|646739623|gb|KK962862.1|	7172-8441	7.8	3.8	1	0.523762	-1	0.415	no
gi|646739633|gb|KK962861.1|	01631-10184	17	7.9	80	24	-2	0.2477	no
gi|646739633|gb|KK962861.1|	12029-11371	17	7.9	32	18	-0.819151	0.4581	no
gi|646739633|gb|KK962861.1|	22757-12370	17	7.9	2	2	0.119891	0.92255	no
gi|646739633|gb|KK962861.1|	35905-13651	17	7.9	5	4	-0.272776	0.81935	no
gi|646739633|gb|KK962861.1|	44058-14442	17	7.9	11	18	0.693166	0.6234	no
gi|646739633|gb|KK962861.1|	58746-16006	17	7.9	6	2	-1	0.38795	no
gi|646739633|gb|KK962861.1|	91497-19246	17	7.9	38	46	0.272308	0.8077	no
gi|646739633|gb|KK962861.1|	96743-19727	17	7.9	3	2	-0.234415	0.87465	no
gi|646739633|gb|KK962861.1|	99361-19962	17	7.9	4	24	2	0.27475	no
gi|646739633|gb|KK962861.1|	01706-20377	17	7.9	0.408238	0.665442	0.704903	1	no
gi|646739633|gb|KK962861.1|	11819-21211	17	7.9	11	11	-0.00503479	0.92625	no
gi|646739633|gb|KK962861.1|	26425-22671	17	7.9	11	11	-0.00297634	0.92625	no
gi|646739633|gb|KK962861.1|	27037-22726	17	7.9	56	24	-1	0.39	no
gi|646739633|gb|KK962861.1|	29061-22968	17	7.9	5	13	1	0.30185	no
gi|646739633|gb|KK962861.1|	38574-24033	17	7.9	21	12	-0.784277	0.4637	no
gi|646739633|gb|KK962861.1|	43257-24394	17	7.9	1	3	1	0.3428	no
gi|646739633|gb|KK962861.1|	52562-25287	17	7.9	10	10	0.0675466	0.9332	no
gi|646739633|gb|KK962861.1|	56087-25638	17	7.9	3	13	2	0.30875	no
gi|646739633|gb|KK962861.1|	63469-26373	17	7.9	24	12	-0.976153	0.5349	no
gi|646739633|gb|KK962861.1|	66754-26706	17	7.9	16	18	0.137881	0.9051	no

gi|646739633|gb|KK962861.1|	80312-28570	17	7.9	8	7	-0.184218	0.86595	no
gi|646739633|gb|KK962861.1|	86316-28873	17	7.9	8	7	-0.239796	0.87625	no
gi|646739633|gb|KK962861.1|	91404-29613	17	7.9	12	14	0.257567	0.822	no
gi|646739633|gb|KK962861.1|	05425-30587	17	7.9	3	8	2	0.29945	no
gi|646739633|gb|KK962861.1|	91491-92122	17	7.9	5	3	-0.456427	0.71675	no
gi|646739633|gb|KK962861.1|	94571-95012	17	7.9	5	4	-0.338872	0.8001	no
gi|646739633|gb|KK962861.1|	›8048-10121(	17	7.9	21	16	-0.424667	0.7088	no
gi|646739640|gb|KK962860.1|	14334-14572	21	6.2	100	0	-inf	0.0033	no
gi|646739640|gb|KK962860.1|	16325-16994	21	6.2	16	2	-3	0.074	no
gi|646739640|gb|KK962860.1|	67881-16819	21	6.2	758	3	-8	0.10535	no
gi|646739640|gb|KK962860.1|	01957-21256	21	6.2	11	7	-0.625342	0.58855	no
gi|646739640|gb|KK962860.1|	17181-21798	21	6.2	2	0.943402	-0.963679	0.54505	no
gi|646739640|gb|KK962860.1|	18283-21904	21	6.2	0.735508	2	1	0.36865	no
gi|646739640|gb|KK962860.1|	19192-22293	21	6.2	12	5	-1	0.3635	no
gi|646739640|gb|KK962860.1|	47945-24857	21	6.2	8	2	-2	0.2808	no
gi|646739640|gb|KK962860.1|	27346-27693	21	6.2	14	0	-inf	0.00195	no
gi|646739640|gb|KK962860.1|	58482-45936	21	6.2	1	0.626923	-1	0.4271	no
gi|646739640|gb|KK962860.1|	61390-46358	21	6.2	4	3	-0.368756	0.80385	no
gi|646739640|gb|KK962860.1|	79434-48015	21	6.2	4	5	0.402101	0.77405	no
gi|646739640|gb|KK962860.1|	85002-48547	21	6.2	0.852952	5	2	0.28805	no
gi|646739640|gb|KK962860.1|	85610-48703	21	6.2	1	1	-0.112082	1	no
gi|646739640|gb|KK962860.1|	4966-5544	21	6.2	6	2	-2	0.28025	no
gi|646739640|gb|KK962860.1|	20800-52247	21	6.2	25	8	-2	0.21615	no
gi|646739640|gb|KK962860.1|	51795-55279	21	6.2	21	7	-2	0.2771	no
gi|646739640|gb|KK962860.1|	81882-58274	21	6.2	2	3	0.179202	0.88665	no
gi|646739640|gb|KK962860.1|	68497-69654	21	6.2	133	1	-7	0.0105	no
gi|646739640|gb|KK962860.1|	75138-75761	21	6.2	12	0	-inf	0.00025	yes
gi|646739640|gb|KK962860.1|	86469-86768	21	6.2	17	0	-inf	0.0041	no
gi|646739679|gb|KK962857.1|	12765-11497	15	6.3	9	13	0.543875	0.6667	no
gi|646739679|gb|KK962857.1|	30750-13135	15	6.3	5	6	0.214814	0.87575	no

gi|646739679|gb|KK962857.1|	64856-65214	15	6.3	8	2	-2	0.26015	no
gi|646739679|gb|KK962857.1|	89895-91097	15	6.3	18	3	-2	0.15305	no
gi|646739687|gb|KK962856.1|	72255-17395	14	8.4	0.768819	2	1	0.48285	no
gi|646739687|gb|KK962856.1|	78274-80813	14	8.4	0	3	inf	0.00025	yes
gi|646739687|gb|KK962856.1|	84909-85445	14	8.4	0.32745	12	5	0.09875	no
gi|646739714|gb|KK962853.1|	27263-12816	22	6.7	1	1	-0.174282	0.9042	no
gi|646739714|gb|KK962853.1|	33395-13456	22	6.7	0.907932	0.866767	-0.0669388	1	no
gi|646739714|gb|KK962853.1|	52430-15310	22	6.7	4	6	0.725593	0.61395	no
gi|646739714|gb|KK962853.1|	16425-16721	22	6.7	1	52	5	0.09985	no
gi|646739714|gb|KK962853.1|	67860-16861	22	6.7	0.554936	5	3	0.1491	no
gi|646739739|gb|KK962850.1|	06252-10765	22	7.8	0.722748	1	0.677	0.62555	no
gi|646739739|gb|KK962850.1|	08826-10989	22	7.8	1	2	0.651406	0.62395	no
gi|646739739|gb|KK962850.1|	15062-11593	22	7.8	14	8	-0.850204	0.5763	no
gi|646739739|gb|KK962850.1|	97172-19830	22	7.8	0	40	inf	5.00E-05	yes
gi|646739739|gb|KK962850.1|	98383-19935	22	7.8	0	3	inf	0.00515	no
gi|646739739|gb|KK962850.1|	17962-21897	22	7.8	9	0.87508	-3	0.06415	no
gi|646739739|gb|KK962850.1|	76772-77012	22	7.8	13	35.15	1	0.38225	no
gi|646739739|gb|KK962850.1|	93197-94422	22	7.8	0.858332	0.546984	-0.650037	1	no
gi|646739746|gb|KK962849.1|	16814-11756	20	5.8	17	3	-3	0.0754	no
gi|646739746|gb|KK962849.1|	32912-13334	20	5.8	2	13	3	0.1776	no
gi|646739746|gb|KK962849.1|	40419-14122	20	5.8	2	3	0.272955	0.8194	no
gi|646739746|gb|KK962849.1|	83090-83572	20	5.8	0	14	inf	0.0017	no
gi|646739758|gb|KK962847.1|	59719-25994	17	8.2	61	127.25	1	0.4551	no
gi|646739758|gb|KK962847.1|	76986-27891	17	8.2	20	28	0.47998	0.66575	no
gi|646739758|gb|KK962847.1|	83905-28416	17	8.2	34	32	-0.0858644	0.94545	no
gi|646739758|gb|KK962847.1|	03631-30464	17	8.2	15	17	0.224115	0.8803	no
gi|646739758|gb|KK962847.1|	08208-30840	17	8.2	254	36	-3	0.1235	no
gi|646739783|gb|KK962846.1|	26361-32697	16	9.1	11	3	-2	0.1969	no
gi|646739790|gb|KK962845.1|	18988-11946	20	7	9	7	-0.33976	0.8061	no
gi|646739790|gb|KK962845.1|	42949-14338	20	7	6	8	0.279987	0.8231	no

gi|646739797|gb|KK962844.1|	52647-52988	13	7.9	7	6	-0.184014	0.9036	no
gi|646739806|gb|KK962843.1|	27773-12864	17	12	1	0.845006	-0.498219	0.73865	no
gi|646739806|gb|KK962843.1|	28716-13036	17	12	1	0.479053	-1	0.3526	no
gi|646739806|gb|KK962843.1|	32478-13345	17	12	1	0.54618	-0.906135	1	no
gi|646739806|gb|KK962843.1|	33508-13395	17	12	5	3	-0.599983	0.6657	no
gi|646739806|gb|KK962843.1|	44789-24558	17	12	1	2	0.716699	0.6352	no
gi|646739806|gb|KK962843.1|	68062-26938	17	12	7	4	-0.682344	0.6555	no
gi|646739806|gb|KK962843.1|	69813-27247	17	12	83	25	-2	0.14475	no
gi|646739806|gb|KK962843.1|	33497-34164	17	12	1	2	0.476598	0.7721	no
gi|646739813|gb|KK962842.1|	13901-11487	19	9.6	2	3	0.338859	0.8072	no
gi|646739813|gb|KK962842.1|	15699-11834	19	9.6	0.503242	2	2	0.21275	no
gi|646739813|gb|KK962842.1|	24121-12617	19	9.6	0.87377	1	0.254744	1	no
gi|646739813|gb|KK962842.1|	26559-12696	19	9.6	4	3	-0.437663	0.76715	no
gi|646739813|gb|KK962842.1|	28424-32910	19	9.6	3	3	-0.198686	0.8743	no
gi|646739813|gb|KK962842.1|	48387-44885	19	9.6	5	3	-0.84954	0.54905	no
gi|646739835|gb|KK962839.1|	16137-18733	10	6.6	9	0.746455	-4	0.0091	no
gi|646739835|gb|KK962839.1|	20569-21318	10	6.6	3	1	-1	0.3737	no
gi|646739835|gb|KK962839.1|	40333-40961	10	6.6	6719.26	2483.96	-1	0.23295	no
gi|646739835|gb|KK962839.1|	44044-46038	10	6.6	775	442	-0.811449	0.502	no
gi|646739835|gb|KK962839.1|	48424-49731	10	6.6	30	8	-2	0.1478	no
gi|646739835|gb|KK962839.1|	54684-55881	10	6.6	5	3	-0.761179	0.63345	no
gi|646739835|gb|KK962839.1|	57642-58716	10	6.6	1	0.645149	-0.655976	1	no
gi|646739835|gb|KK962839.1|	58865-61038	10	6.6	80	137	0.769055	0.5124	no
gi|646739835|gb|KK962839.1|	7562-8027	10	6.6	71	3	-4	0.02475	no
gi|646739835|gb|KK962839.1|	8880-11576	10	6.6	179	52	-2	0.1506	no
gi|646739841|gb|KK962838.1|	21787-12647	21	5.7	3	0.456878	-3	0.1363	no
gi|646739841|gb|KK962838.1|	27119-12833	21	5.7	0.865469	1	0.51962	0.7038	no
gi|646739841|gb|KK962838.1|	28588-12999	21	5.7	1	0.348144	-2	0.27245	no
gi|646739841|gb|KK962838.1|	30144-13154	21	5.7	1	0.695693	-0.590575	1	no
gi|646739841|gb|KK962838.1|	31661-13326	21	5.7	1	0.595899	-0.886138	1	no

gi|646739841|gb|KK962838.1|	33697-13619	21	5.7	2	1	-0.354789	0.82165	no
gi|646739841|gb|KK962838.1|	36263-13733	21	5.7	2	1	-0.808119	0.51635	no
gi|646739841|gb|KK962838.1|	49674-15010	21	5.7	577	208	-1	0.20615	no
gi|646739841|gb|KK962838.1|	52717-15296	21	5.7	123	87	-0.495741	0.7511	no
gi|646739841|gb|KK962838.1|	71491-17235	21	5.7	0.607548	1	1	0.48525	no
gi|646739841|gb|KK962838.1|	76518-17672	21	5.7	229	211	-0.118269	0.939	no
gi|646739841|gb|KK962838.1|	84284-18502	21	5.7	3	3	-0.0143415	0.9587	no
gi|646739841|gb|KK962838.1|	89474-19124	21	5.7	4	3	-0.303324	0.84015	no
gi|646739841|gb|KK962838.1|	93299-19377	21	5.7	5	7	0.532671	0.67195	no
gi|646739841|gb|KK962838.1|	93897-19510	21	5.7	2	4	1	0.36595	no
gi|646739841|gb|KK962838.1|	01752-20519	21	5.7	2	3	0.843705	0.5847	no
gi|646739841|gb|KK962838.1|	05290-20850	21	5.7	2	2	0.144437	0.92025	no
gi|646739841|gb|KK962838.1|	08717-21333	21	5.7	1	1	0.299062	0.85035	no
gi|646739841|gb|KK962838.1|	14658-21885	21	5.7	397	237	-0.742049	0.5327	no
gi|646739841|gb|KK962838.1|	20196-22221	21	5.7	2	1	-0.465477	0.7704	no
gi|646739841|gb|KK962838.1|	32932-23360	21	5.7	8	8	0.062275	0.96905	no
gi|646739841|gb|KK962838.1|	35999-23730	21	5.7	0.791439	0.757855	-0.0625559	1	no
gi|646739841|gb|KK962838.1|	55349-25667	21	5.7	0.953718	0	-inf	1	no
gi|646739841|gb|KK962838.1|	71893-27233	21	5.7	129	100	-0.366695	0.735	no
gi|646739841|gb|KK962838.1|	82970-28692	21	5.7	5	0.801394	-3	0.06375	no
gi|646739841|gb|KK962838.1|	88199-28854	21	5.7	21	1	-4	0.18555	no
gi|646739841|gb|KK962838.1|	98201-29882	21	5.7	2	7	2	0.27665	no
gi|646739841|gb|KK962838.1|	65828-36610	21	5.7	151	0	-inf	0.00035	yes
gi|646739841|gb|KK962838.1|	69363-36977	21	5.7	77	41	-0.911134	0.5548	no
gi|646739841|gb|KK962838.1|	90444-39132	21	5.7	2	0.626923	-1	0.3882	no
gi|646739841|gb|KK962838.1|	98190-39841	21	5.7	34	15	-1	0.47085	no
gi|646739841|gb|KK962838.1|	99283-40000	21	5.7	3	1	-0.893803	0.53405	no
gi|646739841|gb|KK962838.1|	10897-41158	21	5.7	10	9	-0.150056	0.9211	no
gi|646739841|gb|KK962838.1|	14006-41593	21	5.7	76	29	-1	0.23455	no
gi|646739841|gb|KK962838.1|	42449-43212	21	5.7	7	7	-0.059401	0.9689	no

gi|646739841|gb|KK962838.1|	26891-52773	21	5.7	3	2	-0.501289	0.72395	no
gi|646739841|gb|KK962838.1|	28711-52926	21	5.7	0.936578	3	2	0.29625	no
gi|646739841|gb|KK962838.1|	93302-59377	21	5.7	7	7	0.0301704	0.96195	no
gi|646739841|gb|KK962838.1|	05611-60586	21	5.7	115	251	1	0.46955	no
gi|646739841|gb|KK962838.1|	08562-60878	21	5.7	13	68	2	0.2124	no
gi|646739841|gb|KK962838.1|	10575-61115	21	5.7	9	18	0.90421	0.56955	no
gi|646739841|gb|KK962838.1|	14698-61540	21	5.7	2	5	1	0.488	no
gi|646739841|gb|KK962838.1|	24072-62771	21	5.7	0.792647	0.836359	0.0774435	1	no
gi|646739841|gb|KK962838.1|	27967-62947	21	5.7	1	0.851223	-0.472802	0.72015	no
gi|646739841|gb|KK962838.1|	29583-63114	21	5.7	5	9	1	0.49435	no
gi|646739841|gb|KK962838.1|	31294-63544	21	5.7	9	7	-0.448282	0.68355	no
gi|646739841|gb|KK962838.1|	40796-64165	21	5.7	2	2	-0.23676	0.86015	no
gi|646739841|gb|KK962838.1|	81645-82479	21	5.7	0.958527	1	0.235221	0.87595	no
gi|646739864|gb|KK962836.1|	55321-15579	17	6.2	8	7	-0.12876	0.9149	no
gi|646739864|gb|KK962836.1|	35152-23566	17	6.2	3	1	-0.789026	0.6307	no
gi|646739864|gb|KK962836.1|	52797-25385	17	6.2	0	102	inf	5.00E-05	yes
gi|646739864|gb|KK962836.1|	45880-46370	17	6.2	5	4	-0.574963	0.6718	no
gi|646739864|gb|KK962836.1|	81235-81577	17	6.2	15	7	-1	0.41495	no
gi|646739875|gb|KK962835.1|	08363-11422	20	9.5	5	4	-0.391766	0.71355	no
gi|646739875|gb|KK962835.1|	25043-13273	20	9.5	47	33	-0.494029	0.6837	no
gi|646739875|gb|KK962835.1|	34803-13658	20	9.5	0.907605	2	1	0.4223	no
gi|646739875|gb|KK962835.1|	38170-14028	20	9.5	1	2	0.350958	0.82465	no
gi|646739875|gb|KK962835.1|	41476-14197	20	9.5	42	49	0.224944	0.8771	no
gi|646739875|gb|KK962835.1|	42119-14238	20	9.5	16	28	0.797143	0.566	no
gi|646739875|gb|KK962835.1|	44943-14569	20	9.5	2	2	-0.247398	0.85975	no
gi|646739875|gb|KK962835.1|	88119-19059	20	9.5	2	0.48436	-2	0.2035	no
gi|646739875|gb|KK962835.1|	90730-19184	20	9.5	3	0.772467	-2	0.24075	no
gi|646739875|gb|KK962835.1|	29977-23021	20	9.5	28	18	-0.610065	0.6687	no
gi|646739875|gb|KK962835.1|	33155-23585	20	9.5	2	0.605484	-1	0.3236	no
gi|646739875|gb|KK962835.1|	36146-23826	20	9.5	4	0.648482	-3	0.08375	no

gi|646739875|gb|KK962835.1|	39041-24082	20	9.5	1	0.348993	-2	1	no
gi|646739875|gb|KK962835.1|	49498-25073	20	9.5	0.094205	2	4	0.09645	no
gi|646739875|gb|KK962835.1|	78475-27891	20	9.5	9	13	0.621132	0.6563	no
gi|646739875|gb|KK962835.1|	90169-29100	20	9.5	0.158116	2	4	0.10345	no
gi|646739875|gb|KK962835.1|	91110-29212	20	9.5	0.12236	2	4	0.1061	no
gi|646739875|gb|KK962835.1|	98858-30119	20	9.5	26	37	0.492728	0.6621	no
gi|646739875|gb|KK962835.1|	12925-31692	20	9.5	3	9	1	0.2676	no
gi|646739875|gb|KK962835.1|	49670-35171	20	9.5	0.413945	2	2	0.13775	no
gi|646739875|gb|KK962835.1|	62899-36495	20	9.5	0.359788	3	3	0.0963	no
gi|646739875|gb|KK962835.1|	74654-37908	20	9.5	0.265868	2	3	0.05635	no
gi|646739875|gb|KK962835.1|	80006-38092	20	9.5	4	19	2	0.1716	no
gi|646739875|gb|KK962835.1|	48960-51236	20	9.5	8	10	0.224562	0.85685	no
gi|646739875|gb|KK962835.1|	51365-52579	20	9.5	3	3	-0.00655083	0.9838	no
gi|646739875|gb|KK962835.1|	56222-57733	20	9.5	2	4	1	0.5177	no
gi|646739875|gb|KK962835.1|	70576-78150	20	9.5	4	6	0.635913	0.56915	no
gi|646739875|gb|KK962835.1|	81201-83160	20	9.5	3	6	0.914134	0.543	no
gi|646739875|gb|KK962835.1|	88328-89114	20	9.5	0.34838	3	3	0.2502	no
gi|646739875|gb|KK962835.1|	99049-99464	20	9.5	38	17	-1	0.4427	no
gi|646739883|gb|KK962834.1|	28536-12963	17	8.3	0.768145	2	1	0.3574	no
gi|646739883|gb|KK962834.1|	31275-13235	17	8.3	1	1	0.151564	0.9186	no
gi|646739883|gb|KK962834.1|	47496-14785	17	8.3	14	3	-2	0.1793	no
gi|646739883|gb|KK962834.1|	48162-14920	17	8.3	1	0.499904	-1	0.38955	no
gi|646739883|gb|KK962834.1|	51945-15255	17	8.3	73	4	-4	0.0054	no
gi|646739883|gb|KK962834.1|	53587-15390	17	8.3	22	10	-1	0.398	no
gi|646739883|gb|KK962834.1|	56556-15866	17	8.3	0.648717	0.723224	0.156852	1	no
gi|646739883|gb|KK962834.1|	64599-16600	17	8.3	1	0.462609	-2	0.2508	no
gi|646739883|gb|KK962834.1|	67559-16814	17	8.3	2	7	2	0.32675	no
gi|646739883|gb|KK962834.1|	74305-17563	17	8.3	22	14	-0.706823	0.57525	no
gi|646739883|gb|KK962834.1|	11878-21218	17	8.3	5	9	0.836577	0.60915	no
gi|646739883|gb|KK962834.1|	20235-22092	17	8.3	14	13	-0.0822263	0.95865	no

gi|646739883|gb|KK962834.1|	23011-22477	17	8.3	11	11	-0.0535109	0.97075	no
gi|646739883|gb|KK962834.1|	24900-22596	17	8.3	9	9	-0.0539693	0.97125	no
gi|646739883|gb|KK962834.1|	35166-23618	17	8.3	18	16	-0.166702	0.91335	no
gi|646739883|gb|KK962834.1|	36890-23827	17	8.3	9	11	0.264175	0.85685	no
gi|646739883|gb|KK962834.1|	45587-24653	17	8.3	0.804368	0.761974	-0.0781135	1	no
gi|646739883|gb|KK962834.1|	48705-24925	17	8.3	14	14	-0.0434843	0.9773	no
gi|646739883|gb|KK962834.1|	57209-25985	17	8.3	3	2	-0.814461	0.58875	no
gi|646739883|gb|KK962834.1|	71382-27162	17	8.3	43	21.09	-1	0.4356	no
gi|646739883|gb|KK962834.1|	72691-27440	17	8.3	4	1	-2	0.156	no
gi|646739883|gb|KK962834.1|	88976-28975	17	8.3	7	4	-0.992991	0.54705	no
gi|646739883|gb|KK962834.1|	48647-49747	17	8.3	3	4	0.635415	0.68845	no
gi|646739883|gb|KK962834.1|	49933-50798	17	8.3	2	1	-0.636539	0.65905	no
gi|646739883|gb|KK962834.1|	56016-56458	17	8.3	2	4	0.661774	0.66225	no
gi|646739883|gb|KK962834.1|	92423-92789	17	8.3	18	12	-0.667103	0.63045	no
gi|646739900|gb|KK962832.1|	19778-21043	25	18	3	2	-0.286188	0.8596	no
gi|646739907|gb|KK962831.1|	63801-16431	17	7.5	4	5	0.436196	0.74185	no
gi|646739907|gb|KK962831.1|	95076-29537	17	7.5	220	28	-3	0.08825	no
gi|646739907|gb|KK962831.1|	97665-29816	17	7.5	41	2	-4	0.03985	no
gi|646739907|gb|KK962831.1|	00206-30065	17	7.5	9	0	-inf	0.0008	yes
gi|646739907|gb|KK962831.1|	03546-30376	17	7.5	44	10	-2	0.21985	no
gi|646739925|gb|KK962829.1|	72964-37337	14	5.6	3	10	1	0.3034	no
gi|646739925|gb|KK962829.1|	03112-40339	14	5.6	12	12	-0.0178078	0.92625	no
gi|646739932|gb|KK962828.1|	70995-17281	15	8.9	292	119	-1	0.29025	no
gi|646739932|gb|KK962828.1|	51155-25274	15	8.9	6	7	0.229092	0.8783	no
gi|646739932|gb|KK962828.1|	61365-26179	15	8.9	69	30	-1	0.43105	no
gi|646739932|gb|KK962828.1|	74504-27511	15	8.9	10	3	-2	0.20635	no
gi|646739932|gb|KK962828.1|	77018-27843	15	8.9	0.954551	1	0.262221	0.8481	no
gi|646739932|gb|KK962828.1|	79248-27988	15	8.9	5	0.673682	-3	0.12235	no
gi|646739932|gb|KK962828.1|	80400-28157	15	8.9	0.7054	0.721538	0.032634	1	no
gi|646739932|gb|KK962828.1|	93211-29388	15	8.9	2	2	-0.174411	0.89575	no

gi|646739944|gb|KK962827.1|	06123-10637	12	7.4	0	38	inf	0.0008	yes
gi|646739944|gb|KK962827.1|	77002-17749	12	7.4	0	10	inf	0.00315	no
gi|646739944|gb|KK962827.1|	85102-18596	12	7.4	0.305277	3	3	0.2688	no
gi|646739944|gb|KK962827.1|	18932-19436	12	7.4	0	20	inf	0.00075	yes
gi|646739944|gb|KK962827.1|	70756-27114	12	7.4	19	14	-0.414487	0.8033	no
gi|646739944|gb|KK962827.1|	12236-31318	12	7.4	1	0.189695	-3	0.21635	no
gi|646739944|gb|KK962827.1|	75318-75581	12	7.4	0	25	inf	0.00255	no
gi|646739944|gb|KK962827.1|	87276-88515	12	7.4	0	4	inf	0.00095	yes
gi|646739955|gb|KK962826.1|	1857-2514	13	7.5	8	2	-2	0.2479	no
gi|646739972|gb|KK962824.1|	10037-11056	16	11	0	56	inf	5.00E-05	yes
gi|646739972|gb|KK962824.1|	77253-17762	16	11	53	10	-2	0.11005	no
gi|646739972|gb|KK962824.1|	)6145-10260:	16	11	35	8	-2	0.0694	no
gi|646740004|gb|KK962821.1|	00921-10133	22	9.6	4	6	0.572188	0.7088	no
gi|646740004|gb|KK962821.1|	02608-10397	22	9.6	1	1	0.523914	0.67915	no
gi|646740004|gb|KK962821.1|	18127-11897	22	9.6	5	4	-0.253002	0.87325	no
gi|646740004|gb|KK962821.1|	32805-13447	22	9.6	11	8	-0.461428	0.76405	no
gi|646740004|gb|KK962821.1|	38469-13923	22	9.6	5	5	-0.146755	0.92755	no
gi|646740004|gb|KK962821.1|	54243-15556	22	9.6	7	8	0.340905	0.82005	no
gi|646740004|gb|KK962821.1|	80580-18120	22	9.6	2	1	-0.947633	0.589	no
gi|646740004|gb|KK962821.1|	84023-18497	22	9.6	3	4	0.571109	0.7294	no
gi|646740004|gb|KK962821.1|	96084-19927	22	9.6	5	14	1	0.2849	no
gi|646740004|gb|KK962821.1|	01298-20243	22	9.6	2	5	0.947104	0.5539	no
gi|646740004|gb|KK962821.1|	08947-21100	22	9.6	1	8	3	0.1353	no
gi|646740004|gb|KK962821.1|	21391-22276	22	9.6	0.247798	1	2	0.186	no
gi|646740004|gb|KK962821.1|	24218-22744	22	9.6	2	6	2	0.16355	no
gi|646740004|gb|KK962821.1|	29477-23070	22	9.6	2	9	2	0.0994	no
gi|646740004|gb|KK962821.1|	30875-23219	22	9.6	0	1	inf	0.0014	yes
gi|646740004|gb|KK962821.1|	32546-23602	22	9.6	0.057431	5	6	0.23275	no
gi|646740004|gb|KK962821.1|	51557-25190	22	9.6	39	31	-0.365862	0.8112	no
gi|646740004|gb|KK962821.1|	55797-25671	22	9.6	0.421738	2	2	0.255	no

gi|646740004|gb|KK962821.1|	82662-28360	22	9.6	56	34	-0.719098	0.51735	no
gi|646740004|gb|KK962821.1|	29024-30908	22	9.6	6	4	-0.566528	0.713	no
gi|646740004|gb|KK962821.1|	91553-29270	22	9.6	56	55	-0.0324617	0.97745	no
gi|646740004|gb|KK962821.1|	45427-34633	22	9.6	6	2	-1	0.35535	no
gi|646740004|gb|KK962821.1|	3677-5492	22	9.6	47	36	-0.407656	0.7223	no
gi|646740004|gb|KK962821.1|	71094-37132	22	9.6	26	19	-0.424781	0.7683	no
gi|646740004|gb|KK962821.1|	75397-37616	22	9.6	4	4	-0.0326457	0.9735	no
gi|646740004|gb|KK962821.1|	76314-37674	22	9.6	7	0.660087	-3	0.1849	no
gi|646740004|gb|KK962821.1|	77959-37847	22	9.6	4	0.965839	-2	0.20435	no
gi|646740004|gb|KK962821.1|	78648-37896	22	9.6	10	8	-0.313303	0.83155	no
gi|646740004|gb|KK962821.1|	79084-37973	22	9.6	3	4	0.125302	0.9232	no
gi|646740004|gb|KK962821.1|	79843-38812	22	9.6	19	10	-0.96551	0.4139	no
gi|646740004|gb|KK962821.1|	40004-41917	22	9.6	1	1	-0.244261	0.85885	no
gi|646740004|gb|KK962821.1|	11424-41510	22	9.6	75	0.826895	-6	0.0057	no
gi|646740004|gb|KK962821.1|	18839-42367	22	9.6	19	14	-0.434474	0.70815	no
gi|646740004|gb|KK962821.1|	42032-45048	22	9.6	1	0.975105	-0.529406	0.73785	no
gi|646740004|gb|KK962821.1|	25871-42822	22	9.6	1	1	0.274331	0.86675	no
gi|646740004|gb|KK962821.1|	28346-43074	22	9.6	0.948463	0.563249	-0.751818	1	no
gi|646740004|gb|KK962821.1|	30869-43257	22	9.6	1	0.919792	-0.234875	1	no
gi|646740004|gb|KK962821.1|	42042-44341	22	9.6	1	1	-0.39342	0.75525	no
gi|646740004|gb|KK962821.1|	52326-45572	22	9.6	0.618375	0.77193	0.319988	1	no
gi|646740004|gb|KK962821.1|	46344-48617	22	9.6	10	5	-1	0.3916	no
gi|646740004|gb|KK962821.1|	81128-48141	22	9.6	5	15	2	0.2907	no
gi|646740004|gb|KK962821.1|	84721-48518	22	9.6	1	7	2	0.18105	no
gi|646740004|gb|KK962821.1|	85434-48901	22	9.6	1	2	0.877371	0.55645	no
gi|646740004|gb|KK962821.1|	00646-50118	22	9.6	10	19	0.881778	0.56505	no
gi|646740004|gb|KK962821.1|	52164-53411	22	9.6	12	7	-0.705464	0.64095	no
gi|646740004|gb|KK962821.1|	54391-57803	22	9.6	3	4	0.259256	0.86675	no
gi|646740004|gb|KK962821.1|	45637-54586	22	9.6	24	37	0.630395	0.6569	no
gi|646740004|gb|KK962821.1|	59746-56047	22	9.6	17	33	0.91729	0.5436	no

gi|646740004|gb|KK962821.1|	65688-56669	22	9.6	14	25	0.769534	0.5407	no
gi|646740004|gb|KK962821.1|	58043-63349	22	9.6	3	3	-0.0344887	0.98065	no
gi|646740004|gb|KK962821.1|	94143-59484	22	9.6	19	9	-1	0.50565	no
gi|646740004|gb|KK962821.1|	99269-60095	22	9.6	1	1	0.0897838	0.943	no
gi|646740004|gb|KK962821.1|	01094-60420	22	9.6	1	0.708422	-1	0.52485	no
gi|646740004|gb|KK962821.1|	04359-60583	22	9.6	19	3	-3	0.0618	no
gi|646740004|gb|KK962821.1|	14201-61464	22	9.6	14	2	-3	0.1399	no
gi|646740004|gb|KK962821.1|	20505-62085	22	9.6	21	7	-2	0.2868	no
gi|646740004|gb|KK962821.1|	21176-62246	22	9.6	3	5	0.873301	0.5803	no
gi|646740004|gb|KK962821.1|	30722-63094	22	9.6	24	29	0.267321	0.8464	no
gi|646740004|gb|KK962821.1|	38589-63889	22	9.6	45	27	-0.755519	0.63895	no
gi|646740004|gb|KK962821.1|	43886-64483	22	9.6	1	1	-0.145033	0.9177	no
gi|646740004|gb|KK962821.1|	44940-64683	22	9.6	2	1	-0.517489	0.70655	no
gi|646740004|gb|KK962821.1|	49256-65433	22	9.6	1	0.56014	-1	0.36665	no
gi|646740004|gb|KK962821.1|	56746-65800	22	9.6	1	0.264949	-2	1	no
gi|646740004|gb|KK962821.1|	60356-66234	22	9.6	8	3	-2	0.323	no
gi|646740004|gb|KK962821.1|	62937-66365	22	9.6	2	0.563661	-2	0.2551	no
gi|646740004|gb|KK962821.1|	64313-66597	22	9.6	1	0.664574	-0.662695	1	no
gi|646740004|gb|KK962821.1|	70738-67209	22	9.6	21	11	-0.953576	0.45655	no
gi|646740004|gb|KK962821.1|	72432-67416	22	9.6	6	8	0.361131	0.81605	no
gi|646740004|gb|KK962821.1|	80627-68122	22	9.6	3	2	-0.861188	0.56515	no
gi|646740004|gb|KK962821.1|	74313-76217	22	9.6	0.504939	2	2	0.19095	no
gi|646740004|gb|KK962821.1|	7551-8861	22	9.6	0.701501	2	1	0.3728	no
gi|646740004|gb|KK962821.1|	76380-78191	22	9.6	0.356743	1	2	0.26735	no
gi|646740004|gb|KK962821.1|	79047-82800	22	9.6	4	5	0.427934	0.78035	no
gi|646740004|gb|KK962821.1|	9067-11253	22	9.6	0.718675	0.90313	0.329594	1	no
gi|646740004|gb|KK962821.1|	91105-91915	22	9.6	0.833303	1	0.758988	0.6148	no
gi|646740015|gb|KK962820.1|	00076-10185	20	8.4	0.791769	0.968448	0.290595	1	no
gi|646740015|gb|KK962820.1|	37128-13811	20	8.4	1	1	-0.365271	0.78495	no
gi|646740015|gb|KK962820.1|	44711-14520	20	8.4	5	6	0.0766466	0.9494	no

gi|646740015|gb|KK962820.1|	49521-15057	20	8.4	2	6	2	0.2433	no
gi|646740015|gb|KK962820.1|	50751-15145	20	8.4	2	5	1	0.3725	no
gi|646740015|gb|KK962820.1|	63382-16380	20	8.4	4	11	2	0.35555	no
gi|646740015|gb|KK962820.1|	65316-16586	20	8.4	3	2	-1	0.46265	no
gi|646740015|gb|KK962820.1|	75961-17668	20	8.4	4	8	0.900749	0.51715	no
gi|646740015|gb|KK962820.1|	94721-19585	20	8.4	2	3	0.69696	0.63145	no
gi|646740015|gb|KK962820.1|	21517-21781	20	8.4	7	20	2	0.3584	no
gi|646740015|gb|KK962820.1|	36240-23678	20	8.4	6	6	-0.0217667	0.9633	no
gi|646740015|gb|KK962820.1|	47920-25093	20	8.4	1	1	-0.15089	0.9231	no
gi|646740015|gb|KK962820.1|	62407-26437	20	8.4	31	10	-2	0.2371	no
gi|646740015|gb|KK962820.1|	74361-27468	20	8.4	458	138	-2	0.1958	no
gi|646740015|gb|KK962820.1|	99757-30040	20	8.4	3	3	0.334538	0.79815	no
gi|646740015|gb|KK962820.1|	08234-30941	20	8.4	28	18	-0.688732	0.5826	no
gi|646740015|gb|KK962820.1|	16832-32352	20	8.4	12	11	-0.0497588	0.9638	no
gi|646740015|gb|KK962820.1|	44814-47449	20	8.4	0.971839	1	0.217214	0.8939	no
gi|646740015|gb|KK962820.1|	48722-49978	20	8.4	12	9	-0.423402	0.7837	no
gi|646740015|gb|KK962820.1|	51638-54460	20	8.4	23	20	-0.231454	0.8366	no
gi|646740015|gb|KK962820.1|	86225-86554	20	8.4	8	4	-1	0.4767	no
gi|646740025|gb|KK962819.1|	33439-13408	16	8.1	1	2	0.694433	0.63935	no
gi|646740025|gb|KK962819.1|	76179-18046	16	8.1	4	4	-0.159924	0.9186	no
gi|646740025|gb|KK962819.1|	90809-19299	16	8.1	2	3	0.840857	0.58055	no
gi|646740035|gb|KK962818.1|	04100-20449	11	6	7	0.825753	-3	0.19445	no
gi|646740046|gb|KK962817.1|	72610-17293	10	6.1	8	12	0.632629	0.653	no
gi|646740054|gb|KK962816.1|	33886-34737	15	6.1	5	0.658351	-3	0.1377	no
gi|646740054|gb|KK962816.1|	9342-10439	15	6.1	0.219731	4	4	0.24765	no
gi|646740073|gb|KK962814.1|	03604-10438	18	8.3	1	5	2	0.17575	no
gi|646740073|gb|KK962814.1|	10676-11241	18	8.3	3	4	0.590524	0.63685	no
gi|646740073|gb|KK962814.1|	13440-11588	18	8.3	2	8	2	0.2082	no
gi|646740073|gb|KK962814.1|	11432-11798	18	8.3	25	0	-inf	0.00225	no
gi|646740073|gb|KK962814.1|	15808-16569	18	8.3	18	26	0.490554	0.74935	no

gi|646740073|gb|KK962814.1|	19316-20166	18	8.3	5	9	0.821616	0.5967	no
gi|646740073|gb|KK962814.1|	31806-34185	18	8.3	122	78	-0.647263	0.59025	no
gi|646740073|gb|KK962814.1|	40975-42718	18	8.3	0.248696	20	6	0.09345	no
gi|646740073|gb|KK962814.1|	4328-5242	18	8.3	2	0.597481	-1	0.3882	no
gi|646740073|gb|KK962814.1|	58616-59288	18	8.3	3	2	-0.329866	0.79045	no
gi|646740073|gb|KK962814.1|	59433-60295	18	8.3	10.21	13	0.341515	0.8196	no
gi|646740073|gb|KK962814.1|	60563-61421	18	8.3	5	6	0.156422	0.91965	no
gi|646740073|gb|KK962814.1|	61568-62315	18	8.3	84	103	0.294772	0.7946	no
gi|646740073|gb|KK962814.1|	70452-70835	18	8.3	15	21	0.476642	0.7674	no
gi|646740073|gb|KK962814.1|	77545-82743	18	8.3	77	46	-0.740076	0.5447	no
gi|646740073|gb|KK962814.1|	89953-92930	18	8.3	31	36	0.230797	0.84305	no
gi|646740073|gb|KK962814.1|	95300-96539	18	8.3	3	2	-0.427185	0.75625	no
gi|646740073|gb|KK962814.1|	9663-9840	18	8.3	172	303	0.818546	0.5802	no
gi|646740073|gb|KK962814.1|	98928-99906	18	8.3	3	2	-0.491097	0.72075	no
gi|646740085|gb|KK962813.1|	65387-16953	20	5.5	6	6	-0.0972052	0.92565	no
gi|646740085|gb|KK962813.1|	71618-17268	20	5.5	3	2	-0.671041	0.6334	no
gi|646740085|gb|KK962813.1|	73351-17383	20	5.5	18	14	-0.371182	0.8123	no
gi|646740085|gb|KK962813.1|	52003-25393	20	5.5	2	2	-0.0741536	0.96135	no
gi|646740085|gb|KK962813.1|	58580-26390	20	5.5	23	27	0.22318	0.85325	no
gi|646740085|gb|KK962813.1|	64089-26669	20	5.5	0.473041	1	1	1	no
gi|646740085|gb|KK962813.1|	71266-27831	20	5.5	3	4	0.477446	0.662	no
gi|646740085|gb|KK962813.1|	80302-28192	20	5.5	0.878511	0.585192	-0.58615	1	no
gi|646740085|gb|KK962813.1|	85912-28637	20	5.5	3	3	-0.0735719	0.92345	no
gi|646740085|gb|KK962813.1|	88923-28919	20	5.5	4	17	2	0.3115	no
gi|646740085|gb|KK962813.1|	97458-29765	20	5.5	34	63	0.894424	0.57995	no
gi|646740085|gb|KK962813.1|	05793-30761	20	5.5	0.412752	2	2	0.2165	no
gi|646740085|gb|KK962813.1|	52740-45344	20	5.5	60	13	-2	0.1151	no
gi|646740085|gb|KK962813.1|	64134-46449	20	5.5	14	5	-1	0.3055	no
gi|646740085|gb|KK962813.1|	71373-47162	20	5.5	22	12	-0.884567	0.5727	no
gi|646740085|gb|KK962813.1|	74298-47478	20	5.5	27	10	-1	0.38805	no

gi|646740085|gb|KK962813.1| 75984-48015	20	5.5	13	14	0.109354	0.92415	no
gi|646740085|gb|KK962813.1| 80931-48112	20	5.5	107	106	-0.0165891	0.94585	no
gi|646740085|gb|KK962813.1| 81347-48226	20	5.5	6	2	-2	0.21815	no
gi|646740085|gb|KK962813.1| 83498-49010	20	5.5	9	12	0.331847	0.77115	no
gi|646740098|gb|KK962812.1| 40996-14135	20	10	8	1	-3	0.1972	no
gi|646740098|gb|KK962812.1| 44331-14555	20	10	5	6	0.21056	0.88955	no
gi|646740098|gb|KK962812.1| 50199-15159	20	10	195	170	-0.199041	0.86625	no
gi|646740098|gb|KK962812.1| 53430-15556	20	10	0.442431	1	2	0.2828	no
gi|646740098|gb|KK962812.1| 56838-15883	20	10	2	0.691045	-2	0.29035	no
gi|646740098|gb|KK962812.1| 58995-16246	20	10	3	0.293335	-4	0.03705	no
gi|646740098|gb|KK962812.1| 62580-16870	20	10	56	0.805521	-6	0.00185	no
gi|646740098|gb|KK962812.1| 68806-18367	20	10	37	3	-4	0.0059	no
gi|646740098|gb|KK962812.1| 86162-18882	20	10	15	16	0.0807876	0.94115	no
gi|646740098|gb|KK962812.1| 88960-19196	20	10	6	6	0.00688857	0.99555	no
gi|646740098|gb|KK962812.1| 93815-19483	20	10	0.120292	2	4	0.0966	no
gi|646740098|gb|KK962812.1| 96073-19751	20	10	4	12	2	0.3285	no
gi|646740098|gb|KK962812.1| 98142-19903	20	10	1	0.818256	-0.303939	1	no
gi|646740098|gb|KK962812.1| 99198-20033	20	10	0.418586	1	2	0.36595	no
gi|646740098|gb|KK962812.1| 17499-21839	20	10	3	6	0.825569	0.60365	no
gi|646740098|gb|KK962812.1| 22090-22646	20	10	3	7	0.990845	0.53105	no
gi|646740098|gb|KK962812.1| 68707-27427	20	10	104	13	-3	0.0183	no
gi|646740098|gb|KK962812.1| 76735-27817	20	10	2	2	0.274151	0.86445	no
gi|646740098|gb|KK962812.1| 80286-28096	20	10	39	17	-1	0.4286	no
gi|646740098|gb|KK962812.1| 95326-29600	20	10	34	16	-1	0.47025	no
gi|646740098|gb|KK962812.1| 12604-31581	20	10	75	51	-0.551741	0.6451	no
gi|646740098|gb|KK962812.1| 37260-34412	20	10	201	104	-0.955084	0.43115	no
gi|646740098|gb|KK962812.1| 55929-35617	20	10	40	23	-0.800457	0.5591	no
gi|646740098|gb|KK962812.1| 84749-38832	20	10	11	15	0.483105	0.6664	no
gi|646740098|gb|KK962812.1| 41233-54392	20	10	75	25	-2	0.1872	no
gi|646740098|gb|KK962812.1| 44125-54560	20	10	5	2	-2	0.3304	no

gi|646740098|gb|KK962812.1|	59596-56102	20	10	1	0.681713	-0.69693	1	no
gi|646740098|gb|KK962812.1|	61225-56265	20	10	1	0.452581	-2	0.2986	no
gi|646740098|gb|KK962812.1|	62907-56349	20	10	4	0	-inf	0.0035	no
gi|646740098|gb|KK962812.1|	63625-56579	20	10	1	1	0.143547	0.91465	no
gi|646740098|gb|KK962812.1|	68114-56953	20	10	2	0.798005	-0.915202	0.4794	no
gi|646740098|gb|KK962812.1|	70271-57121	20	10	0.945277	1	0.284128	0.8455	no
gi|646740098|gb|KK962812.1|	71674-57311	20	10	0.783337	0.676615	-0.211298	1	no
gi|646740098|gb|KK962812.1|	73284-57434	20	10	1	2	0.665145	0.6258	no
gi|646740098|gb|KK962812.1|	74707-57662	20	10	1	2	0.533347	0.73695	no
gi|646740098|gb|KK962812.1|	76695-57774	20	10	0.929703	0.829101	-0.165222	1	no
gi|646740098|gb|KK962812.1|	77983-58028	20	10	45	18	-1	0.23875	no
gi|646740098|gb|KK962812.1|	80429-58098	20	10	2	3	0.22757	0.87175	no
gi|646740098|gb|KK962812.1|	81118-58331	20	10	1	3	1	0.5009	no
gi|646740098|gb|KK962812.1|	83595-58572	20	10	1	5	2	0.1947	no
gi|646740098|gb|KK962812.1|	87938-58965	20	10	0.569127	0.27408	-1	1	no
gi|646740098|gb|KK962812.1|	89968-59858	20	10	7	6	-0.301944	0.78435	no
gi|646740098|gb|KK962812.1|	03424-60514	20	10	4	4	-0.213251	0.88625	no
gi|646740098|gb|KK962812.1|	05548-60646	20	10	13	28	1	0.38595	no
gi|646740098|gb|KK962812.1|	06842-60924	20	10	1	0.686516	-0.909729	0.5153	no
gi|646740098|gb|KK962812.1|	09709-61034	20	10	1	2	0.733028	0.64505	no
gi|646740098|gb|KK962812.1|	12041-61250	20	10	128	141	0.136995	0.8977	no
gi|646740098|gb|KK962812.1|	30133-63055	20	10	3	6	1	0.46165	no
gi|646740098|gb|KK962812.1|	39305-64641	20	10	2	2	0.633123	0.67865	no
gi|646740098|gb|KK962812.1|	46616-64851	20	10	0.898759	1	0.703144	0.62405	no
gi|646740098|gb|KK962812.1|	)9417-10003(	20	10	0.515839	5	3	0.24145	no
gi|646740116|gb|KK962810.1|	33570-34322	12	9.4	11.91	4	-2	0.34395	no
gi|646740116|gb|KK962810.1|	35252-38488	12	9.4	24	13	-0.896109	0.4275	no
gi|646740131|gb|KK962808.1|	54279-15506	9.6	7.7	2	2	-0.506889	0.69735	no
gi|646740131|gb|KK962808.1|	00896-20134	9.6	7.7	4	2	-1	0.4557	no
gi|646740131|gb|KK962808.1|	05171-20575	9.6	7.7	2	1	-0.764276	0.6333	no

gi|646740149|gb|KK962806.1|	74201-74760	12	7	7	0.414395	-4	0.1898	no
gi|646740158|gb|KK962805.1|	08483-10881	24	6.5	7	5	-0.520312	0.7398	no
gi|646740158|gb|KK962805.1|	49756-15215	24	6.5	0.734272	2	1	0.3734	no
gi|646740158|gb|KK962805.1|	54264-15579	24	6.5	0.436726	0.629533	0.527554	1	no
gi|646740158|gb|KK962805.1|	10540-21102	24	6.5	12	4	-1	0.32035	no
gi|646740158|gb|KK962805.1|	21586-25314	24	6.5	2	2	0.543345	0.7213	no
gi|646740158|gb|KK962805.1|	39623-23986	24	6.5	51	11	-2	0.1807	no
gi|646740158|gb|KK962805.1|	55395-25578	24	6.5	3	10	2	0.26745	no
gi|646740158|gb|KK962805.1|	71047-27139	24	6.5	46	45	-0.0208753	0.987	no
gi|646740158|gb|KK962805.1|	95153-29560	24	6.5	3	4	0.4019	0.7939	no
gi|646740158|gb|KK962805.1|	96157-29830	24	6.5	0.440325	1	1	0.3822	no
gi|646740158|gb|KK962805.1|	98466-29974	24	6.5	0.811491	0.906092	0.159081	1	no
gi|646740158|gb|KK962805.1|	00730-30261	24	6.5	0.284664	0.906134	2	1	no
gi|646740158|gb|KK962805.1|	12126-31282	24	6.5	1	2	0.260219	0.86925	no
gi|646740158|gb|KK962805.1|	13440-31589	24	6.5	1	0.733742	-0.520862	1	no
gi|646740158|gb|KK962805.1|	17440-32605	24	6.5	25	20	-0.324167	0.78155	no
gi|646740158|gb|KK962805.1|	27025-32750	24	6.5	2	4	1	0.3784	no
gi|646740158|gb|KK962805.1|	27656-33099	24	6.5	0.99097	1	0.252673	0.8728	no
gi|646740158|gb|KK962805.1|	32770-33817	24	6.5	2	4	0.802158	0.5638	no
gi|646740158|gb|KK962805.1|	31124-33239	24	6.5	1	1	-0.478589	0.7198	no
gi|646740158|gb|KK962805.1|	35517-33655	24	6.5	0.823838	1	0.512352	0.7286	no
gi|646740158|gb|KK962805.1|	36656-33802	24	6.5	6	3	-1	0.50845	no
gi|646740158|gb|KK962805.1|	41448-34234	24	6.5	2	1	-0.596044	0.6374	no
gi|646740158|gb|KK962805.1|	42481-34292	24	6.5	4	4	-0.0182492	0.9298	no
gi|646740158|gb|KK962805.1|	43009-34427	24	6.5	0.827622	1	0.81055	0.5352	no
gi|646740158|gb|KK962805.1|	48613-35060	24	6.5	13	41	2	0.14215	no
gi|646740158|gb|KK962805.1|	83987-38422	24	6.5	4	48	4	0.10085	no
gi|646740158|gb|KK962805.1|	98251-49956	24	6.5	1	2	0.342031	0.8061	no
gi|646740158|gb|KK962805.1|	99789-50378	24	6.5	0.86538	2	1	0.4879	no
gi|646740158|gb|KK962805.1|	09450-61340	24	6.5	49	21	-1	0.30145	no

gi|646740158|gb|KK962805.1|	21033-62243	24	6.5	1	0.813727	-0.83774	0.51815	no
gi|646740158|gb|KK962805.1|	22656-62336	24	6.5	0.613743	2	2	0.25705	no
gi|646740158|gb|KK962805.1|	24841-62537	24	6.5	4	3	-0.430812	0.74425	no
gi|646740158|gb|KK962805.1|	32722-63313	24	6.5	3	3	-0.207	0.88655	no
gi|646740158|gb|KK962805.1|	44474-64725	24	6.5	1	1	0.207353	0.88895	no
gi|646740158|gb|KK962805.1|	49459-65041	24	6.5	3	4	0.362847	0.78825	no
gi|646740158|gb|KK962805.1|	55271-66210	24	6.5	0.702852	0.945192	0.427387	1	no
gi|646740158|gb|KK962805.1|	63375-66452	24	6.5	1	1	0.00265153	1	no
gi|646740158|gb|KK962805.1|	66574-66867	24	6.5	0.652943	0.436727	-0.580225	1	no
gi|646740158|gb|KK962805.1|	68863-67009	24	6.5	2	0.408182	-2	0.23485	no
gi|646740158|gb|KK962805.1|	83345-68464	24	6.5	3	3	-0.062727	0.9687	no
gi|646740158|gb|KK962805.1|	88848-68980	24	6.5	3	2	-0.73314	0.60715	no
gi|646740158|gb|KK962805.1|	18774-71987	24	6.5	16	10	-0.720507	0.63555	no
gi|646740158|gb|KK962805.1|	20447-72164	24	6.5	0.688162	0.704312	0.0334669	1	no
gi|646740158|gb|KK962805.1|	25479-72928	24	6.5	3	3	-0.31894	0.83315	no
gi|646740158|gb|KK962805.1|	29391-73418	24	6.5	61	38	-0.67931	0.5717	no
gi|646740158|gb|KK962805.1|	80420-78076	24	6.5	13	5	-1	0.37415	no
gi|646740158|gb|KK962805.1|	87072-78852	24	6.5	0.772223	0.555991	-0.473955	1	no
gi|646740158|gb|KK962805.1|	91623-79202	24	6.5	18	13	-0.451467	0.7733	no
gi|646740158|gb|KK962805.1|	00803-80112	24	6.5	108	11	-3	0.04635	no
gi|646740158|gb|KK962805.1|	81432-84604	24	6.5	16	21	0.378995	0.7387	no
gi|646740158|gb|KK962805.1|	34006-83667	24	6.5	6	4	-0.564511	0.70865	no
gi|646740158|gb|KK962805.1|	36838-84002	24	6.5	4	3	-0.334621	0.8191	no
gi|646740158|gb|KK962805.1|	93713-93997	24	6.5	15	14	-0.160189	0.9113	no
gi|646740158|gb|KK962805.1|	9525-11559	24	6.5	16	9	-0.939798	0.4665	no
gi|646740211|gb|KK962799.1|	42341-24303	14	5.6	2	1	-0.65647	0.64775	no
gi|646740218|gb|KK962798.1|	24225-22461	14	6.5	23	9	-1	0.36055	no
gi|646740225|gb|KK962797.1|	04379-10780	16	8.8	43	2	-4	0.01005	no
gi|646740225|gb|KK962797.1|	12194-12802	16	8.8	4	1	-2	0.30325	no
gi|646740225|gb|KK962797.1|	25631-12733	16	8.8	1	0.092544	-4	0.1745	no

gi|646740225|gb|KK962797.1|	28355-12870	16	8.8	11	1	-3	0.18235	no
gi|646740225|gb|KK962797.1|	31774-13214	16	8.8	25	0	-inf	0.002	no
gi|646740225|gb|KK962797.1|	37938-13856	16	8.8	2	8	2	0.2749	no
gi|646740225|gb|KK962797.1|	38842-13954	16	8.8	6	5	-0.22335	0.88285	no
gi|646740225|gb|KK962797.1|	42803-14366	16	8.8	2	0	-inf	0.00655	no
gi|646740225|gb|KK962797.1|	56877-15888	16	8.8	4	2	-0.766769	0.6153	no
gi|646740225|gb|KK962797.1|	60789-16166	16	8.8	0.745965	1	0.501781	1	no
gi|646740225|gb|KK962797.1|	61764-16391	16	8.8	12	7	-0.777054	0.53935	no
gi|646740225|gb|KK962797.1|	65983-16673	16	8.8	0.745874	2	1	0.36865	no
gi|646740225|gb|KK962797.1|	72844-17398	16	8.8	1	1	0.253717	0.8481	no
gi|646740225|gb|KK962797.1|	1741-3275	16	8.8	2	1	-0.957718	0.51215	no
gi|646740225|gb|KK962797.1|	74322-17497	16	8.8	2	2	0.251414	0.86925	no
gi|646740225|gb|KK962797.1|	78979-18148	16	8.8	1	1	-0.461597	0.7693	no
gi|646740225|gb|KK962797.1|	88451-19098	16	8.8	6	2	-2	0.3081	no
gi|646740225|gb|KK962797.1|	91101-19227	16	8.8	0.501128	1	1	0.36645	no
gi|646740225|gb|KK962797.1|	93075-19364	16	8.8	7	4	-0.898861	0.54035	no
gi|646740225|gb|KK962797.1|	02198-20259	16	8.8	5	4	-0.312759	0.84645	no
gi|646740225|gb|KK962797.1|	03234-20645	16	8.8	2	2	-0.288288	0.8487	no
gi|646740225|gb|KK962797.1|	21518-22186	16	8.8	10	9	-0.124673	0.93465	no
gi|646740225|gb|KK962797.1|	25675-22812	16	8.8	1	0.551798	-1	0.3963	no
gi|646740225|gb|KK962797.1|	28583-22946	16	8.8	29	20	-0.535374	0.7271	no
gi|646740225|gb|KK962797.1|	30567-23114	16	8.8	2	2	-0.542426	0.7295	no
gi|646740225|gb|KK962797.1|	31337-23234	16	8.8	1	0.696471	-0.948597	0.54505	no
gi|646740225|gb|KK962797.1|	32959-23402	16	8.8	1	5	2	0.2084	no
gi|646740225|gb|KK962797.1|	34533-23541	16	8.8	1	1	0.502651	0.7286	no
gi|646740225|gb|KK962797.1|	35581-23617	16	8.8	7	13	0.98807	0.5437	no
gi|646740225|gb|KK962797.1|	36292-23938	16	8.8	2	3	0.737216	0.62225	no
gi|646740225|gb|KK962797.1|	47975-24958	16	8.8	3	4	0.306646	0.83775	no
gi|646740225|gb|KK962797.1|	26583-26931	16	8.8	15	10	-0.6067	0.62885	no
gi|646740225|gb|KK962797.1|	28762-29996	16	8.8	3	0.406551	-3	0.16625	no

gi|646740225|gb|KK962797.1|	3542-4113	16	8.8	4	2	-1	0.42345	no
gi|646740225|gb|KK962797.1|	5070-6927	16	8.8	132	55	-1	0.2914	no
gi|646740225|gb|KK962797.1|	94962-95685	16	8.8	20	6	-2	0.2969	no
gi|646740225|gb|KK962797.1|	96219-96787	16	8.8	116	35	-2	0.1944	no
gi|646740225|gb|KK962797.1|	97475-98571	16	8.8	29	5	-3	0.0721	no
gi|646740225|gb|KK962797.1|	98686-99961	16	8.8	8	2	-2	0.25115	no
gi|646740232|gb|KK962796.1|	71218-71638	9.6	5.2	8	0	-inf	0.0011	yes
gi|646740232|gb|KK962796.1|	78305-79453	9.6	5.2	0.828736	0.444746	-0.89793	1	no
gi|646740261|gb|KK962793.1|	27337-12781	19	12	7	2	-2	0.18625	no
gi|646740261|gb|KK962793.1|	53552-15382	19	12	0	25	inf	0.0014	yes
gi|646740261|gb|KK962793.1|	53923-15482	19	12	0	6	inf	0.001	yes
gi|646740261|gb|KK962793.1|	97460-19854	19	12	1	3	1	0.4012	no
gi|646740270|gb|KK962792.1|	53440-15455	14	8.5	0	4	inf	0.00175	no
gi|646740270|gb|KK962792.1|	54623-15576	14	8.5	0	3	inf	0.00435	no
gi|646740270|gb|KK962792.1|	57100-15834	14	8.5	0.186888	2	4	0.2663	no
gi|646740270|gb|KK962792.1|	60152-16092	14	8.5	0.178903	2	4	0.1061	no
gi|646740270|gb|KK962792.1|	02797-20372	14	8.5	2	0.972954	-0.872661	0.5465	no
gi|646740270|gb|KK962792.1|	42139-42941	14	8.5	1	2	1	0.39835	no
gi|646740270|gb|KK962792.1|	59429-60017	14	8.5	0	5	inf	0.001	yes
gi|646740270|gb|KK962792.1|	60585-61665	14	8.5	0	7	inf	0.0002	yes
gi|646740278|gb|KK962791.1|	94247-94864	9.9	5.3	2	2	0.6889	0.6395	no
gi|646740287|gb|KK962790.1|	01254-10295	20	8.5	1	2	0.254965	0.8721	no
gi|646740287|gb|KK962790.1|	03230-10545	20	8.5	0.704091	1	0.536378	1	no
gi|646740287|gb|KK962790.1|	24632-13196	20	8.5	3	3	-0.0862433	0.9362	no
gi|646740287|gb|KK962790.1|	40981-14326	20	8.5	5	3	-0.855496	0.5725	no
gi|646740287|gb|KK962790.1|	49345-15138	20	8.5	18	14	-0.324296	0.7701	no
gi|646740287|gb|KK962790.1|	59864-16112	20	8.5	6	4	-0.526355	0.7282	no
gi|646740287|gb|KK962790.1|	62399-16316	20	8.5	0.90856	2	0.753951	0.6148	no
gi|646740287|gb|KK962790.1|	71849-17220	20	8.5	15	48	2	0.2341	no
gi|646740287|gb|KK962790.1|	52402-25266	20	8.5	28	88	2	0.2643	no

gi|646740287|gb|KK962790.1|	91473-29180	20	8.5	17	25	0.605979	0.66905	no
gi|646740287|gb|KK962790.1|	96586-29745	20	8.5	9	26	1	0.3438	no
gi|646740287|gb|KK962790.1|	03279-30397	20	8.5	41	136	2	0.20255	no
gi|646740287|gb|KK962790.1|	06226-30671	20	8.5	22	103	2	0.1062	no
gi|646740287|gb|KK962790.1|	08856-31011	20	8.5	0.459333	1	1	1	no
gi|646740287|gb|KK962790.1|	11975-31236	20	8.5	10	31	2	0.25135	no
gi|646740287|gb|KK962790.1|	13452-31467	20	8.5	0.19074	2	3	0.24675	no
gi|646740287|gb|KK962790.1|	14982-31660	20	8.5	99	144	0.538147	0.6469	no
gi|646740287|gb|KK962790.1|	21178-32991	20	8.5	3	3	0.0363173	0.9718	no
gi|646740287|gb|KK962790.1|	33411-33410	20	8.5	14	6	-1	0.4217	no
gi|646740287|gb|KK962790.1|	39394-34027	20	8.5	9	10	0.109784	0.9365	no
gi|646740287|gb|KK962790.1|	44974-34519	20	8.5	82	71	-0.197173	0.87065	no
gi|646740287|gb|KK962790.1|	46235-34659	20	8.5	9	14	0.560788	0.666	no
gi|646740287|gb|KK962790.1|	48710-35183	20	8.5	0.774228	2	1	0.4641	no
gi|646740287|gb|KK962790.1|	52106-35296	20	8.5	2	3	0.50063	0.72945	no
gi|646740287|gb|KK962790.1|	53195-35654	20	8.5	3	3	0.0568095	0.97015	no
gi|646740287|gb|KK962790.1|	73330-37484	20	8.5	29	16	-0.881536	0.41555	no
gi|646740287|gb|KK962790.1|	75734-37852	20	8.5	1	1	-0.182498	0.91165	no
gi|646740287|gb|KK962790.1|	78658-37959	20	8.5	0.956445	3	1	0.31275	no
gi|646740287|gb|KK962790.1|	79694-38158	20	8.5	0.283096	1	2	0.18085	no
gi|646740287|gb|KK962790.1|	94667-39564	20	8.5	2	0.18133	-3	0.1863	no
gi|646740287|gb|KK962790.1|	96650-39734	20	8.5	1	2	0.966781	0.5626	no
gi|646740287|gb|KK962790.1|	98269-39942	20	8.5	0.817749	0.585322	-0.482428	1	no
gi|646740287|gb|KK962790.1|	99493-39996	20	8.5	3	1	-2	0.3832	no
gi|646740287|gb|KK962790.1|	07905-40829	20	8.5	17	8	-1	0.4759	no
gi|646740287|gb|KK962790.1|	12317-41308	20	8.5	524	13	-5	0.0032	no
gi|646740287|gb|KK962790.1|	14262-42088	20	8.5	52	6	-3	0.01315	no
gi|646740287|gb|KK962790.1|	21439-42212	20	8.5	4	0	-inf	0.00045	yes
gi|646740287|gb|KK962790.1|	23100-42412	20	8.5	5	0	-inf	0.00135	yes
gi|646740287|gb|KK962790.1|	25191-42649	20	8.5	16	2	-3	0.04095	no

gi|646740287|gb|KK962790.1|	77328-78794	20	8.5	20	26	0.381064	0.72535	no
gi|646740287|gb|KK962790.1|	85294-86890	20	8.5	19	14	-0.368059	0.73055	no
gi|646740287|gb|KK962790.1|	90014-99796	20	8.5	12	10	-0.292215	0.8011	no
gi|646740296|gb|KK962789.1|	67419-16770	24	9	15	9	-0.636934	0.6676	no
gi|646740304|gb|KK962788.1|	10811-15691	24	9.6	5	4	-0.36436	0.7771	no
gi|646740304|gb|KK962788.1|	08806-10928	24	9.6	3	3	0.19684	0.88975	no
gi|646740304|gb|KK962788.1|	09436-11001	24	9.6	32	22	-0.531193	0.7232	no
gi|646740304|gb|KK962788.1|	14167-11459	24	9.6	119	39	-2	0.2217	no
gi|646740304|gb|KK962788.1|	17562-11979	24	9.6	1	1	0.398915	0.8084	no
gi|646740304|gb|KK962788.1|	24747-12667	24	9.6	2	2	0.102861	0.94665	no
gi|646740304|gb|KK962788.1|	28270-12949	24	9.6	0.762176	0.683042	-0.15815	1	no
gi|646740304|gb|KK962788.1|	29638-13081	24	9.6	1	1	0.058777	0.94695	no
gi|646740304|gb|KK962788.1|	35730-13604	24	9.6	10	6	-0.739808	0.6124	no
gi|646740304|gb|KK962788.1|	38596-14210	24	9.6	75	8	-3	0.00995	no
gi|646740304|gb|KK962788.1|	42508-14322	24	9.6	2	0.280668	-3	0.1923	no
gi|646740304|gb|KK962788.1|	50470-15122	24	9.6	31	0.262266	-7	0.1666	no
gi|646740304|gb|KK962788.1|	55671-15621	24	9.6	6	3	-0.722206	0.5734	no
gi|646740304|gb|KK962788.1|	81675-18221	24	9.6	4	2	-0.932451	0.52635	no
gi|646740304|gb|KK962788.1|	85913-18648	24	9.6	3	4	0.606779	0.64685	no
gi|646740304|gb|KK962788.1|	89162-18967	24	9.6	6	4	-0.565813	0.6717	no
gi|646740304|gb|KK962788.1|	90354-19318	24	9.6	1	0.627685	-1	0.40245	no
gi|646740304|gb|KK962788.1|	96537-19710	24	9.6	3	3	0.091917	0.93525	no
gi|646740304|gb|KK962788.1|	19960-22712	24	9.6	0.778096	0.700234	-0.152111	1	no
gi|646740304|gb|KK962788.1|	00071-20030	24	9.6	92	0	-inf	0.0047	no
gi|646740304|gb|KK962788.1|	01367-20223	24	9.6	0.300811	2	3	0.2655	no
gi|646740304|gb|KK962788.1|	20609-22146	24	9.6	13	7	-0.972207	0.52595	no
gi|646740304|gb|KK962788.1|	27041-22812	24	9.6	0	3	inf	0.00515	no
gi|646740304|gb|KK962788.1|	22930-26872	24	9.6	12	9	-0.408426	0.7168	no
gi|646740304|gb|KK962788.1|	35289-33566	24	9.6	0	16	inf	0.00435	no
gi|646740304|gb|KK962788.1|	36708-37330	24	9.6	11	6	-0.821881	0.6058	no

gi|646740304|gb|KK962788.1|	33166-43400	24	9.6	2	2	0.635942	0.62995	no
gi|646740304|gb|KK962788.1|	07163-50759	24	9.6	14	17	0.229943	0.8865	no
gi|646740304|gb|KK962788.1|	57697-58142	24	9.6	59	28	-1	0.47595	no
gi|646740304|gb|KK962788.1|	00227-60796	24	9.6	9	2	-2	0.13745	no
gi|646740304|gb|KK962788.1|	36583-63696	24	9.6	17	3	-3	0.16295	no
gi|646740304|gb|KK962788.1|	93627-69418	24	9.6	1	2	1	0.48625	no
gi|646740304|gb|KK962788.1|	70091-70582	24	9.6	3	3	-0.252572	0.87285	no
gi|646740304|gb|KK962788.1|	70974-72114	24	9.6	2	2	0.217231	0.86235	no
gi|646740304|gb|KK962788.1|	72237-76101	24	9.6	2	1	-0.281222	0.85085	no
gi|646740304|gb|KK962788.1|	78448-84034	24	9.6	5	3	-0.839735	0.51235	no
gi|646740304|gb|KK962788.1|	84229-85619	24	9.6	2	1	-0.545801	0.6602	no
gi|646740304|gb|KK962788.1|	85844-87149	24	9.6	33	10	-2	0.21025	no
gi|646740304|gb|KK962788.1|	89751-90090	24	9.6	23	16	-0.478984	0.7303	no
gi|646740304|gb|KK962788.1|	91621-92181	24	9.6	2	3	0.451513	0.7474	no
gi|646740304|gb|KK962788.1|	92364-92857	24	9.6	8	9	0.204026	0.88065	no
gi|646740313|gb|KK962787.1|	85650-18643	13	7.6	14	0	-inf	0.0003	yes
gi|646740313|gb|KK962787.1|	88753-20308	13	7.6	84	3	-5	0.00085	yes
gi|646740313|gb|KK962787.1|	03672-20489	13	7.6	2	0.274321	-3	0.1299	no
gi|646740313|gb|KK962787.1|	05852-20788	13	7.6	2	0.151171	-4	0.1308	no
gi|646740313|gb|KK962787.1|	08029-20961	13	7.6	1	0.500613	-1	0.292	no
gi|646740313|gb|KK962787.1|	04745-30525	13	7.6	4	2	-0.888976	0.5584	no
gi|646740313|gb|KK962787.1|	59761-60649	13	7.6	4	0	-inf	0.0031	no
gi|646740313|gb|KK962787.1|	70672-71990	13	7.6	14	0.250058	-6	0.10695	no
gi|646740313|gb|KK962787.1|	72095-72821	13	7.6	16	0	-inf	0.0004	yes
gi|646740337|gb|KK962784.1|	05855-10630	22	9.6	3	3	-0.0835264	0.92325	no
gi|646740337|gb|KK962784.1|	10151-11428	22	9.6	10	7	-0.505571	0.6468	no
gi|646740337|gb|KK962784.1|	12266-13288	22	9.6	0.482423	1	1	1	no
gi|646740337|gb|KK962784.1|	01508-20350	22	9.6	2	3	0.491229	0.7464	no
gi|646740337|gb|KK962784.1|	50286-35073	22	9.6	0.475119	26	6	0.09695	no
gi|646740337|gb|KK962784.1|	47472-54848	22	9.6	2	6	1	0.3126	no

gi|646740337|gb|KK962784.1|	81560-82052	22	9.6	0	7	inf	0.0008	yes
gi|646740337|gb|KK962784.1|	87463-95246	22	9.6	6	3	-1	0.3119	no
gi|646740344|gb|KK962783.1|	50622-25115	14	6.7	2	3	0.704187	0.64555	no
gi|646740350|gb|KK962782.1|	06416-10732	12	6.1	0	2	inf	0.001	yes
gi|646740357|gb|KK962781.1|	64923-16667	13	6.4	6	3	-0.909247	0.5459	no
gi|646740357|gb|KK962781.1|	67286-16757	13	6.4	36	49	0.449932	0.7825	no
gi|646740357|gb|KK962781.1|	69457-17002	13	6.4	4	2	-1	0.3446	no
gi|646740357|gb|KK962781.1|	80082-18147	13	6.4	1	0.467389	-2	0.2508	no
gi|646740357|gb|KK962781.1|	83199-18358	13	6.4	10	5	-0.964936	0.49115	no
gi|646740357|gb|KK962781.1|	99209-20024	13	6.4	3	3	0.0789332	0.96195	no
gi|646740357|gb|KK962781.1|	09176-20969	13	6.4	3	7	1	0.3627	no
gi|646740357|gb|KK962781.1|	12352-21299	13	6.4	2	0.654313	-2	0.29735	no
gi|646740365|gb|KK962780.1|	64336-16464	21	7.9	12	0	-inf	0.00655	no
gi|646740365|gb|KK962780.1|	65332-16600	21	7.9	16	0	-inf	5.00E-05	yes
gi|646740365|gb|KK962780.1|	67097-16952	21	7.9	13	0	-inf	5.00E-05	yes
gi|646740365|gb|KK962780.1|	70407-17210	21	7.9	38	0	-inf	5.00E-05	yes
gi|646740365|gb|KK962780.1|	80093-18739	21	7.9	5	5	-0.0460726	0.9638	no
gi|646740365|gb|KK962780.1|	91281-19252	21	7.9	6	6	-0.107427	0.9449	no
gi|646740365|gb|KK962780.1|	05789-20687	21	7.9	2	0.958609	-0.708075	0.5885	no
gi|646740365|gb|KK962780.1|	14507-21528	21	7.9	3	2	-0.94791	0.46895	no
gi|646740365|gb|KK962780.1|	17443-21850	21	7.9	0	8	inf	0.00015	yes
gi|646740365|gb|KK962780.1|	30028-23081	21	7.9	19	7	-1	0.3396	no
gi|646740365|gb|KK962780.1|	37163-23794	21	7.9	20	27	0.453247	0.7667	no
gi|646740365|gb|KK962780.1|	43223-24355	21	7.9	36	0	-inf	0.002	no
gi|646740365|gb|KK962780.1|	57822-25827	21	7.9	5	14	1	0.3612	no
gi|646740365|gb|KK962780.1|	80186-28083	21	7.9	3	0.324821	-3	0.1923	no
gi|646740365|gb|KK962780.1|	12060-31317	21	7.9	1	0.767227	-0.48415	1	no
gi|646740365|gb|KK962780.1|	25762-32636	21	7.9	0.786811	3	2	0.29365	no
gi|646740365|gb|KK962780.1|	26641-32777	21	7.9	3	3	-0.120839	0.94	no
gi|646740365|gb|KK962780.1|	34679-33620	21	7.9	1	0.840677	-0.559948	0.6642	no

gi|646740365|gb|KK962780.1|	88352-89318	21	7.9	9	2	-2	0.1216	no
gi|646740371|gb|KK962779.1|	11137-11156	14	9.1	12	4	-2	0.33195	no
gi|646740371|gb|KK962779.1|	13308-14622	14	9.1	0.262069	16	6	0.1062	no
gi|646740371|gb|KK962779.1|	17044-17470	14	9.1	0	29	inf	0.00015	yes
gi|646740371|gb|KK962779.1|	90846-19660	14	9.1	0.151401	82	9	0.01635	no
gi|646740371|gb|KK962779.1|	99569-20020	14	9.1	0	54	inf	5.00E-05	yes
gi|646740371|gb|KK962779.1|	04094-20517	14	9.1	0	10	inf	0.00045	yes
gi|646740371|gb|KK962779.1|	05389-20741	14	9.1	0	14	inf	5.00E-05	yes
gi|646740371|gb|KK962779.1|	31716-23281	14	9.1	0	10	inf	0.00025	yes
gi|646740371|gb|KK962779.1|	36273-23710	14	9.1	0.160031	21	7	0.0948	no
gi|646740371|gb|KK962779.1|	39715-24034	14	9.1	0	13	inf	0.00055	yes
gi|646740371|gb|KK962779.1|	66315-26883	14	9.1	0.408291	192	9	0.0126	no
gi|646740371|gb|KK962779.1|	76431-27690	14	9.1	0	7	inf	0.001	yes
gi|646740371|gb|KK962779.1|	77009-27758	14	9.1	0	7	inf	0.00435	no
gi|646740371|gb|KK962779.1|	78214-27839	14	9.1	28	11805	9	0.2322	no
gi|646740371|gb|KK962779.1|	90490-29139	14	9.1	0.568362	181	8	0.09345	no
gi|646740371|gb|KK962779.1|	14804-31958	14	9.1	0	8	inf	5.00E-05	yes
gi|646740371|gb|KK962779.1|	19944-32092	14	9.1	0	5	inf	0.0009	yes
gi|646740371|gb|KK962779.1|	21178-32297	14	9.1	0	46	inf	5.00E-05	yes
gi|646740371|gb|KK962779.1|	35736-36324	14	9.1	1	424	8	0.05805	no
gi|646740371|gb|KK962779.1|	36455-38284	14	9.1	0.117555	36	8	0.2322	no
gi|646740371|gb|KK962779.1|	88733-38967	14	9.1	1	4	2	0.2559	no
gi|646740371|gb|KK962779.1|	41161-42839	14	9.1	0	12	inf	0.0001	yes
gi|646740371|gb|KK962779.1|	43048-43364	14	9.1	0	357	inf	5.00E-05	yes
gi|646740371|gb|KK962779.1|	48822-50547	14	9.1	0.251741	236	10	0.09345	no
gi|646740371|gb|KK962779.1|	58822-60861	14	9.1	0.051859	40	10	0.09445	no
gi|646740371|gb|KK962779.1|	71159-72896	14	9.1	0.312129	238	10	0.05805	no
gi|646740371|gb|KK962779.1|	95439-95869	14	9.1	10	7	-0.47182	0.6954	no
gi|646740395|gb|KK962776.1|	34217-13465	16	6.3	5	5	0.0761655	0.94195	no
gi|646740395|gb|KK962776.1|	43748-14428	16	6.3	4	0.893594	-2	0.19275	no

gi|646740395|gb|KK962776.1|	50523-15191	16	6.3	0.894224	0.350844	-1	1	no
gi|646740395|gb|KK962776.1|	52436-15345	16	6.3	38	11	-2	0.18995	no
gi|646740395|gb|KK962776.1|	54755-15604	16	6.3	2	0.642307	-2	0.3192	no
gi|646740395|gb|KK962776.1|	80370-18221	16	6.3	1	2	0.471093	0.76815	no
gi|646740395|gb|KK962776.1|	82333-18503	16	6.3	2	2	0.169139	0.90755	no
gi|646740395|gb|KK962776.1|	85779-18647	16	6.3	163	80	-1	0.36655	no
gi|646740395|gb|KK962776.1|	04292-20513	16	6.3	0	4	inf	0.00315	no
gi|646740395|gb|KK962776.1|	27268-22748	16	6.3	65	218	2	0.24375	no
gi|646740395|gb|KK962776.1|	32788-23317	16	6.3	4	20	2	0.1707	no
gi|646740395|gb|KK962776.1|	37256-23772	16	6.3	0.852952	6	3	0.26275	no
gi|646740395|gb|KK962776.1|	40658-24317	16	6.3	2	3	0.765863	0.6143	no
gi|646740405|gb|KK962775.1|	32208-13417	20	5.8	0.812497	0.86267	0.086446	1	no
gi|646740405|gb|KK962775.1|	34703-13568	20	5.8	2	3	0.616172	0.625	no
gi|646740405|gb|KK962775.1|	53030-15341	20	5.8	6	4	-0.821859	0.58385	no
gi|646740405|gb|KK962775.1|	61882-16218	20	5.8	9	11	0.244481	0.8678	no
gi|646740405|gb|KK962775.1|	64138-16533	20	5.8	1	0.990148	-0.133325	1	no
gi|646740405|gb|KK962775.1|	87493-18798	20	5.8	4	1	-2	0.2639	no
gi|646740405|gb|KK962775.1|	89279-18971	20	5.8	21	10	-1	0.49945	no
gi|646740405|gb|KK962775.1|	92344-19355	20	5.8	0.674544	0.552552	-0.287803	1	no
gi|646740405|gb|KK962775.1|	00249-20169	20	5.8	0.854437	1	0.651422	0.61505	no
gi|646740405|gb|KK962775.1|	11537-21229	20	5.8	3	1	-2	0.30685	no
gi|646740405|gb|KK962775.1|	12420-21369	20	5.8	0.727708	2	1	0.41115	no
gi|646740405|gb|KK962775.1|	14315-21816	20	5.8	5	3	-0.654762	0.6634	no
gi|646740405|gb|KK962775.1|	25702-22715	20	5.8	0.619329	1	0.985411	0.4966	no
gi|646740405|gb|KK962775.1|	37810-23922	20	5.8	4	2	-0.948793	0.54825	no
gi|646740405|gb|KK962775.1|	44846-24664	20	5.8	1	2	0.599067	0.6595	no
gi|646740405|gb|KK962775.1|	51759-25218	20	5.8	25	26	0.0404993	0.9805	no
gi|646740405|gb|KK962775.1|	58677-26676	20	5.8	6	6	-0.126964	0.9145	no
gi|646740405|gb|KK962775.1|	66842-27331	20	5.8	6	3	-0.91668	0.48375	no
gi|646740405|gb|KK962775.1|	04387-30517	20	5.8	4	4	0.271268	0.83955	no

gi|646740405|gb|KK962775.1|	20470-32132	20	5.8	2	0	-inf	0.00655	no
gi|646740405|gb|KK962775.1|	53973-35454	20	5.8	12	9	-0.442773	0.77435	no
gi|646740405|gb|KK962775.1|	62360-36286	20	5.8	3	8	2	0.3502	no
gi|646740405|gb|KK962775.1|	63426-36397	20	5.8	4	2	-0.815973	0.56425	no
gi|646740405|gb|KK962775.1|	71085-37229	20	5.8	5	5	0.0123525	0.9895	no
gi|646740405|gb|KK962775.1|	75234-37831	20	5.8	1	1	0.341928	0.82565	no
gi|646740405|gb|KK962775.1|	90617-39133	20	5.8	1	2	0.707101	0.63615	no
gi|646740405|gb|KK962775.1|	23140-42344	20	5.8	508	467	-0.119912	0.9117	no
gi|646740405|gb|KK962775.1|	47004-44793	20	5.8	1	1	-0.230912	0.86285	no
gi|646740405|gb|KK962775.1|	64498-46549	20	5.8	1	0.534702	-1	0.47685	no
gi|646740405|gb|KK962775.1|	96800-98009	20	5.8	21	8	-1	0.28655	no
gi|646740420|gb|KK962773.1|	98931-19970	20	7.5	3	0.998936	-2	0.30685	no
gi|646740420|gb|KK962773.1|	22412-22424	20	7.5	6	0.507818	-3	0.04945	no
gi|646740420|gb|KK962773.1|	38675-23951	20	7.5	13	11	-0.28646	0.84495	no
gi|646740420|gb|KK962773.1|	39906-24094	20	7.5	0.945135	0.50549	-0.902838	1	no
gi|646740420|gb|KK962773.1|	54767-25577	20	7.5	0.614238	1	0.77365	1	no
gi|646740420|gb|KK962773.1|	56628-25709	20	7.5	3	3	-0.0724753	0.92345	no
gi|646740430|gb|KK962772.1|	14021-14819	13	7.3	6	1	-2	0.18495	no
gi|646740430|gb|KK962772.1|	18633-19302	13	7.3	2	2	-0.37104	0.80365	no
gi|646740430|gb|KK962772.1|	3303-3715	13	7.3	13	31	1	0.41125	no
gi|646740430|gb|KK962772.1|	9329-10010	13	7.3	3	3	0.100536	0.9357	no
gi|646740448|gb|KK962770.1|	24-2783	20	8	2	1	-0.460197	0.7629	no
gi|646740483|gb|KK962766.1|	22181-12268	16	5.9	5	3	-0.567047	0.6718	no
gi|646740483|gb|KK962766.1|	22933-12378	16	5.9	0.307816	3	3	0.24735	no
gi|646740483|gb|KK962766.1|	48798-14933	16	5.9	4	9	1	0.437	no
gi|646740483|gb|KK962766.1|	66740-16919	16	5.9	1	0.979205	-0.506375	0.7553	no
gi|646740483|gb|KK962766.1|	70627-17257	16	5.9	0.874341	0.791103	-0.144331	1	no
gi|646740483|gb|KK962766.1|	74012-17513	16	5.9	2	0.910378	-1	0.4155	no
gi|646740483|gb|KK962766.1|	76936-17845	16	5.9	1	1	-0.320561	0.807	no
gi|646740483|gb|KK962766.1|	70358-27089	16	5.9	4	13	2	0.29645	no

gi|646740490|gb|KK962765.1|	04846-10532	11	6.2	0	7	inf	0.001	yes
gi|646740490|gb|KK962765.1|	10636-11113	11	6.2	2	3	0.427326	0.77485	no
gi|646740490|gb|KK962765.1|	15627-11710	11	6.2	0.606143	0.436565	-0.47346	1	no
gi|646740490|gb|KK962765.1|	20598-12186	11	6.2	21	44	1	0.32255	no
gi|646740490|gb|KK962765.1|	34885-13631	11	6.2	807	0.22629	-12	0.10535	no
gi|646740490|gb|KK962765.1|	38881-14034	11	6.2	63	0.110205	-9	0.1665	no
gi|646740490|gb|KK962765.1|	42480-14390	11	6.2	9	0	-inf	0.00045	yes
gi|646740490|gb|KK962765.1|	56974-15882	11	6.2	29	0	-inf	5.00E-05	yes
gi|646740490|gb|KK962765.1|	64713-16537	11	6.2	23	0	-inf	0.00025	yes
gi|646740490|gb|KK962765.1|	76909-78342	11	6.2	0.235599	0.791357	2	1	no
gi|646740490|gb|KK962765.1|	79786-81735	11	6.2	0	1	inf	1	no
gi|646740508|gb|KK962763.1|	09759-11102	14	5.4	18	13	-0.501201	0.73805	no
gi|646740508|gb|KK962763.1|	74507-76484	14	5.4	13	9	-0.447948	0.72445	no
gi|646740508|gb|KK962763.1|	78795-80476	14	5.4	16	10	-0.751523	0.5538	no
gi|646740508|gb|KK962763.1|	83451-83784	14	5.4	14	17	0.289996	0.80405	no
gi|646740508|gb|KK962763.1|	96121-98276	14	5.4	20	13	-0.660915	0.54785	no
gi|646740529|gb|KK962761.1|	41922-42903	11	7.9	1	2	0.50908	0.695	no
gi|646740544|gb|KK962759.1|	09468-10995	20	8	45	6	-3	0.0612	no
gi|646740544|gb|KK962759.1|	69830-17055	20	8	21	4	-2	0.09385	no
gi|646740544|gb|KK962759.1|	25797-26070	20	8	0	284.55	inf	0.00015	yes
gi|646740544|gb|KK962759.1|	31179-32741	20	8	0	6	inf	0.00035	yes
gi|646740544|gb|KK962759.1|	4381-6625	20	8	0.279088	93	8	0.0463	no
gi|646740544|gb|KK962759.1|	45577-47668	20	8	84	34	-1	0.2707	no
gi|646740544|gb|KK962759.1|	55605-56562	20	8	0	2	inf	0.0008	yes
gi|646740544|gb|KK962759.1|	56670-57552	20	8	225	185	-0.282459	0.81285	no
gi|646740544|gb|KK962759.1|	70030-70559	20	8	3	5	0.441152	0.7403	no
gi|646740544|gb|KK962759.1|	78945-80600	20	8	13	7	-0.897844	0.5509	no
gi|646740544|gb|KK962759.1|	96659-96900	20	8	471	664	0.497335	0.69155	no
gi|646740544|gb|KK962759.1|	98840-99020	20	8	539	696	0.369048	0.80785	no
gi|646740570|gb|KK962756.1|	42668-14400	16	6.4	0.858171	0.246649	-2	1	no

gi|646740570|gb|KK962756.1|	44888-14530	16	6.4	4	6	0.745205	0.59135	no
gi|646740570|gb|KK962756.1|	46998-14725	16	6.4	32	49	0.624135	0.673	no
gi|646740570|gb|KK962756.1|	50323-15070	16	6.4	27	45	0.724455	0.6319	no
gi|646740570|gb|KK962756.1|	51280-15169	16	6.4	3	4	0.379543	0.79685	no
gi|646740570|gb|KK962756.1|	52387-15255	16	6.4	358	305	-0.231729	0.87075	no
gi|646740570|gb|KK962756.1|	52701-15373	16	6.4	5	6	0.355722	0.8144	no
gi|646740570|gb|KK962756.1|	96645-19711	16	6.4	4	1	-2	0.2618	no
gi|646740570|gb|KK962756.1|	22167-23977	16	6.4	2	0.086031	-5	0.1761	no
gi|646740570|gb|KK962756.1|	31845-23589	16	6.4	3	1	-0.946205	0.5269	no
gi|646740570|gb|KK962756.1|	41379-24281	16	6.4	1	0.224791	-2	1	no
gi|646740570|gb|KK962756.1|	43507-24485	16	6.4	1	1	0.108567	1	no
gi|646740570|gb|KK962756.1|	45322-24630	16	6.4	2	2	0.130803	0.9189	no
gi|646740570|gb|KK962756.1|	47808-24830	16	6.4	5	3	-1	0.4359	no
gi|646740570|gb|KK962756.1|	48385-24941	16	6.4	2	0.856306	-1	0.43875	no
gi|646740570|gb|KK962756.1|	86861-87905	16	6.4	4	0	-inf	0.0026	no
gi|646740570|gb|KK962756.1|	87958-89217	16	6.4	6	0.396649	-4	0.0867	no
gi|646740570|gb|KK962756.1|	97492-98688	16	6.4	6	0.140862	-5	0.16985	no
gi|646740619|gb|KK962751.1|	07570-10968	21	9.4	2	2	-0.165012	0.91805	no
gi|646740619|gb|KK962751.1|	19879-12138	21	9.4	0.813462	2	1	0.36255	no
gi|646740619|gb|KK962751.1|	25359-12602	21	9.4	0.668238	3	2	0.2051	no
gi|646740619|gb|KK962751.1|	26677-12787	21	9.4	0.782314	1	0.519069	0.7091	no
gi|646740619|gb|KK962751.1|	14139-14998	21	9.4	1	0.649943	-0.914576	0.58965	no
gi|646740619|gb|KK962751.1|	47914-14874	21	9.4	1	3	1	0.37615	no
gi|646740619|gb|KK962751.1|	17556-18250	21	9.4	1	1	-0.103768	0.9035	no
gi|646740619|gb|KK962751.1|	22870-24391	21	9.4	6	4	-0.50731	0.73465	no
gi|646740619|gb|KK962751.1|	28798-29189	21	9.4	63	26	-1	0.4057	no
gi|646740619|gb|KK962751.1|	30200-30988	21	9.4	5	2	-2	0.2844	no
gi|646740619|gb|KK962751.1|	39184-39559	21	9.4	12	5	-1	0.3389	no
gi|646740619|gb|KK962751.1|	51940-52263	21	9.4	21	9	-1	0.3781	no
gi|646740619|gb|KK962751.1|	56983-59463	21	9.4	7	3	-1	0.43055	no

gi|646740619|gb|KK962751.1|	61151-63285	21	9.4	124	50	-1	0.27855	no
gi|646740619|gb|KK962751.1|	66246-69054	21	9.4	24	7	-2	0.1722	no
gi|646740619|gb|KK962751.1|	6702-9769	21	9.4	6	5	-0.436088	0.7712	no
gi|646740619|gb|KK962751.1|	73766-74102	21	9.4	11	6	-0.84315	0.567	no
gi|646740619|gb|KK962751.1|	75149-75497	21	9.4	4	12	1	0.3365	no
gi|646740619|gb|KK962751.1|	93665-95033	21	9.4	4	4	-0.045324	0.9788	no
gi|646740619|gb|KK962751.1|	98846-99411	21	9.4	2	2	-0.354334	0.81835	no
gi|646740637|gb|KK962749.1|	159-783	20	7.3	20	23	0.248753	0.86965	no
gi|646740637|gb|KK962749.1|	21276-22202	20	7.3	3	21	3	0.07595	no
gi|646740637|gb|KK962749.1|	45122-24569	20	7.3	2	25	4	0.05155	no
gi|646740645|gb|KK962748.1|	70832-17102	11	6.7	2714.48	1950.51	-0.476823	0.6588	no
gi|646740661|gb|KK962746.1|	07849-31033	14	5	2	2	0.244533	0.87435	no
gi|646740661|gb|KK962746.1|	31275-32161	14	5	2	3	0.802211	0.5875	no
gi|646740677|gb|KK962744.1|	96105-19802	14	8.1	0.668388	1	1	0.4823	no
gi|646740677|gb|KK962744.1|	99281-20017	14	8.1	0.867192	2	1	0.4476	no
gi|646740677|gb|KK962744.1|	29217-30150	14	8.1	11	8	-0.459999	0.7631	no
gi|646740677|gb|KK962744.1|	92813-29349	14	8.1	1	2	0.894925	0.545	no
gi|646740677|gb|KK962744.1|	58353-35980	14	8.1	10	11	0.165091	0.9083	no
gi|646740677|gb|KK962744.1|	64277-36504	14	8.1	2	1	-0.509204	0.7312	no
gi|646740677|gb|KK962744.1|	66127-36683	14	8.1	45	21	-1	0.37595	no
gi|646740677|gb|KK962744.1|	72570-37306	14	8.1	5	3	-0.689516	0.60995	no
gi|646740677|gb|KK962744.1|	09373-41077	14	8.1	2	0.93157	-0.723145	0.5707	no
gi|646740686|gb|KK962743.1|	15590-11621	12	5.5	2	1	-0.75346	0.6333	no
gi|646740686|gb|KK962743.1|	17012-11877	12	5.5	1	2	0.646873	0.6852	no
gi|646740686|gb|KK962743.1|	53605-15462	12	5.5	0	2	inf	0.00255	no
gi|646740686|gb|KK962743.1|	36545-37906	12	5.5	0.585514	3	2	0.1393	no
gi|646740694|gb|KK962742.1|	01783-10204	20	7.9	15	54	2	0.2648	no
gi|646740694|gb|KK962742.1|	05338-10579	20	7.9	6	8	0.406731	0.74225	no
gi|646740694|gb|KK962742.1|	24601-12484	20	7.9	20	42	1	0.4342	no
gi|646740694|gb|KK962742.1|	25042-12680	20	7.9	1	0.176885	-3	1	no

gi|646740694|gb|KK962742.1|	27093-12740	20	7.9	20	26	0.339078	0.8092	no
gi|646740694|gb|KK962742.1|	33117-13328	20	7.9	238	35	-3	0.1511	no
gi|646740694|gb|KK962742.1|	34835-13678	20	7.9	14	5	-2	0.2344	no
gi|646740694|gb|KK962742.1|	51478-15252	20	7.9	6	5	-0.51387	0.73585	no
gi|646740694|gb|KK962742.1|	11021-21428	20	7.9	19	13	-0.592322	0.59495	no
gi|646740694|gb|KK962742.1|	21609-21886	20	7.9	4	19	2	0.28375	no
gi|646740694|gb|KK962742.1|	20589-22294	20	7.9	0.925622	2	1	0.3917	no
gi|646740694|gb|KK962742.1|	23054-22375	20	7.9	0.624767	3	2	0.1897	no
gi|646740694|gb|KK962742.1|	25172-22593	20	7.9	2	2	-0.624681	0.64225	no
gi|646740694|gb|KK962742.1|	26320-22738	20	7.9	2	0.982911	-0.709057	0.5885	no
gi|646740694|gb|KK962742.1|	33242-23403	20	7.9	1	0.730398	-0.92116	0.5894	no
gi|646740694|gb|KK962742.1|	35278-23598	20	7.9	1	2	0.7458	0.6148	no
gi|646740694|gb|KK962742.1|	37433-23964	20	7.9	0.378976	2	3	0.1232	no
gi|646740694|gb|KK962742.1|	40182-24048	20	7.9	7	12	0.716793	0.643	no
gi|646740694|gb|KK962742.1|	50118-25196	20	7.9	0.058076	1	4	0.11275	no
gi|646740694|gb|KK962742.1|	52113-25463	20	7.9	0.122271	3	4	0.11245	no
gi|646740694|gb|KK962742.1|	54770-25551	20	7.9	0.748889	5	3	0.1737	no
gi|646740694|gb|KK962742.1|	72743-27349	20	7.9	3	4	0.581916	0.6939	no
gi|646740694|gb|KK962742.1|	01604-30230	20	7.9	38	9	-2	0.1975	no
gi|646740694|gb|KK962742.1|	06892-30952	20	7.9	6	8	0.457825	0.7142	no
gi|646740694|gb|KK962742.1|	12093-31416	20	7.9	0.71481	1	0.727613	0.607	no
gi|646740694|gb|KK962742.1|	39075-33992	20	7.9	3	3	0.306603	0.7997	no
gi|646740694|gb|KK962742.1|	52519-52872	20	7.9	2	8	2	0.30685	no
gi|646740694|gb|KK962742.1|	58847-59728	20	7.9	0.738817	4	2	0.21835	no
gi|646740694|gb|KK962742.1|	63393-63807	20	7.9	23	5	-2	0.1617	no
gi|646740694|gb|KK962742.1|	64119-69492	20	7.9	3	3	0.111357	0.9399	no
gi|646740694|gb|KK962742.1|	92817-93987	20	7.9	6	3	-0.897088	0.557	no
gi|646740712|gb|KK962740.1|	09434-21001	14	8.5	3	2	-0.127388	0.9221	no
gi|646740712|gb|KK962740.1|	43279-34371	14	8.5	4	3	-0.093358	0.92295	no
gi|646740712|gb|KK962740.1|	[illegible]9944-10089[illegible]	14	8.5	0.935718	0.569881	-0.715413	1	no

gi|646740720|gb|KK962739.1|	42859-14327	12	13	0	29	inf	0.00045	yes
gi|646740720|gb|KK962739.1|	94820-19521	12	13	0	19	inf	0.00175	no
gi|646740720|gb|KK962739.1|	57468-58180	12	13	0	8	inf	0.0009	yes
gi|646740720|gb|KK962739.1|	76111-76571	12	13	0	20	inf	0.0003	yes
gi|646740756|gb|KK962735.1|	05013-10613	23	10	46	16	-2	0.24745	no
gi|646740756|gb|KK962735.1|	12714-11372	23	10	518	98	-2	0.0509	no
gi|646740756|gb|KK962735.1|	16997-11851	23	10	0.87968	0.845259	-0.0575856	1	no
gi|646740756|gb|KK962735.1|	22956-12359	23	10	44	24	-0.89216	0.5536	no
gi|646740756|gb|KK962735.1|	24582-12607	23	10	0.522671	0.430335	-0.280441	1	no
gi|646740756|gb|KK962735.1|	37179-13755	23	10	21	14	-0.608256	0.71255	no
gi|646740756|gb|KK962735.1|	13745-19417	23	10	9	8	-0.157051	0.8878	no
gi|646740756|gb|KK962735.1|	44837-14691	23	10	224	90	-1	0.2803	no
gi|646740756|gb|KK962735.1|	54747-15533	23	10	3	0.766186	-2	0.2673	no
gi|646740756|gb|KK962735.1|	55482-15613	23	10	2	2	0.111236	0.93055	no
gi|646740756|gb|KK962735.1|	72518-17275	23	10	29	17	-0.765699	0.605	no
gi|646740756|gb|KK962735.1|	77513-17818	23	10	0.878348	2	1	0.4335	no
gi|646740756|gb|KK962735.1|	90827-19987	23	10	17	12	-0.472525	0.6857	no
gi|646740756|gb|KK962735.1|	09750-21323	23	10	13	12	-0.0357641	0.97375	no
gi|646740756|gb|KK962735.1|	23124-22339	23	10	42	17	-1	0.40095	no
gi|646740756|gb|KK962735.1|	38132-23849	23	10	9	25	1	0.3302	no
gi|646740756|gb|KK962735.1|	54132-35563	23	10	2	4	0.977718	0.5318	no
gi|646740756|gb|KK962735.1|	56514-36059	23	10	3	4	0.252242	0.8665	no
gi|646740756|gb|KK962735.1|	61573-36230	23	10	0.971448	2	1	0.36955	no
gi|646740756|gb|KK962735.1|	87938-39580	23	10	14	13	-0.103384	0.92655	no
gi|646740756|gb|KK962735.1|	64785-46660	23	10	4	2	-0.540516	0.719	no
gi|646740756|gb|KK962735.1|	70707-47766	23	10	3	3	0.003445	0.9962	no
gi|646740756|gb|KK962735.1|	77993-48010	23	10	0.69674	1	1	0.4593	no
gi|646740756|gb|KK962735.1|	83293-48353	23	10	16	37	1	0.39625	no
gi|646740756|gb|KK962735.1|	88456-48902	23	10	11	40	2	0.24685	no
gi|646740756|gb|KK962735.1|	91044-49124	23	10	60	50	-0.275847	0.8403	no

gi|646740756|gb|KK962735.1|	09666-50986	23	10	55	68	0.291861	0.83945	no
gi|646740756|gb|KK962735.1|	10563-51086	23	10	22	41	0.859265	0.53685	no
gi|646740756|gb|KK962735.1|	13431-51366	23	10	21	70	2	0.3033	no
gi|646740756|gb|KK962735.1|	15351-51569	23	10	9	22	1	0.41175	no
gi|646740756|gb|KK962735.1|	18419-51903	23	10	3	10	2	0.2314	no
gi|646740756|gb|KK962735.1|	8616-10959	23	10	0.886188	0.964549	0.122241	1	no
gi|646740756|gb|KK962735.1|	)3489-10490(	23	10	2	3	0.365902	0.7405	no
gi|646740768|gb|KK962733.1|	59030-15953	13	6.8	46	34	-0.447208	0.7731	no
gi|646740768|gb|KK962733.1|	59833-16212	13	6.8	7	9	0.313025	0.80625	no
gi|646740782|gb|KK962731.1|	88767-89352	23	12	4	5	0.458652	0.71725	no
gi|646740823|gb|KK962727.1|	33656-23404	20	10	50	6	-3	0.05745	no
gi|646740823|gb|KK962727.1|	35355-23596	20	10	119	79	-0.589318	0.5896	no
gi|646740823|gb|KK962727.1|	42479-24276	20	10	17	10	-0.795415	0.5934	no
gi|646740823|gb|KK962727.1|	45233-24553	20	10	128	57	-1	0.44795	no
gi|646740823|gb|KK962727.1|	45740-24596	20	10	9	36	2	0.32265	no
gi|646740823|gb|KK962727.1|	46798-24699	20	10	211.02	118	-0.842542	0.61345	no
gi|646740823|gb|KK962727.1|	47490-25048	20	10	9	3	-2	0.20355	no
gi|646740823|gb|KK962727.1|	52755-25399	20	10	1	0.539911	-1	0.45565	no
gi|646740823|gb|KK962727.1|	44845-34560	20	10	0	24	inf	0.0002	yes
gi|646740823|gb|KK962727.1|	46546-34708	20	10	0	29	inf	5.00E-05	yes
gi|646740823|gb|KK962727.1|	64381-46777	20	10	3	2	-0.646654	0.6671	no
gi|646740832|gb|KK962726.1|	02225-10290	8	9.5	5	4	-0.345107	0.8003	no
gi|646740832|gb|KK962726.1|	03788-10483	8	9.5	2	0.669003	-2	0.24935	no
gi|646740832|gb|KK962726.1|	17524-11897	8	9.5	2	0.782234	-2	0.2896	no
gi|646740832|gb|KK962726.1|	19934-12077	8	9.5	2	2	-0.408452	0.76285	no
gi|646740832|gb|KK962726.1|	23251-12451	8	9.5	1	1	0.035617	0.95325	no
gi|646740832|gb|KK962726.1|	27779-12837	8	9.5	2	3	0.460995	0.73365	no
gi|646740832|gb|KK962726.1|	28568-12928	8	9.5	7	3	-1	0.3786	no
gi|646740832|gb|KK962726.1|	66170-66747	8	9.5	0	7	inf	0.00435	no
gi|646740861|gb|KK962723.1|	11876-11271	11	6.2	2	3	0.739355	0.5522	no

gi|646740870|gb|KK962722.1|	54146-15460	15	6.4	7	2	-2	0.19495	no
gi|646740870|gb|KK962722.1|	49383-24986	15	6.4	100	0	-inf	5.00E-05	yes
gi|646740870|gb|KK962722.1|	51511-25179	15	6.4	28	0	-inf	0.00195	no
gi|646740870|gb|KK962722.1|	81250-28153	15	6.4	34	2	-4	0.1695	no
gi|646740879|gb|KK962721.1|	38937-14404	24	6.4	10	8	-0.30627	0.7849	no
gi|646740879|gb|KK962721.1|	47009-14939	24	6.4	15	9	-0.813329	0.45325	no
gi|646740879|gb|KK962721.1|	53599-15402	24	6.4	13	9	-0.498888	0.7197	no
gi|646740879|gb|KK962721.1|	58241-15887	24	6.4	11	11	0.0437284	0.9769	no
gi|646740879|gb|KK962721.1|	59572-16015	24	6.4	4	4	0.216566	0.86295	no
gi|646740879|gb|KK962721.1|	64278-16457	24	6.4	12	15	0.399347	0.78235	no
gi|646740879|gb|KK962721.1|	77031-17723	24	6.4	717	1677.68	1	0.3431	no
gi|646740879|gb|KK962721.1|	84251-18449	24	6.4	72	190	1	0.38625	no
gi|646740879|gb|KK962721.1|	89538-19074	24	6.4	0.777385	0.557089	-0.480722	1	no
gi|646740879|gb|KK962721.1|	94920-19573	24	6.4	21	35	0.716922	0.5731	no
gi|646740879|gb|KK962721.1|	96769-19857	24	6.4	13	52	2	0.08665	no
gi|646740879|gb|KK962721.1|	00467-20221	24	6.4	200	151	-0.403778	0.7362	no
gi|646740879|gb|KK962721.1|	04737-20735	24	6.4	2	2	-0.391708	0.7948	no
gi|646740879|gb|KK962721.1|	17256-32042	24	6.4	2	2	0.101004	0.94325	no
gi|646740879|gb|KK962721.1|	20837-32222	24	6.4	2	2	0.273026	0.86445	no
gi|646740879|gb|KK962721.1|	56132-35666	24	6.4	5	2	-2	0.3025	no
gi|646740879|gb|KK962721.1|	67386-36797	24	6.4	3	0.764047	-2	0.2673	no
gi|646740879|gb|KK962721.1|	04874-40540	24	6.4	1	8	3	0.1554	no
gi|646740879|gb|KK962721.1|	58041-45965	24	6.4	2	0.098309	-5	0.1764	no
gi|646740879|gb|KK962721.1|	98725-49889	24	6.4	179.12	183	0.0313054	0.9381	no
gi|646740879|gb|KK962721.1|	06429-60668	24	6.4	19	37	0.94676	0.4939	no
gi|646740879|gb|KK962721.1|	65388-66202	24	6.4	0.330937	3	3	0.25	no
gi|646740879|gb|KK962721.1|	68237-66941	24	6.4	0.904972	0.288003	-2	1	no
gi|646740879|gb|KK962721.1|	72136-67237	24	6.4	66	80	0.283137	0.8342	no
gi|646740879|gb|KK962721.1|	74397-67552	24	6.4	3	6	0.867942	0.5814	no
gi|646740879|gb|KK962721.1|	76886-67711	24	6.4	28	65	1	0.4426	no

gi|646740879|gb|KK962721.1|	77281-68110	24	6.4	5	2	-1	0.48855	no
gi|646740879|gb|KK962721.1|	82916-68684	24	6.4	121	80	-0.590359	0.62295	no
gi|646740879|gb|KK962721.1|	00899-70308	24	6.4	13	53	2	0.08475	no
gi|646740879|gb|KK962721.1|	04486-70508	24	6.4	48	339.72	3	0.0577	no
gi|646740879|gb|KK962721.1|	06239-70749	24	6.4	0.09251	2	4	0.09905	no
gi|646740879|gb|KK962721.1|	70916-71550	24	6.4	2	4	1	0.41625	no
gi|646740879|gb|KK962721.1|	13030-71450	24	6.4	0.455727	9	4	0.0528	no
gi|646740879|gb|KK962721.1|	17677-71799	24	6.4	10	79	3	0.07175	no
gi|646740879|gb|KK962721.1|	56108-75880	24	6.4	86	62	-0.467499	0.69145	no
gi|646740879|gb|KK962721.1|	65840-76639	24	6.4	6	5	-0.412534	0.74285	no
gi|646740879|gb|KK962721.1|	85372-78608	24	6.4	1	2	1	0.4427	no
gi|646740879|gb|KK962721.1|	97128-79734	24	6.4	16	42	1	0.379	no
gi|646740879|gb|KK962721.1|	05510-80583	24	6.4	75	38	-0.985487	0.5239	no
gi|646740879|gb|KK962721.1|	82831-83287	24	6.4	7	3	-1	0.40185	no
gi|646740879|gb|KK962721.1|	31812-83345	24	6.4	1	2	0.764108	0.58465	no
gi|646740879|gb|KK962721.1|	65564-86585	24	6.4	19	4	-2	0.17305	no
gi|646740879|gb|KK962721.1|	73602-87530	24	6.4	0.832311	0.554884	-0.584938	1	no
gi|646740879|gb|KK962721.1|	81357-88292	24	6.4	51	24	-1	0.3447	no
gi|646740879|gb|KK962721.1|	95654-96444	24	6.4	6	7	0.307465	0.84455	no
gi|646740888|gb|KK962720.1|	93648-19498	12	7.7	1	1	0.0757208	0.9507	no
gi|646740917|gb|KK962717.1|	11588-11290	16	7	0.611495	0.878441	0.522604	1	no
gi|646740917|gb|KK962717.1|	26338-12717	16	7	20	11	-0.893971	0.55315	no
gi|646740917|gb|KK962717.1|	30038-13029	16	7	23	6	-2	0.22445	no
gi|646740917|gb|KK962717.1|	33121-13339	16	7	20	13	-0.685788	0.6142	no
gi|646740917|gb|KK962717.1|	37582-13851	16	7	3	3	-0.00851974	0.96475	no
gi|646740917|gb|KK962717.1|	38791-13978	16	7	1	0.358825	-2	0.29945	no
gi|646740937|gb|KK962715.1|	63689-16484	21	8	0.923027	2	0.932494	0.48725	no
gi|646740937|gb|KK962715.1|	77130-17749	21	8	2	10	2	0.202	no
gi|646740937|gb|KK962715.1|	90895-19138	21	8	6	15	1	0.3536	no
gi|646740945|gb|KK962714.1|	06905-40756	22	7.1	0.693147	2	2	0.2936	no

gi|646740945|gb|KK962714.1|	14549-41536	22	7.1	1	2	0.303408	0.83465	no
gi|646740945|gb|KK962714.1|	51627-45233	22	7.1	2	0.576799	-2	0.38325	no
gi|646740955|gb|KK962713.1|	49575-15040	28	13	4	3	-0.218323	0.8741	no
gi|646740955|gb|KK962713.1|	50522-15154	28	13	3	2	-0.595651	0.66215	no
gi|646740955|gb|KK962713.1|	51665-15230	28	13	2	2	0.055373	0.9298	no
gi|646740955|gb|KK962713.1|	68947-17171	28	13	5	5	0.00932336	0.9939	no
gi|646740955|gb|KK962713.1|	00383-20104	28	13	4	2	-0.93862	0.49045	no
gi|646740955|gb|KK962713.1|	06837-20829	28	13	1	2	0.686662	0.624	no
gi|646740955|gb|KK962713.1|	16852-21868	28	13	0.994791	0.931	-0.0956127	1	no
gi|646740955|gb|KK962713.1|	32667-23432	28	13	1	2	0.447392	0.74885	no
gi|646740955|gb|KK962713.1|	69641-27066	28	13	2	3	0.330851	0.8105	no
gi|646740955|gb|KK962713.1|	28065-28959	28	13	2	2	-0.0373035	0.95635	no
gi|646740955|gb|KK962713.1|	24962-32560	28	13	4	0	-inf	0.0012	yes
gi|646740955|gb|KK962713.1|	25740-32765	28	13	19	7	-1	0.28865	no
gi|646740955|gb|KK962713.1|	27742-32985	28	13	16	6	-1	0.2727	no
gi|646740955|gb|KK962713.1|	31587-33288	28	13	5	3	-0.761615	0.62385	no
gi|646740955|gb|KK962713.1|	33020-33398	28	13	19	7	-1	0.38555	no
gi|646740955|gb|KK962713.1|	49176-34993	28	13	1	2	0.490554	0.72995	no
gi|646740955|gb|KK962713.1|	86-297	28	13	172	173	0.00870141	0.98255	no
gi|646740972|gb|KK962711.1|	15465-16864	22	6.3	2	2	0.598807	0.6609	no
gi|646740972|gb|KK962711.1|	60198-16076	22	6.3	3	0.818884	-2	0.2673	no
gi|646740972|gb|KK962711.1|	63824-16572	22	6.3	2	2	0.170661	0.91415	no
gi|646740972|gb|KK962711.1|	68713-16928	22	6.3	3	8	2	0.32745	no
gi|646740972|gb|KK962711.1|	72127-17354	22	6.3	2	3	0.271545	0.85875	no
gi|646740972|gb|KK962711.1|	17328-19732	22	6.3	1	2	0.966553	0.5415	no
gi|646740972|gb|KK962711.1|	82274-18701	22	6.3	1	2	0.595163	0.6975	no
gi|646740972|gb|KK962711.1|	87131-18977	22	6.3	0.696214	0.786894	0.176639	1	no
gi|646740972|gb|KK962711.1|	90002-19160	22	6.3	0.958693	3	2	0.20295	no
gi|646740972|gb|KK962711.1|	94268-19513	22	6.3	1	0.852973	-0.306061	1	no
gi|646740972|gb|KK962711.1|	95273-19786	22	6.3	0.237736	1	2	0.17545	no

gi|646740972|gb|KK962711.1|	97986-19916	22	6.3	0.797486	1	0.518428	0.7091	no
gi|646740972|gb|KK962711.1|	57162-25823	22	6.3	3	3	-0.393035	0.80305	no
gi|646740972|gb|KK962711.1|	58290-25897	22	6.3	3	3	0.0641799	0.9489	no
gi|646740972|gb|KK962711.1|	79904-28085	22	6.3	1	0.379389	-2	0.29945	no
gi|646740972|gb|KK962711.1|	40155-44122	22	6.3	6	9	0.569021	0.71155	no
gi|646740972|gb|KK962711.1|	41454-44264	22	6.3	2	0.993254	-1	0.39885	no
gi|646740981|gb|KK962710.1|	56107-35671	15	7.8	5	4	-0.199494	0.8652	no
gi|646740981|gb|KK962710.1|	71554-76379	15	7.8	16	10	-0.67924	0.548	no
gi|646740996|gb|KK962708.1|	52930-15418	12	6.6	2	0	-inf	0.0012	yes
gi|646741006|gb|KK962707.1|	06006-30640	17	9.3	0.604519	57	7	0.0948	no
gi|646741006|gb|KK962707.1|	21463-32247	17	9.3	2	0	-inf	0.0011	yes
gi|646741015|gb|KK962706.1|	90026-19128	18	12	4	7	0.643682	0.67705	no
gi|646741015|gb|KK962706.1|	62457-26328	18	12	0.161709	2	4	0.10345	no
gi|646741015|gb|KK962706.1|	36446-37204	18	12	2	2	-0.509812	0.7106	no
gi|646741024|gb|KK962705.1|	26902-12717	10	5.6	117	86	-0.448335	0.76075	no
gi|646741024|gb|KK962705.1|	63311-16350	10	5.6	237	355	0.584503	0.71045	no
gi|646741024|gb|KK962705.1|	20006-20126	10	5.6	3620.04	2544.53	-0.508605	0.7318	no
gi|646741024|gb|KK962705.1|	31406-31956	10	5.6	10	28	1	0.34185	no
gi|646741024|gb|KK962705.1|	39307-39533	10	5.6	48	40	-0.264058	0.8348	no
gi|646741024|gb|KK962705.1|	58135-58289	10	5.6	568	487	-0.222202	0.8661	no
gi|646741024|gb|KK962705.1|	69192-69441	10	5.6	80	86	0.109096	0.94725	no
gi|646741034|gb|KK962704.1|	07436-11272	24	10	4	3	-0.305882	0.8037	no
gi|646741034|gb|KK962704.1|	12978-11362	24	10	15	11	-0.48238	0.7488	no
gi|646741034|gb|KK962704.1|	14064-11563	24	10	146	77	-0.934177	0.4284	no
gi|646741034|gb|KK962704.1|	21378-12202	24	10	263	123.21	-1	0.3517	no
gi|646741034|gb|KK962704.1|	12594-13064	24	10	6	3	-1	0.43465	no
gi|646741034|gb|KK962704.1|	15735-17263	24	10	1	2	0.527592	0.71595	no
gi|646741034|gb|KK962704.1|	71336-17287	24	10	98	66	-0.578387	0.621	no
gi|646741034|gb|KK962704.1|	73352-17524	24	10	28	20	-0.504722	0.6403	no
gi|646741034|gb|KK962704.1|	95225-19776	24	10	109	62	-0.818785	0.48915	no

gi|646741034|gb|KK962704.1|	99173-20153	24	10	1	0.701354	-1	0.43635	no
gi|646741034|gb|KK962704.1|	20017-20635	24	10	123	99	-0.31472	0.77565	no
gi|646741034|gb|KK962704.1|	05810-20671	24	10	1	0.605478	-0.910953	0.58965	no
gi|646741034|gb|KK962704.1|	06911-20985	24	10	2.27	2	-0.503714	0.73965	no
gi|646741034|gb|KK962704.1|	11349-21808	24	10	1	1	-0.0162957	0.9917	no
gi|646741034|gb|KK962704.1|	18408-22165	24	10	0.680297	0.675285	-0.0106684	1	no
gi|646741034|gb|KK962704.1|	220-2157	24	10	23	18	-0.364979	0.73665	no
gi|646741034|gb|KK962704.1|	22116-22234	24	10	23	46	1	0.45985	no
gi|646741034|gb|KK962704.1|	30696-23113	24	10	110	65	-0.765956	0.5353	no
gi|646741034|gb|KK962704.1|	43498-24448	24	10	0.76344	0.905441	0.246105	1	no
gi|646741034|gb|KK962704.1|	52035-25341	24	10	4	0.237374	-4	0.1212	no
gi|646741034|gb|KK962704.1|	26101-29257	24	10	1	1	0.278505	0.861	no
gi|646741034|gb|KK962704.1|	66856-26777	24	10	4	0.19916	-4	0.1812	no
gi|646741034|gb|KK962704.1|	69428-27006	24	10	6	0.338511	-4	0.18795	no
gi|646741034|gb|KK962704.1|	2933-3967	24	10	17	12	-0.467827	0.7538	no
gi|646741034|gb|KK962704.1|	54849-35574	24	10	3	2	-0.496285	0.7247	no
gi|646741034|gb|KK962704.1|	60681-36151	24	10	8	7	-0.210028	0.89255	no
gi|646741034|gb|KK962704.1|	36994-37692	24	10	6	6	-0.00359591	0.9807	no
gi|646741034|gb|KK962704.1|	45839-48732	24	10	2	2	-0.0810413	0.9576	no
gi|646741034|gb|KK962704.1|	5189-5920	24	10	2	1	-0.361312	0.812	no
gi|646741034|gb|KK962704.1|	68854-70921	24	10	1	2	0.136419	0.93125	no
gi|646741034|gb|KK962704.1|	79649-82242	24	10	4	2	-1	0.43925	no
gi|646741034|gb|KK962704.1|	84303-84457	24	10	236.51	313	0.403402	0.79515	no
gi|646741034|gb|KK962704.1|	85206-85603	24	10	6	5	-0.372548	0.78525	no
gi|646741034|gb|KK962704.1|	87261-88005	24	10	1	2	1	0.4512	no
gi|646741034|gb|KK962704.1|	8935-11474	24	10	0.729132	0.353032	-1	1	no
gi|646741034|gb|KK962704.1|	91982-92201	24	10	82	31	-1	0.2942	no
gi|646741038|gb|KK962703.1|	15328-15762	14	9.4	4	13	2	0.29935	no
gi|646741038|gb|KK962703.1|	42629-34310	14	9.4	0	7	inf	0.001	yes
gi|646741048|gb|KK962702.1|	56255-57117	17	6.4	0	280	inf	5.00E-05	yes

gi|646741048|gb|KK962702.1|	95351-95716	17	6.4	6	6	-0.0832862	0.92955	no
gi|646741057|gb|KK962701.1|	55169-15559	21	8.8	8	5	-0.615854	0.63065	no
gi|646741057|gb|KK962701.1|	69593-17038	21	8.8	36.62	42	0.210702	0.83685	no
gi|646741057|gb|KK962701.1|	85769-18929	21	8.8	18	18	-0.000874727	0.99835	no
gi|646741057|gb|KK962701.1|	89765-18994	21	8.8	94	47	-1	0.47705	no
gi|646741057|gb|KK962701.1|	95397-19887	21	8.8	10	9	-0.236145	0.8255	no
gi|646741057|gb|KK962701.1|	03167-20370	21	8.8	0.972176	4	2	0.26115	no
gi|646741057|gb|KK962701.1|	05111-20613	21	8.8	2	0.85415	-0.866879	0.5465	no
gi|646741057|gb|KK962701.1|	27671-22821	21	8.8	2	2	0.182349	0.8995	no
gi|646741057|gb|KK962701.1|	38025-23851	21	8.8	52	31	-0.753698	0.6139	no
gi|646741057|gb|KK962701.1|	45558-24720	21	8.8	63	34	-0.892056	0.43475	no
gi|646741057|gb|KK962701.1|	62328-26287	21	8.8	0.949223	4	2	0.19065	no
gi|646741057|gb|KK962701.1|	74557-27588	21	8.8	7	5	-0.495443	0.7429	no
gi|646741057|gb|KK962701.1|	80963-28227	21	8.8	0.961829	2	0.648107	0.61505	no
gi|646741057|gb|KK962701.1|	90531-29436	21	8.8	1	0.94334	-0.336101	0.82935	no
gi|646741057|gb|KK962701.1|	97482-30103	21	8.8	0.645038	0.694583	0.106763	1	no
gi|646741057|gb|KK962701.1|	06668-30757	21	8.8	0.569236	2	2	0.26425	no
gi|646741057|gb|KK962701.1|	22689-32344	21	8.8	2	0.782269	-2	0.35195	no
gi|646741057|gb|KK962701.1|	31672-33200	21	8.8	12	5	-1	0.39165	no
gi|646741057|gb|KK962701.1|	32939-33330	21	8.8	82	35	-1	0.4273	no
gi|646741065|gb|KK962700.1|	85878-28682	18	7.8	0.934368	0.758779	-0.300311	1	no
gi|646741065|gb|KK962700.1|	87383-28829	18	7.8	0.97959	1	0.282485	0.8455	no
gi|646741083|gb|KK962698.1|	28109-23345	18	8	5	7	0.529456	0.62765	no
gi|646741093|gb|KK962697.1|	67990-68761	16	13	0	3	inf	0.001	yes
gi|646741153|gb|KK962692.1|	54485-55773	14	4.9	2	0	-inf	0.00595	no
gi|646741164|gb|KK962691.1|	07675-10947	18	8.4	0.841048	0.173715	-2	1	no
gi|646741164|gb|KK962691.1|	10743-11160	18	8.4	0.917346	1	0.237511	1	no
gi|646741164|gb|KK962691.1|	21140-12254	18	8.4	6	8	0.48605	0.7369	no
gi|646741164|gb|KK962691.1|	23102-12639	18	8.4	9	5	-0.784537	0.5417	no
gi|646741164|gb|KK962691.1|	19865-20574	18	8.4	1	4	2	0.21275	no

gi|646741164|gb|KK962691.1|	66457-26660	18	8.4	7042.9	6824.45	-0.0454573	0.9752	no
gi|646741164|gb|KK962691.1|	87785-38885	18	8.4	0.339264	2	2	0.1611	no
gi|646741164|gb|KK962691.1|	18466-51929	18	8.4	2	3	0.945529	0.53955	no
gi|646741164|gb|KK962691.1|	26670-52703	18	8.4	6	3	-0.896508	0.5997	no
gi|646741164|gb|KK962691.1|	53468-55431	18	8.4	14	10	-0.421544	0.77745	no
gi|646741177|gb|KK962690.1|	46092-24695	15	6.2	0.920387	1	0.500376	0.73295	no
gi|646741177|gb|KK962690.1|	47153-24866	15	6.2	1	2	0.816864	0.55455	no
gi|646741177|gb|KK962690.1|	48767-25039	15	6.2	0.942647	0.485914	-0.956015	1	no
gi|646741177|gb|KK962690.1|	50502-25377	15	6.2	1	2	0.504503	0.7444	no
gi|646741185|gb|KK962689.1|	32045-23359	20	7.9	0.498916	1	1	0.39575	no
gi|646741185|gb|KK962689.1|	35460-23614	20	7.9	7	1	-3	0.15385	no
gi|646741185|gb|KK962689.1|	66123-26911	20	7.9	0.372109	0.885869	1	1	no
gi|646741185|gb|KK962689.1|	69845-27110	20	7.9	0.644988	0.925513	0.520982	1	no
gi|646741185|gb|KK962689.1|	73676-27471	20	7.9	2	2	-0.22497	0.86175	no
gi|646741185|gb|KK962689.1|	90777-29130	20	7.9	37	20	-0.915424	0.54845	no
gi|646741207|gb|KK962687.1|	30141-13224	17	7.6	0.60076	3	2	0.1575	no
gi|646741207|gb|KK962687.1|	33462-13526	17	7.6	0.238442	0.948149	2	1	no
gi|646741207|gb|KK962687.1|	43460-14466	17	7.6	0.778202	0.975907	0.326598	1	no
gi|646741207|gb|KK962687.1|	45935-14665	17	7.6	0.792104	4	2	0.19115	no
gi|646741207|gb|KK962687.1|	46826-14848	17	7.6	0.264515	1	2	0.21305	no
gi|646741207|gb|KK962687.1|	64706-16516	17	7.6	2	10	2	0.2084	no
gi|646741207|gb|KK962687.1|	67768-16857	17	7.6	9	24	1	0.34985	no
gi|646741207|gb|KK962687.1|	69021-16972	17	7.6	3	7	1	0.34785	no
gi|646741207|gb|KK962687.1|	72590-17305	17	7.6	6	8	0.413494	0.74225	no
gi|646741207|gb|KK962687.1|	73649-17520	17	7.6	0.212838	1	3	0.1521	no
gi|646741207|gb|KK962687.1|	88771-19234	17	7.6	0.808896	2	1	0.47145	no
gi|646741207|gb|KK962687.1|	93161-19458	17	7.6	3	3	0.0399441	0.9785	no
gi|646741207|gb|KK962687.1|	01447-20176	17	7.6	5	12	1	0.4286	no
gi|646741207|gb|KK962687.1|	02725-20293	17	7.6	68	117	0.785976	0.6018	no
gi|646741207|gb|KK962687.1|	41370-24182	17	7.6	21	17	-0.317934	0.83785	no

gi|646741207|gb|KK962687.1|	48346-25044	17	7.6	14	5	-1	0.2795	no
gi|646741207|gb|KK962687.1|	50562-25156	17	7.6	1	0.175696	-3	0.1932	no
gi|646741207|gb|KK962687.1|	51680-25198	17	7.6	17	5	-2	0.2741	no
gi|646741207|gb|KK962687.1|	52204-25480	17	7.6	0.868352	0.688162	-0.335532	1	no
gi|646741207|gb|KK962687.1|	66326-26747	17	7.6	11	5	-1	0.46545	no
gi|646741207|gb|KK962687.1|	66428-68968	17	7.6	23	12	-0.886718	0.42815	no
gi|646741207|gb|KK962687.1|	70432-72199	17	7.6	6	3	-0.917251	0.54615	no
gi|646741233|gb|KK962685.1|	09204-11079	18	8.2	16	9	-0.777902	0.53615	no
gi|646741233|gb|KK962685.1|	45949-14667	18	8.2	1	2	0.485993	0.73295	no
gi|646741233|gb|KK962685.1|	59127-16054	18	8.2	3	2	-0.697254	0.6699	no
gi|646741233|gb|KK962685.1|	84622-18587	18	8.2	0.09251	62	9	0.09445	no
gi|646741233|gb|KK962685.1|	13519-21390	18	8.2	3	7	1	0.39565	no
gi|646741233|gb|KK962685.1|	57186-25761	18	8.2	48	22	-1	0.46615	no
gi|646741233|gb|KK962685.1|	66319-26669	18	8.2	3	6	0.924563	0.591	no
gi|646741233|gb|KK962685.1|	68212-26971	18	8.2	0.962134	1	0.286129	0.8199	no
gi|646741233|gb|KK962685.1|	86321-28795	18	8.2	10	5	-0.982454	0.5139	no
gi|646741233|gb|KK962685.1|	47994-49039	18	8.2	8	0.33409	-5	0.111	no
gi|646741233|gb|KK962685.1|	77443-77798	18	8.2	0	11	inf	0.0014	yes
gi|646741233|gb|KK962685.1|	79301-79819	18	8.2	4	3	-0.563015	0.69125	no
gi|646741233|gb|KK962685.1|	97242-98174	18	8.2	2	3	0.76902	0.5924	no
gi|646741233|gb|KK962685.1|	99195-99651	18	8.2	5	5	0.085388	0.94115	no
gi|646741247|gb|KK962684.1|	45814-14619	22	19	0	34	inf	0.00045	yes
gi|646741247|gb|KK962684.1|	39182-24048	22	19	0	2	inf	0.0008	yes
gi|646741265|gb|KK962682.1|	44620-14482	17	13	37	42	0.185232	0.89465	no
gi|646741265|gb|KK962682.1|	22436-22800	17	13	0	84	inf	5.00E-05	yes
gi|646741265|gb|KK962682.1|	89680-90395	17	13	0	7	inf	0.00155	yes
gi|646741289|gb|KK962680.1|	11219-11747	14	6	4	11	1	0.3561	no
gi|646741289|gb|KK962680.1|	29137-29566	14	6	8	10	0.197327	0.87065	no
gi|646741289|gb|KK962680.1|	31291-32309	14	6	0.242476	2	3	0.24675	no
gi|646741289|gb|KK962680.1|	44029-44626	14	6	2	5	2	0.2949	no

gi|646741289|gb|KK962680.1|	49211-50121	14	6	0.706115	1	0.989875	0.5626	no
gi|646741289|gb|KK962680.1|	59252-60395	14	6	0.416701	1	2	0.36595	no
gi|646741289|gb|KK962680.1|	61284-61710	14	6	19	23	0.305365	0.845	no
gi|646741289|gb|KK962680.1|	7974-8596	14	6	0.503308	3	3	0.26125	no
gi|646741289|gb|KK962680.1|	82912-83216	14	6	0	19	inf	0.001	yes
gi|646741289|gb|KK962680.1|	86415-87261	14	6	0	6	inf	0.0009	yes
gi|646741289|gb|KK962680.1|	9916-10351	14	6	4	22	2	0.13715	no
gi|646741308|gb|KK962678.1|	28538-22892	15	6.8	5	9	0.672032	0.6285	no
gi|646741318|gb|KK962677.1|	81361-18172	31	19	2	10	3	0.26545	no
gi|646741342|gb|KK962675.1|	05915-10724	18	7.3	4	0.864058	-2	0.15445	no
gi|646741342|gb|KK962675.1|	34327-13510	18	7.3	14	8	-0.784984	0.6082	no
gi|646741342|gb|KK962675.1|	45887-14606	18	7.3	132	140	0.0775642	0.93885	no
gi|646741342|gb|KK962675.1|	52731-15295	18	7.3	286	132	-1	0.479	no
gi|646741342|gb|KK962675.1|	56900-15746	18	7.3	4	5	0.132269	0.9156	no
gi|646741342|gb|KK962675.1|	77196-17744	18	7.3	21	29	0.458565	0.74985	no
gi|646741342|gb|KK962675.1|	84264-18812	18	7.3	10	7	-0.522162	0.6305	no
gi|646741342|gb|KK962675.1|	97765-19925	18	7.3	47	33	-0.496211	0.6613	no
gi|646741342|gb|KK962675.1|	99913-20115	18	7.3	8	6	-0.362646	0.8101	no
gi|646741342|gb|KK962675.1|	11668-21182	18	7.3	406	122	-2	0.2608	no
gi|646741342|gb|KK962675.1|	16046-21928	18	7.3	1	2	0.579322	0.71045	no
gi|646741342|gb|KK962675.1|	28967-22933	18	7.3	69	76	0.126447	0.9325	no
gi|646741342|gb|KK962675.1|	43514-34389	18	7.3	20	0	-inf	0.0031	no
gi|646741342|gb|KK962675.1|	82575-84399	18	7.3	10	3	-2	0.24625	no
gi|646741355|gb|KK962674.1|	52624-15574	21	10	12	9	-0.428541	0.6935	no
gi|646741355|gb|KK962674.1|	58214-15990	21	10	8	5	-0.552739	0.717	no
gi|646741355|gb|KK962674.1|	15977-16600	21	10	18	15	-0.235172	0.87385	no
gi|646741355|gb|KK962674.1|	34625-23496	21	10	10	5	-1	0.46035	no
gi|646741365|gb|KK962673.1|	06943-10757	17	8.8	85	37	-1	0.3601	no
gi|646741365|gb|KK962673.1|	16851-17410	17	8.8	32	18	-0.850449	0.576	no
gi|646741365|gb|KK962673.1|	19361-19571	17	8.8	76	49	-0.631822	0.6364	no

gi|646741365|gb|KK962673.1|	58665-63223	17	8.8	19	20	0.111247	0.91845	no
gi|646741365|gb|KK962673.1|	65541-67240	17	8.8	4	1	-2	0.206	no
gi|646741365|gb|KK962673.1|	89009-89612	17	8.8	2	1	-0.706696	0.64185	no
gi|646741376|gb|KK962672.1|	97069-19735	12	7.2	18	27	0.552508	0.66865	no
gi|646741376|gb|KK962672.1|	06682-20749	12	7.2	2	3	0.603167	0.6268	no
gi|646741387|gb|KK962671.1|	87139-19020	15	6	6	0.04798	-7	0.16695	no
gi|646741398|gb|KK962670.1|	87524-88059	22	11	4	5	0.443305	0.7254	no
gi|646741410|gb|KK962669.1|	02779-10333	21	9.3	27	18	-0.584617	0.6956	no
gi|646741410|gb|KK962669.1|	08243-11842	21	9.3	3	3	-0.137408	0.897	no
gi|646741410|gb|KK962669.1|	21934-12328	21	9.3	1	1	-0.00680644	1	no
gi|646741410|gb|KK962669.1|	25096-12681	21	9.3	2	2	-0.206114	0.8965	no
gi|646741410|gb|KK962669.1|	33281-13776	21	9.3	1	1	-0.0169012	0.98895	no
gi|646741410|gb|KK962669.1|	44910-14588	21	9.3	3	4	0.640028	0.64085	no
gi|646741410|gb|KK962669.1|	15779-16835	21	9.3	1	0.824066	-0.334903	1	no
gi|646741410|gb|KK962669.1|	59079-15984	21	9.3	2	1	-0.509446	0.7312	no
gi|646741410|gb|KK962669.1|	59946-16062	21	9.3	2	0.905458	-0.743175	0.6333	no
gi|646741410|gb|KK962669.1|	76422-18022	21	9.3	5	2	-1	0.42185	no
gi|646741410|gb|KK962669.1|	83198-18430	21	9.3	2	1	-0.584241	0.67605	no
gi|646741410|gb|KK962669.1|	92577-19524	21	9.3	2	1	-0.926317	0.55445	no
gi|646741410|gb|KK962669.1|	06789-21108	21	9.3	1	0.701508	-0.554903	1	no
gi|646741410|gb|KK962669.1|	21287-22110	21	9.3	0.488542	2	2	0.25505	no
gi|646741410|gb|KK962669.1|	13939-21448	21	9.3	2	1	-0.775997	0.633	no
gi|646741410|gb|KK962669.1|	23965-27445	21	9.3	25	15	-0.783369	0.49385	no
gi|646741410|gb|KK962669.1|	27823-29201	21	9.3	2	0	-inf	0.0047	no
gi|646741410|gb|KK962669.1|	90541-29170	21	9.3	1	0.878931	-0.597944	0.64385	no
gi|646741410|gb|KK962669.1|	01955-30278	21	9.3	5	7	0.407687	0.79725	no
gi|646741410|gb|KK962669.1|	06057-30688	21	9.3	2	0	-inf	0.0035	no
gi|646741410|gb|KK962669.1|	31183-34960	21	9.3	78	29	-1	0.2384	no
gi|646741410|gb|KK962669.1|	15503-31642	21	9.3	2	0.39427	-2	0.2551	no
gi|646741410|gb|KK962669.1|	17464-31862	21	9.3	1	0	-inf	0.00225	no

gi|646741410|gb|KK962669.1|	35504-37144	21	9.3	26	57	1	0.32415	no
gi|646741410|gb|KK962669.1|	37429-38949	21	9.3	29	40	0.459797	0.6758	no
gi|646741410|gb|KK962669.1|	62874-63785	21	9.3	1	6	2	0.17635	no
gi|646741410|gb|KK962669.1|	66510-68362	21	9.3	0.347586	2	2	0.17565	no
gi|646741410|gb|KK962669.1|	76169-77632	21	9.3	2	0.441178	-2	0.2048	no
gi|646741410|gb|KK962669.1|	79363-81257	21	9.3	1	6	2	0.19805	no
gi|646741410|gb|KK962669.1|	93645-94609	21	9.3	1	4	2	0.1935	no
gi|646741422|gb|KK962668.1|	00652-10424	19	11	0	2	inf	0.00025	yes
gi|646741422|gb|KK962668.1|	57068-15771	19	11	2	1	-0.848314	0.56515	no
gi|646741422|gb|KK962668.1|	14030-21477	19	11	2	2	0.166361	0.91025	no
gi|646741422|gb|KK962668.1|	21476-22230	19	11	3	2	-0.865156	0.50275	no
gi|646741422|gb|KK962668.1|	26681-22772	19	11	1	3	1	0.3929	no
gi|646741422|gb|KK962668.1|	29702-23200	19	11	20	14	-0.466338	0.6736	no
gi|646741422|gb|KK962668.1|	32584-23571	19	11	12	6	-0.916982	0.4755	no
gi|646741422|gb|KK962668.1|	37911-23856	19	11	2	2	-0.0122012	0.92345	no
gi|646741422|gb|KK962668.1|	43731-24562	19	11	13	7	-0.861565	0.4984	no
gi|646741422|gb|KK962668.1|	61370-26391	19	11	32	16	-1	0.3643	no
gi|646741422|gb|KK962668.1|	71080-27204	19	11	0.782818	0.927724	0.24502	1	no
gi|646741422|gb|KK962668.1|	78510-28398	19	11	4	4	-0.158577	0.8779	no
gi|646741422|gb|KK962668.1|	84192-28922	19	11	5	4	-0.507787	0.68635	no
gi|646741422|gb|KK962668.1|	89424-29590	19	11	0.817081	1	0.493967	0.73725	no
gi|646741422|gb|KK962668.1|	96784-29783	19	11	4	2	-0.874174	0.52245	no
gi|646741422|gb|KK962668.1|	01718-30380	19	11	15	10	-0.688816	0.5847	no
gi|646741422|gb|KK962668.1|	3589-4175	19	11	3	3	0.458918	0.7419	no
gi|646741422|gb|KK962668.1|	44441-44499	19	11	0	34	inf	0.00025	yes
gi|646741422|gb|KK962668.1|	45645-44589	19	11	0	37	inf	0.001	yes
gi|646741422|gb|KK962668.1|	69277-69749	19	11	0.420827	9	4	0.11205	no
gi|646741444|gb|KK962666.1|	86885-28736	14	6.5	2	2	-0.166235	0.8987	no
gi|646741444|gb|KK962666.1|	87460-88093	14	6.5	4	4	0.263542	0.82625	no
gi|646741444|gb|KK962666.1|	99823-10031[illegible]	14	6.5	4	1	-2	0.2163	no

gi|646741488|gb|KK962662.1|	56938-25757	13	5	6	0	-inf	0.0047	no
gi|646741506|gb|KK962660.1|	36815-38085	15	5.7	2	8	2	0.14275	no
gi|646741506|gb|KK962660.1|	61649-62422	15	5.7	0.535675	2	2	0.2903	no
gi|646741506|gb|KK962660.1|	65399-68082	15	5.7	0.457261	2	2	0.19325	no
gi|646741540|gb|KK962657.1|	13000-12062	19	7	3	4	0.264406	0.80575	no
gi|646741540|gb|KK962657.1|	24294-12478	19	7	5	2	-1	0.38755	no
gi|646741540|gb|KK962657.1|	33966-13443	19	7	70	15	-2	0.1662	no
gi|646741540|gb|KK962657.1|	36742-13708	19	7	31	11	-1	0.3357	no
gi|646741540|gb|KK962657.1|	10010-21169	19	7	2	5	1	0.43155	no
gi|646741540|gb|KK962657.1|	82666-38312	19	7	4	4	-0.178821	0.90115	no
gi|646741540|gb|KK962657.1|	94763-39581	19	7	1	0.988879	-0.223872	0.86285	no
gi|646741540|gb|KK962657.1|	03974-40431	19	7	27	3	-3	0.13865	no
gi|646741540|gb|KK962657.1|	83872-85557	19	7	0.905736	3	2	0.2564	no
gi|646741540|gb|KK962657.1|	86898-88227	19	7	0.689093	0.247549	-1	1	no
gi|646741540|gb|KK962657.1|	95024-96444	19	7	7	7	0.17263	0.9062	no
gi|646741553|gb|KK962656.1|	12726-11295	17	7.9	85	0	-inf	0.0008	yes
gi|646741553|gb|KK962656.1|	59107-26182	17	7.9	8	3	-1	0.3195	no
gi|646741553|gb|KK962656.1|	61932-26923	17	7.9	2	2	0.596095	0.6969	no
gi|646741553|gb|KK962656.1|	31283-33141	17	7.9	1507.89	543	-1	0.3319	no
gi|646741553|gb|KK962656.1|	43135-44547	17	7.9	13	12	-0.0350983	0.973	no
gi|646741553|gb|KK962656.1|	45581-44702	17	7.9	0.390347	0.899164	1	1	no
gi|646741553|gb|KK962656.1|	47250-44834	17	7.9	0.775503	2	2	0.284	no
gi|646741553|gb|KK962656.1|	53224-45411	17	7.9	35	31	-0.177422	0.86655	no
gi|646741553|gb|KK962656.1|	53384-55376	17	7.9	9	0	-inf	0.0035	no
gi|646741553|gb|KK962656.1|	53707-65387	17	7.9	208	0	-inf	0.00655	no
gi|646741582|gb|KK962654.1|	69646-17055	16	8.4	6	3	-0.817264	0.6096	no
gi|646741582|gb|KK962654.1|	70642-17116	16	8.4	5	1	-2	0.281	no
gi|646741582|gb|KK962654.1|	20999-22168	16	8.4	5	0.897507	-3	0.1849	no
gi|646741582|gb|KK962654.1|	58595-25896	16	8.4	26	17	-0.658235	0.68145	no
gi|646741594|gb|KK962653.1|	59679-26434	17	6.6	2	2	-0.454704	0.75575	no

gi|646741594|gb|KK962653.1|	73155-27443	17	6.6	8	6	-0.543848	0.7184	no
gi|646741594|gb|KK962653.1|	85090-28826	17	6.6	11	9	-0.365819	0.73745	no
gi|646741606|gb|KK962652.1|	26356-32667	12	6.9	5	10	1	0.4813	no
gi|646741606|gb|KK962652.1|	27995-32893	12	6.9	2	2	0.0212259	0.95325	no
gi|646741606|gb|KK962652.1|	34038-33502	12	6.9	3	4	0.398631	0.8001	no
gi|646741621|gb|KK962651.1|	02304-10323	18	7.2	12	6	-1	0.51025	no
gi|646741621|gb|KK962651.1|	03960-10489	18	7.2	23	17	-0.468427	0.7606	no
gi|646741621|gb|KK962651.1|	16314-11884	18	7.2	21	16	-0.363779	0.74475	no
gi|646741621|gb|KK962651.1|	19172-11956	18	7.2	7	4	-0.773086	0.59415	no
gi|646741621|gb|KK962651.1|	25013-12672	18	7.2	15	5	-2	0.23445	no
gi|646741621|gb|KK962651.1|	28056-13026	18	7.2	18	11	-0.676215	0.534	no
gi|646741621|gb|KK962651.1|	34668-13612	18	7.2	19	9	-1	0.4122	no
gi|646741621|gb|KK962651.1|	40866-15241	18	7.2	5	5	0.0938768	0.93325	no
gi|646741621|gb|KK962651.1|	54278-15558	18	7.2	2	1	-1	0.45235	no
gi|646741621|gb|KK962651.1|	56344-15788	18	7.2	15	21	0.513539	0.6366	no
gi|646741621|gb|KK962651.1|	58498-15868	18	7.2	214	58	-2	0.1743	no
gi|646741621|gb|KK962651.1|	74424-17493	18	7.2	12	4	-2	0.27475	no
gi|646741621|gb|KK962651.1|	95272-19616	18	7.2	4	3	-0.703262	0.60455	no
gi|646741621|gb|KK962651.1|	01693-20249	18	7.2	33	10	-2	0.26755	no
gi|646741621|gb|KK962651.1|	03887-20751	18	7.2	1	4	2	0.2338	no
gi|646741621|gb|KK962651.1|	07782-20962	18	7.2	33	6	-2	0.0965	no
gi|646741621|gb|KK962651.1|	24172-22494	18	7.2	1	4	2	0.2665	no
gi|646741621|gb|KK962651.1|	25176-22658	18	7.2	2	4	0.805058	0.61225	no
gi|646741621|gb|KK962651.1|	19827-32064	18	7.2	39	21	-0.856506	0.49715	no
gi|646741621|gb|KK962651.1|	22583-32305	18	7.2	3	5	0.830138	0.57025	no
gi|646741621|gb|KK962651.1|	26031-32653	18	7.2	4	5	0.294191	0.8218	no
gi|646741621|gb|KK962651.1|	26923-32823	18	7.2	0.613232	2	1	0.30095	no
gi|646741621|gb|KK962651.1|	29513-32974	18	7.2	29	127	2	0.19535	no
gi|646741621|gb|KK962651.1|	30954-33149	18	7.2	5	18	2	0.18865	no
gi|646741630|gb|KK962650.1|	77699-27853	12	8.2	2	3	0.398719	0.7465	no

gi|646741630|gb|KK962650.1|	41080-43331	12	8.2	1	2	0.910974	0.56465	no
gi|646741641|gb|KK962649.1|	14833-16685	19	7.3	1	0.502801	-1	1	no
gi|646741641|gb|KK962649.1|	27155-29609	19	7.3	1	0.18327	-3	0.1624	no
gi|646741641|gb|KK962649.1|	31163-32458	19	7.3	6	2	-2	0.19065	no
gi|646741641|gb|KK962649.1|	33634-35287	19	7.3	2	0.477704	-2	0.3102	no
gi|646741641|gb|KK962649.1|	43129-34408	19	7.3	1	1	0.0482492	0.94695	no
gi|646741656|gb|KK962648.1|	0-777	24	9.7	39	40	0.010903	0.9906	no
gi|646741656|gb|KK962648.1|	42209-14247	24	9.7	13	27	1	0.4437	no
gi|646741656|gb|KK962648.1|	43964-14428	24	9.7	13	14	0.126025	0.92305	no
gi|646741656|gb|KK962648.1|	44809-14531	24	9.7	4	2	-0.706234	0.628	no
gi|646741656|gb|KK962648.1|	55414-15639	24	9.7	2	0.180847	-4	0.17455	no
gi|646741656|gb|KK962648.1|	57513-15810	24	9.7	3	2	-0.540555	0.71825	no
gi|646741656|gb|KK962648.1|	58225-16045	24	9.7	22	6	-2	0.17605	no
gi|646741656|gb|KK962648.1|	67063-16865	24	9.7	17	9	-0.923151	0.46165	no
gi|646741656|gb|KK962648.1|	74774-17547	24	9.7	3	6	0.941104	0.50735	no
gi|646741656|gb|KK962648.1|	78583-17919	24	9.7	6	8	0.464189	0.7372	no
gi|646741656|gb|KK962648.1|	79809-18004	24	9.7	41	33	-0.343405	0.7952	no
gi|646741656|gb|KK962648.1|	82023-18228	24	9.7	21	26	0.297069	0.8262	no
gi|646741656|gb|KK962648.1|	06703-20860	24	9.7	2	2	0.118159	0.9428	no
gi|646741656|gb|KK962648.1|	08892-21430	24	9.7	2	3	0.27071	0.85825	no
gi|646741656|gb|KK962648.1|	14789-21641	24	9.7	21	19	-0.135553	0.8995	no
gi|646741656|gb|KK962648.1|	2154-4661	24	9.7	4	3	-0.409148	0.78495	no
gi|646741656|gb|KK962648.1|	21604-22222	24	9.7	2	3	0.659153	0.6445	no
gi|646741656|gb|KK962648.1|	23529-22431	24	9.7	9	5	-0.902929	0.56375	no
gi|646741656|gb|KK962648.1|	24530-22523	24	9.7	4	3	-0.305272	0.79815	no
gi|646741656|gb|KK962648.1|	29580-23017	24	9.7	1	2	0.460484	0.7722	no
gi|646741656|gb|KK962648.1|	31076-23232	24	9.7	1	1	-0.216457	0.87515	no
gi|646741656|gb|KK962648.1|	35719-23633	24	9.7	3	2	-0.271142	0.83675	no
gi|646741656|gb|KK962648.1|	38895-24073	24	9.7	48	29	-0.70707	0.5421	no
gi|646741656|gb|KK962648.1|	64320-26608	24	9.7	0	23	inf	5.00E-05	yes

gi|646741656|gb|KK962648.1|	70956-27258	24	9.7	0.201409	10	6	0.10595	no
gi|646741656|gb|KK962648.1|	74893-27542	24	9.7	0	6	inf	0.00035	yes
gi|646741656|gb|KK962648.1|	81232-28387	24	9.7	30	41	0.466474	0.67915	no
gi|646741656|gb|KK962648.1|	83994-28610	24	9.7	0.449597	0.579196	0.36542	1	no
gi|646741656|gb|KK962648.1|	86866-28771	24	9.7	1	2	1	0.44735	no
gi|646741656|gb|KK962648.1|	89491-29015	24	9.7	2	0.93442	-0.93826	0.589	no
gi|646741656|gb|KK962648.1|	95775-29778	24	9.7	0.315936	0.686384	1	1	no
gi|646741656|gb|KK962648.1|	57743-35851	24	9.7	14.38	31	1	0.46545	no
gi|646741656|gb|KK962648.1|	90446-39116	24	9.7	5	5	0.0113543	0.9754	no
gi|646741656|gb|KK962648.1|	87853-58813	24	9.7	15	31	1	0.4918	no
gi|646741656|gb|KK962648.1|	90021-59155	24	9.7	0	40	inf	5.00E-05	yes
gi|646741656|gb|KK962648.1|	11290-61375	24	9.7	7	0	-inf	0.00015	yes
gi|646741656|gb|KK962648.1|	14493-61642	24	9.7	4	0.079668	-6	0.16885	no
gi|646741656|gb|KK962648.1|	28205-62915	24	9.7	9	0	-inf	0.00035	yes
gi|646741656|gb|KK962648.1|	29677-63519	24	9.7	4	0.02568	-7	0.1665	no
gi|646741656|gb|KK962648.1|	35325-63634	24	9.7	8	0.17213	-6	0.1683	no
gi|646741656|gb|KK962648.1|	43604-64475	24	9.7	1	0.296822	-2	0.1855	no
gi|646741656|gb|KK962648.1|	44858-64848	24	9.7	22	1	-4	0.01	no
gi|646741656|gb|KK962648.1|	61140-66966	24	9.7	3	1	-1	0.42235	no
gi|646741656|gb|KK962648.1|	90985-80093	24	9.7	3	3	0.171218	0.8729	no
gi|646741656|gb|KK962648.1|	47420-85280	24	9.7	19	13	-0.55618	0.6278	no
gi|646741656|gb|KK962648.1|	59039-85968	24	9.7	1	2	0.73626	0.64505	no
gi|646741656|gb|KK962648.1|	59998-86170	24	9.7	4	7	0.652635	0.6607	no
gi|646741656|gb|KK962648.1|	63861-86565	24	9.7	0.602705	0.871362	0.53182	1	no
gi|646741656|gb|KK962648.1|	66057-86669	24	9.7	16	15	-0.156191	0.919	no
gi|646741656|gb|KK962648.1|	79161-88758	24	9.7	66	52	-0.341027	0.7775	no
gi|646741656|gb|KK962648.1|	02508-90345	24	9.7	7	8	0.178096	0.90625	no
gi|646741656|gb|KK962648.1|	06484-90822	24	9.7	0.249871	0.631863	1	1	no
gi|646741656|gb|KK962648.1|	26971-92787	24	9.7	1	2	0.919195	0.48975	no
gi|646741656|gb|KK962648.1|	38600-93923	24	9.7	4	1	-2	0.2449	no

gi|646741656|gb|KK962648.1|	49069-94985	24	9.7	2	3	0.36813	0.76905	no
gi|646741656|gb|KK962648.1|	59210-95969	24	9.7	1	4	2	0.2626	no
gi|646741656|gb|KK962648.1|	62451-96327	24	9.7	2	0.684934	-2	0.35375	no
gi|646741656|gb|KK962648.1|	94223-99603	24	9.7	1	1	0.0960244	0.95125	no
gi|646741667|gb|KK962647.1|	40127-24069	22	7.8	8	5	-0.76878	0.58175	no
gi|646741667|gb|KK962647.1|	38233-33836	22	7.8	3135.19	627	-2	0.13605	no
gi|646741667|gb|KK962647.1|	99158-40541	22	7.8	13	11	-0.227319	0.83965	no
gi|646741667|gb|KK962647.1|	08407-41293	22	7.8	7	6	-0.244579	0.82375	no
gi|646741699|gb|KK962644.1|	02413-10380	20	9.2	2	5	1	0.3876	no
gi|646741699|gb|KK962644.1|	17534-11882	20	9.2	0.358914	2	3	0.2073	no
gi|646741699|gb|KK962644.1|	19854-12018	20	9.2	5	13	1	0.399	no
gi|646741699|gb|KK962644.1|	12038-13158	20	9.2	3	3	-0.121455	0.93995	no
gi|646741699|gb|KK962644.1|	61322-16225	20	9.2	0.959278	2	0.86842	0.5462	no
gi|646741699|gb|KK962644.1|	63810-16489	20	9.2	0.777364	0.63459	-0.292766	1	no
gi|646741699|gb|KK962644.1|	65260-16635	20	9.2	8	2	-2	0.17815	no
gi|646741699|gb|KK962644.1|	68323-16879	20	9.2	4	4	0.0556494	0.93645	no
gi|646741699|gb|KK962644.1|	84701-18495	20	9.2	18	14	-0.360743	0.81925	no
gi|646741699|gb|KK962644.1|	85448-18565	20	9.2	26	56	1	0.4599	no
gi|646741699|gb|KK962644.1|	00779-20210	20	9.2	3	0	-inf	0.0026	no
gi|646741699|gb|KK962644.1|	11842-21357	20	9.2	14	0	-inf	5.00E-05	yes
gi|646741699|gb|KK962644.1|	34676-23689	20	9.2	60	0.068352	-10	0.1665	no
gi|646741699|gb|KK962644.1|	70550-27269	20	9.2	27	0	-inf	5.00E-05	yes
gi|646741699|gb|KK962644.1|	77806-28012	20	9.2	113	0.065105	-11	0.1665	no
gi|646741699|gb|KK962644.1|	32900-33444	20	9.2	102	0	-inf	5.00E-05	yes
gi|646741699|gb|KK962644.1|	34567-33959	20	9.2	14	0	-inf	5.00E-05	yes
gi|646741699|gb|KK962644.1|	77294-37766	20	9.2	83	108	0.372426	0.8017	no
gi|646741699|gb|KK962644.1|	05615-40589	20	9.2	133	69	-0.945722	0.5362	no
gi|646741699|gb|KK962644.1|	4160-4507	20	9.2	4	6	0.309522	0.8529	no
gi|646741699|gb|KK962644.1|	91471-49183	20	9.2	4	7	0.817178	0.5898	no
gi|646741699|gb|KK962644.1|	23167-52441	20	9.2	3	0	-inf	0.00315	no

gi|646741699|gb|KK962644.1|	24701-52922	20	9.2	28	0.063275	-9	0.10535	no
gi|646741699|gb|KK962644.1|	34267-53502	20	9.2	0.732599	3	2	0.26425	no
gi|646741699|gb|KK962644.1|	44461-54475	20	9.2	11	18	0.736029	0.61405	no
gi|646741699|gb|KK962644.1|	53001-55327	20	9.2	68	29	-1	0.36925	no
gi|646741699|gb|KK962644.1|	9905-10576	20	9.2	120	42	-2	0.25995	no
gi|646741709|gb|KK962643.1|	0-601	15	6	0	32	inf	0.00025	yes
gi|646741709|gb|KK962643.1|	09191-20969	15	6	5	0.505991	-3	0.179	no
gi|646741709|gb|KK962643.1|	35815-23651	15	6	3	2	-0.518486	0.685	no
gi|646741709|gb|KK962643.1|	40170-40707	15	6	2	2	0.444006	0.77485	no
gi|646741719|gb|KK962642.1|	01808-10199	21	8.9	112.87	121	0.102322	0.93355	no
gi|646741719|gb|KK962642.1|	04802-10518	21	8.9	3	9	2	0.29455	no
gi|646741719|gb|KK962642.1|	08484-11011	21	8.9	0.603353	0.580602	-0.0554513	1	no
gi|646741719|gb|KK962642.1|	14733-11523	21	8.9	159	55	-2	0.2542	no
gi|646741719|gb|KK962642.1|	14163-22872	21	8.9	16	8	-0.974806	0.40565	no
gi|646741719|gb|KK962642.1|	47579-14851	21	8.9	1	0.19184	-3	0.19945	no
gi|646741719|gb|KK962642.1|	48625-15272	21	8.9	3	2	-0.784089	0.6104	no
gi|646741719|gb|KK962642.1|	57540-15853	21	8.9	3	1	-0.812152	0.51635	no
gi|646741719|gb|KK962642.1|	98272-19894	21	8.9	8	7	-0.340179	0.83305	no
gi|646741719|gb|KK962642.1|	08958-20925	21	8.9	37	31	-0.278084	0.864	no
gi|646741719|gb|KK962642.1|	46856-24757	21	8.9	2	1	-0.192786	0.89315	no
gi|646741719|gb|KK962642.1|	48870-24909	21	8.9	22	36	0.703521	0.63725	no
gi|646741719|gb|KK962642.1|	76205-27777	21	8.9	1	2	0.187291	0.896	no
gi|646741719|gb|KK962642.1|	77904-27843	21	8.9	2	4	0.802615	0.58155	no
gi|646741719|gb|KK962642.1|	81000-28414	21	8.9	62	46	-0.42082	0.7205	no
gi|646741719|gb|KK962642.1|	2990-3587	21	8.9	3	4	0.599255	0.63685	no
gi|646741719|gb|KK962642.1|	03526-30443	21	8.9	11	11	-0.00737857	0.99175	no
gi|646741719|gb|KK962642.1|	57383-35927	21	8.9	3	5	0.76629	0.61335	no
gi|646741719|gb|KK962642.1|	88352-38881	21	8.9	8	9	0.148821	0.92065	no
gi|646741719|gb|KK962642.1|	89257-39384	21	8.9	11	12	0.0256199	0.9793	no
gi|646741719|gb|KK962642.1|	02795-40529	21	8.9	55	54	-0.0198948	0.98665	no

gi|646741719|gb|KK962642.1|	14167-41470	21	8.9	2	3	0.930469	0.56315	no
gi|646741719|gb|KK962642.1|	15679-41668	21	8.9	1	1	-0.141406	0.9179	no
gi|646741719|gb|KK962642.1|	17417-42314	21	8.9	3	3	-0.192907	0.90015	no
gi|646741719|gb|KK962642.1|	56042-45683	21	8.9	3	9	1	0.29335	no
gi|646741719|gb|KK962642.1|	65825-46674	21	8.9	19	19	0.0324067	0.98135	no
gi|646741719|gb|KK962642.1|	51152-52541	21	8.9	3	2	-0.434794	0.7828	no
gi|646741719|gb|KK962642.1|	54713-55638	21	8.9	1	0.587988	-1	0.4271	no
gi|646741719|gb|KK962642.1|	5694-6180	21	8.9	31	14	-1	0.4538	no
gi|646741719|gb|KK962642.1|	65352-65797	21	8.9	20	7	-2	0.28215	no
gi|646741719|gb|KK962642.1|	66335-67727	21	8.9	0.895011	0.702293	-0.349832	1	no
gi|646741719|gb|KK962642.1|	88839-89351	21	8.9	6	19	2	0.24445	no
gi|646741719|gb|KK962642.1|	92274-92474	21	8.9	41	38	-0.099864	0.90895	no
gi|646741719|gb|KK962642.1|	94144-94505	21	8.9	57	158	1	0.251	no
gi|646741719|gb|KK962642.1|	95102-96769	21	8.9	0.3276	0.946083	2	1	no
gi|646741719|gb|KK962642.1|	96944-97249	21	8.9	15	39	1	0.3439	no
gi|646741719|gb|KK962642.1|	98608-99541	21	8.9	1	4	2	0.23665	no
gi|646741744|gb|KK962640.1|	37609-13923	17	6.8	9	23	1	0.28965	no
gi|646741744|gb|KK962640.1|	48130-15215	17	6.8	34	36	0.114909	0.9229	no
gi|646741744|gb|KK962640.1|	73786-17452	17	6.8	671	62	-3	0.00735	no
gi|646741744|gb|KK962640.1|	07315-20808	17	6.8	7	3	-0.978449	0.4817	no
gi|646741744|gb|KK962640.1|	22842-22352	17	6.8	31	5	-3	0.11715	no
gi|646741744|gb|KK962640.1|	47870-24894	17	6.8	5	5	-0.105148	0.94555	no
gi|646741744|gb|KK962640.1|	99038-99411	17	6.8	49	109	1	0.4506	no
gi|646741754|gb|KK962639.1|	72746-27309	17	7.3	6	3	-0.907665	0.5995	no
gi|646741767|gb|KK962638.1|	38492-23908	21	10	37	4	-3	0.0421	no
gi|646741767|gb|KK962638.1|	50538-25167	21	10	3	4	0.78977	0.615	no
gi|646741767|gb|KK962638.1|	60809-26118	21	10	7	11	0.603721	0.63375	no
gi|646741767|gb|KK962638.1|	69807-27017	21	10	21	7	-2	0.29235	no
gi|646741767|gb|KK962638.1|	73948-27452	21	10	101	41	-1	0.31375	no
gi|646741767|gb|KK962638.1|	94960-29794	21	10	63	27	-1	0.28905	no

gi|646741767|gb|KK962638.1|	14299-32021	21	10	45	36	-0.318793	0.7873	no
gi|646741767|gb|KK962638.1|	20425-32448	21	10	6	3	-0.848165	0.50035	no
gi|646741767|gb|KK962638.1|	29138-32967	21	10	0.978935	6	3	0.13525	no
gi|646741767|gb|KK962638.1|	32201-33464	21	10	0.887568	3	2	0.20395	no
gi|646741767|gb|KK962638.1|	56584-35709	21	10	26	23	-0.139316	0.929	no
gi|646741767|gb|KK962638.1|	36280-37350	21	10	0	27	inf	5.00E-05	yes
gi|646741767|gb|KK962638.1|	72231-37279	21	10	3	0.821339	-2	0.29375	no
gi|646741767|gb|KK962638.1|	88639-48889	21	10	42	22	-0.953845	0.4649	no
gi|646741767|gb|KK962638.1|	94478-49604	21	10	23	20	-0.164953	0.878	no
gi|646741767|gb|KK962638.1|	97551-50617	21	10	9	6	-0.522047	0.6431	no
gi|646741778|gb|KK962637.1|	04775-10829	19	6.1	15	19	0.394809	0.72325	no
gi|646741778|gb|KK962637.1|	11537-11322	19	6.1	25	35	0.495745	0.6539	no
gi|646741778|gb|KK962637.1|	13620-11479	19	6.1	21	30	0.52519	0.6214	no
gi|646741778|gb|KK962637.1|	27078-13253	19	6.1	38	32	-0.274209	0.8137	no
gi|646741778|gb|KK962637.1|	32623-13639	19	6.1	36	34	-0.0516227	0.9657	no
gi|646741778|gb|KK962637.1|	55515-15634	19	6.1	12	3	-2	0.2844	no
gi|646741778|gb|KK962637.1|	59574-16014	19	6.1	77	49	-0.650586	0.6127	no
gi|646741778|gb|KK962637.1|	82839-18329	19	6.1	13	33	1	0.4216	no
gi|646741778|gb|KK962637.1|	84130-18507	19	6.1	3	3	0.0915494	0.94845	no
gi|646741778|gb|KK962637.1|	94826-19542	19	6.1	10	11	0.0358082	0.97975	no
gi|646741778|gb|KK962637.1|	04818-20515	19	6.1	23	12	-0.961622	0.51425	no
gi|646741778|gb|KK962637.1|	16695-21728	19	6.1	2	3	0.822537	0.5637	no
gi|646741778|gb|KK962637.1|	25146-25534	19	6.1	5	6	0.163386	0.9115	no
gi|646741778|gb|KK962637.1|	27685-28358	19	6.1	6	2	-2	0.26375	no
gi|646741778|gb|KK962637.1|	67519-67997	19	6.1	20	3	-3	0.10095	no
gi|646741778|gb|KK962637.1|	8955-18756	19	6.1	8	5	-0.735668	0.51665	no
gi|646741778|gb|KK962637.1|	90020-90294	19	6.1	16	18	0.179092	0.9007	no
gi|646741815|gb|KK962634.1|	02186-10298	22	10	0.676389	2	2	0.26425	no
gi|646741815|gb|KK962634.1|	04257-10669	22	10	0.67748	0.24591	-1	1	no
gi|646741815|gb|KK962634.1|	24882-12611	22	10	0.755949	0.948619	0.32754	1	no

gi|646741815|gb|KK962634.1|	57795-16310	22	10	0.67695	0.667918	-0.0193779	1	no
gi|646741815|gb|KK962634.1|	64730-16715	22	10	0.768496	0.929745	0.274798	1	no
gi|646741815|gb|KK962634.1|	69717-17131	22	10	0.413315	0.596336	0.528882	1	no
gi|646741815|gb|KK962634.1|	23485-22511	22	10	0.940593	1	0.422546	0.7207	no
gi|646741815|gb|KK962634.1|	25415-22585	22	10	4	3	-0.603212	0.6685	no
gi|646741815|gb|KK962634.1|	26146-22652	22	10	5	9	0.861608	0.54295	no
gi|646741815|gb|KK962634.1|	41385-24247	22	10	0.661548	1	0.929894	0.53925	no
gi|646741815|gb|KK962634.1|	47670-24869	22	10	0.720816	1	0.733921	0.6352	no
gi|646741815|gb|KK962634.1|	23050-32382	22	10	2	3	0.617623	0.6266	no
gi|646741815|gb|KK962634.1|	32531-33094	22	10	159	38	-2	0.1311	no
gi|646741815|gb|KK962634.1|	39389-39620	22	10	55	33	-0.761922	0.5531	no
gi|646741815|gb|KK962634.1|	45724-46542	22	10	1	0.927286	-0.503258	0.7384	no
gi|646741815|gb|KK962634.1|	53108-55116	22	10	21	17	-0.340089	0.7593	no
gi|646741815|gb|KK962634.1|	67973-69553	22	10	216	109.58	-0.979698	0.41105	no
gi|646741815|gb|KK962634.1|	81960-83486	22	10	14	10	-0.382249	0.80095	no
gi|646741815|gb|KK962634.1|	86756-91709	22	10	12	14	0.136774	0.90215	no
gi|646741838|gb|KK962632.1|	38231-13890	20	8.5	113	92	-0.29662	0.788	no
gi|646741838|gb|KK962632.1|	40759-14157	20	8.5	80	76	-0.0779864	0.94515	no
gi|646741838|gb|KK962632.1|	52655-15289	20	8.5	40	67	0.741744	0.6051	no
gi|646741838|gb|KK962632.1|	59710-16001	20	8.5	29	44	0.586034	0.7017	no
gi|646741838|gb|KK962632.1|	61498-16173	20	8.5	10	29	1	0.36225	no
gi|646741838|gb|KK962632.1|	62646-16291	20	8.5	30	38	0.353996	0.7971	no
gi|646741838|gb|KK962632.1|	55264-55540	20	8.5	432	9	-6	0.0442	no
gi|646741908|gb|KK962627.1|	94677-29485	12	5.7	232	0	-inf	0.0035	no
gi|646741908|gb|KK962627.1|	96091-29688	12	5.7	2	0	-inf	0.00655	no
gi|646741913|gb|KK962626.1|	31000-13161	30	13	20	4	-2	0.10775	no
gi|646741913|gb|KK962626.1|	81544-18234	30	13	3	2	-0.352649	0.77885	no
gi|646741913|gb|KK962626.1|	2-384	30	13	13	11	-0.197384	0.86635	no
gi|646741926|gb|KK962624.1|	23051-12437	17	9.1	0.086621	3	5	0.10155	no
gi|646741926|gb|KK962624.1|	24496-12603	17	9.1	0.216828	2	3	0.152	no

gi|646741926|gb|KK962624.1| 32949-13425	17	9.1	0	1	inf	0.00255	no
gi|646741926|gb|KK962624.1| 58261-15845	17	9.1	143	168	0.226492	0.86955	no
gi|646741926|gb|KK962624.1| 63487-26399	17	9.1	5	0.965839	-2	0.19235	no
gi|646741926|gb|KK962624.1| 64098-26820	17	9.1	12	8	-0.657933	0.5517	no
gi|646741926|gb|KK962624.1| 79374-27965	17	9.1	86	39	-1	0.47615	no
gi|646741926|gb|KK962624.1| 31207-33533	17	9.1	0.402058	21	6	0.01635	no
gi|646741926|gb|KK962624.1| 79749-38040	17	9.1	3	2	-0.400663	0.7788	no
gi|646741926|gb|KK962624.1| 80685-38207	17	9.1	0.812931	0.818636	0.0100885	1	no
gi|646741926|gb|KK962624.1| 84652-38570	17	9.1	0.464268	1	2	0.29375	no
gi|646741926|gb|KK962624.1| 86807-38851	17	9.1	0.508672	1	1	1	no
gi|646741926|gb|KK962624.1| 91140-39198	17	9.1	2	2	0.362009	0.79135	no
gi|646741945|gb|KK962622.1| 33781-13435	19	12	2	2	-0.223296	0.87465	no
gi|646741945|gb|KK962622.1| 45903-14762	19	12	5	4	-0.396969	0.789	no
gi|646741945|gb|KK962622.1| 18785-19477	19	12	0.63474	3	2	0.1897	no
gi|646741945|gb|KK962622.1| 60152-26065	19	12	1	3	1	0.48625	no
gi|646741970|gb|KK962620.1| 04939-10534	24	6	154	0	-inf	5.00E-05	yes
gi|646741970|gb|KK962620.1| 06942-10783	24	6	218.98	0.617415	-8	0.07395	no
gi|646741970|gb|KK962620.1| 11325-11160	24	6	1618.83	4	-9	0.10535	no
gi|646741970|gb|KK962620.1| 15842-11620	24	6	30	0	-inf	0.0023	no
gi|646741970|gb|KK962620.1| 18610-11904	24	6	12	0	-inf	0.00495	no
gi|646741970|gb|KK962620.1| 21703-12191	24	6	119	0	-inf	0.00095	yes
gi|646741970|gb|KK962620.1| 24786-12496	24	6	244	0	-inf	0.0012	yes
gi|646741970|gb|KK962620.1| 83618-28454	24	6	2	0.195432	-3	0.1757	no
gi|646741970|gb|KK962620.1| 84765-28616	24	6	2	0.698078	-1	0.3883	no
gi|646741970|gb|KK962620.1| 34593-35391	24	6	1	1	0.231694	0.87595	no
gi|646741970|gb|KK962620.1| 49974-35049	24	6	3	5	0.761599	0.58035	no
gi|646741970|gb|KK962620.1| 50640-35414	24	6	0.768871	2	1	0.3882	no
gi|646741970|gb|KK962620.1| 54231-35518	24	6	1	2	0.885876	0.4909	no
gi|646741970|gb|KK962620.1| 36249-37460	24	6	1	4	2	0.21525	no
gi|646741970|gb|KK962620.1| 95123-39572	24	6	1	2	0.725284	0.64505	no

gi|646741970|gb|KK962620.1|	97976-39829	24	6	8	13	0.626098	0.65485	no
gi|646741970|gb|KK962620.1|	04092-40468	24	6	2	3	0.098425	0.9345	no
gi|646741970|gb|KK962620.1|	46505-46875	24	6	32	28	-0.181973	0.9055	no
gi|646741970|gb|KK962620.1|	06660-60698	24	6	121	63	-0.94824	0.5327	no
gi|646741970|gb|KK962620.1|	63632-64136	24	6	118	0.993732	-7	0.10535	no
gi|646741970|gb|KK962620.1|	64445-66694	24	6	11	0.538408	-4	0.0095	no
gi|646741970|gb|KK962620.1|	67738-70738	24	6	6	1	-2	0.1622	no
gi|646741970|gb|KK962620.1|	81181-68208	24	6	10	6	-0.671149	0.66005	no
gi|646741970|gb|KK962620.1|	94309-69452	24	6	37	42	0.180468	0.89585	no
gi|646741970|gb|KK962620.1|	14221-71686	24	6	14	6	-1	0.35505	no
gi|646741970|gb|KK962620.1|	22065-72508	24	6	23	11	-1	0.353	no
gi|646741970|gb|KK962620.1|	28992-72945	24	6	19.63	13	-0.620567	0.692	no
gi|646741970|gb|KK962620.1|	72908-74332	24	6	7	0.341715	-4	0.0802	no
gi|646741970|gb|KK962620.1|	74439-76447	24	6	2	0.917241	-1	0.35935	no
gi|646741970|gb|KK962620.1|	58048-75930	24	6	27	10	-1	0.2878	no
gi|646741970|gb|KK962620.1|	76719-77212	24	6	26	1	-5	0.11015	no
gi|646741970|gb|KK962620.1|	77767-80477	24	6	3	0.054777	-6	0.168	no
gi|646741970|gb|KK962620.1|	81164-81460	24	6	546	2	-8	0.1665	no
gi|646741970|gb|KK962620.1|	83146-84188	24	6	21	0	-inf	0.00015	yes
gi|646741970|gb|KK962620.1|	85984-86534	24	6	370	0.851972	-9	0.10535	no
gi|646741970|gb|KK962620.1|	87828-88711	24	6	10	0	-inf	0.00035	yes
gi|646741970|gb|KK962620.1|	88826-89122	24	6	300	2	-7	0.1665	no
gi|646741970|gb|KK962620.1|	92563-93087	24	6	60	0	-inf	5.00E-05	yes
gi|646741970|gb|KK962620.1|	94973-96789	24	6	24	0	-inf	5.00E-05	yes
gi|646741992|gb|KK962618.1|	36232-13763	13	6.8	2	0	-inf	0.00525	no
gi|646741992|gb|KK962618.1|	60973-16147	13	6.8	8	2	-2	0.14795	no
gi|646741992|gb|KK962618.1|	63801-16606	13	6.8	36	2	-4	0.01275	no
gi|646741992|gb|KK962618.1|	70587-27141	13	6.8	2	2	-0.395387	0.76995	no
gi|646742017|gb|KK962616.1|	20827-12588	18	7.5	93	3	-5	0.0031	no
gi|646742017|gb|KK962616.1|	41361-14217	18	7.5	4	4	-0.240912	0.8852	no

gi|646742017|gb|KK962616.1|	43105-14387	18	7.5	3	3	0.104369	0.9336	no
gi|646742017|gb|KK962616.1|	44935-14682	18	7.5	2	2	0.00703336	0.9838	no
gi|646742017|gb|KK962616.1|	53744-15490	18	7.5	0.918953	2	0.932647	0.48725	no
gi|646742017|gb|KK962616.1|	55566-15701	18	7.5	1	1	0.110964	0.92895	no
gi|646742017|gb|KK962616.1|	57303-15876	18	7.5	2	3	0.580602	0.71845	no
gi|646742017|gb|KK962616.1|	62673-16371	18	7.5	3	3	-0.0113115	0.9741	no
gi|646742017|gb|KK962616.1|	65661-16670	18	7.5	0.822791	2	1	0.3574	no
gi|646742017|gb|KK962616.1|	67182-16879	18	7.5	0.815101	0.588151	-0.470791	1	no
gi|646742017|gb|KK962616.1|	70859-17212	18	7.5	4	5	0.484549	0.754	no
gi|646742017|gb|KK962616.1|	35955-23621	18	7.5	62	79	0.35009	0.8245	no
gi|646742017|gb|KK962616.1|	61596-26591	18	7.5	8	7	-0.195127	0.86185	no
gi|646742017|gb|KK962616.1|	72173-27336	18	7.5	15	15	-0.0892619	0.95025	no
gi|646742017|gb|KK962616.1|	86328-28668	18	7.5	5	7	0.542172	0.71965	no
gi|646742017|gb|KK962616.1|	49417-49946	18	7.5	0	6	inf	0.0008	yes
gi|646742017|gb|KK962616.1|	50976-52023	18	7.5	0.934397	1	0.512652	0.71995	no
gi|646742030|gb|KK962615.1|	76613-18501	23	6.1	2	2	-0.0465436	0.9763	no
gi|646742030|gb|KK962615.1|	87043-19548	23	6.1	14	11	-0.328633	0.7812	no
gi|646742030|gb|KK962615.1|	47179-24763	23	6.1	2	9	2	0.24155	no
gi|646742030|gb|KK962615.1|	49252-24953	23	6.1	4	21	3	0.2738	no
gi|646742030|gb|KK962615.1|	51610-25239	23	6.1	0.874242	2	1	0.43255	no
gi|646742030|gb|KK962615.1|	53169-25447	23	6.1	20	32	0.684458	0.5239	no
gi|646742030|gb|KK962615.1|	74725-27535	23	6.1	3	3	0.00971509	0.94695	no
gi|646742030|gb|KK962615.1|	79251-28005	23	6.1	3	3	0.196092	0.8697	no
gi|646742030|gb|KK962615.1|	76539-38174	23	6.1	42	37	-0.187436	0.87355	no
gi|646742030|gb|KK962615.1|	89636-38990	23	6.1	25	16	-0.611056	0.6444	no
gi|646742030|gb|KK962615.1|	88457-88853	23	6.1	7	2	-2	0.3592	no
gi|646742040|gb|KK962614.1|	47486-14826	15	6.9	4	3	-0.382033	0.7814	no
gi|646742040|gb|KK962614.1|	07178-30782	15	6.9	4	3	-0.527959	0.67825	no
gi|646742040|gb|KK962614.1|	08024-31167	15	6.9	3	5	0.71306	0.64225	no
gi|646742040|gb|KK962614.1|	19166-31993	15	6.9	0.726848	3	2	0.26425	no

gi|646742040|gb|KK962614.1|	25584-32628	15	6.9	2	1	-0.687937	0.6459	no
gi|646742054|gb|KK962613.1|	10067-11034	13	7	10	26	1	0.3644	no
gi|646742054|gb|KK962613.1|	43457-44206	13	7	4	9	1	0.49055	no
gi|646742078|gb|KK962611.1|	15345-15533	18	8.1	82	83	0.0189696	0.93585	no
gi|646742078|gb|KK962611.1|	18538-19886	18	8.1	0.931035	1	0.649056	0.61505	no
gi|646742078|gb|KK962611.1|	21851-22211	18	8.1	303	117	-1	0.28945	no
gi|646742078|gb|KK962611.1|	36492-38829	18	8.1	4	3	-0.447968	0.76745	no
gi|646742078|gb|KK962611.1|	39257-43525	18	8.1	38	33	-0.179748	0.87885	no
gi|646742078|gb|KK962611.1|	45316-45880	18	8.1	6	10	0.645368	0.63855	no
gi|646742078|gb|KK962611.1|	46503-46848	18	8.1	131.66	76	-0.785585	0.60335	no
gi|646742078|gb|KK962611.1|	46950-47779	18	8.1	0.805728	1	0.760825	0.61475	no
gi|646742078|gb|KK962611.1|	48552-49398	18	8.1	19	16	-0.221947	0.88125	no
gi|646742078|gb|KK962611.1|	98868-99126	18	8.1	316	81	-2	0.21975	no
gi|646742090|gb|KK962610.1|	23495-12399	15	17	0	11	inf	0.0023	no
gi|646742090|gb|KK962610.1|	07444-20768	15	17	893	945	0.0816246	0.9396	no
gi|646742109|gb|KK962608.1|	00795-30596	14	6	2	2	0.0575414	0.96985	no
gi|646742109|gb|KK962608.1|	06084-30679	14	6	4	1	-2	0.2419	no
gi|646742133|gb|KK962606.1|	17110-21879	17	9.5	5	0	-inf	0.00015	yes
gi|646742133|gb|KK962606.1|	64198-26452	17	9.5	29	0	-inf	0.00315	no
gi|646742133|gb|KK962606.1|	76681-27766	17	9.5	11	5	-1	0.4229	no
gi|646742133|gb|KK962606.1|	23497-32432	17	9.5	2	1	-0.639619	0.65475	no
gi|646742145|gb|KK962605.1|	66258-16721	13	8	12	19	0.629117	0.6783	no
gi|646742145|gb|KK962605.1|	80518-18127	13	8	9	6	-0.659296	0.6778	no
gi|646742145|gb|KK962605.1|	84743-18495	13	8	40	56	0.487098	0.7219	no
gi|646742145|gb|KK962605.1|	12457-31356	13	8	2	0.154318	-4	0.17415	no
gi|646742208|gb|KK962600.1|	63799-16457	24	11	2	1	-0.829721	0.5782	no
gi|646742208|gb|KK962600.1|	65521-16587	24	11	16	4	-2	0.1965	no
gi|646742208|gb|KK962600.1|	68324-16945	24	11	5	3	-0.638809	0.6838	no
gi|646742208|gb|KK962600.1|	17723-18110	24	11	2	7	2	0.27495	no
gi|646742208|gb|KK962600.1|	85817-18640	24	11	4	2	-1	0.3937	no

gi|646742208|gb|KK962600.1|	97441-19782	24	11	16	0	-inf	0.00595	no
gi|646742208|gb|KK962600.1|	07746-20799	24	11	129	88	-0.549459	0.72345	no
gi|646742208|gb|KK962600.1|	24118-22497	24	11	19	7	-1	0.37085	no
gi|646742208|gb|KK962600.1|	29091-23174	24	11	16	5	-2	0.17885	no
gi|646742208|gb|KK962600.1|	57419-25784	24	11	4	5	0.388548	0.7941	no
gi|646742208|gb|KK962600.1|	86714-28754	24	11	5	6	0.235036	0.8775	no
gi|646742208|gb|KK962600.1|	88635-28907	24	11	11	3	-2	0.21945	no
gi|646742208|gb|KK962600.1|	99374-29978	24	11	15	18	0.268512	0.8673	no
gi|646742208|gb|KK962600.1|	36556-36770	24	11	160	17	-3	0.11555	no
gi|646742208|gb|KK962600.1|	69453-37037	24	11	15	7	-1	0.4615	no
gi|646742208|gb|KK962600.1|	70490-37191	24	11	8	7	-0.201588	0.89505	no
gi|646742208|gb|KK962600.1|	73578-37734	24	11	15	16	0.113245	0.91995	no
gi|646742208|gb|KK962600.1|	77623-37903	24	11	8	10	0.28658	0.84975	no
gi|646742208|gb|KK962600.1|	01822-40294	24	11	16	7	-1	0.45115	no
gi|646742208|gb|KK962600.1|	03183-40368	24	11	5	2	-1	0.43605	no
gi|646742208|gb|KK962600.1|	05107-40535	24	11	23	9	-1	0.43905	no
gi|646742208|gb|KK962600.1|	05525-40969	24	11	16	6	-1	0.23395	no
gi|646742208|gb|KK962600.1|	09862-41141	24	11	2	1	-0.536155	0.69885	no
gi|646742208|gb|KK962600.1|	18936-41932	24	11	16	9	-0.767585	0.5849	no
gi|646742208|gb|KK962600.1|	27275-42813	24	11	1	0.867976	-0.499624	0.73865	no
gi|646742208|gb|KK962600.1|	41431-44313	24	11	2	1	-0.847852	0.60535	no
gi|646742208|gb|KK962600.1|	77728-47902	24	11	0.622067	0.382847	-0.700301	1	no
gi|646742208|gb|KK962600.1|	82685-83564	24	11	0	4	inf	0.00315	no
gi|646742208|gb|KK962600.1|	84559-85682	24	11	0	5	inf	0.00085	yes
gi|646742228|gb|KK962598.1|	71839-17231	11	5.8	3	2	-0.803665	0.6307	no
gi|646742228|gb|KK962598.1|	39722-34023	11	5.8	4	3	-0.0799793	0.9361	no
gi|646742228|gb|KK962598.1|	58670-35967	11	5.8	5	1	-2	0.19805	no
gi|646742237|gb|KK962597.1|	30019-13116	21	6	7	8	0.301858	0.84385	no
gi|646742237|gb|KK962597.1|	37107-13967	21	6	0.080034	5	6	0.2333	no
gi|646742237|gb|KK962597.1|	79608-18447	21	6	22	19	-0.160371	0.8903	no

gi|646742237|gb|KK962597.1|	85804-18691	21	6	1	0.309692	-2	0.20995	no
gi|646742237|gb|KK962597.1|	87136-19162	21	6	1	0.223388	-2	0.13245	no
gi|646742237|gb|KK962597.1|	97089-19738	21	6	32	32	0.00108299	0.9693	no
gi|646742237|gb|KK962597.1|	28749-22892	21	6	83	221	1	0.3593	no
gi|646742237|gb|KK962597.1|	35385-33594	21	6	968	960.56	-0.0114331	0.9908	no
gi|646742237|gb|KK962597.1|	12871-41315	21	6	7	14	1	0.4822	no
gi|646742237|gb|KK962597.1|	46209-44662	21	6	3	7	1	0.4453	no
gi|646742237|gb|KK962597.1|	59301-46022	21	6	19	17	-0.207641	0.8922	no
gi|646742237|gb|KK962597.1|	65384-46835	21	6	12	13	0.0940725	0.9318	no
gi|646742237|gb|KK962597.1|	72305-47342	21	6	0.64241	1	1	0.44445	no
gi|646742237|gb|KK962597.1|	73513-47715	21	6	6	8	0.494664	0.64625	no
gi|646742237|gb|KK962597.1|	83567-48744	21	6	17	2	-3	0.02895	no
gi|646742237|gb|KK962597.1|	27996-53224	21	6	0.046354	2	5	0.2348	no
gi|646742237|gb|KK962597.1|	32419-53405	21	6	0	2	inf	0.00155	yes
gi|646742237|gb|KK962597.1|	37098-63877	21	6	0.652429	1	0.667627	1	no
gi|646742237|gb|KK962597.1|	38885-63993	21	6	0.579608	0.992495	0.775982	1	no
gi|646742259|gb|KK962595.1|	91680-29309	16	8.5	0.398415	1	1	1	no
gi|646742259|gb|KK962595.1|	45306-34602	16	8.5	3	3	-0.099677	0.94015	no
gi|646742259|gb|KK962595.1|	98862-50007	16	8.5	14	4	-2	0.2607	no
gi|646742259|gb|KK962595.1|	09350-51167	16	8.5	32	24	-0.460889	0.6812	no
gi|646742259|gb|KK962595.1|	11928-51288	16	8.5	9	12	0.440629	0.77065	no
gi|646742259|gb|KK962595.1|	22439-52308	16	8.5	2	3	0.473226	0.7268	no
gi|646742259|gb|KK962595.1|	25564-52670	16	8.5	0.520876	1	1	0.36645	no
gi|646742259|gb|KK962595.1|	30187-53045	16	8.5	46	17	-1	0.2789	no
gi|646742259|gb|KK962595.1|	44527-54532	16	8.5	5	3	-0.55972	0.6866	no
gi|646742259|gb|KK962595.1|	56418-55748	16	8.5	0	4	inf	0.0023	no
gi|646742259|gb|KK962595.1|	60621-56175	16	8.5	0.210244	158	10	0.2322	no
gi|646742259|gb|KK962595.1|	75401-57569	16	8.5	0	92	inf	0.00045	yes
gi|646742259|gb|KK962595.1|	75798-57618	16	8.5	0	16	inf	0.00315	no
gi|646742270|gb|KK962594.1|	36144-13664	21	10	13	49	2	0.23475	no

gi|646742270|gb|KK962594.1|	64814-16522	21	10	24	60	1	0.40545	no
gi|646742270|gb|KK962594.1|	65339-16728	21	10	1	1	0.455683	0.7401	no
gi|646742270|gb|KK962594.1|	67442-16973	21	10	1	2	0.937429	0.55725	no
gi|646742270|gb|KK962594.1|	72243-17415	21	10	1	4	1	0.39975	no
gi|646742270|gb|KK962594.1|	74282-18034	21	10	19	22	0.177013	0.8749	no
gi|646742270|gb|KK962594.1|	81973-18653	21	10	9	5	-0.87869	0.42415	no
gi|646742270|gb|KK962594.1|	00721-20100	21	10	45	14	-2	0.2613	no
gi|646742270|gb|KK962594.1|	19681-22073	21	10	81	60	-0.438062	0.69775	no
gi|646742270|gb|KK962594.1|	22730-22290	21	10	130	169	0.385046	0.7809	no
gi|646742270|gb|KK962594.1|	25127-22578	21	10	2	1	-0.332976	0.81865	no
gi|646742270|gb|KK962594.1|	32989-23421	21	10	8	12	0.625432	0.6844	no
gi|646742270|gb|KK962594.1|	34662-23816	21	10	2	2	0.431682	0.77095	no
gi|646742270|gb|KK962594.1|	39191-24120	21	10	0.945655	2	1	0.3702	no
gi|646742270|gb|KK962594.1|	41582-24271	21	10	2	0.452658	-2	0.26095	no
gi|646742270|gb|KK962594.1|	64059-26451	21	10	61	14	-2	0.1839	no
gi|646742270|gb|KK962594.1|	77338-28223	21	10	1	0.669731	-0.861526	0.57865	no
gi|646742270|gb|KK962594.1|	82362-28344	21	10	0.671958	0.799783	0.251237	1	no
gi|646742270|gb|KK962594.1|	18223-31846	21	10	18	22	0.331041	0.81255	no
gi|646742270|gb|KK962594.1|	19147-32041	21	10	0.458878	1	1	1	no
gi|646742270|gb|KK962594.1|	22691-32326	21	10	10	4	-1	0.37335	no
gi|646742270|gb|KK962594.1|	36198-33965	21	10	10	7	-0.546971	0.60975	no
gi|646742270|gb|KK962594.1|	40113-34129	21	10	0.397886	0.997425	1	1	no
gi|646742270|gb|KK962594.1|	41428-34651	21	10	9	11	0.25642	0.822	no
gi|646742270|gb|KK962594.1|	47348-34814	21	10	5	9	0.836076	0.59505	no
gi|646742270|gb|KK962594.1|	48301-35051	21	10	6	4	-0.610554	0.6927	no
gi|646742270|gb|KK962594.1|	55635-35614	21	10	4	6	0.547172	0.6664	no
gi|646742270|gb|KK962594.1|	70310-37188	21	10	0.912501	1	0.287429	1	no
gi|646742270|gb|KK962594.1|	21659-42218	21	10	11	17	0.604354	0.6991	no
gi|646742270|gb|KK962594.1|	39179-43945	21	10	15	8	-0.813682	0.5891	no
gi|646742270|gb|KK962594.1|	64452-48017	21	10	4	3	-0.158245	0.8868	no

gi|646742270|gb|KK962594.1|	91655-49207	21	10	54	15	-2	0.255	no
gi|646742270|gb|KK962594.1|	12806-51502	21	10	4	3	-0.444596	0.76655	no
gi|646742270|gb|KK962594.1|	15350-51553	21	10	80	51	-0.652481	0.66735	no
gi|646742270|gb|KK962594.1|	17395-51873	21	10	1	0.122545	-3	1	no
gi|646742270|gb|KK962594.1|	27662-52803	21	10	29	23	-0.337385	0.8229	no
gi|646742270|gb|KK962594.1|	35950-53650	21	10	9	5	-0.998768	0.4939	no
gi|646742298|gb|KK962592.1|	10105-21062	10	7	1	6	2	0.19645	no
gi|646742298|gb|KK962592.1|	27014-22825	10	7	1	3	2	0.2742	no
gi|646742298|gb|KK962592.1|	27871-28279	10	7	0	36	inf	5.00E-05	yes
gi|646742298|gb|KK962592.1|	08738-30897	10	7	0	232	inf	0.0002	yes
gi|646742298|gb|KK962592.1|	20491-32119	10	7	0	4	inf	0.0008	yes
gi|646742298|gb|KK962592.1|	69698-37008	10	7	5	8	0.677959	0.6285	no
gi|646742307|gb|KK962591.1|	03907-10848	23	7.7	20	9	-1	0.32045	no
gi|646742307|gb|KK962591.1|	09049-10994	23	7.7	1	0.612769	-1	0.4766	no
gi|646742307|gb|KK962591.1|	10758-11324	23	7.7	0.74545	0.841995	0.175701	1	no
gi|646742307|gb|KK962591.1|	14292-11529	23	7.7	0.990673	0.529142	-0.904753	1	no
gi|646742307|gb|KK962591.1|	30819-13180	23	7.7	1	0.718599	-0.660436	0.6593	no
gi|646742307|gb|KK962591.1|	32694-13325	23	7.7	3	1	-1	0.4702	no
gi|646742307|gb|KK962591.1|	37488-13932	23	7.7	0.642875	2	1	0.41485	no
gi|646742307|gb|KK962591.1|	47454-14805	23	7.7	5	2	-1	0.29235	no
gi|646742307|gb|KK962591.1|	16918-17437	23	7.7	6	0.471331	-4	0.1755	no
gi|646742307|gb|KK962591.1|	18686-20147	23	7.7	27	0.331422	-6	0.07395	no
gi|646742307|gb|KK962591.1|	97465-19785	23	7.7	7	11	0.598954	0.62905	no
gi|646742307|gb|KK962591.1|	13183-21354	23	7.7	9	4	-1	0.4507	no
gi|646742307|gb|KK962591.1|	32541-23333	23	7.7	17	11	-0.623318	0.682	no
gi|646742307|gb|KK962591.1|	41587-42515	23	7.7	40	98	1	0.24735	no
gi|646742307|gb|KK962591.1|	44663-45896	23	7.7	11.54	33	2	0.2471	no
gi|646742307|gb|KK962591.1|	53558-54145	23	7.7	0.83365	4	2	0.16245	no
gi|646742307|gb|KK962591.1|	59943-61332	23	7.7	0.978958	4	2	0.19365	no
gi|646742307|gb|KK962591.1|	72989-73535	23	7.7	16	35	1	0.45685	no

gi|646742307|gb|KK962591.1|	87410-87624	23	7.7	40	17	-1	0.4523	no
gi|646742319|gb|KK962590.1|	00355-10069	18	8.5	8	11	0.465714	0.73605	no
gi|646742319|gb|KK962590.1|	01077-10151	18	8.5	8	6	-0.344208	0.79035	no
gi|646742319|gb|KK962590.1|	02712-10313	18	8.5	8	11	0.47948	0.73915	no
gi|646742319|gb|KK962590.1|	03428-10560	18	8.5	4	3	-0.594429	0.6888	no
gi|646742319|gb|KK962590.1|	22466-12334	18	8.5	6	3	-0.924323	0.5624	no
gi|646742319|gb|KK962590.1|	29850-13155	18	8.5	0.511484	0.554128	0.115532	1	no
gi|646742319|gb|KK962590.1|	34805-13744	18	8.5	18	10	-0.819939	0.45595	no
gi|646742319|gb|KK962590.1|	40938-14207	18	8.5	31	14	-1	0.37985	no
gi|646742319|gb|KK962590.1|	42788-14395	18	8.5	0.815947	0.730078	-0.160424	1	no
gi|646742319|gb|KK962590.1|	48711-15047	18	8.5	0.307359	0.533086	0.794444	1	no
gi|646742319|gb|KK962590.1|	56396-15796	18	8.5	0.493217	0.609733	0.305954	1	no
gi|646742319|gb|KK962590.1|	62816-16417	18	8.5	24	10	-1	0.2994	no
gi|646742319|gb|KK962590.1|	73617-17393	18	8.5	108.11	40	-1	0.356	no
gi|646742319|gb|KK962590.1|	74817-17569	18	8.5	0.74717	1	0.987128	0.5626	no
gi|646742319|gb|KK962590.1|	77533-17782	18	8.5	16	7	-1	0.4551	no
gi|646742319|gb|KK962590.1|	84981-18522	18	8.5	62	35	-0.843702	0.5041	no
gi|646742319|gb|KK962590.1|	29371-23358	18	8.5	17	5	-2	0.1702	no
gi|646742319|gb|KK962590.1|	33725-23511	18	8.5	2	0.4686	-2	0.19895	no
gi|646742319|gb|KK962590.1|	36313-23739	18	8.5	3	0.803571	-2	0.2686	no
gi|646742319|gb|KK962590.1|	56166-25885	18	8.5	13	8	-0.762312	0.48615	no
gi|646742319|gb|KK962590.1|	59478-26377	18	8.5	7	5	-0.275393	0.79615	no
gi|646742319|gb|KK962590.1|	64451-26588	18	8.5	0.782674	0.676053	-0.211276	1	no
gi|646742319|gb|KK962590.1|	67296-26812	18	8.5	1	0.904118	-0.671762	0.6592	no
gi|646742319|gb|KK962590.1|	69994-27091	18	8.5	0.975165	0.790838	-0.302265	1	no
gi|646742319|gb|KK962590.1|	71517-27739	18	8.5	12	8	-0.582547	0.61215	no
gi|646742319|gb|KK962590.1|	88665-28975	18	8.5	15	1	-3	0.0373	no
gi|646742319|gb|KK962590.1|	32827-33627	18	8.5	2	1	-0.620541	0.64315	no
gi|646742319|gb|KK962590.1|	43847-34415	18	8.5	4	14	2	0.25825	no
gi|646742319|gb|KK962590.1|	62162-64715	18	8.5	1	0.818771	-0.698373	0.61635	no

gi|646742319|gb|KK962590.1|	65166-66507	18	8.5	1	2	0.22378	0.84925	no
gi|646742319|gb|KK962590.1|	73770-74586	18	8.5	2	2	-0.602989	0.6374	no
gi|646742319|gb|KK962590.1|	91776-92090	18	8.5	15	22	0.58212	0.7107	no
gi|646742319|gb|KK962590.1|	92512-95142	18	8.5	7	6	-0.184912	0.9047	no
gi|646742319|gb|KK962590.1|	95908-96613	18	8.5	7	3	-1	0.3173	no
gi|646742319|gb|KK962590.1|	98063-98528	18	8.5	25	21	-0.211032	0.88785	no
gi|646742332|gb|KK962589.1|	48317-15047	17	8.1	2	0.9847	-0.74427	0.5837	no
gi|646742332|gb|KK962589.1|	60551-16132	17	8.1	4	0.754752	-2	0.1718	no
gi|646742343|gb|KK962588.1|	01279-10187	20	9.4	5	2	-1	0.3407	no
gi|646742343|gb|KK962588.1|	04394-10491	20	9.4	11	0.463382	-5	0.17835	no
gi|646742343|gb|KK962588.1|	10872-11322	20	9.4	0.465916	44	7	0.09475	no
gi|646742343|gb|KK962588.1|	20357-12181	20	9.4	0.767738	0.110564	-3	1	no
gi|646742343|gb|KK962588.1|	39678-14059	20	9.4	2	6	1	0.3826	no
gi|646742343|gb|KK962588.1|	47038-15226	20	9.4	3	2	-0.53096	0.72195	no
gi|646742343|gb|KK962588.1|	76297-18303	20	9.4	3	3	0.0852619	0.95645	no
gi|646742343|gb|KK962588.1|	18183-18734	20	9.4	0	13	inf	0.001	yes
gi|646742343|gb|KK962588.1|	89021-19228	20	9.4	16	14	-0.198788	0.8628	no
gi|646742343|gb|KK962588.1|	98840-19926	20	9.4	10	12	0.237969	0.8605	no
gi|646742343|gb|KK962588.1|	12687-21326	20	9.4	3	2	-0.543249	0.71625	no
gi|646742343|gb|KK962588.1|	26018-22680	20	9.4	2	0.246114	-3	0.21605	no
gi|646742343|gb|KK962588.1|	27920-22833	20	9.4	3	4	0.64179	0.6668	no
gi|646742343|gb|KK962588.1|	31182-23197	20	9.4	1	0.722622	-0.727874	0.66415	no
gi|646742343|gb|KK962588.1|	46415-24759	20	9.4	0.501672	2	2	0.21905	no
gi|646742343|gb|KK962588.1|	55204-25680	20	9.4	1	2	0.792021	0.58225	no
gi|646742343|gb|KK962588.1|	56922-25763	20	9.4	2	4	1	0.41885	no
gi|646742343|gb|KK962588.1|	25709-27454	20	9.4	1	0.179458	-3	0.1619	no
gi|646742343|gb|KK962588.1|	64373-26625	20	9.4	11	5	-1	0.4284	no
gi|646742343|gb|KK962588.1|	66899-26767	20	9.4	1	1	-0.508023	0.7384	no
gi|646742343|gb|KK962588.1|	68570-26930	20	9.4	2	2	0.487094	0.72185	no
gi|646742343|gb|KK962588.1|	72904-27398	20	9.4	5	1	-2	0.1781	no

gi|646742343|gb|KK962588.1|	75105-28359	20	9.4	5	5	-0.0601522	0.9562	no
gi|646742343|gb|KK962588.1|	85395-28691	20	9.4	11	8	-0.426859	0.77695	no
gi|646742343|gb|KK962588.1|	30454-32006	20	9.4	54	25	-1	0.3179	no
gi|646742343|gb|KK962588.1|	15791-31664	20	9.4	2	4	0.799248	0.58765	no
gi|646742343|gb|KK962588.1|	18195-31907	20	9.4	43	13	-2	0.18955	no
gi|646742343|gb|KK962588.1|	24711-32675	20	9.4	4	4	0.183261	0.8997	no
gi|646742343|gb|KK962588.1|	30150-33044	20	9.4	34	36	0.0962139	0.95185	no
gi|646742343|gb|KK962588.1|	33109-34350	20	9.4	11	8	-0.437822	0.7093	no
gi|646742343|gb|KK962588.1|	54691-35518	20	9.4	2	3	0.427326	0.75545	no
gi|646742343|gb|KK962588.1|	55968-35621	20	9.4	54	40	-0.413278	0.73945	no
gi|646742343|gb|KK962588.1|	60659-36132	20	9.4	47	40	-0.237468	0.84775	no
gi|646742343|gb|KK962588.1|	70280-37060	20	9.4	12	11	-0.187772	0.8943	no
gi|646742343|gb|KK962588.1|	73328-37453	20	9.4	0.680212	0.557089	-0.288077	1	no
gi|646742343|gb|KK962588.1|	75302-37639	20	9.4	1	0.941974	-0.35948	0.79735	no
gi|646742343|gb|KK962588.1|	76602-37819	20	9.4	2	0.900439	-1	0.44795	no
gi|646742343|gb|KK962588.1|	82471-38314	20	9.4	17	16	-0.1066	0.9453	no
gi|646742343|gb|KK962588.1|	87895-38836	20	9.4	5	16	2	0.24765	no
gi|646742343|gb|KK962588.1|	88523-38945	20	9.4	2	5	1	0.3593	no
gi|646742343|gb|KK962588.1|	91820-39422	20	9.4	1	0.937884	-0.555407	0.7341	no
gi|646742343|gb|KK962588.1|	95205-39707	20	9.4	0.975058	1	0.445352	0.7487	no
gi|646742343|gb|KK962588.1|	98023-39881	20	9.4	79	81	0.0415317	0.9694	no
gi|646742343|gb|KK962588.1|	21205-42378	20	9.4	19	13	-0.584214	0.5926	no
gi|646742343|gb|KK962588.1|	24534-42664	20	9.4	11	6	-0.841766	0.4972	no
gi|646742343|gb|KK962588.1|	29549-42980	20	9.4	12	13	0.139405	0.9047	no
gi|646742343|gb|KK962588.1|	49582-45089	20	9.4	0.435549	1	1	1	no
gi|646742343|gb|KK962588.1|	78804-47978	20	9.4	6	10	0.705359	0.64555	no
gi|646742343|gb|KK962588.1|	90526-49221	20	9.4	12	11	-0.0894023	0.95275	no
gi|646742343|gb|KK962588.1|	97859-49937	20	9.4	7	6	-0.254502	0.86745	no
gi|646742343|gb|KK962588.1|	08687-50934	20	9.4	25	17	-0.564584	0.7049	no
gi|646742343|gb|KK962588.1|	10379-51170	20	9.4	2	3	0.464015	0.76845	no

gi|646742343|gb|KK962588.1|	11880-51265	20	9.4	1	2	0.299905	0.83465	no
gi|646742343|gb|KK962588.1|	17256-51784	20	9.4	2	2	0.46074	0.7494	no
gi|646742343|gb|KK962588.1|	18712-52515	20	9.4	17	13	-0.439027	0.7115	no
gi|646742343|gb|KK962588.1|	32532-53322	20	9.4	0.852148	2	1	0.4335	no
gi|646742343|gb|KK962588.1|	50683-55093	20	9.4	23	31	0.431024	0.7468	no
gi|646742343|gb|KK962588.1|	56222-56893	20	9.4	8	9	0.0367399	0.98045	no
gi|646742343|gb|KK962588.1|	58608-59131	20	9.4	11	19	0.752074	0.6279	no
gi|646742343|gb|KK962588.1|	63482-63731	20	9.4	9	34	2	0.2327	no
gi|646742343|gb|KK962588.1|	68229-68763	20	9.4	3	9	2	0.2906	no
gi|646742343|gb|KK962588.1|	91780-91976	20	9.4	64	51	-0.345569	0.8096	no
gi|646742353|gb|KK962587.1|	77785-17829	13	8.6	3	4	0.264262	0.84125	no
gi|646742367|gb|KK962586.1|	22476-32304	12	6	1589.5	1141.53	-0.477614	0.69515	no
gi|646742367|gb|KK962586.1|	48824-49256	12	6	7	0	-inf	0.0035	no
gi|646742378|gb|KK962585.1|	21584-12179	13	7.1	403	94	-2	0.20205	no
gi|646742378|gb|KK962585.1|	84512-28585	13	7.1	13	8	-0.731811	0.62745	no
gi|646742378|gb|KK962585.1|	89490-29090	13	7.1	1	1	-0.474838	0.708	no
gi|646742390|gb|KK962584.1|	35260-23565	43	17	6	8	0.518206	0.70115	no
gi|646742390|gb|KK962584.1|	22227-32251	43	17	0	65	inf	0.0007	yes
gi|646742390|gb|KK962584.1|	92079-39278	43	17	9	8	-0.233135	0.88375	no
gi|646742390|gb|KK962584.1|	68663-47115	43	17	4	3	-0.247617	0.8637	no
gi|646742390|gb|KK962584.1|	97832-49804	43	17	90	129	0.515491	0.7098	no
gi|646742390|gb|KK962584.1|	98449-50044	43	17	3	2	-0.623254	0.6889	no
gi|646742390|gb|KK962584.1|	02662-50581	43	17	0.927121	2	1	0.4451	no
gi|646742390|gb|KK962584.1|	11877-51253	43	17	3	3	0.0158858	0.94695	no
gi|646742390|gb|KK962584.1|	12937-51407	43	17	3	3	0.324106	0.83615	no
gi|646742390|gb|KK962584.1|	64931-66122	43	17	4	7	0.826838	0.5876	no
gi|646742390|gb|KK962584.1|	81219-83572	43	17	0.705572	0.704	-0.00321761	1	no
gi|646742390|gb|KK962584.1|	83828-84637	43	17	13	9	-0.411037	0.782	no
gi|646742403|gb|KK962583.1|	70995-71681	11	8.5	0	83	inf	5.00E-05	yes
gi|646742403|gb|KK962583.1|	72526-72863	11	8.5	0.972567	112	7	0.09505	no

gi|646742403|gb|KK962583.1|	94428-94857	11	8.5	0	33	inf	0.0004	yes
gi|646742413|gb|KK962582.1|	02188-10235	23	10	238	88	-1	0.3356	no
gi|646742413|gb|KK962582.1|	04737-10620	23	10	0.765198	0.330615	-1	1	no
gi|646742413|gb|KK962582.1|	08167-10851	23	10	3	9	2	0.2835	no
gi|646742413|gb|KK962582.1|	11106-12470	23	10	0.750767	2	1	0.42295	no
gi|646742413|gb|KK962582.1|	12825-11342	23	10	90	47	-0.945178	0.4669	no
gi|646742413|gb|KK962582.1|	14520-11527	23	10	2	0.785282	-1	0.47555	no
gi|646742413|gb|KK962582.1|	15472-11651	23	10	2	3	0.775686	0.59125	no
gi|646742413|gb|KK962582.1|	22288-12475	23	10	88	46	-0.933881	0.4295	no
gi|646742413|gb|KK962582.1|	27361-12763	23	10	59	48	-0.31324	0.83925	no
gi|646742413|gb|KK962582.1|	30236-13066	23	10	4	7	0.7119	0.6065	no
gi|646742413|gb|KK962582.1|	32772-13305	23	10	7	17	1	0.44955	no
gi|646742413|gb|KK962582.1|	52288-15281	23	10	21	13	-0.707546	0.6439	no
gi|646742413|gb|KK962582.1|	53024-15509	23	10	2	1	-0.509426	0.754	no
gi|646742413|gb|KK962582.1|	57069-15796	23	10	2	3	0.116692	0.9226	no
gi|646742413|gb|KK962582.1|	15958-18168	23	10	0.615206	1	0.742697	1	no
gi|646742413|gb|KK962582.1|	72567-17333	23	10	3	1	-1	0.3737	no
gi|646742413|gb|KK962582.1|	76286-17674	23	10	50	33	-0.606566	0.6863	no
gi|646742413|gb|KK962582.1|	74777-27548	23	10	4	7	1	0.4554	no
gi|646742413|gb|KK962582.1|	27785-28280	23	10	5	2	-1	0.38755	no
gi|646742413|gb|KK962582.1|	89532-29353	23	10	6	5	-0.320166	0.80355	no
gi|646742413|gb|KK962582.1|	30639-32173	23	10	18	12	-0.624939	0.6289	no
gi|646742413|gb|KK962582.1|	06792-30802	23	10	5	3	-0.76351	0.6224	no
gi|646742413|gb|KK962582.1|	32232-32731	23	10	7	11	0.573496	0.68115	no
gi|646742413|gb|KK962582.1|	23932-32426	23	10	7	11	0.649613	0.65	no
gi|646742413|gb|KK962582.1|	24804-32555	23	10	2	2	0.126493	0.9235	no
gi|646742413|gb|KK962582.1|	25685-32754	23	10	9	16	0.79427	0.5374	no
gi|646742413|gb|KK962582.1|	27978-32883	23	10	3	5	0.853843	0.535	no
gi|646742413|gb|KK962582.1|	34937-33555	23	10	1	5	2	0.24695	no
gi|646742413|gb|KK962582.1|	40238-34083	23	10	2	3	0.460995	0.74835	no

gi|646742413|gb|KK962582.1|	45714-34654	23	10	2	1	-0.502209	0.7124	no
gi|646742413|gb|KK962582.1|	52496-35302	23	10	1	3	1	0.4404	no
gi|646742413|gb|KK962582.1|	53244-35596	23	10	0.901894	1	0.202551	1	no
gi|646742413|gb|KK962582.1|	56583-36005	23	10	0.920033	1	0.297507	0.852	no
gi|646742413|gb|KK962582.1|	60225-36119	23	10	0.259112	2	3	0.2655	no
gi|646742413|gb|KK962582.1|	61411-36241	23	10	2	2	-0.127035	0.9237	no
gi|646742413|gb|KK962582.1|	62517-36333	23	10	3	2	-0.503748	0.7002	no
gi|646742413|gb|KK962582.1|	73419-37482	23	10	40	21	-0.904794	0.40945	no
gi|646742413|gb|KK962582.1|	85867-38763	23	10	52	44	-0.243242	0.83135	no
gi|646742413|gb|KK962582.1|	92723-39295	23	10	144	139	-0.047314	0.9748	no
gi|646742413|gb|KK962582.1|	99621-40055	23	10	2	1	-1	0.41125	no
gi|646742413|gb|KK962582.1|	01463-40433	23	10	1	2	1	0.43335	no
gi|646742413|gb|KK962582.1|	04604-40700	23	10	2	4	1	0.48325	no
gi|646742413|gb|KK962582.1|	07146-40797	23	10	2	3	0.497603	0.69045	no
gi|646742413|gb|KK962582.1|	08370-40930	23	10	3	10	2	0.3072	no
gi|646742413|gb|KK962582.1|	09721-41304	23	10	72	71	-0.0131399	0.991	no
gi|646742413|gb|KK962582.1|	75184-57975	23	10	5	6	0.357147	0.73865	no
gi|646742413|gb|KK962582.1|	83806-59107	23	10	3	5	0.674057	0.52845	no
gi|646742413|gb|KK962582.1|	18813-61925	23	10	3	5	0.815055	0.57265	no
gi|646742413|gb|KK962582.1|	52027-65273	23	10	5	4	-0.2542	0.8778	no
gi|646742413|gb|KK962582.1|	50361-75185	23	10	0	6	inf	0.00025	yes
gi|646742413|gb|KK962582.1|	32616-84143	23	10	3	5	0.705674	0.5224	no
gi|646742413|gb|KK962582.1|	97374-97946	23	10	8	9	0.056513	0.97135	no
gi|646742424|gb|KK962581.1|	34112-13478	11	7	4	2	-0.885792	0.4927	no
gi|646742424|gb|KK962581.1|	89899-39014	11	7	0	121	inf	0.00075	yes
gi|646742424|gb|KK962581.1|	95957-39614	11	7	41	56	0.43722	0.7663	no
gi|646742424|gb|KK962581.1|	69012-46980	11	7	2	1	-0.380591	0.7808	no
gi|646742424|gb|KK962581.1|	70688-47145	11	7	3	0.757558	-2	0.268	no
gi|646742438|gb|KK962580.1|	76539-27732	12	4.4	2	0.246114	-3	0.1863	no
gi|646742438|gb|KK962580.1|	52206-52750	12	4.4	6	2	-1	0.3191	no

gi|646742438|gb|KK962580.1|	54861-55187	12	4.4	10	1	-3	0.20685	no
gi|646742450|gb|KK962579.1|	45349-14752	19	7.6	18	15	-0.344827	0.74815	no
gi|646742450|gb|KK962579.1|	50242-15196	19	7.6	10	7	-0.61913	0.6758	no
gi|646742450|gb|KK962579.1|	64253-16681	19	7.6	0.68324	0.700596	0.03619	1	no
gi|646742450|gb|KK962579.1|	67037-16850	19	7.6	14	14	0.033059	0.98375	no
gi|646742450|gb|KK962579.1|	77642-17974	19	7.6	1	0.9422	-0.0861616	1	no
gi|646742450|gb|KK962579.1|	10948-21417	19	7.6	135	48	-1	0.22125	no
gi|646742450|gb|KK962579.1|	44277-24446	19	7.6	117	68	-0.779	0.5811	no
gi|646742450|gb|KK962579.1|	48192-24963	19	7.6	1	1	-0.322151	0.78865	no
gi|646742450|gb|KK962579.1|	49806-25166	19	7.6	0.403495	1	1	1	no
gi|646742450|gb|KK962579.1|	59402-25984	19	7.6	7	10	0.398739	0.7837	no
gi|646742450|gb|KK962579.1|	60665-26334	19	7.6	1	1	0.108777	0.943	no
gi|646742450|gb|KK962579.1|	68291-27011	19	7.6	18	16	-0.134375	0.90165	no
gi|646742450|gb|KK962579.1|	71321-27233	19	7.6	5	5	-0.173341	0.9129	no
gi|646742450|gb|KK962579.1|	75375-27606	19	7.6	2	6	1	0.3544	no
gi|646742450|gb|KK962579.1|	77630-27812	19	7.6	2	5	1	0.43	no
gi|646742450|gb|KK962579.1|	80585-28173	19	7.6	12	19	0.666917	0.6608	no
gi|646742450|gb|KK962579.1|	84398-28535	19	7.6	0.922392	3	2	0.31715	no
gi|646742450|gb|KK962579.1|	88404-88905	19	7.6	4	6	0.42998	0.7319	no
gi|646742471|gb|KK962577.1|	01901-10253	22	5.9	4	1	-2	0.24415	no
gi|646742471|gb|KK962577.1|	23147-12405	22	5.9	2	2	0.125864	0.9189	no
gi|646742471|gb|KK962577.1|	26987-12757	22	5.9	5	2	-1	0.4247	no
gi|646742471|gb|KK962577.1|	42327-14255	22	5.9	69	50	-0.460975	0.6988	no
gi|646742471|gb|KK962577.1|	17774-22307	22	5.9	6	4	-0.698912	0.58135	no
gi|646742471|gb|KK962577.1|	35786-24140	22	5.9	1	0.75582	-0.868457	0.5728	no
gi|646742471|gb|KK962577.1|	23978-24945	22	5.9	2	0.554361	-2	0.19185	no
gi|646742471|gb|KK962577.1|	41525-24268	22	5.9	0.306318	1	2	0.186	no
gi|646742471|gb|KK962577.1|	42853-24348	22	5.9	11	8	-0.404686	0.79355	no
gi|646742471|gb|KK962577.1|	43956-24487	22	5.9	2	1	-0.495265	0.7125	no
gi|646742471|gb|KK962577.1|	45177-24662	22	5.9	1	1	-0.239666	0.8481	no

gi|646742471|gb|KK962577.1|	47515-24827	22	5.9	1	2	1	0.4512	no
gi|646742471|gb|KK962577.1|	49821-25007	22	5.9	22	12	-0.881449	0.5727	no
gi|646742471|gb|KK962577.1|	50664-25140	22	5.9	5	7	0.657821	0.6768	no
gi|646742471|gb|KK962577.1|	53978-25498	22	5.9	2	1	-0.382689	0.77245	no
gi|646742471|gb|KK962577.1|	70835-27111	22	5.9	25	4	-3	0.1429	no
gi|646742471|gb|KK962577.1|	80605-28106	22	5.9	5	4	-0.24246	0.8558	no
gi|646742471|gb|KK962577.1|	01615-30192	22	5.9	50	27	-0.898645	0.5756	no
gi|646742471|gb|KK962577.1|	15029-31569	22	5.9	2	3	0.338184	0.7979	no
gi|646742471|gb|KK962577.1|	31995-33234	22	5.9	11	13	0.309522	0.8084	no
gi|646742471|gb|KK962577.1|	93251-39402	22	5.9	2	2	0.0320784	0.94695	no
gi|646742471|gb|KK962577.1|	05126-40625	22	5.9	16	17	0.117439	0.93525	no
gi|646742471|gb|KK962577.1|	11644-41295	22	5.9	0.52513	0.62862	0.259513	1	no
gi|646742471|gb|KK962577.1|	13885-41463	22	5.9	1	2	0.488678	0.72995	no
gi|646742471|gb|KK962577.1|	26314-42714	22	5.9	2	3	0.497884	0.69045	no
gi|646742471|gb|KK962577.1|	48597-51435	22	5.9	1	1	0.33374	0.8307	no
gi|646742471|gb|KK962577.1|	06560-50776	22	5.9	7	6	-0.154563	0.91405	no
gi|646742471|gb|KK962577.1|	41721-54341	22	5.9	86	46	-0.886004	0.45005	no
gi|646742471|gb|KK962577.1|	66824-67171	22	5.9	12	1	-3	0.18235	no
gi|646742471|gb|KK962577.1|	71362-73013	22	5.9	0.795251	0.382702	-1	1	no
gi|646742471|gb|KK962577.1|	82012-83348	22	5.9	21	11	-0.919016	0.4676	no
gi|646742488|gb|KK962576.1|	73735-17739	19	9.1	7	2	-2	0.23745	no
gi|646742488|gb|KK962576.1|	78339-18055	19	9.1	4	3	-0.33246	0.83045	no
gi|646742488|gb|KK962576.1|	84307-18521	19	9.1	6	2	-2	0.1973	no
gi|646742488|gb|KK962576.1|	94424-19633	19	9.1	6	3	-1	0.49105	no
gi|646742488|gb|KK962576.1|	96619-19925	19	9.1	4	2	-1	0.51015	no
gi|646742488|gb|KK962576.1|	08003-21027	19	9.1	7	4	-0.658838	0.66045	no
gi|646742488|gb|KK962576.1|	12066-21932	19	9.1	2	2	-0.65604	0.6674	no
gi|646742488|gb|KK962576.1|	21111-22220	19	9.1	9	3	-1	0.408	no
gi|646742488|gb|KK962576.1|	26819-22934	19	9.1	10	3	-2	0.23585	no
gi|646742488|gb|KK962576.1|	34832-23575	19	9.1	1	0.989989	-0.494981	0.73195	no

gi|646742488|gb|KK962576.1|	36503-23776	19	9.1	1	0.659154	-0.74196	1	no
gi|646742488|gb|KK962576.1|	62830-26358	19	9.1	40	22	-0.86581	0.49205	no
gi|646742488|gb|KK962576.1|	75484-27617	19	9.1	29	5	-3	0.13995	no
gi|646742488|gb|KK962576.1|	80177-28140	19	9.1	0.762176	0.81965	0.104884	1	no
gi|646742488|gb|KK962576.1|	92542-29330	19	9.1	1	2	0.0761177	0.92975	no
gi|646742488|gb|KK962576.1|	94590-29594	19	9.1	2	3	0.272247	0.865	no
gi|646742488|gb|KK962576.1|	96861-29889	19	9.1	1	1	0.119597	0.94085	no
gi|646742488|gb|KK962576.1|	00072-30160	19	9.1	0.506327	0.72994	0.527708	1	no
gi|646742488|gb|KK962576.1|	16687-31749	19	9.1	2	3	0.621485	0.62655	no
gi|646742488|gb|KK962576.1|	24611-32657	19	9.1	0.757444	1	0.974499	0.50205	no
gi|646742488|gb|KK962576.1|	26683-32903	19	9.1	0.48485	2	2	0.2719	no
gi|646742488|gb|KK962576.1|	50219-35227	19	9.1	10	4	-1	0.3788	no
gi|646742488|gb|KK962576.1|	59077-36059	19	9.1	17	10	-0.754596	0.55105	no
gi|646742488|gb|KK962576.1|	61561-36300	19	9.1	25	9	-1	0.277	no
gi|646742488|gb|KK962576.1|	63277-36553	19	9.1	11	4	-2	0.23615	no
gi|646742488|gb|KK962576.1|	68284-37358	19	9.1	14	5	-2	0.1775	no
gi|646742488|gb|KK962576.1|	84884-38592	19	9.1	56	28	-1	0.362	no
gi|646742488|gb|KK962576.1|	87372-39104	19	9.1	7	3	-1	0.2858	no
gi|646742488|gb|KK962576.1|	91257-39435	19	9.1	5	2	-1	0.3743	no
gi|646742488|gb|KK962576.1|	94520-39623	19	9.1	0.252773	1	3	0.20675	no
gi|646742488|gb|KK962576.1|	97634-39791	19	9.1	0	24	inf	0.001	yes
gi|646742488|gb|KK962576.1|	6293-6738	19	9.1	9	10	0.230725	0.86825	no
gi|646742488|gb|KK962576.1|	7013-7192	19	9.1	303.43	271	-0.162949	0.909	no
gi|646742529|gb|KK962573.1|	24285-22507	21	7.3	13	15	0.223445	0.8811	no
gi|646742541|gb|KK962572.1|	20836-21370	19	10	3	0.896512	-2	0.3832	no
gi|646742541|gb|KK962572.1|	22530-32480	19	10	5	3	-0.83136	0.58135	no
gi|646742541|gb|KK962572.1|	70919-37207	19	10	10	7	-0.467939	0.75795	no
gi|646742541|gb|KK962572.1|	07317-41178	19	10	2	3	0.498157	0.7428	no
gi|646742541|gb|KK962572.1|	11888-41376	19	10	4	5	0.293688	0.84855	no
gi|646742541|gb|KK962572.1|	19427-42080	19	10	1	1	0.109355	0.92895	no

gi|646742541|gb|KK962572.1|	67959-47208	19	10	59	140	1	0.31345	no
gi|646742541|gb|KK962572.1|	72377-47587	19	10	144	359	1	0.27335	no
gi|646742541|gb|KK962572.1|	81865-48296	19	10	2	1	-0.29219	0.81635	no
gi|646742541|gb|KK962572.1|	90146-49864	19	10	28	14	-1	0.3871	no
gi|646742541|gb|KK962572.1|	04418-50553	19	10	11	7	-0.766228	0.61395	no
gi|646742541|gb|KK962572.1|	06199-50676	19	10	7	4	-0.96172	0.51835	no
gi|646742541|gb|KK962572.1|	09027-50926	19	10	44	0	-inf	0.0041	no
gi|646742541|gb|KK962572.1|	14987-51553	19	10	2	3	0.223668	0.87175	no
gi|646742541|gb|KK962572.1|	23990-52448	19	10	6	2	-1	0.32	no
gi|646742541|gb|KK962572.1|	30978-53182	19	10	1	0.659417	-0.91535	0.58965	no
gi|646742541|gb|KK962572.1|	32858-53419	19	10	0.770923	0.738606	-0.0617827	1	no
gi|646742541|gb|KK962572.1|	35683-53751	19	10	0.765077	1	0.416827	1	no
gi|646742541|gb|KK962572.1|	48933-54924	19	10	151	59	-1	0.37885	no
gi|646742541|gb|KK962572.1|	69332-57007	19	10	33	5	-3	0.0949	no
gi|646742541|gb|KK962572.1|	70528-57267	19	10	23	18	-0.399623	0.71925	no
gi|646742541|gb|KK962572.1|	79488-58019	19	10	229	115	-0.997194	0.39665	no
gi|646742541|gb|KK962572.1|	80326-58178	19	10	3	3	0.232466	0.87985	no
gi|646742541|gb|KK962572.1|	82650-58338	19	10	3	12	2	0.1935	no
gi|646742541|gb|KK962572.1|	83482-58558	19	10	2	3	0.455147	0.7696	no
gi|646742541|gb|KK962572.1|	86421-59829	19	10	15	9	-0.718427	0.5492	no
gi|646742541|gb|KK962572.1|	29399-62968	19	10	44	45	0.0255032	0.9789	no
gi|646742541|gb|KK962572.1|	31237-63269	19	10	60	19	-2	0.1387	no
gi|646742541|gb|KK962572.1|	32866-63386	19	10	0.992001	0.529831	-0.904809	1	no
gi|646742541|gb|KK962572.1|	34035-63528	19	10	0.928811	1	0.198803	1	no
gi|646742541|gb|KK962572.1|	36894-64555	19	10	10	9	-0.169289	0.8804	no
gi|646742541|gb|KK962572.1|	45830-64815	19	10	0.848379	1	0.289234	1	no
gi|646742541|gb|KK962572.1|	48402-64998	19	10	1	0.704497	-0.570954	1	no
gi|646742541|gb|KK962572.1|	69599-70437	19	10	0.951952	2	1	0.3983	no
gi|646742541|gb|KK962572.1|	87375-91053	19	10	3	5	0.435408	0.7724	no
gi|646742553|gb|KK962571.1|	45566-14608	20	8.4	7	4	-0.956519	0.46115	no

gi|646742553|gb|KK962571.1|	82369-18291	20	8.4	0.942859	4	2	0.20605	no
gi|646742553|gb|KK962571.1|	33498-23416	20	8.4	9	2	-2	0.2264	no
gi|646742553|gb|KK962571.1|	41125-24201	20	8.4	1	0.206431	-3	0.2033	no
gi|646742553|gb|KK962571.1|	43565-24454	20	8.4	11	12	0.0827566	0.95535	no
gi|646742553|gb|KK962571.1|	51020-25183	20	8.4	39	27	-0.497035	0.69135	no
gi|646742553|gb|KK962571.1|	52893-25534	20	8.4	4	3	-0.33075	0.8309	no
gi|646742553|gb|KK962571.1|	96247-29697	20	8.4	3	5	0.485852	0.72195	no
gi|646742553|gb|KK962571.1|	08894-30925	20	8.4	11	22	0.97676	0.49815	no
gi|646742553|gb|KK962571.1|	19174-31997	20	8.4	23	9	-1	0.39215	no
gi|646742553|gb|KK962571.1|	21814-32317	20	8.4	1	0.722868	-0.798116	0.5562	no
gi|646742553|gb|KK962571.1|	23676-32533	20	8.4	11	8	-0.470034	0.7497	no
gi|646742553|gb|KK962571.1|	36465-33830	20	8.4	51	21	-1	0.2505	no
gi|646742553|gb|KK962571.1|	70727-37200	20	8.4	18	20	0.225489	0.8537	no
gi|646742553|gb|KK962571.1|	72648-37527	20	8.4	11	17	0.681461	0.53385	no
gi|646742553|gb|KK962571.1|	75677-37815	20	8.4	7	13	0.88988	0.48775	no
gi|646742553|gb|KK962571.1|	79288-38078	20	8.4	11	12	0.1826	0.90535	no
gi|646742553|gb|KK962571.1|	90020-39158	20	8.4	35	28	-0.336017	0.75575	no
gi|646742553|gb|KK962571.1|	98247-39930	20	8.4	5	5	0.00814777	0.98525	no
gi|646742553|gb|KK962571.1|	00122-40244	20	8.4	3	2	-0.424409	0.7772	no
gi|646742553|gb|KK962571.1|	14977-41550	20	8.4	4	1	-2	0.2828	no
gi|646742553|gb|KK962571.1|	44331-44877	20	8.4	24	18	-0.38944	0.7863	no
gi|646742576|gb|KK962569.1|	82555-18319	16	6.3	1	2	0.734471	0.64505	no
gi|646742576|gb|KK962569.1|	83805-18666	16	6.3	1	0.618859	-0.874724	0.528	no
gi|646742608|gb|KK962566.1|	73037-17377	14	8.8	0.955421	2	0.97319	0.5626	no
gi|646742608|gb|KK962566.1|	80154-18107	14	8.8	1	1	0.14259	0.9186	no
gi|646742608|gb|KK962566.1|	42149-42406	14	8.8	15	27	0.870583	0.54995	no
gi|646742618|gb|KK962565.1|	16623-21715	10	5.9	0	5	inf	0.00255	no
gi|646742618|gb|KK962565.1|	18790-21941	10	5.9	0	37	inf	5.00E-05	yes
gi|646742632|gb|KK962564.1|	53320-35478	15	8.9	0.914456	1	0.526391	0.6791	no
gi|646742647|gb|KK962563.1|	58811-15912	21	10	9	27	2	0.3159	no

gi|646742647|gb|KK962563.1|	85166-18605	21	10	4	5	0.0555008	0.9719	no
gi|646742647|gb|KK962563.1|	87421-18801	21	10	4	4	0.114078	0.9298	no
gi|646742647|gb|KK962563.1|	91970-19225	21	10	34	47	0.460463	0.72965	no
gi|646742647|gb|KK962563.1|	89789-39065	21	10	0.911322	3	2	0.28735	no
gi|646742647|gb|KK962563.1|	69704-70672	21	10	8	6	-0.467452	0.76135	no
gi|646742685|gb|KK962560.1|	14375-18726	21	9.5	28	26	-0.116404	0.92525	no
gi|646742685|gb|KK962560.1|	2076-2451	21	9.5	17	21	0.342834	0.8276	no
gi|646742685|gb|KK962560.1|	22477-23725	21	9.5	0.838455	0.93554	0.158066	1	no
gi|646742685|gb|KK962560.1|	23875-26036	21	9.5	0.825181	0.63335	-0.381708	1	no
gi|646742685|gb|KK962560.1|	58327-25920	21	9.5	0	12	inf	0.00025	yes
gi|646742685|gb|KK962560.1|	26146-26879	21	9.5	2	1	-0.972473	0.54505	no
gi|646742685|gb|KK962560.1|	87173-29006	21	9.5	0	9	inf	5.00E-05	yes
gi|646742685|gb|KK962560.1|	33461-34156	21	9.5	0.840571	2	1	0.4862	no
gi|646742685|gb|KK962560.1|	37808-39278	21	9.5	0.989846	0.767694	-0.366672	1	no
gi|646742685|gb|KK962560.1|	02146-40546	21	9.5	2	15	3	0.0359	no
gi|646742685|gb|KK962560.1|	43174-59879	21	9.5	2	4	0.91278	0.42265	no
gi|646742685|gb|KK962560.1|	4739-5285	21	9.5	9	13	0.495971	0.75675	no
gi|646742685|gb|KK962560.1|	65325-70524	21	9.5	23	16	-0.545536	0.636	no
gi|646742685|gb|KK962560.1|	7340-8264	21	9.5	13	26	0.938948	0.5364	no
gi|646742685|gb|KK962560.1|	91097-92074	21	9.5	24	17	-0.483379	0.7567	no
gi|646742685|gb|KK962560.1|	92724-93773	21	9.5	0.932044	0.997358	0.0977134	1	no
gi|646742685|gb|KK962560.1|	99108-99475	21	9.5	10	9	-0.196479	0.87605	no
gi|646742706|gb|KK962559.1|	35467-13700	22	5.8	15	16	0.0917624	0.9314	no
gi|646742706|gb|KK962559.1|	53171-15400	22	5.8	4	1	-2	0.27165	no
gi|646742706|gb|KK962559.1|	88198-18878	22	5.8	2	3	0.265201	0.84035	no
gi|646742706|gb|KK962559.1|	20748-22156	22	5.8	0.481414	2	2	0.24775	no
gi|646742706|gb|KK962559.1|	3281-4015	22	5.8	2	6	1	0.32645	no
gi|646742706|gb|KK962559.1|	35870-33668	22	5.8	0.831804	1	0.759088	0.61475	no
gi|646742706|gb|KK962559.1|	40064-34138	22	5.8	0.60521	1	1	0.44735	no
gi|646742706|gb|KK962559.1|	43450-34441	22	5.8	0.393072	2	2	0.18785	no

gi|646742706|gb|KK962559.1|	34392-35310	22	5.8	3	4	0.505019	0.71415	no
gi|646742706|gb|KK962559.1|	48656-35008	22	5.8	32	16	-1	0.35095	no
gi|646742706|gb|KK962559.1|	51639-35214	22	5.8	4	5	0.430843	0.73175	no
gi|646742706|gb|KK962559.1|	63375-36437	22	5.8	2	2	0.131605	0.9183	no
gi|646742706|gb|KK962559.1|	69893-37147	22	5.8	5	3	-0.760943	0.62085	no
gi|646742706|gb|KK962559.1|	73286-37346	22	5.8	173	32	-2	0.18175	no
gi|646742706|gb|KK962559.1|	80514-38697	22	5.8	2	1	-0.91452	0.54455	no
gi|646742706|gb|KK962559.1|	01365-40159	22	5.8	13	40	2	0.2843	no
gi|646742706|gb|KK962559.1|	19850-42071	22	5.8	2	3	0.607713	0.6265	no
gi|646742706|gb|KK962559.1|	44588-45852	22	5.8	4	0.657876	-3	0.1332	no
gi|646742706|gb|KK962559.1|	47689-48773	22	5.8	1	0.637572	-0.80752	1	no
gi|646742706|gb|KK962559.1|	51426-52022	22	5.8	2	2	0.461501	0.7494	no
gi|646742706|gb|KK962559.1|	60655-63986	22	5.8	2	2	0.339346	0.81865	no
gi|646742706|gb|KK962559.1|	69862-71120	22	5.8	1	0.26469	-2	1	no
gi|646742706|gb|KK962559.1|	92949-93613	22	5.8	3	0.314345	-3	0.179	no
gi|646742706|gb|KK962559.1|	95736-95945	22	5.8	123	31	-2	0.17995	no
gi|646742717|gb|KK962558.1|	49871-50776	12	6.2	3	2	-0.888233	0.48915	no
gi|646742726|gb|KK962557.1|	14324-14834	20	9.1	9	24	1	0.3928	no
gi|646742726|gb|KK962557.1|	72815-17581	20	9.1	17	12	-0.42737	0.7018	no
gi|646742726|gb|KK962557.1|	80934-18315	20	9.1	0.563845	0.477021	-0.241246	1	no
gi|646742726|gb|KK962557.1|	18138-19054	20	9.1	4	2	-1	0.4171	no
gi|646742726|gb|KK962557.1|	83270-18712	20	9.1	6	6	-0.110085	0.91565	no
gi|646742726|gb|KK962557.1|	87392-18763	20	9.1	134	238.72	0.83327	0.59185	no
gi|646742726|gb|KK962557.1|	89224-19023	20	9.1	1	2	0.358722	0.79375	no
gi|646742726|gb|KK962557.1|	93535-19497	20	9.1	0.548801	0.564315	0.0402178	1	no
gi|646742726|gb|KK962557.1|	95682-19721	20	9.1	0.579112	0.730501	0.335044	1	no
gi|646742726|gb|KK962557.1|	97641-19888	20	9.1	9	11	0.216566	0.88705	no
gi|646742726|gb|KK962557.1|	02632-20370	20	9.1	90	268	2	0.18895	no
gi|646742726|gb|KK962557.1|	05565-20623	20	9.1	54	269	2	0.049	no
gi|646742726|gb|KK962557.1|	07125-20743	20	9.1	276	915	2	0.1253	no

gi|646742726|gb|KK962557.1|	09605-21026	20	9.1	2	1	-0.694054	0.64585	no
gi|646742726|gb|KK962557.1|	11675-21192	20	9.1	64	242	2	0.24165	no
gi|646742726|gb|KK962557.1|	15003-21630	20	9.1	45	162	2	0.11675	no
gi|646742726|gb|KK962557.1|	16425-21716	20	9.1	228	117	-0.961572	0.3985	no
gi|646742726|gb|KK962557.1|	29691-23016	20	9.1	24	49	1	0.4923	no
gi|646742726|gb|KK962557.1|	60330-26092	20	9.1	4	11	1	0.3381	no
gi|646742726|gb|KK962557.1|	63079-26399	20	9.1	1	2	0.183161	0.89685	no
gi|646742726|gb|KK962557.1|	26388-29144	20	9.1	15	31	1	0.3352	no
gi|646742726|gb|KK962557.1|	67747-26794	20	9.1	10	115	4	0.1022	no
gi|646742726|gb|KK962557.1|	71264-27161	20	9.1	4	7	0.801432	0.6065	no
gi|646742726|gb|KK962557.1|	01305-30219	20	9.1	2	5	2	0.30965	no
gi|646742726|gb|KK962557.1|	13954-31425	20	9.1	16	52	2	0.25335	no
gi|646742726|gb|KK962557.1|	15066-31561	20	9.1	1	4	2	0.2794	no
gi|646742726|gb|KK962557.1|	32831-33307	20	9.1	13	41	2	0.27795	no
gi|646742726|gb|KK962557.1|	47924-34811	20	9.1	104	208	0.996338	0.52125	no
gi|646742726|gb|KK962557.1|	55713-35631	20	9.1	2	3	0.463712	0.74835	no
gi|646742726|gb|KK962557.1|	57006-35720	20	9.1	8	102	4	0.10175	no
gi|646742726|gb|KK962557.1|	57808-35839	20	9.1	2	7	2	0.3079	no
gi|646742726|gb|KK962557.1|	60127-36079	20	9.1	0.448718	4	3	0.24135	no
gi|646742726|gb|KK962557.1|	62167-36242	20	9.1	15	22	0.548655	0.71465	no
gi|646742726|gb|KK962557.1|	63736-36432	20	9.1	0.274615	5	4	0.0994	no
gi|646742726|gb|KK962557.1|	64803-36527	20	9.1	5	9	0.71519	0.6263	no
gi|646742726|gb|KK962557.1|	72100-37233	20	9.1	25	56	1	0.476	no
gi|646742726|gb|KK962557.1|	73402-37374	20	9.1	16	39	1	0.35535	no
gi|646742726|gb|KK962557.1|	74161-37463	20	9.1	10	25	1	0.40085	no
gi|646742726|gb|KK962557.1|	77130-37764	20	9.1	3	8	1	0.40175	no
gi|646742726|gb|KK962557.1|	37824-40312	20	9.1	0.248594	0.481353	0.953302	1	no
gi|646742726|gb|KK962557.1|	82780-38313	20	9.1	8	32	2	0.20555	no
gi|646742726|gb|KK962557.1|	84260-38459	20	9.1	16	61	2	0.1874	no
gi|646742726|gb|KK962557.1|	90019-39028	20	9.1	22	54	1	0.39185	no

gi|646742726|gb|KK962557.1|	91084-39133	20	9.1	25	42	0.752862	0.5633	no
gi|646742726|gb|KK962557.1|	92251-39319	20	9.1	0.267735	2	3	0.2578	no
gi|646742726|gb|KK962557.1|	99614-40096	20	9.1	0.931035	4	2	0.2016	no
gi|646742726|gb|KK962557.1|	08853-40904	20	9.1	41	75	0.852258	0.59005	no
gi|646742726|gb|KK962557.1|	12867-41311	20	9.1	15	82	2	0.1674	no
gi|646742726|gb|KK962557.1|	16394-41767	20	9.1	0.62932	3	2	0.15045	no
gi|646742726|gb|KK962557.1|	20487-42069	20	9.1	28	80	2	0.3404	no
gi|646742726|gb|KK962557.1|	30025-43190	20	9.1	0.739672	2	1	0.3286	no
gi|646742726|gb|KK962557.1|	33562-43619	20	9.1	0.233634	1	2	1	no
gi|646742726|gb|KK962557.1|	50623-50843	20	9.1	5	100	4	0.11035	no
gi|646742736|gb|KK962556.1|	92465-19322	17	8.1	1	2	0.904855	0.54495	no
gi|646742736|gb|KK962556.1|	22050-23269	17	8.1	8	5	-0.592787	0.6939	no
gi|646742736|gb|KK962556.1|	27549-22782	17	8.1	97	52	-0.895383	0.56135	no
gi|646742736|gb|KK962556.1|	23378-24397	17	8.1	1	0.690271	-0.658714	1	no
gi|646742736|gb|KK962556.1|	26048-29144	17	8.1	9	6	-0.436997	0.7243	no
gi|646742745|gb|KK962555.1|	40820-14254	13	5.6	7	0.27316	-5	0.078	no
gi|646742745|gb|KK962555.1|	39262-39551	13	5.6	177	4	-6	0.10695	no
gi|646742745|gb|KK962555.1|	64003-64645	13	5.6	6	4	-0.823615	0.55975	no
gi|646742745|gb|KK962555.1|	67375-68556	13	5.6	31	0.715452	-5	0.0281	no
gi|646742745|gb|KK962555.1|	71282-71917	13	5.6	20	0.673682	-5	0.10915	no
gi|646742761|gb|KK962554.1|	26456-26971	16	8.3	0	9	inf	0.00435	no
gi|646742761|gb|KK962554.1|	13215-31453	16	8.3	2	0.749484	-1	0.4168	no
gi|646742761|gb|KK962554.1|	15900-31692	16	8.3	1	0.855226	-0.488419	0.73195	no
gi|646742761|gb|KK962554.1|	17098-31880	16	8.3	1	0.921666	-0.23492	1	no
gi|646742761|gb|KK962554.1|	19597-32345	16	8.3	1	0.673958	-1	0.49225	no
gi|646742761|gb|KK962554.1|	23666-32410	16	8.3	5	0.650875	-3	0.20135	no
gi|646742761|gb|KK962554.1|	72011-37255	16	8.3	3	3	0.0303459	0.9298	no
gi|646742775|gb|KK962553.1|	84927-28535	12	4.9	7	3	-1	0.32335	no
gi|646742775|gb|KK962553.1|	08350-30903	12	4.9	0	3	inf	0.0014	yes
gi|646742775|gb|KK962553.1|	11912-31238	12	4.9	0	59	inf	5.00E-05	yes

gi|646742789|gb|KK962552.1|	02681-10573	17	7.3	16	20	0.261028	0.8199	no
gi|646742789|gb|KK962552.1|	16949-11772	17	7.3	2	4	1	0.4627	no
gi|646742789|gb|KK962552.1|	19594-11985	17	7.3	23	50	1	0.4656	no
gi|646742789|gb|KK962552.1|	20051-12043	17	7.3	7	23	2	0.2713	no
gi|646742789|gb|KK962552.1|	34785-13514	17	7.3	4	9	1	0.4374	no
gi|646742789|gb|KK962552.1|	41897-14220	17	7.3	4	32	3	0.14815	no
gi|646742789|gb|KK962552.1|	78173-17845	17	7.3	56	82	0.545567	0.7225	no
gi|646742789|gb|KK962552.1|	89397-19059	17	7.3	9	4	-1	0.379	no
gi|646742789|gb|KK962552.1|	98345-19871	17	7.3	18	5	-2	0.25335	no
gi|646742789|gb|KK962552.1|	21627-22232	17	7.3	3	8	1	0.38015	no
gi|646742789|gb|KK962552.1|	28221-22891	17	7.3	0	31	inf	5.00E-05	yes
gi|646742789|gb|KK962552.1|	80402-38077	17	7.3	5	5	-0.148026	0.91555	no
gi|646742789|gb|KK962552.1|	92076-39302	17	7.3	3	4	0.506914	0.714	no
gi|646742789|gb|KK962552.1|	01609-40252	17	7.3	0.978111	0.594862	-0.717444	1	no
gi|646742789|gb|KK962552.1|	15346-41577	17	7.3	5	0.669552	-3	0.21305	no
gi|646742789|gb|KK962552.1|	90078-92997	17	7.3	62	59	-0.0513389	0.9649	no
gi|646742827|gb|KK962549.1|	40852-24144	16	6.5	2	2	0.460484	0.7494	no
gi|646742827|gb|KK962549.1|	42438-24349	16	6.5	0.1152	3	5	0.11005	no
gi|646742827|gb|KK962549.1|	52560-25374	16	6.5	0.198303	3	4	0.2606	no
gi|646742827|gb|KK962549.1|	64189-26553	16	6.5	0.169434	2	4	0.2606	no
gi|646742827|gb|KK962549.1|	69545-27024	16	6.5	0.205944	3	4	0.09985	no
gi|646742827|gb|KK962549.1|	38109-34093	16	6.5	0	2	inf	0.00025	yes
gi|646742842|gb|KK962548.1|	07437-10823	22	10	0.680118	3	2	0.1908	no
gi|646742842|gb|KK962548.1|	08611-11090	22	10	13	16	0.241443	0.82615	no
gi|646742842|gb|KK962548.1|	11179-11144	22	10	25	8	-2	0.28835	no
gi|646742842|gb|KK962548.1|	13744-11449	22	10	57	48	-0.24884	0.82115	no
gi|646742842|gb|KK962548.1|	57935-15836	22	10	7	4	-0.835291	0.5286	no
gi|646742842|gb|KK962548.1|	58719-15896	22	10	30	10	-2	0.2984	no
gi|646742842|gb|KK962548.1|	63206-16355	22	10	29	9	-2	0.26405	no
gi|646742842|gb|KK962548.1|	71340-17203	22	10	1	2	0.481988	0.7489	no

gi|646742842|gb|KK962548.1|	15350-21554	22	10	143	39	-2	0.22655	no
gi|646742842|gb|KK962548.1|	67463-27042	22	10	1	0.596201	-0.961738	0.4902	no
gi|646742842|gb|KK962548.1|	71160-27418	22	10	0.93522	0.680373	-0.458979	1	no
gi|646742842|gb|KK962548.1|	74426-27646	22	10	0.778324	0.751666	-0.0502786	1	no
gi|646742842|gb|KK962548.1|	76633-28275	22	10	20	10	-1	0.36165	no
gi|646742842|gb|KK962548.1|	85888-28916	22	10	52	35.48	-0.543233	0.6432	no
gi|646742842|gb|KK962548.1|	00049-30116	22	10	0.646902	2	1	0.3407	no
gi|646742842|gb|KK962548.1|	12994-31669	22	10	34	26	-0.385308	0.7394	no
gi|646742842|gb|KK962548.1|	29781-33058	22	10	3	3	0.0476836	0.95565	no
gi|646742842|gb|KK962548.1|	31007-33182	22	10	2	4	0.998949	0.5252	no
gi|646742842|gb|KK962548.1|	33998-34831	22	10	2	0.904118	-0.961269	0.54505	no
gi|646742842|gb|KK962548.1|	46094-34795	22	10	0.461713	0.667926	0.532693	1	no
gi|646742842|gb|KK962548.1|	50105-35059	22	10	2	4	0.646962	0.6597	no
gi|646742842|gb|KK962548.1|	39312-39568	22	10	30	64	1	0.4656	no
gi|646742842|gb|KK962548.1|	89495-90174	22	10	3	9	2	0.2526	no
gi|646742842|gb|KK962548.1|	98569-99109	22	10	2	1	-0.77735	0.633	no
gi|646742855|gb|KK962547.1|	09634-21108	14	5.2	1	2	0.760444	0.5849	no
gi|646742855|gb|KK962547.1|	18584-21903	14	5.2	23	7	-2	0.24795	no
gi|646742855|gb|KK962547.1|	45619-24731	14	5.2	2	2	0.14712	0.92335	no
gi|646742855|gb|KK962547.1|	47490-24796	14	5.2	5	6	0.291663	0.81415	no
gi|646742855|gb|KK962547.1|	77282-27772	14	5.2	1	6	2	0.19685	no
gi|646742855|gb|KK962547.1|	78053-27948	14	5.2	0.392	2	2	0.22455	no
gi|646742869|gb|KK962546.1|	37897-13824	14	6.5	5	6	0.306861	0.8529	no
gi|646742869|gb|KK962546.1|	41937-43189	14	6.5	1	0.931869	-0.479237	0.71555	no
gi|646742869|gb|KK962546.1|	44359-46383	14	6.5	0.836782	0.606032	-0.465456	1	no
gi|646742869|gb|KK962546.1|	46516-48473	14	6.5	1	0.865672	-0.591691	0.6633	no
gi|646742869|gb|KK962546.1|	48557-50399	14	6.5	1	1	-0.274861	0.81675	no
gi|646742902|gb|KK962544.1|	28958-22990	18	7.1	0.133289	2	4	0.1061	no
gi|646742902|gb|KK962544.1|	92614-29529	18	7.1	5	5	-0.0346017	0.9823	no
gi|646742902|gb|KK962544.1|	96118-29658	18	7.1	3	5	0.776603	0.5884	no

gi|646742902|gb|KK962544.1|	96934-29895	18	7.1	5	4	-0.465477	0.76145	no
gi|646742902|gb|KK962544.1|	66507-36790	18	7.1	11	6	-0.883254	0.56285	no
gi|646742902|gb|KK962544.1|	69204-37048	18	7.1	4	4	0.208374	0.8903	no
gi|646742902|gb|KK962544.1|	88452-38922	18	7.1	2	2	-0.400649	0.7698	no
gi|646742902|gb|KK962544.1|	89345-39079	18	7.1	1	0.555536	-0.95936	1	no
gi|646742914|gb|KK962543.1|	61295-26198	15	6.3	0.633296	2	2	0.25705	no
gi|646742927|gb|KK962542.1|	02547-10355	21	6.4	1	0.880759	-0.337657	1	no
gi|646742927|gb|KK962542.1|	50558-15136	21	6.4	0.665444	2	1	0.36865	no
gi|646742927|gb|KK962542.1|	03701-40393	21	6.4	51	38	-0.424781	0.7292	no
gi|646742927|gb|KK962542.1|	57492-58100	21	6.4	1	3	1	0.43555	no
gi|646742927|gb|KK962542.1|	01143-60164	21	6.4	43	12	-2	0.2298	no
gi|646742927|gb|KK962542.1|	64844-65693	21	6.4	29	10	-2	0.3359	no
gi|646742927|gb|KK962542.1|	73621-75416	21	6.4	6	2	-2	0.28965	no
gi|646742927|gb|KK962542.1|	76092-77221	21	6.4	3	0.605564	-2	0.1985	no
gi|646742927|gb|KK962542.1|	80747-81035	21	6.4	15	2	-3	0.20535	no
gi|646742941|gb|KK962541.1|	15552-31766	20	6.1	18	0.072361	-8	0.1665	no
gi|646742941|gb|KK962541.1|	42956-34354	20	6.1	1	2	0.459967	0.7722	no
gi|646742941|gb|KK962541.1|	65000-36576	20	6.1	2	0	-inf	0.00655	no
gi|646742941|gb|KK962541.1|	68822-37079	20	6.1	1	0	-inf	1	no
gi|646742941|gb|KK962541.1|	70867-37220	20	6.1	1	0	-inf	0.0011	yes
gi|646742941|gb|KK962541.1|	72382-37428	20	6.1	1	0	-inf	0.00525	no
gi|646742941|gb|KK962541.1|	74777-37616	20	6.1	1	0	-inf	0.00095	yes
gi|646742941|gb|KK962541.1|	77646-37855	20	6.1	2	0	-inf	0.0041	no
gi|646742941|gb|KK962541.1|	86622-38769	20	6.1	2	0.162446	-4	0.17455	no
gi|646742941|gb|KK962541.1|	92174-39410	20	6.1	1	0	-inf	1	no
gi|646742941|gb|KK962541.1|	94218-39561	20	6.1	1	0	-inf	0.00095	yes
gi|646742941|gb|KK962541.1|	98473-39963	20	6.1	1	0	-inf	0.0035	no
gi|646742941|gb|KK962541.1|	99702-40105	20	6.1	2	0	-inf	0.00045	yes
gi|646742941|gb|KK962541.1|	02698-40470	20	6.1	3	0	-inf	0.00085	yes
gi|646742941|gb|KK962541.1|	04753-40549	20	6.1	2	0	-inf	0.00475	no

gi|646742941|gb|KK962541.1|	78948-47953	20	6.1	6	2	-2	0.29145	no
gi|646742941|gb|KK962541.1|	94196-59518	20	6.1	1	1	-0.00531658	0.9367	no
gi|646742969|gb|KK962539.1|	62227-16252	12	6.7	3	24	3	0.27805	no
gi|646742969|gb|KK962539.1|	53997-25634	12	6.7	2	1	-0.344048	0.82725	no
gi|646743020|gb|KK962535.1|	80813-18166	18	5.6	6	0.662637	-3	0.11695	no
gi|646743020|gb|KK962535.1|	12908-21355	18	5.6	1	2	0.472637	0.7721	no
gi|646743020|gb|KK962535.1|	39361-23996	18	5.6	6	5	-0.35941	0.79345	no
gi|646743020|gb|KK962535.1|	2866-3279	18	5.6	5	4	-0.207	0.8859	no
gi|646743020|gb|KK962535.1|	44501-46191	18	5.6	0.580231	0.372461	-0.639538	1	no
gi|646743020|gb|KK962535.1|	453-1036	18	5.6	3	0.386338	-3	0.2136	no
gi|646743020|gb|KK962535.1|	71569-72935	18	5.6	0.999221	0.838138	-0.253617	1	no
gi|646743020|gb|KK962535.1|	92234-98713	18	5.6	14	9	-0.686705	0.5507	no
gi|646743045|gb|KK962533.1|	28868-13149	18	5.1	6	5	-0.258416	0.86225	no
gi|646743045|gb|KK962533.1|	33823-13600	18	5.1	67	73	0.12441	0.91665	no
gi|646743045|gb|KK962533.1|	38934-13993	18	5.1	1	3	0.957406	0.53785	no
gi|646743045|gb|KK962533.1|	40178-14669	18	5.1	3	5	0.7303	0.5676	no
gi|646743045|gb|KK962533.1|	46850-14775	18	5.1	1	0.809712	-0.496062	0.73865	no
gi|646743045|gb|KK962533.1|	49369-14964	18	5.1	15	56	2	0.2391	no
gi|646743058|gb|KK962532.1|	46568-14689	17	8	4	8	0.870514	0.60045	no
gi|646743084|gb|KK962530.1|	18838-12091	24	6.2	1	0.294149	-2	1	no
gi|646743084|gb|KK962530.1|	24527-12695	24	6.2	4	5	0.439442	0.7781	no
gi|646743084|gb|KK962530.1|	32364-13376	24	6.2	0.807257	0.813031	0.0102812	1	no
gi|646743084|gb|KK962530.1|	34145-13618	24	6.2	18	9	-1	0.43415	no
gi|646743084|gb|KK962530.1|	46298-14890	24	6.2	3	5	0.809078	0.58945	no
gi|646743084|gb|KK962530.1|	50362-15164	24	6.2	3	5	0.769579	0.62345	no
gi|646743084|gb|KK962530.1|	52494-15273	24	6.2	3	42	4	0.10085	no
gi|646743084|gb|KK962530.1|	15314-16124	24	6.2	0.833303	2	1	0.33895	no
gi|646743084|gb|KK962530.1|	56852-15721	24	6.2	6	4	-0.675317	0.66215	no
gi|646743084|gb|KK962530.1|	58463-15868	24	6.2	31	40	0.388019	0.7888	no
gi|646743084|gb|KK962530.1|	59853-16097	24	6.2	4	9	1	0.4507	no

gi|646743084|gb|KK962530.1|	63287-16405	24	6.2	9	11	0.336114	0.8292	no
gi|646743084|gb|KK962530.1|	64906-16554	24	6.2	9	15	0.720802	0.6469	no
gi|646743084|gb|KK962530.1|	67917-16817	24	6.2	63	137	1	0.4769	no
gi|646743084|gb|KK962530.1|	68280-16853	24	6.2	22	29	0.428912	0.7372	no
gi|646743084|gb|KK962530.1|	69425-16972	24	6.2	60	93	0.643714	0.6729	no
gi|646743084|gb|KK962530.1|	70228-17098	24	6.2	2	6	2	0.2253	no
gi|646743084|gb|KK962530.1|	77816-17829	24	6.2	2	4	0.644098	0.6597	no
gi|646743084|gb|KK962530.1|	88806-18900	24	6.2	40	45	0.168803	0.90065	no
gi|646743084|gb|KK962530.1|	96470-19666	24	6.2	83	196.46	1	0.4237	no
gi|646743084|gb|KK962530.1|	97533-19770	24	6.2	128	142	0.154162	0.90065	no
gi|646743084|gb|KK962530.1|	98375-19864	24	6.2	31	66	1	0.44435	no
gi|646743084|gb|KK962530.1|	00510-20158	24	6.2	1	1	0.151239	0.9186	no
gi|646743084|gb|KK962530.1|	02722-20318	24	6.2	5	6	0.285212	0.82155	no
gi|646743084|gb|KK962530.1|	05101-20545	24	6.2	23	50	1	0.4748	no
gi|646743084|gb|KK962530.1|	07862-20864	24	6.2	2	3	0.782617	0.5545	no
gi|646743084|gb|KK962530.1|	09523-21041	24	6.2	3	6	0.933823	0.5585	no
gi|646743084|gb|KK962530.1|	16812-21730	24	6.2	2	6	1	0.3215	no
gi|646743084|gb|KK962530.1|	17847-21815	24	6.2	6	29	2	0.21465	no
gi|646743084|gb|KK962530.1|	23800-22418	24	6.2	7	5	-0.518438	0.7055	no
gi|646743084|gb|KK962530.1|	30654-23109	24	6.2	23	12	-0.909218	0.5684	no
gi|646743084|gb|KK962530.1|	41277-24215	24	6.2	1	1	0.0873007	0.92955	no
gi|646743084|gb|KK962530.1|	54214-25566	24	6.2	0.619848	2	2	0.2545	no
gi|646743084|gb|KK962530.1|	58407-25907	24	6.2	1	2	0.214101	0.89085	no
gi|646743084|gb|KK962530.1|	67583-26878	24	6.2	8	4	-0.997919	0.51885	no
gi|646743084|gb|KK962530.1|	07955-31061	24	6.2	12	0	-inf	5.00E-05	yes
gi|646743084|gb|KK962530.1|	33003-33341	24	6.2	6	0	-inf	0.00655	no
gi|646743084|gb|KK962530.1|	39397-34007	24	6.2	2	2	0.0631519	0.9298	no
gi|646743084|gb|KK962530.1|	51421-35221	24	6.2	1	0.720066	-0.727664	0.66415	no
gi|646743084|gb|KK962530.1|	67473-36935	24	6.2	44	19	-1	0.2803	no
gi|646743084|gb|KK962530.1|	69464-37128	24	6.2	0.708931	0.683485	-0.0527354	1	no

gi|646743084|gb|KK962530.1|	73648-37524	24	6.2	1	0.696746	-0.833723	0.51905	no
gi|646743084|gb|KK962530.1|	75481-37758	24	6.2	4	2	-0.756449	0.62195	no
gi|646743084|gb|KK962530.1|	79691-38065	24	6.2	2	2	-0.0770941	0.9502	no
gi|646743084|gb|KK962530.1|	92225-39252	24	6.2	31	30	-0.0543073	0.9696	no
gi|646743084|gb|KK962530.1|	22318-42290	24	6.2	3	3	-0.0553908	0.93665	no
gi|646743084|gb|KK962530.1|	27896-42841	24	6.2	5	0.942662	-2	0.16345	no
gi|646743084|gb|KK962530.1|	35594-43619	24	6.2	6	11	1	0.4562	no
gi|646743084|gb|KK962530.1|	40549-44079	24	6.2	31	7	-2	0.2052	no
gi|646743084|gb|KK962530.1|	44741-44933	24	6.2	18	13	-0.490699	0.6644	no
gi|646743084|gb|KK962530.1|	57830-45817	24	6.2	89	96	0.102986	0.9471	no
gi|646743084|gb|KK962530.1|	59248-45956	24	6.2	14	7	-0.991375	0.52725	no
gi|646743084|gb|KK962530.1|	61039-46933	24	6.2	4	3	-0.438653	0.68195	no
gi|646743084|gb|KK962530.1|	75058-47546	24	6.2	6	7	0.376358	0.7794	no
gi|646743084|gb|KK962530.1|	84438-48480	24	6.2	30	26	-0.254367	0.86895	no
gi|646743084|gb|KK962530.1|	93377-49435	24	6.2	2	3	0.167806	0.90825	no
gi|646743084|gb|KK962530.1|	22355-52353	24	6.2	1	2	0.295963	0.8034	no
gi|646743084|gb|KK962530.1|	23668-52473	24	6.2	0.687775	1	0.735776	0.6352	no
gi|646743084|gb|KK962530.1|	24800-52649	24	6.2	1	1	0.282597	0.835	no
gi|646743084|gb|KK962530.1|	03582-60409	24	6.2	29	22	-0.415679	0.78225	no
gi|646743084|gb|KK962530.1|	15865-61619	24	6.2	6	10	0.694525	0.6337	no
gi|646743125|gb|KK962527.1|	04297-10789	16	8.2	43	2	-4	0.0078	no
gi|646743125|gb|KK962527.1|	10475-11136	16	8.2	2	0	-inf	0.00475	no
gi|646743125|gb|KK962527.1|	11734-12296	16	8.2	2	1	-0.962916	0.589	no
gi|646743125|gb|KK962527.1|	13793-14010	16	8.2	21	37	0.793811	0.62195	no
gi|646743125|gb|KK962527.1|	14165-14942	16	8.2	10	10	-0.018526	0.98715	no
gi|646743125|gb|KK962527.1|	48488-14902	16	8.2	3	4	0.291654	0.82735	no
gi|646743125|gb|KK962527.1|	33483-34307	16	8.2	2	4	0.859889	0.5453	no
gi|646743125|gb|KK962527.1|	45967-34636	16	8.2	8	13	0.664874	0.64845	no
gi|646743125|gb|KK962527.1|	91923-92550	16	8.2	3	0	-inf	0.00475	no
gi|646743125|gb|KK962527.1|	97696-98788	16	8.2	2	0	-inf	0.00225	no

gi|646743164|gb|KK962524.1|	00359-10086	19	7.1	7	10	0.501659	0.7182	no
gi|646743164|gb|KK962524.1|	01511-10253	19	7.1	6	3	-1	0.4044	no
gi|646743164|gb|KK962524.1|	03189-10436	19	7.1	50	15	-2	0.2053	no
gi|646743164|gb|KK962524.1|	20227-12055	19	7.1	27	18	-0.607533	0.65945	no
gi|646743164|gb|KK962524.1|	53941-15417	19	7.1	24	21	-0.221509	0.89085	no
gi|646743164|gb|KK962524.1|	56489-15677	19	7.1	60	10	-3	0.1305	no
gi|646743164|gb|KK962524.1|	59931-16048	19	7.1	20	5	-2	0.1422	no
gi|646743164|gb|KK962524.1|	64421-16596	19	7.1	11	6	-0.775292	0.61165	no
gi|646743164|gb|KK962524.1|	75153-17584	19	7.1	2	1	-0.367614	0.80365	no
gi|646743164|gb|KK962524.1|	85731-18693	19	7.1	12	16	0.39984	0.79135	no
gi|646743164|gb|KK962524.1|	91456-19246	19	7.1	1	1	-0.22635	0.86285	no
gi|646743164|gb|KK962524.1|	93313-19862	19	7.1	5	5	0.0441765	0.9674	no
gi|646743164|gb|KK962524.1|	08281-21150	19	7.1	15	21	0.535632	0.63435	no
gi|646743164|gb|KK962524.1|	14411-21513	19	7.1	2	0.83171	-1	0.4263	no
gi|646743164|gb|KK962524.1|	16599-21817	19	7.1	2	0.911187	-1	0.4295	no
gi|646743164|gb|KK962524.1|	28567-22900	19	7.1	6	0	-inf	0.0041	no
gi|646743164|gb|KK962524.1|	29569-23125	19	7.1	52	46	-0.170386	0.88675	no
gi|646743164|gb|KK962524.1|	61442-26251	19	7.1	1	0	-inf	0.00475	no
gi|646743164|gb|KK962524.1|	49335-49673	19	7.1	4	14	2	0.24325	no
gi|646743164|gb|KK962524.1|	64290-67104	19	7.1	0.614627	2	2	0.1822	no
gi|646743164|gb|KK962524.1|	82406-84244	19	7.1	3	7	1	0.4456	no
gi|646743164|gb|KK962524.1|	86374-88499	19	7.1	5	9	0.80299	0.59925	no
gi|646743164|gb|KK962524.1|	94543-98784	19	7.1	9	8	-0.164358	0.88135	no
gi|646743164|gb|KK962524.1|	98958-99516	19	7.1	4	2	-0.549102	0.6919	no
gi|646743179|gb|KK962523.1|	29222-13027	18	8.3	0.807406	0.49384	-0.70925	1	no
gi|646743179|gb|KK962523.1|	30334-13186	18	8.3	67	0.626626	-7	0.01665	no
gi|646743179|gb|KK962523.1|	32129-13303	18	8.3	5	1	-2	0.2402	no
gi|646743179|gb|KK962523.1|	33433-13377	18	8.3	7	2	-2	0.30625	no
gi|646743179|gb|KK962523.1|	35553-13611	18	8.3	11	9	-0.285759	0.85735	no
gi|646743179|gb|KK962523.1|	37771-13870	18	8.3	2	3	0.74679	0.5522	no

gi|646743179|gb|KK962523.1|	38864-14060	18	8.3	15	9	-0.793698	0.5321	no
gi|646743179|gb|KK962523.1|	42889-14353	18	8.3	2	2	0.249649	0.86925	no
gi|646743179|gb|KK962523.1|	84600-18749	18	8.3	3	1	-2	0.30485	no
gi|646743179|gb|KK962523.1|	92113-19343	18	8.3	4	0.993823	-2	0.20865	no
gi|646743179|gb|KK962523.1|	21335-22172	18	8.3	0.635725	2	1	0.43305	no
gi|646743179|gb|KK962523.1|	50874-51122	18	8.3	87	106	0.286448	0.8532	no
gi|646743179|gb|KK962523.1|	54832-58531	18	8.3	0	34	inf	5.00E-05	yes
gi|646743179|gb|KK962523.1|	62328-63607	18	8.3	0.542051	0.648445	0.258557	1	no
gi|646743179|gb|KK962523.1|	66982-67491	18	8.3	10	3	-2	0.28995	no
gi|646743179|gb|KK962523.1|	74131-75289	18	8.3	88	37	-1	0.2582	no
gi|646743195|gb|KK962522.1|	64773-16521	14	6.4	5	0.638971	-3	0.1955	no
gi|646743195|gb|KK962522.1|	07775-20839	14	6.4	3	7	1	0.35545	no
gi|646743195|gb|KK962522.1|	12735-21370	14	6.4	0.90944	3	2	0.30205	no
gi|646743195|gb|KK962522.1|	13942-21462	14	6.4	0	3	inf	0.0014	yes
gi|646743195|gb|KK962522.1|	22245-22247	14	6.4	26	88	2	0.2954	no
gi|646743195|gb|KK962522.1|	24219-22567	14	6.4	3	9	1	0.3498	no
gi|646743195|gb|KK962522.1|	27653-22836	14	6.4	1	3	2	0.27405	no
gi|646743195|gb|KK962522.1|	38248-24027	14	6.4	56	44	-0.353341	0.75645	no
gi|646743195|gb|KK962522.1|	49656-25007	14	6.4	30	30	0.00380286	0.99005	no
gi|646743195|gb|KK962522.1|	50619-25141	14	6.4	1	2	0.4942	0.72185	no
gi|646743195|gb|KK962522.1|	56374-25684	14	6.4	3	2	-0.998883	0.589	no
gi|646743195|gb|KK962522.1|	05997-30645	14	6.4	7	6	-0.175915	0.89145	no
gi|646743195|gb|KK962522.1|	21033-32169	14	6.4	1	2	0.21302	0.89085	no
gi|646743195|gb|KK962522.1|	32853-33324	14	6.4	4	6	0.361687	0.80815	no
gi|646743195|gb|KK962522.1|	41405-34175	14	6.4	8	2	-2	0.27365	no
gi|646743195|gb|KK962522.1|	49703-35197	14	6.4	16	7	-1	0.367	no
gi|646743195|gb|KK962522.1|	55945-35627	14	6.4	10	9	-0.22753	0.86095	no
gi|646743195|gb|KK962522.1|	95147-95914	14	6.4	39	28	-0.495551	0.69345	no
gi|646743195|gb|KK962522.1|	97244-98438	14	6.4	12	16	0.385929	0.79745	no
gi|646743195|gb|KK962522.1|	99701-10023[illegible]	14	6.4	9	26	2	0.3321	no

gi|646743207|gb|KK962521.1|	91425-19842	30	18	16	17	0.0226515	0.9832	no
gi|646743207|gb|KK962521.1|	06372-20806	30	18	0.834043	0.18534	-2	1	no
gi|646743207|gb|KK962521.1|	57977-26034	30	18	7	2	-2	0.20405	no
gi|646743207|gb|KK962521.1|	61672-26236	30	18	0.83678	4	2	0.21685	no
gi|646743207|gb|KK962521.1|	95681-29609	30	18	5	0.7517	-3	0.21305	no
gi|646743207|gb|KK962521.1|	98433-29941	30	18	0	4	inf	0.00315	no
gi|646743236|gb|KK962519.1|	14738-11563	22	9.1	1	1	0.141145	0.9186	no
gi|646743236|gb|KK962519.1|	59385-16051	22	9.1	0.741753	0.454681	-0.706086	1	no
gi|646743236|gb|KK962519.1|	60631-16220	22	9.1	27	14	-0.965264	0.4579	no
gi|646743236|gb|KK962519.1|	69808-17579	22	9.1	15	13	-0.208514	0.85945	no
gi|646743236|gb|KK962519.1|	77561-18152	22	9.1	1	1	0.239081	0.876	no
gi|646743236|gb|KK962519.1|	81652-18587	22	9.1	0.862293	0.509863	-0.758069	1	no
gi|646743236|gb|KK962519.1|	86092-18972	22	9.1	0.657149	0.518659	-0.341432	1	no
gi|646743236|gb|KK962519.1|	92983-19511	22	9.1	0.887692	0.50039	-0.827006	1	no
gi|646743236|gb|KK962519.1|	97041-19797	22	9.1	0.677175	0.96192	0.506388	1	no
gi|646743236|gb|KK962519.1|	98978-20062	22	9.1	2	1	-0.207209	0.89815	no
gi|646743236|gb|KK962519.1|	08731-20903	22	9.1	77.1	17	-2	0.14305	no
gi|646743236|gb|KK962519.1|	31821-23282	22	9.1	0.994667	0.531214	-0.904921	1	no
gi|646743236|gb|KK962519.1|	47154-24818	22	9.1	0.118596	2	4	0.10345	no
gi|646743236|gb|KK962519.1|	55005-25592	22	9.1	1	0.592265	-1	0.4766	no
gi|646743236|gb|KK962519.1|	70036-27034	22	9.1	33	11	-2	0.29415	no
gi|646743236|gb|KK962519.1|	94688-29541	22	9.1	0.393529	2	2	0.2836	no
gi|646743236|gb|KK962519.1|	01661-30259	22	9.1	2	0.579617	-2	0.2858	no
gi|646743236|gb|KK962519.1|	05553-30683	22	9.1	0.450829	0.906092	1	1	no
gi|646743236|gb|KK962519.1|	07787-30801	22	9.1	78	23	-2	0.2237	no
gi|646743236|gb|KK962519.1|	08166-31382	22	9.1	25	8	-2	0.1606	no
gi|646743236|gb|KK962519.1|	14225-32199	22	9.1	4	6	0.660395	0.54835	no
gi|646743236|gb|KK962519.1|	22760-32312	22	9.1	4	6	0.594786	0.669	no
gi|646743236|gb|KK962519.1|	35977-33661	22	9.1	1	13	3	0.0855	no
gi|646743236|gb|KK962519.1|	39304-33978	22	9.1	0.397924	21	6	0.09745	no

gi|646743236|gb|KK962519.1|	46643-34718	22	9.1	0.324059	8	5	0.1093	no
gi|646743236|gb|KK962519.1|	70250-37062	22	9.1	8	5	-0.796817	0.58775	no
gi|646743236|gb|KK962519.1|	84119-38440	22	9.1	35	47	0.397268	0.7701	no
gi|646743236|gb|KK962519.1|	86463-38700	22	9.1	21	8	-1	0.39295	no
gi|646743236|gb|KK962519.1|	87631-39263	22	9.1	6	5	-0.186562	0.86225	no
gi|646743236|gb|KK962519.1|	04313-40534	22	9.1	12	6	-0.869796	0.5731	no
gi|646743236|gb|KK962519.1|	61038-56148	22	9.1	3	5	0.813777	0.57265	no
gi|646743236|gb|KK962519.1|	70531-57143	22	9.1	2	1	-0.6332	0.65905	no
gi|646743248|gb|KK962518.1|	01673-20204	11	5.9	35	0.950521	-5	0.1713	no
gi|646743261|gb|KK962517.1|	00577-10154	19	8.9	0.645972	1	0.771517	1	no
gi|646743261|gb|KK962517.1|	01648-10562	19	8.9	5	4	-0.270745	0.8634	no
gi|646743261|gb|KK962517.1|	08351-10929	19	8.9	8	2	-2	0.1525	no
gi|646743261|gb|KK962517.1|	09550-11191	19	8.9	1	1	0.060963	0.96835	no
gi|646743261|gb|KK962517.1|	15538-12268	19	8.9	1	1	0.137809	0.92415	no
gi|646743261|gb|KK962517.1|	31882-13339	19	8.9	2	1	-0.0577447	0.9618	no
gi|646743261|gb|KK962517.1|	33630-13634	19	8.9	11	11	-0.0751444	0.94205	no
gi|646743261|gb|KK962517.1|	50553-15096	19	8.9	5	15	2	0.32885	no
gi|646743261|gb|KK962517.1|	76537-17720	19	8.9	22	9	-1	0.41835	no
gi|646743261|gb|KK962517.1|	08909-20987	19	8.9	5	6	0.298601	0.8467	no
gi|646743261|gb|KK962517.1|	11081-21618	19	8.9	6	6	0.00342153	0.99705	no
gi|646743261|gb|KK962517.1|	24739-22499	19	8.9	10	16	0.71858	0.6574	no
gi|646743261|gb|KK962517.1|	27427-22768	19	8.9	36	39	0.14225	0.9243	no
gi|646743261|gb|KK962517.1|	29642-23154	19	8.9	0.728032	0.243142	-2	1	no
gi|646743261|gb|KK962517.1|	34232-23575	19	8.9	0.658195	0.210822	-2	1	no
gi|646743261|gb|KK962517.1|	56035-25693	19	8.9	5	4	-0.0369275	0.979	no
gi|646743261|gb|KK962517.1|	63451-26424	19	8.9	2	0.722622	-1	0.3853	no
gi|646743261|gb|KK962517.1|	66019-26717	19	8.9	3	1	-1	0.38635	no
gi|646743261|gb|KK962517.1|	67462-26911	19	8.9	1	0.478715	-1	0.292	no
gi|646743261|gb|KK962517.1|	74891-27582	19	8.9	1	0.583773	-1	0.4271	no
gi|646743261|gb|KK962517.1|	75986-27748	19	8.9	2	0.857273	-1	0.3405	no

gi|646743261|gb|KK962517.1|	82565-28801	19	8.9	33	11	-2	0.2011	no
gi|646743261|gb|KK962517.1|	91187-29238	19	8.9	1	1	0.0595326	0.9469	no
gi|646743261|gb|KK962517.1|	95869-29666	19	8.9	2	7	2	0.1702	no
gi|646743261|gb|KK962517.1|	97867-30013	19	8.9	78	23	-2	0.14785	no
gi|646743261|gb|KK962517.1|	02653-30587	19	8.9	38	51	0.421551	0.7194	no
gi|646743261|gb|KK962517.1|	07298-30792	19	8.9	1	5	2	0.2746	no
gi|646743261|gb|KK962517.1|	14431-31483	19	8.9	9	13	0.554477	0.69815	no
gi|646743261|gb|KK962517.1|	17885-31860	19	8.9	3	0	-inf	0.0011	yes
gi|646743261|gb|KK962517.1|	21091-32173	19	8.9	8	3	-1	0.32505	no
gi|646743261|gb|KK962517.1|	21892-33315	19	8.9	4	10	2	0.19045	no
gi|646743261|gb|KK962517.1|	33623-33590	19	8.9	0.41038	4	3	0.0583	no
gi|646743261|gb|KK962517.1|	34237-35511	19	8.9	4	4	-0.0635212	0.96735	no
gi|646743261|gb|KK962517.1|	46076-34727	19	8.9	3	6	0.841033	0.58795	no
gi|646743261|gb|KK962517.1|	51446-35413	19	8.9	0.265642	3	4	0.0524	no
gi|646743261|gb|KK962517.1|	77376-37798	19	8.9	0.518707	3	3	0.26695	no
gi|646743261|gb|KK962517.1|	83837-38460	19	8.9	1	6	2	0.16645	no
gi|646743261|gb|KK962517.1|	94355-39494	19	8.9	23	16	-0.505569	0.741	no
gi|646743261|gb|KK962517.1|	19646-42299	19	8.9	3	3	-0.0243543	0.9872	no
gi|646743261|gb|KK962517.1|	32052-43566	19	8.9	3	1	-1	0.3545	no
gi|646743261|gb|KK962517.1|	35795-43731	19	8.9	1	2	1	0.45835	no
gi|646743261|gb|KK962517.1|	42180-44268	19	8.9	2	3	0.693014	0.6482	no
gi|646743261|gb|KK962517.1|	49941-45102	19	8.9	36	22	-0.687045	0.5226	no
gi|646743261|gb|KK962517.1|	51129-45162	19	8.9	18	7	-1	0.3045	no
gi|646743261|gb|KK962517.1|	52662-45435	19	8.9	20	15	-0.47423	0.6605	no
gi|646743261|gb|KK962517.1|	55696-45726	19	8.9	12	7	-0.840175	0.57655	no
gi|646743261|gb|KK962517.1|	57972-45942	19	8.9	28	20	-0.509863	0.63415	no
gi|646743261|gb|KK962517.1|	67831-46840	19	8.9	5	0.402228	-4	0.17385	no
gi|646743261|gb|KK962517.1|	69839-71365	19	8.9	2	3	0.227993	0.88355	no
gi|646743261|gb|KK962517.1|	71697-72424	19	8.9	2	2	0.316207	0.8299	no
gi|646743261|gb|KK962517.1|	73777-74178	19	8.9	7	9	0.493492	0.69715	no

gi|646743261|gb|KK962517.1|	74380-75384	19	8.9	4	4	-0.135968	0.93145	no
gi|646743261|gb|KK962517.1|	78361-79099	19	8.9	11	10	-0.110271	0.94365	no
gi|646743261|gb|KK962517.1|	80730-81609	19	8.9	0.592948	2	2	0.2964	no
gi|646743261|gb|KK962517.1|	98958-99510	19	8.9	0.620253	6	3	0.245	no
gi|646743292|gb|KK962515.1|	03770-20431	14	8.9	3	2	-0.554277	0.72945	no
gi|646743292|gb|KK962515.1|	33308-23408	14	8.9	6	2	-2	0.2393	no
gi|646743302|gb|KK962514.1|	02028-10240	23	8.4	2	8	2	0.24905	no
gi|646743302|gb|KK962514.1|	36788-13705	23	8.4	55	105	0.920441	0.56	no
gi|646743302|gb|KK962514.1|	40166-14149	23	8.4	103	114	0.154794	0.899	no
gi|646743302|gb|KK962514.1|	43324-14385	23	8.4	10	13	0.392326	0.80485	no
gi|646743302|gb|KK962514.1|	56504-15668	23	8.4	86	130	0.596153	0.66505	no
gi|646743302|gb|KK962514.1|	60621-16097	23	8.4	302	153	-0.975186	0.36835	no
gi|646743302|gb|KK962514.1|	63020-16328	23	8.4	13	17	0.3811	0.7963	no
gi|646743302|gb|KK962514.1|	68485-16966	23	8.4	0.603314	1	0.933172	0.53855	no
gi|646743302|gb|KK962514.1|	69969-17137	23	8.4	0.967021	0.347846	-1	1	no
gi|646743302|gb|KK962514.1|	78355-18176	23	8.4	6	6	0.0166404	0.99035	no
gi|646743302|gb|KK962514.1|	82382-18390	23	8.4	12	14	0.229232	0.87675	no
gi|646743302|gb|KK962514.1|	01384-20196	23	8.4	121	86	-0.496263	0.6505	no
gi|646743302|gb|KK962514.1|	45156-24888	23	8.4	0.931064	0.814138	-0.193607	1	no
gi|646743302|gb|KK962514.1|	49104-25078	23	8.4	5	2	-2	0.30885	no
gi|646743302|gb|KK962514.1|	95459-29628	23	8.4	10	11	0.12799	0.92935	no
gi|646743302|gb|KK962514.1|	97675-29796	23	8.4	60	38	-0.640331	0.68675	no
gi|646743302|gb|KK962514.1|	04302-30451	23	8.4	12	59	2	0.29635	no
gi|646743302|gb|KK962514.1|	11110-31201	23	8.4	22	35	0.664476	0.60245	no
gi|646743302|gb|KK962514.1|	24477-32969	23	8.4	10	7	-0.551562	0.6157	no
gi|646743302|gb|KK962514.1|	30898-33254	23	8.4	1	2	0.866678	0.53065	no
gi|646743302|gb|KK962514.1|	32784-33333	23	8.4	85	64	-0.404575	0.69685	no
gi|646743302|gb|KK962514.1|	34742-33496	23	8.4	224	156.62	-0.519275	0.73385	no
gi|646743302|gb|KK962514.1|	35231-33902	23	8.4	2	4	0.885426	0.5575	no
gi|646743302|gb|KK962514.1|	44031-34462	23	8.4	7	12	0.659276	0.68095	no

gi|646743302|gb|KK962514.1| 45939-34661	23	8.4	9	14	0.64794	0.676	no
gi|646743302|gb|KK962514.1| 47674-34785	23	8.4	65	102	0.653959	0.65375	no
gi|646743302|gb|KK962514.1| 48068-34832	23	8.4	132	111	-0.248231	0.87405	no
gi|646743302|gb|KK962514.1| 35999-37996	23	8.4	3	5	0.701376	0.64775	no
gi|646743302|gb|KK962514.1| 62346-36313	23	8.4	7	7	0.0665644	0.9654	no
gi|646743302|gb|KK962514.1| 63379-36359	23	8.4	65	54.88	-0.241102	0.84115	no
gi|646743302|gb|KK962514.1| 66617-36736	23	8.4	4	0.796011	-2	0.1733	no
gi|646743302|gb|KK962514.1| 67494-36768	23	8.4	47	61	0.356234	0.8111	no
gi|646743302|gb|KK962514.1| 68084-37014	23	8.4	3	4	0.196751	0.89165	no
gi|646743302|gb|KK962514.1| 71995-37344	23	8.4	111	37	-2	0.17935	no
gi|646743302|gb|KK962514.1| 99903-40017	23	8.4	84	30	-1	0.2822	no
gi|646743315|gb|KK962513.1| 99152-99609	10	6	8	0.597838	-4	0.1731	no
gi|646743341|gb|KK962511.1| 50164-15050	19	5	16	7	-1	0.373	no
gi|646743341|gb|KK962511.1| 75288-17560	19	5	11	1	-3	0.2056	no
gi|646743341|gb|KK962511.1| 12581-21320	19	5	1	3	1	0.4438	no
gi|646743341|gb|KK962511.1| 15912-22215	19	5	16	15	-0.0291738	0.9815	no
gi|646743341|gb|KK962511.1| 61444-26214	19	5	2	3	0.34401	0.7962	no
gi|646743341|gb|KK962511.1| 67539-26799	19	5	12	7	-0.832534	0.55315	no
gi|646743341|gb|KK962511.1| 71845-27218	19	5	11	18	0.620551	0.6776	no
gi|646743341|gb|KK962511.1| 04596-30699	19	5	21	19	-0.167204	0.88535	no
gi|646743341|gb|KK962511.1| 26426-42828	19	5	0.115717	0.920664	3	1	no
gi|646743341|gb|KK962511.1| 37072-43758	19	5	17	64	2	0.22015	no
gi|646743341|gb|KK962511.1| 77887-78250	19	5	0.787063	22	5	0.10445	no
gi|646743341|gb|KK962511.1| 80825-81598	19	5	0	17	inf	5.00E-05	yes
gi|646743352|gb|KK962510.1| 36798-13974	20	5.4	1	2	0.750142	0.62775	no
gi|646743352|gb|KK962510.1| 43668-24495	20	5.4	0.896416	1	0.199901	1	no
gi|646743352|gb|KK962510.1| 08560-40873	20	5.4	303	0	-inf	0.00225	no
gi|646743352|gb|KK962510.1| 26744-42691	20	5.4	222	0	-inf	0.0041	no
gi|646743352|gb|KK962510.1| 42958-44354	20	5.4	4	0	-inf	0.00225	no
gi|646743352|gb|KK962510.1| 46898-44823	20	5.4	3	0	-inf	0.0023	no

gi|646743352|gb|KK962510.1|	54511-45536	20	5.4	3	0	-inf	0.00045	yes
gi|646743352|gb|KK962510.1|	55758-45885	20	5.4	2	0	-inf	0.00015	yes
gi|646743352|gb|KK962510.1|	58969-46036	20	5.4	1	0	-inf	0.0012	yes
gi|646743352|gb|KK962510.1|	60603-46122	20	5.4	59	0	-inf	5.00E-05	yes
gi|646743352|gb|KK962510.1|	00702-50115	20	5.4	15	5	-2	0.27935	no
gi|646743352|gb|KK962510.1|	16249-51662	20	5.4	15	17	0.189762	0.88925	no
gi|646743352|gb|KK962510.1|	28601-52889	20	5.4	13	9	-0.620538	0.66815	no
gi|646743352|gb|KK962510.1|	38541-53893	20	5.4	6	6	0.0415859	0.9428	no
gi|646743352|gb|KK962510.1|	44228-54655	20	5.4	34	24	-0.472432	0.67765	no
gi|646743363|gb|KK962509.1|	58657-15940	11	6.4	1	1	0.225644	0.87595	no
gi|646743377|gb|KK962508.1|	17954-11847	19	8	1	4	1	0.37825	no
gi|646743377|gb|KK962508.1|	20878-22150	19	8	6	6	0.113989	0.94275	no
gi|646743377|gb|KK962508.1|	37817-23832	19	8	4	1	-1	0.3707	no
gi|646743377|gb|KK962508.1|	00193-30448	19	8	18	10	-0.811448	0.4755	no
gi|646743377|gb|KK962508.1|	05500-30644	19	8	0.673233	2	1	0.43245	no
gi|646743377|gb|KK962508.1|	24168-32577	19	8	0.273104	0.788187	2	1	no
gi|646743377|gb|KK962508.1|	29157-33095	19	8	0.360215	1	2	0.2839	no
gi|646743377|gb|KK962508.1|	34111-33632	19	8	0.473236	1	2	0.317	no
gi|646743377|gb|KK962508.1|	68992-70281	19	8	6	5	-0.184381	0.90695	no
gi|646743394|gb|KK962507.1|	11738-12001	20	8.8	32	43	0.430718	0.7542	no
gi|646743394|gb|KK962507.1|	20941-12398	20	8.8	0.764354	0.580268	-0.397521	1	no
gi|646743394|gb|KK962507.1|	25126-12533	20	8.8	30	29	-0.0370499	0.90935	no
gi|646743394|gb|KK962507.1|	33347-13537	20	8.8	8	7	-0.150895	0.92045	no
gi|646743394|gb|KK962507.1|	55355-15661	20	8.8	59	40	-0.56015	0.62475	no
gi|646743394|gb|KK962507.1|	67393-16795	20	8.8	5	8	0.612586	0.66145	no
gi|646743394|gb|KK962507.1|	86063-18672	20	8.8	6	4	-0.417214	0.755	no
gi|646743394|gb|KK962507.1|	00616-20088	20	8.8	12	14	0.166551	0.9048	no
gi|646743394|gb|KK962507.1|	22050-22367	20	8.8	6	11	0.943252	0.5535	no
gi|646743394|gb|KK962507.1|	20868-22191	20	8.8	0.351731	2	2	0.2023	no
gi|646743394|gb|KK962507.1|	22637-22291	20	8.8	57	22	-1	0.33715	no

gi|646743394|gb|KK962507.1|	26729-22793	20	8.8	10	8	-0.398191	0.79155	no
gi|646743394|gb|KK962507.1|	34844-23504	20	8.8	119.57	76	-0.653691	0.6185	no
gi|646743394|gb|KK962507.1|	57412-25878	20	8.8	0.825257	0.474747	-0.797685	1	no
gi|646743394|gb|KK962507.1|	70740-27357	20	8.8	0.645736	1	0.762564	1	no
gi|646743394|gb|KK962507.1|	73682-27607	20	8.8	1	0.944411	-0.140473	1	no
gi|646743394|gb|KK962507.1|	82472-28279	20	8.8	9	8	-0.140213	0.91755	no
gi|646743394|gb|KK962507.1|	99595-30020	20	8.8	7	5	-0.329598	0.84085	no
gi|646743394|gb|KK962507.1|	02490-30815	20	8.8	39	22	-0.792843	0.51385	no
gi|646743394|gb|KK962507.1|	24859-32542	20	8.8	18	18	0.000769097	0.9913	no
gi|646743394|gb|KK962507.1|	32211-33544	20	8.8	0.683955	0.316815	-1	1	no
gi|646743394|gb|KK962507.1|	37159-34039	20	8.8	54	45	-0.262609	0.8248	no
gi|646743394|gb|KK962507.1|	53156-35439	20	8.8	117	184	0.652248	0.5844	no
gi|646743394|gb|KK962507.1|	57347-35818	20	8.8	2	9	2	0.1287	no
gi|646743394|gb|KK962507.1|	60517-36081	20	8.8	48	221	2	0.1792	no
gi|646743394|gb|KK962507.1|	62918-36311	20	8.8	63	297	2	0.17825	no
gi|646743394|gb|KK962507.1|	63645-36403	20	8.8	10	19	0.880554	0.5333	no
gi|646743394|gb|KK962507.1|	68060-36921	20	8.8	1	3	1	0.3425	no
gi|646743394|gb|KK962507.1|	70682-37116	20	8.8	132	231	0.805671	0.47405	no
gi|646743394|gb|KK962507.1|	72910-37435	20	8.8	16	3	-2	0.08055	no
gi|646743394|gb|KK962507.1|	87941-38847	20	8.8	11	0.887814	-4	0.12965	no
gi|646743394|gb|KK962507.1|	38840-39070	20	8.8	56	166	2	0.2734	no
gi|646743394|gb|KK962507.1|	92375-39319	20	8.8	142	27	-2	0.0961	no
gi|646743394|gb|KK962507.1|	94517-39512	20	8.8	9	12	0.380008	0.80435	no
gi|646743394|gb|KK962507.1|	96119-39645	20	8.8	24	9	-1	0.35335	no
gi|646743394|gb|KK962507.1|	00044-40094	20	8.8	49	13	-2	0.15845	no
gi|646743394|gb|KK962507.1|	03757-40402	20	8.8	255	98	-1	0.36935	no
gi|646743394|gb|KK962507.1|	04784-40736	20	8.8	4	0.519193	-3	0.0678	no
gi|646743394|gb|KK962507.1|	18258-41863	20	8.8	391	51	-3	0.0456	no
gi|646743394|gb|KK962507.1|	20372-42077	20	8.8	0	75	inf	0.00035	yes
gi|646743394|gb|KK962507.1|	41172-44179	20	8.8	22	3	-3	0.0644	no

gi|646743394|gb|KK962507.1|	46507-46730	20	8.8	28	19	-0.572065	0.7432	no
gi|646743394|gb|KK962507.1|	72510-47311	20	8.8	51	13	-2	0.2156	no
gi|646743394|gb|KK962507.1|	18102-51880	20	8.8	146	252.47	0.794391	0.49365	no
gi|646743394|gb|KK962507.1|	53717-54243	20	8.8	21	23	0.106621	0.94145	no
gi|646743394|gb|KK962507.1|	71939-72313	20	8.8	13	5	-2	0.2882	no
gi|646743394|gb|KK962507.1|	85883-86174	20	8.8	6	11	0.80232	0.62595	no
gi|646743394|gb|KK962507.1|	89115-90440	20	8.8	0.864586	1	0.522908	0.69625	no
gi|646743394|gb|KK962507.1|	91559-92041	20	8.8	2	3	0.158183	0.9062	no
gi|646743394|gb|KK962507.1|	92329-93250	20	8.8	1	3	1	0.41885	no
gi|646743394|gb|KK962507.1|	99557-10055[illegible]	20	8.8	2.48	7	2	0.2724	no
gi|646743405|gb|KK962506.1|	52597-15294	12	5.7	9	10	0.157519	0.9097	no
gi|646743416|gb|KK962505.1|	51325-15188	26	12	6	1	-2	0.1692	no
gi|646743416|gb|KK962505.1|	53252-15459	26	12	2	1	-0.83035	0.5713	no
gi|646743416|gb|KK962505.1|	37107-23765	26	12	3	2	-0.400606	0.8003	no
gi|646743416|gb|KK962505.1|	40006-24075	26	12	2	2	-0.025638	0.9367	no
gi|646743416|gb|KK962505.1|	59281-25977	26	12	6	2	-2	0.3025	no
gi|646743416|gb|KK962505.1|	17792-31865	26	12	17	13	-0.3364	0.822	no
gi|646743416|gb|KK962505.1|	32483-33445	26	12	1	1	-0.00664254	0.9367	no
gi|646743416|gb|KK962505.1|	28317-32908	26	12	3	0.758968	-2	0.24625	no
gi|646743416|gb|KK962505.1|	56284-56542	26	12	49	22	-1	0.35435	no
gi|646743416|gb|KK962505.1|	68617-68913	26	12	15	9	-0.772542	0.60035	no
gi|646743444|gb|KK962503.1|	73043-27434	16	10	0.531668	0.509027	-0.0627844	1	no
gi|646743457|gb|KK962502.1|	47585-34808	15	8.8	3	3	0.0136279	0.9298	no
gi|646743457|gb|KK962502.1|	80555-38106	15	8.8	22	0.479552	-5	0.16895	no
gi|646743457|gb|KK962502.1|	20183-42066	15	8.8	5	2	-1	0.38755	no
gi|646743479|gb|KK962500.1|	61642-16328	19	9.3	1	2	0.529659	0.70285	no
gi|646743479|gb|KK962500.1|	69275-17243	19	9.3	2	3	0.400191	0.7852	no
gi|646743479|gb|KK962500.1|	73822-17758	19	9.3	9	6	-0.557372	0.60435	no
gi|646743479|gb|KK962500.1|	95646-19614	19	9.3	9	3	-2	0.2688	no
gi|646743479|gb|KK962500.1|	99756-20257	19	9.3	22	22	0.00673839	0.99495	no

gi|646743479|gb|KK962500.1|	24128-25075	19	9.3	6	3	-0.92912	0.5583	no
gi|646743479|gb|KK962500.1|	6620-7044	19	9.3	4	2	-0.836021	0.6128	no
gi|646743492|gb|KK962499.1|	02986-10592	24	11	1	1	-0.285326	0.8535	no
gi|646743492|gb|KK962499.1|	06212-10765	24	11	2	2	-0.174638	0.91215	no
gi|646743492|gb|KK962499.1|	14548-11583	24	11	1	1	-0.156126	0.90205	no
gi|646743492|gb|KK962499.1|	13084-14061	24	11	2	0	-inf	0.00095	yes
gi|646743492|gb|KK962499.1|	37145-13850	24	11	0.336404	1	2	0.2025	no
gi|646743492|gb|KK962499.1|	38807-14079	24	11	0.26708	2	3	0.12495	no
gi|646743492|gb|KK962499.1|	14283-15207	24	11	19	0.19628	-7	0.16745	no
gi|646743492|gb|KK962499.1|	51447-15222	24	11	1	3	1	0.31555	no
gi|646743492|gb|KK962499.1|	52825-15398	24	11	2	13	2	0.14135	no
gi|646743492|gb|KK962499.1|	15310-17177	24	11	1	0.415155	-2	0.28285	no
gi|646743492|gb|KK962499.1|	56617-15735	24	11	2	1	-0.682831	0.6459	no
gi|646743492|gb|KK962499.1|	171-1714	24	11	8	0.310683	-5	0.07895	no
gi|646743492|gb|KK962499.1|	18776-19826	24	11	11	1	-3	0.05855	no
gi|646743492|gb|KK962499.1|	92141-19294	24	11	3	3	0.0478885	0.95565	no
gi|646743492|gb|KK962499.1|	94968-19710	24	11	1	0.428004	-1	1	no
gi|646743492|gb|KK962499.1|	97409-19991	24	11	36	13	-1	0.20665	no
gi|646743492|gb|KK962499.1|	20636-22008	24	11	1	0.595517	-0.854369	1	no
gi|646743492|gb|KK962499.1|	06447-20725	24	11	1	2	1	0.39835	no
gi|646743492|gb|KK962499.1|	11537-21178	24	11	212	32	-3	0.0735	no
gi|646743492|gb|KK962499.1|	15213-21649	24	11	2	0.515309	-2	0.2986	no
gi|646743492|gb|KK962499.1|	16676-21692	24	11	49	36	-0.444573	0.7208	no
gi|646743492|gb|KK962499.1|	19890-22022	24	11	198	60	-1.71	0.27845	no
gi|646743492|gb|KK962499.1|	22182-22703	24	11	3	0.936238	-2	0.26405	no
gi|646743492|gb|KK962499.1|	25219-23803	24	11	5	3	-0.454453	0.684	no
gi|646743492|gb|KK962499.1|	40433-24148	24	11	8	8	0.0275207	0.9842	no
gi|646743492|gb|KK962499.1|	60082-26038	24	11	9	7	-0.351671	0.8176	no
gi|646743492|gb|KK962499.1|	3092-4135	24	11	1	0.837286	-0.625051	0.66015	no
gi|646743492|gb|KK962499.1|	10930-31133	24	11	14	3	-2	0.20715	no

gi|646743492|gb|KK962499.1|	25458-32608	24	11	3	4	0.594463	0.6289	no
gi|646743492|gb|KK962499.1|	33589-33437	24	11	1	3	1	0.34525	no
gi|646743492|gb|KK962499.1|	38086-33860	24	11	1	4	1	0.37825	no
gi|646743492|gb|KK962499.1|	44817-34595	24	11	26	24	-0.13615	0.89695	no
gi|646743492|gb|KK962499.1|	57820-36122	24	11	31	38	0.273128	0.81975	no
gi|646743492|gb|KK962499.1|	68850-36904	24	11	134	82	-0.702195	0.5819	no
gi|646743492|gb|KK962499.1|	69973-37022	24	11	11	17	0.706597	0.65785	no
gi|646743492|gb|KK962499.1|	70335-37067	24	11	22	24	0.0955783	0.95285	no
gi|646743492|gb|KK962499.1|	71135-37142	24	11	16	9	-0.784721	0.5939	no
gi|646743492|gb|KK962499.1|	72983-37324	24	11	82	81	-0.0090209	0.9851	no
gi|646743492|gb|KK962499.1|	78862-37916	24	11	20	40	0.979623	0.49895	no
gi|646743492|gb|KK962499.1|	12889-41314	24	11	82	66	-0.325814	0.83345	no
gi|646743492|gb|KK962499.1|	51759-45334	24	11	2	3	0.0850282	0.9538	no
gi|646743492|gb|KK962499.1|	74030-47604	24	11	0.946193	2	1	0.36115	no
gi|646743492|gb|KK962499.1|	28332-52910	24	11	2	1	-0.967922	0.54505	no
gi|646743492|gb|KK962499.1|	65492-65916	24	11	4	2	-0.836021	0.6128	no
gi|646743492|gb|KK962499.1|	78979-67914	24	11	970	334	-2	0.27845	no
gi|646743492|gb|KK962499.1|	80031-68045	24	11	6	5	-0.1957	0.879	no
gi|646743492|gb|KK962499.1|	80753-68138	24	11	3	4	0.121649	0.9232	no
gi|646743492|gb|KK962499.1|	83699-68418	24	11	28	22	-0.386629	0.79775	no
gi|646743492|gb|KK962499.1|	84084-85763	24	11	7	3	-1	0.4004	no
gi|646743492|gb|KK962499.1|	93534-93842	24	11	10	8	-0.428217	0.76235	no
gi|646743492|gb|KK962499.1|	)7386-10286(	24	11	1	0.982732	-0.5455	0.71625	no
gi|646743501|gb|KK962498.1|	67483-16814	19	9.5	0.677987	4	2	0.1548	no
gi|646743501|gb|KK962498.1|	85559-28627	19	9.5	41	35	-0.238847	0.84605	no
gi|646743501|gb|KK962498.1|	03187-30584	19	9.5	5	5	-0.180611	0.90715	no
gi|646743501|gb|KK962498.1|	08883-31203	19	9.5	5	3	-0.969442	0.51815	no
gi|646743501|gb|KK962498.1|	20272-32378	19	9.5	2	2	0.173513	0.90575	no
gi|646743501|gb|KK962498.1|	24595-32748	19	9.5	28	22	-0.32039	0.7816	no
gi|646743501|gb|KK962498.1|	33401-33514	19	9.5	17	6	-2	0.2416	no

gi|646743501|gb|KK962498.1|	35489-33626	19	9.5	3	5	0.740135	0.599	no
gi|646743501|gb|KK962498.1|	37569-33852	19	9.5	1	4	1	0.3449	no
gi|646743501|gb|KK962498.1|	39968-34040	19	9.5	21	32	0.648982	0.67235	no
gi|646743501|gb|KK962498.1|	42680-34334	19	9.5	3	3	0.212837	0.87135	no
gi|646743501|gb|KK962498.1|	45042-34552	19	9.5	2	12	2	0.1972	no
gi|646743501|gb|KK962498.1|	47068-34761	19	9.5	2	2	0.447719	0.7747	no
gi|646743501|gb|KK962498.1|	48528-34884	19	9.5	34	50	0.53483	0.7337	no
gi|646743501|gb|KK962498.1|	50110-35065	19	9.5	3	5	1	0.42775	no
gi|646743501|gb|KK962498.1|	51428-35177	19	9.5	15	15	0.00323076	0.96045	no
gi|646743501|gb|KK962498.1|	53079-35333	19	9.5	16	40	1	0.3421	no
gi|646743501|gb|KK962498.1|	02623-40328	19	9.5	3	2	-0.902094	0.5339	no
gi|646743501|gb|KK962498.1|	31314-43207	19	9.5	8	2	-2	0.2303	no
gi|646743501|gb|KK962498.1|	77616-48068	19	9.5	0.361921	133	9	0.0099	no
gi|646743501|gb|KK962498.1|	81964-48336	19	9.5	0.56508	151	8	0.02865	no
gi|646743501|gb|KK962498.1|	07673-50818	19	9.5	1	3	1	0.44135	no
gi|646743538|gb|KK962497.1|	18435-42094	12	7	2	0.955505	-0.709556	0.65785	no
gi|646743550|gb|KK962496.1|	95293-29655	12	5.4	12	0.792525	-4	0.03045	no
gi|646743550|gb|KK962496.1|	79198-82972	12	5.4	9	4	-1	0.35255	no
gi|646743576|gb|KK962494.1|	11223-11144	19	8.2	60	40	-0.585056	0.64125	no
gi|646743576|gb|KK962494.1|	12275-11253	19	8.2	5	22	2	0.31645	no
gi|646743576|gb|KK962494.1|	27185-12750	19	8.2	335	161	-1	0.41035	no
gi|646743576|gb|KK962494.1|	40135-14084	19	8.2	2	10	2	0.13385	no
gi|646743576|gb|KK962494.1|	42304-14343	19	8.2	0.844816	2	1	0.41115	no
gi|646743576|gb|KK962494.1|	43881-14536	19	8.2	0.678577	1	0.678669	1	no
gi|646743576|gb|KK962494.1|	49808-15551	19	8.2	3	5	0.445135	0.6767	no
gi|646743576|gb|KK962494.1|	56228-15659	19	8.2	8	9	0.178593	0.89745	no
gi|646743576|gb|KK962494.1|	57412-15760	19	8.2	332	281	-0.240852	0.87925	no
gi|646743576|gb|KK962494.1|	60202-16037	19	8.2	1811.05	946	-0.937616	0.53515	no
gi|646743576|gb|KK962494.1|	95325-19627	19	8.2	2	1	-0.685473	0.60255	no
gi|646743576|gb|KK962494.1|	06617-20915	19	8.2	2	3	0.641669	0.6622	no

gi|646743576|gb|KK962494.1|	19154-22030	19	8.2	29	46	0.666841	0.5421	no
gi|646743576|gb|KK962494.1|	22601-22293	19	8.2	5	17	2	0.2445	no
gi|646743576|gb|KK962494.1|	27242-22876	19	8.2	0.436382	1	2	0.3018	no
gi|646743576|gb|KK962494.1|	32089-23540	19	8.2	36	30	-0.281848	0.80625	no
gi|646743576|gb|KK962494.1|	36014-23642	19	8.2	5	14	1	0.34895	no
gi|646743576|gb|KK962494.1|	51643-25183	19	8.2	232	148	-0.645958	0.6478	no
gi|646743576|gb|KK962494.1|	68286-26930	19	8.2	5	3	-0.723469	0.6544	no
gi|646743576|gb|KK962494.1|	01257-30154	19	8.2	5	24	2	0.18335	no
gi|646743576|gb|KK962494.1|	20924-32115	19	8.2	34	30	-0.159066	0.91175	no
gi|646743576|gb|KK962494.1|	31959-33332	19	8.2	14	15	0.0677637	0.9646	no
gi|646743576|gb|KK962494.1|	40789-34263	19	8.2	3	4	0.368983	0.80625	no
gi|646743576|gb|KK962494.1|	46938-34748	19	8.2	2	3	0.448045	0.75425	no
gi|646743576|gb|KK962494.1|	48841-35034	19	8.2	1	1	-0.26146	0.8321	no
gi|646743576|gb|KK962494.1|	48246-48728	19	8.2	7	6	-0.289276	0.81485	no
gi|646743576|gb|KK962494.1|	59607-59998	19	8.2	5	7	0.551537	0.70915	no
gi|646743576|gb|KK962494.1|	62168-63341	19	8.2	2	2	0.0034877	0.96475	no
gi|646743576|gb|KK962494.1|	77613-79226	19	8.2	4	2	-1	0.4216	no
gi|646743576|gb|KK962494.1|	79352-82840	19	8.2	3	2	-0.71299	0.63865	no
gi|646743598|gb|KK962492.1|	86125-38755	17	8.9	1	0	-inf	1	no
gi|646743598|gb|KK962492.1|	00243-41040	17	8.9	353	0.068314	-12	0.02795	no
gi|646743598|gb|KK962492.1|	23178-42415	17	8.9	337	0.726291	-9	0.0442	no
gi|646743598|gb|KK962492.1|	26514-42703	17	8.9	165	0.466529	-8	0.1665	no
gi|646743598|gb|KK962492.1|	27999-42853	17	8.9	117	0.442476	-8	0.1665	no
gi|646743598|gb|KK962492.1|	35504-43734	17	8.9	22	0	-inf	5.00E-05	yes
gi|646743598|gb|KK962492.1|	38161-43900	17	8.9	13	0	-inf	0.00025	yes
gi|646743612|gb|KK962491.1|	25647-12696	12	7.2	0.955509	2	0.763779	0.5491	no
gi|646743612|gb|KK962491.1|	27311-12782	12	7.2	2	2	-0.371159	0.811	no
gi|646743612|gb|KK962491.1|	27942-12980	12	7.2	1	2	0.532754	0.73695	no
gi|646743612|gb|KK962491.1|	31072-13261	12	7.2	2	2	-0.0851344	0.95605	no
gi|646743612|gb|KK962491.1|	34568-13560	12	7.2	5	7	0.446611	0.77215	no

gi|646743627|gb|KK962490.1|	31886-43269	15	7.5	8	0.234627	-5	0.1711	no
gi|646743650|gb|KK962489.1|	28066-12936	17	6.5	2	3	0.281227	0.85845	no
gi|646743677|gb|KK962488.1|	21279-32159	14	11	68	70	0.035594	0.9814	no
gi|646743690|gb|KK962487.1|	10591-19279	20	10	8	6	-0.425113	0.7043	no
gi|646743690|gb|KK962487.1|	19575-20046	20	10	3	3	0.152595	0.9062	no
gi|646743690|gb|KK962487.1|	20145-21418	20	10	4	2	-1	0.38995	no
gi|646743690|gb|KK962487.1|	21567-23753	20	10	6	3	-0.917673	0.54595	no
gi|646743690|gb|KK962487.1|	37359-37701	20	10	12	10	-0.227724	0.8582	no
gi|646743690|gb|KK962487.1|	38099-43884	20	10	2	0	-inf	0.0035	no
gi|646743690|gb|KK962487.1|	5902-6902	20	10	6	3	-1	0.5224	no
gi|646743690|gb|KK962487.1|	39619-63999	20	10	18	2	-3	0.14185	no
gi|646743690|gb|KK962487.1|	49823-65009	20	10	6	24	2	0.1951	no
gi|646743690|gb|KK962487.1|	87306-68805	20	10	0.559405	3	2	0.18805	no
gi|646743690|gb|KK962487.1|	88488-68950	20	10	0.848666	2	0.873728	0.5462	no
gi|646743690|gb|KK962487.1|	97384-69781	20	10	2	7	2	0.1969	no
gi|646743690|gb|KK962487.1|	99827-70231	20	10	5	10	0.853354	0.4977	no
gi|646743703|gb|KK962486.1|	35909-33697	13	7	0	4	inf	0.00155	yes
gi|646743703|gb|KK962486.1|	37551-33837	13	7	0.162561	2	4	0.10345	no
gi|646743717|gb|KK962485.1|	29858-31016	13	5.9	0.922005	0.586587	-0.65243	1	no
gi|646743729|gb|KK962484.1|	04095-30437	15	11	18	2	-3	0.19615	no
gi|646743729|gb|KK962484.1|	05020-30553	15	11	14	3	-2	0.1818	no
gi|646743729|gb|KK962484.1|	07508-30901	15	11	12	8	-0.52725	0.72265	no
gi|646743729|gb|KK962484.1|	09925-31216	15	11	8	8	-0.117873	0.93925	no
gi|646743729|gb|KK962484.1|	43967-34450	15	11	41	3	-4	0.03115	no
gi|646743729|gb|KK962484.1|	58151-35868	15	11	35	2	-4	0.05135	no
gi|646743729|gb|KK962484.1|	58910-35953	15	11	3	0.3419	-3	0.1994	no
gi|646743729|gb|KK962484.1|	63245-36345	15	11	258	7	-5	0.1725	no
gi|646743729|gb|KK962484.1|	65190-65589	15	11	6	2	-2	0.26015	no
gi|646743729|gb|KK962484.1|	77868-82371	15	11	31	19	-0.702909	0.5473	no
gi|646743729|gb|KK962484.1|	84377-84585	15	11	40	32	-0.319939	0.85005	no

gi|646743744|gb|KK962483.1|	22414-32439	16	9	2	2	-0.395481	0.80285	no
gi|646743744|gb|KK962483.1|	24664-32689	16	9	0.84192	0.610553	-0.463566	1	no
gi|646743744|gb|KK962483.1|	32863-33334	16	9	4	5	0.131711	0.927	no
gi|646743744|gb|KK962483.1|	33647-33392	16	9	15	12	-0.224016	0.86765	no
gi|646743744|gb|KK962483.1|	36671-33736	16	9	6	9	0.481673	0.7587	no
gi|646743744|gb|KK962483.1|	42068-34307	16	9	4	1	-2	0.31065	no
gi|646743744|gb|KK962483.1|	51116-35161	16	9	2	10	2	0.22275	no
gi|646743744|gb|KK962483.1|	56655-35761	16	9	1	0.93411	-0.492256	0.73195	no
gi|646743744|gb|KK962483.1|	64250-36464	16	9	4	9	1	0.3859	no
gi|646743744|gb|KK962483.1|	70323-37062	16	9	16	3	-2	0.20235	no
gi|646743744|gb|KK962483.1|	91547-39172	16	9	1660.88	257	-3	0.1058	no
gi|646743744|gb|KK962483.1|	02627-40395	16	9	2	0.992007	-0.724965	0.5707	no
gi|646743744|gb|KK962483.1|	28935-42939	16	9	3	2	-0.813345	0.6134	no
gi|646743744|gb|KK962483.1|	47765-54892	16	9	3	1	-1	0.44215	no
gi|646743744|gb|KK962483.1|	02430-60583	16	9	0.29381	2	3	0.06535	no
gi|646743744|gb|KK962483.1|	06029-60664	16	9	0.510194	10	4	0.24375	no
gi|646743744|gb|KK962483.1|	24000-62557	16	9	0.140385	1	3	0.24065	no
gi|646743744|gb|KK962483.1|	25719-62802	16	9	0.361575	2	2	0.13585	no
gi|646743744|gb|KK962483.1|	37758-63814	16	9	0	12	inf	0.00035	yes
gi|646743744|gb|KK962483.1|	42756-64319	16	9	4	11	1	0.3594	no
gi|646743744|gb|KK962483.1|	82292-83279	16	9	1	0.719552	-0.660494	0.6593	no
gi|646743772|gb|KK962481.1|	14615-15927	22	8.9	6	5	-0.234882	0.8749	no
gi|646743772|gb|KK962481.1|	30244-34032	22	8.9	4	3	-0.401357	0.7857	no
gi|646743772|gb|KK962481.1|	34140-38126	22	8.9	5	4	-0.426273	0.77555	no
gi|646743772|gb|KK962481.1|	78306-37871	22	8.9	3	8	2	0.3259	no
gi|646743772|gb|KK962481.1|	35895-43621	22	8.9	6	7	0.274825	0.85615	no
gi|646743772|gb|KK962481.1|	46610-47429	22	8.9	3	4	0.414377	0.774	no
gi|646743772|gb|KK962481.1|	56905-60475	22	8.9	9	2	-2	0.0944	no
gi|646743772|gb|KK962481.1|	61207-62828	22	8.9	0.677262	0.390971	-0.792651	1	no
gi|646743772|gb|KK962481.1|	62947-63865	22	8.9	3	1	-1	0.3511	no

gi|646743772|gb|KK962481.1|	30940-63564	22	8.9	5	6	0.237237	0.8259	no
gi|646743772|gb|KK962481.1|	36597-63959	22	8.9	8	4	-0.962578	0.4533	no
gi|646743772|gb|KK962481.1|	53032-65343	22	8.9	4	6	0.561475	0.7088	no
gi|646743772|gb|KK962481.1|	59167-65952	22	8.9	9	6	-0.419694	0.74615	no
gi|646743772|gb|KK962481.1|	68761-66968	22	8.9	50	55	0.138658	0.9006	no
gi|646743772|gb|KK962481.1|	70324-67118	22	8.9	6	16	1	0.37685	no
gi|646743772|gb|KK962481.1|	72458-67265	22	8.9	50	82	0.706523	0.63575	no
gi|646743772|gb|KK962481.1|	74806-67553	22	8.9	2	1	-0.362285	0.812	no
gi|646743772|gb|KK962481.1|	82371-68321	22	8.9	2	0.671379	-1	0.3882	no
gi|646743772|gb|KK962481.1|	03993-70434	22	8.9	2	9	2	0.2774	no
gi|646743772|gb|KK962481.1|	46903-74716	22	8.9	37.46	41	0.145066	0.91815	no
gi|646743772|gb|KK962481.1|	55058-75543	22	8.9	42	40	-0.0910294	0.9523	no
gi|646743772|gb|KK962481.1|	57004-75809	22	8.9	4	3	-0.52985	0.74555	no
gi|646743772|gb|KK962481.1|	67217-76786	22	8.9	46	28	-0.693753	0.64965	no
gi|646743772|gb|KK962481.1|	73351-77424	22	8.9	8	7	-0.137345	0.92565	no
gi|646743772|gb|KK962481.1|	04379-80641	22	8.9	15	5	-2	0.21055	no
gi|646743772|gb|KK962481.1|	08803-80942	22	8.9	3	2	-0.796301	0.5828	no
gi|646743772|gb|KK962481.1|	31008-83132	22	8.9	49	39.82	-0.310238	0.8409	no
gi|646743772|gb|KK962481.1|	36999-83768	22	8.9	1	3	1	0.45245	no
gi|646743772|gb|KK962481.1|	43171-84400	22	8.9	1	1	-0.179542	0.9042	no
gi|646743772|gb|KK962481.1|	51499-85167	22	8.9	259	139	-0.904461	0.4827	no
gi|646743772|gb|KK962481.1|	54914-85517	22	8.9	9	15	0.732814	0.6574	no
gi|646743772|gb|KK962481.1|	59055-85955	22	8.9	80	42	-0.923775	0.46625	no
gi|646743772|gb|KK962481.1|	69616-87512	22	8.9	32	30	-0.119622	0.9198	no
gi|646743772|gb|KK962481.1|	92378-89323	22	8.9	5	4	-0.236589	0.87815	no
gi|646743826|gb|KK962478.1|	17514-11831	15	8.5	11	4	-2	0.30385	no
gi|646743826|gb|KK962478.1|	56753-15702	15	8.5	52	23	-1	0.4142	no
gi|646743826|gb|KK962478.1|	62106-16264	15	8.5	15	0	-inf	0.0015	yes
gi|646743826|gb|KK962478.1|	85478-18682	15	8.5	11	14	0.264712	0.86095	no
gi|646743826|gb|KK962478.1|	87413-18797	15	8.5	8	6	-0.448343	0.7875	no

gi|646743826|gb|KK962478.1|	88158-18976	15	8.5	10	9	-0.156802	0.91625	no
gi|646743826|gb|KK962478.1|	90802-19335	15	8.5	5	5	-0.12809	0.93095	no
gi|646743826|gb|KK962478.1|	94259-19587	15	8.5	10	7	-0.564675	0.71195	no
gi|646743826|gb|KK962478.1|	96022-19930	15	8.5	7	6	-0.144203	0.91015	no
gi|646743826|gb|KK962478.1|	99948-20026	15	8.5	17	5	-2	0.2616	no
gi|646743826|gb|KK962478.1|	02407-20305	15	8.5	1	2	0.473806	0.74895	no
gi|646743826|gb|KK962478.1|	04549-20538	15	8.5	51	33	-0.635785	0.54875	no
gi|646743826|gb|KK962478.1|	06102-20630	15	8.5	108	20	-2	0.1547	no
gi|646743826|gb|KK962478.1|	17411-21827	15	8.5	17	9	-0.958384	0.5237	no
gi|646743826|gb|KK962478.1|	26316-22720	15	8.5	20	12	-0.787794	0.608	no
gi|646743826|gb|KK962478.1|	35610-23801	15	8.5	10	9	-0.163471	0.88115	no
gi|646743826|gb|KK962478.1|	39747-24034	15	8.5	16	7	-1	0.4751	no
gi|646743826|gb|KK962478.1|	67169-26742	15	8.5	0	40	inf	0.00605	no
gi|646743826|gb|KK962478.1|	27762-28644	15	8.5	5	23	2	0.16865	no
gi|646743826|gb|KK962478.1|	31630-32482	15	8.5	2	1	-0.500138	0.7124	no
gi|646743826|gb|KK962478.1|	35989-36807	15	8.5	2	6	2	0.27825	no
gi|646743826|gb|KK962478.1|	37234-39018	15	8.5	0.907605	0.962206	0.0842801	1	no
gi|646743826|gb|KK962478.1|	41189-41576	15	8.5	23	49	1	0.48035	no
gi|646743826|gb|KK962478.1|	44127-45115	15	8.5	0.504795	1	1	1	no
gi|646743826|gb|KK962478.1|	50566-50744	15	8.5	232	28	-3	0.12415	no
gi|646743826|gb|KK962478.1|	58476-59062	15	8.5	22	7	-2	0.282	no
gi|646743826|gb|KK962478.1|	63674-66834	15	8.5	68	61	-0.14552	0.90505	no
gi|646743826|gb|KK962478.1|	82271-83374	15	8.5	0.327264	1	2	0.25365	no
gi|646743826|gb|KK962478.1|	92872-93119	15	8.5	35	0	-inf	0.0041	no
gi|646743826|gb|KK962478.1|	)9452-10062[illegible]	15	8.5	1	0.866767	-0.704369	0.5887	no
gi|646743836|gb|KK962477.1|	08661-10930	14	7.1	2	2	-0.0133858	0.92345	no
gi|646743836|gb|KK962477.1|	09371-10994	14	7.1	7	1	-3	0.19185	no
gi|646743836|gb|KK962477.1|	70182-27126	14	7.1	8	6	-0.424328	0.7744	no
gi|646743836|gb|KK962477.1|	61223-62228	14	7.1	1	0.878479	-0.489549	0.73195	no
gi|646743836|gb|KK962477.1|	73199-74899	14	7.1	1	0.554884	-1	0.4182	no

gi|646743859|gb|KK962475.1|	14006-11636	20	7.9	0.749313	2	1	0.38875	no
gi|646743859|gb|KK962475.1|	41555-14680	20	7.9	104	96	-0.104573	0.93035	no
gi|646743859|gb|KK962475.1|	51204-15187	20	7.9	11	24	1	0.46265	no
gi|646743859|gb|KK962475.1|	55386-15586	20	7.9	47	118.33	1	0.3123	no
gi|646743859|gb|KK962475.1|	76713-17704	20	7.9	86	58	-0.565073	0.7059	no
gi|646743859|gb|KK962475.1|	79494-17982	20	7.9	16	25	0.622056	0.6614	no
gi|646743859|gb|KK962475.1|	87912-18817	20	7.9	534.83	333	-0.681439	0.58965	no
gi|646743859|gb|KK962475.1|	22762-22390	20	7.9	96	42	-1	0.3043	no
gi|646743859|gb|KK962475.1|	24136-22536	20	7.9	1	1	0.0725288	0.9517	no
gi|646743859|gb|KK962475.1|	29740-22999	20	7.9	45	20	-1	0.38535	no
gi|646743859|gb|KK962475.1|	31501-23179	20	7.9	80	88	0.124451	0.9375	no
gi|646743859|gb|KK962475.1|	34042-23447	20	7.9	10	6	-0.827598	0.5175	no
gi|646743859|gb|KK962475.1|	45872-24725	20	7.9	15	1	-3	0.03185	no
gi|646743859|gb|KK962475.1|	50574-25177	20	7.9	0.389511	0.97691	1	1	no
gi|646743859|gb|KK962475.1|	55706-25765	20	7.9	12	7	-0.821398	0.51585	no
gi|646743859|gb|KK962475.1|	63132-26334	20	7.9	120	73	-0.709824	0.5587	no
gi|646743859|gb|KK962475.1|	75730-27600	20	7.9	20	17	-0.26841	0.83765	no
gi|646743859|gb|KK962475.1|	77971-27875	20	7.9	61	41	-0.575531	0.5913	no
gi|646743859|gb|KK962475.1|	87717-28826	20	7.9	5	5	-0.0547818	0.95765	no
gi|646743859|gb|KK962475.1|	92158-29235	20	7.9	148	46	-2	0.2205	no
gi|646743859|gb|KK962475.1|	93353-29376	20	7.9	2	10	3	0.1367	no
gi|646743859|gb|KK962475.1|	05638-30586	20	7.9	9	45	2	0.28065	no
gi|646743859|gb|KK962475.1|	07631-30803	20	7.9	120	77	-0.638438	0.67225	no
gi|646743859|gb|KK962475.1|	08924-31065	20	7.9	2	0.908705	-1	0.44925	no
gi|646743859|gb|KK962475.1|	20908-32120	20	7.9	59	51	-0.203946	0.8948	no
gi|646743859|gb|KK962475.1|	24496-32736	20	7.9	4	3	-0.291257	0.8485	no
gi|646743859|gb|KK962475.1|	27625-32898	20	7.9	11	10	-0.188715	0.8993	no
gi|646743859|gb|KK962475.1|	39604-34065	20	7.9	0.69553	0.661664	-0.072015	1	no
gi|646743859|gb|KK962475.1|	41096-34159	20	7.9	17	3	-2	0.1242	no
gi|646743859|gb|KK962475.1|	44210-34481	20	7.9	2	1	-0.757966	0.6333	no

gi|646743859|gb|KK962475.1|	57134-35826	20	7.9	3	0.604216	-2	0.19295	no
gi|646743859|gb|KK962475.1|	94913-39519	20	7.9	18	6	-2	0.357	no
gi|646743859|gb|KK962475.1|	98245-39887	20	7.9	5	0.682092	-3	0.1287	no
gi|646743859|gb|KK962475.1|	12790-41323	20	7.9	30	4	-3	0.07285	no
gi|646743859|gb|KK962475.1|	14900-41586	20	7.9	0.523354	1	1	0.43305	no
gi|646743859|gb|KK962475.1|	18923-42110	20	7.9	23	6	-2	0.14455	no
gi|646743859|gb|KK962475.1|	22280-42468	20	7.9	6	0.498826	-4	0.03385	no
gi|646743859|gb|KK962475.1|	25478-43354	20	7.9	8	5	-0.769235	0.48795	no
gi|646743859|gb|KK962475.1|	34063-43432	20	7.9	162	152	-0.0937938	0.9489	no
gi|646743859|gb|KK962475.1|	38841-43950	20	7.9	8	6	-0.485655	0.7609	no
gi|646743859|gb|KK962475.1|	40556-44251	20	7.9	53	32	-0.724735	0.5339	no
gi|646743859|gb|KK962475.1|	43282-44486	20	7.9	16	8	-0.963254	0.4372	no
gi|646743859|gb|KK962475.1|	45048-44578	20	7.9	2	1	-0.834164	0.57665	no
gi|646743859|gb|KK962475.1|	45896-44696	20	7.9	12	10	-0.251632	0.8706	no
gi|646743859|gb|KK962475.1|	49059-44940	20	7.9	197	148	-0.411309	0.74165	no
gi|646743859|gb|KK962475.1|	49599-45137	20	7.9	11	2	-2	0.18465	no
gi|646743859|gb|KK962475.1|	53359-45468	20	7.9	2	0.373367	-2	0.20465	no
gi|646743859|gb|KK962475.1|	57408-45856	20	7.9	1	0.738019	-0.482736	1	no
gi|646743859|gb|KK962475.1|	61763-46336	20	7.9	49	22	-1	0.2881	no
gi|646743859|gb|KK962475.1|	71211-47235	20	7.9	14	9	-0.658571	0.6548	no
gi|646743859|gb|KK962475.1|	73976-47563	20	7.9	21	12	-0.8099	0.53165	no
gi|646743859|gb|KK962475.1|	80891-48466	20	7.9	12	0.536324	-4	0.00205	no
gi|646743859|gb|KK962475.1|	85075-48596	20	7.9	2	0	-inf	0.00225	no
gi|646743859|gb|KK962475.1|	86143-48678	20	7.9	8	3	-2	0.3105	no
gi|646743859|gb|KK962475.1|	89107-49012	20	7.9	5	3	-0.980536	0.5449	no
gi|646743859|gb|KK962475.1|	15508-51576	20	7.9	0	123	inf	0.00025	yes
gi|646743859|gb|KK962475.1|	21767-52260	20	7.9	0	54	inf	5.00E-05	yes
gi|646743859|gb|KK962475.1|	23252-52510	20	7.9	2	7	2	0.2443	no
gi|646743859|gb|KK962475.1|	27202-52761	20	7.9	2	142	6	0.1049	no
gi|646743859|gb|KK962475.1|	32953-53330	20	7.9	0	129	inf	5.00E-05	yes

gi|646743859|gb|KK962475.1|	85637-58674	20	7.9	8	10	0.314856	0.8339	no
gi|646743873|gb|KK962474.1|	08822-10927	20	9.4	4	4	-0.179414	0.90115	no
gi|646743873|gb|KK962474.1|	09759-11142	20	9.4	57	16	-2	0.11005	no
gi|646743873|gb|KK962474.1|	23185-12340	20	9.4	313	297	-0.0759119	0.95955	no
gi|646743873|gb|KK962474.1|	36422-13677	20	9.4	22	7	-2	0.27	no
gi|646743873|gb|KK962474.1|	38421-13886	20	9.4	5	6	0.263774	0.8324	no
gi|646743873|gb|KK962474.1|	50607-15170	20	9.4	0.327651	1	2	0.20215	no
gi|646743873|gb|KK962474.1|	51990-15281	20	9.4	73	39	-0.909443	0.4081	no
gi|646743873|gb|KK962474.1|	64467-16475	20	9.4	436	276	-0.662229	0.53315	no
gi|646743873|gb|KK962474.1|	70129-17111	20	9.4	1	1	0.0940429	1	no
gi|646743873|gb|KK962474.1|	86837-18760	20	9.4	5	5	-0.0445429	0.97635	no
gi|646743873|gb|KK962474.1|	93926-20892	20	9.4	7	7	-0.0678757	0.95265	no
gi|646743873|gb|KK962474.1|	12259-21349	20	9.4	0.663488	1	1	0.39575	no
gi|646743873|gb|KK962474.1|	13721-21414	20	9.4	0.532557	6	4	0.10725	no
gi|646743873|gb|KK962474.1|	14276-21827	20	9.4	6	25	2	0.05935	no
gi|646743873|gb|KK962474.1|	27389-23513	20	9.4	4	17	2	0.0656	no
gi|646743873|gb|KK962474.1|	38910-24609	20	9.4	2	11	2	0.11365	no
gi|646743873|gb|KK962474.1|	47884-25220	20	9.4	2	31	4	0.01425	no
gi|646743873|gb|KK962474.1|	53447-25377	20	9.4	8	113	4	0.0394	no
gi|646743873|gb|KK962474.1|	30390-31195	20	9.4	2	0.948346	-0.674476	0.6592	no
gi|646743873|gb|KK962474.1|	31351-32706	20	9.4	83	77	-0.122971	0.91805	no
gi|646743873|gb|KK962474.1|	61760-36239	20	9.4	13	6	-1	0.49855	no
gi|646743873|gb|KK962474.1|	64196-36550	20	9.4	22	13	-0.776167	0.5354	no
gi|646743873|gb|KK962474.1|	83253-38663	20	9.4	31	10	-2	0.15925	no
gi|646743873|gb|KK962474.1|	41869-50678	20	9.4	5	2	-1	0.30995	no
gi|646743873|gb|KK962474.1|	61282-46178	20	9.4	0.367047	7	4	0.09685	no
gi|646743873|gb|KK962474.1|	63633-46397	20	9.4	0	20	inf	0.00435	no
gi|646743873|gb|KK962474.1|	6264-6682	20	9.4	11	4	-2	0.2441	no
gi|646743873|gb|KK962474.1|	88121-93284	20	9.4	3	2	-0.393431	0.7937	no
gi|646743873|gb|KK962474.1|	93343-97076	20	9.4	3	3	0.0385664	0.97865	no

gi|646743886|gb|KK962473.1|	57300-15797	21	9.3	0	9	inf	0.0009	yes
gi|646743886|gb|KK962473.1|	69325-17084	21	9.3	0	2	inf	0.0026	no
gi|646743886|gb|KK962473.1|	17-488	21	9.3	9	8	-0.236447	0.86395	no
gi|646743886|gb|KK962473.1|	94402-19626	21	9.3	1	2	0.664011	0.62905	no
gi|646743886|gb|KK962473.1|	19060-21969	21	9.3	1	2	0.207994	0.8909	no
gi|646743886|gb|KK962473.1|	25111-27922	21	9.3	5	5	-0.152616	0.9191	no
gi|646743886|gb|KK962473.1|	60331-26059	21	9.3	26	16	-0.729403	0.6076	no
gi|646743886|gb|KK962473.1|	00607-30118	21	9.3	3	2	-0.712351	0.6413	no
gi|646743886|gb|KK962473.1|	09121-31534	21	9.3	7	6	-0.267374	0.8132	no
gi|646743886|gb|KK962473.1|	22798-32429	21	9.3	0	39	inf	5.00E-05	yes
gi|646743886|gb|KK962473.1|	42780-34344	21	9.3	6	4	-0.523402	0.7044	no
gi|646743886|gb|KK962473.1|	59988-36619	21	9.3	47	38	-0.290466	0.8099	no
gi|646743886|gb|KK962473.1|	72967-37355	21	9.3	1	3	2	0.29945	no
gi|646743886|gb|KK962473.1|	75140-37575	21	9.3	2	0.704966	-2	0.29375	no
gi|646743886|gb|KK962473.1|	75950-37831	21	9.3	1	1	0.00134812	0.9797	no
gi|646743886|gb|KK962473.1|	80133-38037	21	9.3	18	42	1	0.39305	no
gi|646743886|gb|KK962473.1|	38276-39816	21	9.3	8	7	-0.155703	0.9178	no
gi|646743886|gb|KK962473.1|	88281-38906	21	9.3	1	2	0.715055	0.6352	no
gi|646743886|gb|KK962473.1|	93109-39353	21	9.3	4	5	0.390676	0.7941	no
gi|646743886|gb|KK962473.1|	94025-39439	21	9.3	12	41	2	0.2231	no
gi|646743886|gb|KK962473.1|	95184-39604	21	9.3	2	5	1	0.39125	no
gi|646743886|gb|KK962473.1|	01437-40196	21	9.3	1	4	2	0.29945	no
gi|646743886|gb|KK962473.1|	06927-40723	21	9.3	156	99	-0.661127	0.66095	no
gi|646743886|gb|KK962473.1|	38765-43907	21	9.3	25	28	0.166478	0.90465	no
gi|646743886|gb|KK962473.1|	4473-5072	21	9.3	0.804907	4	2	0.1906	no
gi|646743886|gb|KK962473.1|	57186-45793	21	9.3	40	30	-0.40003	0.74705	no
gi|646743886|gb|KK962473.1|	74277-47449	21	9.3	139	63	-1	0.4261	no
gi|646743886|gb|KK962473.1|	90369-49197	21	9.3	0.480057	0.395818	-0.278369	1	no
gi|646743886|gb|KK962473.1|	13436-51524	21	9.3	2	3	0.43579	0.7774	no
gi|646743886|gb|KK962473.1|	16785-52218	21	9.3	5	3	-0.598616	0.63565	no

gi|646743886|gb|KK962473.1|	22337-52399	21	9.3	7	1	-3	0.06305	no
gi|646743886|gb|KK962473.1|	24499-52639	21	9.3	0.732465	0.081534	-3	1	no
gi|646743886|gb|KK962473.1|	30154-53097	21	9.3	3	4	0.41874	0.75275	no
gi|646743886|gb|KK962473.1|	32213-53282	21	9.3	11	2	-2	0.1395	no
gi|646743886|gb|KK962473.1|	43100-54432	21	9.3	10	9	-0.248329	0.8734	no
gi|646743886|gb|KK962473.1|	52540-55456	21	9.3	10	9	-0.0618843	0.9699	no
gi|646743886|gb|KK962473.1|	87127-58736	21	9.3	362	145	-1	0.39645	no
gi|646743886|gb|KK962473.1|	04760-60561	21	9.3	2	2	-0.392624	0.76995	no
gi|646743886|gb|KK962473.1|	06501-60950	21	9.3	19	14	-0.43918	0.7014	no
gi|646743886|gb|KK962473.1|	10347-61104	21	9.3	4	0.578024	-3	0.129	no
gi|646743886|gb|KK962473.1|	11809-61387	21	9.3	2	1	-0.171339	0.90905	no
gi|646743886|gb|KK962473.1|	14511-61528	21	9.3	1	2	0.491861	0.73295	no
gi|646743886|gb|KK962473.1|	82384-83830	21	9.3	5	5	0.0214182	0.9875	no
gi|646743886|gb|KK962473.1|	97201-97709	21	9.3	3	2	-0.0524743	0.92345	no
gi|646743900|gb|KK962472.1|	62958-26399	16	8	1	1	-0.139826	0.9179	no
gi|646743944|gb|KK962469.1|	07730-10873	20	9.1	3	3	-0.10611	0.9495	no
gi|646743944|gb|KK962469.1|	08848-11311	20	9.1	57	35	-0.718347	0.5475	no
gi|646743944|gb|KK962469.1|	17708-12078	20	9.1	2	0.381141	-2	0.1471	no
gi|646743944|gb|KK962469.1|	21074-12135	20	9.1	37	36	-0.0481469	0.9671	no
gi|646743944|gb|KK962469.1|	21465-12400	20	9.1	6	2	-2	0.3258	no
gi|646743944|gb|KK962469.1|	25497-12804	20	9.1	47	34	-0.479882	0.67995	no
gi|646743944|gb|KK962469.1|	28233-13053	20	9.1	1	0.655456	-0.664652	1	no
gi|646743944|gb|KK962469.1|	30780-13314	20	9.1	2	0.381481	-2	0.17585	no
gi|646743944|gb|KK962469.1|	38014-13915	20	9.1	0.625756	0.895361	0.516871	1	no
gi|646743944|gb|KK962469.1|	39264-14011	20	9.1	1	0.879223	-0.307667	1	no
gi|646743944|gb|KK962469.1|	40471-14146	20	9.1	2	0.715756	-1	0.45505	no
gi|646743944|gb|KK962469.1|	44347-14482	20	9.1	3	3	0.197341	0.88975	no
gi|646743944|gb|KK962469.1|	48548-15001	20	9.1	1	0.768315	-0.473609	1	no
gi|646743944|gb|KK962469.1|	50155-15215	20	9.1	0.797098	0.538698	-0.56528	1	no
gi|646743944|gb|KK962469.1|	15833-16336	20	9.1	11	0.99733	-4	0.13385	no

gi|646743944|gb|KK962469.1|	63771-16425	20	9.1	25	14	-0.855134	0.57845	no
gi|646743944|gb|KK962469.1|	68013-16883	20	9.1	4	0.457392	-3	0.1566	no
gi|646743944|gb|KK962469.1|	93846-19588	20	9.1	2	3	0.0986146	0.94965	no
gi|646743944|gb|KK962469.1|	96119-19813	20	9.1	1	1	0.322938	0.83695	no
gi|646743944|gb|KK962469.1|	00619-20279	20	9.1	11	5	-1	0.33795	no
gi|646743944|gb|KK962469.1|	03656-20433	20	9.1	18	14	-0.341064	0.82065	no
gi|646743944|gb|KK962469.1|	07132-20823	20	9.1	0.437901	1	2	0.36595	no
gi|646743944|gb|KK962469.1|	09943-21073	20	9.1	4	4	0.0574525	0.9677	no
gi|646743944|gb|KK962469.1|	16824-21737	20	9.1	3	3	-0.288597	0.83625	no
gi|646743944|gb|KK962469.1|	18465-21933	20	9.1	3	3	0.221512	0.85885	no
gi|646743944|gb|KK962469.1|	22868-22345	20	9.1	2	2	-0.347388	0.81865	no
gi|646743944|gb|KK962469.1|	28031-22850	20	9.1	6	4	-0.390679	0.76035	no
gi|646743944|gb|KK962469.1|	32570-23390	20	9.1	2	1	-0.13992	0.90355	no
gi|646743944|gb|KK962469.1|	23469-23805	20	9.1	19	6	-2	0.24115	no
gi|646743944|gb|KK962469.1|	44309-24464	20	9.1	20	14	-0.507332	0.6726	no
gi|646743944|gb|KK962469.1|	24662-24939	20	9.1	24	0	-inf	0.0035	no
gi|646743944|gb|KK962469.1|	53784-25571	20	9.1	0.664389	2	2	0.24255	no
gi|646743944|gb|KK962469.1|	27055-27538	20	9.1	30	7	-2	0.13815	no
gi|646743944|gb|KK962469.1|	91029-29196	20	9.1	8	5	-0.75658	0.62535	no
gi|646743944|gb|KK962469.1|	98408-29888	20	9.1	13	3	-2	0.2187	no
gi|646743944|gb|KK962469.1|	13624-31768	20	9.1	378	346.4	-0.125782	0.9167	no
gi|646743944|gb|KK962469.1|	17942-32119	20	9.1	3	5	0.857666	0.57655	no
gi|646743944|gb|KK962469.1|	21333-32185	20	9.1	19	38	1	0.49235	no
gi|646743944|gb|KK962469.1|	38993-33939	20	9.1	302	614	1	0.3749	no
gi|646743944|gb|KK962469.1|	88910-38922	20	9.1	96	79	-0.278702	0.85235	no
gi|646743944|gb|KK962469.1|	00072-40146	20	9.1	35	28	-0.325282	0.76665	no
gi|646743944|gb|KK962469.1|	22141-42240	20	9.1	71	18	-2	0.21435	no
gi|646743944|gb|KK962469.1|	22742-42358	20	9.1	2	0.884957	-1	0.41185	no
gi|646743944|gb|KK962469.1|	33944-43635	20	9.1	2	0.623819	-1	0.3126	no
gi|646743944|gb|KK962469.1|	50044-45021	20	9.1	347	140.97	-1	0.32755	no

gi|646743944|gb|KK962469.1|	90871-49284	20	9.1	15	9	-0.654148	0.60585	no
gi|646743944|gb|KK962469.1|	92958-49381	20	9.1	0.917346	1	0.500546	0.73295	no
gi|646743944|gb|KK962469.1|	93949-49786	20	9.1	3	3	-0.0909018	0.94675	no
gi|646743944|gb|KK962469.1|	06730-50694	20	9.1	114	59	-0.963049	0.4492	no
gi|646743944|gb|KK962469.1|	50905-51978	20	9.1	0	9	inf	0.0001	yes
gi|646743944|gb|KK962469.1|	59642-61883	20	9.1	3	2	-0.734803	0.62765	no
gi|646743944|gb|KK962469.1|	69369-70419	20	9.1	3	1	-1	0.3295	no
gi|646743944|gb|KK962469.1|	71657-80587	20	9.1	3	1	-2	0.20485	no
gi|646743944|gb|KK962469.1|	80805-83277	20	9.1	2	1	-0.719636	0.6399	no
gi|646743944|gb|KK962469.1|	83842-85030	20	9.1	23	13	-0.85624	0.49915	no
gi|646743944|gb|KK962469.1|	88901-90746	20	9.1	12	4	-2	0.3155	no
gi|646743944|gb|KK962469.1|	94398-96377	20	9.1	31	9	-2	0.19345	no
gi|646743944|gb|KK962469.1|	97096-10758	20	9.1	11	7	-0.745834	0.5237	no
gi|646743956|gb|KK962468.1|	01634-10366	15	5.5	0.884581	4	2	0.11965	no
gi|646743956|gb|KK962468.1|	14825-15463	15	5.5	0	13	inf	0.00055	yes
gi|646743956|gb|KK962468.1|	49592-15022	15	5.5	4	3	-0.699101	0.5862	no
gi|646743956|gb|KK962468.1|	16340-19117	15	5.5	0.513589	8	4	0.00775	no
gi|646743956|gb|KK962468.1|	19523-22663	15	5.5	0.577249	8	4	0.00725	no
gi|646743956|gb|KK962468.1|	99167-19957	15	5.5	0	12	inf	0.00515	no
gi|646743956|gb|KK962468.1|	19227-21991	15	5.5	0	97	inf	5.00E-05	yes
gi|646743956|gb|KK962468.1|	22779-23912	15	5.5	0.526807	8	4	0.07355	no
gi|646743956|gb|KK962468.1|	32092-23297	15	5.5	0.444711	120	8	0.1049	no
gi|646743956|gb|KK962468.1|	2411-2880	15	5.5	4	0.56845	-3	0.21305	no
gi|646743956|gb|KK962468.1|	24156-25307	15	5.5	2	9	3	0.08805	no
gi|646743956|gb|KK962468.1|	27693-29060	15	5.5	0.998323	6	3	0.1037	no
gi|646743956|gb|KK962468.1|	23339-32370	15	5.5	2	51	5	0.236	no
gi|646743956|gb|KK962468.1|	34731-35430	15	5.5	15	26	0.764388	0.6086	no
gi|646743956|gb|KK962468.1|	60648-36087	15	5.5	0	136	inf	0.001	yes
gi|646743956|gb|KK962468.1|	37793-39041	15	5.5	5	4	-0.407811	0.7892	no
gi|646743956|gb|KK962468.1|	45256-45932	15	5.5	0	13	inf	0.00015	yes

gi|646743956|gb|KK962468.1|	47784-47958	15	5.5	77	111	0.532603	0.73815	no
gi|646743956|gb|KK962468.1|	57481-57697	15	5.5	98	81	-0.281169	0.84385	no
gi|646743956|gb|KK962468.1|	6220-6507	15	5.5	0	21	inf	0.0014	yes
gi|646743956|gb|KK962468.1|	95326-97152	15	5.5	0.647785	2	2	0.2183	no
gi|646743956|gb|KK962468.1|	98387-99619	15	5.5	0.378748	3	3	0.1625	no
gi|646743956|gb|KK962468.1|	99692-10149	15	5.5	2	3	1	0.49935	no
gi|646743994|gb|KK962465.1|	79091-58028	20	10	10	8	-0.376386	0.8016	no
gi|646743994|gb|KK962465.1|	84393-58549	20	10	2	0.313991	-2	0.16215	no
gi|646743994|gb|KK962465.1|	86949-58835	20	10	0.967864	1	0.524878	0.67915	no
gi|646743994|gb|KK962465.1|	88478-58938	20	10	2	2	0.388534	0.75195	no
gi|646743994|gb|KK962465.1|	91333-59609	20	10	2	3	0.759579	0.6094	no
gi|646743994|gb|KK962465.1|	98902-59946	20	10	2	2	-0.549102	0.72945	no
gi|646743994|gb|KK962465.1|	00068-60097	20	10	2	1	-0.147919	0.9177	no
gi|646743994|gb|KK962465.1|	04261-60641	20	10	0.731169	6	3	0.05415	no
gi|646743994|gb|KK962465.1|	07158-60753	20	10	0.696685	13	4	0.11435	no
gi|646744020|gb|KK962463.1|	01580-10232	15	5	0	3	inf	0.0014	yes
gi|646744020|gb|KK962463.1|	21700-12218	15	5	0.392977	84	8	0.09445	no
gi|646744020|gb|KK962463.1|	39896-14045	15	5	0.304086	5	4	0.0989	no
gi|646744020|gb|KK962463.1|	91909-29258	15	5	2	2	-0.396628	0.7788	no
gi|646744029|gb|KK962462.1|	98979-40449	19	7.9	12	11	-0.192582	0.867	no
gi|646744029|gb|KK962462.1|	28330-42872	19	7.9	3	7	1	0.42445	no
gi|646744029|gb|KK962462.1|	32804-43323	19	7.9	13	5	-1	0.40315	no
gi|646744029|gb|KK962462.1|	33990-43462	19	7.9	2	2	-0.0150164	0.92345	no
gi|646744029|gb|KK962462.1|	35349-43591	19	7.9	3	5	0.868649	0.51375	no
gi|646744029|gb|KK962462.1|	38561-43887	19	7.9	75	27	-1	0.36655	no
gi|646744029|gb|KK962462.1|	55919-45612	19	7.9	36	34	-0.0724658	0.90935	no
gi|646744029|gb|KK962462.1|	64116-46496	19	7.9	32	26	-0.312853	0.7978	no
gi|646744029|gb|KK962462.1|	75296-47835	19	7.9	5	2	-0.877345	0.5604	no
gi|646744057|gb|KK962460.1|	07487-20865	14	6.5	18	15	-0.285622	0.8456	no
gi|646744057|gb|KK962460.1|	09769-21060	14	6.5	24	13	-0.891417	0.5498	no

gi|646744057|gb|KK962460.1|	16712-21775	14	6.5	1	1	0.149781	0.9186	no
gi|646744057|gb|KK962460.1|	37740-23977	14	6.5	3	6	0.702071	0.6369	no
gi|646744057|gb|KK962460.1|	39874-24391	14	6.5	4	4	0.10629	0.94365	no
gi|646744057|gb|KK962460.1|	44034-24459	14	6.5	18	12	-0.620334	0.6823	no
gi|646744057|gb|KK962460.1|	82910-28394	14	6.5	1	2	0.734337	0.56995	no
gi|646744072|gb|KK962459.1|	02620-10549	18	7.1	8	6	-0.3089	0.8007	no
gi|646744072|gb|KK962459.1|	92461-29269	18	7.1	24	20	-0.217601	0.89085	no
gi|646744072|gb|KK962459.1|	12092-31234	18	7.1	19	15	-0.369994	0.81185	no
gi|646744072|gb|KK962459.1|	13495-31416	18	7.1	2	2	-0.00793743	0.92345	no
gi|646744072|gb|KK962459.1|	16745-31776	18	7.1	16	10	-0.626507	0.6727	no
gi|646744072|gb|KK962459.1|	27970-32993	18	7.1	40	26	-0.647353	0.5651	no
gi|646744072|gb|KK962459.1|	33817-33951	18	7.1	5	10	0.995766	0.3719	no
gi|646744072|gb|KK962459.1|	71828-37452	18	7.1	6	3	-0.733554	0.62765	no
gi|646744072|gb|KK962459.1|	77816-37800	18	7.1	66	138	1	0.4379	no
gi|646744072|gb|KK962459.1|	95560-39574	18	7.1	181	204	0.168533	0.90655	no
gi|646744072|gb|KK962459.1|	95862-39640	18	7.1	9	3	-2	0.24725	no
gi|646744086|gb|KK962458.1|	06700-20797	20	8.2	2	4	1	0.4065	no
gi|646744086|gb|KK962458.1|	08258-21030	20	8.2	1	2	0.470653	0.76695	no
gi|646744086|gb|KK962458.1|	90234-90961	20	8.2	12	20	0.769066	0.61075	no
gi|646744086|gb|KK962458.1|	91012-96206	20	8.2	3	7	1	0.33395	no
gi|646744099|gb|KK962457.1|	15007-11564	20	7.4	0.969941	3	1	0.3719	no
gi|646744099|gb|KK962457.1|	16507-11709	20	7.4	2	3	0.821752	0.5637	no
gi|646744099|gb|KK962457.1|	17191-11766	20	7.4	5	4	-0.0401996	0.94695	no
gi|646744099|gb|KK962457.1|	30208-13132	20	7.4	2	2	-0.176028	0.88275	no
gi|646744099|gb|KK962457.1|	91776-19218	20	7.4	7	1	-2	0.169	no
gi|646744099|gb|KK962457.1|	43867-24422	20	7.4	42	0	-inf	0.00075	yes
gi|646744099|gb|KK962457.1|	34468-35712	20	7.4	0.56123	0.536708	-0.064454	1	no
gi|646744099|gb|KK962457.1|	37952-43837	20	7.4	2	9	2	0.151	no
gi|646744129|gb|KK962455.1|	00731-10262	19	9	0.565501	0.572806	0.0185172	1	no
gi|646744129|gb|KK962455.1|	04674-10866	19	9	0.866306	0.613495	-0.497825	1	no

gi|646744129|gb|KK962455.1|	08768-11205	19	9	20	13	-0.548271	0.62755	no
gi|646744129|gb|KK962455.1|	12693-11529	19	9	1	0.458194	-1	0.38515	no
gi|646744129|gb|KK962455.1|	32007-32818	19	9	4	7	0.710178	0.65435	no
gi|646744129|gb|KK962455.1|	3663-4483	19	9	0.98227	2	0.719328	0.6352	no
gi|646744129|gb|KK962455.1|	76391-37688	19	9	0.386562	7	4	0.099	no
gi|646744129|gb|KK962455.1|	77001-37841	19	9	0.400138	10	5	0.0631	no
gi|646744129|gb|KK962455.1|	78541-37959	19	9	0.1152	4	5	0.0981	no
gi|646744129|gb|KK962455.1|	84919-38627	19	9	4	2	-0.891226	0.5783	no
gi|646744129|gb|KK962455.1|	98264-39947	19	9	3	2	-0.828645	0.55855	no
gi|646744129|gb|KK962455.1|	4673-6082	19	9	12	7	-0.749578	0.6203	no
gi|646744129|gb|KK962455.1|	56-3466	19	9	0.879753	0.683352	-0.364469	1	no
gi|646744129|gb|KK962455.1|	41930-64540	19	9	2	2	-0.109457	0.94015	no
gi|646744129|gb|KK962455.1|	44829-74529	19	9	4	7	0.675986	0.60925	no
gi|646744129|gb|KK962455.1|	95762-97384	19	9	0.947473	0.78138	-0.278061	1	no
gi|646744140|gb|KK962454.1|	18142-11840	21	9.3	143	53	-1	0.365	no
gi|646744140|gb|KK962454.1|	35646-13608	21	9.3	18	5	-2	0.1958	no
gi|646744140|gb|KK962454.1|	56050-15656	21	9.3	4	7	1	0.48725	no
gi|646744140|gb|KK962454.1|	59487-15984	21	9.3	6	6	-0.0879613	0.9294	no
gi|646744140|gb|KK962454.1|	61863-16406	21	9.3	6	3	-0.90924	0.5515	no
gi|646744140|gb|KK962454.1|	77445-17828	21	9.3	1	2	0.691175	0.6295	no
gi|646744140|gb|KK962454.1|	78378-17946	21	9.3	0.663922	2	1	0.3407	no
gi|646744140|gb|KK962454.1|	79663-18285	21	9.3	0.756039	1	0.416143	1	no
gi|646744140|gb|KK962454.1|	83004-18417	21	9.3	1	1	0.00341332	1	no
gi|646744140|gb|KK962454.1|	89149-19034	21	9.3	2	2	-0.269461	0.8181	no
gi|646744140|gb|KK962454.1|	02720-20311	21	9.3	22	17	-0.374362	0.81495	no
gi|646744140|gb|KK962454.1|	10487-21077	21	9.3	15	6	-1	0.3917	no
gi|646744140|gb|KK962454.1|	34379-23510	21	9.3	16	7	-1	0.4834	no
gi|646744140|gb|KK962454.1|	51217-25244	21	9.3	0.378748	3	3	0.17255	no
gi|646744140|gb|KK962454.1|	71499-27267	21	9.3	11	0	-inf	0.00015	yes
gi|646744140|gb|KK962454.1|	86279-28838	21	9.3	10	0.941194	-3	0.0095	no

gi|646744140|gb|KK962454.1|	94831-29685	21	9.3	6	0	-inf	0.0002	yes
gi|646744140|gb|KK962454.1|	11443-31560	21	9.3	7	0.034536	-8	0.1665	no
gi|646744140|gb|KK962454.1|	16225-31719	21	9.3	2	0	-inf	0.0011	yes
gi|646744140|gb|KK962454.1|	22722-32334	21	9.3	0.750907	26	5	0.10835	no
gi|646744140|gb|KK962454.1|	23587-32643	21	9.3	13	10	-0.36263	0.7361	no
gi|646744140|gb|KK962454.1|	27313-32825	21	9.3	2	2	-0.493347	0.6951	no
gi|646744140|gb|KK962454.1|	28344-32869	21	9.3	7	2	-2	0.30625	no
gi|646744140|gb|KK962454.1|	19315-42130	21	9.3	33	0.077264	-9	0.1665	no
gi|646744140|gb|KK962454.1|	26573-42779	21	9.3	4	0.137438	-5	0.17385	no
gi|646744140|gb|KK962454.1|	33714-43662	21	9.3	33	0	-inf	5.00E-05	yes
gi|646744140|gb|KK962454.1|	69015-46967	21	9.3	6	29	2	0.1565	no
gi|646744140|gb|KK962454.1|	71886-47229	21	9.3	17	54	2	0.28595	no
gi|646744140|gb|KK962454.1|	80273-48099	21	9.3	0.393529	3	3	0.2578	no
gi|646744140|gb|KK962454.1|	85018-48560	21	9.3	0.841156	5	2	0.15625	no
gi|646744140|gb|KK962454.1|	85923-48754	21	9.3	0.742813	1	0.976803	0.53675	no
gi|646744140|gb|KK962454.1|	93108-49330	21	9.3	25	76.59	2	0.28335	no
gi|646744140|gb|KK962454.1|	01925-50630	21	9.3	17	12	-0.420346	0.712	no
gi|646744140|gb|KK962454.1|	06758-51201	21	9.3	2	0.648866	-1	0.3441	no
gi|646744140|gb|KK962454.1|	54610-55708	21	9.3	2	0.604371	-1	0.3015	no
gi|646744140|gb|KK962454.1|	74022-57588	21	9.3	0.86302	0.749113	-0.204212	1	no
gi|646744140|gb|KK962454.1|	76041-57686	21	9.3	2	3	0.845051	0.51475	no
gi|646744140|gb|KK962454.1|	77761-58069	21	9.3	43	16	-1	0.2193	no
gi|646744140|gb|KK962454.1|	86740-58751	21	9.3	0.179597	3	4	0.10345	no
gi|646744140|gb|KK962454.1|	91271-69178	21	9.3	31	10	-2	0.30575	no
gi|646744140|gb|KK962454.1|	98477-69864	21	9.3	282	38	-3	0.1389	no
gi|646744140|gb|KK962454.1|	04201-70438	21	9.3	35.25	73	1	0.4643	no
gi|646744140|gb|KK962454.1|	06379-70700	21	9.3	7	7	0.105714	0.95005	no
gi|646744140|gb|KK962454.1|	11727-71288	21	9.3	81	26	-2	0.14415	no
gi|646744168|gb|KK962452.1|	59698-16026	22	8.7	24	35	0.538536	0.71965	no
gi|646744168|gb|KK962452.1|	70811-37160	22	8.7	0.170342	4	4	0.1128	no

gi|646744168|gb|KK962452.1|	61829-46244	22	8.7	22	18	-0.299254	0.84225	no
gi|646744168|gb|KK962452.1|	65415-46580	22	8.7	6	7	0.187067	0.8989	no
gi|646744168|gb|KK962452.1|	76054-47762	22	8.7	0.632691	1	0.680404	1	no
gi|646744168|gb|KK962452.1|	81904-48262	22	8.7	10	4	-1	0.3282	no
gi|646744168|gb|KK962452.1|	85686-48623	22	8.7	2	2	0.183695	0.8995	no
gi|646744168|gb|KK962452.1|	13433-51568	22	8.7	10	12	0.213079	0.8356	no
gi|646744168|gb|KK962452.1|	16136-51694	22	8.7	2	2	0.495551	0.7158	no
gi|646744168|gb|KK962452.1|	17052-51871	22	8.7	2	2	-0.107464	0.9456	no
gi|646744168|gb|KK962452.1|	32210-53288	22	8.7	24	4	-3	0.07995	no
gi|646744168|gb|KK962452.1|	51000-55484	22	8.7	14	7	-0.919186	0.407	no
gi|646744168|gb|KK962452.1|	57583-55810	22	8.7	2	4	0.855083	0.55795	no
gi|646744168|gb|KK962452.1|	60397-56123	22	8.7	1	0.898135	-0.50147	0.73865	no
gi|646744168|gb|KK962452.1|	61370-56263	22	8.7	1	0.131448	-3	1	no
gi|646744168|gb|KK962452.1|	95724-59983	22	8.7	9	6	-0.419629	0.70295	no
gi|646744168|gb|KK962452.1|	10380-71337	22	8.7	433	502	0.2128	0.8614	no
gi|646744168|gb|KK962452.1|	13819-71411	22	8.7	276	807	2	0.25575	no
gi|646744168|gb|KK962452.1|	14748-71505	22	8.7	219	567	1	0.30415	no
gi|646744168|gb|KK962452.1|	15755-71680	22	8.7	12	55	2	0.11285	no
gi|646744168|gb|KK962452.1|	17121-71766	22	8.7	42	79	0.925668	0.4695	no
gi|646744168|gb|KK962452.1|	18674-71899	22	8.7	337	713	1	0.33505	no
gi|646744168|gb|KK962452.1|	19598-72022	22	8.7	207	584	1	0.21305	no
gi|646744168|gb|KK962452.1|	20827-72127	22	8.7	39	111	2	0.24575	no
gi|646744168|gb|KK962452.1|	21791-72291	22	8.7	8	30	2	0.1563	no
gi|646744168|gb|KK962452.1|	23508-72400	22	8.7	18	69	2	0.21525	no
gi|646744168|gb|KK962452.1|	24176-72437	22	8.7	45	178	2	0.2531	no
gi|646744168|gb|KK962452.1|	24635-72529	22	8.7	172	418	1	0.28335	no
gi|646744168|gb|KK962452.1|	25732-72610	22	8.7	206	568	1	0.19935	no
gi|646744168|gb|KK962452.1|	26519-72761	22	8.7	125	333.59	1	0.24095	no
gi|646744168|gb|KK962452.1|	27951-72839	22	8.7	123	298	1	0.2593	no
gi|646744168|gb|KK962452.1|	28719-72911	22	8.7	46	190	2	0.1305	no

gi|646744168|gb|KK962452.1|	29315-73015	22	8.7	83	336	2	0.0936	no
gi|646744168|gb|KK962452.1|	31116-73177	22	8.7	96	358	2	0.1089	no
gi|646744168|gb|KK962452.1|	32657-73296	22	8.7	313	1005.58	2	0.14145	no
gi|646744168|gb|KK962452.1|	33660-73411	22	8.7	2	13	3	0.16995	no
gi|646744168|gb|KK962452.1|	34282-73454	22	8.7	248	779	2	0.20965	no
gi|646744168|gb|KK962452.1|	35206-73550	22	8.7	573	1975.18	2	0.1307	no
gi|646744168|gb|KK962452.1|	36258-73656	22	8.7	111	381	2	0.17615	no
gi|646744168|gb|KK962452.1|	36671-73776	22	8.7	7	20	1	0.349	no
gi|646744168|gb|KK962452.1|	38332-73875	22	8.7	47	187	2	0.141	no
gi|646744168|gb|KK962452.1|	38909-73988	22	8.7	4	24	3	0.1155	no
gi|646744168|gb|KK962452.1|	41882-74219	22	8.7	94	570	3	0.0668	no
gi|646744168|gb|KK962452.1|	57558-75820	22	8.7	1	5	2	0.24625	no
gi|646744168|gb|KK962452.1|	81788-78267	22	8.7	0.879557	0.83078	-0.0823115	1	no
gi|646744179|gb|KK962451.1|	26619-12780	24	11	0.29938	1	2	0.25365	no
gi|646744179|gb|KK962451.1|	28131-12932	24	11	0.593637	0.992216	0.741073	1	no
gi|646744179|gb|KK962451.1|	29429-13293	24	11	400	252	-0.66621	0.58745	no
gi|646744179|gb|KK962451.1|	13534-16722	24	11	32	16	-1	0.3793	no
gi|646744179|gb|KK962451.1|	81269-18162	24	11	21	51	1	0.4093	no
gi|646744179|gb|KK962451.1|	18188-20976	24	11	39	60	0.609631	0.6029	no
gi|646744179|gb|KK962451.1|	86515-18686	24	11	3	10	2	0.2517	no
gi|646744179|gb|KK962451.1|	86966-18741	24	11	11	2	-2	0.21945	no
gi|646744179|gb|KK962451.1|	1872-3622	24	11	3	2	-0.348436	0.8225	no
gi|646744179|gb|KK962451.1|	92650-19347	24	11	0.324552	3	3	0.25	no
gi|646744179|gb|KK962451.1|	95083-19531	24	11	7	43	3	0.26905	no
gi|646744179|gb|KK962451.1|	96452-19667	24	11	26	46	0.843469	0.5786	no
gi|646744179|gb|KK962451.1|	98666-20069	24	11	0.886075	2	0.839459	0.5471	no
gi|646744179|gb|KK962451.1|	04770-20511	24	11	19	45	1	0.4509	no
gi|646744179|gb|KK962451.1|	09164-20993	24	11	3	4	0.33128	0.80865	no
gi|646744179|gb|KK962451.1|	10479-21149	24	11	4	2	-0.887607	0.5301	no
gi|646744179|gb|KK962451.1|	21092-22963	24	11	29	32	0.114052	0.9221	no

gi|646744179|gb|KK962451.1|	14006-21725	24	11	1	2	0.745413	0.6379	no
gi|646744179|gb|KK962451.1|	17637-21926	24	11	3	2	-0.7186	0.6531	no
gi|646744179|gb|KK962451.1|	22004-22375	24	11	2	3	0.688867	0.66035	no
gi|646744179|gb|KK962451.1|	26210-22814	24	11	51	46	-0.136946	0.9085	no
gi|646744179|gb|KK962451.1|	23927-26415	24	11	9	10	0.112595	0.9158	no
gi|646744179|gb|KK962451.1|	58338-25929	24	11	3	0.937984	-2	0.27815	no
gi|646744179|gb|KK962451.1|	59633-26099	24	11	14	3	-2	0.1672	no
gi|646744179|gb|KK962451.1|	62356-26272	24	11	43	5	-3	0.071	no
gi|646744179|gb|KK962451.1|	26980-31877	24	11	21	30	0.526065	0.65175	no
gi|646744179|gb|KK962451.1|	89525-29008	24	11	4	4	-0.033609	0.95325	no
gi|646744179|gb|KK962451.1|	10031-31142	24	11	1	0.699878	-0.590743	1	no
gi|646744179|gb|KK962451.1|	11576-31253	24	11	2	1	-0.492256	0.70055	no
gi|646744179|gb|KK962451.1|	17979-31875	24	11	2	3	0.732985	0.57435	no
gi|646744179|gb|KK962451.1|	32390-33696	24	11	7	7	-0.0251096	0.98555	no
gi|646744179|gb|KK962451.1|	25421-32833	24	11	5	0.708539	-3	0.06515	no
gi|646744179|gb|KK962451.1|	32614-33431	24	11	2	1	-1	0.4165	no
gi|646744179|gb|KK962451.1|	34386-33618	24	11	2	0.956043	-1	0.43525	no
gi|646744179|gb|KK962451.1|	36284-33815	24	11	2	0.746366	-1	0.36135	no
gi|646744179|gb|KK962451.1|	39165-34044	24	11	14	12	-0.245778	0.87585	no
gi|646744179|gb|KK962451.1|	41783-34209	24	11	7	17	1	0.35135	no
gi|646744179|gb|KK962451.1|	46364-34682	24	11	3	2	-0.174219	0.89395	no
gi|646744179|gb|KK962451.1|	51444-35199	24	11	11	5	-1	0.4182	no
gi|646744179|gb|KK962451.1|	61520-36237	24	11	18	10	-0.839938	0.57895	no
gi|646744179|gb|KK962451.1|	75682-37640	24	11	2	2	0.0710949	0.9485	no
gi|646744179|gb|KK962451.1|	89529-38986	24	11	31	57	0.870339	0.5825	no
gi|646744179|gb|KK962451.1|	96825-39749	24	11	15	13	-0.217833	0.88035	no
gi|646744179|gb|KK962451.1|	4109-4824	24	11	4	3	-0.593586	0.62625	no
gi|646744179|gb|KK962451.1|	22084-42491	24	11	6	5	-0.13966	0.9239	no
gi|646744179|gb|KK962451.1|	61548-62197	24	11	2	0.325596	-3	0.2033	no
gi|646744205|gb|KK962450.1|	41968-24241	14	7.1	7	5	-0.403611	0.75895	no

gi|646744205|gb|KK962450.1|	44359-44690	14	7.1	0	23	inf	0.00435	no
gi|646744247|gb|KK962447.1|	69039-16970	13	5.5	2	3	0.153759	0.91085	no
gi|646744247|gb|KK962447.1|	79238-28000	13	5.5	0	3	inf	0.0014	yes
gi|646744247|gb|KK962447.1|	16885-31804	13	5.5	2	0	-inf	0.0008	yes
gi|646744247|gb|KK962447.1|	19670-32002	13	5.5	9	1	-3	0.1972	no
gi|646744259|gb|KK962446.1|	48113-14910	15	7.1	0.502738	1	2	0.3671	no
gi|646744259|gb|KK962446.1|	51643-15269	15	7.1	1	0.828089	-0.335098	1	no
gi|646744259|gb|KK962446.1|	77006-27958	15	7.1	3	4	0.538838	0.7241	no
gi|646744259|gb|KK962446.1|	79864-28238	15	7.1	2	3	0.673426	0.6667	no
gi|646744270|gb|KK962445.1|	06766-10752	21	7.9	8	6	-0.408055	0.7928	no
gi|646744270|gb|KK962445.1|	07710-10792	21	7.9	6	67	4	0.1029	no
gi|646744270|gb|KK962445.1|	09563-11014	21	7.9	1	3	0.943543	0.5627	no
gi|646744270|gb|KK962445.1|	19521-11990	21	7.9	10	29	1	0.30135	no
gi|646744270|gb|KK962445.1|	25772-12709	21	7.9	0.691023	0.496457	-0.477064	1	no
gi|646744270|gb|KK962445.1|	27719-12795	21	7.9	59	167	2	0.28215	no
gi|646744270|gb|KK962445.1|	28667-13210	21	7.9	13	31	1	0.26815	no
gi|646744270|gb|KK962445.1|	38120-13833	21	7.9	59	11	-2	0.17855	no
gi|646744270|gb|KK962445.1|	87011-18848	21	7.9	4	0.329278	-4	0.0981	no
gi|646744270|gb|KK962445.1|	90574-19195	21	7.9	0.902151	0.471858	-0.935015	1	no
gi|646744270|gb|KK962445.1|	93111-19449	21	7.9	1	0.94701	-0.475682	0.71985	no
gi|646744270|gb|KK962445.1|	21770-22374	21	7.9	12	8	-0.506576	0.6816	no
gi|646744270|gb|KK962445.1|	28324-22964	21	7.9	6	3	-0.692187	0.65605	no
gi|646744270|gb|KK962445.1|	30441-23088	21	7.9	22	27	0.330329	0.8224	no
gi|646744270|gb|KK962445.1|	32853-23439	21	7.9	1	1	0.0803262	0.9493	no
gi|646744270|gb|KK962445.1|	48180-24841	21	7.9	51	15	-2	0.2386	no
gi|646744270|gb|KK962445.1|	65335-26839	21	7.9	0.726165	0.768488	0.0817245	1	no
gi|646744270|gb|KK962445.1|	81649-28273	21	7.9	31	7	-2	0.11655	no
gi|646744270|gb|KK962445.1|	99441-30143	21	7.9	23	3	-3	0.03835	no
gi|646744270|gb|KK962445.1|	02986-30327	21	7.9	27	2	-4	0.1722	no
gi|646744270|gb|KK962445.1|	33873-33489	21	7.9	19	2	-3	0.04985	no

gi|646744270|gb|KK962445.1|	36987-33723	21	7.9	73	6	-4	0.1378	no
gi|646744270|gb|KK962445.1|	48050-34830	21	7.9	71	17	-2	0.20185	no
gi|646744270|gb|KK962445.1|	35110-36045	21	7.9	42	55	0.403964	0.7096	no
gi|646744270|gb|KK962445.1|	57091-35773	21	7.9	7	1	-3	0.1435	no
gi|646744270|gb|KK962445.1|	62140-36240	21	7.9	25	8	-2	0.28835	no
gi|646744270|gb|KK962445.1|	74525-37685	21	7.9	11	10	-0.145817	0.89275	no
gi|646744270|gb|KK962445.1|	41042-41551	21	7.9	77	88	0.191746	0.8547	no
gi|646744270|gb|KK962445.1|	60259-68185	21	7.9	6	5	-0.297459	0.78445	no
gi|646744270|gb|KK962445.1|	6335-7054	21	7.9	0.996517	2	0.970425	0.5626	no
gi|646744312|gb|KK962442.1|	28263-12854	12	6.5	396	201	-0.977619	0.43815	no
gi|646744324|gb|KK962441.1|	02276-10266	20	7.2	28	21	-0.453268	0.76895	no
gi|646744324|gb|KK962441.1|	06132-10695	20	7.2	0.820009	1	0.496839	0.7568	no
gi|646744324|gb|KK962441.1|	12674-13417	20	7.2	297	79	-2	0.1095	no
gi|646744324|gb|KK962441.1|	58656-15975	20	7.2	0.981793	0.935481	-0.0697108	1	no
gi|646744324|gb|KK962441.1|	70412-17111	20	7.2	2	1	-0.518169	0.73195	no
gi|646744324|gb|KK962441.1|	18583-19957	20	7.2	3	6	0.890975	0.56755	no
gi|646744324|gb|KK962441.1|	87715-18801	20	7.2	26	29	0.162815	0.90545	no
gi|646744324|gb|KK962441.1|	20098-21094	20	7.2	5	8	0.575535	0.70745	no
gi|646744324|gb|KK962441.1|	03929-20432	20	7.2	21	11	-1	0.45145	no
gi|646744324|gb|KK962441.1|	08491-20933	20	7.2	2	2	0.346978	0.79725	no
gi|646744324|gb|KK962441.1|	32013-23255	20	7.2	7	2	-2	0.22575	no
gi|646744324|gb|KK962441.1|	34616-23530	20	7.2	2	2	0.117151	0.92795	no
gi|646744324|gb|KK962441.1|	45597-24701	20	7.2	0.636915	0.687469	0.110194	1	no
gi|646744324|gb|KK962441.1|	49561-25004	20	7.2	6	3	-0.900711	0.5046	no
gi|646744324|gb|KK962441.1|	50940-26107	20	7.2	12	10	-0.208624	0.8566	no
gi|646744324|gb|KK962441.1|	29439-29884	20	7.2	0.954948	5	2	0.28805	no
gi|646744324|gb|KK962441.1|	31060-32106	20	7.2	20	40	1	0.4347	no
gi|646744324|gb|KK962441.1|	16370-31706	20	7.2	0.630428	2	2	0.25705	no
gi|646744324|gb|KK962441.1|	19891-32013	20	7.2	887	2763.63	2	0.154	no
gi|646744324|gb|KK962441.1|	50404-35135	20	7.2	20	53	1	0.2723	no

gi|646744324|gb|KK962441.1|	53581-35491	20	7.2	0.428688	1	2	0.2702	no
gi|646744324|gb|KK962441.1|	55664-35679	20	7.2	0.212416	2	3	0.25	no
gi|646744324|gb|KK962441.1|	65320-36671	20	7.2	52	41	-0.357536	0.74865	no
gi|646744324|gb|KK962441.1|	87341-38849	20	7.2	0.514504	1	1	1	no
gi|646744324|gb|KK962441.1|	94964-39860	20	7.2	3	7	1	0.3802	no
gi|646744324|gb|KK962441.1|	99070-40007	20	7.2	0.988029	0.52777	-0.904642	1	no
gi|646744324|gb|KK962441.1|	04437-40590	20	7.2	0.381337	1	2	0.26895	no
gi|646744324|gb|KK962441.1|	06501-40902	20	7.2	3	3	0.188541	0.8963	no
gi|646744324|gb|KK962441.1|	09296-41162	20	7.2	49	66	0.421605	0.72005	no
gi|646744324|gb|KK962441.1|	11990-41380	20	7.2	0.4154	0.68651	0.724782	1	no
gi|646744324|gb|KK962441.1|	18188-41931	20	7.2	0.316806	1	2	0.20215	no
gi|646744324|gb|KK962441.1|	42749-43414	20	7.2	1	2	0.476236	0.7489	no
gi|646744324|gb|KK962441.1|	28846-43076	20	7.2	0.778849	1	0.895974	0.52715	no
gi|646744324|gb|KK962441.1|	37551-43874	20	7.2	0.889505	0.707986	-0.329281	1	no
gi|646744324|gb|KK962441.1|	38850-43980	20	7.2	0.922392	1	0.285225	0.8455	no
gi|646744324|gb|KK962441.1|	40285-46184	20	7.2	46	98	1	0.3646	no
gi|646744324|gb|KK962441.1|	64568-46544	20	7.2	3	4	0.646091	0.63835	no
gi|646744324|gb|KK962441.1|	54633-55757	20	7.2	82	88	0.106721	0.92265	no
gi|646744324|gb|KK962441.1|	56905-57156	20	7.2	22	33	0.574864	0.68235	no
gi|646744324|gb|KK962441.1|	57312-58176	20	7.2	5	8	0.630164	0.6839	no
gi|646744324|gb|KK962441.1|	587-903	20	7.2	80.58	75	-0.0960142	0.94635	no
gi|646744324|gb|KK962441.1|	61507-62568	20	7.2	0.918171	0.982911	0.0982977	1	no
gi|646744324|gb|KK962441.1|	62681-64744	20	7.2	3	7	1	0.3894	no
gi|646744324|gb|KK962441.1|	71067-71917	20	7.2	16	17	0.0892231	0.9536	no
gi|646744324|gb|KK962441.1|	73064-74231	20	7.2	24	40	0.761214	0.4867	no
gi|646744324|gb|KK962441.1|	9101-9965	20	7.2	5	8	0.841918	0.59095	no
gi|646744339|gb|KK962440.1|	35873-13695	12	7.5	12	17	0.447158	0.7693	no
gi|646744339|gb|KK962440.1|	50985-15121	12	7.5	4	333	6	0.0951	no
gi|646744365|gb|KK962438.1|	06625-10790	17	7.9	3	4	0.180674	0.90825	no
gi|646744365|gb|KK962438.1|	12158-11301	17	7.9	1	1	-0.0843325	0.93265	no

gi|646744365|gb|KK962438.1|	24279-12611	17	7.9	2	4	0.862567	0.5779	no
gi|646744365|gb|KK962438.1|	04947-20538	17	7.9	7	6	-0.342171	0.7904	no
gi|646744365|gb|KK962438.1|	09062-21018	17	7.9	0.964732	0.306544	-2	1	no
gi|646744365|gb|KK962438.1|	12153-21300	17	7.9	1	0.655172	-0.722358	1	no
gi|646744365|gb|KK962438.1|	30479-23160	17	7.9	1	2	0.516388	0.66675	no
gi|646744365|gb|KK962438.1|	25030-32597	17	7.9	1	3	1	0.35935	no
gi|646744365|gb|KK962438.1|	26030-32655	17	7.9	2	2	0.175117	0.89965	no
gi|646744365|gb|KK962438.1|	34053-35272	17	7.9	2	3	0.382184	0.8157	no
gi|646744365|gb|KK962438.1|	35489-35991	17	7.9	1	4	1	0.37835	no
gi|646744365|gb|KK962438.1|	11604-51208	17	7.9	4	2	-1	0.46445	no
gi|646744365|gb|KK962438.1|	53410-54357	17	7.9	1	0.949801	-0.630525	0.65905	no
gi|646744391|gb|KK962436.1|	03993-20449	17	11	5	0.99733	-2	0.19235	no
gi|646744391|gb|KK962436.1|	21355-22215	17	11	1	1	0.272231	0.8456	no
gi|646744391|gb|KK962436.1|	815-1472	17	11	5	2	-2	0.3147	no
gi|646744403|gb|KK962435.1|	90807-19137	16	7.2	3	2	-0.869505	0.56495	no
gi|646744415|gb|KK962434.1|	07437-30780	13	9.3	13	11	-0.163996	0.89025	no
gi|646744427|gb|KK962433.1|	63880-64301	12	7.1	0	67	inf	0.0002	yes
gi|646744441|gb|KK962432.1|	07435-10797	24	11	4	1	-1	0.37175	no
gi|646744441|gb|KK962432.1|	08733-11009	24	11	17	8	-1	0.473	no
gi|646744441|gb|KK962432.1|	11411-11287	24	11	0.613172	0.441537	-0.473759	1	no
gi|646744441|gb|KK962432.1|	45743-14722	24	11	0.832764	1	0.652092	0.6075	no
gi|646744441|gb|KK962432.1|	47801-14896	24	11	2	2	0.283106	0.8375	no
gi|646744441|gb|KK962432.1|	80332-18088	24	11	0.93038	3	2	0.26115	no
gi|646744441|gb|KK962432.1|	81755-18197	24	11	29	24	-0.254415	0.8749	no
gi|646744441|gb|KK962432.1|	83165-18420	24	11	5	7	0.405436	0.7894	no
gi|646744441|gb|KK962432.1|	89196-18996	24	11	1	4	1	0.28925	no
gi|646744441|gb|KK962432.1|	29569-22995	24	11	6	7	0.179163	0.9	no
gi|646744441|gb|KK962432.1|	59996-36081	24	11	8	12	0.525018	0.7244	no
gi|646744441|gb|KK962432.1|	73757-37488	24	11	3	5	0.885334	0.58125	no
gi|646744441|gb|KK962432.1|	75000-37702	24	11	6	7	0.316659	0.83265	no

gi|646744441|gb|KK962432.1|	78822-37913	24	11	14	10	-0.515742	0.69785	no
gi|646744441|gb|KK962432.1|	86285-38649	24	11	42	23	-0.844405	0.59745	no
gi|646744453|gb|KK962431.1|	10149-10501	19	8.5	53	37	-0.508908	0.7325	no
gi|646744453|gb|KK962431.1|	07498-10837	19	8.5	2	0.848971	-0.957893	0.54505	no
gi|646744453|gb|KK962431.1|	17462-11988	19	8.5	7	3	-1	0.43775	no
gi|646744453|gb|KK962431.1|	26116-13070	19	8.5	7	7	-0.0644121	0.95165	no
gi|646744453|gb|KK962431.1|	12626-14192	19	8.5	0.987939	0.915971	-0.10912	1	no
gi|646744453|gb|KK962431.1|	57957-15820	19	8.5	41	32	-0.328908	0.7864	no
gi|646744453|gb|KK962431.1|	60178-16060	19	8.5	2	4	0.975639	0.5906	no
gi|646744453|gb|KK962431.1|	88039-18995	19	8.5	6	6	0.00445269	0.99435	no
gi|646744453|gb|KK962431.1|	98059-19918	19	8.5	4	2	-0.706045	0.6654	no
gi|646744453|gb|KK962431.1|	20133-21025	19	8.5	52	27	-0.981515	0.45235	no
gi|646744453|gb|KK962431.1|	22190-22717	19	8.5	2	3	0.703451	0.64555	no
gi|646744453|gb|KK962431.1|	31532-23178	19	8.5	17	8	-1	0.487	no
gi|646744453|gb|KK962431.1|	23640-24821	19	8.5	47	33	-0.506627	0.6418	no
gi|646744453|gb|KK962431.1|	28455-29164	19	8.5	8	3	-2	0.2874	no
gi|646744453|gb|KK962431.1|	29854-31483	19	8.5	7	4	-0.67368	0.66095	no
gi|646744453|gb|KK962431.1|	51119-35237	19	8.5	0.18539	6	5	0.23485	no
gi|646744453|gb|KK962431.1|	58807-36038	19	8.5	2	1	-0.96631	0.4969	no
gi|646744453|gb|KK962431.1|	45871-46385	19	8.5	12	13	0.102824	0.94505	no
gi|646744453|gb|KK962431.1|	49965-51803	19	8.5	3	3	-0.102272	0.94875	no
gi|646744453|gb|KK962431.1|	57382-58579	19	8.5	3	5	0.774186	0.617	no
gi|646744453|gb|KK962431.1|	58746-59547	19	8.5	12	8	-0.674885	0.6605	no
gi|646744453|gb|KK962431.1|	65743-66168	19	8.5	9	8	-0.115398	0.92645	no
gi|646744453|gb|KK962431.1|	66358-67639	19	8.5	1	1	0.280643	0.8201	no
gi|646744453|gb|KK962431.1|	74843-75273	19	8.5	73	81	0.158015	0.9155	no
gi|646744453|gb|KK962431.1|	76929-79664	19	8.5	1	3	1	0.4266	no
gi|646744453|gb|KK962431.1|	79859-82793	19	8.5	1	4	1	0.3757	no
gi|646744453|gb|KK962431.1|	85310-85746	19	8.5	30	44	0.530147	0.72495	no
gi|646744453|gb|KK962431.1|	85912-86210	19	8.5	96	145	0.591542	0.694	no

gi|646744453|gb|KK962431.1|	89024-89664	19	8.5	12	8	-0.587457	0.6988	no
gi|646744453|gb|KK962431.1|	92724-94388	19	8.5	0.590936	1	0.945019	0.48355	no
gi|646744453|gb|KK962431.1|	94968-95980	19	8.5	81	36	-1	0.3015	no
gi|646744497|gb|KK962428.1|	10375-11223	16	8.8	17	4	-2	0.235	no
gi|646744497|gb|KK962428.1|	14690-16458	16	8.8	9	3	-1	0.35875	no
gi|646744521|gb|KK962426.1|	42405-42712	18	8.4	7	9	0.511113	0.74235	no
gi|646744534|gb|KK962425.1|	22125-12404	19	8.6	0.668791	0.645297	-0.0515928	1	no
gi|646744534|gb|KK962425.1|	24847-12592	19	8.6	0.904704	1	0.513903	0.71995	no
gi|646744534|gb|KK962425.1|	26385-12908	19	8.6	1	0.878921	-0.215375	1	no
gi|646744534|gb|KK962425.1|	29317-13122	19	8.6	0.839021	1	0.32685	1	no
gi|646744534|gb|KK962425.1|	31383-13377	19	8.6	1	0.943973	-0.19936	1	no
gi|646744534|gb|KK962425.1|	34927-13879	19	8.6	13	10	-0.337812	0.76265	no
gi|646744534|gb|KK962425.1|	48437-14955	19	8.6	73	26	-2	0.18315	no
gi|646744534|gb|KK962425.1|	51144-15181	19	8.6	13	9	-0.498263	0.74615	no
gi|646744534|gb|KK962425.1|	51930-15366	19	8.6	0.814281	0.814562	0.000497572	1	no
gi|646744534|gb|KK962425.1|	94762-29584	19	8.6	2	1	-0.485683	0.70055	no
gi|646744534|gb|KK962425.1|	29793-32360	19	8.6	4	4	0.229065	0.8787	no
gi|646744534|gb|KK962425.1|	00131-30078	19	8.6	3	3	0.184107	0.8935	no
gi|646744534|gb|KK962425.1|	02408-30728	19	8.6	9	7	-0.319219	0.77525	no
gi|646744534|gb|KK962425.1|	09993-31304	19	8.6	4	6	0.7009	0.6455	no
gi|646744534|gb|KK962425.1|	27780-42826	19	8.6	2	9	2	0.2845	no
gi|646744534|gb|KK962425.1|	05155-50643	19	8.6	0	56	inf	5.00E-05	yes
gi|646744534|gb|KK962425.1|	07824-50797	19	8.6	0	441	inf	0.0014	yes
gi|646744534|gb|KK962425.1|	11529-51191	19	8.6	0.69195	137	8	0.09445	no
gi|646744534|gb|KK962425.1|	14152-51455	19	8.6	0	8	inf	0.0014	yes
gi|646744534|gb|KK962425.1|	14697-51521	19	8.6	0	36	inf	0.00025	yes
gi|646744534|gb|KK962425.1|	21312-52193	19	8.6	0	41	inf	5.00E-05	yes
gi|646744534|gb|KK962425.1|	22883-52422	19	8.6	0	25	inf	5.00E-05	yes
gi|646744534|gb|KK962425.1|	29061-52953	19	8.6	0	27	inf	0.0004	yes
gi|646744534|gb|KK962425.1|	33240-53399	19	8.6	0	13	inf	0.00015	yes

gi|646744534|gb|KK962425.1|	35679-53672	19	8.6	0.116652	16	7	0.09495	no
gi|646744534|gb|KK962425.1|	43667-54390	19	8.6	0	79	inf	0.0023	no
gi|646744534|gb|KK962425.1|	55098-62800	19	8.6	22	10	-1	0.3384	no
gi|646744534|gb|KK962425.1|	58034-56218	19	8.6	0	4	inf	5.00E-05	yes
gi|646744534|gb|KK962425.1|	63513-56500	19	8.6	0.074849	34	9	0.09445	no
gi|646744534|gb|KK962425.1|	65621-56662	19	8.6	0.124	41	8	0.09445	no
gi|646744534|gb|KK962425.1|	67490-56808	19	8.6	2	1	-0.760641	0.6333	no
gi|646744534|gb|KK962425.1|	68400-56955	19	8.6	1	1	0.254461	0.8481	no
gi|646744534|gb|KK962425.1|	02235-60386	19	8.6	46	0.097322	-9	0.1665	no
gi|646744534|gb|KK962425.1|	21378-62208	19	8.6	3	0	-inf	0.0012	yes
gi|646744534|gb|KK962425.1|	22298-62492	19	8.6	5	0	-inf	5.00E-05	yes
gi|646744534|gb|KK962425.1|	69395-67054	19	8.6	39	0	-inf	5.00E-05	yes
gi|646744534|gb|KK962425.1|	67007-68077	19	8.6	0.454017	1	2	0.3671	no
gi|646744534|gb|KK962425.1|	70783-67469	19	8.6	6	0	-inf	5.00E-05	yes
gi|646744534|gb|KK962425.1|	95702-69618	19	8.6	3	1	-2	0.3832	no
gi|646744534|gb|KK962425.1|	19098-72169	19	8.6	5	8	0.853296	0.4943	no
gi|646744534|gb|KK962425.1|	24630-72527	19	8.6	4	4	0.0858109	0.9457	no
gi|646744534|gb|KK962425.1|	37824-74024	19	8.6	37	30	-0.267866	0.81735	no
gi|646744534|gb|KK962425.1|	75338-75814	19	8.6	2	4	0.833257	0.57025	no
gi|646744534|gb|KK962425.1|	61120-76139	19	8.6	68	19	-2	0.2247	no
gi|646744534|gb|KK962425.1|	)9538-10081(	19	8.6	5	1	-2	0.16375	no
gi|646744567|gb|KK962423.1|	10132-10916	12	8.5	4	4	0.0571225	0.9677	no
gi|646744567|gb|KK962423.1|	03523-30429	12	8.5	6	0.756152	-3	0.12745	no
gi|646744567|gb|KK962423.1|	43521-34457	12	8.5	10	0.498069	-4	0.0839	no
gi|646744567|gb|KK962423.1|	46484-35134	12	8.5	8	0.381326	-4	0.0042	no
gi|646744567|gb|KK962423.1|	67148-56750	12	8.5	8	1	-3	0.20705	no
gi|646744567|gb|KK962423.1|	70950-57137	12	8.5	11	7	-0.616607	0.6224	no
gi|646744579|gb|KK962422.1|	82609-18334	22	9.9	2	3	0.809292	0.5504	no
gi|646744579|gb|KK962422.1|	88098-18927	22	9.9	2	3	0.8401	0.557	no
gi|646744579|gb|KK962422.1|	01713-21282	22	9.9	2	3	0.66655	0.5316	no

gi|646744579|gb|KK962422.1|	15234-21590	22	9.9	5	3	-0.593254	0.6238	no
gi|646744579|gb|KK962422.1|	26441-22726	22	9.9	0.812806	2	1	0.36955	no
gi|646744579|gb|KK962422.1|	45507-24646	22	9.9	1	3	0.992982	0.4717	no
gi|646744579|gb|KK962422.1|	61473-26188	22	9.9	5	2	-2	0.3081	no
gi|646744579|gb|KK962422.1|	03237-30370	22	9.9	16	7	-1	0.38955	no
gi|646744579|gb|KK962422.1|	05049-30591	22	9.9	13	7	-0.863621	0.5722	no
gi|646744579|gb|KK962422.1|	08113-30932	22	9.9	13	10	-0.353308	0.8136	no
gi|646744579|gb|KK962422.1|	24338-32528	22	9.9	1	0.569084	-0.907994	1	no
gi|646744579|gb|KK962422.1|	25426-32807	22	9.9	14	9	-0.752017	0.4886	no
gi|646744579|gb|KK962422.1|	45610-34683	22	9.9	15	13	-0.141694	0.9256	no
gi|646744579|gb|KK962422.1|	47137-35124	22	9.9	1	1	0.0523901	0.97295	no
gi|646744579|gb|KK962422.1|	53612-36327	22	9.9	15	7	-1	0.32845	no
gi|646744579|gb|KK962422.1|	11098-41199	22	9.9	2	0.612769	-2	0.2263	no
gi|646744579|gb|KK962422.1|	12228-41344	22	9.9	2	0.412733	-3	0.1859	no
gi|646744579|gb|KK962422.1|	13682-41434	22	9.9	2	0.9496	-0.939535	0.589	no
gi|646744579|gb|KK962422.1|	14424-41507	22	9.9	2	0.326374	-3	0.21605	no
gi|646744579|gb|KK962422.1|	37165-44183	22	9.9	5	3	-0.670643	0.59075	no
gi|646744579|gb|KK962422.1|	79777-48071	22	9.9	1	0.193757	-3	0.21605	no
gi|646744579|gb|KK962422.1|	18011-52586	22	9.9	12	11.59	-0.0165468	0.9889	no
gi|646744579|gb|KK962422.1|	26005-52733	22	9.9	0.860565	1	0.523045	0.69625	no
gi|646744579|gb|KK962422.1|	27510-52887	22	9.9	0.918432	1	0.386411	0.77315	no
gi|646744579|gb|KK962422.1|	29103-53032	22	9.9	1	1	0.060222	1	no
gi|646744579|gb|KK962422.1|	31376-53242	22	9.9	0.936763	0.501135	-0.902485	1	no
gi|646744579|gb|KK962422.1|	42992-54637	22	9.9	0.858592	1	0.684344	0.6619	no
gi|646744579|gb|KK962422.1|	46630-54773	22	9.9	0.649931	2	1	0.3407	no
gi|646744579|gb|KK962422.1|	36214-63796	22	9.9	2	2	-0.253016	0.8708	no
gi|646744579|gb|KK962422.1|	38413-64070	22	9.9	0.637113	0.924335	0.536868	1	no
gi|646744579|gb|KK962422.1|	40758-64201	22	9.9	0.370409	2	2	0.21355	no
gi|646744579|gb|KK962422.1|	06898-70756	22	9.9	12	5	-1	0.394	no
gi|646744598|gb|KK962421.1|	78594-18004	17	7.1	3	0.556447	-2	0.1837	no

gi|646744598|gb|KK962421.1|	53866-25407	17	7.1	51	12	-2	0.2311	no
gi|646744640|gb|KK962418.1|	94852-29571	15	7.5	15	10	-0.498953	0.7459	no
gi|646744640|gb|KK962418.1|	09115-31038	15	7.5	0.90785	0.78187	-0.215524	1	no
gi|646744640|gb|KK962418.1|	14148-31524	15	7.5	1	2	0.652449	0.6155	no
gi|646744652|gb|KK962417.1|	79405-17990	25	11	5	21.33	2	0.16035	no
gi|646744652|gb|KK962417.1|	81749-18225	25	11	3	4	0.432536	0.75085	no
gi|646744652|gb|KK962417.1|	85755-18686	25	11	0.433287	1	2	0.36595	no
gi|646744652|gb|KK962417.1|	91667-19387	25	11	0.569923	0.482105	-0.24142	1	no
gi|646744652|gb|KK962417.1|	98163-19842	25	11	17	40	1	0.4464	no
gi|646744652|gb|KK962417.1|	04551-20487	25	11	3	10	2	0.2864	no
gi|646744652|gb|KK962417.1|	06220-20695	25	11	0.573252	3	2	0.18805	no
gi|646744652|gb|KK962417.1|	17146-21774	25	11	2	13	3	0.11195	no
gi|646744652|gb|KK962417.1|	27930-22958	25	11	40	50	0.31841	0.7757	no
gi|646744652|gb|KK962417.1|	82612-28419	25	11	2	1	-0.187626	0.89145	no
gi|646744652|gb|KK962417.1|	96094-29805	25	11	2	0.866174	-0.864721	0.5404	no
gi|646744652|gb|KK962417.1|	11722-31577	25	11	3	2	-0.360017	0.80965	no
gi|646744652|gb|KK962417.1|	15851-31722	25	11	5	4	-0.426822	0.78385	no
gi|646744652|gb|KK962417.1|	22186-32281	25	11	2	2	0.276287	0.8403	no
gi|646744652|gb|KK962417.1|	23014-32399	25	11	0.50896	1	1	0.43305	no
gi|646744652|gb|KK962417.1|	32746-33340	25	11	1	4	1	0.34755	no
gi|646744652|gb|KK962417.1|	44971-34517	25	11	151	41	-2	0.17025	no
gi|646744652|gb|KK962417.1|	50070-35258	25	11	6	5	-0.15577	0.91785	no
gi|646744652|gb|KK962417.1|	52851-35382	25	11	1	1	0.186494	0.8968	no
gi|646744652|gb|KK962417.1|	74835-37577	25	11	0.269686	6	4	0.24185	no
gi|646744652|gb|KK962417.1|	59141-66256	25	11	5	10	0.917794	0.4725	no
gi|646744652|gb|KK962417.1|	63446-66784	25	11	2	5	1	0.34265	no
gi|646744652|gb|KK962417.1|	72010-67386	25	11	33	25	-0.417895	0.71265	no
gi|646744652|gb|KK962417.1|	75896-67660	25	11	0.404697	3	3	0.26125	no
gi|646744652|gb|KK962417.1|	76916-67969	25	11	9	11	0.271175	0.8016	no
gi|646744652|gb|KK962417.1|	81120-68226	25	11	0.206952	54	8	0.2322	no

gi|646744652|gb|KK962417.1|	91177-69234	25	11	35	12	-2	0.25245	no
gi|646744652|gb|KK962417.1|	95750-69938	25	11	0.629395	0.917089	0.543096	1	no
gi|646744652|gb|KK962417.1|	11243-71335	25	11	0.599463	0.506797	-0.242262	1	no
gi|646744652|gb|KK962417.1|	29953-73026	25	11	15	6	-1	0.361	no
gi|646744666|gb|KK962416.1|	41711-24213	23	9.1	4	3	-0.419528	0.76715	no
gi|646744666|gb|KK962416.1|	29607-30291	23	9.1	0.86204	3	2	0.2986	no
gi|646744666|gb|KK962416.1|	19688-32100	23	9.1	2	5	2	0.2607	no
gi|646744666|gb|KK962416.1|	21784-32239	23	9.1	4	9	1	0.34245	no
gi|646744675|gb|KK962415.1|	59477-16054	13	7.3	0.571023	1	1	0.36855	no
gi|646744675|gb|KK962415.1|	97794-19825	13	7.3	3	5	0.997368	0.53295	no
gi|646744675|gb|KK962415.1|	07548-40837	13	7.3	1	2	0.690436	0.6295	no
gi|646744686|gb|KK962414.1|	44068-34502	12	7.3	2	2	-0.12054	0.92125	no
gi|646744686|gb|KK962414.1|	71482-47283	12	7.3	5	6	0.320735	0.82985	no
gi|646744699|gb|KK962413.1|	08462-10959	14	6.9	16	13	-0.217129	0.88375	no
gi|646744699|gb|KK962413.1|	21423-12173	14	6.9	5	15	2	0.35435	no
gi|646744699|gb|KK962413.1|	31398-13185	14	6.9	2	4	0.672651	0.662	no
gi|646744699|gb|KK962413.1|	33485-13414	14	6.9	6	9	0.63863	0.6894	no
gi|646744699|gb|KK962413.1|	13943-15520	14	6.9	16	3	-2	0.09715	no
gi|646744699|gb|KK962413.1|	56525-15680	14	6.9	23	13	-0.818476	0.54625	no
gi|646744699|gb|KK962413.1|	57224-15743	14	6.9	65	12	-2	0.17855	no
gi|646744699|gb|KK962413.1|	60340-16068	14	6.9	34	37	0.104662	0.94705	no
gi|646744699|gb|KK962413.1|	16933-17480	14	6.9	26	11	-1	0.44515	no
gi|646744699|gb|KK962413.1|	18789-19946	14	6.9	6	4	-0.509015	0.7368	no
gi|646744699|gb|KK962413.1|	25933-22683	14	6.9	3	3	0.269472	0.84325	no
gi|646744699|gb|KK962413.1|	30535-30786	14	6.9	63	36	-0.823167	0.55785	no
gi|646744699|gb|KK962413.1|	30927-31143	14	6.9	1049.36	766	-0.453349	0.7191	no
gi|646744699|gb|KK962413.1|	39910-41212	14	6.9	2	2	0.135241	0.91405	no
gi|646744699|gb|KK962413.1|	41297-42018	14	6.9	2	2	-0.099677	0.9387	no
gi|646744699|gb|KK962413.1|	64805-65991	14	6.9	0.695552	1	1	0.44735	no
gi|646744699|gb|KK962413.1|	66163-66352	14	6.9	91	51	-0.845126	0.5825	no

gi|646744699|gb|KK962413.1|	70329-71410	14	6.9	363	284	-0.35731	0.767	no
gi|646744699|gb|KK962413.1|	87780-89593	14	6.9	3	5	0.633407	0.67445	no
gi|646744699|gb|KK962413.1|	96428-97167	14	6.9	3	1	-2	0.24545	no
gi|646744699|gb|KK962413.1|	98026-10053	14	6.9	15	15	0.0537599	0.96115	no
gi|646744719|gb|KK962412.1|	84263-48603	15	9.5	2	0.879266	-1	0.38185	no
gi|646744719|gb|KK962412.1|	11238-51140	15	9.5	896	1098.29	0.29434	0.8452	no
gi|646744719|gb|KK962412.1|	76130-57765	15	9.5	1	3	1	0.32055	no
gi|646744731|gb|KK962411.1|	79209-38023	13	6.5	0.845343	28	5	0.0288	no
gi|646744731|gb|KK962411.1|	92104-39285	13	6.5	0.188364	17	7	0.09485	no
gi|646744731|gb|KK962411.1|	93332-39456	13	6.5	0.472468	2	2	0.24085	no
gi|646744731|gb|KK962411.1|	94712-39556	13	6.5	0.154939	6	5	0.0987	no
gi|646744731|gb|KK962411.1|	96187-39762	13	6.5	0.156403	4	5	0.238	no
gi|646744731|gb|KK962411.1|	10854-41113	13	6.5	0	57	inf	0.0012	yes
gi|646744731|gb|KK962411.1|	24278-42454	13	6.5	0	55	inf	0.00175	no
gi|646744731|gb|KK962411.1|	28915-42914	13	6.5	0	47	inf	0.0014	yes
gi|646744743|gb|KK962410.1|	00337-10133	17	8.9	2	0.353681	-2	0.1971	no
gi|646744743|gb|KK962410.1|	26023-12629	17	8.9	28	7	-2	0.19715	no
gi|646744743|gb|KK962410.1|	44144-14440	17	8.9	27	5	-2	0.1703	no
gi|646744743|gb|KK962410.1|	46261-14822	17	8.9	33	15	-1	0.28825	no
gi|646744743|gb|KK962410.1|	49004-14948	17	8.9	187	128	-0.55021	0.61475	no
gi|646744743|gb|KK962410.1|	49584-15043	17	8.9	30	15	-1	0.5079	no
gi|646744743|gb|KK962410.1|	32473-23346	17	8.9	1	0.177767	-3	0.21635	no
gi|646744743|gb|KK962410.1|	50122-25116	17	8.9	100	31	-2	0.14125	no
gi|646744743|gb|KK962410.1|	54307-25694	17	8.9	9	4	-1	0.33685	no
gi|646744743|gb|KK962410.1|	67466-26903	17	8.9	25	18	-0.479951	0.6597	no
gi|646744743|gb|KK962410.1|	41418-42270	17	8.9	1	1	-0.0851008	0.93265	no
gi|646744756|gb|KK962409.1|	10595-10978	18	8.1	4	7	0.766149	0.59565	no
gi|646744756|gb|KK962409.1|	11119-12631	18	8.1	0.662384	2	2	0.28315	no
gi|646744756|gb|KK962409.1|	26394-12763	18	8.1	11	5	-1	0.4233	no
gi|646744756|gb|KK962409.1|	57130-16187	18	8.1	6	5	-0.468741	0.66305	no

gi|646744756|gb|KK962409.1|	62580-16457	18	8.1	10	10	0.014133	0.99205	no
gi|646744756|gb|KK962409.1|	17096-19145	18	8.1	1	2	0.409262	0.8	no
gi|646744756|gb|KK962409.1|	1749-2374	18	8.1	0	4	inf	0.001	yes
gi|646744756|gb|KK962409.1|	74917-17695	18	8.1	3	1	-1	0.41625	no
gi|646744756|gb|KK962409.1|	90843-19131	18	8.1	6	6	-0.169862	0.90125	no
gi|646744756|gb|KK962409.1|	91492-19292	18	8.1	6	5	-0.17751	0.90475	no
gi|646744756|gb|KK962409.1|	06499-20880	18	8.1	22	15	-0.599903	0.5898	no
gi|646744756|gb|KK962409.1|	10379-21111	18	8.1	13	19	0.507581	0.73015	no
gi|646744756|gb|KK962409.1|	11464-21207	18	8.1	7	6	-0.0914966	0.9545	no
gi|646744756|gb|KK962409.1|	21171-21395	18	8.1	82	120	0.547641	0.68995	no
gi|646744756|gb|KK962409.1|	12211-21295	18	8.1	2	1	-0.775271	0.59035	no
gi|646744756|gb|KK962409.1|	28248-22924	18	8.1	2	4	0.902434	0.52445	no
gi|646744756|gb|KK962409.1|	29349-23247	18	8.1	2	1	-0.729866	0.62825	no
gi|646744756|gb|KK962409.1|	36720-23824	18	8.1	0.581843	0.419364	-0.472427	1	no
gi|646744756|gb|KK962409.1|	23921-25413	18	8.1	0.897456	2	1	0.395	no
gi|646744756|gb|KK962409.1|	39509-24002	18	8.1	7	3	-1	0.3508	no
gi|646744756|gb|KK962409.1|	2492-8092	18	8.1	19	19	-0.0708832	0.9497	no
gi|646744756|gb|KK962409.1|	62048-26421	18	8.1	2	2	0.107064	0.9433	no
gi|646744756|gb|KK962409.1|	66588-26682	18	8.1	15	62	2	0.2676	no
gi|646744756|gb|KK962409.1|	70816-27105	18	8.1	20	17	-0.180737	0.8995	no
gi|646744756|gb|KK962409.1|	71949-27309	18	8.1	2	6	2	0.2244	no
gi|646744756|gb|KK962409.1|	29576-30630	18	8.1	0.578883	1	1	0.43245	no
gi|646744756|gb|KK962409.1|	37489-33830	18	8.1	2	0.694278	-2	0.35195	no
gi|646744756|gb|KK962409.1|	41845-34210	18	8.1	40	31	-0.357434	0.766	no
gi|646744756|gb|KK962409.1|	50177-35083	18	8.1	32	18	-0.873734	0.5667	no
gi|646744756|gb|KK962409.1|	55003-35598	18	8.1	12	5	-1	0.40455	no
gi|646744756|gb|KK962409.1|	57749-35874	18	8.1	7	4	-0.923069	0.55325	no
gi|646744756|gb|KK962409.1|	66316-36696	18	8.1	19	11	-0.787146	0.6101	no
gi|646744756|gb|KK962409.1|	67480-36822	18	8.1	2	1	-0.970625	0.54505	no
gi|646744756|gb|KK962409.1|	92379-39362	18	8.1	0.749064	0.805859	0.105439	1	no

gi|646744756|gb|KK962409.1|	13308-41446	18	8.1	5	5	-0.143018	0.92535	no
gi|646744756|gb|KK962409.1|	27573-42832	18	8.1	3	0.785282	-2	0.268	no
gi|646744756|gb|KK962409.1|	29599-43114	18	8.1	3	1	-1	0.40225	no
gi|646744756|gb|KK962409.1|	34631-43676	18	8.1	2	2	0.161177	0.9187	no
gi|646744756|gb|KK962409.1|	43528-45308	18	8.1	1	0.876994	-0.23385	1	no
gi|646744756|gb|KK962409.1|	45470-46852	18	8.1	0.738778	0.944538	0.354467	1	no
gi|646744756|gb|KK962409.1|	65252-65456	18	8.1	52	70	0.436771	0.7522	no
gi|646744756|gb|KK962409.1|	76695-77704	18	8.1	2	2	-0.254864	0.83805	no
gi|646744756|gb|KK962409.1|	96943-99804	18	8.1	3	3	-0.258788	0.86165	no
gi|646744768|gb|KK962408.1|	18234-11837	23	5.9	874	829	-0.075655	0.9353	no
gi|646744768|gb|KK962408.1|	26330-12680	23	5.9	3	3	-0.0682333	0.92345	no
gi|646744768|gb|KK962408.1|	70308-17066	23	5.9	22	32	0.52498	0.74005	no
gi|646744768|gb|KK962408.1|	79167-17967	23	5.9	6	0.495079	-4	0.1737	no
gi|646744768|gb|KK962408.1|	75949-37680	23	5.9	2	2	-0.599674	0.6374	no
gi|646744768|gb|KK962408.1|	83912-38486	23	5.9	2	0.18943	-3	0.1757	no
gi|646744768|gb|KK962408.1|	95994-39722	23	5.9	1	0.135517	-3	0.1932	no
gi|646744768|gb|KK962408.1|	42427-42549	23	5.9	6331.13	4139.3	-0.613076	0.625	no
gi|646744768|gb|KK962408.1|	92146-49282	23	5.9	2	10	3	0.12335	no
gi|646744768|gb|KK962408.1|	86246-86725	23	5.9	24	20	-0.277704	0.85575	no
gi|646744782|gb|KK962407.1|	25886-12642	13	7.1	6	9	0.519384	0.7058	no
gi|646744805|gb|KK962405.1|	52633-25325	15	9.3	5	5	-0.080306	0.94955	no
gi|646744805|gb|KK962405.1|	22298-32340	15	9.3	1	0.622938	-1	0.45565	no
gi|646744805|gb|KK962405.1|	26912-32705	15	9.3	1257.66	521	-1	0.3357	no
gi|646744805|gb|KK962405.1|	28918-42948	15	9.3	3	3	-0.00581963	0.94695	no
gi|646744805|gb|KK962405.1|	30018-43070	15	9.3	2	2	-0.393759	0.7788	no
gi|646744805|gb|KK962405.1|	41822-44235	15	9.3	2	2	-0.363349	0.811	no
gi|646744819|gb|KK962404.1|	68065-16956	20	8.6	45	34	-0.406217	0.71725	no
gi|646744819|gb|KK962404.1|	71788-17300	20	8.6	2	2	-0.0209486	0.95635	no
gi|646744819|gb|KK962404.1|	98160-19838	20	8.6	13	36	1	0.3711	no
gi|646744819|gb|KK962404.1|	36185-23749	20	8.6	0.613232	0.880885	0.52252	1	no

gi|646744833|gb|KK962403.1|	14425-11506	22	9.4	0	4	inf	0.0014	yes
gi|646744833|gb|KK962403.1|	30075-13131	22	9.4	42	39	-0.0944271	0.9317	no
gi|646744833|gb|KK962403.1|	33965-13463	22	9.4	0.221683	5	5	0.11045	no
gi|646744833|gb|KK962403.1|	64816-16718	22	9.4	155	113.47	-0.446289	0.70845	no
gi|646744833|gb|KK962403.1|	37971-23843	22	9.4	7	6	-0.175915	0.89145	no
gi|646744833|gb|KK962403.1|	39535-24078	22	9.4	7	10	0.427558	0.7763	no
gi|646744833|gb|KK962403.1|	41067-24404	22	9.4	1	0.742262	-0.45919	1	no
gi|646744833|gb|KK962403.1|	44229-25090	22	9.4	47	24	-0.944905	0.43255	no
gi|646744833|gb|KK962403.1|	54685-25997	22	9.4	31	25	-0.311362	0.79475	no
gi|646744833|gb|KK962403.1|	21913-32264	22	9.4	0	8	inf	0.0012	yes
gi|646744833|gb|KK962403.1|	28355-32865	22	9.4	12	5	-1	0.4638	no
gi|646744833|gb|KK962403.1|	37762-33992	22	9.4	0.631352	0.281635	-1	1	no
gi|646744833|gb|KK962403.1|	42012-34233	22	9.4	7	25	2	0.2597	no
gi|646744833|gb|KK962403.1|	57640-36043	22	9.4	1	1	0.0963857	0.94765	no
gi|646744833|gb|KK962403.1|	64786-36558	22	9.4	2	1	-0.504857	0.7312	no
gi|646744833|gb|KK962403.1|	65773-36910	22	9.4	1	1	-0.205078	0.8996	no
gi|646744833|gb|KK962403.1|	80522-38228	22	9.4	0.798603	1	0.737875	0.61065	no
gi|646744833|gb|KK962403.1|	82648-38495	22	9.4	0.811166	1	0.367095	1	no
gi|646744833|gb|KK962403.1|	86392-38696	22	9.4	2	2	0.192597	0.8995	no
gi|646744833|gb|KK962403.1|	90058-39182	22	9.4	0.613089	0.974772	0.668969	1	no
gi|646744833|gb|KK962403.1|	91964-40011	22	9.4	0.937306	0.856787	-0.129582	1	no
gi|646744833|gb|KK962403.1|	02842-40530	22	9.4	0.501909	1	1	0.41855	no
gi|646744833|gb|KK962403.1|	20283-42165	22	9.4	0.914307	2	1	0.3631	no
gi|646744833|gb|KK962403.1|	22635-42284	22	9.4	100	96	-0.0569041	0.9631	no
gi|646744833|gb|KK962403.1|	27758-42839	22	9.4	2	2	0.055373	0.9298	no
gi|646744833|gb|KK962403.1|	31843-44087	22	9.4	17	17	0.0643888	0.9569	no
gi|646744833|gb|KK962403.1|	41434-44228	22	9.4	1	0.877801	-0.67015	0.6592	no
gi|646744833|gb|KK962403.1|	42390-44383	22	9.4	0.777413	0.671592	-0.211097	1	no
gi|646744833|gb|KK962403.1|	44070-44727	22	9.4	15	8	-0.895453	0.4143	no
gi|646744833|gb|KK962403.1|	51257-45314	22	9.4	2	0.739139	-1	0.3316	no

gi|646744833|gb|KK962403.1|	53281-45451	22	9.4	0.941092	3	2	0.26265	no
gi|646744833|gb|KK962403.1|	54724-45531	22	9.4	3	5	0.701492	0.57905	no
gi|646744833|gb|KK962403.1|	58047-45894	22	9.4	3	2	-0.634239	0.66525	no
gi|646744833|gb|KK962403.1|	63262-46377	22	9.4	29	37	0.378637	0.8002	no
gi|646744833|gb|KK962403.1|	81056-48135	22	9.4	13	13	0.0652743	0.9424	no
gi|646744833|gb|KK962403.1|	84151-48488	22	9.4	0.973489	2	1	0.36955	no
gi|646744833|gb|KK962403.1|	86138-48708	22	9.4	2	2	0.0591937	0.9549	no
gi|646744833|gb|KK962403.1|	87434-48823	22	9.4	0.675155	4	2	0.2155	no
gi|646744833|gb|KK962403.1|	89461-49141	22	9.4	5	4	-0.203148	0.88985	no
gi|646744833|gb|KK962403.1|	96141-49647	22	9.4	67	76	0.167929	0.91365	no
gi|646744833|gb|KK962403.1|	04857-50566	22	9.4	5	13	1	0.39695	no
gi|646744833|gb|KK962403.1|	07383-50759	22	9.4	68	30	-1	0.42055	no
gi|646744833|gb|KK962403.1|	08178-50952	22	9.4	0.844079	2	1	0.41785	no
gi|646744833|gb|KK962403.1|	11378-51164	22	9.4	18	30	0.699626	0.59665	no
gi|646744833|gb|KK962403.1|	12262-51421	22	9.4	1	0.949324	-0.525153	0.70235	no
gi|646744833|gb|KK962403.1|	17759-51803	22	9.4	18	18	0.00422801	0.9426	no
gi|646744833|gb|KK962403.1|	18566-51969	22	9.4	0.633612	3	2	0.23745	no
gi|646744833|gb|KK962403.1|	22301-52285	22	9.4	19	25	0.376816	0.80035	no
gi|646744833|gb|KK962403.1|	26433-52824	22	9.4	2	2	0.0373067	0.9795	no
gi|646744833|gb|KK962403.1|	28679-52980	22	9.4	2	3	0.857425	0.54965	no
gi|646744833|gb|KK962403.1|	54286-55511	22	9.4	1	1	0.0824714	0.92955	no
gi|646744833|gb|KK962403.1|	55812-55757	22	9.4	0.612277	0.531	-0.205472	1	no
gi|646744833|gb|KK962403.1|	59023-56139	22	9.4	42	24	-0.816791	0.4795	no
gi|646744833|gb|KK962403.1|	66749-56706	22	9.4	7	8	0.270059	0.84425	no
gi|646744833|gb|KK962403.1|	67360-56920	22	9.4	0.638439	0.923512	0.532581	1	no
gi|646744833|gb|KK962403.1|	75734-57696	22	9.4	2	2	-0.0650411	0.95855	no
gi|646744833|gb|KK962403.1|	82231-58261	22	9.4	48	36	-0.407529	0.7917	no
gi|646744833|gb|KK962403.1|	27144-62821	22	9.4	4	5	0.304262	0.84415	no
gi|646744833|gb|KK962403.1|	70325-67098	22	9.4	0	9	inf	0.0012	yes
gi|646744833|gb|KK962403.1|	03437-70386	22	9.4	4	3	-0.613629	0.6685	no

gi|646744833|gb|KK962403.1|	10351-71092	22	9.4	5	4	-0.254299	0.83525	no
gi|646744833|gb|KK962403.1|	39293-74097	22	9.4	12	6	-0.903446	0.5521	no
gi|646744833|gb|KK962403.1|	46776-74750	22	9.4	347	382	0.137897	0.90275	no
gi|646744833|gb|KK962403.1|	48281-74859	22	9.4	67	127	0.916633	0.5513	no
gi|646744833|gb|KK962403.1|	52224-75243	22	9.4	209	162	-0.3745	0.80735	no
gi|646744833|gb|KK962403.1|	57681-75968	22	9.4	6	13	1	0.3957	no
gi|646744833|gb|KK962403.1|	62424-76617	22	9.4	6	1	-2	0.1371	no
gi|646744833|gb|KK962403.1|	80395-78211	22	9.4	76	61	-0.302543	0.79245	no
gi|646744833|gb|KK962403.1|	07690-80880	22	9.4	4	3	-0.136269	0.93285	no
gi|646744833|gb|KK962403.1|	45894-84656	22	9.4	1	2	0.477313	0.7489	no
gi|646744833|gb|KK962403.1|	46711-84779	22	9.4	8	2	-2	0.17025	no
gi|646744833|gb|KK962403.1|	62503-86422	22	9.4	30	38	0.314431	0.78035	no
gi|646744833|gb|KK962403.1|	70426-87099	22	9.4	3	0.397557	-3	0.2136	no
gi|646744833|gb|KK962403.1|	85972-88844	22	9.4	0.83275	1	0.303729	1	no
gi|646744833|gb|KK962403.1|	88565-89024	22	9.4	0.90637	0.374	-1	1	no
gi|646744833|gb|KK962403.1|	90359-89171	22	9.4	1	0.605076	-0.854829	1	no
gi|646744833|gb|KK962403.1|	07523-90809	22	9.4	2	2	0.192879	0.89115	no
gi|646744833|gb|KK962403.1|	10503-91072	22	9.4	43	14	-2	0.3672	no
gi|646744874|gb|KK962400.1|	02971-10673	19	8.3	8	10	0.355117	0.74215	no
gi|646744874|gb|KK962400.1|	09292-11002	19	8.3	3	2	-0.73557	0.5852	no
gi|646744874|gb|KK962400.1|	13501-11617	19	8.3	6	4	-0.506756	0.7364	no
gi|646744874|gb|KK962400.1|	18513-11918	19	8.3	20	16	-0.357704	0.81335	no
gi|646744874|gb|KK962400.1|	35597-13595	19	8.3	9	2	-2	0.2103	no
gi|646744874|gb|KK962400.1|	46929-14722	19	8.3	78	59	-0.394238	0.79125	no
gi|646744874|gb|KK962400.1|	60036-16079	19	8.3	70	44	-0.685017	0.53085	no
gi|646744874|gb|KK962400.1|	68894-16993	19	8.3	14	11	-0.405186	0.78915	no
gi|646744874|gb|KK962400.1|	80668-18110	19	8.3	20	14	-0.500027	0.7459	no
gi|646744874|gb|KK962400.1|	95505-19571	19	8.3	93	20	-2	0.19045	no
gi|646744874|gb|KK962400.1|	14059-21532	19	8.3	0.455714	0.915699	1	1	no
gi|646744874|gb|KK962400.1|	30905-23126	19	8.3	30	21	-0.525745	0.74085	no

gi|646744874|gb|KK962400.1|	40671-24101	19	8.3	6	8	0.521016	0.7234	no
gi|646744874|gb|KK962400.1|	86731-28727	19	8.3	1	4	2	0.2794	no
gi|646744874|gb|KK962400.1|	94028-29592	19	8.3	0.563435	2	1	0.34165	no
gi|646744874|gb|KK962400.1|	29937-30684	19	8.3	0.939911	1	0.488807	0.7568	no
gi|646744874|gb|KK962400.1|	07440-30766	19	8.3	50	74	0.557694	0.7122	no
gi|646744874|gb|KK962400.1|	21100-32139	19	8.3	6	12	1	0.4822	no
gi|646744874|gb|KK962400.1|	25497-32903	19	8.3	22	21	-0.0133695	0.9894	no
gi|646744874|gb|KK962400.1|	33293-34901	19	8.3	6	4	-0.488018	0.739	no
gi|646744874|gb|KK962400.1|	35938-33739	19	8.3	0.845213	0.331963	-1	1	no
gi|646744874|gb|KK962400.1|	37504-34028	19	8.3	33	18	-0.868228	0.44445	no
gi|646744874|gb|KK962400.1|	46956-35016	19	8.3	1	1	-0.0432121	1	no
gi|646744874|gb|KK962400.1|	35049-38872	19	8.3	4	3	-0.415188	0.78025	no
gi|646744874|gb|KK962400.1|	52270-35540	19	8.3	1	28	4	0.0123	no
gi|646744874|gb|KK962400.1|	63686-36549	19	8.3	10	8	-0.262134	0.8676	no
gi|646744874|gb|KK962400.1|	66809-36799	19	8.3	10	7	-0.581108	0.70715	no
gi|646744874|gb|KK962400.1|	76224-37653	19	8.3	24	12	-0.994569	0.45795	no
gi|646744874|gb|KK962400.1|	85556-38758	19	8.3	2	4	0.696835	0.64995	no
gi|646744874|gb|KK962400.1|	87723-38795	19	8.3	39	31	-0.333031	0.80345	no
gi|646744874|gb|KK962400.1|	89455-38971	19	8.3	28	13	-1	0.4418	no
gi|646744874|gb|KK962400.1|	91252-39153	19	8.3	26	34	0.380727	0.7864	no
gi|646744874|gb|KK962400.1|	98649-39889	19	8.3	56	24	-1	0.3691	no
gi|646744874|gb|KK962400.1|	40799-41273	19	8.3	18	6	-2	0.2408	no
gi|646744874|gb|KK962400.1|	13552-41474	19	8.3	37	10	-2	0.16555	no
gi|646744874|gb|KK962400.1|	21280-42247	19	8.3	0.986232	0.423907	-1	1	no
gi|646744874|gb|KK962400.1|	44417-45099	19	8.3	5	3	-0.93582	0.52415	no
gi|646744874|gb|KK962400.1|	46139-44648	19	8.3	9	12	0.449648	0.72775	no
gi|646744874|gb|KK962400.1|	50883-45115	19	8.3	112	82	-0.446802	0.76455	no
gi|646744874|gb|KK962400.1|	61914-46223	19	8.3	29	21	-0.425615	0.7902	no
gi|646744874|gb|KK962400.1|	66857-47026	19	8.3	36	18	-0.952575	0.4141	no
gi|646744874|gb|KK962400.1|	79293-47997	19	8.3	3	2	-0.221562	0.8729	no

gi|646744874|gb|KK962400.1|	80134-48204	19	8.3	5	6	0.18135	0.90095	no
gi|646744874|gb|KK962400.1|	89860-49170	19	8.3	3	2	-0.332551	0.83655	no
gi|646744874|gb|KK962400.1|	95812-49894	19	8.3	4	5	0.0663783	0.964	no
gi|646744874|gb|KK962400.1|	69677-70318	19	8.3	15	7	-1	0.49995	no
gi|646744874|gb|KK962400.1|	90856-91370	19	8.3	7	4	-0.642603	0.60975	no
gi|646744874|gb|KK962400.1|	91576-91875	19	8.3	18	13	-0.467148	0.72555	no
gi|646744874|gb|KK962400.1|	92671-93225	19	8.3	3	2	-0.398349	0.8003	no
gi|646744874|gb|KK962400.1|	93511-94708	19	8.3	42	22	-0.928834	0.3926	no
gi|646744874|gb|KK962400.1|	97748-98835	19	8.3	3	2	-0.807384	0.5543	no
gi|646744890|gb|KK962399.1|	93312-19461	18	8.5	0.530145	1	1	0.33975	no
gi|646744890|gb|KK962399.1|	39598-23999	18	8.5	44	26	-0.748693	0.6135	no
gi|646744890|gb|KK962399.1|	63411-27050	18	8.5	24	20	-0.254153	0.828	no
gi|646744890|gb|KK962399.1|	75938-27724	18	8.5	4	3	-0.477707	0.7567	no
gi|646744890|gb|KK962399.1|	77384-27912	18	8.5	1	2	0.38678	0.77835	no
gi|646744890|gb|KK962399.1|	80890-28320	18	8.5	2	2	-0.00726338	0.9827	no
gi|646744890|gb|KK962399.1|	88816-29014	18	8.5	1	0.618873	-0.855494	1	no
gi|646744890|gb|KK962399.1|	94212-29612	18	8.5	4	3	-0.612141	0.6943	no
gi|646744890|gb|KK962399.1|	02395-30409	18	8.5	0.383348	0.738335	0.945622	1	no
gi|646744890|gb|KK962399.1|	04214-30505	18	8.5	1	1	0.0834008	0.92955	no
gi|646744890|gb|KK962399.1|	06696-30783	18	8.5	3	3	-0.171506	0.9098	no
gi|646744890|gb|KK962399.1|	08139-31182	18	8.5	3	3	-0.0682016	0.96365	no
gi|646744890|gb|KK962399.1|	13548-32204	18	8.5	16	4	-2	0.11945	no
gi|646744890|gb|KK962399.1|	30884-33297	18	8.5	3	0.364526	-3	0.0978	no
gi|646744890|gb|KK962399.1|	33444-33397	18	8.5	11	3	-2	0.2342	no
gi|646744890|gb|KK962399.1|	43134-34557	18	8.5	4	3	-0.783989	0.6015	no
gi|646744890|gb|KK962399.1|	53215-35487	18	8.5	10	4	-1	0.4141	no
gi|646744890|gb|KK962399.1|	56507-35759	18	8.5	3	2	-0.807339	0.5826	no
gi|646744890|gb|KK962399.1|	44218-44435	18	8.5	80	58	-0.461003	0.7077	no
gi|646744904|gb|KK962398.1|	14331-11886	18	7.8	3	18	3	0.0692	no
gi|646744904|gb|KK962398.1|	20725-12098	18	7.8	0	45	inf	0.00435	no

gi|646744904|gb|KK962398.1|	40566-14131	18	7.8	0.189134	3	4	0.09875	no
gi|646744904|gb|KK962398.1|	42291-14260	18	7.8	0	17	inf	0.001	yes
gi|646744904|gb|KK962398.1|	96500-49691	18	7.8	47	37	-0.336533	0.8227	no
gi|646744904|gb|KK962398.1|	51972-52394	18	7.8	5	8	0.6744	0.61735	no
gi|646744916|gb|KK962397.1|	07508-10871	18	8.3	3	4	0.326494	0.8363	no
gi|646744916|gb|KK962397.1|	08797-10917	18	8.3	4	5	0.34495	0.823	no
gi|646744916|gb|KK962397.1|	21525-12410	18	8.3	4	6	0.440239	0.76965	no
gi|646744916|gb|KK962397.1|	25067-12826	18	8.3	6	4	-0.450785	0.76455	no
gi|646744916|gb|KK962397.1|	39306-14278	18	8.3	4	3	-0.133087	0.9301	no
gi|646744916|gb|KK962397.1|	63582-16613	18	8.3	3	4	0.326098	0.8314	no
gi|646744916|gb|KK962397.1|	89695-19078	18	8.3	0.667916	0.954116	0.514499	1	no
gi|646744916|gb|KK962397.1|	92348-19356	18	8.3	0.674544	0.69069	0.0341252	1	no
gi|646744916|gb|KK962397.1|	20016-22044	18	8.3	3	5	0.873974	0.5771	no
gi|646744916|gb|KK962397.1|	46531-24740	18	8.3	0.602602	1	1	0.43305	no
gi|646744916|gb|KK962397.1|	47536-24849	18	8.3	9	4	-1	0.4704	no
gi|646744916|gb|KK962397.1|	90438-39074	18	8.3	12	5	-1	0.39645	no
gi|646744916|gb|KK962397.1|	64221-46729	18	8.3	230	119	-0.943943	0.4359	no
gi|646744916|gb|KK962397.1|	84866-48505	18	8.3	70	27	-1	0.41275	no
gi|646744916|gb|KK962397.1|	85340-48649	18	8.3	13	11	-0.223377	0.88305	no
gi|646744916|gb|KK962397.1|	91493-49204	18	8.3	8	5	-0.608931	0.6577	no
gi|646744916|gb|KK962397.1|	95670-49611	18	8.3	5	5	0.0793492	0.9416	no
gi|646744916|gb|KK962397.1|	20456-52145	18	8.3	1	2	0.510284	0.7139	no
gi|646744916|gb|KK962397.1|	22397-52344	18	8.3	1	0.999808	-0.361817	0.79735	no
gi|646744916|gb|KK962397.1|	27274-52789	18	8.3	23	11	-1	0.5031	no
gi|646744916|gb|KK962397.1|	30619-53447	18	8.3	3	2	-0.667828	0.6544	no
gi|646744916|gb|KK962397.1|	39843-54891	18	8.3	11	7	-0.679118	0.55585	no
gi|646744916|gb|KK962397.1|	59561-56033	18	8.3	12	10	-0.282813	0.85445	no
gi|646744916|gb|KK962397.1|	85343-58597	18	8.3	83	39	-1	0.40495	no
gi|646744930|gb|KK962396.1|	14946-15842	18	8.5	5	3	-0.837621	0.60665	no
gi|646744930|gb|KK962396.1|	19490-20940	18	8.5	3	2	-0.696413	0.67005	no

gi|646744930|gb|KK962396.1|	28-1839	18	8.5	2	2	0.368599	0.8165	no
gi|646744930|gb|KK962396.1|	29235-30221	18	8.5	0.885808	3	2	0.30205	no
gi|646744930|gb|KK962396.1|	96995-29860	18	8.5	12	8	-0.556233	0.71725	no
gi|646744930|gb|KK962396.1|	30328-31430	18	8.5	0.655301	0.624371	-0.0697543	1	no
gi|646744930|gb|KK962396.1|	31530-32125	18	8.5	6	4	-0.602882	0.66355	no
gi|646744930|gb|KK962396.1|	55716-57080	18	8.5	11	9	-0.307306	0.8406	no
gi|646744930|gb|KK962396.1|	59929-60464	18	8.5	20	3	-3	0.0927	no
gi|646744930|gb|KK962396.1|	70755-71379	18	8.5	2	2	0.105496	0.93295	no
gi|646744930|gb|KK962396.1|	71651-71965	18	8.5	15	4	-2	0.2743	no
gi|646744930|gb|KK962396.1|	78414-79012	18	8.5	17	19	0.134427	0.92435	no
gi|646744930|gb|KK962396.1|	82298-84444	18	8.5	2	2	-0.0492647	0.9746	no
gi|646744930|gb|KK962396.1|	88807-90140	18	8.5	0.686536	0.493299	-0.476874	1	no
gi|646744930|gb|KK962396.1|	90452-92157	18	8.5	5	2	-1	0.47335	no
gi|646744930|gb|KK962396.1|	94280-95678	18	8.5	83	35	-1	0.28075	no
gi|646744957|gb|KK962394.1|	07162-10839	21	9.8	1	0.818003	-0.702405	0.5887	no
gi|646744957|gb|KK962394.1|	08570-11115	21	9.8	0.793813	0.922617	0.216934	1	no
gi|646744957|gb|KK962394.1|	13073-11812	21	9.8	0.771021	1	0.650218	0.67625	no
gi|646744957|gb|KK962394.1|	18489-11940	21	9.8	2	3	0.745672	0.5522	no
gi|646744957|gb|KK962394.1|	19509-12482	21	9.8	42	31	-0.456877	0.6993	no
gi|646744957|gb|KK962394.1|	31977-13760	21	9.8	9	20	1	0.28155	no
gi|646744957|gb|KK962394.1|	39211-13967	21	9.8	0.872443	7	3	0.24955	no
gi|646744957|gb|KK962394.1|	39845-14615	21	9.8	1	2	0.562859	0.712	no
gi|646744957|gb|KK962394.1|	51620-15276	21	9.8	0.836226	1	0.838719	0.54675	no
gi|646744957|gb|KK962394.1|	22068-22568	21	9.8	15	9	-0.758082	0.6336	no
gi|646744957|gb|KK962394.1|	89841-29047	21	9.8	4	2	-1	0.3261	no
gi|646744957|gb|KK962394.1|	31919-33399	21	9.8	4	3	-0.61088	0.68525	no
gi|646744957|gb|KK962394.1|	69942-57016	21	9.8	0	181	inf	0.00055	yes
gi|646744957|gb|KK962394.1|	58096-58293	21	9.8	71	41	-0.795304	0.60525	no
gi|646744957|gb|KK962394.1|	27192-62819	21	9.8	0	5	inf	0.00105	yes
gi|646744957|gb|KK962394.1|	40132-64030	21	9.8	0	411	inf	0.00155	yes

gi|646744957|gb|KK962394.1|	67462-67688	21	9.8	96	67	-0.527092	0.75495	no
gi|646744957|gb|KK962394.1|	06028-70811	21	9.8	4	4	0.268064	0.86295	no
gi|646744957|gb|KK962394.1|	16599-72362	21	9.8	11	7.41	-0.558959	0.62315	no
gi|646744957|gb|KK962394.1|	26006-72801	21	9.8	1	0.917758	-0.340099	0.80625	no
gi|646744957|gb|KK962394.1|	28624-72985	21	9.8	0.755178	0.676909	-0.157854	1	no
gi|646744957|gb|KK962394.1|	31685-73620	21	9.8	0.86814	1	0.393042	0.7956	no
gi|646744957|gb|KK962394.1|	36573-74102	21	9.8	24	13	-0.809011	0.48655	no
gi|646744957|gb|KK962394.1|	52331-75420	21	9.8	3	7	1	0.45075	no
gi|646744957|gb|KK962394.1|	86770-78784	21	9.8	9	15	0.713258	0.6356	no
gi|646744957|gb|KK962394.1|	78948-79164	21	9.8	77	173	1	0.39345	no
gi|646744957|gb|KK962394.1|	89703-79173	21	9.8	0.574314	0.453764	-0.339895	1	no
gi|646744957|gb|KK962394.1|	95362-79656	21	9.8	0.585498	3	2	0.1647	no
gi|646744957|gb|KK962394.1|	96665-79810	21	9.8	0.62561	1	0.84764	0.54675	no
gi|646744957|gb|KK962394.1|	99128-80389	21	9.8	20	20	0.036379	0.97445	no
gi|646744957|gb|KK962394.1|	13150-81503	21	9.8	0.96489	1	0.445556	0.7487	no
gi|646744957|gb|KK962394.1|	15307-81915	21	9.8	0.46325	0.937864	1	1	no
gi|646744957|gb|KK962394.1|	24111-82472	21	9.8	1	3	0.950324	0.56265	no
gi|646744957|gb|KK962394.1|	8248-8540	21	9.8	3	18	3	0.273	no
gi|646744957|gb|KK962394.1|	59160-85973	21	9.8	8	17	1	0.48055	no
gi|646744957|gb|KK962394.1|	72186-87255	21	9.8	0	17	inf	0.00435	no
gi|646744957|gb|KK962394.1|	95422-96989	21	9.8	1	1	-0.319669	0.807	no
gi|646744970|gb|KK962393.1|	87144-28732	12	7.7	89	76	-0.222295	0.885	no
gi|646744970|gb|KK962393.1|	6746-7311	12	7.7	1	3	1	0.4404	no
gi|646744983|gb|KK962392.1|	07947-20857	16	8.3	5	3	-0.780081	0.53545	no
gi|646744983|gb|KK962392.1|	26865-22759	16	8.3	7	6	-0.208597	0.88985	no
gi|646744983|gb|KK962392.1|	39569-24211	16	8.3	3	2	-0.991897	0.52005	no
gi|646744983|gb|KK962392.1|	43185-24471	16	8.3	4	5	0.161426	0.91345	no
gi|646744983|gb|KK962392.1|	45712-24832	16	8.3	3	3	0.0681213	0.96255	no
gi|646744983|gb|KK962392.1|	48798-25082	16	8.3	5	3	-0.673022	0.65225	no
gi|646744983|gb|KK962392.1|	52858-25431	16	8.3	19	7	-1	0.26875	no

gi|646744983|gb|KK962392.1|	64800-26564	16	8.3	2	3	0.499248	0.7295	no
gi|646744983|gb|KK962392.1|	73358-27382	16	8.3	468	467	-0.0010041	0.99885	no
gi|646744983|gb|KK962392.1|	79914-28032	16	8.3	3	11	2	0.2834	no
gi|646744983|gb|KK962392.1|	80476-28110	16	8.3	91	22	-2	0.12645	no
gi|646744983|gb|KK962392.1|	82200-28275	16	8.3	17	20	0.226299	0.8781	no
gi|646744983|gb|KK962392.1|	99222-29981	16	8.3	0	5	inf	0.0008	yes
gi|646744983|gb|KK962392.1|	00681-30399	16	8.3	0.030245	10	8	0.09445	no
gi|646745008|gb|KK962390.1|	41175-14202	20	7.2	1	4	1	0.3594	no
gi|646745008|gb|KK962390.1|	44898-14627	20	7.2	0.911034	0.952828	0.0647111	1	no
gi|646745008|gb|KK962390.1|	46708-14769	20	7.2	2	3	0.596602	0.67255	no
gi|646745008|gb|KK962390.1|	48137-14951	20	7.2	0.823788	1	0.37229	1	no
gi|646745008|gb|KK962390.1|	49625-15035	20	7.2	4	3	-0.09911	0.9386	no
gi|646745008|gb|KK962390.1|	56692-15761	20	7.2	1	1	0.313	0.8346	no
gi|646745008|gb|KK962390.1|	77368-17911	20	7.2	1	2	0.964187	0.47935	no
gi|646745008|gb|KK962390.1|	79446-18059	20	7.2	5	5	-0.0679335	0.96575	no
gi|646745008|gb|KK962390.1|	81209-18321	20	7.2	4	3	-0.0937129	0.95155	no
gi|646745008|gb|KK962390.1|	35589-23576	20	7.2	43	194	2	0.29955	no
gi|646745008|gb|KK962390.1|	46159-46623	20	7.2	0	16	inf	0.0012	yes
gi|646745008|gb|KK962390.1|	53820-54533	20	7.2	0	36	inf	0.00015	yes
gi|646745008|gb|KK962390.1|	03170-60515	20	7.2	7	11	0.766261	0.54455	no
gi|646745008|gb|KK962390.1|	06383-60655	20	7.2	37	139	2	0.3562	no
gi|646745008|gb|KK962390.1|	19929-62243	20	7.2	11	23	1	0.3586	no
gi|646745008|gb|KK962390.1|	72457-72888	20	7.2	3	3	-0.193588	0.88665	no
gi|646745008|gb|KK962390.1|	75596-77566	20	7.2	2	5	0.893062	0.5594	no
gi|646745008|gb|KK962390.1|	77673-78095	20	7.2	2	4	0.969856	0.5906	no
gi|646745008|gb|KK962390.1|	78195-82493	20	7.2	0.82384	2	0.989423	0.53195	no
gi|646745008|gb|KK962390.1|	84860-85909	20	7.2	0.815539	2	1	0.40695	no
gi|646745022|gb|KK962389.1|	82867-18343	22	9.4	6	10	0.774651	0.57085	no
gi|646745022|gb|KK962389.1|	83643-18422	22	9.4	6	5	-0.235405	0.8431	no
gi|646745034|gb|KK962388.1|	40087-34093	16	7.8	3	0	-inf	0.00195	no

gi|646745034|gb|KK962388.1| 41940-34324	16	7.8	0.88442	0	-inf	1	no
gi|646745053|gb|KK962387.1| 71740-27252	19	8	2	0.988036	-1	0.3737	no
gi|646745053|gb|KK962387.1| 94112-29540	19	8	0.623865	1	0.71465	1	no
gi|646745053|gb|KK962387.1| 74728-37595	19	8	0.758272	0.815545	0.105049	1	no
gi|646745053|gb|KK962387.1| 29178-52959	19	8	6	7	0.242821	0.85015	no
gi|646745053|gb|KK962387.1| 89166-58956	19	8	6	0	-inf	0.00655	no
gi|646745053|gb|KK962387.1| 14586-61559	19	8	2	24	4	0.02295	no
gi|646745053|gb|KK962387.1| 16485-61676	19	8	2	83	5	0.09755	no
gi|646745053|gb|KK962387.1| 17677-61805	19	8	2	44	4	0.11285	no
gi|646745053|gb|KK962387.1| 19263-62067	19	8	0.321221	12	5	0.0958	no
gi|646745053|gb|KK962387.1| 61482-66417	19	8	42	7	-3	0.07155	no
gi|646745053|gb|KK962387.1| 64589-66682	19	8	2	2	-0.667924	0.66375	no
gi|646745053|gb|KK962387.1| 69316-66970	19	8	7	8	0.2011	0.8827	no
gi|646745053|gb|KK962387.1| 70181-67200	19	8	1	0.342173	-2	0.20495	no
gi|646745053|gb|KK962387.1| 76974-67739	19	8	30	8	-2	0.19775	no
gi|646745053|gb|KK962387.1| 82089-68323	19	8	0.827809	0.888522	0.102109	1	no
gi|646745053|gb|KK962387.1| 83430-68524	19	8	2	0.773302	-1	0.4082	no
gi|646745053|gb|KK962387.1| 85403-68773	19	8	3	2	-0.595252	0.69975	no
gi|646745053|gb|KK962387.1| 88017-69019	19	8	2	0.905448	-0.807952	0.5543	no
gi|646745053|gb|KK962387.1| 91267-69373	19	8	1	0.910205	-0.509163	0.75275	no
gi|646745053|gb|KK962387.1| 93963-69646	19	8	3	2	-0.889074	0.5602	no
gi|646745053|gb|KK962387.1| 02276-70317	19	8	3	2	-0.574213	0.65795	no
gi|646745053|gb|KK962387.1| 04017-70507	19	8	1	0.981725	-0.602094	0.64385	no
gi|646745053|gb|KK962387.1| 05236-70757	19	8	2	1	-0.199694	0.89865	no
gi|646745053|gb|KK962387.1| 09189-71627	19	8	3	1	-1	0.41945	no
gi|646745053|gb|KK962387.1| 28574-72881	19	8	34	7	-2	0.2052	no
gi|646745053|gb|KK962387.1| 37408-73774	19	8	11	4	-2	0.2835	no
gi|646745053|gb|KK962387.1| 40109-74041	19	8	23	13	-0.842982	0.514	no
gi|646745053|gb|KK962387.1| 54858-75585	19	8	2	2	-0.0202863	0.9504	no
gi|646745053|gb|KK962387.1| 78243-78008	19	8	31	24	-0.393246	0.72715	no

gi|646745053|gb|KK962387.1|	80292-78069	19	8	9	4	-1	0.3625	no
gi|646745053|gb|KK962387.1|	96179-79677	19	8	16	6	-1	0.4183	no
gi|646745053|gb|KK962387.1|	97201-79874	19	8	17	8	-1	0.4239	no
gi|646745053|gb|KK962387.1|	38384-83903	19	8	11	3	-2	0.24495	no
gi|646745053|gb|KK962387.1|	39115-83991	19	8	3	2	-0.674578	0.60105	no
gi|646745053|gb|KK962387.1|	41253-84279	19	8	5	2	-1	0.4288	no
gi|646745053|gb|KK962387.1|	44858-84677	19	8	4	2	-1	0.35795	no
gi|646745080|gb|KK962385.1|	03649-10390	20	7.2	0	38	inf	0.00035	yes
gi|646745080|gb|KK962385.1|	16936-17557	20	7.2	4	5	0.274759	0.81975	no
gi|646745080|gb|KK962385.1|	19130-21427	20	7.2	0.72492	2	1	0.2971	no
gi|646745080|gb|KK962385.1|	22179-23481	20	7.2	61	70	0.199295	0.861	no
gi|646745080|gb|KK962385.1|	24310-22495	20	7.2	24	4	-2	0.08685	no
gi|646745080|gb|KK962385.1|	29794-32419	20	7.2	7	11	0.631204	0.6241	no
gi|646745080|gb|KK962385.1|	52509-54241	20	7.2	28	20	-0.481812	0.661	no
gi|646745080|gb|KK962385.1|	55202-56991	20	7.2	24	14	-0.764651	0.47915	no
gi|646745080|gb|KK962385.1|	59590-59947	20	7.2	5	8	0.737289	0.5987	no
gi|646745080|gb|KK962385.1|	60890-63870	20	7.2	12	8	-0.662452	0.5458	no
gi|646745080|gb|KK962385.1|	6858-7394	20	7.2	8	12	0.50024	0.75205	no
gi|646745080|gb|KK962385.1|	74831-75878	20	7.2	2	1	-0.0178629	0.9504	no
gi|646745080|gb|KK962385.1|	76494-78047	20	7.2	1	1	-0.260427	0.8321	no
gi|646745080|gb|KK962385.1|	78750-82420	20	7.2	6	2	-2	0.1688	no
gi|646745080|gb|KK962385.1|	84438-91555	20	7.2	6	8	0.437492	0.69305	no
gi|646745080|gb|KK962385.1|	92456-93117	20	7.2	2	4	0.738537	0.56005	no
gi|646745080|gb|KK962385.1|	93239-95223	20	7.2	4	6	0.420174	0.77565	no
gi|646745091|gb|KK962384.1|	02107-10234	19	7.9	27	32	0.248704	0.86165	no
gi|646745091|gb|KK962384.1|	06791-11055	19	7.9	4	4	0.163013	0.9159	no
gi|646745091|gb|KK962384.1|	11461-11392	19	7.9	1	0.851047	-0.608725	0.6548	no
gi|646745091|gb|KK962384.1|	17460-12232	19	7.9	4	2	-1	0.41965	no
gi|646745091|gb|KK962384.1|	23422-12648	19	7.9	59	4	-4	0.0187	no
gi|646745091|gb|KK962384.1|	50464-15180	19	7.9	22	27	0.265781	0.80455	no

gi|646745091|gb|KK962384.1|	54038-15537	19	7.9	0.771638	0.492851	-0.646772	1	no
gi|646745091|gb|KK962384.1|	58105-15881	19	7.9	1	1	0.220189	0.89085	no
gi|646745091|gb|KK962384.1|	62205-16248	19	7.9	58	114	0.986402	0.53375	no
gi|646745091|gb|KK962384.1|	63253-16442	19	7.9	5	9	0.90139	0.5477	no
gi|646745091|gb|KK962384.1|	65966-16619	19	7.9	9	46	2	0.28065	no
gi|646745091|gb|KK962384.1|	68703-16899	19	7.9	63	31	-1	0.52405	no
gi|646745091|gb|KK962384.1|	71328-17161	19	7.9	32	35	0.13918	0.9147	no
gi|646745091|gb|KK962384.1|	76781-17710	19	7.9	45	39	-0.190699	0.9014	no
gi|646745091|gb|KK962384.1|	79696-17997	19	7.9	14	20	0.517562	0.7043	no
gi|646745091|gb|KK962384.1|	25977-22658	19	7.9	0	7	inf	0.00315	no
gi|646745091|gb|KK962384.1|	65794-26693	19	7.9	1	2	0.838839	0.57515	no
gi|646745091|gb|KK962384.1|	70480-27193	19	7.9	23	14	-0.706019	0.57505	no
gi|646745091|gb|KK962384.1|	84283-28573	19	7.9	17	2	-3	0.00815	no
gi|646745091|gb|KK962384.1|	92478-29348	19	7.9	15	3	-2	0.14885	no
gi|646745091|gb|KK962384.1|	97414-29764	19	7.9	29	13	-1	0.47135	no
gi|646745091|gb|KK962384.1|	24614-32488	19	7.9	24	39	0.674146	0.65085	no
gi|646745091|gb|KK962384.1|	26405-32671	19	7.9	3	15	3	0.27295	no
gi|646745091|gb|KK962384.1|	31784-33311	19	7.9	3	5	0.641635	0.6796	no
gi|646745091|gb|KK962384.1|	33216-33903	19	7.9	1	2	0.148244	0.9197	no
gi|646745091|gb|KK962384.1|	39139-34061	19	7.9	2	2	-0.420909	0.7566	no
gi|646745091|gb|KK962384.1|	42918-34369	19	7.9	1	2	1	0.44735	no
gi|646745091|gb|KK962384.1|	43829-34412	19	7.9	15	20	0.492456	0.6995	no
gi|646745091|gb|KK962384.1|	46514-35045	19	7.9	88	65	-0.429084	0.7208	no
gi|646745091|gb|KK962384.1|	60094-36089	19	7.9	0.85486	3	2	0.2245	no
gi|646745091|gb|KK962384.1|	67171-36745	19	7.9	299	154	-0.961546	0.52585	no
gi|646745091|gb|KK962384.1|	90184-39057	19	7.9	30	24	-0.285094	0.8578	no
gi|646745091|gb|KK962384.1|	00534-40169	19	7.9	10	5	-0.872812	0.56335	no
gi|646745091|gb|KK962384.1|	10943-41176	19	7.9	0.812806	2	0.982746	0.5626	no
gi|646745091|gb|KK962384.1|	22583-42279	19	7.9	103	79	-0.390046	0.74655	no
gi|646745091|gb|KK962384.1|	39883-44036	19	7.9	27	12	-1	0.44065	no

gi|646745091|gb|KK962384.1|	50179-45685	19	7.9	50	31	-0.682723	0.57895	no
gi|646745091|gb|KK962384.1|	62019-46358	19	7.9	12	9	-0.505675	0.7381	no
gi|646745091|gb|KK962384.1|	47957-48404	19	7.9	22	2	-4	0.09815	no
gi|646745091|gb|KK962384.1|	83924-48452	19	7.9	164	133	-0.301142	0.79105	no
gi|646745091|gb|KK962384.1|	97813-49811	19	7.9	11	6	-0.759182	0.61235	no
gi|646745091|gb|KK962384.1|	99359-49964	19	7.9	35	26	-0.438896	0.7539	no
gi|646745091|gb|KK962384.1|	10117-51050	19	7.9	5	5	0.137439	0.91875	no
gi|646745091|gb|KK962384.1|	17590-51810	19	7.9	5	3	-0.678887	0.6101	no
gi|646745091|gb|KK962384.1|	51911-53522	19	7.9	68	5	-4	0.0186	no
gi|646745091|gb|KK962384.1|	53602-56356	19	7.9	1	0.592036	-1	0.4604	no
gi|646745091|gb|KK962384.1|	41768-54280	19	7.9	0	11	inf	5.00E-05	yes
gi|646745091|gb|KK962384.1|	69104-69856	19	7.9	2	1	-0.969989	0.54505	no
gi|646745091|gb|KK962384.1|	72211-73874	19	7.9	24	7	-2	0.20565	no
gi|646745091|gb|KK962384.1|	75199-76348	19	7.9	1	0.296174	-2	1	no
gi|646745091|gb|KK962384.1|	77231-77516	19	7.9	47	33	-0.515307	0.7507	no
gi|646745091|gb|KK962384.1|	79284-80260	19	7.9	1	0.912749	-0.62872	0.65905	no
gi|646745091|gb|KK962384.1|	81131-82232	19	7.9	1	0.312545	-2	1	no
gi|646745091|gb|KK962384.1|	88341-89556	19	7.9	2	0.552552	-2	0.2986	no
gi|646745091|gb|KK962384.1|	92151-96397	19	7.9	1	0.642447	-1	0.4687	no
gi|646745105|gb|KK962383.1|	37546-43799	14	7.6	7	2	-2	0.2364	no
gi|646745120|gb|KK962382.1|	16218-11661	16	8.5	0	15	inf	0.00315	no
gi|646745120|gb|KK962382.1|	45497-14577	16	8.5	9	16	0.875978	0.56375	no
gi|646745120|gb|KK962382.1|	72425-27308	16	8.5	2	4	1	0.3682	no
gi|646745120|gb|KK962382.1|	93225-39361	16	8.5	7	8	0.0674855	0.94855	no
gi|646745120|gb|KK962382.1|	06463-40768	16	8.5	2	2	-0.143278	0.9035	no
gi|646745120|gb|KK962382.1|	08901-41016	16	8.5	10	5	-1	0.48015	no
gi|646745135|gb|KK962381.1|	01167-10249	20	8.7	0	2	inf	0.00035	yes
gi|646745135|gb|KK962381.1|	30154-13047	20	8.7	126	13	-3	0.04195	no
gi|646745135|gb|KK962381.1|	32900-13384	20	8.7	10	25	1	0.4103	no
gi|646745135|gb|KK962381.1|	34386-13463	20	8.7	17	63	2	0.25765	no

gi|646745135|gb|KK962381.1|	37873-14000	20	8.7	16.06	7	-1	0.39355	no
gi|646745135|gb|KK962381.1|	56879-16119	20	8.7	2	2	0.040621	0.9792	no
gi|646745135|gb|KK962381.1|	63110-16346	20	8.7	38	62	0.699621	0.65115	no
gi|646745135|gb|KK962381.1|	76565-17771	20	8.7	1	0	-inf	0.0035	no
gi|646745135|gb|KK962381.1|	83314-18379	20	8.7	0.7992	5	3	0.26175	no
gi|646745135|gb|KK962381.1|	95797-19669	20	8.7	1	0.817025	-0.496509	0.73865	no
gi|646745135|gb|KK962381.1|	96997-19768	20	8.7	2	9	2	0.1406	no
gi|646745135|gb|KK962381.1|	98264-19873	20	8.7	1	14	3	0.1351	no
gi|646745135|gb|KK962381.1|	00502-20221	20	8.7	0.317484	1	2	0.21685	no
gi|646745135|gb|KK962381.1|	07093-20867	20	8.7	205	244.66	0.254837	0.83135	no
gi|646745135|gb|KK962381.1|	10324-21075	20	8.7	7	21	2	0.26255	no
gi|646745135|gb|KK962381.1|	11017-21121	20	8.7	295	555	0.911322	0.5589	no
gi|646745135|gb|KK962381.1|	15912-21670	20	8.7	5	12	1	0.40015	no
gi|646745135|gb|KK962381.1|	16935-21739	20	8.7	14	32	1	0.46175	no
gi|646745135|gb|KK962381.1|	19182-21962	20	8.7	3	20	3	0.11875	no
gi|646745135|gb|KK962381.1|	20732-22092	20	8.7	218	338	0.632647	0.6866	no
gi|646745135|gb|KK962381.1|	21605-22194	20	8.7	37	41	0.145293	0.925	no
gi|646745135|gb|KK962381.1|	23349-22563	20	8.7	3	3	0.298089	0.841	no
gi|646745135|gb|KK962381.1|	25744-22735	20	8.7	0.818707	0.49226	-0.733927	1	no
gi|646745135|gb|KK962381.1|	51778-25324	20	8.7	4	0.110205	-5	0.171	no
gi|646745135|gb|KK962381.1|	54556-25538	20	8.7	66	12	-2	0.0827	no
gi|646745135|gb|KK962381.1|	64004-26539	20	8.7	1	0.117251	-4	0.17455	no
gi|646745135|gb|KK962381.1|	65737-27163	20	8.7	24.91	8	-2	0.1428	no
gi|646745135|gb|KK962381.1|	93809-29442	20	8.7	6	6	0.0505563	0.97125	no
gi|646745135|gb|KK962381.1|	14752-31777	20	8.7	41	25	-0.716845	0.53825	no
gi|646745135|gb|KK962381.1|	23497-32465	20	8.7	1	0.292977	-2	0.1971	no
gi|646745135|gb|KK962381.1|	25034-32631	20	8.7	2	3	0.351845	0.82435	no
gi|646745135|gb|KK962381.1|	36585-33706	20	8.7	12	3	-2	0.2431	no
gi|646745135|gb|KK962381.1|	44426-34551	20	8.7	3	1	-0.959342	0.51975	no
gi|646745135|gb|KK962381.1|	91794-39687	20	8.7	22	22	-0.0452716	0.96735	no

gi|646745135|gb|KK962381.1|	00891-40136	20	8.7	0	16	inf	0.0009	yes
gi|646745135|gb|KK962381.1|	12917-41433	20	8.7	19	12	-0.638767	0.61695	no
gi|646745135|gb|KK962381.1|	15522-41701	20	8.7	1	0.323521	-2	0.2263	no
gi|646745135|gb|KK962381.1|	42099-44284	20	8.7	2	2	-0.164049	0.90845	no
gi|646745135|gb|KK962381.1|	55592-45606	20	8.7	3	11	2	0.28375	no
gi|646745135|gb|KK962381.1|	56861-45729	20	8.7	5	3	-0.458025	0.7599	no
gi|646745135|gb|KK962381.1|	58320-55979	20	8.7	1	3	1	0.3401	no
gi|646745135|gb|KK962381.1|	)9801-10099(	20	8.7	0	2	inf	0.0014	yes
gi|646745171|gb|KK962378.1|	07966-20840	19	8.1	18	17	-0.0760436	0.96055	no
gi|646745171|gb|KK962378.1|	55396-25559	19	8.1	117	222	0.923603	0.524	no
gi|646745171|gb|KK962378.1|	95792-49677	19	8.1	2	0.359776	-3	0.1387	no
gi|646745171|gb|KK962378.1|	25284-62634	19	8.1	4	5	0.380069	0.80205	no
gi|646745171|gb|KK962378.1|	35142-63585	19	8.1	10	4	-1	0.32805	no
gi|646745171|gb|KK962378.1|	59991-66131	19	8.1	7	3	-1	0.45235	no
gi|646745171|gb|KK962378.1|	74293-67497	19	8.1	128.26	113	-0.188808	0.8669	no
gi|646745171|gb|KK962378.1|	79869-68075	19	8.1	2	3	0.396045	0.7418	no
gi|646745171|gb|KK962378.1|	68199-68818	19	8.1	4	3	-0.340397	0.79	no
gi|646745171|gb|KK962378.1|	85160-68545	19	8.1	49	39	-0.334479	0.8258	no
gi|646745171|gb|KK962378.1|	94025-69449	19	8.1	14	14	0.0578896	0.9701	no
gi|646745171|gb|KK962378.1|	00182-70064	19	8.1	5	16	2	0.2553	no
gi|646745171|gb|KK962378.1|	04905-70528	19	8.1	19	34	0.829159	0.59825	no
gi|646745171|gb|KK962378.1|	10299-71153	19	8.1	2	4	1	0.36685	no
gi|646745171|gb|KK962378.1|	12408-71271	19	8.1	62	54	-0.218844	0.88405	no
gi|646745171|gb|KK962378.1|	14529-71476	19	8.1	37	54	0.52268	0.7302	no
gi|646745171|gb|KK962378.1|	17281-71753	19	8.1	51	34	-0.609268	0.61335	no
gi|646745171|gb|KK962378.1|	18233-71864	19	8.1	6	4	-0.359803	0.7972	no
gi|646745171|gb|KK962378.1|	19028-71933	19	8.1	47	97	1	0.5033	no
gi|646745171|gb|KK962378.1|	20573-72084	19	8.1	57	73	0.364505	0.81325	no
gi|646745171|gb|KK962378.1|	21516-72190	19	8.1	31	41	0.416463	0.7808	no
gi|646745171|gb|KK962378.1|	24097-72496	19	8.1	4	9	1	0.4833	no

gi|646745171|gb|KK962378.1|	27826-72840	19	8.1	24	42	0.79512	0.60515	no
gi|646745171|gb|KK962378.1|	31769-73222	19	8.1	4	2	-0.765576	0.60695	no
gi|646745171|gb|KK962378.1|	32524-73291	19	8.1	11	14	0.357946	0.7956	no
gi|646745171|gb|KK962378.1|	33867-73434	19	8.1	8	11	0.494678	0.7217	no
gi|646745171|gb|KK962378.1|	36413-73680	19	8.1	58	63	0.120679	0.9388	no
gi|646745171|gb|KK962378.1|	37159-73766	19	8.1	15	37	1	0.3973	no
gi|646745171|gb|KK962378.1|	39271-73977	19	8.1	48	71	0.579727	0.646	no
gi|646745171|gb|KK962378.1|	42538-74301	19	8.1	5	4	-0.473352	0.7249	no
gi|646745171|gb|KK962378.1|	43318-74455	19	8.1	6	9	0.627263	0.6772	no
gi|646745171|gb|KK962378.1|	45509-74616	19	8.1	8	7	-0.235736	0.8794	no
gi|646745171|gb|KK962378.1|	47791-74879	19	8.1	7	9	0.39953	0.78785	no
gi|646745171|gb|KK962378.1|	48993-75424	19	8.1	5	4	-0.319933	0.8004	no
gi|646745171|gb|KK962378.1|	55012-75961	19	8.1	18	7	-1	0.22	no
gi|646745171|gb|KK962378.1|	61978-76402	19	8.1	0.309248	2	2	0.17285	no
gi|646745171|gb|KK962378.1|	65808-76665	19	8.1	0.626047	1	1	0.48525	no
gi|646745171|gb|KK962378.1|	67544-76885	19	8.1	0.263813	2	3	0.15285	no
gi|646745171|gb|KK962378.1|	13493-81382	19	8.1	14	21	0.571565	0.6908	no
gi|646745171|gb|KK962378.1|	31038-83251	19	8.1	19	13	-0.496835	0.6969	no
gi|646745186|gb|KK962377.1|	15939-11915	21	9.8	6	5	-0.220079	0.8865	no
gi|646745186|gb|KK962377.1|	25868-12696	21	9.8	0.994693	1	0.514768	0.7038	no
gi|646745186|gb|KK962377.1|	30098-13115	21	9.8	6	3	-0.766773	0.63005	no
gi|646745186|gb|KK962377.1|	32764-13370	21	9.8	2	1	-0.592883	0.6374	no
gi|646745186|gb|KK962377.1|	34166-13446	21	9.8	11	5	-1	0.4638	no
gi|646745186|gb|KK962377.1|	44822-14568	21	9.8	6	3	-0.89127	0.5763	no
gi|646745186|gb|KK962377.1|	45989-14647	21	9.8	4	3	-0.714352	0.62725	no
gi|646745186|gb|KK962377.1|	48412-14932	21	9.8	9	4	-0.990316	0.52675	no
gi|646745186|gb|KK962377.1|	70957-17163	21	9.8	2	0.915596	-0.936675	0.589	no
gi|646745186|gb|KK962377.1|	83044-18472	21	9.8	126	66	-0.929447	0.4314	no
gi|646745186|gb|KK962377.1|	84833-18531	21	9.8	8	13	0.615797	0.65415	no
gi|646745186|gb|KK962377.1|	88085-19038	21	9.8	2	3	0.607326	0.68755	no

gi|646745186|gb|KK962377.1|	92499-19306	21	9.8	13	6	-1	0.38835	no
gi|646745186|gb|KK962377.1|	93553-19562	21	9.8	0.816926	0.665805	-0.295107	1	no
gi|646745186|gb|KK962377.1|	95744-19645	21	9.8	3	4	0.483826	0.7434	no
gi|646745186|gb|KK962377.1|	04964-20985	21	9.8	2	3	0.0411755	0.97765	no
gi|646745186|gb|KK962377.1|	10200-22433	21	9.8	8	7	-0.214219	0.85565	no
gi|646745186|gb|KK962377.1|	29136-23072	21	9.8	0.830961	3	2	0.17615	no
gi|646745186|gb|KK962377.1|	34950-23620	21	9.8	0.830925	0.397423	-1	1	no
gi|646745186|gb|KK962377.1|	36699-23868	21	9.8	2	1	-0.663799	0.6796	no
gi|646745186|gb|KK962377.1|	60252-26100	21	9.8	1	1	0.00363588	0.92345	no
gi|646745186|gb|KK962377.1|	82628-28319	21	9.8	4	2	-1	0.32725	no
gi|646745186|gb|KK962377.1|	90117-29055	21	9.8	7	0	-inf	0.00225	no
gi|646745186|gb|KK962377.1|	10795-31159	21	9.8	1	2	0.301873	0.83465	no
gi|646745186|gb|KK962377.1|	26452-32761	21	9.8	5	1	-2	0.1447	no
gi|646745186|gb|KK962377.1|	27723-32866	21	9.8	1	0.953807	-0.341213	0.8138	no
gi|646745186|gb|KK962377.1|	35395-33965	21	9.8	15	4	-2	0.13645	no
gi|646745186|gb|KK962377.1|	42407-34615	21	9.8	12	5	-1	0.3162	no
gi|646745186|gb|KK962377.1|	52008-35226	21	9.8	23	11	-1	0.4668	no
gi|646745186|gb|KK962377.1|	55227-35615	21	9.8	5	3	-0.896453	0.51795	no
gi|646745186|gb|KK962377.1|	72045-37274	21	9.8	3	5	0.868854	0.5576	no
gi|646745186|gb|KK962377.1|	74986-37531	21	9.8	23	25	0.14954	0.93125	no
gi|646745186|gb|KK962377.1|	75845-37702	21	9.8	0.803546	0.719244	-0.1599	1	no
gi|646745186|gb|KK962377.1|	78504-37962	21	9.8	3	3	0.331383	0.83255	no
gi|646745186|gb|KK962377.1|	79819-38537	21	9.8	1	2	0.422775	0.78355	no
gi|646745186|gb|KK962377.1|	88297-38930	21	9.8	6	1	-3	0.1202	no
gi|646745186|gb|KK962377.1|	14435-41557	21	9.8	4	5	0.22306	0.8827	no
gi|646745186|gb|KK962377.1|	20137-42065	21	9.8	1	6	3	0.14855	no
gi|646745186|gb|KK962377.1|	21699-42250	21	9.8	2	1	-0.379122	0.7808	no
gi|646745186|gb|KK962377.1|	22614-42371	21	9.8	2	4	1	0.43655	no
gi|646745186|gb|KK962377.1|	38238-43846	21	9.8	55	16	-2	0.23825	no
gi|646745186|gb|KK962377.1|	40225-44221	21	9.8	0.800316	6	3	0.06465	no

gi|646745186|gb|KK962377.1|	58977-46094	21	9.8	55	11	-2	0.0971	no
gi|646745186|gb|KK962377.1|	84040-48508	21	9.8	1	1	0.342477	0.8037	no
gi|646745186|gb|KK962377.1|	90012-49104	21	9.8	7	3	-1	0.4692	no
gi|646745186|gb|KK962377.1|	98677-50060	21	9.8	3	2	-0.554002	0.7261	no
gi|646745186|gb|KK962377.1|	10851-51186	21	9.8	1	1	0.341179	0.8037	no
gi|646745186|gb|KK962377.1|	17561-51787	21	9.8	0	16	inf	0.0014	yes
gi|646745186|gb|KK962377.1|	24744-53204	21	9.8	26	24	-0.119295	0.92115	no
gi|646745186|gb|KK962377.1|	36110-53742	21	9.8	0.262565	1	2	0.20215	no
gi|646745186|gb|KK962377.1|	47550-54884	21	9.8	1	2	0.40644	0.74905	no
gi|646745186|gb|KK962377.1|	52087-55235	21	9.8	26	15	-0.843962	0.54175	no
gi|646745186|gb|KK962377.1|	56379-55719	21	9.8	1	1	-0.0888097	0.9324	no
gi|646745186|gb|KK962377.1|	70040-57039	21	9.8	46	34	-0.417186	0.7777	no
gi|646745186|gb|KK962377.1|	73108-57435	21	9.8	2	1	-0.631304	0.61695	no
gi|646745186|gb|KK962377.1|	81728-58214	21	9.8	4	9	0.967597	0.4927	no
gi|646745186|gb|KK962377.1|	84756-58563	21	9.8	1	1	-0.17613	0.9042	no
gi|646745186|gb|KK962377.1|	91609-59241	21	9.8	4	4	0.351161	0.79655	no
gi|646745186|gb|KK962377.1|	59380-59807	21	9.8	2	6	2	0.35625	no
gi|646745186|gb|KK962377.1|	99167-59986	21	9.8	2	3	0.651119	0.643	no
gi|646745186|gb|KK962377.1|	09073-60929	21	9.8	73	78	0.0905276	0.9505	no
gi|646745186|gb|KK962377.1|	15919-61756	21	9.8	0.86524	1	0.288667	1	no
gi|646745186|gb|KK962377.1|	17714-61874	21	9.8	1	1	0.189861	0.8968	no
gi|646745186|gb|KK962377.1|	18871-61992	21	9.8	0.810436	0.495644	-0.709396	1	no
gi|646745186|gb|KK962377.1|	20025-62176	21	9.8	0.933237	0.269722	-2	1	no
gi|646745186|gb|KK962377.1|	62116-62353	21	9.8	18	18	0.068007	0.90935	no
gi|646745186|gb|KK962377.1|	22258-62338	21	9.8	10	5	-1	0.45565	no
gi|646745186|gb|KK962377.1|	72594-67283	21	9.8	19	20	0.0926273	0.9177	no
gi|646745186|gb|KK962377.1|	79672-68284	21	9.8	0.825507	7	3	0.04965	no
gi|646745186|gb|KK962377.1|	83466-68815	21	9.8	20	18	-0.155103	0.8921	no
gi|646745186|gb|KK962377.1|	93729-69445	21	9.8	13	19	0.570206	0.6983	no
gi|646745186|gb|KK962377.1|	00069-70084	21	9.8	6	11	0.831009	0.5921	no

gi|646745186|gb|KK962377.1|	03332-70372	21	9.8	6	18	1	0.3238	no
gi|646745186|gb|KK962377.1|	06149-70747	21	9.8	14	14	0.0391964	0.9777	no
gi|646745186|gb|KK962377.1|	15743-71635	21	9.8	8	3	-1	0.33125	no
gi|646745186|gb|KK962377.1|	26452-72771	21	9.8	0.368198	1	2	0.26385	no
gi|646745186|gb|KK962377.1|	29813-73069	21	9.8	3	8	1	0.28935	no
gi|646745186|gb|KK962377.1|	54031-75440	21	9.8	1	11	3	0.2507	no
gi|646745186|gb|KK962377.1|	65800-77951	21	9.8	19	17	-0.189612	0.87495	no
gi|646745186|gb|KK962377.1|	20702-82121	21	9.8	3	4	0.432953	0.75085	no
gi|646745186|gb|KK962377.1|	21490-82247	21	9.8	0.633586	2	1	0.36855	no
gi|646745186|gb|KK962377.1|	26815-82770	21	9.8	3	2	-0.234315	0.844	no
gi|646745186|gb|KK962377.1|	27866-82893	21	9.8	2	2	-0.178304	0.8816	no
gi|646745186|gb|KK962377.1|	34195-83457	21	9.8	37	22	-0.75892	0.61715	no
gi|646745186|gb|KK962377.1|	40463-84065	21	9.8	68	71	0.055338	0.9215	no
gi|646745186|gb|KK962377.1|	69212-87026	21	9.8	0.465436	1	2	0.29375	no
gi|646745186|gb|KK962377.1|	70410-87187	21	9.8	0.613172	0.662305	0.111203	1	no
gi|646745186|gb|KK962377.1|	74685-87568	21	9.8	21	5	-2	0.21925	no
gi|646745186|gb|KK962377.1|	86106-88689	21	9.8	1	1	0.230951	0.87595	no
gi|646745186|gb|KK962377.1|	91442-89221	21	9.8	0.889361	1	0.492207	0.7568	no
gi|646745186|gb|KK962377.1|	93199-89861	21	9.8	3	2	-0.742225	0.61875	no
gi|646745186|gb|KK962377.1|	01934-90380	21	9.8	30	9	-1.8	0.1843	no
gi|646745186|gb|KK962377.1|	05236-90605	21	9.8	19	17	-0.179214	0.90725	no
gi|646745186|gb|KK962377.1|	28616-92953	21	9.8	1	2	0.504735	0.71995	no
gi|646745186|gb|KK962377.1|	29690-93095	21	9.8	5	2	-1	0.4487	no
gi|646745186|gb|KK962377.1|	33034-93343	21	9.8	5	5	-0.0444903	0.9298	no
gi|646745186|gb|KK962377.1|	37376-93808	21	9.8	0.814728	2	1	0.4332	no
gi|646745186|gb|KK962377.1|	44266-94531	21	9.8	1	1	0.223244	0.8612	no
gi|646745186|gb|KK962377.1|	45931-94732	21	9.8	0.892656	0.35024	-1	1	no
gi|646745186|gb|KK962377.1|	47683-94844	21	9.8	1	1	-0.316319	0.8509	no
gi|646745186|gb|KK962377.1|	54395-95712	21	9.8	14	14	-0.00577482	0.9965	no
gi|646745186|gb|KK962377.1|	59425-96247	21	9.8	10	8	-0.373333	0.726	no

gi|646745186|gb|KK962377.1|	67467-96839	21	9.8	11	8	-0.443267	0.77045	no
gi|646745186|gb|KK962377.1|	71135-97165	21	9.8	14	15	0.125758	0.9344	no
gi|646745186|gb|KK962377.1|	75365-97674	21	9.8	2	2	-0.0118098	0.9797	no
gi|646745186|gb|KK962377.1|	83558-98506	21	9.8	1	0.748341	-0.572459	1	no
gi|646745186|gb|KK962377.1|	89498-98993	21	9.8	27	7	-2	0.18205	no
gi|646745197|gb|KK962376.1|	16650-11711	21	8.9	61	59	-0.0450028	0.9743	no
gi|646745197|gb|KK962376.1|	18370-12026	21	8.9	10	14	0.460393	0.71545	no
gi|646745197|gb|KK962376.1|	12283-24182	21	8.9	5	4	-0.232068	0.8397	no
gi|646745197|gb|KK962376.1|	25418-12763	21	8.9	7	6	-0.225118	0.88335	no
gi|646745197|gb|KK962376.1|	28769-13394	21	8.9	9	6	-0.656204	0.55725	no
gi|646745197|gb|KK962376.1|	41787-14209	21	8.9	8	8	-0.0167467	0.91525	no
gi|646745197|gb|KK962376.1|	53957-15488	21	8.9	6	8	0.299057	0.8477	no
gi|646745197|gb|KK962376.1|	57674-15808	21	8.9	7	3	-1	0.3543	no
gi|646745197|gb|KK962376.1|	63422-16365	21	8.9	187	45	-2	0.15595	no
gi|646745197|gb|KK962376.1|	65533-16643	21	8.9	14	12	-0.296073	0.8437	no
gi|646745197|gb|KK962376.1|	67156-16765	21	8.9	48	30	-0.667311	0.66265	no
gi|646745197|gb|KK962376.1|	85791-18726	21	8.9	16	7	-1	0.33625	no
gi|646745197|gb|KK962376.1|	24306-26421	21	8.9	0.547144	0.865102	0.66095	1	no
gi|646745197|gb|KK962376.1|	28511-42822	21	8.9	16	16	-0.000258472	0.99965	no
gi|646745197|gb|KK962376.1|	53941-35478	21	8.9	4	9	1	0.38605	no
gi|646745197|gb|KK962376.1|	92664-39697	21	8.9	5	6	0.341979	0.7487	no
gi|646745197|gb|KK962376.1|	43049-45120	21	8.9	0.611345	2	1	0.3627	no
gi|646745197|gb|KK962376.1|	34771-43742	21	8.9	0.423516	0.783325	0.887193	1	no
gi|646745197|gb|KK962376.1|	49968-45088	21	8.9	0.700767	0.994342	0.504807	1	no
gi|646745197|gb|KK962376.1|	58663-45896	21	8.9	21	21	0.0287484	0.96	no
gi|646745197|gb|KK962376.1|	63930-46589	21	8.9	12	14	0.258616	0.8032	no
gi|646745197|gb|KK962376.1|	01369-50373	21	8.9	1	3	0.940814	0.54755	no
gi|646745197|gb|KK962376.1|	14060-51725	21	8.9	25	17	-0.526134	0.6406	no
gi|646745197|gb|KK962376.1|	19656-52093	21	8.9	0.725568	1	0.521471	1	no
gi|646745197|gb|KK962376.1|	29651-53001	21	8.9	6	7	0.142521	0.9183	no

gi|646745197|gb|KK962376.1|	43767-54460	21	8.9	2	0.678089	-1	0.4271	no
gi|646745197|gb|KK962376.1|	52971-55346	21	8.9	104	47	-1	0.36925	no
gi|646745197|gb|KK962376.1|	68806-57332	21	8.9	9	7	-0.308559	0.7781	no
gi|646745197|gb|KK962376.1|	73782-57410	21	8.9	22	3	-3	0.1185	no
gi|646745197|gb|KK962376.1|	75216-57554	21	8.9	13	4	-2	0.2827	no
gi|646745197|gb|KK962376.1|	94703-59543	21	8.9	11	2	-2	0.1452	no
gi|646745197|gb|KK962376.1|	05361-60569	21	8.9	24	2	-3	0.1481	no
gi|646745197|gb|KK962376.1|	76104-76330	21	8.9	61	62	0.0254487	0.95995	no
gi|646745197|gb|KK962376.1|	80963-83394	21	8.9	9	11	0.277657	0.7973	no
gi|646745197|gb|KK962376.1|	8807-9247	21	8.9	4	3	-0.602557	0.6685	no
gi|646745197|gb|KK962376.1|	96098-97205	21	8.9	1	2	0.515424	0.6803	no
gi|646745213|gb|KK962375.1|	17867-11889	12	7.3	2	8	2	0.20775	no
gi|646745213|gb|KK962375.1|	38035-13822	12	7.3	86	58	-0.56913	0.7095	no
gi|646745213|gb|KK962375.1|	73450-17496	12	7.3	2	0.423288	-2	0.2048	no
gi|646745213|gb|KK962375.1|	76549-17895	12	7.3	2	1	-0.611175	0.7009	no
gi|646745213|gb|KK962375.1|	79724-18572	12	7.3	8	6	-0.392397	0.71515	no
gi|646745213|gb|KK962375.1|	88340-18888	12	7.3	4	2	-1	0.4366	no
gi|646745213|gb|KK962375.1|	89988-19065	12	7.3	6	6	0.00302545	0.9754	no
gi|646745213|gb|KK962375.1|	90829-19270	12	7.3	0.513343	0.825261	0.684927	1	no
gi|646745213|gb|KK962375.1|	16943-21711	12	7.3	716	244	-2	0.2786	no
gi|646745213|gb|KK962375.1|	47868-45202	12	7.3	1	4	2	0.248	no
gi|646745213|gb|KK962375.1|	07030-50740	12	7.3	5	4	-0.463459	0.7629	no
gi|646745213|gb|KK962375.1|	10163-51124	12	7.3	3	1	-1	0.40445	no
gi|646745213|gb|KK962375.1|	23399-52381	12	7.3	13	4	-2	0.3317	no
gi|646745213|gb|KK962375.1|	36151-53658	12	7.3	4	3	-0.2881	0.85095	no
gi|646745213|gb|KK962375.1|	49274-54970	12	7.3	3	3	0.126937	0.91245	no
gi|646745213|gb|KK962375.1|	02845-60351	12	7.3	2	2	-0.107828	0.9288	no
gi|646745213|gb|KK962375.1|	19734-62240	12	7.3	2	3	0.539335	0.72455	no
gi|646745213|gb|KK962375.1|	85885-86003	12	7.3	12916.1	455	-5	0.1689	no
gi|646745227|gb|KK962374.1|	91460-29180	11	6.1	5	8	0.788218	0.607	no

gi|646745227|gb|KK962374.1|	91890-29233	11	6.1	2	8	2	0.26185	no
gi|646745242|gb|KK962373.1|	85941-48662	17	7.8	2	0.911514	-0.743686	0.6333	no
gi|646745242|gb|KK962373.1|	86921-48779	17	7.8	2	0.423822	-2	0.197	no
gi|646745242|gb|KK962373.1|	90448-49111	17	7.8	3	2	-0.6387	0.6351	no
gi|646745242|gb|KK962373.1|	95188-49571	17	7.8	5	0.455695	-3	0.179	no
gi|646745289|gb|KK962370.1|	38229-13843	14	8.1	194	94	-1	0.5212	no
gi|646745289|gb|KK962370.1|	41343-14222	14	8.1	13	5	-1	0.39855	no
gi|646745289|gb|KK962370.1|	12131-41289	14	8.1	2	1	-1	0.4518	no
gi|646745304|gb|KK962369.1|	14277-15518	15	8.4	1	2	0.700984	0.62685	no
gi|646745304|gb|KK962369.1|	29805-23116	15	8.4	11	17	0.652084	0.67045	no
gi|646745304|gb|KK962369.1|	17343-41761	15	8.4	10	23	1	0.42745	no
gi|646745327|gb|KK962367.1|	14607-11514	18	8.3	4	0.876472	-2	0.2045	no
gi|646745327|gb|KK962367.1|	13819-14236	18	8.3	18	14	-0.341366	0.8329	no
gi|646745327|gb|KK962367.1|	46879-14769	18	8.3	1	4	2	0.3172	no
gi|646745327|gb|KK962367.1|	57993-15942	18	8.3	19	6	-2	0.1868	no
gi|646745327|gb|KK962367.1|	20661-21267	18	8.3	256	139	-0.877382	0.44575	no
gi|646745327|gb|KK962367.1|	28196-29109	18	8.3	0.140579	3	5	0.11005	no
gi|646745327|gb|KK962367.1|	80569-38097	18	8.3	4	6	0.564041	0.7088	no
gi|646745327|gb|KK962367.1|	31825-43254	18	8.3	2	2	0.0705327	0.9485	no
gi|646745327|gb|KK962367.1|	32894-43497	18	8.3	9	13	0.608645	0.6262	no
gi|646745327|gb|KK962367.1|	56760-45760	18	8.3	2	3	0.761836	0.55745	no
gi|646745327|gb|KK962367.1|	47608-51019	18	8.3	18	15	-0.250004	0.82325	no
gi|646745327|gb|KK962367.1|	51410-51650	18	8.3	33	7.03	-2	0.2052	no
gi|646745327|gb|KK962367.1|	53101-55194	18	8.3	22	20	-0.134937	0.9027	no
gi|646745327|gb|KK962367.1|	58648-62543	18	8.3	16	17	0.0551994	0.95885	no
gi|646745327|gb|KK962367.1|	96890-97412	18	8.3	31	13	-1	0.4273	no
gi|646745342|gb|KK962366.1|	41620-14220	7.8	4.8	4	0.777062	-2	0.1596	no
gi|646745342|gb|KK962366.1|	45910-14658	7.8	4.8	1	2	0.478015	0.7489	no
gi|646745342|gb|KK962366.1|	51176-15201	7.8	4.8	3	4	0.338246	0.80565	no
gi|646745342|gb|KK962366.1|	58621-59659	7.8	4.8	2	2	-0.165872	0.89995	no

gi|646745342|gb|KK962366.1|	59723-60327	7.8	4.8	2	1	-0.536529	0.73	no
gi|646745372|gb|KK962364.1|	07028-10097	23	10	0.563913	0.65574	0.217653	1	no
gi|646745372|gb|KK962364.1|	11606-10130	23	10	0.37822	0.762762	1	1	no
gi|646745372|gb|KK962364.1|	13205-10139	23	10	0.523556	3	3	0.13425	no
gi|646745372|gb|KK962364.1|	15007-10162	23	10	0.453036	1	2	0.3354	no
gi|646745372|gb|KK962364.1|	16644-10177	23	10	0.641668	0.764633	0.252941	1	no
gi|646745372|gb|KK962364.1|	33672-10347	23	10	0.580336	1	1	0.36855	no
gi|646745372|gb|KK962364.1|	74709-10756	23	10	1	6	2	0.17945	no
gi|646745372|gb|KK962364.1|	76632-10778	23	10	0.468639	2	2	0.21645	no
gi|646745372|gb|KK962364.1|	78003-10794	23	10	80	84	0.0731946	0.9478	no
gi|646745372|gb|KK962364.1|	79706-10805	23	10	3	0.858368	-2	0.22645	no
gi|646745372|gb|KK962364.1|	80629-10815	23	10	1	0.756663	-0.952259	0.54505	no
gi|646745372|gb|KK962364.1|	91697-10921	23	10	14	0	-inf	0.0031	no
gi|646745372|gb|KK962364.1|	62606-16476	23	10	3	3	0.0149887	0.9914	no
gi|646745372|gb|KK962364.1|	69034-17065	23	10	0.542206	0.684693	0.336615	1	no
gi|646745372|gb|KK962364.1|	72375-17310	23	10	2	6	2	0.2845	no
gi|646745372|gb|KK962364.1|	80815-18208	23	10	1	0.783375	-0.893657	0.50335	no
gi|646745372|gb|KK962364.1|	82192-18640	23	10	4	6	0.578408	0.6468	no
gi|646745372|gb|KK962364.1|	97070-19810	23	10	12	23	0.937654	0.4581	no
gi|646745372|gb|KK962364.1|	21128-22874	23	10	18	11	-0.777268	0.50885	no
gi|646745372|gb|KK962364.1|	29219-22949	23	10	15	15	-0.00632671	0.9333	no
gi|646745372|gb|KK962364.1|	31279-23364	23	10	2	0.826543	-0.971563	0.48405	no
gi|646745372|gb|KK962364.1|	35252-23861	23	10	1	0.649846	-0.680117	1	no
gi|646745372|gb|KK962364.1|	38731-24236	23	10	2	1	-0.433445	0.7764	no
gi|646745372|gb|KK962364.1|	42478-24435	23	10	0.400501	0.496667	0.310471	1	no
gi|646745372|gb|KK962364.1|	45913-24623	23	10	14	23	0.663413	0.6518	no
gi|646745372|gb|KK962364.1|	46827-24847	23	10	3	0.862904	-2	0.2173	no
gi|646745372|gb|KK962364.1|	72397-27349	23	10	4	8	1	0.51055	no
gi|646745372|gb|KK962364.1|	31687-33209	23	10	5	2	-1	0.4814	no
gi|646745372|gb|KK962364.1|	50552-35100	23	10	57	45	-0.350189	0.8139	no

gi|646745372|gb|KK962364.1|	69657-37265	23	10	16	2	-3	0.04995	no
gi|646745372|gb|KK962364.1|	07679-41080	23	10	3	3	-0.0576323	0.9681	no
gi|646745372|gb|KK962364.1|	13867-41500	23	10	1	1	0.101593	0.94	no
gi|646745372|gb|KK962364.1|	16834-41805	23	10	58	67	0.196461	0.8604	no
gi|646745372|gb|KK962364.1|	31379-43270	23	10	0.861366	0.247549	-2	1	no
gi|646745372|gb|KK962364.1|	44496-45083	23	10	0.277883	3	4	0.1063	no
gi|646745372|gb|KK962364.1|	51528-45239	23	10	5	7	0.375183	0.80765	no
gi|646745372|gb|KK962364.1|	55866-45674	23	10	4	7	0.801977	0.60765	no
gi|646745372|gb|KK962364.1|	58011-45819	23	10	47.91	121.96	1	0.37885	no
gi|646745372|gb|KK962364.1|	62110-46278	23	10	2	3	0.188159	0.8935	no
gi|646745372|gb|KK962364.1|	62956-46602	23	10	36	18	-0.993911	0.38845	no
gi|646745372|gb|KK962364.1|	66660-46955	23	10	4	2	-0.944966	0.5378	no
gi|646745372|gb|KK962364.1|	71122-47165	23	10	4	4	0.153445	0.90915	no
gi|646745372|gb|KK962364.1|	79310-48007	23	10	4	5	0.289521	0.85525	no
gi|646745372|gb|KK962364.1|	84916-49092	23	10	1	2	1	0.4839	no
gi|646745372|gb|KK962364.1|	94324-49697	23	10	92	69	-0.402378	0.7344	no
gi|646745372|gb|KK962364.1|	20844-52145	23	10	10	4	-1	0.3389	no
gi|646745372|gb|KK962364.1|	65778-56630	23	10	3	9	2	0.2952	no
gi|646745372|gb|KK962364.1|	92883-59344	23	10	4	4	-0.186532	0.8883	no
gi|646745372|gb|KK962364.1|	96219-59886	23	10	1	0.900053	-0.548265	0.72785	no
gi|646745372|gb|KK962364.1|	98972-60627	23	10	1	1	-0.314495	0.82945	no
gi|646745372|gb|KK962364.1|	06520-60802	23	10	0.740695	1	0.790152	0.54425	no
gi|646745372|gb|KK962364.1|	12722-61635	23	10	3	2	-0.827278	0.5823	no
gi|646745372|gb|KK962364.1|	29384-62968	23	10	33	12.97	-1	0.36845	no
gi|646745372|gb|KK962364.1|	48960-64999	23	10	3	3	0.0524094	0.9705	no
gi|646745372|gb|KK962364.1|	55141-65698	23	10	3	8	2	0.28755	no
gi|646745372|gb|KK962364.1|	11604-71272	23	10	0.4263	1	2	0.29375	no
gi|646745372|gb|KK962364.1|	14197-71525	23	10	1	0.992495	-0.072015	1	no
gi|646745372|gb|KK962364.1|	17118-71798	23	10	1	0.858368	-0.306391	1	no
gi|646745372|gb|KK962364.1|	18644-71918	23	10	3	4	0.123114	0.9305	no

gi|646745372|gb|KK962364.1|	19541-72053	23	10	2	4	0.732396	0.6025	no
gi|646745372|gb|KK962364.1|	25984-72746	23	10	0.529508	0.762762	0.526582	1	no
gi|646745372|gb|KK962364.1|	27588-72904	23	10	0.462947	0.77775	0.748459	1	no
gi|646745372|gb|KK962364.1|	31605-73298	23	10	0.74207	0.948665	0.354343	1	no
gi|646745372|gb|KK962364.1|	33287-74151	23	10	9	8	-0.185932	0.8703	no
gi|646745372|gb|KK962364.1|	53825-75435	23	10	3	2	-0.407952	0.8003	no
gi|646745372|gb|KK962364.1|	62324-76287	23	10	5	11	1	0.4593	no
gi|646745372|gb|KK962364.1|	63184-76449	23	10	54	90	0.733201	0.5172	no
gi|646745372|gb|KK962364.1|	64824-76778	23	10	3	2	-0.707173	0.6425	no
gi|646745372|gb|KK962364.1|	68318-77013	23	10	0.768008	1	0.41675	1	no
gi|646745372|gb|KK962364.1|	71040-77203	23	10	0.746	2	1	0.4476	no
gi|646745372|gb|KK962364.1|	77889-77886	23	10	3	2	-0.713549	0.57365	no
gi|646745372|gb|KK962364.1|	82754-78501	23	10	4	6	0.748154	0.62415	no
gi|646745372|gb|KK962364.1|	85164-78615	23	10	46	19	-1	0.31915	no
gi|646745372|gb|KK962364.1|	93408-79405	23	10	3	3	-0.112921	0.93575	no
gi|646745372|gb|KK962364.1|	95305-79837	23	10	39	27	-0.501113	0.66515	no
gi|646745372|gb|KK962364.1|	15781-81600	23	10	18	27.25	0.606273	0.67725	no
gi|646745372|gb|KK962364.1|	21366-82259	23	10	13	8	-0.723872	0.63365	no
gi|646745372|gb|KK962364.1|	41816-84622	23	10	13	7	-0.921536	0.4106	no
gi|646745372|gb|KK962364.1|	51728-85226	23	10	21	14	-0.602852	0.6914	no
gi|646745372|gb|KK962364.1|	81892-88261	23	10	5	2	-1	0.34075	no
gi|646745372|gb|KK962364.1|	87917-88850	23	10	4	5	0.566098	0.6557	no
gi|646745372|gb|KK962364.1|	88989-89344	23	10	51	26	-0.978712	0.41045	no
gi|646745372|gb|KK962364.1|	94990-89562	23	10	1	2	0.732394	0.64505	no
gi|646745372|gb|KK962364.1|	97281-89903	23	10	0.864626	0.357027	-1	1	no
gi|646745372|gb|KK962364.1|	01469-90248	23	10	1	0.86836	-0.337054	1	no
gi|646745372|gb|KK962364.1|	08049-90904	23	10	1	0.177071	-3	0.19945	no
gi|646745372|gb|KK962364.1|	10840-91156	23	10	2	1	-0.684783	0.6459	no
gi|646745372|gb|KK962364.1|	43934-94480	23	10	21	15	-0.477835	0.75145	no
gi|646745372|gb|KK962364.1|	48959-95644	23	10	7	4	-0.579216	0.59925	no

gi|646745372|gb|KK962364.1|	62681-96291	23	10	53	16	-2	0.2386	no
gi|646745372|gb|KK962364.1|	67523-96813	23	10	11	6	-0.839261	0.6004	no
gi|646745372|gb|KK962364.1|	82452-98630	23	10	4	5	0.202617	0.8955	no
gi|646745372|gb|KK962364.1|	91974-99709	23	10	23	21	-0.15023	0.9	no
gi|646745372|gb|KK962364.1|	)9716-10011	23	10	1	3	0.99944	0.48265	no
gi|646745387|gb|KK962363.1|	00037-20121	20	8.6	2	3	0.228885	0.88705	no
gi|646745387|gb|KK962363.1|	01274-20199	20	8.6	4	2	-1	0.45385	no
gi|646745387|gb|KK962363.1|	91506-29171	20	8.6	565	416	-0.441892	0.77215	no
gi|646745387|gb|KK962363.1|	70652-37089	20	8.6	263	179	-0.552577	0.71255	no
gi|646745387|gb|KK962363.1|	80918-38116	20	8.6	25	33	0.404939	0.76825	no
gi|646745387|gb|KK962363.1|	96320-39773	20	8.6	1	0.114772	-3	0.1757	no
gi|646745387|gb|KK962363.1|	99126-40020	20	8.6	1	0.642857	-0.945344	0.5451	no
gi|646745387|gb|KK962363.1|	04649-40568	20	8.6	1	0	-inf	0.00475	no
gi|646745387|gb|KK962363.1|	13729-41499	20	8.6	2	0	-inf	0.0008	yes
gi|646745387|gb|KK962363.1|	15181-41538	20	8.6	72	90	0.320806	0.8153	no
gi|646745387|gb|KK962363.1|	25868-42677	20	8.6	1	0.807304	-0.66584	0.6592	no
gi|646745387|gb|KK962363.1|	34597-43579	20	8.6	0.393654	0.846053	1	1	no
gi|646745387|gb|KK962363.1|	55464-45619	20	8.6	1	1	-0.0978686	0.9036	no
gi|646745387|gb|KK962363.1|	56300-45690	20	8.6	2	2	-0.215576	0.8925	no
gi|646745387|gb|KK962363.1|	59488-46100	20	8.6	0.585525	0.632958	0.112379	1	no
gi|646745387|gb|KK962363.1|	62260-46442	20	8.6	0.966744	1	0.301434	0.8277	no
gi|646745387|gb|KK962363.1|	64927-46551	20	8.6	1	2	1	0.48625	no
gi|646745387|gb|KK962363.1|	70480-47128	20	8.6	7	19	1	0.33625	no
gi|646745387|gb|KK962363.1|	73100-47452	20	8.6	17	48	2	0.1844	no
gi|646745387|gb|KK962363.1|	81884-48217	20	8.6	63	107.66	0.768755	0.6119	no
gi|646745387|gb|KK962363.1|	87185-48917	20	8.6	0.907024	0.8499	-0.0938486	1	no
gi|646745387|gb|KK962363.1|	89456-49121	20	8.6	26	38	0.504183	0.65065	no
gi|646745387|gb|KK962363.1|	10655-51211	20	8.6	0.61419	0.331692	-0.88884	1	no
gi|646745387|gb|KK962363.1|	34075-53639	20	8.6	45	36	-0.299377	0.7911	no
gi|646745412|gb|KK962361.1|	05005-10530	14	7.2	9	7	-0.351671	0.8176	no

gi|646745412|gb|KK962361.1|	02716-30323	14	7.2	2	4	0.799161	0.58155	no
gi|646745412|gb|KK962361.1|	38648-33997	14	7.2	0.346806	0.74742	1	1	no
gi|646745412|gb|KK962361.1|	40272-34174	14	7.2	0.913704	1	0.263378	1	no
gi|646745412|gb|KK962361.1|	20742-42127	14	7.2	5	0.455695	-3	0.17715	no
gi|646745412|gb|KK962361.1|	76567-77410	14	7.2	2	3	0.762105	0.55745	no
gi|646745434|gb|KK962359.1|	04312-10586	22	10	2	2	0.343644	0.82775	no
gi|646745434|gb|KK962359.1|	10451-10802	22	10	6	6	0.0923353	0.92585	no
gi|646745434|gb|KK962359.1|	06060-10734	22	10	0.898156	0.773698	-0.215196	1	no
gi|646745434|gb|KK962359.1|	07483-10965	22	10	3	2	-0.747868	0.6295	no
gi|646745434|gb|KK962359.1|	10394-11527	22	10	76	50	-0.592867	0.62325	no
gi|646745434|gb|KK962359.1|	37463-13797	22	10	13	12	-0.0336866	0.98175	no
gi|646745434|gb|KK962359.1|	38458-13979	22	10	1	2	0.885696	0.53285	no
gi|646745434|gb|KK962359.1|	43744-14405	22	10	34	62	0.867776	0.5787	no
gi|646745434|gb|KK962359.1|	52183-15242	22	10	31	36	0.223636	0.86215	no
gi|646745434|gb|KK962359.1|	84341-18632	22	10	0.855149	0.464375	-0.880887	1	no
gi|646745434|gb|KK962359.1|	93080-19361	22	10	5	0.893594	-2	0.1572	no
gi|646745434|gb|KK962359.1|	19350-23090	22	10	11	6	-0.872854	0.42185	no
gi|646745434|gb|KK962359.1|	20234-22114	22	10	0.988561	4	2	0.24515	no
gi|646745434|gb|KK962359.1|	32693-23322	22	10	11	12	0.0548557	0.97065	no
gi|646745434|gb|KK962359.1|	37154-23755	22	10	22	19	-0.155522	0.92025	no
gi|646745434|gb|KK962359.1|	40806-24101	22	10	95	31	-2	0.25655	no
gi|646745434|gb|KK962359.1|	44625-24833	22	10	8	6	-0.427569	0.6838	no
gi|646745434|gb|KK962359.1|	58283-25881	22	10	4	4	-0.0882761	0.9432	no
gi|646745434|gb|KK962359.1|	87573-29181	22	10	21	24	0.234776	0.8387	no
gi|646745434|gb|KK962359.1|	02074-30298	22	10	0.425618	2	2	0.255	no
gi|646745434|gb|KK962359.1|	03186-30341	22	10	27	39	0.567696	0.70155	no
gi|646745434|gb|KK962359.1|	04612-30716	22	10	2	5	1	0.41155	no
gi|646745434|gb|KK962359.1|	08609-30892	22	10	23	37	0.71604	0.60155	no
gi|646745434|gb|KK962359.1|	22198-32336	22	10	0.908923	0.723076	-0.33001	1	no
gi|646745434|gb|KK962359.1|	56657-35682	22	10	400.71	127	-2	0.23695	no

gi|646745434|gb|KK962359.1|	79733-38022	22	10	4	5	0.423628	0.74255	no
gi|646745434|gb|KK962359.1|	92425-39297	22	10	1	5	2	0.1963	no
gi|646745434|gb|KK962359.1|	42552-44492	22	10	27	16	-0.763908	0.49455	no
gi|646745434|gb|KK962359.1|	29142-43121	22	10	0.509174	0.811549	0.67252	1	no
gi|646745434|gb|KK962359.1|	38283-44409	22	10	8	16	0.958881	0.40535	no
gi|646745434|gb|KK962359.1|	46593-44830	22	10	0.380719	3	3	0.11105	no
gi|646745434|gb|KK962359.1|	4471-5448	22	10	3	2	-0.375679	0.7836	no
gi|646745434|gb|KK962359.1|	60944-46449	22	10	0.786217	0.981771	0.320458	1	no
gi|646745434|gb|KK962359.1|	76666-47955	22	10	3	6	1	0.49915	no
gi|646745434|gb|KK962359.1|	79807-48784	22	10	18	18	-0.0202206	0.9859	no
gi|646745434|gb|KK962359.1|	97755-50028	22	10	18	25	0.434805	0.6988	no
gi|646745434|gb|KK962359.1|	03181-50390	22	10	1	2	0.708664	0.63615	no
gi|646745434|gb|KK962359.1|	04100-50607	22	10	0.698846	1	0.921068	0.53425	no
gi|646745434|gb|KK962359.1|	06585-50978	22	10	0.597717	1	1	0.3683	no
gi|646745434|gb|KK962359.1|	14931-51819	22	10	1	11	3	0.04205	no
gi|646745434|gb|KK962359.1|	49929-55014	22	10	61	36	-0.782135	0.56155	no
gi|646745434|gb|KK962359.1|	50650-55264	22	10	2	1	-0.465807	0.764	no
gi|646745434|gb|KK962359.1|	55642-55601	22	10	10	9	-0.140477	0.90915	no
gi|646745434|gb|KK962359.1|	59673-56007	22	10	7	2	-2	0.3592	no
gi|646745434|gb|KK962359.1|	63907-56474	22	10	3	2	-0.289966	0.8051	no
gi|646745434|gb|KK962359.1|	66169-56718	22	10	1	0.517046	-1	0.4271	no
gi|646745434|gb|KK962359.1|	72450-57282	22	10	16	12	-0.414042	0.7724	no
gi|646745434|gb|KK962359.1|	81348-58188	22	10	5	6	0.344818	0.7794	no
gi|646745434|gb|KK962359.1|	82904-58350	22	10	1	3	0.947428	0.5627	no
gi|646745434|gb|KK962359.1|	86943-58826	22	10	2	2	0.0533681	0.9623	no
gi|646745434|gb|KK962359.1|	94610-59569	22	10	0.335573	1	2	0.25365	no
gi|646745434|gb|KK962359.1|	11182-61148	22	10	13	0	-inf	0.00655	no
gi|646745434|gb|KK962359.1|	16097-61867	22	10	1	0.173729	-3	0.12875	no
gi|646745434|gb|KK962359.1|	67603-67859	22	10	13	19	0.616101	0.66825	no
gi|646745434|gb|KK962359.1|	91606-69290	22	10	0.353768	0.88913	1	1	no

gi|646745434|gb|KK962359.1|	92968-69374	22	10	2	1	-0.355334	0.812	no
gi|646745434|gb|KK962359.1|	11566-71210	22	10	3	5	0.709095	0.5867	no
gi|646745434|gb|KK962359.1|	24429-72888	22	10	7	12	0.793101	0.47155	no
gi|646745434|gb|KK962359.1|	30058-73282	22	10	0.957986	2	0.926861	0.5597	no
gi|646745434|gb|KK962359.1|	33165-73423	22	10	0.568917	1	0.999095	0.5626	no
gi|646745434|gb|KK962359.1|	34443-73500	22	10	1	4	2	0.29945	no
gi|646745434|gb|KK962359.1|	35124-73773	22	10	0.627779	0.912152	0.539018	1	no
gi|646745434|gb|KK962359.1|	43753-74504	22	10	0.535773	1	0.936984	1	no
gi|646745434|gb|KK962359.1|	50865-75386	22	10	8	7	-0.202065	0.8502	no
gi|646745434|gb|KK962359.1|	55297-75671	22	10	11	10	-0.0598239	0.9666	no
gi|646745434|gb|KK962359.1|	76790-79971	22	10	0	13	inf	5.00E-05	yes
gi|646745434|gb|KK962359.1|	68391-77331	22	10	13	18	0.488294	0.67525	no
gi|646745434|gb|KK962359.1|	76227-77773	22	10	0.518072	0.746577	0.527137	1	no
gi|646745434|gb|KK962359.1|	78821-77913	22	10	17	12	-0.547163	0.67125	no
gi|646745434|gb|KK962359.1|	87108-79028	22	10	0.094925	11	7	0.1049	no
gi|646745434|gb|KK962359.1|	90635-79089	22	10	0	75	inf	0.00105	yes
gi|646745434|gb|KK962359.1|	06196-80849	22	10	0.045263	7	7	0.0946	no
gi|646745434|gb|KK962359.1|	82494-82920	22	10	14	10	-0.559705	0.6863	no
gi|646745434|gb|KK962359.1|	84812-85333	22	10	12	7	-0.649312	0.68465	no
gi|646745434|gb|KK962359.1|	90095-90372	22	10	50	28	-0.868129	0.5315	no
gi|646745448|gb|KK962358.1|	57209-35809	17	9.1	1	0.62789	-1	0.4766	no
gi|646745448|gb|KK962358.1|	92826-39298	17	9.1	86967.7	92150.5	0.0835114	0.9453	no
gi|646745448|gb|KK962358.1|	61829-62473	17	9.1	2	0.329526	-3	0.2033	no
gi|646745462|gb|KK962357.1|	12319-21282	23	11	4	0.97959	-2	0.2163	no
gi|646745462|gb|KK962357.1|	33532-23413	23	11	17	8	-1	0.45665	no
gi|646745462|gb|KK962357.1|	38551-23934	23	11	0	4	inf	0.00515	no
gi|646745462|gb|KK962357.1|	45552-24571	23	11	351	271	-0.373656	0.7717	no
gi|646745462|gb|KK962357.1|	05746-30597	23	11	34	44	0.351467	0.7969	no
gi|646745462|gb|KK962357.1|	21525-32222	23	11	2	3	0.357235	0.78075	no
gi|646745462|gb|KK962357.1|	35352-33686	23	11	11	6	-0.922815	0.5449	no

gi|646745462|gb|KK962357.1|	39379-34028	23	11	44	16	-1	0.2806	no
gi|646745462|gb|KK962357.1|	63485-36415	23	11	5	0.620114	-3	0.1548	no
gi|646745462|gb|KK962357.1|	71188-37310	23	11	16	6	-1	0.26035	no
gi|646745462|gb|KK962357.1|	82918-38613	23	11	19	7	-1	0.1961	no
gi|646745462|gb|KK962357.1|	01547-40316	23	11	6	2	-2	0.3001	no
gi|646745462|gb|KK962357.1|	03282-40594	23	11	4	3	-0.417612	0.77985	no
gi|646745462|gb|KK962357.1|	13294-41467	23	11	17	11	-0.666441	0.5938	no
gi|646745462|gb|KK962357.1|	15964-41795	23	11	7	4	-0.930961	0.5439	no
gi|646745462|gb|KK962357.1|	18014-42112	23	11	4	3	-0.34316	0.8171	no
gi|646745462|gb|KK962357.1|	29173-42957	23	11	16	3	-2	0.1764	no
gi|646745462|gb|KK962357.1|	42776-44340	23	11	4	2	-0.629964	0.63115	no
gi|646745462|gb|KK962357.1|	49306-45012	23	11	4	3	-0.181623	0.9151	no
gi|646745462|gb|KK962357.1|	50701-45098	23	11	31	2	-4	0.17175	no
gi|646745462|gb|KK962357.1|	52294-45389	23	11	4	3	-0.259527	0.86335	no
gi|646745462|gb|KK962357.1|	89034-49127	23	11	2	0.202808	-3	0.12875	no
gi|646745462|gb|KK962357.1|	47010-55090	23	11	13	6	-1	0.3064	no
gi|646745462|gb|KK962357.1|	55864-56371	23	11	190	238.89	0.326681	0.77425	no
gi|646745462|gb|KK962357.1|	63739-56517	23	11	0.234802	0.901404	2	1	no
gi|646745462|gb|KK962357.1|	72042-57323	23	11	0.099152	22	8	0.09445	no
gi|646745462|gb|KK962357.1|	87127-58935	23	11	0.797161	0.544074	-0.551069	1	no
gi|646745462|gb|KK962357.1|	06661-60730	23	11	4	5	0.192922	0.8676	no
gi|646745462|gb|KK962357.1|	10106-61279	23	11	1	2	0.539443	0.73695	no
gi|646745462|gb|KK962357.1|	12984-61469	23	11	2	2	0.0781323	0.95885	no
gi|646745462|gb|KK962357.1|	14882-61654	23	11	3	3	-0.277469	0.8613	no
gi|646745462|gb|KK962357.1|	34954-63689	23	11	0.657309	0.475771	-0.466303	1	no
gi|646745462|gb|KK962357.1|	37586-63898	23	11	1	2	0.804914	0.57675	no
gi|646745462|gb|KK962357.1|	41207-64834	23	11	10	8	-0.450238	0.6973	no
gi|646745462|gb|KK962357.1|	49815-65068	23	11	2	5	2	0.25	no
gi|646745462|gb|KK962357.1|	65224-67582	23	11	4	2	-0.926014	0.55	no
gi|646745462|gb|KK962357.1|	53442-65462	23	11	0.400036	1	1	1	no

gi|646745462|gb|KK962357.1|	55990-65751	23	11	0.727304	0.419364	-0.794355	1	no
gi|646745462|gb|KK962357.1|	58193-66023	23	11	0.931898	2	0.949761	0.4911	no
gi|646745462|gb|KK962357.1|	60339-66113	23	11	2	2	-0.0199496	0.9367	no
gi|646745462|gb|KK962357.1|	61249-66314	23	11	1	0.16356	-3	0.1238	no
gi|646745462|gb|KK962357.1|	63262-66628	23	11	1	2	0.43411	0.7756	no
gi|646745462|gb|KK962357.1|	71877-67229	23	11	9	1	-3	0.13995	no
gi|646745462|gb|KK962357.1|	68060-70101	23	11	1	1	-0.0502487	1	no
gi|646745462|gb|KK962357.1|	84865-68545	23	11	2	1	-1	0.47035	no
gi|646745462|gb|KK962357.1|	16585-71895	23	11	2	2	-0.163003	0.9194	no
gi|646745462|gb|KK962357.1|	42001-74295	23	11	1	0.748319	-0.492319	1	no
gi|646745462|gb|KK962357.1|	53072-75488	23	11	27	14	-0.935187	0.39285	no
gi|646745462|gb|KK962357.1|	65318-76630	23	11	29	13	-1	0.3397	no
gi|646745462|gb|KK962357.1|	81660-78202	23	11	0.793027	11	4	0.1007	no
gi|646745462|gb|KK962357.1|	78202-79298	23	11	0.219992	2	3	0.2578	no
gi|646745462|gb|KK962357.1|	87975-78946	23	11	0.975397	2	1	0.43335	no
gi|646745462|gb|KK962357.1|	91291-79241	23	11	2	2	-0.249311	0.83865	no
gi|646745462|gb|KK962357.1|	09318-80991	23	11	4	3	-0.444149	0.7345	no
gi|646745462|gb|KK962357.1|	11089-81135	23	11	11	17	0.638765	0.66495	no
gi|646745462|gb|KK962357.1|	14366-81483	23	11	11	9	-0.230905	0.88435	no
gi|646745462|gb|KK962357.1|	87584-89073	23	11	2	3	0.476185	0.7679	no
gi|646745487|gb|KK962355.1|	81022-28130	19	9.5	2	28	4	0.0976	no
gi|646745487|gb|KK962355.1|	92493-29284	19	9.5	0.830554	9	3	0.1075	no
gi|646745487|gb|KK962355.1|	28533-32904	19	9.5	5	0.479552	-3	0.17715	no
gi|646745487|gb|KK962355.1|	39097-33939	19	9.5	8	8	-0.024073	0.91525	no
gi|646745487|gb|KK962355.1|	42295-34261	19	9.5	34	11	-2	0.2773	no
gi|646745487|gb|KK962355.1|	61728-36264	19	9.5	3	2	-0.23223	0.844	no
gi|646745487|gb|KK962355.1|	92934-39622	19	9.5	7	8	0.236393	0.829	no
gi|646745487|gb|KK962355.1|	30614-43503	19	9.5	3	2	-0.927875	0.54355	no
gi|646745487|gb|KK962355.1|	48421-45737	19	9.5	10	9	-0.166338	0.88675	no
gi|646745487|gb|KK962355.1|	97611-49906	19	9.5	1	2	0.443495	0.74935	no

gi|646745487|gb|KK962355.1|	04115-50463	19	9.5	15	11	-0.371556	0.8079	no
gi|646745487|gb|KK962355.1|	15752-51821	19	9.5	0.167986	5	5	0.097	no
gi|646745487|gb|KK962355.1|	23091-52404	19	9.5	0.264671	5	4	0.24605	no
gi|646745487|gb|KK962355.1|	38415-53939	19	9.5	21	10	-1	0.4661	no
gi|646745487|gb|KK962355.1|	50318-55166	19	9.5	22	16	-0.482129	0.7032	no
gi|646745487|gb|KK962355.1|	68156-57100	19	9.5	0.9979	1	0.256459	0.87415	no
gi|646745487|gb|KK962355.1|	81032-58235	19	9.5	1	1	0.0372883	0.95325	no
gi|646745487|gb|KK962355.1|	12038-61266	19	9.5	10	5	-1	0.4481	no
gi|646745487|gb|KK962355.1|	42055-64242	19	9.5	168	4	-5	0.0443	no
gi|646745487|gb|KK962355.1|	42945-64383	19	9.5	42	7	-3	0.06355	no
gi|646745487|gb|KK962355.1|	43887-64602	19	9.5	14	4	-2	0.18685	no
gi|646745487|gb|KK962355.1|	46789-64730	19	9.5	3	2	-0.563409	0.72945	no
gi|646745487|gb|KK962355.1|	47494-64784	19	9.5	11	9	-0.210735	0.8723	no
gi|646745487|gb|KK962355.1|	59226-65984	19	9.5	17	5	-2	0.1881	no
gi|646745487|gb|KK962355.1|	65984-66674	19	9.5	3	2	-0.78992	0.54305	no
gi|646745487|gb|KK962355.1|	17227-72010	19	9.5	28	17	-0.720512	0.52455	no
gi|646745487|gb|KK962355.1|	20876-72207	19	9.5	1	2	0.906162	0.54305	no
gi|646745499|gb|KK962354.1|	03154-10509	20	9.3	1	2	0.422676	0.78685	no
gi|646745499|gb|KK962354.1|	05219-10771	20	9.3	2	3	0.682711	0.65395	no
gi|646745499|gb|KK962354.1|	07990-11015	20	9.3	0.533381	1	1	0.3611	no
gi|646745499|gb|KK962354.1|	18207-11930	20	9.3	4	3	-0.372668	0.8173	no
gi|646745499|gb|KK962354.1|	26719-12797	20	9.3	3	3	0.137653	0.9267	no
gi|646745499|gb|KK962354.1|	13667-16233	20	9.3	1	0.174474	-3	0.1554	no
gi|646745499|gb|KK962354.1|	17020-18750	20	9.3	0.752663	0.634394	-0.246624	1	no
gi|646745499|gb|KK962354.1|	18949-21009	20	9.3	1	0.148523	-3	0.151	no
gi|646745499|gb|KK962354.1|	2105-4688	20	9.3	11	8	-0.482689	0.69975	no
gi|646745499|gb|KK962354.1|	21154-22719	20	9.3	2	0.509255	-2	0.2662	no
gi|646745499|gb|KK962354.1|	27667-22835	20	9.3	7	1	-3	0.1387	no
gi|646745499|gb|KK962354.1|	22870-24514	20	9.3	1	0.9615	-0.607844	0.6756	no
gi|646745499|gb|KK962354.1|	25117-27740	20	9.3	12	10	-0.239899	0.82435	no

gi|646745499|gb|KK962354.1|	28902-30128	20	9.3	0.381088	0.81965	1	1	no
gi|646745499|gb|KK962354.1|	16800-31769	20	9.3	1	1	-0.34496	0.8138	no
gi|646745499|gb|KK962354.1|	41006-34272	20	9.3	3	0.182474	-4	0.12165	no
gi|646745499|gb|KK962354.1|	35274-35972	20	9.3	0.834898	3	2	0.205	no
gi|646745499|gb|KK962354.1|	53896-35593	20	9.3	3	0.525584	-2	0.1133	no
gi|646745499|gb|KK962354.1|	56275-35965	20	9.3	2	0.473831	-2	0.10905	no
gi|646745499|gb|KK962354.1|	70120-37121	20	9.3	6	0	-inf	0.00025	yes
gi|646745499|gb|KK962354.1|	05615-40668	20	9.3	1	1	0.151332	0.9186	no
gi|646745499|gb|KK962354.1|	41622-43037	20	9.3	1	0.114772	-3	1	no
gi|646745499|gb|KK962354.1|	31050-43168	20	9.3	2	1	-0.75219	0.6333	no
gi|646745499|gb|KK962354.1|	50250-45383	20	9.3	31	20	-0.633463	0.58285	no
gi|646745499|gb|KK962354.1|	54034-45471	20	9.3	2	2	-0.00637894	0.92345	no
gi|646745499|gb|KK962354.1|	55873-45611	20	9.3	32	37	0.22001	0.87835	no
gi|646745499|gb|KK962354.1|	57086-45776	20	9.3	2	2	0.286741	0.83965	no
gi|646745499|gb|KK962354.1|	54234-55007	20	9.3	2	6	1	0.32915	no
gi|646745499|gb|KK962354.1|	5949-7676	20	9.3	2	0	-inf	0.0047	no
gi|646745499|gb|KK962354.1|	91929-94737	20	9.3	11	15	0.44051	0.6885	no
gi|646745499|gb|KK962354.1|	96156-96571	20	9.3	67	85	0.35509	0.81875	no
gi|646745499|gb|KK962354.1|	9765-12436	20	9.3	3	0.500881	-2	0.11395	no
gi|646745513|gb|KK962353.1|	66774-26777	15	5.7	4	7	1	0.4993	no
gi|646745522|gb|KK962352.1|	20016-20263	18	9.3	32	47	0.550077	0.71855	no
gi|646745522|gb|KK962352.1|	25849-26106	18	9.3	47	25	-0.944385	0.45615	no
gi|646745522|gb|KK962352.1|	36895-33744	18	9.3	3	3	-0.205016	0.878	no
gi|646745522|gb|KK962352.1|	90957-49145	18	9.3	3	2	-0.793286	0.6307	no
gi|646745522|gb|KK962352.1|	38359-53890	18	9.3	4	0	-inf	0.00475	no
gi|646745522|gb|KK962352.1|	47386-54878	18	9.3	5	0	-inf	5.00E-05	yes
gi|646745522|gb|KK962352.1|	50290-55191	18	9.3	1	0	-inf	0.00525	no
gi|646745522|gb|KK962352.1|	68769-57028	18	9.3	0.367406	1	2	0.2453	no
gi|646745522|gb|KK962352.1|	75579-57735	18	9.3	1	0.964693	-0.256811	0.8321	no
gi|646745522|gb|KK962352.1|	79246-58009	18	9.3	2	0.88065	-0.959831	0.54505	no

gi|646745522|gb|KK962352.1|	82415-58372	18	9.3	0.527626	0.505237	-0.0625559	1	no
gi|646745522|gb|KK962352.1|	99146-60061	18	9.3	0.38071	0.658023	0.789447	1	no
gi|646745522|gb|KK962352.1|	03692-60484	18	9.3	2	6	2	0.2431	no
gi|646745522|gb|KK962352.1|	13948-61456	18	9.3	11	4	-2	0.2625	no
gi|646745522|gb|KK962352.1|	18391-62026	18	9.3	0.912048	6	3	0.0808	no
gi|646745522|gb|KK962352.1|	63837-66452	18	9.3	14	8	-0.764729	0.61495	no
gi|646745522|gb|KK962352.1|	84395-85742	18	9.3	0.169434	1	3	0.2578	no
gi|646745522|gb|KK962352.1|	95452-97758	18	9.3	1	2	1	0.4619	no
gi|646745533|gb|KK962351.1|	10450-11204	22	9.7	0.69195	0.199656	-2	1	no
gi|646745533|gb|KK962351.1|	12855-11443	22	9.7	1	0.70502	-0.664082	1	no
gi|646745533|gb|KK962351.1|	16936-11958	22	9.7	3	2	-0.7545	0.62665	no
gi|646745533|gb|KK962351.1|	26835-12722	22	9.7	83	11	-3	0.0529	no
gi|646745533|gb|KK962351.1|	45061-14537	22	9.7	16	16	0.0191836	0.9512	no
gi|646745533|gb|KK962351.1|	70970-17179	22	9.7	3	2	-0.580683	0.65795	no
gi|646745533|gb|KK962351.1|	73590-17515	22	9.7	0.141242	0.916659	3	1	no
gi|646745533|gb|KK962351.1|	82522-18413	22	9.7	2	1	-0.363978	0.78875	no
gi|646745533|gb|KK962351.1|	87601-18849	22	9.7	6	2	-2	0.1973	no
gi|646745533|gb|KK962351.1|	90427-19550	22	9.7	6	5	-0.109687	0.91665	no
gi|646745533|gb|KK962351.1|	99605-19998	22	9.7	56	20	-1	0.34515	no
gi|646745533|gb|KK962351.1|	20883-22133	22	9.7	0.922882	6	3	0.2628	no
gi|646745533|gb|KK962351.1|	23239-22357	22	9.7	17	17	0.0128005	0.96385	no
gi|646745533|gb|KK962351.1|	24045-22472	22	9.7	2	3	0.93848	0.49995	no
gi|646745533|gb|KK962351.1|	29337-22965	22	9.7	81	59	-0.468045	0.752	no
gi|646745533|gb|KK962351.1|	23675-24821	22	9.7	2	2	-0.35744	0.79815	no
gi|646745533|gb|KK962351.1|	47445-25075	22	9.7	13	11	-0.290575	0.78865	no
gi|646745533|gb|KK962351.1|	25035-26081	22	9.7	10	8	-0.325127	0.82935	no
gi|646745533|gb|KK962351.1|	28620-29858	22	9.7	0.470546	0.809867	0.78335	1	no
gi|646745533|gb|KK962351.1|	70527-37330	22	9.7	1	1	0.191995	0.90715	no
gi|646745533|gb|KK962351.1|	73449-37767	22	9.7	0.908904	2	0.782653	0.6114	no
gi|646745533|gb|KK962351.1|	77765-38093	22	9.7	1	2	0.507633	0.7291	no

gi|646745533|gb|KK962351.1|	84992-38528	22	9.7	24	23	-0.0740833	0.95295	no
gi|646745533|gb|KK962351.1|	85623-38608	22	9.7	26	40	0.613867	0.6819	no
gi|646745533|gb|KK962351.1|	93053-39386	22	9.7	15	24	0.739209	0.62495	no
gi|646745533|gb|KK962351.1|	40070-40504	22	9.7	19	6	-2	0.2533	no
gi|646745533|gb|KK962351.1|	43893-44467	22	9.7	3	0.792813	-2	0.20515	no
gi|646745533|gb|KK962351.1|	67274-46795	22	9.7	2	0.909486	-0.743515	0.6333	no
gi|646745533|gb|KK962351.1|	80396-48178	22	9.7	0.489911	0.58727	0.261505	1	no
gi|646745533|gb|KK962351.1|	49259-53961	22	9.7	6	3	-1	0.4338	no
gi|646745533|gb|KK962351.1|	05610-50624	22	9.7	3	4	0.470618	0.7049	no
gi|646745533|gb|KK962351.1|	07504-50885	22	9.7	0.930184	0.243115	-2	1	no
gi|646745533|gb|KK962351.1|	27581-52810	22	9.7	4	8	0.942172	0.53015	no
gi|646745533|gb|KK962351.1|	58159-60190	22	9.7	2	0.603673	-2	0.3169	no
gi|646745533|gb|KK962351.1|	01459-60175	22	9.7	22	48	1	0.4069	no
gi|646745533|gb|KK962351.1|	03653-60412	22	9.7	6	13	1	0.4653	no
gi|646745533|gb|KK962351.1|	07525-61223	22	9.7	0.9138	2	0.758361	0.61865	no
gi|646745533|gb|KK962351.1|	20816-62133	22	9.7	5	14	1	0.3293	no
gi|646745533|gb|KK962351.1|	23202-62546	22	9.7	6	5	-0.287265	0.84645	no
gi|646745533|gb|KK962351.1|	46420-64710	22	9.7	108.15	103.74	-0.060065	0.95375	no
gi|646745533|gb|KK962351.1|	75167-67730	22	9.7	3	16	2	0.1018	no
gi|646745533|gb|KK962351.1|	88642-90920	22	9.7	27	8	-2	0.2136	no
gi|646745543|gb|KK962350.1|	18554-11931	21	10	3	2	-0.509935	0.7	no
gi|646745543|gb|KK962350.1|	20463-12072	21	10	65	82	0.340069	0.8331	no
gi|646745543|gb|KK962350.1|	28312-12952	21	10	2	0	-inf	0.00195	no
gi|646745543|gb|KK962350.1|	32376-13362	21	10	0.467219	0.804267	0.783575	1	no
gi|646745543|gb|KK962350.1|	68505-16979	21	10	1	0.89923	-0.25572	1	no
gi|646745543|gb|KK962350.1|	70687-17172	21	10	0.117542	3	5	0.10595	no
gi|646745543|gb|KK962350.1|	96734-19728	21	10	199	176	-0.180433	0.87205	no
gi|646745543|gb|KK962350.1|	10460-21074	21	10	7	28	2	0.20375	no
gi|646745543|gb|KK962350.1|	26034-22674	21	10	1	1	-0.00100985	0.92345	no
gi|646745543|gb|KK962350.1|	32527-23365	21	10	1	2	0.623344	0.621	no

gi|646745543|gb|KK962350.1|	35601-23813	21	10	119	115.38	-0.0461774	0.96635	no
gi|646745543|gb|KK962350.1|	45767-25318	21	10	0.78837	1	0.639703	0.6718	no
gi|646745543|gb|KK962350.1|	53286-25968	21	10	125	89	-0.486383	0.6874	no
gi|646745543|gb|KK962350.1|	86989-38989	21	10	1	3	1	0.42305	no
gi|646745543|gb|KK962350.1|	97348-39797	21	10	3	1	-2	0.285	no
gi|646745543|gb|KK962350.1|	02444-40409	21	10	4	2	-1	0.46655	no
gi|646745543|gb|KK962350.1|	04307-40672	21	10	1	0.559636	-0.877253	1	no
gi|646745543|gb|KK962350.1|	09531-41067	21	10	1	1	0.364907	0.78175	no
gi|646745543|gb|KK962350.1|	10797-41178	21	10	1	0.54254	-0.905839	1	no
gi|646745543|gb|KK962350.1|	26514-42677	21	10	15	14	-0.13236	0.91245	no
gi|646745543|gb|KK962350.1|	38523-44042	21	10	1	0.650711	-0.636717	1	no
gi|646745543|gb|KK962350.1|	47552-44817	21	10	4	2	-1	0.359	no
gi|646745543|gb|KK962350.1|	73355-47356	21	10	49	20	-1	0.44885	no
gi|646745543|gb|KK962350.1|	02911-50310	21	10	38	52	0.455769	0.76525	no
gi|646745543|gb|KK962350.1|	06628-50688	21	10	46	50	0.130674	0.92625	no
gi|646745543|gb|KK962350.1|	13177-51358	21	10	5	6	0.373075	0.79075	no
gi|646745543|gb|KK962350.1|	17299-51764	21	10	7	4	-0.691804	0.64855	no
gi|646745543|gb|KK962350.1|	27816-52829	21	10	13	8	-0.727609	0.5956	no
gi|646745543|gb|KK962350.1|	28439-53016	21	10	0.881693	2	1	0.4278	no
gi|646745543|gb|KK962350.1|	74030-57499	21	10	5	5	-0.0259752	0.98275	no
gi|646745543|gb|KK962350.1|	58206-59091	21	10	0.293651	2	3	0.2821	no
gi|646745543|gb|KK962350.1|	94712-59642	21	10	0.697506	2	1	0.36115	no
gi|646745543|gb|KK962350.1|	06366-60663	21	10	39	24	-0.689289	0.5817	no
gi|646745543|gb|KK962350.1|	10302-61080	21	10	13	11	-0.239439	0.8773	no
gi|646745543|gb|KK962350.1|	12302-61417	21	10	2	4	1	0.4662	no
gi|646745543|gb|KK962350.1|	61576-64253	21	10	0.305596	1	2	0.17965	no
gi|646745543|gb|KK962350.1|	19016-61981	21	10	3	2	-0.335347	0.7905	no
gi|646745543|gb|KK962350.1|	78514-67871	21	10	179	132	-0.442315	0.7817	no
gi|646745543|gb|KK962350.1|	94899-69526	21	10	45	59	0.379505	0.8012	no
gi|646745543|gb|KK962350.1|	97294-69806	21	10	9	4	-1	0.32615	no

gi|646745543|gb|KK962350.1|	03427-70909	21	10	0.99156	1	0.44354	0.7688	no
gi|646745543|gb|KK962350.1|	12235-71557	21	10	35	20	-0.828381	0.47815	no
gi|646745543|gb|KK962350.1|	19184-71988	21	10	4	1	-1	0.3613	no
gi|646745543|gb|KK962350.1|	25478-72595	21	10	2	5	1	0.4025	no
gi|646745543|gb|KK962350.1|	30898-73216	21	10	1	2	0.414283	0.74125	no
gi|646745543|gb|KK962350.1|	37156-73786	21	10	2	1	-0.516928	0.72175	no
gi|646745543|gb|KK962350.1|	38413-73955	21	10	0.943953	0.75026	-0.331326	1	no
gi|646745543|gb|KK962350.1|	39705-74011	21	10	2	7	2	0.28975	no
gi|646745543|gb|KK962350.1|	45217-74544	21	10	30	39	0.392109	0.7888	no
gi|646745543|gb|KK962350.1|	56009-75629	21	10	14	24	0.780597	0.558	no
gi|646745543|gb|KK962350.1|	66622-76789	21	10	0.819477	1	0.52135	0.7038	no
gi|646745543|gb|KK962350.1|	68784-77036	21	10	0.419246	1	1	1	no
gi|646745543|gb|KK962350.1|	76767-77741	21	10	10	11	0.0911597	0.9507	no
gi|646745543|gb|KK962350.1|	78733-78113	21	10	0.946699	1	0.663247	0.6846	no
gi|646745543|gb|KK962350.1|	81837-78325	21	10	0.238024	2	3	0.152	no
gi|646745543|gb|KK962350.1|	85387-78684	21	10	4	8	0.998864	0.5175	no
gi|646745543|gb|KK962350.1|	79167-80089	21	10	0.554732	1	1	0.4847	no
gi|646745543|gb|KK962350.1|	93319-79511	21	10	1	4	2	0.22065	no
gi|646745543|gb|KK962350.1|	96777-79723	21	10	4	7	0.672082	0.60925	no
gi|646745543|gb|KK962350.1|	00086-80074	21	10	6	13	1	0.4815	no
gi|646745543|gb|KK962350.1|	09473-80988	21	10	8	21	1	0.31455	no
gi|646745543|gb|KK962350.1|	43530-84393	21	10	63	40	-0.681373	0.6485	no
gi|646745543|gb|KK962350.1|	56779-85980	21	10	0.667773	1	0.80406	0.56015	no
gi|646745543|gb|KK962350.1|	59947-86084	21	10	1	1	0.281099	0.8455	no
gi|646745543|gb|KK962350.1|	64231-86523	21	10	0.985398	0.701874	-0.489494	1	no
gi|646745543|gb|KK962350.1|	65383-86607	21	10	1	1	0.21832	0.89085	no
gi|646745543|gb|KK962350.1|	70823-87138	21	10	4	5	0.103895	0.93535	no
gi|646745543|gb|KK962350.1|	77502-87833	21	10	0.797395	1	0.761381	0.61475	no
gi|646745543|gb|KK962350.1|	88748-89132	21	10	0.876578	0.810389	-0.113268	1	no
gi|646745543|gb|KK962350.1|	01185-90233	21	10	2	1	-0.40047	0.75465	no

gi|646745543|gb|KK962350.1|	03121-90342	21	10	7	8	0.233292	0.8757	no
gi|646745543|gb|KK962350.1|	03740-90455	21	10	1	0.936889	-0.503847	0.7384	no
gi|646745543|gb|KK962350.1|	05480-90640	21	10	0.833347	0.985675	0.242195	1	no
gi|646745543|gb|KK962350.1|	09303-90975	21	10	5	3	-0.596866	0.6657	no
gi|646745543|gb|KK962350.1|	19165-92058	21	10	0.792054	1	0.373368	1	no
gi|646745543|gb|KK962350.1|	26824-92714	21	10	20	4	-2	0.17575	no
gi|646745543|gb|KK962350.1|	32314-93262	21	10	12	10.23	-0.256069	0.8575	no
gi|646745543|gb|KK962350.1|	93925-94997	21	10	20	40	0.982005	0.44555	no
gi|646745543|gb|KK962350.1|	39386-93972	21	10	27	11	-1	0.3998	no
gi|646745543|gb|KK962350.1|	55249-95615	21	10	0.282878	4	4	0.2504	no
gi|646745543|gb|KK962350.1|	57847-95802	21	10	45	89	1	0.4644	no
gi|646745543|gb|KK962350.1|	66866-96788	21	10	17	0.346457	-6	0.10715	no
gi|646745556|gb|KK962349.1|	26131-12762	14	6.9	1	3	0.959955	0.4802	no
gi|646745556|gb|KK962349.1|	23451-22377	14	6.9	15	9	-0.619837	0.64235	no
gi|646745556|gb|KK962349.1|	66631-26693	14	6.9	15	14	-0.165183	0.90625	no
gi|646745579|gb|KK962347.1|	18091-11859	15	7.6	6	2	-2	0.3025	no
gi|646745579|gb|KK962347.1|	53680-15417	15	7.6	57	5	-4	0.02785	no
gi|646745579|gb|KK962347.1|	92421-19350	15	7.6	2	4	1	0.3872	no
gi|646745579|gb|KK962347.1|	02513-20357	15	7.6	3	8	2	0.33235	no
gi|646745579|gb|KK962347.1|	03921-20623	15	7.6	4	12	2	0.18905	no
gi|646745579|gb|KK962347.1|	11121-21217	15	7.6	3	0.997358	-1	0.3416	no
gi|646745579|gb|KK962347.1|	60851-26122	15	7.6	19	6	-2	0.27775	no
gi|646745579|gb|KK962347.1|	29054-30032	15	7.6	0.895607	1	0.701548	0.62425	no
gi|646745579|gb|KK962347.1|	87303-38781	15	7.6	3	1	-0.789857	0.6307	no
gi|646745579|gb|KK962347.1|	98852-49960	15	7.6	2	3	1	0.41885	no
gi|646745579|gb|KK962347.1|	46717-64847	15	7.6	3	2	-0.275959	0.85545	no
gi|646745579|gb|KK962347.1|	54220-65520	15	7.6	455	81	-2	0.04195	no
gi|646745579|gb|KK962347.1|	58575-65932	15	7.6	1	1	-0.0952697	1	no
gi|646745579|gb|KK962347.1|	99893-10153	15	7.6	11	4	-1	0.3929	no
gi|646745589|gb|KK962346.1|	13202-11430	14	6.3	2	2	0.308887	0.8041	no

gi|646745589|gb|KK962346.1|	44918-14537	14	6.3	8	5	-0.681914	0.59115	no
gi|646745589|gb|KK962346.1|	96354-19702	14	6.3	4	0	-inf	0.0008	yes
gi|646745589|gb|KK962346.1|	4071-4724	14	6.3	4	3	-0.613465	0.62785	no
gi|646745601|gb|KK962345.1|	95585-29892	23	17	0.06	62	10	0.2322	no
gi|646745601|gb|KK962345.1|	99119-29961	23	17	0	15	inf	0.0012	yes
gi|646745601|gb|KK962345.1|	76621-77335	23	17	2	26	3	0.0404	no
gi|646745612|gb|KK962344.1|	20302-10206	20	8.2	7	13	0.84005	0.5686	no
gi|646745612|gb|KK962344.1|	15831-11596	20	8.2	670	760	0.18117	0.8829	no
gi|646745612|gb|KK962344.1|	27346-12918	20	8.2	4	4	0.0179083	0.98915	no
gi|646745612|gb|KK962344.1|	29289-13132	20	8.2	1	2	0.596025	0.70635	no
gi|646745612|gb|KK962344.1|	70180-17056	20	8.2	3	6	0.833493	0.58765	no
gi|646745612|gb|KK962344.1|	70762-17193	20	8.2	1	3	1	0.45895	no
gi|646745612|gb|KK962344.1|	72172-17303	20	8.2	1	2	0.330705	0.8043	no
gi|646745612|gb|KK962344.1|	74372-17531	20	8.2	1	1	-0.36762	0.78495	no
gi|646745612|gb|KK962344.1|	75491-17677	20	8.2	5	2	-0.902728	0.5734	no
gi|646745612|gb|KK962344.1|	88196-18939	20	8.2	9	8	-0.207331	0.89145	no
gi|646745612|gb|KK962344.1|	89970-19178	20	8.2	1	0.685211	-0.555287	1	no
gi|646745612|gb|KK962344.1|	92965-19398	20	8.2	45	64	0.499635	0.6518	no
gi|646745612|gb|KK962344.1|	98102-20030	20	8.2	24	25	0.0313522	0.97655	no
gi|646745612|gb|KK962344.1|	16699-21702	20	8.2	39	4	-3	0.11085	no
gi|646745612|gb|KK962344.1|	23992-22455	20	8.2	12	4	-2	0.28975	no
gi|646745612|gb|KK962344.1|	26609-22740	20	8.2	21	3	-3	0.07285	no
gi|646745612|gb|KK962344.1|	71640-27415	20	8.2	8	2	-2	0.2513	no
gi|646745612|gb|KK962344.1|	24283-32521	20	8.2	2	0.390301	-2	0.197	no
gi|646745612|gb|KK962344.1|	97756-39834	20	8.2	1	2	0.458918	0.7747	no
gi|646745612|gb|KK962344.1|	02942-40568	20	8.2	1	3	1	0.41415	no
gi|646745612|gb|KK962344.1|	6152-6677	20	8.2	2	2	0.176637	0.89965	no
gi|646745612|gb|KK962344.1|	64424-64655	20	8.2	12	33	1	0.36545	no
gi|646745612|gb|KK962344.1|	7151-12189	20	8.2	4	3	-0.393388	0.7959	no
gi|646745612|gb|KK962344.1|	48982-95050	20	8.2	1	0	-inf	0.00095	yes

gi|646745612|gb|KK962344.1|	51558-95249	20	8.2	18	0.577971	-5	0.0772	no
gi|646745631|gb|KK962343.1|	14478-11497	18	8.6	4	9	1	0.45165	no
gi|646745631|gb|KK962343.1|	1419-1971	18	8.6	22	25	0.185977	0.904	no
gi|646745631|gb|KK962343.1|	14728-14970	18	8.6	13	27	1	0.4731	no
gi|646745631|gb|KK962343.1|	55525-15768	18	8.6	2	2	-0.362265	0.81605	no
gi|646745631|gb|KK962343.1|	65794-16604	18	8.6	57	14	-2	0.22395	no
gi|646745631|gb|KK962343.1|	79885-18129	18	8.6	1	1	-0.392517	0.75525	no
gi|646745631|gb|KK962343.1|	99141-20021	18	8.6	6	0.967724	-3	0.11955	no
gi|646745631|gb|KK962343.1|	18715-22004	18	8.6	1	0.496457	-1	0.31375	no
gi|646745631|gb|KK962343.1|	41706-24435	18	8.6	4	4	0.109849	0.9441	no
gi|646745631|gb|KK962343.1|	44471-24790	18	8.6	1	2	0.612887	0.6855	no
gi|646745631|gb|KK962343.1|	48052-25110	18	8.6	2	2	0.602544	0.69	no
gi|646745631|gb|KK962343.1|	51444-25231	18	8.6	61	32	-0.941562	0.38745	no
gi|646745631|gb|KK962343.1|	54417-25467	18	8.6	19	32	0.724368	0.59415	no
gi|646745631|gb|KK962343.1|	62630-26286	18	8.6	19	48	1	0.37905	no
gi|646745631|gb|KK962343.1|	80471-28074	18	8.6	113.96	80	-0.517588	0.7312	no
gi|646745631|gb|KK962343.1|	93949-29424	18	8.6	14	19	0.36887	0.77305	no
gi|646745631|gb|KK962343.1|	31158-31436	18	8.6	37	34	-0.133247	0.92	no
gi|646745631|gb|KK962343.1|	31985-32231	18	8.6	21	19	-0.123067	0.92165	no
gi|646745631|gb|KK962343.1|	35352-37680	18	8.6	0.4463	0.971454	1	1	no
gi|646745631|gb|KK962343.1|	3647-5958	18	8.6	53	62	0.208703	0.85585	no
gi|646745631|gb|KK962343.1|	18016-41877	18	8.6	14	0	-inf	0.00035	yes
gi|646745631|gb|KK962343.1|	26671-42754	18	8.6	4	0	-inf	0.0042	no
gi|646745631|gb|KK962343.1|	40352-44182	18	8.6	3	0	-inf	0.00135	yes
gi|646745631|gb|KK962343.1|	49342-51670	18	8.6	3	3	0.105452	0.94445	no
gi|646745631|gb|KK962343.1|	52720-53075	18	8.6	10	7	-0.457828	0.7391	no
gi|646745631|gb|KK962343.1|	6095-6581	18	8.6	148	72	-1	0.4259	no
gi|646745631|gb|KK962343.1|	62768-66228	18	8.6	3	3	-0.171666	0.9136	no
gi|646745631|gb|KK962343.1|	66318-68376	18	8.6	4	4	0.0148737	0.9898	no
gi|646745631|gb|KK962343.1|	79742-82280	18	8.6	1	1	-0.0463868	0.9765	no

gi|646745644|gb|KK962342.1|	35939-13637	31	14	3	4	0.394795	0.7964	no
gi|646745644|gb|KK962342.1|	16554-18617	31	14	0.102342	1	4	0.25525	no
gi|646745644|gb|KK962342.1|	75884-27644	31	14	2	2	-0.226254	0.87465	no
gi|646745644|gb|KK962342.1|	31803-32705	31	14	1	4	2	0.3388	no
gi|646745644|gb|KK962342.1|	18767-42022	31	14	0.076774	7	7	0.09485	no
gi|646745644|gb|KK962342.1|	54885-45562	31	14	0	12	inf	0.0002	yes
gi|646745644|gb|KK962342.1|	58023-45820	31	14	15.97	217	4	0.12215	no
gi|646745644|gb|KK962342.1|	63447-46522	31	14	0.060586	12	8	0.09475	no
gi|646745644|gb|KK962342.1|	82522-48286	31	14	10	11	0.169357	0.9034	no
gi|646745644|gb|KK962342.1|	83829-48436	31	14	11	9	-0.251139	0.8695	no
gi|646745644|gb|KK962342.1|	86430-48796	31	14	0.651501	16	5	0.01685	no
gi|646745644|gb|KK962342.1|	10112-51067	31	14	7	20	2	0.3361	no
gi|646745644|gb|KK962342.1|	52053-52656	31	14	7	3	-1	0.3644	no
gi|646745644|gb|KK962342.1|	30479-53143	31	14	5	5	-0.0579793	0.9696	no
gi|646745644|gb|KK962342.1|	32423-53345	31	14	3	3	-0.0731215	0.96285	no
gi|646745644|gb|KK962342.1|	35478-53671	31	14	3	4	0.520218	0.7408	no
gi|646745644|gb|KK962342.1|	5876-10225	31	14	5	11	1	0.34815	no
gi|646745644|gb|KK962342.1|	69730-70044	31	14	0	26	inf	0.00435	no
gi|646745644|gb|KK962342.1|	83559-84303	31	14	0	7	inf	0.0017	no
gi|646745644|gb|KK962342.1|	89664-90022	31	14	0	34	inf	0.0007	yes
gi|646745659|gb|KK962341.1|	42084-24237	18	7.1	19	9	-1	0.45155	no
gi|646745659|gb|KK962341.1|	66475-46672	18	7.1	17	12	-0.475867	0.76265	no
gi|646745675|gb|KK962340.1|	16194-31723	14	5.9	0.824887	0.840397	0.0268742	1	no
gi|646745689|gb|KK962339.1|	52961-15330	14	7.2	12	1	-3	0.18235	no
gi|646745689|gb|KK962339.1|	95766-19649	14	7.2	3	4	0.306386	0.8266	no
gi|646745689|gb|KK962339.1|	01039-50253	14	7.2	2	1	-0.280339	0.81125	no
gi|646745689|gb|KK962339.1|	80704-68118	14	7.2	4	9	1	0.38665	no
gi|646745689|gb|KK962339.1|	83025-68330	14	7.2	19	40	1	0.46175	no
gi|646745702|gb|KK962338.1|	09626-11061	13	7.6	3	1	-1	0.3582	no
gi|646745702|gb|KK962338.1|	92232-92880	13	7.6	5	5	0.151102	0.91235	no

gi|646745732|gb|KK962336.1|	35386-23701	14	8.8	1	0.876529	-0.548641	0.6616	no
gi|646745732|gb|KK962336.1|	37072-23761	14	8.8	6	4	-0.402274	0.75295	no
gi|646745732|gb|KK962336.1|	44011-24570	14	8.8	2	0.927334	-0.791449	0.5842	no
gi|646745732|gb|KK962336.1|	40330-34078	14	8.8	3	4	0.405548	0.7964	no
gi|646745732|gb|KK962336.1|	91398-39191	14	8.8	3	4	0.438151	0.7507	no
gi|646745732|gb|KK962336.1|	95055-39580	14	8.8	0.372188	2	3	0.2662	no
gi|646745732|gb|KK962336.1|	84660-58542	14	8.8	0.726848	3	2	0.2413	no
gi|646745732|gb|KK962336.1|	80798-68169	14	8.8	4	5	0.558089	0.7241	no
gi|646745778|gb|KK962333.1|	93349-19448	17	7.7	3	3	0.253435	0.87805	no
gi|646745778|gb|KK962333.1|	26901-22790	17	7.7	21	33	0.666071	0.59315	no
gi|646745778|gb|KK962333.1|	28522-32895	17	7.7	184	466	1	0.23755	no
gi|646745778|gb|KK962333.1|	36663-33710	17	7.7	6	78	4	0.0305	no
gi|646745778|gb|KK962333.1|	42504-34365	17	7.7	0.513932	2	2	0.18085	no
gi|646745778|gb|KK962333.1|	44360-34495	17	7.7	3	12	2	0.2005	no
gi|646745778|gb|KK962333.1|	45178-35194	17	7.7	6	11	0.963538	0.3952	no
gi|646745792|gb|KK962332.1|	93445-19453	12	5.9	61	15	-2	0.13825	no
gi|646745792|gb|KK962332.1|	44860-46091	12	5.9	5	3	-0.479913	0.7573	no
gi|646745792|gb|KK962332.1|	75838-47636	12	5.9	2	3	0.664271	0.65435	no
gi|646745792|gb|KK962332.1|	51591-57600	12	5.9	3	5	0.797307	0.53035	no
gi|646745792|gb|KK962332.1|	61062-62626	12	5.9	0.777424	0.713492	-0.123804	1	no
gi|646745792|gb|KK962332.1|	66482-76709	12	5.9	6	9	0.52332	0.741	no
gi|646745792|gb|KK962332.1|	95520-79639	12	5.9	0.750808	1	0.764492	0.61475	no
gi|646745792|gb|KK962332.1|	09379-81145	12	5.9	2	2	-0.112615	0.9431	no
gi|646745809|gb|KK962331.1|	35217-14476	21	9	5	5	-0.0883321	0.93645	no
gi|646745809|gb|KK962331.1|	76465-17714	21	9	31	19	-0.727312	0.63365	no
gi|646745809|gb|KK962331.1|	92859-20108	21	9	14	12	-0.280235	0.8087	no
gi|646745809|gb|KK962331.1|	02047-20240	21	9	8	5	-0.530725	0.7233	no
gi|646745809|gb|KK962331.1|	16308-21687	21	9	1	6	2	0.2149	no
gi|646745809|gb|KK962331.1|	48090-24916	21	9	0	20	inf	0.0001	yes
gi|646745809|gb|KK962331.1|	62856-36328	21	9	0	15	inf	0.0026	no

gi|646745809|gb|KK962331.1|	93469-39983	21	9	10	8	-0.345172	0.7586	no
gi|646745809|gb|KK962331.1|	82551-48503	21	9	0.455757	1	1	1	no
gi|646745809|gb|KK962331.1|	96692-49803	21	9	0.847947	0.853194	0.00889967	1	no
gi|646745824|gb|KK962330.1|	66941-26744	11	6.8	4	2	-0.704968	0.628	no
gi|646745824|gb|KK962330.1|	67661-26857	11	6.8	0.698651	1	0.767983	0.6143	no
gi|646745851|gb|KK962328.1|	21252-12206	15	6.6	7	3	-1	0.39675	no
gi|646745851|gb|KK962328.1|	55769-35660	15	6.6	5	0	-inf	0.00225	no
gi|646745851|gb|KK962328.1|	36167-43692	15	6.6	6	4	-0.657024	0.63005	no
gi|646745851|gb|KK962328.1|	72051-67245	15	6.6	5	0.767971	-3	0.21305	no
gi|646745851|gb|KK962328.1|	84920-85766	15	6.6	1	3	1	0.41885	no
gi|646745867|gb|KK962327.1|	32435-13529	16	8.4	48	40	-0.270287	0.81645	no
gi|646745867|gb|KK962327.1|	14153-14446	16	8.4	8	12	0.706882	0.64395	no
gi|646745867|gb|KK962327.1|	44542-14621	16	8.4	9	46	2	0.0853	no
gi|646745867|gb|KK962327.1|	21250-21570	16	8.4	17	16	-0.0518688	0.95635	no
gi|646745867|gb|KK962327.1|	77305-77853	16	8.4	47	26	-0.859949	0.57005	no
gi|646745881|gb|KK962326.1|	48613-14907	16	8.1	3	2	-0.587048	0.6685	no
gi|646745881|gb|KK962326.1|	51584-15283	16	8.1	0.838455	1	0.350711	1	no
gi|646745881|gb|KK962326.1|	53050-15367	16	8.1	4	1	-2	0.25335	no
gi|646745881|gb|KK962326.1|	15821-16856	16	8.1	3	1	-1	0.3416	no
gi|646745881|gb|KK962326.1|	69816-17119	16	8.1	1	0.118687	-3	0.1791	no
gi|646745881|gb|KK962326.1|	77058-17835	16	8.1	0.355809	0.766415	1	1	no
gi|646745881|gb|KK962326.1|	79211-18019	16	8.1	0.89068	0.543264	-0.713253	1	no
gi|646745881|gb|KK962326.1|	87869-19003	16	8.1	0.970292	1	0.301373	0.8277	no
gi|646745881|gb|KK962326.1|	95564-19582	16	8.1	489	260	-0.910457	0.467	no
gi|646745881|gb|KK962326.1|	97395-19833	16	8.1	1	0.193206	-3	0.2033	no
gi|646745881|gb|KK962326.1|	18602-21884	16	8.1	13	37	2	0.34375	no
gi|646745881|gb|KK962326.1|	33410-23484	16	8.1	9	9	0.0481108	0.9742	no
gi|646745881|gb|KK962326.1|	37808-23905	16	8.1	0.560098	1	1	0.33975	no
gi|646745881|gb|KK962326.1|	39437-24008	16	8.1	8	3	-2	0.27995	no
gi|646745881|gb|KK962326.1|	40416-24157	16	8.1	2	0.878931	-1	0.3859	no

gi|646745881|gb|KK962326.1|	57020-25756	16	8.1	93	68	-0.462625	0.66445	no
gi|646745881|gb|KK962326.1|	59758-26481	16	8.1	4	9	1	0.2876	no
gi|646745881|gb|KK962326.1|	65495-26653	16	8.1	5	8	0.635056	0.6804	no
gi|646745881|gb|KK962326.1|	87751-28937	16	8.1	38	16	-1	0.2604	no
gi|646745881|gb|KK962326.1|	01620-30244	16	8.1	1	0.683783	-0.724695	0.66415	no
gi|646745881|gb|KK962326.1|	06686-30774	16	8.1	10	0.655274	-4	0.0583	no
gi|646745881|gb|KK962326.1|	07805-30922	16	8.1	6	0.456386	-4	0.05835	no
gi|646745881|gb|KK962326.1|	10380-31176	16	8.1	4	0.117454	-5	0.17155	no
gi|646745881|gb|KK962326.1|	19852-32066	16	8.1	231	114	-1	0.38345	no
gi|646745881|gb|KK962326.1|	20798-32285	16	8.1	2	0.745063	-1	0.31235	no
gi|646745881|gb|KK962326.1|	27295-33107	16	8.1	5	6	0.223292	0.8377	no
gi|646745881|gb|KK962326.1|	31313-33847	16	8.1	15	30	0.978545	0.41065	no
gi|646745881|gb|KK962326.1|	45424-34725	16	8.1	0.588521	5	3	0.06575	no
gi|646745881|gb|KK962326.1|	47357-34914	16	8.1	3	6	1	0.48785	no
gi|646745881|gb|KK962326.1|	34910-36232	16	8.1	0.867016	0.24914	-2	1	no
gi|646745881|gb|KK962326.1|	49339-35164	16	8.1	3	5	0.734974	0.623	no
gi|646745881|gb|KK962326.1|	56431-35958	16	8.1	22	28	0.370822	0.73875	no
gi|646745881|gb|KK962326.1|	60852-36147	16	8.1	7	9	0.41251	0.801	no
gi|646745881|gb|KK962326.1|	66314-36775	16	8.1	0.544647	6	4	0.05565	no
gi|646745881|gb|KK962326.1|	68541-36887	16	8.1	3	15	2	0.17815	no
gi|646745881|gb|KK962326.1|	69046-36964	16	8.1	5	8	0.531384	0.69865	no
gi|646745881|gb|KK962326.1|	71773-37208	16	8.1	91	77	-0.244141	0.87095	no
gi|646745881|gb|KK962326.1|	94777-39518	16	8.1	5	5	-0.0411328	0.9298	no
gi|646745881|gb|KK962326.1|	95420-39655	16	8.1	0.419535	1	1	1	no
gi|646745881|gb|KK962326.1|	96685-39799	16	8.1	0.881051	1	0.370347	0.78175	no
gi|646745881|gb|KK962326.1|	98178-40098	16	8.1	5	2	-0.991669	0.5127	no
gi|646745881|gb|KK962326.1|	01848-40305	16	8.1	3	5	0.997325	0.53345	no
gi|646745881|gb|KK962326.1|	19567-42027	16	8.1	4	2	-1	0.4043	no
gi|646745881|gb|KK962326.1|	26248-42731	16	8.1	17	16	-0.076381	0.95935	no
gi|646745881|gb|KK962326.1|	44746-45235	16	8.1	167	208	0.315834	0.77935	no

gi|646745881|gb|KK962326.1|	64439-46495	16	8.1	2	3	0.656986	0.6549	no
gi|646745881|gb|KK962326.1|	67106-46776	16	8.1	19	36	0.963441	0.5282	no
gi|646745881|gb|KK962326.1|	70923-47200	16	8.1	9	22	1	0.30935	no
gi|646745881|gb|KK962326.1|	76977-47727	16	8.1	66	5	-4	0.0935	no
gi|646745881|gb|KK962326.1|	93104-49617	16	8.1	6	7	0.121088	0.92195	no
gi|646745881|gb|KK962326.1|	97188-49864	16	8.1	4	11	1	0.38555	no
gi|646745881|gb|KK962326.1|	10561-51184	16	8.1	3	2	-0.529005	0.7035	no
gi|646745881|gb|KK962326.1|	11950-51428	16	8.1	1	2	0.888692	0.57125	no
gi|646745881|gb|KK962326.1|	25435-52555	16	8.1	2927.91	4644.96	0.665794	0.651	no
gi|646745881|gb|KK962326.1|	92538-93278	16	8.1	2	6	2	0.2702	no
gi|646745929|gb|KK962323.1|	08622-10927	20	8.8	0	9	inf	0.0012	yes
gi|646745929|gb|KK962323.1|	19066-20166	20	8.8	0.875802	0.469358	-0.899916	1	no
gi|646745929|gb|KK962323.1|	88347-28876	20	8.8	4	4	-0.104327	0.9229	no
gi|646745929|gb|KK962323.1|	89270-28977	20	8.8	4	3	-0.569587	0.7155	no
gi|646745929|gb|KK962323.1|	35882-33636	20	8.8	6	7	0.324092	0.785	no
gi|646745929|gb|KK962323.1|	35655-53654	20	8.8	4	3	-0.252237	0.87795	no
gi|646745941|gb|KK962322.1|	58068-15912	21	10	0.460227	2	2	0.2641	no
gi|646745941|gb|KK962322.1|	62086-26696	21	10	0.780526	0.759532	-0.0393352	1	no
gi|646745941|gb|KK962322.1|	67172-26876	21	10	0.969455	0.999015	0.0433323	1	no
gi|646745941|gb|KK962322.1|	69275-27020	21	10	1	1	0.0904699	0.9294	no
gi|646745941|gb|KK962322.1|	70387-27120	21	10	2	2	-0.0887106	0.94285	no
gi|646745941|gb|KK962322.1|	95708-29617	21	10	3	8	2	0.2977	no
gi|646745941|gb|KK962322.1|	09895-31834	21	10	9	11	0.250413	0.83245	no
gi|646745941|gb|KK962322.1|	24480-42473	21	10	18	42	1	0.4466	no
gi|646745941|gb|KK962322.1|	30997-43327	21	10	0.779387	2	1	0.41535	no
gi|646745941|gb|KK962322.1|	33423-43680	21	10	1	2	0.576266	0.70945	no
gi|646745941|gb|KK962322.1|	39635-44000	21	10	4	6	0.604799	0.669	no
gi|646745941|gb|KK962322.1|	45407-44811	21	10	3	3	-0.222328	0.88515	no
gi|646745941|gb|KK962322.1|	89177-49005	21	10	2	3	0.386465	0.75545	no
gi|646745941|gb|KK962322.1|	90960-49277	21	10	2	2	0.47554	0.7609	no

gi|646745941|gb|KK962322.1|	93134-49829	21	10	1	2	0.546025	0.7153	no
gi|646745941|gb|KK962322.1|	99950-50233	21	10	0.738755	0.820011	0.150548	1	no
gi|646745941|gb|KK962322.1|	03895-50486	21	10	0.905625	2	0.870992	0.5462	no
gi|646745941|gb|KK962322.1|	08934-51288	21	10	8	7	-0.085384	0.93575	no
gi|646745941|gb|KK962322.1|	28248-52915	21	10	0.425618	1	2	0.2897	no
gi|646745941|gb|KK962322.1|	29290-53003	21	10	7	2	-2	0.2385	no
gi|646745941|gb|KK962322.1|	31891-53281	21	10	7	10	0.478747	0.74585	no
gi|646745941|gb|KK962322.1|	32987-53654	21	10	2	2	0.0715709	0.9632	no
gi|646745941|gb|KK962322.1|	47065-54745	21	10	9	6	-0.536093	0.68715	no
gi|646745941|gb|KK962322.1|	52992-55319	21	10	88	112	0.353227	0.81695	no
gi|646745941|gb|KK962322.1|	53850-55531	21	10	0.762047	0.768315	0.0118179	1	no
gi|646745941|gb|KK962322.1|	60265-56196	21	10	2	1	-0.354029	0.79385	no
gi|646745941|gb|KK962322.1|	66409-56702	21	10	3	0.708621	-2	0.2673	no
gi|646745941|gb|KK962322.1|	68035-56871	21	10	10	8	-0.368443	0.8104	no
gi|646745941|gb|KK962322.1|	70002-57051	21	10	9	8	-0.0633123	0.9671	no
gi|646745941|gb|KK962322.1|	70943-57149	21	10	8	10	0.334799	0.8329	no
gi|646745941|gb|KK962322.1|	71821-57202	21	10	96	22	-2	0.20475	no
gi|646745941|gb|KK962322.1|	75067-57546	21	10	7	2	-2	0.29135	no
gi|646745941|gb|KK962322.1|	04620-60478	21	10	316	80	-2	0.2625	no
gi|646745941|gb|KK962322.1|	36056-63657	21	10	4	0.469719	-3	0.1921	no
gi|646745941|gb|KK962322.1|	64128-66560	21	10	3	1	-1	0.38275	no
gi|646745941|gb|KK962322.1|	67503-66781	21	10	7	9	0.260192	0.86695	no
gi|646745941|gb|KK962322.1|	75398-67581	21	10	19	6	-2	0.2608	no
gi|646745941|gb|KK962322.1|	77399-67922	21	10	2	0.340243	-2	0.2149	no
gi|646745941|gb|KK962322.1|	80169-68156	21	10	1	0.701688	-0.890376	0.50965	no
gi|646745941|gb|KK962322.1|	86664-69835	21	10	21	12	-0.884089	0.46085	no
gi|646745941|gb|KK962322.1|	05450-70850	21	10	16	34	1	0.3741	no
gi|646745941|gb|KK962322.1|	09596-71056	21	10	4	10	1	0.40015	no
gi|646745941|gb|KK962322.1|	23741-72397	21	10	7	35	2	0.3025	no
gi|646745941|gb|KK962322.1|	31461-73230	21	10	2	6	1	0.37065	no

gi|646745941|gb|KK962322.1|	32497-73463	21	10	12	6	-1	0.43435	no
gi|646745941|gb|KK962322.1|	41507-74295	21	10	0.467233	0.672702	0.525824	1	no
gi|646745941|gb|KK962322.1|	49302-74996	21	10	4	6	0.627135	0.65375	no
gi|646745941|gb|KK962322.1|	57759-75815	21	10	8	10	0.244354	0.84205	no
gi|646745941|gb|KK962322.1|	77258-77771	21	10	5	2	-1	0.3648	no
gi|646745941|gb|KK962322.1|	95336-79549	21	10	236.51	243	0.0408319	0.91565	no
gi|646745941|gb|KK962322.1|	96846-79801	21	10	2	2	0.337414	0.8088	no
gi|646745941|gb|KK962322.1|	02151-80258	21	10	7	8	0.172402	0.8827	no
gi|646745941|gb|KK962322.1|	14053-81596	21	10	22	10	-1	0.3547	no
gi|646745941|gb|KK962322.1|	26176-83566	21	10	5	8	0.56218	0.62025	no
gi|646745941|gb|KK962322.1|	45648-84714	21	10	0.597338	0.645503	0.111876	1	no
gi|646745941|gb|KK962322.1|	57807-85876	21	10	0.917167	2	1	0.31275	no
gi|646745941|gb|KK962322.1|	60375-86280	21	10	3	2	-0.564483	0.71645	no
gi|646745941|gb|KK962322.1|	67509-86898	21	10	0.90847	0.654322	-0.473439	1	no
gi|646745941|gb|KK962322.1|	90819-89468	21	10	12	7	-0.818879	0.4628	no
gi|646745941|gb|KK962322.1|	96339-89764	21	10	1	1	-0.0626983	1	no
gi|646745941|gb|KK962322.1|	98544-90033	21	10	0.901123	0.955431	0.0844277	1	no
gi|646745941|gb|KK962322.1|	00466-90313	21	10	0.690155	1	0.617327	1	no
gi|646745941|gb|KK962322.1|	18337-92280	21	10	44	32	-0.475257	0.6941	no
gi|646745959|gb|KK962321.1|	03883-10505	20	6.9	0.503857	3	3	0.13935	no
gi|646745959|gb|KK962321.1|	17273-12090	20	6.9	13	28	1	0.35195	no
gi|646745959|gb|KK962321.1|	21034-12144	20	6.9	22	376	4	0.01515	no
gi|646745959|gb|KK962321.1|	22062-12276	20	6.9	4	104	5	0.0016	no
gi|646745959|gb|KK962321.1|	24347-12526	20	6.9	0.41919	2	2	0.255	no
gi|646745959|gb|KK962321.1|	28519-12956	20	6.9	1	0.665721	-0.657224	1	no
gi|646745959|gb|KK962321.1|	47205-14760	20	6.9	4	18	2	0.20215	no
gi|646745959|gb|KK962321.1|	70325-17237	20	6.9	52	23	-1	0.2972	no
gi|646745959|gb|KK962321.1|	73259-17424	20	6.9	9	20	1	0.46525	no
gi|646745959|gb|KK962321.1|	74350-17525	20	6.9	4	17	2	0.20035	no
gi|646745959|gb|KK962321.1|	75852-17787	20	6.9	0.576591	0.987006	0.775511	1	no

gi|646745959|gb|KK962321.1|	78160-17935	20	6.9	0.788985	0.706512	-0.159285	1	no
gi|646745959|gb|KK962321.1|	79475-18059	20	6.9	0.214137	1	3	0.2655	no
gi|646745959|gb|KK962321.1|	91298-19247	20	6.9	77	108	0.495605	0.6682	no
gi|646745959|gb|KK962321.1|	92722-19539	20	6.9	9	18	0.905176	0.40875	no
gi|646745959|gb|KK962321.1|	95847-19615	20	6.9	32	270	3	0.04895	no
gi|646745959|gb|KK962321.1|	08743-21207	20	6.9	2	2	-0.194806	0.8912	no
gi|646745959|gb|KK962321.1|	12960-21579	20	6.9	2	10	2	0.12275	no
gi|646745959|gb|KK962321.1|	15924-21705	20	6.9	0.633612	1	0.738821	1	no
gi|646745959|gb|KK962321.1|	18304-21936	20	6.9	0.228409	1	3	0.2655	no
gi|646745959|gb|KK962321.1|	24931-22723	20	6.9	3	3	0.0656913	0.96585	no
gi|646745959|gb|KK962321.1|	67396-26825	20	6.9	2	0.870761	-0.821722	0.57835	no
gi|646745959|gb|KK962321.1|	73664-27432	20	6.9	3	3	-0.0542787	0.9503	no
gi|646745959|gb|KK962321.1|	86773-28747	20	6.9	2	1	-0.688252	0.6459	no
gi|646745959|gb|KK962321.1|	89888-29171	20	6.9	2	1	-0.637595	0.64585	no
gi|646745959|gb|KK962321.1|	02318-30552	20	6.9	1	0.411173	-2	0.2934	no
gi|646745959|gb|KK962321.1|	06407-30778	20	6.9	0.825257	0.118687	-3	1	no
gi|646745959|gb|KK962321.1|	08223-31037	20	6.9	1	0.282514	-2	0.2276	no
gi|646745959|gb|KK962321.1|	10516-31154	20	6.9	2	0.510562	-2	0.2463	no
gi|646745959|gb|KK962321.1|	19480-32547	20	6.9	1	1	-0.390297	0.7916	no
gi|646745959|gb|KK962321.1|	25524-32689	20	6.9	3	3	-0.161774	0.91505	no
gi|646745959|gb|KK962321.1|	30749-33113	20	6.9	3	5	0.620027	0.66895	no
gi|646745959|gb|KK962321.1|	39574-39836	20	6.9	14	10	-0.437111	0.7662	no
gi|646745959|gb|KK962321.1|	23635-42829	20	6.9	4	2	-0.888472	0.55815	no
gi|646745959|gb|KK962321.1|	91404-49198	20	6.9	4	2	-1	0.39475	no
gi|646745959|gb|KK962321.1|	00051-50248	20	6.9	76	25	-2	0.17605	no
gi|646745959|gb|KK962321.1|	04392-51116	20	6.9	6	4	-0.542344	0.62035	no
gi|646745959|gb|KK962321.1|	52233-52606	20	6.9	20	6	-2	0.2495	no
gi|646745959|gb|KK962321.1|	53527-65428	20	6.9	1	3	1	0.3075	no
gi|646745959|gb|KK962321.1|	66194-66758	20	6.9	2	2	-0.298509	0.82695	no
gi|646745974|gb|KK962320.1|	48020-14825	15	8.4	54	0	-inf	0.0011	yes

gi|646745974|gb|KK962320.1|	51350-15153	15	8.4	125	0	-inf	0.00475	no
gi|646745974|gb|KK962320.1|	65216-16564	15	8.4	6	0	-inf	0.0041	no
gi|646745974|gb|KK962320.1|	98620-19889	15	8.4	22	13	-0.813682	0.54625	no
gi|646745974|gb|KK962320.1|	99609-20043	15	8.4	5	4	-0.273199	0.86345	no
gi|646745974|gb|KK962320.1|	09578-21052	15	8.4	0.798583	1	0.729565	0.6352	no
gi|646745974|gb|KK962320.1|	21098-22169	15	8.4	63	20	-2	0.2117	no
gi|646745974|gb|KK962320.1|	22519-22472	15	8.4	0.899147	0.960797	0.0956734	1	no
gi|646745974|gb|KK962320.1|	30539-23143	15	8.4	28	13	-1	0.47335	no
gi|646745974|gb|KK962320.1|	59446-25985	15	8.4	4	5	0.373075	0.79925	no
gi|646745974|gb|KK962320.1|	62281-26309	15	8.4	1	2	0.688599	0.6295	no
gi|646745974|gb|KK962320.1|	72919-27330	15	8.4	4	4	0.092028	0.91495	no
gi|646745974|gb|KK962320.1|	79256-27981	15	8.4	2	3	0.0355504	0.9298	no
gi|646745974|gb|KK962320.1|	80190-28079	15	8.4	4	1	-1	0.3276	no
gi|646745974|gb|KK962320.1|	93866-49467	15	8.4	0.828824	1	0.759286	0.61475	no
gi|646745974|gb|KK962320.1|	42081-54260	15	8.4	9	0.926764	-3	0.1474	no
gi|646745974|gb|KK962320.1|	43995-54469	15	8.4	11	7	-0.628266	0.6756	no
gi|646745974|gb|KK962320.1|	11306-61294	15	8.4	1	2	0.529643	0.7073	no
gi|646745974|gb|KK962320.1|	64242-86493	15	8.4	0	3	inf	0.0014	yes
gi|646745997|gb|KK962319.1|	04158-10439	21	8.5	11	59	2	0.21065	no
gi|646745997|gb|KK962319.1|	05543-10584	21	8.5	7	59	3	0.1021	no
gi|646745997|gb|KK962319.1|	07001-10805	21	8.5	0.348201	1	2	0.25365	no
gi|646745997|gb|KK962319.1|	17079-11794	21	8.5	4	31	3	0.0589	no
gi|646745997|gb|KK962319.1|	25172-13041	21	8.5	0.482372	1	2	0.31635	no
gi|646745997|gb|KK962319.1|	1282-1431	21	8.5	871	576	-0.596965	0.61425	no
gi|646745997|gb|KK962319.1|	30851-13721	21	8.5	9	22	1	0.26755	no
gi|646745997|gb|KK962319.1|	70170-17108	21	8.5	0	14	inf	0.00015	yes
gi|646745997|gb|KK962319.1|	72178-17273	21	8.5	0	24	inf	0.0003	yes
gi|646745997|gb|KK962319.1|	90591-19168	21	8.5	0	3	inf	0.00435	no
gi|646745997|gb|KK962319.1|	96665-19732	21	8.5	0	5	inf	0.00515	no
gi|646745997|gb|KK962319.1|	98691-19942	21	8.5	0	9	inf	0.0007	yes

gi|646745997|gb|KK962319.1|	20560-21257	21	8.5	3	4	0.374758	0.75635	no
gi|646745997|gb|KK962319.1|	11151-21196	21	8.5	0.165173	11	6	0.0949	no
gi|646745997|gb|KK962319.1|	20941-22119	21	8.5	35	20	-0.77145	0.56995	no
gi|646745997|gb|KK962319.1|	22513-25018	21	8.5	9	10	0.15986	0.87875	no
gi|646745997|gb|KK962319.1|	28013-22902	21	8.5	0.121877	6	6	0.09655	no
gi|646745997|gb|KK962319.1|	34060-23429	21	8.5	0	39	inf	0.0014	yes
gi|646745997|gb|KK962319.1|	38905-24039	21	8.5	63	119	0.922959	0.4354	no
gi|646745997|gb|KK962319.1|	47688-25167	21	8.5	15	12	-0.34259	0.7608	no
gi|646745997|gb|KK962319.1|	26333-27994	21	8.5	48	31	-0.607968	0.59495	no
gi|646745997|gb|KK962319.1|	29750-31056	21	8.5	8	6	-0.462192	0.7583	no
gi|646745997|gb|KK962319.1|	33800-35714	21	8.5	13	7	-0.927616	0.469	no
gi|646745997|gb|KK962319.1|	40246-41470	21	8.5	2	1	-0.31022	0.8116	no
gi|646745997|gb|KK962319.1|	42263-42719	21	8.5	9	4	-1	0.347	no
gi|646745997|gb|KK962319.1|	44830-45552	21	8.5	2	0.83171	-1	0.4263	no
gi|646745997|gb|KK962319.1|	18379-51862	21	8.5	33	46	0.443918	0.72705	no
gi|646745997|gb|KK962319.1|	22268-52252	21	8.5	31	40	0.347077	0.78425	no
gi|646745997|gb|KK962319.1|	27010-52731	21	8.5	32	32	-0.0203766	0.9778	no
gi|646745997|gb|KK962319.1|	28132-52848	21	8.5	13	10	-0.424821	0.73385	no
gi|646745997|gb|KK962319.1|	30505-53086	21	8.5	5	15	2	0.32365	no
gi|646745997|gb|KK962319.1|	31713-53309	21	8.5	1	2	0.524343	0.7133	no
gi|646745997|gb|KK962319.1|	34675-53524	21	8.5	2	3	0.455062	0.7474	no
gi|646745997|gb|KK962319.1|	36384-53893	21	8.5	41	50	0.268245	0.81615	no
gi|646745997|gb|KK962319.1|	42008-54267	21	8.5	7	9	0.238648	0.8806	no
gi|646745997|gb|KK962319.1|	9477-9632	21	8.5	225	199	-0.172548	0.8948	no
gi|646746012|gb|KK962318.1|	21202-12135	11	6.3	896	0	-inf	0.0004	yes
gi|646746012|gb|KK962318.1|	21590-12173	11	6.3	659	0	-inf	0.00655	no
gi|646746012|gb|KK962318.1|	22830-12338	11	6.3	20	0	-inf	0.00035	yes
gi|646746012|gb|KK962318.1|	27685-12782	11	6.3	3245.47	83	-5	0.17705	no
gi|646746012|gb|KK962318.1|	29683-12989	11	6.3	91	0	-inf	0.00225	no
gi|646746012|gb|KK962318.1|	30727-13098	11	6.3	120	3	-5	0.1705	no

gi|646746012|gb|KK962318.1|	34063-13460	11	6.3	150.43	0.443907	-8	0.1665	no
gi|646746012|gb|KK962318.1|	45559-14608	11	6.3	52	1	-5	0.0766	no
gi|646746012|gb|KK962318.1|	74541-17467	11	6.3	2337.52	0	-inf	0.00475	no
gi|646746012|gb|KK962318.1|	85921-18610	11	6.3	874	11	-6	0.1674	no
gi|646746025|gb|KK962317.1|	128-1379	19	8.4	2	4	1	0.4342	no
gi|646746025|gb|KK962317.1|	07765-21209	19	8.4	211.65	325	0.616782	0.6117	no
gi|646746025|gb|KK962317.1|	40966-24204	19	8.4	0.336794	1	2	0.25365	no
gi|646746025|gb|KK962317.1|	64343-26497	19	8.4	5	6	0.234895	0.86155	no
gi|646746025|gb|KK962317.1|	77827-27845	19	8.4	0.248965	13	6	0.0976	no
gi|646746025|gb|KK962317.1|	11972-31212	19	8.4	611.3	160	-2	0.22455	no
gi|646746025|gb|KK962317.1|	43529-34446	19	8.4	1	1	0.0911505	0.9294	no
gi|646746025|gb|KK962317.1|	58438-35877	19	8.4	10	7	-0.574446	0.69605	no
gi|646746025|gb|KK962317.1|	60868-36172	19	8.4	2	2	-0.692697	0.60075	no
gi|646746025|gb|KK962317.1|	48929-44950	19	8.4	36	12	-2	0.2945	no
gi|646746025|gb|KK962317.1|	61344-46192	19	8.4	0	5	inf	0.0008	yes
gi|646746025|gb|KK962317.1|	62169-46305	19	8.4	0	3	inf	0.00515	no
gi|646746025|gb|KK962317.1|	64914-46712	19	8.4	0.283651	1	2	0.22635	no
gi|646746025|gb|KK962317.1|	67283-46923	19	8.4	0.436398	0.789722	0.855702	1	no
gi|646746025|gb|KK962317.1|	73168-47539	19	8.4	0.234936	2	3	0.11705	no
gi|646746025|gb|KK962317.1|	06450-50717	19	8.4	0.390215	3	3	0.24735	no
gi|646746025|gb|KK962317.1|	64818-56508	19	8.4	26	10	-1	0.3397	no
gi|646746025|gb|KK962317.1|	79688-58007	19	8.4	11	4	-1	0.3389	no
gi|646746025|gb|KK962317.1|	28044-62880	19	8.4	21	12	-0.805701	0.59625	no
gi|646746040|gb|KK962316.1|	07868-10893	19	6.9	0.799433	0.815156	0.0280993	1	no
gi|646746040|gb|KK962316.1|	24546-12560	19	6.9	0.693792	1	0.928083	0.53925	no
gi|646746040|gb|KK962316.1|	29946-13057	19	6.9	3	2	-0.423513	0.73935	no
gi|646746040|gb|KK962316.1|	31873-14041	19	6.9	13	10	-0.432179	0.70445	no
gi|646746040|gb|KK962316.1|	45325-14571	19	6.9	6	3	-0.811033	0.58385	no
gi|646746040|gb|KK962316.1|	47040-14729	19	6.9	82	19	-2	0.17455	no
gi|646746040|gb|KK962316.1|	47422-14793	19	6.9	10	0.97959	-3	0.1406	no

gi|646746040|gb|KK962316.1|	49828-15113	19	6.9	13	4	-2	0.27555	no
gi|646746040|gb|KK962316.1|	51248-15204	19	6.9	5	0.240022	-4	0.1819	no
gi|646746040|gb|KK962316.1|	54153-15453	19	6.9	74	5	-4	0.0337	no
gi|646746040|gb|KK962316.1|	63145-16570	19	6.9	1	0.116666	-4	0.13225	no
gi|646746040|gb|KK962316.1|	72877-17897	19	6.9	30	21	-0.535615	0.65415	no
gi|646746040|gb|KK962316.1|	80221-18311	19	6.9	1	1	-0.160006	0.92175	no
gi|646746040|gb|KK962316.1|	84562-18550	19	6.9	1	2	1	0.41615	no
gi|646746040|gb|KK962316.1|	85965-19120	19	6.9	2	3	0.50348	0.73975	no
gi|646746040|gb|KK962316.1|	94230-19445	19	6.9	63	29	-1	0.42285	no
gi|646746040|gb|KK962316.1|	38297-24163	19	6.9	2	0.65613	-2	0.28545	no
gi|646746040|gb|KK962316.1|	42850-24304	19	6.9	265	217.04	-0.286062	0.8573	no
gi|646746040|gb|KK962316.1|	43414-24536	19	6.9	0.763246	1	0.974358	0.50205	no
gi|646746040|gb|KK962316.1|	45902-24733	19	6.9	0.784666	1	0.904134	0.48345	no
gi|646746040|gb|KK962316.1|	48073-24855	19	6.9	30	73	1	0.4028	no
gi|646746040|gb|KK962316.1|	52246-25262	19	6.9	8	7	-0.114482	0.93605	no
gi|646746040|gb|KK962316.1|	52756-25318	19	6.9	5	5	0.0708296	0.9423	no
gi|646746040|gb|KK962316.1|	25511-32567	19	6.9	491	451	-0.121307	0.9273	no
gi|646746040|gb|KK962316.1|	94696-39493	19	6.9	17	25	0.560648	0.72965	no
gi|646746040|gb|KK962316.1|	22194-42253	19	6.9	7	8	0.301412	0.8386	no
gi|646746040|gb|KK962316.1|	10691-51181	19	6.9	1	4	1	0.3294	no
gi|646746040|gb|KK962316.1|	15963-51662	19	6.9	3	11	2	0.21665	no
gi|646746040|gb|KK962316.1|	17355-52118	19	6.9	51	53	0.0567191	0.96195	no
gi|646746040|gb|KK962316.1|	40223-54394	19	6.9	17	14	-0.271898	0.8038	no
gi|646746040|gb|KK962316.1|	53121-55419	19	6.9	0.681862	0.973509	0.513715	1	no
gi|646746040|gb|KK962316.1|	55846-55646	19	6.9	2	2	-0.21089	0.89255	no
gi|646746040|gb|KK962316.1|	59189-56044	19	6.9	1	1	0.0353358	0.95325	no
gi|646746040|gb|KK962316.1|	71551-72038	19	6.9	16	6	-1	0.3118	no
gi|646746040|gb|KK962316.1|	77329-77855	19	6.9	99	70	-0.487442	0.6487	no
gi|646746040|gb|KK962316.1|	81018-81571	19	6.9	30	12	-1	0.38605	no
gi|646746040|gb|KK962316.1|	82420-83143	19	6.9	0.988019	2	1	0.4427	no

gi|646746074|gb|KK962314.1|	38629-33921	17	7	4	2	-1	0.4235	no
gi|646746074|gb|KK962314.1|	40486-34168	17	7	6	6	-0.167808	0.90945	no
gi|646746074|gb|KK962314.1|	57923-35885	17	7	9	14	0.613036	0.6848	no
gi|646746074|gb|KK962314.1|	83256-38605	17	7	7	4	-0.919449	0.5458	no
gi|646746074|gb|KK962314.1|	89338-39115	17	7	4	4	0.00429556	0.99025	no
gi|646746074|gb|KK962314.1|	68360-46865	17	7	98	49	-0.98403	0.51925	no
gi|646746074|gb|KK962314.1|	75129-47537	17	7	435	363	-0.258739	0.8645	no
gi|646746074|gb|KK962314.1|	15020-51531	17	7	11	20	0.818254	0.53805	no
gi|646746074|gb|KK962314.1|	16292-51788	17	7	0.277195	0.799801	2	1	no
gi|646746074|gb|KK962314.1|	19847-52954	17	7	34	35	0.0389091	0.9742	no
gi|646746074|gb|KK962314.1|	29669-53413	17	7	67	56	-0.262761	0.8242	no
gi|646746074|gb|KK962314.1|	53256-55352	17	7	158	45	-2	0.2556	no
gi|646746074|gb|KK962314.1|	64365-56463	17	7	21	16	-0.348022	0.79725	no
gi|646746074|gb|KK962314.1|	67597-56857	17	7	83	18	-2	0.11205	no
gi|646746074|gb|KK962314.1|	71414-57409	17	7	3	7	1	0.4862	no
gi|646746074|gb|KK962314.1|	01797-60199	17	7	279.07	23	-4	0.0993	no
gi|646746074|gb|KK962314.1|	84554-78523	17	7	4	2	-0.96221	0.46765	no
gi|646746087|gb|KK962313.1|	11861-31286	20	10	1	2	0.510616	0.70715	no
gi|646746087|gb|KK962313.1|	22552-32302	20	10	3	3	0.194801	0.88975	no
gi|646746087|gb|KK962313.1|	27333-32895	20	10	2	2	-0.0557023	0.9719	no
gi|646746087|gb|KK962313.1|	44962-34533	20	10	7	6	-0.0218832	0.9365	no
gi|646746101|gb|KK962312.1|	88008-18896	13	6.3	2	1	-0.192634	0.88995	no
gi|646746101|gb|KK962312.1|	72163-72760	13	6.3	25	26	0.0569099	0.9704	no
gi|646746101|gb|KK962312.1|	73596-74726	13	6.3	13	9	-0.483612	0.74865	no
gi|646746115|gb|KK962311.1|	18072-31889	19	6.7	8	14	0.842517	0.58235	no
gi|646746115|gb|KK962311.1|	20464-32164	19	6.7	0.199371	2	3	0.2438	no
gi|646746115|gb|KK962311.1|	23298-32449	19	6.7	0.097789	3	5	0.10595	no
gi|646746115|gb|KK962311.1|	34885-33528	19	6.7	11	33	2	0.249	no
gi|646746115|gb|KK962311.1|	52474-35279	19	6.7	9	3	-2	0.30365	no
gi|646746115|gb|KK962311.1|	57457-35794	19	6.7	4	0.523273	-3	0.20305	no

gi|646746115|gb|KK962311.1|	64604-36796	19	6.7	46	28	-0.702756	0.55105	no
gi|646746115|gb|KK962311.1|	68370-37152	19	6.7	0.318925	0.742726	1	1	no
gi|646746115|gb|KK962311.1|	92916-39392	19	6.7	7	6	-0.327854	0.82735	no
gi|646746115|gb|KK962311.1|	98545-39874	19	6.7	74	23	-2	0.3027	no
gi|646746115|gb|KK962311.1|	06791-40705	19	6.7	15	12	-0.329388	0.82015	no
gi|646746115|gb|KK962311.1|	15760-41602	19	6.7	18	8	-1	0.4401	no
gi|646746115|gb|KK962311.1|	04587-50515	19	6.7	9	42	2	0.1671	no
gi|646746128|gb|KK962310.1|	94743-96230	13	7.1	0.150183	13	6	0.23275	no
gi|646746144|gb|KK962309.1|	50110-45052	12	7.8	7	6	-0.323277	0.7969	no
gi|646746158|gb|KK962308.1|	06276-10677	19	6.6	1	6	2	0.15685	no
gi|646746158|gb|KK962308.1|	43194-14371	19	6.6	7	10	0.582925	0.6811	no
gi|646746158|gb|KK962308.1|	49039-14983	19	6.6	1	1	-0.183136	0.90405	no
gi|646746158|gb|KK962308.1|	49944-15089	19	6.6	0.79288	2	1	0.4476	no
gi|646746158|gb|KK962308.1|	55515-16008	19	6.6	8	7	-0.0796766	0.94185	no
gi|646746158|gb|KK962308.1|	66185-16713	19	6.6	1	0.75772	-0.81482	0.57835	no
gi|646746158|gb|KK962308.1|	16873-18065	19	6.6	0.59237	401	9	0.0463	no
gi|646746158|gb|KK962308.1|	70149-17271	19	6.6	14	13	-0.138545	0.8996	no
gi|646746158|gb|KK962308.1|	19575-20720	19	6.6	0	2	inf	0.001	yes
gi|646746158|gb|KK962308.1|	96528-19718	19	6.6	1	4	1	0.34755	no
gi|646746158|gb|KK962308.1|	12818-21400	19	6.6	0.99258	1	0.196645	0.88295	no
gi|646746158|gb|KK962308.1|	17835-21850	19	6.6	2	4	0.891994	0.50445	no
gi|646746158|gb|KK962308.1|	18974-21988	19	6.6	2	3	0.745744	0.5522	no
gi|646746158|gb|KK962308.1|	35603-23692	19	6.6	0.52021	1	1	0.4444	no
gi|646746158|gb|KK962308.1|	24079-24568	19	6.6	0	8	inf	0.00515	no
gi|646746158|gb|KK962308.1|	72946-27394	19	6.6	0.993332	0.884202	-0.167899	1	no
gi|646746158|gb|KK962308.1|	36875-37898	19	6.6	11	0.515083	-4	0.0803	no
gi|646746158|gb|KK962308.1|	14980-41669	19	6.6	0.885914	0.182843	-2	1	no
gi|646746158|gb|KK962308.1|	41817-45373	19	6.6	0.952957	604	9	0.0036	no
gi|646746158|gb|KK962308.1|	47100-44741	19	6.6	11	12	0.088579	0.94055	no
gi|646746158|gb|KK962308.1|	54080-45439	19	6.6	16.59	9	-0.958864	0.48655	no

gi|646746158|gb|KK962308.1|	58025-45829	19	6.6	30	50	0.726694	0.61145	no
gi|646746158|gb|KK962308.1|	62151-46264	19	6.6	4	0.525264	-3	0.20305	no
gi|646746158|gb|KK962308.1|	16499-51673	19	6.6	28	26	-0.124638	0.9266	no
gi|646746158|gb|KK962308.1|	25608-52584	19	6.6	72	106	0.560083	0.6787	no
gi|646746158|gb|KK962308.1|	29057-52957	19	6.6	21	17	-0.260061	0.8653	no
gi|646746158|gb|KK962308.1|	40072-54029	19	6.6	44	67	0.610411	0.68605	no
gi|646746158|gb|KK962308.1|	49388-55008	19	6.6	2	1	-0.196717	0.89315	no
gi|646746158|gb|KK962308.1|	50375-55083	19	6.6	3	2	-0.400624	0.8098	no
gi|646746158|gb|KK962308.1|	55928-56095	19	6.6	0	264	inf	0.0008	yes
gi|646746158|gb|KK962308.1|	56209-56973	19	6.6	0	3	inf	0.0014	yes
gi|646746158|gb|KK962308.1|	6-364	19	6.6	25	3	-3	0.1373	no
gi|646746158|gb|KK962308.1|	67348-67953	19	6.6	8	7	-0.21436	0.89495	no
gi|646746158|gb|KK962308.1|	79515-83420	19	6.6	7	5	-0.445322	0.7216	no
gi|646746171|gb|KK962307.1|	22910-22417	15	11	2	2	0.309786	0.84905	no
gi|646746171|gb|KK962307.1|	51331-45201	15	11	5	7	0.415424	0.78735	no
gi|646746171|gb|KK962307.1|	64123-46471	15	11	67	2	-5	0.0442	no
gi|646746171|gb|KK962307.1|	02424-50287	15	11	6	2	-2	0.2782	no
gi|646746171|gb|KK962307.1|	03674-50418	15	11	10	0.983088	-3	0.1406	no
gi|646746171|gb|KK962307.1|	16440-51754	15	11	63	1	-6	0.00855	no
gi|646746189|gb|KK962306.1|	00714-10145	16	8.8	3	0.800699	-2	0.19015	no
gi|646746189|gb|KK962306.1|	04198-10938	16	8.8	4	3	-0.686607	0.5836	no
gi|646746189|gb|KK962306.1|	10340-12074	16	8.8	12	6	-1	0.34985	no
gi|646746189|gb|KK962306.1|	20861-12125	16	8.8	19	23	0.267299	0.85965	no
gi|646746189|gb|KK962306.1|	21872-12243	16	8.8	2	2	-0.355842	0.81835	no
gi|646746189|gb|KK962306.1|	22770-12449	16	8.8	0.630639	0.820031	0.378864	1	no
gi|646746189|gb|KK962306.1|	25504-12704	16	8.8	1	1	0.305393	0.7979	no
gi|646746189|gb|KK962306.1|	29464-13285	16	8.8	2	2	-0.255153	0.86365	no
gi|646746189|gb|KK962306.1|	22122-42321	16	8.8	2	0	-inf	0.00525	no
gi|646746189|gb|KK962306.1|	51911-53655	16	8.8	0.994116	0.269365	-2	1	no
gi|646746189|gb|KK962306.1|	85707-87237	16	8.8	0.943271	0.313796	-2	1	no

gi|646746189|gb|KK962306.1|	90177-90800	16	8.8	2	0.347111	-3	0.2136	no
gi|646746202|gb|KK962305.1|	32023-32317	21	6.4	12	11	-0.191562	0.90165	no
gi|646746202|gb|KK962305.1|	82804-38346	21	6.4	24	21	-0.173399	0.90815	no
gi|646746202|gb|KK962305.1|	86623-38687	21	6.4	25	6	-2	0.22445	no
gi|646746202|gb|KK962305.1|	95704-39619	21	6.4	44	23.37	-0.927676	0.54215	no
gi|646746202|gb|KK962305.1|	97074-39996	21	6.4	7	8	0.151226	0.88335	no
gi|646746202|gb|KK962305.1|	07521-40824	21	6.4	2	2	0.333567	0.7982	no
gi|646746202|gb|KK962305.1|	26506-42692	21	6.4	2	6	2	0.2744	no
gi|646746202|gb|KK962305.1|	42276-44270	21	6.4	8	3	-1	0.39915	no
gi|646746202|gb|KK962305.1|	57991-45837	21	6.4	37	24	-0.645914	0.6788	no
gi|646746202|gb|KK962305.1|	76312-78811	21	6.4	16	12	-0.398688	0.7218	no
gi|646746202|gb|KK962305.1|	79176-84551	21	6.4	7	5	-0.357734	0.7407	no
gi|646746202|gb|KK962305.1|	84740-87979	21	6.4	0.806143	0.36111	-1	1	no
gi|646746216|gb|KK962304.1|	13420-14157	130	33	1	1	0.224463	0.87595	no
gi|646746216|gb|KK962304.1|	36558-13841	130	33	0.634855	0.333963	-0.926739	1	no
gi|646746216|gb|KK962304.1|	38614-13952	130	33	2	1	-0.273087	0.83355	no
gi|646746216|gb|KK962304.1|	140-2383	130	33	4	2	-1	0.47085	no
gi|646746216|gb|KK962304.1|	42333-14744	130	33	2	0.974013	-0.86392	0.56595	no
gi|646746216|gb|KK962304.1|	87604-18845	130	33	1	0.437486	-2	0.2991	no
gi|646746216|gb|KK962304.1|	01255-20187	130	33	2	4	0.981307	0.5187	no
gi|646746216|gb|KK962304.1|	23285-24601	130	33	0.871918	2	1	0.41785	no
gi|646746216|gb|KK962304.1|	54135-25734	130	33	1	0.820412	-0.497789	0.7574	no
gi|646746216|gb|KK962304.1|	25851-26873	130	33	2	0.343824	-2	0.1971	no
gi|646746216|gb|KK962304.1|	58794-25998	130	33	1	0.564622	-1	0.41495	no
gi|646746216|gb|KK962304.1|	27244-28039	130	33	2	0.723906	-1	0.427	no
gi|646746216|gb|KK962304.1|	28129-29194	130	33	2	3	0.683451	0.6338	no
gi|646746216|gb|KK962304.1|	86428-28739	130	33	5	2	-1	0.38245	no
gi|646746216|gb|KK962304.1|	29899-30719	130	33	2	3	0.496936	0.69895	no
gi|646746216|gb|KK962304.1|	10670-31186	130	33	2	2	0.255859	0.8611	no
gi|646746216|gb|KK962304.1|	12262-31331	130	33	2	2	-0.072015	0.9531	no

gi|646746216|gb|KK962304.1|	17497-31856	130	33	4	2	-0.848808	0.53525	no
gi|646746216|gb|KK962304.1|	28065-33095	130	33	4	2	-0.972389	0.5225	no
gi|646746216|gb|KK962304.1|	66724-36785	130	33	2	0.606919	-1	0.31375	no
gi|646746216|gb|KK962304.1|	75326-37748	130	33	12	6	-1	0.39565	no
gi|646746216|gb|KK962304.1|	82085-38284	130	33	2	0.770444	-1	0.427	no
gi|646746216|gb|KK962304.1|	01953-40294	130	33	2	2	-0.568571	0.65795	no
gi|646746216|gb|KK962304.1|	14289-41491	130	33	2	1	-0.946972	0.589	no
gi|646746216|gb|KK962304.1|	32476-43321	130	33	4	3	-0.133991	0.91105	no
gi|646746216|gb|KK962304.1|	43268-44908	130	33	4	2	-1	0.4868	no
gi|646746216|gb|KK962304.1|	34592-43515	130	33	2	0.814019	-2	0.3832	no
gi|646746216|gb|KK962304.1|	47051-44886	130	33	6	4	-0.423061	0.77905	no
gi|646746216|gb|KK962304.1|	50789-45186	130	33	2	2	-0.0711066	0.9548	no
gi|646746216|gb|KK962304.1|	55857-45717	130	33	0.871918	1	0.200731	1	no
gi|646746216|gb|KK962304.1|	58298-45892	130	33	3	2	-0.532374	0.71825	no
gi|646746216|gb|KK962304.1|	61023-46248	130	33	0.767101	0.55237	-0.473781	1	no
gi|646746216|gb|KK962304.1|	75893-47656	130	33	2	0	-inf	0.00655	no
gi|646746216|gb|KK962304.1|	80680-48112	130	33	4	1	-2	0.2882	no
gi|646746216|gb|KK962304.1|	81450-48301	130	33	1	1	0.0808321	1	no
gi|646746216|gb|KK962304.1|	00605-50139	130	33	2	2	-0.0205876	0.9367	no
gi|646746216|gb|KK962304.1|	09964-51102	130	33	3	3	-0.124143	0.9397	no
gi|646746216|gb|KK962304.1|	28358-52991	130	33	1	1	-0.542265	0.6602	no
gi|646746216|gb|KK962304.1|	48572-54903	130	33	4	0.580347	-3	0.21305	no
gi|646746216|gb|KK962304.1|	5502-6385	130	33	3	2	-0.819511	0.51525	no
gi|646746216|gb|KK962304.1|	51289-55232	130	33	2	0.674825	-1	0.37415	no
gi|646746216|gb|KK962304.1|	54095-55536	130	33	0.63989	0.524774	-0.286126	1	no
gi|646746216|gb|KK962304.1|	80483-58115	130	33	1	1	-0.107828	0.9034	no
gi|646746216|gb|KK962304.1|	88596-58905	130	33	5	4	-0.589257	0.6453	no
gi|646746216|gb|KK962304.1|	02840-60339	130	33	3	0.833834	-2	0.2673	no
gi|646746216|gb|KK962304.1|	15530-61670	130	33	2	1	-0.674473	0.6172	no
gi|646746216|gb|KK962304.1|	43697-64440	130	33	2	0.849009	-0.931063	0.58905	no

gi|646746216|gb|KK962304.1|	46736-64845	130	33	24	13	-0.953139	0.37915	no
gi|646746216|gb|KK962304.1|	48825-65027	130	33	20	15	-0.4303	0.72915	no
gi|646746216|gb|KK962304.1|	54711-65533	130	33	2	1	-0.75346	0.6333	no
gi|646746216|gb|KK962304.1|	56414-65742	130	33	0.977589	0.870589	-0.167238	1	no
gi|646746216|gb|KK962304.1|	68460-66951	130	33	6	6	0.175961	0.9118	no
gi|646746216|gb|KK962304.1|	86783-68972	130	33	6	5	-0.22986	0.8787	no
gi|646746216|gb|KK962304.1|	02365-70295	130	33	3	1	-1	0.3848	no
gi|646746216|gb|KK962304.1|	70479-71425	130	33	1	0.760906	-0.952518	0.54505	no
gi|646746216|gb|KK962304.1|	05050-70601	130	33	0.910719	0.740156	-0.299177	1	no
gi|646746216|gb|KK962304.1|	10190-71177	130	33	252	109	-1	0.3189	no
gi|646746216|gb|KK962304.1|	12617-71365	130	33	2	2	-0.48775	0.69515	no
gi|646746216|gb|KK962304.1|	21156-72238	130	33	5	2	-1	0.36785	no
gi|646746216|gb|KK962304.1|	24253-72607	130	33	2	2	-0.342229	0.8297	no
gi|646746216|gb|KK962304.1|	72891-74095	130	33	1	0.558809	-1	0.41495	no
gi|646746216|gb|KK962304.1|	44403-74587	130	33	4	2	-0.94754	0.5483	no
gi|646746216|gb|KK962304.1|	49364-75004	130	33	1	1	-0.1066	0.9035	no
gi|646746216|gb|KK962304.1|	52404-75284	130	33	8	4	-0.794551	0.55085	no
gi|646746216|gb|KK962304.1|	61055-76214	130	33	2	3	0.601962	0.6722	no
gi|646746216|gb|KK962304.1|	82238-78321	130	33	1	0.36412	-1	1	no
gi|646746216|gb|KK962304.1|	96441-79868	130	33	1	0.674012	-0.948888	0.5142	no
gi|646746216|gb|KK962304.1|	18923-81983	130	33	2	2	-0.232159	0.8616	no
gi|646746216|gb|KK962304.1|	82078-83557	130	33	0.604657	0.653272	0.111565	1	no
gi|646746216|gb|KK962304.1|	21633-82334	130	33	2	0.736332	-1	0.3534	no
gi|646746216|gb|KK962304.1|	36335-83709	130	33	6	6	0.121077	0.9378	no
gi|646746216|gb|KK962304.1|	46268-84898	130	33	306	142	-1	0.36085	no
gi|646746216|gb|KK962304.1|	84838-90491	130	33	4	3	-0.543019	0.67355	no
gi|646746216|gb|KK962304.1|	50295-85069	130	33	10	9	-0.260946	0.8321	no
gi|646746216|gb|KK962304.1|	54168-85692	130	33	2	0.913878	-0.927333	0.56555	no
gi|646746216|gb|KK962304.1|	60188-87056	130	33	4	3	-0.582598	0.5955	no
gi|646746216|gb|KK962304.1|	72382-87451	130	33	2	0.855108	-1	0.43625	no

gi|646746216|gb|KK962304.1|	75857-87659	130	33	3	1	-1	0.33	no
gi|646746216|gb|KK962304.1|	80559-88118	130	33	3	4	0.468284	0.70815	no
gi|646746216|gb|KK962304.1|	91500-89258	130	33	2	1	-0.315531	0.8115	no
gi|646746216|gb|KK962304.1|	00853-90193	130	33	1	0.639826	-0.807656	1	no
gi|646746216|gb|KK962304.1|	25702-92667	130	33	1	0.552855	-1	0.3882	no
gi|646746216|gb|KK962304.1|	93121-93961	130	33	1	0.446849	-2	0.38425	no
gi|646746216|gb|KK962304.1|	35109-93577	130	33	13	5	-1	0.3653	no
gi|646746216|gb|KK962304.1|	55351-95611	130	33	1	1	-0.0940463	1	no
gi|646746216|gb|KK962304.1|	59985-96138	130	33	0.727166	0.581234	-0.323166	1	no
gi|646746216|gb|KK962304.1|	98327-10051	130	33	0.478126	0.623966	0.384075	1	no
gi|646746216|gb|KK962304.1|	86412-98757	130	33	4	3	-0.152128	0.9197	no
gi|646746216|gb|KK962304.1|	88524-99017	130	33	2	0.958106	-0.955686	0.5125	no
gi|646746216|gb|KK962304.1|	92132-99324	130	33	7	5	-0.327721	0.83065	no
gi|646746216|gb|KK962304.1|	93477-99446	130	33	3	3	-0.0141887	0.9741	no
gi|646746216|gb|KK962304.1|	98603-99929	130	33	6	4	-0.412866	0.77145	no
gi|646746231|gb|KK962303.1|	06651-10726	21	6	2	2	0.465594	0.7494	no
gi|646746231|gb|KK962303.1|	20223-12133	21	6	16	13	-0.301455	0.83705	no
gi|646746231|gb|KK962303.1|	35101-13558	21	6	0	36	inf	0.0001	yes
gi|646746231|gb|KK962303.1|	40052-14057	21	6	7	0.926764	-3	0.12305	no
gi|646746231|gb|KK962303.1|	49943-15058	21	6	2	4	1	0.41625	no
gi|646746231|gb|KK962303.1|	62440-16363	21	6	0.593003	1	1	0.3963	no
gi|646746231|gb|KK962303.1|	67196-16914	21	6	0.709983	1	0.533752	1	no
gi|646746231|gb|KK962303.1|	71141-17173	21	6	3	1	-1	0.4217	no
gi|646746231|gb|KK962303.1|	91171-19163	21	6	19	13	-0.491402	0.7489	no
gi|646746231|gb|KK962303.1|	95070-19544	21	6	7	10	0.4835	0.7179	no
gi|646746231|gb|KK962303.1|	09639-21216	21	6	6	0.709139	-3	0.0424	no
gi|646746231|gb|KK962303.1|	05726-30589	21	6	102	142	0.47609	0.7599	no
gi|646746231|gb|KK962303.1|	16447-31668	21	6	45	102	1	0.39905	no
gi|646746231|gb|KK962303.1|	17149-31748	21	6	4	14	2	0.24325	no
gi|646746231|gb|KK962303.1|	37056-33776	21	6	1	1	0.220189	0.89085	no

gi|646746231|gb|KK962303.1|	46041-35047	21	6	0.730786	0.872307	0.255386	1	no
gi|646746231|gb|KK962303.1|	50620-35185	21	6	0.850443	1	0.35026	1	no
gi|646746231|gb|KK962303.1|	52549-35354	21	6	2	1	-0.297351	0.8162	no
gi|646746231|gb|KK962303.1|	53723-35530	21	6	0.556473	0.602068	0.113615	1	no
gi|646746231|gb|KK962303.1|	56413-35757	21	6	1	3	1	0.4342	no
gi|646746231|gb|KK962303.1|	58355-35921	21	6	1	0.65307	-0.914832	0.58965	no
gi|646746231|gb|KK962303.1|	59346-36148	21	6	2	0.995536	-0.656992	0.63065	no
gi|646746231|gb|KK962303.1|	62677-36551	21	6	55	51	-0.10948	0.9278	no
gi|646746231|gb|KK962303.1|	83654-38442	21	6	11	4	-2	0.24425	no
gi|646746231|gb|KK962303.1|	86361-38790	21	6	34	23	-0.586521	0.5904	no
gi|646746231|gb|KK962303.1|	05609-40635	21	6	3	2	-0.418601	0.73725	no
gi|646746231|gb|KK962303.1|	15012-41518	21	6	161	45	-2	0.27165	no
gi|646746231|gb|KK962303.1|	39910-44013	21	6	38	58	0.593582	0.6368	no
gi|646746231|gb|KK962303.1|	49291-44962	21	6	14	10	-0.416389	0.7404	no
gi|646746231|gb|KK962303.1|	53815-45419	21	6	16	14	-0.233851	0.887	no
gi|646746231|gb|KK962303.1|	97473-97759	21	6	8	19	1	0.423	no
gi|646746243|gb|KK962302.1|	04257-20920	12	7.7	8	7	-0.0612587	0.957	no
gi|646746243|gb|KK962302.1|	18438-21883	12	7.7	6	3	-0.808238	0.58385	no
gi|646746243|gb|KK962302.1|	20033-22210	12	7.7	21	16	-0.344101	0.7491	no
gi|646746243|gb|KK962302.1|	33687-23445	12	7.7	0.905008	3	2	0.2489	no
gi|646746243|gb|KK962302.1|	35128-23627	12	7.7	3	4	0.789889	0.615	no
gi|646746243|gb|KK962302.1|	69009-26927	12	7.7	15	29	0.936315	0.50625	no
gi|646746243|gb|KK962302.1|	72582-27362	12	7.7	2	3	0.33203	0.8104	no
gi|646746243|gb|KK962302.1|	91109-29144	12	7.7	16	11	-0.620326	0.6262	no
gi|646746243|gb|KK962302.1|	95663-95988	12	7.7	0	68	inf	0.00025	yes
gi|646746257|gb|KK962301.1|	57447-65815	13	7.3	1	2	0.898261	0.545	no
gi|646746257|gb|KK962301.1|	13366-71500	13	7.3	0.939229	1	0.62903	0.65845	no
gi|646746284|gb|KK962299.1|	16646-11704	16	6.7	9	11	0.263978	0.82545	no
gi|646746284|gb|KK962299.1|	06426-20771	16	6.7	117	207	0.821556	0.4925	no
gi|646746284|gb|KK962299.1|	08901-20997	16	6.7	0.2259	2	3	0.2578	no

gi|646746284|gb|KK962299.1|	12414-21286	16	6.7	3	16	3	0.15445	no
gi|646746284|gb|KK962299.1|	13351-21378	16	6.7	2	9	2	0.18795	no
gi|646746284|gb|KK962299.1|	16985-21811	16	6.7	1	8	3	0.10175	no
gi|646746284|gb|KK962299.1|	20988-22125	16	6.7	39	129	2	0.22005	no
gi|646746284|gb|KK962299.1|	22522-22274	16	6.7	13	168	4	0.1239	no
gi|646746284|gb|KK962299.1|	31160-23177	16	6.7	9	36	2	0.21385	no
gi|646746284|gb|KK962299.1|	33115-23825	16	6.7	0.302918	3	3	0.0397	no
gi|646746284|gb|KK962299.1|	38365-23957	16	6.7	0.097071	2	5	0.11005	no
gi|646746284|gb|KK962299.1|	39936-24172	16	6.7	0.240612	2	3	0.153	no
gi|646746284|gb|KK962299.1|	42465-24386	16	6.7	41	139	2	0.13745	no
gi|646746284|gb|KK962299.1|	45545-24762	16	6.7	2	7	2	0.19875	no
gi|646746284|gb|KK962299.1|	51105-25130	16	6.7	776.89	752	-0.0460337	0.97325	no
gi|646746284|gb|KK962299.1|	34778-35140	16	6.7	112	32	-2	0.2479	no
gi|646746284|gb|KK962299.1|	95332-39563	16	6.7	0	21	inf	0.0008	yes
gi|646746284|gb|KK962299.1|	02629-40328	16	6.7	0	9	inf	0.0012	yes
gi|646746284|gb|KK962299.1|	67733-46788	16	6.7	3632.8	2406.62	-0.594074	0.7013	no
gi|646746284|gb|KK962299.1|	83031-58334	16	6.7	60	101	0.752319	0.6281	no
gi|646746298|gb|KK962298.1|	26547-12698	18	8.3	75	57	-0.383561	0.79665	no
gi|646746298|gb|KK962298.1|	54412-15590	18	8.3	0	2	inf	0.00315	no
gi|646746298|gb|KK962298.1|	1576-1996	18	8.3	32	62	0.968356	0.5279	no
gi|646746298|gb|KK962298.1|	67800-16936	18	8.3	9	2	-2	0.1444	no
gi|646746298|gb|KK962298.1|	97927-19824	18	8.3	197	21	-3	0.06935	no
gi|646746298|gb|KK962298.1|	00066-20046	18	8.3	29	0.798103	-5	0.1715	no
gi|646746298|gb|KK962298.1|	21081-21453	18	8.3	3	10	2	0.26885	no
gi|646746298|gb|KK962298.1|	18404-21864	18	8.3	39	15	-1	0.36285	no
gi|646746298|gb|KK962298.1|	35199-23557	18	8.3	1	10	3	0.25645	no
gi|646746298|gb|KK962298.1|	31619-31875	18	8.3	190	85.9	-1	0.45955	no
gi|646746310|gb|KK962297.1|	15188-16356	23	11	3	8	1	0.3675	no
gi|646746310|gb|KK962297.1|	64311-16485	23	11	13	14	0.169857	0.91395	no
gi|646746310|gb|KK962297.1|	66797-16701	23	11	33	65	0.981866	0.5129	no

gi|646746310|gb|KK962297.1|	72660-17343	23	11	1	3	1	0.4088	no
gi|646746310|gb|KK962297.1|	18328-19233	23	11	1	34	5	0.01675	no
gi|646746310|gb|KK962297.1|	99784-20022	23	11	8	5	-0.797181	0.55065	no
gi|646746310|gb|KK962297.1|	02131-20294	23	11	7	15	1	0.51495	no
gi|646746310|gb|KK962297.1|	09660-21184	23	11	2	3	0.505654	0.7401	no
gi|646746310|gb|KK962297.1|	11954-21309	23	11	0.937577	1	0.15434	1	no
gi|646746310|gb|KK962297.1|	14292-21589	23	11	3	3	0.0733242	0.9621	no
gi|646746310|gb|KK962297.1|	20746-22371	23	11	2	2	0.210623	0.8887	no
gi|646746310|gb|KK962297.1|	33465-23759	23	11	1	1	-0.104242	0.94465	no
gi|646746310|gb|KK962297.1|	23591-28334	23	11	1	0.84244	-0.261935	1	no
gi|646746310|gb|KK962297.1|	37806-23924	23	11	3	3	-0.0217531	0.9838	no
gi|646746310|gb|KK962297.1|	41911-24602	23	11	14.33	7	-1	0.3121	no
gi|646746310|gb|KK962297.1|	63578-26383	23	11	7	36	2	0.17705	no
gi|646746310|gb|KK962297.1|	73927-27413	23	11	108	64	-0.747543	0.53965	no
gi|646746310|gb|KK962297.1|	82928-28319	23	11	10	14	0.43468	0.7629	no
gi|646746310|gb|KK962297.1|	88467-28873	23	11	17	26	0.623396	0.63695	no
gi|646746310|gb|KK962297.1|	91628-29214	23	11	4	3	-0.563804	0.69125	no
gi|646746310|gb|KK962297.1|	97649-30049	23	11	7	5	-0.61897	0.618	no
gi|646746310|gb|KK962297.1|	3-320	23	11	4	83	5	0.1104	no
gi|646746310|gb|KK962297.1|	00918-30170	23	11	13	9	-0.586949	0.6935	no
gi|646746310|gb|KK962297.1|	02625-30351	23	11	1	3	1	0.3462	no
gi|646746310|gb|KK962297.1|	03766-30500	23	11	1	0.403329	-2	0.2858	no
gi|646746310|gb|KK962297.1|	09620-31177	23	11	0.878792	1	0.365808	0.78985	no
gi|646746310|gb|KK962297.1|	21206-32252	23	11	6	2	-2	0.16775	no
gi|646746310|gb|KK962297.1|	23513-32407	23	11	4	2	-1	0.43665	no
gi|646746310|gb|KK962297.1|	32173-33245	23	11	71	17	-2	0.17585	no
gi|646746310|gb|KK962297.1|	36057-33654	23	11	32	49	0.639775	0.66995	no
gi|646746310|gb|KK962297.1|	39479-34063	23	11	1	2	0.89593	0.48395	no
gi|646746310|gb|KK962297.1|	43852-34462	23	11	11	9	-0.20523	0.8931	no
gi|646746310|gb|KK962297.1|	46210-34673	23	11	4	7	0.853954	0.55865	no

gi|646746310|gb|KK962297.1|	46860-34740	23	11	3	7	1	0.44965	no
gi|646746310|gb|KK962297.1|	58811-35996	23	11	28	42	0.574002	0.592	no
gi|646746310|gb|KK962297.1|	61148-36296	23	11	17	27	0.699867	0.51885	no
gi|646746310|gb|KK962297.1|	36598-37362	23	11	2	1	-0.357081	0.812	no
gi|646746310|gb|KK962297.1|	69333-37404	23	11	45	49	0.13166	0.91665	no
gi|646746310|gb|KK962297.1|	74767-37737	23	11	11	13	0.272866	0.79775	no
gi|646746310|gb|KK962297.1|	38007-39646	23	11	5	2	-0.935091	0.5371	no
gi|646746310|gb|KK962297.1|	84234-38607	23	11	24	11	-1	0.39965	no
gi|646746310|gb|KK962297.1|	401-853	23	11	2	4	1	0.45825	no
gi|646746310|gb|KK962297.1|	4057-4483	23	11	7	5	-0.612173	0.6308	no
gi|646746310|gb|KK962297.1|	77978-47891	23	11	0.679163	2	2	0.27275	no
gi|646746310|gb|KK962297.1|	80413-48093	23	11	4	1	-1	0.3715	no
gi|646746310|gb|KK962297.1|	82401-48298	23	11	1	3	1	0.43555	no
gi|646746310|gb|KK962297.1|	00623-50117	23	11	3	1	-0.967647	0.589	no
gi|646746310|gb|KK962297.1|	01385-50286	23	11	0.681353	0.545268	-0.321436	1	no
gi|646746310|gb|KK962297.1|	13477-51368	23	11	42	33	-0.330241	0.8193	no
gi|646746310|gb|KK962297.1|	32544-53376	23	11	3	0.965983	-2	0.23245	no
gi|646746310|gb|KK962297.1|	42632-54447	23	11	2	0.590613	-2	0.27025	no
gi|646746310|gb|KK962297.1|	44674-54694	23	11	2	0.864621	-1	0.44495	no
gi|646746310|gb|KK962297.1|	50666-56015	23	11	8	7	-0.239577	0.83695	no
gi|646746310|gb|KK962297.1|	64218-56489	23	11	15	4	-2	0.1491	no
gi|646746310|gb|KK962297.1|	76540-57715	23	11	2	2	0.466053	0.7483	no
gi|646746310|gb|KK962297.1|	61585-64498	23	11	12	12	0.00151143	0.9981	no
gi|646746310|gb|KK962297.1|	6455-7495	23	11	0.824887	7	3	0.076	no
gi|646746310|gb|KK962297.1|	7613-8603	23	11	0.251711	3	3	0.26825	no
gi|646746310|gb|KK962297.1|	77974-78686	23	11	2	9	2	0.215	no
gi|646746310|gb|KK962297.1|	90567-94438	23	11	9	16	0.853776	0.4498	no
gi|646746310|gb|KK962297.1|	9231-9767	23	11	3	15	2	0.13745	no
gi|646746327|gb|KK962296.1|	61194-26237	13	6.8	0.301003	1	2	0.186	no
gi|646746327|gb|KK962296.1|	63217-26432	13	6.8	0.979483	0.466668	-1	1	no

gi|646746327|gb|KK962296.1| 69509-27102	13	6.8	0.585525	0.843944	0.527416	1	no
gi|646746327|gb|KK962296.1| 71397-27271	13	6.8	0.608622	0.999312	0.715388	1	no
gi|646746327|gb|KK962296.1| 75054-27582	13	6.8	0.534644	2	2	0.2903	no
gi|646746327|gb|KK962296.1| 76807-27764	13	6.8	1	2	0.787762	0.55445	no
gi|646746327|gb|KK962296.1| 90694-29334	13	6.8	0.889606	2	0.922538	0.56455	no
gi|646746339|gb|KK962295.1| 02772-10399	20	8.2	1	3	2	0.30355	no
gi|646746339|gb|KK962295.1| 04529-10597	20	8.2	1	3	1	0.4232	no
gi|646746339|gb|KK962295.1| 36748-13767	20	8.2	2	0	-inf	0.0012	yes
gi|646746339|gb|KK962295.1| 50651-15255	20	8.2	2	0.162873	-3	0.1408	no
gi|646746339|gb|KK962295.1| 16632-21733	20	8.2	0.415579	10	5	0.23975	no
gi|646746339|gb|KK962295.1| 18250-21900	20	8.2	3	3	-0.435811	0.7269	no
gi|646746339|gb|KK962295.1| 20302-22080	20	8.2	18	5	-2	0.2215	no
gi|646746339|gb|KK962295.1| 45855-25205	20	8.2	14	0.819364	-4	0.01425	no
gi|646746339|gb|KK962295.1| 89814-39066	20	8.2	0.462494	2	2	0.18785	no
gi|646746339|gb|KK962295.1| 41216-54418	20	8.2	22	30	0.407646	0.723	no
gi|646746339|gb|KK962295.1| 44781-54654	20	8.2	10	11	0.0719922	0.9613	no
gi|646746339|gb|KK962295.1| 51425-55220	20	8.2	0.889361	4	2	0.21275	no
gi|646746355|gb|KK962294.1| 01362-10022	22	10	2	0	-inf	0.0041	no
gi|646746355|gb|KK962294.1| 02369-10080	22	10	1	0.445064	-1	1	no
gi|646746355|gb|KK962294.1| 08364-10113	22	10	3	1	-1	0.42755	no
gi|646746355|gb|KK962294.1| 11489-10246	22	10	51	28	-0.847796	0.48425	no
gi|646746355|gb|KK962294.1| 01715-10267	22	10	1	0.935398	-0.340316	0.8138	no
gi|646746355|gb|KK962294.1| 30159-10311	22	10	2	2	-0.407647	0.75455	no
gi|646746355|gb|KK962294.1| 03564-10569	22	10	1	1	-0.0494346	0.9749	no
gi|646746355|gb|KK962294.1| 48510-10492	22	10	2	5	2	0.30345	no
gi|646746355|gb|KK962294.1| 08153-10895	22	10	0.677627	2	1	0.4331	no
gi|646746355|gb|KK962294.1| 09058-10967	22	10	7	4	-0.806738	0.5686	no
gi|646746355|gb|KK962294.1| 13771-11443	22	10	13	6	-1	0.4543	no
gi|646746355|gb|KK962294.1| 14882-11515	22	10	24	29	0.28963	0.8138	no
gi|646746355|gb|KK962294.1| 27095-12751	22	10	7	14	1	0.47565	no

gi|646746355|gb|KK962294.1|	40280-14089	22	10	3	1	-2	0.34995	no
gi|646746355|gb|KK962294.1|	43485-15025	22	10	27	21	-0.35811	0.76045	no
gi|646746355|gb|KK962294.1|	14809-15602	22	10	3	0.242163	-3	0.1787	no
gi|646746355|gb|KK962294.1|	60081-16080	22	10	12	10	-0.321785	0.82645	no
gi|646746355|gb|KK962294.1|	70367-17445	22	10	5	0.422494	-3	0.03145	no
gi|646746355|gb|KK962294.1|	17864-18144	22	10	95	31	-2	0.31395	no
gi|646746355|gb|KK962294.1|	83117-18465	22	10	16	5	-2	0.1718	no
gi|646746355|gb|KK962294.1|	86010-18662	22	10	2	1	-1	0.47035	no
gi|646746355|gb|KK962294.1|	89002-19092	22	10	2	0.643003	-2	0.25575	no
gi|646746355|gb|KK962294.1|	92602-19829	22	10	16	3	-3	0.0766	no
gi|646746355|gb|KK962294.1|	05745-20741	22	10	0.459291	0.757923	0.722642	1	no
gi|646746355|gb|KK962294.1|	08032-21393	22	10	15	10	-0.561596	0.6269	no
gi|646746355|gb|KK962294.1|	21176-21751	22	10	0.57633	4	3	0.2672	no
gi|646746355|gb|KK962294.1|	17210-21798	22	10	0.882577	2	1	0.36955	no
gi|646746355|gb|KK962294.1|	69633-27008	22	10	0	7	inf	0.001	yes
gi|646746355|gb|KK962294.1|	83798-28444	22	10	7	5	-0.385542	0.8108	no
gi|646746355|gb|KK962294.1|	96753-29786	22	10	4	4	-0.109023	0.9435	no
gi|646746355|gb|KK962294.1|	01498-30237	22	10	7	8	0.295169	0.84935	no
gi|646746355|gb|KK962294.1|	11335-31366	22	10	0.536336	0.843134	0.652624	1	no
gi|646746355|gb|KK962294.1|	31493-32083	22	10	16	5	-2	0.24625	no
gi|646746355|gb|KK962294.1|	15095-31629	22	10	0.390332	0.839078	1	1	no
gi|646746355|gb|KK962294.1|	24166-32520	22	10	2	2	0.232294	0.8447	no
gi|646746355|gb|KK962294.1|	36306-34040	22	10	6	2	-2	0.15795	no
gi|646746355|gb|KK962294.1|	40535-34205	22	10	1	0.105493	-4	0.1745	no
gi|646746355|gb|KK962294.1|	43123-34564	22	10	1	0.059353	-4	0.18145	no
gi|646746355|gb|KK962294.1|	45354-45847	22	10	3	5	0.748347	0.5809	no
gi|646746355|gb|KK962294.1|	59378-46021	22	10	0.634635	2	1	0.36865	no
gi|646746355|gb|KK962294.1|	45996-46750	22	10	2	1	-1	0.4518	no
gi|646746355|gb|KK962294.1|	65604-46635	22	10	2	1	-0.510937	0.7312	no
gi|646746355|gb|KK962294.1|	86826-48769	22	10	2	2	-0.19931	0.8762	no

gi|646746355|gb|KK962294.1|	92607-49597	22	10	1	0.909495	-0.491668	0.7521	no
gi|646746355|gb|KK962294.1|	96658-50083	22	10	102.92	71	-0.531255	0.6562	no
gi|646746355|gb|KK962294.1|	02820-50339	22	10	3	3	0.134545	0.92595	no
gi|646746355|gb|KK962294.1|	22379-52363	22	10	1	0.132087	-3	1	no
gi|646746355|gb|KK962294.1|	26305-52659	22	10	23	4	-3	0.1404	no
gi|646746355|gb|KK962294.1|	41461-54391	22	10	2	3	1	0.49475	no
gi|646746355|gb|KK962294.1|	44208-54504	22	10	0.158931	2	4	0.10345	no
gi|646746355|gb|KK962294.1|	45518-54592	22	10	3	5	0.632726	0.6686	no
gi|646746355|gb|KK962294.1|	49297-55075	22	10	0.848737	2	1	0.4157	no
gi|646746355|gb|KK962294.1|	54724-55679	22	10	161	124	-0.373026	0.75805	no
gi|646746355|gb|KK962294.1|	26508-62709	22	10	6	0	-inf	0.00495	no
gi|646746355|gb|KK962294.1|	27297-62959	22	10	0.362283	0.262817	-0.463056	1	no
gi|646746355|gb|KK962294.1|	30048-63171	22	10	1	0.378697	-2	0.2334	no
gi|646746355|gb|KK962294.1|	37367-63787	22	10	7	3	-1	0.4156	no
gi|646746355|gb|KK962294.1|	54245-65486	22	10	1	2	0.466508	0.7722	no
gi|646746355|gb|KK962294.1|	96342-69683	22	10	4	22	2	0.1236	no
gi|646746355|gb|KK962294.1|	26983-72809	22	10	2	3	0.433005	0.75435	no
gi|646746355|gb|KK962294.1|	30340-73174	22	10	0.961994	2	0.747437	0.5505	no
gi|646746355|gb|KK962294.1|	32132-73283	22	10	3	3	0.161296	0.89535	no
gi|646746355|gb|KK962294.1|	73317-74437	22	10	27	19	-0.48983	0.70785	no
gi|646746355|gb|KK962294.1|	33392-73358	22	10	73	47	-0.633169	0.6744	no
gi|646746355|gb|KK962294.1|	33994-73431	22	10	1	13	4	0.1063	no
gi|646746355|gb|KK962294.1|	35220-73651	22	10	1	2	0.522149	0.6663	no
gi|646746355|gb|KK962294.1|	36653-74209	22	10	14	15	0.0777165	0.94645	no
gi|646746355|gb|KK962294.1|	75497-77592	22	10	4	6	0.751118	0.59135	no
gi|646746355|gb|KK962294.1|	88554-78910	22	10	47	65	0.487437	0.69675	no
gi|646746355|gb|KK962294.1|	90027-79055	22	10	30	63	1	0.408	no
gi|646746355|gb|KK962294.1|	07693-80797	22	10	270	297	0.135692	0.9107	no
gi|646746355|gb|KK962294.1|	09688-81016	22	10	2	3	0.157197	0.9062	no
gi|646746355|gb|KK962294.1|	10402-81222	22	10	18	23	0.36413	0.73815	no

gi|646746355|gb|KK962294.1|	25975-82658	22	10	14	13	-0.106313	0.94445	no
gi|646746355|gb|KK962294.1|	27663-82863	22	10	39	47	0.260796	0.8119	no
gi|646746355|gb|KK962294.1|	28822-83040	22	10	131	50	-1	0.2366	no
gi|646746355|gb|KK962294.1|	31319-83179	22	10	3	6	0.876625	0.5168	no
gi|646746355|gb|KK962294.1|	35471-83670	22	10	40	16	-1	0.3029	no
gi|646746355|gb|KK962294.1|	57003-85774	22	10	12	13	0.102999	0.94445	no
gi|646746355|gb|KK962294.1|	86840-87665	22	10	2	1	-0.724978	0.5852	no
gi|646746355|gb|KK962294.1|	91254-89164	22	10	31	23	-0.444751	0.77875	no
gi|646746355|gb|KK962294.1|	09485-91198	22	10	2	4	1	0.42065	no
gi|646746371|gb|KK962293.1|	32167-13261	13	6.7	5	10	0.984343	0.5409	no
gi|646746371|gb|KK962293.1|	49248-34975	13	6.7	1	3	2	0.29695	no
gi|646746371|gb|KK962293.1|	63195-46374	13	6.7	20	20	-0.0063273	0.9913	no
gi|646746371|gb|KK962293.1|	65785-46703	13	6.7	3	1	-1	0.45435	no
gi|646746371|gb|KK962293.1|	67103-46786	13	6.7	1	1	-0.187396	0.90405	no
gi|646746371|gb|KK962293.1|	68523-46934	13	6.7	5	2	-1	0.47215	no
gi|646746390|gb|KK962292.1|	10912-12224	18	6.4	8	5	-0.791561	0.60205	no
gi|646746390|gb|KK962292.1|	13038-14640	18	6.4	7	3	-1	0.35385	no
gi|646746390|gb|KK962292.1|	14759-15559	18	6.4	18	17	-0.0967837	0.9483	no
gi|646746390|gb|KK962292.1|	57604-15822	18	6.4	2	1	-1	0.47035	no
gi|646746390|gb|KK962292.1|	16018-16744	18	6.4	8	4	-1	0.36865	no
gi|646746390|gb|KK962292.1|	63022-16421	18	6.4	2	0.566389	-2	0.2986	no
gi|646746390|gb|KK962292.1|	67924-16847	18	6.4	4	2	-0.930793	0.52635	no
gi|646746390|gb|KK962292.1|	70121-17139	18	6.4	2	3	0.91749	0.5047	no
gi|646746390|gb|KK962292.1|	73154-17560	18	6.4	20	12	-0.712645	0.5175	no
gi|646746390|gb|KK962292.1|	76534-18092	18	6.4	53	50	-0.0942466	0.9349	no
gi|646746390|gb|KK962292.1|	84163-18440	18	6.4	21	29	0.48667	0.73095	no
gi|646746390|gb|KK962292.1|	93366-19367	18	6.4	21	34	0.705719	0.61805	no
gi|646746390|gb|KK962292.1|	23360-23599	18	6.4	64	18	-2	0.1967	no
gi|646746390|gb|KK962292.1|	71884-27343	18	6.4	2	2	-0.472063	0.762	no
gi|646746390|gb|KK962292.1|	73676-27451	18	6.4	0.632466	1	1	0.48525	no

gi|646746390|gb|KK962292.1| 79225-27980	18	6.4	18	6	-2	0.3386	no
gi|646746390|gb|KK962292.1| 96430-29744	18	6.4	11	7	-0.742868	0.622	no
gi|646746390|gb|KK962292.1| 02473-30807	18	6.4	13	9	-0.620985	0.58535	no
gi|646746390|gb|KK962292.1| 77372-37768	18	6.4	64	33	-0.944167	0.5427	no
gi|646746390|gb|KK962292.1| 81150-38139	18	6.4	85	3	-5	0.1777	no
gi|646746390|gb|KK962292.1| 03131-40388	18	6.4	9	1	-3	0.09815	no
gi|646746390|gb|KK962292.1| 05213-40583	18	6.4	12	21	0.851394	0.5694	no
gi|646746390|gb|KK962292.1| 11544-41272	18	6.4	2	1	-0.704074	0.5769	no
gi|646746390|gb|KK962292.1| 18417-41947	18	6.4	0.574569	2	2	0.33695	no
gi|646746390|gb|KK962292.1| 21639-42212	18	6.4	22	22	-0.0637211	0.96705	no
gi|646746390|gb|KK962292.1| 33679-43404	18	6.4	15	14	-0.109977	0.92775	no
gi|646746390|gb|KK962292.1| 36596-43728	18	6.4	76	26	-2	0.24255	no
gi|646746390|gb|KK962292.1| 39211-44031	18	6.4	0.985278	2	0.804628	0.5518	no
gi|646746390|gb|KK962292.1| 95562-95714	18	6.4	842	76	-3	0.11455	no
gi|646746407|gb|KK962291.1| 11804-12574	21	9.9	0.897987	2	0.754662	0.6148	no
gi|646746407|gb|KK962291.1| 07131-20761	21	9.9	112	83.53	-0.42169	0.69685	no
gi|646746407|gb|KK962291.1| 10124-21110	21	9.9	0.769792	1	0.508784	1	no
gi|646746407|gb|KK962291.1| 15246-21623	21	9.9	1	3	0.957178	0.53785	no
gi|646746407|gb|KK962291.1| 18133-21933	21	9.9	2	4	1	0.396	no
gi|646746407|gb|KK962291.1| 19786-22151	21	9.9	3	3	-0.0540374	0.97215	no
gi|646746407|gb|KK962291.1| 22530-22439	21	9.9	2	2	0.301502	0.8524	no
gi|646746407|gb|KK962291.1| 25282-22853	21	9.9	2	3	0.343954	0.81495	no
gi|646746407|gb|KK962291.1| 31397-23413	21	9.9	2	3	0.426018	0.7784	no
gi|646746407|gb|KK962291.1| 34397-23963	21	9.9	2	2	-0.00884341	0.99385	no
gi|646746407|gb|KK962291.1| 39807-24018	21	9.9	4	4	0.0850313	0.91505	no
gi|646746407|gb|KK962291.1| 40984-24264	21	9.9	5	4	-0.213727	0.88935	no
gi|646746407|gb|KK962291.1| 43227-24419	21	9.9	1	2	0.508055	0.7139	no
gi|646746407|gb|KK962291.1| 44775-24605	21	9.9	90	64	-0.497246	0.6553	no
gi|646746407|gb|KK962291.1| 64633-26632	21	9.9	0.902581	3	2	0.19695	no
gi|646746407|gb|KK962291.1| 69168-26954	21	9.9	6	4	-0.650912	0.66675	no

gi|646746407|gb|KK962291.1|	73112-27367	21	9.9	6	6	-0.0321097	0.96975	no
gi|646746407|gb|KK962291.1|	78769-27962	21	9.9	6	2	-2	0.2444	no
gi|646746407|gb|KK962291.1|	85223-28590	21	9.9	2	2	0.478878	0.7389	no
gi|646746407|gb|KK962291.1|	94704-29494	21	9.9	31	29	-0.0982923	0.93945	no
gi|646746407|gb|KK962291.1|	18859-31909	21	9.9	28	12	-1	0.47135	no
gi|646746407|gb|KK962291.1|	40634-34114	21	9.9	4	5	0.435399	0.7425	no
gi|646746407|gb|KK962291.1|	44718-34498	21	9.9	19	19	-0.00337363	0.9426	no
gi|646746407|gb|KK962291.1|	48720-35056	21	9.9	0.116739	1	3	1	no
gi|646746407|gb|KK962291.1|	50693-35121	21	9.9	9	6	-0.616296	0.659	no
gi|646746407|gb|KK962291.1|	75575-37853	21	9.9	16	10	-0.67268	0.5434	no
gi|646746407|gb|KK962291.1|	40727-44742	21	9.9	8	6	-0.597683	0.57775	no
gi|646746407|gb|KK962291.1|	24614-42520	21	9.9	972	990	0.0258629	0.9821	no
gi|646746407|gb|KK962291.1|	25337-43588	21	9.9	7	19	1	0.22275	no
gi|646746407|gb|KK962291.1|	36178-43671	21	9.9	440	1285.11	2	0.2084	no
gi|646746407|gb|KK962291.1|	37471-44120	21	9.9	10	34	2	0.127	no
gi|646746407|gb|KK962291.1|	41704-44192	21	9.9	183	654	2	0.25305	no
gi|646746407|gb|KK962291.1|	42818-45029	21	9.9	6	12	0.908045	0.42265	no
gi|646746407|gb|KK962291.1|	52037-45321	21	9.9	1	5	2	0.1531	no
gi|646746407|gb|KK962291.1|	54754-45814	21	9.9	0.766583	3	2	0.2883	no
gi|646746407|gb|KK962291.1|	58284-45918	21	9.9	0.570113	2	2	0.2406	no
gi|646746407|gb|KK962291.1|	59354-45980	21	9.9	3	5	0.821179	0.57265	no
gi|646746407|gb|KK962291.1|	53493-55857	21	9.9	15	9	-0.737989	0.49495	no
gi|646746407|gb|KK962291.1|	57359-58402	21	9.9	10	12	0.161545	0.91305	no
gi|646746407|gb|KK962291.1|	64699-65180	21	9.9	699	607.84	-0.20147	0.8627	no
gi|646746407|gb|KK962291.1|	67088-67851	21	9.9	1	1	-0.187276	0.90405	no
gi|646746407|gb|KK962291.1|	76169-76637	21	9.9	154	108.45	-0.507964	0.63975	no
gi|646746418|gb|KK962290.1|	20661-42134	12	8.4	2	1	-0.328307	0.81865	no
gi|646746432|gb|KK962289.1|	55316-15878	14	7.8	4	4	-0.0198254	0.9893	no
gi|646746432|gb|KK962289.1|	59674-16077	14	7.8	4	4	0.269921	0.86475	no
gi|646746432|gb|KK962289.1|	79354-27953	14	7.8	147	58	-1	0.35895	no

gi|646746432|gb|KK962289.1|	81511-48378	14	7.8	0.823601	0.199138	-2	1	no
gi|646746432|gb|KK962289.1|	31993-73267	14	7.8	4	2	-1	0.32625	no
gi|646746432|gb|KK962289.1|	84720-78547	14	7.8	2	3	0.111181	0.93025	no
gi|646746432|gb|KK962289.1|	86346-78764	14	7.8	43	8	-3	0.0728	no
gi|646746432|gb|KK962289.1|	88807-79072	14	7.8	2	0	-inf	0.0017	no
gi|646746448|gb|KK962288.1|	12286-11250	11	5.9	0	90	inf	0.00435	no
gi|646746448|gb|KK962288.1|	37288-13788	11	5.9	2	4	1	0.416	no
gi|646746448|gb|KK962288.1|	99339-99742	11	5.9	3	8	1	0.3675	no
gi|646746464|gb|KK962287.1|	78076-17921	25	12	3	4	0.56582	0.7216	no
gi|646746464|gb|KK962287.1|	59238-26013	25	12	3	5	1	0.427	no
gi|646746464|gb|KK962287.1|	83457-28389	25	12	12	19	0.667813	0.66915	no
gi|646746475|gb|KK962286.1|	12642-13057	19	8.8	44	7	-3	0.07895	no
gi|646746475|gb|KK962286.1|	32731-13416	19	8.8	9	13	0.475959	0.75325	no
gi|646746475|gb|KK962286.1|	44659-14689	19	8.8	20	13	-0.595728	0.58435	no
gi|646746475|gb|KK962286.1|	59135-15942	19	8.8	13	20	0.674711	0.6203	no
gi|646746475|gb|KK962286.1|	80570-18168	19	8.8	5	2	-1	0.34265	no
gi|646746475|gb|KK962286.1|	86330-18745	19	8.8	0	14	inf	0.00015	yes
gi|646746475|gb|KK962286.1|	93533-20957	19	8.8	205	151	-0.445253	0.6947	no
gi|646746475|gb|KK962286.1|	10486-21333	19	8.8	15	19	0.391753	0.7237	no
gi|646746475|gb|KK962286.1|	15278-21668	19	8.8	0.640774	1	1	0.41045	no
gi|646746475|gb|KK962286.1|	82444-28370	19	8.8	29	15	-0.948765	0.4657	no
gi|646746475|gb|KK962286.1|	01355-40194	19	8.8	2	2	0.460226	0.7494	no
gi|646746475|gb|KK962286.1|	09484-40996	19	8.8	29	39	0.421706	0.77895	no
gi|646746475|gb|KK962286.1|	31636-43188	19	8.8	13	20	0.607062	0.66825	no
gi|646746475|gb|KK962286.1|	33177-43342	19	8.8	5	35	3	0.25735	no
gi|646746475|gb|KK962286.1|	34013-43454	19	8.8	2	6	2	0.2279	no
gi|646746475|gb|KK962286.1|	39733-44071	19	8.8	1	1	0.316612	0.8346	no
gi|646746475|gb|KK962286.1|	40891-44401	19	8.8	34	30	-0.182833	0.8748	no
gi|646746475|gb|KK962286.1|	54397-45509	19	8.8	7	10	0.564919	0.7109	no
gi|646746475|gb|KK962286.1|	57817-46282	19	8.8	40	37	-0.128777	0.9114	no

gi|646746475|gb|KK962286.1|	46130-47112	19	8.8	2	3	0.924177	0.5333	no
gi|646746475|gb|KK962286.1|	47794-49531	19	8.8	1	3	1	0.45795	no
gi|646746475|gb|KK962286.1|	94990-49540	19	8.8	6	2	-1	0.389	no
gi|646746475|gb|KK962286.1|	50666-52344	19	8.8	2	5	1	0.4527	no
gi|646746475|gb|KK962286.1|	58593-60984	19	8.8	0.259883	1	2	1	no
gi|646746475|gb|KK962286.1|	61158-65306	19	8.8	60	89	0.57895	0.6273	no
gi|646746475|gb|KK962286.1|	66127-67102	19	8.8	1	5	2	0.20705	no
gi|646746475|gb|KK962286.1|	70284-72311	19	8.8	2	3	0.816974	0.60235	no
gi|646746475|gb|KK962286.1|	78138-83210	19	8.8	4	6	0.599844	0.56875	no
gi|646746475|gb|KK962286.1|	84208-86016	19	8.8	4	4	0.117021	0.93615	no
gi|646746475|gb|KK962286.1|	88879-90992	19	8.8	1	2	0.749525	0.63505	no
gi|646746489|gb|KK962285.1|	59420-26033	17	6.8	0.982562	2	1	0.40715	no
gi|646746489|gb|KK962285.1|	65640-26635	17	6.8	0.400328	4	3	0.2413	no
gi|646746489|gb|KK962285.1|	35297-43554	17	6.8	40	62	0.643483	0.6596	no
gi|646746489|gb|KK962285.1|	83406-68370	17	6.8	17	0	-inf	0.00475	no
gi|646746505|gb|KK962284.1|	59690-26011	20	7.6	7	23	2	0.24435	no
gi|646746505|gb|KK962284.1|	81161-28225	20	7.6	3	2	-0.20135	0.8828	no
gi|646746505|gb|KK962284.1|	04595-30514	20	7.6	13	0.422052	-5	0.17345	no
gi|646746505|gb|KK962284.1|	05373-30669	20	7.6	5	6	0.146732	0.92055	no
gi|646746505|gb|KK962284.1|	15576-31583	20	7.6	111	0	-inf	0.00125	yes
gi|646746505|gb|KK962284.1|	16466-31952	20	7.6	63	30	-1	0.36935	no
gi|646746505|gb|KK962284.1|	21363-32166	20	7.6	22	21	-0.0605989	0.95845	no
gi|646746505|gb|KK962284.1|	40522-34091	20	7.6	7	4	-0.643008	0.66075	no
gi|646746505|gb|KK962284.1|	48509-34913	20	7.6	58	37	-0.649944	0.6054	no
gi|646746505|gb|KK962284.1|	75485-37734	20	7.6	51	28	-0.841582	0.45825	no
gi|646746505|gb|KK962284.1|	83303-38354	20	7.6	82	46	-0.851537	0.5418	no
gi|646746505|gb|KK962284.1|	87081-38755	20	7.6	268	147	-0.868573	0.43355	no
gi|646746505|gb|KK962284.1|	98173-39855	20	7.6	16	15	-0.0870107	0.956	no
gi|646746505|gb|KK962284.1|	32108-43644	20	7.6	9	12	0.383012	0.72815	no
gi|646746505|gb|KK962284.1|	36952-44698	20	7.6	5	7	0.495227	0.65725	no

gi|646746505|gb|KK962284.1|	51582-45237	20	7.6	27	7	-2	0.2183	no
gi|646746505|gb|KK962284.1|	60427-46060	20	7.6	339	197	-0.78213	0.6417	no
gi|646746505|gb|KK962284.1|	01699-50196	20	7.6	18	281	4	0.02965	no
gi|646746505|gb|KK962284.1|	09035-50991	20	7.6	2	1	-1	0.43695	no
gi|646746505|gb|KK962284.1|	12884-51308	20	7.6	279	110	-1	0.3451	no
gi|646746505|gb|KK962284.1|	14645-51648	20	7.6	2	1	-0.665534	0.67955	no
gi|646746505|gb|KK962284.1|	17791-52048	20	7.6	1	1	-0.102108	0.94815	no
gi|646746505|gb|KK962284.1|	20580-52134	20	7.6	96	90	-0.103193	0.92775	no
gi|646746505|gb|KK962284.1|	21663-52285	20	7.6	84	16	-2	0.097	no
gi|646746505|gb|KK962284.1|	42924-54542	20	7.6	27	26	-0.0807715	0.94005	no
gi|646746505|gb|KK962284.1|	99976-60093	20	7.6	0.92635	2	1	0.35905	no
gi|646746525|gb|KK962283.1|	10780-10114	21	6.8	3	0.617307	-2	0.19405	no
gi|646746525|gb|KK962283.1|	16665-10192	21	6.8	5	5	0.0240571	0.9861	no
gi|646746525|gb|KK962283.1|	23419-10256	21	6.8	6	4	-0.549626	0.7167	no
gi|646746525|gb|KK962283.1|	26172-10271	21	6.8	1	2	0.886192	0.4909	no
gi|646746525|gb|KK962283.1|	28488-10292	21	6.8	2	4	1	0.42735	no
gi|646746525|gb|KK962283.1|	11501-11307	21	6.8	206	148	-0.476944	0.6932	no
gi|646746525|gb|KK962283.1|	21515-12175	21	6.8	67	75	0.152893	0.906	no
gi|646746525|gb|KK962283.1|	52175-15355	21	6.8	1	0.82719	-0.475633	0.71555	no
gi|646746525|gb|KK962283.1|	57447-15828	21	6.8	2	3	0.598427	0.66775	no
gi|646746525|gb|KK962283.1|	60064-16095	21	6.8	3	2	-0.66666	0.6016	no
gi|646746525|gb|KK962283.1|	99112-19975	21	6.8	11	8	-0.495852	0.75005	no
gi|646746525|gb|KK962283.1|	11383-21618	21	6.8	7	7	0.0537958	0.9586	no
gi|646746525|gb|KK962283.1|	26250-33089	21	6.8	23	15	-0.603836	0.60635	no
gi|646746525|gb|KK962283.1|	06794-30791	21	6.8	2	4	0.577334	0.72195	no
gi|646746525|gb|KK962283.1|	42601-43536	21	6.8	1	1	-0.368281	0.78495	no
gi|646746525|gb|KK962283.1|	44366-46418	21	6.8	1	2	0.534822	0.7368	no
gi|646746525|gb|KK962283.1|	46622-48273	21	6.8	0.530167	0.574053	0.114736	1	no
gi|646746525|gb|KK962283.1|	17704-51837	21	6.8	3	4	0.476778	0.6946	no
gi|646746525|gb|KK962283.1|	53180-54265	21	6.8	1	0.477618	-1	0.38955	no

gi|646746525|gb|KK962283.1|	59996-60469	21	6.8	4	1	-2	0.28945	no
gi|646746525|gb|KK962283.1|	84269-84853	21	6.8	15	8	-0.810104	0.6003	no
gi|646746525|gb|KK962283.1|	85299-87046	21	6.8	0.434051	1	2	0.31585	no
gi|646746525|gb|KK962283.1|	33863-93424	21	6.8	7	3	-0.969817	0.5492	no
gi|646746525|gb|KK962283.1|	93842-94332	21	6.8	4	7	1	0.41955	no
gi|646746525|gb|KK962283.1|	41033-94186	21	6.8	4	1	-2	0.26815	no
gi|646746525|gb|KK962283.1|	41985-94268	21	6.8	1	2	0.259438	0.86925	no
gi|646746525|gb|KK962283.1|	43755-94440	21	6.8	4	2	-1	0.42505	no
gi|646746525|gb|KK962283.1|	44608-94501	21	6.8	7	4	-0.893343	0.5347	no
gi|646746525|gb|KK962283.1|	53411-95361	21	6.8	84	16	-2	0.1757	no
gi|646746543|gb|KK962282.1|	46456-14700	12	8.1	3	2	-0.399306	0.8003	no
gi|646746543|gb|KK962282.1|	89558-39047	12	8.1	12	4	-2	0.2767	no
gi|646746543|gb|KK962282.1|	27256-42791	12	8.1	2	2	-0.372865	0.80365	no
gi|646746556|gb|KK962281.1|	04424-10059	20	9.1	3	0.85323	-2	0.19465	no
gi|646746556|gb|KK962281.1|	56261-15677	20	9.1	0	12	inf	0.0017	no
gi|646746556|gb|KK962281.1|	69202-26959	20	9.1	13	8	-0.637396	0.6123	no
gi|646746556|gb|KK962281.1|	98809-29899	20	9.1	94	35	-1	0.397	no
gi|646746556|gb|KK962281.1|	01198-30309	20	9.1	4	3	-0.301839	0.841	no
gi|646746556|gb|KK962281.1|	15250-31714	20	9.1	1	0.733369	-0.54483	1	no
gi|646746556|gb|KK962281.1|	17833-32094	20	9.1	4	3	-0.564683	0.71325	no
gi|646746556|gb|KK962281.1|	26469-32715	20	9.1	18	19	0.0817946	0.9568	no
gi|646746556|gb|KK962281.1|	28437-32871	20	9.1	20	11	-0.823365	0.57265	no
gi|646746556|gb|KK962281.1|	29004-32926	20	9.1	29	18	-0.635357	0.6401	no
gi|646746556|gb|KK962281.1|	33894-33412	20	9.1	24	25	0.0415255	0.91805	no
gi|646746556|gb|KK962281.1|	39038-34007	20	9.1	2	2	0.270936	0.8231	no
gi|646746556|gb|KK962281.1|	40278-34128	20	9.1	0.739048	1	0.732898	0.6352	no
gi|646746556|gb|KK962281.1|	45458-34692	20	9.1	10	2	-2	0.176	no
gi|646746556|gb|KK962281.1|	53531-35398	20	9.1	11	5	-1	0.40795	no
gi|646746556|gb|KK962281.1|	63356-36364	20	9.1	63	31	-1	0.52405	no
gi|646746556|gb|KK962281.1|	65019-36549	20	9.1	31	48	0.633487	0.676	no

gi|646746556|gb|KK962281.1|	66396-36659	20	9.1	80	124	0.619733	0.68705	no
gi|646746556|gb|KK962281.1|	66823-36710	20	9.1	28	13	-1	0.4153	no
gi|646746556|gb|KK962281.1|	68915-36986	20	9.1	1	0.564351	-1	0.47685	no
gi|646746556|gb|KK962281.1|	71516-37178	20	9.1	14	23	0.686219	0.623	no
gi|646746556|gb|KK962281.1|	72031-37364	20	9.1	0.68075	0.785905	0.20723	1	no
gi|646746556|gb|KK962281.1|	78802-38078	20	9.1	0.69763	0.543942	-0.359008	1	no
gi|646746556|gb|KK962281.1|	91686-39212	20	9.1	6	3	-1	0.3845	no
gi|646746556|gb|KK962281.1|	93176-39348	20	9.1	312	44	-3	0.0518	no
gi|646746556|gb|KK962281.1|	08893-40962	20	9.1	104	67	-0.634784	0.5702	no
gi|646746556|gb|KK962281.1|	21058-42163	20	9.1	11	5	-1	0.3855	no
gi|646746556|gb|KK962281.1|	36132-43661	20	9.1	2	6	2	0.3219	no
gi|646746556|gb|KK962281.1|	51308-45247	20	9.1	1	1	-0.0672389	0.9467	no
gi|646746556|gb|KK962281.1|	55085-46136	20	9.1	7	6	-0.328222	0.76895	no
gi|646746556|gb|KK962281.1|	62093-46580	20	9.1	11	9	-0.30833	0.77495	no
gi|646746556|gb|KK962281.1|	76726-47770	20	9.1	13	12	-0.205402	0.8964	no
gi|646746556|gb|KK962281.1|	80500-48120	20	9.1	3	4	0.38399	0.7547	no
gi|646746556|gb|KK962281.1|	81632-48257	20	9.1	1	2	0.35465	0.79375	no
gi|646746556|gb|KK962281.1|	96131-49643	20	9.1	1	14	3	0.109	no
gi|646746556|gb|KK962281.1|	06939-50778	20	9.1	3	3	-0.0860712	0.9464	no
gi|646746556|gb|KK962281.1|	08910-51401	20	9.1	17	15	-0.203414	0.8589	no
gi|646746556|gb|KK962281.1|	19444-52731	20	9.1	15	14	-0.0728794	0.9499	no
gi|646746556|gb|KK962281.1|	30511-53201	20	9.1	1	1	0.320646	0.80365	no
gi|646746556|gb|KK962281.1|	35307-53611	20	9.1	3	1	-1	0.42505	no
gi|646746556|gb|KK962281.1|	61147-56204	20	9.1	2	0.205183	-3	0.1863	no
gi|646746556|gb|KK962281.1|	93039-59444	20	9.1	17	9	-0.892298	0.48145	no
gi|646746556|gb|KK962281.1|	66370-66815	20	9.1	1	1	0.144675	0.9055	no
gi|646746556|gb|KK962281.1|	69882-67035	20	9.1	45	22.93	-0.967239	0.52235	no
gi|646746556|gb|KK962281.1|	70456-67217	20	9.1	1	0.732853	-0.861569	0.502	no
gi|646746556|gb|KK962281.1|	72283-67250	20	9.1	45	32	-0.493263	0.71765	no
gi|646746556|gb|KK962281.1|	73287-67590	20	9.1	1	0.738951	-0.930428	0.4964	no

gi|646746556|gb|KK962281.1|	78962-68045	20	9.1	0.969892	0.322496	-2	1	no
gi|646746556|gb|KK962281.1|	81879-68281	20	9.1	2	5	1	0.42035	no
gi|646746556|gb|KK962281.1|	84088-68432	20	9.1	21	22	0.0716327	0.918	no
gi|646746556|gb|KK962281.1|	86065-68653	20	9.1	5	8	0.737029	0.61105	no
gi|646746556|gb|KK962281.1|	87721-68820	20	9.1	20	29	0.503797	0.73405	no
gi|646746556|gb|KK962281.1|	88379-68867	20	9.1	13	30	1	0.4227	no
gi|646746556|gb|KK962281.1|	90762-69290	20	9.1	2	3	0.383632	0.79915	no
gi|646746556|gb|KK962281.1|	03361-70463	20	9.1	0.453036	1	2	0.2702	no
gi|646746556|gb|KK962281.1|	05431-70634	20	9.1	0.42046	3	3	0.1381	no
gi|646746556|gb|KK962281.1|	10107-71037	20	9.1	22	11	-0.965858	0.5349	no
gi|646746556|gb|KK962281.1|	10736-71140	20	9.1	1	2	0.213383	0.89085	no
gi|646746556|gb|KK962281.1|	13919-71460	20	9.1	1	1	-0.00504355	0.92345	no
gi|646746556|gb|KK962281.1|	14814-71719	20	9.1	1	1	0.286011	0.85925	no
gi|646746556|gb|KK962281.1|	18960-71923	20	9.1	23	37	0.70968	0.6341	no
gi|646746556|gb|KK962281.1|	22338-72303	20	9.1	0.42316	4	3	0.24735	no
gi|646746556|gb|KK962281.1|	52022-75226	20	9.1	17	21.09	0.341813	0.81245	no
gi|646746556|gb|KK962281.1|	66980-76769	20	9.1	6	1	-2	0.1725	no
gi|646746556|gb|KK962281.1|	71959-77244	20	9.1	8	10	0.271624	0.84165	no
gi|646746556|gb|KK962281.1|	75869-77623	20	9.1	12	9	-0.493787	0.7022	no
gi|646746556|gb|KK962281.1|	12908-81337	20	9.1	3	2	-0.396612	0.8098	no
gi|646746556|gb|KK962281.1|	31399-83261	20	9.1	0.771712	0.553115	-0.480482	1	no
gi|646746556|gb|KK962281.1|	42695-84399	20	9.1	0.708213	0.381413	-0.892831	1	no
gi|646746556|gb|KK962281.1|	47435-84938	20	9.1	3	2	-0.441102	0.7695	no
gi|646746556|gb|KK962281.1|	49619-85042	20	9.1	2	2	-0.31211	0.8121	no
gi|646746556|gb|KK962281.1|	79031-87927	20	9.1	44	13	-2	0.2462	no
gi|646746556|gb|KK962281.1|	83100-88352	20	9.1	12	7	-0.813086	0.57325	no
gi|646746556|gb|KK962281.1|	90029-89237	20	9.1	0.842686	0.708007	-0.251232	1	no
gi|646746556|gb|KK962281.1|	10856-91237	20	9.1	0.953742	0.422959	-1	1	no
gi|646746556|gb|KK962281.1|	27255-92777	20	9.1	2	5	1	0.4188	no
gi|646746556|gb|KK962281.1|	29367-92979	20	9.1	4	7	0.8515	0.5268	no

gi|646746556|gb|KK962281.1|	33883-93551	20	9.1	0.806809	0.097044	-3	1	no
gi|646746556|gb|KK962281.1|	36944-94010	20	9.1	3	4	0.55734	0.71445	no
gi|646746556|gb|KK962281.1|	47982-95510	20	9.1	16	16	0.0550675	0.9605	no
gi|646746556|gb|KK962281.1|	94891-96057	20	9.1	2	0.872339	-0.897238	0.50335	no
gi|646746556|gb|KK962281.1|	57319-95916	20	9.1	66	72	0.109398	0.9236	no
gi|646746556|gb|KK962281.1|	59264-96222	20	9.1	111	89	-0.320574	0.7903	no
gi|646746566|gb|KK962280.1|	13825-14158	13	6.9	31	12	-1	0.36135	no
gi|646746566|gb|KK962280.1|	26143-22725	13	6.9	6	4	-0.534861	0.7311	no
gi|646746566|gb|KK962280.1|	54582-25487	13	6.9	8	16	1	0.4558	no
gi|646746566|gb|KK962280.1|	24214-42489	13	6.9	0	24	inf	5.00E-05	yes
gi|646746566|gb|KK962280.1|	25376-42732	13	6.9	0.382299	257	9	0.02865	no
gi|646746566|gb|KK962280.1|	33910-43467	13	6.9	0	45	inf	5.00E-05	yes
gi|646746566|gb|KK962280.1|	43478-44420	13	6.9	0.197604	27	7	0.0952	no
gi|646746566|gb|KK962280.1|	51850-45244	13	6.9	0.268302	64	8	0.09445	no
gi|646746566|gb|KK962280.1|	55700-45611	13	6.9	6	1683.31	8	0.0126	no
gi|646746566|gb|KK962280.1|	83834-48457	13	6.9	2	0	-inf	0.00475	no
gi|646746566|gb|KK962280.1|	91182-49206	13	6.9	0.890849	0.841078	-0.0829409	1	no
gi|646746566|gb|KK962280.1|	35508-53615	13	6.9	3	2	-0.904898	0.5339	no
gi|646746581|gb|KK962279.1|	00976-10129	21	9.1	12	4	-1	0.36425	no
gi|646746581|gb|KK962279.1|	04542-10692	21	9.1	18	9	-0.984295	0.36395	no
gi|646746581|gb|KK962279.1|	34348-13465	21	9.1	9	15	0.76966	0.5851	no
gi|646746581|gb|KK962279.1|	48220-14851	21	9.1	19	42	1.11	0.4442	no
gi|646746581|gb|KK962279.1|	49455-15068	21	9.1	0.856566	0.272942	-2	1	no
gi|646746581|gb|KK962279.1|	50953-15205	21	9.1	0.548025	1	1	1	no
gi|646746581|gb|KK962279.1|	63513-16477	21	9.1	34	23	-0.591727	0.58635	no
gi|646746581|gb|KK962279.1|	80644-18112	21	9.1	3	7	1	0.42775	no
gi|646746581|gb|KK962279.1|	18382-18856	21	9.1	18	3	-3	0.11815	no
gi|646746581|gb|KK962279.1|	91923-19470	21	9.1	7	8	0.122402	0.9195	no
gi|646746581|gb|KK962279.1|	12385-21272	21	9.1	13	26	1	0.45985	no
gi|646746581|gb|KK962279.1|	23017-23312	21	9.1	9	52	3	0.15165	no

gi|646746581|gb|KK962279.1|	42722-24297	21	9.1	32	27	-0.244894	0.84665	no
gi|646746581|gb|KK962279.1|	60776-26155	21	9.1	0.885956	1	0.75547	0.6148	no
gi|646746581|gb|KK962279.1|	64659-26709	21	9.1	46	40	-0.204298	0.8619	no
gi|646746581|gb|KK962279.1|	26853-28259	21	9.1	1	2	1	0.46085	no
gi|646746581|gb|KK962279.1|	71561-27327	21	9.1	0.761438	0.733348	-0.0542286	1	no
gi|646746581|gb|KK962279.1|	74318-27625	21	9.1	19	19	0.0363105	0.9733	no
gi|646746581|gb|KK962279.1|	86369-28795	21	9.1	0.069559	28	9	0.09445	no
gi|646746581|gb|KK962279.1|	90620-29115	21	9.1	63	23	-1	0.3418	no
gi|646746581|gb|KK962279.1|	97898-29816	21	9.1	10	11	0.171645	0.89715	no
gi|646746581|gb|KK962279.1|	08275-31538	21	9.1	12	6	-1	0.3754	no
gi|646746581|gb|KK962279.1|	15882-31729	21	9.1	2	0.460256	-2	0.2261	no
gi|646746581|gb|KK962279.1|	18163-31878	21	9.1	3	0.703153	-2	0.2673	no
gi|646746581|gb|KK962279.1|	18942-32055	21	9.1	1	1	-0.0183466	0.9627	no
gi|646746581|gb|KK962279.1|	25429-32648	21	9.1	1	2	0.387708	0.75975	no
gi|646746581|gb|KK962279.1|	26631-32723	21	9.1	3	4	0.588273	0.6289	no
gi|646746581|gb|KK962279.1|	29016-32987	21	9.1	0.612576	2	2	0.36865	no
gi|646746581|gb|KK962279.1|	42618-34387	21	9.1	0.277807	1	2	1	no
gi|646746581|gb|KK962279.1|	43977-34603	21	9.1	0.922095	5	3	0.087	no
gi|646746581|gb|KK962279.1|	35151-35333	21	9.1	230	297	0.369875	0.7872	no
gi|646746581|gb|KK962279.1|	53479-35420	21	9.1	2	2	0.122715	0.92795	no
gi|646746581|gb|KK962279.1|	81203-38223	21	9.1	7	2	-2	0.26785	no
gi|646746581|gb|KK962279.1|	83293-38387	21	9.1	3	0.772675	-2	0.2673	no
gi|646746581|gb|KK962279.1|	83990-38722	21	9.1	16	11	-0.635658	0.56255	no
gi|646746581|gb|KK962279.1|	00778-40183	21	9.1	1	0.164613	-3	0.1932	no
gi|646746581|gb|KK962279.1|	02033-40500	21	9.1	2	1	-0.301674	0.84375	no
gi|646746581|gb|KK962279.1|	41217-41461	21	9.1	19	50	1	0.4079	no
gi|646746581|gb|KK962279.1|	17702-41799	21	9.1	19	0	-inf	0.0041	no
gi|646746581|gb|KK962279.1|	18089-41913	21	9.1	1	1	-0.0724103	1	no
gi|646746581|gb|KK962279.1|	42122-43158	21	9.1	2	6	2	0.25565	no
gi|646746581|gb|KK962279.1|	25670-42702	21	9.1	5	3	-0.868687	0.5724	no

gi|646746581|gb|KK962279.1|	28390-43001	21	9.1	3	2	-0.58023	0.71635	no
gi|646746581|gb|KK962279.1|	43606-45107	21	9.1	1	1	0.304664	0.7998	no
gi|646746581|gb|KK962279.1|	38919-44067	21	9.1	6	6	0.141956	0.9265	no
gi|646746581|gb|KK962279.1|	49858-45156	21	9.1	2	9	2	0.23615	no
gi|646746581|gb|KK962279.1|	45382-45589	21	9.1	218	117	-0.892096	0.5839	no
gi|646746581|gb|KK962279.1|	93081-49434	21	9.1	56	63	0.164366	0.88305	no
gi|646746581|gb|KK962279.1|	95370-49725	21	9.1	1	0.823258	-0.393028	1	no
gi|646746581|gb|KK962279.1|	14859-51649	21	9.1	166	133	-0.324861	0.78355	no
gi|646746581|gb|KK962279.1|	36140-53642	21	9.1	5	17	2	0.2677	no
gi|646746581|gb|KK962279.1|	54881-55541	21	9.1	4	2	-0.934158	0.52525	no
gi|646746581|gb|KK962279.1|	78765-57898	21	9.1	40	80	0.999906	0.5541	no
gi|646746581|gb|KK962279.1|	19512-61983	21	9.1	10	7	-0.568509	0.7215	no
gi|646746581|gb|KK962279.1|	62119-63467	21	9.1	2	3	0.456411	0.78285	no
gi|646746581|gb|KK962279.1|	76487-78205	21	9.1	0.6956	1	0.978267	0.53675	no
gi|646746581|gb|KK962279.1|	68833-76954	21	9.1	2	10	2	0.1644	no
gi|646746581|gb|KK962279.1|	75099-77976	21	9.1	17	6	-1	0.20585	no
gi|646746581|gb|KK962279.1|	83617-78522	21	9.1	13	5	-1	0.41225	no
gi|646746581|gb|KK962279.1|	01017-80185	21	9.1	4	1	-2	0.2717	no
gi|646746581|gb|KK962279.1|	04916-80547	21	9.1	6	0.841513	-3	0.12165	no
gi|646746581|gb|KK962279.1|	19698-82056	21	9.1	26.45	25	-0.0797166	0.9592	no
gi|646746581|gb|KK962279.1|	20691-82217	21	9.1	2	2	-0.430203	0.788	no
gi|646746581|gb|KK962279.1|	35489-83722	21	9.1	4	3	-0.221031	0.88595	no
gi|646746581|gb|KK962279.1|	47062-84903	21	9.1	0.268943	0.70096	1	1	no
gi|646746581|gb|KK962279.1|	52078-85315	21	9.1	1	1	-0.186214	0.88995	no
gi|646746581|gb|KK962279.1|	94561-97656	21	9.1	0.65155	0.805964	0.306839	1	no
gi|646746581|gb|KK962279.1|	97882-98274	21	9.1	23	20	-0.184489	0.9052	no
gi|646746595|gb|KK962278.1|	55809-12574	20	11	5	6	0.508286	0.73445	no
gi|646746595|gb|KK962278.1|	19614-13206	20	11	6	4	-0.634592	0.6905	no
gi|646746595|gb|KK962278.1|	00804-30144	20	11	1	2	0.472041	0.74895	no
gi|646746595|gb|KK962278.1|	33353-43396	20	11	2	1	-0.119139	0.9032	no

gi|646746595|gb|KK962278.1|	77005-57714	20	11	544	731	0.427936	0.75935	no
gi|646746595|gb|KK962278.1|	72458-67266	20	11	33	70	1	0.44225	no
gi|646746595|gb|KK962278.1|	01947-70385	20	11	5	11	1	0.4523	no
gi|646746595|gb|KK962278.1|	04267-70588	20	11	0.951649	2	1	0.40605	no
gi|646746595|gb|KK962278.1|	06494-70801	20	11	0.513553	0.422959	-0.279998	1	no
gi|646746595|gb|KK962278.1|	10160-71258	20	11	0.681535	2	1	0.384	no
gi|646746595|gb|KK962278.1|	12729-71382	20	11	12	6	-1	0.5024	no
gi|646746595|gb|KK962278.1|	40914-74195	20	11	2	1	-0.70989	0.5885	no
gi|646746595|gb|KK962278.1|	45997-74817	20	11	266	72	-2	0.12445	no
gi|646746595|gb|KK962278.1|	48346-74978	20	11	0.860214	0.112582	-3	1	no
gi|646746595|gb|KK962278.1|	53700-75499	20	11	1	1	-0.156067	0.90205	no
gi|646746595|gb|KK962278.1|	55138-75586	20	11	1	1	-0.321362	0.8509	no
gi|646746595|gb|KK962278.1|	58600-76206	20	11	0.952632	0.546622	-0.801375	1	no
gi|646746595|gb|KK962278.1|	62825-76580	20	11	31	32	0.069617	0.95045	no
gi|646746595|gb|KK962278.1|	17443-81822	20	11	17	32	0.885787	0.5665	no
gi|646746595|gb|KK962278.1|	35964-83675	20	11	2	2	-0.0201605	0.9367	no
gi|646746595|gb|KK962278.1|	09860-91014	20	11	6	19	2	0.35485	no
gi|646746622|gb|KK962276.1|	07876-20816	11	5	23	8	-2	0.25655	no
gi|646746622|gb|KK962276.1|	11620-21333	11	5	14	6	-1	0.3711	no
gi|646746636|gb|KK962275.1|	08080-11057	18	9.6	27	28	0.0471858	0.9644	no
gi|646746636|gb|KK962275.1|	29952-13056	18	9.6	9	3	-2	0.30635	no
gi|646746636|gb|KK962275.1|	32494-13352	18	9.6	5	3	-0.777562	0.6244	no
gi|646746636|gb|KK962275.1|	34390-13651	18	9.6	19	1	-4	0.0266	no
gi|646746636|gb|KK962275.1|	17855-18982	18	9.6	0.318259	1	2	1	no
gi|646746636|gb|KK962275.1|	79232-17976	18	9.6	13	3	-2	0.1866	no
gi|646746636|gb|KK962275.1|	80362-18397	18	9.6	79	4	-4	0.011	no
gi|646746636|gb|KK962275.1|	97668-19899	18	9.6	2	0.123888	-4	0.19065	no
gi|646746636|gb|KK962275.1|	00374-20125	18	9.6	97	42	-1	0.2771	no
gi|646746636|gb|KK962275.1|	39067-24311	18	9.6	37	14	-1	0.2166	no
gi|646746636|gb|KK962275.1|	64820-26589	18	9.6	265	18	-4	0.0149	no

gi|646746636|gb|KK962275.1|	67709-26878	18	9.6	1	0.642857	-0.945344	0.5451	no
gi|646746636|gb|KK962275.1|	69443-27010	18	9.6	22	13	-0.701851	0.6347	no
gi|646746636|gb|KK962275.1|	87727-28888	18	9.6	6	9	0.564152	0.7055	no
gi|646746636|gb|KK962275.1|	89550-29162	18	9.6	56	27	-1	0.3732	no
gi|646746636|gb|KK962275.1|	52711-59025	18	9.6	3	3	-0.310293	0.80585	no
gi|646746636|gb|KK962275.1|	67935-68912	18	9.6	5	3	-0.976583	0.54835	no
gi|646746636|gb|KK962275.1|	77539-77867	18	9.6	60	71	0.23101	0.8776	no
gi|646746636|gb|KK962275.1|	78335-80343	18	9.6	0.474985	3	2	0.12875	no
gi|646746636|gb|KK962275.1|	80448-81242	18	9.6	1	0.966928	-0.505693	0.7384	no
gi|646746636|gb|KK962275.1|	84029-85277	18	9.6	52	24	-1	0.32885	no
gi|646746651|gb|KK962274.1|	05843-10628	16	6.6	24	12	-0.969994	0.54175	no
gi|646746651|gb|KK962274.1|	10726-12693	16	6.6	1	2	1.07	0.43135	no
gi|646746651|gb|KK962274.1|	20203-12041	16	6.6	50	94	0.894759	0.5622	no
gi|646746651|gb|KK962274.1|	20882-12148	16	6.6	28	16	-0.765068	0.6168	no
gi|646746651|gb|KK962274.1|	23630-12408	16	6.6	8	3	-1	0.30405	no
gi|646746651|gb|KK962274.1|	36112-13782	16	6.6	216	151	-0.510982	0.6718	no
gi|646746651|gb|KK962274.1|	14301-15440	16	6.6	1	3	0.986237	0.52035	no
gi|646746651|gb|KK962274.1|	16274-17923	16	6.6	0.199098	2	3	0.152	no
gi|646746651|gb|KK962274.1|	71279-17392	16	6.6	7	12	0.816285	0.52645	no
gi|646746651|gb|KK962274.1|	74966-17572	16	6.6	0.913941	2	0.753589	0.6148	no
gi|646746651|gb|KK962274.1|	87914-19101	16	6.6	10	8	-0.380437	0.72365	no
gi|646746651|gb|KK962274.1|	19284-19984	16	6.6	2	8	2	0.182	no
gi|646746651|gb|KK962274.1|	96735-19689	16	6.6	193.12	262	0.439313	0.75955	no
gi|646746651|gb|KK962274.1|	99070-19972	16	6.6	5	4	-0.285186	0.8381	no
gi|646746651|gb|KK962274.1|	99860-20183	16	6.6	3	3	0.411209	0.79255	no
gi|646746651|gb|KK962274.1|	01942-20321	16	6.6	1	3	1	0.38265	no
gi|646746651|gb|KK962274.1|	04859-20574	16	6.6	1	2	0.962315	0.49675	no
gi|646746651|gb|KK962274.1|	21838-22929	16	6.6	2	3	1	0.46225	no
gi|646746651|gb|KK962274.1|	18484-22035	16	6.6	0.39976	1	1	1	no
gi|646746651|gb|KK962274.1|	28090-22991	16	6.6	28	10	-2	0.2644	no

gi|646746651|gb|KK962274.1|	24461-27962	16	6.6	135	204	0.597514	0.6175	no
gi|646746651|gb|KK962274.1|	69850-27145	16	6.6	2	1	-0.230006	0.86825	no
gi|646746651|gb|KK962274.1|	35662-36004	16	6.6	30	5	-3	0.1347	no
gi|646746651|gb|KK962274.1|	4171-4792	16	6.6	1	3	1	0.3434	no
gi|646746651|gb|KK962274.1|	44821-45073	16	6.6	24	9	-1	0.37075	no
gi|646746651|gb|KK962274.1|	77671-78544	16	6.6	3	2	-0.431266	0.71965	no
gi|646746651|gb|KK962274.1|	98207-98657	16	6.6	6	3	-0.974768	0.5242	no
gi|646746663|gb|KK962273.1|	34542-13478	21	10	26	45	0.782737	0.5535	no
gi|646746663|gb|KK962273.1|	36557-13740	21	10	2	3	0.17776	0.88665	no
gi|646746663|gb|KK962273.1|	37843-13922	21	10	1	2	0.417378	0.7207	no
gi|646746663|gb|KK962273.1|	42449-14476	21	10	79	50	-0.661454	0.57285	no
gi|646746663|gb|KK962273.1|	45650-14635	21	10	1	0.86337	-0.739627	0.6333	no
gi|646746663|gb|KK962273.1|	48969-15026	21	10	20	13	-0.59987	0.63135	no
gi|646746663|gb|KK962273.1|	52476-15342	21	10	0.937072	0.57068	-0.715478	1	no
gi|646746663|gb|KK962273.1|	61229-16324	21	10	0.474174	1	1	0.3822	no
gi|646746663|gb|KK962273.1|	66112-16990	21	10	2	2	0.496964	0.73675	no
gi|646746663|gb|KK962273.1|	70127-17134	21	10	0.869082	3	2	0.28595	no
gi|646746663|gb|KK962273.1|	84488-19061	21	10	2	2	0.151169	0.91495	no
gi|646746663|gb|KK962273.1|	95666-19660	21	10	0.679163	3	2	0.22	no
gi|646746663|gb|KK962273.1|	00723-20169	21	10	1	3	1	0.48525	no
gi|646746663|gb|KK962273.1|	02753-20328	21	10	2	3	0.440786	0.75535	no
gi|646746663|gb|KK962273.1|	17141-21776	21	10	2	3	0.78911	0.56915	no
gi|646746663|gb|KK962273.1|	35778-23612	21	10	15	13	-0.190356	0.8762	no
gi|646746663|gb|KK962273.1|	47361-24757	21	10	30	35	0.225985	0.8687	no
gi|646746663|gb|KK962273.1|	68600-27139	21	10	16	13	-0.245223	0.82745	no
gi|646746663|gb|KK962273.1|	74107-27926	21	10	1	1	0.311553	0.83705	no
gi|646746663|gb|KK962273.1|	88626-29022	21	10	11	6	-0.734065	0.62805	no
gi|646746663|gb|KK962273.1|	02915-30333	21	10	3	4	0.11729	0.9137	no
gi|646746663|gb|KK962273.1|	04344-30537	21	10	1	0.848807	-0.625611	0.66015	no
gi|646746663|gb|KK962273.1|	14172-31475	21	10	6	3	-0.863276	0.49435	no

gi|646746663|gb|KK962273.1|	33997-33507	21	10	1	1	0.151379	0.9186	no
gi|646746663|gb|KK962273.1|	35176-33622	21	10	0.696403	0.993707	0.512899	1	no
gi|646746663|gb|KK962273.1|	36354-33687	21	10	18	20	0.0933079	0.94855	no
gi|646746663|gb|KK962273.1|	41166-34274	21	10	1	1	-0.319488	0.807	no
gi|646746663|gb|KK962273.1|	43786-34542	21	10	0.937191	2	0.722189	0.60775	no
gi|646746663|gb|KK962273.1|	35450-36300	21	10	55	15	-2	0.1577	no
gi|646746663|gb|KK962273.1|	36566-36961	21	10	9	2	-2	0.1604	no
gi|646746663|gb|KK962273.1|	75531-37582	21	10	665	197	-2	0.2006	no
gi|646746663|gb|KK962273.1|	87154-38816	21	10	7	9	0.224115	0.8801	no
gi|646746663|gb|KK962273.1|	91536-39222	21	10	0.852148	3	2	0.27835	no
gi|646746663|gb|KK962273.1|	94329-39479	21	10	994	259	-2	0.1069	no
gi|646746663|gb|KK962273.1|	40032-40504	21	10	6	0	-inf	0.00225	no
gi|646746663|gb|KK962273.1|	41319-41730	21	10	14	4	-2	0.24635	no
gi|646746663|gb|KK962273.1|	65508-46653	21	10	2	3	0.604795	0.6684	no
gi|646746663|gb|KK962273.1|	69485-47043	21	10	0.92635	0.564351	-0.714964	1	no
gi|646746663|gb|KK962273.1|	73797-47485	21	10	0.114629	3	5	0.1111	no
gi|646746663|gb|KK962273.1|	75211-47558	21	10	7	24	2	0.2355	no
gi|646746663|gb|KK962273.1|	79342-48044	21	10	2	7	2	0.2362	no
gi|646746663|gb|KK962273.1|	19237-51982	21	10	2	2	-0.349239	0.81835	no
gi|646746663|gb|KK962273.1|	31480-53179	21	10	14	20	0.484258	0.70805	no
gi|646746663|gb|KK962273.1|	32317-53300	21	10	12	6	-0.908988	0.5565	no
gi|646746663|gb|KK962273.1|	54930-55776	21	10	19	25	0.352495	0.82275	no
gi|646746663|gb|KK962273.1|	56225-56517	21	10	23	13	-0.88012	0.5069	no
gi|646746663|gb|KK962273.1|	67153-56982	21	10	14	1	-3	0.0364	no
gi|646746663|gb|KK962273.1|	88828-58983	21	10	3	0.176381	-4	0.19065	no
gi|646746663|gb|KK962273.1|	61601-62058	21	10	2	5	2	0.35625	no
gi|646746663|gb|KK962273.1|	6314-7584	21	10	4	1	-2	0.2362	no
gi|646746663|gb|KK962273.1|	63477-64230	21	10	10	11	0.215393	0.88785	no
gi|646746663|gb|KK962273.1|	67614-70050	21	10	2	2	0.377461	0.8086	no
gi|646746663|gb|KK962273.1|	70113-71778	21	10	0.65613	0.947404	0.529997	1	no

gi|646746663|gb|KK962273.1|	71959-73404	21	10	3	3	-0.041172	0.97795	no
gi|646746663|gb|KK962273.1|	73543-75138	21	10	1	1	0.0882569	0.943	no
gi|646746663|gb|KK962273.1|	76101-77262	21	10	2	4	0.643141	0.6938	no
gi|646746663|gb|KK962273.1|	89373-90746	21	10	3	0.951992	-1	0.3232	no
gi|646746680|gb|KK962272.1|	60130-26153	12	6	0.080655	13	7	0.09455	no
gi|646746680|gb|KK962272.1|	67178-26762	12	6	2	4	0.984343	0.5906	no
gi|646746680|gb|KK962272.1|	28715-32944	12	6	10	5	-0.986973	0.53875	no
gi|646746680|gb|KK962272.1|	43423-54383	12	6	41	13	-2	0.29535	no
gi|646746694|gb|KK962271.1|	78821-28082	19	7.6	3	9	2	0.3062	no
gi|646746694|gb|KK962271.1|	01950-30412	19	7.6	2	8	2	0.26475	no
gi|646746694|gb|KK962271.1|	07643-30793	19	7.6	3	19	3	0.25945	no
gi|646746694|gb|KK962271.1|	27844-32857	19	7.6	9	9	0.0498936	0.97535	no
gi|646746694|gb|KK962271.1|	98412-39877	19	7.6	51	10	-2	0.11165	no
gi|646746694|gb|KK962271.1|	45632-44695	19	7.6	5	3	-0.88446	0.5683	no
gi|646746694|gb|KK962271.1|	61252-46409	19	7.6	2	2	0.0129579	0.9864	no
gi|646746694|gb|KK962271.1|	64206-46615	19	7.6	1	3	0.778875	0.61785	no
gi|646746694|gb|KK962271.1|	70123-47109	19	7.6	10	5	-1	0.4922	no
gi|646746694|gb|KK962271.1|	86612-48711	19	7.6	4	2	-0.830499	0.56075	no
gi|646746694|gb|KK962271.1|	64863-56568	19	7.6	4	2	-1	0.4551	no
gi|646746694|gb|KK962271.1|	75222-57691	19	7.6	0.707691	0.371696	-0.928999	1	no
gi|646746706|gb|KK962270.1|	19776-10215	23	11	0.500757	1	2	0.349	no
gi|646746706|gb|KK962270.1|	21626-10258	23	11	0.91212	1	0.544465	0.72115	no
gi|646746706|gb|KK962270.1|	27525-10291	23	11	0.589681	0.662258	0.167459	1	no
gi|646746706|gb|KK962270.1|	51677-10524	23	11	4	1	-2	0.3017	no
gi|646746706|gb|KK962270.1|	58787-10592	23	11	0	11	inf	0.0026	no
gi|646746706|gb|KK962270.1|	62724-10646	23	11	0.454623	1	1	0.32365	no
gi|646746706|gb|KK962270.1|	67576-10684	23	11	3	2	-0.693397	0.60075	no
gi|646746706|gb|KK962270.1|	76012-10820	23	11	4	4	0.161869	0.87905	no
gi|646746706|gb|KK962270.1|	83269-10860	23	11	0.516662	0.268256	-0.945609	1	no
gi|646746706|gb|KK962270.1|	86148-10885	23	11	2	0.637594	-1	0.339	no

gi|646746706|gb|KK962270.1|	92026-10926	23	11	2	0.747353	-2	0.29375	no
gi|646746706|gb|KK962270.1|	97330-11005	23	11	6	8	0.278569	0.82745	no
gi|646746706|gb|KK962270.1|	03711-11044	23	11	7	0.738341	-3	0.11075	no
gi|646746706|gb|KK962270.1|	06408-11083	23	11	7	5	-0.341111	0.82095	no
gi|646746706|gb|KK962270.1|	09694-11101	23	11	5	1	-2	0.19175	no
gi|646746706|gb|KK962270.1|	31531-13211	23	11	18	26	0.524415	0.7229	no
gi|646746706|gb|KK962270.1|	23572-23161	23	11	16	13	-0.321689	0.7792	no
gi|646746706|gb|KK962270.1|	43500-24416	23	11	2	1	-0.693689	0.64585	no
gi|646746706|gb|KK962270.1|	45805-24672	23	11	10	12	0.279138	0.85185	no
gi|646746706|gb|KK962270.1|	24719-28535	23	11	4	3	-0.561224	0.7047	no
gi|646746706|gb|KK962270.1|	51060-25193	23	11	2	4	0.781647	0.58135	no
gi|646746706|gb|KK962270.1|	52042-25324	23	11	1	2	0.419429	0.72465	no
gi|646746706|gb|KK962270.1|	54575-25549	23	11	11	12	0.174232	0.9089	no
gi|646746706|gb|KK962270.1|	56523-25718	23	11	5	3	-0.662001	0.6519	no
gi|646746706|gb|KK962270.1|	69474-26982	23	11	15	26	0.812227	0.5555	no
gi|646746706|gb|KK962270.1|	79306-28091	23	11	65	48	-0.443774	0.69825	no
gi|646746706|gb|KK962270.1|	83217-28375	23	11	52	25	-1	0.49695	no
gi|646746706|gb|KK962270.1|	86722-28764	23	11	3	4	0.282767	0.8338	no
gi|646746706|gb|KK962270.1|	28847-29621	23	11	1	3	0.81402	0.5504	no
gi|646746706|gb|KK962270.1|	90886-29126	23	11	11	8	-0.38993	0.76265	no
gi|646746706|gb|KK962270.1|	92313-29253	23	11	47	83	0.821067	0.5898	no
gi|646746706|gb|KK962270.1|	16597-31689	23	11	65	46	-0.510146	0.7487	no
gi|646746706|gb|KK962270.1|	25123-32579	23	11	3	5	0.669958	0.6335	no
gi|646746706|gb|KK962270.1|	44640-34487	23	11	27	32	0.245146	0.86255	no
gi|646746706|gb|KK962270.1|	45944-34611	23	11	212	151	-0.48461	0.73635	no
gi|646746706|gb|KK962270.1|	53823-35491	23	11	2	3	0.192616	0.89005	no
gi|646746706|gb|KK962270.1|	36308-36598	23	11	5	31	3	0.18	no
gi|646746706|gb|KK962270.1|	68852-36981	23	11	1	1	0.186311	0.8968	no
gi|646746706|gb|KK962270.1|	70560-37254	23	11	0.533544	0.540845	0.0196094	1	no
gi|646746706|gb|KK962270.1|	72757-37420	23	11	0.699085	0.894718	0.355966	1	no

gi|646746706|gb|KK962270.1|	79344-38048	23	11	0.727588	1	1	0.44735	no
gi|646746706|gb|KK962270.1|	85494-38659	23	11	2	2	0.0677057	0.95335	no
gi|646746706|gb|KK962270.1|	86856-38846	23	11	36	19	-0.898921	0.4223	no
gi|646746706|gb|KK962270.1|	95953-39660	23	11	0	17	inf	0.00025	yes
gi|646746706|gb|KK962270.1|	40056-40272	23	11	27	43	0.659938	0.6552	no
gi|646746706|gb|KK962270.1|	02735-40850	23	11	22	19	-0.243746	0.83385	no
gi|646746706|gb|KK962270.1|	08817-41011	23	11	0.620874	1	0.714794	1	no
gi|646746706|gb|KK962270.1|	10970-41181	23	11	1	0.89223	-0.501109	0.73865	no
gi|646746706|gb|KK962270.1|	14313-41579	23	11	0.377294	0.652225	0.789679	1	no
gi|646746706|gb|KK962270.1|	15900-41645	23	11	6	10	0.803284	0.5621	no
gi|646746706|gb|KK962270.1|	19911-42010	23	11	46	51	0.139858	0.90425	no
gi|646746706|gb|KK962270.1|	27034-42729	23	11	14	13	-0.115183	0.9144	no
gi|646746706|gb|KK962270.1|	43480-43635	23	11	314	199	-0.657975	0.65155	no
gi|646746706|gb|KK962270.1|	97433-49781	23	11	18	29	0.740126	0.63515	no
gi|646746706|gb|KK962270.1|	01481-60182	23	11	14	28	0.980863	0.50085	no
gi|646746706|gb|KK962270.1|	15636-61610	23	11	54	41	-0.391498	0.79735	no
gi|646746706|gb|KK962270.1|	30728-63167	23	11	0.934368	1	0.507044	0.7286	no
gi|646746706|gb|KK962270.1|	41959-64254	23	11	2	3	0.459445	0.74015	no
gi|646746706|gb|KK962270.1|	50075-65141	23	11	0.937898	1	0.523314	0.686	no
gi|646746706|gb|KK962270.1|	51587-65245	23	11	3	4	0.73576	0.60565	no
gi|646746706|gb|KK962270.1|	54437-65548	23	11	0.117392	29	8	0.09445	no
gi|646746706|gb|KK962270.1|	56764-65754	23	11	2	3	0.782617	0.5545	no
gi|646746706|gb|KK962270.1|	70574-67192	23	11	10	9	-0.209457	0.88845	no
gi|646746706|gb|KK962270.1|	74840-68131	23	11	8	6	-0.502643	0.65155	no
gi|646746706|gb|KK962270.1|	82497-68377	23	11	10	7	-0.491705	0.74215	no
gi|646746706|gb|KK962270.1|	86079-68727	23	11	0.987284	0.282899	-2	1	no
gi|646746706|gb|KK962270.1|	94444-70547	23	11	6	4	-0.697853	0.53425	no
gi|646746706|gb|KK962270.1|	06641-70707	23	11	9	2	-2	0.189	no
gi|646746706|gb|KK962270.1|	19236-72019	23	11	1	2	0.677543	0.6345	no
gi|646746706|gb|KK962270.1|	20311-72153	23	11	0.666904	0.546433	-0.287433	1	no

gi|646746706|gb|KK962270.1|	21641-72267	23	11	1	1	-0.0727628	0.94655	no
gi|646746706|gb|KK962270.1|	64484-77006	23	11	10	8	-0.34726	0.7611	no
gi|646746706|gb|KK962270.1|	72809-77589	23	11	0.424854	0.523151	0.30026	1	no
gi|646746706|gb|KK962270.1|	76136-77637	23	11	72	91	0.320547	0.84385	no
gi|646746706|gb|KK962270.1|	78346-77882	23	11	137	105	-0.384011	0.7278	no
gi|646746706|gb|KK962270.1|	81834-78271	23	11	9	11	0.220488	0.88105	no
gi|646746706|gb|KK962270.1|	96428-79675	23	11	38	42	0.148661	0.9224	no
gi|646746706|gb|KK962270.1|	04758-80550	23	11	1	2	0.296545	0.83555	no
gi|646746706|gb|KK962270.1|	80950-86147	23	11	15	22	0.567341	0.61925	no
gi|646746706|gb|KK962270.1|	16515-81769	23	11	0.400036	1	2	0.36595	no
gi|646746706|gb|KK962270.1|	21570-82197	23	11	11	16	0.519088	0.7079	no
gi|646746706|gb|KK962270.1|	25601-82582	23	11	26	26	0.0295392	0.91805	no
gi|646746706|gb|KK962270.1|	26172-82673	23	11	6	7	0.148757	0.91235	no
gi|646746706|gb|KK962270.1|	36781-83751	23	11	5	7	0.323999	0.83495	no
gi|646746706|gb|KK962270.1|	37745-83834	23	11	4	4	-0.0236752	0.95325	no
gi|646746706|gb|KK962270.1|	38696-84841	23	11	4	5	0.326231	0.771	no
gi|646746706|gb|KK962270.1|	52005-85271	23	11	1	1	0.483375	0.7721	no
gi|646746706|gb|KK962270.1|	53496-85422	23	11	1	2	0.849118	0.548	no
gi|646746706|gb|KK962270.1|	55355-86129	23	11	19	19	0.0145993	0.9896	no
gi|646746706|gb|KK962270.1|	70957-87257	23	11	0.408751	0.491546	0.266107	1	no
gi|646746706|gb|KK962270.1|	85898-88665	23	11	2	0.782269	-1	0.427	no
gi|646746706|gb|KK962270.1|	09567-91104	23	11	0.605647	0.654322	0.111523	1	no
gi|646746706|gb|KK962270.1|	16993-91823	23	11	3	14	2	0.1487	no
gi|646746723|gb|KK962269.1|	23628-22380	16	5.2	30	115	2	0.3508	no
gi|646746723|gb|KK962269.1|	75815-27684	16	5.2	0.835528	1	0.704434	0.62425	no
gi|646746723|gb|KK962269.1|	78181-28077	16	5.2	39	49	0.352163	0.766	no
gi|646746723|gb|KK962269.1|	64549-36502	16	5.2	0	5	inf	0.00255	no
gi|646746723|gb|KK962269.1|	88929-49089	16	5.2	0.054103	3	6	0.0973	no
gi|646746723|gb|KK962269.1|	00323-50227	16	5.2	0.109293	33	8	0.2322	no
gi|646746723|gb|KK962269.1|	03145-50523	16	5.2	0.151089	10	6	0.10595	no

gi|646746723|gb|KK962269.1|	31580-53268	16	5.2	0.759142	0.930137	0.293075	1	no
gi|646746723|gb|KK962269.1|	54369-55558	16	5.2	38	4	-3	0.0298	no
gi|646746723|gb|KK962269.1|	83139-58332	16	5.2	91	61	-0.582092	0.7095	no
gi|646746723|gb|KK962269.1|	38611-63912	16	5.2	4	4	0.020362	0.9493	no
gi|646746723|gb|KK962269.1|	69840-67015	16	5.2	36	12	-2	0.2842	no
gi|646746723|gb|KK962269.1|	87168-68989	16	5.2	1	2	0.177089	0.91175	no
gi|646746723|gb|KK962269.1|	90007-69099	16	5.2	1	1	-0.00508392	0.9367	no
gi|646746723|gb|KK962269.1|	95265-69593	16	5.2	2	7	2	0.20435	no
gi|646746723|gb|KK962269.1|	96512-69736	16	5.2	4	3	-0.347877	0.83735	no
gi|646746723|gb|KK962269.1|	99096-69994	16	5.2	0.619757	2	2	0.2407	no
gi|646746735|gb|KK962268.1|	54862-15552	18	13	2	2	0.254025	0.86925	no
gi|646746735|gb|KK962268.1|	94424-29503	18	13	0	7	inf	0.00315	no
gi|646746752|gb|KK962267.1|	09128-10955	20	7.9	6	1	-2	0.2159	no
gi|646746752|gb|KK962267.1|	13266-14109	20	7.9	7	4	-0.872898	0.59015	no
gi|646746752|gb|KK962267.1|	97049-19737	20	7.9	38	29	-0.362312	0.8198	no
gi|646746752|gb|KK962267.1|	16954-31768	20	7.9	0	30	inf	5.00E-05	yes
gi|646746752|gb|KK962267.1|	29033-33073	20	7.9	0.127871	13	7	0.2322	no
gi|646746752|gb|KK962267.1|	34691-33852	20	7.9	0.02583	21	10	0.09445	no
gi|646746752|gb|KK962267.1|	84725-38731	20	7.9	15	11	-0.422856	0.6956	no
gi|646746752|gb|KK962267.1|	93472-39394	20	7.9	5	7	0.416676	0.7279	no
gi|646746752|gb|KK962267.1|	02885-40313	20	7.9	23	22	-0.0867697	0.93275	no
gi|646746752|gb|KK962267.1|	25526-42642	20	7.9	0.576327	2	2	0.3671	no
gi|646746752|gb|KK962267.1|	31387-43249	20	7.9	32	18	-0.823961	0.51655	no
gi|646746752|gb|KK962267.1|	37199-43759	20	7.9	11	22	1	0.45395	no
gi|646746752|gb|KK962267.1|	39673-44004	20	7.9	5	9	0.856338	0.5431	no
gi|646746752|gb|KK962267.1|	42331-44259	20	7.9	33	16	-1	0.4196	no
gi|646746752|gb|KK962267.1|	84716-48752	20	7.9	16	19	0.232865	0.83455	no
gi|646746752|gb|KK962267.1|	89812-49153	20	7.9	1	1	-0.331601	0.78235	no
gi|646746752|gb|KK962267.1|	93700-49656	20	7.9	32	22	-0.533786	0.6429	no
gi|646746752|gb|KK962267.1|	00282-50178	20	7.9	2	4	1	0.42455	no

gi|646746752|gb|KK962267.1|	5013-6815	20	7.9	6	3	-0.996949	0.51505	no
gi|646746752|gb|KK962267.1|	02777-50505	20	7.9	2	3	0.427127	0.7795	no
gi|646746752|gb|KK962267.1|	28537-52875	20	7.9	46	22	-1	0.471	no
gi|646746752|gb|KK962267.1|	47364-54848	20	7.9	0.644648	2	2	0.3034	no
gi|646746752|gb|KK962267.1|	51120-55133	20	7.9	38	31	-0.309894	0.85005	no
gi|646746752|gb|KK962267.1|	61672-56390	20	7.9	1	1	-0.556683	0.7334	no
gi|646746752|gb|KK962267.1|	72670-57331	20	7.9	0.235732	4	4	0.0994	no
gi|646746752|gb|KK962267.1|	73871-57609	20	7.9	0.704807	0.681459	-0.0486013	1	no
gi|646746752|gb|KK962267.1|	86209-58651	20	7.9	3	15	2	0.28235	no
gi|646746752|gb|KK962267.1|	92567-59286	20	7.9	165	105	-0.657729	0.6708	no
gi|646746752|gb|KK962267.1|	93885-59442	20	7.9	38	13	-2	0.31755	no
gi|646746752|gb|KK962267.1|	11965-61219	20	7.9	42.91	13	-2	0.29155	no
gi|646746752|gb|KK962267.1|	7873-10170	20	7.9	20	8	-1	0.33775	no
gi|646746766|gb|KK962266.1|	15162-15607	18	5.4	5	4	-0.336316	0.8001	no
gi|646746766|gb|KK962266.1|	19301-20138	18	5.4	3	7	1	0.3849	no
gi|646746766|gb|KK962266.1|	85677-28672	18	5.4	0.818631	1	0.29021	1	no
gi|646746766|gb|KK962266.1|	97008-29731	18	5.4	10	16	0.714519	0.59915	no
gi|646746766|gb|KK962266.1|	23318-42560	18	5.4	2	2	0.185418	0.9033	no
gi|646746766|gb|KK962266.1|	37880-43859	18	5.4	2	0.850778	-1	0.4263	no
gi|646746766|gb|KK962266.1|	38714-44018	18	5.4	9	8	-0.263125	0.85935	no
gi|646746766|gb|KK962266.1|	48405-45017	18	5.4	2	3	0.582229	0.71555	no
gi|646746766|gb|KK962266.1|	51350-45227	18	5.4	2	3	1	0.4858	no
gi|646746766|gb|KK962266.1|	81904-82566	18	5.4	4	0.947401	-2	0.2306	no
gi|646746781|gb|KK962265.1|	55869-15647	17	7.2	53	69	0.375595	0.72535	no
gi|646746781|gb|KK962265.1|	21027-42137	17	7.2	9	18	0.984933	0.5352	no
gi|646746781|gb|KK962265.1|	24069-42659	17	7.2	1	1	0.0645649	1	no
gi|646746781|gb|KK962265.1|	26722-42743	17	7.2	1	2	0.706068	0.63615	no
gi|646746781|gb|KK962265.1|	30827-43133	17	7.2	3	4	0.604519	0.6504	no
gi|646746781|gb|KK962265.1|	33294-43808	17	7.2	51	32	-0.662838	0.5764	no
gi|646746812|gb|KK962263.1|	14194-14420	16	6.1	44	9	-2	0.2039	no

gi|646746812|gb|KK962263.1|	22978-25590	16	6.1	11	12	0.0738339	0.9444	no
gi|646746812|gb|KK962263.1|	25767-26202	16	6.1	4	5	0.586766	0.6563	no
gi|646746812|gb|KK962263.1|	26608-29530	16	6.1	29	24	-0.290645	0.79665	no
gi|646746812|gb|KK962263.1|	33497-35368	16	6.1	19	13	-0.524599	0.63005	no
gi|646746812|gb|KK962263.1|	98708-39897	16	6.1	16	54	2	0.2852	no
gi|646746812|gb|KK962263.1|	21967-42233	16	6.1	2	15	3	0.275	no
gi|646746812|gb|KK962263.1|	26344-42685	16	6.1	1	8	3	0.20695	no
gi|646746812|gb|KK962263.1|	33310-43538	16	6.1	21	37	0.790699	0.4883	no
gi|646746812|gb|KK962263.1|	8615-8823	16	6.1	66	26	-1	0.36235	no
gi|646746828|gb|KK962262.1|	25025-12616	20	9.2	0.735019	1	0.516631	1	no
gi|646746828|gb|KK962262.1|	26746-12879	20	9.2	0.412102	1	2	0.2933	no
gi|646746828|gb|KK962262.1|	29853-13145	20	9.2	3	7	1	0.3754	no
gi|646746828|gb|KK962262.1|	35063-13603	20	9.2	3	3	0.0934453	0.95135	no
gi|646746828|gb|KK962262.1|	44864-14506	20	9.2	170	58	-2	0.25165	no
gi|646746828|gb|KK962262.1|	58249-15844	20	9.2	54	33	-0.702195	0.6602	no
gi|646746828|gb|KK962262.1|	73268-17354	20	9.2	13	34	1	0.39355	no
gi|646746828|gb|KK962262.1|	74374-17520	20	9.2	0.322291	3	3	0.2689	no
gi|646746828|gb|KK962262.1|	75488-17678	20	9.2	0.80134	0.894994	0.159463	1	no
gi|646746828|gb|KK962262.1|	86076-18886	20	9.2	30	22	-0.444298	0.6933	no
gi|646746828|gb|KK962262.1|	00096-20240	20	9.2	6	5	-0.327303	0.8252	no
gi|646746828|gb|KK962262.1|	10586-21280	20	9.2	7	10	0.411649	0.74145	no
gi|646746828|gb|KK962262.1|	14507-21769	20	9.2	16	12	-0.39958	0.7156	no
gi|646746828|gb|KK962262.1|	23378-22368	20	9.2	17	13	-0.382343	0.7614	no
gi|646746828|gb|KK962262.1|	37461-23783	20	9.2	32	21	-0.596598	0.70115	no
gi|646746828|gb|KK962262.1|	53624-25389	20	9.2	32	19	-0.74554	0.5625	no
gi|646746828|gb|KK962262.1|	34882-43519	20	9.2	5	18	2	0.2494	no
gi|646746828|gb|KK962262.1|	53321-45410	20	9.2	20	14	-0.558531	0.70745	no
gi|646746828|gb|KK962262.1|	11914-51220	20	9.2	5	13	1	0.37975	no
gi|646746828|gb|KK962262.1|	13151-51340	20	9.2	13	17	0.390732	0.8017	no
gi|646746828|gb|KK962262.1|	27057-52751	20	9.2	3	6	0.921889	0.52385	no

gi|646746828|gb|KK962262.1|	31158-53144	20	9.2	21	11	-0.904415	0.5122	no
gi|646746828|gb|KK962262.1|	34863-53512	20	9.2	31	34	0.156038	0.8979	no
gi|646746828|gb|KK962262.1|	38495-53873	20	9.2	45	31	-0.551651	0.667	no
gi|646746828|gb|KK962262.1|	39707-54068	20	9.2	1	3	1	0.46055	no
gi|646746828|gb|KK962262.1|	40820-54855	20	9.2	9	11	0.281161	0.80625	no
gi|646746828|gb|KK962262.1|	48647-54972	20	9.2	2	0	-inf	0.00225	no
gi|646746828|gb|KK962262.1|	52702-55456	20	9.2	8	4	-0.916578	0.5424	no
gi|646746828|gb|KK962262.1|	64574-56514	20	9.2	310	278	-0.154561	0.89495	no
gi|646746828|gb|KK962262.1|	65807-56624	20	9.2	55	55	-0.00596975	0.9946	no
gi|646746828|gb|KK962262.1|	81891-58235	20	9.2	2	6	2	0.322	no
gi|646746828|gb|KK962262.1|	07010-60837	20	9.2	2	0.119418	-4	0.18715	no
gi|646746828|gb|KK962262.1|	14047-61492	20	9.2	4	3	-0.704831	0.60455	no
gi|646746828|gb|KK962262.1|	14992-61627	20	9.2	1	0.516289	-1	0.34475	no
gi|646746828|gb|KK962262.1|	71551-67202	20	9.2	16	53	2	0.27625	no
gi|646746841|gb|KK962261.1|	06237-41408	20	9.6	13	13	-0.0147978	0.9901	no
gi|646746841|gb|KK962261.1|	15894-41733	20	9.6	0.779378	0.336629	-1	1	no
gi|646746841|gb|KK962261.1|	18138-41906	20	9.6	1	2	0.505299	0.71995	no
gi|646746841|gb|KK962261.1|	40045-44032	20	9.6	5	24	2	0.18335	no
gi|646746841|gb|KK962261.1|	46127-44713	20	9.6	3	2	-0.7522	0.60005	no
gi|646746841|gb|KK962261.1|	54734-45948	20	9.6	2	4	0.616391	0.6915	no
gi|646746841|gb|KK962261.1|	59559-46102	20	9.6	0.532994	1	1	0.35635	no
gi|646746841|gb|KK962261.1|	62862-46352	20	9.6	4	1	-2	0.2449	no
gi|646746841|gb|KK962261.1|	63693-46450	20	9.6	1	1	-0.0887106	0.9324	no
gi|646746841|gb|KK962261.1|	65435-46626	20	9.6	1	2	0.498163	0.7299	no
gi|646746841|gb|KK962261.1|	73667-47475	20	9.6	4	2	-0.954806	0.48615	no
gi|646746841|gb|KK962261.1|	77088-48048	20	9.6	6	5	-0.334472	0.789	no
gi|646746841|gb|KK962261.1|	82100-48468	20	9.6	3	3	-0.231668	0.87225	no
gi|646746841|gb|KK962261.1|	86553-48757	20	9.6	0.481795	1	1	1	no
gi|646746841|gb|KK962261.1|	92806-49425	20	9.6	0	1	inf	0.0008	yes
gi|646746841|gb|KK962261.1|	05807-50657	20	9.6	1	1	-0.187036	0.90405	no

gi|646746841|gb|KK962261.1|	29279-53451	20	9.6	31	23	-0.388986	0.7405	no
gi|646746841|gb|KK962261.1|	34634-53509	20	9.6	2	4	0.671509	0.662	no
gi|646746841|gb|KK962261.1|	35246-53682	20	9.6	1	0.90983	-0.693919	0.5833	no
gi|646746841|gb|KK962261.1|	37056-53769	20	9.6	50	12	-2	0.13615	no
gi|646746841|gb|KK962261.1|	37829-53864	20	9.6	0.665444	2	1	0.43305	no
gi|646746841|gb|KK962261.1|	54416-56068	20	9.6	22	13	-0.805524	0.4889	no
gi|646746841|gb|KK962261.1|	91942-59352	20	9.6	0.559417	0.403467	-0.471473	1	no
gi|646746841|gb|KK962261.1|	96496-59731	20	9.6	0.984011	1	0.233805	0.87595	no
gi|646746841|gb|KK962261.1|	45745-64839	20	9.6	1	0.842054	-0.460774	0.7732	no
gi|646746841|gb|KK962261.1|	12776-71350	20	9.6	9	2	-2	0.13095	no
gi|646746841|gb|KK962261.1|	16040-71682	20	9.6	10	1	-3	0.0994	no
gi|646746841|gb|KK962261.1|	73300-97408	20	9.6	2	2	-0.313913	0.8121	no
gi|646746857|gb|KK962260.1|	02009-40274	20	6.5	0.762763	3	2	0.26425	no
gi|646746857|gb|KK962260.1|	07571-40784	20	6.5	2	32	4	0.09805	no
gi|646746857|gb|KK962260.1|	27666-42903	20	6.5	34	31	-0.163745	0.8804	no
gi|646746868|gb|KK962259.1|	01043-10178	21	10	87	76	-0.193336	0.85815	no
gi|646746868|gb|KK962259.1|	07622-10808	21	10	25	31	0.281241	0.84895	no
gi|646746868|gb|KK962259.1|	08434-10894	21	10	2	2	0.171964	0.89965	no
gi|646746868|gb|KK962259.1|	09240-11108	21	10	0.524663	0.421604	-0.315503	1	no
gi|646746868|gb|KK962259.1|	13819-11691	21	10	2	0.806527	-1	0.405	no
gi|646746868|gb|KK962259.1|	18046-11961	21	10	2	1	-0.641561	0.64565	no
gi|646746868|gb|KK962259.1|	20451-12204	21	10	0.624155	0.200098	-2	1	no
gi|646746868|gb|KK962259.1|	27799-13822	21	10	27	16	-0.805207	0.5	no
gi|646746868|gb|KK962259.1|	52214-15874	21	10	28	15	-0.899536	0.44615	no
gi|646746868|gb|KK962259.1|	67267-16841	21	10	0.518541	1	1	0.4305	no
gi|646746868|gb|KK962259.1|	84266-18466	21	10	23	33	0.513412	0.74155	no
gi|646746868|gb|KK962259.1|	84866-18683	21	10	43	20	-1	0.34645	no
gi|646746868|gb|KK962259.1|	89704-19021	21	10	4	1	-2	0.3498	no
gi|646746868|gb|KK962259.1|	10682-21125	21	10	23	19	-0.276326	0.8535	no
gi|646746868|gb|KK962259.1|	16066-21647	21	10	5	2	-1	0.45395	no

gi|646746868|gb|KK962259.1|	32014-23255	21	10	4	2	-0.817992	0.56425	no
gi|646746868|gb|KK962259.1|	53797-25450	21	10	2	1	-0.516324	0.73195	no
gi|646746868|gb|KK962259.1|	61370-26155	21	10	80	30	-1	0.40995	no
gi|646746868|gb|KK962259.1|	62360-26433	21	10	4	3	-0.381066	0.80115	no
gi|646746868|gb|KK962259.1|	65709-26760	21	10	0.5074	0.652667	0.363224	1	no
gi|646746868|gb|KK962259.1|	68821-27448	21	10	22	16	-0.465955	0.6868	no
gi|646746868|gb|KK962259.1|	05669-30672	21	10	22	14	-0.676297	0.65555	no
gi|646746868|gb|KK962259.1|	40960-41584	21	10	3	1	-2	0.34995	no
gi|646746868|gb|KK962259.1|	42797-54356	21	10	1	2	0.268644	0.8456	no
gi|646746868|gb|KK962259.1|	55134-56169	21	10	2	0.338253	-2	0.15265	no
gi|646746868|gb|KK962259.1|	20511-62380	21	10	14	18	0.355744	0.7514	no
gi|646746868|gb|KK962259.1|	65836-66206	21	10	218	110	-0.989494	0.43355	no
gi|646746868|gb|KK962259.1|	79207-80211	21	10	23	24	0.10322	0.93575	no
gi|646746868|gb|KK962259.1|	00853-80128	21	10	46	13	-2	0.26605	no
gi|646746868|gb|KK962259.1|	82514-83119	21	10	4	0.363738	-3	0.1786	no
gi|646746868|gb|KK962259.1|	92497-92687	21	10	44	59	0.424432	0.76635	no
gi|646746897|gb|KK962257.1|	16642-10219	21	9.2	15	15	0.0415486	0.97395	no
gi|646746897|gb|KK962257.1|	81801-28241	21	9.2	3	7	1	0.4739	no
gi|646746897|gb|KK962257.1|	93581-29458	21	9.2	1	1	-0.0745671	0.94595	no
gi|646746897|gb|KK962257.1|	33487-35906	21	9.2	8	6	-0.289609	0.84435	no
gi|646746897|gb|KK962257.1|	24770-52543	21	9.2	14	5	-1	0.37575	no
gi|646746897|gb|KK962257.1|	48732-55029	21	9.2	0.353645	29	6	0.05805	no
gi|646746897|gb|KK962257.1|	66717-56755	21	9.2	3	2	-0.267372	0.8229	no
gi|646746897|gb|KK962257.1|	68209-57364	21	9.2	20	7	-2	0.1928	no
gi|646746897|gb|KK962257.1|	11044-61199	21	9.2	1	0.375713	-1	1	no
gi|646746897|gb|KK962257.1|	62762-66579	21	9.2	20	21	0.0604889	0.95685	no
gi|646746897|gb|KK962257.1|	77506-67856	21	9.2	2	0.985289	-0.709153	0.5885	no
gi|646746897|gb|KK962257.1|	79300-67981	21	9.2	5	4	-0.472297	0.71885	no
gi|646746897|gb|KK962257.1|	81065-68197	21	9.2	2	0.201526	-3	0.1932	no
gi|646746897|gb|KK962257.1|	96288-69783	21	9.2	5	1	-2	0.19675	no

gi|646746897|gb|KK962257.1|	97953-69912	21	9.2	3	1	-1	0.39885	no
gi|646746897|gb|KK962257.1|	08500-71358	21	9.2	0.095799	6.07	6	0.05805	no
gi|646746897|gb|KK962257.1|	20633-72178	21	9.2	0.513362	1	1	0.36645	no
gi|646746897|gb|KK962257.1|	27559-73092	21	9.2	46	40	-0.191863	0.867	no
gi|646746897|gb|KK962257.1|	56713-75761	21	9.2	2	1	-0.148511	0.9177	no
gi|646746897|gb|KK962257.1|	53713-85448	21	9.2	5	13	1	0.3656	no
gi|646746897|gb|KK962257.1|	72480-87326	21	9.2	418	304	-0.456382	0.7012	no
gi|646746897|gb|KK962257.1|	76116-87912	21	9.2	19	14	-0.471711	0.6692	no
gi|646746897|gb|KK962257.1|	79312-88069	21	9.2	0.655529	1	0.694367	1	no
gi|646746897|gb|KK962257.1|	93319-89359	21	9.2	27	13	-1	0.42475	no
gi|646746897|gb|KK962257.1|	97544-89845	21	9.2	1	1	-0.173696	0.9042	no
gi|646746897|gb|KK962257.1|	98589-90090	21	9.2	2	2	0.0109883	0.9838	no
gi|646746897|gb|KK962257.1|	15251-91700	21	9.2	3	3	-0.0369298	0.97875	no
gi|646746897|gb|KK962257.1|	70289-97051	21	9.2	21	52	1	0.3797	no
gi|646746911|gb|KK962256.1|	10008-10313	17	7	35	39	0.187897	0.9039	no
gi|646746911|gb|KK962256.1|	12696-13330	17	7	8	11	0.5604	0.7165	no
gi|646746911|gb|KK962256.1|	13980-14409	17	7	4	3	-0.0954549	0.92295	no
gi|646746911|gb|KK962256.1|	94958-29518	17	7	443	342	-0.373334	0.8024	no
gi|646746911|gb|KK962256.1|	11462-31439	17	7	24	18	-0.4047	0.7175	no
gi|646746911|gb|KK962256.1|	31632-31851	17	7	51	25	-1	0.45515	no
gi|646746911|gb|KK962256.1|	19522-32184	17	7	3	3	0.0873583	0.9543	no
gi|646746911|gb|KK962256.1|	327-722	17	7	17	2	-3	0.1577	no
gi|646746911|gb|KK962256.1|	28662-32941	17	7	79	77	-0.0371801	0.97255	no
gi|646746911|gb|KK962256.1|	39797-40299	17	7	686	0.500477	-10	0.1665	no
gi|646746911|gb|KK962256.1|	42525-43070	17	7	43	0	-inf	0.00015	yes
gi|646746911|gb|KK962256.1|	29796-53123	17	7	0.938415	3	2	0.3043	no
gi|646746911|gb|KK962256.1|	35072-53643	17	7	0.334882	2	2	0.16265	no
gi|646746911|gb|KK962256.1|	37606-53868	17	7	1	2	0.651453	0.62395	no
gi|646746911|gb|KK962256.1|	38809-53979	17	7	14	13	-0.168525	0.90955	no
gi|646746911|gb|KK962256.1|	39907-54051	17	7	3	1	-0.857266	0.56515	no

gi|646746911|gb|KK962256.1|	44468-54516	17	7	46	26	-0.81062	0.521	no
gi|646746911|gb|KK962256.1|	53098-55408	17	7	3	3	-0.0755893	0.96125	no
gi|646746911|gb|KK962256.1|	63115-56396	17	7	2	0.662637	-1	0.4271	no
gi|646746911|gb|KK962256.1|	66415-56717	17	7	1	1	-0.509204	0.7384	no
gi|646746911|gb|KK962256.1|	70018-57094	17	7	1	0.393698	-2	0.2991	no
gi|646746911|gb|KK962256.1|	9290-9854	17	7	2	2	0.23033	0.87175	no
gi|646746911|gb|KK962256.1|	97361-97545	17	7	1287.1	828	-0.636196	0.6735	no
gi|646746942|gb|KK962254.1|	29901-10306	23	9.8	0.823775	3	2	0.2422	no
gi|646746942|gb|KK962254.1|	45593-10474	23	9.8	4	7	0.60702	0.6872	no
gi|646746942|gb|KK962254.1|	47648-10482	23	9.8	2	5	2	0.2949	no
gi|646746942|gb|KK962254.1|	50100-10515	23	9.8	0.309664	1	2	1	no
gi|646746942|gb|KK962254.1|	61065-10620	23	9.8	4	5	0.284844	0.85735	no
gi|646746942|gb|KK962254.1|	65795-10675	23	9.8	4	3	-0.103181	0.94805	no
gi|646746942|gb|KK962254.1|	67712-10688	23	9.8	0.303637	1	2	0.186	no
gi|646746942|gb|KK962254.1|	80197-10837	23	9.8	1	0.619772	-0.720282	1	no
gi|646746942|gb|KK962254.1|	86963-10879	23	9.8	1	0.904234	-0.628305	0.65905	no
gi|646746942|gb|KK962254.1|	96569-11022	23	9.8	2	0.6927	-1	0.4681	no
gi|646746942|gb|KK962254.1|	02442-11100	23	9.8	2	1	-0.551341	0.71695	no
gi|646746942|gb|KK962254.1|	11021-11129	23	9.8	0.836997	1	0.713919	0.6232	no
gi|646746942|gb|KK962254.1|	18859-11199	23	9.8	0.757366	1	0.878123	0.5385	no
gi|646746942|gb|KK962254.1|	35476-11357	23	9.8	42	3	-4	0.1743	no
gi|646746942|gb|KK962254.1|	38623-13992	23	9.8	2	2	-0.647805	0.6445	no
gi|646746942|gb|KK962254.1|	40299-14204	23	9.8	2	1	-0.971302	0.48845	no
gi|646746942|gb|KK962254.1|	44113-14517	23	9.8	13	10	-0.352792	0.8121	no
gi|646746942|gb|KK962254.1|	48447-14946	23	9.8	0.969904	2	0.681083	0.619	no
gi|646746942|gb|KK962254.1|	61182-16158	23	9.8	5	6	0.199767	0.88545	no
gi|646746942|gb|KK962254.1|	62400-16288	23	9.8	13	6	-1	0.4252	no
gi|646746942|gb|KK962254.1|	63656-17037	23	9.8	8	5	-0.688745	0.5342	no
gi|646746942|gb|KK962254.1|	72012-17246	23	9.8	145	39	-2	0.1585	no
gi|646746942|gb|KK962254.1|	74320-17592	23	9.8	4	0.494772	-3	0.09275	no

gi|646746942|gb|KK962254.1|	77172-17799	23	9.8	2	0.463643	-2	0.2551	no
gi|646746942|gb|KK962254.1|	79338-17990	23	9.8	3	0.404605	-3	0.19565	no
gi|646746942|gb|KK962254.1|	80312-18231	23	9.8	4	0.688707	-2	0.10625	no
gi|646746942|gb|KK962254.1|	86708-18767	23	9.8	7	1	-3	0.09485	no
gi|646746942|gb|KK962254.1|	88427-19393	23	9.8	7	2	-2	0.1725	no
gi|646746942|gb|KK962254.1|	95768-19651	23	9.8	2	0.264305	-3	0.1923	no
gi|646746942|gb|KK962254.1|	99667-20023	23	9.8	8	0.408222	-4	0.1812	no
gi|646746942|gb|KK962254.1|	19709-22046	23	9.8	13	3	-2	0.1642	no
gi|646746942|gb|KK962254.1|	20638-22190	23	9.8	11	0.919244	-4	0.0377	no
gi|646746942|gb|KK962254.1|	24795-22664	23	9.8	90	12	-3	0.0135	no
gi|646746942|gb|KK962254.1|	27453-22973	23	9.8	2	2	-0.278746	0.85655	no
gi|646746942|gb|KK962254.1|	23987-30585	23	9.8	0.029173	11	9	0.2322	no
gi|646746942|gb|KK962254.1|	32980-33450	23	9.8	0	23	inf	0.00075	yes
gi|646746942|gb|KK962254.1|	34934-35179	23	9.8	21	20	-0.126397	0.91875	no
gi|646746942|gb|KK962254.1|	35293-36074	23	9.8	0.703379	1	1	0.4859	no
gi|646746942|gb|KK962254.1|	11202-41151	23	9.8	6	12	0.934874	0.5538	no
gi|646746942|gb|KK962254.1|	18701-42066	23	9.8	35	15	-1	0.2763	no
gi|646746942|gb|KK962254.1|	71930-47236	23	9.8	2	5	1	0.45855	no
gi|646746942|gb|KK962254.1|	57991-55844	23	9.8	0	9	inf	0.00035	yes
gi|646746942|gb|KK962254.1|	81618-58215	23	9.8	17	9	-0.835754	0.5993	no
gi|646746942|gb|KK962254.1|	82420-58457	23	9.8	2	1	-0.551677	0.7276	no
gi|646746942|gb|KK962254.1|	88118-58831	23	9.8	92	16.89	-2	0.17565	no
gi|646746942|gb|KK962254.1|	90206-59040	23	9.8	78	56	-0.473936	0.7381	no
gi|646746942|gb|KK962254.1|	02050-60233	23	9.8	7	32	2	0.2506	no
gi|646746942|gb|KK962254.1|	09340-60964	23	9.8	8	6	-0.342305	0.8178	no
gi|646746942|gb|KK962254.1|	16620-61688	23	9.8	16	13	-0.340361	0.82015	no
gi|646746942|gb|KK962254.1|	42217-64245	23	9.8	33	14.06	-1	0.40885	no
gi|646746942|gb|KK962254.1|	58347-66145	23	9.8	19	7	-1	0.19935	no
gi|646746942|gb|KK962254.1|	65979-67082	23	9.8	46	33	-0.467715	0.67445	no
gi|646746942|gb|KK962254.1|	67764-66833	23	9.8	6	5	-0.236815	0.8431	no

gi|646746942|gb|KK962254.1|	70694-67131	23	9.8	1	2	1	0.4355	no
gi|646746942|gb|KK962254.1|	75806-68308	23	9.8	14	13	-0.0773465	0.9439	no
gi|646746942|gb|KK962254.1|	90756-69136	23	9.8	2	2	-0.121491	0.9259	no
gi|646746942|gb|KK962254.1|	92099-69253	23	9.8	12	16	0.393442	0.8018	no
gi|646746942|gb|KK962254.1|	99721-70018	23	9.8	14	5	-2	0.3109	no
gi|646746942|gb|KK962254.1|	17869-71809	23	9.8	48	18	-1	0.34675	no
gi|646746942|gb|KK962254.1|	50922-75137	23	9.8	2	4	1	0.45825	no
gi|646746942|gb|KK962254.1|	60828-76198	23	9.8	1	4	2	0.29275	no
gi|646746942|gb|KK962254.1|	00876-80182	23	9.8	0.93168	0.567498	-0.71522	1	no
gi|646746942|gb|KK962254.1|	27473-82848	23	9.8	0.978882	0.697366	-0.48922	1	no
gi|646746942|gb|KK962254.1|	10834-91167	23	9.8	3	2	-0.211782	0.86015	no
gi|646746942|gb|KK962254.1|	56300-95728	23	9.8	14	14	-0.044312	0.97575	no
gi|646746942|gb|KK962254.1|	87646-98787	23	9.8	47	4	-4	0.1839	no
gi|646746942|gb|KK962254.1|	94418-10050	23	9.8	13	10	-0.345034	0.7695	no
gi|646746958|gb|KK962253.1|	71628-37407	13	5.9	8	6	-0.485841	0.7487	no
gi|646746958|gb|KK962253.1|	83178-38530	13	5.9	6	4	-0.389184	0.79165	no
gi|646746974|gb|KK962252.1|	13531-11168	23	6.1	3	11	2	0.1889	no
gi|646746974|gb|KK962252.1|	27620-11358	23	6.1	11	3	-2	0.0758	no
gi|646746974|gb|KK962252.1|	36520-11379	23	6.1	2	0.339153	-2	0.16805	no
gi|646746974|gb|KK962252.1|	38060-11396	23	6.1	2	0.306242	-3	0.1864	no
gi|646746974|gb|KK962252.1|	39731-11410	23	6.1	1	0.251215	-2	0.1971	no
gi|646746974|gb|KK962252.1|	41758-11429	23	6.1	3	5	0.933098	0.5583	no
gi|646746974|gb|KK962252.1|	43638-11445	23	6.1	0.890849	1	0.238987	1	no
gi|646746974|gb|KK962252.1|	44619-11477	23	6.1	2	0.329565	-3	0.08215	no
gi|646746974|gb|KK962252.1|	47959-11502	23	6.1	1	0.464654	-2	0.32725	no
gi|646746974|gb|KK962252.1|	50369-11510	23	6.1	3	0.273887	-4	0.17385	no
gi|646746974|gb|KK962252.1|	52327-11538	23	6.1	1	0.739026	-0.913176	0.4794	no
gi|646746974|gb|KK962252.1|	53968-11556	23	6.1	1	0.190278	-3	0.1238	no
gi|646746974|gb|KK962252.1|	56543-11574	23	6.1	1	0.571481	-1	0.3882	no
gi|646746974|gb|KK962252.1|	59209-11642	23	6.1	1	0.622059	-0.994719	0.52435	no

gi|646746974|gb|KK962252.1|	70320-11713	23	6.1	1	1	0.188797	0.8968	no
gi|646746974|gb|KK962252.1|	72275-11754	23	6.1	6	2	-1	0.37035	no
gi|646746974|gb|KK962252.1|	76313-11775	23	6.1	4	2	-1	0.49435	no
gi|646746974|gb|KK962252.1|	77697-11784	23	6.1	3	2	-0.832993	0.53485	no
gi|646746974|gb|KK962252.1|	80155-11846	23	6.1	5	1	-2	0.16095	no
gi|646746974|gb|KK962252.1|	92857-11980	23	6.1	7	3	-1	0.42605	no
gi|646746974|gb|KK962252.1|	98170-12001	23	6.1	3	1	-1	0.37335	no
gi|646746974|gb|KK962252.1|	06866-12092	23	6.1	4	2	-1	0.5058	no
gi|646746974|gb|KK962252.1|	18708-12195	23	6.1	2	0.237918	-3	0.2033	no
gi|646746974|gb|KK962252.1|	22985-12372	23	6.1	2	1	-0.833769	0.57665	no
gi|646746974|gb|KK962252.1|	24973-13230	23	6.1	17	11	-0.614519	0.59975	no
gi|646746974|gb|KK962252.1|	69535-12732	23	6.1	1	0.236187	-2	1	no
gi|646746974|gb|KK962252.1|	14482-13157	23	6.1	1	0.393962	-2	0.2858	no
gi|646746974|gb|KK962252.1|	15879-13173	23	6.1	1	0.452204	-2	0.2986	no
gi|646746974|gb|KK962252.1|	18841-13199	23	6.1	1	0.459297	-1	0.35685	no
gi|646746974|gb|KK962252.1|	20226-13208	23	6.1	18	2	-3	0.0658	no
gi|646746974|gb|KK962252.1|	20988-13223	23	6.1	2	0.813727	-1	0.4362	no
gi|646746974|gb|KK962252.1|	22531-13240	23	6.1	3	0.217061	-4	0.1308	no
gi|646746974|gb|KK962252.1|	25612-13282	23	6.1	1	0.67004	-0.829937	0.55005	no
gi|646746974|gb|KK962252.1|	30111-13314	23	6.1	2	0.884164	-1	0.4132	no
gi|646746974|gb|KK962252.1|	31894-13330	23	6.1	4	2	-1	0.3132	no
gi|646746974|gb|KK962252.1|	40506-14139	23	6.1	1	0.825725	-0.49704	0.73865	no
gi|646746974|gb|KK962252.1|	41679-14258	23	6.1	3	1	-1	0.45725	no
gi|646746974|gb|KK962252.1|	46445-14669	23	6.1	54	28	-0.968907	0.4529	no
gi|646746974|gb|KK962252.1|	86140-18667	23	6.1	71	25	-1	0.25275	no
gi|646746974|gb|KK962252.1|	95212-19589	23	6.1	13	5	-1	0.4527	no
gi|646746974|gb|KK962252.1|	02046-20276	23	6.1	6	7	0.16307	0.91615	no
gi|646746974|gb|KK962252.1|	08541-20889	23	6.1	6	9	0.50865	0.7261	no
gi|646746974|gb|KK962252.1|	32772-23329	23	6.1	5	9	0.779572	0.5847	no
gi|646746974|gb|KK962252.1|	44525-24490	23	6.1	15	34	1	0.4631	no

gi|646746974|gb|KK962252.1|	89250-29022	23	6.1	4	4	-0.119188	0.9355	no
gi|646746974|gb|KK962252.1|	24639-32538	23	6.1	4	9	1	0.46105	no
gi|646746974|gb|KK962252.1|	28058-33190	23	6.1	2	2	-0.176248	0.90355	no
gi|646746974|gb|KK962252.1|	34431-33529	23	6.1	1	3	2	0.2884	no
gi|646746974|gb|KK962252.1|	37143-33804	23	6.1	38	26	-0.512687	0.68525	no
gi|646746974|gb|KK962252.1|	41501-34365	23	6.1	6	0.778535	-3	0.0525	no
gi|646746974|gb|KK962252.1|	52809-35365	23	6.1	1	0.443926	-2	0.38425	no
gi|646746974|gb|KK962252.1|	69082-38045	23	6.1	6	7	0.0513875	0.9658	no
gi|646746974|gb|KK962252.1|	80703-38477	23	6.1	18	9	-1	0.3668	no
gi|646746974|gb|KK962252.1|	84872-38638	23	6.1	0.661332	1	0.679321	1	no
gi|646746974|gb|KK962252.1|	86660-38816	23	6.1	0.518072	0.85323	0.719783	1	no
gi|646746974|gb|KK962252.1|	89804-39091	23	6.1	0.863542	0.925907	0.100601	1	no
gi|646746974|gb|KK962252.1|	91317-39177	23	6.1	4	1	-2	0.28945	no
gi|646746974|gb|KK962252.1|	93934-39587	23	6.1	0.440029	0.87583	0.99305	1	no
gi|646746974|gb|KK962252.1|	04647-40580	23	6.1	2	2	-0.0676601	0.95445	no
gi|646746974|gb|KK962252.1|	30462-43099	23	6.1	52	15	-2	0.26225	no
gi|646746974|gb|KK962252.1|	61543-46314	23	6.1	2	2	0.3915	0.812	no
gi|646746974|gb|KK962252.1|	63689-46442	23	6.1	1	4	1	0.31555	no
gi|646746974|gb|KK962252.1|	66254-46710	23	6.1	69	5	-4	0.0238	no
gi|646746974|gb|KK962252.1|	77980-48949	23	6.1	22	9	-1	0.3008	no
gi|646746974|gb|KK962252.1|	04140-50465	23	6.1	7	8	0.214984	0.8744	no
gi|646746974|gb|KK962252.1|	09922-51018	23	6.1	30	28	-0.0931565	0.93745	no
gi|646746974|gb|KK962252.1|	52231-53389	23	6.1	0.61467	2	1	0.3407	no
gi|646746974|gb|KK962252.1|	36136-54025	23	6.1	2	2	0.0227339	0.98685	no
gi|646746974|gb|KK962252.1|	56265-55822	23	6.1	2	0	-inf	0.0032	no
gi|646746974|gb|KK962252.1|	66051-56798	23	6.1	11	0.079949	-7	0.16675	no
gi|646746974|gb|KK962252.1|	68110-56895	23	6.1	6	0.220162	-5	0.1759	no
gi|646746974|gb|KK962252.1|	94644-59704	23	6.1	11	15	0.472215	0.6609	no
gi|646746974|gb|KK962252.1|	60692-66175	23	6.1	2	0.655274	-1	0.34325	no
gi|646746974|gb|KK962252.1|	76341-67791	23	6.1	8	8	-0.0750688	0.9606	no

gi|646746974|gb|KK962252.1|	09419-71220	23	6.1	2	8	2	0.12265	no
gi|646746974|gb|KK962252.1|	98083-80107	23	6.1	1	3	1	0.40535	no
gi|646746974|gb|KK962252.1|	94059-89449	23	6.1	3	4	0.394795	0.7964	no
gi|646746974|gb|KK962252.1|	99649-90412	23	6.1	33	17	-0.972744	0.4087	no
gi|646746974|gb|KK962252.1|	19103-91960	23	6.1	19	10	-0.848413	0.59115	no
gi|646746974|gb|KK962252.1|	9901-10561	23	6.1	2	3	0.837888	0.55995	no
gi|646746991|gb|KK962251.1|	24430-22678	22	9.2	4	1	-2	0.25975	no
gi|646746991|gb|KK962251.1|	34466-23577	22	9.2	1	18	4	0.01105	no
gi|646746991|gb|KK962251.1|	43835-24436	22	9.2	8	6	-0.498554	0.71705	no
gi|646746991|gb|KK962251.1|	50829-25369	22	9.2	5	4	-0.169232	0.9119	no
gi|646746991|gb|KK962251.1|	54348-25690	22	9.2	14	11	-0.362355	0.7378	no
gi|646746991|gb|KK962251.1|	21268-32196	22	9.2	3	1	-1	0.4308	no
gi|646746991|gb|KK962251.1|	22764-32424	22	9.2	10	4	-1	0.35765	no
gi|646746991|gb|KK962251.1|	29350-33162	22	9.2	0.412004	0.464882	0.174205	1	no
gi|646746991|gb|KK962251.1|	38253-35093	22	9.2	5	6	0.0861587	0.9399	no
gi|646746991|gb|KK962251.1|	66686-37025	22	9.2	60	78	0.382589	0.7475	no
gi|646746991|gb|KK962251.1|	15228-41550	22	9.2	44	51	0.195634	0.8988	no
gi|646746991|gb|KK962251.1|	19274-42020	22	9.2	70	40	-0.811917	0.4641	no
gi|646746991|gb|KK962251.1|	38197-43853	22	9.2	28	19	-0.559358	0.73235	no
gi|646746991|gb|KK962251.1|	38741-43906	22	9.2	25	25	-0.0194473	0.97385	no
gi|646746991|gb|KK962251.1|	40891-44109	22	9.2	34	94	1	0.34665	no
gi|646746991|gb|KK962251.1|	45191-44553	22	9.2	29	24	-0.275441	0.86135	no
gi|646746991|gb|KK962251.1|	71114-47448	22	9.2	0.682481	0.302489	-1	1	no
gi|646746991|gb|KK962251.1|	74851-47736	22	9.2	8	5	-0.681513	0.662	no
gi|646746991|gb|KK962251.1|	77891-47966	22	9.2	111.65	53	-1	0.3613	no
gi|646746991|gb|KK962251.1|	96773-49727	22	9.2	18	26	0.51571	0.7355	no
gi|646746991|gb|KK962251.1|	54053-54657	22	9.2	0	4	inf	0.0014	yes
gi|646746991|gb|KK962251.1|	59018-60103	22	9.2	0	3	inf	0.00315	no
gi|646746991|gb|KK962251.1|	15826-61611	22	9.2	12	16	0.389341	0.78285	no
gi|646746991|gb|KK962251.1|	36466-63691	22	9.2	7	9	0.502669	0.7231	no

gi|646746991|gb|KK962251.1|	41523-64178	22	9.2	21	31	0.560103	0.6757	no
gi|646746991|gb|KK962251.1|	64598-64885	22	9.2	0	89	inf	5.00E-05	yes
gi|646746991|gb|KK962251.1|	50933-65192	22	9.2	0.504795	1	1	1	no
gi|646746991|gb|KK962251.1|	62014-66594	22	9.2	24	24	0.03319	0.97635	no
gi|646746991|gb|KK962251.1|	68641-66971	22	9.2	1	0.648231	-1	0.4145	no
gi|646746991|gb|KK962251.1|	69770-67272	22	9.2	1	0.947106	-0.298812	0.8445	no
gi|646746991|gb|KK962251.1|	89139-69040	22	9.2	2	1	-0.5491	0.65745	no
gi|646746991|gb|KK962251.1|	80844-78171	22	9.2	1	1	0.279389	0.8456	no
gi|646747006|gb|KK962250.1|	70104-37100	17	5.3	16	5	-2	0.3097	no
gi|646747006|gb|KK962250.1|	74627-37510	17	5.3	6	3	-0.902195	0.5046	no
gi|646747006|gb|KK962250.1|	01504-40545	17	5.3	10	12	0.14378	0.89665	no
gi|646747006|gb|KK962250.1|	16911-41709	17	5.3	108	53	-1	0.47705	no
gi|646747006|gb|KK962250.1|	18439-41891	17	5.3	2	9	2	0.21525	no
gi|646747006|gb|KK962250.1|	27904-52847	17	5.3	5	4	-0.544087	0.668	no
gi|646747019|gb|KK962249.1|	03000-10369	13	8.7	22	18	-0.241646	0.87205	no
gi|646747019|gb|KK962249.1|	03768-10659	13	8.7	5	4	-0.429469	0.7777	no
gi|646747019|gb|KK962249.1|	06695-10871	13	8.7	7	8	0.136439	0.9293	no
gi|646747019|gb|KK962249.1|	26498-12756	13	8.7	12	10	-0.181667	0.9024	no
gi|646747019|gb|KK962249.1|	79620-18032	13	8.7	2	2	-0.517543	0.70145	no
gi|646747019|gb|KK962249.1|	19366-19967	13	8.7	86	50	-0.786872	0.54205	no
gi|646747019|gb|KK962249.1|	38427-23861	13	8.7	148	71	-1	0.43035	no
gi|646747019|gb|KK962249.1|	24201-24415	13	8.7	1300.36	646	-1	0.4232	no
gi|646747019|gb|KK962249.1|	37060-38726	13	8.7	0.590099	0.473371	-0.317984	1	no
gi|646747019|gb|KK962249.1|	53071-54045	13	8.7	1	0.732169	-0.661261	0.6593	no
gi|646747019|gb|KK962249.1|	55727-56336	13	8.7	13	5	-2	0.28075	no
gi|646747019|gb|KK962249.1|	61561-66110	13	8.7	6	5	-0.386421	0.70915	no
gi|646747019|gb|KK962249.1|	68968-69777	13	8.7	1	2	0.303208	0.83465	no
gi|646747019|gb|KK962249.1|	74328-74876	13	8.7	14	10	-0.426751	0.7812	no
gi|646747019|gb|KK962249.1|	83878-85711	13	8.7	2	2	0.390361	0.8003	no
gi|646747019|gb|KK962249.1|	90018-91425	13	8.7	1	1	0.146486	0.9094	no

gi|646747032|gb|KK962248.1|	16013-10172	20	8.7	0.634872	1	0.714117	1	no
gi|646747032|gb|KK962248.1|	22346-32403	20	8.7	22	11	-1	0.4195	no
gi|646747032|gb|KK962248.1|	24417-32525	20	8.7	1	1	-0.0866157	0.93265	no
gi|646747032|gb|KK962248.1|	26197-32871	20	8.7	1	2	0.453533	0.7681	no
gi|646747032|gb|KK962248.1|	28841-33158	20	8.7	6	5	-0.375907	0.7995	no
gi|646747032|gb|KK962248.1|	32676-33658	20	8.7	17	15	-0.139468	0.9	no
gi|646747032|gb|KK962248.1|	37572-34064	20	8.7	1	1	0.208625	0.88875	no
gi|646747032|gb|KK962248.1|	62639-36780	20	8.7	15	10	-0.533981	0.63955	no
gi|646747032|gb|KK962248.1|	77628-37813	20	8.7	10	23	1	0.4645	no
gi|646747032|gb|KK962248.1|	83399-38359	20	8.7	96	16	-3	0.17045	no
gi|646747032|gb|KK962248.1|	85067-38530	20	8.7	65	28	-1	0.33475	no
gi|646747032|gb|KK962248.1|	92605-39280	20	8.7	22	126	3	0.1997	no
gi|646747032|gb|KK962248.1|	96087-39637	20	8.7	16	38	1	0.39245	no
gi|646747032|gb|KK962248.1|	00736-40112	20	8.7	59	0.835372	-6	0.16745	no
gi|646747032|gb|KK962248.1|	08104-40861	20	8.7	12	19	0.592581	0.6993	no
gi|646747032|gb|KK962248.1|	14666-41492	20	8.7	5	22	2	0.31645	no
gi|646747032|gb|KK962248.1|	19635-42014	20	8.7	5	1	-2	0.281	no
gi|646747032|gb|KK962248.1|	34139-43438	20	8.7	23	22	-0.0867697	0.93275	no
gi|646747032|gb|KK962248.1|	41266-44149	20	8.7	16	29	0.84888	0.5964	no
gi|646747032|gb|KK962248.1|	47480-44770	20	8.7	20	29	0.589249	0.67725	no
gi|646747032|gb|KK962248.1|	52619-45308	20	8.7	18	6	-2	0.2643	no
gi|646747032|gb|KK962248.1|	53959-45419	20	8.7	23	20	-0.213735	0.89085	no
gi|646747032|gb|KK962248.1|	55621-45705	20	8.7	0.472402	0.680005	0.525531	1	no
gi|646747032|gb|KK962248.1|	57549-45879	20	8.7	0.558408	1	0.935705	1	no
gi|646747032|gb|KK962248.1|	59403-46073	20	8.7	1	0.494197	-1	1	no
gi|646747032|gb|KK962248.1|	70978-47331	20	8.7	21	13	-0.699599	0.5268	no
gi|646747032|gb|KK962248.1|	92781-49353	20	8.7	18	8	-1	0.45465	no
gi|646747032|gb|KK962248.1|	99934-50190	20	8.7	12	8	-0.562663	0.64595	no
gi|646747032|gb|KK962248.1|	07410-51153	20	8.7	1	0.871603	-0.512298	0.74125	no
gi|646747032|gb|KK962248.1|	34952-53649	20	8.7	0.93739	2	0.734236	0.6303	no

gi|646747032|gb|KK962248.1|	54768-55170	20	8.7	27	16	-0.698284	0.6568	no
gi|646747032|gb|KK962248.1|	48414-56172	20	8.7	4	3	-0.392939	0.7288	no
gi|646747032|gb|KK962248.1|	67134-56972	20	8.7	6	5	-0.194912	0.89525	no
gi|646747032|gb|KK962248.1|	86225-58789	20	8.7	4	5	0.160905	0.9119	no
gi|646747032|gb|KK962248.1|	47335-74907	20	8.7	8	9	0.149271	0.9199	no
gi|646747032|gb|KK962248.1|	06142-80667	20	8.7	8	6	-0.395716	0.7729	no
gi|646747032|gb|KK962248.1|	90219-99093	20	8.7	1	1	0.221529	0.89085	no
gi|646747046|gb|KK962247.1|	01449-10192	22	9	37	24	-0.611478	0.6866	no
gi|646747046|gb|KK962247.1|	12179-11154	22	9	2	0.845821	-1	0.3682	no
gi|646747046|gb|KK962247.1|	15649-11182	22	9	2	2	-0.214557	0.88505	no
gi|646747046|gb|KK962247.1|	23739-11326	22	9	3	6	1	0.3287	no
gi|646747046|gb|KK962247.1|	32777-11373	22	9	1	4	1	0.34265	no
gi|646747046|gb|KK962247.1|	13386-11389	22	9	8	7	-0.151596	0.9017	no
gi|646747046|gb|KK962247.1|	37421-11389	22	9	0.150549	2	4	0.252	no
gi|646747046|gb|KK962247.1|	41256-11419	22	9	4	3	-0.179741	0.88055	no
gi|646747046|gb|KK962247.1|	49394-11503	22	9	0.743005	1	0.925322	0.5404	no
gi|646747046|gb|KK962247.1|	50598-11516	22	9	0.364191	2	2	0.2023	no
gi|646747046|gb|KK962247.1|	51856-11526	22	9	0.915748	2	1	0.4427	no
gi|646747046|gb|KK962247.1|	53959-11595	22	9	74	48	-0.621934	0.5995	no
gi|646747046|gb|KK962247.1|	15453-11589	22	9	21	6	-2	0.21275	no
gi|646747046|gb|KK962247.1|	60479-11612	22	9	1	0.751967	-0.7303	0.66415	no
gi|646747046|gb|KK962247.1|	64794-11661	22	9	4	9	1	0.42405	no
gi|646747046|gb|KK962247.1|	67271-11675	22	9	10	3	-2	0.2986	no
gi|646747046|gb|KK962247.1|	11887-12663	22	9	1	2	0.492322	0.72185	no
gi|646747046|gb|KK962247.1|	25881-12610	22	9	101	56	-0.850944	0.53645	no
gi|646747046|gb|KK962247.1|	26864-12707	22	9	148	108	-0.456962	0.7786	no
gi|646747046|gb|KK962247.1|	41058-14141	22	9	8	6	-0.417186	0.74615	no
gi|646747046|gb|KK962247.1|	44476-14484	22	9	8	12	0.580608	0.6655	no
gi|646747046|gb|KK962247.1|	49466-14966	22	9	129	16.89	-3	0.12805	no
gi|646747046|gb|KK962247.1|	52416-15263	22	9	29	28	-0.0274755	0.90935	no

gi|646747046|gb|KK962247.1|	15278-19926	22	9	2	1	-0.570211	0.7039	no
gi|646747046|gb|KK962247.1|	56045-15623	22	9	75	29	-1	0.41275	no
gi|646747046|gb|KK962247.1|	70807-17138	22	9	12	3	-2	0.1761	no
gi|646747046|gb|KK962247.1|	71746-17196	22	9	42	10	-2	0.2311	no
gi|646747046|gb|KK962247.1|	74036-17454	22	9	4	0.489795	-3	0.1921	no
gi|646747046|gb|KK962247.1|	80149-18115	22	9	0.247668	4	4	0.25035	no
gi|646747046|gb|KK962247.1|	88342-18871	22	9	163	70	-1	0.34735	no
gi|646747046|gb|KK962247.1|	20066-20406	22	9	9	10	0.14802	0.918	no
gi|646747046|gb|KK962247.1|	05317-20596	22	9	3	3	-0.335114	0.7904	no
gi|646747046|gb|KK962247.1|	10842-21142	22	9	2	2	0.194267	0.89115	no
gi|646747046|gb|KK962247.1|	12140-21381	22	9	0.587186	0.471071	-0.317873	1	no
gi|646747046|gb|KK962247.1|	13915-21507	22	9	0.713953	0.584062	-0.289707	1	no
gi|646747046|gb|KK962247.1|	16553-21773	22	9	2	1	-0.559757	0.6584	no
gi|646747046|gb|KK962247.1|	32071-23266	22	9	9	4	-0.99743	0.4941	no
gi|646747046|gb|KK962247.1|	43494-24370	22	9	76.27	52	-0.562747	0.67515	no
gi|646747046|gb|KK962247.1|	44813-24515	22	9	10	5	-1	0.46035	no
gi|646747046|gb|KK962247.1|	73440-27385	22	9	15	8	-0.914356	0.52095	no
gi|646747046|gb|KK962247.1|	76100-28053	22	9	12	7	-0.730167	0.50985	no
gi|646747046|gb|KK962247.1|	28834-29087	22	9	43	43	0.0285253	0.96625	no
gi|646747046|gb|KK962247.1|	88853-29755	22	9	7	5	-0.446772	0.6899	no
gi|646747046|gb|KK962247.1|	07033-30735	22	9	42	21	-0.973103	0.54875	no
gi|646747046|gb|KK962247.1|	30466-33146	22	9	0.993332	4	2	0.2557	no
gi|646747046|gb|KK962247.1|	3504-4610	22	9	0.434814	0.932267	1	1	no
gi|646747046|gb|KK962247.1|	36858-37417	22	9	2	3	0.451206	0.7474	no
gi|646747046|gb|KK962247.1|	71223-37203	22	9	1	6	2	0.17635	no
gi|646747046|gb|KK962247.1|	85348-39715	22	9	6	6	0.0346451	0.9747	no
gi|646747046|gb|KK962247.1|	01112-40154	22	9	29	9	-2	0.21025	no
gi|646747046|gb|KK962247.1|	06622-40703	22	9	39	32	-0.320417	0.8249	no
gi|646747046|gb|KK962247.1|	76882-47727	22	9	0	22	inf	0.0017	no
gi|646747046|gb|KK962247.1|	4794-5396	22	9	2	3	0.825556	0.56315	no

gi|646747046|gb|KK962247.1|	51603-51870	22	9	88	45	-0.948274	0.5489	no
gi|646747046|gb|KK962247.1|	52389-52694	22	9	21	13	-0.755519	0.56185	no
gi|646747046|gb|KK962247.1|	39595-54041	22	9	0.985759	5	2	0.20515	no
gi|646747046|gb|KK962247.1|	42655-54291	22	9	0	28	inf	0.00255	no
gi|646747046|gb|KK962247.1|	07868-60845	22	9	5	0	-inf	0.0012	yes
gi|646747046|gb|KK962247.1|	08805-61003	22	9	5	0.542068	-3	0.0793	no
gi|646747046|gb|KK962247.1|	12277-61417	22	9	1	2	0.808772	0.5524	no
gi|646747046|gb|KK962247.1|	22117-62779	22	9	10	11	0.0679958	0.95305	no
gi|646747046|gb|KK962247.1|	59180-66003	22	9	26	12	-1	0.4709	no
gi|646747046|gb|KK962247.1|	66046-66259	22	9	76.27	40	-0.925317	0.4977	no
gi|646747046|gb|KK962247.1|	68123-68337	22	9	46	34	-0.442513	0.7503	no
gi|646747046|gb|KK962247.1|	71215-71365	22	9	529	126	-2	0.2212	no
gi|646747046|gb|KK962247.1|	17857-72465	22	9	6	8	0.455685	0.68745	no
gi|646747046|gb|KK962247.1|	7186-8834	22	9	39	27	-0.510214	0.6444	no
gi|646747046|gb|KK962247.1|	72792-73021	22	9	25	21	-0.22546	0.87595	no
gi|646747046|gb|KK962247.1|	31254-73323	22	9	20	11	-0.901603	0.40005	no
gi|646747046|gb|KK962247.1|	73612-73871	22	9	51	27	-0.930984	0.46115	no
gi|646747046|gb|KK962247.1|	38709-73923	22	9	0.673686	6	3	0.245	no
gi|646747046|gb|KK962247.1|	41977-74222	22	9	16	20	0.352256	0.81125	no
gi|646747046|gb|KK962247.1|	50095-75077	22	9	4	3	-0.310306	0.79735	no
gi|646747046|gb|KK962247.1|	52022-75410	22	9	45	18	-1	0.2524	no
gi|646747046|gb|KK962247.1|	62744-76403	22	9	7	2	-2	0.1348	no
gi|646747046|gb|KK962247.1|	64558-76492	22	9	19	19	-0.0641773	0.96675	no
gi|646747046|gb|KK962247.1|	66473-76869	22	9	0.754091	0.75187	-0.00425633	1	no
gi|646747046|gb|KK962247.1|	70757-77145	22	9	11	8	-0.598775	0.6956	no
gi|646747046|gb|KK962247.1|	74954-77523	22	9	12	12	-0.0178078	0.92625	no
gi|646747046|gb|KK962247.1|	75391-77573	22	9	11	11	0.0424805	0.9494	no
gi|646747046|gb|KK962247.1|	76211-77704	22	9	3	3	0.0131027	0.96985	no
gi|646747046|gb|KK962247.1|	84651-78486	22	9	44	37.78	-0.210779	0.8833	no
gi|646747046|gb|KK962247.1|	05730-80595	22	9	34	13	-1	0.40345	no

gi|646747046|gb|KK962247.1|	16057-81633	22	9	21	14	-0.579701	0.687	no
gi|646747046|gb|KK962247.1|	19980-82205	22	9	9	17	0.817435	0.52495	no
gi|646747046|gb|KK962247.1|	82431-83687	22	9	0.462086	0.795623	0.783923	1	no
gi|646747046|gb|KK962247.1|	42834-84329	22	9	2	4	1	0.45825	no
gi|646747046|gb|KK962247.1|	90034-90527	22	9	11	2	-3	0.14475	no
gi|646747062|gb|KK962246.1|	11734-11341	12	8	1	0.938233	-0.607286	0.67585	no
gi|646747062|gb|KK962246.1|	49577-35038	12	8	1	2	0.910385	0.5424	no
gi|646747076|gb|KK962245.1|	17026-11801	23	9.6	3	1	-1	0.43135	no
gi|646747076|gb|KK962245.1|	21848-12311	23	9.6	0.365651	1	2	1	no
gi|646747076|gb|KK962245.1|	31955-13726	23	9.6	21	19	-0.10081	0.92625	no
gi|646747076|gb|KK962245.1|	37693-13903	23	9.6	15	16	0.0508315	0.9722	no
gi|646747076|gb|KK962245.1|	56762-15694	23	9.6	236	156	-0.595016	0.61555	no
gi|646747076|gb|KK962245.1|	60220-16053	23	9.6	5	13	1	0.37175	no
gi|646747076|gb|KK962245.1|	63655-16402	23	9.6	13	4	-2	0.2333	no
gi|646747076|gb|KK962245.1|	67170-16798	23	9.6	1	3	0.911289	0.49355	no
gi|646747076|gb|KK962245.1|	72233-17246	23	9.6	56	13	-2	0.20225	no
gi|646747076|gb|KK962245.1|	75634-17735	23	9.6	0.819273	2	1	0.47595	no
gi|646747076|gb|KK962245.1|	08463-20887	23	9.6	4	3	-0.428599	0.76715	no
gi|646747076|gb|KK962245.1|	31993-23234	23	9.6	5	5	0.0579865	0.91525	no
gi|646747076|gb|KK962245.1|	70215-27070	23	9.6	3	2	-0.989076	0.589	no
gi|646747076|gb|KK962245.1|	79573-28052	23	9.6	2	3	0.729436	0.56995	no
gi|646747076|gb|KK962245.1|	91824-29254	23	9.6	3	1	-0.892941	0.53905	no
gi|646747076|gb|KK962245.1|	52879-35339	23	9.6	15	16	0.0870746	0.9552	no
gi|646747076|gb|KK962245.1|	92793-39398	23	9.6	4	5	0.268774	0.8588	no
gi|646747076|gb|KK962245.1|	41091-42153	23	9.6	2	0.818105	-1	0.43875	no
gi|646747076|gb|KK962245.1|	17830-41835	23	9.6	2	3	0.923192	0.5637	no
gi|646747076|gb|KK962245.1|	47960-50054	23	9.6	2	3	0.502803	0.74455	no
gi|646747076|gb|KK962245.1|	80909-48230	23	9.6	0.727166	0.34874	-1	1	no
gi|646747076|gb|KK962245.1|	86551-49073	23	9.6	13	7	-0.869787	0.42705	no
gi|646747076|gb|KK962245.1|	99975-50351	23	9.6	10	7	-0.651471	0.54365	no

gi|646747076|gb|KK962245.1|	04404-50507	23	9.6	2	2	-0.200759	0.89315	no
gi|646747076|gb|KK962245.1|	06880-50875	23	9.6	0.572145	0.165556	-2	1	no
gi|646747076|gb|KK962245.1|	09553-51015	23	9.6	9	2	-2	0.19635	no
gi|646747076|gb|KK962245.1|	10214-51787	23	9.6	7	2	-2	0.21815	no
gi|646747076|gb|KK962245.1|	19487-52139	23	9.6	0.898214	0.974899	0.118194	1	no
gi|646747076|gb|KK962245.1|	52174-53286	23	9.6	0.971484	0.462953	-1	1	no
gi|646747076|gb|KK962245.1|	48417-54911	23	9.6	3	1	-1	0.3953	no
gi|646747076|gb|KK962245.1|	50269-55127	23	9.6	3	1	-1	0.38195	no
gi|646747076|gb|KK962245.1|	52484-55361	23	9.6	0.637248	0.759497	0.253189	1	no
gi|646747076|gb|KK962245.1|	53747-55523	23	9.6	0.374545	0.755483	1	1	no
gi|646747076|gb|KK962245.1|	56856-55958	23	9.6	32	13	-1	0.26355	no
gi|646747076|gb|KK962245.1|	61256-57075	23	9.6	27	21	-0.400257	0.74245	no
gi|646747076|gb|KK962245.1|	88349-59276	23	9.6	19	13	-0.538468	0.6359	no
gi|646747076|gb|KK962245.1|	59082-59779	23	9.6	3	0.584224	-2	0.19615	no
gi|646747076|gb|KK962245.1|	93332-59374	23	9.6	12	4	-2	0.23205	no
gi|646747076|gb|KK962245.1|	94093-59467	23	9.6	4	3	-0.34897	0.7831	no
gi|646747076|gb|KK962245.1|	95492-60030	23	9.6	0.994209	0.769975	-0.368738	1	no
gi|646747076|gb|KK962245.1|	00476-60336	23	9.6	1	0.561525	-1	0.46115	no
gi|646747076|gb|KK962245.1|	05797-60674	23	9.6	8	0.951133	-3	0.07675	no
gi|646747076|gb|KK962245.1|	15596-61700	23	9.6	0.96618	0.926798	-0.0600365	1	no
gi|646747076|gb|KK962245.1|	21162-62160	23	9.6	3	4	0.4019	0.7964	no
gi|646747076|gb|KK962245.1|	22029-62262	23	9.6	5	2	-1	0.29235	no
gi|646747076|gb|KK962245.1|	40169-64047	23	9.6	15	6	-1	0.42735	no
gi|646747076|gb|KK962245.1|	54592-65542	23	9.6	3	0.683783	-2	0.19355	no
gi|646747076|gb|KK962245.1|	76720-67780	23	9.6	0.556596	0.795097	0.514499	1	no
gi|646747076|gb|KK962245.1|	85055-68753	23	9.6	0.956802	0.422872	-1	1	no
gi|646747076|gb|KK962245.1|	88087-69077	23	9.6	2	0.553809	-2	0.2601	no
gi|646747076|gb|KK962245.1|	70162-71036	23	9.6	8	0.846323	-3	0.08555	no
gi|646747076|gb|KK962245.1|	02075-70275	23	9.6	13	2	-2	0.1118	no
gi|646747076|gb|KK962245.1|	08018-70896	23	9.6	4	0.19103	-4	0.18315	no

gi|646747076|gb|KK962245.1|	17948-72143	23	9.6	3	3	-0.323333	0.82585	no
gi|646747076|gb|KK962245.1|	72945-73351	23	9.6	4	8	0.886812	0.53255	no
gi|646747076|gb|KK962245.1|	30600-73159	23	9.6	6	2	-1	0.37565	no
gi|646747076|gb|KK962245.1|	34778-73565	23	9.6	11	13	0.28261	0.85315	no
gi|646747076|gb|KK962245.1|	8-463	23	9.6	6	0.603024	-3	0.1849	no
gi|646747076|gb|KK962245.1|	82193-83153	23	9.6	3	0.186565	-4	0.1869	no
gi|646747076|gb|KK962245.1|	88086-89537	23	9.6	0.464108	1	1	0.33945	no
gi|646747094|gb|KK962244.1|	20430-23061	20	10	0.506207	0.67898	0.423642	1	no
gi|646747094|gb|KK962244.1|	22204-22519	20	10	23	25	0.0649699	0.9519	no
gi|646747094|gb|KK962244.1|	29011-23188	20	10	22	25	0.190919	0.8631	no
gi|646747094|gb|KK962244.1|	33737-23616	20	10	0.895339	0.742778	-0.269503	1	no
gi|646747094|gb|KK962244.1|	46268-24700	20	10	1	2	0.70989	0.63615	no
gi|646747094|gb|KK962244.1|	49622-25318	20	10	32	30	-0.09028	0.9337	no
gi|646747094|gb|KK962244.1|	61609-26317	20	10	0.63462	3	2	0.1801	no
gi|646747094|gb|KK962244.1|	70523-27105	20	10	4	3	-0.334302	0.79845	no
gi|646747094|gb|KK962244.1|	33499-34621	20	10	0.853584	1	0.516058	0.7091	no
gi|646747094|gb|KK962244.1|	36768-37660	20	10	20	26	0.37383	0.8106	no
gi|646747094|gb|KK962244.1|	41813-54285	20	10	288	286	-0.0120877	0.99045	no
gi|646747094|gb|KK962244.1|	49015-54920	20	10	2761.37	1699.91	-0.699928	0.5019	no
gi|646747094|gb|KK962244.1|	50278-55162	20	10	771	708.72	-0.122382	0.91645	no
gi|646747094|gb|KK962244.1|	54362-55463	20	10	1777.03	1065.26	-0.738271	0.52905	no
gi|646747094|gb|KK962244.1|	69760-57048	20	10	1	1	-0.000284545	0.92345	no
gi|646747094|gb|KK962244.1|	71669-87206	20	10	8	0	-inf	0.0035	no
gi|646747094|gb|KK962244.1|	9977-10219	20	10	42	34	-0.292733	0.81755	no
gi|646747111|gb|KK962243.1|	06821-20744	21	9.4	2	1	-0.117558	0.9032	no
gi|646747111|gb|KK962243.1|	08978-21015	21	9.4	2	1	-0.247216	0.84115	no
gi|646747111|gb|KK962243.1|	26590-22730	21	9.4	26	6	-2	0.18675	no
gi|646747111|gb|KK962243.1|	45413-24595	21	9.4	7	3	-1	0.3102	no
gi|646747111|gb|KK962243.1|	61876-26325	21	9.4	18	3	-2	0.08065	no
gi|646747111|gb|KK962243.1|	69662-27132	21	9.4	1	0	-inf	0.0004	yes

gi|646747111|gb|KK962243.1|	80839-28182	21	9.4	496	0.537526	-10	0.07395	no
gi|646747111|gb|KK962243.1|	83759-28683	21	9.4	4	0	-inf	0.00015	yes
gi|646747111|gb|KK962243.1|	90299-29137	21	9.4	2	0	-inf	0.00095	yes
gi|646747111|gb|KK962243.1|	04599-30568	21	9.4	1	2	0.13576	0.9183	no
gi|646747111|gb|KK962243.1|	08012-30838	21	9.4	8	2	-2	0.21095	no
gi|646747111|gb|KK962243.1|	17523-32288	21	9.4	2	1	-0.551015	0.7168	no
gi|646747111|gb|KK962243.1|	23637-32646	21	9.4	3	4	0.559786	0.7144	no
gi|646747111|gb|KK962243.1|	26528-32948	21	9.4	2	2	-0.0156741	0.9871	no
gi|646747111|gb|KK962243.1|	54498-35526	21	9.4	3	1	-0.993799	0.50955	no
gi|646747111|gb|KK962243.1|	86717-38728	21	9.4	8	3	-1	0.33515	no
gi|646747111|gb|KK962243.1|	00730-40685	21	9.4	8	7	-0.228863	0.8397	no
gi|646747111|gb|KK962243.1|	08717-41054	21	9.4	0.82393	0.851141	0.0468773	1	no
gi|646747111|gb|KK962243.1|	10652-41289	21	9.4	1	2	0.378966	0.8107	no
gi|646747111|gb|KK962243.1|	14405-41481	21	9.4	5	8	0.523399	0.70115	no
gi|646747111|gb|KK962243.1|	22446-42340	21	9.4	2	1	-0.492572	0.7125	no
gi|646747111|gb|KK962243.1|	23853-42471	21	9.4	4	5	0.227443	0.88335	no
gi|646747111|gb|KK962243.1|	25186-42543	21	9.4	11	23	1	0.4646	no
gi|646747111|gb|KK962243.1|	27133-42735	21	9.4	37	38	0.0128974	0.9394	no
gi|646747111|gb|KK962243.1|	54961-45517	21	9.4	69	98	0.493709	0.73815	no
gi|646747111|gb|KK962243.1|	58891-45914	21	9.4	19	27	0.478003	0.74185	no
gi|646747111|gb|KK962243.1|	59400-46034	21	9.4	2	4	1	0.4396	no
gi|646747111|gb|KK962243.1|	62802-46336	21	9.4	1	4	2	0.27935	no
gi|646747111|gb|KK962243.1|	68183-46844	21	9.4	5	24	2	0.30035	no
gi|646747111|gb|KK962243.1|	71105-47138	21	9.4	18	12	-0.545944	0.7002	no
gi|646747111|gb|KK962243.1|	77255-47753	21	9.4	10	26	1	0.3312	no
gi|646747111|gb|KK962243.1|	80691-48095	21	9.4	17	32	0.917013	0.51305	no
gi|646747111|gb|KK962243.1|	83216-48347	21	9.4	18	11	-0.676681	0.66965	no
gi|646747111|gb|KK962243.1|	09833-51051	21	9.4	11	9	-0.341476	0.8213	no
gi|646747111|gb|KK962243.1|	32794-53298	21	9.4	121	64	-0.919082	0.49955	no
gi|646747111|gb|KK962243.1|	38587-53889	21	9.4	31	36	0.251615	0.8745	no

gi|646747111|gb|KK962243.1|	39065-53939	21	9.4	6	13	1	0.4553	no
gi|646747111|gb|KK962243.1|	53759-55449	21	9.4	4	3	-0.301674	0.8004	no
gi|646747111|gb|KK962243.1|	64647-56574	21	9.4	0.329988	1	2	1	no
gi|646747111|gb|KK962243.1|	70081-57124	21	9.4	0.7116	0.727729	0.0323346	1	no
gi|646747111|gb|KK962243.1|	75512-57658	21	9.4	3	4	0.754676	0.64305	no
gi|646747111|gb|KK962243.1|	84551-58607	21	9.4	2	5	1	0.50055	no
gi|646747111|gb|KK962243.1|	11486-61484	21	9.4	4	4	0.0841499	0.9528	no
gi|646747111|gb|KK962243.1|	19791-62091	21	9.4	7	7	0.0646791	0.9653	no
gi|646747111|gb|KK962243.1|	21688-62355	21	9.4	27	11	-1	0.34065	no
gi|646747111|gb|KK962243.1|	24559-62648	21	9.4	0.443989	0.321311	-0.46655	1	no
gi|646747111|gb|KK962243.1|	34508-63617	21	9.4	5	5	0.0484327	0.9713	no
gi|646747111|gb|KK962243.1|	41750-64868	21	9.4	1	1	0.163088	0.9165	no
gi|646747111|gb|KK962243.1|	50162-65316	21	9.4	2	2	0.509212	0.7414	no
gi|646747111|gb|KK962243.1|	59659-66505	21	9.4	1	0.762624	-0.97074	0.5241	no
gi|646747111|gb|KK962243.1|	79000-68020	21	9.4	4	3	-0.406756	0.802	no
gi|646747111|gb|KK962243.1|	82191-68377	21	9.4	1	0.700858	-1	0.4158	no
gi|646747111|gb|KK962243.1|	87975-68913	21	9.4	1	0.440893	-1	0.38955	no
gi|646747111|gb|KK962243.1|	89552-69125	21	9.4	0.768819	0.555262	-0.469476	1	no
gi|646747111|gb|KK962243.1|	91603-69250	21	9.4	2	3	0.397022	0.7418	no
gi|646747111|gb|KK962243.1|	10341-71085	21	9.4	6	4	-0.565813	0.6619	no
gi|646747111|gb|KK962243.1|	11143-71182	21	9.4	2	3	0.630366	0.64195	no
gi|646747111|gb|KK962243.1|	13911-71433	21	9.4	4	6	0.557027	0.69815	no
gi|646747111|gb|KK962243.1|	15054-71717	21	9.4	0.844678	1	0.255329	1	no
gi|646747111|gb|KK962243.1|	19118-72122	21	9.4	0.299247	0.795123	1	1	no
gi|646747124|gb|KK962242.1|	20721-21641	17	8.7	2	0	-inf	0.0035	no
gi|646747124|gb|KK962242.1|	29367-22962	17	8.7	5	149	5	0.2367	no
gi|646747124|gb|KK962242.1|	33751-23442	17	8.7	13	3	-2	0.17815	no
gi|646747124|gb|KK962242.1|	45953-24621	17	8.7	17	26	0.656823	0.6381	no
gi|646747124|gb|KK962242.1|	48207-24895	17	8.7	2	4	0.810607	0.5827	no
gi|646747124|gb|KK962242.1|	24865-26470	17	8.7	0.685288	0.692177	0.0144305	1	no

gi|646747124|gb|KK962242.1|	50935-25129	17	8.7	6	15	1	0.3809	no
gi|646747124|gb|KK962242.1|	53999-25441	17	8.7	7	3	-1	0.4184	no
gi|646747124|gb|KK962242.1|	60048-26307	17	8.7	21	10	-1	0.3383	no
gi|646747124|gb|KK962242.1|	78380-27893	17	8.7	3	3	0.129278	0.926	no
gi|646747124|gb|KK962242.1|	80257-28132	17	8.7	3	0.97003	-1	0.3416	no
gi|646747124|gb|KK962242.1|	84247-28622	17	8.7	1	1	-0.0826052	0.9581	no
gi|646747124|gb|KK962242.1|	91616-29209	17	8.7	11	7	-0.580768	0.6818	no
gi|646747124|gb|KK962242.1|	07081-30792	17	8.7	4	3	-0.50093	0.7186	no
gi|646747124|gb|KK962242.1|	11559-31282	17	8.7	5	4	-0.338748	0.8303	no
gi|646747124|gb|KK962242.1|	52273-35412	17	8.7	7	5	-0.350037	0.82295	no
gi|646747124|gb|KK962242.1|	54902-35657	17	8.7	0.523419	0.850289	0.699986	1	no
gi|646747124|gb|KK962242.1|	59969-36402	17	8.7	7	6	-0.376082	0.72205	no
gi|646747124|gb|KK962242.1|	36963-37482	17	8.7	18	0.471331	-5	0.1698	no
gi|646747124|gb|KK962242.1|	74374-37505	17	8.7	9	11	0.261378	0.86395	no
gi|646747124|gb|KK962242.1|	40911-41354	17	8.7	24	0	-inf	0.00085	yes
gi|646747124|gb|KK962242.1|	44887-45193	17	8.7	485.24	14	-5	0.00505	no
gi|646747124|gb|KK962242.1|	45602-46136	17	8.7	7	2	-2	0.23	no
gi|646747136|gb|KK962241.1|	12123-31233	13	7.2	0	59	inf	0.00255	no
gi|646747149|gb|KK962240.1|	04145-10441	21	10	26	0	-inf	0.0035	no
gi|646747149|gb|KK962240.1|	16690-22017	21	10	37	81.63	1	0.34395	no
gi|646747149|gb|KK962240.1|	39482-24023	21	10	0.742883	2	1	0.4859	no
gi|646747149|gb|KK962240.1|	49968-25144	21	10	0.456101	1	1	1	no
gi|646747149|gb|KK962240.1|	51629-27128	21	10	9	10	0.137911	0.9126	no
gi|646747149|gb|KK962240.1|	33456-33499	21	10	1	0	-inf	1	no
gi|646747149|gb|KK962240.1|	43636-34423	21	10	27	13	-1	0.4924	no
gi|646747149|gb|KK962240.1|	56269-35769	21	10	0.872006	0.570483	-0.612156	1	no
gi|646747149|gb|KK962240.1|	59693-36091	21	10	3	2	-0.61785	0.7087	no
gi|646747149|gb|KK962240.1|	64399-36504	21	10	4	2	-0.720206	0.59125	no
gi|646747149|gb|KK962240.1|	65276-37372	21	10	3	4	0.46248	0.667	no
gi|646747149|gb|KK962240.1|	06650-70776	21	10	3	1	-1	0.4214	no

gi|646747149|gb|KK962240.1|	11612-81219	21	10	2	6	1	0.3683	no
gi|646747161|gb|KK962239.1|	13311-11417	24	11	1	1	0.0157853	1	no
gi|646747161|gb|KK962239.1|	21349-12216	24	11	1	0.697849	-1	0.47555	no
gi|646747161|gb|KK962239.1|	27860-12923	24	11	2	0.238626	-3	0.1297	no
gi|646747161|gb|KK962239.1|	31358-13352	24	11	6	4	-0.428173	0.77755	no
gi|646747161|gb|KK962239.1|	49830-15021	24	11	16	14	-0.199401	0.8989	no
gi|646747161|gb|KK962239.1|	51479-15289	24	11	2	2	-0.245263	0.87655	no
gi|646747161|gb|KK962239.1|	53022-15446	24	11	1	1	-0.304206	0.81285	no
gi|646747161|gb|KK962239.1|	79026-18005	24	11	2	0.678197	-2	0.2986	no
gi|646747161|gb|KK962239.1|	80191-18146	24	11	0.813871	1	0.351637	1	no
gi|646747161|gb|KK962239.1|	1928-3427	24	11	0.892406	2	0.942054	0.52865	no
gi|646747161|gb|KK962239.1|	01283-20204	24	11	41	27	-0.621148	0.62265	no
gi|646747161|gb|KK962239.1|	220-756	24	11	2	3	0.706691	0.6455	no
gi|646747161|gb|KK962239.1|	22160-22360	24	11	1	2	0.443406	0.74935	no
gi|646747161|gb|KK962239.1|	35144-23570	24	11	10	9	-0.153298	0.91905	no
gi|646747161|gb|KK962239.1|	38104-23900	24	11	2	3	0.619043	0.62405	no
gi|646747161|gb|KK962239.1|	39277-23954	24	11	20	32	0.65958	0.6119	no
gi|646747161|gb|KK962239.1|	43393-24437	24	11	1	0.363632	-2	0.2551	no
gi|646747161|gb|KK962239.1|	46995-24765	24	11	3	0.960744	-1	0.3848	no
gi|646747161|gb|KK962239.1|	63479-26472	24	11	0.653447	0.937386	0.520572	1	no
gi|646747161|gb|KK962239.1|	74777-27547	24	11	13	12	-0.114188	0.938	no
gi|646747161|gb|KK962239.1|	81979-28374	24	11	4	4	0.100817	0.9478	no
gi|646747161|gb|KK962239.1|	83817-28426	24	11	4	6	0.721945	0.60555	no
gi|646747161|gb|KK962239.1|	90458-29182	24	11	8	6	-0.255934	0.86305	no
gi|646747161|gb|KK962239.1|	92604-29317	24	11	3	4	0.455062	0.7419	no
gi|646747161|gb|KK962239.1|	10111-31237	24	11	49	39	-0.340928	0.76725	no
gi|646747161|gb|KK962239.1|	24359-32565	24	11	0.623264	1	0.714679	1	no
gi|646747161|gb|KK962239.1|	37408-33809	24	11	2	6	2	0.24435	no
gi|646747161|gb|KK962239.1|	23473-42479	24	11	0	2	inf	0.00435	no
gi|646747161|gb|KK962239.1|	66305-46672	24	11	4	17	2	0.2254	no

gi|646747161|gb|KK962239.1|	5423-6324	24	11	0.57276	2	2	0.20295	no
gi|646747161|gb|KK962239.1|	51717-65230	24	11	0	4	inf	0.0014	yes
gi|646747161|gb|KK962239.1|	92647-69346	24	11	1	3	1	0.3622	no
gi|646747161|gb|KK962239.1|	26249-72679	24	11	0	38	inf	0.0001	yes
gi|646747161|gb|KK962239.1|	27522-72789	24	11	3	9	2	0.35885	no
gi|646747161|gb|KK962239.1|	29405-72979	24	11	0.660366	19	5	0.1025	no
gi|646747161|gb|KK962239.1|	80461-92639	24	11	17	11.19	-0.578296	0.6303	no
gi|646747161|gb|KK962239.1|	9078-10000	24	11	34	42	0.302483	0.77795	no
gi|646747175|gb|KK962238.1|	13206-11433	20	8.4	1	1	0.22684	0.8612	no
gi|646747175|gb|KK962238.1|	15899-12141	20	8.4	16	17	0.110929	0.92495	no
gi|646747175|gb|KK962238.1|	22542-12337	20	8.4	1	1	0.276046	0.8456	no
gi|646747175|gb|KK962238.1|	12344-16064	20	8.4	1	1	-0.134748	0.9292	no
gi|646747175|gb|KK962238.1|	33901-13427	20	8.4	7	2	-2	0.26015	no
gi|646747175|gb|KK962238.1|	45638-14616	20	8.4	28	17	-0.764698	0.61805	no
gi|646747175|gb|KK962238.1|	63962-16559	20	8.4	3	5	0.46929	0.76105	no
gi|646747175|gb|KK962238.1|	17488-18221	20	8.4	2	1	-0.83497	0.57665	no
gi|646747175|gb|KK962238.1|	89909-19323	20	8.4	0.844111	1	0.378641	1	no
gi|646747175|gb|KK962238.1|	95294-19746	20	8.4	0.434354	0.909649	1	1	no
gi|646747175|gb|KK962238.1|	98712-20125	20	8.4	7	4	-0.657809	0.66475	no
gi|646747175|gb|KK962238.1|	03268-20437	20	8.4	1	2	0.863305	0.50195	no
gi|646747175|gb|KK962238.1|	05934-20710	20	8.4	2	1	-0.481976	0.7079	no
gi|646747175|gb|KK962238.1|	19658-22028	20	8.4	2	3	0.982419	0.5187	no
gi|646747175|gb|KK962238.1|	35879-23606	20	8.4	121	12	-3	0.1936	no
gi|646747175|gb|KK962238.1|	39845-24017	20	8.4	37	34	-0.127994	0.93615	no
gi|646747175|gb|KK962238.1|	70321-27206	20	8.4	8	6	-0.602039	0.68715	no
gi|646747175|gb|KK962238.1|	85448-28593	20	8.4	3	3	-0.255399	0.87285	no
gi|646747175|gb|KK962238.1|	96163-29849	20	8.4	2	3	0.0677347	0.96385	no
gi|646747175|gb|KK962238.1|	04632-30497	20	8.4	7	16	1	0.4107	no
gi|646747175|gb|KK962238.1|	05074-30695	20	8.4	2	2	0.301658	0.8524	no
gi|646747175|gb|KK962238.1|	12634-31333	20	8.4	41	16	-1	0.2849	no

gi|646747175|gb|KK962238.1|	13610-31473	20	8.4	21	22	0.108359	0.93075	no
gi|646747175|gb|KK962238.1|	16448-32104	20	8.4	1	0.715318	-0.545969	1	no
gi|646747175|gb|KK962238.1|	31873-33171	20	8.4	2	2	-0.285206	0.81015	no
gi|646747175|gb|KK962238.1|	31220-33175	20	8.4	24	29	0.295039	0.8475	no
gi|646747175|gb|KK962238.1|	13975-41444	20	8.4	3	10	2	0.3447	no
gi|646747175|gb|KK962238.1|	64880-46729	20	8.4	7	5	-0.362045	0.80735	no
gi|646747175|gb|KK962238.1|	67958-47002	20	8.4	5	5	0.176901	0.90845	no
gi|646747175|gb|KK962238.1|	78672-47969	20	8.4	12	14	0.213825	0.8899	no
gi|646747175|gb|KK962238.1|	79804-48119	20	8.4	14	14	0.0865648	0.9558	no
gi|646747175|gb|KK962238.1|	89148-48982	20	8.4	6	3	-0.76196	0.58885	no
gi|646747175|gb|KK962238.1|	90100-49357	20	8.4	6	5	-0.175613	0.8913	no
gi|646747175|gb|KK962238.1|	98632-50012	20	8.4	41	4	-3	0.0276	no
gi|646747175|gb|KK962238.1|	09393-51053	20	8.4	20	12	-0.763437	0.6151	no
gi|646747175|gb|KK962238.1|	12806-51423	20	8.4	0.788679	0.567619	-0.474514	1	no
gi|646747175|gb|KK962238.1|	17859-51910	20	8.4	36	5	-3	0.04625	no
gi|646747175|gb|KK962238.1|	29761-53243	20	8.4	29	2	-4	0.01695	no
gi|646747175|gb|KK962238.1|	51722-55520	20	8.4	10	1	-3	0.0439	no
gi|646747175|gb|KK962238.1|	66002-66222	20	8.4	30	25	-0.263128	0.854	no
gi|646747175|gb|KK962238.1|	69631-69812	20	8.4	550	344	-0.67686	0.6693	no
gi|646747175|gb|KK962238.1|	84417-85780	20	8.4	13	9	-0.619044	0.68225	no
gi|646747175|gb|KK962238.1|	99064-10127[illegible]	20	8.4	22	17	-0.433593	0.6953	no
gi|646747189|gb|KK962237.1|	24953-22558	19	11	0.247642	3	4	0.1063	no
gi|646747189|gb|KK962237.1|	96834-29728	19	11	3	8	2	0.29975	no
gi|646747189|gb|KK962237.1|	51579-51982	19	11	13	2	-2	0.14425	no
gi|646747201|gb|KK962236.1|	34067-13433	18	7.3	4	20	2	0.31615	no
gi|646747201|gb|KK962236.1|	35011-13536	18	7.3	11	16	0.621812	0.66905	no
gi|646747201|gb|KK962236.1|	36499-13692	18	7.3	4	8	1	0.4023	no
gi|646747201|gb|KK962236.1|	47670-14803	18	7.3	10	16	0.742114	0.6064	no
gi|646747201|gb|KK962236.1|	68504-16987	18	7.3	2	3	0.421074	0.79035	no
gi|646747201|gb|KK962236.1|	70235-17092	18	7.3	15	4	-2	0.1658	no

gi|646747201|gb|KK962236.1|	72643-17342	18	7.3	16	13	-0.211769	0.88455	no
gi|646747201|gb|KK962236.1|	77018-17870	18	7.3	91	45	-1	0.38745	no
gi|646747201|gb|KK962236.1|	79272-18080	18	7.3	19	23	0.265743	0.8044	no
gi|646747201|gb|KK962236.1|	81982-18268	18	7.3	1	1	-0.102044	0.9035	no
gi|646747201|gb|KK962236.1|	84341-18519	18	7.3	0.928081	2	1	0.4498	no
gi|646747201|gb|KK962236.1|	85444-18570	18	7.3	15	22	0.545712	0.7154	no
gi|646747201|gb|KK962236.1|	90956-19259	18	7.3	26	36	0.472977	0.67335	no
gi|646747201|gb|KK962236.1|	98042-20029	18	7.3	12	10	-0.332415	0.7547	no
gi|646747201|gb|KK962236.1|	14232-21483	18	7.3	4	2	-1	0.43665	no
gi|646747201|gb|KK962236.1|	34400-23563	18	7.3	9	20	1	0.3908	no
gi|646747201|gb|KK962236.1|	96296-29668	18	7.3	1	8	3	0.276	no
gi|646747201|gb|KK962236.1|	17040-31721	18	7.3	154.65	87	-0.836806	0.5575	no
gi|646747201|gb|KK962236.1|	92574-39285	18	7.3	9	16	0.878227	0.56375	no
gi|646747201|gb|KK962236.1|	94181-39464	18	7.3	2	4	0.672082	0.662	no
gi|646747226|gb|KK962234.1|	91654-39199	12	7.8	7	7	0.0748198	0.9262	no
gi|646747226|gb|KK962234.1|	88005-48878	12	7.8	4	3	-0.128489	0.9125	no
gi|646747247|gb|KK962233.1|	48396-51510	11	6.7	2	6	2	0.3151	no
gi|646747247|gb|KK962233.1|	51618-52102	11	6.7	6	13	1	0.39695	no
gi|646747247|gb|KK962233.1|	59001-59813	11	6.7	1	7	2	0.1398	no
gi|646747247|gb|KK962233.1|	71977-72878	11	6.7	0.28638	4	4	0.25595	no
gi|646747263|gb|KK962232.1|	06955-10714	18	8.6	52	56	0.115292	0.9101	no
gi|646747263|gb|KK962232.1|	73241-17691	18	8.6	0.947407	0.867781	-0.126654	1	no
gi|646747263|gb|KK962232.1|	77036-18344	18	8.6	0.751854	0.857028	0.188889	1	no
gi|646747263|gb|KK962232.1|	86508-18763	18	8.6	1	1	-0.136018	0.92415	no
gi|646747263|gb|KK962232.1|	87980-18846	18	8.6	4	3	-0.57543	0.7155	no
gi|646747263|gb|KK962232.1|	88618-19003	18	8.6	1	2	0.525139	0.7133	no
gi|646747263|gb|KK962232.1|	66274-27187	18	8.6	11	8	-0.451229	0.6857	no
gi|646747263|gb|KK962232.1|	81381-28299	18	8.6	0.751035	1	0.877013	0.4936	no
gi|646747263|gb|KK962232.1|	83165-28666	18	8.6	0.913222	1	0.630155	0.6853	no
gi|646747263|gb|KK962232.1|	88333-29383	18	8.6	1	2	0.396425	0.7842	no

gi|646747263|gb|KK962232.1|	99664-30239	18	8.6	55	25	-1	0.3244	no
gi|646747263|gb|KK962232.1|	13378-31429	18	8.6	1	2	0.50516	0.71995	no
gi|646747263|gb|KK962232.1|	15834-31627	18	8.6	3	2	-0.821743	0.6134	no
gi|646747263|gb|KK962232.1|	41941-34216	18	8.6	37	51	0.476557	0.72385	no
gi|646747263|gb|KK962232.1|	54354-35499	18	8.6	16	8	-0.92992	0.54785	no
gi|646747263|gb|KK962232.1|	76464-37968	18	8.6	1	1	-0.059664	0.9668	no
gi|646747263|gb|KK962232.1|	80394-38283	18	8.6	41	28	-0.525084	0.65195	no
gi|646747263|gb|KK962232.1|	86832-38896	18	8.6	32	16	-1	0.34395	no
gi|646747263|gb|KK962232.1|	89446-39063	18	8.6	13	8	-0.637079	0.67725	no
gi|646747263|gb|KK962232.1|	91331-39244	18	8.6	0.861532	0.61587	-0.484277	1	no
gi|646747263|gb|KK962232.1|	92549-39482	18	8.6	1	1	-0.0954775	0.9523	no
gi|646747263|gb|KK962232.1|	20295-42086	18	8.6	75	62	-0.273662	0.79555	no
gi|646747263|gb|KK962232.1|	25739-42597	18	8.6	20	24	0.308467	0.84205	no
gi|646747263|gb|KK962232.1|	85203-48538	18	8.6	447	92	-2	0.1598	no
gi|646747263|gb|KK962232.1|	95018-49585	18	8.6	1	2	0.861281	0.54775	no
gi|646747263|gb|KK962232.1|	97040-49731	18	8.6	32	29	-0.125407	0.9241	no
gi|646747263|gb|KK962232.1|	98011-50277	18	8.6	29	22.98	-0.348214	0.7626	no
gi|646747263|gb|KK962232.1|	46686-54749	18	8.6	1	0.935274	-0.673673	0.6592	no
gi|646747263|gb|KK962232.1|	81178-58391	18	8.6	15	9	-0.636696	0.55235	no
gi|646747279|gb|KK962231.1|	42285-14246	17	9.7	86	87	0.0111909	0.91215	no
gi|646747279|gb|KK962231.1|	05332-80594	17	9.7	11	4	-1	0.34595	no
gi|646747293|gb|KK962230.1|	32638-33347	11	6.6	3	11	2	0.3194	no
gi|646747293|gb|KK962230.1|	43234-34414	11	6.6	0.707194	4	3	0.16655	no
gi|646747293|gb|KK962230.1|	57339-35880	11	6.6	0.918997	4	2	0.16725	no
gi|646747317|gb|KK962228.1|	46437-24763	16	8.5	0.097686	19	8	0.09445	no
gi|646747317|gb|KK962228.1|	62084-26451	16	8.5	0	3	inf	0.0002	yes
gi|646747317|gb|KK962228.1|	67279-26879	16	8.5	0	2	inf	0.00515	no
gi|646747317|gb|KK962228.1|	69455-27174	16	8.5	0	3	inf	0.00045	yes
gi|646747317|gb|KK962228.1|	73747-27529	16	8.5	0	3	inf	0.0017	no
gi|646747317|gb|KK962228.1|	76703-27789	16	8.5	0	9	inf	5.00E-05	yes

gi|646747317|gb|KK962228.1|	06033-60754	16	8.5	0.073892	54	10	0.09445	no
gi|646747317|gb|KK962228.1|	18585-61943	16	8.5	0.931194	1	0.499775	0.73295	no
gi|646747317|gb|KK962228.1|	26502-62692	16	8.5	4	2	-0.836758	0.6128	no
gi|646747317|gb|KK962228.1|	32075-63591	16	8.5	6	5	-0.407456	0.73875	no
gi|646747317|gb|KK962228.1|	64466-66552	16	8.5	0.693792	2	1	0.3964	no
gi|646747317|gb|KK962228.1|	72360-67271	16	8.5	12	2	-3	0.14175	no
gi|646747342|gb|KK962226.1|	00035-10062	30	12	6	2	-2	0.19445	no
gi|646747342|gb|KK962226.1|	09546-11021	30	12	2	0.308653	-3	0.1994	no
gi|646747342|gb|KK962226.1|	17141-11851	30	12	10	5	-1	0.47085	no
gi|646747342|gb|KK962226.1|	21370-12193	30	12	3	0.814019	-2	0.2673	no
gi|646747342|gb|KK962226.1|	80183-28123	30	12	0.699915	1	0.735094	0.6352	no
gi|646747342|gb|KK962226.1|	82022-28344	30	12	1	2	0.52544	0.71615	no
gi|646747342|gb|KK962226.1|	83609-28490	30	12	1	2	0.802228	0.5768	no
gi|646747342|gb|KK962226.1|	88888-29058	30	12	0.448168	0.832326	0.893109	1	no
gi|646747342|gb|KK962226.1|	96299-29985	30	12	2	3	0.0876761	0.9542	no
gi|646747342|gb|KK962226.1|	00985-30177	30	12	10	10	-0.0538271	0.9725	no
gi|646747342|gb|KK962226.1|	02057-30344	30	12	0.819411	1	0.524442	0.6954	no
gi|646747342|gb|KK962226.1|	08458-30867	30	12	52	57	0.128495	0.91735	no
gi|646747342|gb|KK962226.1|	31354-33171	30	12	4	7	0.807679	0.5898	no
gi|646747342|gb|KK962226.1|	35263-33612	30	12	3	3	0.319973	0.8254	no
gi|646747342|gb|KK962226.1|	40392-34122	30	12	2	0.225279	-3	0.1932	no
gi|646747342|gb|KK962226.1|	43848-34515	30	12	2	0.884988	-0.840192	0.5181	no
gi|646747342|gb|KK962226.1|	54936-35516	30	12	27	45	0.758276	0.593	no
gi|646747342|gb|KK962226.1|	73346-47398	30	12	3	19	3	0.0887	no
gi|646747342|gb|KK962226.1|	02440-50402	30	12	9	9	0.0864304	0.95635	no
gi|646747342|gb|KK962226.1|	85175-85578	30	12	4	6	0.562337	0.7088	no
gi|646747342|gb|KK962226.1|	98895-99921	30	12	9	3	-2	0.26475	no
gi|646747355|gb|KK962225.1|	53432-25397	17	10	6	8	0.294057	0.8303	no
gi|646747369|gb|KK962224.1|	78306-17861	13	7.3	47	24	-0.956095	0.55775	no
gi|646747369|gb|KK962224.1|	52455-25288	13	7.3	12	7	-0.670689	0.63525	no

gi|646747369|gb|KK962224.1|	75499-27603	13	7.3	4	3	-0.333601	0.79845	no
gi|646747369|gb|KK962224.1|	02689-30373	13	7.3	2	4	0.649855	0.6371	no
gi|646747369|gb|KK962224.1|	80098-80567	13	7.3	3	4	0.221924	0.88325	no
gi|646747385|gb|KK962223.1|	08041-21885	21	5.4	12	27	1	0.30375	no
gi|646747385|gb|KK962223.1|	21418-22101	21	5.4	4	2	-0.883184	0.49395	no
gi|646747385|gb|KK962223.1|	36442-23674	21	5.4	7	13	0.913271	0.55755	no
gi|646747385|gb|KK962223.1|	23706-24307	21	5.4	10	11	0.121705	0.93785	no
gi|646747385|gb|KK962223.1|	43439-24365	21	5.4	27	32	0.2449	0.8667	no
gi|646747385|gb|KK962223.1|	60952-26114	21	5.4	63	25	-1	0.41275	no
gi|646747385|gb|KK962223.1|	73234-27363	21	5.4	13	4	-2	0.28935	no
gi|646747385|gb|KK962223.1|	34214-33632	21	5.4	5	5	0.0561774	0.97135	no
gi|646747385|gb|KK962223.1|	38016-33894	21	5.4	7	7	-0.0126169	0.9889	no
gi|646747385|gb|KK962223.1|	56841-35986	21	5.4	0.334248	0.583595	0.804047	1	no
gi|646747385|gb|KK962223.1|	81034-38165	21	5.4	4	5	0.0797103	0.94885	no
gi|646747385|gb|KK962223.1|	84897-38607	21	5.4	3	1	-0.914751	0.5316	no
gi|646747385|gb|KK962223.1|	04566-41158	21	5.4	15	16	0.116657	0.92015	no
gi|646747385|gb|KK962223.1|	20787-42261	21	5.4	1	1	0.0297525	1	no
gi|646747385|gb|KK962223.1|	40177-44048	21	5.4	19	14	-0.487111	0.6994	no
gi|646747385|gb|KK962223.1|	62499-62756	21	5.4	10	30	2	0.29035	no
gi|646747385|gb|KK962223.1|	42805-64329	21	5.4	10	3	-2	0.24785	no
gi|646747385|gb|KK962223.1|	98643-107753	21	5.4	12	12	0.0198342	0.98505	no
gi|646747398|gb|KK962222.1|	29546-12981	11	6	4	31	3	0.2499	no
gi|646747398|gb|KK962222.1|	30936-31220	11	6	5	14	1	0.3798	no
gi|646747398|gb|KK962222.1|	33992-34860	11	6	35	36	0.0651131	0.9497	no
gi|646747398|gb|KK962222.1|	6716-8006	11	6	1	0.898379	-0.255691	1	no
gi|646747398|gb|KK962222.1|	726-1636	11	6	26	35	0.455814	0.7162	no
gi|646747411|gb|KK962221.1|	48584-15051	17	6.6	8	0	-inf	0.0001	yes
gi|646747411|gb|KK962221.1|	50583-15313	17	6.6	21	0	-inf	5.00E-05	yes
gi|646747411|gb|KK962221.1|	87105-38743	17	6.6	1	99	7	0.0948	no
gi|646747411|gb|KK962221.1|	88549-38893	17	6.6	0.65182	19	5	0.1025	no

gi|646747411|gb|KK962221.1|	97892-39871	17	6.6	11	17	0.636722	0.67035	no
gi|646747411|gb|KK962221.1|	00678-40174	17	6.6	7	2	-2	0.26285	no
gi|646747411|gb|KK962221.1|	03150-40336	17	6.6	28	55	0.977222	0.54415	no
gi|646747411|gb|KK962221.1|	09256-41122	17	6.6	1	1	0.229072	0.8655	no
gi|646747411|gb|KK962221.1|	12923-41374	17	6.6	3	2	-0.268407	0.8229	no
gi|646747411|gb|KK962221.1|	41721-44344	17	6.6	6	7	0.120553	0.9349	no
gi|646747411|gb|KK962221.1|	44501-44597	17	6.6	2	3	0.157306	0.9185	no
gi|646747424|gb|KK962220.1|	04474-10475	22	5.7	13	10	-0.294405	0.8508	no
gi|646747424|gb|KK962220.1|	06327-10681	22	5.7	0.7992	5	3	0.26725	no
gi|646747424|gb|KK962220.1|	18752-11261	22	5.7	9	9	-0.026188	0.98155	no
gi|646747424|gb|KK962220.1|	54861-11552	22	5.7	15	10	-0.573725	0.6818	no
gi|646747424|gb|KK962220.1|	59617-11599	22	5.7	20	14	-0.509008	0.67525	no
gi|646747424|gb|KK962220.1|	63094-11640	22	5.7	1	1	0.317245	0.8346	no
gi|646747424|gb|KK962220.1|	65043-11653	22	5.7	11	10	-0.0674001	0.91525	no
gi|646747424|gb|KK962220.1|	85503-11860	22	5.7	3	3	0.0300045	0.9298	no
gi|646747424|gb|KK962220.1|	88453-11893	22	5.7	3	2	-0.426299	0.72	no
gi|646747424|gb|KK962220.1|	91058-11914	22	5.7	10	2	-2	0.169	no
gi|646747424|gb|KK962220.1|	94375-12006	22	5.7	13	7	-0.874799	0.4484	no
gi|646747424|gb|KK962220.1|	20886-12196	22	5.7	3	2	-0.222694	0.88895	no
gi|646747424|gb|KK962220.1|	15808-12321	22	5.7	5	4	-0.533451	0.6393	no
gi|646747424|gb|KK962220.1|	64265-12646	22	5.7	88	10	-3	0.0445	no
gi|646747424|gb|KK962220.1|	94227-12944	22	5.7	35	13	-1	0.40345	no
gi|646747424|gb|KK962220.1|	04009-13042	22	5.7	34	39	0.208908	0.8791	no
gi|646747424|gb|KK962220.1|	12666-13135	22	5.7	3	0.813352	-2	0.199	no
gi|646747424|gb|KK962220.1|	32415-13338	22	5.7	0.901842	1	0.286211	1	no
gi|646747424|gb|KK962220.1|	50301-13506	22	5.7	21	12	-0.866244	0.5155	no
gi|646747424|gb|KK962220.1|	60304-13605	22	5.7	23	5	-2	0.201	no
gi|646747424|gb|KK962220.1|	62447-13626	22	5.7	37	14	-1	0.40325	no
gi|646747424|gb|KK962220.1|	70069-13716	22	5.7	14	4	-2	0.17	no
gi|646747424|gb|KK962220.1|	71844-13844	22	5.7	8	8	0.0262942	0.9817	no

gi|646747424|gb|KK962220.1|	38849-13959	22	5.7	3	0.265856	-4	0.1757	no
gi|646747424|gb|KK962220.1|	12227-14182	22	5.7	10	7	-0.481406	0.66925	no
gi|646747424|gb|KK962220.1|	18290-14189	22	5.7	2	2	-0.210006	0.89255	no
gi|646747424|gb|KK962220.1|	23456-14248	22	5.7	0.92176	1	0.386308	0.77315	no
gi|646747424|gb|KK962220.1|	24961-14265	22	5.7	0.336415	0.87402	1	1	no
gi|646747424|gb|KK962220.1|	26905-14286	22	5.7	2	2	-0.00897889	0.98095	no
gi|646747424|gb|KK962220.1|	31296-14325	22	5.7	0.838455	0.668243	-0.327361	1	no
gi|646747424|gb|KK962220.1|	33529-14353	22	5.7	0.744709	1	0.832399	0.5688	no
gi|646747424|gb|KK962220.1|	35540-14374	22	5.7	2	2	0.53309	0.73915	no
gi|646747424|gb|KK962220.1|	63729-16446	22	5.7	62	24	-1	0.2976	no
gi|646747424|gb|KK962220.1|	82538-18789	22	5.7	0.85266	5	3	0.0719	no
gi|646747424|gb|KK962220.1|	32190-23268	22	5.7	0.757646	8	3	0.26865	no
gi|646747424|gb|KK962220.1|	33017-23356	22	5.7	3	15	2	0.1334	no
gi|646747424|gb|KK962220.1|	44923-24670	22	5.7	18	32	0.843871	0.4464	no
gi|646747424|gb|KK962220.1|	47754-24810	22	5.7	19	36	0.962914	0.48365	no
gi|646747424|gb|KK962220.1|	48412-24877	22	5.7	23	89	2	0.2245	no
gi|646747424|gb|KK962220.1|	49591-24993	22	5.7	8	50	3	0.0994	no
gi|646747424|gb|KK962220.1|	51090-25370	22	5.7	0.783727	0.967942	0.30457	1	no
gi|646747424|gb|KK962220.1|	59727-26011	22	5.7	6	10	0.7701	0.5591	no
gi|646747424|gb|KK962220.1|	61776-26425	22	5.7	4	11	1	0.2656	no
gi|646747424|gb|KK962220.1|	64390-26603	22	5.7	0.73055	2	2	0.2833	no
gi|646747424|gb|KK962220.1|	66208-26879	22	5.7	0.396566	1	1	1	no
gi|646747424|gb|KK962220.1|	68977-27056	22	5.7	0.972366	0.701376	-0.471312	1	no
gi|646747424|gb|KK962220.1|	72565-27456	22	5.7	0.529271	0.843179	0.671832	1	no
gi|646747424|gb|KK962220.1|	75521-27739	22	5.7	0.457999	1	1	0.3716	no
gi|646747424|gb|KK962220.1|	81977-28309	22	5.7	0.962501	1	0.515975	0.7038	no
gi|646747424|gb|KK962220.1|	97070-29730	22	5.7	14	79	3	0.19885	no
gi|646747424|gb|KK962220.1|	02596-30475	22	5.7	0.631021	2	1	0.3513	no
gi|646747424|gb|KK962220.1|	36076-34218	22	5.7	5	3	-0.830522	0.5178	no
gi|646747424|gb|KK962220.1|	43861-34526	22	5.7	0.402575	0.810948	1	1	no

gi|646747424|gb|KK962220.1|	60678-36093	22	5.7	32	3	-4	0.1801	no
gi|646747424|gb|KK962220.1|	92728-39297	22	5.7	21	28	0.465188	0.74325	no
gi|646747424|gb|KK962220.1|	20781-42728	22	5.7	14	10	-0.471641	0.68555	no
gi|646747424|gb|KK962220.1|	64223-46533	22	5.7	1	1	-0.0692818	0.9467	no
gi|646747424|gb|KK962220.1|	92811-49493	22	5.7	3	4	0.511609	0.7316	no
gi|646747424|gb|KK962220.1|	95232-49814	22	5.7	2	3	0.413149	0.7845	no
gi|646747424|gb|KK962220.1|	17737-51977	22	5.7	2	1	-0.943403	0.49485	no
gi|646747424|gb|KK962220.1|	19971-52167	22	5.7	0.447239	0.922919	1	1	no
gi|646747424|gb|KK962220.1|	21849-52356	22	5.7	3	5	0.97141	0.5246	no
gi|646747424|gb|KK962220.1|	23740-52678	22	5.7	7	13	0.846255	0.4283	no
gi|646747424|gb|KK962220.1|	28247-52995	22	5.7	44	33	-0.430489	0.69905	no
gi|646747424|gb|KK962220.1|	32232-54876	22	5.7	7	4	-0.914917	0.4346	no
gi|646747424|gb|KK962220.1|	87455-58872	22	5.7	10	0	-inf	0.0002	yes
gi|646747424|gb|KK962220.1|	61850-65562	22	5.7	24	15	-0.694531	0.5402	no
gi|646747424|gb|KK962220.1|	37699-63851	22	5.7	3	5	0.563465	0.6808	no
gi|646747424|gb|KK962220.1|	39318-64036	22	5.7	1	2	0.775537	0.55405	no
gi|646747424|gb|KK962220.1|	41170-64223	22	5.7	8	3	-2	0.34	no
gi|646747424|gb|KK962220.1|	50490-65090	22	5.7	5	1	-2	0.28785	no
gi|646747424|gb|KK962220.1|	54540-65540	22	5.7	2	0.214253	-3	0.1994	no
gi|646747424|gb|KK962220.1|	66361-73294	22	5.7	10	8	-0.390848	0.7364	no
gi|646747424|gb|KK962220.1|	73568-67828	22	5.7	9	5	-0.807871	0.4563	no
gi|646747424|gb|KK962220.1|	73457-74525	22	5.7	11	4	-2	0.3303	no
gi|646747424|gb|KK962220.1|	86141-78699	22	5.7	1	1	0.0142611	1	no
gi|646747424|gb|KK962220.1|	87850-79035	22	5.7	0.78266	0.658068	-0.250149	1	no
gi|646747424|gb|KK962220.1|	90912-79166	22	5.7	2	0.788318	-1	0.47555	no
gi|646747424|gb|KK962220.1|	93350-79724	22	5.7	69	33	-1	0.37415	no
gi|646747424|gb|KK962220.1|	21941-82280	22	5.7	5	3	-1	0.4385	no
gi|646747424|gb|KK962220.1|	84409-84804	22	5.7	9	8	-0.122823	0.9264	no
gi|646747424|gb|KK962220.1|	51169-85543	22	5.7	6	5	-0.21175	0.84095	no
gi|646747424|gb|KK962220.1|	57874-86051	22	5.7	9	6	-0.55866	0.65645	no

gi|646747424|gb|KK962220.1|	61721-86254	22	5.7	1	0.925704	-0.673086	0.6592	no
gi|646747424|gb|KK962220.1|	64739-86680	22	5.7	9	8	-0.157533	0.91745	no
gi|646747424|gb|KK962220.1|	69821-87360	22	5.7	11	8	-0.453191	0.67725	no
gi|646747424|gb|KK962220.1|	17034-91753	22	5.7	6	5	-0.246377	0.8441	no
gi|646747424|gb|KK962220.1|	94362-95233	22	5.7	2	0.637725	-1	0.4271	no
gi|646747424|gb|KK962220.1|	48578-95038	22	5.7	0.478116	1	1	0.3716	no
gi|646747424|gb|KK962220.1|	50830-95132	22	5.7	3	5	0.790345	0.5817	no
gi|646747424|gb|KK962220.1|	54266-95617	22	5.7	0.783086	0.323803	-1	1	no
gi|646747424|gb|KK962220.1|	57478-96351	22	5.7	17	18	0.101141	0.9319	no
gi|646747438|gb|KK962219.1|	04714-10579	17	7.9	3	2	-0.436819	0.7923	no
gi|646747438|gb|KK962219.1|	06293-10710	17	7.9	1	4	2	0.30665	no
gi|646747438|gb|KK962219.1|	07381-10778	17	7.9	5	3	-0.632915	0.6683	no
gi|646747438|gb|KK962219.1|	10021-12025	17	7.9	5	4	-0.445504	0.6906	no
gi|646747438|gb|KK962219.1|	42286-14273	17	7.9	10	2	-2	0.22785	no
gi|646747438|gb|KK962219.1|	51270-15224	17	7.9	0.900588	1	0.701309	0.62425	no
gi|646747438|gb|KK962219.1|	82125-83369	17	7.9	1	2	0.635986	0.6172	no
gi|646747438|gb|KK962219.1|	89027-89595	17	7.9	7	8	0.202367	0.8973	no
gi|646747438|gb|KK962219.1|	99496-104069	17	7.9	19	20	0.0190714	0.9852	no
gi|646747449|gb|KK962218.1|	18618-12592	22	10	3	6	0.898036	0.4088	no
gi|646747449|gb|KK962218.1|	06610-30769	22	10	0	4	inf	0.0017	no
gi|646747449|gb|KK962218.1|	48213-55081	22	10	0.078807	19	8	0.2322	no
gi|646747449|gb|KK962218.1|	59098-56011	22	10	0	2	inf	0.0014	yes
gi|646747449|gb|KK962218.1|	65270-57046	22	10	0.056142	11	8	0.1049	no
gi|646747449|gb|KK962218.1|	75694-57680	22	10	0	21	inf	0.00015	yes
gi|646747449|gb|KK962218.1|	80891-58281	22	10	0.055532	13	8	0.09445	no
gi|646747449|gb|KK962218.1|	11083-61940	22	10	3	5	0.623677	0.56445	no
gi|646747449|gb|KK962218.1|	21957-62348	22	10	3	4	0.0719324	0.963	no
gi|646747449|gb|KK962218.1|	23614-62412	22	10	5	4	-0.265846	0.83475	no
gi|646747449|gb|KK962218.1|	24181-62502	22	10	4	4	-0.220555	0.89485	no
gi|646747449|gb|KK962218.1|	25637-63096	22	10	3	2	-0.365813	0.81345	no

gi|646747449|gb|KK962218.1|	35401-63744	22	10	4	3	-0.465237	0.7612	no
gi|646747449|gb|KK962218.1|	43509-64427	22	10	0.545136	2	2	0.2903	no
gi|646747449|gb|KK962218.1|	79111-67964	22	10	5	2	-1	0.41455	no
gi|646747449|gb|KK962218.1|	94755-69544	22	10	1	3	0.843604	0.5599	no
gi|646747449|gb|KK962218.1|	55134-75747	22	10	3	4	0.141299	0.9229	no
gi|646747449|gb|KK962218.1|	59602-77653	22	10	4	5	0.22089	0.84955	no
gi|646747449|gb|KK962218.1|	77595-77834	22	10	13	10	-0.410136	0.78095	no
gi|646747449|gb|KK962218.1|	81352-78314	22	10	0.841595	0.782217	-0.105557	1	no
gi|646747449|gb|KK962218.1|	83276-78529	22	10	2	2	-0.00161105	0.9797	no
gi|646747449|gb|KK962218.1|	86153-78754	22	10	1	0.939633	-0.382351	0.7684	no
gi|646747449|gb|KK962218.1|	89055-79027	22	10	0.863673	0.275156	-2	1	no
gi|646747449|gb|KK962218.1|	91758-79227	22	10	2	5	0.951558	0.5192	no
gi|646747449|gb|KK962218.1|	94686-79620	22	10	0.365953	1	2	0.18085	no
gi|646747449|gb|KK962218.1|	08130-81040	22	10	3	4	0.606022	0.69345	no
gi|646747449|gb|KK962218.1|	12390-81311	22	10	43	37	-0.232015	0.852	no
gi|646747449|gb|KK962218.1|	14077-81430	22	10	27	28	0.0171258	0.91805	no
gi|646747449|gb|KK962218.1|	22044-82486	22	10	2	2	0.384836	0.80335	no
gi|646747449|gb|KK962218.1|	34571-83544	22	10	0.593899	1	1	0.43305	no
gi|646747449|gb|KK962218.1|	35571-83944	22	10	6	3	-1	0.348	no
gi|646747449|gb|KK962218.1|	43007-84458	22	10	5	11	1	0.46855	no
gi|646747449|gb|KK962218.1|	61966-86298	22	10	0	2	inf	0.0008	yes
gi|646747449|gb|KK962218.1|	71424-87499	22	10	24	18	-0.445622	0.701	no
gi|646747449|gb|KK962218.1|	75535-87669	22	10	3	2	-0.930041	0.5076	no
gi|646747449|gb|KK962218.1|	77057-87769	22	10	2	1	-0.336737	0.81865	no
gi|646747449|gb|KK962218.1|	20428-92248	22	10	0.25742	0.596706	1	1	no
gi|646747449|gb|KK962218.1|	23712-92481	22	10	0.652222	1	0.737774	1	no
gi|646747459|gb|KK962217.1|	12032-10129	21	5.9	2	0.381523	-2	0.197	no
gi|646747459|gb|KK962217.1|	45976-10462	21	5.9	17	36	1	0.481	no
gi|646747459|gb|KK962217.1|	74692-10749	21	5.9	14	12	-0.316316	0.8504	no
gi|646747459|gb|KK962217.1|	30775-13781	21	5.9	15	12	-0.249066	0.82545	no

gi|646747459|gb|KK962217.1|	49100-14947	21	5.9	4	7	0.754638	0.59865	no
gi|646747459|gb|KK962217.1|	59759-16022	21	5.9	6	0.570792	-3	0.1849	no
gi|646747459|gb|KK962217.1|	70421-17093	21	5.9	8	3	-1	0.3608	no
gi|646747459|gb|KK962217.1|	72861-17324	21	5.9	16	0.892243	-4	0.18585	no
gi|646747459|gb|KK962217.1|	74585-17622	21	5.9	1	0.870283	-0.300547	1	no
gi|646747459|gb|KK962217.1|	81754-18550	21	5.9	3	0.231421	-4	0.0351	no
gi|646747459|gb|KK962217.1|	96486-19811	21	5.9	2	0.486262	-2	0.3102	no
gi|646747459|gb|KK962217.1|	10785-21095	21	5.9	40	132	2	0.3831	no
gi|646747459|gb|KK962217.1|	15314-21552	21	5.9	34	28	-0.285813	0.8537	no
gi|646747459|gb|KK962217.1|	29058-22998	21	5.9	1	4	2	0.32315	no
gi|646747459|gb|KK962217.1|	31352-23165	21	5.9	10	11	0.240818	0.86965	no
gi|646747459|gb|KK962217.1|	32874-23316	21	5.9	31	45	0.539286	0.69025	no
gi|646747459|gb|KK962217.1|	35481-23600	21	5.9	2	5	1	0.35005	no
gi|646747459|gb|KK962217.1|	48674-24972	21	5.9	32	50	0.653761	0.55475	no
gi|646747459|gb|KK962217.1|	91169-49268	21	5.9	21	27	0.366892	0.7357	no
gi|646747459|gb|KK962217.1|	49580-55042	21	5.9	4	2	-0.822328	0.571	no
gi|646747459|gb|KK962217.1|	62524-63184	21	5.9	1	2	0.475317	0.7489	no
gi|646747459|gb|KK962217.1|	81324-78223	21	5.9	18	13	-0.506924	0.74125	no
gi|646747459|gb|KK962217.1|	34935-83517	21	5.9	10	44	2	0.19125	no
gi|646747459|gb|KK962217.1|	57120-85850	21	5.9	1	2	0.524515	0.6647	no
gi|646747459|gb|KK962217.1|	58958-86156	21	5.9	1	2	0.736894	0.63475	no
gi|646747459|gb|KK962217.1|	61887-86254	21	5.9	3	2	-0.662001	0.64775	no
gi|646747459|gb|KK962217.1|	62649-86375	21	5.9	2	3	0.515553	0.7099	no
gi|646747459|gb|KK962217.1|	74269-88038	21	5.9	1	2	0.425098	0.7725	no
gi|646747459|gb|KK962217.1|	85647-88822	21	5.9	19	14	-0.470341	0.67475	no
gi|646747459|gb|KK962217.1|	98473-90073	21	5.9	19	11	-0.804882	0.4611	no
gi|646747459|gb|KK962217.1|	19199-91978	21	5.9	25	8	-2	0.2914	no
gi|646747459|gb|KK962217.1|	46555-94675	21	5.9	78	56	-0.473936	0.7381	no
gi|646747459|gb|KK962217.1|	50595-95093	21	5.9	98	18	-2	0.14065	no
gi|646747459|gb|KK962217.1|	80478-98140	21	5.9	6	4	-0.593618	0.7043	no

gi|646747459|gb|KK962217.1|	9809-10441	21	5.9	3	2	-0.530217	0.7013	no
gi|646747459|gb|KK962217.1|	87168-99660	21	5.9	4	3	-0.212616	0.84485	no
gi|646747471|gb|KK962216.1|	04034-10644	24	6.4	3	3	0.184148	0.89885	no
gi|646747471|gb|KK962216.1|	06906-10767	24	6.4	1	1	-0.186328	0.90405	no
gi|646747471|gb|KK962216.1|	16189-11818	24	6.4	0.691215	0.616005	-0.166194	1	no
gi|646747471|gb|KK962216.1|	20754-12174	24	6.4	3	3	0.0736203	0.9624	no
gi|646747471|gb|KK962216.1|	25830-12694	24	6.4	0.540976	0.928017	0.778587	1	no
gi|646747471|gb|KK962216.1|	27341-13265	24	6.4	16	11	-0.641401	0.57315	no
gi|646747471|gb|KK962216.1|	1348-2429	24	6.4	1	1	0.192344	0.8968	no
gi|646747471|gb|KK962216.1|	36721-13811	24	6.4	63	41	-0.620896	0.58375	no
gi|646747471|gb|KK962216.1|	39052-14014	24	6.4	7	7	0.186535	0.9009	no
gi|646747471|gb|KK962216.1|	72291-17642	24	6.4	86	88	0.0252212	0.982	no
gi|646747471|gb|KK962216.1|	78340-17904	24	6.4	3	3	0.367288	0.7632	no
gi|646747471|gb|KK962216.1|	84553-18505	24	6.4	6	2	-2	0.2499	no
gi|646747471|gb|KK962216.1|	85968-18752	24	6.4	3	2	-0.270316	0.8612	no
gi|646747471|gb|KK962216.1|	93416-19407	24	6.4	21	90	2	0.1243	no
gi|646747471|gb|KK962216.1|	12519-21290	24	6.4	200	313	0.646598	0.5597	no
gi|646747471|gb|KK962216.1|	17304-21756	24	6.4	567	1130.55	0.995745	0.369	no
gi|646747471|gb|KK962216.1|	30405-23073	24	6.4	32	19	-0.71746	0.66105	no
gi|646747471|gb|KK962216.1|	42392-24335	24	6.4	23	11	-1	0.4655	no
gi|646747471|gb|KK962216.1|	48501-24927	24	6.4	0.179946	2	4	0.1061	no
gi|646747471|gb|KK962216.1|	50639-25259	24	6.4	0.925163	0.945466	0.0313175	1	no
gi|646747471|gb|KK962216.1|	52709-25333	24	6.4	2	1	-0.338208	0.81865	no
gi|646747471|gb|KK962216.1|	53673-25423	24	6.4	4	3	-0.134065	0.9271	no
gi|646747471|gb|KK962216.1|	54311-25510	24	6.4	1	1	0.0792691	0.92975	no
gi|646747471|gb|KK962216.1|	55370-25633	24	6.4	0.520411	1	1	1	no
gi|646747471|gb|KK962216.1|	56787-25762	24	6.4	1	0.675837	-0.916691	0.58965	no
gi|646747471|gb|KK962216.1|	57874-25913	24	6.4	0.552847	1	1	0.3963	no
gi|646747471|gb|KK962216.1|	63641-26426	24	6.4	4	1	-2	0.25335	no
gi|646747471|gb|KK962216.1|	69813-27330	24	6.4	3	3	-0.00594037	0.9936	no

gi|646747471|gb|KK962216.1|	75316-27642	24	6.4	5	5	0.210741	0.89045	no
gi|646747471|gb|KK962216.1|	97635-29857	24	6.4	12	1	-3	0.04895	no
gi|646747471|gb|KK962216.1|	01971-30315	24	6.4	6	10	0.67759	0.6594	no
gi|646747471|gb|KK962216.1|	40880-34271	24	6.4	5	6	0.21441	0.8855	no
gi|646747471|gb|KK962216.1|	63675-36421	24	6.4	3	3	0.0313582	0.9298	no
gi|646747471|gb|KK962216.1|	11335-41187	24	6.4	3	3	0.0286193	0.9298	no
gi|646747471|gb|KK962216.1|	44754-45373	24	6.4	6	6	-0.160268	0.8891	no
gi|646747471|gb|KK962216.1|	47157-47399	24	6.4	3	34	3	0.1082	no
gi|646747471|gb|KK962216.1|	72657-47473	24	6.4	2	2	-0.20194	0.89805	no
gi|646747471|gb|KK962216.1|	92088-49284	24	6.4	2	0.515578	-2	0.26845	no
gi|646747471|gb|KK962216.1|	51890-52578	24	6.4	0.427054	2	2	0.28375	no
gi|646747471|gb|KK962216.1|	39721-54065	24	6.4	4	2	-0.716542	0.60775	no
gi|646747471|gb|KK962216.1|	59724-56023	24	6.4	1	4	1	0.37825	no
gi|646747471|gb|KK962216.1|	71229-57149	24	6.4	29	9	-2	0.256	no
gi|646747471|gb|KK962216.1|	87520-59246	24	6.4	8	14	0.802305	0.4758	no
gi|646747471|gb|KK962216.1|	97778-59885	24	6.4	2	0.808358	-1	0.37145	no
gi|646747471|gb|KK962216.1|	99476-60061	24	6.4	0.835282	2	0.976263	0.4966	no
gi|646747471|gb|KK962216.1|	02074-60318	24	6.4	0.86759	4	2	0.2016	no
gi|646747471|gb|KK962216.1|	61071-62441	24	6.4	7	6	-0.248497	0.86615	no
gi|646747471|gb|KK962216.1|	70511-71898	24	6.4	0.980687	2	0.639993	0.6091	no
gi|646747471|gb|KK962216.1|	11654-71200	24	6.4	9	2	-2	0.21775	no
gi|646747471|gb|KK962216.1|	12267-71295	24	6.4	2	4	0.606078	0.6288	no
gi|646747471|gb|KK962216.1|	22805-72464	24	6.4	0.874439	0.168642	-2	1	no
gi|646747471|gb|KK962216.1|	39609-74041	24	6.4	31	7	-2	0.1676	no
gi|646747471|gb|KK962216.1|	45040-74693	24	6.4	2	2	-0.283998	0.85915	no
gi|646747471|gb|KK962216.1|	49292-75034	24	6.4	1	0.330832	-2	1	no
gi|646747471|gb|KK962216.1|	54294-75599	24	6.4	0.8982	0.46335	-0.954933	1	no
gi|646747471|gb|KK962216.1|	56603-75762	24	6.4	1	2	0.859187	0.50475	no
gi|646747471|gb|KK962216.1|	64589-76504	24	6.4	3	6	1	0.4321	no
gi|646747471|gb|KK962216.1|	65813-76605	24	6.4	28	23	-0.269943	0.83825	no

gi|646747471|gb|KK962216.1|	70324-77142	24	6.4	0.870652	0.622224	-0.484662	1	no
gi|646747471|gb|KK962216.1|	74248-77563	24	6.4	0.900554	4	2	0.2016	no
gi|646747471|gb|KK962216.1|	8084-8714	24	6.4	6	5	-0.174496	0.91215	no
gi|646747471|gb|KK962216.1|	14065-81488	24	6.4	2	3	0.274736	0.8192	no
gi|646747471|gb|KK962216.1|	26786-82737	24	6.4	1	5	2	0.2149	no
gi|646747471|gb|KK962216.1|	40278-84123	24	6.4	3	3	-0.208464	0.8769	no
gi|646747471|gb|KK962216.1|	44639-84574	24	6.4	4	5	0.267281	0.86275	no
gi|646747471|gb|KK962216.1|	53842-85417	24	6.4	25	15	-0.770369	0.5812	no
gi|646747471|gb|KK962216.1|	55106-85565	24	6.4	2	3	0.448369	0.75415	no
gi|646747471|gb|KK962216.1|	55853-85630	24	6.4	5	5	-0.00830651	0.9493	no
gi|646747471|gb|KK962216.1|	63814-86411	24	6.4	19	5	-2	0.23205	no
gi|646747471|gb|KK962216.1|	92601-89350	24	6.4	6	6	0.111546	0.94225	no
gi|646747471|gb|KK962216.1|	95055-89574	24	6.4	1	1	-0.326972	0.81865	no
gi|646747471|gb|KK962216.1|	97113-90036	24	6.4	0.648724	1	0.956921	0.49115	no
gi|646747471|gb|KK962216.1|	11759-91266	24	6.4	2	2	0.629616	0.6212	no
gi|646747471|gb|KK962216.1|	91795-93120	24	6.4	0.864586	0.496912	-0.79902	1	no
gi|646747471|gb|KK962216.1|	97568-98443	24	6.4	1	1	0.0163546	1	no
gi|646747482|gb|KK962215.1|	01173-10486	21	9.2	1	1	-0.256463	0.87135	no
gi|646747482|gb|KK962215.1|	06195-10759	21	9.2	0.730355	2	1	0.38585	no
gi|646747482|gb|KK962215.1|	20225-12816	21	9.2	1	2	0.878223	0.4836	no
gi|646747482|gb|KK962215.1|	28270-12981	21	9.2	43	27	-0.657884	0.55525	no
gi|646747482|gb|KK962215.1|	58985-15919	21	9.2	211	34	-3	0.1125	no
gi|646747482|gb|KK962215.1|	60563-16119	21	9.2	129	108	-0.247654	0.83135	no
gi|646747482|gb|KK962215.1|	70990-17445	21	9.2	1	1	-0.00759326	0.9852	no
gi|646747482|gb|KK962215.1|	74944-17586	21	9.2	4	4	0.0577706	0.97095	no
gi|646747482|gb|KK962215.1|	93102-19340	21	9.2	15	64	2	0.16955	no
gi|646747482|gb|KK962215.1|	45909-24607	21	9.2	146	524	2	0.28455	no
gi|646747482|gb|KK962215.1|	73821-27407	21	9.2	10	30	2	0.29035	no
gi|646747482|gb|KK962215.1|	84870-28550	21	9.2	1	5	2	0.21345	no
gi|646747482|gb|KK962215.1|	89858-29248	21	9.2	13	22	0.805102	0.4727	no

gi|646747482|gb|KK962215.1|	99774-30028	21	9.2	14	13	-0.0768971	0.9592	no
gi|646747482|gb|KK962215.1|	00675-30145	21	9.2	9	7	-0.480597	0.7512	no
gi|646747482|gb|KK962215.1|	05821-30621	21	9.2	119	58	-1	0.4969	no
gi|646747482|gb|KK962215.1|	18207-31879	21	9.2	15	3	-2	0.1262	no
gi|646747482|gb|KK962215.1|	21871-32297	21	9.2	2	2	0.128272	0.9153	no
gi|646747482|gb|KK962215.1|	33436-33517	21	9.2	48	24	-1	0.3662	no
gi|646747482|gb|KK962215.1|	64844-36515	21	9.2	92	56.56	-0.706439	0.64315	no
gi|646747482|gb|KK962215.1|	68090-36874	21	9.2	29	39	0.438856	0.77815	no
gi|646747482|gb|KK962215.1|	77750-37807	21	9.2	3	13	2	0.2031	no
gi|646747482|gb|KK962215.1|	78843-37928	21	9.2	6	1	-2	0.2003	no
gi|646747482|gb|KK962215.1|	81955-38229	21	9.2	4	7	0.880752	0.59645	no
gi|646747482|gb|KK962215.1|	03853-40470	21	9.2	6	8	0.355558	0.81755	no
gi|646747482|gb|KK962215.1|	12859-41368	21	9.2	2	2	-0.203408	0.87595	no
gi|646747482|gb|KK962215.1|	20293-42240	21	9.2	0.646973	0.504911	-0.357675	1	no
gi|646747482|gb|KK962215.1|	22517-42771	21	9.2	30	29	-0.0635111	0.95615	no
gi|646747482|gb|KK962215.1|	28731-43484	21	9.2	5	6	0.0253469	0.9807	no
gi|646747482|gb|KK962215.1|	39711-44003	21	9.2	15	38	1	0.3385	no
gi|646747482|gb|KK962215.1|	40899-44219	21	9.2	2	2	0.0151026	0.97905	no
gi|646747482|gb|KK962215.1|	46305-44781	21	9.2	0.960597	0.745405	-0.365907	1	no
gi|646747482|gb|KK962215.1|	48127-45139	21	9.2	5	3	-0.799741	0.58905	no
gi|646747482|gb|KK962215.1|	56399-46155	21	9.2	3	2	-0.222653	0.88725	no
gi|646747482|gb|KK962215.1|	29062-52965	21	9.2	1	3	1	0.3719	no
gi|646747482|gb|KK962215.1|	47665-54934	21	9.2	27	13	-1	0.3873	no
gi|646747482|gb|KK962215.1|	71886-57221	21	9.2	9	5	-0.882882	0.5642	no
gi|646747482|gb|KK962215.1|	83440-58548	21	9.2	2	2	-0.430521	0.7841	no
gi|646747482|gb|KK962215.1|	74272-75527	21	9.2	0.832587	0.929134	0.158287	1	no
gi|646747482|gb|KK962215.1|	76622-76856	21	9.2	22	19	-0.206129	0.89115	no
gi|646747482|gb|KK962215.1|	95558-96177	21	9.2	3	7	1	0.35965	no
gi|646747482|gb|KK962215.1|	96554-98294	21	9.2	0.622996	0.810239	0.379124	1	no
gi|646747482|gb|KK962215.1|	)8944-10046[illegible]	21	9.2	0.585989	2	2	0.2716	no

gi|646747494|gb|KK962214.1|	29122-22949	18	8.5	2	8	2	0.2893	no
gi|646747494|gb|KK962214.1|	93339-29459	18	8.5	0.18465	37	8	0.2322	no
gi|646747494|gb|KK962214.1|	00102-30058	18	8.5	22	9	-1	0.41985	no
gi|646747494|gb|KK962214.1|	02161-30240	18	8.5	14	46	2	0.26085	no
gi|646747494|gb|KK962214.1|	48119-34941	18	8.5	0.267628	2	3	0.1465	no
gi|646747494|gb|KK962214.1|	80011-38027	18	8.5	300	119	-1	0.379	no
gi|646747494|gb|KK962214.1|	84893-38514	18	8.5	8	20	1	0.39505	no
gi|646747494|gb|KK962214.1|	06123-40861	18	8.5	40	41	0.0422875	0.9729	no
gi|646747494|gb|KK962214.1|	08969-40965	18	8.5	0.649546	3	2	0.2051	no
gi|646747494|gb|KK962214.1|	10486-41105	18	8.5	21	19	-0.130766	0.9282	no
gi|646747494|gb|KK962214.1|	16864-41731	18	8.5	0.468185	8	4	0.099	no
gi|646747494|gb|KK962214.1|	24200-42443	18	8.5	27	32	0.245146	0.86255	no
gi|646747494|gb|KK962214.1|	24716-42628	18	8.5	3	6	1	0.49695	no
gi|646747494|gb|KK962214.1|	30957-43225	18	8.5	0.971933	0	-inf	1	no
gi|646747494|gb|KK962214.1|	33607-43465	18	8.5	22	3	-3	0.10975	no
gi|646747494|gb|KK962214.1|	79096-79549	18	8.5	6	2	-2	0.2447	no
gi|646747500|gb|KK962213.1|	88911-19078	21	9.4	0.05725	18	8	0.09445	no
gi|646747500|gb|KK962213.1|	91839-19329	21	9.4	0.077094	11	7	0.09495	no
gi|646747500|gb|KK962213.1|	04238-20544	21	9.4	0.774133	2	1	0.3744	no
gi|646747500|gb|KK962213.1|	42416-24480	21	9.4	9	16	0.758066	0.4818	no
gi|646747500|gb|KK962213.1|	47069-24738	21	9.4	5	15	2	0.27835	no
gi|646747500|gb|KK962213.1|	54417-25627	21	9.4	4	3	-0.730342	0.6379	no
gi|646747500|gb|KK962213.1|	56406-26631	21	9.4	4	0.799138	-2	0.11555	no
gi|646747500|gb|KK962213.1|	67905-26894	21	9.4	0.940333	1	0.512402	0.71995	no
gi|646747500|gb|KK962213.1|	73393-27370	21	9.4	11	5	-1	0.39645	no
gi|646747500|gb|KK962213.1|	77750-27805	21	9.4	8	7	-0.349763	0.8176	no
gi|646747500|gb|KK962213.1|	22130-42249	21	9.4	5	4	-0.475604	0.76275	no
gi|646747500|gb|KK962213.1|	24261-42536	21	9.4	0.65763	2	1	0.3964	no
gi|646747500|gb|KK962213.1|	25605-42624	21	9.4	4	10	1	0.3435	no
gi|646747500|gb|KK962213.1|	27512-42933	21	9.4	2	4	1	0.43015	no

gi|646747500|gb|KK962213.1|	29529-43118	21	9.4	23	31	0.438154	0.69085	no
gi|646747500|gb|KK962213.1|	49420-44962	21	9.4	120	7	-4	0.1722	no
gi|646747500|gb|KK962213.1|	53252-45513	21	9.4	1	1	-0.145124	0.9326	no
gi|646747500|gb|KK962213.1|	62499-46449	21	9.4	5	2	-0.89203	0.55285	no
gi|646747500|gb|KK962213.1|	64733-46604	21	9.4	1	1	-0.0623309	0.9541	no
gi|646747500|gb|KK962213.1|	67247-46869	21	9.4	2	4	0.781214	0.61395	no
gi|646747500|gb|KK962213.1|	76631-47927	21	9.4	4	2	-1	0.50485	no
gi|646747500|gb|KK962213.1|	85269-48821	21	9.4	1	1	0.162186	0.91875	no
gi|646747500|gb|KK962213.1|	91846-49245	21	9.4	27	20	-0.481601	0.74875	no
gi|646747500|gb|KK962213.1|	97391-49825	21	9.4	0.306288	2	3	0.2655	no
gi|646747500|gb|KK962213.1|	04877-50519	21	9.4	9.48	6	-0.736472	0.64635	no
gi|646747500|gb|KK962213.1|	06447-50762	21	9.4	2	2	0.0587033	0.96765	no
gi|646747500|gb|KK962213.1|	27843-52911	21	9.4	2	1	-0.648605	0.6128	no
gi|646747500|gb|KK962213.1|	32983-53367	21	9.4	1	2	0.257991	0.86925	no
gi|646747500|gb|KK962213.1|	33919-53516	21	9.4	10	7	-0.47946	0.75095	no
gi|646747500|gb|KK962213.1|	67428-67376	21	9.4	6	3	-1	0.3789	no
gi|646747513|gb|KK962212.1|	24719-22529	12	6.4	49	32	-0.6324	0.67485	no
gi|646747513|gb|KK962212.1|	05320-30575	12	6.4	0	9	inf	0.00035	yes
gi|646747528|gb|KK962211.1|	13999-21486	18	8.1	2	10	3	0.0971	no
gi|646747528|gb|KK962211.1|	44448-24621	18	8.1	49	38	-0.388598	0.73265	no
gi|646747528|gb|KK962211.1|	48975-24963	18	8.1	41	17	-1	0.394	no
gi|646747528|gb|KK962211.1|	55548-25626	18	8.1	2	2	0.0689368	0.92975	no
gi|646747528|gb|KK962211.1|	59711-26011	18	8.1	4	7	0.734809	0.6207	no
gi|646747528|gb|KK962211.1|	14135-51493	18	8.1	1	1	-0.183032	0.90405	no
gi|646747528|gb|KK962211.1|	15255-51941	18	8.1	1	1	0.0995644	0.94635	no
gi|646747528|gb|KK962211.1|	19477-52056	18	8.1	2	2	0.215029	0.86245	no
gi|646747528|gb|KK962211.1|	25883-52784	18	8.1	2	2	0.584498	0.71555	no
gi|646747528|gb|KK962211.1|	28011-52886	18	8.1	1	2	0.862171	0.54775	no
gi|646747528|gb|KK962211.1|	29017-53111	18	8.1	0.553091	1	1	0.3744	no
gi|646747528|gb|KK962211.1|	44403-54530	18	8.1	2	0.609102	-1	0.3882	no

gi|646747528|gb|KK962211.1|	46734-54792	18	8.1	0.788145	0.564622	-0.481177	1	no
gi|646747528|gb|KK962211.1|	05104-60669	18	8.1	2	2	-0.123353	0.93885	no
gi|646747547|gb|KK962210.1|	08186-10884	19	7.5	3	0	-inf	0.00225	no
gi|646747547|gb|KK962210.1|	47605-14787	19	7.5	33	15	-1	0.3979	no
gi|646747547|gb|KK962210.1|	79065-28452	19	7.5	7	8	0.146719	0.89475	no
gi|646747547|gb|KK962210.1|	84907-28838	19	7.5	4	4	-0.00926966	0.994	no
gi|646747547|gb|KK962210.1|	07863-30828	19	7.5	17	9	-0.915414	0.5052	no
gi|646747547|gb|KK962210.1|	23199-32501	19	7.5	0.059267	1	4	0.09645	no
gi|646747547|gb|KK962210.1|	44216-34614	19	7.5	0.663198	0.799964	0.270495	1	no
gi|646747547|gb|KK962210.1|	49405-35013	19	7.5	2	1	-0.361587	0.812	no
gi|646747547|gb|KK962210.1|	51823-35253	19	7.5	3	0.281831	-3	0.179	no
gi|646747547|gb|KK962210.1|	65617-36711	19	7.5	0	3	inf	0.00085	yes
gi|646747547|gb|KK962210.1|	71202-37260	19	7.5	2	3	0.787984	0.56695	no
gi|646747547|gb|KK962210.1|	79340-38017	19	7.5	1	0.905627	-0.50193	0.73865	no
gi|646747547|gb|KK962210.1|	93458-39516	19	7.5	5	3	-0.449757	0.7688	no
gi|646747547|gb|KK962210.1|	98766-39970	19	7.5	39	28	-0.483366	0.6538	no
gi|646747547|gb|KK962210.1|	03714-40468	19	7.5	5	5	-0.0767234	0.9602	no
gi|646747547|gb|KK962210.1|	05628-40698	19	7.5	1	0.725445	-0.476292	1	no
gi|646747547|gb|KK962210.1|	07739-40881	19	7.5	22	19	-0.230443	0.8777	no
gi|646747547|gb|KK962210.1|	19748-42016	19	7.5	28	24	-0.222299	0.88525	no
gi|646747547|gb|KK962210.1|	24368-42576	19	7.5	0.811505	0.817227	0.0101369	1	no
gi|646747547|gb|KK962210.1|	4252-4670	19	7.5	15	9	-0.840522	0.549	no
gi|646747547|gb|KK962210.1|	33592-43466	19	7.5	0.905811	0.97003	0.0988184	1	no
gi|646747547|gb|KK962210.1|	34998-43658	19	7.5	23	18	-0.349387	0.74435	no
gi|646747547|gb|KK962210.1|	40470-44070	19	7.5	726	224.8	-2	0.27755	no
gi|646747547|gb|KK962210.1|	76902-47789	19	7.5	6	6	-0.208225	0.89555	no
gi|646747547|gb|KK962210.1|	99462-50125	19	7.5	2	7	2	0.264	no
gi|646747559|gb|KK962209.1|	07899-10830	19	9.5	7	8	0.245016	0.8443	no
gi|646747559|gb|KK962209.1|	09517-11045	19	9.5	0	3	inf	0.00035	yes
gi|646747559|gb|KK962209.1|	17655-11958	19	9.5	50	46	-0.132574	0.90975	no

gi|646747559|gb|KK962209.1|	20705-12477	19	9.5	15	12	-0.364792	0.7432	no
gi|646747559|gb|KK962209.1|	28028-12836	19	9.5	23	23	-0.0412459	0.9757	no
gi|646747559|gb|KK962209.1|	64950-16585	19	9.5	13	5	-1	0.3989	no
gi|646747559|gb|KK962209.1|	80219-18081	19	9.5	4	1	-2	0.2436	no
gi|646747559|gb|KK962209.1|	18147-21527	19	9.5	1	2	0.404821	0.7903	no
gi|646747559|gb|KK962209.1|	96518-19817	19	9.5	4	1	-2	0.2136	no
gi|646747559|gb|KK962209.1|	01373-20346	19	9.5	2	2	-0.0955418	0.9515	no
gi|646747559|gb|KK962209.1|	08151-21011	19	9.5	8	0.547706	-4	0.0277	no
gi|646747559|gb|KK962209.1|	11101-21474	19	9.5	3	0.357705	-3	0.0451	no
gi|646747559|gb|KK962209.1|	15541-22452	19	9.5	3	3	0.280259	0.7932	no
gi|646747559|gb|KK962209.1|	36302-23778	19	9.5	7	0.653796	-3	0.04465	no
gi|646747559|gb|KK962209.1|	42514-24342	19	9.5	3	0.796641	-2	0.2124	no
gi|646747559|gb|KK962209.1|	44319-24509	19	9.5	2	0.248364	-3	0.1994	no
gi|646747559|gb|KK962209.1|	61789-26625	19	9.5	2	0.288571	-3	0.0657	no
gi|646747559|gb|KK962209.1|	67704-27904	19	9.5	11	4	-1	0.27055	no
gi|646747559|gb|KK962209.1|	27326-29169	19	9.5	1	1	-0.352016	0.79515	no
gi|646747559|gb|KK962209.1|	83529-28441	19	9.5	0	5	inf	0.0017	no
gi|646747559|gb|KK962209.1|	92190-29378	19	9.5	0.137874	2	4	0.2606	no
gi|646747559|gb|KK962209.1|	06958-30760	19	9.5	0	5	inf	0.00035	yes
gi|646747559|gb|KK962209.1|	30798-32151	19	9.5	3	2	-0.685797	0.6704	no
gi|646747559|gb|KK962209.1|	32069-33356	19	9.5	2	0.536643	-2	0.28235	no
gi|646747559|gb|KK962209.1|	41769-42368	19	9.5	3	2	-0.537751	0.69565	no
gi|646747559|gb|KK962209.1|	49291-44975	19	9.5	52	102	0.9779	0.44155	no
gi|646747559|gb|KK962209.1|	50101-45243	19	9.5	1	3	2	0.29915	no
gi|646747559|gb|KK962209.1|	56553-45685	19	9.5	8	19	1	0.38115	no
gi|646747559|gb|KK962209.1|	58827-45901	19	9.5	57	142	1	0.3788	no
gi|646747559|gb|KK962209.1|	59161-45934	19	9.5	38	165	2	0.24765	no
gi|646747559|gb|KK962209.1|	59762-46004	19	9.5	22	103	2	0.1374	no
gi|646747559|gb|KK962209.1|	46338-46566	19	9.5	29	39	0.396149	0.7883	no
gi|646747559|gb|KK962209.1|	64011-46426	19	9.5	17	74	2	0.2161	no

gi|646747559|gb|KK962209.1|	65064-46624	19	9.5	0.600703	3	2	0.1757	no
gi|646747559|gb|KK962209.1|	92307-49276	19	9.5	0.450603	7	4	0.09965	no
gi|646747559|gb|KK962209.1|	49993-52042	19	9.5	1	1	0.534793	0.6982	no
gi|646747559|gb|KK962209.1|	06222-50644	19	9.5	103	241	1	0.4405	no
gi|646747559|gb|KK962209.1|	23423-52384	19	9.5	2	5	2	0.29775	no
gi|646747559|gb|KK962209.1|	60025-60498	19	9.5	2	4	0.902102	0.5676	no
gi|646747559|gb|KK962209.1|	52843-65321	19	9.5	9	0	-inf	0.0041	no
gi|646747559|gb|KK962209.1|	49911-75321	19	9.5	5	8	0.634631	0.61615	no
gi|646747559|gb|KK962209.1|	75992-76733	19	9.5	7	6	-0.0562929	0.9727	no
gi|646747559|gb|KK962209.1|	81787-82399	19	9.5	0.258635	4	4	0.0994	no
gi|646747559|gb|KK962209.1|	92800-94489	19	9.5	1	2	0.650666	0.6825	no
gi|646747559|gb|KK962209.1|	)9851-10014:	19	9.5	0	60	inf	0.0007	yes
gi|646747574|gb|KK962208.1|	00638-10127	27	10	4	4	0.0547456	0.9562	no
gi|646747574|gb|KK962208.1|	04218-10443	27	10	60	32	-0.892603	0.52625	no
gi|646747574|gb|KK962208.1|	12689-11296	27	10	26	26	-0.0384594	0.9548	no
gi|646747574|gb|KK962208.1|	36682-13722	27	10	3	4	0.446396	0.7403	no
gi|646747574|gb|KK962208.1|	70287-17082	27	10	2	4	1	0.4503	no
gi|646747574|gb|KK962208.1|	72265-17252	27	10	23	3	-3	0.2001	no
gi|646747574|gb|KK962208.1|	75233-17626	27	10	2	3	0.604847	0.6684	no
gi|646747574|gb|KK962208.1|	77012-17754	27	10	2	5	1	0.38605	no
gi|646747574|gb|KK962208.1|	90910-19222	27	10	1	2	0.522715	0.7175	no
gi|646747574|gb|KK962208.1|	93497-19522	27	10	1	2	0.296489	0.8313	no
gi|646747574|gb|KK962208.1|	97286-20427	27	10	5	2	-1	0.37165	no
gi|646747574|gb|KK962208.1|	23373-22389	27	10	2	3	0.214984	0.88855	no
gi|646747574|gb|KK962208.1|	24553-22497	27	10	3	4	0.649405	0.6668	no
gi|646747574|gb|KK962208.1|	41698-24219	27	10	2	2	-0.158544	0.8987	no
gi|646747574|gb|KK962208.1|	01402-30175	27	10	15	4	-2	0.2308	no
gi|646747574|gb|KK962208.1|	02030-30263	27	10	2	2	-0.385011	0.8011	no
gi|646747574|gb|KK962208.1|	19091-31996	27	10	2	2	0.0424301	0.94695	no
gi|646747574|gb|KK962208.1|	38132-33859	27	10	7	4	-0.869365	0.50645	no

gi|646747574|gb|KK962208.1|	42224-34361	27	10	11	7	-0.621465	0.68005	no
gi|646747574|gb|KK962208.1|	49897-35029	27	10	233	86	-1	0.27115	no
gi|646747574|gb|KK962208.1|	65709-36646	27	10	43	3	-4	0.00715	no
gi|646747574|gb|KK962208.1|	72377-37327	27	10	3	2	-0.496434	0.7247	no
gi|646747574|gb|KK962208.1|	84897-38658	27	10	56	41	-0.43486	0.701	no
gi|646747574|gb|KK962208.1|	87780-38898	27	10	1	1	-0.118291	0.9313	no
gi|646747574|gb|KK962208.1|	98997-39926	27	10	659	358.68	-0.878139	0.4163	no
gi|646747574|gb|KK962208.1|	99419-39996	27	10	6	6	0.107332	0.9392	no
gi|646747574|gb|KK962208.1|	08807-40935	27	10	130	64	-1	0.35175	no
gi|646747574|gb|KK962208.1|	09717-41089	27	10	12	7	-0.80967	0.6003	no
gi|646747574|gb|KK962208.1|	11097-41199	27	10	6	8	0.612889	0.68895	no
gi|646747574|gb|KK962208.1|	12149-41457	27	10	6	8	0.442668	0.7691	no
gi|646747574|gb|KK962208.1|	14851-41646	27	10	0.40845	0.589429	0.529158	1	no
gi|646747574|gb|KK962208.1|	30137-43123	27	10	0.655301	0.780464	0.252174	1	no
gi|646747574|gb|KK962208.1|	36263-43720	27	10	2	0.955149	-1	0.37095	no
gi|646747574|gb|KK962208.1|	37383-43857	27	10	0.393654	1	2	0.36595	no
gi|646747574|gb|KK962208.1|	41314-44275	27	10	0.545565	0.561048	0.0403749	1	no
gi|646747574|gb|KK962208.1|	63584-46488	27	10	3	4	0.0948143	0.95045	no
gi|646747574|gb|KK962208.1|	66097-46678	27	10	14	10	-0.520112	0.7334	no
gi|646747574|gb|KK962208.1|	78867-47933	27	10	6	9	0.60505	0.66195	no
gi|646747574|gb|KK962208.1|	89933-49066	27	10	0.782078	2	1	0.36865	no
gi|646747574|gb|KK962208.1|	98039-49837	27	10	27	10	-2	0.3141	no
gi|646747574|gb|KK962208.1|	03169-50422	27	10	15	12	-0.297277	0.8389	no
gi|646747574|gb|KK962208.1|	09989-51064	27	10	15	6	-1	0.3761	no
gi|646747574|gb|KK962208.1|	14335-51459	27	10	0	46.46	inf	0.00435	no
gi|646747574|gb|KK962208.1|	15298-51557	27	10	0	28	inf	0.0008	yes
gi|646747574|gb|KK962208.1|	24800-52552	27	10	0	60	inf	5.00E-05	yes
gi|646747574|gb|KK962208.1|	28481-52887	27	10	84	73	-0.206974	0.8877	no
gi|646747574|gb|KK962208.1|	57603-55858	27	10	8	8	0.0551254	0.96955	no
gi|646747574|gb|KK962208.1|	59294-55997	27	10	32	10	-2	0.28755	no

gi|646747574|gb|KK962208.1|	06215-60751	27	10	7	7	-0.0102309	0.9936	no
gi|646747574|gb|KK962208.1|	61690-62098	27	10	38	36	-0.0855269	0.95135	no
gi|646747574|gb|KK962208.1|	67846-68450	27	10	2	5	1	0.406	no
gi|646747574|gb|KK962208.1|	71978-72474	27	10	9	8	-0.188896	0.9104	no
gi|646747574|gb|KK962208.1|	80595-80869	27	10	19	24	0.316595	0.81385	no
gi|646747574|gb|KK962208.1|	82932-86632	27	10	27	23	-0.269067	0.8175	no
gi|646747574|gb|KK962208.1|	88678-90312	27	10	22	14	-0.631739	0.55635	no
gi|646747574|gb|KK962208.1|	)9735-10048	27	10	1	1	0.00363588	0.92345	no
gi|646747586|gb|KK962207.1|	00172-10107	20	8.8	10	5	-1	0.51595	no
gi|646747586|gb|KK962207.1|	02464-10352	20	8.8	16	10	-0.619931	0.679	no
gi|646747586|gb|KK962207.1|	05479-10962	20	8.8	9	9	0.145239	0.8894	no
gi|646747586|gb|KK962207.1|	12051-11580	20	8.8	9	8	-0.152752	0.8865	no
gi|646747586|gb|KK962207.1|	12045-13194	20	8.8	30	3	-3	0.0371	no
gi|646747586|gb|KK962207.1|	26213-12682	20	8.8	69	27	-1	0.2931	no
gi|646747586|gb|KK962207.1|	14939-15327	20	8.8	13	6	-1	0.40585	no
gi|646747586|gb|KK962207.1|	54836-15541	20	8.8	14	17	0.268856	0.8616	no
gi|646747586|gb|KK962207.1|	69798-17063	20	8.8	1	2	0.498891	0.7299	no
gi|646747586|gb|KK962207.1|	82707-18431	20	8.8	2	3	0.229461	0.8833	no
gi|646747586|gb|KK962207.1|	86338-18970	20	8.8	6	2	-2	0.2267	no
gi|646747586|gb|KK962207.1|	90188-19185	20	8.8	4	3	-0.265104	0.8607	no
gi|646747586|gb|KK962207.1|	06287-20946	20	8.8	3	3	-0.29791	0.84625	no
gi|646747586|gb|KK962207.1|	09575-21062	20	8.8	0.81247	0.828089	0.0274717	1	no
gi|646747586|gb|KK962207.1|	16136-21700	20	8.8	2	2	-0.0395212	0.95635	no
gi|646747586|gb|KK962207.1|	20867-22251	20	8.8	0.530547	0.382972	-0.470243	1	no
gi|646747586|gb|KK962207.1|	23521-22804	20	8.8	5	3	-0.67539	0.5893	no
gi|646747586|gb|KK962207.1|	40173-24270	20	8.8	0.732983	0.709757	-0.0464541	1	no
gi|646747586|gb|KK962207.1|	53854-25478	20	8.8	1	2	0.675911	0.6345	no
gi|646747586|gb|KK962207.1|	56226-25688	20	8.8	3	0.9496	-2	0.35005	no
gi|646747586|gb|KK962207.1|	62822-26372	20	8.8	1	1	-0.0810248	0.93285	no
gi|646747586|gb|KK962207.1|	65334-26645	20	8.8	0.854569	1	0.516017	0.7091	no

gi|646747586|gb|KK962207.1|	72121-27499	20	8.8	96	42	-1	0.3284	no
gi|646747586|gb|KK962207.1|	78163-27954	20	8.8	22	12	-0.885257	0.4837	no
gi|646747586|gb|KK962207.1|	80784-28138	20	8.8	1	3	0.948656	0.56265	no
gi|646747586|gb|KK962207.1|	82495-28326	20	8.8	0.178558	129	10	0.09445	no
gi|646747586|gb|KK962207.1|	89524-29002	20	8.8	12	8	-0.56873	0.72365	no
gi|646747586|gb|KK962207.1|	93320-29397	20	8.8	6	4	-0.58528	0.65915	no
gi|646747586|gb|KK962207.1|	96829-29761	20	8.8	1	12	3	0.0632	no
gi|646747586|gb|KK962207.1|	05391-30566	20	8.8	20	11	-0.828355	0.57265	no
gi|646747586|gb|KK962207.1|	33360-33934	20	8.8	2	6	2	0.2819	no
gi|646747586|gb|KK962207.1|	38819-33946	20	8.8	7	5	-0.574866	0.7268	no
gi|646747586|gb|KK962207.1|	37746-39245	20	8.8	1	0.857273	-0.280339	1	no
gi|646747586|gb|KK962207.1|	40042-40878	20	8.8	0.796023	1	0.761473	0.61475	no
gi|646747586|gb|KK962207.1|	05419-40598	20	8.8	2	4	0.913337	0.5059	no
gi|646747586|gb|KK962207.1|	07192-40745	20	8.8	13	19	0.576388	0.71435	no
gi|646747586|gb|KK962207.1|	11565-41295	20	8.8	2	2	0.446488	0.74595	no
gi|646747586|gb|KK962207.1|	24683-42563	20	8.8	0.665486	2	1	0.43245	no
gi|646747586|gb|KK962207.1|	25733-42622	20	8.8	2	4	1	0.3784	no
gi|646747586|gb|KK962207.1|	26716-42735	20	8.8	1	3	0.886676	0.5491	no
gi|646747586|gb|KK962207.1|	28258-43215	20	8.8	0.991301	2	0.873074	0.5747	no
gi|646747586|gb|KK962207.1|	43224-43566	20	8.8	16	9	-0.784672	0.53905	no
gi|646747586|gb|KK962207.1|	36416-43748	20	8.8	1	2	0.250821	0.8482	no
gi|646747586|gb|KK962207.1|	44357-44900	20	8.8	18	23	0.370712	0.7488	no
gi|646747586|gb|KK962207.1|	56904-45932	20	8.8	0	48	inf	5.00E-05	yes
gi|646747586|gb|KK962207.1|	45869-46303	20	8.8	4	9	1	0.38095	no
gi|646747586|gb|KK962207.1|	47040-47243	20	8.8	91	29	-2	0.26835	no
gi|646747586|gb|KK962207.1|	49436-49828	20	8.8	79	38	-1	0.49215	no
gi|646747586|gb|KK962207.1|	70979-71252	20	8.8	24	40	0.761597	0.6039	no
gi|646747586|gb|KK962207.1|	74101-74614	20	8.8	17	20	0.212605	0.8861	no
gi|646747586|gb|KK962207.1|	857-1797	20	8.8	1	0.9592	-0.493479	0.73195	no
gi|646747586|gb|KK962207.1|	87369-87671	20	8.8	89	92	0.0494836	0.97405	no

gi|646747597|gb|KK962206.1|	10186-21112	20	8.1	1	2	0.921492	0.48975	no
gi|646747597|gb|KK962206.1|	20769-22113	20	8.1	27	17	-0.670568	0.6764	no
gi|646747597|gb|KK962206.1|	28025-22853	20	8.1	4	5	0.170744	0.89035	no
gi|646747597|gb|KK962206.1|	47814-24845	20	8.1	2	3	0.47184	0.7323	no
gi|646747597|gb|KK962206.1|	49190-25109	20	8.1	14	15	0.129666	0.90635	no
gi|646747597|gb|KK962206.1|	55646-25607	20	8.1	7	0.682582	-3	0.1785	no
gi|646747597|gb|KK962206.1|	61399-26194	20	8.1	2	3	0.709095	0.6455	no
gi|646747597|gb|KK962206.1|	63373-26354	20	8.1	94	170	0.85524	0.58935	no
gi|646747597|gb|KK962206.1|	63957-26439	20	8.1	7	10	0.588109	0.66265	no
gi|646747597|gb|KK962206.1|	76688-27720	20	8.1	8	11	0.376751	0.81175	no
gi|646747597|gb|KK962206.1|	78199-28132	20	8.1	1	1	-0.327335	0.8303	no
gi|646747597|gb|KK962206.1|	91658-30176	20	8.1	2	2	0.110248	0.91575	no
gi|646747597|gb|KK962206.1|	05039-30623	20	8.1	6	2	-1	0.4138	no
gi|646747597|gb|KK962206.1|	23950-32512	20	8.1	8	7	-0.133942	0.9278	no
gi|646747597|gb|KK962206.1|	36368-33707	20	8.1	18	8	-1	0.4611	no
gi|646747597|gb|KK962206.1|	92278-49279	20	8.1	16	0.959104	-4	0.1182	no
gi|646747597|gb|KK962206.1|	17231-51859	20	8.1	0.831936	0.119628	-3	1	no
gi|646747597|gb|KK962206.1|	38239-53858	20	8.1	7	5	-0.504564	0.74455	no
gi|646747597|gb|KK962206.1|	40016-64150	20	8.1	4	2	-1	0.4866	no
gi|646747597|gb|KK962206.1|	53190-65372	20	8.1	2	3	0.664271	0.65435	no
gi|646747597|gb|KK962206.1|	56549-65791	20	8.1	0.751445	0.72031	-0.0610481	1	no
gi|646747597|gb|KK962206.1|	59621-66018	20	8.1	3	2	-0.133145	0.9221	no
gi|646747597|gb|KK962206.1|	75878-67761	20	8.1	1	0.814562	-0.822625	0.57685	no
gi|646747597|gb|KK962206.1|	79281-68026	20	8.1	1	0.721464	-0.812613	0.57835	no
gi|646747597|gb|KK962206.1|	01843-70207	20	8.1	0	64	inf	0.00515	no
gi|646747614|gb|KK962205.1|	22505-12335	21	8.9	1	3	1	0.3975	no
gi|646747614|gb|KK962205.1|	12591-13512	21	8.9	0.555564	1	1	0.43305	no
gi|646747614|gb|KK962205.1|	50989-15126	21	8.9	0	25	inf	0.001	yes
gi|646747614|gb|KK962205.1|	53446-15373	21	8.9	0	55	inf	0.00105	yes
gi|646747614|gb|KK962205.1|	57959-15843	21	8.9	0.418976	8	4	0.11345	no

gi|646747614|gb|KK962205.1|	61070-16156	21	8.9	0	7	inf	0.0008	yes
gi|646747614|gb|KK962205.1|	62379-16305	21	8.9	0	14	inf	0.00025	yes
gi|646747614|gb|KK962205.1|	26717-28043	21	8.9	4	4	-0.21403	0.8892	no
gi|646747614|gb|KK962205.1|	72766-27317	21	8.9	38	15	-1	0.40525	no
gi|646747614|gb|KK962205.1|	78571-27897	21	8.9	2	7	2	0.2461	no
gi|646747614|gb|KK962205.1|	79844-28023	21	8.9	3	8	1	0.4246	no
gi|646747614|gb|KK962205.1|	29212-30961	21	8.9	5	5	0.0348296	0.98075	no
gi|646747614|gb|KK962205.1|	93175-29369	21	8.9	3	2	-0.731009	0.64125	no
gi|646747614|gb|KK962205.1|	99654-29983	21	8.9	123	92	-0.422234	0.7498	no
gi|646747614|gb|KK962205.1|	31182-37503	21	8.9	4	5	0.180775	0.8619	no
gi|646747614|gb|KK962205.1|	15392-31565	21	8.9	10	18	0.949605	0.5828	no
gi|646747614|gb|KK962205.1|	25115-32646	21	8.9	4	18	2	0.1008	no
gi|646747614|gb|KK962205.1|	14541-41477	21	8.9	83	8	-3	0.11385	no
gi|646747614|gb|KK962205.1|	26215-42678	21	8.9	5	3	-0.737014	0.56855	no
gi|646747614|gb|KK962205.1|	62932-46393	21	8.9	0.738066	0.87621	0.247527	1	no
gi|646747614|gb|KK962205.1|	81230-48428	21	8.9	1	1	-0.022765	0.98235	no
gi|646747614|gb|KK962205.1|	85376-48662	21	8.9	81	17	-2	0.10925	no
gi|646747614|gb|KK962205.1|	87503-48873	21	8.9	57	35	-0.702372	0.53365	no
gi|646747614|gb|KK962205.1|	98729-50018	21	8.9	2	2	-0.6666	0.62615	no
gi|646747614|gb|KK962205.1|	04168-50462	21	8.9	19	29	0.632959	0.6736	no
gi|646747614|gb|KK962205.1|	06682-50722	21	8.9	5	3	-0.77735	0.5577	no
gi|646747614|gb|KK962205.1|	23325-52372	21	8.9	0	11	inf	0.00035	yes
gi|646747614|gb|KK962205.1|	29249-53244	21	8.9	0.409103	3	3	0.0867	no
gi|646747614|gb|KK962205.1|	32568-53296	21	8.9	19	18	-0.0866962	0.9547	no
gi|646747614|gb|KK962205.1|	34718-53501	21	8.9	16	15	-0.0620481	0.9489	no
gi|646747614|gb|KK962205.1|	44238-54447	21	8.9	46	66	0.52287	0.7074	no
gi|646747614|gb|KK962205.1|	51119-55210	21	8.9	22	5	-2	0.16635	no
gi|646747614|gb|KK962205.1|	14016-61416	21	8.9	294	253	-0.217845	0.88365	no
gi|646747614|gb|KK962205.1|	44356-64745	21	8.9	3	3	0.114505	0.94085	no
gi|646747614|gb|KK962205.1|	69198-67036	21	8.9	2	2	-0.145279	0.90155	no

gi|646747614|gb|KK962205.1|	72333-67405	21	8.9	2	2	0.0782956	0.95885	no
gi|646747614|gb|KK962205.1|	74159-67551	21	8.9	0.757598	1	0.813189	0.5352	no
gi|646747614|gb|KK962205.1|	89071-68949	21	8.9	3	8	1	0.38315	no
gi|646747614|gb|KK962205.1|	94475-69507	21	8.9	3	5	0.947178	0.475	no
gi|646747614|gb|KK962205.1|	14256-71520	21	8.9	1	1	-0.144776	0.9177	no
gi|646747614|gb|KK962205.1|	72750-73212	21	8.9	5	4	-0.589257	0.6453	no
gi|646747614|gb|KK962205.1|	38369-74219	21	8.9	3	4	0.271931	0.8518	no
gi|646747614|gb|KK962205.1|	42332-74503	21	8.9	1	1	0.00343853	1	no
gi|646747614|gb|KK962205.1|	50279-75415	21	8.9	34	26	-0.393623	0.73615	no
gi|646747614|gb|KK962205.1|	60905-76329	21	8.9	6	5	-0.238477	0.8751	no
gi|646747614|gb|KK962205.1|	65665-76734	21	8.9	7	6	-0.183951	0.90835	no
gi|646747614|gb|KK962205.1|	80147-78157	21	8.9	8	9	0.100965	0.94675	no
gi|646747614|gb|KK962205.1|	7883-8628	21	8.9	18	14	-0.423988	0.77945	no
gi|646747614|gb|KK962205.1|	89249-78968	21	8.9	6	9	0.618521	0.6247	no
gi|646747614|gb|KK962205.1|	9896-11047	21	8.9	5	3	-0.6527	0.6791	no
gi|646747627|gb|KK962204.1|	07405-10784	18	7.5	15	11	-0.51252	0.75145	no
gi|646747627|gb|KK962204.1|	14742-11636	18	7.5	5	8	0.729941	0.6241	no
gi|646747627|gb|KK962204.1|	29920-13399	18	7.5	5	5	0.0368388	0.97725	no
gi|646747627|gb|KK962204.1|	34951-13580	18	7.5	0.154939	29	8	0.0947	no
gi|646747627|gb|KK962204.1|	42611-14342	18	7.5	1	2	1	0.4498	no
gi|646747627|gb|KK962204.1|	46310-14676	18	7.5	1	12	3	0.15115	no
gi|646747627|gb|KK962204.1|	14703-15929	18	7.5	11	9	-0.33806	0.8261	no
gi|646747627|gb|KK962204.1|	61259-16523	18	7.5	0.49676	3	3	0.0702	no
gi|646747627|gb|KK962204.1|	65303-16728	18	7.5	0.053571	6	7	0.0948	no
gi|646747627|gb|KK962204.1|	16820-20084	18	7.5	11	9	-0.207524	0.84795	no
gi|646747627|gb|KK962204.1|	72262-27270	18	7.5	7	9	0.299204	0.81195	no
gi|646747627|gb|KK962204.1|	05479-30596	18	7.5	4	4	-0.0919002	0.9348	no
gi|646747627|gb|KK962204.1|	24181-32523	18	7.5	2	0.16582	-3	0.1863	no
gi|646747627|gb|KK962204.1|	26915-32783	18	7.5	3	2	-0.301986	0.798	no
gi|646747627|gb|KK962204.1|	28645-32887	18	7.5	12	29	1	0.42395	no

gi|646747627|gb|KK962204.1|	33128-33493	18	7.5	1	0.948149	-0.532085	0.6991	no
gi|646747627|gb|KK962204.1|	36411-33679	18	7.5	8	10	0.224573	0.8517	no
gi|646747627|gb|KK962204.1|	37563-33888	18	7.5	9	3	-2	0.33665	no
gi|646747627|gb|KK962204.1|	40279-34191	18	7.5	11	0.483835	-5	0.03315	no
gi|646747627|gb|KK962204.1|	63155-36337	18	7.5	0	122	inf	0.00175	no
gi|646747627|gb|KK962204.1|	71921-37213	18	7.5	0	199.17	inf	0.0003	yes
gi|646747627|gb|KK962204.1|	78392-37993	18	7.5	0	26	inf	5.00E-05	yes
gi|646747627|gb|KK962204.1|	08533-40874	18	7.5	0	102	inf	0.00435	no
gi|646747627|gb|KK962204.1|	26093-42648	18	7.5	0	16	inf	0.00315	no
gi|646747627|gb|KK962204.1|	28885-42921	18	7.5	4	12	2	0.2988	no
gi|646747627|gb|KK962204.1|	42585-44297	18	7.5	5	3	-0.643969	0.66675	no
gi|646747627|gb|KK962204.1|	70699-47148	18	7.5	35	10	-2	0.18065	no
gi|646747627|gb|KK962204.1|	73508-47436	18	7.5	3	0.867976	-2	0.2477	no
gi|646747627|gb|KK962204.1|	48754-50923	18	7.5	15	21	0.516663	0.63735	no
gi|646747627|gb|KK962204.1|	09186-51043	18	7.5	1	0.931869	-0.372322	0.77685	no
gi|646747627|gb|KK962204.1|	24990-52613	18	7.5	0.730048	1	0.516871	1	no
gi|646747627|gb|KK962204.1|	27699-52920	18	7.5	2	2	0.315674	0.84775	no
gi|646747627|gb|KK962204.1|	34407-53545	18	7.5	45	8	-2	0.0791	no
gi|646747627|gb|KK962204.1|	36430-53720	18	7.5	9	0.504102	-4	0.1171	no
gi|646747627|gb|KK962204.1|	37309-53844	18	7.5	3	2	-0.630332	0.64375	no
gi|646747627|gb|KK962204.1|	41746-54213	18	7.5	68	64	-0.0817555	0.9567	no
gi|646747627|gb|KK962204.1|	42358-54372	18	7.5	14	9	-0.615258	0.68405	no
gi|646747627|gb|KK962204.1|	57713-55843	18	7.5	26	38	0.549842	0.65845	no
gi|646747627|gb|KK962204.1|	79718-57992	18	7.5	65	106	0.713684	0.63985	no
gi|646747627|gb|KK962204.1|	96697-59716	18	7.5	3	3	-0.0735719	0.92345	no
gi|646747627|gb|KK962204.1|	97911-59808	18	7.5	34	159	2	0.2965	no
gi|646747627|gb|KK962204.1|	65671-66238	18	7.5	9	10	0.239487	0.879	no
gi|646747643|gb|KK962203.1|	35410-33635	13	8.9	4	3	-0.341279	0.83785	no
gi|646747643|gb|KK962203.1|	02275-40335	13	8.9	5	5	0.00407705	0.98765	no
gi|646747658|gb|KK962202.1|	02706-10326	15	7.1	0.610153	5	3	0.2509	no

gi|646747658|gb|KK962202.1| 20504-22126	15	7.1	1	0.514602	-2	0.38345	no
gi|646747658|gb|KK962202.1| 74327-76856	15	7.1	18	19	0.0874139	0.93595	no
gi|646747669|gb|KK962201.1| 52073-15255	19	6	7	4	-0.860375	0.50815	no
gi|646747669|gb|KK962201.1| 57060-15890	19	6	4	8	0.942657	0.53745	no
gi|646747669|gb|KK962201.1| 64523-16508	19	6	8	9	0.21081	0.8938	no
gi|646747669|gb|KK962201.1| 74670-17523	19	6	3	0	-inf	0.00655	no
gi|646747669|gb|KK962201.1| 91567-19407	19	6	0.246936	3	4	0.06865	no
gi|646747669|gb|KK962201.1| 94534-19474	19	6	52	44	-0.244496	0.86345	no
gi|646747669|gb|KK962201.1| 94912-19731	19	6	0.47291	2	2	0.1636	no
gi|646747669|gb|KK962201.1| 98739-19966	19	6	32	14	-1	0.32975	no
gi|646747669|gb|KK962201.1| 05525-20569	19	6	0	1583.16	inf	0.00015	yes
gi|646747669|gb|KK962201.1| 06455-20685	19	6	1	62	6	0.23365	no
gi|646747669|gb|KK962201.1| 52394-25339	19	6	2	0	-inf	0.00225	no
gi|646747669|gb|KK962201.1| 72483-27297	19	6	19	0	-inf	0.00085	yes
gi|646747669|gb|KK962201.1| 73076-27612	19	6	5	0.096471	-6	0.10705	no
gi|646747669|gb|KK962201.1| 76312-27685	19	6	5	0	-inf	0.00195	no
gi|646747669|gb|KK962201.1| 79333-27953	19	6	89	74	-0.269235	0.83565	no
gi|646747669|gb|KK962201.1| 89060-28956	19	6	10	0	-inf	0.0047	no
gi|646747669|gb|KK962201.1| 91667-29187	19	6	147	42	-2	0.18505	no
gi|646747669|gb|KK962201.1| 11928-31292	19	6	0.124	2	4	0.10345	no
gi|646747669|gb|KK962201.1| 15976-31705	19	6	0.44743	2	2	0.20295	no
gi|646747669|gb|KK962201.1| 17888-31930	19	6	0.239875	1	3	0.13755	no
gi|646747669|gb|KK962201.1| 19448-31973	19	6	165.82	105	-0.655731	0.65925	no
gi|646747669|gb|KK962201.1| 21359-32153	19	6	361	317	-0.184729	0.8895	no
gi|646747669|gb|KK962201.1| 24243-32443	19	6	423	583	0.461786	0.76505	no
gi|646747669|gb|KK962201.1| 34496-33523	19	6	0	3	inf	0.0008	yes
gi|646747669|gb|KK962201.1| 43302-34476	19	6	0	1	inf	0.001	yes
gi|646747669|gb|KK962201.1| 43391-44400	19	6	5	0	-inf	0.00045	yes
gi|646747669|gb|KK962201.1| 52924-45368	19	6	6	0.25877	-5	0.1763	no
gi|646747682|gb|KK962200.1| 57504-35776	19	7.1	28	36	0.371682	0.7699	no

gi|646747682|gb|KK962200.1|	61613-36185	19	7.1	30	67	1	0.4526	no
gi|646747682|gb|KK962200.1|	25526-42579	19	7.1	49	38	-0.359598	0.82275	no
gi|646747682|gb|KK962200.1|	35603-43619	19	7.1	3	5	0.945911	0.475	no
gi|646747682|gb|KK962200.1|	39755-44016	19	7.1	4	4	0.11004	0.9144	no
gi|646747682|gb|KK962200.1|	66608-46813	19	7.1	44	36	-0.3026	0.78625	no
gi|646747682|gb|KK962200.1|	58791-60478	19	7.1	0.064605	19	8	0.09445	no
gi|646747682|gb|KK962200.1|	60579-61294	19	7.1	0	7	inf	0.00155	yes
gi|646747682|gb|KK962200.1|	77697-78670	19	7.1	20	18	-0.109967	0.9398	no
gi|646747697|gb|KK962199.1|	73627-17420	12	8.4	6	8	0.457027	0.7402	no
gi|646747697|gb|KK962199.1|	36505-73719	12	8.4	1	3	1	0.34675	no
gi|646747710|gb|KK962198.1|	92057-49258	10	9	3	0.920553	-2	0.3832	no
gi|646747725|gb|KK962197.1|	48013-14840	11	6.2	10	2	-3	0.154	no
gi|646747738|gb|KK962196.1|	13357-21394	20	8.3	1	4	2	0.2446	no
gi|646747738|gb|KK962196.1|	36236-23780	20	8.3	0.14135	10	6	0.2331	no
gi|646747738|gb|KK962196.1|	54517-25578	20	8.3	27	16	-0.737706	0.5594	no
gi|646747738|gb|KK962196.1|	70322-27536	20	8.3	13	11	-0.303411	0.79075	no
gi|646747738|gb|KK962196.1|	32082-33230	20	8.3	1800.74	2686.89	0.577348	0.60985	no
gi|646747738|gb|KK962196.1|	37154-33763	20	8.3	683	603	-0.181525	0.8788	no
gi|646747738|gb|KK962196.1|	67492-36984	20	8.3	18	22	0.272156	0.80525	no
gi|646747738|gb|KK962196.1|	76660-37892	20	8.3	0.644402	3	2	0.1514	no
gi|646747738|gb|KK962196.1|	83333-38455	20	8.3	1	3	1	0.36345	no
gi|646747738|gb|KK962196.1|	84668-38623	20	8.3	0.422115	1	1	1	no
gi|646747738|gb|KK962196.1|	86358-38919	20	8.3	309.39	583	0.91376	0.44795	no
gi|646747738|gb|KK962196.1|	97876-40160	20	8.3	6	5	-0.270629	0.8338	no
gi|646747738|gb|KK962196.1|	46117-44659	20	8.3	6	6	0.0718099	0.95065	no
gi|646747738|gb|KK962196.1|	55441-45618	20	8.3	2	2	0.1993	0.8791	no
gi|646747738|gb|KK962196.1|	71991-47234	20	8.3	8	8	0.149855	0.92045	no
gi|646747738|gb|KK962196.1|	73765-47451	20	8.3	0.934223	2	0.974617	0.5626	no
gi|646747738|gb|KK962196.1|	44184-54635	20	8.3	4	0.209811	-4	0.08355	no
gi|646747738|gb|KK962196.1|	46524-54711	20	8.3	5	0.764047	-3	0.13945	no

gi|646747738|gb|KK962196.1|	66569-66767	20	8.3	18	8	-1	0.41715	no
gi|646747738|gb|KK962196.1|	04588-70483	20	8.3	92	9	-3	0.1134	no
gi|646747754|gb|KK962195.1|	22984-23447	21	5.3	4	2	-0.588699	0.6685	no
gi|646747754|gb|KK962195.1|	65084-26528	21	5.3	239.14	236.46	-0.0162611	0.98105	no
gi|646747754|gb|KK962195.1|	76553-28140	21	5.3	2	2	0.0386328	0.9785	no
gi|646747754|gb|KK962195.1|	86194-28740	21	5.3	3	1	-1	0.47445	no
gi|646747754|gb|KK962195.1|	90408-29454	21	5.3	2	1	-0.815595	0.5912	no
gi|646747754|gb|KK962195.1|	03694-30891	21	5.3	9	4	-1	0.2789	no
gi|646747754|gb|KK962195.1|	07114-40769	21	5.3	2	5	1	0.42745	no
gi|646747754|gb|KK962195.1|	92526-59874	21	5.3	3	2	-0.411717	0.783	no
gi|646747754|gb|KK962195.1|	33042-63338	21	5.3	17	70	2	0.14885	no
gi|646747754|gb|KK962195.1|	44148-64516	21	5.3	159	244	0.619622	0.60665	no
gi|646747754|gb|KK962195.1|	02431-70446	21	5.3	91	54	-0.761594	0.52105	no
gi|646747754|gb|KK962195.1|	09692-71021	21	5.3	10	12	0.336952	0.8323	no
gi|646747754|gb|KK962195.1|	13735-71470	21	5.3	8	6	-0.245121	0.8691	no
gi|646747754|gb|KK962195.1|	15621-71641	21	5.3	3	5	0.90945	0.5256	no
gi|646747754|gb|KK962195.1|	18013-71853	21	5.3	1	3	1	0.48625	no
gi|646747754|gb|KK962195.1|	43604-74433	21	5.3	8	7	-0.238731	0.8752	no
gi|646747754|gb|KK962195.1|	85122-78558	21	5.3	3	2	-0.174781	0.89395	no
gi|646747754|gb|KK962195.1|	13986-82198	21	5.3	6	4	-0.680409	0.5363	no
gi|646747771|gb|KK962194.1|	81974-18261	18	7	3	3	0.33332	0.79825	no
gi|646747771|gb|KK962194.1|	05202-20570	18	7	0.73696	4	3	0.26725	no
gi|646747771|gb|KK962194.1|	13369-21382	18	7	82	92	0.166164	0.8943	no
gi|646747771|gb|KK962194.1|	14187-21490	18	7	2	0.563661	-2	0.29735	no
gi|646747771|gb|KK962194.1|	45157-24741	18	7	2	3	0.437083	0.76665	no
gi|646747786|gb|KK962193.1|	10233-15377	21	5.6	3	3	-0.0528991	0.97105	no
gi|646747786|gb|KK962193.1|	18626-11894	21	5.6	13	3	-2	0.2047	no
gi|646747786|gb|KK962193.1|	32738-12339	21	5.6	14	7	-0.999181	0.51415	no
gi|646747786|gb|KK962193.1|	48127-12483	21	5.6	56	60	0.0984134	0.93295	no
gi|646747786|gb|KK962193.1|	49725-12514	21	5.6	0.638032	1	0.668118	1	no

gi|646747786|gb|KK962193.1|	58924-12591	21	5.6	33	11	-2	0.3145	no
gi|646747786|gb|KK962193.1|	66679-12669	21	5.6	20	33	0.719597	0.63545	no
gi|646747786|gb|KK962193.1|	93529-12947	21	5.6	10	4	-1	0.3725	no
gi|646747786|gb|KK962193.1|	94829-12977	21	5.6	0.700376	1	0.742099	0.5858	no
gi|646747786|gb|KK962193.1|	99360-13000	21	5.6	2	1	-0.331668	0.81865	no
gi|646747786|gb|KK962193.1|	40874-14110	21	5.6	43	8	-2	0.17695	no
gi|646747786|gb|KK962193.1|	55930-15621	21	5.6	27	0	-inf	0.00195	no
gi|646747786|gb|KK962193.1|	66535-16850	21	5.6	0.323861	0.625228	0.949007	1	no
gi|646747786|gb|KK962193.1|	68769-17005	21	5.6	0.991823	0.517767	-0.93778	1	no
gi|646747786|gb|KK962193.1|	70249-17313	21	5.6	5	4	-0.428343	0.7775	no
gi|646747786|gb|KK962193.1|	73849-17767	21	5.6	6	4	-0.808697	0.51775	no
gi|646747786|gb|KK962193.1|	29426-29698	21	5.6	8	18	1	0.44965	no
gi|646747786|gb|KK962193.1|	17435-31781	21	5.6	17721.9	6533.98	-1	0.2307	no
gi|646747786|gb|KK962193.1|	69164-36950	21	5.6	497	400.69	-0.31211	0.78065	no
gi|646747786|gb|KK962193.1|	24982-82954	21	5.6	1	0.250992	-3	0.0957	no
gi|646747786|gb|KK962193.1|	32802-83362	21	5.6	3	2	-0.865061	0.50275	no
gi|646747802|gb|KK962192.1|	34974-13562	19	7.8	2	6	1	0.37425	no
gi|646747802|gb|KK962192.1|	36678-14268	19	7.8	10	11	0.202101	0.85725	no
gi|646747802|gb|KK962192.1|	54820-15658	19	7.8	60	66	0.126142	0.91405	no
gi|646747802|gb|KK962192.1|	61912-16271	19	7.8	2	5	1	0.4713	no
gi|646747802|gb|KK962192.1|	66168-16765	19	7.8	7	10	0.386226	0.8	no
gi|646747802|gb|KK962192.1|	67782-16872	19	7.8	1	0.765197	-0.493347	1	no
gi|646747802|gb|KK962192.1|	68841-17243	19	7.8	2	2	-0.214446	0.88485	no
gi|646747802|gb|KK962192.1|	78905-18036	19	7.8	0.919759	0.110384	-3	1	no
gi|646747802|gb|KK962192.1|	80587-18200	19	7.8	1	3	0.814785	0.55495	no
gi|646747802|gb|KK962192.1|	90073-19150	19	7.8	5	7	0.597039	0.69355	no
gi|646747802|gb|KK962192.1|	193-384	19	7.8	192	173	-0.154093	0.91085	no
gi|646747802|gb|KK962192.1|	94688-19561	19	7.8	16	37	1	0.3466	no
gi|646747802|gb|KK962192.1|	98647-19965	19	7.8	29	40	0.424439	0.69175	no
gi|646747802|gb|KK962192.1|	01271-20201	19	7.8	31	14	-1	0.46055	no

gi|646747802|gb|KK962192.1|	07440-20767	19	7.8	33	42	0.350153	0.78865	no
gi|646747802|gb|KK962192.1|	07842-20942	19	7.8	1	2	0.680638	0.6223	no
gi|646747802|gb|KK962192.1|	10100-21081	19	7.8	4	6	0.483826	0.76785	no
gi|646747802|gb|KK962192.1|	13768-21455	19	7.8	2	0.739682	-1	0.47555	no
gi|646747802|gb|KK962192.1|	14744-21536	19	7.8	3	2	-0.533267	0.6957	no
gi|646747802|gb|KK962192.1|	17733-21822	19	7.8	11	13	0.262462	0.87295	no
gi|646747802|gb|KK962192.1|	19648-22145	19	7.8	67	37	-0.846775	0.46285	no
gi|646747802|gb|KK962192.1|	32354-23360	19	7.8	1	0.265467	-2	1	no
gi|646747802|gb|KK962192.1|	52417-25329	19	7.8	2	2	0.238827	0.8563	no
gi|646747802|gb|KK962192.1|	65197-26696	19	7.8	2	3	0.664801	0.66825	no
gi|646747802|gb|KK962192.1|	69466-27077	19	7.8	4	3	-0.203306	0.89315	no
gi|646747802|gb|KK962192.1|	06259-30673	19	7.8	5	0	-inf	0.00475	no
gi|646747802|gb|KK962192.1|	17128-31735	19	7.8	161	77	-1	0.5154	no
gi|646747802|gb|KK962192.1|	19375-32034	19	7.8	89	67	-0.415805	0.7068	no
gi|646747802|gb|KK962192.1|	25195-32668	19	7.8	0.896007	0.537919	-0.73612	1	no
gi|646747802|gb|KK962192.1|	29744-33032	19	7.8	4	0.783736	-2	0.194	no
gi|646747802|gb|KK962192.1|	30423-33151	19	7.8	1	0.950774	-0.222332	1	no
gi|646747802|gb|KK962192.1|	40034-34215	19	7.8	8	4	-1	0.4945	no
gi|646747802|gb|KK962192.1|	48578-34927	19	7.8	2	1	-0.655515	0.64775	no
gi|646747802|gb|KK962192.1|	60288-36076	19	7.8	4	8	1	0.48725	no
gi|646747802|gb|KK962192.1|	61409-36237	19	7.8	1	2	0.370674	0.77965	no
gi|646747802|gb|KK962192.1|	73316-37585	19	7.8	1	1	-0.19836	0.89915	no
gi|646747802|gb|KK962192.1|	89338-38967	19	7.8	9	12	0.452026	0.7408	no
gi|646747802|gb|KK962192.1|	24877-42548	19	7.8	21	21	0.0107172	0.9929	no
gi|646747802|gb|KK962192.1|	28357-63009	19	7.8	3	2	-0.299019	0.8495	no
gi|646747817|gb|KK962191.1|	01559-10179	22	5.3	13	42.18	2	0.2782	no
gi|646747817|gb|KK962191.1|	14545-11538	22	5.3	4	7	0.691175	0.6595	no
gi|646747817|gb|KK962191.1|	28448-12869	22	5.3	14	34	1	0.3954	no
gi|646747817|gb|KK962191.1|	44655-14505	22	5.3	5	14	2	0.3471	no
gi|646747817|gb|KK962191.1|	51848-15209	22	5.3	157	161	0.03743	0.9808	no

gi|646747817|gb|KK962191.1|	52433-15293	22	5.3	3	3	-0.418891	0.76145	no
gi|646747817|gb|KK962191.1|	53723-15590	22	5.3	65	53	-0.297029	0.79885	no
gi|646747817|gb|KK962191.1|	58383-16203	22	5.3	4	6	0.808043	0.5209	no
gi|646747817|gb|KK962191.1|	62722-16439	22	5.3	2	4	1	0.44015	no
gi|646747817|gb|KK962191.1|	82047-28241	22	5.3	4	8	1	0.4623	no
gi|646747817|gb|KK962191.1|	29346-29629	22	5.3	28	16	-0.799845	0.5433	no
gi|646747817|gb|KK962191.1|	30418-32192	22	5.3	16	12	-0.419487	0.73935	no
gi|646747817|gb|KK962191.1|	29221-32939	22	5.3	447	19	-5	0.1688	no
gi|646747817|gb|KK962191.1|	31267-33151	22	5.3	41	0	-inf	0.0035	no
gi|646747817|gb|KK962191.1|	36033-33638	22	5.3	393	120	-2	0.20535	no
gi|646747817|gb|KK962191.1|	58776-35899	22	5.3	11	59	2	0.27355	no
gi|646747817|gb|KK962191.1|	3951-5983	22	5.3	4	6	0.390234	0.7973	no
gi|646747817|gb|KK962191.1|	44435-45139	22	5.3	12	5	-1	0.38635	no
gi|646747817|gb|KK962191.1|	08789-51239	22	5.3	5	29	2	0.08655	no
gi|646747817|gb|KK962191.1|	26797-52761	22	5.3	6	18	2	0.31295	no
gi|646747817|gb|KK962191.1|	29992-53234	22	5.3	8	33	2	0.1429	no
gi|646747817|gb|KK962191.1|	6706-8336	22	5.3	16	11	-0.571855	0.6487	no
gi|646747817|gb|KK962191.1|	70104-72689	22	5.3	8	2	-2	0.2484	no
gi|646747817|gb|KK962191.1|	72850-74166	22	5.3	4	3	-0.538741	0.72985	no
gi|646747817|gb|KK962191.1|	99142-99620	22	5.3	47	48	0.0331735	0.9818	no
gi|646747841|gb|KK962189.1|	00569-10130	23	10	0.388579	19	6	0.2332	no
gi|646747841|gb|KK962189.1|	06062-10639	23	10	43	98	1	0.43525	no
gi|646747841|gb|KK962189.1|	07400-10775	23	10	81	231	2	0.2469	no
gi|646747841|gb|KK962189.1|	08728-10898	23	10	29	142	2	0.14095	no
gi|646747841|gb|KK962189.1|	11829-11206	23	10	69	164	1	0.36615	no
gi|646747841|gb|KK962189.1|	12539-11350	23	10	4	11	2	0.3231	no
gi|646747841|gb|KK962189.1|	18123-12189	23	10	2	4	0.998018	0.5116	no
gi|646747841|gb|KK962189.1|	23486-12398	23	10	91	113	0.302244	0.7775	no
gi|646747841|gb|KK962189.1|	31737-13663	23	10	3	4	0.379643	0.8044	no
gi|646747841|gb|KK962189.1|	38789-14568	23	10	4	3	-0.0287742	0.9789	no

gi|646747841|gb|KK962189.1|	46374-14694	23	10	5	4	-0.546683	0.66795	no
gi|646747841|gb|KK962189.1|	60130-16103	23	10	1	1	0.503715	0.7286	no
gi|646747841|gb|KK962189.1|	86687-18696	23	10	29	2	-4	0.17335	no
gi|646747841|gb|KK962189.1|	05891-20612	23	10	40	54	0.420037	0.73405	no
gi|646747841|gb|KK962189.1|	20306-22067	23	10	18	16	-0.103202	0.9476	no
gi|646747841|gb|KK962189.1|	54668-25561	23	10	2	0.758779	-2	0.2986	no
gi|646747841|gb|KK962189.1|	85485-28577	23	10	36	4	-3	0.1469	no
gi|646747841|gb|KK962189.1|	92135-29723	23	10	16	7	-1	0.2857	no
gi|646747841|gb|KK962189.1|	99568-30133	23	10	38.86	18.19	-1	0.32675	no
gi|646747841|gb|KK962189.1|	01946-30268	23	10	2	3	0.61276	0.62835	no
gi|646747841|gb|KK962189.1|	05068-30583	23	10	2	4	1	0.4808	no
gi|646747841|gb|KK962189.1|	17967-31841	23	10	3	4	0.176988	0.90135	no
gi|646747841|gb|KK962189.1|	22325-32258	23	10	23	8	-1	0.37675	no
gi|646747841|gb|KK962189.1|	31995-33250	23	10	5	5	-0.0149231	0.9559	no
gi|646747841|gb|KK962189.1|	36187-33642	23	10	33	28.12	-0.24315	0.85775	no
gi|646747841|gb|KK962189.1|	89088-38984	23	10	2	1	-0.679984	0.6459	no
gi|646747841|gb|KK962189.1|	13980-41585	23	10	0.343499	2	3	0.15485	no
gi|646747841|gb|KK962189.1|	17525-41893	23	10	3	5	0.559833	0.70515	no
gi|646747841|gb|KK962189.1|	22046-42665	23	10	14	10	-0.40202	0.7206	no
gi|646747841|gb|KK962189.1|	26948-42736	23	10	7	4	-0.879641	0.54005	no
gi|646747841|gb|KK962189.1|	52522-45534	23	10	0.755702	1	0.607247	0.6579	no
gi|646747841|gb|KK962189.1|	57202-46127	23	10	0.824354	1	0.501101	0.743	no
gi|646747841|gb|KK962189.1|	62209-46339	23	10	6	7	0.155785	0.91365	no
gi|646747841|gb|KK962189.1|	65320-46664	23	10	1	1	-0.307304	0.8119	no
gi|646747841|gb|KK962189.1|	78217-47949	23	10	0.63363	0.779625	0.299139	1	no
gi|646747841|gb|KK962189.1|	81313-48530	23	10	9	7	-0.436907	0.6924	no
gi|646747841|gb|KK962189.1|	94164-49828	23	10	61	67	0.139597	0.9081	no
gi|646747841|gb|KK962189.1|	99393-50060	23	10	0.86999	0.692806	-0.328547	1	no
gi|646747841|gb|KK962189.1|	10732-51209	23	10	0.500963	0.72031	0.523914	1	no
gi|646747841|gb|KK962189.1|	30216-53038	23	10	306	226.93	-0.430163	0.72615	no

gi|646747841|gb|KK962189.1|	97248-59836	23	10	16	20	0.357025	0.81425	no
gi|646747841|gb|KK962189.1|	63688-66566	23	10	1	0.778844	-0.603469	0.6764	no
gi|646747841|gb|KK962189.1|	70848-67185	23	10	14	7	-1	0.47755	no
gi|646747841|gb|KK962189.1|	72180-67291	23	10	2	5	0.989186	0.5252	no
gi|646747841|gb|KK962189.1|	85896-68618	23	10	10	9	-0.0498539	0.91525	no
gi|646747841|gb|KK962189.1|	91705-69281	23	10	4	5	0.312105	0.8391	no
gi|646747841|gb|KK962189.1|	93029-69647	23	10	0.492903	1	1	1	no
gi|646747841|gb|KK962189.1|	96610-70004	23	10	2	6	2	0.20715	no
gi|646747841|gb|KK962189.1|	03421-70661	23	10	2	4	0.892599	0.549	no
gi|646747841|gb|KK962189.1|	06763-70925	23	10	0.372391	1	2	1	no
gi|646747841|gb|KK962189.1|	09533-71026	23	10	1	2	0.264566	0.8456	no
gi|646747841|gb|KK962189.1|	71026-72568	23	10	9	5	-0.919598	0.54415	no
gi|646747841|gb|KK962189.1|	10430-71175	23	10	0.781046	0.249369	-2	1	no
gi|646747841|gb|KK962189.1|	29263-73063	23	10	6	4	-0.597873	0.69195	no
gi|646747841|gb|KK962189.1|	31119-73210	23	10	1	0.54399	-0.905957	1	no
gi|646747841|gb|KK962189.1|	39036-73993	23	10	1	2	0.351787	0.794	no
gi|646747841|gb|KK962189.1|	49627-75294	23	10	63	33	-0.931575	0.43525	no
gi|646747841|gb|KK962189.1|	77517-80654	23	10	8	5	-0.640283	0.6175	no
gi|646747841|gb|KK962189.1|	99646-99889	23	10	170	80	-1	0.4866	no
gi|646747857|gb|KK962188.1|	81983-18229	14	7.6	31	0	-inf	0.00595	no
gi|646747857|gb|KK962188.1|	84486-18478	14	7.6	271	3	-6	0.1064	no
gi|646747857|gb|KK962188.1|	95783-19706	14	7.6	49	0	-inf	5.00E-05	yes
gi|646747857|gb|KK962188.1|	09432-20986	14	7.6	681	2	-8	0.07395	no
gi|646747857|gb|KK962188.1|	84287-28445	14	7.6	5658.82	159	-5	0.0131	no
gi|646747857|gb|KK962188.1|	87278-28768	14	7.6	170	13	-4	0.02995	no
gi|646747857|gb|KK962188.1|	90186-29095	14	7.6	276	10	-5	0.0066	no
gi|646747857|gb|KK962188.1|	10154-41039	14	7.6	3	36	3	0.1082	no
gi|646747857|gb|KK962188.1|	92892-93159	14	7.6	2496.73	129	-4	0.0093	no
gi|646747857|gb|KK962188.1|	93711-93885	14	7.6	3445.77	175	-4	0.0116	no
gi|646747870|gb|KK962187.1|	21939-22222	13	5.8	12	8	-0.6072	0.67445	no

gi|646747870|gb|KK962187.1|	24299-32638	13	5.8	1	1	-0.0815221	0.9603	no
gi|646747870|gb|KK962187.1|	33966-34255	13	5.8	10	15	0.628218	0.6584	no
gi|646747870|gb|KK962187.1|	59124-35992	13	5.8	3	2	-0.090339	0.94565	no
gi|646747870|gb|KK962187.1|	46241-47226	13	5.8	12	9	-0.475888	0.7547	no
gi|646747870|gb|KK962187.1|	48455-49125	13	5.8	3	2	-0.107828	0.9358	no
gi|646747870|gb|KK962187.1|	50412-51652	13	5.8	0.845263	0.673549	-0.327617	1	no
gi|646747870|gb|KK962187.1|	66969-67779	13	5.8	1	0.940134	-0.311401	0.8509	no
gi|646747870|gb|KK962187.1|	69539-70671	13	5.8	1	3	1	0.40135	no
gi|646747884|gb|KK962186.1|	47390-10486	22	11	0.888667	2	1	0.34205	no
gi|646747884|gb|KK962186.1|	48979-10498	22	11	0.915832	0.863832	-0.0843325	1	no
gi|646747884|gb|KK962186.1|	50782-10517	22	11	0.686215	2	1	0.43255	no
gi|646747884|gb|KK962186.1|	52331-10562	22	11	1	1	0.0443321	1	no
gi|646747884|gb|KK962186.1|	56516-10572	22	11	1	3	1	0.39835	no
gi|646747884|gb|KK962186.1|	57332-10587	22	11	0.900554	1	0.649997	0.61505	no
gi|646747884|gb|KK962186.1|	80488-10811	22	11	2	0.600977	-2	0.29735	no
gi|646747884|gb|KK962186.1|	09304-10956	22	11	323	71	-2	0.175	no
gi|646747884|gb|KK962186.1|	99905-11003	22	11	15	0.515452	-5	0.1736	no
gi|646747884|gb|KK962186.1|	07152-11082	22	11	2	2	0.428512	0.7547	no
gi|646747884|gb|KK962186.1|	09544-11098	22	11	12	8	-0.470146	0.73585	no
gi|646747884|gb|KK962186.1|	19571-11202	22	11	2	2	-0.514148	0.7106	no
gi|646747884|gb|KK962186.1|	30776-11310	22	11	34	2	-4	0.17335	no
gi|646747884|gb|KK962186.1|	41014-11418	22	11	12	4	-2	0.29045	no
gi|646747884|gb|KK962186.1|	41959-11465	22	11	1	0.712216	-0.516189	1	no
gi|646747884|gb|KK962186.1|	86451-11918	22	11	8	6	-0.443779	0.68275	no
gi|646747884|gb|KK962186.1|	05611-12062	22	11	2	1	-1	0.47035	no
gi|646747884|gb|KK962186.1|	15707-12167	22	11	2	2	-0.275161	0.81735	no
gi|646747884|gb|KK962186.1|	18831-12235	22	11	40	27	-0.559645	0.6408	no
gi|646747884|gb|KK962186.1|	23725-12261	22	11	1	1	-0.214127	0.8918	no
gi|646747884|gb|KK962186.1|	28630-12291	22	11	3	0.920553	-2	0.29375	no
gi|646747884|gb|KK962186.1|	29366-12304	22	11	0.86759	0.775115	-0.162604	1	no

gi|646747884|gb|KK962186.1|	30598-12355	22	11	4	3	-0.792242	0.52755	no
gi|646747884|gb|KK962186.1|	35878-12371	22	11	1	0	-inf	0.0041	no
gi|646747884|gb|KK962186.1|	39020-12407	22	11	3	4	0.329393	0.8309	no
gi|646747884|gb|KK962186.1|	50710-12510	22	11	2	10	2	0.3069	no
gi|646747884|gb|KK962186.1|	53498-12537	22	11	12	14	0.133618	0.90715	no
gi|646747884|gb|KK962186.1|	73125-12734	22	11	23	30	0.369739	0.8174	no
gi|646747884|gb|KK962186.1|	74757-12752	22	11	3	8	1	0.39125	no
gi|646747884|gb|KK962186.1|	80325-12805	22	11	35	48	0.473597	0.76105	no
gi|646747884|gb|KK962186.1|	00359-13013	22	11	2	0.37728	-2	0.197	no
gi|646747884|gb|KK962186.1|	08590-13100	22	11	0.525214	0.648588	0.304398	1	no
gi|646747884|gb|KK962186.1|	11741-13126	22	11	3	4	0.340665	0.8056	no
gi|646747884|gb|KK962186.1|	12709-13135	22	11	2	0.226407	-3	0.1932	no
gi|646747884|gb|KK962186.1|	14419-13153	22	11	7	3	-1	0.4757	no
gi|646747884|gb|KK962186.1|	16016-13167	22	11	9	7	-0.196602	0.90215	no
gi|646747884|gb|KK962186.1|	18836-13214	22	11	0.899382	0.79546	-0.177144	1	no
gi|646747884|gb|KK962186.1|	23846-13257	22	11	0.771427	0.717852	-0.103845	1	no
gi|646747884|gb|KK962186.1|	27986-13316	22	11	7	4	-0.919085	0.47165	no
gi|646747884|gb|KK962186.1|	20813-22118	22	11	10	7	-0.559049	0.6826	no
gi|646747884|gb|KK962186.1|	24063-24961	22	11	1	0.611848	-0.911471	0.58965	no
gi|646747884|gb|KK962186.1|	30239-34287	22	11	66	57	-0.214914	0.86185	no
gi|646747884|gb|KK962186.1|	02081-40233	22	11	17	25	0.496188	0.72495	no
gi|646747884|gb|KK962186.1|	14017-41447	22	11	10	9	-0.205928	0.902	no
gi|646747884|gb|KK962186.1|	54082-45474	22	11	4	0.624373	-3	0.15175	no
gi|646747884|gb|KK962186.1|	55878-45629	22	11	13	2	-3	0.17415	no
gi|646747884|gb|KK962186.1|	72289-47459	22	11	13	5	-1	0.31175	no
gi|646747884|gb|KK962186.1|	74921-47631	22	11	0.652639	0.704115	0.109527	1	no
gi|646747884|gb|KK962186.1|	79970-48252	22	11	6	4	-0.690486	0.65035	no
gi|646747884|gb|KK962186.1|	83968-48566	22	11	0.897578	1	0.308117	1	no
gi|646747884|gb|KK962186.1|	06966-51332	22	11	24	22	-0.184021	0.87665	no
gi|646747884|gb|KK962186.1|	19987-52042	22	11	30	40	0.393442	0.7975	no

gi|646747884|gb|KK962186.1|	21885-52272	22	11	6	4	-0.536823	0.73145	no
gi|646747884|gb|KK962186.1|	24511-52520	22	11	1	4	2	0.2493	no
gi|646747884|gb|KK962186.1|	25718-52674	22	11	0.4793	1	2	0.3671	no
gi|646747884|gb|KK962186.1|	30002-53126	22	11	2	1	-0.107081	0.92875	no
gi|646747884|gb|KK962186.1|	32640-54075	22	11	0.835881	2	0.937882	0.53895	no
gi|646747884|gb|KK962186.1|	40972-54258	22	11	1	0.982381	-0.0558042	1	no
gi|646747884|gb|KK962186.1|	42849-54587	22	11	0.86936	2	0.928032	0.55955	no
gi|646747884|gb|KK962186.1|	45999-54887	22	11	0.775698	1	0.604513	0.71035	no
gi|646747884|gb|KK962186.1|	50580-55227	22	11	0.643803	0.278963	-1	1	no
gi|646747884|gb|KK962186.1|	56512-55763	22	11	1	0.764633	-0.484025	1	no
gi|646747884|gb|KK962186.1|	5784-6079	22	11	13	28	1	0.4882	no
gi|646747884|gb|KK962186.1|	82516-58355	22	11	3	3	0.0530205	0.9697	no
gi|646747884|gb|KK962186.1|	83822-58454	22	11	2	2	-0.291475	0.82545	no
gi|646747884|gb|KK962186.1|	98771-59912	22	11	19	44	1	0.4475	no
gi|646747884|gb|KK962186.1|	10512-61093	22	11	7	4	-0.995119	0.5242	no
gi|646747884|gb|KK962186.1|	17902-61816	22	11	14	20	0.562889	0.71435	no
gi|646747884|gb|KK962186.1|	48632-64884	22	11	17	61	2	0.2432	no
gi|646747884|gb|KK962186.1|	91824-69533	22	11	29	16	-0.820838	0.47765	no
gi|646747884|gb|KK962186.1|	00078-70057	22	11	2	4	0.649718	0.6597	no
gi|646747884|gb|KK962186.1|	04610-70499	22	11	4	4	-0.235874	0.87385	no
gi|646747884|gb|KK962186.1|	18646-72018	22	11	0.938121	1	0.286758	0.8199	no
gi|646747884|gb|KK962186.1|	22403-72283	22	11	3	7	1	0.36265	no
gi|646747884|gb|KK962186.1|	24756-73289	22	11	6	6	-0.0605384	0.95475	no
gi|646747884|gb|KK962186.1|	38663-73892	22	11	125.55	43	-2	0.3434	no
gi|646747884|gb|KK962186.1|	44593-74569	22	11	43	16	-1	0.27805	no
gi|646747884|gb|KK962186.1|	52174-75296	22	11	3	3	-0.0466907	0.9562	no
gi|646747884|gb|KK962186.1|	75707-76025	22	11	10	6	-0.903106	0.56075	no
gi|646747884|gb|KK962186.1|	46794-84710	22	11	194	86	-1	0.4379	no
gi|646747884|gb|KK962186.1|	71534-87215	22	11	2	4	1	0.4268	no
gi|646747884|gb|KK962186.1|	72918-87311	22	11	45	49	0.145802	0.9042	no

gi|646747884|gb|KK962186.1|	73938-87475	22	11	2	0.699047	-1	0.3882	no
gi|646747884|gb|KK962186.1|	75023-87588	22	11	5	5	0.237257	0.87675	no
gi|646747884|gb|KK962186.1|	77211-87755	22	11	13	7	-0.912871	0.4938	no
gi|646747884|gb|KK962186.1|	78095-88120	22	11	4	1	-2	0.3257	no
gi|646747884|gb|KK962186.1|	81328-88925	22	11	27	11	-1	0.2823	no
gi|646747898|gb|KK962185.1|	08506-11034	17	7.9	2	2	0.269447	0.861	no
gi|646747898|gb|KK962185.1|	35483-13602	17	7.9	3	3	0.0316917	0.9298	no
gi|646747898|gb|KK962185.1|	47662-14867	17	7.9	2	2	0.00811567	0.9587	no
gi|646747898|gb|KK962185.1|	79934-18039	17	7.9	2	3	0.410743	0.81675	no
gi|646747898|gb|KK962185.1|	83485-18385	17	7.9	8	3	-2	0.35815	no
gi|646747898|gb|KK962185.1|	91561-19247	17	7.9	11	5	-1	0.49145	no
gi|646747898|gb|KK962185.1|	92926-19384	17	7.9	4	4	0.126857	0.93825	no
gi|646747898|gb|KK962185.1|	19659-21082	17	7.9	9	8	-0.248748	0.8708	no
gi|646747898|gb|KK962185.1|	22016-22754	17	7.9	19	7	-1	0.35045	no
gi|646747898|gb|KK962185.1|	25069-22530	17	7.9	14	21	0.660184	0.67095	no
gi|646747898|gb|KK962185.1|	23680-24606	17	7.9	11	6	-0.85432	0.57445	no
gi|646747898|gb|KK962185.1|	38636-23888	17	7.9	14	15	0.118551	0.90815	no
gi|646747898|gb|KK962185.1|	50703-25117	17	7.9	8	5	-0.906827	0.47665	no
gi|646747898|gb|KK962185.1|	58772-25895	17	7.9	97	60	-0.693419	0.6538	no
gi|646747898|gb|KK962185.1|	82499-28273	17	7.9	47	18	-1	0.3247	no
gi|646747898|gb|KK962185.1|	86325-28669	17	7.9	17	10	-0.804606	0.58755	no
gi|646747898|gb|KK962185.1|	87413-28796	17	7.9	7	5	-0.615119	0.6607	no
gi|646747898|gb|KK962185.1|	19006-31988	17	7.9	7	9	0.439681	0.77865	no
gi|646747898|gb|KK962185.1|	20002-32173	17	7.9	8	2	-2	0.19075	no
gi|646747898|gb|KK962185.1|	29881-33173	17	7.9	2	3	0.57841	0.7091	no
gi|646747898|gb|KK962185.1|	31948-33312	17	7.9	6	4	-0.506453	0.73815	no
gi|646747898|gb|KK962185.1|	33228-33396	17	7.9	4	4	0.165301	0.90365	no
gi|646747898|gb|KK962185.1|	45057-34617	17	7.9	58	35	-0.716451	0.51635	no
gi|646747898|gb|KK962185.1|	60007-36028	17	7.9	31.47	38	0.264097	0.8445	no
gi|646747898|gb|KK962185.1|	42652-44292	17	7.9	0.601175	0.674963	0.167024	1	no

gi|646747898|gb|KK962185.1|	47155-44828	17	7.9	0	6	inf	0.00055	yes
gi|646747898|gb|KK962185.1|	54638-45586	17	7.9	0.47587	2	2	0.22555	no
gi|646747898|gb|KK962185.1|	06673-50804	17	7.9	1	2	0.704614	0.6267	no
gi|646747898|gb|KK962185.1|	27339-52780	17	7.9	10	5	-1	0.47975	no
gi|646747898|gb|KK962185.1|	10654-61164	17	7.9	8	8	-0.108582	0.9417	no
gi|646747898|gb|KK962185.1|	12243-61249	17	7.9	19	21	0.118551	0.9237	no
gi|646747898|gb|KK962185.1|	19655-62045	17	7.9	3	0.721342	-2	0.17925	no
gi|646747898|gb|KK962185.1|	69542-67086	17	7.9	2	3	0.352818	0.82425	no
gi|646747898|gb|KK962185.1|	88643-89216	17	7.9	10	9	-0.191015	0.90495	no
gi|646747914|gb|KK962184.1|	80134-10809	23	10	0.458673	2	2	0.20395	no
gi|646747914|gb|KK962184.1|	97861-10989	23	10	11	1	-3	0.0497	no
gi|646747914|gb|KK962184.1|	08990-11094	23	10	4	2	-0.752217	0.6076	no
gi|646747914|gb|KK962184.1|	26522-22685	23	10	9	20	1	0.46945	no
gi|646747914|gb|KK962184.1|	36176-83758	23	10	2	2	0.302628	0.82235	no
gi|646747914|gb|KK962184.1|	48923-84913	23	10	189	17	-3	0.1037	no
gi|646747914|gb|KK962184.1|	67559-86802	23	10	14	56	2	0.2103	no
gi|646747914|gb|KK962184.1|	74015-87657	23	10	2	6	2	0.2492	no
gi|646747928|gb|KK962183.1|	76653-17835	13	5.9	1	2	0.656115	0.68815	no
gi|646747928|gb|KK962183.1|	26860-42860	13	5.9	0.310245	0.986366	2	1	no
gi|646747928|gb|KK962183.1|	28925-42944	13	5.9	1	3	1	0.48625	no
gi|646747928|gb|KK962183.1|	90689-93539	13	5.9	20	17	-0.259358	0.82045	no
gi|646747942|gb|KK962182.1|	85886-18663	16	6.6	0.750406	2	2	0.2977	no
gi|646747942|gb|KK962182.1|	51028-35197	16	6.6	0.267348	2	3	0.25	no
gi|646747942|gb|KK962182.1|	46501-47074	16	6.6	6	16	1	0.2763	no
gi|646747956|gb|KK962181.1|	34116-13464	22	9.7	3	3	-0.0726943	0.93645	no
gi|646747956|gb|KK962181.1|	68328-16918	22	9.7	0	6	inf	0.0012	yes
gi|646747956|gb|KK962181.1|	03988-20564	22	9.7	0.993352	1	0.207861	0.86735	no
gi|646747956|gb|KK962181.1|	06398-20792	22	9.7	1	1	0.179669	0.88385	no
gi|646747956|gb|KK962181.1|	08857-21210	22	9.7	10	3	-2	0.2366	no
gi|646747956|gb|KK962181.1|	12494-21372	22	9.7	8	9	0.172097	0.9112	no

gi|646747956|gb|KK962181.1|	19316-22010	22	9.7	16	9	-0.765989	0.6207	no
gi|646747956|gb|KK962181.1|	24607-22522	22	9.7	2	1	-0.756568	0.6333	no
gi|646747956|gb|KK962181.1|	27812-22846	22	9.7	4	5	0.0328475	0.9646	no
gi|646747956|gb|KK962181.1|	30366-23147	22	9.7	0.755599	1	0.708283	0.6242	no
gi|646747956|gb|KK962181.1|	33343-23408	22	9.7	4	8	1	0.42315	no
gi|646747956|gb|KK962181.1|	43896-24446	22	9.7	1	3	1	0.4404	no
gi|646747956|gb|KK962181.1|	45331-24608	22	9.7	1	3	1	0.44735	no
gi|646747956|gb|KK962181.1|	48105-24840	22	9.7	218	88	-1	0.391	no
gi|646747956|gb|KK962181.1|	60281-26067	22	9.7	19	7	-1	0.34695	no
gi|646747956|gb|KK962181.1|	60787-26316	22	9.7	14	3	-2	0.0794	no
gi|646747956|gb|KK962181.1|	94545-29602	22	9.7	12	8	-0.722425	0.63705	no
gi|646747956|gb|KK962181.1|	06484-30757	22	9.7	3	2	-0.68691	0.6331	no
gi|646747956|gb|KK962181.1|	17437-31832	22	9.7	0.730649	1	0.76584	0.6143	no
gi|646747956|gb|KK962181.1|	26978-32725	22	9.7	25	15	-0.709152	0.60215	no
gi|646747956|gb|KK962181.1|	52411-35375	22	9.7	0.425142	0.977713	1	1	no
gi|646747956|gb|KK962181.1|	60460-36338	22	9.7	24	20	-0.307397	0.78585	no
gi|646747956|gb|KK962181.1|	67204-36951	22	9.7	18	12	-0.616596	0.57135	no
gi|646747956|gb|KK962181.1|	72358-37548	22	9.7	8	4	-0.799199	0.5263	no
gi|646747956|gb|KK962181.1|	84572-38575	22	9.7	2	0	-inf	0.0042	no
gi|646747956|gb|KK962181.1|	56828-85719	22	9.7	13	24	0.825758	0.55225	no
gi|646747990|gb|KK962179.1|	01773-10613	21	9.3	11	14	0.361735	0.74745	no
gi|646747990|gb|KK962179.1|	08335-11005	21	9.3	4	7	0.740276	0.61825	no
gi|646747990|gb|KK962179.1|	10155-11210	21	9.3	69	39	-0.831269	0.47135	no
gi|646747990|gb|KK962179.1|	15364-11596	21	9.3	2	4	0.783929	0.57095	no
gi|646747990|gb|KK962179.1|	52248-15348	21	9.3	8	9	0.0939529	0.95045	no
gi|646747990|gb|KK962179.1|	54614-15909	21	9.3	13	9	-0.537333	0.6233	no
gi|646747990|gb|KK962179.1|	85176-18555	21	9.3	9	5	-1	0.4473	no
gi|646747990|gb|KK962179.1|	20939-23052	21	9.3	17	10	-0.71185	0.53915	no
gi|646747990|gb|KK962179.1|	31276-23368	21	9.3	0.60049	0.684941	0.189841	1	no
gi|646747990|gb|KK962179.1|	42363-24309	21	9.3	1	2	0.487363	0.73295	no

gi|646747990|gb|KK962179.1|	43281-24497	21	9.3	1	0.744921	-0.639538	0.61365	no
gi|646747990|gb|KK962179.1|	49056-25583	21	9.3	4	1	-2	0.2005	no
gi|646747990|gb|KK962179.1|	58461-25930	21	9.3	2	0.223057	-3	0.1863	no
gi|646747990|gb|KK962179.1|	60965-26283	21	9.3	0.804485	0.332531	-1	1	no
gi|646747990|gb|KK962179.1|	63364-26466	21	9.3	0.619686	0.762825	0.299814	1	no
gi|646747990|gb|KK962179.1|	65657-26670	21	9.3	2	0.668179	-1	0.37415	no
gi|646747990|gb|KK962179.1|	66947-26838	21	9.3	0.863132	0	-inf	1	no
gi|646747990|gb|KK962179.1|	68638-26998	21	9.3	2	2	0.00897857	0.96475	no
gi|646747990|gb|KK962179.1|	83909-28601	21	9.3	4	0.726321	-2	0.12145	no
gi|646747990|gb|KK962179.1|	11853-31263	21	9.3	58	8	-3	0.04755	no
gi|646747990|gb|KK962179.1|	13254-31533	21	9.3	1	0.367487	-2	1	no
gi|646747990|gb|KK962179.1|	15448-31594	21	9.3	4	0.511626	-3	0.20305	no
gi|646747990|gb|KK962179.1|	18412-32218	21	9.3	0.893417	0.421278	-1	1	no
gi|646747990|gb|KK962179.1|	29106-33023	21	9.3	3	2	-0.27708	0.86675	no
gi|646747990|gb|KK962179.1|	32172-33249	21	9.3	18	3	-3	0.1362	no
gi|646747990|gb|KK962179.1|	47368-34788	21	9.3	16	3	-3	0.12285	no
gi|646747990|gb|KK962179.1|	53528-35463	21	9.3	0.764518	0.936557	0.292816	1	no
gi|646747990|gb|KK962179.1|	55209-35764	21	9.3	26	15	-0.788757	0.47345	no
gi|646747990|gb|KK962179.1|	57859-35917	21	9.3	0.783988	0.375433	-1	1	no
gi|646747990|gb|KK962179.1|	66262-36787	21	9.3	1	1	0.248982	0.8327	no
gi|646747990|gb|KK962179.1|	69634-37068	21	9.3	1	3	1	0.35885	no
gi|646747990|gb|KK962179.1|	70836-37196	21	9.3	0.844816	1	0.323783	1	no
gi|646747990|gb|KK962179.1|	02383-40265	21	9.3	79	55	-0.528794	0.7355	no
gi|646747990|gb|KK962179.1|	26800-42924	21	9.3	1	0.244912	-2	1	no
gi|646747990|gb|KK962179.1|	52610-45739	21	9.3	8	3	-1	0.34525	no
gi|646747990|gb|KK962179.1|	64188-46472	21	9.3	4	3	-0.332566	0.79845	no
gi|646747990|gb|KK962179.1|	65681-46677	21	9.3	44	25	-0.845114	0.43915	no
gi|646747990|gb|KK962179.1|	72845-47459	21	9.3	2	0.357497	-2	0.19895	no
gi|646747990|gb|KK962179.1|	91327-49156	21	9.3	30	16	-0.946877	0.5681	no
gi|646747990|gb|KK962179.1|	16026-51678	21	9.3	9	3	-2	0.21185	no

gi|646747990|gb|KK962179.1|	25162-52630	21	9.3	7	4	-0.912052	0.55225	no
gi|646747990|gb|KK962179.1|	36730-53754	21	9.3	0.821466	1	0.759777	0.61475	no
gi|646747990|gb|KK962179.1|	73933-57484	21	9.3	94	8	-3	0.0207	no
gi|646747990|gb|KK962179.1|	82813-58385	21	9.3	24	2	-3	0.01005	no
gi|646747990|gb|KK962179.1|	04517-60604	21	9.3	4	0	-inf	0.00045	yes
gi|646747990|gb|KK962179.1|	13635-61484	21	9.3	13	0	-inf	0.0002	yes
gi|646747990|gb|KK962179.1|	19898-62056	21	9.3	5	0	-inf	0.00495	no
gi|646747990|gb|KK962179.1|	61005-66243	21	9.3	3	2	-0.1265	0.9362	no
gi|646747990|gb|KK962179.1|	63302-66389	21	9.3	6	3	-1	0.4416	no
gi|646747990|gb|KK962179.1|	66857-66924	21	9.3	6	1	-2	0.159	no
gi|646747990|gb|KK962179.1|	69466-67044	21	9.3	1	0.180847	-3	0.19945	no
gi|646747990|gb|KK962179.1|	03510-80700	21	9.3	4	7	0.85122	0.50095	no
gi|646747990|gb|KK962179.1|	99213-10114[illegible]	21	9.3	4	3	-0.189875	0.902	no
gi|646748002|gb|KK962178.1|	00376-10011	22	6.3	1	1	-0.187396	0.90405	no
gi|646748002|gb|KK962178.1|	18664-10196	22	6.3	1	2	0.508664	0.7139	no
gi|646748002|gb|KK962178.1|	34526-10349	22	6.3	9	0	-inf	0.00225	no
gi|646748002|gb|KK962178.1|	35503-10362	22	6.3	59	14	-2	0.1346	no
gi|646748002|gb|KK962178.1|	43503-10462	22	6.3	15	5	-2	0.25275	no
gi|646748002|gb|KK962178.1|	71502-10724	22	6.3	208	58	-2	0.11695	no
gi|646748002|gb|KK962178.1|	74265-10753	22	6.3	0.675227	1	0.73648	0.6352	no
gi|646748002|gb|KK962178.1|	92161-10925	22	6.3	7	12	0.717016	0.57915	no
gi|646748002|gb|KK962178.1|	94279-10958	22	6.3	0.698743	0.503959	-0.471455	1	no
gi|646748002|gb|KK962178.1|	97444-10992	22	6.3	2	0.422129	-2	0.2533	no
gi|646748002|gb|KK962178.1|	58594-11587	22	6.3	185	18	-3	0.11345	no
gi|646748002|gb|KK962178.1|	75923-11767	22	6.3	2	0.715007	-1	0.3853	no
gi|646748002|gb|KK962178.1|	82149-11825	22	6.3	82	9	-3	0.04545	no
gi|646748002|gb|KK962178.1|	05584-12126	22	6.3	55	39	-0.522076	0.66275	no
gi|646748002|gb|KK962178.1|	24611-12248	22	6.3	150	124.87	-0.261008	0.836	no
gi|646748002|gb|KK962178.1|	51909-12539	22	6.3	2	2	0.450289	0.7698	no
gi|646748002|gb|KK962178.1|	84405-12850	22	6.3	4	4	0.0257667	0.9559	no

gi|646748002|gb|KK962178.1|	06152-13079	22	6.3	2	2	0.153281	0.9248	no
gi|646748002|gb|KK962178.1|	17383-13267	22	6.3	6	7	0.181391	0.87375	no
gi|646748002|gb|KK962178.1|	49056-13501	22	6.3	5	3	-0.517432	0.73945	no
gi|646748002|gb|KK962178.1|	51470-13529	22	6.3	1	1	0.0782532	0.9493	no
gi|646748002|gb|KK962178.1|	53097-13542	22	6.3	0.955872	1	0.346299	0.8037	no
gi|646748002|gb|KK962178.1|	56154-13571	22	6.3	2	0.34166	-2	0.1855	no
gi|646748002|gb|KK962178.1|	02182-14025	22	6.3	5	6	0.0438264	0.91525	no
gi|646748002|gb|KK962178.1|	13950-14159	22	6.3	2	2	-0.352851	0.8279	no
gi|646748002|gb|KK962178.1|	16116-14165	22	6.3	13	7	-0.982383	0.5121	no
gi|646748002|gb|KK962178.1|	23240-14235	22	6.3	15	20	0.383106	0.78595	no
gi|646748002|gb|KK962178.1|	26245-14266	22	6.3	6	7	0.212414	0.87975	no
gi|646748002|gb|KK962178.1|	40936-14429	22	6.3	0.907024	0.618109	-0.55328	1	no
gi|646748002|gb|KK962178.1|	44421-14460	22	6.3	0.590936	0.568839	-0.054981	1	no
gi|646748002|gb|KK962178.1|	46189-14559	22	6.3	12	10	-0.288383	0.8037	no
gi|646748002|gb|KK962178.1|	59618-14603	22	6.3	2	1	-0.833508	0.57665	no
gi|646748002|gb|KK962178.1|	67410-14733	22	6.3	12	5	-1	0.2064	no
gi|646748002|gb|KK962178.1|	77880-14792	22	6.3	0.94927	0.248002	-2	1	no
gi|646748002|gb|KK962178.1|	80959-14816	22	6.3	2	0.271157	-3	0.1994	no
gi|646748002|gb|KK962178.1|	15173-16922	22	6.3	3	2	-0.804348	0.6064	no
gi|646748002|gb|KK962178.1|	30219-15314	22	6.3	1	2	0.199362	0.8723	no
gi|646748002|gb|KK962178.1|	35728-15499	22	6.3	5	1	-2	0.11825	no
gi|646748002|gb|KK962178.1|	50396-15550	22	6.3	5	2	-2	0.20955	no
gi|646748002|gb|KK962178.1|	59112-15596	22	6.3	2	2	-0.542154	0.7295	no
gi|646748002|gb|KK962178.1|	59983-15611	22	6.3	1	0	-inf	0.0041	no
gi|646748002|gb|KK962178.1|	64072-15648	22	6.3	4	3	-0.317662	0.794	no
gi|646748002|gb|KK962178.1|	69957-15721	22	6.3	16	16	0.00438941	0.99665	no
gi|646748002|gb|KK962178.1|	72940-15745	22	6.3	0.623688	0.299925	-1	1	no
gi|646748002|gb|KK962178.1|	78878-15800	22	6.3	0.831529	0.743681	-0.161082	1	no
gi|646748002|gb|KK962178.1|	80157-15804	22	6.3	116.13	128	0.140671	0.92545	no
gi|646748002|gb|KK962178.1|	91785-15923	22	6.3	3	3	0.168878	0.9081	no

gi|646748002|gb|KK962178.1|	01284-16021	22	6.3	0.589172	1	1	0.48525	no
gi|646748002|gb|KK962178.1|	02359-16026	22	6.3	8	18	1	0.44965	no
gi|646748002|gb|KK962178.1|	26622-16270	22	6.3	7	3	-1	0.43205	no
gi|646748002|gb|KK962178.1|	31861-16332	22	6.3	2	5	1	0.4163	no
gi|646748002|gb|KK962178.1|	36442-16369	22	6.3	4	3	-0.206016	0.878	no
gi|646748002|gb|KK962178.1|	51085-16514	22	6.3	9	5	-0.874136	0.5704	no
gi|646748002|gb|KK962178.1|	61839-16624	22	6.3	1	4	2	0.25505	no
gi|646748002|gb|KK962178.1|	84266-16851	22	6.3	0.964995	0.978567	0.0201496	1	no
gi|646748002|gb|KK962178.1|	86408-16871	22	6.3	48	12	-2	0.1265	no
gi|646748002|gb|KK962178.1|	91083-17041	22	6.3	5	4	-0.304345	0.78035	no
gi|646748002|gb|KK962178.1|	04300-17203	22	6.3	13	7	-0.839135	0.48325	no
gi|646748002|gb|KK962178.1|	27751-17283	22	6.3	9	8	-0.168324	0.9104	no
gi|646748002|gb|KK962178.1|	28948-17292	22	6.3	382	297	-0.361728	0.7326	no
gi|646748002|gb|KK962178.1|	29805-17301	22	6.3	221	192	-0.201946	0.89065	no
gi|646748002|gb|KK962178.1|	17360-31893	22	6.3	0.703525	0.507351	-0.471617	1	no
gi|646748002|gb|KK962178.1|	19572-32135	22	6.3	0.303528	3	3	0.08265	no
gi|646748002|gb|KK962178.1|	52950-35393	22	6.3	0.126027	5	5	0.10045	no
gi|646748002|gb|KK962178.1|	87319-38883	22	6.3	1	0.316479	-2	0.2359	no
gi|646748002|gb|KK962178.1|	89435-39061	22	6.3	1	0.429271	-1	0.35685	no
gi|646748002|gb|KK962178.1|	41785-43877	22	6.3	45	50	0.138182	0.906	no
gi|646748002|gb|KK962178.1|	27033-42929	22	6.3	0.046121	4	6	0.09565	no
gi|646748002|gb|KK962178.1|	29350-43267	22	6.3	0.030176	3	7	0.09485	no
gi|646748002|gb|KK962178.1|	32789-43412	22	6.3	0	5	inf	0.00045	yes
gi|646748002|gb|KK962178.1|	43913-44498	22	6.3	310	1	-8	0.00855	no
gi|646748002|gb|KK962178.1|	45166-44781	22	6.3	1	0	-inf	0.0014	yes
gi|646748002|gb|KK962178.1|	48917-45008	22	6.3	2	0	-inf	0.0008	yes
gi|646748002|gb|KK962178.1|	51055-45232	22	6.3	18	0.260623	-6	0.10535	no
gi|646748002|gb|KK962178.1|	76472-48793	22	6.3	4	0	-inf	5.00E-05	yes
gi|646748002|gb|KK962178.1|	89183-49736	22	6.3	5	0	-inf	5.00E-05	yes
gi|646748002|gb|KK962178.1|	02755-50402	22	6.3	98	0.130437	-10	0.1665	no

gi|646748002|gb|KK962178.1|	20197-52168	22	6.3	24	12	-0.947672	0.4634	no
gi|646748002|gb|KK962178.1|	32171-53269	22	6.3	7	0	-inf	0.0004	yes
gi|646748002|gb|KK962178.1|	43573-54526	22	6.3	0.644252	0	-inf	1	no
gi|646748002|gb|KK962178.1|	46184-55167	22	6.3	2	0	-inf	5.00E-05	yes
gi|646748002|gb|KK962178.1|	52289-55301	22	6.3	21	0	-inf	0.0002	yes
gi|646748002|gb|KK962178.1|	78606-58003	22	6.3	3	2	-0.126523	0.9362	no
gi|646748002|gb|KK962178.1|	99237-60041	22	6.3	2	0.863092	-0.896866	0.50335	no
gi|646748002|gb|KK962178.1|	35943-63636	22	6.3	41	1	-5	0.11015	no
gi|646748002|gb|KK962178.1|	79120-68020	22	6.3	0	2	inf	0.001	yes
gi|646748002|gb|KK962178.1|	93012-69331	22	6.3	0	223	inf	0.00015	yes
gi|646748002|gb|KK962178.1|	96399-69855	22	6.3	0.243467	8	5	0.0609	no
gi|646748002|gb|KK962178.1|	02966-70471	22	6.3	0.619244	0.715938	0.209327	1	no
gi|646748002|gb|KK962178.1|	05033-70574	22	6.3	0.407364	2	2	0.28375	no
gi|646748002|gb|KK962178.1|	09404-71094	22	6.3	202	12	-4	0.01035	no
gi|646748002|gb|KK962178.1|	31390-73246	22	6.3	95	1	-6	0.0049	no
gi|646748002|gb|KK962178.1|	32852-73421	22	6.3	3	0.239891	-3	0.1371	no
gi|646748002|gb|KK962178.1|	36607-73734	22	6.3	2	1	-0.360633	0.812	no
gi|646748002|gb|KK962178.1|	47936-74905	22	6.3	18	0.45878	-5	0.07615	no
gi|646748002|gb|KK962178.1|	52565-75308	22	6.3	4	0	-inf	0.00475	no
gi|646748002|gb|KK962178.1|	86871-88835	22	6.3	0.828022	0.759105	-0.125369	1	no
gi|646748002|gb|KK962178.1|	88536-88952	22	6.3	2	2	0.0617976	0.95485	no
gi|646748002|gb|KK962178.1|	89758-89074	22	6.3	0.631856	1	0.772466	1	no
gi|646748002|gb|KK962178.1|	92168-89239	22	6.3	20	29	0.526952	0.73335	no
gi|646748002|gb|KK962178.1|	94642-89571	22	6.3	6	8	0.26109	0.8635	no
gi|646748002|gb|KK962178.1|	97430-89908	22	6.3	2	0.859262	-0.885216	0.54695	no
gi|646748002|gb|KK962178.1|	00022-90100	22	6.3	2	0.544718	-2	0.2858	no
gi|646748002|gb|KK962178.1|	01202-90273	22	6.3	2	1	-0.8509	0.5532	no
gi|646748002|gb|KK962178.1|	04283-90534	22	6.3	1	1	-0.00128605	0.9367	no
gi|646748002|gb|KK962178.1|	08651-91038	22	6.3	4	0.361305	-3	0.0675	no
gi|646748002|gb|KK962178.1|	13750-91757	22	6.3	88	57	-0.636526	0.59845	no

gi|646748002|gb|KK962178.1|	17829-91946	22	6.3	1	2	0.596592	0.6597	no
gi|646748002|gb|KK962178.1|	21250-92149	22	6.3	134.53	199	0.561669	0.71215	no
gi|646748002|gb|KK962178.1|	24971-92550	22	6.3	1	3	1	0.4404	no
gi|646748002|gb|KK962178.1|	26347-92971	22	6.3	0.564513	0.735553	0.381824	1	no
gi|646748002|gb|KK962178.1|	30464-93092	22	6.3	6	8	0.300401	0.79895	no
gi|646748002|gb|KK962178.1|	36288-93649	22	6.3	91	207	1	0.41	no
gi|646748002|gb|KK962178.1|	36787-93703	22	6.3	19	29	0.633124	0.64875	no
gi|646748002|gb|KK962178.1|	41748-94358	22	6.3	0.46725	0.675826	0.532456	1	no
gi|646748002|gb|KK962178.1|	44056-94486	22	6.3	2	8	2	0.2087	no
gi|646748002|gb|KK962178.1|	46282-94855	22	6.3	0.641848	0.532075	-0.270603	1	no
gi|646748002|gb|KK962178.1|	50149-95139	22	6.3	0.8393	0.936462	0.158034	1	no
gi|646748002|gb|KK962178.1|	54415-95603	22	6.3	2	3	0.44432	0.773	no
gi|646748002|gb|KK962178.1|	96819-97063	22	6.3	22	26	0.285272	0.8462	no
gi|646748014|gb|KK962177.1|	54596-15473	15	6.4	3259.36	3734.87	0.196471	0.89825	no
gi|646748014|gb|KK962177.1|	15334-21547	15	6.4	536	708	0.400679	0.80065	no
gi|646748014|gb|KK962177.1|	18167-21963	15	6.4	2	5	1	0.37615	no
gi|646748014|gb|KK962177.1|	23469-22367	15	6.4	59	49	-0.270447	0.84035	no
gi|646748014|gb|KK962177.1|	26744-22784	15	6.4	2	2	0.707897	0.61375	no
gi|646748014|gb|KK962177.1|	27991-22944	15	6.4	1	0.44334	-2	0.2519	no
gi|646748014|gb|KK962177.1|	29567-23083	15	6.4	1	0.654702	-0.616214	1	no
gi|646748014|gb|KK962177.1|	36229-23714	15	6.4	1	1	0.142165	0.9186	no
gi|646748014|gb|KK962177.1|	37657-23894	15	6.4	0.717828	2	1	0.3239	no
gi|646748014|gb|KK962177.1|	46483-24673	15	6.4	21	43	1	0.4977	no
gi|646748014|gb|KK962177.1|	52106-25402	15	6.4	8	7	-0.206717	0.8887	no
gi|646748014|gb|KK962177.1|	61917-26559	15	6.4	11	13	0.228999	0.83605	no
gi|646748014|gb|KK962177.1|	65727-27044	15	6.4	30	35	0.236535	0.84515	no
gi|646748014|gb|KK962177.1|	26608-26843	15	6.4	29	34.32	0.230571	0.8672	no
gi|646748014|gb|KK962177.1|	84798-28507	15	6.4	677.77	384	-0.820091	0.4486	no
gi|646748014|gb|KK962177.1|	94668-29546	15	6.4	8	10	0.365982	0.81445	no
gi|646748014|gb|KK962177.1|	09184-30962	15	6.4	4	2	-1	0.45555	no

gi|646748014|gb|KK962177.1|	09878-31054	15	6.4	39	13	-2	0.32395	no
gi|646748014|gb|KK962177.1|	23381-32482	15	6.4	13	7	-0.8835	0.5681	no
gi|646748014|gb|KK962177.1|	24927-32823	15	6.4	9	4	-1	0.33035	no
gi|646748014|gb|KK962177.1|	33563-34121	15	6.4	6	8	0.524899	0.70395	no
gi|646748014|gb|KK962177.1|	26219-42647	15	6.4	75	25	-2	0.29955	no
gi|646748014|gb|KK962177.1|	46604-44687	15	6.4	23	18	-0.405871	0.77105	no
gi|646748014|gb|KK962177.1|	47234-44778	15	6.4	4	3	-0.136271	0.9271	no
gi|646748014|gb|KK962177.1|	44865-46138	15	6.4	1	2	0.405934	0.74905	no
gi|646748014|gb|KK962177.1|	49499-44973	15	6.4	33	35.15	0.0787785	0.9412	no
gi|646748014|gb|KK962177.1|	50093-45148	15	6.4	3	2	-0.259756	0.86925	no
gi|646748014|gb|KK962177.1|	57850-45859	15	6.4	7	2	-2	0.19775	no
gi|646748014|gb|KK962177.1|	62163-46315	15	6.4	30	10	-2	0.2197	no
gi|646748014|gb|KK962177.1|	09295-51257	15	6.4	36	17	-1	0.35175	no
gi|646748014|gb|KK962177.1|	27660-52899	15	6.4	1	1	-0.306815	0.8119	no
gi|646748014|gb|KK962177.1|	52956-53300	15	6.4	5	10	0.890477	0.5478	no
gi|646748014|gb|KK962177.1|	33004-53429	15	6.4	8	4	-1	0.4874	no
gi|646748014|gb|KK962177.1|	61579-61787	15	6.4	20	70	2	0.24645	no
gi|646748014|gb|KK962177.1|	62611-64015	15	6.4	2	2	0.00136403	0.9741	no
gi|646748014|gb|KK962177.1|	71736-73187	15	6.4	15	7	-1	0.4916	no
gi|646748014|gb|KK962177.1|	74268-77702	15	6.4	20	18	-0.182599	0.86985	no
gi|646748014|gb|KK962177.1|	89873-90103	15	6.4	0	46	inf	0.0014	yes
gi|646748014|gb|KK962177.1|	96942-97182	15	6.4	103	217.93	1	0.48925	no
gi|646748030|gb|KK962176.1|	99948-40090	14	7.3	1	0.94189	-0.630139	0.65905	no
gi|646748030|gb|KK962176.1|	11121-41203	14	7.3	4	7	0.767483	0.62325	no
gi|646748030|gb|KK962176.1|	12082-41282	14	7.3	4	7	0.672583	0.6786	no
gi|646748030|gb|KK962176.1|	40307-44302	14	7.3	9	5	-1	0.42165	no
gi|646748044|gb|KK962175.1|	10225-10991	19	7.2	1	1	0.00572834	0.92345	no
gi|646748044|gb|KK962175.1|	1403-1844	19	7.2	4	12	2	0.3447	no
gi|646748044|gb|KK962175.1|	14599-15696	19	7.2	34	46	0.414128	0.70455	no
gi|646748044|gb|KK962175.1|	27915-22886	19	7.2	8	7	-0.246123	0.86905	no

gi|646748044|gb|KK962175.1|	36858-23792	19	7.2	1	1	-0.264129	0.8336	no
gi|646748044|gb|KK962175.1|	54591-25490	19	7.2	6	15	1	0.40665	no
gi|646748044|gb|KK962175.1|	66858-26739	19	7.2	7	0.443907	-4	0.1707	no
gi|646748044|gb|KK962175.1|	27159-27384	19	7.2	228	9	-5	0.11205	no
gi|646748044|gb|KK962175.1|	84368-28519	19	7.2	4	0.456623	-3	0.15655	no
gi|646748044|gb|KK962175.1|	85302-28632	19	7.2	6	5	-0.353542	0.8261	no
gi|646748044|gb|KK962175.1|	96627-29752	19	7.2	2	1	-0.718901	0.5855	no
gi|646748044|gb|KK962175.1|	29855-30371	19	7.2	7	3	-1	0.3349	no
gi|646748044|gb|KK962175.1|	98970-30116	19	7.2	1	2	0.188181	0.90745	no
gi|646748044|gb|KK962175.1|	01280-30238	19	7.2	1	2	0.962624	0.53735	no
gi|646748044|gb|KK962175.1|	03507-30533	19	7.2	4	3	-0.57964	0.7023	no
gi|646748044|gb|KK962175.1|	06870-30963	19	7.2	9	4	-1	0.28085	no
gi|646748044|gb|KK962175.1|	33481-34849	19	7.2	214	76	-1	0.21685	no
gi|646748044|gb|KK962175.1|	66869-36742	19	7.2	4	1	-2	0.2601	no
gi|646748044|gb|KK962175.1|	70322-37077	19	7.2	6	1	-2	0.16235	no
gi|646748044|gb|KK962175.1|	73070-37355	19	7.2	29	15	-0.888792	0.5658	no
gi|646748044|gb|KK962175.1|	80377-38111	19	7.2	2	1	-0.682967	0.6459	no
gi|646748044|gb|KK962175.1|	39448-39637	19	7.2	125	30	-2	0.2132	no
gi|646748044|gb|KK962175.1|	94718-39614	19	7.2	3	0.907432	-2	0.20885	no
gi|646748044|gb|KK962175.1|	96265-39708	19	7.2	3	0.231032	-4	0.17385	no
gi|646748044|gb|KK962175.1|	27713-42852	19	7.2	45	30	-0.607525	0.63345	no
gi|646748044|gb|KK962175.1|	43512-45040	19	7.2	38	13	-2	0.2495	no
gi|646748044|gb|KK962175.1|	62294-46260	19	7.2	17	4	-2	0.23205	no
gi|646748044|gb|KK962175.1|	64760-46512	19	7.2	4	12	2	0.26755	no
gi|646748044|gb|KK962175.1|	76629-47792	19	7.2	17	20	0.19287	0.87315	no
gi|646748044|gb|KK962175.1|	89316-49087	19	7.2	24	7	-2	0.17015	no
gi|646748044|gb|KK962175.1|	49094-50272	19	7.2	52	40	-0.381659	0.7305	no
gi|646748044|gb|KK962175.1|	98143-50360	19	7.2	3	3	-0.389148	0.79595	no
gi|646748044|gb|KK962175.1|	04288-50691	19	7.2	3	3	-0.0351389	0.98135	no
gi|646748044|gb|KK962175.1|	07113-50818	19	7.2	0.906921	0.647458	-0.486191	1	no

gi|646748044|gb|KK962175.1|	52785-53748	19	7.2	101	29	-2	0.1046	no
gi|646748044|gb|KK962175.1|	54985-55869	19	7.2	7	1	-3	0.1125	no
gi|646748044|gb|KK962175.1|	56379-57490	19	7.2	3	0.926961	-2	0.27405	no
gi|646748044|gb|KK962175.1|	65910-67065	19	7.2	3	0.588495	-3	0.15635	no
gi|646748057|gb|KK962174.1|	80594-18315	14	7.3	49	42	-0.222185	0.8484	no
gi|646748072|gb|KK962173.1|	02044-10297	27	13	0	4	inf	0.00175	no
gi|646748072|gb|KK962173.1|	03667-10401	27	13	0	15	inf	0.0008	yes
gi|646748072|gb|KK962173.1|	44234-14450	27	13	424	145	-2	0.2285	no
gi|646748072|gb|KK962173.1|	61424-16212	27	13	28	4	-3	0.05605	no
gi|646748072|gb|KK962173.1|	63099-16345	27	13	8	2	-2	0.21775	no
gi|646748072|gb|KK962173.1|	53423-35511	27	13	2	0	-inf	0.0047	no
gi|646748072|gb|KK962173.1|	71698-37342	27	13	2	0.090871	-4	0.1839	no
gi|646748072|gb|KK962173.1|	74398-37544	27	13	2	0	-inf	0.00195	no
gi|646748072|gb|KK962173.1|	75566-37966	27	13	2	0	-inf	0.00065	yes
gi|646748072|gb|KK962173.1|	89442-39051	27	13	2	0	-inf	0.0012	yes
gi|646748072|gb|KK962173.1|	92282-39257	27	13	271	136	-0.992323	0.51535	no
gi|646748072|gb|KK962173.1|	41638-44255	27	13	1	0.592265	-1	0.4766	no
gi|646748072|gb|KK962173.1|	62523-46297	27	13	3	4	0.626742	0.66265	no
gi|646748072|gb|KK962173.1|	63781-46518	27	13	2	1	-0.138159	0.90355	no
gi|646748072|gb|KK962173.1|	58447-55940	27	13	0	4	inf	0.00315	no
gi|646748072|gb|KK962173.1|	64890-56551	27	13	0	4	inf	0.001	yes
gi|646748072|gb|KK962173.1|	86207-86778	27	13	0	10	inf	0.00155	yes
gi|646748072|gb|KK962173.1|	92789-93234	27	13	0	9	inf	0.00605	no
gi|646748086|gb|KK962172.1|	54021-35773	20	10	3	0.390137	-3	0.0784	no
gi|646748086|gb|KK962172.1|	59298-36178	20	10	9	6	-0.670801	0.59655	no
gi|646748086|gb|KK962172.1|	63165-36601	20	10	81	72	-0.175701	0.8818	no
gi|646748086|gb|KK962172.1|	77234-37973	20	10	21	18	-0.245761	0.8234	no
gi|646748086|gb|KK962172.1|	82217-38288	20	10	2	0.617307	-2	0.38325	no
gi|646748086|gb|KK962172.1|	88919-38972	20	10	2	5	1	0.4631	no
gi|646748086|gb|KK962172.1|	95899-39662	20	10	0.99013	1	0.485428	0.7568	no

gi|646748086|gb|KK962172.1|	97250-39780	20	10	2	3	0.712046	0.64515	no
gi|646748086|gb|KK962172.1|	02044-40727	20	10	14	5	-1	0.22825	no
gi|646748086|gb|KK962172.1|	57872-45865	20	10	3	4	0.586551	0.6691	no
gi|646748086|gb|KK962172.1|	29969-53168	20	10	1	1	0.11577	0.93125	no
gi|646748086|gb|KK962172.1|	32598-53384	20	10	0.747551	0.938312	0.327895	1	no
gi|646748086|gb|KK962172.1|	34333-53548	20	10	0.720305	0.589134	-0.290014	1	no
gi|646748086|gb|KK962172.1|	35773-53746	20	10	12	11	-0.141333	0.92435	no
gi|646748086|gb|KK962172.1|	47166-54796	20	10	4	3	-0.443767	0.74595	no
gi|646748086|gb|KK962172.1|	51658-55194	20	10	10	14	0.424773	0.7548	no
gi|646748086|gb|KK962172.1|	52926-55443	20	10	0.442999	0.851223	0.942235	1	no
gi|646748086|gb|KK962172.1|	60839-56716	20	10	2	2	-0.0879633	0.95495	no
gi|646748086|gb|KK962172.1|	67427-56836	20	10	4	6	0.70583	0.6602	no
gi|646748086|gb|KK962172.1|	69435-57055	20	10	0.64241	0.765495	0.252899	1	no
gi|646748086|gb|KK962172.1|	80997-58436	20	10	5	4	-0.416145	0.78725	no
gi|646748086|gb|KK962172.1|	92012-59320	20	10	6	5	-0.269602	0.8575	no
gi|646748086|gb|KK962172.1|	35514-63612	20	10	16	4	-2	0.14945	no
gi|646748086|gb|KK962172.1|	74042-67479	20	10	5	4	-0.317662	0.84755	no
gi|646748086|gb|KK962172.1|	92420-69310	20	10	8	9	0.052962	0.97185	no
gi|646748086|gb|KK962172.1|	99101-69940	20	10	15	24	0.688277	0.64735	no
gi|646748086|gb|KK962172.1|	03355-70354	20	10	68	132	0.948423	0.50755	no
gi|646748086|gb|KK962172.1|	04275-70473	20	10	17	6	-1	0.31695	no
gi|646748086|gb|KK962172.1|	07885-70827	20	10	8	3	-1	0.40155	no
gi|646748086|gb|KK962172.1|	11976-71223	20	10	13	17	0.387719	0.8017	no
gi|646748086|gb|KK962172.1|	17546-71809	20	10	8	15	0.950545	0.5509	no
gi|646748086|gb|KK962172.1|	18309-71868	20	10	23	27	0.245549	0.87275	no
gi|646748086|gb|KK962172.1|	21120-72136	20	10	13	23	0.896577	0.58765	no
gi|646748086|gb|KK962172.1|	23729-72411	20	10	10	13	0.357946	0.7993	no
gi|646748086|gb|KK962172.1|	24711-72503	20	10	7	7	0.00862496	0.91525	no
gi|646748086|gb|KK962172.1|	32934-73395	20	10	0.603029	1	0.774405	1	no
gi|646748086|gb|KK962172.1|	34131-73455	20	10	4	6	0.556296	0.6983	no

gi|646748086|gb|KK962172.1|	34668-73672	20	10	1	1	-0.132583	0.93545	no
gi|646748086|gb|KK962172.1|	38898-73927	20	10	40	9	-2	0.14965	no
gi|646748086|gb|KK962172.1|	52463-75544	20	10	1	1	0.403328	0.8004	no
gi|646748086|gb|KK962172.1|	55924-76109	20	10	1	1	0.418284	0.7784	no
gi|646748086|gb|KK962172.1|	61613-76711	20	10	1	1	0.117609	0.9372	no
gi|646748086|gb|KK962172.1|	74261-77575	20	10	14	15	0.0760811	0.9521	no
gi|646748086|gb|KK962172.1|	84983-78574	20	10	1	0.763232	-0.923882	0.5894	no
gi|646748086|gb|KK962172.1|	96814-79701	20	10	129	27	-2	0.1496	no
gi|646748086|gb|KK962172.1|	97888-79863	20	10	2	1	-0.970753	0.54505	no
gi|646748086|gb|KK962172.1|	99703-80191	20	10	0	2	inf	0.0017	no
gi|646748086|gb|KK962172.1|	02910-80494	20	10	3	2	-0.813319	0.60035	no
gi|646748086|gb|KK962172.1|	05487-80757	20	10	1	1	-0.10866	0.9428	no
gi|646748086|gb|KK962172.1|	16232-81719	20	10	0.653269	1	1	0.43245	no
gi|646748086|gb|KK962172.1|	25408-82602	20	10	62	59	-0.083239	0.93715	no
gi|646748086|gb|KK962172.1|	32534-83359	20	10	638	331	-0.945131	0.4308	no
gi|646748086|gb|KK962172.1|	36747-83699	20	10	384	535	0.478281	0.69825	no
gi|646748086|gb|KK962172.1|	38047-83822	20	10	159.7	136	-0.236949	0.85725	no
gi|646748086|gb|KK962172.1|	39673-84022	20	10	11	11	0.0204873	0.98315	no
gi|646748086|gb|KK962172.1|	42541-84312	20	10	24	18	-0.445676	0.7694	no
gi|646748086|gb|KK962172.1|	43270-84405	20	10	2	1	-0.643843	0.65475	no
gi|646748086|gb|KK962172.1|	48908-84964	20	10	2	0.800699	-0.92701	0.58905	no
gi|646748086|gb|KK962172.1|	50986-85123	20	10	209	186	-0.164486	0.9149	no
gi|646748086|gb|KK962172.1|	51815-85218	20	10	114	142	0.314539	0.80215	no
gi|646748086|gb|KK962172.1|	52714-85441	20	10	2	3	0.874524	0.58015	no
gi|646748086|gb|KK962172.1|	64767-87180	20	10	124	58	-1	0.3634	no
gi|646748086|gb|KK962172.1|	76265-87784	20	10	2	0.602514	-2	0.24485	no
gi|646748086|gb|KK962172.1|	86365-88660	20	10	15	20	0.355662	0.81125	no
gi|646748086|gb|KK962172.1|	91198-89743	20	10	5	5	0.0553392	0.9598	no
gi|646748086|gb|KK962172.1|	97630-89942	20	10	8	5	-0.561223	0.7094	no
gi|646748086|gb|KK962172.1|	48773-94924	20	10	2	3	0.155185	0.9062	no

gi|646748086|gb|KK962172.1|	49891-95023	20	10	13	14	0.104387	0.93725	no
gi|646748086|gb|KK962172.1|	60504-96089	20	10	11	9	-0.267268	0.8165	no
gi|646748103|gb|KK962171.1|	21893-12385	23	10	0.973856	3	2	0.2402	no
gi|646748103|gb|KK962171.1|	26876-12808	23	10	0.680212	0.417816	-0.703114	1	no
gi|646748103|gb|KK962171.1|	33999-13479	23	10	0.68898	3	2	0.2413	no
gi|646748103|gb|KK962171.1|	38982-14060	23	10	0.338631	1	2	0.24075	no
gi|646748103|gb|KK962171.1|	44206-14483	23	10	0.750907	3	2	0.1897	no
gi|646748103|gb|KK962171.1|	30078-33579	23	10	3	3	0.0342401	0.9842	no
gi|646748103|gb|KK962171.1|	33709-34450	23	10	3	2	-0.511972	0.68925	no
gi|646748103|gb|KK962171.1|	43918-46908	23	10	26	21	-0.296421	0.79715	no
gi|646748103|gb|KK962171.1|	07604-60818	23	10	46	115	1	0.31345	no
gi|646748103|gb|KK962171.1|	10653-61131	23	10	53	132	1	0.23615	no
gi|646748103|gb|KK962171.1|	65251-67263	23	10	0.157968	1	3	0.1631	no
gi|646748103|gb|KK962171.1|	67683-70926	23	10	5	15	2	0.2474	no
gi|646748103|gb|KK962171.1|	73874-74386	23	10	6	6	-0.0348914	0.96325	no
gi|646748103|gb|KK962171.1|	78602-79156	23	10	1	6	2	0.1846	no
gi|646748103|gb|KK962171.1|	82771-84439	23	10	5	14	1	0.2497	no
gi|646748103|gb|KK962171.1|	84544-86208	23	10	3	13	2	0.13515	no
gi|646748116|gb|KK962170.1|	04539-10486	20	8.3	2	12	2	0.2808	no
gi|646748116|gb|KK962170.1|	40810-14144	20	8.3	10	11	0.156414	0.9188	no
gi|646748116|gb|KK962170.1|	44252-14468	20	8.3	0	34	inf	0.00015	yes
gi|646748116|gb|KK962170.1|	51991-15879	20	8.3	3	4	0.58888	0.5865	no
gi|646748116|gb|KK962170.1|	17559-31866	20	8.3	0.765422	1	0.877735	0.5385	no
gi|646748116|gb|KK962170.1|	19714-32093	20	8.3	2	2	-0.364802	0.78625	no
gi|646748116|gb|KK962170.1|	45573-34612	20	8.3	0	11	inf	0.00095	yes
gi|646748116|gb|KK962170.1|	59750-36083	20	8.3	0	2	inf	0.0008	yes
gi|646748116|gb|KK962170.1|	61004-36126	20	8.3	0	27	inf	0.00255	no
gi|646748116|gb|KK962170.1|	63889-36622	20	8.3	0	5	inf	0.00025	yes
gi|646748116|gb|KK962170.1|	66464-36752	20	8.3	0	14	inf	0.0002	yes
gi|646748116|gb|KK962170.1|	87253-38875	20	8.3	0.965997	0.428298	-1	1	no

gi|646748116|gb|KK962170.1|	95342-39577	20	8.3	2	6	2	0.35625	no
gi|646748116|gb|KK962170.1|	14223-41451	20	8.3	5	15	2	0.29085	no
gi|646748116|gb|KK962170.1|	15263-41560	20	8.3	12	17	0.518181	0.6796	no
gi|646748116|gb|KK962170.1|	16633-41698	20	8.3	9	11	0.309522	0.8205	no
gi|646748116|gb|KK962170.1|	17981-41881	20	8.3	2	6	2	0.28965	no
gi|646748116|gb|KK962170.1|	30078-43086	20	8.3	15	9	-0.752535	0.62005	no
gi|646748116|gb|KK962170.1|	33578-43406	20	8.3	39	16	-1	0.39335	no
gi|646748116|gb|KK962170.1|	68933-46945	20	8.3	15	8	-0.899507	0.5761	no
gi|646748116|gb|KK962170.1|	75571-47588	20	8.3	26	15	-0.813587	0.51565	no
gi|646748116|gb|KK962170.1|	07525-51034	20	8.3	10	5	-0.865893	0.49355	no
gi|646748116|gb|KK962170.1|	10639-51220	20	8.3	8	6	-0.471746	0.75685	no
gi|646748116|gb|KK962170.1|	15241-51627	20	8.3	5	4	-0.36268	0.8115	no
gi|646748116|gb|KK962170.1|	30443-53205	20	8.3	0.885626	0.688165	-0.363944	1	no
gi|646748116|gb|KK962170.1|	38635-54014	20	8.3	0.960597	0.958378	-0.00333705	1	no
gi|646748116|gb|KK962170.1|	40436-54432	20	8.3	0.917315	0.594318	-0.626181	1	no
gi|646748116|gb|KK962170.1|	50403-55392	20	8.3	3	3	-0.0422069	0.97865	no
gi|646748116|gb|KK962170.1|	54209-55766	20	8.3	3	3	-0.147484	0.9208	no
gi|646748116|gb|KK962170.1|	59696-56138	20	8.3	6	5	-0.28901	0.84455	no
gi|646748116|gb|KK962170.1|	68549-56883	20	8.3	55	57	0.069262	0.9658	no
gi|646748116|gb|KK962170.1|	70664-57180	20	8.3	1	0.892417	-0.219976	1	no
gi|646748116|gb|KK962170.1|	72022-57223	20	8.3	45	17	-1	0.3894	no
gi|646748116|gb|KK962170.1|	73609-57404	20	8.3	3	5	0.61515	0.6634	no
gi|646748116|gb|KK962170.1|	79476-80801	20	8.3	5	4	-0.144516	0.92095	no
gi|646748116|gb|KK962170.1|	83449-84327	20	8.3	4	3	-0.548604	0.69025	no
gi|646748116|gb|KK962170.1|	95435-95812	20	8.3	75	62	-0.274446	0.8563	no
gi|646748129|gb|KK962169.1|	13230-10134	21	9.8	0	36	inf	0.00035	yes
gi|646748129|gb|KK962169.1|	16678-10212	21	9.8	9	92	3	0.00995	no
gi|646748129|gb|KK962169.1|	01770-10237	21	9.8	6	6	-0.0591356	0.96665	no
gi|646748129|gb|KK962169.1|	02868-10513	21	9.8	0.367864	0.466938	0.34406	1	no
gi|646748129|gb|KK962169.1|	30096-10304	21	9.8	0	246.42	inf	5.00E-05	yes

gi|646748129|gb|KK962169.1|	31463-10317	21	9.8	0	85	inf	0.00045	yes
gi|646748129|gb|KK962169.1|	32014-10329	21	9.8	0	6	inf	0.00105	yes
gi|646748129|gb|KK962169.1|	36633-10368	21	9.8	0	145	inf	0.00015	yes
gi|646748129|gb|KK962169.1|	40199-10403	21	9.8	0	181	inf	0.0014	yes
gi|646748129|gb|KK962169.1|	05585-10833	21	9.8	2	4	1	0.4389	no
gi|646748129|gb|KK962169.1|	67377-10679	21	9.8	18	8	-1	0.48625	no
gi|646748129|gb|KK962169.1|	95328-10959	21	9.8	2	2	-0.221878	0.8925	no
gi|646748129|gb|KK962169.1|	98916-11036	21	9.8	16	12	-0.359595	0.7495	no
gi|646748129|gb|KK962169.1|	04613-11063	21	9.8	0.632796	1	1	0.43385	no
gi|646748129|gb|KK962169.1|	19899-11212	21	9.8	1	2	0.416075	0.7207	no
gi|646748129|gb|KK962169.1|	25171-11268	21	9.8	1	2	0.529428	0.70225	no
gi|646748129|gb|KK962169.1|	36899-12373	21	9.8	10	8	-0.200069	0.86705	no
gi|646748129|gb|KK962169.1|	24648-12522	21	9.8	3	0.396407	-3	0.2033	no
gi|646748129|gb|KK962169.1|	26793-13217	21	9.8	3	3	-0.35468	0.8138	no
gi|646748129|gb|KK962169.1|	34078-13487	21	9.8	38	28	-0.460761	0.70655	no
gi|646748129|gb|KK962169.1|	53081-15394	21	9.8	0.759435	2	0.986308	0.5626	no
gi|646748129|gb|KK962169.1|	59438-16482	21	9.8	3	2	-0.495997	0.7394	no
gi|646748129|gb|KK962169.1|	70262-17116	21	9.8	1	2	0.641661	0.62475	no
gi|646748129|gb|KK962169.1|	94592-19563	21	9.8	10	5	-1	0.4958	no
gi|646748129|gb|KK962169.1|	07294-21022	21	9.8	3	2	-0.51383	0.7291	no
gi|646748129|gb|KK962169.1|	10403-21300	21	9.8	25	19	-0.374767	0.73695	no
gi|646748129|gb|KK962169.1|	30772-23126	21	9.8	3	2	-0.798292	0.6307	no
gi|646748129|gb|KK962169.1|	33754-23642	21	9.8	44	26	-0.748358	0.5242	no
gi|646748129|gb|KK962169.1|	36593-23761	21	9.8	3	3	-0.189336	0.90635	no
gi|646748129|gb|KK962169.1|	38511-23895	21	9.8	0	7	inf	0.0014	yes
gi|646748129|gb|KK962169.1|	39977-24115	21	9.8	5	5	0.211474	0.89645	no
gi|646748129|gb|KK962169.1|	46206-24644	21	9.8	115	71	-0.687726	0.66615	no
gi|646748129|gb|KK962169.1|	47865-24855	21	9.8	2	3	0.480872	0.7265	no
gi|646748129|gb|KK962169.1|	48660-24918	21	9.8	4	4	0.149028	0.9183	no
gi|646748129|gb|KK962169.1|	52265-25352	21	9.8	0.832587	0.796401	-0.0641056	1	no

gi|646748129|gb|KK962169.1|	58575-25990	21	9.8	0.689735	1	1	0.4105	no
gi|646748129|gb|KK962169.1|	63485-26386	21	9.8	20	7	-1	0.3255	no
gi|646748129|gb|KK962169.1|	64095-26526	21	9.8	0.911909	0.870474	-0.0670882	1	no
gi|646748129|gb|KK962169.1|	68946-27014	21	9.8	4	8	0.781857	0.61035	no
gi|646748129|gb|KK962169.1|	76972-27789	21	9.8	0.553074	1	1	0.43305	no
gi|646748129|gb|KK962169.1|	92042-29249	21	9.8	88	45	-0.977994	0.5222	no
gi|646748129|gb|KK962169.1|	65514-36696	21	9.8	1	0	-inf	0.0012	yes
gi|646748129|gb|KK962169.1|	67717-36857	21	9.8	2	0	-inf	0.0035	no
gi|646748129|gb|KK962169.1|	32120-43296	21	9.8	0	2	inf	0.00255	no
gi|646748129|gb|KK962169.1|	44181-45206	21	9.8	0.120136	2	4	0.09975	no
gi|646748129|gb|KK962169.1|	54229-45453	21	9.8	26	20	-0.373409	0.75465	no
gi|646748129|gb|KK962169.1|	89740-49030	21	9.8	7	9	0.454197	0.7746	no
gi|646748129|gb|KK962169.1|	91359-49311	21	9.8	0.432025	0.891981	1	1	no
gi|646748129|gb|KK962169.1|	93800-49563	21	9.8	0.52799	0.593936	0.169798	1	no
gi|646748129|gb|KK962169.1|	13995-51506	21	9.8	1	2	0.513715	0.6858	no
gi|646748129|gb|KK962169.1|	27885-53115	21	9.8	3	3	0.117445	0.93685	no
gi|646748129|gb|KK962169.1|	33549-53649	21	9.8	1	2	0.583753	0.71665	no
gi|646748129|gb|KK962169.1|	39756-53996	21	9.8	45	44	-0.0518506	0.9297	no
gi|646748129|gb|KK962169.1|	43034-54419	21	9.8	1	0.729293	-0.619818	0.66065	no
gi|646748129|gb|KK962169.1|	62364-56452	21	9.8	0.872348	0.772892	-0.174638	1	no
gi|646748129|gb|KK962169.1|	64648-56831	21	9.8	49	37	-0.40457	0.73165	no
gi|646748129|gb|KK962169.1|	69483-56997	21	9.8	2	5	2	0.2822	no
gi|646748129|gb|KK962169.1|	73819-57463	21	9.8	3	2	-0.503947	0.68985	no
gi|646748129|gb|KK962169.1|	74934-57704	21	9.8	3	2	-0.464655	0.76615	no
gi|646748129|gb|KK962169.1|	81922-58355	21	9.8	0.671365	0.678337	0.0149049	1	no
gi|646748129|gb|KK962169.1|	83649-58581	21	9.8	0.970292	1	0.535838	0.6982	no
gi|646748129|gb|KK962169.1|	03758-60848	21	9.8	24	24	0.0473012	0.96715	no
gi|646748129|gb|KK962169.1|	08799-61542	21	9.8	104	70	-0.565367	0.6407	no
gi|646748129|gb|KK962169.1|	20765-62490	21	9.8	7	11	0.637128	0.5629	no
gi|646748129|gb|KK962169.1|	25076-63176	21	9.8	1	0.294216	-2	0.13485	no

gi|646748129|gb|KK962169.1|	31944-64703	21	9.8	2	1	-1	0.42395	no
gi|646748129|gb|KK962169.1|	56498-65883	21	9.8	4	2	-1	0.3607	no
gi|646748129|gb|KK962169.1|	78162-67841	21	9.8	0	36	inf	0.0008	yes
gi|646748129|gb|KK962169.1|	79249-67953	21	9.8	0	33	inf	0.00435	no
gi|646748129|gb|KK962169.1|	81065-68178	21	9.8	0	16	inf	0.0003	yes
gi|646748129|gb|KK962169.1|	82533-68312	21	9.8	2	6	1	0.4458	no
gi|646748129|gb|KK962169.1|	87218-68990	21	9.8	8	8	0.0154286	0.9883	no
gi|646748129|gb|KK962169.1|	90505-69461	21	9.8	0.672361	0.910444	0.437334	1	no
gi|646748129|gb|KK962169.1|	96766-69851	21	9.8	2	1	-0.516093	0.74705	no
gi|646748129|gb|KK962169.1|	98642-69983	21	9.8	1	1	-0.0662107	0.9531	no
gi|646748129|gb|KK962169.1|	00552-70191	21	9.8	1	0.481058	-2	0.2986	no
gi|646748129|gb|KK962169.1|	08056-70855	21	9.8	14	7	-1	0.45685	no
gi|646748129|gb|KK962169.1|	13312-71430	21	9.8	7	4	-0.627841	0.6778	no
gi|646748129|gb|KK962169.1|	21540-72289	21	9.8	1	2	0.704255	0.62675	no
gi|646748129|gb|KK962169.1|	54542-75526	21	9.8	0	3	inf	0.001	yes
gi|646748129|gb|KK962169.1|	62972-76661	21	9.8	3	3	-0.378283	0.80305	no
gi|646748129|gb|KK962169.1|	66839-76836	21	9.8	0.801294	0.420013	-0.931898	1	no
gi|646748129|gb|KK962169.1|	05842-80671	21	9.8	0.744764	1	0.764896	0.61475	no
gi|646748129|gb|KK962169.1|	15338-81579	21	9.8	6	6	0.0270368	0.9505	no
gi|646748129|gb|KK962169.1|	39384-84006	21	9.8	3	4	0.479721	0.7091	no
gi|646748129|gb|KK962169.1|	19277-91969	21	9.8	2	6	2	0.2461	no
gi|646748142|gb|KK962168.1|	72933-17415	20	9.3	19	23	0.25682	0.8406	no
gi|646748142|gb|KK962168.1|	75834-17731	20	9.3	5	6	0.220604	0.882	no
gi|646748142|gb|KK962168.1|	84800-18525	20	9.3	0.454879	62	7	0.0948	no
gi|646748142|gb|KK962168.1|	97609-19832	20	9.3	0.201032	26	7	0.09495	no
gi|646748142|gb|KK962168.1|	20094-22284	20	9.3	9	7	-0.307018	0.80575	no
gi|646748142|gb|KK962168.1|	28801-22968	20	9.3	1	2	0.350335	0.794	no
gi|646748142|gb|KK962168.1|	29856-23347	20	9.3	5	3	-0.694528	0.64725	no
gi|646748142|gb|KK962168.1|	58850-25939	20	9.3	8	5	-0.667433	0.62895	no
gi|646748142|gb|KK962168.1|	68574-27192	20	9.3	2	0.347806	-2	0.11735	no

gi|646748142|gb|KK962168.1|	72142-27324	20	9.3	29	3	-3	0.0423	no
gi|646748142|gb|KK962168.1|	86617-28690	20	9.3	43	0	-inf	0.0042	no
gi|646748142|gb|KK962168.1|	87401-28800	20	9.3	5	2	-1	0.3215	no
gi|646748142|gb|KK962168.1|	89784-29078	20	9.3	31	2	-4	0.007	no
gi|646748142|gb|KK962168.1|	03511-30374	20	9.3	24	25	0.0415255	0.91805	no
gi|646748142|gb|KK962168.1|	04931-30561	20	9.3	0.429028	3	3	0.2662	no
gi|646748142|gb|KK962168.1|	06215-30665	20	9.3	6	5	-0.185582	0.893	no
gi|646748142|gb|KK962168.1|	15061-31579	20	9.3	5	3	-0.455919	0.7403	no
gi|646748142|gb|KK962168.1|	25387-32644	20	9.3	0.927373	0.496248	-0.90209	1	no
gi|646748142|gb|KK962168.1|	27004-32727	20	9.3	22	2	-3	0.1901	no
gi|646748142|gb|KK962168.1|	29511-32981	20	9.3	16	12.97	-0.346001	0.7982	no
gi|646748142|gb|KK962168.1|	30518-33072	20	9.3	76	93	0.295309	0.81745	no
gi|646748142|gb|KK962168.1|	36407-33818	20	9.3	3	2	-0.611327	0.6899	no
gi|646748142|gb|KK962168.1|	39950-34138	20	9.3	11	4	-1	0.37145	no
gi|646748142|gb|KK962168.1|	41497-34499	20	9.3	1	0.955261	-0.291289	0.84775	no
gi|646748142|gb|KK962168.1|	53581-37150	20	9.3	3	3	0.0648249	0.95505	no
gi|646748142|gb|KK962168.1|	71699-37492	20	9.3	2	2	-0.0525637	0.9701	no
gi|646748142|gb|KK962168.1|	76037-37637	20	9.3	8	16	0.940221	0.48355	no
gi|646748142|gb|KK962168.1|	77910-37816	20	9.3	15	19	0.353067	0.8076	no
gi|646748142|gb|KK962168.1|	83150-38362	20	9.3	8	5	-0.832827	0.511	no
gi|646748142|gb|KK962168.1|	85621-38619	20	9.3	6	6	0.0644332	0.9611	no
gi|646748142|gb|KK962168.1|	96909-39750	20	9.3	3	1	-2	0.34985	no
gi|646748142|gb|KK962168.1|	99267-39965	20	9.3	19	7	-1	0.3622	no
gi|646748142|gb|KK962168.1|	12810-41344	20	9.3	45	36	-0.326663	0.8323	no
gi|646748142|gb|KK962168.1|	14603-41545	20	9.3	3	2	-0.940711	0.47075	no
gi|646748142|gb|KK962168.1|	22543-42451	20	9.3	13	8	-0.785009	0.53055	no
gi|646748142|gb|KK962168.1|	27614-42787	20	9.3	86	110	0.364643	0.8068	no
gi|646748142|gb|KK962168.1|	30665-43094	20	9.3	7	16	1	0.44765	no
gi|646748142|gb|KK962168.1|	45778-44873	20	9.3	23	9	-1	0.2298	no
gi|646748142|gb|KK962168.1|	48852-45042	20	9.3	2	2	0.220278	0.89215	no

gi|646748142|gb|KK962168.1|	58029-46005	20	9.3	1	1	0.32309	0.83695	no
gi|646748142|gb|KK962168.1|	60170-46236	20	9.3	2	2	0.320358	0.8386	no
gi|646748142|gb|KK962168.1|	63217-46383	20	9.3	7	5	-0.426351	0.75475	no
gi|646748142|gb|KK962168.1|	74469-47482	20	9.3	55	36	-0.613334	0.6808	no
gi|646748142|gb|KK962168.1|	77339-47792	20	9.3	16	11	-0.565217	0.70885	no
gi|646748142|gb|KK962168.1|	90913-49248	20	9.3	1	0.710823	-0.834206	0.519	no
gi|646748142|gb|KK962168.1|	92639-49302	20	9.3	38	5	-3	0.08055	no
gi|646748142|gb|KK962168.1|	83615-68400	20	9.3	8	0.816339	-3	0.1825	no
gi|646748142|gb|KK962168.1|	00219-70122	20	9.3	4	0.698263	-3	0.15075	no
gi|646748156|gb|KK962167.1|	27650-28411	14	7.8	12	7	-0.896469	0.5586	no
gi|646748156|gb|KK962167.1|	28663-29614	14	7.8	5	0.566708	-3	0.11105	no
gi|646748156|gb|KK962167.1|	95014-39590	14	7.8	0.727204	2	2	0.33695	no
gi|646748172|gb|KK962166.1|	23502-25870	14	9.7	7	4	-0.908342	0.54655	no
gi|646748172|gb|KK962166.1|	62877-63763	14	9.7	10	7	-0.393513	0.7917	no
gi|646748185|gb|KK962165.1|	18119-23216	22	9.9	9	6	-0.472838	0.6684	no
gi|646748185|gb|KK962165.1|	31431-32522	22	9.9	0.663922	0.632372	-0.070239	1	no
gi|646748185|gb|KK962165.1|	85242-48665	22	9.9	226	118	-0.934246	0.43605	no
gi|646748185|gb|KK962165.1|	88113-48975	22	9.9	3	4	0.596691	0.69675	no
gi|646748185|gb|KK962165.1|	08947-50949	22	9.9	26	27	0.0610755	0.9672	no
gi|646748185|gb|KK962165.1|	10590-51110	22	9.9	66	76	0.189057	0.8812	no
gi|646748185|gb|KK962165.1|	93949-59516	22	9.9	4	5	0.441651	0.7744	no
gi|646748185|gb|KK962165.1|	09271-61036	22	9.9	0.879967	0.62871	-0.485055	1	no
gi|646748185|gb|KK962165.1|	19603-62012	22	9.9	3	6	1	0.3942	no
gi|646748185|gb|KK962165.1|	22701-62293	22	9.9	25	15	-0.743008	0.6194	no
gi|646748185|gb|KK962165.1|	35380-63597	22	9.9	3	1	-1	0.4217	no
gi|646748185|gb|KK962165.1|	6361-6938	22	9.9	17	20	0.198161	0.891	no
gi|646748185|gb|KK962165.1|	37701-63829	22	9.9	24	10	-1	0.4	no
gi|646748185|gb|KK962165.1|	58432-66031	22	9.9	19	23	0.263907	0.8099	no
gi|646748185|gb|KK962165.1|	61343-66167	22	9.9	7	6	-0.199875	0.8947	no
gi|646748185|gb|KK962165.1|	79496-68095	22	9.9	5	0.994267	-2	0.10205	no

gi|646748185|gb|KK962165.1|	81094-68238	22	9.9	0.538371	0.901791	0.744192	1	no
gi|646748185|gb|KK962165.1|	85209-68603	22	9.9	10	4	-1	0.367	no
gi|646748185|gb|KK962165.1|	90133-69039	22	9.9	48	29	-0.720246	0.55605	no
gi|646748185|gb|KK962165.1|	94289-69529	22	9.9	17	7	-1	0.3976	no
gi|646748185|gb|KK962165.1|	85353-78639	22	9.9	0	2	inf	0.001	yes
gi|646748185|gb|KK962165.1|	93409-79433	22	9.9	0.275713	5	4	0.24765	no
gi|646748185|gb|KK962165.1|	96957-79765	22	9.9	4	4	-0.0662934	0.95625	no
gi|646748185|gb|KK962165.1|	06298-80725	22	9.9	0	3	inf	0.00035	yes
gi|646748185|gb|KK962165.1|	14224-81457	22	9.9	17	8	-1	0.40925	no
gi|646748185|gb|KK962165.1|	16127-81713	22	9.9	7	4	-0.823803	0.5996	no
gi|646748185|gb|KK962165.1|	17743-81810	22	9.9	9	5	-0.806908	0.58775	no
gi|646748185|gb|KK962165.1|	35294-83573	22	9.9	0	9	inf	0.00035	yes
gi|646748185|gb|KK962165.1|	51892-85226	22	9.9	5	5	0.0721319	0.91525	no
gi|646748185|gb|KK962165.1|	8529-9511	22	9.9	2	3	0.509139	0.725	no
gi|646748185|gb|KK962165.1|	60634-86203	22	9.9	17	16	-0.0812176	0.948	no
gi|646748185|gb|KK962165.1|	66090-86673	22	9.9	0.470273	60	7	0.2322	no
gi|646748185|gb|KK962165.1|	69048-86986	22	9.9	3	3	-0.131289	0.9126	no
gi|646748185|gb|KK962165.1|	82353-88274	22	9.9	0	23	inf	0.0012	yes
gi|646748185|gb|KK962165.1|	88228-88884	22	9.9	0.25302	6	5	0.10925	no
gi|646748185|gb|KK962165.1|	99364-90059	22	9.9	3	2	-0.579416	0.67725	no
gi|646748185|gb|KK962165.1|	03398-90366	22	9.9	8	14	0.754074	0.63005	no
gi|646748185|gb|KK962165.1|	06929-90730	22	9.9	12	18	0.578361	0.68425	no
gi|646748185|gb|KK962165.1|	07913-90814	22	9.9	29	19	-0.613725	0.6687	no
gi|646748185|gb|KK962165.1|	20916-92115	22	9.9	13	32	1	0.42505	no
gi|646748185|gb|KK962165.1|	32549-93477	22	9.9	1	0.407647	-2	0.2588	no
gi|646748185|gb|KK962165.1|	36982-93749	22	9.9	7	8	0.113471	0.93225	no
gi|646748200|gb|KK962164.1|	26251-12693	19	7.7	8	6	-0.520279	0.74315	no
gi|646748200|gb|KK962164.1|	40390-14105	19	7.7	2	5	1	0.44335	no
gi|646748200|gb|KK962164.1|	44945-14703	19	7.7	9	5	-1	0.50125	no
gi|646748200|gb|KK962164.1|	52981-15339	19	7.7	3	7	0.965287	0.53295	no

gi|646748200|gb|KK962164.1|	57608-15863	19	7.7	1	5	2	0.25165	no
gi|646748200|gb|KK962164.1|	66735-17376	19	7.7	17	8	-1	0.31165	no
gi|646748200|gb|KK962164.1|	03689-20427	19	7.7	3	2	-0.804918	0.5828	no
gi|646748200|gb|KK962164.1|	62831-26711	19	7.7	6	4	-0.570868	0.64645	no
gi|646748200|gb|KK962164.1|	82642-28298	19	7.7	167	442	1	0.30075	no
gi|646748200|gb|KK962164.1|	08889-31039	19	7.7	11	16	0.469034	0.7054	no
gi|646748200|gb|KK962164.1|	32503-32763	19	7.7	24	123	2	0.1262	no
gi|646748200|gb|KK962164.1|	56581-35696	19	7.7	9	16	0.818574	0.5743	no
gi|646748200|gb|KK962164.1|	68669-37034	19	7.7	25	23	-0.135048	0.89865	no
gi|646748200|gb|KK962164.1|	94289-39452	19	7.7	38	39	0.0531232	0.94515	no
gi|646748200|gb|KK962164.1|	98755-39920	19	7.7	29	19	-0.551417	0.72155	no
gi|646748200|gb|KK962164.1|	06361-40806	19	7.7	9	5	-1	0.49665	no
gi|646748200|gb|KK962164.1|	12097-41259	19	7.7	0.372845	28	6	0.09525	no
gi|646748200|gb|KK962164.1|	74819-47621	19	7.7	13	32	1	0.321	no
gi|646748200|gb|KK962164.1|	47527-47747	19	7.7	35	234	3	0.10205	no
gi|646748200|gb|KK962164.1|	76370-47900	19	7.7	38	47	0.317987	0.78895	no
gi|646748200|gb|KK962164.1|	82737-48438	19	7.7	0.132923	2	4	0.25535	no
gi|646748200|gb|KK962164.1|	90187-49077	19	7.7	0	14	inf	0.00055	yes
gi|646748200|gb|KK962164.1|	09177-51060	19	7.7	30	0	-inf	5.00E-05	yes
gi|646748200|gb|KK962164.1|	11954-51526	19	7.7	3	0.044097	-6	0.1675	no
gi|646748200|gb|KK962164.1|	18640-51978	19	7.7	57	0.149063	-9	0.1665	no
gi|646748200|gb|KK962164.1|	24690-52555	19	7.7	2	0	-inf	0.0041	no
gi|646748200|gb|KK962164.1|	25679-52733	19	7.7	1	0	-inf	1	no
gi|646748200|gb|KK962164.1|	27466-52871	19	7.7	1	0	-inf	1	no
gi|646748200|gb|KK962164.1|	28832-52936	19	7.7	110	0	-inf	5.00E-05	yes
gi|646748200|gb|KK962164.1|	61055-56147	19	7.7	0	37	inf	0.00045	yes
gi|646748200|gb|KK962164.1|	7891-9398	19	7.7	0	1	inf	0.00035	yes
gi|646748216|gb|KK962163.1|	15502-15973	15	5.8	12	0	-inf	0.0031	no
gi|646748216|gb|KK962163.1|	16755-17713	15	5.8	6	0	-inf	0.0015	yes
gi|646748216|gb|KK962163.1|	18634-20677	15	5.8	280	0.074959	-12	0.1665	no

gi|646748216|gb|KK962163.1|	22277-22829	15	5.8	121.57	0.423355	-8	0.1665	no
gi|646748216|gb|KK962163.1|	29161-30417	15	5.8	18	0	-inf	0.00015	yes
gi|646748216|gb|KK962163.1|	94472-29518	15	5.8	1	3	1	0.34675	no
gi|646748216|gb|KK962163.1|	95371-29585	15	5.8	0.789225	4	2	0.28615	no
gi|646748216|gb|KK962163.1|	35954-33651	15	5.8	2	3	0.642921	0.6513	no
gi|646748216|gb|KK962163.1|	41360-43348	15	5.8	303	0.773076	-9	0.00485	no
gi|646748216|gb|KK962163.1|	53500-53938	15	5.8	4	5	0.0335586	0.9365	no
gi|646748216|gb|KK962163.1|	75162-57722	15	5.8	2	0.148199	-4	0.12985	no
gi|646748216|gb|KK962163.1|	58514-59635	15	5.8	2	0.305509	-3	0.16105	no
gi|646748216|gb|KK962163.1|	61952-62283	15	5.8	12	14	0.161513	0.8986	no
gi|646748216|gb|KK962163.1|	7100-7714	15	5.8	22	0	-inf	0.0003	yes
gi|646748232|gb|KK962162.1|	13172-21387	13	9.6	13	19	0.550033	0.7094	no
gi|646748232|gb|KK962162.1|	13785-31449	13	9.6	14	15	0.1508	0.92025	no
gi|646748232|gb|KK962162.1|	06306-70663	13	9.6	5	13	1	0.39905	no
gi|646748245|gb|KK962161.1|	97724-49971	18	8.6	2	10	2	0.2023	no
gi|646748245|gb|KK962161.1|	42629-54310	18	8.6	3	2	-0.997844	0.589	no
gi|646748245|gb|KK962161.1|	67837-56964	18	8.6	200	159	-0.330073	0.7809	no
gi|646748245|gb|KK962161.1|	70860-57167	18	8.6	2	2	0.344348	0.79725	no
gi|646748245|gb|KK962161.1|	72601-57602	18	8.6	2	3	0.377417	0.80115	no
gi|646748245|gb|KK962161.1|	79264-57965	18	8.6	24	46	0.930799	0.55145	no
gi|646748245|gb|KK962161.1|	80192-58073	18	8.6	4	6	0.447391	0.72325	no
gi|646748245|gb|KK962161.1|	83645-58424	18	8.6	3	4	0.222221	0.86295	no
gi|646748245|gb|KK962161.1|	88381-58869	18	8.6	5	22	2	0.2587	no
gi|646748245|gb|KK962161.1|	91425-59194	18	8.6	128	79	-0.695405	0.51925	no
gi|646748245|gb|KK962161.1|	94442-59472	18	8.6	20	19	-0.100825	0.9401	no
gi|646748245|gb|KK962161.1|	97755-59890	18	8.6	2	1	-0.0679729	0.9524	no
gi|646748245|gb|KK962161.1|	04006-60423	18	8.6	270	148	-0.865517	0.5695	no
gi|646748245|gb|KK962161.1|	05543-60578	18	8.6	18	18	0.068007	0.90935	no
gi|646748261|gb|KK962160.1|	10546-10899	12	7.8	0	13	inf	0.001	yes
gi|646748261|gb|KK962160.1|	82933-18321	12	7.8	27	16	-0.797619	0.5433	no

gi|646748261|gb|KK962160.1|	06846-30697	12	7.8	3771.83	475	-3	0.1109	no
gi|646748261|gb|KK962160.1|	44815-45547	12	7.8	1	1	-0.0981405	0.9036	no
gi|646748295|gb|KK962158.1|	17871-11905	18	8.2	1	0.429724	-1	1	no
gi|646748295|gb|KK962158.1|	84246-18448	18	8.2	31	33	0.0716327	0.94415	no
gi|646748295|gb|KK962158.1|	98793-50282	18	8.2	0.049031	45	10	0.2322	no
gi|646748295|gb|KK962158.1|	05956-50695	18	8.2	0	27	inf	5.00E-05	yes
gi|646748295|gb|KK962158.1|	11505-51165	18	8.2	0	1242.27	inf	0.001	yes
gi|646748295|gb|KK962158.1|	82636-83118	18	8.2	8	2	-2	0.20255	no
gi|646748295|gb|KK962158.1|	83815-84152	18	8.2	9	5	-0.874136	0.5704	no
gi|646748295|gb|KK962158.1|	85227-85499	18	8.2	22	34	0.621612	0.67795	no
gi|646748295|gb|KK962158.1|	90370-90660	18	8.2	47	44	-0.106649	0.94555	no
gi|646748295|gb|KK962158.1|	93364-93539	18	8.2	148	92	-0.682069	0.6278	no
gi|646748310|gb|KK962157.1|	1-1307	23	6.3	0.264064	3	3	0.1438	no
gi|646748310|gb|KK962157.1|	01395-10272	23	6.3	0.945742	0.741296	-0.351397	1	no
gi|646748310|gb|KK962157.1|	03347-10509	23	6.3	0.681168	0.80543	0.241749	1	no
gi|646748310|gb|KK962157.1|	06530-10752	23	6.3	0.374005	1	2	0.255	no
gi|646748310|gb|KK962157.1|	11514-11573	23	6.3	37	23	-0.701029	0.54775	no
gi|646748310|gb|KK962157.1|	15938-11637	23	6.3	45	7	-3	0.06875	no
gi|646748310|gb|KK962157.1|	29246-13301	23	6.3	12	12	0.0459629	0.96725	no
gi|646748310|gb|KK962157.1|	33093-13602	23	6.3	4	3	-0.437347	0.7729	no
gi|646748310|gb|KK962157.1|	71899-17803	23	6.3	12	11	-0.134111	0.90515	no
gi|646748310|gb|KK962157.1|	83805-18457	23	6.3	2	3	0.491744	0.6943	no
gi|646748310|gb|KK962157.1|	17626-21785	23	6.3	4	50	4	0.1019	no
gi|646748310|gb|KK962157.1|	25104-22550	23	6.3	5	7	0.535238	0.70235	no
gi|646748310|gb|KK962157.1|	30384-23064	23	6.3	34	38	0.15061	0.9227	no
gi|646748310|gb|KK962157.1|	38169-38712	23	6.3	9	5	-0.952489	0.50215	no
gi|646748310|gb|KK962157.1|	47209-47716	23	6.3	7	6	-0.152427	0.8957	no
gi|646748310|gb|KK962157.1|	5063-5392	23	6.3	56	4	-4	0.0888	no
gi|646748310|gb|KK962157.1|	51584-53338	23	6.3	2	1	-0.305175	0.8216	no
gi|646748310|gb|KK962157.1|	62557-56325	23	6.3	4	3	-0.518486	0.6801	no

gi|646748310|gb|KK962157.1|	61749-65290	23	6.3	14	11	-0.319673	0.77765	no
gi|646748310|gb|KK962157.1|	6295-6568	23	6.3	130	0	-inf	0.00025	yes
gi|646748310|gb|KK962157.1|	51580-65198	23	6.3	6	4	-0.480912	0.75385	no
gi|646748310|gb|KK962157.1|	62354-66300	23	6.3	2	2	0.0587687	0.9298	no
gi|646748310|gb|KK962157.1|	19634-71997	23	6.3	79	64	-0.317576	0.8271	no
gi|646748310|gb|KK962157.1|	22852-72366	23	6.3	4	8	0.88357	0.5776	no
gi|646748310|gb|KK962157.1|	23763-72410	23	6.3	32	35	0.131774	0.9311	no
gi|646748310|gb|KK962157.1|	72777-77320	23	6.3	2	5	2	0.29775	no
gi|646748310|gb|KK962157.1|	77384-77942	23	6.3	2	2	0.450898	0.75415	no
gi|646748310|gb|KK962157.1|	78289-78683	23	6.3	3	5	0.624721	0.66895	no
gi|646748310|gb|KK962157.1|	32606-83321	23	6.3	3	4	0.338658	0.78895	no
gi|646748310|gb|KK962157.1|	38727-83926	23	6.3	5	6	0.336742	0.7785	no
gi|646748310|gb|KK962157.1|	45017-84600	23	6.3	2	0.90183	-1	0.33035	no
gi|646748310|gb|KK962157.1|	46384-84719	23	6.3	2	2	-0.281955	0.832	no
gi|646748310|gb|KK962157.1|	48355-85086	23	6.3	3	2	-1	0.45845	no
gi|646748310|gb|KK962157.1|	51063-85392	23	6.3	2	2	0.0130475	0.9864	no
gi|646748310|gb|KK962157.1|	54579-85741	23	6.3	0.968979	0.730609	-0.407366	1	no
gi|646748310|gb|KK962157.1|	57533-85910	23	6.3	3	3	-0.28712	0.85175	no
gi|646748310|gb|KK962157.1|	73696-87416	23	6.3	3	2	-0.585431	0.6685	no
gi|646748310|gb|KK962157.1|	97556-89823	23	6.3	8	4	-1	0.43725	no
gi|646748327|gb|KK962156.1|	24318-12613	19	8.9	4	0	-inf	0.0002	yes
gi|646748327|gb|KK962156.1|	27284-12845	19	8.9	5	0	-inf	0.00045	yes
gi|646748327|gb|KK962156.1|	29448-12983	19	8.9	98	0	-inf	5.00E-05	yes
gi|646748327|gb|KK962156.1|	35775-13758	19	8.9	3	0	-inf	0.00025	yes
gi|646748327|gb|KK962156.1|	39866-14087	19	8.9	2	0	-inf	0.0008	yes
gi|646748327|gb|KK962156.1|	46355-14720	19	8.9	2	0	-inf	0.0035	no
gi|646748327|gb|KK962156.1|	49512-15210	19	8.9	3	0	-inf	5.00E-05	yes
gi|646748327|gb|KK962156.1|	52424-15318	19	8.9	5	0	-inf	0.00315	no
gi|646748327|gb|KK962156.1|	54195-15489	19	8.9	6	0	-inf	0.0031	no
gi|646748327|gb|KK962156.1|	61166-16258	19	8.9	0.957019	0	-inf	1	no

gi|646748327|gb|KK962156.1|	64023-16540	19	8.9	3	0	-inf	0.0019	no
gi|646748327|gb|KK962156.1|	65532-16803	19	8.9	174	0.119491	-11	0.10535	no
gi|646748327|gb|KK962156.1|	68199-17010	19	8.9	1	0	-inf	0.00525	no
gi|646748327|gb|KK962156.1|	71291-17275	19	8.9	2	0	-inf	0.00525	no
gi|646748327|gb|KK962156.1|	73040-17403	19	8.9	2	0	-inf	0.0041	no
gi|646748327|gb|KK962156.1|	74171-17577	19	8.9	1	0.098667	-4	0.17175	no
gi|646748327|gb|KK962156.1|	75900-17651	19	8.9	3	0	-inf	0.00655	no
gi|646748327|gb|KK962156.1|	81280-18343	19	8.9	0.438933	0.353511	-0.312246	1	no
gi|646748327|gb|KK962156.1|	85161-18636	19	8.9	2	1	-0.328823	0.78865	no
gi|646748327|gb|KK962156.1|	12580-21310	19	8.9	5	0	-inf	0.0011	yes
gi|646748327|gb|KK962156.1|	13983-21583	19	8.9	3	0	-inf	0.00025	yes
gi|646748327|gb|KK962156.1|	48407-24875	19	8.9	133	0	-inf	5.00E-05	yes
gi|646748327|gb|KK962156.1|	48872-24947	19	8.9	3	0	-inf	0.0041	no
gi|646748327|gb|KK962156.1|	78971-27919	19	8.9	937	60	-4	0.00385	no
gi|646748327|gb|KK962156.1|	81910-28296	19	8.9	5	0	-inf	0.0015	yes
gi|646748327|gb|KK962156.1|	83095-28380	19	8.9	17	11	-0.643737	0.67455	no
gi|646748327|gb|KK962156.1|	03869-31073	19	8.9	12	0	-inf	5.00E-05	yes
gi|646748327|gb|KK962156.1|	10931-31297	19	8.9	3	0	-inf	0.00085	yes
gi|646748327|gb|KK962156.1|	14399-31504	19	8.9	90	0	-inf	5.00E-05	yes
gi|646748327|gb|KK962156.1|	91855-39249	19	8.9	7	10	0.518945	0.73825	no
gi|646748327|gb|KK962156.1|	21940-42239	19	8.9	58	46	-0.315167	0.83715	no
gi|646748327|gb|KK962156.1|	33638-43744	19	8.9	13	10	-0.315533	0.77325	no
gi|646748327|gb|KK962156.1|	46528-44761	19	8.9	0.781114	1	0.514408	1	no
gi|646748327|gb|KK962156.1|	49112-45046	19	8.9	1	0.484926	-1	1	no
gi|646748327|gb|KK962156.1|	50565-45260	19	8.9	20	13	-0.606552	0.574	no
gi|646748327|gb|KK962156.1|	52820-45422	19	8.9	0.967021	1	0.109865	1	no
gi|646748327|gb|KK962156.1|	61471-46248	19	8.9	0.853137	0.521016	-0.71145	1	no
gi|646748327|gb|KK962156.1|	63729-46515	19	8.9	17	11.94	-0.528499	0.67445	no
gi|646748327|gb|KK962156.1|	65627-46814	19	8.9	25	28	0.160852	0.8851	no
gi|646748327|gb|KK962156.1|	68554-47068	19	8.9	18	19	0.0736598	0.9477	no

gi|646748327|gb|KK962156.1|	72377-47271	19	8.9	6	8	0.522415	0.7234	no
gi|646748327|gb|KK962156.1|	72955-47424	19	8.9	0.624466	1	1	0.44735	no
gi|646748327|gb|KK962156.1|	74695-47594	19	8.9	1	1	-0.0641369	1	no
gi|646748327|gb|KK962156.1|	76110-47718	19	8.9	1	0.644383	-1	0.45505	no
gi|646748327|gb|KK962156.1|	82437-48545	19	8.9	14	8	-0.859398	0.4285	no
gi|646748327|gb|KK962156.1|	91002-49188	19	8.9	2	3	0.724809	0.57015	no
gi|646748327|gb|KK962156.1|	04008-50562	19	8.9	0.40785	0.980961	1	1	no
gi|646748327|gb|KK962156.1|	06498-50721	19	8.9	6	8	0.292497	0.8544	no
gi|646748327|gb|KK962156.1|	07325-50895	19	8.9	0.335682	1	2	0.18085	no
gi|646748327|gb|KK962156.1|	12187-51242	19	8.9	53	74	0.483045	0.7374	no
gi|646748327|gb|KK962156.1|	14011-51440	19	8.9	29	28	-0.0617918	0.96835	no
gi|646748327|gb|KK962156.1|	23861-52522	19	8.9	3	2	-0.213	0.8909	no
gi|646748327|gb|KK962156.1|	43144-54380	19	8.9	5	6	0.401687	0.7712	no
gi|646748327|gb|KK962156.1|	45083-54551	19	8.9	0.502295	7	4	0.1047	no
gi|646748327|gb|KK962156.1|	56274-55739	19	8.9	8	3	-1	0.3858	no
gi|646748327|gb|KK962156.1|	79768-58894	19	8.9	15	15	-0.0404059	0.9726	no
gi|646748327|gb|KK962156.1|	69265-66978	19	8.9	16	18	0.131104	0.9386	no
gi|646748327|gb|KK962156.1|	71538-67194	19	8.9	11	12	0.126801	0.924	no
gi|646748327|gb|KK962156.1|	82179-68426	19	8.9	9	8	-0.136515	0.9265	no
gi|646748327|gb|KK962156.1|	84765-68582	19	8.9	0.923901	0.49444	-0.901944	1	no
gi|646748327|gb|KK962156.1|	87966-69379	19	8.9	4	4	0.0650388	0.95015	no
gi|646748327|gb|KK962156.1|	03038-70406	19	8.9	1	3	0.926461	0.5314	no
gi|646748327|gb|KK962156.1|	04411-70476	19	8.9	49	6	-3	0.0776	no
gi|646748327|gb|KK962156.1|	25779-72642	19	8.9	1	4	1	0.348	no
gi|646748327|gb|KK962156.1|	27036-72800	19	8.9	0.646875	1	0.993849	0.5626	no
gi|646748327|gb|KK962156.1|	30350-73178	19	8.9	0.1568	1	3	0.24365	no
gi|646748327|gb|KK962156.1|	34910-74217	19	8.9	24	20	-0.231462	0.8487	no
gi|646748327|gb|KK962156.1|	54851-75520	19	8.9	6	3	-0.903863	0.5997	no
gi|646748327|gb|KK962156.1|	74553-77562	19	8.9	1	0.643619	-0.807886	0.57835	no
gi|646748327|gb|KK962156.1|	81841-78215	19	8.9	70	14	-2	0.1256	no

gi|646748327|gb|KK962156.1|	84863-78575	19	8.9	9	6	-0.612364	0.69245	no
gi|646748327|gb|KK962156.1|	86602-78690	19	8.9	20	37	0.885369	0.52805	no
gi|646748327|gb|KK962156.1|	90639-79115	19	8.9	0.706587	5	3	0.26145	no
gi|646748327|gb|KK962156.1|	92039-79291	19	8.9	2	3	0.395106	0.7425	no
gi|646748344|gb|KK962155.1|	11153-11140	12	6.5	12	25	1	0.46825	no
gi|646748344|gb|KK962155.1|	80826-28228	12	6.5	73	0	-inf	5.00E-05	yes
gi|646748344|gb|KK962155.1|	53112-45392	12	6.5	0.998165	2	1	0.4498	no
gi|646748344|gb|KK962155.1|	54826-45795	12	6.5	1	0.750427	-0.458575	1	no
gi|646748360|gb|KK962154.1|	37604-33831	14	7.5	31	5	-3	0.12295	no
gi|646748360|gb|KK962154.1|	44345-34546	14	7.5	2	0.306544	-2	0.1855	no
gi|646748360|gb|KK962154.1|	47679-34904	14	7.5	2	0	-inf	0.00595	no
gi|646748360|gb|KK962154.1|	57942-36019	14	7.5	1	0.067135	-4	1	no
gi|646748379|gb|KK962153.1|	12146-13170	14	8.9	3	2	-0.204731	0.88085	no
gi|646748379|gb|KK962153.1|	20936-22146	14	8.9	2	2	-0.239611	0.86555	no
gi|646748379|gb|KK962153.1|	22279-26644	14	8.9	2	1	-0.455233	0.7643	no
gi|646748379|gb|KK962153.1|	40680-24139	14	8.9	2	0.563661	-2	0.29735	no
gi|646748379|gb|KK962153.1|	47466-44791	14	8.9	4	8	1	0.40765	no
gi|646748379|gb|KK962153.1|	05202-60582	14	8.9	2	0.687239	-2	0.2673	no
gi|646748379|gb|KK962153.1|	33075-63375	14	8.9	16	3	-2	0.1272	no
gi|646748379|gb|KK962153.1|	80293-68089	14	8.9	4	2	-1	0.3937	no
gi|646748394|gb|KK962152.1|	69689-26998	13	8.5	18	5	-2	0.272	no
gi|646748407|gb|KK962151.1|	03333-10352	22	9.1	251	355	0.500072	0.7471	no
gi|646748407|gb|KK962151.1|	20605-12236	22	9.1	0.245235	0.797541	2	1	no
gi|646748407|gb|KK962151.1|	38420-13858	22	9.1	212	151	-0.48461	0.73635	no
gi|646748407|gb|KK962151.1|	47349-16433	22	9.1	10	15	0.486012	0.6853	no
gi|646748407|gb|KK962151.1|	69538-17377	22	9.1	19	19	-0.0145492	0.9908	no
gi|646748407|gb|KK962151.1|	17287-17523	22	9.1	190	289	0.603213	0.6872	no
gi|646748407|gb|KK962151.1|	74253-17686	22	9.1	3	4	0.764543	0.61605	no
gi|646748407|gb|KK962151.1|	18198-19065	22	9.1	1	0.641746	-0.721263	1	no
gi|646748407|gb|KK962151.1|	84952-18649	22	9.1	14	24	0.843618	0.51015	no

gi|646748407|gb|KK962151.1|	90092-19063	22	9.1	1	4	1	0.3775	no
gi|646748407|gb|KK962151.1|	02716-20538	22	9.1	31	24	-0.380397	0.74045	no
gi|646748407|gb|KK962151.1|	05563-20624	22	9.1	2	3	1	0.4155	no
gi|646748407|gb|KK962151.1|	16287-22691	22	9.1	18	18	0.0165048	0.9891	no
gi|646748407|gb|KK962151.1|	33520-23535	22	9.1	0.997314	2	1	0.43785	no
gi|646748407|gb|KK962151.1|	40330-24114	22	9.1	2	3	0.496839	0.69895	no
gi|646748407|gb|KK962151.1|	63730-26503	22	9.1	22	10	-1	0.3702	no
gi|646748407|gb|KK962151.1|	69625-37042	22	9.1	4	6	0.672123	0.67705	no
gi|646748407|gb|KK962151.1|	90662-39587	22	9.1	0	3	inf	0.00015	yes
gi|646748407|gb|KK962151.1|	03176-40510	22	9.1	2	3	0.335601	0.82765	no
gi|646748407|gb|KK962151.1|	07367-40818	22	9.1	0.99281	3	2	0.24925	no
gi|646748407|gb|KK962151.1|	12295-41281	22	9.1	2	3	0.661688	0.6549	no
gi|646748407|gb|KK962151.1|	14882-41517	22	9.1	38	37	-0.0498539	0.9737	no
gi|646748407|gb|KK962151.1|	16211-41667	22	9.1	88	140	0.676308	0.59065	no
gi|646748407|gb|KK962151.1|	16932-41828	22	9.1	2	2	0.108645	0.94555	no
gi|646748407|gb|KK962151.1|	19243-42072	22	9.1	4	8	0.872925	0.5686	no
gi|646748407|gb|KK962151.1|	26895-42784	22	9.1	1	2	0.507173	0.70715	no
gi|646748407|gb|KK962151.1|	29873-43164	22	9.1	4	7	0.760408	0.61155	no
gi|646748407|gb|KK962151.1|	34964-43588	22	9.1	1	1	0.312303	0.8346	no
gi|646748407|gb|KK962151.1|	36004-43762	22	9.1	0.879796	0.879052	-0.0012207	1	no
gi|646748407|gb|KK962151.1|	40240-44059	22	9.1	3	27	3	0.14985	no
gi|646748407|gb|KK962151.1|	47311-44755	22	9.1	9	44	2	0.17965	no
gi|646748407|gb|KK962151.1|	73181-47408	22	9.1	83	0.602788	-7	0.07395	no
gi|646748407|gb|KK962151.1|	84824-48590	22	9.1	4	0.159768	-5	0.174	no
gi|646748407|gb|KK962151.1|	86578-48716	22	9.1	6	0.755609	-3	0.1552	no
gi|646748407|gb|KK962151.1|	05856-50616	22	9.1	41	8	-2	0.16165	no
gi|646748407|gb|KK962151.1|	06862-50754	22	9.1	3	0	-inf	0.0041	no
gi|646748407|gb|KK962151.1|	12903-51370	22	9.1	0	5	inf	0.0026	no
gi|646748407|gb|KK962151.1|	14397-51533	22	9.1	1	0.386411	-2	0.2551	no
gi|646748407|gb|KK962151.1|	28063-52988	22	9.1	0.76654	3	2	0.20615	no

gi|646748407|gb|KK962151.1|	34601-53554	22	9.1	8	8	0.00615789	0.9901	no
gi|646748407|gb|KK962151.1|	44601-54492	22	9.1	0	21	inf	0.00605	no
gi|646748407|gb|KK962151.1|	76567-57712	22	9.1	2	5	1	0.37115	no
gi|646748407|gb|KK962151.1|	83819-58451	22	9.1	5	9	0.688439	0.6612	no
gi|646748407|gb|KK962151.1|	86543-58684	22	9.1	12	19	0.653999	0.61675	no
gi|646748407|gb|KK962151.1|	91245-59146	22	9.1	65	30	-1	0.41645	no
gi|646748407|gb|KK962151.1|	92055-59233	22	9.1	20	42	1	0.4805	no
gi|646748407|gb|KK962151.1|	02356-60306	22	9.1	5	5	0.0471246	0.9718	no
gi|646748407|gb|KK962151.1|	04577-60482	22	9.1	23	12	-0.894125	0.57035	no
gi|646748407|gb|KK962151.1|	06024-60654	22	9.1	21	24	0.174342	0.9095	no
gi|646748407|gb|KK962151.1|	17463-62115	22	9.1	0.996588	0.431757	-1	1	no
gi|646748407|gb|KK962151.1|	22796-62459	22	9.1	0.841048	0.173715	-2	1	no
gi|646748407|gb|KK962151.1|	25659-62719	22	9.1	2	0.834858	-0.864628	0.4995	no
gi|646748407|gb|KK962151.1|	27303-62825	22	9.1	144.66	73	-0.982899	0.402	no
gi|646748407|gb|KK962151.1|	38513-64021	22	9.1	63	19	-2	0.1265	no
gi|646748407|gb|KK962151.1|	18243-71921	22	9.1	4	4	-0.228182	0.88505	no
gi|646748407|gb|KK962151.1|	20161-72117	22	9.1	3	1	-1	0.33475	no
gi|646748407|gb|KK962151.1|	22793-72459	22	9.1	1	0.346764	-2	1	no
gi|646748407|gb|KK962151.1|	25387-72700	22	9.1	5	1	-2	0.1685	no
gi|646748407|gb|KK962151.1|	31361-73161	22	9.1	23	14	-0.717323	0.63775	no
gi|646748407|gb|KK962151.1|	44816-74513	22	9.1	8	6	-0.545489	0.73775	no
gi|646748407|gb|KK962151.1|	55707-75597	22	9.1	25	20	-0.306093	0.8249	no
gi|646748407|gb|KK962151.1|	59961-76343	22	9.1	17	8	-1	0.2981	no
gi|646748407|gb|KK962151.1|	73928-77538	22	9.1	7	2	-2	0.2582	no
gi|646748407|gb|KK962151.1|	98736-79965	22	9.1	0	3	inf	0.00435	no
gi|646748428|gb|KK962150.1|	39643-34019	22	11	51	18	-2	0.3241	no
gi|646748428|gb|KK962150.1|	58210-35894	22	11	2	0.811853	-0.927944	0.58905	no
gi|646748428|gb|KK962150.1|	40613-41186	22	11	2	15	3	0.10695	no
gi|646748428|gb|KK962150.1|	43903-46535	22	11	13	22	0.751206	0.49575	no
gi|646748428|gb|KK962150.1|	56304-45700	22	11	3	0.87822	-2	0.2312	no

gi|646748428|gb|KK962150.1|	60084-46077	22	11	3	1	-0.783146	0.5838	no
gi|646748428|gb|KK962150.1|	62971-46345	22	11	4	2	-1	0.41965	no
gi|646748428|gb|KK962150.1|	64783-46559	22	11	1	0.7051	-0.919084	0.5894	no
gi|646748428|gb|KK962150.1|	65797-46634	22	11	3	2	-0.874867	0.5585	no
gi|646748428|gb|KK962150.1|	81159-48188	22	11	4	1	-1	0.30195	no
gi|646748428|gb|KK962150.1|	82101-48474	22	11	1	0.338361	-2	0.24645	no
gi|646748428|gb|KK962150.1|	84887-48537	22	11	3	2	-0.578294	0.72945	no
gi|646748428|gb|KK962150.1|	90005-49138	22	11	2	0.590336	-2	0.2129	no
gi|646748428|gb|KK962150.1|	49645-50498	22	11	11	18	0.783741	0.5974	no
gi|646748428|gb|KK962150.1|	01106-50185	22	11	15	9	-0.727898	0.63645	no
gi|646748428|gb|KK962150.1|	20216-52214	22	11	16	15	-0.131085	0.90995	no
gi|646748428|gb|KK962150.1|	23469-52404	22	11	4	2	-0.76733	0.55795	no
gi|646748442|gb|KK962149.1|	82918-38344	17	9.4	0	8	inf	0.00435	no
gi|646748442|gb|KK962149.1|	09768-81118	17	9.4	4	1	-2	0.1911	no
gi|646748457|gb|KK962148.1|	42789-43088	13	10	24	12	-1	0.42055	no
gi|646748457|gb|KK962148.1|	84071-58463	13	10	16	6	-1	0.3852	no
gi|646748457|gb|KK962148.1|	80234-81039	13	10	1	2	1	0.39835	no
gi|646748474|gb|KK962147.1|	15035-11676	20	9	1	0.998907	-0.46906	0.74175	no
gi|646748474|gb|KK962147.1|	26261-12716	20	9	2	1	-1	0.37095	no
gi|646748474|gb|KK962147.1|	51086-15153	20	9	17	14	-0.267951	0.8626	no
gi|646748474|gb|KK962147.1|	71054-17525	20	9	0.961813	0.752701	-0.35368	1	no
gi|646748474|gb|KK962147.1|	75480-17670	20	9	1	2	0.197893	0.8725	no
gi|646748474|gb|KK962147.1|	76827-17775	20	9	3	5	0.958089	0.4884	no
gi|646748474|gb|KK962147.1|	77933-17904	20	9	72	43	-0.737409	0.51425	no
gi|646748474|gb|KK962147.1|	96687-20041	20	9	37	31	-0.248106	0.8302	no
gi|646748474|gb|KK962147.1|	08166-20862	20	9	46	15	-2	0.3078	no
gi|646748474|gb|KK962147.1|	14311-21979	20	9	51	44	-0.225517	0.84815	no
gi|646748474|gb|KK962147.1|	20058-22655	20	9	1	1	0.255956	0.866	no
gi|646748474|gb|KK962147.1|	26662-22904	20	9	1	2	0.238031	0.8783	no
gi|646748474|gb|KK962147.1|	29197-23008	20	9	1	1.25	-0.234471	0.8627	no

gi|646748474|gb|KK962147.1|	31941-23266	20	9	1	2	0.485286	0.7489	no
gi|646748474|gb|KK962147.1|	35131-23533	20	9	54	44	-0.31107	0.83955	no
gi|646748474|gb|KK962147.1|	44213-24452	20	9	264	138	-0.934078	0.4595	no
gi|646748474|gb|KK962147.1|	46515-24763	20	9	2	5	1	0.32965	no
gi|646748474|gb|KK962147.1|	64745-26694	20	9	7	6	-0.256016	0.86335	no
gi|646748474|gb|KK962147.1|	68134-26988	20	9	0.557692	0.985064	0.820748	1	no
gi|646748474|gb|KK962147.1|	70263-27155	20	9	4	3	-0.419044	0.78315	no
gi|646748474|gb|KK962147.1|	72382-27264	20	9	17	24	0.483279	0.73245	no
gi|646748474|gb|KK962147.1|	78649-28611	20	9	11	7	-0.683506	0.5559	no
gi|646748474|gb|KK962147.1|	86434-29040	20	9	43	10	-2	0.0817	no
gi|646748474|gb|KK962147.1|	91026-29146	20	9	19	9	-1	0.38855	no
gi|646748474|gb|KK962147.1|	12048-31229	20	9	39	6	-3	0.14865	no
gi|646748474|gb|KK962147.1|	37475-33970	20	9	18	11	-0.687206	0.52275	no
gi|646748474|gb|KK962147.1|	51429-35296	20	9	13	9	-0.504391	0.7415	no
gi|646748474|gb|KK962147.1|	58660-36302	20	9	20	15	-0.471353	0.6766	no
gi|646748474|gb|KK962147.1|	65051-36545	20	9	8	6	-0.334237	0.7969	no
gi|646748474|gb|KK962147.1|	66700-36749	20	9	36	19	-0.897799	0.4732	no
gi|646748474|gb|KK962147.1|	57900-45909	20	9	2	3	0.781963	0.5688	no
gi|646748474|gb|KK962147.1|	69275-47010	20	9	3	4	0.105567	0.9385	no
gi|646748474|gb|KK962147.1|	71184-47159	20	9	11	8	-0.413767	0.7435	no
gi|646748474|gb|KK962147.1|	72653-47362	20	9	3	3	-0.384361	0.7806	no
gi|646748474|gb|KK962147.1|	80945-48165	20	9	28	10	-2	0.3328	no
gi|646748474|gb|KK962147.1|	94768-50000	20	9	8	9	0.178613	0.87075	no
gi|646748474|gb|KK962147.1|	02044-50297	20	9	3	0.971561	-2	0.31835	no
gi|646748474|gb|KK962147.1|	21611-52307	20	9	0.61014	0.878785	0.52637	1	no
gi|646748474|gb|KK962147.1|	68296-56882	20	9	4	2	-1	0.46265	no
gi|646748474|gb|KK962147.1|	79001-58631	20	9	8	6	-0.562836	0.61735	no
gi|646748474|gb|KK962147.1|	87282-58756	20	9	18	19	0.036678	0.94505	no
gi|646748474|gb|KK962147.1|	88159-58983	20	9	2	2	-0.206795	0.89445	no
gi|646748474|gb|KK962147.1|	91779-60262	20	9	18	10	-0.80174	0.49655	no

gi|646748474|gb|KK962147.1|	67582-68285	20	9	86	6	-4	0.02285	no
gi|646748474|gb|KK962147.1|	62997-76321	20	9	85	11	-3	0.12215	no
gi|646748490|gb|KK962146.1|	64040-16540	19	7.7	2	3	0.420795	0.7904	no
gi|646748490|gb|KK962146.1|	66356-16739	19	7.7	3	2	-0.625202	0.6691	no
gi|646748490|gb|KK962146.1|	16962-17666	19	7.7	0.411888	3	3	0.2662	no
gi|646748490|gb|KK962146.1|	74053-17550	19	7.7	8	6	-0.556373	0.7085	no
gi|646748490|gb|KK962146.1|	77270-17944	19	7.7	11	14	0.381108	0.73055	no
gi|646748490|gb|KK962146.1|	98397-19888	19	7.7	3	6	0.881137	0.5168	no
gi|646748490|gb|KK962146.1|	01723-20227	19	7.7	2	2	0.448369	0.7747	no
gi|646748490|gb|KK962146.1|	04483-20474	19	7.7	29	27	-0.0876697	0.94065	no
gi|646748490|gb|KK962146.1|	23205-22386	19	7.7	1	2	0.961114	0.5626	no
gi|646748490|gb|KK962146.1|	31237-23207	19	7.7	14	12	-0.237985	0.87215	no
gi|646748490|gb|KK962146.1|	2710-3121	19	7.7	2	6	1	0.3935	no
gi|646748490|gb|KK962146.1|	14298-31459	19	7.7	40	32	-0.326136	0.83745	no
gi|646748490|gb|KK962146.1|	56537-59027	19	7.7	2	3	0.601013	0.69415	no
gi|646748490|gb|KK962146.1|	65280-71934	19	7.7	3	3	0.110275	0.91875	no
gi|646748490|gb|KK962146.1|	72046-74990	19	7.7	5	3	-0.817516	0.5942	no
gi|646748490|gb|KK962146.1|	86493-87048	19	7.7	9	16	0.828474	0.5927	no
gi|646748490|gb|KK962146.1|	92273-92974	19	7.7	4	4	0.095278	0.93855	no
gi|646748508|gb|KK962145.1|	43774-44840	11	7.6	1	4	2	0.30355	no
gi|646748508|gb|KK962145.1|	47321-47804	11	7.6	3	8	2	0.3616	no
gi|646748530|gb|KK962144.1|	67667-16998	16	8.1	43	55	0.337384	0.77355	no
gi|646748558|gb|KK962142.1|	25190-32574	21	9.9	3	2	-0.686916	0.6321	no
gi|646748558|gb|KK962142.1|	68145-36968	21	9.9	0.431297	0.82912	0.942899	1	no
gi|646748558|gb|KK962142.1|	10503-41144	21	9.9	1	3	1	0.4754	no
gi|646748558|gb|KK962142.1|	12422-41288	21	9.9	4	4	-0.005421	0.9298	no
gi|646748558|gb|KK962142.1|	13961-41545	21	9.9	4	4	-0.0205534	0.98825	no
gi|646748558|gb|KK962142.1|	47448-54782	21	9.9	4	5	0.607984	0.669	no
gi|646748558|gb|KK962142.1|	17546-61843	21	9.9	3	3	-0.149117	0.91705	no
gi|646748558|gb|KK962142.1|	83311-68352	21	9.9	188	25	-3	0.11645	no

gi|646748558|gb|KK962142.1|	28991-73027	21	9.9	0.990857	1	0.384177	0.77315	no
gi|646748558|gb|KK962142.1|	60905-76260	21	9.9	1	2	1	0.40825	no
gi|646748558|gb|KK962142.1|	87216-79054	21	9.9	0.120469	4	5	0.0975	no
gi|646748558|gb|KK962142.1|	03776-80543	21	9.9	0.991224	4	2	0.15735	no
gi|646748558|gb|KK962142.1|	05674-80891	21	9.9	0	4	inf	0.00025	yes
gi|646748558|gb|KK962142.1|	09036-81247	21	9.9	0	4	inf	0.0001	yes
gi|646748558|gb|KK962142.1|	29535-83021	21	9.9	2	2	-0.035019	0.9367	no
gi|646748580|gb|KK962141.1|	58434-16001	20	5.6	32	23	-0.477633	0.66485	no
gi|646748580|gb|KK962141.1|	64339-16639	20	5.6	19	16	-0.244993	0.8239	no
gi|646748580|gb|KK962141.1|	68719-16926	20	5.6	27	23	-0.223659	0.87765	no
gi|646748580|gb|KK962141.1|	71359-17912	20	5.6	1	1	0.0628554	0.966	no
gi|646748580|gb|KK962141.1|	79411-18489	20	5.6	2	1	-0.164071	0.9119	no
gi|646748580|gb|KK962141.1|	85664-18696	20	5.6	0.532689	0.892471	0.744513	1	no
gi|646748580|gb|KK962141.1|	10037-21031	20	5.6	16	10	-0.652363	0.657	no
gi|646748580|gb|KK962141.1|	17485-21781	20	5.6	5	10	0.963624	0.5533	no
gi|646748580|gb|KK962141.1|	20246-22076	20	5.6	4	3	-0.0787745	0.9361	no
gi|646748580|gb|KK962141.1|	56124-25664	20	5.6	3	3	0.0748061	0.9363	no
gi|646748580|gb|KK962141.1|	14411-31512	20	5.6	2	1	-0.363727	0.80365	no
gi|646748580|gb|KK962141.1|	23336-32369	20	5.6	10	6	-0.672924	0.6255	no
gi|646748580|gb|KK962141.1|	36994-33876	20	5.6	82	28	-2	0.1952	no
gi|646748580|gb|KK962141.1|	41613-34398	20	5.6	35	12	-2	0.1715	no
gi|646748580|gb|KK962141.1|	47208-34775	20	5.6	53	32	-0.708156	0.63705	no
gi|646748580|gb|KK962141.1|	51511-35324	20	5.6	2	0.180532	-4	0.12385	no
gi|646748580|gb|KK962141.1|	53389-35493	20	5.6	1	0.724926	-0.47212	1	no
gi|646748580|gb|KK962141.1|	56978-35771	20	5.6	2	0.802273	-1	0.47555	no
gi|646748580|gb|KK962141.1|	70609-37150	20	5.6	2	1	-0.718826	0.5855	no
gi|646748580|gb|KK962141.1|	72897-37443	20	5.6	1	1	-0.150325	0.9101	no
gi|646748580|gb|KK962141.1|	76230-37815	20	5.6	2	1	-0.566109	0.6781	no
gi|646748580|gb|KK962141.1|	79305-37999	20	5.6	2	0.899482	-0.935316	0.589	no
gi|646748580|gb|KK962141.1|	80448-38202	20	5.6	1	0.604302	-0.886474	1	no

gi|646748580|gb|KK962141.1|	82137-38334	20	5.6	0.780664	0.559385	-0.480861	1	no
gi|646748580|gb|KK962141.1|	83472-38892	20	5.6	1	0.364211	-2	0.1644	no
gi|646748580|gb|KK962141.1|	89024-38965	20	5.6	6	4	-0.41432	0.76945	no
gi|646748580|gb|KK962141.1|	97666-39880	20	5.6	0.732525	0.748604	0.0313245	1	no
gi|646748580|gb|KK962141.1|	08663-41459	20	5.6	6	5	-0.285188	0.788	no
gi|646748580|gb|KK962141.1|	14764-41928	20	5.6	22	13	-0.721432	0.52965	no
gi|646748580|gb|KK962141.1|	31094-43512	20	5.6	10	5	-1	0.38945	no
gi|646748580|gb|KK962141.1|	50343-45062	20	5.6	216	44	-2	0.16315	no
gi|646748580|gb|KK962141.1|	64970-46701	20	5.6	0.620224	1	0.949791	0.5268	no
gi|646748580|gb|KK962141.1|	70512-47094	20	5.6	5	4	-0.190842	0.9009	no
gi|646748580|gb|KK962141.1|	77091-47735	20	5.6	53	0	-inf	0.00525	no
gi|646748580|gb|KK962141.1|	10807-51440	20	5.6	5	4	-0.097212	0.9472	no
gi|646748580|gb|KK962141.1|	14489-52190	20	5.6	3	4	0.370023	0.7342	no
gi|646748580|gb|KK962141.1|	22988-53046	20	5.6	7	9	0.238564	0.8359	no
gi|646748580|gb|KK962141.1|	52487-55276	20	5.6	17	4	-2	0.2666	no
gi|646748580|gb|KK962141.1|	56169-55635	20	5.6	141	63	-1	0.3968	no
gi|646748580|gb|KK962141.1|	65645-56646	20	5.6	1	0.689574	-1	0.47555	no
gi|646748580|gb|KK962141.1|	71619-57190	20	5.6	41	59	0.517106	0.74125	no
gi|646748580|gb|KK962141.1|	94779-59885	20	5.6	38	29	-0.397878	0.7279	no
gi|646748580|gb|KK962141.1|	32363-63295	20	5.6	1	3	1	0.3774	no
gi|646748580|gb|KK962141.1|	74932-67528	20	5.6	3	6	0.902234	0.5935	no
gi|646748580|gb|KK962141.1|	05359-70594	20	5.6	13	14	0.0952759	0.9495	no
gi|646748580|gb|KK962141.1|	06138-70696	20	5.6	25	23	-0.134949	0.93095	no
gi|646748580|gb|KK962141.1|	08081-71058	20	5.6	11	15	0.398744	0.7134	no
gi|646748580|gb|KK962141.1|	13878-71443	20	5.6	11	7	-0.679952	0.6688	no
gi|646748580|gb|KK962141.1|	26095-72661	20	5.6	40	26	-0.612091	0.68565	no
gi|646748580|gb|KK962141.1|	47793-75204	20	5.6	2	2	0.0763927	0.9598	no
gi|646748580|gb|KK962141.1|	57367-75858	20	5.6	0.385053	1	2	1	no
gi|646748580|gb|KK962141.1|	75421-77906	20	5.6	7	6	-0.389295	0.71215	no
gi|646748580|gb|KK962141.1|	77622-79375	20	5.6	0.185277	5	5	0.10985	no

gi|646748580|gb|KK962141.1|	81849-78602	20	5.6	14	6	-1	0.293	no
gi|646748580|gb|KK962141.1|	65592-86589	20	5.6	102	18	-3	0.08815	no
gi|646748580|gb|KK962141.1|	90917-89130	20	5.6	7	6	-0.299966	0.8259	no
gi|646748580|gb|KK962141.1|	91429-89199	20	5.6	3	3	-0.199354	0.87805	no
gi|646748599|gb|KK962140.1|	43054-24339	21	9.8	3	10	2	0.28405	no
gi|646748599|gb|KK962140.1|	46357-24675	21	9.8	19	13	-0.545204	0.7323	no
gi|646748599|gb|KK962140.1|	50444-25300	21	9.8	6	2	-2	0.22315	no
gi|646748599|gb|KK962140.1|	53158-25368	21	9.8	16	2	-3	0.0938	no
gi|646748599|gb|KK962140.1|	56475-25688	21	9.8	31	7	-2	0.14175	no
gi|646748599|gb|KK962140.1|	57455-25847	21	9.8	9	2	-2	0.13205	no
gi|646748599|gb|KK962140.1|	66170-26721	21	9.8	50	6	-3	0.04195	no
gi|646748599|gb|KK962140.1|	69481-27302	21	9.8	11	8	-0.543265	0.6168	no
gi|646748599|gb|KK962140.1|	73805-27526	21	9.8	1	0.77458	-0.473824	1	no
gi|646748599|gb|KK962140.1|	87383-28794	21	9.8	4	7	0.954013	0.52785	no
gi|646748599|gb|KK962140.1|	91153-29198	21	9.8	1	0.910182	-0.502209	0.73865	no
gi|646748599|gb|KK962140.1|	01094-30294	21	9.8	0.925736	0.669574	-0.467357	1	no
gi|646748599|gb|KK962140.1|	03309-31795	21	9.8	3	2	-0.409975	0.71065	no
gi|646748599|gb|KK962140.1|	82540-38297	21	9.8	0	9	inf	0.0008	yes
gi|646748599|gb|KK962140.1|	85362-48604	21	9.8	2	0.907468	-0.93599	0.589	no
gi|646748599|gb|KK962140.1|	01830-50200	21	9.8	65	123	0.923978	0.5824	no
gi|646748599|gb|KK962140.1|	03251-50374	21	9.8	8	10	0.282936	0.83365	no
gi|646748599|gb|KK962140.1|	14641-51517	21	9.8	0.992734	4	2	0.20605	no
gi|646748599|gb|KK962140.1|	18946-51951	21	9.8	2	5	1	0.392	no
gi|646748599|gb|KK962140.1|	21027-52255	21	9.8	1	0.733321	-0.665053	0.6175	no
gi|646748599|gb|KK962140.1|	24219-52763	21	9.8	42	23	-0.861959	0.45735	no
gi|646748599|gb|KK962140.1|	27780-52814	21	9.8	267	56	-2	0.1033	no
gi|646748599|gb|KK962140.1|	52795-55312	21	9.8	3	9	2	0.3712	no
gi|646748599|gb|KK962140.1|	77913-57903	21	9.8	0.424832	10	5	0.098	no
gi|646748599|gb|KK962140.1|	84681-58583	21	9.8	0.308359	1	2	0.20215	no
gi|646748599|gb|KK962140.1|	98411-60015	21	9.8	0.993449	2	1	0.3615	no

gi|646748599|gb|KK962140.1|	00416-60212	21	9.8	3	3	0.26054	0.8646	no
gi|646748599|gb|KK962140.1|	17462-61762	21	9.8	240	169	-0.509583	0.71685	no
gi|646748599|gb|KK962140.1|	38066-63884	21	9.8	0.887655	2	1	0.36955	no
gi|646748599|gb|KK962140.1|	40592-64149	21	9.8	2	1	-0.688487	0.60225	no
gi|646748599|gb|KK962140.1|	47801-64902	21	9.8	1	0.959155	-0.257786	0.8545	no
gi|646748599|gb|KK962140.1|	59284-66077	21	9.8	87	70	-0.301642	0.7957	no
gi|646748599|gb|KK962140.1|	00350-80148	21	9.8	0	30	inf	5.00E-05	yes
gi|646748615|gb|KK962139.1|	23626-42462	19	7.8	0.625026	1	0.995317	0.5626	no
gi|646748615|gb|KK962139.1|	27459-42894	19	7.8	0.603179	0.76032	0.33402	1	no
gi|646748615|gb|KK962139.1|	31428-43330	19	7.8	0.512711	1	1	1	no
gi|646748615|gb|KK962139.1|	38528-43936	19	7.8	2	0.452059	-2	0.20925	no
gi|646748615|gb|KK962139.1|	40750-44287	19	7.8	1	1	0.39112	0.7754	no
gi|646748615|gb|KK962139.1|	46428-44688	19	7.8	3	2	-0.180011	0.89395	no
gi|646748615|gb|KK962139.1|	53054-45353	19	7.8	0	25	inf	0.0002	yes
gi|646748615|gb|KK962139.1|	56342-45670	19	7.8	0.799072	24	5	0.1022	no
gi|646748615|gb|KK962139.1|	57029-45840	19	7.8	86	69	-0.301398	0.79225	no
gi|646748615|gb|KK962139.1|	58525-46074	19	7.8	12	20	0.745056	0.50165	no
gi|646748615|gb|KK962139.1|	60852-46321	19	7.8	3	3	0.052017	0.97055	no
gi|646748615|gb|KK962139.1|	63353-46384	19	7.8	9	3	-2	0.26755	no
gi|646748615|gb|KK962139.1|	67805-46854	19	7.8	5	3	-0.681897	0.625	no
gi|646748615|gb|KK962139.1|	68963-46946	19	7.8	15	20	0.429545	0.77695	no
gi|646748615|gb|KK962139.1|	70976-47173	19	7.8	10	10	0.0101951	0.99045	no
gi|646748615|gb|KK962139.1|	13640-51462	19	7.8	0	27	inf	5.00E-05	yes
gi|646748615|gb|KK962139.1|	08817-61128	19	7.8	3	2	-0.27425	0.85635	no
gi|646748615|gb|KK962139.1|	11572-61273	19	7.8	3	2	-0.301933	0.84815	no
gi|646748615|gb|KK962139.1|	12894-61325	19	7.8	7	3	-1	0.4325	no
gi|646748615|gb|KK962139.1|	13583-61669	19	7.8	4	3	-0.559708	0.70875	no
gi|646748615|gb|KK962139.1|	18043-62012	19	7.8	3	4	0.393102	0.78615	no
gi|646748615|gb|KK962139.1|	24424-62536	19	7.8	4	4	0.107839	0.94385	no
gi|646748615|gb|KK962139.1|	47779-64922	19	7.8	0.310184	1	2	0.18825	no

gi|646748615|gb|KK962139.1|	53896-65590	19	7.8	0.3172	0.99536	2	1	no
gi|646748615|gb|KK962139.1|	63979-66571	19	7.8	2	3	0.946136	0.55355	no
gi|646748615|gb|KK962139.1|	66029-66706	19	7.8	6	6	0.002005	0.98765	no
gi|646748615|gb|KK962139.1|	82472-68428	19	7.8	0.533743	2	2	0.2176	no
gi|646748615|gb|KK962139.1|	85628-68715	19	7.8	5	3	-0.74371	0.6212	no
gi|646748631|gb|KK962138.1|	15643-16116	16	8.7	7	2	-2	0.22915	no
gi|646748631|gb|KK962138.1|	31124-31894	16	8.7	11	12	0.163573	0.91335	no
gi|646748631|gb|KK962138.1|	69599-37060	16	8.7	0	2	inf	0.0008	yes
gi|646748631|gb|KK962138.1|	41264-43030	16	8.7	0.918416	2	0.87253	0.5449	no
gi|646748663|gb|KK962136.1|	31036-13189	18	6.5	0.153398	27	7	0.0948	no
gi|646748663|gb|KK962136.1|	38234-13981	18	6.5	0	7	inf	0.0002	yes
gi|646748663|gb|KK962136.1|	14954-41519	18	6.5	35	15	-1	0.40815	no
gi|646748663|gb|KK962136.1|	30659-43111	18	6.5	570	188	-2	0.17265	no
gi|646748663|gb|KK962136.1|	37862-44681	18	6.5	3	3	-0.108065	0.9251	no
gi|646748663|gb|KK962136.1|	54874-45506	18	6.5	293	133	-1	0.4918	no
gi|646748663|gb|KK962136.1|	48237-65042	18	6.5	4	3	-0.573605	0.7091	no
gi|646748663|gb|KK962136.1|	54458-65547	18	6.5	2	0.174118	-3	0.1757	no
gi|646748663|gb|KK962136.1|	55583-65619	18	6.5	3	2	-0.270685	0.83675	no
gi|646748663|gb|KK962136.1|	56889-65790	18	6.5	1	0.523024	-1	1	no
gi|646748663|gb|KK962136.1|	69112-67047	18	6.5	1	0.480632	-1	0.34475	no
gi|646748663|gb|KK962136.1|	70639-67171	18	6.5	1	0.323729	-2	0.26845	no
gi|646748663|gb|KK962136.1|	78095-67958	18	6.5	0.97303	1	0.285844	0.8199	no
gi|646748663|gb|KK962136.1|	81481-68276	18	6.5	1	1	0.280762	0.8201	no
gi|646748663|gb|KK962136.1|	83166-68638	18	6.5	6	2	-1	0.42085	no
gi|646748663|gb|KK962136.1|	86684-68714	18	6.5	5	2	-1	0.42175	no
gi|646748663|gb|KK962136.1|	89171-69141	18	6.5	5	14	2	0.22475	no
gi|646748663|gb|KK962136.1|	91727-69531	18	6.5	2	17	3	0.02765	no
gi|646748663|gb|KK962136.1|	95752-69700	18	6.5	2	26	4	0.00515	no
gi|646748663|gb|KK962136.1|	76572-77095	18	6.5	11	10	-0.168766	0.9176	no
gi|646748663|gb|KK962136.1|	88964-90512	18	6.5	2	7	2	0.30845	no

gi|646748686|gb|KK962135.1|	30739-13215	12	6.3	5	6	0.175243	0.90225	no
gi|646748686|gb|KK962135.1|	47840-14909	12	6.3	0.929744	1	0.198771	1	no
gi|646748686|gb|KK962135.1|	70047-17027	12	6.3	40	58	0.526952	0.68325	no
gi|646748686|gb|KK962135.1|	38377-38556	12	6.3	143.73	136	-0.0849462	0.9405	no
gi|646748686|gb|KK962135.1|	89458-58993	12	6.3	3	2	-0.583846	0.72945	no
gi|646748695|gb|KK962134.1|	09445-11096	20	5.8	1	2	0.597766	0.6627	no
gi|646748695|gb|KK962134.1|	11211-17081	20	5.8	86	12	-3	0.0249	no
gi|646748695|gb|KK962134.1|	14960-11954	20	5.8	7	5	-0.280812	0.78995	no
gi|646748695|gb|KK962134.1|	19706-12731	20	5.8	12	13	0.18584	0.87405	no
gi|646748695|gb|KK962134.1|	30409-13071	20	5.8	66	204	2	0.29725	no
gi|646748695|gb|KK962134.1|	39299-13958	20	5.8	17	67	2	0.2057	no
gi|646748695|gb|KK962134.1|	54576-15480	20	5.8	348	990	2	0.2522	no
gi|646748695|gb|KK962134.1|	63619-16394	20	5.8	95	255	1	0.2766	no
gi|646748695|gb|KK962134.1|	18871-19218	20	5.8	13	7	-0.912871	0.4938	no
gi|646748695|gb|KK962134.1|	93021-19427	20	5.8	6	4	-0.456612	0.7567	no
gi|646748695|gb|KK962134.1|	15557-21586	20	5.8	17	2	-3	0.182	no
gi|646748695|gb|KK962134.1|	48259-24848	20	5.8	43	38	-0.161305	0.91195	no
gi|646748695|gb|KK962134.1|	26449-26906	20	5.8	6	2	-2	0.27825	no
gi|646748695|gb|KK962134.1|	05411-30556	20	5.8	368	229	-0.68503	0.63595	no
gi|646748695|gb|KK962134.1|	4944-5455	20	5.8	3	1	-0.98085	0.589	no
gi|646748695|gb|KK962134.1|	59698-66131	20	5.8	0.885626	0.983093	0.15063	1	no
gi|646748695|gb|KK962134.1|	63999-66497	20	5.8	1	2	0.830411	0.5472	no
gi|646748695|gb|KK962134.1|	09854-71136	20	5.8	4	4	0.10272	0.9518	no
gi|646748695|gb|KK962134.1|	12863-71389	20	5.8	1	1	-0.00268054	0.9367	no
gi|646748695|gb|KK962134.1|	19361-72135	20	5.8	4	2	-0.946892	0.53945	no
gi|646748695|gb|KK962134.1|	26783-72820	20	5.8	1	0.56857	-1	0.33035	no
gi|646748695|gb|KK962134.1|	28402-72956	20	5.8	2	1	-1	0.4328	no
gi|646748695|gb|KK962134.1|	731-995	20	5.8	168	35	-2	0.1129	no
gi|646748695|gb|KK962134.1|	7550-7763	20	5.8	76.27	6	-4	0.17805	no
gi|646748695|gb|KK962134.1|	80136-80602	20	5.8	2	3	0.675986	0.662	no

gi|646748695|gb|KK962134.1|	84067-85396	20	5.8	0.602956	0.742648	0.300625	1	no
gi|646748695|gb|KK962134.1|	8526-9306	20	5.8	2	0.743733	-1	0.3853	no
gi|646748695|gb|KK962134.1|	87632-88596	20	5.8	78	2	-6	0.0049	no
gi|646748695|gb|KK962134.1|	90473-90868	20	5.8	13	0.811707	-4	0.17025	no
gi|646748695|gb|KK962134.1|	92048-93204	20	5.8	7	0.587858	-4	0.06265	no
gi|646748695|gb|KK962134.1|	97931-10910	20	5.8	11	19	0.874552	0.4665	no
gi|646748712|gb|KK962133.1|	68177-27006	13	4.5	0.113169	94	10	0.2322	no
gi|646748712|gb|KK962133.1|	83167-48356	13	4.5	22	60	1	0.3637	no
gi|646748712|gb|KK962133.1|	06095-50668	13	4.5	2	3	0.267062	0.84035	no
gi|646748712|gb|KK962133.1|	06963-51080	13	4.5	0.852374	1	0.543624	0.73115	no
gi|646748712|gb|KK962133.1|	13327-51455	13	4.5	19	16	-0.190374	0.902	no
gi|646748712|gb|KK962133.1|	66089-66342	13	4.5	50	95	0.918101	0.49795	no
gi|646748728|gb|KK962132.1|	26896-22921	19	8.1	8	9	0.0728916	0.9551	no
gi|646748728|gb|KK962132.1|	39476-24029	19	8.1	3	9	1	0.30555	no
gi|646748728|gb|KK962132.1|	47043-24793	19	8.1	0.584519	1	1	0.43305	no
gi|646748728|gb|KK962132.1|	51567-35347	19	8.1	6	5	-0.189131	0.90245	no
gi|646748728|gb|KK962132.1|	54418-35773	19	8.1	7	6	-0.1599	0.88325	no
gi|646748728|gb|KK962132.1|	61956-36228	19	8.1	23	10	-1	0.4331	no
gi|646748728|gb|KK962132.1|	13441-41534	19	8.1	3	3	0.435945	0.778	no
gi|646748728|gb|KK962132.1|	26277-42807	19	8.1	0.419707	1	2	0.2813	no
gi|646748728|gb|KK962132.1|	28401-42926	19	8.1	3	6	0.899095	0.57475	no
gi|646748728|gb|KK962132.1|	30379-43236	19	8.1	8	13	0.63126	0.61485	no
gi|646748728|gb|KK962132.1|	32917-43393	19	8.1	14	12	-0.284023	0.84805	no
gi|646748728|gb|KK962132.1|	40542-44608	19	8.1	7	5	-0.630232	0.56925	no
gi|646748728|gb|KK962132.1|	71551-47176	19	8.1	62	110	0.839718	0.57255	no
gi|646748728|gb|KK962132.1|	40478-54070	19	8.1	46	56	0.274587	0.82705	no
gi|646748728|gb|KK962132.1|	42868-54310	19	8.1	58	50	-0.204886	0.86565	no
gi|646748728|gb|KK962132.1|	44273-54553	19	8.1	0.82598	0.658515	-0.326891	1	no
gi|646748728|gb|KK962132.1|	45688-54743	19	8.1	0.372543	2	2	0.173	no
gi|646748728|gb|KK962132.1|	47602-54886	19	8.1	0.916846	0.921026	0.00656328	1	no

gi|646748728|gb|KK962132.1|	48988-55069	19	8.1	0.890788	2	1	0.36055	no
gi|646748728|gb|KK962132.1|	51136-55404	19	8.1	49	42	-0.211277	0.85895	no
gi|646748728|gb|KK962132.1|	04306-60447	19	8.1	467	91	-2	0.1274	no
gi|646748728|gb|KK962132.1|	48441-64953	19	8.1	2	3	0.354392	0.79625	no
gi|646748746|gb|KK962131.1|	37420-10379	23	9.6	6	1	-3	0.151	no
gi|646748746|gb|KK962131.1|	13797-21422	23	9.6	13	10	-0.391237	0.7755	no
gi|646748746|gb|KK962131.1|	70850-27201	23	9.6	0.815048	1	0.325041	1	no
gi|646748746|gb|KK962131.1|	84392-28607	23	9.6	5	2	-1	0.4396	no
gi|646748746|gb|KK962131.1|	36114-33818	23	9.6	8	8	0.129463	0.9304	no
gi|646748746|gb|KK962131.1|	55603-35579	23	9.6	55	148	1	0.40885	no
gi|646748746|gb|KK962131.1|	85651-38684	23	9.6	1	0.283787	-2	1	no
gi|646748746|gb|KK962131.1|	92432-39318	23	9.6	2	0.788318	-1	0.3853	no
gi|646748746|gb|KK962131.1|	94708-39719	23	9.6	0.952945	0.240677	-2	1	no
gi|646748746|gb|KK962131.1|	21208-42207	23	9.6	27	44	0.713169	0.57565	no
gi|646748746|gb|KK962131.1|	22616-42301	23	9.6	43	61	0.524416	0.72555	no
gi|646748746|gb|KK962131.1|	23931-42826	23	9.6	2	2	0.0800395	0.9564	no
gi|646748746|gb|KK962131.1|	28318-43094	23	9.6	2	1	-0.576369	0.714	no
gi|646748746|gb|KK962131.1|	32175-44265	23	9.6	12	11	-0.236429	0.84115	no
gi|646748746|gb|KK962131.1|	52210-45521	23	9.6	11	0.049019	-8	0.1665	no
gi|646748746|gb|KK962131.1|	03308-50412	23	9.6	2	3	0.818475	0.58245	no
gi|646748746|gb|KK962131.1|	29836-53051	23	9.6	2	2	-0.395934	0.7788	no
gi|646748746|gb|KK962131.1|	72033-57367	23	9.6	7	4	-0.961409	0.52605	no
gi|646748746|gb|KK962131.1|	73842-57619	23	9.6	1	0.96	-0.140721	1	no
gi|646748746|gb|KK962131.1|	76408-57885	23	9.6	1	0.980974	-0.416204	0.787	no
gi|646748746|gb|KK962131.1|	83165-58388	23	9.6	1	1	0.000284776	0.92345	no
gi|646748746|gb|KK962131.1|	84194-58603	23	9.6	0.757847	0.252962	-2	1	no
gi|646748746|gb|KK962131.1|	86243-58729	23	9.6	6	5	-0.314118	0.8345	no
gi|646748746|gb|KK962131.1|	87594-59119	23	9.6	14	12	-0.169566	0.87395	no
gi|646748746|gb|KK962131.1|	31136-63195	23	9.6	20	14	-0.503453	0.7357	no
gi|646748746|gb|KK962131.1|	57677-65786	23	9.6	33	69	1	0.4641	no

gi|646748746|gb|KK962131.1|	65850-66603	23	9.6	75	132	0.812815	0.55525	no
gi|646748746|gb|KK962131.1|	72874-67333	23	9.6	19.72	15	-0.380112	0.8071	no
gi|646748746|gb|KK962131.1|	80903-68416	23	9.6	19	20	0.0933771	0.9356	no
gi|646748746|gb|KK962131.1|	32694-83300	23	9.6	26	17	-0.612642	0.61595	no
gi|646748746|gb|KK962131.1|	87693-88881	23	9.6	0.63945	2	2	0.3034	no
gi|646748746|gb|KK962131.1|	89036-88974	23	9.6	2	1	-0.516174	0.72175	no
gi|646748771|gb|KK962130.1|	91095-19209	21	8.3	2	0.888836	-1	0.43875	no
gi|646748771|gb|KK962130.1|	37100-23897	21	8.3	0.057215	3	6	0.0973	no
gi|646748771|gb|KK962130.1|	60528-66169	21	8.3	0.808814	0.289539	-1	1	no
gi|646748771|gb|KK962130.1|	73215-67350	21	8.3	44	14	-2	0.2804	no
gi|646748771|gb|KK962130.1|	78635-67895	21	8.3	11	1	-3	0.19715	no
gi|646748771|gb|KK962130.1|	84658-68559	21	8.3	3	0.770628	-2	0.26335	no
gi|646748771|gb|KK962130.1|	10532-71304	21	8.3	1	0.297678	-2	1	no
gi|646748771|gb|KK962130.1|	13190-71526	21	8.3	0.457501	0.441942	-0.0499178	1	no
gi|646748771|gb|KK962130.1|	42274-74276	21	8.3	3	6	0.886305	0.51595	no
gi|646748771|gb|KK962130.1|	45038-74961	21	8.3	18	11	-0.753485	0.5057	no
gi|646748771|gb|KK962130.1|	52251-75288	21	8.3	3	2	-0.52959	0.6841	no
gi|646748771|gb|KK962130.1|	54729-75747	21	8.3	0.815802	3	2	0.21355	no
gi|646748771|gb|KK962130.1|	62510-76463	21	8.3	0.098896	39	9	0.2322	no
gi|646748771|gb|KK962130.1|	66184-76833	21	8.3	0	16	inf	5.00E-05	yes
gi|646748771|gb|KK962130.1|	07236-80969	21	8.3	11	8	-0.353424	0.74065	no
gi|646748771|gb|KK962130.1|	11347-81439	21	8.3	1	1	-0.284847	0.8535	no
gi|646748771|gb|KK962130.1|	14663-81675	21	8.3	0.7047	0.437666	-0.687177	1	no
gi|646748771|gb|KK962130.1|	16876-81854	21	8.3	0.52379	0.567254	0.115007	1	no
gi|646748771|gb|KK962130.1|	24301-82500	21	8.3	2	1	-0.65428	0.64775	no
gi|646748771|gb|KK962130.1|	28297-82905	21	8.3	17	30	0.820899	0.5885	no
gi|646748771|gb|KK962130.1|	29166-82975	21	8.3	1	3	1	0.4438	no
gi|646748771|gb|KK962130.1|	29900-83574	21	8.3	7	11	0.587268	0.6053	no
gi|646748771|gb|KK962130.1|	38798-84924	21	8.3	16	32	0.948531	0.4292	no
gi|646748771|gb|KK962130.1|	49932-85239	21	8.3	13	26	1	0.357	no

gi|646748771|gb|KK962130.1|	52550-85935	21	8.3	10	15	0.571275	0.62175	no
gi|646748771|gb|KK962130.1|	59864-86168	21	8.3	0.592668	5	3	0.0652	no
gi|646748771|gb|KK962130.1|	62099-86289	21	8.3	13	19	0.550372	0.71575	no
gi|646748771|gb|KK962130.1|	63125-87008	21	8.3	7	6	-0.0755828	0.94755	no
gi|646748771|gb|KK962130.1|	70854-87212	21	8.3	2	2	0.184346	0.91035	no
gi|646748771|gb|KK962130.1|	86449-88727	21	8.3	1015.05	977	-0.0555354	0.96375	no
gi|646748785|gb|KK962129.1|	81725-18297	20	8.2	4	6	0.589412	0.6989	no
gi|646748785|gb|KK962129.1|	83147-18366	20	8.2	5	8	0.61557	0.66805	no
gi|646748785|gb|KK962129.1|	84588-18650	20	8.2	14	8	-0.780107	0.5341	no
gi|646748785|gb|KK962129.1|	87619-18917	20	8.2	6	6	-0.0739859	0.9628	no
gi|646748785|gb|KK962129.1|	97480-19813	20	8.2	4	1	-2	0.2449	no
gi|646748785|gb|KK962129.1|	98280-19970	20	8.2	18	10	-0.783957	0.53275	no
gi|646748785|gb|KK962129.1|	02376-20454	20	8.2	6	7	0.120887	0.9366	no
gi|646748785|gb|KK962129.1|	11754-21448	20	8.2	10	8	-0.339184	0.74825	no
gi|646748785|gb|KK962129.1|	16279-21835	20	8.2	0.355834	0.515599	0.535045	1	no
gi|646748785|gb|KK962129.1|	18728-22073	20	8.2	1	0.766959	-0.536071	1	no
gi|646748785|gb|KK962129.1|	20904-22232	20	8.2	0.554438	0.798005	0.525371	1	no
gi|646748785|gb|KK962129.1|	25911-22656	20	8.2	0.466735	4	3	0.2509	no
gi|646748785|gb|KK962129.1|	27386-22858	20	8.2	1	0.703582	-0.966498	0.50955	no
gi|646748785|gb|KK962129.1|	23134-23449	20	8.2	11	6	-0.908059	0.559	no
gi|646748785|gb|KK962129.1|	54046-25472	20	8.2	52	24	-1	0.3783	no
gi|646748785|gb|KK962129.1|	83647-28431	20	8.2	0.890984	2	1	0.4335	no
gi|646748785|gb|KK962129.1|	89275-29016	20	8.2	6	12	0.883663	0.56525	no
gi|646748785|gb|KK962129.1|	95923-30694	20	8.2	7	8	0.202896	0.85655	no
gi|646748785|gb|KK962129.1|	14649-31593	20	8.2	0.715054	1	0.691842	0.6189	no
gi|646748785|gb|KK962129.1|	21993-32389	20	8.2	0.618648	0.895265	0.533196	1	no
gi|646748785|gb|KK962129.1|	24111-32982	20	8.2	5	3	-0.757879	0.552	no
gi|646748785|gb|KK962129.1|	30797-33244	20	8.2	1	1	0.0271753	0.9587	no
gi|646748785|gb|KK962129.1|	43785-34665	20	8.2	13	10	-0.409976	0.7011	no
gi|646748785|gb|KK962129.1|	54266-35520	20	8.2	28	21	-0.417681	0.73955	no

gi|646748785|gb|KK962129.1|	62654-36368	20	8.2	0.834452	0.84987	0.0264142	1	no
gi|646748785|gb|KK962129.1|	65471-36791	20	8.2	13	10	-0.408575	0.70335	no
gi|646748785|gb|KK962129.1|	75918-37629	20	8.2	15	8	-0.873304	0.4975	no
gi|646748785|gb|KK962129.1|	77089-37853	20	8.2	21	13	-0.69109	0.58405	no
gi|646748785|gb|KK962129.1|	80947-38237	20	8.2	9	7	-0.20708	0.88825	no
gi|646748785|gb|KK962129.1|	82529-38715	20	8.2	4	4	0.0528283	0.9706	no
gi|646748785|gb|KK962129.1|	87397-38800	20	8.2	5	4	-0.246542	0.83875	no
gi|646748785|gb|KK962129.1|	90185-39415	20	8.2	6	3	-1	0.36395	no
gi|646748785|gb|KK962129.1|	95895-39781	20	8.2	1	3	1	0.4009	no
gi|646748785|gb|KK962129.1|	98534-40027	20	8.2	2	5	1	0.3818	no
gi|646748785|gb|KK962129.1|	33441-43396	20	8.2	23	6	-2	0.15275	no
gi|646748785|gb|KK962129.1|	60425-46113	20	8.2	3	2	-0.516776	0.685	no
gi|646748785|gb|KK962129.1|	64154-46739	20	8.2	58	34	-0.765493	0.51375	no
gi|646748785|gb|KK962129.1|	67576-46922	20	8.2	1	0.095946	-4	0.1745	no
gi|646748785|gb|KK962129.1|	80260-48045	20	8.2	55	59	0.0992161	0.91985	no
gi|646748785|gb|KK962129.1|	83238-48351	20	8.2	19	7	-2	0.357	no
gi|646748785|gb|KK962129.1|	84076-48495	20	8.2	3	5	0.810064	0.55695	no
gi|646748785|gb|KK962129.1|	00498-50162	20	8.2	1	0	-inf	0.00225	no
gi|646748785|gb|KK962129.1|	01801-50226	20	8.2	10	9	-0.142925	0.9325	no
gi|646748785|gb|KK962129.1|	04572-50486	20	8.2	16	15	-0.0600848	0.94895	no
gi|646748785|gb|KK962129.1|	07819-50894	20	8.2	94	47	-1	0.3792	no
gi|646748785|gb|KK962129.1|	33545-53464	20	8.2	5	3	-0.580212	0.71895	no
gi|646748785|gb|KK962129.1|	45032-54803	20	8.2	4	4	0.0829521	0.9551	no
gi|646748785|gb|KK962129.1|	49544-55111	20	8.2	5	3	-0.866441	0.57225	no
gi|646748785|gb|KK962129.1|	53983-55699	20	8.2	9	5	-0.827832	0.50965	no
gi|646748785|gb|KK962129.1|	57856-55843	20	8.2	12	10	-0.246428	0.87265	no
gi|646748785|gb|KK962129.1|	63085-56461	20	8.2	20	20	-0.0573507	0.959	no
gi|646748785|gb|KK962129.1|	77856-57815	20	8.2	3	162	6	0.23385	no
gi|646748785|gb|KK962129.1|	79390-57998	20	8.2	0	13	inf	0.0006	yes
gi|646748785|gb|KK962129.1|	04908-60542	20	8.2	14	33	1	0.4211	no

gi|646748785|gb|KK962129.1|	07029-60759	20	8.2	0.596545	47	6	0.2328	no
gi|646748785|gb|KK962129.1|	19490-62093	20	8.2	0.624028	1	0.695704	1	no
gi|646748785|gb|KK962129.1|	23132-62451	20	8.2	0.163448	1	3	0.2578	no
gi|646748785|gb|KK962129.1|	25805-62986	20	8.2	6	6	0.0932691	0.93245	no
gi|646748785|gb|KK962129.1|	39084-63996	20	8.2	12	7	-0.750049	0.61705	no
gi|646748785|gb|KK962129.1|	41926-64375	20	8.2	2	3	0.174803	0.9079	no
gi|646748785|gb|KK962129.1|	46438-65599	20	8.2	10	5	-0.959363	0.4045	no
gi|646748785|gb|KK962129.1|	74582-67513	20	8.2	2	4	1	0.45385	no
gi|646748785|gb|KK962129.1|	83781-68409	20	8.2	16	17	0.0123501	0.9512	no
gi|646748785|gb|KK962129.1|	94421-69463	20	8.2	48	23	-1	0.471	no
gi|646748785|gb|KK962129.1|	81619-81943	20	8.2	19	7	-1	0.29295	no
gi|646748785|gb|KK962129.1|	82060-82401	20	8.2	6	6	0.038378	0.91525	no
gi|646748785|gb|KK962129.1|	96824-98638	20	8.2	2	0.171628	-4	0.128	no
gi|646748801|gb|KK962128.1|	70381-37103	12	9.2	3	0.319498	-3	0.1786	no
gi|646748819|gb|KK962127.1|	73444-17437	13	7.8	1	1	-0.0085873	0.9367	no
gi|646748819|gb|KK962127.1|	75283-17576	13	7.8	2	4	0.64361	0.6597	no
gi|646748819|gb|KK962127.1|	06271-40669	13	7.8	11	12	0.0777799	0.9554	no
gi|646748819|gb|KK962127.1|	49965-51505	13	7.8	3	1	-1	0.4486	no
gi|646748819|gb|KK962127.1|	58247-65846	13	7.8	190	121	-0.65081	0.68	no
gi|646748838|gb|KK962126.1|	54133-35456	13	5.6	6	1	-2	0.20015	no
gi|646748838|gb|KK962126.1|	02323-40322	13	5.6	2	0.202127	-3	0.18	no
gi|646748838|gb|KK962126.1|	09483-41077	13	5.6	2	2	0.159434	0.8995	no
gi|646748838|gb|KK962126.1|	25620-52606	13	5.6	5	2	-1	0.3894	no
gi|646748838|gb|KK962126.1|	64247-56543	13	5.6	0.6963	1	1	0.44735	no
gi|646748838|gb|KK962126.1|	65494-56632	13	5.6	2	0.90714	-1	0.3737	no
gi|646748838|gb|KK962126.1|	11707-61279	13	5.6	6	7	0.140995	0.92495	no
gi|646748838|gb|KK962126.1|	12904-61369	13	5.6	2	4	0.856556	0.54615	no
gi|646748838|gb|KK962126.1|	11894-71362	13	5.6	2	2	0.361102	0.8211	no
gi|646748855|gb|KK962125.1|	04269-10455	22	5.8	24	21	-0.143339	0.906	no
gi|646748855|gb|KK962125.1|	17361-11755	22	5.8	27	74	1	0.35755	no

gi|646748855|gb|KK962125.1|	18387-11889	22	5.8	3	2	-0.246377	0.8729	no
gi|646748855|gb|KK962125.1|	28469-12908	22	5.8	4	6	0.746161	0.5951	no
gi|646748855|gb|KK962125.1|	34644-14199	22	5.8	3	2	-0.511469	0.7414	no
gi|646748855|gb|KK962125.1|	42475-14487	22	5.8	78	56	-0.475923	0.69325	no
gi|646748855|gb|KK962125.1|	57303-15791	22	5.8	2	1	-0.535574	0.73	no
gi|646748855|gb|KK962125.1|	60555-16112	22	5.8	15	17	0.209471	0.895	no
gi|646748855|gb|KK962125.1|	61233-16292	22	5.8	3	2	-0.725999	0.64835	no
gi|646748855|gb|KK962125.1|	66352-16956	22	5.8	5	2	-0.996204	0.511	no
gi|646748855|gb|KK962125.1|	86082-19897	22	5.8	8	9	0.125441	0.9109	no
gi|646748855|gb|KK962125.1|	02704-20358	22	5.8	1	2	0.695218	0.629	no
gi|646748855|gb|KK962125.1|	04508-20658	22	5.8	1	0.956503	-0.0864245	1	no
gi|646748855|gb|KK962125.1|	13473-21819	22	5.8	5	10	0.974556	0.3701	no
gi|646748855|gb|KK962125.1|	47393-34797	22	5.8	2	3	0.62831	0.64705	no
gi|646748855|gb|KK962125.1|	55765-35626	22	5.8	9	5	-0.771221	0.59595	no
gi|646748855|gb|KK962125.1|	81471-38183	22	5.8	82	28	-2	0.31825	no
gi|646748855|gb|KK962125.1|	98597-39932	22	5.8	2	0.830018	-1	0.47555	no
gi|646748855|gb|KK962125.1|	11205-41378	22	5.8	457	67	-3	0.02775	no
gi|646748855|gb|KK962125.1|	15037-41587	22	5.8	908	197	-2	0.07635	no
gi|646748855|gb|KK962125.1|	81515-48220	22	5.8	2	0.899482	-1	0.47355	no
gi|646748855|gb|KK962125.1|	86849-48744	22	5.8	0.270636	4	4	0.0994	no
gi|646748855|gb|KK962125.1|	98188-50182	22	5.8	6	2	-1	0.3026	no
gi|646748855|gb|KK962125.1|	19879-52060	22	5.8	0.3969	3	3	0.26125	no
gi|646748855|gb|KK962125.1|	75874-67637	22	5.8	14	3	-2	0.1765	no
gi|646748855|gb|KK962125.1|	85410-85714	22	5.8	30	19	-0.631811	0.6535	no
gi|646748855|gb|KK962125.1|	95086-95543	22	5.8	2	4	0.670932	0.662	no
gi|646748871|gb|KK962124.1|	35940-13743	21	8.1	2	0.967488	-0.946997	0.52245	no
gi|646748871|gb|KK962124.1|	41684-14240	21	8.1	2	2	0.0706741	0.9485	no
gi|646748871|gb|KK962124.1|	57301-15756	21	8.1	12	18	0.630493	0.667	no
gi|646748871|gb|KK962124.1|	68063-16884	21	8.1	2	4	0.68542	0.6239	no
gi|646748871|gb|KK962124.1|	76059-17678	21	8.1	5	1	-2	0.2135	no

gi|646748871|gb|KK962124.1|	80202-28115	21	8.1	1	0.936689	-0.629886	0.65905	no
gi|646748871|gb|KK962124.1|	35715-43613	21	8.1	0	8	inf	0.001	yes
gi|646748871|gb|KK962124.1|	66154-66513	21	8.1	95	88	-0.102738	0.94385	no
gi|646748871|gb|KK962124.1|	85154-85379	21	8.1	546	690	0.338501	0.78645	no
gi|646748888|gb|KK962123.1|	08843-10909	20	8.1	9	25	2	0.35955	no
gi|646748888|gb|KK962123.1|	22230-12286	20	8.1	8	13	0.71121	0.6486	no
gi|646748888|gb|KK962123.1|	25640-12638	20	8.1	0.189134	21	7	0.0951	no
gi|646748888|gb|KK962123.1|	44601-14510	20	8.1	0	8	inf	0.00435	no
gi|646748888|gb|KK962123.1|	22372-22306	20	8.1	12	1	-3	0.0828	no
gi|646748888|gb|KK962123.1|	24400-22524	20	8.1	2	2	0.0394597	0.94695	no
gi|646748888|gb|KK962123.1|	49896-25111	20	8.1	1	0.55199	-1	0.41495	no
gi|646748888|gb|KK962123.1|	51279-25208	20	8.1	55	33	-0.71987	0.4986	no
gi|646748888|gb|KK962123.1|	74283-27486	20	8.1	0	4	inf	0.0014	yes
gi|646748888|gb|KK962123.1|	36159-36947	20	8.1	11	6	-0.965771	0.535	no
gi|646748888|gb|KK962123.1|	99680-40130	20	8.1	41	38	-0.104083	0.92625	no
gi|646748888|gb|KK962123.1|	34318-43588	20	8.1	0	2	inf	0.00435	no
gi|646748888|gb|KK962123.1|	51717-45192	20	8.1	227	300	0.403829	0.791	no
gi|646748888|gb|KK962123.1|	54182-45438	20	8.1	39	51	0.396749	0.8008	no
gi|646748888|gb|KK962123.1|	55417-45565	20	8.1	0	43	inf	0.0014	yes
gi|646748888|gb|KK962123.1|	60782-46376	20	8.1	1	0.640724	-0.665594	1	no
gi|646748888|gb|KK962123.1|	63937-46532	20	8.1	1	1	-0.241118	0.84115	no
gi|646748888|gb|KK962123.1|	65421-46605	20	8.1	105	70	-0.586689	0.5914	no
gi|646748888|gb|KK962123.1|	78161-47842	20	8.1	23	3	-3	0.1932	no
gi|646748888|gb|KK962123.1|	78609-47920	20	8.1	1	4	2	0.27865	no
gi|646748888|gb|KK962123.1|	79679-48583	20	8.1	15	11	-0.448329	0.70125	no
gi|646748888|gb|KK962123.1|	97081-50016	20	8.1	8	4	-0.944186	0.4557	no
gi|646748888|gb|KK962123.1|	49920-50737	20	8.1	3	0.928873	-2	0.2476	no
gi|646748888|gb|KK962123.1|	18665-52032	20	8.1	6	4	-0.636172	0.6701	no
gi|646748888|gb|KK962123.1|	25996-52665	20	8.1	3	2	-1	0.43055	no
gi|646748888|gb|KK962123.1|	34865-53565	20	8.1	1	1	0.271593	0.8456	no

gi|646748888|gb|KK962123.1|	59274-55971	20	8.1	7	9	0.398739	0.74265	no
gi|646748888|gb|KK962123.1|	98600-59998	20	8.1	3	1	-1	0.41455	no
gi|646748888|gb|KK962123.1|	13967-61554	20	8.1	0.83976	0.504709	-0.734525	1	no
gi|646748888|gb|KK962123.1|	15659-61754	20	8.1	0.568279	0.740035	0.380992	1	no
gi|646748888|gb|KK962123.1|	18260-61931	20	8.1	5	2	-1	0.3021	no
gi|646748888|gb|KK962123.1|	21354-62512	20	8.1	2	2	0.0580177	0.9696	no
gi|646748888|gb|KK962123.1|	25220-62707	20	8.1	0.75169	0.669161	-0.167784	1	no
gi|646748888|gb|KK962123.1|	39401-64059	20	8.1	22	5	-2	0.1153	no
gi|646748888|gb|KK962123.1|	52121-65489	20	8.1	9	5	-0.947911	0.45475	no
gi|646748888|gb|KK962123.1|	55619-65769	20	8.1	1	2	0.857011	0.53235	no
gi|646748888|gb|KK962123.1|	58191-65876	20	8.1	0.5728	4	3	0.2672	no
gi|646748888|gb|KK962123.1|	90409-69061	20	8.1	0	268	inf	0.0002	yes
gi|646748888|gb|KK962123.1|	04106-70450	20	8.1	0	35	inf	0.00025	yes
gi|646748888|gb|KK962123.1|	60784-76158	20	8.1	0	3	inf	0.0014	yes
gi|646748888|gb|KK962123.1|	61840-76293	20	8.1	0	2	inf	0.0014	yes
gi|646748888|gb|KK962123.1|	64179-76664	20	8.1	0.961557	42	5	0.011	no
gi|646748888|gb|KK962123.1|	23007-82551	20	8.1	0.698719	117	7	0.00225	no
gi|646748888|gb|KK962123.1|	31600-83225	20	8.1	0.229907	4	4	0.0999	no
gi|646748888|gb|KK962123.1|	68884-87014	20	8.1	2	185	7	0.00135	yes
gi|646748888|gb|KK962123.1|	71100-87212	20	8.1	0.241842	2	3	0.2655	no
gi|646748888|gb|KK962123.1|	94617-95712	20	8.1	8	3	-1	0.4121	no
gi|646748903|gb|KK962122.1|	64908-16762	17	8.2	1	2	0.298568	0.84595	no
gi|646748922|gb|KK962121.1|	25695-52641	12	7.5	2	1	-0.515291	0.72175	no
gi|646748937|gb|KK962120.1|	41274-34347	18	6.5	5	5	-0.0225515	0.9885	no
gi|646748955|gb|KK962119.1|	12969-13677	19	9.9	4	1	-2	0.2831	no
gi|646748955|gb|KK962119.1|	14860-16043	19	9.9	0.498429	2	2	0.2458	no
gi|646748955|gb|KK962119.1|	73690-17400	19	9.9	169	3	-6	0.1067	no
gi|646748955|gb|KK962119.1|	75562-17663	19	9.9	35	26	-0.426171	0.69595	no
gi|646748955|gb|KK962119.1|	94636-19653	19	9.9	336	45	-3	0.02345	no
gi|646748955|gb|KK962119.1|	00822-20182	19	9.9	27	7	-2	0.1486	no

gi|646748955|gb|KK962119.1|	03665-20470	19	9.9	25	8	-2	0.2414	no
gi|646748955|gb|KK962119.1|	06144-20627	19	9.9	49937.9	14740.7	-2	0.17855	no
gi|646748955|gb|KK962119.1|	20762-21532	19	9.9	3	2	-0.730765	0.5852	no
gi|646748955|gb|KK962119.1|	12227-21235	19	9.9	6696.74	1168.76	-3	0.1192	no
gi|646748955|gb|KK962119.1|	13313-21363	19	9.9	96	8	-4	0.03925	no
gi|646748955|gb|KK962119.1|	18192-21934	19	9.9	286	152	-0.909732	0.44655	no
gi|646748955|gb|KK962119.1|	23733-22385	19	9.9	37829	1622.27	-5	0.05085	no
gi|646748955|gb|KK962119.1|	25137-22524	19	9.9	86279.8	2200.11	-5	0.17775	no
gi|646748955|gb|KK962119.1|	32344-23333	19	9.9	118	20	-3	0.07915	no
gi|646748955|gb|KK962119.1|	33857-23498	19	9.9	1	2	0.653769	0.6155	no
gi|646748955|gb|KK962119.1|	40949-24137	19	9.9	48	1	-5	0.1085	no
gi|646748955|gb|KK962119.1|	42472-24309	19	9.9	4	0.350674	-4	0.1755	no
gi|646748955|gb|KK962119.1|	78422-27904	19	9.9	3	4	0.592489	0.6289	no
gi|646748955|gb|KK962119.1|	93852-29493	19	9.9	4	4	0.0364065	0.9806	no
gi|646748955|gb|KK962119.1|	98536-29918	19	9.9	69	16	-2	0.1216	no
gi|646748955|gb|KK962119.1|	99999-30049	19	9.9	8	10	0.4255	0.7555	no
gi|646748955|gb|KK962119.1|	04446-30459	19	9.9	7462.12	809.68	-3	0.0374	no
gi|646748955|gb|KK962119.1|	32309-34367	19	9.9	2	2	-0.117196	0.94045	no
gi|646748955|gb|KK962119.1|	23486-32360	19	9.9	167729	39985.5	-2	0.12985	no
gi|646748955|gb|KK962119.1|	24033-32459	19	9.9	227.03	145	-0.644068	0.5693	no
gi|646748955|gb|KK962119.1|	30455-33057	19	9.9	31856	3415.2	-3	0.0361	no
gi|646748955|gb|KK962119.1|	39045-34033	19	9.9	164.87	99	-0.739453	0.53465	no
gi|646748955|gb|KK962119.1|	42977-34313	19	9.9	22112	2540.77	-3	0.03465	no
gi|646748955|gb|KK962119.1|	48290-34861	19	9.9	11	3	-2	0.21775	no
gi|646748955|gb|KK962119.1|	48773-34959	19	9.9	0.329171	2	3	0.2578	no
gi|646748955|gb|KK962119.1|	64015-36475	19	9.9	7	5	-0.433708	0.7829	no
gi|646748955|gb|KK962119.1|	66542-36675	19	9.9	406	69	-3	0.087	no
gi|646748955|gb|KK962119.1|	78463-37862	19	9.9	187191	13424.1	-4	0.0037	no
gi|646748955|gb|KK962119.1|	38160-39417	19	9.9	0.55395	0.529897	-0.064043	1	no
gi|646748955|gb|KK962119.1|	89758-39037	19	9.9	57	45	-0.327908	0.79645	no

gi|646748955|gb|KK962119.1|	97648-39944	19	9.9	165	93	-0.82359	0.49065	no
gi|646748955|gb|KK962119.1|	08491-41009	19	9.9	463	219	-1	0.37785	no
gi|646748955|gb|KK962119.1|	16238-41671	19	9.9	6	13	1	0.42415	no
gi|646748955|gb|KK962119.1|	17354-41751	19	9.9	6875.07	204	-5	0.0125	no
gi|646748955|gb|KK962119.1|	43315-44438	19	9.9	4	3	-0.23831	0.8791	no
gi|646748955|gb|KK962119.1|	46593-44709	19	9.9	43	23	-0.91639	0.5481	no
gi|646748955|gb|KK962119.1|	45024-51854	19	9.9	11	7	-0.694641	0.53755	no
gi|646748955|gb|KK962119.1|	59641-46004	19	9.9	862	244	-2	0.1257	no
gi|646748955|gb|KK962119.1|	75516-47570	19	9.9	4853.4	3734.11	-0.378233	0.7307	no
gi|646748955|gb|KK962119.1|	76767-47736	19	9.9	36	1	-5	0.0772	no
gi|646748955|gb|KK962119.1|	97708-49803	19	9.9	20	16	-0.261936	0.8324	no
gi|646748955|gb|KK962119.1|	02745-50347	19	9.9	265	100	-1	0.2283	no
gi|646748955|gb|KK962119.1|	03745-50397	19	9.9	4815.39	1757.08	-1	0.218	no
gi|646748955|gb|KK962119.1|	09617-51171	19	9.9	40	19	-1	0.3353	no
gi|646748955|gb|KK962119.1|	19262-52007	19	9.9	8	10	0.283159	0.8582	no
gi|646748955|gb|KK962119.1|	52428-55330	19	9.9	12	11	-0.168463	0.87755	no
gi|646748955|gb|KK962119.1|	35937-53674	19	9.9	43	5	-3	0.04935	no
gi|646748955|gb|KK962119.1|	48987-55322	19	9.9	86	10	-3	0.0151	no
gi|646748955|gb|KK962119.1|	59474-56035	19	9.9	5	0.208974	-5	0.1763	no
gi|646748955|gb|KK962119.1|	60477-56178	19	9.9	5	0.378928	-4	0.0908	no
gi|646748955|gb|KK962119.1|	66546-56700	19	9.9	6	0	-inf	0.00225	no
gi|646748955|gb|KK962119.1|	82746-58709	19	9.9	7	6	-0.187791	0.8629	no
gi|646748955|gb|KK962119.1|	58900-59434	19	9.9	7	9	0.375838	0.81905	no
gi|646748955|gb|KK962119.1|	06070-60770	19	9.9	0.806223	0.096975	-3	1	no
gi|646748955|gb|KK962119.1|	37221-63834	19	9.9	3	4	0.369341	0.8116	no
gi|646748955|gb|KK962119.1|	38776-63911	19	9.9	5	6	0.302791	0.85295	no
gi|646748955|gb|KK962119.1|	45154-64585	19	9.9	3	5	0.823648	0.56455	no
gi|646748955|gb|KK962119.1|	66328-66919	19	9.9	9	4	-1	0.41715	no
gi|646748955|gb|KK962119.1|	69316-67654	19	9.9	14	7	-0.971143	0.39285	no
gi|646748955|gb|KK962119.1|	84401-68558	19	9.9	1	0	-inf	1	no

gi|646748955|gb|KK962119.1|	68984-71592	19	9.9	3	1	-1	0.3834	no
gi|646748955|gb|KK962119.1|	30321-73185	19	9.9	8	22	1	0.2637	no
gi|646748955|gb|KK962119.1|	37760-73930	19	9.9	1	2	0.293453	0.8322	no
gi|646748955|gb|KK962119.1|	42774-74465	19	9.9	20.99	25	0.223681	0.8373	no
gi|646748955|gb|KK962119.1|	54124-75695	19	9.9	2	0.784001	-2	0.33155	no
gi|646748955|gb|KK962119.1|	57202-75887	19	9.9	0.651052	0.658128	0.0155973	1	no
gi|646748955|gb|KK962119.1|	59515-76045	19	9.9	3	3	-0.209952	0.87685	no
gi|646748955|gb|KK962119.1|	76653-76997	19	9.9	8	6	-0.542482	0.7222	no
gi|646748955|gb|KK962119.1|	78479-78693	19	9.9	57	28	-1	0.45515	no
gi|646748955|gb|KK962119.1|	88637-78888	19	9.9	42	12	-2	0.2372	no
gi|646748955|gb|KK962119.1|	00982-80161	19	9.9	12	8	-0.500694	0.7514	no
gi|646748955|gb|KK962119.1|	14944-81524	19	9.9	9	8	-0.0297426	0.91525	no
gi|646748955|gb|KK962119.1|	25610-83125	19	9.9	15	10	-0.485964	0.67255	no
gi|646748955|gb|KK962119.1|	83477-83817	19	9.9	34	2	-4	0.1241	no
gi|646748955|gb|KK962119.1|	39110-84038	19	9.9	0.90696	0.520747	-0.800457	1	no
gi|646748955|gb|KK962119.1|	41428-84272	19	9.9	0.445189	0.894994	1	1	no
gi|646748955|gb|KK962119.1|	49913-85135	19	9.9	7	3	-1	0.40645	no
gi|646748955|gb|KK962119.1|	53927-85499	19	9.9	14	7	-1	0.48235	no
gi|646748971|gb|KK962118.1|	36111-13652	22	11	40	16	-1	0.4076	no
gi|646748971|gb|KK962118.1|	42285-14256	22	11	95	49	-0.961118	0.53715	no
gi|646748971|gb|KK962118.1|	60993-16239	22	11	1	2	0.137783	0.9072	no
gi|646748971|gb|KK962118.1|	62612-16368	22	11	0.112675	2	4	0.09905	no
gi|646748971|gb|KK962118.1|	72771-17351	22	11	4	3	-0.318676	0.794	no
gi|646748971|gb|KK962118.1|	05311-20670	22	11	2	2	-0.119243	0.93875	no
gi|646748971|gb|KK962118.1|	09824-21085	22	11	1	1	-0.225021	0.86285	no
gi|646748971|gb|KK962118.1|	23547-22587	22	11	1	1	-0.507218	0.75435	no
gi|646748971|gb|KK962118.1|	23269-23565	22	11	21	41	1	0.45965	no
gi|646748971|gb|KK962118.1|	78392-27949	22	11	6	0	-inf	0.00025	yes
gi|646748971|gb|KK962118.1|	85951-28704	22	11	16	0.158277	-7	0.1673	no
gi|646748971|gb|KK962118.1|	29783-31321	22	11	16	4	-2	0.1136	no

gi|646748971|gb|KK962118.1|	01363-30177	22	11	2	6	2	0.2744	no
gi|646748971|gb|KK962118.1|	16048-31834	22	11	0.814339	0.459485	-0.825611	1	no
gi|646748971|gb|KK962118.1|	27385-32836	22	11	1	1	-0.142937	0.9179	no
gi|646748971|gb|KK962118.1|	30835-33120	22	11	5	3	-0.888357	0.6013	no
gi|646748971|gb|KK962118.1|	32603-33308	22	11	2	7	2	0.21735	no
gi|646748971|gb|KK962118.1|	34433-33523	22	11	2	2	0.173111	0.8983	no
gi|646748971|gb|KK962118.1|	40683-34219	22	11	0.734813	1	0.527317	1	no
gi|646748971|gb|KK962118.1|	50725-35413	22	11	1	2	1	0.4522	no
gi|646748971|gb|KK962118.1|	56798-35988	22	11	0.782963	2	1	0.4541	no
gi|646748971|gb|KK962118.1|	69297-36941	22	11	3435.12	5123.08	0.576649	0.7246	no
gi|646748971|gb|KK962118.1|	46336-46931	22	11	2	3	0.823819	0.56315	no
gi|646748971|gb|KK962118.1|	58540-59364	22	11	6	16	1	0.38575	no
gi|646748971|gb|KK962118.1|	62389-64032	22	11	6	4	-0.667394	0.6603	no
gi|646748989|gb|KK962117.1|	00755-40145	13	6.4	3	0.876336	-2	0.24535	no
gi|646748989|gb|KK962117.1|	23481-42387	13	6.4	1	7	3	0.276	no
gi|646748989|gb|KK962117.1|	24051-42699	13	6.4	0.310122	2	3	0.11655	no
gi|646748989|gb|KK962117.1|	27211-42867	13	6.4	0.763304	2	1	0.4342	no
gi|646749005|gb|KK962116.1|	60944-16172	21	8.5	0.529546	2	2	0.2903	no
gi|646749005|gb|KK962116.1|	67853-16902	21	8.5	1	1	-0.13409	1	no
gi|646749005|gb|KK962116.1|	70069-17077	21	8.5	3	8	2	0.29205	no
gi|646749005|gb|KK962116.1|	85415-18596	21	8.5	5	17	2	0.2323	no
gi|646749005|gb|KK962116.1|	46224-24737	21	8.5	0.722717	1	0.517225	1	no
gi|646749005|gb|KK962116.1|	39610-33999	21	8.5	0	9	inf	0.0014	yes
gi|646749005|gb|KK962116.1|	52774-35482	21	8.5	2	0.298353	-2	0.16875	no
gi|646749005|gb|KK962116.1|	55308-35632	21	8.5	1	0.522353	-1	1	no
gi|646749005|gb|KK962116.1|	62704-36397	21	8.5	1	0.786401	-0.701133	0.5887	no
gi|646749005|gb|KK962116.1|	69110-36933	21	8.5	25	56	1	0.4107	no
gi|646749005|gb|KK962116.1|	70846-37143	21	8.5	5	2	-2	0.2223	no
gi|646749005|gb|KK962116.1|	20878-42120	21	8.5	12	4	-2	0.29795	no
gi|646749005|gb|KK962116.1|	35870-43819	21	8.5	2	2	0.367229	0.81035	no

gi|646749005|gb|KK962116.1|	44623-44520	21	8.5	3	4	0.456473	0.72525	no
gi|646749005|gb|KK962116.1|	69054-46924	21	8.5	199.75	167	-0.261138	0.85305	no
gi|646749005|gb|KK962116.1|	14841-51622	21	8.5	5	2	-1	0.40835	no
gi|646749020|gb|KK962115.1|	15335-11588	19	6.3	29	19	-0.598937	0.6926	no
gi|646749020|gb|KK962115.1|	30906-13111	19	6.3	42	35	-0.259442	0.86925	no
gi|646749020|gb|KK962115.1|	31915-13211	19	6.3	72	45	-0.679194	0.62515	no
gi|646749020|gb|KK962115.1|	39354-13956	19	6.3	23	57	1	0.4163	no
gi|646749020|gb|KK962115.1|	00999-20148	19	6.3	10	7	-0.638168	0.64265	no
gi|646749020|gb|KK962115.1|	83450-28415	19	6.3	3	4	0.589216	0.62695	no
gi|646749020|gb|KK962115.1|	02840-40354	19	6.3	2	0.85613	-1	0.4263	no
gi|646749020|gb|KK962115.1|	05634-40622	19	6.3	2	2	-0.346871	0.81865	no
gi|646749020|gb|KK962115.1|	06665-40705	19	6.3	7	8	0.205943	0.8825	no
gi|646749020|gb|KK962115.1|	26366-42973	19	6.3	28	29	0.0281426	0.9808	no
gi|646749020|gb|KK962115.1|	33799-43458	19	6.3	4	4	0.188256	0.8903	no
gi|646749020|gb|KK962115.1|	37027-43742	19	6.3	47	30	-0.655851	0.66205	no
gi|646749020|gb|KK962115.1|	61683-46194	19	6.3	316	0	-inf	0.0004	yes
gi|646749020|gb|KK962115.1|	97582-49990	19	6.3	37	6	-3	0.0757	no
gi|646749020|gb|KK962115.1|	10507-51095	19	6.3	3	7	1	0.4347	no
gi|646749020|gb|KK962115.1|	12230-51310	19	6.3	29	1	-4	0.01915	no
gi|646749020|gb|KK962115.1|	16504-51753	19	6.3	4	7	1	0.49605	no
gi|646749020|gb|KK962115.1|	27891-52821	19	6.3	18	11	-0.728341	0.5833	no
gi|646749020|gb|KK962115.1|	53603-54155	19	6.3	0	12	inf	0.0012	yes
gi|646749020|gb|KK962115.1|	55748-56105	19	6.3	0	10	inf	0.00255	no
gi|646749020|gb|KK962115.1|	16546-61676	19	6.3	84.82	109.76	0.371875	0.78475	no
gi|646749020|gb|KK962115.1|	22409-62370	19	6.3	0.531668	1	1	0.3963	no
gi|646749020|gb|KK962115.1|	62421-63309	19	6.3	11	8	-0.45872	0.7599	no
gi|646749020|gb|KK962115.1|	69334-70068	19	6.3	2	5	0.989594	0.5252	no
gi|646749020|gb|KK962115.1|	74762-75574	19	6.3	5	5	0.21236	0.8942	no
gi|646749020|gb|KK962115.1|	77888-78555	19	6.3	18	2	-3	0.0659	no
gi|646749036|gb|KK962114.1|	10464-11041	20	7.5	1	3	1	0.44985	no

gi|646749036|gb|KK962114.1|	78438-18093	20	7.5	2	2	0.610459	0.68735	no
gi|646749036|gb|KK962114.1|	81003-18214	20	7.5	1	2	0.516871	0.6803	no
gi|646749036|gb|KK962114.1|	82199-18368	20	7.5	0.901097	2	1	0.36395	no
gi|646749036|gb|KK962114.1|	86911-19034	20	7.5	9	7	-0.431194	0.6866	no
gi|646749036|gb|KK962114.1|	91532-19427	20	7.5	1	1	-0.0679512	0.96495	no
gi|646749036|gb|KK962114.1|	03824-20495	20	7.5	2	0.754431	-1	0.41105	no
gi|646749036|gb|KK962114.1|	52866-25300	20	7.5	4772.63	1400.58	-2	0.28285	no
gi|646749036|gb|KK962114.1|	67383-46749	20	7.5	7810.22	5297.32	-0.560101	0.7088	no
gi|646749036|gb|KK962114.1|	78274-47869	20	7.5	54	29	-0.902911	0.54665	no
gi|646749036|gb|KK962114.1|	81676-48195	20	7.5	76	51	-0.558837	0.7206	no
gi|646749036|gb|KK962114.1|	83189-48359	20	7.5	1	6	2	0.2893	no
gi|646749036|gb|KK962114.1|	87382-48777	20	7.5	98	27	-2	0.23315	no
gi|646749036|gb|KK962114.1|	89595-49190	20	7.5	10	10	-0.0114634	0.99065	no
gi|646749036|gb|KK962114.1|	12578-51522	20	7.5	0.116133	5	5	0.10675	no
gi|646749036|gb|KK962114.1|	24374-52473	20	7.5	0.77537	21	5	0.10445	no
gi|646749036|gb|KK962114.1|	35235-53632	20	7.5	12	17	0.462211	0.75845	no
gi|646749036|gb|KK962114.1|	56132-55638	20	7.5	34	67	0.983565	0.5154	no
gi|646749036|gb|KK962114.1|	71211-57137	20	7.5	117	205	0.813365	0.59985	no
gi|646749036|gb|KK962114.1|	74404-57471	20	7.5	17	10	-0.739808	0.56085	no
gi|646749036|gb|KK962114.1|	25186-62540	20	7.5	54	49	-0.133217	0.9225	no
gi|646749036|gb|KK962114.1|	58528-65867	20	7.5	1351.39	1263.03	-0.0975512	0.95025	no
gi|646749036|gb|KK962114.1|	36187-73650	20	7.5	1	12	3	0.10895	no
gi|646749036|gb|KK962114.1|	44258-74472	20	7.5	4	6	0.412405	0.77005	no
gi|646749036|gb|KK962114.1|	47998-74997	20	7.5	6	5	-0.441708	0.76835	no
gi|646749036|gb|KK962114.1|	50590-75263	20	7.5	101	54	-0.890385	0.4509	no
gi|646749036|gb|KK962114.1|	57272-75754	20	7.5	9	19	1	0.4584	no
gi|646749036|gb|KK962114.1|	59242-76114	20	7.5	7	6	-0.293291	0.8472	no
gi|646749036|gb|KK962114.1|	66421-76803	20	7.5	0.681251	1	1	0.47045	no
gi|646749036|gb|KK962114.1|	68240-76860	20	7.5	11	8	-0.479096	0.7138	no
gi|646749036|gb|KK962114.1|	72209-77251	20	7.5	33	72	1	0.4706	no

gi|646749051|gb|KK962113.1|	03291-10405	19	8.4	59	34	-0.778505	0.54185	no
gi|646749051|gb|KK962113.1|	21211-12307	19	8.4	14	7	-0.944404	0.46155	no
gi|646749051|gb|KK962113.1|	50136-15371	19	8.4	1	1	-0.0420366	0.97845	no
gi|646749051|gb|KK962113.1|	58724-16182	19	8.4	33	20	-0.748012	0.51435	no
gi|646749051|gb|KK962113.1|	61974-16993	19	8.4	20	24	0.249626	0.83655	no
gi|646749051|gb|KK962113.1|	84989-18761	19	8.4	4	1	-2	0.23965	no
gi|646749051|gb|KK962113.1|	93260-19582	19	8.4	2	1	-0.429535	0.78295	no
gi|646749051|gb|KK962113.1|	97749-19913	19	8.4	4	1	-1	0.304	no
gi|646749051|gb|KK962113.1|	99352-20047	19	8.4	3	0.455189	-3	0.1859	no
gi|646749051|gb|KK962113.1|	02669-20369	19	8.4	1	0.51639	-1	1	no
gi|646749051|gb|KK962113.1|	68441-26870	19	8.4	34	17	-0.946197	0.4842	no
gi|646749051|gb|KK962113.1|	74746-27653	19	8.4	3	0	-inf	0.0008	yes
gi|646749051|gb|KK962113.1|	07437-30931	19	8.4	139	10	-4	0.01775	no
gi|646749051|gb|KK962113.1|	22110-32233	19	8.4	164	112	-0.551383	0.7256	no
gi|646749051|gb|KK962113.1|	90442-39083	19	8.4	15	12	-0.317309	0.85005	no
gi|646749051|gb|KK962113.1|	03786-40518	19	8.4	6	2	-2	0.31745	no
gi|646749051|gb|KK962113.1|	12670-41296	19	8.4	24	20	-0.23218	0.85155	no
gi|646749051|gb|KK962113.1|	32638-43495	19	8.4	37	14	-1	0.2164	no
gi|646749051|gb|KK962113.1|	36442-43771	19	8.4	0.91053	0.261376	-2	1	no
gi|646749051|gb|KK962113.1|	40452-44361	19	8.4	39	9	-2	0.07085	no
gi|646749051|gb|KK962113.1|	44540-44526	19	8.4	8	7	-0.215154	0.8902	no
gi|646749051|gb|KK962113.1|	46096-44707	19	8.4	1	0.544718	-0.906016	1	no
gi|646749051|gb|KK962113.1|	58141-45843	19	8.4	14	14	0.0515305	0.9426	no
gi|646749051|gb|KK962113.1|	79714-48111	19	8.4	6	4	-0.496303	0.73955	no
gi|646749051|gb|KK962113.1|	50006-50417	19	8.4	9	7	-0.301714	0.809	no
gi|646749051|gb|KK962113.1|	31513-53185	19	8.4	25	13	-0.915532	0.51375	no
gi|646749051|gb|KK962113.1|	57972-56508	19	8.4	34	36	0.0766593	0.94785	no
gi|646749051|gb|KK962113.1|	73232-57723	19	8.4	2	2	0.173176	0.90385	no
gi|646749051|gb|KK962113.1|	77386-57971	19	8.4	0.80334	1	0.759561	0.5829	no
gi|646749051|gb|KK962113.1|	83085-58537	19	8.4	0.81834	0.725557	-0.17361	1	no

gi|646749051|gb|KK962113.1|	90856-59615	19	8.4	1	1	0.162873	0.91685	no
gi|646749051|gb|KK962113.1|	96984-60015	19	8.4	0.921074	1	0.268566	1	no
gi|646749051|gb|KK962113.1|	01944-60557	19	8.4	2	3	0.720861	0.6366	no
gi|646749051|gb|KK962113.1|	06961-60727	19	8.4	16	27.27	0.809354	0.5733	no
gi|646749051|gb|KK962113.1|	32819-63306	19	8.4	14	31	1	0.4504	no
gi|646749051|gb|KK962113.1|	54569-65592	19	8.4	0.502323	1	1	0.3963	no
gi|646749051|gb|KK962113.1|	65575-65854	19	8.4	47	4	-4	0.1378	no
gi|646749051|gb|KK962113.1|	56236-65687	19	8.4	2	1	-0.336326	0.81865	no
gi|646749051|gb|KK962113.1|	66752-67134	19	8.4	12	6	-1	0.41555	no
gi|646749051|gb|KK962113.1|	72110-67283	19	8.4	32	45	0.494871	0.6895	no
gi|646749051|gb|KK962113.1|	85040-85330	19	8.4	14	11	-0.369683	0.79025	no
gi|646749051|gb|KK962113.1|	88061-91041	19	8.4	0.984615	0.642123	-0.61671	1	no
gi|646749051|gb|KK962113.1|	95985-98351	19	8.4	11	4	-2	0.2043	no
gi|646749065|gb|KK962112.1|	25555-12621	20	7.9	0.453093	6	4	0.2563	no
gi|646749065|gb|KK962112.1|	26319-12720	20	7.9	3	6	1	0.427	no
gi|646749065|gb|KK962112.1|	36905-13756	20	7.9	4	6	0.397868	0.77175	no
gi|646749065|gb|KK962112.1|	42022-14229	20	7.9	103	78	-0.406352	0.7921	no
gi|646749065|gb|KK962112.1|	62366-16260	20	7.9	37	35	-0.0950978	0.93945	no
gi|646749065|gb|KK962112.1|	64704-16523	20	7.9	24	12	-0.992341	0.52295	no
gi|646749065|gb|KK962112.1|	18926-19081	20	7.9	5299.33	2357.62	-1	0.4411	no
gi|646749065|gb|KK962112.1|	60860-26242	20	7.9	5	0.203549	-5	0.11085	no
gi|646749065|gb|KK962112.1|	68249-26911	20	7.9	42	30	-0.504088	0.63765	no
gi|646749065|gb|KK962112.1|	73202-27347	20	7.9	32	31	-0.0424867	0.95985	no
gi|646749065|gb|KK962112.1|	76200-27666	20	7.9	11	14	0.353511	0.8221	no
gi|646749065|gb|KK962112.1|	77603-27827	20	7.9	3	4	0.584756	0.6557	no
gi|646749065|gb|KK962112.1|	01211-30139	20	7.9	24	86	2	0.3674	no
gi|646749065|gb|KK962112.1|	02310-30260	20	7.9	10	23	1	0.37235	no
gi|646749065|gb|KK962112.1|	14469-31745	20	7.9	12	11	-0.150079	0.8838	no
gi|646749065|gb|KK962112.1|	24373-32807	20	7.9	68	14	-2	0.05805	no
gi|646749065|gb|KK962112.1|	30029-33432	20	7.9	34	5	-3	0.02145	no

gi|646749065|gb|KK962112.1|	41339-34225	20	7.9	0.843476	0.797812	-0.0802985	1	no
gi|646749082|gb|KK962111.1|	06517-10077	20	9.3	1	2	0.761029	0.54915	no
gi|646749082|gb|KK962111.1|	11518-10135	20	9.3	0.926197	2	1	0.39715	no
gi|646749082|gb|KK962111.1|	16312-10287	20	9.3	8	7	-0.123818	0.9107	no
gi|646749082|gb|KK962111.1|	30780-10327	20	9.3	17	9	-0.86728	0.49985	no
gi|646749082|gb|KK962111.1|	46238-10468	20	9.3	3	1	-2	0.3498	no
gi|646749082|gb|KK962111.1|	53162-10533	20	9.3	15	118	3	0.1182	no
gi|646749082|gb|KK962111.1|	09044-11117	20	9.3	2	2	-0.0784082	0.95765	no
gi|646749082|gb|KK962111.1|	76101-17683	20	9.3	3	4	0.679603	0.6241	no
gi|646749082|gb|KK962111.1|	18754-21935	20	9.3	3	5	0.842223	0.51495	no
gi|646749082|gb|KK962111.1|	19036-42058	20	9.3	0	12	inf	5.00E-05	yes
gi|646749082|gb|KK962111.1|	23031-42415	20	9.3	1	0	-inf	0.00475	no
gi|646749082|gb|KK962111.1|	89861-59085	20	9.3	0.872679	1	0.287611	1	no
gi|646749082|gb|KK962111.1|	98077-79891	20	9.3	0	8	inf	0.00065	yes
gi|646749082|gb|KK962111.1|	85133-99682	20	9.3	2	2	0.457092	0.66525	no
gi|646749098|gb|KK962110.1|	00526-10232	20	7.7	16	12	-0.415195	0.6964	no
gi|646749098|gb|KK962110.1|	13129-11145	20	7.7	62	28	-1	0.304	no
gi|646749098|gb|KK962110.1|	15872-11218	20	7.7	7	3	-1	0.32655	no
gi|646749098|gb|KK962110.1|	26412-11278	20	7.7	3	0.779666	-2	0.2505	no
gi|646749098|gb|KK962110.1|	28079-11319	20	7.7	2	0.792406	-2	0.3209	no
gi|646749098|gb|KK962110.1|	37831-11413	20	7.7	2	0.747138	-2	0.3131	no
gi|646749098|gb|KK962110.1|	54860-11562	20	7.7	2	0	-inf	0.0047	no
gi|646749098|gb|KK962110.1|	59716-11609	20	7.7	2	0	-inf	0.00045	yes
gi|646749098|gb|KK962110.1|	61464-11621	20	7.7	2	0	-inf	0.00475	no
gi|646749098|gb|KK962110.1|	62706-11646	20	7.7	2	0	-inf	0.0017	no
gi|646749098|gb|KK962110.1|	70140-11724	20	7.7	2	0.258069	-3	0.143	no
gi|646749098|gb|KK962110.1|	73528-11759	20	7.7	1	0.247819	-2	0.16925	no
gi|646749098|gb|KK962110.1|	83664-11873	20	7.7	1	0.195462	-2	1	no
gi|646749098|gb|KK962110.1|	87489-11886	20	7.7	5	0	-inf	0.00025	yes
gi|646749098|gb|KK962110.1|	92822-11951	20	7.7	2	0	-inf	0.00055	yes

gi|646749098|gb|KK962110.1|	31885-12340	20	7.7	1	0.844466	-0.40277	1	no
gi|646749098|gb|KK962110.1|	34452-13589	20	7.7	5	4	-0.0467324	0.97615	no
gi|646749098|gb|KK962110.1|	71555-13721	20	7.7	3	1	-0.996445	0.54505	no
gi|646749098|gb|KK962110.1|	43897-14431	20	7.7	11	11	-0.0316445	0.96865	no
gi|646749098|gb|KK962110.1|	83297-14844	20	7.7	0.970352	0.462427	-1	1	no
gi|646749098|gb|KK962110.1|	89715-14915	20	7.7	1	0.601457	-1	0.46485	no
gi|646749098|gb|KK962110.1|	91635-14929	20	7.7	1	0.603999	-0.7393	1	no
gi|646749098|gb|KK962110.1|	71615-17418	20	7.7	15	8	-0.903437	0.409	no
gi|646749098|gb|KK962110.1|	78540-17967	20	7.7	2	5	1	0.40085	no
gi|646749098|gb|KK962110.1|	18956-19351	20	7.7	42	27	-0.637396	0.6762	no
gi|646749098|gb|KK962110.1|	91745-19214	20	7.7	40	21	-0.976869	0.53055	no
gi|646749098|gb|KK962110.1|	56076-25637	20	7.7	76	34	-1	0.461	no
gi|646749098|gb|KK962110.1|	92869-29326	20	7.7	6	6	0.00274329	0.9365	no
gi|646749098|gb|KK962110.1|	96479-30392	20	7.7	35	16	-1	0.3649	no
gi|646749098|gb|KK962110.1|	19723-32082	20	7.7	0.434814	0.932267	1	1	no
gi|646749098|gb|KK962110.1|	23260-32478	20	7.7	0.873452	2	0.849482	0.5654	no
gi|646749098|gb|KK962110.1|	32648-33351	20	7.7	1	4	2	0.3098	no
gi|646749098|gb|KK962110.1|	33695-33436	20	7.7	2	8	2	0.25985	no
gi|646749098|gb|KK962110.1|	35385-33804	20	7.7	24	26	0.125006	0.911	no
gi|646749098|gb|KK962110.1|	43393-34377	20	7.7	7	4	-0.785393	0.58875	no
gi|646749098|gb|KK962110.1|	69756-37081	20	7.7	0.802404	0.818105	0.0279563	1	no
gi|646749098|gb|KK962110.1|	75035-37563	20	7.7	5	4	-0.463257	0.7078	no
gi|646749098|gb|KK962110.1|	90563-39127	20	7.7	1	1	0.221529	0.89085	no
gi|646749098|gb|KK962110.1|	95110-39907	20	7.7	2	2	0.0864582	0.95375	no
gi|646749098|gb|KK962110.1|	99359-40224	20	7.7	4	3	-0.572814	0.70545	no
gi|646749098|gb|KK962110.1|	06445-40684	20	7.7	12	14	0.217689	0.8721	no
gi|646749098|gb|KK962110.1|	23722-42420	20	7.7	3	3	0.200759	0.88975	no
gi|646749098|gb|KK962110.1|	63656-46394	20	7.7	18	20	0.0938852	0.9386	no
gi|646749098|gb|KK962110.1|	68015-46876	20	7.7	2	2	-0.096284	0.941	no
gi|646749098|gb|KK962110.1|	72889-47351	20	7.7	1	2	0.206325	0.89115	no

gi|646749098|gb|KK962110.1|	74264-47526	20	7.7	19	10	-0.864685	0.57335	no
gi|646749098|gb|KK962110.1|	98924-49953	20	7.7	2	1	-0.757966	0.6333	no
gi|646749098|gb|KK962110.1|	01607-50232	20	7.7	0.802389	2	1	0.3715	no
gi|646749098|gb|KK962110.1|	04798-50592	20	7.7	0.638715	1	0.738534	1	no
gi|646749098|gb|KK962110.1|	06032-50780	20	7.7	0.549958	0.706618	0.36161	1	no
gi|646749098|gb|KK962110.1|	09814-51064	20	7.7	2	2	-0.0873592	0.9446	no
gi|646749098|gb|KK962110.1|	10749-51135	20	7.7	2	2	0.241319	0.8693	no
gi|646749098|gb|KK962110.1|	14645-51522	20	7.7	2	4	1	0.4212	no
gi|646749098|gb|KK962110.1|	51498-52156	20	7.7	2	0.956255	-0.747448	0.6333	no
gi|646749098|gb|KK962110.1|	16382-52288	20	7.7	12	9	-0.383314	0.7378	no
gi|646749098|gb|KK962110.1|	45668-54854	20	7.7	13	9	-0.511653	0.6376	no
gi|646749098|gb|KK962110.1|	51108-55171	20	7.7	1	4	1	0.3434	no
gi|646749098|gb|KK962110.1|	75098-57817	20	7.7	0.951785	1	0.438127	0.7844	no
gi|646749098|gb|KK962110.1|	78293-57888	20	7.7	2	2	-0.124222	0.9259	no
gi|646749098|gb|KK962110.1|	79220-58254	20	7.7	0.60274	1	0.920654	0.50335	no
gi|646749098|gb|KK962110.1|	82759-58467	20	7.7	1	1	-0.158542	0.89385	no
gi|646749098|gb|KK962110.1|	88920-59119	20	7.7	0.457104	0.663143	0.536798	1	no
gi|646749098|gb|KK962110.1|	04088-60558	20	7.7	23	7	-2	0.2042	no
gi|646749098|gb|KK962110.1|	06432-60750	20	7.7	31	7	-2	0.1171	no
gi|646749098|gb|KK962110.1|	19903-62027	20	7.7	18	16	-0.156813	0.9239	no
gi|646749098|gb|KK962110.1|	28821-62903	20	7.7	53	17	-2	0.30525	no
gi|646749098|gb|KK962110.1|	33281-63423	20	7.7	4	3	-0.350426	0.8253	no
gi|646749098|gb|KK962110.1|	39766-64081	20	7.7	4	3	-0.413397	0.8018	no
gi|646749098|gb|KK962110.1|	50123-65074	20	7.7	2	2	-0.209571	0.89255	no
gi|646749098|gb|KK962110.1|	52170-65454	20	7.7	41	37	-0.130092	0.9133	no
gi|646749098|gb|KK962110.1|	54603-65649	20	7.7	0.509262	0.65503	0.363154	1	no
gi|646749098|gb|KK962110.1|	56594-65822	20	7.7	0.403404	0.485219	0.26641	1	no
gi|646749098|gb|KK962110.1|	64100-66452	20	7.7	7	3	-1	0.3845	no
gi|646749098|gb|KK962110.1|	73391-67400	20	7.7	1	3	0.950324	0.56265	no
gi|646749098|gb|KK962110.1|	80176-68217	20	7.7	5	3	-0.780276	0.60435	no

gi|646749098|gb|KK962110.1|	90652-69124	20	7.7	5	5	-0.0427932	0.9586	no
gi|646749098|gb|KK962110.1|	96436-69725	20	7.7	2	4	1	0.4874	no
gi|646749098|gb|KK962110.1|	15328-71575	20	7.7	49	38	-0.384682	0.80265	no
gi|646749098|gb|KK962110.1|	16522-71745	20	7.7	1	0.771724	-0.66367	0.6592	no
gi|646749098|gb|KK962110.1|	35372-73605	20	7.7	8	20	1	0.374	no
gi|646749098|gb|KK962110.1|	45845-74645	20	7.7	18	12	-0.621177	0.68175	no
gi|646749098|gb|KK962110.1|	48990-74995	20	7.7	8	7	-0.0642785	0.96655	no
gi|646749098|gb|KK962110.1|	75231-77648	20	7.7	0.742303	8	3	0.04755	no
gi|646749098|gb|KK962110.1|	76617-77791	20	7.7	0.266344	6	4	0.1125	no
gi|646749098|gb|KK962110.1|	78043-78585	20	7.7	0.514361	8	4	0.01745	no
gi|646749098|gb|KK962110.1|	86438-79285	20	7.7	0.63084	8	4	0.024	no
gi|646749098|gb|KK962110.1|	93491-79538	20	7.7	0.056447	5	6	0.0952	no
gi|646749098|gb|KK962110.1|	80282-80605	20	7.7	63	35	-0.857626	0.57915	no
gi|646749098|gb|KK962110.1|	86177-86526	20	7.7	8	7	-0.272818	0.8395	no
gi|646749098|gb|KK962110.1|	90330-90631	20	7.7	13	7	-0.932837	0.5576	no
gi|646749098|gb|KK962110.1|	92527-92753	20	7.7	22	45	1	0.45985	no
gi|646749098|gb|KK962110.1|	94901-96899	20	7.7	0.636947	1	0.856205	0.5652	no
gi|646749098|gb|KK962110.1|	97032-98321	20	7.7	0.805214	1	0.521888	0.7038	no
gi|646749098|gb|KK962110.1|	99160-10026[illegible]	20	7.7	3	6	0.856289	0.5844	no
gi|646749114|gb|KK962109.1|	97158-10976	20	8.8	5	0	-inf	0.00655	no
gi|646749114|gb|KK962109.1|	98375-19939	20	8.8	0.602244	1	0.774458	1	no
gi|646749114|gb|KK962109.1|	03190-20491	20	8.8	1	2	0.37049	0.7861	no
gi|646749114|gb|KK962109.1|	42312-24291	20	8.8	11	7	-0.537504	0.73585	no
gi|646749114|gb|KK962109.1|	60096-26033	20	8.8	79	135	0.77484	0.63025	no
gi|646749114|gb|KK962109.1|	67954-36849	20	8.8	4	2	-0.93347	0.52525	no
gi|646749114|gb|KK962109.1|	72271-37283	20	8.8	2	2	0.451513	0.7747	no
gi|646749114|gb|KK962109.1|	48480-55181	20	8.8	22	27	0.27758	0.81395	no
gi|646749114|gb|KK962109.1|	52626-55544	20	8.8	2	7	2	0.1836	no
gi|646749114|gb|KK962109.1|	55608-55593	20	8.8	4	12	1	0.36975	no
gi|646749114|gb|KK962109.1|	58878-56017	20	8.8	6	12	1	0.44325	no

gi|646749114|gb|KK962109.1|	60428-56113	20	8.8	1	4	2	0.24925	no
gi|646749114|gb|KK962109.1|	61418-56185	20	8.8	0.989193	6	3	0.2756	no
gi|646749114|gb|KK962109.1|	61995-56308	20	8.8	3	7	1	0.50385	no
gi|646749114|gb|KK962109.1|	66344-56661	20	8.8	70	116	0.73428	0.6435	no
gi|646749114|gb|KK962109.1|	20573-82073	20	8.8	368	530	0.526147	0.72485	no
gi|646749114|gb|KK962109.1|	24297-82457	20	8.8	14	18	0.36928	0.7961	no
gi|646749114|gb|KK962109.1|	27958-92850	20	8.8	6	4	-0.629634	0.6156	no
gi|646749155|gb|KK962107.1|	97102-19759	22	9.2	0	13	inf	0.00185	no
gi|646749155|gb|KK962107.1|	44141-45170	22	9.2	5	7	0.374234	0.8055	no
gi|646749155|gb|KK962107.1|	51846-56344	22	9.2	5	9	0.949555	0.38245	no
gi|646749155|gb|KK962107.1|	76829-77300	22	9.2	3	7	1	0.3215	no
gi|646749155|gb|KK962107.1|	73530-77376	22	9.2	41	70	0.767399	0.6041	no
gi|646749155|gb|KK962107.1|	77081-77731	22	9.2	37	19	-0.943095	0.53935	no
gi|646749155|gb|KK962107.1|	81413-78194	22	9.2	4	7	0.647603	0.66395	no
gi|646749155|gb|KK962107.1|	85923-78624	22	9.2	26	36	0.446937	0.7807	no
gi|646749155|gb|KK962107.1|	94981-79555	22	9.2	3	4	0.192313	0.88805	no
gi|646749155|gb|KK962107.1|	10919-81312	22	9.2	18	16	-0.182341	0.8697	no
gi|646749155|gb|KK962107.1|	11944-91276	22	9.2	3	1	-1	0.33355	no
gi|646749173|gb|KK962106.1|	06503-12434	21	5.7	9	14	0.626859	0.6028	no
gi|646749173|gb|KK962106.1|	74232-11840	21	5.7	0.618438	13	4	0.0075	no
gi|646749173|gb|KK962106.1|	88955-11940	21	5.7	1	22	4	0.0097	no
gi|646749173|gb|KK962106.1|	94286-11948	21	5.7	0.353293	8	5	0.1113	no
gi|646749173|gb|KK962106.1|	05222-12067	21	5.7	3	2	-0.9957	0.53945	no
gi|646749173|gb|KK962106.1|	10186-12118	21	5.7	0.320563	0.926068	2	1	no
gi|646749173|gb|KK962106.1|	20807-12223	21	5.7	3	35	4	0.022	no
gi|646749173|gb|KK962106.1|	34419-12350	21	5.7	1	7	3	0.17545	no
gi|646749173|gb|KK962106.1|	46865-12517	21	5.7	3	7	1	0.31595	no
gi|646749173|gb|KK962106.1|	52348-12535	21	5.7	103	53	-0.96554	0.40835	no
gi|646749173|gb|KK962106.1|	63321-12653	21	5.7	0.927745	1	0.364878	0.78985	no
gi|646749173|gb|KK962106.1|	65467-12661	21	5.7	0.785392	2	1	0.4332	no

gi|646749173|gb|KK962106.1| 98971-20355	21	5.7	58	78	0.4403	0.7177	no
gi|646749173|gb|KK962106.1| 03672-20632	21	5.7	0.540821	2	2	0.2544	no
gi|646749173|gb|KK962106.1| 07454-20871	21	5.7	0.641157	2	2	0.3011	no
gi|646749173|gb|KK962106.1| 08907-21515	21	5.7	6	5	-0.427826	0.6908	no
gi|646749173|gb|KK962106.1| 15494-21956	21	5.7	0.72718	0.91898	0.337721	1	no
gi|646749173|gb|KK962106.1| 22268-22359	21	5.7	0.173079	1	3	1	no
gi|646749173|gb|KK962106.1| 31721-24087	21	5.7	3	4	0.269561	0.8013	no
gi|646749173|gb|KK962106.1| 45625-24593	21	5.7	11	16	0.548011	0.6797	no
gi|646749173|gb|KK962106.1| 67484-26825	21	5.7	1	2	0.298629	0.83555	no
gi|646749173|gb|KK962106.1| 37161-39556	21	5.7	15	9	-0.790407	0.5396	no
gi|646749173|gb|KK962106.1| 92024-49338	21	5.7	1	1	0.145403	0.9094	no
gi|646749173|gb|KK962106.1| 18855-51963	21	5.7	16	1	-3	0.0477	no
gi|646749173|gb|KK962106.1| 23421-52389	21	5.7	5	1	-2	0.21625	no
gi|646749173|gb|KK962106.1| 52804-56147	21	5.7	47	109	1	0.31745	no
gi|646749173|gb|KK962106.1| 33192-53351	21	5.7	6.39	8	0.281019	0.8442	no
gi|646749173|gb|KK962106.1| 36828-53719	21	5.7	13	0	-inf	0.00195	no
gi|646749173|gb|KK962106.1| 51240-55178	21	5.7	4	3	-0.331212	0.79845	no
gi|646749173|gb|KK962106.1| 60266-56091	21	5.7	2	3	0.302909	0.83035	no
gi|646749173|gb|KK962106.1| 63970-56496	21	5.7	2	0.888836	-0.97548	0.50955	no
gi|646749173|gb|KK962106.1| 81672-58953	21	5.7	7	8	0.16387	0.88465	no
gi|646749173|gb|KK962106.1| 61928-63037	21	5.7	0.649931	0.77423	0.252476	1	no
gi|646749173|gb|KK962106.1| 21658-62243	21	5.7	9	4	-1	0.45675	no
gi|646749173|gb|KK962106.1| 37175-63841	21	5.7	0.842697	0.26862	-2	1	no
gi|646749173|gb|KK962106.1| 39281-63958	21	5.7	4	19	2	0.1792	no
gi|646749173|gb|KK962106.1| 42867-64353	21	5.7	1	2	0.476778	0.7489	no
gi|646749173|gb|KK962106.1| 51339-65303	21	5.7	25	16	-0.682243	0.5308	no
gi|646749173|gb|KK962106.1| 66046-68291	21	5.7	3	3	-0.000497013	0.98955	no
gi|646749173|gb|KK962106.1| 87103-68819	21	5.7	0.111186	11	7	0.0947	no
gi|646749173|gb|KK962106.1| 90604-69270	21	5.7	49	16	-2	0.1652	no
gi|646749173|gb|KK962106.1| 94313-69689	21	5.7	26	12	-1	0.33535	no

gi|646749173|gb|KK962106.1|	71083-71326	21	5.7	331	899	1	0.2701	no
gi|646749173|gb|KK962106.1|	71875-73151	21	5.7	0.815465	0.260124	-2	1	no
gi|646749173|gb|KK962106.1|	74018-74663	21	5.7	2	0.657464	-2	0.2673	no
gi|646749173|gb|KK962106.1|	76229-77022	21	5.7	85	237	1	0.21365	no
gi|646749173|gb|KK962106.1|	77814-78901	21	5.7	21	55	1	0.29995	no
gi|646749173|gb|KK962106.1|	79687-81681	21	5.7	0.266005	1	2	0.1889	no
gi|646749173|gb|KK962106.1|	81911-84114	21	5.7	1	2	0.313795	0.84475	no
gi|646749173|gb|KK962106.1|	84265-90899	21	5.7	0.986296	2	1	0.48775	no
gi|646749173|gb|KK962106.1|	91261-94614	21	5.7	6	8	0.479158	0.7015	no
gi|646749173|gb|KK962106.1|	95215-96406	21	5.7	0.988339	0.991181	0.00414294	1	no
gi|646749173|gb|KK962106.1|	97630-99104	21	5.7	0.834814	0.546586	-0.611006	1	no
gi|646749173|gb|KK962106.1|	99267-10094	21	5.7	5	4	-0.175118	0.90665	no
gi|646749188|gb|KK962105.1|	10766-11255	18	7.9	12	8	-0.608475	0.62455	no
gi|646749188|gb|KK962105.1|	16780-11841	18	7.9	7	5	-0.5859	0.6925	no
gi|646749188|gb|KK962105.1|	28021-12832	18	7.9	30	47	0.648329	0.63835	no
gi|646749188|gb|KK962105.1|	83542-18420	18	7.9	0.450895	3	3	0.2662	no
gi|646749188|gb|KK962105.1|	01431-20243	18	7.9	0.247007	2	3	0.2578	no
gi|646749188|gb|KK962105.1|	04878-20626	18	7.9	14	6	-1	0.4813	no
gi|646749188|gb|KK962105.1|	07191-20820	18	7.9	0.733192	2	2	0.2385	no
gi|646749188|gb|KK962105.1|	14866-21618	18	7.9	0.259861	4	4	0.122	no
gi|646749188|gb|KK962105.1|	17372-21898	18	7.9	0.955863	0.788187	-0.278265	1	no
gi|646749188|gb|KK962105.1|	22335-22267	18	7.9	5	7	0.558823	0.73385	no
gi|646749188|gb|KK962105.1|	26852-22710	18	7.9	11	17	0.706597	0.65785	no
gi|646749188|gb|KK962105.1|	28408-22864	18	7.9	43	38	-0.198688	0.87595	no
gi|646749188|gb|KK962105.1|	35409-23576	18	7.9	7	8	0.151096	0.92045	no
gi|646749188|gb|KK962105.1|	52268-25260	18	7.9	5	9	0.776884	0.6126	no
gi|646749188|gb|KK962105.1|	06846-31101	18	7.9	1	2	0.577541	0.706	no
gi|646749188|gb|KK962105.1|	11789-31431	18	7.9	1	2	0.719099	0.6451	no
gi|646749188|gb|KK962105.1|	17749-31864	18	7.9	1	3	1	0.47555	no
gi|646749188|gb|KK962105.1|	19282-32252	18	7.9	36	36	-0.0114114	0.99145	no

gi|646749188|gb|KK962105.1|	33858-33411	18	7.9	33	23	-0.492364	0.69565	no
gi|646749188|gb|KK962105.1|	42342-34285	18	7.9	21	37	0.80379	0.60105	no
gi|646749188|gb|KK962105.1|	43145-35071	18	7.9	7	7	-0.087836	0.9356	no
gi|646749188|gb|KK962105.1|	53987-35705	18	7.9	3	5	0.732015	0.63225	no
gi|646749188|gb|KK962105.1|	67332-36846	18	7.9	1	2	0.516306	0.69625	no
gi|646749188|gb|KK962105.1|	68996-36965	18	7.9	4	3	-0.443159	0.72525	no
gi|646749188|gb|KK962105.1|	70044-37085	18	7.9	36	16	-1	0.37585	no
gi|646749188|gb|KK962105.1|	71030-37652	18	7.9	2	3	0.32405	0.8293	no
gi|646749188|gb|KK962105.1|	76713-37735	18	7.9	3	1	-1	0.44845	no
gi|646749188|gb|KK962105.1|	81881-38454	18	7.9	1	1	-0.0225707	0.9828	no
gi|646749188|gb|KK962105.1|	88311-38855	18	7.9	24	22	-0.0900275	0.93225	no
gi|646749188|gb|KK962105.1|	90071-39111	18	7.9	1	2	0.649905	0.62395	no
gi|646749188|gb|KK962105.1|	40894-41755	18	7.9	2	0.215958	-3	0.18	no
gi|646749188|gb|KK962105.1|	13245-41397	18	7.9	2	1	-0.513178	0.7384	no
gi|646749188|gb|KK962105.1|	25441-42640	18	7.9	6	6	-0.0613111	0.97015	no
gi|646749188|gb|KK962105.1|	42118-54330	18	7.9	0.299057	2	3	0.164	no
gi|646749188|gb|KK962105.1|	43422-54480	18	7.9	0.411528	8	4	0.0654	no
gi|646749188|gb|KK962105.1|	50984-55124	18	7.9	2	47	4	0.10915	no
gi|646749188|gb|KK962105.1|	82466-78338	18	7.9	0	6	inf	0.00105	yes
gi|646749188|gb|KK962105.1|	90588-90890	18	7.9	11	6	-0.761039	0.61235	no
gi|646749209|gb|KK962104.1|	38913-24042	21	9.2	0.807012	2	1	0.39205	no
gi|646749209|gb|KK962104.1|	67466-56917	21	9.2	2	7	2	0.2593	no
gi|646749209|gb|KK962104.1|	75267-57564	21	9.2	7	5	-0.50731	0.73475	no
gi|646749209|gb|KK962104.1|	75989-57751	21	9.2	3	2	-0.674178	0.66835	no
gi|646749209|gb|KK962104.1|	78528-57979	21	9.2	0.641157	0.525791	-0.286188	1	no
gi|646749209|gb|KK962104.1|	96917-59835	21	9.2	18	9	-1	0.4073	no
gi|646749209|gb|KK962104.1|	04733-60635	21	9.2	0.744988	0.586457	-0.345192	1	no
gi|646749209|gb|KK962104.1|	31360-63216	21	9.2	0.83935	2	0.980978	0.5626	no
gi|646749209|gb|KK962104.1|	04253-70640	21	9.2	4	5	0.153845	0.91855	no
gi|646749209|gb|KK962104.1|	06548-70750	21	9.2	0.786143	0.745236	-0.0770941	1	no

gi|646749209|gb|KK962104.1|	09741-71071	21	9.2	5	5	0.0938058	0.9517	no
gi|646749209|gb|KK962104.1|	11135-71145	21	9.2	16	12	-0.365771	0.7688	no
gi|646749209|gb|KK962104.1|	15578-71660	21	9.2	151	83	-0.866911	0.46295	no
gi|646749209|gb|KK962104.1|	68543-77156	21	9.2	27	5	-2	0.08325	no
gi|646749209|gb|KK962104.1|	83631-78427	21	9.2	109.38	78	-0.490329	0.66515	no
gi|646749209|gb|KK962104.1|	97346-80039	21	9.2	970	303	-2	0.1625	no
gi|646749209|gb|KK962104.1|	03404-80394	21	9.2	65	62	-0.0526173	0.96035	no
gi|646749209|gb|KK962104.1|	70995-87316	21	9.2	28	5	-2	0.0758	no
gi|646749209|gb|KK962104.1|	75750-87790	21	9.2	0.438471	157	8	0.01635	no
gi|646749209|gb|KK962104.1|	79986-88104	21	9.2	0.918171	1	0.32069	0.8346	no
gi|646749209|gb|KK962104.1|	81424-88371	21	9.2	0.63774	0.726971	0.188929	1	no
gi|646749209|gb|KK962104.1|	89451-89014	21	9.2	3	0.87822	-2	0.3507	no
gi|646749209|gb|KK962104.1|	91959-89410	21	9.2	1	0.637315	-1	0.47175	no
gi|646749209|gb|KK962104.1|	94212-89782	21	9.2	2	0.642696	-1	0.3822	no
gi|646749209|gb|KK962104.1|	00206-90125	21	9.2	1	0.833173	-0.487348	0.73195	no
gi|646749209|gb|KK962104.1|	11796-91505	21	9.2	2	1	-0.409189	0.7923	no
gi|646749209|gb|KK962104.1|	18980-91934	21	9.2	15	4	-2	0.19685	no
gi|646749209|gb|KK962104.1|	22498-92365	21	9.2	35	49	0.500306	0.64905	no
gi|646749230|gb|KK962103.1|	64029-26448	13	7.9	2	3	0.406731	0.81675	no
gi|646749230|gb|KK962103.1|	73756-27420	13	7.9	5	2	-0.918794	0.55025	no
gi|646749230|gb|KK962103.1|	27885-32825	13	7.9	4	9	1	0.371	no
gi|646749230|gb|KK962103.1|	36692-33795	13	7.9	0.916846	1	0.199208	1	no
gi|646749230|gb|KK962103.1|	38202-33926	13	7.9	1	0.981725	-0.602094	0.64385	no
gi|646749230|gb|KK962103.1|	39816-34042	13	7.9	2	1	-0.121251	0.9032	no
gi|646749248|gb|KK962102.1|	21252-21706	20	7.5	3	4	0.406142	0.7939	no
gi|646749248|gb|KK962102.1|	83095-28344	20	7.5	13	9	-0.499159	0.6979	no
gi|646749248|gb|KK962102.1|	90081-29407	20	7.5	3	7	1	0.32475	no
gi|646749248|gb|KK962102.1|	02199-30242	20	7.5	42	34	-0.288349	0.83695	no
gi|646749248|gb|KK962102.1|	06327-30676	20	7.5	2	7	2	0.19685	no
gi|646749248|gb|KK962102.1|	22355-32275	20	7.5	2	6	1	0.3935	no

gi|646749248|gb|KK962102.1|	23466-32393	20	7.5	3	6	0.929674	0.5221	no
gi|646749248|gb|KK962102.1|	26950-33422	20	7.5	32	35	0.108386	0.9263	no
gi|646749248|gb|KK962102.1|	45029-34669	20	7.5	4	6	0.368294	0.80915	no
gi|646749248|gb|KK962102.1|	64375-36564	20	7.5	0.998635	4	2	0.2253	no
gi|646749248|gb|KK962102.1|	56457-57444	20	7.5	7	8	0.173828	0.9025	no
gi|646749248|gb|KK962102.1|	70675-73090	20	7.5	12	21	0.79473	0.4716	no
gi|646749248|gb|KK962102.1|	89654-89983	20	7.5	16	14	-0.163408	0.89835	no
gi|646749264|gb|KK962101.1|	192-676	13	8.6	10	9	-0.134202	0.9334	no
gi|646749264|gb|KK962101.1|	2660-3143	13	8.6	4	0	-inf	0.00475	no
gi|646749264|gb|KK962101.1|	39346-39555	13	8.6	52	75	0.533112	0.6874	no
gi|646749264|gb|KK962101.1|	9825-10460	13	8.6	3	1	-1	0.3848	no
gi|646749283|gb|KK962100.1|	19633-11996	12	8.6	10.65	6	-0.718981	0.6309	no
gi|646749283|gb|KK962100.1|	89064-18951	12	8.6	5	5	-0.056298	0.94695	no
gi|646749283|gb|KK962100.1|	17807-51918	12	8.6	0.985038	0.472269	-1	1	no
gi|646749283|gb|KK962100.1|	40044-54099	12	8.6	2	0	-inf	0.0012	yes
gi|646749283|gb|KK962100.1|	55598-56305	12	8.6	207	126.75	-0.705888	0.5552	no
gi|646749283|gb|KK962100.1|	76334-57739	12	8.6	1	1	0.361475	0.79375	no
gi|646749302|gb|KK962099.1|	69384-17079	17	7	11	3	-2	0.24385	no
gi|646749302|gb|KK962099.1|	08434-21097	17	7	25	12	-1	0.33375	no
gi|646749302|gb|KK962099.1|	46952-24714	17	7	53	58	0.108942	0.9101	no
gi|646749302|gb|KK962099.1|	62636-26332	17	7	1	5	2	0.3097	no
gi|646749302|gb|KK962099.1|	02604-30289	17	7	8	22	1	0.3254	no
gi|646749302|gb|KK962099.1|	03572-30421	17	7	2	6	2	0.2698	no
gi|646749302|gb|KK962099.1|	06443-30849	17	7	1	3	0.806134	0.6107	no
gi|646749302|gb|KK962099.1|	09336-30988	17	7	3	3	0.0864417	0.93585	no
gi|646749302|gb|KK962099.1|	10028-31147	17	7	297	287	-0.0479897	0.96665	no
gi|646749302|gb|KK962099.1|	12172-31555	17	7	54	71	0.413322	0.7332	no
gi|646749302|gb|KK962099.1|	16627-31798	17	7	19	37	0.940416	0.3913	no
gi|646749302|gb|KK962099.1|	18942-32094	17	7	24	43	0.830104	0.47245	no
gi|646749302|gb|KK962099.1|	21103-32332	17	7	37	29	-0.348361	0.7613	no

gi|646749302|gb|KK962099.1|	27974-32846	17	7	2	4	0.648354	0.6597	no
gi|646749302|gb|KK962099.1|	28935-32918	17	7	73	42	-0.780803	0.5729	no
gi|646749302|gb|KK962099.1|	41394-34156	17	7	340	164.54	-1	0.4115	no
gi|646749302|gb|KK962099.1|	42270-34252	17	7	23	25	0.130674	0.92645	no
gi|646749302|gb|KK962099.1|	26897-42787	17	7	0.382246	2	2	0.18785	no
gi|646749302|gb|KK962099.1|	49526-45169	17	7	6	1	-2	0.18375	no
gi|646749302|gb|KK962099.1|	58840-45952	17	7	2	0.298513	-3	0.1994	no
gi|646749302|gb|KK962099.1|	69887-47182	17	7	2	1	-0.186273	0.9093	no
gi|646749302|gb|KK962099.1|	71933-47288	17	7	1	1	-0.077598	0.9329	no
gi|646749302|gb|KK962099.1|	73030-47462	17	7	1	1	0.0814405	1	no
gi|646749302|gb|KK962099.1|	76935-47827	17	7	0.855789	1	0.66071	0.6152	no
gi|646749302|gb|KK962099.1|	15918-51646	17	7	3	3	0.126441	0.926	no
gi|646749302|gb|KK962099.1|	09025-60959	17	7	0.891975	4	2	0.19065	no
gi|646749302|gb|KK962099.1|	11175-61421	17	7	18	10	-0.894845	0.42535	no
gi|646749317|gb|KK962098.1|	27697-22990	20	5.6	24	3	-3	0.04875	no
gi|646749317|gb|KK962098.1|	48380-24890	20	5.6	21	8	-1	0.37515	no
gi|646749317|gb|KK962098.1|	54933-26059	20	5.6	29	39	0.420409	0.72905	no
gi|646749317|gb|KK962098.1|	61108-26959	20	5.6	2	3	0.767547	0.54735	no
gi|646749317|gb|KK962098.1|	69971-27142	20	5.6	2	4	0.668086	0.6688	no
gi|646749317|gb|KK962098.1|	72439-27676	20	5.6	2	4	0.714625	0.6445	no
gi|646749317|gb|KK962098.1|	85420-28695	20	5.6	0.869348	2	1	0.42335	no
gi|646749317|gb|KK962098.1|	87759-28880	20	5.6	1	2	0.512602	0.70715	no
gi|646749317|gb|KK962098.1|	93770-29752	20	5.6	0.686712	1	0.972267	0.5391	no
gi|646749317|gb|KK962098.1|	00648-30179	20	5.6	0.519122	1	1	1	no
gi|646749317|gb|KK962098.1|	02568-30428	20	5.6	0.189709	1	2	1	no
gi|646749317|gb|KK962098.1|	04961-30650	20	5.6	1	2	1	0.4066	no
gi|646749317|gb|KK962098.1|	08257-30924	20	5.6	2	6	1	0.29735	no
gi|646749317|gb|KK962098.1|	14048-31518	20	5.6	3	10	2	0.2635	no
gi|646749317|gb|KK962098.1|	21618-32181	20	5.6	227	701	2	0.31515	no
gi|646749317|gb|KK962098.1|	23238-32363	20	5.6	14	50	2	0.253	no

gi|646749317|gb|KK962098.1|	36128-33674	20	5.6	5	13	2	0.2802	no
gi|646749317|gb|KK962098.1|	39135-33936	20	5.6	51	158	2	0.2711	no
gi|646749317|gb|KK962098.1|	48740-34916	20	5.6	10	4	-1	0.2953	no
gi|646749317|gb|KK962098.1|	60077-36074	20	5.6	10	7	-0.461822	0.7661	no
gi|646749317|gb|KK962098.1|	86365-38658	20	5.6	29	29	0.00861982	0.91805	no
gi|646749317|gb|KK962098.1|	59174-46015	20	5.6	0.891907	1	0.286688	1	no
gi|646749317|gb|KK962098.1|	06249-50757	20	5.6	0.693613	3	2	0.1875	no
gi|646749317|gb|KK962098.1|	45994-54647	20	5.6	13	28	1	0.4944	no
gi|646749334|gb|KK962097.1|	64859-46510	11	8	27	19	-0.485637	0.72085	no
gi|646749357|gb|KK962096.1|	15868-11611	19	8.8	0	425.72	inf	0.00015	yes
gi|646749357|gb|KK962096.1|	11810-13057	19	8.8	5	12	1	0.4221	no
gi|646749357|gb|KK962096.1|	26088-26388	19	8.8	27	26	-0.0127282	0.96835	no
gi|646749357|gb|KK962096.1|	88315-28904	19	8.8	2	0.818367	-0.92849	0.58905	no
gi|646749357|gb|KK962096.1|	95102-29599	19	8.8	1	1	0.0179891	1	no
gi|646749357|gb|KK962096.1|	65893-36602	19	8.8	0	1898.16	inf	0.001	yes
gi|646749357|gb|KK962096.1|	45399-47612	19	8.8	1	1	-0.107585	0.9428	no
gi|646749357|gb|KK962096.1|	73859-47456	19	8.8	1	3	1	0.34675	no
gi|646749357|gb|KK962096.1|	80711-48277	19	8.8	0.613372	0.888708	0.534949	1	no
gi|646749357|gb|KK962096.1|	89829-49035	19	8.8	1	12	4	0.1291	no
gi|646749357|gb|KK962096.1|	93963-49522	19	8.8	0.459333	0.922816	1	1	no
gi|646749357|gb|KK962096.1|	98635-50068	19	8.8	111	125	0.178043	0.8829	no
gi|646749357|gb|KK962096.1|	50209-53009	19	8.8	18	17.39	-0.066716	0.9514	no
gi|646749357|gb|KK962096.1|	02161-50473	19	8.8	0.598957	1	0.801814	1	no
gi|646749357|gb|KK962096.1|	04911-50600	19	8.8	1	0.471533	-1	1	no
gi|646749357|gb|KK962096.1|	07134-50993	19	8.8	0.943336	1	0.540032	0.73535	no
gi|646749357|gb|KK962096.1|	10479-51123	19	8.8	1	2	0.490433	0.73295	no
gi|646749357|gb|KK962096.1|	11869-51544	19	8.8	1	2	0.482793	0.75385	no
gi|646749357|gb|KK962096.1|	15495-51686	19	8.8	0.66257	0.476414	-0.475857	1	no
gi|646749357|gb|KK962096.1|	17006-51826	19	8.8	1	1	-0.478925	0.7198	no
gi|646749357|gb|KK962096.1|	20844-52231	19	8.8	9	5	-0.825783	0.5851	no

gi|646749357|gb|KK962096.1|	22579-52530	19	8.8	5	5	0.00301827	0.99545	no
gi|646749357|gb|KK962096.1|	53196-53517	19	8.8	33	16	-1	0.4609	no
gi|646749357|gb|KK962096.1|	56769-57439	19	8.8	6	5	-0.259831	0.8774	no
gi|646749357|gb|KK962096.1|	60103-60430	19	8.8	20	5	-2	0.1927	no
gi|646749357|gb|KK962096.1|	85251-85720	19	8.8	5	6	0.277065	0.8317	no
gi|646749379|gb|KK962095.1|	38258-74093	14	7.3	2	4	1	0.4513	no
gi|646749402|gb|KK962094.1|	89874-29049	14	7.4	20	25	0.369646	0.80455	no
gi|646749402|gb|KK962094.1|	06330-30723	14	7.4	1	1	-0.495769	0.73195	no
gi|646749402|gb|KK962094.1|	53711-35394	14	7.4	0	220	inf	0.00025	yes
gi|646749402|gb|KK962094.1|	61428-36170	14	7.4	0	76	inf	0.00055	yes
gi|646749402|gb|KK962094.1|	48052-48343	14	7.4	6	11	0.80232	0.62595	no
gi|646749402|gb|KK962094.1|	83184-48377	14	7.4	3	2	-1	0.44495	no
gi|646749402|gb|KK962094.1|	87170-48783	14	7.4	4	3	-0.289847	0.8104	no
gi|646749402|gb|KK962094.1|	50977-51499	14	7.4	4	0.933059	-2	0.19275	no
gi|646749424|gb|KK962093.1|	07279-60835	15	8.9	3	3	0.176821	0.91365	no
gi|646749424|gb|KK962093.1|	09376-60989	15	8.9	11	19	0.71645	0.64565	no
gi|646749424|gb|KK962093.1|	10102-61397	15	8.9	4	4	-0.0851858	0.9535	no
gi|646749424|gb|KK962093.1|	32742-63343	15	8.9	2	3	0.650635	0.643	no
gi|646749424|gb|KK962093.1|	42559-64435	15	8.9	4	8	0.817862	0.59655	no
gi|646749424|gb|KK962093.1|	66374-67552	15	8.9	0.902031	0.287089	-2	1	no
gi|646749424|gb|KK962093.1|	67700-69562	15	8.9	68	56	-0.276762	0.8088	no
gi|646749424|gb|KK962093.1|	72651-73266	15	8.9	7	18	1	0.3989	no
gi|646749424|gb|KK962093.1|	76178-77302	15	8.9	0.958072	0.608961	-0.653784	1	no
gi|646749424|gb|KK962093.1|	78273-78512	15	8.9	61	57	-0.0947035	0.94625	no
gi|646749424|gb|KK962093.1|	88948-89644	15	8.9	1	4	1	0.36795	no
gi|646749444|gb|KK962092.1|	45486-14701	15	9	2	2	0.296306	0.85495	no
gi|646749444|gb|KK962092.1|	69460-16990	15	9	10	9	-0.0150201	0.96865	no
gi|646749444|gb|KK962092.1|	70124-17279	15	9	3	4	0.159047	0.9149	no
gi|646749444|gb|KK962092.1|	74195-17626	15	9	17	18	0.0770981	0.94245	no
gi|646749444|gb|KK962092.1|	47375-44774	15	9	4	6	0.599322	0.669	no

gi|646749444|gb|KK962092.1|	47894-44862	15	9	11	8	-0.564216	0.71735	no
gi|646749444|gb|KK962092.1|	50466-45126	15	9	1	1	0.494518	0.73295	no
gi|646749444|gb|KK962092.1|	57675-45841	15	9	2	0.805442	-0.927407	0.58905	no
gi|646749444|gb|KK962092.1|	79010-48065	15	9	5	4	-0.43078	0.776	no
gi|646749444|gb|KK962092.1|	00329-50172	15	9	40	41	0.0597232	0.9583	no
gi|646749444|gb|KK962092.1|	02029-50386	15	9	4	3	-0.648141	0.67015	no
gi|646749444|gb|KK962092.1|	04672-50596	15	9	67	80	0.25353	0.833	no
gi|646749444|gb|KK962092.1|	11082-51214	15	9	5	7	0.479484	0.74195	no
gi|646749444|gb|KK962092.1|	64791-66558	15	9	7	6	-0.277966	0.8529	no
gi|646749444|gb|KK962092.1|	85622-88772	15	9	4	3	-0.0496821	0.9723	no
gi|646749464|gb|KK962091.1|	07804-10901	18	8.8	105	171	0.702688	0.5548	no
gi|646749464|gb|KK962091.1|	18100-11835	18	8.8	209	151	-0.469324	0.75385	no
gi|646749464|gb|KK962091.1|	47491-14793	18	8.8	11	5	-1	0.44955	no
gi|646749464|gb|KK962091.1|	52880-15443	18	8.8	13	7	-0.923683	0.54345	no
gi|646749464|gb|KK962091.1|	56817-16418	18	8.8	9	5	-0.704726	0.52485	no
gi|646749464|gb|KK962091.1|	66034-17104	18	8.8	11	8	-0.469444	0.6725	no
gi|646749464|gb|KK962091.1|	08297-20871	18	8.8	3	14	2	0.1858	no
gi|646749464|gb|KK962091.1|	17052-21791	18	8.8	2	3	0.878973	0.49255	no
gi|646749464|gb|KK962091.1|	56754-25780	18	8.8	6	2	-2	0.203	no
gi|646749464|gb|KK962091.1|	71130-27173	18	8.8	3	5	0.812585	0.5233	no
gi|646749464|gb|KK962091.1|	71842-27303	18	8.8	5	9	0.895007	0.5614	no
gi|646749464|gb|KK962091.1|	87926-28823	18	8.8	87	11	-3	0.06955	no
gi|646749464|gb|KK962091.1|	22300-32517	18	8.8	0.705449	0.872077	0.305913	1	no
gi|646749464|gb|KK962091.1|	30686-33287	18	8.8	0.525667	0.692942	0.398584	1	no
gi|646749464|gb|KK962091.1|	33109-33348	18	8.8	17	3	-3	0.1708	no
gi|646749464|gb|KK962091.1|	45896-34742	18	8.8	3	2	-0.716314	0.6518	no
gi|646749464|gb|KK962091.1|	47582-34944	18	8.8	1	2	0.458778	0.7383	no
gi|646749464|gb|KK962091.1|	49905-35175	18	8.8	0.463447	0.419001	-0.145453	1	no
gi|646749464|gb|KK962091.1|	52342-35682	18	8.8	0.678538	0.734508	0.114347	1	no
gi|646749464|gb|KK962091.1|	61957-36248	18	8.8	8	8	0.0050533	0.97445	no

gi|646749464|gb|KK962091.1|	68856-36909	18	8.8	22	39	0.793871	0.5834	no
gi|646749464|gb|KK962091.1|	94824-39814	18	8.8	2	3	0.438307	0.77395	no
gi|646749464|gb|KK962091.1|	19361-41955	18	8.8	101	34	-2	0.33035	no
gi|646749464|gb|KK962091.1|	24027-42761	18	8.8	36	21	-0.819615	0.48205	no
gi|646749464|gb|KK962091.1|	29302-43043	18	8.8	1	0.752757	-0.746484	0.59275	no
gi|646749464|gb|KK962091.1|	30663-43488	18	8.8	1	1	-0.33815	0.82065	no
gi|646749464|gb|KK962091.1|	37727-43826	18	8.8	6	12	0.974521	0.4848	no
gi|646749464|gb|KK962091.1|	4581-4824	18	8.8	13	23	0.896577	0.58765	no
gi|646749464|gb|KK962091.1|	61960-46230	18	8.8	25	17	-0.592269	0.7157	no
gi|646749464|gb|KK962091.1|	56400-57460	18	8.8	4	4	0.223781	0.88575	no
gi|646749464|gb|KK962091.1|	58199-59949	18	8.8	18	11	-0.638655	0.61415	no
gi|646749482|gb|KK962090.1|	05245-10552	19	7.1	89	34	-1	0.3943	no
gi|646749482|gb|KK962090.1|	21943-12292	19	7.1	7	4	-0.763704	0.62695	no
gi|646749482|gb|KK962090.1|	23566-12551	19	7.1	15	8	-0.967855	0.45135	no
gi|646749482|gb|KK962090.1|	26389-12786	19	7.1	2	0.766455	-2	0.2896	no
gi|646749482|gb|KK962090.1|	36595-13725	19	7.1	47	47	-0.00075441	0.99695	no
gi|646749482|gb|KK962090.1|	40002-14071	19	7.1	13	2	-3	0.073	no
gi|646749482|gb|KK962090.1|	47524-14971	19	7.1	7	2	-2	0.25795	no
gi|646749482|gb|KK962090.1|	17884-19158	19	7.1	0.90785	0.912182	0.00686812	1	no
gi|646749482|gb|KK962090.1|	86794-28710	19	7.1	149	18	-3	0.04785	no
gi|646749482|gb|KK962090.1|	87814-28875	19	7.1	2	2	-0.323687	0.79055	no
gi|646749482|gb|KK962090.1|	92104-29230	19	7.1	83	16.89	-2	0.2112	no
gi|646749482|gb|KK962090.1|	98632-29901	19	7.1	29	17	-0.761559	0.6292	no
gi|646749482|gb|KK962090.1|	00407-30068	19	7.1	63	52	-0.271579	0.86255	no
gi|646749482|gb|KK962090.1|	09752-31008	19	7.1	5	24	2	0.2162	no
gi|646749482|gb|KK962090.1|	16664-31685	19	7.1	94	21	-2	0.2214	no
gi|646749482|gb|KK962090.1|	31592-33361	19	7.1	9	6	-0.633214	0.67065	no
gi|646749482|gb|KK962090.1|	37366-33768	19	7.1	15	11	-0.43362	0.74035	no
gi|646749482|gb|KK962090.1|	45417-34645	19	7.1	1	0.672317	-0.657624	1	no
gi|646749482|gb|KK962090.1|	49258-34957	19	7.1	9	5	-0.729941	0.64635	no

gi|646749482|gb|KK962090.1|	35486-36033	19	7.1	44	18	-1	0.39055	no
gi|646749482|gb|KK962090.1|	45389-46947	19	7.1	0.497002	1	1	0.39575	no
gi|646749482|gb|KK962090.1|	5193-6081	19	7.1	102	79	-0.372353	0.7392	no
gi|646749482|gb|KK962090.1|	53880-55556	19	7.1	73	62	-0.248352	0.83115	no
gi|646749482|gb|KK962090.1|	61046-56219	19	7.1	0.30939	3	3	0.1373	no
gi|646749482|gb|KK962090.1|	70394-57188	19	7.1	0.674182	6	3	0.05265	no
gi|646749482|gb|KK962090.1|	06811-60712	19	7.1	23	8	-1	0.27315	no
gi|646749482|gb|KK962090.1|	71505-74816	19	7.1	63	43	-0.538932	0.6527	no
gi|646749482|gb|KK962090.1|	76021-77659	19	7.1	0.334469	0.675923	1	1	no
gi|646749499|gb|KK962089.1|	32783-10332	20	8.7	9	1	-3	0.13465	no
gi|646749499|gb|KK962089.1|	14474-13148	20	8.7	16	16	0.0240501	0.96385	no
gi|646749499|gb|KK962089.1|	76363-14768	20	8.7	4	3	-0.338691	0.79845	no
gi|646749499|gb|KK962089.1|	77243-17761	20	8.7	16	0.926388	-4	0.18845	no
gi|646749499|gb|KK962089.1|	89754-19009	20	8.7	3	8	2	0.36955	no
gi|646749499|gb|KK962089.1|	96282-19688	20	8.7	2	4	0.877534	0.50595	no
gi|646749499|gb|KK962089.1|	97192-19784	20	8.7	3	9	1	0.362	no
gi|646749499|gb|KK962089.1|	09109-21558	20	8.7	11	8	-0.587828	0.6019	no
gi|646749499|gb|KK962089.1|	23677-22417	20	8.7	17	12	-0.541872	0.72825	no
gi|646749499|gb|KK962089.1|	84921-28597	20	8.7	0.233011	4	4	0.2454	no
gi|646749499|gb|KK962089.1|	92276-29269	20	8.7	0.563179	10	4	0.09685	no
gi|646749499|gb|KK962089.1|	15265-31570	20	8.7	21	7	-2	0.26195	no
gi|646749499|gb|KK962089.1|	27349-32876	20	8.7	3	2	-0.709327	0.6615	no
gi|646749499|gb|KK962089.1|	29202-33255	20	8.7	16	5	-2	0.22635	no
gi|646749499|gb|KK962089.1|	17279-41779	20	8.7	1	3	2	0.29695	no
gi|646749499|gb|KK962089.1|	15580-51620	20	8.7	1	4	2	0.27405	no
gi|646749499|gb|KK962089.1|	02338-70551	20	8.7	0.601809	1	0.753118	1	no
gi|646749499|gb|KK962089.1|	19573-72121	20	8.7	0.936513	2	0.970133	0.5026	no
gi|646749499|gb|KK962089.1|	34771-73599	20	8.7	0.855686	0.681669	-0.328009	1	no
gi|646749499|gb|KK962089.1|	36174-73765	20	8.7	0.755822	1	0.526603	1	no
gi|646749499|gb|KK962089.1|	40625-74199	20	8.7	0.331879	0.715877	1	1	no

gi|646749499|gb|KK962089.1|	43479-74424	20	8.7	1	2	1	0.4512	no
gi|646749499|gb|KK962089.1|	51806-75208	20	8.7	15	15	-0.0111207	0.9333	no
gi|646749499|gb|KK962089.1|	79880-78028	20	8.7	17	23	0.469228	0.77125	no
gi|646749499|gb|KK962089.1|	84068-78496	20	8.7	9	10	0.0886034	0.9498	no
gi|646749499|gb|KK962089.1|	86738-78751	20	8.7	18	18	-0.0438658	0.97725	no
gi|646749499|gb|KK962089.1|	88445-78909	20	8.7	1	2	0.211155	0.8909	no
gi|646749499|gb|KK962089.1|	98852-79924	20	8.7	18	13	-0.443841	0.78585	no
gi|646749499|gb|KK962089.1|	01907-80238	20	8.7	5	2	-2	0.2807	no
gi|646749499|gb|KK962089.1|	11598-81499	20	8.7	33	20	-0.709903	0.5444	no
gi|646749499|gb|KK962089.1|	17058-81951	20	8.7	29	18	-0.677324	0.54425	no
gi|646749499|gb|KK962089.1|	19750-82082	20	8.7	1	2	0.776844	0.55405	no
gi|646749499|gb|KK962089.1|	21480-82346	20	8.7	2	1	-0.202794	0.89525	no
gi|646749499|gb|KK962089.1|	23662-82467	20	8.7	2	3	0.980428	0.5204	no
gi|646749499|gb|KK962089.1|	24817-82537	20	8.7	6	6	0.109861	0.93915	no
gi|646749499|gb|KK962089.1|	43329-84667	20	8.7	0.838917	1	0.435288	0.78825	no
gi|646749499|gb|KK962089.1|	59148-85953	20	8.7	6	2	-2	0.2745	no
gi|646749499|gb|KK962089.1|	69412-87199	20	8.7	31	27	-0.199347	0.86375	no
gi|646749499|gb|KK962089.1|	84948-98512	20	8.7	308	95	-2	0.2248	no
gi|646749499|gb|KK962089.1|	96589-100329	20	8.7	14	10	-0.55683	0.62885	no
gi|646749512|gb|KK962088.1|	00544-10198	18	7.2	3	2	-0.644101	0.68495	no
gi|646749512|gb|KK962088.1|	13198-11518	18	7.2	1	0.850869	-0.254334	1	no
gi|646749512|gb|KK962088.1|	23540-12383	18	7.2	18	23	0.334893	0.7874	no
gi|646749512|gb|KK962088.1|	24416-12485	18	7.2	13	11	-0.360093	0.825	no
gi|646749512|gb|KK962088.1|	44467-14495	18	7.2	63	24	-1	0.3666	no
gi|646749512|gb|KK962088.1|	56232-15642	18	7.2	241	128	-0.919082	0.51135	no
gi|646749512|gb|KK962088.1|	63925-16519	18	7.2	2	0.391311	-3	0.17015	no
gi|646749512|gb|KK962088.1|	67015-16808	18	7.2	16	5	-2	0.2982	no
gi|646749512|gb|KK962088.1|	17645-19533	18	7.2	8	18	1	0.3605	no
gi|646749512|gb|KK962088.1|	38070-23822	18	7.2	555	191	-2	0.28075	no
gi|646749512|gb|KK962088.1|	44897-24752	18	7.2	67	32	-1	0.35775	no

gi|646749512|gb|KK962088.1|	48317-25137	18	7.2	54	32	-0.732153	0.5362	no
gi|646749512|gb|KK962088.1|	70300-27128	18	7.2	0.762392	2	2	0.249	no
gi|646749512|gb|KK962088.1|	75180-27588	18	7.2	0.207325	4	4	0.09875	no
gi|646749512|gb|KK962088.1|	93589-29380	18	7.2	84.82	20	-2	0.16495	no
gi|646749512|gb|KK962088.1|	23150-32340	18	7.2	58	74	0.365692	0.8195	no
gi|646749512|gb|KK962088.1|	24584-32518	18	7.2	7	6	-0.292886	0.8569	no
gi|646749512|gb|KK962088.1|	32861-33507	18	7.2	9	7	-0.365771	0.8101	no
gi|646749512|gb|KK962088.1|	39191-34033	18	7.2	119.99	70	-0.773408	0.508	no
gi|646749512|gb|KK962088.1|	41763-34237	18	7.2	73	79	0.11183	0.91835	no
gi|646749512|gb|KK962088.1|	58851-35919	18	7.2	162	81	-0.993732	0.51635	no
gi|646749512|gb|KK962088.1|	73842-37482	18	7.2	1	0.90908	-0.628541	0.65905	no
gi|646749512|gb|KK962088.1|	04648-40535	18	7.2	7	3	-1	0.43875	no
gi|646749512|gb|KK962088.1|	06386-40808	18	7.2	9	6	-0.631732	0.67555	no
gi|646749512|gb|KK962088.1|	09180-40974	18	7.2	7	6	-0.323411	0.8063	no
gi|646749512|gb|KK962088.1|	19131-42202	18	7.2	16	12	-0.412768	0.7062	no
gi|646749512|gb|KK962088.1|	22379-42389	18	7.2	0.950727	0.9487	-0.00307869	1	no
gi|646749512|gb|KK962088.1|	53056-45373	18	7.2	0	51	inf	5.00E-05	yes
gi|646749512|gb|KK962088.1|	66155-46683	18	7.2	2	0.907468	-0.93599	0.589	no
gi|646749512|gb|KK962088.1|	80746-48170	18	7.2	30	10	-2	0.2209	no
gi|646749512|gb|KK962088.1|	83440-48379	18	7.2	231	167	-0.467181	0.6595	no
gi|646749512|gb|KK962088.1|	97447-49815	18	7.2	4	5	0.249509	0.8539	no
gi|646749512|gb|KK962088.1|	98362-49910	18	7.2	28	33	0.211636	0.88495	no
gi|646749512|gb|KK962088.1|	24646-52748	18	7.2	6	3	-1	0.41815	no
gi|646749512|gb|KK962088.1|	31921-53293	18	7.2	5	1	-2	0.212	no
gi|646749512|gb|KK962088.1|	47156-54829	18	7.2	5	3	-0.849951	0.58905	no
gi|646749512|gb|KK962088.1|	59530-56051	18	7.2	3	0.17965	-4	0.17035	no
gi|646749512|gb|KK962088.1|	76759-57697	18	7.2	68	0	-inf	0.0035	no
gi|646749512|gb|KK962088.1|	86681-59152	18	7.2	2	3	0.947675	0.53385	no
gi|646749512|gb|KK962088.1|	46285-64833	18	7.2	2	4	1	0.4438	no
gi|646749512|gb|KK962088.1|	88032-88545	18	7.2	12	7	-0.84511	0.539	no

gi|646749512|gb|KK962088.1|	98342-98931	18	7.2	2	2	0.459707	0.7494	no
gi|646749512|gb|KK962088.1|	99037-99857	18	7.2	2	2	-0.280672	0.832	no
gi|646749532|gb|KK962087.1|	05132-10579	14	6	5	1	-2	0.22175	no
gi|646749532|gb|KK962087.1|	08028-10905	14	6	2	0.681604	-1	0.374	no
gi|646749532|gb|KK962087.1|	14215-11510	14	6	8	2	-2	0.20245	no
gi|646749532|gb|KK962087.1|	22629-12361	14	6	19	4	-2	0.17995	no
gi|646749532|gb|KK962087.1|	24335-12501	14	6	39	9	-2	0.19985	no
gi|646749532|gb|KK962087.1|	28989-12927	14	6	153	72	-1	0.47685	no
gi|646749532|gb|KK962087.1|	07359-20764	14	6	82	16	-2	0.1284	no
gi|646749532|gb|KK962087.1|	09131-20946	14	6	58	29	-1	0.51275	no
gi|646749532|gb|KK962087.1|	16179-21745	14	6	0.814667	0.649687	-0.326465	1	no
gi|646749532|gb|KK962087.1|	21138-22233	14	6	208	49	-2	0.08075	no
gi|646749532|gb|KK962087.1|	24318-22491	14	6	25	19	-0.389135	0.79085	no
gi|646749532|gb|KK962087.1|	32526-23292	14	6	4	7	0.554332	0.70885	no
gi|646749532|gb|KK962087.1|	33021-23383	14	6	2	2	0.233019	0.8571	no
gi|646749532|gb|KK962087.1|	44380-24738	14	6	0.842316	1	0.420627	0.7872	no
gi|646749532|gb|KK962087.1|	24671-25554	14	6	0.441879	362	10	0.1049	no
gi|646749532|gb|KK962087.1|	48000-24905	14	6	2	1	-0.224067	0.86175	no
gi|646749532|gb|KK962087.1|	51629-25373	14	6	0.551293	0.508424	-0.116787	1	no
gi|646749532|gb|KK962087.1|	61117-26135	14	6	39	23	-0.810123	0.5522	no
gi|646749532|gb|KK962087.1|	81753-28284	14	6	2	2	-0.177244	0.8816	no
gi|646749532|gb|KK962087.1|	88290-28905	14	6	1	0.510734	-2	0.38345	no
gi|646749532|gb|KK962087.1|	91973-29222	14	6	159	208	0.390605	0.79245	no
gi|646749532|gb|KK962087.1|	27320-32807	14	6	18	21	0.235871	0.8775	no
gi|646749532|gb|KK962087.1|	38112-33885	14	6	698	410	-0.7674	0.51905	no
gi|646749532|gb|KK962087.1|	58037-35904	14	6	2	1	-0.811094	0.5394	no
gi|646749532|gb|KK962087.1|	61012-36323	14	6	2	2	-0.141752	0.92665	no
gi|646749532|gb|KK962087.1|	64282-36489	14	6	0.767391	3	2	0.2057	no
gi|646749532|gb|KK962087.1|	66454-36704	14	6	1	2	0.723518	0.64505	no
gi|646749532|gb|KK962087.1|	70798-37225	14	6	21	10	-1	0.37845	no

gi|646749532|gb|KK962087.1|	37216-37523	14	6	16	3	-2	0.17305	no
gi|646749532|gb|KK962087.1|	77309-37787	14	6	11	14	0.373735	0.8097	no
gi|646749532|gb|KK962087.1|	78979-38025	14	6	21	14	-0.628469	0.6224	no
gi|646749532|gb|KK962087.1|	82286-38360	14	6	10	7	-0.400608	0.7863	no
gi|646749532|gb|KK962087.1|	88141-39223	14	6	12	7	-0.652668	0.55785	no
gi|646749532|gb|KK962087.1|	93597-39442	14	6	32	7	-2	0.1702	no
gi|646749532|gb|KK962087.1|	39378-41183	14	6	4	4	0.203965	0.8905	no
gi|646749532|gb|KK962087.1|	24131-42452	14	6	288	41	-3	0.0513	no
gi|646749532|gb|KK962087.1|	42757-44057	14	6	2	3	0.415292	0.79215	no
gi|646749532|gb|KK962087.1|	35325-43585	14	6	9	36	2	0.22415	no
gi|646749532|gb|KK962087.1|	61830-46205	14	6	34	69	1	0.43455	no
gi|646749532|gb|KK962087.1|	69202-47041	14	6	0.776569	0.834776	0.104275	1	no
gi|646749532|gb|KK962087.1|	72602-47523	14	6	64	87	0.445664	0.7067	no
gi|646749532|gb|KK962087.1|	47273-47522	14	6	20	9	-1	0.4864	no
gi|646749532|gb|KK962087.1|	81115-48208	14	6	1	0.552105	-0.906615	1	no
gi|646749532|gb|KK962087.1|	82919-48370	14	6	1	2	0.492888	0.72995	no
gi|646749532|gb|KK962087.1|	84781-48508	14	6	5	16	2	0.35435	no
gi|646749532|gb|KK962087.1|	86847-48763	14	6	57	21	-1	0.2798	no
gi|646749532|gb|KK962087.1|	88520-48954	14	6	12	8	-0.594266	0.69685	no
gi|646749532|gb|KK962087.1|	93428-49570	14	6	26	14	-0.958632	0.396	no
gi|646749532|gb|KK962087.1|	00690-50131	14	6	3	2	-0.668786	0.64675	no
gi|646749532|gb|KK962087.1|	03001-50368	14	6	1	3	1	0.45245	no
gi|646749532|gb|KK962087.1|	90241-90748	14	6	23	13	-0.821221	0.5942	no
gi|646749532|gb|KK962087.1|	96478-96849	14	6	24	7	-2	0.2299	no
gi|646749551|gb|KK962086.1|	18625-11981	15	7.6	2	3	0.644283	0.6925	no
gi|646749573|gb|KK962085.1|	02798-10310	23	10	8	14	0.834817	0.5691	no
gi|646749573|gb|KK962085.1|	11039-11262	23	10	0.697689	0.603854	-0.208384	1	no
gi|646749573|gb|KK962085.1|	19274-11990	23	10	2	3	0.984566	0.5187	no
gi|646749573|gb|KK962085.1|	21110-12264	23	10	0.725024	2	2	0.26165	no
gi|646749573|gb|KK962085.1|	25184-12654	23	10	0.501416	0.961264	0.938926	1	no

gi|646749573|gb|KK962085.1|	38345-14023	23	10	9	7	-0.295923	0.8449	no
gi|646749573|gb|KK962085.1|	47858-14841	23	10	12	13	0.107975	0.94515	no
gi|646749573|gb|KK962085.1|	49926-15066	23	10	2	2	0.294986	0.83555	no
gi|646749573|gb|KK962085.1|	83965-18523	23	10	0.821093	0.785643	-0.0636729	1	no
gi|646749573|gb|KK962085.1|	02492-20391	23	10	6	4	-0.576945	0.7103	no
gi|646749573|gb|KK962085.1|	17384-21946	23	10	7	6	-0.198291	0.89265	no
gi|646749573|gb|KK962085.1|	19830-22010	23	10	24	11	-1	0.44845	no
gi|646749573|gb|KK962085.1|	24186-22600	23	10	4	5	0.34869	0.81665	no
gi|646749573|gb|KK962085.1|	39271-23998	23	10	8	4	-0.918422	0.5069	no
gi|646749573|gb|KK962085.1|	53023-25344	23	10	0.535478	8	4	0.10035	no
gi|646749573|gb|KK962085.1|	70458-27182	23	10	0.497373	1	2	0.3018	no
gi|646749573|gb|KK962085.1|	74036-27462	23	10	3	10	2	0.24175	no
gi|646749573|gb|KK962085.1|	76845-27740	23	10	7	4	-0.687227	0.6374	no
gi|646749573|gb|KK962085.1|	82792-28556	23	10	2	2	0.11538	0.94575	no
gi|646749573|gb|KK962085.1|	86840-28782	23	10	3	5.96	0.909795	0.5671	no
gi|646749573|gb|KK962085.1|	15273-31658	23	10	0.612652	0.880069	0.522548	1	no
gi|646749573|gb|KK962085.1|	18107-31834	23	10	549	695	0.340352	0.78565	no
gi|646749573|gb|KK962085.1|	36011-33689	23	10	50	74	0.556393	0.6166	no
gi|646749573|gb|KK962085.1|	39333-33992	23	10	10	12	0.290559	0.8494	no
gi|646749573|gb|KK962085.1|	40543-34109	23	10	92	52	-0.813072	0.52575	no
gi|646749573|gb|KK962085.1|	10770-41118	23	10	6	10	0.728248	0.5761	no
gi|646749573|gb|KK962085.1|	13085-41709	23	10	34	29	-0.232092	0.8433	no
gi|646749573|gb|KK962085.1|	23878-42477	23	10	2	1	-0.148888	0.9177	no
gi|646749573|gb|KK962085.1|	31824-43394	23	10	0.596244	0.504107	-0.242171	1	no
gi|646749573|gb|KK962085.1|	34265-43609	23	10	13	10	-0.336735	0.78935	no
gi|646749573|gb|KK962085.1|	39055-43952	23	10	15	8	-0.865539	0.52805	no
gi|646749573|gb|KK962085.1|	40229-44156	23	10	0.773071	0.370311	-1	1	no
gi|646749573|gb|KK962085.1|	42980-44465	23	10	13	12	-0.0951957	0.95055	no
gi|646749573|gb|KK962085.1|	56565-45730	23	10	6	4	-0.704881	0.60615	no
gi|646749573|gb|KK962085.1|	58578-45959	23	10	1	2	0.358993	0.79375	no

gi|646749573|gb|KK962085.1|	66708-46802	23	10	2	1	-0.555427	0.6596	no
gi|646749573|gb|KK962085.1|	79315-48039	23	10	0.562006	1	0.999561	0.5626	no
gi|646749573|gb|KK962085.1|	85495-48741	23	10	12	5	-1	0.3276	no
gi|646749573|gb|KK962085.1|	34440-53466	23	10	56	0	-inf	0.0041	no
gi|646749573|gb|KK962085.1|	61044-56215	23	10	1	0.462427	-1	1	no
gi|646749573|gb|KK962085.1|	78949-58825	23	10	14	8	-0.92032	0.4319	no
gi|646749573|gb|KK962085.1|	13133-61388	23	10	8	4	-0.903001	0.5702	no
gi|646749573|gb|KK962085.1|	40374-64134	23	10	2	0.735144	-2	0.24935	no
gi|646749573|gb|KK962085.1|	57730-65884	23	10	3	0.76636	-2	0.2505	no
gi|646749573|gb|KK962085.1|	64696-66624	23	10	0.930861	4	2	0.16615	no
gi|646749597|gb|KK962084.1|	20269-12064	16	7.7	0.726368	12	4	0.0993	no
gi|646749597|gb|KK962084.1|	22082-12231	16	7.7	369	85	-2	0.193	no
gi|646749597|gb|KK962084.1|	36822-23713	16	7.7	19	17	-0.153172	0.8966	no
gi|646749597|gb|KK962084.1|	37690-24084	16	7.7	0.82892	0.742472	-0.158897	1	no
gi|646749597|gb|KK962084.1|	46435-24716	16	7.7	3	2	-0.834834	0.53485	no
gi|646749597|gb|KK962084.1|	50856-25153	16	7.7	3	3	-0.257748	0.85185	no
gi|646749597|gb|KK962084.1|	52424-25360	16	7.7	1	0.717722	-0.481755	1	no
gi|646749597|gb|KK962084.1|	54026-25482	16	7.7	1	1	0.231798	0.87595	no
gi|646749597|gb|KK962084.1|	59211-26115	16	7.7	15	12	-0.300966	0.78225	no
gi|646749597|gb|KK962084.1|	61595-26548	16	7.7	9	9	-0.0253498	0.98215	no
gi|646749597|gb|KK962084.1|	67850-27011	16	7.7	8	10	0.303191	0.8097	no
gi|646749597|gb|KK962084.1|	80160-28152	16	7.7	10	12	0.169682	0.91285	no
gi|646749597|gb|KK962084.1|	08828-30994	16	7.7	2	2	0.615427	0.6657	no
gi|646749597|gb|KK962084.1|	23088-32335	16	7.7	19	10	-1	0.4668	no
gi|646749597|gb|KK962084.1|	30474-33148	16	7.7	8	5.92	-0.424071	0.7821	no
gi|646749597|gb|KK962084.1|	32793-33295	16	7.7	397	118	-2	0.23805	no
gi|646749597|gb|KK962084.1|	62792-36381	16	7.7	0.720816	0.856306	0.248494	1	no
gi|646749597|gb|KK962084.1|	64170-36677	16	7.7	12	9	-0.417971	0.69515	no
gi|646749597|gb|KK962084.1|	67051-36975	16	7.7	14	16	0.127012	0.9099	no
gi|646749597|gb|KK962084.1|	79071-38026	16	7.7	28	23	-0.275825	0.7952	no

gi|646749597|gb|KK962084.1|	88057-38924	16	7.7	0.691837	0.424792	-0.703676	1	no
gi|646749597|gb|KK962084.1|	98074-39880	16	7.7	2	2	0.0717845	0.92975	no
gi|646749597|gb|KK962084.1|	72593-47381	16	7.7	1	0.412733	-2	0.269	no
gi|646749597|gb|KK962084.1|	97599-59869	16	7.7	26	12	-1	0.3573	no
gi|646749597|gb|KK962084.1|	99688-60477	16	7.7	14	12	-0.187079	0.86915	no
gi|646749597|gb|KK962084.1|	13738-61434	16	7.7	1	5	2	0.2179	no
gi|646749597|gb|KK962084.1|	15653-61834	16	7.7	13	11	-0.247489	0.8138	no
gi|646749597|gb|KK962084.1|	26133-62714	16	7.7	5	5	0.0328412	0.98205	no
gi|646749597|gb|KK962084.1|	38668-63891	16	7.7	15	33	1	0.45245	no
gi|646749597|gb|KK962084.1|	40557-64259	16	7.7	0.518882	0.676499	0.382681	1	no
gi|646749597|gb|KK962084.1|	44813-64633	16	7.7	2	4	0.954897	0.54145	no
gi|646749616|gb|KK962083.1|	01061-10356	22	10	7	3	-1	0.49645	no
gi|646749616|gb|KK962083.1|	15177-10156	22	10	6	4	-0.76398	0.56735	no
gi|646749616|gb|KK962083.1|	17162-10182	22	10	0.576718	0.98768	0.776177	1	no
gi|646749616|gb|KK962083.1|	04340-10718	22	10	0.572434	0.832421	0.540205	1	no
gi|646749616|gb|KK962083.1|	51596-10519	22	10	1	16	4	0.10085	no
gi|646749616|gb|KK962083.1|	75267-10767	22	10	0.52564	0.75729	0.52677	1	no
gi|646749616|gb|KK962083.1|	03206-11034	22	10	185	6	-5	0.17335	no
gi|646749616|gb|KK962083.1|	18345-11187	22	10	64	59	-0.108264	0.9411	no
gi|646749616|gb|KK962083.1|	13285-11582	22	10	1	2	0.538612	0.7371	no
gi|646749616|gb|KK962083.1|	33424-11345	22	10	0.544798	0.934407	0.778329	1	no
gi|646749616|gb|KK962083.1|	43090-11440	22	10	0.694456	0.985675	0.50523	1	no
gi|646749616|gb|KK962083.1|	44108-11456	22	10	42	15	-1	0.17945	no
gi|646749616|gb|KK962083.1|	47103-11494	22	10	0.666557	1	1	0.46435	no
gi|646749616|gb|KK962083.1|	51679-11526	22	10	10	18	0.870748	0.55935	no
gi|646749616|gb|KK962083.1|	53891-11544	22	10	21	18	-0.220575	0.8792	no
gi|646749616|gb|KK962083.1|	54700-11556	22	10	0.967879	0.981398	0.0200115	1	no
gi|646749616|gb|KK962083.1|	56593-11578	22	10	14	12	-0.262354	0.86355	no
gi|646749616|gb|KK962083.1|	59921-11601	22	10	119	88	-0.435386	0.7856	no
gi|646749616|gb|KK962083.1|	62838-11633	22	10	13	17	0.342097	0.82335	no

gi|646749616|gb|KK962083.1|	77980-11785	22	10	2	6	2	0.34605	no
gi|646749616|gb|KK962083.1|	79776-11800	22	10	72	64	-0.163709	0.924	no
gi|646749616|gb|KK962083.1|	82256-11827	22	10	13	3	-2	0.15885	no
gi|646749616|gb|KK962083.1|	88117-11883	22	10	786	511	-0.62204	0.5584	no
gi|646749616|gb|KK962083.1|	97367-11978	22	10	2	6	2	0.35625	no
gi|646749616|gb|KK962083.1|	10633-12148	22	10	10	9	-0.0317451	0.97615	no
gi|646749616|gb|KK962083.1|	23149-12240	22	10	3	2	-0.78813	0.59575	no
gi|646749616|gb|KK962083.1|	24091-12254	22	10	0.525628	0.755044	0.52252	1	no
gi|646749616|gb|KK962083.1|	35627-12361	22	10	2	4	1	0.4024	no
gi|646749616|gb|KK962083.1|	38455-12394	22	10	0.62672	1	0.772811	1	no
gi|646749616|gb|KK962083.1|	43148-12443	22	10	1	0.717722	-0.481755	1	no
gi|646749616|gb|KK962083.1|	44819-12463	22	10	2	1	-0.571226	0.6774	no
gi|646749616|gb|KK962083.1|	50020-12510	22	10	0.6827	0.649783	-0.0712944	1	no
gi|646749616|gb|KK962083.1|	68834-12692	22	10	4	3	-0.442448	0.76125	no
gi|646749616|gb|KK962083.1|	26964-12914	22	10	0.817887	0.488301	-0.744131	1	no
gi|646749616|gb|KK962083.1|	69734-12756	22	10	4	3	-0.152733	0.90245	no
gi|646749616|gb|KK962083.1|	12779-13562	22	10	2	0.986243	-0.828817	0.5782	no
gi|646749616|gb|KK962083.1|	88804-12902	22	10	5	7	0.480512	0.753	no
gi|646749616|gb|KK962083.1|	90364-12912	22	10	4	2	-0.682086	0.6255	no
gi|646749616|gb|KK962083.1|	91386-12937	22	10	4	2	-0.94828	0.53395	no
gi|646749616|gb|KK962083.1|	29294-13046	22	10	1	1	-0.134016	1	no
gi|646749616|gb|KK962083.1|	14577-13168	22	10	1	1	0.174143	0.9163	no
gi|646749616|gb|KK962083.1|	23695-13242	22	10	0.645887	6	3	0.24155	no
gi|646749616|gb|KK962083.1|	40136-13406	22	10	6	9	0.511941	0.72225	no
gi|646749616|gb|KK962083.1|	70197-13704	22	10	15	23	0.581422	0.72965	no
gi|646749616|gb|KK962083.1|	98883-13999	22	10	0	3	inf	0.0023	no
gi|646749616|gb|KK962083.1|	06085-14062	22	10	497	42	-4	0.1033	no
gi|646749616|gb|KK962083.1|	08594-14088	22	10	26	12	-1	0.4807	no
gi|646749616|gb|KK962083.1|	09487-14096	22	10	297	18	-4	0.0878	no
gi|646749616|gb|KK962083.1|	16060-14162	22	10	198.74	14	-4	0.18015	no

gi|646749616|gb|KK962083.1|	27173-14282	22	10	19	14	-0.436189	0.7733	no
gi|646749616|gb|KK962083.1|	42895-14383	22	10	2	1	-0.71574	0.58605	no
gi|646749616|gb|KK962083.1|	41472-14426	22	10	9	3	-1	0.3551	no
gi|646749616|gb|KK962083.1|	43552-14445	22	10	20	7	-2	0.32145	no
gi|646749616|gb|KK962083.1|	45052-14452	22	10	22	17	-0.3928	0.80985	no
gi|646749616|gb|KK962083.1|	74448-14753	22	10	11	0.766277	-4	0.05885	no
gi|646749616|gb|KK962083.1|	82941-14836	22	10	7	0	-inf	0.0014	yes
gi|646749616|gb|KK962083.1|	68130-16947	22	10	3	2	-0.242194	0.87515	no
gi|646749616|gb|KK962083.1|	95195-19600	22	10	12	4	-2	0.3323	no
gi|646749616|gb|KK962083.1|	02982-20346	22	10	29	13	-1	0.4712	no
gi|646749616|gb|KK962083.1|	09552-21008	22	10	2	2	0.177751	0.89965	no
gi|646749616|gb|KK962083.1|	13386-21654	22	10	1	2	0.541558	0.72855	no
gi|646749616|gb|KK962083.1|	26695-22748	22	10	1	0.973864	-0.506122	0.7384	no
gi|646749616|gb|KK962083.1|	28657-22893	22	10	18	13	-0.548284	0.69945	no
gi|646749616|gb|KK962083.1|	30977-23120	22	10	36	41	0.197436	0.8824	no
gi|646749616|gb|KK962083.1|	51423-25573	22	10	27	26	-0.043355	0.96915	no
gi|646749616|gb|KK962083.1|	32669-33244	22	10	6	11	0.818484	0.55125	no
gi|646749616|gb|KK962083.1|	30666-33381	22	10	31	1	-5	0.0078	no
gi|646749616|gb|KK962083.1|	55774-35677	22	10	0.873856	1	0.702592	0.62425	no
gi|646749616|gb|KK962083.1|	05270-41071	22	10	20	19	-0.0959433	0.93285	no
gi|646749616|gb|KK962083.1|	40871-41413	22	10	9	7	-0.402274	0.8099	no
gi|646749616|gb|KK962083.1|	28272-42944	22	10	3	2	-0.829788	0.55855	no
gi|646749616|gb|KK962083.1|	34632-43539	22	10	1	0.774836	-0.732204	0.66415	no
gi|646749616|gb|KK962083.1|	49298-45084	22	10	1	0.62184	-0.794067	1	no
gi|646749616|gb|KK962083.1|	52293-45353	22	10	0.933496	0.267825	-2	1	no
gi|646749616|gb|KK962083.1|	61183-46202	22	10	5	5	-0.153275	0.92245	no
gi|646749616|gb|KK962083.1|	62489-46285	22	10	145	83	-0.80308	0.5989	no
gi|646749616|gb|KK962083.1|	74557-47780	22	10	2	2	0.54188	0.71525	no
gi|646749616|gb|KK962083.1|	78391-47935	22	10	3	3	0.0720786	0.9625	no
gi|646749616|gb|KK962083.1|	83563-48398	22	10	5	4	-0.463104	0.7542	no

gi|646749616|gb|KK962083.1|	91727-49211	22	10	16	19	0.2305	0.88555	no
gi|646749616|gb|KK962083.1|	06494-50736	22	10	0.301301	2	3	0.2655	no
gi|646749616|gb|KK962083.1|	5117-6044	22	10	0.825908	3	2	0.2385	no
gi|646749616|gb|KK962083.1|	13482-51623	22	10	0.481293	0.807322	0.746228	1	no
gi|646749616|gb|KK962083.1|	16437-52633	22	10	6	10	0.648904	0.56725	no
gi|646749616|gb|KK962083.1|	30197-53170	22	10	1	2	1	0.4337	no
gi|646749616|gb|KK962083.1|	40973-54283	22	10	3	3	0.117704	0.9386	no
gi|646749616|gb|KK962083.1|	52982-55387	22	10	1	3	2	0.2862	no
gi|646749616|gb|KK962083.1|	54198-55451	22	10	34	23	-0.547768	0.73055	no
gi|646749616|gb|KK962083.1|	57376-56787	22	10	27	33	0.293426	0.8092	no
gi|646749616|gb|KK962083.1|	70029-57212	22	10	1	2	0.535196	0.7424	no
gi|646749616|gb|KK962083.1|	58238-59733	22	10	1	0.752491	-0.572601	1	no
gi|646749616|gb|KK962083.1|	93768-59462	22	10	0.929635	2	1	0.34485	no
gi|646749616|gb|KK962083.1|	61676-63166	22	10	1	3	2	0.25685	no
gi|646749616|gb|KK962083.1|	25526-62608	22	10	2	5	1	0.4277	no
gi|646749616|gb|KK962083.1|	28661-62946	22	10	3	2	-0.422602	0.72885	no
gi|646749616|gb|KK962083.1|	37507-63807	22	10	4	2	-1	0.43665	no
gi|646749616|gb|KK962083.1|	64241-64505	22	10	4	35	3	0.24665	no
gi|646749616|gb|KK962083.1|	65132-65508	22	10	6	16	2	0.3515	no
gi|646749616|gb|KK962083.1|	70797-73191	22	10	50	36	-0.50003	0.66325	no
gi|646749616|gb|KK962083.1|	73723-77577	22	10	0.92547	1	0.327938	0.83165	no
gi|646749616|gb|KK962083.1|	40674-74688	22	10	25	17	-0.595184	0.6112	no
gi|646749616|gb|KK962083.1|	50380-75196	22	10	0.696115	0.702933	0.0140617	1	no
gi|646749616|gb|KK962083.1|	54932-75563	22	10	3	4	0.483072	0.7434	no
gi|646749616|gb|KK962083.1|	55785-75654	22	10	5	5	0.239016	0.8803	no
gi|646749616|gb|KK962083.1|	85083-78575	22	10	3	2	-0.786437	0.5838	no
gi|646749616|gb|KK962083.1|	86955-78717	22	10	47	9	-2	0.2039	no
gi|646749616|gb|KK962083.1|	80206-82357	22	10	2	1	-0.983632	0.5499	no
gi|646749616|gb|KK962083.1|	83706-85524	22	10	0.473528	0.599181	0.339543	1	no
gi|646749616|gb|KK962083.1|	37330-83820	22	10	2	0.422503	-2	0.2551	no

gi|646749616|gb|KK962083.1|	85661-87434	22	10	0.487563	0.440489	-0.146482	1	no
gi|646749616|gb|KK962083.1|	88530-92135	22	10	0.46951	0.764559	0.703474	1	no
gi|646749616|gb|KK962083.1|	08088-91241	22	10	2	3	0.490756	0.74655	no
gi|646749616|gb|KK962083.1|	93243-93504	22	10	42	23	-0.854934	0.49825	no
gi|646749616|gb|KK962083.1|	40037-94286	22	10	6	4	-0.532906	0.7233	no
gi|646749616|gb|KK962083.1|	45979-94738	22	10	0.801662	1	0.903556	0.48345	no
gi|646749616|gb|KK962083.1|	47524-94971	22	10	2	2	-0.23263	0.8834	no
gi|646749616|gb|KK962083.1|	58446-95914	22	10	4	3	-0.305885	0.79815	no
gi|646749616|gb|KK962083.1|	71886-97644	22	10	17	15	-0.105093	0.9243	no
gi|646749616|gb|KK962083.1|	77461-97917	22	10	0.697029	0.732359	0.0713319	1	no
gi|646749632|gb|KK962082.1|	71873-17218	20	8.6	4	20	2	0.1498	no
gi|646749632|gb|KK962082.1|	74815-17559	20	8.6	9	7	-0.301227	0.8452	no
gi|646749632|gb|KK962082.1|	86476-18682	20	8.6	57	6	-3	0.05995	no
gi|646749632|gb|KK962082.1|	90055-19302	20	8.6	66	44	-0.598067	0.61825	no
gi|646749632|gb|KK962082.1|	93683-19523	20	8.6	0.356094	0.616193	0.791122	1	no
gi|646749632|gb|KK962082.1|	06371-20781	20	8.6	40	30	-0.41408	0.70655	no
gi|646749632|gb|KK962082.1|	18016-21829	20	8.6	53	36	-0.581837	0.7284	no
gi|646749632|gb|KK962082.1|	26751-22696	20	8.6	42	23	-0.844405	0.59745	no
gi|646749632|gb|KK962082.1|	29984-23367	20	8.6	57	78	0.466377	0.69125	no
gi|646749632|gb|KK962082.1|	50812-25304	20	8.6	35	58	0.741705	0.52925	no
gi|646749632|gb|KK962082.1|	60096-26061	20	8.6	3	3	-0.147197	0.9178	no
gi|646749632|gb|KK962082.1|	61504-26169	20	8.6	61	139	1	0.40345	no
gi|646749632|gb|KK962082.1|	26655-29201	20	8.6	2	3	0.235232	0.87595	no
gi|646749632|gb|KK962082.1|	67315-26832	20	8.6	4	11	2	0.34055	no
gi|646749632|gb|KK962082.1|	34088-33643	20	8.6	2	2	0.0677859	0.96505	no
gi|646749632|gb|KK962082.1|	37705-34030	20	8.6	23	28	0.27691	0.80675	no
gi|646749632|gb|KK962082.1|	43223-34543	20	8.6	1	0.274097	-2	0.1936	no
gi|646749632|gb|KK962082.1|	55011-35628	20	8.6	1	4	2	0.25775	no
gi|646749632|gb|KK962082.1|	56338-35795	20	8.6	1	1	-0.259236	0.8321	no
gi|646749632|gb|KK962082.1|	59900-36228	20	8.6	273	204	-0.419808	0.7279	no

gi|646749632|gb|KK962082.1|	62602-36301	20	8.6	23	1	-4	0.11935	no
gi|646749632|gb|KK962082.1|	94955-39540	20	8.6	25	5	-2	0.14255	no
gi|646749632|gb|KK962082.1|	21314-42162	20	8.6	101	89	-0.185927	0.9031	no
gi|646749632|gb|KK962082.1|	47266-45312	20	8.6	15	14	-0.0780631	0.94325	no
gi|646749632|gb|KK962082.1|	54838-45709	20	8.6	15	19	0.359574	0.74375	no
gi|646749632|gb|KK962082.1|	4618-5582	20	8.6	2	4	0.783689	0.5676	no
gi|646749632|gb|KK962082.1|	66314-46836	20	8.6	16	13	-0.341975	0.7511	no
gi|646749632|gb|KK962082.1|	77595-48022	20	8.6	58	61	0.0592791	0.958	no
gi|646749632|gb|KK962082.1|	00950-50127	20	8.6	51	20	-1	0.4043	no
gi|646749632|gb|KK962082.1|	14754-51541	20	8.6	1	3	2	0.27405	no
gi|646749632|gb|KK962082.1|	18667-51974	20	8.6	1	0.798841	-0.333679	1	no
gi|646749632|gb|KK962082.1|	29787-53023	20	8.6	2	5	1	0.4595	no
gi|646749632|gb|KK962082.1|	33625-53411	20	8.6	6	10	0.748347	0.5983	no
gi|646749632|gb|KK962082.1|	38709-53941	20	8.6	2	0.867036	-1	0.4263	no
gi|646749632|gb|KK962082.1|	65149-56561	20	8.6	46	71	0.628347	0.6847	no
gi|646749632|gb|KK962082.1|	81302-58206	20	8.6	95	139	0.547413	0.6252	no
gi|646749632|gb|KK962082.1|	82287-58294	20	8.6	290	287	-0.0125863	0.992	no
gi|646749632|gb|KK962082.1|	89062-59039	20	8.6	229	188	-0.284365	0.8082	no
gi|646749632|gb|KK962082.1|	04627-60961	20	8.6	0.039113	4	7	0.2322	no
gi|646749649|gb|KK962081.1|	01091-10308	19	7.6	0.585885	0.231396	-1	1	no
gi|646749649|gb|KK962081.1|	08297-10895	19	7.6	2	2	-0.525621	0.72155	no
gi|646749649|gb|KK962081.1|	10331-11108	19	7.6	3	0.516557	-3	0.13745	no
gi|646749649|gb|KK962081.1|	11849-11276	19	7.6	0.835856	1	0.727482	0.6352	no
gi|646749649|gb|KK962081.1|	38666-13961	19	7.6	1	0	-inf	0.00475	no
gi|646749649|gb|KK962081.1|	89709-39142	19	7.6	4	2	-1	0.49255	no
gi|646749649|gb|KK962081.1|	91491-39300	19	7.6	2	3	0.227657	0.88355	no
gi|646749649|gb|KK962081.1|	00815-40158	19	7.6	2	4	1	0.37175	no
gi|646749649|gb|KK962081.1|	01870-40293	19	7.6	5	5	0.0345003	0.98225	no
gi|646749649|gb|KK962081.1|	03688-40751	19	7.6	2	3	0.456139	0.76165	no
gi|646749649|gb|KK962081.1|	19153-41983	19	7.6	0	12	inf	0.00045	yes

gi|646749649|gb|KK962081.1|	32160-43278	19	7.6	0.252335	29	7	0.09495	no
gi|646749649|gb|KK962081.1|	43760-44439	19	7.6	0.980146	2	1	0.4355	no
gi|646749649|gb|KK962081.1|	45595-44683	19	7.6	2	3	0.2795	0.8604	no
gi|646749649|gb|KK962081.1|	47870-44916	19	7.6	2	2	-0.325992	0.84235	no
gi|646749649|gb|KK962081.1|	69339-47034	19	7.6	3	2	-0.422435	0.7201	no
gi|646749649|gb|KK962081.1|	78343-47962	19	7.6	0.99279	2	0.96908	0.53685	no
gi|646749649|gb|KK962081.1|	93336-49400	19	7.6	0	6	inf	0.00435	no
gi|646749649|gb|KK962081.1|	57144-58267	19	7.6	2	0.914468	-1	0.4155	no
gi|646749649|gb|KK962081.1|	82302-58315	19	7.6	1	2	0.500206	0.72155	no
gi|646749649|gb|KK962081.1|	67569-69582	19	7.6	4	4	0.0869726	0.95275	no
gi|646749649|gb|KK962081.1|	71702-72439	19	7.6	1	1	-0.319858	0.8509	no
gi|646749649|gb|KK962081.1|	73518-75529	19	7.6	7	4	-0.761982	0.6168	no
gi|646749649|gb|KK962081.1|	75644-77432	19	7.6	12	9	-0.413241	0.74625	no
gi|646749649|gb|KK962081.1|	99689-10092[illegible]	19	7.6	10	2	-2	0.2099	no
gi|646749672|gb|KK962080.1|	01373-10158	20	7.6	52	90	0.8026	0.60475	no
gi|646749672|gb|KK962080.1|	10009-11029	20	7.6	28	14	-0.99249	0.48	no
gi|646749672|gb|KK962080.1|	11498-11193	20	7.6	6	6	0.104614	0.9401	no
gi|646749672|gb|KK962080.1|	14564-11482	20	7.6	39	53	0.461333	0.74455	no
gi|646749672|gb|KK962080.1|	19523-12130	20	7.6	0.607454	0.790316	0.379655	1	no
gi|646749672|gb|KK962080.1|	24810-12567	20	7.6	6	0.855662	-3	0.10925	no
gi|646749672|gb|KK962080.1|	54877-15609	20	7.6	3	3	0.224028	0.88665	no
gi|646749672|gb|KK962080.1|	56779-16150	20	7.6	6	2	-1	0.2972	no
gi|646749672|gb|KK962080.1|	62262-16579	20	7.6	23	7	-2	0.1221	no
gi|646749672|gb|KK962080.1|	68322-16908	20	7.6	18	7	-1	0.34585	no
gi|646749672|gb|KK962080.1|	99925-20438	20	7.6	17	12	-0.584565	0.6003	no
gi|646749672|gb|KK962080.1|	12839-21635	20	7.6	0.993627	1	0.220816	0.8905	no
gi|646749672|gb|KK962080.1|	16659-21743	20	7.6	1	2	0.492092	0.72185	no
gi|646749672|gb|KK962080.1|	19013-21994	20	7.6	0.408097	3	3	0.134	no
gi|646749672|gb|KK962080.1|	02140-30253	20	7.6	4	3	-0.442042	0.76715	no
gi|646749672|gb|KK962080.1|	91170-49155	20	7.6	136	88	-0.621765	0.6796	no

gi|646749672|gb|KK962080.1|	20199-62166	20	7.6	11	5	-0.977883	0.52295	no
gi|646749672|gb|KK962080.1|	27844-62833	20	7.6	65	56	-0.205821	0.891	no
gi|646749672|gb|KK962080.1|	40445-64086	20	7.6	27	40	0.546892	0.7188	no
gi|646749672|gb|KK962080.1|	43659-64390	20	7.6	101	121	0.259147	0.86745	no
gi|646749672|gb|KK962080.1|	46976-64766	20	7.6	2	2	0.157793	0.91085	no
gi|646749672|gb|KK962080.1|	50889-65123	20	7.6	80	58	-0.481892	0.74605	no
gi|646749672|gb|KK962080.1|	53671-65539	20	7.6	19	12	-0.651249	0.60915	no
gi|646749672|gb|KK962080.1|	58227-65865	20	7.6	4	7	0.7119	0.6065	no
gi|646749672|gb|KK962080.1|	61221-66138	20	7.6	31	237	3	0.1167	no
gi|646749672|gb|KK962080.1|	72356-67284	20	7.6	11	9	-0.244273	0.88125	no
gi|646749692|gb|KK962079.1|	21481-10226	18	9.1	0	16	inf	5.00E-05	yes
gi|646749692|gb|KK962079.1|	46466-55910	18	9.1	4	3	-0.383827	0.72365	no
gi|646749692|gb|KK962079.1|	88263-58859	18	9.1	24	45	0.917399	0.5616	no
gi|646749692|gb|KK962079.1|	64508-77060	18	9.1	6	6	-0.119691	0.9141	no
gi|646749692|gb|KK962079.1|	76211-77647	18	9.1	2	47	4	0.10915	no
gi|646749692|gb|KK962079.1|	01091-80231	18	9.1	0.571044	1	1	0.3963	no
gi|646749692|gb|KK962079.1|	23925-82436	18	9.1	4	8	0.984343	0.49265	no
gi|646749692|gb|KK962079.1|	65461-86659	18	9.1	0.952592	0.605564	-0.653578	1	no
gi|646749692|gb|KK962079.1|	16445-91752	18	9.1	1	2.25	0.999515	0.4717	no
gi|646749692|gb|KK962079.1|	35421-93858	18	9.1	0.859321	1	0.430531	0.78505	no
gi|646749692|gb|KK962079.1|	38706-93994	18	9.1	0.749064	2	1	0.3239	no
gi|646749692|gb|KK962079.1|	66259-96924	18	9.1	0.135461	14	7	0.09345	no
gi|646749709|gb|KK962078.1|	46766-14720	18	12	0	8	inf	0.0008	yes
gi|646749709|gb|KK962078.1|	54608-35511	18	12	11	18	0.782742	0.61885	no
gi|646749709|gb|KK962078.1|	27223-52749	18	12	21	19	-0.157964	0.91085	no
gi|646749709|gb|KK962078.1|	31190-53484	18	12	14	13	-0.108923	0.9242	no
gi|646749727|gb|KK962077.1|	17761-12096	21	8.9	2	2	-0.336288	0.8195	no
gi|646749727|gb|KK962077.1|	21070-12297	21	8.9	5	2	-1	0.4012	no
gi|646749727|gb|KK962077.1|	25045-12549	21	8.9	3	4	0.627343	0.66265	no
gi|646749727|gb|KK962077.1|	27254-12841	21	8.9	3	3	0.0650594	0.96555	no

gi|646749727|gb|KK962077.1|	45009-14526	21	8.9	29	49	0.749666	0.6125	no
gi|646749727|gb|KK962077.1|	86558-18747	21	8.9	3	2	-0.887228	0.4892	no
gi|646749727|gb|KK962077.1|	90936-19152	21	8.9	4	8	0.944079	0.50745	no
gi|646749727|gb|KK962077.1|	96555-19746	21	8.9	6	5	-0.431206	0.7803	no
gi|646749727|gb|KK962077.1|	02701-20301	21	8.9	9	12	0.451149	0.7539	no
gi|646749727|gb|KK962077.1|	04136-21160	21	8.9	7	10	0.550854	0.6218	no
gi|646749727|gb|KK962077.1|	11802-21316	21	8.9	4	4	-0.101614	0.9445	no
gi|646749727|gb|KK962077.1|	18012-22134	21	8.9	12	5	-1	0.3234	no
gi|646749727|gb|KK962077.1|	33864-23747	21	8.9	10	10	-0.0728945	0.94795	no
gi|646749727|gb|KK962077.1|	87262-38855	21	8.9	2	0.128461	-4	0.18865	no
gi|646749727|gb|KK962077.1|	88728-38962	21	8.9	2	0.61093	-2	0.2263	no
gi|646749727|gb|KK962077.1|	24548-42482	21	8.9	30	17	-0.816067	0.5284	no
gi|646749727|gb|KK962077.1|	25721-42601	21	8.9	2	16	3	0.10955	no
gi|646749727|gb|KK962077.1|	30322-43508	21	8.9	4	4	-0.22546	0.8526	no
gi|646749727|gb|KK962077.1|	37702-43996	21	8.9	4	7	0.794154	0.59755	no
gi|646749727|gb|KK962077.1|	42732-44307	21	8.9	9	9	-0.0137324	0.9428	no
gi|646749727|gb|KK962077.1|	43280-44496	21	8.9	4	4	-0.0652534	0.96375	no
gi|646749727|gb|KK962077.1|	47641-44860	21	8.9	11	9	-0.411575	0.7862	no
gi|646749727|gb|KK962077.1|	56041-46228	21	8.9	49	42	-0.248341	0.8334	no
gi|646749727|gb|KK962077.1|	62421-46354	21	8.9	1	2	0.253024	0.8481	no
gi|646749727|gb|KK962077.1|	65191-46575	21	8.9	3	9	2	0.31515	no
gi|646749727|gb|KK962077.1|	65866-46669	21	8.9	1	0.682637	-0.917246	0.58965	no
gi|646749727|gb|KK962077.1|	68333-46848	21	8.9	474	382	-0.310634	0.82335	no
gi|646749727|gb|KK962077.1|	74035-47542	21	8.9	1	0.469816	-1	0.34475	no
gi|646749727|gb|KK962077.1|	81503-48425	21	8.9	64	36	-0.817094	0.4893	no
gi|646749727|gb|KK962077.1|	92266-49365	21	8.9	0.408259	0.704725	0.787574	1	no
gi|646749727|gb|KK962077.1|	94052-49658	21	8.9	19	23	0.271728	0.81515	no
gi|646749727|gb|KK962077.1|	03891-50461	21	8.9	7	10	0.604916	0.69675	no
gi|646749727|gb|KK962077.1|	04830-50774	21	8.9	19	22	0.267185	0.8193	no
gi|646749727|gb|KK962077.1|	10173-51136	21	8.9	7	19	1	0.40145	no

gi|646749727|gb|KK962077.1|	11770-51272	21	8.9	1	0.939283	-0.340505	0.8138	no
gi|646749727|gb|KK962077.1|	28202-52850	21	8.9	19	23	0.237088	0.8443	no
gi|646749727|gb|KK962077.1|	34010-53550	21	8.9	7	7	0.0207695	0.99005	no
gi|646749727|gb|KK962077.1|	47587-54828	21	8.9	0.421239	3	3	0.2662	no
gi|646749727|gb|KK962077.1|	66666-56753	21	8.9	5	8	0.794778	0.61095	no
gi|646749727|gb|KK962077.1|	68388-56888	21	8.9	4	7	0.777468	0.547	no
gi|646749727|gb|KK962077.1|	77526-79786	21	8.9	3	2	-0.486433	0.7439	no
gi|646749727|gb|KK962077.1|	80296-84400	21	8.9	5	4	-0.386294	0.75825	no
gi|646749727|gb|KK962077.1|	21450-82164	21	8.9	0	86	inf	0.0014	yes
gi|646749727|gb|KK962077.1|	)6939-10039:	21	8.9	7	5	-0.594943	0.6358	no
gi|646749741|gb|KK962076.1|	61032-17408	17	7.6	15	12	-0.370972	0.7554	no
gi|646749741|gb|KK962076.1|	99392-20023	17	7.6	1	1	0.0135542	1	no
gi|646749741|gb|KK962076.1|	07400-20777	17	7.6	36	7	-2	0.1167	no
gi|646749741|gb|KK962076.1|	92077-29314	17	7.6	0.569617	0.813202	0.513621	1	no
gi|646749741|gb|KK962076.1|	06457-30764	17	7.6	1	0	-inf	0.0041	no
gi|646749741|gb|KK962076.1|	09857-31108	17	7.6	1	0	-inf	0.00225	no
gi|646749741|gb|KK962076.1|	16900-31830	17	7.6	1	0	-inf	0.0012	yes
gi|646749741|gb|KK962076.1|	34215-33462	17	7.6	9	0	-inf	0.00195	no
gi|646749741|gb|KK962076.1|	40181-34054	17	7.6	31	27	-0.20463	0.89625	no
gi|646749741|gb|KK962076.1|	48172-34883	17	7.6	13	4	-2	0.22795	no
gi|646749741|gb|KK962076.1|	86375-38837	17	7.6	6	3	-0.833906	0.5806	no
gi|646749741|gb|KK962076.1|	69849-47019	17	7.6	29	49	0.739534	0.62315	no
gi|646749741|gb|KK962076.1|	73270-47348	17	7.6	36	111	2	0.31875	no
gi|646749741|gb|KK962076.1|	75420-47633	17	7.6	4	7	0.837382	0.59925	no
gi|646749741|gb|KK962076.1|	12569-51300	17	7.6	3	5	0.811826	0.57265	no
gi|646749741|gb|KK962076.1|	18891-51986	17	7.6	0	3	inf	0.00605	no
gi|646749741|gb|KK962076.1|	23909-52544	17	7.6	0.793795	2	1	0.48175	no
gi|646749741|gb|KK962076.1|	41649-54604	17	7.6	2	5	2	0.2348	no
gi|646749741|gb|KK962076.1|	54007-55505	17	7.6	0.117244	3	5	0.1111	no
gi|646749741|gb|KK962076.1|	64233-56551	17	7.6	1	3	1	0.4043	no

gi|646749741|gb|KK962076.1|	68214-57111	17	7.6	0.139758	1	3	0.1406	no
gi|646749741|gb|KK962076.1|	73046-57438	17	7.6	3	6	1	0.4557	no
gi|646749741|gb|KK962076.1|	74548-57814	17	7.6	5	9	0.91303	0.4747	no
gi|646749741|gb|KK962076.1|	78941-58045	17	7.6	0	3	inf	0.00105	yes
gi|646749741|gb|KK962076.1|	80525-58261	17	7.6	0	6	inf	0.0001	yes
gi|646749741|gb|KK962076.1|	85076-58536	17	7.6	40	46	0.204585	0.8997	no
gi|646749741|gb|KK962076.1|	01330-60183	17	7.6	7	9	0.508845	0.71425	no
gi|646749741|gb|KK962076.1|	12446-61309	17	7.6	9	5	-0.848122	0.594	no
gi|646749755|gb|KK962075.1|	01520-10292	22	9.2	1	0	-inf	0.0012	yes
gi|646749755|gb|KK962075.1|	03603-10573	22	9.2	1	0	-inf	1	no
gi|646749755|gb|KK962075.1|	40960-10432	22	9.2	11	8	-0.445414	0.72885	no
gi|646749755|gb|KK962075.1|	07243-10876	22	9.2	2	0	-inf	0.00495	no
gi|646749755|gb|KK962075.1|	84148-10902	22	9.2	11	9	-0.287324	0.7995	no
gi|646749755|gb|KK962075.1|	08941-11044	22	9.2	0.966774	0	-inf	1	no
gi|646749755|gb|KK962075.1|	94816-10971	22	9.2	5	3	-0.953128	0.52375	no
gi|646749755|gb|KK962075.1|	97741-10986	22	9.2	1	0.383138	-2	0.26845	no
gi|646749755|gb|KK962075.1|	02901-11038	22	9.2	0.74902	1	0.924985	0.5404	no
gi|646749755|gb|KK962075.1|	10613-11148	22	9.2	2	0	-inf	0.0041	no
gi|646749755|gb|KK962075.1|	69510-11699	22	9.2	8	42	2	0.1225	no
gi|646749755|gb|KK962075.1|	78653-11795	22	9.2	22	93	2	0.14115	no
gi|646749755|gb|KK962075.1|	80311-11805	22	9.2	230	735	2	0.2946	no
gi|646749755|gb|KK962075.1|	82095-11823	22	9.2	403	1872.39	2	0.11015	no
gi|646749755|gb|KK962075.1|	85500-11865	22	9.2	0.235382	2	3	0.2438	no
gi|646749755|gb|KK962075.1|	86924-12032	22	9.2	76	84	0.134307	0.9132	no
gi|646749755|gb|KK962075.1|	03396-12084	22	9.2	3	31	3	0.0287	no
gi|646749755|gb|KK962075.1|	07962-13083	22	9.2	8	10	0.197663	0.87625	no
gi|646749755|gb|KK962075.1|	67192-13675	22	9.2	8	14	0.838321	0.56865	no
gi|646749755|gb|KK962075.1|	70167-13718	22	9.2	0.335439	0.774689	1	1	no
gi|646749755|gb|KK962075.1|	71939-13727	22	9.2	1	4	1	0.3559	no
gi|646749755|gb|KK962075.1|	76066-13779	22	9.2	20	30	0.627053	0.5709	no

gi|646749755|gb|KK962075.1|	95129-13953	22	9.2	20	50	1	0.4158	no
gi|646749755|gb|KK962075.1|	81408-18246	22	9.2	6	0	-inf	0.00045	yes
gi|646749755|gb|KK962075.1|	07530-21272	22	9.2	11	0.027352	-9	0.1665	no
gi|646749755|gb|KK962075.1|	12883-21538	22	9.2	34	0.059772	-9	0.1665	no
gi|646749755|gb|KK962075.1|	19518-22425	22	9.2	3	0.030161	-6	0.1674	no
gi|646749755|gb|KK962075.1|	27634-22838	22	9.2	3	0	-inf	0.00225	no
gi|646749755|gb|KK962075.1|	54324-25460	22	9.2	23	6	-2	0.2561	no
gi|646749755|gb|KK962075.1|	66179-26641	22	9.2	16	28	0.856655	0.5964	no
gi|646749755|gb|KK962075.1|	76511-27667	22	9.2	100	179.65	0.840051	0.6021	no
gi|646749755|gb|KK962075.1|	80019-28277	22	9.2	0.704274	2	2	0.2588	no
gi|646749755|gb|KK962075.1|	99226-29970	22	9.2	14	11	-0.344317	0.8256	no
gi|646749755|gb|KK962075.1|	08223-30985	22	9.2	0.736897	0	-inf	1	no
gi|646749755|gb|KK962075.1|	15559-31615	22	9.2	2	4	0.876286	0.50595	no
gi|646749755|gb|KK962075.1|	31117-33346	22	9.2	24	29	0.290222	0.7938	no
gi|646749755|gb|KK962075.1|	35534-33692	22	9.2	0.569552	0.936391	0.717282	1	no
gi|646749755|gb|KK962075.1|	38422-33932	22	9.2	0.574538	1	1	0.43305	no
gi|646749755|gb|KK962075.1|	40254-34072	22	9.2	5	7	0.654502	0.60405	no
gi|646749755|gb|KK962075.1|	43643-34466	22	9.2	7	8	0.152447	0.9208	no
gi|646749755|gb|KK962075.1|	50308-35053	22	9.2	13	39	2	0.2843	no
gi|646749755|gb|KK962075.1|	60599-36126	22	9.2	0	3	inf	0.0014	yes
gi|646749755|gb|KK962075.1|	63764-36409	22	9.2	8	15	0.87642	0.51445	no
gi|646749755|gb|KK962075.1|	73036-37323	22	9.2	30	107	2	0.3206	no
gi|646749755|gb|KK962075.1|	86653-38692	22	9.2	16	20	0.34656	0.80155	no
gi|646749755|gb|KK962075.1|	06396-40674	22	9.2	12	16	0.41243	0.74965	no
gi|646749755|gb|KK962075.1|	08071-40905	22	9.2	1	1	0.0939247	1	no
gi|646749755|gb|KK962075.1|	15604-41685	22	9.2	2	3	0.453648	0.7835	no
gi|646749755|gb|KK962075.1|	17017-42669	22	9.2	18	15	-0.294539	0.80145	no
gi|646749755|gb|KK962075.1|	38721-43917	22	9.2	4	4	0.042381	0.9365	no
gi|646749755|gb|KK962075.1|	42937-44319	22	9.2	201.9	367	0.862392	0.5759	no
gi|646749755|gb|KK962075.1|	44959-44587	22	9.2	11	17	0.645811	0.6721	no

gi|646749755|gb|KK962075.1|	46379-44902	22	9.2	1	5	2	0.26245	no
gi|646749755|gb|KK962075.1|	49947-45126	22	9.2	974	634.57	-0.618117	0.60375	no
gi|646749755|gb|KK962075.1|	52249-45385	22	9.2	2	2	-0.207945	0.8925	no
gi|646749755|gb|KK962075.1|	53933-45543	22	9.2	0.888834	2	1	0.45745	no
gi|646749755|gb|KK962075.1|	55614-45747	22	9.2	5	5	-0.13026	0.93205	no
gi|646749755|gb|KK962075.1|	60448-46098	22	9.2	37	88	1	0.32435	no
gi|646749755|gb|KK962075.1|	65507-46618	22	9.2	20	62	2	0.2045	no
gi|646749755|gb|KK962075.1|	74868-47509	22	9.2	735.55	365.68	-1	0.5134	no
gi|646749755|gb|KK962075.1|	78326-48005	22	9.2	0.629781	0.727939	0.208968	1	no
gi|646749755|gb|KK962075.1|	84901-48615	22	9.2	0.555058	0.663667	0.257823	1	no
gi|646749755|gb|KK962075.1|	87729-48817	22	9.2	15	8	-0.969853	0.49215	no
gi|646749755|gb|KK962075.1|	88909-48920	22	9.2	63	13	-2	0.14895	no
gi|646749755|gb|KK962075.1|	89312-49058	22	9.2	31	24	-0.347375	0.7474	no
gi|646749755|gb|KK962075.1|	90746-49178	22	9.2	1	3	1	0.48385	no
gi|646749755|gb|KK962075.1|	92952-49526	22	9.2	2	0.457273	-2	0.2347	no
gi|646749755|gb|KK962075.1|	95370-49575	22	9.2	25	16	-0.650912	0.6859	no
gi|646749755|gb|KK962075.1|	97606-49800	22	9.2	69	46	-0.606948	0.6795	no
gi|646749755|gb|KK962075.1|	90445-59056	22	9.2	17919	4634.91	-2	0.23075	no
gi|646749755|gb|KK962075.1|	91261-59239	22	9.2	7	7	0.0310017	0.9829	no
gi|646749755|gb|KK962075.1|	72549-67309	22	9.2	10	7	-0.419673	0.7863	no
gi|646749755|gb|KK962075.1|	73314-67408	22	9.2	2	2	-0.624322	0.64225	no
gi|646749755|gb|KK962075.1|	88729-68979	22	9.2	1	1	0.343649	0.8037	no
gi|646749755|gb|KK962075.1|	04620-70488	22	9.2	16	20	0.343255	0.81005	no
gi|646749755|gb|KK962075.1|	29496-73126	22	9.2	3	2	-0.407134	0.7932	no
gi|646749755|gb|KK962075.1|	31364-73222	22	9.2	2	3	0.0851685	0.94745	no
gi|646749755|gb|KK962075.1|	32390-73438	22	9.2	0.690026	1	0.740728	0.61065	no
gi|646749755|gb|KK962075.1|	37159-73797	22	9.2	0.663059	3	2	0.2407	no
gi|646749755|gb|KK962075.1|	41027-74155	22	9.2	2	3	0.219124	0.88855	no
gi|646749755|gb|KK962075.1|	42945-74816	22	9.2	33	36	0.107614	0.92995	no
gi|646749755|gb|KK962075.1|	75028-75727	22	9.2	0.416512	5	3	0.2671	no

gi|646749755|gb|KK962075.1|	57521-75818	22	9.2	2	2	-0.0387022	0.9367	no
gi|646749755|gb|KK962075.1|	63064-76460	22	9.2	37	35	-0.110745	0.92265	no
gi|646749755|gb|KK962075.1|	05052-80563	22	9.2	9	8	-0.150108	0.92595	no
gi|646749755|gb|KK962075.1|	80875-82620	22	9.2	0.745087	0	-inf	1	no
gi|646749755|gb|KK962075.1|	82978-83663	22	9.2	2	8	2	0.1463	no
gi|646749755|gb|KK962075.1|	63464-96452	22	9.2	1	0.49444	-1	1	no
gi|646749755|gb|KK962075.1|	96683-97098	22	9.2	7	0	-inf	0.0041	no
gi|646749755|gb|KK962075.1|	99019-99790	22	9.2	2	0	-inf	0.00475	no
gi|646749773|gb|KK962074.1|	10994-11151	18	5.6	84	61	-0.458287	0.71255	no
gi|646749773|gb|KK962074.1|	33015-13421	18	5.6	0.586736	1	1	0.44445	no
gi|646749773|gb|KK962074.1|	43307-14423	18	5.6	6	0.582103	-3	0.10285	no
gi|646749773|gb|KK962074.1|	50436-15179	18	5.6	2	2	-0.0976509	0.936	no
gi|646749773|gb|KK962074.1|	52238-15889	18	5.6	23	12	-0.968747	0.4091	no
gi|646749773|gb|KK962074.1|	60140-16066	18	5.6	7	1	-2	0.14795	no
gi|646749773|gb|KK962074.1|	77344-17895	18	5.6	0.478638	0.592	0.306663	1	no
gi|646749773|gb|KK962074.1|	82003-18249	18	5.6	8	2	-2	0.1924	no
gi|646749773|gb|KK962074.1|	91077-19173	18	5.6	6	4	-0.468472	0.73335	no
gi|646749773|gb|KK962074.1|	95147-19604	18	5.6	3	3	-0.212641	0.87525	no
gi|646749773|gb|KK962074.1|	07492-20929	18	5.6	5	3	-0.684845	0.6497	no
gi|646749773|gb|KK962074.1|	10380-22204	18	5.6	11	9	-0.202802	0.86435	no
gi|646749773|gb|KK962074.1|	42915-24418	18	5.6	2	0.521247	-2	0.2257	no
gi|646749773|gb|KK962074.1|	50189-25121	18	5.6	9	9	-0.082572	0.953	no
gi|646749773|gb|KK962074.1|	51372-25321	18	5.6	307	140	-1	0.34885	no
gi|646749773|gb|KK962074.1|	53345-26994	18	5.6	4	3	-0.496865	0.6614	no
gi|646749773|gb|KK962074.1|	26702-31184	18	5.6	4	3	-0.377016	0.805	no
gi|646749773|gb|KK962074.1|	70341-27306	18	5.6	1	1	0.0541805	0.97335	no
gi|646749773|gb|KK962074.1|	73250-27502	18	5.6	13	15	0.279717	0.7964	no
gi|646749773|gb|KK962074.1|	76814-27769	18	5.6	2	2	0.123983	0.92535	no
gi|646749773|gb|KK962074.1|	81148-28187	18	5.6	24	19	-0.368642	0.8064	no
gi|646749773|gb|KK962074.1|	82061-28277	18	5.6	6	7	0.0698165	0.9648	no

gi|646749773|gb|KK962074.1|	86594-28745	18	5.6	2	3	0.0855082	0.94745	no
gi|646749773|gb|KK962074.1|	88214-28836	18	5.6	359	166.03	-1	0.46135	no
gi|646749773|gb|KK962074.1|	89151-29079	18	5.6	0.333986	0.57854	0.792628	1	no
gi|646749773|gb|KK962074.1|	92041-29331	18	5.6	2	3	0.843369	0.53865	no
gi|646749773|gb|KK962074.1|	98020-29825	18	5.6	138	57	-1	0.36605	no
gi|646749773|gb|KK962074.1|	31382-36820	18	5.6	5	5	0.0122239	0.99075	no
gi|646749773|gb|KK962074.1|	64386-36467	18	5.6	36	39	0.143948	0.92965	no
gi|646749773|gb|KK962074.1|	94955-39905	18	5.6	15	12	-0.283311	0.8007	no
gi|646749773|gb|KK962074.1|	04412-40540	18	5.6	0.249673	2	3	0.2438	no
gi|646749773|gb|KK962074.1|	84092-84531	18	5.6	3	5	0.589433	0.6563	no
gi|646749773|gb|KK962074.1|	49519-85046	18	5.6	2	1	-0.270824	0.8336	no
gi|646749773|gb|KK962074.1|	74411-87571	18	5.6	2	5	1	0.3883	no
gi|646749773|gb|KK962074.1|	88725-88862	18	5.6	1608.65	708	-1	0.3813	no
gi|646749794|gb|KK962073.1|	05810-10630	20	8.4	19	2	-4	0.09535	no
gi|646749794|gb|KK962073.1|	21580-12200	20	8.4	6	3	-1	0.46155	no
gi|646749794|gb|KK962073.1|	30219-13063	20	8.4	4	5	0.189989	0.89945	no
gi|646749794|gb|KK962073.1|	32140-13384	20	8.4	17	15	-0.173885	0.868	no
gi|646749794|gb|KK962073.1|	34801-13499	20	8.4	37	51	0.461786	0.7615	no
gi|646749794|gb|KK962073.1|	35165-13543	20	8.4	16	12	-0.508961	0.73785	no
gi|646749794|gb|KK962073.1|	46075-14664	20	8.4	13	4	-2	0.27715	no
gi|646749794|gb|KK962073.1|	04279-20517	20	8.4	22	6	-2	0.25075	no
gi|646749794|gb|KK962073.1|	05323-20574	20	8.4	10	10	-0.0571796	0.9612	no
gi|646749794|gb|KK962073.1|	13240-21400	20	8.4	49	0	-inf	5.00E-05	yes
gi|646749794|gb|KK962073.1|	14784-21548	20	8.4	1	3	1	0.34675	no
gi|646749794|gb|KK962073.1|	15661-21639	20	8.4	3	2	-0.320945	0.8121	no
gi|646749794|gb|KK962073.1|	34209-23471	20	8.4	8	4	-0.920684	0.53045	no
gi|646749794|gb|KK962073.1|	54105-25434	20	8.4	0	69	inf	0.00435	no
gi|646749794|gb|KK962073.1|	55809-25672	20	8.4	1	1	-0.0094837	0.9367	no
gi|646749794|gb|KK962073.1|	56897-25726	20	8.4	231	31	-3	0.0474	no
gi|646749794|gb|KK962073.1|	59022-25929	20	8.4	0	24	inf	0.0014	yes

gi|646749794|gb|KK962073.1|	61617-26248	20	8.4	0.150896	25	7	0.09465	no
gi|646749794|gb|KK962073.1|	75626-27592	20	8.4	0	43	inf	0.0017	no
gi|646749794|gb|KK962073.1|	76508-27718	20	8.4	0	26	inf	5.00E-05	yes
gi|646749794|gb|KK962073.1|	93679-29431	20	8.4	1	24	4	0.06595	no
gi|646749794|gb|KK962073.1|	12916-31398	20	8.4	0.678528	0.968875	0.513903	1	no
gi|646749794|gb|KK962073.1|	44197-34471	20	8.4	6	9	0.595463	0.66355	no
gi|646749794|gb|KK962073.1|	58078-35906	20	8.4	0	19	inf	5.00E-05	yes
gi|646749794|gb|KK962073.1|	66742-36839	20	8.4	11	6	-0.747745	0.625	no
gi|646749794|gb|KK962073.1|	81259-38266	20	8.4	9	7	-0.225691	0.8794	no
gi|646749794|gb|KK962073.1|	07673-40830	20	8.4	14	9	-0.554918	0.71965	no
gi|646749794|gb|KK962073.1|	47081-48960	20	8.4	12	5	-1	0.3245	no
gi|646749794|gb|KK962073.1|	24013-62435	20	8.4	6	6	-0.181269	0.9036	no
gi|646749794|gb|KK962073.1|	46059-64634	20	8.4	31	36	0.208924	0.8764	no
gi|646749794|gb|KK962073.1|	66251-67412	20	8.4	12	10	-0.247053	0.8345	no
gi|646749794|gb|KK962073.1|	94209-95244	20	8.4	0.94877	2	1	0.41595	no
gi|646749814|gb|KK962072.1|	16874-21712	16	8.2	53	58	0.121635	0.93105	no
gi|646749832|gb|KK962071.1|	49510-10498	22	6	9	7	-0.244276	0.85495	no
gi|646749832|gb|KK962071.1|	54165-10543	22	6	80	61	-0.389447	0.79485	no
gi|646749832|gb|KK962071.1|	64259-10652	22	6	2	1	-0.682194	0.60255	no
gi|646749832|gb|KK962071.1|	65410-10665	22	6	2	1	-0.874632	0.4908	no
gi|646749832|gb|KK962071.1|	66901-10705	22	6	2	1	-0.885789	0.5646	no
gi|646749832|gb|KK962071.1|	71096-10761	22	6	1	0.984715	-0.534287	0.7207	no
gi|646749832|gb|KK962071.1|	76448-10813	22	6	67	28	-1	0.2951	no
gi|646749832|gb|KK962071.1|	36222-11364	22	6	20	5	-2	0.22445	no
gi|646749832|gb|KK962071.1|	52340-11526	22	6	23	12	-0.911638	0.53075	no
gi|646749832|gb|KK962071.1|	03632-12144	22	6	7	8	0.129021	0.9112	no
gi|646749832|gb|KK962071.1|	29842-12303	22	6	5	7	0.426419	0.7279	no
gi|646749832|gb|KK962071.1|	16560-13175	22	6	7	9	0.36415	0.8018	no
gi|646749832|gb|KK962071.1|	31314-13323	22	6	0.619171	2	1	0.36855	no
gi|646749832|gb|KK962071.1|	50182-13509	22	6	3	5	0.567461	0.6863	no

gi|646749832|gb|KK962071.1|	61748-13631	22	6	0.869771	3	2	0.27365	no
gi|646749832|gb|KK962071.1|	67948-13691	22	6	3	5	0.519988	0.7333	no
gi|646749832|gb|KK962071.1|	73662-13758	22	6	38	24	-0.652232	0.57	no
gi|646749832|gb|KK962071.1|	86617-13961	22	6	13	6	-1	0.34205	no
gi|646749832|gb|KK962071.1|	15295-14160	22	6	4	4	-0.27272	0.84435	no
gi|646749832|gb|KK962071.1|	28557-14298	22	6	23	28	0.26213	0.8062	no
gi|646749832|gb|KK962071.1|	36607-14370	22	6	19	3	-3	0.1235	no
gi|646749832|gb|KK962071.1|	67993-14681	22	6	445	120.22	-2	0.2752	no
gi|646749832|gb|KK962071.1|	76317-14775	22	6	43	30	-0.484955	0.66045	no
gi|646749832|gb|KK962071.1|	85625-14862	22	6	100	28	-2	0.1695	no
gi|646749832|gb|KK962071.1|	87021-14877	22	6	5	7	0.414576	0.79985	no
gi|646749832|gb|KK962071.1|	35346-15359	22	6	2	2	-0.124736	0.9032	no
gi|646749832|gb|KK962071.1|	41249-15417	22	6	2	3	0.161066	0.9062	no
gi|646749832|gb|KK962071.1|	66356-15670	22	6	128	27	-2	0.10475	no
gi|646749832|gb|KK962071.1|	71552-15733	22	6	0.553614	0.177812	-2	1	no
gi|646749832|gb|KK962071.1|	75387-15755	22	6	198.74	42	-2	0.19015	no
gi|646749832|gb|KK962071.1|	77579-15799	22	6	12	4	-1	0.2718	no
gi|646749832|gb|KK962071.1|	81733-15831	22	6	2	2	-0.0271011	0.9744	no
gi|646749832|gb|KK962071.1|	30196-16307	22	6	2	1	-0.779441	0.63075	no
gi|646749832|gb|KK962071.1|	62824-16630	22	6	67	57	-0.224754	0.88755	no
gi|646749832|gb|KK962071.1|	80612-16820	22	6	2	1	-0.0948019	0.9362	no
gi|646749832|gb|KK962071.1|	90737-16913	22	6	69	15	-2	0.0982	no
gi|646749832|gb|KK962071.1|	00278-17005	22	6	19	2	-3	0.2001	no
gi|646749832|gb|KK962071.1|	01476-17037	22	6	2	2	-0.222178	0.88595	no
gi|646749832|gb|KK962071.1|	05988-17063	22	6	11	2	-2	0.169	no
gi|646749832|gb|KK962071.1|	11061-17123	22	6	19	16	-0.252795	0.84015	no
gi|646749832|gb|KK962071.1|	16502-17186	22	6	15	8	-0.959018	0.44685	no
gi|646749832|gb|KK962071.1|	20547-17213	22	6	7	2	-2	0.1567	no
gi|646749832|gb|KK962071.1|	38568-17414	22	6	13	11	-0.201764	0.8491	no
gi|646749832|gb|KK962071.1|	41565-17446	22	6	1	2	0.674345	0.6652	no

gi|646749832|gb|KK962071.1|	39358-24044	22	6	2	3	1	0.4579	no
gi|646749832|gb|KK962071.1|	66191-26832	22	6	85	29	-2	0.19385	no
gi|646749832|gb|KK962071.1|	71440-27252	22	6	0.888416	0.475943	-0.900448	1	no
gi|646749832|gb|KK962071.1|	73484-27411	22	6	3	7	1	0.36335	no
gi|646749832|gb|KK962071.1|	78117-27861	22	6	19	25	0.372961	0.8044	no
gi|646749832|gb|KK962071.1|	10660-31197	22	6	3	0.499656	-2	0.1683	no
gi|646749832|gb|KK962071.1|	44066-34467	22	6	26	6	-2	0.1974	no
gi|646749832|gb|KK962071.1|	48710-34910	22	6	20	22	0.117491	0.94075	no
gi|646749832|gb|KK962071.1|	50825-35204	22	6	43	63	0.548223	0.6291	no
gi|646749832|gb|KK962071.1|	72176-37316	22	6	65	32	-0.993364	0.36905	no
gi|646749832|gb|KK962071.1|	77077-37782	22	6	6	8	0.30887	0.83845	no
gi|646749832|gb|KK962071.1|	78112-38048	22	6	6	4	-0.5636	0.70705	no
gi|646749832|gb|KK962071.1|	10968-41199	22	6	13	9	-0.638979	0.6707	no
gi|646749832|gb|KK962071.1|	12871-42076	22	6	6	5	-0.310206	0.7741	no
gi|646749832|gb|KK962071.1|	22435-42314	22	6	2	0.571952	-2	0.29735	no
gi|646749832|gb|KK962071.1|	38128-43834	22	6	63	0	-inf	0.0041	no
gi|646749832|gb|KK962071.1|	24659-52542	22	6	2	1	-0.509446	0.7312	no
gi|646749832|gb|KK962071.1|	37339-53754	22	6	42	27	-0.652169	0.67415	no
gi|646749832|gb|KK962071.1|	45357-54565	22	6	14	10	-0.498004	0.71765	no
gi|646749832|gb|KK962071.1|	51279-55182	22	6	3	3	0.446396	0.75025	no
gi|646749832|gb|KK962071.1|	67975-56850	22	6	4	4	-0.260023	0.85035	no
gi|646749832|gb|KK962071.1|	68856-56941	22	6	2	3	1	0.44985	no
gi|646749832|gb|KK962071.1|	71543-57189	22	6	14	30	1	0.42945	no
gi|646749832|gb|KK962071.1|	72213-57318	22	6	1	0.920176	-0.491578	0.73195	no
gi|646749832|gb|KK962071.1|	76969-57807	22	6	0.976039	0.465069	-1	1	no
gi|646749832|gb|KK962071.1|	78862-58024	22	6	0.494273	0.947836	0.93933	1	no
gi|646749832|gb|KK962071.1|	84862-58631	22	6	0.461792	0.775845	0.748525	1	no
gi|646749832|gb|KK962071.1|	86530-58810	22	6	76	27	-2	0.1937	no
gi|646749832|gb|KK962071.1|	95987-60081	22	6	2	2	0.00745597	0.9939	no
gi|646749832|gb|KK962071.1|	00878-60165	22	6	6	10	0.805019	0.6084	no

gi|646749832|gb|KK962071.1|	01716-60295	22	6	4	2	-0.946746	0.55885	no
gi|646749832|gb|KK962071.1|	03892-60645	22	6	16	4	-2	0.11455	no
gi|646749832|gb|KK962071.1|	06570-60734	22	6	2	0.249276	-3	0.2033	no
gi|646749832|gb|KK962071.1|	08326-61491	22	6	3	1	-1	0.46845	no
gi|646749832|gb|KK962071.1|	41882-64302	22	6	1	0.149391	-3	0.18	no
gi|646749832|gb|KK962071.1|	56011-65661	22	6	8	0	-inf	0.0033	no
gi|646749832|gb|KK962071.1|	69956-67072	22	6	14	11	-0.345227	0.80945	no
gi|646749832|gb|KK962071.1|	80262-68299	22	6	4	7	0.756172	0.62205	no
gi|646749832|gb|KK962071.1|	83104-68473	22	6	4	7	0.85134	0.5728	no
gi|646749832|gb|KK962071.1|	98228-69882	22	6	3	7	1	0.4381	no
gi|646749832|gb|KK962071.1|	48415-74906	22	6	3	0	-inf	0.00225	no
gi|646749832|gb|KK962071.1|	46938-84898	22	6	0.776582	0.225001	-2	1	no
gi|646749832|gb|KK962071.1|	49078-85083	22	6	26	19	-0.447917	0.68305	no
gi|646749832|gb|KK962071.1|	51554-85959	22	6	20	14	-0.548524	0.64055	no
gi|646749832|gb|KK962071.1|	79512-88007	22	6	4	3	-0.247419	0.8518	no
gi|646749832|gb|KK962071.1|	87094-88795	22	6	55	20	-1	0.2651	no
gi|646749832|gb|KK962071.1|	16837-91932	22	6	0.497189	0.541522	0.123227	1	no
gi|646749832|gb|KK962071.1|	19834-92137	22	6	0.649469	0.728261	0.165196	1	no
gi|646749832|gb|KK962071.1|	21607-92752	22	6	0.849434	1	0.753346	0.6217	no
gi|646749832|gb|KK962071.1|	28150-92994	22	6	0.83887	3	2	0.24405	no
gi|646749832|gb|KK962071.1|	30791-93440	22	6	1	2	0.719906	0.6344	no
gi|646749832|gb|KK962071.1|	39011-93933	22	6	5	6	0.28254	0.85585	no
gi|646749832|gb|KK962071.1|	47641-94882	22	6	9	4	-1	0.4423	no
gi|646749832|gb|KK962071.1|	50973-95416	22	6	11	9	-0.332098	0.75965	no
gi|646749832|gb|KK962071.1|	72014-97279	22	6	8	5	-0.473946	0.75625	no
gi|646749832|gb|KK962071.1|	82644-98339	22	6	3	2	-0.791173	0.54305	no
gi|646749852|gb|KK962070.1|	13976-21527	19	8.6	11	8	-0.54824	0.7132	no
gi|646749852|gb|KK962070.1|	16473-21716	19	8.6	2	3	0.633037	0.64195	no
gi|646749852|gb|KK962070.1|	24700-22527	19	8.6	7	1	-3	0.1694	no
gi|646749852|gb|KK962070.1|	30619-23127	19	8.6	16	4	-2	0.14565	no

gi|646749852|gb|KK962070.1|	32489-23338	19	8.6	13	2	-2	0.10275	no
gi|646749852|gb|KK962070.1|	54353-25615	19	8.6	2	2	-0.293952	0.84955	no
gi|646749852|gb|KK962070.1|	57071-25976	19	8.6	5	2	-1	0.3406	no
gi|646749852|gb|KK962070.1|	59926-26114	19	8.6	5	1	-2	0.2107	no
gi|646749852|gb|KK962070.1|	61271-26613	19	8.6	1	3	1	0.48595	no
gi|646749852|gb|KK962070.1|	76189-27708	19	8.6	53	17	-2	0.2164	no
gi|646749852|gb|KK962070.1|	86778-29011	19	8.6	0.721174	2	1	0.4965	no
gi|646749852|gb|KK962070.1|	01827-30642	19	8.6	240	85	-2	0.22225	no
gi|646749852|gb|KK962070.1|	15665-31890	19	8.6	126	31	-2	0.09995	no
gi|646749852|gb|KK962070.1|	34949-33576	19	8.6	0.166062	2	4	0.10345	no
gi|646749852|gb|KK962070.1|	57140-35753	19	8.6	2597.24	4469.86	0.783248	0.5168	no
gi|646749852|gb|KK962070.1|	57653-35850	19	8.6	0.462494	2	2	0.20395	no
gi|646749852|gb|KK962070.1|	70799-37185	19	8.6	6	3	-0.911259	0.5706	no
gi|646749852|gb|KK962070.1|	71922-37293	19	8.6	0.849779	1	0.288712	1	no
gi|646749852|gb|KK962070.1|	92809-39435	19	8.6	16	18	0.19117	0.85515	no
gi|646749852|gb|KK962070.1|	05337-41301	19	8.6	2	22	4	0.02095	no
gi|646749852|gb|KK962070.1|	14017-41570	19	8.6	0.518288	4	3	0.0876	no
gi|646749852|gb|KK962070.1|	16489-41697	19	8.6	1	9	3	0.16865	no
gi|646749852|gb|KK962070.1|	20987-42230	19	8.6	0.784727	11	4	0.0349	no
gi|646749852|gb|KK962070.1|	80153-49216	19	8.6	16	22	0.454756	0.7017	no
gi|646749852|gb|KK962070.1|	02535-50330	19	8.6	0.543005	2	2	0.20395	no
gi|646749852|gb|KK962070.1|	13191-51337	19	8.6	57	99	0.784913	0.61595	no
gi|646749852|gb|KK962070.1|	13341-61396	19	8.6	39.48	55	0.486751	0.699	no
gi|646749852|gb|KK962070.1|	40817-64158	19	8.6	26	27	0.0431525	0.9785	no
gi|646749852|gb|KK962070.1|	82841-83697	19	8.6	2	0	-inf	0.00655	no
gi|646749852|gb|KK962070.1|	85132-86359	19	8.6	1	0	-inf	0.00225	no
gi|646749852|gb|KK962070.1|	88851-90084	19	8.6	2	0	-inf	0.00045	yes
gi|646749852|gb|KK962070.1|	98149-98900	19	8.6	2	0	-inf	0.00475	no
gi|646749868|gb|KK962069.1|	11439-12157	19	7.1	6	4	-0.515146	0.74795	no
gi|646749868|gb|KK962069.1|	18691-11893	19	7.1	19	44	1	0.39355	no

gi|646749868|gb|KK962069.1|	15187-15372	19	7.1	117	102	-0.194037	0.88525	no
gi|646749868|gb|KK962069.1|	65354-16582	19	7.1	2	4	0.899996	0.56765	no
gi|646749868|gb|KK962069.1|	75896-17608	19	7.1	59	83	0.504396	0.7389	no
gi|646749868|gb|KK962069.1|	87378-18831	19	7.1	4	10	1	0.4409	no
gi|646749868|gb|KK962069.1|	90571-19083	19	7.1	10	31	2	0.272	no
gi|646749868|gb|KK962069.1|	08860-21018	19	7.1	17	8	-0.989433	0.51685	no
gi|646749868|gb|KK962069.1|	10476-21118	19	7.1	18	16	-0.142866	0.9232	no
gi|646749868|gb|KK962069.1|	12723-21366	19	7.1	1	0.577971	-1	0.4766	no
gi|646749868|gb|KK962069.1|	16074-21720	19	7.1	1	2	0.0311638	0.95325	no
gi|646749868|gb|KK962069.1|	18911-21911	19	7.1	57	23	-1	0.41275	no
gi|646749868|gb|KK962069.1|	19882-22054	19	7.1	2	2	-0.202754	0.89315	no
gi|646749868|gb|KK962069.1|	25936-22639	19	7.1	4	3	-0.436696	0.76145	no
gi|646749868|gb|KK962069.1|	34550-23582	19	7.1	7	6	-0.300252	0.84235	no
gi|646749868|gb|KK962069.1|	36707-23732	19	7.1	4	1	-1	0.32925	no
gi|646749868|gb|KK962069.1|	38382-23868	19	7.1	44	23	-0.955557	0.4936	no
gi|646749868|gb|KK962069.1|	24099-24894	19	7.1	18	14	-0.400618	0.7908	no
gi|646749868|gb|KK962069.1|	44017-24513	19	7.1	2	0.305853	-2	0.15305	no
gi|646749868|gb|KK962069.1|	45401-24629	19	7.1	2	0.204564	-3	0.1787	no
gi|646749868|gb|KK962069.1|	61064-26266	19	7.1	18	16	-0.125569	0.90345	no
gi|646749868|gb|KK962069.1|	62822-26452	19	7.1	18	19	0.0359887	0.97405	no
gi|646749868|gb|KK962069.1|	64832-26781	19	7.1	7	10	0.382294	0.7179	no
gi|646749868|gb|KK962069.1|	75231-27654	19	7.1	2	2	-0.177864	0.9111	no
gi|646749868|gb|KK962069.1|	87297-28846	19	7.1	0.911909	2	1	0.41795	no
gi|646749868|gb|KK962069.1|	2879-3537	19	7.1	2	2	-0.525055	0.72155	no
gi|646749868|gb|KK962069.1|	89760-29063	19	7.1	12	13	0.110185	0.94245	no
gi|646749868|gb|KK962069.1|	00529-30140	19	7.1	3	19	2	0.1463	no
gi|646749868|gb|KK962069.1|	02881-30355	19	7.1	0.880429	3	2	0.2986	no
gi|646749868|gb|KK962069.1|	30442-30757	19	7.1	22	19	-0.207619	0.8613	no
gi|646749868|gb|KK962069.1|	05870-30672	19	7.1	0.774696	2	1	0.43255	no
gi|646749868|gb|KK962069.1|	08236-30953	19	7.1	0.443057	0.763541	0.785213	1	no

gi|646749868|gb|KK962069.1|	24953-32521	19	7.1	14	26	0.879216	0.54265	no
gi|646749868|gb|KK962069.1|	50518-35074	19	7.1	9	36	2	0.32265	no
gi|646749868|gb|KK962069.1|	68590-36906	19	7.1	4	1	-2	0.2639	no
gi|646749868|gb|KK962069.1|	69215-37062	19	7.1	3	5	0.454702	0.7637	no
gi|646749868|gb|KK962069.1|	70733-37153	19	7.1	5	4	-0.458645	0.7727	no
gi|646749868|gb|KK962069.1|	71604-37520	19	7.1	2	3	0.806032	0.60085	no
gi|646749868|gb|KK962069.1|	76544-37857	19	7.1	2	2	0.134086	0.9311	no
gi|646749868|gb|KK962069.1|	80021-38051	19	7.1	2	4	0.649718	0.6597	no
gi|646749868|gb|KK962069.1|	81681-38269	19	7.1	4	3	-0.308757	0.84505	no
gi|646749868|gb|KK962069.1|	90475-39297	19	7.1	24	31	0.368397	0.74375	no
gi|646749868|gb|KK962069.1|	03975-40897	19	7.1	22	22	-0.0448263	0.97045	no
gi|646749868|gb|KK962069.1|	09177-41173	19	7.1	5	6	0.410911	0.78885	no
gi|646749868|gb|KK962069.1|	25781-42692	19	7.1	0.522641	1	1	0.36645	no
gi|646749868|gb|KK962069.1|	36653-43682	19	7.1	113	109	-0.0434621	0.91215	no
gi|646749868|gb|KK962069.1|	37750-43802	19	7.1	10	18	0.852225	0.5711	no
gi|646749868|gb|KK962069.1|	44977-44535	19	7.1	12	10	-0.198804	0.8702	no
gi|646749868|gb|KK962069.1|	44696-45015	19	7.1	0	53	inf	0.00055	yes
gi|646749868|gb|KK962069.1|	53632-45391	19	7.1	13	15	0.204585	0.88595	no
gi|646749868|gb|KK962069.1|	4632-5081	19	7.1	3	9	2	0.2515	no
gi|646749868|gb|KK962069.1|	66177-66514	19	7.1	2	11	2	0.27785	no
gi|646749868|gb|KK962069.1|	65219-66561	19	7.1	29	32	0.114967	0.9439	no
gi|646749868|gb|KK962069.1|	71068-67141	19	7.1	0	23	inf	0.00315	no
gi|646749887|gb|KK962068.1|	67104-46813	20	9.5	0.711577	0.845634	0.249012	1	no
gi|646749887|gb|KK962068.1|	62232-66260	20	9.5	0	14	inf	0.00605	no
gi|646749887|gb|KK962068.1|	63996-66542	20	9.5	0.079003	11	7	0.0947	no
gi|646749887|gb|KK962068.1|	85466-68838	20	9.5	3	1	-1	0.5125	no
gi|646749887|gb|KK962068.1|	92108-69398	20	9.5	23	10	-1	0.3825	no
gi|646749887|gb|KK962068.1|	97935-69888	20	9.5	2	3	0.594442	0.67255	no
gi|646749887|gb|KK962068.1|	11248-71148	20	9.5	34	21	-0.661744	0.63705	no
gi|646749887|gb|KK962068.1|	12199-71280	20	9.5	1	2	0.726264	0.64505	no

gi|646749887|gb|KK962068.1|	16419-71819	20	9.5	3	3	-0.391803	0.79325	no
gi|646749887|gb|KK962068.1|	36129-73718	20	9.5	1	0.660055	-0.946386	0.5451	no
gi|646749887|gb|KK962068.1|	38056-73905	20	9.5	3	0.17684	-4	0.1839	no
gi|646749887|gb|KK962068.1|	44620-74495	20	9.5	17	0	-inf	0.0012	yes
gi|646749887|gb|KK962068.1|	47530-74796	20	9.5	17	0	-inf	0.002	no
gi|646749887|gb|KK962068.1|	49092-74940	20	9.5	104	46	-1	0.43975	no
gi|646749887|gb|KK962068.1|	54931-75616	20	9.5	0.284936	1	2	0.20215	no
gi|646749887|gb|KK962068.1|	75839-77608	20	9.5	32	9	-2	0.27855	no
gi|646749887|gb|KK962068.1|	77941-79010	20	9.5	2	2	0.191787	0.88265	no
gi|646749887|gb|KK962068.1|	92462-79298	20	9.5	2	2	0.174731	0.89965	no
gi|646749887|gb|KK962068.1|	87820-89206	20	9.5	1	3	1	0.4595	no
gi|646749906|gb|KK962067.1|	15446-16642	21	8.2	3	3	-0.0171592	0.9797	no
gi|646749906|gb|KK962067.1|	17186-19133	21	8.2	20	16	-0.379945	0.7253	no
gi|646749906|gb|KK962067.1|	13638-21569	21	8.2	2	1	-0.295243	0.8527	no
gi|646749906|gb|KK962067.1|	16224-21731	21	8.2	1	2	0.862422	0.50475	no
gi|646749906|gb|KK962067.1|	18031-21948	21	8.2	2	3	0.657355	0.63255	no
gi|646749906|gb|KK962067.1|	19545-22042	21	8.2	3	2	-0.649982	0.61035	no
gi|646749906|gb|KK962067.1|	23762-22425	21	8.2	4	5	0.138715	0.91885	no
gi|646749906|gb|KK962067.1|	24603-26723	21	8.2	11	11	0.0180652	0.98585	no
gi|646749906|gb|KK962067.1|	48761-24920	21	8.2	6	4	-0.718034	0.60145	no
gi|646749906|gb|KK962067.1|	50198-25077	21	8.2	4	3	-0.451259	0.7303	no
gi|646749906|gb|KK962067.1|	2571-4597	21	8.2	8	7	-0.329553	0.82805	no
gi|646749906|gb|KK962067.1|	64900-26542	21	8.2	3	3	-0.0764219	0.9363	no
gi|646749906|gb|KK962067.1|	69413-57733	21	8.2	7	6	-0.383027	0.73365	no
gi|646749906|gb|KK962067.1|	79324-58233	21	8.2	4	5	0.303936	0.8401	no
gi|646749906|gb|KK962067.1|	10635-61389	21	8.2	5	5	0.215115	0.88615	no
gi|646749906|gb|KK962067.1|	15946-61859	21	8.2	11	5	-1	0.42235	no
gi|646749906|gb|KK962067.1|	33951-63445	21	8.2	14	7	-1	0.4489	no
gi|646749906|gb|KK962067.1|	40766-64097	21	8.2	68	78	0.199083	0.87155	no
gi|646749928|gb|KK962066.1|	06262-10712	14	6.4	2	0.215273	-3	0.18	no

gi|646749928|gb|KK962066.1|	10479-11126	14	6.4	4	0.487807	-3	0.1551	no
gi|646749928|gb|KK962066.1|	11403-11408	14	6.4	6	0.886095	-3	0.1106	no
gi|646749928|gb|KK962066.1|	22923-12401	14	6.4	4	0	-inf	0.0014	yes
gi|646749928|gb|KK962066.1|	67976-26914	14	6.4	16	25	0.622398	0.62965	no
gi|646749928|gb|KK962066.1|	88678-48892	14	6.4	12	26	1	0.4712	no
gi|646749928|gb|KK962066.1|	33840-53439	14	6.4	47	35	-0.423881	0.77415	no
gi|646749928|gb|KK962066.1|	54424-55509	14	6.4	1	2	0.476417	0.7489	no
gi|646749928|gb|KK962066.1|	70588-71361	14	6.4	170	57	-2	0.17215	no
gi|646749928|gb|KK962066.1|	78837-79518	14	6.4	38	15	-1	0.35795	no
gi|646749928|gb|KK962066.1|	79704-80711	14	6.4	2	0.87621	-1	0.371	no
gi|646749928|gb|KK962066.1|	89529-90516	14	6.4	4	2	-0.712961	0.6095	no
gi|646749945|gb|KK962065.1|	09684-10115	22	6.1	0.759282	0.929261	0.291448	1	no
gi|646749945|gb|KK962065.1|	12025-10134	22	6.1	0.409343	0.824319	1	1	no
gi|646749945|gb|KK962065.1|	13581-10154	22	6.1	0.821839	0.509407	-0.690037	1	no
gi|646749945|gb|KK962065.1|	44476-10458	22	6.1	4	3	-0.292076	0.8487	no
gi|646749945|gb|KK962065.1|	07243-10795	22	6.1	4	3	-0.59388	0.62625	no
gi|646749945|gb|KK962065.1|	91645-10924	22	6.1	1	1	0.0143481	1	no
gi|646749945|gb|KK962065.1|	08187-11088	22	6.1	2	1	-0.94632	0.589	no
gi|646749945|gb|KK962065.1|	23144-11233	22	6.1	104	38	-1	0.3968	no
gi|646749945|gb|KK962065.1|	43857-11442	22	6.1	104.42	54	-0.953667	0.52425	no
gi|646749945|gb|KK962065.1|	63467-11642	22	6.1	25	0.763232	-5	0.0771	no
gi|646749945|gb|KK962065.1|	80432-11811	22	6.1	33	21	-0.67713	0.65335	no
gi|646749945|gb|KK962065.1|	20077-12202	22	6.1	44	29	-0.580676	0.7052	no
gi|646749945|gb|KK962065.1|	33407-12336	22	6.1	56	61	0.114725	0.92845	no
gi|646749945|gb|KK962065.1|	34050-12358	22	6.1	5	4	-0.119798	0.93525	no
gi|646749945|gb|KK962065.1|	37278-12401	22	6.1	1	0.865533	-0.581521	0.72645	no
gi|646749945|gb|KK962065.1|	40361-12438	22	6.1	2	1	-0.357718	0.8173	no
gi|646749945|gb|KK962065.1|	43999-12454	22	6.1	19	14	-0.467915	0.7087	no
gi|646749945|gb|KK962065.1|	59538-12598	22	6.1	8	39	2	0.2186	no
gi|646749945|gb|KK962065.1|	95969-12962	22	6.1	39	125.44	2	0.2598	no

gi|646749945|gb|KK962065.1|	98972-12992	22	6.1	6	57	3	0.13685	no
gi|646749945|gb|KK962065.1|	00713-13022	22	6.1	1	4	2	0.2395	no
gi|646749945|gb|KK962065.1|	04887-13065	22	6.1	22	34	0.673317	0.53655	no
gi|646749945|gb|KK962065.1|	14826-13224	22	6.1	16	11	-0.456396	0.6911	no
gi|646749945|gb|KK962065.1|	34213-13345	22	6.1	14	10	-0.517359	0.68285	no
gi|646749945|gb|KK962065.1|	34175-13514	22	6.1	46	27	-0.765278	0.4817	no
gi|646749945|gb|KK962065.1|	74120-13745	22	6.1	171.62	72	-1	0.3297	no
gi|646749945|gb|KK962065.1|	83555-13877	22	6.1	6	2	-1	0.31075	no
gi|646749945|gb|KK962065.1|	94275-13945	22	6.1	21	7	-2	0.357	no
gi|646749945|gb|KK962065.1|	29488-14302	22	6.1	12	11	-0.0594303	0.9663	no
gi|646749945|gb|KK962065.1|	39159-14403	22	6.1	0.377589	0.812291	1	1	no
gi|646749945|gb|KK962065.1|	43608-14462	22	6.1	2	2	0.0284654	0.98415	no
gi|646749945|gb|KK962065.1|	67274-14685	22	6.1	2	0.813915	-0.894885	0.50335	no
gi|646749945|gb|KK962065.1|	69274-14711	22	6.1	0.620912	0.571771	-0.118951	1	no
gi|646749945|gb|KK962065.1|	83247-14844	22	6.1	0.18841	1	3	1	no
gi|646749945|gb|KK962065.1|	85852-14865	22	6.1	5	2	-2	0.3356	no
gi|646749945|gb|KK962065.1|	87068-14922	22	6.1	22	11	-0.979304	0.39465	no
gi|646749945|gb|KK962065.1|	93376-14946	22	6.1	0.532178	0.764257	0.522149	1	no
gi|646749945|gb|KK962065.1|	94802-14965	22	6.1	2	1	-0.468301	0.7359	no
gi|646749945|gb|KK962065.1|	10135-15106	22	6.1	3	2	-0.994806	0.589	no
gi|646749945|gb|KK962065.1|	49584-15501	22	6.1	2	2	-0.0221974	0.92345	no
gi|646749945|gb|KK962065.1|	58838-15956	22	6.1	8	6	-0.412128	0.7927	no
gi|646749945|gb|KK962065.1|	64290-16570	22	6.1	13	15	0.19454	0.8942	no
gi|646749945|gb|KK962065.1|	66676-16670	22	6.1	4	4	-0.100534	0.9229	no
gi|646749945|gb|KK962065.1|	84486-16860	22	6.1	251	269	0.0966513	0.9356	no
gi|646749945|gb|KK962065.1|	87136-16945	22	6.1	8	14	0.853068	0.45615	no
gi|646749945|gb|KK962065.1|	21220-17217	22	6.1	18	6	-2	0.2297	no
gi|646749945|gb|KK962065.1|	72837-17355	22	6.1	2	1	-0.193363	0.89315	no
gi|646749945|gb|KK962065.1|	42642-17431	22	6.1	3	1	-1	0.4702	no
gi|646749945|gb|KK962065.1|	49650-17564	22	6.1	28	18	-0.623883	0.60105	no

gi|646749945|gb|KK962065.1|	57101-17587	22	6.1	4	0.495857	-3	0.09745	no
gi|646749945|gb|KK962065.1|	72445-17739	22	6.1	9	5	-0.964987	0.528	no
gi|646749945|gb|KK962065.1|	84523-17849	22	6.1	12	4	-2	0.24385	no
gi|646749945|gb|KK962065.1|	18630-20583	22	6.1	0.490369	0.473282	-0.0511666	1	no
gi|646749945|gb|KK962065.1|	17835-19181	22	6.1	26.07	7	-2	0.2672	no
gi|646749945|gb|KK962065.1|	44748-19454	22	6.1	2	2	0.485993	0.72185	no
gi|646749945|gb|KK962065.1|	46401-19470	22	6.1	5	10	0.840761	0.54095	no
gi|646749945|gb|KK962065.1|	47845-19487	22	6.1	2	2	0.638961	0.62475	no
gi|646749945|gb|KK962065.1|	53907-19554	22	6.1	0.512335	0.738451	0.527416	1	no
gi|646749945|gb|KK962065.1|	55564-19562	22	6.1	1	2	0.472637	0.74895	no
gi|646749945|gb|KK962065.1|	59547-19612	22	6.1	1	1	-0.054318	0.96365	no
gi|646749945|gb|KK962065.1|	61387-19636	22	6.1	0.183022	0.663792	2	1	no
gi|646749945|gb|KK962065.1|	73589-19741	22	6.1	70	69	-0.0186807	0.9869	no
gi|646749945|gb|KK962065.1|	80713-19810	22	6.1	9	15	0.846396	0.5711	no
gi|646749945|gb|KK962065.1|	82557-19827	22	6.1	31	29	-0.0982923	0.93945	no
gi|646749945|gb|KK962065.1|	88861-19920	22	6.1	3	3	0.144049	0.92275	no
gi|646749945|gb|KK962065.1|	93444-19962	22	6.1	2	1	-0.428201	0.78355	no
gi|646749945|gb|KK962065.1|	10417-20127	22	6.1	0.764611	0.783143	0.0345493	1	no
gi|646749945|gb|KK962065.1|	12854-20133	22	6.1	4	4	-0.181829	0.9009	no
gi|646749945|gb|KK962065.1|	16858-20184	22	6.1	0.53515	0.772484	0.529561	1	no
gi|646749945|gb|KK962065.1|	18625-20210	22	6.1	25	15	-0.747822	0.50005	no
gi|646749945|gb|KK962065.1|	23035-20234	22	6.1	5	6	0.166247	0.9115	no
gi|646749945|gb|KK962065.1|	21756-22655	22	6.1	5	5	0.0272029	0.98145	no
gi|646749945|gb|KK962065.1|	53295-25488	22	6.1	0.833456	1	0.528688	0.6791	no
gi|646749945|gb|KK962065.1|	56259-25860	22	6.1	0.79757	1	0.759671	0.5829	no
gi|646749945|gb|KK962065.1|	58964-26209	22	6.1	19	9	-1	0.3613	no
gi|646749945|gb|KK962065.1|	62192-26369	22	6.1	0.371836	0.535796	0.527016	1	no
gi|646749945|gb|KK962065.1|	69825-27211	22	6.1	15	8	-0.984537	0.44305	no
gi|646749945|gb|KK962065.1|	29721-32484	22	6.1	47	31	-0.582101	0.61575	no
gi|646749945|gb|KK962065.1|	42802-34335	22	6.1	2	1	-0.966669	0.589	no

gi|646749945|gb|KK962065.1|	43723-44075	22	6.1	118	95	-0.316786	0.8357	no
gi|646749945|gb|KK962065.1|	50489-51860	22	6.1	10	7	-0.488483	0.7474	no
gi|646749945|gb|KK962065.1|	31749-53207	22	6.1	46	28	-0.715949	0.64695	no
gi|646749945|gb|KK962065.1|	35491-53632	22	6.1	3	3	-0.308734	0.79435	no
gi|646749945|gb|KK962065.1|	48899-55021	22	6.1	0.608622	0.874398	0.522743	1	no
gi|646749945|gb|KK962065.1|	50652-55236	22	6.1	0.887735	0.82446	-0.106681	1	no
gi|646749945|gb|KK962065.1|	52498-55392	22	6.1	1	0.454095	-2	0.2986	no
gi|646749945|gb|KK962065.1|	54519-55840	22	6.1	0.992664	0.630773	-0.654185	1	no
gi|646749945|gb|KK962065.1|	59272-56056	22	6.1	1	0.894151	-0.255545	1	no
gi|646749945|gb|KK962065.1|	60951-56184	22	6.1	74	37	-1	0.36465	no
gi|646749945|gb|KK962065.1|	80317-58115	22	6.1	2	4	1	0.4054	no
gi|646749945|gb|KK962065.1|	59462-59767	22	6.1	396	250	-0.661805	0.5392	no
gi|646749945|gb|KK962065.1|	63136-63636	22	6.1	18	13	-0.484726	0.7602	no
gi|646749945|gb|KK962065.1|	34484-63582	22	6.1	0.340427	0.733946	1	1	no
gi|646749945|gb|KK962065.1|	66960-68201	22	6.1	27	24	-0.147606	0.89205	no
gi|646749945|gb|KK962065.1|	71641-71946	22	6.1	11	8	-0.433591	0.76235	no
gi|646749945|gb|KK962065.1|	72077-77339	22	6.1	3	5	0.844588	0.58625	no
gi|646749945|gb|KK962065.1|	78440-78685	22	6.1	15	16	0.0959952	0.909	no
gi|646749945|gb|KK962065.1|	84626-88023	22	6.1	10	12	0.190341	0.86485	no
gi|646749945|gb|KK962065.1|	20438-92141	22	6.1	4	0.182796	-5	0.17685	no
gi|646749945|gb|KK962065.1|	65699-96677	22	6.1	2	1	-0.585633	0.6374	no
gi|646749945|gb|KK962065.1|	67830-96929	22	6.1	0.533433	0.768315	0.526391	1	no
gi|646749945|gb|KK962065.1|	69519-97063	22	6.1	0.539714	0.925907	0.778673	1	no
gi|646749945|gb|KK962065.1|	)2532-10094!	22	6.1	6	5	-0.347815	0.7587	no
gi|646749964|gb|KK962064.1|	01111-10151	18	8.7	0	15	inf	0.00315	no
gi|646749964|gb|KK962064.1|	13672-11423	18	8.7	0	7	inf	0.00435	no
gi|646749964|gb|KK962064.1|	81768-18391	18	8.7	2	1	-0.586293	0.71155	no
gi|646749964|gb|KK962064.1|	86236-18714	18	8.7	3	2	-0.49548	0.69495	no
gi|646749964|gb|KK962064.1|	90280-19660	18	8.7	1	0.757332	-0.757582	0.62055	no
gi|646749964|gb|KK962064.1|	08096-20896	18	8.7	1	1	0.0161122	1	no

gi|646749964|gb|KK962064.1|	67678-26853	18	8.7	7	4	-0.762573	0.6306	no
gi|646749964|gb|KK962064.1|	33493-33652	18	8.7	6	4	-0.57696	0.7037	no
gi|646749964|gb|KK962064.1|	41008-34229	18	8.7	4	3	-0.539454	0.72985	no
gi|646749964|gb|KK962064.1|	46235-34842	18	8.7	6	4	-0.378683	0.8006	no
gi|646749964|gb|KK962064.1|	91634-39292	18	8.7	3	3	0.0745746	0.96005	no
gi|646749964|gb|KK962064.1|	01360-40223	18	8.7	1	0.424485	-2	0.26845	no
gi|646749964|gb|KK962064.1|	03349-40491	18	8.7	0.423403	0.610647	0.528309	1	no
gi|646749964|gb|KK962064.1|	36158-43813	18	8.7	20	12	-0.720542	0.50385	no
gi|646749964|gb|KK962064.1|	53074-45329	18	8.7	25	35	0.485333	0.75635	no
gi|646749964|gb|KK962064.1|	57127-45837	18	8.7	3	2	-0.479269	0.7674	no
gi|646749964|gb|KK962064.1|	62408-46316	18	8.7	0.741396	2	1	0.4331	no
gi|646749964|gb|KK962064.1|	74913-47596	18	8.7	0.932044	1	0.682676	0.619	no
gi|646749964|gb|KK962064.1|	76105-47633	18	8.7	34	30	-0.159066	0.91175	no
gi|646749964|gb|KK962064.1|	08873-51005	18	8.7	0.902031	2	1	0.41795	no
gi|646749964|gb|KK962064.1|	13272-51388	18	8.7	16	30	0.904629	0.5456	no
gi|646749964|gb|KK962064.1|	71075-57229	18	8.7	30	25	-0.262537	0.8101	no
gi|646749964|gb|KK962064.1|	75600-57584	18	8.7	23	21	-0.0835494	0.93275	no
gi|646749964|gb|KK962064.1|	86591-58681	18	8.7	101	89	-0.176185	0.91425	no
gi|646749964|gb|KK962064.1|	10153-61534	18	8.7	28	32	0.225603	0.8488	no
gi|646749964|gb|KK962064.1|	33792-63454	18	8.7	3	4	0.209082	0.86095	no
gi|646749964|gb|KK962064.1|	55973-65618	18	8.7	158	143	-0.143264	0.92855	no
gi|646749984|gb|KK962063.1|	30106-23121	15	7.3	20	19	-0.0338712	0.9816	no
gi|646749984|gb|KK962063.1|	11452-41159	15	7.3	1257.66	463	-1	0.2837	no
gi|646749984|gb|KK962063.1|	88690-48997	15	7.3	12	12	-0.0110451	0.99375	no
gi|646749984|gb|KK962063.1|	15089-51701	15	7.3	17	14	-0.360235	0.73305	no
gi|646749984|gb|KK962063.1|	55617-55625	15	7.3	1	2	0.471233	0.7721	no
gi|646749984|gb|KK962063.1|	10969-61120	15	7.3	31	16	-0.9507	0.5681	no
gi|646749984|gb|KK962063.1|	68292-66876	15	7.3	6	2	-1	0.33665	no
gi|646750002|gb|KK962062.1|	1163-1819	24	12	11	29	1	0.36065	no
gi|646750002|gb|KK962062.1|	42485-14330	24	12	2	0.919433	-0.824704	0.5782	no

gi|646750002|gb|KK962062.1|	43582-14376	24	12	97	180	0.891543	0.58615	no
gi|646750002|gb|KK962062.1|	44719-14492	24	12	35	80	1	0.40765	no
gi|646750002|gb|KK962062.1|	75114-17546	24	12	6	16	1	0.3928	no
gi|646750002|gb|KK962062.1|	89802-19142	24	12	0.543002	0.293863	-0.885811	1	no
gi|646750002|gb|KK962062.1|	21237-22205	24	12	0.989272	3	2	0.2875	no
gi|646750002|gb|KK962062.1|	28539-22972	24	12	2	2	-0.0664982	0.95855	no
gi|646750002|gb|KK962062.1|	37655-23831	24	12	8	7	-0.17732	0.9113	no
gi|646750002|gb|KK962062.1|	39198-23967	24	12	4	7	0.835269	0.52685	no
gi|646750002|gb|KK962062.1|	52790-25350	24	12	20	19	-0.0866261	0.9553	no
gi|646750002|gb|KK962062.1|	54368-25731	24	12	66	83	0.327294	0.78225	no
gi|646750002|gb|KK962062.1|	66060-26678	24	12	2	0.828334	-0.929326	0.58905	no
gi|646750002|gb|KK962062.1|	79425-28230	24	12	40	32	-0.297419	0.79545	no
gi|646750002|gb|KK962062.1|	82696-28374	24	12	151	149	-0.0154741	0.9894	no
gi|646750002|gb|KK962062.1|	89829-29063	24	12	4	11	1	0.37385	no
gi|646750002|gb|KK962062.1|	22644-32303	24	12	6	7	0.186106	0.8989	no
gi|646750002|gb|KK962062.1|	30098-33029	24	12	29	80	1	0.3625	no
gi|646750002|gb|KK962062.1|	41721-34207	24	12	835	685	-0.284118	0.8023	no
gi|646750002|gb|KK962062.1|	43358-34369	24	12	13	23	0.737785	0.5913	no
gi|646750002|gb|KK962062.1|	34343-35434	24	12	2	3	0.37722	0.8197	no
gi|646750002|gb|KK962062.1|	44884-34544	24	12	6	5	-0.168468	0.89105	no
gi|646750002|gb|KK962062.1|	53609-35421	24	12	37	58	0.626483	0.62365	no
gi|646750002|gb|KK962062.1|	54456-35512	24	12	17	10	-0.854428	0.5809	no
gi|646750002|gb|KK962062.1|	65480-36615	24	12	1	4	2	0.21285	no
gi|646750002|gb|KK962062.1|	73050-37590	24	12	129	84	-0.619641	0.6069	no
gi|646750002|gb|KK962062.1|	76228-37843	24	12	1	1	0.334571	0.8354	no
gi|646750002|gb|KK962062.1|	38204-39672	24	12	3	6	0.854992	0.57785	no
gi|646750002|gb|KK962062.1|	96070-40053	24	12	16	19	0.295742	0.7991	no
gi|646750002|gb|KK962062.1|	39842-41682	24	12	1	3	1	0.36175	no
gi|646750002|gb|KK962062.1|	02333-40313	24	12	20	26	0.366038	0.8095	no
gi|646750002|gb|KK962062.1|	42174-48009	24	12	0.596922	0.920461	0.624813	1	no

gi|646750002|gb|KK962062.1|	33557-43401	24	12	8	5	-0.677281	0.6037	no
gi|646750002|gb|KK962062.1|	34561-43487	24	12	19	23	0.251615	0.86705	no
gi|646750002|gb|KK962062.1|	71365-47255	24	12	0.692577	1	0.711325	0.62255	no
gi|646750002|gb|KK962062.1|	99107-50033	24	12	0.948812	2	1	0.4011	no
gi|646750002|gb|KK962062.1|	03354-50494	24	12	42	22	-0.942819	0.39465	no
gi|646750002|gb|KK962062.1|	19585-51982	24	12	145.78	71	-1	0.5164	no
gi|646750002|gb|KK962062.1|	25897-52623	24	12	5	11	1	0.439	no
gi|646750002|gb|KK962062.1|	33494-53465	24	12	1	2	0.633239	0.6172	no
gi|646750002|gb|KK962062.1|	00525-60125	24	12	1	2	0.486271	0.73295	no
gi|646750002|gb|KK962062.1|	12721-61397	24	12	5	6	0.231288	0.87885	no
gi|646750002|gb|KK962062.1|	14094-61478	24	12	2	2	0.118624	0.92795	no
gi|646750002|gb|KK962062.1|	64290-65755	24	12	5	7	0.504932	0.7359	no
gi|646750002|gb|KK962062.1|	66827-67826	24	12	2	1	-0.852454	0.5053	no
gi|646750002|gb|KK962062.1|	68606-71757	24	12	73	108	0.571709	0.63045	no
gi|646750002|gb|KK962062.1|	83375-84153	24	12	40	37	-0.134327	0.90045	no
gi|646750002|gb|KK962062.1|	99826-10199[illegible]	24	12	0.5306	0.559496	0.0765019	1	no
gi|646750021|gb|KK962061.1|	07752-10899	16	7.5	63	64	0.0287512	0.9796	no
gi|646750021|gb|KK962061.1|	23262-22393	16	7.5	3	1	-1	0.44855	no
gi|646750021|gb|KK962061.1|	24629-22594	16	7.5	2	3	0.300155	0.84955	no
gi|646750021|gb|KK962061.1|	26017-22694	16	7.5	2	2	0.631176	0.6212	no
gi|646750021|gb|KK962061.1|	49615-25152	16	7.5	2	5	1	0.4177	no
gi|646750021|gb|KK962061.1|	54670-35740	16	7.5	15	7	-1	0.3831	no
gi|646750021|gb|KK962061.1|	57547-35847	16	7.5	11	7	-0.568424	0.70965	no
gi|646750021|gb|KK962061.1|	58579-36038	16	7.5	10	7	-0.637906	0.67365	no
gi|646750021|gb|KK962061.1|	61581-36413	16	7.5	10	5	-1	0.40515	no
gi|646750021|gb|KK962061.1|	64754-36522	16	7.5	8	3	-1	0.3475	no
gi|646750021|gb|KK962061.1|	96936-39718	16	7.5	23	12	-0.894125	0.57035	no
gi|646750021|gb|KK962061.1|	03300-40435	16	7.5	0.811452	0.661664	-0.294407	1	no
gi|646750021|gb|KK962061.1|	21609-42311	16	7.5	0.889547	0.64093	-0.472903	1	no
gi|646750021|gb|KK962061.1|	25037-42551	16	7.5	3	2	-0.580267	0.72945	no

gi|646750021|gb|KK962061.1|	26531-42738	16	7.5	2	2	0.210614	0.87615	no
gi|646750021|gb|KK962061.1|	36771-43718	16	7.5	2	4	0.963718	0.5906	no
gi|646750021|gb|KK962061.1|	66878-56745	16	7.5	4	0	-inf	0.0035	no
gi|646750021|gb|KK962061.1|	67570-56805	16	7.5	8	0	-inf	0.0004	yes
gi|646750021|gb|KK962061.1|	68677-56975	16	7.5	5	0	-inf	0.0008	yes
gi|646750021|gb|KK962061.1|	72632-57443	16	7.5	1	0	-inf	1	no
gi|646750021|gb|KK962061.1|	00639-70144	16	7.5	0	2	inf	0.00255	no
gi|646750043|gb|KK962060.1|	00290-10023	21	8.3	74	3	-5	0.00605	no
gi|646750043|gb|KK962060.1|	05650-10072	21	8.3	8	1	-3	0.06655	no
gi|646750043|gb|KK962060.1|	07618-10102	21	8.3	13	0.576391	-4	0.006	no
gi|646750043|gb|KK962060.1|	10526-10111	21	8.3	10	0.371646	-5	0.1756	no
gi|646750043|gb|KK962060.1|	10911-11124	21	8.3	33	22	-0.584473	0.7263	no
gi|646750043|gb|KK962060.1|	48694-14928	21	8.3	5	3	-0.540033	0.67245	no
gi|646750043|gb|KK962060.1|	52437-15297	21	8.3	3	2	-0.556343	0.72945	no
gi|646750043|gb|KK962060.1|	53121-15926	21	8.3	6	4	-0.563765	0.59725	no
gi|646750043|gb|KK962060.1|	61545-16524	21	8.3	27	11	-1	0.2716	no
gi|646750043|gb|KK962060.1|	72090-17233	21	8.3	1165.19	706	-0.723654	0.50585	no
gi|646750043|gb|KK962060.1|	23279-23960	21	8.3	3	6	1	0.4166	no
gi|646750043|gb|KK962060.1|	78568-27888	21	8.3	80.58	44	-0.869738	0.5694	no
gi|646750043|gb|KK962060.1|	87946-28925	21	8.3	222.73	122	-0.864059	0.4669	no
gi|646750043|gb|KK962060.1|	90263-29154	21	8.3	2	2.98	0.652866	0.63305	no
gi|646750043|gb|KK962060.1|	08991-31058	21	8.3	1	1	0.336079	0.7815	no
gi|646750043|gb|KK962060.1|	11056-31125	21	8.3	695	209	-2	0.28025	no
gi|646750043|gb|KK962060.1|	11538-31238	21	8.3	21	14	-0.568044	0.70245	no
gi|646750043|gb|KK962060.1|	12486-31359	21	8.3	3	3	-0.162604	0.9215	no
gi|646750043|gb|KK962060.1|	13849-31464	21	8.3	1	0.972121	-0.313369	0.8509	no
gi|646750043|gb|KK962060.1|	27149-33408	21	8.3	7	5	-0.554529	0.60845	no
gi|646750043|gb|KK962060.1|	53433-35637	21	8.3	7	6	-0.146167	0.9228	no
gi|646750043|gb|KK962060.1|	83860-38764	21	8.3	11	9	-0.338745	0.7579	no
gi|646750043|gb|KK962060.1|	87901-38924	21	8.3	5	3	-0.739747	0.6311	no

gi|646750043|gb|KK962060.1|	67115-46735	21	8.3	0	38	inf	0.00255	no
gi|646750043|gb|KK962060.1|	82646-48297	21	8.3	0	15	inf	0.0014	yes
gi|646750043|gb|KK962060.1|	90616-49173	21	8.3	18	13	-0.432799	0.7714	no
gi|646750043|gb|KK962060.1|	91871-49288	21	8.3	0.848666	1	0.511158	0.7286	no
gi|646750043|gb|KK962060.1|	93982-49444	21	8.3	1	11	3	0.1509	no
gi|646750043|gb|KK962060.1|	98638-49887	21	8.3	143	11	-4	0.0935	no
gi|646750043|gb|KK962060.1|	01655-50331	21	8.3	12	8	-0.630062	0.6805	no
gi|646750043|gb|KK962060.1|	11861-51240	21	8.3	15	13	-0.279921	0.85585	no
gi|646750043|gb|KK962060.1|	34206-53498	21	8.3	36	18	-1	0.42315	no
gi|646750043|gb|KK962060.1|	61092-56293	21	8.3	15	11	-0.512182	0.6837	no
gi|646750043|gb|KK962060.1|	63071-56508	21	8.3	8	8	-0.00444471	0.9953	no
gi|646750043|gb|KK962060.1|	65196-56637	21	8.3	1	2	0.759106	0.54915	no
gi|646750043|gb|KK962060.1|	71616-57183	21	8.3	44	108	1	0.4139	no
gi|646750043|gb|KK962060.1|	75208-57564	21	8.3	4	6	0.565389	0.698	no
gi|646750043|gb|KK962060.1|	85233-58566	21	8.3	3	5	1	0.4607	no
gi|646750043|gb|KK962060.1|	60529-61396	21	8.3	0.755713	2	1	0.43255	no
gi|646750043|gb|KK962060.1|	57678-75799	21	8.3	1	13	3	0.10895	no
gi|646750043|gb|KK962060.1|	69435-77310	21	8.3	8	12	0.605812	0.57875	no
gi|646750043|gb|KK962060.1|	77269-77853	21	8.3	1	3	0.943812	0.5627	no
gi|646750043|gb|KK962060.1|	76853-78187	21	8.3	6	9	0.419117	0.69925	no
gi|646750043|gb|KK962060.1|	99470-80068	21	8.3	0.096063	6	6	0.09675	no
gi|646750043|gb|KK962060.1|	79957-82131	21	8.3	1	0.76891	-0.396944	1	no
gi|646750043|gb|KK962060.1|	44496-84630	21	8.3	0.65319	0.257604	-1	1	no
gi|646750043|gb|KK962060.1|	46601-84820	21	8.3	29	21	-0.447537	0.6852	no
gi|646750043|gb|KK962060.1|	53108-85687	21	8.3	5	2	-1	0.4363	no
gi|646750043|gb|KK962060.1|	60489-86141	21	8.3	5	3	-0.935273	0.49795	no
gi|646750043|gb|KK962060.1|	87236-92354	21	8.3	10	10	-0.00394786	0.9959	no
gi|646750043|gb|KK962060.1|	80099-88055	21	8.3	7	15	1	0.42355	no
gi|646750043|gb|KK962060.1|	94428-89525	21	8.3	2	1	-0.824325	0.53835	no
gi|646750043|gb|KK962060.1|	02198-90305	21	8.3	1	2	0.330109	0.8043	no

gi|646750043|gb|KK962060.1|	25211-92561	21	8.3	5	5	0.18126	0.89945	no
gi|646750043|gb|KK962060.1|	52530-95272	21	8.3	109.73	0	-inf	0.0035	no
gi|646750043|gb|KK962060.1|	55441-95562	21	8.3	277	12	-5	0.16855	no
gi|646750043|gb|KK962060.1|	57665-95821	21	8.3	21	0.429993	-6	0.1682	no
gi|646750043|gb|KK962060.1|	)6116-10103[illegible]	21	8.3	6	7	0.217424	0.83825	no
gi|646750043|gb|KK962060.1|	65755-96635	21	8.3	68	3	-4	0.00655	no
gi|646750043|gb|KK962060.1|	70800-97106	21	8.3	208	7	-5	0.07845	no
gi|646750043|gb|KK962060.1|	81535-98179	21	8.3	297	10	-5	0.04835	no
gi|646750043|gb|KK962060.1|	84081-98634	21	8.3	5	0.600054	-3	0.0483	no
gi|646750043|gb|KK962060.1|	86498-98870	21	8.3	4	0.137396	-5	0.1091	no
gi|646750043|gb|KK962060.1|	88981-98959	21	8.3	59	2	-5	0.01075	no
gi|646750061|gb|KK962059.1|	09600-40987	15	7.1	17	24	0.471031	0.7218	no
gi|646750061|gb|KK962059.1|	22023-42334	15	7.1	0.260593	2	3	0.1332	no
gi|646750061|gb|KK962059.1|	23685-42408	15	7.1	2	7	2	0.2461	no
gi|646750061|gb|KK962059.1|	43052-44438	15	7.1	61	110	0.840129	0.4687	no
gi|646750061|gb|KK962059.1|	46949-44749	15	7.1	148	227	0.615624	0.58975	no
gi|646750061|gb|KK962059.1|	63007-46369	15	7.1	113	115	0.0154911	0.9892	no
gi|646750061|gb|KK962059.1|	67408-46788	15	7.1	0.819899	8	3	0.27075	no
gi|646750061|gb|KK962059.1|	72953-47355	15	7.1	0.263753	4	4	0.0999	no
gi|646750061|gb|KK962059.1|	95234-49619	15	7.1	1	0.930268	-0.340066	0.8138	no
gi|646750061|gb|KK962059.1|	04555-50474	15	7.1	107	48	-1	0.43485	no
gi|646750061|gb|KK962059.1|	05247-50577	15	7.1	12	6	-0.988427	0.48165	no
gi|646750061|gb|KK962059.1|	22627-52292	15	7.1	15	14	-0.0944696	0.93845	no
gi|646750061|gb|KK962059.1|	24168-52511	15	7.1	2	2	-0.0782442	0.94535	no
gi|646750061|gb|KK962059.1|	32555-53465	15	7.1	30	16	-0.853072	0.43955	no
gi|646750061|gb|KK962059.1|	35140-53632	15	7.1	55	20	-1	0.2836	no
gi|646750061|gb|KK962059.1|	47706-54789	15	7.1	176	94	-0.913277	0.47135	no
gi|646750061|gb|KK962059.1|	70668-57414	15	7.1	2	1	-0.388121	0.796	no
gi|646750061|gb|KK962059.1|	75317-57639	15	7.1	9	4	-1	0.4076	no
gi|646750061|gb|KK962059.1|	59492-60298	15	7.1	37	14	-1	0.27245	no

gi|646750061|gb|KK962059.1|	13220-61404	15	7.1	2	1	-0.239551	0.8627	no
gi|646750061|gb|KK962059.1|	14287-61478	15	7.1	19	0.513532	-5	0.16995	no
gi|646750061|gb|KK962059.1|	15005-61620	15	7.1	9	3	-2	0.3016	no
gi|646750081|gb|KK962058.1|	07628-10834	19	8.6	0	32	inf	5.00E-05	yes
gi|646750081|gb|KK962058.1|	15569-11622	19	8.6	6	3	-1	0.4522	no
gi|646750081|gb|KK962058.1|	37234-13805	19	8.6	185	35	-2	0.03835	no
gi|646750081|gb|KK962058.1|	48982-15097	19	8.6	2	1	-0.610176	0.69795	no
gi|646750081|gb|KK962058.1|	51463-15265	19	8.6	4	1	-2	0.2737	no
gi|646750081|gb|KK962058.1|	64190-16626	19	8.6	2	0.955467	-0.85194	0.52915	no
gi|646750081|gb|KK962058.1|	69461-17049	19	8.6	12	8	-0.473352	0.75455	no
gi|646750081|gb|KK962058.1|	75811-17690	19	8.6	2	0.792311	-1	0.33035	no
gi|646750081|gb|KK962058.1|	78764-18062	19	8.6	4	2	-0.888746	0.56665	no
gi|646750081|gb|KK962058.1|	82312-18289	19	8.6	2	2	0.0422638	0.9298	no
gi|646750081|gb|KK962058.1|	10348-21118	19	8.6	8	3	-1	0.32805	no
gi|646750081|gb|KK962058.1|	45469-24631	19	8.6	0.312495	3	3	0.24735	no
gi|646750081|gb|KK962058.1|	46791-24796	19	8.6	0.101102	4	5	0.09815	no
gi|646750081|gb|KK962058.1|	68279-27003	19	8.6	0.184907	3	4	0.1147	no
gi|646750081|gb|KK962058.1|	75767-27641	19	8.6	0	71	inf	5.00E-05	yes
gi|646750081|gb|KK962058.1|	78563-28149	19	8.6	0	3	inf	5.00E-05	yes
gi|646750081|gb|KK962058.1|	83130-28394	19	8.6	0	6	inf	0.00185	no
gi|646750081|gb|KK962058.1|	84050-28727	19	8.6	1	96	6	0.0033	no
gi|646750081|gb|KK962058.1|	35799-33639	19	8.6	2	10	2	0.1874	no
gi|646750081|gb|KK962058.1|	41465-34177	19	8.6	17	12	-0.454152	0.73465	no
gi|646750081|gb|KK962058.1|	43543-34645	19	8.6	33	2	-4	0.01675	no
gi|646750081|gb|KK962058.1|	49437-35044	19	8.6	0.488795	1	1	0.43305	no
gi|646750081|gb|KK962058.1|	50566-35099	19	8.6	6	0.685916	-3	0.1891	no
gi|646750081|gb|KK962058.1|	53862-35416	19	8.6	13	2	-3	0.197	no
gi|646750081|gb|KK962058.1|	55854-35781	19	8.6	89	6	-4	0.01225	no
gi|646750081|gb|KK962058.1|	66539-36673	19	8.6	269	0	-inf	0.0047	no
gi|646750081|gb|KK962058.1|	74233-37448	19	8.6	335	18	-4	0.02635	no

gi|646750081|gb|KK962058.1|	17053-42133	19	8.6	24	19	-0.321223	0.78285	no
gi|646750081|gb|KK962058.1|	26859-62907	19	8.6	4	5	0.478962	0.74585	no
gi|646750100|gb|KK962057.1|	72397-27470	16	8.2	3	0.196352	-4	0.08365	no
gi|646750100|gb|KK962057.1|	75379-27653	16	8.2	25	15	-0.766808	0.5387	no
gi|646750100|gb|KK962057.1|	78313-27870	16	8.2	9	5	-0.866361	0.514	no
gi|646750100|gb|KK962057.1|	79210-28076	16	8.2	0.641464	1	0.817575	0.5351	no
gi|646750100|gb|KK962057.1|	86979-28725	16	8.2	24	11	-1	0.44585	no
gi|646750100|gb|KK962057.1|	87800-28833	16	8.2	7	2	-2	0.19315	no
gi|646750100|gb|KK962057.1|	97851-29844	16	8.2	4	3	-0.276739	0.83245	no
gi|646750100|gb|KK962057.1|	99100-30121	16	8.2	12	6	-0.936423	0.45925	no
gi|646750100|gb|KK962057.1|	01464-30359	16	8.2	2	0.573082	-2	0.2559	no
gi|646750100|gb|KK962057.1|	03822-30520	16	8.2	3	1	-1	0.39385	no
gi|646750100|gb|KK962057.1|	19112-31974	16	8.2	1	2	0.469572	0.7721	no
gi|646750100|gb|KK962057.1|	84806-38598	16	8.2	0	1	inf	0.00255	no
gi|646750100|gb|KK962057.1|	57847-45844	16	8.2	1	3	1	0.3719	no
gi|646750100|gb|KK962057.1|	67177-46774	16	8.2	20	32	0.688077	0.64325	no
gi|646750100|gb|KK962057.1|	67942-46844	16	8.2	5	17	2	0.246	no
gi|646750100|gb|KK962057.1|	70216-47698	16	8.2	2	4	1	0.3345	no
gi|646750100|gb|KK962057.1|	86441-48797	16	8.2	0.576855	2	2	0.2327	no
gi|646750100|gb|KK962057.1|	97745-59812	16	8.2	101	15	-3	0.10555	no
gi|646750100|gb|KK962057.1|	16230-61676	16	8.2	5	3	-0.779796	0.5516	no
gi|646750100|gb|KK962057.1|	54776-65712	16	8.2	40	13	-2	0.15505	no
gi|646750100|gb|KK962057.1|	67551-66823	16	8.2	23	4	-3	0.07395	no
gi|646750100|gb|KK962057.1|	69554-67045	16	8.2	5	4	-0.455942	0.7734	no
gi|646750100|gb|KK962057.1|	81006-68200	16	8.2	0.499347	1	1	1	no
gi|646750100|gb|KK962057.1|	92606-69289	16	8.2	19	22	0.213181	0.8658	no
gi|646750100|gb|KK962057.1|	06122-70703	16	8.2	11.51	12	0.054074	0.9721	no
gi|646750115|gb|KK962056.1|	96410-97385	14	8	0.642384	1	0.994151	0.5626	no
gi|646750135|gb|KK962055.1|	40722-14142	16	7.6	3	4	0.482457	0.70425	no
gi|646750135|gb|KK962055.1|	44254-14615	16	7.6	5	5	0.123563	0.9354	no

gi|646750135|gb|KK962055.1|	55465-15582	16	7.6	5	7	0.547075	0.71965	no
gi|646750135|gb|KK962055.1|	64840-16577	16	7.6	0	18	inf	0.00035	yes
gi|646750135|gb|KK962055.1|	72088-17484	16	7.6	20	18	-0.190187	0.8654	no
gi|646750135|gb|KK962055.1|	80580-18353	16	7.6	15	14	-0.130906	0.9078	no
gi|646750135|gb|KK962055.1|	86696-18901	16	7.6	33	17	-0.953877	0.39595	no
gi|646750135|gb|KK962055.1|	89171-19042	16	7.6	6	10	0.814494	0.58925	no
gi|646750135|gb|KK962055.1|	95181-19763	16	7.6	15	14	-0.140007	0.89455	no
gi|646750135|gb|KK962055.1|	99102-20033	16	7.6	2	2	-0.142682	0.9035	no
gi|646750135|gb|KK962055.1|	09077-21043	16	7.6	3	2	-0.905057	0.50825	no
gi|646750135|gb|KK962055.1|	10598-21177	16	7.6	4	2	-0.691171	0.66915	no
gi|646750135|gb|KK962055.1|	24860-22673	16	7.6	0.455743	0.247277	-0.88209	1	no
gi|646750135|gb|KK962055.1|	34691-23497	16	7.6	14	8	-0.804366	0.6083	no
gi|646750135|gb|KK962055.1|	35101-23588	16	7.6	3	0.245669	-4	0.17385	no
gi|646750135|gb|KK962055.1|	42400-24349	16	7.6	1	0.473727	-1	0.38955	no
gi|646750135|gb|KK962055.1|	43661-24604	16	7.6	0.912152	1	0.14526	1	no
gi|646750135|gb|KK962055.1|	65854-26804	16	7.6	5	3	-0.419936	0.7785	no
gi|646750135|gb|KK962055.1|	68884-26977	16	7.6	1	2	0.35111	0.794	no
gi|646750135|gb|KK962055.1|	84972-29126	16	7.6	91	57	-0.665038	0.57745	no
gi|646750135|gb|KK962055.1|	91409-29450	16	7.6	3	2	-0.213599	0.88235	no
gi|646750135|gb|KK962055.1|	98443-30079	16	7.6	7	3	-1	0.43795	no
gi|646750135|gb|KK962055.1|	12288-31383	16	7.6	3	2	-0.0164021	0.98265	no
gi|646750135|gb|KK962055.1|	16453-31836	16	7.6	0.891721	0.725959	-0.296705	1	no
gi|646750135|gb|KK962055.1|	51922-35265	16	7.6	19	19	-0.0189564	0.9907	no
gi|646750135|gb|KK962055.1|	84110-38494	16	7.6	2	3	0.276228	0.81805	no
gi|646750135|gb|KK962055.1|	89346-38963	16	7.6	10	14	0.424773	0.7548	no
gi|646750135|gb|KK962055.1|	03564-40613	16	7.6	11	9	-0.272553	0.7935	no
gi|646750135|gb|KK962055.1|	47701-48657	16	7.6	529	224	-1	0.3093	no
gi|646750135|gb|KK962055.1|	49586-51367	16	7.6	2	0.96407	-0.763757	0.5909	no
gi|646750135|gb|KK962055.1|	13180-51367	16	7.6	2	5	1	0.43	no
gi|646750135|gb|KK962055.1|	51848-52918	16	7.6	1	0.810289	-0.334235	1	no

gi|646750135|gb|KK962055.1|	53253-54930	16	7.6	1	2	0.682189	0.62225	no
gi|646750135|gb|KK962055.1|	34817-53583	16	7.6	0.242476	2	3	0.2655	no
gi|646750135|gb|KK962055.1|	55032-56094	16	7.6	21	12	-0.741614	0.62515	no
gi|646750135|gb|KK962055.1|	57460-65778	16	7.6	0	62	inf	5.00E-05	yes
gi|646750135|gb|KK962055.1|	69575-70054	16	7.6	37	60	0.70924	0.63655	no
gi|646750135|gb|KK962055.1|	42968-74410	16	7.6	1	1	-0.260936	0.8545	no
gi|646750135|gb|KK962055.1|	76320-76611	16	7.6	875	1017.29	0.217358	0.8489	no
gi|646750135|gb|KK962055.1|	23016-82395	16	7.6	0	8	inf	0.0002	yes
gi|646750135|gb|KK962055.1|	24121-82447	16	7.6	0	27	inf	0.00155	yes
gi|646750135|gb|KK962055.1|	82575-84957	16	7.6	15	13	-0.164475	0.87705	no
gi|646750135|gb|KK962055.1|	37703-83878	16	7.6	0	3	inf	0.00315	no
gi|646750135|gb|KK962055.1|	40729-84145	16	7.6	0.197183	6	5	0.1055	no
gi|646750135|gb|KK962055.1|	46610-84719	16	7.6	0	22	inf	0.00035	yes
gi|646750135|gb|KK962055.1|	48271-84873	16	7.6	0	17	inf	0.0012	yes
gi|646750135|gb|KK962055.1|	88521-89709	16	7.6	1	0.852251	-0.596869	0.64385	no
gi|646750150|gb|KK962054.1|	10603-21106	13	7.7	5	10	0.914905	0.5393	no
gi|646750150|gb|KK962054.1|	77874-27917	13	7.7	0.088442	4	5	0.09865	no
gi|646750150|gb|KK962054.1|	50407-35128	13	7.7	17	14	-0.260939	0.86295	no
gi|646750150|gb|KK962054.1|	86514-38715	13	7.7	2	3	0.472241	0.7268	no
gi|646750150|gb|KK962054.1|	28526-42908	13	7.7	2	1	-0.96539	0.589	no
gi|646750150|gb|KK962054.1|	04967-50549	13	7.7	3	0.463382	-3	0.2136	no
gi|646750165|gb|KK962053.1|	27327-12923	25	10	0.560364	0.567671	0.0186927	1	no
gi|646750165|gb|KK962053.1|	33542-13472	25	10	17	16	-0.108701	0.9429	no
gi|646750165|gb|KK962053.1|	55012-15872	25	10	0.882427	0.974222	0.142773	1	no
gi|646750165|gb|KK962053.1|	66343-17013	25	10	6	3	-0.844931	0.5054	no
gi|646750165|gb|KK962053.1|	85830-18632	25	10	5	5	0.0127378	0.9493	no
gi|646750165|gb|KK962053.1|	93503-19528	25	10	13	8	-0.659012	0.59765	no
gi|646750165|gb|KK962053.1|	98909-19965	25	10	2	2	-0.404795	0.7698	no
gi|646750165|gb|KK962053.1|	00674-20128	25	10	3	3	0.312423	0.82475	no
gi|646750165|gb|KK962053.1|	07400-20853	25	10	3	0.902307	-2	0.25785	no

gi|646750165|gb|KK962053.1|	16417-21767	25	10	2	2	0.368885	0.7857	no
gi|646750165|gb|KK962053.1|	19793-22010	25	10	11	16	0.549698	0.6797	no
gi|646750165|gb|KK962053.1|	26059-22729	25	10	5	8	0.758972	0.61905	no
gi|646750165|gb|KK962053.1|	66890-36748	25	10	4	4	-0.0795733	0.94965	no
gi|646750165|gb|KK962053.1|	76703-37778	25	10	2	2	-0.0706451	0.9531	no
gi|646750165|gb|KK962053.1|	96878-40079	25	10	5	7	0.420969	0.73995	no
gi|646750165|gb|KK962053.1|	08640-41183	25	10	5	8	0.550298	0.65935	no
gi|646750165|gb|KK962053.1|	12208-41522	25	10	1	1	0.493674	0.7516	no
gi|646750165|gb|KK962053.1|	15491-42161	25	10	9	9	-0.0378693	0.97455	no
gi|646750165|gb|KK962053.1|	98838-50573	25	10	12	8	-0.618889	0.59635	no
gi|646750165|gb|KK962053.1|	06132-50641	25	10	31	32	0.0302299	0.9688	no
gi|646750165|gb|KK962053.1|	08396-50948	25	10	6	5	-0.129222	0.9336	no
gi|646750165|gb|KK962053.1|	09562-51075	25	10	0.495227	0.993254	1	1	no
gi|646750165|gb|KK962053.1|	17220-61866	25	10	11	10	-0.0537332	0.9679	no
gi|646750165|gb|KK962053.1|	19925-62105	25	10	2	2	0.268337	0.84175	no
gi|646750165|gb|KK962053.1|	21873-62552	25	10	6	6	0.0276795	0.9796	no
gi|646750165|gb|KK962053.1|	26667-62939	25	10	7	6	-0.154714	0.91695	no
gi|646750165|gb|KK962053.1|	37594-63835	25	10	2	3	0.642314	0.6367	no
gi|646750165|gb|KK962053.1|	39908-64032	25	10	4	6	0.379543	0.78665	no
gi|646750165|gb|KK962053.1|	41981-64222	25	10	24	6	-2	0.27905	no
gi|646750165|gb|KK962053.1|	51848-65326	25	10	0.477686	1	2	0.3018	no
gi|646750165|gb|KK962053.1|	58770-65910	25	10	6	8	0.287043	0.8442	no
gi|646750165|gb|KK962053.1|	65933-66660	25	10	0.663466	3	2	0.2051	no
gi|646750165|gb|KK962053.1|	88846-68915	25	10	18	5	-2	0.23205	no
gi|646750165|gb|KK962053.1|	92964-69376	25	10	1	1	0.231694	0.87595	no
gi|646750165|gb|KK962053.1|	95570-69798	25	10	19	4	-2	0.12195	no
gi|646750165|gb|KK962053.1|	06954-70722	25	10	15	10	-0.648647	0.67375	no
gi|646750165|gb|KK962053.1|	23667-72413	25	10	3	2	-0.396054	0.8098	no
gi|646750165|gb|KK962053.1|	41292-74297	25	10	165	35	-2	0.0669	no
gi|646750165|gb|KK962053.1|	61179-76468	25	10	9	5	-0.798311	0.5278	no

gi|646750165|gb|KK962053.1|	73108-77333	25	10	87	171	0.978319	0.53785	no
gi|646750165|gb|KK962053.1|	73878-77471	25	10	4	4	0.065661	0.96805	no
gi|646750165|gb|KK962053.1|	82147-78446	25	10	1	2	1	0.4768	no
gi|646750165|gb|KK962053.1|	87032-78750	25	10	4	6	0.708234	0.59645	no
gi|646750165|gb|KK962053.1|	87649-78838	25	10	3	0.271699	-3	0.18625	no
gi|646750165|gb|KK962053.1|	05696-80626	25	10	1	3	0.939914	0.5627	no
gi|646750165|gb|KK962053.1|	13896-81498	25	10	12	7	-0.699096	0.6423	no
gi|646750165|gb|KK962053.1|	46149-84864	25	10	1	0.839762	-0.608532	0.6548	no
gi|646750165|gb|KK962053.1|	51498-85298	25	10	1	0.325591	-2	0.2359	no
gi|646750165|gb|KK962053.1|	53945-85439	25	10	8	6	-0.516261	0.67995	no
gi|646750165|gb|KK962053.1|	63355-86425	25	10	6	2	-2	0.2859	no
gi|646750165|gb|KK962053.1|	72633-87330	25	10	2	0.923869	-0.937372	0.589	no
gi|646750165|gb|KK962053.1|	79294-88053	25	10	1	2	0.520508	0.6755	no
gi|646750165|gb|KK962053.1|	96893-97145	25	10	30	6	-2	0.17765	no
gi|646750165|gb|KK962053.1|	97839-10110	25	10	14	9	-0.570367	0.60535	no
gi|646750185|gb|KK962052.1|	42233-14386	18	7.1	0.943333	0.38901	-1	1	no
gi|646750185|gb|KK962052.1|	49127-15037	18	7.1	1	0.133386	-3	1	no
gi|646750185|gb|KK962052.1|	53918-15454	18	7.1	0.998528	15	4	0.1075	no
gi|646750185|gb|KK962052.1|	58760-15979	18	7.1	1	0	-inf	0.00475	no
gi|646750185|gb|KK962052.1|	90121-19042	18	7.1	3	28	3	0.27165	no
gi|646750185|gb|KK962052.1|	91621-19185	18	7.1	0	128	inf	0.0003	yes
gi|646750185|gb|KK962052.1|	92352-19891	18	7.1	1	20	4	0.016	no
gi|646750185|gb|KK962052.1|	99015-20038	18	7.1	0.082821	2	4	0.09875	no
gi|646750185|gb|KK962052.1|	00500-20271	18	7.1	0.800814	1	0.53632	0.7012	no
gi|646750185|gb|KK962052.1|	18428-21861	18	7.1	105.75	177	0.743862	0.6222	no
gi|646750185|gb|KK962052.1|	21188-22144	18	7.1	10	35	2	0.24635	no
gi|646750185|gb|KK962052.1|	22755-22349	18	7.1	29	124	2	0.12855	no
gi|646750185|gb|KK962052.1|	26062-22765	18	7.1	27	62	1	0.2977	no
gi|646750185|gb|KK962052.1|	48526-24948	18	7.1	3	0.939283	-2	0.28865	no
gi|646750185|gb|KK962052.1|	49535-25094	18	7.1	51	17	-2	0.15185	no

gi|646750185|gb|KK962052.1|	51064-25259	18	7.1	0.507517	0.52259	0.0422237	1	no
gi|646750185|gb|KK962052.1|	58236-25984	18	7.1	0.27371	0.789908	2	1	no
gi|646750185|gb|KK962052.1|	59993-26113	18	7.1	6	5	-0.256774	0.86275	no
gi|646750185|gb|KK962052.1|	62353-26404	18	7.1	0.901952	0.558308	-0.69199	1	no
gi|646750185|gb|KK962052.1|	65033-26927	18	7.1	4	2	-1	0.33675	no
gi|646750185|gb|KK962052.1|	69483-27195	18	7.1	4	2	-0.75305	0.6207	no
gi|646750185|gb|KK962052.1|	86820-28705	18	7.1	60	35	-0.77317	0.5549	no
gi|646750185|gb|KK962052.1|	17059-31752	18	7.1	5	0.580347	-3	0.19565	no
gi|646750185|gb|KK962052.1|	31725-33199	18	7.1	20	5	-2	0.22445	no
gi|646750185|gb|KK962052.1|	32297-33345	18	7.1	35	10	-2	0.1846	no
gi|646750185|gb|KK962052.1|	35462-33944	18	7.1	17	17	0.0272453	0.98075	no
gi|646750185|gb|KK962052.1|	40663-34162	18	7.1	3	5	0.722055	0.64745	no
gi|646750185|gb|KK962052.1|	47793-34812	18	7.1	16	23	0.542281	0.7021	no
gi|646750185|gb|KK962052.1|	24067-42443	18	7.1	35	28	-0.36032	0.81605	no
gi|646750185|gb|KK962052.1|	65160-46597	18	7.1	4	2	-1	0.455	no
gi|646750185|gb|KK962052.1|	81342-48205	18	7.1	11	5	-0.996317	0.53505	no
gi|646750185|gb|KK962052.1|	34567-53561	18	7.1	2	2	0.512849	0.6858	no
gi|646750185|gb|KK962052.1|	55751-55603	18	7.1	17	14	-0.314418	0.8277	no
gi|646750185|gb|KK962052.1|	71658-57194	18	7.1	22	38	0.80232	0.5697	no
gi|646750204|gb|KK962051.1|	00181-10062	19	7.5	7	5	-0.508151	0.69735	no
gi|646750204|gb|KK962051.1|	33147-13354	19	7.5	19	9	-1	0.42385	no
gi|646750204|gb|KK962051.1|	84950-18525	19	7.5	127.43	61	-1	0.4856	no
gi|646750204|gb|KK962051.1|	86693-18703	19	7.5	0	199	inf	5.00E-05	yes
gi|646750204|gb|KK962051.1|	96560-19674	19	7.5	0	180	inf	0.00605	no
gi|646750204|gb|KK962051.1|	07540-20848	19	7.5	4	0.569881	-3	0.1369	no
gi|646750204|gb|KK962051.1|	10888-21149	19	7.5	2	4	0.978044	0.5187	no
gi|646750204|gb|KK962051.1|	22074-24260	19	7.5	18	16	-0.223522	0.8388	no
gi|646750204|gb|KK962051.1|	25861-22627	19	7.5	3	5	0.860155	0.57745	no
gi|646750204|gb|KK962051.1|	28651-23021	19	7.5	2	0.508111	-2	0.3195	no
gi|646750204|gb|KK962051.1|	47859-24830	19	7.5	4	1	-2	0.2882	no

gi|646750204|gb|KK962051.1|	55563-25830	19	7.5	12	8	-0.614176	0.56345	no
gi|646750204|gb|KK962051.1|	65835-27016	19	7.5	9	5	-0.872863	0.41625	no
gi|646750204|gb|KK962051.1|	70342-27209	19	7.5	0.679803	0.357262	-0.928134	1	no
gi|646750204|gb|KK962051.1|	28309-29133	19	7.5	0.975367	3	2	0.2875	no
gi|646750204|gb|KK962051.1|	98743-29943	19	7.5	2	1	-0.519617	0.73195	no
gi|646750204|gb|KK962051.1|	37172-38153	19	7.5	34	13	-1	0.2971	no
gi|646750204|gb|KK962051.1|	98163-39953	19	7.5	0.249356	44	7	0.1049	no
gi|646750204|gb|KK962051.1|	07050-41070	19	7.5	9	9	-0.0706161	0.9475	no
gi|646750204|gb|KK962051.1|	40860-43296	19	7.5	29	13	-1	0.30475	no
gi|646750204|gb|KK962051.1|	11774-41381	19	7.5	3	0.526458	-2	0.1317	no
gi|646750204|gb|KK962051.1|	15000-41730	19	7.5	5	3	-0.791626	0.60265	no
gi|646750204|gb|KK962051.1|	18707-42056	19	7.5	0.636443	0.25109	-1	1	no
gi|646750204|gb|KK962051.1|	20870-43124	19	7.5	4	7	0.696114	0.533	no
gi|646750204|gb|KK962051.1|	34186-43829	19	7.5	22	24	0.0834521	0.94195	no
gi|646750204|gb|KK962051.1|	41384-44331	19	7.5	0.606121	0.717852	0.244079	1	no
gi|646750204|gb|KK962051.1|	45410-44612	19	7.5	16	18	0.139326	0.92695	no
gi|646750204|gb|KK962051.1|	57899-46094	19	7.5	0.497063	1	1	1	no
gi|646750204|gb|KK962051.1|	61147-46183	19	7.5	2	0.899482	-1	0.47355	no
gi|646750204|gb|KK962051.1|	66456-46661	19	7.5	314	199	-0.657975	0.65155	no
gi|646750204|gb|KK962051.1|	69331-47069	19	7.5	30	32	0.129004	0.90855	no
gi|646750204|gb|KK962051.1|	71662-47238	19	7.5	1	2	0.48557	0.73295	no
gi|646750204|gb|KK962051.1|	16071-51637	19	7.5	25	30	0.246288	0.85675	no
gi|646750204|gb|KK962051.1|	37808-54258	19	7.5	2	1	-0.854459	0.57385	no
gi|646750204|gb|KK962051.1|	54150-54463	19	7.5	22	4	-2	0.15335	no
gi|646750204|gb|KK962051.1|	46574-54691	19	7.5	18	38	1	0.4486	no
gi|646750204|gb|KK962051.1|	47407-54863	19	7.5	0.758272	0.407773	-0.894951	1	no
gi|646750204|gb|KK962051.1|	48789-54984	19	7.5	0.580336	1	0.998325	0.5626	no
gi|646750204|gb|KK962051.1|	51977-55294	19	7.5	2	0.185039	-3	0.1932	no
gi|646750204|gb|KK962051.1|	55493-55711	19	7.5	26	36	0.476419	0.75635	no
gi|646750204|gb|KK962051.1|	60821-56152	19	7.5	0.8293	2	1	0.4332	no

gi|646750204|gb|KK962051.1|	66652-56746	19	7.5	0.494636	2	2	0.20395	no
gi|646750204|gb|KK962051.1|	73506-57470	19	7.5	10.64	2	-2	0.09355	no
gi|646750204|gb|KK962051.1|	85117-58538	19	7.5	313	353	0.171369	0.8883	no
gi|646750204|gb|KK962051.1|	88650-58906	19	7.5	6	15	1	0.36295	no
gi|646750204|gb|KK962051.1|	89678-59006	19	7.5	505	286	-0.822718	0.46785	no
gi|646750204|gb|KK962051.1|	91515-59605	19	7.5	10	7	-0.589436	0.5986	no
gi|646750204|gb|KK962051.1|	05185-60587	19	7.5	7	4	-0.711773	0.60205	no
gi|646750204|gb|KK962051.1|	29104-63087	19	7.5	0.244426	0.706618	2	1	no
gi|646750204|gb|KK962051.1|	33747-63609	19	7.5	1	5	2	0.25035	no
gi|646750204|gb|KK962051.1|	38521-63935	19	7.5	0.798772	2	1	0.36955	no
gi|646750204|gb|KK962051.1|	41129-64134	19	7.5	197	97	-1	0.52355	no
gi|646750204|gb|KK962051.1|	64910-65267	19	7.5	7	4	-0.847673	0.58245	no
gi|646750204|gb|KK962051.1|	56030-65665	19	7.5	10	12	0.263648	0.863	no
gi|646750204|gb|KK962051.1|	61855-66408	19	7.5	1	0.543529	-1	0.45805	no
gi|646750204|gb|KK962051.1|	66181-66859	19	7.5	0.770273	0.186375	-2	1	no
gi|646750204|gb|KK962051.1|	97700-69890	19	7.5	109	34	-2	0.1483	no
gi|646750204|gb|KK962051.1|	01006-70270	19	7.5	6	6	0.0419637	0.97705	no
gi|646750204|gb|KK962051.1|	06962-70747	19	7.5	4	0	-inf	0.00655	no
gi|646750204|gb|KK962051.1|	14886-71526	19	7.5	641	387	-0.727703	0.53185	no
gi|646750204|gb|KK962051.1|	55158-75538	19	7.5	44	89	1	0.4812	no
gi|646750204|gb|KK962051.1|	69621-76987	19	7.5	20	33	0.715637	0.5956	no
gi|646750204|gb|KK962051.1|	80046-78102	19	7.5	154	42	-2	0.1074	no
gi|646750204|gb|KK962051.1|	7873-9332	19	7.5	21	18	-0.234045	0.82835	no
gi|646750204|gb|KK962051.1|	82140-83460	19	7.5	2	1	-0.892267	0.5465	no
gi|646750232|gb|KK962050.1|	25099-25491	14	7.6	4	3	-0.225131	0.87385	no
gi|646750252|gb|KK962049.1|	17164-21781	22	10	2	0.98147	-0.942203	0.589	no
gi|646750252|gb|KK962049.1|	97214-30595	22	10	3	4	0.0540048	0.9599	no
gi|646750252|gb|KK962049.1|	29223-33165	22	10	0.679658	0.370039	-0.877132	1	no
gi|646750252|gb|KK962049.1|	35443-34003	22	10	1	0.31115	-2	1	no
gi|646750252|gb|KK962049.1|	40187-34397	22	10	18	5	-2	0.1159	no

gi|646750252|gb|KK962049.1|	45945-34672	22	10	3	1	-1	0.42505	no
gi|646750252|gb|KK962049.1|	56631-35909	22	10	5	2	-1	0.3908	no
gi|646750252|gb|KK962049.1|	66589-36786	22	10	0.636745	0.652812	0.035954	1	no
gi|646750252|gb|KK962049.1|	71785-37324	22	10	0.847324	0.332777	-1	1	no
gi|646750252|gb|KK962049.1|	76465-38523	22	10	6	3	-0.907139	0.41385	no
gi|646750252|gb|KK962049.1|	87909-38896	22	10	0.925056	1	0.68297	0.619	no
gi|646750252|gb|KK962049.1|	96526-39865	22	10	5	3	-0.495472	0.74925	no
gi|646750252|gb|KK962049.1|	00688-40111	22	10	13	10	-0.349138	0.8307	no
gi|646750252|gb|KK962049.1|	01169-40205	22	10	1	0.622133	-1	0.4766	no
gi|646750252|gb|KK962049.1|	03703-40548	22	10	21	9	-1	0.37525	no
gi|646750252|gb|KK962049.1|	05630-40749	22	10	31	15	-1	0.35375	no
gi|646750252|gb|KK962049.1|	08825-40935	22	10	6	5	-0.323296	0.78535	no
gi|646750252|gb|KK962049.1|	22567-42381	22	10	1	0.936462	-0.131472	1	no
gi|646750252|gb|KK962049.1|	35771-43640	22	10	46	27	-0.765806	0.61395	no
gi|646750252|gb|KK962049.1|	55679-45831	22	10	15	9	-0.740454	0.489	no
gi|646750252|gb|KK962049.1|	25527-62600	22	10	3	2	-0.387622	0.80985	no
gi|646750252|gb|KK962049.1|	29980-63158	22	10	1	0.792213	-0.558494	0.66445	no
gi|646750252|gb|KK962049.1|	31701-63295	22	10	1	1	0.142314	0.91015	no
gi|646750252|gb|KK962049.1|	33228-63423	22	10	0.866839	1	0.510284	0.7286	no
gi|646750252|gb|KK962049.1|	34458-63545	22	10	0.870334	0.885356	0.0246895	1	no
gi|646750252|gb|KK962049.1|	39165-63964	22	10	3	2	-0.99631	0.589	no
gi|646750252|gb|KK962049.1|	39971-64043	22	10	5	2	-2	0.2915	no
gi|646750252|gb|KK962049.1|	42468-64314	22	10	2	3	1	0.4155	no
gi|646750252|gb|KK962049.1|	46461-64740	22	10	0.537023	2	2	0.20295	no
gi|646750252|gb|KK962049.1|	47567-64896	22	10	0.564587	1	1	0.39575	no
gi|646750252|gb|KK962049.1|	49075-65566	22	10	24	25	0.0894019	0.93915	no
gi|646750252|gb|KK962049.1|	12060-81279	22	10	4	7	0.728102	0.6563	no
gi|646750252|gb|KK962049.1|	78385-87888	22	10	2	4	0.914092	0.56505	no
gi|646750252|gb|KK962049.1|	86377-88667	22	10	15	5	-2	0.3575	no
gi|646750252|gb|KK962049.1|	97858-90053	22	10	0.34337	0.443591	0.369465	1	no

gi|646750252|gb|KK962049.1|	02411-90775	22	10	12	12	0.00548044	0.995	no
gi|646750267|gb|KK962048.1|	11584-11264	16	8.2	0	7	inf	0.0002	yes
gi|646750267|gb|KK962048.1|	12995-11377	16	8.2	0	11	inf	0.00025	yes
gi|646750267|gb|KK962048.1|	23887-12496	16	8.2	10	7	-0.453537	0.7627	no
gi|646750267|gb|KK962048.1|	26593-12687	16	8.2	67	10	-3	0.11195	no
gi|646750267|gb|KK962048.1|	29310-13108	16	8.2	0.243141	0.878697	2	1	no
gi|646750267|gb|KK962048.1|	14020-15698	16	8.2	3	1	-0.884836	0.58625	no
gi|646750267|gb|KK962048.1|	64656-16602	16	8.2	0.333374	0.838878	1	1	no
gi|646750267|gb|KK962048.1|	17391-18882	16	8.2	2	2	0.0907532	0.95345	no
gi|646750267|gb|KK962048.1|	19078-19971	16	8.2	3	2	-0.648814	0.61035	no
gi|646750267|gb|KK962048.1|	97249-19750	16	8.2	5	44	3	0.2789	no
gi|646750267|gb|KK962048.1|	98848-20071	16	8.2	21	50	1	0.27105	no
gi|646750267|gb|KK962048.1|	07450-21293	16	8.2	22	19	-0.189767	0.86825	no
gi|646750267|gb|KK962048.1|	29582-23112	16	8.2	0.504749	0.831661	0.72043	1	no
gi|646750267|gb|KK962048.1|	31269-23198	16	8.2	2	2	-0.51573	0.70145	no
gi|646750267|gb|KK962048.1|	33536-23469	16	8.2	0.820469	0.880832	0.102419	1	no
gi|646750267|gb|KK962048.1|	42905-24317	16	8.2	10	42	2	0.24675	no
gi|646750267|gb|KK962048.1|	45163-24705	16	8.2	0.563778	0.734251	0.381146	1	no
gi|646750267|gb|KK962048.1|	64232-26451	16	8.2	9	16	0.871412	0.56985	no
gi|646750267|gb|KK962048.1|	65207-26580	16	8.2	1	4	2	0.27405	no
gi|646750267|gb|KK962048.1|	77548-27780	16	8.2	22	27	0.291408	0.8262	no
gi|646750267|gb|KK962048.1|	87115-28722	16	8.2	24124.2	12390.7	-0.961229	0.52705	no
gi|646750267|gb|KK962048.1|	34921-35549	16	8.2	18	4	-2	0.1636	no
gi|646750267|gb|KK962048.1|	98147-39832	16	8.2	46	92	0.992803	0.5813	no
gi|646750267|gb|KK962048.1|	34214-43464	16	8.2	18	18	0.0581007	0.96905	no
gi|646750267|gb|KK962048.1|	38450-44118	16	8.2	2	2	0.573643	0.71035	no
gi|646750267|gb|KK962048.1|	41323-44313	16	8.2	0.713945	0.430157	-0.73095	1	no
gi|646750267|gb|KK962048.1|	44384-44501	16	8.2	3	3	0.0105616	0.94695	no
gi|646750267|gb|KK962048.1|	58888-45935	16	8.2	13	11	-0.247108	0.87355	no
gi|646750267|gb|KK962048.1|	61458-46346	16	8.2	7	6	-0.221982	0.8816	no

gi|646750267|gb|KK962048.1|	46260-46544	16	8.2	15	8	-0.967544	0.5573	no
gi|646750267|gb|KK962048.1|	63591-46635	16	8.2	1	1	-0.353257	0.82325	no
gi|646750267|gb|KK962048.1|	81411-48293	16	8.2	6	4	-0.365609	0.80265	no
gi|646750267|gb|KK962048.1|	85097-48841	16	8.2	12	11	-0.179635	0.872	no
gi|646750267|gb|KK962048.1|	90399-49068	16	8.2	9	31	2	0.2894	no
gi|646750267|gb|KK962048.1|	07688-50955	16	8.2	2	2	0.0134043	0.9854	no
gi|646750267|gb|KK962048.1|	18784-51902	16	8.2	39	62	0.66726	0.6512	no
gi|646750267|gb|KK962048.1|	22632-52408	16	8.2	0.54146	0.779666	0.526001	1	no
gi|646750267|gb|KK962048.1|	34297-53465	16	8.2	4	8	0.997852	0.5533	no
gi|646750267|gb|KK962048.1|	49555-55393	16	8.2	55	44	-0.3507	0.77555	no
gi|646750267|gb|KK962048.1|	67940-56912	16	8.2	2	2	0.126074	0.92185	no
gi|646750267|gb|KK962048.1|	76237-57769	16	8.2	3	5	0.908111	0.56675	no
gi|646750267|gb|KK962048.1|	63943-86440	16	8.2	7	3	-1	0.3683	no
gi|646750267|gb|KK962048.1|	67242-86744	16	8.2	113	80	-0.489877	0.7005	no
gi|646750267|gb|KK962048.1|	75344-87560	16	8.2	96	102	0.0990534	0.9521	no
gi|646750267|gb|KK962048.1|	76157-87699	16	8.2	4	4	0.161856	0.9212	no
gi|646750267|gb|KK962048.1|	99566-99984	16	8.2	8	11	0.574515	0.68965	no
gi|646750285|gb|KK962047.1|	01154-10154	20	8.3	17	5	-2	0.26925	no
gi|646750285|gb|KK962047.1|	01695-10358	20	8.3	0.338679	0.735135	1	1	no
gi|646750285|gb|KK962047.1|	04341-10481	20	8.3	5	3	-0.697769	0.60965	no
gi|646750285|gb|KK962047.1|	07256-10819	20	8.3	2	2	-0.024193	0.9504	no
gi|646750285|gb|KK962047.1|	10903-11213	20	8.3	81	7	-3	0.0248	no
gi|646750285|gb|KK962047.1|	21390-12198	20	8.3	5	2	-2	0.24145	no
gi|646750285|gb|KK962047.1|	32435-13529	20	8.3	40	5	-3	0.042	no
gi|646750285|gb|KK962047.1|	41202-14141	20	8.3	135	115	-0.233866	0.886	no
gi|646750285|gb|KK962047.1|	51247-25137	20	8.3	3520.38	1898.16	-0.891132	0.47315	no
gi|646750285|gb|KK962047.1|	65491-26611	20	8.3	3	3	0.315179	0.82475	no
gi|646750285|gb|KK962047.1|	71854-27487	20	8.3	0.033401	9	8	0.09445	no
gi|646750285|gb|KK962047.1|	77084-27756	20	8.3	84	29	-2	0.24155	no
gi|646750285|gb|KK962047.1|	90523-29207	20	8.3	27	11	-1	0.329	no

gi|646750285|gb|KK962047.1|	02334-30298	20	8.3	0	28	inf	0.0005	yes
gi|646750285|gb|KK962047.1|	04043-30592	20	8.3	0.056828	25	9	0.09445	no
gi|646750285|gb|KK962047.1|	16316-31711	20	8.3	3	0	-inf	0.0008	yes
gi|646750285|gb|KK962047.1|	21033-32155	20	8.3	7	0	-inf	0.0004	yes
gi|646750285|gb|KK962047.1|	27877-32810	20	8.3	41	0	-inf	0.00655	no
gi|646750285|gb|KK962047.1|	37222-33770	20	8.3	29	1	-5	0.10945	no
gi|646750285|gb|KK962047.1|	48620-34971	20	8.3	0.87684	0.783166	-0.162995	1	no
gi|646750285|gb|KK962047.1|	54633-35485	20	8.3	921	130	-3	0.0906	no
gi|646750285|gb|KK962047.1|	76384-37662	20	8.3	24	47	0.964718	0.49575	no
gi|646750285|gb|KK962047.1|	78357-37876	20	8.3	5	16	2	0.28965	no
gi|646750285|gb|KK962047.1|	81672-38283	20	8.3	2	3	0.380068	0.81585	no
gi|646750285|gb|KK962047.1|	86363-38740	20	8.3	20	9	-1	0.4158	no
gi|646750285|gb|KK962047.1|	93646-39418	20	8.3	8	14	0.698799	0.6606	no
gi|646750285|gb|KK962047.1|	61348-46254	20	8.3	2	5	0.962465	0.5471	no
gi|646750285|gb|KK962047.1|	63909-46491	20	8.3	5	6	0.306695	0.8422	no
gi|646750285|gb|KK962047.1|	66479-46723	20	8.3	0.745874	4	2	0.1831	no
gi|646750285|gb|KK962047.1|	85573-48588	20	8.3	10	24	1	0.4312	no
gi|646750285|gb|KK962047.1|	55318-55536	20	8.3	42	16	-1	0.3894	no
gi|646750285|gb|KK962047.1|	91703-70152	20	8.3	10	8	-0.309977	0.7928	no
gi|646750285|gb|KK962047.1|	04758-70514	20	8.3	12	4	-1	0.321	no
gi|646750285|gb|KK962047.1|	10636-71224	20	8.3	15	10	-0.505146	0.67705	no
gi|646750285|gb|KK962047.1|	25026-72607	20	8.3	0.698154	0.830115	0.249766	1	no
gi|646750285|gb|KK962047.1|	30299-73102	20	8.3	3	1	-0.893226	0.53405	no
gi|646750285|gb|KK962047.1|	51862-75247	20	8.3	3	1	-1	0.44495	no
gi|646750285|gb|KK962047.1|	60746-76127	20	8.3	3	0.899449	-2	0.26405	no
gi|646750285|gb|KK962047.1|	03334-80378	20	8.3	4	2	-0.593858	0.6685	no
gi|646750304|gb|KK962046.1|	14865-15715	16	6.3	3	1	-1	0.3839	no
gi|646750304|gb|KK962046.1|	64043-46531	16	6.3	2	2	0.061858	0.966	no
gi|646750304|gb|KK962046.1|	83535-48431	16	6.3	1	2	0.269815	0.8456	no
gi|646750304|gb|KK962046.1|	84644-48636	16	6.3	6	12	1	0.51355	no

gi|646750304|gb|KK962046.1|	12255-51479	16	6.3	10	11	0.0611355	0.9547	no
gi|646750304|gb|KK962046.1|	18019-51896	16	6.3	24	19	-0.348817	0.8175	no
gi|646750304|gb|KK962046.1|	79124-58151	16	6.3	2	2	0.613534	0.6934	no
gi|646750319|gb|KK962045.1|	66277-10673	16	8.4	0.989963	0.471533	-1	1	no
gi|646750319|gb|KK962045.1|	16785-31750	16	8.4	2	0.276111	-3	0.2033	no
gi|646750319|gb|KK962045.1|	74369-37545	16	8.4	1	0.158648	-3	1	no
gi|646750319|gb|KK962045.1|	84070-38669	16	8.4	126	24	-2	0.0511	no
gi|646750319|gb|KK962045.1|	89584-39164	16	8.4	9	0	-inf	5.00E-05	yes
gi|646750319|gb|KK962045.1|	58228-45880	16	8.4	7	10	0.514241	0.74705	no
gi|646750319|gb|KK962045.1|	66804-46707	16	8.4	17	49	1	0.3529	no
gi|646750319|gb|KK962045.1|	74132-47455	16	8.4	1	9	3	0.24555	no
gi|646750319|gb|KK962045.1|	76081-47663	16	8.4	20	68	2	0.1905	no
gi|646750319|gb|KK962045.1|	86878-48732	16	8.4	8	8	-0.103761	0.93245	no
gi|646750319|gb|KK962045.1|	30281-53127	16	8.4	2	0	-inf	0.00225	no
gi|646750319|gb|KK962045.1|	31422-53489	16	8.4	73	0.837842	-6	0.0067	no
gi|646750319|gb|KK962045.1|	10400-71161	16	8.4	1	1	-0.118014	0.9313	no
gi|646750319|gb|KK962045.1|	19324-71987	16	8.4	0.933469	4	2	0.20605	no
gi|646750319|gb|KK962045.1|	20116-72108	16	8.4	2	5	1	0.359	no
gi|646750319|gb|KK962045.1|	70018-87252	16	8.4	2	2	-0.309588	0.83905	no
gi|646750319|gb|KK962045.1|	88252-88932	16	8.4	2	0.805479	-1	0.33035	no
gi|646750319|gb|KK962045.1|	89599-89122	16	8.4	0.406357	0.6842	0.75167	1	no
gi|646750319|gb|KK962045.1|	99081-89943	16	8.4	6	7	0.31978	0.83855	no
gi|646750319|gb|KK962045.1|	37976-93848	16	8.4	0	14.59	inf	0.00105	yes
gi|646750341|gb|KK962044.1|	29363-12300	19	5.6	4	4	0.175757	0.88885	no
gi|646750341|gb|KK962044.1|	37293-12392	19	5.6	29	15	-0.96952	0.39095	no
gi|646750341|gb|KK962044.1|	47452-12483	19	5.6	0.750808	1	0.986885	0.5626	no
gi|646750341|gb|KK962044.1|	63331-12640	19	5.6	4	6	0.558712	0.6883	no
gi|646750341|gb|KK962044.1|	86795-12870	19	5.6	52	26	-1	0.45515	no
gi|646750341|gb|KK962044.1|	07286-13076	19	5.6	10	7	-0.571607	0.7215	no
gi|646750341|gb|KK962044.1|	26640-13280	19	5.6	0.859488	0.89991	0.066303	1	no

gi|646750341|gb|KK962044.1|	29292-13309	19	5.6	0.384676	0.648248	0.752901	1	no
gi|646750341|gb|KK962044.1|	31098-13321	19	5.6	1	0.332861	-2	0.26845	no
gi|646750341|gb|KK962044.1|	35671-13379	19	5.6	1	0.88191	-0.518013	0.7075	no
gi|646750341|gb|KK962044.1|	38123-13393	19	5.6	2	2	0.239308	0.842	no
gi|646750341|gb|KK962044.1|	40245-13418	19	5.6	0.748274	0.098167	-3	1	no
gi|646750341|gb|KK962044.1|	53763-13554	19	5.6	3	2	-0.67361	0.6617	no
gi|646750341|gb|KK962044.1|	72576-13728	19	5.6	17	10	-0.665105	0.66965	no
gi|646750341|gb|KK962044.1|	74349-13750	19	5.6	2	1	-0.516324	0.73195	no
gi|646750341|gb|KK962044.1|	13242-21437	19	5.6	2	2	-0.290967	0.803	no
gi|646750341|gb|KK962044.1|	18376-21877	19	5.6	123	3	-5	0.04625	no
gi|646750341|gb|KK962044.1|	81645-28204	19	5.6	6	7	0.215958	0.8797	no
gi|646750341|gb|KK962044.1|	82651-28383	19	5.6	1	0.286785	-2	1	no
gi|646750341|gb|KK962044.1|	18763-31934	19	5.6	10	5	-0.929996	0.49505	no
gi|646750341|gb|KK962044.1|	41424-34242	19	5.6	2	0.879617	-1	0.41105	no
gi|646750341|gb|KK962044.1|	47140-34811	19	5.6	16	7	-1	0.40115	no
gi|646750341|gb|KK962044.1|	84482-38523	19	5.6	0.375203	4	3	0.26695	no
gi|646750341|gb|KK962044.1|	70559-47199	19	5.6	17	4	-2	0.0985	no
gi|646750341|gb|KK962044.1|	75205-47633	19	5.6	1	1	0.194378	0.88295	no
gi|646750341|gb|KK962044.1|	87282-48775	19	5.6	7	6	-0.122349	0.92355	no
gi|646750341|gb|KK962044.1|	89725-49087	19	5.6	1	0.886587	-0.219741	1	no
gi|646750341|gb|KK962044.1|	49774-50373	19	5.6	1	3	0.947676	0.56265	no
gi|646750341|gb|KK962044.1|	57844-55867	19	5.6	1	2	0.498163	0.7299	no
gi|646750341|gb|KK962044.1|	61174-56270	19	5.6	1	0.836144	-0.720277	0.57085	no
gi|646750341|gb|KK962044.1|	00430-60101	19	5.6	3	2	-0.389881	0.80035	no
gi|646750341|gb|KK962044.1|	06398-60677	19	5.6	10	9	-0.143662	0.90915	no
gi|646750341|gb|KK962044.1|	62661-63091	19	5.6	7	3	-0.987836	0.5242	no
gi|646750341|gb|KK962044.1|	63217-63810	19	5.6	52	38	-0.466011	0.759	no
gi|646750341|gb|KK962044.1|	36200-63650	19	5.6	35	21	-0.697678	0.6205	no
gi|646750341|gb|KK962044.1|	50519-65130	19	5.6	4	2	-1	0.47345	no
gi|646750341|gb|KK962044.1|	65618-66810	19	5.6	2	3	0.0907928	0.954	no

gi|646750341|gb|KK962044.1|	76259-67681	19	5.6	3	0	-inf	0.00655	no
gi|646750341|gb|KK962044.1|	77886-67866	19	5.6	20	17	-0.198233	0.8927	no
gi|646750341|gb|KK962044.1|	84552-68622	19	5.6	1	2	0.447598	0.74445	no
gi|646750341|gb|KK962044.1|	02037-70422	19	5.6	0.477879	0.485059	0.0215139	1	no
gi|646750341|gb|KK962044.1|	06233-70733	19	5.6	8	7	-0.287259	0.8469	no
gi|646750341|gb|KK962044.1|	17683-71930	19	5.6	6	7	0.283521	0.8493	no
gi|646750341|gb|KK962044.1|	20553-72194	19	5.6	0.812218	0.701084	-0.21228	1	no
gi|646750341|gb|KK962044.1|	23037-72464	19	5.6	0.685288	0.791059	0.207076	1	no
gi|646750341|gb|KK962044.1|	26848-72750	19	5.6	2	1	-0.332598	0.81865	no
gi|646750341|gb|KK962044.1|	39708-74089	19	5.6	1	0.852251	-0.703784	0.5887	no
gi|646750341|gb|KK962044.1|	43308-74456	19	5.6	0.643706	1	0.713689	1	no
gi|646750341|gb|KK962044.1|	46299-74700	19	5.6	5	8	0.713937	0.65545	no
gi|646750341|gb|KK962044.1|	53464-75413	19	5.6	10	3	-2	0.2195	no
gi|646750341|gb|KK962044.1|	58407-75935	19	5.6	1	0.572284	-1	0.3882	no
gi|646750341|gb|KK962044.1|	71680-77232	19	5.6	2	4	1	0.41625	no
gi|646750341|gb|KK962044.1|	72535-77288	19	5.6	4	9	0.990216	0.5533	no
gi|646750341|gb|KK962044.1|	77611-77987	19	5.6	4	2	-1	0.4941	no
gi|646750341|gb|KK962044.1|	29063-83099	19	5.6	4	2	-1	0.40115	no
gi|646750341|gb|KK962044.1|	31107-83222	19	5.6	5	2	-1	0.30215	no
gi|646750341|gb|KK962044.1|	32288-83311	19	5.6	5	4	-0.360853	0.8229	no
gi|646750341|gb|KK962044.1|	33195-83388	19	5.6	5	3	-0.704206	0.61635	no
gi|646750341|gb|KK962044.1|	33988-83748	19	5.6	2	1	-0.752773	0.6184	no
gi|646750341|gb|KK962044.1|	38004-84643	19	5.6	2	2	-0.0108008	0.99355	no
gi|646750341|gb|KK962044.1|	56315-85773	19	5.6	31	38	0.29559	0.789	no
gi|646750341|gb|KK962044.1|	59629-86147	19	5.6	0.696916	0.672061	-0.0523935	1	no
gi|646750341|gb|KK962044.1|	63208-86473	19	5.6	1	0.839377	-0.472446	0.72015	no
gi|646750341|gb|KK962044.1|	65968-86795	19	5.6	1	0.541153	-0.906401	1	no
gi|646750341|gb|KK962044.1|	68071-86906	19	5.6	2	0.356468	-2	0.1971	no
gi|646750341|gb|KK962044.1|	72164-87234	19	5.6	194	252	0.37697	0.78805	no
gi|646750341|gb|KK962044.1|	74539-87657	19	5.6	5	9	0.844974	0.57915	no

gi|646750341|gb|KK962044.1|	79042-88318	19	5.6	1	1	0.168823	0.91135	no
gi|646750341|gb|KK962044.1|	84517-88579	19	5.6	0.900779	1	0.369678	0.78175	no
gi|646750341|gb|KK962044.1|	85960-88638	19	5.6	23	51	1	0.45275	no
gi|646750341|gb|KK962044.1|	87839-89209	19	5.6	3	4	0.544557	0.7259	no
gi|646750341|gb|KK962044.1|	97746-90821	19	5.6	29	37	0.359882	0.76055	no
gi|646750341|gb|KK962044.1|	12705-91365	19	5.6	3	1	-1	0.3837	no
gi|646750341|gb|KK962044.1|	14716-91533	19	5.6	55	30	-0.881341	0.486	no
gi|646750341|gb|KK962044.1|	15682-91644	19	5.6	2	0.258279	-3	0.1994	no
gi|646750341|gb|KK962044.1|	16714-91692	19	5.6	14	91	3	0.25275	no
gi|646750341|gb|KK962044.1|	31070-93133	19	5.6	11	27	1	0.3852	no
gi|646750341|gb|KK962044.1|	44918-94506	19	5.6	235	253	0.104083	0.90055	no
gi|646750341|gb|KK962044.1|	54661-95539	19	5.6	2	0.269545	-3	0.1923	no
gi|646750354|gb|KK962043.1|	01344-10206	20	9.2	0	58	inf	5.00E-05	yes
gi|646750354|gb|KK962043.1|	26354-12848	20	9.2	2	2	-0.188802	0.90495	no
gi|646750354|gb|KK962043.1|	29003-13044	20	9.2	14	11	-0.310611	0.8356	no
gi|646750354|gb|KK962043.1|	30982-13949	20	9.2	23	12	-0.912196	0.43965	no
gi|646750354|gb|KK962043.1|	49146-14981	20	9.2	0	100	inf	5.00E-05	yes
gi|646750354|gb|KK962043.1|	54080-15700	20	9.2	0.24238	80	8	0.02865	no
gi|646750354|gb|KK962043.1|	76625-17721	20	9.2	2	2	-0.0246866	0.92345	no
gi|646750354|gb|KK962043.1|	92706-19370	20	9.2	13	10	-0.381062	0.80135	no
gi|646750354|gb|KK962043.1|	93841-19561	20	9.2	0.91118	0.61469	-0.567878	1	no
gi|646750354|gb|KK962043.1|	97088-19729	20	9.2	42	40	-0.0672065	0.918	no
gi|646750354|gb|KK962043.1|	97511-19812	20	9.2	79	162.78	1	0.34765	no
gi|646750354|gb|KK962043.1|	98444-19895	20	9.2	99	111	0.166744	0.87505	no
gi|646750354|gb|KK962043.1|	12496-21340	20	9.2	23	8	-1	0.3469	no
gi|646750354|gb|KK962043.1|	14709-21494	20	9.2	27	7.03	-2	0.2773	no
gi|646750354|gb|KK962043.1|	17377-21760	20	9.2	40	24	-0.691533	0.61855	no
gi|646750354|gb|KK962043.1|	28330-22988	20	9.2	19	12	-0.659207	0.59975	no
gi|646750354|gb|KK962043.1|	33417-23785	20	9.2	17	12	-0.496242	0.6613	no
gi|646750354|gb|KK962043.1|	43723-24497	20	9.2	0.831755	0.795623	-0.0640743	1	no

gi|646750354|gb|KK962043.1|	58022-25883	20	9.2	0.493756	2	2	0.18805	no
gi|646750354|gb|KK962043.1|	62882-26338	20	9.2	3	11	2	0.2719	no
gi|646750354|gb|KK962043.1|	76412-27699	20	9.2	3	1	-1	0.4702	no
gi|646750354|gb|KK962043.1|	87905-28857	20	9.2	6	9	0.579691	0.7169	no
gi|646750354|gb|KK962043.1|	97549-29920	20	9.2	65	24	-1	0.2137	no
gi|646750354|gb|KK962043.1|	08086-31018	20	9.2	0.301686	0.36433	0.272199	1	no
gi|646750354|gb|KK962043.1|	17035-31785	20	9.2	4	4	-0.0880267	0.9551	no
gi|646750354|gb|KK962043.1|	24387-32495	20	9.2	78	20	-2	0.1507	no
gi|646750354|gb|KK962043.1|	26751-32702	20	9.2	33	43	0.41599	0.7562	no
gi|646750354|gb|KK962043.1|	29821-33086	20	9.2	0.94393	0.504863	-0.902787	1	no
gi|646750354|gb|KK962043.1|	10221-41044	20	9.2	124	188.33	0.597823	0.705	no
gi|646750354|gb|KK962043.1|	14842-41684	20	9.2	0.528364	0.918275	0.797394	1	no
gi|646750354|gb|KK962043.1|	18435-42012	20	9.2	1	3	1	0.3659	no
gi|646750354|gb|KK962043.1|	22000-42229	20	9.2	8	9	0.215279	0.8895	no
gi|646750354|gb|KK962043.1|	25007-43011	20	9.2	70	70	0.00123231	0.9991	no
gi|646750354|gb|KK962043.1|	38022-43828	20	9.2	14	21	0.554443	0.7144	no
gi|646750354|gb|KK962043.1|	39519-43996	20	9.2	12	14	0.280983	0.8576	no
gi|646750354|gb|KK962043.1|	54733-45509	20	9.2	28	36	0.364072	0.8078	no
gi|646750354|gb|KK962043.1|	55584-45615	20	9.2	3	2	-0.546095	0.71625	no
gi|646750354|gb|KK962043.1|	64824-46542	20	9.2	3	2	-0.800785	0.5828	no
gi|646750354|gb|KK962043.1|	73997-47424	20	9.2	52	85	0.708972	0.61035	no
gi|646750354|gb|KK962043.1|	80132-48064	20	9.2	24	26	0.151069	0.9186	no
gi|646750354|gb|KK962043.1|	93400-49363	20	9.2	50	49	-0.0425686	0.95795	no
gi|646750354|gb|KK962043.1|	96978-49731	20	9.2	48	53	0.145209	0.92285	no
gi|646750354|gb|KK962043.1|	97682-49915	20	9.2	6	6	-0.0237634	0.98885	no
gi|646750354|gb|KK962043.1|	13276-51449	20	9.2	1	1	0.230181	0.8612	no
gi|646750354|gb|KK962043.1|	22536-52355	20	9.2	1	2	0.221916	0.8758	no
gi|646750354|gb|KK962043.1|	23663-52481	20	9.2	0.931285	0.296174	-2	1	no
gi|646750354|gb|KK962043.1|	26855-52899	20	9.2	0.834373	1	0.840499	0.54645	no
gi|646750354|gb|KK962043.1|	29823-53249	20	9.2	2	4	1	0.4789	no

gi|646750354|gb|KK962043.1|	38194-53921	20	9.2	82	49	-0.756559	0.50365	no
gi|646750354|gb|KK962043.1|	45317-54675	20	9.2	1	3	0.801279	0.55965	no
gi|646750354|gb|KK962043.1|	51041-55417	20	9.2	18	15	-0.305035	0.78745	no
gi|646750354|gb|KK962043.1|	64757-56742	20	9.2	0.995653	1	0.00882618	1	no
gi|646750354|gb|KK962043.1|	67544-57223	20	9.2	1	2	0.419807	0.78325	no
gi|646750354|gb|KK962043.1|	80195-58092	20	9.2	1	2	0.485852	0.73295	no
gi|646750354|gb|KK962043.1|	82587-58425	20	9.2	0.5876	1	0.945145	0.48355	no
gi|646750354|gb|KK962043.1|	72381-77391	20	9.2	2	2	0.0207485	0.97905	no
gi|646750354|gb|KK962043.1|	84653-78890	20	9.2	2	4	0.882964	0.5648	no
gi|646750354|gb|KK962043.1|	14444-81612	20	9.2	0.585123	0.844994	0.530202	1	no
gi|646750372|gb|KK962042.1|	96647-19704	18	9.5	37	17	-1	0.4914	no
gi|646750372|gb|KK962042.1|	77672-27849	18	9.5	3	6	0.994339	0.49085	no
gi|646750372|gb|KK962042.1|	73519-37513	18	9.5	4	4	0.150595	0.9201	no
gi|646750372|gb|KK962042.1|	68489-77094	18	9.5	10	13	0.394336	0.7086	no
gi|646750372|gb|KK962042.1|	71805-77380	18	9.5	12	13	0.144361	0.8927	no
gi|646750372|gb|KK962042.1|	75159-77723	18	9.5	6	5	-0.397879	0.79455	no
gi|646750389|gb|KK962041.1|	11073-11459	21	9.5	5	3	-0.699809	0.64095	no
gi|646750389|gb|KK962041.1|	17384-11898	21	9.5	3	4	0.175332	0.9108	no
gi|646750389|gb|KK962041.1|	1201-3037	21	9.5	2	3	0.610421	0.6936	no
gi|646750389|gb|KK962041.1|	35376-13683	21	9.5	5	10	0.92466	0.54475	no
gi|646750389|gb|KK962041.1|	47294-14801	21	9.5	13	31	1	0.4283	no
gi|646750389|gb|KK962041.1|	49188-14991	21	9.5	69	87	0.335769	0.75925	no
gi|646750389|gb|KK962041.1|	12260-21429	21	9.5	0.674547	4	2	0.11115	no
gi|646750389|gb|KK962041.1|	15164-21547	21	9.5	2	74	5	0.2369	no
gi|646750389|gb|KK962041.1|	76836-27728	21	9.5	5	6	0.262514	0.84835	no
gi|646750389|gb|KK962041.1|	79289-28129	21	9.5	17	13	-0.369091	0.7324	no
gi|646750389|gb|KK962041.1|	81829-28360	21	9.5	6	4	-0.571938	0.70755	no
gi|646750389|gb|KK962041.1|	84707-28634	21	9.5	0.670878	0.387344	-0.792433	1	no
gi|646750389|gb|KK962041.1|	00820-30142	21	9.5	6	27	2	0.19275	no
gi|646750389|gb|KK962041.1|	01783-30350	21	9.5	42	57	0.436992	0.69805	no

gi|646750389|gb|KK962041.1|	03733-30547	21	9.5	2	6	2	0.2827	no
gi|646750389|gb|KK962041.1|	05796-30708	21	9.5	0.269709	1	2	1	no
gi|646750389|gb|KK962041.1|	16787-31738	21	9.5	2	4	1	0.31165	no
gi|646750389|gb|KK962041.1|	22582-32283	21	9.5	11	21	0.919636	0.58385	no
gi|646750389|gb|KK962041.1|	26170-32862	21	9.5	2	4	0.742865	0.62195	no
gi|646750389|gb|KK962041.1|	30313-33097	21	9.5	4	7	0.86782	0.54085	no
gi|646750389|gb|KK962041.1|	32080-33231	21	9.5	20	45	1	0.41505	no
gi|646750389|gb|KK962041.1|	33010-33322	21	9.5	15	46	2	0.34985	no
gi|646750389|gb|KK962041.1|	39938-34017	21	9.5	17	76	2	0.2256	no
gi|646750389|gb|KK962041.1|	42006-34226	21	9.5	55	91	0.732367	0.58975	no
gi|646750389|gb|KK962041.1|	44928-34521	21	9.5	32	74	1	0.3815	no
gi|646750389|gb|KK962041.1|	51626-35293	21	9.5	14	5	-1	0.3387	no
gi|646750389|gb|KK962041.1|	54314-35471	21	9.5	38	22	-0.811433	0.5947	no
gi|646750389|gb|KK962041.1|	64603-36586	21	9.5	3	3	-0.107112	0.94075	no
gi|646750389|gb|KK962041.1|	66180-36948	21	9.5	0.51535	0.397264	-0.375456	1	no
gi|646750389|gb|KK962041.1|	75929-37678	21	9.5	24	17	-0.444567	0.7627	no
gi|646750389|gb|KK962041.1|	95131-39674	21	9.5	50	15	-2	0.1178	no
gi|646750389|gb|KK962041.1|	97049-39834	21	9.5	3	4	0.60967	0.6954	no
gi|646750389|gb|KK962041.1|	98814-40037	21	9.5	1	1	-0.334205	0.7821	no
gi|646750389|gb|KK962041.1|	00455-40554	21	9.5	1	0.670507	-1	0.50935	no
gi|646750389|gb|KK962041.1|	11844-41229	21	9.5	15	13	-0.203333	0.89845	no
gi|646750389|gb|KK962041.1|	21686-42187	21	9.5	112.87	55	-1	0.4616	no
gi|646750389|gb|KK962041.1|	24223-42444	21	9.5	29	49	0.741252	0.59315	no
gi|646750389|gb|KK962041.1|	34544-43489	21	9.5	50	46	-0.104682	0.9457	no
gi|646750389|gb|KK962041.1|	38933-43919	21	9.5	23	49	1	0.47305	no
gi|646750389|gb|KK962041.1|	44933-44541	21	9.5	2	3	0.158671	0.9062	no
gi|646750389|gb|KK962041.1|	46072-45453	21	9.5	35	30	-0.20217	0.872	no
gi|646750389|gb|KK962041.1|	64552-46597	21	9.5	2	2	-0.118393	0.939	no
gi|646750389|gb|KK962041.1|	98520-60219	21	9.5	0.622218	0.354795	-0.810434	1	no
gi|646750389|gb|KK962041.1|	16104-61737	21	9.5	17	12	-0.463413	0.7626	no

gi|646750389|gb|KK962041.1|	18498-61932	21	9.5	11	12	0.164744	0.91185	no
gi|646750389|gb|KK962041.1|	21944-62218	21	9.5	31	33	0.0716327	0.94415	no
gi|646750389|gb|KK962041.1|	22440-62283	21	9.5	21	16	-0.402931	0.79765	no
gi|646750389|gb|KK962041.1|	25334-62751	21	9.5	16	8	-1	0.43495	no
gi|646750389|gb|KK962041.1|	28694-62932	21	9.5	6	6	0.055373	0.9696	no
gi|646750389|gb|KK962041.1|	32142-63236	21	9.5	45	27.25	-0.715655	0.61175	no
gi|646750389|gb|KK962041.1|	32713-63303	21	9.5	52	34	-0.640071	0.68325	no
gi|646750389|gb|KK962041.1|	36545-63932	21	9.5	13	7	-0.920504	0.4723	no
gi|646750389|gb|KK962041.1|	52714-65349	21	9.5	3	3	0.0448628	0.95575	no
gi|646750389|gb|KK962041.1|	55633-65661	21	9.5	1	0.546914	-1	0.3882	no
gi|646750389|gb|KK962041.1|	73085-67374	21	9.5	4	7	0.847472	0.5465	no
gi|646750389|gb|KK962041.1|	76726-67686	21	9.5	4475.77	1751.35	-1	0.38855	no
gi|646750389|gb|KK962041.1|	80293-68207	21	9.5	1	1	0.0463393	0.96875	no
gi|646750389|gb|KK962041.1|	83562-68595	21	9.5	84	32	-1	0.2414	no
gi|646750389|gb|KK962041.1|	97404-101434	21	9.5	6	0.964881	-3	0.07205	no
gi|646750409|gb|KK962040.1|	25718-10298	21	8.4	0.625959	21	5	0.0115	no
gi|646750409|gb|KK962040.1|	44063-10466	21	8.4	1	3	0.788811	0.6154	no
gi|646750409|gb|KK962040.1|	51311-10527	21	8.4	41	34	-0.29128	0.7943	no
gi|646750409|gb|KK962040.1|	59120-10600	21	8.4	0.57276	2	2	0.26425	no
gi|646750409|gb|KK962040.1|	63600-10645	21	8.4	7	1	-3	0.09485	no
gi|646750409|gb|KK962040.1|	73783-10751	21	8.4	1	1	-0.394197	0.75525	no
gi|646750409|gb|KK962040.1|	80615-10810	21	8.4	4	3	-0.578783	0.7155	no
gi|646750409|gb|KK962040.1|	84763-10851	21	8.4	6	2	-1	0.38765	no
gi|646750409|gb|KK962040.1|	55376-11566	21	8.4	2	0	-inf	0.0012	yes
gi|646750409|gb|KK962040.1|	76755-11777	21	8.4	3	3	0.174909	0.9141	no
gi|646750409|gb|KK962040.1|	12046-13692	21	8.4	28	36	0.361455	0.74345	no
gi|646750409|gb|KK962040.1|	05891-12065	21	8.4	3	2	-0.790595	0.5829	no
gi|646750409|gb|KK962040.1|	1343-2026	21	8.4	2	2	0.116816	0.92795	no
gi|646750409|gb|KK962040.1|	48000-14922	21	8.4	0.476851	1	1	1	no
gi|646750409|gb|KK962040.1|	02867-15031	21	8.4	18	7	-1	0.39135	no

gi|646750409|gb|KK962040.1|	85206-15853	21	8.4	58	366	3	0.1724	no
gi|646750409|gb|KK962040.1|	88466-15887	21	8.4	12	14	0.166551	0.9048	no
gi|646750409|gb|KK962040.1|	12349-16130	21	8.4	2	2	0.0738977	0.92975	no
gi|646750409|gb|KK962040.1|	66958-16722	21	8.4	93	23	-2	0.16995	no
gi|646750409|gb|KK962040.1|	72134-17243	21	8.4	6	17	2	0.35435	no
gi|646750409|gb|KK962040.1|	73988-17424	21	8.4	15	25	0.71858	0.6261	no
gi|646750409|gb|KK962040.1|	97572-19880	21	8.4	0.757496	0.678941	-0.157952	1	no
gi|646750409|gb|KK962040.1|	20715-21410	21	8.4	10	3	-2	0.2539	no
gi|646750409|gb|KK962040.1|	2131-5108	21	8.4	0.373886	1	2	0.26105	no
gi|646750409|gb|KK962040.1|	50720-25158	21	8.4	0.45417	1	2	0.2897	no
gi|646750409|gb|KK962040.1|	58708-36314	21	8.4	7	8	0.126908	0.90645	no
gi|646750409|gb|KK962040.1|	84793-38533	21	8.4	0.972176	4	2	0.19065	no
gi|646750409|gb|KK962040.1|	06896-40745	21	8.4	5	2	-1	0.32725	no
gi|646750409|gb|KK962040.1|	51082-45183	21	8.4	0.747378	12	4	0.1061	no
gi|646750409|gb|KK962040.1|	83692-48698	21	8.4	2	5	2	0.317	no
gi|646750409|gb|KK962040.1|	01119-50170	21	8.4	23	2	-4	0.05835	no
gi|646750409|gb|KK962040.1|	10676-61213	21	8.4	6	2	-2	0.29055	no
gi|646750409|gb|KK962040.1|	17486-61791	21	8.4	9	7	-0.456622	0.71955	no
gi|646750409|gb|KK962040.1|	22957-62367	21	8.4	2	29	4	0.03025	no
gi|646750409|gb|KK962040.1|	24077-62477	21	8.4	0.633296	4	3	0.13815	no
gi|646750409|gb|KK962040.1|	24885-62766	21	8.4	0.22011	5	5	0.05215	no
gi|646750409|gb|KK962040.1|	32285-63440	21	8.4	0.049554	2	5	0.09865	no
gi|646750409|gb|KK962040.1|	34603-63672	21	8.4	0.596244	2	2	0.2156	no
gi|646750409|gb|KK962040.1|	36833-63785	21	8.4	1	2	0.359314	0.79375	no
gi|646750409|gb|KK962040.1|	44648-64606	21	8.4	0.715913	0.457926	-0.64467	1	no
gi|646750409|gb|KK962040.1|	52977-65400	21	8.4	0.958599	0.68332	-0.488367	1	no
gi|646750409|gb|KK962040.1|	54109-65560	21	8.4	0.523093	0.646015	0.304501	1	no
gi|646750409|gb|KK962040.1|	6645-8263	21	8.4	10	16	0.593357	0.6318	no
gi|646750409|gb|KK962040.1|	88985-68964	21	8.4	0.462098	8	4	0.2466	no
gi|646750409|gb|KK962040.1|	94914-69647	21	8.4	0.566698	3	2	0.13805	no

gi|646750409|gb|KK962040.1|	22027-72377	21	8.4	9	0	-inf	5.00E-05	yes
gi|646750409|gb|KK962040.1|	25743-72609	21	8.4	14	0	-inf	0.0012	yes
gi|646750409|gb|KK962040.1|	28824-72925	21	8.4	11	0	-inf	0.0004	yes
gi|646750409|gb|KK962040.1|	32515-73540	21	8.4	13	0	-inf	5.00E-05	yes
gi|646750409|gb|KK962040.1|	38908-74541	21	8.4	26	0.129791	-8	0.01665	no
gi|646750409|gb|KK962040.1|	49214-75042	21	8.4	60	0.139415	-9	0.1665	no
gi|646750409|gb|KK962040.1|	52151-75378	21	8.4	43	0.096767	-9	0.1665	no
gi|646750409|gb|KK962040.1|	54691-75557	21	8.4	6	0	-inf	0.0006	yes
gi|646750409|gb|KK962040.1|	76739-77827	21	8.4	0.941057	0.521787	-0.850822	1	no
gi|646750409|gb|KK962040.1|	78593-78003	21	8.4	0.780693	0.786769	0.0111839	1	no
gi|646750409|gb|KK962040.1|	89182-78981	21	8.4	1	1	-0.114969	0.9034	no
gi|646750409|gb|KK962040.1|	04530-80540	21	8.4	4	9	1	0.42165	no
gi|646750409|gb|KK962040.1|	06621-80762	21	8.4	0.864525	2	0.872965	0.5462	no
gi|646750409|gb|KK962040.1|	08239-81084	21	8.4	6	10	0.88544	0.48665	no
gi|646750409|gb|KK962040.1|	15381-81617	21	8.4	1	2	1	0.4498	no
gi|646750409|gb|KK962040.1|	16236-82109	21	8.4	8	6	-0.52803	0.6271	no
gi|646750409|gb|KK962040.1|	21296-82250	21	8.4	0.584881	0.977916	0.741566	1	no
gi|646750409|gb|KK962040.1|	26392-82697	21	8.4	15	4	-2	0.1935	no
gi|646750409|gb|KK962040.1|	49604-85254	21	8.4	22	13	-0.745296	0.49675	no
gi|646750409|gb|KK962040.1|	58953-86278	21	8.4	3	2	-0.822033	0.582	no
gi|646750409|gb|KK962040.1|	63509-86558	21	8.4	3	1	-0.95042	0.5467	no
gi|646750409|gb|KK962040.1|	8697-9049	21	8.4	8	17	1	0.46055	no
gi|646750409|gb|KK962040.1|	81243-88166	21	8.4	4	40	3	0.0632	no
gi|646750409|gb|KK962040.1|	85890-88793	21	8.4	0.15549	6	5	0.10815	no
gi|646750409|gb|KK962040.1|	10892-91418	21	8.4	0.914535	4	2	0.1646	no
gi|646750409|gb|KK962040.1|	20814-92674	21	8.4	2	0.619288	-2	0.2254	no
gi|646750409|gb|KK962040.1|	29375-93177	21	8.4	3	0.437679	-3	0.1046	no
gi|646750409|gb|KK962040.1|	32689-93939	21	8.4	12	1	-3	0.03465	no
gi|646750409|gb|KK962040.1|	39598-94209	21	8.4	2	2	-0.103256	0.94725	no
gi|646750409|gb|KK962040.1|	43692-94664	21	8.4	2	1	-0.0759478	0.96355	no

gi|646750409|gb|KK962040.1|	46939-94831	21	8.4	0.497373	2	2	0.2329	no
gi|646750409|gb|KK962040.1|	49785-95089	21	8.4	0.651456	0.620801	-0.069538	1	no
gi|646750409|gb|KK962040.1|	54037-95508	21	8.4	2	0	-inf	0.00045	yes
gi|646750409|gb|KK962040.1|	55470-95853	21	8.4	12	0.095722	-7	0.10535	no
gi|646750409|gb|KK962040.1|	58839-95992	21	8.4	1	0	-inf	0.00475	no
gi|646750409|gb|KK962040.1|	60020-96180	21	8.4	2	0	-inf	0.0019	no
gi|646750409|gb|KK962040.1|	62680-96350	21	8.4	2	0	-inf	0.0041	no
gi|646750409|gb|KK962040.1|	64757-96567	21	8.4	97	0.787396	-7	0.0442	no
gi|646750409|gb|KK962040.1|	9806-10107	21	8.4	21	43	1	0.4659	no
gi|646750428|gb|KK962039.1|	101-880	21	7.8	1	0.496728	-2	0.38345	no
gi|646750428|gb|KK962039.1|	03174-10532	21	7.8	3	1	-1	0.46105	no
gi|646750428|gb|KK962039.1|	05522-10642	21	7.8	5	4	-0.457736	0.7761	no
gi|646750428|gb|KK962039.1|	07361-10889	21	7.8	3	2	-0.554715	0.72845	no
gi|646750428|gb|KK962039.1|	13892-11573	21	7.8	30	22	-0.434642	0.69125	no
gi|646750428|gb|KK962039.1|	16369-11743	21	7.8	1	2	0.376165	0.779	no
gi|646750428|gb|KK962039.1|	17532-11814	21	7.8	3	2	-0.53487	0.6957	no
gi|646750428|gb|KK962039.1|	12376-14141	21	7.8	1	0.885578	-0.731555	0.61545	no
gi|646750428|gb|KK962039.1|	24123-12440	21	7.8	19	14	-0.451922	0.7404	no
gi|646750428|gb|KK962039.1|	47398-14841	21	7.8	0.977589	0.870589	-0.167238	1	no
gi|646750428|gb|KK962039.1|	48575-14917	21	7.8	3	2	-0.652491	0.627	no
gi|646750428|gb|KK962039.1|	53051-15438	21	7.8	4	2	-1	0.5108	no
gi|646750428|gb|KK962039.1|	19664-20014	21	7.8	401	555.45	0.470766	0.6796	no
gi|646750428|gb|KK962039.1|	20719-22707	21	7.8	22	17	-0.349252	0.7457	no
gi|646750428|gb|KK962039.1|	56207-25664	21	7.8	4	3	-0.413234	0.8098	no
gi|646750428|gb|KK962039.1|	29533-30169	21	7.8	9	6	-0.529382	0.7403	no
gi|646750428|gb|KK962039.1|	48383-48968	21	7.8	2	1	-0.76374	0.6333	no
gi|646750428|gb|KK962039.1|	49164-50469	21	7.8	14	13	-0.0955631	0.9495	no
gi|646750428|gb|KK962039.1|	57573-59566	21	7.8	0.904937	0.308352	-2	1	no
gi|646750428|gb|KK962039.1|	61637-63903	21	7.8	6	2	-2	0.2921	no
gi|646750428|gb|KK962039.1|	6284-7704	21	7.8	0.476456	0.571443	0.262267	1	no

gi|646750428|gb|KK962039.1|	41054-64157	21	7.8	3	5	0.588988	0.64695	no
gi|646750428|gb|KK962039.1|	53299-65356	21	7.8	25	10	-1	0.4	no
gi|646750428|gb|KK962039.1|	54137-65629	21	7.8	0.825614	0.352043	-1	1	no
gi|646750428|gb|KK962039.1|	56408-65788	21	7.8	1	0.653796	-1	0.4176	no
gi|646750428|gb|KK962039.1|	59763-66481	21	7.8	37	19	-0.940609	0.4259	no
gi|646750428|gb|KK962039.1|	66207-67892	21	7.8	3	2	-0.536804	0.7327	no
gi|646750428|gb|KK962039.1|	68073-70162	21	7.8	3	2	-1	0.5042	no
gi|646750428|gb|KK962039.1|	75336-75891	21	7.8	7	12	0.722981	0.6459	no
gi|646750428|gb|KK962039.1|	79761-81278	21	7.8	5	3	-0.824095	0.58655	no
gi|646750428|gb|KK962039.1|	83293-84342	21	7.8	5	4	-0.124679	0.93815	no
gi|646750428|gb|KK962039.1|	88998-89695	21	7.8	6	3	-1	0.4534	no
gi|646750428|gb|KK962039.1|	90984-92266	21	7.8	2	1	-0.741353	0.60125	no
gi|646750428|gb|KK962039.1|	92396-93182	21	7.8	9	6	-0.710916	0.6445	no
gi|646750428|gb|KK962039.1|	93668-95397	21	7.8	3	3	-0.02362	0.98675	no
gi|646750428|gb|KK962039.1|	95720-96558	21	7.8	3	2	-0.349376	0.77885	no
gi|646750452|gb|KK962038.1|	28480-62926	17	9.1	0.879224	2	1	0.36955	no
gi|646750452|gb|KK962038.1|	31445-63168	17	9.1	32	34	0.0892219	0.93905	no
gi|646750452|gb|KK962038.1|	48441-64886	17	9.1	11	10	-0.202328	0.86465	no
gi|646750467|gb|KK962037.1|	31417-13266	21	5.4	1	2	0.842628	0.57475	no
gi|646750467|gb|KK962037.1|	86700-48724	21	5.4	16	8	-0.949868	0.5449	no
gi|646750467|gb|KK962037.1|	15276-82130	21	5.4	7	5	-0.641153	0.56165	no
gi|646750467|gb|KK962037.1|	22314-82355	21	5.4	9	7	-0.267955	0.8552	no
gi|646750484|gb|KK962036.1|	41011-14142	20	5.5	16	11	-0.571547	0.7334	no
gi|646750484|gb|KK962036.1|	49477-15439	20	5.5	12	8	-0.57761	0.60955	no
gi|646750484|gb|KK962036.1|	54586-15774	20	5.5	14	9	-0.568419	0.6021	no
gi|646750484|gb|KK962036.1|	58006-16081	20	5.5	9	8	-0.19501	0.85585	no
gi|646750484|gb|KK962036.1|	87223-18762	20	5.5	36	13	-1	0.3678	no
gi|646750484|gb|KK962036.1|	22730-23471	20	5.5	47	30	-0.664619	0.60415	no
gi|646750484|gb|KK962036.1|	43837-24432	20	5.5	10	4	-1	0.42015	no
gi|646750484|gb|KK962036.1|	74772-27528	20	5.5	5	1	-2	0.23925	no

gi|646750484|gb|KK962036.1|	29777-30248	20	5.5	23	11	-1	0.4878	no
gi|646750484|gb|KK962036.1|	09233-31007	20	5.5	5	4	-0.340376	0.8249	no
gi|646750484|gb|KK962036.1|	20920-32117	20	5.5	41	6	-3	0.1377	no
gi|646750484|gb|KK962036.1|	25850-32607	20	5.5	169.64	50	-2	0.2352	no
gi|646750484|gb|KK962036.1|	36265-33652	20	5.5	21	44	1	0.4886	no
gi|646750484|gb|KK962036.1|	43125-34377	20	5.5	3	6	1	0.4169	no
gi|646750484|gb|KK962036.1|	58789-35911	20	5.5	5	11	0.954462	0.5533	no
gi|646750484|gb|KK962036.1|	40389-42011	20	5.5	31	26	-0.232407	0.83195	no
gi|646750484|gb|KK962036.1|	10236-41221	20	5.5	0.913344	0.700156	-0.383482	1	no
gi|646750484|gb|KK962036.1|	4132-4615	20	5.5	2	5	1	0.35065	no
gi|646750484|gb|KK962036.1|	19124-42410	20	5.5	9	6	-0.535262	0.62525	no
gi|646750484|gb|KK962036.1|	25118-42630	20	5.5	2	2	-0.356041	0.7982	no
gi|646750484|gb|KK962036.1|	27443-43123	20	5.5	2	2	-0.359189	0.8122	no
gi|646750484|gb|KK962036.1|	36216-44123	20	5.5	11	7	-0.595299	0.5938	no
gi|646750484|gb|KK962036.1|	41339-44206	20	5.5	2	1	-1	0.4518	no
gi|646750484|gb|KK962036.1|	43330-44459	20	5.5	3	1	-1	0.3593	no
gi|646750484|gb|KK962036.1|	49375-45056	20	5.5	39	22	-0.819763	0.45275	no
gi|646750484|gb|KK962036.1|	56035-46284	20	5.5	13	12	-0.143124	0.90375	no
gi|646750484|gb|KK962036.1|	67745-48226	20	5.5	3	3	-0.0332949	0.9753	no
gi|646750484|gb|KK962036.1|	82372-48507	20	5.5	2	2	-0.234196	0.87485	no
gi|646750484|gb|KK962036.1|	06512-50672	20	5.5	19	50	1	0.3823	no
gi|646750484|gb|KK962036.1|	10901-51289	20	5.5	0.53201	0.693399	0.382232	1	no
gi|646750484|gb|KK962036.1|	15552-51640	20	5.5	0.777299	2	1	0.36955	no
gi|646750484|gb|KK962036.1|	16633-52034	20	5.5	1	1	0.229192	0.88205	no
gi|646750484|gb|KK962036.1|	41863-54209	20	5.5	32	8	-2	0.277	no
gi|646750484|gb|KK962036.1|	60638-61650	20	5.5	2	8	2	0.152	no
gi|646750484|gb|KK962036.1|	17676-61945	20	5.5	0.546708	0.526877	-0.0533047	1	no
gi|646750484|gb|KK962036.1|	62081-63209	20	5.5	0.741753	0.454681	-0.706086	1	no
gi|646750484|gb|KK962036.1|	21679-62794	20	5.5	5	3	-0.903439	0.47715	no
gi|646750484|gb|KK962036.1|	41008-64194	20	5.5	14	11	-0.29411	0.8446	no

gi|646750484|gb|KK962036.1|	43340-64355	20	5.5	77	102	0.41051	0.7799	no
gi|646750484|gb|KK962036.1|	59406-66167	20	5.5	5	2	-1	0.43765	no
gi|646750484|gb|KK962036.1|	62839-66675	20	5.5	2	2	-0.349269	0.81095	no
gi|646750484|gb|KK962036.1|	67220-67000	20	5.5	2	3	0.375536	0.8003	no
gi|646750484|gb|KK962036.1|	78219-67938	20	5.5	12	3	-2	0.1847	no
gi|646750484|gb|KK962036.1|	90392-69114	20	5.5	135	41	-2	0.13155	no
gi|646750484|gb|KK962036.1|	69672-76612	20	5.5	20	15	-0.418806	0.71995	no
gi|646750484|gb|KK962036.1|	05630-70908	20	5.5	26	15	-0.848165	0.4552	no
gi|646750484|gb|KK962036.1|	10313-71080	20	5.5	42	0	-inf	0.00045	yes
gi|646750484|gb|KK962036.1|	23116-72374	20	5.5	5	0	-inf	0.00525	no
gi|646750484|gb|KK962036.1|	25712-72652	20	5.5	2	0	-inf	0.0041	no
gi|646750484|gb|KK962036.1|	35184-73572	20	5.5	16	0	-inf	0.00045	yes
gi|646750484|gb|KK962036.1|	50460-76082	20	5.5	17	15	-0.212466	0.8589	no
gi|646750484|gb|KK962036.1|	62809-76562	20	5.5	3	4	0.636956	0.6768	no
gi|646750484|gb|KK962036.1|	68489-76878	20	5.5	10	12	0.229422	0.86985	no
gi|646750484|gb|KK962036.1|	69994-77030	20	5.5	10	10	0.062442	0.93325	no
gi|646750484|gb|KK962036.1|	83273-78476	20	5.5	0.894563	0.751895	-0.250653	1	no
gi|646750484|gb|KK962036.1|	86721-79036	20	5.5	0.956078	0.995081	0.0576858	1	no
gi|646750484|gb|KK962036.1|	03368-80429	20	5.5	0.967879	0.785118	-0.301917	1	no
gi|646750484|gb|KK962036.1|	14283-81456	20	5.5	15	15	-0.00632671	0.9333	no
gi|646750484|gb|KK962036.1|	89341-89016	20	5.5	10	11	0.169643	0.9113	no
gi|646750484|gb|KK962036.1|	94106-89477	20	5.5	11	10	-0.0926503	0.9517	no
gi|646750484|gb|KK962036.1|	99149-89973	20	5.5	2	1	-0.762947	0.6333	no
gi|646750484|gb|KK962036.1|	03194-90367	20	5.5	9	11	0.290461	0.85355	no
gi|646750505|gb|KK962035.1|	47314-11489	19	9.9	14	10.31	-0.415423	0.74365	no
gi|646750505|gb|KK962035.1|	53068-11541	19	9.9	0.422408	1	2	0.29375	no
gi|646750505|gb|KK962035.1|	54392-11578	19	9.9	2	2	0.621859	0.68055	no
gi|646750505|gb|KK962035.1|	67320-16846	19	9.9	0.725957	0.890466	0.294676	1	no
gi|646750505|gb|KK962035.1|	95531-19650	19	9.9	5	6	0.214933	0.8888	no
gi|646750505|gb|KK962035.1|	17183-21780	19	9.9	8	4	-0.991148	0.4941	no

gi|646750505|gb|KK962035.1|	36319-23925	19	9.9	18	13	-0.487506	0.65705	no
gi|646750505|gb|KK962035.1|	44133-24445	19	9.9	9	11	0.271659	0.84335	no
gi|646750505|gb|KK962035.1|	48949-24927	19	9.9	9	17	0.97683	0.4856	no
gi|646750505|gb|KK962035.1|	44345-44550	19	9.9	5	4	-0.570006	0.7192	no
gi|646750505|gb|KK962035.1|	45727-47100	19	9.9	2	3	0.661671	0.6302	no
gi|646750505|gb|KK962035.1|	35222-53718	19	9.9	1	1	0.459883	0.7335	no
gi|646750505|gb|KK962035.1|	65822-66119	19	9.9	73	27	-1	0.3841	no
gi|646750505|gb|KK962035.1|	76520-77142	19	9.9	3	1	-1	0.42175	no
gi|646750520|gb|KK962034.1|	45879-24606	13	7.3	208	168	-0.307528	0.80505	no
gi|646750520|gb|KK962034.1|	17265-52108	13	7.3	4	2	-1	0.41735	no
gi|646750542|gb|KK962033.1|	14063-14563	15	8.2	5	4	-0.37781	0.76955	no
gi|646750542|gb|KK962033.1|	70502-17157	15	8.2	27	2	-4	0.0058	no
gi|646750542|gb|KK962033.1|	17099-17847	15	8.2	3	1	-1	0.3737	no
gi|646750542|gb|KK962033.1|	73760-17441	15	8.2	19	0.958494	-4	0.0811	no
gi|646750542|gb|KK962033.1|	20814-52212	15	8.2	17	8	-1	0.48225	no
gi|646750542|gb|KK962033.1|	42652-54327	15	8.2	2	2	0.205031	0.89115	no
gi|646750542|gb|KK962033.1|	53988-85525	15	8.2	2	4	1	0.404	no
gi|646750557|gb|KK962032.1|	88516-11896	20	6.1	6	3	-1	0.45525	no
gi|646750557|gb|KK962032.1|	90199-11911	20	6.1	7	5	-0.577477	0.70405	no
gi|646750557|gb|KK962032.1|	26026-13265	20	6.1	2	3	1	0.48625	no
gi|646750557|gb|KK962032.1|	60894-13612	20	6.1	21	25	0.206775	0.8945	no
gi|646750557|gb|KK962032.1|	66035-13664	20	6.1	8	0	-inf	0.0041	no
gi|646750557|gb|KK962032.1|	86863-58768	20	6.1	2	0.925704	-1	0.4112	no
gi|646750557|gb|KK962032.1|	88363-58907	20	6.1	1	2	0.484122	0.7479	no
gi|646750557|gb|KK962032.1|	95090-59603	20	6.1	1	0.56829	-1	0.3882	no
gi|646750557|gb|KK962032.1|	76710-67762	20	6.1	6	13	1	0.4752	no
gi|646750557|gb|KK962032.1|	77808-68325	20	6.1	7	9	0.376458	0.73015	no
gi|646750557|gb|KK962032.1|	84557-68969	20	6.1	8	8	-0.0524512	0.9621	no
gi|646750557|gb|KK962032.1|	93144-69489	20	6.1	4	5	0.10237	0.9439	no
gi|646750557|gb|KK962032.1|	09828-71314	20	6.1	82	56	-0.558727	0.6439	no

gi|646750557|gb|KK962032.1|	16547-71951	20	6.1	0.477962	0.94364	0.98134	1	no
gi|646750557|gb|KK962032.1|	25926-72620	20	6.1	27	26	-0.0433004	0.9547	no
gi|646750557|gb|KK962032.1|	27683-72792	20	6.1	52	36	-0.51333	0.6912	no
gi|646750557|gb|KK962032.1|	28749-72895	20	6.1	63	67	0.0847966	0.9405	no
gi|646750557|gb|KK962032.1|	43272-74369	20	6.1	20	32	0.628819	0.6853	no
gi|646750557|gb|KK962032.1|	48459-74884	20	6.1	87	43	-1	0.5002	no
gi|646750557|gb|KK962032.1|	54070-75456	20	6.1	11	12	0.155647	0.9217	no
gi|646750557|gb|KK962032.1|	71243-77586	20	6.1	54	27	-0.975296	0.4127	no
gi|646750557|gb|KK962032.1|	83303-78399	20	6.1	12	4	-2	0.2625	no
gi|646750557|gb|KK962032.1|	96343-79707	20	6.1	2	0	-inf	0.0041	no
gi|646750557|gb|KK962032.1|	32278-83314	20	6.1	8	2	-2	0.2109	no
gi|646750557|gb|KK962032.1|	33272-83437	20	6.1	3	1	-1	0.35015	no
gi|646750557|gb|KK962032.1|	60084-86212	20	6.1	44	25	-0.780379	0.49315	no
gi|646750557|gb|KK962032.1|	63969-86474	20	6.1	1	1	0.00712276	0.92345	no
gi|646750557|gb|KK962032.1|	82351-88298	20	6.1	2	0.335188	-3	0.21605	no
gi|646750600|gb|KK962030.1|	00881-10015	23	8.6	4	0.627244	-3	0.15135	no
gi|646750600|gb|KK962030.1|	02843-10039	23	8.6	2	0.592348	-2	0.26425	no
gi|646750600|gb|KK962030.1|	06543-10071	23	8.6	17	0.370639	-6	0.16885	no
gi|646750600|gb|KK962030.1|	43880-10442	23	8.6	45	20	-1	0.45455	no
gi|646750600|gb|KK962030.1|	45138-10457	23	8.6	2	3	0.292571	0.83225	no
gi|646750600|gb|KK962030.1|	46255-10468	23	8.6	6	4	-0.592897	0.6637	no
gi|646750600|gb|KK962030.1|	59353-10596	23	8.6	40	32	-0.325824	0.7864	no
gi|646750600|gb|KK962030.1|	65024-10653	23	8.6	47	13	-2	0.23275	no
gi|646750600|gb|KK962030.1|	79712-10806	23	8.6	0.523354	5	3	0.12865	no
gi|646750600|gb|KK962030.1|	06938-11072	23	8.6	1	31	5	0.10755	no
gi|646750600|gb|KK962030.1|	21577-11235	23	8.6	5	13	1	0.2565	no
gi|646750600|gb|KK962030.1|	27498-11277	23	8.6	29	9	-2	0.29845	no
gi|646750600|gb|KK962030.1|	18278-12194	23	8.6	1	2	0.866568	0.50195	no
gi|646750600|gb|KK962030.1|	41802-12433	23	8.6	13	10	-0.308355	0.8361	no
gi|646750600|gb|KK962030.1|	58299-12588	23	8.6	4	1	-2	0.2829	no

gi|646750600|gb|KK962030.1|	90641-12909	23	8.6	8	20	1	0.33665	no
gi|646750600|gb|KK962030.1|	15541-13158	23	8.6	9	5	-0.885874	0.5642	no
gi|646750600|gb|KK962030.1|	19139-13194	23	8.6	83	49	-0.777481	0.60905	no
gi|646750600|gb|KK962030.1|	23561-13244	23	8.6	0.138062	5	5	0.1016	no
gi|646750600|gb|KK962030.1|	24625-13262	23	8.6	0.069403	3	6	0.0977	no
gi|646750600|gb|KK962030.1|	26395-13271	23	8.6	0.173212	4	5	0.11045	no
gi|646750600|gb|KK962030.1|	31541-13321	23	8.6	0	7	inf	0.00435	no
gi|646750600|gb|KK962030.1|	59474-13604	23	8.6	2	24	4	0.02875	no
gi|646750600|gb|KK962030.1|	71995-13732	23	8.6	2	6	2	0.20225	no
gi|646750600|gb|KK962030.1|	73339-13741	23	8.6	3	3	-0.170873	0.88095	no
gi|646750600|gb|KK962030.1|	46626-14475	23	8.6	13	6	-0.985256	0.52685	no
gi|646750600|gb|KK962030.1|	48843-14492	23	8.6	3	5	1	0.46125	no
gi|646750600|gb|KK962030.1|	68305-14697	23	8.6	20	22	0.165542	0.87355	no
gi|646750600|gb|KK962030.1|	29750-15305	23	8.6	1	0.696654	-0.725748	0.66415	no
gi|646750600|gb|KK962030.1|	55102-15575	23	8.6	7	8	0.176599	0.9074	no
gi|646750600|gb|KK962030.1|	57791-15598	23	8.6	1	0.374175	-2	0.31965	no
gi|646750600|gb|KK962030.1|	70057-15721	23	8.6	24.41	18	-0.429558	0.6965	no
gi|646750600|gb|KK962030.1|	72521-15735	23	8.6	1	0	-inf	0.0035	no
gi|646750600|gb|KK962030.1|	73788-15755	23	8.6	1	0	-inf	0.00045	yes
gi|646750600|gb|KK962030.1|	75753-15780	23	8.6	4	5	0.328939	0.82775	no
gi|646750600|gb|KK962030.1|	57851-15901	23	8.6	4	3	-0.192808	0.8996	no
gi|646750600|gb|KK962030.1|	79109-15824	23	8.6	0.940064	0.92725	-0.0198008	1	no
gi|646750600|gb|KK962030.1|	02276-16029	23	8.6	2	2	-0.254821	0.8594	no
gi|646750600|gb|KK962030.1|	13888-16148	23	8.6	2	0.582103	-1	0.3538	no
gi|646750600|gb|KK962030.1|	41781-18437	23	8.6	0	6	inf	0.0001	yes
gi|646750600|gb|KK962030.1|	79906-18809	23	8.6	0	2	inf	0.001	yes
gi|646750600|gb|KK962030.1|	81011-18816	23	8.6	0	3	inf	0.00255	no
gi|646750600|gb|KK962030.1|	98464-19936	23	8.6	7	1	-3	0.0989	no
gi|646750600|gb|KK962030.1|	81345-38177	23	8.6	6	29	2	0.1427	no
gi|646750600|gb|KK962030.1|	88484-38876	23	8.6	9	25	1	0.3308	no

gi|646750600|gb|KK962030.1|	93450-39612	23	8.6	19	29	0.584264	0.60695	no
gi|646750600|gb|KK962030.1|	54445-55494	23	8.6	6	0	-inf	0.0011	yes
gi|646750600|gb|KK962030.1|	17188-61774	23	8.6	16	8	-0.946917	0.54665	no
gi|646750600|gb|KK962030.1|	22484-62344	23	8.6	1	0.560466	-1	0.47685	no
gi|646750600|gb|KK962030.1|	29797-63029	23	8.6	21	2	-3	0.07415	no
gi|646750600|gb|KK962030.1|	49744-65087	23	8.6	2	2	-0.193943	0.86845	no
gi|646750600|gb|KK962030.1|	51056-65183	23	8.6	4	3	-0.128939	0.9125	no
gi|646750600|gb|KK962030.1|	58744-65926	23	8.6	32	8	-2	0.21885	no
gi|646750600|gb|KK962030.1|	60632-66197	23	8.6	0.170057	2	3	0.26815	no
gi|646750600|gb|KK962030.1|	64953-66777	23	8.6	0.539787	1	1	0.3787	no
gi|646750600|gb|KK962030.1|	70666-67224	23	8.6	9	5	-0.82758	0.58935	no
gi|646750600|gb|KK962030.1|	78232-67919	23	8.6	18	4	-2	0.15885	no
gi|646750600|gb|KK962030.1|	11423-71169	23	8.6	21	26	0.304055	0.81505	no
gi|646750600|gb|KK962030.1|	39580-74075	23	8.6	1	2	0.392456	0.75895	no
gi|646750600|gb|KK962030.1|	78378-77875	23	8.6	5	3	-0.880628	0.6013	no
gi|646750600|gb|KK962030.1|	87088-78810	23	8.6	14	10	-0.501603	0.74125	no
gi|646750600|gb|KK962030.1|	27445-82865	23	8.6	1	0.277689	-2	0.1971	no
gi|646750600|gb|KK962030.1|	40786-84225	23	8.6	9	10	0.103158	0.94335	no
gi|646750600|gb|KK962030.1|	54118-85625	23	8.6	4	3	-0.0493448	0.97245	no
gi|646750600|gb|KK962030.1|	56600-85978	23	8.6	5	7	0.55057	0.6562	no
gi|646750600|gb|KK962030.1|	59849-86249	23	8.6	11	13	0.31508	0.7697	no
gi|646750600|gb|KK962030.1|	76037-87970	23	8.6	11	14	0.282908	0.7945	no
gi|646750600|gb|KK962030.1|	13191-91338	23	8.6	74	16.89	-2	0.22755	no
gi|646750600|gb|KK962030.1|	13920-91626	23	8.6	2	0.449484	-2	0.18445	no
gi|646750600|gb|KK962030.1|	16529-91825	23	8.6	1	0.454049	-1	1	no
gi|646750600|gb|KK962030.1|	19948-92120	23	8.6	4	9	1	0.49105	no
gi|646750600|gb|KK962030.1|	22263-92420	23	8.6	14	2	-3	0.0737	no
gi|646750600|gb|KK962030.1|	31244-93179	23	8.6	27	13	-1	0.48945	no
gi|646750600|gb|KK962030.1|	35694-93800	23	8.6	8	5	-0.830174	0.58775	no
gi|646750600|gb|KK962030.1|	38200-93940	23	8.6	11	11	-0.00703433	0.99385	no

gi|646750600|gb|KK962030.1|	39593-93995	23	8.6	10	12	0.212781	0.8625	no
gi|646750600|gb|KK962030.1|	40722-94105	23	8.6	20	16	-0.260444	0.8324	no
gi|646750600|gb|KK962030.1|	41574-94208	23	8.6	14	16	0.184608	0.9057	no
gi|646750600|gb|KK962030.1|	43649-94420	23	8.6	4	5	0.56699	0.6607	no
gi|646750600|gb|KK962030.1|	47961-94868	23	8.6	10	12	0.17787	0.90325	no
gi|646750600|gb|KK962030.1|	51858-95250	23	8.6	2	2	-0.0118132	0.92345	no
gi|646750600|gb|KK962030.1|	52837-96078	23	8.6	5	2	-0.973279	0.4505	no
gi|646750600|gb|KK962030.1|	73551-97420	23	8.6	6	3	-1	0.45715	no
gi|646750600|gb|KK962030.1|	)5286-10007(	23	8.6	66	8	-3	0.01635	no
gi|646750622|gb|KK962029.1|	36811-43745	17	10	4	4	0.0249803	0.9559	no
gi|646750622|gb|KK962029.1|	82968-68378	17	10	5	0.691918	-3	0.15925	no
gi|646750622|gb|KK962029.1|	48439-74925	17	10	0	40	inf	5.00E-05	yes
gi|646750622|gb|KK962029.1|	52012-75245	17	10	11	0	-inf	0.00495	no
gi|646750622|gb|KK962029.1|	89506-78973	17	10	21	25	0.300609	0.84205	no
gi|646750622|gb|KK962029.1|	91286-79229	17	10	0	12	inf	5.00E-05	yes
gi|646750622|gb|KK962029.1|	92358-79285	17	10	6	2	-2	0.23925	no
gi|646750643|gb|KK962028.1|	05476-10672	21	7.5	0.56123	0.93924	0.742901	1	no
gi|646750643|gb|KK962028.1|	25527-12586	21	7.5	16	30	0.874959	0.53375	no
gi|646750643|gb|KK962028.1|	29119-12943	21	7.5	44	42	-0.0896064	0.9517	no
gi|646750643|gb|KK962028.1|	13659-14554	21	7.5	2	0.614619	-1	0.35375	no
gi|646750643|gb|KK962028.1|	36678-13750	21	7.5	3	4	0.192369	0.8902	no
gi|646750643|gb|KK962028.1|	39500-13998	21	7.5	5	9	0.837228	0.5687	no
gi|646750643|gb|KK962028.1|	45670-14594	21	7.5	247.32	122	-1	0.50115	no
gi|646750643|gb|KK962028.1|	15802-16788	21	7.5	1	1	0.146803	0.9186	no
gi|646750643|gb|KK962028.1|	64964-16670	21	7.5	0.749108	1	0.646562	0.60805	no
gi|646750643|gb|KK962028.1|	16993-20482	21	7.5	43	18	-1	0.2773	no
gi|646750643|gb|KK962028.1|	90534-19149	21	7.5	2	2	-0.570259	0.65795	no
gi|646750643|gb|KK962028.1|	42213-24291	21	7.5	0.620588	5	3	0.1622	no
gi|646750643|gb|KK962028.1|	26113-27850	21	7.5	4	3	-0.638841	0.6766	no
gi|646750643|gb|KK962028.1|	62260-26290	21	7.5	3	0.990968	-2	0.26205	no

gi|646750643|gb|KK962028.1|	80320-28104	21	7.5	1	1	-0.0989698	0.9036	no
gi|646750643|gb|KK962028.1|	28180-28622	21	7.5	4	2	-1	0.45555	no
gi|646750643|gb|KK962028.1|	88869-28997	21	7.5	2	7	2	0.15805	no
gi|646750643|gb|KK962028.1|	02754-30423	21	7.5	0.678577	0.651703	-0.0582969	1	no
gi|646750643|gb|KK962028.1|	05907-30889	21	7.5	48	25	-0.929634	0.4242	no
gi|646750643|gb|KK962028.1|	13602-31400	21	7.5	17	13	-0.399971	0.8045	no
gi|646750643|gb|KK962028.1|	31097-33193	21	7.5	6	4	-0.616766	0.69825	no
gi|646750643|gb|KK962028.1|	35012-33521	21	7.5	152	75	-1	0.40685	no
gi|646750643|gb|KK962028.1|	34212-36360	21	7.5	1	0.779348	-0.464285	1	no
gi|646750643|gb|KK962028.1|	50969-35121	21	7.5	20	15	-0.376331	0.81185	no
gi|646750643|gb|KK962028.1|	77696-37810	21	7.5	29	18	-0.7103	0.65565	no
gi|646750643|gb|KK962028.1|	3779-6680	21	7.5	0.664352	2	1	0.3707	no
gi|646750643|gb|KK962028.1|	98337-39904	21	7.5	0.823775	3	2	0.2977	no
gi|646750643|gb|KK962028.1|	05166-40617	21	7.5	1	2	0.373222	0.779	no
gi|646750643|gb|KK962028.1|	45964-44876	21	7.5	4	2	-0.816667	0.5907	no
gi|646750643|gb|KK962028.1|	48972-45141	21	7.5	1	0.735399	-0.520895	1	no
gi|646750643|gb|KK962028.1|	55516-45795	21	7.5	0.339206	0.615612	0.859861	1	no
gi|646750643|gb|KK962028.1|	74798-48610	21	7.5	4	5	0.556145	0.62175	no
gi|646750643|gb|KK962028.1|	47678-49072	21	7.5	0.812218	0.584236	-0.475314	1	no
gi|646750643|gb|KK962028.1|	12733-51347	21	7.5	19	12	-0.73852	0.6285	no
gi|646750643|gb|KK962028.1|	17451-51889	21	7.5	21	18	-0.231181	0.8253	no
gi|646750643|gb|KK962028.1|	24400-52486	21	7.5	21	20	-0.0730217	0.96025	no
gi|646750643|gb|KK962028.1|	41942-54261	21	7.5	79	91	0.209202	0.8471	no
gi|646750643|gb|KK962028.1|	65655-67648	21	7.5	1	0.616704	-1	0.45765	no
gi|646750643|gb|KK962028.1|	83337-68448	21	7.5	0.622249	0.742055	0.254034	1	no
gi|646750643|gb|KK962028.1|	86657-68706	21	7.5	15	31	1	0.49405	no
gi|646750643|gb|KK962028.1|	87619-68793	21	7.5	44	67	0.607001	0.69635	no
gi|646750643|gb|KK962028.1|	89484-68989	21	7.5	2	12	2	0.21895	no
gi|646750643|gb|KK962028.1|	90966-69128	21	7.5	28	45	0.673786	0.67045	no
gi|646750643|gb|KK962028.1|	92840-69438	21	7.5	2	6	1	0.3452	no

gi|646750643|gb|KK962028.1|	95251-69593	21	7.5	10	21	1	0.45025	no
gi|646750643|gb|KK962028.1|	70058-71388	21	7.5	0.774509	0.494648	-0.64688	1	no
gi|646750643|gb|KK962028.1|	00611-70395	21	7.5	85	68	-0.30458	0.7925	no
gi|646750643|gb|KK962028.1|	06082-71453	21	7.5	7	6	-0.19152	0.8639	no
gi|646750643|gb|KK962028.1|	15014-71625	21	7.5	1	0.537772	-1	0.45565	no
gi|646750643|gb|KK962028.1|	72878-73806	21	7.5	0.412341	2	2	0.2023	no
gi|646750643|gb|KK962028.1|	32615-73289	21	7.5	48	4	-3	0.1391	no
gi|646750643|gb|KK962028.1|	35798-73823	21	7.5	19	0.615054	-5	0.0051	no
gi|646750643|gb|KK962028.1|	61288-76178	21	7.5	18	8	-1	0.49345	no
gi|646750643|gb|KK962028.1|	65202-76571	21	7.5	6	6	0.133806	0.91655	no
gi|646750643|gb|KK962028.1|	70248-77079	21	7.5	3	5	0.708757	0.5867	no
gi|646750643|gb|KK962028.1|	77771-77982	21	7.5	148	54	-1	0.33235	no
gi|646750643|gb|KK962028.1|	87009-78906	21	7.5	0.924655	1	0.364936	0.78985	no
gi|646750643|gb|KK962028.1|	90449-79195	21	7.5	10	5	-0.984283	0.51295	no
gi|646750643|gb|KK962028.1|	32509-83397	21	7.5	24	11	-1	0.3673	no
gi|646750643|gb|KK962028.1|	83978-84273	21	7.5	217	42	-2	0.14425	no
gi|646750643|gb|KK962028.1|	40495-84080	21	7.5	24	8	-2	0.2357	no
gi|646750643|gb|KK962028.1|	56704-85769	21	7.5	1	2	0.372273	0.779	no
gi|646750643|gb|KK962028.1|	71231-87152	21	7.5	11	27	1	0.4319	no
gi|646750643|gb|KK962028.1|	72159-87246	21	7.5	28	18	-0.625408	0.60935	no
gi|646750643|gb|KK962028.1|	73390-87504	21	7.5	22	7	-2	0.2337	no
gi|646750643|gb|KK962028.1|	03750-90439	21	7.5	0.235732	6	5	0.1105	no
gi|646750643|gb|KK962028.1|	17711-91800	21	7.5	0	20	inf	0.0014	yes
gi|646750643|gb|KK962028.1|	98846-10529[illegible]	21	7.5	56	42	-0.412112	0.7339	no
gi|646750660|gb|KK962027.1|	71829-67289	13	6.7	7	9	0.317842	0.82615	no
gi|646750660|gb|KK962027.1|	85662-86176	13	6.7	3	10	2	0.2827	no
gi|646750680|gb|KK962026.1|	43594-34451	11	6.2	1	2	0.50523	0.7139	no
gi|646750702|gb|KK962025.1|	71992-47268	12	6.5	1	4	1	0.31035	no
gi|646750702|gb|KK962025.1|	10372-61129	12	6.5	1	0.58714	-1	0.4766	no
gi|646750702|gb|KK962025.1|	11890-61268	12	6.5	2	2	0.0789481	0.94685	no

gi|646750724|gb|KK962024.1|	09300-11029	11	7.4	0	3	inf	0.00435	no
gi|646750724|gb|KK962024.1|	17568-21823	11	7.4	1	2	0.213743	0.89085	no
gi|646750724|gb|KK962024.1|	18311-21935	11	7.4	2	2	0.0648421	0.954	no
gi|646750724|gb|KK962024.1|	99568-50004	11	7.4	4	2	-1	0.41965	no
gi|646750745|gb|KK962023.1|	01338-10032	18	9.4	0.330809	1	2	1	no
gi|646750745|gb|KK962023.1|	14352-10147	18	9.4	0.742074	10	4	0.1007	no
gi|646750745|gb|KK962023.1|	21247-10221	18	9.4	10	7	-0.475636	0.75555	no
gi|646750745|gb|KK962023.1|	22759-10239	18	9.4	0.66828	0.547536	-0.2875	1	no
gi|646750745|gb|KK962023.1|	01190-11024	18	9.4	1	0.524774	-0.938203	1	no
gi|646750745|gb|KK962023.1|	53619-11617	18	9.4	28	22	-0.326605	0.7827	no
gi|646750745|gb|KK962023.1|	61876-11627	18	9.4	0.742374	1	0.987448	0.5626	no
gi|646750745|gb|KK962023.1|	63440-11677	18	9.4	8	5	-0.606009	0.57225	no
gi|646750745|gb|KK962023.1|	80687-11819	18	9.4	2	2	-0.31418	0.8195	no
gi|646750745|gb|KK962023.1|	83475-11847	18	9.4	0.666904	0.81965	0.297529	1	no
gi|646750745|gb|KK962023.1|	90908-11933	18	9.4	2	0.84349	-1	0.3586	no
gi|646750745|gb|KK962023.1|	19954-72009	18	9.4	838	983	0.229752	0.87325	no
gi|646750745|gb|KK962023.1|	82185-78364	18	9.4	0.383551	0.883793	1	1	no
gi|646750745|gb|KK962023.1|	29863-83019	18	9.4	18	37	1	0.45195	no
gi|646750745|gb|KK962023.1|	40056-84164	18	9.4	26	47	0.863602	0.4506	no
gi|646750745|gb|KK962023.1|	63155-86493	18	9.4	24	18	-0.422327	0.6959	no
gi|646750745|gb|KK962023.1|	21081-92576	18	9.4	8	4	-1	0.41505	no
gi|646750745|gb|KK962023.1|	25902-93763	18	9.4	4	4	-0.0284961	0.97865	no
gi|646750745|gb|KK962023.1|	43842-94561	18	9.4	0.304727	0.880979	2	1	no
gi|646750745|gb|KK962023.1|	54743-95590	18	9.4	2	3	0.447067	0.74755	no
gi|646750745|gb|KK962023.1|	77874-97855	18	9.4	6	4	-0.679352	0.6194	no
gi|646750745|gb|KK962023.1|	81017-98209	18	9.4	14	12	-0.268137	0.8588	no
gi|646750745|gb|KK962023.1|	84676-98827	18	9.4	2	1	-0.145056	0.9229	no
gi|646750745|gb|KK962023.1|	89056-99091	18	9.4	1	2	0.458643	0.7383	no
gi|646750745|gb|KK962023.1|	91073-99272	18	9.4	1	0.476697	-1	0.292	no
gi|646750745|gb|KK962023.1|	94006-99718	18	9.4	0.791042	0.967218	0.290087	1	no

gi|646750745|gb|KK962023.1|	97452-99824	18	9.4	6	2	-2	0.2393	no
gi|646750745|gb|KK962023.1|	98594-99977	18	9.4	3	11	2	0.21035	no
gi|646750770|gb|KK962022.1|	39735-14020	14	7.7	5	5	-0.169333	0.90765	no
gi|646750770|gb|KK962022.1|	73386-37452	14	7.7	1	1	0.379288	0.7733	no
gi|646750770|gb|KK962022.1|	05212-40544	14	7.7	26	43	0.708982	0.6096	no
gi|646750795|gb|KK962021.1|	97623-19886	19	8.9	2	3	0.640706	0.6854	no
gi|646750795|gb|KK962021.1|	98918-19946	19	8.9	2	3	1	0.4503	no
gi|646750795|gb|KK962021.1|	26981-42777	19	8.9	0.685154	1	1	0.48525	no
gi|646750795|gb|KK962021.1|	82448-48458	19	8.9	8	3	-1	0.45765	no
gi|646750795|gb|KK962021.1|	84825-48605	19	8.9	2	1	-0.480012	0.7079	no
gi|646750795|gb|KK962021.1|	87664-48832	19	8.9	3	2	-0.846241	0.53425	no
gi|646750795|gb|KK962021.1|	94147-49520	19	8.9	0.808413	0.824066	0.027667	1	no
gi|646750795|gb|KK962021.1|	11143-51138	19	8.9	18	13	-0.488967	0.74775	no
gi|646750795|gb|KK962021.1|	15342-51699	19	8.9	6	5	-0.14154	0.92375	no
gi|646750795|gb|KK962021.1|	44991-54561	19	8.9	16	12	-0.444253	0.76695	no
gi|646750795|gb|KK962021.1|	49622-55089	19	8.9	3	3	-0.16442	0.91435	no
gi|646750795|gb|KK962021.1|	52907-55956	19	8.9	41	26	-0.628662	0.60085	no
gi|646750795|gb|KK962021.1|	60641-56109	19	8.9	3	6	1	0.4321	no
gi|646750795|gb|KK962021.1|	62001-56385	19	8.9	0.812559	0.503734	-0.689811	1	no
gi|646750795|gb|KK962021.1|	67429-56785	19	8.9	7	11	0.583321	0.68965	no
gi|646750795|gb|KK962021.1|	91224-59323	19	8.9	0.579546	0.610462	0.0749795	1	no
gi|646750795|gb|KK962021.1|	95446-59776	19	8.9	35	37	0.0958445	0.9344	no
gi|646750795|gb|KK962021.1|	02811-60418	19	8.9	0.577165	0.474332	-0.283087	1	no
gi|646750795|gb|KK962021.1|	04300-60660	19	8.9	0.496196	0.654508	0.399501	1	no
gi|646750795|gb|KK962021.1|	06827-60758	19	8.9	2	2	-0.0950211	0.93195	no
gi|646750795|gb|KK962021.1|	11990-61296	19	8.9	11	12	0.126612	0.93355	no
gi|646750795|gb|KK962021.1|	15123-61612	19	8.9	3	4	0.439614	0.7512	no
gi|646750795|gb|KK962021.1|	18719-61977	19	8.9	0.919311	2	1	0.3744	no
gi|646750795|gb|KK962021.1|	24310-62563	19	8.9	0.606343	0.497823	-0.284502	1	no
gi|646750795|gb|KK962021.1|	28590-62900	19	8.9	10	10	0.0986478	0.9378	no

gi|646750795|gb|KK962021.1|	30568-63167	19	8.9	0.54288	0.931201	0.778459	1	no
gi|646750795|gb|KK962021.1|	35988-63761	19	8.9	0.743899	1	0.654842	0.6075	no
gi|646750795|gb|KK962021.1|	98540-70070	19	8.9	9	5	-0.933664	0.53795	no
gi|646750795|gb|KK962021.1|	86620-88831	19	8.9	1	4	2	0.26735	no
gi|646750795|gb|KK962021.1|	92626-89312	19	8.9	3	20	3	0.0977	no
gi|646750795|gb|KK962021.1|	04607-90605	19	8.9	0.778067	3	2	0.17515	no
gi|646750795|gb|KK962021.1|	07677-90790	19	8.9	18	56	2	0.28385	no
gi|646750795|gb|KK962021.1|	10031-91134	19	8.9	6	11	0.932023	0.53835	no
gi|646750795|gb|KK962021.1|	20646-92127	19	8.9	5	13	1	0.45135	no
gi|646750820|gb|KK962020.1|	67256-26799	18	7	2	1	-0.681897	0.6459	no
gi|646750820|gb|KK962020.1|	68791-27025	18	7	12	8	-0.445313	0.7672	no
gi|646750820|gb|KK962020.1|	39832-34023	18	7	4	16	2	0.24065	no
gi|646750820|gb|KK962020.1|	52162-45536	18	7	1	3	1	0.48845	no
gi|646750820|gb|KK962020.1|	45339-45947	18	7	4	0.360858	-4	0.1755	no
gi|646750820|gb|KK962020.1|	56687-45739	18	7	2	5	1	0.3809	no
gi|646750820|gb|KK962020.1|	57519-45915	18	7	1	4	2	0.24325	no
gi|646750820|gb|KK962020.1|	63498-46453	18	7	1	0.842484	-0.750835	0.59275	no
gi|646750820|gb|KK962020.1|	67138-46845	18	7	1	1	-0.307332	0.8119	no
gi|646750820|gb|KK962020.1|	92522-49274	18	7	965	534	-0.853505	0.49505	no
gi|646750820|gb|KK962020.1|	01883-50218	18	7	75	51	-0.564697	0.71	no
gi|646750820|gb|KK962020.1|	07422-50801	18	7	22	8	-2	0.32885	no
gi|646750820|gb|KK962020.1|	10077-51027	18	7	140	54	-1	0.3006	no
gi|646750820|gb|KK962020.1|	10599-51080	18	7	29	98	2	0.2696	no
gi|646750820|gb|KK962020.1|	13702-51389	18	7	159	61	-1	0.3082	no
gi|646750820|gb|KK962020.1|	14222-51634	18	7	56	16	-2	0.1242	no
gi|646750820|gb|KK962020.1|	16893-51720	18	7	16	17	0.144714	0.9037	no
gi|646750820|gb|KK962020.1|	17623-51841	18	7	11	4	-2	0.3472	no
gi|646750820|gb|KK962020.1|	20361-52049	18	7	932	506	-0.880442	0.5828	no
gi|646750820|gb|KK962020.1|	57836-55984	18	7	0.528063	1	1	1	no
gi|646750820|gb|KK962020.1|	84266-58910	18	7	57	38	-0.581531	0.6209	no

gi|646750820|gb|KK962020.1|	93450-59447	18	7	29	27	-0.118559	0.9105	no
gi|646750820|gb|KK962020.1|	96380-59864	18	7	50	27	-0.867349	0.45515	no
gi|646750820|gb|KK962020.1|	06241-61022	18	7	35	46	0.39877	0.7364	no
gi|646750820|gb|KK962020.1|	14713-61550	18	7	1	3	1	0.34525	no
gi|646750820|gb|KK962020.1|	15620-61648	18	7	2	3	0.848469	0.50535	no
gi|646750820|gb|KK962020.1|	17529-61786	18	7	16	85	2	0.0948	no
gi|646750820|gb|KK962020.1|	19994-62081	18	7	0.814237	4	2	0.2192	no
gi|646750820|gb|KK962020.1|	27256-62762	18	7	0	11	inf	0.001	yes
gi|646750820|gb|KK962020.1|	37984-63887	18	7	0.28997	2	3	0.2655	no
gi|646750820|gb|KK962020.1|	40347-64065	18	7	71	105	0.575286	0.7019	no
gi|646750820|gb|KK962020.1|	42439-64540	18	7	37	45	0.254917	0.82775	no
gi|646750845|gb|KK962019.1|	28580-63010	15	7.3	9	20	1	0.4005	no
gi|646750869|gb|KK962018.1|	00740-10375	20	10	4	8	0.817096	0.5219	no
gi|646750869|gb|KK962018.1|	03873-10405	20	10	72	173	1	0.38535	no
gi|646750869|gb|KK962018.1|	04822-11034	20	10	88	103.54	0.241365	0.8407	no
gi|646750869|gb|KK962018.1|	20352-12068	20	10	53	2	-4	0.1131	no
gi|646750869|gb|KK962018.1|	35675-13594	20	10	12	9	-0.410809	0.8114	no
gi|646750869|gb|KK962018.1|	50879-15111	20	10	37	14.06	-1	0.36285	no
gi|646750869|gb|KK962018.1|	66342-16681	20	10	183	57	-2	0.2143	no
gi|646750869|gb|KK962018.1|	66921-16729	20	10	20	8	-1	0.3998	no
gi|646750869|gb|KK962018.1|	70006-17036	20	10	18	5	-2	0.2724	no
gi|646750869|gb|KK962018.1|	03253-20457	20	10	5	3	-0.397795	0.79265	no
gi|646750869|gb|KK962018.1|	21023-22290	20	10	8	3	-1	0.4196	no
gi|646750869|gb|KK962018.1|	24728-22613	20	10	4	5	0.146556	0.92185	no
gi|646750869|gb|KK962018.1|	67394-37078	20	10	2	0	-inf	0.00025	yes
gi|646750869|gb|KK962018.1|	38921-39095	20	10	135	461	2	0.26575	no
gi|646750869|gb|KK962018.1|	91211-39273	20	10	1	0	-inf	0.00095	yes
gi|646750869|gb|KK962018.1|	94959-39543	20	10	5	3	-0.456761	0.74105	no
gi|646750869|gb|KK962018.1|	95728-39730	20	10	0.770946	0.202184	-2	1	no
gi|646750869|gb|KK962018.1|	97600-39859	20	10	2	1	-1	0.41345	no

gi|646750869|gb|KK962018.1|	99448-39972	20	10	35	4	-3	0.11735	no
gi|646750869|gb|KK962018.1|	38785-43929	20	10	4	3	-0.440691	0.74355	no
gi|646750869|gb|KK962018.1|	57840-45914	20	10	2	0.883342	-1	0.4637	no
gi|646750869|gb|KK962018.1|	75114-47686	20	10	2	3	0.673289	0.66745	no
gi|646750869|gb|KK962018.1|	47569-58292	20	10	5	6	0.0475686	0.9664	no
gi|646750869|gb|KK962018.1|	47494-54791	20	10	2	6	2	0.35625	no
gi|646750869|gb|KK962018.1|	58683-58969	20	10	22	38	0.828254	0.5699	no
gi|646750869|gb|KK962018.1|	59241-59534	20	10	242	415.5	0.778937	0.54235	no
gi|646750869|gb|KK962018.1|	76264-76645	20	10	84	197	1	0.34035	no
gi|646750869|gb|KK962018.1|	7931-8299	20	10	14	48	2	0.20355	no
gi|646750869|gb|KK962018.1|	79709-80457	20	10	11	21	0.92822	0.53975	no
gi|646750869|gb|KK962018.1|	83952-84794	20	10	4	10	1	0.4151	no
gi|646750869|gb|KK962018.1|	85403-85602	20	10	160	188	0.231459	0.8635	no
gi|646750869|gb|KK962018.1|	89710-99716	20	10	6	12	0.928268	0.4256	no
gi|646750889|gb|KK962017.1|	06657-10074	22	6.1	3	4	0.578714	0.6859	no
gi|646750889|gb|KK962017.1|	12885-10170	22	6.1	36	26	-0.458389	0.69785	no
gi|646750889|gb|KK962017.1|	44652-10454	22	6.1	2	2	0.646835	0.6364	no
gi|646750889|gb|KK962017.1|	52130-10527	22	6.1	2	3	0.320238	0.82475	no
gi|646750889|gb|KK962017.1|	32328-11329	22	6.1	6	8	0.331252	0.8355	no
gi|646750889|gb|KK962017.1|	20503-12341	22	6.1	15	4	-2	0.17925	no
gi|646750889|gb|KK962017.1|	26672-12734	22	6.1	2	0.614524	-2	0.2684	no
gi|646750889|gb|KK962017.1|	41465-14223	22	6.1	8	4	-1	0.5147	no
gi|646750889|gb|KK962017.1|	27578-14280	22	6.1	3	2	-0.811649	0.6134	no
gi|646750889|gb|KK962017.1|	19847-16202	22	6.1	3	4	0.400017	0.7939	no
gi|646750889|gb|KK962017.1|	34076-16359	22	6.1	9	13	0.504569	0.6861	no
gi|646750889|gb|KK962017.1|	36572-16383	22	6.1	1	0.960967	-0.33073	0.78235	no
gi|646750889|gb|KK962017.1|	39367-16461	22	6.1	26	18	-0.527049	0.66215	no
gi|646750889|gb|KK962017.1|	55016-16565	22	6.1	26	12	-1	0.3652	no
gi|646750889|gb|KK962017.1|	59280-16601	22	6.1	2	4	0.967463	0.5212	no
gi|646750889|gb|KK962017.1|	64848-16650	22	6.1	107	59	-0.870182	0.54145	no

gi|646750889|gb|KK962017.1|	67389-16697	22	6.1	0.536336	0.518852	-0.0478161	1	no
gi|646750889|gb|KK962017.1|	75309-16761	22	6.1	78	39	-0.997734	0.3712	no
gi|646750889|gb|KK962017.1|	22044-17232	22	6.1	7	5	-0.438459	0.7688	no
gi|646750889|gb|KK962017.1|	55483-17565	22	6.1	1	0.328828	-2	1	no
gi|646750889|gb|KK962017.1|	99683-18005	22	6.1	3	2	-0.495408	0.69035	no
gi|646750889|gb|KK962017.1|	11696-18137	22	6.1	2	2	0.630809	0.69155	no
gi|646750889|gb|KK962017.1|	13867-18151	22	6.1	2	3	0.875554	0.52225	no
gi|646750889|gb|KK962017.1|	22155-18256	22	6.1	2	3	0.609647	0.682	no
gi|646750889|gb|KK962017.1|	89584-19211	22	6.1	1	1	-0.0464296	0.97645	no
gi|646750889|gb|KK962017.1|	01824-19029	22	6.1	2	2	0.252731	0.8614	no
gi|646750889|gb|KK962017.1|	04888-19076	22	6.1	25	20	-0.297539	0.7904	no
gi|646750889|gb|KK962017.1|	23924-19248	22	6.1	1	0.948474	-0.171028	1	no
gi|646750889|gb|KK962017.1|	92491-19547	22	6.1	2	2	-0.196138	0.8985	no
gi|646750889|gb|KK962017.1|	29785-19300	22	6.1	85	62	-0.443901	0.75225	no
gi|646750889|gb|KK962017.1|	49375-19522	22	6.1	0.430157	0.521262	0.277147	1	no
gi|646750889|gb|KK962017.1|	52701-19531	22	6.1	3	2	-0.392254	0.80985	no
gi|646750889|gb|KK962017.1|	56903-19607	22	6.1	3	2	-0.335622	0.82445	no
gi|646750889|gb|KK962017.1|	96494-20160	22	6.1	2	2	0.0553218	0.9709	no
gi|646750889|gb|KK962017.1|	76373-19785	22	6.1	2	0.829835	-1	0.4589	no
gi|646750889|gb|KK962017.1|	78693-19801	22	6.1	1	1	-0.184849	0.8801	no
gi|646750889|gb|KK962017.1|	82568-19847	22	6.1	0.885803	0.998675	0.17303	1	no
gi|646750889|gb|KK962017.1|	85648-19881	22	6.1	1.78	1	-0.718063	0.65155	no
gi|646750889|gb|KK962017.1|	90102-19910	22	6.1	0.685199	1	0.768885	0.6143	no
gi|646750889|gb|KK962017.1|	99095-20019	22	6.1	13	13	-0.0329486	0.97605	no
gi|646750889|gb|KK962017.1|	27584-28366	22	6.1	2	0.494018	-2	0.1855	no
gi|646750889|gb|KK962017.1|	79994-28987	22	6.1	2	3	0.380818	0.72435	no
gi|646750889|gb|KK962017.1|	31249-33052	22	6.1	4	3	-0.601274	0.69315	no
gi|646750889|gb|KK962017.1|	33920-36389	22	6.1	9	4	-1	0.324	no
gi|646750889|gb|KK962017.1|	94349-49486	22	6.1	3	4	0.435399	0.7508	no
gi|646750889|gb|KK962017.1|	50447-51787	22	6.1	3	1	-1	0.3594	no

gi|646750889|gb|KK962017.1|	85520-58732	22	6.1	54	68	0.349159	0.76215	no
gi|646750889|gb|KK962017.1|	87957-58893	22	6.1	1	0.733158	-0.813324	0.57835	no
gi|646750889|gb|KK962017.1|	52578-65307	22	6.1	2	6	2	0.3219	no
gi|646750889|gb|KK962017.1|	65773-66636	22	6.1	0	15	inf	0.00075	yes
gi|646750889|gb|KK962017.1|	99880-70075	22	6.1	4	8	0.916979	0.56775	no
gi|646750889|gb|KK962017.1|	11247-71191	22	6.1	2	2	-0.00882902	0.92345	no
gi|646750889|gb|KK962017.1|	24060-72501	22	6.1	3	3	0.166391	0.90825	no
gi|646750889|gb|KK962017.1|	77276-77766	22	6.1	33	5	-3	0.0845	no
gi|646750889|gb|KK962017.1|	09438-81071	22	6.1	3	7	1	0.37175	no
gi|646750889|gb|KK962017.1|	12377-82050	22	6.1	37	30	-0.315973	0.7903	no
gi|646750889|gb|KK962017.1|	20862-82568	22	6.1	2	0.857134	-1	0.3676	no
gi|646750889|gb|KK962017.1|	29186-83094	22	6.1	0.984203	0.622341	-0.661249	1	no
gi|646750889|gb|KK962017.1|	31365-83257	22	6.1	0.964639	0.276558	-2	1	no
gi|646750889|gb|KK962017.1|	22812-92318	22	6.1	68	38	-0.848466	0.5772	no
gi|646750889|gb|KK962017.1|	92479-92745	22	6.1	43	22	-0.993295	0.45035	no
gi|646750889|gb|KK962017.1|	29783-93009	22	6.1	110	22	-2	0.16795	no
gi|646750889|gb|KK962017.1|	33600-93669	22	6.1	13	8	-0.812745	0.45455	no
gi|646750889|gb|KK962017.1|	58366-95879	22	6.1	13	3	-2	0.2649	no
gi|646750889|gb|KK962017.1|	70762-97157	22	6.1	3	1	-1	0.3294	no
gi|646750889|gb|KK962017.1|	90680-99118	22	6.1	4	1	-1	0.41965	no
gi|646750889|gb|KK962017.1|	94299-99451	22	6.1	34	28	-0.29051	0.8504	no
gi|646750910|gb|KK962016.1|	74893-47584	13	8.5	0.263916	25	7	0.23255	no
gi|646750910|gb|KK962016.1|	52179-53293	13	8.5	0.969223	2	0.80523	0.5518	no
gi|646750910|gb|KK962016.1|	53694-55498	13	8.5	2	2	-0.0894826	0.9528	no
gi|646750910|gb|KK962016.1|	56841-57185	13	8.5	7	9	0.305515	0.83395	no
gi|646750927|gb|KK962015.1|	24623-12498	15	5.4	12	8	-0.667103	0.6115	no
gi|646750927|gb|KK962015.1|	94278-39461	15	5.4	3	12	2	0.2031	no
gi|646750927|gb|KK962015.1|	20439-72111	15	5.4	7	3	-1	0.41045	no
gi|646750948|gb|KK962014.1|	09978-71101	14	8.4	10.37	6	-0.699203	0.64415	no
gi|646750948|gb|KK962014.1|	94802-79675	14	8.4	0.598283	0.944917	0.65936	1	no

gi|646750948|gb|KK962014.1|	65864-96634	14	8.4	2	3	0.159639	0.9062	no
gi|646750969|gb|KK962013.1|	36063-38233	16	7.4	13	7	-0.982418	0.43875	no
gi|646750969|gb|KK962013.1|	41123-46141	16	7.4	11	7	-0.651086	0.5579	no
gi|646750969|gb|KK962013.1|	37810-63800	16	7.4	481	0	-inf	0.0003	yes
gi|646750969|gb|KK962013.1|	43874-64499	16	7.4	3	0	-inf	0.00595	no
gi|646750969|gb|KK962013.1|	66681-66822	16	7.4	3	0	-inf	0.0023	no
gi|646750969|gb|KK962013.1|	68346-66932	16	7.4	4	0	-inf	0.0032	no
gi|646750969|gb|KK962013.1|	69461-66985	16	7.4	113	0	-inf	5.00E-05	yes
gi|646750969|gb|KK962013.1|	70049-67100	16	7.4	1	0	-inf	0.00655	no
gi|646750990|gb|KK962012.1|	74521-17558	14	10	1	0.984099	-0.0716754	1	no
gi|646750990|gb|KK962012.1|	85814-18652	14	10	2	1	-0.516928	0.73195	no
gi|646750990|gb|KK962012.1|	11043-21151	14	10	8	11	0.568157	0.68535	no
gi|646750990|gb|KK962012.1|	09364-51116	14	10	5	3	-0.642037	0.6685	no
gi|646750990|gb|KK962012.1|	11335-51246	14	10	2	3	0.144255	0.9295	no
gi|646750990|gb|KK962012.1|	35987-53672	14	10	12	8	-0.583431	0.7012	no
gi|646750990|gb|KK962012.1|	41385-54165	14	10	28	17	-0.731734	0.59925	no
gi|646750990|gb|KK962012.1|	09488-61000	14	10	26	14	-0.915674	0.5469	no
gi|646750990|gb|KK962012.1|	08950-80931	14	10	19	0	-inf	0.00495	no
gi|646751012|gb|KK962011.1|	80221-18861	22	5.6	3	4	0.39541	0.7183	no
gi|646751012|gb|KK962011.1|	91972-19878	22	5.6	19	17.23	-0.123269	0.913	no
gi|646751012|gb|KK962011.1|	05540-20754	22	5.6	1	2	0.654613	0.67895	no
gi|646751012|gb|KK962011.1|	11588-21343	22	5.6	0.697792	0.925229	0.407013	1	no
gi|646751012|gb|KK962011.1|	53849-25406	22	5.6	52	41	-0.330936	0.80105	no
gi|646751012|gb|KK962011.1|	59002-26187	22	5.6	1	1	-0.173364	0.90905	no
gi|646751012|gb|KK962011.1|	62007-26400	22	5.6	1	1	-0.117769	0.92995	no
gi|646751012|gb|KK962011.1|	64750-26551	22	5.6	1	1	-0.186917	0.90405	no
gi|646751012|gb|KK962011.1|	87028-28742	22	5.6	3	10	2	0.2381	no
gi|646751012|gb|KK962011.1|	36002-33619	22	5.6	96	77	-0.324018	0.8043	no
gi|646751012|gb|KK962011.1|	41826-34376	22	5.6	5	3	-0.556018	0.71235	no
gi|646751012|gb|KK962011.1|	57292-35752	22	5.6	32	37	0.211451	0.8835	no

gi|646751012|gb|KK962011.1|	76161-37644	22	5.6	20	17	-0.210453	0.8757	no
gi|646751012|gb|KK962011.1|	80276-38184	22	5.6	2	2	0.113345	0.94425	no
gi|646751012|gb|KK962011.1|	85481-38658	22	5.6	1	0.468278	-2	0.2858	no
gi|646751012|gb|KK962011.1|	3887-7704	22	5.6	0.623389	1	0.765967	1	no
gi|646751012|gb|KK962011.1|	97501-39802	22	5.6	4	8	1	0.48255	no
gi|646751012|gb|KK962011.1|	06732-40897	22	5.6	8	6	-0.523826	0.7304	no
gi|646751012|gb|KK962011.1|	16877-41774	22	5.6	0.900969	2	1	0.3983	no
gi|646751012|gb|KK962011.1|	26012-42625	22	5.6	68	71	0.0752215	0.9567	no
gi|646751012|gb|KK962011.1|	34791-43527	22	5.6	15	17	0.172304	0.91175	no
gi|646751012|gb|KK962011.1|	62596-46300	22	5.6	24	9	-1	0.3059	no
gi|646751012|gb|KK962011.1|	66451-46671	22	5.6	42	19	-1	0.3673	no
gi|646751012|gb|KK962011.1|	72470-47312	22	5.6	9	6	-0.676872	0.6757	no
gi|646751012|gb|KK962011.1|	74619-47579	22	5.6	2	1.88	-0.241005	0.86555	no
gi|646751012|gb|KK962011.1|	85629-48723	22	5.6	4	2	-1	0.38405	no
gi|646751012|gb|KK962011.1|	94432-49609	22	5.6	1	0.662258	-0.832541	0.51905	no
gi|646751012|gb|KK962011.1|	96213-49777	22	5.6	1	1	-0.182276	0.8806	no
gi|646751012|gb|KK962011.1|	04259-50577	22	5.6	0.586453	0.633944	0.112339	1	no
gi|646751012|gb|KK962011.1|	05977-50862	22	5.6	0.309947	0.563	0.861115	1	no
gi|646751012|gb|KK962011.1|	10227-51963	22	5.6	9	7	-0.314322	0.78445	no
gi|646751012|gb|KK962011.1|	25917-52827	22	5.6	4	14	2	0.18465	no
gi|646751012|gb|KK962011.1|	54495-55505	22	5.6	2	2	-0.0345289	0.92345	no
gi|646751012|gb|KK962011.1|	595-1118	22	5.6	2	2	-0.0465105	0.92345	no
gi|646751012|gb|KK962011.1|	97111-59760	22	5.6	1	5	2	0.20605	no
gi|646751012|gb|KK962011.1|	15218-61563	22	5.6	2	6	2	0.35625	no
gi|646751012|gb|KK962011.1|	15914-61623	22	5.6	80	120	0.579056	0.7072	no
gi|646751012|gb|KK962011.1|	18521-61885	22	5.6	27	53	0.97743	0.5315	no
gi|646751012|gb|KK962011.1|	21610-62246	22	5.6	0.465597	3	2	0.1544	no
gi|646751012|gb|KK962011.1|	40336-64060	22	5.6	9	27	2	0.29035	no
gi|646751012|gb|KK962011.1|	44914-64576	22	5.6	6	14	1	0.40845	no
gi|646751012|gb|KK962011.1|	46844-64718	22	5.6	41	87	1	0.48035	no

gi|646751012|gb|KK962011.1|	47803-64821	22	5.6	38	46	0.2718	0.8596	no
gi|646751012|gb|KK962011.1|	65658-66592	22	5.6	40	57	0.500999	0.71365	no
gi|646751012|gb|KK962011.1|	81796-68227	22	5.6	8	28	2	0.2064	no
gi|646751012|gb|KK962011.1|	82782-68475	22	5.6	128	115	-0.153458	0.8975	no
gi|646751012|gb|KK962011.1|	7980-8705	22	5.6	9	40	2	0.11365	no
gi|646751012|gb|KK962011.1|	98059-79845	22	5.6	8	5	-0.750164	0.57515	no
gi|646751012|gb|KK962011.1|	61953-96262	22	5.6	21	44	1	0.38005	no
gi|646751012|gb|KK962011.1|	63418-96384	22	5.6	5	12	1	0.4158	no
gi|646751012|gb|KK962011.1|	67173-96774	22	5.6	3	4	0.329817	0.78955	no
gi|646751031|gb|KK962010.1|	06476-10071	23	7.5	1	2	0.736648	0.64505	no
gi|646751031|gb|KK962010.1|	55515-10564	23	7.5	1	2	0.966346	0.49665	no
gi|646751031|gb|KK962010.1|	69333-10698	23	7.5	32	0.566127	-6	0.1688	no
gi|646751031|gb|KK962010.1|	49304-15002	23	7.5	1	3	1	0.4155	no
gi|646751031|gb|KK962010.1|	56095-15633	23	7.5	29	57	0.967537	0.56005	no
gi|646751031|gb|KK962010.1|	65207-16609	23	7.5	2	1	-0.371818	0.78495	no
gi|646751031|gb|KK962010.1|	70796-17116	23	7.5	7	8	0.337401	0.80135	no
gi|646751031|gb|KK962010.1|	72027-17249	23	7.5	43	39	-0.130039	0.9318	no
gi|646751031|gb|KK962010.1|	76731-17764	23	7.5	2	5	1	0.44325	no
gi|646751031|gb|KK962010.1|	78325-18114	23	7.5	8	7	-0.0279303	0.9796	no
gi|646751031|gb|KK962010.1|	19335-19956	23	7.5	2	2	-0.0180225	0.92345	no
gi|646751031|gb|KK962010.1|	96861-19873	23	7.5	0.570381	0.41263	-0.467076	1	no
gi|646751031|gb|KK962010.1|	71421-27439	23	7.5	33	20	-0.759005	0.51175	no
gi|646751031|gb|KK962010.1|	74946-27548	23	7.5	3	3	0.0817834	0.93615	no
gi|646751031|gb|KK962010.1|	78057-27849	23	7.5	14	8	-0.825604	0.5561	no
gi|646751031|gb|KK962010.1|	82683-28379	23	7.5	43	32	-0.420859	0.6944	no
gi|646751031|gb|KK962010.1|	37191-33833	23	7.5	5	3	-0.789791	0.6204	no
gi|646751031|gb|KK962010.1|	57663-35815	23	7.5	22	25	0.183148	0.90405	no
gi|646751031|gb|KK962010.1|	97131-39983	23	7.5	3	5	0.437622	0.7678	no
gi|646751031|gb|KK962010.1|	23081-42365	23	7.5	11	15	0.492961	0.75505	no
gi|646751031|gb|KK962010.1|	48977-44926	23	7.5	13	13	0.0141204	0.9333	no

gi|646751031|gb|KK962010.1|	45357-49750	23	7.5	1	1	0.264711	0.86025	no
gi|646751031|gb|KK962010.1|	62217-46300	23	7.5	1	3	2	0.24	no
gi|646751031|gb|KK962010.1|	73820-47402	23	7.5	49	33	-0.552633	0.73405	no
gi|646751031|gb|KK962010.1|	87054-48724	23	7.5	62	84	0.43722	0.75205	no
gi|646751031|gb|KK962010.1|	71568-57179	23	7.5	16	118	3	0.1472	no
gi|646751031|gb|KK962010.1|	73076-57370	23	7.5	2	4	0.7595	0.5662	no
gi|646751031|gb|KK962010.1|	76423-57665	23	7.5	19	52	1	0.3287	no
gi|646751031|gb|KK962010.1|	78328-57895	23	7.5	21	37	0.845439	0.57995	no
gi|646751031|gb|KK962010.1|	80170-58046	23	7.5	41	62	0.584764	0.71485	no
gi|646751031|gb|KK962010.1|	86061-58784	23	7.5	0.545991	0.350797	-0.63824	1	no
gi|646751031|gb|KK962010.1|	91723-59264	23	7.5	1	1	0.312303	0.8346	no
gi|646751031|gb|KK962010.1|	93096-59338	23	7.5	18	70	2	0.19635	no
gi|646751031|gb|KK962010.1|	94883-59516	23	7.5	33	81	1	0.342	no
gi|646751031|gb|KK962010.1|	95811-59627	23	7.5	4	14	2	0.22405	no
gi|646751031|gb|KK962010.1|	96676-59857	23	7.5	159	196	0.304325	0.79645	no
gi|646751031|gb|KK962010.1|	13390-61421	23	7.5	5	3	-0.871443	0.5328	no
gi|646751031|gb|KK962010.1|	61409-66210	23	7.5	1	1	0.48071	0.7721	no
gi|646751031|gb|KK962010.1|	15508-71639	23	7.5	0.878166	3	2	0.2385	no
gi|646751031|gb|KK962010.1|	17015-71935	23	7.5	2	2	0.161762	0.9141	no
gi|646751031|gb|KK962010.1|	28233-72870	23	7.5	6	0	-inf	0.0011	yes
gi|646751031|gb|KK962010.1|	44334-74599	23	7.5	6	9	0.605405	0.68325	no
gi|646751031|gb|KK962010.1|	01283-80221	23	7.5	6	1	-2	0.1538	no
gi|646751031|gb|KK962010.1|	74084-87539	23	7.5	2	2	0.444545	0.74595	no
gi|646751031|gb|KK962010.1|	77410-87845	23	7.5	1	2	0.222895	0.8612	no
gi|646751031|gb|KK962010.1|	80930-88145	23	7.5	7	2	-2	0.19315	no
gi|646751031|gb|KK962010.1|	87219-88786	23	7.5	1	2	0.694233	0.63935	no
gi|646751031|gb|KK962010.1|	88034-88973	23	7.5	1	0.647364	-0.832031	0.51905	no
gi|646751031|gb|KK962010.1|	90203-89125	23	7.5	1	1	0.150426	0.9186	no
gi|646751031|gb|KK962010.1|	97172-89806	23	7.5	2	0.825725	-1	0.4538	no
gi|646751031|gb|KK962010.1|	98162-89931	23	7.5	2	0.739628	-1	0.37145	no

gi|646751031|gb|KK962010.1|	99470-90069	23	7.5	2	2	-0.544376	0.69505	no
gi|646751031|gb|KK962010.1|	09896-91078	23	7.5	2.5	2	-0.584968	0.62895	no
gi|646751031|gb|KK962010.1|	61485-96215	23	7.5	2	0.930171	-0.745258	0.6333	no
gi|646751056|gb|KK962009.1|	47406-54844	14	8.5	0.707046	0.672317	-0.0726614	1	no
gi|646751056|gb|KK962009.1|	83776-84478	14	8.5	6	0.578024	-3	0.13485	no
[truncated: 475,936 more chars]
