# Supplementary material for: The X Chromosome of Hemipteran Insects: Conservation, Dosage Compensation and Sex-Biased Expression
Source: Genome Biol Evol. 2015 Nov 10;7(12):3259–68. doi: 10.1093/gbe/evv215 (PMC4700948; doi:10.1093/gbe/evv215)
Supplement: Supplementary Data [file S1 Data (rev) O.fasciatus.pdf › S1 Data (rev) O.fasciatus.pdf]

scaffold	gene	covF	covM	expF	expM	log2(fold_change)	p-value	significance
gi|641542060|gb|KK868928.1|	2985-3309	9.3	29	7	4	-0.805709	0.6299	no
gi|641542062|gb|KK868927.1|	2968-3581	7.1	34	0.508206	2	2	0.3323	no
gi|641542070|gb|KK868923.1|	141-506	6.9	27	6	1	-2	0.2477	no
gi|641542075|gb|KK868922.1|	12-537	9.7	18	1	2	0.594568	0.70555	no
gi|641542084|gb|KK868918.1|	2675-3093	9.9	37	142	16	-3	0.0269	no
gi|641542140|gb|KK868891.1|	143-825	8.6	24	10	9	-0.227594	0.8593	no
gi|641542140|gb|KK868891.1|	3320-3969	8.6	24	3	3	-0.208007	0.88325	no
gi|641542168|gb|KK868880.1|	241-636	8.4	24	6	6	-0.126309	0.92925	no
gi|641542176|gb|KK868877.1|	1200-1838	15	72	14	13	-0.0290044	0.9831	no
gi|641542181|gb|KK868875.1|	4034-4226	9.8	32	591	445	-0.407978	0.74325	no
gi|641542181|gb|KK868875.1|	93-437	9.8	32	17	22	0.367194	0.7803	no
gi|641542187|gb|KK868873.1|	189-1171	11	35	0.269998	0.958094	2	0.3323	no
gi|641542193|gb|KK868871.1|	393-1426	10	30	1	1	-0.0951568	0.9478	no
gi|641542202|gb|KK868867.1|	50-1879	9.6	36	2	1	-0.613676	0.655	no
gi|641542224|gb|KK868857.1|	2663-3422	8	25	1	1	0.186833	0.91015	no
gi|641542226|gb|KK868856.1|	0-1460	9.7	36	7	4	-0.775196	0.53945	no
gi|641542238|gb|KK868850.1|	1487-1962	8.2	31	15	23	0.664314	0.60885	no
gi|641542238|gb|KK868850.1|	91-313	8.2	31	22	55	1	0.3552	no
gi|641542275|gb|KK868832.1|	0-1120	10	43	0.535977	7	4	0.06515	no
gi|641542279|gb|KK868830.1|	0-2708	12	41	15	5	-1	0.3968	no
gi|641542279|gb|KK868830.1|	5481-6054	12	41	4	0.849558	-2	0.21545	no
gi|641542289|gb|KK868825.1|	3364-3881	8.3	27	151	101	-0.581551	0.71625	no
gi|641542289|gb|KK868825.1|	655-1230	8.3	27	0.931359	2	1	0.51085	no
gi|641542291|gb|KK868824.1|	453-644	8.7	20	33	30	-0.17495	0.90955	no
gi|641542305|gb|KK868817.1|	2782-3521	9.9	31	0.260434	4	4	0.24305	no
gi|641542321|gb|KK868809.1|	2689-3127	7.4	21	3	2	-0.87079	0.6061	no
gi|641542321|gb|KK868809.1|	389-1093	7.4	21	0.69583	1	0.863782	0.62725	no
gi|641542323|gb|KK868808.1|	3-499	7.9	9.8	2	1	-0.822106	0.6492	no
gi|641542357|gb|KK868793.1|	7-879	5.8	20	14	4	-2	0.2262	no

gi|641542359|gb|KK868792.1|	562-1429	8	20	13	3	-2	0.13615	no
gi|641542359|gb|KK868792.1|	5817-6147	8	20	3	5	0.796414	0.6409	no
gi|641542365|gb|KK868789.1|	76-658	14	40	1	0.830098	-0.817915	0.6492	no
gi|641542367|gb|KK868788.1|	250-415	6.5	34	0	517	inf	5.00E-05	yes
gi|641542389|gb|KK868779.1|	438-997	11	45	2	3	0.596179	0.6863	no
gi|641542418|gb|KK868766.1|	165-952	7.5	27	1	0.363331	-2	0.4266	no
gi|641542420|gb|KK868765.1|	34-621	8.8	22	2	1	-1	0.5005	no
gi|641542420|gb|KK868765.1|	3425-3932	8.8	22	3	3	-0.21377	0.89225	no
gi|641542423|gb|KK868764.1|	3250-3980	18	63	0.926847	2	1	0.39245	no
gi|641542429|gb|KK868761.1|	542-737	8.2	32	14	14	-0.0573317	0.91115	no
gi|641542437|gb|KK868757.1|	369-1353	10	36	0.359084	0.955677	1	0.44255	no
gi|641542447|gb|KK868752.1|	1274-1802	7.6	29	1	3	1	0.4611	no
gi|641542481|gb|KK868735.1|	11209-12057	11	39	2	4	1	0.42355	no
gi|641542481|gb|KK868735.1|	12171-13403	11	39	2	2	0.42221	0.7548	no
gi|641542481|gb|KK868735.1|	1500-3890	11	39	2	2	0.251188	0.8455	no
gi|641542491|gb|KK868730.1|	1-1235	7.1	21	15	1	-4	0.02485	no
gi|641542491|gb|KK868730.1|	2562-3630	7.1	21	10	1	-3	0.06155	no
gi|641542511|gb|KK868720.1|	2422-2977	10	36	0.589686	3	2	0.256	no
gi|641542526|gb|KK868713.1|	5712-6792	9.8	27	7	1	-2	0.1039	no
gi|641542541|gb|KK868706.1|	1-533	12	44	3	5	0.787569	0.58375	no
gi|641542541|gb|KK868706.1|	619-1265	12	44	0.785187	1	0.862296	0.62755	no
gi|641542543|gb|KK868705.1|	2816-3821	11	23	2	2	0.020077	0.9647	no
gi|641542543|gb|KK868705.1|	3950-4638	11	23	1	2	0.737876	0.6176	no
gi|641542543|gb|KK868705.1|	44-570	11	23	1	1	-0.212735	0.8945	no
gi|641542543|gb|KK868705.1|	624-2705	11	23	0.826041	0.687302	-0.265269	1	no
gi|641542556|gb|KK868699.1|	2060-2340	7.9	13	8	8	-0.0899614	0.94215	no
gi|641542564|gb|KK868695.1|	58-1284	11	35	2	1	-0.393398	0.78035	no
gi|641542564|gb|KK868695.1|	7443-7989	11	35	122	10	-4	0.0821	no
gi|641542570|gb|KK868692.1|	156-675	8.8	23	27	52	0.96462	0.45245	no
gi|641542578|gb|KK868688.1|	2661-4241	12	35	3	2	-1	0.44075	no

gi|641542578|gb|KK868688.1|	38-2517	12	35	7	5	-0.430146	0.7319	no
gi|641542581|gb|KK868687.1|	319-558	5.8	21	7	10	0.548367	0.7576	no
gi|641542586|gb|KK868685.1|	1571-2117	11	36	1	2	1	0.51085	no
gi|641542586|gb|KK868685.1|	2243-2431	11	36	292	328	0.171004	0.89155	no
gi|641542588|gb|KK868684.1|	1642-2140	8.7	35	2	0.703505	-2	0.3455	no
gi|641542596|gb|KK868680.1|	789-1291	13	40	0.920746	2	1	0.51315	no
gi|641542608|gb|KK868674.1|	7795-8553	12	21	0.880763	2	1	0.44	no
gi|641542622|gb|KK868668.1|	885-1171	6.4	26	12	2	-3	0.20825	no
gi|641542627|gb|KK868666.1|	20-498	8	28	3	2	-0.67127	0.68185	no
gi|641542629|gb|KK868665.1|	2632-3584	9.4	32	0.467836	1	1	0.3994	no
gi|641542643|gb|KK868658.1|	2198-2390	7.8	26	0	36	inf	0.0068	no
gi|641542655|gb|KK868652.1|	18-1165	11	26	4	2	-0.792423	0.5461	no
gi|641542655|gb|KK868652.1|	3139-3758	11	26	8	8	-0.0162753	0.98645	no
gi|641542657|gb|KK868651.1|	6132-7166	11	33	0.422097	0.898981	1	0.50945	no
gi|641542657|gb|KK868651.1|	8424-9606	11	33	15	31	1	0.3929	no
gi|641542663|gb|KK868648.1|	44-768	11	37	4	2	-1	0.4141	no
gi|641542667|gb|KK868646.1|	10529-11181	11	37	2	1	-0.779079	0.6308	no
gi|641542667|gb|KK868646.1|	6536-8233	11	37	6	5	-0.0140386	0.99175	no
gi|641542667|gb|KK868646.1|	8341-9420	11	37	0.961446	0.731499	-0.394351	0.80865	no
gi|641542697|gb|KK868631.1|	8780-9909	6.4	31	2	5	2	0.2327	no
gi|641542703|gb|KK868628.1|	1734-3149	5.3	28	0.580493	0.884442	0.607489	0.6779	no
gi|641542705|gb|KK868627.1|	2645-3560	13	44	4	3	-0.0422337	0.9744	no
gi|641542713|gb|KK868623.1|	33-709	9.3	38	2	23	3	0.0367	no
gi|641542735|gb|KK868613.1|	4057-4266	9.2	24	25	47	0.897453	0.53645	no
gi|641542743|gb|KK868609.1|	1404-1933	7.6	35	1	4	2	0.28545	no
gi|641542743|gb|KK868609.1|	4-595	7.6	35	12	6	-0.926099	0.48785	no
gi|641542761|gb|KK868601.1|	114-631	14	37	0.658714	4	3	0.19335	no
gi|641542772|gb|KK868596.1|	5975-6916	8.7	27	0.664633	1	1	0.48415	no
gi|641542772|gb|KK868596.1|	96-951	8.7	27	3	2	-0.692071	0.62735	no
gi|641542795|gb|KK868585.1|	119-742	6.9	29	6	2	-2	0.3008	no

gi|641542795|gb|KK868585.1|	2704-3722	6.9	29	2	0.654556	-2	0.2712	no
gi|641542809|gb|KK868578.1|	0-235	12	34	33	39	0.251284	0.8555	no
gi|641542825|gb|KK868570.1|	1357-1585	12	36	13	4	-2	0.3394	no
gi|641542825|gb|KK868570.1|	1921-2172	12	36	746	698	-0.0969735	0.9645	no
gi|641542837|gb|KK868564.1|	3330-4108	5.9	31	6	4	-0.51327	0.6995	no
gi|641542847|gb|KK868559.1|	3702-3959	11	49	317	105	-2	0.21235	no
gi|641542865|gb|KK868550.1|	141-332	8.6	12	31	7	-2	0.2746	no
gi|641542869|gb|KK868548.1|	12-720	10	28	0.690408	4	3	0.1484	no
gi|641542869|gb|KK868548.1|	3007-3780	10	28	1	19	4	0.03605	no
gi|641542873|gb|KK868546.1|	2452-3035	8	24	5	2	-1	0.4618	no
gi|641542881|gb|KK868542.1|	3005-4078	7.3	14	4	2	-0.809434	0.55465	no
gi|641542881|gb|KK868542.1|	8-912	7.3	14	3	3	0.156543	0.9073	no
gi|641542883|gb|KK868541.1|	3342-3586	7.9	30	33	9	-2	0.24475	no
gi|641542887|gb|KK868539.1|	28-310	9.5	41	10	10	-0.0747559	0.95335	no
gi|641542904|gb|KK868531.1|	2812-3073	3.7	28	11	20	0.87825	0.5548	no
gi|641542937|gb|KK868515.1|	2913-3538	7.7	25	1	8	3	0.10365	no
gi|641542937|gb|KK868515.1|	89-258	7.7	25	41	125	2	0.29585	no
gi|641542947|gb|KK868510.1|	5833-6572	8.5	31	73	21	-2	0.3115	no
gi|641542949|gb|KK868509.1|	0-1313	9	27	25	26	0.0728381	0.95395	no
gi|641542949|gb|KK868509.1|	2844-3023	9	27	52	64	0.312607	0.835	no
gi|641542963|gb|KK868502.1|	115-1023	9.3	38	1	1.51	0.466545	0.75495	no
gi|641542963|gb|KK868502.1|	3059-4170	9.3	38	2	2	-0.260851	0.8498	no
gi|641542965|gb|KK868501.1|	271-922	13	35	1	0.941289	-0.20795	0.8945	no
gi|641542969|gb|KK868499.1|	2685-3095	8	13	2	5	1	0.41185	no
gi|641542969|gb|KK868499.1|	7-643	8	13	3	3	-0.333919	0.8145	no
gi|641542971|gb|KK868498.1|	2327-3201	7.5	26	2	2	-0.112572	0.9368	no
gi|641542994|gb|KK868487.1|	551-985	9.1	10	1	3	0.851315	0.62755	no
gi|641543004|gb|KK868482.1|	111-850	7.8	32	2	2	0.0165283	0.9597	no
gi|641543004|gb|KK868482.1|	2712-3150	7.8	32	2	2	-0.411359	0.82205	no
gi|641543010|gb|KK868479.1|	75-418	8.9	44	9	17	0.913504	0.5114	no

gi|641543028|gb|KK868470.1|	7618-8416	4.9	11	0.822324	0.71349	-0.204814	1	no
gi|641543030|gb|KK868469.1|	33-643	9	34	2	8	2	0.15975	no
gi|641543064|gb|KK868452.1|	3303-3661	11	41	2	3	0.579008	0.76735	no
gi|641543064|gb|KK868452.1|	4-1329	11	41	4	3	-0.200252	0.8784	no
gi|641543068|gb|KK868450.1|	48-284	9.5	37	35	21	-0.725868	0.6068	no
gi|641543079|gb|KK868445.1|	335-759	12	58	0.306838	17	6	0.10515	no
gi|641543081|gb|KK868444.1|	35-496	9.1	31	3	0	-inf	0.0102	no
gi|641543091|gb|KK868439.1|	28-301	7	30	11	34	2	0.267	no
gi|641543115|gb|KK868427.1|	5896-6247	8.5	25	8	10	0.236821	0.86455	no
gi|641543136|gb|KK868417.1|	92-654	5.8	8.7	0.385669	2	3	0.2991	no
gi|641543197|gb|KK868387.1|	3032-3490	5.5	15	3	0	-inf	0.0075	no
gi|641543213|gb|KK868379.1|	207-1068	9	29	0.212715	2	3	0.27515	no
gi|641543215|gb|KK868378.1|	124-497	4.8	23	0	13	inf	0.0005	yes
gi|641543219|gb|KK868376.1|	41-950	6.4	13	30	34	0.173011	0.8827	no
gi|641543219|gb|KK868376.1|	6125-6542	6.4	13	15	5	-2	0.2899	no
gi|641543223|gb|KK868374.1|	201-1158	6.9	24	2	0.98937	-0.909992	0.54665	no
gi|641543236|gb|KK868368.1|	20-716	8.5	29	0.989704	0.857541	-0.206792	0.8945	no
gi|641543242|gb|KK868365.1|	3090-3476	9.2	32	1	4	2	0.3454	no
gi|641543267|gb|KK868354.1|	2263-2985	12	37	3	3	0.16057	0.90595	no
gi|641543269|gb|KK868353.1|	1-428	7.4	23	0.605972	5	3	0.27515	no
gi|641543286|gb|KK868345.1|	3-438	9.9	30	0	43	inf	5.00E-05	yes
gi|641543288|gb|KK868344.1|	385-628	9.2	13	7	6	-0.0350218	0.91115	no
gi|641543292|gb|KK868342.1|	1000-1199	6.6	15	26	3	-3	0.23065	no
gi|641543335|gb|KK868321.1|	2243-3592	3.5	1.30E+01	4	4	-0.260684	0.8447	no
gi|641543350|gb|KK868314.1|	3464-3870	8.8	27	2	5	2	0.31505	no
gi|641543362|gb|KK868308.1|	3466-6894	6.9	20	5	3	-0.734989	0.5631	no
gi|641543364|gb|KK868307.1|	263-1475	9.5	43	4	3	-0.666496	0.61005	no
gi|641543370|gb|KK868304.1|	1234-2609	12	56	2	0.548596	-2	0.29105	no
gi|641543386|gb|KK868296.1|	3021-3328	12	35	7	2	-2	0.31725	no
gi|641543394|gb|KK868292.1|	2311-3312	14	56	9	6	-0.710199	0.57735	no

gi|641543394|gb|KK868292.1|	45-1963	14	56	87	36	-1	0.55875	no
gi|641543435|gb|KK868272.1|	3006-3703	6.3	15	49	27	-0.834041	0.50255	no
gi|641543441|gb|KK868269.1|	1822-2290	8.8	36	0	4	inf	0.0068	no
gi|641543453|gb|KK868263.1|	2188-4064	11	34	45	29	-0.612218	0.7837	no
gi|641543453|gb|KK868263.1|	5-1680	11	34	25	19	-0.427695	0.7921	no
gi|641543479|gb|KK868250.1|	286-493	7.9	22	56	54	-0.0510835	0.9702	no
gi|641543497|gb|KK868241.1|	2667-3447	5	21	6	6	-0.125679	0.9212	no
gi|641543513|gb|KK868233.1|	224-908	9.1	21	2	4	0.693384	0.62925	no
gi|641543551|gb|KK868216.1|	0-1130	10	33	6	2	-2	0.236	no
gi|641543555|gb|KK868214.1|	2256-2578	15	25	6	2	-2	0.27505	no
gi|641543555|gb|KK868214.1|	94-424	15	25	12	3	-2	0.21665	no
gi|641543572|gb|KK868206.1|	84-325	8.9	22	7	23	2	0.295	no
gi|641543588|gb|KK868198.1|	8307-9036	8.4	23	1	0.804986	-0.398711	0.82205	no
gi|641543598|gb|KK868193.1|	62-220	5.2	16	54	58	0.0924408	0.93345	no
gi|641543618|gb|KK868183.1|	93-245	11	37	44	62	0.503998	0.7741	no
gi|641543622|gb|KK868181.1|	0-1212	7.2	28	3	5	1	0.3984	no
gi|641543622|gb|KK868181.1|	4151-4429	7.2	28	14	7	-1	0.4761	no
gi|641543622|gb|KK868181.1|	5687-6179	7.2	28	0	8	inf	0.00115	yes
gi|641543638|gb|KK868173.1|	0-1323	8.8	11	7	6	-0.269523	0.83485	no
gi|641543665|gb|KK868160.1|	1797-3263	10	34	5	4	-0.195625	0.87625	no
gi|641543669|gb|KK868158.1|	4000-4205	10	37	25	8	-2	0.4022	no
gi|641543678|gb|KK868154.1|	1334-1937	9.4	27	0.69416	3	2	0.2441	no
gi|641543699|gb|KK868144.1|	21-844	9.5	34	18	11	-0.777342	0.5511	no
gi|641543699|gb|KK868144.1|	7432-7597	9.5	34	268	127	-1	0.4337	no
gi|641543709|gb|KK868139.1|	369-569	9.3	32	0	200	inf	5.00E-05	yes
gi|641543713|gb|KK868137.1|	243-874	11	51	1	1	0.183777	0.91015	no
gi|641543719|gb|KK868134.1|	28-762	15	54	33	43	0.373285	0.76495	no
gi|641543719|gb|KK868134.1|	3048-3413	15	54	18	26	0.580101	0.65785	no
gi|641543723|gb|KK868132.1|	1607-2160	5.7	18	4	7	0.595915	0.66385	no
gi|641543723|gb|KK868132.1|	51-525	5.7	18	4	1	-2	0.3398	no

gi|641543754|gb|KK868117.1|	7481-8342	5	25	0.106358	3	5	0.11025	no
gi|641543784|gb|KK868102.1|	90-662	10	38	2	4	1	0.38675	no
gi|641543809|gb|KK868090.1|	1458-1971	5	18	5	0	-inf	0.0032	yes
gi|641543809|gb|KK868090.1|	47-1155	5	18	19	6	-2	0.18415	no
gi|641543824|gb|KK868083.1|	2309-2835	6.8	35	0.427305	6	4	0.24305	no
gi|641543863|gb|KK868064.1|	1332-1673	4.5	17	7	5	-0.423891	0.7699	no
gi|641543865|gb|KK868063.1|	4799-5664	11	38	3	0.642545	-2	0.2443	no
gi|641543899|gb|KK868046.1|	2709-2959	7.6	21	10	0	-inf	0.01175	no
gi|641543917|gb|KK868037.1|	77-858	6.1	20	0	3	inf	0.0016	yes
gi|641543925|gb|KK868033.1|	2025-2825	8.3	20	2	0.711146	-2	0.3134	no
gi|641543927|gb|KK868032.1|	1865-2582	8.4	23	4	10	1	0.39745	no
gi|641543929|gb|KK868031.1|	2541-3157	8.2	23	0.168199	3	4	0.12385	no
gi|641543953|gb|KK868020.1|	605-1283	8.7	23	3	2	-0.247872	0.8669	no
gi|641543997|gb|KK868001.1|	166-1026	11	41	0.42607	1	1	0.44255	no
gi|641544005|gb|KK867997.1|	2684-2997	9	41	16	17	0.0343762	0.97465	no
gi|641544009|gb|KK867995.1|	134-559	11	32	3	4	0.43543	0.7686	no
gi|641544009|gb|KK867995.1|	1567-1869	11	32	8	10	0.326854	0.8199	no
gi|641544047|gb|KK867976.1|	2436-2732	11	43	1	25	4	0.2375	no
gi|641544054|gb|KK867973.1|	0-638	9.7	20	17	21	0.330538	0.7908	no
gi|641544054|gb|KK867973.1|	2833-3136	9.7	20	15	0	-inf	0.0021	yes
gi|641544074|gb|KK867963.1|	3655-4174	6.8	28	22	42	0.961622	0.4517	no
gi|641544078|gb|KK867961.1|	2849-3166	9.6	28	126.51	58	-1	0.376	no
gi|641544104|gb|KK867948.1|	3118-3529	7.7	39	6	10	0.808398	0.5635	no
gi|641544135|gb|KK867933.1|	45-698	11	29	24	11	-1	0.37215	no
gi|641544151|gb|KK867925.1|	120-357	11	40	9	3	-1	0.4427	no
gi|641544159|gb|KK867921.1|	2601-3161	10	12	3	5	0.776794	0.5926	no
gi|641544159|gb|KK867921.1|	78-717	10	12	0.957048	2	1	0.41185	no
gi|641544168|gb|KK867917.1|	2850-3728	11	17	11	23	1	0.3728	no
gi|641544168|gb|KK867917.1|	32-586	11	17	0.394212	3	3	0.2674	no
gi|641544184|gb|KK867909.1|	2928-3088	2.4	8.2	0	98	inf	0.00455	no

gi|641544185|gb|KK867908.1|	3292-3928	7.5	35	0.321181	3	3	0.2674	no
gi|641544185|gb|KK867908.1|	4773-5409	7.5	35	1	1	0.183929	0.91015	no
gi|641544191|gb|KK867904.1|	0-907	10	22	4	5	0.236306	0.85555	no
gi|641544193|gb|KK867903.1|	1112-2034	7	34	1	2	0.729739	0.63585	no
gi|641544225|gb|KK867886.1|	179-957	6.5	24	2	3	0.709123	0.6357	no
gi|641544229|gb|KK867882.1|	185-618	7.8	22	3	4	0.266261	0.85625	no
gi|641544229|gb|KK867882.1|	3183-3914	7.8	22	19	22	0.21886	0.86715	no
gi|641544235|gb|KK867879.1|	884-2121	12	50	2	4	0.969232	0.4717	no
gi|641544271|gb|KK867862.1|	7-1016	14	69	0.696104	321	9	0.04645	no
gi|641544277|gb|KK867859.1|	1635-1831	4.5	38	104	30	-2	0.2208	no
gi|641544277|gb|KK867859.1|	68-350	4.5	38	17	6	-1	0.3753	no
gi|641544313|gb|KK867842.1|	43-711	13	51	1	2	0.27794	0.8559	no
gi|641544319|gb|KK867839.1|	35-1395	12	39	0.851067	0.370424	-1	1	no
gi|641544321|gb|KK867838.1|	3590-3982	6.3	16	23	29	0.338705	0.7917	no
gi|641544334|gb|KK867832.1|	1811-2114	7.5	29	6	9	0.568105	0.71515	no
gi|641544374|gb|KK867812.1|	144-447	11	37	1	7	3	0.30875	no
gi|641544374|gb|KK867812.1|	3348-4339	11	37	2	5	1	0.2868	no
gi|641544388|gb|KK867805.1|	5424-5843	8.3	11	2	2	0.586839	0.70505	no
gi|641544394|gb|KK867802.1|	2396-3956	20	59	29	8	-2	0.29665	no
gi|641544410|gb|KK867794.1|	2542-3347	8.6	34	9	2	-2	0.10805	no
gi|641544452|gb|KK867773.1|	2006-3010	9.9	13	4	1	-2	0.2521	no
gi|641544478|gb|KK867760.1|	259-854	11	30	7	57	3	0.033	no
gi|641544480|gb|KK867759.1|	3086-3444	8.9	33	2	5	1	0.4655	no
gi|641544482|gb|KK867758.1|	1310-1453	6.5	26	110	77	-0.500983	0.7686	no
gi|641544484|gb|KK867757.1|	21-432	10	37	2	3	0.363613	0.82765	no
gi|641544503|gb|KK867748.1|	396-730	6	11	91	61	-0.589317	0.64015	no
gi|641544518|gb|KK867741.1|	64-274	10	13	23	0	-inf	0.0075	no
gi|641544529|gb|KK867736.1|	2293-2953	11	26	2	2	0.0850808	0.94	no
gi|641544539|gb|KK867731.1|	11041-11568	11	35	3	0.643358	-2	0.2185	no
gi|641544539|gb|KK867731.1|	4271-5256	11	35	2	0	-inf	0.00495	no

gi|641544539|gb|KK867731.1|	5419-6152	11	35	2	0.199762	-3	0.2348	no
gi|641544539|gb|KK867731.1|	6271-8373	11	35	1	0.39652	-2	0.27895	no
gi|641544539|gb|KK867731.1|	75-1238	11	35	138	6	-4	0.0354	no
gi|641544539|gb|KK867731.1|	8902-9538	11	35	1	0.729706	-0.985996	0.62775	no
gi|641544539|gb|KK867731.1|	9664-10025	11	35	56	3	-4	0.04705	no
gi|641544545|gb|KK867728.1|	372-1601	12	21	1	2	0.751008	0.5889	no
gi|641544552|gb|KK867725.1|	1913-2828	8.9	42	1	2	0.72966	0.63585	no
gi|641544591|gb|KK867706.1|	701-1090	10	15	5	0.539276	-3	0.21195	no
gi|641544604|gb|KK867700.1|	2014-3204	8.8	22	3	2	-0.696168	0.6092	no
gi|641544604|gb|KK867700.1|	77-378	8.8	22	13	8	-0.654774	0.6518	no
gi|641544616|gb|KK867694.1|	2838-3881	9.5	23	29	17	-0.798531	0.52605	no
gi|641544637|gb|KK867684.1|	3668-4167	12	27	1	2	0.815507	0.6234	no
gi|641544639|gb|KK867683.1|	30-545	12	46	4	1	-1	0.4012	no
gi|641544639|gb|KK867683.1|	3148-3626	12	46	6	2	-2	0.2507	no
gi|641544641|gb|KK867682.1|	23-762	7.9	19	5	9	0.851469	0.5115	no
gi|641544641|gb|KK867682.1|	2764-3153	7.9	19	3	4	0.390797	0.8091	no
gi|641544669|gb|KK867669.1|	29-1770	7.9	23	8	6	-0.391489	0.7521	no
gi|641544669|gb|KK867669.1|	3409-4158	7.9	23	5	6	0.198329	0.8775	no
gi|641544684|gb|KK867662.1|	262-622	9.1	21	3	2	-0.643066	0.71075	no
gi|641544684|gb|KK867662.1|	2989-3204	9.1	21	16	14	-0.199144	0.89755	no
gi|641544686|gb|KK867661.1|	6472-6985	9.5	37	3	3	-0.0435061	0.9565	no
gi|641544743|gb|KK867636.1|	3515-4157	7.7	34	44.68	50	0.153081	0.9022	no
gi|641544762|gb|KK867630.1|	1101-1963	7.4	28	2	2	-0.107015	0.9412	no
gi|641544781|gb|KK867620.1|	11030-11463	13	48	0.886673	3	2	0.3323	no
gi|641544781|gb|KK867620.1|	9922-10504	13	48	1	0.830098	-0.817915	0.6492	no
gi|641544783|gb|KK867618.1|	0-523	9	32	3	5	0.687573	0.631	no
gi|641544783|gb|KK867618.1|	593-1352	9	32	1	3	0.949794	0.5394	no
gi|641544789|gb|KK867615.1|	5-1051	6.7	22	5	6	0.300536	0.81025	no
gi|641544791|gb|KK867614.1|	1103-1557	16	33	76	47	-0.68346	0.58565	no
gi|641544791|gb|KK867614.1|	3292-4265	16	33	11	8	-0.407233	0.7469	no

gi|641544809|gb|KK867605.1|	294-1157	11	32	0.530195	0.805573	0.603492	1	no
gi|641544809|gb|KK867605.1|	3061-3861	11	32	2	4	1	0.4131	no
gi|641544811|gb|KK867604.1|	5323-6090	9.9	24	10	3	-2	0.18515	no
gi|641544817|gb|KK867601.1|	1444-2073	5.1	44	6	0	-inf	0.0006	yes
gi|641544817|gb|KK867601.1|	21-564	5.1	44	2	0	-inf	0.01	no
gi|641544853|gb|KK867583.1|	10217-10569	8.5	29	11	7	-0.685008	0.62985	no
gi|641544894|gb|KK867563.1|	3699-4196	7.8	23	2	1	-0.407007	0.82205	no
gi|641544929|gb|KK867546.1|	2718-3169	7	37	3	5	0.727252	0.6396	no
gi|641544946|gb|KK867539.1|	281-671	6.9	21	3	2	-0.416432	0.7868	no
gi|641544971|gb|KK867527.1|	24-6038	12	33	7	6	-0.122226	0.95645	no
gi|641544979|gb|KK867523.1|	0-817	9.1	31	4	2	-0.719089	0.6088	no
gi|641544981|gb|KK867522.1|	4102-4324	11	49	0	23	inf	0.00455	no
gi|641545015|gb|KK867506.1|	0-1330	9.4	32	14	13	-0.0892573	0.94245	no
gi|641545019|gb|KK867504.1|	2-681	7.6	23	3	3	0.159578	0.90795	no
gi|641545029|gb|KK867499.1|	353-765	9.1	27	2	4	1	0.47105	no
gi|641545039|gb|KK867494.1|	1073-1390	7	17	4	2	-0.65108	0.71075	no
gi|641545043|gb|KK867492.1|	306-486	7.7	25	44	5	-3	0.21995	no
gi|641545047|gb|KK867490.1|	41-1116	10	28	4	3	-0.453275	0.7252	no
gi|641545056|gb|KK867486.1|	2511-3107	7.9	21	0.88279	2	1	0.4611	no
gi|641545072|gb|KK867478.1|	9541-10243	8.5	32	16	10	-0.758379	0.5535	no
gi|641545098|gb|KK867466.1|	2836-3123	6.2	22	2	9	2	0.2624	no
gi|641545100|gb|KK867465.1|	5429-6077	12	47	0.312691	2	3	0.2991	no
gi|641545100|gb|KK867465.1|	6263-7268	12	47	0.262328	0.931016	2	0.3323	no
gi|641545116|gb|KK867457.1|	863-1032	8.9	35	74	53	-0.48643	0.74365	no
gi|641545152|gb|KK867440.1|	1471-2026	10	44	2	1	-0.725924	0.67005	no
gi|641545152|gb|KK867440.1|	286-546	10	44	5	7	0.292959	0.86155	no
gi|641545174|gb|KK867429.1|	96-223	7.1	24	211	297	0.495105	0.77425	no
gi|641545182|gb|KK867425.1|	2777-3455	7.6	12	1	1	0.377733	0.8276	no
gi|641545182|gb|KK867425.1|	58-1269	7.6	12	9	5	-0.835705	0.5195	no
gi|641545206|gb|KK867413.1|	767-4217	17	31	4	1	-2	0.1858	no

gi|641545210|gb|KK867411.1|	1-248	8.6	13	11	6	-0.907981	0.59485	no
gi|641545216|gb|KK867408.1|	329-958	12	46	2	4	1	0.4339	no
gi|641545252|gb|KK867390.1|	929-1163	7	13	4	18	2	0.24555	no
gi|641545254|gb|KK867389.1|	0-979	6.8	27	0	90	inf	0.00015	yes
gi|641545254|gb|KK867389.1|	2664-3104	6.8	27	0	6	inf	0.0025	yes
gi|641545280|gb|KK867376.1|	17-1191	6.7	37	3	3	-0.10991	0.93025	no
gi|641545282|gb|KK867375.1|	2071-3648	17	62	19	27	0.505985	0.7508	no
gi|641545282|gb|KK867375.1|	53-1308	17	62	2	2	-0.300132	0.82655	no
gi|641545284|gb|KK867374.1|	3190-3485	10	31	13	4	-2	0.2907	no
gi|641545299|gb|KK867367.1|	1242-1703	6.8	25	4	2	-0.731391	0.652	no
gi|641545299|gb|KK867367.1|	37-856	6.8	25	5	6	0.230901	0.8613	no
gi|641545307|gb|KK867363.1|	2638-3928	9.8	25	1	9	3	0.0544	no
gi|641545314|gb|KK867360.1|	753-1100	8.1	14	34	7	-2	0.11415	no
gi|641545329|gb|KK867353.1|	20-971	6.5	23	3	2	-0.542322	0.69675	no
gi|641545329|gb|KK867353.1|	3120-3796	6.5	23	4	2	-0.935978	0.513	no
gi|641545333|gb|KK867351.1|	148-1035	9.5	25	0.921238	1	0.603798	0.6902	no
gi|641545339|gb|KK867348.1|	2539-3283	12	31	18	5	-2	0.1876	no
gi|641545347|gb|KK867344.1|	53-638	9.9	25	64	19	-2	0.16665	no
gi|641545349|gb|KK867343.1|	11329-12226	11	34	3	2	-0.618473	0.6561	no
gi|641545349|gb|KK867343.1|	13841-14860	11	34	0.601696	0.915271	0.605163	0.69715	no
gi|641545365|gb|KK867336.1|	527-743	11	20	14	2	-3	0.2588	no
gi|641545369|gb|KK867334.1|	339-774	5.4	17	3	2	-0.87106	0.6061	no
gi|641545388|gb|KK867325.1|	1848-2073	6.2	23	14	22	0.67997	0.65015	no
gi|641545400|gb|KK867319.1|	1953-2713	8.8	14	2	2	0.486412	0.7378	no
gi|641545412|gb|KK867313.1|	181-553	9	28	35	11	-2	0.22425	no
gi|641545422|gb|KK867308.1|	1849-2025	8	28	45	16	-1	0.4117	no
gi|641545430|gb|KK867304.1|	3344-3521	9	27	47	21	-1	0.4762	no
gi|641545447|gb|KK867296.1|	383-975	10	39	9	0.539659	-4	0.13505	no
gi|641545451|gb|KK867294.1|	211-366	8.3	23	207	76	-1	0.34055	no
gi|641545462|gb|KK867289.1|	1940-3742	10	30	1	0.73915	-0.455475	0.7554	no

gi|641545462|gb|KK867289.1|	330-1217	10	30	1	0.466672	-1	0.4312	no
gi|641545462|gb|KK867289.1|	4284-6599	10	30	1	2	0.685495	0.59675	no
gi|641545462|gb|KK867289.1|	6706-7236	10	30	1	1	0.00986151	0.91115	no
gi|641545482|gb|KK867279.1|	0-892	9.2	14	66	31	-1	0.50645	no
gi|641545482|gb|KK867279.1|	2386-3081	9.2	14	14	12	-0.229535	0.85405	no
gi|641545541|gb|KK867251.1|	5192-8304	12	42	2	2	0.128157	0.9175	no
gi|641545546|gb|KK867249.1|	55-595	8.3	23	7	2	-2	0.17965	no
gi|641545546|gb|KK867249.1|	5910-6447	8.3	23	2	2	-0.252826	0.88485	no
gi|641545548|gb|KK867248.1|	6577-6780	9.5	43	26	6	-2	0.26405	no
gi|641545552|gb|KK867246.1|	5140-6059	9.7	21	0.684837	2	1	0.44	no
gi|641545552|gb|KK867246.1|	531-882	9.7	21	0.44142	12	5	0.109	no
gi|641545558|gb|KK867243.1|	99-566	8.5	20	2	2	-0.409014	0.82205	no
gi|641545575|gb|KK867235.1|	3481-4026	14	32	1	2	0.373158	0.8276	no
gi|641545583|gb|KK867231.1|	3093-3418	8.4	18	10	2	-3	0.18255	no
gi|641545587|gb|KK867229.1|	2225-3493	11	22	4	5	0.104325	0.9383	no
gi|641545587|gb|KK867229.1|	774-1201	11	22	4	4	0.172589	0.9057	no
gi|641545589|gb|KK867228.1|	2202-2766	13	38	0.383591	3	3	0.2674	no
gi|641545610|gb|KK867218.1|	1571-2891	10	31	2	4	0.958553	0.48425	no
gi|641545616|gb|KK867215.1|	4222-5171	8.1	42	0.187879	15	6	0.2322	no
gi|641545616|gb|KK867215.1|	5885-6622	8.1	42	0.130697	11	6	0.10465	no
gi|641545626|gb|KK867210.1|	2600-3427	10	38	0.672492	0.851184	0.339955	1	no
gi|641545634|gb|KK867206.1|	1-446	8.2	26	7	9	0.348241	0.7987	no
gi|641545643|gb|KK867202.1|	21-910	12	38	0.816518	0.775558	-0.0742493	1	no
gi|641545653|gb|KK867197.1|	2289-3248	12	34	7	5	-0.454293	0.72365	no
gi|641545665|gb|KK867191.1|	1355-2069	8.3	33	0.818918	2	1	0.3778	no
gi|641545687|gb|KK867180.1|	2313-2735	8.9	23	0.309459	5	4	0.1196	no
gi|641545693|gb|KK867177.1|	5611-5976	5.9	20	19	5	-2	0.20235	no
gi|641545703|gb|KK867172.1|	3604-4135	8.5	46	3	4	0.594874	0.6749	no
gi|641545703|gb|KK867172.1|	635-1317	8.5	46	1	2	0.737729	0.6176	no
gi|641545743|gb|KK867152.1|	5638-6079	8.5	33	12	8	-0.520719	0.69965	no

gi|641545757|gb|KK867145.1|	2935-3229	7.8	30	6	14	1	0.4466	no
gi|641545759|gb|KK867144.1|	1-734	9.5	20	53	3	-4	0.0112	no
gi|641545759|gb|KK867144.1|	2842-3352	9.5	20	126	11	-4	0.08855	no
gi|641545781|gb|KK867136.1|	26-525	7.5	22	4	0	-inf	0.00435	no
gi|641545781|gb|KK867136.1|	2782-3322	7.5	22	12	0.309789	-5	0.1843	no
gi|641545786|gb|KK867134.1|	1539-1954	9.2	27	4	0.95792	-2	0.3216	no
gi|641545786|gb|KK867134.1|	3732-4410	9.2	27	3	3	0.13064	0.92865	no
gi|641545792|gb|KK867131.1|	3866-4571	8.7	28	24	6	-2	0.15205	no
gi|641545792|gb|KK867131.1|	5666-6356	8.7	28	1	1	0.185383	0.91015	no
gi|641545800|gb|KK867127.1|	2795-4729	9.6	34	25	17	-0.544635	0.7305	no
gi|641545816|gb|KK867119.1|	17-733	8.5	42	1	0	-inf	0.01175	no
gi|641545819|gb|KK867118.1|	9252-9513	8.9	38	7	39	2	0.1409	no
gi|641545830|gb|KK867113.1|	3463-3657	12	55	34	115	2	0.22675	no
gi|641545851|gb|KK867104.1|	7781-8367	10	34	0.905275	3	2	0.31505	no
gi|641545851|gb|KK867104.1|	8557-10364	10	34	0.702889	1	0.856595	0.5529	no
gi|641545862|gb|KK867099.1|	21558-21997	11	36	8	7	-0.131162	0.92575	no
gi|641545862|gb|KK867099.1|	3479-3722	11	36	16	30	0.890978	0.5412	no
gi|641545862|gb|KK867099.1|	6959-7521	11	36	5	7	0.537414	0.6895	no
gi|641545862|gb|KK867099.1|	8209-8702	11	36	15	30	0.985063	0.44695	no
gi|641545862|gb|KK867099.1|	9975-10392	11	36	38	51	0.409239	0.7481	no
gi|641545874|gb|KK867093.1|	2131-2397	8.2	32	8	5	-0.611995	0.70485	no
gi|641545895|gb|KK867083.1|	16-271	5.8	26	8	4	-0.860729	0.64085	no
gi|641545899|gb|KK867081.1|	3854-3968	10	32	2402.3	778	-2	0.34625	no
gi|641545899|gb|KK867081.1|	64-1131	10	32	3	2	-0.628908	0.63635	no
gi|641545903|gb|KK867079.1|	3585-4217	11	22	2	0.736307	-1	0.50365	no
gi|641545903|gb|KK867079.1|	82-868	11	22	2	2	0.223832	0.87445	no
gi|641545905|gb|KK867078.1|	2904-3151	8.8	30	125.06	57	-1	0.3794	no
gi|641545910|gb|KK867076.1|	1-1114	13	35	11	13	0.167455	0.8938	no
gi|641545918|gb|KK867072.1|	3173-3538	11	34	2	4	1	0.5126	no
gi|641545931|gb|KK867066.1|	5018-5591	6.7	28	2	3	0.066251	0.9548	no

gi|641545931|gb|KK867066.1|	73-394	6.7	28	115	80	-0.517808	0.67735	no
gi|641545937|gb|KK867063.1|	120-803	8.6	24	0.725744	1	0.60025	0.70505	no
gi|641545940|gb|KK867062.1|	3333-3964	6.5	21	0	12	inf	0.0001	yes
gi|641545960|gb|KK867056.1|	11-253	7	11	2	13	3	0.30875	no
gi|641545960|gb|KK867056.1|	392-701	7	11	3	8	1	0.482	no
gi|641545975|gb|KK867049.1|	14-752	12	45	0.130457	25	8	0.10435	no
gi|641545979|gb|KK867047.1|	354-630	14	46	6	7	0.145931	0.92285	no
gi|641545987|gb|KK867043.1|	1509-2056	6.8	22	7	6	-0.291882	0.825	no
gi|641545996|gb|KK867039.1|	2316-3182	9.5	28	13	7	-0.981431	0.44565	no
gi|641546021|gb|KK867027.1|	1678-1854	10	31	45	54	0.254366	0.8678	no
gi|641546029|gb|KK867023.1|	1538-1737	6.2	27	65	16	-2	0.2083	no
gi|641546031|gb|KK867022.1|	1368-1764	9.6	27	3	3	-0.0937673	0.9262	no
gi|641546033|gb|KK867021.1|	3389-3659	11	19	28	5	-3	0.14475	no
gi|641546039|gb|KK867018.1|	3903-4172	17	8.30E+01	53	58	0.14383	0.91045	no
gi|641546039|gb|KK867018.1|	814-1261	17	8.30E+01	5	4	-0.410582	0.791	no
gi|641546070|gb|KK867003.1|	126-487	7.2	24	48	34	-0.511119	0.689	no
gi|641546110|gb|KK866985.1|	565-795	7.7	28	21	8	-1	0.3869	no
gi|641546128|gb|KK866976.1|	2680-2890	5.5	22	94	18	-2	0.1267	no
gi|641546142|gb|KK866969.1|	2738-2991	10	35	14	14	0.0681872	0.95625	no
gi|641546142|gb|KK866969.1|	5324-5557	10	35	5	15	2	0.41735	no
gi|641546160|gb|KK866960.1|	2-1348	3.3	12	14	0.187457	-6	0.13015	no
gi|641546168|gb|KK866956.1|	2098-2719	6.1	17	1	0.503392	-1	0.4534	no
gi|641546187|gb|KK866947.1|	126-1177	8.9	29	0.248227	2	3	0.1719	no
gi|641546199|gb|KK866941.1|	2619-2784	6	23	11	60	3	0.3098	no
gi|641546201|gb|KK866940.1|	6792-6990	7.4	22	53	3	-4	0.1925	no
gi|641546207|gb|KK866937.1|	3403-4278	5.5	17	1.25	0.316574	-2	0.31075	no
gi|641546209|gb|KK866936.1|	113-588	9.2	47	2	2	0.106131	0.92335	no
gi|641546209|gb|KK866936.1|	1521-1937	9.2	47	3	0.953799	-1	0.4534	no
gi|641546216|gb|KK866933.1|	3035-3648	6.2	33	15	7	-1	0.4079	no
gi|641546222|gb|KK866930.1|	2166-2506	6.6	29	25	18	-0.508138	0.70965	no

gi|641546224|gb|KK866929.1|	2340-3329	8.9	27	0.356822	0.949687	1	0.44255	no
gi|641546227|gb|KK866928.1|	38-919	8.4	27	0.826044	0.784552	-0.0743484	1	no
gi|641546238|gb|KK866923.1|	0-467	8.6	28	3	2	-0.409014	0.8125	no
gi|641546238|gb|KK866923.1|	2576-3116	8.6	28	2	0.929368	-0.989648	0.62775	no
gi|641546242|gb|KK866921.1|	35-2538	8.3	24	4	2	-0.743863	0.564	no
gi|641546248|gb|KK866918.1|	1337-2657	8.5	29	9	8	-0.0986345	0.93445	no
gi|641546248|gb|KK866918.1|	194-1236	8.5	29	6	5	-0.301533	0.81225	no
gi|641546248|gb|KK866918.1|	2792-6438	8.5	29	2	1	-1	0.40065	no
gi|641546248|gb|KK866918.1|	6531-7507	8.5	29	0.725527	0.551668	-0.395231	1	no
gi|641546248|gb|KK866918.1|	8449-10260	8.5	29	0.744971	0.200466	-2	1	no
gi|641546250|gb|KK866917.1|	3097-3445	9.1	14	4	0	-inf	0.01175	no
gi|641546258|gb|KK866913.1|	2166-3005	10	32	11	30	1	0.27565	no
gi|641546260|gb|KK866912.1|	8-264	6.8	25	19	6	-2	0.2883	no
gi|641546262|gb|KK866911.1|	664-880	13	18	26	7	-2	0.29405	no
gi|641546274|gb|KK866905.1|	4055-4593	11	38	22	10	-1	0.3926	no
gi|641546276|gb|KK866904.1|	1-870	6.9	23	33	22	-0.614235	0.6259	no
gi|641546278|gb|KK866903.1|	155-658	7.4	27	1	1	0.00839005	0.91115	no
gi|641546305|gb|KK866890.1|	1048-1901	9.7	15	1	0	-inf	0.01	no
gi|641546313|gb|KK866886.1|	0-420	4.8	13	7	56	3	0.04645	no
gi|641546327|gb|KK866879.1|	3297-3672	6.7	28	6	1	-2	0.2169	no
gi|641546343|gb|KK866871.1|	1181-1329	8	29	137	75	-0.876819	0.59135	no
gi|641546359|gb|KK866863.1|	3262-3930	10	20	3	15	2	0.1146	no
gi|641546393|gb|KK866847.1|	2526-3432	7.6	21	2	3	0.106526	0.9402	no
gi|641546403|gb|KK866842.1|	2383-3413	8.6	29	7	2	-2	0.18645	no
gi|641546405|gb|KK866841.1|	119-613	7.4	34	13	11	-0.228222	0.8634	no
gi|641546405|gb|KK866841.1|	4347-5119	7.4	34	8	9	0.17987	0.88685	no
gi|641546437|gb|KK866826.1|	42-578	12	36	2	5	0.91705	0.5415	no
gi|641546462|gb|KK866814.1|	7905-8079	11	33	2244.72	1409.96	-0.670883	0.6814	no
gi|641546464|gb|KK866813.1|	61-1001	9.5	25	3	2	-0.936164	0.5111	no
gi|641546464|gb|KK866813.1|	9280-13378	9.5	25	11	8	-0.391605	0.80755	no

gi|641546468|gb|KK866811.1|	2563-3930	7.1	17	1	0.368219	-2	0.24435	no
gi|641546468|gb|KK866811.1|	94-275	7.1	17	281	300	0.0938736	0.9429	no
gi|641546507|gb|KK866793.1|	0-1129	10	40	14	25	0.870384	0.4859	no
gi|641546507|gb|KK866793.1|	6050-7153	10	40	10	21	1	0.41205	no
gi|641546511|gb|KK866791.1|	3302-3960	10	28	6	6	-0.0633657	0.96125	no
gi|641546511|gb|KK866791.1|	47-1037	10	28	6	2.71	-1	0.37125	no
gi|641546513|gb|KK866790.1|	5695-6363	7.8	23	1	2	0.429943	0.7802	no
gi|641546525|gb|KK866784.1|	43-1261	10	34	5	4	-0.453985	0.7298	no
gi|641546525|gb|KK866784.1|	4320-4666	10	34	5	9	0.692476	0.64805	no
gi|641546531|gb|KK866781.1|	2816-3214	7	17	7	2	-2	0.24245	no
gi|641546531|gb|KK866781.1|	3333-3793	7	17	8	2	-2	0.18465	no
gi|641546537|gb|KK866778.1|	1998-2872	9.5	24	0	3	inf	0.0016	yes
gi|641546561|gb|KK866766.1|	554-1179	8.9	15	4	2	-1	0.48275	no
gi|641546571|gb|KK866761.1|	225-382	5.9	26	141	170.75	0.272464	0.84435	no
gi|641546577|gb|KK866758.1|	146-497	9.5	26	63	31	-1	0.4315	no
gi|641546596|gb|KK866749.1|	1183-1371	9.5	18	55	12	-2	0.22245	no
gi|641546622|gb|KK866736.1|	324-920	5.6	19	16	30	0.8685	0.49825	no
gi|641546626|gb|KK866734.1|	109-670	6.5	21	2	2	0.274337	0.85615	no
gi|641546632|gb|KK866731.1|	3158-4074	11	33	0.785928	4	2	0.1741	no
gi|641546638|gb|KK866728.1|	3610-4682	9.9	50	0	152	inf	0.0002	yes
gi|641546649|gb|KK866723.1|	10343-10817	9.8	27	4	0	-inf	0.0059	no
gi|641546649|gb|KK866723.1|	153-475	9.8	27	4	2	-1	0.44305	no
gi|641546649|gb|KK866723.1|	2198-2949	9.8	27	19	7	-2	0.24405	no
gi|641546649|gb|KK866723.1|	530-2143	9.8	27	0.898957	0.532893	-0.754405	0.6172	no
gi|641546661|gb|KK866717.1|	2484-3396	6.6	24	3	2	-0.446531	0.7504	no
gi|641546661|gb|KK866717.1|	360-887	6.6	24	3	0.965037	-2	0.33985	no
gi|641546684|gb|KK866707.1|	3-902	8.8	28	17	2	-3	0.0614	no
gi|641546696|gb|KK866700.1|	2048-3176	6.1	25	1	2	0.136515	0.9277	no
gi|641546704|gb|KK866696.1|	4-764	17	100	2	2	-0.32411	0.8097	no
gi|641546706|gb|KK866694.1|	2279-2817	11	45	20	0.311562	-6	0.1832	no

gi|641546710|gb|KK866690.1|	0-682	7.2	20	24	29	0.230276	0.8541	no
gi|641546724|gb|KK866683.1|	323-888	14	50	4	6	0.526046	0.706	no
gi|641546728|gb|KK866681.1|	2631-3416	5.2	28	1	4	1	0.32805	no
gi|641546728|gb|KK866681.1|	49-666	5.2	28	2	1	-0.861062	0.60675	no
gi|641546742|gb|KK866674.1|	2828-3431	10	30	2	8	2	0.18015	no
gi|641546758|gb|KK866666.1|	3181-3870	10	16	1	1	0.378004	0.8276	no
gi|641546770|gb|KK866660.1|	3085-3341	5.2	14	0	166	inf	5.00E-05	yes
gi|641546776|gb|KK866657.1|	79-239	10	20	44	81	0.87114	0.5931	no
gi|641546780|gb|KK866655.1|	2500-2902	8.8	26	2	6	2	0.32035	no
gi|641546788|gb|KK866651.1|	215-375	10	31	126	72	-0.813359	0.5843	no
gi|641546788|gb|KK866651.1|	3388-3636	10	31	251	201	-0.323635	0.7946	no
gi|641546822|gb|KK866635.1|	0-450	10	32	9	4	-1	0.4313	no
gi|641546822|gb|KK866635.1|	2733-3907	10	32	2	3	0.5161	0.6957	no
gi|641546835|gb|KK866629.1|	1130-1315	7	32	27	8	-2	0.363	no
gi|641546841|gb|KK866626.1|	935-1773	11	39	0.330491	2	3	0.18845	no
gi|641546847|gb|KK866623.1|	3368-4122	6.8	23	90	7	-4	0.08025	no
gi|641546847|gb|KK866623.1|	70-1175	6.8	23	8	4	-1	0.44265	no
gi|641546863|gb|KK866616.1|	0-670	12	55	1	2	0.23735	0.88205	no
gi|641546863|gb|KK866616.1|	2364-3306	12	55	0.853383	39	6	0.0318	no
gi|641546880|gb|KK866608.1|	10007-10421	9.2	33	13	11	-0.311797	0.8131	no
gi|641546880|gb|KK866608.1|	4931-5720	9.2	33	2	0.905289	-1	0.39825	no
gi|641546880|gb|KK866608.1|	6630-8719	9.2	33	0.972106	1	0.156701	0.91335	no
gi|641546880|gb|KK866608.1|	9095-9272	9.2	33	51	36	-0.48205	0.75545	no
gi|641546900|gb|KK866598.1|	135-515	5.7	10	6	1	-2	0.2169	no
gi|641546906|gb|KK866595.1|	1020-1223	8.3	22	52	3	-4	0.19105	no
gi|641546906|gb|KK866595.1|	2452-2870	8.3	22	3	2	-0.413263	0.82205	no
gi|641546910|gb|KK866593.1|	221-667	9.7	41	2	2	-0.218021	0.8945	no
gi|641546914|gb|KK866591.1|	52-1495	8.9	30	4	3	-0.215524	0.8653	no
gi|641546975|gb|KK866561.1|	1216-1482	10	34	9	6	-0.579573	0.74505	no
gi|641546977|gb|KK866560.1|	0-171	nan	14	43	544	4	0.0393	no

gi|641546988|gb|KK866555.1|	3189-4951	9.5	30	0.858655	2	1	0.45915	no
gi|641546996|gb|KK866551.1|	114-366	12	41	50	27	-0.871236	0.5225	no
gi|641547008|gb|KK866545.1|	2743-3487	13	41	0.645157	1	0.864623	0.62725	no
gi|641547008|gb|KK866545.1|	5147-6434	13	41	4	2	-1	0.2965	no
gi|641547017|gb|KK866541.1|	1463-1957	12	43	8	5	-0.751147	0.5978	no
gi|641547017|gb|KK866541.1|	651-1075	12	43	139	26	-2	0.0715	no
gi|641547017|gb|KK866541.1|	8614-9198	12	43	2	3	0.597199	0.6792	no
gi|641547038|gb|KK866531.1|	1513-1877	7.3	24	1	6	2	0.2624	no
gi|641547038|gb|KK866531.1|	88-556	7.3	24	2	3	0.591059	0.69715	no
gi|641547052|gb|KK866524.1|	274-1073	9.1	30	26	9	-2	0.23405	no
gi|641547052|gb|KK866524.1|	3712-5114	9.1	30	8	2	-2	0.1709	no
gi|641547056|gb|KK866522.1|	3274-3945	8.7	27	36	10	-2	0.1661	no
gi|641547062|gb|KK866519.1|	4930-5306	9.2	28	11	1	-3	0.1503	no
gi|641547064|gb|KK866518.1|	3156-3321	11	57	137	52	-1	0.35655	no
gi|641547094|gb|KK866504.1|	3992-4378	13	45	6	8	0.489949	0.7326	no
gi|641547098|gb|KK866502.1|	1511-1994	11	35	9	4	-1	0.37655	no
gi|641547098|gb|KK866502.1|	343-758	11	35	2	3	0.849463	0.62755	no
gi|641547104|gb|KK866499.1|	0-320	5.3	24	5	3	-0.749968	0.63835	no
gi|641547104|gb|KK866499.1|	2303-2993	5.3	24	2	2	-0.177187	0.90465	no
gi|641547108|gb|KK866497.1|	90-616	12	38	9	10	0.143958	0.91275	no
gi|641547141|gb|KK866482.1|	10-1103	7.8	22	17	14	-0.335619	0.78715	no
gi|641547141|gb|KK866482.1|	2719-3633	7.8	22	0.197029	16	6	0.2323	no
gi|641547141|gb|KK866482.1|	3881-4793	7.8	22	13	11	-0.281262	0.8271	no
gi|641547147|gb|KK866479.1|	0-756	11	14	92	69	-0.412182	0.85515	no
gi|641547149|gb|KK866478.1|	265-749	9.1	24	4	1	-2	0.30255	no
gi|641547157|gb|KK866474.1|	0-253	7.2	23	8	19	1	0.43925	no
gi|641547161|gb|KK866472.1|	251-981	6.8	30	5	7	0.360302	0.7866	no
gi|641547163|gb|KK866471.1|	2636-3265	8.2	19	10	6	-0.711588	0.5853	no
gi|641547163|gb|KK866471.1|	60-1215	8.2	19	5	7	0.298996	0.8203	no
gi|641547167|gb|KK866469.1|	52-808	11	27	1	0.958166	-0.0762563	0.92685	no

gi|641547198|gb|KK866454.1|	0-1665	12	43	1	3	0.970883	0.47085	no
gi|641547198|gb|KK866454.1|	3058-5030	12	43	2	2	0.098813	0.9439	no
gi|641547212|gb|KK866447.1|	36-423	11	14	8	5	-0.77045	0.59645	no
gi|641547226|gb|KK866441.1|	19-711	8.7	20	9	6	-0.667012	0.613	no
gi|641547226|gb|KK866441.1|	2152-2575	8.7	20	2	4	0.587238	0.6936	no
gi|641547256|gb|KK866427.1|	137-1339	7.5	26	3	3	0.13698	0.9191	no
gi|641547284|gb|KK866413.1|	2879-3827	8.3	43	1	1	0.145057	0.9285	no
gi|641547298|gb|KK866406.1|	5060-5341	7.5	31	0	11	inf	0.0068	no
gi|641547308|gb|KK866401.1|	4513-5167	8.7	26	106	70	-0.589545	0.70945	no
gi|641547312|gb|KK866399.1|	13-653	7.9	32	0.954902	2	1	0.41185	no
gi|641547359|gb|KK866376.1|	21-1122	3.9	11	1	2	0.786384	0.5866	no
gi|641547404|gb|KK866353.1|	2178-3020	6.2	37	0.656894	1	1	0.5082	no
gi|641547411|gb|KK866352.1|	0-1444	7.7	20	8	2	-2	0.1458	no
gi|641547420|gb|KK866348.1|	83-1012	11	50	0	5	inf	0.00035	yes
gi|641547427|gb|KK866345.1|	2268-2791	8.3	21	66	32	-1	0.4144	no
gi|641547433|gb|KK866342.1|	80-845	nan	19	0	6	inf	0.0003	yes
gi|641547458|gb|KK866330.1|	129-790	5.8	25	0	3	inf	0.0023	yes
gi|641547458|gb|KK866330.1|	6217-6676	5.8	25	1	2	0.590384	0.70505	no
gi|641547464|gb|KK866327.1|	1477-2518	11	20	0.920926	0.63684	-0.532154	0.7478	no
gi|641547464|gb|KK866327.1|	6144-7280	11	20	1	0.343684	-2	0.33985	no
gi|641547466|gb|KK866326.1|	2969-3781	3.3	15	0	8	inf	0.00025	yes
gi|641547468|gb|KK866325.1|	163-686	9.4	28	6	8	0.372071	0.77625	no
gi|641547468|gb|KK866325.1|	1693-2923	9.4	28	13	5	-1	0.3091	no
gi|641547470|gb|KK866324.1|	1633-1944	5	26	26	12	-1	0.42395	no
gi|641547470|gb|KK866324.1|	174-787	5	26	17	16	-0.0538401	0.96605	no
gi|641547481|gb|KK866322.1|	11-600	5.8	18	6	4	-0.402611	0.77275	no
gi|641547483|gb|KK866321.1|	18-814	12	44	1	0.536887	-1	0.50885	no
gi|641547498|gb|KK866314.1|	1010-2464	10	36	4	0.085713	-5	0.18405	no
gi|641547500|gb|KK866313.1|	1715-1957	11	32	0	183	inf	5.00E-05	yes
gi|641547500|gb|KK866313.1|	26-1208	11	32	2	1	-0.896154	0.52785	no

gi|641547500|gb|KK866313.1|	8464-8998	11	32	2	3	0.305517	0.84285	no
gi|641547526|gb|KK866300.1|	1561-2170	8.7	24	2	0.776692	-1	0.50365	no
gi|641547579|gb|KK866275.1|	2529-2684	10	39	0	882	inf	0.0001	yes
gi|641547581|gb|KK866274.1|	2190-2903	7.8	32	0.683749	1	0.863983	0.62725	no
gi|641547593|gb|KK866268.1|	75-1303	13	42	29	19	-0.579682	0.6427	no
gi|641547636|gb|KK866247.1|	105-995	10	29	2	6	1	0.3644	no
gi|641547643|gb|KK866244.1|	3064-3635	8.2	19	5	3	-0.877247	0.5419	no
gi|641547649|gb|KK866241.1|	2511-2989	8.1	44	0	73	inf	5.00E-05	yes
gi|641547653|gb|KK866239.1|	0-1413	13	35	1	0.88589	-0.655553	0.6446	no
gi|641547653|gb|KK866239.1|	3798-4218	13	35	4	2	-0.676095	0.68185	no
gi|641547667|gb|KK866232.1|	0-1458	9.3	25	1	6	2	0.13815	no
gi|641547671|gb|KK866230.1|	0-534	9	30	40	114.09	2	0.3763	no
gi|641547690|gb|KK866221.1|	3754-4621	10	19	1	0.480483	-1	0.47135	no
gi|641547699|gb|KK866217.1|	16069-16842	10	28	1	1	0.0875476	0.9392	no
gi|641547699|gb|KK866217.1|	1887-2287	10	28	53	28	-0.945738	0.4598	no
gi|641547699|gb|KK866217.1|	2464-3974	10	28	3	0.738659	-2	0.2273	no
gi|641547699|gb|KK866217.1|	528-895	10	28	10	10	-0.120037	0.9341	no
gi|641547699|gb|KK866217.1|	5619-6841	10	28	12	8	-0.472372	0.7084	no
gi|641547699|gb|KK866217.1|	8070-9988	10	28	6	3	-1	0.4061	no
gi|641547715|gb|KK866209.1|	3-592	11	56	2	3	0.749392	0.6263	no
gi|641547727|gb|KK866203.1|	10-529	7.4	40	2	2	0.371859	0.82765	no
gi|641547739|gb|KK866197.1|	3743-4473	6.8	11	2	5	1	0.384	no
gi|641547745|gb|KK866194.1|	2652-3026	4.2	26	7	5	-0.496576	0.7276	no
gi|641547747|gb|KK866193.1|	2790-3290	8.4	30	96	41	-1	0.3203	no
gi|641547792|gb|KK866172.1|	30-277	7	21	9	14	0.55145	0.7313	no
gi|641547796|gb|KK866170.1|	5822-6525	10	33	0.13944	2	4	0.1276	no
gi|641547796|gb|KK866170.1|	589-762	10	33	114	117	0.0303605	0.9763	no
gi|641547800|gb|KK866168.1|	8-779	13	25	3	0	-inf	0.0013	yes
gi|641547804|gb|KK866166.1|	2360-3268	13	29	14	16	0.185548	0.88405	no
gi|641547814|gb|KK866161.1|	395-800	8	35	0	6	inf	0.00375	no

gi|641547816|gb|KK866160.1|	136-1060	11	39	3	0.44311	-3	0.15805	no
gi|641547816|gb|KK866160.1|	5699-6185	11	39	47	25	-0.89909	0.4744	no
gi|641547826|gb|KK866155.1|	2286-2997	7.7	26	0.686397	3	2	0.2128	no
gi|641547829|gb|KK866154.1|	0-498	6.6	19	9	9	-0.0850184	0.94945	no
gi|641547829|gb|KK866154.1|	1665-2104	6.6	19	16	7	-1	0.38795	no
gi|641547869|gb|KK866133.1|	4536-4786	9.9	35	13	0	-inf	0.0075	no
gi|641547869|gb|KK866133.1|	664-895	9.9	35	12	2	-3	0.2588	no
gi|641547879|gb|KK866128.1|	2185-3073	7.9	32	0.817696	3	2	0.2901	no
gi|641547879|gb|KK866128.1|	935-1966	7.9	32	0.677779	2	2	0.34655	no
gi|641547881|gb|KK866127.1|	4187-4613	12	43	9	19	0.990886	0.46805	no
gi|641547881|gb|KK866127.1|	85-1426	12	43	13	49	2	0.26705	no
gi|641547906|gb|KK866115.1|	68-971	8.9	23	0.900411	1	0.434066	0.78015	no
gi|641547922|gb|KK866108.1|	1660-2362	8.6	28	1	0.635612	-0.98426	0.62775	no
gi|641547922|gb|KK866108.1|	3003-4791	8.6	28	13	4	-2	0.2096	no
gi|641547922|gb|KK866108.1|	304-1471	8.6	28	4	0.998298	-2	0.18465	no
gi|641547922|gb|KK866108.1|	7134-8468	8.6	28	69	205	2	0.45265	no
gi|641547934|gb|KK866102.1|	7583-7833	12	33	26	37	0.495964	0.7169	no
gi|641547943|gb|KK866098.1|	1783-2127	8.1	25	17	22	0.406722	0.7579	no
gi|641547956|gb|KK866092.1|	1870-2366	6	18	2	1	-0.629461	0.71175	no
gi|641547962|gb|KK866089.1|	74-982	6.6	21	6	0.151	-5	0.18375	no
gi|641547981|gb|KK866080.1|	97-799	9	28	11	17	0.619318	0.6241	no
gi|641547983|gb|KK866079.1|	31-786	8.7	33	2	2	0.0168346	0.95145	no
gi|641547991|gb|KK866075.1|	3321-3576	8.7	31	0	46	inf	0.00065	yes
gi|641548005|gb|KK866068.1|	3-704	9.8	29	5	3	-0.443715	0.7469	no
gi|641548016|gb|KK866063.1|	17-1030	6.9	22	1	0.52683	-0.979852	0.5872	no
gi|641548020|gb|KK866061.1|	72-726	7	34	4	0	-inf	0.0013	yes
gi|641548029|gb|KK866057.1|	3673-3953	14	50	22	5	-2	0.21125	no
gi|641548043|gb|KK866050.1|	0-928	8.6	33	2	1	-1	0.41135	no
gi|641548103|gb|KK866020.1|	31-613	7.7	31	4	1	-1	0.3596	no
gi|641548144|gb|KK866004.1|	2928-3556	9.8	45	2	2	-0.500814	0.74225	no

gi|641548160|gb|KK865998.1|	80-289	8.1	20	11	18	0.757275	0.64735	no
gi|641548167|gb|KK865995.1|	0-1342	8.7	29	26	33	0.376615	0.8112	no
gi|641548167|gb|KK865995.1|	4237-4582	8.7	29	30	25	-0.258117	0.84345	no
gi|641548169|gb|KK865994.1|	6269-6954	13	37	1	1	-0.0777726	0.92685	no
gi|641548185|gb|KK865986.1|	16-1344	15	22	11	0	-inf	5.00E-05	yes
gi|641548189|gb|KK865984.1|	3047-3706	8.7	15	0.610583	1	1	0.51305	no
gi|641548210|gb|KK865974.1|	2500-2884	9.3	36	9	3	-2	0.2807	no
gi|641548223|gb|KK865968.1|	1-549	7.9	20	4	2	-1	0.38115	no
gi|641548223|gb|KK865968.1|	2492-3168	7.9	20	4	3	-0.399926	0.77305	no
gi|641548229|gb|KK865965.1|	2278-2622	6.8	25	3	3	0.313613	0.86115	no
gi|641548262|gb|KK865949.1|	1852-2561	11	36	0.826879	0.836041	0.015898	1	no
gi|641548268|gb|KK865946.1|	0-722	5.8	14	0	3	inf	0.00255	yes
gi|641548277|gb|KK865942.1|	2328-3057	8.7	27	1	6	2	0.1981	no
gi|641548279|gb|KK865941.1|	140-1149	9.4	29	2	0.397055	-2	0.28145	no
gi|641548279|gb|KK865941.1|	4589-5293	9.4	29	2	3	0.700284	0.6288	no
gi|641548315|gb|KK865923.1|	17-368	6.2	24	0.88284	7	3	0.27615	no
gi|641548315|gb|KK865923.1|	2763-3315	6.2	24	1	3	0.95844	0.57355	no
gi|641548320|gb|KK865921.1|	23-672	9.1	30	0.780009	3	2	0.23645	no
gi|641548348|gb|KK865907.1|	232-715	11	40	4	0.739173	-2	0.24735	no
gi|641548352|gb|KK865905.1|	4006-4410	8.7	29	8	8	-0.0928361	0.94695	no
gi|641548352|gb|KK865905.1|	465-1347	8.7	29	28	20	-0.484395	0.7	no
gi|641548368|gb|KK865897.1|	331-1101	9.2	41	72	71	-0.00785514	0.9969	no
gi|641548370|gb|KK865896.1|	66-1141	7.2	21	1	1	0.0206565	0.9647	no
gi|641548372|gb|KK865895.1|	2257-3288	5.4	22	1	1	0.479735	0.75585	no
gi|641548389|gb|KK865887.1|	20-1129	7.4	26	105	49	-1	0.6212	no
gi|641548394|gb|KK865885.1|	4025-4195	9.7	33	27	38	0.51414	0.7671	no
gi|641548396|gb|KK865884.1|	3028-3761	8.7	24	3	0.599286	-2	0.21545	no
gi|641548396|gb|KK865884.1|	950-1148	8.7	24	18	64	2	0.24945	no
gi|641548398|gb|KK865883.1|	2035-2391	6.8	14	46	26	-0.854274	0.5085	no
gi|641548398|gb|KK865883.1|	209-760	6.8	14	21	17	-0.364282	0.77565	no

gi|641548398|gb|KK865883.1|	2522-2896	6.8	14	3	1	-1	0.44305	no
gi|641548404|gb|KK865880.1|	1755-2823	9.7	17	1	4	2	0.26155	no
gi|641548404|gb|KK865880.1|	3411-4162	9.7	17	0.89185	0.773375	-0.205633	0.89485	no
gi|641548415|gb|KK865875.1|	271-579	11	56	0	10	inf	0.00375	no
gi|641548415|gb|KK865875.1|	3879-4117	11	56	0	20	inf	0.00375	no
gi|641548442|gb|KK865859.1|	0-1038	12	48	0.924205	2	0.730856	0.63585	no
gi|641548444|gb|KK865858.1|	4478-6193	13	45	0.931801	0.852382	-0.12852	0.9259	no
gi|641548444|gb|KK865858.1|	563-766	13	45	34	67	0.967927	0.4924	no
gi|641548444|gb|KK865858.1|	990-1225	13	45	118	154	0.389771	0.7637	no
gi|641548454|gb|KK865853.1|	1942-3881	7.8	21	2	2	0.241213	0.8518	no
gi|641548456|gb|KK865852.1|	1-476	10	32	4	5	0.291997	0.8393	no
gi|641548456|gb|KK865852.1|	2634-3671	10	32	3	3	-0.0727555	0.9581	no
gi|641548460|gb|KK865850.1|	209-457	7.3	11	91	66	-0.448181	0.72135	no
gi|641548478|gb|KK865841.1|	1423-2012	6.6	18	2	3	0.218877	0.8867	no
gi|641548478|gb|KK865841.1|	57-733	6.6	18	3	2	-0.399926	0.78745	no
gi|641548498|gb|KK865831.1|	416-812	6.7	19	3	0.521648	-3	0.2588	no
gi|641548508|gb|KK865826.1|	0-636	6.5	34	24	0.243235	-7	0.18295	no
gi|641548508|gb|KK865826.1|	3704-4127	6.5	34	4	0.462944	-3	0.2348	no
gi|641548521|gb|KK865820.1|	217-875	7.7	13	1	0.695406	-0.815438	0.6492	no
gi|641548528|gb|KK865815.1|	6622-7099	8.2	45	0	23	inf	5.00E-05	yes
gi|641548554|gb|KK865798.1|	120-1775	12	43	0.630566	1	0.814736	0.57955	no
gi|641548563|gb|KK865792.1|	107-579	4.6	13	3	8	2	0.2527	no
gi|641548569|gb|KK865789.1|	146-758	12	41	46	22	-1	0.39515	no
gi|641548571|gb|KK865788.1|	3-354	7.8	32	23	11	-1	0.4546	no
gi|641548571|gb|KK865788.1|	779-1732	7.8	32	12	7	-0.706248	0.57945	no
gi|641548599|gb|KK865774.1|	16-837	7.4	26	9	1	-3	0.0902	no
gi|641548607|gb|KK865770.1|	2927-3593	9.5	26	0.601523	2	1	0.44255	no
gi|641548619|gb|KK865764.1|	848-1542	15	44	0	19	inf	0.0001	yes
gi|641548629|gb|KK865759.1|	2-1322	9.4	27	4	3	-0.34401	0.7926	no
gi|641548640|gb|KK865754.1|	6417-6912	7.2	28	19	8	-1	0.34695	no

gi|641548648|gb|KK865750.1|	2240-2937	16	19	14	14	-0.00640202	0.99385	no
gi|641548678|gb|KK865735.1|	3167-3619	9.3	26	21	3	-3	0.0938	no
gi|641548682|gb|KK865733.1|	1-1039	11	48	0.504112	1	1	0.47015	no
gi|641548705|gb|KK865723.1|	6407-7201	6.8	31	2	1	-0.49706	0.7427	no
gi|641548709|gb|KK865721.1|	24-648	10	43	11	6	-0.801943	0.54265	no
gi|641548731|gb|KK865710.1|	2430-3087	5	13	3	4	0.51711	0.71615	no
gi|641548737|gb|KK865707.1|	2986-3544	7.3	30	0.974736	3	1	0.39995	no
gi|641548742|gb|KK865705.1|	1154-1299	9.4	31	135	0	-inf	0.0102	no
gi|641548744|gb|KK865704.1|	0-272	9.8	21	2	14	3	0.2064	no
gi|641548744|gb|KK865704.1|	1386-2002	9.8	21	3	2	-0.986626	0.5501	no
gi|641548753|gb|KK865700.1|	2892-3196	11	30	27	32	0.278838	0.834	no
gi|641548755|gb|KK865699.1|	34-937	9.3	36	8	6	-0.267276	0.8366	no
gi|641548755|gb|KK865699.1|	8681-9654	9.3	36	4	7	0.635767	0.62565	no
gi|641548757|gb|KK865698.1|	920-1296	9	24	5	3	-0.681325	0.6692	no
gi|641548791|gb|KK865681.1|	237-555	7.8	26	8	6	-0.42847	0.7699	no
gi|641548795|gb|KK865679.1|	89-1020	10	31	2	1	-0.855124	0.56515	no
gi|641548799|gb|KK865677.1|	4034-5030	7.2	22	3	2	-0.94253	0.5005	no
gi|641548848|gb|KK865654.1|	1791-3245	7.4	20	82	67	-0.287841	0.8934	no
gi|641548884|gb|KK865636.1|	3152-3885	8.2	44	0	2	inf	0.00375	no
gi|641548902|gb|KK865628.1|	7986-8636	12	42	0.466979	3	3	0.19335	no
gi|641548911|gb|KK865624.1|	35-1352	9.2	31	0.252484	15	6	0.11615	no
gi|641548935|gb|KK865613.1|	624-1109	5.2	22	22	31	0.49383	0.70025	no
gi|641548947|gb|KK865607.1|	3571-3779	9	12	18	0	-inf	0.01175	no
gi|641548959|gb|KK865601.1|	2546-2895	12	38	4	3	-0.74441	0.63835	no
gi|641548959|gb|KK865601.1|	58-998	12	38	28	15	-0.839004	0.5049	no
gi|641548988|gb|KK865587.1|	620-969	9.7	30	8	4	-0.924982	0.56085	no
gi|641548992|gb|KK865585.1|	3348-3753	7.2	19	2	2	0.000385715	0.91115	no
gi|641549004|gb|KK865579.1|	2539-2912	4.1	13	0.780892	5	3	0.30875	no
gi|641549026|gb|KK865569.1|	0-319	9	25	12	3	-2	0.2489	no
gi|641549026|gb|KK865569.1|	2422-2948	9	25	3	1	-1	0.5005	no

gi|641549066|gb|KK865549.1|	185-1147	9.8	12	2	2	0.217608	0.882	no
gi|641549092|gb|KK865537.1|	3056-3870	7.7	26	0.801059	1	0.795439	0.63025	no
gi|641549092|gb|KK865537.1|	42-796	7.7	26	1	5	2	0.2392	no
gi|641549092|gb|KK865537.1|	5974-6283	7.7	26	5	4	-0.108552	0.9242	no
gi|641549092|gb|KK865537.1|	893-1876	7.7	26	0.629195	1	1	0.48415	no
gi|641549100|gb|KK865534.1|	3173-3841	10	27	8	7	-0.137098	0.9188	no
gi|641549114|gb|KK865528.1|	5433-5857	9.1	25	3	4	0.417411	0.78025	no
gi|641549116|gb|KK865527.1|	2593-3072	14	57	0.994311	2	1	0.51315	no
gi|641549145|gb|KK865513.1|	3307-3594	8.2	15	21	9	-1	0.4148	no
gi|641549163|gb|KK865504.1|	24-410	7.8	27	4	1	-2	0.31725	no
gi|641549198|gb|KK865487.1|	3101-4061	7.4	28	0.462942	2	2	0.3084	no
gi|641549198|gb|KK865487.1|	4843-5969	7.4	28	0.760745	0.578934	-0.394014	1	no
gi|641549200|gb|KK865486.1|	0-1251	9.8	28	11	4	-2	0.2363	no
gi|641549200|gb|KK865486.1|	3064-3344	9.8	28	15	8	-0.952458	0.53525	no
gi|641549202|gb|KK865485.1|	1292-4026	9.3	30	9	2	-2	0.1235	no
gi|641549202|gb|KK865485.1|	4152-4987	9.3	30	9	2	-2	0.09865	no
gi|641549202|gb|KK865485.1|	48-778	9.3	30	5	3	-0.720618	0.6074	no
gi|641549202|gb|KK865485.1|	7194-7530	9.3	30	0	9	inf	0.00375	no
gi|641549204|gb|KK865484.1|	58-1165	9.8	25	23	2	-3	0.0222	no
gi|641549208|gb|KK865482.1|	3978-4547	11	38	0.946224	2	1	0.51085	no
gi|641549208|gb|KK865482.1|	7398-9953	11	38	11	16	0.464632	0.77695	no
gi|641549213|gb|KK865480.1|	2573-3501	nan	12	0.869689	0.293803	-2	1	no
gi|641549222|gb|KK865476.1|	236-831	5.7	19	5	0.267834	-4	0.1901	no
gi|641549228|gb|KK865473.1|	1807-3052	9.1	29	9	7	-0.301502	0.8132	no
gi|641549228|gb|KK865473.1|	36-1695	9.1	29	4	3	-0.440067	0.7303	no
gi|641549234|gb|KK865470.1|	3474-3691	14	51	0	952	inf	0.00055	yes
gi|641549243|gb|KK865465.1|	7292-7803	5.2	12	8	3	-1	0.3303	no
gi|641549255|gb|KK865458.1|	520-1316	8.7	32	0.117866	3	5	0.1123	no
gi|641549283|gb|KK865442.1|	1953-2477	11	39	2	0.649105	-1	0.4534	no
gi|641549292|gb|KK865438.1|	78-312	13	21	15	4	-2	0.27505	no

gi|641549306|gb|KK865431.1|	7341-7652	8.8	21	7	8	0.15494	0.91535	no
gi|641549306|gb|KK865431.1|	7747-8299	8.8	21	2	2	0.180833	0.91015	no
gi|641549310|gb|KK865429.1|	25-706	9.3	34	0.437236	2	2	0.3323	no
gi|641549325|gb|KK865422.1|	2479-3290	6.3	20	17	2	-3	0.05175	no
gi|641549325|gb|KK865422.1|	36-771	6.3	20	23	13	-0.835653	0.50855	no
gi|641549340|gb|KK865415.1|	6098-6859	3.2	13	0.500626	13	5	0.1177	no
gi|641549342|gb|KK865414.1|	0-1871	9.1	25	4	4	0.00889439	0.9935	no
gi|641549342|gb|KK865414.1|	3464-3787	9.1	25	4	5	0.379949	0.8102	no
gi|641549352|gb|KK865409.1|	11541-11758	7.8	13	27	27	0.0362788	0.96365	no
gi|641549358|gb|KK865406.1|	1556-2211	6.5	20	11	7	-0.622876	0.63405	no
gi|641549363|gb|KK865404.1|	6706-7075	8.6	13	12	13	0.133242	0.9211	no
gi|641549363|gb|KK865404.1|	88-3285	8.6	13	70	69	-0.0118339	0.9949	no
gi|641549367|gb|KK865402.1|	1274-1792	5.9	17	1	1	0.00923504	0.91115	no
gi|641549384|gb|KK865394.1|	527-716	8.3	36	11	35	2	0.34335	no
gi|641549392|gb|KK865390.1|	1824-2031	11	36	1873.69	721.42	-1	0.40915	no
gi|641549392|gb|KK865390.1|	7216-9171	11	36	363.45	189	-0.942363	0.5999	no
gi|641549416|gb|KK865379.1|	2175-3538	8.8	20	0.727612	0.092369	-3	1	no
gi|641549451|gb|KK865363.1|	0-782	11	29	0	128	inf	0.00015	yes
gi|641549472|gb|KK865354.1|	0-665	9.4	34	19	9	-1	0.40905	no
gi|641549472|gb|KK865354.1|	2514-3170	9.4	34	19	14	-0.400456	0.75085	no
gi|641549488|gb|KK865347.1|	1-2169	8	30	5	8	0.748741	0.54825	no
gi|641549488|gb|KK865347.1|	2560-5574	8	30	4	2	-0.602306	0.6404	no
gi|641549488|gb|KK865347.1|	8825-9257	8	30	11	8	-0.494365	0.7125	no
gi|641549497|gb|KK865343.1|	5800-5969	4.9	21	41	79	0.928608	0.5574	no
gi|641549497|gb|KK865343.1|	9241-9484	4.9	21	20	19	-0.0350218	0.9646	no
gi|641549501|gb|KK865341.1|	11-1399	8.9	17	3	4	0.639088	0.6261	no
gi|641549501|gb|KK865341.1|	7748-7911	8.9	17	51	16	-2	0.3627	no
gi|641549505|gb|KK865339.1|	124-526	9.7	33	4	7	0.700485	0.64285	no
gi|641549505|gb|KK865339.1|	3508-4370	9.7	33	0.743386	1	0.966049	0.5733	no
gi|641549512|gb|KK865336.1|	127-1085	7	19	2	0.98808	-0.675517	0.6649	no

gi|641549516|gb|KK865334.1|	2-1922	8.7	33	8	12	0.556166	0.65375	no
gi|641549516|gb|KK865334.1|	3605-3992	8.7	33	1	6	2	0.26275	no
gi|641549522|gb|KK865331.1|	115-775	6.3	20	14	12	-0.251483	0.84385	no
gi|641549524|gb|KK865330.1|	6658-7516	8.8	27	3	4	0.542025	0.68935	no
gi|641549542|gb|KK865322.1|	2458-3287	12	39	0.782098	0.678812	-0.204336	1	no
gi|641549542|gb|KK865322.1|	8113-8702	12	39	10	19	0.939781	0.46825	no
gi|641549558|gb|KK865315.1|	3157-4140	4.9	17	0.17977	1	3	0.2991	no
gi|641549581|gb|KK865306.1|	5089-5270	11	37	62	14	-2	0.23555	no
gi|641549602|gb|KK865296.1|	1552-1772	7.9	29	0	26	inf	0.00375	no
gi|641549604|gb|KK865295.1|	174-1171	5.9	12	2	2	-0.231534	0.8652	no
gi|641549606|gb|KK865294.1|	1433-1681	3.6	10	87	75	-0.196642	0.87525	no
gi|641549615|gb|KK865290.1|	57-1415	12	24	35.38	8	-2	0.2314	no
gi|641549615|gb|KK865290.1|	8144-8399	12	24	16	1	-4	0.20495	no
gi|641549623|gb|KK865287.1|	2565-2958	9.5	36	4	7	0.806333	0.598	no
gi|641549623|gb|KK865287.1|	47-1251	9.5	36	4	2	-1	0.42625	no
gi|641549627|gb|KK865285.1|	22-408	9	15	6	4	-0.609587	0.6847	no
gi|641549627|gb|KK865285.1|	5072-6042	9	15	0.82254	1	0.434786	0.78015	no
gi|641549633|gb|KK865282.1|	4633-4868	7.1	13	8	7	-0.260615	0.88385	no
gi|641549653|gb|KK865272.1|	2945-4180	11	47	1	4	2	0.2311	no
gi|641549655|gb|KK865271.1|	19-763	6	25	0.903219	3	1	0.36635	no
gi|641549659|gb|KK865269.1|	2698-6353	24	120	2701.44	1	-11	0.2544	no
gi|641549659|gb|KK865269.1|	7574-11577	24	120	6323.29	3	-11	5.00E-05	yes
gi|641549661|gb|KK865268.1|	1493-2332	9.1	27	78	10	-3	0.123	no
gi|641549661|gb|KK865268.1|	32-1097	9.1	27	0.814103	2	1	0.3975	no
gi|641549663|gb|KK865267.1|	1638-3280	9.6	34	3	2	-0.440661	0.74275	no
gi|641549663|gb|KK865267.1|	3741-4790	9.6	34	1	2	0.190377	0.8991	no
gi|641549663|gb|KK865267.1|	41-314	9.6	34	12	7	-0.761846	0.62905	no
gi|641549667|gb|KK865265.1|	108-815	8.4	14	10	8	-0.337784	0.7943	no
gi|641549682|gb|KK865258.1|	1777-2324	9.5	36	2.01	2	-0.141323	0.9254	no
gi|641549682|gb|KK865258.1|	4436-4797	9.5	36	14	3	-2	0.1912	no

gi|641549687|gb|KK865256.1|	0-774	10	29	106	37	-2	0.3741	no
gi|641549689|gb|KK865255.1|	10121-11441	12	41	3	2	-0.947509	0.48845	no
gi|641549689|gb|KK865255.1|	2352-2739	12	41	3	2	-0.586738	0.70485	no
gi|641549689|gb|KK865255.1|	2880-3826	12	41	1	1	-0.202887	0.8956	no
gi|641549689|gb|KK865255.1|	797-1801	12	41	1	0.798996	-0.510447	0.7498	no
gi|641549724|gb|KK865237.1|	8454-9053	6.3	23	8	10	0.456891	0.72625	no
gi|641549726|gb|KK865236.1|	903-1304	11	28	17	15	-0.122926	0.92395	no
gi|641549728|gb|KK865235.1|	4679-5488	9.6	25	1	1	0.0881429	0.9392	no
gi|641549730|gb|KK865234.1|	0-960	4.1	16	7	6	-0.235911	0.8496	no
gi|641549730|gb|KK865234.1|	1471-2047	4.1	16	2	3	0.596886	0.6792	no
gi|641549730|gb|KK865234.1|	4140-4791	4.1	16	2	3	0.392954	0.78845	no
gi|641549732|gb|KK865233.1|	4141-4944	7.6	21	1	0.707659	-0.85681	0.60675	no
gi|641549734|gb|KK865232.1|	2985-3698	8.5	39	0.820498	1	0.600949	0.70555	no
gi|641549740|gb|KK865229.1|	5435-5630	10	34	12	68	3	0.1641	no
gi|641549744|gb|KK865227.1|	237-586	7	22	5	3	-0.881914	0.60075	no
gi|641549753|gb|KK865223.1|	2609-2928	4.7	33	121	107	-0.180327	0.88235	no
gi|641549753|gb|KK865223.1|	5439-5575	4.7	33	126	119	-0.0872973	0.91115	no
gi|641549755|gb|KK865222.1|	11-805	12	43	11	9	-0.251673	0.8397	no
gi|641549755|gb|KK865222.1|	2713-4682	12	43	1	2	0.85693	0.52105	no
gi|641549755|gb|KK865222.1|	4794-5135	12	43	10	25	1	0.33285	no
gi|641549755|gb|KK865222.1|	5265-6404	12	43	0.675239	0.342596	-0.978889	1	no
gi|641549755|gb|KK865222.1|	6758-7655	12	43	1	2	0.225408	0.87445	no
gi|641549757|gb|KK865221.1|	1151-1369	10	39	0	25	inf	0.00455	no
gi|641549759|gb|KK865220.1|	582-895	8.8	34	4	4	0.0850023	0.9242	no
gi|641549777|gb|KK865211.1|	190-878	8.5	31	3	1	-1	0.48745	no
gi|641549789|gb|KK865205.1|	16-2146	7.1	25	10	0.055828	-8	0.18285	no
gi|641549789|gb|KK865205.1|	2390-3396	7.1	25	52	10	-2	0.197	no
gi|641549789|gb|KK865205.1|	5313-5900	7.1	25	24	0	-inf	5.00E-05	yes
gi|641549806|gb|KK865197.1|	9289-9862	9.6	30	8	7	-0.151696	0.91535	no
gi|641549819|gb|KK865191.1|	2697-2992	9.3	38	5	8	0.566122	0.7371	no

gi|641549827|gb|KK865187.1|	7292-8275	8.8	14	7	8	0.183699	0.88705	no
gi|641549839|gb|KK865181.1|	1211-1390	10	36	28	20	-0.480942	0.7824	no
gi|641549884|gb|KK865160.1|	422-877	10	33	38	30	-0.339046	0.78845	no
gi|641549900|gb|KK865152.1|	12408-13310	10	34	2	2	0.0514377	0.96935	no
gi|641549910|gb|KK865147.1|	5097-5462	5.8	21	4	5	0.580101	0.73035	no
gi|641549916|gb|KK865144.1|	23-228	9	25	19	28	0.532858	0.7207	no
gi|641549921|gb|KK865142.1|	7505-7878	6.2	22	3	8	1	0.4308	no
gi|641549938|gb|KK865134.1|	1089-1365	11	32	41	24	-0.801601	0.55095	no
gi|641549942|gb|KK865132.1|	262-759	7.7	28	6	9	0.70847	0.6059	no
gi|641549942|gb|KK865132.1|	3158-3616	7.7	28	2	3	0.812698	0.6234	no
gi|641549942|gb|KK865132.1|	8001-8617	7.7	28	3	6	1	0.4141	no
gi|641549942|gb|KK865132.1|	815-1243	7.7	28	2	5	1	0.48925	no
gi|641549942|gb|KK865132.1|	8758-9099	7.7	28	5	7	0.438605	0.7689	no
gi|641549980|gb|KK865114.1|	862-1004	11	37	145	0	-inf	0.01175	no
gi|641550012|gb|KK865099.1|	7033-7589	10	40	0.588062	4	3	0.19335	no
gi|641550018|gb|KK865096.1|	6660-7342	7.7	31	1	0.44098	-2	0.4266	no
gi|641550022|gb|KK865094.1|	2710-3107	13	50	16	1	-4	0.13735	no
gi|641550057|gb|KK865077.1|	2871-3261	14	47	5	2	-1	0.4158	no
gi|641550060|gb|KK865076.1|	45-639	7.2	29	0.354879	3	3	0.27515	no
gi|641550063|gb|KK865075.1|	9441-9825	9	32	1	4	2	0.3198	no
gi|641550067|gb|KK865073.1|	2313-3132	4.9	7.2	0.567598	2	2	0.2717	no
gi|641550073|gb|KK865070.1|	166-362	6.7	21	30	13	-1	0.4762	no
gi|641550077|gb|KK865068.1|	2460-3609	7.1	16	4	4	-0.00683757	0.9848	no
gi|641550081|gb|KK865066.1|	14009-14980	4.2	18	6	0	-inf	5.00E-05	yes
gi|641550085|gb|KK865064.1|	3796-4438	11	38	5	4	-0.493964	0.7227	no
gi|641550085|gb|KK865064.1|	4689-5180	11	38	2	2	0.370227	0.82765	no
gi|641550085|gb|KK865064.1|	8644-8899	11	38	5	8	0.817343	0.6381	no
gi|641550085|gb|KK865064.1|	9889-10785	11	38	0.808361	0.767855	-0.0741645	1	no
gi|641550096|gb|KK865057.1|	3772-4144	8.1	20	24	23	-0.0511283	0.9684	no
gi|641550105|gb|KK865053.1|	10660-11266	7.2	24	1	1	0.0130365	0.91115	no

gi|641550105|gb|KK865053.1|	14226-14432	7.2	24	17	6	-2	0.3632	no
gi|641550113|gb|KK865049.1|	2200-3140	11	48	11	12	0.136899	0.9103	no
gi|641550114|gb|KK865048.1|	2049-2539	12	37	29	19	-0.586414	0.6497	no
gi|641550114|gb|KK865048.1|	38-1938	12	37	30	14	-1	0.50275	no
gi|641550114|gb|KK865048.1|	4035-5482	12	37	108	66	-0.727919	0.7272	no
gi|641550148|gb|KK865032.1|	282-819	10	17	2	2	-0.667864	0.68185	no
gi|641550172|gb|KK865020.1|	3562-3830	9	31	8	10	0.236629	0.8785	no
gi|641550185|gb|KK865014.1|	2609-4295	9.7	36	1	2	1	0.39205	no
gi|641550185|gb|KK865014.1|	4599-5531	9.7	36	0.576645	1	0.826711	0.6232	no
gi|641550196|gb|KK865007.1|	7907-8123	7.4	30	18	26	0.538302	0.72795	no
gi|641550200|gb|KK865004.1|	375-778	7.3	12	3	0	-inf	0.01175	no
gi|641550200|gb|KK865004.1|	4162-4431	7.3	12	10	2	-2	0.27505	no
gi|641550237|gb|KK864987.1|	4320-4870	9.8	43	0.199313	17	6	0.1046	no
gi|641550241|gb|KK864985.1|	32-398	10	46	15	17	0.217683	0.86695	no
gi|641550243|gb|KK864984.1|	3529-3968	6.6	23	1	3	1	0.4611	no
gi|641550245|gb|KK864983.1|	0-1785	12	34	36	25	-0.497883	0.8246	no
gi|641550245|gb|KK864983.1|	3507-4004	12	34	33	22	-0.579475	0.65845	no
gi|641550245|gb|KK864983.1|	6124-6456	12	34	9	4	-1	0.39705	no
gi|641550251|gb|KK864980.1|	3854-4661	9.1	33	43	23	-0.880398	0.4835	no
gi|641550251|gb|KK864980.1|	8872-9422	9.1	33	2	2	0.403135	0.8089	no
gi|641550251|gb|KK864980.1|	92-1246	9.1	33	3	6	1	0.4393	no
gi|641550262|gb|KK864975.1|	0-785	6.8	29	7	17	1	0.33815	no
gi|641550262|gb|KK864975.1|	8418-8809	6.8	29	4	35	3	0.0575	no
gi|641550296|gb|KK864961.1|	4428-5224	10	33	2	5	1	0.34375	no
gi|641550325|gb|KK864947.1|	131-1865	8.6	22	76	48	-0.65524	0.75945	no
gi|641550350|gb|KK864936.1|	10541-10784	6.1	23	7	10	0.549941	0.7576	no
gi|641550350|gb|KK864936.1|	9114-9862	6.1	23	0.128098	2	4	0.1175	no
gi|641550369|gb|KK864928.1|	8-667	6.8	27	3	6	1	0.448	no
gi|641550379|gb|KK864924.1|	140-895	6.2	38	2	2	0.338763	0.81785	no
gi|641550379|gb|KK864924.1|	5842-6326	6.2	38	3	1	-1	0.46975	no

gi|641550397|gb|KK864917.1|	0-526	5.6	21	18	14	-0.303501	0.8083	no
gi|641550404|gb|KK864914.1|	2640-3636	7.4	28	47	0.537958	-6	0.07275	no
gi|641550410|gb|KK864911.1|	2178-2647	13	43	4	0	-inf	0.0056	no
gi|641550425|gb|KK864905.1|	11670-11857	12	38	42	32	-0.383545	0.8004	no
gi|641550425|gb|KK864905.1|	12392-12563	12	38	30	25	-0.292651	0.86415	no
gi|641550425|gb|KK864905.1|	13-764	12	38	80	254	2	0.46275	no
gi|641550425|gb|KK864905.1|	14126-14331	12	38	29	25	-0.204107	0.8927	no
gi|641550425|gb|KK864905.1|	15161-15530	12	38	5	8	0.687616	0.64365	no
gi|641550425|gb|KK864905.1|	21307-22031	12	38	5	7	0.516296	0.7012	no
gi|641550425|gb|KK864905.1|	22148-26612	12	38	2	2	-0.282133	0.8245	no
gi|641550430|gb|KK864903.1|	5817-6672	7.9	32	0.21465	2	3	0.25475	no
gi|641550438|gb|KK864900.1|	2548-2891	9.4	43	7	12	0.663932	0.64235	no
gi|641550447|gb|KK864896.1|	3315-3991	13	23	3	0.446428	-3	0.19	no
gi|641550447|gb|KK864896.1|	4769-5837	13	23	0.567913	0.61723	0.120138	1	no
gi|641550463|gb|KK864889.1|	0-327	5	18	0	1617.78	inf	0.00015	yes
gi|641550474|gb|KK864885.1|	8933-9404	8.1	18	178	226	0.341017	0.8798	no
gi|641550477|gb|KK864884.1|	3375-3641	9.7	24	9	0	-inf	0.0102	no
gi|641550477|gb|KK864884.1|	669-924	9.7	24	23	3	-3	0.16155	no
gi|641550503|gb|KK864873.1|	664-856	9.1	39	33	0	-inf	0.0075	no
gi|641550528|gb|KK864861.1|	1984-2471	7.2	15	3	2	-0.786148	0.63075	no
gi|641550530|gb|KK864860.1|	1378-1766	4.4	37	0	6	inf	0.00455	no
gi|641550545|gb|KK864853.1|	5508-6520	11	21	0.953627	0.923076	-0.0469747	0.94545	no
gi|641550545|gb|KK864853.1|	588-870	11	21	4	5	0.562674	0.76735	no
gi|641550545|gb|KK864853.1|	6765-7006	11	21	12	3	-2	0.3145	no
gi|641550561|gb|KK864845.1|	588-2023	10	34	2	13	3	0.0696	no
gi|641550563|gb|KK864844.1|	3667-4200	7.9	26	0	4	inf	0.0025	yes
gi|641550582|gb|KK864835.1|	5445-5892	7	27	0	4	inf	0.0068	no
gi|641550600|gb|KK864827.1|	247-456	6.6	16	41	21	-0.98868	0.523	no
gi|641550602|gb|KK864826.1|	43-1262	7.9	28	3	5	0.808196	0.5349	no
gi|641550602|gb|KK864826.1|	6091-6918	7.9	28	4	12	2	0.2288	no

gi|641550602|gb|KK864826.1|	9533-10069	7.9	28	1	5	2	0.25895	no
gi|641550609|gb|KK864823.1|	4854-5312	11	38	1	3	1	0.4611	no
gi|641550627|gb|KK864815.1|	5572-6357	8	21	1	2	0.727857	0.63585	no
gi|641550637|gb|KK864810.1|	437-1183	9.1	36	1	3	1	0.3594	no
gi|641550645|gb|KK864806.1|	0-554	9.9	29	7	2	-2	0.26455	no
gi|641550645|gb|KK864806.1|	3420-4918	9.9	29	8	3	-2	0.24765	no
gi|641550645|gb|KK864806.1|	6381-7070	9.9	29	4	3	-0.136569	0.92235	no
gi|641550656|gb|KK864801.1|	116-957	7.4	27	13	5	-1	0.2832	no
gi|641550656|gb|KK864801.1|	3876-4586	7.4	27	10	3	-2	0.1581	no
gi|641550656|gb|KK864801.1|	6128-6749	7.4	27	2	2	-0.623893	0.686	no
gi|641550662|gb|KK864798.1|	354-3896	16	53	3	0.032303	-6	0.1829	no
gi|641550662|gb|KK864798.1|	6425-6949	16	53	3	0	-inf	0.0056	no
gi|641550674|gb|KK864792.1|	1479-1874	8.4	28	16	11	-0.54706	0.685	no
gi|641550724|gb|KK864775.1|	6055-6890	12	41	3	1	-1	0.4571	no
gi|641550726|gb|KK864774.1|	540-815	6.1	24	0	17	inf	0.0023	yes
gi|641550733|gb|KK864771.1|	0-516	7	23	6	4	-0.349327	0.8033	no
gi|641550733|gb|KK864771.1|	1316-2632	7	23	6	9	0.63877	0.6105	no
gi|641550755|gb|KK864761.1|	115-1031	8.7	27	0.98241	0	-inf	0.01175	no
gi|641550755|gb|KK864761.1|	2420-2851	8.7	27	75	0.447935	-7	0.18285	no
gi|641550770|gb|KK864754.1|	287-959	6.4	27	1	2	0.599972	0.67805	no
gi|641550770|gb|KK864754.1|	3159-3930	6.4	27	1	3	0.950009	0.5394	no
gi|641550801|gb|KK864740.1|	2079-2910	2.5	16	1	3	1	0.3975	no
gi|641550803|gb|KK864739.1|	3984-4818	11	44	0.443414	1	1	0.44255	no
gi|641550853|gb|KK864719.1|	41-938	5.7	19	4	5	0.211602	0.87575	no
gi|641550855|gb|KK864718.1|	0-360	9.4	20	8	3	-2	0.3099	no
gi|641550855|gb|KK864718.1|	5149-5984	9.4	20	5	3	-0.781558	0.57275	no
gi|641550861|gb|KK864715.1|	3376-3838	5	21	75	60	-0.330315	0.7906	no
gi|641550901|gb|KK864700.1|	1980-3104	9.4	22	0.91486	0.23207	-2	0.31075	no
gi|641550922|gb|KK864692.1|	4-127	11	18	185	607	2	0.35335	no
gi|641550944|gb|KK864684.1|	5012-5182	8	32	80	19	-2	0.2438	no

gi|641550948|gb|KK864682.1|	5438-6537	9.5	37	0.861821	0	-inf	1	no
gi|641550995|gb|KK864659.1|	2276-2530	6.4	15	13	14	0.175451	0.90375	no
gi|641550995|gb|KK864659.1|	3452-3830	6.4	15	51	0	-inf	5.00E-05	yes
gi|641551029|gb|KK864642.1|	4675-5025	7	27	10	7	-0.623945	0.6634	no
gi|641551040|gb|KK864637.1|	3072-4457	6.9	29	1	1	0.220344	0.882	no
gi|641551040|gb|KK864637.1|	64-1257	6.9	29	2	2	0.0828502	0.95285	no
gi|641551042|gb|KK864636.1|	1961-2272	1	2.80E+01	0	10	inf	0.00375	no
gi|641551059|gb|KK864628.1|	143-282	11	38	191	24	-3	0.2348	no
gi|641551059|gb|KK864628.1|	1658-2142	11	38	32	12	-1	0.28535	no
gi|641551059|gb|KK864628.1|	8063-8421	11	38	12	9	-0.420992	0.7568	no
gi|641551069|gb|KK864623.1|	283-583	9.9	15	6	5	-0.280624	0.8649	no
gi|641551071|gb|KK864622.1|	3292-3611	8.4	18	16	26	0.713765	0.59675	no
gi|641551079|gb|KK864618.1|	3550-4797	11	34	2	2	0.606716	0.65265	no
gi|641551079|gb|KK864618.1|	6775-7672	11	34	1	0.460061	-1	0.47135	no
gi|641551093|gb|KK864611.1|	72-802	10	21	0.662034	2	2	0.32945	no
gi|641551099|gb|KK864608.1|	837-1440	12	45	1	2	0.597895	0.69715	no
gi|641551146|gb|KK864585.1|	17-909	8.1	27	8	5	-0.799497	0.53645	no
gi|641551146|gb|KK864585.1|	4589-4794	8.1	27	19	8	-1	0.4938	no
gi|641551152|gb|KK864582.1|	3507-3714	7.5	24	83.98	27	-2	0.2513	no
gi|641551174|gb|KK864571.1|	3288-3519	10	44	5	11	1	0.52535	no
gi|641551180|gb|KK864568.1|	2651-3559	11	42	4	1	-2	0.24755	no
gi|641551204|gb|KK864556.1|	7199-7856	11	24	8	7	-0.236929	0.8567	no
gi|641551208|gb|KK864554.1|	12-564	8.1	33	2	1	-0.40413	0.82205	no
gi|641551221|gb|KK864550.1|	573-1144	8.3	34	1	3	1	0.41185	no
gi|641551223|gb|KK864549.1|	158-650	8.9	21	16	12	-0.364249	0.7781	no
gi|641551229|gb|KK864546.1|	136-1194	9.3	36	0.656567	3	2	0.22535	no
gi|641551231|gb|KK864545.1|	1543-2441	5.7	17	129	0.153137	-10	0.18375	no
gi|641551237|gb|KK864542.1|	3251-3685	4.7	9.1	1	3	0.851315	0.62755	no
gi|641551239|gb|KK864541.1|	11033-11444	6.3	29	2	2	0.586006	0.70505	no
gi|641551245|gb|KK864538.1|	2384-3579	8.1	11	8	7	-0.311114	0.80745	no

gi|641551245|gb|KK864538.1|	3757-4865	8.1	11	8	5	-0.598497	0.643	no
gi|641551245|gb|KK864538.1|	449-599	8.1	11	67	41	-0.719549	0.6976	no
gi|641551247|gb|KK864537.1|	2455-2726	8.4	23	14	7	-0.943022	0.55575	no
gi|641551247|gb|KK864537.1|	27-1086	8.4	23	4	5	0.353956	0.7841	no
gi|641551247|gb|KK864537.1|	7862-8655	8.4	23	4	1	-2	0.2371	no
gi|641551251|gb|KK864535.1|	0-752	5.1	43	11	1	-3	0.06185	no
gi|641551251|gb|KK864535.1|	3110-4653	5.1	43	4	0	-inf	5.00E-05	yes
gi|641551251|gb|KK864535.1|	6813-7226	5.1	43	5	0	-inf	0.0056	no
gi|641551280|gb|KK864521.1|	2170-2614	7.6	29	3	3	-0.147801	0.92525	no
gi|641551284|gb|KK864519.1|	2888-3418	8.2	29	37	15	-1	0.32425	no
gi|641551286|gb|KK864518.1|	5851-6013	8.6	40	29	75	1	0.41995	no
gi|641551288|gb|KK864517.1|	2051-2313	7.6	15	11	3	-2	0.26435	no
gi|641551292|gb|KK864515.1|	127-2150	18	71	2	2	0.0589035	0.9637	no
gi|641551306|gb|KK864508.1|	9705-9938	8.8	39	22	5	-2	0.24665	no
gi|641551308|gb|KK864507.1|	54-272	8.8	29	0	311	inf	5.00E-05	yes
gi|641551310|gb|KK864506.1|	254-1265	8.6	29	0	2	inf	0.00255	yes
gi|641551314|gb|KK864504.1|	0-366	8.2	24	1	7	3	0.2064	no
gi|641551316|gb|KK864503.1|	3031-3756	11	17	3	2	-0.398794	0.77825	no
gi|641551322|gb|KK864500.1|	2864-3132	2.9	6.1	35	15	-1	0.371	no
gi|641551324|gb|KK864499.1|	2780-3567	5	16	0.717952	1	1	0.5082	no
gi|641551326|gb|KK864498.1|	2787-3815	6.8	23	1	3	1	0.31595	no
gi|641551332|gb|KK864495.1|	50-525	12	34	6	3	-0.697949	0.63565	no
gi|641551332|gb|KK864495.1|	7715-8097	12	34	33	12	-1	0.28065	no
gi|641551352|gb|KK864485.1|	0-606	11	47	2	2	-0.179609	0.90465	no
gi|641551358|gb|KK864482.1|	3905-4049	7.2	21	65	110	0.762565	0.6588	no
gi|641551397|gb|KK864463.1|	9388-10131	8.9	35	0.646334	2	2	0.28495	no
gi|641551455|gb|KK864435.1|	1079-1472	4.2	11	2	5	1	0.4021	no
gi|641551461|gb|KK864432.1|	1586-2568	10	37	1	2	1	0.45475	no
gi|641551469|gb|KK864428.1|	21-1265	9.9	46	0.337573	0.719666	1	1	no
gi|641551475|gb|KK864425.1|	2723-3200	5.1	20	2	2	-0.215659	0.8945	no

gi|641551501|gb|KK864412.1|	4131-4663	6.8	38	0	17	inf	0.00015	yes
gi|641551501|gb|KK864412.1|	6972-7390	6.8	38	0	9	inf	0.00135	yes
gi|641551530|gb|KK864398.1|	5-400	10	14	21	9	-1	0.401	no
gi|641551530|gb|KK864398.1|	5912-6318	10	14	21	7	-1	0.28625	no
gi|641551534|gb|KK864396.1|	2825-3296	11	21	3	0.77037	-2	0.3455	no
gi|641551534|gb|KK864396.1|	6337-6676	11	21	4	3	-0.424257	0.7868	no
gi|641551536|gb|KK864395.1|	3184-3775	9.2	29	3	2	-0.572462	0.7144	no
gi|641551536|gb|KK864395.1|	361-778	9.2	29	2	2	0.00167243	0.91115	no
gi|641551544|gb|KK864391.1|	1141-2175	9.9	47	48	49	0.0419474	0.9846	no
gi|641551544|gb|KK864391.1|	4323-4761	9.9	47	2	2	0.325607	0.83525	no
gi|641551554|gb|KK864386.1|	6242-7098	8.4	39	2	2	0.496483	0.73345	no
gi|641551587|gb|KK864370.1|	3662-3926	13	41	9	1	-3	0.2472	no
gi|641551595|gb|KK864366.1|	5185-5351	9	28	41	14	-1	0.4362	no
gi|641551603|gb|KK864362.1|	2389-3254	5.7	25	0.740056	0.803182	0.118091	1	no
gi|641551605|gb|KK864361.1|	141-1169	4	10	2	2	0.0202782	0.9781	no
gi|641551605|gb|KK864361.1|	7791-8236	4	10	3	4	0.299743	0.84335	no
gi|641551609|gb|KK864359.1|	6374-6977	7.1	23	9	4	-1	0.3733	no
gi|641551611|gb|KK864358.1|	382-1264	5.8	19	1	0.783417	-0.396264	0.81255	no
gi|641551624|gb|KK864352.1|	2317-2825	4.3	21	2	1	-1	0.46975	no
gi|641551651|gb|KK864339.1|	1837-2267	10	33	14	16	0.162605	0.90275	no
gi|641551651|gb|KK864339.1|	7842-8212	10	33	6	4	-0.611797	0.6844	no
gi|641551655|gb|KK864337.1|	2673-3174	11	35	9	10.8	0.189878	0.8836	no
gi|641551655|gb|KK864337.1|	8246-8598	11	35	28	36	0.382106	0.7664	no
gi|641551693|gb|KK864319.1|	7939-8665	12	41	6	41	3	0.03915	no
gi|641551707|gb|KK864312.1|	6077-6353	9.3	20	41	2	-4	0.1337	no
gi|641551709|gb|KK864311.1|	8783-9055	9.1	31	11	21	0.922352	0.5259	no
gi|641551768|gb|KK864285.1|	144-796	9.1	18	3	3	0.0969323	0.94755	no
gi|641551768|gb|KK864285.1|	2970-3523	9.1	18	4	2	-0.863517	0.56245	no
gi|641551768|gb|KK864285.1|	5654-6144	9.1	18	2	0.722096	-1	0.4534	no
gi|641551770|gb|KK864284.1|	26-409	9.9	30	2	5	1	0.48155	no

gi|641551788|gb|KK864275.1|	2047-3392	9	27	3	2	-1	0.44925	no
gi|641551788|gb|KK864275.1|	96-863	9	27	3	3	0.0170525	0.9781	no
gi|641551790|gb|KK864274.1|	901-1148	11	17	18	9	-0.95105	0.55415	no
gi|641551804|gb|KK864267.1|	20-577	9.4	19	6	4	-0.703469	0.62395	no
gi|641551804|gb|KK864267.1|	2287-3996	9.4	19	1	0.570501	-1	0.4394	no
gi|641551819|gb|KK864260.1|	0-418	7.8	40	1	3	1	0.51315	no
gi|641551833|gb|KK864253.1|	5528-6193	7.5	33	0.3014	5	4	0.2392	no
gi|641551847|gb|KK864246.1|	2980-3506	7.5	38	1	2	0.331585	0.83525	no
gi|641551860|gb|KK864240.1|	11037-12116	9.9	29	6	6	0.0297891	0.9806	no
gi|641551879|gb|KK864231.1|	2410-2697	12	41	0	13	inf	0.0025	yes
gi|641551879|gb|KK864231.1|	2923-3923	12	41	0.527917	0.802947	0.604994	1	no
gi|641551879|gb|KK864231.1|	5997-6946	12	41	0.751518	0.571323	-0.395501	1	no
gi|641551879|gb|KK864231.1|	7044-8840	12	41	4	5	0.234827	0.8534	no
gi|641551879|gb|KK864231.1|	9112-13748	12	41	6	6	-0.200843	0.8711	no
gi|641551879|gb|KK864231.1|	99-345	12	41	72	34	-1	0.41745	no
gi|641551888|gb|KK864227.1|	263-704	6.2	17	0.572372	5	3	0.25965	no
gi|641551888|gb|KK864227.1|	3055-3242	6.2	17	42	211	2	0.1249	no
gi|641551896|gb|KK864223.1|	4279-4780	8.7	26	13	7	-0.892193	0.51165	no
gi|641551943|gb|KK864205.1|	2006-2838	8.5	38	0.222404	10	5	0.23305	no
gi|641551948|gb|KK864202.1|	59-736	10	37	1	0.222755	-3	0.271	no
gi|641551958|gb|KK864197.1|	1942-2084	8.1	33	145	287	0.98397	0.5348	no
gi|641551958|gb|KK864197.1|	307-548	8.1	33	2	16	3	0.2768	no
gi|641551968|gb|KK864192.1|	137-1383	9.6	40	0.87602	2	1	0.46885	no
gi|641551976|gb|KK864188.1|	12967-15021	10	17	1	1	0.377829	0.78285	no
gi|641551976|gb|KK864188.1|	15233-16493	10	17	0.266	0.911434	2	0.33105	no
gi|641551976|gb|KK864188.1|	232-1043	10	17	4	6	0.446628	0.73445	no
gi|641551995|gb|KK864178.1|	3485-4054	9.7	33	0.37849	5	4	0.24305	no
gi|641551997|gb|KK864177.1|	0-398	4.8	15	4	0	-inf	0.01	no
gi|641552007|gb|KK864172.1|	2855-3203	8.7	21	9	3	-1	0.36935	no
gi|641552009|gb|KK864171.1|	3350-3818	7.1	12	14	18	0.301552	0.8146	no

gi|641552009|gb|KK864171.1|	69-811	7.1	12	5	4	-0.135416	0.91855	no
gi|641552017|gb|KK864167.1|	2662-3225	6.8	23	0.769255	2	1	0.51315	no
gi|641552017|gb|KK864167.1|	9-1217	6.8	23	9	7	-0.382005	0.7676	no
gi|641552030|gb|KK864161.1|	2-292	18	60	9	36	2	0.17605	no
gi|641552038|gb|KK864157.1|	100-664	6.8	27	0.191795	7	5	0.1079	no
gi|641552046|gb|KK864153.1|	14670-16876	57	46	2	0.483496	-2	0.1218	no
gi|641552046|gb|KK864153.1|	324-2028	57	46	2	0.143097	-3	0.144	no
gi|641552046|gb|KK864153.1|	5024-5633	57	46	2	0.517795	-2	0.4266	no
gi|641552046|gb|KK864153.1|	5772-9582	57	46	3	0.687935	-2	0.1345	no
gi|641552050|gb|KK864151.1|	167-435	5.3	23	0	15	inf	0.00375	no
gi|641552061|gb|KK864146.1|	3339-3649	9.7	28	13	24	0.853542	0.5368	no
gi|641552069|gb|KK864142.1|	108-299	5	37	3	285	7	0.1044	no
gi|641552079|gb|KK864137.1|	2967-4064	10	24	10	8	-0.305408	0.8102	no
gi|641552079|gb|KK864137.1|	412-1223	10	24	13	17	0.419525	0.73925	no
gi|641552090|gb|KK864130.1|	1-335	6.8	27	12	23	0.930946	0.49635	no
gi|641552090|gb|KK864130.1|	3757-4305	6.8	27	2	0.908687	-0.819349	0.6492	no
gi|641552098|gb|KK864126.1|	2857-3296	8.9	29	3	0	-inf	0.01175	no
gi|641552108|gb|KK864121.1|	5348-6342	12	34	0.265941	2	3	0.19335	no
gi|641552110|gb|KK864120.1|	0-619	5.8	26	16	15	-0.00998697	0.99215	no
gi|641552124|gb|KK864113.1|	3149-3816	8.2	19	3	2	-0.478161	0.73945	no
gi|641552124|gb|KK864113.1|	581-1434	8.2	19	0.430605	0.981294	1	0.51305	no
gi|641552141|gb|KK864105.1|	3007-3295	6.8	15	0	12	inf	0.00375	no
gi|641552145|gb|KK864103.1|	3027-3469	7.3	26	0.57011	7	4	0.24715	no
gi|641552158|gb|KK864097.1|	10782-11626	11	29	15	13	-0.186199	0.8796	no
gi|641552158|gb|KK864097.1|	8482-9316	11	29	1	0.505149	-1	0.47135	no
gi|641552170|gb|KK864091.1|	2832-4025	7.2	24	5	16	2	0.22355	no
gi|641552172|gb|KK864090.1|	1487-2569	14	46	2	1	-0.716256	0.61465	no
gi|641552182|gb|KK864085.1|	2468-2959	8.2	16	7	1	-3	0.1479	no
gi|641552188|gb|KK864082.1|	5064-6265	8.8	30	4	2	-0.799533	0.5604	no
gi|641552202|gb|KK864075.1|	1372-1900	10	29	2	4	0.720253	0.63605	no

gi|641552204|gb|KK864074.1|	20-286	11	21	8	9	0.19536	0.8977	no
gi|641552204|gb|KK864074.1|	818-1011	11	21	770	191.37	-2	0.1262	no
gi|641552207|gb|KK864073.1|	457-621	10	28	93	93	0.00827668	0.9637	no
gi|641552207|gb|KK864073.1|	7507-8355	10	28	4	6	0.481299	0.7121	no
gi|641552219|gb|KK864067.1|	6418-7218	6.9	34	0	3	inf	0.0023	yes
gi|641552221|gb|KK864066.1|	167-862	9.5	38	0.425006	2	2	0.21365	no
gi|641552234|gb|KK864060.1|	4742-5028	8.2	25	0	30	inf	0.0004	yes
gi|641552263|gb|KK864046.1|	3087-3303	7.6	18	96	500	2	0.0796	no
gi|641552269|gb|KK864045.1|	912-4034	8.9	48	0.556419	320	9	0.00645	no
gi|641552289|gb|KK864038.1|	2877-3552	7	22	6	4	-0.721879	0.5916	no
gi|641552291|gb|KK864037.1|	208-661	8.5	27	1	3	1	0.4427	no
gi|641552307|gb|KK864029.1|	1686-2368	4	21	1	0.881961	-0.721703	0.67005	no
gi|641552340|gb|KK864014.1|	2478-3771	6.4	17	12	2	-2	0.07185	no
gi|641552340|gb|KK864014.1|	36-643	6.4	17	4	3	-0.340566	0.81335	no
gi|641552354|gb|KK864007.1|	140-1341	9.1	31	6	6	-0.00359427	0.9925	no
gi|641552354|gb|KK864007.1|	7157-7695	9.1	31	2	3	1	0.49835	no
gi|641552386|gb|KK863993.1|	0-149	44	46	4296.7	12157.8	2	0.5125	no
gi|641552386|gb|KK863993.1|	380-2578	44	46	6	58	3	0.0987	no
gi|641552402|gb|KK863985.1|	165-1526	9.4	21	0.72886	0.370108	-0.977698	1	no
gi|641552408|gb|KK863982.1|	2255-3077	7.1	41	2	0.857931	-0.882512	0.5831	no
gi|641552430|gb|KK863971.1|	1348-4565	9.9	39	7	5	-0.527124	0.67835	no
gi|641552430|gb|KK863971.1|	15760-17006	9.9	39	2	2	-0.0607141	0.9661	no
gi|641552430|gb|KK863971.1|	720-1283	9.9	39	1	1	-0.211004	0.8945	no
gi|641552433|gb|KK863970.1|	19-859	7.1	31	2	0.667304	-1	0.46585	no
gi|641552433|gb|KK863970.1|	942-1175	7.1	31	5	13	1	0.4482	no
gi|641552435|gb|KK863969.1|	17-914	6.8	26	3	7	1	0.41365	no
gi|641552435|gb|KK863969.1|	2828-3451	6.8	26	2	7	2	0.2393	no
gi|641552442|gb|KK863966.1|	99-1159	8.4	37	4	9	1	0.43605	no
gi|641552458|gb|KK863959.1|	3232-3724	1.8	nan	1	1	0.00772017	0.91115	no
gi|641552469|gb|KK863954.1|	10196-10682	11	36	8	9	0.236155	0.8641	no

gi|641552469|gb|KK863954.1|	253-1192	11	36	0.761623	0.289481	-1	1	no
gi|641552484|gb|KK863947.1|	15891-16751	11	45	0.42607	1	2	0.3613	no
gi|641552484|gb|KK863947.1|	239-1955	11	45	5	2	-1	0.4053	no
gi|641552484|gb|KK863947.1|	2641-3344	11	45	0.278879	2	3	0.2851	no
gi|641552488|gb|KK863945.1|	2775-4308	8.6	40	5	0	-inf	5.00E-05	yes
gi|641552496|gb|KK863941.1|	3117-4314	9.2	19	0.989554	1	0.384043	0.79305	no
gi|641552502|gb|KK863938.1|	11250-11997	12	43	1	2	0.891153	0.57435	no
gi|641552506|gb|KK863936.1|	19-1015	8	29	1	2	0.698067	0.6288	no
gi|641552512|gb|KK863933.1|	613-1846	8.3	33	8	5	-0.745662	0.5631	no
gi|641552529|gb|KK863924.1|	1380-1732	6.9	19	6	5	-0.229329	0.8776	no
gi|641552535|gb|KK863921.1|	3653-4670	11	37	0.775492	2	1	0.43005	no
gi|641552535|gb|KK863921.1|	43-1969	11	37	0.90002	1	0.608919	0.6608	no
gi|641552539|gb|KK863919.1|	6203-6666	5.4	20	2	5	1	0.45715	no
gi|641552566|gb|KK863906.1|	1955-2821	12	42	0.422259	14	5	0.11675	no
gi|641552574|gb|KK863902.1|	115-719	9.3	14	2	0.524041	-2	0.4266	no
gi|641552600|gb|KK863890.1|	5029-5379	7.2	27	4	1	-2	0.3414	no
gi|641552608|gb|KK863886.1|	1699-2226	7.5	24	3	0	-inf	0.0059	no
gi|641552608|gb|KK863886.1|	2339-2766	7.5	24	7	10	0.456382	0.74445	no
gi|641552615|gb|KK863883.1|	2720-3175	6.5	19	3	5	0.715601	0.63725	no
gi|641552615|gb|KK863883.1|	5586-6115	6.5	19	4	2	-0.685335	0.65225	no
gi|641552617|gb|KK863882.1|	11870-12069	6.1	25	148	354.31	1	0.325	no
gi|641552626|gb|KK863878.1|	253-1002	7.1	26	2	0.776147	-1	0.44155	no
gi|641552645|gb|KK863869.1|	1280-1787	9.2	37	8	5	-0.57634	0.6725	no
gi|641552659|gb|KK863863.1|	24-723	9.1	27	3	2	-0.647294	0.66025	no
gi|641552659|gb|KK863863.1|	9374-10593	9.1	27	2	2	-0.486547	0.72355	no
gi|641552688|gb|KK863849.1|	2721-5941	9.7	33	3	2	-0.507188	0.69265	no
gi|641552692|gb|KK863847.1|	6785-7026	5.3	12	7	7	-0.0358009	0.91115	no
gi|641552700|gb|KK863843.1|	1409-1964	11	54	17	55	2	0.2025	no
gi|641552700|gb|KK863843.1|	3953-4374	11	54	13	14	0.0874687	0.9467	no
gi|641552712|gb|KK863837.1|	2623-3386	5.8	32	2	0.567865	-2	0.24335	no

gi|641552719|gb|KK863834.1|	2338-3228	9.2	30	18	8	-1	0.3304	no
gi|641552719|gb|KK863834.1|	46-556	9.2	30	5	2	-1	0.43995	no
gi|641552733|gb|KK863828.1|	2518-3498	8.8	27	1	2	0.0642392	0.9579	no
gi|641552782|gb|KK863804.1|	6717-7172	10	35	2	1	-0.824967	0.64915	no
gi|641552782|gb|KK863804.1|	7616-9215	10	35	1	0.922616	-0.391876	0.78285	no
gi|641552803|gb|KK863793.1|	5476-5983	13	36	2	1	-0.728343	0.67005	no
gi|641552805|gb|KK863792.1|	140-1507	14	44	3	0.368219	-3	0.097	no
gi|641552805|gb|KK863792.1|	6056-6265	14	44	482.65	73	-3	0.0483	no
gi|641552812|gb|KK863788.1|	104-859	7.8	15	0.758983	1	0.601797	0.70555	no
gi|641552828|gb|KK863780.1|	128-1135	8.8	29	4	5	0.382665	0.7773	no
gi|641552828|gb|KK863780.1|	6326-6955	8.8	29	5	4	-0.220675	0.87585	no
gi|641552845|gb|KK863772.1|	6331-6577	8.3	34	28	8	-2	0.2445	no
gi|641552845|gb|KK863772.1|	6812-8677	8.3	34	2	3	0.637364	0.6226	no
gi|641552845|gb|KK863772.1|	714-1028	8.3	34	2	7	2	0.3198	no
gi|641552848|gb|KK863771.1|	0-511	5.9	16	9	2	-2	0.1742	no
gi|641552848|gb|KK863771.1|	5075-5266	5.9	16	0	222	inf	5.00E-05	yes
gi|641552859|gb|KK863766.1|	0-909	11	41	1	0.45237	-2	0.40415	no
gi|641552861|gb|KK863765.1|	1133-2191	8.4	30	0	2	inf	0.00135	yes
gi|641552861|gb|KK863765.1|	4300-4455	8.4	30	0	512	inf	0.0001	yes
gi|641552891|gb|KK863750.1|	1558-1956	6.1	20	0	52	inf	5.00E-05	yes
gi|641552891|gb|KK863750.1|	3043-3796	6.1	20	0.380852	3	3	0.1811	no
gi|641552910|gb|KK863741.1|	146-751	5.9	16	4	0	-inf	0.0024	yes
gi|641552935|gb|KK863732.1|	453-1196	13	21	11	5	-1	0.33945	no
gi|641552935|gb|KK863732.1|	4895-5183	13	21	7	1	-3	0.2472	no
gi|641552940|gb|KK863730.1|	20-811	11	34	0.831987	2	0.964997	0.5733	no
gi|641552940|gb|KK863730.1|	2632-3392	11	34	1	3	2	0.3469	no
gi|641552944|gb|KK863728.1|	3493-3787	7.2	27	6	5	-0.28213	0.8649	no
gi|641552952|gb|KK863725.1|	1259-1673	8.7	34	0	15	inf	0.0003	yes
gi|641552954|gb|KK863724.1|	502-691	2.3	9.3	22	12	-0.890619	0.63655	no
gi|641552958|gb|KK863722.1|	3282-4198	7.2	22	121	11.2	-3	0.08215	no

gi|641552958|gb|KK863722.1|	6055-6364	7.2	22	6	0.865333	-3	0.2375	no
gi|641552958|gb|KK863722.1|	68-561	7.2	22	6	1	-3	0.1746	no
gi|641552960|gb|KK863721.1|	12955-13641	11	16	8	2	-2	0.21595	no
gi|641552960|gb|KK863721.1|	2085-3555	11	16	19	15	-0.38815	0.75725	no
gi|641552962|gb|KK863720.1|	2222-2865	7	35	1	2	0.599174	0.6936	no
gi|641552983|gb|KK863712.1|	133-731	12	57	1	1	0.334684	0.83525	no
gi|641552998|gb|KK863705.1|	416-896	8.8	30	1	2	0.591901	0.70555	no
gi|641553015|gb|KK863697.1|	8371-8736	11	17	22	5	-2	0.1529	no
gi|641553018|gb|KK863696.1|	0-326	5	8.2	42	40	-0.0838972	0.94535	no
gi|641553056|gb|KK863681.1|	4413-5176	8.8	31	0.87301	0.757154	-0.205412	1	no
gi|641553064|gb|KK863677.1|	106-929	8.7	38	0.676777	4	3	0.1324	no
gi|641553083|gb|KK863668.1|	3226-4005	7.7	32	1	0.920688	-0.245772	0.88495	no
gi|641553095|gb|KK863662.1|	2675-4275	7.8	25	63	25	-1	0.422	no
gi|641553101|gb|KK863659.1|	106-521	7	12	10	7	-0.460877	0.7336	no
gi|641553119|gb|KK863650.1|	7438-8231	7.5	29	55	60	0.126291	0.9353	no
gi|641553128|gb|KK863646.1|	1948-3341	26	150	0	21	inf	5.00E-05	yes
gi|641553130|gb|KK863645.1|	2841-3690	9.8	25	17	12	-0.472646	0.7065	no
gi|641553130|gb|KK863645.1|	5463-5644	9.8	25	20	183	3	0.09	no
gi|641553142|gb|KK863639.1|	0-445	6	15	6	14	1	0.3723	no
gi|641553144|gb|KK863638.1|	0-578	13	22	5	4	-0.157922	0.90935	no
gi|641553146|gb|KK863637.1|	2290-3377	14	40	0.714616	0.604139	-0.242288	1	no
gi|641553146|gb|KK863637.1|	3743-4073	14	40	7	6	-0.126418	0.9347	no
gi|641553146|gb|KK863637.1|	6042-6533	14	40	0	6	inf	0.0016	yes
gi|641553152|gb|KK863634.1|	852-1159	4.3	17	1	7	3	0.30875	no
gi|641553168|gb|KK863626.1|	107-1397	12	33	1	0.394022	-2	0.362	no
gi|641553168|gb|KK863626.1|	3518-4103	12	33	2	0.274602	-3	0.271	no
gi|641553168|gb|KK863626.1|	5454-5593	12	33	589	98	-3	0.1318	no
gi|641553170|gb|KK863625.1|	222-999	11	62	1	0.369532	-2	0.4266	no
gi|641553170|gb|KK863625.1|	3250-4308	11	62	1	0.24974	-2	0.26805	no
gi|641553172|gb|KK863624.1|	10807-12535	10	41	5	3	-0.600975	0.64095	no

gi|641553172|gb|KK863624.1|	6066-6270	10	41	4	57	4	0.24115	no
gi|641553172|gb|KK863624.1|	8071-8396	10	41	3	5	0.57301	0.7575	no
gi|641553176|gb|KK863622.1|	10339-11039	11	19	47	54	0.189919	0.87385	no
gi|641553176|gb|KK863622.1|	2565-2927	11	19	11	9	-0.268357	0.84635	no
gi|641553189|gb|KK863616.1|	6620-6991	10	40	2	4	1	0.5126	no
gi|641553189|gb|KK863616.1|	7109-8358	10	40	0.739137	3	2	0.2143	no
gi|641553239|gb|KK863593.1|	2433-3281	5.6	21	2	6	2	0.21335	no
gi|641553257|gb|KK863584.1|	6394-7029	6.5	33	37	36	-0.0504426	0.9672	no
gi|641553263|gb|KK863581.1|	3510-3765	8.8	30	21	15	-0.445691	0.76325	no
gi|641553263|gb|KK863581.1|	5927-6301	8.8	30	3	2	-0.225928	0.88445	no
gi|641553265|gb|KK863580.1|	11-507	3.1	7.6	2	5	2	0.29615	no
gi|641553277|gb|KK863574.1|	9083-10138	11	32	2	5	0.917407	0.5039	no
gi|641553288|gb|KK863569.1|	3283-3847	9.8	37	2	8	2	0.18745	no
gi|641553288|gb|KK863569.1|	5764-6511	9.8	37	61	53	-0.193534	0.87775	no
gi|641553288|gb|KK863569.1|	679-937	9.8	37	27	38	0.504703	0.7092	no
gi|641553292|gb|KK863567.1|	157-460	10	33	5	3	-0.846933	0.64855	no
gi|641553292|gb|KK863567.1|	3972-4838	10	33	0.844518	0.641593	-0.396469	1	no
gi|641553307|gb|KK863560.1|	2752-3036	4.7	15	9	6	-0.552253	0.73315	no
gi|641553315|gb|KK863556.1|	1580-2279	9.8	18	1	0.852482	-0.721295	0.67005	no
gi|641553326|gb|KK863551.1|	3269-3841	9.3	27	16	8	-1	0.42115	no
gi|641553326|gb|KK863551.1|	56-1233	9.3	27	11	9	-0.384524	0.7587	no
gi|641553332|gb|KK863548.1|	5913-6958	11	44	0.916591	0.633857	-0.532121	0.7478	no
gi|641553332|gb|KK863548.1|	7736-7940	11	44	24	34	0.532344	0.7197	no
gi|641553338|gb|KK863545.1|	6438-6999	6.7	18	3	2	-0.59638	0.68495	no
gi|641553338|gb|KK863545.1|	9029-10776	6.7	18	13	11	-0.248517	0.83955	no
gi|641553397|gb|KK863516.1|	2876-3102	10	33	3	20	3	0.2768	no
gi|641553397|gb|KK863516.1|	5880-6631	10	33	1	4	2	0.2622	no
gi|641553408|gb|KK863511.1|	5034-5587	5.6	24	2	0	-inf	0.01175	no
gi|641553442|gb|KK863495.1|	1-1336	14	52	5	0	-inf	0.00025	yes
gi|641553442|gb|KK863495.1|	2481-2971	14	52	67	0	-inf	5.00E-05	yes

gi|641553442|gb|KK863495.1|	4964-6327	14	52	14	0	-inf	5.00E-05	yes
gi|641553461|gb|KK863487.1|	10117-11285	9.5	33	5	12	1	0.3365	no
gi|641553461|gb|KK863487.1|	12689-13332	9.5	33	13	21	0.667887	0.5992	no
gi|641553461|gb|KK863487.1|	3265-3694	9.5	33	3	3	0.00285422	0.9394	no
gi|641553461|gb|KK863487.1|	8774-9924	9.5	33	15	25	0.74235	0.55155	no
gi|641553467|gb|KK863484.1|	9442-9783	8.8	32	13	1	-3	0.1503	no
gi|641553554|gb|KK863442.1|	2303-3451	7.1	22	0.891814	1	0.606133	0.67895	no
gi|641553554|gb|KK863442.1|	69-744	7.1	22	1	2	0.448046	0.7632	no
gi|641553586|gb|KK863426.1|	11035-12281	6.9	24	11	7	-0.636744	0.61565	no
gi|641553586|gb|KK863426.1|	114-815	6.9	24	3	1	-1	0.38125	no
gi|641553590|gb|KK863424.1|	8-1394	11	43	7	4	-0.803824	0.53305	no
gi|641553596|gb|KK863421.1|	3860-4202	9.6	16	39	42	0.108141	0.9332	no
gi|641553603|gb|KK863418.1|	3191-4087	9.1	27	3	1	-1	0.47655	no
gi|641553603|gb|KK863418.1|	6620-6920	9.1	27	4	5	0.0819464	0.9243	no
gi|641553623|gb|KK863408.1|	2-1393	11	43	5	18	2	0.1272	no
gi|641553623|gb|KK863408.1|	9811-10294	11	43	3	11	2	0.1802	no
gi|641553676|gb|KK863383.1|	153-644	6	22	23	12	-0.992343	0.44805	no
gi|641553676|gb|KK863383.1|	3991-4179	6	22	0	40	inf	0.0068	no
gi|641553680|gb|KK863381.1|	3257-3968	8.1	16	7	5	-0.56902	0.66465	no
gi|641553680|gb|KK863381.1|	9918-10106	8.1	16	44	71	0.69381	0.63175	no
gi|641553695|gb|KK863374.1|	1028-1316	9	26	33	56	0.760697	0.56455	no
gi|641553712|gb|KK863366.1|	12668-12850	7.5	27	32	9	-2	0.33925	no
gi|641553712|gb|KK863366.1|	15042-15254	7.5	27	60	50	-0.27099	0.8372	no
gi|641553714|gb|KK863365.1|	8782-9362	7.2	22	0.735456	13	4	0.1195	no
gi|641553748|gb|KK863349.1|	3454-3740	7.7	33	4	4	-0.021197	0.91115	no
gi|641553756|gb|KK863345.1|	6524-7070	9.8	13	0.403138	3	3	0.2674	no
gi|641553766|gb|KK863340.1|	9078-9383	6.4	23	12	57	2	0.1219	no
gi|641553774|gb|KK863336.1|	25-938	12	56	0	7	inf	5.00E-05	yes
gi|641553774|gb|KK863336.1|	7122-7374	12	56	0	36	inf	0.00045	yes
gi|641553784|gb|KK863331.1|	7687-8158	11	36	0.511337	3	3	0.2991	no

gi|641553788|gb|KK863329.1|	88-448	4.7	19	3	6	0.941896	0.5742	no
gi|641553794|gb|KK863326.1|	1049-1212	8.4	28	135	185	0.448821	0.74035	no
gi|641553794|gb|KK863326.1|	24142-24507	8.4	28	3	2	-0.227254	0.88445	no
gi|641553794|gb|KK863326.1|	2913-3345	8.4	28	52	51	-0.0219562	0.98525	no
gi|641553794|gb|KK863326.1|	4196-5409	8.4	28	0	4	inf	0.00015	yes
gi|641553817|gb|KK863315.1|	11516-11677	8.1	33	30	43	0.509123	0.7741	no
gi|641553827|gb|KK863310.1|	2403-2670	8.2	32	15	17	0.195675	0.88865	no
gi|641553829|gb|KK863309.1|	10144-11254	9.2	34	36	58	0.711892	0.6477	no
gi|641553869|gb|KK863290.1|	0-809	14	26	24	32	0.380324	0.75545	no
gi|641553869|gb|KK863290.1|	2169-2872	14	26	3	9	1	0.29265	no
gi|641553869|gb|KK863290.1|	3410-3966	14	26	3	6	1	0.45595	no
gi|641553882|gb|KK863284.1|	7039-7296	10	7.00E+00	7	5	-0.252363	0.88385	no
gi|641553888|gb|KK863281.1|	250-1478	6.5	19	8	6	-0.477921	0.70795	no
gi|641553888|gb|KK863281.1|	2794-3270	6.5	19	23	19	-0.301458	0.81495	no
gi|641553888|gb|KK863281.1|	3643-4084	6.5	19	55	26	-1	0.40405	no
gi|641553920|gb|KK863267.1|	50-678	7	19	21	3	-3	0.05655	no
gi|641553931|gb|KK863260.1|	1-541	7.2	11	6	7	0.247392	0.85555	no
gi|641553931|gb|KK863260.1|	595-1675	7.2	11	11	5	-1	0.4029	no
gi|641553931|gb|KK863260.1|	6107-6499	7.2	11	9	4	-1	0.4783	no
gi|641553937|gb|KK863257.1|	2967-3904	9.5	39	0.190919	2	4	0.2456	no
gi|641553945|gb|KK863253.1|	1650-2000	2.6	15	2	8	2	0.2502	no
gi|641553945|gb|KK863253.1|	3599-3963	2.6	15	12	27	1	0.38435	no
gi|641553947|gb|KK863252.1|	3490-3719	7.4	31	14	40	1	0.3254	no
gi|641553949|gb|KK863251.1|	2778-3523	9.2	32	3	2	-0.661426	0.64395	no
gi|641553951|gb|KK863250.1|	4089-4452	12	36	6	2	-2	0.3256	no
gi|641553974|gb|KK863240.1|	12124-12371	11	36	15	11	-0.44855	0.76435	no
gi|641553976|gb|KK863239.1|	10552-11320	8.3	24	9	5	-0.780437	0.5564	no
gi|641553976|gb|KK863239.1|	7501-8766	8.3	24	111	4	-5	0.02865	no
gi|641553979|gb|KK863238.1|	8072-8818	9	36	3	7	1	0.3731	no
gi|641553979|gb|KK863238.1|	996-1258	9	36	0.882064	13	4	0.12515	no

gi|641553981|gb|KK863237.1|	2262-2949	7	19	2	0.436541	-2	0.31075	no
gi|641553984|gb|KK863236.1|	101-438	7.2	17	0.48087	8	4	0.11975	no
gi|641553991|gb|KK863233.1|	6334-6587	8.5	31	44.73	71	0.673908	0.60165	no
gi|641554005|gb|KK863226.1|	10288-10758	4.7	16	2	1	-0.631189	0.71175	no
gi|641554007|gb|KK863225.1|	31-569	8.1	21	2	4	1	0.38865	no
gi|641554009|gb|KK863224.1|	0-711	9.5	30	88	2	-5	0.03365	no
gi|641554034|gb|KK863212.1|	1-1420	12	18	2	4	1	0.3451	no
gi|641554034|gb|KK863212.1|	10217-16275	12	18	5	2	-0.924724	0.45655	no
gi|641554034|gb|KK863212.1|	2692-3217	12	18	3	0.323589	-3	0.2348	no
gi|641554034|gb|KK863212.1|	3270-4060	12	18	1	0.180756	-3	0.2382	no
gi|641554034|gb|KK863212.1|	8210-9374	12	18	1	1	-0.13073	0.9258	no
gi|641554034|gb|KK863212.1|	9661-10060	12	18	3	3	-0.263337	0.8654	no
gi|641554057|gb|KK863202.1|	8721-9183	7	39	3	2	-0.868818	0.6061	no
gi|641554069|gb|KK863196.1|	1160-1884	8.5	27	4	3	-0.556356	0.6937	no
gi|641554069|gb|KK863196.1|	3056-3538	8.5	27	1	2	0.592035	0.70555	no
gi|641554073|gb|KK863194.1|	67-445	4.4	14	4	2	-0.877444	0.60075	no
gi|641554085|gb|KK863188.1|	3154-3522	6.9	31	2	6	2	0.30385	no
gi|641554088|gb|KK863187.1|	2209-3038	9.7	13	5	2	-1	0.3331	no
gi|641554088|gb|KK863187.1|	47-1007	9.7	13	9	2	-2	0.10735	no
gi|641554105|gb|KK863179.1|	5756-6206	8.8	21	4	8	0.911595	0.5273	no
gi|641554105|gb|KK863179.1|	7700-8171	8.8	21	1	5	2	0.2362	no
gi|641554109|gb|KK863177.1|	0-672	7.4	18	9	1	-3	0.08965	no
gi|641554109|gb|KK863177.1|	3477-4243	7.4	18	19	26	0.481703	0.7005	no
gi|641554117|gb|KK863173.1|	3537-4377	8.2	31	1	1	-0.0488994	0.94545	no
gi|641554117|gb|KK863173.1|	7030-7817	8.2	31	0.837611	2	1	0.36605	no
gi|641554119|gb|KK863172.1|	1849-2091	7	24	189	399	1	0.38765	no
gi|641554119|gb|KK863172.1|	4461-4725	7	24	13	28	1	0.44235	no
gi|641554136|gb|KK863164.1|	251-683	11	45	6	0	-inf	0.00495	no
gi|641554136|gb|KK863164.1|	6428-6868	11	45	5	0.864284	-3	0.1899	no
gi|641554136|gb|KK863164.1|	882-1641	11	45	11	3	-2	0.21135	no

gi|641554167|gb|KK863149.1|	40-334	7.7	28	5	10	1	0.4944	no
gi|641554171|gb|KK863147.1|	1-693	8.2	30	28	1	-4	0.0311	no
gi|641554180|gb|KK863143.1|	110-1200	9.3	34	8	0.602122	-4	0.0533	no
gi|641554185|gb|KK863141.1|	0-190	14	41	0	38	inf	0.0068	no
gi|641554198|gb|KK863135.1|	2461-2882	11	36	1	3	1	0.4427	no
gi|641554198|gb|KK863135.1|	3343-3984	11	36	1	0.962158	-0.208239	0.8945	no
gi|641554198|gb|KK863135.1|	530-1620	11	36	3	3	-0.131234	0.92195	no
gi|641554203|gb|KK863133.1|	12-2311	8.8	33	0.706785	2	1	0.3216	no
gi|641554207|gb|KK863131.1|	5361-6271	10	27	4	3	-0.258424	0.84635	no
gi|641554227|gb|KK863122.1|	1632-1904	10	36	14	9	-0.610144	0.6813	no
gi|641554227|gb|KK863122.1|	3377-3688	10	36	18	18	-0.0377051	0.978	no
gi|641554233|gb|KK863119.1|	4277-4609	12	43	0.496596	26	6	0.10555	no
gi|641554233|gb|KK863119.1|	9271-9444	12	43	8	47	3	0.3098	no
gi|641554246|gb|KK863113.1|	3510-4302	9.3	13	1	1	-0.134523	0.92965	no
gi|641554246|gb|KK863113.1|	47-804	9.3	13	62	83	0.423419	0.79155	no
gi|641554260|gb|KK863106.1|	10921-11264	8	28	4	4	0.161431	0.91775	no
gi|641554260|gb|KK863106.1|	670-1068	8	28	9	3	-1	0.34505	no
gi|641554278|gb|KK863097.1|	4874-5848	8.5	30	0	4	inf	0.00065	yes
gi|641554278|gb|KK863097.1|	7917-8230	8.5	30	0	18	inf	0.0005	yes
gi|641554300|gb|KK863086.1|	1966-2557	7.6	21	1	2	0.182426	0.91015	no
gi|641554305|gb|KK863084.1|	1755-2497	5.3	22	1	5	2	0.2044	no
gi|641554305|gb|KK863084.1|	25-741	5.3	22	18	47	1	0.27515	no
gi|641554307|gb|KK863083.1|	3420-4206	10	26	0.719167	4	3	0.13615	no
gi|641554340|gb|KK863067.1|	2583-2959	5.3	14	0	7	inf	0.0025	yes
gi|641554340|gb|KK863067.1|	3260-3526	5.3	14	0	67	inf	0.00015	yes
gi|641554346|gb|KK863064.1|	5972-6300	9.3	32	24	5	-2	0.15805	no
gi|641554373|gb|KK863051.1|	449-1066	11	36	2	0.762155	-0.986593	0.62775	no
gi|641554382|gb|KK863047.1|	6033-6956	6.4	25	4	2	-0.565706	0.6742	no
gi|641554387|gb|KK863045.1|	1807-3470	8.9	34	1	1	0.286379	0.83235	no
gi|641554387|gb|KK863045.1|	3649-3802	8.9	34	59	107	0.867148	0.59315	no

gi|641554387|gb|KK863045.1|	5189-5858	8.9	34	1	2	0.0853207	0.94	no
gi|641554387|gb|KK863045.1|	5974-6906	8.9	34	2	0.87665	-1	0.41765	no
gi|641554416|gb|KK863031.1|	2426-2893	9.3	27	6	11	0.702017	0.61075	no
gi|641554422|gb|KK863028.1|	8089-8702	9.8	47	0	98	inf	0.00075	yes
gi|641554427|gb|KK863026.1|	5231-5996	14	53	0.124278	3	5	0.1123	no
gi|641554427|gb|KK863026.1|	6126-6551	14	53	0	8	inf	0.00135	yes
gi|641554429|gb|KK863025.1|	0-317	6.8	19	7	0	-inf	0.0075	no
gi|641554431|gb|KK863024.1|	194-774	12	21	1	1	-0.402956	0.82205	no
gi|641554431|gb|KK863024.1|	2706-3887	12	21	3	3	0.165768	0.8973	no
gi|641554441|gb|KK863019.1|	0-681	7.2	26	3	6	1	0.46105	no
gi|641554464|gb|KK863008.1|	210-772	8.3	28	38	97	1	0.42595	no
gi|641554468|gb|KK863006.1|	1135-2562	11	36	1	0.087585	-4	0.1972	no
gi|641554468|gb|KK863006.1|	14512-15228	11	36	2	0.824904	-1	0.50315	no
gi|641554468|gb|KK863006.1|	16742-17554	11	36	1	1	0.0178003	0.95145	no
gi|641554468|gb|KK863006.1|	5328-7937	11	36	68	62	-0.141071	0.9408	no
gi|641554493|gb|KK862995.1|	2986-4214	5.8	26	0.685607	0.52198	-0.393387	1	no
gi|641554497|gb|KK862993.1|	0-508	8.7	32	21	11	-1	0.4361	no
gi|641554497|gb|KK862993.1|	1076-1424	8.7	32	7	10	0.577346	0.699	no
gi|641554501|gb|KK862991.1|	178-318	3.7	18	147	1081.27	3	0.08505	no
gi|641554513|gb|KK862985.1|	3064-3313	2.8	12	6	6	-0.0327776	0.91115	no
gi|641554515|gb|KK862984.1|	14755-15749	13	47	2	0.943773	-0.757631	0.61675	no
gi|641554517|gb|KK862983.1|	15565-16329	9.4	14	10	4	-1	0.277	no
gi|641554521|gb|KK862981.1|	3907-4504	8	15	0.880603	2	1	0.51085	no
gi|641554525|gb|KK862979.1|	22-566	6.9	30	0.810864	3	2	0.3014	no
gi|641554529|gb|KK862977.1|	2520-3978	11	41	22	10	-1	0.3937	no
gi|641554529|gb|KK862977.1|	4848-5358	11	41	20	12	-0.794515	0.54395	no
gi|641554542|gb|KK862971.1|	26-399	4.9	22	8	0.584177	-4	0.19895	no
gi|641554546|gb|KK862969.1|	8-296	7.3	20	7	0	-inf	0.01175	no
gi|641554550|gb|KK862967.1|	833-1126	11	46	3	7	1	0.44675	no
gi|641554552|gb|KK862966.1|	26-376	7.1	23	5	0	-inf	0.0102	no

gi|641554552|gb|KK862966.1|	3470-4507	7.1	23	2	0	-inf	0.0013	yes
gi|641554552|gb|KK862966.1|	6120-6488	7.1	23	4	0	-inf	0.0102	no
gi|641554558|gb|KK862963.1|	4673-5568	5.8	24	2	2	-0.161639	0.91175	no
gi|641554560|gb|KK862962.1|	1297-1437	10	45	196	23	-3	0.23015	no
gi|641554568|gb|KK862958.1|	5315-5658	7.8	23	5	9	0.817477	0.59255	no
gi|641554576|gb|KK862954.1|	5069-5996	12	34	0.483821	1	2	0.32945	no
gi|641554581|gb|KK862952.1|	12-1476	7.8	23	77	64	-0.258442	0.90785	no
gi|641554581|gb|KK862952.1|	2802-3028	7.8	23	25	18	-0.45709	0.7601	no
gi|641554581|gb|KK862952.1|	8708-9345	7.8	23	2	3	0.376604	0.7978	no
gi|641554583|gb|KK862951.1|	80-364	6.2	25	6	6	0.148187	0.92285	no
gi|641554591|gb|KK862947.1|	2554-3565	13	23	1	1	-0.0323368	0.95655	no
gi|641554591|gb|KK862947.1|	90-970	13	23	1	0.314277	-2	0.3455	no
gi|641554597|gb|KK862944.1|	2909-4331	10	40	11	19	0.850922	0.4939	no
gi|641554601|gb|KK862942.1|	0-1817	10	28	28	22	-0.327861	0.88615	no
gi|641554601|gb|KK862942.1|	4160-4687	10	28	2	1	-0.575254	0.7586	no
gi|641554601|gb|KK862942.1|	7304-7783	10	28	5	3	-0.800485	0.5932	no
gi|641554609|gb|KK862938.1|	3283-4056	10	24	13	38	2	0.2138	no
gi|641554636|gb|KK862925.1|	2-1393	11	37	13	89	3	0.12845	no
gi|641554644|gb|KK862921.1|	9899-10939	8.6	40	0.586738	2	2	0.20985	no
gi|641554646|gb|KK862920.1|	1879-2350	6.1	19	0.255669	15	6	0.1049	no
gi|641554646|gb|KK862920.1|	2899-3081	6.1	19	0	434	inf	5.00E-05	yes
gi|641554659|gb|KK862914.1|	2312-3341	6.4	20	6	3	-1	0.40505	no
gi|641554659|gb|KK862914.1|	6323-6763	6.4	20	9	4	-1	0.43125	no
gi|641554670|gb|KK862909.1|	11866-12232	7.7	18	13	20	0.624647	0.6507	no
gi|641554670|gb|KK862909.1|	75-308	7.7	18	45	85	0.930575	0.47755	no
gi|641554686|gb|KK862901.1|	2862-3840	8	35	14	1	-3	0.03655	no
gi|641554702|gb|KK862893.1|	1405-1864	9.3	23	8	8	0.0285047	0.9774	no
gi|641554702|gb|KK862893.1|	19-537	9.3	23	2	0.991365	-0.628195	0.71175	no
gi|641554702|gb|KK862893.1|	2069-2946	9.3	23	3	6	1	0.4325	no
gi|641554724|gb|KK862882.1|	59-488	5.6	20	3	3	0.225247	0.88235	no

gi|641554726|gb|KK862881.1|	28-1281	10	29	12	7	-0.792522	0.5284	no
gi|641554726|gb|KK862881.1|	7495-8467	10	29	11	3	-2	0.17995	no
gi|641554732|gb|KK862878.1|	8046-9062	5.5	19	3	0.393686	-3	0.12655	no
gi|641554739|gb|KK862875.1|	173-382	8.7	33	20	23	0.245376	0.87255	no
gi|641554757|gb|KK862866.1|	7263-7765	7.7	24	1	3	2	0.32945	no
gi|641554769|gb|KK862860.1|	11136-11460	11	42	3	4	0.309768	0.86115	no
gi|641554769|gb|KK862860.1|	657-2769	11	42	4	5	0.505628	0.6857	no
gi|641554769|gb|KK862860.1|	91-579	11	42	2	0.726895	-2	0.4266	no
gi|641554782|gb|KK862854.1|	101-305	7.8	21	73	37	-0.976669	0.48805	no
gi|641554782|gb|KK862854.1|	3836-4021	7.8	21	44	8	-2	0.2223	no
gi|641554798|gb|KK862846.1|	0-1198	7.6	24	2	0.430027	-2	0.24435	no
gi|641554798|gb|KK862846.1|	10642-11284	7.6	24	12	5	-1	0.34725	no
gi|641554798|gb|KK862846.1|	2769-3383	7.6	24	5	2	-2	0.3259	no
gi|641554804|gb|KK862843.1|	3154-3699	9.5	31	2	13	3	0.10595	no
gi|641554818|gb|KK862836.1|	10445-10851	11	39	84	52	-0.683288	0.589	no
gi|641554818|gb|KK862836.1|	106-1609	11	39	0.487287	1	1	0.4299	no
gi|641554818|gb|KK862836.1|	2234-3079	11	39	2	0.49665	-2	0.29	no
gi|641554818|gb|KK862836.1|	3577-4006	11	39	21	25	0.239893	0.85475	no
gi|641554822|gb|KK862834.1|	6687-7351	8.5	45	1	2	0.237192	0.88205	no
gi|641554822|gb|KK862834.1|	7401-8850	8.5	45	0.395325	0.602375	0.60762	1	no
gi|641554828|gb|KK862831.1|	2870-4030	7.7	18	3	1	-2	0.28615	no
gi|641554828|gb|KK862831.1|	35-428	7.7	18	4	0	-inf	0.01175	no
gi|641554828|gb|KK862831.1|	5294-5908	7.7	18	25	21	-0.271333	0.82545	no
gi|641554830|gb|KK862830.1|	143-881	6.2	28	0.39137	2	2	0.2235	no
gi|641554854|gb|KK862819.1|	8182-8662	8.4	29	9	8	-0.0779507	0.95095	no
gi|641554856|gb|KK862818.1|	3374-3994	6.4	38	0	7	inf	0.00065	yes
gi|641554860|gb|KK862816.1|	3465-3968	9.3	26	31	18	-0.76619	0.54785	no
gi|641554864|gb|KK862814.1|	9015-9640	9.3	40	0.988129	1	0.598627	0.70555	no
gi|641554868|gb|KK862812.1|	5760-5914	13	66	32	80	1	0.47755	no
gi|641554878|gb|KK862808.1|	13930-17005	8.4	27	13	4	-2	0.34015	no

gi|641554886|gb|KK862804.1|	1396-1899	9.2	29	143	158	0.144001	0.94525	no
gi|641554886|gb|KK862804.1|	5459-6044	9.2	29	1	2	0.959808	0.57345	no
gi|641554886|gb|KK862804.1|	982-1197	9.2	29	2	31	4	0.11405	no
gi|641554896|gb|KK862799.1|	4322-5171	7.1	30	0.64986	1	1	0.47015	no
gi|641554917|gb|KK862789.1|	5721-6225	8.9	22	30	25	-0.268069	0.83025	no
gi|641554921|gb|KK862787.1|	3635-4479	9.1	35	7	48	3	0.04935	no
gi|641554921|gb|KK862787.1|	689-885	9.1	35	0	50	inf	0.0023	yes
gi|641554946|gb|KK862775.1|	13171-13693	8.3	17	2	5	1	0.3889	no
gi|641554946|gb|KK862775.1|	14477-15220	8.3	17	20	11	-0.876478	0.49365	no
gi|641554946|gb|KK862775.1|	7809-8509	8.3	17	1	0.850809	-0.721272	0.67005	no
gi|641554956|gb|KK862771.1|	100-840	7	23	0.519912	4	3	0.15255	no
gi|641554956|gb|KK862771.1|	1759-3575	7	23	1	2	1	0.4501	no
gi|641554956|gb|KK862771.1|	3999-4535	7	23	0.62231	2	2	0.3323	no
gi|641554956|gb|KK862771.1|	4588-5737	7	23	0.519679	2	2	0.2915	no
gi|641554956|gb|KK862771.1|	6005-6217	7	23	147	317	1	0.38495	no
gi|641554956|gb|KK862771.1|	6461-8424	7	23	2	6	2	0.17135	no
gi|641554968|gb|KK862765.1|	1-794	9.1	31	0.47383	1	2	0.3613	no
gi|641554974|gb|KK862762.1|	6789-7819	7.9	46	1	3	1	0.345	no
gi|641554974|gb|KK862762.1|	9692-10369	7.9	46	2	0.668266	-1	0.47135	no
gi|641554984|gb|KK862757.1|	4989-5334	7.1	14	32	10	-2	0.247	no
gi|641555000|gb|KK862749.1|	2073-2525	9.3	46	3	5	0.727333	0.6396	no
gi|641555000|gb|KK862749.1|	4326-5243	9.3	46	2	1	-0.321847	0.82575	no
gi|641555000|gb|KK862749.1|	7637-8557	9.3	46	1	0.594056	-0.980777	0.5872	no
gi|641555012|gb|KK862743.1|	13427-14130	9.6	17	2	3	0.716203	0.64045	no
gi|641555012|gb|KK862743.1|	3027-3275	9.6	17	53	29	-0.872678	0.5224	no
gi|641555012|gb|KK862743.1|	55-711	9.6	17	2	1	-0.400456	0.8125	no
gi|641555016|gb|KK862741.1|	22-415	11	34	0	6	inf	0.00375	no
gi|641555023|gb|KK862738.1|	2-414	5.8	11	72	64	-0.181364	0.88345	no
gi|641555023|gb|KK862738.1|	3259-3637	5.8	11	11	5	-1	0.44965	no
gi|641555023|gb|KK862738.1|	5417-5879	5.8	11	14	18	0.349605	0.7894	no

gi|641555060|gb|KK862721.1|	0-988	10	20	14	2	-3	0.061	no
gi|641555060|gb|KK862721.1|	11566-11991	10	20	4	1	-2	0.40265	no
gi|641555080|gb|KK862711.1|	14021-14384	12	41	1	6	2	0.2624	no
gi|641555084|gb|KK862709.1|	260-639	8.3	27	22	30	0.477156	0.7129	no
gi|641555084|gb|KK862709.1|	8398-8739	8.3	27	5	9	0.95462	0.53935	no
gi|641555088|gb|KK862707.1|	811-984	4.1	16	16	41	1	0.44845	no
gi|641555104|gb|KK862700.1|	4071-4346	13	47	5	5	-0.0242864	0.91115	no
gi|641555109|gb|KK862698.1|	5273-6001	nan	17	13	0	-inf	5.00E-05	yes
gi|641555129|gb|KK862689.1|	5999-6448	7	23	2	1	-0.995378	0.62775	no
gi|641555135|gb|KK862686.1|	5164-5780	6	14	3	0	-inf	0.0059	no
gi|641555143|gb|KK862682.1|	223-737	13	39	0.886466	3	2	0.3613	no
gi|641555143|gb|KK862682.1|	7575-7948	13	39	10	0	-inf	0.0013	yes
gi|641555147|gb|KK862680.1|	2339-3556	7.2	20	2	3	0.232156	0.85735	no
gi|641555147|gb|KK862680.1|	5359-6368	7.2	20	4	5	0.210796	0.8697	no
gi|641555147|gb|KK862680.1|	8079-8745	7.2	20	4	2	-0.9307	0.51735	no
gi|641555151|gb|KK862678.1|	3093-3629	6	16	1	2	1	0.51085	no
gi|641555153|gb|KK862677.1|	4914-5887	11	38	0.455204	1	1	0.4595	no
gi|641555162|gb|KK862673.1|	1221-1993	12	36	0.859392	1	0.379711	0.82465	no
gi|641555162|gb|KK862673.1|	2229-5208	12	36	2	3	0.659681	0.60415	no
gi|641555162|gb|KK862673.1|	416-1130	12	36	2	1	-0.761599	0.61575	no
gi|641555166|gb|KK862671.1|	5857-6371	8.2	13	2	1	-0.628413	0.71175	no
gi|641555174|gb|KK862667.1|	142-686	7.5	27	154	142	-0.120703	0.958	no
gi|641555183|gb|KK862663.1|	25-415	11	35	8	13	0.703862	0.60995	no
gi|641555183|gb|KK862663.1|	3453-4688	11	35	0.74909	3	2	0.23885	no
gi|641555183|gb|KK862663.1|	5083-5707	11	35	0.495213	2	2	0.2812	no
gi|641555183|gb|KK862663.1|	9042-9650	11	35	0.514339	3	3	0.1889	no
gi|641555185|gb|KK862662.1|	13721-14260	9.8	34	2	7	2	0.3109	no
gi|641555185|gb|KK862662.1|	2319-3974	9.8	34	20	10	-0.96267	0.4436	no
gi|641555198|gb|KK862656.1|	5016-6121	15	59	0.155661	3	4	0.2392	no
gi|641555219|gb|KK862647.1|	2150-2692	9.2	28	5	41	3	0.0411	no

gi|641555219|gb|KK862647.1|	3311-3700	9.2	28	2	13	3	0.12845	no
gi|641555221|gb|KK862646.1|	7717-7875	3.3	20	115	10	-4	0.2048	no
gi|641555227|gb|KK862643.1|	9554-11136	7.9	29	0.714819	2	1	0.4088	no
gi|641555237|gb|KK862638.1|	4569-5270	8.6	25	8	2	-2	0.18115	no
gi|641555262|gb|KK862628.1|	10550-10889	12	40	29	31	0.104437	0.9329	no
gi|641555270|gb|KK862624.1|	0-1136	9.7	33	40	37	-0.1005	0.94925	no
gi|641555272|gb|KK862623.1|	0-1135	9.3	32	0.979497	1	0.365039	0.8038	no
gi|641555274|gb|KK862622.1|	3799-4910	4.6	19	2	2	0.120456	0.93065	no
gi|641555289|gb|KK862615.1|	2511-2998	7.5	25	5	3	-0.55964	0.7056	no
gi|641555289|gb|KK862615.1|	8664-9646	7.5	25	0.449996	0.684353	0.604827	1	no
gi|641555335|gb|KK862598.1|	10674-11045	7.6	23	88	37	-1	0.3328	no
gi|641555335|gb|KK862598.1|	11268-11809	7.6	23	39	20	-0.922486	0.4647	no
gi|641555372|gb|KK862581.1|	17848-19175	7.8	27	2	2	0.130675	0.92385	no
gi|641555378|gb|KK862578.1|	2877-4280	7.8	33	0.293131	0.62524	1	1	no
gi|641555378|gb|KK862578.1|	812-1423	7.8	33	0.851069	1	0.59817	0.70505	no
gi|641555380|gb|KK862577.1|	7599-7822	6.9	28	65	0	-inf	0.00015	yes
gi|641555380|gb|KK862577.1|	9565-9806	6.9	28	155	0	-inf	5.00E-05	yes
gi|641555416|gb|KK862560.1|	8910-9157	4.1	15	9	23	1	0.41035	no
gi|641555418|gb|KK862559.1|	4935-5435	11	39	3	4	0.593174	0.6792	no
gi|641555418|gb|KK862559.1|	6-628	11	39	2	4	1	0.3654	no
gi|641555418|gb|KK862559.1|	8185-8761	11	39	2	3	0.471355	0.7564	no
gi|641555420|gb|KK862558.1|	10430-10956	6.7	25	12	59	2	0.09125	no
gi|641555420|gb|KK862558.1|	3-1228	6.7	25	0.481323	1	2	0.3164	no
gi|641555422|gb|KK862557.1|	6238-9377	14	32	31	23	-0.443091	0.83685	no
gi|641555442|gb|KK862548.1|	7865-8800	7.7	61	1	2	0.784923	0.5866	no
gi|641555468|gb|KK862535.1|	8472-9428	8.8	41	1	5	2	0.1743	no
gi|641555478|gb|KK862530.1|	2041-2255	8.5	27	182	58	-2	0.21295	no
gi|641555485|gb|KK862527.1|	3260-4131	11	16	0.733485	0.955312	0.381204	0.82465	no
gi|641555485|gb|KK862527.1|	4287-5213	11	16	0.775173	0.589205	-0.395747	1	no
gi|641555485|gb|KK862527.1|	5314-5870	11	16	7	2	-2	0.3008	no

gi|641555485|gb|KK862527.1|	619-1222	11	16	5	2	-1	0.4521	no
gi|641555489|gb|KK862525.1|	5974-7118	11	38	6	6	0.0466793	0.9707	no
gi|641555492|gb|KK862524.1|	5718-8268	8.6	36	1	0.50476	-1	0.41425	no
gi|641555499|gb|KK862521.1|	10474-11320	10	30	0.435237	1	2	0.3613	no
gi|641555499|gb|KK862521.1|	4647-9600	10	30	16	12	-0.411187	0.8569	no
gi|641555521|gb|KK862510.1|	2804-2992	8.5	23	55	24	-1	0.44025	no
gi|641555525|gb|KK862508.1|	6755-7257	9.8	32	0	4	inf	0.00375	no
gi|641555547|gb|KK862498.1|	5315-6022	7.9	20	5	3	-0.839757	0.5438	no
gi|641555551|gb|KK862496.1|	2183-3669	8.7	44	0	6	inf	5.00E-05	yes
gi|641555565|gb|KK862489.1|	1987-2610	6.5	27	0.992733	2	0.820956	0.62335	no
gi|641555565|gb|KK862489.1|	2944-3404	6.5	27	3	3	0.00549814	0.95145	no
gi|641555569|gb|KK862487.1|	135-1483	50	290	0.982774	0.654967	-0.585437	0.68495	no
gi|641555569|gb|KK862487.1|	2438-3614	50	290	6	3	-0.865759	0.5099	no
gi|641555569|gb|KK862487.1|	8255-8856	50	290	1	2	0.820217	0.62335	no
gi|641555569|gb|KK862487.1|	9157-9993	50	290	6	4	-0.585913	0.6561	no
gi|641555571|gb|KK862486.1|	11791-13343	11	17	21	9	-1	0.33715	no
gi|641555571|gb|KK862486.1|	40-999	11	17	0.834387	3	2	0.28425	no
gi|641555571|gb|KK862486.1|	9416-9804	11	17	51	31	-0.723346	0.57075	no
gi|641555573|gb|KK862485.1|	4358-5457	9.3	20	2	1	-0.546206	0.7107	no
gi|641555573|gb|KK862485.1|	6080-6875	9.3	20	1	0.537776	-1	0.50885	no
gi|641555591|gb|KK862476.1|	1-380	12	21	11	8	-0.417875	0.7584	no
gi|641555591|gb|KK862476.1|	11026-11865	12	21	5	4	-0.212412	0.87585	no
gi|641555591|gb|KK862476.1|	5154-5775	12	21	2	0.755087	-1	0.50365	no
gi|641555604|gb|KK862470.1|	15081-16426	7.7	26	19	24	0.297767	0.8035	no
gi|641555604|gb|KK862470.1|	6848-7567	7.7	26	2	2	0.186041	0.89915	no
gi|641555610|gb|KK862467.1|	3380-3631	7.4	26	0	22	inf	0.0023	yes
gi|641555627|gb|KK862459.1|	10639-11377	6.8	22	3	4	0.186434	0.89225	no
gi|641555627|gb|KK862459.1|	65-441	6.8	22	0.768697	7	3	0.2558	no
gi|641555627|gb|KK862459.1|	8938-9273	6.8	22	2	4	0.574994	0.76735	no
gi|641555635|gb|KK862455.1|	2404-3194	7.7	25	40	25	-0.673449	0.5887	no

gi|641555635|gb|KK862455.1|	32-856	7.7	25	42	39	-0.104746	0.92955	no
gi|641555635|gb|KK862455.1|	5425-5997	7.7	25	2	2	0.0821519	0.9406	no
gi|641555645|gb|KK862450.1|	10213-11265	6.6	22	5	9	0.834258	0.51625	no
gi|641555653|gb|KK862446.1|	6236-6824	6.9	24	1	2	0.597351	0.69715	no
gi|641555659|gb|KK862443.1|	6420-7314	9.5	16	2	4	0.796528	0.56215	no
gi|641555667|gb|KK862439.1|	3282-4511	8.4	26	8	5	-0.696774	0.58345	no
gi|641555667|gb|KK862439.1|	4715-5375	8.4	26	10	9	-0.213261	0.8702	no
gi|641555667|gb|KK862439.1|	740-2216	8.4	26	7	3	-1	0.3598	no
gi|641555671|gb|KK862437.1|	11376-11754	10	17	13	9	-0.598585	0.66705	no
gi|641555671|gb|KK862437.1|	9152-9648	10	17	10	1	-3	0.11515	no
gi|641555677|gb|KK862434.1|	1253-2014	9.9	35	1	2	0.923835	0.5636	no
gi|641555677|gb|KK862434.1|	2902-4307	9.9	35	23	42	0.842279	0.6047	no
gi|641555677|gb|KK862434.1|	6446-7212	9.9	35	30	52	0.779101	0.53695	no
gi|641555682|gb|KK862432.1|	1172-1437	8.3	29	82	193	1	0.32955	no
gi|641555682|gb|KK862432.1|	3007-3185	8.3	29	25	81	2	0.29635	no
gi|641555684|gb|KK862431.1|	12670-13433	13	21	8	1	-3	0.1107	no
gi|641555684|gb|KK862431.1|	15085-15969	13	21	6	4	-0.765473	0.5567	no
gi|641555688|gb|KK862429.1|	11097-11323	8.7	31	22	16	-0.45709	0.76465	no
gi|641555688|gb|KK862429.1|	2341-3902	8.7	31	2	2	-0.357228	0.7864	no
gi|641555688|gb|KK862429.1|	66-875	8.7	31	3	5	0.843724	0.54365	no
gi|641555694|gb|KK862426.1|	1471-2406	7.3	28	0.478588	1	1	0.4595	no
gi|641555696|gb|KK862425.1|	2569-3367	3.8	19	0	17	inf	5.00E-05	yes
gi|641555704|gb|KK862421.1|	170-541	9.1	27	3	3	0.095568	0.92385	no
gi|641555704|gb|KK862421.1|	2006-2288	9.1	27	4	9	1	0.46685	no
gi|641555740|gb|KK862404.1|	6408-7166	10	49	8	0.954787	-3	0.08065	no
gi|641555766|gb|KK862392.1|	1234-2223	7.2	25	8	2	-2	0.2059	no
gi|641555766|gb|KK862392.1|	4046-4751	7.2	25	8	2	-2	0.15635	no
gi|641555770|gb|KK862390.1|	2889-3050	7.2	24	73	26	-1	0.40875	no
gi|641555784|gb|KK862383.1|	1-423	230	5.50E+02	41	10	-2	0.1348	no
gi|641555784|gb|KK862383.1|	4201-4444	230	5.50E+02	60	43	-0.476531	0.71905	no

gi|641555784|gb|KK862383.1|	5712-5994	230	5.50E+02	71	34	-1	0.4271	no
gi|641555799|gb|KK862376.1|	1-599	6.7	25	0.878426	2	0.860752	0.62755	no
gi|641555799|gb|KK862376.1|	10940-11594	6.7	25	5	4	-0.318049	0.81845	no
gi|641555799|gb|KK862376.1|	2668-3819	6.7	25	3	14	2	0.08575	no
gi|641555807|gb|KK862373.1|	2652-3090	9.4	48	0	10	inf	0.0005	yes
gi|641555811|gb|KK862371.1|	5855-6400	7.8	37	23	52	1	0.35725	no
gi|641555821|gb|KK862366.1|	12251-12791	8.4	27	1	1	0.0103524	0.91115	no
gi|641555823|gb|KK862365.1|	6633-7121	9.3	40	2	1	-0.407572	0.82205	no
gi|641555833|gb|KK862360.1|	0-395	6.2	13	9	5	-0.94633	0.51265	no
gi|641555833|gb|KK862360.1|	10255-10422	6.2	13	1086.37	778	-0.481024	0.70085	no
gi|641555835|gb|KK862359.1|	7426-7926	7.3	19	8	9	0.164331	0.90055	no
gi|641555839|gb|KK862357.1|	367-1176	10	14	25	7	-2	0.1734	no
gi|641555839|gb|KK862357.1|	7118-8337	10	14	11	16	0.505117	0.6849	no
gi|641555861|gb|KK862346.1|	13671-13869	8.9	28	91	185	1	0.43585	no
gi|641555861|gb|KK862346.1|	2877-3669	8.9	28	6	9	0.51498	0.6926	no
gi|641555867|gb|KK862343.1|	4601-4778	9.7	15	44	10	-2	0.2746	no
gi|641555867|gb|KK862343.1|	7951-8241	9.7	15	37	20	-0.868131	0.5243	no
gi|641555871|gb|KK862341.1|	8099-8659	8.8	34	5	6	0.470691	0.7311	no
gi|641555875|gb|KK862339.1|	10998-11755	7.5	19	1	0.573884	-0.983128	0.62775	no
gi|641555875|gb|KK862339.1|	3015-3486	7.5	19	27	21	-0.355286	0.78435	no
gi|641555875|gb|KK862339.1|	6167-6600	7.5	19	45	28	-0.659738	0.60075	no
gi|641555879|gb|KK862337.1|	78-1037	10	40	3	2	-0.262132	0.8498	no
gi|641555912|gb|KK862321.1|	2156-3230	4.2	22	2	0.858249	-1	0.4114	no
gi|641555934|gb|KK862311.1|	2233-2497	9.2	17	13	5	-1	0.41545	no
gi|641555934|gb|KK862311.1|	2741-3031	9.2	17	116	65	-0.830656	0.50755	no
gi|641555965|gb|KK862297.1|	7-656	6.9	23	2	0.236348	-3	0.2481	no
gi|641555974|gb|KK862293.1|	10612-11038	11	28	2	3	0.809923	0.6234	no
gi|641555974|gb|KK862293.1|	34-602	11	28	2	1	-0.403439	0.82205	no
gi|641555974|gb|KK862293.1|	9510-10502	11	28	2	3	0.756924	0.59325	no
gi|641555983|gb|KK862289.1|	3488-3723	9.6	30	13	2	-3	0.23545	no

gi|641555987|gb|KK862287.1|	6624-6797	11	37	49	23	-1	0.5073	no
gi|641556012|gb|KK862275.1|	3120-3362	8.9	27	2	21	3	0.2564	no
gi|641556014|gb|KK862274.1|	15060-16121	9.7	32	5	0.871076	-2	0.1181	no
gi|641556014|gb|KK862274.1|	20341-21265	9.7	32	7	6	-0.235305	0.8581	no
gi|641556022|gb|KK862270.1|	4293-4567	6.2	37	22	27	0.276587	0.83655	no
gi|641556028|gb|KK862267.1|	3052-3421	8.1	14	7	3	-1	0.42815	no
gi|641556028|gb|KK862267.1|	3596-4066	8.1	14	11	2	-3	0.11055	no
gi|641556039|gb|KK862262.1|	1945-4927	8.9	30	19	8	-1	0.44095	no
gi|641556039|gb|KK862262.1|	201-481	8.9	30	5	5	0.0766885	0.92435	no
gi|641556039|gb|KK862262.1|	6004-6635	8.9	30	36	20	-0.880157	0.48265	no
gi|641556039|gb|KK862262.1|	7066-7339	8.9	30	32	34	0.060511	0.96425	no
gi|641556041|gb|KK862261.1|	189-785	9.5	28	8	18	1	0.4092	no
gi|641556041|gb|KK862261.1|	5282-5995	9.5	28	0.546999	1	1	0.51305	no
gi|641556046|gb|KK862259.1|	4745-5875	10	34	2	0.691761	-1	0.4177	no
gi|641556046|gb|KK862259.1|	6020-6246	10	34	12	12	-0.0420523	0.9394	no
gi|641556046|gb|KK862259.1|	7185-8241	10	34	3	4	0.509256	0.7039	no
gi|641556046|gb|KK862259.1|	8566-10330	10	34	7	8	0.261548	0.8362	no
gi|641556053|gb|KK862256.1|	12-612	7.7	13	24	18	-0.423584	0.7313	no
gi|641556053|gb|KK862256.1|	6020-8310	7.7	13	18	12	-0.611086	0.69985	no
gi|641556053|gb|KK862256.1|	8537-9107	7.7	13	36	17	-1	0.39285	no
gi|641556057|gb|KK862254.1|	1187-1537	10	29	4	9	1	0.4458	no
gi|641556057|gb|KK862254.1|	234-960	10	29	0.800422	2	1	0.4118	no
gi|641556057|gb|KK862254.1|	3242-4376	10	29	10	8	-0.204135	0.86925	no
gi|641556065|gb|KK862250.1|	291-1423	8.3	25	18	23	0.333261	0.7878	no
gi|641556067|gb|KK862249.1|	0-172	8.2	25	109	48	-1	0.4232	no
gi|641556067|gb|KK862249.1|	542-821	8.2	25	8	2	-2	0.31705	no
gi|641556079|gb|KK862243.1|	1532-2110	5.4	14	1	0.838637	-0.818072	0.6492	no
gi|641556109|gb|KK862229.1|	7292-7504	10	41	27	25	-0.141707	0.92145	no
gi|641556113|gb|KK862227.1|	2764-2944	3.6	12	50	34	-0.579936	0.7195	no
gi|641556121|gb|KK862223.1|	3063-3383	8.9	42	30	12	-1	0.34655	no

gi|641556121|gb|KK862223.1|	3696-5289	8.9	42	70	15	-2	0.2079	no
gi|641556121|gb|KK862223.1|	6821-7213	8.9	42	29	7	-2	0.1497	no
gi|641556121|gb|KK862223.1|	8349-8850	8.9	42	3	0	-inf	0.0059	no
gi|641556148|gb|KK862211.1|	6717-8565	11	39	2	0.457309	-2	0.1292	no
gi|641556152|gb|KK862209.1|	3363-4008	9.6	32	0.314771	3	3	0.25475	no
gi|641556156|gb|KK862207.1|	10383-11090	9.1	34	0.276702	2	3	0.27515	no
gi|641556156|gb|KK862207.1|	7763-8708	9.1	34	0.661086	0.717943	0.11903	1	no
gi|641556189|gb|KK862191.1|	9847-10268	4.9	23	3	5	0.724543	0.6396	no
gi|641556197|gb|KK862187.1|	0-1301	7	24	7	4	-1	0.4389	no
gi|641556216|gb|KK862178.1|	2181-3553	10	30	28.22	10	-1	0.2442	no
gi|641556216|gb|KK862178.1|	4586-5941	10	30	11	3	-2	0.2202	no
gi|641556216|gb|KK862178.1|	6929-7281	10	30	9	7	-0.421974	0.76465	no
gi|641556216|gb|KK862178.1|	7-389	10	30	16	13	-0.258771	0.84415	no
gi|641556227|gb|KK862173.1|	0-973	11	19	0.546245	2	2	0.20395	no
gi|641556227|gb|KK862173.1|	6156-6920	11	19	0.746979	2	1	0.4702	no
gi|641556235|gb|KK862169.1|	7121-7972	20	59	3	0.656179	-2	0.2443	no
gi|641556237|gb|KK862168.1|	7355-7829	28	31	2	2	0.328453	0.83525	no
gi|641556237|gb|KK862168.1|	7996-8542	28	31	4	9	1	0.4264	no
gi|641556247|gb|KK862163.1|	3035-3780	8.2	31	0.128797	8	6	0.10475	no
gi|641556258|gb|KK862158.1|	1132-1408	8.8	29	0	25	inf	0.0005	yes
gi|641556258|gb|KK862158.1|	8121-8908	8.8	29	0	4	inf	0.0005	yes
gi|641556290|gb|KK862142.1|	23-651	12	40	16	1	-4	0.0554	no
gi|641556290|gb|KK862142.1|	8000-8367	12	40	15	1	-4	0.141	no
gi|641556292|gb|KK862141.1|	10662-10999	9.2	28	7	9	0.468456	0.7498	no
gi|641556292|gb|KK862141.1|	14-1072	9.2	28	37	49	0.406889	0.7981	no
gi|641556325|gb|KK862125.1|	14-613	6.6	20	2	1	-0.572171	0.7586	no
gi|641556327|gb|KK862124.1|	2907-3103	8.2	19	217	333	0.617432	0.61955	no
gi|641556327|gb|KK862124.1|	8741-9376	8.2	19	3	0.731345	-2	0.28995	no
gi|641556330|gb|KK862123.1|	10-825	13	29	12	0	-inf	5.00E-05	yes
gi|641556330|gb|KK862123.1|	10135-11305	13	29	7	0.221164	-5	0.1309	no

gi|641556330|gb|KK862123.1|	4121-4588	13	29	9	0	-inf	0.0003	yes
gi|641556330|gb|KK862123.1|	974-1199	13	29	21	0	-inf	0.0059	no
gi|641556336|gb|KK862120.1|	1945-2227	8.9	32	2	30	4	0.1416	no
gi|641556336|gb|KK862120.1|	2404-2790	8.9	32	1	5	2	0.30385	no
gi|641556336|gb|KK862120.1|	9642-10186	8.9	32	6	1	-2	0.1841	no
gi|641556351|gb|KK862113.1|	3881-4038	9.1	20	49	40	-0.300426	0.8639	no
gi|641556351|gb|KK862113.1|	5210-5635	9.1	20	2	3	0.365041	0.82765	no
gi|641556363|gb|KK862107.1|	4780-5013	7.9	26	31	24	-0.397505	0.779	no
gi|641556367|gb|KK862105.1|	375-797	5	11	4	7	0.886699	0.54535	no
gi|641556369|gb|KK862104.1|	4798-5153	3.7	13	2	8	2	0.2502	no
gi|641556372|gb|KK862103.1|	17394-17873	13	33	4	4	-0.0361983	0.96295	no
gi|641556372|gb|KK862103.1|	20432-20785	13	33	18	18	-0.0592371	0.96485	no
gi|641556372|gb|KK862103.1|	21686-22589	13	33	3	3	0.320198	0.8152	no
gi|641556378|gb|KK862100.1|	16301-17070	8.9	36	25	28	0.122243	0.92045	no
gi|641556378|gb|KK862100.1|	19294-19898	8.9	36	12	20	0.798627	0.5319	no
gi|641556378|gb|KK862100.1|	7642-8545	8.9	36	1	2	0.118564	0.93605	no
gi|641556378|gb|KK862100.1|	8598-11167	8.9	36	2	2	-0.7208	0.583	no
gi|641556390|gb|KK862094.1|	11154-11449	12	39	25	7	-2	0.212	no
gi|641556390|gb|KK862094.1|	5052-6088	12	39	4	2	-1	0.45605	no
gi|641556390|gb|KK862094.1|	7050-8021	12	39	2	2	-0.338696	0.80955	no
gi|641556396|gb|KK862091.1|	10776-11122	11	32	3	3	-0.00796346	0.91115	no
gi|641556413|gb|KK862083.1|	87-346	6.2	28	5	11	1	0.46685	no
gi|641556422|gb|KK862079.1|	2916-3607	8.8	22	0.999671	0.649579	-0.621948	0.71195	no
gi|641556422|gb|KK862079.1|	5297-6194	8.8	22	2	2	0.241349	0.86475	no
gi|641556435|gb|KK862073.1|	12007-13698	13	42	21	1	-4	0.0107	no
gi|641556435|gb|KK862073.1|	2261-3093	13	42	1	0	-inf	0.01	no
gi|641556435|gb|KK862073.1|	4834-5307	13	42	22	0.764992	-5	0.1317	no
gi|641556447|gb|KK862067.1|	2373-3204	4	8.3	0.66826	1	0.603048	0.70555	no
gi|641556451|gb|KK862065.1|	16-829	7.2	26	1	4	1	0.35165	no
gi|641556457|gb|KK862062.1|	6106-6800	8.9	31	28	13	-1	0.3698	no

gi|641556459|gb|KK862061.1|	11-454	9.6	33	12	6	-1	0.43385	no
gi|641556466|gb|KK862058.1|	2001-2488	7.3	20	8	4	-0.992599	0.49945	no
gi|641556478|gb|KK862052.1|	552-1150	7.8	33	0.35137	3	3	0.2674	no
gi|641556480|gb|KK862051.1|	544-1238	5.7	26	0.425857	3	3	0.1811	no
gi|641556480|gb|KK862051.1|	9926-10612	5.7	26	0	5	inf	0.00075	yes
gi|641556484|gb|KK862049.1|	3328-3974	9.5	42	0.62815	1	1	0.51315	no
gi|641556489|gb|KK862047.1|	467-681	7.1	27	12	10	-0.270016	0.8666	no
gi|641556495|gb|KK862044.1|	1-740	6.8	16	15	7	-1	0.4357	no
gi|641556501|gb|KK862041.1|	2708-3188	8.1	31	11	13	0.18751	0.88415	no
gi|641556501|gb|KK862041.1|	9056-9306	8.1	31	15	4	-2	0.3233	no
gi|641556501|gb|KK862041.1|	98-422	8.1	31	4	8	1	0.49435	no
gi|641556511|gb|KK862036.1|	6207-6880	7.9	26	0.444545	2	2	0.2812	no
gi|641556516|gb|KK862034.1|	0-238	8.4	24	70	53	-0.40473	0.7596	no
gi|641556516|gb|KK862034.1|	2831-3539	8.4	24	1	0.628243	-1	0.50885	no
gi|641556530|gb|KK862027.1|	8314-9360	8.6	35	0.915513	2	0.846399	0.58475	no
gi|641556538|gb|KK862023.1|	6071-6257	9.4	36	35	46	0.397278	0.79345	no
gi|641556544|gb|KK862020.1|	2698-3235	8.9	18	2	2	0.402525	0.8089	no
gi|641556544|gb|KK862020.1|	5323-5678	8.9	18	3	3	0.093096	0.9241	no
gi|641556547|gb|KK862019.1|	9332-9752	9.5	19	3	2	-0.582985	0.7586	no
gi|641556549|gb|KK862018.1|	630-833	12	41	22	0	-inf	0.0102	no
gi|641556576|gb|KK862005.1|	3129-3318	8	30	0	46	inf	0.00375	no
gi|641556602|gb|KK861995.1|	1268-3323	8.2	37	3	2	-0.390844	0.75955	no
gi|641556602|gb|KK861995.1|	168-824	8.2	37	18	14	-0.388177	0.7627	no
gi|641556602|gb|KK861995.1|	3495-4552	8.2	37	3	4	0.445014	0.7334	no
gi|641556611|gb|KK861991.1|	13812-14152	9.1	29	4	6	0.575927	0.73035	no
gi|641556611|gb|KK861991.1|	7697-7931	9.1	29	15	0	-inf	0.01	no
gi|641556617|gb|KK861988.1|	1378-1618	9.5	35	11	15	0.396763	0.7973	no
gi|641556633|gb|KK861980.1|	3-887	8	31	0.514028	2	2	0.28495	no
gi|641556638|gb|KK861978.1|	8352-8687	5.7	27	0.974094	7	3	0.28555	no
gi|641556646|gb|KK861974.1|	2960-3271	6.6	14	3	6	1	0.5132	no

gi|641556654|gb|KK861970.1|	1580-2191	10	13	0.851069	2	1	0.51085	no
gi|641556654|gb|KK861970.1|	31-302	10	13	2	9	2	0.3198	no
gi|641556654|gb|KK861970.1|	9275-9641	10	13	7	13	0.869759	0.537	no
gi|641556673|gb|KK861961.1|	11426-11857	8.9	35	20	20	-0.0400269	0.97595	no
gi|641556673|gb|KK861961.1|	2161-5162	8.9	35	3	2	-0.284802	0.823	no
gi|641556673|gb|KK861961.1|	6120-9537	8.9	35	1	0.637529	-1	0.3881	no
gi|641556681|gb|KK861957.1|	12180-12867	9.6	38	8	5	-0.683445	0.6009	no
gi|641556681|gb|KK861957.1|	16025-16331	9.6	38	18	11	-0.800413	0.5695	no
gi|641556681|gb|KK861957.1|	22776-23603	9.6	38	6	5	-0.0866705	0.9466	no
gi|641556681|gb|KK861957.1|	26226-26841	9.6	38	7	4	-0.954238	0.49255	no
gi|641556681|gb|KK861957.1|	3811-6381	9.6	38	21	5	-2	0.2332	no
gi|641556681|gb|KK861957.1|	534-1208	9.6	38	1	0.448273	-1	0.4534	no
gi|641556681|gb|KK861957.1|	7029-12038	9.6	38	20	18	-0.184377	0.9348	no
gi|641556722|gb|KK861938.1|	2421-3124	7.5	20	21	4	-2	0.1015	no
gi|641556722|gb|KK861938.1|	4573-5348	7.5	20	13	4	-2	0.17515	no
gi|641556722|gb|KK861938.1|	76-970	7.5	20	10	7	-0.48358	0.7092	no
gi|641556724|gb|KK861937.1|	7719-8995	8.2	33	0.655156	1	0.74437	0.6176	no
gi|641556736|gb|KK861931.1|	4213-4767	4.4	15	0.197106	10	6	0.10555	no
gi|641556738|gb|KK861930.1|	4259-5739	13	45	0.936909	0	-inf	0.00435	no
gi|641556742|gb|KK861928.1|	4344-4743	6.5	31	2	4	1	0.5126	no
gi|641556744|gb|KK861927.1|	4115-5719	10	34	14	14	-0.0231173	0.98365	no
gi|641556744|gb|KK861927.1|	5914-8783	10	34	2	3	0.715477	0.57365	no
gi|641556744|gb|KK861927.1|	73-494	10	34	3	6	0.965551	0.5384	no
gi|641556744|gb|KK861927.1|	785-1165	10	34	4	0.563686	-3	0.2376	no
gi|641556744|gb|KK861927.1|	8929-9402	10	34	29	25	-0.244196	0.8431	no
gi|641556744|gb|KK861927.1|	9521-11066	10	34	5	5	0.122529	0.9242	no
gi|641556748|gb|KK861925.1|	12472-13384	12	74	0.889104	0.750811	-0.243902	0.88495	no
gi|641556750|gb|KK861924.1|	16321-17974	9.4	16	38	13	-2	0.3608	no
gi|641556750|gb|KK861924.1|	50-1074	9.4	16	2	0.909776	-0.835366	0.57785	no
gi|641556752|gb|KK861923.1|	10802-11405	8.4	28	4	2	-1	0.47525	no

gi|641556752|gb|KK861923.1|	5419-6620	8.4	28	0.704013	1	0.743963	0.6176	no
gi|641556752|gb|KK861923.1|	9230-10309	8.4	28	6	1	-2	0.12735	no
gi|641556756|gb|KK861921.1|	12084-12668	11	34	76	83	0.139934	0.9068	no
gi|641556756|gb|KK861921.1|	2575-3145	11	34	3	0.570832	-2	0.24735	no
gi|641556756|gb|KK861921.1|	5108-5685	11	34	3	4	0.234356	0.87065	no
gi|641556764|gb|KK861917.1|	4608-5779	8.2	21	0.943357	0.994222	0.0757641	0.95315	no
gi|641556768|gb|KK861915.1|	8949-9494	6.8	29	5	4	-0.343049	0.81055	no
gi|641556768|gb|KK861915.1|	9552-11192	6.8	29	2	2	-0.643295	0.62945	no
gi|641556774|gb|KK861912.1|	3086-3967	7	36	1	3	2	0.32045	no
gi|641556784|gb|KK861907.1|	1803-2153	8.4	17	3	5	0.770334	0.6361	no
gi|641556784|gb|KK861907.1|	6220-6813	8.4	17	2	0.269161	-3	0.2481	no
gi|641556786|gb|KK861906.1|	7395-7634	7.7	23	17	17	-0.0365959	0.9597	no
gi|641556793|gb|KK861903.1|	11572-11939	10	40	71	86	0.270719	0.8196	no
gi|641556793|gb|KK861903.1|	17021-18366	10	40	2	2	-0.678208	0.61415	no
gi|641556793|gb|KK861903.1|	18593-19727	10	40	0.905114	0.803631	-0.171567	0.90465	no
gi|641556793|gb|KK861903.1|	19923-20191	10	40	142.72	90	-0.658254	0.5982	no
gi|641556793|gb|KK861903.1|	20348-21175	10	40	8	7	-0.22483	0.86085	no
gi|641556793|gb|KK861903.1|	8127-8989	10	40	0.849584	0.806774	-0.0745932	1	no
gi|641556798|gb|KK861901.1|	2673-3133	6.7	20	2	2	0.105034	0.92335	no
gi|641556800|gb|KK861900.1|	7097-7860	6.1	20	1	0.378577	-2	0.3455	no
gi|641556804|gb|KK861898.1|	140-700	9	35	1	1	0.110795	0.92265	no
gi|641556810|gb|KK861895.1|	11751-12062	11	18	5	0.852841	-3	0.2588	no
gi|641556810|gb|KK861895.1|	3094-3377	11	18	31	1	-5	0.18485	no
gi|641556815|gb|KK861893.1|	13259-13633	3	12	0.776786	5	3	0.28555	no
gi|641556819|gb|KK861891.1|	11214-11998	7.1	24	38	38	0.0127304	0.99125	no
gi|641556819|gb|KK861891.1|	8680-9261	7.1	24	2	2	-0.139882	0.9254	no
gi|641556825|gb|KK861888.1|	10865-11386	10	32	48	75	0.658775	0.59645	no
gi|641556825|gb|KK861888.1|	12184-12497	10	32	22	37	0.781933	0.5457	no
gi|641556825|gb|KK861888.1|	4077-4623	10	32	26	51	0.990616	0.43525	no
gi|641556825|gb|KK861888.1|	6371-7572	10	32	28	50	0.826789	0.6138	no

gi|641556825|gb|KK861888.1|	8726-10748	10	32	0.697681	0.532088	-0.390901	1	no
gi|641556832|gb|KK861885.1|	2-294	8.4	22	4	18	2	0.2117	no
gi|641556832|gb|KK861885.1|	2153-2518	8.4	22	0.815276	6	3	0.27615	no
gi|641556832|gb|KK861885.1|	2901-3576	8.4	22	5	11	1	0.4259	no
gi|641556832|gb|KK861885.1|	6834-7530	8.4	22	10	23	1	0.35155	no
gi|641556838|gb|KK861882.1|	10279-11740	12	38	2	0.426201	-2	0.23555	no
gi|641556838|gb|KK861882.1|	3251-4347	12	38	2	2	-0.153217	0.91575	no
gi|641556838|gb|KK861882.1|	5608-7262	12	38	2	3	0.315501	0.8086	no
gi|641556838|gb|KK861882.1|	7334-9122	12	38	1	0.406707	-2	0.32595	no
gi|641556846|gb|KK861878.1|	16911-17777	7.5	29	8	9	0.102633	0.9356	no
gi|641556851|gb|KK861876.1|	0-566	8.2	28	1	0.865331	-0.625915	0.71195	no
gi|641556855|gb|KK861874.1|	4881-5426	10	15	3	0	-inf	0.0075	no
gi|641556863|gb|KK861870.1|	17505-18068	10	35	0.576941	6	3	0.1465	no
gi|641556871|gb|KK861866.1|	4538-4714	9.3	28	19	27	0.517401	0.7699	no
gi|641556882|gb|KK861861.1|	1952-3285	13	24	0.497866	0.947959	0.929068	0.56225	no
gi|641556882|gb|KK861861.1|	6734-7261	13	24	0.426029	3	3	0.2991	no
gi|641556886|gb|KK861859.1|	1975-2512	9.4	32	0	9	inf	0.0004	yes
gi|641556890|gb|KK861857.1|	178-1035	6	27	10.7	37	2	0.171	no
gi|641556890|gb|KK861857.1|	3041-3463	6	27	4	16	2	0.17595	no
gi|641556890|gb|KK861857.1|	3592-4513	6	27	32	76	1	0.45105	no
gi|641556908|gb|KK861848.1|	1168-1460	12	46	3	10	2	0.3046	no
gi|641556932|gb|KK861836.1|	30-356	2.2	10	4	2	-0.841822	0.64855	no
gi|641556932|gb|KK861836.1|	3502-3723	2.2	10	214	120	-0.831902	0.51445	no
gi|641556938|gb|KK861833.1|	1551-1870	4.7	13	0	46	inf	5.00E-05	yes
gi|641556941|gb|KK861832.1|	1-475	7.1	29	3	6	1	0.45155	no
gi|641556941|gb|KK861832.1|	6137-7114	7.1	29	3	0.413224	-3	0.13385	no
gi|641556951|gb|KK861827.1|	700-1118	7.1	25	3	6	1	0.47835	no
gi|641556951|gb|KK861827.1|	8-362	7.1	25	15	16	0.0929316	0.94505	no
gi|641556975|gb|KK861815.1|	14494-14863	7.5	27	52	2	-4	0.0629	no
gi|641556975|gb|KK861815.1|	2027-2278	7.5	27	117	10	-4	0.04515	no

gi|641556977|gb|KK861814.1|	3248-3505	11	44	0	29	inf	0.00105	yes
gi|641556992|gb|KK861807.1|	12788-13352	8.1	33	2	9	2	0.21675	no
gi|641556997|gb|KK861805.1|	9-357	19	68	8	1	-3	0.1909	no
gi|641557001|gb|KK861803.1|	9-1138	6.5	32	12	9	-0.422562	0.7331	no
gi|641557003|gb|KK861802.1|	3146-3310	6.7	26	71	108.54	0.617692	0.676	no
gi|641557009|gb|KK861799.1|	9059-9393	8.1	27	11	4	-1	0.3758	no
gi|641557013|gb|KK861797.1|	0-823	5.5	11	14	6	-1	0.34405	no
gi|641557013|gb|KK861797.1|	7600-8012	5.5	11	9	11	0.238189	0.86045	no
gi|641557013|gb|KK861797.1|	8266-8727	5.5	11	60.71	50	-0.271387	0.82495	no
gi|641557031|gb|KK861788.1|	3800-4633	7.4	28	0.333082	2	2	0.2235	no
gi|641557043|gb|KK861782.1|	2591-3176	6.7	28	3	4	0.789883	0.5808	no
gi|641557070|gb|KK861769.1|	1434-1580	7.6	27	874	1533.66	0.810941	0.5204	no
gi|641557070|gb|KK861769.1|	2581-2894	7.6	27	74	120	0.696877	0.57375	no
gi|641557070|gb|KK861769.1|	3295-3839	7.6	27	1	2	0.595504	0.70505	no
gi|641557070|gb|KK861769.1|	62-257	7.6	27	292	799	1	0.259	no
gi|641557070|gb|KK861769.1|	942-1169	7.6	27	196	416	1	0.38565	no
gi|641557074|gb|KK861767.1|	4852-4968	11	39	278	2542.1	3	0.2581	no
gi|641557079|gb|KK861765.1|	10015-11585	9.9	35	3	1	-0.976927	0.47475	no
gi|641557079|gb|KK861765.1|	90-503	9.9	35	4	0.966265	-2	0.2679	no
gi|641557087|gb|KK861761.1|	10619-11026	9.5	37	3	9	2	0.2764	no
gi|641557087|gb|KK861761.1|	521-702	9.5	37	29	9	-2	0.363	no
gi|641557087|gb|KK861761.1|	7110-8415	9.5	37	0.701838	0.777431	0.147578	1	no
gi|641557087|gb|KK861761.1|	8742-10074	9.5	37	0.560591	1	1	0.49595	no
gi|641557091|gb|KK861759.1|	2205-2382	10	40	58	36	-0.674695	0.6657	no
gi|641557096|gb|KK861757.1|	145-555	8.1	17	2	2	0.322864	0.83525	no
gi|641557096|gb|KK861757.1|	3070-4163	8.1	17	0.867581	4	2	0.1431	no
gi|641557096|gb|KK861757.1|	9227-9886	8.1	17	1	3	1	0.4275	no
gi|641557100|gb|KK861755.1|	2708-3332	8.8	30	8	9	0.0840222	0.9486	no
gi|641557108|gb|KK861751.1|	11740-12449	7.6	17	20	7	-1	0.2669	no
gi|641557108|gb|KK861751.1|	3433-3936	7.6	17	3	0.346178	-3	0.2348	no

gi|641557108|gb|KK861751.1|	6837-7154	7.6	17	3	4	0.571313	0.76735	no
gi|641557121|gb|KK861745.1|	130-789	12	39	2	1	-0.778885	0.6308	no
gi|641557121|gb|KK861745.1|	2515-2939	12	39	7	12	0.64623	0.63875	no
gi|641557121|gb|KK861745.1|	4948-5708	12	39	0.501512	2	2	0.33105	no
gi|641557125|gb|KK861743.1|	256-1046	9.6	41	0.595276	0.903778	0.60241	0.70505	no
gi|641557169|gb|KK861725.1|	3004-3636	6.7	24	4	2	-1	0.4338	no
gi|641557171|gb|KK861724.1|	6715-7325	10	37	4	12	2	0.2755	no
gi|641557180|gb|KK861720.1|	3478-3982	11	18	0.914852	2	1	0.51315	no
gi|641557182|gb|KK861719.1|	8756-9180	11	43	19	12	-0.611973	0.6413	no
gi|641557184|gb|KK861718.1|	2845-3836	6.2	13	1	0.270661	-2	0.21865	no
gi|641557184|gb|KK861718.1|	6876-7331	6.2	13	98	28	-2	0.1621	no
gi|641557186|gb|KK861717.1|	4228-4465	11	34	41	81	0.972864	0.46305	no
gi|641557186|gb|KK861717.1|	750-1253	11	34	2	1	-0.821685	0.6492	no
gi|641557231|gb|KK861695.1|	2884-3410	11	36	40	19	-1	0.39365	no
gi|641557231|gb|KK861695.1|	3543-3861	11	36	5	0.811638	-3	0.2588	no
gi|641557233|gb|KK861694.1|	24-251	13	42	34	10	-2	0.2754	no
gi|641557275|gb|KK861674.1|	13-2727	11	31	4	5	0.317207	0.79475	no
gi|641557275|gb|KK861674.1|	5278-6292	11	31	2	1	-0.579305	0.6957	no
gi|641557275|gb|KK861674.1|	6976-7194	11	31	54	16	-2	0.2436	no
gi|641557275|gb|KK861674.1|	7335-7773	11	31	19	3	-3	0.1003	no
gi|641557281|gb|KK861671.1|	8689-11010	10	48	0.766086	3	2	0.19275	no
gi|641557297|gb|KK861663.1|	13097-13450	12	50	2	5	1	0.4433	no
gi|641557315|gb|KK861654.1|	212-1376	11	39	7	4	-0.700426	0.5837	no
gi|641557315|gb|KK861654.1|	6365-7060	11	39	3	0.85924	-2	0.2844	no
gi|641557315|gb|KK861654.1|	8249-8916	11	39	2	2	-0.400159	0.79645	no
gi|641557342|gb|KK861641.1|	3543-3905	7.8	26	39	1	-5	0.13065	no
gi|641557342|gb|KK861641.1|	579-1325	7.8	26	3	2	-0.75201	0.6028	no
gi|641557344|gb|KK861640.1|	2994-3456	6.5	14	29	13	-1	0.3802	no
gi|641557344|gb|KK861640.1|	35-209	6.5	14	99	23	-2	0.2092	no
gi|641557383|gb|KK861621.1|	381-696	7.7	27	7	5	-0.429126	0.77855	no

gi|641557383|gb|KK861621.1|	8266-8875	7.7	27	80	30	-1	0.2731	no
gi|641557385|gb|KK861620.1|	8465-9016	9.5	26	1	5	2	0.2254	no
gi|641557387|gb|KK861619.1|	1119-1890	9.7	35	0.614917	1	1	0.50945	no
gi|641557387|gb|KK861619.1|	1986-3041	9.7	35	0.5765	3	2	0.18265	no
gi|641557387|gb|KK861619.1|	5891-6880	9.7	35	2	0.542678	-2	0.362	no
gi|641557387|gb|KK861619.1|	7348-8214	9.7	35	0.950082	0.801992	-0.244465	0.88495	no
gi|641557389|gb|KK861618.1|	10177-11150	9	32	0.182082	1	3	0.2991	no
gi|641557389|gb|KK861618.1|	9344-9984	9	32	2	2	-0.307804	0.84435	no
gi|641557391|gb|KK861617.1|	0-2207	6.5	21	126.81	164	0.36948	0.8451	no
gi|641557391|gb|KK861617.1|	2414-2980	6.5	21	3	2	-0.81856	0.5995	no
gi|641557391|gb|KK861617.1|	8623-8939	6.5	21	4	2	-0.651298	0.71075	no
gi|641557423|gb|KK861601.1|	2062-2921	9.2	32	2	0	-inf	0.0034	yes
gi|641557423|gb|KK861601.1|	4589-5773	9.2	32	2	3	0.503265	0.7091	no
gi|641557423|gb|KK861601.1|	7311-8279	9.2	32	11	5	-1	0.3353	no
gi|641557431|gb|KK861597.1|	25-537	10	34	0.446002	4	3	0.25965	no
gi|641557433|gb|KK861596.1|	6696-7342	7.4	27	17	18	0.0978638	0.93575	no
gi|641557435|gb|KK861595.1|	12087-13331	7.6	32	3	13	2	0.12425	no
gi|641557435|gb|KK861595.1|	6508-7258	7.6	32	1	46	5	0.023	no
gi|641557435|gb|KK861595.1|	9786-10407	7.6	32	0.16623	17	7	0.1044	no
gi|641557441|gb|KK861592.1|	0-371	8	38	0	17	inf	0.0004	yes
gi|641557441|gb|KK861592.1|	2792-3470	8	38	44	3	-4	0.01485	no
gi|641557441|gb|KK861592.1|	4302-5041	8	38	25	4	-3	0.0566	no
gi|641557441|gb|KK861592.1|	7652-7989	8	38	26	4	-3	0.10835	no
gi|641557449|gb|KK861588.1|	6731-7730	8.8	28	1	2	0.827378	0.58405	no
gi|641557449|gb|KK861588.1|	9662-10653	8.8	28	1	2	0.505376	0.72975	no
gi|641557452|gb|KK861587.1|	16810-16979	9.2	32	83	92	0.151	0.91885	no
gi|641557452|gb|KK861587.1|	17431-17591	9.2	32	38	36	-0.0763931	0.91115	no
gi|641557452|gb|KK861587.1|	19608-19817	9.2	32	18	34	0.913394	0.5496	no
gi|641557458|gb|KK861584.1|	374-1840	8.9	31	0.334317	2	2	0.1663	no
gi|641557467|gb|KK861580.1|	2118-3681	9.1	23	11	12	0.0327658	0.979	no

gi|641557483|gb|KK861573.1|	8986-9841	8.5	14	3	6	1	0.43015	no
gi|641557487|gb|KK861571.1|	2442-4001	10	18	3	2	-1	0.40695	no
gi|641557502|gb|KK861564.1|	1676-2045	11	38	6	0	-inf	0.0059	no
gi|641557502|gb|KK861564.1|	2317-2951	11	38	8	0.732992	-4	0.1081	no
gi|641557504|gb|KK861563.1|	7776-8220	8	33	0	103	inf	5.00E-05	yes
gi|641557506|gb|KK861562.1|	4470-4895	6.9	30	3	3	0.224863	0.88235	no
gi|641557522|gb|KK861554.1|	6030-6626	7.8	26	0.88279	3	2	0.2851	no
gi|641557538|gb|KK861546.1|	12969-13636	9.1	40	5	0.227427	-5	0.1859	no
gi|641557548|gb|KK861541.1|	2730-3463	9	33	1	0.799049	-0.858061	0.60675	no
gi|641557548|gb|KK861541.1|	5052-5860	9	33	5	6	0.346361	0.793	no
gi|641557548|gb|KK861541.1|	5999-6926	9	33	2	4	0.663157	0.62845	no
gi|641557579|gb|KK861526.1|	12636-13775	11	16	0.825292	1	0.46857	0.75495	no
gi|641557593|gb|KK861519.1|	2959-3396	7.1	16	3	4	0.325518	0.8186	no
gi|641557597|gb|KK861517.1|	37-1227	11	25	0.355899	2	2	0.181	no
gi|641557605|gb|KK861513.1|	3-619	9.6	42	4	8	0.861371	0.52745	no
gi|641557617|gb|KK861507.1|	2702-3056	8.1	33	0	8	inf	0.0025	yes
gi|641557619|gb|KK861506.1|	1486-2339	7.4	27	0.107651	5	5	0.1054	no
gi|641557619|gb|KK861506.1|	2459-3138	7.4	27	1	11	3	0.07525	no
gi|641557645|gb|KK861493.1|	249-586	8.6	27	4	1	-1	0.44305	no
gi|641557655|gb|KK861488.1|	2-740	6.2	24	4	0.395874	-3	0.14385	no
gi|641557657|gb|KK861487.1|	28-382	10	40	2	5	1	0.4655	no
gi|641557657|gb|KK861487.1|	8280-8706	10	40	8	2	-2	0.20445	no
gi|641557657|gb|KK861487.1|	9862-11275	10	40	5	3	-0.723164	0.582	no
gi|641557666|gb|KK861483.1|	4649-5947	7	19	4	4	-0.158559	0.9038	no
gi|641557668|gb|KK861482.1|	1696-2563	9.3	32	3	1	-1	0.434	no
gi|641557668|gb|KK861482.1|	17369-18043	9.3	32	5	4	-0.52926	0.70065	no
gi|641557668|gb|KK861482.1|	251-1076	9.3	32	11	8	-0.507464	0.6906	no
gi|641557668|gb|KK861482.1|	3446-4408	9.3	32	5	3	-0.980331	0.47305	no
gi|641557668|gb|KK861482.1|	8674-9746	9.3	32	1	3	2	0.291	no
gi|641557676|gb|KK861478.1|	3047-4120	8.9	21	2	1	-0.394396	0.78685	no

gi|641557690|gb|KK861471.1|	2246-3193	10	31	0.09419	33	8	0.10435	no
gi|641557692|gb|KK861470.1|	3487-5166	8.8	32	2	4	0.579204	0.6578	no
gi|641557692|gb|KK861470.1|	5784-6102	8.8	32	9	11	0.378885	0.7926	no
gi|641557692|gb|KK861470.1|	714-3191	8.8	32	3	6	0.897989	0.47565	no
gi|641557700|gb|KK861466.1|	10082-10677	9	13	38	17	-1	0.38105	no
gi|641557700|gb|KK861466.1|	10903-11303	9	13	5	3	-0.90065	0.57745	no
gi|641557700|gb|KK861466.1|	30-797	9	13	3	3	0.116588	0.93195	no
gi|641557706|gb|KK861463.1|	14-554	5.8	31	20.71	10	-1	0.4382	no
gi|641557706|gb|KK861463.1|	2144-2949	5.8	31	1	0.176338	-3	0.271	no
gi|641557706|gb|KK861463.1|	6029-7099	5.8	31	0.890394	1	0.731112	0.63585	no
gi|641557708|gb|KK861462.1|	5484-6145	10	41	2	0.460651	-2	0.3455	no
gi|641557731|gb|KK861451.1|	9648-9794	8.1	30	47	478	3	0.1318	no
gi|641557749|gb|KK861442.1|	11416-11891	11	38	11	8	-0.440864	0.73725	no
gi|641557749|gb|KK861442.1|	2289-4461	11	38	22	15	-0.516401	0.82395	no
gi|641557749|gb|KK861442.1|	4564-7286	11	38	13	5	-1	0.287	no
gi|641557749|gb|KK861442.1|	8252-8842	11	38	39	28	-0.476921	0.6967	no
gi|641557751|gb|KK861441.1|	3136-3744	7.8	21	1	2	0.598068	0.69715	no
gi|641557751|gb|KK861441.1|	36-586	7.8	21	8	17	1	0.39465	no
gi|641557751|gb|KK861441.1|	5718-6042	7.8	21	25	2	-4	0.13645	no
gi|641557751|gb|KK861441.1|	6233-6684	7.8	21	3	2	-0.673286	0.68185	no
gi|641557753|gb|KK861440.1|	4108-4360	7.3	17	2	12	3	0.30875	no
gi|641557767|gb|KK861433.1|	0-814	8.9	28	8	5	-0.78423	0.55225	no
gi|641557767|gb|KK861433.1|	1784-3438	8.9	28	3	2	-0.534675	0.68895	no
gi|641557767|gb|KK861433.1|	5038-5262	8.9	28	13	6	-1	0.5278	no
gi|641557767|gb|KK861433.1|	5913-6278	8.9	28	4	5	0.580101	0.73035	no
gi|641557789|gb|KK861422.1|	33-682	9.5	26	0.156002	3	4	0.1175	no
gi|641557789|gb|KK861422.1|	3309-3937	9.5	26	0.981301	18	4	0.06625	no
gi|641557789|gb|KK861422.1|	952-1740	9.5	26	0.358371	2	3	0.18845	no
gi|641557798|gb|KK861418.1|	466-1021	9.3	39	0.98281	1	0.596004	0.70505	no
gi|641557798|gb|KK861418.1|	5430-5877	9.3	39	2	3	0.589418	0.70555	no

gi|641557819|gb|KK861409.1|	1010-1504	8.5	29	0	16	inf	0.00025	yes
gi|641557819|gb|KK861409.1|	10947-11588	8.5	29	2	1	-0.722812	0.652	no
gi|641557819|gb|KK861409.1|	7351-7863	8.5	29	19	50	1	0.26935	no
gi|641557821|gb|KK861408.1|	3788-3985	8.2	34	131	124	-0.0813571	0.9491	no
gi|641557821|gb|KK861408.1|	7446-7617	8.2	34	74	19	-2	0.28355	no
gi|641557827|gb|KK861405.1|	0-1019	2.8	8.2	2	1	-0.684343	0.63875	no
gi|641557832|gb|KK861403.1|	14337-14562	8.6	19	4	14	2	0.3484	no
gi|641557836|gb|KK861401.1|	0-263	7.8	31	41	99	1	0.3321	no
gi|641557847|gb|KK861397.1|	5673-6196	9.7	30	12	12	0.0350363	0.97725	no
gi|641557875|gb|KK861388.1|	149-410	9.4	28	14	41	2	0.29735	no
gi|641557875|gb|KK861388.1|	3252-3495	9.4	28	11	19	0.812975	0.58315	no
gi|641557875|gb|KK861388.1|	5568-5783	9.4	28	16	21	0.385818	0.80025	no
gi|641557896|gb|KK861377.1|	3851-4203	7.7	27	2	4	0.84106	0.63695	no
gi|641557912|gb|KK861370.1|	1771-2611	7.5	26	1	0.333652	-2	0.26805	no
gi|641557912|gb|KK861370.1|	23-707	7.5	26	1	3	1	0.47445	no
gi|641557912|gb|KK861370.1|	3188-3869	7.5	26	3	5	0.533086	0.7052	no
gi|641557912|gb|KK861370.1|	5035-5420	7.5	26	3	2	-1	0.5278	no
gi|641557912|gb|KK861370.1|	8111-8950	7.5	26	0.769948	2	0.965733	0.5733	no
gi|641557914|gb|KK861369.1|	10396-10944	9.3	27	9	8	-0.0756883	0.954	no
gi|641557914|gb|KK861369.1|	11-736	9.3	27	1	0.608259	-1	0.50885	no
gi|641557914|gb|KK861369.1|	7511-8408	9.3	27	20	22	0.12431	0.9167	no
gi|641557935|gb|KK861359.1|	1732-2736	7.3	27	1	0	-inf	0.0075	no
gi|641557935|gb|KK861359.1|	2881-4026	7.3	27	0.745526	0.907843	0.284185	0.85455	no
gi|641557935|gb|KK861359.1|	4803-5703	7.3	27	1	1	0.0734404	0.95375	no
gi|641557935|gb|KK861359.1|	839-1619	7.3	27	2	0.919125	-0.77627	0.63565	no
gi|641557939|gb|KK861357.1|	2119-3447	6.4	24	3	4	0.711454	0.58415	no
gi|641557939|gb|KK861357.1|	618-1396	6.4	24	1	0.553354	-0.982755	0.62775	no
gi|641557941|gb|KK861356.1|	1424-2699	11	33	0.590186	0.399475	-0.563064	1	no
gi|641557941|gb|KK861356.1|	3082-5034	11	33	2	0.184414	-4	0.10225	no
gi|641557941|gb|KK861356.1|	8725-10285	11	33	43	3	-4	0.0647	no

gi|641557943|gb|KK861355.1|	10775-12022	12	47	0.471255	0.820139	0.799361	1	no
gi|641557943|gb|KK861355.1|	22969-23291	12	47	44	18	-1	0.3404	no
gi|641557954|gb|KK861350.1|	1713-2576	7.3	33	0.848312	2	1	0.4562	no
gi|641557956|gb|KK861349.1|	8510-9159	8	14	10	7	-0.493762	0.70945	no
gi|641557960|gb|KK861347.1|	10565-10873	11	38	32	18	-0.793282	0.5556	no
gi|641557960|gb|KK861347.1|	7613-8506	11	38	24	14	-0.754429	0.54155	no
gi|641557968|gb|KK861343.1|	7553-8364	11	40	0.574973	2	2	0.3084	no
gi|641557970|gb|KK861342.1|	2931-3513	13	27	1	6	2	0.2009	no
gi|641557976|gb|KK861339.1|	12235-13411	11	57	0.938548	2	0.993333	0.51835	no
gi|641557976|gb|KK861339.1|	13492-14890	11	57	0.765308	1	0.813872	0.5797	no
gi|641557978|gb|KK861338.1|	1797-2409	9	37	10	13	0.391752	0.76575	no
gi|641557978|gb|KK861338.1|	209-702	9	37	3	3	-0.407255	0.7964	no
gi|641557991|gb|KK861333.1|	8542-9142	8.8	27	0.349642	2	3	0.2851	no
gi|641558009|gb|KK861324.1|	8300-8599	9.1	10	4	14	2	0.2623	no
gi|641558017|gb|KK861320.1|	108-837	13	46	20	14	-0.509355	0.67995	no
gi|641558017|gb|KK861320.1|	4683-6386	13	46	19	10	-0.951205	0.44625	no
gi|641558017|gb|KK861320.1|	8134-8525	13	46	34	19	-0.831344	0.52135	no
gi|641558022|gb|KK861318.1|	515-1853	8.2	34	1	2	0.912017	0.52255	no
gi|641558022|gb|KK861318.1|	6802-7272	8.2	34	18	34	0.900888	0.47925	no
gi|641558022|gb|KK861318.1|	8412-9144	8.2	34	0.527655	1	1	0.44255	no
gi|641558022|gb|KK861318.1|	9395-12108	8.2	34	30	70	1	0.5675	no
gi|641558044|gb|KK861307.1|	1139-1511	6.1	28	1	5	2	0.3198	no
gi|641558044|gb|KK861307.1|	477-1066	6.1	28	0.718729	2	1	0.44255	no
gi|641558053|gb|KK861303.1|	2775-3413	120	270	0.479601	2	2	0.2812	no
gi|641558057|gb|KK861301.1|	9594-9968	13	51	2	5	0.996464	0.5903	no
gi|641558059|gb|KK861300.1|	2631-3573	12	47	0.758563	0.576649	-0.395575	1	no
gi|641558059|gb|KK861300.1|	7062-8622	12	47	0.726342	1	0.707539	0.6229	no
gi|641558068|gb|KK861296.1|	2531-4552	12	42	3	3	-0.356956	0.77735	no
gi|641558068|gb|KK861296.1|	5738-6004	12	42	7	5	-0.44207	0.7866	no
gi|641558078|gb|KK861291.1|	4970-5457	5.6	31	7	0.729319	-3	0.15065	no

gi|641558097|gb|KK861282.1|	0-357	5.1	11	7	1	-2	0.2169	no
gi|641558097|gb|KK861282.1|	2049-2448	5.1	11	4	3	-0.552843	0.7452	no
gi|641558097|gb|KK861282.1|	7423-7833	5.1	11	5	0.97905	-2	0.21845	no
gi|641558099|gb|KK861281.1|	2775-3373	11	43	1	3	1	0.4402	no
gi|641558099|gb|KK861281.1|	3537-4319	11	43	2	2	0.361268	0.8122	no
gi|641558105|gb|KK861278.1|	11781-13578	13	47	16	8	-1	0.4149	no
gi|641558105|gb|KK861278.1|	21550-21909	13	47	10	6	-0.683866	0.62995	no
gi|641558105|gb|KK861278.1|	24446-24716	13	47	7	2	-2	0.3632	no
gi|641558118|gb|KK861272.1|	9482-9655	12	40	77	52	-0.562215	0.69945	no
gi|641558122|gb|KK861270.1|	2721-3299	13	62	3	4	0.427041	0.76565	no
gi|641558134|gb|KK861264.1|	10016-11198	5.9	41	0.789327	1	0.731877	0.63585	no
gi|641558136|gb|KK861263.1|	10769-11444	5.7	27	3	0.894697	-1	0.37245	no
gi|641558136|gb|KK861263.1|	2323-3240	5.7	27	1	0.298256	-2	0.3217	no
gi|641558136|gb|KK861263.1|	519-839	5.7	27	6	0	-inf	0.0102	no
gi|641558136|gb|KK861263.1|	9026-9365	5.7	27	58	53	-0.137953	0.91235	no
gi|641558138|gb|KK861262.1|	3780-4527	8.3	34	2	1	-0.175961	0.90465	no
gi|641558142|gb|KK861260.1|	8888-9098	10	40	44	3	-4	0.19355	no
gi|641558167|gb|KK861248.1|	13657-14132	13	47	0.504149	4	3	0.2674	no
gi|641558167|gb|KK861248.1|	9849-11449	13	47	1	3	1	0.4553	no
gi|641558179|gb|KK861242.1|	109-904	10	37	5	2	-1	0.3622	no
gi|641558183|gb|KK861240.1|	14881-15192	13	49	3	42	4	0.0747	no
gi|641558185|gb|KK861239.1|	114-1598	9.2	20	0.824119	63	6	0.02285	no
gi|641558185|gb|KK861239.1|	13003-13467	9.2	20	0.524412	4	3	0.27515	no
gi|641558187|gb|KK861238.1|	25-1047	9.4	26	3	2	-0.674919	0.63545	no
gi|641558187|gb|KK861238.1|	9385-9839	9.4	26	13	10	-0.410009	0.7525	no
gi|641558197|gb|KK861233.1|	3280-3925	8.1	28	4	5	0.406588	0.76885	no
gi|641558197|gb|KK861233.1|	5071-6466	8.1	28	2	1	-0.695154	0.6092	no
gi|641558199|gb|KK861232.1|	110-589	6.3	31	0	169	inf	0.0005	yes
gi|641558213|gb|KK861225.1|	15581-15812	7.3	32	14	9	-0.592433	0.69735	no
gi|641558227|gb|KK861218.1|	13619-16144	12	37	45	42	-0.0966249	0.96455	no

gi|641558227|gb|KK861218.1|	1610-2132	12	37	11	2	-2	0.1528	no
gi|641558227|gb|KK861218.1|	2258-7603	12	37	12	5	-1	0.4714	no
gi|641558229|gb|KK861217.1|	10665-11427	7.2	26	0.749614	2	1	0.4118	no
gi|641558251|gb|KK861206.1|	29-719	5.8	34	1	1	-0.0776511	0.92685	no
gi|641558256|gb|KK861204.1|	12434-13951	11	39	0.910681	0.816399	-0.157672	0.91355	no
gi|641558256|gb|KK861204.1|	27845-28076	11	39	12	6	-1	0.5278	no
gi|641558279|gb|KK861194.1|	2819-3124	9.5	35	23	14	-0.679344	0.6089	no
gi|641558279|gb|KK861194.1|	4156-4975	9.5	35	0.908157	0.517218	-0.812168	0.6492	no
gi|641558299|gb|KK861184.1|	2313-2595	8.6	22	4	13	2	0.28805	no
gi|641558306|gb|KK861181.1|	15950-17314	9.3	37	1	0.461451	-1	0.38185	no
gi|641558306|gb|KK861181.1|	17396-18274	9.3	37	3	2	-1	0.4503	no
gi|641558308|gb|KK861180.1|	1-688	8.7	26	2	0.654811	-2	0.33985	no
gi|641558308|gb|KK861180.1|	4843-5804	8.7	26	1	0.562417	-1	0.50315	no
gi|641558310|gb|KK861179.1|	11180-11656	7.2	15	4	3	-0.215728	0.89225	no
gi|641558310|gb|KK861179.1|	5912-7030	7.2	15	4	0.93429	-2	0.14655	no
gi|641558310|gb|KK861179.1|	8020-8413	7.2	15	9	1	-3	0.1613	no
gi|641558332|gb|KK861170.1|	3906-4270	12	37	0.819776	13	4	0.24015	no
gi|641558336|gb|KK861168.1|	8224-9212	8.1	35	0.089318	213	11	0.10465	no
gi|641558357|gb|KK861159.1|	7357-7840	12	46	0.735525	3	2	0.3323	no
gi|641558400|gb|KK861139.1|	9421-10576	11	46	2	6	2	0.22835	no
gi|641558402|gb|KK861138.1|	2364-3607	7.6	30	6	8	0.430537	0.73805	no
gi|641558402|gb|KK861138.1|	3722-4100	7.6	30	16	28.47	0.833526	0.5241	no
gi|641558414|gb|KK861133.1|	11374-11966	9.6	36	191	0.539659	-8	0.1321	no
gi|641558414|gb|KK861133.1|	2803-3597	9.6	36	10	11	0.151814	0.90075	no
gi|641558414|gb|KK861133.1|	7170-8026	9.6	36	2	3	0.401765	0.77255	no
gi|641558418|gb|KK861131.1|	3208-3786	9.9	39	1	2	1	0.5082	no
gi|641558418|gb|KK861131.1|	3899-4934	9.9	39	1	2	0.517837	0.7188	no
gi|641558418|gb|KK861131.1|	39-1201	9.9	39	0.878898	0.557466	-0.656812	0.6826	no
gi|641558434|gb|KK861123.1|	0-280	11	38	4	40	3	0.09855	no
gi|641558438|gb|KK861121.1|	3481-3900	11	39	7	0.47082	-4	0.1972	no

gi|641558440|gb|KK861120.1|	3884-4638	9	35	0.887065	0.961569	0.116352	0.92175	no
gi|641558453|gb|KK861114.1|	13248-13639	9.8	35	48	44	-0.12873	0.91535	no
gi|641558453|gb|KK861114.1|	89-814	9.8	35	47	23	-1	0.4211	no
gi|641558473|gb|KK861105.1|	11051-12224	8.7	35	16	58	2	0.2944	no
gi|641558488|gb|KK861098.1|	1268-1665	11	17	4	2	-1	0.46955	no
gi|641558488|gb|KK861098.1|	20-691	11	17	1	0.902141	-0.721982	0.67005	no
gi|641558488|gb|KK861098.1|	3488-4323	11	17	0.774762	1	0.603106	0.69715	no
gi|641558488|gb|KK861098.1|	4631-5127	11	17	2	0.708064	-2	0.4266	no
gi|641558494|gb|KK861095.1|	5444-6175	7.5	33	0.13216	2	4	0.1276	no
gi|641558494|gb|KK861095.1|	8171-8922	7.5	33	0.254814	2	3	0.25965	no
gi|641558494|gb|KK861095.1|	9030-9585	7.5	33	1	3	1	0.47095	no
gi|641558507|gb|KK861089.1|	15095-16051	7.9	26	8	7	-0.288514	0.82325	no
gi|641558513|gb|KK861086.1|	2867-3406	7.3	19	4	4	-0.067698	0.95985	no
gi|641558525|gb|KK861081.1|	12423-12940	7.6	26	2	3	0.594143	0.6902	no
gi|641558527|gb|KK861080.1|	0-323	4.7	19	46	5	-3	0.06125	no
gi|641558538|gb|KK861075.1|	5011-5225	10	18	12	22	0.899909	0.5829	no
gi|641558542|gb|KK861073.1|	7018-7197	10	41	69	0	-inf	0.00345	yes
gi|641558574|gb|KK861058.1|	15307-15712	7.2	15	7	3	-1	0.41465	no
gi|641558578|gb|KK861056.1|	14120-14607	9.3	34	1	4	2	0.28545	no
gi|641558578|gb|KK861056.1|	15203-15432	9.3	34	11	35	2	0.28575	no
gi|641558578|gb|KK861056.1|	16605-17322	9.3	34	0.949917	2	0.793681	0.63025	no
gi|641558578|gb|KK861056.1|	17949-18183	9.3	34	22	27	0.283292	0.8442	no
gi|641558597|gb|KK861048.1|	1098-1416	12	33	27	0	-inf	0.00025	yes
gi|641558597|gb|KK861048.1|	4-205	12	33	41.76	0	-inf	0.00345	yes
gi|641558597|gb|KK861048.1|	7169-8714	12	33	15	0	-inf	5.00E-05	yes
gi|641558599|gb|KK861047.1|	17-541	8.2	23	3	1	-1	0.5005	no
gi|641558599|gb|KK861047.1|	8152-8386	8.2	23	27	2	-4	0.19605	no
gi|641558599|gb|KK861047.1|	8923-9601	8.2	23	7	1	-3	0.1179	no
gi|641558601|gb|KK861046.1|	14292-14660	8.8	26	4	1	-2	0.3414	no
gi|641558601|gb|KK861046.1|	2508-2981	8.8	26	0	62	inf	5.00E-05	yes

gi|641558617|gb|KK861039.1|	12755-13123	7.4	26	4	9	1	0.40675	no
gi|641558617|gb|KK861039.1|	13304-13598	7.4	26	6	17	1	0.34495	no
gi|641558617|gb|KK861039.1|	2475-3609	7.4	26	2	0.574022	-2	0.23555	no
gi|641558617|gb|KK861039.1|	45-517	7.4	26	2	2	0.176309	0.9106	no
gi|641558617|gb|KK861039.1|	9889-10583	7.4	26	3	3	0.159944	0.90795	no
gi|641558636|gb|KK861031.1|	4383-4800	5.6	2.40E+01	1	3	1	0.51315	no
gi|641558648|gb|KK861026.1|	6460-9337	12	44	0.211219	2	3	0.0826	no
gi|641558648|gb|KK861026.1|	9653-10314	12	44	1	0.921302	-0.207674	0.8945	no
gi|641558671|gb|KK861016.1|	14626-14786	8.6	24	0	116	inf	0.0025	yes
gi|641558673|gb|KK861015.1|	3319-4428	11	42	2	2	-0.586777	0.67295	no
gi|641558673|gb|KK861015.1|	54-258	11	42	20	34	0.795379	0.5997	no
gi|641558673|gb|KK861015.1|	5516-7246	11	42	2	3	0.993147	0.45415	no
gi|641558675|gb|KK861014.1|	8580-9578	5.3	15	0.793848	0.804937	0.0200136	1	no
gi|641558683|gb|KK861010.1|	14694-15202	6.3	28	2	6	2	0.21075	no
gi|641558706|gb|KK861001.1|	4983-5887	10	17	0.799236	0.60739	-0.395998	1	no
gi|641558706|gb|KK861001.1|	6271-7132	10	17	0.531788	0.969574	0.8665	0.62725	no
gi|641558708|gb|KK861000.1|	6497-6856	7.3	30	10	35	2	0.1969	no
gi|641558713|gb|KK860998.1|	3295-3559	5.5	30	0.863992	295	8	0.10435	no
gi|641558713|gb|KK860998.1|	5745-5958	5.5	30	2	220	7	0.10435	no
gi|641558717|gb|KK860996.1|	18660-20143	11	19	3	5	0.907305	0.48555	no
gi|641558717|gb|KK860996.1|	6133-6966	11	19	11	7	-0.609917	0.63085	no
gi|641558727|gb|KK860991.1|	5355-6275	10	39	0.488494	1	1	0.50945	no
gi|641558744|gb|KK860983.1|	14874-15337	9.9	36	3	10	2	0.2406	no
gi|641558744|gb|KK860983.1|	2723-2978	9.9	36	9	29	2	0.2544	no
gi|641558744|gb|KK860983.1|	6310-6509	9.9	36	24	22	-0.122328	0.93375	no
gi|641558751|gb|KK860980.1|	14512-15304	7.9	26	18	17	-0.125185	0.9187	no
gi|641558751|gb|KK860980.1|	3087-3299	7.9	26	0	45	inf	0.00135	yes
gi|641558751|gb|KK860980.1|	7095-7867	7.9	26	0.368311	3	3	0.1563	no
gi|641558763|gb|KK860974.1|	0-600	5.4	22	23	12	-0.923043	0.47435	no
gi|641558765|gb|KK860973.1|	12262-12454	9.2	43	10	43	2	0.25545	no

gi|641558765|gb|KK860973.1|	134-969	9.2	43	0.332041	2	3	0.18845	no
gi|641558765|gb|KK860973.1|	3766-4006	9.2	43	6	96	4	0.08505	no
gi|641558765|gb|KK860973.1|	8185-10070	9.2	43	1	12	3	0.0237	no
gi|641558767|gb|KK860972.1|	11108-12073	8.4	16	1	0.699387	-1	0.4973	no
gi|641558767|gb|KK860972.1|	7862-8417	8.4	16	0.589686	2	2	0.2812	no
gi|641558767|gb|KK860972.1|	8499-9075	8.4	16	3	1	-1	0.4012	no
gi|641558771|gb|KK860970.1|	1847-3653	7.8	30	1	1	-0.308873	0.82085	no
gi|641558771|gb|KK860970.1|	4682-5249	7.8	30	4	0.863042	-2	0.22115	no
gi|641558771|gb|KK860970.1|	9441-10267	7.8	30	3	2	-0.335625	0.8148	no
gi|641558777|gb|KK860967.1|	11423-11788	5.8	22	4	5	0.258173	0.87415	no
gi|641558780|gb|KK860966.1|	71-615	10	31	1	2	1	0.4611	no
gi|641558787|gb|KK860963.1|	10198-10830	16	140	34	28	-0.257419	0.83465	no
gi|641558787|gb|KK860963.1|	15090-15774	16	140	4	0.658791	-2	0.1864	no
gi|641558787|gb|KK860963.1|	18595-19641	16	140	2	2	-0.0112809	0.97395	no
gi|641558796|gb|KK860959.1|	2092-2837	7.6	37	0.257594	3	3	0.2556	no
gi|641558804|gb|KK860955.1|	3816-4244	9.6	32	8	3	-2	0.3268	no
gi|641558804|gb|KK860955.1|	4497-5017	9.6	32	8	5	-0.691097	0.61145	no
gi|641558804|gb|KK860955.1|	6899-7482	9.6	32	1	2	0.182123	0.91015	no
gi|641558809|gb|KK860953.1|	44-620	12	51	0.185785	4	4	0.11365	no
gi|641558822|gb|KK860947.1|	10135-10422	9.7	31	8	5	-0.699001	0.66875	no
gi|641558822|gb|KK860947.1|	13542-14014	9.7	31	7	5	-0.515569	0.7102	no
gi|641558822|gb|KK860947.1|	14402-15320	9.7	31	0.979692	0.893536	-0.132802	0.92965	no
gi|641558822|gb|KK860947.1|	3571-3976	9.7	31	3	2	-0.999614	0.62775	no
gi|641558822|gb|KK860947.1|	4-387	9.7	31	4	0.555318	-3	0.2376	no
gi|641558822|gb|KK860947.1|	6423-7175	9.7	31	4	2	-0.934312	0.5133	no
gi|641558824|gb|KK860946.1|	4477-4966	13	46	0	21	inf	5.00E-05	yes
gi|641558826|gb|KK860945.1|	5437-5760	8.7	46	5	6	0.402669	0.81025	no
gi|641558841|gb|KK860941.1|	4923-5366	3.3	5.8	9	4	-0.995884	0.49955	no
gi|641558851|gb|KK860935.1|	10448-11100	9.8	35	0.15498	3	4	0.11365	no
gi|641558851|gb|KK860935.1|	12878-13639	9.8	35	0	59	inf	5.00E-05	yes

gi|641558863|gb|KK860929.1|	7343-8502	17	57	15	11	-0.42873	0.72635	no
gi|641558863|gb|KK860929.1|	9693-9945	17	57	16	25	0.640725	0.6544	no
gi|641558874|gb|KK860923.1|	5758-6319	8.2	24	0.966793	1	0.596265	0.70505	no
gi|641558887|gb|KK860917.1|	2181-2995	10	40	0.686622	3	2	0.19145	no
gi|641558887|gb|KK860917.1|	395-589	10	40	10	35	2	0.30935	no
gi|641558896|gb|KK860913.1|	18245-19429	12	49	0.429671	0.981204	1	0.47015	no
gi|641558905|gb|KK860909.1|	616-1318	8.3	33	0.558858	1	1	0.51305	no
gi|641558910|gb|KK860907.1|	5519-5858	8.8	31	3	3	-0.231612	0.88445	no
gi|641558918|gb|KK860903.1|	10080-11055	5.7	25	0.454036	0.96665	1	0.50945	no
gi|641558921|gb|KK860902.1|	10228-12315	10	32	2	1	-0.52324	0.6957	no
gi|641558921|gb|KK860902.1|	12887-13560	10	32	12	9	-0.329613	0.7971	no
gi|641558921|gb|KK860902.1|	7220-7751	10	32	34	49	0.530334	0.6754	no
gi|641558921|gb|KK860902.1|	8829-9917	10	32	4	4	-0.131249	0.92015	no
gi|641558930|gb|KK860898.1|	4530-5076	8.8	34	2	2	0.595597	0.6936	no
gi|641558930|gb|KK860898.1|	6439-6688	8.8	34	52	15	-2	0.2203	no
gi|641558930|gb|KK860898.1|	7197-8430	8.8	34	3	0.311685	-3	0.12655	no
gi|641558954|gb|KK860886.1|	8908-9815	9.9	39	2	3	0.838503	0.5603	no
gi|641558966|gb|KK860880.1|	14341-15451	15	40	0.309621	2	2	0.21345	no
gi|641558966|gb|KK860880.1|	15598-16589	15	40	1	0.405991	-1	0.4312	no
gi|641558966|gb|KK860880.1|	16858-19403	15	40	3	1	-0.875607	0.5036	no
gi|641558966|gb|KK860880.1|	646-2278	15	40	0.837787	0.60099	-0.479242	1	no
gi|641558977|gb|KK860875.1|	7030-7178	7.8	37	53	90	0.764727	0.6588	no
gi|641558979|gb|KK860874.1|	5332-5500	9.1	36	62	0	-inf	0.0075	no
gi|641558979|gb|KK860874.1|	7588-8433	9.1	36	1	0	-inf	0.01	no
gi|641559015|gb|KK860857.1|	5983-6264	10	34	19	5	-2	0.2554	no
gi|641559015|gb|KK860857.1|	8732-9229	10	34	3	0.352888	-3	0.217	no
gi|641559031|gb|KK860849.1|	152-330	12	33	103	218	1	0.42275	no
gi|641559031|gb|KK860849.1|	2607-2798	12	33	13	48	2	0.2801	no
gi|641559031|gb|KK860849.1|	6370-6581	12	33	59	108	0.874674	0.51465	no
gi|641559039|gb|KK860845.1|	10159-10958	8.4	33	8	1	-2	0.11465	no

gi|641559039|gb|KK860845.1|	1179-1731	8.4	33	2	2	-0.40413	0.80855	no
gi|641559039|gb|KK860845.1|	3992-5317	8.4	33	0.752087	0.477324	-0.655931	1	no
gi|641559039|gb|KK860845.1|	6010-6328	8.4	33	7	0.811638	-3	0.2309	no
gi|641559039|gb|KK860845.1|	7723-8219	8.4	33	14	1	-4	0.1031	no
gi|641559041|gb|KK860844.1|	3446-4803	11	39	0.548528	2	2	0.2366	no
gi|641559041|gb|KK860844.1|	4863-6159	11	39	0.578926	2	2	0.284	no
gi|641559043|gb|KK860843.1|	10097-10641	10	32	2	0.306304	-3	0.2481	no
gi|641559043|gb|KK860843.1|	11556-11959	10	32	2	2	0.00015996	0.91115	no
gi|641559043|gb|KK860843.1|	12013-12727	10	32	1	1	0.185933	0.91015	no
gi|641559043|gb|KK860843.1|	13653-14491	10	32	0.550818	1	0.866183	0.62725	no
gi|641559043|gb|KK860843.1|	14693-15430	10	32	0.784184	1	0.601451	0.70555	no
gi|641559043|gb|KK860843.1|	15921-16648	10	32	2	2	-0.31629	0.83735	no
gi|641559043|gb|KK860843.1|	18995-19327	10	32	3	3	-0.0105471	0.91115	no
gi|641559043|gb|KK860843.1|	19387-19819	10	32	2	2	0.00313476	0.91115	no
gi|641559045|gb|KK860842.1|	13559-14048	10	34	13	12	-0.101405	0.9387	no
gi|641559045|gb|KK860842.1|	15152-15485	10	34	7	7	-0.162356	0.9149	no
gi|641559045|gb|KK860842.1|	4083-4535	10	34	4	0	-inf	0.0056	no
gi|641559061|gb|KK860835.1|	10351-11062	8	35	1	4	2	0.29755	no
gi|641559061|gb|KK860835.1|	13377-13650	8	35	13	20	0.647545	0.65245	no
gi|641559079|gb|KK860826.1|	14508-15128	12	40	3	8	1	0.36205	no
gi|641559079|gb|KK860826.1|	1518-3512	12	40	33	100	2	0.46165	no
gi|641559079|gb|KK860826.1|	3647-5344	12	40	1	2	0.501483	0.71175	no
gi|641559097|gb|KK860817.1|	2952-3729	11	45	0.852007	0.923831	0.116763	0.92175	no
gi|641559099|gb|KK860816.1|	13976-14236	7.4	30	8	4	-1	0.5278	no
gi|641559099|gb|KK860816.1|	4197-4546	7.4	30	8	7	-0.0505132	0.9627	no
gi|641559112|gb|KK860810.1|	2493-4247	9.2	29	3	3	0.177908	0.8911	no
gi|641559112|gb|KK860810.1|	84-1368	9.2	29	3	3	0.0923334	0.94455	no
gi|641559116|gb|KK860808.1|	127-804	7.8	28	0.146954	2	4	0.1276	no
gi|641559122|gb|KK860805.1|	12659-13237	6.1	26	11	6	-0.917607	0.4958	no
gi|641559126|gb|KK860803.1|	212-832	7	38	1	1	-0.401533	0.82205	no

gi|641559135|gb|KK860799.1|	14996-15477	7.6	24	8	4	-0.9486	0.5066	no
gi|641559135|gb|KK860799.1|	7892-8301	7.6	24	27	20	-0.396508	0.76235	no
gi|641559141|gb|KK860796.1|	10848-11005	5.7	26	42	40	-0.0780337	0.91115	no
gi|641559141|gb|KK860796.1|	12900-13865	5.7	26	2	0.699387	-1	0.3598	no
gi|641559145|gb|KK860794.1|	24050-24401	9.1	14	11	12	0.103927	0.9409	no
gi|641559154|gb|KK860790.1|	9281-9589	8.7	26	3	6	1	0.5132	no
gi|641559158|gb|KK860788.1|	1808-2484	8.1	31	0.441776	2	2	0.2812	no
gi|641559160|gb|KK860787.1|	7982-8523	12	40	6	4	-0.504173	0.7186	no
gi|641559165|gb|KK860785.1|	5440-5960	7.2	27	2	0.985392	-0.820733	0.6492	no
gi|641559171|gb|KK860782.1|	1889-2271	6.1	20	2	4	1	0.5126	no
gi|641559171|gb|KK860782.1|	4657-5117	6.1	20	2	1	-0.824577	0.64915	no
gi|641559217|gb|KK860761.1|	13818-14864	10	35	1	0.506492	-1	0.46585	no
gi|641559217|gb|KK860761.1|	6444-6814	10	35	4	2	-1	0.46955	no
gi|641559217|gb|KK860761.1|	7057-7376	10	35	5	2	-2	0.3632	no
gi|641559227|gb|KK860756.1|	13562-14397	9.1	17	343.33	169	-1	0.62485	no
gi|641559227|gb|KK860756.1|	5683-6022	9.1	17	106	42	-1	0.2984	no
gi|641559230|gb|KK860755.1|	13084-13580	7.7	22	3	1	-2	0.403	no
gi|641559230|gb|KK860755.1|	15716-16065	7.7	22	9	5	-0.937055	0.5384	no
gi|641559230|gb|KK860755.1|	2682-3192	7.7	22	3	4	0.468225	0.75865	no
gi|641559230|gb|KK860755.1|	5129-5900	7.7	22	1	0.373355	-2	0.31075	no
gi|641559232|gb|KK860754.1|	16633-17217	8.9	15	0.363964	2	3	0.2851	no
gi|641559232|gb|KK860754.1|	8366-9186	8.9	15	0.680027	2	1	0.47015	no
gi|641559254|gb|KK860744.1|	6630-7352	3.7	18	5	0	-inf	0.00085	yes
gi|641559258|gb|KK860742.1|	709-7025	9.2	31	6	2	-1	0.35175	no
gi|641559274|gb|KK860734.1|	2867-3374	9.1	31	1	2	0.85662	0.62755	no
gi|641559295|gb|KK860724.1|	13654-14546	11	37	0.914627	2	0.755862	0.62565	no
gi|641559295|gb|KK860724.1|	31-782	11	37	1	0.966719	-0.0763496	0.92685	no
gi|641559295|gb|KK860724.1|	3344-3715	11	37	7	9	0.31796	0.8298	no
gi|641559295|gb|KK860724.1|	5442-6555	11	37	1	0.587099	-0.979066	0.57205	no
gi|641559295|gb|KK860724.1|	8613-10315	11	37	2	1	-0.391588	0.77105	no

gi|641559298|gb|KK860723.1|	11255-11574	8.4	18	50	52	0.0549547	0.96525	no
gi|641559298|gb|KK860723.1|	12221-12433	8.4	18	197	119	-0.724163	0.57725	no
gi|641559298|gb|KK860723.1|	15081-15332	8.4	18	77	50	-0.626417	0.6264	no
gi|641559298|gb|KK860723.1|	83-699	8.4	18	19	21	0.122998	0.92195	no
gi|641559300|gb|KK860722.1|	5255-6110	8.5	38	2	2	0.396934	0.787	no
gi|641559304|gb|KK860720.1|	235-627	6.8	21	4	0.531583	-3	0.2309	no
gi|641559341|gb|KK860704.1|	3486-3710	8.7	27	17	8	-1	0.5083	no
gi|641559353|gb|KK860697.1|	0-604	8.1	28	91	70	-0.361275	0.82445	no
gi|641559353|gb|KK860697.1|	16582-18346	8.1	28	1	2	0.547166	0.6888	no
gi|641559353|gb|KK860697.1|	20682-21257	8.1	28	3	0.563437	-3	0.20575	no
gi|641559353|gb|KK860697.1|	5483-5797	8.1	28	4	8	1	0.49435	no
gi|641559362|gb|KK860694.1|	12362-12647	7.1	34	97	11.51	-3	0.0462	no
gi|641559373|gb|KK860689.1|	1709-2273	9.5	18	5	3	-0.818645	0.57245	no
gi|641559373|gb|KK860689.1|	3019-3843	9.5	18	1	2	0.740448	0.6176	no
gi|641559373|gb|KK860689.1|	4114-4915	9.5	18	0.818251	1	0.795235	0.63025	no
gi|641559375|gb|KK860688.1|	3216-3419	8.3	26	78	84	0.104406	0.93775	no
gi|641559375|gb|KK860688.1|	8067-8479	8.3	26	27	18	-0.613825	0.64525	no
gi|641559381|gb|KK860685.1|	2773-3237	6.5	23	3	0.789788	-2	0.3216	no
gi|641559414|gb|KK860669.1|	8620-8889	9.1	28	8	5	-0.763061	0.63785	no
gi|641559426|gb|KK860663.1|	3502-4311	11	4.00E+01	1	1	0.0177536	0.95145	no
gi|641559430|gb|KK860661.1|	6-696	9.2	21	3	0	-inf	0.0024	yes
gi|641559430|gb|KK860661.1|	6035-6557	9.2	21	9	4	-1	0.40375	no
gi|641559432|gb|KK860660.1|	11070-11485	7.4	17	15	57	2	0.14475	no
gi|641559432|gb|KK860660.1|	7713-8009	7.4	17	1	45	5	0.2334	no
gi|641559453|gb|KK860650.1|	45-419	6.8	18	5	0	-inf	0.01	no
gi|641559453|gb|KK860650.1|	7262-7446	6.8	18	500	565	0.177783	0.8846	no
gi|641559460|gb|KK860647.1|	6359-6787	11	38	1	5	2	0.26255	no
gi|641559460|gb|KK860647.1|	6844-7565	11	38	2	3	0.716599	0.6393	no
gi|641559465|gb|KK860645.1|	11671-12162	10	27	10	8	-0.276136	0.8371	no
gi|641559465|gb|KK860645.1|	184-709	10	27	2	2	-0.253429	0.88485	no

gi|641559465|gb|KK860645.1|	4327-4562	10	27	21	4	-3	0.20805	no
gi|641559465|gb|KK860645.1|	864-1271	10	27	1	3	1	0.4427	no
gi|641559469|gb|KK860643.1|	2886-3123	5	24	8	28	2	0.28635	no
gi|641559473|gb|KK860641.1|	10482-11126	8.2	56	2	0	-inf	0.0102	no
gi|641559473|gb|KK860641.1|	2461-2913	8.2	56	2	0.825399	-1	0.4534	no
gi|641559473|gb|KK860641.1|	8498-10338	8.2	56	1	0.525174	-1	0.4394	no
gi|641559485|gb|KK860636.1|	3006-3564	6	37	0	3	inf	0.0068	no
gi|641559485|gb|KK860636.1|	8007-8725	6	37	7	5	-0.51442	0.69945	no
gi|641559505|gb|KK860627.1|	2236-2976	7.8	17	0	5	inf	0.00065	yes
gi|641559505|gb|KK860627.1|	7712-8001	7.8	17	0	22	inf	0.0005	yes
gi|641559518|gb|KK860621.1|	6073-6992	11	39	1	0.594872	-1	0.50315	no
gi|641559521|gb|KK860620.1|	2593-2836	9.3	37	7	35	2	0.1584	no
gi|641559521|gb|KK860620.1|	5224-5447	9.3	37	0	29	inf	0.00255	yes
gi|641559523|gb|KK860619.1|	11156-12242	7.9	23	1	0.483851	-1	0.46585	no
gi|641559523|gb|KK860619.1|	1319-1636	7.9	23	39	35	-0.131705	0.9179	no
gi|641559523|gb|KK860619.1|	7908-9110	7.9	23	1	0.963731	-0.311073	0.8374	no
gi|641559539|gb|KK860611.1|	551-1522	18	57	0.456377	3	3	0.1279	no
gi|641559544|gb|KK860609.1|	21280-21786	9.6	45	2	1	-0.728401	0.67005	no
gi|641559550|gb|KK860606.1|	10854-11715	10	40	0.531788	1	1	0.3994	no
gi|641559555|gb|KK860604.1|	3771-4210	8.8	29	12	4	-1	0.3241	no
gi|641559607|gb|KK860578.1|	14-486	9.4	16	14	13	-0.09218	0.9453	no
gi|641559609|gb|KK860577.1|	11783-13021	8.7	17	0.814838	0.930589	0.19163	0.90355	no
gi|641559609|gb|KK860577.1|	16037-16255	8.7	17	20	14	-0.576225	0.73235	no
gi|641559609|gb|KK860577.1|	3224-3680	8.7	17	3	4	0.727653	0.6396	no
gi|641559611|gb|KK860576.1|	1130-2181	16	44	5	6	0.315924	0.80595	no
gi|641559611|gb|KK860576.1|	15148-16934	16	44	18	5	-2	0.1717	no
gi|641559651|gb|KK860557.1|	2300-2629	9.1	19	9	0	-inf	0.0034	yes
gi|641559651|gb|KK860557.1|	9300-9701	9.1	19	1	5	2	0.2624	no
gi|641559663|gb|KK860552.1|	16618-17350	8.4	23	2	4	0.890857	0.52765	no
gi|641559663|gb|KK860552.1|	17407-18061	8.4	23	4	6	0.429564	0.7497	no

gi|641559663|gb|KK860552.1|	18341-18790	8.4	23	7	4	-0.884347	0.54105	no
gi|641559663|gb|KK860552.1|	334-920	8.4	23	33	59	0.843127	0.5035	no
gi|641559663|gb|KK860552.1|	6670-6964	8.4	23	10	7	-0.626779	0.6819	no
gi|641559663|gb|KK860552.1|	7822-8363	8.4	23	8	7	-0.238627	0.8554	no
gi|641559667|gb|KK860550.1|	1490-2061	7.4	43	0.56473	3	2	0.256	no
gi|641559671|gb|KK860548.1|	14357-14584	9.2	17	31	10	-2	0.2967	no
gi|641559671|gb|KK860548.1|	351-921	9.2	17	11	4	-2	0.2588	no
gi|641559671|gb|KK860548.1|	5687-6155	9.2	17	4	2	-0.993904	0.57205	no
gi|641559679|gb|KK860544.1|	10214-11116	8.4	34	5	8	0.719456	0.582	no
gi|641559696|gb|KK860536.1|	182-377	10	38	389	737	0.92286	0.4616	no
gi|641559696|gb|KK860536.1|	2523-3970	10	38	2	2	0.393488	0.77285	no
gi|641559727|gb|KK860523.1|	3043-3273	9.6	38	79	62	-0.341697	0.79425	no
gi|641559727|gb|KK860523.1|	3672-4624	9.6	38	1	0.853605	-0.39547	0.80865	no
gi|641559727|gb|KK860523.1|	6034-7502	9.6	38	3	3	0.0357844	0.9799	no
gi|641559727|gb|KK860523.1|	9258-9484	9.6	38	30	28	-0.109166	0.93915	no
gi|641559740|gb|KK860517.1|	417-1372	5.8	22	38	3	-4	0.0123	no
gi|641559749|gb|KK860513.1|	73-741	7.1	29	0.299492	3	3	0.2556	no
gi|641559751|gb|KK860512.1|	104-665	9.8	31	5	6	0.200336	0.8863	no
gi|641559751|gb|KK860512.1|	3467-5080	9.8	31	0.998841	0.609021	-0.713763	0.633	no
gi|641559751|gb|KK860512.1|	5202-5461	9.8	31	7	7	-0.122409	0.9229	no
gi|641559751|gb|KK860512.1|	7782-8012	9.8	31	10	9	-0.133426	0.9206	no
gi|641559757|gb|KK860509.1|	12605-13644	11	36	5	0.638343	-3	0.0838	no
gi|641559763|gb|KK860506.1|	219-1529	7.4	27	187	0.193489	-10	0.1307	no
gi|641559763|gb|KK860506.1|	3293-3647	7.4	27	5	0	-inf	0.01	no
gi|641559763|gb|KK860506.1|	3788-4576	7.4	27	4	0.181361	-4	0.1875	no
gi|641559765|gb|KK860505.1|	11849-12671	9.1	14	0	2	inf	0.00375	no
gi|641559776|gb|KK860500.1|	73-1457	6.4	30	1205.32	972	-0.30966	0.8414	no
gi|641559778|gb|KK860499.1|	82-684	14	46	75	23	-2	0.1806	no
gi|641559786|gb|KK860495.1|	160-311	9.6	22	64	117	0.865986	0.59325	no
gi|641559786|gb|KK860495.1|	3249-3526	9.6	22	8	22	1	0.3408	no

gi|641559786|gb|KK860495.1|	636-842	9.6	22	15	17	0.118332	0.929	no
gi|641559791|gb|KK860493.1|	6183-6863	8.8	23	0.730228	3	2	0.28495	no
gi|641559799|gb|KK860489.1|	3127-4864	10	34	1	0.560168	-1	0.45755	no
gi|641559799|gb|KK860489.1|	32-1443	10	34	2	0.354937	-2	0.18425	no
gi|641559799|gb|KK860489.1|	7176-8533	10	34	47	36	-0.377956	0.8681	no
gi|641559801|gb|KK860488.1|	12533-13030	6.7	27	48	19	-1	0.28985	no
gi|641559801|gb|KK860488.1|	4262-5220	6.7	27	1	0.846926	-0.395409	0.80865	no
gi|641559805|gb|KK860486.1|	113-944	10	35	7	6	-0.171392	0.8922	no
gi|641559805|gb|KK860486.1|	3126-3377	10	35	5	9	0.815941	0.6381	no
gi|641559807|gb|KK860485.1|	7699-8306	11	47	16	10	-0.700308	0.59055	no
gi|641559809|gb|KK860484.1|	14449-14848	9.2	37	56	67.39	0.278233	0.825	no
gi|641559809|gb|KK860484.1|	333-1098	9.2	37	0.124278	2	4	0.12385	no
gi|641559817|gb|KK860480.1|	5459-5873	5.9	15	5	0	-inf	0.00435	no
gi|641559821|gb|KK860478.1|	2853-3861	8.9	19	13	2	-3	0.07595	no
gi|641559821|gb|KK860478.1|	5775-7036	8.9	19	8	6	-0.482215	0.7085	no
gi|641559823|gb|KK860477.1|	4429-5022	12	42	0	3	inf	0.0068	no
gi|641559830|gb|KK860474.1|	5690-6351	6.2	41	1	0.230325	-3	0.271	no
gi|641559830|gb|KK860474.1|	9585-10626	6.2	41	7	0.63684	-3	0.06665	no
gi|641559834|gb|KK860472.1|	0-378	9.2	32	17	7	-1	0.4104	no
gi|641559836|gb|KK860471.1|	2895-3520	9.2	32	8	1	-3	0.11975	no
gi|641559836|gb|KK860471.1|	372-740	9.2	32	4	2	-0.741376	0.63835	no
gi|641559836|gb|KK860471.1|	6022-7732	9.2	32	10	5	-0.882554	0.4828	no
gi|641559836|gb|KK860471.1|	9488-9775	9.2	32	28	23.66	-0.234332	0.86055	no
gi|641559838|gb|KK860470.1|	12301-13021	9.5	39	0	80	inf	0.00065	yes
gi|641559856|gb|KK860461.1|	8195-8887	9.5	17	0.712616	2	1	0.3994	no
gi|641559858|gb|KK860460.1|	3871-4315	6.8	26	64	18	-2	0.1569	no
gi|641559874|gb|KK860453.1|	2907-3553	11	31	1	2	0.236692	0.88205	no
gi|641559874|gb|KK860453.1|	5419-6405	11	31	1	0.272363	-2	0.31075	no
gi|641559874|gb|KK860453.1|	57-752	11	31	3	3	0.131055	0.92865	no
gi|641559897|gb|KK860442.1|	14242-15014	7.6	23	0.613851	2	2	0.3084	no

gi|641559923|gb|KK860431.1|	9481-9716	7.7	23	5	16	2	0.33985	no
gi|641559950|gb|KK860418.1|	325-1364	9.5	21	0.587433	2	1	0.36605	no
gi|641559962|gb|KK860413.1|	16478-16696	6	22	9	16	0.761644	0.64725	no
gi|641559972|gb|KK860408.1|	1215-2526	8.8	12	2	4	0.68504	0.6012	no
gi|641559972|gb|KK860408.1|	3427-3835	8.8	12	0.987265	5	2	0.2235	no
gi|641559972|gb|KK860408.1|	4800-5229	8.8	12	4	9	1	0.41305	no
gi|641559972|gb|KK860408.1|	6044-6783	8.8	12	4	5	0.267072	0.83875	no
gi|641559972|gb|KK860408.1|	8340-8911	8.8	12	1	1	0.0117218	0.91115	no
gi|641559978|gb|KK860405.1|	10435-11232	11	41	0.23534	5	5	0.23595	no
gi|641559978|gb|KK860405.1|	5215-6011	11	41	0.825062	0.536887	-0.619884	1	no
gi|641559993|gb|KK860398.1|	15143-15900	8.4	13	28	5	-3	0.07205	no
gi|641559993|gb|KK860398.1|	2536-2734	8.4	13	38	3	-4	0.20485	no
gi|641560006|gb|KK860392.1|	558-1073	7.9	36	0.441861	3	3	0.28535	no
gi|641560012|gb|KK860389.1|	1160-2170	7.9	27	0.695249	2	2	0.3067	no
gi|641560012|gb|KK860389.1|	2238-3440	7.9	27	0.281326	1	2	0.2441	no
gi|641560012|gb|KK860389.1|	3792-4682	7.9	27	1	3	1	0.34865	no
gi|641560012|gb|KK860389.1|	570-1058	7.9	27	1	3	1	0.47105	no
gi|641560020|gb|KK860385.1|	0-1098	8.4	34	0.235303	1	3	0.19335	no
gi|641560028|gb|KK860381.1|	9-604	10	40	6	2	-1	0.3819	no
gi|641560034|gb|KK860378.1|	11313-11533	8.7	38	48	20	-1	0.3715	no
gi|641560039|gb|KK860376.1|	1132-1582	8.6	28	3	2	-0.869765	0.6061	no
gi|641560039|gb|KK860376.1|	11432-11878	8.6	28	5	3	-0.851239	0.5661	no
gi|641560039|gb|KK860376.1|	1692-2887	8.6	28	4	2	-1	0.3325	no
gi|641560039|gb|KK860376.1|	3576-4950	8.6	28	2	3	0.852433	0.5306	no
gi|641560039|gb|KK860376.1|	7697-7995	8.6	28	34	16	-1	0.43525	no
gi|641560039|gb|KK860376.1|	8522-11329	8.6	28	23	15	-0.613452	0.7875	no
gi|641560051|gb|KK860370.1|	1942-3042	11	37	0.939129	2	0.828197	0.58405	no
gi|641560051|gb|KK860370.1|	6220-6681	11	37	2	2	-0.216818	0.8945	no
gi|641560055|gb|KK860368.1|	3674-4169	11	21	5	2	-2	0.34595	no
gi|641560062|gb|KK860365.1|	6556-6980	10	33	0	9	inf	0.00145	yes

gi|641560066|gb|KK860363.1|	5738-5960	4.5	15	0	40	inf	0.0015	yes
gi|641560074|gb|KK860359.1|	10819-11248	9.7	28	4	4	0.00285422	0.95145	no
gi|641560074|gb|KK860359.1|	13307-14052	9.7	28	1	1	-0.135358	0.92965	no
gi|641560074|gb|KK860359.1|	336-865	9.7	28	0.423499	3	3	0.2991	no
gi|641560081|gb|KK860356.1|	18-833	5	22	1	0	-inf	0.0102	no
gi|641560087|gb|KK860353.1|	104-1159	9.3	36	0.741215	2	1	0.47685	no
gi|641560087|gb|KK860353.1|	2255-2886	9.3	36	0.324855	2	3	0.27515	no
gi|641560087|gb|KK860353.1|	3051-3364	9.3	36	18	30	0.740354	0.5823	no
gi|641560097|gb|KK860348.1|	0-1100	9.9	32	1.8	5	1	0.27755	no
gi|641560097|gb|KK860348.1|	13204-13956	9.9	32	2	3	0.441276	0.74845	no
gi|641560097|gb|KK860348.1|	5084-5231	9.9	32	78	203.8	1	0.394	no
gi|641560097|gb|KK860348.1|	7942-8255	9.9	32	97	28	-2	0.1682	no
gi|641560101|gb|KK860346.1|	3731-4410	3.9	12	2	0.221844	-3	0.2348	no
gi|641560124|gb|KK860336.1|	6784-7304	9.7	35	3	0.328464	-3	0.2242	no
gi|641560126|gb|KK860335.1|	13451-14055	11	52	0.865586	42	6	0.08155	no
gi|641560126|gb|KK860335.1|	16369-16942	11	52	2	49	4	0.0231	no
gi|641560147|gb|KK860325.1|	11182-11642	9.8	32	6	2	-1	0.3795	no
gi|641560147|gb|KK860325.1|	13619-14052	9.8	32	3	2	-0.581736	0.7586	no
gi|641560147|gb|KK860325.1|	16318-16821	9.8	32	5	2	-1	0.43995	no
gi|641560160|gb|KK860319.1|	9997-10835	12	31	0.440655	1	1	0.51305	no
gi|641560166|gb|KK860316.1|	0-189	8.2	26	62	58	-0.0922525	0.94955	no
gi|641560166|gb|KK860316.1|	2730-3819	8.2	26	8	9	0.218701	0.86415	no
gi|641560171|gb|KK860314.1|	1014-1985	7.8	15	0.912755	1	0.60472	0.67795	no
gi|641560171|gb|KK860314.1|	22319-23009	7.8	15	9	15	0.66651	0.6021	no
gi|641560173|gb|KK860313.1|	2511-3234	10	18	2	2	-0.568761	0.71445	no
gi|641560173|gb|KK860313.1|	308-691	10	18	3	6	0.904593	0.5694	no
gi|641560178|gb|KK860311.1|	6511-6972	6.6	19	38	25	-0.58193	0.6576	no
gi|641560184|gb|KK860308.1|	5928-6601	4.8	12	1	0.449202	-2	0.4266	no
gi|641560184|gb|KK860308.1|	81-484	4.8	12	1	4	2	0.2812	no
gi|641560184|gb|KK860308.1|	8749-9200	4.8	12	1	5	2	0.2855	no

gi|641560201|gb|KK860300.1|	2788-3194	14	50	3	3	0.170422	0.9106	no
gi|641560201|gb|KK860300.1|	9399-9891	14	50	6	5	-0.29184	0.83095	no
gi|641560203|gb|KK860299.1|	2904-3191	6.3	23	6	8	0.394109	0.8104	no
gi|641560205|gb|KK860298.1|	2585-3050	6.4	28	3	4	0.34983	0.81305	no
gi|641560208|gb|KK860297.1|	9046-9553	5.4	23	5	29	3	0.07105	no
gi|641560218|gb|KK860293.1|	16770-17002	59	300	15	4	-2	0.27505	no
gi|641560218|gb|KK860293.1|	4831-6170	59	300	9	7	-0.307103	0.80425	no
gi|641560226|gb|KK860289.1|	2859-3171	8.7	31	0	16	inf	0.0015	yes
gi|641560245|gb|KK860281.1|	0-4568	9	32	0.582916	7	4	0.01575	no
gi|641560245|gb|KK860281.1|	15480-16406	9	32	0.096897	1	4	0.12385	no
gi|641560245|gb|KK860281.1|	4786-5389	9	32	0.8677	13	4	0.08555	no
gi|641560251|gb|KK860278.1|	3910-4860	7.9	10	9	11	0.344642	0.78625	no
gi|641560251|gb|KK860278.1|	5916-7670	7.9	10	7	7.48	0.050547	0.9664	no
gi|641560251|gb|KK860278.1|	9269-10043	7.9	10	1	2	0.602138	0.6902	no
gi|641560267|gb|KK860271.1|	17685-18921	11	47	0.884438	1	0.491179	0.7378	no
gi|641560277|gb|KK860266.1|	180-422	10	36	19	2	-4	0.20495	no
gi|641560277|gb|KK860266.1|	4643-4774	10	36	363	128	-2	0.4066	no
gi|641560277|gb|KK860266.1|	9859-10353	10	36	2	0.35634	-3	0.271	no
gi|641560279|gb|KK860265.1|	2292-2711	7.5	32	0.626944	20	5	0.2337	no
gi|641560285|gb|KK860263.1|	6232-6505	12	45	9	2	-2	0.27505	no
gi|641560287|gb|KK860262.1|	20251-21235	7.3	25	1	2	0.604846	0.67915	no
gi|641560287|gb|KK860262.1|	3075-3303	7.3	25	33	39	0.221858	0.87145	no
gi|641560287|gb|KK860262.1|	8425-9243	7.3	25	3	3	-0.0410015	0.9713	no
gi|641560295|gb|KK860258.1|	12467-16804	11	38	2	0.70492	-1	0.36545	no
gi|641560295|gb|KK860258.1|	17308-17548	11	38	1050.27	1582.88	0.591788	0.7881	no
gi|641560295|gb|KK860258.1|	8695-9629	11	38	0.191694	2	3	0.25965	no
gi|641560295|gb|KK860258.1|	9798-10984	11	38	0.285869	3	4	0.12815	no
gi|641560302|gb|KK860255.1|	11134-11907	8	25	2	3	0.450118	0.75195	no
gi|641560337|gb|KK860240.1|	7114-7505	7.8	34	0	6	inf	0.00455	no
gi|641560343|gb|KK860237.1|	2-959	13	34	0.650672	1	0.797226	0.63025	no

gi|641560352|gb|KK860233.1|	12356-12755	13	23	11	6	-0.910104	0.52	no
gi|641560352|gb|KK860233.1|	3305-3786	13	23	7	6	-0.215387	0.87675	no
gi|641560359|gb|KK860230.1|	386-601	9.8	32	10	38	2	0.25655	no
gi|641560361|gb|KK860229.1|	9827-10147	5.9	28	6	2	-1	0.4694	no
gi|641560363|gb|KK860228.1|	11466-11828	13	37	19	324	4	0.05675	no
gi|641560363|gb|KK860228.1|	4789-5356	13	37	13	156	4	0.0807	no
gi|641560363|gb|KK860228.1|	7437-7786	13	37	2	44	4	0.08395	no
gi|641560371|gb|KK860224.1|	11763-12613	9.6	37	1	1	-0.289768	0.8456	no
gi|641560371|gb|KK860224.1|	12815-13553	9.6	37	1	1	-0.272998	0.86195	no
gi|641560371|gb|KK860224.1|	18317-18568	9.6	37	10	3	-2	0.3397	no
gi|641560407|gb|KK860208.1|	10572-11307	8.6	32	0	2	inf	0.00375	no
gi|641560407|gb|KK860208.1|	15761-16202	8.6	32	0	21	inf	0.00025	yes
gi|641560423|gb|KK860202.1|	10786-11535	7.5	16	10	16	0.689147	0.58945	no
gi|641560425|gb|KK860201.1|	14576-16073	8.6	30	41	48	0.230248	0.9173	no
gi|641560425|gb|KK860201.1|	22612-23437	8.6	30	24	24	-0.0195661	0.98795	no
gi|641560427|gb|KK860200.1|	6774-7259	9.2	33	4	5	0.385782	0.79325	no
gi|641560427|gb|KK860200.1|	9982-11620	9.2	33	0.834303	0.897746	0.105737	0.94435	no
gi|641560432|gb|KK860198.1|	1-387	7.9	15	6	18	1	0.2953	no
gi|641560439|gb|KK860195.1|	8603-9585	9.1	34	0.089999	3	5	0.10725	no
gi|641560457|gb|KK860186.1|	16716-16866	13	19	86	14	-3	0.25845	no
gi|641560460|gb|KK860185.1|	18313-18671	9.4	26	32	21	-0.605416	0.6434	no
gi|641560460|gb|KK860185.1|	3153-3487	9.4	26	5	1	-2	0.3414	no
gi|641560460|gb|KK860185.1|	4563-6032	9.4	26	2	1	-1	0.42135	no
gi|641560474|gb|KK860179.1|	15003-15367	7.9	29	0	37	inf	5.00E-05	yes
gi|641560491|gb|KK860171.1|	11524-12378	10	36	3	18	3	0.0592	no
gi|641560491|gb|KK860171.1|	15191-15389	10	36	27	205	3	0.07005	no
gi|641560491|gb|KK860171.1|	16-397	10	36	3	3	0.0969702	0.92385	no
gi|641560491|gb|KK860171.1|	3133-4972	10	36	5	6	0.143075	0.906	no
gi|641560491|gb|KK860171.1|	708-2353	10	36	0.976793	2	0.809891	0.55395	no
gi|641560491|gb|KK860171.1|	7346-8565	10	36	17	13	-0.423185	0.73555	no

gi|641560491|gb|KK860171.1|	9953-11388	10	36	0.628241	2	2	0.2143	no
gi|641560502|gb|KK860166.1|	11100-12769	11	17	23	21	-0.100213	0.93585	no
gi|641560502|gb|KK860166.1|	24599-25308	11	17	5	4	-0.44096	0.7453	no
gi|641560502|gb|KK860166.1|	7137-7331	11	17	24	24	0.0125234	0.94155	no
gi|641560520|gb|KK860157.1|	15149-15642	11	45	3	2	-0.670289	0.68185	no
gi|641560524|gb|KK860155.1|	1161-1466	10	27	45	21	-1	0.41895	no
gi|641560531|gb|KK860152.1|	1297-3722	9.6	24	1	0.77528	-0.816581	0.54815	no
gi|641560531|gb|KK860152.1|	17265-17861	9.6	24	1	1	0.0126835	0.91115	no
gi|641560531|gb|KK860152.1|	419-736	9.6	24	20	10	-1	0.45825	no
gi|641560539|gb|KK860148.1|	2411-3871	5.6	22	17	10	-0.812114	0.51625	no
gi|641560539|gb|KK860148.1|	8238-8659	5.6	22	10	2	-2	0.19735	no
gi|641560545|gb|KK860145.1|	6634-7228	7.9	34	27	1	-5	0.0624	no
gi|641560547|gb|KK860144.1|	2006-2339	11	39	56	109	0.960888	0.44655	no
gi|641560547|gb|KK860144.1|	4730-5483	11	39	3	2	-0.776752	0.58825	no
gi|641560547|gb|KK860144.1|	5828-9090	11	39	18	31	0.772796	0.7276	no
gi|641560547|gb|KK860144.1|	72-246	11	39	582	1024.91	0.816493	0.5109	no
gi|641560549|gb|KK860143.1|	8991-9126	11	31	90	254	1	0.42175	no
gi|641560551|gb|KK860142.1|	156-677	10	44	2	0	-inf	0.0102	no
gi|641560551|gb|KK860142.1|	16450-17469	10	44	2	0.392259	-3	0.1748	no
gi|641560551|gb|KK860142.1|	19434-20203	10	44	1	0.749295	-0.857381	0.60675	no
gi|641560558|gb|KK860139.1|	18828-19063	8.6	25	7	12	0.769133	0.64635	no
gi|641560558|gb|KK860139.1|	3139-3572	8.6	25	4	4	0.0576748	0.9556	no
gi|641560558|gb|KK860139.1|	3666-4558	8.6	25	1	2	0.340825	0.81785	no
gi|641560558|gb|KK860139.1|	8196-10278	8.6	25	0.938185	3	1	0.2907	no
gi|641560560|gb|KK860138.1|	14007-15379	8.4	26	0.902559	0.36666	-1	0.4417	no
gi|641560560|gb|KK860138.1|	1660-1881	8.4	26	348	637	0.873589	0.47845	no
gi|641560560|gb|KK860138.1|	241-480	8.4	26	46	111	1	0.3308	no
gi|641560560|gb|KK860138.1|	7959-8097	8.4	26	74	182.29	1	0.47755	no
gi|641560560|gb|KK860138.1|	8237-8655	8.4	26	49	104	1	0.3798	no
gi|641560562|gb|KK860137.1|	7551-7865	6.7	30	4	3	-0.65174	0.71075	no

gi|641560566|gb|KK860135.1|	2020-2198	6.2	19	28	15	-0.896532	0.6344	no
gi|641560575|gb|KK860131.1|	2554-3415	30	170	4	6	0.603465	0.64705	no
gi|641560575|gb|KK860131.1|	5948-7257	30	170	13	12	-0.108133	0.93045	no
gi|641560584|gb|KK860127.1|	2959-3699	6.5	25	1	2	0.601511	0.6936	no
gi|641560584|gb|KK860127.1|	30-574	6.5	25	0.405432	3	3	0.28535	no
gi|641560584|gb|KK860127.1|	982-1829	6.5	25	0.760496	0.825226	0.117849	1	no
gi|641560589|gb|KK860125.1|	11446-11669	10	38	0	65	inf	0.0003	yes
gi|641560589|gb|KK860125.1|	13446-13633	10	38	0	40	inf	0.0068	no
gi|641560595|gb|KK860123.1|	67-370	6.4	27	107	0	-inf	5.00E-05	yes
gi|641560595|gb|KK860123.1|	9274-10236	6.4	27	2	0.140422	-4	0.1991	no
gi|641560602|gb|KK860120.1|	4826-5505	11	41	0.731735	2	2	0.32945	no
gi|641560616|gb|KK860113.1|	14863-15182	12	44	3	9	2	0.31615	no
gi|641560616|gb|KK860113.1|	15352-16059	12	44	2	6	2	0.2395	no
gi|641560616|gb|KK860113.1|	16761-18189	12	44	0.976431	1	0.520077	0.71875	no
gi|641560616|gb|KK860113.1|	18267-20624	12	44	0.851588	2	0.996624	0.4718	no
gi|641560616|gb|KK860113.1|	733-987	12	44	13	21	0.760414	0.60445	no
gi|641560623|gb|KK860110.1|	11854-12315	7	30	2	2	-0.216818	0.8945	no
gi|641560625|gb|KK860109.1|	11-226	8.9	25	34	31	-0.154056	0.91325	no
gi|641560625|gb|KK860109.1|	16219-16408	8.9	25	38	12	-2	0.3343	no
gi|641560625|gb|KK860109.1|	1756-2375	8.9	25	1	0.505737	-1	0.4534	no
gi|641560625|gb|KK860109.1|	19320-19523	8.9	25	125	38	-2	0.2239	no
gi|641560633|gb|KK860105.1|	15167-15405	9.9	43	61	44	-0.452036	0.7386	no
gi|641560640|gb|KK860102.1|	11556-12744	10	29	8	0.108585	-6	0.18295	no
gi|641560640|gb|KK860102.1|	14594-15287	10	29	5	0	-inf	0.00105	yes
gi|641560640|gb|KK860102.1|	8070-9784	10	29	16	0.071079	-8	0.18285	no
gi|641560643|gb|KK860101.1|	1882-2665	7.8	27	0.843311	2	0.964863	0.5733	no
gi|641560643|gb|KK860101.1|	6736-6926	7.8	27	24	8	-2	0.363	no
gi|641560645|gb|KK860100.1|	80-277	8.1	35	18	10	-0.886341	0.63655	no
gi|641560647|gb|KK860099.1|	12302-12710	10	35	1	6	2	0.2441	no
gi|641560647|gb|KK860099.1|	12763-13568	10	35	0.929009	2	1	0.4562	no

gi|641560647|gb|KK860099.1|	4586-5130	10	35	1	3	1	0.4891	no
gi|641560647|gb|KK860099.1|	5497-6911	10	35	0.639066	0.442583	-0.530018	1	no
gi|641560647|gb|KK860099.1|	7719-8047	10	35	4	5	0.380977	0.8102	no
gi|641560647|gb|KK860099.1|	9327-9728	10	35	3	9	2	0.32805	no
gi|641560649|gb|KK860098.1|	10321-10543	9.6	28	38	25	-0.574365	0.6895	no
gi|641560649|gb|KK860098.1|	617-1059	9.6	28	27	46	0.745497	0.5501	no
gi|641560649|gb|KK860098.1|	9063-9818	9.6	28	1	2	0.142365	0.929	no
gi|641560666|gb|KK860090.1|	1493-3133	9.8	27	1	2	0.871278	0.52235	no
gi|641560683|gb|KK860082.1|	0-907	12	39	2	4	0.604037	0.6544	no
gi|641560683|gb|KK860082.1|	2306-3646	12	39	4	12	2	0.17425	no
gi|641560691|gb|KK860079.1|	11607-12040	8.2	25	2	3	0.395544	0.80895	no
gi|641560691|gb|KK860079.1|	214-530	8.2	25	2	12	2	0.21565	no
gi|641560691|gb|KK860079.1|	2272-2982	8.2	25	0.687729	2	1	0.4611	no
gi|641560697|gb|KK860076.1|	8353-9183	11	20	1	0.169437	-3	0.22425	no
gi|641560703|gb|KK860073.1|	13518-14078	11	37	2	1	-0.725706	0.67005	no
gi|641560703|gb|KK860073.1|	14657-14902	11	37	17	13	-0.449296	0.7651	no
gi|641560703|gb|KK860073.1|	22244-22469	11	37	14	0	-inf	0.01175	no
gi|641560703|gb|KK860073.1|	9288-10305	11	37	1	9	3	0.07365	no
gi|641560710|gb|KK860070.1|	12762-13299	8.3	35	2	14	3	0.10955	no
gi|641560712|gb|KK860069.1|	10308-10954	6.6	37	0	5	inf	0.0015	yes
gi|641560712|gb|KK860069.1|	403-827	6.6	37	0.306838	7	4	0.1123	no
gi|641560715|gb|KK860068.1|	11408-12049	7.2	28	30	0.24054	-7	0.1829	no
gi|641560715|gb|KK860068.1|	1505-1964	7.2	28	3	0	-inf	0.01175	no
gi|641560715|gb|KK860068.1|	4338-4759	7.2	28	3	0	-inf	0.01175	no
gi|641560715|gb|KK860068.1|	5428-5772	7.2	28	5	0	-inf	0.01175	no
gi|641560741|gb|KK860057.1|	5254-5772	6.7	26	0	23	inf	5.00E-05	yes
gi|641560753|gb|KK860052.1|	10582-12026	7.2	27	6	3	-0.890649	0.4931	no
gi|641560753|gb|KK860052.1|	13838-14618	7.2	27	15	19	0.399425	0.75435	no
gi|641560783|gb|KK860039.1|	1083-2289	8.3	23	6	8	0.477755	0.7038	no
gi|641560783|gb|KK860039.1|	5267-5944	8.3	23	2	3	0.278172	0.8498	no

gi|641560785|gb|KK860038.1|	3078-3507	8.6	35	0	5	inf	0.00375	no
gi|641560788|gb|KK860037.1|	478-1218	9.4	39	2	1	-0.398489	0.7965	no
gi|641560803|gb|KK860030.1|	6828-7390	6	25	0	10	inf	0.00065	yes
gi|641560815|gb|KK860024.1|	14779-15030	9.1	35	61	32	-0.918399	0.4834	no
gi|641560815|gb|KK860024.1|	15869-16138	9.1	35	22	8	-1	0.3581	no
gi|641560815|gb|KK860024.1|	3193-4138	9.1	35	1	0	-inf	0.0075	no
gi|641560823|gb|KK860020.1|	14598-15294	7.1	24	7	8	0.0859902	0.94725	no
gi|641560823|gb|KK860020.1|	3692-4901	7.1	24	0.97784	2	1	0.468	no
gi|641560823|gb|KK860020.1|	8817-12477	7.1	24	2	2	0.491985	0.70055	no
gi|641560826|gb|KK860019.1|	1383-2199	9.3	13	1	4	2	0.29475	no
gi|641560826|gb|KK860019.1|	14404-15016	9.3	13	2	2	-0.276266	0.86185	no
gi|641560826|gb|KK860019.1|	4078-4561	9.3	13	193	341	0.822339	0.7036	no
gi|641560836|gb|KK860014.1|	28-368	9	39	160	229	0.515264	0.67655	no
gi|641560836|gb|KK860014.1|	6937-7497	9	39	66	116	0.824076	0.61405	no
gi|641560836|gb|KK860014.1|	9428-9927	9	39	42	67	0.670282	0.5923	no
gi|641560848|gb|KK860009.1|	16714-16917	8	24	20	9	-1	0.4938	no
gi|641560850|gb|KK860008.1|	13691-14265	9.4	17	3	1	-1	0.4658	no
gi|641560850|gb|KK860008.1|	6242-6439	9.4	17	23	62	1	0.3401	no
gi|641560854|gb|KK860006.1|	14289-14603	9.6	17	26	6	-2	0.1667	no
gi|641560854|gb|KK860006.1|	4-413	9.6	17	17	9	-0.91671	0.5049	no
gi|641560854|gb|KK860006.1|	708-1292	9.6	17	35	13	-1	0.2889	no
gi|641560861|gb|KK860003.1|	10081-10699	10	26	5	1	-2	0.15635	no
gi|641560861|gb|KK860003.1|	1480-2038	10	26	12	35	2	0.25325	no
gi|641560886|gb|KK859993.1|	4289-4887	8.4	39	2	3	0.597718	0.6863	no
gi|641560920|gb|KK859977.1|	0-935	9.3	34	7	1	-3	0.09185	no
gi|641560920|gb|KK859977.1|	4712-5898	9.3	34	0.643205	2	2	0.33265	no
gi|641560920|gb|KK859977.1|	6817-7341	9.3	34	9	7	-0.33837	0.8021	no
gi|641560920|gb|KK859977.1|	8310-8636	9.3	34	2	6	2	0.41725	no
gi|641560930|gb|KK859973.1|	4809-5059	5.2	25	6	9	0.552548	0.7576	no
gi|641560930|gb|KK859973.1|	6890-8295	5.2	25	0.409704	1	2	0.3164	no

gi|641560932|gb|KK859972.1|	10719-12534	11	23	4	2	-1	0.3908	no
gi|641560932|gb|KK859972.1|	2866-3375	11	23	6	3	-0.936815	0.5155	no
gi|641560932|gb|KK859972.1|	6112-7060	11	23	0.752516	0.429058	-0.810549	1	no
gi|641560932|gb|KK859972.1|	7170-7518	11	23	13	3	-2	0.18365	no
gi|641560932|gb|KK859972.1|	7955-8704	11	23	4	3	-0.315853	0.81855	no
gi|641560936|gb|KK859970.1|	11165-13734	11	34	1	0.864891	-0.271511	0.84005	no
gi|641560936|gb|KK859970.1|	15741-16923	11	34	8	8	0.0276427	0.98285	no
gi|641560936|gb|KK859970.1|	18560-19360	11	34	65	55	-0.234488	0.88305	no
gi|641560936|gb|KK859970.1|	6343-8518	11	34	9	5	-0.824747	0.51405	no
gi|641560936|gb|KK859970.1|	8661-9187	11	34	18	17	-0.114331	0.93185	no
gi|641560960|gb|KK859960.1|	10968-11226	10	34	25	0	-inf	0.00185	yes
gi|641560960|gb|KK859960.1|	11815-12026	10	34	28	8	-2	0.29405	no
gi|641560960|gb|KK859960.1|	1465-1682	10	34	24	0	-inf	0.0059	no
gi|641560960|gb|KK859960.1|	3714-3963	10	34	42	10	-2	0.20105	no
gi|641560960|gb|KK859960.1|	435-695	10	34	32	3	-4	0.14105	no
gi|641560960|gb|KK859960.1|	6590-7352	10	34	15	2	-3	0.05895	no
gi|641560960|gb|KK859960.1|	7934-8329	10	34	5	1	-2	0.26505	no
gi|641560984|gb|KK859949.1|	13872-14636	10	31	0.622483	34	6	0.08155	no
gi|641560984|gb|KK859949.1|	2019-2461	10	31	50	16	-2	0.21405	no
gi|641560984|gb|KK859949.1|	26240-26596	10	31	6	3	-1	0.4158	no
gi|641560984|gb|KK859949.1|	4504-5314	10	31	0.921453	0.699654	-0.397268	0.82205	no
gi|641560996|gb|KK859944.1|	6414-6712	10	36	24	19	-0.359121	0.786	no
gi|641561000|gb|KK859942.1|	14032-14453	9.1	20	5	7	0.487504	0.73535	no
gi|641561000|gb|KK859942.1|	16268-16754	9.1	20	9	8	-0.0540645	0.9691	no
gi|641561000|gb|KK859942.1|	5820-6621	9.1	20	0.935144	0.35499	-1	0.4534	no
gi|641561000|gb|KK859942.1|	8187-8578	9.1	20	10	2	-3	0.1613	no
gi|641561002|gb|KK859941.1|	11108-12542	8.1	30	7	0.609645	-3	0.04615	no
gi|641561002|gb|KK859941.1|	13953-14845	8.1	30	6	2	-2	0.2387	no
gi|641561002|gb|KK859941.1|	278-487	8.1	30	14	5	-1	0.4383	no
gi|641561011|gb|KK859937.1|	13049-14235	6.7	15	0.78614	1	0.847378	0.58475	no

gi|641561018|gb|KK859934.1|	14275-14539	10	21	16	11	-0.520709	0.71535	no
gi|641561018|gb|KK859934.1|	2884-3095	10	21	14	8	-0.879165	0.63655	no
gi|641561018|gb|KK859934.1|	6914-7129	10	21	13	9	-0.462178	0.78255	no
gi|641561022|gb|KK859932.1|	25-611	8.5	29	2	3	0.597276	0.6792	no
gi|641561022|gb|KK859932.1|	5996-6466	8.5	29	2	3	0.591203	0.6902	no
gi|641561030|gb|KK859928.1|	1319-1658	8.4	13	2	5	1	0.4433	no
gi|641561040|gb|KK859924.1|	20771-21749	8.7	34	5	2	-1	0.30535	no
gi|641561045|gb|KK859922.1|	13091-13355	11	17	10	0	-inf	0.0102	no
gi|641561045|gb|KK859922.1|	3457-3668	11	17	80	27.74	-2	0.2863	no
gi|641561045|gb|KK859922.1|	5878-6602	11	17	7	4	-0.804807	0.55925	no
gi|641561045|gb|KK859922.1|	6844-7080	11	17	23	31	0.469148	0.73605	no
gi|641561053|gb|KK859918.1|	3282-3899	10	49	1	2	0.598369	0.70555	no
gi|641561105|gb|KK859894.1|	12792-14146	7.3	29	15	18	0.297132	0.8102	no
gi|641561105|gb|KK859894.1|	14269-15608	7.3	29	58	66	0.187533	0.93035	no
gi|641561105|gb|KK859894.1|	7109-7944	7.3	29	2	0.672486	-1	0.4015	no
gi|641561105|gb|KK859894.1|	9431-10512	7.3	29	2	2	0.283736	0.83405	no
gi|641561121|gb|KK859887.1|	10091-10634	9.2	25	7	2	-2	0.3089	no
gi|641561121|gb|KK859887.1|	12018-12251	9.2	25	0	22	inf	0.00375	no
gi|641561121|gb|KK859887.1|	6814-7263	9.2	25	7	2	-2	0.2534	no
gi|641561130|gb|KK859883.1|	13425-13666	8.3	28	164.82	130	-0.34673	0.78195	no
gi|641561136|gb|KK859880.1|	1-551	8.6	25	12	7	-0.773454	0.55205	no
gi|641561138|gb|KK859879.1|	10561-10963	12	42	15	5	-2	0.29235	no
gi|641561138|gb|KK859879.1|	12436-15522	12	42	8	6	-0.358198	0.77305	no
gi|641561144|gb|KK859876.1|	21-621	12	21	0.699283	2	2	0.3613	no
gi|641561150|gb|KK859873.1|	5200-5631	9.3	42	2	1	-0.634388	0.71175	no
gi|641561154|gb|KK859871.1|	3645-4426	7.9	33	0.483537	2	2	0.2441	no
gi|641561162|gb|KK859867.1|	4098-4315	8	29	0	176	inf	5.00E-05	yes
gi|641561162|gb|KK859867.1|	9307-10287	8	29	0	34	inf	5.00E-05	yes
gi|641561183|gb|KK859857.1|	19325-19846	6.6	25	117	34	-2	0.31155	no
gi|641561192|gb|KK859853.1|	804-1152	16	74	5	0	-inf	0.01	no

gi|641561199|gb|KK859850.1|	18363-19190	10	35	0.896656	1	0.602989	0.6936	no
gi|641561199|gb|KK859850.1|	25-728	10	35	2	2	0.311219	0.84265	no
gi|641561199|gb|KK859850.1|	3315-3777	10	35	12	11	-0.113931	0.92915	no
gi|641561199|gb|KK859850.1|	816-3243	10	35	1	2	0.365761	0.77595	no
gi|641561199|gb|KK859850.1|	9994-12154	10	35	24	19	-0.369488	0.87505	no
gi|641561201|gb|KK859849.1|	2342-3120	7.4	24	132	10	-4	0.0697	no
gi|641561201|gb|KK859849.1|	3220-3560	7.4	24	32	0.70339	-6	0.1831	no
gi|641561201|gb|KK859849.1|	6394-6858	7.4	24	45	2	-4	0.03335	no
gi|641561203|gb|KK859848.1|	6116-6587	11	52	7	5	-0.408725	0.77665	no
gi|641561208|gb|KK859846.1|	12296-12927	8.5	32	0.812138	1	0.598814	0.70505	no
gi|641561208|gb|KK859846.1|	2515-3152	8.5	32	0.80114	2	1	0.39995	no
gi|641561208|gb|KK859846.1|	4773-6520	8.5	32	2	2	0.257053	0.8467	no
gi|641561208|gb|KK859846.1|	782-1098	8.5	32	7	2	-2	0.265	no
gi|641561208|gb|KK859846.1|	7981-9161	8.5	32	10	17	0.831754	0.5136	no
gi|641561236|gb|KK859834.1|	12100-12963	7.4	29	1	0.161115	-3	0.2382	no
gi|641561236|gb|KK859834.1|	8832-9777	7.4	29	0.944409	0	-inf	0.01175	no
gi|641561240|gb|KK859832.1|	11100-11823	6.3	29	0	24	inf	5.00E-05	yes
gi|641561248|gb|KK859828.1|	713-1004	7.7	28	106	130	0.303893	0.80605	no
gi|641561261|gb|KK859822.1|	5111-5648	11	40	3	0	-inf	0.0075	no
gi|641561261|gb|KK859822.1|	5835-6332	11	40	2	0	-inf	0.01175	no
gi|641561263|gb|KK859821.1|	5936-6854	11	37	0.685784	2	1	0.44	no
gi|641561267|gb|KK859819.1|	125-630	9.1	46	0.911933	5	3	0.19915	no
gi|641561267|gb|KK859819.1|	1320-1902	9.1	46	7	1	-2	0.15585	no
gi|641561267|gb|KK859819.1|	2498-2973	9.1	46	4	0.759688	-2	0.24735	no
gi|641561267|gb|KK859819.1|	8256-8626	9.1	46	6	1	-2	0.2169	no
gi|641561283|gb|KK859812.1|	5400-5653	8.4	36	0	37	inf	0.00065	yes
gi|641561283|gb|KK859812.1|	5761-6309	8.4	36	0	3	inf	0.00455	no
gi|641561297|gb|KK859806.1|	2401-2764	7.7	20	5	3	-0.798717	0.6299	no
gi|641561305|gb|KK859802.1|	27167-27871	11	40	69	22	-2	0.18515	no
gi|641561305|gb|KK859802.1|	8276-8747	11	40	0.767006	4	2	0.21375	no

gi|641561307|gb|KK859801.1|	13586-13777	13	38	139	41	-2	0.21375	no
gi|641561307|gb|KK859801.1|	3854-4262	13	38	3	2	-0.414319	0.8125	no
gi|641561307|gb|KK859801.1|	7145-7608	13	38	4	1	-2	0.2877	no
gi|641561321|gb|KK859795.1|	6375-8439	9.3	48	0.075778	9	7	0.23225	no
gi|641561321|gb|KK859795.1|	9120-9673	9.3	48	1	2	0.595915	0.70555	no
gi|641561323|gb|KK859794.1|	11872-12122	8.5	15	25	1	-4	0.19355	no
gi|641561323|gb|KK859794.1|	2738-3318	8.5	15	8	0	-inf	0.0003	yes
gi|641561323|gb|KK859794.1|	5485-5807	8.5	15	8	0	-inf	0.0059	no
gi|641561323|gb|KK859794.1|	6854-7226	8.5	15	25	3	-3	0.07985	no
gi|641561326|gb|KK859793.1|	22-822	8.7	32	0	2	inf	0.0068	no
gi|641561335|gb|KK859789.1|	11661-13094	10	37	3	3	-0.0580215	0.96455	no
gi|641561335|gb|KK859789.1|	14648-15201	10	37	3	5	0.689024	0.63045	no
gi|641561335|gb|KK859789.1|	8946-9958	10	37	2	2	-0.0578631	0.963	no
gi|641561346|gb|KK859784.1|	12072-12872	5.5	25	1	0.53336	-1	0.50885	no
gi|641561352|gb|KK859781.1|	24392-25038	7.4	12	2	0	-inf	0.0075	no
gi|641561359|gb|KK859778.1|	1-259	9.5	33	9	0	-inf	0.01175	no
gi|641561359|gb|KK859778.1|	15220-16489	9.5	33	8	11	0.47677	0.7072	no
gi|641561359|gb|KK859778.1|	23606-24189	9.5	33	38	54	0.497132	0.6883	no
gi|641561359|gb|KK859778.1|	7250-7812	9.5	33	0	36	inf	5.00E-05	yes
gi|641561367|gb|KK859775.1|	19-482	9.8	19	6	4	-0.610945	0.66815	no
gi|641561369|gb|KK859774.1|	3285-4119	8.1	32	0.110854	2	4	0.1175	no
gi|641561369|gb|KK859774.1|	5521-5764	8.1	32	13	27	1	0.4736	no
gi|641561369|gb|KK859774.1|	7652-8379	8.1	32	1	2	0.463744	0.7552	no
gi|641561369|gb|KK859774.1|	9431-9798	8.1	32	3	4	0.358011	0.83635	no
gi|641561398|gb|KK859762.1|	6838-7493	5.3	16	1	3	2	0.2961	no
gi|641561401|gb|KK859761.1|	10738-11180	8.9	36	44	43	-0.0101841	0.99405	no
gi|641561401|gb|KK859761.1|	13255-14249	8.9	36	27	24	-0.160595	0.89395	no
gi|641561419|gb|KK859753.1|	10561-11168	9	19	2	1	-0.249963	0.88485	no
gi|641561419|gb|KK859753.1|	16-932	9	19	2	3	0.250504	0.86015	no
gi|641561419|gb|KK859753.1|	3087-4650	9	19	1	0.946828	-0.128952	0.9259	no

gi|641561419|gb|KK859753.1|	6621-7122	9	19	2	1	-0.821803	0.6492	no
gi|641561423|gb|KK859751.1|	4570-4994	8.8	17	87	33	-1	0.27455	no
gi|641561423|gb|KK859751.1|	5746-6205	8.8	17	43	12	-2	0.1816	no
gi|641561440|gb|KK859743.1|	3686-4075	7.6	22	5	5	-0.0539877	0.95635	no
gi|641561454|gb|KK859736.1|	12104-12607	8.9	34	167	149	-0.162898	0.94585	no
gi|641561454|gb|KK859736.1|	15195-15444	8.9	34	76	33	-1	0.3637	no
gi|641561454|gb|KK859736.1|	1622-2339	8.9	34	0.949917	3	1	0.36635	no
gi|641561469|gb|KK859729.1|	3314-3758	8.1	35	4	0	-inf	0.0075	no
gi|641561487|gb|KK859721.1|	10410-12172	6.9	24	2	4	0.997604	0.45745	no
gi|641561495|gb|KK859717.1|	6522-6980	3.3	11	19	6	-2	0.25595	no
gi|641561503|gb|KK859713.1|	12029-12334	7.2	20	14	3	-2	0.18995	no
gi|641561515|gb|KK859707.1|	10194-10460	3.8	17	29	46	0.679921	0.615	no
gi|641561526|gb|KK859702.1|	1860-2805	8.8	29	0.283323	1	2	0.2812	no
gi|641561526|gb|KK859702.1|	19037-19234	8.8	29	50	20	-1	0.38965	no
gi|641561526|gb|KK859702.1|	3294-5558	8.8	29	0.855646	1	0.720509	0.60105	no
gi|641561529|gb|KK859701.1|	0-343	6.6	30	8	3	-2	0.31735	no
gi|641561529|gb|KK859701.1|	16052-17291	6.6	30	4	3	-0.540169	0.68005	no
gi|641561529|gb|KK859701.1|	3674-3932	6.6	30	10	3	-2	0.31705	no
gi|641561529|gb|KK859701.1|	446-1368	6.6	30	16	5	-2	0.22455	no
gi|641561531|gb|KK859700.1|	13109-13538	9.7	31	3	8	1	0.4231	no
gi|641561537|gb|KK859697.1|	13932-15228	9.7	16	11	8	-0.51405	0.6785	no
gi|641561537|gb|KK859697.1|	16911-17166	9.7	16	13	7	-0.931118	0.57015	no
gi|641561537|gb|KK859697.1|	5871-6213	9.7	16	10	4	-1	0.41145	no
gi|641561537|gb|KK859697.1|	9140-10268	9.7	16	2	1	-0.394	0.78685	no
gi|641561548|gb|KK859692.1|	12014-12363	8.3	31	5	15	2	0.30175	no
gi|641561548|gb|KK859692.1|	3121-3343	8.3	31	4	30	3	0.1886	no
gi|641561548|gb|KK859692.1|	3454-4439	8.3	31	4	5	0.372194	0.7809	no
gi|641561552|gb|KK859690.1|	2703-2884	5.8	21	10	33	2	0.3486	no
gi|641561558|gb|KK859687.1|	2897-3421	8	22	0	4	inf	0.00375	no
gi|641561558|gb|KK859687.1|	3826-4810	8	22	0	2	inf	0.0015	yes

gi|641561566|gb|KK859683.1|	6465-8188	9.4	29	1	2	1	0.4379	no
gi|641561570|gb|KK859681.1|	0-383	14	20	2445.07	0	-inf	0.0003	yes
gi|641561570|gb|KK859681.1|	11254-11843	14	20	7	0	-inf	0.00115	yes
gi|641561570|gb|KK859681.1|	13185-15249	14	20	5	0	-inf	5.00E-05	yes
gi|641561570|gb|KK859681.1|	19959-20676	14	20	3	0	-inf	0.00345	yes
gi|641561570|gb|KK859681.1|	22399-23930	14	20	2	0.161574	-4	0.13985	no
gi|641561570|gb|KK859681.1|	2449-3808	14	20	989	0.092685	-13	0.18295	no
gi|641561578|gb|KK859677.1|	316-1027	9.2	35	1	2	1	0.49595	no
gi|641561578|gb|KK859677.1|	4592-5370	9.2	35	0.972052	7	3	0.1029	no
gi|641561581|gb|KK859676.1|	1-9962	10	38	9	6	-0.682259	0.75455	no
gi|641561581|gb|KK859676.1|	12059-12700	10	38	46	30	-0.60848	0.62775	no
gi|641561581|gb|KK859676.1|	14780-16700	10	38	24	9	-1	0.4227	no
gi|641561581|gb|KK859676.1|	18618-19341	10	38	12	3	-2	0.17025	no
gi|641561585|gb|KK859674.1|	10205-10880	11	35	1	2	0.430124	0.7802	no
gi|641561585|gb|KK859674.1|	7450-8583	11	35	1	2	1	0.41185	no
gi|641561587|gb|KK859673.1|	12746-13300	12	36	34	6	-3	0.06995	no
gi|641561587|gb|KK859673.1|	14563-16181	12	36	26	9	-2	0.3681	no
gi|641561587|gb|KK859673.1|	16568-17093	12	36	27	11	-1	0.33325	no
gi|641561587|gb|KK859673.1|	19779-22398	12	36	12	6	-0.943357	0.45045	no
gi|641561589|gb|KK859672.1|	6235-6590	8.6	41	26	45	0.800915	0.54165	no
gi|641561589|gb|KK859672.1|	6932-7436	8.6	41	4	9	1	0.381	no
gi|641561594|gb|KK859670.1|	16671-17355	8.7	28	1	2	0.43035	0.7802	no
gi|641561602|gb|KK859666.1|	19253-21752	13	37	0	18	inf	0.00055	yes
gi|641561602|gb|KK859666.1|	22813-22979	13	37	0	80	inf	0.00455	no
gi|641561602|gb|KK859666.1|	23043-24187	13	37	0	24	inf	5.00E-05	yes
gi|641561602|gb|KK859666.1|	28500-28963	13	37	2	1	-0.824349	0.64915	no
gi|641561605|gb|KK859665.1|	3284-3735	6.4	33	2	9	2	0.161	no
gi|641561605|gb|KK859665.1|	6380-6976	6.4	33	10	30	2	0.2128	no
gi|641561609|gb|KK859663.1|	12358-12790	12	36	2	2	-0.411903	0.82205	no
gi|641561609|gb|KK859663.1|	24055-24313	12	36	19	23	0.250474	0.8579	no

gi|641561609|gb|KK859663.1|	3269-3628	12	36	6	2	-2	0.3256	no
gi|641561609|gb|KK859663.1|	9273-10635	12	36	0.364118	30	6	0.06285	no
gi|641561615|gb|KK859660.1|	17432-18609	6.3	23	0.865469	1	0.480785	0.75585	no
gi|641561621|gb|KK859657.1|	3709-4264	7.5	25	6	3	-1	0.39145	no
gi|641561621|gb|KK859657.1|	6987-7797	7.5	25	0.806271	0.874568	0.117305	1	no
gi|641561639|gb|KK859649.1|	8350-8977	5.9	20	30	23	-0.385368	0.7605	no
gi|641561656|gb|KK859641.1|	0-856	6.5	28	0.642975	3	2	0.2254	no
gi|641561660|gb|KK859639.1|	15689-16057	8.2	38	8	1	-3	0.1714	no
gi|641561664|gb|KK859637.1|	16630-16827	8.7	14	27	7	-2	0.27465	no
gi|641561664|gb|KK859637.1|	7978-8655	8.7	14	7	3	-1	0.3365	no
gi|641561664|gb|KK859637.1|	9092-9455	8.7	14	3	2	-0.642598	0.71075	no
gi|641561666|gb|KK859636.1|	11151-11366	5.4	25	33	14	-1	0.44195	no
gi|641561666|gb|KK859636.1|	11661-13410	5.4	25	5	2	-2	0.20705	no
gi|641561666|gb|KK859636.1|	18498-18873	5.4	25	63	46	-0.454055	0.7227	no
gi|641561666|gb|KK859636.1|	18995-20650	5.4	25	2	2	0.240554	0.8539	no
gi|641561666|gb|KK859636.1|	5203-5407	5.4	25	10	31	2	0.3222	no
gi|641561668|gb|KK859635.1|	20073-20837	8.5	24	2	0.188958	-4	0.20475	no
gi|641561674|gb|KK859632.1|	12556-13918	38	430	10	2	-3	0.07285	no
gi|641561674|gb|KK859632.1|	1423-1558	38	430	180.16	127	-0.50237	0.7686	no
gi|641561674|gb|KK859632.1|	14681-19318	38	430	9	5	-0.976513	0.5476	no
gi|641561674|gb|KK859632.1|	181-1287	38	430	4	0	-inf	0.0002	yes
gi|641561674|gb|KK859632.1|	1874-3116	38	430	10	0.412021	-5	0.06295	no
gi|641561674|gb|KK859632.1|	20902-22368	38	430	1	0	-inf	0.0024	yes
gi|641561674|gb|KK859632.1|	23042-24205	38	430	4	0.111379	-5	0.18465	no
gi|641561674|gb|KK859632.1|	9954-10494	38	430	5	0	-inf	0.0024	yes
gi|641561681|gb|KK859629.1|	14814-15628	8.7	18	0.915496	0.521371	-0.812244	0.6492	no
gi|641561681|gb|KK859629.1|	2623-3021	8.7	18	4	2	-0.874889	0.60075	no
gi|641561687|gb|KK859626.1|	11860-12381	6	23	15	23	0.656642	0.607	no
gi|641561687|gb|KK859626.1|	5265-6756	6	23	5	6	0.249319	0.8447	no
gi|641561687|gb|KK859626.1|	8160-9950	6	23	3	5	1	0.39375	no

gi|641561708|gb|KK859617.1|	4801-5008	9.4	41	9	14	0.533879	0.768	no
gi|641561718|gb|KK859612.1|	5331-5899	4.8	20	3	1	-0.988402	0.57205	no
gi|641561722|gb|KK859610.1|	12935-13636	6.6	25	3	0.636857	-2	0.21545	no
gi|641561727|gb|KK859608.1|	5183-6495	6.2	18	0.063404	24	9	0.10435	no
gi|641561727|gb|KK859608.1|	6908-8005	6.2	18	3	5	0.902764	0.4976	no
gi|641561731|gb|KK859606.1|	9262-10112	5.1	18	0.432578	0.985762	1	0.51305	no
gi|641561738|gb|KK859603.1|	1332-3672	11	44	38	20	-0.953016	0.66435	no
gi|641561738|gb|KK859603.1|	14476-15076	11	44	3	4	0.317681	0.8318	no
gi|641561738|gb|KK859603.1|	17998-18185	11	44	20	16	-0.284009	0.8664	no
gi|641561738|gb|KK859603.1|	3834-4401	11	44	5	3	-0.573406	0.6915	no
gi|641561738|gb|KK859603.1|	7701-7854	11	44	84	24	-2	0.3266	no
gi|641561741|gb|KK859602.1|	13017-13960	9.7	38	0.757548	0.719853	-0.073636	1	no
gi|641561741|gb|KK859602.1|	16800-17412	9.7	38	1	1	-0.0798688	0.92675	no
gi|641561741|gb|KK859602.1|	17575-18577	9.7	38	0.526608	0.800966	0.605012	1	no
gi|641561741|gb|KK859602.1|	2414-2744	9.7	38	6	2	-1	0.4191	no
gi|641561748|gb|KK859599.1|	11056-11793	11	36	0	118.98	inf	0.00035	yes
gi|641561750|gb|KK859598.1|	14746-15190	6.5	25	9	10	0.112726	0.9351	no
gi|641561750|gb|KK859598.1|	16496-16780	6.5	25	5	6	0.340832	0.8379	no
gi|641561750|gb|KK859598.1|	2639-3077	6.5	25	25	36	0.55429	0.67145	no
gi|641561750|gb|KK859598.1|	7443-8801	6.5	25	10	16	0.780237	0.5337	no
gi|641561750|gb|KK859598.1|	985-1180	6.5	25	26	54	1	0.4715	no
gi|641561752|gb|KK859597.1|	10163-10653	9.8	32	46	121	1	0.40995	no
gi|641561752|gb|KK859597.1|	12835-13226	9.8	32	12	43	2	0.1786	no
gi|641561752|gb|KK859597.1|	843-1342	9.8	32	4	17	2	0.17825	no
gi|641561752|gb|KK859597.1|	9676-9985	9.8	32	26	41	0.664677	0.6178	no
gi|641561754|gb|KK859596.1|	13152-13469	7.1	26	54	0	-inf	5.00E-05	yes
gi|641561758|gb|KK859594.1|	6750-7608	5.6	18	0.641034	1	0.825818	0.6232	no
gi|641561799|gb|KK859576.1|	4314-5669	9.8	35	2.32	1	-0.940249	0.50105	no
gi|641561799|gb|KK859576.1|	5789-6657	9.8	35	1	0.639697	-1	0.46585	no
gi|641561799|gb|KK859576.1|	973-2155	9.8	35	2	0.764699	-2	0.27805	no

gi|641561801|gb|KK859575.1|	129-1121	7.4	26	4	4	0.0903476	0.9443	no
gi|641561801|gb|KK859575.1|	9520-10295	7.4	26	2	5	1	0.384	no
gi|641561803|gb|KK859574.1|	10363-10912	12	39	3	0.906165	-2	0.3026	no
gi|641561803|gb|KK859574.1|	11082-14519	12	39	23	18	-0.385414	0.86615	no
gi|641561803|gb|KK859574.1|	18693-19875	12	39	0.645813	0.655456	0.0213837	1	no
gi|641561803|gb|KK859574.1|	21954-22181	12	39	14	6	-1	0.4939	no
gi|641561803|gb|KK859574.1|	24347-24895	12	39	6	4	-0.561852	0.6913	no
gi|641561803|gb|KK859574.1|	31249-31832	12	39	0.547346	3	2	0.21375	no
gi|641561811|gb|KK859570.1|	4077-4811	14	46	6	2	-2	0.2746	no
gi|641561811|gb|KK859570.1|	894-1720	14	46	21	25	0.249338	0.8396	no
gi|641561829|gb|KK859562.1|	12665-13360	8.5	31	172	86	-0.999929	0.6578	no
gi|641561829|gb|KK859562.1|	20776-21669	8.5	31	6	2	-2	0.2166	no
gi|641561829|gb|KK859562.1|	23917-24374	8.5	31	33	23	-0.495745	0.6985	no
gi|641561829|gb|KK859562.1|	544-1000	8.5	31	68	35	-0.944187	0.4624	no
gi|641561838|gb|KK859558.1|	7824-8343	11	32	0.872912	64	6	0.1159	no
gi|641561838|gb|KK859558.1|	9217-9813	11	32	1	12	4	0.07685	no
gi|641561845|gb|KK859555.1|	11415-11961	11	39	2	3	0.595597	0.6902	no
gi|641561845|gb|KK859555.1|	5583-6473	11	39	2	2	0.0632664	0.9586	no
gi|641561845|gb|KK859555.1|	8754-9568	11	39	2	2	0.0178311	0.9647	no
gi|641561863|gb|KK859547.1|	10991-11890	9.2	39	3	2	-0.890821	0.53155	no
gi|641561863|gb|KK859547.1|	2938-3437	9.2	39	4	6	0.680577	0.6314	no
gi|641561863|gb|KK859547.1|	4260-4889	9.2	39	0.652697	2	2	0.3613	no
gi|641561863|gb|KK859547.1|	4992-5489	9.2	39	1	2	0.592993	0.70555	no
gi|641561873|gb|KK859542.1|	4428-4670	5.8	23	0	32	inf	0.00145	yes
gi|641561882|gb|KK859538.1|	2782-2999	6.6	19	11	14	0.316387	0.86275	no
gi|641561884|gb|KK859537.1|	4521-5405	10	37	0	15	inf	5.00E-05	yes
gi|641561904|gb|KK859528.1|	12679-13284	9.6	39	0	10	inf	0.00015	yes
gi|641561919|gb|KK859522.1|	11395-12509	9	31	3	2	-0.879523	0.52505	no
gi|641561919|gb|KK859522.1|	4807-7325	9	31	0.640145	2	1	0.33355	no
gi|641561919|gb|KK859522.1|	7555-8234	9	31	0.731735	2	2	0.3084	no

gi|641561924|gb|KK859520.1|	13292-13699	7.6	35	0	85	inf	5.00E-05	yes
gi|641561924|gb|KK859520.1|	18972-19257	7.6	35	2	7	2	0.34545	no
gi|641561948|gb|KK859509.1|	1-410	9.5	31	10	6	-0.667967	0.6373	no
gi|641561948|gb|KK859509.1|	3148-3463	9.5	31	21	36	0.787685	0.54715	no
gi|641561958|gb|KK859507.1|	818-977	7.9	32	46	65	0.508021	0.75565	no
gi|641561961|gb|KK859505.1|	12334-13567	9.8	34	1	0.83116	-0.30025	0.84515	no
gi|641561961|gb|KK859505.1|	13868-14184	9.8	34	14	6	-1	0.40095	no
gi|641561961|gb|KK859505.1|	6419-6704	9.8	34	8	1	-3	0.2375	no
gi|641561961|gb|KK859505.1|	7619-8326	9.8	34	4	3	-0.154072	0.9102	no
gi|641561961|gb|KK859505.1|	8469-11165	9.8	34	2	2	0.326178	0.80245	no
gi|641561979|gb|KK859500.1|	4205-4573	6.4	29	4	8	0.959064	0.53905	no
gi|641561993|gb|KK859493.1|	15680-16335	9.9	22	2	1	-0.400483	0.8086	no
gi|641561999|gb|KK859490.1|	11108-11873	8.8	34	23	6	-2	0.15805	no
gi|641561999|gb|KK859490.1|	13148-16393	8.8	34	4	3	-0.553427	0.6607	no
gi|641561999|gb|KK859490.1|	2864-3293	8.8	34	4	5	0.10239	0.94445	no
gi|641561999|gb|KK859490.1|	4805-5544	8.8	34	1	2	0.601491	0.67805	no
gi|641562003|gb|KK859488.1|	10676-13132	11	41	15	11	-0.461577	0.71115	no
gi|641562003|gb|KK859488.1|	14917-15283	11	41	6	5	-0.326638	0.82445	no
gi|641562003|gb|KK859488.1|	17343-18096	11	41	0.888654	0.770624	-0.205595	0.89485	no
gi|641562003|gb|KK859488.1|	25591-26185	11	41	2	2	0.235003	0.88205	no
gi|641562003|gb|KK859488.1|	3292-3549	11	41	18	1	-4	0.2035	no
gi|641562014|gb|KK859483.1|	626-1012	9.9	39	0	38	inf	5.00E-05	yes
gi|641562022|gb|KK859479.1|	10436-11736	9	38	1	0.195234	-3	0.1718	no
gi|641562022|gb|KK859479.1|	9330-10030	9	38	2	0.212702	-3	0.22425	no
gi|641562025|gb|KK859478.1|	3512-3990	11	29	0	13	inf	5.00E-05	yes
gi|641562025|gb|KK859478.1|	7466-7674	11	29	222	21	-3	0.0438	no
gi|641562039|gb|KK859471.1|	1964-2131	5.1	18	34	42	0.29007	0.8636	no
gi|641562049|gb|KK859467.1|	0-523	8.3	25	8	2	-2	0.2192	no
gi|641562049|gb|KK859467.1|	1510-1725	8.3	25	21	0	-inf	0.0075	no
gi|641562049|gb|KK859467.1|	7233-7972	8.3	25	20	12	-0.682718	0.58885	no

gi|641562052|gb|KK859466.1|	76-713	8.4	33	3	0.485383	-3	0.19	no
gi|641562063|gb|KK859461.1|	1343-2398	13	45	5	0.751823	-3	0.1007	no
gi|641562063|gb|KK859461.1|	20281-20526	13	45	7	30	2	0.2374	no
gi|641562063|gb|KK859461.1|	4654-4909	13	45	52	8	-3	0.10755	no
gi|641562063|gb|KK859461.1|	9021-9634	13	45	5	0.256452	-4	0.1915	no
gi|641562063|gb|KK859461.1|	9779-12208	13	45	25	4	-3	0.16965	no
gi|641562065|gb|KK859460.1|	18019-18601	8.6	29	1	0.830098	-0.62527	0.71195	no
gi|641562078|gb|KK859454.1|	11-501	6.6	31	2	2	-0.255441	0.88485	no
gi|641562103|gb|KK859442.1|	17049-17799	11	23	0.638184	0.968448	0.601703	0.70505	no
gi|641562103|gb|KK859442.1|	18053-19001	11	23	0.470323	1	1	0.50945	no
gi|641562108|gb|KK859440.1|	13892-14460	8.8	16	0.569249	2	2	0.2812	no
gi|641562108|gb|KK859440.1|	2303-3686	8.8	16	3	3	0.270324	0.835	no
gi|641562108|gb|KK859440.1|	37-466	8.8	16	6	7	0.24678	0.8584	no
gi|641562108|gb|KK859440.1|	7108-7774	8.8	16	2	10	2	0.1416	no
gi|641562121|gb|KK859434.1|	517-867	8	32	5	0	-inf	0.01	no
gi|641562130|gb|KK859430.1|	14566-14916	8	16	28	4	-3	0.0882	no
gi|641562133|gb|KK859429.1|	16244-16777	5.5	15	0	48	inf	5.00E-05	yes
gi|641562133|gb|KK859429.1|	3690-4175	5.5	15	3	2	-0.523244	0.7412	no
gi|641562150|gb|KK859422.1|	16767-17001	7.2	25	16	4	-2	0.26405	no
gi|641562154|gb|KK859420.1|	9934-10397	6.4	26	1	2	1	0.51315	no
gi|641562156|gb|KK859419.1|	20774-21541	8.5	36	1	5	2	0.22615	no
gi|641562156|gb|KK859419.1|	4049-4513	8.5	36	0	4	inf	0.00455	no
gi|641562170|gb|KK859413.1|	10079-10977	7.4	26	3	4	0.216908	0.8722	no
gi|641562170|gb|KK859413.1|	15309-16699	7.4	26	13	12	-0.160089	0.8974	no
gi|641562170|gb|KK859413.1|	16936-17671	7.4	26	20	19	-0.114135	0.92665	no
gi|641562170|gb|KK859413.1|	18842-19419	7.4	26	16	12	-0.33596	0.79085	no
gi|641562197|gb|KK859400.1|	9858-10224	9	26	0	13	inf	0.00105	yes
gi|641562209|gb|KK859395.1|	14720-16329	7.8	25	29	7	-2	0.25075	no
gi|641562209|gb|KK859395.1|	16808-17093	7.8	25	28	9	-2	0.2889	no
gi|641562209|gb|KK859395.1|	5839-6341	7.8	25	3	1	-1	0.4657	no

gi|641562209|gb|KK859395.1|	9769-10493	7.8	25	16	4	-2	0.1549	no
gi|641562217|gb|KK859391.1|	7337-7578	7.8	24	11	10	-0.187804	0.90945	no
gi|641562219|gb|KK859390.1|	10764-11208	9	27	1	3	1	0.4427	no
gi|641562219|gb|KK859390.1|	17322-17531	9	27	23	295	4	0.03315	no
gi|641562227|gb|KK859387.1|	11443-12295	9.9	19	2	0.491389	-2	0.28145	no
gi|641562227|gb|KK859387.1|	7596-7776	9.9	19	30	10	-2	0.363	no
gi|641562229|gb|KK859386.1|	12263-12909	5.6	20	0.62815	1	1	0.51315	no
gi|641562240|gb|KK859381.1|	2397-2727	6.5	21	6	11	1	0.4816	no
gi|641562240|gb|KK859381.1|	4856-5502	6.5	21	7	9	0.387758	0.7692	no
gi|641562247|gb|KK859378.1|	14362-17201	9.4	38	2	1	-0.47479	0.7177	no
gi|641562247|gb|KK859378.1|	21503-21772	9.4	38	8	23	1	0.33255	no
gi|641562247|gb|KK859378.1|	22952-23236	9.4	38	1	11	3	0.27615	no
gi|641562269|gb|KK859368.1|	10658-11162	7.7	35	2	1	-0.821626	0.6492	no
gi|641562274|gb|KK859366.1|	13764-15090	6.3	22	3	0.572284	-2	0.1424	no
gi|641562274|gb|KK859366.1|	16928-17713	6.3	22	1	0.182277	-3	0.2382	no
gi|641562274|gb|KK859366.1|	19314-19767	6.3	22	13	9	-0.41009	0.7581	no
gi|641562274|gb|KK859366.1|	6581-7070	6.3	22	2	0	-inf	0.01175	no
gi|641562274|gb|KK859366.1|	8980-10156	6.3	22	4	1	-2	0.1867	no
gi|641562279|gb|KK859364.1|	10736-12056	11	22	0.944288	2	1	0.45235	no
gi|641562279|gb|KK859364.1|	18733-19147	11	22	0.961168	3	2	0.3323	no
gi|641562279|gb|KK859364.1|	7922-8582	11	22	2	0.230816	-3	0.24815	no
gi|641562287|gb|KK859360.1|	14051-15056	26	66	0	4	inf	0.0004	yes
gi|641562301|gb|KK859354.1|	10690-11263	9.2	39	2	29	4	0.03725	no
gi|641562301|gb|KK859354.1|	13006-13339	9.2	39	0	10	inf	0.0025	yes
gi|641562301|gb|KK859354.1|	1315-1914	9.2	39	0.350504	8	4	0.23615	no
gi|641562301|gb|KK859354.1|	17420-19649	9.2	39	1	0.424924	-1	0.35235	no
gi|641562311|gb|KK859350.1|	16020-16346	8.6	24	8	0.768873	-3	0.21195	no
gi|641562313|gb|KK859349.1|	4445-5231	7.6	28	0.479445	2	2	0.26155	no
gi|641562315|gb|KK859348.1|	0-704	6.3	20	2	4	0.963318	0.5128	no
gi|641562322|gb|KK859345.1|	10349-10820	1.8	21	2	1	-0.993687	0.62775	no

gi|641562327|gb|KK859343.1|	10844-11382	7.7	27	8	6	-0.368255	0.77995	no
gi|641562327|gb|KK859343.1|	11458-11859	7.7	27	1	6	2	0.26275	no
gi|641562327|gb|KK859343.1|	13523-14093	7.7	27	2	0	-inf	0.01	no
gi|641562327|gb|KK859343.1|	18549-19537	7.7	27	3	2	-0.593056	0.65665	no
gi|641562329|gb|KK859342.1|	17218-17768	6.5	21	0.199313	3	4	0.1276	no
gi|641562333|gb|KK859340.1|	10553-11092	7.9	27	2	0.932019	-0.819771	0.6492	no
gi|641562333|gb|KK859340.1|	12476-13022	7.9	27	2	0	-inf	0.01	no
gi|641562333|gb|KK859340.1|	13122-14213	7.9	27	0.869519	1	0.73127	0.63585	no
gi|641562333|gb|KK859340.1|	9059-9622	7.9	27	5	4	-0.0817214	0.95345	no
gi|641562337|gb|KK859338.1|	12635-14584	5.7	22	1	0	-inf	0.00055	yes
gi|641562341|gb|KK859336.1|	13132-13440	8.9	26	5	10	0.984326	0.552	no
gi|641562341|gb|KK859336.1|	13545-14433	8.9	26	0.613272	0.776671	0.340776	1	no
gi|641562341|gb|KK859336.1|	20914-21522	8.9	26	4	5	0.375675	0.79055	no
gi|641562341|gb|KK859336.1|	21588-22124	8.9	26	2	0.940062	-1	0.50365	no
gi|641562344|gb|KK859335.1|	10869-11269	7.9	35	6	36	3	0.08335	no
gi|641562344|gb|KK859335.1|	17684-17875	7.9	35	8	96	4	0.14575	no
gi|641562351|gb|KK859332.1|	11208-11393	9.1	19	71	4	-4	0.1925	no
gi|641562351|gb|KK859332.1|	19354-19514	9.1	19	69	0	-inf	0.0102	no
gi|641562351|gb|KK859332.1|	8117-8297	9.1	19	37	0	-inf	0.0102	no
gi|641562354|gb|KK859331.1|	2602-3651	8.8	29	2	4	1	0.3681	no
gi|641562354|gb|KK859331.1|	497-642	8.8	29	111	87	-0.347933	0.83385	no
gi|641562354|gb|KK859331.1|	6303-7288	8.8	29	30	27	-0.175832	0.8848	no
gi|641562354|gb|KK859331.1|	8045-9602	8.8	29	26	44	0.773686	0.6317	no
gi|641562376|gb|KK859321.1|	116-272	4.4	16	0	125	inf	0.00375	no
gi|641562378|gb|KK859320.1|	11861-12379	10	37	1	3	1	0.39995	no
gi|641562378|gb|KK859320.1|	13315-14017	10	37	0.419143	2	2	0.2812	no
gi|641562378|gb|KK859320.1|	2719-4041	10	37	0.942612	1	0.507554	0.72935	no
gi|641562378|gb|KK859320.1|	5486-6543	10	37	0.492996	0.8751	0.827871	1	no
gi|641562387|gb|KK859316.1|	16047-18287	8.8	36	11	7	-0.551021	0.6666	no
gi|641562419|gb|KK859303.1|	18028-18176	8.5	36	53	119	1	0.48565	no

gi|641562419|gb|KK859303.1|	21688-22173	8.5	36	2	4	0.744236	0.6263	no
gi|641562419|gb|KK859303.1|	24035-25190	8.5	36	1	1	0.258255	0.8632	no
gi|641562419|gb|KK859303.1|	25784-26250	8.5	36	4	5	0.291352	0.8393	no
gi|641562434|gb|KK859297.1|	9776-10087	8.5	37	0.574497	11	4	0.1137	no
gi|641562454|gb|KK859288.1|	15405-15918	8.2	25	3	29	3	0.04175	no
gi|641562456|gb|KK859287.1|	23696-24307	8	26	5	3	-0.456278	0.7504	no
gi|641562456|gb|KK859287.1|	31993-32319	8	26	4	4	-0.104857	0.92555	no
gi|641562459|gb|KK859286.1|	10361-10704	4.8	22	350	238	-0.559509	0.8046	no
gi|641562471|gb|KK859281.1|	14450-15580	9.3	20	2	1	-1	0.4635	no
gi|641562471|gb|KK859281.1|	23818-24915	9.3	20	9	10	0.144809	0.9106	no
gi|641562479|gb|KK859277.1|	18599-19557	7.4	29	3	0.846926	-2	0.22025	no
gi|641562482|gb|KK859276.1|	21403-22204	10	42	0	5	inf	0.00065	yes
gi|641562487|gb|KK859274.1|	18397-18909	8.5	27	1	2	0.856903	0.62755	no
gi|641562495|gb|KK859270.1|	15341-15838	7	25	2	1	-0.6294	0.71175	no
gi|641562501|gb|KK859267.1|	8980-9522	1.2	5.10E+01	0	3	inf	0.0068	no
gi|641562503|gb|KK859266.1|	14328-14844	11	46	16	16	0.0388738	0.9756	no
gi|641562503|gb|KK859266.1|	21056-21550	11	46	0	5	inf	0.00255	yes
gi|641562508|gb|KK859264.1|	15477-16319	9.3	24	3	3	-0.151682	0.9166	no
gi|641562514|gb|KK859261.1|	10973-12273	9.8	42	1	0.780937	-0.393015	0.7971	no
gi|641562514|gb|KK859261.1|	12393-12812	9.8	42	2	3	0.809231	0.6234	no
gi|641562514|gb|KK859261.1|	13023-14169	9.8	42	2	0.680169	-2	0.2762	no
gi|641562514|gb|KK859261.1|	14222-16606	9.8	42	2	1	-0.337898	0.79935	no
gi|641562516|gb|KK859260.1|	3734-3911	5.7	17	36	21	-0.803978	0.633	no
gi|641562528|gb|KK859255.1|	6751-7423	7.8	23	2	0.450134	-2	0.31075	no
gi|641562541|gb|KK859250.1|	15069-15962	9.7	45	0	4	inf	0.00075	yes
gi|641562558|gb|KK859242.1|	4371-4590	6.5	27	67	31	-1	0.431	no
gi|641562560|gb|KK859241.1|	17009-17264	9.9	17	20	4	-2	0.2165	no
gi|641562560|gb|KK859241.1|	17594-18170	9.9	17	2	0.561981	-2	0.2679	no
gi|641562560|gb|KK859241.1|	20010-20624	9.9	17	2	0.255847	-3	0.271	no
gi|641562560|gb|KK859241.1|	66-1444	9.9	17	3	1	-1	0.3705	no

gi|641562560|gb|KK859241.1|	8994-9202	9.9	17	55	3	-4	0.1889	no
gi|641562568|gb|KK859240.1|	11737-12040	8.3	15	31	7	-2	0.1744	no
gi|641562572|gb|KK859238.1|	7766-8383	6.5	31	3	0	-inf	0.00435	no
gi|641562574|gb|KK859237.1|	3397-3947	8.7	39	2	0	-inf	0.01175	no
gi|641562606|gb|KK859223.1|	18563-19147	13	38	2	0.550596	-2	0.3216	no
gi|641562610|gb|KK859221.1|	18441-18800	8	31	6	0	-inf	0.0059	no
gi|641562614|gb|KK859219.1|	12073-12748	13	46	0.442695	2	2	0.256	no
gi|641562622|gb|KK859216.1|	16746-17389	44	36	2	0	-inf	0.0102	no
gi|641562622|gb|KK859216.1|	17785-18647	44	36	1	0	-inf	0.0075	no
gi|641562622|gb|KK859216.1|	1804-2694	44	36	1	0.619559	-0.718093	0.6701	no
gi|641562622|gb|KK859216.1|	23634-24154	44	36	3	0	-inf	0.0056	no
gi|641562622|gb|KK859216.1|	24213-25845	44	36	2	0	-inf	0.0006	yes
gi|641562622|gb|KK859216.1|	26295-27837	44	36	17	0	-inf	5.00E-05	yes
gi|641562624|gb|KK859215.1|	17057-17500	10	30	3	0	-inf	0.0102	no
gi|641562627|gb|KK859212.1|	4231-4709	4.7	18	4	3	-0.145201	0.9233	no
gi|641562630|gb|KK859211.1|	793-1208	6.1	22	22	3	-3	0.09425	no
gi|641562633|gb|KK859210.1|	13478-13858	4.9	16	2	3	0.582262	0.76735	no
gi|641562637|gb|KK859208.1|	101-1199	7.4	24	8	4	-0.78467	0.5504	no
gi|641562645|gb|KK859205.1|	15552-16387	8.3	17	4	4	-0.174502	0.8944	no
gi|641562645|gb|KK859205.1|	22392-22896	8.3	17	2	2	-0.0846607	0.9267	no
gi|641562645|gb|KK859205.1|	26399-26874	8.3	17	2	0.379844	-3	0.271	no
gi|641562645|gb|KK859205.1|	3220-6859	8.3	17	2	3	0.524032	0.67615	no
gi|641562645|gb|KK859205.1|	33583-34087	8.3	17	3	2	-0.299674	0.84385	no
gi|641562649|gb|KK859203.1|	8171-9063	4	16	0.609751	2	1	0.4118	no
gi|641562671|gb|KK859193.1|	14591-14928	8.1	33	3	14	2	0.21165	no
gi|641562671|gb|KK859193.1|	15436-16927	8.1	33	5	11	1	0.39755	no
gi|641562671|gb|KK859193.1|	21259-21767	8.1	33	0.677464	5	3	0.1666	no
gi|641562692|gb|KK859184.1|	11318-11500	8.4	26	16	41	1	0.4186	no
gi|641562692|gb|KK859184.1|	2455-2775	8.4	26	11	51	2	0.12075	no
gi|641562692|gb|KK859184.1|	6-276	8.4	26	4	17	2	0.2396	no

gi|641562695|gb|KK859183.1|	11128-12070	8.5	29	4	1	-2	0.2619	no
gi|641562695|gb|KK859183.1|	5-940	8.5	29	56	14	-2	0.26205	no
gi|641562695|gb|KK859183.1|	7507-8273	8.5	29	1193.09	344	-2	0.35015	no
gi|641562695|gb|KK859183.1|	8595-9243	8.5	29	33	4	-3	0.038	no
gi|641562705|gb|KK859178.1|	21974-23880	7.6	28	8	17	1	0.3417	no
gi|641562705|gb|KK859178.1|	2823-3033	7.6	28	524	880	0.74799	0.6527	no
gi|641562705|gb|KK859178.1|	5212-5519	7.6	28	24	68	1	0.26095	no
gi|641562705|gb|KK859178.1|	6973-8067	7.6	28	38	73	0.948707	0.5594	no
gi|641562705|gb|KK859178.1|	9279-10452	7.6	28	25	47	0.901126	0.58575	no
gi|641562724|gb|KK859169.1|	12137-12413	8.3	37	9	12	0.435438	0.7696	no
gi|641562724|gb|KK859169.1|	7403-7676	8.3	37	24	16	-0.539454	0.70225	no
gi|641562726|gb|KK859168.1|	14008-19110	9.3	20	7	4	-0.812748	0.50865	no
gi|641562739|gb|KK859162.1|	9127-9653	8	30	0.640958	7	3	0.1488	no
gi|641562741|gb|KK859161.1|	0-186	7.8	31	35	8	-2	0.2746	no
gi|641562741|gb|KK859161.1|	599-781	7.8	31	19	50	1	0.40035	no
gi|641562741|gb|KK859161.1|	6214-6622	7.8	31	11	5	-1	0.4631	no
gi|641562741|gb|KK859161.1|	9065-10512	7.8	31	158	130	-0.279964	0.8903	no
gi|641562747|gb|KK859158.1|	23256-24149	8.5	18	6	3	-1	0.41185	no
gi|641562750|gb|KK859157.1|	17550-18647	6.9	32	4	2	-0.839002	0.53355	no
gi|641562774|gb|KK859149.1|	3252-4412	10	38	0.44036	0.446894	0.0212474	1	no
gi|641562785|gb|KK859145.1|	22801-23373	11	48	2	0.283925	-3	0.2348	no
gi|641562785|gb|KK859145.1|	23491-27379	11	48	1	0.439187	-1	0.31675	no
gi|641562785|gb|KK859145.1|	39255-40098	11	48	3	2	-0.659815	0.64395	no
gi|641562785|gb|KK859145.1|	40783-42082	11	48	0.833954	0.488526	-0.771531	1	no
gi|641562785|gb|KK859145.1|	43127-43708	11	48	2	0.277406	-3	0.2381	no
gi|641562785|gb|KK859145.1|	43992-44807	11	48	0.914018	0.347023	-1	0.4534	no
gi|641562787|gb|KK859144.1|	13125-13521	7.5	30	1	20	4	0.12315	no
gi|641562787|gb|KK859144.1|	17705-19103	7.5	30	8	2	-2	0.107	no
gi|641562787|gb|KK859144.1|	3055-4193	7.5	30	0	11	inf	5.00E-05	yes
gi|641562817|gb|KK859131.1|	12934-13080	7.1	39	152	82	-0.877911	0.59135	no

gi|641562819|gb|KK859130.1|	14924-15973	4.6	14	4	3	-0.365439	0.7825	no
gi|641562821|gb|KK859129.1|	186-485	7.3	28	116	0	-inf	5.00E-05	yes
gi|641562821|gb|KK859129.1|	3335-4490	7.3	28	9	0	-inf	5.00E-05	yes
gi|641562821|gb|KK859129.1|	4552-5419	7.3	28	18	0	-inf	5.00E-05	yes
gi|641562821|gb|KK859129.1|	7902-8780	7.3	28	17	0	-inf	5.00E-05	yes
gi|641562823|gb|KK859128.1|	25159-26249	6.3	26	0.158271	2	3	0.2556	no
gi|641562830|gb|KK859125.1|	4948-5245	5.9	23	5	7	0.373983	0.8127	no
gi|641562836|gb|KK859122.1|	1847-2003	11	20	132	52	-1	0.3996	no
gi|641562852|gb|KK859115.1|	15599-16344	10	35	2	2	-0.108885	0.9412	no
gi|641562852|gb|KK859115.1|	17635-21421	10	35	13	28	1	0.60865	no
gi|641562852|gb|KK859115.1|	2418-3197	10	35	52	14	-2	0.29255	no
gi|641562852|gb|KK859115.1|	4932-6125	10	35	2	2	-0.037444	0.9722	no
gi|641562856|gb|KK859113.1|	11483-13087	9.2	28	9	14	0.712669	0.56705	no
gi|641562856|gb|KK859113.1|	13591-14115	9.2	28	1	2	0.594516	0.70555	no
gi|641562856|gb|KK859113.1|	9108-9704	9.2	28	1	3	1	0.4284	no
gi|641562863|gb|KK859110.1|	2-646	13	5.10E+01	6	5	-0.362322	0.78885	no
gi|641562873|gb|KK859106.1|	10661-11857	9	18	26	10	-1	0.2674	no
gi|641562875|gb|KK859105.1|	0-165	6.4	22	95	202	1	0.4428	no
gi|641562875|gb|KK859105.1|	13092-13324	6.4	22	5	44	3	0.14495	no
gi|641562875|gb|KK859105.1|	15331-16638	6.4	22	2	6	2	0.2365	no
gi|641562875|gb|KK859105.1|	20512-20764	6.4	22	13	10	-0.339823	0.8219	no
gi|641562881|gb|KK859102.1|	12621-13158	9.8	37	3	2	-0.627222	0.686	no
gi|641562881|gb|KK859102.1|	4519-7438	9.8	37	0.779856	1	0.703275	0.6003	no
gi|641562883|gb|KK859101.1|	10283-10505	8	34	0	28	inf	0.0025	yes
gi|641562890|gb|KK859098.1|	7851-8776	10	37	0.873264	2	1	0.49595	no
gi|641562896|gb|KK859095.1|	3091-3891	7.4	15	2	7	2	0.2314	no
gi|641562896|gb|KK859095.1|	6641-7146	7.4	15	5	21	2	0.13815	no
gi|641562898|gb|KK859094.1|	18644-18841	12	45	23	3	-3	0.2472	no
gi|641562900|gb|KK859093.1|	13812-15043	9	40	0	1	inf	0.00375	no
gi|641562900|gb|KK859093.1|	22769-23200	9	40	0	6	inf	0.0025	yes

gi|641562900|gb|KK859093.1|	3384-3891	9	40	12	7	-0.714537	0.5947	no
gi|641562908|gb|KK859090.1|	20091-20457	9.4	37	0.810822	5	3	0.30875	no
gi|641562938|gb|KK859085.1|	954-1939	10	42	0.717259	1	0.604855	0.6936	no
gi|641562939|gb|KK859084.1|	128-349	7.1	29	0	22	inf	0.0068	no
gi|641562945|gb|KK859080.1|	17696-18137	7.7	40	1	3	1	0.4611	no
gi|641562951|gb|KK859078.1|	11794-12398	7.5	32	5	3	-1	0.46305	no
gi|641562951|gb|KK859078.1|	18772-18957	7.5	32	47	4	-3	0.20685	no
gi|641562952|gb|KK859077.1|	201-1597	8.5	33	6	2	-2	0.24885	no
gi|641562952|gb|KK859077.1|	2788-4498	8.5	33	179	108	-0.724283	0.7213	no
gi|641562952|gb|KK859077.1|	6104-8298	8.5	33	91	66	-0.46993	0.81905	no
gi|641562961|gb|KK859073.1|	5717-6118	6.9	26	10	11	0.186344	0.8853	no
gi|641562964|gb|KK859072.1|	15251-15624	10	36	43	29	-0.543044	0.66855	no
gi|641562964|gb|KK859072.1|	24227-24838	10	36	6	7	0.265594	0.83985	no
gi|641562966|gb|KK859071.1|	5638-7004	6.3	21	0.786227	0.829198	0.0767717	1	no
gi|641562968|gb|KK859070.1|	13877-14304	6.5	17	0	10	inf	0.00115	yes
gi|641562970|gb|KK859069.1|	6709-7716	5.7	17	10	12	0.237326	0.848	no
gi|641562973|gb|KK859068.1|	10248-10822	7.7	14	13.26	9	-0.552941	0.67055	no
gi|641562973|gb|KK859068.1|	11664-12099	7.7	14	10	11	0.0858717	0.94905	no
gi|641562973|gb|KK859068.1|	13046-13523	7.7	14	3	2	-0.730232	0.67005	no
gi|641562973|gb|KK859068.1|	19837-20398	7.7	14	1	2	0.78891	0.63035	no
gi|641562973|gb|KK859068.1|	8518-9651	7.7	14	15	10	-0.608979	0.62935	no
gi|641562976|gb|KK859067.1|	26071-26356	6.5	21	5	6	0.341103	0.8376	no
gi|641562983|gb|KK859064.1|	15204-16012	9	19	0.46223	2	2	0.2441	no
gi|641562986|gb|KK859063.1|	2390-2735	7	24	0.915116	5	3	0.30875	no
gi|641562986|gb|KK859063.1|	5820-6308	7	24	1	5	2	0.23675	no
gi|641562986|gb|KK859063.1|	7341-8084	7	24	5	8	0.529419	0.68565	no
gi|641562986|gb|KK859063.1|	8202-9053	7	24	3	9	2	0.25835	no
gi|641562992|gb|KK859061.1|	102-941	7.7	28	2	2	-0.396837	0.78615	no
gi|641562992|gb|KK859061.1|	11182-12856	7.7	28	0.622464	0.729959	0.229826	1	no
gi|641562992|gb|KK859061.1|	12920-13951	7.7	28	0.423612	25	6	0.08155	no

gi|641562992|gb|KK859061.1|	14600-15374	7.7	28	0	66	inf	0.00095	yes
gi|641562992|gb|KK859061.1|	19195-19993	7.7	28	0	2	inf	0.0068	no
gi|641562992|gb|KK859061.1|	3102-4531	7.7	28	11	3	-2	0.1775	no
gi|641562992|gb|KK859061.1|	5460-6543	7.7	28	29	9	-2	0.18365	no
gi|641562992|gb|KK859061.1|	8094-9581	7.7	28	9	4	-1	0.3649	no
gi|641562996|gb|KK859059.1|	10984-13674	11	37	0.596009	0.866327	0.539576	1	no
gi|641562996|gb|KK859059.1|	9413-9597	11	37	33	13	-1	0.44165	no
gi|641562996|gb|KK859059.1|	974-1244	11	37	99	72	-0.464673	0.7138	no
gi|641562999|gb|KK859058.1|	12857-13244	8.5	25	13	9	-0.626266	0.6419	no
gi|641563001|gb|KK859057.1|	18400-18949	11	46	0	78	inf	5.00E-05	yes
gi|641563001|gb|KK859057.1|	19673-20103	11	46	0	6	inf	0.0025	yes
gi|641563001|gb|KK859057.1|	20429-21657	11	46	0	31	inf	5.00E-05	yes
gi|641563001|gb|KK859057.1|	22675-22938	11	46	0	48	inf	0.0004	yes
gi|641563001|gb|KK859057.1|	24094-25967	11	46	0	18	inf	0.0011	yes
gi|641563013|gb|KK859052.1|	8544-8897	5.8	22	0	10	inf	0.0015	yes
gi|641563015|gb|KK859051.1|	14357-15348	11	34	0.800832	0.270661	-2	1	no
gi|641563017|gb|KK859050.1|	10796-11680	12	40	1	1	-0.289324	0.85105	no
gi|641563017|gb|KK859050.1|	12971-13800	12	40	2	3	0.509909	0.7255	no
gi|641563017|gb|KK859050.1|	14742-15507	12	40	14	4	-2	0.17945	no
gi|641563017|gb|KK859050.1|	19898-21853	12	40	7	10	0.385254	0.75895	no
gi|641563017|gb|KK859050.1|	25090-25570	12	40	15	25	0.682098	0.59025	no
gi|641563017|gb|KK859050.1|	26198-27050	12	40	0.646891	0.982779	0.603344	0.70555	no
gi|641563017|gb|KK859050.1|	27732-29867	12	40	8	12	0.643395	0.60475	no
gi|641563017|gb|KK859050.1|	3652-3879	12	40	11	8	-0.456649	0.78585	no
gi|641563017|gb|KK859050.1|	4840-6127	12	40	0.583699	0.592651	0.021959	1	no
gi|641563017|gb|KK859050.1|	6247-6760	12	40	2	1	-0.821114	0.6492	no
gi|641563017|gb|KK859050.1|	7027-9072	12	40	2	3	0.0812075	0.9506	no
gi|641563017|gb|KK859050.1|	9715-10658	12	40	2	1	-0.758134	0.61675	no
gi|641563030|gb|KK859044.1|	14014-14820	8.4	26	2	1	-0.397331	0.7965	no
gi|641563030|gb|KK859044.1|	21926-24311	8.4	26	6	4	-0.448579	0.72325	no

gi|641563030|gb|KK859044.1|	30559-31585	8.4	26	11	13	0.328383	0.7952	no
gi|641563033|gb|KK859043.1|	13121-13605	8.4	16	5	3	-0.630225	0.6654	no
gi|641563033|gb|KK859043.1|	18493-18787	8.4	16	7	4	-0.893565	0.5974	no
gi|641563033|gb|KK859043.1|	2047-3733	8.4	16	0.474964	2	2	0.24655	no
gi|641563033|gb|KK859043.1|	26881-27418	8.4	16	9	3	-2	0.2948	no
gi|641563033|gb|KK859043.1|	4675-5259	8.4	16	1	2	1	0.5082	no
gi|641563033|gb|KK859043.1|	5949-6367	8.4	16	61	55	-0.155195	0.90095	no
gi|641563042|gb|KK859040.1|	10619-11577	7	29	0.649819	0.705772	0.119164	1	no
gi|641563044|gb|KK859039.1|	6029-6807	6.4	25	3	1	-1	0.44105	no
gi|641563044|gb|KK859039.1|	6962-7388	6.4	25	2	2	-0.41247	0.82205	no
gi|641563044|gb|KK859039.1|	7509-8581	6.4	25	1	0.983083	-0.394404	0.7971	no
gi|641563044|gb|KK859039.1|	9621-10168	6.4	25	1	3	2	0.32945	no
gi|641563048|gb|KK859037.1|	40-366	8.5	33	49	4	-4	0.0561	no
gi|641563048|gb|KK859037.1|	8572-9688	8.5	33	0	43	inf	0.00065	yes
gi|641563050|gb|KK859036.1|	13971-14635	12	48	2	0.457735	-2	0.3455	no
gi|641563050|gb|KK859036.1|	142-324	12	48	389	425	0.129094	0.91695	no
gi|641563050|gb|KK859036.1|	14797-15013	12	48	176	172	-0.0336042	0.97925	no
gi|641563050|gb|KK859036.1|	17074-17285	12	48	115	119	0.0460668	0.9713	no
gi|641563050|gb|KK859036.1|	2503-3875	12	48	2	0.824985	-0.923203	0.52095	no
gi|641563095|gb|KK859016.1|	13677-14607	8.5	24	2	1	-0.395704	0.78685	no
gi|641563095|gb|KK859016.1|	4404-4696	8.5	24	21	9	-1	0.40555	no
gi|641563095|gb|KK859016.1|	5105-6140	8.5	24	2	3	0.428422	0.75275	no
gi|641563095|gb|KK859016.1|	6504-12376	8.5	24	7	7	-0.13471	0.95385	no
gi|641563095|gb|KK859016.1|	839-1130	8.5	24	23	21	-0.130055	0.9244	no
gi|641563101|gb|KK859013.1|	10199-10413	9.7	36	0	24	inf	0.0068	no
gi|641563117|gb|KK859006.1|	12677-13453	10	41	2	0	-inf	0.0059	no
gi|641563117|gb|KK859006.1|	821-1408	10	41	3	0	-inf	0.0056	no
gi|641563136|gb|KK859001.1|	18112-18311	14	25	15	41	1	0.3819	no
gi|641563138|gb|KK859000.1|	14497-15046	8.9	29	4	0.60411	-3	0.20575	no
gi|641563138|gb|KK859000.1|	2744-3397	8.9	29	4	2	-1	0.3895	no

gi|641563138|gb|KK859000.1|	3800-4701	8.9	29	128	129	0.0116596	0.9951	no
gi|641563138|gb|KK859000.1|	8708-9194	8.9	29	3	0.365879	-3	0.2381	no
gi|641563138|gb|KK859000.1|	9374-9947	8.9	29	4	3	-0.473624	0.7407	no
gi|641563148|gb|KK858996.1|	18420-18645	5.5	26	8	22	1	0.3871	no
gi|641563163|gb|KK858989.1|	5216-5755	8.7	34	7	5	-0.449127	0.7434	no
gi|641563163|gb|KK858989.1|	584-1457	8.7	34	7	7	-0.153808	0.90415	no
gi|641563185|gb|KK858979.1|	13124-14077	6.3	24	2	2	-0.269929	0.84935	no
gi|641563192|gb|KK858976.1|	16222-16591	6.7	26	4	5	0.258773	0.87415	no
gi|641563192|gb|KK858976.1|	17124-17898	6.7	26	2	3	0.509029	0.7255	no
gi|641563194|gb|KK858975.1|	12241-12819	7	31	5	6	0.111539	0.93425	no
gi|641563204|gb|KK858971.1|	9478-10090	6.3	25	2	2	-0.294882	0.8456	no
gi|641563207|gb|KK858970.1|	359-1603	9.8	20	16	8	-1	0.42315	no
gi|641563209|gb|KK858969.1|	15081-15972	10	17	1	3	1	0.41195	no
gi|641563209|gb|KK858969.1|	21316-21838	10	17	1	2	0.816803	0.6234	no
gi|641563209|gb|KK858969.1|	9870-10214	10	17	2	3	0.576647	0.76735	no
gi|641563223|gb|KK858963.1|	14779-15011	8.3	31	6	13	1	0.5133	no
gi|641563223|gb|KK858963.1|	15121-15653	8.3	31	5	8	0.538341	0.69395	no
gi|641563223|gb|KK858963.1|	17275-17631	8.3	31	3	3	-0.00627666	0.91115	no
gi|641563227|gb|KK858961.1|	10008-10213	10	22	21	20	-0.119218	0.93375	no
gi|641563229|gb|KK858960.1|	12047-12434	8.7	30	4	3	-0.416813	0.77915	no
gi|641563229|gb|KK858960.1|	15225-16413	8.7	30	10	6	-0.58779	0.64475	no
gi|641563229|gb|KK858960.1|	17363-18027	8.7	30	9	14	0.599762	0.64515	no
gi|641563229|gb|KK858960.1|	2871-3104	8.7	30	142	42	-2	0.18765	no
gi|641563229|gb|KK858960.1|	8839-9926	8.7	30	14	1	-3	0.03405	no
gi|641563231|gb|KK858959.1|	2904-3401	8.2	27	3	2	-0.785519	0.63075	no
gi|641563231|gb|KK858959.1|	291-826	8.2	27	2	5	1	0.46	no
gi|641563236|gb|KK858957.1|	23474-24020	10	27	45	33	-0.457352	0.7121	no
gi|641563236|gb|KK858957.1|	25935-26505	10	27	8	4	-1	0.4593	no
gi|641563245|gb|KK858953.1|	2107-2764	7.9	28	0.459916	3	3	0.19335	no
gi|641563252|gb|KK858950.1|	8802-10301	8.8	39	0.760328	0.413818	-0.877626	1	no

gi|641563262|gb|KK858946.1|	3702-4659	11	44	0	3	inf	0.00145	yes
gi|641563262|gb|KK858946.1|	4737-6065	11	44	0	6	inf	5.00E-05	yes
gi|641563273|gb|KK858941.1|	2487-3112	8.2	34	2	0.498766	-2	0.3455	no
gi|641563273|gb|KK858941.1|	663-839	8.2	34	34	5	-3	0.2587	no
gi|641563276|gb|KK858940.1|	14255-15280	4.2	17	4	0.129812	-5	0.18485	no
gi|641563276|gb|KK858940.1|	19285-20041	4.2	17	3	0	-inf	0.0021	yes
gi|641563279|gb|KK858939.1|	12316-12604	7.4	31	6	16	1	0.37385	no
gi|641563290|gb|KK858934.1|	15091-15429	4.7	20	6	0	-inf	0.0075	no
gi|641563303|gb|KK858928.1|	14556-14988	11	33	5	2	-1	0.4929	no
gi|641563303|gb|KK858928.1|	418-1223	11	33	2	3	1	0.46925	no
gi|641563306|gb|KK858927.1|	688-1061	9.7	35	7	0	-inf	0.00495	no
gi|641563317|gb|KK858923.1|	2157-2285	8.1	31	7879.07	4465.56	-0.819183	0.5209	no
gi|641563320|gb|KK858922.1|	18016-18959	12	23	0.757548	0.719853	-0.073636	1	no
gi|641563335|gb|KK858916.1|	10057-10759	9.3	31	0.558858	2	2	0.26155	no
gi|641563335|gb|KK858916.1|	3190-3644	9.3	31	4	0	-inf	0.0059	no
gi|641563335|gb|KK858916.1|	3863-4712	9.3	31	0.75817	2	2	0.2915	no
gi|641563335|gb|KK858916.1|	6662-7365	9.3	31	2	2	0.0157628	0.9597	no
gi|641563348|gb|KK858910.1|	496-1297	9.6	39	8	2	-2	0.1257	no
gi|641563350|gb|KK858909.1|	9812-10043	3.5	14	13	142	3	0.04265	no
gi|641563352|gb|KK858908.1|	3030-3825	7.8	26	0.236125	3	4	0.2456	no
gi|641563402|gb|KK858886.1|	32690-32949	10	39	11	4	-1	0.4178	no
gi|641563404|gb|KK858885.1|	11954-12286	8.8	16	116	41	-1	0.25425	no
gi|641563408|gb|KK858883.1|	12800-13051	9.5	21	28	0	-inf	0.0013	yes
gi|641563408|gb|KK858883.1|	20948-21848	9.5	21	0.703301	1	0.603955	0.69715	no
gi|641563408|gb|KK858883.1|	3729-4211	9.5	21	8	9	0.0895346	0.94635	no
gi|641563408|gb|KK858883.1|	6357-6620	9.5	21	31	36	0.194402	0.8797	no
gi|641563413|gb|KK858881.1|	21290-22269	12	41	0.632406	2	2	0.24985	no
gi|641563419|gb|KK858879.1|	14762-15205	9.6	38	2	2	0.103652	0.92335	no
gi|641563423|gb|KK858877.1|	12002-12409	9.2	35	3	6	1	0.49595	no
gi|641563423|gb|KK858877.1|	15485-16038	9.2	35	1	2	1	0.5082	no

gi|641563423|gb|KK858877.1|	5377-5722	9.2	35	5	3	-0.882608	0.60075	no
gi|641563423|gb|KK858877.1|	9177-9657	9.2	35	1	4	2	0.32035	no
gi|641563470|gb|KK858857.1|	18565-19179	11	34	1	2	0.790915	0.63035	no
gi|641563470|gb|KK858857.1|	21287-21624	11	34	2	4	1	0.5206	no
gi|641563470|gb|KK858857.1|	22750-24059	11	34	0.381446	0.580987	0.607028	1	no
gi|641563470|gb|KK858857.1|	25335-26309	11	34	0.181848	2	3	0.2672	no
gi|641563470|gb|KK858857.1|	28370-29317	11	34	0.847707	1	0.241908	0.8798	no
gi|641563470|gb|KK858857.1|	29421-30367	11	34	4	5	0.444003	0.7337	no
gi|641563470|gb|KK858857.1|	31224-33000	11	34	2	1	-0.843917	0.5368	no
gi|641563470|gb|KK858857.1|	33071-35462	11	34	4	3	-0.311747	0.8042	no
gi|641563470|gb|KK858857.1|	35761-36476	11	34	16	3	-2	0.09685	no
gi|641563470|gb|KK858857.1|	36896-37837	11	34	16	9	-0.874239	0.49525	no
gi|641563470|gb|KK858857.1|	38733-39972	11	34	0.542705	0.4132	-0.393327	1	no
gi|641563470|gb|KK858857.1|	47426-48086	11	34	7	3	-1	0.42365	no
gi|641563470|gb|KK858857.1|	50097-50254	11	34	262	20	-4	0.1397	no
gi|641563470|gb|KK858857.1|	550-4372	11	34	17	9	-0.936654	0.556	no
gi|641563470|gb|KK858857.1|	7545-7887	11	34	5	0	-inf	0.01175	no
gi|641563474|gb|KK858855.1|	0-400	11	42	3	5	0.754702	0.6288	no
gi|641563479|gb|KK858853.1|	8380-8557	5	17	22	26	0.254916	0.8823	no
gi|641563481|gb|KK858852.1|	17703-18247	8.7	35	0.810864	5	3	0.19915	no
gi|641563481|gb|KK858852.1|	20966-21424	8.7	35	9	8	-0.0877659	0.9478	no
gi|641563481|gb|KK858852.1|	647-1152	8.7	35	1	2	0.330435	0.83525	no
gi|641563494|gb|KK858846.1|	10740-11584	11	40	1	1	0.188196	0.90355	no
gi|641563494|gb|KK858846.1|	1441-1687	11	40	9	25	1	0.3763	no
gi|641563494|gb|KK858846.1|	19675-20722	11	40	2	8	2	0.1535	no
gi|641563494|gb|KK858846.1|	20972-21936	11	40	0	2	inf	0.00255	yes
gi|641563494|gb|KK858846.1|	2182-3619	11	40	2	5	1	0.3012	no
gi|641563494|gb|KK858846.1|	24-987	11	40	2	5	2	0.2133	no
gi|641563494|gb|KK858846.1|	24067-24483	11	40	2	2	0.101105	0.92335	no
gi|641563494|gb|KK858846.1|	4072-4926	11	40	0.859903	0.816511	-0.0747005	1	no

gi|641563494|gb|KK858846.1|	5002-9361	11	40	1	4	2	0.216	no
gi|641563494|gb|KK858846.1|	9942-10504	11	40	1	2	0.788953	0.63035	no
gi|641563507|gb|KK858840.1|	9132-10202	7.1	38	3	0	-inf	0.00115	yes
gi|641563511|gb|KK858838.1|	30617-30836	9.6	39	31	0	-inf	0.00345	yes
gi|641563516|gb|KK858836.1|	11116-12217	11	19	0.938093	0.356905	-1	0.4312	no
gi|641563516|gb|KK858836.1|	2931-3418	11	19	5	5	0.0398225	0.97235	no
gi|641563516|gb|KK858836.1|	4194-5154	11	19	10	9	-0.0789152	0.9511	no
gi|641563516|gb|KK858836.1|	6631-7026	11	19	13	13	0.0581154	0.9652	no
gi|641563516|gb|KK858836.1|	68-445	11	19	2	2	-0.00311345	0.91115	no
gi|641563516|gb|KK858836.1|	9804-10704	11	19	2	0.763522	-1	0.38165	no
gi|641563533|gb|KK858829.1|	482-948	6.7	29	0.780916	3	2	0.3323	no
gi|641563541|gb|KK858826.1|	16839-17271	8.2	16	7	0	-inf	0.0022	yes
gi|641563541|gb|KK858826.1|	21882-22827	8.2	16	9	0	-inf	0.0001	yes
gi|641563541|gb|KK858826.1|	8011-11395	8.2	16	8	0.033901	-8	0.18285	no
gi|641563543|gb|KK858825.1|	1006-1449	8.6	18	2	2	-0.410921	0.82205	no
gi|641563543|gb|KK858825.1|	6848-7324	8.6	18	1	2	1	0.51315	no
gi|641563559|gb|KK858818.1|	13018-14191	9.4	34	0.651758	1	1	0.49595	no
gi|641563559|gb|KK858818.1|	82-762	9.4	34	3	2	-0.399825	0.78565	no
gi|641563570|gb|KK858813.1|	26633-28109	11	43	24	28	0.222498	0.887	no
gi|641563570|gb|KK858813.1|	29225-29735	11	43	18	13	-0.424623	0.7436	no
gi|641563570|gb|KK858813.1|	35573-36190	11	43	4	3	-0.780142	0.5821	no
gi|641563570|gb|KK858813.1|	9690-10909	11	43	0.760798	0	-inf	1	no
gi|641563578|gb|KK858810.1|	3948-4317	5.7	21	4	4	0.218131	0.8957	no
gi|641563582|gb|KK858808.1|	107-2762	10	16	2	2	-0.143428	0.9121	no
gi|641563582|gb|KK858808.1|	16761-18293	10	16	3	0.887992	-2	0.2224	no
gi|641563582|gb|KK858808.1|	20477-20798	10	16	23	32	0.467836	0.72355	no
gi|641563582|gb|KK858808.1|	3607-4253	10	16	2	5	1	0.43805	no
gi|641563582|gb|KK858808.1|	6155-7449	10	16	2	1	-0.186593	0.8943	no
gi|641563586|gb|KK858807.1|	0-1859	8.2	25	25	22	-0.182161	0.93725	no
gi|641563586|gb|KK858807.1|	2933-4190	8.2	25	9	8	-0.17564	0.88955	no

gi|641563586|gb|KK858807.1|	5289-6285	8.2	25	5	3	-0.705382	0.58865	no
gi|641563586|gb|KK858807.1|	6705-7045	8.2	25	4	6	0.745852	0.629	no
gi|641563594|gb|KK858803.1|	12701-13537	12	30	1	0.167861	-3	0.24815	no
gi|641563594|gb|KK858803.1|	15243-15943	12	30	0.981871	1	0.378264	0.8276	no
gi|641563596|gb|KK858802.1|	12416-13056	6.8	470	0	14	inf	5.00E-05	yes
gi|641563607|gb|KK858797.1|	12739-13286	8.7	38	1	2	1	0.5082	no
gi|641563607|gb|KK858797.1|	14104-14556	8.7	38	4	7	0.88939	0.545	no
gi|641563607|gb|KK858797.1|	15424-16286	8.7	38	0.53099	2	2	0.32945	no
gi|641563607|gb|KK858797.1|	22810-23289	8.7	38	2	4	1	0.48925	no
gi|641563614|gb|KK858794.1|	3027-3952	11	37	1	3	1	0.4102	no
gi|641563614|gb|KK858794.1|	33452-35414	11	37	12	0.916861	-4	0.0178	no
gi|641563624|gb|KK858790.1|	23709-24499	8.6	32	0.595276	3	2	0.20805	no
gi|641563624|gb|KK858790.1|	28619-29025	8.6	32	2	4	1	0.5082	no
gi|641563636|gb|KK858785.1|	9950-10651	8.3	33	4	4	-0.0873765	0.95065	no
gi|641563645|gb|KK858781.1|	79-399	9.7	43	2	6	2	0.41725	no
gi|641563661|gb|KK858774.1|	10796-11510	12	45	21	0	-inf	5.00E-05	yes
gi|641563661|gb|KK858774.1|	11814-12349	12	45	41	0	-inf	5.00E-05	yes
gi|641563661|gb|KK858774.1|	12944-14290	12	45	2	0.093729	-4	0.18685	no
gi|641563661|gb|KK858774.1|	14504-15120	12	45	11	0	-inf	0.0002	yes
gi|641563685|gb|KK858763.1|	3211-3678	6.4	16	0	6	inf	0.0015	yes
gi|641563691|gb|KK858760.1|	16757-17034	9.7	40	13	0	-inf	0.00435	no
gi|641563691|gb|KK858760.1|	17253-21758	9.7	40	8	0.100366	-6	0.06105	no
gi|641563691|gb|KK858760.1|	24424-24935	9.7	40	0	30	inf	5.00E-05	yes
gi|641563691|gb|KK858760.1|	6067-6887	9.7	40	7	6	-0.279758	0.83335	no
gi|641563691|gb|KK858760.1|	7816-11619	9.7	40	9	10	0.18306	0.9114	no
gi|641563706|gb|KK858754.1|	2011-2947	7.9	30	0	3	inf	0.00105	yes
gi|641563712|gb|KK858751.1|	14563-15901	11	43	0.309804	0.755061	1	1	no
gi|641563712|gb|KK858751.1|	27189-27834	11	43	2	0.715276	-1	0.47135	no
gi|641563721|gb|KK858747.1|	0-497	6.3	27	4	0.352888	-3	0.2089	no
gi|641563736|gb|KK858741.1|	28458-28879	6.8	23	5	8	0.674503	0.6406	no

gi|641563738|gb|KK858740.1|	10873-11583	9.5	35	0.550183	2	2	0.33105	no
gi|641563738|gb|KK858740.1|	19706-20323	9.5	35	3	0.508103	-3	0.1832	no
gi|641563738|gb|KK858740.1|	20964-21628	9.5	35	2	0.228867	-3	0.2089	no
gi|641563741|gb|KK858739.1|	10606-11754	6.2	19	0.891814	0.452498	-0.97883	0.5872	no
gi|641563741|gb|KK858739.1|	1215-1578	6.2	19	19	26	0.514206	0.69235	no
gi|641563753|gb|KK858734.1|	10512-11056	7.5	28	5	4	-0.347913	0.80665	no
gi|641563753|gb|KK858734.1|	12418-13948	7.5	28	5	5	-0.00984612	0.99185	no
gi|641563753|gb|KK858734.1|	7143-7996	7.5	28	0.430605	1	2	0.3613	no
gi|641563753|gb|KK858734.1|	9410-10317	7.5	28	19	13	-0.506387	0.6816	no
gi|641563764|gb|KK858729.1|	14023-14230	7.8	16	52	100	0.935977	0.4869	no
gi|641563764|gb|KK858729.1|	14669-15055	7.8	16	4	3	-0.291411	0.8545	no
gi|641563764|gb|KK858729.1|	17166-18151	7.8	16	0.179315	1	3	0.2851	no
gi|641563769|gb|KK858727.1|	131-1305	7	26	1	4	2	0.24115	no
gi|641563769|gb|KK858727.1|	15107-15506	7	26	2	4	0.777305	0.6361	no
gi|641563769|gb|KK858727.1|	22243-22426	7	26	28	31	0.158634	0.9178	no
gi|641563769|gb|KK858727.1|	5702-6585	7	26	0.617731	2	2	0.32035	no
gi|641563769|gb|KK858727.1|	8629-9155	7	26	6	11	0.775192	0.5645	no
gi|641563788|gb|KK858720.1|	10356-10713	9.6	33	4	2	-1	0.5278	no
gi|641563788|gb|KK858720.1|	10872-11277	9.6	33	3	2	-0.999614	0.62775	no
gi|641563788|gb|KK858720.1|	19792-20306	9.6	33	10	7	-0.568292	0.6753	no
gi|641563802|gb|KK858714.1|	12729-14092	7.3	37	0.363806	1	1	0.37775	no
gi|641563802|gb|KK858714.1|	2811-3595	7.3	37	1	3	2	0.33365	no
gi|641563802|gb|KK858714.1|	3892-4252	7.3	37	3	4	0.164289	0.91775	no
gi|641563804|gb|KK858713.1|	13458-13703	11	39	17	11	-0.641942	0.6753	no
gi|641563804|gb|KK858713.1|	18801-19995	11	39	1	0.863484	-0.785899	0.59675	no
gi|641563804|gb|KK858713.1|	30171-30729	11	39	3	0	-inf	0.0059	no
gi|641563804|gb|KK858713.1|	34764-35467	11	39	0.557759	2	2	0.3613	no
gi|641563814|gb|KK858709.1|	10440-11355	7.1	36	1	0.59816	-1	0.50315	no
gi|641563814|gb|KK858709.1|	6779-7373	7.1	36	2	5	2	0.28165	no
gi|641563814|gb|KK858709.1|	8161-9296	7.1	36	1	4	2	0.27305	no

gi|641563816|gb|KK858708.1|	15494-16270	7.4	32	0.609624	1	1	0.4595	no
gi|641563821|gb|KK858706.1|	3181-3927	8.3	27	2	0.780341	-1	0.4015	no
gi|641563823|gb|KK858705.1|	10633-11155	9.2	30	7	0.97949	-3	0.1479	no
gi|641563823|gb|KK858705.1|	12462-13400	9.2	30	15	14	-0.150505	0.90085	no
gi|641563823|gb|KK858705.1|	13903-15130	9.2	30	1	0.626981	-1	0.4423	no
gi|641563823|gb|KK858705.1|	16215-16826	9.2	30	4	3	-0.40183	0.77785	no
gi|641563823|gb|KK858705.1|	16978-17985	9.2	30	1	0.663381	-0.773454	0.63565	no
gi|641563823|gb|KK858705.1|	19599-19947	9.2	30	15	17	0.176808	0.8967	no
gi|641563823|gb|KK858705.1|	3040-3966	9.2	30	2	1	-0.855179	0.56515	no
gi|641563823|gb|KK858705.1|	5625-7968	9.2	30	39	9	-2	0.23455	no
gi|641563840|gb|KK858698.1|	16519-16930	12	39	5	2	-1	0.49285	no
gi|641563840|gb|KK858698.1|	18113-18469	12	39	7	3	-1	0.4588	no
gi|641563843|gb|KK858697.1|	18426-19498	9	33	0.565322	0.983083	0.798241	0.63025	no
gi|641563849|gb|KK858694.1|	7299-7914	6.8	22	0.505793	2	2	0.2812	no
gi|641563851|gb|KK858693.1|	1686-2224	6	25	0.824945	2	2	0.3613	no
gi|641563858|gb|KK858690.1|	12706-16699	13	45	1	1	0.0555644	0.96515	no
gi|641563858|gb|KK858690.1|	17746-18368	13	45	2	2	-0.101908	0.9456	no
gi|641563858|gb|KK858690.1|	20466-21431	13	45	0.735896	2	1	0.38845	no
gi|641563858|gb|KK858690.1|	9150-11597	13	45	1	1	0.194672	0.879	no
gi|641563864|gb|KK858688.1|	7434-8334	10	46	0	10	inf	5.00E-05	yes
gi|641563868|gb|KK858686.1|	18239-19592	9.4	38	0.795055	0.093164	-3	1	no
gi|641563868|gb|KK858686.1|	22788-23057	9.4	38	8	19	1	0.41145	no
gi|641563868|gb|KK858686.1|	23307-24072	9.4	38	0.124278	2	4	0.1136	no
gi|641563868|gb|KK858686.1|	2929-4269	9.4	38	0.804084	2	1	0.3486	no
gi|641563868|gb|KK858686.1|	5462-6358	9.4	38	1	1	-0.1737	0.90465	no
gi|641563875|gb|KK858683.1|	17737-18300	13	36	3	4	0.333316	0.81655	no
gi|641563875|gb|KK858683.1|	19542-19739	13	36	50	75	0.592827	0.6631	no
gi|641563875|gb|KK858683.1|	20995-21144	13	36	40	228	3	0.20215	no
gi|641563875|gb|KK858683.1|	22732-23424	13	36	1	3	1	0.4329	no
gi|641563875|gb|KK858683.1|	24230-24802	13	36	2	1	-0.725203	0.67005	no

gi|641563875|gb|KK858683.1|	24938-25788	13	36	1	1	0.31381	0.84265	no
gi|641563875|gb|KK858683.1|	26521-26757	13	36	6	12	1	0.5133	no
gi|641563875|gb|KK858683.1|	27638-29263	13	36	16	31	1	0.5401	no
gi|641563875|gb|KK858683.1|	32945-33429	13	36	4	3	-0.495295	0.74245	no
gi|641563875|gb|KK858683.1|	34286-35622	13	36	16	4	-2	0.16585	no
gi|641563875|gb|KK858683.1|	36792-38583	13	36	21	11	-0.914556	0.46295	no
gi|641563875|gb|KK858683.1|	39366-40113	13	36	107	8	-4	0.07505	no
gi|641563875|gb|KK858683.1|	4756-4995	13	36	139	182	0.384391	0.75915	no
gi|641563875|gb|KK858683.1|	7316-7539	13	36	13	29	1	0.45215	no
gi|641563889|gb|KK858677.1|	10553-10760	9.5	29	58	105	0.865085	0.5137	no
gi|641563889|gb|KK858677.1|	11033-11455	9.5	29	3	7	0.935062	0.5433	no
gi|641563889|gb|KK858677.1|	13771-13978	9.5	29	11	35	2	0.31825	no
gi|641563889|gb|KK858677.1|	14533-17457	9.5	29	10	18	0.883188	0.5841	no
gi|641563889|gb|KK858677.1|	17914-18244	9.5	29	20	24	0.28862	0.8282	no
gi|641563889|gb|KK858677.1|	19642-20526	9.5	29	2	3	0.521299	0.7153	no
gi|641563889|gb|KK858677.1|	27147-27966	9.5	29	2	7	2	0.2743	no
gi|641563889|gb|KK858677.1|	29046-30480	9.5	29	3	4	0.492086	0.7122	no
gi|641563889|gb|KK858677.1|	8994-9463	9.5	29	9	15	0.706609	0.58575	no
gi|641563891|gb|KK858676.1|	10784-11078	13	43	17	2	-3	0.15505	no
gi|641563891|gb|KK858676.1|	18903-19671	13	43	3	2	-0.28249	0.83825	no
gi|641563891|gb|KK858676.1|	20424-21716	13	43	0.06456	4	6	0.10485	no
gi|641563891|gb|KK858676.1|	24752-25473	13	43	8	10	0.348356	0.7856	no
gi|641563891|gb|KK858676.1|	26699-27087	13	43	6	5	-0.24676	0.8699	no
gi|641563891|gb|KK858676.1|	36348-41195	13	43	3	3	0.321559	0.79765	no
gi|641563891|gb|KK858676.1|	4080-4337	13	43	18	4	-2	0.23955	no
gi|641563891|gb|KK858676.1|	41542-42552	13	43	0.782155	0.26438	-2	1	no
gi|641563891|gb|KK858676.1|	44121-44718	13	43	2	3	0.460179	0.7552	no
gi|641563891|gb|KK858676.1|	47502-48243	13	43	2	0.196858	-3	0.2348	no
gi|641563891|gb|KK858676.1|	78-881	13	43	4	1	-2	0.275	no
gi|641563910|gb|KK858668.1|	14444-15912	9.9	36	3	1	-2	0.21255	no

gi|641563912|gb|KK858667.1|	748-1923	5.7	18	0.144539	1	3	0.2672	no
gi|641563918|gb|KK858664.1|	13523-14122	8.9	44	0.175252	2	4	0.1276	no
gi|641563922|gb|KK858662.1|	17990-18712	7.2	29	2	0	-inf	0.01	no
gi|641563922|gb|KK858662.1|	20279-20732	7.2	29	2	0.822312	-2	0.4266	no
gi|641563932|gb|KK858657.1|	10730-12769	4.9	16	2	0	-inf	0.00025	yes
gi|641563932|gb|KK858657.1|	12828-13425	4.9	16	3	0	-inf	0.0056	no
gi|641563932|gb|KK858657.1|	13923-15898	4.9	16	2	0.121357	-4	0.1341	no
gi|641563932|gb|KK858657.1|	16043-18161	4.9	16	3	0.056175	-6	0.18325	no
gi|641563932|gb|KK858657.1|	2047-3484	4.9	16	4	0	-inf	5.00E-05	yes
gi|641563932|gb|KK858657.1|	26240-26915	4.9	16	2	0	-inf	0.0056	no
gi|641563932|gb|KK858657.1|	30105-31333	4.9	16	5	0	-inf	0.0001	yes
gi|641563932|gb|KK858657.1|	33-509	4.9	16	13	0	-inf	0.0002	yes
gi|641563932|gb|KK858657.1|	8418-8961	4.9	16	3	0	-inf	0.0056	no
gi|641563950|gb|KK858649.1|	5669-6245	11	44	2	2	0.426961	0.78025	no
gi|641563950|gb|KK858649.1|	9162-9771	11	44	2	6	1	0.3132	no
gi|641563952|gb|KK858648.1|	21198-21676	8.9	38	4	1	-2	0.2877	no
gi|641563954|gb|KK858647.1|	17571-17975	12	44	3	8	1	0.4346	no
gi|641563956|gb|KK858646.1|	3858-4437	11	21	1	2	0.597005	0.69715	no
gi|641563961|gb|KK858644.1|	11444-12091	7.7	29	2	0	-inf	0.0102	no
gi|641563965|gb|KK858642.1|	11250-13404	10	18	5	1	-2	0.12525	no
gi|641563965|gb|KK858642.1|	14151-16126	10	18	7	0.485429	-4	0.0319	no
gi|641563965|gb|KK858642.1|	16678-17893	10	18	17	3	-2	0.077	no
gi|641563965|gb|KK858642.1|	19380-19830	10	18	9	3	-2	0.2749	no
gi|641563965|gb|KK858642.1|	21672-23968	10	18	2	0.668521	-1	0.3408	no
gi|641563965|gb|KK858642.1|	25425-26185	10	18	4	0	-inf	0.0013	yes
gi|641563967|gb|KK858641.1|	5830-6531	10	40	9	2	-2	0.11855	no
gi|641563973|gb|KK858638.1|	19516-20920	9.7	19	2	0.089246	-4	0.1892	no
gi|641563973|gb|KK858638.1|	39-2092	9.7	19	3	0.523229	-3	0.093	no
gi|641563985|gb|KK858633.1|	20942-21429	11	43	1238.82	2152.22	0.79686	0.659	no
gi|641563985|gb|KK858633.1|	22335-23862	11	43	293	446	0.606323	0.7376	no

gi|641563985|gb|KK858633.1|	28090-28496	11	43	4	4	0.0549452	0.9557	no
gi|641563985|gb|KK858633.1|	31137-31821	11	43	1	1	0.377882	0.8276	no
gi|641563989|gb|KK858631.1|	21972-22376	8.2	44	10	11	0.186686	0.88445	no
gi|641563989|gb|KK858631.1|	24961-26223	8.2	44	4	13	2	0.22585	no
gi|641563989|gb|KK858631.1|	26350-26753	8.2	44	3	10	2	0.28165	no
gi|641563989|gb|KK858631.1|	27014-28576	8.2	44	0.466251	1	2	0.29745	no
gi|641563991|gb|KK858630.1|	6416-6782	7	29	56	81	0.516594	0.678	no
gi|641563993|gb|KK858629.1|	2693-3320	8.3	39	0.819638	2	1	0.39995	no
gi|641563993|gb|KK858629.1|	6074-6447	8.3	39	2	5	1	0.4655	no
gi|641563998|gb|KK858627.1|	21218-21723	7.6	26	3	0.687995	-2	0.2679	no
gi|641564000|gb|KK858626.1|	38-258	7.2	32	11	17	0.732834	0.66305	no
gi|641564006|gb|KK858623.1|	417-1188	7.1	27	2	1	-0.513392	0.7498	no
gi|641564008|gb|KK858622.1|	4114-4851	5.9	21	0.784184	0.991497	0.338417	0.8352	no
gi|641564015|gb|KK858619.1|	7556-7997	7.1	33	2	2	0.103479	0.92335	no
gi|641564015|gb|KK858619.1|	9446-10099	7.1	33	2	1	-0.985502	0.57205	no
gi|641564017|gb|KK858618.1|	12291-13323	6.7	21	0.846211	0.128731	-3	1	no
gi|641564032|gb|KK858612.1|	30384-31160	10	35	6	3	-1	0.40785	no
gi|641564032|gb|KK858612.1|	32867-33755	10	35	0.817696	2	0.925738	0.5636	no
gi|641564032|gb|KK858612.1|	33847-34933	10	35	0.317963	0.846739	1	1	no
gi|641564037|gb|KK858610.1|	3496-3703	12	25	160	113	-0.500068	0.6977	no
gi|641564051|gb|KK858604.1|	2287-2910	7.6	27	2	0	-inf	0.01175	no
gi|641564051|gb|KK858604.1|	2981-3443	7.6	27	8	0	-inf	0.0015	yes
gi|641564051|gb|KK858604.1|	4744-5884	7.6	27	4	0	-inf	0.0002	yes
gi|641564051|gb|KK858604.1|	7977-9128	7.6	27	1	0	-inf	0.00435	no
gi|641564097|gb|KK858584.1|	26379-27149	8.3	33	2	0	-inf	0.0056	no
gi|641564097|gb|KK858584.1|	27793-28313	8.3	33	2	0.328464	-3	0.2381	no
gi|641564114|gb|KK858577.1|	17035-17933	9.8	37	3	5	0.875233	0.51735	no
gi|641564120|gb|KK858574.1|	11281-12177	9.6	28	2	3	0.18887	0.89225	no
gi|641564120|gb|KK858574.1|	16759-17714	9.6	28	0.652385	0.566835	-0.202794	1	no
gi|641564125|gb|KK858572.1|	26256-26784	8.8	33	0.21238	4	4	0.1196	no

gi|641564125|gb|KK858572.1|	635-1716	8.8	33	0	5	inf	0.00015	yes
gi|641564125|gb|KK858572.1|	7426-7589	8.8	33	28.18	450	4	0.0862	no
gi|641564125|gb|KK858572.1|	7827-8570	8.8	33	0	2	inf	0.00455	no
gi|641564125|gb|KK858572.1|	8688-9880	8.8	33	0	2	inf	0.0015	yes
gi|641564127|gb|KK858571.1|	19592-20250	10	40	2	1	-0.622793	0.686	no
gi|641564135|gb|KK858568.1|	18378-19992	8	33	34	14	-1	0.4597	no
gi|641564135|gb|KK858568.1|	21161-21342	8	33	65	9	-3	0.18145	no
gi|641564135|gb|KK858568.1|	7413-8552	8	33	0.525186	2	2	0.24245	no
gi|641564135|gb|KK858568.1|	9747-11260	8	33	8	21	1	0.25425	no
gi|641564137|gb|KK858567.1|	33851-35242	11	40	0	405	inf	0.00035	yes
gi|641564137|gb|KK858567.1|	944-1135	11	40	54	70	0.3811	0.78345	no
gi|641564142|gb|KK858565.1|	25434-25600	4.1	16	244	51	-2	0.1487	no
gi|641564142|gb|KK858565.1|	8613-9517	4.1	16	21	5	-2	0.14385	no
gi|641564145|gb|KK858564.1|	9627-10548	10	40	0.487821	1	1	0.50945	no
gi|641564150|gb|KK858562.1|	11642-12458	11	39	2	0.173234	-3	0.217	no
gi|641564150|gb|KK858562.1|	18115-19808	11	39	0.75639	0.432434	-0.806649	1	no
gi|641564150|gb|KK858562.1|	19967-21690	11	39	0.463461	1	2	0.3192	no
gi|641564150|gb|KK858562.1|	27512-28270	11	39	2	0.572872	-1	0.4312	no
gi|641564150|gb|KK858562.1|	28835-29852	11	39	0.603161	0.655348	0.119719	1	no
gi|641564150|gb|KK858562.1|	31726-32445	11	39	1	0.615166	-0.813959	0.6492	no
gi|641564150|gb|KK858562.1|	40509-41279	11	39	1	0.561	-1	0.4312	no
gi|641564150|gb|KK858562.1|	47099-48037	11	39	5	4	-0.422089	0.7478	no
gi|641564150|gb|KK858562.1|	50126-50337	11	39	28	0	-inf	0.0056	no
gi|641564173|gb|KK858552.1|	15190-15962	8.7	33	71	19	-2	0.27545	no
gi|641564173|gb|KK858552.1|	17394-19856	8.7	33	40	0.52432	-6	0.019	no
gi|641564173|gb|KK858552.1|	20309-21332	8.7	33	5	0.390373	-4	0.1059	no
gi|641564173|gb|KK858552.1|	324-596	8.7	33	24	8	-2	0.3143	no
gi|641564173|gb|KK858552.1|	7239-8095	8.7	33	6	5	-0.210188	0.87605	no
gi|641564173|gb|KK858552.1|	8495-10291	8.7	33	57	0.40468	-7	0.03455	no
gi|641564184|gb|KK858547.1|	11145-11418	7.3	28	9	7	-0.314387	0.85305	no

gi|641564191|gb|KK858544.1|	37314-37929	9.2	14	1	0.510492	-1	0.4534	no
gi|641564196|gb|KK858542.1|	0-543	7.7	24	5	5	0.0104945	0.9728	no
gi|641564196|gb|KK858542.1|	10146-10553	7.7	24	11	10	-0.109575	0.9359	no
gi|641564196|gb|KK858542.1|	11247-11630	7.7	24	4	3	-0.68037	0.6692	no
gi|641564196|gb|KK858542.1|	13566-14075	7.7	24	3	2	-0.669335	0.68185	no
gi|641564196|gb|KK858542.1|	16128-17539	7.7	24	0.931845	0.177469	-2	0.21865	no
gi|641564196|gb|KK858542.1|	17740-19182	7.7	24	2	0.605746	-1	0.3507	no
gi|641564196|gb|KK858542.1|	7312-8367	7.7	24	0.823572	2	1	0.3975	no
gi|641564200|gb|KK858540.1|	14-850	47	37	228	1	-8	0.0329	no
gi|641564200|gb|KK858540.1|	16369-17105	47	37	1	0.595989	-1	0.47135	no
gi|641564200|gb|KK858540.1|	2896-3257	47	37	128	0	-inf	5.00E-05	yes
gi|641564200|gb|KK858540.1|	7888-9119	47	37	14	0.416379	-5	0.06165	no
gi|641564209|gb|KK858536.1|	11762-12278	11	34	2	0	-inf	0.01175	no
gi|641564209|gb|KK858536.1|	14186-14622	11	34	3	0.439025	-3	0.271	no
gi|641564209|gb|KK858536.1|	17118-18057	11	34	2	4	1	0.45945	no
gi|641564209|gb|KK858536.1|	19906-20161	11	34	80	71	-0.165583	0.89465	no
gi|641564209|gb|KK858536.1|	23274-23639	11	34	2	4	0.802493	0.6409	no
gi|641564209|gb|KK858536.1|	25300-25613	11	34	5	6	0.377784	0.8127	no
gi|641564209|gb|KK858536.1|	4195-4550	11	34	30	31	0.0342023	0.9804	no
gi|641564209|gb|KK858536.1|	423-1218	11	34	0.9445	0.537776	-0.812545	0.6492	no
gi|641564211|gb|KK858535.1|	10611-10960	9.5	36	13	2	-3	0.1529	no
gi|641564211|gb|KK858535.1|	19410-20815	9.5	36	18	1	-4	0.01915	no
gi|641564211|gb|KK858535.1|	21140-21497	9.5	36	17	6	-1	0.33445	no
gi|641564211|gb|KK858535.1|	24249-26809	9.5	36	11	11	0.018948	0.98705	no
gi|641564211|gb|KK858535.1|	26883-27925	9.5	36	0.418108	1	1	0.4595	no
gi|641564211|gb|KK858535.1|	3127-3372	9.5	36	49	9	-2	0.1388	no
gi|641564211|gb|KK858535.1|	35006-35659	9.5	36	2	0.468611	-2	0.2679	no
gi|641564211|gb|KK858535.1|	4420-4578	9.5	36	68	10	-3	0.23985	no
gi|641564211|gb|KK858535.1|	7569-8034	9.5	36	4	2	-1	0.3722	no
gi|641564213|gb|KK858534.1|	9045-9546	10	36	3	1	-1	0.4311	no

gi|641564217|gb|KK858532.1|	10606-11460	9.5	41	0.537439	1	1	0.3994	no
gi|641564222|gb|KK858530.1|	35212-36171	8.1	32	0	3	inf	0.0015	yes
gi|641564241|gb|KK858522.1|	34349-34827	6.7	28	1	2	0.32873	0.83525	no
gi|641564246|gb|KK858520.1|	12383-13758	47	410	4	5	0.349013	0.7833	no
gi|641564246|gb|KK858520.1|	15417-16492	47	410	13	0.489873	-5	0.06235	no
gi|641564246|gb|KK858520.1|	18181-19025	47	410	3	0.331607	-3	0.1507	no
gi|641564246|gb|KK858520.1|	19134-21004	47	410	2	0.064474	-5	0.18595	no
gi|641564246|gb|KK858520.1|	30231-30612	47	410	7	0	-inf	0.00495	no
gi|641564246|gb|KK858520.1|	3456-4503	47	410	10	0.126475	-6	0.18295	no
gi|641564265|gb|KK858512.1|	33893-34088	11	39	42	44	0.0581455	0.96475	no
gi|641564265|gb|KK858512.1|	34821-37181	11	39	0.719576	0.548979	-0.390396	1	no
gi|641564265|gb|KK858512.1|	37302-37776	11	39	1	2	0.591487	0.70505	no
gi|641564265|gb|KK858512.1|	38884-40182	11	39	0.706293	0.586761	-0.267494	1	no
gi|641564265|gb|KK858512.1|	40792-42395	11	39	0.100594	4	5	0.23275	no
gi|641564265|gb|KK858512.1|	4458-5466	11	39	15	19	0.403859	0.7461	no
gi|641564265|gb|KK858512.1|	46333-49113	11	39	2	5	2	0.22805	no
gi|641564265|gb|KK858512.1|	50306-50855	11	39	15	34	1	0.3551	no
gi|641564280|gb|KK858506.1|	14248-14780	6.3	21	7	0	-inf	0.00175	yes
gi|641564280|gb|KK858506.1|	17353-18331	6.3	21	9	0	-inf	0.0001	yes
gi|641564280|gb|KK858506.1|	19754-20245	6.3	21	14	0	-inf	5.00E-05	yes
gi|641564280|gb|KK858506.1|	21813-22351	6.3	21	15	0	-inf	5.00E-05	yes
gi|641564286|gb|KK858503.1|	9448-9861	7.2	27	12	3	-2	0.22595	no
gi|641564294|gb|KK858500.1|	16111-16548	10	42	0	6	inf	0.00255	yes
gi|641564296|gb|KK858499.1|	12371-16846	9.7	39	38	40	0.0720301	0.9722	no
gi|641564296|gb|KK858499.1|	21968-23496	9.7	39	2	2	0.157238	0.90445	no
gi|641564296|gb|KK858499.1|	25963-27569	9.7	39	0.652468	0.765043	0.229633	1	no
gi|641564296|gb|KK858499.1|	27698-28908	9.7	39	0.348885	2	3	0.14835	no
gi|641564296|gb|KK858499.1|	29129-30356	9.7	39	0.411763	2	2	0.1527	no
gi|641564309|gb|KK858494.1|	10648-11155	9.1	19	3	0	-inf	0.0075	no
gi|641564309|gb|KK858494.1|	11274-11875	9.1	19	2	0	-inf	0.0102	no

gi|641564309|gb|KK858494.1|	15021-15855	9.1	19	2	0	-inf	0.0034	yes
gi|641564309|gb|KK858494.1|	21673-22366	9.1	19	2	0	-inf	0.0075	no
gi|641564309|gb|KK858494.1|	22935-23829	9.1	19	2	0	-inf	0.00315	yes
gi|641564309|gb|KK858494.1|	24058-26185	9.1	19	2	0	-inf	5.00E-05	yes
gi|641564309|gb|KK858494.1|	26850-27851	9.1	19	1	0	-inf	0.00435	no
gi|641564311|gb|KK858493.1|	94-820	7.9	24	7	15	1	0.34295	no
gi|641564317|gb|KK858491.1|	14319-14850	11	45	1	3	2	0.31505	no
gi|641564323|gb|KK858488.1|	13391-14075	6.5	20	2	3	0.600275	0.68145	no
gi|641564325|gb|KK858487.1|	9301-9715	5.5	22	4	4	-0.0511061	0.9564	no
gi|641564332|gb|KK858484.1|	14110-14857	7.4	25	0.898312	7	3	0.09695	no
gi|641564332|gb|KK858484.1|	15933-17164	7.4	25	31	4	-3	0.0268	no
gi|641564332|gb|KK858484.1|	18650-19095	7.4	25	5	26	3	0.09215	no
gi|641564332|gb|KK858484.1|	4780-5735	7.4	25	1	0.283417	-2	0.31075	no
gi|641564332|gb|KK858484.1|	7071-8274	7.4	25	3	0.427902	-3	0.10835	no
gi|641564334|gb|KK858483.1|	3715-4056	5.2	18	20	24	0.271254	0.8377	no
gi|641564341|gb|KK858480.1|	11115-11764	11	19	1	0.236348	-3	0.271	no
gi|641564341|gb|KK858480.1|	14373-15640	11	19	0.594592	1	1	0.49595	no
gi|641564341|gb|KK858480.1|	16222-16844	11	19	13	6	-1	0.38155	no
gi|641564341|gb|KK858480.1|	17487-17807	11	19	31	12	-1	0.32585	no
gi|641564341|gb|KK858480.1|	18605-19065	11	19	2	2	0.175423	0.9106	no
gi|641564341|gb|KK858480.1|	2113-6182	11	19	2	3	0.528348	0.6792	no
gi|641564341|gb|KK858480.1|	22047-23075	11	19	1	2	0.811692	0.5797	no
gi|641564341|gb|KK858480.1|	23228-25353	11	19	0.587059	2	1	0.31125	no
gi|641564341|gb|KK858480.1|	28004-28626	11	19	2	0.251114	-3	0.2348	no
gi|641564341|gb|KK858480.1|	6408-6943	11	19	4	5	0.425148	0.7658	no
gi|641564341|gb|KK858480.1|	7019-8845	11	19	1	1	-0.0793461	0.95365	no
gi|641564349|gb|KK858477.1|	11750-12517	7.5	27	0.495374	3	2	0.21345	no
gi|641564349|gb|KK858477.1|	14654-15807	7.5	27	7	9	0.422453	0.73595	no
gi|641564349|gb|KK858477.1|	16700-17116	7.5	27	3	16	2	0.1437	no
gi|641564349|gb|KK858477.1|	20088-21366	7.5	27	0.326973	1	2	0.3084	no

gi|641564349|gb|KK858477.1|	26638-27185	7.5	27	9	27	2	0.22215	no
gi|641564349|gb|KK858477.1|	29660-30632	7.5	27	0.45579	2	2	0.28495	no
gi|641564349|gb|KK858477.1|	5325-6320	7.5	27	5	11	1	0.3749	no
gi|641564349|gb|KK858477.1|	6488-6844	7.5	27	11	28	1	0.335	no
gi|641564349|gb|KK858477.1|	8444-9791	7.5	27	0.73772	2	1	0.39595	no
gi|641564354|gb|KK858475.1|	6922-7351	8	30	19	22	0.218583	0.86255	no
gi|641564361|gb|KK858472.1|	10490-11770	9.9	40	76	2	-6	0.0182	no
gi|641564361|gb|KK858472.1|	13296-13607	9.9	40	6	0.852841	-3	0.2476	no
gi|641564367|gb|KK858470.1|	11994-13016	8.2	33	0.685157	0.260562	-1	1	no
gi|641564367|gb|KK858470.1|	13112-15467	8.2	33	0.360621	6	4	0.0269	no
gi|641564367|gb|KK858470.1|	28048-28727	8.2	33	1	0.887375	-0.207205	0.8945	no
gi|641564369|gb|KK858469.1|	27493-27692	8.4	23	35	13	-1	0.3803	no
gi|641564369|gb|KK858469.1|	5673-6515	8.4	23	2	2	-0.0488711	0.9693	no
gi|641564381|gb|KK858464.1|	26017-29199	13	34	29	14	-1	0.63075	no
gi|641564381|gb|KK858464.1|	5928-6064	13	34	2607.97	148	-4	0.04745	no
gi|641564387|gb|KK858462.1|	15982-16742	9.9	22	111	126.16	0.188484	0.9303	no
gi|641564387|gb|KK858462.1|	18441-19275	9.9	22	0.997682	0.336766	-2	0.4266	no
gi|641564387|gb|KK858462.1|	22007-22337	9.9	22	65	115	0.820183	0.51425	no
gi|641564387|gb|KK858462.1|	22620-23001	9.9	22	66	108	0.716148	0.56405	no
gi|641564389|gb|KK858461.1|	19553-20041	54	39	25	66	1	0.26835	no
gi|641564389|gb|KK858461.1|	20482-23068	54	39	0.029616	7	8	0.10445	no
gi|641564389|gb|KK858461.1|	2077-3930	54	39	4	5	0.407135	0.74985	no
gi|641564389|gb|KK858461.1|	230-1876	54	39	2	3	0.688431	0.6082	no
gi|641564389|gb|KK858461.1|	23609-24315	54	39	182	880.43	2	0.27685	no
gi|641564389|gb|KK858461.1|	27007-27800	54	39	0	3	inf	0.00255	yes
gi|641564389|gb|KK858461.1|	31800-32174	54	39	42	22	-0.964007	0.46105	no
gi|641564389|gb|KK858461.1|	37036-37524	54	39	3	11	2	0.20595	no
gi|641564389|gb|KK858461.1|	47718-47912	54	39	2	62	5	0.10925	no
gi|641564389|gb|KK858461.1|	49154-49711	54	39	0.586448	2	2	0.2812	no
gi|641564389|gb|KK858461.1|	7820-8472	54	39	16	13	-0.265638	0.83655	no

gi|641564389|gb|KK858461.1|	8606-10104	54	39	0.869613	0.828269	-0.0702743	1	no
gi|641564395|gb|KK858458.1|	6450-7150	8.9	32	0.701337	1	0.600656	0.70505	no
gi|641564397|gb|KK858457.1|	11260-14677	9	32	7	0	-inf	5.00E-05	yes
gi|641564397|gb|KK858457.1|	15245-15887	9	32	17	0	-inf	5.00E-05	yes
gi|641564397|gb|KK858457.1|	6256-6789	9	32	3	0	-inf	0.0056	no
gi|641564397|gb|KK858457.1|	8822-9702	9	32	2	0	-inf	0.0032	yes
gi|641564397|gb|KK858457.1|	9861-10752	9	32	6	0	-inf	5.00E-05	yes
gi|641564420|gb|KK858447.1|	0-2372	13	42	16	3	-2	0.0649	no
gi|641564420|gb|KK858447.1|	12414-13327	13	42	3	0.149954	-4	0.1868	no
gi|641564420|gb|KK858447.1|	14646-15202	13	42	3	1	-1	0.5005	no
gi|641564420|gb|KK858447.1|	20660-21975	13	42	13	2	-3	0.05325	no
gi|641564420|gb|KK858447.1|	25260-27733	13	42	1	0.426921	-2	0.23385	no
gi|641564420|gb|KK858447.1|	3016-5840	13	42	17	3	-2	0.2012	no
gi|641564420|gb|KK858447.1|	43743-44985	13	42	1	0.20601	-2	0.21865	no
gi|641564420|gb|KK858447.1|	6075-6655	13	42	4	0.556231	-3	0.1793	no
gi|641564422|gb|KK858446.1|	20919-22201	9	16	2	1	-0.833676	0.54675	no
gi|641564422|gb|KK858446.1|	7357-8433	9	16	1	0.489319	-1	0.4015	no
gi|641564425|gb|KK858445.1|	12102-13308	8.8	37	0.770584	0.959933	0.316982	0.84265	no
gi|641564425|gb|KK858445.1|	23953-24611	8.8	37	0.917849	2	1	0.5082	no
gi|641564425|gb|KK858445.1|	3026-3775	8.8	37	1	3	1	0.39085	no
gi|641564425|gb|KK858445.1|	6479-7180	8.8	37	0.559961	1	1	0.44255	no
gi|641564425|gb|KK858445.1|	66-1056	8.8	37	0.712746	3	2	0.2529	no
gi|641564425|gb|KK858445.1|	8455-10031	8.8	37	0.923047	3	2	0.2205	no
gi|641564435|gb|KK858441.1|	13-532	9.1	36	0	13	inf	5.00E-05	yes
gi|641564440|gb|KK858439.1|	10765-11643	11	36	0.725964	2	1	0.44	no
gi|641564440|gb|KK858439.1|	11962-13210	11	36	0.672581	1	0.606721	0.6779	no
gi|641564440|gb|KK858439.1|	14459-17433	11	36	15	10	-0.60506	0.70525	no
gi|641564440|gb|KK858439.1|	20207-20566	11	36	34	25	-0.47528	0.7118	no
gi|641564440|gb|KK858439.1|	22983-23248	11	36	77	59	-0.379652	0.769	no
gi|641564440|gb|KK858439.1|	24293-25273	11	36	33	32	-0.0343753	0.97675	no

gi|641564440|gb|KK858439.1|	27824-28189	11	36	19	16	-0.2196	0.8693	no
gi|641564440|gb|KK858439.1|	33085-33527	11	36	109	75	-0.544759	0.66005	no
gi|641564440|gb|KK858439.1|	36161-36320	11	36	412	269.47	-0.611278	0.64115	no
gi|641564440|gb|KK858439.1|	37222-37805	11	36	1	2	0.819553	0.62335	no
gi|641564440|gb|KK858439.1|	378-2822	11	36	2	2	0.399489	0.7562	no
gi|641564440|gb|KK858439.1|	38330-38770	11	36	26	24	-0.137617	0.915	no
gi|641564440|gb|KK858439.1|	4005-6406	11	36	28	93	2	0.43115	no
gi|641564440|gb|KK858439.1|	40754-41521	11	36	57	44	-0.367091	0.7635	no
gi|641564440|gb|KK858439.1|	6686-8989	11	36	21	97	2	0.31955	no
gi|641564440|gb|KK858439.1|	9139-10195	11	36	0.411308	2	2	0.18135	no
gi|641564449|gb|KK858435.1|	10955-11819	1.3	83	0	8	inf	0.00025	yes
gi|641564449|gb|KK858435.1|	15950-16421	1.3	83	0	4	inf	0.0068	no
gi|641564449|gb|KK858435.1|	5948-7818	1.3	83	0	3	inf	5.00E-05	yes
gi|641564451|gb|KK858434.1|	12058-12266	9.6	19	95	101	0.0818716	0.95025	no
gi|641564451|gb|KK858434.1|	13205-13650	9.6	19	51	72	0.498795	0.6917	no
gi|641564457|gb|KK858431.1|	13861-15207	7.8	24	3	1	-1	0.3062	no
gi|641564457|gb|KK858431.1|	16552-17027	7.8	24	3	0.379844	-3	0.2348	no
gi|641564461|gb|KK858429.1|	18972-19325	8.1	33	0.436276	10	4	0.11235	no
gi|641564461|gb|KK858429.1|	20700-20894	8.1	33	0	83	inf	0.00075	yes
gi|641564461|gb|KK858429.1|	24039-24456	8.1	33	0.316204	24	6	0.10475	no
gi|641564461|gb|KK858429.1|	27650-27951	8.1	33	4	75	4	0.0648	no
gi|641564461|gb|KK858429.1|	30013-30367	8.1	33	4	36	3	0.0638	no
gi|641564466|gb|KK858427.1|	10589-11627	5.2	20	2	0	-inf	0.0024	yes
gi|641564466|gb|KK858427.1|	13759-14940	5.2	20	2	0	-inf	0.00315	yes
gi|641564466|gb|KK858427.1|	16819-17527	5.2	20	2	0	-inf	0.0102	no
gi|641564466|gb|KK858427.1|	19996-20283	5.2	20	8	0	-inf	0.0102	no
gi|641564466|gb|KK858427.1|	20354-21326	5.2	20	5	0	-inf	0.0002	yes
gi|641564466|gb|KK858427.1|	3868-4958	5.2	20	2	0	-inf	0.0013	yes
gi|641564466|gb|KK858427.1|	5845-7197	5.2	20	2	0	-inf	0.00185	yes
gi|641564466|gb|KK858427.1|	90-964	5.2	20	14	0	-inf	5.00E-05	yes

gi|641564468|gb|KK858426.1|	0-573	8.1	35	2	2	-0.296319	0.8456	no
gi|641564471|gb|KK858425.1|	0-1296	13	54	0.385951	31	6	0.06285	no
gi|641564471|gb|KK858425.1|	11862-12384	13	54	7	3	-1	0.3557	no
gi|641564471|gb|KK858425.1|	17345-18818	13	54	3	4	0.464751	0.73025	no
gi|641564471|gb|KK858425.1|	20587-20917	13	54	23	17	-0.425978	0.74765	no
gi|641564471|gb|KK858425.1|	21174-22635	13	54	4	2	-1	0.3422	no
gi|641564471|gb|KK858425.1|	24159-25984	13	54	1	2	0.778633	0.57205	no
gi|641564471|gb|KK858425.1|	26756-28227	13	54	3	2	-0.872291	0.52795	no
gi|641564471|gb|KK858425.1|	28582-32506	13	54	11	4	-1	0.3954	no
gi|641564493|gb|KK858415.1|	16323-16931	9.7	36	2	2	0.598068	0.6902	no
gi|641564493|gb|KK858415.1|	21206-21907	9.7	36	1	1	0.238109	0.88205	no
gi|641564493|gb|KK858415.1|	23645-24292	9.7	36	0.940144	1	0.59929	0.70555	no
gi|641564493|gb|KK858415.1|	27647-28857	9.7	36	0.488438	1	1	0.36605	no
gi|641564495|gb|KK858414.1|	10892-11603	8.6	15	4	3	-0.446401	0.7456	no
gi|641564495|gb|KK858414.1|	12749-13634	8.6	15	12	2	-3	0.0635	no
gi|641564495|gb|KK858414.1|	13974-15032	8.6	15	10	0.874091	-3	0.04615	no
gi|641564495|gb|KK858414.1|	17005-17536	8.6	15	7	2	-2	0.2192	no
gi|641564495|gb|KK858414.1|	2472-4001	8.6	15	8	7	-0.186983	0.87955	no
gi|641564502|gb|KK858411.1|	13965-14683	8.9	30	2	2	-0.206297	0.8956	no
gi|641564502|gb|KK858411.1|	15939-17070	8.9	30	0.681012	0.691025	0.0210578	1	no
gi|641564502|gb|KK858411.1|	17945-18684	8.9	30	1	0.790304	-0.568434	0.7586	no
gi|641564502|gb|KK858411.1|	19394-20178	8.9	30	0.962147	0.36517	-1	0.4534	no
gi|641564502|gb|KK858411.1|	23285-25263	8.9	30	11	13	0.181952	0.88295	no
gi|641564515|gb|KK858405.1|	15403-15725	13	41	12	9	-0.302085	0.83355	no
gi|641564518|gb|KK858404.1|	17836-18532	7.3	31	1	1	-0.247434	0.88495	no
gi|641564530|gb|KK858398.1|	26649-27502	7.5	23	0.968861	2	1	0.431	no
gi|641564530|gb|KK858398.1|	30113-30706	7.5	23	2	6	2	0.238	no
gi|641564541|gb|KK858393.1|	2-445	9.1	18	3	12	2	0.21555	no
gi|641564541|gb|KK858393.1|	4422-4812	9.1	18	1	5	2	0.2624	no
gi|641564550|gb|KK858389.1|	12016-12362	5.8	22	0.454792	6	4	0.12785	no

gi|641564550|gb|KK858389.1|	3190-3382	5.8	22	38	62	0.706597	0.6222	no
gi|641564550|gb|KK858389.1|	50-986	5.8	22	3	0.581295	-2	0.20505	no
gi|641564550|gb|KK858389.1|	5913-6653	5.8	22	31	40	0.339673	0.77805	no
gi|641564560|gb|KK858385.1|	16241-16493	9.9	35	155	13	-4	0.032	no
gi|641564560|gb|KK858385.1|	20624-22452	9.9	35	0.650483	0.066128	-3	1	no
gi|641564560|gb|KK858385.1|	24322-24995	9.9	35	5	1	-2	0.21535	no
gi|641564560|gb|KK858385.1|	31841-32852	9.9	35	1	0.396087	-2	0.4042	no
gi|641564560|gb|KK858385.1|	33445-34016	9.9	35	11	5	-1	0.35645	no
gi|641564560|gb|KK858385.1|	34978-35842	9.9	35	6	6	-0.0793047	0.9504	no
gi|641564560|gb|KK858385.1|	36069-37504	9.9	35	1	2	0.299445	0.8312	no
gi|641564560|gb|KK858385.1|	38857-39735	9.9	35	0.725964	0.630383	-0.203669	1	no
gi|641564560|gb|KK858385.1|	44898-45294	9.9	35	14	9	-0.613635	0.6499	no
gi|641564570|gb|KK858380.1|	17591-17768	9.4	39	0	83	inf	0.0015	yes
gi|641564573|gb|KK858379.1|	25819-26885	13	51	0.487901	2	2	0.2254	no
gi|641564573|gb|KK858379.1|	2639-5331	13	51	0.680608	0.865634	0.346933	1	no
gi|641564573|gb|KK858379.1|	28857-30130	13	51	0.262792	1	2	0.29935	no
gi|641564573|gb|KK858379.1|	30389-30735	13	51	0	96	inf	5.00E-05	yes
gi|641564573|gb|KK858379.1|	35868-36125	13	51	23	29	0.303453	0.8238	no
gi|641564573|gb|KK858379.1|	43198-50668	13	51	29	38	0.403761	0.8377	no
gi|641564573|gb|KK858379.1|	68049-68583	13	51	0	38	inf	5.00E-05	yes
gi|641564573|gb|KK858379.1|	82108-82710	13	51	4	2	-0.817178	0.57245	no
gi|641564577|gb|KK858377.1|	16188-17456	10	37	0.396025	2	2	0.20395	no
gi|641564577|gb|KK858377.1|	3256-4391	10	37	0.602767	0.458731	-0.393953	1	no
gi|641564580|gb|KK858375.1|	23720-24527	9.9	36	1	1	-0.397315	0.8086	no
gi|641564580|gb|KK858375.1|	26829-27376	9.9	36	2	3	1	0.49835	no
gi|641564580|gb|KK858375.1|	28664-29017	9.9	36	5	14	1	0.32465	no
gi|641564580|gb|KK858375.1|	29123-29846	9.9	36	4	13	2	0.2184	no
gi|641564582|gb|KK858374.1|	15170-16119	10	40	0.563638	0.571323	0.0195362	1	no
gi|641564585|gb|KK858372.1|	12856-13470	11	20	4	3	-0.209085	0.88325	no
gi|641564586|gb|KK858371.1|	15621-16349	9.6	36	5	3	-1	0.4566	no

gi|641564586|gb|KK858371.1|	20456-21881	9.6	36	14	5	-1	0.2779	no
gi|641564586|gb|KK858371.1|	21982-24166	9.6	36	15	4	-2	0.12315	no
gi|641564586|gb|KK858371.1|	29030-30129	9.6	36	0	6	inf	0.0001	yes
gi|641564586|gb|KK858371.1|	31196-31390	9.6	36	0	326	inf	5.00E-05	yes
gi|641564586|gb|KK858371.1|	37002-37482	9.6	36	0	87	inf	5.00E-05	yes
gi|641564586|gb|KK858371.1|	7046-7655	9.6	36	1	0.776692	-0.624291	0.71195	no
gi|641564593|gb|KK858369.1|	11003-12817	8.4	37	0.218703	1	3	0.16775	no
gi|641564593|gb|KK858369.1|	217-535	8.4	37	22	40	0.828688	0.53195	no
gi|641564593|gb|KK858369.1|	43333-43694	8.4	37	5	6	0.200972	0.89045	no
gi|641564594|gb|KK858368.1|	0-1122	8.5	35	0.152806	3	4	0.23765	no
gi|641564594|gb|KK858368.1|	13698-13918	8.5	35	5	31	3	0.1886	no
gi|641564594|gb|KK858368.1|	14432-14636	8.5	35	28	34	0.309952	0.83185	no
gi|641564594|gb|KK858368.1|	29753-31322	8.5	35	0	46	inf	0.00015	yes
gi|641564594|gb|KK858368.1|	37727-38216	8.5	35	0	4	inf	0.00375	no
gi|641564595|gb|KK858367.1|	19006-19734	9.5	40	0.79742	3	2	0.2254	no
gi|641564605|gb|KK858363.1|	2931-3835	8.4	32	0.89914	1	0.241432	0.8798	no
gi|641564605|gb|KK858363.1|	30028-30323	8.4	32	8	5	-0.696912	0.66875	no
gi|641564625|gb|KK858355.1|	19722-19882	9.4	37	63	18	-2	0.3266	no
gi|641564627|gb|KK858354.1|	24999-25242	8.9	35	51	171	2	0.1862	no
gi|641564630|gb|KK858353.1|	112-701	8.6	31	7	3	-1	0.3175	no
gi|641564630|gb|KK858353.1|	6473-7065	8.6	31	2	0.809489	-0.987462	0.62775	no
gi|641564633|gb|KK858352.1|	81-902	6.7	28	1	2	0.728431	0.63585	no
gi|641564635|gb|KK858351.1|	36605-37250	11	40	0.944314	0.953701	0.0142702	0.91115	no
gi|641564635|gb|KK858351.1|	39672-40713	11	40	195	11	-4	0.04495	no
gi|641564635|gb|KK858351.1|	41813-42393	11	40	6	3	-0.905456	0.5258	no
gi|641564635|gb|KK858351.1|	43783-44559	11	40	33	3	-4	0.02205	no
gi|641564635|gb|KK858351.1|	48538-48815	11	40	20	1	-4	0.19105	no
gi|641564635|gb|KK858351.1|	50106-50439	11	40	8	0	-inf	0.0056	no
gi|641564635|gb|KK858351.1|	8462-9170	11	40	0.414245	1	2	0.3323	no
gi|641564658|gb|KK858340.1|	12643-13770	10	35	2	0.231326	-3	0.18365	no

gi|641564658|gb|KK858340.1|	24773-25090	10	35	0.550043	80	7	0.10445	no
gi|641564663|gb|KK858338.1|	10776-10995	7.6	31	44	9	-2	0.18295	no
gi|641564663|gb|KK858338.1|	21446-21731	7.6	31	21	14	-0.642955	0.65295	no
gi|641564673|gb|KK858334.1|	25826-26607	11	45	0	3	inf	0.0015	yes
gi|641564682|gb|KK858331.1|	20511-20824	8.4	39	7	0	-inf	0.01	no
gi|641564682|gb|KK858331.1|	7498-7684	8.4	39	0	54	inf	0.0025	yes
gi|641564689|gb|KK858328.1|	23298-24640	8.4	33	1	0.188109	-3	0.18365	no
gi|641564695|gb|KK858325.1|	11039-11996	9	31	21	46	1	0.38635	no
gi|641564695|gb|KK858325.1|	13363-17001	9	31	25	58	1	0.5664	no
gi|641564695|gb|KK858325.1|	18206-18839	9	31	2	3	0.357868	0.8122	no
gi|641564695|gb|KK858325.1|	19688-19973	9	31	8	0	-inf	0.01	no
gi|641564695|gb|KK858325.1|	20270-20643	9	31	8	9	0.259356	0.8556	no
gi|641564695|gb|KK858325.1|	21908-22955	9	31	1	2	0.489921	0.7378	no
gi|641564695|gb|KK858325.1|	8597-9439	9	31	6	8	0.463028	0.72085	no
gi|641564707|gb|KK858320.1|	7698-9358	8.9	39	3	6	0.970869	0.4472	no
gi|641564707|gb|KK858320.1|	9558-10418	8.9	39	0.745623	0.971023	0.38106	0.82465	no
gi|641564718|gb|KK858315.1|	10864-12195	11	42	6	4	-0.470871	0.7115	no
gi|641564718|gb|KK858315.1|	34145-34787	11	42	0	24	inf	5.00E-05	yes
gi|641564718|gb|KK858315.1|	36327-37861	11	42	0.105778	5	6	0.23245	no
gi|641564718|gb|KK858315.1|	38851-39102	11	42	0	34	inf	0.00115	yes
gi|641564718|gb|KK858315.1|	52448-53418	11	42	13	18	0.495363	0.69185	no
gi|641564720|gb|KK858314.1|	19988-20445	9.7	45	2	1	-0.632164	0.71175	no
gi|641564725|gb|KK858312.1|	27477-27760	8.2	4.50E+01	17	0	-inf	0.0021	yes
gi|641564728|gb|KK858311.1|	15636-16597	11	43	1	2	0.382229	0.7933	no
gi|641564731|gb|KK858310.1|	32184-33257	8.2	47	1	2	0.698713	0.6288	no
gi|641564733|gb|KK858309.1|	10659-11342	11	58	44	0	-inf	5.00E-05	yes
gi|641564733|gb|KK858309.1|	9170-10224	11	58	7	0	-inf	5.00E-05	yes
gi|641564742|gb|KK858305.1|	2921-3141	13	68	15	11	-0.459811	0.77155	no
gi|641564742|gb|KK858305.1|	82-660	13	68	4	0	-inf	0.00315	yes
gi|641564742|gb|KK858305.1|	9925-10535	13	68	15	0	-inf	5.00E-05	yes

gi|641564748|gb|KK858302.1|	8586-8803	10	48	0	34	inf	0.0023	yes
gi|641564751|gb|KK858301.1|	11535-12353	9.7	34	0.454808	2	2	0.33105	no
gi|641564751|gb|KK858301.1|	18919-19312	9.7	34	2	4	1	0.5126	no
gi|641564751|gb|KK858301.1|	19424-20121	9.7	34	1	2	0.278659	0.8559	no
gi|641564751|gb|KK858301.1|	29131-29768	9.7	34	3	4	0.446994	0.75555	no
gi|641564751|gb|KK858301.1|	30003-31972	9.7	34	3	6	1	0.398	no
gi|641564751|gb|KK858301.1|	32513-32816	9.7	34	4	5	0.568105	0.7575	no
gi|641564753|gb|KK858300.1|	10352-10969	7.6	25	3	2	-0.723559	0.652	no
gi|641564753|gb|KK858300.1|	11056-11716	7.6	25	3	4	0.512191	0.72575	no
gi|641564753|gb|KK858300.1|	16000-17942	7.6	25	0.040535	2	5	0.1059	no
gi|641564753|gb|KK858300.1|	32388-32769	7.6	25	0	119	inf	5.00E-05	yes
gi|641564753|gb|KK858300.1|	3873-4507	7.6	25	6	5	-0.475094	0.7223	no
gi|641564753|gb|KK858300.1|	7999-8411	7.6	25	0.969717	3	2	0.3323	no
gi|641564756|gb|KK858299.1|	12703-13042	10	41	0	19	inf	0.00075	yes
gi|641564756|gb|KK858299.1|	13474-14054	10	41	0	75	inf	5.00E-05	yes
gi|641564756|gb|KK858299.1|	27979-28290	10	41	5	12	1	0.4461	no
gi|641564756|gb|KK858299.1|	9965-10535	10	41	0	4	inf	0.0023	yes
gi|641564761|gb|KK858297.1|	10258-12700	13	44	0.252114	5	4	0.04865	no
gi|641564761|gb|KK858297.1|	13086-15111	13	44	0.580447	2	2	0.2243	no
gi|641564761|gb|KK858297.1|	16862-17735	13	44	0.522372	2	2	0.3084	no
gi|641564761|gb|KK858297.1|	18949-19223	13	44	0	53	inf	0.00025	yes
gi|641564761|gb|KK858297.1|	28118-28350	13	44	1	61	6	0.10555	no
gi|641564761|gb|KK858297.1|	28893-29626	13	44	0.263337	10	5	0.23325	no
gi|641564761|gb|KK858297.1|	32803-33136	13	44	7	0	-inf	0.0059	no
gi|641564766|gb|KK858295.1|	27672-28824	5.6	27	1	2	1	0.4227	no
gi|641564775|gb|KK858291.1|	17836-18334	9.8	31	5	0	-inf	0.00345	yes
gi|641564775|gb|KK858291.1|	7781-8086	9.8	31	17	0	-inf	0.0013	yes
gi|641564778|gb|KK858290.1|	20203-21022	6.3	23	1	0.517218	-2	0.40415	no
gi|641564781|gb|KK858289.1|	5147-5965	8.8	35	1	0.172681	-3	0.22425	no
gi|641564793|gb|KK858284.1|	12347-13570	8.7	32	3	4	0.41394	0.7497	no

gi|641564793|gb|KK858284.1|	16133-16721	8.7	32	0.540412	2	2	0.3323	no
gi|641564793|gb|KK858284.1|	2553-3969	8.7	32	11	6	-0.896273	0.47355	no
gi|641564793|gb|KK858284.1|	29669-30067	8.7	32	5	2	-1	0.451	no
gi|641564793|gb|KK858284.1|	46-909	8.7	32	261.28	115	-1	0.58665	no
gi|641564796|gb|KK858283.1|	1263-1439	10	39	157	80	-0.968026	0.48525	no
gi|641564796|gb|KK858283.1|	4756-4996	10	39	67	30	-1	0.39845	no
gi|641564796|gb|KK858283.1|	5073-6381	10	39	25	14	-0.876961	0.4813	no
gi|641564801|gb|KK858281.1|	13649-14720	5.7	24	0.404262	2	2	0.2717	no
gi|641564801|gb|KK858281.1|	1862-2897	5.7	24	0.168638	1	3	0.2991	no
gi|641564801|gb|KK858281.1|	36440-36671	5.7	24	29	2	-4	0.1967	no
gi|641564809|gb|KK858278.1|	25119-25915	9	35	0	2	inf	0.0068	no
gi|641564822|gb|KK858272.1|	19169-19629	7.2	32	2	3	0.227891	0.88235	no
gi|641564822|gb|KK858272.1|	27292-28783	7.2	32	3	3	0.240991	0.8536	no
gi|641564826|gb|KK858270.1|	6025-6553	7.8	34	0.42476	5	4	0.2497	no
gi|641564833|gb|KK858267.1|	2798-3864	7.3	21	2	2	0.0694968	0.95855	no
gi|641564833|gb|KK858267.1|	52-836	7.3	21	4	0.547755	-3	0.13665	no
gi|641564836|gb|KK858266.1|	30598-30970	9.5	39	1	4	2	0.3454	no
gi|641564841|gb|KK858264.1|	12115-13521	6.6	22	6	2	-2	0.1771	no
gi|641564841|gb|KK858264.1|	2313-3433	6.6	22	4	11	1	0.28785	no
gi|641564845|gb|KK858262.1|	14128-14648	9	41	0.87025	17	4	0.11805	no
gi|641564847|gb|KK858261.1|	0-715	11	22	4	4	-0.265741	0.841	no
gi|641564847|gb|KK858261.1|	1193-1887	11	22	5	4	-0.317022	0.81845	no
gi|641564847|gb|KK858261.1|	18336-18824	11	22	4	1	-2	0.28145	no
gi|641564847|gb|KK858261.1|	20585-22195	11	22	0.150144	0.991752	3	0.1879	no
gi|641564847|gb|KK858261.1|	23065-23367	11	22	6	0.91184	-3	0.2588	no
gi|641564847|gb|KK858261.1|	3881-4424	11	22	5	2	-2	0.28755	no
gi|641564847|gb|KK858261.1|	8640-9110	11	22	3	3	0.0606887	0.95525	no
gi|641564850|gb|KK858260.1|	6656-7484	8.7	37	0.447619	1	1	0.51305	no
gi|641564861|gb|KK858256.1|	0-999	11	43	6	3	-1	0.40985	no
gi|641564861|gb|KK858256.1|	14441-15735	11	43	2	1	-0.734081	0.59225	no

gi|641564861|gb|KK858256.1|	3295-4399	11	43	0.389581	0.711465	0.868867	1	no
gi|641564861|gb|KK858256.1|	6925-8010	11	43	0.07958	5	6	0.10485	no
gi|641564877|gb|KK858250.1|	6121-6791	10	22	3	14	2	0.1169	no
gi|641564877|gb|KK858250.1|	9422-9748	10	22	6	24	2	0.1755	no
gi|641564877|gb|KK858250.1|	9887-10196	10	22	3	6	1	0.5132	no
gi|641564883|gb|KK858248.1|	24593-25252	10	21	0.152646	3	4	0.1175	no
gi|641564883|gb|KK858248.1|	33221-34082	10	21	0.212715	2	4	0.24945	no
gi|641564883|gb|KK858248.1|	9658-11027	10	21	0.301622	1	2	0.28495	no
gi|641564885|gb|KK858247.1|	35186-35688	2.8	150	3	2	-0.406706	0.7964	no
gi|641564894|gb|KK858244.1|	5976-6734	7.4	26	0.880763	1	0.601852	0.69715	no
gi|641564897|gb|KK858243.1|	17470-19078	12	44	3	2	-0.546177	0.66955	no
gi|641564897|gb|KK858243.1|	23269-25258	12	44	6	2	-2	0.2378	no
gi|641564904|gb|KK858240.1|	25664-27310	7.9	38	0	35	inf	0.0006	yes
gi|641564911|gb|KK858237.1|	36497-36759	8.3	37	0	114.17	inf	0.0001	yes
gi|641564915|gb|KK858235.1|	16646-17158	12	21	1	2	0.330834	0.83525	no
gi|641564915|gb|KK858235.1|	18670-20000	12	21	1	0.285137	-2	0.28145	no
gi|641564915|gb|KK858235.1|	22131-23271	12	21	1	0.912627	-0.563845	0.7148	no
gi|641564915|gb|KK858235.1|	2475-2931	12	21	3	0.406593	-3	0.2348	no
gi|641564915|gb|KK858235.1|	30255-30522	12	21	26	9	-2	0.28235	no
gi|641564915|gb|KK858235.1|	5016-5558	12	21	3	0.308037	-3	0.217	no
gi|641564915|gb|KK858235.1|	7030-8291	12	21	2	2	-0.293675	0.83	no
gi|641564920|gb|KK858233.1|	10737-11545	11	23	0.924461	0.877404	-0.0753715	0.92685	no
gi|641564920|gb|KK858233.1|	13828-14154	11	23	52	82	0.657279	0.60625	no
gi|641564920|gb|KK858233.1|	21771-23001	11	23	1	0.520975	-1	0.47035	no
gi|641564920|gb|KK858233.1|	23169-24596	11	23	2	2	-0.32535	0.8079	no
gi|641564920|gb|KK858233.1|	24668-26428	11	23	1	1	0.0521637	0.969	no
gi|641564920|gb|KK858233.1|	27113-27569	11	23	12	9	-0.442272	0.7371	no
gi|641564920|gb|KK858233.1|	29911-30204	11	23	34	23	-0.549868	0.6781	no
gi|641564920|gb|KK858233.1|	30334-31244	11	23	3	2	-0.440322	0.74975	no
gi|641564920|gb|KK858233.1|	31487-32794	11	23	0.636887	1	1	0.4325	no

gi|641564920|gb|KK858233.1|	3632-4114	11	23	3	0.370839	-3	0.2381	no
gi|641564920|gb|KK858233.1|	42029-42814	11	23	0.600322	1	1	0.50945	no
gi|641564920|gb|KK858233.1|	44591-45204	11	23	4	5	0.527847	0.70535	no
gi|641564926|gb|KK858230.1|	11515-12079	9.4	14	1	5	2	0.2254	no
gi|641564930|gb|KK858228.1|	16232-17296	6	16	0	6	inf	0.0001	yes
gi|641564930|gb|KK858228.1|	23457-23627	6	16	80	38	-1	0.48965	no
gi|641564942|gb|KK858224.1|	14411-14601	9.2	31	32	170	2	0.11775	no
gi|641564942|gb|KK858224.1|	5523-6786	9.2	31	1	4	2	0.2508	no
gi|641564942|gb|KK858224.1|	8852-9681	9.2	31	3	8	1	0.3744	no
gi|641564948|gb|KK858221.1|	17457-17893	6.9	31	3	3	-0.286007	0.86025	no
gi|641564963|gb|KK858215.1|	27186-27765	9.8	42	5	332	6	0.0153	no
gi|641564963|gb|KK858215.1|	35460-35978	9.8	42	3	49	4	0.02195	no
gi|641564963|gb|KK858215.1|	37350-37653	9.8	42	4	76	4	0.0649	no
gi|641564966|gb|KK858214.1|	14528-15636	9.6	39	1	0.472241	-1	0.50315	no
gi|641564966|gb|KK858214.1|	26418-27577	9.6	39	7	3	-1	0.437	no
gi|641564966|gb|KK858214.1|	27627-28236	9.6	39	7	1	-2	0.1354	no
gi|641564972|gb|KK858211.1|	10169-10791	9.2	36	2	0.251114	-3	0.22425	no
gi|641564972|gb|KK858211.1|	10899-12531	9.2	36	1	0.225371	-2	0.1864	no
gi|641564972|gb|KK858211.1|	15544-16376	9.2	36	1	0	-inf	0.0102	no
gi|641564972|gb|KK858211.1|	20038-20615	9.2	36	2	0	-inf	0.01	no
gi|641564972|gb|KK858211.1|	35706-36948	9.2	36	0.135287	2	4	0.23955	no
gi|641564972|gb|KK858211.1|	7790-8652	9.2	36	1	2	0.224967	0.87445	no
gi|641564972|gb|KK858211.1|	8765-9533	9.2	36	0.865392	0.938241	0.116606	0.92175	no
gi|641565001|gb|KK858200.1|	18786-19412	7.8	39	3	2	-0.841914	0.5768	no
gi|641565004|gb|KK858199.1|	20312-21005	9.1	43	2	2	-0.0515841	0.94545	no
gi|641565012|gb|KK858195.1|	14560-15139	12	5.00E+01	0.737362	2	1	0.51315	no
gi|641565015|gb|KK858194.1|	11648-12800	7.6	32	0.740071	2	1	0.4713	no
gi|641565015|gb|KK858194.1|	13844-14372	7.6	32	3	3	0.0642078	0.9548	no
gi|641565015|gb|KK858194.1|	21312-21702	7.6	32	5	4	-0.416432	0.7699	no
gi|641565017|gb|KK858193.1|	15683-16981	5.7	18	7	4	-0.676818	0.60885	no

gi|641565017|gb|KK858193.1|	17387-17876	5.7	18	6	10	0.64694	0.6376	no
gi|641565017|gb|KK858193.1|	24711-25287	5.7	18	8	3	-2	0.27645	no
gi|641565021|gb|KK858191.1|	11241-11501	10	36	71	13	-2	0.1029	no
gi|641565021|gb|KK858191.1|	13539-14640	10	36	2	0.356905	-2	0.2338	no
gi|641565021|gb|KK858191.1|	24810-24985	10	36	92	11	-3	0.1604	no
gi|641565021|gb|KK858191.1|	26408-26740	10	36	5	3	-0.885016	0.60065	no
gi|641565021|gb|KK858191.1|	29249-29525	10	36	15	0	-inf	0.00345	yes
gi|641565021|gb|KK858191.1|	3-3849	10	36	2	1	-0.287404	0.81815	no
gi|641565021|gb|KK858191.1|	39543-39745	10	36	61	15	-2	0.20845	no
gi|641565021|gb|KK858191.1|	43373-44113	10	36	1	2	0.279582	0.8559	no
gi|641565021|gb|KK858191.1|	46513-48276	10	36	2	1	-0.748988	0.58305	no
gi|641565021|gb|KK858191.1|	48397-49260	10	36	2	0.161115	-4	0.20475	no
gi|641565021|gb|KK858191.1|	6216-6380	10	36	60	23	-1	0.43985	no
gi|641565021|gb|KK858191.1|	62412-62818	10	36	7	29	2	0.1363	no
gi|641565021|gb|KK858191.1|	8499-9803	10	36	2	0.77813	-1	0.3656	no
gi|641565024|gb|KK858190.1|	22758-23187	7.8	33	0.90141	3	2	0.3323	no
gi|641565024|gb|KK858190.1|	23316-24061	7.8	33	0.643984	1	1	0.51085	no
gi|641565032|gb|KK858187.1|	27036-27275	2.4	21	20	10	-0.954134	0.55365	no
gi|641565035|gb|KK858186.1|	19086-19707	8.5	35	1	0.503392	-1	0.4534	no
gi|641565038|gb|KK858185.1|	10690-12143	9.8	35	0.900727	0.514684	-0.807402	0.5996	no
gi|641565038|gb|KK858185.1|	12259-13623	9.8	35	0.666407	0.461451	-0.530226	1	no
gi|641565038|gb|KK858185.1|	14455-14921	9.8	35	22	9	-1	0.313	no
gi|641565038|gb|KK858185.1|	19048-19467	9.8	35	5	2	-1	0.4599	no
gi|641565038|gb|KK858185.1|	20842-22387	9.8	35	76	39	-0.948496	0.66315	no
gi|641565038|gb|KK858185.1|	22489-23018	9.8	35	19	13	-0.539528	0.67635	no
gi|641565038|gb|KK858185.1|	2436-3782	9.8	35	14	4	-2	0.206	no
gi|641565038|gb|KK858185.1|	24447-29645	9.8	35	1	0.929593	-0.591937	0.6411	no
gi|641565038|gb|KK858185.1|	29790-35163	9.8	35	4	3	-0.241099	0.84995	no
gi|641565038|gb|KK858185.1|	35298-36369	9.8	35	5	5	0.158129	0.90345	no
gi|641565038|gb|KK858185.1|	36638-39012	9.8	35	3	6	0.798656	0.5306	no

gi|641565038|gb|KK858185.1|	46872-48128	9.8	35	0.600758	0.304953	-0.978199	1	no
gi|641565038|gb|KK858185.1|	48620-50739	9.8	35	5	4	-0.348303	0.7822	no
gi|641565038|gb|KK858185.1|	51191-52776	9.8	35	15	17	0.140814	0.9096	no
gi|641565038|gb|KK858185.1|	5325-5846	9.8	35	34	8	-2	0.1304	no
gi|641565038|gb|KK858185.1|	56433-57328	9.8	35	3	0.461368	-2	0.1864	no
gi|641565038|gb|KK858185.1|	58426-58819	9.8	35	10	8	-0.170947	0.90085	no
gi|641565038|gb|KK858185.1|	600-2222	9.8	35	2	0.60519	-2	0.27425	no
gi|641565038|gb|KK858185.1|	6403-6559	9.8	35	861.2	146	-3	0.0845	no
gi|641565038|gb|KK858185.1|	9758-10048	9.8	35	9	4	-1	0.45045	no
gi|641565041|gb|KK858184.1|	14690-14922	7.7	29	19	7	-1	0.4143	no
gi|641565041|gb|KK858184.1|	4959-5216	7.7	29	16	4	-2	0.2851	no
gi|641565056|gb|KK858179.1|	172-1104	9.1	17	2	3	0.68678	0.6336	no
gi|641565056|gb|KK858179.1|	7133-8150	9.1	17	1	1	-0.394854	0.7971	no
gi|641565056|gb|KK858179.1|	9421-9986	9.1	17	0.956399	1	0.596435	0.70505	no
gi|641565060|gb|KK858177.1|	1672-2463	9.3	40	20	11	-0.809699	0.5242	no
gi|641565060|gb|KK858177.1|	2871-5222	9.3	40	3	2	-0.133249	0.91365	no
gi|641565060|gb|KK858177.1|	6659-7589	9.3	40	8	11	0.416669	0.74215	no
gi|641565072|gb|KK858172.1|	14939-15556	9.8	21	0	16	inf	5.00E-05	yes
gi|641565075|gb|KK858171.1|	10499-11045	8.8	34	1	2	0.817989	0.6234	no
gi|641565075|gb|KK858171.1|	11162-11690	8.8	34	1	2	0.594723	0.70505	no
gi|641565075|gb|KK858171.1|	12595-13689	8.8	34	0.23635	1	3	0.19335	no
gi|641565075|gb|KK858171.1|	13927-15395	8.8	34	0.667583	2	2	0.2964	no
gi|641565075|gb|KK858171.1|	7768-8402	8.8	34	0.645281	3	2	0.262	no
gi|641565080|gb|KK858169.1|	10625-11413	11	39	5	4	-0.361098	0.78565	no
gi|641565080|gb|KK858169.1|	11836-12800	11	39	3	0.700292	-2	0.23545	no
gi|641565080|gb|KK858169.1|	12956-13794	11	39	0.330491	2	2	0.25595	no
gi|641565080|gb|KK858169.1|	15703-16446	11	39	1	0.980725	-0.535934	0.7478	no
gi|641565080|gb|KK858169.1|	19185-20529	11	39	0.493098	0.65724	0.414545	1	no
gi|641565080|gb|KK858169.1|	20697-25184	11	39	12	6	-1	0.5073	no
gi|641565080|gb|KK858169.1|	25817-27120	11	39	24	10	-1	0.3036	no

gi|641565080|gb|KK858169.1|	28939-29434	11	39	17	8	-1	0.3927	no
gi|641565080|gb|KK858169.1|	29667-31487	11	39	18	13	-0.521888	0.67405	no
gi|641565080|gb|KK858169.1|	33217-35582	11	39	0.424224	204	9	0.01525	no
gi|641565082|gb|KK858168.1|	10438-10971	9.7	33	0.837054	3	2	0.33255	no
gi|641565082|gb|KK858168.1|	12248-12703	9.7	33	14	26	0.946214	0.4649	no
gi|641565082|gb|KK858168.1|	14815-15547	9.7	33	0.263828	2	3	0.2672	no
gi|641565085|gb|KK858167.1|	20383-21825	8.8	33	3	0.865351	-2	0.18185	no
gi|641565085|gb|KK858167.1|	22600-23841	8.8	33	3	1	-1	0.45995	no
gi|641565088|gb|KK858166.1|	1933-3112	7.4	34	5	6	0.436403	0.73105	no
gi|641565088|gb|KK858166.1|	21035-21420	7.4	34	3	2	-0.639464	0.71075	no
gi|641565088|gb|KK858166.1|	22074-22692	7.4	34	1	5	2	0.1921	no
gi|641565088|gb|KK858166.1|	29-285	7.4	34	12	6	-1	0.48875	no
gi|641565088|gb|KK858166.1|	35127-35352	7.4	34	71	132	0.892409	0.49495	no
gi|641565088|gb|KK858166.1|	37408-37623	7.4	34	24	52	1	0.44805	no
gi|641565091|gb|KK858165.1|	16868-17308	9.5	38	0.574652	8	4	0.2432	no
gi|641565091|gb|KK858165.1|	20348-20780	9.5	38	2	4	1	0.47105	no
gi|641565091|gb|KK858165.1|	23647-24163	9.5	38	0.880996	2	1	0.51315	no
gi|641565106|gb|KK858159.1|	20822-22022	8.6	32	0	9	inf	5.00E-05	yes
gi|641565109|gb|KK858158.1|	270-1157	7.5	37	2	2	0.0563102	0.9652	no
gi|641565109|gb|KK858158.1|	9333-9544	7.5	37	111	138.7	0.317232	0.8075	no
gi|641565112|gb|KK858157.1|	12768-13118	9.7	34	8	21	1	0.3238	no
gi|641565112|gb|KK858157.1|	15630-16853	9.7	34	3	7	1	0.39665	no
gi|641565112|gb|KK858157.1|	19181-19604	9.7	34	16	31	0.952887	0.4639	no
gi|641565112|gb|KK858157.1|	25941-26170	9.7	34	22	67	2	0.25945	no
gi|641565112|gb|KK858157.1|	26307-26554	9.7	34	30	103.86	2	0.1951	no
gi|641565112|gb|KK858157.1|	28318-28756	9.7	34	10	21	1	0.40925	no
gi|641565112|gb|KK858157.1|	29680-30103	9.7	34	15	50	2	0.19005	no
gi|641565112|gb|KK858157.1|	30532-32217	9.7	34	2	2	-0.069705	0.95925	no
gi|641565112|gb|KK858157.1|	34664-35563	9.7	34	2	5	1	0.39515	no
gi|641565112|gb|KK858157.1|	3469-4156	9.7	34	3	0.21827	-4	0.20475	no

gi|641565112|gb|KK858157.1|	37592-38643	9.7	34	9	22	1	0.2929	no
gi|641565112|gb|KK858157.1|	39710-40933	9.7	34	5	17	2	0.21515	no
gi|641565112|gb|KK858157.1|	47480-48430	9.7	34	2	4	1	0.3291	no
gi|641565112|gb|KK858157.1|	5870-6915	9.7	34	1	0.760628	-0.510095	0.7498	no
gi|641565112|gb|KK858157.1|	7832-12055	9.7	34	4	2	-0.732616	0.5633	no
gi|641565112|gb|KK858157.1|	9-307	9.7	34	5	18	2	0.25775	no
gi|641565117|gb|KK858155.1|	33299-34032	10	40	0.395006	2	2	0.2235	no
gi|641565129|gb|KK858151.1|	13534-13701	2.8	20	15	91	3	0.1934	no
gi|641565138|gb|KK858148.1|	10278-11645	10	31	2	2	0.0508974	0.969	no
gi|641565138|gb|KK858148.1|	21607-22296	10	31	0.143388	2	4	0.1276	no
gi|641565152|gb|KK858143.1|	16344-17622	8.3	28	3	2	-0.627589	0.63825	no
gi|641565152|gb|KK858143.1|	33293-33573	8.3	28	10	9	-0.138324	0.92835	no
gi|641565158|gb|KK858141.1|	27163-28680	9.7	45	0.642834	2	1	0.36145	no
gi|641565158|gb|KK858141.1|	2780-3553	9.7	45	0.612789	2	2	0.32945	no
gi|641565158|gb|KK858141.1|	36750-37265	9.7	45	2	1	-0.405966	0.82205	no
gi|641565160|gb|KK858140.1|	25884-26699	6.2	32	2	0.867558	-1	0.4703	no
gi|641565162|gb|KK858139.1|	29789-31113	8.4	31	3	0	-inf	0.00015	yes
gi|641565165|gb|KK858138.1|	15038-16115	8	29	1	0.610959	-0.979328	0.57205	no
gi|641565172|gb|KK858135.1|	68-378	6.8	21	1	9	3	0.27615	no
gi|641565172|gb|KK858135.1|	9408-9641	6.8	21	50	0	-inf	0.00095	yes
gi|641565175|gb|KK858134.1|	1-1280	12	46	2	2	0.606882	0.65855	no
gi|641565175|gb|KK858134.1|	10817-11524	12	46	0	47	inf	5.00E-05	yes
gi|641565175|gb|KK858134.1|	12720-13176	12	46	0	27	inf	5.00E-05	yes
gi|641565175|gb|KK858134.1|	14683-15420	12	46	0	17	inf	5.00E-05	yes
gi|641565175|gb|KK858134.1|	15669-16270	12	46	2	1	-0.402175	0.8125	no
gi|641565175|gb|KK858134.1|	17978-19711	12	46	0	25	inf	0.0009	yes
gi|641565175|gb|KK858134.1|	23407-24681	12	46	0.131274	12	7	0.2326	no
gi|641565175|gb|KK858134.1|	30064-30235	12	46	30	19	-0.707689	0.6977	no
gi|641565175|gb|KK858134.1|	32434-34026	12	46	0.405544	0.849958	1	1	no
gi|641565175|gb|KK858134.1|	35574-36641	12	46	0.487341	1	1	0.47015	no

gi|641565175|gb|KK858134.1|	5259-6696	12	46	2	2	0.312119	0.8213	no
gi|641565175|gb|KK858134.1|	8597-9081	12	46	111	81	-0.449653	0.71565	no
gi|641565183|gb|KK858131.1|	19430-19760	8.1	36	7	4	-0.541455	0.7341	no
gi|641565183|gb|KK858131.1|	3121-5829	8.1	36	23	28	0.30077	0.891	no
gi|641565183|gb|KK858131.1|	32352-32534	8.1	36	32	18	-0.801255	0.633	no
gi|641565183|gb|KK858131.1|	85-849	8.1	36	0.37349	2	2	0.256	no
gi|641565194|gb|KK858126.1|	10841-11793	11	35	0.093567	2	5	0.11	no
gi|641565194|gb|KK858126.1|	12543-13443	11	35	0.100472	2	4	0.11365	no
gi|641565194|gb|KK858126.1|	15265-15979	11	35	0	8	inf	5.00E-05	yes
gi|641565194|gb|KK858126.1|	16080-16651	11	35	0.376487	6	4	0.2387	no
gi|641565194|gb|KK858126.1|	21210-22053	11	35	0.437253	2	2	0.3012	no
gi|641565194|gb|KK858126.1|	28490-29430	11	35	1	1	0.0194417	0.95145	no
gi|641565194|gb|KK858126.1|	29542-29913	11	35	2	5	2	0.3332	no
gi|641565194|gb|KK858126.1|	30930-31093	11	35	28.18	56	0.995648	0.60445	no
gi|641565194|gb|KK858126.1|	3195-3395	11	35	9	68	3	0.15175	no
gi|641565194|gb|KK858126.1|	7091-8261	11	35	0.217921	2	3	0.1666	no
gi|641565194|gb|KK858126.1|	8424-9066	11	35	0.792199	3	2	0.31505	no
gi|641565194|gb|KK858126.1|	9311-9855	11	35	0	4	inf	0.00255	yes
gi|641565202|gb|KK858122.1|	13869-14619	9.2	35	0.255273	3	4	0.24715	no
gi|641565204|gb|KK858121.1|	22835-24747	7.9	35	135	216.88	0.680955	0.72205	no
gi|641565208|gb|KK858119.1|	21364-22160	9.8	48	1	4	2	0.2242	no
gi|641565211|gb|KK858118.1|	11584-12696	9.6	16	2	4	0.995836	0.46925	no
gi|641565211|gb|KK858118.1|	13889-14846	9.6	16	1	1	-0.202774	0.8956	no
gi|641565211|gb|KK858118.1|	31-831	9.6	16	16	12	-0.471974	0.7107	no
gi|641565211|gb|KK858118.1|	3567-4191	9.6	16	17	13	-0.401405	0.7468	no
gi|641565211|gb|KK858118.1|	5629-7013	9.6	16	3	2	-0.764606	0.57995	no
gi|641565211|gb|KK858118.1|	8346-8760	9.6	16	9	6	-0.520591	0.71	no
gi|641565211|gb|KK858118.1|	9318-9994	9.6	16	2	2	-0.177533	0.90465	no
gi|641565213|gb|KK858117.1|	35624-36428	7	29	0.116317	4	5	0.1072	no
gi|641565220|gb|KK858114.1|	12065-12837	9.6	37	2	5	1	0.45035	no

gi|641565220|gb|KK858114.1|	29126-30084	9.6	37	0	2	inf	0.0023	yes
gi|641565220|gb|KK858114.1|	40484-41160	9.6	37	0.147259	2	4	0.12385	no
gi|641565220|gb|KK858114.1|	41643-42464	9.6	37	0.226313	3	4	0.24315	no
gi|641565220|gb|KK858114.1|	43874-44465	9.6	37	0	3	inf	0.00375	no
gi|641565220|gb|KK858114.1|	44531-46232	9.6	37	0	2	inf	0.00025	yes
gi|641565220|gb|KK858114.1|	54692-55184	9.6	37	0	5	inf	0.0025	yes
gi|641565223|gb|KK858113.1|	17978-18726	9.1	38	1	0.971924	-0.398335	0.81255	no
gi|641565225|gb|KK858112.1|	23511-24546	8.9	29	0.337275	1	2	0.26155	no
gi|641565228|gb|KK858111.1|	14886-15041	5	100	0	109	inf	0.0068	no
gi|641565228|gb|KK858111.1|	18771-20236	5	100	2	2	-0.31832	0.8114	no
gi|641565231|gb|KK858110.1|	10000-10386	8.1	1.50E+01	15	12	-0.279438	0.83515	no
gi|641565233|gb|KK858109.1|	16207-16614	8.3	24	9	2	-2	0.1912	no
gi|641565233|gb|KK858109.1|	28808-29045	8.3	24	2	26	3	0.2508	no
gi|641565233|gb|KK858109.1|	37048-37485	8.3	24	10	51	2	0.09225	no
gi|641565233|gb|KK858109.1|	39090-39758	8.3	24	0.449237	3	3	0.19335	no
gi|641565235|gb|KK858108.1|	12494-13322	9.5	34	0.671429	0.679878	0.0180416	1	no
gi|641565235|gb|KK858108.1|	19375-20052	9.5	34	1	0.891021	-0.207255	0.8945	no
gi|641565240|gb|KK858106.1|	6907-7519	8.3	22	1	2	0.768128	0.6262	no
gi|641565240|gb|KK858106.1|	7859-8031	8.3	22	29	48	0.707898	0.6643	no
gi|641565245|gb|KK858104.1|	10847-12084	8.2	33	1	3	0.84767	0.5436	no
gi|641565251|gb|KK858102.1|	10761-11141	11	30	3	3	0.0968348	0.92385	no
gi|641565251|gb|KK858102.1|	14485-14700	11	30	16	40	1	0.39115	no
gi|641565251|gb|KK858102.1|	23610-24228	11	30	0.167406	3	4	0.1196	no
gi|641565251|gb|KK858102.1|	27889-28954	11	30	1	2	1	0.468	no
gi|641565251|gb|KK858102.1|	46073-47057	11	30	0.359084	1	2	0.33105	no
gi|641565251|gb|KK858102.1|	48015-49799	11	30	0.089133	37	9	0.23555	no
gi|641565251|gb|KK858102.1|	52258-53358	11	30	2	2	-0.178467	0.89445	no
gi|641565253|gb|KK858101.1|	44095-45068	8.4	35	0.546245	3	2	0.19145	no
gi|641565253|gb|KK858101.1|	46347-46992	8.4	35	1	5	2	0.2545	no
gi|641565264|gb|KK858097.1|	19623-22577	7.9	39	0	18	inf	0.0007	yes

gi|641565279|gb|KK858092.1|	32229-32845	7.4	32	13	12	-0.04552	0.97015	no
gi|641565288|gb|KK858089.1|	12770-13328	8.5	36	110.34	409	2	0.40675	no
gi|641565288|gb|KK858089.1|	17357-18546	8.5	36	0.49876	2	2	0.2915	no
gi|641565293|gb|KK858087.1|	10592-12116	12	39	0.852596	0.812111	-0.0701856	1	no
gi|641565293|gb|KK858087.1|	12356-12771	12	39	2	2	-0.220927	0.8945	no
gi|641565293|gb|KK858087.1|	13596-14182	12	39	2	0.821731	-1	0.47135	no
gi|641565293|gb|KK858087.1|	16062-16849	12	39	1	0.363331	-2	0.3455	no
gi|641565293|gb|KK858087.1|	17357-17790	12	39	2	0.888658	-1	0.4534	no
gi|641565293|gb|KK858087.1|	18842-20891	12	39	1	1	-0.230389	0.86755	no
gi|641565293|gb|KK858087.1|	21065-21885	12	39	0.793365	1	0.602885	0.69715	no
gi|641565293|gb|KK858087.1|	21943-22363	12	39	3	0.937654	-2	0.3216	no
gi|641565293|gb|KK858087.1|	4889-5736	12	39	0.869139	0.660181	-0.396725	1	no
gi|641565293|gb|KK858087.1|	8763-10540	12	39	2	2	-0.48656	0.715	no
gi|641565307|gb|KK858081.1|	23370-23720	18	87	16	8	-0.966632	0.49595	no
gi|641565310|gb|KK858080.1|	11454-11694	8.6	18	40	2	-5	0.18655	no
gi|641565310|gb|KK858080.1|	32235-32807	8.6	18	2	0.283925	-3	0.2481	no
gi|641565310|gb|KK858080.1|	482-711	8.6	18	26	6	-2	0.22345	no
gi|641565321|gb|KK858076.1|	23493-23743	8.6	37	2	49	5	0.23505	no
gi|641565332|gb|KK858072.1|	1449-1942	10	36	3	0.357506	-3	0.2381	no
gi|641565348|gb|KK858065.1|	16222-17475	7	34	1	2	1	0.45425	no
gi|641565351|gb|KK858064.1|	5117-5521	7.2	18	3	4	0.415311	0.78025	no
gi|641565356|gb|KK858062.1|	26809-27035	12	47	0	22	inf	0.00455	no
gi|641565356|gb|KK858062.1|	8595-9041	12	47	4	1	-2	0.40265	no
gi|641565358|gb|KK858061.1|	13431-15510	13	50	1	3	1	0.338	no
gi|641565358|gb|KK858061.1|	16497-18060	13	50	0.569446	0.236707	-1	1	no
gi|641565358|gb|KK858061.1|	39454-40653	13	50	0.070542	2	5	0.10875	no
gi|641565358|gb|KK858061.1|	4879-5239	13	50	29	11	-1	0.32535	no
gi|641565358|gb|KK858061.1|	5531-6208	13	50	2	1	-0.778412	0.6308	no
gi|641565361|gb|KK858060.1|	20900-21919	11	44	4	6	0.539575	0.6777	no
gi|641565361|gb|KK858060.1|	2161-2730	11	44	2	1	-0.0814699	0.92675	no

gi|641565361|gb|KK858060.1|	23750-24531	11	44	2	0.55054	-2	0.40415	no
gi|641565361|gb|KK858060.1|	24665-28836	11	44	1	2	0.610849	0.63265	no
gi|641565361|gb|KK858060.1|	42357-43997	11	44	0.147026	1	3	0.1719	no
gi|641565361|gb|KK858060.1|	4428-6399	11	44	4	7	1	0.41355	no
gi|641565361|gb|KK858060.1|	7692-10746	11	44	0.916401	2	0.758366	0.56705	no
gi|641565368|gb|KK858057.1|	23455-24373	8.8	31	57	33	-0.800227	0.6214	no
gi|641565368|gb|KK858057.1|	27060-27321	8.8	31	9	7	-0.443678	0.7786	no
gi|641565368|gb|KK858057.1|	47887-48087	8.8	31	26	25	-0.0546906	0.95135	no
gi|641565374|gb|KK858055.1|	1-5310	11	38	3	0.571038	-2	0.0738	no
gi|641565374|gb|KK858055.1|	16349-16654	11	38	0	17	inf	0.0015	yes
gi|641565374|gb|KK858055.1|	16975-17555	11	38	0	6	inf	0.00115	yes
gi|641565374|gb|KK858055.1|	23704-25054	11	38	12	4	-2	0.19215	no
gi|641565374|gb|KK858055.1|	26208-27586	11	38	2	3	0.994361	0.4718	no
gi|641565374|gb|KK858055.1|	27705-28167	11	38	6	2	-1	0.3584	no
gi|641565374|gb|KK858055.1|	29453-29677	11	38	18	4	-2	0.26405	no
gi|641565374|gb|KK858055.1|	36909-37943	11	38	15	12	-0.277352	0.8174	no
gi|641565374|gb|KK858055.1|	38771-40298	11	38	11	8	-0.461145	0.7076	no
gi|641565374|gb|KK858055.1|	41206-42473	11	38	1	0.100611	-3	0.206	no
gi|641565374|gb|KK858055.1|	7573-8834	11	38	7	2	-2	0.1657	no
gi|641565378|gb|KK858053.1|	16345-16525	11	35	0	87	inf	0.00135	yes
gi|641565378|gb|KK858053.1|	21401-21719	11	35	3	20	3	0.13395	no
gi|641565378|gb|KK858053.1|	24-222	11	35	20	3	-3	0.2588	no
gi|641565378|gb|KK858053.1|	37340-37636	11	35	65	26	-1	0.3166	no
gi|641565378|gb|KK858053.1|	39030-39549	11	35	1	2	0.331217	0.83525	no
gi|641565378|gb|KK858053.1|	41534-43271	11	35	19	3	-3	0.04725	no
gi|641565378|gb|KK858053.1|	4331-4572	11	35	24	8	-2	0.34895	no
gi|641565378|gb|KK858053.1|	43527-45207	11	35	2	2	0.295196	0.8232	no
gi|641565378|gb|KK858053.1|	45270-47219	11	35	1	0.985218	-0.478499	0.7302	no
gi|641565378|gb|KK858053.1|	7617-7874	11	35	5	10	1	0.51325	no
gi|641565383|gb|KK858051.1|	14296-14499	9.8	51	88	26	-2	0.2328	no

gi|641565383|gb|KK858051.1|	35436-36629	9.8	51	0.425795	2	2	0.25645	no
gi|641565383|gb|KK858051.1|	39514-40790	9.8	51	0.786187	1	0.481336	0.75585	no
gi|641565386|gb|KK858050.1|	11386-12271	11	44	2	3	0.603773	0.6624	no
gi|641565386|gb|KK858050.1|	12601-13092	11	44	1	4	1	0.41185	no
gi|641565386|gb|KK858050.1|	14149-16339	11	44	0.425947	1	1	0.3614	no
gi|641565386|gb|KK858050.1|	24301-25486	11	44	0.643855	0.980217	0.606364	0.6902	no
gi|641565386|gb|KK858050.1|	37309-38043	11	44	1	0.398789	-2	0.3455	no
gi|641565386|gb|KK858050.1|	45439-46442	11	44	2	3	0.749411	0.5894	no
gi|641565389|gb|KK858049.1|	3982-4294	9.4	20	4	4	0.0847773	0.9242	no
gi|641565392|gb|KK858048.1|	2379-2770	6.9	32	6	0.534124	-4	0.2056	no
gi|641565401|gb|KK858045.1|	14829-15045	10	19	14	9	-0.631623	0.70385	no
gi|641565401|gb|KK858045.1|	24108-24322	10	19	79	31	-1	0.34585	no
gi|641565401|gb|KK858045.1|	25368-26140	10	19	0.859392	0.559069	-0.620289	1	no
gi|641565407|gb|KK858043.1|	25643-27200	11	20	2	3	0.788566	0.5569	no
gi|641565407|gb|KK858043.1|	2784-3981	11	20	2	4	1	0.4553	no
gi|641565407|gb|KK858043.1|	30234-30584	11	20	12	5	-1	0.4263	no
gi|641565407|gb|KK858043.1|	31717-33205	11	20	1	0.918121	-0.254734	0.85915	no
gi|641565407|gb|KK858043.1|	48302-48937	11	20	2	2	0.461433	0.7552	no
gi|641565410|gb|KK858042.1|	12890-13890	87	45	0.967847	0	-inf	0.0102	no
gi|641565410|gb|KK858042.1|	14031-15499	87	45	3	0	-inf	5.00E-05	yes
gi|641565410|gb|KK858042.1|	17583-20223	87	45	1315.59	0	-inf	0.06715	no
gi|641565410|gb|KK858042.1|	23642-25353	87	45	2	0	-inf	0.00115	yes
gi|641565410|gb|KK858042.1|	25593-27689	87	45	902	0	-inf	5.00E-05	yes
gi|641565410|gb|KK858042.1|	3-2715	87	45	3869.68	0.042938	-16	0.43635	no
gi|641565410|gb|KK858042.1|	32278-35993	87	45	429	0	-inf	5.00E-05	yes
gi|641565410|gb|KK858042.1|	43397-44242	87	45	6	0	-inf	0.0002	yes
gi|641565410|gb|KK858042.1|	44569-44861	87	45	20	15	-0.434649	0.7544	no
gi|641565410|gb|KK858042.1|	49879-50241	87	45	4	7	0.869146	0.5832	no
gi|641565410|gb|KK858042.1|	52060-52333	87	45	9	3	-1	0.4189	no
gi|641565410|gb|KK858042.1|	53992-54567	87	45	18	16	-0.170176	0.89595	no

gi|641565410|gb|KK858042.1|	5932-7212	87	45	2	0	-inf	0.0013	yes
gi|641565410|gb|KK858042.1|	60689-61102	87	45	6	23	2	0.1997	no
gi|641565410|gb|KK858042.1|	63322-63499	87	45	25	47	0.88052	0.58725	no
gi|641565410|gb|KK858042.1|	7414-8312	87	45	3	0	-inf	0.0013	yes
gi|641565410|gb|KK858042.1|	8456-10278	87	45	2	0	-inf	0.00095	yes
gi|641565418|gb|KK858039.1|	11311-12826	8.8	33	0.107301	35	8	0.2342	no
gi|641565418|gb|KK858039.1|	14914-15390	8.8	33	4	7	1	0.47075	no
gi|641565418|gb|KK858039.1|	16102-16725	8.8	33	2	8	2	0.2634	no
gi|641565418|gb|KK858039.1|	17110-17458	8.8	33	2	6	2	0.33325	no
gi|641565418|gb|KK858039.1|	18212-19855	8.8	33	3	5	0.654794	0.6151	no
gi|641565418|gb|KK858039.1|	28039-29057	8.8	33	1	1	-0.22492	0.87645	no
gi|641565418|gb|KK858039.1|	30700-31925	8.8	33	0.893886	0.104699	-3	0.22425	no
gi|641565418|gb|KK858039.1|	32109-33935	8.8	33	1	53	5	0.0244	no
gi|641565418|gb|KK858039.1|	36826-37103	8.8	33	5	11	1	0.43635	no
gi|641565418|gb|KK858039.1|	8335-9024	8.8	33	0.716939	1	0.600397	0.70505	no
gi|641565420|gb|KK858038.1|	11627-12508	9.8	35	0.516277	2	2	0.3084	no
gi|641565420|gb|KK858038.1|	13464-15145	9.8	35	0.476562	0.871835	0.871391	1	no
gi|641565432|gb|KK858033.1|	15777-16625	7.5	19	3	3	0.266254	0.8441	no
gi|641565432|gb|KK858033.1|	32109-32959	7.5	19	1	0	-inf	0.01	no
gi|641565432|gb|KK858033.1|	3472-4401	7.5	19	0.675503	0.733512	0.118859	1	no
gi|641565432|gb|KK858033.1|	5941-6637	7.5	19	0.989704	0.643156	-0.621829	0.71195	no
gi|641565434|gb|KK858032.1|	26939-27304	8.1	33	6	0	-inf	0.0059	no
gi|641565434|gb|KK858032.1|	27519-27871	8.1	33	3	2	-0.644366	0.71075	no
gi|641565439|gb|KK858030.1|	21329-22332	9.9	39	3	0	-inf	0.0013	yes
gi|641565439|gb|KK858030.1|	23094-23400	9.9	39	552	2	-8	0.13295	no
gi|641565439|gb|KK858030.1|	23584-26691	9.9	39	50	0.111365	-9	0.0961	no
gi|641565439|gb|KK858030.1|	27448-28631	9.9	39	67	0	-inf	0.0001	yes
gi|641565439|gb|KK858030.1|	30744-31913	9.9	39	2	0	-inf	0.0013	yes
gi|641565439|gb|KK858030.1|	33143-33621	9.9	39	4	5	0.385314	0.79325	no
gi|641565447|gb|KK858027.1|	17466-19010	9.4	37	1	0	-inf	0.00185	yes

gi|641565447|gb|KK858027.1|	24411-24781	9.4	37	19	1	-4	0.13545	no
gi|641565451|gb|KK858025.1|	30339-32487	9.1	17	19	13	-0.540527	0.7438	no
gi|641565451|gb|KK858025.1|	33443-33970	9.1	17	18	14	-0.388051	0.76305	no
gi|641565451|gb|KK858025.1|	34068-35007	9.1	17	0.571217	0.723703	0.341359	1	no
gi|641565451|gb|KK858025.1|	36048-37381	9.1	17	3	6	0.899321	0.47865	no
gi|641565454|gb|KK858024.1|	1-607	5.4	20	5	0	-inf	0.0013	yes
gi|641565454|gb|KK858024.1|	12135-14234	5.4	20	10	0	-inf	5.00E-05	yes
gi|641565454|gb|KK858024.1|	14689-15404	5.4	20	9	0	-inf	5.00E-05	yes
gi|641565454|gb|KK858024.1|	3413-4380	5.4	20	5	0	-inf	0.0002	yes
gi|641565454|gb|KK858024.1|	5559-6953	5.4	20	4	0	-inf	0.0003	yes
gi|641565454|gb|KK858024.1|	8730-11102	5.4	20	5	0	-inf	5.00E-05	yes
gi|641565456|gb|KK858023.1|	14820-16106	7.4	27	0.908809	0.889789	-0.0305134	0.95655	no
gi|641565456|gb|KK858023.1|	17535-18213	7.4	27	24	10	-1	0.31615	no
gi|641565456|gb|KK858023.1|	20111-21394	7.4	27	18	16	-0.248225	0.84255	no
gi|641565456|gb|KK858023.1|	6284-7370	7.4	27	0.476945	1	1	0.37775	no
gi|641565471|gb|KK858017.1|	12750-12941	9.8	40	39	7	-2	0.2223	no
gi|641565473|gb|KK858016.1|	11689-12704	9.1	37	2	11	2	0.11785	no
gi|641565473|gb|KK858016.1|	13919-14665	9.1	37	2	6	2	0.2884	no
gi|641565473|gb|KK858016.1|	34497-34689	9.1	37	93	14	-3	0.12615	no
gi|641565473|gb|KK858016.1|	8682-8980	9.1	37	2	10	2	0.21715	no
gi|641565478|gb|KK858014.1|	5773-5926	7.6	42	125	0	-inf	0.0059	no
gi|641565482|gb|KK858012.1|	39851-41030	12	32	0.647782	1	1	0.47685	no
gi|641565482|gb|KK858012.1|	48161-48969	12	32	0.346673	3	3	0.1723	no
gi|641565485|gb|KK858011.1|	6036-6968	8.8	30	1	2	1	0.44525	no
gi|641565491|gb|KK858009.1|	17335-17590	4.9	16	7	18	1	0.37825	no
gi|641565497|gb|KK858007.1|	14787-15462	10	37	4	2	-0.662985	0.6411	no
gi|641565497|gb|KK858007.1|	27469-28056	10	37	5	2	-1	0.47525	no
gi|641565497|gb|KK858007.1|	31723-32528	10	37	26	11	-1	0.32955	no
gi|641565497|gb|KK858007.1|	33195-34650	10	37	1	1	0.373178	0.78995	no
gi|641565497|gb|KK858007.1|	35054-36029	10	37	0.544843	2	2	0.214	no

gi|641565497|gb|KK858007.1|	36148-37012	10	37	1	0.6435	-0.718423	0.6701	no
gi|641565497|gb|KK858007.1|	37301-38785	10	37	0.769177	0.920962	0.259825	0.8632	no
gi|641565497|gb|KK858007.1|	38978-39803	10	37	2	3	0.422388	0.7653	no
gi|641565497|gb|KK858007.1|	43452-44488	10	37	3	2	-0.897192	0.5277	no
gi|641565497|gb|KK858007.1|	44922-45844	10	37	1	0.59243	-1	0.46585	no
gi|641565497|gb|KK858007.1|	45995-46874	10	37	1	0.4721	-2	0.40415	no
gi|641565497|gb|KK858007.1|	47223-48628	10	37	2	1	-0.48566	0.72355	no
gi|641565497|gb|KK858007.1|	50460-50761	10	37	12	10	-0.294878	0.83625	no
gi|641565497|gb|KK858007.1|	54041-54811	10	37	0.739184	2	2	0.3067	no
gi|641565497|gb|KK858007.1|	57723-58104	10	37	2	6	2	0.38055	no
gi|641565497|gb|KK858007.1|	58217-58804	10	37	1	2	0.959884	0.57345	no
gi|641565500|gb|KK858006.1|	12011-13302	9.6	19	3	1	-2	0.27545	no
gi|641565500|gb|KK858006.1|	18315-18683	9.6	19	4	2	-1	0.50345	no
gi|641565503|gb|KK858005.1|	11484-12262	9.7	36	3	12	2	0.18375	no
gi|641565503|gb|KK858005.1|	13825-14391	9.7	36	4	2	-0.927085	0.5316	no
gi|641565503|gb|KK858005.1|	15-630	9.7	36	5	10	0.892034	0.505	no
gi|641565511|gb|KK858002.1|	14603-14830	6.4	31	69	154	1	0.37795	no
gi|641565511|gb|KK858002.1|	16305-16587	6.4	31	19	56	2	0.24955	no
gi|641565511|gb|KK858002.1|	8372-8582	6.4	31	11	23	1	0.4899	no
gi|641565517|gb|KK858001.1|	10607-11742	9.5	31	0.904151	0.917462	0.0210846	0.95145	no
gi|641565517|gb|KK858001.1|	33431-33873	9.5	31	2	2	-0.411008	0.82205	no
gi|641565527|gb|KK857997.1|	10536-10879	9	30	15	8	-1	0.4662	no
gi|641565527|gb|KK857997.1|	14756-16446	9	30	5	7	0.32736	0.7913	no
gi|641565527|gb|KK857997.1|	16623-17023	9	30	2	48	4	0.05455	no
gi|641565527|gb|KK857997.1|	18097-18622	9	30	3	3	-0.235507	0.87505	no
gi|641565527|gb|KK857997.1|	20434-21009	9	30	7	15	1	0.39195	no
gi|641565527|gb|KK857997.1|	24555-24778	9	30	19	19	0.0110524	0.95595	no
gi|641565527|gb|KK857997.1|	26194-26892	9	30	0.844947	1	0.337576	0.8352	no
gi|641565527|gb|KK857997.1|	30603-30898	9	30	6	6	-0.0188401	0.9394	no
gi|641565527|gb|KK857997.1|	31481-34590	9	30	4	6	0.550364	0.65635	no

gi|641565527|gb|KK857997.1|	36513-37480	9	30	4	2	-0.767288	0.5776	no
gi|641565527|gb|KK857997.1|	38529-38719	9	30	126	121	-0.06001	0.9626	no
gi|641565527|gb|KK857997.1|	40385-40881	9	30	20	12	-0.711923	0.583	no
gi|641565527|gb|KK857997.1|	5500-5901	9	30	12	4	-2	0.307	no
gi|641565532|gb|KK857995.1|	10515-11323	9.5	46	1	2	0.6027	0.67915	no
gi|641565532|gb|KK857995.1|	11966-13067	9.5	46	2	0.832779	-0.908762	0.54665	no
gi|641565532|gb|KK857995.1|	18785-19698	9.5	46	1	0	-inf	0.0102	no
gi|641565532|gb|KK857995.1|	19837-20487	9.5	46	2	0.471668	-2	0.3216	no
gi|641565532|gb|KK857995.1|	3069-3744	9.5	46	0.29513	2	3	0.2991	no
gi|641565532|gb|KK857995.1|	38935-39320	9.5	46	3	2	-0.417072	0.7868	no
gi|641565532|gb|KK857995.1|	39375-39873	9.5	46	3	1	-1	0.44155	no
gi|641565532|gb|KK857995.1|	53391-53953	9.5	46	0.192835	4	5	0.1123	no
gi|641565545|gb|KK857990.1|	14516-15030	11	17	4	3	-0.575946	0.7144	no
gi|641565545|gb|KK857990.1|	15082-15720	11	17	2	1	-0.275442	0.8619	no
gi|641565545|gb|KK857990.1|	16557-18213	11	17	2	2	0.267251	0.83665	no
gi|641565545|gb|KK857990.1|	18691-19282	11	17	3	2	-0.650464	0.66015	no
gi|641565545|gb|KK857990.1|	23935-24772	11	17	1	1	0.281207	0.85465	no
gi|641565545|gb|KK857990.1|	34286-35319	11	17	2	2	-0.131683	0.92475	no
gi|641565545|gb|KK857990.1|	37920-38355	11	17	14	20	0.497224	0.7024	no
gi|641565552|gb|KK857987.1|	16017-16484	8.8	24	3	3	0.269058	0.85625	no
gi|641565552|gb|KK857987.1|	4978-5152	8.8	24	55	17	-2	0.33105	no
gi|641565552|gb|KK857987.1|	9748-10308	8.8	24	12	6	-1	0.39335	no
gi|641565555|gb|KK857986.1|	17870-18365	9.3	29	0	4	inf	0.00455	no
gi|641565555|gb|KK857986.1|	28358-29016	9.3	29	0	5	inf	0.0005	yes
gi|641565564|gb|KK857983.1|	15594-16032	11	44	0.289632	8	5	0.10875	no
gi|641565564|gb|KK857983.1|	29678-29865	11	44	0	57	inf	0.00255	yes
gi|641565566|gb|KK857982.1|	9854-10025	9.2	44	30	31	0.0292771	0.92445	no
gi|641565579|gb|KK857977.1|	19584-20388	8.7	25	0.930536	5	2	0.17345	no
gi|641565579|gb|KK857977.1|	320-939	8.7	25	2	0	-inf	0.0075	no
gi|641565608|gb|KK857969.1|	16588-17626	13	42	12	38	2	0.18775	no

gi|641565608|gb|KK857969.1|	17748-18295	13	42	20.1	50	1	0.30295	no
gi|641565608|gb|KK857969.1|	18550-18992	13	42	15	47	2	0.218	no
gi|641565608|gb|KK857969.1|	19258-22595	13	42	19	12	-0.667661	0.76915	no
gi|641565608|gb|KK857969.1|	30906-31665	13	42	41	30	-0.420497	0.7371	no
gi|641565608|gb|KK857969.1|	35463-36537	13	42	5	4	-0.25237	0.85005	no
gi|641565608|gb|KK857969.1|	39819-40409	13	42	29	31	0.0853641	0.9443	no
gi|641565608|gb|KK857969.1|	45226-46424	13	42	14	8	-0.730593	0.56245	no
gi|641565608|gb|KK857969.1|	49823-51571	13	42	9	5	-0.790022	0.52815	no
gi|641565610|gb|KK857968.1|	16170-18214	5	18	5	6	0.428565	0.73595	no
gi|641565610|gb|KK857968.1|	19918-20481	5	18	3	2	-0.31054	0.8441	no
gi|641565610|gb|KK857968.1|	21816-23056	5	18	1	4	1	0.28665	no
gi|641565610|gb|KK857968.1|	24271-25389	5	18	7	8	0.296247	0.8108	no
gi|641565610|gb|KK857968.1|	2600-4404	5	18	39	57	0.539398	0.80505	no
gi|641565610|gb|KK857968.1|	27420-27927	5	18	0.90615	3	2	0.3613	no
gi|641565610|gb|KK857968.1|	4733-6336	5	18	10	13	0.365775	0.76835	no
gi|641565612|gb|KK857967.1|	129-268	10	40	37239.7	55903	0.586082	0.77175	no
gi|641565612|gb|KK857967.1|	6728-7218	10	40	1	3	1	0.4014	no
gi|641565622|gb|KK857963.1|	17459-18088	8.3	31	2	0	-inf	0.0059	no
gi|641565623|gb|KK857962.1|	5908-6502	8	33	2	1	-0.572352	0.7586	no
gi|641565631|gb|KK857959.1|	30599-31120	6.9	19	0.867604	7	3	0.15545	no
gi|641565634|gb|KK857958.1|	12-344	10	20	3	3	-0.0105471	0.91115	no
gi|641565634|gb|KK857958.1|	12399-12870	10	20	12	4	-2	0.2518	no
gi|641565634|gb|KK857958.1|	1645-2162	10	20	1	4	2	0.32035	no
gi|641565634|gb|KK857958.1|	27949-28326	10	20	3	2	-0.640543	0.71075	no
gi|641565634|gb|KK857958.1|	28750-29969	10	20	1	1	0.104062	0.94435	no
gi|641565634|gb|KK857958.1|	32215-33489	10	20	1	1	-0.326029	0.8148	no
gi|641565634|gb|KK857958.1|	3698-4203	10	20	2	1	-0.728459	0.67005	no
gi|641565634|gb|KK857958.1|	4346-5171	10	20	4	6	0.768019	0.568	no
gi|641565634|gb|KK857958.1|	5391-5701	10	20	1	10	3	0.26155	no
gi|641565634|gb|KK857958.1|	6799-10116	10	20	4	2	-0.916805	0.47085	no

gi|641565634|gb|KK857958.1|	686-1314	10	20	3	4	0.276793	0.8425	no
gi|641565639|gb|KK857956.1|	19095-19424	7.9	34	4	2	-1	0.44305	no
gi|641565642|gb|KK857955.1|	3053-3480	6.3	31	3	2	-0.260371	0.88485	no
gi|641565642|gb|KK857955.1|	4094-4502	6.3	31	3	1	-0.999282	0.62775	no
gi|641565651|gb|KK857952.1|	5203-5701	7.8	26	0	14	inf	5.00E-05	yes
gi|641565659|gb|KK857949.1|	6916-8542	9.3	37	1	0.603503	-1	0.4146	no
gi|641565670|gb|KK857945.1|	10075-10884	7.6	37	0.807585	3	2	0.24985	no
gi|641565670|gb|KK857945.1|	10969-11914	7.6	37	0.566646	3	2	0.1527	no
gi|641565670|gb|KK857945.1|	13660-14973	7.6	37	0.633468	3	2	0.20405	no
gi|641565670|gb|KK857945.1|	9231-9867	7.6	37	0.963543	1	0.335932	0.83525	no
gi|641565672|gb|KK857944.1|	36906-37223	9	34	3	7	1	0.40225	no
gi|641565672|gb|KK857944.1|	6090-6428	9	34	0	10	inf	0.00255	yes
gi|641565679|gb|KK857941.1|	1-1116	7.7	29	2	18	3	0.02945	no
gi|641565679|gb|KK857941.1|	9469-9994	7.7	29	0	32	inf	5.00E-05	yes
gi|641565681|gb|KK857940.1|	25233-25834	23	51	1	2	0.182787	0.91015	no
gi|641565681|gb|KK857940.1|	37490-38347	23	51	2	4	0.723706	0.6003	no
gi|641565681|gb|KK857940.1|	39962-40263	23	51	22	15	-0.602306	0.6541	no
gi|641565690|gb|KK857937.1|	4416-4991	6	100	1	6	2	0.16045	no
gi|641565694|gb|KK857935.1|	20485-21065	8.7	27	2	4	0.975556	0.53815	no
gi|641565694|gb|KK857935.1|	23187-24558	8.7	27	4	4	0.0894596	0.94405	no
gi|641565694|gb|KK857935.1|	25473-25671	8.7	27	24	29	0.239719	0.87725	no
gi|641565694|gb|KK857935.1|	25821-26636	8.7	27	5	5	-0.0816892	0.9514	no
gi|641565694|gb|KK857935.1|	27350-27600	8.7	27	18	16	-0.157946	0.91535	no
gi|641565694|gb|KK857935.1|	3610-4532	8.7	27	320	0	-inf	0.00025	yes
gi|641565694|gb|KK857935.1|	6666-7694	8.7	27	512	0	-inf	0.0003	yes
gi|641565694|gb|KK857935.1|	9742-11333	8.7	27	146	0.231972	-9	0.0958	no
gi|641565696|gb|KK857934.1|	27094-29088	8.5	38	0.275544	19	6	0.05155	no
gi|641565696|gb|KK857934.1|	39096-40041	8.5	38	0.472205	1	1	0.3994	no
gi|641565699|gb|KK857933.1|	37485-37971	7.1	18	25	37	0.549654	0.6623	no
gi|641565699|gb|KK857933.1|	39138-39359	7.1	18	4	15	2	0.3484	no

gi|641565701|gb|KK857932.1|	11339-12095	11	42	0.126272	5	5	0.1059	no
gi|641565701|gb|KK857932.1|	20406-20871	11	42	2	8	2	0.22535	no
gi|641565701|gb|KK857932.1|	2598-2857	11	42	56	118	1	0.3976	no
gi|641565701|gb|KK857932.1|	26170-26582	11	42	0	6	inf	0.00375	no
gi|641565701|gb|KK857932.1|	27772-29492	11	42	0.464372	0.637196	0.456455	1	no
gi|641565701|gb|KK857932.1|	50481-51386	11	42	0.598582	0.758174	0.34098	1	no
gi|641565701|gb|KK857932.1|	8489-9306	11	42	0.455539	1	2	0.3613	no
gi|641565704|gb|KK857931.1|	10403-12169	11	36	166	47	-2	0.3951	no
gi|641565704|gb|KK857931.1|	12507-14049	11	36	0.736051	2	2	0.26725	no
gi|641565704|gb|KK857931.1|	14258-14811	11	36	93	78	-0.26436	0.83005	no
gi|641565704|gb|KK857931.1|	15730-16896	11	36	0.729397	0.444143	-0.71568	1	no
gi|641565704|gb|KK857931.1|	17394-17654	11	36	33	28	-0.261142	0.8469	no
gi|641565704|gb|KK857931.1|	17832-18156	11	36	23	15	-0.638702	0.64415	no
gi|641565704|gb|KK857931.1|	19816-20280	11	36	81	66	-0.288382	0.81535	no
gi|641565704|gb|KK857931.1|	22777-23315	11	36	126	115	-0.130788	0.95025	no
gi|641565704|gb|KK857931.1|	28276-29998	11	36	5	11	0.995486	0.42935	no
gi|641565704|gb|KK857931.1|	35124-37620	11	36	29	34	0.238427	0.91065	no
gi|641565704|gb|KK857931.1|	38991-41761	11	36	28	14	-0.995001	0.6651	no
gi|641565704|gb|KK857931.1|	41923-46486	11	36	16	7	-1	0.4592	no
gi|641565704|gb|KK857931.1|	46622-48888	11	36	0.889012	1	0.60948	0.65585	no
gi|641565704|gb|KK857931.1|	49390-50563	11	36	0.86901	3	2	0.2964	no
gi|641565704|gb|KK857931.1|	50683-54563	11	36	0.979988	0.99765	0.0257692	0.9836	no
gi|641565704|gb|KK857931.1|	54874-57802	11	36	0.62183	1	0.725649	0.59755	no
gi|641565704|gb|KK857931.1|	58279-59697	11	36	0.347441	0.352912	0.0225385	1	no
gi|641565708|gb|KK857929.1|	12979-13538	9.2	45	6	7	0.240035	0.8629	no
gi|641565711|gb|KK857928.1|	10581-12515	11	36	0.447939	0.745273	0.734466	1	no
gi|641565711|gb|KK857928.1|	13652-13841	11	36	24	35	0.524419	0.7391	no
gi|641565711|gb|KK857928.1|	14293-14638	11	36	2	5	1	0.513	no
gi|641565711|gb|KK857928.1|	16549-18304	11	36	2	6	1	0.314	no
gi|641565711|gb|KK857928.1|	18450-20905	11	36	4	2	-0.778207	0.5425	no

gi|641565711|gb|KK857928.1|	21570-32362	11	36	7	8	0.221598	0.92335	no
gi|641565711|gb|KK857928.1|	2341-3810	11	36	0.611471	0.76236	0.318188	1	no
gi|641565711|gb|KK857928.1|	32-1190	11	36	0.956096	1	0.365189	0.8038	no
gi|641565711|gb|KK857928.1|	32549-34001	11	36	1	0.429244	-2	0.2987	no
gi|641565711|gb|KK857928.1|	34132-34855	11	36	1	2	0.738668	0.6176	no
gi|641565711|gb|KK857928.1|	35346-36870	11	36	0.639447	0.812111	0.344852	1	no
gi|641565711|gb|KK857928.1|	37026-38271	11	36	44	30	-0.567097	0.72315	no
gi|641565711|gb|KK857928.1|	38394-39629	11	36	8	4	-0.9656	0.45535	no
gi|641565711|gb|KK857928.1|	44264-45055	11	36	17	19	0.207567	0.8726	no
gi|641565711|gb|KK857928.1|	4646-6760	11	36	3	5	0.796881	0.5398	no
gi|641565711|gb|KK857928.1|	49251-49458	11	36	113.84	224	0.978181	0.44295	no
gi|641565711|gb|KK857928.1|	52409-52669	11	36	14	33	1	0.3687	no
gi|641565711|gb|KK857928.1|	56846-59227	11	36	1	0.840286	-0.550834	0.68155	no
gi|641565711|gb|KK857928.1|	59301-61797	11	36	1	3	1	0.42395	no
gi|641565714|gb|KK857927.1|	21551-21903	9.3	38	0	50	inf	5.00E-05	yes
gi|641565717|gb|KK857926.1|	12307-13523	14	110	1	0	-inf	0.0059	no
gi|641565722|gb|KK857924.1|	18677-19421	9.8	31	1	0.587371	-1	0.47135	no
gi|641565722|gb|KK857924.1|	23833-24271	9.8	31	4	0.435557	-3	0.2242	no
gi|641565722|gb|KK857924.1|	30007-30533	9.8	31	2	2	0.401975	0.8089	no
gi|641565722|gb|KK857924.1|	30939-31808	9.8	31	0.840757	2	1	0.49765	no
gi|641565722|gb|KK857924.1|	32091-33520	9.8	31	0.918249	0.612107	-0.585101	0.68495	no
gi|641565722|gb|KK857924.1|	35270-36309	9.8	31	0.83919	2	1	0.47135	no
gi|641565722|gb|KK857924.1|	38006-38254	9.8	31	16	0	-inf	0.0056	no
gi|641565722|gb|KK857924.1|	41796-42237	9.8	31	4	0	-inf	0.0059	no
gi|641565722|gb|KK857924.1|	47542-47860	9.8	31	9	7	-0.346008	0.81605	no
gi|641565722|gb|KK857924.1|	48931-49807	9.8	31	1	0.790281	-0.533843	0.7478	no
gi|641565722|gb|KK857924.1|	51922-58637	9.8	31	10	4	-1	0.44995	no
gi|641565727|gb|KK857922.1|	21408-22319	8.6	30	0	4	inf	0.00075	yes
gi|641565734|gb|KK857919.1|	1519-2051	12	45	3	2	-0.783587	0.6308	no
gi|641565734|gb|KK857919.1|	18597-19056	12	45	0	13	inf	0.00065	yes

gi|641565734|gb|KK857919.1|	2663-2927	12	45	140	97	-0.534629	0.6743	no
gi|641565734|gb|KK857919.1|	29303-29917	12	45	0.168999	4	5	0.1123	no
gi|641565734|gb|KK857919.1|	34-687	12	45	2	2	-0.0786112	0.95295	no
gi|641565734|gb|KK857919.1|	4002-4528	12	45	7	5	-0.449774	0.7434	no
gi|641565734|gb|KK857919.1|	5156-5367	12	45	23	3	-3	0.2236	no
gi|641565734|gb|KK857919.1|	8468-9449	12	45	0.090114	1	4	0.12385	no
gi|641565739|gb|KK857917.1|	3213-4386	6.8	24	0.28967	3	3	0.1441	no
gi|641565739|gb|KK857917.1|	455-1028	6.8	24	1	0.849558	-0.625627	0.71195	no
gi|641565745|gb|KK857915.1|	23335-23603	11	40	6	5	-0.248797	0.88385	no
gi|641565745|gb|KK857915.1|	29453-31022	11	40	32	30	-0.0564978	0.9803	no
gi|641565745|gb|KK857915.1|	32605-34229	11	40	15	11	-0.492664	0.68735	no
gi|641565745|gb|KK857915.1|	35334-36083	11	40	8	4	-0.983278	0.45605	no
gi|641565745|gb|KK857915.1|	37224-38326	11	40	2	1	-0.827141	0.56065	no
gi|641565745|gb|KK857915.1|	38449-40617	11	40	2	1	-0.85533	0.5205	no
gi|641565745|gb|KK857915.1|	41408-42029	11	40	1	0.503392	-2	0.4266	no
gi|641565745|gb|KK857915.1|	42224-42611	11	40	10	2	-2	0.19605	no
gi|641565745|gb|KK857915.1|	48531-48869	11	40	5	2	-1	0.46945	no
gi|641565745|gb|KK857915.1|	54528-55569	11	40	0.586044	0.63684	0.119923	1	no
gi|641565745|gb|KK857915.1|	57311-57991	11	40	2	0.664172	-2	0.403	no
gi|641565748|gb|KK857914.1|	18064-22042	10	37	15	12	-0.348081	0.88085	no
gi|641565748|gb|KK857914.1|	22550-24485	10	37	11	5	-1	0.32275	no
gi|641565761|gb|KK857909.1|	41035-41480	11	54	647	723	0.160962	0.9365	no
gi|641565761|gb|KK857909.1|	53546-54046	11	54	0	12	inf	0.00065	yes
gi|641565772|gb|KK857905.1|	11537-14784	10	18	2	1	-0.224542	0.86235	no
gi|641565772|gb|KK857905.1|	14977-15317	10	18	5	1	-2	0.31725	no
gi|641565772|gb|KK857905.1|	23809-24460	10	18	1	0.941289	-0.400596	0.82205	no
gi|641565772|gb|KK857905.1|	2584-4579	10	18	2	2	-0.316949	0.80965	no
gi|641565772|gb|KK857905.1|	45449-46126	10	18	2	1	-0.9024	0.5689	no
gi|641565772|gb|KK857905.1|	512-1718	10	18	2	2	-0.0815678	0.9533	no
gi|641565772|gb|KK857905.1|	5937-9979	10	18	2	2	0.070231	0.95685	no

gi|641565777|gb|KK857903.1|	1-321	11	42	149	0	-inf	5.00E-05	yes
gi|641565777|gb|KK857903.1|	12975-13992	11	42	1	0	-inf	0.01	no
gi|641565777|gb|KK857903.1|	14071-15447	11	42	4	0.091356	-5	0.18365	no
gi|641565777|gb|KK857903.1|	15573-16183	11	42	109	0	-inf	0.0002	yes
gi|641565777|gb|KK857903.1|	16601-18854	11	42	107	0	-inf	0.0002	yes
gi|641565777|gb|KK857903.1|	19035-19740	11	42	2	0	-inf	0.0102	no
gi|641565777|gb|KK857903.1|	20030-20987	11	42	1	0	-inf	0.0075	no
gi|641565777|gb|KK857903.1|	21949-22395	11	42	5	0	-inf	0.00495	no
gi|641565777|gb|KK857903.1|	22790-23163	11	42	12	0	-inf	0.0013	yes
gi|641565777|gb|KK857903.1|	23367-24260	11	42	214	0	-inf	0.0004	yes
gi|641565777|gb|KK857903.1|	24376-27019	11	42	252	0	-inf	5.00E-05	yes
gi|641565777|gb|KK857903.1|	27742-31368	11	42	67	0.031513	-11	0.1839	no
gi|641565777|gb|KK857903.1|	32233-33128	11	42	2	0	-inf	0.0032	yes
gi|641565777|gb|KK857903.1|	33675-34364	11	42	2	2	-0.0370332	0.95655	no
gi|641565777|gb|KK857903.1|	34766-36400	11	42	19	0.150039	-7	0.13015	no
gi|641565777|gb|KK857903.1|	40014-40689	11	42	1	4	2	0.30675	no
gi|641565777|gb|KK857903.1|	5788-9250	11	42	10	0	-inf	5.00E-05	yes
gi|641565777|gb|KK857903.1|	9587-12533	11	42	52	0	-inf	0.00035	yes
gi|641565780|gb|KK857902.1|	1120-2080	9.9	40	54	54	-0.00413601	0.9984	no
gi|641565780|gb|KK857902.1|	18436-18732	9.9	40	5	18	2	0.21165	no
gi|641565780|gb|KK857902.1|	18987-19656	9.9	40	0.448291	4	3	0.15395	no
gi|641565780|gb|KK857902.1|	19730-22032	9.9	40	0.302476	4	4	0.04695	no
gi|641565780|gb|KK857902.1|	23990-25261	9.9	40	0	19	inf	5.00E-05	yes
gi|641565780|gb|KK857902.1|	26130-28343	9.9	40	0	12	inf	5.00E-05	yes
gi|641565780|gb|KK857902.1|	30427-31590	9.9	40	42	1309.14	5	0.0368	no
gi|641565780|gb|KK857902.1|	35139-36911	9.9	40	11	44	2	0.24805	no
gi|641565780|gb|KK857902.1|	38206-39720	9.9	40	0.536914	2	2	0.3192	no
gi|641565780|gb|KK857902.1|	41066-41494	9.9	40	8	30	2	0.179	no
gi|641565782|gb|KK857901.1|	13213-14345	12	24	1	3	1	0.38135	no
gi|641565782|gb|KK857901.1|	14541-15125	12	24	0.545945	2	2	0.256	no

gi|641565782|gb|KK857901.1|	15219-17829	12	24	4	6	0.414491	0.74475	no
gi|641565782|gb|KK857901.1|	30370-32070	12	24	5	11	0.991735	0.43705	no
gi|641565782|gb|KK857901.1|	32231-33162	12	24	2	0.292612	-2	0.21865	no
gi|641565782|gb|KK857901.1|	33820-34170	12	24	5	1	-2	0.31725	no
gi|641565782|gb|KK857901.1|	35794-36056	12	24	3	10	2	0.3198	no
gi|641565782|gb|KK857901.1|	39212-40087	12	24	0.833335	0.633149	-0.396352	1	no
gi|641565782|gb|KK857901.1|	43423-44671	12	24	0.605323	0.307261	-0.978241	1	no
gi|641565785|gb|KK857900.1|	14165-15132	11	37	1	0	-inf	0.01	no
gi|641565785|gb|KK857900.1|	17354-19333	11	37	1	1	0.261098	0.84985	no
gi|641565785|gb|KK857900.1|	28819-29118	11	37	1	21	4	0.2398	no
gi|641565785|gb|KK857900.1|	30479-31048	11	37	0	32	inf	5.00E-05	yes
gi|641565788|gb|KK857899.1|	10279-10937	8.5	35	11	19	0.813003	0.5293	no
gi|641565788|gb|KK857899.1|	12352-14399	8.5	35	12	15	0.310986	0.79865	no
gi|641565788|gb|KK857899.1|	14985-15221	8.5	35	16	51	2	0.2514	no
gi|641565788|gb|KK857899.1|	16205-16918	8.5	35	8	17	1	0.41345	no
gi|641565788|gb|KK857899.1|	17375-17794	8.5	35	0.313472	6	4	0.1175	no
gi|641565788|gb|KK857899.1|	20217-20625	8.5	35	0.658177	4	3	0.28535	no
gi|641565788|gb|KK857899.1|	43739-44332	8.5	35	0	5	inf	0.0015	yes
gi|641565801|gb|KK857894.1|	21666-22360	9.2	29	0.141952	3	4	0.11365	no
gi|641565801|gb|KK857894.1|	46833-47280	9.2	29	0.838585	4	2	0.256	no
gi|641565803|gb|KK857893.1|	10478-11714	10	35	1	0	-inf	0.00495	no
gi|641565803|gb|KK857893.1|	14244-15266	10	35	4	0.260562	-4	0.13555	no
gi|641565803|gb|KK857893.1|	16002-18166	10	35	1	0.109731	-3	0.14235	no
gi|641565803|gb|KK857893.1|	18861-21243	10	35	18	1	-4	0.08195	no
gi|641565803|gb|KK857893.1|	22364-25271	10	35	1	0	-inf	0.00025	yes
gi|641565803|gb|KK857893.1|	7380-8730	10	35	2	0	-inf	0.00105	yes
gi|641565811|gb|KK857890.1|	1298-2845	13	23	1	2	0.607962	0.66295	no
gi|641565811|gb|KK857890.1|	21-380	13	23	5	5	-0.121272	0.93965	no
gi|641565811|gb|KK857890.1|	24440-25098	13	23	3	3	0.184562	0.8926	no
gi|641565811|gb|KK857890.1|	4426-5339	13	23	0.887865	1	0.434182	0.78015	no

gi|641565817|gb|KK857888.1|	18212-18689	9.2	40	0	8	inf	0.00145	yes
gi|641565820|gb|KK857887.1|	18522-18948	9	34	3	0.914405	-2	0.3216	no
gi|641565820|gb|KK857887.1|	20151-20947	9	34	4	8	0.746898	0.5648	no
gi|641565820|gb|KK857887.1|	23462-24042	9	34	2	65	5	0.0209	no
gi|641565820|gb|KK857887.1|	26932-27317	9	34	0	37	inf	5.00E-05	yes
gi|641565820|gb|KK857887.1|	34334-34737	9	34	0.673385	107	7	0.2322	no
gi|641565820|gb|KK857887.1|	3486-3730	9	34	12	9	-0.324144	0.832	no
gi|641565825|gb|KK857885.1|	13871-15120	12	41	3	1	-1	0.37865	no
gi|641565825|gb|KK857885.1|	16894-17394	12	41	3	7	1	0.43855	no
gi|641565825|gb|KK857885.1|	8359-8898	12	41	20	53	1	0.2575	no
gi|641565827|gb|KK857884.1|	40583-41101	7	27	0.218898	3	4	0.1276	no
gi|641565829|gb|KK857883.1|	19210-19616	6.6	2.10E+02	0	18	inf	0.0004	yes
gi|641565829|gb|KK857883.1|	19823-21797	6.6	2.10E+02	6	4	-0.411166	0.74495	no
gi|641565832|gb|KK857882.1|	3876-4454	6.5	32	0	3	inf	0.0068	no
gi|641565838|gb|KK857880.1|	13209-13782	9.8	33	20	7	-2	0.2303	no
gi|641565838|gb|KK857880.1|	14340-15365	9.8	33	0.938685	5	2	0.11465	no
gi|641565838|gb|KK857880.1|	24569-25375	9.8	33	2	2	0.0721543	0.95375	no
gi|641565838|gb|KK857880.1|	26887-28219	9.8	33	5	3	-0.9949	0.46385	no
gi|641565838|gb|KK857880.1|	29779-30484	9.8	33	9	4	-0.984192	0.4646	no
gi|641565841|gb|KK857879.1|	20995-22534	10	17	1	2	0.669337	0.6241	no
gi|641565841|gb|KK857879.1|	24137-24662	10	17	3	6	1	0.4385	no
gi|641565841|gb|KK857879.1|	25489-26362	10	17	2	4	1	0.35155	no
gi|641565841|gb|KK857879.1|	28234-28738	10	17	3	8	1	0.42755	no
gi|641565841|gb|KK857879.1|	30890-32784	10	17	2	4	1	0.3605	no
gi|641565844|gb|KK857878.1|	2-1499	7.1	33	15	13	-0.240203	0.8458	no
gi|641565844|gb|KK857878.1|	8733-9043	7.1	33	8	15	0.956772	0.52125	no
gi|641565844|gb|KK857878.1|	9681-10687	7.1	33	5	0.265678	-4	0.13405	no
gi|641565847|gb|KK857877.1|	2932-3264	7	29	11	246	4	0.0547	no
gi|641565849|gb|KK857876.1|	21595-22393	10	37	3	9	2	0.25305	no
gi|641565849|gb|KK857876.1|	9936-10560	10	37	77	21	-2	0.1557	no

gi|641565855|gb|KK857874.1|	18991-19529	11	39	0.412473	3	3	0.28535	no
gi|641565855|gb|KK857874.1|	29679-30050	11	39	6	0	-inf	0.0056	no
gi|641565855|gb|KK857874.1|	39822-40692	11	39	2	0	-inf	0.0032	yes
gi|641565855|gb|KK857874.1|	41031-43594	11	39	3	0.365086	-3	0.0839	no
gi|641565855|gb|KK857874.1|	43739-44374	11	39	2	0.243782	-3	0.22425	no
gi|641565855|gb|KK857874.1|	44926-45480	11	39	4	0.595843	-3	0.20575	no
gi|641565855|gb|KK857874.1|	52106-53255	11	39	38	18	-1	0.5198	no
gi|641565855|gb|KK857874.1|	54606-55129	11	39	1	5	2	0.2365	no
gi|641565855|gb|KK857874.1|	57467-58697	11	39	295	132	-1	0.56025	no
gi|641565855|gb|KK857874.1|	60198-61159	11	39	1	0.703021	-0.658413	0.6826	no
gi|641565861|gb|KK857872.1|	8354-9125	10	34	0	3	inf	0.0016	yes
gi|641565884|gb|KK857864.1|	0-1497	9.4	38	34	37	0.126044	0.9546	no
gi|641565884|gb|KK857864.1|	12548-14640	9.4	38	21	3	-3	0.17345	no
gi|641565884|gb|KK857864.1|	17248-17585	9.4	38	10	6	-0.816946	0.58055	no
gi|641565884|gb|KK857864.1|	4075-4838	9.4	38	20	18	-0.165396	0.89325	no
gi|641565884|gb|KK857864.1|	6423-8449	9.4	38	10	7	-0.662567	0.60295	no
gi|641565884|gb|KK857864.1|	9545-10996	9.4	38	15	8	-0.944545	0.45355	no
gi|641565894|gb|KK857860.1|	1002-1485	11	20	2	2	-0.144864	0.92525	no
gi|641565894|gb|KK857860.1|	22552-23596	11	20	2	1	-0.642553	0.6611	no
gi|641565894|gb|KK857860.1|	25519-26069	11	20	2	2	0.0108175	0.95145	no
gi|641565894|gb|KK857860.1|	2869-4209	11	20	0.742232	5	3	0.09145	no
gi|641565894|gb|KK857860.1|	34119-34995	11	20	1	2	0.362652	0.8038	no
gi|641565894|gb|KK857860.1|	36577-36984	11	20	2	9	2	0.19235	no
gi|641565894|gb|KK857860.1|	38826-39871	11	20	2	13	3	0.04925	no
gi|641565894|gb|KK857860.1|	43376-44309	11	20	2	1	-0.483134	0.755	no
gi|641565907|gb|KK857855.1|	10900-11109	10	800	0	55	inf	0.00105	yes
gi|641565907|gb|KK857855.1|	11417-11859	10	800	0	9	inf	0.00145	yes
gi|641565907|gb|KK857855.1|	17044-17848	10	800	5	4	-0.467752	0.7306	no
gi|641565907|gb|KK857855.1|	25082-25580	10	800	0.23319	4	4	0.1175	no
gi|641565907|gb|KK857855.1|	27349-28451	10	800	0	4	inf	5.00E-05	yes

gi|641565907|gb|KK857855.1|	30187-30843	10	800	0	4	inf	0.0015	yes
gi|641565907|gb|KK857855.1|	32275-32805	10	800	0	3	inf	0.0068	no
gi|641565907|gb|KK857855.1|	35448-35960	10	800	0	13	inf	0.00015	yes
gi|641565907|gb|KK857855.1|	36131-37031	10	800	0	7	inf	0.0001	yes
gi|641565907|gb|KK857855.1|	39346-40353	10	800	0	6	inf	0.00025	yes
gi|641565907|gb|KK857855.1|	43080-43474	10	800	0	13	inf	0.00075	yes
gi|641565907|gb|KK857855.1|	51-484	10	800	0	9	inf	0.00145	yes
gi|641565910|gb|KK857854.1|	44439-44853	12	41	0.320389	71	8	0.10445	no
gi|641565910|gb|KK857854.1|	47828-48013	12	41	6	115	4	0.23715	no
gi|641565912|gb|KK857853.1|	27095-27745	12	40	0	5	inf	0.00145	yes
gi|641565912|gb|KK857853.1|	48875-49162	12	40	9	0	-inf	0.0075	no
gi|641565912|gb|KK857853.1|	53546-54037	12	40	3	0	-inf	0.0075	no
gi|641565912|gb|KK857853.1|	56367-57868	12	40	3	0	-inf	0.0002	yes
gi|641565912|gb|KK857853.1|	61569-62113	12	40	4	0.306304	-4	0.20475	no
gi|641565919|gb|KK857850.1|	11312-11616	6.5	35	0	77	inf	5.00E-05	yes
gi|641565919|gb|KK857850.1|	22164-22490	6.5	35	0	26	inf	0.00025	yes
gi|641565919|gb|KK857850.1|	24037-24272	6.5	35	0	88	inf	0.0001	yes
gi|641565930|gb|KK857848.1|	40892-41093	7.5	31	15	21	0.530791	0.7472	no
gi|641565931|gb|KK857847.1|	18135-19229	9.8	38	0.393916	2	2	0.181	no
gi|641565931|gb|KK857847.1|	7834-9689	9.8	38	0.895861	2	1	0.32235	no
gi|641565933|gb|KK857846.1|	24679-25733	9.4	35	0	84	inf	0.00035	yes
gi|641565933|gb|KK857846.1|	28818-30306	9.4	35	0.109542	2	4	0.2373	no
gi|641565933|gb|KK857846.1|	31389-32385	9.4	35	0.088426	4	5	0.10595	no
gi|641565933|gb|KK857846.1|	33079-33785	9.4	35	0.138622	3	4	0.1136	no
gi|641565933|gb|KK857846.1|	35437-35758	9.4	35	0	30	inf	0.00015	yes
gi|641565933|gb|KK857846.1|	37377-37520	9.4	35	0	213	inf	0.00455	no
gi|641565933|gb|KK857846.1|	38594-39145	9.4	35	0.198757	8	5	0.10655	no
gi|641565933|gb|KK857846.1|	40931-42236	9.4	35	6	9	0.655372	0.60035	no
gi|641565933|gb|KK857846.1|	42404-43223	9.4	35	0.794637	2	0.96544	0.5733	no
gi|641565933|gb|KK857846.1|	44852-45435	9.4	35	13	27	1	0.3942	no

gi|641565939|gb|KK857844.1|	10651-11801	8.8	16	3	3	-0.0105256	0.98515	no
gi|641565939|gb|KK857844.1|	1644-1946	8.8	16	11	5	-1	0.4252	no
gi|641565939|gb|KK857844.1|	21593-22409	8.8	16	5	1	-2	0.1844	no
gi|641565939|gb|KK857844.1|	23596-24198	8.8	16	3	0	-inf	0.0056	no
gi|641565939|gb|KK857844.1|	4053-5290	8.8	16	12	6	-0.938334	0.4697	no
gi|641565939|gb|KK857844.1|	42054-42872	8.8	16	2	1	-0.982108	0.5501	no
gi|641565939|gb|KK857844.1|	46479-47211	8.8	16	3	0	-inf	0.0013	yes
gi|641565939|gb|KK857844.1|	9359-10323	8.8	16	3	4	0.217628	0.8719	no
gi|641565949|gb|KK857840.1|	11271-12704	9.7	20	6	5	-0.339973	0.78585	no
gi|641565949|gb|KK857840.1|	1130-1948	9.7	20	4	3	-0.436674	0.7453	no
gi|641565949|gb|KK857840.1|	14598-15695	9.7	20	2	0.955951	-1	0.477	no
gi|641565949|gb|KK857840.1|	17781-18663	9.7	20	2	0.783417	-2	0.34745	no
gi|641565949|gb|KK857840.1|	24812-25108	9.7	20	40	18	-1	0.39795	no
gi|641565949|gb|KK857840.1|	25556-25726	9.7	20	36	13	-1	0.4362	no
gi|641565949|gb|KK857840.1|	41764-42971	9.7	20	0.769822	2	1	0.44465	no
gi|641565949|gb|KK857840.1|	5366-5762	9.7	20	6	3	-0.830733	0.59595	no
gi|641565949|gb|KK857840.1|	8241-8823	9.7	20	10	6	-0.643886	0.628	no
gi|641565949|gb|KK857840.1|	9385-9819	9.7	20	9	8	-0.117988	0.93075	no
gi|641565952|gb|KK857839.1|	15248-15674	10	32	7	8	0.298024	0.83445	no
gi|641565952|gb|KK857839.1|	15848-19923	10	32	32	24	-0.380872	0.8586	no
gi|641565952|gb|KK857839.1|	21122-22296	10	32	12	7	-0.840386	0.5097	no
gi|641565952|gb|KK857839.1|	25081-25589	10	32	4	2	-0.768928	0.6131	no
gi|641565952|gb|KK857839.1|	30944-32286	10	32	0.370469	0.658382	0.829573	1	no
gi|641565952|gb|KK857839.1|	38864-39351	10	32	3	4	0.45486	0.76285	no
gi|641565952|gb|KK857839.1|	43620-43910	10	32	36	0	-inf	0.0002	yes
gi|641565959|gb|KK857836.1|	13751-14288	11	40	4	1	-1	0.37245	no
gi|641565959|gb|KK857836.1|	16417-17033	11	40	0.672794	4	3	0.18535	no
gi|641565959|gb|KK857836.1|	26522-27311	11	40	5	28	2	0.0773	no
gi|641565959|gb|KK857836.1|	34092-34310	11	40	6	20	2	0.34065	no
gi|641565959|gb|KK857836.1|	35945-36938	11	40	0.266275	2	3	0.19335	no

gi|641565959|gb|KK857836.1|	37041-37287	11	40	13	12	-0.0338841	0.95145	no
gi|641565959|gb|KK857836.1|	38812-39019	11	40	2	30	4	0.1204	no
gi|641565959|gb|KK857836.1|	4-2347	11	40	2	9	2	0.1305	no
gi|641565959|gb|KK857836.1|	72144-73811	11	40	2	5	2	0.2356	no
gi|641565974|gb|KK857831.1|	11123-14186	9.1	38	3	4	0.244625	0.84665	no
gi|641565974|gb|KK857831.1|	279-630	9.1	38	0.88284	5	3	0.30875	no
gi|641565974|gb|KK857831.1|	40157-40637	9.1	38	68	38	-0.858412	0.4942	no
gi|641565974|gb|KK857831.1|	48590-49345	9.1	38	3	3	-0.15309	0.9102	no
gi|641565974|gb|KK857831.1|	4968-5808	9.1	38	0.54911	4	3	0.1279	no
gi|641565976|gb|KK857830.1|	33086-34007	10	39	1	7	3	0.09135	no
gi|641565978|gb|KK857829.1|	19563-22280	10	31	5	0.814213	-3	0.0595	no
gi|641565978|gb|KK857829.1|	22427-22974	10	31	3	0.303741	-3	0.21435	no
gi|641565978|gb|KK857829.1|	23107-28488	10	31	10	6	-0.868088	0.59455	no
gi|641565978|gb|KK857829.1|	2765-3599	10	31	0.332561	2	3	0.1811	no
gi|641565978|gb|KK857829.1|	28752-31223	10	31	7	2	-2	0.12805	no
gi|641565978|gb|KK857829.1|	31978-32373	10	31	3	2	-0.585741	0.70485	no
gi|641565978|gb|KK857829.1|	32526-34006	10	31	0.771572	1	0.385342	0.793	no
gi|641565978|gb|KK857829.1|	47724-50871	10	31	2	0.073238	-5	0.13115	no
gi|641565981|gb|KK857828.1|	64176-64482	11	44	2	8	2	0.2624	no
gi|641565993|gb|KK857824.1|	11580-12335	9.4	32	2	0	-inf	0.01	no
gi|641565993|gb|KK857824.1|	13597-13984	9.4	32	4	0.544524	-3	0.2309	no
gi|641565993|gb|KK857824.1|	40890-42114	9.4	32	0.550618	0.838403	0.60659	1	no
gi|641565993|gb|KK857824.1|	862-1973	9.4	32	0.850527	0.353025	-1	1	no
gi|641565998|gb|KK857822.1|	1021-1316	11	38	0	13	inf	0.00255	yes
gi|641565998|gb|KK857822.1|	20196-22770	11	38	1	3	0.943274	0.47365	no
gi|641565998|gb|KK857822.1|	23635-25853	11	38	11	26	1	0.44665	no
gi|641565998|gb|KK857822.1|	26875-27512	11	38	0.961368	1	0.598997	0.70555	no
gi|641565998|gb|KK857822.1|	28329-28907	11	38	1	2	0.181928	0.91015	no
gi|641565998|gb|KK857822.1|	3797-8151	11	38	11	2	-3	0.1421	no
gi|641565998|gb|KK857822.1|	52287-52921	11	38	24	26	0.098008	0.93795	no

gi|641565998|gb|KK857822.1|	8350-11616	11	38	14	6	-1	0.45095	no
gi|641566001|gb|KK857821.1|	25015-26075	9.8	33	0.982572	0.498331	-0.97946	0.5872	no
gi|641566001|gb|KK857821.1|	32292-32966	9.8	33	2	0.448273	-2	0.3216	no
gi|641566001|gb|KK857821.1|	34037-34527	9.8	33	2	1	-0.629836	0.71175	no
gi|641566001|gb|KK857821.1|	48220-48915	9.8	33	2	0.21481	-3	0.2381	no
gi|641566001|gb|KK857821.1|	52379-53152	9.8	33	2	2	-0.315417	0.83735	no
gi|641566003|gb|KK857820.1|	11873-13556	5.9	19	0.475922	1	1	0.36435	no
gi|641566003|gb|KK857820.1|	126-726	5.9	19	1	2	0.597789	0.70555	no
gi|641566003|gb|KK857820.1|	22905-23526	5.9	19	1	1	-0.208856	0.8945	no
gi|641566003|gb|KK857820.1|	38321-38774	5.9	19	17	5	-2	0.244	no
gi|641566003|gb|KK857820.1|	5592-6978	5.9	19	0	7	inf	5.00E-05	yes
gi|641566006|gb|KK857819.1|	126-2250	11	24	0.807625	1	0.397766	0.77935	no
gi|641566006|gb|KK857819.1|	20320-21031	11	24	0.686397	2	1	0.3994	no
gi|641566006|gb|KK857819.1|	27040-27887	11	24	0.325927	2	3	0.1811	no
gi|641566006|gb|KK857819.1|	44331-44588	11	24	7	46	3	0.10395	no
gi|641566006|gb|KK857819.1|	47927-49512	11	24	29	29	-0.0224436	0.9917	no
gi|641566006|gb|KK857819.1|	51401-51567	11	24	25	51	0.997328	0.60445	no
gi|641566006|gb|KK857819.1|	55867-56886	11	24	0.687653	1	0.927091	0.56225	no
gi|641566006|gb|KK857819.1|	5855-6335	11	24	10	18	0.814293	0.5408	no
gi|641566006|gb|KK857819.1|	59250-59658	11	24	2	1	-0.636712	0.71175	no
gi|641566006|gb|KK857819.1|	6492-7753	11	24	0.597939	2	2	0.19345	no
gi|641566006|gb|KK857819.1|	9547-11333	11	24	0.267063	0.407218	0.608619	1	no
gi|641566011|gb|KK857817.1|	11989-12602	7.8	33	0.338804	3	3	0.27515	no
gi|641566011|gb|KK857817.1|	18803-19168	7.8	33	0	133	inf	5.00E-05	yes
gi|641566011|gb|KK857817.1|	27150-28101	7.8	33	0	5	inf	0.0004	yes
gi|641566011|gb|KK857817.1|	34311-34663	7.8	33	0	26	inf	5.00E-05	yes
gi|641566014|gb|KK857816.1|	45256-45907	8.8	41	0	3	inf	0.00455	no
gi|641566026|gb|KK857812.1|	11372-12302	10	36	25	98	2	0.26005	no
gi|641566026|gb|KK857812.1|	16384-16684	10	36	2	8	2	0.33325	no
gi|641566026|gb|KK857812.1|	17805-19382	10	36	1	2	0.406423	0.7718	no

gi|641566026|gb|KK857812.1|	20425-21529	10	36	0.467498	1	1	0.37775	no
gi|641566026|gb|KK857812.1|	23-504	10	36	1	3	1	0.47105	no
gi|641566026|gb|KK857812.1|	5482-5684	10	36	4	30	3	0.27705	no
gi|641566026|gb|KK857812.1|	5853-7696	10	36	0.55862	0.851859	0.608747	1	no
gi|641566026|gb|KK857812.1|	8093-9319	10	36	4	15	2	0.16285	no
gi|641566035|gb|KK857809.1|	16982-17345	26	44	22	7	-2	0.2281	no
gi|641566035|gb|KK857809.1|	17568-18689	26	44	1	0.232819	-2	0.24735	no
gi|641566035|gb|KK857809.1|	18948-19666	26	44	2	0.205444	-3	0.20595	no
gi|641566035|gb|KK857809.1|	19893-24920	26	44	7	1	-2	0.08975	no
gi|641566035|gb|KK857809.1|	25370-25920	26	44	38	11	-2	0.1733	no
gi|641566035|gb|KK857809.1|	26366-28107	26	44	8	2	-2	0.10115	no
gi|641566035|gb|KK857809.1|	34031-34654	26	44	2	0.250534	-3	0.2381	no
gi|641566035|gb|KK857809.1|	34803-36585	26	44	11	2	-2	0.08935	no
gi|641566035|gb|KK857809.1|	36814-38019	26	44	2	0.533822	-2	0.3475	no
gi|641566035|gb|KK857809.1|	43697-44001	26	44	16	0	-inf	0.0013	yes
gi|641566035|gb|KK857809.1|	55131-56559	26	44	1	0.087515	-4	0.20475	no
gi|641566035|gb|KK857809.1|	58515-59464	26	44	1	0.142831	-3	0.21435	no
gi|641566035|gb|KK857809.1|	62087-63071	26	44	6	0.27305	-4	0.1339	no
gi|641566035|gb|KK857809.1|	64312-64939	26	44	22	2	-3	0.0425	no
gi|641566038|gb|KK857808.1|	17160-20870	13	44	12	3	-2	0.228	no
gi|641566038|gb|KK857808.1|	33564-34532	13	44	5	0	-inf	0.00025	yes
gi|641566038|gb|KK857808.1|	52129-53051	13	44	3	0	-inf	0.0015	yes
gi|641566038|gb|KK857808.1|	9628-9972	13	44	11	0	-inf	0.00315	yes
gi|641566041|gb|KK857807.1|	20334-21082	7.7	30	3	3	-0.452782	0.75065	no
gi|641566041|gb|KK857807.1|	22896-23951	7.7	30	0.905929	0.877126	-0.0466136	0.94545	no
gi|641566041|gb|KK857807.1|	24847-25535	7.7	30	0.431035	3	3	0.19335	no
gi|641566041|gb|KK857807.1|	29828-30546	7.7	30	0.677216	2	1	0.3994	no
gi|641566041|gb|KK857807.1|	32010-32798	7.7	30	0.955655	4	2	0.1909	no
gi|641566041|gb|KK857807.1|	32917-33404	7.7	30	2	2	0.106937	0.92265	no
gi|641566041|gb|KK857807.1|	37729-38996	7.7	30	2	3	1	0.4553	no

gi|641566055|gb|KK857802.1|	17388-18043	8.6	28	0.923825	1	0.336482	0.83525	no
gi|641566055|gb|KK857802.1|	2921-3670	8.6	28	7	12	0.804501	0.5475	no
gi|641566055|gb|KK857802.1|	9471-9755	8.6	28	10	16	0.66276	0.6471	no
gi|641566060|gb|KK857800.1|	18267-20065	9.5	37	2	13	3	0.03655	no
gi|641566060|gb|KK857800.1|	20264-20513	9.5	37	13	82	3	0.0913	no
gi|641566060|gb|KK857800.1|	20728-22105	9.5	37	0.119832	1	3	0.2672	no
gi|641566060|gb|KK857800.1|	22667-23898	9.5	37	0.615259	3	2	0.1801	no
gi|641566060|gb|KK857800.1|	24675-25030	9.5	37	3	46	4	0.06535	no
gi|641566060|gb|KK857800.1|	38880-39636	9.5	37	3	15	2	0.0983	no
gi|641566065|gb|KK857798.1|	6348-7512	7.5	29	0.365452	0.667584	0.86927	1	no
gi|641566074|gb|KK857795.1|	36-336	10	36	7	7	-0.0175892	0.95145	no
gi|641566077|gb|KK857794.1|	11814-12597	9.9	19	2	0	-inf	0.0059	no
gi|641566077|gb|KK857794.1|	20936-21448	9.9	19	2	1	-0.406132	0.82205	no
gi|641566077|gb|KK857794.1|	25987-27130	9.9	19	0.597679	1	1	0.4562	no
gi|641566077|gb|KK857794.1|	28012-29364	9.9	19	2	1	-0.0366308	0.9722	no
gi|641566077|gb|KK857794.1|	37771-37947	9.9	19	19	37	1	0.5133	no
gi|641566077|gb|KK857794.1|	40274-41823	9.9	19	0.418425	0.876875	1	0.49695	no
gi|641566077|gb|KK857794.1|	49143-49969	9.9	19	0.898077	1	0.602975	0.6936	no
gi|641566080|gb|KK857793.1|	28494-28768	17	35	9	13	0.56038	0.7212	no
gi|641566083|gb|KK857792.1|	11870-12176	9.6	37	1	8	3	0.28555	no
gi|641566083|gb|KK857792.1|	12431-13384	9.6	37	0.467219	1	1	0.3994	no
gi|641566085|gb|KK857791.1|	15559-15773	11	38	12	36	2	0.30555	no
gi|641566085|gb|KK857791.1|	16745-17258	11	38	4	4	0.00896136	0.9647	no
gi|641566085|gb|KK857791.1|	24184-24797	11	38	4	6	0.598237	0.6601	no
gi|641566085|gb|KK857791.1|	25616-27197	11	38	4	15	2	0.13495	no
gi|641566085|gb|KK857791.1|	30755-32998	11	38	0.311192	2	3	0.08545	no
gi|641566085|gb|KK857791.1|	34332-34959	11	38	2	0.99297	-0.723238	0.67005	no
gi|641566085|gb|KK857791.1|	35421-35931	11	38	1	3	1	0.39995	no
gi|641566091|gb|KK857789.1|	14831-15088	7.3	23	60	12	-2	0.128	no
gi|641566091|gb|KK857789.1|	171-357	7.3	23	514	170	-2	0.22885	no

gi|641566091|gb|KK857789.1|	30172-30560	7.3	23	3	0.541888	-3	0.2588	no
gi|641566091|gb|KK857789.1|	39027-40459	7.3	23	0.229005	3	4	0.12815	no
gi|641566091|gb|KK857789.1|	491-626	7.3	23	405	95	-2	0.24355	no
gi|641566094|gb|KK857788.1|	10636-10860	9.6	18	14	14	0.0274465	0.94155	no
gi|641566094|gb|KK857788.1|	19764-20485	9.6	18	2	2	-0.398878	0.7915	no
gi|641566094|gb|KK857788.1|	25392-26228	9.6	18	0.221015	2	3	0.2672	no
gi|641566094|gb|KK857788.1|	26504-28499	9.6	18	1	2	0.60905	0.6587	no
gi|641566094|gb|KK857788.1|	29317-29967	9.6	18	0.466979	2	2	0.2812	no
gi|641566094|gb|KK857788.1|	38507-39584	9.6	18	3	4	0.734917	0.5902	no
gi|641566094|gb|KK857788.1|	41154-42119	9.6	18	0.91987	2	1	0.4325	no
gi|641566094|gb|KK857788.1|	43096-43572	9.6	18	4	3	-0.045803	0.9565	no
gi|641566094|gb|KK857788.1|	50900-51997	9.6	18	6	4	-0.453111	0.7245	no
gi|641566094|gb|KK857788.1|	59222-59523	9.6	18	4	4	-0.0173439	0.91115	no
gi|641566094|gb|KK857788.1|	6166-6530	9.6	18	3	2	-0.420052	0.7868	no
gi|641566094|gb|KK857788.1|	6970-7697	9.6	18	5	5	-0.160593	0.90315	no
gi|641566094|gb|KK857788.1|	78-416	9.6	18	5	9	0.701089	0.64045	no
gi|641566136|gb|KK857772.1|	13274-14742	8.7	36	0.945743	0	-inf	0.00435	no
gi|641566136|gb|KK857772.1|	14846-15522	8.7	36	3	0	-inf	0.00435	no
gi|641566136|gb|KK857772.1|	21226-21915	8.7	36	2	0	-inf	0.0075	no
gi|641566136|gb|KK857772.1|	23259-24438	8.7	36	1	0	-inf	0.00345	yes
gi|641566136|gb|KK857772.1|	29663-30707	8.7	36	1	0	-inf	0.0056	no
gi|641566136|gb|KK857772.1|	32227-33295	8.7	36	2	0	-inf	0.00495	no
gi|641566136|gb|KK857772.1|	35896-36393	8.7	36	3	0	-inf	0.01	no
gi|641566151|gb|KK857767.1|	36108-36575	8.2	36	2	2	-0.257011	0.88485	no
gi|641566160|gb|KK857763.1|	11929-13667	10	21	4	7	0.912509	0.4716	no
gi|641566160|gb|KK857763.1|	35468-36498	10	21	0.424119	0.903268	1	0.50945	no
gi|641566160|gb|KK857763.1|	37848-38905	10	21	0.821661	1	0.605479	0.67795	no
gi|641566162|gb|KK857762.1|	407-1247	9.3	19	12	9	-0.44929	0.72835	no
gi|641566162|gb|KK857762.1|	54160-54531	9.3	19	4	0.590295	-3	0.2476	no
gi|641566167|gb|KK857760.1|	14492-14984	8.9	40	5	7	0.391049	0.7778	no

gi|641566167|gb|KK857760.1|	17077-18022	8.9	40	0.661086	0.574354	-0.202898	1	no
gi|641566167|gb|KK857760.1|	18807-19849	8.9	40	0.668973	1	0.605358	0.6936	no
gi|641566167|gb|KK857760.1|	26947-28494	8.9	40	2	1	-0.211466	0.88	no
gi|641566167|gb|KK857760.1|	29686-30245	8.9	40	2	2	0.181141	0.91015	no
gi|641566167|gb|KK857760.1|	33016-33948	8.9	40	5	5	-0.274375	0.8326	no
gi|641566167|gb|KK857760.1|	36615-37107	8.9	40	0	33	inf	5.00E-05	yes
gi|641566170|gb|KK857759.1|	10499-11014	8.5	36	0.220931	4	4	0.1196	no
gi|641566170|gb|KK857759.1|	27061-27575	8.5	36	0	36	inf	5.00E-05	yes
gi|641566170|gb|KK857759.1|	32025-32845	8.5	36	0.793365	1	0.380493	0.82465	no
gi|641566170|gb|KK857759.1|	5240-6528	8.5	36	1	5	2	0.13645	no
gi|641566170|gb|KK857759.1|	77-1329	8.5	36	0.603032	2	2	0.26135	no
gi|641566170|gb|KK857759.1|	8172-9245	8.5	36	0.161336	2	4	0.243	no
gi|641566172|gb|KK857758.1|	28368-29051	11	43	0.870893	0.880171	0.0152875	0.91115	no
gi|641566172|gb|KK857758.1|	30758-32530	11	43	0.763398	2	1	0.3948	no
gi|641566172|gb|KK857758.1|	32886-33585	11	43	1	3	2	0.3469	no
gi|641566172|gb|KK857758.1|	34434-35013	11	43	1	3	1	0.47095	no
gi|641566172|gb|KK857758.1|	35129-35559	11	43	2	4	1	0.47105	no
gi|641566172|gb|KK857758.1|	35653-36961	11	43	0.954471	1	0.400573	0.78645	no
gi|641566176|gb|KK857756.1|	13922-14485	12	42	0.576941	2	2	0.2812	no
gi|641566178|gb|KK857755.1|	51475-51762	9.4	36	0	86	inf	0.0001	yes
gi|641566181|gb|KK857754.1|	11805-13066	8.8	33	12	34	1	0.39125	no
gi|641566181|gb|KK857754.1|	14931-16455	8.8	33	9	18	0.911563	0.4612	no
gi|641566181|gb|KK857754.1|	1641-2070	8.8	33	4	17	2	0.16395	no
gi|641566181|gb|KK857754.1|	16566-18984	8.8	33	9	20	1	0.47525	no
gi|641566181|gb|KK857754.1|	243-653	8.8	33	3	6	0.848933	0.5678	no
gi|641566181|gb|KK857754.1|	24547-25874	8.8	33	2	2	-0.305425	0.8236	no
gi|641566181|gb|KK857754.1|	26018-26531	8.8	33	2	1	-1	0.50365	no
gi|641566181|gb|KK857754.1|	26644-28873	8.8	33	2	0.796732	-1	0.328	no
gi|641566181|gb|KK857754.1|	5061-5480	8.8	33	1	3	1	0.51315	no
gi|641566184|gb|KK857753.1|	41646-42361	7.3	32	0.272448	4	4	0.24065	no

gi|641566187|gb|KK857752.1|	10944-11458	8.8	21	1	2	0.593979	0.70555	no
gi|641566189|gb|KK857751.1|	34189-34574	7.4	32	0	353	inf	5.00E-05	yes
gi|641566189|gb|KK857751.1|	36072-36485	7.4	32	0	5	inf	0.0068	no
gi|641566189|gb|KK857751.1|	36574-37557	7.4	32	0	2	inf	0.00135	yes
gi|641566189|gb|KK857751.1|	43541-44110	7.4	32	0	9	inf	0.0003	yes
gi|641566198|gb|KK857747.1|	1040-1481	11	20	2	4	1	0.48925	no
gi|641566198|gb|KK857747.1|	10774-11821	11	20	0.415654	1	1	0.3994	no
gi|641566198|gb|KK857747.1|	14148-15741	11	20	0.810507	0.463283	-0.806932	1	no
gi|641566198|gb|KK857747.1|	15873-16394	11	20	2	2	-0.0837138	0.9267	no
gi|641566198|gb|KK857747.1|	18174-19678	11	20	6	4	-0.601635	0.6402	no
gi|641566198|gb|KK857747.1|	20044-21730	11	20	2	1	-0.143703	0.92105	no
gi|641566198|gb|KK857747.1|	21921-23475	11	20	1	0.635387	-0.851447	0.5652	no
gi|641566198|gb|KK857747.1|	23629-27968	11	20	3	5	0.647069	0.6065	no
gi|641566198|gb|KK857747.1|	28580-29696	11	20	12	25	1	0.4112	no
gi|641566198|gb|KK857747.1|	2953-4686	11	20	3	1	-1	0.3536	no
gi|641566198|gb|KK857747.1|	30187-33369	11	20	1	1	-0.171003	0.8949	no
gi|641566198|gb|KK857747.1|	540-898	11	20	1	5	2	0.3198	no
gi|641566198|gb|KK857747.1|	54392-56498	11	20	1	2	0.872276	0.521	no
gi|641566198|gb|KK857747.1|	80205-81421	11	20	10	15	0.557636	0.65395	no
gi|641566198|gb|KK857747.1|	8022-8940	11	20	0.685784	0.595691	-0.203192	1	no
gi|641566198|gb|KK857747.1|	83776-84347	11	20	43	4	-3	0.02885	no
gi|641566198|gb|KK857747.1|	9941-10282	11	20	4	2	-1	0.5278	no
gi|641566217|gb|KK857740.1|	10578-11306	11	22	0.664517	2	1	0.4611	no
gi|641566217|gb|KK857740.1|	16479-17749	11	22	0.527051	3	2	0.1732	no
gi|641566217|gb|KK857740.1|	18378-20888	11	22	2	2	-0.23074	0.8532	no
gi|641566217|gb|KK857740.1|	20956-22668	11	22	2	0.854064	-1	0.32795	no
gi|641566217|gb|KK857740.1|	22728-23727	11	22	2	0.669951	-1	0.43755	no
gi|641566217|gb|KK857740.1|	25290-25909	11	22	2	0.252868	-3	0.271	no
gi|641566217|gb|KK857740.1|	2720-3510	11	22	1	0.723022	-0.719518	0.6701	no
gi|641566217|gb|KK857740.1|	27322-28175	11	22	2	3	0.298503	0.8318	no

gi|641566217|gb|KK857740.1|	4348-5975	11	22	0.692352	0.98001	0.501291	0.73235	no
gi|641566217|gb|KK857740.1|	7248-9261	11	22	0.895895	2	1	0.3065	no
gi|641566217|gb|KK857740.1|	87-911	11	22	0.675701	2	1	0.4118	no
gi|641566220|gb|KK857739.1|	22718-23779	12	40	1	0.248879	-2	0.25005	no
gi|641566220|gb|KK857739.1|	24228-25489	12	40	0.86369	0	-inf	1	no
gi|641566220|gb|KK857739.1|	26455-28061	12	40	1	0.153009	-3	0.1621	no
gi|641566220|gb|KK857739.1|	28169-28699	12	40	3	0	-inf	0.01	no
gi|641566220|gb|KK857739.1|	29047-30590	12	40	1	0	-inf	0.0021	yes
gi|641566220|gb|KK857739.1|	33875-34440	12	40	2	0	-inf	0.01	no
gi|641566220|gb|KK857739.1|	36109-37555	12	40	1	0.086259	-4	0.1977	no
gi|641566220|gb|KK857739.1|	38275-39251	12	40	3	0.551668	-2	0.1916	no
gi|641566220|gb|KK857739.1|	39467-46645	12	40	14	0.759107	-4	0.0488	no
gi|641566220|gb|KK857739.1|	46992-47614	12	40	18	2	-3	0.05605	no
gi|641566220|gb|KK857739.1|	49354-49587	12	40	289	16	-4	0.0211	no
gi|641566220|gb|KK857739.1|	50128-50461	12	40	24	0.73477	-5	0.18455	no
gi|641566220|gb|KK857739.1|	51345-52600	12	40	0.534511	0.610478	0.191721	1	no
gi|641566220|gb|KK857739.1|	54456-55045	12	40	6	5	-0.315148	0.82045	no
gi|641566220|gb|KK857739.1|	55803-56467	12	40	23	8	-2	0.2446	no
gi|641566220|gb|KK857739.1|	58013-59157	12	40	13	5	-1	0.29215	no
gi|641566220|gb|KK857739.1|	62289-63582	12	40	0.70951	2	1	0.3575	no
gi|641566220|gb|KK857739.1|	7842-8960	12	40	0.690607	0.817504	0.243361	1	no
gi|641566230|gb|KK857737.1|	17030-17423	6.3	20	114	198.4	0.80377	0.62355	no
gi|641566236|gb|KK857735.1|	36801-37009	8.8	34	13	11	-0.272971	0.8666	no
gi|641566239|gb|KK857734.1|	12790-13007	9.7	38	0	140	inf	5.00E-05	yes
gi|641566239|gb|KK857734.1|	30735-31410	9.7	38	0	105	inf	0.0003	yes
gi|641566239|gb|KK857734.1|	6322-6925	9.7	38	0	7	inf	0.00045	yes
gi|641566242|gb|KK857733.1|	29612-29774	8.3	32	35	33	-0.0752897	0.91115	no
gi|641566242|gb|KK857733.1|	32513-33315	8.3	32	1	2	0.602605	0.6902	no
gi|641566242|gb|KK857733.1|	34806-36091	8.3	32	0.519796	2	2	0.2528	no
gi|641566242|gb|KK857733.1|	37991-38843	8.3	32	0.21563	2	3	0.2556	no

gi|641566247|gb|KK857731.1|	0-486	9.4	35	25	42	0.724749	0.5627	no
gi|641566247|gb|KK857731.1|	15393-15861	9.4	35	4	11	1	0.3812	no
gi|641566247|gb|KK857731.1|	22465-23118	9.4	35	2	2	-0.500075	0.74225	no
gi|641566247|gb|KK857731.1|	23652-24125	9.4	35	2	4	0.913345	0.56635	no
gi|641566247|gb|KK857731.1|	29034-29272	9.4	35	35	17	-1	0.46355	no
gi|641566247|gb|KK857731.1|	2906-4651	9.4	35	25	14	-0.799564	0.6264	no
gi|641566247|gb|KK857731.1|	30239-31226	9.4	35	0.715447	0.544042	-0.395126	1	no
gi|641566247|gb|KK857731.1|	33437-34217	9.4	35	5	4	-0.159599	0.90485	no
gi|641566247|gb|KK857731.1|	36057-36255	9.4	35	11	19	0.79226	0.6531	no
gi|641566247|gb|KK857731.1|	36627-37447	9.4	35	4	4	-0.174723	0.8944	no
gi|641566247|gb|KK857731.1|	37793-38931	9.4	35	1	1	0.103567	0.94435	no
gi|641566247|gb|KK857731.1|	39053-43768	9.4	35	4	3	-0.44388	0.7176	no
gi|641566247|gb|KK857731.1|	45470-46939	9.4	35	0.444706	0.338826	-0.392306	1	no
gi|641566247|gb|KK857731.1|	47424-47850	9.4	35	4	3	-0.41247	0.80855	no
gi|641566247|gb|KK857731.1|	51309-52161	9.4	35	8	5	-0.756552	0.5606	no
gi|641566247|gb|KK857731.1|	5979-6917	9.4	35	0.476655	0.724672	0.604383	1	no
gi|641566247|gb|KK857731.1|	8651-10252	9.4	35	1	1	0.332495	0.81315	no
gi|641566250|gb|KK857730.1|	8882-9547	8.7	35	2	1	-0.137177	0.92965	no
gi|641566253|gb|KK857729.1|	10619-10844	9.6	35	8	22	1	0.3871	no
gi|641566253|gb|KK857729.1|	11169-11394	9.6	35	13	34	1.46	0.347	no
gi|641566253|gb|KK857729.1|	12159-12551	9.6	35	3	7	1	0.3905	no
gi|641566253|gb|KK857729.1|	20260-20771	9.6	35	1	10	3	0.11265	no
gi|641566253|gb|KK857729.1|	21213-22164	9.6	35	0.093691	1	4	0.12385	no
gi|641566253|gb|KK857729.1|	22368-22819	9.6	35	0.825765	14	4	0.14015	no
gi|641566253|gb|KK857729.1|	23031-24004	9.6	35	0.364163	7	4	0.11855	no
gi|641566253|gb|KK857729.1|	26975-28077	9.6	35	1	6	2	0.1518	no
gi|641566253|gb|KK857729.1|	28749-29089	9.6	35	3	5	0.798319	0.6409	no
gi|641566255|gb|KK857728.1|	11964-15850	8.3	32	13.64	9	-0.541043	0.73185	no
gi|641566255|gb|KK857728.1|	20108-21630	8.3	32	5	6	0.335507	0.79355	no
gi|641566255|gb|KK857728.1|	22036-22832	8.3	32	6	9	0.661402	0.6107	no

gi|641566255|gb|KK857728.1|	23559-24669	8.3	32	9	7	-0.356971	0.7832	no
gi|641566255|gb|KK857728.1|	24884-25558	8.3	32	2	0.224137	-3	0.2089	no
gi|641566255|gb|KK857728.1|	26559-27761	8.3	32	2	0.749569	-1	0.46025	no
gi|641566255|gb|KK857728.1|	35588-35830	8.3	32	16.61	5	-2	0.3233	no
gi|641566267|gb|KK857724.1|	17352-17816	11	39	1	2	1	0.51315	no
gi|641566267|gb|KK857724.1|	37388-39787	11	39	1	0.980512	-0.112813	0.9324	no
gi|641566267|gb|KK857724.1|	40528-41409	11	39	10	28	2	0.2365	no
gi|641566267|gb|KK857724.1|	44013-44162	11	39	131	242	0.889287	0.54545	no
gi|641566269|gb|KK857723.1|	10095-11185	9.4	38	1	2	0.605731	0.67415	no
gi|641566269|gb|KK857723.1|	11349-12510	9.4	38	0.513222	0.558041	0.120789	1	no
gi|641566269|gb|KK857723.1|	15460-16787	9.4	38	0.938447	0.57178	-0.714815	0.6527	no
gi|641566269|gb|KK857723.1|	2402-2829	9.4	38	5	2	-1	0.43485	no
gi|641566269|gb|KK857723.1|	29619-30520	9.4	38	0.501646	1	1	0.4595	no
gi|641566269|gb|KK857723.1|	6119-8127	9.4	38	0.66401	0.595765	-0.156461	1	no
gi|641566269|gb|KK857723.1|	8295-8794	9.4	38	3	4	0.214602	0.8867	no
gi|641566269|gb|KK857723.1|	86-917	9.4	38	1	2	1	0.47685	no
gi|641566269|gb|KK857723.1|	9520-9973	9.4	38	0.819497	5	3	0.19335	no
gi|641566272|gb|KK857722.1|	12721-13089	9	37	4	2	-1	0.5278	no
gi|641566272|gb|KK857722.1|	16632-17100	9	37	2	3	1	0.5082	no
gi|641566272|gb|KK857722.1|	17975-18681	9	37	2	4	0.922721	0.52235	no
gi|641566272|gb|KK857722.1|	18837-20445	9	37	0.551307	0.61117	0.148719	1	no
gi|641566272|gb|KK857722.1|	20567-21039	9	37	2	3	0.761271	0.6278	no
gi|641566272|gb|KK857722.1|	21372-21910	9	37	5	7	0.479742	0.7192	no
gi|641566272|gb|KK857722.1|	22066-22547	9	37	3	3	0.0070055	0.95145	no
gi|641566272|gb|KK857722.1|	25566-26028	9	37	0	54	inf	5.00E-05	yes
gi|641566272|gb|KK857722.1|	26434-27188	9	37	0	5	inf	0.00065	yes
gi|641566272|gb|KK857722.1|	29840-30585	9	37	0	2	inf	0.00375	no
gi|641566272|gb|KK857722.1|	36393-37893	9	37	0	1	inf	0.0016	yes
gi|641566272|gb|KK857722.1|	40924-41116	9	37	0	87	inf	0.00075	yes
gi|641566272|gb|KK857722.1|	47992-48512	9	37	0.87025	12	4	0.12375	no

gi|641566272|gb|KK857722.1|	72398-73149	9	37	13.76	5	-2	0.2402	no
gi|641566274|gb|KK857721.1|	3089-3633	11	46	0	11	inf	5.00E-05	yes
gi|641566274|gb|KK857721.1|	6513-7455	11	46	0	5	inf	0.0004	yes
gi|641566277|gb|KK857720.1|	0-613	3	25	4	0.512903	-3	0.16885	no
gi|641566277|gb|KK857720.1|	13732-14717	3	25	1	4	2	0.22225	no
gi|641566277|gb|KK857720.1|	6115-6345	3	25	10	34	2	0.28575	no
gi|641566280|gb|KK857719.1|	1-557	11	23	0.784083	2	1	0.44255	no
gi|641566293|gb|KK857714.1|	11811-12412	7.6	29	2	2	0.0128621	0.9394	no
gi|641566293|gb|KK857714.1|	19752-20151	7.6	29	3	2	-1	0.5278	no
gi|641566293|gb|KK857714.1|	22423-22896	7.6	29	3	3	0.00645461	0.95145	no
gi|641566296|gb|KK857713.1|	12535-13723	9.7	34	3	1	-1	0.3511	no
gi|641566296|gb|KK857713.1|	13905-14859	9.7	34	3	2	-0.565375	0.6925	no
gi|641566296|gb|KK857713.1|	14-834	9.7	34	1	2	1	0.49595	no
gi|641566296|gb|KK857713.1|	15640-17589	9.7	34	6	5	-0.0401287	0.97195	no
gi|641566296|gb|KK857713.1|	20385-20918	9.7	34	2	0.632162	-2	0.3216	no
gi|641566296|gb|KK857713.1|	20979-21938	9.7	34	1	0.986793	-0.494935	0.7427	no
gi|641566296|gb|KK857713.1|	22446-25110	9.7	34	11	8	-0.332878	0.7895	no
gi|641566296|gb|KK857713.1|	26137-27370	9.7	34	0.955225	0.935055	-0.0307898	0.95655	no
gi|641566296|gb|KK857713.1|	27499-28132	9.7	34	20	16	-0.355321	0.77645	no
gi|641566296|gb|KK857713.1|	4996-7386	9.7	34	0.677479	0.886073	0.387249	0.7817	no
gi|641566296|gb|KK857713.1|	7512-9181	9.7	34	7	10	0.576846	0.64635	no
gi|641566302|gb|KK857711.1|	0-649	9.3	36	2	1	-1	0.4955	no
gi|641566302|gb|KK857711.1|	14562-15159	9.3	36	2	0.53304	-2	0.4266	no
gi|641566302|gb|KK857711.1|	24181-25672	9.3	36	5	1	-2	0.22255	no
gi|641566302|gb|KK857711.1|	27040-38640	9.3	36	20	11	-0.937964	0.6446	no
gi|641566302|gb|KK857711.1|	39073-39865	9.3	36	61	20	-2	0.3466	no
gi|641566302|gb|KK857711.1|	41441-43496	9.3	36	5	8	0.598185	0.62985	no
gi|641566302|gb|KK857711.1|	43713-44564	9.3	36	7	17	1	0.34175	no
gi|641566302|gb|KK857711.1|	46743-47272	9.3	36	2	2	-0.0832985	0.9267	no
gi|641566302|gb|KK857711.1|	48415-48686	9.3	36	21	46	1	0.3939	no

gi|641566302|gb|KK857711.1|	50940-51665	9.3	36	6	6	0.0162435	0.9856	no
gi|641566302|gb|KK857711.1|	57607-60164	9.3	36	0.659553	0.915029	0.472329	0.73935	no
gi|641566302|gb|KK857711.1|	6754-7607	9.3	36	3	0.327098	-3	0.1461	no
gi|641566302|gb|KK857711.1|	68418-68794	9.3	36	12	0	-inf	0.0013	yes
gi|641566302|gb|KK857711.1|	75930-76801	9.3	36	0.209567	2	3	0.2672	no
gi|641566312|gb|KK857707.1|	314-731	9.9	32	6	2	-2	0.362	no
gi|641566316|gb|KK857706.1|	29392-29668	7.7	33	29	39	0.400504	0.76125	no
gi|641566316|gb|KK857706.1|	30952-31279	7.7	33	6	2	-2	0.27505	no
gi|641566316|gb|KK857706.1|	41614-41842	7.7	33	11	17	0.713711	0.65605	no
gi|641566331|gb|KK857700.1|	27271-28389	9.9	20	1	3	1	0.445	no
gi|641566331|gb|KK857700.1|	29605-30239	9.9	20	0.806602	1	0.861941	0.62755	no
gi|641566334|gb|KK857699.1|	2728-3151	9.8	37	3	5	0.587238	0.6863	no
gi|641566340|gb|KK857697.1|	12584-13681	11	22	18	29	0.70575	0.565	no
gi|641566340|gb|KK857697.1|	15015-15997	11	22	0.269998	2	3	0.1666	no
gi|641566340|gb|KK857697.1|	17376-19478	11	22	45	89	0.992443	0.6396	no
gi|641566340|gb|KK857697.1|	19905-21158	11	22	31	63	1	0.5164	no
gi|641566340|gb|KK857697.1|	21240-21734	11	22	5	11	1	0.40665	no
gi|641566340|gb|KK857697.1|	21902-22171	11	22	3	11	2	0.33975	no
gi|641566340|gb|KK857697.1|	37370-38301	11	22	0.673666	3	2	0.1741	no
gi|641566348|gb|KK857694.1|	20379-20905	12	23	7	11	0.59462	0.65855	no
gi|641566348|gb|KK857694.1|	20956-23098	12	23	3	4	0.629762	0.619	no
gi|641566358|gb|KK857690.1|	14005-15667	12	33	176	97	-0.857076	0.66945	no
gi|641566358|gb|KK857690.1|	15928-19849	12	33	15	11	-0.363121	0.87125	no
gi|641566358|gb|KK857690.1|	20026-25161	12	33	4	2	-0.557314	0.6635	no
gi|641566358|gb|KK857690.1|	25294-26852	12	33	1	0.316764	-2	0.22015	no
gi|641566358|gb|KK857690.1|	27005-28176	12	33	3	3	0.0213163	0.98325	no
gi|641566358|gb|KK857690.1|	31793-32329	12	33	3	1	-1	0.44155	no
gi|641566358|gb|KK857690.1|	33513-34498	12	33	0.268972	1	2	0.2812	no
gi|641566358|gb|KK857690.1|	34623-37770	12	33	1	1	0.0391762	0.9763	no
gi|641566358|gb|KK857690.1|	38003-38772	12	33	1	1	-0.272419	0.86195	no

gi|641566358|gb|KK857690.1|	40934-42541	12	33	0.902777	0.458702	-0.976815	0.5501	no
gi|641566358|gb|KK857690.1|	44542-45685	12	33	3	3	-0.262655	0.8486	no
gi|641566367|gb|KK857687.1|	32037-32477	7.4	35	5	1	-2	0.2877	no
gi|641566375|gb|KK857686.1|	37361-38029	10	39	60	28	-1	0.382	no
gi|641566375|gb|KK857686.1|	39728-40145	10	39	59	28	-1	0.3878	no
gi|641566375|gb|KK857686.1|	40807-42646	10	39	1	1	0.3572	0.79455	no
gi|641566375|gb|KK857686.1|	42991-43603	10	39	0.849035	3	2	0.2851	no
gi|641566380|gb|KK857684.1|	1-226	10	38	4	14	2	0.3484	no
gi|641566380|gb|KK857684.1|	26324-30689	10	38	14	11	-0.366846	0.87405	no
gi|641566380|gb|KK857684.1|	32032-34121	10	38	7	2	-2	0.20345	no
gi|641566380|gb|KK857684.1|	35836-36343	10	38	2	1	-0.821452	0.6492	no
gi|641566380|gb|KK857684.1|	41959-42431	10	38	4	3	-0.238729	0.87495	no
gi|641566380|gb|KK857684.1|	51845-52033	10	38	0	47	inf	0.00375	no
gi|641566380|gb|KK857684.1|	53587-53800	10	38	0	68	inf	0.00035	yes
gi|641566380|gb|KK857684.1|	62696-62989	10	38	1	32	4.61	0.235	no
gi|641566380|gb|KK857684.1|	63102-64009	10	38	0.198967	7	5	0.23345	no
gi|641566380|gb|KK857684.1|	70373-70790	10	38	3	6	0.849669	0.5678	no
gi|641566385|gb|KK857682.1|	17083-18418	9.3	43	7	0.473151	-4	0.04955	no
gi|641566385|gb|KK857682.1|	18783-19890	9.3	43	9	0	-inf	5.00E-05	yes
gi|641566385|gb|KK857682.1|	20018-20625	9.3	43	6	0	-inf	0.00085	yes
gi|641566385|gb|KK857682.1|	21024-22574	9.3	43	2	0	-inf	0.0015	yes
gi|641566385|gb|KK857682.1|	23011-26576	9.3	43	2	0.064165	-5	0.1305	no
gi|641566385|gb|KK857682.1|	27415-27817	9.3	43	5	0	-inf	0.0056	no
gi|641566385|gb|KK857682.1|	27936-28535	9.3	43	3	0	-inf	0.00495	no
gi|641566385|gb|KK857682.1|	29650-31665	9.3	43	2	0	-inf	0.0003	yes
gi|641566385|gb|KK857682.1|	31815-32555	9.3	43	5	0	-inf	0.00115	yes
gi|641566385|gb|KK857682.1|	33578-37242	9.3	43	5	0.062335	-6	0.1302	no
gi|641566385|gb|KK857682.1|	37313-41638	9.3	43	6	2	-2	0.21285	no
gi|641566385|gb|KK857682.1|	42539-43022	9.3	43	7	0	-inf	0.0013	yes
gi|641566385|gb|KK857682.1|	46493-48771	9.3	43	1	0.051868	-5	0.18485	no

gi|641566385|gb|KK857682.1|	54583-56341	9.3	43	2	0	-inf	0.0006	yes
gi|641566385|gb|KK857682.1|	56466-56882	9.3	43	4	0	-inf	0.0059	no
gi|641566385|gb|KK857682.1|	57241-58106	9.3	43	2	0	-inf	0.00435	no
gi|641566385|gb|KK857682.1|	6444-7573	9.3	43	47	0.115416	-9	0.1856	no
gi|641566385|gb|KK857682.1|	64874-65244	9.3	43	5	0	-inf	0.0075	no
gi|641566385|gb|KK857682.1|	9724-16850	9.3	43	4	0.280947	-4	0.01455	no
gi|641566390|gb|KK857680.1|	23021-23318	9.2	29	0	18	inf	0.0015	yes
gi|641566395|gb|KK857678.1|	12879-15205	10	19	13	15	0.265752	0.8269	no
gi|641566395|gb|KK857678.1|	31336-32440	10	19	10	2	-3	0.0746	no
gi|641566395|gb|KK857678.1|	3226-4112	10	19	0.820064	1	0.773711	0.62605	no
gi|641566395|gb|KK857678.1|	32979-33548	10	19	5	0.572334	-3	0.15585	no
gi|641566395|gb|KK857678.1|	40110-40863	10	19	5	9	0.864794	0.5102	no
gi|641566395|gb|KK857678.1|	45082-47062	10	19	8	8	0.0425328	0.9733	no
gi|641566395|gb|KK857678.1|	50-519	10	19	2	1	-0.823906	0.64915	no
gi|641566397|gb|KK857677.1|	12988-13371	10	43	0	418.71	inf	0.0002	yes
gi|641566397|gb|KK857677.1|	14375-14712	10	43	0	228	inf	5.00E-05	yes
gi|641566397|gb|KK857677.1|	17317-17633	10	43	0	249	inf	5.00E-05	yes
gi|641566397|gb|KK857677.1|	20849-21353	10	43	0	38	inf	5.00E-05	yes
gi|641566403|gb|KK857674.1|	29908-31657	10	44	0	17	inf	5.00E-05	yes
gi|641566405|gb|KK857673.1|	20221-21435	8.8	37	0.48652	2	2	0.20985	no
gi|641566405|gb|KK857673.1|	36630-37584	8.8	37	0	146	inf	0.0001	yes
gi|641566405|gb|KK857673.1|	41095-41448	8.8	37	0	131	inf	5.00E-05	yes
gi|641566408|gb|KK857672.1|	40044-40699	11	43	1	3	1	0.49595	no
gi|641566408|gb|KK857672.1|	41183-42103	11	43	0.586193	1	0.826578	0.6232	no
gi|641566408|gb|KK857672.1|	47810-48620	11	43	1	1	-0.097708	0.9478	no
gi|641566408|gb|KK857672.1|	62359-63935	11	43	0.974327	1	0.608054	0.66095	no
gi|641566408|gb|KK857672.1|	64913-65887	11	43	0.909238	1	0.282822	0.85465	no
gi|641566408|gb|KK857672.1|	68515-70443	11	43	0.081726	2	5	0.2335	no
gi|641566408|gb|KK857672.1|	71043-71554	11	43	0.671098	3	2	0.2812	no
gi|641566411|gb|KK857671.1|	3603-6581	7.5	23	0.305328	0.893317	2	0.296	no

gi|641566413|gb|KK857670.1|	27274-28649	9	33	2	0.182865	-4	0.14205	no
gi|641566413|gb|KK857670.1|	29474-31914	9	33	0.977812	0.625713	-0.644055	0.64875	no
gi|641566413|gb|KK857670.1|	31988-33032	9	33	1	0.12692	-3	0.206	no
gi|641566413|gb|KK857670.1|	33223-34370	9	33	3	1	-0.938194	0.50615	no
gi|641566413|gb|KK857670.1|	37271-43091	9	33	2	3	0.267633	0.82855	no
gi|641566413|gb|KK857670.1|	53448-53660	9	33	0	2274.13	inf	5.00E-05	yes
gi|641566419|gb|KK857668.1|	15606-16328	11	20	4	3	-0.652614	0.6429	no
gi|641566419|gb|KK857668.1|	19797-20320	11	20	4	0.651043	-3	0.1832	no
gi|641566419|gb|KK857668.1|	2-666	11	20	3	4	0.429837	0.76505	no
gi|641566419|gb|KK857668.1|	22300-23123	11	20	0.676777	1	0.825322	0.6232	no
gi|641566419|gb|KK857668.1|	24891-25699	11	20	9	2	-2	0.12255	no
gi|641566419|gb|KK857668.1|	35874-38454	11	20	1	0.634353	-0.670252	0.6364	no
gi|641566419|gb|KK857668.1|	46692-47085	11	20	17	21	0.254633	0.84165	no
gi|641566419|gb|KK857668.1|	48694-50204	11	20	11	6	-0.76497	0.5445	no
gi|641566419|gb|KK857668.1|	52245-52650	11	20	7	3	-1	0.4601	no
gi|641566419|gb|KK857668.1|	52838-53280	11	20	4	2	-0.789519	0.63015	no
gi|641566419|gb|KK857668.1|	54819-55624	11	20	0.696757	0.705353	0.0176907	1	no
gi|641566419|gb|KK857668.1|	7711-8076	11	20	3	2	-0.227254	0.88445	no
gi|641566419|gb|KK857668.1|	8487-10235	11	20	0.683998	1	0.701637	0.62415	no
gi|641566422|gb|KK857667.1|	11752-12120	12	51	4	0	-inf	0.0102	no
gi|641566422|gb|KK857667.1|	13947-20452	12	51	9	0.034287	-8	0.1339	no
gi|641566422|gb|KK857667.1|	20896-22341	12	51	4	0	-inf	5.00E-05	yes
gi|641566422|gb|KK857667.1|	22391-24542	12	51	5	0	-inf	5.00E-05	yes
gi|641566422|gb|KK857667.1|	2348-2983	12	51	13	0	-inf	5.00E-05	yes
gi|641566422|gb|KK857667.1|	28145-28954	12	51	2	0	-inf	0.0032	yes
gi|641566422|gb|KK857667.1|	8661-9188	12	51	6	0	-inf	0.0015	yes
gi|641566429|gb|KK857664.1|	13122-14390	12	37	3	9	1	0.27165	no
gi|641566429|gb|KK857664.1|	17081-18381	12	37	14	10	-0.413287	0.74195	no
gi|641566429|gb|KK857664.1|	22524-23969	12	37	0.339935	0.86328	1	1	no
gi|641566429|gb|KK857664.1|	24072-24299	12	37	23	30	0.362778	0.7961	no

gi|641566429|gb|KK857664.1|	25662-26454	12	37	6	10	0.737373	0.56925	no
gi|641566429|gb|KK857664.1|	29009-29585	12	37	4	2	-1	0.4334	no
gi|641566429|gb|KK857664.1|	34190-34440	12	37	164	40	-2	0.13385	no
gi|641566429|gb|KK857664.1|	34671-34924	12	37	88	26	-2	0.20055	no
gi|641566429|gb|KK857664.1|	35948-36137	12	37	153	50	-2	0.25615	no
gi|641566429|gb|KK857664.1|	38703-39485	12	37	10	4	-1	0.37535	no
gi|641566429|gb|KK857664.1|	40281-40788	12	37	5	2	-1	0.43995	no
gi|641566429|gb|KK857664.1|	40994-41256	12	37	8	8	-0.028314	0.9394	no
gi|641566429|gb|KK857664.1|	45324-46722	12	37	1	2	0.317914	0.82675	no
gi|641566429|gb|KK857664.1|	48482-49904	12	37	4	0.791471	-2	0.1068	no
gi|641566429|gb|KK857664.1|	50258-51813	12	37	24	3	-3	0.0359	no
gi|641566429|gb|KK857664.1|	53961-54144	12	37	25	53	1	0.48435	no
gi|641566429|gb|KK857664.1|	56759-57591	12	37	0.444807	2	2	0.26155	no
gi|641566429|gb|KK857664.1|	57723-57933	12	37	27	0	-inf	0.0059	no
gi|641566429|gb|KK857664.1|	58678-59428	12	37	12	1	-3	0.0588	no
gi|641566429|gb|KK857664.1|	61611-62571	12	37	0.555531	0.98551	0.827004	0.6232	no
gi|641566429|gb|KK857664.1|	64111-64710	12	37	0.175252	3	4	0.1175	no
gi|641566429|gb|KK857664.1|	65405-65926	12	37	1	2	0.594358	0.70505	no
gi|641566429|gb|KK857664.1|	66413-67063	12	37	110.83	202	0.868477	0.6995	no
gi|641566429|gb|KK857664.1|	68005-69886	12	37	33	59	0.8375	0.6956	no
gi|641566434|gb|KK857662.1|	31324-32766	5.6	24	2	3	0.948631	0.478	no
gi|641566434|gb|KK857662.1|	34746-35427	5.6	24	0.874473	3	2	0.30775	no
gi|641566437|gb|KK857661.1|	22420-24579	9.2	34	0.288447	4	4	0.0565	no
gi|641566437|gb|KK857661.1|	25043-27178	9.2	34	2	5	1	0.36	no
gi|641566437|gb|KK857661.1|	28004-29549	9.2	34	4	2	-0.546373	0.6695	no
gi|641566437|gb|KK857661.1|	29683-31705	9.2	34	0.620161	0.472967	-0.390901	1	no
gi|641566437|gb|KK857661.1|	31829-32219	9.2	34	27	23	-0.252933	0.84075	no
gi|641566437|gb|KK857661.1|	32564-33093	9.2	34	9	7	-0.341096	0.799	no
gi|641566437|gb|KK857661.1|	34263-34501	9.2	34	29	17	-0.773964	0.5887	no
gi|641566437|gb|KK857661.1|	35780-37355	9.2	34	0.821081	0.860399	0.0674823	1	no

gi|641566437|gb|KK857661.1|	37494-38113	9.2	34	3	0.252868	-3	0.2089	no
gi|641566437|gb|KK857661.1|	48349-48629	9.2	34	23	3	-3	0.1474	no
gi|641566437|gb|KK857661.1|	55883-56341	9.2	34	3	1	-1	0.4311	no
gi|641566440|gb|KK857660.1|	11329-11902	13	49	13	13	-0.0199056	0.98775	no
gi|641566440|gb|KK857660.1|	13277-13689	13	49	39	16	-1	0.33745	no
gi|641566440|gb|KK857660.1|	14082-14864	13	49	13	4	-2	0.1819	no
gi|641566440|gb|KK857660.1|	17695-17989	13	49	28	5	-3	0.1326	no
gi|641566440|gb|KK857660.1|	19453-20750	13	49	5	0.587292	-3	0.0704	no
gi|641566440|gb|KK857660.1|	28881-29264	13	49	64	49	-0.392532	0.75105	no
gi|641566440|gb|KK857660.1|	31585-32607	13	49	20	14	-0.489138	0.6977	no
gi|641566440|gb|KK857660.1|	42677-43286	13	49	7	3	-1	0.42495	no
gi|641566442|gb|KK857659.1|	10858-11287	9.1	18	13	3	-2	0.1615	no
gi|641566442|gb|KK857659.1|	23871-24369	9.1	18	6	2	-1	0.4097	no
gi|641566442|gb|KK857659.1|	47077-47750	9.1	18	0.88909	1	0.336963	0.83525	no
gi|641566442|gb|KK857659.1|	47934-50445	9.1	18	0.794956	0.419928	-0.920732	1	no
gi|641566442|gb|KK857659.1|	64053-64647	9.1	18	4	2	-0.987389	0.5308	no
gi|641566442|gb|KK857659.1|	70554-71078	9.1	18	3	0.324552	-3	0.21435	no
gi|641566442|gb|KK857659.1|	8626-8904	9.1	18	8	4	-0.897885	0.5974	no
gi|641566447|gb|KK857657.1|	26111-27649	9	33	91	0.401835	-8	0.046	no
gi|641566447|gb|KK857657.1|	30667-31035	9	33	9	0.599703	-4	0.19705	no
gi|641566447|gb|KK857657.1|	31457-32270	9	33	253	1	-8	0.0229	no
gi|641566447|gb|KK857657.1|	33032-33230	9	33	73	0	-inf	0.00105	yes
gi|641566447|gb|KK857657.1|	42708-43283	9	33	40	0	-inf	5.00E-05	yes
gi|641566447|gb|KK857657.1|	52336-52952	9	33	3	0.509295	-3	0.19	no
gi|641566455|gb|KK857654.1|	22725-22958	6.1	29	20	25	0.353266	0.8082	no
gi|641566473|gb|KK857648.1|	34309-34661	8.5	35	4	3	-0.269971	0.8654	no
gi|641566476|gb|KK857647.1|	4702-5325	10	39	0.992733	2	1	0.5082	no
gi|641566482|gb|KK857645.1|	14366-14885	9.7	38	4	4	-0.194245	0.8871	no
gi|641566482|gb|KK857645.1|	47360-48581	9.7	38	3	0.315317	-3	0.10995	no
gi|641566485|gb|KK857644.1|	29322-29753	12	40	8	5	-0.527473	0.70835	no

gi|641566485|gb|KK857644.1|	3227-3678	12	40	5	9	0.961717	0.49765	no
gi|641566485|gb|KK857644.1|	353-1165	12	40	2	0	-inf	0.0059	no
gi|641566485|gb|KK857644.1|	57284-57961	12	40	1	2	0.23753	0.88205	no
gi|641566485|gb|KK857644.1|	58507-59480	12	40	1	2	0.256817	0.8632	no
gi|641566488|gb|KK857643.1|	49725-50038	9.6	41	7	18	1	0.35775	no
gi|641566493|gb|KK857641.1|	33449-34230	9.7	32	2	0.183513	-3	0.21435	no
gi|641566493|gb|KK857641.1|	37485-38139	9.7	32	2	0.701402	-1	0.47135	no
gi|641566493|gb|KK857641.1|	38345-39231	9.7	32	0.51254	1	1	0.3994	no
gi|641566496|gb|KK857640.1|	22223-22583	7.2	26	13	3	-2	0.21145	no
gi|641566496|gb|KK857640.1|	25967-27229	7.2	26	0	2	inf	0.0016	yes
gi|641566496|gb|KK857640.1|	28652-28892	7.2	26	19	2	-4	0.20495	no
gi|641566499|gb|KK857639.1|	21874-22597	11	43	2	1	-0.884263	0.5831	no
gi|641566499|gb|KK857639.1|	24559-25522	11	43	0.645586	2	1	0.36605	no
gi|641566499|gb|KK857639.1|	27657-28117	11	43	2	4	1	0.47105	no
gi|641566499|gb|KK857639.1|	48165-48585	11	43	7	6	-0.28753	0.8439	no
gi|641566502|gb|KK857638.1|	15308-16162	11	36	1	1	0.313865	0.84265	no
gi|641566502|gb|KK857638.1|	22093-22425	11	36	15	12	-0.332475	0.81035	no
gi|641566502|gb|KK857638.1|	27351-32293	11	36	32	51	0.679428	0.7336	no
gi|641566502|gb|KK857638.1|	32840-33529	11	36	2	0	-inf	0.0059	no
gi|641566502|gb|KK857638.1|	33819-37686	11	36	32	14	-1	0.5988	no
gi|641566502|gb|KK857638.1|	38371-39329	11	36	14	3	-2	0.11755	no
gi|641566502|gb|KK857638.1|	39860-40689	11	36	0.893826	0.509109	-0.812019	0.6492	no
gi|641566502|gb|KK857638.1|	41253-42719	11	36	1	0.169812	-3	0.1689	no
gi|641566502|gb|KK857638.1|	43346-44034	11	36	12.5	4	-2	0.24455	no
gi|641566502|gb|KK857638.1|	56626-57271	11	36	4	2	-0.663802	0.6411	no
gi|641566502|gb|KK857638.1|	58025-58490	11	36	3	0	-inf	0.01175	no
gi|641566509|gb|KK857636.1|	11-3710	11	40	10	21	1	0.63445	no
gi|641566509|gb|KK857636.1|	12387-12848	11	40	1	9	3	0.1385	no
gi|641566509|gb|KK857636.1|	14106-16171	11	40	0.378686	2	2	0.1587	no
gi|641566509|gb|KK857636.1|	19812-21244	11	40	0.057251	4	6	0.10475	no

gi|641566509|gb|KK857636.1|	21366-21939	11	40	0	5	inf	0.0015	yes
gi|641566509|gb|KK857636.1|	22505-23135	11	40	0.1628	2	4	0.12385	no
gi|641566509|gb|KK857636.1|	24369-25370	11	40	0.351508	2	3	0.1746	no
gi|641566509|gb|KK857636.1|	27435-28077	11	40	0.31688	4	4	0.24715	no
gi|641566509|gb|KK857636.1|	33143-34022	11	40	0	2	inf	0.0023	yes
gi|641566509|gb|KK857636.1|	4077-7658	11	40	72	232	2	0.36335	no
gi|641566509|gb|KK857636.1|	42604-44425	11	40	0	116	inf	5.00E-05	yes
gi|641566509|gb|KK857636.1|	44943-45271	11	40	0	8	inf	0.00455	no
gi|641566509|gb|KK857636.1|	55554-60703	11	40	23	2	-4	0.07635	no
gi|641566509|gb|KK857636.1|	64324-65721	11	40	34	106	2	0.45965	no
gi|641566509|gb|KK857636.1|	74708-75751	11	40	4	12	1	0.27465	no
gi|641566509|gb|KK857636.1|	85707-88958	11	40	28	28	0.0296792	0.98805	no
gi|641566509|gb|KK857636.1|	89353-90561	11	40	2	1	-0.109707	0.9376	no
gi|641566509|gb|KK857636.1|	90683-92392	11	40	2	2	-0.118552	0.9282	no
gi|641566509|gb|KK857636.1|	9472-9997	11	40	1	2	0.331534	0.83525	no
gi|641566512|gb|KK857635.1|	32703-33990	11	43	3	5	0.819915	0.53525	no
gi|641566512|gb|KK857635.1|	34701-38719	11	43	3	2	-0.279333	0.825	no
gi|641566512|gb|KK857635.1|	38841-44922	11	43	11	6	-0.937219	0.5597	no
gi|641566516|gb|KK857633.1|	24780-26073	10	39	34	28	-0.25103	0.87185	no
gi|641566519|gb|KK857632.1|	10562-11898	11	40	8	7	-0.226836	0.8543	no
gi|641566519|gb|KK857632.1|	12507-13280	11	40	8	5	-0.517178	0.69095	no
gi|641566519|gb|KK857632.1|	16238-18494	11	40	13	11	-0.266849	0.8266	no
gi|641566519|gb|KK857632.1|	19945-20265	11	40	38	14	-1	0.27575	no
gi|641566519|gb|KK857632.1|	31743-32584	11	40	2	6	2	0.22255	no
gi|641566519|gb|KK857632.1|	32796-35396	11	40	7	3	-1	0.40705	no
gi|641566519|gb|KK857632.1|	37659-38793	11	40	0.829688	0.918435	0.146609	0.9285	no
gi|641566519|gb|KK857632.1|	40619-41175	11	40	13	11	-0.256395	0.8405	no
gi|641566519|gb|KK857632.1|	4831-8215	11	40	177	122	-0.53386	0.77225	no
gi|641566521|gb|KK857631.1|	49963-51128	12	46	0.14603	2	4	0.243	no
gi|641566521|gb|KK857631.1|	55248-55712	12	46	0	152	inf	0.00075	yes

gi|641566521|gb|KK857631.1|	64105-64640	12	46	0.416084	28	6	0.2322	no
gi|641566521|gb|KK857631.1|	65019-65950	12	46	0.192476	44	8	0.2322	no
gi|641566521|gb|KK857631.1|	66411-66634	12	46	0	1227.95	inf	0.0002	yes
gi|641566521|gb|KK857631.1|	73080-73668	12	46	0	21	inf	5.00E-05	yes
gi|641566523|gb|KK857630.1|	16312-16584	8.7	38	0	29	inf	0.00045	yes
gi|641566531|gb|KK857627.1|	18350-18974	10	37	4	7	0.76852	0.57895	no
gi|641566531|gb|KK857627.1|	39152-41330	10	37	2	0.817183	-1	0.4194	no
gi|641566533|gb|KK857626.1|	30739-31312	9	31	3	0.849558	-2	0.33985	no
gi|641566533|gb|KK857626.1|	31645-31856	9	31	23	30	0.420395	0.76945	no
gi|641566533|gb|KK857626.1|	36191-36385	9	31	19	17	-0.150975	0.91755	no
gi|641566533|gb|KK857626.1|	41687-41940	9	31	12	7	-0.70942	0.66485	no
gi|641566533|gb|KK857626.1|	47766-48499	9	31	0	3	inf	0.0023	yes
gi|641566533|gb|KK857626.1|	50551-50765	9	31	7	19	2	0.41765	no
gi|641566536|gb|KK857625.1|	18118-18418	9.2	35	2	8	2	0.33325	no
gi|641566545|gb|KK857622.1|	37890-38223	9.8	38	26	46	0.800886	0.5452	no
gi|641566545|gb|KK857622.1|	38381-39218	9.8	38	0.772347	1	0.603135	0.69715	no
gi|641566548|gb|KK857621.1|	21707-23032	10	44	0	21	inf	5.00E-05	yes
gi|641566548|gb|KK857621.1|	50682-51872	10	44	0.21354	6	5	0.13565	no
gi|641566548|gb|KK857621.1|	67160-67512	10	44	6	3	-1	0.49575	no
gi|641566553|gb|KK857619.1|	10113-13634	9.1	38	39	49	0.330225	0.87435	no
gi|641566553|gb|KK857619.1|	14096-15109	9.1	38	7	10	0.586963	0.64175	no
gi|641566553|gb|KK857619.1|	39658-39873	9.1	38	18	26	0.537822	0.72795	no
gi|641566553|gb|KK857619.1|	43788-45498	9.1	38	0.046744	17	9	0.10435	no
gi|641566553|gb|KK857619.1|	58308-59048	9.1	38	3	6	0.807961	0.54625	no
gi|641566553|gb|KK857619.1|	8601-9492	9.1	38	19	16	-0.239428	0.8493	no
gi|641566553|gb|KK857619.1|	9606-9926	9.1	38	32	22	-0.555795	0.67875	no
gi|641566559|gb|KK857617.1|	41912-43025	10	40	0.154304	2	3	0.2556	no
gi|641566559|gb|KK857617.1|	43255-43801	10	40	0.806276	2	2	0.3613	no
gi|641566559|gb|KK857617.1|	44127-45306	10	40	0.86371	2	1	0.45475	no
gi|641566559|gb|KK857617.1|	46272-46744	10	40	2	1	-0.993616	0.62775	no

gi|641566559|gb|KK857617.1|	47172-50813	10	40	43	28	-0.582324	0.7824	no
gi|641566559|gb|KK857617.1|	62070-62750	10	40	2	3	0.841183	0.585	no
gi|641566559|gb|KK857617.1|	64560-65731	10	40	4	0.994222	-2	0.2013	no
gi|641566563|gb|KK857616.1|	33464-34099	7.3	33	2	0	-inf	0.01175	no
gi|641566568|gb|KK857614.1|	13312-14250	9.3	39	1	1	0.0194204	0.95145	no
gi|641566568|gb|KK857614.1|	3862-4995	9.3	39	0.604053	1	0.927962	0.56225	no
gi|641566568|gb|KK857614.1|	47-781	9.3	39	0.788547	2	1	0.5082	no
gi|641566572|gb|KK857612.1|	12472-12683	11	35	17	15	-0.201094	0.89755	no
gi|641566572|gb|KK857612.1|	15610-15864	11	35	18	6	-2	0.30685	no
gi|641566572|gb|KK857612.1|	24297-27451	11	35	1	0.767186	-0.389662	0.7745	no
gi|641566572|gb|KK857612.1|	28190-28838	11	35	0.625382	2	2	0.33105	no
gi|641566572|gb|KK857612.1|	29311-30080	11	35	2	1	-0.39795	0.7965	no
gi|641566572|gb|KK857612.1|	30226-32972	11	35	30	1	-5	0.031	no
gi|641566572|gb|KK857612.1|	35958-36959	11	35	7	1	-2	0.11795	no
gi|641566572|gb|KK857612.1|	40210-41020	11	35	5	1	-2	0.16325	no
gi|641566572|gb|KK857612.1|	42360-42689	11	35	34	10	-2	0.2116	no
gi|641566572|gb|KK857612.1|	45464-46084	11	35	2	1	-0.780045	0.6308	no
gi|641566572|gb|KK857612.1|	59744-60301	11	35	2	0	-inf	0.01	no
gi|641566572|gb|KK857612.1|	61675-62171	11	35	11	4	-2	0.288	no
gi|641566572|gb|KK857612.1|	64635-64946	11	35	9	3	-1	0.4158	no
gi|641566572|gb|KK857612.1|	66083-66321	11	35	23	5	-2	0.22415	no
gi|641566572|gb|KK857612.1|	77421-77684	11	35	32	8	-2	0.187	no
gi|641566581|gb|KK857609.1|	21664-22623	8.2	34	0.09271	1	4	0.12385	no
gi|641566581|gb|KK857609.1|	23736-24860	8.2	34	0.762383	1	0.869007	0.56565	no
gi|641566581|gb|KK857609.1|	37452-37778	8.2	34	0	14	inf	0.00135	yes
gi|641566581|gb|KK857609.1|	45829-46154	8.2	34	0	403	inf	0.00105	yes
gi|641566588|gb|KK857606.1|	12758-13639	22	43	1	0.15691	-3	0.22425	no
gi|641566588|gb|KK857606.1|	19228-20300	22	43	5	0.614427	-3	0.08425	no
gi|641566588|gb|KK857606.1|	25480-26421	22	43	121	10	-4	0.0726	no
gi|641566588|gb|KK857606.1|	26537-27941	22	43	17	2	-3	0.0227	no

gi|641566588|gb|KK857606.1|	28409-29562	22	43	2	0.112537	-4	0.1879	no
gi|641566588|gb|KK857606.1|	29985-33225	22	43	38	2	-4	0.0471	no
gi|641566588|gb|KK857606.1|	33366-35996	22	43	5	1	-2	0.1129	no
gi|641566593|gb|KK857604.1|	0-1464	9.2	34	11	5	-1	0.3373	no
gi|641566593|gb|KK857604.1|	11458-12298	9.2	34	0.439288	3	3	0.1746	no
gi|641566593|gb|KK857604.1|	23557-23728	9.2	34	61	12	-2	0.24495	no
gi|641566593|gb|KK857604.1|	2961-5088	9.2	34	1	2	0.765394	0.56155	no
gi|641566593|gb|KK857604.1|	36892-37641	9.2	34	75	42	-0.836437	0.60955	no
gi|641566593|gb|KK857604.1|	42759-43978	9.2	34	24	19	-0.401612	0.7488	no
gi|641566593|gb|KK857604.1|	51094-51494	9.2	34	47	58	0.298726	0.8147	no
gi|641566599|gb|KK857602.1|	16644-17939	10	54	0.257535	0.588356	1	1	no
gi|641566599|gb|KK857602.1|	18941-19865	10	54	0.874463	1	0.24166	0.8798	no
gi|641566599|gb|KK857602.1|	8231-8823	10	54	1	5	2	0.20435	no
gi|641566601|gb|KK857601.1|	16766-17082	11	48	2	12	3	0.17355	no
gi|641566606|gb|KK857599.1|	16408-20773	10	40	2	2	0.528456	0.67815	no
gi|641566606|gb|KK857599.1|	22773-24331	10	40	0.46762	0.712719	0.607997	1	no
gi|641566606|gb|KK857599.1|	25244-25771	10	40	9	12	0.482197	0.7115	no
gi|641566606|gb|KK857599.1|	29334-30014	10	40	1	0.442781	-2	0.4266	no
gi|641566606|gb|KK857599.1|	30605-30973	10	40	2	4	0.580553	0.7575	no
gi|641566606|gb|KK857599.1|	32824-33433	10	40	17	17	0.0205567	0.98845	no
gi|641566606|gb|KK857599.1|	35833-38704	10	40	2	1	-0.747426	0.5648	no
gi|641566606|gb|KK857599.1|	41311-42662	10	40	0.673903	0.746597	0.147789	1	no
gi|641566606|gb|KK857599.1|	44282-45293	10	40	24	22	-0.119504	0.92045	no
gi|641566606|gb|KK857599.1|	45434-45768	10	40	5	7	0.574803	0.71515	no
gi|641566606|gb|KK857599.1|	45894-46483	10	40	2	2	-0.295696	0.8456	no
gi|641566606|gb|KK857599.1|	49053-49434	10	40	3	3	0.0969702	0.92385	no
gi|641566606|gb|KK857599.1|	50099-50497	10	40	11	4	-2	0.30775	no
gi|641566606|gb|KK857599.1|	53066-53260	10	40	26	7	-2	0.31355	no
gi|641566606|gb|KK857599.1|	57091-57786	10	40	0.850013	1	0.60054	0.70555	no
gi|641566606|gb|KK857599.1|	60142-60657	10	40	1	2	0.594034	0.70505	no

gi|641566606|gb|KK857599.1|	62996-63582	10	40	14	4	-2	0.17875	no
gi|641566611|gb|KK857597.1|	13191-13668	11	40	14	23	0.691232	0.58775	no
gi|641566611|gb|KK857597.1|	15632-16658	11	40	0.767091	1	0.435299	0.78015	no
gi|641566611|gb|KK857597.1|	18096-19764	11	40	0.673077	0.513047	-0.391679	1	no
gi|641566611|gb|KK857597.1|	20537-23023	11	40	1	0.424503	-1	0.38335	no
gi|641566611|gb|KK857597.1|	30851-34367	11	40	4	0.292995	-4	0.0306	no
gi|641566611|gb|KK857597.1|	36780-37536	11	40	332	180	-0.887232	0.67535	no
gi|641566611|gb|KK857597.1|	37911-38716	11	40	141	62	-1	0.5997	no
gi|641566611|gb|KK857597.1|	38973-41539	11	40	1	0.638093	-0.940355	0.50355	no
gi|641566611|gb|KK857597.1|	42932-44468	11	40	12	5	-1	0.3426	no
gi|641566611|gb|KK857597.1|	44588-45996	11	40	0.642228	0.88953	0.469958	0.7528	no
gi|641566611|gb|KK857597.1|	46971-47584	11	40	17	11	-0.63371	0.6212	no
gi|641566611|gb|KK857597.1|	48710-49535	11	40	2	0.341548	-2	0.2115	no
gi|641566611|gb|KK857597.1|	49688-50895	11	40	1	0.532771	-1	0.4973	no
gi|641566611|gb|KK857597.1|	51092-52478	11	40	0.475705	0.543545	0.192334	1	no
gi|641566611|gb|KK857597.1|	53770-54362	11	40	6	4	-0.624892	0.65185	no
gi|641566611|gb|KK857597.1|	59389-59584	11	40	528	245	-1	0.385	no
gi|641566611|gb|KK857597.1|	59672-60295	11	40	2	2	0.44656	0.7665	no
gi|641566611|gb|KK857597.1|	60651-62320	11	40	1	1	0.0233613	0.9697	no
gi|641566611|gb|KK857597.1|	63354-64332	11	40	1	0.275133	-2	0.31075	no
gi|641566611|gb|KK857597.1|	66457-67228	11	40	1	0.373355	-2	0.3455	no
gi|641566611|gb|KK857597.1|	72934-75393	11	40	2	2	-0.197631	0.88005	no
gi|641566611|gb|KK857597.1|	81150-81814	11	40	0.906126	1	0.336727	0.83525	no
gi|641566611|gb|KK857597.1|	8136-8772	11	40	3	0	-inf	0.0056	no
gi|641566611|gb|KK857597.1|	93450-94029	11	40	2	0.278829	-3	0.2381	no
gi|641566611|gb|KK857597.1|	94598-95865	11	40	1	0	-inf	0.0024	yes
gi|641566614|gb|KK857596.1|	33507-33678	9.8	42	4	68	4	0.1204	no
gi|641566623|gb|KK857593.1|	16108-16859	8.6	32	8	5	-0.526033	0.6929	no
gi|641566623|gb|KK857593.1|	19117-19321	8.6	32	8	17	1	0.52555	no
gi|641566623|gb|KK857593.1|	1991-2486	8.6	32	43	26	-0.705472	0.57815	no

gi|641566623|gb|KK857593.1|	22864-23977	8.6	32	0.925826	0.11742	-3	0.2348	no
gi|641566623|gb|KK857593.1|	27277-27515	8.6	32	12	10	-0.189002	0.90945	no
gi|641566623|gb|KK857593.1|	3321-4962	8.6	32	12	9	-0.362129	0.7708	no
gi|641566623|gb|KK857593.1|	34256-34559	8.6	32	32	29	-0.132335	0.9212	no
gi|641566623|gb|KK857593.1|	40327-40761	8.6	32	1	5	2	0.2441	no
gi|641566623|gb|KK857593.1|	42-1298	8.6	32	6	4	-0.442705	0.7303	no
gi|641566623|gb|KK857593.1|	43248-43589	8.6	32	2	15	3	0.14055	no
gi|641566623|gb|KK857593.1|	63268-63608	8.6	32	2	9	2	0.27245	no
gi|641566623|gb|KK857593.1|	68081-68249	8.6	32	24	95	2	0.27195	no
gi|641566626|gb|KK857592.1|	32167-32825	10	41	0	24	inf	5.00E-05	yes
gi|641566632|gb|KK857590.1|	21336-22396	12	22	5	0.373748	-4	0.1026	no
gi|641566632|gb|KK857590.1|	28380-29418	12	22	1	0.511277	-0.979638	0.5872	no
gi|641566632|gb|KK857590.1|	29677-30145	12	22	2	2	-0.256938	0.88485	no
gi|641566637|gb|KK857588.1|	39179-39551	12	49	3	3	0.0957131	0.92385	no
gi|641566637|gb|KK857588.1|	39726-40497	12	49	2	0.373355	-2	0.21865	no
gi|641566643|gb|KK857586.1|	21645-22249	9.4	36	2	2	0.138498	0.929	no
gi|641566649|gb|KK857584.1|	16381-17169	11	42	8	2	-2	0.15375	no
gi|641566649|gb|KK857584.1|	23534-24064	11	42	3	6	0.764749	0.599	no
gi|641566649|gb|KK857584.1|	43214-43398	11	42	45	35	-0.385153	0.8004	no
gi|641566649|gb|KK857584.1|	52643-53561	11	42	1	2	0.282235	0.84775	no
gi|641566649|gb|KK857584.1|	53771-54809	11	42	1	0.639096	-1	0.4973	no
gi|641566649|gb|KK857584.1|	56321-56992	11	42	4	9	1	0.35585	no
gi|641566654|gb|KK857582.1|	3131-3723	8.1	34	0	16	inf	5.00E-05	yes
gi|641566657|gb|KK857581.1|	5111-5948	9.6	38	1	0	-inf	0.01175	no
gi|641566659|gb|KK857580.1|	0-834	9.3	35	5	4	-0.522439	0.69455	no
gi|641566659|gb|KK857580.1|	12981-13666	9.3	35	2	2	-0.0517774	0.94545	no
gi|641566659|gb|KK857580.1|	18210-18648	9.3	35	4	6	0.695557	0.6417	no
gi|641566659|gb|KK857580.1|	22826-23709	9.3	35	1	1	0.451745	0.7632	no
gi|641566659|gb|KK857580.1|	24260-24890	9.3	35	3	5	0.669173	0.63245	no
gi|641566664|gb|KK857578.1|	15748-17221	9.8	38	25	92	2	0.2646	no

gi|641566664|gb|KK857578.1|	17335-18951	9.8	38	0.847222	0.835641	-0.019857	1	no
gi|641566664|gb|KK857578.1|	34856-35751	9.8	38	1	5	2	0.19615	no
gi|641566664|gb|KK857578.1|	42335-42855	9.8	38	234	752	2	0.42825	no
gi|641566664|gb|KK857578.1|	52906-53634	9.8	38	108	522	2	0.2978	no
gi|641566664|gb|KK857578.1|	62040-63366	9.8	38	5	0	-inf	5.00E-05	yes
gi|641566664|gb|KK857578.1|	64901-65771	9.8	38	2	0	-inf	0.00495	no
gi|641566664|gb|KK857578.1|	67927-68483	9.8	38	7	0	-inf	0.00045	yes
gi|641566667|gb|KK857577.1|	41794-42332	8.1	34	0	9	inf	0.0004	yes
gi|641566670|gb|KK857576.1|	22343-22633	9.6	20	43	27	-0.680284	0.60985	no
gi|641566670|gb|KK857576.1|	31363-32159	9.6	20	15	15	0.00640551	0.99425	no
gi|641566670|gb|KK857576.1|	33731-35323	9.6	20	12	13	0.117627	0.92185	no
gi|641566670|gb|KK857576.1|	35518-35725	9.6	20	30	24	-0.296196	0.84275	no
gi|641566670|gb|KK857576.1|	64002-64287	9.6	20	6	5	-0.114576	0.9242	no
gi|641566673|gb|KK857575.1|	11861-12977	11	34	0.845909	0.351117	-1	1	no
gi|641566673|gb|KK857575.1|	14309-18078	11	34	15	16	0.14539	0.94875	no
gi|641566676|gb|KK857574.1|	19906-20758	11	41	0.107815	2	4	0.12385	no
gi|641566676|gb|KK857574.1|	25612-26525	11	41	0.59191	0.749771	0.341072	1	no
gi|641566676|gb|KK857574.1|	26676-28049	11	41	0.841673	0.641112	-0.392684	1	no
gi|641566676|gb|KK857574.1|	29549-29772	11	41	33	15	-1	0.43305	no
gi|641566676|gb|KK857574.1|	32374-39828	11	41	13	6	-1	0.61935	no
gi|641566676|gb|KK857574.1|	46500-47278	11	41	3	1	-1	0.36935	no
gi|641566676|gb|KK857574.1|	48408-50346	11	41	4	2	-0.608288	0.6426	no
gi|641566676|gb|KK857574.1|	54591-55857	11	41	0.661275	0.704936	0.0922423	1	no
gi|641566676|gb|KK857574.1|	59939-61667	11	41	0.323364	1	2	0.2914	no
gi|641566676|gb|KK857574.1|	62551-64214	11	41	12	12	0.0292215	0.98135	no
gi|641566676|gb|KK857574.1|	64372-65883	11	41	4	3	-0.412622	0.7505	no
gi|641566676|gb|KK857574.1|	67536-68583	11	41	0	2	inf	0.0016	yes
gi|641566676|gb|KK857574.1|	70357-70853	11	41	4	0.708064	-2	0.21085	no
gi|641566676|gb|KK857574.1|	72199-73555	11	41	35	25	-0.502381	0.7543	no
gi|641566676|gb|KK857574.1|	75407-77022	11	41	29	22	-0.367167	0.8193	no

gi|641566676|gb|KK857574.1|	79050-79547	11	41	3	3	-0.0444373	0.9565	no
gi|641566681|gb|KK857572.1|	43351-44066	7.2	30	1	1	-0.0770793	0.92685	no
gi|641566681|gb|KK857572.1|	44378-45297	7.2	30	0.587003	0.74359	0.34114	1	no
gi|641566684|gb|KK857571.1|	10988-12202	8.6	39	0.069503	14	8	0.10435	no
gi|641566684|gb|KK857571.1|	18555-19435	8.6	39	0.620438	0.785691	0.340677	1	no
gi|641566684|gb|KK857571.1|	27163-27938	8.6	39	1	0.556197	-1	0.50885	no
gi|641566687|gb|KK857570.1|	0-518	8.3	35	2	3	0.304691	0.84335	no
gi|641566687|gb|KK857570.1|	2790-3667	8.3	35	0.830891	2	1	0.49765	no
gi|641566687|gb|KK857570.1|	5641-6926	8.3	35	6	14	1	0.37155	no
gi|641566693|gb|KK857568.1|	10684-11995	8.5	33	2	5	2	0.2374	no
gi|641566693|gb|KK857568.1|	16917-17480	8.5	33	2	1	-0.541153	0.7461	no
gi|641566693|gb|KK857568.1|	43803-44746	8.5	33	9	4	-1	0.381	no
gi|641566695|gb|KK857567.1|	11381-12567	11	36	2	3	0.667771	0.6242	no
gi|641566695|gb|KK857567.1|	13449-14114	11	36	2	1	-0.815249	0.5996	no
gi|641566695|gb|KK857567.1|	14214-15312	11	36	0.862776	1	0.60579	0.68625	no
gi|641566695|gb|KK857567.1|	15830-17438	11	36	1	1	0.456148	0.75005	no
gi|641566695|gb|KK857567.1|	26583-29839	11	36	5	2	-0.917696	0.46555	no
gi|641566695|gb|KK857567.1|	31338-32389	11	36	2	2	-0.165087	0.9024	no
gi|641566695|gb|KK857567.1|	32808-34341	11	36	1	0.887331	-0.740007	0.5925	no
gi|641566695|gb|KK857567.1|	35815-36690	11	36	1	1	0.451645	0.7632	no
gi|641566695|gb|KK857567.1|	43787-44687	11	36	12	6	-0.981007	0.44405	no
gi|641566695|gb|KK857567.1|	52797-53331	11	36	0.417302	3	3	0.2674	no
gi|641566695|gb|KK857567.1|	56192-57360	11	36	0.509528	1	1	0.39245	no
gi|641566695|gb|KK857567.1|	70049-70387	11	36	16	11	-0.468836	0.73415	no
gi|641566695|gb|KK857567.1|	7553-10945	11	36	2	4	0.880247	0.4858	no
gi|641566697|gb|KK857566.1|	26289-27464	6.4	26	0.650427	2	1	0.43005	no
gi|641566697|gb|KK857566.1|	32278-32680	6.4	26	0	38	inf	5.00E-05	yes
gi|641566697|gb|KK857566.1|	32844-33440	6.4	26	1	1	0.112219	0.92265	no
gi|641566697|gb|KK857566.1|	33512-33894	6.4	26	1	61	6	0.13465	no
gi|641566702|gb|KK857564.1|	20090-20493	11	19	0.336693	7	4	0.11365	no

gi|641566702|gb|KK857564.1|	21193-22709	11	19	0.21444	1	2	0.23615	no
gi|641566702|gb|KK857564.1|	23378-24555	11	19	0.721224	2	1	0.3418	no
gi|641566702|gb|KK857564.1|	24943-25748	11	19	0.580631	3	3	0.1678	no
gi|641566702|gb|KK857564.1|	44646-46414	11	19	4	0	-inf	5.00E-05	yes
gi|641566702|gb|KK857564.1|	63603-64927	11	19	14	17	0.303012	0.8023	no
gi|641566707|gb|KK857562.1|	28130-28275	7.4	35	98	174	0.821992	0.599	no
gi|641566710|gb|KK857561.1|	10605-11855	9.7	23	0.469914	0.511134	0.121305	1	no
gi|641566713|gb|KK857560.1|	115-726	8.8	41	2	1	-0.861262	0.60675	no
gi|641566713|gb|KK857560.1|	16397-17490	8.8	41	0.473226	1	1	0.47015	no
gi|641566713|gb|KK857560.1|	25206-26433	8.8	41	0.274509	174	9	0.11695	no
gi|641566713|gb|KK857560.1|	26543-27503	8.8	41	0.925884	6	3	0.09095	no
gi|641566713|gb|KK857560.1|	30416-31312	8.8	41	1	0.153571	-3	0.24815	no
gi|641566713|gb|KK857560.1|	35084-35888	8.8	41	5	8	0.602637	0.6416	no
gi|641566713|gb|KK857560.1|	36616-37062	8.8	41	6	8	0.515333	0.7125	no
gi|641566713|gb|KK857560.1|	59553-59897	8.8	41	2	5	1	0.4433	no
gi|641566713|gb|KK857560.1|	6616-7911	8.8	41	1	0.098059	-4	0.19735	no
gi|641566713|gb|KK857560.1|	69821-70032	8.8	41	17	13	-0.464128	0.77155	no
gi|641566715|gb|KK857559.1|	22572-23174	11	50	0.34793	3	3	0.2674	no
gi|641566715|gb|KK857559.1|	24065-25157	11	50	0	3	inf	0.00105	yes
gi|641566715|gb|KK857559.1|	46476-46740	11	50	46	13	-2	0.2017	no
gi|641566720|gb|KK857557.1|	27547-28562	9	33	0.691008	1	0.605128	0.6936	no
gi|641566720|gb|KK857557.1|	32252-33110	9	33	1	0.649291	-1	0.50315	no
gi|641566720|gb|KK857557.1|	35355-36442	9	33	0.555812	0.362483	-0.616683	1	no
gi|641566729|gb|KK857554.1|	15651-16476	8.6	31	2	0.683096	-2	0.362	no
gi|641566729|gb|KK857554.1|	18414-19204	8.6	31	25	23	-0.113797	0.92655	no
gi|641566729|gb|KK857554.1|	20836-21938	8.6	31	23	0.594189	-5	0.04295	no
gi|641566729|gb|KK857554.1|	23868-24298	8.6	31	3	1	-1	0.50365	no
gi|641566729|gb|KK857554.1|	25034-25251	8.6	31	22	0	-inf	0.0059	no
gi|641566729|gb|KK857554.1|	28094-28660	8.6	31	3	0	-inf	0.0059	no
gi|641566729|gb|KK857554.1|	29366-29732	8.6	31	25	0	-inf	0.0002	yes

gi|641566738|gb|KK857551.1|	12086-12447	11	39	3	14	3	0.15005	no
gi|641566738|gb|KK857551.1|	13728-15118	11	39	4	4	-0.129578	0.9213	no
gi|641566738|gb|KK857551.1|	149-815	11	39	0.751904	2	2	0.32945	no
gi|641566738|gb|KK857551.1|	21404-21774	11	39	0	8	inf	0.00255	yes
gi|641566738|gb|KK857551.1|	2303-3053	11	39	1	3	1	0.3594	no
gi|641566738|gb|KK857551.1|	26660-27000	11	39	0	222	inf	5.00E-05	yes
gi|641566738|gb|KK857551.1|	31119-32856	11	39	0.045925	160	12	0.1051	no
gi|641566738|gb|KK857551.1|	37414-37544	11	39	0	1753.72	inf	0.00015	yes
gi|641566738|gb|KK857551.1|	43659-45515	11	39	0	37	inf	0.00045	yes
gi|641566738|gb|KK857551.1|	48194-48751	11	39	1	4	1	0.3664	no
gi|641566738|gb|KK857551.1|	48861-49546	11	39	2	4	1	0.45565	no
gi|641566738|gb|KK857551.1|	49613-50283	11	39	1	3	1	0.36635	no
gi|641566738|gb|KK857551.1|	50679-52264	11	39	0.458535	2	2	0.22345	no
gi|641566738|gb|KK857551.1|	5238-5401	11	39	118	201	0.76176	0.58275	no
gi|641566738|gb|KK857551.1|	59360-60577	11	39	2	0.527577	-2	0.1893	no
gi|641566738|gb|KK857551.1|	60627-63007	11	39	3	2	-0.88787	0.49065	no
gi|641566738|gb|KK857551.1|	63147-64005	11	39	2	1	-0.981537	0.5308	no
gi|641566738|gb|KK857551.1|	73171-73499	11	39	4	4	0.0881949	0.9241	no
gi|641566742|gb|KK857550.1|	16006-16533	7.5	28	0	9	inf	0.0004	yes
gi|641566748|gb|KK857548.1|	1196-2229	11	42	3	2	-0.434245	0.7477	no
gi|641566748|gb|KK857548.1|	12101-15480	11	42	2	1	-0.786857	0.5434	no
gi|641566748|gb|KK857548.1|	15611-16901	11	42	1	0	-inf	0.00345	yes
gi|641566748|gb|KK857548.1|	20661-21454	11	42	2	0.899272	-0.776052	0.63565	no
gi|641566748|gb|KK857548.1|	22010-23114	11	42	0.779163	0.118577	-3	1	no
gi|641566748|gb|KK857548.1|	2345-4736	11	42	0.870648	0.688855	-0.33789	1	no
gi|641566748|gb|KK857548.1|	24367-27234	11	42	0.901039	0.242706	-2	0.22675	no
gi|641566748|gb|KK857548.1|	6011-6970	11	42	1	0.14097	-3	0.217	no
gi|641566748|gb|KK857548.1|	79744-80207	11	42	11	11	0.0563522	0.9684	no
gi|641566748|gb|KK857548.1|	8298-9300	11	42	2	0.667472	-1	0.38185	no
gi|641566750|gb|KK857547.1|	12693-13099	7.1	29	0.66418	27	5	0.233	no

gi|641566750|gb|KK857547.1|	21723-21899	7.1	29	15	578	5	0.11595	no
gi|641566750|gb|KK857547.1|	47215-47472	7.1	29	0	141	inf	5.00E-05	yes
gi|641566752|gb|KK857546.1|	30643-31140	12	45	0.467906	3	3	0.28535	no
gi|641566752|gb|KK857546.1|	5091-5940	12	45	67	16	-2	0.2422	no
gi|641566755|gb|KK857545.1|	21454-22273	8.8	34	1	0	-inf	0.01	no
gi|641566755|gb|KK857545.1|	25366-25691	8.8	34	3	15	2	0.1937	no
gi|641566755|gb|KK857545.1|	3624-4001	8.8	34	0	6	inf	0.0068	no
gi|641566755|gb|KK857545.1|	36651-36883	8.8	34	0	22	inf	0.00375	no
gi|641566755|gb|KK857545.1|	38770-39022	8.8	34	2	12	3	0.30875	no
gi|641566755|gb|KK857545.1|	41394-41837	8.8	34	0.567866	12	4	0.23685	no
gi|641566755|gb|KK857545.1|	4871-5307	8.8	34	0	18	inf	5.00E-05	yes
gi|641566761|gb|KK857543.1|	19355-19995	9.5	41	2	0.241074	-3	0.2481	no
gi|641566761|gb|KK857543.1|	53162-53621	9.5	41	1	2	0.590384	0.70505	no
gi|641566767|gb|KK857541.1|	11975-12173	9.5	40	24	6	-2	0.31355	no
gi|641566767|gb|KK857541.1|	14694-15252	9.5	40	0.974736	1	0.596135	0.70505	no
gi|641566767|gb|KK857541.1|	33961-34853	9.5	40	0	2	inf	0.0068	no
gi|641566778|gb|KK857537.1|	26911-27315	12	39	16	0	-inf	0.0002	yes
gi|641566778|gb|KK857537.1|	28388-29532	12	39	0.970205	0	-inf	0.0075	no
gi|641566778|gb|KK857537.1|	36764-38030	12	39	1	0	-inf	0.0032	yes
gi|641566778|gb|KK857537.1|	39126-40300	12	39	1	0	-inf	0.0034	yes
gi|641566778|gb|KK857537.1|	41292-42521	12	39	0.753438	0	-inf	1	no
gi|641566778|gb|KK857537.1|	64944-66594	12	39	0.973436	0.074197	-4	0.1991	no
gi|641566784|gb|KK857535.1|	10401-11101	9.2	33	2	16	3	0.0591	no
gi|641566784|gb|KK857535.1|	13022-13380	9.2	33	3	29	3	0.078	no
gi|641566784|gb|KK857535.1|	14033-14279	9.2	33	5	48	3	0.10715	no
gi|641566784|gb|KK857535.1|	17214-17447	9.2	33	1	62	6	0.1054	no
gi|641566784|gb|KK857535.1|	19049-19264	9.2	33	11	43	2	0.25105	no
gi|641566784|gb|KK857535.1|	20235-20570	9.2	33	0.487047	7	4	0.12785	no
gi|641566784|gb|KK857535.1|	20939-26960	9.2	33	5	1	-2	0.10075	no
gi|641566784|gb|KK857535.1|	3191-3729	9.2	33	0.206236	10	6	0.1054	no

gi|641566784|gb|KK857535.1|	3855-4038	9.2	33	25	45	0.843132	0.59505	no
gi|641566784|gb|KK857535.1|	43065-43243	9.2	33	18	66	2	0.28035	no
gi|641566784|gb|KK857535.1|	45763-48860	9.2	33	1	2	0.944717	0.46895	no
gi|641566784|gb|KK857535.1|	5-251	9.2	33	5	28	2	0.1844	no
gi|641566784|gb|KK857535.1|	52176-53396	9.2	33	1	0.420831	-2	0.30015	no
gi|641566784|gb|KK857535.1|	6190-7395	9.2	33	4	26	3	0.0573	no
gi|641566784|gb|KK857535.1|	62375-63610	9.2	33	0.476694	0.414784	-0.200704	1	no
gi|641566784|gb|KK857535.1|	65351-66387	9.2	33	0.252655	1	2	0.25595	no
gi|641566784|gb|KK857535.1|	67750-68888	9.2	33	0.751061	0.457278	-0.715861	1	no
gi|641566784|gb|KK857535.1|	75693-76604	9.2	33	0.296782	2	3	0.1666	no
gi|641566784|gb|KK857535.1|	76725-77579	9.2	33	0.322463	2	2	0.2235	no
gi|641566784|gb|KK857535.1|	77926-78544	9.2	33	2	11	3	0.08445	no
gi|641566784|gb|KK857535.1|	83764-84411	9.2	33	3	1	-0.815747	0.5996	no
gi|641566784|gb|KK857535.1|	84550-88778	9.2	33	13	15	0.170161	0.93775	no
gi|641566784|gb|KK857535.1|	9712-9990	9.2	33	8	62	3	0.06465	no
gi|641566790|gb|KK857533.1|	5327-6502	9	32	0.722697	2	2	0.24675	no
gi|641566793|gb|KK857532.1|	1504-1919	11	21	23	12	-0.95954	0.4654	no
gi|641566793|gb|KK857532.1|	18223-19466	11	21	0.608212	0.411629	-0.563231	1	no
gi|641566793|gb|KK857532.1|	24524-24709	11	21	24	8	-1	0.4383	no
gi|641566793|gb|KK857532.1|	3023-4048	11	21	0.85335	0.389436	-1	1	no
gi|641566793|gb|KK857532.1|	37346-37590	11	21	161	66	-1	0.3177	no
gi|641566793|gb|KK857532.1|	38281-38781	11	21	19	29	0.610876	0.63495	no
gi|641566793|gb|KK857532.1|	4100-4660	11	21	2	0.87932	-0.98874	0.62775	no
gi|641566796|gb|KK857531.1|	17933-19198	8.9	42	1	1	0.30725	0.83475	no
gi|641566796|gb|KK857531.1|	20352-21034	8.9	42	1	2	0.0856521	0.94	no
gi|641566796|gb|KK857531.1|	22095-23224	8.9	42	0.985791	0.923332	-0.0944329	0.9478	no
gi|641566796|gb|KK857531.1|	31528-32227	8.9	42	0.140545	2	4	0.12385	no
gi|641566796|gb|KK857531.1|	33325-34361	8.9	42	0	6	inf	5.00E-05	yes
gi|641566796|gb|KK857531.1|	34542-36251	8.9	42	0.327423	2	2	0.14665	no
gi|641566796|gb|KK857531.1|	40967-44619	8.9	42	0.14338	2	4	0.06305	no

gi|641566796|gb|KK857531.1|	6045-6905	8.9	42	0.852141	1	0.603452	0.6936	no
gi|641566796|gb|KK857531.1|	75158-75822	8.9	42	2	0.915469	-1	0.44155	no
gi|641566796|gb|KK857531.1|	75895-78246	8.9	42	1	2	0.948395	0.47955	no
gi|641566796|gb|KK857531.1|	78507-80085	8.9	42	1	4	2	0.2564	no
gi|641566796|gb|KK857531.1|	8960-10238	8.9	42	0.326973	0.597559	0.869911	1	no
gi|641566802|gb|KK857529.1|	10091-11240	8.8	29	0.965118	3	2	0.2242	no
gi|641566802|gb|KK857529.1|	13288-13622	8.8	29	6	16	1	0.3253	no
gi|641566802|gb|KK857529.1|	26904-29071	8.8	29	1	1	0.16573	0.90265	no
gi|641566802|gb|KK857529.1|	33361-33818	8.8	29	6	12	0.988777	0.49015	no
gi|641566802|gb|KK857529.1|	35084-35747	8.8	29	11	5	-1	0.40365	no
gi|641566802|gb|KK857529.1|	7771-8304	8.8	29	63	285	2	0.21755	no
gi|641566806|gb|KK857527.1|	10607-11983	9.4	39	34	13	-1	0.4154	no
gi|641566806|gb|KK857527.1|	13262-13615	9.4	39	8	3	-1	0.42815	no
gi|641566806|gb|KK857527.1|	17523-18613	9.4	39	18	10	-0.930321	0.46085	no
gi|641566806|gb|KK857527.1|	2070-2404	9.4	39	22	19	-0.216611	0.8686	no
gi|641566806|gb|KK857527.1|	35159-35611	9.4	39	2	2	0.58983	0.70555	no
gi|641566806|gb|KK857527.1|	69362-70533	9.4	39	0.435396	2	2	0.28005	no
gi|641566809|gb|KK857526.1|	15796-16672	9.8	22	2	3	0.975629	0.4947	no
gi|641566809|gb|KK857526.1|	33036-34166	9.8	22	0.378745	1	2	0.2717	no
gi|641566809|gb|KK857526.1|	41926-42113	9.8	22	17	20	0.260311	0.8823	no
gi|641566812|gb|KK857525.1|	11297-11607	11	42	34	46	0.439689	0.7335	no
gi|641566812|gb|KK857525.1|	19633-20672	11	42	0.923109	2	1	0.44525	no
gi|641566812|gb|KK857525.1|	24014-24417	11	42	5	40	3	0.0575	no
gi|641566812|gb|KK857525.1|	6076-7189	11	42	2	0.939358	-0.716031	0.633	no
gi|641566812|gb|KK857525.1|	66293-66835	11	42	0.815505	9	3	0.13065	no
gi|641566812|gb|KK857525.1|	7480-8041	11	42	1	1	0.333231	0.83525	no
gi|641566812|gb|KK857525.1|	8743-9237	11	42	4	5	0.499698	0.73105	no
gi|641566815|gb|KK857524.1|	24830-25030	9.9	37	34	15	-1	0.47445	no
gi|641566818|gb|KK857523.1|	38058-39304	10	37	0.87602	3	2	0.19185	no
gi|641566818|gb|KK857523.1|	47410-47892	10	37	0	4	inf	0.00375	no

gi|641566824|gb|KK857521.1|	31906-32134	11	45	12	6	-1	0.5278	no
gi|641566824|gb|KK857521.1|	40336-43855	11	45	2	3	0.628705	0.62485	no
gi|641566824|gb|KK857521.1|	53331-53545	11	45	12	17	0.537339	0.7472	no
gi|641566827|gb|KK857520.1|	55607-56125	8.7	36	11	18	0.621678	0.63475	no
gi|641566833|gb|KK857518.1|	21312-21851	2.5	47	0	3	inf	0.00455	no
gi|641566833|gb|KK857518.1|	53569-54435	2.5	47	0	8	inf	0.0001	yes
gi|641566833|gb|KK857518.1|	54569-55131	2.5	47	0.385669	3	3	0.27515	no
gi|641566833|gb|KK857518.1|	57041-57633	2.5	47	0	13	inf	0.0001	yes
gi|641566833|gb|KK857518.1|	60825-61459	2.5	47	0	8	inf	0.00065	yes
gi|641566833|gb|KK857518.1|	61514-62533	2.5	47	0	4	inf	0.0003	yes
gi|641566836|gb|KK857517.1|	34534-35330	12	42	0	3	inf	0.00255	yes
gi|641566839|gb|KK857516.1|	10886-11866	10	42	2	2	0.522345	0.7153	no
gi|641566839|gb|KK857516.1|	13619-14381	10	42	181	118	-0.612838	0.7799	no
gi|641566839|gb|KK857516.1|	14909-18502	10	42	0.812703	1	0.610596	0.63715	no
gi|641566839|gb|KK857516.1|	1882-3587	10	42	12	5	-1	0.2957	no
gi|641566839|gb|KK857516.1|	19090-19959	10	42	2	2	0.188533	0.8991	no
gi|641566839|gb|KK857516.1|	20853-21146	10	42	11	12	0.0631093	0.96095	no
gi|641566839|gb|KK857516.1|	23662-24330	10	42	1	0.453903	-2	0.4266	no
gi|641566839|gb|KK857516.1|	3-1125	10	42	2	0.348853	-2	0.22235	no
gi|641566839|gb|KK857516.1|	30234-30429	10	42	52	54	0.0681992	0.96195	no
gi|641566839|gb|KK857516.1|	32709-33030	10	42	96	108	0.17582	0.89285	no
gi|641566839|gb|KK857516.1|	3759-5090	10	42	0.748113	1	0.607131	0.67895	no
gi|641566839|gb|KK857516.1|	39729-39945	10	42	10	14	0.538302	0.7589	no
gi|641566839|gb|KK857516.1|	5238-6087	10	42	0.86648	1	0.773228	0.62605	no
gi|641566839|gb|KK857516.1|	7184-7388	10	42	16	11	-0.467656	0.78255	no
gi|641566839|gb|KK857516.1|	7697-8661	10	42	4	3	-0.532852	0.69785	no
gi|641566839|gb|KK857516.1|	8734-10788	10	42	2	2	-0.117827	0.92855	no
gi|641566848|gb|KK857513.1|	15468-16163	16	72	0.141669	2	4	0.12385	no
gi|641566848|gb|KK857513.1|	17152-19169	16	72	3	2	-0.544715	0.67735	no
gi|641566848|gb|KK857513.1|	20409-25284	16	72	1	1	-0.299659	0.809	no

gi|641566848|gb|KK857513.1|	23-659	16	72	2	4	0.898527	0.54035	no
gi|641566848|gb|KK857513.1|	25825-28336	16	72	14	9	-0.60523	0.62735	no
gi|641566848|gb|KK857513.1|	28419-31291	16	72	0.476129	1	1	0.3122	no
gi|641566848|gb|KK857513.1|	31742-32847	16	72	0.077831	6	6	0.1051	no
gi|641566848|gb|KK857513.1|	3208-4213	16	72	0.174886	2	4	0.2456	no
gi|641566848|gb|KK857513.1|	33610-34340	16	72	0.132407	9	6	0.10485	no
gi|641566848|gb|KK857513.1|	35066-35568	16	72	0.460373	24	6	0.23255	no
gi|641566848|gb|KK857513.1|	36347-38255	16	72	10	10	-0.0952263	0.9394	no
gi|641566848|gb|KK857513.1|	38370-41781	16	72	5	3	-0.616138	0.6197	no
gi|641566848|gb|KK857513.1|	42773-43291	16	72	5	5	-0.0578792	0.9671	no
gi|641566848|gb|KK857513.1|	4346-4542	16	72	14	23	0.750557	0.6475	no
gi|641566848|gb|KK857513.1|	44591-45598	16	72	4	0.398029	-3	0.10835	no
gi|641566848|gb|KK857513.1|	47060-47748	16	72	3	2	-0.569552	0.71445	no
gi|641566848|gb|KK857513.1|	49806-51127	16	72	0.817656	0.287408	-2	1	no
gi|641566848|gb|KK857513.1|	57551-58635	16	72	2	4	1	0.44725	no
gi|641566848|gb|KK857513.1|	70322-70697	16	72	24	8	-2	0.2372	no
gi|641566848|gb|KK857513.1|	70869-71664	16	72	3	1	-1	0.35055	no
gi|641566848|gb|KK857513.1|	78684-78886	16	72	18	24	0.361385	0.82705	no
gi|641566848|gb|KK857513.1|	8569-10058	16	72	3	3	-0.0156706	0.9881	no
gi|641566851|gb|KK857512.1|	23774-24083	11	20	79	22	-2	0.1807	no
gi|641566851|gb|KK857512.1|	31471-34179	11	20	3	3	-0.155552	0.901	no
gi|641566851|gb|KK857512.1|	37279-37811	11	20	8	4	-0.990038	0.49165	no
gi|641566851|gb|KK857512.1|	38237-39171	11	20	3	2	-0.588305	0.67295	no
gi|641566851|gb|KK857512.1|	54327-54605	11	20	32	13	-1	0.37385	no
gi|641566851|gb|KK857512.1|	56957-58610	11	20	0.922861	0.962579	0.060792	0.9639	no
gi|641566851|gb|KK857512.1|	58748-61819	11	20	1	1	-0.38972	0.76675	no
gi|641566851|gb|KK857512.1|	69767-72362	11	20	2	2	-0.390128	0.76275	no
gi|641566851|gb|KK857512.1|	73524-73916	11	20	6	6	-0.00114521	0.9647	no
gi|641566854|gb|KK857511.1|	28786-29412	8.8	36	9	3	-1	0.3044	no
gi|641566854|gb|KK857511.1|	30714-30922	8.8	36	20	16	-0.340085	0.8319	no

gi|641566854|gb|KK857511.1|	34002-34175	8.8	36	82	58	-0.484213	0.74215	no
gi|641566854|gb|KK857511.1|	35089-36045	8.8	36	8	2	-2	0.21035	no
gi|641566854|gb|KK857511.1|	42528-42974	8.8	36	19	4	-2	0.1444	no
gi|641566854|gb|KK857511.1|	49187-49725	8.8	36	2	2	0.0102563	0.9394	no
gi|641566854|gb|KK857511.1|	50768-51445	8.8	36	1	2	0.430175	0.7802	no
gi|641566857|gb|KK857510.1|	15610-18212	9.4	18	2	2	0.357113	0.7801	no
gi|641566857|gb|KK857510.1|	18351-20010	9.4	18	14	16	0.239271	0.8434	no
gi|641566857|gb|KK857510.1|	20639-21694	9.4	18	13	8	-0.684044	0.59255	no
gi|641566857|gb|KK857510.1|	22663-23279	9.4	18	1	1	0.112909	0.92265	no
gi|641566857|gb|KK857510.1|	23427-25200	9.4	18	1	0.889556	-0.275935	0.8412	no
gi|641566857|gb|KK857510.1|	29992-31215	9.4	18	0.96452	0.524509	-0.878842	0.5832	no
gi|641566857|gb|KK857510.1|	42106-44643	9.4	18	0.725658	1	0.832203	0.54705	no
gi|641566857|gb|KK857510.1|	66065-67136	9.4	18	0.404262	2	2	0.2717	no
gi|641566857|gb|KK857510.1|	67187-68847	9.4	18	0.628413	0.589533	-0.0921405	1	no
gi|641566868|gb|KK857506.1|	14140-14362	6	22	16	0	-inf	0.0102	no
gi|641566891|gb|KK857497.1|	4473-4929	10	40	1	2	0.590149	0.70505	no
gi|641566894|gb|KK857496.1|	17985-18210	11	39	0	274	inf	5.00E-05	yes
gi|641566894|gb|KK857496.1|	19377-19728	11	39	0	36	inf	0.00015	yes
gi|641566894|gb|KK857496.1|	20967-21922	11	39	0	157	inf	5.00E-05	yes
gi|641566894|gb|KK857496.1|	31616-34067	11	39	21	13	-0.68815	0.6757	no
gi|641566894|gb|KK857496.1|	34273-34476	11	39	42	12	-2	0.26735	no
gi|641566894|gb|KK857496.1|	35179-36189	11	39	1	0.660951	-1	0.4973	no
gi|641566894|gb|KK857496.1|	36956-37186	11	39	38	17	-1	0.43355	no
gi|641566894|gb|KK857496.1|	39146-39435	11	39	31	16	-0.958997	0.4827	no
gi|641566894|gb|KK857496.1|	49672-49887	11	39	26	17	-0.654824	0.6685	no
gi|641566894|gb|KK857496.1|	50850-51553	11	39	25	8	-2	0.21385	no
gi|641566897|gb|KK857495.1|	13386-17929	11	41	18	10	-0.943771	0.67225	no
gi|641566897|gb|KK857495.1|	21704-25194	11	41	13	11	-0.321501	0.8364	no
gi|641566897|gb|KK857495.1|	25394-26879	11	41	13	9	-0.562173	0.6502	no
gi|641566897|gb|KK857495.1|	4358-4789	11	41	0.595988	7	3	0.2497	no

gi|641566897|gb|KK857495.1|	44553-45015	11	41	7	3	-1	0.41065	no
gi|641566897|gb|KK857495.1|	5806-8359	11	41	0.690703	1	1	0.4496	no
gi|641566897|gb|KK857495.1|	58512-58921	11	41	7	1	-2	0.221	no
gi|641566897|gb|KK857495.1|	78400-78647	11	41	0	20	inf	0.0025	yes
gi|641566900|gb|KK857494.1|	16324-17329	9.9	40	0.262328	2	3	0.18845	no
gi|641566900|gb|KK857494.1|	18773-19346	9.9	40	1	1	0.333731	0.83525	no
gi|641566900|gb|KK857494.1|	26468-29274	9.9	40	0.406805	7	4	0.01855	no
gi|641566900|gb|KK857494.1|	38694-39377	9.9	40	11	6	-0.789696	0.54715	no
gi|641566900|gb|KK857494.1|	42313-42799	9.9	40	4	1	-2	0.33195	no
gi|641566900|gb|KK857494.1|	45639-45889	9.9	40	95	65	-0.54261	0.665	no
gi|641566900|gb|KK857494.1|	51994-52881	9.9	40	2	1	-0.483665	0.755	no
gi|641566900|gb|KK857494.1|	56800-57707	9.9	40	1	1	0.189	0.90355	no
gi|641566900|gb|KK857494.1|	59699-60451	9.9	40	2	3	0.302181	0.8363	no
gi|641566903|gb|KK857493.1|	34783-34955	7.8	31	21	42	1	0.5133	no
gi|641566903|gb|KK857493.1|	47606-47773	7.8	31	5	77	4	0.1204	no
gi|641566903|gb|KK857493.1|	51056-51225	7.8	31	83	184	1	0.4193	no
gi|641566907|gb|KK857492.1|	56-221	11	47	5	112	4	0.1125	no
gi|641566910|gb|KK857491.1|	19681-20843	8.2	34	0	13	inf	5.00E-05	yes
gi|641566912|gb|KK857490.1|	12179-16478	11	39	4	6	0.389453	0.75835	no
gi|641566912|gb|KK857490.1|	17615-18355	11	39	0	12	inf	5.00E-05	yes
gi|641566912|gb|KK857490.1|	18915-20222	11	39	0	2	inf	0.0016	yes
gi|641566912|gb|KK857490.1|	24205-24700	11	39	0	9	inf	0.00045	yes
gi|641566912|gb|KK857490.1|	25564-25858	11	39	0	260	inf	5.00E-05	yes
gi|641566912|gb|KK857490.1|	28985-29213	11	39	0	25	inf	0.0025	yes
gi|641566912|gb|KK857490.1|	29730-31640	11	39	0.041292	2	5	0.10635	no
gi|641566912|gb|KK857490.1|	32227-33079	11	39	0	16	inf	5.00E-05	yes
gi|641566912|gb|KK857490.1|	33489-34342	11	39	0	3	inf	0.0015	yes
gi|641566912|gb|KK857490.1|	38817-39534	11	39	0	4	inf	0.00105	yes
gi|641566912|gb|KK857490.1|	47948-48404	11	39	0	33	inf	5.00E-05	yes
gi|641566912|gb|KK857490.1|	52282-52911	11	39	0	5	inf	0.0005	yes

gi|641566912|gb|KK857490.1|	55893-57032	11	39	0.300106	1	2	0.29935	no
gi|641566912|gb|KK857490.1|	59516-59824	11	39	0	52	inf	5.00E-05	yes
gi|641566912|gb|KK857490.1|	67864-68087	11	39	0	25	inf	0.00375	no
gi|641566921|gb|KK857487.1|	11624-12199	9.6	20	2	0	-inf	0.0102	no
gi|641566921|gb|KK857487.1|	12327-13083	9.6	20	1	0.958166	-0.246181	0.88495	no
gi|641566921|gb|KK857487.1|	13455-14007	9.6	20	65	30	-1	0.37365	no
gi|641566921|gb|KK857487.1|	19419-20560	9.6	20	1	0.68375	-0.715841	0.6527	no
gi|641566921|gb|KK857487.1|	2195-3512	9.6	20	1	2	0.462676	0.74035	no
gi|641566921|gb|KK857487.1|	22526-22820	9.6	20	20	7	-2	0.3077	no
gi|641566921|gb|KK857487.1|	25539-29342	9.6	20	0.745772	0.98895	0.407164	0.76005	no
gi|641566921|gb|KK857487.1|	29619-30414	9.6	20	2	1	-0.677616	0.6649	no
gi|641566921|gb|KK857487.1|	33776-33940	9.6	20	114	93	-0.296578	0.835	no
gi|641566921|gb|KK857487.1|	34121-35521	9.6	20	0.705265	0.716334	0.0224663	1	no
gi|641566921|gb|KK857487.1|	35658-36778	9.6	20	1	0.932279	-0.300945	0.84515	no
gi|641566921|gb|KK857487.1|	37730-37896	9.6	20	71	29	-1	0.44065	no
gi|641566921|gb|KK857487.1|	38313-38677	9.6	20	138	71	-0.954388	0.43795	no
gi|641566921|gb|KK857487.1|	44151-45478	9.6	20	0.625631	0.57178	-0.129853	1	no
gi|641566921|gb|KK857487.1|	5125-6342	9.6	20	7	7	-0.0600252	0.96365	no
gi|641566921|gb|KK857487.1|	52-1873	9.6	20	48	60	0.313659	0.88835	no
gi|641566921|gb|KK857487.1|	8010-8457	9.6	20	3	4	0.174381	0.9057	no
gi|641566921|gb|KK857487.1|	8963-9860	9.6	20	23	13	-0.813238	0.52085	no
gi|641566930|gb|KK857484.1|	11554-12295	11	21	0.648701	1	1	0.51085	no
gi|641566930|gb|KK857484.1|	1420-2250	11	21	0.223104	2	3	0.2851	no
gi|641566930|gb|KK857484.1|	15429-16035	11	21	3	3	0.0130365	0.9647	no
gi|641566930|gb|KK857484.1|	16152-17707	11	21	2	2	-0.153853	0.9076	no
gi|641566930|gb|KK857484.1|	17983-19818	11	21	2	0.724311	-1	0.4333	no
gi|641566930|gb|KK857484.1|	2924-3984	11	21	0.163762	0.996661	3	0.2991	no
gi|641566930|gb|KK857484.1|	9279-9879	11	21	1	2	1	0.5082	no
gi|641566932|gb|KK857483.1|	77800-78479	27	36	0	2	inf	0.0068	no
gi|641566938|gb|KK857481.1|	14226-15383	11	39	0.073622	2	5	0.10875	no

gi|641566938|gb|KK857481.1|	17021-17588	11	39	1	1	0.0115564	0.91115	no
gi|641566938|gb|KK857481.1|	21093-21453	11	39	37	9	-2	0.1569	no
gi|641566938|gb|KK857481.1|	22368-22699	11	39	13	4	-2	0.2686	no
gi|641566938|gb|KK857481.1|	23179-24259	11	39	0.8003	0.852455	0.0910837	1	no
gi|641566938|gb|KK857481.1|	24944-26897	11	39	0.684787	1	0.843437	0.55945	no
gi|641566938|gb|KK857481.1|	29824-30287	11	39	3	0.39632	-3	0.2381	no
gi|641566938|gb|KK857481.1|	33039-33280	11	39	8	7	-0.258193	0.88385	no
gi|641566938|gb|KK857481.1|	35096-35794	11	39	1	1	0.23804	0.88205	no
gi|641566938|gb|KK857481.1|	36803-38769	11	39	1	2	0.864254	0.52335	no
gi|641566938|gb|KK857481.1|	39634-41065	11	39	0.974061	2	0.979521	0.4944	no
gi|641566938|gb|KK857481.1|	41216-41836	11	39	0.33324	4	4	0.2456	no
gi|641566944|gb|KK857479.1|	27163-28331	13	49	2	3	0.805569	0.55335	no
gi|641566944|gb|KK857479.1|	54665-55327	13	49	1	6	3	0.14055	no
gi|641566944|gb|KK857479.1|	64556-65818	13	49	26	271	3	0.06625	no
gi|641566947|gb|KK857478.1|	13237-14862	9.7	16	1	0.377453	-2	0.25505	no
gi|641566947|gb|KK857478.1|	16773-17502	9.7	16	1	0.201246	-3	0.271	no
gi|641566947|gb|KK857478.1|	18461-19095	9.7	16	3	0.732992	-2	0.24335	no
gi|641566947|gb|KK857478.1|	19352-23377	9.7	16	3	0.310561	-3	0.0351	no
gi|641566947|gb|KK857478.1|	23726-24882	9.7	16	9	1	-3	0.0489	no
gi|641566947|gb|KK857478.1|	33187-33461	9.7	16	9	0	-inf	0.0102	no
gi|641566947|gb|KK857478.1|	34343-35866	9.7	16	1	2	0.332249	0.81315	no
gi|641566947|gb|KK857478.1|	36690-38183	9.7	16	0.381921	0.748305	0.97035	1	no
gi|641566947|gb|KK857478.1|	39913-42561	9.7	16	0.750552	0.704909	-0.0905146	1	no
gi|641566947|gb|KK857478.1|	44362-44901	9.7	16	2	0	-inf	0.0102	no
gi|641566947|gb|KK857478.1|	45676-46344	9.7	16	6	0.226951	-5	0.1857	no
gi|641566947|gb|KK857478.1|	47295-49309	9.7	16	0.973263	1	0.213156	0.87885	no
gi|641566947|gb|KK857478.1|	5361-6248	9.7	16	4	1	-2	0.22315	no
gi|641566947|gb|KK857478.1|	8257-8914	9.7	16	6	3	-1	0.3618	no
gi|641566949|gb|KK857477.1|	25351-25762	11	39	20	7	-1	0.2934	no
gi|641566949|gb|KK857477.1|	37703-40088	11	39	131	101	-0.370216	0.84755	no

gi|641566949|gb|KK857477.1|	42665-44515	11	39	9	19	1	0.42215	no
gi|641566949|gb|KK857477.1|	53322-53702	11	39	4	11	1	0.33675	no
gi|641566949|gb|KK857477.1|	54064-54322	11	39	6	24	2	0.2124	no
gi|641566949|gb|KK857477.1|	55450-55738	11	39	14	43	2	0.24125	no
gi|641566949|gb|KK857477.1|	64252-64430	11	39	14	56	2	0.29495	no
gi|641566949|gb|KK857477.1|	65123-65329	11	39	19	17	-0.203596	0.8975	no
gi|641566949|gb|KK857477.1|	73178-73517	11	39	13	16	0.22782	0.8653	no
gi|641566949|gb|KK857477.1|	74427-74693	11	39	0	50	inf	5.00E-05	yes
gi|641566949|gb|KK857477.1|	76174-76573	11	39	6	5	-0.263337	0.85105	no
gi|641566949|gb|KK857477.1|	77542-77806	11	39	2	10	3	0.30875	no
gi|641566949|gb|KK857477.1|	87234-87456	11	39	71	117	0.707762	0.5849	no
gi|641566949|gb|KK857477.1|	87590-87961	11	39	24	43	0.816623	0.5199	no
gi|641566955|gb|KK857475.1|	14101-14295	10	37	120	0	-inf	0.00025	yes
gi|641566955|gb|KK857475.1|	16044-16350	10	37	12	3	-2	0.2319	no
gi|641566955|gb|KK857475.1|	17908-18128	10	37	39	4	-3	0.1548	no
gi|641566955|gb|KK857475.1|	18564-19741	10	37	22	0	-inf	5.00E-05	yes
gi|641566955|gb|KK857475.1|	21804-21999	10	37	26	0	-inf	0.0102	no
gi|641566955|gb|KK857475.1|	225-438	10	37	98	0	-inf	5.00E-05	yes
gi|641566955|gb|KK857475.1|	23400-23724	10	37	6	0	-inf	0.0102	no
gi|641566955|gb|KK857475.1|	25170-25383	10	37	47	0	-inf	0.0013	yes
gi|641566955|gb|KK857475.1|	34210-35226	10	37	2	0.656143	-2	0.2547	no
gi|641566955|gb|KK857475.1|	35399-36091	10	37	7	0.648284	-3	0.10835	no
gi|641566955|gb|KK857475.1|	38713-39480	10	37	2	0	-inf	0.0059	no
gi|641566955|gb|KK857475.1|	40703-41048	10	37	32	0	-inf	5.00E-05	yes
gi|641566955|gb|KK857475.1|	42300-42525	10	37	28	0	-inf	0.00345	yes
gi|641566955|gb|KK857475.1|	46085-47395	10	37	0.508138	1	1	0.49695	no
gi|641566955|gb|KK857475.1|	48379-49121	10	37	1	0.393002	-2	0.3217	no
gi|641566955|gb|KK857475.1|	4948-5616	10	37	32	0.907806	-5	0.06195	no
gi|641566955|gb|KK857475.1|	52851-53083	10	37	40	0	-inf	0.001	yes
gi|641566955|gb|KK857475.1|	55327-55676	10	37	19	0	-inf	0.0003	yes

gi|641566955|gb|KK857475.1|	56853-57256	10	37	68	0	-inf	5.00E-05	yes
gi|641566955|gb|KK857475.1|	60356-60581	10	37	32	0	-inf	0.00315	yes
gi|641566955|gb|KK857475.1|	64155-64737	10	37	5	7	0.542674	0.695	no
gi|641566955|gb|KK857475.1|	66884-67603	10	37	64	0.205055	-8	0.18285	no
gi|641566955|gb|KK857475.1|	7623-8241	10	37	58	0.506917	-7	0.1301	no
gi|641566957|gb|KK857474.1|	39572-39786	8.3	34	0	36	inf	0.0023	yes
gi|641566960|gb|KK857473.1|	51707-52329	10	20	3	4	0.318424	0.8318	no
gi|641566960|gb|KK857473.1|	56387-56797	10	20	3	2	-0.0921736	0.92655	no
gi|641566964|gb|KK857472.1|	54929-55831	11	22	0.400749	1	1	0.44255	no
gi|641566967|gb|KK857471.1|	74328-74758	9	39	4	2	-0.997052	0.5872	no
gi|641566967|gb|KK857471.1|	76569-76758	9	39	13	39	2	0.38185	no
gi|641566967|gb|KK857471.1|	77506-77766	9	39	18	13	-0.444006	0.7595	no
gi|641566967|gb|KK857471.1|	82004-82236	9	39	61	55	-0.13258	0.9183	no
gi|641566967|gb|KK857471.1|	83824-84002	9	39	120	86	-0.481495	0.72845	no
gi|641566967|gb|KK857471.1|	88621-89315	9	39	3	0.215237	-4	0.20475	no
gi|641566967|gb|KK857471.1|	92630-94381	9	39	0.95575	1	0.608535	0.66295	no
gi|641566967|gb|KK857471.1|	94539-95170	9	39	2	2	0.0842413	0.94	no
gi|641566967|gb|KK857471.1|	95237-95774	9	39	2	2	-0.182437	0.9035	no
gi|641566970|gb|KK857470.1|	13890-14068	11	39	110	10	-3	0.14445	no
gi|641566970|gb|KK857470.1|	1470-1954	11	39	22	1	-4	0.06615	no
gi|641566970|gb|KK857470.1|	17276-17430	11	39	104	0	-inf	0.0075	no
gi|641566970|gb|KK857470.1|	20993-21268	11	39	49	6	-3	0.0822	no
gi|641566970|gb|KK857470.1|	2263-4788	11	39	13	1	-3	0.02275	no
gi|641566970|gb|KK857470.1|	24193-26756	11	39	0.867208	14	4	0.01105	no
gi|641566970|gb|KK857470.1|	26989-27503	11	39	2	2	-0.254018	0.88485	no
gi|641566970|gb|KK857470.1|	27603-27959	11	39	3	3	-0.421314	0.7868	no
gi|641566970|gb|KK857470.1|	29305-30425	11	39	3	0.932279	-2	0.22585	no
gi|641566970|gb|KK857470.1|	30540-34270	11	39	49	13	-2	0.3852	no
gi|641566970|gb|KK857470.1|	66801-67191	11	39	0	6	inf	0.00455	no
gi|641566970|gb|KK857470.1|	7335-7721	11	39	7	3	-1	0.42815	no

gi|641566970|gb|KK857470.1|	9378-9604	11	39	25	4	-3	0.19055	no
gi|641566975|gb|KK857468.1|	25734-26489	12	52	2	0.767892	-1	0.50315	no
gi|641566975|gb|KK857468.1|	30241-30769	12	52	2	1	-0.405277	0.82205	no
gi|641566978|gb|KK857467.1|	1154-1512	12	50	31	1	-5	0.1312	no
gi|641566978|gb|KK857467.1|	118-1019	12	50	5	0.457468	-4	0.1081	no
gi|641566978|gb|KK857467.1|	2204-3904	12	50	9	1	-3	0.04605	no
gi|641566978|gb|KK857467.1|	45022-45271	12	50	87	21	-2	0.1577	no
gi|641566978|gb|KK857467.1|	9967-10573	12	50	6	6	0.0574306	0.9667	no
gi|641566986|gb|KK857464.1|	29768-30837	11	41	0.081038	1	4	0.12385	no
gi|641566986|gb|KK857464.1|	30928-32104	11	41	0.144392	2	4	0.243	no
gi|641566986|gb|KK857464.1|	40289-41477	11	41	0.499263	0.868681	0.799027	1	no
gi|641566986|gb|KK857464.1|	45745-46502	11	41	0	5	inf	0.00065	yes
gi|641566986|gb|KK857464.1|	48303-52837	11	41	12	9	-0.478416	0.83515	no
gi|641566986|gb|KK857464.1|	57695-58949	11	41	0.468139	0.916579	0.969323	0.5733	no
gi|641566986|gb|KK857464.1|	64586-65693	11	41	12	103	3	0.1003	no
gi|641566989|gb|KK857463.1|	20050-21337	9.4	34	2	2	0.258998	0.84985	no
gi|641566989|gb|KK857463.1|	38373-39040	9.4	34	3	0.909709	-2	0.26755	no
gi|641566989|gb|KK857463.1|	47423-47971	9.4	34	1	3	2	0.31505	no
gi|641566989|gb|KK857463.1|	50132-50818	9.4	34	2	1	-0.515153	0.74125	no
gi|641566989|gb|KK857463.1|	51892-52264	9.4	34	70	38	-0.872226	0.49505	no
gi|641566998|gb|KK857460.1|	13584-13760	11	41	41	11	-2	0.31325	no
gi|641566998|gb|KK857460.1|	23183-23379	11	41	9	33	2	0.30935	no
gi|641566998|gb|KK857460.1|	27453-28140	11	41	2	2	-0.31719	0.83735	no
gi|641566998|gb|KK857460.1|	34862-37495	11	41	85	87	0.0347354	0.9866	no
gi|641566998|gb|KK857460.1|	37753-38916	11	41	56	38	-0.560382	0.80175	no
gi|641566998|gb|KK857460.1|	39502-40064	11	41	46	26	-0.833004	0.5119	no
gi|641566998|gb|KK857460.1|	41046-41695	11	41	2	0.472695	-2	0.3455	no
gi|641566998|gb|KK857460.1|	44615-45103	11	41	6	1	-2	0.2184	no
gi|641566998|gb|KK857460.1|	45531-45891	11	41	40	24	-0.742602	0.5664	no
gi|641566998|gb|KK857460.1|	50449-50970	11	41	4	1	-2	0.31215	no

gi|641566998|gb|KK857460.1|	51301-52301	11	41	0.967847	0	-inf	0.0102	no
gi|641567000|gb|KK857459.1|	10357-11314	8.7	37	24	12	-0.924672	0.4678	no
gi|641567000|gb|KK857459.1|	13026-14043	8.7	37	0.430829	0.655348	0.605146	1	no
gi|641567000|gb|KK857459.1|	1696-2500	8.7	37	14	10	-0.45929	0.7218	no
gi|641567000|gb|KK857459.1|	20043-20260	8.7	37	0	48	inf	0.00105	yes
gi|641567000|gb|KK857459.1|	58355-60380	8.7	37	0.425661	0.885363	1	0.47825	no
gi|641567000|gb|KK857459.1|	67693-69097	8.7	37	6	8	0.305789	0.8082	no
gi|641567000|gb|KK857459.1|	73838-74133	8.7	37	6	6	-0.0188401	0.9394	no
gi|641567000|gb|KK857459.1|	75121-75897	8.7	37	6	9	0.632546	0.63685	no
gi|641567000|gb|KK857459.1|	76038-76263	8.7	37	443	724	0.709364	0.66	no
gi|641567000|gb|KK857459.1|	80901-81231	8.7	37	4	7	0.743947	0.629	no
gi|641567000|gb|KK857459.1|	88-849	8.7	37	0.625782	1	0.864942	0.62725	no
gi|641567002|gb|KK857458.1|	12730-12964	8.3	33	9	11	0.323934	0.8379	no
gi|641567002|gb|KK857458.1|	14733-15492	8.3	33	6	3	-0.983092	0.47975	no
gi|641567002|gb|KK857458.1|	18463-18893	8.3	33	2	4	0.950481	0.5736	no
gi|641567002|gb|KK857458.1|	19914-20290	8.3	33	2	9	2	0.2616	no
gi|641567002|gb|KK857458.1|	20763-21500	8.3	33	2	1	-0.398549	0.8086	no
gi|641567002|gb|KK857458.1|	21893-22722	8.3	33	0.446913	1	2	0.3613	no
gi|641567002|gb|KK857458.1|	24415-24817	8.3	33	4	3	-0.678026	0.68185	no
gi|641567002|gb|KK857458.1|	28781-29682	8.3	33	3	3	0.0420881	0.9735	no
gi|641567002|gb|KK857458.1|	3478-3770	8.3	33	0	906	inf	0.0002	yes
gi|641567005|gb|KK857457.1|	19874-20212	7.9	40	0	83	inf	5.00E-05	yes
gi|641567005|gb|KK857457.1|	20616-21432	7.9	40	0	2	inf	0.00375	no
gi|641567005|gb|KK857457.1|	23759-23970	7.9	40	0	73	inf	0.0004	yes
gi|641567005|gb|KK857457.1|	31770-31990	7.9	40	0	46	inf	0.00105	yes
gi|641567005|gb|KK857457.1|	32315-33064	7.9	40	0	56	inf	5.00E-05	yes
gi|641567008|gb|KK857456.1|	15289-16490	12	42	0.563211	0.750311	0.413814	1	no
gi|641567008|gb|KK857456.1|	25305-26313	12	42	0.08712	2	5	0.10875	no
gi|641567008|gb|KK857456.1|	32103-32438	12	42	0	28	inf	0.00015	yes
gi|641567008|gb|KK857456.1|	39934-40657	12	42	0.402484	2	2	0.2235	no

gi|641567008|gb|KK857456.1|	47808-48610	12	42	0.583502	2	1	0.3994	no
gi|641567008|gb|KK857456.1|	55909-57705	12	42	0.751952	3	2	0.2013	no
gi|641567008|gb|KK857456.1|	63192-64007	12	42	0.914018	0.694046	-0.397191	0.82205	no
gi|641567008|gb|KK857456.1|	65158-65767	12	42	1	1	-0.209253	0.8945	no
gi|641567008|gb|KK857456.1|	65929-66784	12	42	1	2	0.340351	0.81785	no
gi|641567008|gb|KK857456.1|	81098-81223	12	42	4301.29	8695.73	1	0.43685	no
gi|641567011|gb|KK857455.1|	12188-13999	11	40	2	4	1	0.39985	no
gi|641567011|gb|KK857455.1|	15979-20903	11	40	3	3	-0.247738	0.84605	no
gi|641567011|gb|KK857455.1|	21039-21690	11	40	63	77	0.282797	0.8175	no
gi|641567011|gb|KK857455.1|	22951-26285	11	40	20	26	0.384024	0.86175	no
gi|641567011|gb|KK857455.1|	26441-29198	11	40	4	5	0.380894	0.7659	no
gi|641567011|gb|KK857455.1|	29313-31177	11	40	140	96	-0.538987	0.78735	no
gi|641567011|gb|KK857455.1|	3470-4409	11	40	1	0.868444	-0.717535	0.6522	no
gi|641567011|gb|KK857455.1|	36903-38430	11	40	0	90	inf	5.00E-05	yes
gi|641567011|gb|KK857455.1|	7379-11731	11	40	27	21	-0.346546	0.8717	no
gi|641567011|gb|KK857455.1|	844-2173	11	40	2	2	-0.129844	0.9265	no
gi|641567023|gb|KK857451.1|	13145-13871	7.7	30	0.667018	3	2	0.2717	no
gi|641567026|gb|KK857450.1|	10690-12254	9.2	36	0.62076	0.709604	0.192979	1	no
gi|641567026|gb|KK857450.1|	12357-20481	9.2	36	3	3	-0.145273	0.90585	no
gi|641567026|gb|KK857450.1|	20622-22276	9.2	36	28	18	-0.667881	0.68045	no
gi|641567026|gb|KK857450.1|	23588-24320	9.2	36	1	0.800525	-0.568575	0.7586	no
gi|641567026|gb|KK857450.1|	26082-26380	9.2	36	4	8	1	0.48315	no
gi|641567026|gb|KK857450.1|	34991-36549	9.2	36	80	86	0.0886231	0.9658	no
gi|641567026|gb|KK857450.1|	37462-40610	9.2	36	8	10	0.329901	0.7872	no
gi|641567026|gb|KK857450.1|	40726-42200	9.2	36	31	27	-0.183399	0.90815	no
gi|641567026|gb|KK857450.1|	45657-46225	9.2	36	1	0.860765	-0.625832	0.71195	no
gi|641567026|gb|KK857450.1|	53928-58665	9.2	36	9	15	0.772292	0.7276	no
gi|641567026|gb|KK857450.1|	63726-66109	9.2	36	0.841468	0.839519	-0.00334388	1	no
gi|641567026|gb|KK857450.1|	7416-10534	9.2	36	0.847883	0.55473	-0.612079	1	no
gi|641567028|gb|KK857449.1|	31516-32605	15	70	8	0	-inf	5.00E-05	yes

gi|641567031|gb|KK857448.1|	2681-3755	9.3	37	0.483459	1	1	0.41175	no
gi|641567031|gb|KK857448.1|	8878-9066	9.3	37	512	348	-0.555843	0.6545	no
gi|641567034|gb|KK857447.1|	21219-21817	8.6	37	1	2	0.790363	0.63035	no
gi|641567034|gb|KK857447.1|	23118-24192	8.6	37	0.161153	2	4	0.24315	no
gi|641567040|gb|KK857445.1|	0-261	11	43	3	13	2	0.2454	no
gi|641567040|gb|KK857445.1|	1048-2921	11	43	3	8	2	0.24915	no
gi|641567040|gb|KK857445.1|	13999-14133	11	43	773	716	-0.110153	0.9374	no
gi|641567040|gb|KK857445.1|	14702-16362	11	43	12	15.77	0.430903	0.72755	no
gi|641567040|gb|KK857445.1|	16615-17213	11	43	1	1	-0.209637	0.8945	no
gi|641567040|gb|KK857445.1|	17452-17751	11	43	7	16	1	0.4259	no
gi|641567040|gb|KK857445.1|	17884-19214	11	43	0.873568	0.190091	-2	1	no
gi|641567040|gb|KK857445.1|	28365-32770	11	43	4	101	5	0.0213	no
gi|641567040|gb|KK857445.1|	61580-62044	11	43	2	1	-0.824274	0.64915	no
gi|641567040|gb|KK857445.1|	6681-6942	11	43	0	24	inf	0.00135	yes
gi|641567049|gb|KK857442.1|	2177-2535	8.2	33	6	6	0.0484937	0.95585	no
gi|641567049|gb|KK857442.1|	5935-6573	8.2	33	2	1	-0.8864	0.5827	no
gi|641567055|gb|KK857440.1|	39665-40500	10	39	1	0.336243	-2	0.31075	no
gi|641567059|gb|KK857439.1|	29548-30502	7	22	0	2	inf	0.00455	no
gi|641567062|gb|KK857438.1|	12577-13344	9.8	36	2	1	-0.838558	0.5768	no
gi|641567062|gb|KK857438.1|	13454-13808	9.8	36	8	11.01	0.417894	0.7563	no
gi|641567062|gb|KK857438.1|	13949-14154	9.8	36	33	31	-0.095173	0.94845	no
gi|641567062|gb|KK857438.1|	14869-15114	9.8	36	11	6	-0.771225	0.63705	no
gi|641567062|gb|KK857438.1|	20743-21114	9.8	36	2	6	1	0.3842	no
gi|641567062|gb|KK857438.1|	27374-27608	9.8	36	18	13	-0.553209	0.72045	no
gi|641567062|gb|KK857438.1|	30061-31435	9.8	36	8	7	-0.284303	0.8206	no
gi|641567062|gb|KK857438.1|	31676-31966	9.8	36	5	7	0.372183	0.8127	no
gi|641567062|gb|KK857438.1|	34618-35051	9.8	36	10	9	-0.177345	0.89315	no
gi|641567062|gb|KK857438.1|	35452-36137	9.8	36	6	4	-0.771669	0.57595	no
gi|641567062|gb|KK857438.1|	36314-36943	9.8	36	2	2	-0.0533241	0.94545	no
gi|641567062|gb|KK857438.1|	39098-40058	9.8	36	1	0.844723	-0.717317	0.6522	no

gi|641567062|gb|KK857438.1|	40728-41743	9.8	36	3	2	-0.10114	0.9397	no
gi|641567062|gb|KK857438.1|	42456-43321	9.8	36	2	4	0.664919	0.62565	no
gi|641567069|gb|KK857435.1|	14307-14939	6.8	28	0	8	inf	0.00025	yes
gi|641567069|gb|KK857435.1|	16021-17335	6.8	28	8	2	-2	0.119	no
gi|641567069|gb|KK857435.1|	24338-24704	6.8	28	16	8	-0.897795	0.5217	no
gi|641567069|gb|KK857435.1|	25386-25599	6.8	28	47	61	0.373354	0.78385	no
gi|641567069|gb|KK857435.1|	32300-33124	6.8	28	3	2	-0.461185	0.74915	no
gi|641567069|gb|KK857435.1|	40075-40765	6.8	28	0.286197	5	4	0.2397	no
gi|641567075|gb|KK857433.1|	37758-38304	10	39	0.806276	3	2	0.33255	no
gi|641567075|gb|KK857433.1|	44208-44871	10	39	3	6	1	0.4401	no
gi|641567075|gb|KK857433.1|	51534-52013	10	39	2	3	0.399188	0.8089	no
gi|641567075|gb|KK857433.1|	55022-58199	10	39	5	2	-1	0.3266	no
gi|641567075|gb|KK857433.1|	59186-66544	10	39	8	8	-0.115173	0.96045	no
gi|641567075|gb|KK857433.1|	74328-75852	10	39	14	6	-1	0.3332	no
gi|641567075|gb|KK857433.1|	77003-77795	10	39	3	2	-0.456451	0.7485	no
gi|641567078|gb|KK857432.1|	59635-60494	9.3	41	4	4	-0.230551	0.86	no
gi|641567078|gb|KK857432.1|	65163-65830	9.3	41	4	5	0.599841	0.6557	no
gi|641567078|gb|KK857432.1|	66820-68202	9.3	41	0.596635	1	0.870389	0.56565	no
gi|641567078|gb|KK857432.1|	68993-69516	9.3	41	22	43	0.949306	0.4516	no
gi|641567087|gb|KK857429.1|	3197-3464	6.6	30	12	1	-3	0.2153	no
gi|641567101|gb|KK857425.1|	73176-74619	9.5	21	0.624213	0.086466	-3	1	no
gi|641567104|gb|KK857424.1|	34327-35248	8.5	38	3	2	-0.189352	0.89425	no
gi|641567109|gb|KK857422.1|	39013-39977	9.5	44	0	4	inf	0.00035	yes
gi|641567111|gb|KK857421.1|	20072-20872	11	39	11	5	-1	0.38895	no
gi|641567111|gb|KK857421.1|	63285-63928	11	39	0.790434	2	1	0.4611	no
gi|641567114|gb|KK857420.1|	17419-18334	10	38	4	11	2	0.21015	no
gi|641567114|gb|KK857420.1|	19341-19566	10	38	11	67	3	0.12135	no
gi|641567114|gb|KK857420.1|	27392-28456	10	38	0.326016	1	2	0.2441	no
gi|641567114|gb|KK857420.1|	35309-35816	10	38	2	8	2	0.1534	no
gi|641567114|gb|KK857420.1|	67662-71213	10	38	3	21	3	0.1646	no

gi|641567120|gb|KK857418.1|	15829-11654	12	45	2	1	-0.399007	0.8086	no
gi|641567120|gb|KK857418.1|	36258-13716	12	45	0.400749	1	2	0.3613	no
gi|641567123|gb|KK857417.1|	20166-22011	11	41	15	12	-0.313906	0.80165	no
gi|641567123|gb|KK857417.1|	40711-41220	11	41	18	14	-0.388599	0.76765	no
gi|641567123|gb|KK857417.1|	45116-45333	11	41	25	23	-0.139293	0.92145	no
gi|641567123|gb|KK857417.1|	48648-48887	11	41	9	5	-0.866671	0.6407	no
gi|641567123|gb|KK857417.1|	68407-68661	11	41	49	16	-2	0.25	no
gi|641567123|gb|KK857417.1|	70034-70735	11	41	21	6	-2	0.1786	no
gi|641567123|gb|KK857417.1|	73339-73928	11	41	23	9	-1	0.3047	no
gi|641567123|gb|KK857417.1|	74650-74893	11	41	42	30	-0.450059	0.7375	no
gi|641567123|gb|KK857417.1|	74994-75838	11	41	2	1	-0.396766	0.7965	no
gi|641567126|gb|KK857416.1|	31593-31815	11	41	0	23	inf	0.00455	no
gi|641567126|gb|KK857416.1|	35196-36029	11	41	4	8	0.917186	0.4871	no
gi|641567126|gb|KK857416.1|	36341-37408	11	41	12	24	0.961701	0.44195	no
gi|641567126|gb|KK857416.1|	37535-41013	11	41	2	3	0.851543	0.5035	no
gi|641567126|gb|KK857416.1|	41191-42750	11	41	2	3	0.262865	0.8487	no
gi|641567126|gb|KK857416.1|	42891-44851	11	41	9	15	0.709748	0.5683	no
gi|641567126|gb|KK857416.1|	49744-52578	11	41	33	21	-0.641947	0.77005	no
gi|641567126|gb|KK857416.1|	52700-53259	11	41	3	0.881695	-2	0.33205	no
gi|641567126|gb|KK857416.1|	56766-56913	11	41	155	0	-inf	0.0059	no
gi|641567130|gb|KK857415.1|	20-1071	7.5	34	3	0.75532	-2	0.19085	no
gi|641567136|gb|KK857413.1|	19976-22458	11	44	45	384.61	3	0.1452	no
gi|641567136|gb|KK857413.1|	28623-28848	11	44	123	16	-3	0.0725	no
gi|641567136|gb|KK857413.1|	32917-33294	11	44	0	339	inf	0.00055	yes
gi|641567139|gb|KK857412.1|	32586-34111	11	45	2	3	0.650958	0.6267	no
gi|641567139|gb|KK857412.1|	65928-66949	11	45	0	4	inf	0.0003	yes
gi|641567139|gb|KK857412.1|	72094-72686	11	45	2	3	1	0.49595	no
gi|641567145|gb|KK857410.1|	26864-27934	9.1	33	0.971339	0	-inf	0.01	no
gi|641567145|gb|KK857410.1|	34328-35128	9.1	33	1	0.355573	-2	0.3217	no
gi|641567157|gb|KK857406.1|	12232-13051	6.5	28	0.794637	0.517218	-0.619523	1	no

gi|641567157|gb|KK857406.1|	13622-14233	6.5	28	0.680855	2	2	0.3613	no
gi|641567160|gb|KK857405.1|	31007-32582	4.9	19	0.564493	1	0.955974	0.5381	no
gi|641567160|gb|KK857405.1|	33548-34548	4.9	19	0.527917	0.669122	0.34196	1	no
gi|641567160|gb|KK857405.1|	36036-36558	4.9	19	1	1	0.00944856	0.91115	no
gi|641567160|gb|KK857405.1|	39505-40068	4.9	19	0.961568	1	0.596351	0.70505	no
gi|641567160|gb|KK857405.1|	50878-51475	4.9	19	41	33	-0.282024	0.8235	no
gi|641567160|gb|KK857405.1|	5749-6469	4.9	19	23	25	0.127646	0.91535	no
gi|641567160|gb|KK857405.1|	61328-61998	4.9	19	3	1	-1	0.39825	no
gi|641567160|gb|KK857405.1|	8613-9333	4.9	19	0.404782	2	2	0.2812	no
gi|641567162|gb|KK857404.1|	18019-18218	7.7	38	237	116	-1	0.4305	no
gi|641567162|gb|KK857404.1|	21563-23436	7.7	38	15	9	-0.773805	0.54035	no
gi|641567162|gb|KK857404.1|	73068-73768	7.7	38	1	0.850809	-0.399344	0.82205	no
gi|641567165|gb|KK857403.1|	10709-12422	11	41	1	1	0.000757483	0.98005	no
gi|641567165|gb|KK857403.1|	12522-15368	11	41	0.854755	1	0.739388	0.58225	no
gi|641567165|gb|KK857403.1|	39850-40648	11	41	0.4699	1	1	0.51305	no
gi|641567165|gb|KK857403.1|	75770-76540	11	41	0.985579	0.374	-1	0.4534	no
gi|641567165|gb|KK857403.1|	92060-93533	11	41	1	0.928865	-0.517822	0.71915	no
gi|641567165|gb|KK857403.1|	95122-96251	11	41	1	0.807915	-0.393993	0.7966	no
gi|641567171|gb|KK857401.1|	14159-14320	10	36	85	336.32	2	0.182	no
gi|641567171|gb|KK857401.1|	14770-15189	10	36	8	8	-0.0805859	0.9549	no
gi|641567171|gb|KK857401.1|	16004-16304	10	36	3	7	1	0.4667	no
gi|641567171|gb|KK857401.1|	18823-20464	10	36	20	17	-0.262767	0.8302	no
gi|641567171|gb|KK857401.1|	21619-23541	10	36	1	1	0.143248	0.9151	no
gi|641567171|gb|KK857401.1|	2347-2525	10	36	25	15	-0.703887	0.6977	no
gi|641567171|gb|KK857401.1|	28428-29512	10	36	0	6	inf	0.0001	yes
gi|641567171|gb|KK857401.1|	40334-40567	10	36	0	22	inf	0.00375	no
gi|641567171|gb|KK857401.1|	42748-42920	10	36	0	84	inf	0.00255	yes
gi|641567171|gb|KK857401.1|	44484-44790	10	36	0.596447	15	5	0.11025	no
gi|641567171|gb|KK857401.1|	47240-47961	10	36	0.134671	2	4	0.1175	no
gi|641567171|gb|KK857401.1|	59159-59605	10	36	2	2	-0.0887378	0.92655	no

gi|641567171|gb|KK857401.1|	611-1129	10	36	29	58	1	0.41525	no
gi|641567171|gb|KK857401.1|	84733-87637	10	36	28	10	-1	0.36445	no
gi|641567171|gb|KK857401.1|	96256-97231	10	36	56	35	-0.681085	0.6744	no
gi|641567174|gb|KK857400.1|	50020-50773	11	43	1	0.385312	-1	0.4534	no
gi|641567180|gb|KK857398.1|	53227-53677	10	41	2	7	1	0.3602	no
gi|641567180|gb|KK857398.1|	75127-75524	10	41	3	4	0.0698509	0.9413	no
gi|641567180|gb|KK857398.1|	79950-83754	10	41	5	3	-0.634189	0.61335	no
gi|641567180|gb|KK857398.1|	84106-85141	10	41	0.590231	1	0.797945	0.63025	no
gi|641567180|gb|KK857398.1|	85529-86462	10	41	1	0.729557	-0.533174	0.7478	no
gi|641567180|gb|KK857398.1|	92333-92575	10	41	0	50	inf	0.0003	yes
gi|641567180|gb|KK857398.1|	95268-96185	10	41	0.19621	2	3	0.2672	no
gi|641567192|gb|KK857394.1|	10195-10928	8.9	41	0.921681	2	0.963941	0.5733	no
gi|641567192|gb|KK857394.1|	13784-15202	8.9	41	0.347441	1	2	0.28005	no
gi|641567192|gb|KK857394.1|	8620-9033	8.9	41	0.643616	6	3	0.25565	no
gi|641567195|gb|KK857393.1|	14386-14741	9.8	40	3	3	0.315488	0.86115	no
gi|641567195|gb|KK857393.1|	8127-8301	9.8	40	28	102	2	0.25305	no
gi|641567198|gb|KK857392.1|	31670-32110	15	35	6	0	-inf	0.0032	yes
gi|641567201|gb|KK857391.1|	31678-32433	7.2	23	15	23	0.589923	0.63735	no
gi|641567201|gb|KK857391.1|	33968-34563	7.2	23	4	11	1	0.30125	no
gi|641567201|gb|KK857391.1|	36676-37557	7.2	23	2	2	0.281795	0.84775	no
gi|641567201|gb|KK857391.1|	38801-39482	7.2	23	2	3	0.278272	0.8498	no
gi|641567204|gb|KK857390.1|	14346-15649	10	41	0	1	inf	0.0023	yes
gi|641567204|gb|KK857390.1|	17455-18456	10	41	0	2	inf	0.0023	yes
gi|641567204|gb|KK857390.1|	22172-22855	10	41	3	8	1	0.294	no
gi|641567204|gb|KK857390.1|	31260-31901	10	41	1	1	-0.248881	0.88485	no
gi|641567204|gb|KK857390.1|	6457-7026	10	41	0.567735	4	3	0.1811	no
gi|641567206|gb|KK857389.1|	16253-18795	10	37	2	1	-0.519467	0.69095	no
gi|641567206|gb|KK857389.1|	18921-19950	10	37	0.679405	1	0.927177	0.56225	no
gi|641567206|gb|KK857389.1|	20187-20856	10	37	0.597721	3	3	0.19845	no
gi|641567206|gb|KK857389.1|	21133-22063	10	37	4	3	-0.495239	0.71145	no

gi|641567206|gb|KK857389.1|	27630-27832	10	37	106	68	-0.645568	0.62485	no
gi|641567206|gb|KK857389.1|	32355-32940	10	37	2	2	-0.0548391	0.9454	no
gi|641567206|gb|KK857389.1|	38315-38508	10	37	98	46	-1	0.4369	no
gi|641567206|gb|KK857389.1|	40682-41895	10	37	0.486999	0.953357	0.969098	0.5733	no
gi|641567206|gb|KK857389.1|	43587-45964	10	37	24	12	-1	0.51785	no
gi|641567206|gb|KK857389.1|	49551-53372	10	37	35	19	-0.877311	0.6855	no
gi|641567206|gb|KK857389.1|	62167-62695	10	37	0.63714	10	4	0.1413	no
gi|641567206|gb|KK857389.1|	70195-70352	10	37	57	40	-0.493071	0.7686	no
gi|641567206|gb|KK857389.1|	71339-74000	10	37	0.71788	0.657374	-0.127028	1	no
gi|641567206|gb|KK857389.1|	75365-75552	10	37	20	69	2	0.2736	no
gi|641567206|gb|KK857389.1|	78851-79949	10	37	0.627473	1	0.775715	0.62605	no
gi|641567206|gb|KK857389.1|	80879-82443	10	37	0.36211	2	3	0.1165	no
gi|641567206|gb|KK857389.1|	83133-83997	10	37	18	18	-0.0245261	0.98435	no
gi|641567206|gb|KK857389.1|	84656-85370	10	37	38	54	0.499967	0.68785	no
gi|641567215|gb|KK857386.1|	24061-24717	11	41	0.768187	1	0.862579	0.62725	no
gi|641567215|gb|KK857386.1|	25450-26796	11	41	0.12306	0.843559	3	1	no
gi|641567215|gb|KK857386.1|	35295-36243	11	41	0.376258	1	2	0.3613	no
gi|641567219|gb|KK857385.1|	16535-17589	11	43	0.906984	2	0.730986	0.63585	no
gi|641567219|gb|KK857385.1|	17784-19168	11	43	1	3	1	0.30395	no
gi|641567219|gb|KK857385.1|	19324-21465	11	43	0.65501	2	1	0.3824	no
gi|641567219|gb|KK857385.1|	21665-22725	11	43	1	2	0.498588	0.73235	no
gi|641567219|gb|KK857385.1|	46340-46919	11	43	0.368681	3	3	0.2851	no
gi|641567219|gb|KK857385.1|	47021-48784	11	43	1	0.964078	-0.338968	0.8091	no
gi|641567219|gb|KK857385.1|	48995-49607	11	43	0.679228	3	2	0.2441	no
gi|641567219|gb|KK857385.1|	49679-50800	11	43	0.305942	1	2	0.29935	no
gi|641567219|gb|KK857385.1|	55300-55846	11	43	0.403138	2	3	0.2991	no
gi|641567222|gb|KK857384.1|	41208-42017	9.8	33	0.692216	2	1	0.3778	no
gi|641567228|gb|KK857382.1|	23499-24155	7.9	37	0	2	inf	0.0068	no
gi|641567231|gb|KK857381.1|	14026-14385	10	37	3	6	0.941738	0.5742	no
gi|641567231|gb|KK857381.1|	14704-15611	10	37	0.497417	1	1	0.3994	no

gi|641567231|gb|KK857381.1|	24106-26830	10	37	1	10	3	0.03185	no
gi|641567231|gb|KK857381.1|	27449-28337	10	37	0.204424	6	5	0.23405	no
gi|641567231|gb|KK857381.1|	28440-29104	10	37	0	11	inf	0.0001	yes
gi|641567231|gb|KK857381.1|	31369-31588	10	37	35	408	4	0.02425	no
gi|641567231|gb|KK857381.1|	33079-33392	10	37	3	56	4	0.0836	no
gi|641567231|gb|KK857381.1|	33652-33898	10	37	5	28	2	0.1844	no
gi|641567231|gb|KK857381.1|	34983-36808	10	37	31	107	2	0.4108	no
gi|641567231|gb|KK857381.1|	37760-38652	10	37	0.609751	1	0.826252	0.6232	no
gi|641567231|gb|KK857381.1|	38893-39109	10	37	14	12	-0.309695	0.8588	no
gi|641567231|gb|KK857381.1|	39186-40062	10	37	2	3	0.60366	0.67295	no
gi|641567231|gb|KK857381.1|	40187-43147	10	37	1	2	0.397204	0.75915	no
gi|641567231|gb|KK857381.1|	43351-44890	10	37	1	2	0.830328	0.54705	no
gi|641567231|gb|KK857381.1|	44966-46190	10	37	2	4	1	0.35235	no
gi|641567231|gb|KK857381.1|	47025-47759	10	37	1	5	2	0.2392	no
gi|641567231|gb|KK857381.1|	53056-53740	10	37	0.724262	1	0.600275	0.70505	no
gi|641567231|gb|KK857381.1|	53907-55049	10	37	0.224366	2	3	0.1622	no
gi|641567231|gb|KK857381.1|	55288-57004	10	37	0.931188	1	0.373982	0.78995	no
gi|641567231|gb|KK857381.1|	58503-58885	10	37	0.745363	4	3	0.30875	no
gi|641567234|gb|KK857380.1|	22373-22811	8.3	32	0	28	inf	5.00E-05	yes
gi|641567243|gb|KK857377.1|	36292-36934	11	43	0.792199	1	0.86218	0.62755	no
gi|641567243|gb|KK857377.1|	42164-42429	11	43	14	1	-3	0.2088	no
gi|641567243|gb|KK857377.1|	52093-52558	11	43	1	2	0.853873	0.62755	no
gi|641567243|gb|KK857377.1|	61435-65043	11	43	22	10	-1	0.465	no
gi|641567243|gb|KK857377.1|	69253-69777	11	43	0.644821	2	2	0.3323	no
gi|641567250|gb|KK857375.1|	0-911	9.9	35	3	3	0.0832514	0.9506	no
gi|641567250|gb|KK857375.1|	15518-17233	9.9	35	0.69885	0.781351	0.160986	1	no
gi|641567250|gb|KK857375.1|	17358-18783	9.9	35	0.806082	1	0.500613	0.73235	no
gi|641567250|gb|KK857375.1|	19123-21353	9.9	35	0.661157	0.796341	0.268393	1	no
gi|641567250|gb|KK857375.1|	21467-23732	9.9	35	4	3	-0.118848	0.9235	no
gi|641567250|gb|KK857375.1|	24143-25513	9.9	35	5	4	-0.247498	0.85	no

gi|641567250|gb|KK857375.1|	25681-28328	9.9	35	9	9	-0.0927786	0.9376	no
gi|641567250|gb|KK857375.1|	31455-32156	9.9	35	0.13999	5	5	0.10725	no
gi|641567250|gb|KK857375.1|	32419-33282	9.9	35	17	28	0.69884	0.57675	no
gi|641567250|gb|KK857375.1|	36700-36961	9.9	35	53	258	2	0.0942	no
gi|641567250|gb|KK857375.1|	38713-38979	9.9	35	9	34	2	0.22325	no
gi|641567250|gb|KK857375.1|	39185-40083	9.9	35	0.705303	0.765683	0.118504	1	no
gi|641567250|gb|KK857375.1|	42231-42565	9.9	35	16	78	2	0.09185	no
gi|641567250|gb|KK857375.1|	42668-44201	9.9	35	0.741003	1	0.607916	0.67385	no
gi|641567250|gb|KK857375.1|	45469-47308	9.9	35	2	3	0.193701	0.88085	no
gi|641567250|gb|KK857375.1|	48866-50167	9.9	35	1	1	-0.200365	0.8877	no
gi|641567250|gb|KK857375.1|	54382-55948	9.9	35	2	1	-0.770489	0.5769	no
gi|641567250|gb|KK857375.1|	58215-58630	9.9	35	4	3	-0.288041	0.86025	no
gi|641567250|gb|KK857375.1|	62417-63167	9.9	35	1	1	0.186666	0.91015	no
gi|641567250|gb|KK857375.1|	63303-65141	9.9	35	1	0.985898	-0.239261	0.8664	no
gi|641567250|gb|KK857375.1|	65314-66306	9.9	35	0.710957	0.94613	0.412276	0.8089	no
gi|641567250|gb|KK857375.1|	67088-70413	9.9	35	2	4	1	0.391	no
gi|641567250|gb|KK857375.1|	70545-72335	9.9	35	1	1	0.172529	0.90195	no
gi|641567250|gb|KK857375.1|	74313-75445	9.9	35	3	2	-0.591912	0.65665	no
gi|641567250|gb|KK857375.1|	76506-78692	9.9	35	2	2	0.024403	0.9849	no
gi|641567250|gb|KK857375.1|	78803-82846	9.9	35	0.993677	0.843028	-0.237197	0.8576	no
gi|641567250|gb|KK857375.1|	83017-84870	9.9	35	1	2	0.52388	0.69635	no
gi|641567250|gb|KK857375.1|	84931-86296	9.9	35	2	2	-0.392718	0.77365	no
gi|641567250|gb|KK857375.1|	8989-14362	9.9	35	15	14	-0.104916	0.9612	no
gi|641567253|gb|KK857374.1|	32092-32693	7.1	30	0	3	inf	0.00375	no
gi|641567259|gb|KK857373.1|	10403-11951	8.6	20	2	4	0.877152	0.5065	no
gi|641567259|gb|KK857373.1|	13336-13648	8.6	20	19	12	-0.709904	0.6174	no
gi|641567259|gb|KK857373.1|	14081-15018	8.6	20	23	8	-2	0.2362	no
gi|641567259|gb|KK857373.1|	25524-25836	8.6	20	17	6	-2	0.316	no
gi|641567259|gb|KK857373.1|	32622-33678	8.6	20	11	4	-1	0.3029	no
gi|641567259|gb|KK857373.1|	35186-35694	8.6	20	4	4	-0.116851	0.9355	no

gi|641567259|gb|KK857373.1|	37039-37823	8.6	20	2	2	-0.134656	0.9246	no
gi|641567259|gb|KK857373.1|	38258-39479	8.6	20	3	3	0.0760589	0.9531	no
gi|641567259|gb|KK857373.1|	44448-45465	8.6	20	7	2	-1	0.2923	no
gi|641567259|gb|KK857373.1|	6116-7293	8.6	20	1	2	0.86935	0.5524	no
gi|641567259|gb|KK857373.1|	71721-72653	8.6	20	0.864967	3	2	0.26135	no
gi|641567259|gb|KK857373.1|	73096-73849	8.6	20	2	0.385312	-2	0.31075	no
gi|641567259|gb|KK857373.1|	7607-8561	8.6	20	24	7	-2	0.18815	no
gi|641567264|gb|KK857371.1|	10593-11171	11	21	6	0.279546	-4	0.1879	no
gi|641567264|gb|KK857371.1|	23262-23981	11	21	5	0.205055	-5	0.18665	no
gi|641567264|gb|KK857371.1|	49493-51167	11	21	0.766109	0.291984	-1	1	no
gi|641567264|gb|KK857371.1|	63-593	11	21	9	2	-3	0.1325	no
gi|641567264|gb|KK857371.1|	7952-8259	11	21	9	0	-inf	0.0059	no
gi|641567264|gb|KK857371.1|	9341-10335	11	21	26	6	-2	0.1191	no
gi|641567265|gb|KK857370.1|	27639-28590	10	37	0.468456	0.997183	1	0.50945	no
gi|641567265|gb|KK857370.1|	50585-51252	10	37	0	5	inf	0.00075	yes
gi|641567267|gb|KK857368.1|	12224-12413	5.6	23	737	998	0.437614	0.72925	no
gi|641567267|gb|KK857368.1|	13881-14742	5.6	23	0.744503	0.484787	-0.618927	1	no
gi|641567267|gb|KK857368.1|	20197-20909	5.6	23	0.959098	0.623424	-0.621466	0.71195	no
gi|641567267|gb|KK857368.1|	3016-3477	5.6	23	6	11	0.760462	0.58275	no
gi|641567267|gb|KK857368.1|	5830-8464	5.6	23	3	7	1	0.3228	no
gi|641567270|gb|KK857366.1|	33353-34106	11	24	0.761704	1	0.60176	0.70555	no
gi|641567276|gb|KK857364.1|	19106-20529	7.9	18	0.288355	2	3	0.1259	no
gi|641567276|gb|KK857364.1|	55848-56594	7.9	18	5	2	-1	0.34895	no
gi|641567279|gb|KK857363.1|	34672-35539	14	77	0.948668	0.640644	-0.566381	0.7586	no
gi|641567279|gb|KK857363.1|	46267-52955	14	77	4	2	-0.918253	0.46095	no
gi|641567279|gb|KK857363.1|	6292-6504	14	77	15	2	-3	0.2588	no
gi|641567282|gb|KK857362.1|	11579-12010	10	36	8	11	0.531421	0.69655	no
gi|641567282|gb|KK857362.1|	15872-16088	10	36	56	46	-0.269053	0.83785	no
gi|641567282|gb|KK857362.1|	17084-17394	10	36	20	15	-0.347789	0.8016	no
gi|641567282|gb|KK857362.1|	17654-18773	10	36	5	5	-0.0494134	0.9703	no

gi|641567282|gb|KK857362.1|	21241-22034	10	36	1	1	-0.175148	0.90465	no
gi|641567282|gb|KK857362.1|	23975-27014	10	36	2	1	-0.408122	0.7533	no
gi|641567282|gb|KK857362.1|	29520-30432	10	36	5	6	0.225583	0.86655	no
gi|641567282|gb|KK857362.1|	30663-33337	10	36	3	2	-0.468052	0.7125	no
gi|641567282|gb|KK857362.1|	34378-35307	10	36	12	4	-2	0.2074	no
gi|641567282|gb|KK857362.1|	38104-42271	10	36	13	6	-1	0.5107	no
gi|641567282|gb|KK857362.1|	42977-44717	10	36	8	8	0.0067699	0.99455	no
gi|641567282|gb|KK857362.1|	48688-49965	10	36	5	12.66	1	0.28005	no
gi|641567282|gb|KK857362.1|	50484-50987	10	36	2	6	2	0.3359	no
gi|641567282|gb|KK857362.1|	57206-57830	10	36	2	5	1	0.3591	no
gi|641567282|gb|KK857362.1|	61522-62441	10	36	0.587003	2	1	0.3778	no
gi|641567282|gb|KK857362.1|	9566-10362	10	36	2	0.71585	-2	0.28465	no
gi|641567288|gb|KK857360.1|	10355-10801	12	36	8	0	-inf	0.0013	yes
gi|641567288|gb|KK857360.1|	12865-14516	12	36	19	0.074146	-8	0.18285	no
gi|641567288|gb|KK857360.1|	15978-16254	12	36	15	0	-inf	0.00345	yes
gi|641567288|gb|KK857360.1|	16492-17194	12	36	3	0	-inf	0.0034	yes
gi|641567288|gb|KK857360.1|	18872-19957	12	36	3	0	-inf	0.0006	yes
gi|641567288|gb|KK857360.1|	40671-41428	12	36	2	2	-0.398166	0.79175	no
gi|641567288|gb|KK857360.1|	43382-46052	12	36	69	21	-2	0.4411	no
gi|641567288|gb|KK857360.1|	48011-49439	12	36	16	6	-1	0.2892	no
gi|641567288|gb|KK857360.1|	49896-51597	12	36	26	10	-1	0.3997	no
gi|641567288|gb|KK857360.1|	5242-5961	12	36	7	0	-inf	0.0003	yes
gi|641567288|gb|KK857360.1|	52917-53820	12	36	2	0	-inf	0.00495	no
gi|641567288|gb|KK857360.1|	54515-55106	12	36	8	0.270501	-5	0.1852	no
gi|641567288|gb|KK857360.1|	57040-58414	12	36	1	0	-inf	0.0034	yes
gi|641567288|gb|KK857360.1|	7971-9470	12	36	14	0	-inf	5.00E-05	yes
gi|641567290|gb|KK857359.1|	10933-11429	9.9	36	2	7	2	0.2676	no
gi|641567290|gb|KK857359.1|	25057-25584	9.9	36	2	0.965037	-0.990291	0.62775	no
gi|641567290|gb|KK857359.1|	25814-26967	9.9	36	4	3	-0.651633	0.6193	no
gi|641567290|gb|KK857359.1|	27469-29241	9.9	36	3	3	0.256284	0.84515	no

gi|641567290|gb|KK857359.1|	30075-31413	9.9	36	2	2	-0.430312	0.75	no
gi|641567290|gb|KK857359.1|	32228-34187	9.9	36	2	0.857189	-1	0.45995	no
gi|641567290|gb|KK857359.1|	34509-36248	9.9	36	0.96318	0.769235	-0.32438	0.8148	no
gi|641567290|gb|KK857359.1|	53240-53533	9.9	36	8	17	1	0.4698	no
gi|641567290|gb|KK857359.1|	6850-7696	9.9	36	3	5	0.892768	0.51445	no
gi|641567290|gb|KK857359.1|	9820-10849	9.9	36	0.679405	2	2	0.2741	no
gi|641567293|gb|KK857358.1|	17612-18002	8.8	38	3	3	-0.0945037	0.9262	no
gi|641567293|gb|KK857358.1|	5658-6314	8.8	38	3	3	0.0970439	0.94755	no
gi|641567296|gb|KK857357.1|	13471-14023	12	45	0	3	inf	0.0068	no
gi|641567296|gb|KK857357.1|	62803-64517	12	45	7	7	-0.165282	0.89285	no
gi|641567296|gb|KK857357.1|	70400-71117	12	45	5	9	0.883436	0.50175	no
gi|641567299|gb|KK857356.1|	12403-12961	11	38	3	0.294694	-3	0.20595	no
gi|641567299|gb|KK857356.1|	20921-21895	11	38	0.181848	1	3	0.2851	no
gi|641567299|gb|KK857356.1|	23606-24733	11	38	0.53195	1	1	0.48415	no
gi|641567299|gb|KK857356.1|	25035-30440	11	38	25	38	0.617488	0.7643	no
gi|641567299|gb|KK857356.1|	31591-31849	11	38	98	139	0.500362	0.6923	no
gi|641567299|gb|KK857356.1|	32681-32877	11	38	28	47	0.750557	0.6144	no
gi|641567299|gb|KK857356.1|	36221-36430	11	38	94	117	0.326296	0.80405	no
gi|641567299|gb|KK857356.1|	37654-38352	11	38	13	16	0.259573	0.8387	no
gi|641567299|gb|KK857356.1|	39234-39895	11	38	7	3	-1	0.4145	no
gi|641567299|gb|KK857356.1|	40028-40709	11	38	6	6	0.0319166	0.9801	no
gi|641567299|gb|KK857356.1|	41369-41619	11	38	46	46	-0.0168179	0.98545	no
gi|641567299|gb|KK857356.1|	43560-44330	11	38	2	2	-0.0608969	0.96245	no
gi|641567299|gb|KK857356.1|	44484-46258	11	38	1	1	0.24602	0.85905	no
gi|641567299|gb|KK857356.1|	47193-48193	11	38	5	6	0.206445	0.8751	no
gi|641567299|gb|KK857356.1|	54125-54465	11	38	12	7	-0.746001	0.5976	no
gi|641567299|gb|KK857356.1|	55487-55853	11	38	11	7	-0.589672	0.6688	no
gi|641567299|gb|KK857356.1|	58641-58871	11	38	26	34	0.392643	0.78045	no
gi|641567299|gb|KK857356.1|	60020-60860	11	38	3	3	-0.013494	0.97395	no
gi|641567299|gb|KK857356.1|	63418-63665	11	38	49	29	-0.755211	0.5837	no

gi|641567299|gb|KK857356.1|	65473-65940	11	38	12	8	-0.610648	0.64985	no
gi|641567299|gb|KK857356.1|	68059-69984	11	38	10	4	-1	0.3485	no
gi|641567299|gb|KK857356.1|	80208-80830	11	38	1	0.753341	-0.986431	0.62775	no
gi|641567299|gb|KK857356.1|	94402-95139	11	38	3	2	-0.85798	0.56505	no
gi|641567311|gb|KK857352.1|	1830-2027	8.1	36	0	62	inf	0.0015	yes
gi|641567311|gb|KK857352.1|	45075-45467	8.1	36	0	106	inf	5.00E-05	yes
gi|641567314|gb|KK857351.1|	13968-14506	7.7	26	3	3	0.216707	0.8867	no
gi|641567314|gb|KK857351.1|	17646-18378	7.7	26	26	38	0.578866	0.64485	no
gi|641567314|gb|KK857351.1|	33981-34444	7.7	26	374	176	-1	0.6252	no
gi|641567314|gb|KK857351.1|	9654-11015	7.7	26	0.789599	0.740215	-0.0931749	1	no
gi|641567321|gb|KK857348.1|	[illegible]03111-10613	19	43	1	2	0.366975	0.7749	no
gi|641567321|gb|KK857348.1|	[illegible]06193-10750	19	43	1	3	1	0.3947	no
gi|641567321|gb|KK857348.1|	26135-26371	19	43	10	5	-0.867887	0.6407	no
gi|641567321|gb|KK857348.1|	26490-26953	19	43	7	4	-0.650319	0.65175	no
gi|641567321|gb|KK857348.1|	35656-36711	19	43	5	3	-0.501452	0.7058	no
gi|641567321|gb|KK857348.1|	40185-40390	19	43	10	14	0.532858	0.768	no
gi|641567321|gb|KK857348.1|	46492-47966	19	43	11	8	-0.510124	0.68215	no
gi|641567321|gb|KK857348.1|	55070-56036	19	43	4	1	-2	0.24965	no
gi|641567321|gb|KK857348.1|	57070-57255	19	43	18	17	-0.0626895	0.91115	no
gi|641567321|gb|KK857348.1|	58858-59848	19	43	0.980026	1	0.145471	0.9285	no
gi|641567321|gb|KK857348.1|	60496-61333	19	43	8	5	-0.759435	0.5673	no
gi|641567321|gb|KK857348.1|	63740-65314	19	43	1	0.547923	-1	0.4606	no
gi|641567321|gb|KK857348.1|	65762-68325	19	43	0.328941	1	2	0.2385	no
gi|641567321|gb|KK857348.1|	70596-71745	19	43	0	2	inf	0.00255	yes
gi|641567321|gb|KK857348.1|	76479-78638	19	43	21	15	-0.545954	0.7322	no
gi|641567321|gb|KK857348.1|	79356-83340	19	43	24	14	-0.796882	0.71255	no
gi|641567321|gb|KK857348.1|	83511-86810	19	43	10	9	-0.0730953	0.96315	no
gi|641567321|gb|KK857348.1|	91023-91409	19	43	7	15	1	0.4167	no
gi|641567321|gb|KK857348.1|	96942-98062	19	43	20	28	0.494235	0.68755	no
gi|641567324|gb|KK857347.1|	62370-63484	9.1	35	1	4	2	0.24815	no

gi|641567327|gb|KK857346.1|	11386-12249	9.5	35	1	2	0.22498	0.87445	no
gi|641567327|gb|KK857346.1|	12445-17085	9.5	35	3	3	-0.0399859	0.97385	no
gi|641567327|gb|KK857346.1|	19744-20507	9.5	35	3	2	-0.639065	0.6539	no
gi|641567327|gb|KK857346.1|	21169-21903	9.5	35	1	0	-inf	0.0102	no
gi|641567327|gb|KK857346.1|	27195-27636	9.5	35	2	2	0.103479	0.92335	no
gi|641567327|gb|KK857346.1|	28757-29714	9.5	35	1	0.98937	-0.173027	0.90465	no
gi|641567327|gb|KK857346.1|	3114-3327	9.5	35	39	44	0.183216	0.89785	no
gi|641567327|gb|KK857346.1|	36311-37049	9.5	35	1	1	-0.0506057	0.94545	no
gi|641567327|gb|KK857346.1|	37787-38525	9.5	35	0.913197	1	0.379079	0.82465	no
gi|641567327|gb|KK857346.1|	51740-52847	9.5	35	0.854259	0.827323	-0.0462223	1	no
gi|641567327|gb|KK857346.1|	76477-77061	9.5	35	0	4	inf	0.0015	yes
gi|641567327|gb|KK857346.1|	83577-83952	9.5	35	7	41	2	0.08945	no
gi|641567327|gb|KK857346.1|	8498-9555	9.5	35	2	2	-0.131487	0.92475	no
gi|641567327|gb|KK857346.1|	90235-90626	9.5	35	6	36	3	0.07695	no
gi|641567327|gb|KK857346.1|	96197-96472	9.5	35	5	3	-0.661716	0.70815	no
gi|641567327|gb|KK857346.1|	9713-11281	9.5	35	2	2	-0.088579	0.94905	no
gi|641567336|gb|KK857343.1|	14468-15126	12	39	2	0.927209	-0.985363	0.5872	no
gi|641567336|gb|KK857343.1|	28587-28942	12	39	5	1	-2	0.27505	no
gi|641567336|gb|KK857343.1|	29043-29689	12	39	5	4	-0.360096	0.7914	no
gi|641567336|gb|KK857343.1|	31430-31759	12	39	7	2	-2	0.2477	no
gi|641567336|gb|KK857343.1|	37016-37612	12	39	2	0.801527	-0.987317	0.62775	no
gi|641567336|gb|KK857343.1|	40649-40934	12	39	9	3	-2	0.4022	no
gi|641567336|gb|KK857343.1|	42275-42561	12	39	20	3	-3	0.15635	no
gi|641567336|gb|KK857343.1|	50346-51371	12	39	21	8	-1	0.2731	no
gi|641567336|gb|KK857343.1|	51869-57791	12	39	49	23	-1	0.5779	no
gi|641567336|gb|KK857343.1|	61200-61412	12	39	113	79	-0.508029	0.69815	no
gi|641567336|gb|KK857343.1|	62153-62672	12	39	2	0.988369	-1	0.50365	no
gi|641567336|gb|KK857343.1|	70349-70965	12	39	0.168199	67	9	0.10435	no
gi|641567336|gb|KK857343.1|	72064-72386	12	39	1	17	4	0.24025	no
gi|641567336|gb|KK857343.1|	75184-75863	12	39	1	0.665531	-1	0.50885	no

gi|641567336|gb|KK857343.1|	85452-86122	12	39	0.745581	1	0.862954	0.62725	no
gi|641567339|gb|KK857342.1|	13131-14986	11	20	0.682561	2	1	0.381	no
gi|641567339|gb|KK857342.1|	22084-22721	11	20	0.961368	1	0.598997	0.70555	no
gi|641567339|gb|KK857342.1|	28262-29413	11	20	3	0	-inf	0.00115	yes
gi|641567339|gb|KK857342.1|	29871-31370	11	20	2	0.082764	-5	0.1848	no
gi|641567339|gb|KK857342.1|	33885-34625	11	20	0.779869	2	2	0.32035	no
gi|641567339|gb|KK857342.1|	35079-35950	11	20	0.419134	2	2	0.26155	no
gi|641567339|gb|KK857342.1|	59766-61191	11	20	0.978813	3	2	0.27745	no
gi|641567342|gb|KK857341.1|	12877-13232	8.8	36	0	326.5	inf	0.0004	yes
gi|641567342|gb|KK857341.1|	1298-1765	8.8	36	0	4	inf	0.0068	no
gi|641567342|gb|KK857341.1|	1870-5294	8.8	36	0	50	inf	0.00055	yes
gi|641567342|gb|KK857341.1|	19548-19877	8.8	36	0.506498	17	5	0.10725	no
gi|641567348|gb|KK857339.1|	64239-65270	8.5	39	0	101.69	inf	5.00E-05	yes
gi|641567350|gb|KK857338.1|	18203-19537	11	20	7	6	-0.138672	0.91155	no
gi|641567350|gb|KK857338.1|	34159-34706	11	20	2	0.607481	-2	0.31075	no
gi|641567350|gb|KK857338.1|	42597-43296	11	20	1	0.639362	-0.814404	0.6492	no
gi|641567358|gb|KK857335.1|	15369-15628	10	36	0	36	inf	0.00075	yes
gi|641567358|gb|KK857335.1|	16404-16756	10	36	0.877667	90	7	0.2323	no
gi|641567358|gb|KK857335.1|	17474-18125	10	36	0.310638	12	5	0.2332	no
gi|641567358|gb|KK857335.1|	18392-19736	10	36	0.246549	2	3	0.15255	no
gi|641567358|gb|KK857335.1|	20072-20416	10	36	0.920711	42	6	0.23285	no
gi|641567358|gb|KK857335.1|	20498-21265	10	36	2	1	-0.397985	0.7965	no
gi|641567358|gb|KK857335.1|	21988-22579	10	36	1	1	0.112036	0.92265	no
gi|641567358|gb|KK857335.1|	27736-29515	10	36	2	2	-0.284482	0.83345	no
gi|641567358|gb|KK857335.1|	32670-33197	10	36	3	0.321679	-3	0.217	no
gi|641567358|gb|KK857335.1|	36895-37395	10	36	20	8	-1	0.30845	no
gi|641567358|gb|KK857335.1|	43904-44187	10	36	250	103	-1	0.31365	no
gi|641567358|gb|KK857335.1|	46989-47339	10	36	19	7	-1	0.3296	no
gi|641567358|gb|KK857335.1|	50936-51573	10	36	81	39	-1	0.529	no
gi|641567358|gb|KK857335.1|	51873-52548	10	36	4	0.223674	-4	0.1933	no

gi|641567358|gb|KK857335.1|	54388-55349	10	36	0.462338	2	2	0.2348	no
gi|641567358|gb|KK857335.1|	55427-55759	10	36	2	4	1	0.5206	no
gi|641567358|gb|KK857335.1|	55885-57599	10	36	12	45	2	0.2616	no
gi|641567358|gb|KK857335.1|	9726-10509	10	36	0.602365	0.914467	0.602293	0.70505	no
gi|641567362|gb|KK857334.1|	34203-34957	14	24	1	0.384628	-2	0.3217	no
gi|641567362|gb|KK857334.1|	35150-36145	14	24	0.973899	0.538628	-0.854483	0.60675	no
gi|641567362|gb|KK857334.1|	36278-37299	14	24	2	0.391313	-2	0.28145	no
gi|641567362|gb|KK857334.1|	41038-41825	14	24	0.598294	1	1	0.4595	no
gi|641567362|gb|KK857334.1|	42257-42950	14	24	3	5	0.784917	0.5732	no
gi|641567362|gb|KK857334.1|	43630-45224	14	24	2	3	0.849117	0.52035	no
gi|641567362|gb|KK857334.1|	46161-48126	14	24	2	0.85429	-0.941202	0.5031	no
gi|641567362|gb|KK857334.1|	55801-57247	14	24	1	1	0.0601213	0.9639	no
gi|641567362|gb|KK857334.1|	60759-61086	14	24	5	6	0.403494	0.81025	no
gi|641567362|gb|KK857334.1|	61315-63490	14	24	2	2	0.574584	0.66595	no
gi|641567362|gb|KK857334.1|	63725-64553	14	24	3	3	-0.190545	0.89425	no
gi|641567362|gb|KK857334.1|	64716-66007	14	24	0.775424	0.688912	-0.170666	1	no
gi|641567362|gb|KK857334.1|	66443-67806	14	24	0.848881	0.831321	-0.0301564	1	no
gi|641567362|gb|KK857334.1|	68176-69957	14	24	13	12	-0.0514184	0.9663	no
gi|641567365|gb|KK857333.1|	15964-16485	10	21	1	2	0.594358	0.70505	no
gi|641567365|gb|KK857333.1|	25374-26123	10	21	2	0	-inf	0.0056	no
gi|641567365|gb|KK857333.1|	35090-35360	10	21	7	6	-0.118898	0.9229	no
gi|641567365|gb|KK857333.1|	46395-49090	10	21	0.934713	0.994286	0.0891383	0.9463	no
gi|641567368|gb|KK857332.1|	14169-15566	10	43	0	30	inf	0.0005	yes
gi|641567368|gb|KK857332.1|	34933-35381	10	43	3	0.418988	-3	0.2348	no
gi|641567368|gb|KK857332.1|	38525-39389	10	43	77	27	-2	0.3732	no
gi|641567371|gb|KK857331.1|	21571-22917	10	42	0.18459	2	4	0.14755	no
gi|641567371|gb|KK857331.1|	5702-6616	10	42	4	1	-2	0.1815	no
gi|641567374|gb|KK857330.1|	15776-16306	9.1	41	2	1	-0.405176	0.82205	no
gi|641567377|gb|KK857329.1|	28839-29977	9.1	31	3	2	-1	0.4363	no
gi|641567377|gb|KK857329.1|	31632-32276	9.1	31	6	3	-0.90981	0.513	no

gi|641567380|gb|KK857328.1|	47438-49550	10	40	0.627981	0.901613	0.521788	0.71825	no
gi|641567380|gb|KK857328.1|	50519-50955	10	40	8	8	0.0579477	0.9684	no
gi|641567380|gb|KK857328.1|	52135-52421	10	40	11	18	0.744338	0.6009	no
gi|641567380|gb|KK857328.1|	55736-56104	10	40	7	8	0.300445	0.83495	no
gi|641567380|gb|KK857328.1|	58382-58621	10	40	16	3	-2	0.24595	no
gi|641567380|gb|KK857328.1|	75568-75770	10	40	6	27	2	0.26345	no
gi|641567380|gb|KK857328.1|	76565-76785	10	40	9	11	0.277155	0.8647	no
gi|641567380|gb|KK857328.1|	84018-84951	10	40	3	2	-0.483134	0.72975	no
gi|641567380|gb|KK857328.1|	85081-86156	10	40	0.885333	1	0.605619	0.6863	no
gi|641567380|gb|KK857328.1|	86207-86412	10	40	6	22	2	0.3201	no
gi|641567380|gb|KK857328.1|	91591-96707	10	40	9	9	-0.059034	0.9791	no
gi|641567383|gb|KK857327.1|	18655-19390	11	46	1	2	0.601411	0.67805	no
gi|641567385|gb|KK857326.1|	35963-36649	8.8	34	0.432789	3	3	0.1889	no
gi|641567391|gb|KK857324.1|	39371-39581	6.2	19	0	167	inf	5.00E-05	yes
gi|641567393|gb|KK857323.1|	37080-37496	11	23	2	3	0.808924	0.6234	no
gi|641567393|gb|KK857323.1|	50723-51114	11	23	5	0.534124	-3	0.21195	no
gi|641567393|gb|KK857323.1|	51572-52102	11	23	13	3	-2	0.12525	no
gi|641567399|gb|KK857321.1|	17112-17375	9.4	37	3	12	2	0.26245	no
gi|641567399|gb|KK857321.1|	49585-50098	9.4	37	3	2	-0.669111	0.68185	no
gi|641567408|gb|KK857318.1|	28333-29075	8.4	34	2	2	0.0165872	0.95145	no
gi|641567408|gb|KK857318.1|	32435-35394	8.4	34	0.537998	0.899449	0.741441	0.58405	no
gi|641567408|gb|KK857318.1|	36444-37070	8.4	34	1	1	-0.249338	0.88485	no
gi|641567408|gb|KK857318.1|	39906-40536	8.4	34	50	23	-1	0.3783	no
gi|641567408|gb|KK857318.1|	40642-41644	8.4	34	0.965448	0.934461	-0.0470642	0.94545	no
gi|641567408|gb|KK857318.1|	43632-44708	8.4	34	7	4	-0.733175	0.57315	no
gi|641567408|gb|KK857318.1|	46915-47201	8.4	34	70	43	-0.722539	0.57905	no
gi|641567408|gb|KK857318.1|	47658-48024	8.4	34	3	2	-0.64214	0.71075	no
gi|641567411|gb|KK857317.1|	18105-19080	9.4	34	0.99888	1	0.467256	0.75495	no
gi|641567411|gb|KK857317.1|	23858-24742	9.4	34	0.925251	3	2	0.2236	no
gi|641567423|gb|KK857312.1|	19587-25554	11	37	5	3	-0.580829	0.63985	no

gi|641567423|gb|KK857312.1|	26551-27738	11	37	15	17	0.219353	0.85695	no
gi|641567429|gb|KK857310.1|	66260-66532	9.4	36	36	48	0.42548	0.7463	no
gi|641567432|gb|KK857309.1|	10214-11028	7.4	36	0	4	inf	0.00105	yes
gi|641567432|gb|KK857309.1|	11744-12319	7.4	36	0	3	inf	0.00455	no
gi|641567432|gb|KK857309.1|	15184-16019	7.4	36	0	4	inf	0.00115	yes
gi|641567435|gb|KK857308.1|	10-237	10	38	241	108.44	-1	0.37495	no
gi|641567435|gb|KK857308.1|	14664-17237	10	38	9	5	-0.870662	0.48745	no
gi|641567435|gb|KK857308.1|	18471-19604	10	38	73	49	-0.578527	0.8005	no
gi|641567435|gb|KK857308.1|	20773-21530	10	38	3	3	-0.175774	0.89825	no
gi|641567435|gb|KK857308.1|	21657-22707	10	38	3	2	-0.394577	0.76905	no
gi|641567435|gb|KK857308.1|	22887-24633	10	38	2	0.556925	-2	0.24635	no
gi|641567435|gb|KK857308.1|	24691-26512	10	38	8	8	0.000277272	0.99595	no
gi|641567435|gb|KK857308.1|	29436-30454	10	38	39	26	-0.568792	0.64085	no
gi|641567435|gb|KK857308.1|	33701-35523	10	38	3	3	0.345667	0.79095	no
gi|641567435|gb|KK857308.1|	38454-38696	10	38	65	41	-0.689233	0.6056	no
gi|641567435|gb|KK857308.1|	40548-41264	10	38	1	2	0.601014	0.6863	no
gi|641567435|gb|KK857308.1|	41845-43359	10	38	0.590605	0.572775	-0.0442241	1	no
gi|641567435|gb|KK857308.1|	43479-44832	10	38	0.978529	0.558984	-0.807808	0.5996	no
gi|641567435|gb|KK857308.1|	65523-66777	10	38	0	35	inf	0.00085	yes
gi|641567435|gb|KK857308.1|	70522-70817	10	38	10	27	1	0.3132	no
gi|641567435|gb|KK857308.1|	71539-72129	10	38	3	79	5	0.01075	no
gi|641567435|gb|KK857308.1|	79822-80075	10	38	2	26	4	0.24355	no
gi|641567435|gb|KK857308.1|	81318-81545	10	38	45	128	2	0.2592	no
gi|641567443|gb|KK857305.1|	61288-62465	10	42	0.721224	2	1	0.4713	no
gi|641567449|gb|KK857303.1|	46381-47431	8.5	39	0.414195	5	3	0.09795	no
gi|641567461|gb|KK857299.1|	29135-29888	10	35	0.888654	1	0.60176	0.69715	no
gi|641567464|gb|KK857298.1|	11804-13023	7.7	30	3	0	-inf	0.00045	yes
gi|641567464|gb|KK857298.1|	17672-19095	7.7	30	2	2	-0.180975	0.88675	no
gi|641567464|gb|KK857298.1|	21113-21754	7.7	30	1	0.721619	-0.815922	0.6492	no
gi|641567464|gb|KK857298.1|	27717-28382	7.7	30	2	0	-inf	0.0102	no

gi|641567464|gb|KK857298.1|	30137-30558	7.7	30	8	0	-inf	0.0022	yes
gi|641567464|gb|KK857298.1|	31549-31943	7.7	30	5	0	-inf	0.0059	no
gi|641567464|gb|KK857298.1|	32100-33433	7.7	30	3	0	-inf	0.0002	yes
gi|641567464|gb|KK857298.1|	35414-36010	7.7	30	2	0.267176	-3	0.2481	no
gi|641567464|gb|KK857298.1|	39559-40274	7.7	30	1	0	-inf	0.01175	no
gi|641567464|gb|KK857298.1|	9587-10045	7.7	30	3	0	-inf	0.01175	no
gi|641567470|gb|KK857296.1|	10317-10976	6.4	27	1	1	-0.0784452	0.92675	no
gi|641567470|gb|KK857296.1|	23270-23470	6.4	27	6	28	2	0.26555	no
gi|641567470|gb|KK857296.1|	2686-2972	6.4	27	4	6	0.563766	0.7575	no
gi|641567470|gb|KK857296.1|	7925-8119	6.4	27	39	56	0.527097	0.71605	no
gi|641567473|gb|KK857295.1|	17406-17724	7.4	33	9	6	-0.621115	0.6835	no
gi|641567473|gb|KK857295.1|	38236-39339	7.4	33	0.390011	0.830953	1	1	no
gi|641567473|gb|KK857295.1|	49438-50282	7.4	33	0.654869	0.663215	0.0182712	1	no
gi|641567473|gb|KK857295.1|	7092-7296	7.4	33	35	17	-1	0.4928	no
gi|641567478|gb|KK857293.1|	65563-66341	9.1	35	0.729039	0.737806	0.0172448	1	no
gi|641567484|gb|KK857291.1|	19447-19873	6.6	28	2	6	1	0.3707	no
gi|641567487|gb|KK857290.1|	15055-15460	7.8	27	3	0	-inf	0.01175	no
gi|641567487|gb|KK857290.1|	20613-21346	7.8	27	1	0	-inf	0.0102	no
gi|641567487|gb|KK857290.1|	27383-29168	7.8	27	0.757155	0	-inf	1	no
gi|641567487|gb|KK857290.1|	31884-32764	7.8	27	1	0	-inf	0.01175	no
gi|641567490|gb|KK857289.1|	27917-28913	11	19	1	1	0.0199952	0.9597	no
gi|641567493|gb|KK857288.1|	11410-11697	8.6	41	8	0	-inf	0.0102	no
gi|641567493|gb|KK857288.1|	11770-12734	8.6	41	2	0.84035	-1	0.3694	no
gi|641567493|gb|KK857288.1|	13074-14153	8.6	41	0.801205	0.487666	-0.716279	1	no
gi|641567493|gb|KK857288.1|	14247-15443	8.6	41	0.91979	0	-inf	0.0075	no
gi|641567493|gb|KK857288.1|	15533-16202	8.6	41	2	0.226477	-3	0.21435	no
gi|641567493|gb|KK857288.1|	29189-30404	8.6	41	2	0.845773	-0.852892	0.5652	no
gi|641567496|gb|KK857287.1|	10428-10618	8.3	28	423	276	-0.61614	0.6216	no
gi|641567496|gb|KK857287.1|	11736-13284	8.3	28	2	2	0.135897	0.91615	no
gi|641567496|gb|KK857287.1|	13459-13756	8.3	28	22	27	0.28652	0.833	no

gi|641567496|gb|KK857287.1|	16316-17110	8.3	28	2	1	-0.290609	0.8456	no
gi|641567496|gb|KK857287.1|	17303-17999	8.3	28	3	2	-0.477439	0.73955	no
gi|641567496|gb|KK857287.1|	23319-23737	8.3	28	2	1	-0.635655	0.71175	no
gi|641567496|gb|KK857287.1|	23896-24811	8.3	28	4	5	0.299275	0.82315	no
gi|641567496|gb|KK857287.1|	25010-25906	8.3	28	3	3	-0.148165	0.91825	no
gi|641567496|gb|KK857287.1|	31416-31785	8.3	28	6	0.596535	-3	0.21195	no
gi|641567496|gb|KK857287.1|	32789-33720	8.3	28	3	3	0.156848	0.9073	no
gi|641567496|gb|KK857287.1|	36505-37331	8.3	28	1	2	0.33994	0.81785	no
gi|641567496|gb|KK857287.1|	44202-45163	8.3	28	133	148	0.146939	0.94455	no
gi|641567496|gb|KK857287.1|	45545-45803	8.3	28	59	78	0.41331	0.74995	no
gi|641567496|gb|KK857287.1|	46229-46927	8.3	28	0.985772	1	0.378218	0.8276	no
gi|641567496|gb|KK857287.1|	49838-51143	8.3	28	61	73	0.261424	0.9073	no
gi|641567496|gb|KK857287.1|	65632-67278	8.3	28	4	4	0.151053	0.9068	no
gi|641567496|gb|KK857287.1|	67393-68290	8.3	28	11	5	-1	0.4156	no
gi|641567496|gb|KK857287.1|	69327-70150	8.3	28	3	2	-0.220192	0.87985	no
gi|641567496|gb|KK857287.1|	71177-71636	8.3	28	2	4	1	0.4421	no
gi|641567496|gb|KK857287.1|	78789-78998	8.3	28	185	201	0.115168	0.92685	no
gi|641567496|gb|KK857287.1|	79326-80221	8.3	28	36	36	-0.0259637	0.98195	no
gi|641567505|gb|KK857284.1|	30808-31672	7.7	32	1	0.482625	-1	0.47135	no
gi|641567505|gb|KK857284.1|	33771-34407	7.7	32	0.802952	2	1	0.51085	no
gi|641567505|gb|KK857284.1|	34587-35323	7.7	32	2	2	-0.0359987	0.95655	no
gi|641567505|gb|KK857284.1|	35464-36265	7.7	32	1	1	0.0880163	0.9392	no
gi|641567505|gb|KK857284.1|	36387-37095	7.7	32	1	3	1	0.4275	no
gi|641567505|gb|KK857284.1|	37373-38864	7.7	32	0.710378	0.749456	0.0772584	1	no
gi|641567510|gb|KK857282.1|	23938-24394	10	43	2	3	0.397504	0.8089	no
gi|641567520|gb|KK857278.1|	34401-34773	9.4	42	0	6	inf	0.0068	no
gi|641567522|gb|KK857277.1|	46081-46260	11	21	17	30	0.782093	0.65375	no
gi|641567522|gb|KK857277.1|	50730-51745	11	21	0.950137	1	0.145697	0.9285	no
gi|641567531|gb|KK857274.1|	15888-16534	10	36	1	0.475805	-1	0.4534	no
gi|641567531|gb|KK857274.1|	17169-19430	10	36	327	195	-0.749279	0.67485	no

gi|641567531|gb|KK857274.1|	19533-21431	10	36	7	3	-1	0.4055	no
gi|641567531|gb|KK857274.1|	22095-23743	10	36	17	11	-0.618162	0.6234	no
gi|641567531|gb|KK857274.1|	27122-27809	10	36	2	0.21827	-3	0.2348	no
gi|641567531|gb|KK857274.1|	30564-31170	10	36	1	1	0.334965	0.83525	no
gi|641567531|gb|KK857274.1|	37161-37788	10	36	1	2	1	0.4891	no
gi|641567531|gb|KK857274.1|	4252-4698	10	36	2	2	0.00437156	0.91115	no
gi|641567531|gb|KK857274.1|	60746-62130	10	36	4	11	1	0.32615	no
gi|641567534|gb|KK857273.1|	1616-2451	7.5	32	6	12	0.91505	0.47985	no
gi|641567534|gb|KK857273.1|	20196-20598	7.5	32	2	11	2	0.1531	no
gi|641567534|gb|KK857273.1|	65927-66568	7.5	32	1	1	-0.248881	0.88485	no
gi|641567534|gb|KK857273.1|	68401-68705	7.5	32	27	23	-0.190647	0.88795	no
gi|641567537|gb|KK857272.1|	26405-27584	8.5	37	0	91	inf	0.0003	yes
gi|641567537|gb|KK857272.1|	29662-30218	8.5	37	0	5	inf	0.00135	yes
gi|641567537|gb|KK857272.1|	3100-3353	8.5	37	0	39	inf	0.00075	yes
gi|641567537|gb|KK857272.1|	4567-4807	8.5	37	0	573	inf	0.0003	yes
gi|641567537|gb|KK857272.1|	69027-69520	8.5	37	0	11	inf	0.0004	yes
gi|641567540|gb|KK857271.1|	19244-21212	10	42	0.279585	2	3	0.0912	no
gi|641567540|gb|KK857271.1|	24988-26265	10	42	0.13091	0.897164	3	0.2851	no
gi|641567540|gb|KK857271.1|	26630-28069	10	42	0.284646	2	3	0.1396	no
gi|641567540|gb|KK857271.1|	6141-7374	10	42	0.204691	1	3	0.18845	no
gi|641567540|gb|KK857271.1|	67729-68433	10	42	1	2	0.600748	0.67805	no
gi|641567540|gb|KK857271.1|	68505-69598	10	42	1	1	0.020791	0.9597	no
gi|641567543|gb|KK857270.1|	19170-19410	9.7	35	9	15	0.718691	0.6556	no
gi|641567543|gb|KK857270.1|	23787-23996	9.7	35	47	50	0.0823702	0.9543	no
gi|641567543|gb|KK857270.1|	29077-33430	9.7	35	19	9	-1	0.6367	no
gi|641567543|gb|KK857270.1|	33991-35810	9.7	35	14	14	-0.00159952	0.9971	no
gi|641567543|gb|KK857270.1|	45799-46643	9.7	35	0	14	inf	5.00E-05	yes
gi|641567543|gb|KK857270.1|	51564-51778	9.7	35	5	82	4	0.1396	no
gi|641567543|gb|KK857270.1|	53255-53751	9.7	35	0.234721	29	7	0.10445	no
gi|641567543|gb|KK857270.1|	53865-54826	9.7	35	0.184935	2	3	0.2556	no

gi|641567543|gb|KK857270.1|	55870-56939	9.7	35	0.729338	13	4	0.0349	no
gi|641567546|gb|KK857269.1|	15143-15679	8.5	40	12	8	-0.667913	0.61665	no
gi|641567546|gb|KK857269.1|	26213-26608	8.5	40	23	26	0.183646	0.88375	no
gi|641567546|gb|KK857269.1|	31844-32322	8.5	40	11	9	-0.315126	0.80695	no
gi|641567546|gb|KK857269.1|	40000-40422	8.5	40	3	3	0.00217656	0.9394	no
gi|641567546|gb|KK857269.1|	42812-44583	8.5	40	0.943619	2	0.916706	0.5087	no
gi|641567546|gb|KK857269.1|	48212-48756	8.5	40	4	4	-0.163488	0.91235	no
gi|641567546|gb|KK857269.1|	50850-51428	8.5	40	13	16	0.214496	0.87025	no
gi|641567546|gb|KK857269.1|	51899-52582	8.5	40	66	90	0.456028	0.77275	no
gi|641567549|gb|KK857268.1|	10019-10629	9.4	35	0.341245	2	3	0.2991	no
gi|641567549|gb|KK857268.1|	1207-1794	9.4	35	0.72238	2	1	0.51315	no
gi|641567549|gb|KK857268.1|	19766-20567	9.4	35	10	9	-0.162945	0.9005	no
gi|641567549|gb|KK857268.1|	24755-25567	9.4	35	0.344424	3	3	0.1622	no
gi|641567549|gb|KK857268.1|	38397-39209	9.4	35	0.57404	2	2	0.2348	no
gi|641567549|gb|KK857268.1|	47682-47890	9.4	35	0	154	inf	5.00E-05	yes
gi|641567552|gb|KK857267.1|	284-1267	9.6	35	2	4	1	0.40925	no
gi|641567568|gb|KK857261.1|	18331-18637	8.7	40	0	16	inf	0.00135	yes
gi|641567568|gb|KK857261.1|	22558-23121	8.7	40	0.192314	14	6	0.10475	no
gi|641567568|gb|KK857261.1|	24903-25532	8.7	40	0	38	inf	5.00E-05	yes
gi|641567568|gb|KK857261.1|	26427-26838	8.7	40	7	8	0.149907	0.9177	no
gi|641567568|gb|KK857261.1|	27291-29217	8.7	40	2	1	-0.795471	0.5576	no
gi|641567568|gb|KK857261.1|	30909-31332	8.7	40	2	5	2	0.32945	no
gi|641567568|gb|KK857261.1|	32113-32727	8.7	40	3	3	0.150811	0.92315	no
gi|641567568|gb|KK857261.1|	34995-35450	8.7	40	4	4	0.242147	0.8654	no
gi|641567568|gb|KK857261.1|	35592-36875	8.7	40	1	1	-0.331698	0.8156	no
gi|641567568|gb|KK857261.1|	38665-39870	8.7	40	3	4	0.402949	0.76195	no
gi|641567568|gb|KK857261.1|	40841-41478	8.7	40	2	1	-0.51648	0.74125	no
gi|641567568|gb|KK857261.1|	41642-45117	8.7	40	1	1	0.315078	0.80755	no
gi|641567568|gb|KK857261.1|	47204-47934	8.7	40	20	24	0.289032	0.816	no
gi|641567568|gb|KK857261.1|	55795-56610	8.7	40	22	24	0.172505	0.88935	no

gi|641567568|gb|KK857261.1|	66117-66809	8.7	40	29	22	-0.392442	0.74425	no
gi|641567573|gb|KK857259.1|	11587-12500	11	41	6	31	2	0.0707	no
gi|641567573|gb|KK857259.1|	14527-16065	11	41	0.210927	2	3	0.1384	no
gi|641567573|gb|KK857259.1|	16806-17126	11	41	2	18	3	0.14585	no
gi|641567573|gb|KK857259.1|	25028-25248	11	41	6	26	2	0.2524	no
gi|641567573|gb|KK857259.1|	28430-30010	11	41	0.357926	4	3	0.06745	no
gi|641567573|gb|KK857259.1|	31073-32182	11	41	0.07749	3	5	0.10725	no
gi|641567573|gb|KK857259.1|	46214-46827	11	41	6	2	-2	0.2293	no
gi|641567573|gb|KK857259.1|	59780-60023	11	41	14	14	0.019426	0.95595	no
gi|641567573|gb|KK857259.1|	67454-68624	11	41	0	6	inf	0.00015	yes
gi|641567573|gb|KK857259.1|	73241-74380	11	41	2	2	0.210145	0.88045	no
gi|641567573|gb|KK857259.1|	90836-91077	11	41	9	5	-0.865876	0.6407	no
gi|641567579|gb|KK857257.1|	27107-29121	9.5	36	2	4	1	0.38905	no
gi|641567579|gb|KK857257.1|	35680-38281	9.5	36	6	4	-0.428392	0.73625	no
gi|641567579|gb|KK857257.1|	39399-40013	9.5	36	5	3	-0.770964	0.58265	no
gi|641567579|gb|KK857257.1|	40950-41637	9.5	36	1	0.654811	-0.984614	0.62775	no
gi|641567579|gb|KK857257.1|	41882-43787	9.5	36	2	3	0.0979155	0.93955	no
gi|641567579|gb|KK857257.1|	44058-46259	9.5	36	12	9	-0.491896	0.6903	no
gi|641567579|gb|KK857257.1|	47069-47511	9.5	36	27	15	-0.780242	0.5529	no
gi|641567579|gb|KK857257.1|	48850-50372	9.5	36	1	0.975998	-0.562045	0.6926	no
gi|641567579|gb|KK857257.1|	50561-51399	9.5	36	2	1	-0.676959	0.6649	no
gi|641567579|gb|KK857257.1|	54022-55992	9.5	36	3	1	-0.995858	0.457	no
gi|641567579|gb|KK857257.1|	56477-59315	9.5	36	3	3	-0.122618	0.9247	no
gi|641567579|gb|KK857257.1|	60868-61548	9.5	36	34	20	-0.782072	0.5341	no
gi|641567579|gb|KK857257.1|	69744-69949	9.5	36	172	118	-0.550558	0.6695	no
gi|641567579|gb|KK857257.1|	71788-72092	9.5	36	36	44	0.300412	0.81735	no
gi|641567579|gb|KK857257.1|	72803-73216	9.5	36	5	7	0.493109	0.73245	no
gi|641567579|gb|KK857257.1|	75968-76359	9.5	36	6	10	0.744158	0.602	no
gi|641567579|gb|KK857257.1|	78559-79171	9.5	36	3	2	-0.723725	0.6309	no
gi|641567584|gb|KK857255.1|	2299-4357	10	19	0.456131	1	1	0.3614	no

gi|641567584|gb|KK857255.1|	24892-26025	10	19	0.45304	1	2	0.32035	no
gi|641567584|gb|KK857255.1|	28037-28602	10	19	2	0.578421	-1	0.4534	no
gi|641567584|gb|KK857255.1|	31748-33053	10	19	0.765641	4	2	0.1277	no
gi|641567584|gb|KK857255.1|	61246-61764	10	19	2	2	0.108771	0.92265	no
gi|641567584|gb|KK857255.1|	76786-77370	10	19	25	0.550596	-5	0.1306	no
gi|641567587|gb|KK857254.1|	6346-6790	11	21	0.848457	3	2	0.3323	no
gi|641567587|gb|KK857254.1|	81692-82423	11	21	0.528639	1	1	0.51305	no
gi|641567591|gb|KK857253.1|	131-1108	10	19	3	2	-0.442527	0.7465	no
gi|641567591|gb|KK857253.1|	13828-14077	10	19	99	37	-1	0.28875	no
gi|641567591|gb|KK857253.1|	15209-15395	10	19	55	8	-3	0.1861	no
gi|641567591|gb|KK857253.1|	16780-17004	10	19	40	12	-2	0.2797	no
gi|641567591|gb|KK857253.1|	22145-23593	10	19	0.847802	0.43061	-0.977346	1	no
gi|641567591|gb|KK857253.1|	23935-24696	10	19	0.750938	2	1	0.4702	no
gi|641567591|gb|KK857253.1|	24826-27225	10	19	0.963875	2	0.656958	0.62365	no
gi|641567591|gb|KK857253.1|	27375-27692	10	19	101	34	-2	0.2324	no
gi|641567591|gb|KK857253.1|	29436-30078	10	19	1	1	0.0141827	0.9394	no
gi|641567591|gb|KK857253.1|	32988-33225	10	19	13	7	-0.911873	0.59485	no
gi|641567591|gb|KK857253.1|	36089-37194	10	19	3	3	0.0017688	0.9823	no
gi|641567591|gb|KK857253.1|	39702-40252	10	19	2	2	0.180743	0.91015	no
gi|641567591|gb|KK857253.1|	41532-41810	10	19	12	10	-0.268528	0.85285	no
gi|641567591|gb|KK857253.1|	47864-49256	10	19	2	1	-0.3468	0.79975	no
gi|641567591|gb|KK857253.1|	51124-52846	10	19	0.834775	2	0.89797	0.52215	no
gi|641567591|gb|KK857253.1|	53678-54409	10	19	1	2	0.738834	0.6176	no
gi|641567591|gb|KK857253.1|	55410-56479	10	19	2	0.123305	-4	0.1901	no
gi|641567591|gb|KK857253.1|	62654-63873	10	19	1	0.947791	-0.47144	0.74025	no
gi|641567591|gb|KK857253.1|	65336-66223	10	19	3	4	0.451795	0.7371	no
gi|641567591|gb|KK857253.1|	7780-8069	10	19	8	5	-0.69847	0.66875	no
gi|641567597|gb|KK857251.1|	36782-37094	9.6	41	0	74	inf	5.00E-05	yes
gi|641567603|gb|KK857249.1|	53765-54351	8.5	34	1	1	0.334241	0.83525	no
gi|641567603|gb|KK857249.1|	59515-59987	8.5	34	53	49	-0.109093	0.9296	no

gi|641567603|gb|KK857249.1|	60590-61306	8.5	34	3	2	-0.720914	0.6313	no
gi|641567603|gb|KK857249.1|	69662-70773	8.5	34	0.231962	1	2	0.2235	no
gi|641567609|gb|KK857247.1|	29663-31582	9.7	19	0.944751	1	0.255269	0.8579	no
gi|641567609|gb|KK857247.1|	31726-33079	9.7	19	0.428107	0.838476	0.9698	1	no
gi|641567609|gb|KK857247.1|	36617-37642	9.7	19	10	5	-0.835358	0.51585	no
gi|641567609|gb|KK857247.1|	41020-41586	9.7	19	0.953835	3	2	0.32945	no
gi|641567609|gb|KK857247.1|	42602-43155	9.7	19	3	0.896218	-2	0.33985	no
gi|641567609|gb|KK857247.1|	44708-45783	9.7	19	0.804848	0.489873	-0.716309	1	no
gi|641567609|gb|KK857247.1|	47111-47828	9.7	19	2	2	-0.13593	0.93	no
gi|641567609|gb|KK857247.1|	53804-53987	9.7	19	87	22	-2	0.2324	no
gi|641567609|gb|KK857247.1|	56529-56747	9.7	19	64	25	-1	0.3401	no
gi|641567609|gb|KK857247.1|	64636-65120	9.7	19	2	1	-0.630225	0.71175	no
gi|641567612|gb|KK857246.1|	75636-76335	10	45	3	3	-0.222489	0.87145	no
gi|641567618|gb|KK857244.1|	46019-47089	8	25	31	0.24633	-7	0.13015	no
gi|641567621|gb|KK857243.1|	22284-22874	11	39	3	5	0.959996	0.51305	no
gi|641567621|gb|KK857243.1|	23036-24334	11	39	0.385251	1	2	0.25645	no
gi|641567621|gb|KK857243.1|	27111-28316	11	39	0.490857	0.427058	-0.200872	1	no
gi|641567621|gb|KK857243.1|	28614-30019	11	39	1	1	-0.335967	0.8109	no
gi|641567621|gb|KK857243.1|	30227-31919	11	39	0.851508	2	0.897892	0.52215	no
gi|641567621|gb|KK857243.1|	32113-32450	11	39	3	2	-0.647021	0.71075	no
gi|641567621|gb|KK857243.1|	48571-49502	11	39	1	0.292612	-2	0.31075	no
gi|641567621|gb|KK857243.1|	50175-50643	11	39	3	0	-inf	0.01	no
gi|641567621|gb|KK857243.1|	50854-51380	11	39	1	0.967894	-0.627773	0.71195	no
gi|641567621|gb|KK857243.1|	51574-52117	11	39	2	0.921504	-1	0.50365	no
gi|641567621|gb|KK857243.1|	52619-54143	11	39	2	1	-0.491649	0.7262	no
gi|641567633|gb|KK857239.1|	11715-12321	9.7	19	0.344555	4	3	0.25495	no
gi|641567633|gb|KK857239.1|	12724-14015	9.7	19	0.387712	2	2	0.19125	no
gi|641567633|gb|KK857239.1|	4031-4445	9.7	19	0.320389	5	4	0.12385	no
gi|641567633|gb|KK857239.1|	4800-5306	9.7	19	0.909032	3	2	0.3014	no
gi|641567633|gb|KK857239.1|	60-718	9.7	19	0.152975	3	4	0.1136	no

gi|641567633|gb|KK857239.1|	69568-73361	9.7	19	0.314882	1	2	0.13925	no
gi|641567633|gb|KK857239.1|	77409-77805	9.7	19	3	7	0.962816	0.53855	no
gi|641567639|gb|KK857237.1|	13-688	13	53	2	5	1	0.3822	no
gi|641567639|gb|KK857237.1|	18675-19279	13	53	0.173117	2	4	0.1276	no
gi|641567642|gb|KK857236.1|	63535-64385	11	43	1	0.657175	-0.718611	0.6701	no
gi|641567645|gb|KK857235.1|	10317-10886	11	37	0	6	inf	0.00145	yes
gi|641567645|gb|KK857235.1|	16971-18248	11	37	0	20	inf	5.00E-05	yes
gi|641567645|gb|KK857235.1|	269-797	11	37	0	3	inf	0.0068	no
gi|641567645|gb|KK857235.1|	3587-4156	11	37	0	7	inf	0.00075	yes
gi|641567645|gb|KK857235.1|	38299-38609	11	37	0	9	inf	0.0068	no
gi|641567645|gb|KK857235.1|	47312-47564	11	37	42	25	-0.78554	0.5606	no
gi|641567645|gb|KK857235.1|	49297-50650	11	37	5	2	-1	0.4099	no
gi|641567645|gb|KK857235.1|	54204-56426	11	37	3	3	0.217101	0.865	no
gi|641567645|gb|KK857235.1|	56594-58002	11	37	0.291922	2	2	0.18135	no
gi|641567645|gb|KK857235.1|	61934-64236	11	37	0.168042	53	8	0.08405	no
gi|641567645|gb|KK857235.1|	70126-70415	11	37	49	39	-0.29978	0.8135	no
gi|641567645|gb|KK857235.1|	76109-76658	11	37	6	3	-0.752189	0.5907	no
gi|641567645|gb|KK857235.1|	85461-85783	11	37	6	6	-0.0125782	0.95145	no
gi|641567645|gb|KK857235.1|	89023-92183	11	37	32	27	-0.240476	0.91165	no
gi|641567657|gb|KK857233.1|	11059-11376	9.9	35	7	3	-1	0.5083	no
gi|641567657|gb|KK857233.1|	11575-11795	9.9	35	42	17	-1	0.3888	no
gi|641567657|gb|KK857233.1|	13844-14612	9.9	35	8	3	-2	0.2331	no
gi|641567657|gb|KK857233.1|	23491-23962	9.9	35	3	0	-inf	0.0102	no
gi|641567657|gb|KK857233.1|	31442-31659	9.9	35	14	2	-3	0.2588	no
gi|641567657|gb|KK857233.1|	33766-34058	9.9	35	17	15	-0.228198	0.87265	no
gi|641567657|gb|KK857233.1|	38626-38770	9.9	35	130	37	-2	0.3266	no
gi|641567657|gb|KK857233.1|	38955-39170	9.9	35	10	14	0.537822	0.7589	no
gi|641567657|gb|KK857233.1|	39787-40474	9.9	35	22	12	-0.86622	0.49685	no
gi|641567657|gb|KK857233.1|	41372-42901	9.9	35	40	36	-0.140019	0.9499	no
gi|641567657|gb|KK857233.1|	43513-43962	9.9	35	5	0.417398	-3	0.20585	no

gi|641567657|gb|KK857233.1|	44559-44915	9.9	35	3	2	-0.643707	0.71075	no
gi|641567657|gb|KK857233.1|	53456-54834	9.9	35	1	0.182403	-3	0.19075	no
gi|641567657|gb|KK857233.1|	56026-56662	9.9	35	4	2	-1	0.41055	no
gi|641567657|gb|KK857233.1|	57657-58200	9.9	35	2	1	-0.404543	0.82205	no
gi|641567657|gb|KK857233.1|	58356-60188	9.9	35	0.95171	0.461768	-1	0.48495	no
gi|641567657|gb|KK857233.1|	61301-62024	9.9	35	2	0.610544	-1	0.4311	no
gi|641567660|gb|KK857232.1|	20865-21038	9.4	41	33	23	-0.484213	0.7824	no
gi|641567660|gb|KK857232.1|	55592-55856	9.4	41	59	13	-2	0.1323	no
gi|641567663|gb|KK857231.1|	32452-33278	10	40	14	0	-inf	5.00E-05	yes
gi|641567663|gb|KK857231.1|	33796-36199	10	40	4	0.048937	-6	0.183	no
gi|641567663|gb|KK857231.1|	40151-42329	10	40	6	0	-inf	5.00E-05	yes
gi|641567663|gb|KK857231.1|	44720-48898	10	40	25	0.027149	-10	0.1832	no
gi|641567663|gb|KK857231.1|	56092-57434	10	40	1	0	-inf	0.00345	yes
gi|641567663|gb|KK857231.1|	58774-59458	10	40	2	0	-inf	0.0102	no
gi|641567663|gb|KK857231.1|	65320-65782	10	40	4	0	-inf	0.0059	no
gi|641567666|gb|KK857230.1|	15509-17264	9.3	35	5	2	-1	0.32025	no
gi|641567666|gb|KK857230.1|	23159-23533	9.3	35	0	7	inf	0.00375	no
gi|641567666|gb|KK857230.1|	30060-30948	9.3	35	2	0.466003	-2	0.29	no
gi|641567666|gb|KK857230.1|	31947-32694	9.3	35	2	2	0.379254	0.7933	no
gi|641567666|gb|KK857230.1|	37073-37339	9.3	35	76	186	1	0.31245	no
gi|641567666|gb|KK857230.1|	46348-46610	9.3	35	8	6	-0.291348	0.86035	no
gi|641567666|gb|KK857230.1|	56457-56704	9.3	35	13	3	-2	0.27505	no
gi|641567666|gb|KK857230.1|	68620-68910	9.3	35	18	13	-0.489619	0.7294	no
gi|641567672|gb|KK857228.1|	13263-13481	10	36	11	11	0.0538249	0.92445	no
gi|641567672|gb|KK857228.1|	20714-21633	10	36	1	4	2	0.2543	no
gi|641567672|gb|KK857228.1|	22450-23849	10	36	1	1	-0.00924663	0.97395	no
gi|641567672|gb|KK857228.1|	25215-26937	10	36	9	9	-0.0101826	0.99295	no
gi|641567672|gb|KK857228.1|	27683-28672	10	36	3	4	0.35978	0.7897	no
gi|641567672|gb|KK857228.1|	29278-30065	10	36	12	8	-0.571288	0.65785	no
gi|641567672|gb|KK857228.1|	33185-33406	10	36	217	181	-0.266703	0.82775	no

gi|641567672|gb|KK857228.1|	37697-38557	10	36	16	11	-0.580972	0.64725	no
gi|641567672|gb|KK857228.1|	44558-45772	10	36	5	2	-1	0.40675	no
gi|641567672|gb|KK857228.1|	50424-50671	10	36	82	32	-1	0.3131	no
gi|641567675|gb|KK857227.1|	11457-12020	11	42	0	3	inf	0.0068	no
gi|641567675|gb|KK857227.1|	27851-29144	11	42	0.322505	0.785899	1	1	no
gi|641567675|gb|KK857227.1|	37243-39308	11	42	0.26508	0.693171	1	1	no
gi|641567675|gb|KK857227.1|	39681-40921	11	42	0.338866	0.619209	0.869713	1	no
gi|641567675|gb|KK857227.1|	42581-48047	11	42	33	29	-0.202	0.91905	no
gi|641567678|gb|KK857226.1|	13195-14113	9.1	30	0.587815	3	2	0.21405	no
gi|641567678|gb|KK857226.1|	28456-29784	9.1	30	0.687587	0.66649	-0.0449594	1	no
gi|641567678|gb|KK857226.1|	6534-7357	9.1	30	0.676777	5	3	0.10065	no
gi|641567684|gb|KK857224.1|	14200-14891	8.5	35	0.856861	2	0.822837	0.62335	no
gi|641567684|gb|KK857224.1|	20276-21464	8.5	35	0.499263	0.977267	0.968952	0.5733	no
gi|641567684|gb|KK857224.1|	30617-30841	8.5	35	1	78	6	0.10495	no
gi|641567684|gb|KK857224.1|	53649-56080	8.5	35	0.855071	2	0.899198	0.51035	no
gi|641567687|gb|KK857223.1|	40048-40608	9	29	0.969426	2	1	0.51085	no
gi|641567687|gb|KK857223.1|	43258-44079	9	29	2	3	0.850828	0.55355	no
gi|641567687|gb|KK857223.1|	52967-53663	9	29	2	3	0.726094	0.63585	no
gi|641567687|gb|KK857223.1|	56601-57215	9	29	1	1	0.0133075	0.91115	no
gi|641567690|gb|KK857222.1|	173-385	14	69	87	0	-inf	0.0004	yes
gi|641567690|gb|KK857222.1|	43743-43960	14	69	33	0	-inf	0.0032	yes
gi|641567693|gb|KK857221.1|	30332-30997	7.1	26	0	3	inf	0.00375	no
gi|641567693|gb|KK857221.1|	45856-47010	7.1	26	0.516969	3	3	0.14025	no
gi|641567696|gb|KK857220.1|	18471-18640	10	33	69	0	-inf	0.0059	no
gi|641567696|gb|KK857220.1|	25516-26140	10	33	53	13	-2	0.12045	no
gi|641567696|gb|KK857220.1|	28956-30872	10	33	3	3	-0.300335	0.81845	no
gi|641567696|gb|KK857220.1|	35841-36984	10	33	7	6	-0.215902	0.8652	no
gi|641567696|gb|KK857220.1|	40109-40617	10	33	1	5	2	0.28055	no
gi|641567696|gb|KK857220.1|	40946-41357	10	33	3	1	-0.829032	0.64915	no
gi|641567696|gb|KK857220.1|	44064-44303	10	33	10	3	-2	0.3632	no

gi|641567696|gb|KK857220.1|	46137-47894	10	33	2	2	-0.137694	0.9158	no
gi|641567696|gb|KK857220.1|	49668-49983	10	33	6	3	-0.888558	0.60065	no
gi|641567696|gb|KK857220.1|	53340-55658	10	33	3	1	-0.922945	0.4828	no
gi|641567696|gb|KK857220.1|	56223-57798	10	33	4	0.547527	-3	0.09	no
gi|641567696|gb|KK857220.1|	57890-59017	10	33	59	13	-2	0.2178	no
gi|641567696|gb|KK857220.1|	6318-6636	10	33	0	8	inf	0.0068	no
gi|641567699|gb|KK857219.1|	10717-11509	11	39	22	25	0.176913	0.8865	no
gi|641567699|gb|KK857219.1|	11694-12055	11	39	28	43	0.621919	0.62095	no
gi|641567699|gb|KK857219.1|	12226-12873	11	39	2	0.712146	-2	0.403	no
gi|641567699|gb|KK857219.1|	13053-16354	11	39	2	2	-0.0474617	0.9713	no
gi|641567699|gb|KK857219.1|	16630-19022	11	39	0.83802	0.63936	-0.390356	1	no
gi|641567699|gb|KK857219.1|	19143-21048	11	39	2	1	-0.642661	0.6295	no
gi|641567699|gb|KK857219.1|	22932-23745	11	39	2	0.17407	-3	0.217	no
gi|641567699|gb|KK857219.1|	23983-27955	11	39	3	4	0.506935	0.6866	no
gi|641567699|gb|KK857219.1|	28189-29104	11	39	5	1	-2	0.19955	no
gi|641567699|gb|KK857219.1|	29966-30488	11	39	2	2	-0.142555	0.92535	no
gi|641567699|gb|KK857219.1|	3030-6781	11	39	2	5	2	0.2238	no
gi|641567699|gb|KK857219.1|	37278-38605	11	39	0.563068	0.28589	-0.97785	1	no
gi|641567699|gb|KK857219.1|	38841-39372	11	39	14	27	0.964824	0.4509	no
gi|641567699|gb|KK857219.1|	41256-44219	11	39	5	6	0.337827	0.78555	no
gi|641567699|gb|KK857219.1|	44364-45222	11	39	1	1	-0.396575	0.7965	no
gi|641567699|gb|KK857219.1|	46391-52827	11	39	7	3	-1	0.4052	no
gi|641567699|gb|KK857219.1|	53034-54717	11	39	0.809067	1	0.427789	0.7651	no
gi|641567699|gb|KK857219.1|	56838-57947	11	39	1	2	0.605869	0.67415	no
gi|641567699|gb|KK857219.1|	66609-67847	11	39	3	1	-2	0.2619	no
gi|641567699|gb|KK857219.1|	68453-70476	11	39	0.736038	1	0.448636	0.74345	no
gi|641567699|gb|KK857219.1|	70947-72470	11	39	0.533274	0.893993	0.745387	0.6176	no
gi|641567699|gb|KK857219.1|	72620-73826	11	39	2	2	0.670619	0.62545	no
gi|641567699|gb|KK857219.1|	74663-76218	11	39	0.416579	1	2	0.3067	no
gi|641567699|gb|KK857219.1|	77643-78486	11	39	1	0.830291	-0.659815	0.6826	no

gi|641567699|gb|KK857219.1|	78576-79062	11	39	0.970729	2	1	0.51315	no
gi|641567699|gb|KK857219.1|	79223-79921	11	39	0.844947	2	1	0.5082	no
gi|641567699|gb|KK857219.1|	9026-9186	11	39	440	215	-1	0.43415	no
gi|641567702|gb|KK857218.1|	3217-3753	13	22	3	0.626708	-2	0.2679	no
gi|641567702|gb|KK857218.1|	40381-41164	13	22	3	1	-1	0.35285	no
gi|641567702|gb|KK857218.1|	42497-45899	13	22	3	2	-0.389508	0.7612	no
gi|641567702|gb|KK857218.1|	45960-47447	13	22	1	0.33412	-2	0.25215	no
gi|641567702|gb|KK857218.1|	49896-51233	13	22	0.868212	0.094465	-3	1	no
gi|641567702|gb|KK857218.1|	5315-6143	13	22	2	0.16997	-3	0.217	no
gi|641567702|gb|KK857218.1|	54101-55132	13	22	2	0.515539	-2	0.362	no
gi|641567702|gb|KK857218.1|	66405-67547	13	22	3	3	-0.0797978	0.95415	no
gi|641567708|gb|KK857216.1|	39188-41067	8.7	19	6	1	-2	0.09625	no
gi|641567708|gb|KK857216.1|	48843-49124	8.7	19	13	7	-1	0.4943	no
gi|641567714|gb|KK857214.1|	45423-45994	7.6	29	0.752974	5	3	0.17715	no
gi|641567714|gb|KK857214.1|	51304-51571	7.6	29	14	19	0.377673	0.79415	no
gi|641567714|gb|KK857214.1|	6007-6639	7.6	29	2	6	2	0.2955	no
gi|641567717|gb|KK857213.1|	17395-17645	9	33	50	28.04	-0.843381	0.5332	no
gi|641567717|gb|KK857213.1|	25608-25943	9	33	9	14	0.574994	0.68325	no
gi|641567717|gb|KK857213.1|	2567-3624	9	33	2	1	-1	0.41275	no
gi|641567723|gb|KK857211.1|	12841-13142	10	43	5	6	0.374974	0.8127	no
gi|641567726|gb|KK857210.1|	23335-23627	10	39	11	0.986822	-3	0.2088	no
gi|641567726|gb|KK857210.1|	57727-58086	10	39	4	4	0.0645949	0.94135	no
gi|641567726|gb|KK857210.1|	60085-65023	10	39	10	2	-3	0.1751	no
gi|641567726|gb|KK857210.1|	66197-66714	10	39	0.878285	2	1	0.4426	no
gi|641567728|gb|KK857209.1|	14282-14493	10	41	17	3	-3	0.2472	no
gi|641567733|gb|KK857207.1|	40994-41886	11	44	0.711376	0.61779	-0.203496	1	no
gi|641567733|gb|KK857207.1|	42038-42969	11	44	1	1	-0.495228	0.7427	no
gi|641567733|gb|KK857207.1|	43127-43859	11	44	4	2	-0.934702	0.5133	no
gi|641567733|gb|KK857207.1|	44421-45197	11	44	0.853474	0.92541	0.116746	0.92175	no
gi|641567733|gb|KK857207.1|	45670-47708	11	44	1	0.879131	-0.341963	0.8007	no

gi|641567733|gb|KK857207.1|	49175-50035	11	44	0.958658	0.647349	-0.566473	0.7586	no
gi|641567733|gb|KK857207.1|	53039-53342	11	44	4	7	0.76075	0.6379	no
gi|641567733|gb|KK857207.1|	53484-54104	11	44	2	1	-0.539037	0.7478	no
gi|641567733|gb|KK857207.1|	57009-57388	11	44	4	2	-1	0.50345	no
gi|641567733|gb|KK857207.1|	60625-61184	11	44	0.972074	1	0.596179	0.70505	no
gi|641567733|gb|KK857207.1|	61454-62453	11	44	0.352382	2	2	0.21345	no
gi|641567733|gb|KK857207.1|	64948-65519	11	44	4	5	0.362219	0.7996	no
gi|641567739|gb|KK857205.1|	.00248-10353	13	38	1	0.943701	-0.389576	0.76675	no
gi|641567739|gb|KK857205.1|	.04448-10617	13	38	2	4	1	0.3594	no
gi|641567739|gb|KK857205.1|	.07377-10757	13	38	55	108	0.957731	0.48465	no
gi|641567739|gb|KK857205.1|	.09816-11002	13	38	18	11	-0.637581	0.70205	no
gi|641567739|gb|KK857205.1|	.14361-11452	13	38	75	134	0.830498	0.5753	no
gi|641567739|gb|KK857205.1|	54718-55737	13	38	48	13	-2	0.2711	no
gi|641567739|gb|KK857205.1|	56091-56378	13	38	31	19	-0.725473	0.60355	no
gi|641567739|gb|KK857205.1|	56789-57025	13	38	47	5	-3	0.12325	no
gi|641567739|gb|KK857205.1|	57714-58222	13	38	62	12	-2	0.07785	no
gi|641567739|gb|KK857205.1|	61084-61349	13	38	13	4	-2	0.32465	no
gi|641567739|gb|KK857205.1|	62852-64076	13	38	0.963582	0.733602	-0.39341	0.7966	no
gi|641567739|gb|KK857205.1|	67656-68022	13	38	9	0.606135	-4	0.19715	no
gi|641567739|gb|KK857205.1|	69713-70021	13	38	68	11	-3	0.0831	no
gi|641567739|gb|KK857205.1|	77837-78114	13	38	25	29	0.217305	0.8725	no
gi|641567739|gb|KK857205.1|	79788-80548	13	38	19	3	-3	0.06115	no
gi|641567739|gb|KK857205.1|	81166-81371	13	38	106	11	-3	0.08815	no
gi|641567739|gb|KK857205.1|	81588-82829	13	38	7	3	-1	0.3241	no
gi|641567739|gb|KK857205.1|	83876-84563	13	38	32	30	-0.0920272	0.94015	no
gi|641567739|gb|KK857205.1|	85530-86322	13	38	3	2	-0.693013	0.62735	no
gi|641567739|gb|KK857205.1|	96053-97763	13	38	8	9	0.208253	0.8672	no
gi|641567739|gb|KK857205.1|	›7844-10010{	13	38	7	7	0.0416697	0.97365	no
gi|641567741|gb|KK857204.1|	83983-84681	8.7	42	0	4	inf	0.0016	yes
gi|641567744|gb|KK857203.1|	32352-33169	9.4	36	8	16	0.901181	0.4807	no

gi|641567744|gb|KK857203.1|	57649-58055	9.4	36	0	5	inf	0.00455	no
gi|641567747|gb|KK857202.1|	70049-70463	11	19	2	2	-0.221031	0.8945	no
gi|641567753|gb|KK857200.1|	34324-35218	9.7	40	0	3	inf	0.0016	yes
gi|641567753|gb|KK857200.1|	66319-66502	9.7	40	40	9	-2	0.258	no
gi|641567753|gb|KK857200.1|	7824-8322	9.7	40	2	1	-0.576871	0.7586	no
gi|641567753|gb|KK857200.1|	9328-10101	9.7	40	3	1	-1	0.43695	no
gi|641567758|gb|KK857198.1|	16094-16349	6.3	23	10	0	-inf	0.01175	no
gi|641567758|gb|KK857198.1|	23520-24231	6.3	23	1	0.416414	-2	0.4266	no
gi|641567758|gb|KK857198.1|	42103-43077	6.3	23	1	0	-inf	0.0102	no
gi|641567758|gb|KK857198.1|	49443-49936	6.3	23	5	0	-inf	0.0032	yes
gi|641567758|gb|KK857198.1|	7730-8003	6.3	23	19	0	-inf	0.0021	yes
gi|641567758|gb|KK857198.1|	8829-9379	6.3	23	3	0	-inf	0.00435	no
gi|641567761|gb|KK857197.1|	32911-33710	11	42	1	0.890395	-0.660477	0.6826	no
gi|641567761|gb|KK857197.1|	46742-47275	11	42	1	1	-0.212381	0.8945	no
gi|641567761|gb|KK857197.1|	48551-49701	11	42	0.667459	0.451555	-0.563779	1	no
gi|641567761|gb|KK857197.1|	51885-52597	11	42	0.822084	1	0.337892	0.8352	no
gi|641567761|gb|KK857197.1|	66100-67158	11	42	0.574496	0.49948	-0.201868	1	no
gi|641567761|gb|KK857197.1|	70646-71318	11	42	0.890951	2	1	0.4702	no
gi|641567764|gb|KK857196.1|	19710-21401	13	61	0.99409	0.938191	-0.0834949	0.9514	no
gi|641567764|gb|KK857196.1|	23492-25291	13	61	4	2	-1	0.3741	no
gi|641567764|gb|KK857196.1|	37861-38246	13	61	1	5	2	0.30385	no
gi|641567764|gb|KK857196.1|	5630-6623	13	61	1	0.269986	-2	0.24735	no
gi|641567764|gb|KK857196.1|	6739-9164	13	61	4	2	-0.894789	0.49095	no
gi|641567770|gb|KK857194.1|	11725-11953	11	39	47	37	-0.337569	0.80205	no
gi|641567770|gb|KK857194.1|	37622-39377	11	39	0.408557	1	2	0.23605	no
gi|641567770|gb|KK857194.1|	40816-44144	11	39	5	15	2	0.34255	no
gi|641567770|gb|KK857194.1|	44427-46023	11	39	0.404385	0.539342	0.41547	1	no
gi|641567770|gb|KK857194.1|	47102-47968	11	39	0.844518	2	1	0.38845	no
gi|641567770|gb|KK857194.1|	48091-50297	11	39	15	25	0.757918	0.6414	no
gi|641567770|gb|KK857194.1|	50480-50890	11	39	4	6	0.711429	0.63725	no

gi|641567770|gb|KK857194.1|	51055-51939	11	39	1	3	1	0.4102	no
gi|641567770|gb|KK857194.1|	52100-53384	11	39	1	2	0.372441	0.78995	no
gi|641567770|gb|KK857194.1|	57754-58446	11	39	6	11	0.929776	0.48345	no
gi|641567770|gb|KK857194.1|	58996-60794	11	39	8	14	0.805338	0.5223	no
gi|641567770|gb|KK857194.1|	61346-67068	11	39	35	24	-0.521142	0.7994	no
gi|641567770|gb|KK857194.1|	67194-67785	11	39	190	151.21	-0.325681	0.885	no
gi|641567770|gb|KK857194.1|	81108-84660	11	39	0.885909	1	0.741819	0.5767	no
gi|641567770|gb|KK857194.1|	84788-85261	11	39	3	3	-0.109023	0.943	no
gi|641567773|gb|KK857193.1|	2706-3134	9.5	36	1	4	2	0.3613	no
gi|641567779|gb|KK857191.1|	39398-39857	9.3	37	0	5	inf	0.00375	no
gi|641567788|gb|KK857188.1|	12063-13100	13	43	0.168237	2	4	0.2456	no
gi|641567788|gb|KK857188.1|	18195-19089	13	43	2	0.924049	-0.718045	0.6522	no
gi|641567788|gb|KK857188.1|	19239-20589	13	43	0.797121	0.840647	0.0767018	1	no
gi|641567788|gb|KK857188.1|	2-1581	13	43	338	285	-0.249086	0.8861	no
gi|641567788|gb|KK857188.1|	2231-3714	13	43	4	3	-0.392255	0.7659	no
gi|641567788|gb|KK857188.1|	22906-23867	13	43	2	2	0.522159	0.7153	no
gi|641567788|gb|KK857188.1|	24510-26529	13	43	0.854123	2	0.904549	0.5195	no
gi|641567788|gb|KK857188.1|	27062-28119	13	43	116	78	-0.578019	0.79075	no
gi|641567788|gb|KK857188.1|	28266-29032	13	43	57	34	-0.718353	0.5578	no
gi|641567788|gb|KK857188.1|	29166-29910	13	43	1	2	0.464085	0.7552	no
gi|641567788|gb|KK857188.1|	30185-30642	13	43	3	6	0.81262	0.58585	no
gi|641567788|gb|KK857188.1|	31380-31775	13	43	7	111	4	0.01715	no
gi|641567788|gb|KK857188.1|	33527-33885	13	43	3	51	4	0.0534	no
gi|641567788|gb|KK857188.1|	36385-37265	13	43	0.310219	2	3	0.1811	no
gi|641567788|gb|KK857188.1|	37342-40503	13	43	32	43	0.3966	0.8518	no
gi|641567788|gb|KK857188.1|	41129-43364	13	43	4	6	0.537884	0.6736	no
gi|641567788|gb|KK857188.1|	43535-44038	13	43	1	2	0.815745	0.6234	no
gi|641567788|gb|KK857188.1|	44209-45505	13	43	0.707577	5	3	0.0846	no
gi|641567788|gb|KK857188.1|	45657-46040	13	43	1	12	3	0.14775	no
gi|641567788|gb|KK857188.1|	51141-53727	13	43	0.266544	0.813554	2	1	no

gi|641567788|gb|KK857188.1|	61486-61806	13	43	0	49	inf	5.00E-05	yes
gi|641567788|gb|KK857188.1|	61997-62775	13	43	0	3	inf	0.00255	yes
gi|641567788|gb|KK857188.1|	6213-7123	13	43	3	1	-1	0.33855	no
gi|641567788|gb|KK857188.1|	63258-63536	13	43	48	63	0.394437	0.7621	no
gi|641567788|gb|KK857188.1|	69751-70577	13	43	2	0.34101	-3	0.19	no
gi|641567788|gb|KK857188.1|	71784-72937	13	43	16	14	-0.241832	0.8435	no
gi|641567788|gb|KK857188.1|	9811-11297	13	43	1	0.919539	-0.456375	0.7554	no
gi|641567803|gb|KK857183.1|	29623-30445	9.3	37	0.903809	0.686345	-0.397085	0.82205	no
gi|641567803|gb|KK857183.1|	44164-44372	9.3	37	18	3	-3	0.2472	no
gi|641567803|gb|KK857183.1|	48562-49131	9.3	37	0	299	inf	0.00015	yes
gi|641567803|gb|KK857183.1|	72395-73255	9.3	37	0.106518	5	6	0.10535	no
gi|641567806|gb|KK857182.1|	10669-11913	9.6	38	39	2	-5	0.04305	no
gi|641567806|gb|KK857182.1|	1637-1968	9.6	38	5	0.744222	-3	0.2476	no
gi|641567806|gb|KK857182.1|	5013-5282	9.6	38	16	0	-inf	0.00345	yes
gi|641567809|gb|KK857181.1|	[illegible]04519-10520	12	46	1	3	1	0.397	no
gi|641567809|gb|KK857181.1|	16643-17279	12	46	1	3	1	0.4284	no
gi|641567809|gb|KK857181.1|	17502-19504	12	46	0.666215	0.657513	-0.0189682	1	no
gi|641567809|gb|KK857181.1|	19695-20127	12	46	0.593542	4	3	0.27515	no
gi|641567809|gb|KK857181.1|	25789-26335	12	46	2	0.30459	-3	0.271	no
gi|641567809|gb|KK857181.1|	26523-27536	12	46	0.606111	0.790245	0.382718	1	no
gi|641567809|gb|KK857181.1|	52742-52992	12	46	28	52	0.874476	0.50945	no
gi|641567816|gb|KK857179.1|	15191-16769	12	37	1	3	0.920004	0.5014	no
gi|641567816|gb|KK857179.1|	16944-18366	12	37	1	0.70353	-0.714411	0.633	no
gi|641567816|gb|KK857179.1|	18470-19763	12	37	0.70951	0.392949	-0.852481	1	no
gi|641567816|gb|KK857179.1|	26570-26795	12	37	11	14	0.349821	0.82145	no
gi|641567816|gb|KK857179.1|	29551-30263	12	37	0.68507	2	1	0.4611	no
gi|641567816|gb|KK857179.1|	3280-3670	12	37	3	2	-1	0.5278	no
gi|641567816|gb|KK857179.1|	37180-38801	12	37	0.794589	0.832719	0.0676204	1	no
gi|641567816|gb|KK857179.1|	39090-39666	12	37	4	1	-1	0.39825	no
gi|641567816|gb|KK857179.1|	45453-46418	12	37	16	10	-0.626293	0.6223	no

gi|641567816|gb|KK857179.1|	76313-76518	12	37	33	8	-2	0.28355	no
gi|641567816|gb|KK857179.1|	84234-84691	12	37	2	4	1	0.47105	no
gi|641567816|gb|KK857179.1|	84795-85208	12	37	5	1	-2	0.28765	no
gi|641567819|gb|KK857178.1|	8860-9608	7.6	34	4	16	2	0.16225	no
gi|641567822|gb|KK857177.1|	11375-12591	10	42	0.346834	0.844947	1	1	no
gi|641567822|gb|KK857177.1|	13607-15883	10	42	0.918768	2	1	0.3971	no
gi|641567822|gb|KK857177.1|	17704-19319	10	42	1	2	0.9915	0.47835	no
gi|641567822|gb|KK857177.1|	19393-20022	10	42	1	0.741336	-0.62364	0.71195	no
gi|641567822|gb|KK857177.1|	20649-21116	10	42	3	1	-1	0.4311	no
gi|641567822|gb|KK857177.1|	24042-28611	10	42	0.437087	1	2	0.2353	no
gi|641567822|gb|KK857177.1|	61340-62200	10	42	2	3	0.603452	0.66485	no
gi|641567822|gb|KK857177.1|	8550-9001	10	42	1	5	2	0.31505	no
gi|641567825|gb|KK857176.1|	25205-25451	4.4	23	20	8	-1	0.39345	no
gi|641567828|gb|KK857175.1|	42938-43200	4.3	89	8	1	-3	0.2588	no
gi|641567828|gb|KK857175.1|	46912-47717	4.3	89	25	20	-0.30005	0.8094	no
gi|641567831|gb|KK857174.1|	11342-11665	10	37	11	16	0.642983	0.6459	no
gi|641567831|gb|KK857174.1|	23706-24237	10	37	6	17	1	0.28375	no
gi|641567831|gb|KK857174.1|	27214-28003	10	37	1	2	0.339359	0.81785	no
gi|641567831|gb|KK857174.1|	32226-32809	10	37	1	4	1	0.38865	no
gi|641567831|gb|KK857174.1|	35864-36035	10	37	48	155	2	0.2599	no
gi|641567831|gb|KK857174.1|	38871-39081	10	37	48	118	1	0.33795	no
gi|641567831|gb|KK857174.1|	41583-41977	10	37	4	9	1	0.34655	no
gi|641567831|gb|KK857174.1|	42129-42506	10	37	12	26	1	0.394	no
gi|641567831|gb|KK857174.1|	43412-43726	10	37	21	76	2	0.1779	no
gi|641567831|gb|KK857174.1|	44688-46487	10	37	3	9	2	0.19755	no
gi|641567831|gb|KK857174.1|	46605-47399	10	37	1	9	3	0.074	no
gi|641567831|gb|KK857174.1|	47553-48289	10	37	0.785633	0.794652	0.0164687	1	no
gi|641567831|gb|KK857174.1|	48431-49799	10	37	29	35	0.250205	0.8731	no
gi|641567831|gb|KK857174.1|	50694-52127	10	37	4	1	-2	0.2728	no
gi|641567831|gb|KK857174.1|	53393-54863	10	37	3	3	-0.0483476	0.9715	no

gi|641567831|gb|KK857174.1|	56571-57058	10	37	28	22	-0.309789	0.809	no
gi|641567831|gb|KK857174.1|	59061-61339	10	37	3	2	-0.481651	0.7145	no
gi|641567831|gb|KK857174.1|	8986-10286	10	37	1	2	0.79963	0.5617	no
gi|641567837|gb|KK857172.1|	27348-28524	7.2	25	0.144392	2	4	0.2494	no
gi|641567837|gb|KK857172.1|	29261-29520	7.2	25	42	51	0.280028	0.8295	no
gi|641567837|gb|KK857172.1|	31089-32830	7.2	25	7	7	-0.0560691	0.963	no
gi|641567837|gb|KK857172.1|	36015-36533	7.2	25	27.8	11	-1	0.31875	no
gi|641567837|gb|KK857172.1|	3633-4509	7.2	25	3	4	0.281732	0.83275	no
gi|641567837|gb|KK857172.1|	38493-38693	7.2	25	136	37	-2	0.18635	no
gi|641567837|gb|KK857172.1|	39561-39940	7.2	25	41	32	-0.365407	0.76965	no
gi|641567837|gb|KK857172.1|	45991-46224	7.2	25	296	78	-2	0.14045	no
gi|641567837|gb|KK857172.1|	47034-47294	7.2	25	32	20	-0.707041	0.60275	no
gi|641567837|gb|KK857172.1|	7027-7465	7.2	25	1	3	1	0.51205	no
gi|641567837|gb|KK857172.1|	8612-9719	7.2	25	0.854259	0.236378	-2	1	no
gi|641567840|gb|KK857171.1|	32468-33067	9.3	37	1	2	0.597754	0.69715	no
gi|641567843|gb|KK857170.1|	12849-13127	11	21	17	24	0.561547	0.68725	no
gi|641567843|gb|KK857170.1|	16610-18507	11	21	47	39	-0.261745	0.9041	no
gi|641567843|gb|KK857170.1|	28669-29516	11	21	1	0.825226	-0.659759	0.6826	no
gi|641567843|gb|KK857170.1|	36648-37922	11	21	4	2	-1	0.29345	no
gi|641567843|gb|KK857170.1|	38680-39468	11	21	22	11	-0.959502	0.4537	no
gi|641567843|gb|KK857170.1|	41727-42756	11	21	42	46	0.14545	0.92235	no
gi|641567849|gb|KK857168.1|	679-4099	11	44	7	6	-0.137766	0.911	no
gi|641567849|gb|KK857168.1|	8-327	11	44	4	3	-0.428254	0.7868	no
gi|641567849|gb|KK857168.1|	80074-81036	11	44	1	0.140422	-3	0.2382	no
gi|641567854|gb|KK857166.1|	01174-10237	11	37	24	4	-3	0.04825	no
gi|641567854|gb|KK857166.1|	04407-10618	11	37	3	0.955567	-2	0.26965	no
gi|641567854|gb|KK857166.1|	08887-10971	11	37	1	0.85387	-0.775552	0.63565	no
gi|641567854|gb|KK857166.1|	11213-11661	11	37	43	41	-0.0479283	0.9692	no
gi|641567854|gb|KK857166.1|	12186-11259	11	37	3	0.487376	-3	0.271	no
gi|641567854|gb|KK857166.1|	17775-12083	11	37	33	11	-2	0.35385	no

gi|641567854|gb|KK857166.1|	203-1019	11	37	3	2	-0.982138	0.4996	no
gi|641567854|gb|KK857166.1|	29057-29571	11	37	0.443233	136.48	8	0.2363	no
gi|641567854|gb|KK857166.1|	36368-37239	11	37	5	12	1	0.31395	no
gi|641567854|gb|KK857166.1|	37551-38173	11	37	1	3	0.750535	0.6262	no
gi|641567854|gb|KK857166.1|	38309-39369	11	37	1	1	-0.0319272	0.95655	no
gi|641567854|gb|KK857166.1|	39590-40892	11	37	0.383858	0.68209	0.829387	1	no
gi|641567854|gb|KK857166.1|	41695-43121	11	37	1	0.96422	-0.392468	0.7888	no
gi|641567854|gb|KK857166.1|	43671-44972	11	37	1	1	-0.0450867	0.9701	no
gi|641567854|gb|KK857166.1|	45259-47325	11	37	1	2	0.131128	0.9196	no
gi|641567854|gb|KK857166.1|	48353-48849	11	37	3	1	-0.992031	0.5872	no
gi|641567854|gb|KK857166.1|	59306-60024	11	37	3	6	0.984386	0.4786	no
gi|641567854|gb|KK857166.1|	62415-63382	11	37	4	15	2	0.18235	no
gi|641567854|gb|KK857166.1|	64044-66285	11	37	0.865266	1	0.287517	0.83235	no
gi|641567854|gb|KK857166.1|	6474-7987	11	37	1	0.900756	-0.254647	0.85915	no
gi|641567854|gb|KK857166.1|	67942-69453	11	37	0.376696	1	2	0.2786	no
gi|641567854|gb|KK857166.1|	76693-78309	11	37	9	31	2	0.307	no
gi|641567854|gb|KK857166.1|	78434-79116	11	37	5	9	0.939027	0.4843	no
gi|641567854|gb|KK857166.1|	93099-94569	11	37	0.111089	1	3	0.2543	no
gi|641567860|gb|KK857164.1|	1417-3170	11	36	3	1	-1	0.3536	no
gi|641567860|gb|KK857164.1|	16793-17844	11	36	0.74468	0.75532	0.0204685	1	no
gi|641567860|gb|KK857164.1|	23065-24703	11	36	2	1	-1	0.44045	no
gi|641567860|gb|KK857164.1|	27285-29481	11	36	1	0.431918	-1	0.32395	no
gi|641567860|gb|KK857164.1|	29749-30463	11	36	2	0.414028	-2	0.25005	no
gi|641567860|gb|KK857164.1|	30976-31228	11	36	10	13	0.401259	0.79705	no
gi|641567860|gb|KK857164.1|	31327-31939	11	36	12	9	-0.422261	0.7429	no
gi|641567860|gb|KK857164.1|	37034-37721	11	36	2	1	-0.885079	0.5827	no
gi|641567860|gb|KK857164.1|	40439-42122	11	36	2	0.798111	-0.932207	0.5136	no
gi|641567860|gb|KK857164.1|	42273-43944	11	36	0.383833	0.950871	1	0.42705	no
gi|641567860|gb|KK857164.1|	44067-45491	11	36	2	2	0.192487	0.88915	no
gi|641567860|gb|KK857164.1|	45610-46004	11	36	3	3	-0.0940091	0.9262	no

gi|641567860|gb|KK857164.1|	52967-56083	11	36	3	3	-0.250285	0.84175	no
gi|641567860|gb|KK857164.1|	54-964	11	36	2	0.90348	-0.898428	0.5692	no
gi|641567860|gb|KK857164.1|	57700-57980	11	36	18	12	-0.563416	0.6987	no
gi|641567860|gb|KK857164.1|	58571-59412	11	36	0.109652	2	4	0.11955	no
gi|641567860|gb|KK857164.1|	61281-61716	11	36	45	21	-1	0.3867	no
gi|641567860|gb|KK857164.1|	65035-65469	11	36	3	2	-0.259716	0.88485	no
gi|641567860|gb|KK857164.1|	65791-66359	11	36	2	1	-0.725368	0.67005	no
gi|641567860|gb|KK857164.1|	67088-67352	11	36	17	11	-0.59471	0.67505	no
gi|641567860|gb|KK857164.1|	73728-74331	11	36	7	3	-1	0.33625	no
gi|641567860|gb|KK857164.1|	79290-79687	11	36	28	17	-0.711032	0.5887	no
gi|641567860|gb|KK857164.1|	79813-80329	11	36	53	33	-0.695418	0.5784	no
gi|641567860|gb|KK857164.1|	83297-83602	11	36	7	8	0.153546	0.91535	no
gi|641567860|gb|KK857164.1|	83716-84956	11	36	3	2	-0.580949	0.67325	no
gi|641567860|gb|KK857164.1|	86041-87737	11	36	2	3	0.745899	0.56425	no
gi|641567860|gb|KK857164.1|	89877-90649	11	36	1	1	0.142672	0.929	no
gi|641567860|gb|KK857164.1|	9408-13638	11	36	0.877428	2	1	0.3702	no
gi|641567863|gb|KK857163.1|	1043-1313	8.6	39	0	16	inf	0.0025	yes
gi|641567863|gb|KK857163.1|	2826-3436	8.6	39	13	11	-0.257474	0.84235	no
gi|641567863|gb|KK857163.1|	46276-46888	8.6	39	10	4	-1	0.35755	no
gi|641567863|gb|KK857163.1|	59455-61546	8.6	39	0.224095	5	4	0.066	no
gi|641567863|gb|KK857163.1|	64079-64698	8.6	39	0.167012	5	5	0.10875	no
gi|641567869|gb|KK857161.1|	22781-26268	11	38	6	0.722513	-3	0.0264	no
gi|641567869|gb|KK857161.1|	27212-28832	11	38	12	2	-3	0.06335	no
gi|641567869|gb|KK857161.1|	29387-35070	11	38	19	5	-2	0.24535	no
gi|641567869|gb|KK857161.1|	3369-4003	11	38	5	0.977323	-2	0.1841	no
gi|641567869|gb|KK857161.1|	41401-43989	11	38	56	80	0.531052	0.7945	no
gi|641567869|gb|KK857161.1|	44510-46672	11	38	20	25	0.332438	0.88515	no
gi|641567869|gb|KK857161.1|	55998-57406	11	38	10	20	0.990509	0.42425	no
gi|641567869|gb|KK857161.1|	57914-59269	11	38	3	5	0.962334	0.46575	no
gi|641567869|gb|KK857161.1|	59471-60748	11	38	14	30	1	0.39125	no

gi|641567869|gb|KK857161.1|	62779-64165	11	38	1	0.634136	-0.672737	0.6649	no
gi|641567869|gb|KK857161.1|	66584-68533	11	38	1	2	0.355208	0.79165	no
gi|641567869|gb|KK857161.1|	69091-69353	11	38	13	6	-1	0.5047	no
gi|641567869|gb|KK857161.1|	9039-9344	11	38	5	4	-0.279413	0.8654	no
gi|641567876|gb|KK857159.1|	16341-18974	9	34	15	22	0.531757	0.8165	no
gi|641567876|gb|KK857159.1|	19228-19932	9	34	5	3	-0.761822	0.5821	no
gi|641567876|gb|KK857159.1|	21469-23287	9	34	4	4	-0.0958521	0.93815	no
gi|641567876|gb|KK857159.1|	24322-25206	9	34	2	2	0.134276	0.92775	no
gi|641567876|gb|KK857159.1|	31021-31768	9	34	1	2	1	0.4977	no
gi|641567876|gb|KK857159.1|	35192-35447	9	34	8	10	0.361664	0.82135	no
gi|641567876|gb|KK857159.1|	41480-43196	9	34	9	7	-0.245701	0.84375	no
gi|641567876|gb|KK857159.1|	46198-46484	9	34	4	12	2	0.36635	no
gi|641567876|gb|KK857159.1|	49776-50160	9	34	3	2	-0.224558	0.88445	no
gi|641567876|gb|KK857159.1|	51769-52045	9	34	12	10	-0.269106	0.85285	no
gi|641567876|gb|KK857159.1|	54084-54370	9	34	4	8	1	0.4667	no
gi|641567876|gb|KK857159.1|	5433-5670	9	34	0	31	inf	0.00135	yes
gi|641567876|gb|KK857159.1|	56072-57790	9	34	1	2	0.415808	0.7568	no
gi|641567876|gb|KK857159.1|	58175-58940	9	34	1	1	-0.134986	0.92965	no
gi|641567876|gb|KK857159.1|	69257-70597	9	34	0.989642	0	-inf	0.0056	no
gi|641567879|gb|KK857158.1|	4-331	9.7	21	6	3	-0.886012	0.60065	no
gi|641567885|gb|KK857156.1|	51522-51669	7.9	35	100	63	-0.668791	0.69655	no
gi|641567888|gb|KK857155.1|	50696-51155	9.6	41	0	6	inf	0.00255	yes
gi|641567893|gb|KK857153.1|	12090-13429	11	34	11	8	-0.511014	0.69285	no
gi|641567893|gb|KK857153.1|	20352-20794	11	34	1	3	0.852027	0.62755	no
gi|641567893|gb|KK857153.1|	22702-23613	11	34	1	0.751854	-0.658951	0.6826	no
gi|641567893|gb|KK857153.1|	32741-33915	11	34	9	17	0.830262	0.50315	no
gi|641567893|gb|KK857153.1|	34097-34525	11	34	40	68	0.792704	0.52475	no
gi|641567893|gb|KK857153.1|	34748-35809	11	34	0.408932	1	2	0.32945	no
gi|641567893|gb|KK857153.1|	36211-37440	11	34	97	8	-4	0.07545	no
gi|641567893|gb|KK857153.1|	39546-40386	11	34	8	3	-1	0.28585	no

gi|641567893|gb|KK857153.1|	43262-44001	11	34	1	0.197576	-3	0.2382	no
gi|641567893|gb|KK857153.1|	44751-45584	11	34	1	0	-inf	0.01	no
gi|641567893|gb|KK857153.1|	46046-48044	11	34	3	1	-1	0.38665	no
gi|641567896|gb|KK857152.1|	18631-19046	11	43	2	4	1	0.40145	no
gi|641567896|gb|KK857152.1|	22177-23591	11	43	0.348582	0.708132	1	1	no
gi|641567896|gb|KK857152.1|	23777-25760	11	43	0.35645	0.966524	1	0.35855	no
gi|641567896|gb|KK857152.1|	26251-27036	11	43	3	4	0.535212	0.7049	no
gi|641567896|gb|KK857152.1|	27443-29355	11	43	3	6	1	0.42625	no
gi|641567896|gb|KK857152.1|	29442-30073	11	43	0.974566	1	0.598814	0.70555	no
gi|641567899|gb|KK857151.1|	38451-39292	9.5	39	5	5	0.0506505	0.97005	no
gi|641567905|gb|KK857149.1|	1331-2145	10	38	12	15	0.328619	0.79025	no
gi|641567905|gb|KK857149.1|	14-1251	10	38	3	1	-1	0.42755	no
gi|641567905|gb|KK857149.1|	18942-19716	10	38	0.611731	2	2	0.28495	no
gi|641567905|gb|KK857149.1|	26566-26728	10	38	64	33	-0.949759	0.59175	no
gi|641567905|gb|KK857149.1|	27353-28300	10	38	2	0.716043	-1	0.38185	no
gi|641567905|gb|KK857149.1|	29991-31194	10	38	2	2	0.354932	0.79535	no
gi|641567905|gb|KK857149.1|	37371-37928	10	38	0.390965	3	3	0.2851	no
gi|641567905|gb|KK857149.1|	40228-40768	10	38	3	4	0.372923	0.7979	no
gi|641567905|gb|KK857149.1|	42972-44945	10	38	0.876225	0.36448	-1	0.4177	no
gi|641567905|gb|KK857149.1|	45591-47557	10	38	0.639774	0.365918	-0.806041	1	no
gi|641567905|gb|KK857149.1|	52602-53315	10	38	1	1	0.185911	0.91015	no
gi|641567905|gb|KK857149.1|	54169-54789	10	38	0.33324	3	3	0.25965	no
gi|641567905|gb|KK857149.1|	58193-58328	10	38	180.16	159	-0.180442	0.9068	no
gi|641567905|gb|KK857149.1|	59424-60832	10	38	0.291922	0.88953	2	0.32945	no
gi|641567905|gb|KK857149.1|	63123-64093	10	38	0.731146	2	1	0.4562	no
gi|641567905|gb|KK857149.1|	65383-65567	10	38	24	43	0.843665	0.59505	no
gi|641567905|gb|KK857149.1|	70667-70852	10	38	24	13	-0.892764	0.63655	no
gi|641567905|gb|KK857149.1|	71471-71686	10	38	13	31	1	0.4425	no
gi|641567905|gb|KK857149.1|	77118-77939	10	38	1	0.859293	-0.3971	0.81255	no
gi|641567905|gb|KK857149.1|	78399-80057	10	38	4	4	-0.12289	0.92455	no

gi|641567905|gb|KK857149.1|	81631-81931	10	38	51	40	-0.363914	0.77115	no
gi|641567905|gb|KK857149.1|	83793-83942	10	38	60	114	0.917301	0.59485	no
gi|641567905|gb|KK857149.1|	84094-85170	10	38	0.803934	0.489319	-0.716301	1	no
gi|641567905|gb|KK857149.1|	87704-89770	10	38	5	5	-0.12431	0.9204	no
gi|641567905|gb|KK857149.1|	91050-91732	10	38	18	15	-0.29947	0.81095	no
gi|641567905|gb|KK857149.1|	93692-94495	10	38	8	7	-0.092524	0.94575	no
gi|641567908|gb|KK857148.1|	13748-14188	9.5	32	20	6	-2	0.23155	no
gi|641567908|gb|KK857148.1|	19476-19983	9.5	32	8	2	-2	0.2643	no
gi|641567908|gb|KK857148.1|	22790-23235	9.5	32	45	9	-2	0.0874	no
gi|641567908|gb|KK857148.1|	2889-3917	9.5	32	6	6	0.0423045	0.9737	no
gi|641567908|gb|KK857148.1|	29601-29818	9.5	32	28	5	-3	0.19055	no
gi|641567908|gb|KK857148.1|	30701-31519	9.5	32	2	0.690725	-1	0.46585	no
gi|641567908|gb|KK857148.1|	32127-33167	9.5	32	0.922016	1	0.14591	0.9285	no
gi|641567908|gb|KK857148.1|	33648-34331	9.5	32	3	0.880171	-2	0.2443	no
gi|641567908|gb|KK857148.1|	37980-39250	9.5	32	40	12	-2	0.30275	no
gi|641567908|gb|KK857148.1|	43802-44838	9.5	32	1	3	1	0.4227	no
gi|641567908|gb|KK857148.1|	47471-47945	9.5	32	1	3	1	0.51205	no
gi|641567908|gb|KK857148.1|	58305-58595	9.5	32	0.678193	9	4	0.12785	no
gi|641567908|gb|KK857148.1|	8744-9030	9.5	32	8	0	-inf	0.01	no
gi|641567914|gb|KK857146.1|	13772-14025	8.1	34	11	3	-2	0.31705	no
gi|641567919|gb|KK857144.1|	56667-57092	9.9	41	3	10	2	0.27725	no
gi|641567919|gb|KK857144.1|	57652-58531	9.9	41	4	18	2	0.09695	no
gi|641567919|gb|KK857144.1|	58634-59198	9.9	41	1	4	1	0.3664	no
gi|641567922|gb|KK857143.1|	10209-10707	9	32	0	464	inf	0.00015	yes
gi|641567922|gb|KK857143.1|	19121-19404	9	32	0	12	inf	0.00455	no
gi|641567922|gb|KK857143.1|	41829-44208	9	32	52	48	-0.118067	0.9553	no
gi|641567922|gb|KK857143.1|	46021-47224	9	32	0.42157	1	1	0.41175	no
gi|641567922|gb|KK857143.1|	53295-54435	9	32	0	3	inf	0.00035	yes
gi|641567922|gb|KK857143.1|	6010-6311	9	32	0	70	inf	5.00E-05	yes
gi|641567925|gb|KK857142.1|	11177-12113	12	40	2	0	-inf	0.00315	yes

gi|641567925|gb|KK857142.1|	1602-1786	12	40	51	0	-inf	0.00435	no
gi|641567925|gb|KK857142.1|	16641-18161	12	40	1	0.244367	-3	0.17485	no
gi|641567925|gb|KK857142.1|	18307-20816	12	40	3	0.186796	-4	0.0691	no
gi|641567925|gb|KK857142.1|	21230-23521	12	40	2	0.154641	-4	0.10375	no
gi|641567925|gb|KK857142.1|	23758-29448	12	40	4	0.334631	-4	0.01725	no
gi|641567925|gb|KK857142.1|	29926-31195	12	40	3	0	-inf	0.00015	yes
gi|641567925|gb|KK857142.1|	31724-32316	12	40	5	0	-inf	0.0013	yes
gi|641567925|gb|KK857142.1|	33079-34572	12	40	5	0.249435	-4	0.09645	no
gi|641567925|gb|KK857142.1|	3786-5200	12	40	2	0	-inf	0.0003	yes
gi|641567925|gb|KK857142.1|	47111-47616	12	40	0	5	inf	0.00255	yes
gi|641567925|gb|KK857142.1|	71237-71424	12	40	23	32	0.523346	0.74475	no
gi|641567925|gb|KK857142.1|	74877-75182	12	40	8	21	1	0.3215	no
gi|641567925|gb|KK857142.1|	82212-82515	12	40	5	10	1	0.5003	no
gi|641567925|gb|KK857142.1|	82670-83332	12	40	2	1	-0.885719	0.5827	no
gi|641567925|gb|KK857142.1|	83452-84291	12	40	3	2	-0.460967	0.74915	no
gi|641567925|gb|KK857142.1|	8535-9754	12	40	0.829961	0.21062	-2	1	no
gi|641567925|gb|KK857142.1|	86452-86820	12	40	3	5	0.773198	0.6361	no
gi|641567925|gb|KK857142.1|	87674-89171	12	40	0.761496	0.414451	-0.877633	1	no
gi|641567934|gb|KK857139.1|	10404-11334	9.4	36	16	28	0.849409	0.4958	no
gi|641567934|gb|KK857139.1|	13109-14611	9.4	36	7	14	1	0.3982	no
gi|641567934|gb|KK857139.1|	16043-17431	9.4	36	4	3	-0.372992	0.774	no
gi|641567934|gb|KK857139.1|	22320-23164	9.4	36	1	4	2	0.2635	no
gi|641567934|gb|KK857139.1|	23725-26241	9.4	36	20	0.931167	-4	0.0494	no
gi|641567934|gb|KK857139.1|	2470-3851	9.4	36	11	16	0.599357	0.63045	no
gi|641567934|gb|KK857139.1|	26700-28322	9.4	36	2	0.075649	-5	0.18485	no
gi|641567934|gb|KK857139.1|	29320-29549	9.4	36	20	0	-inf	0.0059	no
gi|641567934|gb|KK857139.1|	31931-32264	9.4	36	20	10	-1	0.45435	no
gi|641567934|gb|KK857139.1|	34232-34836	9.4	36	4	0	-inf	0.0032	yes
gi|641567934|gb|KK857139.1|	36395-38142	9.4	36	1	2	0.991854	0.47835	no
gi|641567934|gb|KK857139.1|	39060-39759	9.4	36	2	2	-0.206722	0.89275	no

gi|641567934|gb|KK857139.1|	40682-42484	9.4	36	3	4	0.517025	0.6922	no
gi|641567934|gb|KK857139.1|	42718-43955	9.4	36	1	2	0.469158	0.7414	no
gi|641567934|gb|KK857139.1|	44055-45472	9.4	36	0.40568	0.882999	1	0.48415	no
gi|641567934|gb|KK857139.1|	46308-48090	9.4	36	1	2	0.659235	0.6231	no
gi|641567934|gb|KK857139.1|	48906-49108	9.4	36	41	68	0.732944	0.5975	no
gi|641567934|gb|KK857139.1|	937-1307	9.4	36	0.396729	7	4	0.11975	no
gi|641567937|gb|KK857138.1|	10288-10564	9	38	0	16	inf	0.00255	yes
gi|641567937|gb|KK857138.1|	62731-63447	9	38	2	8	2	0.18315	no
gi|641567940|gb|KK857137.1|	14594-14883	10	18	14	13	-0.127313	0.9272	no
gi|641567940|gb|KK857137.1|	16342-18665	10	18	39	24	-0.690099	0.74975	no
gi|641567940|gb|KK857137.1|	19558-20148	10	18	8	3	-1	0.30355	no
gi|641567940|gb|KK857137.1|	21081-22772	10	18	0.946752	0.144337	-3	0.18365	no
gi|641567940|gb|KK857137.1|	35330-37522	10	18	2	1	-0.901587	0.50585	no
gi|641567940|gb|KK857137.1|	4492-5355	10	18	0.318117	1	2	0.3323	no
gi|641567940|gb|KK857137.1|	61913-62711	10	18	1	2	1	0.431	no
gi|641567940|gb|KK857137.1|	63393-64844	10	18	0.507466	2	2	0.18715	no
gi|641567940|gb|KK857137.1|	70155-72468	10	18	0.93615	0.714178	-0.390456	0.7774	no
gi|641567940|gb|KK857137.1|	73558-75473	10	18	1	0.879069	-0.67121	0.6364	no
gi|641567940|gb|KK857137.1|	78777-79296	10	18	1	2	0.594251	0.70505	no
gi|641567940|gb|KK857137.1|	86905-87294	10	18	2	5	1	0.4021	no
gi|641567940|gb|KK857137.1|	88009-88689	10	18	1	2	0.889682	0.57435	no
gi|641567940|gb|KK857137.1|	92535-93179	10	18	1	2	0.599204	0.6936	no
gi|641567940|gb|KK857137.1|	95906-96386	10	18	24	22	-0.0710644	0.9568	no
gi|641567949|gb|KK857134.1|	46087-47526	7.8	31	0.455433	2	2	0.23515	no
gi|641567952|gb|KK857133.1|	45760-47314	9.7	40	12	0.079423	-7	0.18285	no
gi|641567952|gb|KK857133.1|	47951-48802	9.7	40	4	0	-inf	0.00045	yes
gi|641567955|gb|KK857132.1|	34222-34812	9.3	37	0.716917	2	2	0.3613	no
gi|641567955|gb|KK857132.1|	6758-7388	9.3	37	0.6512	2	2	0.33105	no
gi|641567967|gb|KK857128.1|	13365-14877	11	43	0.806594	1	0.700955	0.62415	no
gi|641567967|gb|KK857128.1|	19816-22044	11	43	2	4	0.944611	0.46265	no

gi|641567967|gb|KK857128.1|	22149-22844	11	43	2	5	1	0.34	no
gi|641567967|gb|KK857128.1|	25235-26267	11	43	6	12	1	0.39735	no
gi|641567967|gb|KK857128.1|	28061-28909	11	43	8	10	0.328667	0.79295	no
gi|641567967|gb|KK857128.1|	31607-31993	11	43	7	13	0.846093	0.54455	no
gi|641567967|gb|KK857128.1|	35296-36047	11	43	0.637036	1	1	0.51085	no
gi|641567967|gb|KK857128.1|	46429-46634	11	43	23	42	0.854786	0.5637	no
gi|641567967|gb|KK857128.1|	47600-48128	11	43	1	2	0.594723	0.70555	no
gi|641567967|gb|KK857128.1|	48266-48720	11	43	4	6	0.490455	0.7353	no
gi|641567967|gb|KK857128.1|	51138-55760	11	43	4	5	0.228144	0.853	no
gi|641567967|gb|KK857128.1|	57507-58511	11	43	3	5	0.780117	0.5606	no
gi|641567967|gb|KK857128.1|	74276-74620	11	43	2	9	2	0.27245	no
gi|641567967|gb|KK857128.1|	8953-9853	11	43	0.602829	0.916227	0.603955	0.70555	no
gi|641567970|gb|KK857127.1|	43880-44557	8.5	33	2	1	-1	0.4973	no
gi|641567970|gb|KK857127.1|	4734-5023	8.5	33	16	11	-0.560966	0.699	no
gi|641567970|gb|KK857127.1|	47952-49104	8.5	33	0.962092	0.112654	-3	0.22425	no
gi|641567973|gb|KK857126.1|	32256-32848	10	39	0	4	inf	0.00255	yes
gi|641567973|gb|KK857126.1|	44399-44652	10	39	52	60	0.218011	0.86965	no
gi|641567973|gb|KK857126.1|	51218-51449	10	39	29	35	0.269436	0.84925	no
gi|641567973|gb|KK857126.1|	52840-53058	10	39	6	18	2	0.41765	no
gi|641567973|gb|KK857126.1|	55335-55499	10	39	38	47	0.288385	0.86665	no
gi|641567973|gb|KK857126.1|	57522-57892	10	39	8	3	-1	0.36935	no
gi|641567973|gb|KK857126.1|	60078-62134	10	39	31	38	0.294808	0.8915	no
gi|641567973|gb|KK857126.1|	62338-63050	10	39	88	140	0.675377	0.76885	no
gi|641567973|gb|KK857126.1|	70974-71785	10	39	49	35	-0.456431	0.71625	no
gi|641567973|gb|KK857126.1|	73606-75435	10	39	16	8	-0.976246	0.4361	no
gi|641567973|gb|KK857126.1|	77701-78006	10	39	15	4	-2	0.28035	no
gi|641567973|gb|KK857126.1|	81133-81308	10	39	73	28	-1	0.38615	no
gi|641567973|gb|KK857126.1|	81905-82140	10	39	22	7	-2	0.317	no
gi|641567973|gb|KK857126.1|	93396-93609	10	39	17	5	-2	0.33935	no
gi|641567979|gb|KK857124.1|	10711-11744	10	41	2	2	0.52728	0.70515	no

gi|641567979|gb|KK857124.1|	32725-33350	10	41	2	1	-0.138339	0.9254	no
gi|641567979|gb|KK857124.1|	44562-44829	10	41	6	6	0.0728181	0.9244	no
gi|641567979|gb|KK857124.1|	48059-49453	10	41	0.708795	0.899884	0.34437	0.8163	no
gi|641567979|gb|KK857124.1|	55578-57376	10	41	1	2	0.853759	0.5302	no
gi|641567982|gb|KK857123.1|	12917-13688	7.3	33	0.36895	2	2	0.256	no
gi|641567982|gb|KK857123.1|	35486-36192	7.3	33	0.415865	3	3	0.1811	no
gi|641567982|gb|KK857123.1|	37831-38008	7.3	33	15	115	3	0.15185	no
gi|641567982|gb|KK857123.1|	6718-7839	7.3	33	14	283	4	0.035	no
gi|641567985|gb|KK857122.1|	13365-13828	8.9	39	2	14	3	0.11085	no
gi|641567985|gb|KK857122.1|	26000-26485	8.9	39	0	4	inf	0.00455	no
gi|641567985|gb|KK857122.1|	27157-28826	8.9	39	0	15	inf	5.00E-05	yes
gi|641567985|gb|KK857122.1|	30427-31224	8.9	39	0	12	inf	5.00E-05	yes
gi|641567985|gb|KK857122.1|	36717-38638	8.9	39	14	27	0.975337	0.54585	no
gi|641567985|gb|KK857122.1|	39624-41747	8.9	39	4	6	0.38276	0.7634	no
gi|641567985|gb|KK857122.1|	43510-43861	8.9	39	11	14	0.269736	0.84695	no
gi|641567985|gb|KK857122.1|	51826-52744	8.9	39	0	6	inf	5.00E-05	yes
gi|641567985|gb|KK857122.1|	55915-57256	8.9	39	1	5	2	0.149	no
gi|641567985|gb|KK857122.1|	59630-60206	8.9	39	3	10	2	0.2128	no
gi|641567985|gb|KK857122.1|	64651-64917	8.9	39	6	14	1	0.45735	no
gi|641567985|gb|KK857122.1|	65076-65363	8.9	39	9	21	1	0.42045	no
gi|641567985|gb|KK857122.1|	66272-66694	8.9	39	5	20	2	0.1765	no
gi|641567985|gb|KK857122.1|	79655-80091	8.9	39	2	2	-0.411538	0.82205	no
gi|641567998|gb|KK857118.1|	13478-14074	8.8	40	2	0.801527	-1	0.47135	no
gi|641568005|gb|KK857116.1|	30595-31539	9.7	22	0.567402	2	2	0.28005	no
gi|641568005|gb|KK857116.1|	32047-32563	9.7	22	0.880996	3	2	0.3014	no
gi|641568005|gb|KK857116.1|	32815-33563	9.7	22	0.512393	2	2	0.26155	no
gi|641568005|gb|KK857116.1|	33950-35202	9.7	22	0.402021	0.918307	1	0.47015	no
gi|641568005|gb|KK857116.1|	66973-67525	9.7	22	0.59461	2	2	0.3323	no
gi|641568005|gb|KK857116.1|	68097-69390	9.7	22	0.129002	1	3	0.2556	no
gi|641568008|gb|KK857115.1|	57998-58214	10	38	21	14	-0.577175	0.73235	no

gi|641568014|gb|KK857113.1|	15611-16041	21	81	14	6	-1	0.40845	no
gi|641568014|gb|KK857113.1|	18810-19483	21	81	15	6	-1	0.3424	no
gi|641568014|gb|KK857113.1|	24354-24829	21	81	3	2	-0.145408	0.92525	no
gi|641568014|gb|KK857113.1|	48220-48844	21	81	7	8	0.151136	0.9099	no
gi|641568014|gb|KK857113.1|	54908-55784	21	81	32	26	-0.333014	0.7851	no
gi|641568017|gb|KK857112.1|	19254-20077	7	44	0.451185	1	2	0.3613	no
gi|641568017|gb|KK857112.1|	3655-4420	7	44	0.372834	4	3	0.1539	no
gi|641568017|gb|KK857112.1|	68780-70693	7	44	0.783173	40	6	0.0282	no
gi|641568020|gb|KK857111.1|	20302-20473	9.5	37	0	74	inf	0.00375	no
gi|641568020|gb|KK857111.1|	33121-33623	9.5	37	0	6	inf	0.00135	yes
gi|641568020|gb|KK857111.1|	3612-4043	9.5	37	0	9	inf	0.00145	yes
gi|641568020|gb|KK857111.1|	38995-39327	9.5	37	0	12	inf	0.0015	yes
gi|641568020|gb|KK857111.1|	40813-41109	9.5	37	0	17	inf	0.00135	yes
gi|641568020|gb|KK857111.1|	46697-46862	9.5	37	0	135	inf	0.00135	yes
gi|641568020|gb|KK857111.1|	50657-51614	9.5	37	0.092953	23	8	0.10435	no
gi|641568020|gb|KK857111.1|	56005-56948	9.5	37	52	35	-0.577363	0.7146	no
gi|641568020|gb|KK857111.1|	57117-60037	9.5	37	15	6	-1	0.46555	no
gi|641568020|gb|KK857111.1|	60655-61263	9.5	37	2	3	0.48259	0.75115	no
gi|641568020|gb|KK857111.1|	61421-62212	9.5	37	11	5	-1	0.34045	no
gi|641568020|gb|KK857111.1|	67632-68465	9.5	37	1	2	0.31357	0.84265	no
gi|641568020|gb|KK857111.1|	68815-69577	9.5	37	2	4	0.679928	0.6316	no
gi|641568020|gb|KK857111.1|	8578-8841	9.5	37	0	17	inf	0.0025	yes
gi|641568026|gb|KK857109.1|	1119-1327	9.2	35	18	45	1	0.39135	no
gi|641568026|gb|KK857109.1|	12425-12999	9.2	35	1	2	0.789451	0.63035	no
gi|641568026|gb|KK857109.1|	18102-18383	9.2	35	4	5	0.299361	0.86155	no
gi|641568026|gb|KK857109.1|	9037-9882	9.2	35	3	5	0.972481	0.4788	no
gi|641568041|gb|KK857104.1|	22525-24397	8.7	38	0.886778	2	1	0.44675	no
gi|641568041|gb|KK857104.1|	24970-25456	8.7	38	0.485364	3	3	0.2991	no
gi|641568041|gb|KK857104.1|	38219-39997	8.7	38	0.581562	1	1	0.43475	no
gi|641568041|gb|KK857104.1|	42411-42854	8.7	38	2	3	0.589079	0.70555	no

gi|641568041|gb|KK857104.1|	58392-58712	8.7	38	1	7	3	0.28555	no
gi|641568041|gb|KK857104.1|	58976-59894	8.7	38	0.685784	2	2	0.2915	no
gi|641568041|gb|KK857104.1|	60466-60863	8.7	38	1	5	2	0.30385	no
gi|641568041|gb|KK857104.1|	60950-61781	8.7	38	0.556884	1	0.866082	0.62725	no
gi|641568044|gb|KK857103.1|	01982-10381	10	36	1	1	-0.0287045	0.9779	no
gi|641568044|gb|KK857103.1|	04997-10521	10	36	5	21	2	0.26345	no
gi|641568044|gb|KK857103.1|	16600-17698	10	36	1	0.716172	-0.716138	0.6527	no
gi|641568044|gb|KK857103.1|	19541-20825	10	36	0.650341	0.297139	-1	1	no
gi|641568044|gb|KK857103.1|	24502-25118	10	36	3	3	0.0133737	0.9647	no
gi|641568044|gb|KK857103.1|	26678-28078	10	36	3	5	0.807727	0.54135	no
gi|641568044|gb|KK857103.1|	32664-33213	10	36	1	4	1	0.3664	no
gi|641568044|gb|KK857103.1|	34166-34688	10	36	7	15	1	0.4376	no
gi|641568044|gb|KK857103.1|	37694-38068	10	36	4	6	0.443923	0.76695	no
gi|641568044|gb|KK857103.1|	38-321	10	36	10	10	-0.0744797	0.95335	no
gi|641568044|gb|KK857103.1|	38257-38394	10	36	98	250	1	0.4209	no
gi|641568044|gb|KK857103.1|	40904-41106	10	36	57	118	1	0.43515	no
gi|641568044|gb|KK857103.1|	42402-42646	10	36	3	13	2	0.3198	no
gi|641568044|gb|KK857103.1|	45000-45834	10	36	0.775975	0.841915	0.117665	1	no
gi|641568044|gb|KK857103.1|	46046-46701	10	36	5	11	1	0.41035	no
gi|641568044|gb|KK857103.1|	48061-49308	10	36	2	3	0.72701	0.59915	no
gi|641568044|gb|KK857103.1|	50338-51204	10	36	3	3	0.266496	0.8441	no
gi|641568044|gb|KK857103.1|	51357-52976	10	36	0.845437	7	3	0.05305	no
gi|641568044|gb|KK857103.1|	53158-54590	10	36	0.286257	2	3	0.14835	no
gi|641568044|gb|KK857103.1|	54754-55999	10	36	1	4	2	0.20005	no
gi|641568044|gb|KK857103.1|	61450-61898	10	36	5	3	-0.658426	0.6532	no
gi|641568044|gb|KK857103.1|	6433-7179	10	36	0.642815	2	1	0.4611	no
gi|641568044|gb|KK857103.1|	67506-70649	10	36	0.672554	1	1	0.4282	no
gi|641568044|gb|KK857103.1|	70779-72256	10	36	0.607654	0.757616	0.318217	1	no
gi|641568044|gb|KK857103.1|	74013-74942	10	36	0.868504	0.58681	-0.565639	1	no
gi|641568044|gb|KK857103.1|	80767-81378	10	36	3	4	0.235599	0.86855	no

gi|641568044|gb|KK857103.1|	82654-83099	10	36	7	6	-0.0888224	0.9474	no
gi|641568044|gb|KK857103.1|	83236-83938	10	36	2	1	-1	0.4973	no
gi|641568044|gb|KK857103.1|	89742-89905	10	36	107	64	-0.737706	0.6097	no
gi|641568044|gb|KK857103.1|	90737-91295	10	36	3	0.884083	-2	0.403	no
gi|641568044|gb|KK857103.1|	9278-10188	10	36	0.396263	23	6	0.11605	no
gi|641568044|gb|KK857103.1|	95884-97179	10	36	4	3	-0.393039	0.7652	no
gi|641568044|gb|KK857103.1|	97344-97998	10	36	0.925834	1	0.336454	0.83525	no
gi|641568044|gb|KK857103.1|	98258-99475	10	36	2	0.527577	-2	0.30235	no
gi|641568050|gb|KK857101.1|	10378-10944	8.9	33	0	4	inf	0.00255	yes
gi|641568050|gb|KK857101.1|	19827-20098	8.9	33	10	0	-inf	0.01	no
gi|641568050|gb|KK857101.1|	21618-21792	8.9	33	166	23	-3	0.109	no
gi|641568050|gb|KK857101.1|	24565-24815	8.9	33	20	0	-inf	0.00345	yes
gi|641568050|gb|KK857101.1|	27780-28212	8.9	33	18	1	-4	0.1027	no
gi|641568050|gb|KK857101.1|	34107-34687	8.9	33	36	8	-2	0.10135	no
gi|641568050|gb|KK857101.1|	36297-36630	8.9	33	21	7	-2	0.25135	no
gi|641568050|gb|KK857101.1|	37549-38331	8.9	33	35	5	-3	0.05095	no
gi|641568050|gb|KK857101.1|	38515-40151	8.9	33	5	3	-0.634129	0.6282	no
gi|641568050|gb|KK857101.1|	41743-42531	8.9	33	0.955655	2	0.772302	0.6261	no
gi|641568050|gb|KK857101.1|	43343-49749	8.9	33	1	0.957901	-0.307695	0.8062	no
gi|641568050|gb|KK857101.1|	52703-53042	8.9	33	7	9	0.468828	0.7498	no
gi|641568050|gb|KK857101.1|	57167-58173	8.9	33	1	1	0.342014	0.8164	no
gi|641568050|gb|KK857101.1|	75521-76434	8.9	33	1	0.449863	-2	0.33235	no
gi|641568050|gb|KK857101.1|	8394-8858	8.9	33	2	4	1	0.47105	no
gi|641568055|gb|KK857099.1|	77836-78631	10	44	0.708375	2	2	0.3067	no
gi|641568055|gb|KK857099.1|	78708-80239	10	44	0.424064	2	2	0.1741	no
gi|641568059|gb|KK857098.1|	17926-23546	12	38	23	12	-0.876004	0.6924	no
gi|641568059|gb|KK857098.1|	23714-25131	12	38	11	3	-2	0.13325	no
gi|641568059|gb|KK857098.1|	26393-27180	12	38	4	8	0.907214	0.49715	no
gi|641568059|gb|KK857098.1|	2719-3957	12	38	10	8	-0.213354	0.8656	no
gi|641568059|gb|KK857098.1|	27487-28290	12	38	4	5	0.365582	0.78775	no

gi|641568059|gb|KK857098.1|	28679-33532	12	38	0.714462	0.951952	0.41403	0.7615	no
gi|641568059|gb|KK857098.1|	34082-36431	12	38	0.821876	0.852747	0.053197	1	no
gi|641568059|gb|KK857098.1|	36801-40242	12	38	1	2	0.362587	0.7796	no
gi|641568059|gb|KK857098.1|	40383-40929	12	38	1	1	-0.211758	0.8945	no
gi|641568059|gb|KK857098.1|	4102-4377	12	38	3	11	2	0.3046	no
gi|641568059|gb|KK857098.1|	41313-42721	12	38	0.817381	0.533718	-0.614931	1	no
gi|641568059|gb|KK857098.1|	4657-9483	12	38	9	6	-0.644037	0.6944	no
gi|641568059|gb|KK857098.1|	46754-47316	12	38	4	5	0.518305	0.70875	no
gi|641568059|gb|KK857098.1|	48903-49092	12	38	46	23	-0.978081	0.55285	no
gi|641568059|gb|KK857098.1|	49215-49588	12	38	2	4	1	0.5126	no
gi|641568059|gb|KK857098.1|	50656-53995	12	38	0.900889	0.85964	-0.0676166	0.9598	no
gi|641568059|gb|KK857098.1|	54741-54978	12	38	13	12	-0.104518	0.9384	no
gi|641568059|gb|KK857098.1|	58439-59335	12	38	3	2	-0.774604	0.58825	no
gi|641568059|gb|KK857098.1|	60435-61813	12	38	0.838115	0.729612	-0.200017	1	no
gi|641568059|gb|KK857098.1|	72894-73375	12	38	2	1	-0.408032	0.82205	no
gi|641568059|gb|KK857098.1|	73522-75015	12	38	0.491042	0.83145	0.759783	1	no
gi|641568059|gb|KK857098.1|	75201-75879	12	38	1	1	0.114698	0.92265	no
gi|641568059|gb|KK857098.1|	77437-78060	12	38	4	3	-0.160428	0.9151	no
gi|641568059|gb|KK857098.1|	86772-87107	12	38	94	65	-0.534235	0.67035	no
gi|641568063|gb|KK857097.1|	19477-19846	9	19	2	5	2	0.41725	no
gi|641568063|gb|KK857097.1|	52999-54866	9	19	1	1	-0.221276	0.8759	no
gi|641568063|gb|KK857097.1|	68012-68661	9	19	3	0.472695	-3	0.20575	no
gi|641568063|gb|KK857097.1|	77252-77413	9	19	139	0	-inf	0.00315	yes
gi|641568066|gb|KK857096.1|	18988-19228	7.6	35	8	13	0.741411	0.6387	no
gi|641568066|gb|KK857096.1|	21295-22688	7.6	35	4	8	1	0.3623	no
gi|641568066|gb|KK857096.1|	24010-25319	7.6	35	0.762892	1	0.607028	0.67895	no
gi|641568066|gb|KK857096.1|	26937-27114	7.6	35	29	36	0.325305	0.8234	no
gi|641568066|gb|KK857096.1|	29887-30326	7.6	35	6	12	1	0.46005	no
gi|641568066|gb|KK857096.1|	38219-38591	7.6	35	3	2	-0.226215	0.88445	no
gi|641568072|gb|KK857094.1|	11381-11698	12	42	7	7	0.156275	0.91535	no

gi|641568072|gb|KK857094.1|	12873-14363	12	42	3	6	0.739014	0.5602	no
gi|641568072|gb|KK857094.1|	16213-16463	12	42	20	24	0.23062	0.86885	no
gi|641568072|gb|KK857094.1|	18153-19163	12	42	5	10	0.975922	0.44865	no
gi|641568072|gb|KK857094.1|	29731-35862	12	42	9	4	-1	0.4669	no
gi|641568072|gb|KK857094.1|	40189-41989	12	42	11	11	0.000697026	0.9972	no
gi|641568072|gb|KK857094.1|	42592-43181	12	42	9	5	-0.665646	0.622	no
gi|641568072|gb|KK857094.1|	4278-4461	12	42	78	67	-0.215762	0.8757	no
gi|641568072|gb|KK857094.1|	43984-45101	12	42	4	4	-0.320075	0.8094	no
gi|641568080|gb|KK857092.1|	123-520	7.7	30	1	3	1	0.5206	no
gi|641568086|gb|KK857090.1|	10080-10989	8.9	35	0	4	inf	0.00075	yes
gi|641568086|gb|KK857090.1|	20390-20841	8.9	35	2	0.828509	-2	0.4266	no
gi|641568086|gb|KK857090.1|	21628-23143	8.9	35	6	2	-1	0.28445	no
gi|641568086|gb|KK857090.1|	24584-25922	8.9	35	4	3	-0.114121	0.93	no
gi|641568089|gb|KK857089.1|	00845-10148	11	37	7	2	-1	0.3038	no
gi|641568089|gb|KK857089.1|	35025-36222	11	37	0	1	inf	0.0025	yes
gi|641568089|gb|KK857089.1|	57458-57783	11	37	8	2	-2	0.21685	no
gi|641568089|gb|KK857089.1|	62451-65947	11	37	7	2	-2	0.0833	no
gi|641568089|gb|KK857089.1|	66362-69488	11	37	5	3	-0.75952	0.54645	no
gi|641568089|gb|KK857089.1|	72385-75944	11	37	8	3	-1	0.30015	no
gi|641568092|gb|KK857088.1|	11324-11868	7.1	28	2	2	-0.0565728	0.9454	no
gi|641568092|gb|KK857088.1|	14498-15614	7.1	28	0.461405	3	3	0.1324	no
gi|641568092|gb|KK857088.1|	18221-19301	7.1	28	0.88033	1	0.468153	0.75495	no
gi|641568092|gb|KK857088.1|	21367-22177	7.1	28	0.575908	1	0.865766	0.62725	no
gi|641568092|gb|KK857088.1|	31639-32978	7.1	28	1	2	0.912022	0.52255	no
gi|641568092|gb|KK857088.1|	5239-6162	7.1	28	0.583776	1	1	0.47015	no
gi|641568092|gb|KK857088.1|	7992-8744	7.1	28	0.890249	0.771997	-0.205614	0.89485	no
gi|641568092|gb|KK857088.1|	9468-10695	7.1	28	0.617644	2	2	0.26135	no
gi|641568095|gb|KK857087.1|	15135-16640	11	43	0.162181	3	4	0.13825	no
gi|641568095|gb|KK857087.1|	19652-21114	11	43	23	8	-1	0.2474	no
gi|641568095|gb|KK857087.1|	24423-25243	11	43	4	0.86066	-2	0.1936	no

gi|641568095|gb|KK857087.1|	27091-28180	11	43	1	0.482234	-1	0.46645	no
gi|641568095|gb|KK857087.1|	28294-30405	11	43	2	1	-0.953687	0.46785	no
gi|641568095|gb|KK857087.1|	31551-34985	11	43	0.721432	0.500674	-0.526993	1	no
gi|641568095|gb|KK857087.1|	35181-35857	11	43	2	0.669642	-1	0.4311	no
gi|641568095|gb|KK857087.1|	36047-37202	11	43	2	1	-1	0.46445	no
gi|641568095|gb|KK857087.1|	37348-37727	11	43	3	1	-2	0.3632	no
gi|641568095|gb|KK857087.1|	41820-42841	11	43	5	2	-1	0.3534	no
gi|641568095|gb|KK857087.1|	42999-44404	11	43	1	0.356692	-2	0.31415	no
gi|641568095|gb|KK857087.1|	45408-46009	11	43	2	0.791791	-1	0.50365	no
gi|641568095|gb|KK857087.1|	57735-58327	11	43	1	2	0.5975	0.70555	no
gi|641568098|gb|KK857086.1|	20296-20885	9.9	18	6	1	-3	0.14185	no
gi|641568098|gb|KK857086.1|	25238-26864	9.9	18	0.395906	0.603503	0.608203	1	no
gi|641568098|gb|KK857086.1|	39700-40575	9.9	18	13	13	-0.103799	0.93275	no
gi|641568098|gb|KK857086.1|	41226-41967	9.9	18	1	0.590574	-0.813507	0.6492	no
gi|641568098|gb|KK857086.1|	42698-43138	9.9	18	3	0.432142	-3	0.271	no
gi|641568098|gb|KK857086.1|	49854-50330	9.9	18	0.753574	3	2	0.3323	no
gi|641568098|gb|KK857086.1|	53705-53991	9.9	18	35	6	-2	0.12635	no
gi|641568098|gb|KK857086.1|	72559-72819	9.9	18	15	15	-0.0720376	0.9575	no
gi|641568098|gb|KK857086.1|	81821-82624	9.9	18	2	0.176915	-3	0.2089	no
gi|641568098|gb|KK857086.1|	83695-84335	9.9	18	3	0	-inf	0.00435	no
gi|641568098|gb|KK857086.1|	84765-85532	9.9	18	1	0.93987	-0.245982	0.88495	no
gi|641568098|gb|KK857086.1|	85714-87076	9.9	18	0.849608	0.277344	-2	1	no
gi|641568098|gb|KK857086.1|	87233-87447	9.9	18	51	5	-3	0.14455	no
gi|641568101|gb|KK857085.1|	11680-11945	9	34	9	8	-0.316856	0.85305	no
gi|641568101|gb|KK857085.1|	14326-14978	9	34	2	3	0.599433	0.6792	no
gi|641568101|gb|KK857085.1|	15668-15910	9	34	8	11	0.549554	0.74525	no
gi|641568101|gb|KK857085.1|	18362-18796	9	34	5	2	-1	0.37215	no
gi|641568101|gb|KK857085.1|	19195-19494	9	34	12	8	-0.510876	0.723	no
gi|641568101|gb|KK857085.1|	19733-20544	9	34	1	2	0.718224	0.6393	no
gi|641568101|gb|KK857085.1|	23059-23354	9	34	18	26	0.566122	0.6815	no

gi|641568101|gb|KK857085.1|	27962-28677	9	34	2	1	-0.292092	0.8456	no
gi|641568101|gb|KK857085.1|	29872-30292	9	34	14	9	-0.688695	0.6103	no
gi|641568101|gb|KK857085.1|	31580-32811	9	34	13	8	-0.60811	0.62285	no
gi|641568101|gb|KK857085.1|	33723-36504	9	34	23	20	-0.252601	0.9109	no
gi|641568101|gb|KK857085.1|	36583-45073	9	34	6	6	0.127476	0.9526	no
gi|641568101|gb|KK857085.1|	45203-45901	9	34	6	17	1	0.2763	no
gi|641568103|gb|KK857084.1|	45290-45737	10	21	2	3	1	0.5082	no
gi|641568103|gb|KK857084.1|	47500-47881	10	21	2	8	2	0.2616	no
gi|641568103|gb|KK857084.1|	51016-51446	10	21	2	2	0.102484	0.92335	no
gi|641568106|gb|KK857083.1|	11240-11915	9.6	22	4	0.894697	-2	0.2046	no
gi|641568106|gb|KK857083.1|	13901-14160	9.6	22	6	9	0.555663	0.74515	no
gi|641568106|gb|KK857083.1|	22633-23037	9.6	22	4	0	-inf	0.0102	no
gi|641568106|gb|KK857083.1|	26823-28162	9.6	22	6	4	-0.496455	0.6985	no
gi|641568112|gb|KK857081.1|	17659-18191	11	38	1	2	0.594924	0.70555	no
gi|641568112|gb|KK857081.1|	18494-19031	11	38	259	137	-0.916771	0.68345	no
gi|641568112|gb|KK857081.1|	19368-20840	11	38	28	17	-0.698808	0.66585	no
gi|641568112|gb|KK857081.1|	21000-21937	11	38	1	0.580515	-1	0.50315	no
gi|641568112|gb|KK857081.1|	23707-24228	11	38	51	41	-0.340189	0.78545	no
gi|641568112|gb|KK857081.1|	25403-25944	11	38	49	28	-0.825674	0.5122	no
gi|641568112|gb|KK857081.1|	26215-28436	11	38	0.978666	1	0.387024	0.7675	no
gi|641568112|gb|KK857081.1|	29465-30065	11	38	78	50	-0.645283	0.59735	no
gi|641568112|gb|KK857081.1|	30184-31146	11	38	9	5	-0.994831	0.4508	no
gi|641568112|gb|KK857081.1|	31584-32460	11	38	79	56	-0.502962	0.8276	no
gi|641568112|gb|KK857081.1|	35391-35687	11	38	10	7	-0.626269	0.6819	no
gi|641568112|gb|KK857081.1|	37525-38031	11	38	17	9	-0.84141	0.5259	no
gi|641568112|gb|KK857081.1|	38779-39542	11	38	20	10	-0.974086	0.4472	no
gi|641568112|gb|KK857081.1|	41757-42133	11	38	28	22	-0.340288	0.78985	no
gi|641568112|gb|KK857081.1|	42993-43257	11	38	98	52	-0.905334	0.4874	no
gi|641568112|gb|KK857081.1|	43746-44432	11	38	28	17	-0.691703	0.58765	no
gi|641568112|gb|KK857081.1|	47393-47572	11	38	24	25	0.0336315	0.92445	no

gi|641568112|gb|KK857081.1|	48832-49166	11	38	6	10	0.681718	0.64385	no
gi|641568112|gb|KK857081.1|	49653-49978	11	38	33	19	-0.783134	0.5562	no
gi|641568112|gb|KK857081.1|	51470-51830	11	38	33	18	-0.848095	0.52255	no
gi|641568112|gb|KK857081.1|	51978-53328	11	38	0.858438	1	0.706752	0.6229	no
gi|641568112|gb|KK857081.1|	55221-55601	11	38	4	1	-2	0.31725	no
gi|641568112|gb|KK857081.1|	56551-56782	11	38	51	32	-0.689395	0.605	no
gi|641568112|gb|KK857081.1|	60818-61202	11	38	19	18	-0.0732485	0.95735	no
gi|641568112|gb|KK857081.1|	62781-63474	11	38	1	1	-0.0775793	0.92685	no
gi|641568112|gb|KK857081.1|	63687-64994	11	38	1	2	0.372554	0.78995	no
gi|641568112|gb|KK857081.1|	65117-65488	11	38	18	10	-0.823395	0.54365	no
gi|641568112|gb|KK857081.1|	66525-66817	11	38	42	18	-1	0.3654	no
gi|641568112|gb|KK857081.1|	67679-67836	11	38	64	10	-3	0.25845	no
gi|641568112|gb|KK857081.1|	71072-71631	11	38	42	23	-0.905219	0.47065	no
gi|641568112|gb|KK857081.1|	72750-74841	11	38	3	0.911579	-2	0.22065	no
gi|641568112|gb|KK857081.1|	76232-76622	11	38	4	2	-1	0.50345	no
gi|641568112|gb|KK857081.1|	79018-79193	11	38	62	44.03	-0.483141	0.7566	no
gi|641568112|gb|KK857081.1|	80028-80508	11	38	39	21	-0.913627	0.4771	no
gi|641568112|gb|KK857081.1|	82657-83130	11	38	11	3	-2	0.23595	no
gi|641568112|gb|KK857081.1|	83551-83702	11	38	73	52	-0.496584	0.7686	no
gi|641568112|gb|KK857081.1|	85261-85459	11	38	91	48	-0.921436	0.50665	no
gi|641568112|gb|KK857081.1|	88336-88513	11	38	76	16	-2	0.2143	no
gi|641568112|gb|KK857081.1|	89352-89850	11	38	3	0.703505	-2	0.25005	no
gi|641568115|gb|KK857080.1|	29398-29862	9.8	37	2	2	0.327729	0.83525	no
gi|641568115|gb|KK857080.1|	62385-62707	9.8	37	5	6	0.0578111	0.94155	no
gi|641568115|gb|KK857080.1|	64641-65066	9.8	37	6	7	0.246396	0.8584	no
gi|641568119|gb|KK857079.1|	0-892	9.8	19	3	3	-0.0912863	0.94545	no
gi|641568119|gb|KK857079.1|	49854-50285	9.8	19	46	54	0.237507	0.8462	no
gi|641568119|gb|KK857079.1|	51701-52123	9.8	19	0.928376	5	2	0.2235	no
gi|641568119|gb|KK857079.1|	55976-56714	9.8	19	0.78274	2	1	0.4702	no
gi|641568119|gb|KK857079.1|	56815-57423	9.8	19	2	4	1	0.4318	no

gi|641568119|gb|KK857079.1|	57722-58799	9.8	19	2	2	-0.0110371	0.97395	no
gi|641568119|gb|KK857079.1|	59413-59991	9.8	19	1	2	0.789611	0.63035	no
gi|641568119|gb|KK857079.1|	60653-61504	9.8	19	6	6	-0.142097	0.9143	no
gi|641568119|gb|KK857079.1|	72554-72848	9.8	19	174	130	-0.417893	0.7404	no
gi|641568119|gb|KK857079.1|	76203-77310	9.8	19	3	1	-2	0.2984	no
gi|641568119|gb|KK857079.1|	84950-86479	9.8	19	4	1	-1	0.293	no
gi|641568119|gb|KK857079.1|	9157-9951	9.8	19	1	0.538668	-1	0.47135	no
gi|641568121|gb|KK857078.1|	04007-10420	11	21	0	57	inf	0.0016	yes
gi|641568121|gb|KK857078.1|	11335-11184	11	21	0	56	inf	5.00E-05	yes
gi|641568121|gb|KK857078.1|	35278-35648	11	21	0.396729	5	4	0.12785	no
gi|641568121|gb|KK857078.1|	84796-86896	11	21	1	1	0.0731785	0.95625	no
gi|641568121|gb|KK857078.1|	87659-88525	11	21	1	2	0.603531	0.678	no
gi|641568121|gb|KK857078.1|	89341-90174	11	21	8	9	0.216054	0.86845	no
gi|641568121|gb|KK857078.1|	90352-91538	11	21	12	15	0.329363	0.7898	no
gi|641568121|gb|KK857078.1|	98835-99328	11	21	0.47411	13	5	0.2353	no
gi|641568121|gb|KK857078.1|	99653-99863	11	21	0	128	inf	0.0001	yes
gi|641568124|gb|KK857077.1|	2815-3394	8.6	36	4	28	3	0.06705	no
gi|641568124|gb|KK857077.1|	75824-76776	8.6	36	0.561404	2	2	0.25645	no
gi|641568124|gb|KK857077.1|	77635-79509	8.6	36	0.295241	2	3	0.11415	no
gi|641568124|gb|KK857077.1|	8250-9149	8.6	36	11	40	2	0.14785	no
gi|641568124|gb|KK857077.1|	83343-83723	8.6	36	6	5	-0.324629	0.82445	no
gi|641568124|gb|KK857077.1|	84289-85279	8.6	36	0.178187	2.71	4	0.24065	no
gi|641568126|gb|KK857076.1|	1191-1497	11	42	30	3	-3	0.10745	no
gi|641568126|gb|KK857076.1|	141-1044	11	42	7	2	-2	0.14965	no
gi|641568126|gb|KK857076.1|	17428-18349	11	42	0.487821	1	1	0.50945	no
gi|641568126|gb|KK857076.1|	2003-7180	11	42	6	2	-2	0.2178	no
gi|641568126|gb|KK857076.1|	29786-30213	11	42	0.605972	4	3	0.28535	no
gi|641568126|gb|KK857076.1|	65027-65345	11	42	9	11	0.184507	0.8996	no
gi|641568132|gb|KK857074.1|	13829-14348	21	88	10	11	0.146792	0.91205	no
gi|641568132|gb|KK857074.1|	15046-15729	21	88	24	21	-0.178623	0.88565	no

gi|641568132|gb|KK857074.1|	18169-19554	21	88	0.416592	2	2	0.24225	no
gi|641568132|gb|KK857074.1|	20106-20876	21	88	1	2	0.98058	0.5381	no
gi|641568132|gb|KK857074.1|	22774-23537	21	88	11	11	0.0494024	0.96755	no
gi|641568132|gb|KK857074.1|	28238-28836	21	88	21	34	0.667539	0.59055	no
gi|641568132|gb|KK857074.1|	30365-32482	21	88	2	1	-0.870734	0.52795	no
gi|641568132|gb|KK857074.1|	34267-36108	21	88	5	5	0.146116	0.90735	no
gi|641568132|gb|KK857074.1|	36226-36907	21	88	9	9	0.0617804	0.9618	no
gi|641568132|gb|KK857074.1|	37418-38604	21	88	48	65	0.428372	0.84785	no
gi|641568132|gb|KK857074.1|	4046-4299	21	88	10	7	-0.446386	0.7786	no
gi|641568132|gb|KK857074.1|	46312-47403	21	88	34	17	-0.999688	0.549	no
gi|641568132|gb|KK857074.1|	51067-52386	21	88	7	4	-0.655959	0.6072	no
gi|641568132|gb|KK857074.1|	52922-54455	21	88	0.264644	3	3	0.09615	no
gi|641568132|gb|KK857074.1|	64147-64432	21	88	11	25	1	0.42035	no
gi|641568132|gb|KK857074.1|	70148-70327	21	88	107	291	1	0.28905	no
gi|641568132|gb|KK857074.1|	72507-72660	21	88	67	118.57	0.826506	0.598	no
gi|641568135|gb|KK857073.1|	17272-19319	9.8	38	4	4	0.0628605	0.96015	no
gi|641568135|gb|KK857073.1|	19469-20025	9.8	38	4	5	0.361583	0.7996	no
gi|641568135|gb|KK857073.1|	24497-24675	9.8	38	0	117	inf	0.00115	yes
gi|641568135|gb|KK857073.1|	27803-28321	9.8	38	0.218898	12	6	0.1052	no
gi|641568135|gb|KK857073.1|	30414-30608	9.8	38	0	45	inf	0.0025	yes
gi|641568135|gb|KK857073.1|	38199-38575	9.8	38	14	315	4	0.0453	no
gi|641568135|gb|KK857073.1|	57640-57990	9.8	38	0	10	inf	0.0023	yes
gi|641568138|gb|KK857072.1|	65926-67341	10	22	11	14	0.29478	0.81595	no
gi|641568138|gb|KK857072.1|	68629-69180	10	22	6	4	-0.404175	0.7714	no
gi|641568138|gb|KK857072.1|	72413-73184	10	22	2	1	-0.812952	0.5996	no
gi|641568138|gb|KK857072.1|	75589-76137	10	22	1	3	1	0.47095	no
gi|641568138|gb|KK857072.1|	78982-80174	10	22	0.426222	1	2	0.32035	no
gi|641568142|gb|KK857071.1|	12004-13127	11	42	0	2	inf	0.0015	yes
gi|641568142|gb|KK857071.1|	18194-19138	11	42	0.094567	2	4	0.11955	no
gi|641568142|gb|KK857071.1|	23025-23973	11	42	0	2	inf	0.0023	yes

gi|641568142|gb|KK857071.1|	24624-25629	11	42	0.174886	330	11	0.2337	no
gi|641568142|gb|KK857071.1|	27403-29446	11	42	2	2	0.0757034	0.9529	no
gi|641568142|gb|KK857071.1|	3072-3960	11	42	2	3	1	0.4556	no
gi|641568142|gb|KK857071.1|	35651-36129	11	42	2	0.751865	-2	0.4266	no
gi|641568142|gb|KK857071.1|	5502-6440	11	42	1	2	0.729914	0.63585	no
gi|641568142|gb|KK857071.1|	6527-7436	11	42	0.099205	73	10	0.1065	no
gi|641568142|gb|KK857071.1|	71004-71753	11	42	0.895069	0.776147	-0.20567	0.89485	no
gi|641568142|gb|KK857071.1|	9704-10397	11	42	0	113.44	inf	0.0007	yes
gi|641568145|gb|KK857070.1|	38687-39544	8.8	36	0.107	3	5	0.10725	no
gi|641568145|gb|KK857070.1|	39658-40884	8.8	36	0.618244	3	2	0.1617	no
gi|641568145|gb|KK857070.1|	43325-44092	8.8	36	0.247687	2	3	0.2991	no
gi|641568145|gb|KK857070.1|	47589-48479	8.8	36	0.611507	0.929338	0.603835	0.70555	no
gi|641568145|gb|KK857070.1|	49896-51411	8.8	36	0.751109	1	0.500941	0.73235	no
gi|641568145|gb|KK857070.1|	51659-52492	8.8	36	0.888219	2	0.925005	0.5636	no
gi|641568145|gb|KK857070.1|	55135-55588	8.8	36	2	6	2	0.29635	no
gi|641568145|gb|KK857070.1|	56018-56845	8.8	36	0.448328	1	1	0.51305	no
gi|641568148|gb|KK857069.1|	72072-72967	7.7	31	0	108	inf	0.00025	yes
gi|641568154|gb|KK857067.1|	35038-36145	10	38	0.465959	0.827323	0.828247	1	no
gi|641568166|gb|KK857063.1|	40584-40956	8.8	37	7	6	-0.266857	0.85105	no
gi|641568169|gb|KK857062.1|	39909-40406	8.6	38	0.233953	5	4	0.1136	no
gi|641568172|gb|KK857061.1|	[illegible]01610-10236	11	40	1	2	0.601852	0.6936	no
gi|641568172|gb|KK857061.1|	[illegible]05866-10964	11	40	9	4	-1	0.3519	no
gi|641568172|gb|KK857061.1|	[illegible]17967-11826	11	40	17	0.955514	-4	0.19125	no
gi|641568172|gb|KK857061.1|	[illegible]19705-12081	11	40	7	3	-1	0.35285	no
gi|641568172|gb|KK857061.1|	47138-47765	11	40	3	0.744727	-2	0.2215	no
gi|641568172|gb|KK857061.1|	47947-48371	11	40	29	50	0.800931	0.52705	no
gi|641568172|gb|KK857061.1|	52598-53588	11	40	0.712746	2	1	0.38845	no
gi|641568172|gb|KK857061.1|	57388-59447	11	40	22	27	0.329781	0.87935	no
gi|641568172|gb|KK857061.1|	61523-62747	11	40	0.619446	2	2	0.33265	no
gi|641568172|gb|KK857061.1|	74470-75829	11	40	7	10	0.456577	0.7207	no

gi|641568172|gb|KK857061.1|	76165-83475	11	40	12	5	-1	0.43315	no
gi|641568172|gb|KK857061.1|	83779-86550	11	40	7	7	-0.0135435	0.99145	no
gi|641568172|gb|KK857061.1|	86984-87749	11	40	0.124278	3	5	0.11025	no
gi|641568172|gb|KK857061.1|	88232-88620	11	40	0	46	inf	5.00E-05	yes
gi|641568178|gb|KK857059.1|	20684-20910	1.9	57	0	20	inf	0.0068	no
gi|641568184|gb|KK857057.1|	41746-43913	9.5	39	5	11	1	0.3514	no
gi|641568186|gb|KK857056.1|	75229-77611	8.3	41	0.518063	0.889309	0.779557	0.5885	no
gi|641568186|gb|KK857056.1|	79288-80838	8.3	41	0.57491	1	1	0.40965	no
gi|641568186|gb|KK857056.1|	82415-83213	8.3	41	0.939799	1	0.602541	0.6936	no
gi|641568189|gb|KK857055.1|	0-524	9.2	39	13	8	-0.587687	0.6577	no
gi|641568189|gb|KK857055.1|	11204-11925	9.2	39	31	25	-0.332327	0.78955	no
gi|641568189|gb|KK857055.1|	2375-3794	9.2	39	3	2	-0.650293	0.6193	no
gi|641568189|gb|KK857055.1|	26683-28565	9.2	39	2	2	0.499206	0.7043	no
gi|641568189|gb|KK857055.1|	29653-29973	9.2	39	2	6	2	0.3454	no
gi|641568189|gb|KK857055.1|	30829-31265	9.2	39	18	37	1	0.4257	no
gi|641568189|gb|KK857055.1|	31523-32440	9.2	39	4	6	0.638099	0.62755	no
gi|641568189|gb|KK857055.1|	32849-34115	9.2	39	1	1	-0.32607	0.8148	no
gi|641568189|gb|KK857055.1|	35785-36725	9.2	39	4	8	1	0.3954	no
gi|641568189|gb|KK857055.1|	37883-40238	9.2	39	0.688458	1	0.802243	0.5614	no
gi|641568189|gb|KK857055.1|	48618-49175	9.2	39	4	7	0.807596	0.5528	no
gi|641568189|gb|KK857055.1|	4944-9603	9.2	39	4	3	-0.289452	0.81755	no
gi|641568189|gb|KK857055.1|	63094-66322	9.2	39	9	10	0.235288	0.84525	no
gi|641568189|gb|KK857055.1|	655-1186	9.2	39	6	8	0.424949	0.75165	no
gi|641568189|gb|KK857055.1|	66901-68686	9.2	39	0.846232	0.814946	-0.0543486	1	no
gi|641568189|gb|KK857055.1|	9848-11011	9.2	39	54	47	-0.198688	0.93055	no
gi|641568192|gb|KK857054.1|	42796-43579	10	37	0.963784	2	1	0.49765	no
gi|641568192|gb|KK857054.1|	43825-45054	10	37	2	2	-0.130347	0.9249	no
gi|641568192|gb|KK857054.1|	45108-45989	10	37	2	1	-0.474279	0.73975	no
gi|641568192|gb|KK857054.1|	46306-46907	10	37	1	1	-0.402175	0.82205	no
gi|641568192|gb|KK857054.1|	50238-51662	10	37	0.691489	0.702388	0.022562	1	no

gi|641568192|gb|KK857054.1|	55464-57044	10	37	0.715851	0.467619	-0.614326	1	no
gi|641568207|gb|KK857049.1|	38954-39520	9.2	28	0	7	inf	0.00065	yes
gi|641568207|gb|KK857049.1|	41175-42024	9.2	28	0	2	inf	0.0025	yes
gi|641568207|gb|KK857049.1|	42481-42902	9.2	28	0	6	inf	0.00375	no
gi|641568213|gb|KK857047.1|	25058-25462	9.2	39	1	4	1	0.4427	no
gi|641568213|gb|KK857047.1|	36789-37636	9.2	39	0.543212	2	2	0.32945	no
gi|641568213|gb|KK857047.1|	37974-38621	9.2	39	2	4	1	0.4339	no
gi|641568213|gb|KK857047.1|	39833-41854	9.2	39	2	3	0.114332	0.9286	no
gi|641568213|gb|KK857047.1|	43008-43865	9.2	39	0	2	inf	0.00255	yes
gi|641568213|gb|KK857047.1|	45197-47412	9.2	39	2	3	0.536652	0.67685	no
gi|641568213|gb|KK857047.1|	52490-53157	9.2	39	1	3	1	0.397	no
gi|641568213|gb|KK857047.1|	54023-55829	9.2	39	0.263747	39	7	0.06965	no
gi|641568213|gb|KK857047.1|	57451-58470	9.2	39	24	33	0.447424	0.7183	no
gi|641568213|gb|KK857047.1|	63438-64877	9.2	39	0.227717	0.867431	2	1	no
gi|641568213|gb|KK857047.1|	68932-69368	9.2	39	0	11	inf	0.00045	yes
gi|641568213|gb|KK857047.1|	78257-78378	9.2	39	0	1389.44	inf	0.0025	yes
gi|641568213|gb|KK857047.1|	80472-80711	9.2	39	0	92	inf	0.00015	yes
gi|641568223|gb|KK857044.1|	10893-13918	11	39	2	0.687522	-1	0.38205	no
gi|641568223|gb|KK857044.1|	14208-15345	11	39	4	0.457761	-3	0.0941	no
gi|641568223|gb|KK857044.1|	15780-20540	11	39	5	0.473819	-3	0.0186	no
gi|641568223|gb|KK857044.1|	20998-23156	11	39	5	0.77046	-3	0.06675	no
gi|641568223|gb|KK857044.1|	23801-29685	11	39	3	10	2	0.32385	no
gi|641568223|gb|KK857044.1|	3015-3279	11	39	6	53	3	0.0855	no
gi|641568223|gb|KK857044.1|	30157-32136	11	39	2	20	3	0.02195	no
gi|641568223|gb|KK857044.1|	32277-36803	11	39	2	5	2	0.19795	no
gi|641568223|gb|KK857044.1|	37220-38639	11	39	0.694315	0.793405	0.192467	1	no
gi|641568223|gb|KK857044.1|	39537-40531	11	39	3	6	0.843099	0.52645	no
gi|641568223|gb|KK857044.1|	42162-42654	11	39	3	0	-inf	0.0075	no
gi|641568223|gb|KK857044.1|	43622-45774	11	39	5	44	3	0.1129	no
gi|641568223|gb|KK857044.1|	46500-47506	11	39	0.786014	0.265678	-2	1	no

gi|641568223|gb|KK857044.1|	4800-5223	11	39	112	0.462944	-8	0.18285	no
gi|641568223|gb|KK857044.1|	52308-53033	11	39	2	0	-inf	0.0059	no
gi|641568223|gb|KK857044.1|	5430-7635	11	39	107	2	-6	0.01615	no
gi|641568223|gb|KK857044.1|	54490-55037	11	39	2	0	-inf	0.0102	no
gi|641568223|gb|KK857044.1|	56167-57091	11	39	2	0	-inf	0.00435	no
gi|641568223|gb|KK857044.1|	57307-60097	11	39	6	2	-1	0.2672	no
gi|641568223|gb|KK857044.1|	60365-62639	11	39	48	29	-0.738617	0.74315	no
gi|641568223|gb|KK857044.1|	64830-66290	11	39	2	2	0.45012	0.7403	no
gi|641568223|gb|KK857044.1|	74639-77931	11	39	39	2	-5	0.03265	no
gi|641568223|gb|KK857044.1|	7887-8417	11	39	3	0	-inf	0.01	no
gi|641568223|gb|KK857044.1|	81796-83116	11	39	14	21	0.62662	0.6097	no
gi|641568223|gb|KK857044.1|	86938-87400	11	39	33	41	0.313773	0.8045	no
gi|641568223|gb|KK857044.1|	8847-10464	11	39	3	0.531398	-2	0.1415	no
gi|641568223|gb|KK857044.1|	90766-91977	11	39	7	11	0.565097	0.65795	no
gi|641568223|gb|KK857044.1|	95434-95737	11	39	7	2	-2	0.27505	no
gi|641568223|gb|KK857044.1|	95867-96488	11	39	4	11	1	0.33545	no
gi|641568226|gb|KK857043.1|	[illegible]00977-10177	9.5	18	0.816903	0.708818	-0.204749	1	no
gi|641568226|gb|KK857043.1|	39820-39995	9.5	18	96	127	0.396564	0.7703	no
gi|641568226|gb|KK857043.1|	50631-51400	9.5	18	11	21	0.911379	0.4741	no
gi|641568226|gb|KK857043.1|	52827-54662	9.5	18	0.863608	0.329232	-1	1	no
gi|641568226|gb|KK857043.1|	54769-55691	9.5	18	1	1	0.466705	0.75495	no
gi|641568226|gb|KK857043.1|	56740-58098	9.5	18	1	2	0.476007	0.73245	no
gi|641568226|gb|KK857043.1|	58780-60013	9.5	18	0.614073	3	2	0.1872	no
gi|641568226|gb|KK857043.1|	61284-61566	9.5	18	9	12	0.437143	0.76915	no
gi|641568226|gb|KK857043.1|	62801-64630	9.5	18	0.606746	0.132175	-2	1	no
gi|641568226|gb|KK857043.1|	65279-65510	9.5	18	8	19	1	0.4376	no
gi|641568226|gb|KK857043.1|	65778-66027	9.5	18	7	24	2	0.2863	no
gi|641568226|gb|KK857043.1|	82443-82892	9.5	18	3	4	0.174547	0.9057	no
gi|641568226|gb|KK857043.1|	89302-89793	9.5	18	10	8	-0.24137	0.8517	no
gi|641568226|gb|KK857043.1|	93939-95396	9.5	18	6	1	-2	0.15285	no

gi|641568232|gb|KK857041.1|	123-1022	11	40	1	2	0.488466	0.7378	no
gi|641568232|gb|KK857041.1|	54766-55893	11	40	2	2	-0.551548	0.69645	no
gi|641568232|gb|KK857041.1|	74023-74504	11	40	13	11	-0.277971	0.83645	no
gi|641568232|gb|KK857041.1|	85042-87082	11	40	49	19	-1	0.4122	no
gi|641568235|gb|KK857040.1|	21987-22550	11	47	4	3	-0.474039	0.74015	no
gi|641568235|gb|KK857040.1|	25858-26613	11	47	0.758983	0.959865	0.338763	0.8352	no
gi|641568235|gb|KK857040.1|	31172-33730	11	47	2	1	-0.0906065	0.94495	no
gi|641568235|gb|KK857040.1|	33817-35296	11	47	0.772173	0.168098	-2	1	no
gi|641568235|gb|KK857040.1|	37214-38870	11	47	0.824022	0.665036	-0.30925	1	no
gi|641568235|gb|KK857040.1|	47439-47864	11	47	0	5	inf	0.0068	no
gi|641568235|gb|KK857040.1|	49169-50800	11	47	0.295895	3	3	0.08785	no
gi|641568235|gb|KK857040.1|	52631-53011	11	47	2	10	2	0.183	no
gi|641568235|gb|KK857040.1|	60187-60428	11	47	8	34	2	0.19425	no
gi|641568235|gb|KK857040.1|	61954-62894	11	47	1	17	4	0.02825	no
gi|641568235|gb|KK857040.1|	63694-65176	11	47	1	14	3	0.02685	no
gi|641568235|gb|KK857040.1|	65438-65785	11	47	4	41	4	0.06455	no
gi|641568244|gb|KK857037.1|	33978-34315	12	52	18	4	-2	0.1553	no
gi|641568244|gb|KK857037.1|	70088-70879	12	52	0.237711	2	3	0.2556	no
gi|641568244|gb|KK857037.1|	88382-88672	12	52	18	25	0.453797	0.74285	no
gi|641568254|gb|KK857035.1|	19034-21371	9.4	40	0.297533	2	3	0.10315	no
gi|641568254|gb|KK857035.1|	2039-3350	9.4	40	1	2	0.882672	0.52165	no
gi|641568254|gb|KK857035.1|	23911-25041	9.4	40	0.378745	2	2	0.181	no
gi|641568254|gb|KK857035.1|	5282-5769	9.4	40	4	7	0.840291	0.5609	no
gi|641568254|gb|KK857035.1|	59821-60956	9.4	40	2	0.802779	-0.908526	0.54665	no
gi|641568254|gb|KK857035.1|	6176-6736	9.4	40	2	2	0.0112595	0.9394	no
gi|641568254|gb|KK857035.1|	63397-63962	9.4	40	2	1	-0.403565	0.82205	no
gi|641568254|gb|KK857035.1|	64564-65616	9.4	40	1	0.754443	-0.897061	0.56925	no
gi|641568254|gb|KK857035.1|	66631-70298	9.4	40	3	5	0.682018	0.58305	no
gi|641568254|gb|KK857035.1|	70476-72670	9.4	40	10	13	0.341342	0.78055	no
gi|641568254|gb|KK857035.1|	72911-74863	9.4	40	48	78	0.712484	0.7438	no

gi|641568254|gb|KK857035.1|	76306-77290	9.4	40	103	151	0.55611	0.7932	no
gi|641568254|gb|KK857035.1|	8-667	9.4	40	1	3	1	0.47685	no
gi|641568254|gb|KK857035.1|	84006-85069	9.4	40	2	3	0.453523	0.73585	no
gi|641568257|gb|KK857034.1|	03418-10403	10	40	0.677608	2	1	0.51315	no
gi|641568257|gb|KK857034.1|	05336-10676	10	40	0.802168	0.873029	0.122125	1	no
gi|641568257|gb|KK857034.1|	18902-12302	10	40	14	28	1	0.6475	no
gi|641568257|gb|KK857034.1|	14105-14855	10	40	2	2	0.116277	0.93605	no
gi|641568257|gb|KK857034.1|	15027-15260	10	40	24	25	0.105338	0.9416	no
gi|641568257|gb|KK857034.1|	15516-15769	10	40	18	14	-0.294383	0.83975	no
gi|641568257|gb|KK857034.1|	19479-19942	10	40	8	6	-0.409311	0.771	no
gi|641568257|gb|KK857034.1|	23160-23469	10	40	6	2	-2	0.3414	no
gi|641568257|gb|KK857034.1|	33542-33801	10	40	5	12	1	0.4132	no
gi|641568257|gb|KK857034.1|	3956-5333	10	40	0.479328	0.456392	-0.0707385	1	no
gi|641568257|gb|KK857034.1|	39668-40672	10	40	0.700407	0.399498	-0.810007	1	no
gi|641568257|gb|KK857034.1|	43751-44484	10	40	17	23	0.435634	0.7269	no
gi|641568257|gb|KK857034.1|	44888-45396	10	40	9	9	0.100602	0.937	no
gi|641568257|gb|KK857034.1|	47312-47519	10	40	49	24	-0.996636	0.50755	no
gi|641568257|gb|KK857034.1|	48045-48468	10	40	2	2	0.101811	0.92335	no
gi|641568257|gb|KK857034.1|	51294-55464	10	40	13	4	-2	0.307	no
gi|641568257|gb|KK857034.1|	56775-59397	10	40	16	15	-0.0212987	0.9923	no
gi|641568257|gb|KK857034.1|	60362-61228	10	40	1	3	1	0.4685	no
gi|641568257|gb|KK857034.1|	62173-64287	10	40	9	10	0.2045	0.8676	no
gi|641568257|gb|KK857034.1|	6349-6917	10	40	0.758998	2	1	0.44255	no
gi|641568257|gb|KK857034.1|	64420-65718	10	40	0.449459	1	1	0.43995	no
gi|641568257|gb|KK857034.1|	74034-74924	10	40	1	2	0.603835	0.678	no
gi|641568257|gb|KK857034.1|	75801-76857	10	40	1	1	-0.0949687	0.9478	no
gi|641568257|gb|KK857034.1|	78051-78452	10	40	4	3	-0.415107	0.77855	no
gi|641568257|gb|KK857034.1|	82757-93141	10	40	4	6	0.629459	0.7826	no
gi|641568257|gb|KK857034.1|	93572-94991	10	40	8	8	-0.0904666	0.94095	no
gi|641568257|gb|KK857034.1|	95845-96824	10	40	0.361375	1	2	0.33105	no

gi|641568257|gb|KK857034.1|	97158-98164	10	40	1	1	-0.225026	0.87645	no
gi|641568257|gb|KK857034.1|	99311-99799	10	40	12	10	-0.272642	0.8386	no
gi|641568260|gb|KK857033.1|	21074-22299	12	44	6	15	1	0.31665	no
gi|641568260|gb|KK857033.1|	24081-25103	12	44	35	106	2	0.34075	no
gi|641568275|gb|KK857028.1|	01249-10179	11	40	1	2	1	0.51085	no
gi|641568275|gb|KK857028.1|	03562-10477	11	40	0.626141	1	1	0.47685	no
gi|641568275|gb|KK857028.1|	06412-10729	11	40	0.309766	4	4	0.14485	no
gi|641568275|gb|KK857028.1|	13582-11417	11	40	2	3	0.597389	0.6863	no
gi|641568275|gb|KK857028.1|	13447-14135	11	40	0.574713	1	1	0.51305	no
gi|641568275|gb|KK857028.1|	1525-3227	11	40	1	2	0.508876	0.709	no
gi|641568275|gb|KK857028.1|	16821-18206	11	40	0.833183	0.544001	-0.615025	1	no
gi|641568275|gb|KK857028.1|	22121-23370	11	40	0.671943	1	0.74423	0.6176	no
gi|641568275|gb|KK857028.1|	25613-26353	11	40	1	0.788865	-0.857921	0.60675	no
gi|641568275|gb|KK857028.1|	27197-28385	11	40	1	2	0.513273	0.7255	no
gi|641568275|gb|KK857028.1|	29205-30642	11	40	0.342125	2	2	0.20395	no
gi|641568275|gb|KK857028.1|	30856-31768	11	40	1	1	-0.173512	0.90465	no
gi|641568275|gb|KK857028.1|	31890-32432	11	40	1	2	1	0.4611	no
gi|641568275|gb|KK857028.1|	3915-4531	11	40	0.840993	4	2	0.18215	no
gi|641568275|gb|KK857028.1|	4611-5158	11	40	2.01	0.303741	-3	0.2481	no
gi|641568275|gb|KK857028.1|	49584-50440	11	40	0.750137	0.651244	-0.203956	1	no
gi|641568275|gb|KK857028.1|	5220-5980	11	40	1	2	0.753892	0.6261	no
gi|641568275|gb|KK857028.1|	59290-59653	11	40	42	17	-1	0.30785	no
gi|641568275|gb|KK857028.1|	6086-7852	11	40	2	3	0.688742	0.6082	no
gi|641568275|gb|KK857028.1|	63426-64743	11	40	70	30	-1	0.45005	no
gi|641568275|gb|KK857028.1|	66224-69212	11	40	108	82	-0.391483	0.8375	no
gi|641568275|gb|KK857028.1|	70976-76010	11	40	13	10	-0.353657	0.87455	no
gi|641568275|gb|KK857028.1|	76362-77562	11	40	0.916128	1	0.227942	0.87445	no
gi|641568275|gb|KK857028.1|	78165-81168	11	40	1	2	0.672966	0.60635	no
gi|641568275|gb|KK857028.1|	791-1407	11	40	1	3	1	0.4402	no
gi|641568275|gb|KK857028.1|	7963-9139	11	40	0.72196	1	0.869344	0.56565	no

gi|641568275|gb|KK857028.1|	9300-10160	11	40	2	3	0.685914	0.6336	no
gi|641568278|gb|KK857027.1|	26692-27268	9.6	38	3	29	3	0.0374	no
gi|641568278|gb|KK857027.1|	28780-29305	9.6	38	32	471	4	0.05185	no
gi|641568278|gb|KK857027.1|	30471-31945	9.6	38	2	15	3	0.02905	no
gi|641568278|gb|KK857027.1|	32138-33850	9.6	38	2	2	-0.312128	0.816	no
gi|641568278|gb|KK857027.1|	34075-35614	9.6	38	2	3	0.842401	0.5337	no
gi|641568278|gb|KK857027.1|	37123-38129	9.6	38	33	54	0.685626	0.6784	no
gi|641568278|gb|KK857027.1|	45644-45934	9.6	38	39	59	0.614582	0.6389	no
gi|641568278|gb|KK857027.1|	50149-50649	9.6	38	2	5	1	0.4319	no
gi|641568278|gb|KK857027.1|	50837-51986	9.6	38	8	14	0.81259	0.5164	no
gi|641568278|gb|KK857027.1|	56972-58793	9.6	38	20	20	-0.022168	0.98455	no
gi|641568278|gb|KK857027.1|	58849-60038	9.6	38	0.427509	1	1	0.41175	no
gi|641568278|gb|KK857027.1|	81934-82980	9.6	38	2	2	0.461001	0.7404	no
gi|641568278|gb|KK857027.1|	84392-84967	9.6	38	2	2	-0.296238	0.8456	no
gi|641568278|gb|KK857027.1|	85118-86066	9.6	38	5	8	0.759211	0.5709	no
gi|641568278|gb|KK857027.1|	92751-94016	9.6	38	1	2	0.818314	0.5517	no
gi|641568281|gb|KK857026.1|	48915-49674	11	42	1	0.381243	-2	0.3455	no
gi|641568281|gb|KK857026.1|	64160-64612	11	42	2	2	-0.258167	0.88485	no
gi|641568281|gb|KK857026.1|	64804-65375	11	42	2	0	-inf	0.01	no
gi|641568284|gb|KK857025.1|	33760-34312	7.9	32	1	2	0.818263	0.6234	no
gi|641568284|gb|KK857025.1|	34788-35678	7.9	32	1	2	0.741338	0.6176	no
gi|641568284|gb|KK857025.1|	66388-66845	7.9	32	1	4	2	0.32945	no
gi|641568289|gb|KK857023.1|	66496-66987	8.1	31	0.477273	3	3	0.2991	no
gi|641568289|gb|KK857023.1|	67973-68765	8.1	31	0.711937	2	1	0.4118	no
gi|641568289|gb|KK857023.1|	71577-72635	8.1	31	0.98485	1	0.479956	0.75585	no
gi|641568291|gb|KK857022.1|	42431-42645	9.4	38	10	14	0.537339	0.7589	no
gi|641568295|gb|KK857021.1|	19653-19813	7.7	27	6	98	4	0.1204	no
gi|641568295|gb|KK857021.1|	24220-25651	7.7	27	0	4	inf	5.00E-05	yes
gi|641568295|gb|KK857021.1|	38124-39182	7.7	27	0.902779	2	1	0.47895	no
gi|641568295|gb|KK857021.1|	39430-40274	7.7	27	0.436579	2	2	0.26155	no

gi|641568295|gb|KK857021.1|	41765-42422	7.7	27	1	2	0.9215	0.56365	no
gi|641568295|gb|KK857021.1|	43662-44181	7.7	27	1	2	1	0.51085	no
gi|641568295|gb|KK857021.1|	44304-44643	7.7	27	6	16	1	0.32515	no
gi|641568295|gb|KK857021.1|	44779-46025	7.7	27	0.808634	2	0.928639	0.53445	no
gi|641568295|gb|KK857021.1|	46656-47413	7.7	27	1	5	2	0.17345	no
gi|641568295|gb|KK857021.1|	49573-50018	7.7	27	1	3	1	0.51315	no
gi|641568295|gb|KK857021.1|	56554-56788	7.7	27	12	4	-2	0.3397	no
gi|641568295|gb|KK857021.1|	58666-59315	7.7	27	28	53	0.930969	0.45675	no
gi|641568295|gb|KK857021.1|	59736-60772	7.7	27	4	7	0.946345	0.4697	no
gi|641568295|gb|KK857021.1|	60934-61226	7.7	27	7	25	2	0.24525	no
gi|641568295|gb|KK857021.1|	61906-65469	7.7	27	2	4	1	0.2931	no
gi|641568298|gb|KK857020.1|	07779-10859	12	46	0.911078	1	0.187802	0.91015	no
gi|641568298|gb|KK857020.1|	09033-11015	12	46	0.687626	0.581422	-0.242038	1	no
gi|641568298|gb|KK857020.1|	67649-69596	12	46	4	5	0.290784	0.81465	no
gi|641568301|gb|KK857019.1|	12031-13412	9.5	36	0.417997	1	1	0.3924	no
gi|641568301|gb|KK857019.1|	14130-14779	9.5	36	59	19	-2	0.2045	no
gi|641568301|gb|KK857019.1|	21136-23409	9.5	36	34	27	-0.328388	0.8842	no
gi|641568301|gb|KK857019.1|	2471-3036	9.5	36	6	4	-0.610016	0.6675	no
gi|641568301|gb|KK857019.1|	27204-27388	9.5	36	118	52	-1	0.40105	no
gi|641568301|gb|KK857019.1|	28878-29751	9.5	36	1	1	0.188585	0.90355	no
gi|641568301|gb|KK857019.1|	29918-30838	9.5	36	1	0.445542	-2	0.33985	no
gi|641568301|gb|KK857019.1|	32696-33540	9.5	36	1	1	0.0727189	0.95375	no
gi|641568301|gb|KK857019.1|	45379-46508	9.5	36	5	7	0.539918	0.6705	no
gi|641568301|gb|KK857019.1|	46631-47308	9.5	36	1	0.891021	-0.3999	0.82205	no
gi|641568301|gb|KK857019.1|	47794-48731	9.5	36	5	3	-1	0.43915	no
gi|641568301|gb|KK857019.1|	51035-51266	9.5	36	78	34	-1	0.36965	no
gi|641568301|gb|KK857019.1|	60382-61223	9.5	36	0.219304	6	5	0.2352	no
gi|641568301|gb|KK857019.1|	64987-66391	9.5	36	0.117155	0.892464	3	0.27505	no
gi|641568301|gb|KK857019.1|	663-1939	9.5	36	1	1	0.104366	0.94435	no
gi|641568301|gb|KK857019.1|	71182-72005	9.5	36	2	5	1	0.34365	no

gi|641568301|gb|KK857019.1|	72310-73840	9.5	36	1	0.970166	-0.330693	0.81565	no
gi|641568301|gb|KK857019.1|	8368-9305	9.5	36	1	0.725644	-0.774139	0.63565	no
gi|641568301|gb|KK857019.1|	94154-94695	9.5	36	10	8	-0.433207	0.7458	no
gi|641568304|gb|KK857018.1|	17843-18028	40	3.70E+01	74	13	-3	0.1792	no
gi|641568304|gb|KK857018.1|	69465-70498	40	3.70E+01	1	0	-inf	0.0056	no
gi|641568319|gb|KK857014.1|	14951-15195	9.5	33	23	16	-0.520065	0.7152	no
gi|641568319|gb|KK857014.1|	15347-15564	9.5	33	49	27	-0.830455	0.55165	no
gi|641568319|gb|KK857014.1|	35920-36596	9.5	33	0	71	inf	5.00E-05	yes
gi|641568319|gb|KK857014.1|	48375-51521	9.5	33	0.023995	99	12	0.1044	no
gi|641568319|gb|KK857014.1|	53357-54210	9.5	33	6	7	0.122732	0.92565	no
gi|641568319|gb|KK857014.1|	54348-55414	9.5	33	3	3	-0.16704	0.8973	no
gi|641568319|gb|KK857014.1|	64024-64669	9.5	33	1	2	1	0.4891	no
gi|641568319|gb|KK857014.1|	66059-67941	9.5	33	7	6	-0.432511	0.7293	no
gi|641568319|gb|KK857014.1|	68423-69772	9.5	33	6	6	0.0222496	0.9867	no
gi|641568319|gb|KK857014.1|	76677-77140	9.5	33	9	13	0.461406	0.7303	no
gi|641568319|gb|KK857014.1|	83-571	9.5	33	4	8	1	0.42755	no
gi|641568321|gb|KK857013.1|	54487-54847	8.1	29	99	309	2	0.33755	no
gi|641568321|gb|KK857013.1|	55689-56731	8.1	29	2	0.127218	-4	0.1991	no
gi|641568326|gb|KK857012.1|	43785-43969	8.6	36	33	48	0.521737	0.7344	no
gi|641568328|gb|KK857011.1|	111-524	9.7	32	4	6	0.586219	0.68145	no
gi|641568328|gb|KK857011.1|	11607-11796	9.7	32	46	0	-inf	0.00435	no
gi|641568328|gb|KK857011.1|	12106-12595	9.7	32	6	0.362244	-4	0.1915	no
gi|641568328|gb|KK857011.1|	27042-27480	9.7	32	7	1	-2	0.1911	no
gi|641568328|gb|KK857011.1|	28729-29981	9.7	32	0.402021	0.714239	0.829135	1	no
gi|641568328|gb|KK857011.1|	3226-3856	9.7	32	2	3	0.598784	0.67485	no
gi|641568328|gb|KK857011.1|	32389-33439	9.7	32	0.911229	0.504133	-0.854009	0.60675	no
gi|641568328|gb|KK857011.1|	35790-36045	9.7	32	10	1	-3	0.2375	no
gi|641568328|gb|KK857011.1|	41090-41852	9.7	32	0.999485	2	0.601925	0.6936	no
gi|641568328|gb|KK857011.1|	46827-47435	9.7	32	0.342892	2	3	0.2991	no
gi|641568328|gb|KK857011.1|	51703-53539	9.7	32	0.604157	0.789677	0.38634	1	no

gi|641568328|gb|KK857011.1|	6478-7666	9.7	32	43	2	-5	0.0382	no
gi|641568328|gb|KK857011.1|	93821-94246	9.7	32	2	2	0.324399	0.83525	no
gi|641568328|gb|KK857011.1|	96817-97377	9.7	32	113	165	0.543481	0.8104	no
gi|641568328|gb|KK857011.1|	9909-10341	9.7	32	13	1	-3	0.1142	no
gi|641568330|gb|KK857010.1|	10333-10811	9.1	35	55	68	0.308831	0.7963	no
gi|641568330|gb|KK857010.1|	11701-14102	9.1	35	21	29	0.453784	0.84165	no
gi|641568330|gb|KK857010.1|	15049-15977	9.1	35	111	140	0.3357	0.87875	no
gi|641568330|gb|KK857010.1|	17279-17681	9.1	35	209	246	0.233054	0.91455	no
gi|641568330|gb|KK857010.1|	21516-21961	9.1	35	3	2	-0.410751	0.8125	no
gi|641568330|gb|KK857010.1|	22886-24599	9.1	35	0.97968	0.497877	-0.976522	0.5308	no
gi|641568330|gb|KK857010.1|	25582-27253	9.1	35	2	4	0.492852	0.71195	no
gi|641568330|gb|KK857010.1|	3223-4418	9.1	35	6	10	0.812875	0.5282	no
gi|641568330|gb|KK857010.1|	73644-73952	9.1	35	5	2	-2	0.3632	no
gi|641568330|gb|KK857010.1|	86380-87451	9.1	35	0.32341	0.73815	1	1	no
gi|641568330|gb|KK857010.1|	88529-89024	9.1	35	29	12	-1	0.33615	no
gi|641568337|gb|KK857009.1|	32711-33176	10	34	3	2	-0.409162	0.80855	no
gi|641568337|gb|KK857009.1|	39558-40127	10	34	0.567735	2	2	0.3323	no
gi|641568337|gb|KK857009.1|	53605-54081	10	34	0	5	inf	0.00375	no
gi|641568337|gb|KK857009.1|	59357-59813	10	34	0.810267	3	2	0.2812	no
gi|641568337|gb|KK857009.1|	60272-60646	10	34	3	5	0.774072	0.6361	no
gi|641568337|gb|KK857009.1|	60823-63157	10	34	0.264845	1	2	0.2039	no
gi|641568337|gb|KK857009.1|	63348-63642	10	34	21	22	0.0894285	0.9481	no
gi|641568337|gb|KK857009.1|	63746-64268	10	34	2	3	0.456908	0.76285	no
gi|641568337|gb|KK857009.1|	64368-67582	10	34	4	5	0.390811	0.75175	no
gi|641568337|gb|KK857009.1|	67682-68120	10	34	4	6	0.588641	0.68145	no
gi|641568337|gb|KK857009.1|	70094-70363	10	34	18	24	0.421364	0.76145	no
gi|641568337|gb|KK857009.1|	70618-71532	10	34	1	1	0.0736032	0.95375	no
gi|641568337|gb|KK857009.1|	72645-73433	10	34	1	0.544084	-0.982586	0.62775	no
gi|641568337|gb|KK857009.1|	76399-76776	10	34	37	41	0.146634	0.9063	no
gi|641568337|gb|KK857009.1|	77731-78248	10	34	2	3	0.916071	0.56635	no

gi|641568339|gb|KK857008.1|	.01199-10140	11	38	71	62	-0.190487	0.88545	no
gi|641568339|gb|KK857008.1|	.02803-10346	11	38	7	6	-0.241621	0.8517	no
gi|641568339|gb|KK857008.1|	.03728-10466	11	38	1	1	-0.288756	0.85105	no
gi|641568339|gb|KK857008.1|	.06176-10663	11	38	3	4	0.300643	0.84335	no
gi|641568339|gb|KK857008.1|	.06766-10779	11	38	2	5	1	0.41475	no
gi|641568339|gb|KK857008.1|	11551-12809	11	38	1	3	1	0.4131	no
gi|641568339|gb|KK857008.1|	14307-15820	11	38	0.859714	3	2	0.1855	no
gi|641568339|gb|KK857008.1|	26489-27704	11	38	4	4	0.191502	0.88535	no
gi|641568339|gb|KK857008.1|	35559-35891	11	38	25	12	-1	0.42445	no
gi|641568339|gb|KK857008.1|	36497-41760	11	38	45	40	-0.192959	0.9205	no
gi|641568339|gb|KK857008.1|	57792-58925	11	38	1	5	2	0.1534	no
gi|641568339|gb|KK857008.1|	5903-9599	11	38	4	3	-0.259045	0.83675	no
gi|641568339|gb|KK857008.1|	69927-72135	11	38	3	6	1	0.37935	no
gi|641568339|gb|KK857008.1|	73343-73760	11	38	3	5	0.586635	0.6863	no
gi|641568339|gb|KK857008.1|	78097-78456	11	38	27	8	-2	0.19775	no
gi|641568339|gb|KK857008.1|	ı9992-10016ï	11	38	53	12	-2	0.258	no
gi|641568341|gb|KK857007.1|	1-637	8.6	37	8	12	0.628113	0.62325	no
gi|641568341|gb|KK857007.1|	14335-14500	8.6	37	32	75	1	0.4455	no
gi|641568341|gb|KK857007.1|	16621-16828	8.6	37	39	51	0.389489	0.7796	no
gi|641568341|gb|KK857007.1|	23281-23727	8.6	37	6	3	-1	0.4809	no
gi|641568341|gb|KK857007.1|	24803-25000	8.6	37	20	20	-0.0562661	0.93935	no
gi|641568341|gb|KK857007.1|	2699-3088	8.6	37	2	2	-0.00152032	0.91115	no
gi|641568341|gb|KK857007.1|	5314-6627	8.6	37	2	0.964859	-0.977915	0.49975	no
gi|641568341|gb|KK857007.1|	59671-60196	8.6	37	2	2	0.109141	0.92265	no
gi|641568344|gb|KK857006.1|	62804-63297	8.9	36	0.711165	3	2	0.3323	no
gi|641568353|gb|KK857004.1|	21934-22957	10	38	0.769871	0.260248	-2	1	no
gi|641568353|gb|KK857004.1|	24680-25912	10	38	2	0.20799	-3	0.1718	no
gi|641568353|gb|KK857004.1|	32643-33923	10	38	1	0.397641	-2	0.362	no
gi|641568353|gb|KK857004.1|	3340-3815	10	38	3	1	-2	0.40265	no
gi|641568353|gb|KK857004.1|	35320-37002	10	38	0.809611	2	0.980328	0.4944	no

gi|641568353|gb|KK857004.1|	65819-66465	10	38	20	28	0.49322	0.7003	no
gi|641568353|gb|KK857004.1|	67350-68368	10	38	4	9	1	0.40205	no
gi|641568353|gb|KK857004.1|	68709-70252	10	38	0.630432	0.720627	0.192912	1	no
gi|641568353|gb|KK857004.1|	70521-71633	10	38	1	0.822831	-0.39411	0.7966	no
gi|641568353|gb|KK857004.1|	71767-72953	10	38	0.428804	0.652821	0.60637	1	no
gi|641568353|gb|KK857004.1|	73168-75192	10	38	4	9	1	0.40225	no
gi|641568353|gb|KK857004.1|	76574-77926	10	38	0	3	inf	0.0003	yes
gi|641568353|gb|KK857004.1|	8113-9901	10	38	21	13	-0.708668	0.56915	no
gi|641568353|gb|KK857004.1|	83204-83816	10	38	0	78	inf	5.00E-05	yes
gi|641568353|gb|KK857004.1|	86056-86260	10	38	0	459.16	inf	5.00E-05	yes
gi|641568353|gb|KK857004.1|	88268-88831	10	38	0	69	inf	5.00E-05	yes
gi|641568359|gb|KK857002.1|	04173-10560	9	44	7	12	0.791002	0.5286	no
gi|641568367|gb|KK857001.1|	58687-59434	7.2	29	0	3	inf	0.0015	yes
gi|641568370|gb|KK857000.1|	23304-23636	12	36	39	12	-2	0.22075	no
gi|641568370|gb|KK857000.1|	67221-68202	12	36	2	4	1	0.3434	no
gi|641568380|gb|KK856996.1|	82930-83576	8.3	34	102	113	0.148971	0.94625	no
gi|641568383|gb|KK856995.1|	45130-46024	9.3	35	0.304003	2	2	0.2136	no
gi|641568386|gb|KK856994.1|	12771-14857	8.9	32	29	44	0.592269	0.7908	no
gi|641568386|gb|KK856994.1|	15186-15573	8.9	32	10	18	0.820227	0.54175	no
gi|641568386|gb|KK856994.1|	19894-20771	8.9	32	1	0.157826	-3	0.24815	no
gi|641568386|gb|KK856994.1|	22619-23364	8.9	32	1	1	0.239038	0.88205	no
gi|641568386|gb|KK856994.1|	23497-25220	8.9	32	20	17	-0.191236	0.87595	no
gi|641568386|gb|KK856994.1|	2401-3417	8.9	32	5	6	0.090564	0.94505	no
gi|641568386|gb|KK856994.1|	6102-7422	8.9	32	0.629525	2	1	0.36435	no
gi|641568386|gb|KK856994.1|	73912-74978	8.9	32	0.081317	2	4	0.11365	no
gi|641568386|gb|KK856994.1|	9907-11245	8.9	32	0.743531	0.943826	0.344128	0.8163	no
gi|641568389|gb|KK856993.1|	02377-10270	10	41	39	12	-2	0.211	no
gi|641568389|gb|KK856993.1|	04290-10492	10	41	3	0.977323	-1	0.4012	no
gi|641568389|gb|KK856993.1|	05183-10624	10	41	0.735228	1	1	0.49595	no
gi|641568389|gb|KK856993.1|	06487-10741	10	41	6	4	-0.588327	0.6535	no

gi|641568389|gb|KK856993.1|	[illegible]08459-10884	10	41	32	17	-0.936977	0.47725	no
gi|641568389|gb|KK856993.1|	23301-23682	10	41	5	4	-0.310688	0.84315	no
gi|641568389|gb|KK856993.1|	32644-32925	10	41	23	8	-2	0.2765	no
gi|641568389|gb|KK856993.1|	33073-34668	10	41	1	0.61683	-0.85132	0.5652	no
gi|641568389|gb|KK856993.1|	34813-36378	10	41	2	0.866662	-0.932549	0.5136	no
gi|641568389|gb|KK856993.1|	36734-40025	10	41	8	0.872952	-3	0.02625	no
gi|641568389|gb|KK856993.1|	50114-50818	10	41	2	1	-0.536755	0.7478	no
gi|641568389|gb|KK856993.1|	51398-52143	10	41	2	0.781749	-1	0.46585	no
gi|641568389|gb|KK856993.1|	52256-53492	10	41	0.884438	0.207193	-2	0.26805	no
gi|641568389|gb|KK856993.1|	53955-55342	10	41	0.950612	0.543091	-0.807662	0.5996	no
gi|641568389|gb|KK856993.1|	55445-57057	10	41	1	0.685631	-0.865769	0.54825	no
gi|641568389|gb|KK856993.1|	57203-62019	10	41	4	3	-0.622433	0.61925	no
gi|641568389|gb|KK856993.1|	64796-65755	10	41	0.927097	0.422911	-1	0.50885	no
gi|641568389|gb|KK856993.1|	67024-69769	10	41	0.971921	0.254301	-2	0.2214	no
gi|641568389|gb|KK856993.1|	71522-72102	10	41	2	0.556231	-2	0.3455	no
gi|641568389|gb|KK856993.1|	73007-74273	10	41	1	0.604231	-1	0.46775	no
gi|641568389|gb|KK856993.1|	79438-79962	10	41	0	16	inf	0.0001	yes
gi|641568389|gb|KK856993.1|	8039-12164	10	41	12	7	-0.861443	0.5903	no
gi|641568389|gb|KK856993.1|	80889-81319	10	41	2	4	1	0.5082	no
gi|641568389|gb|KK856993.1|	94846-95242	10	41	65	32	-1	0.4222	no
gi|641568389|gb|KK856993.1|	95560-97314	10	41	3	16	2	0.0884	no
gi|641568394|gb|KK856991.1|	13479-13697	10	41	8	20	1	0.41455	no
gi|641568396|gb|KK856990.1|	13592-14648	12	44	0.904877	2	0.953394	0.53865	no
gi|641568396|gb|KK856990.1|	15132-15981	12	44	0.75817	1	0.795948	0.63025	no
gi|641568396|gb|KK856990.1|	16095-16894	12	44	0.938242	1	0.602557	0.6936	no
gi|641568396|gb|KK856990.1|	16946-17528	12	44	0.548754	3	3	0.19335	no
gi|641568396|gb|KK856990.1|	22200-23561	12	44	0.971814	4	2	0.19965	no
gi|641568396|gb|KK856990.1|	29826-31026	12	44	0.4933	1	1	0.43995	no
gi|641568396|gb|KK856990.1|	40448-41136	12	44	1	33	5	0.026	no
gi|641568396|gb|KK856990.1|	70057-71305	12	44	6	3	-0.995943	0.45745	no

gi|641568396|gb|KK856990.1|	72753-75507	12	44	1095.74	513	-1	0.3552	no
gi|641568399|gb|KK856989.1|	1304-3115	12	40	0.350574	0.334111	-0.0693957	1	no
gi|641568399|gb|KK856989.1|	13756-14455	12	40	0	4	inf	0.00145	yes
gi|641568399|gb|KK856989.1|	15982-16529	12	40	0.402	6	4	0.24015	no
gi|641568399|gb|KK856989.1|	18651-20916	12	40	4	1	-2	0.1853	no
gi|641568399|gb|KK856989.1|	21696-22968	12	40	3	1	-1	0.305	no
gi|641568399|gb|KK856989.1|	24-1126	12	40	1	2	0.306258	0.83475	no
gi|641568399|gb|KK856989.1|	29739-30547	12	40	0.924461	2	0.772625	0.62605	no
gi|641568399|gb|KK856989.1|	36469-39586	12	40	0.872407	0.332952	-1	1	no
gi|641568399|gb|KK856989.1|	40188-42546	12	40	1	1	0.265647	0.8414	no
gi|641568399|gb|KK856989.1|	43888-47569	12	40	14	3	-2	0.2613	no
gi|641568399|gb|KK856989.1|	47751-51077	12	40	26	1	-4	0.0429	no
gi|641568399|gb|KK856989.1|	51255-56804	12	40	71	0.707076	-7	0.00385	no
gi|641568405|gb|KK856987.1|	70503-71267	13	38	0.622483	2	1	0.4611	no
gi|641568408|gb|KK856986.1|	10571-11240	8	32	7	4	-0.78477	0.5708	no
gi|641568408|gb|KK856986.1|	12102-13529	8	32	2	3	0.648178	0.6288	no
gi|641568408|gb|KK856986.1|	19873-21381	8	32	5	4	-0.466936	0.71305	no
gi|641568408|gb|KK856986.1|	4086-4382	8	32	0	14	inf	0.0023	yes
gi|641568411|gb|KK856985.1|	43781-44453	9	34	0	35	inf	5.00E-05	yes
gi|641568411|gb|KK856985.1|	45265-45522	9	34	0	16	inf	0.00375	no
gi|641568411|gb|KK856985.1|	68613-69306	9	34	1	1	-0.247504	0.88495	no
gi|641568414|gb|KK856984.1|	10499-10831	10	39	4	0.739468	-3	0.2588	no
gi|641568414|gb|KK856984.1|	12427-12894	10	39	15	9	-0.756938	0.56505	no
gi|641568414|gb|KK856984.1|	15298-15516	10	39	33	9	-2	0.26795	no
gi|641568414|gb|KK856984.1|	17946-18279	10	39	6	3	-1	0.5083	no
gi|641568414|gb|KK856984.1|	19852-20295	10	39	5	2	-1	0.43485	no
gi|641568414|gb|KK856984.1|	2080-2458	10	39	42	30	-0.511989	0.6891	no
gi|641568414|gb|KK856984.1|	26507-27635	10	39	0.910937	0.462159	-0.978962	0.5872	no
gi|641568414|gb|KK856984.1|	30865-31594	10	39	32	14	-1	0.37655	no
gi|641568414|gb|KK856984.1|	31768-32793	10	39	50	33	-0.574036	0.7214	no

gi|641568414|gb|KK856984.1|	34717-35782	10	39	0.814103	0.867089	0.0909688	1	no
gi|641568414|gb|KK856984.1|	47279-48174	10	39	1	0.461368	-1	0.50885	no
gi|641568414|gb|KK856984.1|	50626-53782	10	39	0.980487	0.36508	-1	0.32125	no
gi|641568414|gb|KK856984.1|	54149-55529	10	39	2	1	-0.186203	0.89465	no
gi|641568414|gb|KK856984.1|	56099-60567	10	39	1	3	0.840435	0.50685	no
gi|641568414|gb|KK856984.1|	60757-62096	10	39	1	3	1	0.437	no
gi|641568414|gb|KK856984.1|	68177-68871	10	39	27	8	-2	0.18415	no
gi|641568414|gb|KK856984.1|	69186-72581	10	39	12	3	-2	0.23225	no
gi|641568414|gb|KK856984.1|	72898-74262	10	39	8	5	-0.742665	0.55825	no
gi|641568414|gb|KK856984.1|	75759-76023	10	39	7	6	-0.120779	0.9229	no
gi|641568414|gb|KK856984.1|	76578-76818	10	39	16	15	-0.0886643	0.9494	no
gi|641568414|gb|KK856984.1|	81282-81645	10	39	5	2	-1	0.4958	no
gi|641568414|gb|KK856984.1|	87807-88907	10	39	11	7	-0.696086	0.5826	no
gi|641568417|gb|KK856983.1|	33180-33521	8.1	32	0	7	inf	0.0068	no
gi|641568417|gb|KK856983.1|	39117-39290	8.1	32	4	64	4	0.1204	no
gi|641568417|gb|KK856983.1|	41111-41714	8.1	32	6	7	0.241751	0.85995	no
gi|641568417|gb|KK856983.1|	41776-42539	8.1	32	0.997726	3	2	0.2743	no
gi|641568417|gb|KK856983.1|	51686-53156	8.1	32	5	7	0.59042	0.645	no
gi|641568417|gb|KK856983.1|	53342-55752	8.1	32	3	3	0.229394	0.8539	no
gi|641568417|gb|KK856983.1|	55900-57626	8.1	32	3	4	0.281308	0.82845	no
gi|641568417|gb|KK856983.1|	58538-58888	8.1	32	25.31	24	-0.0852765	0.94895	no
gi|641568417|gb|KK856983.1|	59897-60759	8.1	32	4	8	1	0.4096	no
gi|641568417|gb|KK856983.1|	60894-61361	8.1	32	4	4	-0.0870862	0.95155	no
gi|641568417|gb|KK856983.1|	61722-62110	8.1	32	15	0	-inf	0.00045	yes
gi|641568417|gb|KK856983.1|	64061-64830	8.1	32	10	0.374647	-5	0.1315	no
gi|641568417|gb|KK856983.1|	65271-66450	8.1	32	15	0	-inf	5.00E-05	yes
gi|641568417|gb|KK856983.1|	72072-73298	8.1	32	7	0.522989	-4	0.0513	no
gi|641568423|gb|KK856981.1|	10642-12897	11	37	1	0.052446	-4	0.1879	no
gi|641568423|gb|KK856981.1|	14514-15551	11	37	0.841184	0.12797	-3	1	no
gi|641568423|gb|KK856981.1|	16219-17537	11	37	3	0.960578	-2	0.23985	no

gi|641568423|gb|KK856981.1|	20363-20808	11	37	3	0	-inf	0.0102	no
gi|641568423|gb|KK856981.1|	25978-26357	11	37	11	0.566529	-4	0.19005	no
gi|641568423|gb|KK856981.1|	44179-45202	11	37	1	0.520497	-0.979765	0.5872	no
gi|641568423|gb|KK856981.1|	50271-50495	11	37	172	39	-2	0.12565	no
gi|641568423|gb|KK856981.1|	54993-56193	11	37	7	8	0.226422	0.86	no
gi|641568423|gb|KK856981.1|	56263-60930	11	37	7	4	-0.572134	0.64205	no
gi|641568423|gb|KK856981.1|	5898-6449	11	37	2	0.600776	-2	0.31075	no
gi|641568423|gb|KK856981.1|	61531-61813	11	37	9	3	-1	0.4189	no
gi|641568423|gb|KK856981.1|	62416-62814	11	37	13	32	1	0.344	no
gi|641568423|gb|KK856981.1|	66310-66903	11	37	6	17	2	0.2336	no
gi|641568423|gb|KK856981.1|	68619-68901	11	37	11	15	0.463138	0.74985	no
gi|641568423|gb|KK856981.1|	73192-73697	11	37	3	7	0.934506	0.5213	no
gi|641568423|gb|KK856981.1|	83257-83689	11	37	4	4	-0.318793	0.8441	no
gi|641568423|gb|KK856981.1|	84736-85333	11	37	4	1	-2	0.30235	no
gi|641568423|gb|KK856981.1|	8654-9778	11	37	2	0	-inf	0.00315	yes
gi|641568423|gb|KK856981.1|	88307-88567	11	37	14	1	-3	0.2088	no
gi|641568430|gb|KK856979.1|	11200-11793	9.7	41	0.355767	2	3	0.2851	no
gi|641568430|gb|KK856979.1|	11956-12686	9.7	41	0.39722	2	2	0.2812	no
gi|641568433|gb|KK856978.1|	10122-10352	9.8	35	19	32	0.725219	0.6159	no
gi|641568433|gb|KK856978.1|	13214-13417	9.8	35	30	20	-0.567708	0.7195	no
gi|641568433|gb|KK856978.1|	15694-15908	9.8	35	60	120	1	0.43505	no
gi|641568433|gb|KK856978.1|	17122-17423	9.8	35	6	12	1	0.4884	no
gi|641568433|gb|KK856978.1|	24944-25339	9.8	35	2	5	1	0.48155	no
gi|641568433|gb|KK856978.1|	3247-3595	9.8	35	8	9	0.297238	0.8387	no
gi|641568433|gb|KK856978.1|	47019-49868	9.8	35	106.19	7	-4	0.0438	no
gi|641568433|gb|KK856978.1|	50732-51575	9.8	35	83	5	-4	0.0626	no
gi|641568433|gb|KK856978.1|	5412-6305	9.8	35	2	2	-0.460259	0.74915	no
gi|641568433|gb|KK856978.1|	55163-55510	9.8	35	170	11	-4	0.01305	no
gi|641568433|gb|KK856978.1|	57583-57884	9.8	35	55	3	-4	0.0967	no
gi|641568433|gb|KK856978.1|	58244-59643	9.8	35	8	0.537695	-4	0.0389	no

gi|641568433|gb|KK856978.1|	60195-60713	9.8	35	41	4	-3	0.028	no
gi|641568433|gb|KK856978.1|	62139-62428	9.8	35	16	0	-inf	0.0021	yes
gi|641568433|gb|KK856978.1|	64047-64724	9.8	35	40	1	-5	0.04265	no
gi|641568433|gb|KK856978.1|	65810-66146	9.8	35	10	0.721006	-4	0.19895	no
gi|641568433|gb|KK856978.1|	68057-68626	9.8	35	43	2	-4	0.0259	no
gi|641568433|gb|KK856978.1|	71290-71447	9.8	35	580	40	-4	0.06775	no
gi|641568433|gb|KK856978.1|	73458-73607	9.8	35	893	14	-6	0.1829	no
gi|641568433|gb|KK856978.1|	7501-8664	9.8	35	5	3	-0.439575	0.7361	no
gi|641568433|gb|KK856978.1|	77726-78299	9.8	35	22	2	-4	0.04275	no
gi|641568433|gb|KK856978.1|	78477-79297	9.8	35	22	2	-4	0.02815	no
gi|641568433|gb|KK856978.1|	81908-83461	9.8	35	3	0.079482	-5	0.18455	no
gi|641568433|gb|KK856978.1|	84033-84827	9.8	35	79	4	-4	0.05405	no
gi|641568436|gb|KK856977.1|	35981-36480	10	43	3	0.350624	-3	0.2242	no
gi|641568436|gb|KK856977.1|	50771-51003	10	43	9	9	0.0600652	0.9244	no
gi|641568439|gb|KK856976.1|	16281-18101	10	41	2	0	-inf	0.00015	yes
gi|641568439|gb|KK856976.1|	19404-20011	10	41	3	0	-inf	0.00495	no
gi|641568439|gb|KK856976.1|	2844-3963	10	41	2	0	-inf	0.0021	yes
gi|641568439|gb|KK856976.1|	47877-48664	10	41	2	0.181666	-3	0.22425	no
gi|641568439|gb|KK856976.1|	7239-8029	10	41	1	0	-inf	0.0102	no
gi|641568439|gb|KK856976.1|	87308-87867	10	41	1	5	2	0.2254	no
gi|641568442|gb|KK856975.1|	13401-17672	9.5	36	2	2	0.372374	0.76755	no
gi|641568442|gb|KK856975.1|	3075-3485	9.5	36	5	5	0.100471	0.94445	no
gi|641568442|gb|KK856975.1|	50617-51198	9.5	36	1	2	0.182046	0.91015	no
gi|641568442|gb|KK856975.1|	5557-6634	9.5	36	2	1	-0.0573308	0.963	no
gi|641568442|gb|KK856975.1|	64686-65061	9.5	36	4	1	-2	0.31725	no
gi|641568442|gb|KK856975.1|	76242-76744	9.5	36	5	2	-1	0.3811	no
gi|641568445|gb|KK856974.1|	62552-63320	11	43	0	50	inf	5.00E-05	yes
gi|641568445|gb|KK856974.1|	64564-65034	11	43	0	7	inf	0.00135	yes
gi|641568445|gb|KK856974.1|	65906-66127	11	43	0	54	inf	0.00045	yes
gi|641568445|gb|KK856974.1|	67344-67731	11	43	0	39	inf	5.00E-05	yes

gi|641568445|gb|KK856974.1|	69637-69861	11	43	38	51	0.430988	0.7561	no
gi|641568445|gb|KK856974.1|	71165-74651	11	43	5	0.65703	-3	0.03505	no
gi|641568451|gb|KK856972.1|	29977-30383	9.7	42	3	0.996614	-2	0.3455	no
gi|641568451|gb|KK856972.1|	48244-48586	9.7	42	0	8	inf	0.00375	no
gi|641568451|gb|KK856972.1|	53898-54184	9.7	42	28	31	0.148728	0.91225	no
gi|641568454|gb|KK856971.1|	30160-30582	11	48	3	3	0.224569	0.88235	no
gi|641568457|gb|KK856970.1|	34250-34502	10	41	18	17	-0.0317003	0.9647	no
gi|641568457|gb|KK856970.1|	38835-39453	10	41	91	41	-1	0.4892	no
gi|641568457|gb|KK856970.1|	39973-41890	10	41	219	139	-0.651965	0.72145	no
gi|641568457|gb|KK856970.1|	41943-42214	10	41	11	6	-0.925948	0.5719	no
gi|641568457|gb|KK856970.1|	42929-43553	10	41	11	7	-0.587818	0.6513	no
gi|641568457|gb|KK856970.1|	54095-55825	10	41	0.184539	76	9	0.1176	no
gi|641568457|gb|KK856970.1|	59775-60092	10	41	0	67	inf	5.00E-05	yes
gi|641568457|gb|KK856970.1|	60364-61061	10	41	0	7	inf	0.0003	yes
gi|641568457|gb|KK856970.1|	61193-61763	10	41	0	4	inf	0.00255	yes
gi|641568457|gb|KK856970.1|	70343-76378	10	41	4	5	0.347305	0.7778	no
gi|641568457|gb|KK856970.1|	76827-77499	10	41	1	0.900269	-0.569953	0.7586	no
gi|641568457|gb|KK856970.1|	77600-80858	10	41	6	2	-2	0.1966	no
gi|641568460|gb|KK856969.1|	13260-13781	11	41	5	3	-0.820679	0.5716	no
gi|641568460|gb|KK856969.1|	14853-15520	11	41	0.900376	1	0.599841	0.70555	no
gi|641568460|gb|KK856969.1|	16021-20053	11	41	14	11	-0.355609	0.87915	no
gi|641568460|gb|KK856969.1|	20484-21553	11	41	3	1	-1	0.4644	no
gi|641568460|gb|KK856969.1|	269-648	11	41	17	46	1	0.269	no
gi|641568460|gb|KK856969.1|	3610-4297	11	41	0.57588	7	4	0.12715	no
gi|641568460|gb|KK856969.1|	37794-38944	11	41	0.519135	2	2	0.19685	no
gi|641568464|gb|KK856968.1|	19553-20094	10	39	3	2	-0.520115	0.74125	no
gi|641568464|gb|KK856968.1|	20319-21220	10	39	1	0	-inf	0.0102	no
gi|641568464|gb|KK856968.1|	21534-22384	10	39	3	1	-1	0.38965	no
gi|641568464|gb|KK856968.1|	23085-23364	10	39	5	3	-0.66056	0.70815	no
gi|641568464|gb|KK856968.1|	24868-25794	10	39	0.775173	1	0.774178	0.62605	no

gi|641568464|gb|KK856968.1|	28360-31901	10	39	4	3	-0.557469	0.6632	no
gi|641568464|gb|KK856968.1|	32008-36540	10	39	13	9	-0.586237	0.79985	no
gi|641568464|gb|KK856968.1|	53861-54854	10	39	13	55	2	0.2526	no
gi|641568464|gb|KK856968.1|	72952-73853	10	39	0	5	inf	0.00025	yes
gi|641568464|gb|KK856968.1|	73935-75447	10	39	0.107546	8	6	0.2324	no
gi|641568464|gb|KK856968.1|	9288-9480	10	39	18	47	1	0.3819	no
gi|641568467|gb|KK856967.1|	22603-23843	9.2	35	16	30	0.953268	0.44685	no
gi|641568467|gb|KK856967.1|	40838-41765	9.2	35	1	0.588405	-0.855168	0.60675	no
gi|641568467|gb|KK856967.1|	43181-46419	9.2	35	1	2	0.519245	0.68955	no
gi|641568467|gb|KK856967.1|	46576-47087	9.2	35	1	5	2	0.2254	no
gi|641568467|gb|KK856967.1|	47483-47930	9.2	35	3	7	1	0.4604	no
gi|641568480|gb|KK856965.1|	0-315	9.3	19	6	14	1	0.3718	no
gi|641568489|gb|KK856962.1|	85311-86433	14	55	3	3	-0.176449	0.89405	no
gi|641568494|gb|KK856961.1|	12724-13623	13	37	1	0	-inf	0.0102	no
gi|641568494|gb|KK856961.1|	17797-19903	13	37	1	1	0.194204	0.8851	no
gi|641568494|gb|KK856961.1|	21655-23214	13	37	0.830714	0.316532	-1	1	no
gi|641568494|gb|KK856961.1|	25615-26934	13	37	0.693094	0.671808	-0.0450012	1	no
gi|641568494|gb|KK856961.1|	44887-46064	13	37	7	3	-1	0.43855	no
gi|641568494|gb|KK856961.1|	47403-49228	13	37	14	8	-0.858685	0.4915	no
gi|641568494|gb|KK856961.1|	49668-51768	13	37	2	0.45364	-2	0.22635	no
gi|641568494|gb|KK856961.1|	53713-54398	13	37	1	0.657459	-0.814738	0.6492	no
gi|641568494|gb|KK856961.1|	54819-56062	13	37	2	2	-0.530809	0.6987	no
gi|641568494|gb|KK856961.1|	58183-58706	13	37	3	0.651043	-2	0.2679	no
gi|641568494|gb|KK856961.1|	58822-59225	13	37	3	2	-0.99984	0.62775	no
gi|641568494|gb|KK856961.1|	60765-64453	13	37	16	12	-0.461184	0.8366	no
gi|641568494|gb|KK856961.1|	65485-66825	13	37	16	17	0.113926	0.92465	no
gi|641568494|gb|KK856961.1|	73168-73402	13	37	22	0	-inf	0.0034	yes
gi|641568498|gb|KK856960.1|	41183-42372	11	22	0.213754	0.976287	2	0.25595	no
gi|641568498|gb|KK856960.1|	52571-52888	11	22	1	7	3	0.28555	no
gi|641568498|gb|KK856960.1|	53171-53818	11	22	2	3	0.358282	0.8122	no

gi|641568498|gb|KK856960.1|	56899-57562	11	22	0.605373	2	1	0.44255	no
gi|641568498|gb|KK856960.1|	60121-61096	11	22	1	2	0.363751	0.8038	no
gi|641568498|gb|KK856960.1|	67436-67737	11	22	6	15	1	0.37375	no
gi|641568498|gb|KK856960.1|	68988-69183	11	22	14	31	1	0.49355	no
gi|641568498|gb|KK856960.1|	70062-70329	11	22	8	14	0.847752	0.5956	no
gi|641568498|gb|KK856960.1|	71421-71687	11	22	7	11	0.727855	0.65545	no
gi|641568498|gb|KK856960.1|	76124-76670	11	22	1	7	3	0.1385	no
gi|641568501|gb|KK856959.1|	22039-22299	8.8	39	0	56	inf	0.0001	yes
gi|641568501|gb|KK856959.1|	27519-27722	8.8	39	0	61	inf	0.00105	yes
gi|641568501|gb|KK856959.1|	31670-31904	8.8	39	0	68	inf	0.00015	yes
gi|641568501|gb|KK856959.1|	55586-55999	8.8	39	0	12	inf	0.00075	yes
gi|641568505|gb|KK856958.1|	:01890-10215	11	43	17	9	-0.88425	0.5574	no
gi|641568505|gb|KK856958.1|	:08264-10868	11	43	11	7	-0.635554	0.64695	no
gi|641568505|gb|KK856958.1|	:09018-10926	11	43	27	17	-0.689558	0.62965	no
gi|641568505|gb|KK856958.1|	23928-24433	11	43	0	21	inf	5.00E-05	yes
gi|641568505|gb|KK856958.1|	82723-83022	11	43	8	44	2	0.11365	no
gi|641568505|gb|KK856958.1|	86576-86826	11	43	11	41	2	0.2125	no
gi|641568505|gb|KK856958.1|	88264-88962	11	43	6	7	0.337576	0.7985	no
gi|641568505|gb|KK856958.1|	91963-95869	11	43	5	7	0.484162	0.6955	no
gi|641568505|gb|KK856958.1|	)7135-10047:	11	43	124	72	-0.777374	0.68605	no
gi|641568509|gb|KK856957.1|	53135-53278	7.2	29	82	77	-0.0859456	0.91115	no
gi|641568509|gb|KK856957.1|	61277-62045	7.2	29	0.370882	2	2	0.2812	no
gi|641568509|gb|KK856957.1|	62924-64432	7.2	29	0.16181	2	3	0.1502	no
gi|641568512|gb|KK856956.1|	2408-4372	8.4	40	7	13	0.776344	0.52285	no
gi|641568512|gb|KK856956.1|	24296-25204	8.4	40	0.596063	0.755001	0.341015	1	no
gi|641568518|gb|KK856954.1|	19102-19884	9.8	34	62	31	-1	0.53805	no
gi|641568518|gb|KK856954.1|	23746-24600	9.8	34	4	4	-0.0664799	0.95765	no
gi|641568518|gb|KK856954.1|	29750-30271	9.8	34	6	5	-0.456268	0.7457	no
gi|641568518|gb|KK856954.1|	31438-32127	9.8	34	2	2	-0.177211	0.90465	no
gi|641568518|gb|KK856954.1|	36628-36963	9.8	34	131	86	-0.601651	0.6385	no

gi|641568518|gb|KK856954.1|	39419-39997	9.8	34	15	17	0.229234	0.85805	no
gi|641568524|gb|KK856952.1|	37541-38456	9.3	37	0.688643	1	0.796774	0.63025	no
gi|641568524|gb|KK856952.1|	40293-40727	9.3	37	2	4	0.780926	0.63115	no
gi|641568527|gb|KK856951.1|	10174-11068	11	24	0.70934	0.924049	0.381491	0.82465	no
gi|641568527|gb|KK856951.1|	1212-1976	11	24	4	12	1	0.2746	no
gi|641568527|gb|KK856951.1|	4613-5072	11	24	3	8	2	0.3217	no
gi|641568527|gb|KK856951.1|	6240-7856	11	24	2	3	1	0.4311	no
gi|641568533|gb|KK856949.1|	02859-10341	9.7	38	0.586448	7	4	0.1449	no
gi|641568533|gb|KK856949.1|	13324-14384	9.7	38	2	2	-0.301388	0.82655	no
gi|641568533|gb|KK856949.1|	16201-17728	9.7	38	9	9	-0.0429541	0.97165	no
gi|641568533|gb|KK856949.1|	19203-21229	9.7	38	15	17	0.161647	0.89495	no
gi|641568533|gb|KK856949.1|	50243-51040	9.7	38	0	24	inf	5.00E-05	yes
gi|641568533|gb|KK856949.1|	56597-57550	9.7	38	0.186888	4	4	0.2367	no
gi|641568533|gb|KK856949.1|	63695-64923	9.7	38	4	6	0.503519	0.6958	no
gi|641568533|gb|KK856949.1|	65836-66023	9.7	38	70	65	-0.12051	0.93025	no
gi|641568533|gb|KK856949.1|	69219-69413	9.7	38	17	17	0.0416698	0.92445	no
gi|641568533|gb|KK856949.1|	70801-72487	9.7	38	2	0.724099	-1	0.42025	no
gi|641568533|gb|KK856949.1|	7337-7607	9.7	38	17	2	-3	0.1714	no
gi|641568533|gb|KK856949.1|	73663-75139	9.7	38	0.221138	0.673961	2	1	no
gi|641568533|gb|KK856949.1|	75396-75628	9.7	38	14	9	-0.592011	0.69735	no
gi|641568533|gb|KK856949.1|	78399-81429	9.7	38	2	2	0.330142	0.80015	no
gi|641568533|gb|KK856949.1|	84669-85357	9.7	38	23	22	-0.054299	0.9659	no
gi|641568541|gb|KK856946.1|	74344-74674	9.3	35	39	41	0.0699794	0.9568	no
gi|641568544|gb|KK856945.1|	56200-56829	11	48	0.326348	3	3	0.2674	no
gi|641568544|gb|KK856945.1|	67571-67942	11	48	17	12	-0.419005	0.7568	no
gi|641568544|gb|KK856945.1|	68573-68979	11	48	3	2	-0.41454	0.8125	no
gi|641568544|gb|KK856945.1|	86645-87649	11	48	0.787958	0.66583	-0.242966	1	no
gi|641568544|gb|KK856945.1|	93019-93533	11	48	12	5	-1	0.39775	no
gi|641568544|gb|KK856945.1|	95184-96048	11	48	10	7	-0.412436	0.74745	no
gi|641568547|gb|KK856944.1|	35716-38441	8.2	33	0.44782	2	2	0.13715	no

gi|641568547|gb|KK856944.1|	38520-39393	8.2	33	0.313423	1	2	0.25595	no
gi|641568549|gb|KK856943.1|	1358-2415	11	21	7	6	-0.255545	0.84165	no
gi|641568549|gb|KK856943.1|	18007-18580	11	21	0.74901	2	1	0.51315	no
gi|641568549|gb|KK856943.1|	25925-26513	11	21	0.900687	2	1	0.39995	no
gi|641568549|gb|KK856943.1|	28081-28813	11	21	0.791483	3	2	0.2803	no
gi|641568549|gb|KK856943.1|	32278-33311	11	21	2	4	1	0.32095	no
gi|641568549|gb|KK856943.1|	3982-4816	11	21	1	1	0.14366	0.929	no
gi|641568549|gb|KK856943.1|	50025-51075	11	21	0.82839	2	0.868457	0.56565	no
gi|641568549|gb|KK856943.1|	51803-53043	11	21	1	2	0.998996	0.5023	no
gi|641568549|gb|KK856943.1|	57348-58624	11	21	0.327578	1	2	0.2126	no
gi|641568549|gb|KK856943.1|	59557-61451	11	21	0.416807	2	2	0.22295	no
gi|641568549|gb|KK856943.1|	61556-62329	11	21	0.735347	6	3	0.11085	no
gi|641568549|gb|KK856943.1|	63006-63459	11	21	4	4	0.104484	0.9444	no
gi|641568549|gb|KK856943.1|	66464-67346	11	21	1	0.313367	-2	0.3455	no
gi|641568549|gb|KK856943.1|	68636-69939	11	21	0.894861	2	1	0.41185	no
gi|641568549|gb|KK856943.1|	70368-72687	11	21	1	1	-0.127414	0.9234	no
gi|641568549|gb|KK856943.1|	77097-77875	11	21	0.486026	1	1	0.51305	no
gi|641568549|gb|KK856943.1|	81611-82781	11	21	0.217921	0.995238	2	0.25595	no
gi|641568549|gb|KK856943.1|	86698-87390	11	21	0.712616	5	3	0.1385	no
gi|641568549|gb|KK856943.1|	89889-90854	11	21	0.643909	0.55951	-0.202694	1	no
gi|641568549|gb|KK856943.1|	98055-99787	11	21	2	4	1	0.3952	no
gi|641568552|gb|KK856942.1|	11431-12511	9	35	1	1	0.12023	0.936	no
gi|641568552|gb|KK856942.1|	20310-21264	9	35	1	1	0.0195877	0.9597	no
gi|641568552|gb|KK856942.1|	22260-23888	9	35	1	2	0.245639	0.85905	no
gi|641568552|gb|KK856942.1|	32539-32696	9	35	0	151	inf	0.0023	yes
gi|641568558|gb|KK856940.1|	00110-10268	11	41	2	0.045425	-5	0.184	no
gi|641568558|gb|KK856940.1|	02822-10346	11	41	3	0.949528	-2	0.26755	no
gi|641568558|gb|KK856940.1|	03622-10405	11	41	4	1	-2	0.40265	no
gi|641568558|gb|KK856940.1|	04128-10470	11	41	2	0.280991	-3	0.2381	no
gi|641568558|gb|KK856940.1|	19574-12079	11	41	3	3	-0.27796	0.8333	no

gi|641568558|gb|KK856940.1|	21554-12250	11	41	2	3	1	0.4642	no
gi|641568558|gb|KK856940.1|	22891-12426	11	41	0.660753	1	1	0.44465	no
gi|641568558|gb|KK856940.1|	24663-25005	11	41	40	24	-0.720692	0.58055	no
gi|641568558|gb|KK856940.1|	27846-33672	11	41	24	13	-0.926809	0.6663	no
gi|641568558|gb|KK856940.1|	308-502	11	41	29	7	-2	0.27465	no
gi|641568558|gb|KK856940.1|	40594-40921	11	41	19	31	0.685894	0.6015	no
gi|641568558|gb|KK856940.1|	41614-42816	11	41	0.773645	0.214162	-2	1	no
gi|641568558|gb|KK856940.1|	43601-49059	11	41	6	11	0.728991	0.65395	no
gi|641568558|gb|KK856940.1|	49412-51163	11	41	0.682679	0.693925	0.0235727	1	no
gi|641568558|gb|KK856940.1|	53116-53582	11	41	1	4	2	0.3014	no
gi|641568558|gb|KK856940.1|	53893-54318	11	41	14	11	-0.380858	0.7712	no
gi|641568558|gb|KK856940.1|	54460-55527	11	41	0.893458	0.617935	-0.531946	0.7478	no
gi|641568558|gb|KK856940.1|	57450-57972	11	41	3	6	0.681874	0.6314	no
gi|641568558|gb|KK856940.1|	58997-59488	11	41	3	3	-0.0594571	0.94525	no
gi|641568558|gb|KK856940.1|	61940-62481	11	41	38	34	-0.14938	0.90245	no
gi|641568558|gb|KK856940.1|	65258-71396	11	41	8	1	-3	0.11805	no
gi|641568558|gb|KK856940.1|	71656-72570	11	41	2	0.299494	-3	0.20575	no
gi|641568558|gb|KK856940.1|	73152-74344	11	41	5	0.540756	-3	0.08085	no
gi|641568558|gb|KK856940.1|	74439-75823	11	41	4	0.362971	-3	0.0865	no
gi|641568558|gb|KK856940.1|	77781-78299	11	41	7	0	-inf	0.00055	yes
gi|641568558|gb|KK856940.1|	80652-81573	11	41	3	0	-inf	0.0015	yes
gi|641568558|gb|KK856940.1|	81734-85065	11	41	3	0.654991	-2	0.1169	no
gi|641568558|gb|KK856940.1|	85127-87627	11	41	3	0.046881	-6	0.18295	no
gi|641568558|gb|KK856940.1|	87804-89837	11	41	2	0.352607	-2	0.1424	no
gi|641568558|gb|KK856940.1|	89923-93954	11	41	3	0.366454	-3	0.04705	no
gi|641568558|gb|KK856940.1|	94200-94847	11	41	4	0	-inf	0.00315	yes
gi|641568558|gb|KK856940.1|	95144-99441	11	41	2	0.10545	-4	0.0666	no
gi|641568561|gb|KK856939.1|	50520-50934	11	46	0	201	inf	0.00035	yes
gi|641568561|gb|KK856939.1|	84296-84654	11	46	11	7	-0.662	0.6447	no
gi|641568561|gb|KK856939.1|	86677-87324	11	46	2	2	-0.500245	0.74225	no

gi|641568568|gb|KK856937.1|	16697-16942	11	41	15	6	-1	0.4486	no
gi|641568568|gb|KK856937.1|	35773-36269	11	41	2	2	0.592931	0.69715	no
gi|641568568|gb|KK856937.1|	36975-37913	11	41	0.285993	2	3	0.1811	no
gi|641568568|gb|KK856937.1|	38187-38858	11	41	0.44641	3	3	0.19335	no
gi|641568568|gb|KK856937.1|	39486-40492	11	41	0.52401	0.929873	0.827441	0.6232	no
gi|641568568|gb|KK856937.1|	61754-62539	11	41	0.600322	1	0.865361	0.62725	no
gi|641568568|gb|KK856937.1|	81464-82947	11	41	0.98971	1	0.43782	0.76105	no
gi|641568568|gb|KK856937.1|	87644-88316	11	41	0.890951	0.900269	0.0150094	0.91115	no
gi|641568571|gb|KK856936.1|	37933-39088	8.6	33	1	0.561515	-1	0.4973	no
gi|641568571|gb|KK856936.1|	43539-50109	8.6	33	3	4	0.684831	0.58485	no
gi|641568571|gb|KK856936.1|	51378-53116	8.6	33	2	5	1	0.3837	no
gi|641568571|gb|KK856936.1|	54055-54759	8.6	33	2	3	0.408103	0.78555	no
gi|641568571|gb|KK856936.1|	54938-56478	8.6	33	0.842452	0.641982	-0.392061	1	no
gi|641568571|gb|KK856936.1|	57357-58040	8.6	33	4	6	0.545802	0.69375	no
gi|641568571|gb|KK856936.1|	60465-60986	8.6	33	2	3	0.594358	0.6902	no
gi|641568571|gb|KK856936.1|	62005-64374	8.6	33	0.912032	2	1	0.34495	no
gi|641568571|gb|KK856936.1|	64524-65204	8.6	33	0.730228	2	2	0.32945	no
gi|641568571|gb|KK856936.1|	65358-66171	8.6	33	0.802356	2	1	0.48415	no
gi|641568571|gb|KK856936.1|	71451-71683	8.6	33	8	41	2	0.15855	no
gi|641568571|gb|KK856936.1|	72877-73496	8.6	33	1	5	2	0.27435	no
gi|641568571|gb|KK856936.1|	74073-74648	8.6	33	3	8	1	0.36105	no
gi|641568571|gb|KK856936.1|	76511-78310	8.6	33	0.927144	1	0.464259	0.74005	no
gi|641568571|gb|KK856936.1|	81092-81766	8.6	33	5	9	0.877557	0.51425	no
gi|641568571|gb|KK856936.1|	83113-84157	8.6	33	4	3	-0.297329	0.82195	no
gi|641568571|gb|KK856936.1|	93704-94484	8.6	33	0.847637	0.7353	-0.205113	1	no
gi|641568574|gb|KK856935.1|	67662-69465	8.9	32	0.440399	0.805845	0.871692	1	no
gi|641568574|gb|KK856935.1|	69764-70528	8.9	32	4	2	-1	0.43175	no
gi|641568574|gb|KK856935.1|	70802-71668	8.9	32	43	31	-0.466142	0.70115	no
gi|641568574|gb|KK856935.1|	73807-74605	8.9	32	4	4	0.280613	0.83275	no
gi|641568574|gb|KK856935.1|	77216-77479	8.9	32	101	64	-0.657153	0.60345	no

gi|641568574|gb|KK856935.1|	80060-80845	8.9	32	2	3	0.280398	0.83965	no
gi|641568574|gb|KK856935.1|	85840-86129	8.9	32	51	19	-1	0.3034	no
gi|641568578|gb|KK856934.1|	11659-11997	11	47	3	4	0.312523	0.86115	no
gi|641568580|gb|KK856933.1|	12669-13310	12	25	1	0.24054	-3	0.271	no
gi|641568580|gb|KK856933.1|	26905-27617	12	25	1	1	0.185889	0.91015	no
gi|641568580|gb|KK856933.1|	52409-58127	12	25	4	1	-2	0.17645	no
gi|641568580|gb|KK856933.1|	59150-61197	12	25	8	4	-0.990104	0.42985	no
gi|641568580|gb|KK856933.1|	8620-9680	12	25	0.736929	0.622913	-0.242494	1	no
gi|641568591|gb|KK856931.1|	11004-12712	9.3	36	0.889307	0.642237	-0.469575	0.74025	no
gi|641568591|gb|KK856931.1|	15898-16941	9.3	36	1	0.635345	-0.979597	0.57205	no
gi|641568591|gb|KK856931.1|	18165-18812	9.3	36	1	0.712146	-0.623102	0.71195	no
gi|641568591|gb|KK856931.1|	20687-21431	9.3	36	2	0	-inf	0.0075	no
gi|641568591|gb|KK856931.1|	21483-22568	9.3	36	2	0.121098	-4	0.1972	no
gi|641568597|gb|KK856929.1|	40448-40731	15	54	2	9	2	0.3198	no
gi|641568600|gb|KK856928.1|	18222-18718	8.7	34	0	10	inf	0.00075	yes
gi|641568606|gb|KK856926.1|	55306-57256	9.3	36	7	13	0.950003	0.448	no
gi|641568606|gb|KK856926.1|	66461-68212	9.3	36	2	2	-0.271171	0.8375	no
gi|641568606|gb|KK856926.1|	70741-71800	9.3	36	0.655806	0.498905	-0.394505	1	no
gi|641568609|gb|KK856925.1|	35706-36153	6.8	30	2	11	3	0.13325	no
gi|641568618|gb|KK856922.1|	.01333-10211	12	42	0.241769	2	3	0.2556	no
gi|641568618|gb|KK856922.1|	.02235-10372	12	42	0.054814	2	5	0.10595	no
gi|641568618|gb|KK856922.1|	66-270	12	42	10	17	0.795379	0.653	no
gi|641568618|gb|KK856922.1|	96397-96977	12	42	1	4	1	0.38865	no
gi|641568621|gb|KK856921.1|	52791-53264	9.5	40	3	4	0.591417	0.67805	no
gi|641568621|gb|KK856921.1|	74119-74786	9.5	40	1	3	1	0.38845	no
gi|641568625|gb|KK856920.1|	28915-29258	11	23	5	8	0.713972	0.64115	no
gi|641568628|gb|KK856919.1|	11248-11622	8.2	34	5	0	-inf	0.01	no
gi|641568628|gb|KK856919.1|	29364-29878	8.2	34	1	3	1	0.41185	no
gi|641568631|gb|KK856918.1|	39220-40968	11	42	0.0912	3	5	0.23345	no
gi|641568631|gb|KK856918.1|	43371-44359	11	42	0	3	inf	0.00045	yes

gi|641568631|gb|KK856918.1|	58363-59183	11	42	0	2	inf	0.0068	no
gi|641568631|gb|KK856918.1|	69130-69853	11	42	0.268323	2	3	0.27515	no
gi|641568631|gb|KK856918.1|	91492-92100	11	42	9	9	-0.000569767	0.98765	no
gi|641568631|gb|KK856918.1|	95239-95779	11	42	0.20505	6	5	0.10875	no
gi|641568637|gb|KK856916.1|	00511-10135	10	38	82	59	-0.47185	0.83505	no
gi|641568637|gb|KK856916.1|	10716-11366	10	38	13	5	-1	0.276	no
gi|641568637|gb|KK856916.1|	12957-14570	10	38	2	4	0.981624	0.4586	no
gi|641568637|gb|KK856916.1|	2633-3849	10	38	0.4162	1	1	0.37775	no
gi|641568637|gb|KK856916.1|	27668-27846	10	38	28	35	0.32586	0.8234	no
gi|641568637|gb|KK856916.1|	31343-31803	10	38	18	42.47	1	0.34185	no
gi|641568637|gb|KK856916.1|	40631-41980	10	38	0.490961	3	3	0.11735	no
gi|641568637|gb|KK856916.1|	51673-52905	10	38	0.068296	1	4	0.12385	no
gi|641568637|gb|KK856916.1|	61364-61692	10	38	31	3	-3	0.0818	no
gi|641568637|gb|KK856916.1|	68866-70717	10	38	1	0.782541	-0.506713	0.71855	no
gi|641568637|gb|KK856916.1|	72399-73412	10	38	15	6	-1	0.33895	no
gi|641568637|gb|KK856916.1|	75125-76167	10	38	4	3	-0.532146	0.6979	no
gi|641568637|gb|KK856916.1|	77005-77666	10	38	8	13	0.65312	0.62265	no
gi|641568637|gb|KK856916.1|	77851-79732	10	38	110	66	-0.749513	0.71835	no
gi|641568637|gb|KK856916.1|	87017-87318	10	38	76	108	0.507747	0.6933	no
gi|641568637|gb|KK856916.1|	89720-89984	10	38	49	47	-0.0661436	0.9608	no
gi|641568637|gb|KK856916.1|	90145-90340	10	38	12	31	1	0.4185	no
gi|641568637|gb|KK856916.1|	96150-97549	10	38	0.529388	0.358463	-0.5625	1	no
gi|641568637|gb|KK856916.1|	98074-98534	10	38	2	2	-0.216894	0.8945	no
gi|641568639|gb|KK856915.1|	13330-22118	11	37	18	17	-0.0669598	0.97255	no
gi|641568639|gb|KK856915.1|	22253-24212	11	37	0.963469	0.795962	-0.27554	0.84275	no
gi|641568639|gb|KK856915.1|	2676-3409	11	37	0	3	inf	0.00255	yes
gi|641568639|gb|KK856915.1|	31090-31606	11	37	5	1	-2	0.2507	no
gi|641568639|gb|KK856915.1|	34892-35976	11	37	0.239009	1	2	0.2235	no
gi|641568639|gb|KK856915.1|	37885-38527	11	37	1	5	2	0.2242	no
gi|641568639|gb|KK856915.1|	56627-57515	11	37	0.51106	2	2	0.2348	no

gi|641568639|gb|KK856915.1|	63148-63751	11	37	2	4	0.70481	0.636	no
gi|641568639|gb|KK856915.1|	6447-6756	11	37	0.583097	49	6	0.1047	no
gi|641568639|gb|KK856915.1|	70831-71796	11	37	0.91987	0.419632	-1	0.50885	no
gi|641568639|gb|KK856915.1|	72475-74072	11	37	0.353585	0.769941	1	1	no
gi|641568639|gb|KK856915.1|	7265-7878	11	37	0	821	inf	5.00E-05	yes
gi|641568639|gb|KK856915.1|	76491-78612	11	37	0.514744	0.729144	0.50235	1	no
gi|641568639|gb|KK856915.1|	81200-82339	11	37	0.450159	0.570994	0.343039	1	no
gi|641568642|gb|KK856914.1|	33586-33720	11	40	0	409	inf	0.00375	no
gi|641568642|gb|KK856914.1|	52670-53452	11	40	1	0.916014	-0.245721	0.88495	no
gi|641568642|gb|KK856914.1|	65615-66744	11	40	11	5	-1	0.4079	no
gi|641568642|gb|KK856914.1|	69424-71774	11	40	0	216	inf	0.0002	yes
gi|641568642|gb|KK856914.1|	73576-74059	11	40	0	7	inf	0.00135	yes
gi|641568642|gb|KK856914.1|	85599-85958	11	40	16	4	-2	0.22655	no
gi|641568646|gb|KK856913.1|	39263-39408	9.1	36	123	35	-2	0.3266	no
gi|641568646|gb|KK856913.1|	40151-42628	9.1	36	16	10	-0.693769	0.6664	no
gi|641568646|gb|KK856913.1|	54150-55034	9.1	36	2	0.468693	-2	0.2338	no
gi|641568646|gb|KK856913.1|	56232-59071	9.1	36	1	0.449667	-2	0.2568	no
gi|641568646|gb|KK856913.1|	60115-60677	9.1	36	6	5	-0.360623	0.79435	no
gi|641568646|gb|KK856913.1|	63078-63719	9.1	36	1	0.962158	-0.208239	0.8945	no
gi|641568646|gb|KK856913.1|	65140-66583	9.1	36	2	1	-0.942599	0.50305	no
gi|641568649|gb|KK856912.1|	14652-14842	11	37	21	23	0.109915	0.93095	no
gi|641568649|gb|KK856912.1|	20666-21431	11	37	4	5	0.395528	0.773	no
gi|641568649|gb|KK856912.1|	36418-37869	11	37	8	6	-0.361998	0.7789	no
gi|641568649|gb|KK856912.1|	38124-39178	11	37	0	24	inf	5.00E-05	yes
gi|641568649|gb|KK856912.1|	39962-40559	11	37	2	0.53304	-2	0.25005	no
gi|641568649|gb|KK856912.1|	40713-42823	11	37	3	3	-0.248733	0.84725	no
gi|641568649|gb|KK856912.1|	44338-45216	11	37	1	0.315192	-2	0.3455	no
gi|641568649|gb|KK856912.1|	49493-50747	11	37	0.735647	0.305526	-1	1	no
gi|641568649|gb|KK856912.1|	52582-52830	11	37	27	8	-2	0.25495	no
gi|641568649|gb|KK856912.1|	53350-54265	11	37	8	5	-0.529726	0.689	no

gi|641568649|gb|KK856912.1|	58656-58832	11	37	60	21	-1	0.37925	no
gi|641568649|gb|KK856912.1|	62553-62829	11	37	48	20	-1	0.36455	no
gi|641568649|gb|KK856912.1|	63824-64811	11	37	0.894309	1	0.452871	0.76315	no
gi|641568649|gb|KK856912.1|	7048-7577	11	37	3	5	0.801224	0.58985	no
gi|641568649|gb|KK856912.1|	72982-73351	11	37	6	10	0.580701	0.6969	no
gi|641568649|gb|KK856912.1|	73990-75294	11	37	6	2	-1	0.28405	no
gi|641568649|gb|KK856912.1|	75477-76343	11	37	5	2	-2	0.25395	no
gi|641568649|gb|KK856912.1|	78087-78627	11	37	5	4	-0.520162	0.71	no
gi|641568649|gb|KK856912.1|	83908-84505	11	37	1	2	0.960252	0.57345	no
gi|641568668|gb|KK856905.1|	11894-12146	9.6	36	6	6	-0.0317003	0.91115	no
gi|641568668|gb|KK856905.1|	12962-13288	9.6	36	20	6	-2	0.2699	no
gi|641568668|gb|KK856905.1|	13991-14208	9.6	36	11	9	-0.268576	0.8666	no
gi|641568668|gb|KK856905.1|	19706-19946	9.6	36	15	0	-inf	0.0075	no
gi|641568668|gb|KK856905.1|	21221-21499	9.6	36	8	2	-2	0.3411	no
gi|641568668|gb|KK856905.1|	22999-23542	9.6	36	3	0.307168	-3	0.21435	no
gi|641568668|gb|KK856905.1|	24487-24988	9.6	36	10	2	-2	0.1557	no
gi|641568668|gb|KK856905.1|	27459-28036	9.6	36	3	1	-2	0.362	no
gi|641568668|gb|KK856905.1|	3121-3381	9.6	36	8	4	-1	0.5278	no
gi|641568676|gb|KK856902.1|	11664-12540	7.8	35	0.520069	0.948337	0.866695	0.62725	no
gi|641568676|gb|KK856902.1|	3280-3797	7.8	35	2	0.33146	-3	0.2481	no
gi|641568676|gb|KK856902.1|	62343-62594	7.8	35	11	15	0.415403	0.7696	no
gi|641568676|gb|KK856902.1|	64023-64279	7.8	35	11	6	-1	0.5083	no
gi|641568676|gb|KK856902.1|	67758-68111	7.8	35	17	8	-1	0.45505	no
gi|641568676|gb|KK856902.1|	68378-68555	7.8	35	145	68	-1	0.4329	no
gi|641568676|gb|KK856902.1|	68820-69404	7.8	35	4	5	0.445196	0.75555	no
gi|641568676|gb|KK856902.1|	70687-70865	7.8	35	50	15	-2	0.33105	no
gi|641568676|gb|KK856902.1|	72408-72933	7.8	35	10	10	-0.00582444	0.9858	no
gi|641568676|gb|KK856902.1|	74796-75743	7.8	35	0.376759	1	2	0.29935	no
gi|641568676|gb|KK856902.1|	79102-80073	7.8	35	0.547653	0.971608	0.827113	0.6232	no
gi|641568679|gb|KK856901.1|	[illegible]00027-10101	11	52	0	14.9	inf	5.00E-05	yes

gi|641568679|gb|KK856901.1|	21022-21440	11	52	4	1	-2	0.33955	no
gi|641568679|gb|KK856901.1|	43414-43876	11	52	0.264134	5	4	0.1175	no
gi|641568679|gb|KK856901.1|	4414-5497	11	52	0.398798	0.728218	0.868714	1	no
gi|641568679|gb|KK856901.1|	52690-53437	11	52	0	2	inf	0.00375	no
gi|641568679|gb|KK856901.1|	70554-71292	11	52	3	5	0.601471	0.65895	no
gi|641568679|gb|KK856901.1|	72736-73635	11	52	0	2	inf	0.0068	no
gi|641568679|gb|KK856901.1|	74584-75120	11	52	0	3	inf	0.00455	no
gi|641568679|gb|KK856901.1|	80775-81310	11	52	1	2	0.595073	0.70555	no
gi|641568682|gb|KK856900.1|	08712-10916	10	38	3	0	-inf	0.0102	no
gi|641568682|gb|KK856900.1|	11822-11254	10	38	9	17	0.829326	0.5204	no
gi|641568682|gb|KK856900.1|	12829-11421	10	38	13	6	-1	0.37	no
gi|641568682|gb|KK856900.1|	14605-11485	10	38	39	48	0.283874	0.8259	no
gi|641568682|gb|KK856900.1|	16238-11740	10	38	51	43	-0.246329	0.91325	no
gi|641568682|gb|KK856900.1|	31107-13208	10	38	2	1	-0.31273	0.8374	no
gi|641568682|gb|KK856900.1|	15766-16382	10	38	1	1	-0.0797357	0.92675	no
gi|641568682|gb|KK856900.1|	18090-18659	10	38	3	3	0.37421	0.7979	no
gi|641568682|gb|KK856900.1|	24418-25073	10	38	1	3	2	0.2961	no
gi|641568682|gb|KK856900.1|	26812-27883	10	38	0.727672	0.73815	0.0206259	1	no
gi|641568682|gb|KK856900.1|	28013-29876	10	38	3	2	-0.229459	0.8647	no
gi|641568682|gb|KK856900.1|	3229-4442	10	38	2	1	-0.393472	0.7888	no
gi|641568682|gb|KK856900.1|	32598-32876	10	38	26	22	-0.245808	0.85165	no
gi|641568682|gb|KK856900.1|	35654-35887	10	38	34	36	0.112952	0.93475	no
gi|641568682|gb|KK856900.1|	38734-39540	10	38	3	4	0.541268	0.68935	no
gi|641568682|gb|KK856900.1|	41403-41696	10	38	11	10	-0.199925	0.88995	no
gi|641568682|gb|KK856900.1|	45635-45877	10	38	14	11	-0.343531	0.82135	no
gi|641568682|gb|KK856900.1|	49110-49317	10	38	30	14	-1	0.47465	no
gi|641568682|gb|KK856900.1|	5174-7710	10	38	26	12	-1	0.4805	no
gi|641568682|gb|KK856900.1|	54435-54747	10	38	4	3	-0.237151	0.88445	no
gi|641568682|gb|KK856900.1|	58483-58752	10	38	7	11	0.728792	0.65545	no
gi|641568682|gb|KK856900.1|	58937-60767	10	38	2	3	0.0492914	0.96745	no

gi|641568682|gb|KK856900.1|	61380-62886	10	38	0.810286	0.987875	0.285897	0.84765	no
gi|641568682|gb|KK856900.1|	63283-66762	10	38	4	6	0.439695	0.7262	no
gi|641568682|gb|KK856900.1|	66905-68780	10	38	0	2	inf	0.0004	yes
gi|641568682|gb|KK856900.1|	72940-73893	10	38	1	63	6	0.03635	no
gi|641568682|gb|KK856900.1|	73998-74415	10	38	4	0	-inf	0.0059	no
gi|641568682|gb|KK856900.1|	76888-78353	10	38	0.669161	0.849726	0.344645	1	no
gi|641568682|gb|KK856900.1|	78517-80252	10	38	0.735758	0.84134	0.193458	1	no
gi|641568686|gb|KK856899.1|	2735-3390	9.8	38	2	0.233298	-3	0.2348	no
gi|641568686|gb|KK856899.1|	50535-51096	9.8	38	0	4	inf	0.00255	yes
gi|641568686|gb|KK856899.1|	51632-52628	9.8	38	1	10	3	0.0514	no
gi|641568686|gb|KK856899.1|	53099-53729	9.8	38	2	0.246551	-3	0.2481	no
gi|641568686|gb|KK856899.1|	55074-57833	9.8	38	2	0.16861	-3	0.0791	no
gi|641568686|gb|KK856899.1|	58081-59485	9.8	38	3	0.178493	-4	0.13625	no
gi|641568686|gb|KK856899.1|	60683-60996	9.8	38	6	3	-0.889003	0.60065	no
gi|641568686|gb|KK856899.1|	61415-61885	9.8	38	4	2	-1	0.46	no
gi|641568686|gb|KK856899.1|	62014-62745	9.8	38	7	1	-3	0.09775	no
gi|641568686|gb|KK856899.1|	72569-72749	9.8	38	0	183	inf	0.00015	yes
gi|641568686|gb|KK856899.1|	73567-74352	9.8	38	0	19	inf	5.00E-05	yes
gi|641568688|gb|KK856898.1|	55003-55626	8.5	36	2	6	1	0.3177	no
gi|641568692|gb|KK856897.1|	55304-55484	8.7	41	34	0	-inf	0.01175	no
gi|641568695|gb|KK856896.1|	67653-67839	13	22	35	8	-2	0.2746	no
gi|641568698|gb|KK856895.1|	26846-28021	9.3	34	0.867236	0.77014	-0.171304	1	no
gi|641568698|gb|KK856895.1|	39936-40813	9.3	34	0.830891	0.473477	-0.811364	1	no
gi|641568698|gb|KK856895.1|	46970-47886	9.3	34	3	0.896001	-2	0.3306	no
gi|641568698|gb|KK856895.1|	49629-50844	9.3	34	2	0.951494	-0.808498	0.5729	no
gi|641568698|gb|KK856895.1|	52944-54313	9.3	34	4	3	-0.516083	0.69475	no
gi|641568698|gb|KK856895.1|	55990-56604	9.3	34	0.675996	2	1	0.51315	no
gi|641568698|gb|KK856895.1|	58869-59257	9.3	34	0	9	inf	0.0015	yes
gi|641568698|gb|KK856895.1|	64139-65199	9.3	34	0.327524	3	3	0.1384	no
gi|641568698|gb|KK856895.1|	70875-71159	9.3	34	0	14	inf	0.0025	yes

gi|641568698|gb|KK856895.1|	81265-82358	9.3	34	22	18	-0.329451	0.78745	no
gi|641568701|gb|KK856894.1|	10714-11097	7.4	35	0	6	inf	0.0068	no
gi|641568704|gb|KK856893.1|	.02019-10414	10	40	2	0.726509	-1	0.3381	no
gi|641568704|gb|KK856893.1|	.04478-10648	10	40	2	0.297555	-2	0.15235	no
gi|641568704|gb|KK856893.1|	.07174-10820	10	40	2	0.258077	-3	0.1571	no
gi|641568704|gb|KK856893.1|	46592-49173	10	40	0.534206	1	1	0.3646	no
gi|641568704|gb|KK856893.1|	52952-53881	10	40	0.868504	2	1	0.47685	no
gi|641568704|gb|KK856893.1|	56473-56862	10	40	5	5	0.204931	0.8897	no
gi|641568704|gb|KK856893.1|	62371-62655	10	40	9	11	0.184712	0.9007	no
gi|641568704|gb|KK856893.1|	63793-64512	10	40	0.675924	2	1	0.3994	no
gi|641568704|gb|KK856893.1|	64751-65307	10	40	2	2	0.136616	0.9341	no
gi|641568704|gb|KK856893.1|	67348-67785	10	40	4	7	0.769124	0.5939	no
gi|641568704|gb|KK856893.1|	68566-69098	10	40	6	7	0.129261	0.9239	no
gi|641568704|gb|KK856893.1|	69218-69899	10	40	4	4	0.278272	0.8373	no
gi|641568704|gb|KK856893.1|	70087-70453	10	40	18	14	-0.388039	0.76575	no
gi|641568704|gb|KK856893.1|	72337-72944	10	40	0.687444	2	1	0.44255	no
gi|641568704|gb|KK856893.1|	73779-74821	10	40	1	0.890528	-0.67475	0.6649	no
gi|641568704|gb|KK856893.1|	75034-76248	10	40	0.695029	0.529125	-0.393466	1	no
gi|641568704|gb|KK856893.1|	77153-79355	10	40	16	16	0.00648451	0.9949	no
gi|641568704|gb|KK856893.1|	83119-85710	10	40	8	7	-0.166974	0.89945	no
gi|641568704|gb|KK856893.1|	90233-91953	10	40	4	3	-0.469545	0.7177	no
gi|641568704|gb|KK856893.1|	92087-96566	10	40	9	4	-1	0.4939	no
gi|641568704|gb|KK856893.1|	97343-98691	10	40	9	3	-1	0.25775	no
gi|641568704|gb|KK856893.1|	98864-10049[illegible]	10	40	6	3	-1	0.4085	no
gi|641568707|gb|KK856892.1|	1278-1604	12	50	4	6	0.573215	0.7371	no
gi|641568710|gb|KK856891.1|	1322-1996	10	38	5	4	-0.247973	0.8477	no
gi|641568710|gb|KK856891.1|	14308-15057	10	38	15	14	-0.167432	0.8926	no
gi|641568710|gb|KK856891.1|	15840-16729	10	38	32	34	0.100544	0.9356	no
gi|641568710|gb|KK856891.1|	20466-21886	10	38	11	6	-0.799667	0.5254	no
gi|641568710|gb|KK856891.1|	2340-4732	10	38	0.41901	0.786905	0.909204	1	no

gi|641568710|gb|KK856891.1|	25269-26394	10	38	1	0.579554	-1	0.4973	no
gi|641568710|gb|KK856891.1|	26591-29720	10	38	3	4	0.191819	0.88015	no
gi|641568710|gb|KK856891.1|	29910-31458	10	38	10	17	0.718997	0.55695	no
gi|641568710|gb|KK856891.1|	31598-32874	10	38	25.42	38	0.595668	0.71635	no
gi|641568710|gb|KK856891.1|	33118-40764	10	38	5	9	0.869144	0.6947	no
gi|641568710|gb|KK856891.1|	52564-53194	10	38	0.9768	1	0.335749	0.83525	no
gi|641568710|gb|KK856891.1|	66520-67636	10	38	0.230702	2	3	0.18845	no
gi|641568713|gb|KK856890.1|	10043-11060	12	42	2	0.393209	-2	0.21615	no
gi|641568713|gb|KK856890.1|	11751-12916	12	42	1	1	-0.130724	0.9304	no
gi|641568713|gb|KK856890.1|	13079-15017	12	42	2	2	0.571468	0.6646	no
gi|641568713|gb|KK856890.1|	16302-16937	12	42	0.643818	2	1	0.44255	no
gi|641568713|gb|KK856890.1|	17068-20285	12	42	24	18	-0.401093	0.8583	no
gi|641568713|gb|KK856890.1|	24997-25604	12	42	1	1	-0.0800385	0.92675	no
gi|641568713|gb|KK856890.1|	28155-28657	12	42	11	8	-0.376333	0.7756	no
gi|641568713|gb|KK856890.1|	31271-31691	12	42	47	38	-0.329537	0.7912	no
gi|641568713|gb|KK856890.1|	32364-32706	12	42	55	40	-0.485638	0.70435	no
gi|641568713|gb|KK856890.1|	55970-57074	12	42	0.934996	1	0.342799	0.8164	no
gi|641568713|gb|KK856890.1|	58211-59716	12	42	0.216241	3	4	0.1269	no
gi|641568713|gb|KK856890.1|	63946-65058	12	42	0.386182	0.822831	1	1	no
gi|641568713|gb|KK856890.1|	66525-67186	12	42	1	0.921302	-0.207674	0.8945	no
gi|641568713|gb|KK856890.1|	68699-70135	12	42	0.798935	0.956474	0.259648	0.8632	no
gi|641568713|gb|KK856890.1|	7643-9337	12	42	0.803128	0.720245	-0.157144	1	no
gi|641568713|gb|KK856890.1|	77630-78976	12	42	2	4	1	0.38475	no
gi|641568713|gb|KK856890.1|	84790-85261	12	42	3	3	0.131843	0.9341	no
gi|641568713|gb|KK856890.1|	91468-93569	12	42	3	2	-0.721973	0.5811	no
gi|641568716|gb|KK856889.1|	04765-10739	10	35	9	8	-0.159485	0.90055	no
gi|641568716|gb|KK856889.1|	07443-10830	10	35	4	3	-0.21367	0.8733	no
gi|641568716|gb|KK856889.1|	10036-11059	10	35	9	1	-3	0.12975	no
gi|641568716|gb|KK856889.1|	10752-11197	10	35	11	5	-0.978367	0.4451	no
gi|641568716|gb|KK856889.1|	11197-11631	10	35	0	8	inf	0.00135	yes

gi|641568716|gb|KK856889.1|	13460-11474	10	35	0.777553	0.690796	-0.170681	1	no
gi|641568716|gb|KK856889.1|	19145-19948	10	35	16	11	-0.526662	0.67715	no
gi|641568716|gb|KK856889.1|	21455-21794	10	35	12	4	-1	0.3447	no
gi|641568716|gb|KK856889.1|	26534-26793	10	35	14	9	-0.543873	0.72285	no
gi|641568716|gb|KK856889.1|	28407-28817	10	35	6	3	-1	0.48475	no
gi|641568716|gb|KK856889.1|	32260-32665	10	35	8	2	-2	0.2505	no
gi|641568716|gb|KK856889.1|	33743-34007	10	35	11	6	-0.821219	0.61345	no
gi|641568716|gb|KK856889.1|	34476-34722	10	35	21	2	-4	0.19865	no
gi|641568716|gb|KK856889.1|	35484-37188	10	35	40	17	-1	0.45205	no
gi|641568716|gb|KK856889.1|	37909-38883	10	35	1	2	0.51164	0.7255	no
gi|641568716|gb|KK856889.1|	39378-41404	10	35	0.309403	1	2	0.2897	no
gi|641568716|gb|KK856889.1|	48149-51487	10	35	4	2	-0.810121	0.519	no
gi|641568716|gb|KK856889.1|	51634-53410	10	35	0.85105	0.341489	-1	1	no
gi|641568716|gb|KK856889.1|	54160-55894	10	35	2	0.4911	-2	0.2683	no
gi|641568716|gb|KK856889.1|	56072-57436	10	35	2	1	-0.615115	0.65815	no
gi|641568716|gb|KK856889.1|	69410-72944	10	35	10	0.939013	-3	0.0195	no
gi|641568716|gb|KK856889.1|	79118-80241	10	35	5	0.696958	-3	0.0968	no
gi|641568716|gb|KK856889.1|	81605-83900	10	35	0.944175	0.565941	-0.738404	0.5925	no
gi|641568716|gb|KK856889.1|	86888-87274	10	35	4	0	-inf	0.01175	no
gi|641568719|gb|KK856888.1|	02379-10295	11	44	24	17	-0.542838	0.67025	no
gi|641568719|gb|KK856888.1|	04547-10652	11	44	1	2	0.649663	0.6286	no
gi|641568719|gb|KK856888.1|	06612-10804	11	44	1	3	1	0.3681	no
gi|641568719|gb|KK856888.1|	30862-33775	11	44	0.677367	1	0.717076	0.5991	no
gi|641568719|gb|KK856888.1|	39344-39892	11	44	1	2	0.595689	0.69715	no
gi|641568719|gb|KK856888.1|	40002-40629	11	44	1	2	0.23612	0.88205	no
gi|641568719|gb|KK856888.1|	40710-41962	11	44	1	2	0.214425	0.87975	no
gi|641568719|gb|KK856888.1|	42076-43684	11	44	2	2	0.655456	0.62395	no
gi|641568719|gb|KK856888.1|	44768-46166	11	44	1	1	-0.0555444	0.96335	no
gi|641568719|gb|KK856888.1|	47167-48418	11	44	1	0.408522	-1	0.4417	no
gi|641568719|gb|KK856888.1|	51511-52709	11	44	0.494284	0.430027	-0.200913	1	no

gi|641568719|gb|KK856888.1|	55929-59522	11	44	1	2	0.740657	0.5729	no
gi|641568719|gb|KK856888.1|	59670-60702	11	44	0.676969	0.90112	0.412629	0.8089	no
gi|641568719|gb|KK856888.1|	69195-69746	11	44	0.993783	3	1	0.39995	no
gi|641568719|gb|KK856888.1|	70368-72833	11	44	0.405534	2	2	0.19155	no
gi|641568719|gb|KK856888.1|	81472-81793	11	44	5	4	-0.427827	0.77915	no
gi|641568719|gb|KK856888.1|	83747-84049	11	44	14	5	-1	0.37545	no
gi|641568719|gb|KK856888.1|	84687-84973	11	44	14	12	-0.1732	0.8996	no
gi|641568719|gb|KK856888.1|	88741-89258	11	44	9	7	-0.335467	0.8016	no
gi|641568719|gb|KK856888.1|	91363-91954	11	44	11	7	-0.656293	0.61495	no
gi|641568719|gb|KK856888.1|	94886-95984	11	44	0.784342	0.59681	-0.39421	1	no
gi|641568719|gb|KK856888.1|	96336-96510	11	44	28	34	0.293927	0.8636	no
gi|641568719|gb|KK856888.1|	97686-98211	11	44	2	1	-0.72736	0.67005	no
gi|641568722|gb|KK856887.1|	21660-22095	11	38	16	11	-0.575127	0.66655	no
gi|641568722|gb|KK856887.1|	26092-26267	11	38	38	22	-0.805069	0.633	no
gi|641568722|gb|KK856887.1|	28256-28485	11	38	49	36	-0.417308	0.75235	no
gi|641568722|gb|KK856887.1|	32802-32964	11	38	64	42	-0.627831	0.69655	no
gi|641568722|gb|KK856887.1|	37664-37972	11	38	15	4	-2	0.28035	no
gi|641568722|gb|KK856887.1|	40909-41146	11	38	33	14	-1	0.3938	no
gi|641568722|gb|KK856887.1|	42041-42423	11	38	34	20	-0.739397	0.5666	no
gi|641568722|gb|KK856887.1|	42590-45163	11	38	1	0.454441	-2	0.25425	no
gi|641568722|gb|KK856887.1|	46221-47320	11	38	1	0.953845	-0.201558	0.8959	no
gi|641568722|gb|KK856887.1|	50528-51791	11	38	4	3	-0.471203	0.7204	no
gi|641568722|gb|KK856887.1|	58446-58846	11	38	5	2	-2	0.3393	no
gi|641568722|gb|KK856887.1|	68891-69525	11	38	0.806602	2	1	0.4611	no
gi|641568722|gb|KK856887.1|	73372-74760	11	38	0.474907	1	1	0.38845	no
gi|641568728|gb|KK856885.1|	32605-33733	7.6	31	0	197	inf	5.00E-05	yes
gi|641568731|gb|KK856884.1|	17512-18613	11	22	3	0	-inf	0.00105	yes
gi|641568740|gb|KK856881.1|	30248-31116	7.7	31	1	0.799622	-0.396442	0.81255	no
gi|641568746|gb|KK856879.1|	11593-12544	9.7	22	0.562147	0.997183	0.826912	0.6232	no
gi|641568746|gb|KK856879.1|	25685-27301	9.7	22	0.548202	2	2	0.3088	no

gi|641568746|gb|KK856879.1|	27913-29145	9.7	22	0.409777	1	1	0.37775	no
gi|641568746|gb|KK856879.1|	3186-3752	9.7	22	1	2	0.374085	0.8276	no
gi|641568746|gb|KK856879.1|	46-674	9.7	22	0.49065	2	2	0.256	no
gi|641568746|gb|KK856879.1|	47591-48294	9.7	22	0.418319	2	2	0.2812	no
gi|641568746|gb|KK856879.1|	50073-50736	9.7	22	13	59	2	0.09765	no
gi|641568746|gb|KK856879.1|	8619-9099	9.7	22	1	2	0.591901	0.70505	no
gi|641568753|gb|KK856877.1|	12-1013	11	40	2	1	-0.5325	0.71245	no
gi|641568753|gb|KK856877.1|	2679-3163	11	40	9	8	-0.144798	0.91155	no
gi|641568753|gb|KK856877.1|	32610-32886	11	40	4	12	2	0.31995	no
gi|641568753|gb|KK856877.1|	36833-37126	11	40	9	5	-0.812902	0.61345	no
gi|641568753|gb|KK856877.1|	3728-4329	11	40	4	3	-0.472565	0.7407	no
gi|641568753|gb|KK856877.1|	46668-49969	11	40	2	3	0.576485	0.6482	no
gi|641568753|gb|KK856877.1|	51783-52709	11	40	0.58138	1	0.826645	0.6232	no
gi|641568753|gb|KK856877.1|	55069-56445	11	40	18	19	0.119931	0.9225	no
gi|641568753|gb|KK856877.1|	6296-6589	11	40	4	14	2	0.29365	no
gi|641568753|gb|KK856877.1|	83790-84899	11	40	0	9	inf	5.00E-05	yes
gi|641568759|gb|KK856875.1|	35929-36746	11	44	1	0.518871	-0.982123	0.62775	no
gi|641568759|gb|KK856875.1|	36981-39006	11	44	0.967412	1	0.42468	0.75445	no
gi|641568759|gb|KK856875.1|	39180-39858	11	44	3	4	0.678128	0.6316	no
gi|641568759|gb|KK856875.1|	40110-40751	11	44	0.635177	2	2	0.3613	no
gi|641568759|gb|KK856875.1|	46107-48094	11	44	1	0.241093	-2	0.2058	no
gi|641568759|gb|KK856875.1|	57367-57678	11	44	5	3	-0.84506	0.64855	no
gi|641568759|gb|KK856875.1|	71832-72597	11	44	0.869947	1	0.601979	0.69715	no
gi|641568759|gb|KK856875.1|	77697-79425	11	44	0.600533	0.70431	0.229967	1	no
gi|641568759|gb|KK856875.1|	79610-81134	11	44	0.479585	0.812111	0.759889	1	no
gi|641568762|gb|KK856874.1|	12254-12905	11	41	3	1	-1	0.4703	no
gi|641568762|gb|KK856874.1|	15907-16370	11	41	2	1	-0.824349	0.64915	no
gi|641568762|gb|KK856874.1|	16450-17841	11	41	28	13	-1	0.37175	no
gi|641568762|gb|KK856874.1|	21487-23222	11	41	26	6	-2	0.25995	no
gi|641568762|gb|KK856874.1|	26330-26640	11	41	3	3	-0.0152133	0.91115	no

gi|641568762|gb|KK856874.1|	26694-31033	11	41	2	2	-0.583263	0.6496	no
gi|641568762|gb|KK856874.1|	2961-3240	11	41	8	3	-1	0.46935	no
gi|641568762|gb|KK856874.1|	32001-40109	11	41	13	6	-1	0.6186	no
gi|641568762|gb|KK856874.1|	41189-41646	11	41	2	2	-0.0878439	0.92655	no
gi|641568762|gb|KK856874.1|	42550-43019	11	41	5	5	-0.0718336	0.95895	no
gi|641568762|gb|KK856874.1|	46279-46617	11	41	11	8	-0.549973	0.7034	no
gi|641568762|gb|KK856874.1|	48887-50210	11	41	2	3	0.326986	0.80765	no
gi|641568762|gb|KK856874.1|	53726-54104	11	41	7	11	0.581988	0.68325	no
gi|641568762|gb|KK856874.1|	56154-56712	11	41	0.974736	2	0.85917	0.62755	no
gi|641568762|gb|KK856874.1|	56855-57992	11	41	1	1	-0.159474	0.91355	no
gi|641568762|gb|KK856874.1|	58212-59663	11	41	0.789391	1	0.385235	0.793	no
gi|641568762|gb|KK856874.1|	60506-62035	11	41	2	0.809076	-1	0.43975	no
gi|641568762|gb|KK856874.1|	6123-6396	11	41	8	1	-3	0.2472	no
gi|641568762|gb|KK856874.1|	63163-63896	11	41	0.131669	2	4	0.12385	no
gi|641568762|gb|KK856874.1|	64054-65967	11	41	5	4	-0.185522	0.8864	no
gi|641568762|gb|KK856874.1|	66078-68172	11	41	2	2	0.233712	0.85845	no
gi|641568762|gb|KK856874.1|	74240-74987	11	41	0	5	inf	0.00075	yes
gi|641568765|gb|KK856873.1|	01886-10269	11	41	15	33	1	0.36065	no
gi|641568765|gb|KK856873.1|	02856-10417	11	41	0.881335	0.767101	-0.200275	0.8959	no
gi|641568765|gb|KK856873.1|	08486-10900	11	41	4	2	-0.768319	0.61315	no
gi|641568765|gb|KK856873.1|	26504-12676	11	41	16	1	-4	0.2029	no
gi|641568765|gb|KK856873.1|	41268-41613	11	41	2	4	0.839858	0.63695	no
gi|641568765|gb|KK856873.1|	42297-44697	11	41	2	5	1	0.29665	no
gi|641568765|gb|KK856873.1|	44836-45741	11	41	0	5	inf	0.00065	yes
gi|641568765|gb|KK856873.1|	52186-52631	11	41	0	7	inf	0.0016	yes
gi|641568765|gb|KK856873.1|	66772-67062	11	41	0	15	inf	0.0023	yes
gi|641568765|gb|KK856873.1|	69483-71237	11	41	19	56	2	0.3531	no
gi|641568765|gb|KK856873.1|	72992-74395	11	41	26	37	0.490084	0.76165	no
gi|641568765|gb|KK856873.1|	75551-75878	11	41	7	7	-0.0640107	0.95555	no
gi|641568765|gb|KK856873.1|	79364-80391	11	41	3	7	1	0.34875	no

gi|641568765|gb|KK856873.1|	81954-82703	11	41	1	2	0.891191	0.57435	no
gi|641568765|gb|KK856873.1|	83481-84010	11	41	9	28	2	0.2423	no
gi|641568765|gb|KK856873.1|	84782-85744	11	41	0.092347	4	6	0.10535	no
gi|641568765|gb|KK856873.1|	85960-86832	11	41	1	2	0.603609	0.67915	no
gi|641568765|gb|KK856873.1|	86898-88995	11	41	0.111694	4	5	0.13555	no
gi|641568765|gb|KK856873.1|	90494-91127	11	41	0.161688	2	4	0.1276	no
gi|641568765|gb|KK856873.1|	91188-91747	11	41	1	1	0.110752	0.92265	no
gi|641568765|gb|KK856873.1|	95104-95726	11	41	0.663368	3	2	0.3012	no
gi|641568765|gb|KK856873.1|	97406-98646	11	41	2	0.619209	-1	0.3694	no
gi|641568777|gb|KK856871.1|	00543-10107	11	43	7	9	0.323622	0.80595	no
gi|641568777|gb|KK856871.1|	19211-12133	11	43	1	1	-0.142806	0.9211	no
gi|641568780|gb|KK856870.1|	11048-11573	8.8	42	0	4	inf	0.00375	no
gi|641568780|gb|KK856870.1|	20970-21332	8.8	42	0	24	inf	0.00015	yes
gi|641568780|gb|KK856870.1|	28187-28730	8.8	42	15	0	-inf	5.00E-05	yes
gi|641568780|gb|KK856870.1|	29707-31228	8.8	42	1	0	-inf	0.0013	yes
gi|641568780|gb|KK856870.1|	38861-39857	8.8	42	1	0.13449	-3	0.21435	no
gi|641568780|gb|KK856870.1|	40126-40578	8.8	42	11	0	-inf	0.0006	yes
gi|641568780|gb|KK856870.1|	47428-48286	8.8	42	2	0	-inf	0.0056	no
gi|641568780|gb|KK856870.1|	52160-54143	8.8	42	1	0.060408	-5	0.1873	no
gi|641568780|gb|KK856870.1|	54259-55902	8.8	42	4	0.074555	-6	0.18335	no
gi|641568780|gb|KK856870.1|	56314-57484	8.8	42	6	0.110582	-6	0.18305	no
gi|641568780|gb|KK856870.1|	57934-59692	8.8	42	3	0.621742	-2	0.1104	no
gi|641568780|gb|KK856870.1|	61728-62131	8.8	42	4	0	-inf	0.01	no
gi|641568780|gb|KK856870.1|	64864-65663	8.8	42	4	0	-inf	0.00045	yes
gi|641568780|gb|KK856870.1|	65804-67472	8.8	42	3	0.439755	-3	0.07605	no
gi|641568780|gb|KK856870.1|	67544-72856	8.8	42	3	0.084549	-5	0.0615	no
gi|641568780|gb|KK856870.1|	84722-86659	8.8	42	5	0	-inf	5.00E-05	yes
gi|641568780|gb|KK856870.1|	86729-89603	8.8	42	5	0	-inf	5.00E-05	yes
gi|641568786|gb|KK856868.1|	05382-10554	11	39	33	46	0.508021	0.7741	no
gi|641568786|gb|KK856868.1|	10560-11090	11	39	5	16	2	0.23955	no

gi|641568786|gb|KK856868.1|	12513-11277	11	39	38	96	1	0.3185	no
gi|641568786|gb|KK856868.1|	16273-11837	11	39	6	3	-1	0.35415	no
gi|641568786|gb|KK856868.1|	24478-12468	11	39	11	14	0.270335	0.8647	no
gi|641568786|gb|KK856868.1|	24928-12587	11	39	1	3	1	0.4739	no
gi|641568786|gb|KK856868.1|	26237-12712	11	39	0.510323	1	1	0.50945	no
gi|641568786|gb|KK856868.1|	33399-13365	11	39	40	74	0.883614	0.4993	no
gi|641568786|gb|KK856868.1|	38029-13840	11	39	3	6	0.903212	0.5694	no
gi|641568786|gb|KK856868.1|	39278-13951	11	39	15	18	0.308153	0.8365	no
gi|641568786|gb|KK856868.1|	16422-16801	11	39	0	23	inf	5.00E-05	yes
gi|641568786|gb|KK856868.1|	38794-39584	11	39	0.952441	1	0.409765	0.8089	no
gi|641568786|gb|KK856868.1|	51396-51648	11	39	14	25	0.83337	0.5608	no
gi|641568786|gb|KK856868.1|	54798-55026	11	39	19	12	-0.678606	0.657	no
gi|641568786|gb|KK856868.1|	74274-74783	11	39	3	3	-0.213656	0.89225	no
gi|641568786|gb|KK856868.1|	75901-76146	11	39	10	9	-0.034259	0.9394	no
gi|641568786|gb|KK856868.1|	82100-82779	11	39	3	5	0.792795	0.56475	no
gi|641568786|gb|KK856868.1|	91487-92047	11	39	2	0.87932	-0.818815	0.6492	no
gi|641568786|gb|KK856868.1|	94338-94610	11	39	17	22	0.415392	0.75765	no
gi|641568786|gb|KK856868.1|	99104-99453	11	39	5	6	0.162481	0.9145	no
gi|641568790|gb|KK856867.1|	12035-13088	11	41	0	2	inf	0.00135	yes
gi|641568790|gb|KK856867.1|	14217-14475	11	41	0	20	inf	0.0023	yes
gi|641568790|gb|KK856867.1|	46933-47370	11	41	2	1	-0.63384	0.71175	no
gi|641568790|gb|KK856867.1|	4930-5416	11	41	0	42	inf	5.00E-05	yes
gi|641568790|gb|KK856867.1|	62552-63326	11	41	2	2	-0.0258931	0.963	no
gi|641568790|gb|KK856867.1|	63415-64534	11	41	0.996463	2	0.605938	0.681	no
gi|641568790|gb|KK856867.1|	64647-65115	11	41	2	2	0.00609629	0.91115	no
gi|641568790|gb|KK856867.1|	66271-68021	11	41	2	0.902681	-0.900481	0.5171	no
gi|641568790|gb|KK856867.1|	68779-72839	11	41	5	3	-0.739436	0.5562	no
gi|641568790|gb|KK856867.1|	73010-73407	11	41	8	6	-0.415576	0.7601	no
gi|641568790|gb|KK856867.1|	73803-77316	11	41	3	2	-0.163886	0.8903	no
gi|641568790|gb|KK856867.1|	77508-78974	11	41	2	2	-0.328186	0.8075	no

gi|641568790|gb|KK856867.1|	79306-81316	11	41	2	0.892665	-0.943464	0.49615	no
gi|641568790|gb|KK856867.1|	83757-84790	11	41	1	0.771471	-0.897217	0.56925	no
gi|641568799|gb|KK856864.1|	16300-16777	9.2	35	0	13	inf	5.00E-05	yes
gi|641568799|gb|KK856864.1|	25245-26919	9.2	35	2	2	0.256865	0.8467	no
gi|641568799|gb|KK856864.1|	46003-47222	9.2	35	1	5	2	0.18675	no
gi|641568799|gb|KK856864.1|	48883-49463	9.2	35	3	5	0.844972	0.56065	no
gi|641568799|gb|KK856864.1|	58915-59108	9.2	35	49	39	-0.335937	0.81535	no
gi|641568799|gb|KK856864.1|	60596-61131	9.2	35	4	3	-0.252924	0.8647	no
gi|641568799|gb|KK856864.1|	64384-64695	9.2	35	7	3	-1	0.49505	no
gi|641568799|gb|KK856864.1|	65762-66048	9.2	35	50	44	-0.193664	0.8797	no
gi|641568799|gb|KK856864.1|	70892-72463	9.2	35	18	8	-1	0.3221	no
gi|641568799|gb|KK856864.1|	77494-77742	9.2	35	58	27	-1	0.42795	no
gi|641568799|gb|KK856864.1|	79577-79818	9.2	35	67	51	-0.403533	0.76055	no
gi|641568799|gb|KK856864.1|	81478-82156	9.2	35	4	4	-0.0373048	0.9769	no
gi|641568799|gb|KK856864.1|	87671-88227	9.2	35	25	17	-0.509994	0.6908	no
gi|641568799|gb|KK856864.1|	90811-91690	9.2	35	14	6	-1	0.34665	no
gi|641568802|gb|KK856863.1|	43586-44824	8.9	35	27	56	1	0.5199	no
gi|641568802|gb|KK856863.1|	51325-51526	8.9	35	33	33	-0.00977691	0.96065	no
gi|641568802|gb|KK856863.1|	53599-54710	8.9	35	50	129	1	0.5373	no
gi|641568802|gb|KK856863.1|	54986-55532	8.9	35	5	10	1	0.42245	no
gi|641568802|gb|KK856863.1|	56025-57033	8.9	35	3	6	0.675456	0.60145	no
gi|641568802|gb|KK856863.1|	57386-58153	8.9	35	4	11	2	0.27365	no
gi|641568802|gb|KK856863.1|	58694-58947	8.9	35	4	9	1	0.52105	no
gi|641568802|gb|KK856863.1|	59129-60081	8.9	35	22	64	2	0.354	no
gi|641568802|gb|KK856863.1|	60593-61322	8.9	35	0.795927	0.804986	0.0163266	1	no
gi|641568802|gb|KK856863.1|	67180-70043	8.9	35	15	12	-0.299052	0.84945	no
gi|641568805|gb|KK856862.1|	75145-76018	9.4	44	0	6	inf	5.00E-05	yes
gi|641568808|gb|KK856861.1|	65381-65559	10	45	18	30.41	0.781539	0.65375	no
gi|641568808|gb|KK856861.1|	65927-68138	10	45	3	37	3	0.0882	no
gi|641568808|gb|KK856861.1|	68655-69709	10	45	7	84	4	0.07995	no

gi|641568808|gb|KK856861.1|	71242-74506	10	45	3	26	3	0.09715	no
gi|641568808|gb|KK856861.1|	76197-76593	10	45	14	138	3	0.02315	no
gi|641568808|gb|KK856861.1|	76719-77520	10	45	13	167	4	0.0692	no
gi|641568810|gb|KK856860.1|	22623-22918	7.6	32	11	3	-2	0.2861	no
gi|641568810|gb|KK856860.1|	23857-25189	7.6	32	0.934319	0.474395	-0.977827	0.57205	no
gi|641568810|gb|KK856860.1|	27099-27472	7.6	32	6	2	-1	0.4158	no
gi|641568810|gb|KK856860.1|	34866-35085	7.6	32	15	20	0.387719	0.79825	no
gi|641568810|gb|KK856860.1|	36586-37673	7.6	32	1	0.120828	-3	0.206	no
gi|641568810|gb|KK856860.1|	37849-39121	7.6	32	2	0.400584	-2	0.18415	no
gi|641568810|gb|KK856860.1|	39809-40588	7.6	32	1	0	-inf	0.01175	no
gi|641568810|gb|KK856860.1|	43568-44511	7.6	32	1	0.287941	-2	0.25005	no
gi|641568810|gb|KK856860.1|	46573-46857	7.6	32	4	18	2	0.21765	no
gi|641568810|gb|KK856860.1|	54170-54653	7.6	32	2	7	1	0.34525	no
gi|641568810|gb|KK856860.1|	55972-56789	7.6	32	0.911078	4	2	0.1909	no
gi|641568810|gb|KK856860.1|	63244-63539	7.6	32	8	26	2	0.2508	no
gi|641568810|gb|KK856860.1|	72108-74255	7.6	32	0.036278	2	6	0.1047	no
gi|641568812|gb|KK856859.1|	69926-70419	7.9	33	7	29	2	0.12345	no
gi|641568812|gb|KK856859.1|	76714-76898	7.9	33	12	130	3	0.13085	no
gi|641568812|gb|KK856859.1|	77151-77366	7.9	33	15	99	3	0.0954	no
gi|641568812|gb|KK856859.1|	80079-80378	7.9	33	26	100	2	0.1445	no
gi|641568815|gb|KK856858.1|	10688-13418	10	34	1	0.170535	-3	0.1224	no
gi|641568815|gb|KK856858.1|	28848-13004	10	34	74	20	-2	0.2631	no
gi|641568815|gb|KK856858.1|	32825-13304	10	34	31.54	11	-2	0.34765	no
gi|641568815|gb|KK856858.1|	37034-13894	10	34	9	7	-0.333008	0.78685	no
gi|641568815|gb|KK856858.1|	39326-14018	10	34	3	2	-0.829507	0.56065	no
gi|641568815|gb|KK856858.1|	41012-14165	10	34	0.790434	2	1	0.51085	no
gi|641568815|gb|KK856858.1|	42330-14326	10	34	4	2	-0.700493	0.6053	no
gi|641568815|gb|KK856858.1|	43399-14376	10	34	6	2	-1	0.38945	no
gi|641568815|gb|KK856858.1|	44879-14734	10	34	1	0.857979	-0.390273	0.77305	no
gi|641568815|gb|KK856858.1|	14591-15411	10	34	0.680027	1	0.825277	0.6232	no

gi|641568815|gb|KK856858.1|	[illegible]47452-14869	10	34	4	6	0.365692	0.77695	no
gi|641568815|gb|KK856858.1|	[illegible]49546-15029	10	34	1	1	0.186551	0.91015	no
gi|641568815|gb|KK856858.1|	[illegible]50600-15235	10	34	15	8	-0.885454	0.47805	no
gi|641568815|gb|KK856858.1|	[illegible]60039-16019	10	34	54	97	0.829406	0.598	no
gi|641568815|gb|KK856858.1|	17849-18261	10	34	5	1	-2	0.33195	no
gi|641568815|gb|KK856858.1|	18639-19270	10	34	5	4	-0.538689	0.697	no
gi|641568815|gb|KK856858.1|	19360-21557	10	34	8	5	-0.722462	0.57045	no
gi|641568815|gb|KK856858.1|	21761-21969	10	34	40	13	-2	0.31095	no
gi|641568815|gb|KK856858.1|	22299-22508	10	34	34	18	-0.90569	0.55025	no
gi|641568815|gb|KK856858.1|	26720-27293	10	34	4	2	-1	0.4862	no
gi|641568815|gb|KK856858.1|	29240-29808	10	34	3	2	-0.988402	0.5501	no
gi|641568815|gb|KK856858.1|	33003-36199	10	34	3	4	0.419543	0.74105	no
gi|641568815|gb|KK856858.1|	36698-37978	10	34	0.65274	1	0.74439	0.6176	no
gi|641568815|gb|KK856858.1|	38319-39823	10	34	0.757423	0.329793	-1	1	no
gi|641568815|gb|KK856858.1|	46016-51455	10	34	10	5	-1	0.52885	no
gi|641568815|gb|KK856858.1|	51577-53891	10	34	1	0.866807	-0.390455	0.7755	no
gi|641568815|gb|KK856858.1|	54001-55040	10	34	1	1	-0.224742	0.87645	no
gi|641568815|gb|KK856858.1|	59200-61301	10	34	0.891678	0.623429	-0.516298	0.71915	no
gi|641568815|gb|KK856858.1|	61999-62358	10	34	4	2	-1	0.50345	no
gi|641568815|gb|KK856858.1|	63283-63988	10	34	0.83336	1	0.337736	0.8352	no
gi|641568815|gb|KK856858.1|	75390-75542	10	34	166	87	-0.936574	0.53805	no
gi|641568815|gb|KK856858.1|	84196-84372	10	34	56	27	-1	0.50195	no
gi|641568815|gb|KK856858.1|	88184-88610	10	34	6	7	0.265602	0.84515	no
gi|641568815|gb|KK856858.1|	8871-10622	10	34	1	0.138785	-3	0.1571	no
gi|641568815|gb|KK856858.1|	91005-91440	10	34	8	4	-0.79014	0.577	no
gi|641568818|gb|KK856857.1|	44709-45359	9.2	35	3	13	2	0.1415	no
gi|641568821|gb|KK856856.1|	18243-19202	12	21	0.370839	0.986793	1	0.44255	no
gi|641568821|gb|KK856856.1|	1996-2453	12	21	2	2	0.0052655	0.91115	no
gi|641568821|gb|KK856856.1|	22160-23037	12	21	0.934752	2	0.755676	0.62565	no
gi|641568821|gb|KK856856.1|	23106-23665	12	21	1	0.881695	-0.626214	0.71195	no

gi|641568821|gb|KK856856.1|	2854-4881	12	21	1	3	1	0.2889	no
gi|641568821|gb|KK856856.1|	31771-33011	12	21	1	2	0.882313	0.52175	no
gi|641568821|gb|KK856856.1|	34060-35943	12	21	0.335619	1	2	0.3067	no
gi|641568821|gb|KK856856.1|	38471-39334	12	21	0.424156	1	2	0.33105	no
gi|641568821|gb|KK856856.1|	47493-48397	12	21	0.099905	2	4	0.1175	no
gi|641568821|gb|KK856856.1|	6594-7384	12	21	1	5	2	0.2102	no
gi|641568821|gb|KK856856.1|	794-1938	12	21	0.522418	0.908796	0.798752	0.63025	no
gi|641568821|gb|KK856856.1|	8790-10612	12	21	1	3	2	0.2359	no
gi|641568824|gb|KK856855.1|	92117-92939	9.9	45	15	3	-2	0.0906	no
gi|641568831|gb|KK856853.1|	23436-24274	10	30	2	3	0.603149	0.6664	no
gi|641568831|gb|KK856853.1|	2525-3475	10	30	2	2	0.189472	0.8991	no
gi|641568831|gb|KK856853.1|	30972-31705	10	30	0	2	inf	0.00455	no
gi|641568831|gb|KK856853.1|	34249-34921	10	30	46	16	-1	0.2461	no
gi|641568831|gb|KK856853.1|	35708-36741	10	30	4	1	-2	0.18085	no
gi|641568831|gb|KK856853.1|	42897-43841	10	30	13	4	-2	0.2332	no
gi|641568831|gb|KK856853.1|	44628-46520	10	30	8	2	-2	0.1416	no
gi|641568831|gb|KK856853.1|	4492-4805	10	30	3	7	0.985467	0.5903	no
gi|641568831|gb|KK856853.1|	60112-60997	10	30	0.513283	33	6	0.0816	no
gi|641568834|gb|KK856852.1|	51523-52379	9.1	40	0.964462	2	0.755402	0.62565	no
gi|641568834|gb|KK856852.1|	59595-60245	9.1	40	2	2	-0.0380538	0.95655	no
gi|641568834|gb|KK856852.1|	63768-65083	9.1	40	0.632336	1	0.870091	0.56565	no
gi|641568834|gb|KK856852.1|	66459-68441	9.1	40	1	1	-0.347905	0.80145	no
gi|641568834|gb|KK856852.1|	68671-69884	9.1	40	0.904426	0.7415	-0.286556	0.85105	no
gi|641568840|gb|KK856850.1|	36051-38431	11	46	12	3	-2	0.1651	no
gi|641568840|gb|KK856850.1|	38764-39101	11	46	2	5	1	0.4433	no
gi|641568840|gb|KK856850.1|	40525-41034	11	46	3	1	-2	0.403	no
gi|641568840|gb|KK856850.1|	51878-52762	11	46	7	2	-1	0.28085	no
gi|641568840|gb|KK856850.1|	62613-63611	11	46	5	2	-1	0.45245	no
gi|641568840|gb|KK856850.1|	63735-64121	11	46	4	2	-1	0.5083	no
gi|641568840|gb|KK856850.1|	65563-67258	11	46	0.944227	2	0.871428	0.53155	no

gi|641568840|gb|KK856850.1|	68293-69796	11	46	77	38	-0.99977	0.6501	no
gi|641568843|gb|KK856849.1|	63972-64192	11	42	42	0	-inf	0.0013	yes
gi|641568846|gb|KK856848.1|	36418-37524	5.1	34	0.310981	0.828228	1	1	no
gi|641568849|gb|KK856847.1|	76883-79332	10	41	2	4	1	0.36095	no
gi|641568849|gb|KK856847.1|	80135-80951	10	41	3	3	-0.397176	0.7742	no
gi|641568849|gb|KK856847.1|	81110-82065	10	41	0.931978	2	0.742064	0.6176	no
gi|641568856|gb|KK856845.1|	63194-63893	11	46	4	5	0.0600649	0.965	no
gi|641568856|gb|KK856845.1|	77187-78063	11	46	0.520069	1	1	0.50945	no
gi|641568859|gb|KK856844.1|	50822-51711	10	43	0.306194	1	2	0.25595	no
gi|641568859|gb|KK856844.1|	89093-93415	10	43	5	0.602668	-3	0.03525	no
gi|641568859|gb|KK856844.1|	95497-96766	10	43	1	0	-inf	0.00345	yes
gi|641568859|gb|KK856844.1|	96833-97900	10	43	0.974682	0.247174	-2	0.31075	no
gi|641568862|gb|KK856843.1|	12991-11414	9.9	35	0.221789	0.787756	2	1	no
gi|641568862|gb|KK856843.1|	19498-12108	9.9	35	1	2	0.29995	0.82805	no
gi|641568862|gb|KK856843.1|	22908-12530	9.9	35	1	2	0.519457	0.6945	no
gi|641568862|gb|KK856843.1|	25891-12697	9.9	35	2	9	2	0.09895	no
gi|641568862|gb|KK856843.1|	1273-2183	9.9	35	0.594395	0.7529	0.341038	1	no
gi|641568862|gb|KK856843.1|	27944-12910	9.9	35	2	4	0.815651	0.5478	no
gi|641568862|gb|KK856843.1|	29303-12983	9.9	35	2	0.962197	-0.99024	0.62775	no
gi|641568862|gb|KK856843.1|	31784-13360	9.9	35	3	5	0.831084	0.5137	no
gi|641568862|gb|KK856843.1|	13193-13569	9.9	35	15	7	-1	0.4077	no
gi|641568862|gb|KK856843.1|	37173-13764	9.9	35	1	7	3	0.17505	no
gi|641568862|gb|KK856843.1|	37741-13878	9.9	35	3	6	1	0.43975	no
gi|641568862|gb|KK856843.1|	39980-14073	9.9	35	2	3	0.901376	0.53955	no
gi|641568862|gb|KK856843.1|	17692-18979	9.9	35	5	5	-0.153408	0.9084	no
gi|641568862|gb|KK856843.1|	19299-20204	9.9	35	5	3	-0.636994	0.6343	no
gi|641568862|gb|KK856843.1|	20933-21527	9.9	35	2	0.805488	-0.987389	0.62775	no
gi|641568862|gb|KK856843.1|	22877-23198	9.9	35	9	13	0.572173	0.6969	no
gi|641568862|gb|KK856843.1|	2997-4072	9.9	35	0.482909	1	2	0.32035	no
gi|641568862|gb|KK856843.1|	30877-32282	9.9	35	3	2	-1	0.45235	no

gi|641568862|gb|KK856843.1|	4175-7197	9.9	35	1	1	-0.268449	0.8365	no
gi|641568862|gb|KK856843.1|	46-1112	9.9	35	7	6	-0.16704	0.89185	no
gi|641568862|gb|KK856843.1|	7493-7669	9.9	35	26	27	0.0319739	0.92445	no
gi|641568862|gb|KK856843.1|	78915-79235	9.9	35	7	6	-0.12848	0.9347	no
gi|641568862|gb|KK856843.1|	88882-89887	9.9	35	7	7	0.0295374	0.981	no
gi|641568862|gb|KK856843.1|	8929-9344	9.9	35	33	25	-0.372152	0.77055	no
gi|641568865|gb|KK856842.1|	81974-82180	9.7	34	13	36	1	0.3819	no
gi|641568865|gb|KK856842.1|	82436-82875	9.7	34	2	2	0.325696	0.83525	no
gi|641568869|gb|KK856841.1|	[illegible]28360-12976	12	50	2	2	0.0224825	0.9781	no
gi|641568869|gb|KK856841.1|	[illegible]32027-13231	12	50	4	5	0.300731	0.8614	no
gi|641568869|gb|KK856841.1|	[illegible]33376-13382	12	50	5	5	-0.0383645	0.96295	no
gi|641568869|gb|KK856841.1|	[illegible]38225-13952	12	50	3	4	0.228454	0.8645	no
gi|641568869|gb|KK856841.1|	87818-88204	12	50	2	3	0.583058	0.7575	no
gi|641568873|gb|KK856840.1|	57924-58715	11	38	0.475421	1	1	0.51305	no
gi|641568873|gb|KK856840.1|	9192-10160	11	38	0.916299	0.557349	-0.717237	0.6701	no
gi|641568875|gb|KK856839.1|	1526-2286	7.9	27	10	8	-0.24246	0.8519	no
gi|641568875|gb|KK856839.1|	2-260	7.9	27	7	15	1	0.5003	no
gi|641568878|gb|KK856838.1|	1089-1308	9	37	11	18	0.732368	0.66305	no
gi|641568878|gb|KK856838.1|	36992-37936	9	37	0.661969	0.431338	-0.617946	1	no
gi|641568878|gb|KK856838.1|	38412-42578	9	37	17	17	0.00689684	0.99715	no
gi|641568878|gb|KK856838.1|	43404-44159	9	37	2	0.767892	-2	0.3134	no
gi|641568878|gb|KK856838.1|	46890-47287	9	37	4	1	-2	0.31725	no
gi|641568878|gb|KK856838.1|	47997-48286	9	37	21	26	0.310808	0.8167	no
gi|641568878|gb|KK856838.1|	53518-53773	9	37	16	21	0.373736	0.79425	no
gi|641568878|gb|KK856838.1|	59951-61549	9	37	2	0.538575	-2	0.23665	no
gi|641568884|gb|KK856836.1|	59258-60099	8.3	33	3	3	-0.0972482	0.9455	no
gi|641568884|gb|KK856836.1|	71920-72560	8.3	33	0.795751	2	1	0.39995	no
gi|641568887|gb|KK856835.1|	29995-30696	8.8	37	0	3	inf	0.00255	yes
gi|641568887|gb|KK856835.1|	30780-31288	8.8	37	0	13	inf	0.0004	yes
gi|641568887|gb|KK856835.1|	47646-51841	8.8	37	0	30	inf	0.0001	yes

gi|641568890|gb|KK856834.1|	10133-10921	11	40	4	6	0.692574	0.6075	no
gi|641568890|gb|KK856834.1|	.06731-10726	11	40	2	0	-inf	0.01	no
gi|641568890|gb|KK856834.1|	.08640-10961	11	40	0.729264	0.277247	-1	1	no
gi|641568890|gb|KK856834.1|	11777-17270	11	40	6	17	1	0.3935	no
gi|641568890|gb|KK856834.1|	17349-18605	11	40	0.467256	2	2	0.2786	no
gi|641568890|gb|KK856834.1|	18822-20926	11	40	1	3	1	0.31455	no
gi|641568890|gb|KK856834.1|	2043-2938	11	40	0.202379	3	4	0.2456	no
gi|641568890|gb|KK856834.1|	26961-29813	11	40	41	13	-2	0.3262	no
gi|641568890|gb|KK856834.1|	29949-31525	11	40	1	0	-inf	0.0021	yes
gi|641568890|gb|KK856834.1|	31631-32726	11	40	1.81	0	-inf	0.00315	yes
gi|641568890|gb|KK856834.1|	32879-33468	11	40	8	3	-2	0.2765	no
gi|641568890|gb|KK856834.1|	37298-38267	11	40	38	0.278316	-7	0.1301	no
gi|641568890|gb|KK856834.1|	38576-39603	11	40	0.510779	0.777009	0.605232	1	no
gi|641568890|gb|KK856834.1|	41473-43695	11	40	52	0	-inf	0.0003	yes
gi|641568890|gb|KK856834.1|	44732-47863	11	40	153	9	-4	0.02875	no
gi|641568890|gb|KK856834.1|	51680-51909	11	40	37	0	-inf	0.0013	yes
gi|641568890|gb|KK856834.1|	58056-59470	11	40	0.929551	0.708132	-0.392515	0.7971	no
gi|641568890|gb|KK856834.1|	73698-74572	11	40	0.938884	2	1	0.3864	no
gi|641568890|gb|KK856834.1|	76803-76953	11	40	172	0	-inf	0.0034	yes
gi|641568902|gb|KK856832.1|	51669-52305	11	43	1	0.486471	-2	0.4266	no
gi|641568902|gb|KK856832.1|	53652-54588	11	43	0.573531	2	2	0.32035	no
gi|641568902|gb|KK856832.1|	54693-55856	11	43	0.512161	1	1	0.39245	no
gi|641568902|gb|KK856832.1|	60393-60644	11	43	83	44	-0.915242	0.48835	no
gi|641568902|gb|KK856832.1|	65883-67446	11	43	0.155303	2	4	0.1435	no
gi|641568902|gb|KK856832.1|	73596-74470	11	43	0.834563	2	0.925563	0.5636	no
gi|641568902|gb|KK856832.1|	75512-79846	11	43	0.479013	3	2	0.08415	no
gi|641568902|gb|KK856832.1|	81325-82082	11	43	2	2	0.379442	0.7933	no
gi|641568902|gb|KK856832.1|	98060-98499	11	43	5	3	-0.91377	0.56415	no
gi|641568902|gb|KK856832.1|	99723-10147[illegible]	11	43	23	11	-1	0.39845	no
gi|641568923|gb|KK856825.1|	17932-18319	8.5	28	2	4	1	0.5126	no

gi|641568923|gb|KK856825.1|	21295-21604	8.5	28	3	3	-0.015443	0.91115	no
gi|641568923|gb|KK856825.1|	26509-26934	8.5	28	12	6	-1	0.4103	no
gi|641568923|gb|KK856825.1|	29673-31414	8.5	28	23	30.59	0.386118	0.8638	no
gi|641568923|gb|KK856825.1|	32474-35941	8.5	28	28	20	-0.459081	0.83315	no
gi|641568923|gb|KK856825.1|	36457-37861	8.5	28	12	7	-0.798547	0.52765	no
gi|641568923|gb|KK856825.1|	52265-53556	8.5	28	2	2	0.13301	0.9231	no
gi|641568923|gb|KK856825.1|	55266-56943	8.5	28	1	2	0.701455	0.6004	no
gi|641568923|gb|KK856825.1|	58470-59193	8.5	28	0.939129	1	0.115737	0.92175	no
gi|641568923|gb|KK856825.1|	64133-65125	8.5	28	1	1	-0.202434	0.8956	no
gi|641568923|gb|KK856825.1|	66526-66856	8.5	28	11	10	-0.0109406	0.9697	no
gi|641568923|gb|KK856825.1|	67069-67937	8.5	28	0.736756	3	2	0.2786	no
gi|641568926|gb|KK856824.1|	58138-58348	10	19	28	3	-3	0.20685	no
gi|641568926|gb|KK856824.1|	60958-61187	10	19	188	10	-4	0.04555	no
gi|641568926|gb|KK856824.1|	61370-61741	10	19	4	2	-1	0.46955	no
gi|641568926|gb|KK856824.1|	62798-63437	10	19	56	9	-3	0.05425	no
gi|641568926|gb|KK856824.1|	64047-65154	10	19	56	7	-3	0.11755	no
gi|641568926|gb|KK856824.1|	66545-67544	10	19	46	12	-2	0.2714	no
gi|641568926|gb|KK856824.1|	67793-69504	10	19	13	7	-0.929733	0.45535	no
gi|641568926|gb|KK856824.1|	70205-73377	10	19	13	12	-0.184599	0.9069	no
gi|641568926|gb|KK856824.1|	79625-80482	10	19	8	5	-0.644516	0.61695	no
gi|641568926|gb|KK856824.1|	89031-89859	10	19	1	0.339939	-2	0.26805	no
gi|641568932|gb|KK856822.1|	16333-17930	9	18	7	8	0.189714	0.88165	no
gi|641568932|gb|KK856822.1|	25057-26271	9	18	0.764532	0.952424	0.317027	0.84265	no
gi|641568932|gb|KK856822.1|	27123-27933	9	18	1	3	2	0.32045	no
gi|641568932|gb|KK856822.1|	28043-29269	9	18	0.54955	0.94138	0.776527	0.62605	no
gi|641568932|gb|KK856822.1|	32285-33262	9	18	0.724599	0.96419	0.412134	0.8089	no
gi|641568932|gb|KK856822.1|	34567-35136	9	18	51	60	0.240886	0.8489	no
gi|641568932|gb|KK856822.1|	35509-36174	9	18	23	27	0.212765	0.8657	no
gi|641568932|gb|KK856822.1|	36898-37489	9	18	3	5	0.597463	0.6632	no
gi|641568932|gb|KK856822.1|	38095-40074	9	18	2	1	-0.115345	0.9313	no

gi|641568932|gb|KK856822.1|	44777-46251	9	18	4	3	-0.205202	0.8755	no
gi|641568932|gb|KK856822.1|	47609-48790	9	18	91	156	0.780447	0.7208	no
gi|641568932|gb|KK856822.1|	49388-50772	9	18	3	4	0.607363	0.64115	no
gi|641568932|gb|KK856822.1|	50931-52165	9	18	0.749811	0.518977	-0.530858	1	no
gi|641568932|gb|KK856822.1|	59796-61281	9	18	0.988176	0.585614	-0.754818	0.6172	no
gi|641568932|gb|KK856822.1|	61844-64414	9	18	3	2	-0.436448	0.73825	no
gi|641568932|gb|KK856822.1|	64960-65816	9	18	3	3	0.0470052	0.97045	no
gi|641568932|gb|KK856822.1|	66518-67376	9	18	9	4	-1	0.3002	no
gi|641568932|gb|KK856822.1|	75304-76409	9	18	1	0	-inf	0.0075	no
gi|641568932|gb|KK856822.1|	82439-83562	9	18	0.839526	0.11616	-3	1	no
gi|641568932|gb|KK856822.1|	83764-85583	9	18	1	0.930912	-0.671414	0.63585	no
gi|641568932|gb|KK856822.1|	85958-88246	9	18	17	8	-1	0.5212	no
gi|641568932|gb|KK856822.1|	8842-9369	9	18	0.639043	3	2	0.2235	no
gi|641568932|gb|KK856822.1|	89654-90514	9	18	0.958658	0.161837	-3	0.271	no
gi|641568941|gb|KK856819.1|	27362-28735	10	43	1	0.549524	-1	0.38115	no
gi|641568941|gb|KK856819.1|	32453-33213	10	43	1	1	0.0169265	0.9394	no
gi|641568941|gb|KK856819.1|	34395-35448	10	43	1	0	-inf	0.0075	no
gi|641568941|gb|KK856819.1|	41579-44301	10	43	0.588461	4	3	0.0662	no
gi|641568941|gb|KK856819.1|	48131-49154	10	43	1	0	-inf	0.0059	no
gi|641568941|gb|KK856819.1|	49452-50989	10	43	0.791564	0	-inf	1	no
gi|641568941|gb|KK856819.1|	52079-53421	10	43	2	0.188109	-3	0.15595	no
gi|641568941|gb|KK856819.1|	53885-55841	10	43	2	0.061332	-5	0.18455	no
gi|641568941|gb|KK856819.1|	55946-59252	10	43	15	32	1	0.6224	no
gi|641568941|gb|KK856819.1|	60349-60613	10	43	28	81	2	0.2491	no
gi|641568941|gb|KK856819.1|	61287-62463	10	43	12	33	1	0.25065	no
gi|641568941|gb|KK856819.1|	72261-73028	10	43	0.123844	2	4	0.11955	no
gi|641568941|gb|KK856819.1|	85840-87557	10	43	2	0.851265	-0.976512	0.48935	no
gi|641568941|gb|KK856819.1|	90310-91271	10	43	23	8	-2	0.2367	no
gi|641568944|gb|KK856818.1|	11197-14088	12	24	8	16	0.972713	0.5528	no
gi|641568950|gb|KK856816.1|	83523-83883	8.9	18	5	9	0.901254	0.54675	no

gi|641568953|gb|KK856815.1|	58825-59321	9.1	41	3	2	-0.281538	0.86025	no
gi|641568953|gb|KK856815.1|	61297-62630	9.1	41	5	10	0.93254	0.46515	no
gi|641568959|gb|KK856813.1|	194-632	8	24	1	2	0.588641	0.70505	no
gi|641568959|gb|KK856813.1|	20766-21195	8	24	3	3	-0.149149	0.92525	no
gi|641568959|gb|KK856813.1|	43154-43495	8	24	3	3	0.090682	0.9241	no
gi|641568959|gb|KK856813.1|	50108-50936	8	24	0.671429	1	0.603004	0.70555	no
gi|641568959|gb|KK856813.1|	52694-54116	8	24	0.692617	1	0.607517	0.67895	no
gi|641568959|gb|KK856813.1|	54551-55721	8	24	1	1	0.120846	0.9357	no
gi|641568959|gb|KK856813.1|	58382-58671	8	24	3	5	0.564565	0.76735	no
gi|641568959|gb|KK856813.1|	59107-59675	8	24	2	7	1	0.3271	no
gi|641568961|gb|KK856812.1|	14760-15324	11	41	3	0.869944	-2	0.33205	no
gi|641568961|gb|KK856812.1|	17243-18204	11	41	1	0	-inf	0.0056	no
gi|641568961|gb|KK856812.1|	19309-19772	11	41	3	0.39632	-3	0.2381	no
gi|641568961|gb|KK856812.1|	20176-20846	11	41	2	0	-inf	0.01	no
gi|641568961|gb|KK856812.1|	25625-26804	11	41	0.359879	2	2	0.2348	no
gi|641568961|gb|KK856812.1|	35181-36261	11	41	2	2	0.0206944	0.9781	no
gi|641568961|gb|KK856812.1|	47666-48421	11	41	0.252994	8	5	0.23355	no
gi|641568961|gb|KK856812.1|	54179-54436	11	41	5	33	3	0.12915	no
gi|641568961|gb|KK856812.1|	54560-55712	11	41	0.370035	1	2	0.32945	no
gi|641568961|gb|KK856812.1|	58925-59676	11	41	0.127407	2	4	0.1276	no
gi|641568961|gb|KK856812.1|	65037-65240	11	41	8	107	4	0.12435	no
gi|641568961|gb|KK856812.1|	86994-87243	11	41	500	579.14	0.212519	0.89255	no
gi|641568967|gb|KK856810.1|	15890-16562	8.6	33	0.296984	3	3	0.25965	no
gi|641568967|gb|KK856810.1|	39659-40256	8.6	33	1	2	0.820074	0.62335	no
gi|641568967|gb|KK856810.1|	67793-68321	8.6	33	1	3	0.787368	0.63115	no
gi|641568967|gb|KK856810.1|	69207-70574	8.6	33	0.785556	1	0.366283	0.8038	no
gi|641568967|gb|KK856810.1|	77972-78650	8.6	33	0.733248	1	0.86316	0.62725	no
gi|641568971|gb|KK856809.1|	54151-54794	8.9	38	0.948521	1	0.599174	0.70555	no
gi|641568980|gb|KK856806.1|	28426-30047	11	39	0.893913	0.302807	-2	0.362	no
gi|641568980|gb|KK856806.1|	36083-38168	11	39	0.711889	0.114308	-3	1	no

gi|641568980|gb|KK856806.1|	53964-54389	11	39	19	5	-2	0.15805	no
gi|641568980|gb|KK856806.1|	70172-73756	11	39	10	9	-0.171128	0.8913	no
gi|641568980|gb|KK856806.1|	74256-76266	11	39	12	7	-0.871658	0.48075	no
gi|641568980|gb|KK856806.1|	77359-78091	11	39	1	0.800525	-0.39865	0.82205	no
gi|641568980|gb|KK856806.1|	84826-88339	11	39	3	3	-0.0399968	0.97365	no
gi|641568980|gb|KK856806.1|	90192-91841	11	39	0.730579	0.222743	-2	1	no
gi|641568980|gb|KK856806.1|	93570-94664	11	39	1	2	0.905321	0.53945	no
gi|641568980|gb|KK856806.1|	95692-95991	11	39	0	12	inf	0.0025	yes
gi|641568980|gb|KK856806.1|	96129-10096[illegible]	11	39	2	4	1	0.3406	no
gi|641568984|gb|KK856805.1|	63940-64366	16	82	6	0	-inf	0.00495	no
gi|641568988|gb|KK856804.1|	32200-32633	8.4	35	7	4	-0.613444	0.6674	no
gi|641568997|gb|KK856801.1|	26066-26548	9	36	3	0	-inf	0.0059	no
gi|641568997|gb|KK856801.1|	30971-31367	9	36	21	15	-0.539078	0.682	no
gi|641568997|gb|KK856801.1|	8615-9948	9	36	1	0.758367	-0.785177	0.59675	no
gi|641569000|gb|KK856800.1|	11537-13474	10	35	2	1	-0.461448	0.73365	no
gi|641569000|gb|KK856800.1|	14290-15055	10	35	3	6	0.985308	0.4785	no
gi|641569000|gb|KK856800.1|	15343-20007	10	35	3	4	0.566619	0.6518	no
gi|641569000|gb|KK856800.1|	20126-22938	10	35	2	3	0.404962	0.755	no
gi|641569000|gb|KK856800.1|	23085-25270	10	35	3	10	2	0.1868	no
gi|641569000|gb|KK856800.1|	29892-31051	10	35	1	4	2	0.25755	no
gi|641569000|gb|KK856800.1|	38477-39901	10	35	0.403369	0.702388	0.80017	1	no
gi|641569000|gb|KK856800.1|	42576-43190	10	35	0.506997	4	3	0.1811	no
gi|641569000|gb|KK856800.1|	45054-46656	10	35	0.553658	2	1	0.32715	no
gi|641569000|gb|KK856800.1|	47082-48521	10	35	2	6	1	0.3228	no
gi|641569000|gb|KK856800.1|	6180-6571	10	35	3	4	0.413768	0.81025	no
gi|641569000|gb|KK856800.1|	69638-70570	10	35	0.672752	0.87665	0.381926	0.82465	no
gi|641569000|gb|KK856800.1|	73578-74140	10	35	0.192835	3	4	0.1276	no
gi|641569000|gb|KK856800.1|	7872-8210	10	35	13	20	0.628025	0.64695	no
gi|641569000|gb|KK856800.1|	85877-86588	10	35	0.411838	3	3	0.1723	no
gi|641569000|gb|KK856800.1|	88532-88960	10	35	0.905169	116	7	0.13455	no

gi|641569000|gb|KK856800.1|	9108-9675	10	35	1	5	2	0.2104	no
gi|641569000|gb|KK856800.1|	95154-95395	10	35	0	179	inf	5.00E-05	yes
gi|641569003|gb|KK856799.1|	67-613	11	41	1	5	2	0.20445	no
gi|641569006|gb|KK856798.1|	1366-1721	8.9	33	0.431243	8	4	0.1137	no
gi|641569006|gb|KK856798.1|	15052-17041	8.9	33	0.039473	12	8	0.10435	no
gi|641569006|gb|KK856798.1|	40873-41305	8.9	33	0	19	inf	0.0001	yes
gi|641569006|gb|KK856798.1|	44589-44848	8.9	33	0	74	inf	0.00015	yes
gi|641569006|gb|KK856798.1|	45589-45754	8.9	33	0	82	inf	0.00455	no
gi|641569006|gb|KK856798.1|	51690-54892	8.9	33	0.141291	59	9	0.06415	no
gi|641569006|gb|KK856798.1|	56276-58289	8.9	33	21	14	-0.575096	0.7235	no
gi|641569006|gb|KK856798.1|	59175-59582	8.9	33	4	0.992166	-2	0.31075	no
gi|641569006|gb|KK856798.1|	61815-65898	8.9	33	0.728484	0.472806	-0.62365	1	no
gi|641569006|gb|KK856798.1|	69580-70007	8.9	33	4	4	-0.112813	0.94295	no
gi|641569006|gb|KK856798.1|	72389-74055	8.9	33	1	0.733919	-0.462074	0.74375	no
gi|641569006|gb|KK856798.1|	76178-79646	8.9	33	2	1	-0.861539	0.51145	no
gi|641569006|gb|KK856798.1|	87195-88085	8.9	33	1	0.619559	-1	0.50315	no
gi|641569009|gb|KK856797.1|	04820-10521	9.4	37	6	3	-1	0.4943	no
gi|641569009|gb|KK856797.1|	05298-10606	9.4	37	24	16	-0.590648	0.6374	no
gi|641569009|gb|KK856797.1|	46869-47966	9.4	37	0.549648	2	2	0.3164	no
gi|641569009|gb|KK856797.1|	53433-53660	9.4	37	23	6	-2	0.284	no
gi|641569009|gb|KK856797.1|	74153-74511	9.4	37	11	6	-0.951506	0.51105	no
gi|641569009|gb|KK856797.1|	87998-89010	9.4	37	1	2	0.605102	0.6744	no
gi|641569009|gb|KK856797.1|	89511-89797	9.4	37	32	18	-0.872334	0.5208	no
gi|641569009|gb|KK856797.1|	89886-90429	9.4	37	18	11	-0.710351	0.5879	no
gi|641569009|gb|KK856797.1|	91187-92559	9.4	37	19	20	0.0312828	0.98155	no
gi|641569009|gb|KK856797.1|	93430-96181	9.4	37	2	2	0.0335214	0.98075	no
gi|641569009|gb|KK856797.1|	96323-97117	9.4	37	2	4	1	0.4527	no
gi|641569012|gb|KK856796.1|	39508-39982	12	44	2	2	-0.215867	0.8945	no
gi|641569012|gb|KK856796.1|	40812-41167	12	44	4	0.643984	-3	0.2476	no
gi|641569012|gb|KK856796.1|	41626-42616	12	44	0.445466	0.948498	1	0.50945	no

gi|641569012|gb|KK856796.1|	44133-45171	12	44	1	0.766916	-0.510153	0.7498	no
gi|641569012|gb|KK856796.1|	69162-70038	12	44	0.520069	0.948337	0.866695	0.62725	no
gi|641569012|gb|KK856796.1|	70145-70722	12	44	0.741205	2	2	0.3613	no
gi|641569012|gb|KK856796.1|	80108-80475	12	44	42	27	-0.641989	0.6143	no
gi|641569021|gb|KK856795.1|	25077-26153	10	41	8	11	0.423149	0.73695	no
gi|641569021|gb|KK856795.1|	47706-48366	10	41	1	0.461631	-1	0.4534	no
gi|641569021|gb|KK856795.1|	49126-49698	10	41	2	1	-0.5732	0.7586	no
gi|641569025|gb|KK856794.1|	19964-21107	9.7	38	0.22413	0.796032	2	1	no
gi|641569028|gb|KK856793.1|	12170-13126	9.7	41	0.930753	1	0.604571	0.67795	no
gi|641569028|gb|KK856793.1|	13929-14306	9.7	41	2	5	0.996887	0.5903	no
gi|641569028|gb|KK856793.1|	15713-16513	9.7	41	2	4	0.532184	0.702	no
gi|641569028|gb|KK856793.1|	27204-28638	9.7	41	0.342954	1	2	0.28005	no
gi|641569028|gb|KK856793.1|	28710-29659	9.7	41	0.845457	0.999815	0.241929	0.8798	no
gi|641569028|gb|KK856793.1|	69784-73361	9.7	41	0.984046	2	0.837359	0.5289	no
gi|641569028|gb|KK856793.1|	73548-74001	9.7	41	2	0.822312	-1	0.4534	no
gi|641569030|gb|KK856792.1|	2-1394	12	45	0.295825	1	2	0.2126	no
gi|641569030|gb|KK856792.1|	27188-27649	12	45	2	0.399203	-3	0.271	no
gi|641569030|gb|KK856792.1|	29345-30642	12	45	0.964001	0.783056	-0.29992	0.84515	no
gi|641569030|gb|KK856792.1|	31260-31893	12	45	1	2	0.406231	0.8089	no
gi|641569030|gb|KK856792.1|	32221-34159	12	45	0.690681	0.743567	0.106443	1	no
gi|641569033|gb|KK856791.1|	00587-10175	11	42	4	1	-2	0.19205	no
gi|641569033|gb|KK856791.1|	02324-10324	11	42	4	6	0.604163	0.6468	no
gi|641569033|gb|KK856791.1|	67674-68622	11	42	0	2	inf	0.00375	no
gi|641569033|gb|KK856791.1|	74611-75008	11	42	0	15	inf	0.0004	yes
gi|641569033|gb|KK856791.1|	9568-10622	11	42	0	37	inf	5.00E-05	yes
gi|641569036|gb|KK856790.1|	01406-10169	10	36	8	8	0.104602	0.94315	no
gi|641569036|gb|KK856790.1|	04497-10470	10	36	16	5	-2	0.3632	no
gi|641569036|gb|KK856790.1|	05471-10598	10	36	4	3	-0.558079	0.7062	no
gi|641569036|gb|KK856790.1|	18605-18953	10	36	6	5	-0.123093	0.93965	no
gi|641569036|gb|KK856790.1|	29591-31743	10	36	3	5	0.657142	0.60755	no

gi|641569036|gb|KK856790.1|	31850-32845	10	36	7	8	0.311217	0.807	no
gi|641569036|gb|KK856790.1|	33856-35692	10	36	2	1	-0.806306	0.5598	no
gi|641569036|gb|KK856790.1|	35860-36506	10	36	2	1	-0.538242	0.7478	no
gi|641569036|gb|KK856790.1|	36762-41117	10	36	2	2	0.458912	0.7135	no
gi|641569036|gb|KK856790.1|	41536-42871	10	36	2	2	-0.177122	0.8947	no
gi|641569036|gb|KK856790.1|	43783-51156	10	36	10	6	-0.865646	0.7043	no
gi|641569036|gb|KK856790.1|	45-1211	10	36	16	11	-0.524683	0.6701	no
gi|641569036|gb|KK856790.1|	52302-53135	10	36	22	21	-0.0343528	0.97765	no
gi|641569036|gb|KK856790.1|	53778-55104	10	36	0.563566	1	1	0.4299	no
gi|641569036|gb|KK856790.1|	57049-57249	10	36	23	9	-1	0.44195	no
gi|641569036|gb|KK856790.1|	60830-61483	10	36	10	6	-0.690046	0.60165	no
gi|641569036|gb|KK856790.1|	67707-68871	10	36	8	3	-1	0.29185	no
gi|641569036|gb|KK856790.1|	82301-87315	10	36	8	7	-0.175144	0.91425	no
gi|641569036|gb|KK856790.1|	87500-88309	10	36	3	3	0.166617	0.9073	no
gi|641569036|gb|KK856790.1|	88821-90105	10	36	0.520273	0.297139	-0.808131	1	no
gi|641569036|gb|KK856790.1|	90260-94753	10	36	1	2	0.434804	0.73535	no
gi|641569036|gb|KK856790.1|	95569-97515	10	36	1	0.740177	-0.560967	0.6932	no
gi|641569042|gb|KK856788.1|	47116-48034	8.5	35	5	9	0.949938	0.47425	no
gi|641569042|gb|KK856788.1|	49983-50615	8.5	35	2	13	2	0.10595	no
gi|641569042|gb|KK856788.1|	66911-67151	8.5	35	27	12	-1	0.41345	no
gi|641569042|gb|KK856788.1|	67387-67640	8.5	35	86	26	-2	0.21105	no
gi|641569042|gb|KK856788.1|	70351-70616	8.5	35	48	11	-2	0.1616	no
gi|641569042|gb|KK856788.1|	72582-73420	8.5	35	4	2	-0.759421	0.58215	no
gi|641569042|gb|KK856788.1|	73852-75050	8.5	35	0.988567	0.215013	-2	0.2515	no
gi|641569042|gb|KK856788.1|	76655-77676	8.5	35	5	3	-0.583853	0.66745	no
gi|641569042|gb|KK856788.1|	79553-80799	8.5	35	3	2	-0.878716	0.52505	no
gi|641569057|gb|KK856783.1|	2618-2895	8.6	36	23	21	-0.097704	0.9464	no
gi|641569060|gb|KK856782.1|	13672-17362	10	39	0.790146	0.959018	0.279439	0.8291	no
gi|641569060|gb|KK856782.1|	17619-18397	10	39	0.486026	73	7	0.12195	no
gi|641569060|gb|KK856782.1|	3980-5598	10	39	0.447899	2	2	0.23605	no

gi|641569060|gb|KK856782.1|	51985-53874	10	39	4	2	-0.705264	0.5896	no
gi|641569063|gb|KK856781.1|	26994-27185	7.7	34	33	33	-0.00502505	0.95595	no
gi|641569070|gb|KK856779.1|	37527-38145	9.7	37	3	1	-1	0.37245	no
gi|641569073|gb|KK856778.1|	63506-66184	9.2	33	5	5	-0.0536826	0.9683	no
gi|641569073|gb|KK856778.1|	68756-69489	9.2	33	3	11	2	0.2324	no
gi|641569073|gb|KK856778.1|	69734-70287	9.2	33	10	27	1	0.27065	no
gi|641569073|gb|KK856778.1|	70732-73407	9.2	33	5	9	0.919435	0.4668	no
gi|641569073|gb|KK856778.1|	81559-82183	9.2	33	7	2	-2	0.2956	no
gi|641569073|gb|KK856778.1|	91737-92243	9.2	33	0	26	inf	5.00E-05	yes
gi|641569077|gb|KK856777.1|	37655-38902	11	43	0.33661	1	2	0.2348	no
gi|641569077|gb|KK856777.1|	47562-48036	11	43	8	0.381166	-4	0.18815	no
gi|641569077|gb|KK856777.1|	51110-51477	11	43	2	4	0.580403	0.7575	no
gi|641569077|gb|KK856777.1|	65418-66883	11	43	0.613397	1	0.733211	0.63585	no
gi|641569077|gb|KK856777.1|	69843-75056	11	43	4	6	0.606309	0.62375	no
gi|641569077|gb|KK856777.1|	79973-81795	11	43	4	3	-0.552762	0.67675	no
gi|641569077|gb|KK856777.1|	82130-82743	11	43	7	10	0.424207	0.74895	no
gi|641569077|gb|KK856777.1|	83047-84220	11	43	11	5	-1	0.36205	no
gi|641569077|gb|KK856777.1|	86107-87078	11	43	4	3	-0.45941	0.73145	no
gi|641569083|gb|KK856775.1|	04609-10510	10	40	0	17	inf	0.00025	yes
gi|641569083|gb|KK856775.1|	06562-10734	10	40	0	64	inf	0.00075	yes
gi|641569083|gb|KK856775.1|	09933-11010	10	40	0	91	inf	0.0016	yes
gi|641569083|gb|KK856775.1|	12233-11342	10	40	0	1	inf	0.00375	no
gi|641569083|gb|KK856775.1|	11378-11697	10	40	0	10	inf	0.0025	yes
gi|641569083|gb|KK856775.1|	15095-11530	10	40	0	35	inf	0.00255	yes
gi|641569083|gb|KK856775.1|	16855-11713	10	40	0	157.4	inf	5.00E-05	yes
gi|641569083|gb|KK856775.1|	19744-12062	10	40	0	8	inf	0.0001	yes
gi|641569083|gb|KK856775.1|	23667-12396	10	40	0	13	inf	0.0025	yes
gi|641569083|gb|KK856775.1|	61334-61718	10	40	1	12	3	0.14575	no
gi|641569083|gb|KK856775.1|	6321-6872	10	40	0	15	inf	0.0001	yes
gi|641569083|gb|KK856775.1|	63744-64002	10	40	0.920292	19	4	0.1141	no

gi|641569083|gb|KK856775.1|	9066-10177	10	40	0	3	inf	0.00065	yes
gi|641569086|gb|KK856774.1|	1021-2946	9.2	37	0.614005	0.873999	0.509382	1	no
gi|641569086|gb|KK856774.1|	10318-10694	9.2	37	3	9	1	0.40675	no
gi|641569086|gb|KK856774.1|	20792-21011	9.2	37	59	9	-3	0.12445	no
gi|641569086|gb|KK856774.1|	3285-5261	9.2	37	31	24	-0.36545	0.8754	no
gi|641569086|gb|KK856774.1|	55855-56820	9.2	37	1	0.699387	-0.532842	0.7478	no
gi|641569086|gb|KK856774.1|	7259-8538	9.2	37	5	10	0.92881	0.46365	no
gi|641569089|gb|KK856773.1|	62692-62883	9.7	20	31	22	-0.47451	0.76935	no
gi|641569089|gb|KK856773.1|	71610-73484	9.7	20	1	1	0.212885	0.8789	no
gi|641569089|gb|KK856773.1|	74203-75918	9.7	20	0.838621	1	0.345411	0.81115	no
gi|641569098|gb|KK856770.1|	44399-45700	8.4	29	1	0	-inf	0.00435	no
gi|641569098|gb|KK856770.1|	48591-49712	8.4	29	1	0	-inf	0.0034	yes
gi|641569104|gb|KK856768.1|	20743-12133	9.5	33	4	2	-1	0.48225	no
gi|641569104|gb|KK856768.1|	66846-67958	9.5	33	2	2	-0.48722	0.72355	no
gi|641569104|gb|KK856768.1|	69881-71616	9.5	33	0.045985	699	14	0.10435	no
gi|641569104|gb|KK856768.1|	71670-72734	9.5	33	0.570528	2	2	0.2786	no
gi|641569104|gb|KK856768.1|	72810-73448	9.5	33	0.159867	2	4	0.12385	no
gi|641569110|gb|KK856766.1|	64618-64850	8.1	30	20	2	-3	0.2088	no
gi|641569117|gb|KK856764.1|	37771-39471	11	39	0.705817	2	1	0.4516	no
gi|641569117|gb|KK856764.1|	45578-46746	11	39	0.727898	0.886467	0.284332	0.85455	no
gi|641569117|gb|KK856764.1|	54718-55279	11	39	3	1	-1	0.37245	no
gi|641569117|gb|KK856764.1|	86687-87438	11	39	2	0.386688	-2	0.21095	no
gi|641569117|gb|KK856764.1|	89074-89623	11	39	1	2	0.3327	0.83525	no
gi|641569117|gb|KK856764.1|	89723-90879	11	39	1	1	-0.015304	0.96665	no
gi|641569121|gb|KK856763.1|	17421-17968	8.8	33	0.603001	2	2	0.3323	no
gi|641569121|gb|KK856763.1|	20898-22190	8.8	33	0.581038	0.884939	0.606946	0.6902	no
gi|641569121|gb|KK856763.1|	28306-29940	8.8	33	0.393705	0.750196	0.930154	1	no
gi|641569121|gb|KK856763.1|	7603-9732	8.8	33	1	4	1	0.26775	no
gi|641569124|gb|KK856762.1|	26876-28003	9.3	37	0.835922	2	0.953917	0.53865	no
gi|641569124|gb|KK856762.1|	3276-3932	9.3	37	0.61455	1	1	0.51305	no

gi|641569124|gb|KK856762.1|	36784-38625	9.3	37	0.774414	4	2	0.13065	no
gi|641569124|gb|KK856762.1|	38778-39596	9.3	37	0.795913	3	2	0.24255	no
gi|641569124|gb|KK856762.1|	40765-41750	9.3	37	1	3	1	0.3964	no
gi|641569124|gb|KK856762.1|	42117-43016	9.3	37	0	3	inf	0.00105	yes
gi|641569124|gb|KK856762.1|	43944-45425	9.3	37	0.550694	1	1	0.4709	no
gi|641569124|gb|KK856762.1|	46843-48485	9.3	37	0.440468	1	1	0.3858	no
gi|641569124|gb|KK856762.1|	50346-51154	9.3	37	1	4	2	0.26665	no
gi|641569124|gb|KK856762.1|	53207-54644	9.3	37	0.399146	1	2	0.2915	no
gi|641569124|gb|KK856762.1|	56294-56601	9.3	37	8	6	-0.324029	0.822	no
gi|641569127|gb|KK856761.1|	14285-15336	10	39	0.579195	0.75532	0.383039	1	no
gi|641569130|gb|KK856760.1|	08507-11002	11	46	0	1	inf	0.0023	yes
gi|641569130|gb|KK856760.1|	67342-69541	11	46	2	0.754727	-2	0.26995	no
gi|641569130|gb|KK856760.1|	70066-72066	11	46	4	0.2992	-4	0.04965	no
gi|641569130|gb|KK856760.1|	76575-77726	11	46	12	2	-3	0.0642	no
gi|641569130|gb|KK856760.1|	77851-79566	11	46	10	1	-3	0.04195	no
gi|641569133|gb|KK856759.1|	05540-10572	11	41	96	187	0.971334	0.47305	no
gi|641569133|gb|KK856759.1|	06694-10704	11	41	6	9	0.685438	0.64385	no
gi|641569133|gb|KK856759.1|	13037-11369	11	41	22	38	0.78374	0.53225	no
gi|641569133|gb|KK856759.1|	14126-11512	11	41	0.439931	2	2	0.28495	no
gi|641569133|gb|KK856759.1|	17194-11749	11	41	13	20	0.634977	0.64745	no
gi|641569133|gb|KK856759.1|	19243-11944	11	41	43	95	1	0.414	no
gi|641569133|gb|KK856759.1|	21196-12228	11	41	57	159	1	0.5063	no
gi|641569133|gb|KK856759.1|	28994-13007	11	41	45	10	-2	0.233	no
gi|641569133|gb|KK856759.1|	56296-57196	11	41	2	2	0.340921	0.81435	no
gi|641569133|gb|KK856759.1|	58380-59594	11	41	9	15	0.853844	0.5029	no
gi|641569133|gb|KK856759.1|	60068-61509	11	41	7	11	0.60759	0.6246	no
gi|641569133|gb|KK856759.1|	67378-68093	11	41	11	15	0.395878	0.7607	no
gi|641569133|gb|KK856759.1|	72533-73423	11	41	1	2	0.826227	0.5841	no
gi|641569133|gb|KK856759.1|	74399-75231	11	41	2	4	0.795708	0.56325	no
gi|641569133|gb|KK856759.1|	75894-77047	11	41	0.591438	31	6	0.0457	no

gi|641569133|gb|KK856759.1|	77328-79396	11	41	0.869583	2	1	0.3168	no
gi|641569133|gb|KK856759.1|	79920-80422	11	41	3	1	-1	0.46975	no
gi|641569133|gb|KK856759.1|	80782-82345	11	41	0.983588	1	0.530011	0.70515	no
gi|641569133|gb|KK856759.1|	83817-85125	11	41	143	113	-0.333131	0.8746	no
gi|641569133|gb|KK856759.1|	86736-93265	11	41	22	21	-0.0380559	0.98445	no
gi|641569136|gb|KK856758.1|	26268-26895	10	41	1	4	2	0.243	no
gi|641569136|gb|KK856758.1|	76265-77414	10	41	3	2	-0.944058	0.5016	no
gi|641569140|gb|KK856757.1|	17916-18525	7.8	35	2	4	1	0.3654	no
gi|641569140|gb|KK856757.1|	19860-20628	7.8	35	0.989019	0.375297	-1	0.4534	no
gi|641569140|gb|KK856757.1|	22196-22949	7.8	35	0.253901	2	3	0.2991	no
gi|641569146|gb|KK856755.1|	1043-1620	10	34	0.926506	4	2	0.20855	no
gi|641569146|gb|KK856755.1|	06579-10711	10	34	1	2	0.595267	0.70555	no
gi|641569146|gb|KK856755.1|	07222-10766	10	34	2	0.850932	-1	0.4534	no
gi|641569146|gb|KK856755.1|	07766-10954	10	34	35	33	-0.0865431	0.9694	no
gi|641569146|gb|KK856755.1|	09685-11391	10	34	18	11	-0.731176	0.74025	no
gi|641569146|gb|KK856755.1|	14079-11570	10	34	3	21	3	0.1725	no
gi|641569146|gb|KK856755.1|	16982-11740	10	34	17	8	-0.998124	0.4767	no
gi|641569146|gb|KK856755.1|	14888-15565	10	34	68	46	-0.572169	0.6437	no
gi|641569146|gb|KK856755.1|	19324-19484	10	34	201	125	-0.684076	0.62985	no
gi|641569146|gb|KK856755.1|	21605-23101	10	34	0.707647	0.912492	0.366783	0.8038	no
gi|641569146|gb|KK856755.1|	23287-23912	10	34	1	1	-0.0794448	0.92675	no
gi|641569146|gb|KK856755.1|	24038-25057	10	34	26	14	-0.890918	0.48135	no
gi|641569146|gb|KK856755.1|	26616-26927	10	34	21	10	-1	0.44775	no
gi|641569146|gb|KK856755.1|	29406-30088	10	34	20	10	-0.995154	0.44135	no
gi|641569146|gb|KK856755.1|	31717-32181	10	34	4	0	-inf	0.0059	no
gi|641569146|gb|KK856755.1|	33-896	10	34	2	2	0.188454	0.8991	no
gi|641569146|gb|KK856755.1|	3311-3657	10	34	4	1	-1	0.44305	no
gi|641569146|gb|KK856755.1|	36296-37546	10	34	0.469914	0.613361	0.384339	1	no
gi|641569146|gb|KK856755.1|	37667-38696	10	34	5	10	1	0.38925	no
gi|641569146|gb|KK856755.1|	39203-39471	10	34	71	20	-2	0.1824	no

gi|641569146|gb|KK856755.1|	45287-46266	10	34	0.903438	0.549567	-0.71713	0.6701	no
gi|641569146|gb|KK856755.1|	4838-6186	10	34	1	0.374267	-1	0.37565	no
gi|641569146|gb|KK856755.1|	48702-49565	10	34	16	12	-0.454611	0.71905	no
gi|641569146|gb|KK856755.1|	49955-50177	10	34	121	59	-1	0.44215	no
gi|641569146|gb|KK856755.1|	52032-52390	10	34	60	26	-1	0.35535	no
gi|641569146|gb|KK856755.1|	54939-55126	10	34	140.87	89	-0.661079	0.6172	no
gi|641569146|gb|KK856755.1|	57543-58089	10	34	7	3	-1	0.31395	no
gi|641569146|gb|KK856755.1|	62828-64133	10	34	2	0.971789	-1	0.41965	no
gi|641569146|gb|KK856755.1|	73365-75427	10	34	0.455154	1	1	0.3959	no
gi|641569146|gb|KK856755.1|	75553-76302	10	34	1	0.970183	-0.0763874	0.92685	no
gi|641569146|gb|KK856755.1|	76479-77387	10	34	0.596063	1.51	1	0.4118	no
gi|641569146|gb|KK856755.1|	78229-79229	10	34	1	2	1	0.42625	no
gi|641569146|gb|KK856755.1|	80219-87815	10	34	14	4	-2	0.31225	no
gi|641569146|gb|KK856755.1|	87885-90335	10	34	6	5	-0.0505373	0.9664	no
gi|641569146|gb|KK856755.1|	93179-93513	10	34	38	37	-0.0481278	0.96825	no
gi|641569146|gb|KK856755.1|	94298-95174	10	34	0.728097	0.632224	-0.203695	1	no
gi|641569146|gb|KK856755.1|	95823-96711	10	34	1	0.621337	-0.981152	0.5872	no
gi|641569146|gb|KK856755.1|	96925-97668	10	34	6	5	-0.301134	0.81945	no
gi|641569146|gb|KK856755.1|	98833-99097	10	34	258	196	-0.399922	0.74245	no
gi|641569149|gb|KK856754.1|	12286-12853	10	37	0.761028	2	1	0.44255	no
gi|641569149|gb|KK856754.1|	12923-13337	10	37	2	2	0.00136133	0.91115	no
gi|641569149|gb|KK856754.1|	31312-32358	10	37	4	3	-0.556881	0.6816	no
gi|641569149|gb|KK856754.1|	39679-39891	10	37	27	35	0.34372	0.812	no
gi|641569149|gb|KK856754.1|	43574-44230	10	37	1	0.931189	-0.207811	0.8945	no
gi|641569149|gb|KK856754.1|	44356-45345	10	37	0.446028	0.949687	1	0.50945	no
gi|641569149|gb|KK856754.1|	55424-56652	10	37	3	4	0.52861	0.68885	no
gi|641569149|gb|KK856754.1|	59970-64331	10	37	9	4	-1	0.41855	no
gi|641569149|gb|KK856754.1|	72591-74160	10	37	12	19	0.651285	0.6063	no
gi|641569149|gb|KK856754.1|	74221-75100	10	37	5	1	-2	0.1727	no
gi|641569149|gb|KK856754.1|	75200-77158	10	37	4	5	0.441037	0.72555	no

gi|641569149|gb|KK856754.1|	77274-79924	10	37	4	3	-0.295608	0.8154	no
gi|641569149|gb|KK856754.1|	80296-82643	10	37	1	1	-0.560337	0.67485	no
gi|641569152|gb|KK856753.1|	34527-35620	9.6	20	8	30	2	0.1327	no
gi|641569152|gb|KK856753.1|	91423-91788	9.6	20	2	4	1	0.4433	no
gi|641569155|gb|KK856752.1|	17856-11807	11	21	15	7	-1	0.4939	no
gi|641569155|gb|KK856752.1|	13986-14937	11	21	0.655838	0.712274	0.119093	1	no
gi|641569155|gb|KK856752.1|	16459-17532	11	21	0.564678	0.61373	0.120177	1	no
gi|641569155|gb|KK856752.1|	20948-21862	11	21	3	2	-0.288967	0.8405	no
gi|641569155|gb|KK856752.1|	33642-33910	11	21	22	6	-2	0.2554	no
gi|641569155|gb|KK856752.1|	43878-44186	11	21	11	2	-3	0.1909	no
gi|641569155|gb|KK856752.1|	44241-46014	11	21	2	2	-0.239409	0.85615	no
gi|641569155|gb|KK856752.1|	46388-47245	11	21	6	4	-0.507619	0.70405	no
gi|641569155|gb|KK856752.1|	48290-49140	11	21	2	0.328587	-2	0.2115	no
gi|641569155|gb|KK856752.1|	49310-50900	11	21	1	0.541656	-0.906476	0.5508	no
gi|641569155|gb|KK856752.1|	52380-54034	11	21	0.922228	0.147988	-3	0.19075	no
gi|641569155|gb|KK856752.1|	58720-59581	11	21	1	0.323191	-2	0.3217	no
gi|641569155|gb|KK856752.1|	60175-61753	11	21	3	2	-0.678821	0.6054	no
gi|641569155|gb|KK856752.1|	63461-64145	11	21	3	1	-2	0.30235	no
gi|641569155|gb|KK856752.1|	66165-67424	11	21	2	1	-1	0.41405	no
gi|641569155|gb|KK856752.1|	68929-70854	11	21	2	0.499428	-2	0.24635	no
gi|641569155|gb|KK856752.1|	72580-72807	11	21	58	8	-3	0.1098	no
gi|641569155|gb|KK856752.1|	73019-73733	11	21	15	3	-2	0.1053	no
gi|641569155|gb|KK856752.1|	74088-79694	11	21	135	48	-1	0.4263	no
gi|641569155|gb|KK856752.1|	7998-9378	11	21	1	0.910481	-0.240651	0.8714	no
gi|641569155|gb|KK856752.1|	80461-81099	11	21	1	0.9686	-0.400973	0.82205	no
gi|641569155|gb|KK856752.1|	82918-85000	11	21	6	3	-1	0.3555	no
gi|641569155|gb|KK856752.1|	85755-87752	11	21	9	8	-0.221709	0.85795	no
gi|641569155|gb|KK856752.1|	89184-90305	11	21	3	3	-0.078546	0.95275	no
gi|641569155|gb|KK856752.1|	91633-92575	11	21	1	0.720812	-0.533078	0.7478	no
gi|641569155|gb|KK856752.1|	97414-98955	11	21	1	0.160377	-3	0.1621	no

gi|641569155|gb|KK856752.1|	9814-12299	11	21	0.742138	1	0.548353	0.6859	no
gi|641569164|gb|KK856749.1|	16542-11706	12	40	0	142.78	inf	0.0005	yes
gi|641569164|gb|KK856749.1|	18794-11943	12	40	0	3	inf	0.00455	no
gi|641569164|gb|KK856749.1|	48531-14927	12	40	3	2	-0.720263	0.61365	no
gi|641569167|gb|KK856748.1|	13912-14995	7.8	31	2	0	-inf	0.00175	yes
gi|641569167|gb|KK856748.1|	1718-3066	7.8	31	19	0	-inf	5.00E-05	yes
gi|641569167|gb|KK856748.1|	19741-20623	7.8	31	21	0	-inf	5.00E-05	yes
gi|641569167|gb|KK856748.1|	5709-6055	7.8	31	7	0	-inf	0.0059	no
gi|641569167|gb|KK856748.1|	9111-10243	7.8	31	5	0	-inf	5.00E-05	yes
gi|641569180|gb|KK856746.1|	78317-78761	9.7	40	1	4	1	0.4014	no
gi|641569180|gb|KK856746.1|	87984-88772	9.7	40	0.597284	0.906806	0.602377	0.70505	no
gi|641569183|gb|KK856745.1|	16458-16692	11	42	26	2	-4	0.19635	no
gi|641569189|gb|KK856743.1|	84815-85115	7.2	30	2	6	1	0.5206	no
gi|641569192|gb|KK856742.1|	12378-13022	8.9	32	45	20	-1	0.3563	no
gi|641569192|gb|KK856742.1|	34164-34390	8.9	32	23	10	-1	0.44045	no
gi|641569195|gb|KK856741.1|	22191-22956	10	20	2	1	-0.719949	0.6521	no
gi|641569195|gb|KK856741.1|	28646-29623	10	20	0.996324	0.550966	-0.854653	0.60675	no
gi|641569195|gb|KK856741.1|	29725-30538	10	20	0.916978	0.696279	-0.397222	0.82205	no
gi|641569195|gb|KK856741.1|	36103-38013	10	20	1	1	0.325094	0.80595	no
gi|641569195|gb|KK856741.1|	38366-39129	10	20	2	1	-0.497592	0.74225	no
gi|641569195|gb|KK856741.1|	40815-42292	10	20	1	0.08418	-4	0.19735	no
gi|641569195|gb|KK856741.1|	46686-47972	10	20	2	0.494327	-2	0.23295	no
gi|641569195|gb|KK856741.1|	48322-50213	10	20	0.876842	1	0.608849	0.66295	no
gi|641569198|gb|KK856740.1|	39650-40147	8.1	34	0.233953	18	6	0.10475	no
gi|641569198|gb|KK856740.1|	44621-45031	8.1	34	0	9	inf	0.00135	yes
gi|641569198|gb|KK856740.1|	45243-45933	8.1	34	0	13	inf	5.00E-05	yes
gi|641569198|gb|KK856740.1|	47229-48361	8.1	34	0	11	inf	5.00E-05	yes
gi|641569198|gb|KK856740.1|	55031-56250	8.1	34	0	2	inf	0.00115	yes
gi|641569198|gb|KK856740.1|	56402-56912	8.1	34	0	120	inf	0.00095	yes
gi|641569198|gb|KK856740.1|	59600-60101	8.1	34	0	4	inf	0.00455	no

gi|641569198|gb|KK856740.1|	62263-63600	8.1	34	0	2	inf	0.00045	yes
gi|641569198|gb|KK856740.1|	65719-66672	8.1	34	0	3	inf	0.0005	yes
gi|641569198|gb|KK856740.1|	67871-68422	8.1	34	0	7	inf	0.00115	yes
gi|641569198|gb|KK856740.1|	68550-68806	8.1	34	0	23	inf	0.0016	yes
gi|641569198|gb|KK856740.1|	71051-71591	8.1	34	0	1531.29	inf	5.00E-05	yes
gi|641569198|gb|KK856740.1|	8900-9252	8.1	34	0	566	inf	0.00015	yes
gi|641569201|gb|KK856739.1|	42383-44057	8.4	36	196	0.218988	-10	0.09525	no
gi|641569201|gb|KK856739.1|	45045-46618	8.4	36	12	0	-inf	5.00E-05	yes
gi|641569201|gb|KK856739.1|	46851-47529	8.4	36	36	0	-inf	5.00E-05	yes
gi|641569201|gb|KK856739.1|	47634-47989	8.4	36	12	0	-inf	0.0013	yes
gi|641569201|gb|KK856739.1|	55389-55685	8.4	36	163	0	-inf	5.00E-05	yes
gi|641569201|gb|KK856739.1|	83317-84331	8.4	36	10	32	2	0.185	no
gi|641569201|gb|KK856739.1|	87953-88226	8.4	36	2	13	3	0.26765	no
gi|641569204|gb|KK856738.1|	23405-23884	8.9	30	5	3	-0.99313	0.5308	no
gi|641569207|gb|KK856737.1|	81284-82144	10	42	0.745623	2	1	0.48415	no
gi|641569210|gb|KK856736.1|	05338-10618	11	39	0.650855	9	4	0.0707	no
gi|641569210|gb|KK856736.1|	06256-10693	11	39	27	20	-0.423207	0.73095	no
gi|641569210|gb|KK856736.1|	08341-10976	11	39	0.520736	0.44078	-0.240492	1	no
gi|641569210|gb|KK856736.1|	09957-11103	11	39	0.643148	2	1	0.38845	no
gi|641569210|gb|KK856736.1|	11162-11531	11	39	10	30	2	0.24315	no
gi|641569210|gb|KK856736.1|	13102-11665	11	39	2	2	-0.439177	0.72925	no
gi|641569210|gb|KK856736.1|	16797-11751	11	39	1	0.41011	-2	0.3455	no
gi|641569210|gb|KK856736.1|	17779-11957	11	39	2	0.405691	-2	0.2213	no
gi|641569210|gb|KK856736.1|	20062-12118	11	39	1	2	0.605993	0.67	no
gi|641569210|gb|KK856736.1|	21343-12398	11	39	0.896717	0.6622	-0.437386	0.7491	no
gi|641569210|gb|KK856736.1|	24175-12592	11	39	1	0.138519	-3	0.15595	no
gi|641569210|gb|KK856736.1|	23392-23742	11	39	5	12	1	0.39545	no
gi|641569210|gb|KK856736.1|	25912-26157	11	39	25	72	2	0.26105	no
gi|641569210|gb|KK856736.1|	30615-33824	11	39	9	13	0.574945	0.72205	no
gi|641569210|gb|KK856736.1|	33941-34596	11	39	0.769854	1	0.862551	0.62725	no

gi|641569210|gb|KK856736.1|	35853-36184	11	39	31	65	1	0.4155	no
gi|641569210|gb|KK856736.1|	38969-39313	11	39	10	32	2	0.24345	no
gi|641569210|gb|KK856736.1|	50827-52084	11	39	0.533504	0.507777	-0.0713032	1	no
gi|641569210|gb|KK856736.1|	54678-55223	11	39	0.404282	3	3	0.27515	no
gi|641569210|gb|KK856736.1|	57238-58040	11	39	3	10	2	0.17425	no
gi|641569210|gb|KK856736.1|	5969-6618	11	39	2	3	0.724879	0.63585	no
gi|641569210|gb|KK856736.1|	7348-7713	11	39	2	7	2	0.36635	no
gi|641569210|gb|KK856736.1|	8521-8769	11	39	14	71	2	0.1259	no
gi|641569210|gb|KK856736.1|	86058-86787	11	39	1	2	0.601289	0.6936	no
gi|641569210|gb|KK856736.1|	86899-88112	11	39	0.973997	0.847428	-0.200827	0.8959	no
gi|641569210|gb|KK856736.1|	88915-89706	11	39	0.831987	1	0.602426	0.69715	no
gi|641569210|gb|KK856736.1|	96237-96649	11	39	0.323239	5	4	0.1196	no
gi|641569223|gb|KK856733.1|	04570-10473	12	42	23	58	1	0.47755	no
gi|641569223|gb|KK856733.1|	05715-10664	12	42	3	32	3	0.0218	no
gi|641569223|gb|KK856733.1|	10881-11418	12	42	2	0.624911	-1	0.4534	no
gi|641569223|gb|KK856733.1|	10506-11081	12	42	5	3	-1	0.5278	no
gi|641569223|gb|KK856733.1|	18496-21330	12	42	2	3	0.676184	0.5969	no
gi|641569223|gb|KK856733.1|	22505-22800	12	42	12	34	1	0.30415	no
gi|641569223|gb|KK856733.1|	24318-24867	12	42	1	2	0.818127	0.6234	no
gi|641569223|gb|KK856733.1|	29058-33884	12	42	3	5	0.821534	0.51285	no
gi|641569223|gb|KK856733.1|	33995-35110	12	42	1	0.937324	-0.394089	0.7971	no
gi|641569223|gb|KK856733.1|	36325-37110	12	42	2	0.911387	-0.776185	0.63565	no
gi|641569223|gb|KK856733.1|	37798-38502	12	42	1	0.422091	-1	0.4534	no
gi|641569223|gb|KK856733.1|	41656-41931	12	42	18	8	-1	0.44345	no
gi|641569223|gb|KK856733.1|	44690-45796	12	42	1	0	-inf	0.0075	no
gi|641569223|gb|KK856733.1|	46062-46661	12	42	2	0.530438	-2	0.3216	no
gi|641569223|gb|KK856733.1|	48197-48552	12	42	24	0	-inf	0.0002	yes
gi|641569223|gb|KK856733.1|	49711-53617	12	42	6	6	0.00193375	0.997	no
gi|641569223|gb|KK856733.1|	53768-58130	12	42	5	0.441178	-4	0.02135	no
gi|641569223|gb|KK856733.1|	62637-64926	12	42	17	0.206385	-6	0.06105	no

gi|641569223|gb|KK856733.1|	73625-76608	12	42	35	19	-0.848952	0.69975	no
gi|641569223|gb|KK856733.1|	78645-82270	12	42	10	7	-0.622335	0.69895	no
gi|641569223|gb|KK856733.1|	82414-82881	12	42	9	47	2	0.08415	no
gi|641569223|gb|KK856733.1|	83048-83772	12	42	0.803447	1	0.601185	0.70555	no
gi|641569223|gb|KK856733.1|	83927-85793	12	42	3	12	2	0.097	no
gi|641569223|gb|KK856733.1|	86466-89983	12	42	3	8	1	0.2882	no
gi|641569223|gb|KK856733.1|	92247-92599	12	42	3	13	2	0.2007	no
gi|641569223|gb|KK856733.1|	95298-95561	12	42	6	125	4	0.05375	no
gi|641569223|gb|KK856733.1|	96423-96866	12	42	1	7	2	0.2089	no
gi|641569233|gb|KK856730.1|	10221-11953	70	42	6	0	-inf	5.00E-05	yes
gi|641569233|gb|KK856730.1|	07123-10852	70	42	70	23	-2	0.3388	no
gi|641569233|gb|KK856730.1|	09761-11099	70	42	3	1	-1	0.30405	no
gi|641569233|gb|KK856730.1|	11961-11384	70	42	7	2	-2	0.21165	no
gi|641569233|gb|KK856730.1|	20841-12203	70	42	2	0.215656	-3	0.1501	no
gi|641569233|gb|KK856730.1|	23106-12551	70	42	3	0.488266	-2	0.1093	no
gi|641569233|gb|KK856730.1|	12338-13645	70	42	4	0.097005	-5	0.1844	no
gi|641569233|gb|KK856730.1|	26330-12809	70	42	4	1	-2	0.2205	no
gi|641569233|gb|KK856730.1|	31707-32723	70	42	6	0.131229	-5	0.18345	no
gi|641569233|gb|KK856730.1|	34670-39627	70	42	58	0	-inf	0.0003	yes
gi|641569233|gb|KK856730.1|	46358-51448	70	42	126	0	-inf	5.00E-05	yes
gi|641569233|gb|KK856730.1|	4741-5207	70	42	5	0	-inf	0.0032	yes
gi|641569233|gb|KK856730.1|	5855-6736	70	42	0.619533	2	1	0.4118	no
gi|641569233|gb|KK856730.1|	58690-60076	70	42	657.9	0	-inf	5.00E-05	yes
gi|641569233|gb|KK856730.1|	62903-68217	70	42	234	0	-inf	5.00E-05	yes
gi|641569233|gb|KK856730.1|	69468-70764	70	42	27	0	-inf	0.0018	yes
gi|641569233|gb|KK856730.1|	7081-7904	70	42	1	0	-inf	0.0102	no
gi|641569233|gb|KK856730.1|	71462-73166	70	42	1	0	-inf	0.00185	yes
gi|641569233|gb|KK856730.1|	73362-74193	70	42	1	0	-inf	0.01175	no
gi|641569233|gb|KK856730.1|	81372-82281	70	42	12	0	-inf	5.00E-05	yes
gi|641569233|gb|KK856730.1|	84401-85612	70	42	0.836498	0	-inf	1	no

gi|641569233|gb|KK856730.1|	8767-9304	70	42	221.52	3	-6	0.0261	no
gi|641569233|gb|KK856730.1|	99-490	70	42	13	0	-inf	0.00045	yes
gi|641569237|gb|KK856729.1|	87567-87951	12	22	12	10	-0.21367	0.8786	no
gi|641569237|gb|KK856729.1|	90965-91441	12	22	9	8	-0.145339	0.91155	no
gi|641569246|gb|KK856728.1|	10501-11084	8.7	32	37	33	-0.142187	0.9054	no
gi|641569246|gb|KK856728.1|	12248-13365	8.7	32	0.768173	0.701475	-0.131041	1	no
gi|641569246|gb|KK856728.1|	16649-16895	8.7	32	65	48	-0.448922	0.72505	no
gi|641569246|gb|KK856728.1|	17703-17903	8.7	32	119	83	-0.522196	0.6923	no
gi|641569246|gb|KK856728.1|	20291-20465	8.7	32	103	23	-2	0.19455	no
gi|641569246|gb|KK856728.1|	22364-23068	8.7	32	17	14	-0.261748	0.83565	no
gi|641569246|gb|KK856728.1|	25986-26361	8.7	32	14	14	0.0961419	0.94275	no
gi|641569246|gb|KK856728.1|	27071-28285	8.7	32	0.417018	1	1	0.41175	no
gi|641569246|gb|KK856728.1|	30447-31152	8.7	32	0.83336	1	0.600771	0.70555	no
gi|641569246|gb|KK856728.1|	32289-34892	8.7	32	2	3	0.777337	0.5396	no
gi|641569246|gb|KK856728.1|	38489-42385	8.7	32	5	4	-0.265687	0.833	no
gi|641569246|gb|KK856728.1|	43083-44299	8.7	32	2	3	0.55924	0.67805	no
gi|641569246|gb|KK856728.1|	44515-46151	8.7	32	1	0.898989	-0.61416	0.65835	no
gi|641569246|gb|KK856728.1|	46540-47719	8.7	32	2	2	0.153816	0.91375	no
gi|641569246|gb|KK856728.1|	47862-48287	8.7	32	2	3	0.587433	0.70555	no
gi|641569246|gb|KK856728.1|	49438-50743	8.7	32	4	9	1	0.30475	no
gi|641569246|gb|KK856728.1|	50843-51942	8.7	32	0.705126	0.834614	0.243227	1	no
gi|641569246|gb|KK856728.1|	52517-56190	8.7	32	3	4	0.47737	0.70455	no
gi|641569246|gb|KK856728.1|	56307-59113	8.7	32	2	1	-0.326735	0.7999	no
gi|641569246|gb|KK856728.1|	59260-59638	8.7	32	4	5	0.292481	0.84525	no
gi|641569246|gb|KK856728.1|	60590-61428	8.7	32	0.330491	1	2	0.3323	no
gi|641569246|gb|KK856728.1|	61533-64963	8.7	32	2	1	-0.479129	0.7121	no
gi|641569246|gb|KK856728.1|	65136-66909	8.7	32	4	5	0.294928	0.81865	no
gi|641569246|gb|KK856728.1|	69175-72410	8.7	32	1	0.568968	-1	0.42795	no
gi|641569247|gb|KK856727.1|	0-7059	13	50	19	32	0.797526	0.70055	no
gi|641569247|gb|KK856727.1|	[illegible]03936-10489	13	50	1	0.141339	-3	0.22425	no

gi|641569247|gb|KK856727.1|	06266-10685	13	50	2	0.811504	-0.987499	0.62775	no
gi|641569247|gb|KK856727.1|	11452-12246	13	50	55	44	-0.328454	0.7903	no
gi|641569247|gb|KK856727.1|	17424-11818	13	50	2	2	0.132494	0.9278	no
gi|641569247|gb|KK856727.1|	12429-13174	13	50	6	0.390874	-4	0.13625	no
gi|641569247|gb|KK856727.1|	14024-15008	13	50	0.538626	0.682626	0.341811	1	no
gi|641569247|gb|KK856727.1|	16267-16600	13	50	24	0	-inf	0.0003	yes
gi|641569247|gb|KK856727.1|	25158-27791	13	50	21	3	-3	0.1193	no
gi|641569247|gb|KK856727.1|	29680-35813	13	50	20	15	-0.397323	0.85305	no
gi|641569247|gb|KK856727.1|	40843-41697	13	50	0.429951	2	2	0.2441	no
gi|641569247|gb|KK856727.1|	41855-48699	13	50	3	5	0.855374	0.49215	no
gi|641569247|gb|KK856727.1|	49581-50320	13	50	5	6	0.289547	0.8243	no
gi|641569247|gb|KK856727.1|	58428-59095	13	50	0	3	inf	0.00375	no
gi|641569247|gb|KK856727.1|	7671-8740	13	50	2	3	1	0.409	no
gi|641569252|gb|KK856726.1|	15857-16169	11	20	5	2	-2	0.3632	no
gi|641569252|gb|KK856726.1|	23032-23604	11	20	2	1	-1	0.5005	no
gi|641569252|gb|KK856726.1|	26780-27044	11	20	10	0	-inf	0.01	no
gi|641569252|gb|KK856726.1|	41194-41694	11	20	1	2	0.33014	0.83525	no
gi|641569252|gb|KK856726.1|	80451-80682	11	20	15	11	-0.45493	0.7758	no
gi|641569252|gb|KK856726.1|	83014-84017	11	20	1	1	0.22651	0.87445	no
gi|641569254|gb|KK856725.1|	73888-74662	11	38	0	10	inf	0.0002	yes
gi|641569257|gb|KK856724.1|	00507-10101	10	36	3	0.687995	-2	0.31075	no
gi|641569257|gb|KK856724.1|	10193-11141	10	36	7	2	-2	0.1665	no
gi|641569257|gb|KK856724.1|	11489-11371	10	36	8	4	-1	0.37805	no
gi|641569257|gb|KK856724.1|	19396-20336	10	36	7	9	0.220261	0.8599	no
gi|641569257|gb|KK856724.1|	22321-23480	10	36	10	20	0.99262	0.4294	no
gi|641569257|gb|KK856724.1|	33462-34248	10	36	3	6	1	0.36835	no
gi|641569257|gb|KK856724.1|	36735-37883	10	36	2	2	0.132202	0.9231	no
gi|641569257|gb|KK856724.1|	41069-42117	10	36	9	6	-0.529523	0.682	no
gi|641569257|gb|KK856724.1|	56064-57059	10	36	0.973899	3	2	0.2515	no
gi|641569257|gb|KK856724.1|	69849-70236	10	36	34	15	-1	0.3603	no

gi|641569257|gb|KK856724.1|	80620-81226	10	36	7	2	-2	0.267	no
gi|641569257|gb|KK856724.1|	8569-14510	10	36	11	9	-0.222967	0.9215	no
gi|641569257|gb|KK856724.1|	93335-93758	10	36	5	1	-2	0.30255	no
gi|641569257|gb|KK856724.1|	95037-95290	10	36	24	16	-0.630811	0.65985	no
gi|641569257|gb|KK856724.1|	97806-99177	10	36	0.602218	0.275228	-1	1	no
gi|641569260|gb|KK856723.1|	13262-13500	9.7	34	0	17	inf	0.0068	no
gi|641569260|gb|KK856723.1|	15981-16619	9.7	34	0	3	inf	0.00455	no
gi|641569260|gb|KK856723.1|	16871-22212	9.7	34	20	15	-0.397715	0.8557	no
gi|641569260|gb|KK856723.1|	22367-23326	9.7	34	0.463548	1	2	0.32945	no
gi|641569260|gb|KK856723.1|	24940-25724	9.7	34	14	9	-0.707546	0.5853	no
gi|641569260|gb|KK856723.1|	27019-27964	9.7	34	1	0.717943	-0.658577	0.6826	no
gi|641569260|gb|KK856723.1|	29770-30011	9.7	34	115	44	-1	0.297	no
gi|641569260|gb|KK856723.1|	30211-31371	9.7	34	60	38	-0.675665	0.7649	no
gi|641569260|gb|KK856723.1|	33623-34463	9.7	34	1	2	0.844185	0.58475	no
gi|641569260|gb|KK856723.1|	39270-40293	9.7	34	3	3	0.261243	0.84845	no
gi|641569260|gb|KK856723.1|	40646-41553	9.7	34	16	7	-1	0.3639	no
gi|641569260|gb|KK856723.1|	42206-43663	9.7	34	10	6	-0.873155	0.486	no
gi|641569260|gb|KK856723.1|	43894-44517	9.7	34	20	19	-0.0483267	0.9682	no
gi|641569260|gb|KK856723.1|	50526-51541	9.7	34	0.259128	2	3	0.1811	no
gi|641569260|gb|KK856723.1|	62593-64400	9.7	34	0	10	inf	5.00E-05	yes
gi|641569260|gb|KK856723.1|	65518-66010	9.7	34	0	63	inf	5.00E-05	yes
gi|641569263|gb|KK856722.1|	57597-58703	7.3	31	0.855197	0.236636	-2	1	no
gi|641569266|gb|KK856721.1|	6595-7292	11	5.60E+01	0	3	inf	0.00375	no
gi|641569266|gb|KK856721.1|	82192-82771	11	5.60E+01	2	0	-inf	0.01175	no
gi|641569266|gb|KK856721.1|	83952-84721	11	5.60E+01	1	0.936618	-0.245946	0.88495	no
gi|641569269|gb|KK856720.1|	1125-1962	7.1	31	1	1	-0.28995	0.8456	no
gi|641569272|gb|KK856719.1|	44604-44987	8.3	34	11	26.1	1	0.35055	no
gi|641569272|gb|KK856719.1|	45117-45545	8.3	34	0.905169	3	2	0.3323	no
gi|641569272|gb|KK856719.1|	50545-51111	8.3	34	3	10	2	0.20885	no
gi|641569272|gb|KK856719.1|	55312-56257	8.3	34	0	577	inf	0.00015	yes

gi|641569275|gb|KK856718.1|	69053-69949	9.9	40	0.606271	1	1	0.47015	no
gi|641569279|gb|KK856717.1|	12115-12900	8.2	31	1	2	0.727857	0.63585	no
gi|641569279|gb|KK856717.1|	23810-25807	8.2	31	14	8	-0.837303	0.49815	no
gi|641569279|gb|KK856717.1|	26497-27650	8.2	31	7	3	-1	0.26075	no
gi|641569279|gb|KK856717.1|	43266-44295	8.2	31	0.849256	0.258385	-2	1	no
gi|641569279|gb|KK856717.1|	47113-47751	8.2	31	2	0.72645	-1	0.47135	no
gi|641569279|gb|KK856717.1|	48009-48390	8.2	31	5	0	-inf	0.0075	no
gi|641569279|gb|KK856717.1|	8504-9151	8.2	31	1	1	0.113864	0.92265	no
gi|641569279|gb|KK856717.1|	9512-10361	8.2	31	0.86648	1	0.410658	0.8089	no
gi|641569288|gb|KK856714.1|	11047-11653	9.8	20	0.861388	5	2	0.18115	no
gi|641569288|gb|KK856714.1|	20627-21555	9.8	20	0.676425	2	2	0.3164	no
gi|641569288|gb|KK856714.1|	41375-41860	9.8	20	2	5	1	0.4292	no
gi|641569291|gb|KK856713.1|	01386-10190	10	21	3	0.669018	-2	0.31075	no
gi|641569291|gb|KK856713.1|	02891-10413	10	21	0.540633	0.823258	0.606694	1	no
gi|641569291|gb|KK856713.1|	06134-10647	10	21	8	1	-3	0.2095	no
gi|641569291|gb|KK856713.1|	14772-11568	10	21	0.790315	0.600649	-0.395905	1	no
gi|641569291|gb|KK856713.1|	18299-12129	10	21	6	0.732699	-3	0.0428	no
gi|641569291|gb|KK856713.1|	22986-12359	10	21	3	2	-0.5538	0.70725	no
gi|641569291|gb|KK856713.1|	28414-13336	10	21	7	3	-1	0.40765	no
gi|641569291|gb|KK856713.1|	35402-13754	10	21	35	5	-3	0.12325	no
gi|641569291|gb|KK856713.1|	43877-14419	10	21	20	2	-4	0.141	no
gi|641569291|gb|KK856713.1|	50326-15056	10	21	74	61	-0.281708	0.82955	no
gi|641569291|gb|KK856713.1|	67387-67721	10	21	9	2	-2	0.2404	no
gi|641569291|gb|KK856713.1|	69037-70270	10	21	5	0.519475	-3	0.0693	no
gi|641569291|gb|KK856713.1|	86833-87288	10	21	13	1	-3	0.11075	no
gi|641569294|gb|KK856712.1|	15118-15765	12	22	12	7	-0.752182	0.5665	no
gi|641569294|gb|KK856712.1|	18729-19475	12	22	16	6	-1	0.26525	no
gi|641569294|gb|KK856712.1|	19645-20512	12	22	1	0	-inf	0.01	no
gi|641569294|gb|KK856712.1|	21208-21496	12	22	12	3	-2	0.2861	no
gi|641569294|gb|KK856712.1|	22537-24129	12	22	1	0.154538	-3	0.1793	no

gi|641569294|gb|KK856712.1|	28254-29032	12	22	2	0.368903	-2	0.21865	no
gi|641569294|gb|KK856712.1|	32020-32253	12	22	143	36	-2	0.1528	no
gi|641569294|gb|KK856712.1|	34043-34713	12	22	1	0.226005	-3	0.271	no
gi|641569294|gb|KK856712.1|	35421-36283	12	22	1	0	-inf	0.01175	no
gi|641569294|gb|KK856712.1|	39540-39933	12	22	8	5	-0.831097	0.566	no
gi|641569294|gb|KK856712.1|	87844-88352	12	22	5	6	0.256607	0.85375	no
gi|641569294|gb|KK856712.1|	88925-89996	12	22	6	5	-0.430937	0.73545	no
gi|641569294|gb|KK856712.1|	91601-92772	12	22	3	1	-1	0.4581	no
gi|641569294|gb|KK856712.1|	95045-95863	12	22	3	0.863406	-2	0.29855	no
gi|641569297|gb|KK856711.1|	36801-37814	10	43	0.432937	1	2	0.32945	no
gi|641569297|gb|KK856711.1|	47956-48924	10	43	0.641409	2	1	0.39245	no
gi|641569297|gb|KK856711.1|	48989-49557	10	43	2	5	1	0.4009	no
gi|641569297|gb|KK856711.1|	49650-52819	10	43	0.619054	2	1	0.3111	no
gi|641569297|gb|KK856711.1|	52905-53609	10	43	2	0.422091	-2	0.31075	no
gi|641569297|gb|KK856711.1|	72235-73140	10	43	0.598582	2	2	0.32035	no
gi|641569297|gb|KK856711.1|	73362-74269	10	43	0.696384	1	0.796682	0.63025	no
gi|641569297|gb|KK856711.1|	74328-75122	10	43	0.827818	2	0.965046	0.5733	no
gi|641569297|gb|KK856711.1|	76527-77832	10	43	0.446624	0.87461	0.969579	1	no
gi|641569297|gb|KK856711.1|	79402-80580	10	43	0.288196	0.767799	1	1	no
gi|641569297|gb|KK856711.1|	97354-98448	10	43	0.315133	2	3	0.1983	no
gi|641569307|gb|KK856708.1|	14851-15597	10	21	1	1	0.408982	0.8089	no
gi|641569307|gb|KK856708.1|	16778-18344	10	21	4	3	-0.266447	0.83255	no
gi|641569307|gb|KK856708.1|	18645-19528	10	21	0.308866	1	2	0.3323	no
gi|641569311|gb|KK856707.1|	01050-10205	11	38	23	18	-0.378463	0.7645	no
gi|641569311|gb|KK856707.1|	05620-10714	11	38	42	19	-1	0.4864	no
gi|641569311|gb|KK856707.1|	20657-12080	11	38	0	591	inf	0.00025	yes
gi|641569311|gb|KK856707.1|	23227-12699	11	38	0.119074	78	9	0.06415	no
gi|641569311|gb|KK856707.1|	27554-13022	11	38	0.314591	6	4	0.02435	no
gi|641569311|gb|KK856707.1|	30335-13140	11	38	0.486225	3	3	0.132	no
gi|641569311|gb|KK856707.1|	32682-13710	11	38	70	365	2	0.1933	no

gi|641569311|gb|KK856707.1|	37756-14018	11	38	8	36	2	0.21665	no
gi|641569311|gb|KK856707.1|	41008-14234	11	38	3	6	0.891879	0.4855	no
gi|641569311|gb|KK856707.1|	44061-14426	11	38	0	36	inf	0.00375	no
gi|641569311|gb|KK856707.1|	33663-35813	11	38	20	9	-1	0.45405	no
gi|641569311|gb|KK856707.1|	35925-36655	11	38	1	0.60262	-1	0.50885	no
gi|641569311|gb|KK856707.1|	38071-38866	11	38	3	1	-1	0.41265	no
gi|641569311|gb|KK856707.1|	39301-39818	11	38	3	0	-inf	0.01	no
gi|641569311|gb|KK856707.1|	40462-40882	11	38	2	3	0.171902	0.9106	no
gi|641569311|gb|KK856707.1|	45380-48026	11	38	1	0.48502	-1	0.37405	no
gi|641569311|gb|KK856707.1|	48167-49389	11	38	3	0.525018	-3	0.11265	no
gi|641569311|gb|KK856707.1|	50129-50540	11	38	29	12	-1	0.34535	no
gi|641569311|gb|KK856707.1|	51700-52589	11	38	12	2	-3	0.0863	no
gi|641569311|gb|KK856707.1|	53582-54020	11	38	0	30	inf	5.00E-05	yes
gi|641569311|gb|KK856707.1|	54606-55028	11	38	0	7	inf	0.0023	yes
gi|641569311|gb|KK856707.1|	59193-59448	11	38	0	59	inf	0.0001	yes
gi|641569311|gb|KK856707.1|	61608-61808	11	38	0	34	inf	0.00455	no
gi|641569311|gb|KK856707.1|	62633-62915	11	38	0	27	inf	0.00045	yes
gi|641569311|gb|KK856707.1|	65848-66093	11	38	0	26.58	inf	0.0016	yes
gi|641569311|gb|KK856707.1|	68620-69091	11	38	0.255669	14	6	0.1052	no
gi|641569311|gb|KK856707.1|	71653-72294	11	38	2	1	-0.400884	0.8086	no
gi|641569311|gb|KK856707.1|	75444-75875	11	38	4	15	2	0.17485	no
gi|641569311|gb|KK856707.1|	77390-79761	11	38	0.357969	2	3	0.0877	no
gi|641569311|gb|KK856707.1|	81620-82253	11	38	0.323376	4	4	0.24715	no
gi|641569311|gb|KK856707.1|	85863-86871	11	38	0.78408	9	4	0.05105	no
gi|641569311|gb|KK856707.1|	88430-88669	11	38	20	138	3	0.06345	no
gi|641569311|gb|KK856707.1|	90726-92454	11	38	0.785313	0.633879	-0.30906	1	no
gi|641569311|gb|KK856707.1|	92571-94542	11	38	2	2	0.407372	0.75805	no
gi|641569311|gb|KK856707.1|	96009-97569	11	38	22	8	-1	0.2654	no
gi|641569314|gb|KK856706.1|	05112-10607	11	47	1	0.55951	-1	0.50315	no
gi|641569314|gb|KK856706.1|	06482-10754	11	47	10	10	0.0205872	0.9875	no

gi|641569314|gb|KK856706.1|	10460-11065	11	47	21	7	-2	0.36305	no
gi|641569314|gb|KK856706.1|	12392-11290	11	47	1	3	1	0.39995	no
gi|641569314|gb|KK856706.1|	14738-11498	11	47	12	10	-0.324916	0.832	no
gi|641569314|gb|KK856706.1|	96871-97787	11	47	0	8	inf	0.0001	yes
gi|641569314|gb|KK856706.1|	99628-10448[illegible]	11	47	4	5	0.37006	0.7692	no
gi|641569318|gb|KK856705.1|	31579-32353	9.5	40	0	5	inf	0.00075	yes
gi|641569318|gb|KK856705.1|	37392-37975	9.5	40	0	15	inf	0.00015	yes
gi|641569318|gb|KK856705.1|	39677-40709	9.5	40	0.084621	30	8	0.10435	no
gi|641569318|gb|KK856705.1|	42799-43053	9.5	40	0	82	inf	5.00E-05	yes
gi|641569318|gb|KK856705.1|	55793-55941	9.5	40	400	1029.59	1	0.301	no
gi|641569318|gb|KK856705.1|	59945-60713	9.5	40	4	13	2	0.22895	no
gi|641569318|gb|KK856705.1|	64052-64987	9.5	40	0	24	inf	5.00E-05	yes
gi|641569325|gb|KK856703.1|	33830-37856	10	36	6	6	-0.0707595	0.9556	no
gi|641569325|gb|KK856703.1|	38211-41038	10	36	6	5	-0.216192	0.8599	no
gi|641569325|gb|KK856703.1|	42399-42943	10	36	3	2	-0.404496	0.79625	no
gi|641569325|gb|KK856703.1|	63003-64038	10	36	0.421594	0.769642	0.868334	1	no
gi|641569325|gb|KK856703.1|	79019-79589	10	36	2	0.856247	-0.988319	0.62775	no
gi|641569328|gb|KK856702.1|	00955-10190	9.6	17	0.750522	0.855855	0.189472	1	no
gi|641569328|gb|KK856702.1|	07294-10833	9.6	17	0.418108	0.890528	1	0.50945	no
gi|641569328|gb|KK856702.1|	24413-25697	9.6	17	0.325171	0.693325	1	1	no
gi|641569328|gb|KK856702.1|	25763-28522	9.6	17	1	2	0.548629	0.67785	no
gi|641569328|gb|KK856702.1|	28813-29504	9.6	17	2	5	2	0.26595	no
gi|641569328|gb|KK856702.1|	37349-37731	9.6	17	9	2	-2	0.21665	no
gi|641569328|gb|KK856702.1|	43117-43974	9.6	17	0.749003	0.812832	0.117985	1	no
gi|641569328|gb|KK856702.1|	46191-47891	9.6	17	2	0.573904	-2	0.27425	no
gi|641569328|gb|KK856702.1|	50862-53507	9.6	17	1	0.970435	-0.0599292	0.9627	no
gi|641569328|gb|KK856702.1|	54975-55935	9.6	17	0.370354	2	2	0.26155	no
gi|641569328|gb|KK856702.1|	57466-60367	9.6	17	0.706539	1	0.762154	0.57495	no
gi|641569328|gb|KK856702.1|	60572-63848	9.6	17	4	2	-1	0.31805	no
gi|641569328|gb|KK856702.1|	64278-69496	9.6	17	7	8	0.146547	0.94885	no

gi|641569328|gb|KK856702.1|	70441-71546	9.6	17	29	26	-0.135019	0.9155	no
gi|641569328|gb|KK856702.1|	73335-73831	9.6	17	6	13	1	0.4422	no
gi|641569328|gb|KK856702.1|	75798-78360	9.6	17	13	14	0.0526671	0.9661	no
gi|641569328|gb|KK856702.1|	80135-81123	9.6	17	16	19	0.293224	0.81235	no
gi|641569328|gb|KK856702.1|	82423-82932	9.6	17	37	28	-0.432773	0.7238	no
gi|641569328|gb|KK856702.1|	83691-84729	9.6	17	21	13	-0.650689	0.60585	no
gi|641569328|gb|KK856702.1|	85298-87105	9.6	17	35	17	-0.994674	0.53905	no
gi|641569328|gb|KK856702.1|	87254-88710	9.6	17	0.336969	1	2	0.28005	no
gi|641569328|gb|KK856702.1|	89249-90195	9.6	17	2	1	-0.243529	0.867	no
gi|641569328|gb|KK856702.1|	97740-98120	9.6	17	16	2	-3	0.1109	no
gi|641569339|gb|KK856699.1|	11952-12809	8	34	33	6	-3	0.0631	no
gi|641569339|gb|KK856699.1|	52238-53324	8	34	4	4	0.0207393	0.9857	no
gi|641569339|gb|KK856699.1|	58867-60031	8	34	2	3	0.86927	0.52295	no
gi|641569339|gb|KK856699.1|	61295-61968	8	34	7	2	-2	0.18	no
gi|641569346|gb|KK856697.1|	23570-24571	9.7	36	4	1	-1	0.32685	no
gi|641569346|gb|KK856697.1|	25906-27093	9.7	36	29	13	-1	0.3614	no
gi|641569346|gb|KK856697.1|	37426-37676	9.7	36	2	46	5	0.2353	no
gi|641569346|gb|KK856697.1|	42258-42464	9.7	36	8	83	3	0.13065	no
gi|641569346|gb|KK856697.1|	51540-52590	9.7	36	0.331356	2	3	0.15965	no
gi|641569346|gb|KK856697.1|	54739-54941	9.7	36	0	986	inf	0.00095	yes
gi|641569346|gb|KK856697.1|	56513-58129	9.7	36	0.099673	4	5	0.233	no
gi|641569346|gb|KK856697.1|	61529-61711	9.7	36	0	589	inf	5.00E-05	yes
gi|641569346|gb|KK856697.1|	66362-66549	9.7	36	0	1668.36	inf	0.0003	yes
gi|641569346|gb|KK856697.1|	67354-67767	9.7	36	0	1017.96	inf	0.0003	yes
gi|641569346|gb|KK856697.1|	71377-71586	9.7	36	20	5	-2	0.3145	no
gi|641569349|gb|KK856696.1|	0-3204	11	38	10	4	-1	0.28635	no
gi|641569349|gb|KK856696.1|	18603-20081	11	38	0.772775	0.672913	-0.199628	1	no
gi|641569349|gb|KK856696.1|	20183-21373	11	38	1	0.433471	-1	0.37565	no
gi|641569349|gb|KK856696.1|	21805-22375	11	38	0.943714	3	1	0.39995	no
gi|641569349|gb|KK856696.1|	23341-25069	11	38	11	4	-1	0.3036	no

gi|641569349|gb|KK856696.1|	60709-61981	11	38	0.591831	1	0.758849	0.6256	no
gi|641569349|gb|KK856696.1|	83781-84986	11	38	0.350612	1	2	0.28495	no
gi|641569352|gb|KK856695.1|	28090-28348	14	63	31	8	-2	0.2193	no
gi|641569352|gb|KK856695.1|	30507-30946	14	63	5	2	-1	0.43485	no
gi|641569352|gb|KK856695.1|	45094-48015	14	63	29	5	-2	0.1767	no
gi|641569352|gb|KK856695.1|	55025-57374	14	63	24	9	-1	0.42695	no
gi|641569352|gb|KK856695.1|	65053-66485	14	63	2	2	-0.12941	0.9265	no
gi|641569352|gb|KK856695.1|	68028-68612	14	63	11	9	-0.269534	0.83225	no
gi|641569352|gb|KK856695.1|	88559-88899	14	63	0.471873	8	4	0.1178	no
gi|641569359|gb|KK856693.1|	59409-60610	8.9	34	2	3	0.559153	0.67805	no
gi|641569359|gb|KK856693.1|	61015-61889	8.9	34	75	168	1	0.59755	no
gi|641569362|gb|KK856692.1|	17832-19396	10	39	1	0.946138	-0.507461	0.7175	no
gi|641569362|gb|KK856692.1|	19843-20934	10	39	0.55333	2	2	0.2915	no
gi|641569362|gb|KK856692.1|	21506-23963	10	39	1	2	0.783751	0.55005	no
gi|641569362|gb|KK856692.1|	24668-26265	10	39	2	5	1	0.3949	no
gi|641569362|gb|KK856692.1|	61166-61517	10	39	4	21	3	0.13465	no
gi|641569362|gb|KK856692.1|	63279-64258	10	39	2	2	0.102298	0.9445	no
gi|641569362|gb|KK856692.1|	71359-72497	10	39	0.600849	0.342958	-0.80897	1	no
gi|641569362|gb|KK856692.1|	81940-82460	10	39	3	7	0.986622	0.5023	no
gi|641569362|gb|KK856692.1|	82653-86582	10	39	0.758531	2	1	0.3386	no
gi|641569362|gb|KK856692.1|	86840-88323	10	39	0.549839	2	2	0.2814	no
gi|641569362|gb|KK856692.1|	95101-95398	10	39	3	6	1	0.52105	no
gi|641569374|gb|KK856690.1|	22532-12288	9.3	36	1	5	2	0.3198	no
gi|641569374|gb|KK856690.1|	33624-13455	9.3	36	0.678276	0.736507	0.118826	1	no
gi|641569374|gb|KK856690.1|	42455-14329	9.3	36	0.21829	1	3	0.2991	no
gi|641569374|gb|KK856690.1|	64862-65531	9.3	36	15	28	0.936322	0.46655	no
gi|641569374|gb|KK856690.1|	65905-66351	9.3	36	0.841851	3	2	0.3323	no
gi|641569374|gb|KK856690.1|	67336-67709	9.3	36	5	211	5	0.035	no
gi|641569374|gb|KK856690.1|	74179-74443	9.3	36	0	81	inf	5.00E-05	yes
gi|641569374|gb|KK856690.1|	78216-78403	9.3	36	0	49	inf	0.00375	no

gi|641569374|gb|KK856690.1|	80158-80396	9.3	36	5	182	5	0.11605	no
gi|641569374|gb|KK856690.1|	81074-81365	9.3	36	0	11	inf	0.00455	no
gi|641569374|gb|KK856690.1|	83268-84353	9.3	36	1	18	4	0.0206	no
gi|641569374|gb|KK856690.1|	88195-88408	9.3	36	2	141	6	0.1046	no
gi|641569374|gb|KK856690.1|	89017-89259	9.3	36	7	96	4	0.0705	no
gi|641569374|gb|KK856690.1|	91756-92128	9.3	36	0	402	inf	0.00035	yes
gi|641569377|gb|KK856689.1|	07934-10967	9.9	21	0.548966	0.558002	0.023553	1	no
gi|641569377|gb|KK856689.1|	115-1155	9.9	21	3	3	0.150775	0.9077	no
gi|641569377|gb|KK856689.1|	17613-18940	9.9	21	16	6	-1	0.27355	no
gi|641569377|gb|KK856689.1|	22204-23307	9.9	21	0.468013	1	1	0.47015	no
gi|641569377|gb|KK856689.1|	24676-24837	9.9	21	115	17	-3	0.185	no
gi|641569377|gb|KK856689.1|	25663-25868	9.9	21	23	6	-2	0.27465	no
gi|641569377|gb|KK856689.1|	29489-31948	9.9	21	0.750662	0.811385	0.112224	1	no
gi|641569377|gb|KK856689.1|	33110-34186	9.9	21	2	0.24466	-3	0.15595	no
gi|641569377|gb|KK856689.1|	37029-37296	9.9	21	37	15	-1	0.3572	no
gi|641569377|gb|KK856689.1|	39865-40340	9.9	21	3	10	2	0.1878	no
gi|641569377|gb|KK856689.1|	40662-41409	9.9	21	7	18	1	0.27645	no
gi|641569377|gb|KK856689.1|	41683-42488	9.9	21	0.812883	1	0.602653	0.69715	no
gi|641569377|gb|KK856689.1|	42703-43217	9.9	21	5	17	2	0.2061	no
gi|641569377|gb|KK856689.1|	43399-44199	9.9	21	0.819605	0.888933	0.117147	0.92175	no
gi|641569377|gb|KK856689.1|	45180-48793	9.9	21	2	3	0.67772	0.59335	no
gi|641569377|gb|KK856689.1|	49512-50435	9.9	21	1	2	0.604219	0.67915	no
gi|641569377|gb|KK856689.1|	51097-55406	9.9	21	2	2	0.252852	0.8437	no
gi|641569377|gb|KK856689.1|	57319-58805	9.9	21	1	1	-0.0926843	0.9492	no
gi|641569377|gb|KK856689.1|	59630-61360	9.9	21	1	0.773734	-0.455647	0.7554	no
gi|641569377|gb|KK856689.1|	62064-62859	9.9	21	0.826438	0.537776	-0.6199	1	no
gi|641569377|gb|KK856689.1|	65400-65857	9.9	21	2	4	0.952798	0.57355	no
gi|641569377|gb|KK856689.1|	65966-67357	9.9	21	1	1	-0.199963	0.8877	no
gi|641569377|gb|KK856689.1|	83464-84183	9.9	21	1	3	0.842087	0.5848	no
gi|641569377|gb|KK856689.1|	84295-84723	9.9	21	3	2	-0.412278	0.8125	no

gi|641569377|gb|KK856689.1|	85030-86228	9.9	21	0.917956	2	0.906002	0.53945	no
gi|641569380|gb|KK856688.1|	00120-10095	9.8	20	1	0.169173	-3	0.2348	no
gi|641569380|gb|KK856688.1|	03127-10340	9.8	20	9	0	-inf	0.0102	no
gi|641569380|gb|KK856688.1|	17809-11894	9.8	20	0.825292	0	-inf	1	no
gi|641569380|gb|KK856688.1|	19133-11983	9.8	20	0.972253	0.631906	-0.621622	0.71195	no
gi|641569380|gb|KK856688.1|	12235-12644	9.8	20	5	2	-1	0.4012	no
gi|641569380|gb|KK856688.1|	37574-37860	9.8	20	20	6	-2	0.2762	no
gi|641569380|gb|KK856688.1|	46889-49146	9.8	20	2	0.157187	-3	0.1118	no
gi|641569380|gb|KK856688.1|	50971-51912	9.8	20	8	4	-1	0.38645	no
gi|641569380|gb|KK856688.1|	52022-53837	9.8	20	8	3	-1	0.31575	no
gi|641569380|gb|KK856688.1|	59954-60389	9.8	20	4	3	-0.189236	0.90345	no
gi|641569380|gb|KK856688.1|	73905-74327	9.8	20	20	1	-4	0.09975	no
gi|641569380|gb|KK856688.1|	88190-92995	9.8	20	3	2	-0.633253	0.6154	no
gi|641569380|gb|KK856688.1|	93278-94114	9.8	20	2	0.335722	-2	0.21865	no
gi|641569380|gb|KK856688.1|	94218-96442	9.8	20	4	0.372723	-3	0.0515	no
gi|641569380|gb|KK856688.1|	96977-99936	9.8	20	9	1	-3	0.05015	no
gi|641569383|gb|KK856687.1|	38797-41800	11	44	0	4	inf	5.00E-05	yes
gi|641569383|gb|KK856687.1|	45442-45987	11	44	0	25	inf	5.00E-05	yes
gi|641569383|gb|KK856687.1|	49289-49786	11	44	0.233953	23	7	0.1045	no
gi|641569383|gb|KK856687.1|	50306-52359	11	44	0.11434	14	7	0.1346	no
gi|641569383|gb|KK856687.1|	52495-53410	11	44	0.098378	3	5	0.10875	no
gi|641569383|gb|KK856687.1|	53702-55355	11	44	0	2	inf	0.00115	yes
gi|641569383|gb|KK856687.1|	55538-56500	11	44	0	2	inf	0.00455	no
gi|641569383|gb|KK856687.1|	62221-63089	11	44	2	2	-0.460573	0.74915	no
gi|641569390|gb|KK856685.1|	0-705	11	41	4	5	0.185733	0.8904	no
gi|641569390|gb|KK856685.1|	13805-11521	11	41	1	2	0.732984	0.5985	no
gi|641569390|gb|KK856685.1|	16751-11852	11	41	4	0.95617	-2	0.18675	no
gi|641569390|gb|KK856685.1|	18695-11957	11	41	0.721736	0.626733	-0.203619	1	no
gi|641569390|gb|KK856685.1|	20780-12143	11	41	6	2	-2	0.2363	no
gi|641569390|gb|KK856685.1|	27701-12792	11	41	40	16	-1	0.3671	no

<table>
<tr><td>gi|641569390|gb|KK856685.1|</td><td>18436-18797</td><td>11</td><td>41</td><td>31</td><td>16</td><td>-0.92953</td><td>0.4804</td><td>no</td></tr>
<tr><td>gi|641569390|gb|KK856685.1|</td><td>25135-25782</td><td>11</td><td>41</td><td>2</td><td>1</td><td>-0.886136</td><td>0.5827</td><td>no</td></tr>
<tr><td>gi|641569390|gb|KK856685.1|</td><td>30362-31802</td><td>11</td><td>41</td><td>35</td><td>8</td><td>-2</td><td>0.25025</td><td>no</td></tr>
<tr><td>gi|641569390|gb|KK856685.1|</td><td>33064-33530</td><td>11</td><td>41</td><td>2</td><td>1</td><td>-0.824125</td><td>0.64915</td><td>no</td></tr>
<tr><td>gi|641569390|gb|KK856685.1|</td><td>34423-35005</td><td>11</td><td>41</td><td>2</td><td>0.830098</td><td>-1</td><td>0.47135</td><td>no</td></tr>
<tr><td>gi|641569390|gb|KK856685.1|</td><td>62831-65045</td><td>11</td><td>41</td><td>0.701471</td><td>0.695621</td><td>-0.0120817</td><td>1</td><td>no</td></tr>
<tr><td>gi|641569390|gb|KK856685.1|</td><td>81047-81451</td><td>11</td><td>41</td><td>33</td><td>9</td><td>-2</td><td>0.1813</td><td>no</td></tr>
<tr><td>gi|641569390|gb|KK856685.1|</td><td>8523-9823</td><td>11</td><td>41</td><td>9</td><td>8</td><td>-0.215372</td><td>0.86375</td><td>no</td></tr>
<tr><td>gi|641569390|gb|KK856685.1|</td><td>87696-90150</td><td>11</td><td>41</td><td>0.564243</td><td>2</td><td>2</td><td>0.27255</td><td>no</td></tr>
<tr><td>gi|641569390|gb|KK856685.1|</td><td>94787-95601</td><td>11</td><td>41</td><td>0.228874</td><td>2</td><td>3</td><td>0.27515</td><td>no</td></tr>
<tr><td>gi|641569394|gb|KK856684.1|</td><td>17371-18368</td><td>10</td><td>43</td><td>0.794838</td><td>0.268645</td><td>-2</td><td>1</td><td>no</td></tr>
<tr><td>gi|641569394|gb|KK856684.1|</td><td>18739-19122</td><td>10</td><td>43</td><td>0.370802</td><td>6</td><td>4</td><td>0.12515</td><td>no</td></tr>
<tr><td>gi|641569394|gb|KK856684.1|</td><td>20969-21572</td><td>10</td><td>43</td><td>2</td><td>2</td><td>0.138463</td><td>0.929</td><td>no</td></tr>
<tr><td>gi|641569394|gb|KK856684.1|</td><td>24164-25882</td><td>10</td><td>43</td><td>0.464982</td><td>1</td><td>1</td><td>0.36435</td><td>no</td></tr>
<tr><td>gi|641569394|gb|KK856684.1|</td><td>29004-29570</td><td>10</td><td>43</td><td>20</td><td>6</td><td>-2</td><td>0.193</td><td>no</td></tr>
<tr><td>gi|641569394|gb|KK856684.1|</td><td>31918-33039</td><td>10</td><td>43</td><td>0.764854</td><td>2</td><td>1</td><td>0.4713</td><td>no</td></tr>
<tr><td>gi|641569394|gb|KK856684.1|</td><td>41034-41966</td><td>10</td><td>43</td><td>31</td><td>12</td><td>-1</td><td>0.27255</td><td>no</td></tr>
<tr><td>gi|641569394|gb|KK856684.1|</td><td>42158-42517</td><td>10</td><td>43</td><td>27</td><td>14</td><td>-0.9614</td><td>0.46805</td><td>no</td></tr>
<tr><td>gi|641569394|gb|KK856684.1|</td><td>80073-80371</td><td>10</td><td>43</td><td>16</td><td>0.940535</td><td>-4</td><td>0.19385</td><td>no</td></tr>
<tr><td>gi|641569394|gb|KK856684.1|</td><td>87975-88216</td><td>10</td><td>43</td><td>13</td><td>0</td><td>-inf</td><td>0.01</td><td>no</td></tr>
<tr><td>gi|641569397|gb|KK856683.1|</td><td>42703-42884</td><td>9.5</td><td>42</td><td>42</td><td>0</td><td>-inf</td><td>0.0075</td><td>no</td></tr>
<tr><td>gi|641569397|gb|KK856683.1|</td><td>43686-45114</td><td>9.5</td><td>42</td><td>7</td><td>0</td><td>-inf</td><td>5.00E-05</td><td>yes</td></tr>
<tr><td>gi|641569397|gb|KK856683.1|</td><td>53595-53953</td><td>9.5</td><td>42</td><td>5</td><td>0</td><td>-inf</td><td>0.0102</td><td>no</td></tr>
<tr><td>gi|641569397|gb|KK856683.1|</td><td>54506-56105</td><td>9.5</td><td>42</td><td>4</td><td>0</td><td>-inf</td><td>5.00E-05</td><td>yes</td></tr>
<tr><td>gi|641569400|gb|KK856682.1|</td><td>00809-10351</td><td>9.7</td><td>34</td><td>2</td><td>2</td><td>0.323099</td><td>0.7983</td><td>no</td></tr>
<tr><td>gi|641569400|gb|KK856682.1|</td><td>10251-10697</td><td>9.7</td><td>34</td><td>6</td><td>0</td><td>-inf</td><td>0.0032</td><td>yes</td></tr>
<tr><td>gi|641569400|gb|KK856682.1|</td><td>03770-10509</td><td>9.7</td><td>34</td><td>10</td><td>9</td><td>-0.0543019</td><td>0.9639</td><td>no</td></tr>
<tr><td>gi|641569400|gb|KK856682.1|</td><td>05564-10950</td><td>9.7</td><td>34</td><td>5</td><td>5</td><td>-0.118886</td><td>0.9264</td><td>no</td></tr>
<tr><td>gi|641569400|gb|KK856682.1|</td><td>10543-11209</td><td>9.7</td><td>34</td><td>12</td><td>14</td><td>0.234504</td><td>0.8487</td><td>no</td></tr>
<tr><td>gi|641569400|gb|KK856682.1|</td><td>11191-12805</td><td>9.7</td><td>34</td><td>3</td><td>4</td><td>0.306999</td><td>0.81085</td><td>no</td></tr>
</table>

gi|641569400|gb|KK856682.1|	13180-11350	9.7	34	13	6	-1	0.42625	no
gi|641569400|gb|KK856682.1|	16800-11710	9.7	34	72	70	-0.0342893	0.9792	no
gi|641569400|gb|KK856682.1|	13757-15217	9.7	34	20	0	-inf	5.00E-05	yes
gi|641569400|gb|KK856682.1|	19680-19982	9.7	34	6	10	0.857369	0.585	no
gi|641569400|gb|KK856682.1|	26002-26393	9.7	34	2	6	2	0.2869	no
gi|641569400|gb|KK856682.1|	30621-30850	9.7	34	1	21	4	0.1198	no
gi|641569400|gb|KK856682.1|	35797-36834	9.7	34	0.168237	4	4	0.236	no
gi|641569400|gb|KK856682.1|	36988-39670	9.7	34	0.3132	2	2	0.14285	no
gi|641569400|gb|KK856682.1|	41364-43254	9.7	34	0.376011	2	3	0.1074	no
gi|641569400|gb|KK856682.1|	56541-56779	9.7	34	1	17	4	0.12515	no
gi|641569400|gb|KK856682.1|	57925-59058	9.7	34	2	5	2	0.21065	no
gi|641569400|gb|KK856682.1|	59674-60285	9.7	34	0.340428	3	3	0.2556	no
gi|641569400|gb|KK856682.1|	60508-63331	9.7	34	0.538898	3	2	0.09175	no
gi|641569400|gb|KK856682.1|	64469-69125	9.7	34	3	3	-0.0390463	0.97445	no
gi|641569400|gb|KK856682.1|	69250-74680	9.7	34	3	2	-0.222783	0.85645	no
gi|641569400|gb|KK856682.1|	75165-77261	9.7	34	250.96	269.29	0.101701	0.95295	no
gi|641569400|gb|KK856682.1|	77383-78900	9.7	34	16	19	0.261687	0.8314	no
gi|641569400|gb|KK856682.1|	7817-8025	9.7	34	27	0	-inf	0.0059	no
gi|641569400|gb|KK856682.1|	79445-80291	9.7	34	3	2	-0.619131	0.65595	no
gi|641569400|gb|KK856682.1|	86433-87240	9.7	34	0.34724	2	2	0.21365	no
gi|641569400|gb|KK856682.1|	87353-88557	9.7	34	2	2	0.606477	0.65695	no
gi|641569400|gb|KK856682.1|	92084-92259	9.7	34	42	66	0.64239	0.67085	no
gi|641569400|gb|KK856682.1|	92646-92826	9.7	34	80	96	0.256565	0.84955	no
gi|641569400|gb|KK856682.1|	93124-94365	9.7	34	4	0.927929	-2	0.1355	no
gi|641569400|gb|KK856682.1|	9730-9939	9.7	34	77	0	-inf	0.0002	yes
gi|641569400|gb|KK856682.1|	9167-10041	9.7	34	3	4	0.104237	0.9366	no
gi|641569403|gb|KK856681.1|	10464-11179	10	45	1	1	0.408347	0.8089	no
gi|641569403|gb|KK856681.1|	11639-12613	10	45	1	1	0.467246	0.75495	no
gi|641569403|gb|KK856681.1|	12841-14413	10	45	7	9	0.283516	0.82055	no
gi|641569403|gb|KK856681.1|	15475-17462	10	45	17	22	0.367415	0.87275	no

gi|641569403|gb|KK856681.1|	19463-20252	10	45	3	3	0.116966	0.93195	no
gi|641569403|gb|KK856681.1|	4570-4928	10	45	9	13	0.579008	0.6832	no
gi|641569403|gb|KK856681.1|	5462-5676	10	45	46	53	0.189415	0.8881	no
gi|641569403|gb|KK856681.1|	6277-6615	10	45	6	5	-0.317527	0.84315	no
gi|641569403|gb|KK856681.1|	7627-8099	10	45	2	1	-0.993616	0.62775	no
gi|641569406|gb|KK856680.1|	25408-26494	10	41	7	12	0.806615	0.52965	no
gi|641569410|gb|KK856679.1|	[illegible]09402-10991	11	45	0	8	inf	0.00075	yes
gi|641569410|gb|KK856679.1|	24307-24583	11	45	0	992	inf	0.0003	yes
gi|641569410|gb|KK856679.1|	58643-60050	11	45	0	11	inf	5.00E-05	yes
gi|641569413|gb|KK856678.1|	14609-15817	9.6	19	2	0.851595	-2	0.31185	no
gi|641569413|gb|KK856678.1|	16097-17734	9.6	19	0.933103	0.149728	-3	0.19075	no
gi|641569413|gb|KK856678.1|	17794-18813	9.6	19	0.945523	0.915271	-0.0469134	0.94545	no
gi|641569413|gb|KK856678.1|	19084-19639	9.6	19	2	0	-inf	0.01175	no
gi|641569413|gb|KK856678.1|	23110-23994	9.6	19	1	0	-inf	0.01	no
gi|641569413|gb|KK856678.1|	33563-34565	9.6	19	3	3	-0.131953	0.92035	no
gi|641569413|gb|KK856678.1|	36109-36702	9.6	19	2	0.538322	-2	0.3216	no
gi|641569413|gb|KK856678.1|	37983-38924	9.6	19	14	18	0.333767	0.7904	no
gi|641569413|gb|KK856678.1|	39863-40394	9.6	19	1	0.953777	-0.627518	0.71195	no
gi|641569413|gb|KK856678.1|	40633-41482	9.6	19	0.64986	1	1	0.47015	no
gi|641569413|gb|KK856678.1|	41850-42489	9.6	19	8	2	-2	0.17975	no
gi|641569413|gb|KK856678.1|	58362-58929	9.6	19	1	2	0.596519	0.70555	no
gi|641569413|gb|KK856678.1|	61508-61729	9.6	19	98	45	-1	0.40595	no
gi|641569416|gb|KK856677.1|	22131-22817	10	35	2	3	0.822716	0.58505	no
gi|641569416|gb|KK856677.1|	23843-24717	10	35	2	3	0.866669	0.5535	no
gi|641569416|gb|KK856677.1|	24860-26044	10	35	7	4	-0.656676	0.6091	no
gi|641569425|gb|KK856674.1|	[illegible]00241-10231	11	42	0.829119	2	1	0.3328	no
gi|641569425|gb|KK856674.1|	[illegible]03176-10403	11	42	0.636234	2	2	0.2582	no
gi|641569425|gb|KK856674.1|	[illegible]11564-11273	11	42	147	102	-0.528235	0.80035	no
gi|641569425|gb|KK856674.1|	13793-14255	11	42	0	14	inf	0.00025	yes
gi|641569425|gb|KK856674.1|	18138-19040	11	42	9	9	-0.00444271	0.99235	no

gi|641569425|gb|KK856674.1|	20915-21757	11	42	1	1	0.143774	0.929	no
gi|641569425|gb|KK856674.1|	24047-25792	11	42	10	2	-3	0.05085	no
gi|641569425|gb|KK856674.1|	33322-33737	11	42	0	29	inf	5.00E-05	yes
gi|641569425|gb|KK856674.1|	36365-37366	11	42	2	1	-1	0.477	no
gi|641569425|gb|KK856674.1|	37628-38318	11	42	0	24	inf	5.00E-05	yes
gi|641569425|gb|KK856674.1|	41383-44714	11	42	26	31	0.216738	0.9187	no
gi|641569425|gb|KK856674.1|	44828-46683	11	42	2	2	-0.136654	0.9174	no
gi|641569425|gb|KK856674.1|	46897-50470	11	42	5	4	-0.0386575	0.97655	no
gi|641569425|gb|KK856674.1|	50976-53449	11	42	3	12	2	0.1001	no
gi|641569425|gb|KK856674.1|	54156-54358	11	42	12	18	0.53131	0.7589	no
gi|641569425|gb|KK856674.1|	55077-55405	11	42	10	3	-2	0.29135	no
gi|641569425|gb|KK856674.1|	55711-57425	11	42	2	3	0.0504472	0.9701	no
gi|641569425|gb|KK856674.1|	57530-58306	11	42	1	1	-0.272296	0.86195	no
gi|641569425|gb|KK856674.1|	58634-59186	11	42	2	3	0.180833	0.9037	no
gi|641569425|gb|KK856674.1|	59287-60262	11	42	0.272422	1	2	0.2812	no
gi|641569425|gb|KK856674.1|	65020-66238	11	42	2	9	2	0.13595	no
gi|641569425|gb|KK856674.1|	66460-66772	11	42	6	12	1	0.4805	no
gi|641569425|gb|KK856674.1|	67003-68025	11	42	1	3	1	0.3264	no
gi|641569425|gb|KK856674.1|	95584-96531	11	42	1	4	2	0.2145	no
gi|641569425|gb|KK856674.1|	97722-10000[illegible]	11	42	1	2	1	0.42285	no
gi|641569429|gb|KK856673.1|	2652-3159	8.4	36	4	9	1	0.4348	no
gi|641569429|gb|KK856673.1|	33742-34169	8.4	36	654.45	540	-0.276085	0.89345	no
gi|641569429|gb|KK856673.1|	37441-40055	8.4	36	20	15	-0.424563	0.84975	no
gi|641569429|gb|KK856673.1|	40437-41156	8.4	36	3	4	0.456689	0.7451	no
gi|641569429|gb|KK856673.1|	42276-42490	8.4	36	20	12	-0.725696	0.6634	no
gi|641569429|gb|KK856673.1|	43894-44427	8.4	36	2	2	0.232404	0.88235	no
gi|641569429|gb|KK856673.1|	44529-45834	8.4	36	2	1	-0.573563	0.68375	no
gi|641569429|gb|KK856673.1|	46171-47580	8.4	36	1	2	0.302611	0.8279	no
gi|641569429|gb|KK856673.1|	47805-49532	8.4	36	0.83205	0.986676	0.245906	0.8641	no
gi|641569429|gb|KK856673.1|	49940-50980	8.4	36	1	2	0.853269	0.5535	no

gi|641569429|gb|KK856673.1|	51472-51824	8.4	36	3	3	0.0925992	0.9241	no
gi|641569429|gb|KK856673.1|	64459-65279	8.4	36	2	0	-inf	0.0059	no
gi|641569432|gb|KK856672.1|	15336-15897	13	47	11	11	0.0743123	0.9548	no
gi|641569432|gb|KK856672.1|	19502-19961	13	47	4	3	-0.316507	0.8441	no
gi|641569432|gb|KK856672.1|	20658-21741	13	47	4	4	0.283751	0.82735	no
gi|641569432|gb|KK856672.1|	28798-29207	13	47	8	6	-0.41421	0.77715	no
gi|641569432|gb|KK856672.1|	40094-40488	13	47	4	3	-0.152903	0.91945	no
gi|641569432|gb|KK856672.1|	4010-5034	13	47	1	3	1	0.38015	no
gi|641569432|gb|KK856672.1|	41013-43765	13	47	2	2	0.0795085	0.9511	no
gi|641569432|gb|KK856672.1|	45444-46089	13	47	14	15	0.0705384	0.95565	no
gi|641569432|gb|KK856672.1|	46231-46856	13	47	7	2	-1	0.32565	no
gi|641569432|gb|KK856672.1|	50355-51021	13	47	2	4	1	0.4454	no
gi|641569432|gb|KK856672.1|	51265-51625	13	47	200	105	-0.923174	0.46245	no
gi|641569432|gb|KK856672.1|	53866-54224	13	47	6	1	-2	0.26505	no
gi|641569432|gb|KK856672.1|	56198-57094	13	47	1	0.307142	-2	0.3455	no
gi|641569432|gb|KK856672.1|	59141-60207	13	47	24	17	-0.434808	0.72745	no
gi|641569432|gb|KK856672.1|	6902-7860	13	47	0.556987	0.98808	0.826983	0.6232	no
gi|641569432|gb|KK856672.1|	70355-71444	13	47	12	7	-0.856007	0.50035	no
gi|641569432|gb|KK856672.1|	71645-74308	13	47	2	1	-0.0305178	0.98085	no
gi|641569432|gb|KK856672.1|	75287-75924	13	47	12	10	-0.144663	0.90885	no
gi|641569432|gb|KK856672.1|	78872-81541	13	47	5	4	-0.236345	0.84865	no
gi|641569432|gb|KK856672.1|	83649-84840	13	47	3	3	0.11336	0.9288	no
gi|641569432|gb|KK856672.1|	8931-9317	13	47	26	21	-0.318761	0.80185	no
gi|641569432|gb|KK856672.1|	92340-93271	13	47	0.48119	1	1	0.4595	no
gi|641569432|gb|KK856672.1|	95342-95450	13	47	4452.62	1543.61	-2	0.4354	no
gi|641569452|gb|KK856668.1|	17427-11764	12	43	10	17	0.759246	0.64725	no
gi|641569452|gb|KK856668.1|	95389-95796	12	43	0	6	inf	0.0025	yes
gi|641569454|gb|KK856667.1|	00501-10162	9.8	34	0.83682	1	0.31648	0.84265	no
gi|641569454|gb|KK856667.1|	22422-29102	9.8	34	14	4	-2	0.31515	no
gi|641569454|gb|KK856667.1|	37468-38817	9.8	34	17	6	-2	0.2276	no

gi|641569454|gb|KK856667.1|	39743-41630	9.8	34	0	5	inf	5.00E-05	yes
gi|641569454|gb|KK856667.1|	44405-45390	9.8	34	37	20	-0.863294	0.4917	no
gi|641569454|gb|KK856667.1|	48523-50261	9.8	34	28	12	-1	0.4596	no
gi|641569454|gb|KK856667.1|	50506-52828	9.8	34	4	1	-2	0.23305	no
gi|641569454|gb|KK856667.1|	52941-54255	9.8	34	3	0.6748	-2	0.2079	no
gi|641569454|gb|KK856667.1|	55920-56488	9.8	34	2	0.286922	-3	0.271	no
gi|641569454|gb|KK856667.1|	56545-58138	9.8	34	2	0.540496	-2	0.3042	no
gi|641569454|gb|KK856667.1|	59195-60929	9.8	34	2	0.701571	-1	0.34415	no
gi|641569454|gb|KK856667.1|	62130-65278	9.8	34	4	2	-0.658155	0.60045	no
gi|641569454|gb|KK856667.1|	65363-67476	9.8	34	5	7	0.644018	0.60765	no
gi|641569454|gb|KK856667.1|	69461-75882	9.8	34	7	3	-1	0.4853	no
gi|641569454|gb|KK856667.1|	75932-79303	9.8	34	5	1	-2	0.1655	no
gi|641569454|gb|KK856667.1|	80007-80955	9.8	34	17	2	-3	0.0511	no
gi|641569454|gb|KK856667.1|	83460-83862	9.8	34	6	2	-2	0.28765	no
gi|641569454|gb|KK856667.1|	85517-85732	9.8	34	15	2	-3	0.2588	no
gi|641569454|gb|KK856667.1|	92062-93588	9.8	34	0.691693	0.648716	-0.0925467	1	no
gi|641569454|gb|KK856667.1|	93778-94485	9.8	34	4	4	-0.0581473	0.96725	no
gi|641569457|gb|KK856666.1|	3070-4562	12	40	8	9	0.233381	0.85315	no
gi|641569457|gb|KK856666.1|	5841-9309	12	40	5	1	-2	0.15545	no
gi|641569457|gb|KK856666.1|	98772-99174	12	40	0	26	inf	0.0001	yes
gi|641569460|gb|KK856665.1|	26148-27418	9.1	19	3	3	0.083274	0.94695	no
gi|641569460|gb|KK856665.1|	27547-28150	9.1	19	12	8	-0.577192	0.6541	no
gi|641569460|gb|KK856665.1|	29249-30426	9.1	19	2	1	-0.393684	0.7888	no
gi|641569460|gb|KK856665.1|	34353-34624	9.1	19	4	8	1	0.51325	no
gi|641569460|gb|KK856665.1|	35547-36231	9.1	19	7	4	-0.956119	0.47955	no
gi|641569460|gb|KK856665.1|	40818-42374	9.1	19	0.988648	0.317228	-2	0.31425	no
gi|641569460|gb|KK856665.1|	48998-49959	9.1	19	22	5	-2	0.12435	no
gi|641569460|gb|KK856665.1|	50240-51621	9.1	19	31	13	-1	0.445	no
gi|641569460|gb|KK856665.1|	56270-56739	9.1	19	14	11	-0.278808	0.83615	no
gi|641569460|gb|KK856665.1|	59622-60133	9.1	19	32	30	-0.0842597	0.94645	no

gi|641569460|gb|KK856665.1|	60608-61397	9.1	19	12	13	0.152228	0.90345	no
gi|641569460|gb|KK856665.1|	6394-7070	9.1	19	1	2	0.962645	0.57335	no
gi|641569460|gb|KK856665.1|	68540-70169	9.1	19	1	1	0.1557	0.91375	no
gi|641569460|gb|KK856665.1|	75482-75695	9.1	19	10	20	0.951891	0.5903	no
gi|641569460|gb|KK856665.1|	83453-83660	9.1	19	17	22	0.363954	0.8166	no
gi|641569463|gb|KK856664.1|	09836-11042	11	46	1	2	0.374883	0.8276	no
gi|641569463|gb|KK856664.1|	15813-16177	11	46	6	7	0.357556	0.8077	no
gi|641569463|gb|KK856664.1|	55469-56190	11	46	1	2	0.753125	0.6261	no
gi|641569469|gb|KK856662.1|	54556-55167	9.1	34	18	18	-0.0148074	0.9918	no
gi|641569469|gb|KK856662.1|	57422-59531	9.1	34	5	6	0.168673	0.89235	no
gi|641569469|gb|KK856662.1|	60833-63671	9.1	34	3	4	0.380231	0.7608	no
gi|641569469|gb|KK856662.1|	65200-65633	9.1	34	18	32	0.827377	0.51915	no
gi|641569469|gb|KK856662.1|	67723-69336	9.1	34	0.799073	0.532893	-0.58448	1	no
gi|641569469|gb|KK856662.1|	69537-70065	9.1	34	2	2	0.402077	0.8089	no
gi|641569469|gb|KK856662.1|	70657-71339	9.1	34	2	3	0.841233	0.585	no
gi|641569469|gb|KK856662.1|	73804-74326	9.1	34	2	3	0.916339	0.56635	no
gi|641569469|gb|KK856662.1|	75329-76110	9.1	34	3	4	0.602259	0.665	no
gi|641569469|gb|KK856662.1|	77151-79086	9.1	34	26	18	-0.523684	0.74285	no
gi|641569469|gb|KK856662.1|	81463-82797	9.1	34	7	5	-0.589252	0.6485	no
gi|641569469|gb|KK856662.1|	83349-84204	9.1	34	2	0.978337	-0.811652	0.5996	no
gi|641569469|gb|KK856662.1|	84333-85501	9.1	34	1	2	0.326152	0.8152	no
gi|641569472|gb|KK856661.1|	40798-41997	11	21	1	2	0.69391	0.62795	no
gi|641569472|gb|KK856661.1|	52870-53647	11	21	1	0.369532	-2	0.31075	no
gi|641569472|gb|KK856661.1|	58365-59160	11	21	2	3	0.339458	0.81435	no
gi|641569472|gb|KK856661.1|	81961-82520	11	21	4	1	-2	0.31335	no
gi|641569478|gb|KK856659.1|	00447-10068	12	42	0	182	inf	5.00E-05	yes
gi|641569478|gb|KK856659.1|	01460-10163	12	42	0	96	inf	0.0015	yes
gi|641569478|gb|KK856659.1|	04505-10478	12	42	0	18	inf	0.0015	yes
gi|641569478|gb|KK856659.1|	07848-10820	12	42	0	8	inf	0.0025	yes
gi|641569478|gb|KK856659.1|	11460-11189	12	42	0	24	inf	0.00015	yes

gi|641569478|gb|KK856659.1|	24142-12438	12	42	0	55	inf	0.00025	yes
gi|641569478|gb|KK856659.1|	28208-29725	12	42	0.696403	0.734759	0.0773481	1	no
gi|641569478|gb|KK856659.1|	30778-32019	12	42	1	0.103103	-4	0.1977	no
gi|641569478|gb|KK856659.1|	32883-33637	12	42	1	0.769255	-0.398222	0.82205	no
gi|641569478|gb|KK856659.1|	34744-35696	12	42	1	0.56907	-1	0.46585	no
gi|641569478|gb|KK856659.1|	36328-37468	12	42	1	0.798549	-0.39392	0.7966	no
gi|641569478|gb|KK856659.1|	74223-74771	12	42	10	0	-inf	0.0003	yes
gi|641569478|gb|KK856659.1|	77532-79757	12	42	1	0.05322	-5	0.1886	no
gi|641569478|gb|KK856659.1|	82609-83211	12	42	2	0	-inf	0.0075	no
gi|641569478|gb|KK856659.1|	85401-88086	12	42	0.910029	0.086806	-3	0.14445	no
gi|641569478|gb|KK856659.1|	89798-90040	12	42	18	2	-3	0.2088	no
gi|641569478|gb|KK856659.1|	93636-95374	12	42	68	0.629781	-7	0.02175	no
gi|641569488|gb|KK856656.1|	13902-14435	9.1	37	39	60	0.641021	0.6103	no
gi|641569488|gb|KK856656.1|	74165-75051	9.1	37	0.307524	1	2	0.2812	no
gi|641569488|gb|KK856656.1|	75174-75881	9.1	37	5	4	-0.514661	0.702	no
gi|641569488|gb|KK856656.1|	76039-76608	9.1	37	2	0.286167	-3	0.22425	no
gi|641569488|gb|KK856656.1|	78356-79136	9.1	37	2	2	-0.134724	0.9246	no
gi|641569488|gb|KK856656.1|	79433-79801	9.1	37	8	5	-0.571451	0.70495	no
gi|641569488|gb|KK856656.1|	81953-82550	9.1	37	2	108	6	0.039	no
gi|641569488|gb|KK856656.1|	82668-85161	9.1	37	0.924442	0.7994	-0.209665	0.88	no
gi|641569488|gb|KK856656.1|	85422-86446	9.1	37	2	2	0.605206	0.6621	no
gi|641569488|gb|KK856656.1|	86618-88691	9.1	37	0.678731	6	3	0.03755	no
gi|641569488|gb|KK856656.1|	90551-91659	9.1	37	2	72	5	0.03115	no
gi|641569488|gb|KK856656.1|	93924-95056	9.1	37	2	4	0.843066	0.52995	no
gi|641569488|gb|KK856656.1|	95989-97391	9.1	37	2	0	-inf	0.00175	yes
gi|641569488|gb|KK856656.1|	98222-99252	9.1	37	2	0	-inf	0.0021	yes
gi|641569495|gb|KK856654.1|	15848-16726	9.5	35	15	6	-1	0.28305	no
gi|641569495|gb|KK856654.1|	17798-18683	9.5	35	13	3	-2	0.1227	no
gi|641569495|gb|KK856654.1|	18877-21500	9.5	35	5	2	-1	0.3958	no
gi|641569495|gb|KK856654.1|	21704-23193	9.5	35	2	0.500408	-2	0.1942	no

gi|641569495|gb|KK856654.1|	24342-24930	9.5	35	8	4	-1	0.38605	no
gi|641569495|gb|KK856654.1|	27164-27909	9.5	35	3	1	-0.912965	0.54625	no
gi|641569495|gb|KK856654.1|	28140-29472	9.5	35	3	0.94879	-2	0.27745	no
gi|641569495|gb|KK856654.1|	31076-32441	9.5	35	5	2	-1	0.4275	no
gi|641569495|gb|KK856654.1|	38376-38829	9.5	35	5	2	-0.91259	0.56415	no
gi|641569495|gb|KK856654.1|	42820-43187	9.5	35	13	13	0.0398347	0.9759	no
gi|641569495|gb|KK856654.1|	49713-50009	9.5	35	5	4	-0.240979	0.88445	no
gi|641569495|gb|KK856654.1|	50915-51194	9.5	35	13	20	0.644294	0.6559	no
gi|641569495|gb|KK856654.1|	57080-58253	9.5	35	0.434505	0.771709	0.828684	1	no
gi|641569495|gb|KK856654.1|	72013-72641	9.5	35	9	3	-1	0.33665	no
gi|641569495|gb|KK856654.1|	74995-75968	9.5	35	4	2	-1	0.45985	no
gi|641569495|gb|KK856654.1|	76018-77115	9.5	35	3	3	0.316276	0.81225	no
gi|641569495|gb|KK856654.1|	78883-79613	9.5	35	6	8	0.534195	0.68795	no
gi|641569495|gb|KK856654.1|	83838-84672	9.5	35	1	0.336766	-2	0.3455	no
gi|641569495|gb|KK856654.1|	84817-85549	9.5	35	2	0	-inf	0.0059	no
gi|641569495|gb|KK856654.1|	86618-88787	9.5	35	2	0.820902	-2	0.2688	no
gi|641569495|gb|KK856654.1|	89529-90283	9.5	35	3	0.192314	-4	0.19145	no
gi|641569495|gb|KK856654.1|	91149-95744	9.5	35	2	1	-0.736026	0.566	no
gi|641569495|gb|KK856654.1|	96131-96963	9.5	35	1	0.844543	-0.534441	0.7478	no
gi|641569495|gb|KK856654.1|	99726-99953	9.5	35	27	14	-0.971223	0.53395	no
gi|641569501|gb|KK856652.1|	00162-10080	9.9	37	1	1	-0.0790451	0.92675	no
gi|641569501|gb|KK856652.1|	03425-10422	9.9	37	2	0.357334	-2	0.21865	no
gi|641569501|gb|KK856652.1|	06560-10931	9.9	37	25	37	0.553568	0.8025	no
gi|641569501|gb|KK856652.1|	10692-11803	9.9	37	58	36.95	-0.652166	0.6834	no
gi|641569501|gb|KK856652.1|	15294-15572	9.9	37	61	43	-0.492901	0.7015	no
gi|641569501|gb|KK856652.1|	17340-17639	9.9	37	7	7	-0.08495	0.9431	no
gi|641569501|gb|KK856652.1|	30655-31568	9.9	37	9	4	-1	0.36575	no
gi|641569501|gb|KK856652.1|	31643-32656	9.9	37	0.692699	15	4	0.04705	no
gi|641569501|gb|KK856652.1|	34039-34552	9.9	37	13	17	0.436383	0.73525	no
gi|641569501|gb|KK856652.1|	34681-35998	9.9	37	14	17	0.294897	0.8084	no

gi|641569501|gb|KK856652.1|	36116-37406	9.9	37	33	35	0.103723	0.9615	no
gi|641569501|gb|KK856652.1|	40685-41564	9.9	37	0.932018	11	3	0.05205	no
gi|641569501|gb|KK856652.1|	43594-43881	9.9	37	11	19	0.733959	0.61885	no
gi|641569501|gb|KK856652.1|	52540-53362	9.9	37	0.790833	2	1	0.48415	no
gi|641569501|gb|KK856652.1|	55974-56521	9.9	37	2	2	-0.181965	0.9035	no
gi|641569501|gb|KK856652.1|	66155-67180	9.9	37	1	2	1	0.42625	no
gi|641569501|gb|KK856652.1|	68478-68996	9.9	37	4	2	-0.68591	0.65225	no
gi|641569501|gb|KK856652.1|	71456-72125	9.9	37	1	1	-0.078178	0.92675	no
gi|641569501|gb|KK856652.1|	79948-81032	9.9	37	8	2	-2	0.20495	no
gi|641569501|gb|KK856652.1|	81597-83386	9.9	37	0.044427	44	10	0.1055	no
gi|641569501|gb|KK856652.1|	84671-88705	9.9	37	1	2	0.1769	0.88825	no
gi|641569501|gb|KK856652.1|	90257-90561	9.9	37	4	7	0.76099	0.6379	no
gi|641569501|gb|KK856652.1|	94407-96523	9.9	37	0.368631	0.393636	0.0946841	1	no
gi|641569501|gb|KK856652.1|	97224-98293	9.9	37	3	5	0.422709	0.75145	no
gi|641569504|gb|KK856651.1|	16288-16946	9.7	36	0.917849	1	0.336565	0.83525	no
gi|641569504|gb|KK856651.1|	17146-26705	9.7	36	106	66	-0.681667	0.6875	no
gi|641569504|gb|KK856651.1|	26817-29361	9.7	36	0.904369	1	0.657124	0.62335	no
gi|641569504|gb|KK856651.1|	29998-31639	9.7	36	0.734622	2	2	0.301	no
gi|641569504|gb|KK856651.1|	34051-38038	9.7	36	7	18	1	0.4532	no
gi|641569504|gb|KK856651.1|	39807-40666	9.7	36	2	6	1	0.3745	no
gi|641569504|gb|KK856651.1|	50564-51474	9.7	36	0.891592	3	2	0.26135	no
gi|641569504|gb|KK856651.1|	53160-54459	9.7	36	0.320751	6	4	0.0852	no
gi|641569504|gb|KK856651.1|	54582-55570	9.7	36	0.625226	1	0.797528	0.63025	no
gi|641569504|gb|KK856651.1|	57017-57988	9.7	36	0.456377	0.832807	0.867755	1	no
gi|641569504|gb|KK856651.1|	59243-59582	9.7	36	9	11	0.253815	0.8559	no
gi|641569504|gb|KK856651.1|	60618-61566	9.7	36	0.376258	1	2	0.33105	no
gi|641569507|gb|KK856650.1|	36631-37226	9.7	39	8	42.05	2	0.07365	no
gi|641569507|gb|KK856650.1|	39231-40113	9.7	39	2	12	2	0.0894	no
gi|641569507|gb|KK856650.1|	45726-46366	9.7	39	18	121	3	0.14405	no
gi|641569507|gb|KK856650.1|	51555-51945	9.7	39	4	18	2	0.1327	no

gi|641569507|gb|KK856650.1|	55351-56023	9.7	39	0	3	inf	0.00375	no
gi|641569507|gb|KK856650.1|	56245-56475	9.7	39	3	26	3	0.25585	no
gi|641569507|gb|KK856650.1|	78225-79093	9.7	39	0.315753	1	2	0.2812	no
gi|641569507|gb|KK856650.1|	79233-80164	9.7	39	0.673666	2	2	0.2915	no
gi|641569507|gb|KK856650.1|	98767-99945	9.7	39	0.072049	5	6	0.10505	no
gi|641569510|gb|KK856649.1|	15475-15661	10	37	38	37	-0.00770594	0.95595	no
gi|641569510|gb|KK856649.1|	28757-29712	10	37	4	3	-0.565365	0.67295	no
gi|641569510|gb|KK856649.1|	30445-31144	10	37	0.140545	7	6	0.10535	no
gi|641569510|gb|KK856649.1|	36736-39621	10	37	66	28	-1	0.55765	no
gi|641569510|gb|KK856649.1|	4-533	10	37	12	5	-1	0.3556	no
gi|641569510|gb|KK856649.1|	41269-42015	10	37	0.771379	0.780341	0.0166647	1	no
gi|641569510|gb|KK856649.1|	5865-6323	10	37	4	4	-0.14666	0.9233	no
gi|641569510|gb|KK856649.1|	72367-72628	10	37	0	20	inf	0.0023	yes
gi|641569510|gb|KK856649.1|	8046-8911	10	37	0.951501	0.642545	-0.566407	0.7586	no
gi|641569510|gb|KK856649.1|	90546-91756	10	37	0	45	inf	0.00065	yes
gi|641569513|gb|KK856648.1|	02069-10456	10	39	2	3	0.511923	0.6915	no
gi|641569513|gb|KK856648.1|	05552-10732	10	39	0	7	inf	5.00E-05	yes
gi|641569513|gb|KK856648.1|	13976-11451	10	39	0.203876	7	5	0.1072	no
gi|641569513|gb|KK856648.1|	54354-54937	10	39	0	6	inf	0.0005	yes
gi|641569513|gb|KK856648.1|	88095-88286	10	39	3	67	5	0.10925	no
gi|641569513|gb|KK856648.1|	93244-95684	10	39	1	1	-0.205874	0.88	no
gi|641569513|gb|KK856648.1|	95739-97516	10	39	0.626693	0.887312	0.501682	0.73235	no
gi|641569513|gb|KK856648.1|	98423-10115	10	39	1	0.851001	-0.389992	0.7668	no
gi|641569516|gb|KK856647.1|	13762-14170	8.6	42	0.987265	3	2	0.3323	no
gi|641569516|gb|KK856647.1|	23228-24120	8.6	42	0.508126	0.772238	0.603859	1	no
gi|641569516|gb|KK856647.1|	40830-41653	8.6	42	0.338389	1	2	0.3323	no
gi|641569519|gb|KK856646.1|	11469-12019	10	39	11	11	-0.107827	0.93435	no
gi|641569519|gb|KK856646.1|	12583-13545	10	39	6	20	2	0.19365	no
gi|641569519|gb|KK856646.1|	166-1453	10	39	1	1	-0.103572	0.94465	no
gi|641569519|gb|KK856646.1|	17836-19228	10	39	3	6	0.929324	0.47725	no

gi|641569519|gb|KK856646.1|	19922-21857	10	39	1	0.744846	-0.850495	0.54315	no
gi|641569519|gb|KK856646.1|	23316-25268	10	39	12	25	0.995718	0.54245	no
gi|641569519|gb|KK856646.1|	30499-31417	10	39	1	2	0.719641	0.6393	no
gi|641569519|gb|KK856646.1|	32358-34181	10	39	24	17	-0.500231	0.7535	no
gi|641569519|gb|KK856646.1|	34453-38585	10	39	28	13	-1	0.6203	no
gi|641569519|gb|KK856646.1|	3613-3921	10	39	2	6	1	0.44675	no
gi|641569519|gb|KK856646.1|	39031-39404	10	39	24	25	0.0533524	0.9672	no
gi|641569519|gb|KK856646.1|	39864-42040	10	39	0.786422	0.599872	-0.39065	1	no
gi|641569519|gb|KK856646.1|	44419-44963	10	39	39	36	-0.103987	0.93645	no
gi|641569519|gb|KK856646.1|	47873-49934	10	39	0.645147	3	2	0.17435	no
gi|641569519|gb|KK856646.1|	51726-52029	10	39	23	222	3	0.02435	no
gi|641569519|gb|KK856646.1|	53292-53474	10	39	3	127.93	5	0.1055	no
gi|641569519|gb|KK856646.1|	54199-54756	10	39	2	37	4	0.0312	no
gi|641569519|gb|KK856646.1|	64063-64538	10	39	1	2	0.854592	0.62755	no
gi|641569519|gb|KK856646.1|	6803-7687	10	39	2	2	-0.106732	0.94465	no
gi|641569519|gb|KK856646.1|	70204-71522	10	39	1	2	0.689533	0.63315	no
gi|641569522|gb|KK856645.1|	38381-38718	10	20	17	3	-3	0.1305	no
gi|641569522|gb|KK856645.1|	42825-43683	10	20	0.320517	2	2	0.21365	no
gi|641569522|gb|KK856645.1|	44867-45526	10	20	0.915874	2	0.822019	0.62335	no
gi|641569522|gb|KK856645.1|	56753-59028	10	20	3	1	-1	0.29355	no
gi|641569522|gb|KK856645.1|	60888-62145	10	20	6	4	-0.620642	0.62945	no
gi|641569522|gb|KK856645.1|	63929-65753	10	20	4	5	0.555594	0.66335	no
gi|641569522|gb|KK856645.1|	83807-84145	10	20	6	6	0.0450432	0.95585	no
gi|641569526|gb|KK856644.1|	.02213-10245	9.4	18	1	30	5	0.109	no
gi|641569526|gb|KK856644.1|	.09425-10962	9.4	18	0	130	inf	5.00E-05	yes
gi|641569526|gb|KK856644.1|	21833-22400	9.4	18	2	2	-0.0555578	0.9454	no
gi|641569526|gb|KK856644.1|	31532-32324	9.4	18	26	51	0.975318	0.44015	no
gi|641569526|gb|KK856644.1|	32579-36508	9.4	18	1	1	-0.0630189	0.9617	no
gi|641569526|gb|KK856644.1|	37014-38207	9.4	18	0.922556	1	0.490935	0.7378	no
gi|641569526|gb|KK856644.1|	41-586	9.4	18	1	2	1	0.4611	no

gi|641569526|gb|KK856644.1|	43864-45419	9.4	18	2	1	-0.491548	0.7262	no
gi|641569526|gb|KK856644.1|	46138-48513	9.4	18	0.77959	0.594773	-0.390377	1	no
gi|641569526|gb|KK856644.1|	48960-50117	9.4	18	5	1	-2	0.16215	no
gi|641569526|gb|KK856644.1|	6085-6361	9.4	18	38	18	-1	0.4261	no
gi|641569526|gb|KK856644.1|	64986-65183	9.4	18	81	7	-4	0.14045	no
gi|641569526|gb|KK856644.1|	69919-70156	9.4	18	84	10	-3	0.0725	no
gi|641569526|gb|KK856644.1|	85140-86349	9.4	18	0.139691	3	4	0.2392	no
gi|641569526|gb|KK856644.1|	87382-88368	9.4	18	0	3	inf	0.0005	yes
gi|641569526|gb|KK856644.1|	93334-93530	9.4	18	0	123	inf	0.0004	yes
gi|641569526|gb|KK856644.1|	9344-9671	9.4	18	150	118	-0.349619	0.77625	no
gi|641569526|gb|KK856644.1|	99072-99304	9.4	18	0	42	inf	0.00115	yes
gi|641569529|gb|KK856643.1|	00305-10111	11	19	0.689967	1	0.82514	0.6232	no
gi|641569529|gb|KK856643.1|	02531-10366	11	19	0.377533	1	2	0.2717	no
gi|641569529|gb|KK856643.1|	13346-11425	11	19	0.89535	0.756056	-0.24396	0.88495	no
gi|641569529|gb|KK856643.1|	15083-11574	11	19	2	2	0.0851885	0.94	no
gi|641569529|gb|KK856643.1|	16938-11769	11	19	1	2	0.601907	0.6936	no
gi|641569529|gb|KK856643.1|	17815-11824	11	19	2	5	2	0.31505	no
gi|641569529|gb|KK856643.1|	18466-11906	11	19	2	2	-0.102722	0.9456	no
gi|641569529|gb|KK856643.1|	16734-16958	11	19	0	95	inf	0.00025	yes
gi|641569529|gb|KK856643.1|	2295-2706	11	19	9	0	-inf	0.0015	yes
gi|641569529|gb|KK856643.1|	29472-29918	11	19	2	3	0.589334	0.70555	no
gi|641569529|gb|KK856643.1|	41554-44109	11	19	5	7	0.437877	0.72805	no
gi|641569529|gb|KK856643.1|	45746-47665	11	19	21	22	0.0967935	0.949	no
gi|641569529|gb|KK856643.1|	48648-49336	11	19	96	69	-0.484124	0.7628	no
gi|641569529|gb|KK856643.1|	54016-54241	11	19	159	83	-0.932871	0.4737	no
gi|641569529|gb|KK856643.1|	57313-57747	11	19	2	2	-0.219074	0.8945	no
gi|641569529|gb|KK856643.1|	62398-63851	11	19	11	13	0.304566	0.8088	no
gi|641569529|gb|KK856643.1|	69440-70168	11	19	6	3	-1	0.39785	no
gi|641569529|gb|KK856643.1|	71015-73100	11	19	11	5	-1	0.3654	no
gi|641569529|gb|KK856643.1|	74546-75670	11	19	6	2	-1	0.3782	no

gi|641569529|gb|KK856643.1|	76368-77179	11	19	1	0.873157	-0.534756	0.7478	no
gi|641569529|gb|KK856643.1|	77284-77641	11	19	4	1	-2	0.3632	no
gi|641569529|gb|KK856643.1|	78121-79036	11	19	0.885398	1	0.756132	0.62565	no
gi|641569529|gb|KK856643.1|	80404-81362	11	19	0.928312	1	0.604591	0.67795	no
gi|641569529|gb|KK856643.1|	83624-83973	11	19	2	5	2	0.41725	no
gi|641569529|gb|KK856643.1|	90380-90705	11	19	5	2	-1	0.5278	no
gi|641569529|gb|KK856643.1|	92194-93455	11	19	0.86369	1	0.60679	0.681	no
gi|641569529|gb|KK856643.1|	94153-95658	11	19	1	2	0.7847	0.5681	no
gi|641569529|gb|KK856643.1|	96862-98190	11	19	1	1	-0.151875	0.9211	no
gi|641569532|gb|KK856642.1|	22399-23348	7.7	34	0	88	inf	0.00015	yes
gi|641569532|gb|KK856642.1|	33197-33745	7.7	34	0	26	inf	5.00E-05	yes
gi|641569535|gb|KK856641.1|	2-596	8.1	35	2	1	-0.402427	0.8125	no
gi|641569535|gb|KK856641.1|	4795-5360	8.1	35	0.38256	4	3	0.2556	no
gi|641569535|gb|KK856641.1|	52480-53573	8.1	35	0.630968	1	0.775678	0.62605	no
gi|641569535|gb|KK856641.1|	55243-55988	8.1	35	0.643984	0.977186	0.601608	0.70505	no
gi|641569535|gb|KK856641.1|	6855-7222	8.1	35	15	54	2	0.16255	no
gi|641569535|gb|KK856641.1|	69762-70903	8.1	35	3	5	0.506551	0.69705	no
gi|641569535|gb|KK856641.1|	71848-73352	8.1	35	2	5	1	0.4197	no
gi|641569535|gb|KK856641.1|	74015-75872	8.1	35	1	3	1	0.33205	no
gi|641569535|gb|KK856641.1|	80871-82555	8.1	35	0.713403	1	0.949401	0.51705	no
gi|641569535|gb|KK856641.1|	82784-83385	8.1	35	0.523175	3	3	0.19335	no
gi|641569539|gb|KK856640.1|	13542-14841	8.3	44	0	6	inf	5.00E-05	yes
gi|641569539|gb|KK856640.1|	25751-26211	8.3	44	0.798272	4	2	0.2235	no
gi|641569542|gb|KK856639.1|	14323-16058	9.3	34	10	3	-2	0.15525	no
gi|641569542|gb|KK856639.1|	1734-4809	9.3	34	4	3	-0.557559	0.6542	no
gi|641569542|gb|KK856639.1|	17440-17705	9.3	34	29	5	-3	0.14465	no
gi|641569542|gb|KK856639.1|	25370-25595	9.3	34	13	2	-3	0.2588	no
gi|641569542|gb|KK856639.1|	29644-29883	9.3	34	20	3	-3	0.20805	no
gi|641569542|gb|KK856639.1|	39451-39778	9.3	34	13	5	-1	0.40095	no
gi|641569542|gb|KK856639.1|	41652-42007	9.3	34	5	0.643984	-3	0.2376	no

gi|641569542|gb|KK856639.1|	5188-9986	9.3	34	8	7	-0.244938	0.88015	no
gi|641569546|gb|KK856638.1|	92462-93408	8.9	34	0.848836	1	0.434542	0.78015	no
gi|641569546|gb|KK856638.1|	95396-97003	8.9	34	42	25	-0.705961	0.7569	no
gi|641569546|gb|KK856638.1|	98345-99502	8.9	34	5	4	-0.260543	0.8382	no
gi|641569549|gb|KK856637.1|	11085-11249	9.4	33	3	2	-0.467823	0.7269	no
gi|641569549|gb|KK856637.1|	11655-12302	9.4	33	13	15	0.241738	0.8472	no
gi|641569549|gb|KK856637.1|	15589-16741	9.4	33	7	4	-0.808879	0.52965	no
gi|641569549|gb|KK856637.1|	17636-18089	9.4	33	15	10	-0.606487	0.64225	no
gi|641569549|gb|KK856637.1|	26115-26762	9.4	33	5	4	-0.313247	0.8207	no
gi|641569549|gb|KK856637.1|	2930-3250	9.4	33	11	0.800538	-4	0.19895	no
gi|641569549|gb|KK856637.1|	30886-31068	9.4	33	51	27	-0.894365	0.5688	no
gi|641569549|gb|KK856637.1|	41158-42243	9.4	33	5	4	-0.0428333	0.97465	no
gi|641569549|gb|KK856637.1|	43009-43233	9.4	33	30	14	-1	0.48115	no
gi|641569549|gb|KK856637.1|	50038-51082	9.4	33	2	1	-0.472628	0.74025	no
gi|641569549|gb|KK856637.1|	5163-5955	9.4	33	11	9	-0.258581	0.83925	no
gi|641569549|gb|KK856637.1|	59930-60996	9.4	33	18	10	-0.868381	0.4843	no
gi|641569549|gb|KK856637.1|	62567-62820	9.4	33	53	21	-1	0.34345	no
gi|641569549|gb|KK856637.1|	64612-64890	9.4	33	67	19	-2	0.18475	no
gi|641569549|gb|KK856637.1|	67760-68348	9.4	33	0	21	inf	5.00E-05	yes
gi|641569549|gb|KK856637.1|	69953-70903	9.4	33	1	0.57057	-1	0.50315	no
gi|641569549|gb|KK856637.1|	8337-9446	9.4	33	16	13	-0.247742	0.84395	no
gi|641569558|gb|KK856634.1|	30404-31282	12	37	1	1	-0.096754	0.9478	no
gi|641569558|gb|KK856634.1|	36745-37845	12	37	0	3	inf	0.00065	yes
gi|641569561|gb|KK856633.1|	11698-12209	11	40	2	1	-1	0.50365	no
gi|641569561|gb|KK856633.1|	12310-13486	11	40	1	0.879266	-0.39369	0.7971	no
gi|641569561|gb|KK856633.1|	13614-14195	11	40	2	3	0.597083	0.67805	no
gi|641569561|gb|KK856633.1|	19056-19517	11	40	28	6	-2	0.13445	no
gi|641569561|gb|KK856633.1|	20652-20843	11	40	64	48	-0.417927	0.7616	no
gi|641569561|gb|KK856633.1|	3652-5542	11	40	1	0.95572	-0.128119	0.92535	no
gi|641569561|gb|KK856633.1|	42190-44217	11	40	15	7	-1	0.3993	no

gi|641569561|gb|KK856633.1|	58866-59744	11	40	10	5	-0.950903	0.4675	no
gi|641569561|gb|KK856633.1|	67218-67577	11	40	22	22.04	-0.0194694	0.98665	no
gi|641569561|gb|KK856633.1|	6998-7657	11	40	3	0.693923	-2	0.24335	no
gi|641569561|gb|KK856633.1|	71364-72099	11	40	1	0.597084	-1	0.50885	no
gi|641569561|gb|KK856633.1|	73981-74238	11	40	20	21	0.00245054	0.9729	no
gi|641569561|gb|KK856633.1|	76015-76379	11	40	5	4	-0.535529	0.73675	no
gi|641569561|gb|KK856633.1|	79271-79813	11	40	28	24	-0.235836	0.85195	no
gi|641569561|gb|KK856633.1|	83250-83498	11	40	57	56	-0.0200869	0.98695	no
gi|641569561|gb|KK856633.1|	84795-85672	11	40	1	0.946954	-0.396327	0.8086	no
gi|641569561|gb|KK856633.1|	87120-88771	11	40	45	24	-0.933574	0.56015	no
gi|641569564|gb|KK856632.1|	24368-26173	9.8	34	4	3	-0.528841	0.67535	no
gi|641569564|gb|KK856632.1|	27078-28335	9.8	34	3	1	-2	0.2363	no
gi|641569564|gb|KK856632.1|	28561-30211	9.8	34	3	0.519378	-3	0.1085	no
gi|641569564|gb|KK856632.1|	31401-35590	9.8	34	5	3	-0.765707	0.54545	no
gi|641569564|gb|KK856632.1|	3284-3930	9.8	34	21	16	-0.347496	0.78365	no
gi|641569564|gb|KK856632.1|	40192-42257	9.8	34	39	12	-2	0.3229	no
gi|641569564|gb|KK856632.1|	44139-45149	9.8	34	2	3	0.296962	0.83145	no
gi|641569564|gb|KK856632.1|	45673-46099	9.8	34	91	35	-1	0.2787	no
gi|641569564|gb|KK856632.1|	47545-47819	9.8	34	12	5	-1	0.4156	no
gi|641569564|gb|KK856632.1|	53108-56151	9.8	34	0.895077	0.872956	-0.0361034	0.97765	no
gi|641569564|gb|KK856632.1|	57417-57578	9.8	34	218	129	-0.753911	0.5795	no
gi|641569564|gb|KK856632.1|	58059-58897	9.8	34	0.991473	0.836709	-0.244848	0.88495	no
gi|641569564|gb|KK856632.1|	59709-60611	9.8	34	8	2	-2	0.1842	no
gi|641569564|gb|KK856632.1|	67393-67591	9.8	34	24	13	-0.930206	0.59265	no
gi|641569564|gb|KK856632.1|	67886-68333	9.8	34	6	3	-0.870014	0.5616	no
gi|641569564|gb|KK856632.1|	71586-71935	9.8	34	17	9	-1	0.4641	no
gi|641569564|gb|KK856632.1|	73856-74053	9.8	34	45	26	-0.793232	0.5973	no
gi|641569564|gb|KK856632.1|	74636-75316	9.8	34	2	0.221391	-3	0.217	no
gi|641569564|gb|KK856632.1|	77381-77811	9.8	34	16	12	-0.493009	0.7145	no
gi|641569564|gb|KK856632.1|	78767-79806	9.8	34	2	2	-0.231168	0.86595	no

gi|641569564|gb|KK856632.1|	80312-81765	9.8	34	17	11	-0.632499	0.6098	no
gi|641569564|gb|KK856632.1|	82008-86499	9.8	34	17	15	-0.145769	0.94515	no
gi|641569564|gb|KK856632.1|	93-831	9.8	34	40	28	-0.555712	0.65825	no
gi|641569564|gb|KK856632.1|	93334-94551	9.8	34	2	0.949639	-1	0.45925	no
gi|641569564|gb|KK856632.1|	9603-10562	9.8	34	0.834387	4	2	0.1801	no
gi|641569568|gb|KK856631.1|	42173-42608	9.8	40	0.586319	4	3	0.28535	no
gi|641569568|gb|KK856631.1|	55540-56433	9.8	40	1	0.462683	-1	0.47135	no
gi|641569574|gb|KK856629.1|	.02175-10348	24	120	8	8	0.077799	0.9505	no
gi|641569574|gb|KK856629.1|	.03619-10417	24	120	2	0.886484	-0.988871	0.62775	no
gi|641569574|gb|KK856629.1|	.05044-10528	24	120	83	31	-1	0.3041	no
gi|641569574|gb|KK856629.1|	.05462-10720	24	120	6	2	-2	0.23245	no
gi|641569574|gb|KK856629.1|	.08502-10942	24	120	0.678276	1	0.966823	0.5733	no
gi|641569574|gb|KK856629.1|	.11712-11224	24	120	67	26	-1	0.27495	no
gi|641569574|gb|KK856629.1|	.12601-11306	24	120	65	32	-1	0.41775	no
gi|641569574|gb|KK856629.1|	.16361-11654	24	120	124	65	-0.936086	0.49695	no
gi|641569574|gb|KK856629.1|	.22221-12250	24	120	16	4	-2	0.27075	no
gi|641569574|gb|KK856629.1|	.22663-12289	24	120	52	9	-3	0.1317	no
gi|641569574|gb|KK856629.1|	.23289-12675	24	120	1	2	0.165118	0.8949	no
gi|641569574|gb|KK856629.1|	12514-13328	24	120	0.457748	1	1	0.51305	no
gi|641569574|gb|KK856629.1|	.29318-13027	24	120	1	2	0.704065	0.6235	no
gi|641569574|gb|KK856629.1|	.30843-13165	24	120	0.231493	2	3	0.2851	no
gi|641569574|gb|KK856629.1|	.34961-13570	24	120	0.387094	2	3	0.19335	no
gi|641569574|gb|KK856629.1|	.35810-13658	24	120	0.975399	1	0.602173	0.6936	no
gi|641569574|gb|KK856629.1|	.42372-14494	24	120	15	4	-2	0.1704	no
gi|641569574|gb|KK856629.1|	.45361-14586	24	120	6	2	-1	0.4097	no
gi|641569574|gb|KK856629.1|	.46364-15503	24	120	9	9	-0.013695	0.9943	no
gi|641569574|gb|KK856629.1|	25227-28986	24	120	13	18	0.528883	0.81655	no
gi|641569574|gb|KK856629.1|	35016-37779	24	120	0.193022	2	3	0.0799	no
gi|641569574|gb|KK856629.1|	55183-56217	24	120	42	39	-0.0940492	0.9378	no
gi|641569574|gb|KK856629.1|	56357-56839	24	120	6	5	-0.231087	0.8711	no

gi|641569574|gb|KK856629.1|	60824-61731	24	120	1	0.453633	-1	0.4312	no
gi|641569574|gb|KK856629.1|	62015-62388	24	120	23	22	-0.0534316	0.96535	no
gi|641569574|gb|KK856629.1|	64066-64549	24	120	32	31	-0.0772533	0.94935	no
gi|641569574|gb|KK856629.1|	66897-68077	24	120	0.503319	1	1	0.43995	no
gi|641569574|gb|KK856629.1|	68221-70378	24	120	1	1	-0.127644	0.9234	no
gi|641569574|gb|KK856629.1|	71942-74895	24	120	4	4	0.170371	0.89385	no
gi|641569574|gb|KK856629.1|	75457-77738	24	120	20	21	0.0293796	0.98895	no
gi|641569574|gb|KK856629.1|	78193-81203	24	120	10	5	-0.931335	0.45805	no
gi|641569574|gb|KK856629.1|	81656-83213	24	120	11	14	0.333819	0.78645	no
gi|641569574|gb|KK856629.1|	83351-85116	24	120	0.541272	0.825302	0.608569	1	no
gi|641569574|gb|KK856629.1|	86745-86954	24	120	67	78	0.23232	0.85945	no
gi|641569574|gb|KK856629.1|	89054-89248	24	120	58	35	-0.735938	0.61385	no
gi|641569574|gb|KK856629.1|	92285-94951	24	120	3	3	0.0249802	0.9847	no
gi|641569577|gb|KK856628.1|	01681-10197	9.7	36	8	10	0.303342	0.83885	no
gi|641569577|gb|KK856628.1|	02498-10353	9.7	36	0.670557	1	0.775266	0.62605	no
gi|641569577|gb|KK856628.1|	06362-10899	9.7	36	1	3	0.955681	0.4713	no
gi|641569577|gb|KK856628.1|	10652-11981	9.7	36	1	1	-0.181374	0.89715	no
gi|641569577|gb|KK856628.1|	11580-11279	9.7	36	3	3	-0.0149714	0.9837	no
gi|641569577|gb|KK856628.1|	12859-11327	9.7	36	6	3	-1	0.4601	no
gi|641569577|gb|KK856628.1|	13692-11496	9.7	36	4	3	-0.489359	0.70445	no
gi|641569577|gb|KK856628.1|	15164-11656	9.7	36	3	4	0.405791	0.7588	no
gi|641569577|gb|KK856628.1|	18566-11899	9.7	36	7	8	0.172298	0.89985	no
gi|641569577|gb|KK856628.1|	13498-14206	9.7	36	3	1	-1	0.4111	no
gi|641569577|gb|KK856628.1|	15287-15505	9.7	36	17	14	-0.335217	0.83195	no
gi|641569577|gb|KK856628.1|	17583-18150	9.7	36	9	3	-2	0.2528	no
gi|641569577|gb|KK856628.1|	19932-20446	9.7	36	8	4	-1	0.42545	no
gi|641569577|gb|KK856628.1|	23694-23863	9.7	36	111	53	-1	0.46875	no
gi|641569577|gb|KK856628.1|	24982-25374	9.7	36	9	5	-0.890114	0.53895	no
gi|641569577|gb|KK856628.1|	28074-29836	9.7	36	8	5	-0.803821	0.52805	no
gi|641569577|gb|KK856628.1|	30540-31458	9.7	36	7	6	-0.395837	0.75745	no

gi|641569577|gb|KK856628.1|	3112-3455	9.7	36	5	0	-inf	0.0102	no
gi|641569577|gb|KK856628.1|	31698-33160	9.7	36	1	3	1	0.37365	no
gi|641569577|gb|KK856628.1|	34992-35752	9.7	36	7	3	-1	0.39115	no
gi|641569577|gb|KK856628.1|	39485-40645	9.7	36	0.807327	0.67034	-0.268259	1	no
gi|641569577|gb|KK856628.1|	40900-41709	9.7	36	2	0.52559	-2	0.29	no
gi|641569577|gb|KK856628.1|	45830-49389	9.7	36	9	9	0.0974732	0.9366	no
gi|641569577|gb|KK856628.1|	4760-5330	9.7	36	4	1	-2	0.28425	no
gi|641569577|gb|KK856628.1|	49928-50899	9.7	36	97	77	-0.318282	0.88315	no
gi|641569577|gb|KK856628.1|	54945-58205	9.7	36	8	8	0.0760704	0.94905	no
gi|641569577|gb|KK856628.1|	59279-60817	9.7	36	2	0.803669	-0.92812	0.5162	no
gi|641569577|gb|KK856628.1|	60967-61858	9.7	36	2	2	0.101347	0.9445	no
gi|641569577|gb|KK856628.1|	62087-69179	9.7	36	71	77	0.111026	0.94755	no
gi|641569577|gb|KK856628.1|	69294-70637	9.7	36	1	1	-0.109022	0.9377	no
gi|641569577|gb|KK856628.1|	71586-72250	9.7	36	3	1	-1	0.3596	no
gi|641569577|gb|KK856628.1|	7842-8223	9.7	36	10	7	-0.53308	0.70345	no
gi|641569577|gb|KK856628.1|	81811-82317	9.7	36	1	2	0.856562	0.62755	no
gi|641569577|gb|KK856628.1|	82480-82975	9.7	36	4	6	0.59287	0.67125	no
gi|641569577|gb|KK856628.1|	8426-9477	9.7	36	2	3	0.403797	0.7721	no
gi|641569577|gb|KK856628.1|	90950-91200	9.7	36	167	100	-0.735029	0.5599	no
gi|641569577|gb|KK856628.1|	99940-10028[illegible]	9.7	36	31	22	-0.486784	0.70855	no
gi|641569580|gb|KK856627.1|	64432-64786	9.4	31	0	85	inf	5.00E-05	yes
gi|641569584|gb|KK856626.1|	[illegible]06446-10810	10	20	0.578888	1	1	0.39595	no
gi|641569584|gb|KK856626.1|	[illegible]11276-11166	10	20	19	50	1	0.2993	no
gi|641569584|gb|KK856626.1|	[illegible]12550-11329	10	20	3	9	2	0.2257	no
gi|641569584|gb|KK856626.1|	[illegible]14566-11515	10	20	9	21	1	0.3786	no
gi|641569584|gb|KK856626.1|	[illegible]16180-11757	10	20	1	3	1	0.4218	no
gi|641569584|gb|KK856626.1|	[illegible]18523-12223	10	20	202	569	1	0.2263	no
gi|641569584|gb|KK856626.1|	[illegible]31025-13253	10	20	4	8	0.789446	0.52975	no
gi|641569584|gb|KK856626.1|	[illegible]34689-13601	10	20	0.687587	1	0.607117	0.68625	no
gi|641569584|gb|KK856626.1|	25023-26902	10	20	2	1	-0.639104	0.6309	no

gi|641569584|gb|KK856626.1|	27069-27879	10	20	3	0.874568	-2	0.23295	no
gi|641569584|gb|KK856626.1|	29555-30914	10	20	7	1	-2	0.11165	no
gi|641569584|gb|KK856626.1|	33813-34762	10	20	3	0.285661	-4	0.14165	no
gi|641569584|gb|KK856626.1|	47109-48756	10	20	1	1	-0.128703	0.9253	no
gi|641569584|gb|KK856626.1|	4969-5115	10	20	116.56	0	-inf	0.01175	no
gi|641569584|gb|KK856626.1|	55469-56287	10	20	10	26	1	0.28625	no
gi|641569584|gb|KK856626.1|	8129-8488	10	20	4	3	-0.420832	0.77915	no
gi|641569584|gb|KK856626.1|	83675-84842	10	20	0.582917	0.443688	-0.393746	1	no
gi|641569584|gb|KK856626.1|	89501-91019	10	20	0.909993	1	0.220843	0.882	no
gi|641569584|gb|KK856626.1|	91694-92426	10	20	1	0.400262	-2	0.3216	no
gi|641569584|gb|KK856626.1|	92735-93703	10	20	1	0.278674	-2	0.24735	no
gi|641569584|gb|KK856626.1|	)4582-10193:	10	20	65	125	0.94399	0.5862	no
gi|641569587|gb|KK856625.1|	55019-55339	8.9	37	7	6	-0.12848	0.9347	no
gi|641569587|gb|KK856625.1|	68778-69105	8.9	37	4	14	2	0.24555	no
gi|641569590|gb|KK856624.1|	33222-34165	8.8	32	3	0	-inf	0.00185	yes
gi|641569590|gb|KK856624.1|	48746-49945	8.8	32	3	5	0.640395	0.62715	no
gi|641569590|gb|KK856624.1|	50923-56612	8.8	32	19	17	-0.198494	0.92455	no
gi|641569590|gb|KK856624.1|	57342-58011	8.8	32	7	6	-0.310839	0.81905	no
gi|641569590|gb|KK856624.1|	58402-63351	8.8	32	4	5	0.356338	0.77855	no
gi|641569590|gb|KK856624.1|	64013-65315	8.8	32	4	3	-0.348611	0.7917	no
gi|641569590|gb|KK856624.1|	65543-67387	8.8	32	7	8	0.196696	0.8719	no
gi|641569590|gb|KK856624.1|	67542-68979	8.8	32	4	5	0.630658	0.6252	no
gi|641569590|gb|KK856624.1|	69088-70641	8.8	32	3	5	0.490624	0.70795	no
gi|641569590|gb|KK856624.1|	71595-74202	8.8	32	11	11	-0.046504	0.9674	no
gi|641569590|gb|KK856624.1|	76286-76572	8.8	32	62	67	0.126702	0.918	no
gi|641569590|gb|KK856624.1|	84848-85039	8.8	32	5	30	3	0.30895	no
gi|641569590|gb|KK856624.1|	85887-86288	8.8	32	19	13	-0.578606	0.6663	no
gi|641569590|gb|KK856624.1|	86694-86990	8.8	32	14	12	-0.125502	0.93185	no
gi|641569593|gb|KK856623.1|	30122-30348	11	44	7	18	1	0.41455	no
gi|641569593|gb|KK856623.1|	35780-36875	11	44	2	0.718549	-2	0.2865	no

gi|641569596|gb|KK856622.1|	08951-10985	12	43	0	6	inf	5.00E-05	yes
gi|641569596|gb|KK856622.1|	11037-11207	12	43	0	2	inf	0.0023	yes
gi|641569596|gb|KK856622.1|	17151-17368	12	43	19	2	-3	0.23065	no
gi|641569596|gb|KK856622.1|	19166-19536	12	43	30	4	-3	0.0706	no
gi|641569596|gb|KK856622.1|	20109-20492	12	43	22	6	-2	0.1782	no
gi|641569596|gb|KK856622.1|	22593-22770	12	43	44	5	-3	0.2302	no
gi|641569596|gb|KK856622.1|	2320-2860	12	43	9	3	-1	0.3033	no
gi|641569596|gb|KK856622.1|	24352-25007	12	43	32	5	-3	0.0538	no
gi|641569596|gb|KK856622.1|	37443-38329	12	43	81	4	-4	0.05415	no
gi|641569596|gb|KK856622.1|	38870-40106	12	43	2	0.31079	-3	0.13665	no
gi|641569596|gb|KK856622.1|	40567-41619	12	43	0.991747	0	-inf	0.01	no
gi|641569596|gb|KK856622.1|	46386-47077	12	43	3	0.216526	-4	0.1933	no
gi|641569596|gb|KK856622.1|	49313-50773	12	43	44	3	-4	0.0761	no
gi|641569596|gb|KK856622.1|	53877-54401	12	43	0.21494	15	6	0.10465	no
gi|641569596|gb|KK856622.1|	57838-58094	12	43	78	19	-2	0.1641	no
gi|641569596|gb|KK856622.1|	58230-59332	12	43	1	0.831865	-0.287266	0.85105	no
gi|641569596|gb|KK856622.1|	65794-66857	12	43	2	0.496616	-2	0.18415	no
gi|641569596|gb|KK856622.1|	68404-68588	12	43	27	9	-2	0.363	no
gi|641569596|gb|KK856622.1|	68827-69876	12	43	38	12	-2	0.19485	no
gi|641569596|gb|KK856622.1|	69995-70793	12	43	13	6	-1	0.3972	no
gi|641569596|gb|KK856622.1|	70919-72431	12	43	0.806594	1	0.607846	0.6735	no
gi|641569596|gb|KK856622.1|	74001-74611	12	43	1	3	1	0.45675	no
gi|641569596|gb|KK856622.1|	8292-9123	12	43	1	0.338345	-2	0.31075	no
gi|641569596|gb|KK856622.1|	9671-10198	12	43	2	0.965037	-0.990291	0.62775	no
gi|641569599|gb|KK856621.1|	01840-10420	11	41	0.68497	4	3	0.08075	no
gi|641569599|gb|KK856621.1|	07316-10902	11	41	0.233719	0.783923	2	1	no
gi|641569599|gb|KK856621.1|	09412-10971	11	41	0	25	inf	0.00035	yes
gi|641569599|gb|KK856621.1|	24210-12451	11	41	5	4	-0.431179	0.7868	no
gi|641569599|gb|KK856621.1|	20243-21571	11	41	8	15	0.898721	0.47705	no
gi|641569599|gb|KK856621.1|	22938-26333	11	41	5	6	0.340399	0.7886	no

gi|641569599|gb|KK856621.1|	49437-50148	11	41	1	3	1	0.40085	no
gi|641569599|gb|KK856621.1|	52408-52629	11	41	7	15	1	0.5133	no
gi|641569599|gb|KK856621.1|	86912-87100	11	41	0	51	inf	0.0025	yes
gi|641569599|gb|KK856621.1|	89800-90641	11	41	0.219304	50	8	0.2322	no
gi|641569599|gb|KK856621.1|	)9505-101306	11	41	0.529137	4	3	0.06945	no
gi|641569603|gb|KK856620.1|	.23240-12472	14	50	0.550266	0.670828	0.285813	1	no
gi|641569603|gb|KK856620.1|	.25256-12664	14	50	0.297316	0.634136	1	1	no
gi|641569603|gb|KK856620.1|	.36066-13661	14	50	2	0.918912	-0.819534	0.6492	no
gi|641569603|gb|KK856620.1|	.41407-14187	14	50	1	2	0.590838	0.70505	no
gi|641569603|gb|KK856620.1|	19956-21406	14	50	0.28215	1	2	0.2077	no
gi|641569603|gb|KK856620.1|	21545-22369	14	50	0.112617	2	4	0.1175	no
gi|641569603|gb|KK856620.1|	40691-41769	14	50	0.882324	3	2	0.26575	no
gi|641569603|gb|KK856620.1|	43295-43928	14	50	1	1	-0.249121	0.88485	no
gi|641569603|gb|KK856620.1|	44165-45165	14	50	0.527917	0.936771	0.827387	0.6232	no
gi|641569603|gb|KK856620.1|	45389-46877	14	50	0.383399	2	3	0.1446	no
gi|641569603|gb|KK856620.1|	46955-47581	14	50	0.985842	2	0.821051	0.62335	no
gi|641569603|gb|KK856620.1|	59537-60325	14	50	0.358371	2	2	0.2235	no
gi|641569603|gb|KK856620.1|	61085-63141	14	50	0.799088	2	1	0.45865	no
gi|641569603|gb|KK856620.1|	70205-71250	14	50	0.249979	0.887399	2	0.3323	no
gi|641569603|gb|KK856620.1|	71896-72939	14	50	0.668184	2	2	0.3067	no
gi|641569603|gb|KK856620.1|	77889-78211	14	50	11	4	-1	0.35335	no
gi|641569603|gb|KK856620.1|	82318-83584	14	50	0.925784	2	0.969386	0.5114	no
gi|641569603|gb|KK856620.1|	83869-84373	14	50	2	3	0.593411	0.6902	no
gi|641569603|gb|KK856620.1|	84630-85346	14	50	1	2	0.601014	0.6863	no
gi|641569603|gb|KK856620.1|	85537-86151	14	50	1	3	1	0.4891	no
gi|641569603|gb|KK856620.1|	86383-87190	14	50	0.694479	0.703063	0.0177222	1	no
gi|641569603|gb|KK856620.1|	88300-89087	14	50	0.478635	1	1	0.44255	no
gi|641569603|gb|KK856620.1|	89745-90377	14	50	0.648227	2	2	0.33105	no
gi|641569606|gb|KK856619.1|	27731-29855	9.9	38	0.256971	1	2	0.1537	no
gi|641569606|gb|KK856619.1|	55983-57680	9.9	38	0.094297	171	11	0.23345	no

gi|641569606|gb|KK856619.1|	64537-64845	9.9	38	0	43	inf	0.0001	yes
gi|641569606|gb|KK856619.1|	74975-75332	9.9	38	70	46	-0.59765	0.632	no
gi|641569606|gb|KK856619.1|	77242-77713	9.9	38	23	9	-1	0.293	no
gi|641569606|gb|KK856619.1|	78224-78488	9.9	38	19	9	-1	0.47095	no
gi|641569606|gb|KK856619.1|	78821-79196	9.9	38	31	68	1	0.3782	no
gi|641569606|gb|KK856619.1|	79417-80744	9.9	38	38	56	0.556317	0.80555	no
gi|641569606|gb|KK856619.1|	82032-82681	9.9	38	33	15	-1	0.37405	no
gi|641569606|gb|KK856619.1|	82799-84920	9.9	38	10	11	0.136846	0.9153	no
gi|641569606|gb|KK856619.1|	90166-90873	9.9	38	2	3	0.507706	0.7258	no
gi|641569606|gb|KK856619.1|	94380-94885	9.9	38	18	34	0.882976	0.48805	no
gi|641569606|gb|KK856619.1|	95019-97511	9.9	38	49	78	0.666414	0.74825	no
gi|641569606|gb|KK856619.1|	97624-99070	9.9	38	0.735937	0.690074	-0.0928306	1	no
gi|641569609|gb|KK856618.1|	38566-39641	7.5	26	2	0	-inf	0.00495	no
gi|641569612|gb|KK856617.1|	01884-10386	11	21	2	1	-0.948967	0.4925	no
gi|641569612|gb|KK856617.1|	07875-10801	11	21	242	269	0.14967	0.9234	no
gi|641569612|gb|KK856617.1|	14514-11482	11	21	5	10	0.98569	0.552	no
gi|641569612|gb|KK856617.1|	20230-12082	11	21	4	2	-0.554393	0.70725	no
gi|641569612|gb|KK856617.1|	12123-14358	11	21	0.763674	0.423676	-0.849995	1	no
gi|641569612|gb|KK856617.1|	21280-12225	11	21	2	2	0.0197872	0.9728	no
gi|641569612|gb|KK856617.1|	30506-13134	11	21	1	1	0.313699	0.84265	no
gi|641569612|gb|KK856617.1|	39559-13997	11	21	0.626944	4	3	0.28535	no
gi|641569612|gb|KK856617.1|	2543-3197	11	21	2	0.233801	-3	0.24815	no
gi|641569612|gb|KK856617.1|	27788-28720	11	21	0.576645	0.584433	0.0193558	1	no
gi|641569612|gb|KK856617.1|	30679-31069	11	21	4	5	0.446065	0.76695	no
gi|641569612|gb|KK856617.1|	45577-49137	11	21	1	1	0.0380002	0.9768	no
gi|641569612|gb|KK856617.1|	56163-56815	11	21	18	1	-4	0.0436	no
gi|641569612|gb|KK856617.1|	6232-7824	11	21	1	0.309076	-2	0.2444	no
gi|641569612|gb|KK856617.1|	65036-65691	11	21	3	0.933192	-2	0.2993	no
gi|641569612|gb|KK856617.1|	74786-76089	11	21	22	6	-2	0.1258	no
gi|641569612|gb|KK856617.1|	76652-77709	11	21	10	2	-2	0.11015	no

gi|641569612|gb|KK856617.1|	82877-83337	11	21	10	3	-2	0.3008	no
gi|641569612|gb|KK856617.1|	87762-89420	11	21	12	9	-0.351064	0.78195	no
gi|641569612|gb|KK856617.1|	92753-93828	11	21	12	8	-0.551922	0.66355	no
gi|641569612|gb|KK856617.1|	94004-94744	11	21	16	6	-2	0.249	no
gi|641569612|gb|KK856617.1|	99056-100391	11	21	22	23	0.112573	0.92895	no
gi|641569624|gb|KK856613.1|	33547-34092	12	42	0.606423	5	3	0.1666	no
gi|641569624|gb|KK856613.1|	36373-37026	12	42	0.154642	2	4	0.1276	no
gi|641569624|gb|KK856613.1|	71597-72013	12	42	1	3	1	0.51315	no
gi|641569630|gb|KK856611.1|	10277-11189	11	39	17	50	2	0.37465	no
gi|641569630|gb|KK856611.1|	03377-10472	11	39	0.740291	0.469864	-0.655849	1	no
gi|641569630|gb|KK856611.1|	11293-14161	11	39	6	14	1	0.4227	no
gi|641569630|gb|KK856611.1|	28836-12957	11	39	1	0.980725	-0.246428	0.88495	no
gi|641569630|gb|KK856611.1|	15344-15757	11	39	34	137.21	2	0.1213	no
gi|641569630|gb|KK856611.1|	1653-2019	11	39	31	79	1	0.28795	no
gi|641569630|gb|KK856611.1|	16877-20428	11	39	6	6	0.201012	0.87315	no
gi|641569630|gb|KK856611.1|	22197-22374	11	39	84	21	-2	0.22065	no
gi|641569630|gb|KK856611.1|	29179-30377	11	39	5	0.752547	-3	0.0796	no
gi|641569630|gb|KK856611.1|	31076-31280	11	39	95	11	-3	0.098	no
gi|641569630|gb|KK856611.1|	34831-35447	11	39	17	2	-3	0.0573	no
gi|641569630|gb|KK856611.1|	37638-37827	11	39	137	27	-2	0.1345	no
gi|641569630|gb|KK856611.1|	40198-40808	11	39	46	13	-2	0.1686	no
gi|641569630|gb|KK856611.1|	42428-43230	11	39	3	0.708818	-2	0.25185	no
gi|641569630|gb|KK856611.1|	47105-48217	11	39	15	7	-1	0.3705	no
gi|641569630|gb|KK856611.1|	48282-54148	11	39	15	6	-1	0.5902	no
gi|641569630|gb|KK856611.1|	5075-6289	11	39	7	13	0.876623	0.49215	no
gi|641569630|gb|KK856611.1|	58440-59492	11	39	2	0.502962	-2	0.2684	no
gi|641569630|gb|KK856611.1|	61324-62074	11	39	0.76582	0.968448	0.338669	0.8352	no
gi|641569630|gb|KK856611.1|	6407-6805	11	39	25	33	0.414618	0.7477	no
gi|641569630|gb|KK856611.1|	8726-9126	11	39	11	39	2	0.16385	no
gi|641569630|gb|KK856611.1|	88687-89273	11	39	1	1	-0.0807963	0.92675	no

gi|641569630|gb|KK856611.1|	90253-90963	11	39	0.275092	2	3	0.2991	no
gi|641569630|gb|KK856611.1|	9918-10157	11	39	15	32	1	0.45955	no
gi|641569630|gb|KK856611.1|	99246-99846	11	39	3	0.529146	-2	0.21095	no
gi|641569630|gb|KK856611.1|	99900-101477	11	39	2	2	0.401606	0.7681	no
gi|641569636|gb|KK856609.1|	22831-23111	9.3	34	10	5	-0.816396	0.61345	no
gi|641569636|gb|KK856609.1|	28171-30298	9.3	34	0.659757	1	1	0.41865	no
gi|641569636|gb|KK856609.1|	57179-58427	9.3	34	0.538065	0.409681	-0.393279	1	no
gi|641569636|gb|KK856609.1|	58934-59608	9.3	34	2	0.448273	-2	0.21865	no
gi|641569636|gb|KK856609.1|	60212-61163	9.3	34	15	5	-2	0.20215	no
gi|641569636|gb|KK856609.1|	61317-62923	9.3	34	19	11	-0.76583	0.53245	no
gi|641569636|gb|KK856609.1|	63124-65373	9.3	34	19	10	-0.829965	0.61595	no
gi|641569636|gb|KK856609.1|	69205-69727	9.3	34	6	49	3	0.0434	no
gi|641569636|gb|KK856609.1|	70009-71144	9.3	34	1	3	2	0.29205	no
gi|641569636|gb|KK856609.1|	71368-73067	9.3	34	5	7	0.525942	0.6714	no
gi|641569636|gb|KK856609.1|	78430-78638	9.3	34	0	512	inf	5.00E-05	yes
gi|641569639|gb|KK856608.1|	21160-22311	10	39	1	0	-inf	0.0056	no
gi|641569639|gb|KK856608.1|	68311-69733	10	39	0.808053	0.263824	-2	1	no
gi|641569642|gb|KK856607.1|	51278-52163	9.6	37	0.821253	0.936035	0.188736	0.91015	no
gi|641569642|gb|KK856607.1|	91268-91720	9.6	37	3	2	-0.41017	0.80855	no
gi|641569642|gb|KK856607.1|	92072-95278	9.6	37	1	0.466733	-1	0.30075	no
gi|641569652|gb|KK856604.1|	12325-11283	8.7	34	4	2	-1	0.4955	no
gi|641569655|gb|KK856603.1|	20383-12066	10	37	18	22	0.312227	0.8184	no
gi|641569655|gb|KK856603.1|	25745-12646	10	37	1	0.615166	-1	0.50885	no
gi|641569655|gb|KK856603.1|	27540-12913	10	37	1	0.773794	-0.529407	0.71695	no
gi|641569655|gb|KK856603.1|	29333-13333	10	37	3	2	-0.614639	0.62825	no
gi|641569655|gb|KK856603.1|	13268-14772	10	37	1	1	0.266781	0.8433	no
gi|641569655|gb|KK856603.1|	34602-13572	10	37	0.997543	1	0.0754168	0.95315	no
gi|641569655|gb|KK856603.1|	41445-14193	10	37	5	1	-2	0.2674	no
gi|641569655|gb|KK856603.1|	49402-14964	10	37	21	16	-0.376059	0.7902	no
gi|641569655|gb|KK856603.1|	15658-18164	10	37	1	1	0.13173	0.91815	no

gi|641569655|gb|KK856603.1|	18608-19222	10	37	1	2	0.59827	0.69715	no
gi|641569655|gb|KK856603.1|	19433-20204	10	37	0.860884	1	0.602086	0.69715	no
gi|641569655|gb|KK856603.1|	20629-21552	10	37	0.778368	0.443715	-0.810818	1	no
gi|641569655|gb|KK856603.1|	22031-25093	10	37	1	2	0.81886	0.5331	no
gi|641569655|gb|KK856603.1|	26505-27058	10	37	1	2	0.78856	0.63035	no
gi|641569655|gb|KK856603.1|	28146-29301	10	37	0.663982	3	2	0.1801	no
gi|641569655|gb|KK856603.1|	3583-5609	10	37	0.541455	1	1	0.43845	no
gi|641569655|gb|KK856603.1|	55055-56187	10	37	0.755872	0.575242	-0.393973	1	no
gi|641569655|gb|KK856603.1|	56774-57113	10	37	8	2	-2	0.28615	no
gi|641569655|gb|KK856603.1|	5825-6589	10	37	0.622483	1	0.864996	0.62725	no
gi|641569655|gb|KK856603.1|	63010-63267	10	37	6	19	2	0.29485	no
gi|641569655|gb|KK856603.1|	6712-7581	10	37	5	9	0.887363	0.4991	no
gi|641569655|gb|KK856603.1|	67139-67750	10	37	1	3	1	0.37835	no
gi|641569655|gb|KK856603.1|	85848-87357	10	37	0.37727	1	2	0.36605	no
gi|641569655|gb|KK856603.1|	90132-90423	10	37	3	10	2	0.38055	no
gi|641569655|gb|KK856603.1|	93025-93413	10	37	15	12	-0.250675	0.84765	no
gi|641569655|gb|KK856603.1|	9793-10206	10	37	3	2	-0.413781	0.8125	no
gi|641569658|gb|KK856602.1|	12791-13699	9.8	38	0.596063	3	2	0.20395	no
gi|641569658|gb|KK856602.1|	26906-27742	9.8	38	0.552538	0.839304	0.60312	1	no
gi|641569658|gb|KK856602.1|	35775-36615	9.8	38	0.109822	2	4	0.1276	no
gi|641569658|gb|KK856602.1|	39656-40982	9.8	38	0.626185	1	1	0.4713	no
gi|641569658|gb|KK856602.1|	57313-58477	9.8	38	0.877084	1	0.343201	0.8164	no
gi|641569658|gb|KK856602.1|	62108-63295	9.8	38	0.428371	2	2	0.28005	no
gi|641569664|gb|KK856600.1|	04633-10512	11	43	0.967449	4	2	0.2441	no
gi|641569664|gb|KK856600.1|	06174-10652	11	43	3	5	0.576999	0.7451	no
gi|641569664|gb|KK856600.1|	10589-11100	11	43	5	4	-0.50135	0.7418	no
gi|641569664|gb|KK856600.1|	11684-11222	11	43	29	11	-1	0.28605	no
gi|641569664|gb|KK856600.1|	18603-12090	11	43	2	3	0.992859	0.44755	no
gi|641569664|gb|KK856600.1|	21367-12333	11	43	0.837338	1	0.801651	0.5614	no
gi|641569664|gb|KK856600.1|	23404-12387	11	43	2	1	-0.631261	0.71175	no

gi|641569664|gb|KK856600.1|	.33553-13447	11	43	0.484483	3	3	0.14425	no
gi|641569664|gb|KK856600.1|	.48954-15143	11	43	0.556847	2	2	0.25035	no
gi|641569664|gb|KK856600.1|	15134-16454	11	43	8	9	0.13676	0.9112	no
gi|641569664|gb|KK856600.1|	.51506-15199	11	43	0.480477	3	3	0.2991	no
gi|641569664|gb|KK856600.1|	.53507-15461	11	43	0.389581	0.592887	0.605833	1	no
gi|641569664|gb|KK856600.1|	16707-19058	11	43	0.919648	1	0.446094	0.7428	no
gi|641569664|gb|KK856600.1|	.75713-17642	11	43	0.408672	2	2	0.256	no
gi|641569664|gb|KK856600.1|	.87510-18854	11	43	0.505913	0.769642	0.6053	1	no
gi|641569664|gb|KK856600.1|	.91993-19285	11	43	0.642003	0.812832	0.340378	1	no
gi|641569664|gb|KK856600.1|	.93944-19522	11	43	0.390205	2	2	0.19125	no
gi|641569664|gb|KK856600.1|	.95379-19578	11	43	1	3	1	0.51315	no
gi|641569664|gb|KK856600.1|	.95858-19725	11	43	0.413809	0.810569	0.96997	1	no
gi|641569664|gb|KK856600.1|	20720-21869	11	43	0.742399	0.678039	-0.130826	1	no
gi|641569664|gb|KK856600.1|	27326-27491	11	43	110	15	-3	0.1699	no
gi|641569664|gb|KK856600.1|	32807-33003	11	43	30	10	-2	0.34625	no
gi|641569664|gb|KK856600.1|	37440-37969	11	43	2	0.319791	-3	0.2481	no
gi|641569664|gb|KK856600.1|	38141-38368	11	43	111	43	-1	0.30795	no
gi|641569664|gb|KK856600.1|	40981-41213	11	43	11	4	-2	0.3632	no
gi|641569664|gb|KK856600.1|	60586-61602	11	43	3	1	-1	0.41465	no
gi|641569664|gb|KK856600.1|	68186-70117	11	43	1	2	1	0.4272	no
gi|641569664|gb|KK856600.1|	84134-85190	11	43	0.493569	0.625794	0.342437	1	no
gi|641569664|gb|KK856600.1|	85305-89259	11	43	22	13	-0.753381	0.73885	no
gi|641569670|gb|KK856598.1|	.04034-10419	9.8	45	6	98	4	0.1204	no
gi|641569670|gb|KK856598.1|	46936-47672	9.8	45	0.261878	2	3	0.2991	no
gi|641569670|gb|KK856598.1|	49642-51155	9.8	45	3	40	4	0.07895	no
gi|641569670|gb|KK856598.1|	79687-80179	9.8	45	2	3	0.785328	0.63115	no
gi|641569673|gb|KK856597.1|	27131-27662	9.2	38	9	2	-2	0.1351	no
gi|641569673|gb|KK856597.1|	27766-28484	9.2	38	1	8	3	0.0961	no
gi|641569673|gb|KK856597.1|	32537-32783	9.2	38	13	17	0.425548	0.77005	no
gi|641569673|gb|KK856597.1|	36391-36762	9.2	38	4	9	1	0.4431	no

gi|641569673|gb|KK856597.1|	37919-38080	9.2	38	18	69	2	0.3223	no
gi|641569673|gb|KK856597.1|	47218-47492	9.2	38	4	10	1	0.4132	no
gi|641569673|gb|KK856597.1|	49023-49239	9.2	38	8	23	2	0.38095	no
gi|641569673|gb|KK856597.1|	60686-60886	9.2	38	11	18	0.793306	0.6531	no
gi|641569673|gb|KK856597.1|	61783-62387	9.2	38	2	6	2	0.22435	no
gi|641569673|gb|KK856597.1|	70168-70378	9.2	38	11	21	0.950416	0.59035	no
gi|641569673|gb|KK856597.1|	74264-75809	9.2	38	3	2	-0.643583	0.62945	no
gi|641569673|gb|KK856597.1|	76132-76647	9.2	38	13	4	-2	0.2035	no
gi|641569673|gb|KK856597.1|	77470-81063	9.2	38	19	12	-0.672921	0.7661	no
gi|641569673|gb|KK856597.1|	83049-84993	9.2	38	109	322	2	0.42425	no
gi|641569673|gb|KK856597.1|	85186-88590	9.2	38	35	91	1	0.50975	no
gi|641569676|gb|KK856596.1|	13555-11417	10	42	0.505793	2	2	0.2812	no
gi|641569676|gb|KK856596.1|	14296-11527	10	42	0.360455	2	3	0.1983	no
gi|641569676|gb|KK856596.1|	20134-12101	10	42	0.92391	2	1	0.35865	no
gi|641569676|gb|KK856596.1|	24526-12512	10	42	0.524462	2	2	0.2812	no
gi|641569676|gb|KK856596.1|	40026-41017	10	42	0.266944	1	2	0.2812	no
gi|641569676|gb|KK856596.1|	64511-65231	10	42	0.404782	2	2	0.2812	no
gi|641569680|gb|KK856595.1|	36539-37069	9.7	36	3	0.637712	-2	0.31075	no
gi|641569680|gb|KK856595.1|	37204-38151	9.7	36	2	0.429626	-2	0.3026	no
gi|641569680|gb|KK856595.1|	38742-39438	9.7	36	1	1	-0.0775085	0.92685	no
gi|641569680|gb|KK856595.1|	40196-41107	9.7	36	2	1	-0.225991	0.87645	no
gi|641569680|gb|KK856595.1|	46742-54854	9.7	36	18	9	-1	0.65005	no
gi|641569680|gb|KK856595.1|	57735-58218	9.7	36	3	0.369587	-3	0.2348	no
gi|641569680|gb|KK856595.1|	64187-65167	9.7	36	0.992515	2	0.845816	0.58475	no
gi|641569680|gb|KK856595.1|	73796-74536	9.7	36	0	10	inf	0.0001	yes
gi|641569680|gb|KK856595.1|	85353-85692	9.7	36	2	4	1	0.5206	no
gi|641569680|gb|KK856595.1|	86851-87825	9.7	36	0.454619	0.691346	0.60475	1	no
gi|641569686|gb|KK856593.1|	01649-10189	9.3	34	18	16	-0.157946	0.91535	no
gi|641569686|gb|KK856593.1|	09835-11091	9.3	34	0.080302	4	6	0.10535	no
gi|641569686|gb|KK856593.1|	13865-14379	9.3	34	0.221616	4	4	0.1175	no

gi|641569686|gb|KK856593.1|	17136-17378	9.3	34	35	204	3	0.06755	no
gi|641569686|gb|KK856593.1|	18504-18754	9.3	34	71	601	3	0.12105	no
gi|641569686|gb|KK856593.1|	28036-28726	9.3	34	3	25	3	0.04945	no
gi|641569686|gb|KK856593.1|	29779-30168	9.3	34	6	51	3	0.04725	no
gi|641569686|gb|KK856593.1|	31285-31537	9.3	34	44	299	3	0.0475	no
gi|641569686|gb|KK856593.1|	33689-34059	9.3	34	7	52	3	0.05775	no
gi|641569686|gb|KK856593.1|	34175-34745	9.3	34	11	68	3	0.04925	no
gi|641569686|gb|KK856593.1|	35737-36787	9.3	34	0.248517	1	2	0.25595	no
gi|641569686|gb|KK856593.1|	57432-63503	9.3	34	3	2	-0.0122933	0.9926	no
gi|641569686|gb|KK856593.1|	63668-65753	9.3	34	6	5	-0.188749	0.87785	no
gi|641569686|gb|KK856593.1|	66046-66958	9.3	34	4	2	-0.788222	0.5679	no
gi|641569686|gb|KK856593.1|	67700-69069	9.3	34	2	1	-0.852132	0.54315	no
gi|641569686|gb|KK856593.1|	69181-71214	9.3	34	3	1	-1	0.3803	no
gi|641569686|gb|KK856593.1|	71926-74090	9.3	34	4	2	-0.827532	0.5277	no
gi|641569686|gb|KK856593.1|	75186-77642	9.3	34	0.939572	2	0.988232	0.46315	no
gi|641569686|gb|KK856593.1|	80151-80469	9.3	34	16	11	-0.479096	0.73315	no
gi|641569686|gb|KK856593.1|	82987-84254	9.3	34	0.330329	0.503057	0.606821	1	no
gi|641569686|gb|KK856593.1|	84399-85074	9.3	34	0.737825	1	0.863083	0.62725	no
gi|641569686|gb|KK856593.1|	8638-9602	9.3	34	3	10	2	0.2417	no
gi|641569686|gb|KK856593.1|	86577-87212	9.3	34	0.160955	5	5	0.1072	no
gi|641569686|gb|KK856593.1|	87960-88212	9.3	34	5	10	1	0.51325	no
gi|641569686|gb|KK856593.1|	97261-97502	9.3	34	105	94	-0.172537	0.89215	no
gi|641569686|gb|KK856593.1|	[illegible]9603-10007[illegible]	9.3	34	8	6	-0.365304	0.78855	no
gi|641569689|gb|KK856592.1|	16283-18266	9	34	0	35	inf	5.00E-05	yes
gi|641569689|gb|KK856592.1|	19529-19967	9	34	0	20	inf	0.0001	yes
gi|641569689|gb|KK856592.1|	50879-53103	9	34	0	32	inf	0.0002	yes
gi|641569692|gb|KK856591.1|	40294-42315	9.4	34	0.465375	2	2	0.1733	no
gi|641569692|gb|KK856591.1|	42604-43406	9.4	34	0	3	inf	0.0023	yes
gi|641569692|gb|KK856591.1|	52507-53525	9.4	34	0.430305	1	1	0.4595	no
gi|641569692|gb|KK856591.1|	53642-54366	9.4	34	0.669539	4	3	0.1484	no

gi|641569692|gb|KK856591.1|	91166-91924	9.4	34	40	0.76383	-6	0.0612	no
gi|641569692|gb|KK856591.1|	92370-92629	9.4	34	13	1	-3	0.2153	no
gi|641569695|gb|KK856590.1|	23497-24438	14	45	0	2	inf	0.0023	yes
gi|641569695|gb|KK856590.1|	24627-25608	14	45	0	3	inf	0.0015	yes
gi|641569695|gb|KK856590.1|	27810-30575	14	45	0	92	inf	0.00035	yes
gi|641569695|gb|KK856590.1|	35010-35514	14	45	0	88	inf	5.00E-05	yes
gi|641569695|gb|KK856590.1|	40124-40761	14	45	2	2	-0.101443	0.9456	no
gi|641569695|gb|KK856590.1|	40886-41863	14	45	1	0.688707	-0.773733	0.63565	no
gi|641569695|gb|KK856590.1|	42057-43164	14	45	1	1	0.283926	0.84765	no
gi|641569695|gb|KK856590.1|	44068-46067	14	45	15	19	0.386027	0.753	no
gi|641569695|gb|KK856590.1|	54382-54879	14	45	7	12	0.864295	0.52335	no
gi|641569695|gb|KK856590.1|	61922-63361	14	45	3	5	0.607583	0.64	no
gi|641569695|gb|KK856590.1|	63584-63911	14	45	3	7	1	0.48165	no
gi|641569698|gb|KK856589.1|	1010-2148	11	41	0.901274	1	0.721544	0.6386	no
gi|641569698|gb|KK856589.1|	.01893-10211	11	41	36	58	0.705066	0.60705	no
gi|641569698|gb|KK856589.1|	.02288-10314	11	41	35	64	0.867121	0.59315	no
gi|641569698|gb|KK856589.1|	.03512-10439	11	41	53	141	1	0.5317	no
gi|641569698|gb|KK856589.1|	.04962-10635	11	41	20	3	-3	0.03925	no
gi|641569698|gb|KK856589.1|	.07198-10793	11	41	1	2	0.771396	0.6261	no
gi|641569698|gb|KK856589.1|	.08106-10832	11	41	87	13	-3	0.09775	no
gi|641569698|gb|KK856589.1|	.10882-11144	11	41	47	4	-3	0.02535	no
gi|641569698|gb|KK856589.1|	.12086-11238	11	41	77	7	-3	0.04515	no
gi|641569698|gb|KK856589.1|	.13160-11504	11	41	0.502832	0.830736	0.724314	1	no
gi|641569698|gb|KK856589.1|	.15478-11569	11	41	105	11	-3	0.0708	no
gi|641569698|gb|KK856589.1|	.20639-12106	11	41	16	2	-3	0.09405	no
gi|641569698|gb|KK856589.1|	.23158-12649	11	41	7	14	1	0.5095	no
gi|641569698|gb|KK856589.1|	.26586-12834	11	41	4	9	1	0.3383	no
gi|641569698|gb|KK856589.1|	20618-21270	11	41	1	1	0.114006	0.92265	no
gi|641569698|gb|KK856589.1|	21355-23735	11	41	0.90744	2	0.931557	0.4835	no
gi|641569698|gb|KK856589.1|	3392-4100	11	41	0.82849	2	1	0.5082	no

gi|641569698|gb|KK856589.1|	34607-35065	11	41	9	10	0.175269	0.8971	no
gi|641569698|gb|KK856589.1|	41961-42221	11	41	14	40	1	0.3084	no
gi|641569698|gb|KK856589.1|	62854-63398	11	41	5	4	-0.46339	0.7433	no
gi|641569698|gb|KK856589.1|	64667-65528	11	41	2	2	0.397015	0.787	no
gi|641569698|gb|KK856589.1|	68314-68573	11	41	131	102	-0.366335	0.7688	no
gi|641569698|gb|KK856589.1|	71109-71317	11	41	75	112	0.569149	0.6613	no
gi|641569698|gb|KK856589.1|	72747-72971	11	41	58	60	0.0424486	0.9745	no
gi|641569698|gb|KK856589.1|	73843-74057	11	41	89	139	0.640432	0.62235	no
gi|641569698|gb|KK856589.1|	76218-78839	11	41	5	7	0.635893	0.61335	no
gi|641569698|gb|KK856589.1|	78986-80365	11	41	2	1	-0.615051	0.65815	no
gi|641569698|gb|KK856589.1|	81244-83595	11	41	0.886803	0.952188	0.102633	0.94225	no
gi|641569698|gb|KK856589.1|	83727-84520	11	41	3	4	0.438961	0.7461	no
gi|641569698|gb|KK856589.1|	85003-85884	11	41	5	10	0.890605	0.49785	no
gi|641569698|gb|KK856589.1|	92404-92701	11	41	65	102	0.663304	0.59795	no
gi|641569698|gb|KK856589.1|	93110-93422	11	41	5	18	2	0.25175	no
gi|641569698|gb|KK856589.1|	95347-96197	11	41	6	9	0.710232	0.5894	no
gi|641569698|gb|KK856589.1|	98368-98894	11	41	5	14	1	0.3224	no
gi|641569698|gb|KK856589.1|	99876-101089	11	41	9	13	0.583069	0.64605	no
gi|641569705|gb|KK856587.1|	40178-41534	9.4	37	2	2	0.607243	0.65855	no
gi|641569705|gb|KK856587.1|	41682-45117	9.4	37	11	9	-0.351592	0.82935	no
gi|641569705|gb|KK856587.1|	46166-47125	9.4	37	2	0.704852	-2	0.2712	no
gi|641569705|gb|KK856587.1|	62013-62699	9.4	37	38	21	-0.840249	0.503	no
gi|641569705|gb|KK856587.1|	62763-63084	9.4	37	41	38	-0.0907921	0.94235	no
gi|641569705|gb|KK856587.1|	64455-65046	9.4	37	25	33	0.366579	0.77105	no
gi|641569705|gb|KK856587.1|	72117-73113	9.4	37	6	10	0.761077	0.5508	no
gi|641569707|gb|KK856586.1|	08895-10993	8.9	17	0.503514	1	1	0.3778	no
gi|641569707|gb|KK856586.1|	12960-11416	8.9	17	0.420318	0.853273	1	1	no
gi|641569707|gb|KK856586.1|	17810-11871	8.9	17	0.399618	1	2	0.3613	no
gi|641569707|gb|KK856586.1|	18917-11961	8.9	17	0.425857	2	2	0.2812	no
gi|641569707|gb|KK856586.1|	20447-12165	8.9	17	0.631102	1	0.758486	0.6256	no

gi|641569707|gb|KK856586.1|	37512-37915	8.9	17	85	186	1	0.5003	no
gi|641569707|gb|KK856586.1|	38976-40034	8.9	17	2	2	0.164914	0.90555	no
gi|641569707|gb|KK856586.1|	41459-41879	8.9	17	12	28	1	0.344	no
gi|641569707|gb|KK856586.1|	44228-48513	8.9	17	2	2	0.323089	0.7951	no
gi|641569707|gb|KK856586.1|	51079-51734	8.9	17	4	12	1	0.28275	no
gi|641569707|gb|KK856586.1|	52423-52801	8.9	17	4	2	-0.877444	0.60075	no
gi|641569707|gb|KK856586.1|	54375-54748	8.9	17	5	14	2	0.2956	no
gi|641569707|gb|KK856586.1|	54955-55796	8.9	17	25	50	1	0.4241	no
gi|641569707|gb|KK856586.1|	57851-59368	8.9	17	2	4	1	0.34815	no
gi|641569707|gb|KK856586.1|	60650-62515	8.9	17	2	3	1	0.44195	no
gi|641569707|gb|KK856586.1|	63066-64612	8.9	17	65	158	1	0.5475	no
gi|641569707|gb|KK856586.1|	67890-68644	8.9	17	3	5	1	0.4401	no
gi|641569707|gb|KK856586.1|	68909-69368	8.9	17	6	11	0.812776	0.55635	no
gi|641569707|gb|KK856586.1|	71640-74709	8.9	17	2	1	-0.96047	0.46395	no
gi|641569707|gb|KK856586.1|	75366-76759	8.9	17	2	0.450316	-2	0.25505	no
gi|641569707|gb|KK856586.1|	77085-77644	8.9	17	9	0.587797	-4	0.1365	no
gi|641569710|gb|KK856585.1|	64489-65229	8.6	33	0.649891	2	2	0.32945	no
gi|641569710|gb|KK856585.1|	74592-75455	8.6	33	0.848312	1	0.603492	0.6936	no
gi|641569713|gb|KK856584.1|	01631-10229	7.6	35	0.152646	2	4	0.12385	no
gi|641569713|gb|KK856584.1|	18783-20641	7.6	35	0.042583	66	11	0.10475	no
gi|641569713|gb|KK856584.1|	73048-75008	7.6	35	0.88268	0.489546	-0.850446	0.5652	no
gi|641569715|gb|KK856583.1|	00108-10043	9	39	3	4	0.572802	0.76735	no
gi|641569715|gb|KK856583.1|	52530-53204	9	39	1	2	0.237453	0.88205	no
gi|641569715|gb|KK856583.1|	71178-71397	9	39	0	89	inf	5.00E-05	yes
gi|641569715|gb|KK856583.1|	71711-74626	9	39	15	4	-2	0.26915	no
gi|641569715|gb|KK856583.1|	75871-76458	9	39	3	0	-inf	0.0059	no
gi|641569715|gb|KK856583.1|	80746-82230	9	39	0.988942	0.083724	-4	0.20475	no
gi|641569715|gb|KK856583.1|	84025-84451	9	39	2	3	1	0.51205	no
gi|641569715|gb|KK856583.1|	86306-86797	9	39	12	15	0.277746	0.8391	no
gi|641569718|gb|KK856582.1|	47041-47599	8.4	36	0.194947	6	5	0.1072	no

gi|641569718|gb|KK856582.1|	47702-48923	8.4	36	0.138057	6	5	0.233	no
gi|641569721|gb|KK856581.1|	55684-56093	10	40	5	38	3	0.06195	no
gi|641569724|gb|KK856580.1|	27020-27662	10	21	2	2	-0.20821	0.89275	no
gi|641569724|gb|KK856580.1|	78151-79126	10	21	0.272422	2	3	0.19335	no
gi|641569727|gb|KK856579.1|	31879-32302	8.3	34	0.924429	4	2	0.256	no
gi|641569727|gb|KK856579.1|	46366-46981	8.3	34	16	21	0.451462	0.7223	no
gi|641569727|gb|KK856579.1|	61444-62053	8.3	34	0	5	inf	0.00145	yes
gi|641569730|gb|KK856578.1|	19261-20014	7.4	32	4	12	1	0.3007	no
gi|641569730|gb|KK856578.1|	20452-21955	7.4	32	3	12	2	0.1312	no
gi|641569730|gb|KK856578.1|	23047-24909	7.4	32	0.169924	8	6	0.1163	no
gi|641569730|gb|KK856578.1|	46473-48622	7.4	32	16	26	0.656409	0.6751	no
gi|641569730|gb|KK856578.1|	48962-50231	7.4	32	14	21	0.620637	0.6056	no
gi|641569730|gb|KK856578.1|	57406-57671	7.4	32	7	28	2	0.20375	no
gi|641569730|gb|KK856578.1|	74693-75333	7.4	32	2	0.723222	-2	0.33205	no
gi|641569730|gb|KK856578.1|	75387-76128	7.4	32	1	2	0.60153	0.6902	no
gi|641569730|gb|KK856578.1|	79118-80924	7.4	32	0.791241	0	-inf	1	no
gi|641569730|gb|KK856578.1|	84153-84352	7.4	32	20	13	-0.640176	0.70205	no
gi|641569730|gb|KK856578.1|	84898-85305	7.4	32	28	2	-4	0.06015	no
gi|641569733|gb|KK856577.1|	2076-2390	8.5	35	187	234	0.320557	0.84205	no
gi|641569742|gb|KK856575.1|	11419-12059	8.8	34	0	2	inf	0.0068	no
gi|641569742|gb|KK856575.1|	41037-41401	8.8	34	0	6	inf	0.0068	no
gi|641569742|gb|KK856575.1|	42846-43352	8.8	34	0	4	inf	0.0025	yes
gi|641569749|gb|KK856573.1|	19213-11943	10	40	0	205	inf	5.00E-05	yes
gi|641569749|gb|KK856573.1|	22868-12309	10	40	0	26	inf	0.0025	yes
gi|641569749|gb|KK856573.1|	28214-12842	10	40	0	92	inf	0.0004	yes
gi|641569749|gb|KK856573.1|	39962-14037	10	40	0	27	inf	0.0002	yes
gi|641569749|gb|KK856573.1|	43673-14387	10	40	11	179	4	0.0865	no
gi|641569749|gb|KK856573.1|	54347-15477	10	40	1	17	4	0.0964	no
gi|641569758|gb|KK856570.1|	18010-11853	7.7	41	0	16	inf	0.0001	yes
gi|641569758|gb|KK856570.1|	40518-41142	7.7	41	0.990426	3	2	0.32035	no

gi|641569758|gb|KK856570.1|	69712-70472	7.7	41	0.250756	2	3	0.2851	no
gi|641569758|gb|KK856570.1|	73414-74537	7.7	41	0.534244	0.464639	-0.201389	1	no
gi|641569761|gb|KK856569.1|	67488-67873	7.9	37	0	125.37	inf	5.00E-05	yes
gi|641569761|gb|KK856569.1|	69958-72861	7.9	37	0	329	inf	5.00E-05	yes
gi|641569761|gb|KK856569.1|	82716-83269	7.9	37	0	138	inf	0.0005	yes
gi|641569763|gb|KK856568.1|	26163-26503	9.2	21	26	6	-2	0.1641	no
gi|641569766|gb|KK856567.1|	54594-55204	8.4	38	10	8	-0.284507	0.83045	no
gi|641569766|gb|KK856567.1|	56819-59220	8.4	38	8	7	-0.279314	0.82465	no
gi|641569766|gb|KK856567.1|	59735-62290	8.4	38	9	5	-0.880273	0.47765	no
gi|641569772|gb|KK856565.1|	32629-33236	8.7	37	0.687444	2	1	0.44255	no
gi|641569775|gb|KK856564.1|	36860-38074	11	40	0.347515	0.529125	0.606534	1	no
gi|641569775|gb|KK856564.1|	61900-62533	11	40	0	4	inf	0.0015	yes
gi|641569779|gb|KK856563.1|	46605-46823	11	40	0	345	inf	5.00E-05	yes
gi|641569779|gb|KK856563.1|	47847-48093	11	40	0	484	inf	5.00E-05	yes
gi|641569779|gb|KK856563.1|	55621-55981	11	40	0	372	inf	0.00045	yes
gi|641569779|gb|KK856563.1|	57402-57893	11	40	0.238636	1598.14	13	0.105	no
gi|641569779|gb|KK856563.1|	60503-62025	11	40	0	143.96	inf	0.0001	yes
gi|641569779|gb|KK856563.1|	64124-64916	11	40	0	39	inf	5.00E-05	yes
gi|641569782|gb|KK856562.1|	21690-21922	6.8	37	0	18	inf	0.0068	no
gi|641569788|gb|KK856560.1|	17465-18880	9.5	37	2	0.795998	-1	0.4151	no
gi|641569788|gb|KK856560.1|	19019-19652	9.5	37	0.808439	1	0.86191	0.62755	no
gi|641569788|gb|KK856560.1|	20780-20948	9.5	37	5	136	5	0.10825	no
gi|641569788|gb|KK856560.1|	2457-7353	9.5	37	18	4	-2	0.19545	no
gi|641569788|gb|KK856560.1|	25785-26779	9.5	37	0.177294	8	5	0.23275	no
gi|641569788|gb|KK856560.1|	28081-28677	9.5	37	0.176558	106.87	9	0.10625	no
gi|641569788|gb|KK856560.1|	28739-29917	9.5	37	32	23	-0.504839	0.68335	no
gi|641569788|gb|KK856560.1|	30689-31881	9.5	37	66	53	-0.325778	0.8843	no
gi|641569788|gb|KK856560.1|	35705-37182	9.5	37	25	26	0.0528345	0.9663	no
gi|641569788|gb|KK856560.1|	37925-39153	9.5	37	14	11	-0.247255	0.84605	no
gi|641569788|gb|KK856560.1|	40604-41250	9.5	37	1	1	-0.0788103	0.92675	no

gi|641569788|gb|KK856560.1|	42683-44073	9.5	37	0.29632	2	2	0.18135	no
gi|641569788|gb|KK856560.1|	44257-44837	9.5	37	0.735456	3	2	0.33105	no
gi|641569788|gb|KK856560.1|	45425-46821	9.5	37	2	1	-0.602041	0.65385	no
gi|641569788|gb|KK856560.1|	49170-50156	9.5	37	0.358176	1	2	0.3613	no
gi|641569788|gb|KK856560.1|	79852-80234	9.5	37	0	7	inf	0.0025	yes
gi|641569788|gb|KK856560.1|	9120-9433	9.5	37	6	3	-0.889003	0.60065	no
gi|641569788|gb|KK856560.1|	9516-9839	9.5	37	24	2	-4	0.1373	no
gi|641569792|gb|KK856559.1|	11586-11187	10	38	10	4	-1	0.4504	no
gi|641569792|gb|KK856559.1|	67208-67552	10	38	0	9	inf	0.0025	yes
gi|641569792|gb|KK856559.1|	71440-73722	10	38	0.305375	2	2	0.1416	no
gi|641569792|gb|KK856559.1|	74770-77958	10	38	0.33122	2	2	0.13705	no
gi|641569792|gb|KK856559.1|	88790-92343	10	38	3	2	-0.261875	0.83435	no
gi|641569792|gb|KK856559.1|	92541-92983	10	38	2	1	-0.826045	0.64915	no
gi|641569792|gb|KK856559.1|	93263-94413	10	38	6	3	-1	0.43825	no
gi|641569792|gb|KK856559.1|	94769-95806	10	38	8	4	-1	0.3692	no
gi|641569795|gb|KK856558.1|	32763-34009	11	23	0.606476	0.307844	-0.978252	1	no
gi|641569795|gb|KK856558.1|	35795-36571	11	23	2	0	-inf	0.0056	no
gi|641569795|gb|KK856558.1|	36669-37797	11	23	0.835025	0.808778	-0.0460767	1	no
gi|641569795|gb|KK856558.1|	38208-40076	11	23	1	1	0.123374	0.92895	no
gi|641569795|gb|KK856558.1|	40353-41049	11	23	1	0.214385	-3	0.24815	no
gi|641569795|gb|KK856558.1|	41766-42654	11	23	0.919908	0.466003	-0.981152	0.62775	no
gi|641569795|gb|KK856558.1|	96731-97219	11	23	1	4	1	0.41185	no
gi|641569798|gb|KK856557.1|	08443-10937	12	46	0.861459	0.291038	-2	1	no
gi|641569798|gb|KK856557.1|	12917-11369	12	46	5	17	2	0.19645	no
gi|641569798|gb|KK856557.1|	14092-11519	12	46	2	5	2	0.24775	no
gi|641569798|gb|KK856557.1|	27810-12914	12	46	0.435632	1	2	0.2915	no
gi|641569798|gb|KK856557.1|	29864-13106	12	46	0.636778	1	0.758433	0.6256	no
gi|641569798|gb|KK856557.1|	35324-13781	12	46	2	9	2	0.13205	no
gi|641569798|gb|KK856557.1|	38048-13889	12	46	1	0.827751	-0.534256	0.7478	no
gi|641569798|gb|KK856557.1|	39407-14111	12	46	0.374445	1	2	0.2897	no

gi|641569798|gb|KK856557.1|	.41487-14175	12	46	78	198	1	0.2955	no
gi|641569798|gb|KK856557.1|	34429-34704	12	46	8	21	1	0.3575	no
gi|641569798|gb|KK856557.1|	38836-39043	12	46	241	0	-inf	5.00E-05	yes
gi|641569798|gb|KK856557.1|	54813-58214	12	46	4	10	1	0.32065	no
gi|641569798|gb|KK856557.1|	58503-62446	12	46	4	9	1	0.4166	no
gi|641569798|gb|KK856557.1|	62841-63198	12	46	6	8	0.46337	0.75555	no
gi|641569798|gb|KK856557.1|	72495-75776	12	46	40	6	-3	0.14695	no
gi|641569798|gb|KK856557.1|	7577-8429	12	46	0.539076	1	1	0.50945	no
gi|641569798|gb|KK856557.1|	77707-78231	12	46	5	0.973657	-2	0.18625	no
gi|641569798|gb|KK856557.1|	79010-79764	12	46	1	2	0.601778	0.6936	no
gi|641569798|gb|KK856557.1|	79966-80582	12	46	0.840993	1	0.598336	0.70505	no
gi|641569798|gb|KK856557.1|	80883-81725	12	46	1	2	0.224694	0.87445	no
gi|641569798|gb|KK856557.1|	88203-88564	12	46	9	38	2	0.1509	no
gi|641569798|gb|KK856557.1|	88695-89383	12	46	10	38	2	0.1358	no
gi|641569798|gb|KK856557.1|	89749-91774	12	46	0.503054	2	2	0.25315	no
gi|641569798|gb|KK856557.1|	91891-92608	12	46	1	3	0.842044	0.5848	no
gi|641569798|gb|KK856557.1|	92796-94401	12	46	0.803607	2	1	0.3653	no
gi|641569798|gb|KK856557.1|	95586-97138	12	46	0.365313	1	2	0.36605	no
gi|641569798|gb|KK856557.1|	97263-10596[illegible]	12	46	1	4	2	0.30275	no
gi|641569801|gb|KK856556.1|	.07266-10903	9.6	36	16	13	-0.326933	0.78845	no
gi|641569801|gb|KK856556.1|	.10224-11123	9.6	36	3	1	-1	0.41385	no
gi|641569801|gb|KK856556.1|	.13083-11393	9.6	36	3	1	-0.981632	0.5088	no
gi|641569801|gb|KK856556.1|	.14256-11717	9.6	36	5	2	-1	0.26855	no
gi|641569801|gb|KK856556.1|	26556-26920	9.6	36	5	4	-0.420052	0.77855	no
gi|641569801|gb|KK856556.1|	29692-29997	9.6	36	8	23	2	0.29655	no
gi|641569801|gb|KK856556.1|	71670-73144	9.6	36	3	3	-0.0230537	0.98505	no
gi|641569801|gb|KK856556.1|	73274-74505	9.6	36	0.205086	2	3	0.1719	no
gi|641569801|gb|KK856556.1|	78794-80079	9.6	36	45	21	-1	0.5134	no
gi|641569801|gb|KK856556.1|	81537-84512	9.6	36	1	1	0.34012	0.79315	no
gi|641569801|gb|KK856556.1|	84692-85316	9.6	36	39	20	-0.974141	0.4415	no

gi|641569801|gb|KK856556.1|	87129-89285	9.6	36	5	3	-0.680187	0.59045	no
gi|641569801|gb|KK856556.1|	89590-89803	9.6	36	44	12	-2	0.2533	no
gi|641569801|gb|KK856556.1|	92889-93082	9.6	36	32	14	-1	0.4762	no
gi|641569801|gb|KK856556.1|	94143-94779	9.6	36	24	11	-1	0.38475	no
gi|641569808|gb|KK856554.1|	.00820-10127	11	21	3	0	-inf	0.01175	no
gi|641569808|gb|KK856554.1|	.05374-10616	11	21	1	0.179854	-3	0.2382	no
gi|641569808|gb|KK856554.1|	.09483-11030	11	21	2	3	0.695949	0.6289	no
gi|641569808|gb|KK856554.1|	.12781-11349	11	21	2	3	0.807356	0.5797	no
gi|641569808|gb|KK856554.1|	.18723-11928	11	21	24	6	-2	0.13545	no
gi|641569808|gb|KK856554.1|	53246-53638	11	21	11	4	-2	0.31785	no
gi|641569808|gb|KK856554.1|	66833-67156	11	21	24	4	-3	0.11855	no
gi|641569808|gb|KK856554.1|	74749-75606	11	21	0.856004	0.487699	-0.811626	1	no
gi|641569808|gb|KK856554.1|	77088-81351	11	21	7	5	-0.488146	0.694	no
gi|641569808|gb|KK856554.1|	81473-85696	11	21	13	5	-1	0.457	no
gi|641569808|gb|KK856554.1|	91795-92007	11	21	27	7	-2	0.29405	no
gi|641569808|gb|KK856554.1|	95906-97366	11	21	2	1	-0.343429	0.8007	no
gi|641569808|gb|KK856554.1|	97621-98508	11	21	4	2	-0.758772	0.5837	no
gi|641569808|gb|KK856554.1|	)8597-100326	11	21	0.87713	0.14077	-3	0.19075	no
gi|641569812|gb|KK856553.1|	11616-13016	9.3	42	1	0.716334	-0.852003	0.5652	no
gi|641569812|gb|KK856553.1|	13757-18020	9.3	42	2	2	0.457078	0.7224	no
gi|641569812|gb|KK856553.1|	18181-18972	9.3	42	2	5	1	0.384	no
gi|641569812|gb|KK856553.1|	19840-21138	9.3	42	3	4	0.606975	0.63935	no
gi|641569812|gb|KK856553.1|	21522-22260	9.3	42	2	2	0.0709563	0.95375	no
gi|641569812|gb|KK856553.1|	28275-29509	9.3	42	0.408988	0.415181	0.0216831	1	no
gi|641569812|gb|KK856553.1|	32902-33923	9.3	42	0.685987	0.521751	-0.394819	1	no
gi|641569812|gb|KK856553.1|	77481-78020	9.3	42	3	3	0.216755	0.8867	no
gi|641569812|gb|KK856553.1|	82597-83712	9.3	42	0.307938	1	2	0.29935	no
gi|641569812|gb|KK856553.1|	84520-85196	9.3	42	0.294517	2	2.77	0.2851	no
gi|641569812|gb|KK856553.1|	85345-85703	9.3	42	2	5	2	0.41725	no
gi|641569812|gb|KK856553.1|	86212-86821	9.3	42	0.855167	2	1	0.51085	no

gi|641569812|gb|KK856553.1|	9039-9659	9.3	42	1	0.504561	-1	0.4534	no
gi|641569812|gb|KK856553.1|	9852-10833	9.3	42	0.811024	0.411131	-0.980145	1	no
gi|641569815|gb|KK856552.1|	02840-10341	9.5	37	2	1	-0.573039	0.7586	no
gi|641569815|gb|KK856552.1|	23101-23692	9.5	37	0	5	inf	0.0016	yes
gi|641569819|gb|KK856551.1|	00437-10066	11	43	8	14	0.764859	0.64725	no
gi|641569819|gb|KK856551.1|	11418-11178	11	43	4	3	-0.556802	0.74515	no
gi|641569819|gb|KK856551.1|	11845-11276	11	43	0.490525	1	1	0.3994	no
gi|641569819|gb|KK856551.1|	73087-73455	11	43	5	2	-1	0.5083	no
gi|641569819|gb|KK856551.1|	80060-80633	11	43	0.561757	2	2	0.3323	no
gi|641569819|gb|KK856551.1|	86390-87841	11	43	0.789391	0.343668	-1	1	no
gi|641569819|gb|KK856551.1|	90826-91506	11	43	3	2	-0.399825	0.78565	no
gi|641569819|gb|KK856551.1|	94942-95681	11	43	27	25	-0.0936546	0.93965	no
gi|641569819|gb|KK856551.1|	95886-96376	11	43	17	21	0.235622	0.8589	no
gi|641569819|gb|KK856551.1|	96721-97811	11	43	0.474813	0.842971	0.828124	1	no
gi|641569819|gb|KK856551.1|	98086-99032	11	43	0.754521	0.86039	0.18943	1	no
gi|641569819|gb|KK856551.1|	99242-99424	11	43	108	73	-0.56679	0.6828	no
gi|641569833|gb|KK856547.1|	10106-11208	8.9	37	0	213.36	inf	5.00E-05	yes
gi|641569833|gb|KK856547.1|	13768-11406	8.9	37	17	12	-0.487569	0.7294	no
gi|641569833|gb|KK856547.1|	23544-12443	8.9	37	0.717556	0.467344	-0.618607	1	no
gi|641569833|gb|KK856547.1|	25640-12748	8.9	37	1	2	0.750768	0.5793	no
gi|641569833|gb|KK856547.1|	13198-13490	8.9	37	7	3	-1	0.49565	no
gi|641569833|gb|KK856547.1|	13925-14153	8.9	37	32	45	0.482386	0.7256	no
gi|641569833|gb|KK856547.1|	14490-14783	8.9	37	17	22	0.381185	0.7802	no
gi|641569833|gb|KK856547.1|	17208-17520	8.9	37	27	19	-0.524953	0.696	no
gi|641569833|gb|KK856547.1|	19325-19561	8.9	37	19	9	-1	0.48305	no
gi|641569833|gb|KK856547.1|	21188-21465	8.9	37	18	15	-0.323264	0.81055	no
gi|641569833|gb|KK856547.1|	29883-30634	8.9	37	1	5	2	0.17185	no
gi|641569833|gb|KK856547.1|	30792-31789	8.9	37	4	0.805936	-2	0.16485	no
gi|641569833|gb|KK856547.1|	31920-33075	8.9	37	2	9	2	0.1133	no
gi|641569833|gb|KK856547.1|	33704-33869	8.9	37	21	52	1	0.47755	no

gi|641569833|gb|KK856547.1|	35616-35886	8.9	37	22	99	2	0.12175	no
gi|641569833|gb|KK856547.1|	38640-39694	8.9	37	0.494719	0.627245	0.342421	1	no
gi|641569833|gb|KK856547.1|	41944-44115	8.9	37	15	7	-1	0.4129	no
gi|641569833|gb|KK856547.1|	44253-47617	8.9	37	12	10	-0.331154	0.83685	no
gi|641569833|gb|KK856547.1|	48870-50615	8.9	37	0.137064	0.835925	3	1	no
gi|641569833|gb|KK856547.1|	53772-54679	8.9	37	2	2	0.241467	0.86475	no
gi|641569833|gb|KK856547.1|	61789-62714	8.9	37	2	0.73751	-1	0.43755	no
gi|641569833|gb|KK856547.1|	63068-63974	8.9	37	3	0	-inf	0.0013	yes
gi|641569833|gb|KK856547.1|	64701-65535	8.9	37	7	1	-3	0.08665	no
gi|641569833|gb|KK856547.1|	65696-66800	8.9	37	6	4	-0.535523	0.67345	no
gi|641569833|gb|KK856547.1|	68155-68462	8.9	37	13	25	0.916979	0.51225	no
gi|641569833|gb|KK856547.1|	72103-73918	8.9	37	7	6	-0.209254	0.8635	no
gi|641569833|gb|KK856547.1|	77312-78012	8.9	37	8	12	0.600656	0.64405	no
gi|641569833|gb|KK856547.1|	79515-80952	8.9	37	9	19	1	0.38665	no
gi|641569833|gb|KK856547.1|	96443-97150	8.9	37	0.968458	2	0.793461	0.63035	no
gi|641569833|gb|KK856547.1|	97312-98227	8.9	37	5	3	-0.717799	0.58705	no
gi|641569836|gb|KK856546.1|	02541-10322	12	19	459.91	326	-0.497015	0.80545	no
gi|641569836|gb|KK856546.1|	14677-11678	12	19	160	103	-0.632276	0.74395	no
gi|641569836|gb|KK856546.1|	11781-13086	12	19	2	0.583074	-2	0.29105	no
gi|641569836|gb|KK856546.1|	17860-11856	12	19	28	16	-0.821395	0.51215	no
gi|641569836|gb|KK856546.1|	23277-12360	12	19	155	59	-1	0.27905	no
gi|641569836|gb|KK856546.1|	23791-12533	12	19	1	1	-0.146938	0.91685	no
gi|641569836|gb|KK856546.1|	25477-12643	12	19	3	0.568323	-3	0.1447	no
gi|641569836|gb|KK856546.1|	26528-12943	12	19	2	0.678789	-2	0.19015	no
gi|641569836|gb|KK856546.1|	32669-13565	12	19	0.709882	0.619216	-0.197136	1	no
gi|641569836|gb|KK856546.1|	26987-27707	12	19	10	7	-0.578223	0.65275	no
gi|641569836|gb|KK856546.1|	2776-2979	12	19	8	26	2	0.34065	no
gi|641569836|gb|KK856546.1|	28276-28977	12	19	12	4	-2	0.2409	no
gi|641569836|gb|KK856546.1|	53774-54412	12	19	2	2	0.0844536	0.94	no
gi|641569836|gb|KK856546.1|	54665-55521	12	19	1	2	0.340364	0.81785	no

gi|641569836|gb|KK856546.1|	55730-56803	12	19	1	0.736476	-0.616789	0.6869	no
gi|641569836|gb|KK856546.1|	60788-61362	12	19	4	1	-2	0.29895	no
gi|641569836|gb|KK856546.1|	61835-63412	12	19	0.768649	0.702947	-0.128909	1	no
gi|641569836|gb|KK856546.1|	69825-70529	12	19	3	1	-1	0.3596	no
gi|641569836|gb|KK856546.1|	75747-76613	12	19	1	1	-0.0969083	0.9478	no
gi|641569836|gb|KK856546.1|	78181-78758	12	19	1	1	0.111499	0.92265	no
gi|641569845|gb|KK856543.1|	02089-10319	8.8	40	1	0.11806	-3	0.21705	no
gi|641569845|gb|KK856543.1|	08524-10932	8.8	40	1	0	-inf	0.01175	no
gi|641569845|gb|KK856543.1|	51827-51994	8.8	40	39	42	0.0974253	0.93295	no
gi|641569845|gb|KK856543.1|	78717-80774	8.8	40	0.83669	0.696143	-0.265309	1	no
gi|641569845|gb|KK856543.1|	84185-86107	8.8	40	10	9	-0.105997	0.9311	no
gi|641569845|gb|KK856543.1|	90149-90976	8.8	40	3	5	0.924918	0.5015	no
gi|641569845|gb|KK856543.1|	91086-91798	8.8	40	2	4	0.781499	0.58755	no
gi|641569845|gb|KK856543.1|	94669-95590	8.8	40	186	0.593242	-8	0.064	no
gi|641569845|gb|KK856543.1|	97570-98809	8.8	40	1	0	-inf	0.0059	no
gi|641569845|gb|KK856543.1|	99301-10013	8.8	40	2	0	-inf	0.0059	no
gi|641569848|gb|KK856542.1|	00404-10086	11	33	2	2	-0.409463	0.82205	no
gi|641569848|gb|KK856542.1|	00962-10220	11	33	0.608793	0.721036	0.244119	1	no
gi|641569848|gb|KK856542.1|	10047-11027	11	33	11	10	-0.133854	0.9206	no
gi|641569848|gb|KK856542.1|	40293-42203	11	33	0.94971	1	0.407254	0.77175	no
gi|641569848|gb|KK856542.1|	46850-47290	11	33	2	0.864284	-1	0.4534	no
gi|641569848|gb|KK856542.1|	64698-65986	11	33	0.453573	0.789481	0.799572	1	no
gi|641569848|gb|KK856542.1|	66170-67007	11	33	0.551677	2	1	0.3994	no
gi|641569848|gb|KK856542.1|	84384-85012	11	33	3	0.990704	-1	0.37245	no
gi|641569848|gb|KK856542.1|	99045-99999	11	33	1	0.141894	-3	0.2382	no
gi|641569851|gb|KK856541.1|	13588-14690	11	21	0.780882	0.713027	-0.131147	1	no
gi|641569851|gb|KK856541.1|	14827-15274	11	21	9	10	0.174381	0.8979	no
gi|641569851|gb|KK856541.1|	18529-19033	11	21	2	1	-0.213944	0.8945	no
gi|641569851|gb|KK856541.1|	19720-21216	11	21	6	4	-0.53726	0.67485	no
gi|641569851|gb|KK856541.1|	30272-30917	11	21	3	1	-1	0.4703	no

gi|641569851|gb|KK856541.1|	31184-32191	11	21	3	0	-inf	0.00175	yes
gi|641569851|gb|KK856541.1|	32337-33103	11	21	3	0	-inf	0.00185	yes
gi|641569851|gb|KK856541.1|	34685-34849	11	21	93	16	-3	0.20775	no
gi|641569851|gb|KK856541.1|	39016-39691	11	21	9	4	-1	0.3848	no
gi|641569851|gb|KK856541.1|	41753-42113	11	21	4	5	0.409401	0.81025	no
gi|641569851|gb|KK856541.1|	50152-50691	11	21	81	28	-2	0.2343	no
gi|641569851|gb|KK856541.1|	59772-60254	11	21	32	56	0.798486	0.5244	no
gi|641569851|gb|KK856541.1|	65316-70785	11	21	10	7	-0.424506	0.8522	no
gi|641569851|gb|KK856541.1|	6630-6870	11	21	28	25	-0.1882	0.89035	no
gi|641569851|gb|KK856541.1|	71771-72310	11	21	440	276	-0.673038	0.7529	no
gi|641569851|gb|KK856541.1|	7193-7496	11	21	28	13	-1	0.4126	no
gi|641569851|gb|KK856541.1|	76725-77080	11	21	44	29	-0.587836	0.64365	no
gi|641569851|gb|KK856541.1|	8808-11118	11	21	3	2	-0.511476	0.6963	no
gi|641569853|gb|KK856540.1|	20326-20931	12	38	0	23	inf	0.0001	yes
gi|641569853|gb|KK856540.1|	55373-56644	12	38	0	599	inf	5.00E-05	yes
gi|641569853|gb|KK856540.1|	60093-66989	12	38	35	41	0.207458	0.9138	no
gi|641569853|gb|KK856540.1|	67091-67884	12	38	76	60	-0.338017	0.88105	no
gi|641569853|gb|KK856540.1|	68683-69287	12	38	3	2	-0.817108	0.5996	no
gi|641569853|gb|KK856540.1|	70960-72405	12	38	0.566558	4	3	0.09085	no
gi|641569853|gb|KK856540.1|	73955-74560	12	38	0.172696	15	6	0.1046	no
gi|641569853|gb|KK856540.1|	77229-77841	12	38	0	8.74	inf	0.00025	yes
gi|641569853|gb|KK856540.1|	78672-79428	12	38	0	2	inf	0.00375	no
gi|641569853|gb|KK856540.1|	81162-81410	12	38	3	382	7	0.13455	no
gi|641569853|gb|KK856540.1|	86268-86792	12	38	0	28	inf	5.00E-05	yes
gi|641569853|gb|KK856540.1|	90336-91581	12	38	0.404702	2	2	0.214	no
gi|641569853|gb|KK856540.1|	93384-94073	12	38	2	5	1	0.45545	no
gi|641569853|gb|KK856540.1|	99807-10026[illegible]	12	38	4	10	1	0.3198	no
gi|641569859|gb|KK856538.1|	30364-31408	9.6	45	11	0.88844	-4	0.041	no
gi|641569865|gb|KK856536.1|	24390-25894	8.7	32	100	62	-0.69464	0.74865	no
gi|641569865|gb|KK856536.1|	27180-27656	8.7	32	6	2	-2	0.29665	no

gi|641569865|gb|KK856536.1|	28427-31254	8.7	32	89	54	-0.720384	0.7171	no
gi|641569865|gb|KK856536.1|	31355-32212	8.7	32	1	0.325133	-2	0.31075	no
gi|641569865|gb|KK856536.1|	5691-6201	8.7	32	2	2	-0.254241	0.88485	no
gi|641569865|gb|KK856536.1|	8631-9232	8.7	32	2	2	0.235254	0.88205	no
gi|641569871|gb|KK856534.1|	46388-46836	10	21	2	2	0.00453907	0.91115	no
gi|641569874|gb|KK856533.1|	41348-14192	11	39	0	4	inf	0.00255	yes
gi|641569874|gb|KK856533.1|	49149-49352	11	39	6	70	4	0.14805	no
gi|641569874|gb|KK856533.1|	50863-51496	11	39	2	0.734646	-2	0.403	no
gi|641569874|gb|KK856533.1|	52944-53325	11	39	0	17	inf	0.0004	yes
gi|641569874|gb|KK856533.1|	59924-60346	11	39	0.309459	6	4	0.1175	no
gi|641569874|gb|KK856533.1|	67973-68370	11	39	0	10	inf	0.0015	yes
gi|641569874|gb|KK856533.1|	79708-80277	11	39	0	6	inf	0.00105	yes
gi|641569874|gb|KK856533.1|	80411-81260	11	39	0	5	inf	0.0004	yes
gi|641569874|gb|KK856533.1|	81567-82044	11	39	0	11	inf	0.0004	yes
gi|641569874|gb|KK856533.1|	83113-83441	11	39	0	11	inf	0.0023	yes
gi|641569874|gb|KK856533.1|	85906-86306	11	39	0	16	inf	0.0003	yes
gi|641569874|gb|KK856533.1|	91171-93751	11	39	0.623501	2	2	0.1798	no
gi|641569874|gb|KK856533.1|	94658-95596	11	39	0.476655	1	1	0.3994	no
gi|641569874|gb|KK856533.1|	96063-97028	11	39	0.459935	1	2	0.32945	no
gi|641569883|gb|KK856530.1|	08083-10888	11	45	0	9	inf	0.0001	yes
gi|641569883|gb|KK856530.1|	11011-11349	11	45	22	7	-2	0.25675	no
gi|641569883|gb|KK856530.1|	44630-14508	11	45	3	3	-0.187778	0.90345	no
gi|641569883|gb|KK856530.1|	15328-15895	11	45	3	3	-0.403481	0.7911	no
gi|641569883|gb|KK856530.1|	68486-16863	11	45	111	31	-2	0.3266	no
gi|641569883|gb|KK856530.1|	17428-19017	11	45	0.812833	0.542043	-0.584551	1	no
gi|641569883|gb|KK856530.1|	19163-20396	11	45	0.682304	1	0.60664	0.6779	no
gi|641569883|gb|KK856530.1|	20500-21372	11	45	5	0.635939	-3	0.1075	no
gi|641569883|gb|KK856530.1|	21466-23102	11	45	0.93375	1	0.682233	0.6235	no
gi|641569883|gb|KK856530.1|	23252-29266	11	45	53	18	-2	0.4549	no
gi|641569883|gb|KK856530.1|	31202-32004	11	45	14	9	-0.679166	0.59165	no

gi|641569883|gb|KK856530.1|	3128-3295	11	45	59	49	-0.265145	0.8546	no
gi|641569883|gb|KK856530.1|	33209-33384	11	45	281	209	-0.425038	0.74375	no
gi|641569883|gb|KK856530.1|	34352-36217	11	45	17	9	-0.940367	0.4538	no
gi|641569883|gb|KK856530.1|	36567-37534	11	45	0.275246	1	2	0.2235	no
gi|641569883|gb|KK856530.1|	42455-43203	11	45	2	3	0.523663	0.70545	no
gi|641569883|gb|KK856530.1|	50768-51349	11	45	6	7	0.307577	0.8162	no
gi|641569883|gb|KK856530.1|	56425-57396	11	45	0.821479	2	0.894227	0.5684	no
gi|641569883|gb|KK856530.1|	57988-59992	11	45	6	4	-0.591631	0.63505	no
gi|641569883|gb|KK856530.1|	61444-61738	11	45	10	26	1	0.36585	no
gi|641569883|gb|KK856530.1|	62819-63993	11	45	2	3	0.549714	0.6871	no
gi|641569883|gb|KK856530.1|	64153-65170	11	45	2	2	-0.449302	0.75275	no
gi|641569883|gb|KK856530.1|	65396-67518	11	45	1	1	0.180424	0.89235	no
gi|641569883|gb|KK856530.1|	67668-70529	11	45	4	2	-0.894113	0.4844	no
gi|641569883|gb|KK856530.1|	71721-72615	11	45	2	0.77004	-2	0.30235	no
gi|641569883|gb|KK856530.1|	74469-76502	11	45	1	2	0.656424	0.62365	no
gi|641569883|gb|KK856530.1|	76620-78204	11	45	1	3	1	0.41245	no
gi|641569883|gb|KK856530.1|	80750-81871	11	45	0.535398	2	2	0.3164	no
gi|641569892|gb|KK856527.1|	03795-10444	9.5	20	6	6	0.0337219	0.98115	no
gi|641569892|gb|KK856527.1|	09979-11553	9.5	20	4	5	0.371968	0.76075	no
gi|641569892|gb|KK856527.1|	15716-11717	9.5	20	3	1	-0.859472	0.52935	no
gi|641569892|gb|KK856527.1|	239-686	9.5	20	15	2	-3	0.09735	no
gi|641569892|gb|KK856527.1|	34814-35145	9.5	20	8	15	0.808685	0.56905	no
gi|641569892|gb|KK856527.1|	43312-44017	9.5	20	1	1	0.0158082	0.9394	no
gi|641569892|gb|KK856527.1|	45097-45918	9.5	20	1	1	-0.134066	0.92965	no
gi|641569892|gb|KK856527.1|	67836-68271	9.5	20	4	2	-1	0.50025	no
gi|641569892|gb|KK856527.1|	99861-10012	9.5	20	33	71	1	0.3928	no
gi|641569897|gb|KK856525.1|	01770-10206	9.2	36	9	3	-2	0.4022	no
gi|641569897|gb|KK856525.1|	06102-10663	9.2	36	4	5	0.253686	0.85475	no
gi|641569897|gb|KK856525.1|	61444-62363	9.2	36	1	2	0.488697	0.7378	no
gi|641569897|gb|KK856525.1|	75461-78989	9.2	36	13	7	-0.943878	0.56425	no

gi|641569897|gb|KK856525.1|	85270-85852	9.2	36	18	7	-1	0.34735	no
gi|641569897|gb|KK856525.1|	85962-88378	9.2	36	16	6	-1	0.43015	no
gi|641569897|gb|KK856525.1|	89518-90104	9.2	36	0.36211	12	5	0.23325	no
gi|641569897|gb|KK856525.1|	90366-91324	9.2	36	0.464156	0.846926	0.867625	1	no
gi|641569904|gb|KK856523.1|	59402-60227	10	21	1	2	0.740463	0.6176	no
gi|641569904|gb|KK856523.1|	71501-73031	10	21	1	0.889319	-0.68755	0.63075	no
gi|641569904|gb|KK856523.1|	77740-77951	10	21	40	20	-0.98769	0.523	no
gi|641569904|gb|KK856523.1|	87666-88776	10	21	0.696647	1	0.605876	0.6902	no
gi|641569907|gb|KK856522.1|	10849-11036	9.9	36	31	0	-inf	0.0102	no
gi|641569907|gb|KK856522.1|	14337-14602	9.9	36	33	6	-2	0.1481	no
gi|641569907|gb|KK856522.1|	16069-16330	9.9	36	31	0	-inf	0.00085	yes
gi|641569907|gb|KK856522.1|	21897-22476	9.9	36	3	0.278829	-3	0.217	no
gi|641569907|gb|KK856522.1|	22906-23377	9.9	36	9	0.77037	-4	0.1412	no
gi|641569907|gb|KK856522.1|	2358-3309	9.9	36	6	4	-0.695041	0.5999	no
gi|641569907|gb|KK856522.1|	27698-28303	9.9	36	15.37	3	-2	0.10175	no
gi|641569907|gb|KK856522.1|	304-603	9.9	36	10	4	-1	0.3893	no
gi|641569907|gb|KK856522.1|	32020-33154	9.9	36	4	1	-1	0.33785	no
gi|641569907|gb|KK856522.1|	33923-35842	9.9	36	6	2	-2	0.2243	no
gi|641569907|gb|KK856522.1|	38924-39131	9.9	36	9	92	3	0.1047	no
gi|641569907|gb|KK856522.1|	42237-42795	9.9	36	9	69	3	0.04055	no
gi|641569907|gb|KK856522.1|	44066-45007	9.9	36	2	13	3	0.06275	no
gi|641569907|gb|KK856522.1|	45313-45621	9.9	36	105	51	-1	0.4115	no
gi|641569907|gb|KK856522.1|	46252-46817	9.9	36	13	13	-0.0734163	0.9533	no
gi|641569907|gb|KK856522.1|	48577-49541	9.9	36	15	6	-1	0.34975	no
gi|641569907|gb|KK856522.1|	50154-50898	9.9	36	0.645157	2	1	0.3994	no
gi|641569907|gb|KK856522.1|	51063-52208	9.9	36	0.447315	2	2	0.16035	no
gi|641569907|gb|KK856522.1|	53847-54237	9.9	36	2	9	2	0.2545	no
gi|641569907|gb|KK856522.1|	58714-60099	9.9	36	0.833183	1	0.500452	0.73235	no
gi|641569907|gb|KK856522.1|	60521-61415	9.9	36	0.70934	0.77004	0.118456	1	no
gi|641569907|gb|KK856522.1|	63139-63594	9.9	36	2	3	1	0.5082	no

gi|641569907|gb|KK856522.1|	6456-8216	9.9	36	0.271501	2	3	0.0887	no
gi|641569907|gb|KK856522.1|	74068-74771	9.9	36	0.557759	2	2	0.33105	no
gi|641569907|gb|KK856522.1|	78204-79037	9.9	36	0.333082	2	2	0.21365	no
gi|641569910|gb|KK856521.1|	21468-21766	9.5	36	14	5	-2	0.34495	no
gi|641569910|gb|KK856521.1|	48302-51002	9.5	36	6	13	1	0.3772	no
gi|641569910|gb|KK856521.1|	51066-55522	9.5	36	4	6	0.571109	0.6446	no
gi|641569910|gb|KK856521.1|	71352-71853	9.5	36	1	2	0.856268	0.62755	no
gi|641569913|gb|KK856520.1|	.01364-10240	10	39	0.334092	3	3	0.15475	no
gi|641569913|gb|KK856520.1|	.03128-10409	10	39	1	0.972856	-0.172897	0.90465	no
gi|641569913|gb|KK856520.1|	16287-17237	10	39	0.375261	1	2	0.33105	no
gi|641569913|gb|KK856520.1|	17395-18480	10	39	0.477481	1	1	0.37775	no
gi|641569913|gb|KK856520.1|	59442-60903	10	39	0.391577	1	2	0.2786	no
gi|641569913|gb|KK856520.1|	66127-67191	10	39	0.896544	2	0.953457	0.53865	no
gi|641569913|gb|KK856520.1|	67948-68282	10	39	0.980383	8	3	0.26765	no
gi|641569913|gb|KK856520.1|	85373-86462	10	39	0.39612	0.964469	1	0.4595	no
gi|641569913|gb|KK856520.1|	87157-88935	10	39	0.805239	3	2	0.15115	no
gi|641569913|gb|KK856520.1|	91718-92535	10	39	0.569424	1	1	0.4595	no
gi|641569913|gb|KK856520.1|	92657-93572	10	39	0.78702	1	0.604129	0.6936	no
gi|641569913|gb|KK856520.1|	94933-95638	10	39	0.694466	1	0.863805	0.62725	no
gi|641569913|gb|KK856520.1|	96839-97532	10	39	0.99566	5	2	0.1745	no
gi|641569913|gb|KK856520.1|	98735-10030[illegible]	10	39	1	2	1	0.4501	no
gi|641569916|gb|KK856519.1|	.04722-10586	12	47	0.970205	2	0.905667	0.53945	no
gi|641569916|gb|KK856519.1|	.07655-11061	12	47	9	3	-1	0.25695	no
gi|641569916|gb|KK856519.1|	.12134-11244	12	47	31	6	-2	0.1297	no
gi|641569916|gb|KK856519.1|	.14818-11644	12	47	12	10	-0.236265	0.85125	no
gi|641569916|gb|KK856519.1|	.17215-11799	12	47	10	2	-2	0.1522	no
gi|641569916|gb|KK856519.1|	35040-36370	12	47	0.249591	2	3	0.18525	no
gi|641569916|gb|KK856519.1|	38501-39970	12	47	0.389118	2	2	0.1822	no
gi|641569916|gb|KK856519.1|	41759-42653	12	47	0.70934	0.462024	-0.618509	1	no
gi|641569916|gb|KK856519.1|	43284-44133	12	47	0.54155	3	3	0.14425	no

gi|641569916|gb|KK856519.1|	44211-44753	12	47	0.815505	2	2	0.3613	no
gi|641569916|gb|KK856519.1|	61445-63551	12	47	8	7	-0.359326	0.77265	no
gi|641569916|gb|KK856519.1|	64595-66043	12	47	6	2	-1	0.27015	no
gi|641569916|gb|KK856519.1|	77884-78797	12	47	8	10	0.31653	0.8051	no
gi|641569916|gb|KK856519.1|	79956-80515	12	47	1	3	1	0.37835	no
gi|641569916|gb|KK856519.1|	80737-81277	12	47	25	28	0.184246	0.8825	no
gi|641569916|gb|KK856519.1|	88940-92693	12	47	3	1	-1	0.27155	no
gi|641569919|gb|KK856518.1|	01717-10229	11	43	1	1	-0.210429	0.8945	no
gi|641569919|gb|KK856518.1|	107-15228	11	43	7	2	-2	0.3275	no
gi|641569919|gb|KK856518.1|	25123-12542	11	43	0	14	inf	0.0015	yes
gi|641569919|gb|KK856518.1|	17659-19642	11	43	26	39	0.629051	0.7758	no
gi|641569919|gb|KK856518.1|	20722-21224	11	43	5	7	0.526179	0.7094	no
gi|641569919|gb|KK856518.1|	41995-42907	11	43	0.296368	2	2	0.2136	no
gi|641569919|gb|KK856518.1|	49188-50083	11	43	0.404758	1	2	0.3613	no
gi|641569919|gb|KK856518.1|	87463-87623	11	43	0	98	inf	0.00455	no
gi|641569921|gb|KK856517.1|	10072-11106	11	43	1	1	0.189855	0.90355	no
gi|641569921|gb|KK856517.1|	11265-11194	11	43	0.879898	2	1	0.3778	no
gi|641569921|gb|KK856517.1|	13025-11727	11	43	0.804244	2	1	0.42645	no
gi|641569921|gb|KK856517.1|	25586-12744	11	43	0.722175	1	0.913643	0.52205	no
gi|641569921|gb|KK856517.1|	14799-14957	11	43	68	19	-2	0.3266	no
gi|641569921|gb|KK856517.1|	2979-3214	11	43	11	16	0.54674	0.7321	no
gi|641569921|gb|KK856517.1|	32602-33631	11	43	0.594479	0.387577	-0.617143	1	no
gi|641569921|gb|KK856517.1|	36259-37031	11	43	2	5	2	0.2916	no
gi|641569921|gb|KK856517.1|	37135-37802	11	43	0.900376	1	0.599841	0.70555	no
gi|641569921|gb|KK856517.1|	37886-38563	11	43	0.293907	2	3	0.2672	no
gi|641569921|gb|KK856517.1|	58047-59008	11	43	2	4	1	0.42385	no
gi|641569921|gb|KK856517.1|	64675-65174	11	43	4	1	-2	0.24335	no
gi|641569921|gb|KK856517.1|	66670-67360	11	43	0	2	inf	0.0068	no
gi|641569921|gb|KK856517.1|	67815-69314	11	43	0.488782	0.827635	0.759804	1	no
gi|641569921|gb|KK856517.1|	80494-81043	11	43	2	2	0.0811614	0.9406	no

gi|641569921|gb|KK856517.1|	82168-83047	11	43	0.207115	3	4	0.24715	no
gi|641569924|gb|KK856516.1|	66514-66808	8.7	41	6	5	-0.28213	0.8649	no
gi|641569924|gb|KK856516.1|	68227-69362	8.7	41	0.452076	1	1	0.41175	no
gi|641569924|gb|KK856516.1|	69506-70001	8.7	41	1	2	0.59287	0.70555	no
gi|641569924|gb|KK856516.1|	71142-71668	8.7	41	1	5	2	0.25895	no
gi|641569926|gb|KK856515.1|	34660-35372	10	44	0.68507	2	2	0.32945	no
gi|641569926|gb|KK856515.1|	37183-37812	10	44	2	2	0.0841794	0.94	no
gi|641569929|gb|KK856514.1|	00584-10139	10	18	1	1	-0.097677	0.9478	no
gi|641569929|gb|KK856514.1|	01544-10266	10	18	50	21	-1	0.4449	no
gi|641569929|gb|KK856514.1|	31949-32802	10	18	7	4	-0.85036	0.5158	no
gi|641569929|gb|KK856514.1|	33159-33471	10	18	140.29	23	-3	0.06325	no
gi|641569929|gb|KK856514.1|	34314-36033	10	18	0.650548	0.991843	0.608455	0.67385	no
gi|641569929|gb|KK856514.1|	37028-37680	10	18	1	1	0.114006	0.92265	no
gi|641569929|gb|KK856514.1|	37893-39132	10	18	0.339191	0.6198	0.869707	1	no
gi|641569929|gb|KK856514.1|	40232-40877	10	18	19	4	-2	0.09125	no
gi|641569929|gb|KK856514.1|	43798-44208	10	18	4	0	-inf	0.0075	no
gi|641569929|gb|KK856514.1|	44672-44865	10	18	76	4	-4	0.188	no
gi|641569929|gb|KK856514.1|	47548-47784	10	18	36	10	-2	0.26325	no
gi|641569929|gb|KK856514.1|	50216-50442	10	18	48	30	-0.679482	0.62015	no
gi|641569929|gb|KK856514.1|	52158-52635	10	18	4	0.377228	-3	0.20585	no
gi|641569929|gb|KK856514.1|	60749-62585	10	18	1	0.460645	-1	0.41145	no
gi|641569929|gb|KK856514.1|	65566-67900	10	18	11	4	-1	0.27135	no
gi|641569929|gb|KK856514.1|	72148-75643	10	18	9	7	-0.38226	0.75635	no
gi|641569929|gb|KK856514.1|	81809-81969	10	18	63	9	-3	0.23985	no
gi|641569929|gb|KK856514.1|	83409-83629	10	18	218	162	-0.430266	0.7349	no
gi|641569929|gb|KK856514.1|	85732-86286	10	18	16	13	-0.252037	0.8453	no
gi|641569929|gb|KK856514.1|	88272-88940	10	18	11	9	-0.284655	0.8238	no
gi|641569929|gb|KK856514.1|	92196-92384	10	18	61	8	-3	0.16975	no
gi|641569932|gb|KK856513.1|	02878-10392	11	22	2	2	-0.0726409	0.9587	no
gi|641569932|gb|KK856513.1|	13809-11413	11	22	3	4	0.309768	0.86115	no

gi|641569932|gb|KK856513.1|	:19043-11988	11	22	8	10	0.267114	0.83145	no
gi|641569932|gb|KK856513.1|	94979-96182	11	22	0.772878	0.213951	-2	1	no
gi|641569932|gb|KK856513.1|	99209-10224	11	22	7	5	-0.513321	0.6866	no
gi|641569942|gb|KK856510.1|	39540-40528	9.2	37	14	11	-0.285492	0.8201	no
gi|641569942|gb|KK856510.1|	42303-42540	9.2	37	6	10	0.810593	0.6381	no
gi|641569942|gb|KK856510.1|	44565-45454	9.2	37	1	0.93067	-0.270647	0.86195	no
gi|641569945|gb|KK856509.1|	16709-18223	10	41	2	0.736425	-2	0.28705	no
gi|641569948|gb|KK856508.1|	:02601-10471	10	37	19	0.788501	-5	0.0458	no
gi|641569948|gb|KK856508.1|	10538-11438	10	37	3	3	0.156496	0.9073	no
gi|641569948|gb|KK856508.1|	:07622-10794	10	37	5	2	-2	0.3632	no
gi|641569948|gb|KK856508.1|	:10338-11060	10	37	8	9	0.194078	0.8977	no
gi|641569948|gb|KK856508.1|	:17560-11937	10	37	6	2	-1	0.3235	no
gi|641569948|gb|KK856508.1|	:23244-12401	10	37	102	99	-0.0526704	0.9795	no
gi|641569948|gb|KK856508.1|	:24176-12754	10	37	2	1	-0.237527	0.8548	no
gi|641569948|gb|KK856508.1|	:40331-14097	10	37	1	1	0.113659	0.92265	no
gi|641569948|gb|KK856508.1|	:44558-14562	10	37	6.35	7	0.0480602	0.9701	no
gi|641569948|gb|KK856508.1|	14857-15517	10	37	8	6	-0.400346	0.7596	no
gi|641569948|gb|KK856508.1|	:49328-14984	10	37	44	41	-0.123924	0.91985	no
gi|641569948|gb|KK856508.1|	:49966-15010	10	37	87	293	2	0.30605	no
gi|641569948|gb|KK856508.1|	:53033-15320	10	37	86	52	-0.706605	0.6288	no
gi|641569948|gb|KK856508.1|	:55116-15562	10	37	8	20	1	0.3275	no
gi|641569948|gb|KK856508.1|	:55981-15659	10	37	4	7	0.88203	0.5238	no
gi|641569948|gb|KK856508.1|	:59769-16263	10	37	10	10	-0.0286143	0.97955	no
gi|641569948|gb|KK856508.1|	:65167-16712	10	37	39	9	-2	0.2354	no
gi|641569948|gb|KK856508.1|	:71432-17460	10	37	6	2	-1	0.34805	no
gi|641569948|gb|KK856508.1|	:92938-19321	10	37	5	3	-0.660277	0.70815	no
gi|641569948|gb|KK856508.1|	23516-23797	10	37	16	12	-0.437604	0.76675	no
gi|641569948|gb|KK856508.1|	27528-27728	10	37	19	9	-1	0.5277	no
gi|641569948|gb|KK856508.1|	28180-28394	10	37	21	24	0.158827	0.9144	no
gi|641569948|gb|KK856508.1|	29551-29992	10	37	3	3	0.00394289	0.95145	no

gi|641569948|gb|KK856508.1|	31547-32007	10	37	6	2	-1	0.3795	no
gi|641569948|gb|KK856508.1|	33848-35081	10	37	0.477612	0.62337	0.384248	1	no
gi|641569948|gb|KK856508.1|	35744-36713	10	37	2	1	-0.243296	0.867	no
gi|641569948|gb|KK856508.1|	38152-38981	10	37	1	0	-inf	0.0102	no
gi|641569948|gb|KK856508.1|	39409-42525	10	37	69	27	-1	0.5274	no
gi|641569948|gb|KK856508.1|	47730-48527	10	37	31	11	-1	0.2587	no
gi|641569948|gb|KK856508.1|	55173-55578	10	37	37	11	-2	0.17795	no
gi|641569948|gb|KK856508.1|	60596-63401	10	37	1	0.7868	-0.842442	0.5371	no
gi|641569948|gb|KK856508.1|	6159-6489	10	37	16	19	0.217878	0.87495	no
gi|641569948|gb|KK856508.1|	63579-63829	10	37	122	38	-2	0.21605	no
gi|641569948|gb|KK856508.1|	64010-65510	10	37	0.596942	0.496202	-0.266665	1	no
gi|641569948|gb|KK856508.1|	68461-69631	10	37	2	0.442328	-2	0.1923	no
gi|641569948|gb|KK856508.1|	69750-69974	10	37	37	6	-3	0.1729	no
gi|641569948|gb|KK856508.1|	74419-77382	10	37	1	0.507651	-1	0.38235	no
gi|641569948|gb|KK856508.1|	78419-79986	10	37	57	20	-2	0.36665	no
gi|641569948|gb|KK856508.1|	80682-80903	10	37	70	28	-1	0.3572	no
gi|641569948|gb|KK856508.1|	83878-84124	10	37	158	65	-1	0.3154	no
gi|641569948|gb|KK856508.1|	85745-86248	10	37	9	4	-1	0.4302	no
gi|641569948|gb|KK856508.1|	86810-87010	10	37	111	74	-0.585205	0.6516	no
gi|641569948|gb|KK856508.1|	90708-91193	10	37	7	4	-0.855226	0.5472	no
gi|641569948|gb|KK856508.1|	94362-94820	10	37	18	9	-0.973595	0.45845	no
gi|641569948|gb|KK856508.1|	96225-98623	10	37	2	2	-0.512339	0.6895	no
gi|641569958|gb|KK856505.1|	46929-47688	8.7	35	0.628001	2	2	0.2717	no
gi|641569958|gb|KK856505.1|	48268-48850	8.7	35	0.365836	2	3	0.2851	no
gi|641569964|gb|KK856503.1|	12814-11321	10	36	2	4	1	0.4655	no
gi|641569964|gb|KK856503.1|	13341-11847	10	36	7	4	-0.994081	0.4253	no
gi|641569964|gb|KK856503.1|	19070-12265	10	36	9	10	0.104702	0.9484	no
gi|641569964|gb|KK856503.1|	24576-12556	10	36	0.71185	0.947313	0.412266	0.8089	no
gi|641569964|gb|KK856503.1|	28212-12909	10	36	0.617731	0.938741	0.603748	0.70555	no
gi|641569964|gb|KK856503.1|	31121-13447	10	36	3	2	-0.868373	0.49395	no

gi|641569964|gb|KK856503.1|	34985-13550	10	36	9	7	-0.54304	0.6917	no
gi|641569964|gb|KK856503.1|	35615-13780	10	36	2	3	0.580796	0.6529	no
gi|641569964|gb|KK856503.1|	47721-14878	10	36	1	2	0.505983	0.72975	no
gi|641569964|gb|KK856503.1|	48913-14931	10	36	3	3	-0.414764	0.8125	no
gi|641569964|gb|KK856503.1|	49513-15170	10	36	2	2	-0.0942326	0.94345	no
gi|641569964|gb|KK856503.1|	64675-16493	10	36	6	8	0.33393	0.8379	no
gi|641569964|gb|KK856503.1|	31152-31738	10	36	1	4	2	0.2254	no
gi|641569964|gb|KK856503.1|	35047-35912	10	36	4	4	0.216495	0.87265	no
gi|641569964|gb|KK856503.1|	36213-45903	10	36	16	12	-0.462924	0.82745	no
gi|641569964|gb|KK856503.1|	46038-48035	10	36	19	5	-2	0.16185	no
gi|641569964|gb|KK856503.1|	51133-52159	10	36	1	0.38897	-2	0.4042	no
gi|641569964|gb|KK856503.1|	74520-75243	10	36	5	2	-1	0.43175	no
gi|641569964|gb|KK856503.1|	75465-76387	10	36	1	0.59243	-0.855223	0.60675	no
gi|641569964|gb|KK856503.1|	77214-78603	10	36	16	6	-1	0.31225	no
gi|641569964|gb|KK856503.1|	84617-85698	10	36	1	0.608212	-0.772847	0.63565	no
gi|641569964|gb|KK856503.1|	87725-88371	10	36	2	0	-inf	0.0102	no
gi|641569964|gb|KK856503.1|	90989-91233	10	36	16	19	0.228397	0.87515	no
gi|641569970|gb|KK856501.1|	07324-10796	12	41	0.639469	3	2	0.2441	no
gi|641569970|gb|KK856501.1|	11816-11236	12	41	0.198203	3	4	0.12385	no
gi|641569970|gb|KK856501.1|	15435-11628	12	41	2	0.991784	-0.811776	0.5996	no
gi|641569970|gb|KK856501.1|	16617-11785	12	41	12	6	-0.842301	0.5071	no
gi|641569970|gb|KK856501.1|	79605-81767	12	41	0.972024	1	0.554881	0.68815	no
gi|641569970|gb|KK856501.1|	85138-87533	12	41	1.03	1	0.00196517	0.98005	no
gi|641569970|gb|KK856501.1|	87584-89306	12	41	0.602893	2	2	0.20195	no
gi|641569970|gb|KK856501.1|	89378-90024	12	41	2	2	0.0846885	0.94	no
gi|641569970|gb|KK856501.1|	90699-92160	12	41	1	1	0.30281	0.8279	no
gi|641569970|gb|KK856501.1|	92244-93459	12	41	0.416609	0.528608	0.343505	1	no
gi|641569970|gb|KK856501.1|	99900-10088	12	41	11	6	-0.806106	0.52875	no
gi|641569973|gb|KK856500.1|	13504-14081	9.8	36	2	1	-0.725002	0.67005	no
gi|641569973|gb|KK856500.1|	18586-18808	9.8	36	12	6	-0.873926	0.6407	no

gi|641569973|gb|KK856500.1|	9981-11348	9.8	36	1	0.092055	-3	0.206	no
gi|641569976|gb|KK856499.1|	04267-10516	10	43	1	0.920121	-0.133046	0.92965	no
gi|641569976|gb|KK856499.1|	06029-10651	10	43	4	14	2	0.23135	no
gi|641569976|gb|KK856499.1|	23204-12347	10	43	14	31	1	0.4333	no
gi|641569976|gb|KK856499.1|	44982-45258	10	43	5	7	0.338576	0.8379	no
gi|641569976|gb|KK856499.1|	88710-89496	10	43	0.839029	1	0.379951	0.82465	no
gi|641569979|gb|KK856498.1|	08544-10989	9.2	33	16	16	0.0222629	0.9852	no
gi|641569979|gb|KK856498.1|	11290-11263	9.2	33	13	10.19	-0.366071	0.77325	no
gi|641569979|gb|KK856498.1|	14488-11560	9.2	33	5	1	-2	0.18955	no
gi|641569979|gb|KK856498.1|	12542-13034	9.2	33	5	1	-2	0.2507	no
gi|641569979|gb|KK856498.1|	165-417	9.2	33	19	26	0.47526	0.73205	no
gi|641569979|gb|KK856498.1|	26610-28525	9.2	33	2	0.502325	-2	0.14655	no
gi|641569979|gb|KK856498.1|	32435-32623	9.2	33	30	4	-3	0.23535	no
gi|641569979|gb|KK856498.1|	33793-34338	9.2	33	2	0.305445	-3	0.2381	no
gi|641569979|gb|KK856498.1|	37386-37630	9.2	33	37	0	-inf	0.00055	yes
gi|641569979|gb|KK856498.1|	38803-39002	9.2	33	126	16	-3	0.0822	no
gi|641569979|gb|KK856498.1|	40645-41003	9.2	33	8	0.633225	-4	0.2035	no
gi|641569979|gb|KK856498.1|	44420-44783	9.2	33	14	1	-3	0.14325	no
gi|641569979|gb|KK856498.1|	47204-47374	9.2	33	49	0	-inf	0.0102	no
gi|641569979|gb|KK856498.1|	54651-55442	9.2	33	4	0.541363	-3	0.1338	no
gi|641569979|gb|KK856498.1|	56279-56881	9.2	33	3	0.789871	-2	0.33205	no
gi|641569979|gb|KK856498.1|	57377-57621	9.2	33	16	2	-3	0.21195	no
gi|641569979|gb|KK856498.1|	59306-59558	9.2	33	14	6	-1	0.4486	no
gi|641569979|gb|KK856498.1|	60731-61717	9.2	33	19	1	-4	0.0236	no
gi|641569979|gb|KK856498.1|	6663-7280	9.2	33	21	3	-3	0.07525	no
gi|641569979|gb|KK856498.1|	68150-68741	9.2	33	1	76	6	0.0516	no
gi|641569979|gb|KK856498.1|	70145-70977	9.2	33	0	5	inf	0.00035	yes
gi|641569979|gb|KK856498.1|	72268-73768	9.2	33	0.162802	0.744303	2	1	no
gi|641569979|gb|KK856498.1|	7795-8532	9.2	33	2	0.198299	-3	0.2348	no
gi|641569979|gb|KK856498.1|	84049-85003	9.2	33	2	2	0.0809883	0.95575	no

gi|641569979|gb|KK856498.1|	85932-86346	9.2	33	2	3	0.363931	0.82765	no
gi|641569979|gb|KK856498.1|	8740-9840	9.2	33	0.704347	0.238199	-2	1	no
gi|641569979|gb|KK856498.1|	92660-94959	9.2	33	3	2	-0.590413	0.65675	no
gi|641569979|gb|KK856498.1|	95069-97415	9.2	33	1	1	0.0950133	0.94155	no
gi|641569979|gb|KK856498.1|	97772-98357	9.2	33	1	1	0.111811	0.92265	no
gi|641569979|gb|KK856498.1|	98525-99211	9.2	33	1	2	0.600324	0.6902	no
gi|641569979|gb|KK856498.1|	9982-11034	9.2	33	0.909102	0.754443	-0.26903	0.86195	no
gi|641569982|gb|KK856497.1|	.09616-11003	9.9	36	0	86	inf	5.00E-05	yes
gi|641569982|gb|KK856497.1|	2-1271	9.9	36	13	13	-0.0225257	0.98595	no
gi|641569982|gb|KK856497.1|	20065-22685	9.9	36	6	9	0.500826	0.69135	no
gi|641569982|gb|KK856497.1|	26104-26976	9.9	36	47	65	0.459051	0.77265	no
gi|641569982|gb|KK856497.1|	27548-33684	9.9	36	5	8	0.604439	0.70925	no
gi|641569982|gb|KK856497.1|	3343-4267	9.9	36	20	35	0.773388	0.53745	no
gi|641569982|gb|KK856497.1|	33924-34763	9.9	36	0.769948	0.668334	-0.204192	1	no
gi|641569982|gb|KK856497.1|	35013-35768	9.9	36	67	131.31	0.964367	0.66625	no
gi|641569982|gb|KK856497.1|	77724-77905	9.9	36	0	183	inf	5.00E-05	yes
gi|641569985|gb|KK856496.1|	.02882-10363	11	39	4	5	0.348059	0.794	no
gi|641569985|gb|KK856496.1|	.09041-11093	11	39	0.706906	1	0.843328	0.55945	no
gi|641569985|gb|KK856496.1|	.17878-11847	11	39	2	3	0.749685	0.6263	no
gi|641569985|gb|KK856496.1|	.31092-13226	11	39	0.433618	3	3	0.132	no
gi|641569985|gb|KK856496.1|	.38058-13841	11	39	16	39	1	0.33125	no
gi|641569985|gb|KK856496.1|	27432-28300	11	39	0.526254	1	1	0.4595	no
gi|641569985|gb|KK856496.1|	44117-47213	11	39	0.561406	0.931553	0.730592	0.5989	no
gi|641569985|gb|KK856496.1|	56732-59586	11	39	10	4	-1	0.3007	no
gi|641569985|gb|KK856496.1|	59777-60497	11	39	1	0	-inf	0.0102	no
gi|641569985|gb|KK856496.1|	90187-90824	11	39	0.961368	0.970766	0.0140343	0.91115	no
gi|641569985|gb|KK856496.1|	96941-101412	11	39	33	24	-0.467371	0.8265	no
gi|641569988|gb|KK856495.1|	.02799-10653	11	43	2	0.793452	-1	0.4272	no
gi|641569988|gb|KK856495.1|	.06831-10793	11	43	21	12	-0.814535	0.5101	no
gi|641569988|gb|KK856495.1|	.08661-11456	11	43	11	4	-1	0.41405	no

gi|641569988|gb|KK856495.1|	15219-11608	11	43	0.734572	1	0.603583	0.69715	no
gi|641569988|gb|KK856495.1|	30090-13043	11	43	8	12	0.494185	0.7295	no
gi|641569988|gb|KK856495.1|	31808-13301	11	43	28	4	-3	0.0496	no
gi|641569988|gb|KK856495.1|	3151-3597	11	43	4	0	-inf	0.0059	no
gi|641569988|gb|KK856495.1|	32260-33052	11	43	0.593281	3	2	0.20805	no
gi|641569988|gb|KK856495.1|	42244-42997	11	43	1	1	0.186722	0.91015	no
gi|641569988|gb|KK856495.1|	43111-45169	11	43	0.646186	1	0.914016	0.52205	no
gi|641569988|gb|KK856495.1|	47780-49543	11	43	1	2	0.853677	0.5302	no
gi|641569988|gb|KK856495.1|	59182-60268	11	43	0.556436	1	0.968272	0.5733	no
gi|641569988|gb|KK856495.1|	65569-66882	11	43	0.823508	0.578916	-0.50843	1	no
gi|641569988|gb|KK856495.1|	67311-68149	11	43	2	0.167342	-3	0.217	no
gi|641569988|gb|KK856495.1|	68966-72688	11	43	0.903434	3	2	0.1735	no
gi|641569988|gb|KK856495.1|	78550-79565	11	43	1	0.919715	-0.287956	0.85105	no
gi|641569988|gb|KK856495.1|	79664-81504	11	43	3	4	0.212812	0.8666	no
gi|641569988|gb|KK856495.1|	81714-83158	11	43	40	93	1	0.5829	no
gi|641569988|gb|KK856495.1|	84550-86027	11	43	11	33	2	0.34455	no
gi|641569988|gb|KK856495.1|	87739-88795	11	43	19	45	1	0.45355	no
gi|641569988|gb|KK856495.1|	88912-89887	11	43	0.181614	2	3	0.2556	no
gi|641569988|gb|KK856495.1|	90835-91667	11	43	43	105	1	0.44065	no
gi|641569988|gb|KK856495.1|	93035-94782	11	43	10	30	2	0.34235	no
gi|641569988|gb|KK856495.1|	98956-102674	11	43	3	1	-1	0.26385	no
gi|641569995|gb|KK856493.1|	6492-6948	8.8	34	1	3	1	0.4427	no
gi|641569999|gb|KK856492.1|	00797-10179	10	38	1	2	0.827322	0.58405	no
gi|641569999|gb|KK856492.1|	01931-10361	10	38	0.571875	2	2	0.1795	no
gi|641569999|gb|KK856492.1|	05108-10538	10	38	12	2	-2	0.21625	no
gi|641569999|gb|KK856492.1|	10652-10963	10	38	11	6	-0.870595	0.56395	no
gi|641569999|gb|KK856492.1|	29710-12992	10	38	10	16	0.760695	0.64725	no
gi|641569999|gb|KK856492.1|	19167-19334	10	38	133	35	-2	0.2394	no
gi|641569999|gb|KK856492.1|	45654-47285	10	38	0.937002	1	0.447753	0.74345	no
gi|641569999|gb|KK856492.1|	48044-48666	10	38	1	2	0.013569	0.9394	no

gi|641569999|gb|KK856492.1|	51224-51479	10	38	30	17	-0.860729	0.5419	no
gi|641569999|gb|KK856492.1|	520-722	10	38	47	30	-0.670324	0.6413	no
gi|641569999|gb|KK856492.1|	53413-54841	10	38	5	5	0.10504	0.93655	no
gi|641569999|gb|KK856492.1|	58960-60792	10	38	2	4	0.583188	0.6579	no
gi|641569999|gb|KK856492.1|	63003-63784	10	38	211	275	0.383724	0.8555	no
gi|641569999|gb|KK856492.1|	65858-72618	10	38	17	21	0.322558	0.8765	no
gi|641569999|gb|KK856492.1|	74420-76274	10	38	32	36	0.179404	0.9373	no
gi|641569999|gb|KK856492.1|	99158-10069[illegible]	10	38	0.528103	0.643892	0.285998	1	no
gi|641570004|gb|KK856490.1|	2674-2961	10	35	0	45	inf	0.00015	yes
gi|641570004|gb|KK856490.1|	35187-36138	10	35	16	12	-0.353417	0.7822	no
gi|641570004|gb|KK856490.1|	38819-39204	10	35	10	15	0.637376	0.64425	no
gi|641570004|gb|KK856490.1|	41089-41718	10	35	20.56	23	0.160631	0.8972	no
gi|641570004|gb|KK856490.1|	45277-47506	10	35	10	15	0.604462	0.6297	no
gi|641570004|gb|KK856490.1|	48538-49058	10	35	4	8	1	0.46135	no
gi|641570004|gb|KK856490.1|	5557-5979	10	35	0	123.66	inf	5.00E-05	yes
gi|641570004|gb|KK856490.1|	6145-6918	10	35	0	15	inf	5.00E-05	yes
gi|641570004|gb|KK856490.1|	7185-7933	10	35	0	9	inf	0.00025	yes
gi|641570007|gb|KK856489.1|	19395-20924	9.5	32	1	0.647261	-0.714025	0.633	no
gi|641570007|gb|KK856489.1|	22821-26472	9.5	32	0.737597	1	0.528163	0.68415	no
gi|641570007|gb|KK856489.1|	26623-27433	9.5	32	3	0	-inf	0.0024	yes
gi|641570007|gb|KK856489.1|	27537-28531	9.5	32	2	0.269649	-3	0.1507	no
gi|641570007|gb|KK856489.1|	28718-32968	9.5	32	3	0.906704	-2	0.17865	no
gi|641570007|gb|KK856489.1|	33098-33979	9.5	32	2	0.784552	-2	0.30235	no
gi|641570007|gb|KK856489.1|	34179-34820	9.5	32	6	7	0.220604	0.86775	no
gi|641570007|gb|KK856489.1|	37223-38085	9.5	32	97	40.5	-1	0.45	no
gi|641570007|gb|KK856489.1|	38858-39927	9.5	32	0.891413	0.123305	-3	0.2382	no
gi|641570007|gb|KK856489.1|	39994-40685	9.5	32	23	10	-1	0.35535	no
gi|641570007|gb|KK856489.1|	47975-51804	9.5	32	3	2	-0.196646	0.87185	no
gi|641570007|gb|KK856489.1|	52423-52893	9.5	32	11	14	0.341225	0.7969	no
gi|641570007|gb|KK856489.1|	58881-59192	9.5	32	36	35	-0.0497504	0.9715	no

gi|641570007|gb|KK856489.1|	59706-60573	9.5	32	7	7	0.0298092	0.9813	no
gi|641570007|gb|KK856489.1|	66763-67104	9.5	32	21	5	-2	0.17095	no
gi|641570007|gb|KK856489.1|	71324-71981	9.5	32	6	0.464597	-4	0.142	no
gi|641570007|gb|KK856489.1|	95928-97028	9.5	32	0.939129	3	1	0.345	no
gi|641570007|gb|KK856489.1|	98735-99088	9.5	32	2	5	1	0.4433	no
gi|641570010|gb|KK856488.1|	75223-77486	9	34	103	12	-3	0.09545	no
gi|641570010|gb|KK856488.1|	77726-80886	9	34	1	2	0.794766	0.54555	no
gi|641570010|gb|KK856488.1|	82978-83732	9	34	2	2	0.186741	0.8991	no
gi|641570010|gb|KK856488.1|	83854-84129	9	34	112	15	-3	0.0503	no
gi|641570010|gb|KK856488.1|	89251-89991	9	34	0.259956	3	4	0.24945	no
gi|641570010|gb|KK856488.1|	90256-91853	9	34	7	6	-0.273238	0.82735	no
gi|641570010|gb|KK856488.1|	92040-92314	9	34	50	6	-3	0.0793	no
gi|641570010|gb|KK856488.1|	92733-93036	9	34	7	8	0.278598	0.8556	no
gi|641570010|gb|KK856488.1|	93701-93889	9	34	135	36	-2	0.18945	no
gi|641570010|gb|KK856488.1|	94010-94519	9	34	53	6	-3	0.0323	no
gi|641570010|gb|KK856488.1|	95194-95485	9	34	37	7	-2	0.12685	no
gi|641570010|gb|KK856488.1|	96117-96567	9	34	5	1	-2	0.24265	no
gi|641570013|gb|KK856487.1|	0-375	9.3	34	5	0	-inf	0.01	no
gi|641570013|gb|KK856487.1|	1057-1605	9.3	34	1	2	0.818081	0.6234	no
gi|641570013|gb|KK856487.1|	2541-3130	9.3	34	3	4	0.497853	0.7333	no
gi|641570016|gb|KK856486.1|	23154-12398	12	20	0.443414	2	2	0.33105	no
gi|641570016|gb|KK856486.1|	13384-13917	12	20	0.627791	2	2	0.3323	no
gi|641570016|gb|KK856486.1|	38369-14258	12	20	5	0.646236	-3	0.0425	no
gi|641570016|gb|KK856486.1|	45758-14622	12	20	3	0.394894	-3	0.2481	no
gi|641570016|gb|KK856486.1|	46921-14733	12	20	3	1	-1	0.50365	no
gi|641570016|gb|KK856486.1|	54291-15503	12	20	3	3	0.0489498	0.97035	no
gi|641570016|gb|KK856486.1|	31759-32197	12	20	4	1	-2	0.40265	no
gi|641570016|gb|KK856486.1|	32904-33106	12	20	31	0	-inf	0.0059	no
gi|641570016|gb|KK856486.1|	36905-37303	12	20	5	1	-2	0.2477	no
gi|641570016|gb|KK856486.1|	38079-39305	12	20	1	0.627587	-0.715327	0.6527	no

gi|641570016|gb|KK856486.1|	40333-41075	12	20	2	0.589502	-2	0.33985	no
gi|641570016|gb|KK856486.1|	42047-42419	12	20	5	2	-1	0.451	no
gi|641570016|gb|KK856486.1|	44030-44627	12	20	7	2	-2	0.25135	no
gi|641570016|gb|KK856486.1|	48541-49006	12	20	7	2	-2	0.2891	no
gi|641570016|gb|KK856486.1|	51105-51349	12	20	42	6	-3	0.1252	no
gi|641570016|gb|KK856486.1|	55283-57857	12	20	29	13	-1	0.4903	no
gi|641570016|gb|KK856486.1|	74373-77918	12	20	6	1	-2	0.12535	no
gi|641570016|gb|KK856486.1|	78019-79119	12	20	0.860869	0.119099	-3	1	no
gi|641570016|gb|KK856486.1|	79648-80806	12	20	0.809004	0.447818	-0.853234	1	no
gi|641570016|gb|KK856486.1|	81820-82707	12	20	0.307079	2	2	0.2235	no
gi|641570016|gb|KK856486.1|	97259-97477	12	20	25	5	-2	0.2132	no
gi|641570019|gb|KK856485.1|	34465-13536	12	43	0.500228	0.760304	0.603991	1	no
gi|641570019|gb|KK856485.1|	19013-19309	12	43	0	32	inf	0.00025	yes
gi|641570019|gb|KK856485.1|	20865-21628	12	43	0	205	inf	0.0001	yes
gi|641570019|gb|KK856485.1|	23131-23899	12	43	0	267	inf	0.00045	yes
gi|641570019|gb|KK856485.1|	24508-24749	12	43	0	267.42	inf	5.00E-05	yes
gi|641570019|gb|KK856485.1|	25157-25856	12	43	0	63	inf	5.00E-05	yes
gi|641570019|gb|KK856485.1|	27388-27633	12	43	0	23	inf	0.0023	yes
gi|641570019|gb|KK856485.1|	28803-29180	12	43	0	8	inf	0.00255	yes
gi|641570019|gb|KK856485.1|	45247-45443	12	43	37	13	-1	0.3803	no
gi|641570019|gb|KK856485.1|	47281-48750	12	43	2	2	-0.188772	0.8921	no
gi|641570019|gb|KK856485.1|	48851-51596	12	43	4	6	0.629253	0.6087	no
gi|641570019|gb|KK856485.1|	51915-52367	12	43	12	17	0.58983	0.6517	no
gi|641570019|gb|KK856485.1|	53922-54408	12	43	21	22	0.0636043	0.9604	no
gi|641570019|gb|KK856485.1|	54508-54911	12	43	4	10	1	0.3639	no
gi|641570019|gb|KK856485.1|	56061-57124	12	43	10	10	0.0789805	0.94645	no
gi|641570019|gb|KK856485.1|	58426-59412	12	43	0.537264	1	1	0.5082	no
gi|641570019|gb|KK856485.1|	59997-61762	12	43	25	14	-0.820475	0.6158	no
gi|641570019|gb|KK856485.1|	99266-10110[illegible]	12	43	0	47	inf	5.00E-05	yes
gi|641570027|gb|KK856483.1|	42394-43327	9.9	37	0	29	inf	5.00E-05	yes

gi|641570027|gb|KK856483.1|	47754-49349	9.9	37	0	7	inf	5.00E-05	yes
gi|641570030|gb|KK856482.1|	15219-15482	11	43	22	24	0.161043	0.9064	no
gi|641570030|gb|KK856482.1|	31573-32070	11	43	2	1	-0.6294	0.71175	no
gi|641570030|gb|KK856482.1|	37935-38297	11	43	5	2	-1	0.4958	no
gi|641570030|gb|KK856482.1|	77156-77772	11	43	0.168199	3	4	0.1175	no
gi|641570033|gb|KK856481.1|	29485-30047	9.8	41	0.964174	2	1	0.51085	no
gi|641570033|gb|KK856481.1|	43900-45306	9.8	41	0.643289	0.445497	-0.53005	1	no
gi|641570033|gb|KK856481.1|	94850-95674	9.8	41	0.675701	1	1	0.5082	no
gi|641570036|gb|KK856480.1|	01718-10244	8.3	33	0.928582	1	0.601289	0.69715	no
gi|641570036|gb|KK856480.1|	16209-18611	8.3	33	4	6	0.757755	0.5488	no
gi|641570036|gb|KK856480.1|	18751-19517	8.3	33	2	4	0.82439	0.5601	no
gi|641570036|gb|KK856480.1|	19592-21051	8.3	33	2	1	-0.299233	0.8299	no
gi|641570036|gb|KK856480.1|	39083-39587	8.3	33	2	4	0.882918	0.5756	no
gi|641570036|gb|KK856480.1|	61913-63262	8.3	33	0.12274	8	6	0.23225	no
gi|641570036|gb|KK856480.1|	65779-66033	8.3	33	7	8	0.331571	0.8379	no
gi|641570039|gb|KK856479.1|	27093-12847	10	40	0.655195	0.544457	-0.267106	1	no
gi|641570039|gb|KK856479.1|	33458-13453	10	40	0.482909	1	1	0.41175	no
gi|641570039|gb|KK856479.1|	13741-14184	10	40	7	18	1	0.30455	no
gi|641570039|gb|KK856479.1|	39434-14059	10	40	0.294796	1	2	0.29935	no
gi|641570039|gb|KK856479.1|	44610-14532	10	40	1	2	0.771004	0.6261	no
gi|641570039|gb|KK856479.1|	14654-17613	10	40	12	27	1	0.4611	no
gi|641570039|gb|KK856479.1|	20496-20980	10	40	6	4	-0.729761	0.6126	no
gi|641570039|gb|KK856479.1|	49293-50761	10	40	0.556319	4	3	0.09085	no
gi|641570039|gb|KK856479.1|	55687-56066	10	40	2	10	3	0.17565	no
gi|641570039|gb|KK856479.1|	59448-60439	10	40	32	8	-2	0.1192	no
gi|641570039|gb|KK856479.1|	65143-66395	10	40	16	6	-2	0.2461	no
gi|641570039|gb|KK856479.1|	72143-72528	10	40	7	0	-inf	0.0034	yes
gi|641570039|gb|KK856479.1|	74452-75433	10	40	18	0	-inf	5.00E-05	yes
gi|641570039|gb|KK856479.1|	77070-79672	10	40	344	0.044898	-13	0.18285	no
gi|641570039|gb|KK856479.1|	86764-87643	10	40	4	0.314733	-4	0.1413	no

gi|641570039|gb|KK856479.1|	90134-90791	10	40	31	0	-inf	5.00E-05	yes
gi|641570039|gb|KK856479.1|	91204-92315	10	40	0.618565	0.588375	-0.0721893	1	no
gi|641570039|gb|KK856479.1|	92555-93316	10	40	1	0.569858	-1	0.47135	no
gi|641570039|gb|KK856479.1|	94887-95537	10	40	35	25	-0.467119	0.70895	no
gi|641570039|gb|KK856479.1|	98521-99757	10	40	12	14	0.246481	0.8424	no
gi|641570042|gb|KK856478.1|	1380-2310	10	36	0.770952	0.439511	-0.810741	1	no
gi|641570042|gb|KK856478.1|	21219-25386	10	36	3	8	2	0.2318	no
gi|641570042|gb|KK856478.1|	26947-27523	10	36	11	6	-0.801663	0.54125	no
gi|641570042|gb|KK856478.1|	27575-32053	10	36	11	6	-0.831452	0.61105	no
gi|641570042|gb|KK856478.1|	40049-41268	10	36	0.691635	0.526551	-0.393438	1	no
gi|641570042|gb|KK856478.1|	46295-47662	10	36	2	0.828493	-1	0.415	no
gi|641570042|gb|KK856478.1|	51357-51912	10	36	1	2	0.373611	0.8276	no
gi|641570042|gb|KK856478.1|	52066-53907	10	36	0.602322	2	2	0.1644	no
gi|641570042|gb|KK856478.1|	54545-56209	10	36	2	0.587932	-2	0.25305	no
gi|641570042|gb|KK856478.1|	56460-57724	10	36	1	1	-0.0561599	0.96335	no
gi|641570042|gb|KK856478.1|	58968-59275	10	36	16	4	-2	0.25045	no
gi|641570042|gb|KK856478.1|	62697-63433	10	36	5	2	-2	0.245	no
gi|641570042|gb|KK856478.1|	72472-72892	10	36	6	0.937654	-3	0.1899	no
gi|641570042|gb|KK856478.1|	79301-79687	10	36	3	1	-2	0.3632	no
gi|641570042|gb|KK856478.1|	82621-83727	10	36	38	9	-2	0.10865	no
gi|641570042|gb|KK856478.1|	85714-86575	10	36	1	0.646383	-0.981497	0.5872	no
gi|641570042|gb|KK856478.1|	88708-89015	10	36	34	3	-4	0.1015	no
gi|641570042|gb|KK856478.1|	90749-91459	10	36	1	0.625824	-0.98408	0.62775	no
gi|641570042|gb|KK856478.1|	92414-95587	10	36	3	0.653398	-2	0.11965	no
gi|641570042|gb|KK856478.1|	95783-98039	10	36	4	2	-1	0.27675	no
gi|641570042|gb|KK856478.1|	98527-99207	10	36	2	0.664172	-2	0.403	no
gi|641570045|gb|KK856477.1|	01915-10213	9.6	43	53	54	0.0325535	0.98185	no
gi|641570045|gb|KK856477.1|	02732-10393	9.6	43	6	4	-0.563477	0.66495	no
gi|641570045|gb|KK856477.1|	04541-10494	9.6	43	19	11	-0.737268	0.572	no
gi|641570045|gb|KK856477.1|	14292-11801	9.6	43	14	15	0.0919724	0.9676	no

gi|641570045|gb|KK856477.1|	21592-12222	9.6	43	12	7	-0.644011	0.62265	no
gi|641570045|gb|KK856477.1|	25191-12583	9.6	43	3	7	1	0.45375	no
gi|641570045|gb|KK856477.1|	32-708	9.6	43	1	2	1	0.4891	no
gi|641570045|gb|KK856477.1|	89636-90058	9.6	43	0	66	inf	5.00E-05	yes
gi|641570045|gb|KK856477.1|	92061-94767	9.6	43	1	4	2	0.2124	no
gi|641570045|gb|KK856477.1|	98398-99066	9.6	43	12	6	-0.914706	0.48655	no
gi|641570045|gb|KK856477.1|	99199-100414	9.6	43	0.902652	0.740051	-0.286545	0.85105	no
gi|641570052|gb|KK856475.1|	27529-28674	13	45	2	1	-0.563812	0.6926	no
gi|641570052|gb|KK856475.1|	32146-35799	13	45	7	4	-0.663135	0.59225	no
gi|641570052|gb|KK856475.1|	36006-38770	13	45	26	23	-0.208327	0.92755	no
gi|641570052|gb|KK856475.1|	44165-44537	13	45	4	4	-0.293329	0.8545	no
gi|641570052|gb|KK856475.1|	49430-50500	13	45	0	2	inf	0.0025	yes
gi|641570052|gb|KK856475.1|	55355-57944	13	45	0.029579	15	9	0.10435	no
gi|641570052|gb|KK856475.1|	58806-59225	13	45	0	6	inf	0.00375	no
gi|641570055|gb|KK856474.1|	11508-12927	8.1	28	0	143.43	inf	0.0005	yes
gi|641570055|gb|KK856474.1|	27681-28339	8.1	28	7	3	-1	0.31015	no
gi|641570058|gb|KK856473.1|	09034-11080	11	40	6	2	-1	0.30415	no
gi|641570058|gb|KK856473.1|	12519-11343	11	40	0.784839	2	1	0.49765	no
gi|641570058|gb|KK856473.1|	13580-11653	11	40	83	89	0.105387	0.95445	no
gi|641570058|gb|KK856473.1|	19298-12133	11	40	6	6	0.119925	0.9239	no
gi|641570058|gb|KK856473.1|	22127-12237	11	40	22	29	0.415403	0.76145	no
gi|641570058|gb|KK856473.1|	31675-13224	11	40	19	18	-0.0328377	0.9788	no
gi|641570058|gb|KK856473.1|	25537-26161	11	40	1	0.499915	-1	0.4534	no
gi|641570058|gb|KK856473.1|	26900-28701	11	40	0.485042	0.336186	-0.52885	1	no
gi|641570058|gb|KK856473.1|	29673-30222	11	40	1	2	0.110308	0.92265	no
gi|641570058|gb|KK856473.1|	55563-55758	11	40	12	17	0.527631	0.7699	no
gi|641570058|gb|KK856473.1|	58249-58765	11	40	0	5	inf	0.00255	yes
gi|641570058|gb|KK856473.1|	64868-65586	11	40	0.677216	2	2	0.32945	no
gi|641570058|gb|KK856473.1|	67446-67670	11	40	3	23	3	0.26765	no
gi|641570058|gb|KK856473.1|	68062-68536	11	40	4	18	2	0.11545	no

gi|641570058|gb|KK856473.1|	71710-76248	11	40	8	7	-0.163958	0.8952	no
gi|641570058|gb|KK856473.1|	76562-79960	11	40	5	3	-0.968523	0.43675	no
gi|641570058|gb|KK856473.1|	85678-88087	11	40	14	7	-0.939594	0.44975	no
gi|641570058|gb|KK856473.1|	88353-88572	11	40	20	16	-0.353362	0.81805	no
gi|641570058|gb|KK856473.1|	91981-92610	11	40	2	0.247112	-3	0.217	no
gi|641570058|gb|KK856473.1|	94443-95149	11	40	2	2	-0.0772786	0.95395	no
gi|641570058|gb|KK856473.1|	99854-10070[illegible]	11	40	0.325927	1	2	0.25595	no
gi|641570064|gb|KK856471.1|	0-277	10	42	0	16	inf	0.00255	yes
gi|641570064|gb|KK856471.1|	53927-55487	10	42	4	0.790753	-2	0.1421	no
gi|641570064|gb|KK856471.1|	56496-56909	10	42	2	4	1	0.5082	no
gi|641570064|gb|KK856471.1|	57053-58084	10	42	0.677779	7	3	0.06965	no
gi|641570064|gb|KK856471.1|	7279-7518	10	42	5	77	4	0.11955	no
gi|641570067|gb|KK856470.1|	32324-32714	10	22	2	3	0.320534	0.86115	no
gi|641570067|gb|KK856470.1|	37739-38316	10	22	1	1	0.111499	0.92265	no
gi|641570067|gb|KK856470.1|	46886-47512	10	22	0.328614	2	3	0.27515	no
gi|641570070|gb|KK856469.1|	27163-28525	9.7	43	57	88	0.620987	0.7774	no
gi|641570070|gb|KK856469.1|	30371-31408	9.7	43	0.757066	2	2	0.2975	no
gi|641570070|gb|KK856469.1|	35989-36965	9.7	43	12	9	-0.51785	0.68205	no
gi|641570070|gb|KK856469.1|	46153-47098	9.7	43	4	3	-0.395543	0.7721	no
gi|641570070|gb|KK856469.1|	50459-51934	9.7	43	0.553276	0.337243	-0.714212	1	no
gi|641570073|gb|KK856468.1|	75309-75985	7.6	33	1	7	2	0.15175	no
gi|641570080|gb|KK856466.1|	[illegible]06712-10805	11	39	1	0.187457	-3	0.20575	no
gi|641570080|gb|KK856466.1|	[illegible]08458-11209	11	39	1	2	0.271132	0.83095	no
gi|641570080|gb|KK856466.1|	[illegible]12439-11346	11	39	1	0.777009	-0.510245	0.7498	no
gi|641570080|gb|KK856466.1|	[illegible]14123-11523	11	39	2	2	-0.252127	0.8512	no
gi|641570080|gb|KK856466.1|	[illegible]15327-11593	11	39	3	2	-0.723759	0.6309	no
gi|641570080|gb|KK856466.1|	[illegible]16040-11765	11	39	2	1	-0.561757	0.6763	no
gi|641570080|gb|KK856466.1|	[illegible]17867-11910	11	39	1	0.20601	-3	0.1793	no
gi|641570080|gb|KK856466.1|	[illegible]19358-12113	11	39	2	4	0.743518	0.56835	no
gi|641570080|gb|KK856466.1|	[illegible]21568-12292	11	39	0.427737	0.744671	0.799879	1	no

gi|641570080|gb|KK856466.1|	26484-12685	11	39	5	0.584177	-3	0.22375	no
gi|641570080|gb|KK856466.1|	28017-13095	11	39	1	0.828118	-0.64136	0.63045	no
gi|641570080|gb|KK856466.1|	26630-27255	11	39	4	5	0.461124	0.74275	no
gi|641570080|gb|KK856466.1|	31467-32371	11	39	22	33	0.565008	0.6456	no
gi|641570080|gb|KK856466.1|	33931-35847	11	39	0.576068	0.878559	0.608899	0.67385	no
gi|641570080|gb|KK856466.1|	37352-38326	11	39	0.818315	0.829616	0.0197872	1	no
gi|641570080|gb|KK856466.1|	42065-42758	11	39	8	12	0.57402	0.65835	no
gi|641570080|gb|KK856466.1|	47492-57216	11	39	13	13	-0.00299261	0.99845	no
gi|641570080|gb|KK856466.1|	64335-65160	11	39	0.787066	1	0.380567	0.82465	no
gi|641570080|gb|KK856466.1|	65284-66475	11	39	32	26	-0.266993	0.8256	no
gi|641570080|gb|KK856466.1|	66775-68745	11	39	47	29	-0.663705	0.76815	no
gi|641570080|gb|KK856466.1|	70864-71565	11	39	30	19	-0.694776	0.57925	no
gi|641570080|gb|KK856466.1|	73696-74983	11	39	12	3	-2	0.1707	no
gi|641570080|gb|KK856466.1|	76737-78230	11	39	1	0.16629	-3	0.1793	no
gi|641570080|gb|KK856466.1|	79953-80296	11	39	12	4	-2	0.32505	no
gi|641570080|gb|KK856466.1|	84780-85192	11	39	3	2	-0.735815	0.67005	no
gi|641570080|gb|KK856466.1|	86864-89598	11	39	0.780906	2	1	0.4471	no
gi|641570080|gb|KK856466.1|	95337-95956	11	39	0	3	inf	0.00375	no
gi|641570080|gb|KK856466.1|	97303-98266	11	39	0.737813	0.841439	0.189604	1	no
gi|641570083|gb|KK856465.1|	31615-32106	9.2	37	1	2	0.329585	0.83525	no
gi|641570086|gb|KK856464.1|	07526-10793	10	35	1	5	2	0.33255	no
gi|641570086|gb|KK856464.1|	50946-51737	10	35	0.950842	3	2	0.2743	no
gi|641570086|gb|KK856464.1|	59059-60562	10	35	0.487287	0.495067	0.0228524	1	no
gi|641570086|gb|KK856464.1|	69861-71092	10	35	0.341811	0.728664	1	1	no
gi|641570086|gb|KK856464.1|	71271-71813	10	35	8	9	0.180372	0.8928	no
gi|641570086|gb|KK856464.1|	78972-80004	10	35	12	22	0.890156	0.484	no
gi|641570086|gb|KK856464.1|	81013-85624	10	35	5	5	-0.0586401	0.96105	no
gi|641570086|gb|KK856464.1|	86034-86553	10	35	3	2	-0.598394	0.68495	no
gi|641570086|gb|KK856464.1|	87861-88701	10	35	3	0.500478	-3	0.1484	no
gi|641570089|gb|KK856463.1|	00375-10075	11	36	0	23	inf	0.0001	yes

<table>
<tr><td>gi|641570089|gb|KK856463.1|</td><td>10142-12608</td><td>11</td><td>36</td><td>3</td><td>2</td><td>-0.103964</td><td>0.93205</td><td>no</td></tr>
<tr><td>gi|641570089|gb|KK856463.1|</td><td>07131-11083</td><td>11</td><td>36</td><td>3</td><td>5</td><td>1</td><td>0.40015</td><td>no</td></tr>
<tr><td>gi|641570089|gb|KK856463.1|</td><td>15898-16880</td><td>11</td><td>36</td><td>10</td><td>6</td><td>-0.68468</td><td>0.5903</td><td>no</td></tr>
<tr><td>gi|641570089|gb|KK856463.1|</td><td>18501-19065</td><td>11</td><td>36</td><td>3</td><td>2</td><td>-0.818645</td><td>0.5995</td><td>no</td></tr>
<tr><td>gi|641570089|gb|KK856463.1|</td><td>19179-19942</td><td>11</td><td>36</td><td>14</td><td>15</td><td>0.0743141</td><td>0.95175</td><td>no</td></tr>
<tr><td>gi|641570089|gb|KK856463.1|</td><td>22636-23270</td><td>11</td><td>36</td><td>2</td><td>4</td><td>0.898467</td><td>0.54035</td><td>no</td></tr>
<tr><td>gi|641570089|gb|KK856463.1|</td><td>26622-27468</td><td>11</td><td>36</td><td>3</td><td>4</td><td>0.0626932</td><td>0.96325</td><td>no</td></tr>
<tr><td>gi|641570089|gb|KK856463.1|</td><td>27651-27979</td><td>11</td><td>36</td><td>25</td><td>17</td><td>-0.546672</td><td>0.6781</td><td>no</td></tr>
<tr><td>gi|641570089|gb|KK856463.1|</td><td>36740-37060</td><td>11</td><td>36</td><td>12</td><td>3</td><td>-2</td><td>0.2617</td><td>no</td></tr>
<tr><td>gi|641570089|gb|KK856463.1|</td><td>40627-40815</td><td>11</td><td>36</td><td>432</td><td>344</td><td>-0.327793</td><td>0.7964</td><td>no</td></tr>
<tr><td>gi|641570089|gb|KK856463.1|</td><td>40947-42728</td><td>11</td><td>36</td><td>4</td><td>3</td><td>-0.391393</td><td>0.7641</td><td>no</td></tr>
<tr><td>gi|641570089|gb|KK856463.1|</td><td>4174-5602</td><td>11</td><td>36</td><td>0.918994</td><td>0.437573</td><td>-1</td><td>0.4973</td><td>no</td></tr>
<tr><td>gi|641570089|gb|KK856463.1|</td><td>44981-46246</td><td>11</td><td>36</td><td>4</td><td>3</td><td>-0.599641</td><td>0.65815</td><td>no</td></tr>
<tr><td>gi|641570089|gb|KK856463.1|</td><td>46718-47379</td><td>11</td><td>36</td><td>22</td><td>24</td><td>0.0906674</td><td>0.94095</td><td>no</td></tr>
<tr><td>gi|641570089|gb|KK856463.1|</td><td>53519-53799</td><td>11</td><td>36</td><td>70</td><td>28</td><td>-1</td><td>0.3256</td><td>no</td></tr>
<tr><td>gi|641570089|gb|KK856463.1|</td><td>53938-54254</td><td>11</td><td>36</td><td>11</td><td>5</td><td>-1</td><td>0.45755</td><td>no</td></tr>
<tr><td>gi|641570089|gb|KK856463.1|</td><td>54411-55488</td><td>11</td><td>36</td><td>0.401511</td><td>2</td><td>2</td><td>0.18135</td><td>no</td></tr>
<tr><td>gi|641570089|gb|KK856463.1|</td><td>60359-60958</td><td>11</td><td>36</td><td>1</td><td>1</td><td>-0.209601</td><td>0.8945</td><td>no</td></tr>
<tr><td>gi|641570089|gb|KK856463.1|</td><td>63239-63566</td><td>11</td><td>36</td><td>4</td><td>3</td><td>-0.233936</td><td>0.88445</td><td>no</td></tr>
<tr><td>gi|641570089|gb|KK856463.1|</td><td>65872-66370</td><td>11</td><td>36</td><td>8</td><td>7</td><td>-0.184554</td><td>0.88795</td><td>no</td></tr>
<tr><td>gi|641570089|gb|KK856463.1|</td><td>71449-72116</td><td>11</td><td>36</td><td>9</td><td>11</td><td>0.237271</td><td>0.85135</td><td>no</td></tr>
<tr><td>gi|641570089|gb|KK856463.1|</td><td>72400-73054</td><td>11</td><td>36</td><td>5</td><td>10</td><td>1</td><td>0.4503</td><td>no</td></tr>
<tr><td>gi|641570089|gb|KK856463.1|</td><td>7709-8427</td><td>11</td><td>36</td><td>20</td><td>12</td><td>-0.79126</td><td>0.53415</td><td>no</td></tr>
<tr><td>gi|641570089|gb|KK856463.1|</td><td>79583-80897</td><td>11</td><td>36</td><td>0</td><td>0.964</td><td>inf</td><td>0.0068</td><td>no</td></tr>
<tr><td>gi|641570089|gb|KK856463.1|</td><td>81002-81647</td><td>11</td><td>36</td><td>22</td><td>9</td><td>-1</td><td>0.34645</td><td>no</td></tr>
<tr><td>gi|641570089|gb|KK856463.1|</td><td>82266-82821</td><td>11</td><td>36</td><td>0</td><td>6</td><td>inf</td><td>0.00145</td><td>yes</td></tr>
<tr><td>gi|641570089|gb|KK856463.1|</td><td>85278-85526</td><td>11</td><td>36</td><td>0</td><td>59</td><td>inf</td><td>5.00E-05</td><td>yes</td></tr>
<tr><td>gi|641570089|gb|KK856463.1|</td><td>86525-87324</td><td>11</td><td>36</td><td>0.586402</td><td>11</td><td>4</td><td>0.0844</td><td>no</td></tr>
<tr><td>gi|641570098|gb|KK856462.1|</td><td>11981-11519</td><td>12</td><td>43</td><td>0.070365</td><td>19</td><td>8</td><td>0.1386</td><td>no</td></tr>
<tr><td>gi|641570098|gb|KK856462.1|</td><td>40004-14082</td><td>12</td><td>43</td><td>1</td><td>2</td><td>0.892392</td><td>0.57435</td><td>no</td></tr>
</table>

gi|641570098|gb|KK856462.1|	20773-21992	12	43	0.622471	1	0.758565	0.6256	no
gi|641570098|gb|KK856462.1|	57320-60177	12	43	20	11	-0.85835	0.5954	no
gi|641570098|gb|KK856462.1|	61095-61858	12	43	2	0.378577	-2	0.25005	no
gi|641570098|gb|KK856462.1|	65856-66052	12	43	16	13	-0.279191	0.8666	no
gi|641570098|gb|KK856462.1|	66263-67697	12	43	86	111	0.375818	0.8575	no
gi|641570098|gb|KK856462.1|	67847-71329	12	43	20	14	-0.493184	0.82805	no
gi|641570098|gb|KK856462.1|	71444-72811	12	43	1	0.644383	-1	0.4842	no
gi|641570098|gb|KK856462.1|	75770-75944	12	43	48	40	-0.261288	0.87105	no
gi|641570098|gb|KK856462.1|	79895-80143	12	43	26	8	-2	0.27655	no
gi|641570098|gb|KK856462.1|	81418-81748	12	43	15	11	-0.377068	0.78365	no
gi|641570098|gb|KK856462.1|	83305-84354	12	43	0	16	inf	5.00E-05	yes
gi|641570104|gb|KK856460.1|	24022-24227	10	41	19	22	0.21093	0.8939	no
gi|641570108|gb|KK856459.1|	16120-16294	9	37	0	79	inf	0.00255	yes
gi|641570108|gb|KK856459.1|	89770-90302	9	37	0	5	inf	0.0023	yes
gi|641570111|gb|KK856458.1|	04411-10540	11	40	5	3	-0.599401	0.6556	no
gi|641570111|gb|KK856458.1|	05758-10605	11	40	18	9	-0.918548	0.511	no
gi|641570111|gb|KK856458.1|	12735-11376	11	40	2	0.642129	-1	0.3598	no
gi|641570111|gb|KK856458.1|	20023-12233	11	40	5	4	-0.419412	0.73685	no
gi|641570111|gb|KK856458.1|	22466-12712	11	40	10	10	0.0191953	0.9927	no
gi|641570111|gb|KK856458.1|	27227-12976	11	40	5	4	-0.424545	0.732	no
gi|641570111|gb|KK856458.1|	29992-13145	11	40	7	11	0.674772	0.59805	no
gi|641570111|gb|KK856458.1|	31628-13288	11	40	5	8	0.774486	0.55075	no
gi|641570111|gb|KK856458.1|	34444-13570	11	40	7	0.303811	-4	0.09595	no
gi|641570111|gb|KK856458.1|	35852-13679	11	40	33	22	-0.577757	0.64345	no
gi|641570111|gb|KK856458.1|	72304-72712	11	40	4	2	-0.792831	0.63015	no
gi|641570111|gb|KK856458.1|	72961-74371	11	40	12	6	-1	0.4025	no
gi|641570111|gb|KK856458.1|	78165-79862	11	40	0.990118	0.862572	-0.198956	0.8879	no
gi|641570111|gb|KK856458.1|	82985-85007	11	40	11	4	-1	0.24725	no
gi|641570111|gb|KK856458.1|	85941-87063	11	40	0.916835	0.697707	-0.394041	0.80865	no
gi|641570111|gb|KK856458.1|	97743-98183	11	40	2	1	-0.82622	0.64915	no

gi|641570111|gb|KK856458.1|	99323-10139	11	40	0.64205	0.403273	-0.670926	1	no
gi|641570114|gb|KK856457.1|	00869-10216	11	39	0.064501	2	5	0.10875	no
gi|641570114|gb|KK856457.1|	11803-11286	11	39	12	9	-0.435155	0.73355	no
gi|641570114|gb|KK856457.1|	14346-11504	11	39	14	13	-0.0599502	0.96305	no
gi|641570114|gb|KK856457.1|	57625-59093	11	39	0	2	inf	0.0015	yes
gi|641570114|gb|KK856457.1|	62015-62577	11	39	0.192835	47	8	0.10435	no
gi|641570114|gb|KK856457.1|	74941-75153	11	39	0	69	inf	0.00035	yes
gi|641570114|gb|KK856457.1|	78000-78195	11	39	0	34	inf	0.0068	no
gi|641570114|gb|KK856457.1|	80986-81281	11	39	0	13	inf	0.00255	yes
gi|641570114|gb|KK856457.1|	99991-10074	11	39	0.127867	2	4	0.1175	no
gi|641570117|gb|KK856456.1|	11979-12899	9.1	30	0.78159	0.74257	-0.0738861	1	no
gi|641570117|gb|KK856456.1|	26354-26775	9.1	30	4	16	2	0.1639	no
gi|641570117|gb|KK856456.1|	28029-28416	9.1	30	19	105	2	0.07155	no
gi|641570117|gb|KK856456.1|	43103-45096	9.1	30	9	6	-0.715916	0.57115	no
gi|641570117|gb|KK856456.1|	46642-47601	9.1	30	0.648968	50	6	0.0648	no
gi|641570117|gb|KK856456.1|	61429-62449	9.1	30	1	0.26119	-2	0.26805	no
gi|641570117|gb|KK856456.1|	62830-64778	9.1	30	43	42	-0.0331238	0.98685	no
gi|641570117|gb|KK856456.1|	72247-72949	9.1	30	8	9	0.245608	0.84875	no
gi|641570117|gb|KK856456.1|	7665-8442	9.1	30	0.121715	7	6	0.10515	no
gi|641570117|gb|KK856456.1|	9571-10122	9.1	30	0.198757	5	5	0.1123	no
gi|641570121|gb|KK856455.1|	00856-10315	10	40	1	3	1	0.3195	no
gi|641570121|gb|KK856455.1|	06867-10798	10	40	7	17	1	0.34185	no
gi|641570121|gb|KK856455.1|	15420-16825	10	40	0.468233	0.802556	0.777374	1	no
gi|641570121|gb|KK856455.1|	17536-20969	10	40	131	334	1	0.44175	no
gi|641570121|gb|KK856455.1|	21196-25901	10	40	3	3	0.0551256	0.9667	no
gi|641570121|gb|KK856455.1|	26763-31270	10	40	26	31	0.274221	0.89515	no
gi|641570121|gb|KK856455.1|	32548-34378	10	40	2	1	-0.391281	0.77215	no
gi|641570121|gb|KK856455.1|	35013-35756	10	40	74	45	-0.725412	0.65715	no
gi|641570121|gb|KK856455.1|	37114-37503	10	40	6	8	0.490333	0.7326	no
gi|641570121|gb|KK856455.1|	38011-38731	10	40	174	135	-0.365682	0.8667	no

gi|641570121|gb|KK856455.1|	39617-40567	10	40	39	72	0.891162	0.57935	no
gi|641570121|gb|KK856455.1|	40829-41447	10	40	40	61	0.604426	0.6287	no
gi|641570121|gb|KK856455.1|	41856-42120	10	40	59	76	0.376721	0.7686	no
gi|641570121|gb|KK856455.1|	43159-43501	10	40	127	218	0.778153	0.52365	no
gi|641570121|gb|KK856455.1|	44865-45441	10	40	64	103	0.681656	0.68305	no
gi|641570121|gb|KK856455.1|	45655-45931	10	40	120	151	0.330849	0.7878	no
gi|641570121|gb|KK856455.1|	47035-47318	10	40	79	109	0.467191	0.70665	no
gi|641570121|gb|KK856455.1|	48307-48634	10	40	91	107	0.226969	0.85515	no
gi|641570121|gb|KK856455.1|	49404-49614	10	40	81	85	0.0562107	0.9663	no
gi|641570121|gb|KK856455.1|	50369-50560	10	40	49	37	-0.40051	0.7797	no
gi|641570121|gb|KK856455.1|	56126-56787	10	40	1	0.921302	-0.207674	0.8945	no
gi|641570121|gb|KK856455.1|	62022-64266	10	40	0.656639	1	1	0.4277	no
gi|641570121|gb|KK856455.1|	77551-80592	10	40	0.845945	2	0.844723	0.5329	no
gi|641570121|gb|KK856455.1|	81115-81313	10	40	168.41	211	0.325692	0.79625	no
gi|641570121|gb|KK856455.1|	83746-84632	10	40	2	4	0.80542	0.56165	no
gi|641570121|gb|KK856455.1|	86126-87699	10	40	0.51392	2	2	0.22295	no
gi|641570121|gb|KK856455.1|	89178-90847	10	40	0.576531	1	1	0.4764	no
gi|641570121|gb|KK856455.1|	91125-92563	10	40	1	2	0.385186	0.78455	no
gi|641570121|gb|KK856455.1|	93070-94638	10	40	0.464213	0.471695	0.0230663	1	no
gi|641570121|gb|KK856455.1|	95048-96435	10	40	1	0.905152	-0.392625	0.78685	no
gi|641570121|gb|KK856455.1|	99276-100252	10	40	0.725527	3	2	0.1998	no
gi|641570123|gb|KK856454.1|	03800-10446	11	38	0.300761	5	4	0.24015	no
gi|641570123|gb|KK856454.1|	06366-10688	11	38	7	9	0.304745	0.81865	no
gi|641570123|gb|KK856454.1|	07758-10840	11	38	2	2	0.599204	0.67805	no
gi|641570123|gb|KK856454.1|	09884-11151	11	38	6	7	0.379585	0.76295	no
gi|641570123|gb|KK856454.1|	11643-11711	11	38	1	1	0.0903841	0.94335	no
gi|641570123|gb|KK856454.1|	18128-11863	11	38	2	2	0.231186	0.88235	no
gi|641570123|gb|KK856454.1|	21885-12218	11	38	2	10	2	0.2454	no
gi|641570123|gb|KK856454.1|	26050-12713	11	38	4	8	1	0.3846	no
gi|641570123|gb|KK856454.1|	28744-12956	11	38	5	3	-1	0.45605	no

gi|641570123|gb|KK856454.1|	34456-13520	11	38	2	3	0.0541776	0.9656	no
gi|641570123|gb|KK856454.1|	36722-13920	11	38	6	2	-1	0.26775	no
gi|641570123|gb|KK856454.1|	39315-13982	11	38	10	4	-1	0.38565	no
gi|641570123|gb|KK856454.1|	39981-14215	11	38	17	13	-0.324659	0.7933	no
gi|641570123|gb|KK856454.1|	14843-15195	11	38	0	38	inf	5.00E-05	yes
gi|641570123|gb|KK856454.1|	48615-15151	11	38	8	6	-0.287971	0.81815	no
gi|641570123|gb|KK856454.1|	15322-16245	11	38	0	3	inf	0.00115	yes
gi|641570123|gb|KK856454.1|	57468-15814	11	38	3	1	-1	0.41725	no
gi|641570123|gb|KK856454.1|	58903-16055	11	38	3	1	-2	0.22735	no
gi|641570123|gb|KK856454.1|	69080-16935	11	38	13	2	-3	0.20825	no
gi|641570123|gb|KK856454.1|	71769-17193	11	38	80	19	-2	0.2438	no
gi|641570123|gb|KK856454.1|	19582-19868	11	38	0	982	inf	0.00025	yes
gi|641570123|gb|KK856454.1|	23926-25559	11	38	1	0.375358	-1	0.3598	no
gi|641570123|gb|KK856454.1|	25825-27092	11	38	2	0.301834	-3	0.13665	no
gi|641570123|gb|KK856454.1|	29080-29474	11	38	4	2	-0.737865	0.63835	no
gi|641570123|gb|KK856454.1|	32582-34839	11	38	18	3	-2	0.1915	no
gi|641570123|gb|KK856454.1|	35272-35926	11	38	1	1	-0.0785832	0.92675	no
gi|641570123|gb|KK856454.1|	37039-40403	11	38	2	1	-0.606341	0.6397	no
gi|641570123|gb|KK856454.1|	41910-42543	11	38	4	3	-0.117331	0.9332	no
gi|641570123|gb|KK856454.1|	43160-43484	11	38	6	3	-1	0.5083	no
gi|641570123|gb|KK856454.1|	4406-5168	11	38	1	23	4	0.03005	no
gi|641570123|gb|KK856454.1|	46955-48567	11	38	0.399818	1	2	0.2733	no
gi|641570123|gb|KK856454.1|	48669-49663	11	38	4	2	-1	0.4599	no
gi|641570123|gb|KK856454.1|	54014-54512	11	38	10	6	-0.811337	0.54905	no
gi|641570123|gb|KK856454.1|	54733-55289	11	38	2	1	-0.251949	0.88485	no
gi|641570123|gb|KK856454.1|	55409-56696	11	38	0.843121	1	0.365913	0.8038	no
gi|641570123|gb|KK856454.1|	63783-64027	11	38	19	9	-1	0.49415	no
gi|641570123|gb|KK856454.1|	71746-74021	11	38	30	14	-1	0.502	no
gi|641570123|gb|KK856454.1|	7457-7816	11	38	0	186	inf	5.00E-05	yes
gi|641570123|gb|KK856454.1|	75527-77785	11	38	1	0.837923	-0.712459	0.5957	no

gi|641570123|gb|KK856454.1|	78107-79288	11	38	2	2	-0.130625	0.9265	no
gi|641570123|gb|KK856454.1|	79422-81565	11	38	15	5	-2	0.21745	no
gi|641570123|gb|KK856454.1|	84132-84560	11	38	117	38	-2	0.20365	no
gi|641570123|gb|KK856454.1|	84943-85383	11	38	126	35	-2	0.1479	no
gi|641570123|gb|KK856454.1|	85815-86757	11	38	1	0.288325	-2	0.3217	no
gi|641570123|gb|KK856454.1|	88131-88682	11	38	62	21	-2	0.21205	no
gi|641570123|gb|KK856454.1|	88888-89670	11	38	5	2	-1	0.378	no
gi|641570123|gb|KK856454.1|	95019-95561	11	38	15	6	-1	0.3135	no
gi|641570123|gb|KK856454.1|	95782-100057	11	38	13	7	-0.886381	0.5844	no
gi|641570123|gb|KK856454.1|	998-1289	11	38	3	17	3	0.17855	no
gi|641570124|gb|KK856453.1|	02946-10400	8.7	40	18	35	0.970259	0.44095	no
gi|641570124|gb|KK856453.1|	04110-10528	8.7	40	6	16	1	0.2918	no
gi|641570124|gb|KK856453.1|	07452-11123	8.7	40	14	3	-2	0.20305	no
gi|641570124|gb|KK856453.1|	10870-11109	8.7	40	41	8	-2	0.1685	no
gi|641570124|gb|KK856453.1|	11487-11250	8.7	40	2	1	-1	0.459	no
gi|641570124|gb|KK856453.1|	14783-11577	8.7	40	1	0.808948	-0.510538	0.7498	no
gi|641570124|gb|KK856453.1|	16562-11690	8.7	40	12	8	-0.538654	0.7023	no
gi|641570124|gb|KK856453.1|	17497-11845	8.7	40	10	6	-0.613274	0.6302	no
gi|641570124|gb|KK856453.1|	22380-12280	8.7	40	146	170	0.225103	0.889	no
gi|641570124|gb|KK856453.1|	28882-13024	8.7	40	0.425899	2	2	0.17405	no
gi|641570124|gb|KK856453.1|	37526-14095	8.7	40	0.065768	17	8	0.13775	no
gi|641570124|gb|KK856453.1|	43046-14407	8.7	40	0.170055	3	4	0.24015	no
gi|641570124|gb|KK856453.1|	55067-15576	8.7	40	63	110	0.791516	0.6228	no
gi|641570124|gb|KK856453.1|	82622-18320	8.7	40	13	25	0.975595	0.447	no
gi|641570124|gb|KK856453.1|	85620-18673	8.7	40	9	9	0.0782603	0.9496	no
gi|641570124|gb|KK856453.1|	86966-18727	8.7	40	29	41	0.507183	0.7002	no
gi|641570124|gb|KK856453.1|	87876-19031	8.7	40	35	11	-2	0.3097	no
gi|641570124|gb|KK856453.1|	91169-19449	8.7	40	4	4	-0.0776061	0.94775	no
gi|641570124|gb|KK856453.1|	94939-19813	8.7	40	2	4	0.803013	0.529	no
gi|641570124|gb|KK856453.1|	98566-19919	8.7	40	1	7	2	0.15525	no

gi|641570124|gb|KK856453.1|	05837-20601	8.7	40	24	15	-0.703334	0.6977	no
gi|641570124|gb|KK856453.1|	3838-4063	8.7	40	19	4	-2	0.24525	no
gi|641570124|gb|KK856453.1|	43463-44120	8.7	40	3	0.696896	-2	0.21545	no
gi|641570124|gb|KK856453.1|	46797-48113	8.7	40	0.252709	1	3	0.1983	no
gi|641570124|gb|KK856453.1|	53220-54070	8.7	40	0.757012	1	0.603317	0.69715	no
gi|641570124|gb|KK856453.1|	55743-56967	8.7	40	0.412964	0.943203	1	0.47015	no
gi|641570124|gb|KK856453.1|	57105-57878	8.7	40	1	0.186036	-3	0.24815	no
gi|641570124|gb|KK856453.1|	57985-58374	8.7	40	1	4	2	0.3454	no
gi|641570124|gb|KK856453.1|	64774-65684	8.7	40	0.198132	2	4	0.24715	no
gi|641570124|gb|KK856453.1|	72790-74625	8.7	40	0.906788	0.526772	-0.783588	0.5969	no
gi|641570124|gb|KK856453.1|	75166-76320	8.7	40	0.81238	2	1	0.47895	no
gi|641570124|gb|KK856453.1|	76815-79465	8.7	40	2	3	0.657233	0.6163	no
gi|641570124|gb|KK856453.1|	79821-82116	8.7	40	1	0.977534	-0.142553	0.9211	no
gi|641570124|gb|KK856453.1|	7994-9295	8.7	40	0.192103	1	3	0.1811	no
gi|641570124|gb|KK856453.1|	82417-83451	8.7	40	0.675354	0.642129	-0.0727806	1	no
gi|641570124|gb|KK856453.1|	85933-86682	8.7	40	1	0.194037	-3	0.2382	no
gi|641570124|gb|KK856453.1|	88037-89185	8.7	40	0.520225	1	0.968703	0.5733	no
gi|641570124|gb|KK856453.1|	90006-91363	8.7	40	0.548528	1	0.896754	0.5684	no
gi|641570124|gb|KK856453.1|	92270-92782	8.7	40	0.446002	3	3	0.28535	no
gi|641570124|gb|KK856453.1|	93493-93758	8.7	40	10	4	-1	0.4178	no
gi|641570124|gb|KK856453.1|	95872-97465	8.7	40	13	8	-0.733683	0.5562	no
gi|641570134|gb|KK856450.1|	01956-10263	10	38	4	3	-0.557518	0.6924	no
gi|641570134|gb|KK856450.1|	03018-10449	10	38	0.554142	1	0.870743	0.56565	no
gi|641570134|gb|KK856450.1|	05119-10782	10	38	0.873521	0.516078	-0.759251	1	no
gi|641570134|gb|KK856450.1|	08382-10859	10	38	54	60	0.151554	0.91265	no
gi|641570134|gb|KK856450.1|	08737-10929	10	38	8	8	-0.00666068	0.98325	no
gi|641570134|gb|KK856450.1|	11003-11207	10	38	4	4	0.11372	0.93065	no
gi|641570134|gb|KK856450.1|	12183-11335	10	38	0.506923	0.661465	0.383899	1	no
gi|641570134|gb|KK856450.1|	13697-11444	10	38	1	2	0.739226	0.6176	no
gi|641570134|gb|KK856450.1|	14582-11515	10	38	2	0.849558	-0.988197	0.62775	no

gi|641570134|gb|KK856450.1|	15910-11672	10	38	0.343311	2	2	0.25595	no
gi|641570134|gb|KK856450.1|	16926-11782	10	38	0.901685	2	0.893485	0.5684	no
gi|641570134|gb|KK856450.1|	11733-12296	10	38	0.576941	2	2	0.3323	no
gi|641570134|gb|KK856450.1|	20564-12272	10	38	22	33	0.566475	0.8033	no
gi|641570134|gb|KK856450.1|	23544-12681	10	38	1	2	0.5035	0.6968	no
gi|641570134|gb|KK856450.1|	28276-12853	10	38	0	80	inf	5.00E-05	yes
gi|641570134|gb|KK856450.1|	29603-13046	10	38	0.317167	13	5	0.13525	no
gi|641570134|gb|KK856450.1|	30898-13140	10	38	0.675329	56	6	0.1346	no
gi|641570134|gb|KK856450.1|	32814-13298	10	38	0	156	inf	0.00065	yes
gi|641570134|gb|KK856450.1|	36516-13675	10	38	0	25	inf	0.0023	yes
gi|641570134|gb|KK856450.1|	39135-14272	10	38	0.835509	25	5	0.0297	no
gi|641570134|gb|KK856450.1|	42845-14322	10	38	15	27	0.819728	0.5372	no
gi|641570134|gb|KK856450.1|	44438-14606	10	38	3	4	0.221192	0.8668	no
gi|641570134|gb|KK856450.1|	24046-25003	10	38	1	4	2	0.2535	no
gi|641570134|gb|KK856450.1|	64515-67939	10	38	7	4	-0.838253	0.5064	no
gi|641570134|gb|KK856450.1|	68134-69173	10	38	0.923109	0.255337	-2	0.3217	no
gi|641570134|gb|KK856450.1|	82624-84708	10	38	9	7	-0.396844	0.7498	no
gi|641570134|gb|KK856450.1|	84906-85297	10	38	4	2	-1	0.5083	no
gi|641570134|gb|KK856450.1|	85425-86193	10	38	2	0.750593	-1	0.50315	no
gi|641570134|gb|KK856450.1|	86360-90058	10	38	2	3	0.700285	0.5851	no
gi|641570134|gb|KK856450.1|	92710-94936	10	38	0.871657	0.691519	-0.333992	1	no
gi|641570134|gb|KK856450.1|	95858-96428	10	38	7	5	-0.688759	0.61165	no
gi|641570134|gb|KK856450.1|	98484-99083	10	38	4	2	-0.817284	0.57245	no
gi|641570135|gb|KK856449.1|	35851-37010	9.7	34	5	7	0.470852	0.7151	no
gi|641570135|gb|KK856449.1|	38459-39609	9.7	34	0.593297	0.451555	-0.393854	1	no
gi|641570135|gb|KK856449.1|	40053-41448	9.7	34	0.826238	1	0.500493	0.73235	no
gi|641570135|gb|KK856449.1|	41549-42071	9.7	34	6	7	0.179374	0.8961	no
gi|641570135|gb|KK856449.1|	42552-43171	9.7	34	3	3	-0.0796374	0.95295	no
gi|641570135|gb|KK856449.1|	43392-45196	9.7	34	1	0.805345	-0.613732	0.65835	no
gi|641570135|gb|KK856449.1|	49764-50106	9.7	34	14	3	-2	0.18365	no

gi|641570135|gb|KK856449.1|	56017-56523	9.7	34	2	2	0.400882	0.8089	no
gi|641570135|gb|KK856449.1|	60611-62709	9.7	34	2	0.908233	-1	0.38315	no
gi|641570135|gb|KK856449.1|	72390-75375	9.7	34	2	2	-0.245394	0.8489	no
gi|641570135|gb|KK856449.1|	76071-77945	9.7	34	1	1	0.023851	0.9781	no
gi|641570135|gb|KK856449.1|	79259-79984	9.7	34	2	1	-0.720722	0.6521	no
gi|641570135|gb|KK856449.1|	80695-82009	9.7	34	2	3	0.370013	0.78515	no
gi|641570135|gb|KK856449.1|	85943-86431	9.7	34	65	74	0.188038	0.87855	no
gi|641570138|gb|KK856448.1|	16655-16919	10	21	4	9	1	0.51325	no
gi|641570138|gb|KK856448.1|	62938-64181	10	21	14	11	-0.319305	0.79825	no
gi|641570138|gb|KK856448.1|	72478-73236	10	21	2	2	-0.108641	0.9412	no
gi|641570138|gb|KK856448.1|	80157-81456	10	21	1	2	0.960617	0.49165	no
gi|641570138|gb|KK856448.1|	89599-89829	10	21	23	6	-2	0.2465	no
gi|641570141|gb|KK856447.1|	03540-10372	11	40	27	9	-2	0.363	no
gi|641570141|gb|KK856447.1|	05818-10619	11	40	6	9	0.581849	0.699	no
gi|641570141|gb|KK856447.1|	11772-12379	11	40	0.515583	2	2	0.256	no
gi|641570141|gb|KK856447.1|	20204-12072	11	40	1	2	0.594516	0.70555	no
gi|641570141|gb|KK856447.1|	23242-12445	11	40	0.347515	183	9	0.08275	no
gi|641570141|gb|KK856447.1|	25410-12845	11	40	34	34	-0.0107441	0.9959	no
gi|641570141|gb|KK856447.1|	28689-12980	11	40	1	0.349605	-2	0.33985	no
gi|641570141|gb|KK856447.1|	36543-13679	11	40	48	27	-0.813168	0.5423	no
gi|641570141|gb|KK856447.1|	37153-13769	11	40	72	36	-1	0.42525	no
gi|641570141|gb|KK856447.1|	45002-14533	11	40	4	4	0.159182	0.918	no
gi|641570141|gb|KK856447.1|	15954-16479	11	40	1	2	0.857603	0.62755	no
gi|641570141|gb|KK856447.1|	37539-38416	11	40	0.934752	2	1	0.47685	no
gi|641570141|gb|KK856447.1|	38467-39504	11	40	0.504711	1	1	0.3778	no
gi|641570141|gb|KK856447.1|	50410-51711	11	40	0.576308	0.877763	0.60699	0.6902	no
gi|641570141|gb|KK856447.1|	57284-57833	11	40	2	1	-0.404265	0.82205	no
gi|641570141|gb|KK856447.1|	60589-61899	11	40	0.69869	0.773957	0.147601	1	no
gi|641570141|gb|KK856447.1|	63294-64979	11	40	0.712924	0.43475	-0.713561	1	no
gi|641570141|gb|KK856447.1|	74670-75363	11	40	0.568949	3	2	0.2135	no

gi|641570141|gb|KK856447.1|	81233-84176	11	40	4	2	-0.933438	0.46625	no
gi|641570141|gb|KK856447.1|	86541-86746	11	40	81	56	-0.537531	0.6926	no
gi|641570141|gb|KK856447.1|	91281-91541	11	40	8	11	0.386069	0.81245	no
gi|641570141|gb|KK856447.1|	93146-93279	11	40	156	183	0.234309	0.88335	no
gi|641570144|gb|KK856446.1|	87163-88017	8.8	43	0.752415	3	2	0.20995	no
gi|641570148|gb|KK856445.1|	05298-10584	9.4	38	2	2	0.180743	0.91015	no
gi|641570148|gb|KK856445.1|	28666-28909	9.4	38	14	0	-inf	0.0075	no
gi|641570152|gb|KK856444.1|	05587-10697	9.9	40	0.415544	0.994835	1	0.43995	no
gi|641570152|gb|KK856444.1|	15396-11647	9.9	40	2	0	-inf	0.0022	yes
gi|641570152|gb|KK856444.1|	16579-11789	9.9	40	2	0.483291	-2	0.2061	no
gi|641570152|gb|KK856444.1|	35052-37174	9.9	40	49	19	-1	0.53455	no
gi|641570152|gb|KK856444.1|	41496-44462	9.9	40	30	22	-0.482907	0.83055	no
gi|641570152|gb|KK856444.1|	46828-47582	9.9	40	3	1	-1	0.36935	no
gi|641570152|gb|KK856444.1|	48565-49583	9.9	40	2	0.785467	-1	0.48805	no
gi|641570152|gb|KK856444.1|	49757-50708	9.9	40	0.936911	1	0.452516	0.76315	no
gi|641570152|gb|KK856444.1|	51010-55425	9.9	40	4	4	-0.0874089	0.9434	no
gi|641570152|gb|KK856444.1|	57375-58171	9.9	40	3	7	1	0.3487	no
gi|641570152|gb|KK856444.1|	58501-58673	9.9	40	38	30	-0.332744	0.83535	no
gi|641570152|gb|KK856444.1|	66687-67286	9.9	40	7	7	-0.00491087	0.98325	no
gi|641570152|gb|KK856444.1|	70668-71018	9.9	40	10	5	-0.945873	0.527	no
gi|641570152|gb|KK856444.1|	74345-74988	9.9	40	0.158087	17	7	0.10435	no
gi|641570152|gb|KK856444.1|	77599-78309	9.9	40	0	17	inf	5.00E-05	yes
gi|641570152|gb|KK856444.1|	90155-90811	9.9	40	2	1	-0.537959	0.7478	no
gi|641570152|gb|KK856444.1|	90867-92183	9.9	40	0.884481	0.769829	-0.200293	0.8959	no
gi|641570152|gb|KK856444.1|	92260-92971	9.9	40	89	90	0.0126143	0.9944	no
gi|641570155|gb|KK856443.1|	23432-12373	10	20	36	29	-0.28628	0.8256	no
gi|641570155|gb|KK856443.1|	26918-12787	10	20	0.648119	2	2	0.3164	no
gi|641570155|gb|KK856443.1|	21-583	10	20	3	0.583071	-3	0.20575	no
gi|641570155|gb|KK856443.1|	3354-3565	10	20	30	13	-1	0.44045	no
gi|641570155|gb|KK856443.1|	42440-43427	10	20	0.804878	0.136011	-3	1	no

gi|641570155|gb|KK856443.1|	51263-52425	10	20	0.732415	0.33448	-1	1	no
gi|641570155|gb|KK856443.1|	52569-53260	10	20	2	0.649579	-2	0.33235	no
gi|641570155|gb|KK856443.1|	61869-63007	10	20	0.450637	0.914555	1	0.5082	no
gi|641570155|gb|KK856443.1|	72787-73378	10	20	0.893892	1	0.597463	0.70505	no
gi|641570155|gb|KK856443.1|	75223-76243	10	20	0.944376	2	0.84618	0.58475	no
gi|641570155|gb|KK856443.1|	94821-96241	10	20	0.520311	2	2	0.2366	no
gi|641570159|gb|KK856442.1|	62026-62760	10	41	2	0	-inf	0.00435	no
gi|641570163|gb|KK856441.1|	.03957-10461	8.4	38	1	6	2	0.1472	no
gi|641570163|gb|KK856441.1|	.14715-11552	8.4	38	13	36	1	0.25325	no
gi|641570163|gb|KK856441.1|	29807-30008	8.4	38	19	9	-1	0.52775	no
gi|641570163|gb|KK856441.1|	36116-36733	8.4	38	0	4	inf	0.0015	yes
gi|641570163|gb|KK856441.1|	5811-6264	8.4	38	5	1	-2	0.23215	no
gi|641570163|gb|KK856441.1|	61403-61650	8.4	38	20	14	-0.526552	0.71375	no
gi|641570163|gb|KK856441.1|	63845-64415	8.4	38	5	4	-0.457805	0.7504	no
gi|641570163|gb|KK856441.1|	8103-8984	8.4	38	26	16	-0.689635	0.5767	no
gi|641570163|gb|KK856441.1|	9184-11043	8.4	38	3	3	0.25648	0.8444	no
gi|641570166|gb|KK856440.1|	90549-90821	11	37	10	0	-inf	0.01	no
gi|641570169|gb|KK856439.1|	.02146-10272	10	36	2	0.280266	-3	0.271	no
gi|641570169|gb|KK856439.1|	12142-14247	10	36	0	52	inf	0.0003	yes
gi|641570169|gb|KK856439.1|	.28470-13051	10	36	0.843943	0.994732	0.237163	0.86645	no
gi|641570169|gb|KK856439.1|	.31883-13286	10	36	0.902286	0.686088	-0.395192	0.81255	no
gi|641570169|gb|KK856439.1|	.35845-13714	10	36	5	7	0.524518	0.684	no
gi|641570169|gb|KK856439.1|	.37364-13961	10	36	6	5	-0.220623	0.86345	no
gi|641570169|gb|KK856439.1|	.41528-14172	10	36	17	56	2	0.2951	no
gi|641570169|gb|KK856439.1|	23085-23572	10	36	2	0.364659	-3	0.2481	no
gi|641570169|gb|KK856439.1|	24539-24771	10	36	39	39	-0.0163869	0.9777	no
gi|641570169|gb|KK856439.1|	32759-33562	10	36	1	0.707659	-0.85681	0.60675	no
gi|641570169|gb|KK856439.1|	34572-35403	10	36	2	2	0.188011	0.8991	no
gi|641570169|gb|KK856439.1|	35600-39179	10	36	0.711442	0.862641	0.278014	1	no
gi|641570169|gb|KK856439.1|	47316-47508	10	36	78	40	-0.96874	0.50155	no

gi|641570169|gb|KK856439.1|	50068-50360	10	36	7	8	0.105919	0.94315	no
gi|641570169|gb|KK856439.1|	55385-55732	10	36	7	10	0.577173	0.699	no
gi|641570169|gb|KK856439.1|	55862-58000	10	36	1	0.94517	-0.1106	0.9375	no
gi|641570169|gb|KK856439.1|	60072-61135	10	36	0.652783	0.993232	0.605526	0.6936	no
gi|641570169|gb|KK856439.1|	63427-64500	10	36	0.806682	0.859222	0.0910306	1	no
gi|641570169|gb|KK856439.1|	64848-65315	10	36	8	9	0.0504174	0.97075	no
gi|641570169|gb|KK856439.1|	65589-66198	10	36	2	2	0.598102	0.6902	no
gi|641570169|gb|KK856439.1|	76750-79131	10	36	5	4	-0.202509	0.87325	no
gi|641570169|gb|KK856439.1|	79316-79917	10	36	6	4	-0.44657	0.7467	no
gi|641570169|gb|KK856439.1|	83567-85527	10	36	3	3	-0.0458794	0.96865	no
gi|641570169|gb|KK856439.1|	87991-88147	10	36	118	125	0.0913088	0.94955	no
gi|641570169|gb|KK856439.1|	94046-94357	10	36	6	11	0.810986	0.59295	no
gi|641570169|gb|KK856439.1|	95673-98854	10	36	0.877421	0.543041	-0.692207	0.61015	no
gi|641570169|gb|KK856439.1|	98978-99580	10	36	2	2	0.138428	0.929	no
gi|641570169|gb|KK856439.1|	99686-10005[illegible]	10	36	9	11	0.227647	0.872	no
gi|641570176|gb|KK856437.1|	[illegible]00036-10057	11	40	1	3	1	0.5082	no
gi|641570176|gb|KK856437.1|	102-724	11	40	1	0.753341	-0.816506	0.6492	no
gi|641570176|gb|KK856437.1|	13808-14134	11	40	6	5	-0.301254	0.8533	no
gi|641570176|gb|KK856437.1|	15761-18251	11	40	6	3	-0.932768	0.4573	no
gi|641570176|gb|KK856437.1|	18306-25317	11	40	10	4	-1	0.4623	no
gi|641570176|gb|KK856437.1|	25395-29878	11	40	12	11	-0.153387	0.9465	no
gi|641570176|gb|KK856437.1|	33161-33982	11	40	25	14	-0.799198	0.5233	no
gi|641570176|gb|KK856437.1|	36927-39013	11	40	33	16	-1	0.53915	no
gi|641570176|gb|KK856437.1|	42938-43981	11	40	0.584661	0.508276	-0.201989	1	no
gi|641570176|gb|KK856437.1|	44769-45952	11	40	1	1	-0.393648	0.78685	no
gi|641570176|gb|KK856437.1|	55375-56900	11	40	7	4	-0.766506	0.55055	no
gi|641570176|gb|KK856437.1|	57013-58412	11	40	5	3	-0.550117	0.66915	no
gi|641570176|gb|KK856437.1|	58589-59341	11	40	0.890249	0.964996	0.116314	0.92175	no
gi|641570176|gb|KK856437.1|	60271-61059	11	40	10	5	-1	0.38555	no
gi|641570176|gb|KK856437.1|	61260-61727	11	40	2	2	-0.216369	0.8945	no

gi|641570176|gb|KK856437.1|	61849-65334	11	40	9	4	-1	0.41485	no
gi|641570176|gb|KK856437.1|	65447-69785	11	40	6	5	-0.161857	0.8946	no
gi|641570176|gb|KK856437.1|	73516-74886	11	40	2	1	-0.615089	0.65815	no
gi|641570176|gb|KK856437.1|	75290-77083	11	40	4	2	-1	0.43465	no
gi|641570176|gb|KK856437.1|	77148-78665	11	40	2	2	-0.0110471	0.9843	no
gi|641570176|gb|KK856437.1|	78879-79520	11	40	2	0.962158	-1	0.5005	no
gi|641570176|gb|KK856437.1|	79961-81065	11	40	2	2	-0.348363	0.7968	no
gi|641570176|gb|KK856437.1|	85726-86445	11	40	0.540739	1	1	0.51305	no
gi|641570176|gb|KK856437.1|	90050-92299	11	40	2	0.946786	-0.68005	0.62365	no
gi|641570176|gb|KK856437.1|	93388-94778	11	40	0.711168	2	1	0.45455	no
gi|641570176|gb|KK856437.1|	95501-96469	11	40	0.824669	0.418012	-0.980272	1	no
gi|641570179|gb|KK856436.1|	17329-11833	9.3	37	1	0.795083	-0.510411	0.7498	no
gi|641570179|gb|KK856436.1|	45271-49868	9.3	37	5	10	0.834001	0.61015	no
gi|641570179|gb|KK856436.1|	50005-52306	9.3	37	4	3	-0.123188	0.92435	no
gi|641570179|gb|KK856436.1|	53028-57022	9.3	37	26	13	-1	0.6396	no
gi|641570179|gb|KK856436.1|	62608-64302	9.3	37	0.614157	2	1	0.3257	no
gi|641570179|gb|KK856436.1|	65891-66512	9.3	37	66	47	-0.510364	0.68495	no
gi|641570179|gb|KK856436.1|	67185-68988	9.3	37	4	6	0.742959	0.5589	no
gi|641570179|gb|KK856436.1|	70826-71334	9.3	37	7	11	0.593642	0.66145	no
gi|641570179|gb|KK856436.1|	74642-74802	9.3	37	415	295	-0.491431	0.70875	no
gi|641570179|gb|KK856436.1|	79352-79490	9.3	37	240	417	0.797068	0.5875	no
gi|641570179|gb|KK856436.1|	84497-85012	9.3	37	0.883722	16	4	0.1195	no
gi|641570179|gb|KK856436.1|	91419-92801	9.3	37	0	2	inf	0.00115	yes
gi|641570182|gb|KK856435.1|	34008-13448	10	36	2	1	-0.577957	0.7586	no
gi|641570182|gb|KK856435.1|	13489-13723	10	36	49	104	1	0.40625	no
gi|641570182|gb|KK856435.1|	14174-16476	10	36	1	0.051279	-5	0.1868	no
gi|641570182|gb|KK856435.1|	55781-15634	10	36	2	4	1	0.38865	no
gi|641570182|gb|KK856435.1|	67305-16783	10	36	1	3	1	0.4611	no
gi|641570182|gb|KK856435.1|	20497-21468	10	36	0.730204	0.277602	-1	1	no
gi|641570182|gb|KK856435.1|	21596-23371	10	36	23	21	-0.144524	0.93105	no

gi|641570182|gb|KK856435.1|	23621-25044	10	36	1	0.351479	-2	0.31425	no
gi|641570182|gb|KK856435.1|	25841-27360	10	36	0.267443	6	5	0.08295	no
gi|641570182|gb|KK856435.1|	28492-32106	10	36	17	7	-1	0.42455	no
gi|641570182|gb|KK856435.1|	3254-4174	10	36	2	3	0.537072	0.70395	no
gi|641570182|gb|KK856435.1|	38887-39565	10	36	0.586599	3	2	0.2441	no
gi|641570182|gb|KK856435.1|	40940-41707	10	36	0.123844	2	4	0.12385	no
gi|641570182|gb|KK856435.1|	41859-43081	10	36	0.758575	1	0.73211	0.63585	no
gi|641570182|gb|KK856435.1|	4291-6656	10	36	0.62002	2	2	0.2813	no
gi|641570182|gb|KK856435.1|	46814-47527	10	36	0.957248	2	0.963519	0.5733	no
gi|641570182|gb|KK856435.1|	50698-51465	10	36	0.743061	2	1	0.4118	no
gi|641570182|gb|KK856435.1|	52451-53378	10	36	0.483821	3	2	0.18135	no
gi|641570182|gb|KK856435.1|	55284-56933	10	36	0.292232	0.96522	2	0.30625	no
gi|641570182|gb|KK856435.1|	61796-62243	10	36	0.279528	13	6	0.1054	no
gi|641570182|gb|KK856435.1|	7023-9090	10	36	0.605251	0.519324	-0.220898	1	no
gi|641570182|gb|KK856435.1|	73093-73602	10	36	3	61	4	0.0172	no
gi|641570182|gb|KK856435.1|	75209-75794	10	36	0.363034	5	4	0.24305	no
gi|641570182|gb|KK856435.1|	77911-81333	10	36	2	0.469031	-2	0.18735	no
gi|641570182|gb|KK856435.1|	83432-83821	10	36	7	92	4	0.023	no
gi|641570182|gb|KK856435.1|	86503-87242	10	36	0.911519	21	4	0.05345	no
gi|641570182|gb|KK856435.1|	89877-90079	10	36	0	77	inf	0.00065	yes
gi|641570182|gb|KK856435.1|	91556-91782	10	36	3	94	5	0.23345	no
gi|641570182|gb|KK856435.1|	92566-92743	10	36	0	78	inf	0.0023	yes
gi|641570182|gb|KK856435.1|	95355-95649	10	36	0	126	inf	5.00E-05	yes
gi|641570182|gb|KK856435.1|	96058-96772	10	36	0	3	inf	0.00255	yes
gi|641570185|gb|KK856434.1|	12861-14839	8.3	32	3	2	-0.82394	0.52965	no
gi|641570185|gb|KK856434.1|	16953-17501	8.3	32	2	2	-0.667345	0.68195	no
gi|641570185|gb|KK856434.1|	18589-19156	8.3	32	2	2	-0.140447	0.9254	no
gi|641570185|gb|KK856434.1|	29094-29337	8.3	32	17	6	-1	0.387	no
gi|641570185|gb|KK856434.1|	29572-29942	8.3	32	12	5	-1	0.4247	no
gi|641570185|gb|KK856434.1|	4776-5084	8.3	32	5	3	-1	0.5278	no

gi|641570185|gb|KK856434.1|	55668-56305	8.3	32	1	2	0.598997	0.69715	no
gi|641570185|gb|KK856434.1|	6262-7007	8.3	32	1	0.586311	-0.81343	0.6492	no
gi|641570185|gb|KK856434.1|	7244-7853	8.3	32	3	4	0.235532	0.86855	no
gi|641570185|gb|KK856434.1|	78517-79265	8.3	32	0.896688	1	0.379273	0.82465	no
gi|641570185|gb|KK856434.1|	81183-84671	8.3	32	30	20	-0.597676	0.78285	no
gi|641570185|gb|KK856434.1|	84871-85296	8.3	32	2	4	1	0.40145	no
gi|641570188|gb|KK856433.1|	27675-12931	10	38	0.785226	0.67331	-0.221837	1	no
gi|641570188|gb|KK856433.1|	37313-37828	10	38	2	1	-0.628358	0.71175	no
gi|641570188|gb|KK856433.1|	50016-50454	10	38	0	6	inf	0.0025	yes
gi|641570188|gb|KK856433.1|	50551-51119	10	38	0	4	inf	0.0023	yes
gi|641570188|gb|KK856433.1|	53838-54436	10	38	0.35137	6	4	0.2397	no
gi|641570188|gb|KK856433.1|	58346-59018	10	38	0.742459	1	0.599972	0.70505	no
gi|641570188|gb|KK856433.1|	62400-64067	10	38	0.865972	2	1	0.2979	no
gi|641570188|gb|KK856433.1|	64245-65379	10	38	1	2	0.24347	0.86475	no
gi|641570188|gb|KK856433.1|	65433-66113	10	38	0.438137	3	3	0.19335	no
gi|641570188|gb|KK856433.1|	66359-68072	10	38	3	8	1	0.2695	no
gi|641570188|gb|KK856433.1|	68150-69470	10	38	1	5	2	0.1806	no
gi|641570188|gb|KK856433.1|	69620-71401	10	38	0	65	inf	0.0002	yes
gi|641570188|gb|KK856433.1|	72597-73638	10	38	0.083721	2	5	0.10875	no
gi|641570188|gb|KK856433.1|	75232-75803	10	38	3	6	0.886191	0.52805	no
gi|641570188|gb|KK856433.1|	76101-77480	10	38	12	12	0.0543222	0.96515	no
gi|641570188|gb|KK856433.1|	77633-79958	10	38	5	6	0.113602	0.92935	no
gi|641570188|gb|KK856433.1|	80173-89136	10	38	6	4	-0.656157	0.67935	no
gi|641570188|gb|KK856433.1|	92575-92893	10	38	7	6	-0.321555	0.84315	no
gi|641570188|gb|KK856433.1|	93688-95212	10	38	1	1	0.303032	0.8279	no
gi|641570188|gb|KK856433.1|	95784-96674	10	38	0.509589	0.774448	0.603835	1	no
gi|641570191|gb|KK856432.1|	11004-14297	9.7	38	7	1	-2.77	0.04575	no
gi|641570191|gb|KK856432.1|	15419-17121	9.7	38	1	0.358215	-2	0.3482	no
gi|641570191|gb|KK856432.1|	26407-26994	9.7	38	7	4	-0.665721	0.6249	no
gi|641570191|gb|KK856432.1|	31056-33795	9.7	38	1	0.424833	-2	0.2556	no

gi|641570191|gb|KK856432.1|	60765-61276	9.7	38	0.894797	3	2	0.33255	no
gi|641570191|gb|KK856432.1|	62223-62550	9.7	38	3	6	1	0.4655	no
gi|641570191|gb|KK856432.1|	6924-8345	9.7	38	22	7	-2	0.21225	no
gi|641570191|gb|KK856432.1|	8791-10397	9.7	38	11	3	-2	0.15585	no
gi|641570194|gb|KK856431.1|	0-230	8.8	34	8	119	4	0.06795	no
gi|641570198|gb|KK856430.1|	01559-10249	10	36	0	19	inf	5.00E-05	yes
gi|641570198|gb|KK856430.1|	03564-10412	10	36	0	14	inf	0.0001	yes
gi|641570198|gb|KK856430.1|	06651-10684	10	36	0	313	inf	0.0001	yes
gi|641570198|gb|KK856430.1|	11956-13789	10	36	3	4	0.379907	0.76035	no
gi|641570198|gb|KK856430.1|	1490-1640	10	36	393	258	-0.606781	0.65315	no
gi|641570198|gb|KK856430.1|	18913-19744	10	36	0.33413	4	4	0.14485	no
gi|641570198|gb|KK856430.1|	21098-21656	10	36	5	15	1	0.2685	no
gi|641570198|gb|KK856430.1|	34152-34942	10	36	2	4	1	0.4556	no
gi|641570198|gb|KK856430.1|	37270-39040	10	36	0.494591	0.342787	-0.528923	1	no
gi|641570198|gb|KK856430.1|	40540-48319	10	36	5	8	0.661176	0.77355	no
gi|641570198|gb|KK856430.1|	48794-50044	10	36	1	3	1	0.36565	no
gi|641570198|gb|KK856430.1|	5091-6354	10	36	25	27	0.0607565	0.9609	no
gi|641570198|gb|KK856430.1|	52477-52931	10	36	0	20	inf	0.00025	yes
gi|641570198|gb|KK856430.1|	53149-53492	10	36	33	9	-2	0.18345	no
gi|641570198|gb|KK856430.1|	58375-58860	10	36	3	6	1	0.4764	no
gi|641570198|gb|KK856430.1|	59552-61177	10	36	4	9	1	0.40805	no
gi|641570198|gb|KK856430.1|	62519-65005	10	36	2	6	1	0.30925	no
gi|641570198|gb|KK856430.1|	67172-67852	10	36	0.292091	3	3	0.25475	no
gi|641570198|gb|KK856430.1|	77272-77566	10	36	12	19	0.71787	0.6129	no
gi|641570198|gb|KK856430.1|	80544-82729	10	36	0.854034	2	0.882382	0.52575	no
gi|641570198|gb|KK856430.1|	8169-8853	10	36	1	0.878388	-0.721653	0.67005	no
gi|641570198|gb|KK856430.1|	84302-84665	10	36	50	81	0.693451	0.58645	no
gi|641570198|gb|KK856430.1|	90623-91451	10	36	3	5	0.657452	0.63225	no
gi|641570198|gb|KK856430.1|	91911-94262	10	36	2	3	0.959535	0.4626	no
gi|641570198|gb|KK856430.1|	9455-10651	10	36	0.566025	1	0.928358	0.56225	no

gi|641570198|gb|KK856430.1|	95946-97009	10	36	0.571185	1514.06	11	0.0516	no
gi|641570198|gb|KK856430.1|	97658-98868	10	36	0.348885	2	3	0.14425	no
gi|641570198|gb|KK856430.1|	99835-100166	10	36	0.499856	103	8	0.10435	no
gi|641570201|gb|KK856429.1|	10606-11144	8.5	42	0.885442	1	0.188068	0.91015	no
gi|641570201|gb|KK856429.1|	15710-11607	8.5	42	2	3	0.580403	0.76735	no
gi|641570201|gb|KK856429.1|	18338-11878	8.5	42	0.279528	6	4	0.11365	no
gi|641570201|gb|KK856429.1|	20899-12155	8.5	42	0.463926	6	4	0.14445	no
gi|641570201|gb|KK856429.1|	76649-77110	8.5	42	0	5	inf	0.0025	yes
gi|641570201|gb|KK856429.1|	78015-78214	8.5	42	22	3	-3	0.2472	no
gi|641570201|gb|KK856429.1|	84242-84719	8.5	42	2	3	0.399051	0.8089	no
gi|641570201|gb|KK856429.1|	92971-93742	8.5	42	0.36895	1	2	0.3323	no
gi|641570207|gb|KK856427.1|	11829-11550	11	40	34	23	-0.552281	0.7956	no
gi|641570207|gb|KK856427.1|	15795-11658	11	40	0.947661	0.539563	-0.812578	0.6492	no
gi|641570207|gb|KK856427.1|	17385-12161	11	40	2	2	0.302748	0.8031	no
gi|641570207|gb|KK856427.1|	23242-12471	11	40	2	2	-0.357518	0.7864	no
gi|641570207|gb|KK856427.1|	56112-15693	11	40	1	2	0.843968	0.58475	no
gi|641570207|gb|KK856427.1|	57208-15832	11	40	4	1	-1	0.344	no
gi|641570207|gb|KK856427.1|	64921-16544	11	40	3	0.652993	-2	0.24735	no
gi|641570207|gb|KK856427.1|	65685-16663	11	40	0.469076	1	1	0.3994	no
gi|641570207|gb|KK856427.1|	88060-89298	11	40	2	1	-0.878759	0.52735	no
gi|641570210|gb|KK856426.1|	04062-10483	11	40	0.737901	0.933389	0.339051	0.8352	no
gi|641570210|gb|KK856426.1|	18542-11955	11	40	1	0.656939	-0.880298	0.5832	no
gi|641570210|gb|KK856426.1|	19643-12049	11	40	1	0.659176	-0.718639	0.6701	no
gi|641570210|gb|KK856426.1|	21466-12276	11	40	0.837749	0.196296	-2	1	no
gi|641570210|gb|KK856426.1|	24201-12501	11	40	1	0.523894	-0.982215	0.62775	no
gi|641570210|gb|KK856426.1|	21693-23931	11	40	0.519922	1	1	0.33615	no
gi|641570210|gb|KK856426.1|	56622-56827	11	40	68	31	-1	0.42505	no
gi|641570210|gb|KK856426.1|	68767-68945	11	40	35	10	-2	0.33925	no
gi|641570210|gb|KK856426.1|	72391-72659	11	40	11	4	-2	0.4022	no
gi|641570210|gb|KK856426.1|	73340-75762	11	40	0.445162	3	3	0.09605	no

gi|641570210|gb|KK856426.1|	92242-94502	11	40	0.96018	1	0.445973	0.7428	no
gi|641570216|gb|KK856424.1|	25999-27087	9.9	37	1	0.844851	-0.674391	0.6649	no
gi|641570216|gb|KK856424.1|	27761-28341	9.9	37	2	1	-0.724884	0.67005	no
gi|641570216|gb|KK856424.1|	37466-39548	9.9	37	1	0.801428	-0.670906	0.6364	no
gi|641570216|gb|KK856424.1|	40042-40382	9.9	37	3	3	-0.00903586	0.91115	no
gi|641570216|gb|KK856424.1|	44313-45293	9.9	37	0.902286	0	-inf	0.01175	no
gi|641570219|gb|KK856423.1|	11949-11422	9.4	20	138	0.883454	-7	0.0077	no
gi|641570219|gb|KK856423.1|	14771-11670	9.4	20	23	1	-4	0.06885	no
gi|641570219|gb|KK856423.1|	37740-13829	9.4	20	0.795026	2	1	0.44255	no
gi|641570219|gb|KK856423.1|	18048-19495	9.4	20	2	2	0.0930397	0.9439	no
gi|641570219|gb|KK856423.1|	25705-27310	9.4	20	1	0.612467	-0.85129	0.5652	no
gi|641570219|gb|KK856423.1|	28134-30030	9.4	20	0.374685	0.952357	1	0.3858	no
gi|641570219|gb|KK856423.1|	41584-41803	9.4	20	12	20	0.709647	0.65735	no
gi|641570219|gb|KK856423.1|	53614-53909	9.4	20	10	16	0.746695	0.6008	no
gi|641570224|gb|KK856421.1|	73166-73639	11	43	1	5	2	0.214	no
gi|641570224|gb|KK856421.1|	75030-76575	11	43	0.734415	1	0.707491	0.6229	no
gi|641570224|gb|KK856421.1|	81170-82624	11	43	0.731259	1	0.607639	0.681	no
gi|641570227|gb|KK856420.1|	07438-10770	10	37	8	4	-1	0.5278	no
gi|641570227|gb|KK856420.1|	10418-11078	10	37	4	4	0.217683	0.8957	no
gi|641570227|gb|KK856420.1|	11537-11210	10	37	1	1	0.111134	0.92265	no
gi|641570227|gb|KK856420.1|	14912-11564	10	37	2	1	-0.884158	0.5831	no
gi|641570227|gb|KK856420.1|	16756-11737	10	37	20	17	-0.211807	0.86415	no
gi|641570227|gb|KK856420.1|	17451-12109	10	37	8	13	0.649003	0.68925	no
gi|641570227|gb|KK856420.1|	21887-12230	10	37	3	0.464889	-3	0.2381	no
gi|641570227|gb|KK856420.1|	28356-12861	10	37	12	3	-2	0.26435	no
gi|641570227|gb|KK856420.1|	29814-13003	10	37	39	21	-0.891848	0.53135	no
gi|641570227|gb|KK856420.1|	31588-13180	10	37	36	46	0.325835	0.8152	no
gi|641570227|gb|KK856420.1|	34960-13518	10	37	20	13	-0.681281	0.657	no
gi|641570227|gb|KK856420.1|	38102-13893	10	37	2	2	-0.686502	0.6386	no
gi|641570227|gb|KK856420.1|	16516-16941	10	37	0	60	inf	5.00E-05	yes

gi|641570227|gb|KK856420.1|	1866-3265	10	37	0.294105	1	2	0.2077	no
gi|641570227|gb|KK856420.1|	39372-39739	10	37	11	6	-0.905024	0.5194	no
gi|641570227|gb|KK856420.1|	41537-44278	10	37	5	3	-0.873919	0.48845	no
gi|641570227|gb|KK856420.1|	44525-47001	10	37	15	14	-0.182452	0.8801	no
gi|641570227|gb|KK856420.1|	63179-65499	10	37	0.633152	1	1	0.409	no
gi|641570227|gb|KK856420.1|	97420-98352	10	37	1	0.438325	-1	0.4312	no
gi|641570238|gb|KK856417.1|	06077-10673	9.3	38	34	8	-2	0.1185	no
gi|641570238|gb|KK856417.1|	07573-10776	9.3	38	105	16	-3	0.12805	no
gi|641570238|gb|KK856417.1|	34255-35997	9.3	38	4	1	-2	0.2428	no
gi|641570238|gb|KK856417.1|	81360-81557	9.3	38	20	16	-0.3193	0.8588	no
gi|641570244|gb|KK856415.1|	00695-10123	10	34	68	32	-1	0.3862	no
gi|641570244|gb|KK856415.1|	03743-10429	10	34	4	0.911222	-2	0.28145	no
gi|641570244|gb|KK856415.1|	07231-10799	10	34	0.995972	0.944791	-0.0761105	0.92685	no
gi|641570244|gb|KK856415.1|	08163-10880	10	34	1	1	0.376664	0.8276	no
gi|641570244|gb|KK856415.1|	09934-11214	10	34	13	11	-0.140979	0.9108	no
gi|641570244|gb|KK856415.1|	13200-11533	10	34	52	4	-4	0.06485	no
gi|641570244|gb|KK856415.1|	19333-20166	10	34	1	0.337291	-2	0.31075	no
gi|641570244|gb|KK856415.1|	20351-20757	10	34	5	2	-0.999503	0.57205	no
gi|641570244|gb|KK856415.1|	22415-25392	10	34	3	2	-0.809015	0.53185	no
gi|641570244|gb|KK856415.1|	25894-26747	10	34	3	3	0.266323	0.8441	no
gi|641570244|gb|KK856415.1|	28470-29612	10	34	2	2	0.173134	0.89795	no
gi|641570244|gb|KK856415.1|	49812-50478	10	34	18	18	-0.0207715	0.9857	no
gi|641570244|gb|KK856415.1|	56163-56704	10	34	1	2	0.332328	0.83525	no
gi|641570244|gb|KK856415.1|	58420-58801	10	34	8	3	-1	0.4264	no
gi|641570244|gb|KK856415.1|	60984-62796	10	34	1	1	-0.391321	0.77885	no
gi|641570244|gb|KK856415.1|	63112-63593	10	34	2	2	0.0070055	0.9394	no
gi|641570244|gb|KK856415.1|	63781-64398	10	34	1	1	0.335335	0.83525	no
gi|641570244|gb|KK856415.1|	64512-65887	10	34	0.660193	1	0.732856	0.63585	no
gi|641570244|gb|KK856415.1|	66238-67248	10	34	7	8	0.165799	0.89935	no
gi|641570244|gb|KK856415.1|	68375-71154	10	34	21	27	0.394258	0.85805	no

gi|641570244|gb|KK856415.1|	71619-75113	10	34	1	1	0.211994	0.87105	no
gi|641570244|gb|KK856415.1|	75217-75812	10	34	2	5	1	0.4102	no
gi|641570244|gb|KK856415.1|	78308-79432	10	34	21	45	1	0.50385	no
gi|641570244|gb|KK856415.1|	81835-83353	10	34	2	2	0.224538	0.86845	no
gi|641570244|gb|KK856415.1|	84712-85743	10	34	2	1	-0.854166	0.56515	no
gi|641570244|gb|KK856415.1|	91360-91593	10	34	29	42	0.545911	0.6914	no
gi|641570245|gb|KK856414.1|	13572-15454	11	41	14	9	-0.639097	0.60745	no
gi|641570245|gb|KK856414.1|	18512-18901	11	41	12	19	0.665904	0.62325	no
gi|641570245|gb|KK856414.1|	20312-21083	11	41	1	0.373355	-2	0.3455	no
gi|641570245|gb|KK856414.1|	21547-21744	11	41	79	39	-1	0.4634	no
gi|641570245|gb|KK856414.1|	23092-23216	11	41	446	235	-0.921225	0.63345	no
gi|641570245|gb|KK856414.1|	27712-27965	11	41	28	33	0.219195	0.8682	no
gi|641570245|gb|KK856414.1|	30540-31000	11	41	17	13	-0.365145	0.7824	no
gi|641570245|gb|KK856414.1|	31361-31741	11	41	56	43	-0.39863	0.74885	no
gi|641570245|gb|KK856414.1|	39476-41641	11	41	4	1	-2	0.1887	no
gi|641570245|gb|KK856414.1|	41785-43775	11	41	5	2	-1	0.29745	no
gi|641570245|gb|KK856414.1|	51959-52885	11	41	15	22	0.537582	0.66475	no
gi|641570245|gb|KK856414.1|	62107-64837	11	41	18	25	0.449538	0.8446	no
gi|641570258|gb|KK856410.1|	10006-11245	8.4	41	0	99	inf	0.00015	yes
gi|641570258|gb|KK856410.1|	03467-10471	8.4	41	0.537045	2	2	0.34655	no
gi|641570258|gb|KK856410.1|	11742-11256	8.4	41	1	0.515576	-1	0.47135	no
gi|641570258|gb|KK856410.1|	11343-12219	8.4	41	0.416056	0.948337	1	0.51305	no
gi|641570258|gb|KK856410.1|	42652-14344	8.4	41	0.596278	5	3	0.11245	no
gi|641570258|gb|KK856410.1|	49949-15017	8.4	41	13	4	-2	0.3632	no
gi|641570258|gb|KK856410.1|	53386-15357	8.4	41	3	38	4	0.12905	no
gi|641570258|gb|KK856410.1|	98623-99584	8.4	41	0.36987	1	2	0.33105	no
gi|641570261|gb|KK856409.1|	06143-10681	11	21	0.887236	1	0.336989	0.83525	no
gi|641570261|gb|KK856409.1|	24957-25387	11	21	76	57	-0.417758	0.73585	no
gi|641570261|gb|KK856409.1|	25723-26976	11	21	34	21	-0.712321	0.6616	no
gi|641570261|gb|KK856409.1|	30217-30990	11	21	2	0.558109	-2	0.3026	no

gi|641570261|gb|KK856409.1|	31372-32010	11	21	4	3	-0.159965	0.9151	no
gi|641570261|gb|KK856409.1|	37524-38648	11	21	25	10	-1	0.3002	no
gi|641570261|gb|KK856409.1|	65008-65282	11	21	21	9	-1	0.42075	no
gi|641570261|gb|KK856409.1|	76185-76825	11	21	2	0.241074	-3	0.22425	no
gi|641570264|gb|KK856408.1|	12860-13649	9.1	20	0.477023	2	2	0.33105	no
gi|641570264|gb|KK856408.1|	19417-19937	9.1	20	0.217563	3	4	0.12385	no
gi|641570264|gb|KK856408.1|	24793-26195	9.1	20	0.469397	1	1	0.38845	no
gi|641570264|gb|KK856408.1|	82647-83087	9.1	20	7	0.432142	-4	0.19145	no
gi|641570267|gb|KK856407.1|	10955-11207	9.5	34	0.536557	0.349981	-0.616454	1	no
gi|641570267|gb|KK856407.1|	14224-11564	9.5	34	2	0.175455	-3	0.1505	no
gi|641570267|gb|KK856407.1|	60688-61427	9.5	34	14	17	0.238921	0.84415	no
gi|641570267|gb|KK856407.1|	64485-66474	9.5	34	0.039473	84	11	0.10535	no
gi|641570267|gb|KK856407.1|	70035-70985	9.5	34	0	25	inf	5.00E-05	yes
gi|641570267|gb|KK856407.1|	75223-76884	9.5	34	0	38	inf	0.00035	yes
gi|641570267|gb|KK856407.1|	83981-85724	9.5	34	0	43	inf	0.00015	yes
gi|641570267|gb|KK856407.1|	87725-88249	9.5	34	0	47	inf	5.00E-05	yes
gi|641570267|gb|KK856407.1|	89323-90012	9.5	34	0	21	inf	5.00E-05	yes
gi|641570267|gb|KK856407.1|	92265-92677	9.5	34	0	52	inf	5.00E-05	yes
gi|641570267|gb|KK856407.1|	95445-95689	9.5	34	0	180.37	inf	5.00E-05	yes
gi|641570267|gb|KK856407.1|	96863-97276	9.5	34	0	204	inf	0.0002	yes
gi|641570267|gb|KK856407.1|	99467-100241	9.5	34	0	130	inf	0.00025	yes
gi|641570271|gb|KK856405.1|	07035-10818	9.8	38	0.668159	0.678039	0.0211768	1	no
gi|641570271|gb|KK856405.1|	08324-10942	9.8	38	4	5	0.471489	0.72145	no
gi|641570271|gb|KK856405.1|	12050-11273	9.8	38	4	7	0.737852	0.58305	no
gi|641570271|gb|KK856405.1|	13515-11508	9.8	38	0.97858	0.863519	-0.180461	0.8996	no
gi|641570271|gb|KK856405.1|	15525-11675	9.8	38	55	15	-2	0.2855	no
gi|641570271|gb|KK856405.1|	17153-11795	9.8	38	16	6	-1	0.27515	no
gi|641570271|gb|KK856405.1|	20704-12093	9.8	38	0	54	inf	0.00035	yes
gi|641570271|gb|KK856405.1|	24509-12516	9.8	38	0	75	inf	5.00E-05	yes
gi|641570271|gb|KK856405.1|	24049-24944	9.8	38	5	2	-1	0.33735	no

gi|641570271|gb|KK856405.1|	26577-26916	9.8	38	2	4	0.575743	0.76735	no
gi|641570271|gb|KK856405.1|	27597-27954	9.8	38	3	3	-0.421152	0.7868	no
gi|641570271|gb|KK856405.1|	30173-30478	9.8	38	10	2	-3	0.20945	no
gi|641570271|gb|KK856405.1|	43124-44955	9.8	38	7	3	-1	0.3558	no
gi|641570271|gb|KK856405.1|	54056-56052	9.8	38	10	7	-0.56959	0.64765	no
gi|641570271|gb|KK856405.1|	59500-60290	9.8	38	3	0.723022	-2	0.18545	no
gi|641570271|gb|KK856405.1|	66365-68594	9.8	38	0.974816	4	2	0.117	no
gi|641570271|gb|KK856405.1|	68808-69224	9.8	38	3	3	-0.287937	0.86025	no
gi|641570271|gb|KK856405.1|	71143-71837	9.8	38	2	0.215237	-3	0.217	no
gi|641570271|gb|KK856405.1|	86755-86964	9.8	38	20	18	-0.117194	0.93375	no
gi|641570271|gb|KK856405.1|	94857-96168	9.8	38	1	2	0.944073	0.49745	no
gi|641570271|gb|KK856405.1|	)8155-101832	9.8	38	13	19	0.547547	0.8116	no
gi|641570274|gb|KK856404.1|	.01732-10198	9.1	35	18	15	-0.213715	0.88455	no
gi|641570274|gb|KK856404.1|	.04309-10509	9.1	35	1	1	0.142962	0.929	no
gi|641570274|gb|KK856404.1|	.07159-10883	9.1	35	46	41	-0.139758	0.94935	no
gi|641570274|gb|KK856404.1|	.12616-11401	9.1	35	0.527635	0.35728	-0.562484	1	no
gi|641570274|gb|KK856404.1|	.20762-12100	9.1	35	33	59	0.844435	0.52595	no
gi|641570274|gb|KK856404.1|	.21900-12263	9.1	35	13	12	-0.121075	0.92485	no
gi|641570274|gb|KK856404.1|	.23811-12442	9.1	35	2	2	-0.0538736	0.94545	no
gi|641570274|gb|KK856404.1|	.41209-14246	9.1	35	1	0.507777	-1	0.4198	no
gi|641570274|gb|KK856404.1|	.44569-14533	9.1	35	0.370236	4	3	0.1487	no
gi|641570274|gb|KK856404.1|	.45922-14669	9.1	35	0.487699	1	1	0.44255	no
gi|641570274|gb|KK856404.1|	23615-24663	9.1	35	16	11	-0.433693	0.72675	no
gi|641570274|gb|KK856404.1|	29828-30384	9.1	35	2	2	-0.278421	0.86185	no
gi|641570274|gb|KK856404.1|	34188-35503	9.1	35	0.442635	1	1	0.43995	no
gi|641570274|gb|KK856404.1|	42264-43402	9.1	35	5	4	-0.393933	0.7605	no
gi|641570274|gb|KK856404.1|	47072-47267	9.1	35	38	58	0.615094	0.6668	no
gi|641570274|gb|KK856404.1|	48218-49119	9.1	35	2	2	-0.280556	0.83825	no
gi|641570274|gb|KK856404.1|	49965-50920	9.1	35	1	1	0.0740457	0.95315	no
gi|641570274|gb|KK856404.1|	55648-56471	9.1	35	1	0.171315	-3	0.24815	no

gi|641570274|gb|KK856404.1|	56571-56927	9.1	35	4	4	-0.00627666	0.9394	no
gi|641570274|gb|KK856404.1|	57452-61485	9.1	35	2	2	-0.00154316	0.9959	no
gi|641570274|gb|KK856404.1|	61995-63456	9.1	35	1	0.852403	-0.318334	0.82575	no
gi|641570274|gb|KK856404.1|	63771-66537	9.1	35	16	12	-0.450506	0.84765	no
gi|641570274|gb|KK856404.1|	66653-66931	9.1	35	8	8	-0.0905297	0.94215	no
gi|641570274|gb|KK856404.1|	70752-71132	9.1	35	0	8	inf	0.00255	yes
gi|641570274|gb|KK856404.1|	73327-73619	9.1	35	1	107	6	0.2322	no
gi|641570274|gb|KK856404.1|	74862-75286	9.1	35	0.306838	7	4	0.1123	no
gi|641570274|gb|KK856404.1|	81919-87139	9.1	35	12	9	-0.28672	0.8996	no
gi|641570274|gb|KK856404.1|	90397-90678	9.1	35	18	10	-0.852642	0.55535	no
gi|641570274|gb|KK856404.1|	93708-94378	9.1	35	1	2	0.59992	0.69715	no
gi|641570280|gb|KK856402.1|	0-259	11	40	6	9	0.555663	0.74515	no
gi|641570280|gb|KK856402.1|	02366-10255	11	40	55	7	-3	0.16975	no
gi|641570280|gb|KK856402.1|	05869-10606	11	40	170.98	4	-6	0.1831	no
gi|641570280|gb|KK856402.1|	06432-10667	11	40	26	5	-3	0.18095	no
gi|641570280|gb|KK856402.1|	07702-10826	11	40	28	6	-2	0.10245	no
gi|641570280|gb|KK856402.1|	09508-10978	11	40	60	15	-2	0.15435	no
gi|641570280|gb|KK856402.1|	11295-11165	11	40	13	7	-0.915438	0.51915	no
gi|641570280|gb|KK856402.1|	12682-11341	11	40	8	4	-1	0.41335	no
gi|641570280|gb|KK856402.1|	14590-11478	11	40	52	3	-4	0.1967	no
gi|641570280|gb|KK856402.1|	15820-11869	11	40	0.87323	0.686641	-0.346805	1	no
gi|641570280|gb|KK856402.1|	19240-12037	11	40	1	1	-0.0220177	0.963	no
gi|641570280|gb|KK856402.1|	20510-12126	11	40	1	0.766533	-0.568109	0.7586	no
gi|641570280|gb|KK856402.1|	26131-12774	11	40	0.796264	0.531026	-0.584465	1	no
gi|641570280|gb|KK856402.1|	27909-12868	11	40	1	0.745426	-0.857328	0.60675	no
gi|641570280|gb|KK856402.1|	30645-13120	11	40	3	0.293898	-4	0.20475	no
gi|641570280|gb|KK856402.1|	31344-13252	11	40	1	1	-0.0216669	0.963	no
gi|641570280|gb|KK856402.1|	33513-13496	11	40	3	1	-1	0.34255	no
gi|641570280|gb|KK856402.1|	35150-13881	11	40	59	15	-2	0.37435	no
gi|641570280|gb|KK856402.1|	49130-14965	11	40	50	34	-0.550745	0.66315	no

gi|641570280|gb|KK856402.1|	49799-15056	11	40	64	37	-0.793732	0.6295	no
gi|641570280|gb|KK856402.1|	55514-15655	11	40	6	2	-1	0.29795	no
gi|641570280|gb|KK856402.1|	58321-15890	11	40	8	1	-3	0.1197	no
gi|641570280|gb|KK856402.1|	59032-15992	11	40	1	0.616032	-0.718045	0.6701	no
gi|641570280|gb|KK856402.1|	60040-16095	11	40	2	0.750811	-2	0.3475	no
gi|641570280|gb|KK856402.1|	25133-25867	11	40	1	0.797578	-0.720537	0.67005	no
gi|641570280|gb|KK856402.1|	3074-3551	11	40	3	1	-2	0.40265	no
gi|641570280|gb|KK856402.1|	33644-34269	11	40	1	1	0.18359	0.91015	no
gi|641570280|gb|KK856402.1|	35686-36087	11	40	2	5	0.947463	0.57385	no
gi|641570280|gb|KK856402.1|	39768-40341	11	40	0.936262	1	0.596766	0.70505	no
gi|641570280|gb|KK856402.1|	63128-63599	11	40	1	3	1	0.51205	no
gi|641570280|gb|KK856402.1|	65040-65965	11	40	0.485147	1	1	0.4595	no
gi|641570280|gb|KK856402.1|	66949-68329	11	40	0.537878	0.273144	-0.977616	1	no
gi|641570280|gb|KK856402.1|	70371-72495	11	40	0.660784	0.336006	-0.975692	1	no
gi|641570280|gb|KK856402.1|	88102-88287	11	40	83	127	0.621809	0.6419	no
gi|641570280|gb|KK856402.1|	89613-89831	11	40	37	5	-3	0.16085	no
gi|641570280|gb|KK856402.1|	91586-91995	11	40	19	3	-3	0.10205	no
gi|641570280|gb|KK856402.1|	96691-96980	11	40	89	11	-3	0.0517	no
gi|641570280|gb|KK856402.1|	99212-99506	11	40	122	24	-2	0.0848	no
gi|641570283|gb|KK856401.1|	28290-29180	9.9	45	2	0.309779	-2	0.2115	no
gi|641570283|gb|KK856401.1|	83598-84482	9.9	45	1	0.937386	-0.270708	0.86195	no
gi|641570286|gb|KK856400.1|	07257-10826	11	41	0.788934	0.79998	0.020059	1	no
gi|641570286|gb|KK856400.1|	08375-10955	11	41	0.645159	0.654795	0.0213898	1	no
gi|641570286|gb|KK856400.1|	10702-11328	11	41	3	3	-0.0139973	0.99175	no
gi|641570286|gb|KK856400.1|	14522-11487	11	41	5	4	-0.296611	0.8545	no
gi|641570286|gb|KK856400.1|	21572-21705	11	41	234	147	-0.672582	0.69655	no
gi|641570286|gb|KK856400.1|	41277-42842	11	41	0.516922	1	1	0.3972	no
gi|641570286|gb|KK856400.1|	44190-45612	11	41	3	1	-1	0.3531	no
gi|641570286|gb|KK856400.1|	69468-69901	11	41	3	0	-inf	0.0102	no
gi|641570286|gb|KK856400.1|	96774-97872	11	41	5	4	-0.213638	0.87225	no

gi|641570292|gb|KK856398.1|	0-498	12	21	25	18	-0.447974	0.72325	no
gi|641570292|gb|KK856398.1|	03330-10408	12	21	0.507802	1	1	0.44255	no
gi|641570292|gb|KK856398.1|	09991-11066	12	21	0.742459	2	1	0.51085	no
gi|641570292|gb|KK856398.1|	2672-3335	12	21	26	8	-2	0.2217	no
gi|641570292|gb|KK856398.1|	31533-33809	12	21	2	3	0.522031	0.68425	no
gi|641570292|gb|KK856398.1|	34223-35559	12	21	0.806904	0.945475	0.228642	0.87445	no
gi|641570292|gb|KK856398.1|	37862-38706	12	21	0.764013	0.994822	0.380841	0.82465	no
gi|641570292|gb|KK856398.1|	76994-79127	12	21	0.694254	3	2	0.14255	no
gi|641570292|gb|KK856398.1|	83437-84296	12	21	0.426712	1	1	0.44255	no
gi|641570292|gb|KK856398.1|	86492-86989	12	21	2	5	1	0.3486	no
gi|641570292|gb|KK856398.1|	89867-90727	12	21	0.42607	2	2	0.26155	no
gi|641570292|gb|KK856398.1|	90830-93377	12	21	0.752679	2	1	0.42295	no
gi|641570292|gb|KK856398.1|	93497-94474	12	21	0.634024	1	0.797424	0.63025	no
gi|641570295|gb|KK856397.1|	07637-10920	10	37	0	3	inf	0.00015	yes
gi|641570295|gb|KK856397.1|	14533-11606	10	37	0.84686	1	0.607916	0.66965	no
gi|641570295|gb|KK856397.1|	18870-11974	10	37	0.627772	3	2	0.21405	no
gi|641570295|gb|KK856397.1|	21872-12245	10	37	1	2	0.959769	0.57345	no
gi|641570295|gb|KK856397.1|	26432-12694	10	37	2	4	1	0.4292	no
gi|641570295|gb|KK856397.1|	16193-18032	10	37	2	0.39412	-2	0.1637	no
gi|641570295|gb|KK856397.1|	18157-19320	10	37	2	2	0.0701759	0.95855	no
gi|641570295|gb|KK856397.1|	22980-24364	10	37	2	0	-inf	0.0013	yes
gi|641570295|gb|KK856397.1|	26181-26491	10	37	2	8	2	0.33325	no
gi|641570295|gb|KK856397.1|	29086-29313	10	37	19	22	0.195427	0.89215	no
gi|641570295|gb|KK856397.1|	46020-46947	10	37	0.677349	1	0.966834	0.5733	no
gi|641570295|gb|KK856397.1|	47954-48121	10	37	25	56	1	0.4854	no
gi|641570295|gb|KK856397.1|	61610-61758	10	37	200	60	-2	0.29355	no
gi|641570295|gb|KK856397.1|	68228-68399	10	37	39	19	-1	0.52735	no
gi|641570295|gb|KK856397.1|	72571-73172	10	37	8	2	-2	0.2012	no
gi|641570299|gb|KK856396.1|	10218-10816	10	33	1	1	-0.402282	0.82205	no
gi|641570299|gb|KK856396.1|	06077-10628	10	33	25	5	-2	0.24525	no

gi|641570299|gb|KK856396.1|	09183-10965	10	33	3	0	-inf	0.0075	no
gi|641570299|gb|KK856396.1|	12118-12764	10	33	2	1	-0.275207	0.8619	no
gi|641570299|gb|KK856396.1|	13126-14549	10	33	0.807395	0.527219	-0.614872	1	no
gi|641570299|gb|KK856396.1|	14758-19609	10	33	2	2	0.161541	0.89715	no
gi|641570299|gb|KK856396.1|	22830-23538	10	33	2	0	-inf	0.0059	no
gi|641570299|gb|KK856396.1|	27915-28545	10	33	0	6	inf	0.00115	yes
gi|641570299|gb|KK856396.1|	44285-45532	10	33	2	1	-0.216406	0.8799	no
gi|641570299|gb|KK856396.1|	46997-48392	10	33	882	1	-9	0.00515	no
gi|641570299|gb|KK856396.1|	53148-53624	10	33	3	3	0.30212	0.84335	no
gi|641570299|gb|KK856396.1|	57320-57571	10	33	66	60	-0.133936	0.9201	no
gi|641570299|gb|KK856396.1|	60337-60822	10	33	16	15	-0.108206	0.9327	no
gi|641570299|gb|KK856396.1|	63087-63760	10	33	13	7	-0.921539	0.4818	no
gi|641570299|gb|KK856396.1|	64711-65407	10	33	0.848317	1	0.600563	0.70555	no
gi|641570299|gb|KK856396.1|	66170-66807	10	33	88	59	-0.577537	0.72	no
gi|641570299|gb|KK856396.1|	67158-70547	10	33	7	6	-0.202552	0.87085	no
gi|641570299|gb|KK856396.1|	77897-78911	10	33	0.864816	2	1	0.47135	no
gi|641570299|gb|KK856396.1|	7886-8591	10	33	1	0.421271	-1	0.4534	no
gi|641570299|gb|KK856396.1|	80189-82221	10	33	6	1	-2	0.0942	no
gi|641570299|gb|KK856396.1|	90986-91289	10	33	28	7	-2	0.20075	no
gi|641570304|gb|KK856395.1|	12226-12442	8.8	33	72	81	0.175732	0.89075	no
gi|641570304|gb|KK856395.1|	12760-13243	8.8	33	63	64	0.0406915	0.97275	no
gi|641570304|gb|KK856395.1|	184-1201	8.8	33	3	3	-0.072926	0.9566	no
gi|641570304|gb|KK856395.1|	18962-19534	8.8	33	21	15	-0.429747	0.73355	no
gi|641570304|gb|KK856395.1|	2318-2917	8.8	33	12	14	0.182716	0.88875	no
gi|641570304|gb|KK856395.1|	24014-24452	8.8	33	55	36	-0.613748	0.6297	no
gi|641570304|gb|KK856395.1|	50341-51179	8.8	33	0.991473	0.502025	-0.981814	0.62775	no
gi|641570304|gb|KK856395.1|	5483-6293	8.8	33	5	6	0.352753	0.79065	no
gi|641570304|gb|KK856395.1|	7655-8202	8.8	33	13	18	0.431256	0.7339	no
gi|641570304|gb|KK856395.1|	9839-10802	8.8	33	11	12	0.126594	0.9185	no
gi|641570307|gb|KK856394.1|	03988-10470	8.9	39	0.407888	1	2	0.3323	no

gi|641570307|gb|KK856394.1|	19338-12083	8.9	39	0.218241	1	2	0.2122	no
gi|641570307|gb|KK856394.1|	21379-12249	8.9	39	0.231205	2	3	0.1502	no
gi|641570307|gb|KK856394.1|	38723-13921	8.9	39	3	4	0.107505	0.94055	no
gi|641570307|gb|KK856394.1|	33261-34428	8.9	39	0.145729	1	3	0.27505	no
gi|641570307|gb|KK856394.1|	6006-7196	8.9	39	0.569439	2	2	0.2528	no
gi|641570316|gb|KK856391.1|	1052-1804	9.1	34	5	8	0.460879	0.72345	no
gi|641570316|gb|KK856391.1|	17032-11786	9.1	34	0.11068	7	6	0.10485	no
gi|641570316|gb|KK856391.1|	19559-12170	9.1	34	0.072704	8	7	0.2322	no
gi|641570316|gb|KK856391.1|	22341-12311	9.1	34	0	4	inf	0.00115	yes
gi|641570316|gb|KK856391.1|	20330-20506	9.1	34	127	155	0.287919	0.83205	no
gi|641570316|gb|KK856391.1|	22932-23171	9.1	34	29	20	-0.510527	0.71815	no
gi|641570316|gb|KK856391.1|	33927-35777	9.1	34	1	2	0.451221	0.73975	no
gi|641570316|gb|KK856391.1|	3600-4124	9.1	34	2	3	1	0.4891	no
gi|641570316|gb|KK856391.1|	44006-44543	9.1	34	7	7	0.135739	0.919	no
gi|641570316|gb|KK856391.1|	58125-58901	9.1	34	4	2	-0.767061	0.58405	no
gi|641570316|gb|KK856391.1|	59189-59615	9.1	34	3	1	-0.997432	0.62775	no
gi|641570316|gb|KK856391.1|	60128-62317	9.1	34	2	0.704326	-1	0.4006	no
gi|641570316|gb|KK856391.1|	62847-63927	9.1	34	55	20	-1	0.38935	no
gi|641570316|gb|KK856391.1|	6294-8171	9.1	34	3	3	-0.235061	0.85325	no
gi|641570316|gb|KK856391.1|	64487-65292	9.1	34	0.929009	2	0.924581	0.5636	no
gi|641570316|gb|KK856391.1|	65544-66139	9.1	34	18	5	-2	0.1767	no
gi|641570316|gb|KK856391.1|	67618-67786	9.1	34	52	14	-2	0.31325	no
gi|641570316|gb|KK856391.1|	70256-70764	9.1	34	3	0.340776	-3	0.2242	no
gi|641570316|gb|KK856391.1|	73137-74252	9.1	34	0.923813	1	0.190873	0.90355	no
gi|641570316|gb|KK856391.1|	75998-77596	9.1	34	0.403809	0.461636	0.193083	1	no
gi|641570316|gb|KK856391.1|	77795-77945	9.1	34	67	68	0.017417	0.92445	no
gi|641570316|gb|KK856391.1|	79356-79595	9.1	34	16	17	0.0629397	0.95685	no
gi|641570316|gb|KK856391.1|	81264-81956	9.1	34	3	5	0.731713	0.59425	no
gi|641570316|gb|KK856391.1|	9017-9425	9.1	34	36	3	-4	0.04485	no
gi|641570316|gb|KK856391.1|	94434-95817	9.1	34	0	4	inf	0.00025	yes

gi|641570316|gb|KK856391.1|	98076-99091	9.1	34	0.086376	3	5	0.1079	no
gi|641570319|gb|KK856390.1|	54761-55127	11	22	7	13	0.952221	0.501	no
gi|641570322|gb|KK856389.1|	04079-10457	11	40	4	5	0.4001	0.7858	no
gi|641570322|gb|KK856389.1|	05680-10648	11	40	1	1	-0.0494553	0.94545	no
gi|641570322|gb|KK856389.1|	09871-11011	11	40	14	10	-0.451633	0.7758	no
gi|641570322|gb|KK856389.1|	21477-12622	11	40	18	7	-1	0.4199	no
gi|641570322|gb|KK856389.1|	26426-13025	11	40	20	15	-0.448067	0.84135	no
gi|641570322|gb|KK856389.1|	34240-13486	11	40	0	6	inf	0.00115	yes
gi|641570322|gb|KK856389.1|	35290-13596	11	40	0.148492	3	4	0.1136	no
gi|641570322|gb|KK856389.1|	38380-13858	11	40	0	87	inf	0.0003	yes
gi|641570322|gb|KK856389.1|	43047-14352	11	40	0	28	inf	5.00E-05	yes
gi|641570322|gb|KK856389.1|	41804-42442	11	40	0	5	inf	0.0015	yes
gi|641570322|gb|KK856389.1|	71343-72493	11	40	0.296648	0.903109	2	0.3613	no
gi|641570322|gb|KK856389.1|	7901-8582	11	40	2	4	0.899761	0.54035	no
gi|641570322|gb|KK856389.1|	83484-83948	11	40	1	2	0.853798	0.62755	no
gi|641570322|gb|KK856389.1|	86475-87918	11	40	2	2	0.0457189	0.97215	no
gi|641570322|gb|KK856389.1|	88117-88642	11	40	0.857178	3	2	0.3613	no
gi|641570337|gb|KK856384.1|	3511-3943	7.8	38	0.593542	4	3	0.27515	no
gi|641570337|gb|KK856384.1|	47825-48924	7.8	38	0.783474	1	0.743301	0.6176	no
gi|641570337|gb|KK856384.1|	6246-7091	7.8	38	11	10	-0.143466	0.912	no
gi|641570340|gb|KK856383.1|	94106-94346	11	40	47	96	1	0.42675	no
gi|641570343|gb|KK856382.1|	14414-15388	8.6	35	0.090924	3	5	0.10875	no
gi|641570343|gb|KK856382.1|	20203-20570	8.6	35	0.806414	7	3	0.2675	no
gi|641570343|gb|KK856382.1|	26736-27271	8.6	35	0.832169	3	2	0.3613	no
gi|641570343|gb|KK856382.1|	34719-35103	8.6	35	6	2	-1	0.38945	no
gi|641570343|gb|KK856382.1|	38315-39235	8.6	35	1	3	0.991209	0.51835	no
gi|641570343|gb|KK856382.1|	42074-42326	8.6	35	41	66	0.684507	0.60665	no
gi|641570343|gb|KK856382.1|	44805-45370	8.6	35	2	5	0.983458	0.51835	no
gi|641570343|gb|KK856382.1|	46081-46294	8.6	35	66	66	0.00633843	0.9829	no
gi|641570343|gb|KK856382.1|	54437-56922	8.6	35	1	2	1	0.43825	no

gi|641570343|gb|KK856382.1|	58739-59515	8.6	35	0.975399	0.92541	-0.0758991	0.92685	no
gi|641570343|gb|KK856382.1|	59826-60066	8.6	35	0	18	inf	0.00455	no
gi|641570343|gb|KK856382.1|	67848-68143	8.6	35	0.650553	10	4	0.12515	no
gi|641570343|gb|KK856382.1|	72026-72249	8.6	35	3	48	4	0.2399	no
gi|641570343|gb|KK856382.1|	78753-78935	8.6	35	10	142	4	0.1409	no
gi|641570343|gb|KK856382.1|	84899-85285	8.6	35	12	8	-0.597514	0.66715	no
gi|641570343|gb|KK856382.1|	85453-85707	8.6	35	10	11	0.232035	0.87905	no
gi|641570343|gb|KK856382.1|	85857-87042	8.6	35	0.715395	0.326739	-1	1	no
gi|641570343|gb|KK856382.1|	87221-88600	8.6	35	0.538333	0.455624	-0.240655	1	no
gi|641570343|gb|KK856382.1|	89144-90628	8.6	35	0.494471	0.837238	0.759751	1	no
gi|641570343|gb|KK856382.1|	95884-96062	8.6	35	25	15	-0.703887	0.6977	no
gi|641570346|gb|KK856381.1|	04983-10651	9.9	20	12	14	0.284347	0.81155	no
gi|641570346|gb|KK856381.1|	10502-11246	9.9	20	8	4	-0.893176	0.4951	no
gi|641570346|gb|KK856381.1|	06726-10771	9.9	20	2	0.546101	-2	0.31415	no
gi|641570346|gb|KK856381.1|	07825-10932	9.9	20	1	0.579788	-0.906776	0.5508	no
gi|641570346|gb|KK856381.1|	12060-11377	9.9	20	19	5	-2	0.1398	no
gi|641570346|gb|KK856381.1|	15020-11528	9.9	20	6	9	0.556648	0.7451	no
gi|641570346|gb|KK856381.1|	15892-11687	9.9	20	38	19	-1	0.5296	no
gi|641570346|gb|KK856381.1|	18187-11867	9.9	20	20	16	-0.373133	0.7676	no
gi|641570346|gb|KK856381.1|	31722-13201	9.9	20	1	8	3	0.30875	no
gi|641570346|gb|KK856381.1|	21916-22139	9.9	20	27	6	-2	0.2373	no
gi|641570346|gb|KK856381.1|	26302-27313	9.9	20	1	0	-inf	0.0056	no
gi|641570346|gb|KK856381.1|	27526-28484	9.9	20	2	0	-inf	0.0034	yes
gi|641570346|gb|KK856381.1|	28590-29150	9.9	20	2	0.293107	-3	0.2381	no
gi|641570346|gb|KK856381.1|	30478-31136	9.9	20	45	11	-2	0.11275	no
gi|641570346|gb|KK856381.1|	32068-32535	9.9	20	3	0	-inf	0.01	no
gi|641570346|gb|KK856381.1|	51117-52604	9.9	20	0.877018	0.66824	-0.392241	0.79825	no
gi|641570346|gb|KK856381.1|	53197-53769	9.9	20	4	1	-2	0.2507	no
gi|641570346|gb|KK856381.1|	59432-60620	9.9	20	0.998526	0.542926	-0.879044	0.5832	no
gi|641570346|gb|KK856381.1|	61753-63547	9.9	20	2	0.675308	-1	0.34415	no

gi|641570346|gb|KK856381.1|	64673-66552	9.9	20	1	0.513039	-1	0.3857	no
gi|641570346|gb|KK856381.1|	69267-69789	9.9	20	4	2	-1	0.4353	no
gi|641570346|gb|KK856381.1|	7371-7744	9.9	20	4	1	-2	0.3414	no
gi|641570346|gb|KK856381.1|	75311-75681	9.9	20	0.793458	5	3	0.28555	no
gi|641570346|gb|KK856381.1|	76484-76793	9.9	20	46	10	-2	0.1193	no
gi|641570346|gb|KK856381.1|	90237-90700	9.9	20	7	1	-3	0.17565	no
gi|641570346|gb|KK856381.1|	91283-92794	9.9	20	3	0.902121	-2	0.2167	no
gi|641570349|gb|KK856380.1|	18132-18293	8.8	38	0	120.73	inf	0.00255	yes
gi|641570349|gb|KK856380.1|	37119-38857	8.8	38	85	87	0.0247055	0.99045	no
gi|641570349|gb|KK856380.1|	8819-9122	8.8	38	24	7	-2	0.2559	no
gi|641570352|gb|KK856379.1|	[illegible]20772-12105	18	68	0	16	inf	0.00255	yes
gi|641570352|gb|KK856379.1|	69247-70688	18	68	19.95	16	-0.363921	0.7661	no
gi|641570355|gb|KK856378.1|	[illegible]04390-10505	9.7	35	2	2	0.0852417	0.94	no
gi|641570355|gb|KK856378.1|	12609-12790	9.7	35	29	9	-2	0.363	no
gi|641570355|gb|KK856378.1|	16529-16710	9.7	35	33	5	-3	0.2472	no
gi|641570355|gb|KK856378.1|	20458-20599	9.7	35	230	65	-2	0.3068	no
gi|641570355|gb|KK856378.1|	2371-2637	9.7	35	10	5	-1	0.5083	no
gi|641570355|gb|KK856378.1|	43285-44586	9.7	35	12	21	0.808288	0.515	no
gi|641570355|gb|KK856378.1|	45480-46741	9.7	35	10	10	-0.00225851	0.9946	no
gi|641570355|gb|KK856378.1|	47525-47954	9.7	35	5	8	0.850851	0.55745	no
gi|641570355|gb|KK856378.1|	48496-49869	9.7	35	6	7	0.183177	0.8853	no
gi|641570355|gb|KK856378.1|	50195-50781	9.7	35	20	18	-0.0915227	0.94155	no
gi|641570355|gb|KK856378.1|	50917-52629	9.7	35	2	0.569376	-1	0.339	no
gi|641570355|gb|KK856378.1|	53705-54048	9.7	35	4	3	-0.593456	0.70485	no
gi|641570355|gb|KK856378.1|	54472-55711	9.7	35	2	1	-0.393327	0.7812	no
gi|641570355|gb|KK856378.1|	6187-6426	9.7	35	14	5	-1	0.4177	no
gi|641570355|gb|KK856378.1|	62185-63841	9.7	35	2	0.812822	-0.93228	0.5136	no
gi|641570355|gb|KK856378.1|	65566-67574	9.7	35	8	4	-0.975889	0.44315	no
gi|641570355|gb|KK856378.1|	69885-70161	9.7	35	5	10	1	0.4832	no
gi|641570355|gb|KK856378.1|	70531-70927	9.7	35	2	6	2	0.3154	no

gi|641570355|gb|KK856378.1|	833-1238	9.7	35	11	3	-2	0.19225	no
gi|641570355|gb|KK856378.1|	91904-92360	9.7	35	0.810267	4	2	0.256	no
gi|641570358|gb|KK856377.1|	28796-29313	8.3	30	15	14	-0.0456811	0.974	no
gi|641570358|gb|KK856377.1|	34405-34589	8.3	30	167	161	-0.050169	0.97015	no
gi|641570358|gb|KK856377.1|	41529-42065	8.3	30	50	47	-0.0889489	0.94215	no
gi|641570358|gb|KK856377.1|	42286-42649	8.3	30	169	262	0.631634	0.6997	no
gi|641570358|gb|KK856377.1|	44364-44766	8.3	30	4	7	0.585008	0.68145	no
gi|641570358|gb|KK856377.1|	46422-46698	8.3	30	50	105	1	0.39865	no
gi|641570358|gb|KK856377.1|	53195-53780	8.3	30	45	67	0.568091	0.64385	no
gi|641570358|gb|KK856377.1|	59485-59994	8.3	30	15	18	0.206676	0.8741	no
gi|641570358|gb|KK856377.1|	61708-62585	8.3	30	2	7	2	0.1978	no
gi|641570358|gb|KK856377.1|	63819-64293	8.3	30	0.505927	3	3	0.2991	no
gi|641570358|gb|KK856377.1|	9992-10578	8.3	30	27	13	-1	0.4107	no
gi|641570361|gb|KK856376.1|	56688-56982	8.6	38	6	7	0.203296	0.8976	no
gi|641570364|gb|KK856375.1|	13670-14169	8.8	39	0	14	inf	5.00E-05	yes
gi|641570364|gb|KK856375.1|	28855-29934	8.8	39	0.560844	0.975332	0.798294	0.63025	no
gi|641570364|gb|KK856375.1|	35844-36472	8.8	39	11	9	-0.261876	0.8409	no
gi|641570377|gb|KK856373.1|	00108-10030	11	39	14	55	2	0.21995	no
gi|641570377|gb|KK856373.1|	01833-10203	11	39	20	70	2	0.239	no
gi|641570377|gb|KK856373.1|	02854-10400	11	39	2	4	1	0.43595	no
gi|641570377|gb|KK856373.1|	05346-10615	11	39	0.687733	0.870348	0.339744	1	no
gi|641570377|gb|KK856373.1|	14508-11607	11	39	1	2	0.608016	0.66085	no
gi|641570377|gb|KK856373.1|	16197-11712	11	39	2	0.29581	-2	0.21865	no
gi|641570377|gb|KK856373.1|	17248-11791	11	39	1	0.919349	-0.207647	0.8945	no
gi|641570377|gb|KK856373.1|	18097-11833	11	39	12	7	-0.775188	0.63705	no
gi|641570377|gb|KK856373.1|	13884-14271	11	39	0.726926	5	3	0.27615	no
gi|641570377|gb|KK856373.1|	24511-33236	11	39	12	9	-0.424182	0.84805	no
gi|641570377|gb|KK856373.1|	33546-34304	11	39	0.880763	2	1	0.44	no
gi|641570377|gb|KK856373.1|	35241-37654	11	39	2	3	0.508386	0.69895	no
gi|641570377|gb|KK856373.1|	39881-41064	11	39	0.430106	0.545663	0.343318	1	no

gi|641570377|gb|KK856373.1|	41760-43050	11	39	58	51	-0.168824	0.94175	no
gi|641570377|gb|KK856373.1|	43349-46917	11	39	15	10	-0.619949	0.6974	no
gi|641570377|gb|KK856373.1|	48351-49215	11	39	0.741163	0.6435	-0.20385	1	no
gi|641570377|gb|KK856373.1|	50699-50860	11	39	67	34	-0.950309	0.59175	no
gi|641570377|gb|KK856373.1|	51009-51693	11	39	2	2	0.140843	0.929	no
gi|641570377|gb|KK856373.1|	53096-53784	11	39	1	1	-0.247624	0.88495	no
gi|641570377|gb|KK856373.1|	57497-58407	11	39	17	10.39	-0.738615	0.5539	no
gi|641570377|gb|KK856373.1|	58866-59655	11	39	4	14	2	0.2037	no
gi|641570377|gb|KK856373.1|	64908-65873	11	39	0.643909	1	0.967231	0.5733	no
gi|641570377|gb|KK856373.1|	65996-66744	11	39	0.512393	2	2	0.3012	no
gi|641570377|gb|KK856373.1|	68253-69144	11	39	1	1	0.144415	0.9285	no
gi|641570377|gb|KK856373.1|	70899-71612	11	39	0.820498	2	1	0.4118	no
gi|641570377|gb|KK856373.1|	85947-89742	11	39	20.81	64	2	0.44465	no
gi|641570377|gb|KK856373.1|	90171-91468	11	39	0.449867	0.685174	0.606971	1	no
gi|641570377|gb|KK856373.1|	93855-94648	11	39	4	11	1	0.27625	no
gi|641570377|gb|KK856373.1|	95114-95567	11	39	2	9	2	0.1758	no
gi|641570377|gb|KK856373.1|	98554-98732	11	39	25	106	2	0.19805	no
gi|641570381|gb|KK856372.1|	35432-35655	8.2	32	42	31	-0.409523	0.7692	no
gi|641570381|gb|KK856372.1|	49234-50076	8.2	32	1	0.49894	-2	0.40415	no
gi|641570381|gb|KK856372.1|	84975-85862	8.2	32	1	0.466672	-2	0.40415	no
gi|641570381|gb|KK856372.1|	94689-95101	8.2	32	33	0.485245	-6	0.1832	no
gi|641570396|gb|KK856368.1|	29590-30533	9.5	38	0.662855	2	1	0.36605	no
gi|641570396|gb|KK856368.1|	31625-32303	9.5	38	0.879898	0.889194	0.0151626	0.91115	no
gi|641570396|gb|KK856368.1|	32589-33423	9.5	38	0.997682	0.505149	-0.981871	0.62775	no
gi|641570396|gb|KK856368.1|	55471-62799	9.5	38	12	8	-0.55985	0.8076	no
gi|641570396|gb|KK856368.1|	63748-64102	9.5	38	3	5	0.993396	0.5903	no
gi|641570396|gb|KK856368.1|	69935-70563	9.5	38	0.6542	1	1	0.51315	no
gi|641570396|gb|KK856368.1|	73847-75169	9.5	38	6	5	-0.192612	0.87705	no
gi|641570399|gb|KK856367.1|	0-384	11	41	4	2	-0.739131	0.63835	no
gi|641570399|gb|KK856367.1|	[illegible]37661-13798	11	41	5	2	-2	0.3414	no

gi|641570399|gb|KK856367.1|	18446-19588	11	41	1	3	1	0.35075	no
gi|641570399|gb|KK856367.1|	20005-20836	11	41	0.779637	2	1	0.39245	no
gi|641570399|gb|KK856367.1|	28079-28313	11	41	0	34	inf	0.0015	yes
gi|641570399|gb|KK856367.1|	31056-31395	11	41	0	23	inf	0.00065	yes
gi|641570399|gb|KK856367.1|	35860-38851	11	41	0	63	inf	5.00E-05	yes
gi|641570399|gb|KK856367.1|	48493-49270	11	41	2	2	-0.134775	0.9246	no
gi|641570399|gb|KK856367.1|	49596-51080	11	41	3	2	-0.71418	0.59445	no
gi|641570399|gb|KK856367.1|	51262-56295	11	41	21	35	0.745616	0.7172	no
gi|641570399|gb|KK856367.1|	58514-58907	11	41	7	54	3	0.0474	no
gi|641570399|gb|KK856367.1|	60377-61101	11	41	3	0.609399	-2	0.2338	no
gi|641570399|gb|KK856367.1|	61811-65289	11	41	9	24	1	0.3903	no
gi|641570399|gb|KK856367.1|	65397-66208	11	41	22	15	-0.484716	0.6976	no
gi|641570399|gb|KK856367.1|	69592-72931	11	41	9	5	-0.896886	0.47435	no
gi|641570399|gb|KK856367.1|	73109-74909	11	41	3	2	-1	0.4393	no
gi|641570399|gb|KK856367.1|	78785-79187	11	41	3	2	-0.414992	0.82205	no
gi|641570399|gb|KK856367.1|	8307-8933	11	41	0.985842	2	1	0.47095	no
gi|641570401|gb|KK856366.1|	.03758-10432	11	40	4	1	-2	0.3463	no
gi|641570401|gb|KK856366.1|	10478-10688	11	40	19	5	-2	0.3145	no
gi|641570401|gb|KK856366.1|	12742-13236	11	40	13.94	5	-1	0.2978	no
gi|641570401|gb|KK856366.1|	14266-18425	11	40	4	4	-0.107179	0.93285	no
gi|641570401|gb|KK856366.1|	3188-3661	11	40	14.47	4	-2	0.21405	no
gi|641570401|gb|KK856366.1|	49573-50742	11	40	0.363575	2	2	0.2126	no
gi|641570401|gb|KK856366.1|	51222-56981	11	40	8	4	-1	0.51805	no
gi|641570401|gb|KK856366.1|	57297-57964	11	40	8	5	-0.459052	0.7233	no
gi|641570401|gb|KK856366.1|	62766-64351	11	40	0.764224	0.543599	-0.491454	1	no
gi|641570401|gb|KK856366.1|	6445-7390	11	40	1	1	-0.495079	0.7427	no
gi|641570401|gb|KK856366.1|	68476-68795	11	40	5	2	-1	0.5278	no
gi|641570401|gb|KK856366.1|	69662-69864	11	40	29	18	-0.691082	0.6568	no
gi|641570401|gb|KK856366.1|	96528-96702	11	40	135	51	-1	0.3371	no
gi|641570404|gb|KK856365.1|	.05885-10620	10	19	2	6	2	0.3454	no

gi|641570404|gb|KK856365.1|	06545-10708	10	19	0.832169	3	2	0.3613	no
gi|641570404|gb|KK856365.1|	07381-11030	10	19	0.727598	1	0.890274	0.5059	no
gi|641570404|gb|KK856365.1|	10416-11375	10	19	12	11	-0.119583	0.95595	no
gi|641570404|gb|KK856365.1|	13914-11485	10	19	0.661969	0.575117	-0.202908	1	no
gi|641570404|gb|KK856365.1|	17657-11913	10	19	0.717577	1	0.99475	0.51835	no
gi|641570404|gb|KK856365.1|	20135-12223	10	19	1	1	0.498202	0.7176	no
gi|641570404|gb|KK856365.1|	23263-12344	10	19	28	4	-3	0.2587	no
gi|641570404|gb|KK856365.1|	25857-12620	10	19	85	67	-0.334126	0.79185	no
gi|641570404|gb|KK856365.1|	25902-26664	10	19	30	18	-0.731975	0.5589	no
gi|641570404|gb|KK856365.1|	32029-32491	10	19	4	0.795513	-2	0.21845	no
gi|641570404|gb|KK856365.1|	33228-35129	10	19	0.99623	0.949572	-0.0692026	0.96075	no
gi|641570404|gb|KK856365.1|	35358-36934	10	19	3	2	-0.713874	0.59445	no
gi|641570404|gb|KK856365.1|	38379-40922	10	19	0.693645	1	0.944236	0.4962	no
gi|641570404|gb|KK856365.1|	41162-41839	10	19	2	2	-0.177508	0.90465	no
gi|641570404|gb|KK856365.1|	43239-44478	10	19	4	1	-2	0.24895	no
gi|641570404|gb|KK856365.1|	45359-45563	10	19	69	26	-1	0.32585	no
gi|641570404|gb|KK856365.1|	69155-69450	10	19	37	9	-2	0.15755	no
gi|641570404|gb|KK856365.1|	70338-71082	10	19	1	0.391581	-2	0.4266	no
gi|641570404|gb|KK856365.1|	71725-73948	10	19	5	1	-2	0.18385	no
gi|641570404|gb|KK856365.1|	77584-77817	10	19	58	15	-2	0.18245	no
gi|641570404|gb|KK856365.1|	78982-79326	10	19	65	12	-2	0.0939	no
gi|641570404|gb|KK856365.1|	80555-81076	10	19	21	9	-1	0.3547	no
gi|641570407|gb|KK856364.1|	26281-12651	11	42	1	18	4	0.12515	no
gi|641570413|gb|KK856362.1|	05524-10572	10	35	39	20	-0.974336	0.55285	no
gi|641570413|gb|KK856362.1|	13445-11405	10	35	2	0.774845	-0.986827	0.62775	no
gi|641570413|gb|KK856362.1|	15823-16396	10	35	3	0	-inf	0.0056	no
gi|641570413|gb|KK856362.1|	1911-2158	10	35	112	9	-4	0.045	no
gi|641570413|gb|KK856362.1|	2-614	10	35	0	4	inf	0.00255	yes
gi|641570413|gb|KK856362.1|	26492-27525	10	35	0	4	inf	0.0003	yes
gi|641570413|gb|KK856362.1|	28567-30438	10	35	0	69	inf	0.0001	yes

gi|641570413|gb|KK856362.1|	35385-36133	10	35	5	5	-0.0764065	0.9547	no
gi|641570413|gb|KK856362.1|	36554-40147	10	35	1	0.572727	-1	0.3148	no
gi|641570413|gb|KK856362.1|	43746-44238	10	35	4	3	-0.599962	0.68495	no
gi|641570413|gb|KK856362.1|	46193-46832	10	35	1	0.241611	-3	0.271	no
gi|641570413|gb|KK856362.1|	48528-48708	10	35	70	24	-2	0.3449	no
gi|641570413|gb|KK856362.1|	52180-52514	10	35	7	0	-inf	0.0059	no
gi|641570413|gb|KK856362.1|	61114-61513	10	35	3	1	-2	0.3632	no
gi|641570413|gb|KK856362.1|	64454-65940	10	35	2	0.501567	-2	0.1762	no
gi|641570413|gb|KK856362.1|	69574-69887	10	35	6	2	-2	0.3414	no
gi|641570413|gb|KK856362.1|	76008-76294	10	35	9	1	-3	0.22365	no
gi|641570413|gb|KK856362.1|	81837-83161	10	35	0.815482	2	0.906659	0.53945	no
gi|641570413|gb|KK856362.1|	99278-99495	10	35	30	16	-0.901793	0.55025	no
gi|641570416|gb|KK856361.1|	22523-23109	10	41	6	3	-1	0.4186	no
gi|641570416|gb|KK856361.1|	23240-23441	10	41	79	24	-2	0.25825	no
gi|641570416|gb|KK856361.1|	28555-28998	10	41	37	12	-2	0.2213	no
gi|641570416|gb|KK856361.1|	30966-31888	10	41	0	104	inf	5.00E-05	yes
gi|641570416|gb|KK856361.1|	33275-33674	10	41	0	209	inf	0.0002	yes
gi|641570416|gb|KK856361.1|	3635-3839	10	41	122	43	-2	0.27035	no
gi|641570416|gb|KK856361.1|	38949-39741	10	41	0	257.26	inf	0.0007	yes
gi|641570416|gb|KK856361.1|	6175-6627	10	41	15	7	-1	0.407	no
gi|641570416|gb|KK856361.1|	86656-87067	10	41	5	0.974752	-2	0.24735	no
gi|641570419|gb|KK856360.1|	00336-10086	12	39	6	4	-0.727257	0.60475	no
gi|641570419|gb|KK856360.1|	02753-10425	12	39	0.5974	0.662108	0.148369	1	no
gi|641570419|gb|KK856360.1|	05211-10755	12	39	0.62608	0.603337	-0.0533811	1	no
gi|641570419|gb|KK856360.1|	12944-11321	12	39	30	2	-4	0.1407	no
gi|641570419|gb|KK856360.1|	14515-11639	12	39	0.42052	9	4	0.0254	no
gi|641570419|gb|KK856360.1|	22414-12263	12	39	2	20	4	0.12785	no
gi|641570419|gb|KK856360.1|	30307-13108	12	39	228	117	-0.967459	0.64905	no
gi|641570419|gb|KK856360.1|	35216-13612	12	39	0.099905	3	5	0.11025	no
gi|641570419|gb|KK856360.1|	40647-14117	12	39	0.20988	6	5	0.1081	no

gi|641570419|gb|KK856360.1|	49197-15120	12	39	0	7	inf	5.00E-05	yes
gi|641570419|gb|KK856360.1|	51519-15286	12	39	0.061423	5	6	0.10455	no
gi|641570419|gb|KK856360.1|	60176-60840	12	39	46	17	-1	0.2615	no
gi|641570419|gb|KK856360.1|	61020-62896	12	39	28	6	-2	0.21035	no
gi|641570419|gb|KK856360.1|	63372-66266	12	39	10	8	-0.423107	0.73685	no
gi|641570419|gb|KK856360.1|	68188-69147	12	39	0.648968	0.563882	-0.202754	1	no
gi|641570419|gb|KK856360.1|	71555-72933	12	39	2	0.912016	-0.771174	0.58845	no
gi|641570419|gb|KK856360.1|	74047-77389	12	39	2	1	-1	0.3845	no
gi|641570419|gb|KK856360.1|	77498-78682	12	39	2	1	-0.457772	0.7554	no
gi|641570419|gb|KK856360.1|	80326-80924	12	39	4	2	-0.916855	0.54625	no
gi|641570419|gb|KK856360.1|	81175-83042	12	39	8	3	-1	0.3015	no
gi|641570419|gb|KK856360.1|	83317-84186	12	39	1	0.159688	-3	0.2382	no
gi|641570419|gb|KK856360.1|	84391-88290	12	39	67	29	-1	0.55485	no
gi|641570419|gb|KK856360.1|	93198-93595	12	39	53	45	-0.23002	0.85615	no
gi|641570423|gb|KK856359.1|	06229-10734	11	43	64	62	-0.045436	0.98245	no
gi|641570423|gb|KK856359.1|	18077-21025	11	43	65	80	0.297948	0.88055	no
gi|641570423|gb|KK856359.1|	24254-24884	11	43	1	20	4	0.05185	no
gi|641570423|gb|KK856359.1|	25694-26055	11	43	0.833563	5	3	0.30875	no
gi|641570423|gb|KK856359.1|	26316-27392	11	43	0.080393	2	5	0.1123	no
gi|641570423|gb|KK856359.1|	28346-28530	11	43	0	48	inf	0.00455	no
gi|641570423|gb|KK856359.1|	2922-3802	11	43	0.723844	0.628553	-0.203644	1	no
gi|641570423|gb|KK856359.1|	34970-36183	11	43	0.626141	13	4	0.03465	no
gi|641570423|gb|KK856359.1|	62709-64991	11	43	3	8	1	0.277	no
gi|641570423|gb|KK856359.1|	6385-7532	11	43	2	1	-0.531377	0.71245	no
gi|641570423|gb|KK856359.1|	65171-67805	11	43	1	0.753259	-0.550553	0.68155	no
gi|641570423|gb|KK856359.1|	68087-70499	11	43	9	24	1	0.4055	no
gi|641570423|gb|KK856359.1|	70928-71877	11	43	17	48	1	0.39345	no
gi|641570423|gb|KK856359.1|	72704-74260	11	43	2	6	1	0.31235	no
gi|641570423|gb|KK856359.1|	74526-75989	11	43	0.781919	2	1	0.3814	no
gi|641570423|gb|KK856359.1|	79946-81161	11	43	0.416609	0.528608	0.343505	1	no

gi|641570423|gb|KK856359.1|	87130-88955	11	43	0	246	inf	5.00E-05	yes
gi|641570431|gb|KK856357.1|	44730-45217	11	22	0	45	inf	5.00E-05	yes
gi|641570431|gb|KK856357.1|	45891-46663	11	22	2	5	2	0.2925	no
gi|641570439|gb|KK856355.1|	01174-10139	11	39	47	156	2	0.21135	no
gi|641570439|gb|KK856355.1|	2224-2775	11	39	0	17	inf	5.00E-05	yes
gi|641570442|gb|KK856354.1|	35239-36496	11	40	4	0	-inf	0.0002	yes
gi|641570442|gb|KK856354.1|	38322-38890	11	40	2	0	-inf	0.01175	no
gi|641570442|gb|KK856354.1|	38984-39951	11	40	1	0	-inf	0.0059	no
gi|641570442|gb|KK856354.1|	41488-41781	11	40	7	0	-inf	0.01175	no
gi|641570442|gb|KK856354.1|	46229-47525	11	40	0.514601	0.783765	0.606966	1	no
gi|641570442|gb|KK856354.1|	51319-52452	11	40	0.302027	1	2	0.23615	no
gi|641570442|gb|KK856354.1|	77294-77898	11	40	2	0	-inf	0.0102	no
gi|641570442|gb|KK856354.1|	79069-80093	11	40	3	0	-inf	0.0013	yes
gi|641570442|gb|KK856354.1|	80499-81536	11	40	2	0	-inf	0.0013	yes
gi|641570442|gb|KK856354.1|	88088-88780	11	40	2	0	-inf	0.0075	no
gi|641570442|gb|KK856354.1|	89052-91359	11	40	1	0	-inf	0.0002	yes
gi|641570446|gb|KK856353.1|	08308-10908	9.9	38	0.122558	4	5	0.10875	no
gi|641570446|gb|KK856353.1|	14809-11865	9.9	38	0.019421	120	13	0.1048	no
gi|641570446|gb|KK856353.1|	17508-21123	9.9	38	0.434794	1	2	0.2324	no
gi|641570446|gb|KK856353.1|	30127-31057	9.9	38	0.289107	1	2	0.3323	no
gi|641570446|gb|KK856353.1|	31151-32070	9.9	38	0.684837	0.74359	0.118748	1	no
gi|641570446|gb|KK856353.1|	38999-39405	9.9	38	2	2	0.000497393	0.91115	no
gi|641570446|gb|KK856353.1|	43651-44301	9.9	38	2	2	0.0144136	0.95145	no
gi|641570446|gb|KK856353.1|	50635-51154	9.9	38	3	2	-0.598394	0.68495	no
gi|641570446|gb|KK856353.1|	70567-71355	9.9	38	12	11	-0.12969	0.9203	no
gi|641570446|gb|KK856353.1|	7211-8272	9.9	38	0.408932	0.746637	0.868545	1	no
gi|641570446|gb|KK856353.1|	96002-97129	9.9	38	0.227979	0.925305	2	0.2812	no
gi|641570453|gb|KK856351.1|	04055-10497	9.6	36	2	0.447383	-2	0.3026	no
gi|641570453|gb|KK856351.1|	12559-11297	9.6	36	7	10	0.384479	0.7815	no
gi|641570453|gb|KK856351.1|	15986-11658	9.6	36	6	3	-0.871839	0.53375	no

gi|641570453|gb|KK856351.1|	19118-11944	9.6	36	16	9	-0.748919	0.59225	no
gi|641570453|gb|KK856351.1|	21814-12242	9.6	36	2	1	-1	0.5005	no
gi|641570453|gb|KK856351.1|	23497-12437	9.6	36	135	3	-5	0.02065	no
gi|641570453|gb|KK856351.1|	29601-30202	9.6	36	0.348784	8	4	0.23615	no
gi|641570453|gb|KK856351.1|	32582-32831	9.6	36	0	34	inf	0.00115	yes
gi|641570453|gb|KK856351.1|	35678-36923	9.6	36	0	6	inf	5.00E-05	yes
gi|641570453|gb|KK856351.1|	45020-45280	9.6	36	25	53	1	0.4233	no
gi|641570453|gb|KK856351.1|	45924-47352	9.6	36	4	4	-0.0954781	0.94035	no
gi|641570453|gb|KK856351.1|	48287-49844	9.6	36	1	1	-0.29247	0.83005	no
gi|641570453|gb|KK856351.1|	50978-53192	9.6	36	6	3	-0.983684	0.44865	no
gi|641570453|gb|KK856351.1|	54422-55937	9.6	36	51	22	-1	0.4522	no
gi|641570453|gb|KK856351.1|	56899-57820	9.6	36	107	43	-1	0.4273	no
gi|641570453|gb|KK856351.1|	63199-65442	9.6	36	34	10	-2	0.3182	no
gi|641570453|gb|KK856351.1|	80306-82672	9.6	36	5	0.597234	-3	0.0403	no
gi|641570453|gb|KK856351.1|	83667-85746	9.6	36	4	0.745366	-2	0.0891	no
gi|641570453|gb|KK856351.1|	86111-86940	9.6	36	8	1	-3	0.08925	no
gi|641570453|gb|KK856351.1|	88902-89453	9.6	36	3	0	-inf	0.0059	no
gi|641570453|gb|KK856351.1|	89553-91937	9.6	36	13	3	-2	0.1088	no
gi|641570453|gb|KK856351.1|	93833-94190	9.6	36	9	4	-1	0.4115	no
gi|641570456|gb|KK856350.1|	48029-49659	10	39	0.987006	3	2	0.2693	no
gi|641570456|gb|KK856350.1|	49830-50864	10	39	1	5	2	0.21145	no
gi|641570456|gb|KK856350.1|	51195-51973	10	39	2	10	3	0.0876	no
gi|641570456|gb|KK856350.1|	61004-61449	10	39	7	5	-0.536281	0.7092	no
gi|641570456|gb|KK856350.1|	61857-65164	10	39	3	1	-1	0.33265	no
gi|641570459|gb|KK856349.1|	02386-10352	9.7	40	1	0.456795	-1	0.46645	no
gi|641570459|gb|KK856349.1|	14412-14643	9.7	40	12	6	-1	0.5278	no
gi|641570459|gb|KK856349.1|	151-969	9.7	40	13	6	-1	0.4006	no
gi|641570459|gb|KK856349.1|	2160-3700	9.7	40	10	4	-1	0.30125	no
gi|641570459|gb|KK856349.1|	25113-26078	9.7	40	5	7	0.575515	0.6587	no
gi|641570459|gb|KK856349.1|	35403-37304	9.7	40	30	25	-0.252358	0.91295	no

gi|641570459|gb|KK856349.1|	38387-38721	9.7	40	24	20	-0.232552	0.8578	no
gi|641570459|gb|KK856349.1|	39177-41992	9.7	40	40	24	-0.724927	0.74235	no
gi|641570459|gb|KK856349.1|	42518-45557	9.7	40	21	8	-1	0.42155	no
gi|641570459|gb|KK856349.1|	47869-48093	9.7	40	20	10	-0.943407	0.56905	no
gi|641570459|gb|KK856349.1|	48603-49266	9.7	40	18	7	-1	0.3046	no
gi|641570459|gb|KK856349.1|	57355-57978	9.7	40	2	2	0.276635	0.8559	no
gi|641570459|gb|KK856349.1|	6285-6597	9.7	40	6	4	-0.429796	0.77915	no
gi|641570462|gb|KK856348.1|	1018-1462	10	37	2	2	-0.410836	0.82205	no
gi|641570462|gb|KK856348.1|	09559-11145	10	37	0.208772	19	6	0.08155	no
gi|641570462|gb|KK856348.1|	13711-14325	10	37	59	26	-1	0.35775	no
gi|641570462|gb|KK856348.1|	15683-15858	10	37	231	143	-0.689592	0.60655	no
gi|641570462|gb|KK856348.1|	1685-4905	10	37	2	7	2	0.2027	no
gi|641570462|gb|KK856348.1|	2-942	10	37	1	0.867284	-0.270065	0.86195	no
gi|641570462|gb|KK856348.1|	20067-21492	10	37	3	2	-0.338024	0.79915	no
gi|641570462|gb|KK856348.1|	21601-23143	10	37	15	12	-0.258788	0.83525	no
gi|641570462|gb|KK856348.1|	23286-24850	10	37	3	3	-0.0924235	0.9448	no
gi|641570462|gb|KK856348.1|	25112-25755	10	37	2	0.957911	-1	0.4658	no
gi|641570462|gb|KK856348.1|	25888-26468	10	37	3	0.556231	-2	0.25005	no
gi|641570462|gb|KK856348.1|	27444-28068	10	37	5	2	-1	0.3486	no
gi|641570462|gb|KK856348.1|	28274-29186	10	37	172	138.45	-0.312154	0.8877	no
gi|641570462|gb|KK856348.1|	33482-33749	10	37	241	44	-2	0.0723	no
gi|641570462|gb|KK856348.1|	39302-40307	10	37	2	0	-inf	0.0032	yes
gi|641570462|gb|KK856348.1|	46407-47002	10	37	8	1	-3	0.11055	no
gi|641570462|gb|KK856348.1|	48507-49548	10	37	5	3	-0.662131	0.61575	no
gi|641570462|gb|KK856348.1|	5019-5243	10	37	143	84	-0.75864	0.5633	no
gi|641570462|gb|KK856348.1|	50427-50665	10	37	38	0	-inf	0.00105	yes
gi|641570462|gb|KK856348.1|	58500-58667	10	37	0	98	inf	0.00255	yes
gi|641570462|gb|KK856348.1|	6344-6693	10	37	73	51	-0.532106	0.6779	no
gi|641570462|gb|KK856348.1|	64234-64555	10	37	0	13	inf	0.0015	yes
gi|641570462|gb|KK856348.1|	84002-85521	10	37	0.053489	1	4	0.11365	no

gi|641570462|gb|KK856348.1|	8871-11166	10	37	1	1	0.380038	0.7786	no
gi|641570465|gb|KK856347.1|	31565-13283	10	42	0.395288	0.902985	1	0.47015	no
gi|641570465|gb|KK856347.1|	32994-13362	10	42	0.3271	3	3	0.2674	no
gi|641570465|gb|KK856347.1|	27475-28054	10	42	0.737362	3	2	0.3012	no
gi|641570465|gb|KK856347.1|	71687-72880	10	42	0.425795	0.972389	1	0.47015	no
gi|641570468|gb|KK856346.1|	27133-27868	9.1	29	2	0.597084	-2	0.40415	no
gi|641570468|gb|KK856346.1|	30932-31557	9.1	29	1	1	0.18359	0.91015	no
gi|641570468|gb|KK856346.1|	32016-33542	9.1	29	1	1	-0.0441837	0.97035	no
gi|641570468|gb|KK856346.1|	84934-85341	9.1	29	2	2	0.100144	0.92335	no
gi|641570474|gb|KK856344.1|	17606-18596	7.5	31	2	0.406499	-2	0.21615	no
gi|641570474|gb|KK856344.1|	20478-20936	7.5	31	3	3	0.0757329	0.941	no
gi|641570474|gb|KK856344.1|	26063-26545	7.5	31	0	5	inf	0.00255	yes
gi|641570474|gb|KK856344.1|	26684-27218	7.5	31	2	3	0.764949	0.62775	no
gi|641570474|gb|KK856344.1|	51398-51994	7.5	31	2	0	-inf	0.0102	no
gi|641570474|gb|KK856344.1|	58008-58617	7.5	31	1	2	1	0.5082	no
gi|641570474|gb|KK856344.1|	59978-61896	7.5	31	1	0.188045	-3	0.15435	no
gi|641570474|gb|KK856344.1|	65830-66880	7.5	31	0.994068	0.882233	-0.172185	0.90465	no
gi|641570474|gb|KK856344.1|	83828-83973	7.5	31	0	747	inf	5.00E-05	yes
gi|641570474|gb|KK856344.1|	90441-90653	7.5	31	0	72	inf	0.0004	yes
gi|641570474|gb|KK856344.1|	990-1325	7.5	31	236	409	0.795682	0.6207	no
gi|641570477|gb|KK856343.1|	44508-45817	9.9	37	2	0	-inf	0.00185	yes
gi|641570477|gb|KK856343.1|	51905-52500	9.9	37	2	0	-inf	0.0059	no
gi|641570477|gb|KK856343.1|	90815-91161	9.9	37	5	0	-inf	0.01	no
gi|641570480|gb|KK856342.1|	16003-17959	7.8	36	0.442342	2	2	0.20295	no
gi|641570480|gb|KK856342.1|	18912-19865	7.8	36	1	0.426242	-1	0.47135	no
gi|641570480|gb|KK856342.1|	22036-22829	7.8	36	1	0	-inf	0.01175	no
gi|641570480|gb|KK856342.1|	46590-47171	7.8	36	3	0.832217	-2	0.24335	no
gi|641570480|gb|KK856342.1|	56409-57373	7.8	36	0.644747	1	0.797296	0.63025	no
gi|641570480|gb|KK856342.1|	58970-60385	7.8	36	1	1	-0.451405	0.7495	no
gi|641570480|gb|KK856342.1|	63458-64724	7.8	36	31	57	0.875937	0.58695	no

gi|641570480|gb|KK856342.1|	69814-70800	7.8	36	39	32	-0.280677	0.81845	no
gi|641570480|gb|KK856342.1|	77704-78223	7.8	36	25	99	2	0.12795	no
gi|641570480|gb|KK856342.1|	87923-88356	7.8	36	5	21	2	0.1544	no
gi|641570480|gb|KK856342.1|	89909-90242	7.8	36	11	27	1	0.34225	no
gi|641570480|gb|KK856342.1|	94409-96114	7.8	36	10	0.357505	-5	0.04355	no
gi|641570484|gb|KK856341.1|	00808-10184	10	37	1	1	-0.224725	0.87645	no
gi|641570484|gb|KK856341.1|	04126-10547	10	37	0.61211	0.186489	-2	1	no
gi|641570484|gb|KK856341.1|	07579-10835	10	37	0.726546	1	0.824634	0.6232	no
gi|641570484|gb|KK856341.1|	08422-10939	10	37	0.54836	0.694897	0.341676	1	no
gi|641570484|gb|KK856341.1|	10394-11136	10	37	0.915114	0.417474	-1	0.50885	no
gi|641570484|gb|KK856341.1|	13211-11424	10	37	1	1	0.257343	0.8632	no
gi|641570484|gb|KK856341.1|	14691-11610	10	37	2	0.6181	-1	0.3708	no
gi|641570484|gb|KK856341.1|	16518-11894	10	37	1	2	0.472182	0.7262	no
gi|641570484|gb|KK856341.1|	19013-12165	10	37	0.520887	0.397482	-0.390081	1	no
gi|641570484|gb|KK856341.1|	21735-12422	10	37	0.803632	0.801839	-0.00322232	1	no
gi|641570484|gb|KK856341.1|	25360-12605	10	37	2	0.43392	-2	0.24735	no
gi|641570484|gb|KK856341.1|	28153-12878	10	37	1	2	0.428828	0.78025	no
gi|641570484|gb|KK856341.1|	29765-13083	10	37	0.322673	0.859222	1	1	no
gi|641570484|gb|KK856341.1|	30972-13257	10	37	0.856262	1	0.427558	0.7651	no
gi|641570484|gb|KK856341.1|	33949-13504	10	37	0.473226	0.600119	0.342719	1	no
gi|641570484|gb|KK856341.1|	35576-13644	10	37	1	0.794924	-0.659425	0.6826	no
gi|641570484|gb|KK856341.1|	40906-14207	10	37	0.803993	0.778848	-0.0458416	1	no
gi|641570484|gb|KK856341.1|	43194-14545	10	37	1	0.731766	-0.627564	0.6541	no
gi|641570484|gb|KK856341.1|	46769-14958	10	37	3	1	-1	0.39675	no
gi|641570484|gb|KK856341.1|	51150-15166	10	37	3	0	-inf	0.0059	no
gi|641570484|gb|KK856341.1|	58047-15858	10	37	2	0.616074	-2	0.3216	no
gi|641570484|gb|KK856341.1|	64348-16472	10	37	5	2	-1	0.5083	no
gi|641570484|gb|KK856341.1|	54338-55501	10	37	0.87799	0.556893	-0.656806	0.6826	no
gi|641570484|gb|KK856341.1|	55691-59895	10	37	1	1	-0.120322	0.9261	no
gi|641570484|gb|KK856341.1|	60065-62217	10	37	3	1	-0.765082	0.56215	no

gi|641570484|gb|KK856341.1|	69241-71419	10	37	0.571366	0.544789	-0.0687184	1	no
gi|641570484|gb|KK856341.1|	75418-76056	10	37	5	5	-0.0599363	0.96605	no
gi|641570484|gb|KK856341.1|	77661-78283	10	37	0.331684	3	3	0.27515	no
gi|641570484|gb|KK856341.1|	78484-79001	10	37	0.658714	3	2	0.2235	no
gi|641570484|gb|KK856341.1|	85036-86094	10	37	1	0.49948	-1	0.37565	no
gi|641570484|gb|KK856341.1|	93081-93643	10	37	2	1	-0.666727	0.68195	no
gi|641570484|gb|KK856341.1|	94928-95390	10	37	2	1	-0.631779	0.71175	no
gi|641570484|gb|KK856341.1|	95836-96573	10	37	0.914881	0.793198	-0.205904	0.89485	no
gi|641570484|gb|KK856341.1|	96948-97755	10	37	0.810226	1	0.380292	0.82465	no
gi|641570484|gb|KK856341.1|	99274-10067	10	37	0.940353	0.805876	-0.222646	0.87645	no
gi|641570488|gb|KK856340.1|	01279-10204	12	45	1	2	0.432108	0.7802	no
gi|641570488|gb|KK856340.1|	05115-10950	12	45	2	2	-0.280696	0.8226	no
gi|641570488|gb|KK856340.1|	10870-11135	12	45	9	9	0.0266361	0.9823	no
gi|641570488|gb|KK856340.1|	14149-11494	12	45	4	4	-0.0495356	0.97035	no
gi|641570488|gb|KK856340.1|	15389-11999	12	45	9	14	0.700001	0.7552	no
gi|641570488|gb|KK856340.1|	20753-12178	12	45	0.510779	2	2	0.20395	no
gi|641570488|gb|KK856340.1|	21843-12270	12	45	0.316694	2	2	0.2235	no
gi|641570488|gb|KK856340.1|	28046-13006	12	45	0.42636	0.886814	1	0.47825	no
gi|641570488|gb|KK856340.1|	33720-13634	12	45	0.407969	2	2	0.17055	no
gi|641570488|gb|KK856340.1|	37632-13928	12	45	3	4	0.127159	0.9218	no
gi|641570488|gb|KK856340.1|	44055-14519	12	45	1	0.683031	-0.616299	0.6875	no
gi|641570488|gb|KK856340.1|	47819-14867	12	45	1	0	-inf	0.0102	no
gi|641570488|gb|KK856340.1|	51504-15279	12	45	10	9	-0.13961	0.9119	no
gi|641570488|gb|KK856340.1|	56372-15873	12	45	22	22	0.00655176	0.99785	no
gi|641570488|gb|KK856340.1|	85080-85829	12	45	0.767202	3	2	0.3067	no
gi|641570488|gb|KK856340.1|	86232-87547	12	45	0.505869	2	2	0.1833	no
gi|641570488|gb|KK856340.1|	89418-96006	12	45	1	1	0.378752	0.7612	no
gi|641570488|gb|KK856340.1|	96534-97783	12	45	1	1	0.244156	0.8647	no
gi|641570488|gb|KK856340.1|	99943-10020	12	45	8	12	0.554992	0.731	no
gi|641570491|gb|KK856339.1|	00420-10160	10	35	99.01	518.42	2	0.25355	no

gi|641570491|gb|KK856339.1|	04608-10538	10	35	165	316	0.935766	0.65985	no
gi|641570491|gb|KK856339.1|	08098-10862	10	35	0	12	inf	5.00E-05	yes
gi|641570491|gb|KK856339.1|	11786-11267	10	35	2	5	2	0.2618	no
gi|641570491|gb|KK856339.1|	13969-11490	10	35	358	1981.58	2	0.18715	no
gi|641570491|gb|KK856339.1|	21828-22219	10	35	0.712792	4	3	0.30875	no
gi|641570491|gb|KK856339.1|	23727-24493	10	35	132	527	2	0.3449	no
gi|641570491|gb|KK856339.1|	43273-44226	10	35	96	165	0.779907	0.7213	no
gi|641570491|gb|KK856339.1|	53625-54415	10	35	4	12	2	0.214	no
gi|641570491|gb|KK856339.1|	59548-60677	10	35	0.15166	2	4	0.2494	no
gi|641570491|gb|KK856339.1|	61853-67910	10	35	4	11	1	0.3907	no
gi|641570491|gb|KK856339.1|	68048-69108	10	35	0.900691	0.996661	0.146071	0.9285	no
gi|641570491|gb|KK856339.1|	70333-70531	10	35	111	716	3	0.0508	no
gi|641570491|gb|KK856339.1|	71305-71917	10	35	0	3	inf	0.00455	no
gi|641570491|gb|KK856339.1|	83937-86302	10	35	0.065265	3	6	0.2328	no
gi|641570491|gb|KK856339.1|	86438-87181	10	35	47	258	2	0.164	no
gi|641570491|gb|KK856339.1|	90068-90260	10	35	337	931	1	0.2513	no
gi|641570491|gb|KK856339.1|	98343-99771	10	35	0.861557	2	1	0.4226	no
gi|641570497|gb|KK856337.1|	42175-42664	11	40	0	6	inf	0.0015	yes
gi|641570501|gb|KK856336.1|	00605-10128	10	37	45	0	-inf	5.00E-05	yes
gi|641570501|gb|KK856336.1|	10136-11163	10	37	12	16	0.42466	0.73	no
gi|641570501|gb|KK856336.1|	07009-10851	10	37	15	0.823856	-4	0.01955	no
gi|641570501|gb|KK856336.1|	09372-11110	10	37	29	0.211293	-7	0.10075	no
gi|641570501|gb|KK856336.1|	13352-11460	10	37	29	0.101555	-8	0.18285	no
gi|641570501|gb|KK856336.1|	15211-11597	10	37	2	0	-inf	0.0059	no
gi|641570501|gb|KK856336.1|	20524-28192	10	37	8	7	-0.167345	0.9386	no
gi|641570501|gb|KK856336.1|	28273-28966	10	37	1	3	0.863527	0.56615	no
gi|641570501|gb|KK856336.1|	29598-30732	10	37	0.377131	1	2	0.3084	no
gi|641570501|gb|KK856336.1|	3696-4530	10	37	39	63	0.674448	0.67555	no
gi|641570501|gb|KK856336.1|	44465-45627	10	37	2	6	1	0.2989	no
gi|641570501|gb|KK856336.1|	46561-46965	10	37	33	59	0.855688	0.50745	no

gi|641570501|gb|KK856336.1|	47320-48391	10	37	12	20	0.782086	0.53545	no
gi|641570501|gb|KK856336.1|	5003-5485	10	37	4	4	-0.0359963	0.96295	no
gi|641570501|gb|KK856336.1|	55696-56631	10	37	0.670024	0.873114	0.381958	1	no
gi|641570501|gb|KK856336.1|	6346-7521	10	37	2	1	-0.278219	0.8412	no
gi|641570501|gb|KK856336.1|	64035-64341	10	37	48	104	1	0.3889	no
gi|641570501|gb|KK856336.1|	73174-74034	10	37	0.745623	3	2	0.2786	no
gi|641570501|gb|KK856336.1|	77595-78192	10	37	1	2	1	0.5082	no
gi|641570501|gb|KK856336.1|	79050-80354	10	37	2	3	0.554537	0.6809	no
gi|641570501|gb|KK856336.1|	81040-81287	10	37	5	12	1	0.46685	no
gi|641570501|gb|KK856336.1|	83950-84638	10	37	1	0.435664	-2	0.4266	no
gi|641570501|gb|KK856336.1|	84775-85933	10	37	1	1	0.158738	0.9153	no
gi|641570501|gb|KK856336.1|	90920-91321	10	37	5	0	-inf	0.0059	no
gi|641570501|gb|KK856336.1|	9544-9959	10	37	10	6	-0.828609	0.5571	no
gi|641570501|gb|KK856336.1|	97653-98155	10	37	90	0	-inf	5.00E-05	yes
gi|641570507|gb|KK856334.1|	14872-15672	10	39	3	2	-0.581851	0.6957	no
gi|641570507|gb|KK856334.1|	88946-90066	10	39	9	6	-0.548777	0.66495	no
gi|641570508|gb|KK856333.1|	.00676-10136	7.7	40	0.570092	2	2	0.26155	no
gi|641570508|gb|KK856333.1|	79004-80117	7.7	40	0	21	inf	5.00E-05	yes
gi|641570511|gb|KK856332.1|	.07296-10898	11	40	0.473693	0.938817	0.986892	0.5381	no
gi|641570511|gb|KK856332.1|	.14256-11548	11	40	0.136989	2	4	0.243	no
gi|641570511|gb|KK856332.1|	.16978-11804	11	40	0.162075	1	3	0.2851	no
gi|641570511|gb|KK856332.1|	.18493-12021	11	40	0.601318	2	1	0.3257	no
gi|641570511|gb|KK856332.1|	13409-14421	11	40	0.433467	3	3	0.1259	no
gi|641570511|gb|KK856332.1|	75983-77651	11	40	0.288461	2	2	0.1589	no
gi|641570511|gb|KK856332.1|	79585-80125	11	40	0.615149	2	2	0.3323	no
gi|641570511|gb|KK856332.1|	8057-9096	11	40	0.167838	1	3	0.27505	no
gi|641570511|gb|KK856332.1|	87523-89181	11	40	0.532461	0.221376	-1	1	no
gi|641570511|gb|KK856332.1|	90757-91371	11	40	1	2	0.59827	0.69715	no
gi|641570511|gb|KK856332.1|	9181-10487	11	40	0.573714	0.873826	0.607014	1	no
gi|641570514|gb|KK856331.1|	.01196-10731	9.8	39	13	8	-0.622004	0.7801	no

gi|641570514|gb|KK856331.1|	:09812-11075	9.8	39	0.761623	3	2	0.22535	no
gi|641570514|gb|KK856331.1|	:27925-13020	9.8	39	21	15	-0.433864	0.78405	no
gi|641570514|gb|KK856331.1|	:36329-13685	9.8	39	2	4	1	0.49595	no
gi|641570514|gb|KK856331.1|	:37979-13834	9.8	39	4	5	0.258475	0.87415	no
gi|641570514|gb|KK856331.1|	44675-44894	9.8	39	78	33	-1	0.37315	no
gi|641570514|gb|KK856331.1|	47054-47345	9.8	39	78	31	-1	0.3082	no
gi|641570514|gb|KK856331.1|	48917-49202	9.8	39	43	27	-0.666802	0.6129	no
gi|641570514|gb|KK856331.1|	54848-55466	9.8	39	1	2	0.820794	0.62335	no
gi|641570514|gb|KK856331.1|	55615-56205	9.8	39	3	2	-0.905074	0.56885	no
gi|641570514|gb|KK856331.1|	56256-56719	9.8	39	4	0.39632	-3	0.21435	no
gi|641570514|gb|KK856331.1|	56873-57809	9.8	39	7	4	-0.698201	0.59055	no
gi|641570514|gb|KK856331.1|	59735-61091	9.8	39	1	0.650469	-0.672865	0.6649	no
gi|641570514|gb|KK856331.1|	64281-65251	9.8	39	26	10	-1	0.30965	no
gi|641570514|gb|KK856331.1|	67184-68369	9.8	39	3	2	-0.978598	0.4756	no
gi|641570514|gb|KK856331.1|	68675-70346	9.8	39	3	2	-0.529174	0.68625	no
gi|641570514|gb|KK856331.1|	70461-70939	9.8	39	3	2	-0.545739	0.7461	no
gi|641570514|gb|KK856331.1|	71083-72731	9.8	39	5	3	-1	0.43955	no
gi|641570514|gb|KK856331.1|	78339-79109	9.8	39	5	1	-2	0.245	no
gi|641570514|gb|KK856331.1|	79276-80071	9.8	39	2	2	-0.260004	0.8591	no
gi|641570514|gb|KK856331.1|	80995-81831	9.8	39	5	3	-0.949421	0.495	no
gi|641570514|gb|KK856331.1|	83050-83357	9.8	39	34	19	-0.804403	0.5441	no
gi|641570514|gb|KK856331.1|	85897-86463	9.8	39	25	9	-1	0.26845	no
gi|641570514|gb|KK856331.1|	90940-91568	9.8	39	12	5	-1	0.36145	no
gi|641570514|gb|KK856331.1|	91828-92565	9.8	39	11	20	0.884623	0.4874	no
gi|641570514|gb|KK856331.1|	93943-94839	9.8	39	0.808361	2	0.925835	0.5636	no
gi|641570514|gb|KK856331.1|	95365-96370	9.8	39	0.961871	0.665011	-0.532464	0.7478	no
gi|641570514|gb|KK856331.1|	96857-97442	9.8	39	2	4	1	0.4339	no
gi|641570514|gb|KK856331.1|	)9236-10022:	9.8	39	0.537944	0.681766	0.341821	1	no
gi|641570517|gb|KK856330.1|	:05131-10538	11	21	11	4	-1	0.4178	no
gi|641570517|gb|KK856330.1|	:07304-10984	11	21	17	18	0.0299225	0.9887	no

gi|641570517|gb|KK856330.1|	14710-11490	11	21	60	13	-2	0.1946	no
gi|641570517|gb|KK856330.1|	15169-11572	11	21	26	2	-4	0.0389	no
gi|641570517|gb|KK856330.1|	23577-12377	11	21	52	0	-inf	0.0032	yes
gi|641570517|gb|KK856330.1|	29378-12969	11	21	9	0	-inf	0.0056	no
gi|641570517|gb|KK856330.1|	33703-13392	11	21	101	2	-6	0.18395	no
gi|641570517|gb|KK856330.1|	34203-13440	11	21	34	0	-inf	0.00435	no
gi|641570517|gb|KK856330.1|	39053-13975	11	21	28	6	-2	0.09555	no
gi|641570517|gb|KK856330.1|	2062-3510	11	21	0.565201	0.344488	-0.714311	1	no
gi|641570517|gb|KK856330.1|	32657-33631	11	21	0.727391	0.967885	0.412105	0.8089	no
gi|641570517|gb|KK856330.1|	4341-4922	11	21	2	2	0.0121209	0.95145	no
gi|641570517|gb|KK856330.1|	58781-60306	11	21	1	0.405751	-1	0.3598	no
gi|641570517|gb|KK856330.1|	64036-64680	11	21	2	1	-0.516274	0.74125	no
gi|641570517|gb|KK856330.1|	65640-66613	11	21	1	3	1	0.3735	no
gi|641570517|gb|KK856330.1|	68706-69560	11	21	0.214976	2	4	0.24945	no
gi|641570517|gb|KK856330.1|	6883-7247	11	21	3	5	0.579948	0.7371	no
gi|641570517|gb|KK856330.1|	85458-85973	11	21	13	3	-2	0.1624	no
gi|641570517|gb|KK856330.1|	93667-94305	11	21	2	0.72645	-2	0.403	no
gi|641570523|gb|KK856328.1|	11038-11270	12	47	1	36	5	0.03675	no
gi|641570523|gb|KK856328.1|	12909-11379	12	47	4	2	-1	0.34895	no
gi|641570523|gb|KK856328.1|	14315-11675	12	47	0.631972	0.530393	-0.2528	1	no
gi|641570523|gb|KK856328.1|	17034-11880	12	47	0.900399	1	0.678966	0.62475	no
gi|641570523|gb|KK856328.1|	25417-12575	12	47	5	1	-2	0.3414	no
gi|641570523|gb|KK856328.1|	31768-13231	12	47	2	2	0.180466	0.9106	no
gi|641570523|gb|KK856328.1|	35094-13564	12	47	2	0.893764	-0.989003	0.62775	no
gi|641570523|gb|KK856328.1|	38378-14238	12	47	11	0.681678	-4	0.06605	no
gi|641570523|gb|KK856328.1|	42491-14338	12	47	3	0.925365	-2	0.2371	no
gi|641570523|gb|KK856328.1|	43493-14959	12	47	5	4	-0.0785605	0.9454	no
gi|641570523|gb|KK856328.1|	56392-15696	12	47	0	54	inf	5.00E-05	yes
gi|641570523|gb|KK856328.1|	57781-15853	12	47	0	48	inf	5.00E-05	yes
gi|641570523|gb|KK856328.1|	58892-16076	12	47	0	9	inf	5.00E-05	yes

gi|641570523|gb|KK856328.1|	62697-16286	12	47	0	283	inf	5.00E-05	yes
gi|641570523|gb|KK856328.1|	65029-16535	12	47	0	24	inf	0.0003	yes
gi|641570523|gb|KK856328.1|	65978-16616	12	47	0	63	inf	0.0016	yes
gi|641570523|gb|KK856328.1|	66657-16900	12	47	0.624336	2	1	0.3532	no
gi|641570523|gb|KK856328.1|	70982-17398	12	47	67	96	0.512166	0.7895	no
gi|641570523|gb|KK856328.1|	74136-17565	12	47	0.912753	2	1	0.37435	no
gi|641570523|gb|KK856328.1|	78142-18115	12	47	2	3	0.66994	0.6014	no
gi|641570523|gb|KK856328.1|	81273-18210	12	47	0.778413	1	0.38067	0.82465	no
gi|641570523|gb|KK856328.1|	86206-18646	12	47	27	19	-0.495635	0.72295	no
gi|641570523|gb|KK856328.1|	88159-18883	12	47	74	89	0.273778	0.8622	no
gi|641570523|gb|KK856328.1|	91638-19225	12	47	2	4	1	0.47995	no
gi|641570523|gb|KK856328.1|	94775-19611	12	47	22	70	2	0.3302	no
gi|641570523|gb|KK856328.1|	52715-53394	12	47	0.439041	2	2	0.21365	no
gi|641570523|gb|KK856328.1|	57354-57571	12	47	11	41	2	0.25105	no
gi|641570523|gb|KK856328.1|	75408-75930	12	47	1	2	0.331377	0.83525	no
gi|641570523|gb|KK856328.1|	90791-92038	12	47	0.942509	0.410069	-1	0.46645	no
gi|641570526|gb|KK856327.1|	00134-10075	9.1	37	542	393	-0.464575	0.8152	no
gi|641570526|gb|KK856327.1|	01532-10169	9.1	37	134.36	141.39	0.0735736	0.96085	no
gi|641570526|gb|KK856327.1|	04233-10498	9.1	37	38	32	-0.238482	0.8447	no
gi|641570526|gb|KK856327.1|	05179-10768	9.1	37	4	3	-0.562858	0.65915	no
gi|641570526|gb|KK856327.1|	08158-11282	9.1	37	4	2	-0.699327	0.58365	no
gi|641570526|gb|KK856327.1|	11434-13308	9.1	37	2	2	-0.117012	0.92815	no
gi|641570526|gb|KK856327.1|	147-982	9.1	37	2	3	0.851033	0.55355	no
gi|641570526|gb|KK856327.1|	16863-20219	9.1	37	4	4	-0.113561	0.9272	no
gi|641570526|gb|KK856327.1|	1966-2441	9.1	37	2	2	-0.0865145	0.9267	no
gi|641570526|gb|KK856327.1|	23200-25229	9.1	37	3	2	-0.580713	0.65845	no
gi|641570526|gb|KK856327.1|	25456-30991	9.1	37	11	3	-2	0.24565	no
gi|641570526|gb|KK856327.1|	33409-33644	9.1	37	28	2	-4	0.1967	no
gi|641570526|gb|KK856327.1|	4434-5065	9.1	37	2	5	1	0.426	no
gi|641570526|gb|KK856327.1|	61600-62546	9.1	37	19	125	3	0.14345	no

gi|641570526|gb|KK856327.1|	62697-63232	9.1	37	0.624127	5	3	0.1725	no
gi|641570526|gb|KK856327.1|	63378-65744	9.1	37	1	1	0.38013	0.7786	no
gi|641570526|gb|KK856327.1|	65851-66475	9.1	37	5	51	3	0.0246	no
gi|641570526|gb|KK856327.1|	80005-80780	9.1	37	0	3	inf	0.00255	yes
gi|641570526|gb|KK856327.1|	8231-8919	9.1	37	0.431035	2	2	0.3323	no
gi|641570526|gb|KK856327.1|	84100-84629	9.1	37	0.635248	3	2	0.2812	no
gi|641570526|gb|KK856327.1|	84803-85082	9.1	37	0	14	inf	0.0025	yes
gi|641570526|gb|KK856327.1|	90613-94577	9.1	37	2	3	0.799801	0.5298	no
gi|641570526|gb|KK856327.1|	9937-11033	9.1	37	5	14	1	0.26455	no
gi|641570529|gb|KK856326.1|	15111-11578	8.9	36	0.747152	1	0.862928	0.62725	no
gi|641570529|gb|KK856326.1|	74319-75021	8.9	36	0.978001	0.635612	-0.62169	0.71195	no
gi|641570529|gb|KK856326.1|	95598-95979	8.9	36	4	10	1	0.3695	no
gi|641570529|gb|KK856326.1|	99867-100338	8.9	36	5	5	0.0437873	0.967	no
gi|641570539|gb|KK856323.1|	20395-21000	10	22	0.345393	9	5	0.2353	no
gi|641570542|gb|KK856322.1|	72270-72707	9.1	40	0	5	inf	0.00455	no
gi|641570542|gb|KK856322.1|	81876-82708	9.1	40	0	4	inf	0.00045	yes
gi|641570555|gb|KK856321.1|	09417-10999	11	38	51	2	-5	0.02945	no
gi|641570555|gb|KK856321.1|	17469-20899	11	38	4	1	-2	0.23305	no
gi|641570555|gb|KK856321.1|	21608-23974	11	38	8	5	-0.748985	0.55415	no
gi|641570555|gb|KK856321.1|	24096-26172	11	38	10	9	-0.150229	0.90405	no
gi|641570555|gb|KK856321.1|	27543-28039	11	38	4	2	-0.728997	0.652	no
gi|641570555|gb|KK856321.1|	48444-49173	11	38	0	17	inf	5.00E-05	yes
gi|641570555|gb|KK856321.1|	76181-77601	11	38	52	0.968928	-6	0.0258	no
gi|641570558|gb|KK856320.1|	09187-11040	9.6	39	0.62369	2	2	0.284	no
gi|641570558|gb|KK856320.1|	16625-11688	9.6	39	2	15	3	0.26765	no
gi|641570558|gb|KK856320.1|	37020-13751	9.6	39	0.472544	7	4	0.24015	no
gi|641570558|gb|KK856320.1|	44671-14593	9.6	39	4	8	0.959451	0.45085	no
gi|641570558|gb|KK856320.1|	53976-15449	9.6	39	2	2	0.594089	0.69715	no
gi|641570558|gb|KK856320.1|	48175-48672	9.6	39	1	4	2	0.32035	no
gi|641570558|gb|KK856320.1|	55177-56462	9.6	39	0.129949	3	4	0.2373	no

gi|641570567|gb|KK856317.1|	00592-10144	10	44	0.865156	2	1	0.38845	no	
gi|641570567|gb|KK856317.1|	14544-11492	10	44	3	2	-0.639995	0.71075	no	
gi|641570567|gb|KK856317.1|	17505-11803	10	44	1	2	0.817317	0.6234	no	
gi|641570567|gb|KK856317.1|	41524-42574	10	44	1	0	-inf	0.0056	no	
gi|641570567|gb|KK856317.1|	42655-44709	10	44	0.952318	0.290527	-2	0.2894	no	
gi|641570567|gb|KK856317.1|	62864-63903	10	44	1	0	-inf	0.0056	no	
gi|641570570|gb|KK856316.1|	04453-10483	10	35	7	1	-3	0.1909	no	
gi|641570570|gb|KK856316.1|	07922-10817	10	35	49	23	-1	0.44115	no	
gi|641570570|gb|KK856316.1|	09973-11070	10	35	2	3	0.601391	0.68145	no	
gi|641570570|gb|KK856316.1|	12532-11295	10	35	5	0	-inf	0.00435	no	
gi|641570570|gb|KK856316.1|	11428-11865	10	35	6	18	2	0.2713	no	
gi|641570570|gb|KK856316.1|	18592-11926	10	35	35	14	-1	0.30365	no	
gi|641570570|gb|KK856316.1|	20358-12159	10	35	3	1	-1	0.45745	no	
gi|641570570|gb|KK856316.1|	29508-13691	10	35	6	7	0.119518	0.9558	no	
gi|641570570|gb|KK856316.1|	37151-13778	10	35	5	4	-0.12114	0.9308	no	
gi|641570570|gb|KK856316.1|	37888-13876	10	35	0.517788	4	3	0.1385	no	
gi|641570570|gb|KK856316.1|	42084-14229	10	35	67	113	0.746883	0.5637	no	
gi|641570570|gb|KK856316.1|	14842-16189	10	35	0.73772	0.655534	-0.170404	1	no	
gi|641570570|gb|KK856316.1|	16712-20136	10	35	5	1	-2	0.18745	no	
gi|641570570|gb|KK856316.1|	20280-22777	10	35	5	3	-0.854514	0.5011	no	
gi|641570570|gb|KK856316.1|	22937-23366	10	35	6	2	-1	0.35835	no	
gi|641570570|gb|KK856316.1|	24-705	10	35	4	3	-0.454247	0.75065	no	
gi|641570570|gb|KK856316.1|	25050-28199	10	35	12	16	0.434484	0.7849	no	
gi|641570570|gb|KK856316.1|	2902-3324	10	35	3	3	-0.149827	0.92525	no	
gi|641570570|gb|KK856316.1|	32650-32970	10	35	2	5	1	0.5206	no	
gi|641570570|gb|KK856316.1|	33179-33636	10	35	24	11	-1	0.3587	no	
gi|641570570|gb|KK856316.1|	37532-38647	10	35	0.923813	0.468662	-0.979052	0.5872	no	
gi|641570570|gb|KK856316.1|	50658-50971	10	35	16	14	-0.200089	0.87995	no	
gi|641570570|gb|KK856316.1|	57452-58720	10	35	0.660042	1	0.606826	0.6779	no	
gi|641570570|gb|KK856316.1|	5747-5963	10	35	77	74	-0.0466604	0.9713	no	

gi|641570570|gb|KK856316.1|	58975-59888	10	35	1	2	0.711022	0.6334	no
gi|641570570|gb|KK856316.1|	60532-62033	10	35	0.650711	1	1	0.45455	no
gi|641570570|gb|KK856316.1|	63862-66401	10	35	7	4	-0.915278	0.47635	no
gi|641570570|gb|KK856316.1|	66783-67559	10	35	2	0.370164	-2	0.26805	no
gi|641570570|gb|KK856316.1|	68639-69526	10	35	1	0	-inf	0.0102	no
gi|641570570|gb|KK856316.1|	73914-75757	10	35	30	23	-0.351895	0.87515	no
gi|641570570|gb|KK856316.1|	76060-77141	10	35	12	5	-1	0.262	no
gi|641570570|gb|KK856316.1|	79105-79503	10	35	5	1	-2	0.2477	no
gi|641570570|gb|KK856316.1|	7941-8939	10	35	0.617437	0.804937	0.382584	1	no
gi|641570570|gb|KK856316.1|	84294-85024	10	35	2	0.60262	-2	0.33235	no
gi|641570570|gb|KK856316.1|	87760-88599	10	35	0.87994	1	0.410518	0.8089	no
gi|641570570|gb|KK856316.1|	89341-89634	10	35	34	21	-0.742513	0.58115	no
gi|641570570|gb|KK856316.1|	90829-91429	10	35	0.349642	7	4	0.23805	no
gi|641570570|gb|KK856316.1|	96183-96518	10	35	3	4	0.311959	0.86115	no
gi|641570570|gb|KK856316.1|	96639-98090	10	35	1	0.429584	-1	0.3598	no
gi|641570573|gb|KK856315.1|	06139-10671	10	45	0	25	inf	5.00E-05	yes
gi|641570573|gb|KK856315.1|	20230-12064	10	45	0	8	inf	0.0016	yes
gi|641570573|gb|KK856315.1|	23348-12365	10	45	0	10	inf	0.00375	no
gi|641570573|gb|KK856315.1|	30959-13109	10	45	0	276	inf	0.00375	no
gi|641570573|gb|KK856315.1|	31503-13198	10	45	0.249443	3	4	0.1276	no
gi|641570573|gb|KK856315.1|	32905-13386	10	45	0.092347	2	4	0.11955	no
gi|641570573|gb|KK856315.1|	34459-14253	10	45	9	29	2	0.4569	no
gi|641570573|gb|KK856315.1|	44230-14649	10	45	0.752969	1	0.904933	0.5195	no
gi|641570573|gb|KK856315.1|	47243-14915	10	45	0.619376	1	0.701997	0.62415	no
gi|641570573|gb|KK856315.1|	49261-15001	10	45	0.511468	1	1	0.44255	no
gi|641570573|gb|KK856315.1|	50741-15118	10	45	19	24	0.282802	0.83065	no
gi|641570573|gb|KK856315.1|	55295-15665	10	45	0.605825	0.461451	-0.392722	1	no
gi|641570573|gb|KK856315.1|	57749-16076	10	45	0.828635	1	0.3732	0.78355	no
gi|641570573|gb|KK856315.1|	62412-16316	10	45	0.630236	1	1	0.51085	no
gi|641570573|gb|KK856315.1|	63511-16423	10	45	25	88	2	0.29855	no

gi|641570573|gb|KK856315.1|	.66039-16714	10	45	0.466471	0.709909	0.605847	1	no
gi|641570573|gb|KK856315.1|	.67341-16935	10	45	0.585568	0.952699	0.702185	0.62415	no
gi|641570573|gb|KK856315.1|	.69685-17258	10	45	0.549734	0.839123	0.61015	1	no
gi|641570573|gb|KK856315.1|	.75410-17680	10	45	0.941135	0.358463	-1	0.4015	no
gi|641570573|gb|KK856315.1|	.77925-17935	10	45	0.457271	0.348368	-0.392437	1	no
gi|641570573|gb|KK856315.1|	.79631-18083	10	45	1	0.746614	-0.834084	0.57785	no
gi|641570573|gb|KK856315.1|	.81591-18200	10	45	2	3	0.363506	0.82765	no
gi|641570573|gb|KK856315.1|	.82122-18542	10	45	3	4	0.829074	0.50795	no
gi|641570573|gb|KK856315.1|	.85924-18634	10	45	3	3	0.224167	0.88235	no
gi|641570573|gb|KK856315.1|	.96283-19689	10	45	2	1	-0.723759	0.67005	no
gi|641570573|gb|KK856315.1|	.97687-20034	10	45	3	2	-0.235088	0.85545	no
gi|641570573|gb|KK856315.1|	.00918-20165	10	45	5	11	1	0.38295	no
gi|641570579|gb|KK856313.1|	.02131-10351	11	38	0.179444	1	2	0.2136	no
gi|641570579|gb|KK856313.1|	.07202-10800	11	38	14	8	-0.773543	0.54775	no
gi|641570579|gb|KK856313.1|	.15560-11655	11	38	3	1	-1	0.45055	no
gi|641570579|gb|KK856313.1|	.17735-11865	11	38	0.7773	1	0.411585	0.8089	no
gi|641570579|gb|KK856313.1|	.19552-12047	11	38	0.683892	0.445542	-0.618207	1	no
gi|641570579|gb|KK856313.1|	.27199-12801	11	38	2	0.864786	-1	0.35975	no
gi|641570579|gb|KK856313.1|	.29452-13117	11	38	3	0.9216	-2	0.28165	no
gi|641570579|gb|KK856313.1|	.32617-13317	11	38	3	0	-inf	0.00435	no
gi|641570579|gb|KK856313.1|	14100-14650	11	38	3	0.903658	-2	0.332	no
gi|641570579|gb|KK856313.1|	.41124-14280	11	38	0.237961	0.798111	2	1	no
gi|641570579|gb|KK856313.1|	.45555-14718	11	38	0.442918	0.675176	0.608226	1	no
gi|641570579|gb|KK856313.1|	.47268-14776	11	38	2	2	0.370539	0.82765	no
gi|641570579|gb|KK856313.1|	.47921-14866	11	38	1	0.586311	-0.983355	0.62775	no
gi|641570579|gb|KK856313.1|	14879-15208	11	38	3	5	0.836857	0.63695	no
gi|641570579|gb|KK856313.1|	.49853-15079	11	38	14	2	-2	0.07915	no
gi|641570579|gb|KK856313.1|	.51026-15387	11	38	2	2	0.486722	0.70395	no
gi|641570579|gb|KK856313.1|	15447-18550	11	38	4	5	0.19792	0.87715	no
gi|641570579|gb|KK856313.1|	.54523-15575	11	38	0.888707	0.312284	-2	0.40425	no

gi|641570579|gb|KK856313.1|	.70151-17084	11	38	17	6	-2	0.24425	no
gi|641570579|gb|KK856313.1|	1713-2565	11	38	37	33	-0.174864	0.888	no
gi|641570579|gb|KK856313.1|	.73340-17376	11	38	12	2	-3	0.13215	no
gi|641570579|gb|KK856313.1|	.73857-17421	11	38	7	0.640359	-3	0.20885	no
gi|641570579|gb|KK856313.1|	18675-19761	11	38	0.476945	0.967702	1	0.5082	no
gi|641570579|gb|KK856313.1|	19876-22502	11	38	0.902989	0.889098	-0.0223649	0.9762	no
gi|641570579|gb|KK856313.1|	23187-24834	11	38	1	0.520448	-1	0.3708	no
gi|641570579|gb|KK856313.1|	25582-27320	11	38	0.688431	0.139951	-2	1	no
gi|641570579|gb|KK856313.1|	27424-28340	11	38	0.785928	1	0.411496	0.8089	no
gi|641570579|gb|KK856313.1|	31056-31765	11	38	10	10	-0.123505	0.92315	no
gi|641570579|gb|KK856313.1|	34967-35488	11	38	4	9	0.97287	0.49105	no
gi|641570579|gb|KK856313.1|	41809-42574	11	38	0.62139	2	2	0.3084	no
gi|641570579|gb|KK856313.1|	43224-43878	11	38	4	5	0.422611	0.75435	no
gi|641570579|gb|KK856313.1|	45083-46545	11	38	7	9	0.249775	0.8431	no
gi|641570579|gb|KK856313.1|	46704-49333	11	38	3	6	1	0.3653	no
gi|641570579|gb|KK856313.1|	51745-52844	11	38	0.548432	1	1	0.39245	no
gi|641570579|gb|KK856313.1|	53915-54298	11	38	0.370802	5	4	0.12785	no
gi|641570579|gb|KK856313.1|	55685-56861	11	38	0.288784	1	2	0.26155	no
gi|641570579|gb|KK856313.1|	56952-57381	11	38	8	10	0.467522	0.7307	no
gi|641570579|gb|KK856313.1|	59175-59720	11	38	3	3	-0.169984	0.9031	no
gi|641570579|gb|KK856313.1|	6094-6526	11	38	1	5	2	0.31505	no
gi|641570579|gb|KK856313.1|	62108-63905	11	38	1	2	0.0878121	0.94635	no
gi|641570579|gb|KK856313.1|	64097-65279	11	38	2	1	-0.278177	0.8412	no
gi|641570579|gb|KK856313.1|	66067-66959	11	38	0.609751	0.772238	0.340825	1	no
gi|641570579|gb|KK856313.1|	69376-69903	11	38	0.426029	3	3	0.27515	no
gi|641570579|gb|KK856313.1|	70235-70598	11	38	14	12	-0.301561	0.8216	no
gi|641570579|gb|KK856313.1|	74577-74777	11	38	17	15	-0.1478	0.91755	no
gi|641570579|gb|KK856313.1|	81726-82346	11	38	20	12	-0.735434	0.56605	no
gi|641570579|gb|KK856313.1|	94570-94974	11	38	18	8	-1	0.3967	no
gi|641570579|gb|KK856313.1|	99752-10023[illegible]	11	38	8	6	-0.362161	0.79265	no

gi|641570587|gb|KK856311.1|	12733-11310	9.9	38	37	2	-4	0.0654	no
gi|641570587|gb|KK856311.1|	36304-13775	9.9	38	0.959311	0.601896	-0.672484	0.6649	no
gi|641570587|gb|KK856311.1|	38037-13879	9.9	38	1	1	0.0168531	0.9394	no
gi|641570587|gb|KK856311.1|	38941-14089	9.9	38	2	0.306312	-2	0.13575	no
gi|641570587|gb|KK856311.1|	44477-14501	9.9	38	7	4	-0.820014	0.56	no
gi|641570587|gb|KK856311.1|	46647-15023	9.9	38	0.896321	1	0.393784	0.7634	no
gi|641570587|gb|KK856311.1|	50364-15148	9.9	38	0.535977	2	1	0.36605	no
gi|641570587|gb|KK856311.1|	52341-15442	9.9	38	5	4	-0.359541	0.7782	no
gi|641570587|gb|KK856311.1|	54538-15622	9.9	38	7	9	0.277161	0.82315	no
gi|641570587|gb|KK856311.1|	61971-16254	9.9	38	0.186761	8	5	0.10635	no
gi|641570587|gb|KK856311.1|	67368-16854	9.9	38	4	12	2	0.2053	no
gi|641570587|gb|KK856311.1|	69167-17105	9.9	38	7	5	-0.593644	0.63345	no
gi|641570587|gb|KK856311.1|	71405-17448	9.9	38	10	3	-2	0.1734	no
gi|641570587|gb|KK856311.1|	74627-17537	9.9	38	5	10	0.971996	0.4632	no
gi|641570587|gb|KK856311.1|	26824-27120	9.9	38	5	6	0.151339	0.92285	no
gi|641570587|gb|KK856311.1|	36-1595	9.9	38	2	1	-0.346196	0.79975	no
gi|641570587|gb|KK856311.1|	45566-46026	9.9	38	6	6	0.0668987	0.9633	no
gi|641570587|gb|KK856311.1|	62954-64150	9.9	38	0.849037	3	2	0.2618	no
gi|641570587|gb|KK856311.1|	64767-66443	9.9	38	0.956343	2	1	0.3924	no
gi|641570587|gb|KK856311.1|	66671-68998	9.9	38	0.431786	0.658818	0.609562	1	no
gi|641570587|gb|KK856311.1|	69514-71247	9.9	38	0.644625	1	0.888599	0.53725	no
gi|641570587|gb|KK856311.1|	71371-73175	9.9	38	1	2	0.345626	0.8013	no
gi|641570587|gb|KK856311.1|	73299-76214	9.9	38	23	72	2	0.45465	no
gi|641570589|gb|KK856310.1|	01863-10223	11	36	8	5	-0.707938	0.63025	no
gi|641570589|gb|KK856310.1|	12784-13115	11	36	90	36	-1	0.3052	no
gi|641570589|gb|KK856310.1|	13650-13899	11	36	20	16	-0.310312	0.8303	no
gi|641570589|gb|KK856310.1|	14686-14882	11	36	229	67	-2	0.1926	no
gi|641570589|gb|KK856310.1|	21456-22120	11	36	14	7	-1	0.3928	no
gi|641570589|gb|KK856310.1|	24544-24726	11	36	105	78	-0.436259	0.74925	no
gi|641570589|gb|KK856310.1|	29995-30191	11	36	35	27	-0.378726	0.80055	no

gi|641570589|gb|KK856310.1|	30307-31328	11	36	40	17	-1	0.32995	no
gi|641570589|gb|KK856310.1|	31545-32004	11	36	63	24	-1	0.2794	no
gi|641570589|gb|KK856310.1|	35784-36573	11	36	2	0	-inf	0.0059	no
gi|641570589|gb|KK856310.1|	37145-37426	11	36	23	3	-3	0.1474	no
gi|641570589|gb|KK856310.1|	37978-38124	11	36	606	362.8	-0.740407	0.57585	no
gi|641570589|gb|KK856310.1|	39504-40081	11	36	5	3	-0.725002	0.6129	no
gi|641570589|gb|KK856310.1|	42582-45454	11	36	37	14	-1	0.5404	no
gi|641570589|gb|KK856310.1|	45819-46802	11	36	28	9	-2	0.20165	no
gi|641570589|gb|KK856310.1|	48922-50508	11	36	4	1	-2	0.25565	no
gi|641570589|gb|KK856310.1|	54533-54858	11	36	17	9	-0.842028	0.55065	no
gi|641570589|gb|KK856310.1|	55135-55352	11	36	20.47	2	-3	0.2236	no
gi|641570589|gb|KK856310.1|	5794-6153	11	36	22	18	-0.290771	0.82695	no
gi|641570589|gb|KK856310.1|	66441-67027	11	36	0	3	inf	0.00375	no
gi|641570589|gb|KK856310.1|	73671-75817	11	36	6	8	0.247905	0.8398	no
gi|641570589|gb|KK856310.1|	76092-84492	11	36	8	7	-0.320305	0.88495	no
gi|641570589|gb|KK856310.1|	88814-91944	11	36	0.386019	38	7	0.01775	no
gi|641570589|gb|KK856310.1|	93668-97606	11	36	25	14	-0.811343	0.7143	no
gi|641570589|gb|KK856310.1|	98335-100355	11	36	15	8	-0.946196	0.44925	no
gi|641570593|gb|KK856309.1|	00379-10119	9.6	33	2	0.173512	-4	0.20475	no
gi|641570593|gb|KK856309.1|	01258-10182	9.6	33	3	0	-inf	0.0059	no
gi|641570593|gb|KK856309.1|	02203-10453	9.6	33	15	4	-2	0.11575	no
gi|641570593|gb|KK856309.1|	04842-10575	9.6	33	2	1	-0.395916	0.7971	no
gi|641570593|gb|KK856309.1|	08225-10913	9.6	33	0.691525	0.900974	0.381703	0.82465	no
gi|641570593|gb|KK856309.1|	09684-10985	9.6	33	44	21	-1	0.52715	no
gi|641570593|gb|KK856309.1|	17711-11822	9.6	33	2	2	-0.0583207	0.9454	no
gi|641570593|gb|KK856309.1|	28777-13357	9.6	33	6	11	0.896747	0.5779	no
gi|641570593|gb|KK856309.1|	34620-13843	9.6	33	4	5	0.500279	0.69	no
gi|641570593|gb|KK856309.1|	45375-45569	9.6	33	99	132	0.417472	0.74735	no
gi|641570593|gb|KK856309.1|	60755-63708	9.6	33	6	12	0.930961	0.45095	no
gi|641570593|gb|KK856309.1|	70490-72936	9.6	33	0.629165	1	0.98822	0.4895	no

gi|641570593|gb|KK856309.1|	73669-74303	9.6	33	8	15	0.938393	0.46655	no
gi|641570593|gb|KK856309.1|	85554-85742	9.6	33	33	63	0.938922	0.52745	no
gi|641570593|gb|KK856309.1|	87010-87869	9.6	33	21	32	0.625243	0.61525	no
gi|641570593|gb|KK856309.1|	90266-92544	9.6	33	0.169979	45	8	0.08395	no
gi|641570593|gb|KK856309.1|	95585-97594	9.6	33	2	5	1	0.26175	no
gi|641570593|gb|KK856309.1|	99187-10014[illegible]	9.6	33	2	0	-inf	0.00435	no
gi|641570596|gb|KK856308.1|	[illegible]00136-10097	9.9	39	7	9	0.3401	0.79265	no
gi|641570596|gb|KK856308.1|	[illegible]01244-10243	9.9	39	1	2	0.326274	0.8152	no
gi|641570596|gb|KK856308.1|	[illegible]16211-11758	9.9	39	3	3	0.270298	0.835	no
gi|641570596|gb|KK856308.1|	[illegible]21462-12489	9.9	39	7	6	-0.220611	0.85805	no
gi|641570596|gb|KK856308.1|	[illegible]25721-12615	9.9	39	12	10	-0.210549	0.875	no
gi|641570596|gb|KK856308.1|	[illegible]27608-12823	9.9	39	3	1	-1	0.3596	no
gi|641570596|gb|KK856308.1|	[illegible]28342-12888	9.9	39	40	27	-0.595977	0.62995	no
gi|641570596|gb|KK856308.1|	[illegible]29097-12957	9.9	39	26	19	-0.464616	0.7099	no
gi|641570596|gb|KK856308.1|	[illegible]34548-13499	9.9	39	0.845142	4	2	0.256	no
gi|641570596|gb|KK856308.1|	[illegible]40070-14072	9.9	39	0.621276	26	5	0.11615	no
gi|641570596|gb|KK856308.1|	[illegible]44473-14502	9.9	39	0	64	inf	5.00E-05	yes
gi|641570596|gb|KK856308.1|	[illegible]49101-14951	9.9	39	0	135	inf	5.00E-05	yes
gi|641570596|gb|KK856308.1|	40424-41451	9.9	39	3	1	-0.889533	0.53165	no
gi|641570596|gb|KK856308.1|	68260-68827	9.9	39	7	3	-1	0.4562	no
gi|641570596|gb|KK856308.1|	71004-72474	9.9	39	0.888715	0.846404	-0.0703739	0.9564	no
gi|641570596|gb|KK856308.1|	79592-80163	9.9	39	2	1	-0.725244	0.67005	no
gi|641570596|gb|KK856308.1|	80268-83261	9.9	39	9	3	-2	0.23445	no
gi|641570596|gb|KK856308.1|	83822-86832	9.9	39	24	15	-0.641522	0.77435	no
gi|641570596|gb|KK856308.1|	92576-93122	9.9	39	7	7	0.031696	0.98115	no
gi|641570596|gb|KK856308.1|	93183-94390	9.9	39	9	8	-0.189973	0.877	no
gi|641570598|gb|KK856307.1|	10919-11291	11	21	27	18	-0.620494	0.638	no
gi|641570598|gb|KK856307.1|	12330-12665	11	21	8	3	-1	0.38945	no
gi|641570598|gb|KK856307.1|	13733-14798	11	21	0.651282	0.867089	0.412897	1	no
gi|641570598|gb|KK856307.1|	23765-24043	11	21	13	4	-2	0.367	no

gi|641570598|gb|KK856307.1|	25829-27521	11	21	1	2	0.733916	0.5985	no
gi|641570598|gb|KK856307.1|	32200-33554	11	21	0.977685	0.186168	-2	0.21865	no
gi|641570598|gb|KK856307.1|	33630-36025	11	21	6	2	-1	0.26585	no
gi|641570598|gb|KK856307.1|	44-682	11	21	8	6	-0.432	0.74835	no
gi|641570598|gb|KK856307.1|	45564-47576	11	21	7	5	-0.518675	0.6757	no
gi|641570598|gb|KK856307.1|	52259-52689	11	21	2	2	0.00294835	0.91115	no
gi|641570598|gb|KK856307.1|	53748-55137	11	21	1	0.271092	-2	0.29015	no
gi|641570598|gb|KK856307.1|	55362-56122	11	21	0.877645	0.951432	0.116462	0.92175	no
gi|641570598|gb|KK856307.1|	62576-62793	11	21	35	11	-2	0.31095	no
gi|641570601|gb|KK856306.1|	44565-14624	10	40	8	2	-2	0.1865	no
gi|641570601|gb|KK856306.1|	46611-14733	10	40	2	1	-1	0.4973	no
gi|641570601|gb|KK856306.1|	50738-15097	10	40	24	25	0.0321669	0.9678	no
gi|641570601|gb|KK856306.1|	57626-15805	10	40	8	2	-2	0.1912	no
gi|641570601|gb|KK856306.1|	59688-15990	10	40	12	7	-0.875786	0.63655	no
gi|641570601|gb|KK856306.1|	27595-27846	10	40	5	9	0.815941	0.6381	no
gi|641570614|gb|KK856303.1|	15661-15960	9.6	34	4	10	1	0.38455	no
gi|641570614|gb|KK856303.1|	16627-17196	9.6	34	0.946224	3	2	0.32945	no
gi|641570614|gb|KK856303.1|	19247-19597	9.6	34	3	10	2	0.30035	no
gi|641570614|gb|KK856303.1|	26671-28832	9.6	34	5	9	0.843793	0.5008	no
gi|641570614|gb|KK856303.1|	30615-32551	9.6	34	9	7	-0.307867	0.79615	no
gi|641570614|gb|KK856303.1|	32861-34895	9.6	34	10	6	-0.736172	0.5582	no
gi|641570614|gb|KK856303.1|	35338-36959	9.6	34	5	2	-1	0.4083	no
gi|641570614|gb|KK856303.1|	38538-38739	9.6	34	23	30	0.393288	0.7976	no
gi|641570614|gb|KK856303.1|	45752-46260	9.6	34	3	4	0.593642	0.6792	no
gi|641570614|gb|KK856303.1|	50013-50896	9.6	34	13	13	0.018786	0.9872	no
gi|641570614|gb|KK856303.1|	55559-56352	9.6	34	2	3	0.762924	0.59575	no
gi|641570614|gb|KK856303.1|	67621-67858	9.6	34	17	29	0.827667	0.56425	no
gi|641570614|gb|KK856303.1|	71396-77140	9.6	34	4	3	-0.334122	0.7887	no
gi|641570614|gb|KK856303.1|	77869-78232	9.6	34	27	12	-1	0.4037	no
gi|641570614|gb|KK856303.1|	78850-80925	9.6	34	22	10	-1	0.5275	no

gi|641570614|gb|KK856303.1|	89976-90772	9.6	34	0	3	inf	0.0015	yes
gi|641570617|gb|KK856302.1|	05807-10687	10	33	15	12	-0.324464	0.79925	no
gi|641570617|gb|KK856302.1|	07141-10922	10	33	74	39	-0.944708	0.6533	no
gi|641570617|gb|KK856302.1|	11790-11311	10	33	20	19	-0.0882666	0.94195	no
gi|641570617|gb|KK856302.1|	17286-11764	10	33	18	18	0.0108843	0.9844	no
gi|641570617|gb|KK856302.1|	19217-12234	10	33	10	15	0.645168	0.695	no
gi|641570617|gb|KK856302.1|	29396-29613	10	33	13	14	0.123742	0.929	no
gi|641570617|gb|KK856302.1|	52717-52955	10	33	49	56	0.200041	0.8844	no
gi|641570617|gb|KK856302.1|	55705-56374	10	33	4	6	0.658788	0.6325	no
gi|641570617|gb|KK856302.1|	56668-57278	10	33	2	0.258282	-3	0.22425	no
gi|641570617|gb|KK856302.1|	57838-59726	10	33	0.418284	0.574108	0.45684	1	no
gi|641570617|gb|KK856302.1|	62446-66293	10	33	1	2	0.610717	0.6385	no
gi|641570617|gb|KK856302.1|	66465-67132	10	33	4	5	0.422964	0.75435	no
gi|641570617|gb|KK856302.1|	68164-68392	10	33	8	12	0.543786	0.75805	no
gi|641570617|gb|KK856302.1|	68751-70127	10	33	3	6	0.836811	0.5183	no
gi|641570617|gb|KK856302.1|	72134-72408	10	33	26	61	1	0.3549	no
gi|641570617|gb|KK856302.1|	85919-86203	10	33	35	113	2	0.196	no
gi|641570617|gb|KK856302.1|	87143-87661	10	33	10	37	2	0.15145	no
gi|641570621|gb|KK856301.1|	01625-10232	10	36	2	5	2	0.27725	no
gi|641570621|gb|KK856301.1|	10234-10937	10	36	2	9	2	0.13155	no
gi|641570621|gb|KK856301.1|	03120-10412	10	36	0.4356	2	2	0.2717	no
gi|641570621|gb|KK856301.1|	06188-10673	10	36	11	17	0.595551	0.6499	no
gi|641570621|gb|KK856301.1|	09496-11032	10	36	2	6	1	0.31365	no
gi|641570621|gb|KK856301.1|	11568-11197	10	36	11	28	1	0.30155	no
gi|641570621|gb|KK856301.1|	12109-11320	10	36	1	2	1	0.4739	no
gi|641570621|gb|KK856301.1|	13753-11577	10	36	5	6	0.273743	0.82655	no
gi|641570621|gb|KK856301.1|	24347-12475	10	36	35	23	-0.587872	0.6441	no
gi|641570621|gb|KK856301.1|	24986-12693	10	36	1	0.308231	-2	0.1973	no
gi|641570621|gb|KK856301.1|	27184-12986	10	36	2	0.607645	-2	0.24955	no
gi|641570621|gb|KK856301.1|	12736-13479	10	36	0.517067	2	2	0.33105	no

gi|641570621|gb|KK856301.1|	.40760-14200	10	36	1	0.307552	-2	0.21625	no
gi|641570621|gb|KK856301.1|	.57535-15926	10	36	0.555426	0.141138	-2	1	no
gi|641570621|gb|KK856301.1|	17083-17984	10	36	2	10	2	0.13605	no
gi|641570621|gb|KK856301.1|	24151-25446	10	36	0.643836	0.588356	-0.130005	1	no
gi|641570621|gb|KK856301.1|	26049-29163	10	36	66	36	-0.900573	0.6647	no
gi|641570621|gb|KK856301.1|	2779-3667	10	36	0.204424	2	3	0.25475	no
gi|641570621|gb|KK856301.1|	29807-30372	10	36	27	0	-inf	5.00E-05	yes
gi|641570621|gb|KK856301.1|	30475-31981	10	36	21	2	-3	0.0194	no
gi|641570621|gb|KK856301.1|	32925-33419	10	36	2	1	-1	0.50365	no
gi|641570621|gb|KK856301.1|	33723-42425	10	36	22	16	-0.458364	0.82595	no
gi|641570621|gb|KK856301.1|	3785-4042	10	36	8	26	2	0.29345	no
gi|641570621|gb|KK856301.1|	63604-65789	10	36	15	9	-0.628433	0.61315	no
gi|641570621|gb|KK856301.1|	66016-67337	10	36	6	2	-2	0.2268	no
gi|641570621|gb|KK856301.1|	70600-71494	10	36	1	0.308016	-2	0.25005	no
gi|641570621|gb|KK856301.1|	8732-9703	10	36	0.547653	4	3	0.1133	no
gi|641570621|gb|KK856301.1|	89760-90507	10	36	51	28	-0.860534	0.4863	no
gi|641570621|gb|KK856301.1|	93825-95341	10	36	26	54	1	0.5231	no
gi|641570621|gb|KK856301.1|	98264-99247	10	36	0.449425	1	1	0.4595	no
gi|641570621|gb|KK856301.1|	99632-10052[illegible]	10	36	5	10	0.94337	0.46425	no
gi|641570624|gb|KK856300.1|	71693-71963	9.3	37	2	10	2	0.3198	no
gi|641570627|gb|KK856299.1|	.03521-10414	11	20	33	9	-2	0.15075	no
gi|641570627|gb|KK856299.1|	.14025-11531	11	20	0.259184	2	3	0.18525	no
gi|641570627|gb|KK856299.1|	.29380-13015	11	20	10	2	-2	0.12515	no
gi|641570627|gb|KK856299.1|	.31407-13317	11	20	4	2	-0.547548	0.6712	no
gi|641570627|gb|KK856299.1|	14700-15338	11	20	1	0.9686	-0.208328	0.8945	no
gi|641570627|gb|KK856299.1|	.48003-14889	11	20	0.408259	1	2	0.3613	no
gi|641570627|gb|KK856299.1|	.49165-14960	11	20	13	6	-1	0.3843	no
gi|641570627|gb|KK856299.1|	20585-20964	11	20	3	3	-0.00283741	0.9394	no
gi|641570627|gb|KK856299.1|	21086-22300	11	20	0.625526	1	1	0.49595	no
gi|641570627|gb|KK856299.1|	24698-25595	11	20	2	0.613414	-1	0.4015	no

gi|641570627|gb|KK856299.1|	31166-31565	11	20	6	9	0.667122	0.6447	no
gi|641570627|gb|KK856299.1|	38657-39947	11	20	2	0.985055	-0.656098	0.64455	no
gi|641570627|gb|KK856299.1|	44676-45977	11	20	0.89648	0.780234	-0.200365	0.8959	no
gi|641570627|gb|KK856299.1|	52017-53889	11	20	0.50673	0.837161	0.724287	1	no
gi|641570627|gb|KK856299.1|	58850-59767	11	20	5	3	-0.862974	0.52625	no
gi|641570627|gb|KK856299.1|	59940-60737	11	20	25	18	-0.481539	0.7006	no
gi|641570627|gb|KK856299.1|	64715-65593	11	20	3	2	-1	0.4351	no
gi|641570627|gb|KK856299.1|	67744-69220	11	20	11	11	-0.104035	0.9311	no
gi|641570627|gb|KK856299.1|	71006-71449	11	20	2	3	0.366686	0.82765	no
gi|641570627|gb|KK856299.1|	73686-75666	11	20	18	6	-2	0.1818	no
gi|641570627|gb|KK856299.1|	78869-79518	11	20	25	9	-2	0.2404	no
gi|641570627|gb|KK856299.1|	79816-80455	11	20	1	1	0.376664	0.8276	no
gi|641570627|gb|KK856299.1|	82173-82645	11	20	3	2	-0.145619	0.92525	no
gi|641570627|gb|KK856299.1|	86557-87435	11	20	2	1	-0.396314	0.79175	no
gi|641570627|gb|KK856299.1|	88523-89127	11	20	2	0	-inf	0.0102	no
gi|641570630|gb|KK856298.1|	1-1431	12	47	1	6	2	0.1245	no
gi|641570630|gb|KK856298.1|	04842-10605	12	47	4	3	-0.24663	0.84985	no
gi|641570630|gb|KK856298.1|	07178-11161	12	47	1	0.714315	-0.626097	0.6324	no
gi|641570630|gb|KK856298.1|	11819-11226	12	47	6	9	0.589584	0.666	no
gi|641570630|gb|KK856298.1|	17425-11768	12	47	15	8	-0.946507	0.55475	no
gi|641570630|gb|KK856298.1|	17923-11840	12	47	1	2	0.329133	0.83525	no
gi|641570630|gb|KK856298.1|	18928-12086	12	47	1	2	0.930863	0.4835	no
gi|641570630|gb|KK856298.1|	21047-12163	12	47	2	2	0.0122751	0.9394	no
gi|641570630|gb|KK856298.1|	25855-12679	12	47	8	10	0.341402	0.78875	no
gi|641570630|gb|KK856298.1|	31803-13216	12	47	5	4	-0.197659	0.8917	no
gi|641570630|gb|KK856298.1|	32641-13452	12	47	1	0.702101	-0.739083	0.5925	no
gi|641570630|gb|KK856298.1|	34705-13604	12	47	0.620152	0.283395	-1	1	no
gi|641570630|gb|KK856298.1|	37559-13833	12	47	5	4	-0.0223527	0.9827	no
gi|641570630|gb|KK856298.1|	38727-14339	12	47	2	2	-0.0577805	0.96295	no
gi|641570630|gb|KK856298.1|	44078-14678	12	47	1	0.12902	-3	0.1287	no

gi|641570630|gb|KK856298.1|	.48442-14919	12	47	2	1	-0.720263	0.6521	no
gi|641570630|gb|KK856298.1|	.50399-15100	12	47	8	3	-1	0.34925	no
gi|641570630|gb|KK856298.1|	.55484-15679	12	47	0.95533	1	0.507483	0.72935	no
gi|641570630|gb|KK856298.1|	.69881-17008	12	47	121	82	-0.555814	0.6674	no
gi|641570630|gb|KK856298.1|	.72509-17307	12	47	0.189245	5	5	0.10875	no
gi|641570630|gb|KK856298.1|	.73179-17395	12	47	0.367673	21	6	0.1348	no
gi|641570630|gb|KK856298.1|	17646-18275	12	47	0.979045	3	1	0.37835	no
gi|641570630|gb|KK856298.1|	.77702-17817	12	47	1	2	0.854592	0.62755	no
gi|641570630|gb|KK856298.1|	.80778-18125	12	47	1	3	1	0.4611	no
gi|641570630|gb|KK856298.1|	.82786-18304	12	47	9	1	-3	0.2472	no
gi|641570630|gb|KK856298.1|	24664-24993	12	47	5	8	0.863329	0.5834	no
gi|641570630|gb|KK856298.1|	31701-32083	12	47	3	5	0.752456	0.6288	no
gi|641570630|gb|KK856298.1|	35682-36027	12	47	2	6	1	0.40225	no
gi|641570630|gb|KK856298.1|	37454-37841	12	47	2	7	2	0.36635	no
gi|641570630|gb|KK856298.1|	48701-49080	12	47	6	0	-inf	0.0059	no
gi|641570630|gb|KK856298.1|	56993-57204	12	47	14	13	-0.1422	0.91755	no
gi|641570630|gb|KK856298.1|	57761-58381	12	47	4	7	0.997016	0.49015	no
gi|641570630|gb|KK856298.1|	61854-62628	12	47	3	5	0.602138	0.6544	no
gi|641570630|gb|KK856298.1|	64199-64597	12	47	2	8	2	0.2545	no
gi|641570630|gb|KK856298.1|	64932-66258	12	47	0.375711	0.476903	0.344074	1	no
gi|641570630|gb|KK856298.1|	67550-68451	12	47	1	1	0.451964	0.7632	no
gi|641570630|gb|KK856298.1|	72509-72895	12	47	3	4	0.220488	0.8957	no
gi|641570630|gb|KK856298.1|	73262-73680	12	47	3	5	0.449234	0.76345	no
gi|641570630|gb|KK856298.1|	77226-78594	12	47	0.362255	0.827789	1	1	no
gi|641570630|gb|KK856298.1|	82029-82982	12	47	2	4	0.781418	0.5694	no
gi|641570630|gb|KK856298.1|	85061-86073	12	47	0.606853	1	1	0.48415	no
gi|641570630|gb|KK856298.1|	88132-89412	12	47	5	11	1	0.3123	no
gi|641570630|gb|KK856298.1|	92755-95104	12	47	2	2	0.579217	0.658	no
gi|641570630|gb|KK856298.1|	96040-97714	12	47	0.430936	0.729959	0.76034	1	no
gi|641570630|gb|KK856298.1|	99817-104677	12	47	3	3	0.181561	0.8801	no

gi|641570633|gb|KK856297.1|	12545-11301	8.2	32	6	10	0.702519	0.61075	no
gi|641570633|gb|KK856297.1|	20751-12095	8.2	32	30	68	1	0.40865	no
gi|641570633|gb|KK856297.1|	19893-22768	8.2	32	4	8	0.864447	0.4887	no
gi|641570633|gb|KK856297.1|	22898-23661	8.2	32	23	44	0.919235	0.46215	no
gi|641570633|gb|KK856297.1|	24023-25051	8.2	32	20	38	0.921069	0.46585	no
gi|641570633|gb|KK856297.1|	25855-27895	8.2	32	1	2	0.60913	0.6479	no
gi|641570633|gb|KK856297.1|	30482-30830	8.2	32	6	10	0.676882	0.64275	no
gi|641570633|gb|KK856297.1|	33259-33835	8.2	32	7	17	1	0.3828	no
gi|641570633|gb|KK856297.1|	34395-34606	8.2	32	284	318	0.164424	0.8951	no
gi|641570633|gb|KK856297.1|	39816-41008	8.2	32	5	5	-0.096612	0.9395	no
gi|641570633|gb|KK856297.1|	41555-42325	8.2	32	62	70	0.175443	0.9119	no
gi|641570633|gb|KK856297.1|	42906-43112	8.2	32	21	25	0.243863	0.8726	no
gi|641570633|gb|KK856297.1|	44883-45198	8.2	32	17	22	0.317117	0.815	no
gi|641570633|gb|KK856297.1|	50690-52137	8.2	32	3	3	-0.113671	0.93005	no
gi|641570633|gb|KK856297.1|	52561-56534	8.2	32	1	2	0.532769	0.6804	no
gi|641570633|gb|KK856297.1|	56653-57580	8.2	32	18	16	-0.162064	0.89575	no
gi|641570633|gb|KK856297.1|	65058-65553	8.2	32	2	2	0.400225	0.8089	no
gi|641570633|gb|KK856297.1|	80258-81437	8.2	32	0.647782	0.438298	-0.563597	1	no
gi|641570633|gb|KK856297.1|	85267-85591	8.2	32	4	4	-0.105269	0.92555	no
gi|641570633|gb|KK856297.1|	91207-91680	8.2	32	27	45	0.759815	0.5533	no
gi|641570641|gb|KK856295.1|	16874-11966	11	44	6	14	1	0.4886	no
gi|641570641|gb|KK856295.1|	24275-12538	11	44	1	2	1	0.38005	no
gi|641570641|gb|KK856295.1|	31149-13662	11	44	6	3	-0.897796	0.4734	no
gi|641570641|gb|KK856295.1|	41783-14197	11	44	22	53	1	0.4143	no
gi|641570641|gb|KK856295.1|	48603-14899	11	44	3	2	-1	0.5278	no
gi|641570641|gb|KK856295.1|	72724-17304	11	44	11	17	0.639708	0.6425	no
gi|641570641|gb|KK856295.1|	74860-17626	11	44	0.758997	0.978483	0.366453	0.8038	no
gi|641570641|gb|KK856295.1|	48179-49400	11	44	6	9	0.525955	0.6828	no
gi|641570641|gb|KK856295.1|	51314-52175	11	44	3	6	0.966036	0.47255	no
gi|641570641|gb|KK856295.1|	57588-61467	11	44	4	5	0.349026	0.77665	no

gi|641570641|gb|KK856295.1|	61617-62165	11	44	2	0.605791	-1	0.4534	no
gi|641570641|gb|KK856295.1|	64846-65136	11	44	84	115	0.456122	0.71625	no
gi|641570641|gb|KK856295.1|	72825-75462	11	44	28	30	0.110913	0.9611	no
gi|641570644|gb|KK856294.1|	.19957-12075	12	45	1	2	0.312953	0.84265	no
gi|641570644|gb|KK856294.1|	.34323-13458	12	45	106	55	-0.935627	0.4648	no
gi|641570644|gb|KK856294.1|	.49564-14984	12	45	34	20	-0.760381	0.5798	no
gi|641570644|gb|KK856294.1|	19980-21040	12	45	1	2	0.705038	0.62345	no
gi|641570644|gb|KK856294.1|	22993-23767	12	45	0.978769	2	0.924066	0.5636	no
gi|641570644|gb|KK856294.1|	38726-40957	12	45	0.52171	1	1	0.32025	no
gi|641570644|gb|KK856294.1|	41697-42528	12	45	0.33413	2	3	0.18845	no
gi|641570644|gb|KK856294.1|	63866-64451	12	45	1	1	-0.402762	0.82205	no
gi|641570644|gb|KK856294.1|	82738-83618	12	45	0.930656	2	0.893218	0.5684	no
gi|641570644|gb|KK856294.1|	83855-84560	12	45	0.83336	2	1	0.4118	no
gi|641570644|gb|KK856294.1|	98277-99229	12	45	0.467836	0.711337	0.60453	1	no
gi|641570644|gb|KK856294.1|	)9550-10138[illegible]	12	45	0.432068	0.922412	1	0.4709	no
gi|641570648|gb|KK856293.1|	.02271-10411	10	21	3	2	-0.865182	0.51185	no
gi|641570648|gb|KK856293.1|	.05333-10612	10	21	1	2	0.602377	0.6902	no
gi|641570648|gb|KK856293.1|	.08366-10904	10	21	51	25	-1	0.39985	no
gi|641570648|gb|KK856293.1|	.09170-10941	10	21	23	8	-2	0.31095	no
gi|641570648|gb|KK856293.1|	.14646-11493	10	21	39	10	-2	0.1871	no
gi|641570648|gb|KK856293.1|	.24034-12460	10	21	2	1	-0.540943	0.7461	no
gi|641570648|gb|KK856293.1|	49493-50438	10	21	1	0.574354	-0.980506	0.5872	no
gi|641570648|gb|KK856293.1|	52131-52765	10	21	4	1	-2	0.2878	no
gi|641570648|gb|KK856293.1|	52965-53144	10	21	69	10	-3	0.18145	no
gi|641570648|gb|KK856293.1|	56062-57010	10	21	33	6	-2	0.06915	no
gi|641570648|gb|KK856293.1|	57180-61803	10	21	3	0.586159	-2	0.10755	no
gi|641570648|gb|KK856293.1|	61864-62546	10	21	1	0.661471	-0.984737	0.62775	no
gi|641570648|gb|KK856293.1|	74095-74888	10	21	17	11	-0.646712	0.61465	no
gi|641570648|gb|KK856293.1|	76910-77052	10	21	203	123	-0.723849	0.64835	no
gi|641570648|gb|KK856293.1|	77836-78936	10	21	0.547826	2	1	0.36605	no

gi|641570648|gb|KK856293.1|	79189-80342	10	21	0.369649	1	2	0.2717	no
gi|641570648|gb|KK856293.1|	81522-82076	10	21	16	8	-0.971725	0.46605	no
gi|641570648|gb|KK856293.1|	83704-84517	10	21	4	3	-0.278577	0.8367	no
gi|641570648|gb|KK856293.1|	89096-89351	10	21	17	13	-0.445691	0.76455	no
gi|641570648|gb|KK856293.1|	89609-91001	10	21	60	46	-0.399728	0.8573	no
gi|641570648|gb|KK856293.1|	97452-98484	10	21	23	17	-0.480882	0.70035	no
gi|641570648|gb|KK856293.1|	99980-102200	10	21	4	2	-1	0.35945	no
gi|641570656|gb|KK856291.1|	01475-10186	11	43	0.719792	4	3	0.30875	no
gi|641570656|gb|KK856291.1|	16155-11684	11	43	0.856861	0.866106	0.0154823	1	no
gi|641570656|gb|KK856291.1|	28456-28850	11	43	18	7	-1	0.32045	no
gi|641570656|gb|KK856291.1|	33119-33417	11	43	10	0	-inf	0.0059	no
gi|641570656|gb|KK856291.1|	34475-34806	11	43	7	0.744222	-3	0.21195	no
gi|641570656|gb|KK856291.1|	49393-51932	11	43	7	2	-2	0.21265	no
gi|641570656|gb|KK856291.1|	52363-55623	11	43	23	6	-2	0.281	no
gi|641570656|gb|KK856291.1|	58005-58262	11	43	11	4	-1	0.4178	no
gi|641570656|gb|KK856291.1|	58371-61503	11	43	51	46	-0.150243	0.9435	no
gi|641570656|gb|KK856291.1|	70823-71974	11	43	1	0.451084	-1	0.4417	no
gi|641570656|gb|KK856291.1|	79777-80339	11	43	0.578504	10	4	0.13935	no
gi|641570660|gb|KK856290.1|	04195-10479	16	69	6	11	1	0.4401	no
gi|641570660|gb|KK856290.1|	10661-11290	16	69	13	0.052727	-8	0.18285	no
gi|641570660|gb|KK856290.1|	14251-12018	16	69	7	0.113048	-6	0.0452	no
gi|641570660|gb|KK856290.1|	20249-12125	16	69	4	0	-inf	0.0002	yes
gi|641570660|gb|KK856290.1|	21421-13135	16	69	5	0.644626	-3	0.1374	no
gi|641570660|gb|KK856290.1|	24495-24722	16	69	3	22	3	0.26765	no
gi|641570660|gb|KK856290.1|	51378-52890	16	69	0.645275	1	1	0.45455	no
gi|641570660|gb|KK856290.1|	54148-54802	16	69	0.462917	2	2	0.3323	no
gi|641570660|gb|KK856290.1|	58061-58619	16	69	0.584842	2	2	0.3323	no
gi|641570660|gb|KK856290.1|	65337-66061	16	69	1	0.812532	-0.858247	0.60675	no
gi|641570660|gb|KK856290.1|	853-1603	16	69	8	6	-0.471053	0.7178	no
gi|641570660|gb|KK856290.1|	87047-88039	16	69	0.533218	0.94613	0.827313	0.6232	no

gi|641570664|gb|KK856289.1|	.06162-10693	9.5	40	10	15	0.674183	0.60325	no
gi|641570664|gb|KK856289.1|	.08367-10928	9.5	40	0.976988	0.445542	-1	0.50885	no
gi|641570664|gb|KK856289.1|	.12712-11332	9.5	40	0.682491	4	2	0.21365	no
gi|641570664|gb|KK856289.1|	26359-26810	9.5	40	1	4	1	0.4014	no
gi|641570674|gb|KK856286.1|	.05956-10616	10	40	0	37	inf	0.00255	yes
gi|641570674|gb|KK856286.1|	.13739-11397	10	40	3	26	3	0.25585	no
gi|641570674|gb|KK856286.1|	.14129-11481	10	40	1	0.21827	-3	0.24815	no
gi|641570674|gb|KK856286.1|	2518-2880	10	40	5	8	0.820648	0.5921	no
gi|641570674|gb|KK856286.1|	26533-26809	10	40	0	56	inf	0.0001	yes
gi|641570674|gb|KK856286.1|	29489-30887	10	40	0	48	inf	0.00025	yes
gi|641570674|gb|KK856286.1|	66410-66911	10	40	0	19	inf	0.00015	yes
gi|641570674|gb|KK856286.1|	77646-77875	10	40	0	63	inf	0.00065	yes
gi|641570674|gb|KK856286.1|	78580-79237	10	40	0	5	inf	0.00115	yes
gi|641570674|gb|KK856286.1|	89065-89495	10	40	0.299227	24	6	0.10455	no
gi|641570677|gb|KK856285.1|	0-1378	11	44	1	192	7	0.00965	no
gi|641570677|gb|KK856285.1|	1510-3381	11	44	0	368	inf	5.00E-05	yes
gi|641570677|gb|KK856285.1|	21307-21915	11	44	2	2	-0.17954	0.90465	no
gi|641570677|gb|KK856285.1|	23578-23890	11	44	57	1231.13	4	0.03425	no
gi|641570677|gb|KK856285.1|	26624-26814	11	44	60	4	-4	0.1967	no
gi|641570677|gb|KK856285.1|	27486-28394	11	44	11	1	-3	0.05145	no
gi|641570677|gb|KK856285.1|	32192-32678	11	44	0	30	inf	5.00E-05	yes
gi|641570677|gb|KK856285.1|	32797-39860	11	44	0	59	inf	5.00E-05	yes
gi|641570677|gb|KK856285.1|	39925-40515	11	44	0	725	inf	0.00015	yes
gi|641570677|gb|KK856285.1|	87615-89218	11	44	12	3	-2	0.16185	no
gi|641570681|gb|KK856284.1|	55808-56206	7.2	31	2	3	0.584543	0.76735	no
gi|641570684|gb|KK856283.1|	.22032-12235	10	38	0	11	inf	0.00255	yes
gi|641570684|gb|KK856283.1|	93606-95713	10	38	0.074073	4	6	0.2323	no
gi|641570684|gb|KK856283.1|	95840-96911	10	38	0.161705	2	4	0.2494	no
gi|641570684|gb|KK856283.1|	97284-97837	10	38	0.59296	3	2	0.256	no
gi|641570684|gb|KK856283.1|	)9650-10080⁴	10	38	0.147705	5	5	0.23325	no

gi|641570686|gb|KK856282.1|	53735-53984	10	40	0	18	inf	0.00375	no
gi|641570686|gb|KK856282.1|	61508-61824	10	40	0	35	inf	0.0001	yes
gi|641570686|gb|KK856282.1|	69087-69432	10	40	0	372	inf	0.00045	yes
gi|641570686|gb|KK856282.1|	71617-73105	10	40	1	3	1	0.3605	no
gi|641570686|gb|KK856282.1|	73226-75421	10	40	0.318661	3	3	0.06855	no
gi|641570686|gb|KK856282.1|	82922-83424	10	40	1	7	3	0.16825	no
gi|641570686|gb|KK856282.1|	85361-86289	10	40	4	5	0.112422	0.9312	no
gi|641570686|gb|KK856282.1|	88071-88927	10	40	0.964462	2	1	0.3864	no
gi|641570686|gb|KK856282.1|	93997-94389	10	40	4	2	-1	0.46955	no
gi|641570691|gb|KK856281.1|	10803-11192	10	42	6	5	-0.303269	0.817	no
gi|641570691|gb|KK856281.1|	1766-2778	10	42	0.78024	1	0.435177	0.78015	no
gi|641570691|gb|KK856281.1|	46-1328	10	42	2	2	0.296557	0.8237	no
gi|641570697|gb|KK856279.1|	10165-11117	8.9	37	6	2	-1	0.30855	no
gi|641570697|gb|KK856279.1|	11556-11205	8.9	37	14	1	-3	0.0864	no
gi|641570697|gb|KK856279.1|	24782-12710	8.9	37	0.498215	0.456105	-0.127404	1	no
gi|641570697|gb|KK856279.1|	30288-13132	8.9	37	10	2	-2	0.13905	no
gi|641570697|gb|KK856279.1|	31469-13992	8.9	37	5	2	-0.957311	0.55965	no
gi|641570697|gb|KK856279.1|	40086-14055	8.9	37	194	4	-6	0.0295	no
gi|641570697|gb|KK856279.1|	42364-14280	8.9	37	185	13	-4	0.07325	no
gi|641570697|gb|KK856279.1|	44473-14507	8.9	37	30	3	-3	0.0406	no
gi|641570697|gb|KK856279.1|	53648-15480	8.9	37	1	0.446894	-2	0.362	no
gi|641570697|gb|KK856279.1|	55217-15677	8.9	37	7	25	2	0.1574	no
gi|641570697|gb|KK856279.1|	59386-16095	8.9	37	0.361318	6	4	0.0565	no
gi|641570697|gb|KK856279.1|	63004-16371	8.9	37	16	27	0.779308	0.5416	no
gi|641570697|gb|KK856279.1|	64403-16474	8.9	37	10	0.707718	-4	0.19635	no
gi|641570697|gb|KK856279.1|	66833-16725	8.9	37	8	12	0.587336	0.6613	no
gi|641570697|gb|KK856279.1|	36443-36882	8.9	37	1	3	1	0.4427	no
gi|641570697|gb|KK856279.1|	37018-37777	8.9	37	0.879201	0.762485	-0.205484	0.89485	no
gi|641570697|gb|KK856279.1|	41600-44606	8.9	37	6	4	-0.457744	0.718	no
gi|641570697|gb|KK856279.1|	45536-45775	8.9	37	17	10	-0.773562	0.6265	no

gi|641570697|gb|KK856279.1|	49761-49961	8.9	37	32	22	-0.569264	0.7195	no
gi|641570697|gb|KK856279.1|	51704-52396	8.9	37	5	4	-0.0776032	0.95575	no
gi|641570697|gb|KK856279.1|	53291-54388	8.9	37	0.471127	0.836457	0.828175	1	no
gi|641570697|gb|KK856279.1|	60141-60408	8.9	37	39	41	0.0480502	0.9721	no
gi|641570697|gb|KK856279.1|	63732-64004	8.9	37	43	32	-0.440219	0.73655	no
gi|641570697|gb|KK856279.1|	67590-68228	8.9	37	23	19	-0.255122	0.8391	no
gi|641570697|gb|KK856279.1|	68718-70225	8.9	37	11	7	-0.621653	0.6268	no
gi|641570697|gb|KK856279.1|	72761-78209	8.9	37	7	5	-0.542594	0.73795	no
gi|641570697|gb|KK856279.1|	78850-82285	8.9	37	6	5	-0.410398	0.74075	no
gi|641570697|gb|KK856279.1|	82448-83349	8.9	37	0.401317	1	2	0.3613	no
gi|641570697|gb|KK856279.1|	89735-89938	8.9	37	8	32	2	0.29495	no
gi|641570700|gb|KK856278.1|	63299-64297	9.6	41	0.705643	0.804937	0.189939	1	no
gi|641570703|gb|KK856277.1|	10010-10655	9.9	36	4	1	-2	0.29695	no
gi|641570703|gb|KK856277.1|	07462-10840	9.9	36	18	7	-1	0.29765	no
gi|641570703|gb|KK856277.1|	09131-10948	9.9	36	8	5	-0.669902	0.64925	no
gi|641570703|gb|KK856277.1|	11026-11119	9.9	36	147	78	-0.922182	0.51635	no
gi|641570703|gb|KK856277.1|	13382-11447	9.9	36	26	13	-1	0.4143	no
gi|641570703|gb|KK856277.1|	14841-11716	9.9	36	0.93175	1	0.261633	0.84985	no
gi|641570703|gb|KK856277.1|	82481-18318	9.9	36	60	45	-0.405933	0.7407	no
gi|641570703|gb|KK856277.1|	19993-20123	9.9	36	0	462	inf	0.0068	no
gi|641570703|gb|KK856277.1|	27851-27995	9.9	36	0	403	inf	0.0005	yes
gi|641570703|gb|KK856277.1|	33384-33726	9.9	36	0	209	inf	5.00E-05	yes
gi|641570703|gb|KK856277.1|	41252-41794	9.9	36	0.815505	2	1	0.51315	no
gi|641570703|gb|KK856277.1|	42710-44046	9.9	36	4	4	0.192116	0.8863	no
gi|641570703|gb|KK856277.1|	46677-48428	9.9	36	0.500631	0.624532	0.319029	1	no
gi|641570703|gb|KK856277.1|	4779-6872	9.9	36	21	14	-0.603774	0.70735	no
gi|641570703|gb|KK856277.1|	50402-51600	9.9	36	4	4	0.210513	0.8714	no
gi|641570703|gb|KK856277.1|	54210-55298	9.9	36	7	4	-0.948872	0.46815	no
gi|641570703|gb|KK856277.1|	55415-56487	9.9	36	1	3	1	0.34975	no
gi|641570703|gb|KK856277.1|	60624-61037	9.9	36	0	42	inf	5.00E-05	yes

gi|641570703|gb|KK856277.1|	61322-61845	9.9	36	0	7	inf	0.00105	yes
gi|641570703|gb|KK856277.1|	74226-75627	9.9	36	2	0.536806	-2	0.2762	no
gi|641570703|gb|KK856277.1|	7445-7630	9.9	36	106	81	-0.399724	0.76485	no
gi|641570703|gb|KK856277.1|	76563-78140	9.9	36	2	0.390526	-2	0.17325	no
gi|641570703|gb|KK856277.1|	7754-9413	9.9	36	13	13	0.0178795	0.98955	no
gi|641570703|gb|KK856277.1|	78304-78478	9.9	36	0	68	inf	0.00375	no
gi|641570703|gb|KK856277.1|	79276-80502	9.9	36	0.686937	0.522989	-0.393398	1	no
gi|641570703|gb|KK856277.1|	80608-81848	9.9	36	0.745505	0.825612	0.147247	1	no
gi|641570703|gb|KK856277.1|	89586-90807	9.9	36	0	2	inf	0.00105	yes
gi|641570703|gb|KK856277.1|	92895-94203	9.9	36	0.381789	0.581507	0.607024	1	no
gi|641570707|gb|KK856276.1|	16068-11689	9.4	20	66	38	-0.815368	0.6192	no
gi|641570707|gb|KK856276.1|	17146-11750	9.4	20	2	6	1	0.48155	no
gi|641570707|gb|KK856276.1|	18172-11921	9.4	20	1	0.892627	-0.394659	0.7966	no
gi|641570707|gb|KK856276.1|	23342-12417	9.4	20	16	18	0.201642	0.87405	no
gi|641570707|gb|KK856276.1|	26335-12719	9.4	20	40	28	-0.515139	0.67495	no
gi|641570707|gb|KK856276.1|	26879-30000	9.4	20	11	18	0.718375	0.6586	no
gi|641570707|gb|KK856276.1|	34062-34904	9.4	20	0.109482	1	4	0.1276	no
gi|641570707|gb|KK856276.1|	35113-36444	9.4	20	0.997485	2	1	0.4263	no
gi|641570707|gb|KK856276.1|	38747-39446	9.4	20	0	2	inf	0.0068	no
gi|641570707|gb|KK856276.1|	46614-47183	9.4	20	0.946224	2	0.859636	0.62755	no
gi|641570707|gb|KK856276.1|	49066-51282	9.4	20	0.91101	2	1	0.3648	no
gi|641570707|gb|KK856276.1|	51333-51688	9.4	20	2	9	2	0.2384	no
gi|641570707|gb|KK856276.1|	51986-52640	9.4	20	0.462917	2	2	0.2812	no
gi|641570707|gb|KK856276.1|	78742-79515	9.4	20	2	1	-0.620272	0.6869	no
gi|641570707|gb|KK856276.1|	95784-96171	9.4	20	4	3	-0.291282	0.8545	no
gi|641570711|gb|KK856275.1|	93259-93634	10	21	2	2	-0.00339378	0.91115	no
gi|641570714|gb|KK856274.1|	00341-10060	10	38	17	18	0.0427199	0.9675	no
gi|641570714|gb|KK856274.1|	03316-10378	10	38	16	19	0.235273	0.85195	no
gi|641570714|gb|KK856274.1|	05977-10673	10	38	0.876095	2	1	0.39245	no
gi|641570714|gb|KK856274.1|	26701-12985	10	38	22	34	0.648054	0.76985	no

gi|641570714|gb|KK856274.1|	18885-19247	10	38	16	10	-0.705763	0.6044	no
gi|641570714|gb|KK856274.1|	22766-25370	10	38	7	2	-2	0.22245	no
gi|641570714|gb|KK856274.1|	37523-38810	10	38	0	4	inf	5.00E-05	yes
gi|641570714|gb|KK856274.1|	67109-67688	10	38	4	0.278829	-4	0.1972	no
gi|641570714|gb|KK856274.1|	67870-68994	10	38	15	20	0.377496	0.76125	no
gi|641570714|gb|KK856274.1|	71983-72978	10	38	0.973899	3	2	0.3092	no
gi|641570714|gb|KK856274.1|	73497-73775	10	38	0	12	inf	0.00455	no
gi|641570714|gb|KK856274.1|	74006-74359	10	38	16	10	-0.72437	0.5933	no
gi|641570714|gb|KK856274.1|	74516-75120	10	38	5	8	0.648556	0.62945	no
gi|641570714|gb|KK856274.1|	80260-80387	10	38	421	0	-inf	0.01175	no
gi|641570714|gb|KK856274.1|	87923-89167	10	38	7	6	-0.0624757	0.96165	no
gi|641570714|gb|KK856274.1|	89343-91064	10	38	4	3	-0.252563	0.84375	no
gi|641570714|gb|KK856274.1|	91609-92974	10	38	5	3	-0.97768	0.46325	no
gi|641570714|gb|KK856274.1|	95830-96838	10	38	2	2	0.135581	0.9277	no
gi|641570717|gb|KK856273.1|	.03416-10363	10	36	0	35	inf	0.0023	yes
gi|641570717|gb|KK856273.1|	.06914-10762	10	36	0	4	inf	0.00105	yes
gi|641570717|gb|KK856273.1|	.08408-10865	10	36	10	13	0.400903	0.79705	no
gi|641570717|gb|KK856273.1|	.08932-11091	10	36	0.871811	2	1	0.45035	no
gi|641570717|gb|KK856273.1|	.11294-11252	10	36	1	3	1	0.437	no
gi|641570717|gb|KK856273.1|	.13199-11544	10	36	1	0.157874	-3	0.11575	no
gi|641570717|gb|KK856273.1|	.15535-11612	10	36	2	0	-inf	0.0059	no
gi|641570717|gb|KK856273.1|	.16549-11784	10	36	1	0.588356	-1	0.48805	no
gi|641570717|gb|KK856273.1|	.17988-11935	10	36	2	0.644383	-1	0.41145	no
gi|641570717|gb|KK856273.1|	.19496-12117	10	36	0.811795	0.873594	0.105848	1	no
gi|641570717|gb|KK856273.1|	.26103-12684	10	36	1	0.780341	-0.568298	0.7586	no
gi|641570717|gb|KK856273.1|	64041-64387	10	36	2	5	1	0.513	no
gi|641570717|gb|KK856273.1|	82510-84071	10	36	2	1	-0.572565	0.68385	no
gi|641570717|gb|KK856273.1|	84323-85560	10	36	2	2	0.22815	0.86845	no
gi|641570717|gb|KK856273.1|	92101-92385	10	36	0	138	inf	5.00E-05	yes
gi|641570717|gb|KK856273.1|	93297-93754	10	36	0.269078	33	7	0.10445	no

gi|641570717|gb|KK856273.1|	94629-95931	10	36	0	10	inf	5.00E-05	yes
gi|641570717|gb|KK856273.1|	97283-97509	10	36	0	212	inf	5.00E-05	yes
gi|641570717|gb|KK856273.1|	98515-98764	10	36	0	60	inf	5.00E-05	yes
gi|641570720|gb|KK856272.1|	01542-10302	9.1	36	0.274706	1	2	0.21205	no
gi|641570720|gb|KK856272.1|	14387-11488	9.1	36	0	7	inf	0.0015	yes
gi|641570720|gb|KK856272.1|	18278-11856	9.1	36	0	20	inf	0.0015	yes
gi|641570720|gb|KK856272.1|	12209-12721	9.1	36	0.223001	3	4	0.1276	no
gi|641570720|gb|KK856272.1|	15149-15547	9.1	36	1	4	1	0.4433	no
gi|641570720|gb|KK856272.1|	17105-18296	9.1	36	0.497759	2	2	0.19685	no
gi|641570720|gb|KK856272.1|	18741-19489	9.1	36	0.512393	2	2	0.3613	no
gi|641570720|gb|KK856272.1|	19619-20210	9.1	36	1	2	0.182426	0.91015	no
gi|641570720|gb|KK856272.1|	20486-21341	9.1	36	16	2	-3	0.0411	no
gi|641570720|gb|KK856272.1|	23333-24207	9.1	36	13	2	-2	0.09085	no
gi|641570720|gb|KK856272.1|	26175-27461	9.1	36	3	2	-0.895584	0.5141	no
gi|641570720|gb|KK856272.1|	27580-29847	9.1	36	3	2	-0.447852	0.7237	no
gi|641570720|gb|KK856272.1|	29964-34085	9.1	36	4	5	0.421006	0.7311	no
gi|641570720|gb|KK856272.1|	34776-36422	9.1	36	0.97612	0.669607	-0.543743	0.71075	no
gi|641570720|gb|KK856272.1|	36830-37512	9.1	36	8	2	-2	0.1949	no
gi|641570720|gb|KK856272.1|	37879-39944	9.1	36	4	0.866463	-2	0.11165	no
gi|641570720|gb|KK856272.1|	40058-45004	9.1	36	24	2	-4	0.0625	no
gi|641570720|gb|KK856272.1|	45236-46184	9.1	36	12	21	0.879111	0.4868	no
gi|641570720|gb|KK856272.1|	49464-50052	9.1	36	6	22	2	0.19265	no
gi|641570720|gb|KK856272.1|	57917-59831	9.1	36	66	158	1	0.5354	no
gi|641570720|gb|KK856272.1|	60016-60752	9.1	36	0.916572	0.794652	-0.205924	0.89485	no
gi|641570720|gb|KK856272.1|	60892-63188	9.1	36	1	1	0.114756	0.9308	no
gi|641570720|gb|KK856272.1|	63320-63981	9.1	36	1	2	0.599681	0.69715	no
gi|641570720|gb|KK856272.1|	64639-65307	9.1	36	132	182	0.457624	0.8344	no
gi|641570720|gb|KK856272.1|	65453-67854	9.1	36	58	53	-0.131039	0.95075	no
gi|641570720|gb|KK856272.1|	68248-68640	9.1	36	20	9	-1	0.37025	no
gi|641570720|gb|KK856272.1|	68943-70264	9.1	36	31	35	0.212721	0.89475	no

gi|641570720|gb|KK856272.1|	74243-74759	9.1	36	39	86	1	0.36095	no
gi|641570720|gb|KK856272.1|	77298-78087	9.1	36	0.954045	0.905289	-0.0756786	0.92685	no
gi|641570720|gb|KK856272.1|	9335-11221	9.1	36	0.167511	2	4	0.12605	no
gi|641570727|gb|KK856270.1|	00344-10163	10	35	0.843121	0.19755	-2	1	no
gi|641570727|gb|KK856270.1|	01778-10600	10	35	42	25	-0.740538	0.7282	no
gi|641570727|gb|KK856270.1|	16381-11713	10	35	1	2	0.464181	0.7552	no
gi|641570727|gb|KK856270.1|	25963-12625	10	35	104	141.96	0.44374	0.72185	no
gi|641570727|gb|KK856270.1|	41146-14173	10	35	31	37	0.2371	0.84765	no
gi|641570727|gb|KK856270.1|	67960-68946	10	35	0.089544	1	4	0.12385	no
gi|641570727|gb|KK856270.1|	84794-86045	10	35	0.335335	3	3	0.1011	no
gi|641570727|gb|KK856270.1|	86411-87354	10	35	4	1	-1	0.31025	no
gi|641570727|gb|KK856270.1|	89279-90273	10	35	32	37	0.212372	0.86225	no
gi|641570727|gb|KK856270.1|	97224-98496	10	35	1	0.801168	-0.715082	0.633	no
gi|641570730|gb|KK856269.1|	50287-15446	9.1	39	6	1	-2	0.06555	no
gi|641570730|gb|KK856269.1|	54646-15492	9.1	39	10	5	-0.922477	0.5719	no
gi|641570730|gb|KK856269.1|	60712-16157	9.1	39	1	0.805573	-0.881935	0.5831	no
gi|641570730|gb|KK856269.1|	61636-16258	9.1	39	0.940645	0.715097	-0.395512	0.81255	no
gi|641570730|gb|KK856269.1|	62828-16390	9.1	39	1	1	-0.394389	0.7924	no
gi|641570730|gb|KK856269.1|	64117-16452	9.1	39	1	5	2	0.2235	no
gi|641570730|gb|KK856269.1|	64684-16579	9.1	39	2	1	-0.201501	0.8877	no
gi|641570730|gb|KK856269.1|	65898-16691	9.1	39	0.862708	1	0.605137	0.67795	no
gi|641570730|gb|KK856269.1|	67581-16819	9.1	39	1	2	0.598402	0.70555	no
gi|641570730|gb|KK856269.1|	69546-17128	9.1	39	0.733376	0.489197	-0.584137	1	no
gi|641570730|gb|KK856269.1|	27540-28450	9.1	39	1	0	-inf	0.01	no
gi|641570730|gb|KK856269.1|	47064-47874	9.1	39	29	20	-0.554369	0.65545	no
gi|641570730|gb|KK856269.1|	48024-48257	9.1	39	9	38	2	0.1945	no
gi|641570730|gb|KK856269.1|	49920-50205	9.1	39	10	11.51	0.215572	0.88695	no
gi|641570730|gb|KK856269.1|	57899-58144	9.1	39	44	108	1	0.3208	no
gi|641570730|gb|KK856269.1|	60477-60726	9.1	39	21	51	1	0.3691	no
gi|641570730|gb|KK856269.1|	63192-64879	9.1	39	37	97	1	0.5223	no

gi|641570730|gb|KK856269.1|	67216-67587	9.1	39	15	55	2	0.1648	no
gi|641570730|gb|KK856269.1|	68970-69630	9.1	39	10	41	2	0.13205	no
gi|641570730|gb|KK856269.1|	72876-74357	9.1	39	0.220278	1	3	0.177	no
gi|641570733|gb|KK856268.1|	55789-56543	10	22	2	0	-inf	0.0075	no
gi|641570740|gb|KK856266.1|	07861-10867	9.5	39	0.457009	2	2	0.33105	no
gi|641570740|gb|KK856266.1|	09277-11008	9.5	39	0.116317	3	5	0.1123	no
gi|641570740|gb|KK856266.1|	13807-11402	9.5	39	6	18	2	0.41765	no
gi|641570740|gb|KK856266.1|	13615-13986	9.5	39	0.394613	8	4	0.1141	no
gi|641570740|gb|KK856266.1|	54286-15546	9.5	39	0.646468	1	0.758343	0.6256	no
gi|641570740|gb|KK856266.1|	2-1339	9.5	39	6	2	-1	0.31505	no
gi|641570740|gb|KK856266.1|	20620-21281	9.5	39	1	5	2	0.18295	no
gi|641570740|gb|KK856266.1|	23601-23855	9.5	39	0.961559	40	5	0.10545	no
gi|641570740|gb|KK856266.1|	44502-45782	9.5	39	9	11	0.221393	0.8568	no
gi|641570740|gb|KK856266.1|	47861-48967	9.5	39	0.855197	0.473273	-0.853584	1	no
gi|641570740|gb|KK856266.1|	49703-52102	9.5	39	0.996004	1	0.410344	0.75835	no
gi|641570740|gb|KK856266.1|	53119-53628	9.5	39	4	5	0.231129	0.87135	no
gi|641570740|gb|KK856266.1|	53836-54828	9.5	39	0.533218	0.675807	0.341886	1	no
gi|641570740|gb|KK856266.1|	74157-75386	9.5	39	0.273978	1	2	0.2441	no
gi|641570743|gb|KK856265.1|	16510-17445	9.6	34	4	1	-2	0.29835	no
gi|641570743|gb|KK856265.1|	18853-22222	9.6	34	2	0.68122	-1	0.33945	no
gi|641570743|gb|KK856265.1|	22880-26589	9.6	34	32	16	-0.998785	0.6546	no
gi|641570743|gb|KK856265.1|	26728-27963	9.6	34	2	0.207392	-3	0.14415	no
gi|641570743|gb|KK856265.1|	28513-28888	9.6	34	6	2	-1	0.4158	no
gi|641570743|gb|KK856265.1|	29074-34960	9.6	34	0.895818	2	1	0.26245	no
gi|641570743|gb|KK856265.1|	35684-37081	9.6	34	2	3	0.607417	0.64895	no
gi|641570743|gb|KK856265.1|	37189-37736	9.6	34	3	8	1	0.30605	no
gi|641570743|gb|KK856265.1|	38743-39403	9.6	34	14	19	0.418047	0.74825	no
gi|641570743|gb|KK856265.1|	39856-40925	9.6	34	1	0.369916	-2	0.33985	no
gi|641570743|gb|KK856265.1|	48696-48936	9.6	34	60	146	1	0.32925	no
gi|641570743|gb|KK856265.1|	5582-6399	9.6	34	2	2	0.254916	0.8632	no

gi|641570743|gb|KK856265.1|	8265-9130	9.6	34	0.105722	2	4	0.1136	no
gi|641570743|gb|KK856265.1|	88455-88948	9.6	34	2	1	-0.729183	0.67005	no
gi|641570743|gb|KK856265.1|	90609-90951	9.6	34	82	30	-1	0.26145	no
gi|641570745|gb|KK856264.1|	12186-12463	9.3	18	27	19	-0.480561	0.7247	no
gi|641570745|gb|KK856264.1|	40829-14100	9.3	18	40	99	1	0.38985	no
gi|641570745|gb|KK856264.1|	41389-14151	9.3	18	360	554	0.622052	0.67755	no
gi|641570745|gb|KK856264.1|	17053-17296	9.3	18	25	3	-3	0.16755	no
gi|641570745|gb|KK856264.1|	27933-28169	9.3	18	77	42	-0.867887	0.5116	no
gi|641570745|gb|KK856264.1|	36594-37014	9.3	18	4	3	-0.190668	0.90345	no
gi|641570745|gb|KK856264.1|	41301-41508	9.3	18	91	65	-0.495868	0.7021	no
gi|641570745|gb|KK856264.1|	42245-42417	9.3	18	34	42	0.322608	0.8234	no
gi|641570745|gb|KK856264.1|	46727-46951	9.3	18	66	56	-0.257682	0.8461	no
gi|641570745|gb|KK856264.1|	49637-50162	9.3	18	114	70	-0.700393	0.65845	no
gi|641570745|gb|KK856264.1|	56526-56882	9.3	18	17	5	-2	0.2441	no
gi|641570745|gb|KK856264.1|	57462-58380	9.3	18	1	0.297845	-2	0.25005	no
gi|641570745|gb|KK856264.1|	60011-61811	9.3	18	0.573589	0.067279	-3	1	no
gi|641570745|gb|KK856264.1|	66959-67288	9.3	18	5	0.753903	-3	0.2476	no
gi|641570745|gb|KK856264.1|	73149-73614	9.3	18	11	3	-2	0.204	no
gi|641570745|gb|KK856264.1|	76585-77622	9.3	18	3	2	-0.942171	0.5005	no
gi|641570745|gb|KK856264.1|	81025-81235	9.3	18	30	8	-2	0.28355	no
gi|641570745|gb|KK856264.1|	87529-88586	9.3	18	2	1	-1	0.41275	no
gi|641570745|gb|KK856264.1|	93458-94072	9.3	18	1	2	0.820662	0.62335	no
gi|641570748|gb|KK856263.1|	168-362	8	36	17	24	0.527097	0.74945	no
gi|641570748|gb|KK856263.1|	64981-65337	8	36	4	6	0.578686	0.71515	no
gi|641570748|gb|KK856263.1|	84464-85067	8	36	13793.5	0	-inf	0.1094	no
gi|641570748|gb|KK856263.1|	9748-10204	8	36	2	3	0.227579	0.88235	no
gi|641570754|gb|KK856261.1|	04971-10624	9.6	38	2	16	3	0.03105	no
gi|641570754|gb|KK856261.1|	15332-15883	9.6	38	0.795026	2	1	0.51315	no
gi|641570754|gb|KK856261.1|	16037-16304	9.6	38	20	46	1	0.3909	no
gi|641570754|gb|KK856261.1|	21466-22674	9.6	38	2	2	-0.187049	0.8943	no

gi|641570754|gb|KK856261.1|	23074-23502	9.6	38	30	25	-0.260275	0.839	no
gi|641570754|gb|KK856261.1|	24036-25297	9.6	38	117	81	-0.532022	0.80125	no
gi|641570754|gb|KK856261.1|	27461-27747	9.6	38	131	129	-0.021197	0.985	no
gi|641570754|gb|KK856261.1|	31497-32503	9.6	38	5	6	0.174414	0.8929	no
gi|641570754|gb|KK856261.1|	37235-37577	9.6	38	56	65	0.212015	0.86255	no
gi|641570754|gb|KK856261.1|	38089-38892	9.6	38	1	0.35383	-2	0.3455	no
gi|641570754|gb|KK856261.1|	39012-39961	9.6	38	0.751518	3	2	0.2741	no
gi|641570754|gb|KK856261.1|	42457-42634	9.6	38	36	224	3	0.09855	no
gi|641570754|gb|KK856261.1|	48418-49161	9.6	38	0.646334	0.980725	0.601569	0.70505	no
gi|641570754|gb|KK856261.1|	70032-70678	9.6	38	16	4	-2	0.1684	no
gi|641570757|gb|KK856260.1|	.39116-13933	10	42	23	22	-0.100091	0.9452	no
gi|641570757|gb|KK856260.1|	.42928-14532	10	42	10	3	-2	0.20805	no
gi|641570757|gb|KK856260.1|	.45556-15021	10	42	10	7	-0.530275	0.7408	no
gi|641570757|gb|KK856260.1|	.53965-15770	10	42	21	13	-0.727657	0.74595	no
gi|641570757|gb|KK856260.1|	.57884-16111	10	42	7	7	0.00552438	0.99695	no
gi|641570757|gb|KK856260.1|	.61345-16174	10	42	11	5	-1	0.3885	no
gi|641570757|gb|KK856260.1|	.62536-16340	10	42	1	0.6435	-0.718423	0.6701	no
gi|641570757|gb|KK856260.1|	.65174-16539	10	42	44	29	-0.605274	0.6655	no
gi|641570757|gb|KK856260.1|	.65516-16569	10	42	22	23	0.0352459	0.92445	no
gi|641570757|gb|KK856260.1|	.86551-18675	10	42	35	58	0.745888	0.58465	no
gi|641570757|gb|KK856260.1|	.87478-18864	10	42	2	0.445973	-2	0.2684	no
gi|641570757|gb|KK856260.1|	.89159-18935	10	42	76	18	-2	0.199	no
gi|641570757|gb|KK856260.1|	.96438-19956	10	42	27	4	-3	0.13605	no
gi|641570757|gb|KK856260.1|	41932-43302	10	42	0	6	inf	5.00E-05	yes
gi|641570760|gb|KK856259.1|	58015-58303	8.9	39	3	17	3	0.1787	no
gi|641570760|gb|KK856259.1|	60409-60655	8.9	39	12	23	0.998537	0.5311	no
gi|641570760|gb|KK856259.1|	63239-63661	8.9	39	4	11	1	0.3419	no
gi|641570760|gb|KK856259.1|	89899-90259	8.9	39	1	10	3	0.1712	no
gi|641570769|gb|KK856258.1|	.00362-10076	9.4	20	11	3	-2	0.26305	no
gi|641570769|gb|KK856258.1|	.01466-10297	9.4	20	7	4	-1	0.42145	no

gi|641570769|gb|KK856258.1|	87536-89772	9.4	20	3	2	-0.810894	0.53045	no
gi|641570769|gb|KK856258.1|	97523-98333	9.4	20	11	5	-1	0.4069	no
gi|641570772|gb|KK856257.1|	.00104-10030	10	21	24	15	-0.731207	0.6634	no
gi|641570772|gb|KK856257.1|	.04311-10487	10	21	3	0.87227	-2	0.28145	no
gi|641570772|gb|KK856257.1|	.25385-12555	10	21	40	23	-0.805609	0.633	no
gi|641570772|gb|KK856257.1|	.28087-12906	10	21	3	7	0.971532	0.4621	no
gi|641570772|gb|KK856257.1|	14054-14297	10	21	0	16	inf	0.0068	no
gi|641570776|gb|KK856256.1|	.07306-10806	8.4	34	0	8	inf	5.00E-05	yes
gi|641570780|gb|KK856255.1|	.07107-10767	8.8	40	0.761028	2	1	0.44255	no
gi|641570780|gb|KK856255.1|	12895-14013	8.8	40	0	217	inf	0.0001	yes
gi|641570780|gb|KK856255.1|	36966-38203	8.8	40	0	264	inf	0.00025	yes
gi|641570780|gb|KK856255.1|	40131-40548	8.8	40	4	3	-0.413365	0.80855	no
gi|641570784|gb|KK856254.1|	20939-21492	11	21	1	4	2	0.32035	no
gi|641570784|gb|KK856254.1|	23916-24907	11	21	2	0.13533	-4	0.19735	no
gi|641570784|gb|KK856254.1|	93543-94500	11	21	4	9	1	0.4293	no
gi|641570784|gb|KK856254.1|	95299-98838	11	21	13	13	-0.0281368	0.99	no
gi|641570784|gb|KK856254.1|	)9479-10004ε	11	21	5	4	-0.196947	0.88765	no
gi|641570788|gb|KK856253.1|	.00224-10057	10	20	9	5	-1	0.4822	no
gi|641570788|gb|KK856253.1|	.01932-10301	10	20	40	47	0.230031	0.9178	no
gi|641570788|gb|KK856253.1|	.03743-10437	10	20	12	18	0.579216	0.6436	no
gi|641570788|gb|KK856253.1|	.11359-11162	10	20	158	63	-1	0.31105	no
gi|641570788|gb|KK856253.1|	.18669-11885	10	20	20	33	0.742531	0.6475	no
gi|641570788|gb|KK856253.1|	.26401-12750	10	20	4	3	-0.485344	0.71225	no
gi|641570788|gb|KK856253.1|	.30303-13084	10	20	1	4	1	0.397	no
gi|641570788|gb|KK856253.1|	.31643-13416	10	20	1	1	0.297845	0.821	no
gi|641570788|gb|KK856253.1|	51134-53158	10	20	6	1	-2	0.14535	no
gi|641570788|gb|KK856253.1|	57088-59178	10	20	0.71001	0.627041	-0.179281	1	no
gi|641570788|gb|KK856253.1|	59481-59977	10	20	3	2	-0.0591454	0.94525	no
gi|641570788|gb|KK856253.1|	69721-70366	10	20	0.786929	2	1	0.51085	no
gi|641570788|gb|KK856253.1|	70602-70830	10	20	29	14	-1	0.46635	no

gi|641570788|gb|KK856253.1|	78937-79257	10	20	3	4	0.308926	0.86115	no
gi|641570788|gb|KK856253.1|	85501-86815	10	20	5	4	-0.426895	0.73805	no
gi|641570788|gb|KK856253.1|	86950-87210	10	20	20	13	-0.58151	0.70095	no
gi|641570788|gb|KK856253.1|	87701-87896	10	20	24	31	0.375628	0.80415	no
gi|641570788|gb|KK856253.1|	89418-89739	10	20	36	19	-0.908954	0.488	no
gi|641570788|gb|KK856253.1|	89873-90428	10	20	8	6	-0.582333	0.67145	no
gi|641570788|gb|KK856253.1|	92753-93850	10	20	11	8	-0.394217	0.75315	no
gi|641570788|gb|KK856253.1|	95301-95701	10	20	3	2	-0.737151	0.63835	no
gi|641570795|gb|KK856251.1|	08935-11299	9.8	38	17	13	-0.387665	0.8607	no
gi|641570795|gb|KK856251.1|	32663-13279	9.8	38	177	350.48	0.981589	0.60445	no
gi|641570795|gb|KK856251.1|	34677-13508	9.8	38	3	1	-0.829248	0.64915	no
gi|641570795|gb|KK856251.1|	35752-13661	9.8	38	0.850861	1	0.77339	0.62605	no
gi|641570795|gb|KK856251.1|	40117-14088	9.8	38	0.990748	1	0.40937	0.8089	no
gi|641570795|gb|KK856251.1|	47013-14772	9.8	38	1	2	0.600993	0.6936	no
gi|641570795|gb|KK856251.1|	3121-3458	9.8	38	0	9	inf	0.00375	no
gi|641570795|gb|KK856251.1|	76987-77769	9.8	38	1	2	0.891782	0.57435	no
gi|641570795|gb|KK856251.1|	78207-81396	9.8	38	8	3	-1	0.2943	no
gi|641570795|gb|KK856251.1|	82007-86492	9.8	38	24	16	-0.589193	0.7793	no
gi|641570795|gb|KK856251.1|	89845-90649	9.8	38	1	0.529878	-1	0.47135	no
gi|641570795|gb|KK856251.1|	90800-92527	9.8	38	2	1	-0.284609	0.83095	no
gi|641570795|gb|KK856251.1|	93667-94844	9.8	38	1	0.219594	-2	0.2515	no
gi|641570795|gb|KK856251.1|	96224-97139	9.8	38	115	157	0.444316	0.8389	no
gi|641570799|gb|KK856250.1|	01713-10189	8.4	30	29	227.99	3	0.07165	no
gi|641570799|gb|KK856250.1|	05694-10588	8.4	30	7	97	4	0.14185	no
gi|641570799|gb|KK856250.1|	60282-60479	8.4	30	38	476.32	4	0.02525	no
gi|641570799|gb|KK856250.1|	92227-92387	8.4	30	44	143.17	2	0.29655	no
gi|641570799|gb|KK856250.1|	97933-98412	8.4	30	2	25	3	0.0513	no
gi|641570803|gb|KK856249.1|	93665-93886	7.3	37	24	28	0.241092	0.86465	no
gi|641570803|gb|KK856249.1|	94042-94332	7.3	37	45	55	0.280099	0.8306	no
gi|641570807|gb|KK856248.1|	07299-10831	11	38	9	7	-0.35267	0.78155	no

gi|641570807|gb|KK856248.1|	08368-10860	11	38	255	139.91	-0.864792	0.4939	no
gi|641570807|gb|KK856248.1|	12149-11247	11	38	61	47	-0.390674	0.763	no
gi|641570807|gb|KK856248.1|	19538-12019	11	38	40	31	-0.389512	0.75595	no
gi|641570807|gb|KK856248.1|	21508-12203	11	38	7	2	-2	0.2056	no
gi|641570807|gb|KK856248.1|	22498-12363	11	38	0.829688	0.574022	-0.531463	1	no
gi|641570807|gb|KK856248.1|	24014-12483	11	38	3	3	0.280896	0.8341	no
gi|641570807|gb|KK856248.1|	26583-12873	11	38	26	18	-0.587006	0.7083	no
gi|641570807|gb|KK856248.1|	13193-14713	11	38	1	2	0.470369	0.7395	no
gi|641570807|gb|KK856248.1|	32124-13484	11	38	8	5	-0.737465	0.56155	no
gi|641570807|gb|KK856248.1|	34992-13592	11	38	11	5	-1	0.42135	no
gi|641570807|gb|KK856248.1|	36130-13655	11	38	32	22	-0.544576	0.67115	no
gi|641570807|gb|KK856248.1|	39650-14000	11	38	4	11	2	0.32805	no
gi|641570807|gb|KK856248.1|	44086-14520	11	38	0.769008	0.351117	-1	1	no
gi|641570807|gb|KK856248.1|	45326-14670	11	38	0.78089	0.73208	-0.093119	1	no
gi|641570807|gb|KK856248.1|	49233-15015	11	38	1	0.741552	-0.980765	0.57205	no
gi|641570807|gb|KK856248.1|	14981-17635	11	38	0.777518	0.439495	-0.823028	1	no
gi|641570807|gb|KK856248.1|	50711-15161	11	38	2	0.609099	-1	0.44155	no
gi|641570807|gb|KK856248.1|	51759-15200	11	38	10	10	-0.0354089	0.9394	no
gi|641570807|gb|KK856248.1|	57672-15866	11	38	0.801839	1	0.894409	0.5684	no
gi|641570807|gb|KK856248.1|	62176-16328	11	38	0.232216	1	2	0.25595	no
gi|641570807|gb|KK856248.1|	63592-16438	11	38	1	1	0.239906	0.88205	no
gi|641570807|gb|KK856248.1|	65868-16811	11	38	0.931755	2	0.761457	0.57555	no
gi|641570807|gb|KK856248.1|	68858-16978	11	38	1	0.888645	-0.395792	0.80865	no
gi|641570807|gb|KK856248.1|	76062-17866	11	38	0.793328	2	0.984278	0.4693	no
gi|641570807|gb|KK856248.1|	17793-19927	11	38	1	1	-0.0536792	0.96725	no
gi|641570807|gb|KK856248.1|	79682-18158	11	38	0.623375	0.633789	0.0239027	1	no
gi|641570807|gb|KK856248.1|	82315-18261	11	38	60.01	49	-0.300357	0.81275	no
gi|641570807|gb|KK856248.1|	83731-18496	11	38	2	0	-inf	0.0021	yes
gi|641570807|gb|KK856248.1|	85030-18841	11	38	9	11	0.303544	0.847	no
gi|641570807|gb|KK856248.1|	89765-19025	11	38	2	10	3	0.1409	no

gi|641570807|gb|KK856248.1|	92643-19320	11	38	2	5	1	0.4102	no
gi|641570807|gb|KK856248.1|	93652-19401	11	38	921	1011.52	0.134974	0.9471	no
gi|641570807|gb|KK856248.1|	99767-20055	11	38	44	49	0.160621	0.8954	no
gi|641570807|gb|KK856248.1|	01752-20272	11	38	26	22	-0.299064	0.81085	no
gi|641570807|gb|KK856248.1|	20460-23676	11	38	4	3	-0.120625	0.9215	no
gi|641570807|gb|KK856248.1|	05255-20562	11	38	0	30	inf	0.0001	yes
gi|641570807|gb|KK856248.1|	23963-26045	11	38	0.675493	0.171735	-2	1	no
gi|641570807|gb|KK856248.1|	26183-26427	11	38	69	66	-0.0573579	0.9641	no
gi|641570807|gb|KK856248.1|	29072-29837	11	38	13	9	-0.546884	0.6701	no
gi|641570807|gb|KK856248.1|	301-574	11	38	17	5	-2	0.25985	no
gi|641570807|gb|KK856248.1|	3226-4671	11	38	1	1	0.0930321	0.94885	no
gi|641570807|gb|KK856248.1|	33240-33481	11	38	35	23	-0.59768	0.66715	no
gi|641570807|gb|KK856248.1|	34515-35370	11	38	0.536624	1	1	0.4595	no
gi|641570807|gb|KK856248.1|	38304-38715	11	38	22	29	0.426807	0.7333	no
gi|641570807|gb|KK856248.1|	40561-40998	11	38	12	19	0.657265	0.6149	no
gi|641570807|gb|KK856248.1|	42927-43572	11	38	0.944314	1	0.599233	0.70555	no
gi|641570807|gb|KK856248.1|	43862-44044	11	38	51	87	0.7686	0.59195	no
gi|641570807|gb|KK856248.1|	47402-47681	11	38	14	4	-2	0.3077	no
gi|641570807|gb|KK856248.1|	5279-6335	11	38	0.822615	0.625794	-0.394529	1	no
gi|641570807|gb|KK856248.1|	65376-65630	11	38	28	14	-0.98209	0.5037	no
gi|641570807|gb|KK856248.1|	6685-6993	11	38	5	0.871706	-3	0.2588	no
gi|641570807|gb|KK856248.1|	75627-76113	11	38	7	2	-2	0.2831	no
gi|641570807|gb|KK856248.1|	82397-82855	11	38	6	4	-0.611328	0.66815	no
gi|641570807|gb|KK856248.1|	91629-91955	11	38	19	25	0.40329	0.75975	no
gi|641570807|gb|KK856248.1|	92076-93728	11	38	0.777679	1	0.515168	0.71885	no
gi|641570807|gb|KK856248.1|	921-2890	11	38	1	0.243534	-2	0.20995	no
gi|641570807|gb|KK856248.1|	95619-99906	11	38	16	13	-0.255935	0.90725	no
gi|641570811|gb|KK856247.1|	12572-13140	9.1	35	22	3	-3	0.06805	no
gi|641570811|gb|KK856247.1|	14743-15000	9.1	35	66	4	-4	0.09945	no
gi|641570811|gb|KK856247.1|	17811-22069	9.1	35	49	0.479076	-7	0.00975	no

gi|641570811|gb|KK856247.1|	27042-27640	9.1	35	3	0.531736	-2	0.24735	no
gi|641570811|gb|KK856247.1|	31529-33366	9.1	35	23	20	-0.153226	0.9215	no
gi|641570811|gb|KK856247.1|	33949-34751	9.1	35	5	4	-0.2771	0.8341	no
gi|641570811|gb|KK856247.1|	36207-37290	9.1	35	13	9	-0.460123	0.71035	no
gi|641570811|gb|KK856247.1|	37621-38137	9.1	35	6	3	-0.990874	0.5088	no
gi|641570811|gb|KK856247.1|	38260-44454	9.1	35	20	11	-0.94203	0.6576	no
gi|641570811|gb|KK856247.1|	46859-47915	9.1	35	0.493569	1	1	0.3778	no
gi|641570811|gb|KK856247.1|	49142-54193	9.1	35	1	3	2	0.23145	no
gi|641570811|gb|KK856247.1|	56833-58200	9.1	35	0.362564	1	2	0.25645	no
gi|641570811|gb|KK856247.1|	58940-59405	9.1	35	0.783757	3	2	0.3323	no
gi|641570811|gb|KK856247.1|	59579-60055	9.1	35	0.753574	4	2	0.2235	no
gi|641570811|gb|KK856247.1|	64225-65761	9.1	35	0.950586	3	2	0.25845	no
gi|641570811|gb|KK856247.1|	9998-11195	9.1	35	2	0.107614	-4	0.1892	no
gi|641570819|gb|KK856245.1|	08452-10877	10	45	1	9	3	0.26155	no
gi|641570819|gb|KK856245.1|	19049-11925	10	45	11	171	4	0.0698	no
gi|641570819|gb|KK856245.1|	30167-13056	10	45	3	3	0.169623	0.91775	no
gi|641570819|gb|KK856245.1|	30735-13187	10	45	2	3	0.502993	0.7091	no
gi|641570819|gb|KK856245.1|	33696-13413	10	45	0	45	inf	5.00E-05	yes
gi|641570819|gb|KK856245.1|	38164-13844	10	45	13	5	-1	0.4252	no
gi|641570819|gb|KK856245.1|	40617-14119	10	45	58	53	-0.123277	0.91815	no
gi|641570819|gb|KK856245.1|	54987-15609	10	45	0	2	inf	0.00135	yes
gi|641570819|gb|KK856245.1|	56156-15716	10	45	0	2	inf	0.0016	yes
gi|641570819|gb|KK856245.1|	58017-15893	10	45	0	2	inf	0.00255	yes
gi|641570819|gb|KK856245.1|	59595-16068	10	45	0	3	inf	0.0005	yes
gi|641570819|gb|KK856245.1|	26545-27899	10	45	0.061105	1	5	0.1123	no
gi|641570819|gb|KK856245.1|	30145-32673	10	45	3	9	1	0.27065	no
gi|641570819|gb|KK856245.1|	33407-35684	10	45	0.544194	0.311359	-0.805542	1	no
gi|641570819|gb|KK856245.1|	36783-37795	10	45	3	3	-0.0271663	0.9761	no
gi|641570819|gb|KK856245.1|	39768-41418	10	45	5	4	-0.418201	0.7399	no
gi|641570819|gb|KK856245.1|	41566-41958	10	45	17	10	-0.782965	0.5566	no

gi|641570819|gb|KK856245.1|	42289-42821	10	45	13	15	0.172233	0.8904	no
gi|641570819|gb|KK856245.1|	42924-43824	10	45	19	17	-0.16158	0.8972	no
gi|641570819|gb|KK856245.1|	45409-45837	10	45	5	2	-0.997241	0.57205	no
gi|641570819|gb|KK856245.1|	47608-48894	10	45	1	2	0.694379	0.62795	no
gi|641570819|gb|KK856245.1|	50229-52543	10	45	1	2	0.575598	0.65785	no
gi|641570819|gb|KK856245.1|	58268-58449	10	45	23	14	-0.702254	0.6977	no
gi|641570819|gb|KK856245.1|	58797-59458	10	45	14	13	-0.122018	0.9239	no
gi|641570819|gb|KK856245.1|	61043-63819	10	45	35	58	0.716078	0.7327	no
gi|641570819|gb|KK856245.1|	65894-67293	10	45	1	2	0.666318	0.6283	no
gi|641570819|gb|KK856245.1|	67404-70431	10	45	12	15	0.414045	0.79335	no
gi|641570819|gb|KK856245.1|	71401-72392	10	45	0.622869	1	0.967482	0.5733	no
gi|641570819|gb|KK856245.1|	72709-76258	10	45	1	0.870348	-0.415899	0.74835	no
gi|641570819|gb|KK856245.1|	7359-9032	10	45	15	12	-0.360733	0.7708	no
gi|641570819|gb|KK856245.1|	76648-77855	10	45	2	2	-0.574078	0.68375	no
gi|641570819|gb|KK856245.1|	77980-80334	10	45	1	0.600548	-0.934724	0.5066	no
gi|641570819|gb|KK856245.1|	80434-81345	10	45	8	70	3	0.1176	no
gi|641570819|gb|KK856245.1|	81508-83672	10	45	0.575429	2	2	0.19395	no
gi|641570819|gb|KK856245.1|	87506-87795	10	45	10	20	0.979602	0.5101	no
gi|641570819|gb|KK856245.1|	88035-89162	10	45	1	2	0.869028	0.5524	no
gi|641570819|gb|KK856245.1|	93859-95088	10	45	0.821933	0.312886	-1	1	no
gi|641570819|gb|KK856245.1|	95802-96258	10	45	4	27	3	0.07375	no
gi|641570819|gb|KK856245.1|	96368-98128	10	45	0.181001	0.827933	2	1	no
gi|641570822|gb|KK856244.1|	00992-10150	11	21	4	2	-1	0.46	no
gi|641570822|gb|KK856244.1|	03449-10373	11	21	41	68	0.727883	0.56985	no
gi|641570822|gb|KK856244.1|	08752-10930	11	21	0.786248	2	1	0.44255	no
gi|641570822|gb|KK856244.1|	10090-11071	11	21	9	12	0.35545	0.78205	no
gi|641570822|gb|KK856244.1|	12183-11254	11	21	3	2	-0.642598	0.71075	no
gi|641570822|gb|KK856244.1|	22557-12325	11	21	72	32	-1	0.4836	no
gi|641570822|gb|KK856244.1|	23321-12442	11	21	0.775748	2	2	0.26655	no
gi|641570822|gb|KK856244.1|	26969-12798	11	21	18	13	-0.423134	0.737	no

gi|641570822|gb|KK856244.1|	31014-13160	11	21	21	17	-0.260444	0.8361	no
gi|641570822|gb|KK856244.1|	35178-13717	11	21	0.901848	0.657876	-0.455069	0.7554	no
gi|641570822|gb|KK856244.1|	37695-13918	11	21	0.658784	0.334636	-0.977211	1	no
gi|641570822|gb|KK856244.1|	39331-13969	11	21	20	9	-1	0.4066	no
gi|641570822|gb|KK856244.1|	40603-14120	11	21	0.523175	3	3	0.1889	no
gi|641570822|gb|KK856244.1|	42366-14277	11	21	6	3	-1	0.4599	no
gi|641570822|gb|KK856244.1|	44944-14749	11	21	42	25	-0.762078	0.73	no
gi|641570822|gb|KK856244.1|	49947-15067	11	21	0.926847	2	0.793955	0.63025	no
gi|641570822|gb|KK856244.1|	51111-15159	11	21	0.482096	4	3	0.27515	no
gi|641570822|gb|KK856244.1|	2391-3321	11	21	0.481845	0.732519	0.604296	1	no
gi|641570822|gb|KK856244.1|	68329-69831	11	21	17	8	-1	0.39295	no
gi|641570822|gb|KK856244.1|	71256-72046	11	21	8	2	-2	0.1895	no
gi|641570822|gb|KK856244.1|	90016-90668	11	21	48	37	-0.381952	0.75765	no
gi|641570822|gb|KK856244.1|	92085-92801	11	21	2	1	-0.621378	0.6869	no
gi|641570822|gb|KK856244.1|	93503-94158	11	21	1	0.699894	-0.815521	0.6492	no
gi|641570822|gb|KK856244.1|	95071-95692	11	21	30	34	0.148889	0.90925	no
gi|641570822|gb|KK856244.1|	96540-96934	11	21	13	22	0.766927	0.55445	no
gi|641570822|gb|KK856244.1|	9675-10007	11	21	4	9	0.971683	0.51885	no
gi|641570830|gb|KK856242.1|	06398-10664	9	31	5	22	2	0.2209	no
gi|641570830|gb|KK856242.1|	06844-10943	9	31	0.533089	1	1	0.3824	no
gi|641570830|gb|KK856242.1|	09498-11053	9	31	0.423105	1	1	0.4595	no
gi|641570830|gb|KK856242.1|	11866-12268	9	31	0.676509	4	3	0.2991	no
gi|641570830|gb|KK856242.1|	13374-14801	9	31	4	7	0.703114	0.584	no
gi|641570830|gb|KK856242.1|	17609-17915	9	31	7	10	0.568821	0.7209	no
gi|641570830|gb|KK856242.1|	19488-21036	9	31	0.785128	1	0.401514	0.78645	no
gi|641570830|gb|KK856242.1|	21575-22931	9	31	0.427	1	1	0.3924	no
gi|641570830|gb|KK856242.1|	23730-27101	9	31	2	2	0.388082	0.7556	no
gi|641570830|gb|KK856242.1|	27407-28877	9	31	2	3	0.707234	0.5965	no
gi|641570830|gb|KK856242.1|	29284-29512	9	31	11	6	-0.871251	0.6407	no
gi|641570830|gb|KK856242.1|	31276-31668	9	31	6	11	0.888672	0.53385	no

gi|641570830|gb|KK856242.1|	32279-33993	9	31	8	18	1	0.3348	no
gi|641570830|gb|KK856242.1|	35196-37106	9	31	1	3	1	0.37495	no
gi|641570830|gb|KK856242.1|	37888-39441	9	31	17	28	0.663231	0.68035	no
gi|641570830|gb|KK856242.1|	39726-40847	9	31	12	10	-0.307633	0.8086	no
gi|641570830|gb|KK856242.1|	41067-43241	9	31	10	7	-0.469222	0.70545	no
gi|641570830|gb|KK856242.1|	44093-45535	9	31	14	15	0.164464	0.8936	no
gi|641570830|gb|KK856242.1|	46572-47136	9	31	0.383591	3	3	0.27515	no
gi|641570830|gb|KK856242.1|	54624-55223	9	31	2	0.530438	-2	0.25005	no
gi|641570830|gb|KK856242.1|	76604-77624	9	31	0.343409	0.914166	1	0.44255	no
gi|641570830|gb|KK856242.1|	85186-85439	9	31	118	341	2	0.2268	no
gi|641570830|gb|KK856242.1|	9806-11192	9	31	0.475705	50	7	0.0555	no
gi|641570833|gb|KK856241.1|	.03893-10643	8.6	43	2	2	0.194779	0.88275	no
gi|641570833|gb|KK856241.1|	.08157-10923	8.6	43	0.07985	2	4	0.1136	no
gi|641570833|gb|KK856241.1|	.39598-13995	8.6	43	0	31	inf	0.0001	yes
gi|641570833|gb|KK856241.1|	.42135-14244	8.6	43	0	44	inf	0.0001	yes
gi|641570833|gb|KK856241.1|	76021-76269	8.6	43	103	85	-0.284682	0.82555	no
gi|641570833|gb|KK856241.1|	77925-78164	8.6	43	10	10	-0.0365959	0.9394	no
gi|641570833|gb|KK856241.1|	84241-86221	8.6	43	38	30	-0.302441	0.89165	no
gi|641570833|gb|KK856241.1|	86345-86987	8.6	43	56	56	0.00805659	0.9939	no
gi|641570833|gb|KK856241.1|	88798-92054	8.6	43	21	20	-0.0692626	0.9737	no
gi|641570833|gb|KK856241.1|	92441-93344	8.6	43	0.600274	2	1	0.4118	no
gi|641570833|gb|KK856241.1|	93453-95355	8.6	43	0.622279	0.822481	0.40242	1	no
gi|641570833|gb|KK856241.1|	96070-10065[illegible]	8.6	43	4	3	-0.527717	0.68	no
gi|641570836|gb|KK856240.1|	.02961-10337	12	44	3	3	0.394092	0.80895	no
gi|641570836|gb|KK856240.1|	10841-11809	12	44	1	0.278674	-2	0.24735	no
gi|641570836|gb|KK856240.1|	.17944-11820	12	44	5	9	0.779686	0.6419	no
gi|641570836|gb|KK856240.1|	164-727	12	44	2	0.581513	-2	0.4266	no
gi|641570836|gb|KK856240.1|	20071-20291	12	44	15	7	-1	0.4939	no
gi|641570836|gb|KK856240.1|	37417-38344	12	44	0.774113	1	0.604264	0.6936	no
gi|641570836|gb|KK856240.1|	5866-7814	12	44	1	0.061611	-4	0.1901	no

gi|641570836|gb|KK856240.1|	65434-65686	12	44	14	3	-2	0.24595	no
gi|641570836|gb|KK856240.1|	7902-8329	12	44	3	0.910638	-2	0.3455	no
gi|641570836|gb|KK856240.1|	82196-82811	12	44	0.505793	2	2	0.2812	no
gi|641570836|gb|KK856240.1|	84994-85207	12	44	17	32	0.915365	0.5496	no
gi|641570839|gb|KK856239.1|	12885-11336	11	44	1	3	1	0.4426	no
gi|641570839|gb|KK856239.1|	31257-13255	11	44	1	0.981482	-0.0711159	0.95545	no
gi|641570839|gb|KK856239.1|	3414-4042	11	44	1	0.743028	-0.623671	0.71195	no
gi|641570839|gb|KK856239.1|	55153-55870	11	44	0.407107	2	2	0.2812	no
gi|641570839|gb|KK856239.1|	87763-88148	11	44	3	2	-0.639464	0.71075	no
gi|641570846|gb|KK856237.1|	11868-12814	8.8	37	1	0	-inf	0.0075	no
gi|641570846|gb|KK856237.1|	14635-16619	8.8	37	33	0	-inf	0.0003	yes
gi|641570846|gb|KK856237.1|	19778-21538	8.8	37	8	0	-inf	5.00E-05	yes
gi|641570846|gb|KK856237.1|	23860-24958	8.8	37	8	0	-inf	5.00E-05	yes
gi|641570846|gb|KK856237.1|	26723-27864	8.8	37	25	0	-inf	5.00E-05	yes
gi|641570846|gb|KK856237.1|	28536-30161	8.8	37	7	0	-inf	5.00E-05	yes
gi|641570846|gb|KK856237.1|	3105-4252	8.8	37	1	0.566215	-0.879301	0.5832	no
gi|641570846|gb|KK856237.1|	33536-33681	8.8	37	135	0	-inf	0.0102	no
gi|641570846|gb|KK856237.1|	4316-5319	8.8	37	3	0.26666	-3	0.1439	no
gi|641570846|gb|KK856237.1|	50583-50791	8.8	37	82	40	-1	0.44525	no
gi|641570846|gb|KK856237.1|	52039-52612	8.8	37	4	2	-1	0.4334	no
gi|641570846|gb|KK856237.1|	55855-56345	8.8	37	2	1	-0.992406	0.62775	no
gi|641570846|gb|KK856237.1|	56643-56800	8.8	37	163	90	-0.846708	0.5635	no
gi|641570846|gb|KK856237.1|	57379-58055	8.8	37	15	3	-2	0.10235	no
gi|641570846|gb|KK856237.1|	7321-8273	8.8	37	1	0	-inf	0.0075	no
gi|641570846|gb|KK856237.1|	8540-10913	8.8	37	2	0.644928	-2	0.2018	no
gi|641570846|gb|KK856237.1|	99142-99521	8.8	37	23	1	-4	0.1336	no
gi|641570849|gb|KK856236.1|	29119-31512	8.7	38	1	1	-0.390355	0.7739	no
gi|641570849|gb|KK856236.1|	32044-32811	8.7	38	2	0.751896	-1	0.44155	no
gi|641570849|gb|KK856236.1|	42986-43477	8.7	38	4	0.35986	-3	0.21435	no
gi|641570849|gb|KK856236.1|	44761-45437	8.7	38	1	0.669642	-0.814963	0.6492	no

gi|641570852|gb|KK856235.1|	34083-34771	8.5	36	0.143678	2	4	0.12385	no
gi|641570852|gb|KK856235.1|	35376-36235	8.5	36	0.106678	1	4	0.1276	no
gi|641570852|gb|KK856235.1|	39039-39861	8.5	36	0	80	inf	0.00045	yes
gi|641570855|gb|KK856234.1|	28886-29549	11	42	0	9	inf	5.00E-05	yes
gi|641570858|gb|KK856233.1|	00434-10176	9.9	38	7	10	0.438724	0.7266	no
gi|641570858|gb|KK856233.1|	05895-10616	9.9	38	19	20	0.098181	0.94585	no
gi|641570858|gb|KK856233.1|	07939-10941	9.9	38	6	6	0.0299599	0.9819	no
gi|641570858|gb|KK856233.1|	10291-11295	9.9	38	0.718171	1	0.551043	0.68205	no
gi|641570858|gb|KK856233.1|	14450-11541	9.9	38	0.460533	0.980409	1	0.50945	no
gi|641570858|gb|KK856233.1|	16628-11703	9.9	38	10	8	-0.365855	0.7865	no
gi|641570858|gb|KK856233.1|	27517-12816	9.9	38	12	23	0.924952	0.46965	no
gi|641570858|gb|KK856233.1|	31088-13202	9.9	38	4	4	0.0699927	0.9606	no
gi|641570858|gb|KK856233.1|	33060-13330	9.9	38	20	11	-0.889494	0.55595	no
gi|641570858|gb|KK856233.1|	35873-13606	9.9	38	23	19	-0.322507	0.83535	no
gi|641570858|gb|KK856233.1|	37306-13841	9.9	38	14	9	-0.665138	0.5994	no
gi|641570858|gb|KK856233.1|	19763-20664	9.9	38	0	2	inf	0.00375	no
gi|641570858|gb|KK856233.1|	25333-26177	9.9	38	0.327434	2	2	0.2235	no
gi|641570858|gb|KK856233.1|	26229-26797	9.9	38	0	3	inf	0.00455	no
gi|641570858|gb|KK856233.1|	49167-51294	9.9	38	5	4	-0.390725	0.7521	no
gi|641570858|gb|KK856233.1|	58509-58914	9.9	38	3	3	-0.0927237	0.92655	no
gi|641570858|gb|KK856233.1|	62086-62637	9.9	38	4	6	0.740215	0.59015	no
gi|641570858|gb|KK856233.1|	63016-64827	9.9	38	4	2	-0.923819	0.4823	no
gi|641570858|gb|KK856233.1|	65972-66232	9.9	38	23	4	-3	0.18095	no
gi|641570858|gb|KK856233.1|	77158-77336	9.9	38	42	10	-2	0.2746	no
gi|641570858|gb|KK856233.1|	80513-81527	9.9	38	1	2	0.60512	0.6744	no
gi|641570858|gb|KK856233.1|	81651-82420	9.9	38	0.61706	1	1	0.50945	no
gi|641570858|gb|KK856233.1|	82558-83235	9.9	38	0.587814	1	1	0.51305	no
gi|641570858|gb|KK856233.1|	83694-84213	9.9	38	0.436456	5	4	0.2497	no
gi|641570858|gb|KK856233.1|	85104-87226	9.9	38	18	10	-0.861776	0.48485	no
gi|641570858|gb|KK856233.1|	96074-96482	9.9	38	109	188	0.784289	0.62515	no

gi|641570858|gb|KK856233.1|	97459-98120	9.9	38	46	49	0.105572	0.93085	no
gi|641570861|gb|KK856232.1|	00774-10127	11	39	2	0.346178	-3	0.2481	no
gi|641570861|gb|KK856232.1|	10367-11204	11	39	0.993018	1	0.0181721	0.9394	no
gi|641570861|gb|KK856232.1|	07683-10797	11	39	16	0.963169	-4	0.19385	no
gi|641570861|gb|KK856232.1|	11336-12798	11	39	0.726641	0.340693	-1	1	no
gi|641570861|gb|KK856232.1|	16537-11843	11	39	16	4	-2	0.14515	no
gi|641570861|gb|KK856232.1|	18566-12214	11	39	24	7	-2	0.30705	no
gi|641570861|gb|KK856232.1|	13108-13832	11	39	19	23	0.307124	0.80765	no
gi|641570861|gb|KK856232.1|	31332-13309	11	39	11	14.53	0.393025	0.75675	no
gi|641570861|gb|KK856232.1|	33303-13591	11	39	23	12	-0.99497	0.54355	no
gi|641570861|gb|KK856232.1|	36854-13865	11	39	10	4	-1	0.28305	no
gi|641570861|gb|KK856232.1|	15304-16301	11	39	0.883154	2	0.868001	0.56565	no
gi|641570861|gb|KK856232.1|	64143-16469	11	39	3	4	0.29631	0.839	no
gi|641570861|gb|KK856232.1|	16704-18637	11	39	77	74	-0.0594318	0.9742	no
gi|641570861|gb|KK856232.1|	80083-18101	11	39	0.670024	0.436557	-0.618042	1	no
gi|641570861|gb|KK856232.1|	18768-19892	11	39	2	3	0.547079	0.68485	no
gi|641570861|gb|KK856232.1|	98977-20051	11	39	0.476003	0.88667	0.897426	0.5684	no
gi|641570861|gb|KK856232.1|	20330-22686	11	39	1	2	1	0.36405	no
gi|641570861|gb|KK856232.1|	05801-20887	11	39	0.590703	1	0.883299	0.52545	no
gi|641570861|gb|KK856232.1|	2195-3235	11	39	8	7	-0.228009	0.86035	no
gi|641570861|gb|KK856232.1|	25796-22686	11	39	0.32415	2	3	0.1983	no
gi|641570861|gb|KK856232.1|	27601-22805	11	39	2	0.834797	-1	0.4534	no
gi|641570861|gb|KK856232.1|	28240-22917	11	39	6	2	-1	0.32465	no
gi|641570861|gb|KK856232.1|	29233-22948	11	39	49	12	-2	0.18245	no
gi|641570861|gb|KK856232.1|	31025-31979	11	39	8	8	-0.0310384	0.9806	no
gi|641570861|gb|KK856232.1|	38469-42415	11	39	3	2	-0.183491	0.88605	no
gi|641570861|gb|KK856232.1|	45731-46512	11	39	0.84619	0.917567	0.116832	0.92175	no
gi|641570861|gb|KK856232.1|	46665-47711	11	39	1	0.759738	-0.617002	0.6869	no
gi|641570861|gb|KK856232.1|	4775-5616	11	39	3	22	3	0.041	no
gi|641570861|gb|KK856232.1|	50520-51018	11	39	1	2	0.593054	0.70505	no

gi|641570861|gb|KK856232.1|	51124-51666	11	39	4	7	0.797044	0.5623	no
gi|641570861|gb|KK856232.1|	54278-55633	11	39	2	0.930039	-0.977724	0.49975	no
gi|641570861|gb|KK856232.1|	55890-56143	11	39	11	21	1	0.4816	no
gi|641570861|gb|KK856232.1|	62176-64474	11	39	2	3	0.500589	0.70025	no
gi|641570861|gb|KK856232.1|	64667-65260	11	39	5	3	-0.51794	0.7103	no
gi|641570861|gb|KK856232.1|	65328-71015	11	39	5	3	-0.55159	0.66045	no
gi|641570861|gb|KK856232.1|	74371-76249	11	39	0.547001	0.577511	0.0783072	1	no
gi|641570861|gb|KK856232.1|	77984-79476	11	39	11	2	-2	0.0798	no
gi|641570867|gb|KK856230.1|	40121-42608	11	41	0.030895	81	11	0.10515	no
gi|641570867|gb|KK856230.1|	64503-64860	11	41	7	3	-1	0.49275	no
gi|641570867|gb|KK856230.1|	64942-65209	11	41	7	6	-0.119827	0.9229	no
gi|641570867|gb|KK856230.1|	65325-66500	11	41	5	4	-0.313526	0.80955	no
gi|641570867|gb|KK856230.1|	75161-75794	11	41	0.970127	4	2	0.21435	no
gi|641570867|gb|KK856230.1|	77380-78526	11	41	0.595793	0.566808	-0.0719521	1	no
gi|641570870|gb|KK856229.1|	24602-12572	9.9	38	4	8	1	0.4333	no
gi|641570870|gb|KK856229.1|	12820-13073	9.9	38	17	0	-inf	0.00435	no
gi|641570870|gb|KK856229.1|	1883-2309	9.9	38	0.608519	6	3	0.25495	no
gi|641570870|gb|KK856229.1|	33359-33915	9.9	38	3	0.296299	-3	0.217	no
gi|641570870|gb|KK856229.1|	4506-8684	9.9	38	7	0.081448	-7	0.09405	no
gi|641570870|gb|KK856229.1|	88254-89056	9.9	38	0.816903	0.708818	-0.204749	1	no
gi|641570876|gb|KK856227.1|	83617-84297	9.3	39	1	2	0.7701	0.6262	no
gi|641570880|gb|KK856226.1|	01527-10206	12	43	12	1	-3	0.0849	no
gi|641570880|gb|KK856226.1|	06443-10708	12	43	0.948521	0.957911	0.014212	0.91115	no
gi|641570880|gb|KK856226.1|	07973-10887	12	43	1	1	-0.173652	0.90465	no
gi|641570880|gb|KK856226.1|	10332-11155	12	43	0.618244	0.627587	0.021639	1	no
gi|641570880|gb|KK856226.1|	14803-11540	12	43	0.17354	7	5	0.10655	no
gi|641570880|gb|KK856226.1|	34002-13450	12	43	1	2	0.856387	0.62755	no
gi|641570880|gb|KK856226.1|	32735-33298	12	43	0	522	inf	0.00015	yes
gi|641570880|gb|KK856226.1|	38299-38572	12	43	0	20	inf	0.0016	yes
gi|641570880|gb|KK856226.1|	48264-48651	12	43	0	769	inf	0.00025	yes

gi|641570880|gb|KK856226.1|	55814-55969	12	43	0	229	inf	0.00105	yes
gi|641570880|gb|KK856226.1|	57562-57942	12	43	0	480	inf	5.00E-05	yes
gi|641570880|gb|KK856226.1|	61160-62899	12	43	0.045866	1	5	0.1072	no
gi|641570880|gb|KK856226.1|	64771-66258	12	43	0	49	inf	0.00015	yes
gi|641570880|gb|KK856226.1|	66755-67137	12	43	0	227	inf	0.0004	yes
gi|641570880|gb|KK856226.1|	68715-70097	12	43	0	1	inf	0.0023	yes
gi|641570880|gb|KK856226.1|	70330-71159	12	43	0	46	inf	5.00E-05	yes
gi|641570880|gb|KK856226.1|	78662-80114	12	43	18	10	-0.906942	0.47005	no
gi|641570883|gb|KK856225.1|	16138-16550	9.2	38	0	5	inf	0.00455	no
gi|641570893|gb|KK856222.1|	05334-10671	9.5	41	3	1	-0.912032	0.50755	no
gi|641570893|gb|KK856222.1|	08198-10963	9.5	41	1	1	0.21525	0.87975	no
gi|641570893|gb|KK856222.1|	16710-11860	9.5	41	0.623741	3	2	0.14775	no
gi|641570893|gb|KK856222.1|	23201-24226	9.5	41	0.34134	2	3	0.177	no
gi|641570893|gb|KK856222.1|	25505-26109	9.5	41	1	8	3	0.11305	no
gi|641570893|gb|KK856222.1|	27794-29153	9.5	41	0.66927	1	0.955179	0.5381	no
gi|641570893|gb|KK856222.1|	42624-43221	9.5	41	0.528362	2	2	0.3323	no
gi|641570893|gb|KK856222.1|	43325-44857	9.5	41	0.900462	4	2	0.1501	no
gi|641570893|gb|KK856222.1|	54750-55278	9.5	41	0.63714	4	3	0.19335	no
gi|641570893|gb|KK856222.1|	59814-60725	9.5	41	0.494637	1	1	0.4595	no
gi|641570893|gb|KK856222.1|	97914-98178	9.5	41	17	34	0.990252	0.4916	no
gi|641570897|gb|KK856221.1|	17516-11778	9.1	35	9	10	0.145045	0.9235	no
gi|641570897|gb|KK856221.1|	21209-22525	9.1	35	3	3	0.0664931	0.9596	no
gi|641570897|gb|KK856221.1|	23405-23729	9.1	35	5	3	-0.749126	0.63835	no
gi|641570897|gb|KK856221.1|	23793-25256	9.1	35	2	1	-0.584973	0.67295	no
gi|641570897|gb|KK856221.1|	25762-26941	9.1	35	18	16	-0.150003	0.9021	no
gi|641570897|gb|KK856221.1|	27852-28639	9.1	35	7	6	-0.374181	0.7719	no
gi|641570897|gb|KK856221.1|	30793-31766	9.1	35	28	12	-1	0.35475	no
gi|641570897|gb|KK856221.1|	42868-43088	9.1	35	31.54	15	-1	0.48085	no
gi|641570897|gb|KK856221.1|	49084-50999	9.1	35	26	14	-0.920096	0.57785	no
gi|641570897|gb|KK856221.1|	51668-52137	9.1	35	1	2	0.854166	0.62755	no

gi|641570897|gb|KK856221.1|	53623-54929	9.1	35	7	4	-0.780009	0.54205	no
gi|641570897|gb|KK856221.1|	55963-56405	9.1	35	4	3	-0.6334	0.68395	no
gi|641570897|gb|KK856221.1|	57982-60103	9.1	35	5	2	-1	0.4366	no
gi|641570897|gb|KK856221.1|	60856-62584	9.1	35	3	4	0.386086	0.7598	no
gi|641570897|gb|KK856221.1|	68845-69346	9.1	35	2	1	-0.406766	0.82205	no
gi|641570897|gb|KK856221.1|	72577-72854	9.1	35	13	7	-0.941241	0.55575	no
gi|641570897|gb|KK856221.1|	81536-82764	9.1	35	0.479925	1	2	0.3164	no
gi|641570897|gb|KK856221.1|	92627-92930	9.1	35	4	20	2	0.15745	no
gi|641570904|gb|KK856219.1|	22547-12271	9.6	20	14	46	2	0.353	no
gi|641570904|gb|KK856219.1|	30055-13062	9.6	20	0.936262	1	0.596766	0.70505	no
gi|641570904|gb|KK856219.1|	61371-63068	9.6	20	0.282891	0.790691	1	1	no
gi|641570904|gb|KK856219.1|	68379-69189	9.6	20	0.115182	2	4	0.11955	no
gi|641570904|gb|KK856219.1|	69600-70183	9.6	20	0.547346	3	2	0.2235	no
gi|641570904|gb|KK856219.1|	81879-82263	9.6	20	1	5	2	0.3332	no
gi|641570904|gb|KK856219.1|	83397-84285	9.6	20	2	4	1	0.4239	no
gi|641570907|gb|KK856218.1|	05823-10650	9	36	2	0.22049	-3	0.217	no
gi|641570907|gb|KK856218.1|	10323-11082	9	36	7	1	-2	0.1557	no
gi|641570907|gb|KK856218.1|	25732-12645	9	36	5	0.409336	-4	0.14165	no
gi|641570907|gb|KK856218.1|	29574-13136	9	36	15	2	-3	0.044	no
gi|641570907|gb|KK856218.1|	91955-92175	9	36	14	2	-3	0.2588	no
gi|641570911|gb|KK856217.1|	36552-37102	9.2	38	4	0	-inf	0.0032	yes
gi|641570911|gb|KK856217.1|	40346-40670	9.2	38	6	0	-inf	0.0102	no
gi|641570911|gb|KK856217.1|	41363-42305	9.2	38	2	0	-inf	0.0034	yes
gi|641570914|gb|KK856216.1|	05124-10616	10	41	0.667397	0.25384	-1	1	no
gi|641570914|gb|KK856216.1|	24346-25067	10	41	5	3	-0.77739	0.57445	no
gi|641570914|gb|KK856216.1|	27139-27892	10	41	1	0.96328	-0.39824	0.81255	no
gi|641570914|gb|KK856216.1|	33243-33872	10	41	2	0.988448	-0.98621	0.5872	no
gi|641570914|gb|KK856216.1|	38992-39600	10	41	1	0.778548	-0.81697	0.6492	no
gi|641570914|gb|KK856216.1|	48326-49153	10	41	0.448328	1	1	0.44255	no
gi|641570914|gb|KK856216.1|	88543-88764	10	41	40	58	0.540652	0.6936	no

gi|641570914|gb|KK856216.1|	99853-10051(	10	41	4	3	-0.584853	0.6957	no
gi|641570921|gb|KK856214.1|	.04860-10559	8.7	39	0.781302	2	1	0.5082	no
gi|641570921|gb|KK856214.1|	.06020-10714	8.7	39	11	97	3	0.10275	no
gi|641570921|gb|KK856214.1|	.09012-10978	8.7	39	1	0.739065	-0.982772	0.5872	no
gi|641570921|gb|KK856214.1|	.10009-11167	8.7	39	0.772377	0.147183	-2	1	no
gi|641570921|gb|KK856214.1|	.13010-11420	8.7	39	1	0.432173	-1	0.4417	no
gi|641570921|gb|KK856214.1|	.19600-12077	8.7	39	11	137	4	0.0654	no
gi|641570921|gb|KK856214.1|	16652-17718	8.7	39	0	9	inf	5.00E-05	yes
gi|641570921|gb|KK856214.1|	98509-10117(	8.7	39	65	59.37	-0.138239	0.94575	no
gi|641570924|gb|KK856213.1|	41477-42839	10	39	0.667549	1	1	0.47825	no
gi|641570924|gb|KK856213.1|	43128-44036	10	39	1	2	0.845057	0.58475	no
gi|641570924|gb|KK856213.1|	93090-93962	10	39	0.313886	1	2	0.25595	no
gi|641570927|gb|KK856212.1|	.60417-16112	10	41	0	5	inf	0.00065	yes
gi|641570927|gb|KK856212.1|	31329-31603	10	41	10	8	-0.332704	0.822	no
gi|641570927|gb|KK856212.1|	38207-38474	10	41	13	1	-3	0.21195	no
gi|641570927|gb|KK856212.1|	39005-39758	10	41	11	6	-0.900741	0.50015	no
gi|641570927|gb|KK856212.1|	41896-42588	10	41	23	8	-2	0.2284	no
gi|641570930|gb|KK856211.1|	0-356	11	39	117	8	-4	0.0166	no
gi|641570930|gb|KK856211.1|	13169-15604	11	39	0.727093	2	1	0.32485	no
gi|641570930|gb|KK856211.1|	17571-18498	11	39	0.967642	1	0.604264	0.67795	no
gi|641570930|gb|KK856211.1|	19190-19991	11	39	0.350679	4	3	0.1487	no
gi|641570930|gb|KK856211.1|	20126-22205	11	39	5	15	2	0.1802	no
gi|641570930|gb|KK856211.1|	22401-23067	11	39	4	13	2	0.2237	no
gi|641570930|gb|KK856211.1|	2773-4711	11	39	2	10	2	0.0991	no
gi|641570930|gb|KK856211.1|	28515-28730	11	39	3	21	3	0.28635	no
gi|641570930|gb|KK856211.1|	29669-30823	11	39	1	2	0.532171	0.70535	no
gi|641570930|gb|KK856211.1|	33388-35726	11	39	0.264351	4	4	0.05675	no
gi|641570930|gb|KK856211.1|	448-2541	11	39	2	5	2	0.2119	no
gi|641570930|gb|KK856211.1|	7542-8208	11	39	0.601523	2	2	0.3012	no
gi|641570930|gb|KK856211.1|	9121-13027	11	39	1	3	1	0.2833	no

gi|641570933|gb|KK856210.1|	46443-50645	9.3	37	7	4	-0.681166	0.5841	no
gi|641570933|gb|KK856210.1|	53294-54502	9.3	37	2	0.958044	-1	0.46365	no
gi|641570933|gb|KK856210.1|	54646-55029	9.3	37	6	7	0.0801645	0.95505	no
gi|641570933|gb|KK856210.1|	55295-56438	9.3	37	10	7	-0.616292	0.62545	no
gi|641570936|gb|KK856209.1|	73006-73622	10	41	2	3	0.723867	0.6359	no
gi|641570940|gb|KK856208.1|	03788-10399	10	35	84	55	-0.612562	0.65445	no
gi|641570940|gb|KK856208.1|	06152-10683	10	35	25	22	-0.188346	0.87755	no
gi|641570940|gb|KK856208.1|	13670-11560	10	35	10	12	0.246363	0.84365	no
gi|641570940|gb|KK856208.1|	15773-11639	10	35	34	24	-0.501868	0.68215	no
gi|641570940|gb|KK856208.1|	18039-11987	10	35	3	2	-0.921787	0.48935	no
gi|641570940|gb|KK856208.1|	20838-12189	10	35	3	0.99781	-2	0.3106	no
gi|641570940|gb|KK856208.1|	22730-12494	10	35	0.980118	0.587441	-0.738511	0.5925	no
gi|641570940|gb|KK856208.1|	25185-12653	10	35	0.981072	0.747242	-0.392783	0.7971	no
gi|641570940|gb|KK856208.1|	29966-13102	10	35	1	2	0.687965	0.6336	no
gi|641570940|gb|KK856208.1|	31277-13333	10	35	2	1	-0.461223	0.73365	no
gi|641570940|gb|KK856208.1|	33500-13545	10	35	3	2	-0.864948	0.51215	no
gi|641570940|gb|KK856208.1|	36171-13810	10	35	3	2	-0.704219	0.58925	no
gi|641570940|gb|KK856208.1|	38845-14103	10	35	1	2	0.372332	0.78355	no
gi|641570940|gb|KK856208.1|	45361-14594	10	35	2	2	0.234513	0.88205	no
gi|641570940|gb|KK856208.1|	48106-14848	10	35	8	10	0.347103	0.8059	no
gi|641570940|gb|KK856208.1|	50191-15164	10	35	0.786261	0.684618	-0.199708	1	no
gi|641570940|gb|KK856208.1|	51802-15296	10	35	2	4	0.766706	0.5675	no
gi|641570940|gb|KK856208.1|	53679-15452	10	35	5	8	0.73445	0.5813	no
gi|641570940|gb|KK856208.1|	55827-15681	10	35	2	2	-0.0955376	0.9461	no
gi|641570940|gb|KK856208.1|	57591-15845	10	35	5	4	-0.299145	0.8218	no
gi|641570940|gb|KK856208.1|	61385-16159	10	35	16	8	-1	0.5278	no
gi|641570940|gb|KK856208.1|	68658-16900	10	35	32	11	-2	0.24425	no
gi|641570940|gb|KK856208.1|	73743-74876	10	35	1	0.459705	-1	0.46645	no
gi|641570940|gb|KK856208.1|	81263-83522	10	35	1	0.418759	-2	0.25315	no
gi|641570940|gb|KK856208.1|	84119-87608	10	35	1	2	0.414144	0.755	no

gi|641570940|gb|KK856208.1|	91044-91865	10	35	0.33947	2	2	0.25595	no
gi|641570940|gb|KK856208.1|	92521-93579	10	35	1	1	-0.031943	0.95655	no
gi|641570940|gb|KK856208.1|	96840-98060	10	35	6	7	0.00617547	0.99345	no
gi|641570940|gb|KK856208.1|	98254-10002	10	35	3	3	-0.115773	0.92775	no
gi|641570944|gb|KK856207.1|	21261-12232	10	41	0	27	inf	5.00E-05	yes
gi|641570944|gb|KK856207.1|	12399-12782	10	41	0.741604	6	3	0.27615	no
gi|641570944|gb|KK856207.1|	13916-14884	10	41	0.824669	1	0.434766	0.78015	no
gi|641570944|gb|KK856207.1|	18585-19966	10	41	0.716566	0.54583	-0.39265	1	no
gi|641570944|gb|KK856207.1|	20972-21894	10	41	0.19486	2	3	0.25965	no
gi|641570944|gb|KK856207.1|	5618-6345	10	41	1	0.403994	-2	0.3216	no
gi|641570944|gb|KK856207.1|	89491-90163	10	41	0	11	inf	0.0001	yes
gi|641570952|gb|KK856204.1|	00705-10276	10	21	2	1	-0.390835	0.767	no
gi|641570952|gb|KK856204.1|	03224-10429	10	21	47	34	-0.46256	0.84135	no
gi|641570952|gb|KK856204.1|	05070-10757	10	21	128	89	-0.526002	0.786	no
gi|641570952|gb|KK856204.1|	07725-11358	10	21	5	3	-0.602972	0.6234	no
gi|641570952|gb|KK856204.1|	14148-11666	10	21	69	53	-0.378165	0.86015	no
gi|641570952|gb|KK856204.1|	19936-12079	10	21	11	5	-0.996091	0.4508	no
gi|641570952|gb|KK856204.1|	23138-12431	10	21	5	3	-0.629325	0.628	no
gi|641570952|gb|KK856204.1|	24448-12489	10	21	6	0	-inf	0.0032	yes
gi|641570952|gb|KK856204.1|	29191-12988	10	21	4	0.64443	-3	0.1578	no
gi|641570952|gb|KK856204.1|	37922-14093	10	21	2	3	0.730533	0.5692	no
gi|641570952|gb|KK856204.1|	41928-14303	10	21	4	3	-0.543038	0.67895	no
gi|641570952|gb|KK856204.1|	45260-14568	10	21	3	2	-0.41306	0.8125	no
gi|641570952|gb|KK856204.1|	50641-15133	10	21	0.987734	1	0.600587	0.69715	no
gi|641570952|gb|KK856204.1|	94153-94834	10	21	4	0.662819	-3	0.1746	no
gi|641570952|gb|KK856204.1|	97439-98983	10	21	6	4	-0.758176	0.55365	no
gi|641570955|gb|KK856203.1|	16235-17317	10	38	0.479097	0.729035	0.605672	1	no
gi|641570955|gb|KK856203.1|	17471-17919	10	38	2	2	0.00453907	0.91115	no
gi|641570955|gb|KK856203.1|	52265-52953	10	38	2	0.653496	-2	0.33235	no
gi|641570955|gb|KK856203.1|	59353-59579	10	38	22	2	-3	0.2088	no

gi|641570955|gb|KK856203.1|	6619-7320	10	38	0	113	inf	5.00E-05	yes
gi|641570955|gb|KK856203.1|	75788-77420	10	38	0	275	inf	5.00E-05	yes
gi|641570958|gb|KK856202.1|	13153-11378	9.8	40	0	3	inf	0.0025	yes
gi|641570958|gb|KK856202.1|	19228-11975	9.8	40	0	4	inf	0.00375	no
gi|641570958|gb|KK856202.1|	97761-98234	9.8	40	0	8	inf	0.0005	yes
gi|641570961|gb|KK856201.1|	10666-11126	8.9	38	1	2	0.597718	0.6936	no
gi|641570961|gb|KK856201.1|	17665-11785	8.9	38	96	149	0.631433	0.6249	no
gi|641570961|gb|KK856201.1|	28357-12916	8.9	38	14	6	-1	0.344	no
gi|641570961|gb|KK856201.1|	29641-13497	8.9	38	8	3	-1	0.41935	no
gi|641570961|gb|KK856201.1|	36379-13704	8.9	38	2	1	-0.274601	0.8619	no
gi|641570961|gb|KK856201.1|	44202-14449	8.9	38	19	3	-3	0.15635	no
gi|641570961|gb|KK856201.1|	47628-14810	8.9	38	0	5	inf	0.00375	no
gi|641570961|gb|KK856201.1|	50450-15117	8.9	38	0.133404	2	4	0.1276	no
gi|641570961|gb|KK856201.1|	54052-15476	8.9	38	7	33	2	0.08875	no
gi|641570961|gb|KK856201.1|	21368-21671	8.9	38	0	17	inf	0.0015	yes
gi|641570961|gb|KK856201.1|	27243-29025	8.9	38	0	2	inf	0.0003	yes
gi|641570961|gb|KK856201.1|	39794-40511	8.9	38	0.949917	4	2	0.24255	no
gi|641570961|gb|KK856201.1|	4040-5546	8.9	38	31	27	-0.212625	0.92415	no
gi|641570961|gb|KK856201.1|	49691-50405	8.9	38	0.409459	4	3	0.1503	no
gi|641570961|gb|KK856201.1|	6514-6947	8.9	38	79	116	0.544468	0.6626	no
gi|641570961|gb|KK856201.1|	68506-69396	8.9	38	0	8	inf	0.0001	yes
gi|641570961|gb|KK856201.1|	7598-7880	8.9	38	55	106.32	0.963212	0.45185	no
gi|641570961|gb|KK856201.1|	80402-81530	8.9	38	0.075911	5	6	0.10475	no
gi|641570967|gb|KK856199.1|	33691-13447	10	44	2	0.182893	-3	0.217	no
gi|641570967|gb|KK856199.1|	77164-17737	10	44	13	7	-0.877216	0.63655	no
gi|641570967|gb|KK856199.1|	29495-30407	10	44	3	3	-0.27726	0.83725	no
gi|641570967|gb|KK856199.1|	38851-39177	10	44	6	5	-0.426785	0.77855	no
gi|641570967|gb|KK856199.1|	47030-47744	10	44	1	0.828056	-0.399029	0.82205	no
gi|641570967|gb|KK856199.1|	64662-65536	10	44	0.730243	1	0.603635	0.69715	no
gi|641570971|gb|KK856198.1|	08599-10922	8.9	39	2	0.248811	-3	0.2381	no

gi|641570971|gb|KK856198.1|	26288-12704	8.9	39	0.379492	1	2	0.3323	no
gi|641570971|gb|KK856198.1|	27880-12861	8.9	39	0.923396	1	0.378958	0.82465	no
gi|641570971|gb|KK856198.1|	13160-15320	8.9	39	0.432451	106	8	0.0224	no
gi|641570971|gb|KK856198.1|	15630-17015	8.9	39	0.714157	1	1	0.4764	no
gi|641570971|gb|KK856198.1|	17239-19794	8.9	39	0.570097	18	5	0.03715	no
gi|641570971|gb|KK856198.1|	21018-22168	8.9	39	0.815783	0.451555	-0.853286	1	no
gi|641570971|gb|KK856198.1|	23625-24786	8.9	39	0.366587	1	2	0.3084	no
gi|641570971|gb|KK856198.1|	25078-25636	8.9	39	0.779789	3	2	0.262	no
gi|641570971|gb|KK856198.1|	26278-27259	8.9	39	0.540683	1	1	0.47015	no
gi|641570971|gb|KK856198.1|	52446-53283	8.9	39	1	1	0.0885615	0.9392	no
gi|641570971|gb|KK856198.1|	9006-9734	8.9	39	1	0.403242	-1	0.4534	no
gi|641570974|gb|KK856197.1|	74926-75737	8.5	43	5	0.523894	-3	0.1224	no
gi|641570981|gb|KK856195.1|	50215-51277	8.2	39	0	5	inf	0.0001	yes
gi|641570981|gb|KK856195.1|	77179-77559	8.2	39	0	15	inf	0.00065	yes
gi|641570987|gb|KK856193.1|	68434-69070	9.5	37	2129.75	0	-inf	5.00E-05	yes
gi|641570987|gb|KK856193.1|	77154-77421	9.5	37	228.8	0	-inf	5.00E-05	yes
gi|641570991|gb|KK856192.1|	02985-10321	11	38	46	20	-1	0.4013	no
gi|641570991|gb|KK856192.1|	04414-10508	11	38	2	2	0.221486	0.8867	no
gi|641570991|gb|KK856192.1|	06732-10743	11	38	3	4	0.389289	0.7835	no
gi|641570991|gb|KK856192.1|	08577-10907	11	38	5	3	-0.406826	0.7851	no
gi|641570991|gb|KK856192.1|	09763-10998	11	38	53	33	-0.667886	0.6184	no
gi|641570991|gb|KK856192.1|	17562-11945	11	38	2	1	-0.713079	0.5955	no
gi|641570991|gb|KK856192.1|	22105-12233	11	38	32	48	0.604311	0.65575	no
gi|641570991|gb|KK856192.1|	22777-12314	11	38	8	5	-0.497901	0.72665	no
gi|641570991|gb|KK856192.1|	23774-12415	11	38	5	3	-0.53308	0.73675	no
gi|641570991|gb|KK856192.1|	24401-12482	11	38	32	32	0.0232382	0.98575	no
gi|641570991|gb|KK856192.1|	29009-13231	11	38	0.704916	1	0.904168	0.4984	no
gi|641570991|gb|KK856192.1|	1324-2108	11	38	2	2	0.60231	0.68135	no
gi|641570991|gb|KK856192.1|	32469-13422	11	38	10	7	-0.377775	0.76505	no
gi|641570991|gb|KK856192.1|	35669-13650	11	38	3	1	-1	0.38965	no

gi|641570991|gb|KK856192.1|	139908-14026	11	38	7	9	0.385878	0.7922	no
gi|641570991|gb|KK856192.1|	141261-14514	11	38	4	3	-0.277477	0.8262	no
gi|641570991|gb|KK856192.1|	145405-14603	11	38	18	16	-0.177032	0.8883	no
gi|641570991|gb|KK856192.1|	150832-15126	11	38	6	7	0.035831	0.9726	no
gi|641570991|gb|KK856192.1|	155318-15631	11	38	1	0.804937	-0.617416	0.6869	no
gi|641570991|gb|KK856192.1|	157322-15784	11	38	7	8	0.340707	0.8018	no
gi|641570991|gb|KK856192.1|	161636-16248	11	38	2	4	0.670431	0.62685	no
gi|641570991|gb|KK856192.1|	166104-16748	11	38	0.059664	62	10	0.1053	no
gi|641570991|gb|KK856192.1|	16611-19114	11	38	1	2	0.609774	0.63785	no
gi|641570991|gb|KK856192.1|	172450-17482	11	38	0	62	inf	0.00015	yes
gi|641570991|gb|KK856192.1|	19240-21309	11	38	0.755756	0.749335	-0.0123084	1	no
gi|641570991|gb|KK856192.1|	2459-4349	11	38	2	2	0.193809	0.8847	no
gi|641570991|gb|KK856192.1|	24644-24836	11	38	30	7	-2	0.2746	no
gi|641570991|gb|KK856192.1|	26805-27029	11	38	28	8	-2	0.2873	no
gi|641570991|gb|KK856192.1|	28450-28692	11	38	30	10	-2	0.29345	no
gi|641570991|gb|KK856192.1|	32457-33482	11	38	4	1	-1	0.33915	no
gi|641570991|gb|KK856192.1|	33913-34158	11	38	16	9	-0.771225	0.6265	no
gi|641570991|gb|KK856192.1|	36218-36442	11	38	30	12	-1	0.4162	no
gi|641570991|gb|KK856192.1|	37746-37986	11	38	12	3	-2	0.3145	no
gi|641570991|gb|KK856192.1|	38358-38814	11	38	2	2	0.327115	0.83525	no
gi|641570991|gb|KK856192.1|	41391-41646	11	38	49	34	-0.561168	0.67175	no
gi|641570991|gb|KK856192.1|	47110-47942	11	38	1	0.844543	-0.244934	0.88495	no
gi|641570991|gb|KK856192.1|	48044-49063	11	38	2	3	0.242593	0.8596	no
gi|641570991|gb|KK856192.1|	49232-50032	11	38	1	0.53336	-0.982389	0.62775	no
gi|641570991|gb|KK856192.1|	50167-51261	11	38	0.787833	0.719345	-0.131205	1	no
gi|641570991|gb|KK856192.1|	51380-51933	11	38	6	3	-0.889512	0.5247	no
gi|641570991|gb|KK856192.1|	52479-53982	11	38	3	1	-1	0.38205	no
gi|641570991|gb|KK856192.1|	58569-59629	11	38	0.327524	1	2	0.26155	no
gi|641570991|gb|KK856192.1|	61441-61823	11	38	28	8	-2	0.21275	no
gi|641570991|gb|KK856192.1|	64039-65559	11	38	7	3	-1	0.3875	no

gi|641570991|gb|KK856192.1|	65793-66457	11	38	3	2	-0.487701	0.7427	no
gi|641570991|gb|KK856192.1|	67661-68492	11	38	3	2	-0.496488	0.72455	no
gi|641570991|gb|KK856192.1|	70348-70813	11	38	5	0.393478	-4	0.20475	no
gi|641570991|gb|KK856192.1|	70916-72417	11	38	1	0.57846	-1	0.4606	no
gi|641570991|gb|KK856192.1|	72875-76444	11	38	4	4	0.176401	0.889	no
gi|641570991|gb|KK856192.1|	77837-78762	11	38	3	1	-1	0.4642	no
gi|641570991|gb|KK856192.1|	81947-83295	11	38	0.859927	0.748534	-0.200147	1	no
gi|641570991|gb|KK856192.1|	83417-83995	11	38	4	3	-0.403034	0.7851	no
gi|641570991|gb|KK856192.1|	84598-86187	11	38	4	2	-0.766302	0.56	no
gi|641570991|gb|KK856192.1|	89630-90045	11	38	15	2	-3	0.11925	no
gi|641570991|gb|KK856192.1|	91144-91441	11	38	20	13	-0.626017	0.66105	no
gi|641570991|gb|KK856192.1|	94782-95177	11	38	11	10	-0.167888	0.9083	no
gi|641570991|gb|KK856192.1|	96490-96819	11	38	35	14	-1	0.33725	no
gi|641571001|gb|KK856189.1|	07316-10775	9.2	37	0	12	inf	0.00035	yes
gi|641571001|gb|KK856189.1|	34591-13593	9.2	37	5	0.093973	-6	0.1838	no
gi|641571004|gb|KK856188.1|	80598-81265	11	41	0	4	inf	0.0015	yes
gi|641571013|gb|KK856185.1|	21065-12199	9.3	20	2	0.73751	-1	0.3598	no
gi|641571013|gb|KK856185.1|	22503-12471	9.3	20	3	1	-1	0.43185	no
gi|641571013|gb|KK856185.1|	84117-84427	9.3	20	8	5	-0.545728	0.7341	no
gi|641571016|gb|KK856184.1|	00254-10093	10	40	4	4	-0.0879057	0.9476	no
gi|641571016|gb|KK856184.1|	29694-13249	10	40	43	28	-0.615364	0.77755	no
gi|641571016|gb|KK856184.1|	39161-13987	10	40	1	1	0.0863096	0.94	no
gi|641571016|gb|KK856184.1|	40268-14092	10	40	4	0.925231	-2	0.21975	no
gi|641571016|gb|KK856184.1|	41233-14263	10	40	2	3	0.887541	0.5062	no
gi|641571016|gb|KK856184.1|	43641-14434	10	40	1	0.634372	-0.984237	0.62775	no
gi|641571016|gb|KK856184.1|	44473-14491	10	40	2	2	-0.411448	0.82205	no
gi|641571016|gb|KK856184.1|	44970-14953	10	40	19	14	-0.433023	0.84085	no
gi|641571016|gb|KK856184.1|	14801-16063	10	40	0.86288	2	0.993818	0.51835	no
gi|641571016|gb|KK856184.1|	50839-15118	10	40	11	12	0.0787899	0.95415	no
gi|641571016|gb|KK856184.1|	53902-15434	10	40	4	4	0.00376746	0.9597	no

gi|641571016|gb|KK856184.1|	55248-15664	10	40	2	1	-0.665618	0.63575	no
gi|641571016|gb|KK856184.1|	56988-15850	10	40	2	3	0.45175	0.73145	no
gi|641571016|gb|KK856184.1|	58616-15909	10	40	0.750942	3	2	0.2812	no
gi|641571016|gb|KK856184.1|	59347-16070	10	40	0.485907	1	1	0.49695	no
gi|641571016|gb|KK856184.1|	61331-16243	10	40	0.782608	0.714596	-0.131161	1	no
gi|641571016|gb|KK856184.1|	62546-16312	10	40	1	1	-0.402839	0.82205	no
gi|641571016|gb|KK856184.1|	75512-17705	10	40	0.839953	1	0.308392	0.83385	no
gi|641571016|gb|KK856184.1|	78369-17967	10	40	0.95533	0.19401	-2	0.24735	no
gi|641571016|gb|KK856184.1|	02095-20375	10	40	0.38698	0.958644	1	0.42705	no
gi|641571016|gb|KK856184.1|	05125-20597	10	40	0.647878	0.984268	0.603331	0.70555	no
gi|641571016|gb|KK856184.1|	4257-5054	10	40	3	2	-0.336075	0.8148	no
gi|641571016|gb|KK856184.1|	47021-48740	10	40	0.650548	0.566767	-0.1989	1	no
gi|641571016|gb|KK856184.1|	50180-50409	10	40	12	10	-0.303779	0.85915	no
gi|641571016|gb|KK856184.1|	52462-58926	10	40	7	5	-0.420616	0.7928	no
gi|641571016|gb|KK856184.1|	5536-6257	10	40	2	1	-0.176486	0.90465	no
gi|641571016|gb|KK856184.1|	59704-63104	10	40	0.618619	0.944494	0.610491	0.65035	no
gi|641571016|gb|KK856184.1|	63225-63864	10	40	1	0.724832	-0.623336	0.71195	no
gi|641571016|gb|KK856184.1|	64362-66107	10	40	0.822386	1	0.526058	0.71365	no
gi|641571016|gb|KK856184.1|	654-2105	10	40	0.563851	0.515501	-0.129338	1	no
gi|641571016|gb|KK856184.1|	66338-67811	10	40	0.3879	0.591096	0.607709	1	no
gi|641571016|gb|KK856184.1|	67961-69482	10	40	1	1	0.529874	0.70515	no
gi|641571016|gb|KK856184.1|	72098-73003	10	40	1	1	0.314507	0.84265	no
gi|641571016|gb|KK856184.1|	78762-79681	10	40	1	3	0.884283	0.5385	no
gi|641571016|gb|KK856184.1|	80772-81805	10	40	1	2	0.605283	0.67915	no
gi|641571016|gb|KK856184.1|	91418-93003	10	40	1	2	0.792506	0.56965	no
gi|641571016|gb|KK856184.1|	94436-95166	10	40	2	2	0.279382	0.8498	no
gi|641571016|gb|KK856184.1|	95329-98237	10	40	0.835219	1	0.780081	0.56485	no
gi|641571016|gb|KK856184.1|	98408-10001	10	40	1	1	0.28622	0.8383	no
gi|641571020|gb|KK856183.1|	08586-11001	15	62	0	1	inf	0.0023	yes
gi|641571020|gb|KK856183.1|	14393-11544	15	62	0.82936	2	1	0.34185	no

gi|641571020|gb|KK856183.1|	16082-11658	15	62	5	52	3	0.02735	no
gi|641571020|gb|KK856183.1|	17023-11733	15	62	10	56	2	0.10125	no
gi|641571020|gb|KK856183.1|	19380-11977	15	62	13	35	1	0.2575	no
gi|641571020|gb|KK856183.1|	23173-12535	15	62	1	0.868167	-0.248616	0.8522	no
gi|641571020|gb|KK856183.1|	26239-12712	15	62	1	2	0.466245	0.75495	no
gi|641571020|gb|KK856183.1|	27562-12941	15	62	1	2	0.324965	0.80595	no
gi|641571020|gb|KK856183.1|	29746-13074	15	62	1	0	-inf	0.01	no
gi|641571020|gb|KK856183.1|	33183-13340	15	62	99	70	-0.497732	0.7025	no
gi|641571020|gb|KK856183.1|	35581-13660	15	62	2	0.391786	-2	0.28145	no
gi|641571020|gb|KK856183.1|	38296-13891	15	62	5	7	0.54771	0.68435	no
gi|641571020|gb|KK856183.1|	40770-14229	15	62	2	5	1	0.299	no
gi|641571020|gb|KK856183.1|	43109-14378	15	62	11	25	1	0.3389	no
gi|641571020|gb|KK856183.1|	14783-15643	15	62	211	150	-0.496603	0.816	no
gi|641571020|gb|KK856183.1|	52255-15307	15	62	5	2	-1	0.32525	no
gi|641571020|gb|KK856183.1|	17744-19175	15	62	12	12	0.060064	0.96015	no
gi|641571020|gb|KK856183.1|	25266-25740	15	62	4	5	0.291927	0.8393	no
gi|641571020|gb|KK856183.1|	35040-36835	15	62	9	16	0.835028	0.5003	no
gi|641571020|gb|KK856183.1|	42808-44872	15	62	1	0.982516	-0.390828	0.77545	no
gi|641571020|gb|KK856183.1|	47750-48336	15	62	4	2	-1	0.41725	no
gi|641571020|gb|KK856183.1|	51552-51998	15	62	14	14	-0.010128	0.9869	no
gi|641571020|gb|KK856183.1|	80976-81865	15	62	0.102065	4	5	0.1059	no
gi|641571020|gb|KK856183.1|	82609-83030	15	62	0.310785	17	6	0.1052	no
gi|641571020|gb|KK856183.1|	83500-83866	15	62	0	8	inf	0.0025	yes
gi|641571020|gb|KK856183.1|	85753-88201	15	62	0.440024	0.767379	0.802356	1	no
gi|641571020|gb|KK856183.1|	92558-93838	15	62	2	11	2	0.08695	no
gi|641571023|gb|KK856182.1|	49374-50509	7.4	31	0.150692	1	3	0.2556	no
gi|641571023|gb|KK856182.1|	50683-52656	7.4	31	0.318627	1421.17	12	0.1308	no
gi|641571023|gb|KK856182.1|	52895-53840	7.4	31	0.094441	53	9	0.1063	no
gi|641571023|gb|KK856182.1|	60371-60583	7.4	31	0	377	inf	5.00E-05	yes
gi|641571023|gb|KK856182.1|	94667-95254	7.4	31	0	43	inf	5.00E-05	yes

gi|641571026|gb|KK856181.1|	12664-11384	7.8	32	8	15	0.941707	0.4575	no
gi|641571029|gb|KK856180.1|	40951-41778	10	42	0	157	inf	0.00015	yes
gi|641571029|gb|KK856180.1|	45874-46517	10	42	0	6	inf	0.00075	yes
gi|641571032|gb|KK856179.1|	02089-10284	9.4	32	2	3	0.88183	0.5386	no
gi|641571032|gb|KK856179.1|	06976-10800	9.4	32	0.760681	1	0.89479	0.5684	no
gi|641571032|gb|KK856179.1|	10011-11081	9.4	32	0.697902	1	0.82503	0.6232	no
gi|641571032|gb|KK856179.1|	11236-11143	9.4	32	14	44	2	0.3063	no
gi|641571032|gb|KK856179.1|	22767-12310	9.4	32	15	50	2	0.2019	no
gi|641571032|gb|KK856179.1|	27906-12814	9.4	32	8	14	0.739796	0.6387	no
gi|641571032|gb|KK856179.1|	31861-13323	9.4	32	1	2	0.738561	0.58405	no
gi|641571032|gb|KK856179.1|	33938-13599	9.4	32	1	3	1	0.3594	no
gi|641571032|gb|KK856179.1|	40848-14110	9.4	32	18	31	0.757297	0.5822	no
gi|641571032|gb|KK856179.1|	42389-14285	9.4	32	11	39	2	0.17565	no
gi|641571032|gb|KK856179.1|	43368-14359	9.4	32	20	76	2	0.1991	no
gi|641571032|gb|KK856179.1|	52658-15433	9.4	32	0.813991	2	1	0.37395	no
gi|641571032|gb|KK856179.1|	67499-16821	9.4	32	4	3	-0.698655	0.6286	no
gi|641571032|gb|KK856179.1|	70224-17070	9.4	32	9	1	-3	0.1418	no
gi|641571032|gb|KK856179.1|	30308-31053	9.4	32	24	20	-0.267808	0.8311	no
gi|641571032|gb|KK856179.1|	31206-33090	9.4	32	1	0.895167	-0.391166	0.7774	no
gi|641571032|gb|KK856179.1|	38901-40506	9.4	32	1	2	0.745645	0.5879	no
gi|641571032|gb|KK856179.1|	40726-41004	9.4	32	13	12	-0.0664843	0.9597	no
gi|641571032|gb|KK856179.1|	41633-42335	9.4	32	7	10	0.507593	0.6964	no
gi|641571032|gb|KK856179.1|	45042-45244	9.4	32	373	373	0.000795259	0.99565	no
gi|641571032|gb|KK856179.1|	4590-4918	9.4	32	47	12	-2	0.1644	no
gi|641571032|gb|KK856179.1|	46847-47833	9.4	32	51	63	0.310803	0.8908	no
gi|641571032|gb|KK856179.1|	59769-60090	9.4	32	4	2	-1	0.44305	no
gi|641571032|gb|KK856179.1|	69666-70845	9.4	32	0.071976	1	4	0.1136	no
gi|641571032|gb|KK856179.1|	71174-71714	9.4	32	0	49	inf	5.00E-05	yes
gi|641571032|gb|KK856179.1|	80006-80908	9.4	32	0.100187	2	4	0.1175	no
gi|641571032|gb|KK856179.1|	81155-81495	9.4	32	0	24	inf	0.00025	yes

gi|641571032|gb|KK856179.1|	81692-83627	9.4	32	0	18	inf	5.00E-05	yes
gi|641571032|gb|KK856179.1|	84493-85217	9.4	32	0.133908	64	9	0.10435	no
gi|641571032|gb|KK856179.1|	93637-93883	9.4	32	14	11	-0.342006	0.82135	no
gi|641571032|gb|KK856179.1|	96237-96802	9.4	32	1	3	1	0.4402	no
gi|641571032|gb|KK856179.1|	97551-97908	9.4	32	7	1	-2	0.2169	no
gi|641571035|gb|KK856178.1|	05233-10576	9.9	42	0	7	inf	0.0005	yes
gi|641571035|gb|KK856178.1|	39921-40310	9.9	42	3	5	0.946012	0.57385	no
gi|641571039|gb|KK856177.1|	00337-10059	8.5	34	9	6	-0.616311	0.70475	no
gi|641571039|gb|KK856177.1|	11387-12213	8.5	34	1	0	-inf	0.01175	no
gi|641571039|gb|KK856177.1|	55378-15596	8.5	34	1	3	0.919166	0.56365	no
gi|641571039|gb|KK856177.1|	58960-15948	8.5	34	1	2	0.109296	0.92265	no
gi|641571039|gb|KK856177.1|	62448-16294	8.5	34	0.692791	4	2	0.21375	no
gi|641571039|gb|KK856177.1|	70640-17125	8.5	34	4	3	-0.209019	0.88325	no
gi|641571039|gb|KK856177.1|	71373-17192	8.5	34	3	2	-0.404265	0.79625	no
gi|641571039|gb|KK856177.1|	17239-18634	8.5	34	1	0.359655	-2	0.31415	no
gi|641571039|gb|KK856177.1|	73008-17356	8.5	34	4	11	2	0.24825	no
gi|641571039|gb|KK856177.1|	73711-17458	8.5	34	0.314351	2	2	0.2235	no
gi|641571039|gb|KK856177.1|	1938-2621	8.5	34	3	0.880171	-2	0.26755	no
gi|641571039|gb|KK856177.1|	19659-20451	8.5	34	1	0.360308	-2	0.3455	no
gi|641571039|gb|KK856177.1|	20624-21628	8.5	34	1	0.798996	-0.510447	0.7498	no
gi|641571039|gb|KK856177.1|	23212-23511	8.5	34	4	4	-0.0178358	0.91115	no
gi|641571039|gb|KK856177.1|	25409-26391	8.5	34	0	2	inf	0.00255	yes
gi|641571039|gb|KK856177.1|	4238-5383	8.5	34	11	8	-0.423231	0.73445	no
gi|641571039|gb|KK856177.1|	54814-54998	8.5	34	30	0	-inf	0.01175	no
gi|641571039|gb|KK856177.1|	68837-69845	8.5	34	12	20	0.75707	0.5472	no
gi|641571039|gb|KK856177.1|	69975-71083	8.5	34	1	0.590301	-1	0.47035	no
gi|641571039|gb|KK856177.1|	72624-72893	8.5	34	42	24	-0.79163	0.5577	no
gi|641571039|gb|KK856177.1|	75864-76041	8.5	34	55	52	-0.0670121	0.95795	no
gi|641571039|gb|KK856177.1|	76192-76919	8.5	34	5	5	0.070733	0.9559	no
gi|641571039|gb|KK856177.1|	81482-81864	8.5	34	21	32	0.633158	0.62385	no

gi|641571039|gb|KK856177.1|	9208-10030	8.5	34	11	0.171586	-6	0.18305	no
gi|641571042|gb|KK856176.1|	05483-10567	10	34	99	186	0.917625	0.49205	no
gi|641571042|gb|KK856176.1|	07854-10850	10	34	8	11	0.368163	0.78065	no
gi|641571042|gb|KK856176.1|	16903-11780	10	34	21	33	0.630867	0.6156	no
gi|641571042|gb|KK856176.1|	26125-12691	10	34	4	0.360308	-3	0.14385	no
gi|641571042|gb|KK856176.1|	36002-13701	10	34	7	13	0.795615	0.53945	no
gi|641571042|gb|KK856176.1|	39455-13982	10	34	95	56	-0.759676	0.54205	no
gi|641571042|gb|KK856176.1|	41835-14212	10	34	266	122	-1	0.3702	no
gi|641571042|gb|KK856176.1|	46155-14642	10	34	172	78	-1	0.36175	no
gi|641571042|gb|KK856176.1|	47172-14747	10	34	5	8	0.569055	0.73035	no
gi|641571042|gb|KK856176.1|	53348-15407	10	34	0.792959	1	0.338296	0.8352	no
gi|641571042|gb|KK856176.1|	55077-15558	10	34	1	2	0.593756	0.70505	no
gi|641571042|gb|KK856176.1|	61920-16260	10	34	2	2	0.0698091	0.9545	no
gi|641571042|gb|KK856176.1|	65105-16542	10	34	76	85	0.156493	0.90165	no
gi|641571042|gb|KK856176.1|	43181-45114	10	34	0	27	inf	0.0005	yes
gi|641571042|gb|KK856176.1|	46617-47143	10	34	0	323	inf	5.00E-05	yes
gi|641571042|gb|KK856176.1|	48386-48605	10	34	0	82	inf	0.0004	yes
gi|641571042|gb|KK856176.1|	49994-50216	10	34	0	598	inf	5.00E-05	yes
gi|641571042|gb|KK856176.1|	52121-53364	10	34	0	42	inf	0.00055	yes
gi|641571042|gb|KK856176.1|	55526-56585	10	34	0.163952	11	6	0.23225	no
gi|641571042|gb|KK856176.1|	58802-58984	10	34	0	302	inf	5.00E-05	yes
gi|641571042|gb|KK856176.1|	60017-62384	10	34	0.163013	1	3	0.12585	no
gi|641571042|gb|KK856176.1|	62730-63634	10	34	0.099905	2	4	0.11955	no
gi|641571042|gb|KK856176.1|	67265-67651	10	34	0	115	inf	5.00E-05	yes
gi|641571042|gb|KK856176.1|	75525-77264	10	34	30	14	-1	0.5056	no
gi|641571042|gb|KK856176.1|	79155-79956	10	34	0.701358	0.887475	0.339555	0.8352	no
gi|641571042|gb|KK856176.1|	80348-81714	10	34	1	0.460666	-1	0.47035	no
gi|641571042|gb|KK856176.1|	84001-85146	10	34	0.820078	0.794363	-0.0459634	1	no
gi|641571042|gb|KK856176.1|	87392-89003	10	34	0.350088	0.762349	1	1	no
gi|641571042|gb|KK856176.1|	92200-92930	10	34	1	0.401747	-2	0.3216	no

gi|641571042|gb|KK856176.1|	93067-94601	10	34	8	7	-0.208773	0.8663	no
gi|641571042|gb|KK856176.1|	99136-99588	10	34	17	33	0.962873	0.45435	no
gi|641571045|gb|KK856175.1|	.04260-10497	9.3	35	1	1	0.238422	0.88205	no
gi|641571045|gb|KK856175.1|	.09910-11017	9.3	35	13	4	-2	0.32465	no
gi|641571045|gb|KK856175.1|	.11342-11153	9.3	35	24	4	-3	0.2587	no
gi|641571045|gb|KK856175.1|	.17496-11792	9.3	35	12	2	-2	0.14395	no
gi|641571045|gb|KK856175.1|	.32042-13224	9.3	35	23	0	-inf	0.01	no
gi|641571045|gb|KK856175.1|	.39512-13986	9.3	35	65	14	-2	0.09955	no
gi|641571045|gb|KK856175.1|	.45242-14582	9.3	35	0.924098	2	0.86	0.62755	no
gi|641571045|gb|KK856175.1|	.46694-14733	9.3	35	1	6	2	0.17355	no
gi|641571045|gb|KK856175.1|	.62424-16299	9.3	35	0.566229	2	2	0.3323	no
gi|641571045|gb|KK856175.1|	19052-20035	9.3	35	0.269655	2	2	0.2136	no
gi|641571045|gb|KK856175.1|	20191-20509	9.3	35	31	6	-2	0.1239	no
gi|641571045|gb|KK856175.1|	20688-22553	9.3	35	2	3	0.680944	0.60915	no
gi|641571045|gb|KK856175.1|	23603-24606	9.3	35	0.438297	0.93331	1	0.50945	no
gi|641571045|gb|KK856175.1|	26500-26788	9.3	35	8	0	-inf	0.0102	no
gi|641571045|gb|KK856175.1|	29330-29707	9.3	35	5	1	-2	0.26505	no
gi|641571045|gb|KK856175.1|	32637-33008	9.3	35	26	10	-1	0.3123	no
gi|641571045|gb|KK856175.1|	34651-36009	9.3	35	81	28	-2	0.36195	no
gi|641571045|gb|KK856175.1|	37818-38216	9.3	35	18	3	-3	0.1257	no
gi|641571045|gb|KK856175.1|	40179-40566	9.3	35	36	10	-2	0.1788	no
gi|641571045|gb|KK856175.1|	42309-42529	9.3	35	60	17	-2	0.2365	no
gi|641571045|gb|KK856175.1|	42760-43100	9.3	35	6	2	-1	0.4191	no
gi|641571045|gb|KK856175.1|	49162-49415	9.3	35	13	4	-2	0.4022	no
gi|641571045|gb|KK856175.1|	50073-52572	9.3	35	8	0.562815	-4	0.0225	no
gi|641571045|gb|KK856175.1|	55498-56412	9.3	35	0.985144	0.44924	-1	0.50885	no
gi|641571045|gb|KK856175.1|	56609-57705	9.3	35	0.864692	0.358878	-1	1	no
gi|641571045|gb|KK856175.1|	58307-60701	9.3	35	160	45	-2	0.3803	no
gi|641571045|gb|KK856175.1|	62322-64324	9.3	35	13	10.58	-0.341184	0.7792	no
gi|641571045|gb|KK856175.1|	65161-66381	9.3	35	11	12	0.172671	0.8933	no

gi|641571045|gb|KK856175.1|	68375-69413	9.3	35	22	28	0.387603	0.7543	no
gi|641571045|gb|KK856175.1|	69496-70784	9.3	35	8	13	0.573503	0.6457	no
gi|641571045|gb|KK856175.1|	72182-75238	9.3	35	15	15	0.0531564	0.9799	no
gi|641571045|gb|KK856175.1|	77987-78274	9.3	35	12	7	-0.716074	0.64315	no
gi|641571045|gb|KK856175.1|	80370-80796	9.3	35	5	3	-0.997432	0.5501	no
gi|641571045|gb|KK856175.1|	80923-81736	9.3	35	10	7	-0.379944	0.7677	no
gi|641571045|gb|KK856175.1|	85234-86808	9.3	35	0.770322	0.234824	-2	1	no
gi|641571045|gb|KK856175.1|	87630-89094	9.3	35	0.948725	0.425197	-1	0.47035	no
gi|641571045|gb|KK856175.1|	90453-91466	9.3	35	0.692699	0.52683	-0.394889	1	no
gi|641571045|gb|KK856175.1|	97557-104022	9.3	35	19	6	-2	0.44055	no
gi|641571048|gb|KK856174.1|	10428-11791	11	39	7	3	-1	0.33105	no
gi|641571048|gb|KK856174.1|	11977-14177	11	39	133	55	-1	0.54405	no
gi|641571048|gb|KK856174.1|	132407-13280	11	39	5	2	-2	0.40245	no
gi|641571048|gb|KK856174.1|	20608-21522	11	39	0.88663	4	2	0.1801	no
gi|641571048|gb|KK856174.1|	21815-22985	11	39	1	3	1	0.445	no
gi|641571048|gb|KK856174.1|	23096-24148	11	39	1	4	1	0.34975	no
gi|641571048|gb|KK856174.1|	24260-26275	11	39	24	26	0.115366	0.9585	no
gi|641571048|gb|KK856174.1|	253-1033	11	39	0.726546	1	0.824634	0.6232	no
gi|641571048|gb|KK856174.1|	27333-28337	11	39	0.437755	1	1	0.4595	no
gi|641571048|gb|KK856174.1|	28764-29826	11	39	1	1	0.383126	0.7933	no
gi|641571048|gb|KK856174.1|	30474-31012	11	39	34	47	0.468107	0.70365	no
gi|641571048|gb|KK856174.1|	34184-35982	11	39	6	9	0.448775	0.72	no
gi|641571048|gb|KK856174.1|	36497-36798	11	39	48	57	0.255028	0.83855	no
gi|641571048|gb|KK856174.1|	37947-38337	11	39	29	39	0.395941	0.7559	no
gi|641571048|gb|KK856174.1|	38762-40724	11	39	1	1	0.287061	0.83085	no
gi|641571048|gb|KK856174.1|	41989-42380	11	39	32	29	-0.142742	0.91195	no
gi|641571048|gb|KK856174.1|	44163-46855	11	39	6	8	0.422152	0.7397	no
gi|641571048|gb|KK856174.1|	47135-49226	11	39	3	3	0.254552	0.8417	no
gi|641571048|gb|KK856174.1|	49402-51914	11	39	0.550116	0.792856	0.527321	1	no
gi|641571048|gb|KK856174.1|	5250-5688	11	39	0.868896	3	2	0.3323	no

<table>
<tr><td>gi|641571048|gb|KK856174.1|</td><td>53718-53950</td><td>11</td><td>39</td><td>26</td><td>22</td><td>-0.261863</td><td>0.8593</td><td>no</td></tr>
<tr><td>gi|641571048|gb|KK856174.1|</td><td>67659-69677</td><td>11</td><td>39</td><td>4</td><td>8</td><td>0.83516</td><td>0.5154</td><td>no</td></tr>
<tr><td>gi|641571048|gb|KK856174.1|</td><td>75582-75819</td><td>11</td><td>39</td><td>0</td><td>21</td><td>inf</td><td>0.00375</td><td>no</td></tr>
<tr><td>gi|641571052|gb|KK856173.1|</td><td>00856-10156</td><td>8.9</td><td>41</td><td>2</td><td>0</td><td>-inf</td><td>0.0059</td><td>no</td></tr>
<tr><td>gi|641571052|gb|KK856173.1|</td><td>01699-10524</td><td>8.9</td><td>41</td><td>2</td><td>11</td><td>3</td><td>0.14885</td><td>no</td></tr>
<tr><td>gi|641571052|gb|KK856173.1|</td><td>05866-10687</td><td>8.9</td><td>41</td><td>2</td><td>0.132676</td><td>-4</td><td>0.19735</td><td>no</td></tr>
<tr><td>gi|641571052|gb|KK856173.1|</td><td>08155-10972</td><td>8.9</td><td>41</td><td>2</td><td>0.078331</td><td>-4</td><td>0.1879</td><td>no</td></tr>
<tr><td>gi|641571052|gb|KK856173.1|</td><td>10851-11927</td><td>8.9</td><td>41</td><td>14.31</td><td>17</td><td>0.289765</td><td>0.8171</td><td>no</td></tr>
<tr><td>gi|641571052|gb|KK856173.1|</td><td>10238-11126</td><td>8.9</td><td>41</td><td>2</td><td>0</td><td>-inf</td><td>0.00185</td><td>yes</td></tr>
<tr><td>gi|641571052|gb|KK856173.1|</td><td>12283-11299</td><td>8.9</td><td>41</td><td>3</td><td>0</td><td>-inf</td><td>0.0032</td><td>yes</td></tr>
<tr><td>gi|641571052|gb|KK856173.1|</td><td>13129-11385</td><td>8.9</td><td>41</td><td>3</td><td>0</td><td>-inf</td><td>0.00495</td><td>no</td></tr>
<tr><td>gi|641571052|gb|KK856173.1|</td><td>14795-11564</td><td>8.9</td><td>41</td><td>2</td><td>0</td><td>-inf</td><td>0.0034</td><td>yes</td></tr>
<tr><td>gi|641571052|gb|KK856173.1|</td><td>15823-11938</td><td>8.9</td><td>41</td><td>11</td><td>0.096332</td><td>-7</td><td>0.09405</td><td>no</td></tr>
<tr><td>gi|641571052|gb|KK856173.1|</td><td>20999-12126</td><td>8.9</td><td>41</td><td>21</td><td>0</td><td>-inf</td><td>0.0021</td><td>yes</td></tr>
<tr><td>gi|641571052|gb|KK856173.1|</td><td>12977-13755</td><td>8.9</td><td>41</td><td>1</td><td>0.737806</td><td>-0.719721</td><td>0.6701</td><td>no</td></tr>
<tr><td>gi|641571052|gb|KK856173.1|</td><td>14403-14766</td><td>8.9</td><td>41</td><td>20</td><td>23</td><td>0.204286</td><td>0.87585</td><td>no</td></tr>
<tr><td>gi|641571052|gb|KK856173.1|</td><td>14895-15551</td><td>8.9</td><td>41</td><td>36</td><td>62</td><td>0.802259</td><td>0.517</td><td>no</td></tr>
<tr><td>gi|641571052|gb|KK856173.1|</td><td>18361-19566</td><td>8.9</td><td>41</td><td>0</td><td>28</td><td>inf</td><td>5.00E-05</td><td>yes</td></tr>
<tr><td>gi|641571052|gb|KK856173.1|</td><td>26004-27641</td><td>8.9</td><td>41</td><td>2</td><td>5</td><td>1</td><td>0.36225</td><td>no</td></tr>
<tr><td>gi|641571052|gb|KK856173.1|</td><td>27873-28307</td><td>8.9</td><td>41</td><td>4</td><td>2</td><td>-0.996681</td><td>0.5872</td><td>no</td></tr>
<tr><td>gi|641571052|gb|KK856173.1|</td><td>28373-29193</td><td>8.9</td><td>41</td><td>0.906703</td><td>1</td><td>0.602885</td><td>0.6936</td><td>no</td></tr>
<tr><td>gi|641571052|gb|KK856173.1|</td><td>29381-32181</td><td>8.9</td><td>41</td><td>47</td><td>34</td><td>-0.473865</td><td>0.8281</td><td>no</td></tr>
<tr><td>gi|641571052|gb|KK856173.1|</td><td>44591-44814</td><td>8.9</td><td>41</td><td>20</td><td>4</td><td>-2</td><td>0.24525</td><td>no</td></tr>
<tr><td>gi|641571052|gb|KK856173.1|</td><td>45539-45740</td><td>8.9</td><td>41</td><td>52.2</td><td>9</td><td>-3</td><td>0.18005</td><td>no</td></tr>
<tr><td>gi|641571052|gb|KK856173.1|</td><td>49792-52256</td><td>8.9</td><td>41</td><td>0.749007</td><td>0.142871</td><td>-2</td><td>1</td><td>no</td></tr>
<tr><td>gi|641571052|gb|KK856173.1|</td><td>52445-53880</td><td>8.9</td><td>41</td><td>0.685354</td><td>0.261066</td><td>-1</td><td>1</td><td>no</td></tr>
<tr><td>gi|641571052|gb|KK856173.1|</td><td>55324-56449</td><td>8.9</td><td>41</td><td>1</td><td>0</td><td>-inf</td><td>0.00495</td><td>no</td></tr>
<tr><td>gi|641571052|gb|KK856173.1|</td><td>59526-61109</td><td>8.9</td><td>41</td><td>0.561239</td><td>0.388843</td><td>-0.529428</td><td>1</td><td>no</td></tr>
<tr><td>gi|641571052|gb|KK856173.1|</td><td>65760-67251</td><td>8.9</td><td>41</td><td>1</td><td>0</td><td>-inf</td><td>0.00495</td><td>no</td></tr>
<tr><td>gi|641571052|gb|KK856173.1|</td><td>67386-68148</td><td>8.9</td><td>41</td><td>2</td><td>0</td><td>-inf</td><td>0.00435</td><td>no</td></tr>
</table>

gi|641571052|gb|KK856173.1|	69335-81874	8.9	41	5	3	-0.868217	0.59175	no
gi|641571052|gb|KK856173.1|	83708-85538	8.9	41	1	0.132095	-3	0.144	no
gi|641571052|gb|KK856173.1|	86718-88023	8.9	41	0.638034	2	2	0.26655	no
gi|641571052|gb|KK856173.1|	88126-90337	8.9	41	0.772767	49	6	0.0181	no
gi|641571052|gb|KK856173.1|	91918-92789	8.9	41	4	0.159219	-4	0.1868	no
gi|641571052|gb|KK856173.1|	94813-98071	8.9	41	3	0.599994	-2	0.1283	no
gi|641571052|gb|KK856173.1|	98270-10075:	8.9	41	1	0.472287	-2	0.2619	no
gi|641571055|gb|KK856172.1|	.00439-10240	10	38	0.518644	0.425944	-0.284081	1	no
gi|641571055|gb|KK856172.1|	.03420-10888	10	38	17	42	1	0.5342	no
gi|641571055|gb|KK856172.1|	10483-12201	10	38	0.511481	0.708923	0.470949	1	no
gi|641571055|gb|KK856172.1|	.09180-11460	10	38	9	19	0.995295	0.6486	no
gi|641571055|gb|KK856172.1|	.14753-11771	10	38	6	16	1	0.42855	no
gi|641571055|gb|KK856172.1|	.17822-11992	10	38	6	15	1	0.32365	no
gi|641571055|gb|KK856172.1|	.20781-12438	10	38	167	492	2	0.353	no
gi|641571055|gb|KK856172.1|	.26057-12709	10	38	757	2044.55	1	0.3805	no
gi|641571055|gb|KK856172.1|	.76979-17806	10	38	1	2	0.885817	0.53755	no
gi|641571055|gb|KK856172.1|	26810-27731	10	38	0.682949	1	0.966767	0.5733	no
gi|641571055|gb|KK856172.1|	30729-32743	10	38	0.545027	0.475043	-0.19827	1	no
gi|641571055|gb|KK856172.1|	32992-35476	10	38	0.494975	3	3	0.08365	no
gi|641571055|gb|KK856172.1|	40139-42372	10	38	0.277972	0.265057	-0.0686379	1	no
gi|641571055|gb|KK856172.1|	44106-47064	10	38	0.71759	4	3	0.0714	no
gi|641571055|gb|KK856172.1|	47195-47767	10	38	1	1	-0.21063	0.8945	no
gi|641571055|gb|KK856172.1|	50579-51571	10	38	0.266609	2	3	0.1811	no
gi|641571055|gb|KK856172.1|	51697-55823	10	38	0.75654	2	2	0.2311	no
gi|641571055|gb|KK856172.1|	58849-61154	10	38	2	5	1	0.3573	no
gi|641571055|gb|KK856172.1|	61440-62563	10	38	2	2	-0.187583	0.8943	no
gi|641571055|gb|KK856172.1|	65908-66800	10	38	12	12	0.0431442	0.9714	no
gi|641571055|gb|KK856172.1|	67991-69079	10	38	7	10	0.426167	0.7334	no
gi|641571055|gb|KK856172.1|	69290-71352	10	38	9	16	0.869169	0.48145	no
gi|641571055|gb|KK856172.1|	71710-73025	10	38	8	22	2	0.236	no

gi|641571055|gb|KK856172.1|	78174-79784	10	38	0.150144	47	8	0.13805	no
gi|641571055|gb|KK856172.1|	94848-96045	10	38	2	4	1	0.33375	no
gi|641571055|gb|KK856172.1|	96156-96914	10	38	1	3	1	0.3594	no
gi|641571055|gb|KK856172.1|	98790-99460	10	38	3	0.904022	-1	0.37245	no
gi|641571063|gb|KK856170.1|	23364-12364	10	20	7	1	-3	0.2588	no
gi|641571063|gb|KK856170.1|	23742-12406	10	20	5	2	-2	0.3632	no
gi|641571063|gb|KK856170.1|	59294-15967	10	20	3	2	-0.640267	0.71075	no
gi|641571063|gb|KK856170.1|	4135-5071	10	20	0.573531	0.726618	0.341327	1	no
gi|641571063|gb|KK856170.1|	5167-6078	10	20	0.494637	0.751854	0.604084	1	no
gi|641571063|gb|KK856170.1|	6-438	10	20	7	4	-0.885834	0.53935	no
gi|641571067|gb|KK856169.1|	04544-10539	11	39	0.217619	2	3	0.2672	no
gi|641571067|gb|KK856169.1|	11075-11656	11	39	4	4	-0.0947943	0.94565	no
gi|641571067|gb|KK856169.1|	11397-11196	11	39	0.377486	2	3	0.2991	no
gi|641571067|gb|KK856169.1|	12087-11345	11	39	0.242537	0.738952	2	1	no
gi|641571067|gb|KK856169.1|	14435-11497	11	39	0.615149	3	2	0.2235	no
gi|641571067|gb|KK856169.1|	12320-17140	11	39	2	2	0.10966	0.9282	no
gi|641571067|gb|KK856169.1|	47503-14837	11	39	0.520069	1	1	0.4595	no
gi|641571067|gb|KK856169.1|	49207-15179	11	39	0.502842	0.9931	0.981834	0.4943	no
gi|641571067|gb|KK856169.1|	17914-18074	11	39	63	18	-2	0.3266	no
gi|641571067|gb|KK856169.1|	18741-21006	11	39	0.821023	0.991675	0.272444	0.8431	no
gi|641571067|gb|KK856169.1|	22552-23225	11	39	1	0.673803	-0.622395	0.71195	no
gi|641571067|gb|KK856169.1|	29148-29687	11	39	3	0.932019	-2	0.33985	no
gi|641571067|gb|KK856169.1|	31161-31774	11	39	2	2	0.0836635	0.94	no
gi|641571067|gb|KK856169.1|	31958-32452	11	39	9	6	-0.446721	0.74175	no
gi|641571067|gb|KK856169.1|	34980-36030	11	39	0.82839	0.3781	-1	1	no
gi|641571067|gb|KK856169.1|	41355-42011	11	39	6	4	-0.439984	0.74455	no
gi|641571067|gb|KK856169.1|	42242-45823	11	39	21	9	-1	0.45035	no
gi|641571067|gb|KK856169.1|	45929-46381	11	39	14	12	-0.252629	0.8527	no
gi|641571067|gb|KK856169.1|	47089-47659	11	39	3	0.285416	-4	0.20475	no
gi|641571067|gb|KK856169.1|	48748-50235	11	39	1	0.33412	-2	0.31425	no

gi|641571067|gb|KK856169.1|	52216-56473	11	39	7	13	0.876312	0.58765	no
gi|641571067|gb|KK856169.1|	56686-57112	11	39	8	7	-0.167357	0.906	no
gi|641571067|gb|KK856169.1|	5990-6726	11	39	2	2	-0.398569	0.7915	no
gi|641571067|gb|KK856169.1|	59972-60622	11	39	0.778298	2	2	0.32945	no
gi|641571067|gb|KK856169.1|	61326-61835	11	39	0.22511	4	4	0.1196	no
gi|641571067|gb|KK856169.1|	7640-10275	11	39	24	22	-0.110901	0.96095	no
gi|641571067|gb|KK856169.1|	86957-87491	11	39	3	2	-0.298061	0.8456	no
gi|641571071|gb|KK856168.1|	32900-33160	11	47	0	768	inf	0.00025	yes
gi|641571071|gb|KK856168.1|	35369-35721	11	47	0	60	inf	0.0001	yes
gi|641571071|gb|KK856168.1|	41959-42171	11	47	0	176	inf	5.00E-05	yes
gi|641571071|gb|KK856168.1|	46897-47221	11	47	0	50	inf	5.00E-05	yes
gi|641571071|gb|KK856168.1|	49938-50782	11	47	0.109145	344	12	0.1051	no
gi|641571071|gb|KK856168.1|	51277-52142	11	47	0	174.29	inf	5.00E-05	yes
gi|641571071|gb|KK856168.1|	54833-55409	11	47	0	10	inf	0.00035	yes
gi|641571074|gb|KK856167.1|	15073-15553	9.3	39	0.990873	3	2	0.33255	no
gi|641571074|gb|KK856167.1|	93679-94094	9.3	39	33	52	0.62707	0.61585	no
gi|641571074|gb|KK856167.1|	98548-98934	9.3	39	5	4	-0.310026	0.84315	no
gi|641571078|gb|KK856166.1|	00112-10152	10	41	0.580493	0.884442	0.607489	0.6779	no
gi|641571078|gb|KK856166.1|	80960-81685	10	41	1	0.608259	-0.983756	0.62775	no
gi|641571078|gb|KK856166.1|	83788-84495	10	41	0.830107	0.839279	0.0158533	1	no
gi|641571086|gb|KK856164.1|	22261-22554	9.8	41	3	11	2	0.2632	no
gi|641571089|gb|KK856163.1|	10488-11533	9.3	37	2	3	0.605382	0.66445	no
gi|641571089|gb|KK856163.1|	15081-11560	9.3	37	2	2	0.109141	0.92265	no
gi|641571089|gb|KK856163.1|	18188-11908	9.3	37	2	0.616032	-2	0.2683	no
gi|641571089|gb|KK856163.1|	19434-12016	9.3	37	1	0.603739	-1	0.47135	no
gi|641571089|gb|KK856163.1|	21793-12234	9.3	37	1	2	0.595915	0.70555	no
gi|641571089|gb|KK856163.1|	22585-12336	9.3	37	1	2	0.450306	0.7632	no
gi|641571089|gb|KK856163.1|	13381-14810	9.3	37	0.860859	0.174888	-2	1	no
gi|641571089|gb|KK856163.1|	46778-14712	9.3	37	0	87	inf	5.00E-05	yes
gi|641571089|gb|KK856163.1|	20044-20454	9.3	37	5	3	-0.636494	0.68395	no

gi|641571089|gb|KK856163.1|	21871-23973	9.3	37	0.668408	0.566457	-0.238762	1	no
gi|641571089|gb|KK856163.1|	24149-25006	9.3	37	11	10	-0.207318	0.86685	no
gi|641571089|gb|KK856163.1|	25275-26569	9.3	37	40	27	-0.582358	0.71765	no
gi|641571089|gb|KK856163.1|	26652-28550	9.3	37	0.91482	0.190248	-2	0.21625	no
gi|641571089|gb|KK856163.1|	28632-29589	9.3	37	1	1	-0.0958589	0.9478	no
gi|641571089|gb|KK856163.1|	3309-3668	9.3	37	74	56	-0.39631	0.7503	no
gi|641571092|gb|KK856162.1|	64502-67034	11	47	2	1	-0.743832	0.57555	no
gi|641571092|gb|KK856162.1|	68418-71719	11	47	4	1	-1	0.291	no
gi|641571092|gb|KK856162.1|	72134-75374	11	47	8	7	-0.146932	0.9059	no
gi|641571096|gb|KK856161.1|	02246-10362	9.8	37	8	34	2	0.2537	no
gi|641571096|gb|KK856161.1|	05093-10591	9.8	37	0.677857	1	0.825307	0.6232	no
gi|641571096|gb|KK856161.1|	07736-10918	9.8	37	0.957022	0.171561	-2	0.2115	no
gi|641571096|gb|KK856161.1|	09357-11356	9.8	37	147.95	6	-5	0.0168	no
gi|641571096|gb|KK856161.1|	21225-22536	9.8	37	0	3	inf	0.00025	yes
gi|641571096|gb|KK856161.1|	69875-70217	9.8	37	0.932096	9	3	0.2558	no
gi|641571096|gb|KK856161.1|	93936-95347	9.8	37	0	0.976077	inf	0.00455	no
gi|641571098|gb|KK856160.1|	13941-11414	11	43	22	17	-0.342125	0.8313	no
gi|641571098|gb|KK856160.1|	15502-11573	11	43	31	2	-4	0.19105	no
gi|641571098|gb|KK856160.1|	17944-11856	11	43	7	0.751602	-3	0.1178	no
gi|641571098|gb|KK856160.1|	22327-12351	11	43	0.642557	0.326083	-0.978586	1	no
gi|641571098|gb|KK856160.1|	26890-12820	11	43	13	2	-2	0.0797	no
gi|641571098|gb|KK856160.1|	28370-13107	11	43	10	2	-2	0.11755	no
gi|641571098|gb|KK856160.1|	12962-13369	11	43	23	24	0.0917561	0.94295	no
gi|641571098|gb|KK856160.1|	45123-14800	11	43	2	0.201352	-3	0.0694	no
gi|641571098|gb|KK856160.1|	48292-14893	11	43	19	3	-3	0.06055	no
gi|641571098|gb|KK856160.1|	49051-15004	11	43	2	0.674964	-2	0.2421	no
gi|641571098|gb|KK856160.1|	53102-15378	11	43	4	1	-2	0.20595	no
gi|641571098|gb|KK856160.1|	55216-15701	11	43	37	17	-1	0.4783	no
gi|641571098|gb|KK856160.1|	58058-15873	11	43	2	0	-inf	0.0056	no
gi|641571098|gb|KK856160.1|	16309-16755	11	43	13	22	0.676797	0.6028	no

gi|641571098|gb|KK856160.1|	73185-17340	11	43	90	38	-1	0.36545	no
gi|641571098|gb|KK856160.1|	20265-20718	11	43	9	2	-2	0.19735	no
gi|641571098|gb|KK856160.1|	23757-24937	11	43	1	0	-inf	0.00435	no
gi|641571098|gb|KK856160.1|	2384-2774	11	43	3	2	-0.831469	0.64855	no
gi|641571098|gb|KK856160.1|	26207-27270	11	43	3	3	-0.0465504	0.9724	no
gi|641571098|gb|KK856160.1|	27419-27950	11	43	20	17	-0.204827	0.86925	no
gi|641571098|gb|KK856160.1|	290-593	11	43	63	52	-0.285506	0.8228	no
gi|641571098|gb|KK856160.1|	29668-29883	11	43	10	12	0.274787	0.8647	no
gi|641571098|gb|KK856160.1|	2985-4195	11	43	0.697769	0.318721	-1	1	no
gi|641571098|gb|KK856160.1|	31638-33379	11	43	8	10	0.234696	0.84585	no
gi|641571098|gb|KK856160.1|	47669-48126	11	43	2	4	1	0.4992	no
gi|641571098|gb|KK856160.1|	77781-78586	11	43	1	1	0.280725	0.85465	no
gi|641571098|gb|KK856160.1|	95427-96501	11	43	0.241729	0.980856	2	0.2812	no
gi|641571098|gb|KK856160.1|	9707-9932	11	43	36	49	0.426989	0.74905	no
gi|641571108|gb|KK856159.1|	17538-11865	8	35	4	6	0.445481	0.72855	no
gi|641571108|gb|KK856159.1|	33476-13506	8	35	0.915094	1	0.345056	0.8112	no
gi|641571108|gb|KK856159.1|	37335-13793	8	35	0.884989	3	2	0.32945	no
gi|641571112|gb|KK856158.1|	02472-10421	11	36	0	3	inf	0.0001	yes
gi|641571112|gb|KK856158.1|	08412-10944	11	36	0.168839	62	9	0.23525	no
gi|641571112|gb|KK856158.1|	10867-11109	11	36	0	35	inf	0.0015	yes
gi|641571112|gb|KK856158.1|	11743-11298	11	36	0.271352	1	2	0.23615	no
gi|641571112|gb|KK856158.1|	13559-11401	11	36	0	21	inf	0.00015	yes
gi|641571112|gb|KK856158.1|	18250-11846	11	36	0	56	inf	0.00115	yes
gi|641571112|gb|KK856158.1|	18835-11965	11	36	0	6	inf	0.00025	yes
gi|641571112|gb|KK856158.1|	21513-12209	11	36	0.18339	7	5	0.1059	no
gi|641571112|gb|KK856158.1|	23064-12424	11	36	0.071757	2	5	0.11025	no
gi|641571112|gb|KK856158.1|	26462-12665	11	36	0	102	inf	0.00075	yes
gi|641571112|gb|KK856158.1|	27407-12825	11	36	0.109313	37	8	0.10435	no
gi|641571112|gb|KK856158.1|	13225-13744	11	36	0.218228	3	4	0.12385	no
gi|641571112|gb|KK856158.1|	33427-13408	11	36	0	4	inf	0.0016	yes

gi|641571112|gb|KK856158.1|	17325-21029	11	36	2	3	0.66895	0.59875	no
gi|641571112|gb|KK856158.1|	21171-21712	11	36	2	2	-0.0567141	0.9454	no
gi|641571112|gb|KK856158.1|	21842-33413	11	36	8	5	-0.599624	0.7907	no
gi|641571112|gb|KK856158.1|	33946-36067	11	36	24	15	-0.709368	0.65865	no
gi|641571112|gb|KK856158.1|	36717-37473	11	36	5	4	-0.542574	0.68975	no
gi|641571112|gb|KK856158.1|	40909-41843	11	36	3	2	-0.343193	0.80655	no
gi|641571112|gb|KK856158.1|	43768-45471	11	36	1	1	0.122987	0.92895	no
gi|641571112|gb|KK856158.1|	45599-46967	11	36	0.603758	2	2	0.1864	no
gi|641571112|gb|KK856158.1|	47963-49487	11	36	2	2	0.238653	0.85495	no
gi|641571112|gb|KK856158.1|	49634-50823	11	36	25	15	-0.803256	0.51965	no
gi|641571112|gb|KK856158.1|	53635-54161	11	36	0	16	inf	0.0001	yes
gi|641571112|gb|KK856158.1|	55632-55889	11	36	0	118	inf	5.00E-05	yes
gi|641571112|gb|KK856158.1|	56293-56670	11	36	0	43	inf	5.00E-05	yes
gi|641571112|gb|KK856158.1|	60064-60380	11	36	0	36	inf	0.00015	yes
gi|641571112|gb|KK856158.1|	62796-63928	11	36	0	4	inf	0.0004	yes
gi|641571112|gb|KK856158.1|	69816-69978	11	36	0	116	inf	0.00255	yes
gi|641571112|gb|KK856158.1|	73151-73334	11	36	0	58	inf	0.0025	yes
gi|641571112|gb|KK856158.1|	74369-74603	11	36	0	74	inf	5.00E-05	yes
gi|641571112|gb|KK856158.1|	81081-81469	11	36	0	31	inf	5.00E-05	yes
gi|641571112|gb|KK856158.1|	88913-89242	11	36	0	78	inf	5.00E-05	yes
gi|641571112|gb|KK856158.1|	90222-91346	11	36	0	23	inf	5.00E-05	yes
gi|641571112|gb|KK856158.1|	93555-93824	11	36	0	88	inf	5.00E-05	yes
gi|641571112|gb|KK856158.1|	96929-97827	11	36	0	10	inf	5.00E-05	yes
gi|641571116|gb|KK856157.1|	07115-10798	11	40	0.105251	4	5	0.1072	no
gi|641571116|gb|KK856157.1|	12748-11714	11	40	1	2	0.23589	0.8561	no
gi|641571116|gb|KK856157.1|	17474-12041	11	40	3	3	0.101165	0.93705	no
gi|641571116|gb|KK856157.1|	22612-12336	11	40	15	14	-0.139097	0.914	no
gi|641571116|gb|KK856157.1|	25649-12588	11	40	60	70	0.217019	0.8648	no
gi|641571116|gb|KK856157.1|	33076-13371	11	40	5	3	-0.681081	0.62805	no
gi|641571116|gb|KK856157.1|	37169-13826	11	40	8	2	-2	0.16555	no

gi|641571116|gb|KK856157.1|	38415-14120	11	40	16	2	-3	0.10285	no
gi|641571116|gb|KK856157.1|	41367-14235	11	40	3	3	0.0429857	0.9735	no
gi|641571116|gb|KK856157.1|	42492-14386	11	40	3	0.91665	-2	0.2363	no
gi|641571116|gb|KK856157.1|	43999-14586	11	40	1	0.258202	-2	0.1536	no
gi|641571116|gb|KK856157.1|	46096-14767	11	40	1	0.156323	-3	0.1571	no
gi|641571116|gb|KK856157.1|	47903-14856	11	40	5	0.925231	-2	0.1841	no
gi|641571116|gb|KK856157.1|	65242-16552	11	40	53	72	0.44096	0.7283	no
gi|641571116|gb|KK856157.1|	89617-19057	11	40	1	0.565354	-0.980382	0.5872	no
gi|641571116|gb|KK856157.1|	94432-19495	11	40	0.85461	2	1	0.51315	no
gi|641571116|gb|KK856157.1|	96044-19710	11	40	0.732692	0.619349	-0.242455	1	no
gi|641571116|gb|KK856157.1|	06097-20686	11	40	0.123844	2	4	0.1136	no
gi|641571116|gb|KK856157.1|	08449-20913	11	40	0.286197	2	3	0.2851	no
gi|641571116|gb|KK856157.1|	10547-21256	11	40	13	10	-0.351731	0.7749	no
gi|641571116|gb|KK856157.1|	14311-21493	11	40	0.495213	2	2	0.3323	no
gi|641571116|gb|KK856157.1|	19447-21957	11	40	677.1	1039.9	0.619007	0.67975	no
gi|641571116|gb|KK856157.1|	20914-22248	11	40	0.669068	0.470669	-0.507439	1	no
gi|641571116|gb|KK856157.1|	24131-22548	11	40	0.489688	18	5	0.046	no
gi|641571116|gb|KK856157.1|	27211-22793	11	40	1	4	2	0.2668	no
gi|641571116|gb|KK856157.1|	28297-22914	11	40	2	2	0.0182851	0.9597	no
gi|641571116|gb|KK856157.1|	29539-23211	11	40	1	1	-0.167755	0.8963	no
gi|641571116|gb|KK856157.1|	36297-23755	11	40	0.531502	0.607055	0.191752	1	no
gi|641571116|gb|KK856157.1|	39815-24131	11	40	22	13	-0.764386	0.53815	no
gi|641571116|gb|KK856157.1|	41537-24378	11	40	2	2	-0.316547	0.8097	no
gi|641571116|gb|KK856157.1|	43894-24437	11	40	1	1	0.00693816	0.91115	no
gi|641571116|gb|KK856157.1|	37359-37706	11	40	81	18	-2	0.10745	no
gi|641571116|gb|KK856157.1|	38346-38712	11	40	4	3	-0.419747	0.77915	no
gi|641571116|gb|KK856157.1|	39664-39925	11	40	18	8	-1	0.44735	no
gi|641571116|gb|KK856157.1|	43252-43901	11	40	6	4	-0.703215	0.6041	no
gi|641571116|gb|KK856157.1|	45422-45827	11	40	3	2	-0.414652	0.82205	no
gi|641571116|gb|KK856157.1|	46156-46568	11	40	4	4	0.00115015	0.95145	no

gi|641571116|gb|KK856157.1|	48237-48746	11	40	5	2	-0.991263	0.5308	no
gi|641571116|gb|KK856157.1|	51604-51802	11	40	20	13	-0.6407	0.70205	no
gi|641571116|gb|KK856157.1|	53863-54116	11	40	8	7	-0.124458	0.9229	no
gi|641571116|gb|KK856157.1|	55361-55603	11	40	12	10	-0.324916	0.832	no
gi|641571116|gb|KK856157.1|	68140-68823	11	40	3	2	-0.622142	0.6689	no
gi|641571116|gb|KK856157.1|	71616-72103	11	40	0	115	inf	5.00E-05	yes
gi|641571116|gb|KK856157.1|	96530-97400	11	40	2	7	2	0.24945	no
gi|641571119|gb|KK856156.1|	11622-12473	11	38	15	7	-1	0.3496	no
gi|641571119|gb|KK856156.1|	12630-20775	11	38	52	36	-0.540055	0.7829	no
gi|641571119|gb|KK856156.1|	26747-12737	11	38	2	8	2	0.21675	no
gi|641571119|gb|KK856156.1|	29383-12982	11	38	19	9	-1	0.4068	no
gi|641571119|gb|KK856156.1|	40269-14131	11	38	3	1	-1	0.4351	no
gi|641571119|gb|KK856156.1|	45753-14703	11	38	1	2	0.536492	0.70035	no
gi|641571119|gb|KK856156.1|	47162-14782	11	38	7	4	-0.737194	0.58545	no
gi|641571119|gb|KK856156.1|	49238-15015	11	38	0.683892	1	0.604186	0.69715	no
gi|641571119|gb|KK856156.1|	51609-15235	11	38	0	18	inf	5.00E-05	yes
gi|641571119|gb|KK856156.1|	52473-15337	11	38	0.300562	11	5	0.1355	no
gi|641571119|gb|KK856156.1|	53975-15420	11	38	0	81	inf	0.00015	yes
gi|641571119|gb|KK856156.1|	55497-15608	11	38	7	0	-inf	0.00115	yes
gi|641571119|gb|KK856156.1|	58356-15859	11	38	16	0	-inf	0.0075	no
gi|641571119|gb|KK856156.1|	61723-16293	11	38	2	0.953357	-0.747109	0.60335	no
gi|641571119|gb|KK856156.1|	66134-16630	11	38	49	0	-inf	0.0102	no
gi|641571119|gb|KK856156.1|	72782-17304	11	38	45	4	-3	0.10645	no
gi|641571119|gb|KK856156.1|	73315-17494	11	38	11	0.828085	-4	0.02275	no
gi|641571119|gb|KK856156.1|	75492-17813	11	38	1	2	0.531914	0.68575	no
gi|641571119|gb|KK856156.1|	84813-18545	11	38	69	7	-3	0.0205	no
gi|641571119|gb|KK856156.1|	86129-18845	11	38	2	0.202618	-3	0.08635	no
gi|641571119|gb|KK856156.1|	90462-19078	11	38	28	3	-3	0.0881	no
gi|641571119|gb|KK856156.1|	92130-19236	11	38	20	2	-3	0.2088	no
gi|641571119|gb|KK856156.1|	94127-19479	11	38	14	3	-2	0.14485	no

gi|641571119|gb|KK856156.1|	95490-19758	11	38	15	1	-3	0.0209	no
gi|641571119|gb|KK856156.1|	99039-20055	11	38	49	37	-0.400098	0.86105	no
gi|641571119|gb|KK856156.1|	02410-20622	11	38	7	8	0.232975	0.8494	no
gi|641571119|gb|KK856156.1|	08310-20862	11	38	3	5	0.569749	0.7575	no
gi|641571119|gb|KK856156.1|	21016-22645	11	38	0.642001	0.828085	0.367204	1	no
gi|641571119|gb|KK856156.1|	11826-21239	11	38	5	2	-1	0.41055	no
gi|641571119|gb|KK856156.1|	18823-21900	11	38	96	56	-0.776951	0.5891	no
gi|641571119|gb|KK856156.1|	25230-22558	11	38	3	4	0.164131	0.91775	no
gi|641571119|gb|KK856156.1|	29414-30250	11	38	21	10	-1	0.41535	no
gi|641571119|gb|KK856156.1|	33931-35087	11	38	1	1	-0.241813	0.867	no
gi|641571119|gb|KK856156.1|	35421-35653	11	38	25	29	0.223564	0.8691	no
gi|641571119|gb|KK856156.1|	36807-38378	11	38	5	8	0.707574	0.5773	no
gi|641571119|gb|KK856156.1|	46201-46901	11	38	2	0	-inf	0.0075	no
gi|641571119|gb|KK856156.1|	47067-47695	11	38	0.6542	2	2	0.3613	no
gi|641571119|gb|KK856156.1|	4778-5102	11	38	0	109	inf	5.00E-05	yes
gi|641571119|gb|KK856156.1|	51240-51497	11	38	4	16	2	0.2511	no
gi|641571119|gb|KK856156.1|	531-3035	11	38	48	54	0.167162	0.9366	no
gi|641571119|gb|KK856156.1|	57859-59204	11	38	9	11	0.237549	0.8508	no
gi|641571119|gb|KK856156.1|	5829-6145	11	38	0	100	inf	5.00E-05	yes
gi|641571119|gb|KK856156.1|	62980-65276	11	38	15	8	-0.904144	0.4708	no
gi|641571119|gb|KK856156.1|	65399-66693	11	38	11	4	-2	0.2428	no
gi|641571119|gb|KK856156.1|	6673-8181	11	38	2	2	0.500917	0.71175	no
gi|641571119|gb|KK856156.1|	67221-68083	11	38	3	0.322709	-3	0.1505	no
gi|641571119|gb|KK856156.1|	70069-70332	11	38	10	3	-2	0.31705	no
gi|641571119|gb|KK856156.1|	76789-77120	11	38	5	4	-0.425781	0.77915	no
gi|641571119|gb|KK856156.1|	8952-11079	11	38	26	15	-0.788713	0.6268	no
gi|641571119|gb|KK856156.1|	92818-93019	11	38	33	0	-inf	0.0056	no
gi|641571123|gb|KK856155.1|	01456-10206	10	42	3	2	-0.55397	0.70725	no
gi|641571123|gb|KK856155.1|	02287-10250	10	42	59	17	-2	0.23675	no
gi|641571123|gb|KK856155.1|	02778-10318	10	42	35	17	-1	0.4188	no

gi|641571123|gb|KK856155.1|	04515-10631	10	42	113.73	36	-2	0.45185	no
gi|641571123|gb|KK856155.1|	13286-11405	10	42	0.741764	1	0.602033	0.70555	no
gi|641571123|gb|KK856155.1|	15530-11594	10	42	7	0	-inf	0.0032	yes
gi|641571123|gb|KK856155.1|	38686-14014	10	42	5	0	-inf	0.0001	yes
gi|641571123|gb|KK856155.1|	40367-14494	10	42	10	0.24657	-5	0.0352	no
gi|641571123|gb|KK856155.1|	45145-14634	10	42	5	0.428325	-4	0.07165	no
gi|641571123|gb|KK856155.1|	48001-15201	10	42	9	0.113197	-6	0.06105	no
gi|641571123|gb|KK856155.1|	52124-15731	10	42	8	0.02164	-9	0.1864	no
gi|641571123|gb|KK856155.1|	57406-15781	10	42	5	0	-inf	0.0059	no
gi|641571123|gb|KK856155.1|	29390-29667	10	42	23	24	0.0466859	0.97345	no
gi|641571123|gb|KK856155.1|	30968-31565	10	42	1	1	0.334648	0.83525	no
gi|641571123|gb|KK856155.1|	60727-62052	10	42	0.564065	0.763718	0.437178	1	no
gi|641571123|gb|KK856155.1|	6195-6669	10	42	7	0.381166	-4	0.1889	no
gi|641571123|gb|KK856155.1|	67167-69522	10	42	0.786809	1	0.725075	0.59755	no
gi|641571123|gb|KK856155.1|	69631-70267	10	42	2	4	0.898527	0.54035	no
gi|641571123|gb|KK856155.1|	85482-86049	10	42	7	4	-0.625874	0.6518	no
gi|641571123|gb|KK856155.1|	88618-93358	10	42	3	4	0.55688	0.64855	no
gi|641571123|gb|KK856155.1|	93947-97582	10	42	2	3	0.424751	0.7393	no
gi|641571123|gb|KK856155.1|	98054-99345	10	42	4	2	-0.836665	0.52595	no
gi|641571127|gb|KK856154.1|	31625-13210	12	48	8	3	-1	0.31845	no
gi|641571127|gb|KK856154.1|	81843-82033	12	48	16	15	-0.06001	0.91115	no
gi|641571130|gb|KK856153.1|	10-335	10	36	19	7	-1	0.3057	no
gi|641571130|gb|KK856153.1|	10112-11210	10	36	0.157191	126	10	0.11675	no
gi|641571130|gb|KK856153.1|	12781-11362	10	36	2	2	-0.0748243	0.95395	no
gi|641571130|gb|KK856153.1|	15023-11524	10	36	21	19	-0.0967776	0.94755	no
gi|641571130|gb|KK856153.1|	11982-13147	10	36	1	1	-0.0311885	0.95655	no
gi|641571130|gb|KK856153.1|	20334-12073	10	36	31	20	-0.675207	0.59865	no
gi|641571130|gb|KK856153.1|	22682-12808	10	36	31	20	-0.638726	0.7633	no
gi|641571130|gb|KK856153.1|	28180-12967	10	36	5	36	3	0.145	no
gi|641571130|gb|KK856153.1|	29836-13023	10	36	3	2	-1	0.5278	no

gi|641571130|gb|KK856153.1|	30439-13144	10	36	0.614376	0.934461	0.605012	0.69715	no
gi|641571130|gb|KK856153.1|	31616-13287	10	36	0.399001	0.607623	0.606784	1	no
gi|641571130|gb|KK856153.1|	37087-14163	10	36	4	2	-0.771491	0.5396	no
gi|641571130|gb|KK856153.1|	14963-15887	10	36	0.38865	3	3	0.1746	no
gi|641571130|gb|KK856153.1|	50109-15110	10	36	3	18	3	0.06565	no
gi|641571130|gb|KK856153.1|	63257-16347	10	36	43	284	3	0.0554	no
gi|641571130|gb|KK856153.1|	64040-16483	10	36	2	6	1	0.32985	no
gi|641571130|gb|KK856153.1|	16907-18120	10	36	0.417427	14	5	0.0632	no
gi|641571130|gb|KK856153.1|	71036-17190	10	36	0.634334	2	2	0.3067	no
gi|641571130|gb|KK856153.1|	72331-17339	10	36	0	25	inf	5.00E-05	yes
gi|641571130|gb|KK856153.1|	84904-18667	10	36	0.49585	2	2	0.2143	no
gi|641571130|gb|KK856153.1|	19453-21314	10	36	0.552583	0.453749	-0.284299	1	no
gi|641571130|gb|KK856153.1|	23200-23834	10	36	2	7	2	0.16705	no
gi|641571130|gb|KK856153.1|	24130-29523	10	36	3	2	-0.224762	0.85965	no
gi|641571130|gb|KK856153.1|	29665-30205	10	36	5	3	-0.694192	0.6359	no
gi|641571130|gb|KK856153.1|	30326-30708	10	36	15	9	-0.739397	0.5799	no
gi|641571130|gb|KK856153.1|	31921-32815	10	36	14	8	-0.845424	0.51295	no
gi|641571130|gb|KK856153.1|	33292-33906	10	36	2	0.511695	-2	0.31075	no
gi|641571130|gb|KK856153.1|	34011-37686	10	36	1	2	0.780562	0.55205	no
gi|641571130|gb|KK856153.1|	37801-38442	10	36	42	18	-1	0.3452	no
gi|641571130|gb|KK856153.1|	38554-41300	10	36	18	2	-3	0.1215	no
gi|641571130|gb|KK856153.1|	41575-43588	10	36	1	0.712956	-0.935238	0.5065	no
gi|641571130|gb|KK856153.1|	43816-44660	10	36	2	1	-0.396766	0.7965	no
gi|641571130|gb|KK856153.1|	45242-48007	10	36	6	2	-1	0.27675	no
gi|641571130|gb|KK856153.1|	48177-51755	10	36	10	6	-0.720423	0.5567	no
gi|641571130|gb|KK856153.1|	4906-6033	10	36	3	1	-1	0.30895	no
gi|641571130|gb|KK856153.1|	54425-55517	10	36	2	1	-0.616646	0.6722	no
gi|641571130|gb|KK856153.1|	55634-56463	10	36	1	0.339406	-2	0.4266	no
gi|641571130|gb|KK856153.1|	65859-68228	10	36	2	0.944318	-1	0.4112	no
gi|641571130|gb|KK856153.1|	68501-70530	10	36	2	1	-1	0.4411	no

gi|641571130|gb|KK856153.1|	70815-72579	10	36	2	0.757008	-1	0.45445	no
gi|641571130|gb|KK856153.1|	72708-74522	10	36	0.874812	0.600287	-0.54332	1	no
gi|641571130|gb|KK856153.1|	75030-77000	10	36	1	1	0.0881719	0.94635	no
gi|641571130|gb|KK856153.1|	77523-79976	10	36	3	3	-0.0154348	0.9905	no
gi|641571130|gb|KK856153.1|	81687-84097	10	36	12	1	-3	0.0194	no
gi|641571130|gb|KK856153.1|	87052-87260	10	36	27	13	-1	0.5041	no
gi|641571130|gb|KK856153.1|	88367-88784	10	36	9	8	-0.0807897	0.9549	no
gi|641571130|gb|KK856153.1|	97405-99616	10	36	26	13	-1	0.52085	no
gi|641571134|gb|KK856152.1|	04539-10555	7.6	34	0.429783	0.784518	0.868198	1	no
gi|641571134|gb|KK856152.1|	17397-18534	7.6	34	28	35	0.314829	0.79725	no
gi|641571134|gb|KK856152.1|	19195-21025	7.6	34	22	15	-0.591219	0.7154	no
gi|641571134|gb|KK856152.1|	22315-24617	7.6	34	9	8	-0.220546	0.8618	no
gi|641571134|gb|KK856152.1|	30076-30268	7.6	34	3	51	4	0.1141	no
gi|641571134|gb|KK856152.1|	3865-4025	7.6	34	63	98	0.646073	0.6721	no
gi|641571134|gb|KK856152.1|	48726-48895	7.6	34	14	138	3	0.1512	no
gi|641571134|gb|KK856152.1|	6707-7239	7.6	34	15	18	0.252532	0.84355	no
gi|641571134|gb|KK856152.1|	8866-9214	7.6	34	0.449353	80	7	0.10435	no
gi|641571138|gb|KK856151.1|	32150-13423	10	45	90	94	0.0716521	0.97315	no
gi|641571138|gb|KK856151.1|	35435-13631	10	45	0.926597	3	2	0.2843	no
gi|641571138|gb|KK856151.1|	54075-15530	10	45	0	72	inf	0.00015	yes
gi|641571138|gb|KK856151.1|	71233-17251	10	45	116	64	-0.861544	0.69255	no
gi|641571138|gb|KK856151.1|	82009-18357	10	45	60	51	-0.227673	0.9206	no
gi|641571138|gb|KK856151.1|	83692-18396	10	45	20.74	9	-1	0.42075	no
gi|641571138|gb|KK856151.1|	84190-18463	10	45	26	25	-0.0450542	0.9727	no
gi|641571138|gb|KK856151.1|	41220-41457	10	45	2	24	3	0.25585	no
gi|641571141|gb|KK856150.1|	69355-69720	9.5	34	5	3	-0.682933	0.6692	no
gi|641571141|gb|KK856150.1|	81019-81301	9.5	34	7	5	-0.285323	0.8649	no
gi|641571141|gb|KK856150.1|	83409-84116	9.5	34	3	1	-1	0.48275	no
gi|641571141|gb|KK856150.1|	85041-86044	9.5	34	13	8	-0.663913	0.6028	no
gi|641571141|gb|KK856150.1|	87992-88292	9.5	34	7	6	-0.210234	0.8895	no

gi|641571144|gb|KK856149.1|	10346-11061	11	20	10	6	-0.704789	0.6649	no
gi|641571144|gb|KK856149.1|	11314-13628	11	20	5	4	-0.410355	0.7491	no
gi|641571144|gb|KK856149.1|	20601-12105	11	20	3	1	-1	0.50365	no
gi|641571144|gb|KK856149.1|	33750-13403	11	20	22	12	-0.805468	0.56945	no
gi|641571144|gb|KK856149.1|	38991-13941	11	20	3	0.453451	-3	0.2481	no
gi|641571144|gb|KK856149.1|	14782-16701	11	20	11	9	-0.254075	0.8397	no
gi|641571144|gb|KK856149.1|	17500-18443	11	20	4	0.575882	-3	0.10835	no
gi|641571144|gb|KK856149.1|	75055-17600	11	20	6	5	-0.243529	0.8483	no
gi|641571144|gb|KK856149.1|	19197-19380	11	20	115	0	-inf	0.00045	yes
gi|641571144|gb|KK856149.1|	20032-22228	11	20	12	0	-inf	5.00E-05	yes
gi|641571144|gb|KK856149.1|	22956-24323	11	20	2	0.276164	-3	0.1224	no
gi|641571144|gb|KK856149.1|	25564-27195	11	20	45	0.37588	-7	0.0525	no
gi|641571144|gb|KK856149.1|	30794-30984	11	20	455	0	-inf	5.00E-05	yes
gi|641571144|gb|KK856149.1|	314-540	11	20	7	20	2	0.3807	no
gi|641571144|gb|KK856149.1|	33821-38339	11	20	2	0.12508	-4	0.0526	no
gi|641571144|gb|KK856149.1|	38479-38895	11	20	18	0	-inf	5.00E-05	yes
gi|641571144|gb|KK856149.1|	39158-39668	11	20	6	0	-inf	0.0022	yes
gi|641571144|gb|KK856149.1|	4242-4457	11	20	20	2	-3	0.23065	no
gi|641571144|gb|KK856149.1|	42641-44658	11	20	6	0.059283	-7	0.18285	no
gi|641571144|gb|KK856149.1|	49197-49699	11	20	54	0	-inf	5.00E-05	yes
gi|641571144|gb|KK856149.1|	49816-50075	11	20	297	0	-inf	5.00E-05	yes
gi|641571144|gb|KK856149.1|	53304-53675	11	20	123	0.590295	-8	0.18285	no
gi|641571144|gb|KK856149.1|	58335-58669	11	20	8	0	-inf	0.00435	no
gi|641571144|gb|KK856149.1|	59014-60212	11	20	0.917956	0	-inf	0.0075	no
gi|641571144|gb|KK856149.1|	60642-61222	11	20	23	0	-inf	5.00E-05	yes
gi|641571144|gb|KK856149.1|	64508-65945	11	20	12	0.434412	-5	0.0432	no
gi|641571144|gb|KK856149.1|	67280-69044	11	20	9	0.068819	-7	0.1829	no
gi|641571144|gb|KK856149.1|	69883-70602	11	20	54	0	-inf	5.00E-05	yes
gi|641571144|gb|KK856149.1|	71447-71980	11	20	24	0	-inf	5.00E-05	yes
gi|641571144|gb|KK856149.1|	73872-74073	11	20	274	0	-inf	5.00E-05	yes

gi|641571144|gb|KK856149.1|	74572-74834	11	20	270	0	-inf	5.00E-05	yes
gi|641571144|gb|KK856149.1|	76535-77176	11	20	4	0	-inf	0.0013	yes
gi|641571144|gb|KK856149.1|	77593-77773	11	20	57	0	-inf	0.00435	no
gi|641571144|gb|KK856149.1|	78467-78664	11	20	118	0	-inf	0.00055	yes
gi|641571144|gb|KK856149.1|	90368-90533	11	20	142	0	-inf	0.00185	yes
gi|641571144|gb|KK856149.1|	93458-93692	11	20	18	0	-inf	0.0059	no
gi|641571144|gb|KK856149.1|	9688-10105	11	20	3	3	0.0720618	0.94125	no
gi|641571147|gb|KK856148.1|	22519-23477	11	42	0.928312	0.564617	-0.717337	0.6701	no
gi|641571147|gb|KK856148.1|	27096-28001	11	42	0.498819	1	1	0.4595	no
gi|641571147|gb|KK856148.1|	40641-41146	11	42	2	0.687995	-1	0.4534	no
gi|641571147|gb|KK856148.1|	41565-42113	11	42	1	1	0.0107264	0.91115	no
gi|641571147|gb|KK856148.1|	58856-59828	11	42	0.182316	65	8	0.2353	no
gi|641571147|gb|KK856148.1|	65529-65795	11	42	44	14	-2	0.2438	no
gi|641571147|gb|KK856148.1|	76695-77007	11	42	6	0	-inf	0.0102	no
gi|641571147|gb|KK856148.1|	77903-80030	11	42	22	5	-2	0.24735	no
gi|641571154|gb|KK856146.1|	1-1458	9.1	35	2	7	2	0.1835	no
gi|641571154|gb|KK856146.1|	01114-10205	9.1	35	8	0.144547	-6	0.1833	no
gi|641571154|gb|KK856146.1|	09594-11056	9.1	35	0.732091	1	0.926629	0.56225	no
gi|641571154|gb|KK856146.1|	10801-11174	9.1	35	1	1	-0.302413	0.84515	no
gi|641571154|gb|KK856146.1|	17405-11866	9.1	35	0.801011	0.914858	0.191726	0.90355	no
gi|641571154|gb|KK856146.1|	22360-23179	9.1	35	1	174	7	0.0198	no
gi|641571154|gb|KK856146.1|	3058-3622	9.1	35	0	6	inf	0.0005	yes
gi|641571154|gb|KK856146.1|	32898-35707	9.1	35	0.081268	8	7	0.1346	no
gi|641571154|gb|KK856146.1|	36340-37096	9.1	35	0.126272	4	5	0.10875	no
gi|641571154|gb|KK856146.1|	39629-40378	9.1	35	0.255734	49	8	0.2322	no
gi|641571154|gb|KK856146.1|	49447-50064	9.1	35	0	12	inf	0.0001	yes
gi|641571154|gb|KK856146.1|	61586-62512	9.1	35	2	0.736507	-1	0.43755	no
gi|641571154|gb|KK856146.1|	66996-67976	9.1	35	92	56	-0.714303	0.7512	no
gi|641571154|gb|KK856146.1|	68806-69771	9.1	35	18	13	-0.449206	0.719	no
gi|641571154|gb|KK856146.1|	73131-75008	9.1	35	6	4	-0.688446	0.59445	no

gi|641571154|gb|KK856146.1|	75602-77796	9.1	35	11	7	-0.753193	0.5422	no
gi|641571154|gb|KK856146.1|	77939-78822	9.1	35	4	1	-2	0.24585	no
gi|641571154|gb|KK856146.1|	81095-81552	9.1	35	9	6	-0.454166	0.7418	no
gi|641571154|gb|KK856146.1|	82199-82627	9.1	35	22	14	-0.628007	0.63035	no
gi|641571154|gb|KK856146.1|	8241-8716	9.1	35	1	40	5	0.1163	no
gi|641571154|gb|KK856146.1|	82734-83564	9.1	35	15	10	-0.53447	0.667	no
gi|641571154|gb|KK856146.1|	85451-87176	9.1	35	2	2	-0.280498	0.82865	no
gi|641571154|gb|KK856146.1|	87808-88103	9.1	35	43	16	-1	0.3076	no
gi|641571154|gb|KK856146.1|	90351-91376	9.1	35	9	3	-2	0.1959	no
gi|641571154|gb|KK856146.1|	91776-93985	9.1	35	15	7	-1	0.39925	no
gi|641571154|gb|KK856146.1|	95201-96195	9.1	35	12	6	-0.926781	0.47105	no
gi|641571154|gb|KK856146.1|	98230-98810	9.1	35	5	3	-0.987918	0.5088	no
gi|641571162|gb|KK856144.1|	25011-25720	9.2	41	0.826879	4	2	0.20405	no
gi|641571162|gb|KK856144.1|	26112-26527	9.2	41	1	4	2	0.33255	no
gi|641571162|gb|KK856144.1|	2808-3512	9.2	41	0.834996	2	1	0.4118	no
gi|641571162|gb|KK856144.1|	29856-31007	9.2	41	0.81493	2	1	0.41005	no
gi|641571162|gb|KK856144.1|	528-885	9.2	41	7	8	0.16381	0.9092	no
gi|641571162|gb|KK856144.1|	8177-8815	9.2	41	5	3	-0.673992	0.6342	no
gi|641571162|gb|KK856144.1|	86631-87121	9.2	41	0	4	inf	0.00455	no
gi|641571169|gb|KK856142.1|	10378-11108	9.6	42	7	1	-3	0.12385	no
gi|641571169|gb|KK856142.1|	12713-11298	9.6	42	25	6	-2	0.19265	no
gi|641571169|gb|KK856142.1|	13688-11609	9.6	42	1	0.929802	-0.271698	0.84005	no
gi|641571169|gb|KK856142.1|	33539-13446	9.6	42	0.68201	0.740538	0.118781	1	no
gi|641571169|gb|KK856142.1|	35584-13680	9.6	42	0.760056	2	1	0.3908	no
gi|641571169|gb|KK856142.1|	38734-13931	9.6	42	1	0.830098	-0.817915	0.6492	no
gi|641571169|gb|KK856142.1|	39463-14171	9.6	42	0.551345	0.9989	0.857386	0.5529	no
gi|641571169|gb|KK856142.1|	48239-14914	9.6	42	15	3	-2	0.0791	no
gi|641571169|gb|KK856142.1|	53952-15435	9.6	42	15	1	-3	0.11395	no
gi|641571169|gb|KK856142.1|	62524-16839	9.6	42	6	6	-0.0167355	0.99345	no
gi|641571169|gb|KK856142.1|	71057-17156	9.6	42	16	22	0.426356	0.73915	no

gi|641571169|gb|KK856142.1|	73086-17328	9.6	42	13	27	1	0.4899	no
gi|641571169|gb|KK856142.1|	73499-17389	9.6	42	7	7	-0.00102203	0.9697	no
gi|641571169|gb|KK856142.1|	56943-58102	9.6	42	0	11	inf	5.00E-05	yes
gi|641571169|gb|KK856142.1|	65230-65810	9.6	42	0	42	inf	5.00E-05	yes
gi|641571169|gb|KK856142.1|	67610-68631	9.6	42	0	26	inf	5.00E-05	yes
gi|641571169|gb|KK856142.1|	78288-81449	9.6	42	42	15	-1	0.51115	no
gi|641571169|gb|KK856142.1|	84034-84372	9.6	42	17	14	-0.272439	0.83565	no
gi|641571169|gb|KK856142.1|	84895-85198	9.6	42	7	5	-0.431895	0.77855	no
gi|641571169|gb|KK856142.1|	86485-86924	9.6	42	24	7	-2	0.181	no
gi|641571169|gb|KK856142.1|	91801-92164	9.6	42	14	4	-2	0.22165	no
gi|641571169|gb|KK856142.1|	94160-94720	9.6	42	5	0.293107	-4	0.194	no
gi|641571176|gb|KK856140.1|	01830-10399	9.9	38	12	10	-0.336993	0.78575	no
gi|641571176|gb|KK856140.1|	04214-10578	9.9	38	4	2	-1	0.43585	no
gi|641571176|gb|KK856140.1|	05917-10747	9.9	38	2	1	-0.391993	0.77885	no
gi|641571176|gb|KK856140.1|	07546-10908	9.9	38	2	0.642936	-1	0.35235	no
gi|641571176|gb|KK856140.1|	17506-11787	9.9	38	0.407638	49	7	0.1045	no
gi|641571176|gb|KK856140.1|	21839-12229	9.9	38	0	27	inf	5.00E-05	yes
gi|641571176|gb|KK856140.1|	12251-13049	9.9	38	1	0.891863	-0.660493	0.6826	no
gi|641571176|gb|KK856140.1|	29463-12970	9.9	38	0	58.86	inf	5.00E-05	yes
gi|641571176|gb|KK856140.1|	14538-16560	9.9	38	3	2	-0.825838	0.5332	no
gi|641571176|gb|KK856140.1|	17401-18980	9.9	38	4	2	-1	0.364	no
gi|641571176|gb|KK856140.1|	19353-20507	9.9	38	0.960085	0.224839	-2	0.26805	no
gi|641571176|gb|KK856140.1|	21703-23723	9.9	38	1	1	0.325302	0.80595	no
gi|641571176|gb|KK856140.1|	24073-24535	9.9	38	3	0.795513	-2	0.3216	no
gi|641571176|gb|KK856140.1|	3096-3644	9.9	38	9	2	-3	0.11815	no
gi|641571176|gb|KK856140.1|	357-1432	9.9	38	0.563394	0.73481	0.383227	1	no
gi|641571176|gb|KK856140.1|	46698-47267	9.9	38	24	27	0.169976	0.88945	no
gi|641571176|gb|KK856140.1|	62160-62371	9.9	38	110	156	0.512788	0.688	no
gi|641571176|gb|KK856140.1|	65180-65444	9.9	38	9	20	1	0.41145	no
gi|641571176|gb|KK856140.1|	68471-68970	9.9	38	0.232432	4	4	0.1175	no

gi|641571176|gb|KK856140.1|	6851-8870	9.9	38	0.698828	1	0.609094	0.6709	no
gi|641571176|gb|KK856140.1|	69418-71545	9.9	38	0.769717	2	1	0.3675	no
gi|641571176|gb|KK856140.1|	73891-74183	9.9	38	51	105.59	1	0.41375	no
gi|641571176|gb|KK856140.1|	82232-83045	9.9	38	0.573111	1	0.865813	0.62725	no
gi|641571176|gb|KK856140.1|	84726-85419	9.9	38	0.99566	2	1	0.4891	no
gi|641571176|gb|KK856140.1|	8994-9838	9.9	38	5	0.994822	-2	0.14005	no
gi|641571176|gb|KK856140.1|	91878-93333	9.9	38	0.449648	1	1	0.4562	no
gi|641571180|gb|KK856139.1|	.05513-10709	10	38	2	2	0.0872401	0.94955	no
gi|641571180|gb|KK856139.1|	.09844-11128	10	38	1	0.260438	-2	0.1911	no
gi|641571180|gb|KK856139.1|	.11620-11244	10	38	2	3	0.515482	0.7188	no
gi|641571180|gb|KK856139.1|	.12552-11354	10	38	2	2	-0.149957	0.9166	no
gi|641571180|gb|KK856139.1|	.13936-11468	10	38	1	0.977186	-0.535896	0.7478	no
gi|641571180|gb|KK856139.1|	.15325-11631	10	38	0.537944	2	2	0.32035	no
gi|641571180|gb|KK856139.1|	.16422-12025	10	38	0.993469	0.297458	-2	0.2304	no
gi|641571180|gb|KK856139.1|	.20624-12305	10	38	2	1	-0.670416	0.6125	no
gi|641571180|gb|KK856139.1|	.23778-12684	10	38	15.01	9	-0.721157	0.6504	no
gi|641571180|gb|KK856139.1|	.36312-13689	10	38	1	0.836486	-0.625387	0.71195	no
gi|641571180|gb|KK856139.1|	.45244-14658	10	38	2	2	0.366173	0.78945	no
gi|641571180|gb|KK856139.1|	.52591-15295	10	38	1	4	2	0.3454	no
gi|641571180|gb|KK856139.1|	.59287-16007	10	38	0.717952	1	0.60236	0.70555	no
gi|641571180|gb|KK856139.1|	.67139-16778	10	38	2	0	-inf	0.0075	no
gi|641571180|gb|KK856139.1|	.72266-17281	10	38	2	1	-0.404357	0.82205	no
gi|641571180|gb|KK856139.1|	.73605-17409	10	38	0	10	inf	0.00035	yes
gi|641571180|gb|KK856139.1|	.09326-20995	10	38	0.3271	8	5	0.2343	no
gi|641571180|gb|KK856139.1|	51963-52200	10	38	0	26	inf	0.0023	yes
gi|641571180|gb|KK856139.1|	63162-63862	10	38	2	4	0.600656	0.67045	no
gi|641571180|gb|KK856139.1|	63987-65823	10	38	0.819928	2	0.945767	0.49725	no
gi|641571180|gb|KK856139.1|	72017-72358	10	38	4	7	0.898037	0.56955	no
gi|641571180|gb|KK856139.1|	74160-74338	10	38	25	25	0.0330782	0.92445	no
gi|641571184|gb|KK856138.1|	.05376-10581	9.9	38	4	2	-1	0.4657	no

gi|641571184|gb|KK856138.1|	24434-12494	9.9	38	0	10	inf	0.0004	yes
gi|641571184|gb|KK856138.1|	42267-14291	9.9	38	0.315471	3	3	0.2556	no
gi|641571184|gb|KK856138.1|	43189-14445	9.9	38	0.330946	102	8	0.0839	no
gi|641571184|gb|KK856138.1|	45455-14676	9.9	38	0.06346	591	13	0.1045	no
gi|641571184|gb|KK856138.1|	47162-14867	9.9	38	0.268661	48	7	0.0859	no
gi|641571184|gb|KK856138.1|	14936-15094	9.9	38	54	29	-0.907559	0.6337	no
gi|641571184|gb|KK856138.1|	50166-15137	9.9	38	0.070053	3	5	0.10635	no
gi|641571184|gb|KK856138.1|	51627-15276	9.9	38	0	195	inf	5.00E-05	yes
gi|641571184|gb|KK856138.1|	18997-20606	9.9	38	35	22	-0.65117	0.68735	no
gi|641571184|gb|KK856138.1|	20990-23313	9.9	38	9	5	-0.698825	0.58095	no
gi|641571184|gb|KK856138.1|	2844-3816	9.9	38	65	6	-3	0.0924	no
gi|641571184|gb|KK856138.1|	39641-40250	9.9	38	1	0.517795	-1	0.4534	no
gi|641571184|gb|KK856138.1|	4013-4310	9.9	38	255	21	-4	0.01515	no
gi|641571184|gb|KK856138.1|	45983-47045	9.9	38	2	2	0.108019	0.93655	no
gi|641571184|gb|KK856138.1|	56-487	9.9	38	12	0	-inf	0.00095	yes
gi|641571184|gb|KK856138.1|	57490-58111	9.9	38	1	0.755087	-0.623893	0.71195	no
gi|641571184|gb|KK856138.1|	59040-59328	9.9	38	8	2	-2	0.27505	no
gi|641571187|gb|KK856137.1|	11753-12635	8.4	35	0.309315	1	2	0.2812	no
gi|641571187|gb|KK856137.1|	62854-64317	8.4	35	0.726068	0.765956	0.0771577	1	no
gi|641571187|gb|KK856137.1|	73159-73902	8.4	35	0.7756	1	0.823962	0.6232	no
gi|641571198|gb|KK856134.1|	10044-11072	8.5	33	0.582982	2	2	0.3012	no
gi|641571198|gb|KK856134.1|	48543-14928	8.5	33	0.904867	3	1	0.36635	no
gi|641571198|gb|KK856134.1|	1613-3812	8.5	33	0.883403	0.269545	-2	0.2894	no
gi|641571198|gb|KK856134.1|	64800-16498	8.5	33	40	17	-1	0.4415	no
gi|641571198|gb|KK856134.1|	65686-16669	8.5	33	0.60984	0.397541	-0.617326	1	no
gi|641571198|gb|KK856134.1|	19061-19411	8.5	33	78	0	-inf	5.00E-05	yes
gi|641571198|gb|KK856134.1|	19532-21235	8.5	33	2	0	-inf	0.00095	yes
gi|641571198|gb|KK856134.1|	21439-23271	8.5	33	0.605634	0	-inf	1	no
gi|641571198|gb|KK856134.1|	23927-24089	8.5	33	268.72	0	-inf	0.00035	yes
gi|641571198|gb|KK856134.1|	25388-27266	8.5	33	2	0.449176	-2	0.2367	no

gi|641571198|gb|KK856134.1|	29135-29643	8.5	33	3	0.340776	-3	0.21435	no
gi|641571198|gb|KK856134.1|	30331-30704	8.5	33	5	0	-inf	0.0075	no
gi|641571198|gb|KK856134.1|	31993-32591	8.5	33	4	0	-inf	0.00345	yes
gi|641571198|gb|KK856134.1|	34787-36788	8.5	33	1	0.179421	-3	0.1554	no
gi|641571198|gb|KK856134.1|	36911-37782	8.5	33	1	0.159219	-3	0.2348	no
gi|641571198|gb|KK856134.1|	4007-5822	8.5	33	0	22	inf	5.00E-05	yes
gi|641571198|gb|KK856134.1|	46530-46708	8.5	33	0	81	inf	0.0015	yes
gi|641571198|gb|KK856134.1|	47506-49028	8.5	33	0	5	inf	5.00E-05	yes
gi|641571198|gb|KK856134.1|	65391-66233	8.5	33	0.218965	2	3	0.25475	no
gi|641571198|gb|KK856134.1|	66343-67201	8.5	33	0.427356	2	2	0.23615	no
gi|641571198|gb|KK856134.1|	67568-68617	8.5	33	0.165872	1	3	0.2991	no
gi|641571198|gb|KK856134.1|	68723-69260	8.5	33	1	2	0.817563	0.6234	no
gi|641571198|gb|KK856134.1|	73447-73726	8.5	33	10	6	-0.923595	0.5719	no
gi|641571202|gb|KK856133.1|	40097-14064	10	42	1	2	0.373158	0.8276	no
gi|641571207|gb|KK856132.1|	05779-10692	10	40	0.890879	0.678039	-0.393861	0.80865	no
gi|641571207|gb|KK856132.1|	11794-14223	10	40	6	5	-0.0927674	0.9434	no
gi|641571207|gb|KK856132.1|	24824-12511	10	40	9	3	-2	0.33815	no
gi|641571207|gb|KK856132.1|	25992-12626	10	40	22	3	-3	0.15635	no
gi|641571207|gb|KK856132.1|	30758-13100	10	40	13	5	-2	0.4022	no
gi|641571207|gb|KK856132.1|	38117-13855	10	40	4	0.881559	-2	0.31075	no
gi|641571207|gb|KK856132.1|	40715-14184	10	40	1	0.114804	-3	0.2089	no
gi|641571207|gb|KK856132.1|	42656-14448	10	40	1	0	-inf	0.0013	yes
gi|641571207|gb|KK856132.1|	14351-15100	10	40	1	0.388073	-2	0.3217	no
gi|641571207|gb|KK856132.1|	44643-14625	10	40	1	0.076235	-4	0.19145	no
gi|641571207|gb|KK856132.1|	46355-14833	10	40	0.99014	0.362446	-1	0.3532	no
gi|641571207|gb|KK856132.1|	49701-15442	10	40	13	6	-1	0.4972	no
gi|641571207|gb|KK856132.1|	16914-19607	10	40	0.822072	0.995081	0.27555	0.8356	no
gi|641571207|gb|KK856132.1|	25449-25825	10	40	8	6	-0.48868	0.72795	no
gi|641571207|gb|KK856132.1|	27406-29593	10	40	0.888726	1	0.424942	0.75445	no
gi|641571207|gb|KK856132.1|	376-1624	10	40	0.067258	282	12	0.1044	no

gi|641571207|gb|KK856132.1|	6925-8832	10	40	0.289549	11	5	0.05195	no
gi|641571207|gb|KK856132.1|	75962-76988	10	40	0.681858	0.518626	-0.394776	1	no
gi|641571210|gb|KK856131.1|	01382-10190	9.5	40	1	2	0.817064	0.6234	no
gi|641571213|gb|KK856130.1|	36593-13693	10	43	113	148	0.391507	0.7531	no
gi|641571213|gb|KK856130.1|	22003-22478	10	43	1	2	0.591557	0.70505	no
gi|641571213|gb|KK856130.1|	22593-23297	10	43	0.974162	1	0.600748	0.69715	no
gi|641571216|gb|KK856129.1|	14885-11598	12	4.10E+01	2	0.237155	-3	0.1718	no
gi|641571216|gb|KK856129.1|	24906-12539	12	4.10E+01	2	1	-0.822804	0.6492	no
gi|641571216|gb|KK856129.1|	26560-12699	12	4.10E+01	16	10	-0.669156	0.6095	no
gi|641571216|gb|KK856129.1|	36830-37548	12	4.10E+01	13	5	-2	0.2585	no
gi|641571216|gb|KK856129.1|	41192-41472	12	4.10E+01	21	3	-3	0.15635	no
gi|641571216|gb|KK856129.1|	4279-4736	12	4.10E+01	4	2	-1	0.44155	no
gi|641571216|gb|KK856129.1|	50145-51857	12	4.10E+01	1	0.640548	-1	0.4349	no
gi|641571216|gb|KK856129.1|	58324-59992	12	4.10E+01	1	1	0.122894	0.93035	no
gi|641571216|gb|KK856129.1|	60860-61685	12	4.10E+01	2	2	-0.0185286	0.96665	no
gi|641571216|gb|KK856129.1|	61913-62839	12	4.10E+01	3	2	-0.189297	0.89425	no
gi|641571216|gb|KK856129.1|	63868-64253	12	4.10E+01	8	5	-0.770709	0.5961	no
gi|641571216|gb|KK856129.1|	64978-65430	12	4.10E+01	5	2	-0.995133	0.5501	no
gi|641571216|gb|KK856129.1|	69863-71476	12	4.10E+01	34	25	-0.489278	0.7616	no
gi|641571224|gb|KK856127.1|	22598-12353	10	21	1	1	-0.0477584	0.94545	no
gi|641571224|gb|KK856127.1|	23726-12479	10	21	1	1	0.490057	0.7378	no
gi|641571224|gb|KK856127.1|	24320-24777	10	21	3	4	0.727732	0.6396	no
gi|641571224|gb|KK856127.1|	45566-48280	10	21	12	11	-0.195717	0.8749	no
gi|641571224|gb|KK856127.1|	49128-49677	10	21	2	2	0.0811614	0.9406	no
gi|641571224|gb|KK856127.1|	57257-57634	10	21	10	6	-0.713607	0.6179	no
gi|641571224|gb|KK856127.1|	70375-70851	10	21	9	4	-1	0.3971	no
gi|641571224|gb|KK856127.1|	77880-78221	10	21	9	0	-inf	0.00345	yes
gi|641571224|gb|KK856127.1|	87966-89609	10	21	0.733607	2	1	0.35035	no
gi|641571224|gb|KK856127.1|	90984-92192	10	21	0.769062	2	1	0.44465	no
gi|641571224|gb|KK856127.1|	92320-92876	10	21	1	1	0.333014	0.83525	no

gi|641571228|gb|KK856126.1|	10334-10553	12	43	6	31	2	0.2169	no
gi|641571228|gb|KK856126.1|	10988-11793	12	43	2	5	2	0.2392	no
gi|641571228|gb|KK856126.1|	19792-12086	12	43	0.321574	2	2	0.23615	no
gi|641571228|gb|KK856126.1|	12045-14979	12	43	2	3	0.81371	0.5247	no
gi|641571228|gb|KK856126.1|	17695-21643	12	43	1	2	0.783597	0.54595	no
gi|641571228|gb|KK856126.1|	21920-22734	12	43	0.801059	2	1	0.44	no
gi|641571228|gb|KK856126.1|	22987-24322	12	43	38	44	0.211592	0.92525	no
gi|641571228|gb|KK856126.1|	25135-25840	12	43	2	0.421271	-2	0.31075	no
gi|641571228|gb|KK856126.1|	32025-32283	12	43	0	330	inf	5.00E-05	yes
gi|641571228|gb|KK856126.1|	33417-34161	12	43	0.258063	30	7	0.2322	no
gi|641571228|gb|KK856126.1|	34887-35084	12	43	0	829	inf	5.00E-05	yes
gi|641571228|gb|KK856126.1|	40527-40773	12	43	0	155	inf	5.00E-05	yes
gi|641571228|gb|KK856126.1|	43943-44172	12	43	0	111	inf	5.00E-05	yes
gi|641571228|gb|KK856126.1|	46382-46684	12	43	0	67	inf	5.00E-05	yes
gi|641571228|gb|KK856126.1|	50544-51259	12	43	0.272448	154	9	0.2333	no
gi|641571228|gb|KK856126.1|	96169-97480	12	43	0.317302	2	2	0.18135	no
gi|641571231|gb|KK856125.1|	04470-10920	25	43	8	3	-1	0.38695	no
gi|641571231|gb|KK856125.1|	11239-11805	25	43	4	0.179724	-4	0.014	no
gi|641571231|gb|KK856125.1|	18617-11937	25	43	2	0.769255	-1	0.37245	no
gi|641571231|gb|KK856125.1|	20778-12184	25	43	1	0.248308	-2	0.21865	no
gi|641571231|gb|KK856125.1|	28106-12835	25	43	8	7	-0.125524	0.9229	no
gi|641571231|gb|KK856125.1|	61822-62149	25	43	18	23	0.310385	0.81235	no
gi|641571231|gb|KK856125.1|	64334-64563	25	43	107	136	0.354115	0.77975	no
gi|641571231|gb|KK856125.1|	66112-66476	25	43	23	35	0.579948	0.65675	no
gi|641571231|gb|KK856125.1|	67366-67762	25	43	5	0	-inf	0.0059	no
gi|641571231|gb|KK856125.1|	70714-71747	25	43	12	17	0.510126	0.6836	no
gi|641571231|gb|KK856125.1|	72365-72712	25	43	42	57	0.463002	0.7181	no
gi|641571231|gb|KK856125.1|	73367-73989	25	43	1	1	0.113105	0.92265	no
gi|641571231|gb|KK856125.1|	75027-76211	25	43	5	9	0.961839	0.4624	no
gi|641571231|gb|KK856125.1|	77301-77988	25	43	20	29	0.558223	0.66145	no

gi|641571231|gb|KK856125.1|	79644-80511	25	43	23	40	0.769131	0.5358	no
gi|641571231|gb|KK856125.1|	80944-81906	25	43	0.831122	2	0.894138	0.5684	no
gi|641571231|gb|KK856125.1|	83436-83825	25	43	3	4	0.390797	0.8091	no
gi|641571231|gb|KK856125.1|	84065-85172	25	43	3	4	0.283926	0.8301	no
gi|641571231|gb|KK856125.1|	98272-99327	25	43	1418.19	0	-inf	5.00E-05	yes
gi|641571231|gb|KK856125.1|	99962-103977	25	43	80	0	-inf	5.00E-05	yes
gi|641571235|gb|KK856124.1|	137348-13801	11	39	0.299492	2	3	0.2851	no
gi|641571235|gb|KK856124.1|	138626-13944	11	39	0.456273	1	1	0.44255	no
gi|641571235|gb|KK856124.1|	139622-14067	11	39	0.577845	3	2	0.17115	no
gi|641571235|gb|KK856124.1|	140776-14116	11	39	3	3	0.360536	0.83635	no
gi|641571235|gb|KK856124.1|	141295-14251	11	39	0.552768	3	2	0.1833	no
gi|641571235|gb|KK856124.1|	143887-14491	11	39	0.678591	1	0.775183	0.62605	no
gi|641571235|gb|KK856124.1|	153182-15377	11	39	1	1	0.334392	0.83525	no
gi|641571235|gb|KK856124.1|	155558-15801	11	39	0.687803	3	2	0.1456	no
gi|641571235|gb|KK856124.1|	158088-15867	11	39	0.893892	3	2	0.32945	no
gi|641571235|gb|KK856124.1|	159070-16077	11	39	0.514862	0.856316	0.733958	1	no
gi|641571235|gb|KK856124.1|	160848-16177	11	39	0.968966	3	1	0.36455	no
gi|641571235|gb|KK856124.1|	162280-16338	11	39	4	7	0.940238	0.4708	no
gi|641571235|gb|KK856124.1|	170724-17277	11	39	0.649323	0.757402	0.222123	1	no
gi|641571235|gb|KK856124.1|	17296-18724	11	39	1	2	0.60754	0.66165	no
gi|641571235|gb|KK856124.1|	173849-17439	11	39	2	0.61089	-1	0.4534	no
gi|641571235|gb|KK856124.1|	175658-17668	11	39	0.678591	1	0.775183	0.62605	no
gi|641571235|gb|KK856124.1|	176860-17795	11	39	0.627473	0.477448	-0.39421	1	no
gi|641571235|gb|KK856124.1|	178792-18678	11	39	25	46	0.898443	0.64845	no
gi|641571235|gb|KK856124.1|	186927-18808	11	39	28	52	0.878021	0.5885	no
gi|641571235|gb|KK856124.1|	188382-18995	11	39	89	155	0.798706	0.70155	no
gi|641571235|gb|KK856124.1|	18938-22151	11	39	13	20	0.643544	0.77805	no
gi|641571235|gb|KK856124.1|	191068-19197	11	39	51	95	0.892213	0.69915	no
gi|641571235|gb|KK856124.1|	34887-35228	11	39	4	5	0.213539	0.8957	no
gi|641571235|gb|KK856124.1|	373-750	11	39	50	26	-0.948666	0.46085	no

gi|641571235|gb|KK856124.1|	41247-41655	11	39	0	24	inf	0.00025	yes
gi|641571235|gb|KK856124.1|	47738-48242	11	39	0	10	inf	0.0004	yes
gi|641571235|gb|KK856124.1|	74-272	11	39	22	35	0.666729	0.6626	no
gi|641571235|gb|KK856124.1|	86834-89263	11	39	1	4	1	0.28015	no
gi|641571235|gb|KK856124.1|	89479-91010	11	39	1	3	1	0.4555	no
gi|641571235|gb|KK856124.1|	92920-94029	11	39	0.852389	4	2	0.1476	no
gi|641571238|gb|KK856123.1|	06247-10655	8.7	34	0	50	inf	5.00E-05	yes
gi|641571238|gb|KK856123.1|	07065-10748	8.7	34	0	11	inf	0.00075	yes
gi|641571238|gb|KK856123.1|	10547-11074	8.7	34	0	159	inf	5.00E-05	yes
gi|641571238|gb|KK856123.1|	13448-11406	8.7	34	0	103	inf	0.00065	yes
gi|641571238|gb|KK856123.1|	33918-34316	8.7	34	0	196	inf	0.00045	yes
gi|641571238|gb|KK856123.1|	35842-36032	8.7	34	0	64	inf	0.0016	yes
gi|641571238|gb|KK856123.1|	39037-39228	8.7	34	0	100	inf	0.00075	yes
gi|641571238|gb|KK856123.1|	50514-50862	8.7	34	0	68	inf	5.00E-05	yes
gi|641571242|gb|KK856122.1|	27583-12810	8.7	21	0.658714	2	2	0.3323	no
gi|641571242|gb|KK856122.1|	98917-99121	8.7	21	2	80	5	0.1055	no
gi|641571245|gb|KK856121.1|	17315-17903	11	41	1	0.81761	-0.817686	0.6492	no
gi|641571245|gb|KK856121.1|	42228-44765	11	41	3	1	-1	0.3979	no
gi|641571245|gb|KK856121.1|	45033-45640	11	41	7	2	-2	0.2849	no
gi|641571245|gb|KK856121.1|	47144-47500	11	41	11	12	0.182757	0.89485	no
gi|641571245|gb|KK856121.1|	47597-48239	11	41	4	3	-0.339454	0.81335	no
gi|641571245|gb|KK856121.1|	49644-52040	11	41	9	10	0.159336	0.89835	no
gi|641571253|gb|KK856119.1|	30315-13150	9	36	0.643855	0.326739	-0.978598	1	no
gi|641571253|gb|KK856119.1|	16162-16923	9	36	1	0.379905	-2	0.4266	no
gi|641571253|gb|KK856119.1|	20467-21132	9	36	2	0.228385	-3	0.2381	no
gi|641571253|gb|KK856119.1|	23857-24462	9	36	2	0.26139	-3	0.2381	no
gi|641571253|gb|KK856119.1|	24768-25631	9	36	2	0.644458	-1	0.44155	no
gi|641571253|gb|KK856119.1|	27217-27771	9	36	2	0.893764	-1	0.50365	no
gi|641571253|gb|KK856119.1|	27943-28448	9	36	4	0.343997	-3	0.2089	no
gi|641571253|gb|KK856119.1|	3732-4359	9	36	1	0.744727	-0.623702	0.71195	no

gi|641571253|gb|KK856119.1|	48289-49124	9	36	2	1	-0.911467	0.5466	no
gi|641571253|gb|KK856119.1|	60854-62766	9	36	2	3	0.608891	0.6437	no
gi|641571253|gb|KK856119.1|	75075-81988	9	36	15	7	-1	0.6184	no
gi|641571253|gb|KK856119.1|	82800-83647	9	36	0.543212	1	1	0.4595	no
gi|641571259|gb|KK856117.1|	54280-54733	12	45	318	21	-4	0.05995	no
gi|641571259|gb|KK856117.1|	79068-80098	12	45	0	3	inf	0.00065	yes
gi|641571259|gb|KK856117.1|	83210-84318	12	45	0.853323	1	0.731392	0.63585	no
gi|641571259|gb|KK856117.1|	90928-91360	12	45	64	4	-4	0.02685	no
gi|641571259|gb|KK856117.1|	91945-92767	12	45	110	9	-4	0.0787	no
gi|641571270|gb|KK856115.1|	08925-11063	11	38	1	0.426471	-1	0.4177	no
gi|641571270|gb|KK856115.1|	12969-11385	11	38	1	1	-0.289274	0.85105	no
gi|641571270|gb|KK856115.1|	14584-11513	11	38	2	2	0.0106344	0.9394	no
gi|641571270|gb|KK856115.1|	18312-11930	11	38	2	0.407009	-2	0.21615	no
gi|641571270|gb|KK856115.1|	21032-12676	11	38	2	0.977453	-1	0.32865	no
gi|641571270|gb|KK856115.1|	27169-12803	11	38	1	1	0.0889843	0.9392	no
gi|641571270|gb|KK856115.1|	28314-13168	11	38	16	5	-2	0.3354	no
gi|641571270|gb|KK856115.1|	31926-13342	11	38	33	16	-1	0.531	no
gi|641571270|gb|KK856115.1|	33530-13439	11	38	55	26	-1	0.5097	no
gi|641571270|gb|KK856115.1|	34548-13492	11	38	9	6	-0.482846	0.74345	no
gi|641571270|gb|KK856115.1|	13762-14336	11	38	9	3	-1	0.32405	no
gi|641571270|gb|KK856115.1|	47727-14792	11	38	144	18	-3	0.07985	no
gi|641571270|gb|KK856115.1|	50535-15187	11	38	1	0.567781	-1	0.48805	no
gi|641571270|gb|KK856115.1|	57983-15824	11	38	36	1	-5	0.18485	no
gi|641571270|gb|KK856115.1|	62151-16252	11	38	38	6	-3	0.08805	no
gi|641571270|gb|KK856115.1|	70207-17041	11	38	22	3	-3	0.23535	no
gi|641571270|gb|KK856115.1|	76542-17812	11	38	5	2	-2	0.225	no
gi|641571270|gb|KK856115.1|	78832-18218	11	38	7	6	-0.150983	0.90655	no
gi|641571270|gb|KK856115.1|	84886-18573	11	38	8	6	-0.29581	0.8208	no
gi|641571270|gb|KK856115.1|	86266-18693	11	38	15	8	-0.885429	0.4961	no
gi|641571270|gb|KK856115.1|	88076-18850	11	38	30	19	-0.672046	0.60265	no

gi|641571270|gb|KK856115.1|	25306-25607	11	38	62	15	-2	0.14185	no
gi|641571270|gb|KK856115.1|	29636-29888	11	38	9	7	-0.294735	0.86035	no
gi|641571270|gb|KK856115.1|	30518-31847	11	38	4	3	-0.418413	0.7503	no
gi|641571270|gb|KK856115.1|	32429-33137	11	38	17	5	-2	0.17125	no
gi|641571270|gb|KK856115.1|	34710-35811	11	38	5	14	2	0.2458	no
gi|641571270|gb|KK856115.1|	35924-36889	11	38	0	4	inf	0.00035	yes
gi|641571270|gb|KK856115.1|	37656-37860	11	38	55	23	-1	0.38815	no
gi|641571270|gb|KK856115.1|	45932-46146	11	38	45	17	-1	0.35455	no
gi|641571270|gb|KK856115.1|	47952-48331	11	38	4	3	-0.154841	0.91945	no
gi|641571270|gb|KK856115.1|	49766-50288	11	38	9	5	-0.797906	0.56205	no
gi|641571270|gb|KK856115.1|	52916-53216	11	38	26	10	-1	0.34635	no
gi|641571270|gb|KK856115.1|	53653-53871	11	38	36	11	-2	0.2967	no
gi|641571270|gb|KK856115.1|	54532-55536	11	38	3	1	-0.979932	0.49965	no
gi|641571270|gb|KK856115.1|	56419-57861	11	38	0.624714	1	1	0.40965	no
gi|641571270|gb|KK856115.1|	57976-58476	11	38	13	8	-0.780284	0.55355	no
gi|641571270|gb|KK856115.1|	59964-60745	11	38	3	3	-0.205096	0.8824	no
gi|641571270|gb|KK856115.1|	61513-61685	11	38	118	36	-2	0.2739	no
gi|641571270|gb|KK856115.1|	6356-6960	11	38	0.692469	4	3	0.19845	no
gi|641571270|gb|KK856115.1|	66966-67922	11	38	18	7	-1	0.27485	no
gi|641571270|gb|KK856115.1|	75681-79141	11	38	8	13	0.738139	0.6511	no
gi|641571270|gb|KK856115.1|	82712-85854	11	38	5	4	-0.144558	0.909	no
gi|641571270|gb|KK856115.1|	90131-90878	11	38	7	0.584203	-4	0.10585	no
gi|641571270|gb|KK856115.1|	91044-94174	11	38	2	2	-0.354055	0.78695	no
gi|641571270|gb|KK856115.1|	94303-95726	11	38	1	0.615089	-0.833052	0.57785	no
gi|641571270|gb|KK856115.1|	98018-98487	11	38	9	6	-0.631261	0.64825	no
gi|641571273|gb|KK856114.1|	.00907-10113	11	40	1	24	4	0.1178	no
gi|641571273|gb|KK856114.1|	.06080-10640	11	40	4	22	3	0.12155	no
gi|641571273|gb|KK856114.1|	.06972-10856	11	40	2	3	0.308549	0.812	no
gi|641571273|gb|KK856114.1|	.09277-11037	11	40	3	2	-0.306733	0.8235	no
gi|641571273|gb|KK856114.1|	.11835-11365	11	40	3	2	-0.503787	0.6958	no

gi|641571273|gb|KK856114.1|	14015-11461	11	40	1	0.786061	-0.817108	0.6492	no
gi|641571273|gb|KK856114.1|	14684-11523	11	40	0.988266	2	0.858949	0.62755	no
gi|641571273|gb|KK856114.1|	15303-11639	11	40	2	0.604139	-1	0.4198	no
gi|641571273|gb|KK856114.1|	12159-12950	11	40	1	0.180454	-3	0.24815	no
gi|641571273|gb|KK856114.1|	30708-13121	11	40	0	5	inf	0.0023	yes
gi|641571273|gb|KK856114.1|	14718-15800	11	40	4	2	-0.657362	0.62775	no
gi|641571273|gb|KK856114.1|	60310-16116	11	40	0.21563	5	4	0.2352	no
gi|641571273|gb|KK856114.1|	91567-19279	11	40	25	10	-1	0.29715	no
gi|641571273|gb|KK856114.1|	24215-24821	11	40	4	3	-0.603635	0.66955	no
gi|641571273|gb|KK856114.1|	25548-25713	11	40	84	37	-1	0.47345	no
gi|641571273|gb|KK856114.1|	30636-30979	11	40	43	29	-0.554776	0.6661	no
gi|641571273|gb|KK856114.1|	32154-32346	11	40	113	91	-0.321973	0.804	no
gi|641571273|gb|KK856114.1|	33054-33393	11	40	15	7	-1	0.457	no
gi|641571273|gb|KK856114.1|	40716-41151	11	40	107	76	-0.4927	0.69115	no
gi|641571273|gb|KK856114.1|	45149-47346	11	40	54	39	-0.44104	0.8398	no
gi|641571273|gb|KK856114.1|	50953-52049	11	40	0.47165	1	1	0.41175	no
gi|641571273|gb|KK856114.1|	52366-54662	11	40	1	1	0.240287	0.85485	no
gi|641571273|gb|KK856114.1|	55097-55964	11	40	2	0.320322	-2	0.21865	no
gi|641571273|gb|KK856114.1|	56072-56892	11	40	8	3	-1	0.3946	no
gi|641571273|gb|KK856114.1|	62678-65701	11	40	4	4	0.198614	0.87665	no
gi|641571273|gb|KK856114.1|	65999-66652	11	40	6	4	-0.851201	0.53585	no
gi|641571273|gb|KK856114.1|	72253-72916	11	40	7	12	0.804093	0.53865	no
gi|641571273|gb|KK856114.1|	7721-8088	11	40	23	19	-0.226952	0.8642	no
gi|641571273|gb|KK856114.1|	84362-85658	11	40	7	12	0.817183	0.52495	no
gi|641571276|gb|KK856113.1|	00028-10095	9.9	34	2	5	1	0.3502	no
gi|641571276|gb|KK856113.1|	02612-10354	9.9	34	11	31	1	0.25565	no
gi|641571276|gb|KK856113.1|	15671-11603	9.9	34	12	10	-0.277578	0.8362	no
gi|641571276|gb|KK856113.1|	26537-12866	9.9	34	13	13	-0.00712182	0.9957	no
gi|641571276|gb|KK856113.1|	29041-13361	9.9	34	5	2	-1	0.34985	no
gi|641571276|gb|KK856113.1|	39417-14046	9.9	34	0.82839	0.504133	-0.716505	1	no

gi|641571276|gb|KK856113.1|	20731-22315	9.9	34	1	0.85484	-0.254418	0.85915	no
gi|641571276|gb|KK856113.1|	24655-25251	9.9	34	4	4	0.0830728	0.9516	no
gi|641571276|gb|KK856113.1|	25394-27127	9.9	34	0.368357	0.28081	-0.391509	1	no
gi|641571276|gb|KK856113.1|	53697-54631	9.9	34	2	1	-0.483123	0.755	no
gi|641571276|gb|KK856113.1|	56297-56645	9.9	34	9	30	2	0.21625	no
gi|641571276|gb|KK856113.1|	58191-58552	9.9	34	28	35	0.367979	0.77385	no
gi|641571276|gb|KK856113.1|	61199-62359	9.9	34	1	0.67034	-0.89629	0.5693	no
gi|641571276|gb|KK856113.1|	63483-66360	9.9	34	2	1	-0.867916	0.5121	no
gi|641571276|gb|KK856113.1|	69139-71932	9.9	34	2	2	0.0437129	0.97205	no
gi|641571276|gb|KK856113.1|	74335-75287	9.9	34	1	0	-inf	0.01	no
gi|641571276|gb|KK856113.1|	77552-79638	9.9	34	1	2	0.563405	0.6753	no
gi|641571276|gb|KK856113.1|	86105-87269	9.9	34	5	6	0.184002	0.88535	no
gi|641571276|gb|KK856113.1|	89298-90175	9.9	34	0.830891	2	1	0.38845	no
gi|641571276|gb|KK856113.1|	97062-97273	9.9	34	12	10	-0.271483	0.8666	no
gi|641571276|gb|KK856113.1|	98351-98563	9.9	34	84	176	1	0.40425	no
gi|641571279|gb|KK856112.1|	17459-11782	9.8	36	9	3	-2	0.302	no
gi|641571279|gb|KK856112.1|	22908-12409	9.8	36	10	6	-0.735291	0.5608	no
gi|641571279|gb|KK856112.1|	12312-14117	9.8	36	1	1	-0.239334	0.8664	no
gi|641571279|gb|KK856112.1|	26481-12774	9.8	36	5	2	-1	0.3864	no
gi|641571279|gb|KK856112.1|	15376-17000	9.8	36	2	3	0.566377	0.6709	no
gi|641571279|gb|KK856112.1|	17424-18773	9.8	36	2	3	0.403679	0.76155	no
gi|641571279|gb|KK856112.1|	85562-18587	9.8	36	0.578768	8	4	0.12785	no
gi|641571279|gb|KK856112.1|	21405-21965	9.8	36	0.387771	8	4	0.23525	no
gi|641571279|gb|KK856112.1|	23833-24275	9.8	36	1	17	4	0.12315	no
gi|641571279|gb|KK856112.1|	28088-28413	9.8	36	0	50	inf	5.00E-05	yes
gi|641571279|gb|KK856112.1|	34257-34849	9.8	36	0	23	inf	5.00E-05	yes
gi|641571279|gb|KK856112.1|	37702-38018	9.8	36	0	11	inf	0.0025	yes
gi|641571279|gb|KK856112.1|	42976-45195	9.8	36	0.489817	1	1	0.3345	no
gi|641571279|gb|KK856112.1|	46091-47248	9.8	36	0.294489	3	4	0.12815	no
gi|641571279|gb|KK856112.1|	64697-65354	9.8	36	1	2	0.599572	0.69715	no

gi|641571279|gb|KK856112.1|	7912-8537	9.8	36	2	1	-0.138339	0.9254	no
gi|641571279|gb|KK856112.1|	8918-10013	9.8	36	13	10	-0.351671	0.7751	no
gi|641571279|gb|KK856112.1|	93572-94277	9.8	36	2	3	0.378378	0.7978	no
gi|641571279|gb|KK856112.1|	96513-97087	9.8	36	0.560282	3	2	0.2235	no
gi|641571290|gb|KK856111.1|	04192-10482	11	41	2	3	0.357806	0.8122	no
gi|641571290|gb|KK856111.1|	05545-10669	11	41	1	0.565032	-1	0.4973	no
gi|641571290|gb|KK856111.1|	06845-10763	11	41	3	1	-1	0.41765	no
gi|641571290|gb|KK856111.1|	09482-11099	11	41	2	1	-0.507625	0.70745	no
gi|641571290|gb|KK856111.1|	14718-11756	11	41	2	0.938794	-1	0.3114	no
gi|641571290|gb|KK856111.1|	17622-11940	11	41	3	3	-0.00805027	0.9896	no
gi|641571290|gb|KK856111.1|	20304-12094	11	41	1	5	2	0.19925	no
gi|641571290|gb|KK856111.1|	22856-12310	11	41	3	16	2	0.2455	no
gi|641571290|gb|KK856111.1|	27025-12729	11	41	6	8	0.367136	0.8128	no
gi|641571290|gb|KK856111.1|	38002-13816	11	41	11	101	3	0.25785	no
gi|641571290|gb|KK856111.1|	49476-15010	11	41	10	4	-1	0.37695	no
gi|641571290|gb|KK856111.1|	53819-15469	11	41	6	6	-0.197794	0.8777	no
gi|641571290|gb|KK856111.1|	60937-16142	11	41	25.31	19	-0.449392	0.721	no
gi|641571290|gb|KK856111.1|	61895-16275	11	41	1	0.978337	-0.133581	0.92965	no
gi|641571290|gb|KK856111.1|	16789-17045	11	41	22	0	-inf	0.00315	yes
gi|641571290|gb|KK856111.1|	71981-17239	11	41	43	27	-0.698617	0.58785	no
gi|641571290|gb|KK856111.1|	76197-17651	11	41	27	17	-0.621551	0.64325	no
gi|641571290|gb|KK856111.1|	76855-17736	11	41	4	4	0.0460397	0.9668	no
gi|641571290|gb|KK856111.1|	79344-18077	11	41	5	3	-0.609267	0.6381	no
gi|641571290|gb|KK856111.1|	83204-18465	11	41	54	41	-0.386378	0.8663	no
gi|641571290|gb|KK856111.1|	89514-19130	11	41	11	13	0.180012	0.88425	no
gi|641571290|gb|KK856111.1|	93939-19469	11	41	164	272	0.727586	0.7274	no
gi|641571290|gb|KK856111.1|	96499-19678	11	41	8	12	0.690096	0.64775	no
gi|641571290|gb|KK856111.1|	27878-28427	11	41	2	0.302055	-3	0.2481	no
gi|641571290|gb|KK856111.1|	37515-39047	11	41	0.688589	0.403633	-0.770599	1	no
gi|641571290|gb|KK856111.1|	39321-39980	11	41	1	3	1	0.47685	no

gi|641571290|gb|KK856111.1|	41961-42150	11	41	32	4	-3	0.2302	no
gi|641571290|gb|KK856111.1|	44089-44799	11	41	10	0.834432	-4	0.07475	no
gi|641571290|gb|KK856111.1|	45788-46736	11	41	16	1	-4	0.03115	no
gi|641571290|gb|KK856111.1|	46811-47771	11	41	2	0.563148	-2	0.22015	no
gi|641571290|gb|KK856111.1|	47947-48952	11	41	2	1	-0.642888	0.6611	no
gi|641571290|gb|KK856111.1|	49080-49679	11	41	2	2	-0.0543231	0.94545	no
gi|641571290|gb|KK856111.1|	49898-54332	11	41	3	3	0.0536103	0.96625	no
gi|641571294|gb|KK856110.1|	04442-10472	10	36	20	26	0.349909	0.79405	no
gi|641571294|gb|KK856110.1|	10012-11031	10	36	48	38	-0.34453	0.7861	no
gi|641571294|gb|KK856110.1|	15169-11604	10	36	0.312042	1	2	0.3323	no
gi|641571294|gb|KK856110.1|	20867-12246	10	36	1	0.230982	-2	0.22235	no
gi|641571294|gb|KK856110.1|	23646-12416	10	36	2	2	-0.253799	0.88485	no
gi|641571294|gb|KK856110.1|	26993-12819	10	36	3	2	-0.463948	0.7328	no
gi|641571294|gb|KK856110.1|	59739-60136	10	36	4	4	-0.0676526	0.9451	no
gi|641571308|gb|KK856106.1|	11924-12459	8.9	36	4	2	-0.767497	0.61315	no
gi|641571308|gb|KK856106.1|	14237-16018	8.9	36	18	15	-0.270087	0.83015	no
gi|641571308|gb|KK856106.1|	16393-18200	8.9	36	2	2	-0.338865	0.79895	no
gi|641571308|gb|KK856106.1|	18390-21270	8.9	36	5	4	-0.493489	0.6905	no
gi|641571308|gb|KK856106.1|	208-397	8.9	36	22	27	0.331774	0.8234	no
gi|641571308|gb|KK856106.1|	22967-24715	8.9	36	2	0.973367	-0.628511	0.65155	no
gi|641571308|gb|KK856106.1|	24841-28051	8.9	36	2	0.752954	-1	0.41785	no
gi|641571308|gb|KK856106.1|	28151-28914	8.9	36	6	5	-0.485519	0.7069	no
gi|641571308|gb|KK856106.1|	29624-30252	8.9	36	1	0.495352	-2	0.4266	no
gi|641571308|gb|KK856106.1|	33543-36369	8.9	36	2	1	-0.452196	0.72615	no
gi|641571308|gb|KK856106.1|	39351-40297	8.9	36	18	13	-0.534509	0.6707	no
gi|641571308|gb|KK856106.1|	46803-50715	8.9	36	11	13	0.221645	0.9245	no
gi|641571308|gb|KK856106.1|	52545-53998	8.9	36	2	2	-0.240362	0.85505	no
gi|641571308|gb|KK856106.1|	63823-64383	8.9	36	1	2	1	0.5082	no
gi|641571308|gb|KK856106.1|	84217-84416	8.9	36	17	13	-0.470251	0.7824	no
gi|641571311|gb|KK856105.1|	31334-13199	9.2	37	1	0.458703	-1	0.4534	no

gi|641571311|gb|KK856105.1|	32642-13377	9.2	37	0.301384	1	2	0.29935	no
gi|641571311|gb|KK856105.1|	34948-13652	9.2	37	0.460856	0.546342	0.24549	1	no
gi|641571311|gb|KK856105.1|	16487-17066	9.2	37	4	3	-0.191491	0.8964	no
gi|641571311|gb|KK856105.1|	20450-21313	9.2	37	2	2	-0.0745799	0.95395	no
gi|641571311|gb|KK856105.1|	22158-23069	9.2	37	0.890346	2	1	0.35865	no
gi|641571311|gb|KK856105.1|	26518-27087	9.2	37	2	1	-0.0814699	0.92675	no
gi|641571311|gb|KK856105.1|	34790-35020	9.2	37	84	74	-0.192319	0.8823	no
gi|641571311|gb|KK856105.1|	37062-37482	9.2	37	4	8	1	0.4741	no
gi|641571311|gb|KK856105.1|	39019-39515	9.2	37	9	7	-0.336679	0.80155	no
gi|641571311|gb|KK856105.1|	41528-41876	9.2	37	28	27	-0.0549219	0.9655	no
gi|641571311|gb|KK856105.1|	5463-6527	9.2	37	3	15	2	0.0974	no
gi|641571311|gb|KK856105.1|	8274-9730	9.2	37	3	13	2	0.1183	no
gi|641571311|gb|KK856105.1|	9831-12428	9.2	37	20	16	-0.260968	0.90745	no
gi|641571315|gb|KK856104.1|	08284-10872	12	46	4	5	0.347454	0.81805	no
gi|641571315|gb|KK856104.1|	12564-11374	12	46	11	15	0.486864	0.69045	no
gi|641571315|gb|KK856104.1|	18159-11894	12	46	0.482713	1	1	0.44255	no
gi|641571315|gb|KK856104.1|	23905-12505	12	46	0.595167	2	2	0.23515	no
gi|641571315|gb|KK856104.1|	28791-13042	12	46	0.197264	2	3	0.147	no
gi|641571315|gb|KK856104.1|	33673-13900	12	46	348.61	251	-0.474347	0.75965	no
gi|641571315|gb|KK856104.1|	39164-14120	12	46	1	0.762326	-0.445323	0.7561	no
gi|641571315|gb|KK856104.1|	41390-14161	12	46	417.53	212	-0.978839	0.43565	no
gi|641571315|gb|KK856104.1|	42296-14376	12	46	75	99	0.405345	0.8491	no
gi|641571315|gb|KK856104.1|	49257-15398	12	46	0	212	inf	0.00015	yes
gi|641571315|gb|KK856104.1|	57384-15916	12	46	3	2	-0.322142	0.8036	no
gi|641571315|gb|KK856104.1|	60114-16059	12	46	11	5	-0.993266	0.48715	no
gi|641571315|gb|KK856104.1|	16677-18311	12	46	0.393705	0.300078	-0.391774	1	no
gi|641571315|gb|KK856104.1|	70046-17028	12	46	19	33	0.783627	0.5842	no
gi|641571315|gb|KK856104.1|	71599-17463	12	46	0.672725	0.684623	0.0252928	1	no
gi|641571315|gb|KK856104.1|	74764-17556	12	46	2	2	-0.259859	0.8591	no
gi|641571315|gb|KK856104.1|	76398-17742	12	46	17	31	0.828639	0.50325	no

gi|641571315|gb|KK856104.1|	77649-17800	12	46	3	6	1	0.49265	no
gi|641571315|gb|KK856104.1|	78505-17962	12	46	0.991099	0.812246	-0.287112	0.85105	no
gi|641571315|gb|KK856104.1|	82300-18281	12	46	3	3	0.0632413	0.9551	no
gi|641571315|gb|KK856104.1|	89551-19135	12	46	0.838322	1	0.753041	0.5882	no
gi|641571315|gb|KK856104.1|	93072-19326	12	46	21	19	-0.153119	0.91315	no
gi|641571315|gb|KK856104.1|	93850-19553	12	46	0.758924	2	1	0.4686	no
gi|641571315|gb|KK856104.1|	97174-19787	12	46	0.699952	2	2	0.32945	no
gi|641571315|gb|KK856104.1|	2482-3259	12	46	9	12	0.335404	0.7886	no
gi|641571315|gb|KK856104.1|	3465-3693	12	46	36	91	1	0.3255	no
gi|641571315|gb|KK856104.1|	40177-40756	12	46	2	0.836486	-1	0.47135	no
gi|641571315|gb|KK856104.1|	474-639	12	46	520	644	0.308249	0.80595	no
gi|641571315|gb|KK856104.1|	55389-56184	12	46	2	0.179259	-4	0.20475	no
gi|641571315|gb|KK856104.1|	56605-57044	12	46	6	0	-inf	0.0024	yes
gi|641571315|gb|KK856104.1|	60577-60963	12	46	7	0	-inf	0.00495	no
gi|641571315|gb|KK856104.1|	62506-63250	12	46	2	0	-inf	0.01	no
gi|641571315|gb|KK856104.1|	64021-64455	12	46	4	0.442547	-3	0.2348	no
gi|641571315|gb|KK856104.1|	66401-68599	12	46	9	0.485424	-4	0.02125	no
gi|641571315|gb|KK856104.1|	69124-72404	12	46	4	0.105127	-5	0.0946	no
gi|641571315|gb|KK856104.1|	72751-73791	12	46	5	0.510072	-3	0.0851	no
gi|641571315|gb|KK856104.1|	74482-75811	12	46	2	0	-inf	0.0013	yes
gi|641571315|gb|KK856104.1|	76795-77556	12	46	2	0	-inf	0.01	no
gi|641571315|gb|KK856104.1|	77620-78339	12	46	2	1	-0.720849	0.6521	no
gi|641571315|gb|KK856104.1|	78481-79408	12	46	2	0.294202	-3	0.1793	no
gi|641571315|gb|KK856104.1|	82163-83500	12	46	2	8	2	0.12	no
gi|641571315|gb|KK856104.1|	84482-85748	12	46	0.595147	1	1	0.49595	no
gi|641571315|gb|KK856104.1|	88912-89244	12	46	11	0.739468	-4	0.1971	no
gi|641571315|gb|KK856104.1|	90012-90488	12	46	110	85	-0.362776	0.76675	no
gi|641571315|gb|KK856104.1|	98835-99302	12	46	39	28	-0.507195	0.6879	no
gi|641571315|gb|KK856104.1|	99796-10076	12	46	0.913933	0.555918	-0.717217	0.6701	no
gi|641571318|gb|KK856103.1|	12003-12916	8.2	40	1	0	-inf	0.0102	no

gi|641571318|gb|KK856103.1|	15053-15339	8.2	40	15	21	0.426262	0.7614	no
gi|641571318|gb|KK856103.1|	16114-16379	8.2	40	12	8	-0.66478	0.67	no
gi|641571318|gb|KK856103.1|	74277-74701	8.2	40	19	172	3	0.11385	no
gi|641571318|gb|KK856103.1|	79177-79687	8.2	40	18	139.19	3	0.1276	no
gi|641571321|gb|KK856102.1|	07338-10784	15	57	3	4	0.245429	0.8653	no
gi|641571321|gb|KK856102.1|	12176-11303	15	57	20	39	0.989119	0.42585	no
gi|641571321|gb|KK856102.1|	15194-11560	15	57	7	0	-inf	0.0032	yes
gi|641571321|gb|KK856102.1|	32638-34779	15	57	3	1	-1	0.4386	no
gi|641571321|gb|KK856102.1|	35230-37166	15	57	3	0.930524	-2	0.2731	no
gi|641571321|gb|KK856102.1|	38483-40437	15	57	4	1	-2	0.23625	no
gi|641571321|gb|KK856102.1|	41443-43174	15	57	40	26	-0.653892	0.7736	no
gi|641571321|gb|KK856102.1|	43558-44890	15	57	1	0.94879	-0.158399	0.91355	no
gi|641571321|gb|KK856102.1|	45132-45728	15	57	2	0.801527	-0.987317	0.62775	no
gi|641571321|gb|KK856102.1|	46318-47163	15	57	1	2	0.340213	0.81785	no
gi|641571321|gb|KK856102.1|	50136-50415	15	57	0.747118	11	4	0.12515	no
gi|641571321|gb|KK856102.1|	65448-66384	15	57	1	2	0.810812	0.5797	no
gi|641571324|gb|KK856101.1|	08384-10948	9.1	39	4	6	0.470875	0.71365	no
gi|641571324|gb|KK856101.1|	36159-13692	9.1	39	3	3	-0.205287	0.8824	no
gi|641571324|gb|KK856101.1|	15439-17389	9.1	39	5	22	2	0.26095	no
gi|641571324|gb|KK856101.1|	17948-19687	9.1	39	6	28	2	0.22705	no
gi|641571324|gb|KK856101.1|	20806-21759	9.1	39	1	4	2	0.2922	no
gi|641571324|gb|KK856101.1|	32457-32930	9.1	39	84	150	0.831755	0.61705	no
gi|641571324|gb|KK856101.1|	59622-60140	9.1	39	2	3	0.594198	0.6936	no
gi|641571324|gb|KK856101.1|	82240-84144	9.1	39	0.870171	1	0.304021	0.8279	no
gi|641571324|gb|KK856101.1|	85702-89118	9.1	39	2	2	0.209138	0.8712	no
gi|641571324|gb|KK856101.1|	95465-95864	9.1	39	1	4	1	0.4433	no
gi|641571327|gb|KK856100.1|	20305-12048	9.6	20	96	0	-inf	0.00185	yes
gi|641571327|gb|KK856100.1|	24529-12488	9.6	20	24	2	-4	0.1024	no
gi|641571327|gb|KK856100.1|	28097-12840	9.6	20	49	0	-inf	0.0001	yes
gi|641571327|gb|KK856100.1|	30236-13045	9.6	20	30	0	-inf	0.00495	no

gi|641571327|gb|KK856100.1|	39962-14106	9.6	20	103.81	4	-5	0.0378	no
gi|641571327|gb|KK856100.1|	14846-16062	9.6	20	1	1	0.159086	0.9153	no
gi|641571327|gb|KK856100.1|	16281-16860	9.6	20	1	2	0.181968	0.91015	no
gi|641571327|gb|KK856100.1|	20203-20738	9.6	20	2	1	-0.726855	0.67005	no
gi|641571327|gb|KK856100.1|	20994-22458	9.6	20	2	0.595276	-2	0.27825	no
gi|641571327|gb|KK856100.1|	24325-24796	9.6	20	8	2	-2	0.1924	no
gi|641571327|gb|KK856100.1|	25772-26746	9.6	20	1	3	1	0.3575	no
gi|641571327|gb|KK856100.1|	30072-30472	9.6	20	10	4	-1	0.4	no
gi|641571327|gb|KK856100.1|	40543-44056	9.6	20	2	1	-0.793836	0.5386	no
gi|641571327|gb|KK856100.1|	45707-46145	9.6	20	7	7	0.0650794	0.9633	no
gi|641571327|gb|KK856100.1|	51541-52067	9.6	20	5	2	-1	0.4334	no
gi|641571327|gb|KK856100.1|	52216-57432	9.6	20	1	1	0.0917851	0.94365	no
gi|641571327|gb|KK856100.1|	58457-59244	9.6	20	2	0.363331	-2	0.25005	no
gi|641571327|gb|KK856100.1|	59614-61215	9.6	20	13	10	-0.425817	0.73215	no
gi|641571327|gb|KK856100.1|	7-739	9.6	20	0.395741	2	2	0.256	no
gi|641571327|gb|KK856100.1|	7473-7856	9.6	20	2	4	1	0.4655	no
gi|641571327|gb|KK856100.1|	9809-10124	9.6	20	11	11	0.023386	0.9675	no
gi|641571330|gb|KK856099.1|	10424-11057	10	39	38	81	1	0.52965	no
gi|641571330|gb|KK856099.1|	16678-17112	10	39	29	110	2	0.14475	no
gi|641571330|gb|KK856099.1|	24714-25419	10	39	0	84	inf	0.0006	yes
gi|641571330|gb|KK856099.1|	28686-30259	10	39	0.051392	1	5	0.10875	no
gi|641571330|gb|KK856099.1|	31548-32157	10	39	0.171033	3	4	0.12385	no
gi|641571330|gb|KK856099.1|	43546-44329	10	39	11	40	2	0.1412	no
gi|641571330|gb|KK856099.1|	46385-46788	10	39	0	339	inf	0.0001	yes
gi|641571334|gb|KK856098.1|	04011-10498	9.3	37	103	79	-0.38134	0.86095	no
gi|641571334|gb|KK856098.1|	05153-10910	9.3	37	4	4	0.17019	0.89085	no
gi|641571334|gb|KK856098.1|	09671-10996	9.3	37	5	11	1	0.5003	no
gi|641571334|gb|KK856098.1|	10923-11207	9.3	37	1	2	0.454123	0.75045	no
gi|641571334|gb|KK856098.1|	12375-11290	9.3	37	2	2	-0.54268	0.7461	no
gi|641571334|gb|KK856098.1|	13287-11410	9.3	37	192.27	141	-0.443087	0.83975	no

gi|641571334|gb|KK856098.1|	14671-11540	9.3	37	2	0.799049	-0.983592	0.5872	no
gi|641571334|gb|KK856098.1|	18242-11914	9.3	37	7	4	-0.980996	0.4607	no
gi|641571334|gb|KK856098.1|	21120-12246	9.3	37	1	2	0.147749	0.9199	no
gi|641571334|gb|KK856098.1|	23567-12714	9.3	37	6	5	-0.255826	0.84195	no
gi|641571334|gb|KK856098.1|	33539-13438	9.3	37	2	0.498174	-2	0.29	no
gi|641571334|gb|KK856098.1|	35929-13643	9.3	37	133	62	-1	0.51125	no
gi|641571334|gb|KK856098.1|	36995-13932	9.3	37	16	8	-1	0.36725	no
gi|641571334|gb|KK856098.1|	39496-13983	9.3	37	62	31	-0.987378	0.43605	no
gi|641571334|gb|KK856098.1|	41192-14311	9.3	37	29	16	-0.853573	0.598	no
gi|641571334|gb|KK856098.1|	44698-14540	9.3	37	17	9	-0.995762	0.4482	no
gi|641571334|gb|KK856098.1|	62913-64105	9.3	37	0.355185	2	2	0.20805	no
gi|641571334|gb|KK856098.1|	66351-67292	9.3	37	0	45	inf	5.00E-05	yes
gi|641571334|gb|KK856098.1|	68683-68883	9.3	37	4	25	3	0.30895	no
gi|641571334|gb|KK856098.1|	70022-70718	9.3	37	2	0.42877	-2	0.25005	no
gi|641571334|gb|KK856098.1|	70976-71891	9.3	37	3	3	-0.115763	0.93255	no
gi|641571334|gb|KK856098.1|	72492-78406	9.3	37	13	17	0.368714	0.8652	no
gi|641571334|gb|KK856098.1|	78629-82215	9.3	37	71.06	90	0.342547	0.86175	no
gi|641571334|gb|KK856098.1|	82865-86693	9.3	37	46	51.31	0.160129	0.93805	no
gi|641571334|gb|KK856098.1|	86932-89481	9.3	37	5	3	-0.573776	0.64995	no
gi|641571334|gb|KK856098.1|	89652-90619	9.3	37	87	81	-0.116726	0.9567	no
gi|641571334|gb|KK856098.1|	93274-93498	9.3	37	469	356	-0.398392	0.74475	no
gi|641571334|gb|KK856098.1|	95182-96449	9.3	37	467	406	-0.203087	0.905	no
gi|641571334|gb|KK856098.1|	96595-99689	9.3	37	44	35	-0.329714	0.88155	no
gi|641571334|gb|KK856098.1|	99889-10380[illegible]	9.3	37	18	14	-0.386262	0.8627	no
gi|641571339|gb|KK856097.1|	58203-58900	10	38	0.141105	2	4	0.11955	no
gi|641571347|gb|KK856095.1|	54352-54764	9	33	2	5	2	0.32945	no
gi|641571350|gb|KK856094.1|	00120-10135	10	36	0.679032	0.723791	0.0920943	1	no
gi|641571350|gb|KK856094.1|	02002-10323	10	36	1	5	2	0.17395	no
gi|641571350|gb|KK856094.1|	05667-10664	10	36	0.452875	0.826448	0.867813	1	no
gi|641571350|gb|KK856094.1|	10304-11127	10	36	1	0.139158	-3	0.2382	no

gi|641571350|gb|KK856094.1|	21724-22073	10	36	21	57	1	0.28685	no
gi|641571350|gb|KK856094.1|	26929-27889	10	36	19	45	1	0.30165	no
gi|641571350|gb|KK856094.1|	28316-29176	10	36	0.852141	0.647349	-0.396548	1	no
gi|641571350|gb|KK856094.1|	29233-31507	10	36	2	3	0.609491	0.6352	no
gi|641571350|gb|KK856094.1|	31743-32078	10	36	8	0	-inf	0.00435	no
gi|641571350|gb|KK856094.1|	32883-33329	10	36	18	12	-0.552685	0.67315	no
gi|641571350|gb|KK856094.1|	33918-34572	10	36	57	30	-0.943208	0.45	no
gi|641571350|gb|KK856094.1|	36177-36743	10	36	9	3	-1	0.3413	no
gi|641571350|gb|KK856094.1|	36927-37724	10	36	4	0.893335	-2	0.1803	no
gi|641571350|gb|KK856094.1|	41239-41474	10	36	28	28	0.0231782	0.9739	no
gi|641571350|gb|KK856094.1|	55479-55838	10	36	10	4	-1	0.4523	no
gi|641571350|gb|KK856094.1|	59998-60201	10	36	20	9	-1	0.4938	no
gi|641571350|gb|KK856094.1|	65454-66697	10	36	2	1	-0.550847	0.69645	no
gi|641571350|gb|KK856094.1|	76309-77689	10	36	10	4	-1	0.2699	no
gi|641571350|gb|KK856094.1|	77906-79311	10	36	6	3	-0.939039	0.46995	no
gi|641571350|gb|KK856094.1|	79413-81665	10	36	5	1	-2	0.15875	no
gi|641571350|gb|KK856094.1|	96197-97539	10	36	0.617448	0.752436	0.285253	1	no
gi|641571350|gb|KK856094.1|	98887-99373	10	36	3	3	0.00733607	0.95145	no
gi|641571354|gb|KK856093.1|	08978-10946	11	42	2	0.729319	-2	0.4266	no
gi|641571354|gb|KK856093.1|	22588-12327	11	42	8	6	-0.426051	0.74595	no
gi|641571354|gb|KK856093.1|	23389-12640	11	42	13	7	-0.922751	0.5694	no
gi|641571354|gb|KK856093.1|	31837-13268	11	42	0.862521	0.655186	-0.396656	1	no
gi|641571354|gb|KK856093.1|	34570-13576	11	42	0.564333	0.6444	0.19141	1	no
gi|641571354|gb|KK856093.1|	35922-13663	11	42	0.679814	1	0.864049	0.62725	no
gi|641571354|gb|KK856093.1|	37091-13843	11	42	0.553771	2	2	0.33265	no
gi|641571354|gb|KK856093.1|	38758-13949	11	42	0.785633	1	0.823824	0.6232	no
gi|641571354|gb|KK856093.1|	40349-14122	11	42	0.523144	1	1	0.3994	no
gi|641571354|gb|KK856093.1|	48668-14946	11	42	4	2	-0.867042	0.5344	no
gi|641571354|gb|KK856093.1|	54581-56942	11	42	0.065386	60	10	0.23295	no
gi|641571360|gb|KK856091.1|	08975-10967	9.5	40	0	2	inf	0.00455	no

gi|641571360|gb|KK856091.1|	12640-11687	9.5	40	26	11	-1	0.59365	no
gi|641571360|gb|KK856091.1|	17272-11894	9.5	40	9	13	0.494795	0.68985	no
gi|641571360|gb|KK856091.1|	19709-12208	9.5	40	7	5	-0.495348	0.68955	no
gi|641571360|gb|KK856091.1|	22236-12383	9.5	40	0.403809	0.769393	0.930049	1	no
gi|641571360|gb|KK856091.1|	12637-13796	9.5	40	1	1	-0.130762	0.9258	no
gi|641571360|gb|KK856091.1|	15271-16005	9.5	40	0.919971	1	0.378999	0.82465	no
gi|641571360|gb|KK856091.1|	19269-20802	9.5	40	0.582216	1	0.95584	0.5381	no
gi|641571360|gb|KK856091.1|	6487-6972	9.5	40	2	0.367107	-3	0.271	no
gi|641571360|gb|KK856091.1|	89369-89648	9.5	40	10	31	2	0.2641	no
gi|641571368|gb|KK856089.1|	03484-10371	8.1	41	26	9	-2	0.34785	no
gi|641571372|gb|KK856088.1|	41773-42267	8.7	37	0.472544	5	3	0.2556	no
gi|641571376|gb|KK856087.1|	11410-11256	11	38	5	4	-0.145927	0.9073	no
gi|641571376|gb|KK856087.1|	36293-13669	11	38	1	4	2	0.3323	no
gi|641571376|gb|KK856087.1|	38454-13950	11	38	0.997569	2	0.605399	0.67915	no
gi|641571376|gb|KK856087.1|	41055-14254	11	38	0.328882	1	2	0.2253	no
gi|641571376|gb|KK856087.1|	55149-55332	11	38	59	13	-2	0.23555	no
gi|641571380|gb|KK856086.1|	00369-10314	9.5	38	8	5	-0.744138	0.5529	no
gi|641571380|gb|KK856086.1|	03281-10393	9.5	38	4	3	-0.0171271	0.97395	no
gi|641571380|gb|KK856086.1|	04117-10510	9.5	38	0.894309	1	0.604874	0.67795	no
gi|641571380|gb|KK856086.1|	05169-10974	9.5	38	6	2	-2	0.1995	no
gi|641571380|gb|KK856086.1|	12951-11365	9.5	38	2	1	-0.679405	0.65225	no
gi|641571380|gb|KK856086.1|	29601-12988	9.5	38	58	35	-0.740554	0.56855	no
gi|641571380|gb|KK856086.1|	31442-13167	9.5	38	88	38	-1	0.3657	no
gi|641571380|gb|KK856086.1|	32133-13238	9.5	38	20	9	-1	0.4473	no
gi|641571380|gb|KK856086.1|	36664-13750	9.5	38	4	3	-0.353811	0.79555	no
gi|641571380|gb|KK856086.1|	39106-14040	9.5	38	0.960514	0.682704	-0.492546	0.7428	no
gi|641571380|gb|KK856086.1|	41732-14230	9.5	38	1	1	0.111257	0.92265	no
gi|641571380|gb|KK856086.1|	42456-14282	9.5	38	6	4	-0.740933	0.6304	no
gi|641571380|gb|KK856086.1|	44323-14570	9.5	38	5	2	-1	0.41315	no
gi|641571380|gb|KK856086.1|	48038-14910	9.5	38	91	0	-inf	5.00E-05	yes

gi|641571380|gb|KK856086.1|	53214-15569	9.5	38	6	0	-inf	5.00E-05	yes
gi|641571380|gb|KK856086.1|	55897-15758	9.5	38	8	0	-inf	5.00E-05	yes
gi|641571380|gb|KK856086.1|	60088-16030	9.5	38	31.54	0	-inf	0.0032	yes
gi|641571380|gb|KK856086.1|	10565-21311	9.5	38	31	0.183705	-7	0.06575	no
gi|641571380|gb|KK856086.1|	13380-21554	9.5	38	12	0.109842	-7	0.1301	no
gi|641571380|gb|KK856086.1|	21547-21678	9.5	38	0	512	inf	0.00375	no
gi|641571380|gb|KK856086.1|	20680-22163	9.5	38	2	0.14097	-4	0.20395	no
gi|641571380|gb|KK856086.1|	25828-26018	9.5	38	0	102	inf	0.00075	yes
gi|641571380|gb|KK856086.1|	27645-27993	9.5	38	0	8	inf	0.00375	no
gi|641571380|gb|KK856086.1|	31700-32005	9.5	38	0.601022	57	7	0.1045	no
gi|641571380|gb|KK856086.1|	37171-39313	9.5	38	15	3	-2	0.06815	no
gi|641571380|gb|KK856086.1|	39439-40537	9.5	38	0.784342	0.358086	-1	1	no
gi|641571380|gb|KK856086.1|	41334-42439	9.5	38	3	1	-2	0.24995	no
gi|641571380|gb|KK856086.1|	58183-59123	9.5	38	4	5	0.274256	0.83665	no
gi|641571380|gb|KK856086.1|	59301-60436	9.5	38	5	8	0.751478	0.55325	no
gi|641571380|gb|KK856086.1|	62306-63151	9.5	38	72	94	0.38413	0.86545	no
gi|641571380|gb|KK856086.1|	64469-68148	9.5	38	1	2	0.440714	0.7298	no
gi|641571380|gb|KK856086.1|	68329-69168	9.5	38	9	5	-0.929332	0.47975	no
gi|641571380|gb|KK856086.1|	74461-76309	9.5	38	2	2	0.130711	0.9196	no
gi|641571380|gb|KK856086.1|	76421-79516	9.5	38	15	10	-0.542225	0.8108	no
gi|641571380|gb|KK856086.1|	79912-84859	9.5	38	2	4	0.912919	0.4665	no
gi|641571380|gb|KK856086.1|	85190-87198	9.5	38	1	1	-0.272282	0.84	no
gi|641571380|gb|KK856086.1|	87335-87612	9.5	38	47	36	-0.392937	0.7623	no
gi|641571380|gb|KK856086.1|	90039-90279	9.5	38	279	350	0.32735	0.7967	no
gi|641571380|gb|KK856086.1|	90690-91742	9.5	38	0.661165	2	2	0.34655	no
gi|641571380|gb|KK856086.1|	98557-99869	9.5	38	12	9	-0.448605	0.7198	no
gi|641571389|gb|KK856085.1|	29526-30123	9.6	39	10	0.799561	-4	0.1026	no
gi|641571389|gb|KK856085.1|	30965-31222	9.6	39	11	3	-2	0.27505	no
gi|641571389|gb|KK856085.1|	31504-32603	9.6	39	1	0.238461	-2	0.24735	no
gi|641571392|gb|KK856084.1|	21317-22100	2.3	27	5	4	-0.272177	0.83735	no

gi|641571392|gb|KK856084.1|	27000-28178	2.3	27	0	3	inf	0.0003	yes
gi|641571396|gb|KK856083.1|	.02897-10310	8.4	35	24	68	1	0.3097	no
gi|641571396|gb|KK856083.1|	.10768-11339	8.4	35	18	104	3	0.2555	no
gi|641571396|gb|KK856083.1|	79117-80047	8.4	35	0.289107	1	2	0.2812	no
gi|641571396|gb|KK856083.1|	83689-84310	8.4	35	0.831151	2	0.861534	0.62755	no
gi|641571396|gb|KK856083.1|	86492-86853	8.4	35	3	17	2	0.15705	no
gi|641571396|gb|KK856083.1|	89995-90272	8.4	35	29	131	2	0.11435	no
gi|641571396|gb|KK856083.1|	91271-91899	8.4	35	4	24	3	0.0793	no
gi|641571399|gb|KK856082.1|	12193-13890	12	43	0.377188	0.71881	0.930327	1	no
gi|641571399|gb|KK856082.1|	.30285-13109	12	43	1	2	0.361755	0.8122	no
gi|641571399|gb|KK856082.1|	14144-15167	12	43	0.855412	0.520497	-0.71673	1	no
gi|641571399|gb|KK856082.1|	.75366-17599	12	43	3	2	-0.763818	0.6156	no
gi|641571399|gb|KK856082.1|	25913-26956	12	43	0.501138	0.508276	0.0204035	1	no
gi|641571399|gb|KK856082.1|	84393-85455	12	43	0	1	inf	0.00375	no
gi|641571402|gb|KK856081.1|	.02176-10306	11	39	0	100.3	inf	5.00E-05	yes
gi|641571402|gb|KK856081.1|	.05692-10617	11	39	0.247718	528	11	0.1052	no
gi|641571402|gb|KK856081.1|	.06999-10795	11	39	0	3	inf	0.00115	yes
gi|641571402|gb|KK856081.1|	.10830-11115	11	39	0	96	inf	5.00E-05	yes
gi|641571402|gb|KK856081.1|	.12190-11278	11	39	2	3	0.471932	0.7564	no
gi|641571402|gb|KK856081.1|	.16673-11700	11	39	0.493374	343	9	0.10615	no
gi|641571402|gb|KK856081.1|	.18505-11939	11	39	0.921238	2	1	0.35865	no
gi|641571402|gb|KK856081.1|	.21699-12386	11	39	0.359278	14	5	0.0228	no
gi|641571402|gb|KK856081.1|	.24158-12572	11	39	0.310607	111	8	0.0664	no
gi|641571402|gb|KK856081.1|	.48445-15073	11	39	0.608128	2	2	0.2139	no
gi|641571402|gb|KK856081.1|	.51194-15211	11	39	1	0.894767	-0.511325	0.7498	no
gi|641571402|gb|KK856081.1|	.60790-16183	11	39	0.669764	0.509472	-0.39465	1	no
gi|641571402|gb|KK856081.1|	.71012-17127	11	39	9	10	0.235365	0.8785	no
gi|641571402|gb|KK856081.1|	.77432-18028	11	39	10	8	-0.219966	0.8554	no
gi|641571402|gb|KK856081.1|	.80362-18197	11	39	7	8	0.143494	0.9079	no
gi|641571402|gb|KK856081.1|	.82028-18445	11	39	4	7	0.609683	0.62795	no

gi|641571402|gb|KK856081.1|	18269-22072	11	39	2	2	0.153225	0.90285	no
gi|641571402|gb|KK856081.1|	85107-18553	11	39	0	14	inf	0.0004	yes
gi|641571402|gb|KK856081.1|	85664-18644	11	39	0.486861	3	3	0.17485	no
gi|641571402|gb|KK856081.1|	90518-19206	11	39	0.052419	2	5	0.10785	no
gi|641571402|gb|KK856081.1|	94337-19593	11	39	0.151645	1	3	0.1563	no
gi|641571402|gb|KK856081.1|	23172-23849	11	39	8	11	0.43345	0.7386	no
gi|641571402|gb|KK856081.1|	24820-27395	11	39	0.952094	1	0.310291	0.82205	no
gi|641571402|gb|KK856081.1|	27572-28602	11	39	2	3	0.679258	0.6235	no
gi|641571402|gb|KK856081.1|	31353-32751	11	39	1	1	0.220398	0.882	no
gi|641571402|gb|KK856081.1|	33114-35092	11	39	0.754617	1	0.820523	0.5505	no
gi|641571402|gb|KK856081.1|	35217-37554	11	39	3	5	0.960072	0.44595	no
gi|641571402|gb|KK856081.1|	3610-3824	11	39	15	31	1	0.49455	no
gi|641571402|gb|KK856081.1|	38217-38973	11	39	3	2	-0.457078	0.7485	no
gi|641571402|gb|KK856081.1|	39095-40276	11	39	1	0.656119	-0.715588	0.6527	no
gi|641571402|gb|KK856081.1|	40386-41304	11	39	1	0.595691	-0.855268	0.60675	no
gi|641571402|gb|KK856081.1|	41420-42373	11	39	38	60	0.684807	0.6757	no
gi|641571402|gb|KK856081.1|	45789-47145	11	39	0.244	11	6	0.11635	no
gi|641571402|gb|KK856081.1|	468-725	11	39	5	14	2	0.38055	no
gi|641571402|gb|KK856081.1|	47475-48572	11	39	0.628169	0.477975	-0.394217	1	no
gi|641571402|gb|KK856081.1|	50990-51629	11	39	2	0.724832	-2	0.33985	no
gi|641571402|gb|KK856081.1|	55066-56699	11	39	0.049247	4	6	0.10475	no
gi|641571402|gb|KK856081.1|	79337-79989	11	39	2	2	-0.178175	0.90465	no
gi|641571402|gb|KK856081.1|	80921-83117	11	39	0.495448	1	1	0.3814	no
gi|641571402|gb|KK856081.1|	85139-86810	11	39	0.287875	140	9	0.0649	no
gi|641571402|gb|KK856081.1|	88430-89390	11	39	0.925884	3	2	0.28165	no
gi|641571402|gb|KK856081.1|	9795-10289	11	39	2	4	0.744811	0.6263	no
gi|641571402|gb|KK856081.1|	99482-100012	11	39	0	92	inf	5.00E-05	yes
gi|641571406|gb|KK856080.1|	28070-12823	9.9	21	17	56	2	0.353	no
gi|641571406|gb|KK856080.1|	12849-13124	9.9	21	43	32	-0.439324	0.7403	no
gi|641571406|gb|KK856080.1|	14089-14372	9.9	21	11	7	-0.536585	0.72615	no

gi|641571406|gb|KK856080.1|	21006-22157	9.9	21	7	3	-1	0.3287	no
gi|641571406|gb|KK856080.1|	23912-24077	9.9	21	53	15	-2	0.3266	no
gi|641571406|gb|KK856080.1|	24425-24989	9.9	21	2	0.869944	-0.818645	0.6492	no
gi|641571406|gb|KK856080.1|	3196-4136	9.9	21	73	39	-0.892763	0.58095	no
gi|641571406|gb|KK856080.1|	380-1423	9.9	21	1	1	0.0204035	0.95145	no
gi|641571406|gb|KK856080.1|	44420-46497	9.9	21	9	17	0.931122	0.45795	no
gi|641571406|gb|KK856080.1|	47015-47219	9.9	21	79	86	0.117307	0.9304	no
gi|641571406|gb|KK856080.1|	59479-59718	9.9	21	16	54	2	0.2317	no
gi|641571406|gb|KK856080.1|	60743-61109	9.9	21	15	36	1	0.3636	no
gi|641571406|gb|KK856080.1|	62784-63040	9.9	21	5	15	2	0.31995	no
gi|641571406|gb|KK856080.1|	63797-64257	9.9	21	5	12	1	0.4254	no
gi|641571406|gb|KK856080.1|	65896-66182	9.9	21	8	13	0.804774	0.6087	no
gi|641571406|gb|KK856080.1|	68125-68379	9.9	21	12	11	-0.0309994	0.95145	no
gi|641571406|gb|KK856080.1|	7210-7410	9.9	21	72	18	-2	0.21635	no
gi|641571406|gb|KK856080.1|	73833-74074	9.9	21	4	10	1	0.52535	no
gi|641571406|gb|KK856080.1|	9625-10155	9.9	21	3	2	-0.520653	0.74125	no
gi|641571410|gb|KK856079.1|	[illegible]26264-12751	9.7	20	6	6	0.0464101	0.9702	no
gi|641571410|gb|KK856079.1|	[illegible]37159-13773	9.7	20	3	1	-1	0.4703	no
gi|641571410|gb|KK856079.1|	[illegible]37931-13917	9.7	20	3	2	-0.326186	0.8079	no
gi|641571410|gb|KK856079.1|	[illegible]40906-14176	9.7	20	6	2	-2	0.2921	no
gi|641571417|gb|KK856077.1|	18165-18346	9.2	19	16	28	0.783173	0.65375	no
gi|641571417|gb|KK856077.1|	75723-76027	9.2	19	8	10	0.220422	0.88695	no
gi|641571421|gb|KK856076.1|	27407-28090	10	43	3	4	0.0766881	0.95805	no
gi|641571421|gb|KK856076.1|	2852-3546	10	43	0.709762	2	2	0.32945	no
gi|641571421|gb|KK856076.1|	28720-28964	10	43	6	6	-0.0346379	0.91115	no
gi|641571421|gb|KK856076.1|	30697-31248	10	43	0.59627	2	2	0.3323	no
gi|641571421|gb|KK856076.1|	41444-42057	10	43	0.508206	4	3	0.1725	no
gi|641571421|gb|KK856076.1|	5546-6893	10	43	0.799197	2	1	0.4006	no
gi|641571421|gb|KK856076.1|	56615-57362	10	43	0.384991	2	2	0.2812	no
gi|641571421|gb|KK856076.1|	7675-8351	10	43	1	1	-0.399926	0.8125	no

gi|641571421|gb|KK856076.1|	82901-83777	10	43	2	0.632224	-2	0.28465	no
gi|641571421|gb|KK856076.1|	85937-86458	10	43	2	6	1	0.34295	no
gi|641571421|gb|KK856076.1|	86807-88619	10	43	12	18	0.562052	0.6491	no
gi|641571425|gb|KK856075.1|	01193-10210	9.3	36	5	9	0.842893	0.52155	no
gi|641571425|gb|KK856075.1|	05265-10635	9.3	36	0.55333	0.601453	0.120312	1	no
gi|641571425|gb|KK856075.1|	43035-43365	9.3	36	6	187	5	0.02045	no
gi|641571425|gb|KK856075.1|	43572-44914	9.3	36	34	48	0.482593	0.83265	no
gi|641571425|gb|KK856075.1|	46663-47267	9.3	36	2	0.524041	-2	0.3455	no
gi|641571425|gb|KK856075.1|	47522-47906	9.3	36	17	25	0.551088	0.6718	no
gi|641571425|gb|KK856075.1|	48929-49228	9.3	36	9	8	-0.169839	0.91475	no
gi|641571425|gb|KK856075.1|	50241-50924	9.3	36	19	30	0.65429	0.60105	no
gi|641571425|gb|KK856075.1|	55241-55480	9.3	36	41	55	0.422836	0.7484	no
gi|641571425|gb|KK856075.1|	60127-61086	9.3	36	2	0.704852	-1	0.4198	no
gi|641571425|gb|KK856075.1|	63737-64262	9.3	36	62	87	0.491475	0.6988	no
gi|641571425|gb|KK856075.1|	67457-68084	9.3	36	123.11	80	-0.618589	0.7008	no
gi|641571425|gb|KK856075.1|	68348-68870	9.3	36	50	53	0.082512	0.94725	no
gi|641571425|gb|KK856075.1|	83034-84242	9.3	36	17	64	2	0.26885	no
gi|641571428|gb|KK856074.1|	11750-11346	11	39	0.558713	0.212956	-1	1	no
gi|641571428|gb|KK856074.1|	15190-11706	11	39	0.63417	0.580263	-0.12816	1	no
gi|641571428|gb|KK856074.1|	17395-11870	11	39	0.380762	0.869924	1	1	no
gi|641571428|gb|KK856074.1|	23364-12442	11	39	13	2	-3	0.0743	no
gi|641571428|gb|KK856074.1|	31132-13272	11	39	2	2	-0.0439679	0.9739	no
gi|641571428|gb|KK856074.1|	34358-13529	11	39	7	9	0.411684	0.75245	no
gi|641571428|gb|KK856074.1|	39368-14014	11	39	2	4	0.89161	0.52765	no
gi|641571428|gb|KK856074.1|	43371-14354	11	39	40	77	0.929177	0.5574	no
gi|641571428|gb|KK856074.1|	48167-14887	11	39	21	38	0.883125	0.4808	no
gi|641571428|gb|KK856074.1|	49865-15039	11	39	20	9	-1	0.3717	no
gi|641571428|gb|KK856074.1|	68253-16848	11	39	100	28.37	-2	0.18745	no
gi|641571428|gb|KK856074.1|	70477-17092	11	39	7	3	-2	0.32695	no
gi|641571428|gb|KK856074.1|	71333-17165	11	39	4	5	0.35041	0.83635	no

gi|641571428|gb|KK856074.1|	.75171-17582	11	39	7	3	-1	0.3278	no
gi|641571428|gb|KK856074.1|	.77054-17721	11	39	38	45	0.245535	0.88235	no
gi|641571428|gb|KK856074.1|	.77571-17779	11	39	40	47	0.2316	0.86855	no
gi|641571428|gb|KK856074.1|	.80165-18036	11	39	148	83	-0.82798	0.5333	no
gi|641571428|gb|KK856074.1|	35112-35535	11	39	0.924429	6	3	0.19335	no
gi|641571428|gb|KK856074.1|	36112-40347	11	39	2	2	0.216959	0.8657	no
gi|641571428|gb|KK856074.1|	40774-42196	11	39	2	1	-0.147371	0.91685	no
gi|641571428|gb|KK856074.1|	43975-45591	11	39	4	1	-2	0.21365	no
gi|641571428|gb|KK856074.1|	47853-48234	11	39	9	2	-2	0.2172	no
gi|641571428|gb|KK856074.1|	5389-6292	11	39	4	11	2	0.22415	no
gi|641571428|gb|KK856074.1|	59028-59977	11	39	0.657578	0.571323	-0.202856	1	no
gi|641571428|gb|KK856074.1|	62170-64206	11	39	0.653909	1	0.913978	0.52205	no
gi|641571428|gb|KK856074.1|	65501-65806	11	39	20	0	-inf	0.00105	yes
gi|641571428|gb|KK856074.1|	70386-70618	11	39	26	0	-inf	0.0032	yes
gi|641571428|gb|KK856074.1|	72294-72642	11	39	16	0.670481	-5	0.1859	no
gi|641571428|gb|KK856074.1|	75751-75918	11	39	216	0	-inf	0.00015	yes
gi|641571428|gb|KK856074.1|	77205-78428	11	39	21	27	0.353919	0.77265	no
gi|641571428|gb|KK856074.1|	78577-79208	11	39	16	22	0.521647	0.677	no
gi|641571428|gb|KK856074.1|	79270-80160	11	39	1	1	0.188797	0.90355	no
gi|641571428|gb|KK856074.1|	83184-85159	11	39	17	13	-0.421431	0.73375	no
gi|641571428|gb|KK856074.1|	86072-87722	11	39	15	8	-0.972104	0.44175	no
gi|641571428|gb|KK856074.1|	89342-90452	11	39	15	5	-2	0.24545	no
gi|641571428|gb|KK856074.1|	94936-95840	11	39	0.999045	2	0.982514	0.5381	no
gi|641571435|gb|KK856072.1|	14952-15436	9.1	36	0.977354	5	2	0.2362	no
gi|641571435|gb|KK856072.1|	52839-54831	9.1	36	0.118222	59	9	0.1354	no
gi|641571435|gb|KK856072.1|	60615-63878	9.1	36	0.207722	114	9	0.0319	no
gi|641571435|gb|KK856072.1|	81234-81921	9.1	36	0	38	inf	5.00E-05	yes
gi|641571435|gb|KK856072.1|	9264-10869	9.1	36	1	0.689026	-0.745495	0.60335	no
gi|641571439|gb|KK856071.1|	.01653-10392	23	43	1015.51	0.104136	-13	0.1659	no
gi|641571439|gb|KK856071.1|	.05849-10805	23	43	2	0	-inf	5.00E-05	yes

gi|641571439|gb|KK856071.1|	08106-10937	23	43	0.726723	0	-inf	1	no
gi|641571439|gb|KK856071.1|	09750-11117	23	43	60	0.262756	-8	0.09805	no
gi|641571439|gb|KK856071.1|	16272-11689	23	43	0.334024	87	8	0.23735	no
gi|641571439|gb|KK856071.1|	19573-12011	23	43	110	80	-0.454014	0.77695	no
gi|641571439|gb|KK856071.1|	20241-12546	23	43	9	13	0.401908	0.8579	no
gi|641571439|gb|KK856071.1|	25670-12760	23	43	0.895879	2	1	0.2953	no
gi|641571439|gb|KK856071.1|	27974-12909	23	43	0.305942	0.931276	2	0.3613	no
gi|641571439|gb|KK856071.1|	29661-13054	23	43	0.410627	2	2	0.26155	no
gi|641571439|gb|KK856071.1|	13501-14689	23	43	4	1	-2	0.24765	no
gi|641571439|gb|KK856071.1|	35124-13611	23	43	0	4	inf	0.0004	yes
gi|641571439|gb|KK856071.1|	16054-17740	23	43	16	6	-2	0.22405	no
gi|641571439|gb|KK856071.1|	25944-26906	23	43	0.646428	2	2	0.2915	no
gi|641571439|gb|KK856071.1|	26986-28542	23	43	2	3	0.975723	0.47185	no
gi|641571439|gb|KK856071.1|	29664-30711	23	43	0.332523	2	2	0.2441	no
gi|641571439|gb|KK856071.1|	30866-33164	23	43	1	4	2	0.2523	no
gi|641571439|gb|KK856071.1|	33731-34042	23	43	5	17	2	0.2709	no
gi|641571439|gb|KK856071.1|	36277-40318	23	43	0.810084	1	0.43677	0.74085	no
gi|641571439|gb|KK856071.1|	41006-41228	23	43	12	30	1	0.3989	no
gi|641571439|gb|KK856071.1|	41589-44043	23	43	2	5	1	0.32625	no
gi|641571439|gb|KK856071.1|	44150-45775	23	43	1	4	1	0.2794	no
gi|641571439|gb|KK856071.1|	45905-47884	23	43	5	13.38	1	0.28295	no
gi|641571439|gb|KK856071.1|	48138-48966	23	43	2	16	3	0.0415	no
gi|641571439|gb|KK856071.1|	49143-50958	23	43	0.874272	15	4	0.01375	no
gi|641571439|gb|KK856071.1|	51073-54433	23	43	0.917284	1	0.382199	0.7687	no
gi|641571439|gb|KK856071.1|	54517-55561	23	43	1	1	0.34234	0.8164	no
gi|641571439|gb|KK856071.1|	56809-57406	23	43	2	0.799561	-2	0.403	no
gi|641571439|gb|KK856071.1|	68026-68844	23	43	432	0	-inf	0.00025	yes
gi|641571439|gb|KK856071.1|	77811-78078	23	43	8	7	-0.0267176	0.9394	no
gi|641571439|gb|KK856071.1|	8944-11921	23	43	6	3	-1	0.4064	no
gi|641571443|gb|KK856070.1|	02612-10355	9.6	33	0.095331	23	8	0.10435	no

gi|641571443|gb|KK856070.1|	07714-10792	9.6	33	0	284	inf	5.00E-05	yes
gi|641571443|gb|KK856070.1|	09936-11055	9.6	33	0	191	inf	0.00015	yes
gi|641571443|gb|KK856070.1|	22608-12337	9.6	33	26	18	-0.468997	0.70515	no
gi|641571443|gb|KK856070.1|	83012-83453	9.6	33	0	21	inf	0.0001	yes
gi|641571443|gb|KK856070.1|	87369-87616	9.6	33	181	148	-0.29158	0.81	no
gi|641571443|gb|KK856070.1|	91572-91767	9.6	33	0	285	inf	0.0001	yes
gi|641571443|gb|KK856070.1|	93412-93553	9.6	33	0	759	inf	5.00E-05	yes
gi|641571450|gb|KK856068.1|	32020-32410	11	48	0	5	inf	0.0068	no
gi|641571450|gb|KK856068.1|	83935-84165	11	48	0	28.37	inf	0.0023	yes
gi|641571450|gb|KK856068.1|	89133-89378	11	48	0	28	inf	0.00135	yes
gi|641571453|gb|KK856067.1|	01227-10294	10	20	0.325061	0.566396	0.801103	1	no
gi|641571453|gb|KK856067.1|	05172-10575	10	20	2	0.836486	-0.987957	0.62775	no
gi|641571453|gb|KK856067.1|	06849-10840	10	20	0.778224	0.553527	-0.491532	1	no
gi|641571453|gb|KK856067.1|	18799-11909	10	20	7	2	-2	0.3411	no
gi|641571453|gb|KK856067.1|	41511-14232	10	20	13	2	-3	0.07155	no
gi|641571453|gb|KK856067.1|	51251-15158	10	20	9	30	2	0.2078	no
gi|641571453|gb|KK856067.1|	63297-16378	10	20	6	6	0.156134	0.91215	no
gi|641571453|gb|KK856067.1|	64165-16461	10	20	2	5	1	0.4421	no
gi|641571453|gb|KK856067.1|	65829-16707	10	20	1	1	-0.267737	0.8494	no
gi|641571453|gb|KK856067.1|	16711-16891	10	20	40	72	0.841528	0.57405	no
gi|641571453|gb|KK856067.1|	67409-16980	10	20	1	0.834191	-0.624815	0.6385	no
gi|641571453|gb|KK856067.1|	69926-17037	10	20	15	12	-0.358114	0.78785	no
gi|641571453|gb|KK856067.1|	17843-18840	10	20	27	15	-0.810071	0.527	no
gi|641571453|gb|KK856067.1|	80055-18067	10	20	2	0.506917	-2	0.4266	no
gi|641571453|gb|KK856067.1|	81176-18196	10	20	3	2	-0.468029	0.74315	no
gi|641571453|gb|KK856067.1|	84711-18597	10	20	0.660657	0.603669	-0.130145	1	no
gi|641571453|gb|KK856067.1|	18962-20676	10	20	2	2	0.0445417	0.97495	no
gi|641571453|gb|KK856067.1|	23494-23732	10	20	82	43	-0.937463	0.47755	no
gi|641571453|gb|KK856067.1|	41283-45656	10	20	5	0.957685	-3	0.0649	no
gi|641571453|gb|KK856067.1|	45784-47772	10	20	1	0.481917	-2	0.2743	no

gi|641571453|gb|KK856067.1|	48097-48744	10	20	2	1	-0.985672	0.57205	no
gi|641571453|gb|KK856067.1|	48953-51463	10	20	2	2	0.0517859	0.96895	no
gi|641571453|gb|KK856067.1|	53412-54753	10	20	0.865181	0.847227	-0.0302535	1	no
gi|641571453|gb|KK856067.1|	54856-55619	10	20	1	2	0.476412	0.75595	no
gi|641571453|gb|KK856067.1|	56299-57101	10	20	5	4	-0.397395	0.769	no
gi|641571453|gb|KK856067.1|	60083-61338	10	20	1	1	0.32665	0.8152	no
gi|641571453|gb|KK856067.1|	63467-68737	10	20	3	4	0.397047	0.7445	no
gi|641571453|gb|KK856067.1|	69307-71173	10	20	0.339034	0.775532	1	1	no
gi|641571453|gb|KK856067.1|	72390-72621	10	20	22	22	0.0425701	0.9635	no
gi|641571453|gb|KK856067.1|	7478-8868	10	20	0.59264	0.63202	0.0928146	1	no
gi|641571453|gb|KK856067.1|	82200-82715	10	20	10	11	0.189644	0.88315	no
gi|641571453|gb|KK856067.1|	85596-86113	10	20	44	40	-0.123618	0.91825	no
gi|641571453|gb|KK856067.1|	90132-91507	10	20	0.420123	0.365731	-0.20003	1	no
gi|641571453|gb|KK856067.1|	92157-92616	10	20	0.267079	4	4	0.12385	no
gi|641571453|gb|KK856067.1|	92742-93331	10	20	6	6	-0.0977567	0.942	no
gi|641571453|gb|KK856067.1|	93599-97480	10	20	7	4	-0.803017	0.51975	no
gi|641571453|gb|KK856067.1|	9608-16256	10	20	3	4	0.327412	0.79115	no
gi|641571453|gb|KK856067.1|	98072-98291	10	20	17	0	-inf	0.0102	no
gi|641571453|gb|KK856067.1|	98480-99695	10	20	6	0.740051	-3	0.06505	no
gi|641571457|gb|KK856066.1|	67361-16894	12	43	3	0.077657	-5	0.1839	no
gi|641571457|gb|KK856066.1|	69049-17037	12	43	17	12	-0.507518	0.6844	no
gi|641571457|gb|KK856066.1|	71680-17859	12	43	9	4	-1	0.45965	no
gi|641571457|gb|KK856066.1|	80145-18358	12	43	2	2	0.330405	0.8024	no
gi|641571457|gb|KK856066.1|	84051-18430	12	43	435	291	-0.580664	0.63575	no
gi|641571457|gb|KK856066.1|	84548-18517	12	43	2	1	-0.516694	0.74125	no
gi|641571457|gb|KK856066.1|	85498-18611	12	43	4	5	0.165508	0.90285	no
gi|641571457|gb|KK856066.1|	86243-18672	12	43	8	8	-0.0405722	0.9737	no
gi|641571457|gb|KK856066.1|	87311-18878	12	43	0.498338	1	1	0.3858	no
gi|641571457|gb|KK856066.1|	88952-19245	12	43	4	4	0.0340488	0.97785	no
gi|641571457|gb|KK856066.1|	92719-19786	12	43	9	9	-0.0265314	0.9912	no

gi|641571457|gb|KK856066.1|	98340-19916	12	43	6	3	-1	0.41185	no
gi|641571457|gb|KK856066.1|	63228-63630	12	43	3	2	-0.999954	0.62775	no
gi|641571461|gb|KK856065.1|	40110-40447	12	46	4	0.716525	-3	0.2588	no
gi|641571465|gb|KK856064.1|	01340-10183	9.2	35	24	6	-2	0.1514	no
gi|641571465|gb|KK856064.1|	02382-10293	9.2	35	5	1	-2	0.27005	no
gi|641571465|gb|KK856064.1|	09705-11110	9.2	35	2	0.17938	-3	0.144	no
gi|641571465|gb|KK856064.1|	12422-11517	9.2	35	166	37	-2	0.3005	no
gi|641571465|gb|KK856064.1|	17455-11951	9.2	35	0.834002	2	1	0.4126	no
gi|641571465|gb|KK856064.1|	19644-12020	9.2	35	38	75	0.992825	0.43005	no
gi|641571465|gb|KK856064.1|	21213-12166	9.2	35	2	2	0.00494795	0.91115	no
gi|641571465|gb|KK856064.1|	22130-12526	9.2	35	0.771775	0.662733	-0.219753	1	no
gi|641571465|gb|KK856064.1|	25621-12786	9.2	35	0.207057	2	3	0.09675	no
gi|641571465|gb|KK856064.1|	28129-12946	9.2	35	0.743531	0.471913	-0.655872	1	no
gi|641571465|gb|KK856064.1|	29746-13002	9.2	35	3	11	2	0.3046	no
gi|641571465|gb|KK856064.1|	30387-13205	9.2	35	2	4	1	0.3717	no
gi|641571465|gb|KK856064.1|	32593-13335	9.2	35	22	40	0.833551	0.50945	no
gi|641571465|gb|KK856064.1|	37988-13830	9.2	35	5	6	0.207634	0.89575	no
gi|641571465|gb|KK856064.1|	44269-14496	9.2	35	0.848317	2	1	0.5082	no
gi|641571465|gb|KK856064.1|	47340-14798	9.2	35	1	1	-0.248678	0.88485	no
gi|641571465|gb|KK856064.1|	37741-38441	9.2	35	0	4	inf	0.00145	yes
gi|641571465|gb|KK856064.1|	72163-72856	9.2	35	2	1	-0.399507	0.8086	no
gi|641571465|gb|KK856064.1|	72951-74115	9.2	35	0.950174	2	1	0.40065	no
gi|641571465|gb|KK856064.1|	78569-79761	9.2	35	7	4	-0.760376	0.55685	no
gi|641571465|gb|KK856064.1|	80035-81031	9.2	35	0.707405	0.537958	-0.395042	1	no
gi|641571465|gb|KK856064.1|	87603-88203	9.2	35	9	3	-2	0.2342	no
gi|641571465|gb|KK856064.1|	88448-88725	9.2	35	61	12	-2	0.12025	no
gi|641571465|gb|KK856064.1|	90904-91148	9.2	35	140	30	-2	0.1075	no
gi|641571465|gb|KK856064.1|	91967-93737	9.2	35	17	5	-2	0.17155	no
gi|641571465|gb|KK856064.1|	95176-95459	9.2	35	49	13	-2	0.1728	no
gi|641571468|gb|KK856063.1|	27917-29028	12	37	1	0	-inf	0.0059	no

gi|641571468|gb|KK856063.1|	34384-36382	12	37	7	0.539156	-4	0.0297	no
gi|641571468|gb|KK856063.1|	36912-41787	12	37	50	7	-3	0.10835	no
gi|641571468|gb|KK856063.1|	44191-45003	12	37	256	324	0.340635	0.86755	no
gi|641571468|gb|KK856063.1|	45963-48011	12	37	1	1	-0.142928	0.9211	no
gi|641571468|gb|KK856063.1|	48372-48668	12	37	6	3	-1	0.49565	no
gi|641571468|gb|KK856063.1|	48854-51275	12	37	2	3	0.359136	0.77655	no
gi|641571468|gb|KK856063.1|	58318-59301	12	37	0.71908	0.273395	-1	1	no
gi|641571472|gb|KK856062.1|	02794-10302	9.9	21	108	53	-1	0.4392	no
gi|641571472|gb|KK856062.1|	05395-10573	9.9	21	70	35	-1	0.42975	no
gi|641571472|gb|KK856062.1|	17671-11789	9.9	21	14	2	-3	0.2588	no
gi|641571472|gb|KK856062.1|	19140-12031	9.9	21	1	2	0.606316	0.67415	no
gi|641571472|gb|KK856062.1|	20808-12205	9.9	21	0.540633	0.411629	-0.393306	1	no
gi|641571472|gb|KK856062.1|	34561-13496	9.9	21	4	3	-0.552726	0.7452	no
gi|641571472|gb|KK856062.1|	38864-13989	9.9	21	3	4	0.302729	0.81775	no
gi|641571472|gb|KK856062.1|	40414-14070	9.9	21	11	5	-1	0.45645	no
gi|641571472|gb|KK856062.1|	43827-14404	9.9	21	25	6	-2	0.2838	no
gi|641571472|gb|KK856062.1|	68189-69522	9.9	21	0.684565	0.379184	-0.852291	1	no
gi|641571472|gb|KK856062.1|	70131-73133	9.9	21	5	5	-0.0418474	0.97265	no
gi|641571472|gb|KK856062.1|	73390-74393	9.9	21	6	5	-0.0597943	0.9649	no
gi|641571472|gb|KK856062.1|	77333-77702	9.9	21	35	24	-0.573104	0.6605	no
gi|641571472|gb|KK856062.1|	78443-79056	9.9	21	57	28	-1	0.41185	no
gi|641571472|gb|KK856062.1|	97143-97587	9.9	21	5	3	-0.825873	0.5984	no
gi|641571472|gb|KK856062.1|	98062-99218	9.9	21	7	1	-2	0.10935	no
gi|641571476|gb|KK856061.1|	16482-11785	12	21	3	2	-0.797061	0.55695	no
gi|641571476|gb|KK856061.1|	18692-12327	12	21	30	7	-2	0.2268	no
gi|641571476|gb|KK856061.1|	23946-12420	12	21	14	3	-2	0.22425	no
gi|641571476|gb|KK856061.1|	13550-13995	12	21	6	4	-0.633143	0.66505	no
gi|641571476|gb|KK856061.1|	40776-14159	12	21	1	0	-inf	0.0075	no
gi|641571476|gb|KK856061.1|	41785-14269	12	21	2	0.599817	-2	0.362	no
gi|641571476|gb|KK856061.1|	15690-16052	12	21	5	1	-2	0.27505	no

gi|641571476|gb|KK856061.1|	16636-17827	12	21	0.497759	0.974334	0.96897	0.5733	no
gi|641571476|gb|KK856061.1|	17934-18920	12	21	3	2	-0.552677	0.69405	no
gi|641571476|gb|KK856061.1|	5532-6049	12	21	0.219571	4	4	0.1196	no
gi|641571476|gb|KK856061.1|	79116-81148	12	21	2	0.176399	-4	0.1059	no
gi|641571476|gb|KK856061.1|	89689-90316	12	21	14	40	2	0.2396	no
gi|641571479|gb|KK856060.1|	06170-10736	11	40	12	7	-0.848765	0.5041	no
gi|641571479|gb|KK856060.1|	07478-10851	11	40	0.843188	0.897916	0.0907265	0.9392	no
gi|641571479|gb|KK856060.1|	08721-10938	11	40	2	2	0.140249	0.929	no
gi|641571479|gb|KK856060.1|	10730-11294	11	40	2	1	-0.786519	0.5601	no
gi|641571479|gb|KK856060.1|	13653-11560	11	40	2	1	-0.654072	0.62895	no
gi|641571479|gb|KK856060.1|	19613-12021	11	40	7	5	-0.554108	0.67995	no
gi|641571479|gb|KK856060.1|	26250-12731	11	40	30	14	-1	0.3874	no
gi|641571479|gb|KK856060.1|	33162-13356	11	40	11	8	-0.502003	0.7185	no
gi|641571479|gb|KK856060.1|	33982-13477	11	40	0.715534	1	0.602393	0.70555	no
gi|641571479|gb|KK856060.1|	41273-14282	11	40	1	2	0.155456	0.91375	no
gi|641571479|gb|KK856060.1|	43955-14416	11	40	33	8	-2	0.24605	no
gi|641571479|gb|KK856060.1|	49387-15014	11	40	2	0.769255	-1	0.46585	no
gi|641571479|gb|KK856060.1|	50329-15182	11	40	2	0.744303	-1	0.3703	no
gi|641571479|gb|KK856060.1|	52259-15250	11	40	24	6	-2	0.21625	no
gi|641571479|gb|KK856060.1|	58123-15937	11	40	0.403166	0.613941	0.606727	1	no
gi|641571479|gb|KK856060.1|	59997-16044	11	40	3	2	-0.673533	0.68185	no
gi|641571479|gb|KK856060.1|	62430-16259	11	40	48	26	-0.905914	0.6344	no
gi|641571479|gb|KK856060.1|	65766-16602	11	40	16	5	-2	0.3662	no
gi|641571479|gb|KK856060.1|	16752-19104	11	40	9	22	1	0.423	no
gi|641571479|gb|KK856060.1|	69920-17013	11	40	28.11	17	-0.742769	0.6213	no
gi|641571479|gb|KK856060.1|	75654-17591	11	40	16	1	-4	0.2029	no
gi|641571479|gb|KK856060.1|	84971-18555	11	40	2	1	-0.781351	0.6308	no
gi|641571479|gb|KK856060.1|	23642-23960	11	40	8	9	0.223607	0.8726	no
gi|641571479|gb|KK856060.1|	24071-36768	11	40	9	18	1	0.6123	no
gi|641571479|gb|KK856060.1|	40717-41643	11	40	15	25	0.693336	0.579	no

gi|641571479|gb|KK856060.1|	43580-45939	11	40	17	39	1	0.59065	no
gi|641571479|gb|KK856060.1|	46119-46335	11	40	16	46	2	0.31235	no
gi|641571479|gb|KK856060.1|	64044-66867	11	40	2	4	0.657914	0.6069	no
gi|641571479|gb|KK856060.1|	67102-67831	11	40	0.530618	2	2	0.3613	no
gi|641571479|gb|KK856060.1|	83595-84806	11	40	0.278833	0.742954	1	1	no
gi|641571479|gb|KK856060.1|	86982-92495	11	40	9	18	1	0.63905	no
gi|641571479|gb|KK856060.1|	9283-9493	11	40	4	28	3	0.27055	no
gi|641571479|gb|KK856060.1|	93034-94305	11	40	0.724021	0.902149	0.317334	0.84265	no
gi|641571479|gb|KK856060.1|	95830-97340	11	40	1	2	0.406205	0.7718	no
gi|641571481|gb|KK856059.1|	02626-10310	9.9	38	9	14	0.671935	0.6171	no
gi|641571481|gb|KK856059.1|	04125-10590	9.9	38	6	5	-0.445043	0.7241	no
gi|641571481|gb|KK856059.1|	06975-10749	9.9	38	0.650703	3	2	0.256	no
gi|641571481|gb|KK856059.1|	07822-11072	9.9	38	0.627803	2	1	0.3172	no
gi|641571481|gb|KK856059.1|	11812-11314	9.9	38	1	2	0.681122	0.6235	no
gi|641571481|gb|KK856059.1|	13328-11482	9.9	38	0.927524	2	1	0.458	no
gi|641571481|gb|KK856059.1|	14951-11620	9.9	38	0.466816	2	2	0.2786	no
gi|641571481|gb|KK856059.1|	16368-11739	9.9	38	0.766168	0.388504	-0.97973	1	no
gi|641571481|gb|KK856059.1|	18264-11887	9.9	38	0.337196	4	3	0.25495	no
gi|641571481|gb|KK856059.1|	19013-11967	9.9	38	0.609272	2	2	0.3613	no
gi|641571481|gb|KK856059.1|	12165-13156	9.9	38	8	0.13533	-6	0.1832	no
gi|641571481|gb|KK856059.1|	24024-12668	9.9	38	1	8	3	0.0362	no
gi|641571481|gb|KK856059.1|	31815-13287	9.9	38	2	1	-0.201821	0.88665	no
gi|641571481|gb|KK856059.1|	41093-14161	9.9	38	0.85461	3	2	0.3613	no
gi|641571481|gb|KK856059.1|	4173-4363	9.9	38	166	0	-inf	5.00E-05	yes
gi|641571481|gb|KK856059.1|	62522-62847	9.9	38	881	2	-9	0.1308	no
gi|641571481|gb|KK856059.1|	77619-81646	9.9	38	1509.34	0.395054	-12	0.22125	no
gi|641571481|gb|KK856059.1|	81877-83277	9.9	38	359	0.626792	-9	0.0266	no
gi|641571481|gb|KK856059.1|	83456-83793	9.9	38	803	0	-inf	0.00015	yes
gi|641571481|gb|KK856059.1|	84563-86039	9.9	38	2	0	-inf	0.00085	yes
gi|641571481|gb|KK856059.1|	88605-88755	9.9	38	440	0	-inf	0.00035	yes

gi|641571484|gb|KK856057.1|	14533-14902	11	39	17	18	0.0952742	0.945	no
gi|641571484|gb|KK856057.1|	17422-17610	11	39	25	16	-0.64604	0.70205	no
gi|641571484|gb|KK856057.1|	80208-80751	11	39	150	117	-0.356545	0.8731	no
gi|641571494|gb|KK856054.1|	59897-16007	11	44	60	0	-inf	0.0034	yes
gi|641571494|gb|KK856054.1|	23185-23680	11	44	13	18	0.429371	0.73825	no
gi|641571498|gb|KK856053.1|	19192-11956	9	42	26	0.596535	-5	0.18415	no
gi|641571498|gb|KK856053.1|	22760-12292	9	42	258	7	-5	0.1843	no
gi|641571501|gb|KK856052.1|	33501-33898	8.6	37	0	10	inf	0.0015	yes
gi|641571501|gb|KK856052.1|	65837-66734	8.6	37	0	90	inf	0.0006	yes
gi|641571505|gb|KK856051.1|	88739-89631	8.6	37	0.914627	2	0.755862	0.62565	no
gi|641571505|gb|KK856051.1|	97037-97956	8.6	37	0.78267	0.892308	0.189137	0.91015	no
gi|641571508|gb|KK856050.1|	02437-10622	9.7	33	9	18	1	0.53275	no
gi|641571508|gb|KK856050.1|	07083-11253	9.7	33	5	7	0.531041	0.73445	no
gi|641571508|gb|KK856050.1|	13090-11521	9.7	33	3	6	1	0.40675	no
gi|641571508|gb|KK856050.1|	17992-12010	9.7	33	0.999467	0.847026	-0.238753	0.8664	no
gi|641571508|gb|KK856050.1|	22679-12283	9.7	33	54	54	0.0203299	0.92445	no
gi|641571508|gb|KK856050.1|	24294-12573	9.7	33	4	4	0.142903	0.9088	no
gi|641571508|gb|KK856050.1|	27326-12751	9.7	33	25	46	0.905071	0.55385	no
gi|641571508|gb|KK856050.1|	29370-12961	9.7	33	24	27	0.156448	0.91105	no
gi|641571508|gb|KK856050.1|	30507-13086	9.7	33	24	29	0.320888	0.8088	no
gi|641571508|gb|KK856050.1|	31907-13216	9.7	33	32	40	0.33327	0.8014	no
gi|641571508|gb|KK856050.1|	34458-13479	9.7	33	26	37	0.522487	0.6901	no
gi|641571508|gb|KK856050.1|	35635-13579	9.7	33	216	224.56	0.0551069	0.967	no
gi|641571508|gb|KK856050.1|	39163-13938	9.7	33	14	12	-0.194499	0.89755	no
gi|641571508|gb|KK856050.1|	41717-14195	9.7	33	43	40	-0.0761243	0.95485	no
gi|641571508|gb|KK856050.1|	43770-14400	9.7	33	14	24	0.769951	0.60965	no
gi|641571508|gb|KK856050.1|	50828-15119	9.7	33	33	47	0.492312	0.7004	no
gi|641571508|gb|KK856050.1|	54757-15555	9.7	33	2	0.357925	-2	0.26805	no
gi|641571508|gb|KK856050.1|	76267-17648	9.7	33	19	26	0.412771	0.79325	no
gi|641571508|gb|KK856050.1|	17632-17848	9.7	33	54	42	-0.379236	0.7819	no

gi|641571508|gb|KK856050.1|	78204-17891	9.7	33	11	10	-0.14232	0.9105	no
gi|641571508|gb|KK856050.1|	81539-18208	9.7	33	2	0.61089	-2	0.4266	no
gi|641571508|gb|KK856050.1|	84973-18515	9.7	33	32	21	-0.614695	0.69715	no
gi|641571508|gb|KK856050.1|	87259-18743	9.7	33	17	60	2	0.3111	no
gi|641571508|gb|KK856050.1|	88224-18849	9.7	33	25	22	-0.225097	0.86505	no
gi|641571508|gb|KK856050.1|	19320-19918	9.7	33	111	45	-1	0.43935	no
gi|641571508|gb|KK856050.1|	96089-19627	9.7	33	29	11	-1	0.44165	no
gi|641571508|gb|KK856050.1|	20089-21323	9.7	33	34	17	-0.992904	0.42655	no
gi|641571508|gb|KK856050.1|	25210-25862	9.7	33	3	3	-0.0635324	0.9621	no
gi|641571508|gb|KK856050.1|	27652-29465	9.7	33	5	3	-0.988882	0.4448	no
gi|641571508|gb|KK856050.1|	36243-37337	9.7	33	2	1	-0.268708	0.8494	no
gi|641571508|gb|KK856050.1|	37440-40376	9.7	33	1	1	0.0252161	0.98455	no
gi|641571508|gb|KK856050.1|	379-1191	9.7	33	8	2	-2	0.12165	no
gi|641571508|gb|KK856050.1|	40497-41102	9.7	33	1	0.522779	-1	0.4534	no
gi|641571508|gb|KK856050.1|	43035-43754	9.7	33	0.675924	2	2	0.28495	no
gi|641571508|gb|KK856050.1|	44218-44824	9.7	33	0.861388	2	1	0.4611	no
gi|641571508|gb|KK856050.1|	45043-51682	9.7	33	4	4	0.108281	0.9317	no
gi|641571508|gb|KK856050.1|	51825-54627	9.7	33	1	2	0.798513	0.5424	no
gi|641571508|gb|KK856050.1|	54737-57244	9.7	33	1	2	0.547042	0.6784	no
gi|641571508|gb|KK856050.1|	57347-58360	9.7	33	2	2	0.212793	0.88035	no
gi|641571508|gb|KK856050.1|	58533-59308	9.7	33	0.488541	2	2	0.3012	no
gi|641571508|gb|KK856050.1|	59605-61585	9.7	33	0.515738	2	2	0.19155	no
gi|641571508|gb|KK856050.1|	61688-62707	9.7	33	0.945523	1	0.605163	0.6863	no
gi|641571508|gb|KK856050.1|	62964-67264	9.7	33	0.948711	3	2	0.2105	no
gi|641571508|gb|KK856050.1|	67415-68003	9.7	33	1	2	0.819744	0.62335	no
gi|641571508|gb|KK856050.1|	69489-70482	9.7	33	1	0.809957	-0.39507	0.80865	no
gi|641571508|gb|KK856050.1|	70607-71188	9.7	33	2	1	-0.250913	0.88485	no
gi|641571508|gb|KK856050.1|	73483-74243	9.7	33	0.376134	1	2	0.3323	no
gi|641571508|gb|KK856050.1|	77784-78924	9.7	33	0.824419	0.798549	-0.0459963	1	no
gi|641571508|gb|KK856050.1|	81066-81512	9.7	33	6	4	-0.562669	0.70545	no

gi|641571508|gb|KK856050.1|	92947-93372	9.7	33	9	38	2	0.1234	no
gi|641571508|gb|KK856050.1|	95787-96702	9.7	33	0.590265	1	1	0.5082	no
gi|641571508|gb|KK856050.1|	96813-101943	9.7	33	2	4	1	0.34075	no
gi|641571512|gb|KK856049.1|	14666-15628	9.8	36	115	145	0.333489	0.87735	no
gi|641571512|gb|KK856049.1|	2-783	9.8	36	7	17	1	0.28	no
gi|641571512|gb|KK856049.1|	7765-8160	9.8	36	11	38	2	0.18195	no
gi|641571515|gb|KK856048.1|	34153-13641	9.9	38	0.650606	1	0.683477	0.6234	no
gi|641571515|gb|KK856048.1|	13698-20413	9.9	38	2	3	0.396774	0.75405	no
gi|641571515|gb|KK856048.1|	37660-13802	9.9	38	6	2	-1	0.4158	no
gi|641571515|gb|KK856048.1|	44304-14497	9.9	38	0.449237	2	2	0.2812	no
gi|641571515|gb|KK856048.1|	54200-15494	9.9	38	2	0.98429	-0.883897	0.5831	no
gi|641571515|gb|KK856048.1|	58228-16014	9.9	38	0.411238	0.501743	0.286973	1	no
gi|641571515|gb|KK856048.1|	60320-16116	9.9	38	0.650855	1	1	0.5082	no
gi|641571515|gb|KK856048.1|	77799-17880	9.9	38	0.964253	0.39999	-1	0.47135	no
gi|641571515|gb|KK856048.1|	80660-18238	9.9	38	0.64927	1	1	0.4385	no
gi|641571515|gb|KK856048.1|	82491-18428	9.9	38	1	3	1	0.3832	no
gi|641571515|gb|KK856048.1|	84650-18604	9.9	38	0.767221	1	0.607408	0.681	no
gi|641571515|gb|KK856048.1|	86204-18810	9.9	38	0.790072	2	1	0.409	no
gi|641571515|gb|KK856048.1|	95493-19655	9.9	38	3	8	1	0.31275	no
gi|641571515|gb|KK856048.1|	09036-21020	9.9	38	19	18	-0.0355337	0.9779	no
gi|641571515|gb|KK856048.1|	10291-21462	9.9	38	20	9	-1	0.61725	no
gi|641571515|gb|KK856048.1|	15016-21557	9.9	38	30.44	5	-3	0.06845	no
gi|641571515|gb|KK856048.1|	17822-21816	9.9	38	3	13	2	0.24555	no
gi|641571515|gb|KK856048.1|	19276-22012	9.9	38	2	2	-0.0748243	0.95395	no
gi|641571515|gb|KK856048.1|	21402-22400	9.9	38	2	2	0.0504496	0.96715	no
gi|641571515|gb|KK856048.1|	22297-22517	9.9	38	59	109	0.898643	0.49225	no
gi|641571515|gb|KK856048.1|	25648-22690	9.9	38	0.803282	0.203876	-2	1	no
gi|641571515|gb|KK856048.1|	28358-22867	9.9	38	3	11	2	0.2932	no
gi|641571515|gb|KK856048.1|	24524-24694	9.9	38	54	57	0.099102	0.9444	no
gi|641571515|gb|KK856048.1|	25492-25730	9.9	38	34	65	0.93791	0.48775	no

gi|641571515|gb|KK856048.1|	31826-32824	9.9	38	0.529232	0.939094	0.827368	0.6232	no
gi|641571515|gb|KK856048.1|	33297-33723	9.9	38	14	92	3	0.0539	no
gi|641571515|gb|KK856048.1|	34376-37147	9.9	38	6	46	3	0.11755	no
gi|641571515|gb|KK856048.1|	37317-41280	9.9	38	36	16	-1	0.6027	no
gi|641571515|gb|KK856048.1|	50328-50660	9.9	38	86	38	-1	0.36515	no
gi|641571515|gb|KK856048.1|	55623-56533	9.9	38	0.792526	2	1	0.42725	no
gi|641571515|gb|KK856048.1|	62125-62381	9.9	38	25	19	-0.392881	0.77955	no
gi|641571515|gb|KK856048.1|	71426-71636	9.9	38	30	8	-2	0.28355	no
gi|641571515|gb|KK856048.1|	72061-72248	9.9	38	37	32	-0.177094	0.9094	no
gi|641571515|gb|KK856048.1|	75076-77236	9.9	38	3	1	-1	0.3777	no
gi|641571515|gb|KK856048.1|	7710-8374	9.9	38	1	2	0.237192	0.88205	no
gi|641571515|gb|KK856048.1|	77515-82023	9.9	38	8	4	-0.954291	0.56315	no
gi|641571515|gb|KK856048.1|	82618-83626	9.9	38	14	12	-0.300718	0.81515	no
gi|641571515|gb|KK856048.1|	91275-92059	9.9	38	15	9	-0.782981	0.53825	no
gi|641571515|gb|KK856048.1|	9748-13550	9.9	38	2	5	0.99484	0.435	no
gi|641571520|gb|KK856047.1|	10891-13363	10	41	0.777474	2	2	0.26565	no
gi|641571520|gb|KK856047.1|	28005-12829	10	41	4	11	1	0.3861	no
gi|641571520|gb|KK856047.1|	13935-14746	10	41	3	3	0.24911	0.8602	no
gi|641571520|gb|KK856047.1|	14976-16094	10	41	2	3	0.664825	0.6284	no
gi|641571520|gb|KK856047.1|	52471-15266	10	41	16	20	0.306304	0.86275	no
gi|641571520|gb|KK856047.1|	16679-18826	10	41	2	5	0.957229	0.4541	no
gi|641571520|gb|KK856047.1|	74318-74630	10	41	10	0.84672	-4	0.2029	no
gi|641571520|gb|KK856047.1|	79138-79596	10	41	1	2	0.590306	0.70505	no
gi|641571520|gb|KK856047.1|	9624-9972	10	41	3	5	0.799739	0.6409	no
gi|641571523|gb|KK856046.1|	28039-12892	10	43	1	0.776671	-0.39619	0.81255	no
gi|641571523|gb|KK856046.1|	36091-13804	10	43	0.443855	3	3	0.07165	no
gi|641571523|gb|KK856046.1|	40937-14170	10	43	0.746979	2	1	0.4702	no
gi|641571523|gb|KK856046.1|	27689-28037	10	43	8	1	-3	0.2095	no
gi|641571523|gb|KK856046.1|	36508-37897	10	43	0.474509	0.45182	-0.0706883	1	no
gi|641571523|gb|KK856046.1|	39123-39590	10	43	1	4	1	0.4014	no

gi|641571523|gb|KK856046.1|	84213-84458	10	43	13	5	-1	0.4177	no
gi|641571526|gb|KK856045.1|	32076-13242	9.6	42	4	1	-2	0.3632	no
gi|641571526|gb|KK856045.1|	37126-13815	9.6	42	0.51201	0.778873	0.605215	1	no
gi|641571526|gb|KK856045.1|	40068-14149	9.6	42	0.690927	1	0.929457	0.53445	no
gi|641571526|gb|KK856045.1|	43180-14412	9.6	42	4	17	2	0.1324	no
gi|641571526|gb|KK856045.1|	48916-14970	9.6	42	0.950842	1	0.409781	0.8089	no
gi|641571526|gb|KK856045.1|	51759-15206	9.6	42	9	38	2	0.1558	no
gi|641571526|gb|KK856045.1|	53683-15400	9.6	42	8	29	2	0.2	no
gi|641571526|gb|KK856045.1|	56973-15730	9.6	42	20	74	2	0.1591	no
gi|641571526|gb|KK856045.1|	58865-15979	9.6	42	10	31	2	0.19895	no
gi|641571530|gb|KK856044.1|	06922-10922	10	38	26	31	0.251166	0.9098	no
gi|641571530|gb|KK856044.1|	09326-11041	10	38	4	4	0.200475	0.87875	no
gi|641571530|gb|KK856044.1|	20319-12050	10	38	50	8	-3	0.1861	no
gi|641571530|gb|KK856044.1|	25529-12600	10	38	9	5	-0.993545	0.4886	no
gi|641571530|gb|KK856044.1|	27826-12827	10	38	4	3	-0.825619	0.5984	no
gi|641571530|gb|KK856044.1|	27502-28232	10	38	3	2	-0.720618	0.61365	no
gi|641571530|gb|KK856044.1|	42184-42634	10	38	6	0.415821	-4	0.19905	no
gi|641571530|gb|KK856044.1|	44327-46105	10	38	5	1	-2	0.14445	no
gi|641571530|gb|KK856044.1|	47239-49036	10	38	2	0.404428	-2	0.13225	no
gi|641571530|gb|KK856044.1|	49813-51177	10	38	2	0.461451	-2	0.2061	no
gi|641571530|gb|KK856044.1|	56222-59712	10	38	31	13	-1	0.5675	no
gi|641571530|gb|KK856044.1|	60052-64187	10	38	15	5	-2	0.31705	no
gi|641571530|gb|KK856044.1|	67815-69984	10	38	2	0.766175	-1	0.438	no
gi|641571530|gb|KK856044.1|	72071-72935	10	38	0.741163	0.965251	0.381113	0.82465	no
gi|641571530|gb|KK856044.1|	75733-76365	10	38	10	3	-2	0.2592	no
gi|641571530|gb|KK856044.1|	76501-77437	10	38	41	16	-1	0.4175	no
gi|641571530|gb|KK856044.1|	84769-88688	10	38	13	7	-0.805692	0.62145	no
gi|641571530|gb|KK856044.1|	90558-92048	10	38	0	3	inf	0.0004	yes
gi|641571533|gb|KK856043.1|	03108-10330	11	40	0	43	inf	0.0025	yes
gi|641571533|gb|KK856043.1|	06004-10678	11	40	0.121091	4	5	0.1079	no

gi|641571533|gb|KK856043.1|	19343-11978	11	40	0	23	inf	0.00015	yes
gi|641571533|gb|KK856043.1|	19917-12094	11	40	0.171082	2	4	0.2456	no
gi|641571533|gb|KK856043.1|	27031-12795	11	40	181	192	0.0843513	0.96665	no
gi|641571533|gb|KK856043.1|	38147-14114	11	40	36	43	0.223894	0.91875	no
gi|641571533|gb|KK856043.1|	41401-14249	11	40	1	0.360072	-2	0.4042	no
gi|641571533|gb|KK856043.1|	47866-14800	11	40	188	20	-3	0.2199	no
gi|641571533|gb|KK856043.1|	50560-15147	11	40	34	0.297436	-7	0.1301	no
gi|641571533|gb|KK856043.1|	55064-15609	11	40	16	0	-inf	5.00E-05	yes
gi|641571533|gb|KK856043.1|	56956-15790	11	40	2	0	-inf	0.0032	yes
gi|641571533|gb|KK856043.1|	61046-16137	11	40	6	0	-inf	0.01	no
gi|641571533|gb|KK856043.1|	63559-16402	11	40	0	11	inf	0.0004	yes
gi|641571533|gb|KK856043.1|	75529-76180	11	40	0	11	inf	0.0001	yes
gi|641571533|gb|KK856043.1|	88657-90741	11	40	0	3	inf	5.00E-05	yes
gi|641571533|gb|KK856043.1|	93062-93235	11	40	0	70	inf	0.00375	no
gi|641571545|gb|KK856040.1|	58012-58805	10	41	0	12	inf	5.00E-05	yes
gi|641571548|gb|KK856039.1|	01183-10181	9.9	18	1	3	1	0.45675	no
gi|641571548|gb|KK856039.1|	03471-10369	9.9	18	5	12	1	0.5254	no
gi|641571548|gb|KK856039.1|	12226-11424	9.9	18	8	10	0.281923	0.81915	no
gi|641571548|gb|KK856039.1|	15875-11868	9.9	18	8	9	0.195041	0.8763	no
gi|641571548|gb|KK856039.1|	18915-11946	9.9	18	3	5	0.683243	0.6314	no
gi|641571548|gb|KK856039.1|	20775-12286	9.9	18	3	4	0.215873	0.8625	no
gi|641571548|gb|KK856039.1|	23289-12493	9.9	18	3	3	0.0233006	0.9859	no
gi|641571548|gb|KK856039.1|	25211-12638	9.9	18	3	0.882852	-2	0.21005	no
gi|641571548|gb|KK856039.1|	27643-12928	9.9	18	2	0.967876	-1	0.45945	no
gi|641571548|gb|KK856039.1|	29356-13079	9.9	18	3	2	-0.222485	0.86035	no
gi|641571548|gb|KK856039.1|	20291-20656	9.9	18	7	1	-3	0.1909	no
gi|641571548|gb|KK856039.1|	34563-37596	9.9	18	27	9	-2	0.31575	no
gi|641571548|gb|KK856039.1|	41100-41796	9.9	18	0.989704	0.857541	-0.206792	0.8945	no
gi|641571548|gb|KK856039.1|	41991-43956	9.9	18	1	2	1	0.4105	no
gi|641571548|gb|KK856039.1|	56697-57100	9.9	18	5	2	-2	0.30255	no

gi|641571548|gb|KK856039.1|	58170-59485	9.9	18	0.822037	0.385257	-1	1	no
gi|641571548|gb|KK856039.1|	62845-63482	9.9	18	2	2	-0.307894	0.84435	no
gi|641571548|gb|KK856039.1|	64561-64927	9.9	18	28	15	-0.964068	0.465	no
gi|641571548|gb|KK856039.1|	65716-67578	9.9	18	13	7	-0.874729	0.4796	no
gi|641571548|gb|KK856039.1|	68007-69000	9.9	18	6	5	-0.273079	0.83725	no
gi|641571548|gb|KK856039.1|	69742-70801	9.9	18	6	5	-0.144527	0.90745	no
gi|641571548|gb|KK856039.1|	94512-95184	9.9	18	15	17	0.252049	0.84315	no
gi|641571551|gb|KK856038.1|	06812-10715	6.8	30	0.477835	32	6	0.10465	no
gi|641571555|gb|KK856037.1|	27756-29075	10	42	13	4	-2	0.17545	no
gi|641571555|gb|KK856037.1|	36259-36572	10	42	8	0.840679	-3	0.21195	no
gi|641571555|gb|KK856037.1|	39399-39698	10	42	13	4	-2	0.2712	no
gi|641571559|gb|KK856036.1|	2899-3547	8.6	36	0.938073	1	0.336285	0.83525	no
gi|641571559|gb|KK856036.1|	34816-35550	8.6	36	0.262849	2	3	0.25965	no
gi|641571559|gb|KK856036.1|	35685-36580	8.6	36	0.303568	2	2	0.2136	no
gi|641571559|gb|KK856036.1|	36758-38901	8.6	36	0.399873	3	3	0.07165	no
gi|641571559|gb|KK856036.1|	39116-39694	8.6	36	29	145	2	0.20225	no
gi|641571559|gb|KK856036.1|	40017-41451	8.6	36	31	166.52	2	0.2795	no
gi|641571559|gb|KK856036.1|	41738-42649	8.6	36	0.791419	3	2	0.22535	no
gi|641571559|gb|KK856036.1|	42851-44114	8.6	36	2	6	2	0.1535	no
gi|641571559|gb|KK856036.1|	44226-44585	8.6	36	13	77	3	0.0704	no
gi|641571559|gb|KK856036.1|	45819-47274	8.6	36	2	12	2	0.0895	no
gi|641571559|gb|KK856036.1|	47543-51335	8.6	36	0.885851	4	2	0.1149	no
gi|641571559|gb|KK856036.1|	51711-52973	8.6	36	0.929256	3	1	0.3046	no
gi|641571559|gb|KK856036.1|	53120-53589	8.6	36	1	4	2	0.26255	no
gi|641571559|gb|KK856036.1|	53716-54408	8.6	36	0	5	inf	0.00045	yes
gi|641571559|gb|KK856036.1|	62993-65430	8.6	36	0.852782	3	2	0.22045	no
gi|641571559|gb|KK856036.1|	66072-67298	8.6	36	3	13	2	0.0886	no
gi|641571562|gb|KK856035.1|	05156-10569	9.8	36	2	0	-inf	0.01	no
gi|641571562|gb|KK856035.1|	05892-10686	9.8	36	3	3	0.0839078	0.9506	no
gi|641571562|gb|KK856035.1|	07048-11067	9.8	36	10	2	-2	0.1207	no

gi|641571562|gb|KK856035.1|	13937-11445	9.8	36	2	1	-0.575727	0.7586	no
gi|641571562|gb|KK856035.1|	21548-12177	9.8	36	20	22	0.0958917	0.94825	no
gi|641571562|gb|KK856035.1|	25138-12583	9.8	36	22	25	0.158069	0.9035	no
gi|641571562|gb|KK856035.1|	18-1273	9.8	36	1	0.406986	-1	0.37565	no
gi|641571562|gb|KK856035.1|	22514-22705	9.8	36	39	11	-2	0.32225	no
gi|641571562|gb|KK856035.1|	23376-24358	9.8	36	0.269998	1	2	0.2235	no
gi|641571562|gb|KK856035.1|	25705-26520	9.8	36	1	0.347023	-2	0.26805	no
gi|641571562|gb|KK856035.1|	27316-27904	9.8	36	5	1	-2	0.23295	no
gi|641571562|gb|KK856035.1|	37454-39608	9.8	36	0	7	inf	5.00E-05	yes
gi|641571562|gb|KK856035.1|	40182-41044	9.8	36	0.106198	3	5	0.11025	no
gi|641571562|gb|KK856035.1|	44975-45409	9.8	36	0	16	inf	0.00035	yes
gi|641571562|gb|KK856035.1|	45570-47382	9.8	36	2	9	2	0.1325	no
gi|641571562|gb|KK856035.1|	47874-48340	9.8	36	0	4	inf	0.00455	no
gi|641571562|gb|KK856035.1|	48523-49151	9.8	36	1	2	0.406076	0.8089	no
gi|641571562|gb|KK856035.1|	53799-54943	9.8	36	0	3	inf	0.00075	yes
gi|641571562|gb|KK856035.1|	56643-58034	9.8	36	0.059215	2	5	0.1059	no
gi|641571562|gb|KK856035.1|	60660-61171	9.8	36	0.894797	6	3	0.1772	no
gi|641571562|gb|KK856035.1|	63681-64722	9.8	36	0	3	inf	0.00105	yes
gi|641571562|gb|KK856035.1|	7565-7828	9.8	36	18	1	-4	0.19635	no
gi|641571562|gb|KK856035.1|	84374-86099	9.8	36	7	7	0.0884635	0.94555	no
gi|641571562|gb|KK856035.1|	86201-90202	9.8	36	9	8	-0.243589	0.84055	no
gi|641571562|gb|KK856035.1|	9102-9363	9.8	36	8	1	-3	0.2588	no
gi|641571562|gb|KK856035.1|	91899-93169	9.8	36	1	3	1	0.42385	no
gi|641571562|gb|KK856035.1|	93734-94556	9.8	36	0.677857	0.857931	0.339881	1	no
gi|641571562|gb|KK856035.1|	95177-95629	9.8	36	14	13	-0.156414	0.9058	no
gi|641571562|gb|KK856035.1|	95884-96131	9.8	36	31	57	0.854013	0.5288	no
gi|641571562|gb|KK856035.1|	97887-98056	9.8	36	32	72	1	0.4722	no
gi|641571562|gb|KK856035.1|	99828-10005	9.8	36	36	49	0.432755	0.75595	no
gi|641571565|gb|KK856034.1|	24222-25949	9.5	42	0.83205	0.775246	-0.102018	1	no
gi|641571565|gb|KK856034.1|	27147-28194	9.5	42	0.581915	0.632375	0.119972	1	no

gi|641571565|gb|KK856034.1|	68112-69428	9.5	42	0.631772	0.769829	0.285133	1	no
gi|641571569|gb|KK856033.1|	00129-10207	9.9	37	3	2	-0.370289	0.7729	no
gi|641571569|gb|KK856033.1|	06063-10622	9.9	37	120.16	191	0.667393	0.6442	no
gi|641571569|gb|KK856033.1|	06685-10711	9.9	37	2	2	0.58753	0.70505	no
gi|641571569|gb|KK856033.1|	12422-11282	9.9	37	1	4	1	0.4433	no
gi|641571569|gb|KK856033.1|	11351-11718	9.9	37	16	34	1	0.4064	no
gi|641571569|gb|KK856033.1|	14301-11490	9.9	37	7	12	0.729069	0.58235	no
gi|641571569|gb|KK856033.1|	14834-16548	9.9	37	4	7	0.745946	0.5538	no
gi|641571569|gb|KK856033.1|	21444-21744	9.9	37	86	2	-6	0.1304	no
gi|641571569|gb|KK856033.1|	26629-27062	9.9	37	93	2	-6	0.0613	no
gi|641571569|gb|KK856033.1|	29734-30011	9.9	37	611	1	-9	0.18535	no
gi|641571569|gb|KK856033.1|	31785-33276	9.9	37	16	0.58291	-5	0.0273	no
gi|641571569|gb|KK856033.1|	33551-34256	9.9	37	2	0.210635	-3	0.2381	no
gi|641571569|gb|KK856033.1|	34360-36056	9.9	37	23	1	-4	0.0088	no
gi|641571569|gb|KK856033.1|	36350-36859	9.9	37	1	3	1	0.39995	no
gi|641571569|gb|KK856033.1|	37403-37690	9.9	37	155	2	-6	0.13015	no
gi|641571569|gb|KK856033.1|	37859-40488	9.9	37	122	1	-7	0.0056	no
gi|641571569|gb|KK856033.1|	44297-45733	9.9	37	28	0.521713	-6	0.0289	no
gi|641571569|gb|KK856033.1|	46545-47130	9.9	37	57	0	-inf	5.00E-05	yes
gi|641571569|gb|KK856033.1|	47719-49172	9.9	37	191	0.343123	-9	0.06225	no
gi|641571569|gb|KK856033.1|	4805-4989	9.9	37	18	61	2	0.29845	no
gi|641571569|gb|KK856033.1|	50369-51599	9.9	37	21	0.83356	-5	0.0221	no
gi|641571569|gb|KK856033.1|	52819-53150	9.9	37	434	0.744222	-9	0.18475	no
gi|641571569|gb|KK856033.1|	55214-56280	9.9	37	1	0.371185	-2	0.28145	no
gi|641571569|gb|KK856033.1|	56402-57521	9.9	37	28	3	-3	0.03235	no
gi|641571569|gb|KK856033.1|	59324-59617	9.9	37	375	0	-inf	0.00065	yes
gi|641571569|gb|KK856033.1|	62668-62957	9.9	37	23	0	-inf	0.00055	yes
gi|641571569|gb|KK856033.1|	64567-64866	9.9	37	430	0	-inf	0.00045	yes
gi|641571569|gb|KK856033.1|	81107-81342	9.9	37	11	14	0.376815	0.8163	no
gi|641571569|gb|KK856033.1|	87113-87350	9.9	37	11	16	0.547559	0.7313	no

gi|641571569|gb|KK856033.1|	88467-88770	9.9	37	11	12	0.0986194	0.94775	no
gi|641571569|gb|KK856033.1|	92097-92699	9.9	37	4	3	-0.539644	0.71205	no
gi|641571569|gb|KK856033.1|	94746-95786	9.9	37	1	2	1	0.45475	no
gi|641571573|gb|KK856032.1|	10458-11137	7.8	36	0.491888	1	1	0.4595	no
gi|641571577|gb|KK856031.1|	05512-10603	8.9	35	0.429881	3	3	0.28535	no
gi|641571577|gb|KK856031.1|	34019-13480	8.9	35	1	0.725445	-0.567548	0.7586	no
gi|641571577|gb|KK856031.1|	14158-14398	8.9	35	6	10	0.8118	0.6381	no
gi|641571577|gb|KK856031.1|	14542-14983	8.9	35	0.858558	4	2	0.256	no
gi|641571577|gb|KK856031.1|	15620-17745	8.9	35	0.476985	2	2	0.1542	no
gi|641571577|gb|KK856031.1|	2620-3063	8.9	35	1	4	2	0.32945	no
gi|641571577|gb|KK856031.1|	29520-30239	8.9	35	0.946294	1	0.378687	0.82465	no
gi|641571577|gb|KK856031.1|	42494-43728	8.9	35	0.408988	0.518977	0.343611	1	no
gi|641571577|gb|KK856031.1|	43853-44503	8.9	35	0.778298	2	1	0.51085	no
gi|641571577|gb|KK856031.1|	45063-46036	8.9	35	0.728326	2	1	0.4562	no
gi|641571577|gb|KK856031.1|	71283-72345	8.9	35	0.571844	0.994372	0.798164	0.63025	no
gi|641571577|gb|KK856031.1|	72960-73401	8.9	35	0.572372	5	3	0.25965	no
gi|641571577|gb|KK856031.1|	75670-76384	8.9	35	0	2	inf	0.00455	no
gi|641571581|gb|KK856030.1|	25391-12882	10	38	2	5	2	0.23505	no
gi|641571581|gb|KK856030.1|	29241-13225	10	38	3	8	1	0.2628	no
gi|641571581|gb|KK856030.1|	37235-13919	10	38	5	35	3	0.1461	no
gi|641571581|gb|KK856030.1|	52126-15295	10	38	0.111552	2	4	0.12385	no
gi|641571581|gb|KK856030.1|	53722-15501	10	38	137	2428.99	4	0.1447	no
gi|641571581|gb|KK856030.1|	60561-16143	10	38	3	18	3	0.0769	no
gi|641571581|gb|KK856030.1|	61700-16296	10	38	2	31	4	0.0123	no
gi|641571581|gb|KK856030.1|	70145-17478	10	38	6	16	1	0.3784	no
gi|641571581|gb|KK856030.1|	94285-19733	10	38	36	92	1	0.52005	no
gi|641571581|gb|KK856030.1|	99915-20033	10	38	28	44	0.634043	0.61225	no
gi|641571581|gb|KK856030.1|	01024-20791	10	38	8	3	-1	0.43025	no
gi|641571581|gb|KK856030.1|	08505-20997	10	38	1	1	0.171606	0.90195	no
gi|641571581|gb|KK856030.1|	10143-21104	10	38	0.800365	2	1	0.42725	no

gi|641571581|gb|KK856030.1|	11828-21478	10	38	2	1	-0.41289	0.74855	no
gi|641571581|gb|KK856030.1|	14939-22125	10	38	24	16	-0.580786	0.78495	no
gi|641571581|gb|KK856030.1|	25840-22781	10	38	25	14	-0.9007	0.58195	no
gi|641571585|gb|KK856029.1|	11143-11333	11	22	37	30	-0.282402	0.8517	no
gi|641571585|gb|KK856029.1|	14259-15014	11	22	7	7	0.0168346	0.98735	no
gi|641571585|gb|KK856029.1|	16192-16449	11	22	17	11	-0.614933	0.68055	no
gi|641571585|gb|KK856029.1|	18422-19011	11	22	3	0.271855	-3	0.21435	no
gi|641571585|gb|KK856029.1|	19851-20589	11	22	11	9	-0.267284	0.83825	no
gi|641571585|gb|KK856029.1|	20810-21571	11	22	8	7	-0.205448	0.87095	no
gi|641571585|gb|KK856029.1|	22774-23766	11	22	17	18	0.0979608	0.93605	no
gi|641571594|gb|KK856027.1|	20876-21410	9.2	37	0.834605	2	1	0.51315	no
gi|641571594|gb|KK856027.1|	23113-24742	9.2	37	2	4	1	0.3683	no
gi|641571594|gb|KK856027.1|	28079-29040	9.2	37	0.647273	0.843625	0.382229	1	no
gi|641571594|gb|KK856027.1|	29469-31191	9.2	37	4	6	0.574516	0.6495	no
gi|641571594|gb|KK856027.1|	31291-34709	9.2	37	47	75	0.663782	0.73685	no
gi|641571594|gb|KK856027.1|	3265-3927	9.2	37	1	3	1	0.397	no
gi|641571594|gb|KK856027.1|	41035-41922	9.2	37	0	5	inf	0.00025	yes
gi|641571594|gb|KK856027.1|	53365-53951	9.2	37	0	62	inf	5.00E-05	yes
gi|641571594|gb|KK856027.1|	691-1636	9.2	37	1	1	-0.0959828	0.9478	no
gi|641571598|gb|KK856026.1|	0-708	9.6	39	6	4	-0.577499	0.6715	no
gi|641571598|gb|KK856026.1|	2660-3285	9.6	39	14	11	-0.287202	0.83235	no
gi|641571598|gb|KK856026.1|	3591-4110	9.6	39	1	1	0.00928888	0.91115	no
gi|641571601|gb|KK856025.1|	04356-10476	7.5	35	0	701	inf	0.00065	yes
gi|641571601|gb|KK856025.1|	05000-10610	7.5	35	0	2	inf	0.0023	yes
gi|641571601|gb|KK856025.1|	06786-10776	7.5	35	0	22	inf	5.00E-05	yes
gi|641571601|gb|KK856025.1|	75944-76329	7.5	35	6	34	3	0.09215	no
gi|641571605|gb|KK856024.1|	32240-13368	10	38	0.960076	0.774482	-0.309917	0.83745	no
gi|641571605|gb|KK856024.1|	33838-13580	10	38	0.520993	0.977985	0.908549	0.53605	no
gi|641571605|gb|KK856024.1|	35882-13656	10	38	1	0.876612	-0.207056	0.8945	no
gi|641571605|gb|KK856024.1|	45899-14683	10	38	0.764707	1	0.604361	0.6936	no

gi|641571605|gb|KK856024.1|	46974-14788	10	38	0.890346	0.451113	-0.980879	0.62775	no
gi|641571605|gb|KK856024.1|	49160-14987	10	38	0.40633	1	2	0.3323	no
gi|641571605|gb|KK856024.1|	52495-15341	10	38	1	0.74359	-0.533329	0.7478	no
gi|641571605|gb|KK856024.1|	15770-16955	10	38	9	9	-0.0761535	0.94795	no
gi|641571605|gb|KK856024.1|	2942-3882	10	38	0.95075	0.578189	-0.717524	0.6701	no
gi|641571605|gb|KK856024.1|	7498-7959	10	38	4	4	-0.146428	0.9233	no
gi|641571609|gb|KK856023.1|	1931-5022	10	41	12	19	0.618924	0.7019	no
gi|641571609|gb|KK856023.1|	41-750	10	41	13	13	-0.00756096	0.99235	no
gi|641571609|gb|KK856023.1|	6018-6573	10	41	12.58	32	1	0.29265	no
gi|641571609|gb|KK856023.1|	60515-61358	10	41	0.437253	2	2	0.2441	no
gi|641571609|gb|KK856023.1|	61736-63386	10	41	2	1	-0.391729	0.76665	no
gi|641571612|gb|KK856022.1|	10981-14316	12	43	1	2	1	0.3998	no
gi|641571612|gb|KK856022.1|	45343-14549	12	43	124.46	68	-0.875668	0.59135	no
gi|641571612|gb|KK856022.1|	49903-15037	12	43	4	6	0.503523	0.7269	no
gi|641571612|gb|KK856022.1|	52834-15318	12	43	4	3	-0.423001	0.7868	no
gi|641571612|gb|KK856022.1|	15740-16942	12	43	2	3	0.557555	0.6801	no
gi|641571612|gb|KK856022.1|	61504-16180	12	43	11	6	-1	0.4943	no
gi|641571612|gb|KK856022.1|	62705-16301	12	43	12	7	-0.821888	0.5797	no
gi|641571612|gb|KK856022.1|	69371-16978	12	43	7	2	-2	0.28425	no
gi|641571612|gb|KK856022.1|	73108-17425	12	43	0.371979	1	2	0.3084	no
gi|641571612|gb|KK856022.1|	74541-17502	12	43	0.492034	3	3	0.2991	no
gi|641571612|gb|KK856022.1|	77926-17997	12	43	1	3	1	0.411	no
gi|641571612|gb|KK856022.1|	18389-18797	12	43	13	24.2	0.914988	0.4921	no
gi|641571612|gb|KK856022.1|	19395-20632	12	43	0.40781	0.517488	0.343628	1	no
gi|641571612|gb|KK856022.1|	31433-31858	12	43	4	3	-0.305651	0.8432	no
gi|641571612|gb|KK856022.1|	33267-33554	12	43	33	60	0.837052	0.52075	no
gi|641571612|gb|KK856022.1|	35967-36165	12	43	27	74	1	0.31615	no
gi|641571612|gb|KK856022.1|	36824-38568	12	43	2	3	0.544388	0.6741	no
gi|641571612|gb|KK856022.1|	39667-40643	12	43	1	2	0.697879	0.6288	no
gi|641571612|gb|KK856022.1|	41934-42676	12	43	3	2	-0.39845	0.779	no

gi|641571612|gb|KK856022.1|	43709-46059	12	43	1	2	0.963229	0.467	no
gi|641571612|gb|KK856022.1|	46279-47263	12	43	2	4	1	0.3963	no
gi|641571612|gb|KK856022.1|	47898-48224	12	43	4	2	-0.841822	0.64855	no
gi|641571612|gb|KK856022.1|	49693-51333	12	43	2	3	0.338154	0.7946	no
gi|641571612|gb|KK856022.1|	51768-52315	12	43	3	7	1	0.40805	no
gi|641571612|gb|KK856022.1|	56577-57410	12	43	1	0.674582	-0.856355	0.60675	no
gi|641571612|gb|KK856022.1|	66325-66786	12	43	15	5	-2	0.25725	no
gi|641571612|gb|KK856022.1|	67802-71257	12	43	2	3	0.951559	0.46	no
gi|641571612|gb|KK856022.1|	73165-74709	12	43	3	3	0.138467	0.9179	no
gi|641571612|gb|KK856022.1|	74824-76554	12	43	1	0.914413	-0.391517	0.7829	no
gi|641571612|gb|KK856022.1|	85078-85574	12	43	0.704163	3	2	0.2812	no
gi|641571612|gb|KK856022.1|	89312-90201	12	43	0.816518	1	0.773748	0.62605	no
gi|641571612|gb|KK856022.1|	9070-10573	12	43	3	3	0.210479	0.87785	no
gi|641571616|gb|KK856021.1|	33280-33888	9.2	37	0	16	inf	5.00E-05	yes
gi|641571616|gb|KK856021.1|	34164-36675	9.2	37	0	2	inf	5.00E-05	yes
gi|641571616|gb|KK856021.1|	37350-38776	9.2	37	0	2	inf	0.00065	yes
gi|641571616|gb|KK856021.1|	38883-39458	9.2	37	0	154	inf	0.00025	yes
gi|641571616|gb|KK856021.1|	46448-46952	9.2	37	0	246	inf	0.00015	yes
gi|641571625|gb|KK856020.1|	.02004-10241	9.2	38	4	6	0.469983	0.7521	no
gi|641571625|gb|KK856020.1|	.11741-11290	9.2	38	0	8	inf	5.00E-05	yes
gi|641571625|gb|KK856020.1|	39886-40457	9.2	38	1	1	0.0117218	0.91115	no
gi|641571625|gb|KK856020.1|	40812-42038	9.2	38	0.755631	0	-inf	1	no
gi|641571629|gb|KK856019.1|	.99203-19954	11	47	0.943746	6	3	0.28555	no
gi|641571629|gb|KK856019.1|	21923-23242	11	47	1	2	0.359148	0.7957	no
gi|641571629|gb|KK856019.1|	29218-29848	11	47	1	1	0.376391	0.8276	no
gi|641571629|gb|KK856019.1|	29981-31121	11	47	0.674524	1	0.60608	0.6902	no
gi|641571629|gb|KK856019.1|	89589-90436	11	47	0.325927	1	2	0.2812	no
gi|641571632|gb|KK856018.1|	.00328-10229	10	36	2	3	0.45699	0.7268	no
gi|641571632|gb|KK856018.1|	.02688-10577	10	36	3	1	-1	0.41845	no
gi|641571632|gb|KK856018.1|	.05936-10664	10	36	3	1	-2	0.30235	no

gi|641571632|gb|KK856018.1|	06701-10922	10	36	1	0.139196	-3	0.1342	no
gi|641571632|gb|KK856018.1|	09370-11155	10	36	2	0.054288	-5	0.1841	no
gi|641571632|gb|KK856018.1|	11650-11266	10	36	2	0.131229	-4	0.19735	no
gi|641571632|gb|KK856018.1|	14783-11668	10	36	2	0.507626	-2	0.2006	no
gi|641571632|gb|KK856018.1|	20178-12048	10	36	12	0.884716	-4	0.19895	no
gi|641571632|gb|KK856018.1|	20573-12110	10	36	6	0.948244	-3	0.15745	no
gi|641571632|gb|KK856018.1|	25137-12564	10	36	1	46	5	0.06355	no
gi|641571632|gb|KK856018.1|	29269-12962	10	36	1	33	5	0.13565	no
gi|641571632|gb|KK856018.1|	36572-13879	10	36	0.418808	0.532461	0.346387	1	no
gi|641571632|gb|KK856018.1|	47105-14747	10	36	0	9	inf	0.0015	yes
gi|641571632|gb|KK856018.1|	49279-14999	10	36	0.135185	3	5	0.1123	no
gi|641571632|gb|KK856018.1|	53144-15356	10	36	1	13	3	0.13045	no
gi|641571632|gb|KK856018.1|	55860-15671	10	36	2	0.648318	-2	0.2683	no
gi|641571632|gb|KK856018.1|	58119-15922	10	36	7	17	1	0.3035	no
gi|641571632|gb|KK856018.1|	60764-16149	10	36	0.790012	0.998811	0.338336	0.8352	no
gi|641571632|gb|KK856018.1|	63075-16352	10	36	16	25	0.614238	0.63365	no
gi|641571632|gb|KK856018.1|	64085-16481	10	36	4	6	0.64701	0.62725	no
gi|641571632|gb|KK856018.1|	24230-24863	10	36	0.485063	2	2	0.2235	no
gi|641571632|gb|KK856018.1|	29973-31020	10	36	0.665046	1	1	0.49695	no
gi|641571632|gb|KK856018.1|	32099-33497	10	36	2	3	0.563027	0.6735	no
gi|641571632|gb|KK856018.1|	34130-34890	10	36	3	5	0.885682	0.52195	no
gi|641571632|gb|KK856018.1|	56648-57183	10	36	2	1	-0.404927	0.82205	no
gi|641571632|gb|KK856018.1|	61518-62937	10	36	11	4	-1	0.26535	no
gi|641571632|gb|KK856018.1|	64459-65357	10	36	0.604545	0.918819	0.603931	0.70555	no
gi|641571632|gb|KK856018.1|	66713-67443	10	36	1	1	-0.536194	0.7478	no
gi|641571632|gb|KK856018.1|	67621-70137	10	36	23	13	-0.8262	0.60575	no
gi|641571632|gb|KK856018.1|	71154-72739	10	36	6	4	-0.505377	0.69565	no
gi|641571632|gb|KK856018.1|	73967-75162	10	36	0.991534	2	0.606424	0.67415	no
gi|641571632|gb|KK856018.1|	75540-76676	10	36	1	1	0.0654854	0.9579	no
gi|641571632|gb|KK856018.1|	78858-79235	10	36	3	6	1	0.4926	no

gi|641571632|gb|KK856018.1|	82500-83724	10	36	0.825927	0.628802	-0.39341	1	no
gi|641571632|gb|KK856018.1|	83874-84998	10	36	0.838622	0.928281	0.146541	0.9285	no
gi|641571632|gb|KK856018.1|	85299-85672	10	36	14	16	0.259356	0.8439	no
gi|641571632|gb|KK856018.1|	86653-87770	10	36	0.614539	1	1	0.49695	no
gi|641571632|gb|KK856018.1|	88173-89641	10	36	1	3	1	0.37365	no
gi|641571632|gb|KK856018.1|	93776-96711	10	36	64	30	-1	0.6144	no
gi|641571632|gb|KK856018.1|	96773-97142	10	36	1	8	3	0.19065	no
gi|641571639|gb|KK856016.1|	06943-11050	11	39	5	4	-0.193694	0.87825	no
gi|641571639|gb|KK856016.1|	1149-1365	11	39	6	23	2	0.30905	no
gi|641571639|gb|KK856016.1|	11586-11938	11	39	32	267	3	0.1172	no
gi|641571639|gb|KK856016.1|	20049-12055	11	39	2	2	0.178605	0.9106	no
gi|641571639|gb|KK856016.1|	12490-12919	11	39	12	88	3	0.0391	no
gi|641571639|gb|KK856016.1|	14336-14624	11	39	0	43	inf	0.0001	yes
gi|641571639|gb|KK856016.1|	46044-14671	11	39	5	2	-2	0.2363	no
gi|641571639|gb|KK856016.1|	49110-14994	11	39	0.556884	2	2	0.28495	no
gi|641571639|gb|KK856016.1|	72320-17310	11	39	0.965426	0.916014	-0.0757962	0.92685	no
gi|641571639|gb|KK856016.1|	79141-18018	11	39	0.583285	0.507085	-0.201973	1	no
gi|641571639|gb|KK856016.1|	80706-18293	11	39	0.52171	0.530634	0.0244687	1	no
gi|641571639|gb|KK856016.1|	11566-21205	11	39	5	2	-1	0.3802	no
gi|641571639|gb|KK856016.1|	26137-27732	11	39	3	7	1	0.2673	no
gi|641571639|gb|KK856016.1|	28582-29365	11	39	0	19	inf	5.00E-05	yes
gi|641571639|gb|KK856016.1|	3099-3586	11	39	15	35	1	0.33265	no
gi|641571639|gb|KK856016.1|	4311-5671	11	39	0.060791	16	8	0.10435	no
gi|641571639|gb|KK856016.1|	49644-50141	11	39	0	8	inf	0.00075	yes
gi|641571639|gb|KK856016.1|	52018-52711	11	39	0	7	inf	0.00065	yes
gi|641571639|gb|KK856016.1|	56273-57149	11	39	0	4	inf	0.00045	yes
gi|641571639|gb|KK856016.1|	57269-59223	11	39	0.040259	64	11	0.1048	no
gi|641571639|gb|KK856016.1|	61500-61745	11	39	0	28	inf	0.00135	yes
gi|641571639|gb|KK856016.1|	65600-66229	11	39	0	62	inf	5.00E-05	yes
gi|641571639|gb|KK856016.1|	68582-69027	11	39	0.845142	11	4	0.1436	no

gi|641571639|gb|KK856016.1|	70312-71733	11	39	0.577652	0.96814	0.745016	0.6176	no
gi|641571639|gb|KK856016.1|	73702-73947	11	39	2	20	3	0.2564	no
gi|641571639|gb|KK856016.1|	7642-8864	11	39	0.068961	4	6	0.10535	no
gi|641571639|gb|KK856016.1|	99310-100148	11	39	0	30	inf	5.00E-05	yes
gi|641571642|gb|KK856015.1|	01719-10226	11	37	5	3	-0.468396	0.74855	no
gi|641571642|gb|KK856015.1|	06094-10686	11	37	4	3.74	-0.0302001	0.97575	no
gi|641571642|gb|KK856015.1|	10332-11061	11	37	6	6	-0.116525	0.9229	no
gi|641571642|gb|KK856015.1|	17249-11861	11	37	0.301879	0.551859	0.870329	1	no
gi|641571642|gb|KK856015.1|	23648-12387	11	37	23	26	0.182076	0.89925	no
gi|641571642|gb|KK856015.1|	26342-12657	11	37	22	9	-1	0.423	no
gi|641571642|gb|KK856015.1|	1315-3859	11	37	0.180874	314	11	0.06345	no
gi|641571642|gb|KK856015.1|	38448-13867	11	37	10	8	-0.264888	0.8666	no
gi|641571642|gb|KK856015.1|	42335-14376	11	37	9	7	-0.494566	0.6935	no
gi|641571642|gb|KK856015.1|	45060-14557	11	37	2	1	-0.575891	0.7586	no
gi|641571642|gb|KK856015.1|	46898-14783	11	37	0.670931	0.58286	-0.203015	1	no
gi|641571642|gb|KK856015.1|	48389-14926	11	37	33	30	-0.150077	0.90495	no
gi|641571642|gb|KK856015.1|	50028-15095	11	37	0.775173	0.736507	-0.0738193	1	no
gi|641571642|gb|KK856015.1|	51141-15214	11	37	0.964253	0.79998	-0.269448	0.86195	no
gi|641571642|gb|KK856015.1|	52318-15293	11	37	3	6	0.994396	0.4878	no
gi|641571642|gb|KK856015.1|	53047-15411	11	37	1	0.74407	-0.716394	0.6522	no
gi|641571642|gb|KK856015.1|	55291-15625	11	37	1	0.839264	-0.395339	0.80865	no
gi|641571642|gb|KK856015.1|	59112-16072	11	37	0.500479	1	1	0.3972	no
gi|641571642|gb|KK856015.1|	60804-16153	11	37	1	0.597084	-1	0.47135	no
gi|641571642|gb|KK856015.1|	66256-16671	11	37	298	271	-0.136687	0.9476	no
gi|641571642|gb|KK856015.1|	69334-17102	11	37	0.426038	0.866022	1	1	no
gi|641571642|gb|KK856015.1|	72769-17433	11	37	0.46523	0.551512	0.245449	1	no
gi|641571642|gb|KK856015.1|	74885-17625	11	37	3	5	0.904976	0.4965	no
gi|641571642|gb|KK856015.1|	76797-17789	11	37	1	2	0.775678	0.5937	no
gi|641571642|gb|KK856015.1|	78100-17892	11	37	28	29	0.0499664	0.96715	no
gi|641571642|gb|KK856015.1|	79168-18231	11	37	128	118	-0.123761	0.946	no

gi|641571642|gb|KK856015.1|	82449-18393	11	37	3	5	0.808058	0.54135	no
gi|641571642|gb|KK856015.1|	84176-18907	11	37	5	8	0.561325	0.7328	no
gi|641571642|gb|KK856015.1|	89388-19090	11	37	2	2	-0.10583	0.93835	no
gi|641571642|gb|KK856015.1|	91006-19647	11	37	2	2	0.34352	0.78565	no
gi|641571642|gb|KK856015.1|	97356-19754	11	37	32	25	-0.35166	0.82885	no
gi|641571642|gb|KK856015.1|	26074-26321	11	37	8	5	-0.863587	0.6407	no
gi|641571642|gb|KK856015.1|	28745-29313	11	37	4	3	-0.473829	0.7407	no
gi|641571642|gb|KK856015.1|	46972-47564	11	37	7	1	-2	0.15135	no
gi|641571642|gb|KK856015.1|	48704-54661	11	37	7	5	-0.571755	0.719	no
gi|641571642|gb|KK856015.1|	59557-59784	11	37	9	10	0.0579238	0.9244	no
gi|641571642|gb|KK856015.1|	59954-63204	11	37	13	11	-0.226285	0.88465	no
gi|641571642|gb|KK856015.1|	64818-65437	11	37	16	9	-0.761968	0.561	no
gi|641571642|gb|KK856015.1|	66063-66663	11	37	14	6	-1	0.36065	no
gi|641571642|gb|KK856015.1|	70859-71351	11	37	3	2	-0.62971	0.68395	no
gi|641571642|gb|KK856015.1|	71625-71880	11	37	12	6	-1	0.48875	no
gi|641571642|gb|KK856015.1|	85183-85409	11	37	21	8	-1	0.41285	no
gi|641571645|gb|KK856014.1|	22230-12240	10	21	58	10	-2	0.21305	no
gi|641571645|gb|KK856014.1|	23996-12449	10	21	3	2	-0.785519	0.63075	no
gi|641571645|gb|KK856014.1|	26670-12899	10	21	0.864384	0.760887	-0.18399	1	no
gi|641571645|gb|KK856014.1|	29769-13007	10	21	9	5	-0.917808	0.5719	no
gi|641571645|gb|KK856014.1|	30755-13116	10	21	14	8	-0.752904	0.57555	no
gi|641571645|gb|KK856014.1|	33010-13317	10	21	103	85	-0.277719	0.84375	no
gi|641571645|gb|KK856014.1|	50860-15184	10	21	0.451143	0.960523	1	0.50945	no
gi|641571645|gb|KK856014.1|	57765-15920	10	21	0.340207	0.431985	0.344567	1	no
gi|641571645|gb|KK856014.1|	69346-17037	10	21	0.929721	0	-inf	0.0102	no
gi|641571645|gb|KK856014.1|	17929-23293	10	21	2	0.146473	-4	0.03285	no
gi|641571645|gb|KK856014.1|	23583-24486	10	21	7	0.304122	-5	0.1313	no
gi|641571645|gb|KK856014.1|	24537-27203	10	21	3	0.087473	-5	0.131	no
gi|641571648|gb|KK856013.1|	37415-13787	10	23	3	0.406593	-3	0.2481	no
gi|641571648|gb|KK856013.1|	89351-89977	10	23	4	0.248811	-4	0.1915	no

gi|641571652|gb|KK856012.1|	02026-10300	9.6	35	3	1	-1	0.41465	no
gi|641571652|gb|KK856012.1|	03271-10657	9.6	35	2	2	0.139542	0.9139	no
gi|641571652|gb|KK856012.1|	06699-10873	9.6	35	4	6	0.734651	0.5559	no
gi|641571652|gb|KK856012.1|	09068-10969	9.6	35	1	2	0.406169	0.8089	no
gi|641571652|gb|KK856012.1|	23945-12551	9.6	35	0.878126	1	0.327915	0.8152	no
gi|641571652|gb|KK856012.1|	25595-12922	9.6	35	4	6	0.641809	0.61085	no
gi|641571652|gb|KK856012.1|	12845-13084	9.6	35	60	0	-inf	0.00055	yes
gi|641571652|gb|KK856012.1|	31850-13313	9.6	35	5	0.591572	-3	0.0657	no
gi|641571652|gb|KK856012.1|	33294-13428	9.6	35	1	0.677499	-0.773609	0.63565	no
gi|641571652|gb|KK856012.1|	37032-13796	9.6	35	1	1	0.466858	0.75495	no
gi|641571652|gb|KK856012.1|	38724-13970	9.6	35	2	1	-0.684699	0.63875	no
gi|641571652|gb|KK856012.1|	40674-14288	9.6	35	1	1	-0.11307	0.9324	no
gi|641571652|gb|KK856012.1|	46360-14727	9.6	35	1	0.749771	-0.88132	0.5832	no
gi|641571652|gb|KK856012.1|	51569-15255	9.6	35	1	2	0.398376	0.787	no
gi|641571652|gb|KK856012.1|	53106-15432	9.6	35	1	2	0.214262	0.87975	no
gi|641571652|gb|KK856012.1|	55822-15658	9.6	35	0.744363	2	1	0.4118	no
gi|641571652|gb|KK856012.1|	56833-15905	9.6	35	38	21	-0.859864	0.70285	no
gi|641571652|gb|KK856012.1|	61756-16215	9.6	35	4	4	-0.192599	0.90345	no
gi|641571652|gb|KK856012.1|	65372-16560	9.6	35	34	6	-3	0.1729	no
gi|641571652|gb|KK856012.1|	16628-16957	9.6	35	17	0.753903	-4	0.1878	no
gi|641571652|gb|KK856012.1|	66582-16677	9.6	35	44	24	-0.891153	0.5752	no
gi|641571652|gb|KK856012.1|	83784-18464	9.6	35	29	9	-2	0.18095	no
gi|641571652|gb|KK856012.1|	20246-20671	9.6	35	12	0.459101	-5	0.18845	no
gi|641571652|gb|KK856012.1|	24086-24271	9.6	35	429	13	-5	0.09455	no
gi|641571652|gb|KK856012.1|	25394-25876	9.6	35	10	0	-inf	0.0006	yes
gi|641571652|gb|KK856012.1|	27841-28379	9.6	35	22	0.623123	-5	0.13135	no
gi|641571652|gb|KK856012.1|	28552-29708	9.6	35	0.810688	0.224373	-2	1	no
gi|641571652|gb|KK856012.1|	32857-33552	9.6	35	16	0	-inf	5.00E-05	yes
gi|641571652|gb|KK856012.1|	46062-47748	9.6	35	4	0.724099	-2	0.10455	no
gi|641571652|gb|KK856012.1|	47875-48800	9.6	35	2	0.885012	-1	0.4411	no

gi|641571652|gb|KK856012.1|	48927-51772	9.6	35	4	2	-1	0.3814	no
gi|641571652|gb|KK856012.1|	51880-54186	9.6	35	4	3	-0.456961	0.72245	no
gi|641571652|gb|KK856012.1|	54368-56342	9.6	35	5	5	0.0729586	0.95315	no
gi|641571652|gb|KK856012.1|	57993-58811	9.6	35	11	0.518044	-4	0.0967	no
gi|641571652|gb|KK856012.1|	6002-6186	9.6	35	42	0	-inf	0.0059	no
gi|641571652|gb|KK856012.1|	62328-62655	9.6	35	61	0	-inf	0.0002	yes
gi|641571652|gb|KK856012.1|	62959-63216	9.6	35	77	0	-inf	0.0001	yes
gi|641571652|gb|KK856012.1|	67848-68003	9.6	35	345	33	-3	0.11095	no
gi|641571652|gb|KK856012.1|	72351-73092	9.6	35	2	0.787432	-0.983432	0.5872	no
gi|641571652|gb|KK856012.1|	7358-7698	9.6	35	31	0	-inf	0.00015	yes
gi|641571652|gb|KK856012.1|	79522-80185	9.6	35	13	0.229351	-6	0.18325	no
gi|641571652|gb|KK856012.1|	82359-82627	9.6	35	63	0	-inf	5.00E-05	yes
gi|641571652|gb|KK856012.1|	86163-86385	9.6	35	163	4	-5	0.1307	no
gi|641571652|gb|KK856012.1|	88726-91487	9.6	35	7	0.589674	-3	0.0264	no
gi|641571652|gb|KK856012.1|	91814-92091	9.6	35	37.29	1	-5	0.18445	no
gi|641571652|gb|KK856012.1|	93017-94798	9.6	35	215	7	-5	0.0168	no
gi|641571652|gb|KK856012.1|	94935-96467	9.6	35	11	6	-0.907676	0.46905	no
gi|641571652|gb|KK856012.1|	96780-98804	9.6	35	94	137	0.542809	0.7903	no
gi|641571652|gb|KK856012.1|	99181-10120	9.6	35	66	88	0.42626	0.8324	no
gi|641571656|gb|KK856011.1|	34363-13485	9.1	39	0.967449	2	1	0.51315	no
gi|641571656|gb|KK856011.1|	15091-15766	9.1	39	1	0.447349	-1	0.4534	no
gi|641571656|gb|KK856011.1|	53745-15427	9.1	39	0	12	inf	0.00015	yes
gi|641571660|gb|KK856010.1|	11273-11149	11	22	41	4	-3	0.14785	no
gi|641571660|gb|KK856010.1|	12893-11310	11	22	20	5	-2	0.2748	no
gi|641571660|gb|KK856010.1|	35965-13697	11	22	2	0.797034	-1	0.4177	no
gi|641571660|gb|KK856010.1|	38886-14075	11	22	0.678473	0.452664	-0.583851	1	no
gi|641571660|gb|KK856010.1|	45097-14533	11	22	99	112	0.177609	0.88785	no
gi|641571660|gb|KK856010.1|	52051-15262	11	22	0.936262	2	0.8598	0.62755	no
gi|641571660|gb|KK856010.1|	52748-15316	11	22	4	1	-1	0.4311	no
gi|641571660|gb|KK856010.1|	53289-15422	11	22	0.476655	1	2	0.32945	no

gi|641571660|gb|KK856010.1|	[illegible]54955-15598	11	22	1	0.902193	-0.49427	0.7428	no
gi|641571660|gb|KK856010.1|	15659-17968	11	22	2	2	0.24341	0.8526	no
gi|641571660|gb|KK856010.1|	[illegible]57754-15839	11	22	2	0.72645	-1	0.50365	no
gi|641571660|gb|KK856010.1|	[illegible]60874-16286	11	22	2	2	0.11154	0.9304	no
gi|641571660|gb|KK856010.1|	[illegible]63145-16434	11	22	1	1	-0.0854361	0.95135	no
gi|641571660|gb|KK856010.1|	[illegible]66075-16976	11	22	3	5	0.715615	0.5674	no
gi|641571660|gb|KK856010.1|	[illegible]73693-17446	11	22	9	11	0.379763	0.7649	no
gi|641571660|gb|KK856010.1|	18406-18770	11	22	27	21	-0.313136	0.809	no
gi|641571660|gb|KK856010.1|	20209-23195	11	22	0.431308	0.542202	0.330109	1	no
gi|641571660|gb|KK856010.1|	23961-24735	11	22	39	31	-0.327041	0.78945	no
gi|641571660|gb|KK856010.1|	24829-25733	11	22	0.999045	0.759238	-0.395998	0.81255	no
gi|641571660|gb|KK856010.1|	25799-26509	11	22	6	2	-2	0.267	no
gi|641571660|gb|KK856010.1|	2900-3160	11	22	40	13	-2	0.2825	no
gi|641571660|gb|KK856010.1|	34538-37204	11	22	28	14	-0.970655	0.6655	no
gi|641571660|gb|KK856010.1|	37469-40560	11	22	24	12	-1	0.5234	no
gi|641571660|gb|KK856010.1|	42110-43274	11	22	10	9	-0.1668	0.89495	no
gi|641571660|gb|KK856010.1|	46199-46790	11	22	0.893892	2	1	0.4611	no
gi|641571660|gb|KK856010.1|	46903-47246	11	22	2	4	0.839503	0.63695	no
gi|641571660|gb|KK856010.1|	47363-48380	11	22	1	2	0.512036	0.7255	no
gi|641571660|gb|KK856010.1|	49044-50535	11	22	14	5	-2	0.24405	no
gi|641571660|gb|KK856010.1|	51819-52532	11	22	23	8	-1	0.2599	no
gi|641571660|gb|KK856010.1|	55465-56314	11	22	3	2	-0.396697	0.77425	no
gi|641571660|gb|KK856010.1|	56775-58593	11	22	2	0.598811	-2	0.19345	no
gi|641571660|gb|KK856010.1|	59195-59668	11	22	3	0	-inf	0.01	no
gi|641571660|gb|KK856010.1|	59878-60891	11	22	2	0.658538	-2	0.2547	no
gi|641571660|gb|KK856010.1|	66031-66259	11	22	35	12	-2	0.3226	no
gi|641571660|gb|KK856010.1|	78476-78710	11	22	17	7	-1	0.4486	no
gi|641571660|gb|KK856010.1|	86427-87516	11	22	0.633792	0.361676	-0.809313	1	no
gi|641571660|gb|KK856010.1|	91829-93037	11	22	1	0.958044	-0.311038	0.8374	no
gi|641571660|gb|KK856010.1|	97344-98432	11	22	0.793128	2	1	0.4325	no

gi|641571660|gb|KK856010.1|	99797-100252	11	22	7	2	-2	0.2873	no
gi|641571667|gb|KK856008.1|	.02326-10279	11	39	3	5	0.969643	0.5382	no
gi|641571667|gb|KK856008.1|	10266-11455	11	39	1	2	0.743892	0.5881	no
gi|641571667|gb|KK856008.1|	.04086-10560	11	39	1	2	0.476628	0.73135	no
gi|641571667|gb|KK856008.1|	.05759-10746	11	39	2	2	-0.00628265	0.9867	no
gi|641571667|gb|KK856008.1|	.15556-11608	11	39	8	7	-0.115569	0.9277	no
gi|641571667|gb|KK856008.1|	11836-13559	11	39	0.787883	0.918592	0.221442	0.882	no
gi|641571667|gb|KK856008.1|	.18401-11995	11	39	0.519959	0.475494	-0.128972	1	no
gi|641571667|gb|KK856008.1|	.20613-12095	11	39	4	4	-0.00831553	0.9394	no
gi|641571667|gb|KK856008.1|	.22234-12277	11	39	0.616924	2	2	0.2812	no
gi|641571667|gb|KK856008.1|	.23122-12377	11	39	4	4	0.146835	0.91985	no
gi|641571667|gb|KK856008.1|	.27038-12839	11	39	12	11	-0.127184	0.91925	no
gi|641571667|gb|KK856008.1|	.29696-13066	11	39	0.459935	0.839264	0.867696	1	no
gi|641571667|gb|KK856008.1|	.34945-13640	11	39	1	1	0.285722	0.8383	no
gi|641571667|gb|KK856008.1|	.36543-13789	11	39	1	0.561886	-1	0.4423	no
gi|641571667|gb|KK856008.1|	13780-14373	11	39	4	2	-0.876394	0.5419	no
gi|641571667|gb|KK856008.1|	.39450-14207	11	39	13	9	-0.612494	0.62	no
gi|641571667|gb|KK856008.1|	.42337-14805	11	39	14	17	0.317579	0.88425	no
gi|641571667|gb|KK856008.1|	.49679-15083	11	39	25	188	3	0.1043	no
gi|641571667|gb|KK856008.1|	.55126-15532	11	39	31	31	-0.00288394	0.95595	no
gi|641571667|gb|KK856008.1|	.58952-16055	11	39	1	1.15	-0.239861	0.8664	no
gi|641571667|gb|KK856008.1|	.60768-16152	11	39	3	3	-0.221344	0.87985	no
gi|641571667|gb|KK856008.1|	.62727-16368	11	39	0.465989	1	1	0.4595	no
gi|641571667|gb|KK856008.1|	.67557-16875	11	39	0.562651	0.321244	-0.808572	1	no
gi|641571667|gb|KK856008.1|	.76092-17672	11	39	0.480684	2	2	0.3323	no
gi|641571667|gb|KK856008.1|	.81761-18342	11	39	0.434166	0.661873	0.608307	1	no
gi|641571667|gb|KK856008.1|	19423-21369	11	39	19	6	-2	0.22375	no
gi|641571667|gb|KK856008.1|	21660-22506	11	39	1	0.495892	-1	0.50885	no
gi|641571667|gb|KK856008.1|	22731-23759	11	39	1	0.77608	-0.617152	0.6869	no
gi|641571667|gb|KK856008.1|	23865-25050	11	39	1	0.980217	-0.0310656	0.95655	no

gi|641571667|gb|KK856008.1|	25281-25502	11	39	55	22	-1	0.3447	no
gi|641571667|gb|KK856008.1|	256-1214	11	39	3	3	-0.395409	0.769	no
gi|641571667|gb|KK856008.1|	26381-26872	11	39	2	2	0.107193	0.92265	no
gi|641571667|gb|KK856008.1|	30220-31660	11	39	0.3413	0.780063	1	1	no
gi|641571667|gb|KK856008.1|	34114-34681	11	39	0.570771	7	4	0.14485	no
gi|641571667|gb|KK856008.1|	34993-35676	11	39	2	1	-0.274219	0.8619	no
gi|641571667|gb|KK856008.1|	43473-44069	11	39	4	4	-0.118561	0.9332	no
gi|641571667|gb|KK856008.1|	44448-48068	11	39	14	4	-2	0.2769	no
gi|641571667|gb|KK856008.1|	4819-5515	11	39	0.989704	2	0.793208	0.63035	no
gi|641571667|gb|KK856008.1|	48528-49546	11	39	0.77455	0.654556	-0.242842	1	no
gi|641571667|gb|KK856008.1|	49844-50949	11	39	0.700476	1	0.60584	0.6902	no
gi|641571667|gb|KK856008.1|	54351-55090	11	39	0.651085	1	1	0.51085	no
gi|641571667|gb|KK856008.1|	57364-57599	11	39	17	2	-3	0.2153	no
gi|641571667|gb|KK856008.1|	69912-70540	11	39	11	13	0.254767	0.8417	no
gi|641571667|gb|KK856008.1|	72216-72938	11	39	44	22	-0.98382	0.42565	no
gi|641571667|gb|KK856008.1|	73966-74258	11	39	2	7	2	0.34545	no
gi|641571667|gb|KK856008.1|	78111-78525	11	39	12	10	-0.339676	0.79735	no
gi|641571667|gb|KK856008.1|	80930-81221	11	39	7	2	-2	0.3411	no
gi|641571667|gb|KK856008.1|	84279-84909	11	39	3	2	-0.623609	0.6689	no
gi|641571667|gb|KK856008.1|	87469-88560	11	39	0.632377	0.721744	0.190701	1	no
gi|641571667|gb|KK856008.1|	90087-90427	11	39	8	5	-0.704181	0.6456	no
gi|641571667|gb|KK856008.1|	94583-95201	11	39	4	3	-0.334484	0.8142	no
gi|641571667|gb|KK856008.1|	96030-96522	11	39	5	5	-0.0288057	0.9666	no
gi|641571667|gb|KK856008.1|	96861-97730	11	39	2	2	-0.396429	0.78765	no
gi|641571670|gb|KK856007.1|	00809-10197	11	38	2	5	1	0.33635	no
gi|641571670|gb|KK856007.1|	06063-10713	11	38	8	19	1	0.3056	no
gi|641571670|gb|KK856007.1|	10677-14820	11	38	4	6	0.521287	0.67865	no
gi|641571670|gb|KK856007.1|	07564-10814	11	38	11	10	-0.204371	0.8734	no
gi|641571670|gb|KK856007.1|	28118-12877	11	38	2	4	1	0.43715	no
gi|641571670|gb|KK856007.1|	14930-18004	11	38	2	3	0.161524	0.9002	no

gi|641571670|gb|KK856007.1|	65757-16640	11	38	0	149.97	inf	0.00035	yes
gi|641571670|gb|KK856007.1|	18152-19073	11	38	23	47	1	0.39145	no
gi|641571670|gb|KK856007.1|	19714-20026	11	38	18	39	1	0.39735	no
gi|641571670|gb|KK856007.1|	20854-22367	11	38	0.537321	1	1	0.4288	no
gi|641571670|gb|KK856007.1|	22530-23084	11	38	6	21	2	0.205	no
gi|641571670|gb|KK856007.1|	24136-26147	11	38	1	2	0.556612	0.6786	no
gi|641571670|gb|KK856007.1|	26783-27039	11	38	50	93	0.892821	0.49455	no
gi|641571670|gb|KK856007.1|	28171-29117	11	38	10	15	0.701143	0.5769	no
gi|641571670|gb|KK856007.1|	32765-33691	11	38	4	4	-0.0671247	0.9584	no
gi|641571670|gb|KK856007.1|	47268-49906	11	38	0.434784	2	2	0.1533	no
gi|641571670|gb|KK856007.1|	59865-60444	11	38	3	10	2	0.2528	no
gi|641571670|gb|KK856007.1|	69456-70551	11	38	4	11	2	0.2011	no
gi|641571670|gb|KK856007.1|	79974-80559	11	38	0.181517	5	5	0.10875	no
gi|641571670|gb|KK856007.1|	81166-82192	11	38	0.511394	1	1	0.5082	no
gi|641571670|gb|KK856007.1|	8519-9191	11	38	54	28	-0.969266	0.44045	no
gi|641571670|gb|KK856007.1|	85907-87016	11	38	0.697409	17	5	0.03275	no
gi|641571670|gb|KK856007.1|	87730-88044	11	38	0	16	inf	0.0015	yes
gi|641571670|gb|KK856007.1|	88727-89401	11	38	1	0.896547	-0.721905	0.67005	no
gi|641571670|gb|KK856007.1|	89509-90504	11	38	0.619754	2	1	0.36605	no
gi|641571670|gb|KK856007.1|	90613-91789	11	38	0.505372	5	3	0.0726	no
gi|641571670|gb|KK856007.1|	92010-92907	11	38	0.403604	2	2	0.23615	no
gi|641571670|gb|KK856007.1|	93183-94306	11	38	0.381603	2	2	0.20805	no
gi|641571670|gb|KK856007.1|	95299-98791	11	38	5	13	1	0.39635	no
gi|641571680|gb|KK856006.1|	01506-10234	12	42	1	0.503582	-1	0.50885	no
gi|641571680|gb|KK856006.1|	08126-10898	12	42	0.642975	1	1	0.47015	no
gi|641571680|gb|KK856006.1|	12501-11270	12	42	9	25	2	0.41825	no
gi|641571680|gb|KK856006.1|	16305-11721	12	42	0.69346	1	0.604072	0.69715	no
gi|641571680|gb|KK856006.1|	20893-12401	12	42	7	7	-0.182921	0.882	no
gi|641571680|gb|KK856006.1|	24683-12547	12	42	4	5	0.246216	0.8587	no
gi|641571680|gb|KK856006.1|	25750-12950	12	42	4	7	0.668261	0.59085	no

gi|641571680|gb|KK856006.1|	29681-13233	12	42	2	2	-0.505545	0.6978	no
gi|641571680|gb|KK856006.1|	32412-13312	12	42	4	0.627031	-3	0.1554	no
gi|641571680|gb|KK856006.1|	33223-13436	12	42	2	0.1136	-4	0.1943	no
gi|641571680|gb|KK856006.1|	13972-15340	12	42	1	0.827789	-0.682212	0.63875	no
gi|641571680|gb|KK856006.1|	42444-14329	12	42	0.859903	2	1	0.49765	no
gi|641571680|gb|KK856006.1|	15469-15984	12	42	3	4	0.246111	0.8653	no
gi|641571680|gb|KK856006.1|	58104-15985	12	42	3	3	0.445029	0.72865	no
gi|641571680|gb|KK856006.1|	73195-17343	12	42	6	10	0.810998	0.6381	no
gi|641571680|gb|KK856006.1|	17439-19199	12	42	4	8	1	0.4243	no
gi|641571680|gb|KK856006.1|	77708-18031	12	42	2	2	0.391701	0.76155	no
gi|641571680|gb|KK856006.1|	80375-18397	12	42	2	2	0.279393	0.82555	no
gi|641571680|gb|KK856006.1|	21241-22100	12	42	0.853425	0.648318	-0.396561	1	no
gi|641571680|gb|KK856006.1|	24000-25337	12	42	1	0.094465	-4	0.20395	no
gi|641571680|gb|KK856006.1|	57663-58369	12	42	0.970352	1	0.115366	0.92265	no
gi|641571680|gb|KK856006.1|	58797-59778	12	42	0.72091	2	1	0.4562	no
gi|641571680|gb|KK856006.1|	72673-73237	12	42	2	3	0.766318	0.6262	no
gi|641571680|gb|KK856006.1|	76420-79840	12	42	3	1	-0.986433	0.4455	no
gi|641571680|gb|KK856006.1|	8318-9260	12	42	1	7	2	0.09805	no
gi|641571680|gb|KK856006.1|	83832-84550	12	42	0.812659	2	1	0.4118	no
gi|641571680|gb|KK856006.1|	85109-85818	12	42	1	0.418021	-1	0.4534	no
gi|641571680|gb|KK856006.1|	87474-87798	12	42	7	2	-2	0.3388	no
gi|641571680|gb|KK856006.1|	91859-92583	12	42	1	1	-0.398815	0.8125	no
gi|641571680|gb|KK856006.1|	95347-96319	12	42	2	1	-0.748907	0.6032	no
gi|641571680|gb|KK856006.1|	97449-97886	12	42	2	3	1	0.5082	no
gi|641571692|gb|KK856004.1|	06825-10758	9.8	37	0.88233	2	0.794479	0.63025	no
gi|641571692|gb|KK856004.1|	11741-11254	9.8	37	9	13	0.44854	0.72715	no
gi|641571692|gb|KK856004.1|	13439-11509	9.8	37	4	10	1	0.3353	no
gi|641571692|gb|KK856004.1|	20386-12118	9.8	37	0	154	inf	0.00015	yes
gi|641571692|gb|KK856004.1|	23524-12536	9.8	37	0	9	inf	5.00E-05	yes
gi|641571692|gb|KK856004.1|	51163-15164	9.8	37	2	22	3	0.05965	no

gi|641571692|gb|KK856004.1|	51800-15570	9.8	37	0.7058	7	3	0.0265	no
gi|641571692|gb|KK856004.1|	21479-21999	9.8	37	50	6	-3	0.039	no
gi|641571692|gb|KK856004.1|	22243-22647	9.8	37	4	3	-0.530241	0.74095	no
gi|641571692|gb|KK856004.1|	28312-28521	9.8	37	13	10	-0.272472	0.8666	no
gi|641571692|gb|KK856004.1|	325-527	9.8	37	35	6	-3	0.2078	no
gi|641571692|gb|KK856004.1|	34910-35679	9.8	37	0.740472	1	0.60205	0.70555	no
gi|641571692|gb|KK856004.1|	38199-39922	9.8	37	0.417115	1	2	0.23605	no
gi|641571692|gb|KK856004.1|	56918-57150	9.8	37	19	24	0.339041	0.8171	no
gi|641571692|gb|KK856004.1|	6145-6323	9.8	37	35	10	-2	0.33925	no
gi|641571692|gb|KK856004.1|	62189-62596	9.8	37	3	5	0.723074	0.6396	no
gi|641571692|gb|KK856004.1|	72170-72792	9.8	37	2	1	-1	0.5005	no
gi|641571692|gb|KK856004.1|	96151-96405	9.8	37	7	24	2	0.2598	no
gi|641571692|gb|KK856004.1|	9795-10043	9.8	37	9	6	-0.618106	0.7047	no
gi|641571695|gb|KK856003.1|	71008-17118	10	40	73	31	-1	0.44025	no
gi|641571695|gb|KK856003.1|	81029-81569	10	40	5	3	-0.9352	0.51565	no
gi|641571708|gb|KK856001.1|	11480-11874	8.6	39	5	3	-0.794449	0.6299	no
gi|641571708|gb|KK856001.1|	38670-13943	8.6	39	0.866905	1	0.602015	0.69715	no
gi|641571712|gb|KK856000.1|	17947-11840	10	39	0	22	inf	0.00015	yes
gi|641571712|gb|KK856000.1|	24829-12521	10	39	0	16	inf	0.0004	yes
gi|641571712|gb|KK856000.1|	28305-12891	10	39	0	4	inf	0.0016	yes
gi|641571712|gb|KK856000.1|	29652-13068	10	39	0	3	inf	0.0005	yes
gi|641571712|gb|KK856000.1|	32190-13243	10	39	0	22	inf	0.0023	yes
gi|641571712|gb|KK856000.1|	35761-13635	10	39	0	5	inf	0.00135	yes
gi|641571712|gb|KK856000.1|	47085-15055	10	39	0	41	inf	0.0001	yes
gi|641571712|gb|KK856000.1|	52268-15550	10	39	13	7	-0.914762	0.58255	no
gi|641571712|gb|KK856000.1|	55894-15749	10	39	12	4	-2	0.2073	no
gi|641571712|gb|KK856000.1|	59509-16006	10	39	25	34	0.465475	0.70785	no
gi|641571712|gb|KK856000.1|	61715-16426	10	39	20	31	0.661436	0.77025	no
gi|641571712|gb|KK856000.1|	66153-16727	10	39	2	1	-0.452921	0.7487	no
gi|641571712|gb|KK856000.1|	67383-16953	10	39	2	2	-0.149684	0.90925	no

gi|641571712|gb|KK856000.1|	70567-17342	10	39	2	1	-1	0.36975	no
gi|641571712|gb|KK856000.1|	77573-17778	10	39	8	14	0.801337	0.653	no
gi|641571712|gb|KK856000.1|	89904-19044	10	39	22	8	-1	0.26665	no
gi|641571712|gb|KK856000.1|	06647-20713	10	39	22	29	0.390794	0.75865	no
gi|641571712|gb|KK856000.1|	07828-20835	10	39	7	8	0.250665	0.8531	no
gi|641571716|gb|KK855999.1|	43978-14472	11	39	0.769981	6	3	0.10065	no
gi|641571716|gb|KK855999.1|	56698-57611	11	39	66	66	0.0126307	0.995	no
gi|641571716|gb|KK855999.1|	57937-59146	11	39	97	53	-0.876223	0.6942	no
gi|641571716|gb|KK855999.1|	59770-60818	11	39	8	5	-0.854025	0.5142	no
gi|641571716|gb|KK855999.1|	61191-62071	11	39	1	2	0.466207	0.75495	no
gi|641571716|gb|KK855999.1|	62377-65274	11	39	2	2	0.136216	0.9125	no
gi|641571716|gb|KK855999.1|	65899-67062	11	39	6	3	-0.853203	0.51555	no
gi|641571716|gb|KK855999.1|	67722-69967	11	39	0.621776	0.737823	0.246881	1	no
gi|641571716|gb|KK855999.1|	70449-72014	11	39	0.878768	0.94545	0.105519	0.94435	no
gi|641571716|gb|KK855999.1|	74752-75117	11	39	11	5	-1	0.4706	no
gi|641571716|gb|KK855999.1|	76771-77285	11	39	11	7	-0.628413	0.63425	no
gi|641571716|gb|KK855999.1|	78611-79014	11	39	17	5	-2	0.2277	no
gi|641571716|gb|KK855999.1|	79700-79959	11	39	101	28	-2	0.1719	no
gi|641571716|gb|KK855999.1|	80360-80773	11	39	17	9	-0.866294	0.52575	no
gi|641571716|gb|KK855999.1|	88843-90144	11	39	0.576308	0.292588	-0.977973	1	no
gi|641571716|gb|KK855999.1|	90289-91270	11	39	1	4	2	0.22585	no
gi|641571716|gb|KK855999.1|	91973-92410	11	39	2	2	-0.218803	0.8945	no
gi|641571726|gb|KK855997.1|	10869-11259	10	39	0.185627	7	5	0.1163	no
gi|641571726|gb|KK855997.1|	44497-14521	10	39	1	1	-0.135951	0.92965	no
gi|641571726|gb|KK855997.1|	46527-14726	10	39	0.778441	4	2	0.15295	no
gi|641571726|gb|KK855997.1|	48245-14946	10	39	0.550618	12	4	0.04775	no
gi|641571726|gb|KK855997.1|	49608-15070	10	39	1	0.477975	-1	0.46645	no
gi|641571726|gb|KK855997.1|	50338-51201	10	39	1	0.161115	-3	0.2382	no
gi|641571726|gb|KK855997.1|	77776-80734	10	39	87	85	-0.037148	0.9838	no
gi|641571726|gb|KK855997.1|	84492-85191	10	39	69	87	0.317602	0.84495	no

gi|641571730|gb|KK855996.1|	04373-10554	10	20	1	1	0.191216	0.8991	no
gi|641571730|gb|KK855996.1|	07453-10924	10	20	3	2	-0.0543277	0.9686	no
gi|641571730|gb|KK855996.1|	09372-11139	10	20	1	1	0.00337068	0.9814	no
gi|641571730|gb|KK855996.1|	11656-11231	10	20	17	17	-0.0709431	0.95465	no
gi|641571730|gb|KK855996.1|	13893-11700	10	20	1	2	0.38001	0.76605	no
gi|641571730|gb|KK855996.1|	17237-12121	10	20	0.768438	1	0.382502	0.7687	no
gi|641571730|gb|KK855996.1|	21396-12236	10	20	6	5	-0.0406245	0.9763	no
gi|641571730|gb|KK855996.1|	22734-12339	10	20	2	0.696896	-1	0.50885	no
gi|641571730|gb|KK855996.1|	23959-12610	10	20	2	2	-0.3058	0.81305	no
gi|641571730|gb|KK855996.1|	28963-13027	10	20	118	152	0.358664	0.86095	no
gi|641571730|gb|KK855996.1|	35447-13618	10	20	26	34	0.355389	0.77345	no
gi|641571730|gb|KK855996.1|	36347-13711	10	20	20	20	0.0126358	0.9922	no
gi|641571730|gb|KK855996.1|	45377-14556	10	20	61	39	-0.630266	0.66615	no
gi|641571730|gb|KK855996.1|	24621-25018	10	20	6	2	-2	0.28755	no
gi|641571730|gb|KK855996.1|	36256-38079	10	20	0.652481	0.862299	0.402252	1	no
gi|641571730|gb|KK855996.1|	40201-41517	10	20	1	1	-0.0450153	0.9701	no
gi|641571730|gb|KK855996.1|	45292-46112	10	20	40	43	0.103455	0.9343	no
gi|641571730|gb|KK855996.1|	46256-48012	10	20	2	2	-0.362306	0.78585	no
gi|641571730|gb|KK855996.1|	49316-49768	10	20	19	9	-1	0.3839	no
gi|641571730|gb|KK855996.1|	54349-54678	10	20	4	4	-0.104249	0.92555	no
gi|641571730|gb|KK855996.1|	69411-72749	10	20	1	2	0.759318	0.5579	no
gi|641571730|gb|KK855996.1|	74075-75887	10	20	0.525536	2	2	0.25355	no
gi|641571730|gb|KK855996.1|	82653-83158	10	20	5	5	-0.0232018	0.97395	no
gi|641571730|gb|KK855996.1|	83293-85292	10	20	0.902848	1	0.670458	0.6232	no
gi|641571730|gb|KK855996.1|	86588-86965	10	20	2	5	1	0.48155	no
gi|641571730|gb|KK855996.1|	87120-87659	10	20	2	0.310673	-3	0.2481	no
gi|641571730|gb|KK855996.1|	88022-89447	10	20	1	2	0.685531	0.6312	no
gi|641571730|gb|KK855996.1|	90854-93450	10	20	4	5	0.209335	0.8664	no
gi|641571730|gb|KK855996.1|	94901-95257	10	20	3	2	-0.643707	0.71075	no
gi|641571730|gb|KK855996.1|	96953-97098	10	20	160	156	-0.0304512	0.95595	no

gi|641571733|gb|KK855995.1|	04033-10580	9.9	37	0.900399	0.617799	-0.543427	0.71075	no
gi|641571733|gb|KK855995.1|	06212-10813	9.9	37	24	63	1	0.54	no
gi|641571733|gb|KK855995.1|	08352-10857	9.9	37	2	90	6	0.1047	no
gi|641571733|gb|KK855995.1|	09245-11073	9.9	37	1	2	0.664343	0.62735	no
gi|641571733|gb|KK855995.1|	10857-11352	9.9	37	15	19	0.367177	0.8746	no
gi|641571733|gb|KK855995.1|	26549-12716	9.9	37	0	5	inf	0.00135	yes
gi|641571733|gb|KK855995.1|	34784-13532	9.9	37	0	4	inf	0.00375	no
gi|641571733|gb|KK855995.1|	40130-14055	9.9	37	0	6	inf	0.0025	yes
gi|641571733|gb|KK855995.1|	47681-14794	9.9	37	0.910463	17	4	0.1137	no
gi|641571733|gb|KK855995.1|	65081-16564	9.9	37	0	16	inf	0.00015	yes
gi|641571733|gb|KK855995.1|	70453-17085	9.9	37	3	142	6	0.06215	no
gi|641571733|gb|KK855995.1|	85930-18626	9.9	37	0.986749	37	5	0.23335	no
gi|641571733|gb|KK855995.1|	87305-18883	9.9	37	0.266236	13	6	0.08165	no
gi|641571733|gb|KK855995.1|	91391-19170	9.9	37	1	21	4	0.2375	no
gi|641571733|gb|KK855995.1|	99463-19971	9.9	37	0	24	inf	0.0016	yes
gi|641571733|gb|KK855995.1|	11689-21221	9.9	37	14	26	0.875929	0.4953	no
gi|641571733|gb|KK855995.1|	43682-44067	9.9	37	2	3	0.845963	0.63695	no
gi|641571733|gb|KK855995.1|	59388-61308	9.9	37	6	6	0.0506129	0.9667	no
gi|641571733|gb|KK855995.1|	62490-63694	9.9	37	3	2	-0.430998	0.74865	no
gi|641571733|gb|KK855995.1|	65146-69217	9.9	37	3	2	-0.573614	0.65125	no
gi|641571733|gb|KK855995.1|	69883-71118	9.9	37	2	3	0.554184	0.6809	no
gi|641571733|gb|KK855995.1|	76193-76399	9.9	37	11	47	2	0.2195	no
gi|641571733|gb|KK855995.1|	89127-90575	9.9	37	6	24	2	0.14015	no
gi|641571733|gb|KK855995.1|	96492-96702	9.9	37	48	136	2	0.26985	no
gi|641571733|gb|KK855995.1|	97731-98054	9.9	37	102	228	1	0.3552	no
gi|641571733|gb|KK855995.1|	99733-101607	9.9	37	30	78	1	0.53525	no
gi|641571737|gb|KK855994.1|	36254-37500	11	23	0.269545	2	3	0.14045	no
gi|641571737|gb|KK855994.1|	39085-40607	11	23	0.853882	3	2	0.16305	no
gi|641571737|gb|KK855994.1|	48703-50287	11	23	0.50985	1	1	0.4709	no
gi|641571737|gb|KK855994.1|	54566-54721	11	23	84	33	-1	0.43985	no

gi|641571737|gb|KK855994.1|	65416-65950	11	23	24	12	-1	0.41255	no
gi|641571737|gb|KK855994.1|	76195-78159	11	23	0.720559	0.488441	-0.560932	1	no
gi|641571737|gb|KK855994.1|	80945-81172	11	23	16	16	-0.0416118	0.95145	no
gi|641571737|gb|KK855994.1|	82134-85431	11	23	15	13	-0.186753	0.93335	no
gi|641571737|gb|KK855994.1|	85958-86298	11	23	36	40	0.14203	0.91015	no
gi|641571737|gb|KK855994.1|	86348-86907	11	23	4	7	0.797813	0.5623	no
gi|641571742|gb|KK855993.1|	12098-12822	10	21	1	1	-0.398815	0.8125	no
gi|641571742|gb|KK855993.1|	45233-14606	10	21	0.893826	1	0.410374	0.8089	no
gi|641571742|gb|KK855993.1|	14837-15646	10	21	0.692216	2	1	0.47015	no
gi|641571742|gb|KK855993.1|	48415-14950	10	21	0.954963	1	0.480163	0.75585	no
gi|641571742|gb|KK855993.1|	2425-3572	10	21	6	2	-1	0.3272	no
gi|641571742|gb|KK855993.1|	369-543	10	21	36	23	-0.653605	0.6967	no
gi|641571742|gb|KK855993.1|	41536-41781	10	21	7	5	-0.671689	0.7081	no
gi|641571742|gb|KK855993.1|	42214-43444	10	21	1	0.62517	-0.808414	0.5996	no
gi|641571742|gb|KK855993.1|	43867-44447	10	21	2	2	-0.0550323	0.9454	no
gi|641571742|gb|KK855993.1|	44519-44951	10	21	3	5	0.877604	0.5758	no
gi|641571742|gb|KK855993.1|	45897-48112	10	21	0.631011	1	0.687411	0.6304	no
gi|641571742|gb|KK855993.1|	49777-51326	10	21	1	0.95659	-0.128997	0.9259	no
gi|641571742|gb|KK855993.1|	58016-59066	10	21	0.497034	1	1	0.41175	no
gi|641571745|gb|KK855992.1|	05520-10664	9.6	42	2	0.696211	-1	0.4423	no
gi|641571745|gb|KK855992.1|	06707-10750	9.6	42	1	0.177495	-3	0.2348	no
gi|641571745|gb|KK855992.1|	08618-11184	9.6	42	3	0.890182	-2	0.1636	no
gi|641571745|gb|KK855992.1|	13324-11462	9.6	42	6	5	-0.267474	0.83175	no
gi|641571745|gb|KK855992.1|	22443-22619	9.6	42	123	16	-3	0.13375	no
gi|641571745|gb|KK855992.1|	25791-25999	9.6	42	143	0	-inf	5.00E-05	yes
gi|641571745|gb|KK855992.1|	39486-39968	9.6	42	12	0	-inf	0.0003	yes
gi|641571745|gb|KK855992.1|	40326-41951	9.6	42	99	0	-inf	5.00E-05	yes
gi|641571745|gb|KK855992.1|	43057-43697	9.6	42	12	0	-inf	5.00E-05	yes
gi|641571745|gb|KK855992.1|	43896-44140	9.6	42	16	0	-inf	0.0059	no
gi|641571745|gb|KK855992.1|	44492-49297	9.6	42	4	0	-inf	5.00E-05	yes

gi|641571745|gb|KK855992.1|	49444-51707	9.6	42	4	0.052244	-6	0.18305	no
gi|641571745|gb|KK855992.1|	52498-53408	9.6	42	4	0	-inf	0.00115	yes
gi|641571745|gb|KK855992.1|	53685-54315	9.6	42	2	0	-inf	0.0059	no
gi|641571745|gb|KK855992.1|	54441-55929	9.6	42	2	0	-inf	0.00015	yes
gi|641571745|gb|KK855992.1|	56255-56577	9.6	42	20	0	-inf	0.00045	yes
gi|641571745|gb|KK855992.1|	57901-58605	9.6	42	2	0	-inf	0.0056	no
gi|641571745|gb|KK855992.1|	59398-59782	9.6	42	5	0	-inf	0.0059	no
gi|641571745|gb|KK855992.1|	60632-62149	9.6	42	2	0	-inf	0.00045	yes
gi|641571745|gb|KK855992.1|	62951-65309	9.6	42	2	0	-inf	5.00E-05	yes
gi|641571745|gb|KK855992.1|	66153-67540	9.6	42	7	0	-inf	5.00E-05	yes
gi|641571745|gb|KK855992.1|	68126-69452	9.6	42	3	0	-inf	0.00025	yes
gi|641571745|gb|KK855992.1|	70209-70533	9.6	42	11	0	-inf	0.0032	yes
gi|641571745|gb|KK855992.1|	71056-76395	9.6	42	5	2	-2	0.2185	no
gi|641571745|gb|KK855992.1|	76457-77279	9.6	42	2	0	-inf	0.00495	no
gi|641571745|gb|KK855992.1|	78028-79647	9.6	42	2	0	-inf	0.00055	yes
gi|641571745|gb|KK855992.1|	9499-9967	9.6	42	3	0	-inf	0.01175	no
gi|641571749|gb|KK855991.1|	02325-10307	9.4	35	10	2	-2	0.1002	no
gi|641571749|gb|KK855991.1|	04486-10464	9.4	35	129	158	0.298169	0.835	no
gi|641571749|gb|KK855991.1|	07763-10861	9.4	35	3	2	-0.603052	0.67015	no
gi|641571749|gb|KK855991.1|	10101-11051	9.4	35	3	0.962075	-1	0.4534	no
gi|641571749|gb|KK855991.1|	11071-11244	9.4	35	0.959468	0.365422	-1	0.4015	no
gi|641571749|gb|KK855991.1|	19679-12007	9.4	35	29	5	-3	0.0867	no
gi|641571749|gb|KK855991.1|	20257-12116	9.4	35	0.993438	1	0.282121	0.85465	no
gi|641571749|gb|KK855991.1|	22562-12355	9.4	35	0.529892	0.671614	0.341932	1	no
gi|641571749|gb|KK855991.1|	40110-14078	9.4	35	0.878082	1	0.337116	0.8352	no
gi|641571749|gb|KK855991.1|	43989-14510	9.4	35	0.997543	2	1	0.38005	no
gi|641571749|gb|KK855991.1|	25805-30083	9.4	35	171	15	-3	0.1141	no
gi|641571749|gb|KK855991.1|	30216-32244	9.4	35	0.888562	0.353567	-1	0.38115	no
gi|641571749|gb|KK855991.1|	33173-33888	9.4	35	1	1	-0.536511	0.7478	no
gi|641571749|gb|KK855991.1|	35471-37756	9.4	35	0.54211	1	1	0.45395	no

gi|641571749|gb|KK855991.1|	43842-48288	9.4	35	8	5	-0.810936	0.51255	no
gi|641571749|gb|KK855991.1|	48447-49619	9.4	35	1	2	0.191247	0.89245	no
gi|641571749|gb|KK855991.1|	49971-51065	9.4	35	2	2	-0.448687	0.75275	no
gi|641571749|gb|KK855991.1|	52658-53715	9.4	35	0.328664	0.8751	1	1	no
gi|641571749|gb|KK855991.1|	54645-54863	9.4	35	2992.51	9391.2	2	0.4115	no
gi|641571749|gb|KK855991.1|	55058-56115	9.4	35	10	30	2	0.2002	no
gi|641571749|gb|KK855991.1|	89104-89323	9.4	35	21	2	-3	0.2153	no
gi|641571749|gb|KK855991.1|	91809-93401	9.4	35	5	0.772689	-3	0.08875	no
gi|641571749|gb|KK855991.1|	97413-99425	9.4	35	15	9	-0.693988	0.5748	no
gi|641571752|gb|KK855990.1|	11665-12777	11	42	32	2	-4	0.01035	no
gi|641571752|gb|KK855990.1|	53007-53616	11	42	27	0.776692	-5	0.09455	no
gi|641571752|gb|KK855990.1|	9363-10169	11	42	71	9	-3	0.13155	no
gi|641571756|gb|KK855989.1|	16760-18728	8.7	40	0	54	inf	0.0001	yes
gi|641571756|gb|KK855989.1|	59395-59637	8.7	40	7	6	-0.0354089	0.91115	no
gi|641571760|gb|KK855988.1|	01580-10304	9.9	37	0.83711	2	1	0.45235	no
gi|641571760|gb|KK855988.1|	05307-10687	9.9	37	56	39	-0.498362	0.8242	no
gi|641571760|gb|KK855988.1|	08845-10914	9.9	37	2	11	3	0.2064	no
gi|641571760|gb|KK855988.1|	11797-11205	9.9	37	0	32	inf	0.00115	yes
gi|641571760|gb|KK855988.1|	14411-11585	9.9	37	13	25	0.90101	0.46485	no
gi|641571760|gb|KK855988.1|	22776-12371	9.9	37	9	3	-1	0.26945	no
gi|641571760|gb|KK855988.1|	24553-12489	9.9	37	7	6	-0.231246	0.8776	no
gi|641571760|gb|KK855988.1|	20927-21429	9.9	37	11	1	-3	0.11075	no
gi|641571760|gb|KK855988.1|	67576-69018	9.9	37	28	57	1	0.51215	no
gi|641571760|gb|KK855988.1|	71103-72312	9.9	37	8	19	1	0.35805	no
gi|641571760|gb|KK855988.1|	72825-74116	9.9	37	5	4	-0.219029	0.863	no
gi|641571760|gb|KK855988.1|	77569-78397	9.9	37	9	12	0.389601	0.75605	no
gi|641571760|gb|KK855988.1|	78839-79938	9.9	37	16	19	0.250213	0.83925	no
gi|641571760|gb|KK855988.1|	80271-84768	9.9	37	10	6	-0.642422	0.6901	no
gi|641571760|gb|KK855988.1|	88154-89507	9.9	37	1	2	0.414585	0.76875	no
gi|641571760|gb|KK855988.1|	90041-90897	9.9	37	0	4	inf	0.00115	yes

gi|641571760|gb|KK855988.1|	90949-92681	9.9	37	2	6	2	0.19185	no
gi|641571760|gb|KK855988.1|	94614-96317	9.9	37	41	34	-0.28092	0.89805	no
gi|641571760|gb|KK855988.1|	96540-97388	9.9	37	16	16	0.023293	0.9853	no
gi|641571760|gb|KK855988.1|	98868-100355	9.9	37	3	2	-0.222316	0.8644	no
gi|641571766|gb|KK855986.1|	65362-67677	9.2	41	0.801657	4	2	0.1139	no
gi|641571766|gb|KK855986.1|	82219-83102	9.2	41	0.514776	0.782284	0.603748	1	no
gi|641571769|gb|KK855985.1|	01512-10274	9	35	0.95707	2	0.969199	0.5114	no
gi|641571769|gb|KK855985.1|	03253-10473	9	35	0.49524	2	2	0.284	no
gi|641571769|gb|KK855985.1|	04874-10629	9	35	0.520736	0.793405	0.607505	1	no
gi|641571769|gb|KK855985.1|	06788-10831	9	35	0.425338	2	2	0.2039	no
gi|641571769|gb|KK855985.1|	08419-10997	9	35	0.415662	2	2	0.1741	no
gi|641571769|gb|KK855985.1|	10109-11140	9	35	0.192625	2	3	0.1666	no
gi|641571769|gb|KK855985.1|	11978-11295	9	35	0.090807	2	5	0.10875	no
gi|641571769|gb|KK855985.1|	13078-11372	9	35	1	3	1	0.4318	no
gi|641571769|gb|KK855985.1|	14537-11558	9	35	0.248808	1	2	0.25595	no
gi|641571769|gb|KK855985.1|	28936-13164	9	35	0.620412	3	2	0.1158	no
gi|641571769|gb|KK855985.1|	31896-13382	9	35	1	4	2	0.23585	no
gi|641571769|gb|KK855985.1|	35980-13762	9	35	0.342114	1	2	0.19685	no
gi|641571769|gb|KK855985.1|	39442-14044	9	35	0.523364	2	2	0.32035	no
gi|641571769|gb|KK855985.1|	41004-14163	9	35	0.812138	3	2	0.31505	no
gi|641571769|gb|KK855985.1|	41948-14304	9	35	0.9433	5	2	0.1252	no
gi|641571769|gb|KK855985.1|	21939-23772	9	35	0	121	inf	0.0005	yes
gi|641571769|gb|KK855985.1|	41416-41769	9	35	0	7	inf	0.0068	no
gi|641571769|gb|KK855985.1|	47623-48296	9	35	0	2	inf	0.0068	no
gi|641571769|gb|KK855985.1|	71704-73517	9	35	5	15	2	0.2006	no
gi|641571769|gb|KK855985.1|	73800-80049	9	35	4	13	2	0.3034	no
gi|641571769|gb|KK855985.1|	84060-85543	9	35	0.219936	0.754098	2	1	no
gi|641571769|gb|KK855985.1|	96761-97158	9	35	3	2	-1	0.5278	no
gi|641571769|gb|KK855985.1|	97920-99040	9	35	0.229704	1	3	0.19335	no
gi|641571769|gb|KK855985.1|	99798-100766	9	35	0.366519	0.975361	1	0.44255	no

gi|641571772|gb|KK855984.1|	20412-21115	9.9	42	0.557759	1	1	0.51305	no
gi|641571772|gb|KK855984.1|	23683-24093	9.9	42	0.978415	3	2	0.3323	no
gi|641571772|gb|KK855984.1|	87818-88597	9.9	42	0.485194	2	2	0.33105	no
gi|641571775|gb|KK855983.1|	08965-10921	9	37	12	3	-2	0.3145	no
gi|641571778|gb|KK855982.1|	14748-14936	11	43	0	40	inf	0.0068	no
gi|641571778|gb|KK855982.1|	28881-29268	11	43	0	13	inf	0.00075	yes
gi|641571778|gb|KK855982.1|	29550-30373	11	43	0	2	inf	0.0025	yes
gi|641571778|gb|KK855982.1|	41878-42099	11	43	0	24	inf	0.00455	no
gi|641571778|gb|KK855982.1|	52420-54899	11	43	2	1	-0.390253	0.7659	no
gi|641571778|gb|KK855982.1|	65350-65785	11	43	5	8	0.588372	0.67365	no
gi|641571778|gb|KK855982.1|	68860-69475	11	43	7	7	-0.00436131	0.98325	no
gi|641571778|gb|KK855982.1|	73810-74489	11	43	1	0.887375	-0.207205	0.8945	no
gi|641571778|gb|KK855982.1|	78818-79726	11	43	1	3	1	0.468	no
gi|641571778|gb|KK855982.1|	79857-81228	11	43	0.481774	0.366971	-0.392692	1	no
gi|641571778|gb|KK855982.1|	81681-82433	11	43	7	9	0.370416	0.7792	no
gi|641571778|gb|KK855982.1|	83462-83798	11	43	3	4	0.312149	0.86115	no
gi|641571778|gb|KK855982.1|	84345-86256	11	43	0.70155	1	0.608889	0.66985	no
gi|641571778|gb|KK855982.1|	86939-89717	11	43	27	19	-0.553394	0.80175	no
gi|641571782|gb|KK855981.1|	34063-34486	11	21	2	6	2	0.2855	no
gi|641571782|gb|KK855981.1|	66161-66634	11	21	2	2	0.00645461	0.91115	no
gi|641571786|gb|KK855980.1|	04792-10726	9.9	19	2	6	1	0.29295	no
gi|641571786|gb|KK855980.1|	15767-11594	9.9	19	4	80	4	0.1125	no
gi|641571786|gb|KK855980.1|	16043-11648	9.9	19	3	4	0.325607	0.8186	no
gi|641571786|gb|KK855980.1|	16547-11711	9.9	19	7	8	0.118472	0.9267	no
gi|641571786|gb|KK855980.1|	18101-11857	9.9	19	9	6	-0.490766	0.7136	no
gi|641571786|gb|KK855980.1|	40139-14177	9.9	19	6	4	-0.719561	0.577	no
gi|641571786|gb|KK855980.1|	42167-14266	9.9	19	7	2	-2	0.2831	no
gi|641571786|gb|KK855980.1|	42779-14435	9.9	19	0.870505	0.70244	-0.309478	1	no
gi|641571786|gb|KK855980.1|	44822-14498	9.9	19	782	336.32	-1	0.3501	no
gi|641571786|gb|KK855980.1|	47397-14776	9.9	19	353	230	-0.615332	0.7894	no

gi|641571786|gb|KK855980.1|	47942-15097	9.9	19	70	52	-0.418506	0.83935	no
gi|641571786|gb|KK855980.1|	51694-15325	9.9	19	0.723691	0.078788	-3	1	no
gi|641571786|gb|KK855980.1|	67538-17155	9.9	19	25	17	-0.53859	0.8047	no
gi|641571786|gb|KK855980.1|	82416-18307	9.9	19	6	22	2	0.18145	no
gi|641571786|gb|KK855980.1|	75800-77566	9.9	19	4	2	-1	0.38825	no
gi|641571786|gb|KK855980.1|	81100-81722	9.9	19	11	3	-2	0.14035	no
gi|641571786|gb|KK855980.1|	96275-98720	9.9	19	12	2	-3	0.0398	no
gi|641571790|gb|KK855979.1|	00460-10067	11	41	9	9	-0.0457108	0.91115	no
gi|641571790|gb|KK855979.1|	01768-10225	11	41	2	8	2	0.1912	no
gi|641571790|gb|KK855979.1|	03129-10400	11	41	1	2	1	0.43255	no
gi|641571790|gb|KK855979.1|	04634-10522	11	41	0.902975	2	1	0.39995	no
gi|641571790|gb|KK855979.1|	15930-11623	11	41	8	18	1	0.4201	no
gi|641571790|gb|KK855979.1|	17284-11882	11	41	0.944247	2	0.961592	0.49115	no
gi|641571790|gb|KK855979.1|	20309-12075	11	41	3	6	1	0.4346	no
gi|641571790|gb|KK855979.1|	21881-12259	11	41	3	7	1	0.29395	no
gi|641571790|gb|KK855979.1|	24002-12430	11	41	8	21	1	0.34075	no
gi|641571790|gb|KK855979.1|	25685-12603	11	41	2	6	1	0.40225	no
gi|641571790|gb|KK855979.1|	28949-12915	11	41	4	41	3	0.25595	no
gi|641571790|gb|KK855979.1|	31450-13210	11	41	3	8	1	0.37505	no
gi|641571790|gb|KK855979.1|	13750-15451	11	41	0.517255	1	1	0.40965	no
gi|641571790|gb|KK855979.1|	56670-15697	11	41	4	9	1	0.49435	no
gi|641571790|gb|KK855979.1|	58023-15816	11	41	37	122	2	0.353	no
gi|641571790|gb|KK855979.1|	60227-16064	11	41	2	5	1	0.4428	no
gi|641571790|gb|KK855979.1|	61374-16158	11	41	13	33	1	0.39925	no
gi|641571790|gb|KK855979.1|	63519-16370	11	41	33	25	-0.352196	0.82885	no
gi|641571790|gb|KK855979.1|	63885-16422	11	41	6	6	-0.00867296	0.95145	no
gi|641571790|gb|KK855979.1|	65226-16547	11	41	35	88	1	0.319	no
gi|641571790|gb|KK855979.1|	77212-18737	11	41	22	56	1	0.47785	no
gi|641571790|gb|KK855979.1|	19152-19982	11	41	0.446209	2	2	0.26155	no
gi|641571790|gb|KK855979.1|	91853-19811	11	41	57	133.41	1	0.49005	no

gi|641571790|gb|KK855979.1|	99348-19979	11	41	20	35	0.811811	0.52705	no
gi|641571790|gb|KK855979.1|	00002-20031	11	41	69	144	1	0.40305	no
gi|641571790|gb|KK855979.1|	20039-20689	11	41	1	2	0.751379	0.6262	no
gi|641571790|gb|KK855979.1|	01890-20228	11	41	74	137.27	0.888878	0.4727	no
gi|641571790|gb|KK855979.1|	02519-20341	11	41	0.710357	0.61691	-0.203484	1	no
gi|641571790|gb|KK855979.1|	03852-20436	11	41	84	170	1	0.53635	no
gi|641571790|gb|KK855979.1|	04465-20482	11	41	74	133	0.852861	0.4904	no
gi|641571790|gb|KK855979.1|	06057-20640	11	41	30	84	1	0.2461	no
gi|641571790|gb|KK855979.1|	06570-20717	11	41	29	56	0.926642	0.4552	no
gi|641571790|gb|KK855979.1|	20759-21228	11	41	2	4	1	0.41185	no
gi|641571790|gb|KK855979.1|	08102-20927	11	41	0.144245	2	4	0.24315	no
gi|641571790|gb|KK855979.1|	21713-22510	11	41	0.706021	1	0.824917	0.6232	no
gi|641571790|gb|KK855979.1|	22641-22974	11	41	5	4	-0.162356	0.91905	no
gi|641571790|gb|KK855979.1|	26801-22793	11	41	4	15	2	0.1727	no
gi|641571790|gb|KK855979.1|	31578-23205	11	41	0.497155	4	3	0.2674	no
gi|641571790|gb|KK855979.1|	24427-27628	11	41	2	3	0.964006	0.4513	no
gi|641571790|gb|KK855979.1|	44754-24637	11	41	2	3	0.476938	0.71405	no
gi|641571790|gb|KK855979.1|	32608-32832	11	41	1	19	4	0.12785	no
gi|641571790|gb|KK855979.1|	36359-36571	11	41	19	17	-0.115712	0.93375	no
gi|641571790|gb|KK855979.1|	39907-40182	11	41	5	10	1	0.4832	no
gi|641571790|gb|KK855979.1|	51951-52210	11	41	15	8	-0.946837	0.55475	no
gi|641571790|gb|KK855979.1|	59840-60731	11	41	0.407085	1	1	0.44255	no
gi|641571790|gb|KK855979.1|	60848-61636	11	41	2	3	0.772302	0.594	no
gi|641571790|gb|KK855979.1|	69581-69991	11	41	2	5	2	0.31505	no
gi|641571790|gb|KK855979.1|	72292-72864	11	41	3	6	1	0.4572	no
gi|641571790|gb|KK855979.1|	73055-75049	11	41	0.708541	0.900587	0.346014	0.81115	no
gi|641571790|gb|KK855979.1|	78993-79415	11	41	2	5	1	0.4428	no
gi|641571790|gb|KK855979.1|	80018-80411	11	41	3	11	2	0.2409	no
gi|641571805|gb|KK855976.1|	14273-11481	11	20	2	0.937366	-0.989792	0.62775	no
gi|641571805|gb|KK855976.1|	15510-11568	11	20	42	0	-inf	0.0102	no

gi|641571805|gb|KK855976.1|	22960-12329	11	20	18	0.707718	-5	0.18845	no
gi|641571805|gb|KK855976.1|	30474-13064	11	20	46	6	-3	0.2302	no
gi|641571805|gb|KK855976.1|	13761-14091	11	20	23	15	-0.627612	0.6428	no
gi|641571805|gb|KK855976.1|	43290-14432	11	20	1	2	0.489847	0.7378	no
gi|641571805|gb|KK855976.1|	54790-15496	11	20	36	0	-inf	0.01175	no
gi|641571805|gb|KK855976.1|	56380-15654	11	20	67	0	-inf	0.0102	no
gi|641571805|gb|KK855976.1|	15795-19067	11	20	0.989545	0.913469	-0.115411	0.93105	no
gi|641571805|gb|KK855976.1|	195-1419	11	20	1	0.314401	-2	0.33295	no
gi|641571805|gb|KK855976.1|	20078-21053	11	20	0.454036	0.96665	1	0.50945	no
gi|641571805|gb|KK855976.1|	21163-22738	11	20	0.769763	1	0.871085	0.5524	no
gi|641571805|gb|KK855976.1|	2358-2594	11	20	14	5	-1	0.4177	no
gi|641571805|gb|KK855976.1|	25346-26921	11	20	1	1	-0.146837	0.91745	no
gi|641571805|gb|KK855976.1|	27309-27768	11	20	5	2	-0.912117	0.56425	no
gi|641571805|gb|KK855976.1|	29057-29968	11	20	2	0.751854	-1	0.43755	no
gi|641571805|gb|KK855976.1|	31938-33024	11	20	0.635927	0.725777	0.190664	1	no
gi|641571805|gb|KK855976.1|	55215-57525	11	20	0.770071	0.408679	-0.914022	1	no
gi|641571805|gb|KK855976.1|	60278-61303	11	20	0.426675	0.778873	0.868249	1	no
gi|641571805|gb|KK855976.1|	87322-87505	11	20	40	9	-2	0.258	no
gi|641571805|gb|KK855976.1|	90807-92576	11	20	9	4	-1	0.3534	no
gi|641571805|gb|KK855976.1|	94226-94624	11	20	3	1	-1	0.44305	no
gi|641571813|gb|KK855975.1|	15152-11535	9.5	18	45	21	-1	0.4608	no
gi|641571813|gb|KK855975.1|	16432-11731	9.5	18	1	0.466672	-2	0.40415	no
gi|641571813|gb|KK855975.1|	22628-12366	9.5	18	1	0.641368	-0.773212	0.63565	no
gi|641571813|gb|KK855975.1|	25257-12632	9.5	18	0.405188	0.739832	0.868607	1	no
gi|641571813|gb|KK855975.1|	26441-12703	9.5	18	2	1	-0.25046	0.88485	no
gi|641571813|gb|KK855975.1|	27225-12814	9.5	18	1	0.148923	-3	0.217	no
gi|641571813|gb|KK855975.1|	28671-12956	9.5	18	1	0.154008	-3	0.22425	no
gi|641571813|gb|KK855975.1|	29756-13158	9.5	18	3	0.860191	-2	0.16735	no
gi|641571813|gb|KK855975.1|	40444-14101	9.5	18	23	8	-2	0.25785	no
gi|641571813|gb|KK855975.1|	41136-14165	9.5	18	10	3	-2	0.27645	no

gi|641571813|gb|KK855975.1|	41873-14474	9.5	18	4	2	-1	0.43535	no
gi|641571813|gb|KK855975.1|	46001-14734	9.5	18	0.987916	0.658382	-0.585464	0.68495	no
gi|641571813|gb|KK855975.1|	47572-14906	9.5	18	2	3	0.285849	0.8252	no
gi|641571813|gb|KK855975.1|	49874-15061	9.5	18	11	10	-0.0799796	0.95285	no
gi|641571813|gb|KK855975.1|	54645-15589	9.5	18	0.672581	0.204841	-2	1	no
gi|641571813|gb|KK855975.1|	57733-15847	9.5	18	16	7	-1	0.37825	no
gi|641571813|gb|KK855975.1|	60714-16113	9.5	18	9	7	-0.360898	0.79965	no
gi|641571813|gb|KK855975.1|	3402-3739	9.5	18	6	7	0.312337	0.83765	no
gi|641571813|gb|KK855975.1|	95304-95647	9.5	18	45	10	-2	0.1253	no
gi|641571817|gb|KK855974.1|	15084-16146	11	22	0.81692	0.124297	-3	1	no
gi|641571817|gb|KK855974.1|	16915-17787	11	22	0.83703	0.794924	-0.0744626	1	no
gi|641571817|gb|KK855974.1|	20471-21438	11	22	1	0.558067	-1	0.46585	no
gi|641571817|gb|KK855974.1|	22433-23577	11	22	1	0.567998	-1	0.43755	no
gi|641571817|gb|KK855974.1|	23855-24690	11	22	0.885442	0.336243	-1	0.4534	no
gi|641571817|gb|KK855974.1|	25629-25866	11	22	61	28	-1	0.4029	no
gi|641571821|gb|KK855973.1|	60305-61244	10	41	0	2	inf	0.0015	yes
gi|641571821|gb|KK855973.1|	69666-69991	10	41	0	10	inf	0.0025	yes
gi|641571833|gb|KK855970.1|	80049-80819	10	22	0.985579	0.748	-0.397932	0.82205	no
gi|641571841|gb|KK855968.1|	03876-10428	10	36	2	3	0.363288	0.82765	no
gi|641571841|gb|KK855968.1|	09957-11014	10	36	45	8	-3	0.2078	no
gi|641571841|gb|KK855968.1|	14672-11539	10	36	1	0.607123	-1	0.50885	no
gi|641571841|gb|KK855968.1|	19191-11948	10	36	11	2	-2	0.21625	no
gi|641571841|gb|KK855968.1|	20537-12136	10	36	4	1	-2	0.2371	no
gi|641571841|gb|KK855968.1|	23325-12586	10	36	23	9	-1	0.4253	no
gi|641571841|gb|KK855968.1|	26002-12725	10	36	0.605323	0.614522	0.0217587	1	no
gi|641571841|gb|KK855968.1|	33529-13858	10	36	6	12	0.912339	0.57625	no
gi|641571841|gb|KK855968.1|	38937-13945	10	36	2	5	1	0.48065	no
gi|641571841|gb|KK855968.1|	39636-14150	10	36	2	3	0.548688	0.6718	no
gi|641571841|gb|KK855968.1|	43785-14452	10	36	14	24	0.789138	0.53045	no
gi|641571841|gb|KK855968.1|	46884-14786	10	36	0.627601	0.545413	-0.2025	1	no

gi|641571841|gb|KK855968.1|	49649-15098	10	36	1	2	0.540026	0.7037	no
gi|641571841|gb|KK855968.1|	60959-16195	10	36	0.177963	7	5	0.23265	no
gi|641571841|gb|KK855968.1|	22860-23302	10	36	4	2	-1	0.50025	no
gi|641571841|gb|KK855968.1|	23354-24168	10	36	5	7	0.396343	0.75725	no
gi|641571841|gb|KK855968.1|	24312-25572	10	36	9	4	-1	0.28355	no
gi|641571841|gb|KK855968.1|	25795-27969	10	36	16	4	-2	0.13135	no
gi|641571841|gb|KK855968.1|	28136-28903	10	36	23	7	-2	0.20025	no
gi|641571841|gb|KK855968.1|	29071-30367	10	36	1	0.685794	-0.977997	0.5308	no
gi|641571841|gb|KK855968.1|	31134-32375	10	36	4	2	-1	0.2965	no
gi|641571841|gb|KK855968.1|	36584-39472	10	36	2	2	-0.427335	0.74075	no
gi|641571841|gb|KK855968.1|	39662-41767	10	36	4	2	-1	0.3871	no
gi|641571841|gb|KK855968.1|	44730-45902	10	36	0.724917	0.551783	-0.393715	1	no
gi|641571841|gb|KK855968.1|	52562-52861	10	36	0	12	inf	0.0025	yes
gi|641571841|gb|KK855968.1|	56679-57496	10	36	0.113885	3	5	0.11	no
gi|641571841|gb|KK855968.1|	57675-59751	10	36	3	4	0.431405	0.73885	no
gi|641571841|gb|KK855968.1|	61744-62403	10	36	0.152646	3	4	0.11365	no
gi|641571841|gb|KK855968.1|	64028-65853	10	36	8	2	-2	0.1706	no
gi|641571841|gb|KK855968.1|	66954-67792	10	36	0.110164	2	4	0.12385	no
gi|641571841|gb|KK855968.1|	70270-70570	10	36	1	8	3	0.28555	no
gi|641571841|gb|KK855968.1|	77431-78253	10	36	5	2	-1	0.4107	no
gi|641571841|gb|KK855968.1|	85834-87111	10	36	1	0.996849	-0.158663	0.91355	no
gi|641571841|gb|KK855968.1|	94391-94797	10	36	9	1	-3	0.157	no
gi|641571841|gb|KK855968.1|	98666-99164	10	36	7	1	-2	0.1557	no
gi|641571846|gb|KK855966.1|	44785-14533	11	23	3	4	0.315717	0.8318	no
gi|641571846|gb|KK855966.1|	71348-72435	11	23	0.714616	0.241655	-2	1	no
gi|641571849|gb|KK855965.1|	35563-35989	11	40	0.30426	5	4	0.1196	no
gi|641571849|gb|KK855965.1|	70269-70914	11	40	22	5	-2	0.1176	no
gi|641571849|gb|KK855965.1|	72919-75368	11	40	79	18	-2	0.34475	no
gi|641571849|gb|KK855965.1|	76720-76958	11	40	12	2	-3	0.2472	no
gi|641571849|gb|KK855965.1|	78910-79295	11	40	4	0	-inf	0.01175	no

gi|641571849|gb|KK855965.1|	79932-80137	11	40	120	14	-3	0.08085	no
gi|641571849|gb|KK855965.1|	80373-81889	11	40	3	2	-0.906714	0.5022	no
gi|641571849|gb|KK855965.1|	82457-83636	11	40	3	3	-0.0400351	0.97565	no
gi|641571849|gb|KK855965.1|	83794-84141	11	40	3	2	-0.645219	0.71075	no
gi|641571849|gb|KK855965.1|	84350-84907	11	40	17	7	-1	0.28455	no
gi|641571849|gb|KK855965.1|	86047-87235	11	40	2	1	-0.578042	0.6957	no
gi|641571849|gb|KK855965.1|	87442-88895	11	40	25	10	-1	0.29085	no
gi|641571849|gb|KK855965.1|	90321-91216	11	40	3	3	0.129964	0.92545	no
gi|641571849|gb|KK855965.1|	97828-98130	11	40	31	17	-0.856635	0.5289	no
gi|641571852|gb|KK855964.1|	42053-14338	10	21	0.31254	1	2	0.2126	no
gi|641571852|gb|KK855964.1|	55647-56663	10	21	0.776437	0.262457	-2	1	no
gi|641571852|gb|KK855964.1|	77581-78271	10	21	0.715492	2	1	0.51085	no
gi|641571852|gb|KK855964.1|	80278-82242	10	21	3	11	2	0.1862	no
gi|641571852|gb|KK855964.1|	82799-85258	10	21	2	6	2	0.19935	no
gi|641571852|gb|KK855964.1|	85315-86264	10	21	0.469699	2	2	0.2348	no
gi|641571852|gb|KK855964.1|	86658-87455	10	21	0.35301	3	3	0.1666	no
gi|641571858|gb|KK855962.1|	23722-12442	9.8	38	0.562182	3	2	0.23615	no
gi|641571858|gb|KK855962.1|	27252-12786	9.8	38	0.509421	2	2	0.256	no
gi|641571866|gb|KK855960.1|	55690-56176	11	45	0	28	inf	5.00E-05	yes
gi|641571870|gb|KK855959.1|	33971-13452	9.7	20	34	11	-2	0.217	no
gi|641571870|gb|KK855959.1|	41971-14263	9.7	20	5	2	-1	0.4314	no
gi|641571870|gb|KK855959.1|	43401-14584	9.7	20	13	8	-0.703553	0.5759	no
gi|641571870|gb|KK855959.1|	46045-14674	9.7	20	1	0.634372	-0.984237	0.62775	no
gi|641571870|gb|KK855959.1|	47552-14846	9.7	20	0.686734	0.894767	0.38176	0.82465	no
gi|641571870|gb|KK855959.1|	48816-15036	9.7	20	12	7	-0.726717	0.5657	no
gi|641571870|gb|KK855959.1|	62055-16228	9.7	20	31	9	-2	0.2761	no
gi|641571870|gb|KK855959.1|	72772-17340	9.7	20	6	3	-0.945414	0.50165	no
gi|641571870|gb|KK855959.1|	73579-17412	9.7	20	5	2	-0.927827	0.5316	no
gi|641571870|gb|KK855959.1|	77041-17744	9.7	20	2	3	0.0999214	0.92335	no
gi|641571870|gb|KK855959.1|	78086-17919	9.7	20	6	2	-2	0.2092	no

gi|641571870|gb|KK855959.1|	45258-48548	9.7	20	13	9	-0.503776	0.7517	no
gi|641571870|gb|KK855959.1|	75805-77619	9.7	20	2	1	-0.815815	0.54745	no
gi|641571870|gb|KK855959.1|	77720-80436	9.7	20	3	3	-0.377937	0.76525	no
gi|641571870|gb|KK855959.1|	85302-86126	9.7	20	1	0.85522	-0.245052	0.88495	no
gi|641571870|gb|KK855959.1|	87197-88332	9.7	20	0.753459	0.344048	-1	1	no
gi|641571870|gb|KK855959.1|	88447-89978	9.7	20	0.53008	0.323147	-0.714018	1	no
gi|641571870|gb|KK855959.1|	91348-91555	9.7	20	35	32	-0.129086	0.92445	no
gi|641571870|gb|KK855959.1|	92555-92724	9.7	20	120	53	-1	0.4232	no
gi|641571870|gb|KK855959.1|	97562-97795	9.7	20	25	5	-2	0.22415	no
gi|641571874|gb|KK855958.1|	10567-11805	11	45	0.746935	0.516994	-0.530836	1	no
gi|641571874|gb|KK855958.1|	16784-11752	11	45	0.778441	1	0.823923	0.6232	no
gi|641571874|gb|KK855958.1|	18022-11890	11	45	0.41242	1	1	0.44255	no
gi|641571874|gb|KK855958.1|	12533-12972	11	45	3	1	-0.996233	0.62775	no
gi|641571874|gb|KK855958.1|	1280-3118	11	45	0.474117	0.19718	-1	1	no
gi|641571874|gb|KK855958.1|	35556-13674	11	45	1	1	0.399961	0.7866	no
gi|641571874|gb|KK855958.1|	13702-14611	11	45	25	17.19	-0.517246	0.6884	no
gi|641571874|gb|KK855958.1|	47467-14850	11	45	1	0.63684	-0.773162	0.63565	no
gi|641571874|gb|KK855958.1|	14967-15708	11	45	50	35	-0.523321	0.67085	no
gi|641571874|gb|KK855958.1|	49954-15030	11	45	2	3	0.578026	0.76735	no
gi|641571874|gb|KK855958.1|	92583-19362	11	45	5	4	-0.148019	0.9098	no
gi|641571874|gb|KK855958.1|	95204-19686	11	45	0.771851	2	1	0.4261	no
gi|641571874|gb|KK855958.1|	20106-20968	11	45	10	0.161355	-6	0.1832	no
gi|641571874|gb|KK855958.1|	06158-20921	11	45	0.938869	1	0.647747	0.6125	no
gi|641571874|gb|KK855958.1|	09454-21043	11	45	1	2	0.827238	0.58405	no
gi|641571874|gb|KK855958.1|	11097-21191	11	45	0.906703	1	0.41024	0.8089	no
gi|641571874|gb|KK855958.1|	11993-21249	11	45	0.695036	2	2	0.3323	no
gi|641571874|gb|KK855958.1|	3205-4474	11	45	0.791312	0.401699	-0.978132	1	no
gi|641571874|gb|KK855958.1|	32611-32812	11	45	184	54	-2	0.20455	no
gi|641571874|gb|KK855958.1|	37888-38085	11	45	215	91	-1	0.35315	no
gi|641571874|gb|KK855958.1|	39487-40049	11	45	1	2	0.788953	0.63035	no

gi|641571874|gb|KK855958.1|	40884-42264	11	45	2	3	0.192309	0.8859	no
gi|641571874|gb|KK855958.1|	44196-44564	11	45	3	2	-0.64184	0.71075	no
gi|641571874|gb|KK855958.1|	4674-8389	11	45	4	2	-1	0.26005	no
gi|641571874|gb|KK855958.1|	51019-51667	11	45	0.469037	3	2	0.21375	no
gi|641571874|gb|KK855958.1|	51770-52550	11	45	0.726546	1	1	0.5082	no
gi|641571874|gb|KK855958.1|	64248-64804	11	45	1	0.888897	-0.626345	0.71195	no
gi|641571874|gb|KK855958.1|	65448-66537	11	45	0.713016	2	2	0.28425	no
gi|641571874|gb|KK855958.1|	84272-84649	11	45	3	7	1	0.4667	no
gi|641571886|gb|KK855956.1|	03070-10396	9.4	35	3	1	-1	0.40315	no
gi|641571886|gb|KK855956.1|	04139-10498	9.4	35	2	3	0.783834	0.58745	no
gi|641571886|gb|KK855956.1|	05235-10651	9.4	35	2	2	0.269826	0.84375	no
gi|641571886|gb|KK855956.1|	06650-10986	9.4	35	2	1	-0.974586	0.45805	no
gi|641571886|gb|KK855956.1|	11004-11406	9.4	35	3	4	0.494793	0.6932	no
gi|641571886|gb|KK855956.1|	14803-11655	9.4	35	2	1	-0.169065	0.90005	no
gi|641571886|gb|KK855956.1|	16918-11874	9.4	35	0.563407	0.594789	0.0782018	1	no
gi|641571886|gb|KK855956.1|	11899-13647	9.4	35	0.866397	0.139052	-3	1	no
gi|641571886|gb|KK855956.1|	19928-12125	9.4	35	2	0.85767	-1	0.415	no
gi|641571886|gb|KK855956.1|	33835-13601	9.4	35	1	2	0.194322	0.8809	no
gi|641571886|gb|KK855956.1|	41138-14168	9.4	35	4	0	-inf	0.0024	yes
gi|641571886|gb|KK855956.1|	46920-14743	9.4	35	10	3	-2	0.23095	no
gi|641571886|gb|KK855956.1|	48104-14901	9.4	35	4	1	-1	0.3199	no
gi|641571886|gb|KK855956.1|	14838-15742	9.4	35	4	0.911085	-2	0.1755	no
gi|641571886|gb|KK855956.1|	50043-15081	9.4	35	2	1	-0.982807	0.5501	no
gi|641571886|gb|KK855956.1|	56850-15800	9.4	35	0.51805	3	2	0.15295	no
gi|641571886|gb|KK855956.1|	58139-15886	9.4	35	8	3	-2	0.26955	no
gi|641571886|gb|KK855956.1|	60946-16198	9.4	35	15	7	-1	0.3832	no
gi|641571886|gb|KK855956.1|	66401-16661	9.4	35	28	26	-0.142693	0.9213	no
gi|641571886|gb|KK855956.1|	72687-17411	9.4	35	2	2	0.556906	0.67935	no
gi|641571886|gb|KK855956.1|	74504-17682	9.4	35	7	9	0.213972	0.8585	no
gi|641571886|gb|KK855956.1|	77544-17806	9.4	35	2	0.651043	-2	0.4266	no

gi|641571886|gb|KK855956.1|	86035-18860	9.4	35	37	13	-1	0.36985	no
gi|641571886|gb|KK855956.1|	19756-19966	9.4	35	23	0	-inf	0.0075	no
gi|641571886|gb|KK855956.1|	00233-20316	9.4	35	0.64632	2	1	0.2982	no
gi|641571886|gb|KK855956.1|	22811-23409	9.4	35	4	0.531736	-3	0.1659	no
gi|641571886|gb|KK855956.1|	55600-56397	9.4	35	9	3	-1	0.2908	no
gi|641571886|gb|KK855956.1|	56640-57402	9.4	35	6	6	0.00153279	0.9858	no
gi|641571886|gb|KK855956.1|	58975-60083	9.4	35	0.853323	0.35418	-1	1	no
gi|641571886|gb|KK855956.1|	60334-61105	9.4	35	5	6	0.187048	0.8869	no
gi|641571886|gb|KK855956.1|	63021-69023	9.4	35	7	5	-0.54128	0.73545	no
gi|641571886|gb|KK855956.1|	70976-73719	9.4	35	55	22	-1	0.55195	no
gi|641571886|gb|KK855956.1|	76521-76742	9.4	35	44	17	-1	0.36205	no
gi|641571886|gb|KK855956.1|	79363-80345	9.4	35	10	4	-1	0.36175	no
gi|641571886|gb|KK855956.1|	80581-81599	9.4	35	0.688489	0.785467	0.190117	1	no
gi|641571886|gb|KK855956.1|	82148-82845	9.4	35	11	3	-2	0.13195	no
gi|641571886|gb|KK855956.1|	8226-11799	9.4	35	10	1	-3	0.02485	no
gi|641571886|gb|KK855956.1|	84220-84578	9.4	35	12	8	-0.643384	0.6436	no
gi|641571886|gb|KK855956.1|	85324-87853	9.4	35	2	1	-0.344394	0.7906	no
gi|641571886|gb|KK855956.1|	88031-90372	9.4	35	3	1	-0.920935	0.48935	no
gi|641571886|gb|KK855956.1|	90673-91877	9.4	35	25	6	-2	0.1325	no
gi|641571886|gb|KK855956.1|	92078-93587	9.4	35	2	0.657084	-1	0.3857	no
gi|641571886|gb|KK855956.1|	94346-96812	9.4	35	0.467717	0.808888	0.790304	1	no
gi|641571886|gb|KK855956.1|	98237-99962	9.4	35	0.231427	2	3	0.1396	no
gi|641571892|gb|KK855954.1|	00660-10430	10	43	4	0	-inf	5.00E-05	yes
gi|641571892|gb|KK855954.1|	06973-10834	10	43	9	0.275461	-5	0.0946	no
gi|641571892|gb|KK855954.1|	08507-11143	10	43	7	0.079012	-6	0.13025	no
gi|641571892|gb|KK855954.1|	19580-12418	10	43	8	1	-2	0.18825	no
gi|641571892|gb|KK855954.1|	24439-12529	10	43	3	0.162566	-4	0.18815	no
gi|641571892|gb|KK855954.1|	26938-12807	10	43	1	0	-inf	0.00435	no
gi|641571892|gb|KK855954.1|	28254-12898	10	43	4	0.601505	-3	0.1554	no
gi|641571892|gb|KK855954.1|	29171-13059	10	43	3	0.087727	-5	0.18445	no

gi|641571892|gb|KK855954.1|	30831-13191	10	43	40	0.604139	-6	0.04245	no
gi|641571892|gb|KK855954.1|	33121-13383	10	43	1	0.618678	-0.814023	0.6492	no
gi|641571892|gb|KK855954.1|	33966-13425	10	43	5	3	-0.850209	0.64085	no
gi|641571892|gb|KK855954.1|	36052-13659	10	43	5	0	-inf	0.0022	yes
gi|641571892|gb|KK855954.1|	37452-13776	10	43	13	0	-inf	0.0024	yes
gi|641571892|gb|KK855954.1|	48686-15089	10	43	3	0	-inf	0.0001	yes
gi|641571892|gb|KK855954.1|	51008-15360	10	43	3	0	-inf	5.00E-05	yes
gi|641571892|gb|KK855954.1|	53811-15521	10	43	0.821446	0	-inf	1	no
gi|641571892|gb|KK855954.1|	57175-16010	10	43	32	0.039535	-10	0.18345	no
gi|641571892|gb|KK855954.1|	61305-16235	10	43	24	0	-inf	5.00E-05	yes
gi|641571892|gb|KK855954.1|	25687-26079	10	43	4	4	-0.00114521	0.95145	no
gi|641571892|gb|KK855954.1|	35858-36825	10	43	21	4	-2	0.09815	no
gi|641571892|gb|KK855954.1|	40349-42128	10	43	1	0	-inf	0.0013	yes
gi|641571892|gb|KK855954.1|	42991-43770	10	43	2	0	-inf	0.0056	no
gi|641571892|gb|KK855954.1|	45246-46973	10	43	1	0	-inf	0.001	yes
gi|641571892|gb|KK855954.1|	47345-48056	10	43	2	0	-inf	0.0034	yes
gi|641571892|gb|KK855954.1|	48658-51319	10	43	3	0.043825	-6	0.183	no
gi|641571892|gb|KK855954.1|	55921-56767	10	43	47	0	-inf	5.00E-05	yes
gi|641571892|gb|KK855954.1|	57065-57592	10	43	10	0	-inf	0.0002	yes
gi|641571892|gb|KK855954.1|	57965-58608	10	43	3	0	-inf	0.00345	yes
gi|641571892|gb|KK855954.1|	63589-65178	10	43	24	0	-inf	5.00E-05	yes
gi|641571892|gb|KK855954.1|	65490-67160	10	43	2	0	-inf	0.00045	yes
gi|641571892|gb|KK855954.1|	68423-73376	10	43	8	0	-inf	0.00065	yes
gi|641571892|gb|KK855954.1|	74258-74683	10	43	5	2	-1	0.44155	no
gi|641571892|gb|KK855954.1|	82819-83539	10	43	72	0.409336	-7	0.1357	no
gi|641571892|gb|KK855954.1|	84237-88453	10	43	3	2	-0.801112	0.53265	no
gi|641571892|gb|KK855954.1|	90576-91686	10	43	0.928862	0	-inf	0.01	no
gi|641571892|gb|KK855954.1|	94595-95890	10	43	3	1	-2	0.28255	no
gi|641571892|gb|KK855954.1|	96417-96951	10	43	4	19	2	0.126	no
gi|641571892|gb|KK855954.1|	97396-98013	10	43	1	1	-0.0797028	0.92675	no

gi|641571896|gb|KK855953.1|	02463-10263	10	34	34	21	-0.652524	0.6967	no
gi|641571896|gb|KK855953.1|	08383-10880	10	34	9	4	-1	0.39755	no
gi|641571896|gb|KK855953.1|	61073-63189	10	34	3	1	-1	0.36625	no
gi|641571896|gb|KK855953.1|	63286-65985	10	34	4	2	-0.782343	0.54865	no
gi|641571896|gb|KK855953.1|	76614-76813	10	34	46	22	-1	0.4792	no
gi|641571896|gb|KK855953.1|	77777-78670	10	34	0.405918	1	1	0.44255	no
gi|641571896|gb|KK855953.1|	79027-79305	10	34	23	9	-1	0.36785	no
gi|641571896|gb|KK855953.1|	95934-96180	10	34	13	23	0.873006	0.5608	no
gi|641571900|gb|KK855952.1|	36757-37608	9.5	35	1	0.328089	-2	0.3455	no
gi|641571900|gb|KK855952.1|	38683-39321	9.5	35	1	1	0.1136	0.92265	no
gi|641571900|gb|KK855952.1|	40095-40777	9.5	35	1	1	-0.399775	0.8125	no
gi|641571900|gb|KK855952.1|	41743-42739	9.5	35	1	3	1	0.4393	no
gi|641571900|gb|KK855952.1|	43003-44948	9.5	35	2	3	0.0357711	0.9787	no
gi|641571900|gb|KK855952.1|	5264-5888	9.5	35	1	0.999829	-0.401405	0.82205	no
gi|641571900|gb|KK855952.1|	86036-86463	9.5	35	0.605972	5	3	0.2674	no
gi|641571904|gb|KK855951.1|	00440-10116	8.9	36	2	4	1	0.3162	no
gi|641571904|gb|KK855951.1|	53896-54413	8.9	36	2	2	-0.0579333	0.9454	no
gi|641571904|gb|KK855951.1|	54518-55731	8.9	36	4	7	0.779365	0.54825	no
gi|641571904|gb|KK855951.1|	56402-57786	8.9	36	1	2	0.663946	0.62735	no
gi|641571904|gb|KK855951.1|	58041-58929	8.9	36	0.919908	1	0.60381	0.6902	no
gi|641571904|gb|KK855951.1|	60036-61039	8.9	36	2	2	0.0524805	0.96895	no
gi|641571904|gb|KK855951.1|	61429-65756	8.9	36	9	3	-2	0.29955	no
gi|641571904|gb|KK855951.1|	67427-68179	8.9	36	4	1	-2	0.2646	no
gi|641571904|gb|KK855951.1|	68431-68730	8.9	36	7	3	-1	0.46935	no
gi|641571904|gb|KK855951.1|	70265-70727	8.9	36	152.67	122	-0.322218	0.84395	no
gi|641571904|gb|KK855951.1|	71813-72952	8.9	36	7	7	0.0137317	0.99205	no
gi|641571904|gb|KK855951.1|	73754-74408	8.9	36	10	9	-0.129864	0.9177	no
gi|641571904|gb|KK855951.1|	74930-77100	8.9	36	13	10	-0.374628	0.7615	no
gi|641571904|gb|KK855951.1|	78290-79108	8.9	36	1	2	0.602855	0.678	no
gi|641571904|gb|KK855951.1|	82509-82816	8.9	36	7	24	2	0.22195	no

gi|641571904|gb|KK855951.1|	83119-83965	8.9	36	0.544046	1	1	0.50945	no
gi|641571904|gb|KK855951.1|	84866-85574	8.9	36	3	7	1	0.31375	no
gi|641571904|gb|KK855951.1|	86079-86763	8.9	36	2	0.658791	-1	0.4311	no
gi|641571904|gb|KK855951.1|	87057-88276	8.9	36	0.484144	3	3	0.1165	no
gi|641571904|gb|KK855951.1|	88458-88668	8.9	36	147	208	0.500189	0.692	no
gi|641571904|gb|KK855951.1|	89406-89953	8.9	36	14	22	0.575465	0.6516	no
gi|641571904|gb|KK855951.1|	90191-90377	8.9	36	35	95	1	0.31665	no
gi|641571907|gb|KK855950.1|	.00617-10149	11	20	0.625002	0.791436	0.340613	1	no
gi|641571907|gb|KK855950.1|	.03519-10366	11	20	144	16	-3	0.2199	no
gi|641571907|gb|KK855950.1|	.04496-10640	11	20	2	1	-0.391125	0.77105	no
gi|641571907|gb|KK855950.1|	.16765-11699	11	20	16	6	-1	0.4177	no
gi|641571907|gb|KK855950.1|	.26755-12774	11	20	89	24	-2	0.28	no
gi|641571907|gb|KK855950.1|	.33788-13428	11	20	2	6	2	0.2556	no
gi|641571907|gb|KK855950.1|	.37040-13726	11	20	19	12	-0.679482	0.657	no
gi|641571907|gb|KK855950.1|	.43796-14434	11	20	4	7	0.885377	0.52805	no
gi|641571907|gb|KK855950.1|	.45774-14634	11	20	3	0.847351	-2	0.33205	no
gi|641571907|gb|KK855950.1|	.46532-14722	11	20	2	0.435664	-2	0.3216	no
gi|641571907|gb|KK855950.1|	.52135-15275	11	20	9	5	-0.961124	0.47925	no
gi|641571907|gb|KK855950.1|	.55144-15553	11	20	61	33	-0.865168	0.4974	no
gi|641571907|gb|KK855950.1|	23320-23817	11	20	21	10	-1	0.43135	no
gi|641571907|gb|KK855950.1|	38558-38762	11	20	854	448	-0.931258	0.4526	no
gi|641571907|gb|KK855950.1|	47337-47822	11	20	8	7	-0.325305	0.8094	no
gi|641571907|gb|KK855950.1|	53294-54219	11	20	2	1	-0.588404	0.68495	no
gi|641571907|gb|KK855950.1|	54345-54906	11	20	3	0.876957	-2	0.33985	no
gi|641571907|gb|KK855950.1|	60081-62962	11	20	1	0.965772	-0.197221	0.88255	no
gi|641571907|gb|KK855950.1|	63163-63929	11	20	19	11	-0.753765	0.5565	no
gi|641571907|gb|KK855950.1|	67915-68787	11	20	0.523144	0.953908	0.866644	0.62725	no
gi|641571907|gb|KK855950.1|	74016-74891	11	20	0.937502	0.949723	0.0186852	0.9394	no
gi|641571907|gb|KK855950.1|	76011-79624	11	20	2	1	-1	0.4038	no
gi|641571907|gb|KK855950.1|	81167-81733	11	20	2	1	-0.403523	0.82205	no

gi|641571907|gb|KK855950.1|	84049-84632	11	20	7	9	0.427236	0.74665	no
gi|641571907|gb|KK855950.1|	85178-87658	11	20	5	5	-0.0717227	0.95505	no
gi|641571907|gb|KK855950.1|	87852-88822	11	20	4	2	-1	0.41645	no
gi|641571907|gb|KK855950.1|	90448-91192	11	20	4	2	-0.620804	0.65595	no
gi|641571907|gb|KK855950.1|	92089-94526	11	20	3	0.86753	-2	0.243	no
gi|641571907|gb|KK855950.1|	94624-95559	11	20	4	0.291038	-4	0.13985	no
gi|641571907|gb|KK855950.1|	95974-98454	11	20	8	4	-1	0.3841	no
gi|641571907|gb|KK855950.1|	99562-10054(	11	20	81	49	-0.714713	0.75235	no
gi|641571911|gb|KK855949.1|	1-317	9	38	14	77	2	0.0836	no
gi|641571911|gb|KK855949.1|	13202-11344	9	38	156	131	-0.251339	0.84315	no
gi|641571911|gb|KK855949.1|	14112-11449	9	38	9	16	0.805057	0.56285	no
gi|641571911|gb|KK855949.1|	16479-11669	9	38	29	49	0.771318	0.58995	no
gi|641571911|gb|KK855949.1|	18780-12031	9	38	2	2	-0.298968	0.8161	no
gi|641571911|gb|KK855949.1|	20452-12352	9	38	0.982796	2	0.988796	0.45295	no
gi|641571911|gb|KK855949.1|	23780-12487	9	38	0.550258	0.717755	0.383383	1	no
gi|641571911|gb|KK855949.1|	26800-12754	9	38	19	46	1	0.31925	no
gi|641571911|gb|KK855949.1|	28108-12945	9	38	0.246335	0.56286	1	1	no
gi|641571911|gb|KK855949.1|	33333-13444	9	38	0.465449	1	1	0.37775	no
gi|641571911|gb|KK855949.1|	41591-14232	9	38	1	0.793198	-0.398549	0.82205	no
gi|641571911|gb|KK855949.1|	44272-14457	9	38	399	160	-1	0.43405	no
gi|641571911|gb|KK855949.1|	47300-14803	9	38	29	10	-2	0.2389	no
gi|641571911|gb|KK855949.1|	49143-14954	9	38	11	19	0.750067	0.57695	no
gi|641571911|gb|KK855949.1|	49898-15077	9	38	25	21	-0.259882	0.8331	no
gi|641571911|gb|KK855949.1|	57921-15811	9	38	418	108	-2	0.13775	no
gi|641571911|gb|KK855949.1|	5121-6094	9	38	2	2	0.212423	0.88035	no
gi|641571911|gb|KK855949.1|	53770-54264	9	38	0	18	inf	0.0001	yes
gi|641571911|gb|KK855949.1|	73732-75478	9	38	5	0.278463	-4	0.0634	no
gi|641571911|gb|KK855949.1|	8070-9068	9	38	326	312	-0.0629078	0.9736	no
gi|641571911|gb|KK855949.1|	82450-83110	9	38	9	18	0.964938	0.4593	no
gi|641571911|gb|KK855949.1|	90246-91216	9	38	0.731146	0.694897	-0.0733613	1	no

gi|641571911|gb|KK855949.1|	93899-95007	9	38	0.543024	0.35418	-0.616531	1	no
gi|641571911|gb|KK855949.1|	97352-98092	9	38	0.779869	3	2	0.21435	no
gi|641571915|gb|KK855948.1|	.01649-10195	8.3	34	7	4	-0.569159	0.74505	no
gi|641571915|gb|KK855948.1|	.02070-10285	8.3	34	1	0.724231	-0.567532	0.7586	no
gi|641571924|gb|KK855947.1|	12906-13076	8	33	80	32	-1	0.39975	no
gi|641571928|gb|KK855946.1|	10472-10902	11	38	1	6	2	0.23675	no
gi|641571928|gb|KK855946.1|	.08353-10985	11	38	13	8	-0.667185	0.59265	no
gi|641571928|gb|KK855946.1|	.10186-11108	11	38	26	23	-0.170557	0.8884	no
gi|641571928|gb|KK855946.1|	.11399-11480	11	38	0.419254	0.539039	0.362566	1	no
gi|641571928|gb|KK855946.1|	.14929-11597	11	38	0.917671	1	0.145943	0.9285	no
gi|641571928|gb|KK855946.1|	.16528-11852	11	38	0.706186	0.8976	0.346025	0.81115	no
gi|641571928|gb|KK855946.1|	.18774-11957	11	38	2	3	0.989596	0.51835	no
gi|641571928|gb|KK855946.1|	.21406-12351	11	38	0.776551	0.902553	0.21693	0.87935	no
gi|641571928|gb|KK855946.1|	.23644-12593	11	38	38	42	0.13297	0.9498	no
gi|641571928|gb|KK855946.1|	13925-14319	11	38	23	9	-1	0.3129	no
gi|641571928|gb|KK855946.1|	18231-18669	11	38	21	5	-2	0.16885	no
gi|641571928|gb|KK855946.1|	.96817-19748	11	38	0.598983	2	2	0.3613	no
gi|641571928|gb|KK855946.1|	.98285-19865	11	38	3	2	-0.641691	0.71075	no
gi|641571928|gb|KK855946.1|	21368-22035	11	38	15	2	-3	0.0763	no
gi|641571928|gb|KK855946.1|	27098-28442	11	38	1	0.469457	-1	0.38185	no
gi|641571928|gb|KK855946.1|	34061-34315	11	38	73	18	-2	0.16295	no
gi|641571928|gb|KK855946.1|	35074-35710	11	38	4	1	-2	0.27005	no
gi|641571928|gb|KK855946.1|	39086-40028	11	38	2	1	-0.758145	0.61675	no
gi|641571928|gb|KK855946.1|	43550-43983	11	38	4	1	-2	0.3396	no
gi|641571928|gb|KK855946.1|	45693-46411	11	38	18	5	-2	0.16355	no
gi|641571928|gb|KK855946.1|	52411-53482	11	38	3	1	-1	0.41385	no
gi|641571928|gb|KK855946.1|	54458-55132	11	38	3	3	0.0150609	0.9647	no
gi|641571928|gb|KK855946.1|	65536-66359	11	38	0	7	inf	0.0001	yes
gi|641571928|gb|KK855946.1|	69-716	11	38	0.783454	1	0.59929	0.70505	no
gi|641571928|gb|KK855946.1|	74734-75520	11	38	1	3	0.924271	0.53715	no

gi|641571928|gb|KK855946.1|	75898-77945	11	38	16	6	-2	0.36835	no
gi|641571928|gb|KK855946.1|	82581-86099	11	38	13	2	-3	0.1627	no
gi|641571928|gb|KK855946.1|	849-1448	11	38	3	1	-1	0.44155	no
gi|641571928|gb|KK855946.1|	88090-88532	11	38	13	6	-1.17	0.411	no
gi|641571928|gb|KK855946.1|	94188-95342	11	38	1	2	0.523709	0.7137	no
gi|641571928|gb|KK855946.1|	9479-10297	11	38	1	2	0.865889	0.56615	no
gi|641571928|gb|KK855946.1|	96066-97320	11	38	1	2	0.882387	0.52175	no
gi|641571928|gb|KK855946.1|	97586-98231	11	38	0.786929	2	1	0.4611	no
gi|641571928|gb|KK855946.1|	98469-101685	11	38	0.796936	1	0.477112	0.72075	no
gi|641571931|gb|KK855945.1|	04967-10534	11	21	16	20	0.31162	0.81025	no
gi|641571931|gb|KK855945.1|	10298-11174	11	21	0.451081	2	2	0.2741	no
gi|641571931|gb|KK855945.1|	13694-11402	11	21	4	2	-0.840818	0.64855	no
gi|641571931|gb|KK855945.1|	19285-11975	11	21	1	2	0.85402	0.62755	no
gi|641571931|gb|KK855945.1|	20566-12192	11	21	0.42444	1	1	0.3924	no
gi|641571931|gb|KK855945.1|	25779-12654	11	21	0.375469	1	2	0.3323	no
gi|641571931|gb|KK855945.1|	26806-12787	11	21	0.405188	2	2	0.2348	no
gi|641571931|gb|KK855945.1|	15158-17629	11	21	9	13	0.540899	0.66305	no
gi|641571931|gb|KK855945.1|	18358-18773	11	21	73	139	0.938414	0.45135	no
gi|641571931|gb|KK855945.1|	21177-21719	11	21	47	71	0.59541	0.62925	no
gi|641571931|gb|KK855945.1|	24214-24767	11	21	3	4	0.208892	0.88465	no
gi|641571931|gb|KK855945.1|	42698-43496	11	21	2	0	-inf	0.0034	yes
gi|641571931|gb|KK855945.1|	431-841	11	21	2	3	0.585898	0.70555	no
gi|641571931|gb|KK855945.1|	56408-58577	11	21	56	0	-inf	0.00015	yes
gi|641571931|gb|KK855945.1|	59912-61178	11	21	34	0.100705	-8	0.1859	no
gi|641571931|gb|KK855945.1|	63057-63723	11	21	0.601523	2	2	0.3613	no
gi|641571936|gb|KK855944.1|	57380-15812	11	44	2	2	-0.305263	0.84435	no
gi|641571936|gb|KK855944.1|	82737-18325	11	44	27	9	-2	0.24385	no
gi|641571936|gb|KK855944.1|	04379-20456	11	44	129	15	-3	0.0975	no
gi|641571936|gb|KK855944.1|	10792-21120	11	44	1	3	1	0.51315	no
gi|641571936|gb|KK855944.1|	33656-34149	11	44	3	3	-0.300339	0.84385	no

gi|641571940|gb|KK855943.1|	.03464-10427	11	42	0.927488	1	0.187631	0.91015	no
gi|641571940|gb|KK855943.1|	.05062-10590	11	42	2	0.334167	-3	0.20575	no
gi|641571940|gb|KK855943.1|	.06768-10706	11	42	8	2	-2	0.265	no
gi|641571940|gb|KK855943.1|	.10874-11199	11	42	0.767341	1	0.743435	0.6176	no
gi|641571940|gb|KK855943.1|	14078-14474	11	42	4	0.521648	-3	0.2376	no
gi|641571940|gb|KK855943.1|	.48013-14889	11	42	0.41423	2	2	0.3012	no
gi|641571940|gb|KK855943.1|	.60556-16150	11	42	0.846581	0.286039	-2	1	no
gi|641571940|gb|KK855943.1|	.62123-16325	11	42	9	2	-2	0.12315	no
gi|641571940|gb|KK855943.1|	.65204-16556	11	42	11	2	-3	0.1613	no
gi|641571940|gb|KK855943.1|	.72158-17279	11	42	8	4	-0.785788	0.5707	no
gi|641571940|gb|KK855943.1|	.78320-17891	11	42	12	4	-2	0.27975	no
gi|641571940|gb|KK855943.1|	18940-19714	11	42	1	0.557151	-1	0.4312	no
gi|641571940|gb|KK855943.1|	48807-49932	11	42	0.83772	0.231822	-2	1	no
gi|641571940|gb|KK855943.1|	57053-58613	11	42	0.415053	0.395376	-0.0700683	1	no
gi|641571940|gb|KK855943.1|	96279-97326	11	42	0.665046	0.25295	-1	1	no
gi|641571943|gb|KK855942.1|	.30182-13071	8.7	40	4	0.951002	-2	0.24335	no
gi|641571943|gb|KK855942.1|	92826-93055	8.7	40	8	12	0.544218	0.75805	no
gi|641571943|gb|KK855942.1|	93599-94193	8.7	40	9	9	-0.0748519	0.95305	no
gi|641571943|gb|KK855942.1|	94407-94843	8.7	40	7	7	0.152363	0.91245	no
gi|641571943|gb|KK855942.1|	96111-97266	8.7	40	4	2	-0.76728	0.5595	no
gi|641571946|gb|KK855941.1|	.08837-10979	11	23	0.73974	0.562417	-0.395379	1	no
gi|641571946|gb|KK855941.1|	.09903-11014	11	23	23	3	-3	0.18155	no
gi|641571946|gb|KK855941.1|	.13465-11461	11	23	0.815783	0.112889	-3	1	no
gi|641571946|gb|KK855941.1|	.17116-11740	11	23	45	14	-2	0.23845	no
gi|641571946|gb|KK855941.1|	.19963-12111	11	23	0.663291	0.897493	0.436259	0.7798	no
gi|641571946|gb|KK855941.1|	.21249-12287	11	23	76	19	-2	0.2422	no
gi|641571946|gb|KK855941.1|	.34585-13491	11	23	4	3	-0.426378	0.7868	no
gi|641571946|gb|KK855941.1|	.37833-13802	11	23	37	23	-0.664481	0.66655	no
gi|641571946|gb|KK855941.1|	.45689-14596	11	23	6	2	-1	0.44305	no
gi|641571946|gb|KK855941.1|	.60239-16096	11	23	1	4	2	0.22535	no

gi|641571946|gb|KK855941.1|	.61365-16213	11	23	0.859392	4	2	0.20995	no
gi|641571946|gb|KK855941.1|	.64009-16545	11	23	0.794453	1	0.607598	0.67415	no
gi|641571946|gb|KK855941.1|	.65798-16676	11	23	0.551922	0.839264	0.604661	1	no
gi|641571946|gb|KK855941.1|	.67235-17005	11	23	1	2	0.280776	0.82565	no
gi|641571946|gb|KK855941.1|	.75244-17637	11	23	0.607943	0.809642	0.413348	1	no
gi|641571946|gb|KK855941.1|	20022-20463	11	23	3	1	-1	0.46975	no
gi|641571946|gb|KK855941.1|	21293-22507	11	23	13	2	-3	0.06395	no
gi|641571946|gb|KK855941.1|	25032-25928	11	23	2	2	0.35598	0.79615	no
gi|641571946|gb|KK855941.1|	26722-28128	11	23	1	0.534597	-0.977509	0.5501	no
gi|641571946|gb|KK855941.1|	28495-29317	11	23	1	0.343172	-2	0.3455	no
gi|641571946|gb|KK855941.1|	33073-35242	11	23	1	0.875629	-0.344856	0.79995	no
gi|641571946|gb|KK855941.1|	49919-50215	11	23	8	6	-0.433624	0.77845	no
gi|641571946|gb|KK855941.1|	5891-6153	11	23	45	14	-2	0.25105	no
gi|641571946|gb|KK855941.1|	61438-62641	11	23	0.702616	0.320926	-1	1	no
gi|641571946|gb|KK855941.1|	62713-63685	11	23	2	0.41587	-2	0.1864	no
gi|641571946|gb|KK855941.1|	63740-65834	11	23	1	2	0.346187	0.79315	no
gi|641571946|gb|KK855941.1|	6382-6563	11	23	59	5	-4	0.2025	no
gi|641571946|gb|KK855941.1|	66998-68996	11	23	1	0.838687	-0.338477	0.80915	no
gi|641571946|gb|KK855941.1|	69092-71697	11	23	1	0.941675	-0.319729	0.81345	no
gi|641571946|gb|KK855941.1|	7479-7672	11	23	91	14	-3	0.12615	no
gi|641571946|gb|KK855941.1|	76956-77261	11	23	4	4	0.305549	0.86115	no
gi|641571946|gb|KK855941.1|	77476-78035	11	23	2	0.881695	-1	0.50365	no
gi|641571946|gb|KK855941.1|	80079-81079	11	23	0.879861	0.936771	0.0904211	0.9392	no
gi|641571946|gb|KK855941.1|	81604-82360	11	23	2	2	0.0168531	0.95145	no
gi|641571946|gb|KK855941.1|	82559-84485	11	23	1	1	0.0683509	0.95865	no
gi|641571946|gb|KK855941.1|	85093-85472	11	23	4	5	0.260197	0.87415	no
gi|641571946|gb|KK855941.1|	85940-86521	11	23	2	0.277406	-3	0.271	no
gi|641571946|gb|KK855941.1|	94165-95200	11	23	2	0.641368	-1	0.43755	no
gi|641571950|gb|KK855940.1|	.02046-10283	10	37	0.119457	3	4	0.11365	no
gi|641571950|gb|KK855940.1|	.07376-10761	10	37	9	10	0.132521	0.92615	no

gi|641571950|gb|KK855940.1|	09527-11039	10	37	0.315284	1	2	0.25595	no
gi|641571950|gb|KK855940.1|	15790-11677	10	37	0.988735	0.136698	-3	0.2382	no
gi|641571950|gb|KK855940.1|	11808-12331	10	37	1	2	0.594464	0.70505	no
gi|641571950|gb|KK855940.1|	25019-12521	10	37	16	36	1	0.4619	no
gi|641571950|gb|KK855940.1|	27558-12796	10	37	0.676509	5	3	0.27515	no
gi|641571950|gb|KK855940.1|	12919-13177	10	37	19	22	0.163011	0.9086	no
gi|641571950|gb|KK855940.1|	31264-13577	10	37	11	9	-0.204001	0.9251	no
gi|641571950|gb|KK855940.1|	40709-14356	10	37	33	18	-0.883607	0.6943	no
gi|641571950|gb|KK855940.1|	43747-14460	10	37	2	1	-0.474618	0.73975	no
gi|641571950|gb|KK855940.1|	46222-14654	10	37	9	5	-0.706689	0.64525	no
gi|641571950|gb|KK855940.1|	49881-15093	10	37	2	2	0.0579351	0.96485	no
gi|641571950|gb|KK855940.1|	51859-15209	10	37	15	9	-0.830548	0.6108	no
gi|641571950|gb|KK855940.1|	56417-15661	10	37	79	31	-1	0.356	no
gi|641571950|gb|KK855940.1|	62984-16371	10	37	0.265309	2	3	0.2991	no
gi|641571950|gb|KK855940.1|	16405-16598	10	37	22	18	-0.321437	0.8588	no
gi|641571950|gb|KK855940.1|	71136-17283	10	37	8	3	-1	0.33815	no
gi|641571950|gb|KK855940.1|	18566-18914	10	37	4	4	-0.00761622	0.9394	no
gi|641571950|gb|KK855940.1|	08947-20912	10	37	98	47	-1	0.4618	no
gi|641571950|gb|KK855940.1|	22091-22880	10	37	10	10	-0.0350367	0.97745	no
gi|641571950|gb|KK855940.1|	24135-24949	10	37	23	16	-0.527405	0.6779	no
gi|641571950|gb|KK855940.1|	45286-24563	10	37	6	0.674435	-3	0.22375	no
gi|641571950|gb|KK855940.1|	54265-25486	10	37	4	2	-0.624783	0.66875	no
gi|641571950|gb|KK855940.1|	25838-26510	10	37	0.148492	2	4	0.11955	no
gi|641571950|gb|KK855940.1|	29006-30728	10	37	1	0.707071	-0.593171	0.6698	no
gi|641571950|gb|KK855940.1|	34693-35376	10	37	2	2	0.0596816	0.95895	no
gi|641571950|gb|KK855940.1|	36146-36743	10	37	10	15	0.572147	0.6611	no
gi|641571950|gb|KK855940.1|	36898-37866	10	37	3	4	0.242121	0.85135	no
gi|641571950|gb|KK855940.1|	47245-47377	10	37	476	829	0.801976	0.57555	no
gi|641571950|gb|KK855940.1|	51068-51718	10	37	282	304	0.107573	0.9597	no
gi|641571950|gb|KK855940.1|	59812-61958	10	37	47	30	-0.669849	0.761	no

gi|641571950|gb|KK855940.1|	62502-66067	10	37	13	7	-0.857248	0.6035	no
gi|641571950|gb|KK855940.1|	67392-67853	10	37	12	18	0.526407	0.68445	no
gi|641571950|gb|KK855940.1|	77044-77275	10	37	6	17	1	0.4135	no
gi|641571950|gb|KK855940.1|	86640-86869	10	37	4	36	3	0.1556	no
gi|641571950|gb|KK855940.1|	90360-90748	10	37	1	17	4	0.14055	no
gi|641571950|gb|KK855940.1|	97526-97740	10	37	13	48	2	0.2483	no
gi|641571953|gb|KK855939.1|	.00896-10131	10	20	20	11	-0.844206	0.51695	no
gi|641571953|gb|KK855939.1|	.03504-10369	10	20	35	17	-1	0.5036	no
gi|641571953|gb|KK855939.1|	.08002-10820	10	20	70	9	-3	0.1341	no
gi|641571953|gb|KK855939.1|	.10521-11067	10	20	141	14	-3	0.21525	no
gi|641571953|gb|KK855939.1|	.21019-12227	10	20	1	0	-inf	0.00435	no
gi|641571953|gb|KK855939.1|	.23509-12462	10	20	2	0.23307	-3	0.1689	no
gi|641571953|gb|KK855939.1|	.25102-12595	10	20	0.965924	0.326112	-2	0.4266	no
gi|641571953|gb|KK855939.1|	.28419-12991	10	20	0.545602	1	1	0.36435	no
gi|641571953|gb|KK855939.1|	.31682-13248	10	20	1	0.528155	-1	0.47135	no
gi|641571953|gb|KK855939.1|	.35717-13832	10	20	8	2	-2	0.16525	no
gi|641571953|gb|KK855939.1|	14132-15142	10	20	1	0.925331	-0.394916	0.7966	no
gi|641571953|gb|KK855939.1|	.45280-14763	10	20	3	0.54974	-2	0.1243	no
gi|641571953|gb|KK855939.1|	.50691-15169	10	20	2	0.668296	-2	0.2547	no
gi|641571953|gb|KK855939.1|	.51937-15253	10	20	2	0.267176	-3	0.2348	no
gi|641571953|gb|KK855939.1|	15336-16144	10	20	74	108	0.545123	0.80755	no
gi|641571953|gb|KK855939.1|	.56241-15765	10	20	5	2	-2	0.23595	no
gi|641571953|gb|KK855939.1|	.58737-15972	10	20	6	5	-0.477616	0.7121	no
gi|641571953|gb|KK855939.1|	.61443-16170	10	20	10	13	0.418819	0.7696	no
gi|641571953|gb|KK855939.1|	17427-18241	10	20	1	2	0.450791	0.7632	no
gi|641571953|gb|KK855939.1|	.74513-17522	10	20	10	15	0.621574	0.63175	no
gi|641571953|gb|KK855939.1|	18318-18718	10	20	3	0.512064	-3	0.2476	no
gi|641571953|gb|KK855939.1|	20392-21558	10	20	1	0.999322	-0.223827	0.87645	no
gi|641571953|gb|KK855939.1|	21868-22655	10	20	0.478635	1	2	0.3613	no
gi|641571953|gb|KK855939.1|	24124-25993	10	20	5	2	-1	0.28825	no

gi|641571953|gb|KK855939.1|	28525-29278	10	20	5	3	-0.720168	0.59165	no
gi|641571953|gb|KK855939.1|	39700-39924	10	20	23	12	-0.873018	0.58325	no
gi|641571953|gb|KK855939.1|	43463-43625	10	20	58	33	-0.812255	0.6158	no
gi|641571953|gb|KK855939.1|	43964-45470	10	20	0.972343	0.411615	-1	0.4383	no
gi|641571953|gb|KK855939.1|	59173-59818	10	20	1	1	0.113806	0.92265	no
gi|641571953|gb|KK855939.1|	59924-60163	10	20	20	13	-0.539096	0.723	no
gi|641571953|gb|KK855939.1|	60274-60910	10	20	0.963543	2	1	0.47095	no
gi|641571953|gb|KK855939.1|	63051-66948	10	20	0.650325	0.642577	-0.0172921	1	no
gi|641571953|gb|KK855939.1|	67328-68873	10	20	7	6	-0.242149	0.84825	no
gi|641571953|gb|KK855939.1|	70062-70821	10	20	3	3	-0.114336	0.9368	no
gi|641571953|gb|KK855939.1|	71524-72424	10	20	0.803772	1	0.603955	0.6936	no
gi|641571953|gb|KK855939.1|	72566-73301	10	20	0.787087	0.995141	0.338377	0.8352	no
gi|641571953|gb|KK855939.1|	7415-8392	10	20	9	10	0.199522	0.8756	no
gi|641571953|gb|KK855939.1|	74363-75921	10	20	0.779366	1	0.701107	0.62415	no
gi|641571953|gb|KK855939.1|	76025-81800	10	20	3	2	-0.49807	0.69545	no
gi|641571953|gb|KK855939.1|	82295-83687	10	20	41	19	-1	0.49485	no
gi|641571953|gb|KK855939.1|	92516-92686	10	20	49	6	-3	0.235	no
gi|641571953|gb|KK855939.1|	94558-95076	10	20	12	4	-2	0.21415	no
gi|641571953|gb|KK855939.1|	9786-10552	10	20	11	19	0.833944	0.51175	no
gi|641571956|gb|KK855938.1|	02303-10282	11	42	5	4	-0.33837	0.8131	no
gi|641571956|gb|KK855938.1|	08040-10864	11	42	5	3	-0.84967	0.54745	no
gi|641571956|gb|KK855938.1|	09671-11061	11	42	0.948204	0.720812	-0.395575	0.81255	no
gi|641571956|gb|KK855938.1|	11063-11202	11	42	6	3	-1	0.3712	no
gi|641571956|gb|KK855938.1|	18971-12181	11	42	8	2	-2	0.09245	no
gi|641571956|gb|KK855938.1|	23701-12428	11	42	8	1	-3	0.1327	no
gi|641571956|gb|KK855938.1|	35095-13560	11	42	10	1	-3	0.1141	no
gi|641571956|gb|KK855938.1|	36321-13681	11	42	9	0.351752	-5	0.1863	no
gi|641571956|gb|KK855938.1|	16573-16846	11	42	22	2	-3	0.15015	no
gi|641571956|gb|KK855938.1|	20154-22049	11	42	0	849	inf	5.00E-05	yes
gi|641571956|gb|KK855938.1|	22726-22322	11	42	0	221	inf	0.00035	yes

gi|641571956|gb|KK855938.1|	23570-22442	11	42	0.107	87	10	0.10555	no
gi|641571956|gb|KK855938.1|	39646-40008	11	42	4	2	-0.590285	0.70485	no
gi|641571956|gb|KK855938.1|	46114-46612	11	42	2	2	-0.406946	0.8125	no
gi|641571956|gb|KK855938.1|	49687-49872	11	42	92	4	-4	0.188	no
gi|641571956|gb|KK855938.1|	54012-54728	11	42	1	0.618678	-1	0.50885	no
gi|641571956|gb|KK855938.1|	55935-56685	11	42	15	5	-2	0.2555	no
gi|641571956|gb|KK855938.1|	56806-56989	11	42	25	22	-0.156868	0.91315	no
gi|641571956|gb|KK855938.1|	79441-80383	11	42	11	3	-2	0.2062	no
gi|641571956|gb|KK855938.1|	93943-94439	11	42	5	5	-0.0989464	0.94545	no
gi|641571956|gb|KK855938.1|	97131-97371	11	42	20	18	-0.161728	0.9133	no
gi|641571960|gb|KK855937.1|	28182-12906	9.4	17	2	0.314733	-2	0.2115	no
gi|641571960|gb|KK855937.1|	36237-13859	9.4	17	0.589834	1	1	0.41865	no
gi|641571960|gb|KK855937.1|	64263-16567	9.4	17	2	0.265333	-3	0.17485	no
gi|641571960|gb|KK855937.1|	66845-16849	9.4	17	3	3	0.132931	0.92125	no
gi|641571960|gb|KK855937.1|	18540-19168	9.4	17	2	2	0.276793	0.8559	no
gi|641571960|gb|KK855937.1|	20253-20840	9.4	17	2	0.546443	-2	0.3455	no
gi|641571960|gb|KK855937.1|	29437-31015	9.4	17	0.256031	0.936587	2	0.28495	no
gi|641571960|gb|KK855937.1|	35397-36546	9.4	17	0.668159	1	0.606139	0.6902	no
gi|641571960|gb|KK855937.1|	36897-38323	9.4	17	1	0.96422	-0.254964	0.85915	no
gi|641571960|gb|KK855937.1|	38833-40578	9.4	17	0.593946	0.139321	-2	1	no
gi|641571960|gb|KK855937.1|	53761-54982	9.4	17	0.552229	0.630634	0.191536	1	no
gi|641571960|gb|KK855937.1|	57483-57932	9.4	17	2	1	-0.995378	0.62775	no
gi|641571960|gb|KK855937.1|	66723-67167	9.4	17	6	4	-0.410836	0.7851	no
gi|641571960|gb|KK855937.1|	81350-81642	9.4	17	12	0.986822	-4	0.2029	no
gi|641571960|gb|KK855937.1|	83507-83758	9.4	17	9	4	-1	0.5278	no
gi|641571964|gb|KK855936.1|	26302-12697	11	41	1	0.894697	-0.207306	0.8945	no
gi|641571964|gb|KK855936.1|	46043-14625	11	41	15	35	1	0.44285	no
gi|641571964|gb|KK855936.1|	68303-16861	11	41	6	0.878167	-3	0.2476	no
gi|641571968|gb|KK855935.1|	03883-10465	9	35	0.863884	3	2	0.2915	no
gi|641571968|gb|KK855935.1|	07270-10776	9	35	0.697296	19	5	0.13615	no

gi|641571968|gb|KK855935.1|	12094-11269	9	35	2	4	0.597682	0.67485	no
gi|641571968|gb|KK855935.1|	13318-11506	9	35	5	7	0.550803	0.6594	no
gi|641571968|gb|KK855935.1|	24359-12490	9	35	91	2	-5	0.0261	no
gi|641571968|gb|KK855935.1|	38327-13854	9	35	38	29	-0.403694	0.777	no
gi|641571968|gb|KK855935.1|	45131-14535	9	35	114	152	0.41782	0.74025	no
gi|641571968|gb|KK855935.1|	48493-14872	9	35	24	29	0.281184	0.84425	no
gi|641571968|gb|KK855935.1|	51522-15172	9	35	46	59	0.340213	0.80215	no
gi|641571968|gb|KK855935.1|	94347-95161	9	35	0.343311	2	2	0.2235	no
gi|641571976|gb|KK855933.1|	25216-12692	10	41	0	2	inf	0.00065	yes
gi|641571976|gb|KK855933.1|	31683-13313	10	41	0	7	inf	5.00E-05	yes
gi|641571976|gb|KK855933.1|	19448-21470	10	41	3	8	1	0.3024	no
gi|641571976|gb|KK855933.1|	21579-25110	10	41	2	2	0.687526	0.59205	no
gi|641571976|gb|KK855933.1|	79053-79456	10	41	0	39	inf	5.00E-05	yes
gi|641571976|gb|KK855933.1|	79816-80484	10	41	0	24	inf	5.00E-05	yes
gi|641571976|gb|KK855933.1|	87272-87436	10	41	0	194	inf	0.00045	yes
gi|641571976|gb|KK855933.1|	91082-91708	10	41	0.164307	12	6	0.1051	no
gi|641571976|gb|KK855933.1|	92079-92938	10	41	0	2	inf	0.0068	no
gi|641571976|gb|KK855933.1|	94232-95261	10	41	0	2	inf	0.00375	no
gi|641571979|gb|KK855932.1|	01555-10194	9.6	38	4	17	2	0.194	no
gi|641571979|gb|KK855932.1|	10348-11087	9.6	38	2	6	2	0.2546	no
gi|641571979|gb|KK855932.1|	20623-12204	9.6	38	0.75156	1	0.907069	0.53605	no
gi|641571979|gb|KK855932.1|	24726-12541	9.6	38	1	5	2	0.17115	no
gi|641571979|gb|KK855932.1|	26162-12777	9.6	38	1	4	2	0.2635	no
gi|641571979|gb|KK855932.1|	28503-12914	9.6	38	2	2	0.0142702	0.9597	no
gi|641571979|gb|KK855932.1|	32742-13330	9.6	38	2	5	2	0.2545	no
gi|641571979|gb|KK855932.1|	40757-14119	9.6	38	8	12	0.535722	0.6905	no
gi|641571979|gb|KK855932.1|	42258-14267	9.6	38	18	61	2	0.1801	no
gi|641571979|gb|KK855932.1|	14368-16411	9.6	38	4	2	-1	0.43125	no
gi|641571979|gb|KK855932.1|	44644-14530	9.6	38	3	3	0.419027	0.7653	no
gi|641571979|gb|KK855932.1|	19772-20592	9.6	38	2	2	-0.619507	0.67055	no

gi|641571979|gb|KK855932.1|	21407-23060	9.6	38	0.29143	0.740445	1	1	no
gi|641571979|gb|KK855932.1|	27068-27362	9.6	38	5	20	2	0.22715	no
gi|641571979|gb|KK855932.1|	30083-30731	9.6	38	1	4	2	0.2961	no
gi|641571979|gb|KK855932.1|	35975-36895	9.6	38	0.390795	1	1	0.44255	no
gi|641571979|gb|KK855932.1|	41888-43620	9.6	38	1	3	1	0.44285	no
gi|641571979|gb|KK855932.1|	423-1322	9.6	38	3	4	0.504408	0.7107	no
gi|641571979|gb|KK855932.1|	45584-49127	9.6	38	3	3	0.210032	0.8657	no
gi|641571979|gb|KK855932.1|	49257-49654	9.6	38	3	3	-0.000538429	0.9394	no
gi|641571979|gb|KK855932.1|	50815-52986	9.6	38	0.967597	2	0.854455	0.5297	no
gi|641571979|gb|KK855932.1|	53814-54569	9.6	38	0.126497	2	4	0.12385	no
gi|641571979|gb|KK855932.1|	55826-58621	9.6	38	1	1	0.0954883	0.94165	no
gi|641571979|gb|KK855932.1|	59556-60394	9.6	38	1	3	1	0.35895	no
gi|641571979|gb|KK855932.1|	71505-71809	9.6	38	10	18	0.890273	0.53895	no
gi|641571979|gb|KK855932.1|	73072-76058	9.6	38	0.71039	2	1	0.4124	no
gi|641571979|gb|KK855932.1|	81260-81720	9.6	38	5	8	0.590461	0.66335	no
gi|641571979|gb|KK855932.1|	84302-85003	9.6	38	5	2	-0.984283	0.49955	no
gi|641571979|gb|KK855932.1|	85970-88267	9.6	38	5	8	0.5916	0.63885	no
gi|641571979|gb|KK855932.1|	89947-90385	9.6	38	8	13	0.637551	0.6351	no
gi|641571979|gb|KK855932.1|	90540-92284	9.6	38	2	0.627348	-1	0.38335	no
gi|641571979|gb|KK855932.1|	92564-94890	9.6	38	0.631369	5	3	0.0508	no
gi|641571979|gb|KK855932.1|	95156-96649	9.6	38	0.10912	9	6	0.23245	no
gi|641571979|gb|KK855932.1|	99627-100382	9.6	38	24.92	41	0.707666	0.57055	no
gi|641571982|gb|KK855931.1|	14899-11542	9.8	35	5	9	0.929039	0.50365	no
gi|641571982|gb|KK855931.1|	38891-13980	9.8	35	0.690562	194	8	0.0545	no
gi|641571982|gb|KK855931.1|	22076-24140	9.8	35	0.985111	6	3	0.0724	no
gi|641571982|gb|KK855931.1|	25552-25793	9.8	35	26	21	-0.273961	0.84865	no
gi|641571982|gb|KK855931.1|	26047-26205	9.8	35	20	77	2	0.3223	no
gi|641571982|gb|KK855931.1|	26487-28759	9.8	35	2	12	3	0.0622	no
gi|641571982|gb|KK855931.1|	30395-31436	9.8	35	0.418603	2	2	0.20805	no
gi|641571982|gb|KK855931.1|	32658-35316	9.8	35	0.460003	3	2	0.0981	no

gi|641571982|gb|KK855931.1|	35756-40075	9.8	35	4	15	2	0.31405	no
gi|641571982|gb|KK855931.1|	41650-42220	9.8	35	0.943714	4	2	0.2128	no
gi|641571982|gb|KK855931.1|	42351-43640	9.8	35	0.453158	3	3	0.12445	no
gi|641571982|gb|KK855931.1|	45137-47375	9.8	35	5	11	1	0.32405	no
gi|641571982|gb|KK855931.1|	53260-53711	9.8	35	6	14	1	0.3163	no
gi|641571982|gb|KK855931.1|	57184-57870	9.8	35	2	3	0.493409	0.73375	no
gi|641571982|gb|KK855931.1|	58213-60368	9.8	35	0.614201	2	2	0.1773	no
gi|641571982|gb|KK855931.1|	64366-65532	9.8	35	0.145879	2	4	0.24715	no
gi|641571982|gb|KK855931.1|	68368-70173	9.8	35	0.571807	1	0.908223	0.53605	no
gi|641571982|gb|KK855931.1|	70396-72088	9.8	35	5	6	0.28006	0.82685	no
gi|641571982|gb|KK855931.1|	73433-74557	9.8	35	0.533668	0.580176	0.120546	1	no
gi|641571982|gb|KK855931.1|	74677-76909	9.8	35	0.834326	1	0.346398	0.79755	no
gi|641571982|gb|KK855931.1|	81872-82080	9.8	35	22	0	-inf	0.01	no
gi|641571982|gb|KK855931.1|	82943-83388	9.8	35	9	4	-1	0.4609	no
gi|641571982|gb|KK855931.1|	84696-85260	9.8	35	2	0.289981	-3	0.2381	no
gi|641571982|gb|KK855931.1|	87880-88231	9.8	35	46	26	-0.814459	0.52965	no
gi|641571982|gb|KK855931.1|	89941-95091	9.8	35	2	7	2	0.2297	no
gi|641571982|gb|KK855931.1|	99929-10034(	9.8	35	4	0.974752	-2	0.31075	no
gi|641571986|gb|KK855930.1|	02066-10247	11	23	4	0.496083	-3	0.2381	no
gi|641571990|gb|KK855929.1|	13347-11382	9.7	19	6	4	-0.64904	0.65175	no
gi|641571990|gb|KK855929.1|	19179-12002	9.7	19	18	6	-1	0.24915	no
gi|641571990|gb|KK855929.1|	21335-12268	9.7	19	20	4	-2	0.0855	no
gi|641571990|gb|KK855929.1|	34301-13486	9.7	19	9	1	-3	0.1082	no
gi|641571990|gb|KK855929.1|	40853-14105	9.7	19	22	9	-1	0.442	no
gi|641571994|gb|KK855928.1|	00784-10185	9.8	39	0	2	inf	0.0023	yes
gi|641571994|gb|KK855928.1|	94265-94673	9.8	39	0	5	inf	0.00455	no
gi|641572001|gb|KK855926.1|	48412-49019	9.1	38	0.343722	3	3	0.27515	no
gi|641572001|gb|KK855926.1|	87736-88483	9.1	38	1	0.973672	-0.398354	0.81255	no
gi|641572001|gb|KK855926.1|	88576-89135	9.1	38	1	1	0.0112163	0.91115	no
gi|641572004|gb|KK855925.1|	36171-13701	10	21	0.652856	3	2	0.20395	no

gi|641572004|gb|KK855925.1|	[illegible]56990-15723	10	21	14	4	-2	0.33635	no
gi|641572004|gb|KK855925.1|	[illegible]87402-18769	10	21	9	5	-0.918799	0.5719	no
gi|641572004|gb|KK855925.1|	20423-20715	10	21	5	6	0.342958	0.8376	no
gi|641572004|gb|KK855925.1|	26886-27586	10	21	0.280535	2	3	0.2851	no
gi|641572004|gb|KK855925.1|	33023-34684	10	21	1	1	0.396798	0.77935	no
gi|641572004|gb|KK855925.1|	34869-36003	10	21	0.377131	1	2	0.2717	no
gi|641572004|gb|KK855925.1|	36570-37203	10	21	2	3	0.977387	0.5381	no
gi|641572004|gb|KK855925.1|	42096-43555	10	21	0.504253	1	2	0.33265	no
gi|641572004|gb|KK855925.1|	43870-44560	10	21	1	3	1	0.4329	no
gi|641572004|gb|KK855925.1|	44749-45284	10	21	0.832169	2	1	0.44255	no
gi|641572004|gb|KK855925.1|	45945-46424	10	21	3	2	-0.671202	0.68185	no
gi|641572004|gb|KK855925.1|	47946-48405	10	21	1	3	1	0.51205	no
gi|641572004|gb|KK855925.1|	51423-52333	10	21	2	3	0.773997	0.59375	no
gi|641572004|gb|KK855925.1|	53545-54197	10	21	3	0.234813	-4	0.20475	no
gi|641572004|gb|KK855925.1|	55017-55627	10	21	1	0.774845	-0.624257	0.71195	no
gi|641572004|gb|KK855925.1|	57457-58659	10	21	0.914308	0.535406	-0.772047	0.63565	no
gi|641572004|gb|KK855925.1|	59374-60537	10	21	1	1	0.0656604	0.9579	no
gi|641572004|gb|KK855925.1|	62701-63722	10	21	3	5	0.936387	0.47945	no
gi|641572004|gb|KK855925.1|	65491-66424	10	21	1	0.729557	-0.533174	0.7478	no
gi|641572004|gb|KK855925.1|	68631-70263	10	21	0.492816	0.751237	0.608221	1	no
gi|641572004|gb|KK855925.1|	72988-75188	10	21	0.423822	0.862116	1	1	no
gi|641572004|gb|KK855925.1|	75695-76466	10	21	0.491934	1	1	0.44255	no
gi|641572004|gb|KK855925.1|	76528-77247	10	21	0.675924	3	2	0.18215	no
gi|641572004|gb|KK855925.1|	77379-78984	10	21	0.753382	0.612467	-0.298749	1	no
gi|641572004|gb|KK855925.1|	79693-83625	10	21	1	2	0.57176	0.6477	no
gi|641572004|gb|KK855925.1|	85881-86372	10	21	15	35	1	0.3329	no
gi|641572004|gb|KK855925.1|	87820-88752	10	21	3	3	0.0973583	0.94525	no
gi|641572004|gb|KK855925.1|	8876-10117	10	21	0.473959	0.412413	-0.200671	1	no
gi|641572004|gb|KK855925.1|	88948-91613	10	21	1	2	0.640315	0.6336	no
gi|641572004|gb|KK855925.1|	92282-93453	10	21	1	4	2	0.2586	no

gi|641572008|gb|KK855924.1|	[illegible]30098-13036	10	41	15	4	-2	0.2469	no
gi|641572008|gb|KK855924.1|	[illegible]31501-13197	10	41	6	9	0.532733	0.691	no
gi|641572008|gb|KK855924.1|	[illegible]53195-15375	10	41	2	2	-0.403735	0.80855	no
gi|641572008|gb|KK855924.1|	[illegible]55090-15565	10	41	4	3	-0.626	0.66875	no
gi|641572011|gb|KK855923.1|	[illegible]01430-10201	10	21	1	4	1	0.4284	no
gi|641572011|gb|KK855923.1|	[illegible]02187-10309	10	21	0.699331	2	2	0.2915	no
gi|641572011|gb|KK855923.1|	[illegible]05032-10573	10	21	1	2	0.770605	0.6261	no
gi|641572011|gb|KK855923.1|	13510-13906	10	21	2	3	0.361912	0.83635	no
gi|641572011|gb|KK855923.1|	14060-15104	10	21	2	2	-0.287711	0.8405	no
gi|641572011|gb|KK855923.1|	[illegible]42784-14378	10	21	0.529232	1	1	0.47015	no
gi|641572011|gb|KK855923.1|	[illegible]43972-14610	10	21	0.658054	1	0.898789	0.52195	no
gi|641572011|gb|KK855923.1|	16416-17855	10	21	2	4	1	0.38715	no
gi|641572011|gb|KK855923.1|	21556-22332	10	21	1	2	0.602173	0.6863	no
gi|641572011|gb|KK855923.1|	24074-24989	10	21	2	4	0.796774	0.56215	no
gi|641572011|gb|KK855923.1|	25646-28325	10	21	2	6	2	0.1572	no
gi|641572011|gb|KK855923.1|	29722-31123	10	21	0.998297	4	2	0.1747	no
gi|641572011|gb|KK855923.1|	31679-32955	10	21	0.720671	2	1	0.3908	no
gi|641572011|gb|KK855923.1|	39805-40443	10	21	0.959203	0.9686	0.0140643	0.91115	no
gi|641572011|gb|KK855923.1|	42629-43725	10	21	0.786083	2	1	0.3975	no
gi|641572011|gb|KK855923.1|	44014-44936	10	21	0.58458	2	2	0.32035	no
gi|641572011|gb|KK855923.1|	49247-50053	10	21	13	14	0.161159	0.89445	no
gi|641572011|gb|KK855923.1|	51070-52653	10	21	7	4	-0.632933	0.62195	no
gi|641572011|gb|KK855923.1|	52806-54181	10	21	0.900264	0.274298	-2	0.33295	no
gi|641572011|gb|KK855923.1|	56105-56589	10	21	2	2	-0.407832	0.8125	no
gi|641572011|gb|KK855923.1|	59751-59975	10	21	11	10	-0.136052	0.9206	no
gi|641572011|gb|KK855923.1|	61808-63195	10	21	0.891198	2	1	0.4739	no
gi|641572011|gb|KK855923.1|	67295-67972	10	21	3	2	-0.840473	0.5768	no
gi|641572011|gb|KK855923.1|	7244-8081	10	21	1	1	-0.271334	0.86195	no
gi|641572011|gb|KK855923.1|	80663-81306	10	21	2	2	0.0846013	0.94	no
gi|641572011|gb|KK855923.1|	81534-82376	10	21	0.875858	0.831566	-0.0748663	0.92685	no

gi|641572011|gb|KK855923.1|	97012-98030	10	21	0.946672	2	1	0.41005	no
gi|641572011|gb|KK855923.1|	98523-99799	10	21	0.58964	1	0.896373	0.5684	no
gi|641572014|gb|KK855922.1|	06479-10682	10	42	0	38	inf	0.00015	yes
gi|641572014|gb|KK855922.1|	10396-11135	10	42	0	35	inf	5.00E-05	yes
gi|641572014|gb|KK855922.1|	11352-12797	10	42	148	39	-2	0.3888	no
gi|641572014|gb|KK855922.1|	13805-14577	10	42	2	1	-0.590542	0.68495	no
gi|641572014|gb|KK855922.1|	14681-18075	10	42	2	1	-0.656299	0.60975	no
gi|641572014|gb|KK855922.1|	18290-19512	10	42	6	2	-2	0.26255	no
gi|641572014|gb|KK855922.1|	22216-23056	10	42	16	4	-2	0.14145	no
gi|641572014|gb|KK855922.1|	23411-25318	10	42	0.744554	0.063083	-4	1	no
gi|641572014|gb|KK855922.1|	26120-27647	10	42	3	1	-1	0.38645	no
gi|641572014|gb|KK855922.1|	32614-33203	10	42	23	6	-2	0.1608	no
gi|641572014|gb|KK855922.1|	36989-37855	10	42	1	0	-inf	0.0075	no
gi|641572014|gb|KK855922.1|	38518-39503	10	42	1	0	-inf	0.0059	no
gi|641572014|gb|KK855922.1|	43251-45360	10	42	22	6	-2	0.29325	no
gi|641572014|gb|KK855922.1|	85494-85779	10	42	0	61	inf	5.00E-05	yes
gi|641572017|gb|KK855921.1|	00893-10206	9.3	35	1	3	1	0.41205	no
gi|641572017|gb|KK855921.1|	02778-10445	9.3	35	2	3	0.425481	0.74975	no
gi|641572017|gb|KK855921.1|	04582-10981	9.3	35	4	3	-0.678344	0.5938	no
gi|641572017|gb|KK855921.1|	10188-11235	9.3	35	1	2	0.713667	0.5835	no
gi|641572017|gb|KK855921.1|	22414-12369	9.3	35	4	0.695233	-2	0.11985	no
gi|641572017|gb|KK855921.1|	42195-14408	9.3	35	0.626316	11	4	0.01855	no
gi|641572017|gb|KK855921.1|	44902-14735	9.3	35	2	26	4	0.08325	no
gi|641572017|gb|KK855921.1|	14505-15107	9.3	35	2	1	-0.572065	0.7586	no
gi|641572017|gb|KK855921.1|	47599-15033	9.3	35	0.836026	14	4	0.0632	no
gi|641572017|gb|KK855921.1|	50435-15244	9.3	35	0.195189	3	4	0.08545	no
gi|641572017|gb|KK855921.1|	52712-15348	9.3	35	0.368311	1	2	0.2812	no
gi|641572017|gb|KK855921.1|	15790-16626	9.3	35	10	6	-0.653219	0.61575	no
gi|641572017|gb|KK855921.1|	71849-17492	9.3	35	10	9	-0.0743459	0.9504	no
gi|641572017|gb|KK855921.1|	76414-17743	9.3	35	7	7	0.0645862	0.9608	no

gi|641572017|gb|KK855921.1|	86537-18968	9.3	35	21	15	-0.47608	0.8346	no
gi|641572017|gb|KK855921.1|	94031-19443	9.3	35	124.96	52	-1	0.3118	no
gi|641572017|gb|KK855921.1|	20184-20406	9.3	35	22	51	1	0.4006	no
gi|641572017|gb|KK855921.1|	24240-25031	9.3	35	0.475421	1	1	0.44255	no
gi|641572017|gb|KK855921.1|	25732-26125	9.3	35	12	12	-0.0859109	0.94575	no
gi|641572017|gb|KK855921.1|	26236-26425	9.3	35	13	39	2	0.38185	no
gi|641572017|gb|KK855921.1|	28716-30024	9.3	35	33	26	-0.338733	0.8323	no
gi|641572017|gb|KK855921.1|	57692-58682	9.3	35	4	6	0.446204	0.7297	no
gi|641572017|gb|KK855921.1|	58798-60164	9.3	35	0.907184	2	0.870321	0.5524	no
gi|641572017|gb|KK855921.1|	60547-61565	9.3	35	1	0.785467	-0.897346	0.56925	no
gi|641572017|gb|KK855921.1|	609-1754	9.3	35	1	1	-0.393887	0.78685	no
gi|641572017|gb|KK855921.1|	6632-7648	9.3	35	0.776437	0.262457	-2	1	no
gi|641572017|gb|KK855921.1|	80984-81375	9.3	35	3	3	-0.264304	0.8654	no
gi|641572017|gb|KK855921.1|	81951-82512	9.3	35	5	6	0.288143	0.8377	no
gi|641572017|gb|KK855921.1|	82871-84062	9.3	35	2	2	-0.109807	0.9376	no
gi|641572017|gb|KK855921.1|	87649-88272	9.3	35	0.661822	2	1	0.44255	no
gi|641572017|gb|KK855921.1|	89399-90263	9.3	35	0.952924	0.6435	-0.56642	0.7586	no
gi|641572017|gb|KK855921.1|	90314-90712	9.3	35	2	3	0.847577	0.63695	no
gi|641572017|gb|KK855921.1|	9064-9661	9.3	35	2	2	-0.179926	0.90465	no
gi|641572017|gb|KK855921.1|	95-280	9.3	35	30	8	-2	0.33925	no
gi|641572017|gb|KK855921.1|	96735-99383	9.3	35	1	3	1	0.4449	no
gi|641572017|gb|KK855921.1|	9801-11728	9.3	35	0.858614	0.623566	-0.461468	1	no
gi|641572017|gb|KK855921.1|	99495-10052(	9.3	35	0.85335	3	2	0.2468	no
gi|641572022|gb|KK855920.1|	01024-10194	12	42	1	2	0.952154	0.53865	no
gi|641572022|gb|KK855920.1|	02845-10754	12	42	8	5	-0.504956	0.6775	no
gi|641572022|gb|KK855920.1|	09153-10954	12	42	7	3	-1	0.398	no
gi|641572022|gb|KK855920.1|	10956-11189	12	42	3	2	-0.743519	0.59245	no
gi|641572022|gb|KK855920.1|	13953-11428	12	42	0.993191	7	3	0.27615	no
gi|641572022|gb|KK855920.1|	18289-11845	12	42	38	27	-0.48699	0.7686	no
gi|641572022|gb|KK855920.1|	24117-12504	12	42	7	6	-0.187954	0.8875	no

gi|641572022|gb|KK855920.1|	25145-12536	12	42	10	15	0.541567	0.7458	no
gi|641572022|gb|KK855920.1|	30647-13175	12	42	0.544815	0.473791	-0.201515	1	no
gi|641572022|gb|KK855920.1|	43895-14477	12	42	0.832111	3	2	0.3067	no
gi|641572022|gb|KK855920.1|	48706-14920	12	42	1	2	0.815446	0.6234	no
gi|641572022|gb|KK855920.1|	55182-15714	12	42	2	0.673506	-1	0.34395	no
gi|641572022|gb|KK855920.1|	57580-15869	12	42	1	0.706819	-0.809162	0.5996	no
gi|641572022|gb|KK855920.1|	59210-16050	12	42	2	0.98416	-0.929112	0.5162	no
gi|641572022|gb|KK855920.1|	60626-16210	12	42	2	1	-0.894784	0.52785	no
gi|641572022|gb|KK855920.1|	62252-16520	12	42	3	2	-0.904378	0.4821	no
gi|641572022|gb|KK855920.1|	21405-22006	12	42	0	4	inf	0.0015	yes
gi|641572022|gb|KK855920.1|	24385-24624	12	42	0	81	inf	0.00025	yes
gi|641572022|gb|KK855920.1|	34019-34914	12	42	0.809516	1	0.41125	0.8089	no
gi|641572022|gb|KK855920.1|	41708-43219	12	42	1	4	2	0.1652	no
gi|641572022|gb|KK855920.1|	45479-45890	12	42	4	6	0.701483	0.64285	no
gi|641572022|gb|KK855920.1|	47579-48295	12	42	2	2	0.115587	0.93605	no
gi|641572022|gb|KK855920.1|	48527-50430	12	42	11	14	0.326897	0.79415	no
gi|641572022|gb|KK855920.1|	50581-51923	12	42	12	10	-0.21536	0.86535	no
gi|641572022|gb|KK855920.1|	52049-52603	12	42	72	101	0.493598	0.7607	no
gi|641572022|gb|KK855920.1|	52784-54177	12	42	74	86	0.223921	0.9196	no
gi|641572022|gb|KK855920.1|	59376-60109	12	42	0	154	inf	5.00E-05	yes
gi|641572022|gb|KK855920.1|	61591-61829	12	42	0	95	inf	0.0002	yes
gi|641572022|gb|KK855920.1|	63712-63980	12	42	0	321	inf	5.00E-05	yes
gi|641572022|gb|KK855920.1|	65306-65569	12	42	0	354	inf	5.00E-05	yes
gi|641572022|gb|KK855920.1|	69349-69745	12	42	0	40	inf	5.00E-05	yes
gi|641572022|gb|KK855920.1|	70412-70671	12	42	0.910463	305	8	0.10435	no
gi|641572022|gb|KK855920.1|	70888-71805	12	42	0.098105	103	10	0.10505	no
gi|641572022|gb|KK855920.1|	72060-72678	12	42	0	24	inf	5.00E-05	yes
gi|641572022|gb|KK855920.1|	72839-73349	12	42	17	9	-0.880175	0.50465	no
gi|641572022|gb|KK855920.1|	74460-75767	12	42	5	6	0.0556089	0.966	no
gi|641572022|gb|KK855920.1|	81362-81951	12	42	9	4	-1	0.3733	no

gi|641572022|gb|KK855920.1|	82747-83003	12	42	104	86	-0.285568	0.82615	no
gi|641572022|gb|KK855920.1|	8737-10013	12	42	1	0.399107	-2	0.362	no
gi|641572022|gb|KK855920.1|	87507-87686	12	42	90	74	-0.274491	0.84685	no
gi|641572022|gb|KK855920.1|	87923-88101	12	42	134	76	-0.822532	0.5478	no
gi|641572022|gb|KK855920.1|	89659-90759	12	42	6	4	-0.629824	0.62775	no
gi|641572022|gb|KK855920.1|	91073-91410	12	42	27	18	-0.613662	0.64465	no
gi|641572022|gb|KK855920.1|	92308-94016	12	42	4	3	-0.0658026	0.9571	no
gi|641572022|gb|KK855920.1|	95371-96062	12	42	28	29	0.0558144	0.9637	no
gi|641572022|gb|KK855920.1|	97330-98492	12	42	14	6	-1	0.34135	no
gi|641572022|gb|KK855920.1|	99455-10091[illegible]	12	42	6	4	-0.586986	0.6434	no
gi|641572025|gb|KK855919.1|	5036-5230	8.6	33	7	42	3	0.20725	no
gi|641572029|gb|KK855918.1|	11258-11966	8.9	32	2	0	-inf	0.0102	no
gi|641572029|gb|KK855918.1|	[illegible]32472-13354	8.9	32	0.971339	0.49266	-0.979382	0.5872	no
gi|641572029|gb|KK855918.1|	3222-3496	8.9	32	8	1	-3	0.2472	no
gi|641572029|gb|KK855918.1|	35140-36359	8.9	32	0.345817	1	2	0.28495	no
gi|641572029|gb|KK855918.1|	40371-40899	8.9	32	1	3	1	0.471	no
gi|641572029|gb|KK855918.1|	46967-47825	8.9	32	0.747873	0.486968	-0.618967	1	no
gi|641572029|gb|KK855918.1|	70411-71061	8.9	32	2	2	-0.593269	0.68495	no
gi|641572029|gb|KK855918.1|	73331-73965	8.9	32	2	0	-inf	0.0059	no
gi|641572029|gb|KK855918.1|	84215-85480	8.9	32	0.529514	3	2	0.1408	no
gi|641572029|gb|KK855918.1|	87626-88440	8.9	32	0.114437	3	5	0.11	no
gi|641572029|gb|KK855918.1|	91181-92518	8.9	32	0.434106	0.661255	0.607158	1	no
gi|641572029|gb|KK855918.1|	96239-97571	8.9	32	0.498304	0.94879	0.929064	0.56225	no
gi|641572033|gb|KK855917.1|	[illegible]41028-14184	10	20	0.803657	0.87175	0.117336	1	no
gi|641572033|gb|KK855917.1|	[illegible]51334-15199	10	20	0.758334	3	2	0.2717	no
gi|641572033|gb|KK855917.1|	32341-32721	10	20	52	2	-5	0.0624	no
gi|641572033|gb|KK855917.1|	34661-35172	10	20	2	0	-inf	0.0102	no
gi|641572033|gb|KK855917.1|	42547-42992	10	20	3	4	0.589249	0.6902	no
gi|641572033|gb|KK855917.1|	43053-43980	10	20	0.870877	0.441303	-0.980699	1	no
gi|641572033|gb|KK855917.1|	44405-45426	10	20	2	2	-0.269288	0.84935	no

gi|641572033|gb|KK855917.1|	6825-7575	10	20	3	7	1	0.4303	no
gi|641572033|gb|KK855917.1|	71724-72262	10	20	3	2	-0.504317	0.73985	no
gi|641572033|gb|KK855917.1|	83866-84342	10	20	0.753574	3	2	0.256	no
gi|641572033|gb|KK855917.1|	94559-94761	10	20	25	15	-0.731724	0.6634	no
gi|641572033|gb|KK855917.1|	99837-10035[illegible]	10	20	4	0.334509	-3	0.2089	no
gi|641572037|gb|KK855916.1|	01807-10382	13	41	6	6	0.074058	0.9529	no
gi|641572037|gb|KK855916.1|	05163-10609	13	41	1	0.587606	-0.855157	0.60675	no
gi|641572037|gb|KK855916.1|	06364-10711	13	41	3	2	-0.398278	0.78765	no
gi|641572037|gb|KK855916.1|	09075-10939	13	41	27	21	-0.371244	0.7804	no
gi|641572037|gb|KK855916.1|	11752-11211	13	41	0.842998	6	3	0.28555	no
gi|641572037|gb|KK855916.1|	12253-11302	13	41	5	6	0.254109	0.8512	no
gi|641572037|gb|KK855916.1|	15035-11566	13	41	12	13	0.10565	0.93215	no
gi|641572037|gb|KK855916.1|	17123-11748	13	41	4	12	2	0.2695	no
gi|641572037|gb|KK855916.1|	18706-11945	13	41	3	3	0.367181	0.79325	no
gi|641572037|gb|KK855916.1|	20159-12316	13	41	3	3	-0.226272	0.85965	no
gi|641572037|gb|KK855916.1|	23624-12386	13	41	10	10	-0.0358009	0.9394	no
gi|641572037|gb|KK855916.1|	28739-12930	13	41	1	2	0.373873	0.8276	no
gi|641572037|gb|KK855916.1|	29474-13041	13	41	0.95075	2	0.982916	0.5381	no
gi|641572037|gb|KK855916.1|	31483-13298	13	41	2	2	-0.0244671	0.97615	no
gi|641572037|gb|KK855916.1|	33222-13449	13	41	2	3	0.906427	0.50735	no
gi|641572037|gb|KK855916.1|	35384-13793	13	41	2	1	-0.364184	0.7832	no
gi|641572037|gb|KK855916.1|	38066-13900	13	41	1	2	0.845433	0.58475	no
gi|641572037|gb|KK855916.1|	39156-14054	13	41	2	2	-0.0206311	0.98035	no
gi|641572037|gb|KK855916.1|	51438-15269	13	41	0.798751	2	1	0.31585	no
gi|641572037|gb|KK855916.1|	54434-15545	13	41	217	4	-6	0.011	no
gi|641572037|gb|KK855916.1|	55740-15733	13	41	0.862397	0.38662	-1	1	no
gi|641572037|gb|KK855916.1|	60969-16278	13	41	151	121	-0.31175	0.87165	no
gi|641572037|gb|KK855916.1|	66406-16671	13	41	56	23	-1	0.32825	no
gi|641572037|gb|KK855916.1|	66932-17050	13	41	5	4	-0.434858	0.72775	no
gi|641572037|gb|KK855916.1|	70733-17308	13	41	2	3	0.753983	0.56485	no

gi|641572037|gb|KK855916.1|	73281-17363	13	41	3	4	0.578026	0.7575	no
gi|641572037|gb|KK855916.1|	75880-17693	13	41	1	0.498905	-1	0.4015	no
gi|641572037|gb|KK855916.1|	78679-17976	13	41	0.877355	0.485479	-0.853752	0.60675	no
gi|641572037|gb|KK855916.1|	80450-18315	13	41	1	1	-0.205597	0.88	no
gi|641572037|gb|KK855916.1|	86125-18655	13	41	52	21	-1	0.3044	no
gi|641572037|gb|KK855916.1|	93333-19417	13	41	0.991473	1	0.240579	0.88205	no
gi|641572037|gb|KK855916.1|	94692-19561	13	41	0.684837	1	0.79682	0.63025	no
gi|641572037|gb|KK855916.1|	95782-19717	13	41	0.651904	0.270866	-1	1	no
gi|641572037|gb|KK855916.1|	04483-20552	13	41	0.668973	2	1	0.4562	no
gi|641572037|gb|KK855916.1|	05852-20663	13	41	1	3	1	0.3653	no
gi|641572037|gb|KK855916.1|	19706-22013	13	41	0	5	inf	0.0068	no
gi|641572037|gb|KK855916.1|	20272-22128	13	41	0.867996	0.792173	-0.131872	1	no
gi|641572037|gb|KK855916.1|	49780-52791	13	41	2	6	2	0.14365	no
gi|641572037|gb|KK855916.1|	56861-58119	13	41	5	4	-0.233748	0.85105	no
gi|641572037|gb|KK855916.1|	60891-61507	13	41	1	2	0.375944	0.8276	no
gi|641572037|gb|KK855916.1|	64357-64884	13	41	3	0.643358	-2	0.31075	no
gi|641572037|gb|KK855916.1|	66054-66615	13	41	4	2	-1	0.45485	no
gi|641572037|gb|KK855916.1|	66791-68172	13	41	2	0.636801	-2	0.2794	no
gi|641572037|gb|KK855916.1|	69597-69924	13	41	10	3	-2	0.3097	no
gi|641572037|gb|KK855916.1|	84074-84295	13	41	58	2	-5	0.1862	no
gi|641572037|gb|KK855916.1|	90744-91390	13	41	6	0	-inf	0.0006	yes
gi|641572037|gb|KK855916.1|	92493-95831	13	41	21	11	-0.896753	0.5786	no
gi|641572037|gb|KK855916.1|	97667-99173	13	41	0.216076	2	3	0.129	no
gi|641572047|gb|KK855913.1|	04114-10435	10	36	10	26	1	0.3763	no
gi|641572047|gb|KK855913.1|	48687-49695	10	36	58	29	-0.995117	0.54455	no
gi|641572047|gb|KK855913.1|	50959-51226	10	36	17.6	2	-3	0.1714	no
gi|641572047|gb|KK855913.1|	53241-55201	10	36	1	0.550739	-1	0.3703	no
gi|641572047|gb|KK855913.1|	61275-61471	10	36	74	10	-3	0.1368	no
gi|641572047|gb|KK855913.1|	62392-62640	10	36	21	6	-2	0.2883	no
gi|641572047|gb|KK855913.1|	63839-65628	10	36	2	0.203226	-3	0.12215	no

gi|641572047|gb|KK855913.1|	68347-68639	10	36	42	16	-1	0.3078	no
gi|641572047|gb|KK855913.1|	73381-73668	10	36	13	1	-4	0.2029	no
gi|641572047|gb|KK855913.1|	83912-84430	10	36	3	0	-inf	0.0059	no
gi|641572047|gb|KK855913.1|	85715-85966	10	36	64	15	-2	0.14665	no
gi|641572047|gb|KK855913.1|	91213-91466	10	36	124	40	-2	0.2209	no
gi|641572047|gb|KK855913.1|	98390-99186	10	36	14	5	-1	0.3054	no
gi|641572054|gb|KK855911.1|	77133-78003	8	35	1	0.159453	-3	0.24815	no
gi|641572057|gb|KK855910.1|	38213-13868	11	27	11	2	-2	0.1449	no
gi|641572057|gb|KK855910.1|	43761-14443	11	27	1	2	0.600023	0.69715	no
gi|641572057|gb|KK855910.1|	48099-14917	11	27	0.642418	0.610959	-0.0724377	1	no
gi|641572057|gb|KK855910.1|	51461-15266	11	27	3	0.854958	-2	0.22585	no
gi|641572057|gb|KK855910.1|	55063-15675	11	27	5	5	-0.195705	0.875	no
gi|641572057|gb|KK855910.1|	58602-16037	11	27	0.581929	2	2	0.1907	no
gi|641572057|gb|KK855910.1|	60946-16166	11	27	0.543851	2	2	0.3613	no
gi|641572057|gb|KK855910.1|	61783-16299	11	27	0.905315	3	2	0.2941	no
gi|641572057|gb|KK855910.1|	63204-16418	11	27	1	2	0.511698	0.7255	no
gi|641572057|gb|KK855910.1|	64839-16652	11	27	0.758416	3	2	0.1807	no
gi|641572057|gb|KK855910.1|	66775-16741	11	27	0.635177	5	3	0.15995	no
gi|641572057|gb|KK855910.1|	68128-16885	11	27	0.806494	4	2	0.19145	no
gi|641572057|gb|KK855910.1|	68950-16933	11	27	2	3	0.582531	0.76735	no
gi|641572064|gb|KK855908.1|	15009-11799	9.6	36	2	2	-0.00791287	0.99075	no
gi|641572064|gb|KK855908.1|	3482-3844	9.6	36	20	17	-0.250435	0.84665	no
gi|641572064|gb|KK855908.1|	4164-4560	9.6	36	2	7	2	0.2382	no
gi|641572064|gb|KK855908.1|	55709-56186	9.6	36	10	7	-0.482305	0.71945	no
gi|641572064|gb|KK855908.1|	57795-58463	9.6	36	3	2	-0.464263	0.7491	no
gi|641572064|gb|KK855908.1|	67211-70392	9.6	36	12	14	0.248198	0.8765	no
gi|641572064|gb|KK855908.1|	8415-8676	9.6	36	0	20	inf	0.0023	yes
gi|641572067|gb|KK855907.1|	10767-11119	12	43	0	161	inf	0.00095	yes
gi|641572067|gb|KK855907.1|	87467-18792	12	43	16	28	0.840693	0.51025	no
gi|641572067|gb|KK855907.1|	20345-21481	12	43	0.752658	0.80193	0.0914806	1	no

gi|641572070|gb|KK855906.1|	45232-14542	9	37	0	48	inf	0.0025	yes
gi|641572070|gb|KK855906.1|	17417-17798	9	37	0	36	inf	5.00E-05	yes
gi|641572070|gb|KK855906.1|	19374-19566	9	37	0	1162.23	inf	5.00E-05	yes
gi|641572070|gb|KK855906.1|	20393-20813	9	37	0	131	inf	0.0011	yes
gi|641572070|gb|KK855906.1|	24496-24752	9	37	0	442	inf	5.00E-05	yes
gi|641572070|gb|KK855906.1|	26490-27130	9	37	0	100	inf	0.00055	yes
gi|641572070|gb|KK855906.1|	28747-29046	9	37	0	659	inf	0.0003	yes
gi|641572070|gb|KK855906.1|	31626-31964	9	37	0	189	inf	5.00E-05	yes
gi|641572070|gb|KK855906.1|	37400-37888	9	37	22	56	1	0.29445	no
gi|641572070|gb|KK855906.1|	39427-40038	9	37	11	1	-3	0.08035	no
gi|641572070|gb|KK855906.1|	41127-42656	9	37	1	0.647261	-0.915659	0.53815	no
gi|641572070|gb|KK855906.1|	44742-47795	9	37	0.99105	0.794262	-0.319344	0.81345	no
gi|641572070|gb|KK855906.1|	48240-48686	9	37	13	5	-1	0.3061	no
gi|641572070|gb|KK855906.1|	57454-57765	9	37	3	13	2	0.22005	no
gi|641572070|gb|KK855906.1|	68502-69141	9	37	0.159508	3	4	0.1175	no
gi|641572080|gb|KK855905.1|	09367-11074	10	37	2	0	-inf	0.0003	yes
gi|641572080|gb|KK855905.1|	11620-11293	10	37	1	0.096486	-3	0.206	no
gi|641572080|gb|KK855905.1|	22373-12314	10	37	2	0.186356	-3	0.21435	no
gi|641572080|gb|KK855905.1|	41610-14376	10	37	4	6	0.666464	0.5976	no
gi|641572080|gb|KK855905.1|	55154-15568	10	37	52	318	3	0.14745	no
gi|641572080|gb|KK855905.1|	56585-15714	10	37	10	120	4	0.0842	no
gi|641572080|gb|KK855905.1|	58697-15956	10	37	6	135	4	0.0421	no
gi|641572080|gb|KK855905.1|	59804-16008	10	37	93	1816.4	4	0.038	no
gi|641572080|gb|KK855905.1|	61198-16250	10	37	10	174	4	0.0417	no
gi|641572080|gb|KK855905.1|	64181-16463	10	37	0	10	inf	0.00045	yes
gi|641572084|gb|KK855904.1|	1516-2958	11	41	14	11	-0.374704	0.7676	no
gi|641572084|gb|KK855904.1|	5521-6055	11	41	59	47	-0.325905	0.79395	no
gi|641572084|gb|KK855904.1|	71818-72016	11	41	0	310	inf	5.00E-05	yes
gi|641572084|gb|KK855904.1|	81744-81918	11	41	28	23	-0.291035	0.86415	no
gi|641572088|gb|KK855903.1|	82606-83216	9	35	0.682491	4	3	0.19845	no

gi|641572088|gb|KK855903.1|	83316-84545	9	35	0.068494	2	5	0.10875	no
gi|641572092|gb|KK855902.1|	31826-13208	8.9	40	28	1	-4	0.18805	no
gi|641572092|gb|KK855902.1|	41143-14137	8.9	40	36	31	-0.211101	0.88065	no
gi|641572092|gb|KK855902.1|	40840-41198	8.9	40	2639.6	1824.96	-0.532458	0.76875	no
gi|641572095|gb|KK855901.1|	04107-10507	7.6	32	2	0	-inf	0.00345	yes
gi|641572095|gb|KK855901.1|	67178-67670	7.6	32	0.713529	4	3	0.19335	no
gi|641572095|gb|KK855901.1|	74060-74465	7.6	32	2	3	0.362956	0.82765	no
gi|641572098|gb|KK855900.1|	09888-11071	9.5	43	0.90237	1	0.60293	0.6936	no
gi|641572098|gb|KK855900.1|	33278-13617	9.5	43	3	6	1	0.3601	no
gi|641572098|gb|KK855900.1|	19230-19803	9.5	43	0.74901	2	1	0.44255	no
gi|641572102|gb|KK855899.1|	10591-11201	10	24	0.750335	0.175882	-2	1	no
gi|641572102|gb|KK855899.1|	19491-12002	10	24	6	2	-2	0.25355	no
gi|641572102|gb|KK855899.1|	27585-12973	10	24	3	1	-1	0.2766	no
gi|641572102|gb|KK855899.1|	29809-13052	10	24	2	2	0.378622	0.7933	no
gi|641572102|gb|KK855899.1|	35820-13636	10	24	2	0.926732	-1	0.50365	no
gi|641572102|gb|KK855899.1|	37639-14027	10	24	1	0.486606	-1	0.4333	no
gi|641572102|gb|KK855899.1|	40931-14126	10	24	121	48	-1	0.30785	no
gi|641572102|gb|KK855899.1|	62843-16444	10	24	2	4	0.79256	0.54675	no
gi|641572102|gb|KK855899.1|	65993-16701	10	24	1	3	1	0.4543	no
gi|641572102|gb|KK855899.1|	74578-17492	10	24	152	256	0.75309	0.6399	no
gi|641572102|gb|KK855899.1|	60706-61382	10	24	2	5	1	0.4122	no
gi|641572102|gb|KK855899.1|	62265-65653	10	24	2	2	0.0064129	0.9891	no
gi|641572102|gb|KK855899.1|	67144-67820	10	24	2	2	0.33704	0.81785	no
gi|641572102|gb|KK855899.1|	71679-72917	10	24	0.475322	0.930589	0.969238	0.5733	no
gi|641572102|gb|KK855899.1|	74603-75506	10	24	0.900411	2	0.893497	0.5684	no
gi|641572102|gb|KK855899.1|	75722-76213	10	24	2	3	0.785265	0.63115	no
gi|641572102|gb|KK855899.1|	76522-78067	10	24	2	1	-0.436439	0.75035	no
gi|641572102|gb|KK855899.1|	78769-80498	10	24	1	1	-0.391519	0.77875	no
gi|641572102|gb|KK855899.1|	81032-83098	10	24	3	2	-0.207961	0.8708	no
gi|641572102|gb|KK855899.1|	83245-85135	10	24	3	5	0.819414	0.51565	no

gi|641572102|gb|KK855899.1|	93379-94346	10	24	0.64224	0.976618	0.604681	0.69715	no
gi|641572102|gb|KK855899.1|	94714-95180	10	24	2	2	0.36852	0.82765	no
gi|641572102|gb|KK855899.1|	95789-98928	10	24	1	2	0.610327	0.64465	no
gi|641572106|gb|KK855898.1|	18971-11935	7.3	40	3	3	0.360272	0.83635	no
gi|641572106|gb|KK855898.1|	78738-18027	7.3	40	19	11	-0.863373	0.48945	no
gi|641572106|gb|KK855898.1|	80808-18204	7.3	40	6	3	-1	0.3975	no
gi|641572106|gb|KK855898.1|	20010-21386	7.3	40	8	3	-2	0.212	no
gi|641572106|gb|KK855898.1|	73940-75435	7.3	40	1	2	0.356248	0.79455	no
gi|641572106|gb|KK855898.1|	81057-81849	7.3	40	1	3	2	0.33365	no
gi|641572106|gb|KK855898.1|	89863-90094	7.3	40	0	34	inf	0.00135	yes
gi|641572106|gb|KK855898.1|	90704-90976	7.3	40	0	40	inf	0.00025	yes
gi|641572110|gb|KK855897.1|	01357-10438	10	40	2	0.496019	-2	0.1171	no
gi|641572110|gb|KK855897.1|	77734-17825	10	40	0	5	inf	0.00255	yes
gi|641572110|gb|KK855897.1|	53869-56217	10	40	0.065781	9	7	0.23225	no
gi|641572110|gb|KK855897.1|	73059-73397	10	40	45	11	-2	0.16065	no
gi|641572110|gb|KK855897.1|	80949-81570	10	40	0.997381	1	0.0135368	0.91115	no
gi|641572110|gb|KK855897.1|	82982-84561	10	40	0.869876	1	0.427491	0.7651	no
gi|641572110|gb|KK855897.1|	87960-88229	10	40	8	11	0.406864	0.79705	no
gi|641572110|gb|KK855897.1|	90237-90458	10	40	10	6	-0.68174	0.6988	no
gi|641572110|gb|KK855897.1|	91193-91503	10	40	8	0.859045	-3	0.21655	no
gi|641572110|gb|KK855897.1|	93345-94017	10	40	15	11	-0.489295	0.70255	no
gi|641572110|gb|KK855897.1|	94612-94859	10	40	18	5	-2	0.2843	no
gi|641572110|gb|KK855897.1|	95204-98449	10	40	50	37	-0.417958	0.8366	no
gi|641572114|gb|KK855896.1|	09261-10963	10	36	1	20	4	0.1393	no
gi|641572114|gb|KK855896.1|	10995-11751	10	36	5	2	-1	0.3209	no
gi|641572114|gb|KK855896.1|	17609-12144	10	36	3	0.386165	-3	0.04705	no
gi|641572114|gb|KK855896.1|	11868-12318	10	36	7	0.831641	-3	0.1659	no
gi|641572114|gb|KK855896.1|	26237-12700	10	36	1	0.943145	-0.398021	0.81255	no
gi|641572114|gb|KK855896.1|	27219-12849	10	36	0.658201	1	1	0.4713	no
gi|641572114|gb|KK855896.1|	45459-14607	10	36	3	0.255246	-4	0.20475	no

gi|641572114|gb|KK855896.1|	61171-16175	10	36	0.91459	2	0.860157	0.62755	no
gi|641572114|gb|KK855896.1|	16641-17945	10	36	3	0.875396	-2	0.2088	no
gi|641572114|gb|KK855896.1|	76700-17703	10	36	31	34	0.111679	0.9309	no
gi|641572114|gb|KK855896.1|	82558-18289	10	36	7	16	1	0.3672	no
gi|641572114|gb|KK855896.1|	90471-19171	10	36	0.677084	1	0.744187	0.6176	no
gi|641572114|gb|KK855896.1|	93165-19359	10	36	5	3	-0.692291	0.65065	no
gi|641572114|gb|KK855896.1|	98479-19885	10	36	3	4	0.358747	0.83635	no
gi|641572114|gb|KK855896.1|	99870-20246	10	36	10	2	-2	0.09295	no
gi|641572114|gb|KK855896.1|	06253-20770	10	36	4	1	-2	0.2487	no
gi|641572114|gb|KK855896.1|	08054-20838	10	36	6	0	-inf	0.01	no
gi|641572114|gb|KK855896.1|	09435-21082	10	36	1	0.452198	-1	0.47035	no
gi|641572114|gb|KK855896.1|	12151-21271	10	36	3	1	-1	0.37245	no
gi|641572114|gb|KK855896.1|	14195-21537	10	36	4	2	-0.838463	0.53355	no
gi|641572114|gb|KK855896.1|	17719-22024	10	36	1	0.509294	-1	0.34395	no
gi|641572114|gb|KK855896.1|	21278-22188	10	36	3	1	-0.986964	0.57205	no
gi|641572114|gb|KK855896.1|	23391-22494	10	36	0.784548	0.558011	-0.491567	1	no
gi|641572114|gb|KK855896.1|	24423-25840	10	36	0.173863	77	9	0.13695	no
gi|641572114|gb|KK855896.1|	26299-27716	10	36	0.927269	2	0.855425	0.55325	no
gi|641572114|gb|KK855896.1|	30153-30590	10	36	6	7	0.340625	0.80465	no
gi|641572114|gb|KK855896.1|	33697-34209	10	36	3	1	-2	0.403	no
gi|641572114|gb|KK855896.1|	41724-42578	10	36	0.644927	0.816511	0.340337	1	no
gi|641572114|gb|KK855896.1|	42746-44167	10	36	11	8	-0.505381	0.68605	no
gi|641572114|gb|KK855896.1|	44470-46626	10	36	0.938888	2	1	0.4084	no
gi|641572114|gb|KK855896.1|	46731-53756	10	36	1	4	1	0.2835	no
gi|641572114|gb|KK855896.1|	54045-54437	10	36	2	12	3	0.1404	no
gi|641572114|gb|KK855896.1|	56114-56493	10	36	1	6	2	0.21705	no
gi|641572114|gb|KK855896.1|	60138-61669	10	36	0.53008	4	3	0.06445	no
gi|641572114|gb|KK855896.1|	62596-64923	10	36	0.332143	3	3	0.05645	no
gi|641572114|gb|KK855896.1|	6646-7440	10	36	2	0	-inf	0.0075	no
gi|641572114|gb|KK855896.1|	69456-70217	10	36	0.250313	2	3	0.2991	no

gi|641572114|gb|KK855896.1|	70954-71653	10	36	0.562182	1	1	0.44255	no
gi|641572118|gb|KK855895.1|	13383-11362	10	43	23	42	0.858063	0.5332	no
gi|641572118|gb|KK855895.1|	170-1060	10	43	35	51	0.543354	0.6562	no
gi|641572118|gb|KK855895.1|	24787-25635	10	43	1	2	1	0.47895	no
gi|641572118|gb|KK855895.1|	32327-33293	10	43	0.275603	1	2	0.25595	no
gi|641572118|gb|KK855895.1|	42197-43136	10	43	0.952028	1	0.282465	0.85465	no
gi|641572118|gb|KK855895.1|	44691-45868	10	43	0.432735	0.658781	0.606316	1	no
gi|641572118|gb|KK855895.1|	74607-75684	10	43	0.240907	166.67	9	0.1362	no
gi|641572118|gb|KK855895.1|	79370-79957	10	43	0	52	inf	5.00E-05	yes
gi|641572118|gb|KK855895.1|	82595-83085	10	43	0	20	inf	0.00015	yes
gi|641572118|gb|KK855895.1|	85393-86559	10	43	0.583518	0.666215	0.19121	1	no
gi|641572118|gb|KK855895.1|	87330-87905	10	43	0.931359	3	2	0.2851	no
gi|641572118|gb|KK855895.1|	89569-90461	10	43	0.508126	0.926686	0.866894	0.62725	no
gi|641572124|gb|KK855893.1|	01803-10239	10	40	0.889418	2	1	0.51085	no
gi|641572124|gb|KK855893.1|	02464-10331	10	40	0.870474	0.495892	-0.811776	1	no
gi|641572124|gb|KK855893.1|	03553-10438	10	40	0.443414	2	2	0.33105	no
gi|641572124|gb|KK855893.1|	05071-10586	10	40	0.713132	2	1	0.4702	no
gi|641572124|gb|KK855893.1|	20159-12174	10	40	4	3	-0.391931	0.76205	no
gi|641572124|gb|KK855893.1|	12856-14371	10	40	149	100	-0.568456	0.78115	no
gi|641572124|gb|KK855893.1|	57946-15914	10	40	0	11	inf	5.00E-05	yes
gi|641572124|gb|KK855893.1|	65539-16574	10	40	15	193	4	0.0625	no
gi|641572124|gb|KK855893.1|	67823-16917	10	40	5	3	-0.977755	0.4599	no
gi|641572124|gb|KK855893.1|	70810-17137	10	40	19	10	-0.930645	0.4778	no
gi|641572124|gb|KK855893.1|	17978-20709	10	40	0.78183	0.596639	-0.389996	1	no
gi|641572124|gb|KK855893.1|	83999-18487	10	40	2	0	-inf	0.0059	no
gi|641572124|gb|KK855893.1|	88593-18967	10	40	0	4	inf	0.00035	yes
gi|641572124|gb|KK855893.1|	95332-19597	10	40	0.792199	2	1	0.4611	no
gi|641572124|gb|KK855893.1|	01680-20234	10	40	0	25	inf	5.00E-05	yes
gi|641572124|gb|KK855893.1|	22586-23157	10	40	5	7	0.712161	0.6036	no
gi|641572124|gb|KK855893.1|	23492-23903	10	40	0	49	inf	5.00E-05	yes

<table>
<tr><td>gi|641572124|gb|KK855893.1|</td><td>24288-24644</td><td>10</td><td>40</td><td>23</td><td>17</td><td>-0.475762</td><td>0.72035</td><td>no</td></tr>
<tr><td>gi|641572124|gb|KK855893.1|</td><td>27626-28887</td><td>10</td><td>40</td><td>0.86369</td><td>0.70823</td><td>-0.286295</td><td>1</td><td>no</td></tr>
<tr><td>gi|641572124|gb|KK855893.1|</td><td>29008-31246</td><td>10</td><td>40</td><td>0.485261</td><td>0.581701</td><td>0.261518</td><td>1</td><td>no</td></tr>
<tr><td>gi|641572124|gb|KK855893.1|</td><td>31503-32211</td><td>10</td><td>40</td><td>2</td><td>3</td><td>0.131353</td><td>0.9278</td><td>no</td></tr>
<tr><td>gi|641572124|gb|KK855893.1|</td><td>37024-37282</td><td>10</td><td>40</td><td>28</td><td>3</td><td>-3</td><td>0.14805</td><td>no</td></tr>
<tr><td>gi|641572124|gb|KK855893.1|</td><td>39285-39455</td><td>10</td><td>40</td><td>71</td><td>38</td><td>-0.900898</td><td>0.56855</td><td>no</td></tr>
<tr><td>gi|641572124|gb|KK855893.1|</td><td>39838-44238</td><td>10</td><td>40</td><td>2</td><td>2</td><td>0.361317</td><td>0.77745</td><td>no</td></tr>
<tr><td>gi|641572124|gb|KK855893.1|</td><td>45156-45712</td><td>10</td><td>40</td><td>29</td><td>10</td><td>-2</td><td>0.2393</td><td>no</td></tr>
<tr><td>gi|641572124|gb|KK855893.1|</td><td>48452-48778</td><td>10</td><td>40</td><td>7</td><td>0.768873</td><td>-3</td><td>0.22375</td><td>no</td></tr>
<tr><td>gi|641572124|gb|KK855893.1|</td><td>48942-49140</td><td>10</td><td>40</td><td>38</td><td>26</td><td>-0.558237</td><td>0.7164</td><td>no</td></tr>
<tr><td>gi|641572124|gb|KK855893.1|</td><td>5666-5916</td><td>10</td><td>40</td><td>16</td><td>4</td><td>-2</td><td>0.29925</td><td>no</td></tr>
<tr><td>gi|641572124|gb|KK855893.1|</td><td>9331-9911</td><td>10</td><td>40</td><td>42</td><td>5</td><td>-3</td><td>0.03685</td><td>no</td></tr>
<tr><td>gi|641572127|gb|KK855892.1|</td><td>21896-12263</td><td>9.8</td><td>39</td><td>2</td><td>3</td><td>0.601431</td><td>0.67045</td><td>no</td></tr>
<tr><td>gi|641572127|gb|KK855892.1|</td><td>24235-12532</td><td>9.8</td><td>39</td><td>0.710631</td><td>0.720942</td><td>0.0207836</td><td>1</td><td>no</td></tr>
<tr><td>gi|641572127|gb|KK855892.1|</td><td>47818-14846</td><td>9.8</td><td>39</td><td>2</td><td>2</td><td>0.447345</td><td>0.7665</td><td>no</td></tr>
<tr><td>gi|641572130|gb|KK855891.1|</td><td>05827-10696</td><td>10</td><td>37</td><td>1</td><td>1</td><td>0.158615</td><td>0.9153</td><td>no</td></tr>
<tr><td>gi|641572130|gb|KK855891.1|</td><td>12207-11358</td><td>10</td><td>37</td><td>0.480956</td><td>2</td><td>2</td><td>0.2897</td><td>no</td></tr>
<tr><td>gi|641572130|gb|KK855891.1|</td><td>13792-11430</td><td>10</td><td>37</td><td>11</td><td>13</td><td>0.188386</td><td>0.8857</td><td>no</td></tr>
<tr><td>gi|641572130|gb|KK855891.1|</td><td>114-603</td><td>10</td><td>37</td><td>7</td><td>4</td><td>-0.892935</td><td>0.52465</td><td>no</td></tr>
<tr><td>gi|641572130|gb|KK855891.1|</td><td>15058-11592</td><td>10</td><td>37</td><td>0.954351</td><td>1</td><td>0.433567</td><td>0.78015</td><td>no</td></tr>
<tr><td>gi|641572130|gb|KK855891.1|</td><td>16034-11814</td><td>10</td><td>37</td><td>2</td><td>1</td><td>-0.613138</td><td>0.63735</td><td>no</td></tr>
<tr><td>gi|641572130|gb|KK855891.1|</td><td>18270-11878</td><td>10</td><td>37</td><td>82</td><td>57</td><td>-0.527217</td><td>0.6698</td><td>no</td></tr>
<tr><td>gi|641572130|gb|KK855891.1|</td><td>18881-12057</td><td>10</td><td>37</td><td>5</td><td>3</td><td>-0.788937</td><td>0.5426</td><td>no</td></tr>
<tr><td>gi|641572130|gb|KK855891.1|</td><td>20739-12276</td><td>10</td><td>37</td><td>3</td><td>1</td><td>-1</td><td>0.31565</td><td>no</td></tr>
<tr><td>gi|641572130|gb|KK855891.1|</td><td>24877-12543</td><td>10</td><td>37</td><td>0.78193</td><td>11</td><td>4</td><td>0.1226</td><td>no</td></tr>
<tr><td>gi|641572130|gb|KK855891.1|</td><td>25986-12914</td><td>10</td><td>37</td><td>0.980156</td><td>2</td><td>0.679052</td><td>0.6041</td><td>no</td></tr>
<tr><td>gi|641572130|gb|KK855891.1|</td><td>29353-13427</td><td>10</td><td>37</td><td>53</td><td>35</td><td>-0.595711</td><td>0.7644</td><td>no</td></tr>
<tr><td>gi|641572130|gb|KK855891.1|</td><td>36836-13826</td><td>10</td><td>37</td><td>1</td><td>1</td><td>0.373063</td><td>0.78995</td><td>no</td></tr>
<tr><td>gi|641572130|gb|KK855891.1|</td><td>41289-14150</td><td>10</td><td>37</td><td>18</td><td>72</td><td>2</td><td>0.17905</td><td>no</td></tr>
<tr><td>gi|641572130|gb|KK855891.1|</td><td>42979-14330</td><td>10</td><td>37</td><td>45</td><td>128</td><td>2</td><td>0.24595</td><td>no</td></tr>
</table>

gi|641572130|gb|KK855891.1|	.47849-15053	10	37	151	124	-0.286379	0.87915	no
gi|641572130|gb|KK855891.1|	.51167-15153	10	37	236	177	-0.414681	0.7918	no
gi|641572130|gb|KK855891.1|	.52362-15284	10	37	10	6	-0.800216	0.561	no
gi|641572130|gb|KK855891.1|	.53232-15350	10	37	6	7	0.144744	0.92285	no
gi|641572130|gb|KK855891.1|	.56476-15725	10	37	0.973723	0.554299	-0.812847	0.6492	no
gi|641572130|gb|KK855891.1|	.59406-16183	10	37	2	1	-0.594667	0.65745	no
gi|641572130|gb|KK855891.1|	.63432-16482	10	37	6	4	-0.452733	0.7296	no
gi|641572130|gb|KK855891.1|	.64934-16556	10	37	73	70	-0.067192	0.95605	no
gi|641572130|gb|KK855891.1|	.67881-16848	10	37	2	1	-0.987209	0.5872	no
gi|641572130|gb|KK855891.1|	.69260-17013	10	37	1	2	0.48817	0.7378	no
gi|641572130|gb|KK855891.1|	.70238-17074	10	37	2	4	1	0.3971	no
gi|641572130|gb|KK855891.1|	.74452-17482	10	37	5	5	0.0506252	0.9558	no
gi|641572130|gb|KK855891.1|	.79575-18004	10	37	3	0.385185	-3	0.2381	no
gi|641572130|gb|KK855891.1|	3228-3936	10	37	6	3	-1	0.3779	no
gi|641572130|gb|KK855891.1|	72057-74002	10	37	0.202329	0.802315	2	1	no
gi|641572130|gb|KK855891.1|	75436-76321	10	37	0.718596	2	1	0.44	no
gi|641572130|gb|KK855891.1|	81178-83247	10	37	4	10	1	0.2977	no
gi|641572130|gb|KK855891.1|	86378-86577	10	37	7	22	2	0.3486	no
gi|641572130|gb|KK855891.1|	92934-93367	10	37	20	16	-0.310882	0.8104	no
gi|641572130|gb|KK855891.1|	93823-95672	10	37	13	9	-0.548423	0.662	no
gi|641572130|gb|KK855891.1|	96130-97833	10	37	0.516569	0.50117	-0.0436625	1	no
gi|641572134|gb|KK855890.1|	.05407-10615	10	36	0.382222	2	2	0.2235	no
gi|641572134|gb|KK855890.1|	.07452-10932	10	36	2	0.642823	-2	0.25425	no
gi|641572134|gb|KK855890.1|	.09674-11219	10	36	2	0.790811	-2	0.23715	no
gi|641572134|gb|KK855890.1|	.12305-11390	10	36	3	2	-0.60487	0.64605	no
gi|641572134|gb|KK855890.1|	.13967-11695	10	36	6	5	-0.217892	0.8627	no
gi|641572134|gb|KK855890.1|	.53375-15352	10	36	268	0	-inf	0.0013	yes
gi|641572134|gb|KK855890.1|	56720-57783	10	36	7	1	-3	0.08085	no
gi|641572134|gb|KK855890.1|	57942-60596	10	36	5	2	-1	0.32345	no
gi|641572134|gb|KK855890.1|	62493-62860	10	36	4	2	-0.879029	0.60075	no

gi|641572134|gb|KK855890.1|	70124-70646	10	36	0	128	inf	0.0005	yes
gi|641572134|gb|KK855890.1|	73689-73955	10	36	0	85	inf	5.00E-05	yes
gi|641572134|gb|KK855890.1|	74867-75506	10	36	0.79754	3	2	0.2717	no
gi|641572134|gb|KK855890.1|	79250-79867	10	36	1	2	0.598369	0.69715	no
gi|641572134|gb|KK855890.1|	80709-84017	10	36	5	4	-0.281707	0.82045	no
gi|641572136|gb|KK855889.1|	01843-10359	11	41	5	6	0.304539	0.8079	no
gi|641572136|gb|KK855889.1|	07891-10849	11	41	3	2	-0.402105	0.7911	no
gi|641572136|gb|KK855889.1|	10206-11224	11	41	0.615778	0.528337	-0.220953	1	no
gi|641572136|gb|KK855889.1|	12379-11393	11	41	2	2	0.114951	0.9288	no
gi|641572136|gb|KK855889.1|	17552-11776	11	41	17	20	0.214925	0.89365	no
gi|641572136|gb|KK855889.1|	17927-11888	11	41	0.370839	2	3	0.177	no
gi|641572136|gb|KK855889.1|	21026-12271	11	41	1	2	0.160911	0.904	no
gi|641572136|gb|KK855889.1|	24972-12544	11	41	5	7	0.517203	0.7125	no
gi|641572136|gb|KK855889.1|	27290-12796	11	41	1	3	1	0.47685	no
gi|641572136|gb|KK855889.1|	28251-12846	11	41	16	47	2	0.3316	no
gi|641572136|gb|KK855889.1|	1304-2820	11	41	2	0.081702	-4	0.18815	no
gi|641572136|gb|KK855889.1|	31205-13176	11	41	1	1	0.333014	0.83525	no
gi|641572136|gb|KK855889.1|	34382-13598	11	41	0.605713	0.692454	0.193083	1	no
gi|641572136|gb|KK855889.1|	38681-14100	11	41	1	1	-0.238443	0.8552	no
gi|641572136|gb|KK855889.1|	41246-14214	11	41	2	2	-0.521648	0.7183	no
gi|641572136|gb|KK855889.1|	44372-14514	11	41	0.490231	1	1	0.44255	no
gi|641572136|gb|KK855889.1|	46529-14771	11	41	1	3	1	0.32035	no
gi|641572136|gb|KK855889.1|	49231-15013	11	41	5	12	1	0.341	no
gi|641572136|gb|KK855889.1|	53330-15359	11	41	5	8	0.819028	0.6381	no
gi|641572136|gb|KK855889.1|	66513-16741	11	41	2	8	2	0.18825	no
gi|641572136|gb|KK855889.1|	67792-17041	11	41	2	5	0.858717	0.50735	no
gi|641572136|gb|KK855889.1|	71251-17402	11	41	0.712773	0.251057	-2	1	no
gi|641572136|gb|KK855889.1|	74169-17449	11	41	29	79	1	0.27025	no
gi|641572136|gb|KK855889.1|	76144-17734	11	41	27	79	2	0.3584	no
gi|641572136|gb|KK855889.1|	78874-18228	11	41	11	31	1	0.36415	no

gi|641572136|gb|KK855889.1|	82791-18415	11	41	38	122	2	0.44685	no
gi|641572136|gb|KK855889.1|	84478-18739	11	41	19	61	2	0.44325	no
gi|641572136|gb|KK855889.1|	91586-19244	11	41	10	11	0.172778	0.89025	no
gi|641572136|gb|KK855889.1|	92581-19284	11	41	10	16	0.673722	0.6619	no
gi|641572136|gb|KK855889.1|	95138-19536	11	41	10	13	0.352001	0.82145	no
gi|641572136|gb|KK855889.1|	00081-20222	11	41	3	2	-0.594226	0.6495	no
gi|641572136|gb|KK855889.1|	05739-20620	11	41	2	2	-0.409162	0.82205	no
gi|641572136|gb|KK855889.1|	3570-4363	11	41	1	0	-inf	0.01	no
gi|641572136|gb|KK855889.1|	43316-44152	11	41	1	3	1	0.43255	no
gi|641572136|gb|KK855889.1|	4464-7008	11	41	2	0.092009	-5	0.1332	no
gi|641572136|gb|KK855889.1|	45212-46476	11	41	0.795014	1	0.829198	0.58405	no
gi|641572136|gb|KK855889.1|	46728-47573	11	41	0.32693	1	2	0.25595	no
gi|641572136|gb|KK855889.1|	702-1144	11	41	8	0	-inf	0.00185	yes
gi|641572136|gb|KK855889.1|	7856-8573	11	41	1	0	-inf	0.01175	no
gi|641572136|gb|KK855889.1|	83098-83863	11	41	0.248556	2	3	0.27515	no
gi|641572136|gb|KK855889.1|	93816-94693	11	41	0.519307	1	1	0.50945	no
gi|641572136|gb|KK855889.1|	9585-10549	11	41	0.736853	0.280117	-1	1	no
gi|641572139|gb|KK855888.1|	01206-10146	9.9	36	22	12	-0.798985	0.5839	no
gi|641572139|gb|KK855888.1|	06078-10663	9.9	36	40	17	-1	0.3241	no
gi|641572139|gb|KK855888.1|	07535-10818	9.9	36	5	4	-0.267245	0.841	no
gi|641572139|gb|KK855888.1|	08581-10884	9.9	36	33	16	-1	0.4433	no
gi|641572139|gb|KK855888.1|	12274-11265	9.9	36	25	14	-0.877307	0.5039	no
gi|641572139|gb|KK855888.1|	15712-11642	9.9	36	4	1	-2	0.27005	no
gi|641572139|gb|KK855888.1|	17638-11791	9.9	36	24	8	-2	0.28235	no
gi|641572139|gb|KK855888.1|	26669-12709	9.9	36	43	20	-1	0.3934	no
gi|641572139|gb|KK855888.1|	27829-12824	9.9	36	12	4	-2	0.2732	no
gi|641572139|gb|KK855888.1|	31918-13225	9.9	36	50	18	-1	0.26105	no
gi|641572139|gb|KK855888.1|	35078-13552	9.9	36	12	2	-3	0.1338	no
gi|641572139|gb|KK855888.1|	36612-13755	9.9	36	3	0.718897	-2	0.2547	no
gi|641572139|gb|KK855888.1|	39767-14000	9.9	36	51	17	-2	0.2628	no

gi|641572139|gb|KK855888.1|	41460-14213	9.9	36	2	0.223674	-3	0.21435	no
gi|641572139|gb|KK855888.1|	43567-14483	9.9	36	8	5	-0.745491	0.5631	no
gi|641572139|gb|KK855888.1|	45474-14571	9.9	36	11	3	-2	0.3397	no
gi|641572139|gb|KK855888.1|	46857-14715	9.9	36	23	11	-1	0.43345	no
gi|641572139|gb|KK855888.1|	47956-14869	9.9	36	16	0	-inf	5.00E-05	yes
gi|641572139|gb|KK855888.1|	49012-14927	9.9	36	72	20	-2	0.19045	no
gi|641572139|gb|KK855888.1|	53271-15355	9.9	36	36	11	-2	0.2301	no
gi|641572139|gb|KK855888.1|	55740-15611	9.9	36	14	3	-2	0.19165	no
gi|641572139|gb|KK855888.1|	57349-15814	9.9	36	0.592288	1	1	0.4595	no
gi|641572139|gb|KK855888.1|	58268-15946	9.9	36	0.777507	0.860908	0.147004	1	no
gi|641572139|gb|KK855888.1|	63202-16378	9.9	36	39	11	-2	0.1607	no
gi|641572139|gb|KK855888.1|	47242-48488	9.9	36	2	3	0.284782	0.82925	no
gi|641572139|gb|KK855888.1|	50569-51283	9.9	36	55	33	-0.739105	0.5559	no
gi|641572139|gb|KK855888.1|	52418-52714	9.9	36	179	127	-0.497282	0.6866	no
gi|641572139|gb|KK855888.1|	53866-54083	9.9	36	46	7	-3	0.15185	no
gi|641572139|gb|KK855888.1|	56072-56323	9.9	36	148	66	-1	0.35945	no
gi|641572139|gb|KK855888.1|	58447-58839	9.9	36	27	19	-0.534827	0.67975	no
gi|641572139|gb|KK855888.1|	60404-61062	9.9	36	0.917849	1	0.599599	0.70555	no
gi|641572139|gb|KK855888.1|	61275-61589	9.9	36	155	88	-0.81839	0.5177	no
gi|641572139|gb|KK855888.1|	64025-64256	9.9	36	32	24	-0.398346	0.779	no
gi|641572139|gb|KK855888.1|	66730-68119	9.9	36	6	3	-1	0.43735	no
gi|641572139|gb|KK855888.1|	69851-70710	9.9	36	5	4	-0.299264	0.8218	no
gi|641572139|gb|KK855888.1|	7173-7999	9.9	36	1	9	3	0.0847	no
gi|641572139|gb|KK855888.1|	77146-77494	9.9	36	22	18	-0.282476	0.8249	no
gi|641572139|gb|KK855888.1|	77979-78913	9.9	36	19	13	-0.578138	0.6434	no
gi|641572139|gb|KK855888.1|	81059-81525	9.9	36	10	8	-0.338698	0.7999	no
gi|641572139|gb|KK855888.1|	82932-83414	9.9	36	58.06	28	-1	0.41295	no
gi|641572139|gb|KK855888.1|	84045-84294	9.9	36	122	66	-0.895274	0.48475	no
gi|641572139|gb|KK855888.1|	88587-88821	9.9	36	112	74	-0.603916	0.6418	no
gi|641572139|gb|KK855888.1|	90759-91124	9.9	36	22	14	-0.651225	0.6215	no

gi|641572139|gb|KK855888.1|	91304-91637	9.9	36	70	27	-1	0.2988	no
gi|641572139|gb|KK855888.1|	96516-96657	9.9	36	123	65	-0.916908	0.6337	no
gi|641572139|gb|KK855888.1|	97734-98280	9.9	36	11	8	-0.511318	0.69955	no
gi|641572142|gb|KK855887.1|	03468-10373	10	36	16	36	1	0.4141	no
gi|641572142|gb|KK855887.1|	07521-11129	10	36	3	3	-0.406389	0.7401	no
gi|641572142|gb|KK855887.1|	12885-11764	10	36	51	2	-5	0.029	no
gi|641572142|gb|KK855887.1|	24360-12750	10	36	5	5	-0.0141614	0.9901	no
gi|641572142|gb|KK855887.1|	28819-13311	10	36	3	2	-0.480252	0.6996	no
gi|641572142|gb|KK855887.1|	33892-13440	10	36	4	2	-1	0.4955	no
gi|641572142|gb|KK855887.1|	34531-13641	10	36	6	1	-2	0.0907	no
gi|641572142|gb|KK855887.1|	36729-13791	10	36	13	5	-1	0.2927	no
gi|641572142|gb|KK855887.1|	43390-14372	10	36	10	4	-1	0.4264	no
gi|641572142|gb|KK855887.1|	43852-14593	10	36	0.711889	0.628696	-0.179289	1	no
gi|641572142|gb|KK855887.1|	146-1711	10	36	2	4	0.576311	0.65775	no
gi|641572142|gb|KK855887.1|	15927-16414	10	36	19	9	-0.973983	0.4607	no
gi|641572142|gb|KK855887.1|	75076-17864	10	36	36	10	-2	0.26655	no
gi|641572142|gb|KK855887.1|	79860-18131	10	36	0.900727	0.428904	-1	0.4973	no
gi|641572142|gb|KK855887.1|	18075-18546	10	36	3	0.77037	-2	0.3216	no
gi|641572142|gb|KK855887.1|	86787-18808	10	36	1	0.586232	-1	0.4423	no
gi|641572142|gb|KK855887.1|	90032-19043	10	36	3	3	-0.262874	0.88485	no
gi|641572142|gb|KK855887.1|	91062-19132	10	36	13	1	-3	0.21195	no
gi|641572142|gb|KK855887.1|	95266-19570	10	36	3	0.860908	-2	0.3216	no
gi|641572142|gb|KK855887.1|	95842-19700	10	36	0.735458	2	1	0.3418	no
gi|641572142|gb|KK855887.1|	98489-19894	10	36	11	4	-2	0.28405	no
gi|641572142|gb|KK855887.1|	07559-20802	10	36	29	10	-2	0.265	no
gi|641572142|gb|KK855887.1|	13512-21413	10	36	0.49869	2	2	0.2812	no
gi|641572142|gb|KK855887.1|	15662-21635	10	36	0.997662	2	0.963039	0.57335	no
gi|641572142|gb|KK855887.1|	16469-21746	10	36	0.975118	2	0.845947	0.58475	no
gi|641572142|gb|KK855887.1|	17571-21842	10	36	0.856004	1	0.603412	0.6936	no
gi|641572142|gb|KK855887.1|	19326-22008	10	36	6	8	0.537777	0.67825	no

gi|641572142|gb|KK855887.1|	22412-22657	10	36	77	59	-0.371294	0.7715	no
gi|641572142|gb|KK855887.1|	26637-26932	10	36	61	30	-1	0.4268	no
gi|641572142|gb|KK855887.1|	32100-32286	10	36	29	8	-2	0.33925	no
gi|641572142|gb|KK855887.1|	35715-35907	10	36	274	69	-2	0.1496	no
gi|641572142|gb|KK855887.1|	41930-42550	10	36	12	5	-1	0.3513	no
gi|641572142|gb|KK855887.1|	4365-5329	10	36	14	6	-1	0.3801	no
gi|641572142|gb|KK855887.1|	43737-45298	10	36	4	1	-2	0.2831	no
gi|641572142|gb|KK855887.1|	48787-49073	10	36	38	7	-2	0.12715	no
gi|641572142|gb|KK855887.1|	49623-50224	10	36	6	2	-1	0.3359	no
gi|641572142|gb|KK855887.1|	53035-53284	10	36	99	31	-2	0.2182	no
gi|641572142|gb|KK855887.1|	6588-7273	10	36	2	2	-0.592346	0.68495	no
gi|641572142|gb|KK855887.1|	66800-67314	10	36	4	2	-0.990983	0.5501	no
gi|641572142|gb|KK855887.1|	70362-70921	10	36	8	2	-2	0.21865	no
gi|641572142|gb|KK855887.1|	7446-7900	10	36	19	4	-2	0.1287	no
gi|641572142|gb|KK855887.1|	75644-75793	10	36	70	43	-0.720129	0.6976	no
gi|641572142|gb|KK855887.1|	78343-78880	10	36	68	102	0.58632	0.62975	no
gi|641572142|gb|KK855887.1|	80586-81780	10	36	0.567158	1	0.928346	0.56225	no
gi|641572142|gb|KK855887.1|	85552-86808	10	36	1	3	1	0.445	no
gi|641572142|gb|KK855887.1|	94215-94945	10	36	1	1	0.0163471	0.9394	no
gi|641572142|gb|KK855887.1|	97536-98908	10	36	0.481365	7	4	0.05405	no
gi|641572147|gb|KK855886.1|	15433-16572	8	31	2026.92	0	-inf	0.00545	no
gi|641572147|gb|KK855886.1|	17570-18559	8	31	3	0	-inf	0.0006	yes
gi|641572147|gb|KK855886.1|	19058-20217	8	31	2	0	-inf	0.0013	yes
gi|641572147|gb|KK855886.1|	20880-21585	8	31	215	0	-inf	5.00E-05	yes
gi|641572147|gb|KK855886.1|	6551-7903	8	31	1470.96	0.372977	-12	0.06105	no
gi|641572147|gb|KK855886.1|	9839-10873	8	31	1	0	-inf	0.0059	no
gi|641572154|gb|KK855884.1|	35727-36465	9.2	38	2	0.593811	-2	0.40415	no
gi|641572154|gb|KK855884.1|	42448-42852	9.2	38	8	0	-inf	0.0022	yes
gi|641572154|gb|KK855884.1|	83888-84219	9.2	38	26	46	0.800495	0.5455	no
gi|641572154|gb|KK855884.1|	86224-87165	9.2	38	1	0.866127	-0.270054	0.86195	no

gi|641572154|gb|KK855884.1|	92137-93331	9.2	38	42	66	0.668633	0.76675	no
gi|641572162|gb|KK855882.1|	06656-10767	12	38	0.345504	1	2	0.26155	no
gi|641572162|gb|KK855882.1|	07859-10854	12	38	3	4	0.185335	0.8927	no
gi|641572162|gb|KK855882.1|	11025-11171	12	38	0.999671	0.866106	-0.20691	0.8945	no
gi|641572162|gb|KK855882.1|	13301-11736	12	38	1	2	0.43797	0.7333	no
gi|641572162|gb|KK855882.1|	11851-16027	12	38	12	14	0.143116	0.94735	no
gi|641572162|gb|KK855882.1|	18598-11939	12	38	2	0.540462	-2	0.33985	no
gi|641572162|gb|KK855882.1|	21866-12332	12	38	2	2	-0.266808	0.8424	no
gi|641572162|gb|KK855882.1|	30376-13091	12	38	1	3	0.957837	0.57355	no
gi|641572162|gb|KK855882.1|	35366-14225	12	38	11	6	-0.918582	0.6775	no
gi|641572162|gb|KK855882.1|	42502-14866	12	38	13	6	-1	0.6451	no
gi|641572162|gb|KK855882.1|	48817-15185	12	38	2	1	-0.656028	0.6169	no
gi|641572162|gb|KK855882.1|	52325-15339	12	38	0.484561	1	1	0.41175	no
gi|641572162|gb|KK855882.1|	53505-15435	12	38	0.43324	2	2	0.3012	no
gi|641572162|gb|KK855882.1|	54514-15658	12	38	2	3	0.959676	0.4618	no
gi|641572162|gb|KK855882.1|	16102-20903	12	38	13	6	-1	0.4687	no
gi|641572162|gb|KK855882.1|	61384-16210	12	38	0.814215	1	0.338002	0.8352	no
gi|641572162|gb|KK855882.1|	64630-16594	12	38	0.698064	5	3	0.07015	no
gi|641572162|gb|KK855882.1|	70963-17158	12	38	1	3	1	0.45675	no
gi|641572162|gb|KK855882.1|	72979-17377	12	38	2	1	-0.497043	0.7427	no
gi|641572162|gb|KK855882.1|	85944-19505	12	38	175	186	0.0852421	0.9552	no
gi|641572162|gb|KK855882.1|	95164-19791	12	38	23	41	0.829199	0.70615	no
gi|641572162|gb|KK855882.1|	03989-20498	12	38	20	46	1	0.47365	no
gi|641572162|gb|KK855882.1|	10745-21163	12	38	20	34	0.755886	0.54635	no
gi|641572162|gb|KK855882.1|	20266-22438	12	38	28	49	0.78461	0.7122	no
gi|641572162|gb|KK855882.1|	28541-22909	12	38	144.08	264	0.874374	0.69125	no
gi|641572162|gb|KK855882.1|	22918-23235	12	38	14	5	-1	0.34045	no
gi|641572162|gb|KK855882.1|	31423-23179	12	38	40	57	0.523568	0.67655	no
gi|641572162|gb|KK855882.1|	32744-23299	12	38	14	15	0.10685	0.9451	no
gi|641572162|gb|KK855882.1|	34388-23606	12	38	414	810	0.970446	0.57065	no

gi|641572162|gb|KK855882.1|	:38124-23848	12	38	60	118	0.974044	0.4347	no
gi|641572162|gb|KK855882.1|	31280-32634	12	38	0.488842	1	2	0.34655	no
gi|641572162|gb|KK855882.1|	32747-33326	12	38	1	1	-0.402995	0.82205	no
gi|641572162|gb|KK855882.1|	36232-36798	12	38	39	11	-2	0.16235	no
gi|641572162|gb|KK855882.1|	37046-37946	12	38	5	2	-1	0.3847	no
gi|641572162|gb|KK855882.1|	380-827	12	38	3	0.420589	-3	0.2348	no
gi|641572162|gb|KK855882.1|	38097-38385	12	38	72	52	-0.463714	0.70955	no
gi|641572162|gb|KK855882.1|	38602-42416	12	38	0.76307	0.328649	-1	1	no
gi|641572162|gb|KK855882.1|	43763-50626	12	38	11	10	-0.174705	0.9357	no
gi|641572162|gb|KK855882.1|	50733-52904	12	38	0.573391	0.71073	0.309783	1	no
gi|641572162|gb|KK855882.1|	59594-60498	12	38	0.89914	2	1	0.3864	no
gi|641572162|gb|KK855882.1|	61325-63964	12	38	4	3	-0.53144	0.6777	no
gi|641572162|gb|KK855882.1|	68019-69179	12	38	0.513754	0.893787	0.798855	0.63025	no
gi|641572162|gb|KK855882.1|	72304-72841	12	38	3	2	-0.989792	0.57205	no
gi|641572162|gb|KK855882.1|	77202-77504	12	38	51	40	-0.347745	0.78645	no
gi|641572162|gb|KK855882.1|	79811-80287	12	38	7	6	-0.359463	0.7929	no
gi|641572162|gb|KK855882.1|	82962-83187	12	38	95	65	-0.544996	0.67785	no
gi|641572162|gb|KK855882.1|	8862-9643	12	38	40	15	-1	0.2595	no
gi|641572162|gb|KK855882.1|	95042-97026	12	38	0.633335	0.724488	0.193993	1	no
gi|641572162|gb|KK855882.1|	9713-10705	12	38	0.622087	2	1	0.39245	no
gi|641572162|gb|KK855882.1|	97278-98183	12	38	0.897873	1	0.604014	0.6902	no
gi|641572162|gb|KK855882.1|	)9184-104075	12	38	0.512662	1	1	0.4007	no
gi|641572166|gb|KK855881.1|	.82631-18838	11	41	2	4	0.945012	0.45615	no
gi|641572166|gb|KK855881.1|	.88615-19113	11	41	2	5	1	0.37895	no
gi|641572166|gb|KK855881.1|	.91255-19227	11	41	0.86376	1	0.605128	0.67795	no
gi|641572166|gb|KK855881.1|	.92335-19327	11	41	2	2	0.217338	0.88205	no
gi|641572166|gb|KK855881.1|	.93519-19471	11	41	2	8	2	0.0992	no
gi|641572166|gb|KK855881.1|	.08399-20877	11	41	0	7	inf	0.00375	no
gi|641572166|gb|KK855881.1|	.17387-21755	11	41	0	91	inf	0.0025	yes
gi|641572170|gb|KK855880.1|	.01901-10385	10	34	1	0.79958	-0.44548	0.7561	no

gi|641572170|gb|KK855880.1|	.05237-10644	10	34	0.627374	0.212272	-2	1	no
gi|641572170|gb|KK855880.1|	.08381-10939	10	34	0.860611	0.130911	-3	1	no
gi|641572170|gb|KK855880.1|	.15325-11713	10	34	0.831585	0.266959	-2	1	no
gi|641572170|gb|KK855880.1|	.17589-11889	10	34	2	0.386979	-2	0.1625	no
gi|641572170|gb|KK855880.1|	.19770-12048	10	34	4	1	-1	0.36985	no
gi|641572170|gb|KK855880.1|	.40879-14135	10	34	2	0	-inf	0.01175	no
gi|641572170|gb|KK855880.1|	.51353-15196	10	34	1	1	0.0132741	0.91115	no
gi|641572170|gb|KK855880.1|	62195-64026	10	34	0.173144	0.660071	2	1	no
gi|641572170|gb|KK855880.1|	65571-65894	10	34	2	6	2	0.41725	no
gi|641572170|gb|KK855880.1|	67218-67920	10	34	0.279429	2	3	0.27515	no
gi|641572170|gb|KK855880.1|	80094-80824	10	34	1	0.200873	-3	0.2381	no
gi|641572170|gb|KK855880.1|	94124-99074	10	34	29	9	-2	0.4432	no
gi|641572170|gb|KK855880.1|	99770-10126	10	34	12	3	-2	0.11965	no
gi|641572177|gb|KK855878.1|	.03305-10357	10	35	13	36	1	0.30795	no
gi|641572177|gb|KK855878.1|	.05493-10599	10	35	49	82	0.747327	0.548	no
gi|641572177|gb|KK855878.1|	.07584-10902	10	35	13	10	-0.405249	0.7441	no
gi|641572177|gb|KK855878.1|	10845-13255	10	35	0.479544	0.926869	0.950703	0.51705	no
gi|641572177|gb|KK855878.1|	.10701-11085	10	35	23	87	2	0.3223	no
gi|641572177|gb|KK855878.1|	.10919-11532	10	35	5	2	-1	0.40665	no
gi|641572177|gb|KK855878.1|	.15464-11673	10	35	6	2	-2	0.14215	no
gi|641572177|gb|KK855878.1|	.23596-12437	10	35	1	0.36765	-2	0.3217	no
gi|641572177|gb|KK855878.1|	.24729-12577	10	35	3	2	-1	0.45745	no
gi|641572177|gb|KK855878.1|	.28478-12959	10	35	2	2	-0.540945	0.69675	no
gi|641572177|gb|KK855878.1|	.29989-13114	10	35	2	0.11207	-4	0.1977	no
gi|641572177|gb|KK855878.1|	.31248-13217	10	35	0.963691	0	-inf	0.01175	no
gi|641572177|gb|KK855878.1|	13831-19125	10	35	22	23	0.0430782	0.98305	no
gi|641572177|gb|KK855878.1|	.68235-16915	10	35	0	29	inf	5.00E-05	yes
gi|641572177|gb|KK855878.1|	.71197-17161	10	35	0	19	inf	5.00E-05	yes
gi|641572177|gb|KK855878.1|	22172-22436	10	35	55	38	-0.535816	0.6842	no
gi|641572177|gb|KK855878.1|	23829-24243	10	35	2	3	0.849358	0.62755	no

gi|641572177|gb|KK855878.1|	28001-28211	10	35	147	67	-1	0.3936	no
gi|641572177|gb|KK855878.1|	32541-34031	10	35	15	7	-1	0.40995	no
gi|641572177|gb|KK855878.1|	34202-35630	10	35	0.861557	0.612602	-0.491996	1	no
gi|641572177|gb|KK855878.1|	39578-40778	10	35	19	28	0.541113	0.6683	no
gi|641572177|gb|KK855878.1|	44334-44775	10	35	0	6	inf	0.0025	yes
gi|641572177|gb|KK855878.1|	5152-5878	10	35	5	8	0.864261	0.51215	no
gi|641572177|gb|KK855878.1|	52244-52538	10	35	4	5	0.302832	0.8614	no
gi|641572177|gb|KK855878.1|	52971-54589	10	35	0.597198	0.758609	0.345146	1	no
gi|641572177|gb|KK855878.1|	55060-57927	10	35	0.424018	2	2	0.1939	no
gi|641572177|gb|KK855878.1|	66147-66528	10	35	15	71	2	0.09485	no
gi|641572177|gb|KK855878.1|	76518-77153	10	35	0.160955	46	8	0.10435	no
gi|641572177|gb|KK855878.1|	86288-86546	10	35	17	68	2	0.1594	no
gi|641572177|gb|KK855878.1|	88050-88443	10	35	28	102.11	2	0.15065	no
gi|641572177|gb|KK855878.1|	90302-92172	10	35	18	65	2	0.2777	no
gi|641572177|gb|KK855878.1|	92293-92984	10	35	6	22	2	0.17485	no
gi|641572177|gb|KK855878.1|	93162-94126	10	35	2	7	2	0.17585	no
gi|641572177|gb|KK855878.1|	95320-96170	10	35	20	80	2	0.25875	no
gi|641572177|gb|KK855878.1|	97359-97809	10	35	8	15	0.991765	0.46705	no
gi|641572177|gb|KK855878.1|	99947-10029[illegible]	10	35	29	46	0.686042	0.5898	no
gi|641572181|gb|KK855877.1|	47352-14810	11	45	0.505087	6	4	0.1278	no
gi|641572181|gb|KK855877.1|	48246-14865	11	45	0.649365	4	3	0.2991	no
gi|641572181|gb|KK855877.1|	62151-16247	11	45	13	6	-1	0.469	no
gi|641572181|gb|KK855877.1|	62864-16465	11	45	1	0.540247	-1	0.339	no
gi|641572181|gb|KK855877.1|	73369-17355	11	45	69	46	-0.602722	0.6724	no
gi|641572181|gb|KK855877.1|	79410-80396	11	45	0.268632	0.953272	2	0.3323	no
gi|641572185|gb|KK855876.1|	00647-10176	8.2	36	0.153136	69	9	0.2345	no
gi|641572185|gb|KK855876.1|	03452-10372	8.2	36	2	201	6	0.1347	no
gi|641572185|gb|KK855876.1|	06698-10789	8.2	36	0.140803	217	11	0.23275	no
gi|641572185|gb|KK855876.1|	26349-12675	8.2	36	4	0.498307	-3	0.2348	no
gi|641572185|gb|KK855876.1|	29311-13195	8.2	36	87	3	-5	0.02765	no

gi|641572185|gb|KK855876.1|	35637-13607	8.2	36	3	0	-inf	0.01	no
gi|641572185|gb|KK855876.1|	36216-14140	8.2	36	85	7	-4	0.1274	no
gi|641572185|gb|KK855876.1|	42505-14325	8.2	36	2	2	0.016816	0.9597	no
gi|641572185|gb|KK855876.1|	45292-14557	8.2	36	21	15	-0.437604	0.75195	no
gi|641572185|gb|KK855876.1|	46442-14714	8.2	36	1	0.638107	-0.814381	0.6492	no
gi|641572185|gb|KK855876.1|	48739-14898	8.2	36	7	6	-0.255904	0.88385	no
gi|641572185|gb|KK855876.1|	51371-15188	8.2	36	3	2	-0.505612	0.73985	no
gi|641572185|gb|KK855876.1|	52477-15279	8.2	36	92	80	-0.19588	0.87625	no
gi|641572185|gb|KK855876.1|	90802-91251	8.2	36	0	10	inf	0.00115	yes
gi|641572185|gb|KK855876.1|	95881-96377	8.2	36	0	119	inf	0.0007	yes
gi|641572185|gb|KK855876.1|	97476-98226	8.2	36	0	3	inf	0.00135	yes
gi|641572185|gb|KK855876.1|	98527-99624	8.2	36	0	25	inf	5.00E-05	yes
gi|641572191|gb|KK855875.1|	02617-10282	9.7	43	10	15	0.536365	0.7589	no
gi|641572191|gb|KK855875.1|	59676-16002	9.7	43	0	23	inf	0.00035	yes
gi|641572191|gb|KK855875.1|	18818-19013	9.7	43	21	54	1	0.383	no
gi|641572191|gb|KK855875.1|	22215-22421	9.7	43	23	30	0.407839	0.79325	no
gi|641572191|gb|KK855875.1|	35330-35616	9.7	43	9	23	1	0.35775	no
gi|641572191|gb|KK855875.1|	520-1760	9.7	43	3	6	1	0.4044	no
gi|641572191|gb|KK855875.1|	57700-59561	9.7	43	0	1072.73	inf	5.00E-05	yes
gi|641572191|gb|KK855875.1|	65336-65581	9.7	43	9	20	1	0.4398	no
gi|641572191|gb|KK855875.1|	71210-71471	9.7	43	7	9	0.363677	0.8128	no
gi|641572191|gb|KK855875.1|	7407-7870	9.7	43	3	6	1	0.4764	no
gi|641572191|gb|KK855875.1|	88910-89128	9.7	43	9	11	0.276217	0.8647	no
gi|641572191|gb|KK855875.1|	91164-91502	9.7	43	0.955671	12	4	0.24575	no
gi|641572195|gb|KK855874.1|	47771-48386	8.8	38	0.505793	2	2	0.256	no
gi|641572195|gb|KK855874.1|	50652-51888	8.8	38	0.680337	2	1	0.4325	no
gi|641572195|gb|KK855874.1|	85121-86492	8.8	38	0.180665	1	3	0.1666	no
gi|641572195|gb|KK855874.1|	86543-88164	8.8	38	0.347633	1	2	0.2786	no
gi|641572195|gb|KK855874.1|	88742-89826	8.8	38	0.796698	0.606169	-0.394313	1	no
gi|641572195|gb|KK855874.1|	91323-91732	8.8	38	3	1	-0.829248	0.64915	no

gi|641572195|gb|KK855874.1|	92604-93745	8.8	38	0.823547	0.797708	-0.0459897	1	no
gi|641572195|gb|KK855874.1|	93819-94861	8.8	38	0.668973	1	0.775283	0.62605	no
gi|641572195|gb|KK855874.1|	94990-97577	8.8	38	6	11	0.844693	0.50025	no
gi|641572199|gb|KK855873.1|	.02726-10324	8.1	36	9	0.666973	-4	0.1398	no
gi|641572199|gb|KK855873.1|	.04530-10539	8.1	36	3	0.161115	-4	0.1933	no
gi|641572199|gb|KK855873.1|	.10941-11113	8.1	36	43	0	-inf	0.00435	no
gi|641572199|gb|KK855873.1|	.17337-11753	8.1	36	82	10	-3	0.1243	no
gi|641572199|gb|KK855873.1|	.47036-14721	8.1	36	59	0	-inf	0.0059	no
gi|641572199|gb|KK855873.1|	22133-22792	8.1	36	20	23	0.246611	0.84275	no
gi|641572199|gb|KK855873.1|	23282-23742	8.1	36	31	58	0.934795	0.46415	no
gi|641572199|gb|KK855873.1|	24889-25273	8.1	36	10	24	1	0.3414	no
gi|641572199|gb|KK855873.1|	68307-68775	8.1	36	0.516862	9	4	0.23975	no
gi|641572199|gb|KK855873.1|	68870-71564	8.1	36	2	0.864942	-0.82299	0.54535	no
gi|641572199|gb|KK855873.1|	72235-74128	8.1	36	4	0.381614	-3	0.0575	no
gi|641572199|gb|KK855873.1|	85170-89569	8.1	36	3	1	-1	0.32035	no
gi|641572199|gb|KK855873.1|	91449-91810	8.1	36	7	7	-0.0485477	0.9628	no
gi|641572199|gb|KK855873.1|	92852-94358	8.1	36	59	60	0.0202156	0.9916	no
gi|641572199|gb|KK855873.1|	9544-10621	8.1	36	1	0	-inf	0.0056	no
gi|641572199|gb|KK855873.1|	97536-98102	8.1	36	8	0.865331	-3	0.11415	no
gi|641572199|gb|KK855873.1|	99807-10010[illegible]	8.1	36	49	7	-3	0.0781	no
gi|641572203|gb|KK855872.1|	.02175-10328	11	41	1	2	0.218859	0.882	no
gi|641572203|gb|KK855872.1|	.26776-12849	11	41	0.696559	1	0.949495	0.51705	no
gi|641572203|gb|KK855872.1|	.56937-15736	11	41	3	0.457202	-3	0.271	no
gi|641572203|gb|KK855872.1|	59429-60165	11	41	0.130939	2	4	0.1276	no
gi|641572203|gb|KK855872.1|	66979-67432	11	41	2	4	0.58991	0.6902	no
gi|641572203|gb|KK855872.1|	73956-74925	11	41	0.274534	1	2	0.25595	no
gi|641572203|gb|KK855872.1|	92292-93178	11	41	1	1	0.314279	0.84265	no
gi|641572203|gb|KK855872.1|	96612-97766	11	41	0.738527	0.562098	-0.393828	1	no
gi|641572203|gb|KK855872.1|	98303-10008[illegible]	11	41	0.891893	1	0.286684	0.83825	no
gi|641572207|gb|KK855871.1|	.00684-10145	10	39	1	2	0.602103	0.6902	no

gi|641572207|gb|KK855871.1|	01937-10272	10	39	17	18	0.080595	0.9498	no
gi|641572207|gb|KK855871.1|	05208-10585	10	39	3	5	0.599348	0.66715	no
gi|641572207|gb|KK855871.1|	14942-11525	10	39	4	22	2	0.15	no
gi|641572207|gb|KK855871.1|	24693-12619	10	39	0.271754	0.911096	2	0.3084	no
gi|641572207|gb|KK855871.1|	32258-13248	10	39	43	113	1	0.3046	no
gi|641572207|gb|KK855871.1|	36732-13742	10	39	9	11	0.215179	0.86585	no
gi|641572207|gb|KK855871.1|	37585-13798	10	39	5	6	0.361912	0.8077	no
gi|641572207|gb|KK855871.1|	39261-14048	10	39	0.620652	2	2	0.33265	no
gi|641572207|gb|KK855871.1|	45899-14621	10	39	12	16	0.426039	0.7574	no
gi|641572207|gb|KK855871.1|	46327-15245	10	39	2	1	-0.530321	0.68095	no
gi|641572207|gb|KK855871.1|	52581-15545	10	39	0.793549	0.807512	0.0251645	1	no
gi|641572207|gb|KK855871.1|	55678-15827	10	39	2	0.991859	-0.738055	0.57035	no
gi|641572207|gb|KK855871.1|	20021-21143	10	39	0.152806	1	3	0.2672	no
gi|641572207|gb|KK855871.1|	22649-22820	10	39	9	340	5	0.2331	no
gi|641572207|gb|KK855871.1|	25466-26603	10	39	0.526301	175	8	0.05345	no
gi|641572207|gb|KK855871.1|	27454-27712	10	39	95	95	-0.00188888	0.9934	no
gi|641572207|gb|KK855871.1|	32959-33883	10	39	25	26	0.076819	0.95	no
gi|641572207|gb|KK855871.1|	34653-36899	10	39	22	15	-0.510243	0.74605	no
gi|641572207|gb|KK855871.1|	38928-39859	10	39	0.577428	0.585223	0.0193449	1	no
gi|641572207|gb|KK855871.1|	39917-41074	10	39	1	3	1	0.38305	no
gi|641572207|gb|KK855871.1|	43078-43736	10	39	6	9	0.561125	0.66695	no
gi|641572207|gb|KK855871.1|	48901-49306	10	39	6	11	0.874855	0.5312	no
gi|641572207|gb|KK855871.1|	54329-55042	10	39	0.820498	1	0.823341	0.6232	no
gi|641572207|gb|KK855871.1|	63081-65006	10	39	4	8	0.965061	0.445	no
gi|641572207|gb|KK855871.1|	85372-86147	10	39	1	0.185399	-3	0.2348	no
gi|641572207|gb|KK855871.1|	9112-9549	10	39	6	38	3	0.0718	no
gi|641572207|gb|KK855871.1|	99224-99648	10	39	2	1	-0.635057	0.71175	no
gi|641572211|gb|KK855870.1|	17827-18692	9.2	37	3	0.963818	-2	0.27545	no
gi|641572215|gb|KK855869.1|	39461-14032	11	22	2	2	-0.460665	0.74915	no
gi|641572215|gb|KK855869.1|	67273-16836	11	22	0.867581	0.240048	-2	1	no

gi|641572219|gb|KK855868.1|	25096-26062	9.8	35	3	0	-inf	0.0013	yes
gi|641572219|gb|KK855868.1|	29555-29947	9.8	35	24	11	-1	0.38705	no
gi|641572219|gb|KK855868.1|	36532-36832	9.8	35	26	14	-0.918054	0.50475	no
gi|641572219|gb|KK855868.1|	40864-41920	9.8	35	24	12	-0.994598	0.43355	no
gi|641572219|gb|KK855868.1|	43141-43447	9.8	35	44	22	-0.996777	0.45425	no
gi|641572219|gb|KK855868.1|	46490-46804	9.8	35	17	15	-0.166313	0.90595	no
gi|641572219|gb|KK855868.1|	53759-53923	9.8	35	38	47	0.288385	0.86665	no
gi|641572219|gb|KK855868.1|	55132-56304	9.8	35	3	1	-1	0.44225	no
gi|641572219|gb|KK855868.1|	60719-62192	9.8	35	2	0.759981	-1	0.31035	no
gi|641572219|gb|KK855868.1|	62623-68729	9.8	35	10	15	0.565308	0.7982	no
gi|641572219|gb|KK855868.1|	70139-70380	9.8	35	29	48	0.706703	0.6036	no
gi|641572219|gb|KK855868.1|	72512-74085	9.8	35	1	2	0.608044	0.6649	no
gi|641572219|gb|KK855868.1|	76667-77457	9.8	35	1	4	2	0.2772	no
gi|641572219|gb|KK855868.1|	86474-87025	9.8	35	2	3	0.76575	0.6262	no
gi|641572223|gb|KK855867.1|	42116-14238	9.8	37	11	14	0.317237	0.83625	no
gi|641572223|gb|KK855867.1|	20663-20820	9.8	37	7	442	6	0.10495	no
gi|641572223|gb|KK855867.1|	29006-29474	9.8	37	0.775293	3	2	0.2812	no
gi|641572223|gb|KK855867.1|	47473-47943	9.8	37	52	107	1	0.4123	no
gi|641572223|gb|KK855867.1|	67836-68316	9.8	37	145	336	1	0.59435	no
gi|641572227|gb|KK855866.1|	46012-47156	7	32	0	3	inf	0.00115	yes
gi|641572227|gb|KK855866.1|	54955-55692	7	32	0	3	inf	0.0025	yes
gi|641572227|gb|KK855866.1|	59326-60596	7	32	0.065881	1	4	0.1136	no
gi|641572227|gb|KK855866.1|	92592-93285	7	32	5	3	-0.70207	0.6041	no
gi|641572230|gb|KK855865.1|	06723-10733	9.8	38	0.504596	3	2	0.21375	no
gi|641572230|gb|KK855865.1|	53142-15370	9.8	38	0.771339	2	2	0.3613	no
gi|641572230|gb|KK855865.1|	2704-2914	9.8	38	62	18	-2	0.24285	no
gi|641572230|gb|KK855865.1|	29722-29982	9.8	38	6	5	-0.251361	0.88385	no
gi|641572230|gb|KK855865.1|	3239-3696	9.8	38	34	9	-2	0.1631	no
gi|641572230|gb|KK855865.1|	8999-9219	9.8	38	39	15	-1	0.3671	no
gi|641572235|gb|KK855864.1|	23102-12501	8.9	39	0.660668	2	2	0.28725	no

gi|641572235|gb|KK855864.1|	36179-13646	8.9	39	25	12	-1	0.44575	no
gi|641572235|gb|KK855864.1|	83218-84692	8.9	39	0.442967	0.675012	0.607712	1	no
gi|641572239|gb|KK855863.1|	01303-10173	11	43	0	48	inf	5.00E-05	yes
gi|641572239|gb|KK855863.1|	02700-10303	11	43	0	144	inf	5.00E-05	yes
gi|641572239|gb|KK855863.1|	04828-10531	11	43	0	13	inf	0.0004	yes
gi|641572239|gb|KK855863.1|	10741-11173	11	43	42	19	-1	0.3722	no
gi|641572239|gb|KK855863.1|	32790-13394	11	43	0.958086	0.224373	-2	0.26805	no
gi|641572239|gb|KK855863.1|	48302-14864	11	43	16	9	-0.810734	0.56975	no
gi|641572239|gb|KK855863.1|	50068-15041	11	43	12	8	-0.592236	0.6688	no
gi|641572239|gb|KK855863.1|	50720-15658	11	43	0.948419	2	0.94833	0.4543	no
gi|641572239|gb|KK855863.1|	58766-16033	11	43	0.668582	0.862269	0.367033	1	no
gi|641572239|gb|KK855863.1|	61414-16196	11	43	3	3	-0.169531	0.9115	no
gi|641572239|gb|KK855863.1|	62130-16322	11	43	0.156868	2	3	0.25475	no
gi|641572239|gb|KK855863.1|	63411-16405	11	43	0.157736	4	5	0.1123	no
gi|641572239|gb|KK855863.1|	70735-17189	11	43	5	3	-0.828727	0.532	no
gi|641572239|gb|KK855863.1|	72591-17493	11	43	1	1	-0.138881	0.9204	no
gi|641572239|gb|KK855863.1|	75064-17739	11	43	0.497982	1	1	0.33615	no
gi|641572239|gb|KK855863.1|	77582-18683	11	43	15	10	-0.59986	0.7787	no
gi|641572239|gb|KK855863.1|	48972-50066	11	43	0.551483	0.359673	-0.616632	1	no
gi|641572239|gb|KK855863.1|	58531-58762	11	43	15	26	0.767463	0.60975	no
gi|641572239|gb|KK855863.1|	64680-64920	11	43	5	12	1	0.44765	no
gi|641572239|gb|KK855863.1|	69605-69828	11	43	11	29	1	0.3989	no
gi|641572239|gb|KK855863.1|	77827-78777	11	43	6	9	0.469157	0.7159	no
gi|641572239|gb|KK855863.1|	84157-86266	11	43	0.517971	2	2	0.2071	no
gi|641572239|gb|KK855863.1|	86818-92460	11	43	22	25	0.222891	0.9147	no
gi|641572243|gb|KK855862.1|	08730-10948	7	39	1	0.571864	-1	0.50885	no
gi|641572247|gb|KK855861.1|	05887-10705	10	37	0.730149	2	1	0.3418	no
gi|641572247|gb|KK855861.1|	07168-10770	10	37	2	2	0.180084	0.9106	no
gi|641572247|gb|KK855861.1|	07864-10897	10	37	1	0.353025	-2	0.33295	no
gi|641572247|gb|KK855861.1|	09842-11114	10	37	1	1	-0.181501	0.89715	no

gi|641572247|gb|KK855861.1|	12039-11408	10	37	2	4	1	0.36555	no
gi|641572247|gb|KK855861.1|	15453-11793	10	37	1	2	0.64292	0.62075	no
gi|641572247|gb|KK855861.1|	20260-12126	10	37	1	3	0.926941	0.51965	no
gi|641572247|gb|KK855861.1|	12238-13650	10	37	0.698309	0.354646	-0.977485	1	no
gi|641572247|gb|KK855861.1|	27050-12905	10	37	8	11	0.499451	0.68945	no
gi|641572247|gb|KK855861.1|	29946-13105	10	37	18	25	0.455935	0.70595	no
gi|641572247|gb|KK855861.1|	31393-13322	10	37	0.520386	0.33064	-0.65432	1	no
gi|641572247|gb|KK855861.1|	36766-13793	10	37	0.798225	1	0.73181	0.63585	no
gi|641572247|gb|KK855861.1|	43033-14318	10	37	84	24	-2	0.3266	no
gi|641572247|gb|KK855861.1|	45795-14686	10	37	13	11	-0.308997	0.8133	no
gi|641572247|gb|KK855861.1|	49781-15107	10	37	35	125	2	0.4159	no
gi|641572247|gb|KK855861.1|	55477-15752	10	37	236	304	0.360934	0.83665	no
gi|641572247|gb|KK855861.1|	15844-17207	10	37	0.848881	0.92369	0.121847	0.9357	no
gi|641572247|gb|KK855861.1|	61135-16191	10	37	6	1	-2	0.18095	no
gi|641572247|gb|KK855861.1|	64375-16464	10	37	9	3	-1	0.4189	no
gi|641572247|gb|KK855861.1|	70045-17095	10	37	5	1	-2	0.15105	no
gi|641572247|gb|KK855861.1|	74978-17513	10	37	78	9	-3	0.23015	no
gi|641572247|gb|KK855861.1|	19950-20535	10	37	10	8	-0.376767	0.77685	no
gi|641572247|gb|KK855861.1|	25880-27021	10	37	1	2	0.676476	0.63115	no
gi|641572247|gb|KK855861.1|	27975-28295	10	37	12	14	0.199991	0.8868	no
gi|641572247|gb|KK855861.1|	28643-29256	10	37	1	2	0.820629	0.62335	no
gi|641572247|gb|KK855861.1|	29445-30469	10	37	2	2	0.020244	0.9647	no
gi|641572247|gb|KK855861.1|	32134-33641	10	37	16	31	0.942248	0.56795	no
gi|641572247|gb|KK855861.1|	35689-36321	10	37	17	30	0.776241	0.53045	no
gi|641572247|gb|KK855861.1|	36807-40354	10	37	4	6	0.47522	0.69805	no
gi|641572247|gb|KK855861.1|	41520-43952	10	37	0.949654	0.869465	-0.127273	0.92735	no
gi|641572247|gb|KK855861.1|	44061-45297	10	37	9	9	0.0600222	0.96095	no
gi|641572247|gb|KK855861.1|	48943-49837	10	37	4	4	-0.133082	0.92065	no
gi|641572247|gb|KK855861.1|	51075-51679	10	37	1	2	0.59793	0.69715	no
gi|641572247|gb|KK855861.1|	60508-61097	10	37	3	2	-0.402611	0.7911	no

gi|641572247|gb|KK855861.1|	63217-64486	10	37	0.72537	0.401699	-0.852601	1	no
gi|641572247|gb|KK855861.1|	6382-6867	10	37	8	4	-0.822804	0.5599	no
gi|641572247|gb|KK855861.1|	65826-68318	10	37	2	3	0.307199	0.80425	no
gi|641572247|gb|KK855861.1|	68448-69740	10	37	2	3	0.606946	0.66025	no
gi|641572247|gb|KK855861.1|	69956-71000	10	37	2	3	0.550926	0.6906	no
gi|641572247|gb|KK855861.1|	71101-76494	10	37	2	2	0.673663	0.5965	no
gi|641572247|gb|KK855861.1|	77712-81243	10	37	2	2	0.0332548	0.9787	no
gi|641572247|gb|KK855861.1|	82950-83184	10	37	47	79	0.757831	0.55745	no
gi|641572247|gb|KK855861.1|	83406-84523	10	37	0.921808	0.116912	-3	0.2348	no
gi|641572247|gb|KK855861.1|	85433-85957	10	37	9	10	0.19116	0.8835	no
gi|641572247|gb|KK855861.1|	87563-88701	10	37	0.525743	0.914555	0.798712	0.63025	no
gi|641572247|gb|KK855861.1|	8786-10323	10	37	2	0.643413	-1	0.3857	no
gi|641572247|gb|KK855861.1|	89599-90775	10	37	0.505372	1	1	0.48415	no
gi|641572247|gb|KK855861.1|	93593-94232	10	37	8	8	0.0844834	0.9481	no
gi|641572247|gb|KK855861.1|	97046-98128	10	37	11	11.3	-0.0150406	0.99075	no
gi|641572247|gb|KK855861.1|	98197-99894	10	37	2	3	0.40404	0.7636	no
gi|641572251|gb|KK855860.1|	0-649	10	19	0.780009	1	0.599348	0.70505	no
gi|641572251|gb|KK855860.1|	20915-12242	10	19	0.429857	1	2	0.2897	no
gi|641572251|gb|KK855860.1|	23856-12511	10	19	3	2	-0.71517	0.5942	no
gi|641572251|gb|KK855860.1|	33145-13347	10	19	3	4	0.311185	0.86115	no
gi|641572251|gb|KK855860.1|	34071-13429	10	19	17	8	-1	0.48735	no
gi|641572251|gb|KK855860.1|	35670-13618	10	19	3	4	0.387305	0.79005	no
gi|641572251|gb|KK855860.1|	39724-14086	10	19	0.978456	1	0.490577	0.7378	no
gi|641572251|gb|KK855860.1|	42966-14366	10	19	4	1	-2	0.26015	no
gi|641572251|gb|KK855860.1|	55912-15623	10	19	26	20	-0.341468	0.79345	no
gi|641572251|gb|KK855860.1|	57244-15811	10	19	0.842007	0.799622	-0.0745144	1	no
gi|641572251|gb|KK855860.1|	59848-16006	10	19	22	18	-0.268103	0.8542	no
gi|641572251|gb|KK855860.1|	61629-16184	10	19	11	26	1	0.4616	no
gi|641572251|gb|KK855860.1|	64037-16482	10	19	9	8	-0.159447	0.9025	no
gi|641572251|gb|KK855860.1|	65902-16607	10	19	36	19	-0.900898	0.6344	no

gi|641572251|gb|KK855860.1|	73742-17428	10	19	8	12	0.523493	0.6888	no
gi|641572251|gb|KK855860.1|	74942-17668	10	19	14	16	0.152028	0.89985	no
gi|641572251|gb|KK855860.1|	79342-17953	10	19	51	27	-0.931267	0.53595	no
gi|641572251|gb|KK855860.1|	1880-3027	10	19	0.818355	3	2	0.23885	no
gi|641572251|gb|KK855860.1|	28378-28783	10	19	6	4	-0.502115	0.7418	no
gi|641572251|gb|KK855860.1|	35994-36529	10	19	3	7	1	0.42885	no
gi|641572251|gb|KK855860.1|	40329-40929	10	19	6	13	1	0.38865	no
gi|641572251|gb|KK855860.1|	41072-41832	10	19	2	1	-0.398111	0.8086	no
gi|641572251|gb|KK855860.1|	43174-44304	10	19	0.605992	3	2	0.1998	no
gi|641572251|gb|KK855860.1|	48291-49525	10	19	0.749811	2	1	0.44465	no
gi|641572251|gb|KK855860.1|	49643-50669	10	19	1	2	0.927152	0.53445	no
gi|641572251|gb|KK855860.1|	50994-51829	10	19	1	1	-0.0489707	0.94545	no
gi|641572251|gb|KK855860.1|	58217-58534	10	19	3	11	2	0.2392	no
gi|641572251|gb|KK855860.1|	59526-59893	10	19	3	4	0.358011	0.83635	no
gi|641572251|gb|KK855860.1|	64154-65263	10	19	1	2	0.0583807	0.96485	no
gi|641572251|gb|KK855860.1|	67427-71646	10	19	1	4	2	0.19	no
gi|641572251|gb|KK855860.1|	95538-96109	10	19	2	1	-0.251313	0.88485	no
gi|641572251|gb|KK855860.1|	96216-96735	10	19	3	3	0.0637367	0.9551	no
gi|641572256|gb|KK855859.1|	11562-11188	9.5	38	4	3	-0.427406	0.7868	no
gi|641572256|gb|KK855859.1|	25767-12672	9.5	38	0.839886	0.425683	-0.980412	1	no
gi|641572256|gb|KK855859.1|	46612-14697	9.5	38	6	0.6194	-3	0.21195	no
gi|641572256|gb|KK855859.1|	48694-15008	9.5	38	2	2	-0.351958	0.79465	no
gi|641572256|gb|KK855859.1|	52883-15386	9.5	38	0.452875	1	2	0.32945	no
gi|641572256|gb|KK855859.1|	31001-32002	9.5	38	0.615138	0.801955	0.382611	1	no
gi|641572256|gb|KK855859.1|	41102-42058	9.5	38	0.744602	3	2	0.2741	no
gi|641572256|gb|KK855859.1|	42853-43655	9.5	38	0.583502	1	1	0.4595	no
gi|641572256|gb|KK855859.1|	58662-59037	9.5	38	3	1	-1	0.44305	no
gi|641572256|gb|KK855859.1|	66278-67203	9.5	38	1	0.147502	-3	0.22425	no
gi|641572256|gb|KK855859.1|	68300-69602	9.5	38	2	0.584648	-1	0.3694	no
gi|641572256|gb|KK855859.1|	97821-98187	9.5	38	4	3	-0.267744	0.8654	no

gi|641572265|gb|KK855857.1|	14670-11534	10	42	0.878082	2	0.822543	0.62335	no
gi|641572265|gb|KK855857.1|	15756-11694	10	42	1	2	0.513279	0.7255	no
gi|641572265|gb|KK855857.1|	17033-12252	10	42	21	65	2	0.4297	no
gi|641572265|gb|KK855857.1|	22717-12550	10	42	38	125	2	0.4068	no
gi|641572265|gb|KK855857.1|	26984-12946	10	42	0.372207	2	3	0.10465	no
gi|641572265|gb|KK855857.1|	30106-13130	10	42	0.352357	0.643761	0.869488	1	no
gi|641572265|gb|KK855857.1|	48616-14893	10	42	15	16	0.0636921	0.96465	no
gi|641572265|gb|KK855857.1|	49187-14941	10	42	11	15	0.375567	0.8163	no
gi|641572265|gb|KK855857.1|	76858-17727	10	42	3	3	0.22437	0.88235	no
gi|641572265|gb|KK855857.1|	57854-58290	10	42	0.875923	3	2	0.3323	no
gi|641572269|gb|KK855856.1|	71407-72240	9	35	0.333082	1	2	0.3323	no
gi|641572273|gb|KK855855.1|	00496-10158	10	39	43	31	-0.487393	0.76105	no
gi|641572273|gb|KK855855.1|	01812-10221	10	39	63	58	-0.129027	0.921	no
gi|641572273|gb|KK855855.1|	05116-10584	10	39	28.15	15	-0.943555	0.44845	no
gi|641572273|gb|KK855855.1|	06020-10687	10	39	13	9	-0.607553	0.63555	no
gi|641572273|gb|KK855855.1|	07067-10833	10	39	5	3	-0.478084	0.7154	no
gi|641572273|gb|KK855855.1|	08860-11300	10	39	2	1	-0.649398	0.61365	no
gi|641572273|gb|KK855855.1|	13197-11450	10	39	0.824246	0.193144	-2	1	no
gi|641572273|gb|KK855855.1|	14888-11508	10	39	9	68	3	0.15175	no
gi|641572273|gb|KK855855.1|	21060-12221	10	39	0.296648	1	2	0.23615	no
gi|641572273|gb|KK855855.1|	24608-12492	10	39	12	58	2	0.10235	no
gi|641572273|gb|KK855855.1|	36173-13702	10	39	0.21563	1	3	0.2851	no
gi|641572273|gb|KK855855.1|	44336-14515	10	39	30	21	-0.53361	0.6663	no
gi|641572273|gb|KK855855.1|	45423-14714	10	39	4	0.706609	-3	0.08875	no
gi|641572273|gb|KK855855.1|	52743-15294	10	39	43	41	-0.0917392	0.9487	no
gi|641572273|gb|KK855855.1|	62263-16263	10	39	2	7	1	0.3842	no
gi|641572273|gb|KK855855.1|	72186-17318	10	39	1	1	-0.0324447	0.95655	no
gi|641572273|gb|KK855855.1|	73334-17401	10	39	2	2	0.474645	0.75595	no
gi|641572273|gb|KK855855.1|	75403-17725	10	39	32	4	-3	0.11355	no
gi|641572273|gb|KK855855.1|	78095-17881	10	39	6	2	-2	0.249	no

gi|641572273|gb|KK855855.1|	81245-18197	10	39	2	0.605991	-2	0.28145	no
gi|641572273|gb|KK855855.1|	84160-18489	10	39	7	5	-0.587583	0.6561	no
gi|641572273|gb|KK855855.1|	39459-40477	10	39	0.602428	2	2	0.3164	no
gi|641572273|gb|KK855855.1|	42154-43004	10	39	0.973301	2	1	0.49595	no
gi|641572273|gb|KK855855.1|	48710-49366	10	39	2	2	0.33651	0.8184	no
gi|641572273|gb|KK855855.1|	52793-53511	10	39	0	6	inf	0.00075	yes
gi|641572273|gb|KK855855.1|	89395-90446	10	39	0.74468	0.881207	0.242861	0.8798	no
gi|641572273|gb|KK855855.1|	90708-91474	10	39	1	0.753204	-0.857434	0.60675	no
gi|641572273|gb|KK855855.1|	92808-93368	10	39	4	2	-1	0.4862	no
gi|641572273|gb|KK855855.1|	93720-95895	10	39	14	4	-2	0.1397	no
gi|641572273|gb|KK855855.1|	96726-100317	10	39	8	6	-0.509366	0.68315	no
gi|641572281|gb|KK855853.1|	13975-11520	10	42	13	6	-1	0.41595	no
gi|641572281|gb|KK855853.1|	21835-12420	10	42	2	2	-0.468392	0.71835	no
gi|641572281|gb|KK855853.1|	40116-14121	10	42	12	17	0.497399	0.68965	no
gi|641572281|gb|KK855853.1|	57773-15880	10	42	2	1	-0.854157	0.56515	no
gi|641572281|gb|KK855853.1|	21248-22885	10	42	6	3	-1	0.42605	no
gi|641572281|gb|KK855853.1|	33799-34056	10	42	7	5	-0.252363	0.88385	no
gi|641572281|gb|KK855853.1|	83322-84828	10	42	9	4	-1	0.3063	no
gi|641572281|gb|KK855853.1|	99274-100191	10	42	0.882944	0.298256	-2	0.4266	no
gi|641572285|gb|KK855852.1|	10874-11599	9.6	39	2	0.608259	-2	0.40415	no
gi|641572285|gb|KK855852.1|	10832-11119	9.6	39	27	22	-0.291077	0.82395	no
gi|641572285|gb|KK855852.1|	16216-11653	9.6	39	6	7	0.157347	0.91535	no
gi|641572285|gb|KK855852.1|	17098-12006	9.6	39	2	2	0.162747	0.9006	no
gi|641572285|gb|KK855852.1|	11774-13719	9.6	39	0.890247	0.555449	-0.68055	0.63875	no
gi|641572285|gb|KK855852.1|	20472-12085	9.6	39	7	11	0.73519	0.6097	no
gi|641572285|gb|KK855852.1|	22698-12294	9.6	39	29	18	-0.691455	0.62965	no
gi|641572285|gb|KK855852.1|	23866-12582	9.6	39	0.722602	2	1	0.3824	no
gi|641572285|gb|KK855852.1|	26772-12845	9.6	39	21	18	-0.207987	0.86255	no
gi|641572285|gb|KK855852.1|	30395-13217	9.6	39	0.044764	2	5	0.1054	no
gi|641572285|gb|KK855852.1|	14704-15420	9.6	39	0.815777	3	2	0.3067	no

gi|641572285|gb|KK855852.1|	43294-43747	9.6	39	1	3	1	0.4427	no
gi|641572285|gb|KK855852.1|	45480-46283	9.6	39	0.582542	1	0.865656	0.62725	no
gi|641572285|gb|KK855852.1|	71571-71857	9.6	39	1	9	3	0.28625	no
gi|641572293|gb|KK855850.1|	00926-10738	11	38	1	2	0.563298	0.66095	no
gi|641572293|gb|KK855850.1|	08603-10889	11	38	10	3	-2	0.32475	no
gi|641572293|gb|KK855850.1|	11574-11181	11	38	118	64	-0.88616	0.50005	no
gi|641572293|gb|KK855850.1|	13376-11352	11	38	287.99	94	-2	0.2918	no
gi|641572293|gb|KK855850.1|	14069-11491	11	38	7	5	-0.489862	0.7112	no
gi|641572293|gb|KK855850.1|	23272-12357	11	38	19	12	-0.639324	0.65325	no
gi|641572293|gb|KK855850.1|	27151-12808	11	38	12	7	-0.803307	0.53125	no
gi|641572293|gb|KK855850.1|	28242-12843	11	38	67	22	-2	0.29215	no
gi|641572293|gb|KK855850.1|	30880-13140	11	38	0.213653	5	5	0.11025	no
gi|641572293|gb|KK855850.1|	39383-13999	11	38	0.504596	5	3	0.14875	no
gi|641572293|gb|KK855850.1|	40261-14131	11	38	0.995232	4	2	0.22585	no
gi|641572293|gb|KK855850.1|	46031-14844	11	38	0.862789	4	2	0.09875	no
gi|641572293|gb|KK855850.1|	48564-14930	11	38	0.520868	18	5	0.1166	no
gi|641572293|gb|KK855850.1|	54543-15482	11	38	212	124	-0.778464	0.5362	no
gi|641572293|gb|KK855850.1|	61817-16216	11	38	19	27	0.542581	0.68495	no
gi|641572293|gb|KK855850.1|	63026-16391	11	38	0.811836	0.61691	-0.396129	1	no
gi|641572293|gb|KK855850.1|	66175-17026	11	38	21	20	-0.0115522	0.9956	no
gi|641572293|gb|KK855850.1|	70543-17241	11	38	10	11	0.0531072	0.96685	no
gi|641572293|gb|KK855850.1|	73015-17442	11	38	1	2	0.0510597	0.969	no
gi|641572293|gb|KK855850.1|	17881-18463	11	38	0.731672	2	1	0.51315	no
gi|641572293|gb|KK855850.1|	85306-18570	11	38	7	25	2	0.1915	no
gi|641572293|gb|KK855850.1|	87957-18849	11	38	6	22	2	0.1976	no
gi|641572293|gb|KK855850.1|	90204-19051	11	38	2	11	3	0.19065	no
gi|641572293|gb|KK855850.1|	96105-19696	11	38	0.954351	0.805573	-0.244505	0.88495	no
gi|641572293|gb|KK855850.1|	02516-20549	11	38	2	3	0.0476149	0.9719	no
gi|641572293|gb|KK855850.1|	06635-20784	11	38	2	1	-0.444144	0.7504	no
gi|641572293|gb|KK855850.1|	09306-20999	11	38	2	0.435664	-2	0.3216	no

gi|641572293|gb|KK855850.1|	10441-21188	11	38	0.565653	0.689524	0.285685	1	no
gi|641572293|gb|KK855850.1|	11999-21247	11	38	2	5	2	0.28055	no
gi|641572293|gb|KK855850.1|	12688-21393	11	38	18	16	-0.201985	0.87445	no
gi|641572293|gb|KK855850.1|	16983-22060	11	38	2	2	0.0643283	0.9592	no
gi|641572293|gb|KK855850.1|	22521-22450	11	38	2	2	-0.139433	0.92035	no
gi|641572293|gb|KK855850.1|	24733-22609	11	38	0.667549	0.739583	0.147838	1	no
gi|641572293|gb|KK855850.1|	35867-23691	11	38	0.500548	0.88844	0.827767	0.6232	no
gi|641572293|gb|KK855850.1|	38310-23974	11	38	2	4	1	0.30815	no
gi|641572293|gb|KK855850.1|	39802-24237	11	38	1	2	0.641553	0.62505	no
gi|641572293|gb|KK855850.1|	42521-24475	11	38	1	1	0.410131	0.75835	no
gi|641572293|gb|KK855850.1|	56795-58242	11	38	0.565653	0.517143	-0.129353	1	no
gi|641572293|gb|KK855850.1|	59892-60359	11	38	9	0.390675	-4	0.1879	no
gi|641572293|gb|KK855850.1|	60727-71564	11	38	25	23	-0.114043	0.95425	no
gi|641572293|gb|KK855850.1|	71775-72346	11	38	15	13	-0.237306	0.85365	no
gi|641572293|gb|KK855850.1|	75160-75431	11	38	8	15	0.93799	0.5432	no
gi|641572293|gb|KK855850.1|	78173-78525	11	38	2	9	2	0.2384	no
gi|641572293|gb|KK855850.1|	81462-82096	11	38	7	8	0.107053	0.93285	no
gi|641572293|gb|KK855850.1|	83794-84724	11	38	60	51	-0.211839	0.92725	no
gi|641572293|gb|KK855850.1|	90589-94688	11	38	5	7	0.405147	0.7438	no
gi|641572293|gb|KK855850.1|	98911-10072	11	38	4	2	-0.721457	0.57515	no
gi|641572297|gb|KK855849.1|	05315-10919	11	21	46.79	7	-3	0.1248	no
gi|641572297|gb|KK855849.1|	17177-11831	11	21	2	1	-0.326792	0.8148	no
gi|641572297|gb|KK855849.1|	18669-11898	11	21	47	38	-0.297882	0.82035	no
gi|641572297|gb|KK855849.1|	19399-12093	11	21	57	31	-0.900979	0.686	no
gi|641572297|gb|KK855849.1|	27507-12789	11	21	13	5	-1	0.37075	no
gi|641572297|gb|KK855849.1|	28120-12915	11	21	3	1	-2	0.28595	no
gi|641572297|gb|KK855849.1|	29425-13140	11	21	1	0.8476	-0.628018	0.6541	no
gi|641572297|gb|KK855849.1|	31986-13303	11	21	98	2	-6	0.0172	no
gi|641572297|gb|KK855849.1|	34035-13433	11	21	11	4	-1	0.4278	no
gi|641572297|gb|KK855849.1|	35946-13656	11	21	7	2	-2	0.26305	no

gi|641572297|gb|KK855849.1|	13609-13860	11	21	3	13	2	0.26245	no
gi|641572297|gb|KK855849.1|	37844-13865	11	21	49	18	-1	0.40325	no
gi|641572297|gb|KK855849.1|	65410-16622	11	21	1	0.86617	-0.397176	0.81255	no
gi|641572297|gb|KK855849.1|	68691-16940	11	21	2	1	-0.66202	0.6826	no
gi|641572297|gb|KK855849.1|	70718-17893	11	21	1	1	0.374994	0.7674	no
gi|641572297|gb|KK855849.1|	80683-18103	11	21	7	0.662703	-3	0.21195	no
gi|641572297|gb|KK855849.1|	41922-42375	11	21	2	2	0.00494795	0.91115	no
gi|641572297|gb|KK855849.1|	55391-56439	11	21	2	0	-inf	0.00315	yes
gi|641572297|gb|KK855849.1|	56872-59297	11	21	3	0	-inf	0.0001	yes
gi|641572297|gb|KK855849.1|	61135-61476	11	21	175	1	-7	0.1301	no
gi|641572297|gb|KK855849.1|	62065-62611	11	21	38	0.609181	-6	0.1306	no
gi|641572297|gb|KK855849.1|	63549-63868	11	21	127	2	-6	0.1301	no
gi|641572297|gb|KK855849.1|	66003-67023	11	21	28	0.522381	-6	0.0612	no
gi|641572297|gb|KK855849.1|	73105-73505	11	21	96	0	-inf	5.00E-05	yes
gi|641572297|gb|KK855849.1|	77497-77869	11	21	538	3	-8	0.0469	no
gi|641572297|gb|KK855849.1|	81212-86323	11	21	2	0.484016	-2	0.14505	no
gi|641572297|gb|KK855849.1|	93371-94932	11	21	1	0.553123	-1	0.4606	no
gi|641572297|gb|KK855849.1|	95190-97330	11	21	123	1	-6	0.0071	no
gi|641572297|gb|KK855849.1|	97959-98398	11	21	274	3	-7	0.0372	no
gi|641572300|gb|KK855848.1|	04787-10515	12	38	13	1	-4	0.14185	no
gi|641572300|gb|KK855848.1|	09068-11000	12	38	0.766778	0.437145	-0.810698	1	no
gi|641572300|gb|KK855848.1|	12913-11350	12	38	2	0	-inf	0.0075	no
gi|641572300|gb|KK855848.1|	13975-11491	12	38	0.765741	0.727595	-0.0737212	1	no
gi|641572300|gb|KK855848.1|	17378-11861	12	38	0.880209	0.61862	-0.508794	0.7498	no
gi|641572300|gb|KK855848.1|	20407-12158	12	38	3	1	-0.938005	0.50615	no
gi|641572300|gb|KK855848.1|	23706-12426	12	38	2	0.294694	-3	0.2381	no
gi|641572300|gb|KK855848.1|	24431-12536	12	38	1	0.731529	-0.881119	0.5832	no
gi|641572300|gb|KK855848.1|	25723-12663	12	38	1	1	-0.289001	0.85105	no
gi|641572300|gb|KK855848.1|	28930-12954	12	38	2	0.771176	-2	0.33985	no
gi|641572300|gb|KK855848.1|	37705-13930	12	38	2	1	-0.691448	0.63295	no

gi|641572300|gb|KK855848.1|	40249-14158	12	38	2	0.662411	-2	0.2794	no
gi|641572300|gb|KK855848.1|	42059-14343	12	38	5	3	-0.80207	0.5493	no
gi|641572300|gb|KK855848.1|	45688-14608	12	38	4	2	-2	0.40245	no
gi|641572300|gb|KK855848.1|	48591-14997	12	38	13	6	-1	0.4039	no
gi|641572300|gb|KK855848.1|	56572-15731	12	38	4	1	-2	0.24115	no
gi|641572300|gb|KK855848.1|	57511-16115	12	38	2	2	0.129089	0.9189	no
gi|641572300|gb|KK855848.1|	61718-16358	12	38	1	1	0.246233	0.85895	no
gi|641572300|gb|KK855848.1|	67188-16853	12	38	0.367584	0.653273	0.829613	1	no
gi|641572300|gb|KK855848.1|	71956-17254	12	38	11	4	-1	0.28775	no
gi|641572300|gb|KK855848.1|	72950-17324	12	38	0	14	inf	0.0023	yes
gi|641572300|gb|KK855848.1|	75925-17636	12	38	66	38	-0.815676	0.51505	no
gi|641572300|gb|KK855848.1|	82110-18252	12	38	5	8	0.679433	0.6313	no
gi|641572300|gb|KK855848.1|	84840-18534	12	38	3	3	0.455849	0.76285	no
gi|641572300|gb|KK855848.1|	90334-19073	12	38	1	5	2	0.3332	no
gi|641572300|gb|KK855848.1|	92630-19415	12	38	13	10	-0.351152	0.77915	no
gi|641572300|gb|KK855848.1|	94266-19500	12	38	5	6	0.160818	0.8997	no
gi|641572300|gb|KK855848.1|	99903-20033	12	38	2	4	1	0.4611	no
gi|641572300|gb|KK855848.1|	01048-20198	12	38	1	3	1	0.40065	no
gi|641572300|gb|KK855848.1|	04646-20514	12	38	3	8	1	0.42755	no
gi|641572300|gb|KK855848.1|	07496-20890	12	38	0.408691	1	2	0.2786	no
gi|641572300|gb|KK855848.1|	16264-21701	12	38	0.767202	2	1	0.5082	no
gi|641572300|gb|KK855848.1|	24909-22522	12	38	3	14	2	0.21625	no
gi|641572300|gb|KK855848.1|	25407-22656	12	38	0.587147	2	2	0.28975	no
gi|641572300|gb|KK855848.1|	26978-22754	12	38	0.961568	3	1	0.39995	no
gi|641572300|gb|KK855848.1|	27908-22889	12	38	1	1	0.119485	0.936	no
gi|641572300|gb|KK855848.1|	28217-28487	12	38	8	1	-3	0.2472	no
gi|641572300|gb|KK855848.1|	29324-29712	12	38	11	4	-2	0.30775	no
gi|641572300|gb|KK855848.1|	31956-32248	12	38	9	6	-0.657042	0.6717	no
gi|641572300|gb|KK855848.1|	43905-44167	12	38	24	12	-1	0.4704	no
gi|641572300|gb|KK855848.1|	52849-53183	12	38	10	4	-1	0.4576	no

gi|641572300|gb|KK855848.1|	56153-57419	12	38	1	0.906346	-0.22326	0.87645	no
gi|641572300|gb|KK855848.1|	57605-58269	12	38	4	3	-0.584663	0.6957	no
gi|641572300|gb|KK855848.1|	63302-63758	12	38	9	6	-0.64689	0.64315	no
gi|641572300|gb|KK855848.1|	68088-68369	12	38	18	23	0.310857	0.81935	no
gi|641572300|gb|KK855848.1|	70984-71205	12	38	75	71	-0.0873789	0.9482	no
gi|641572300|gb|KK855848.1|	85593-85780	12	38	14	53	2	0.2801	no
gi|641572300|gb|KK855848.1|	93161-93384	12	38	11	10	-0.136505	0.9206	no
gi|641572300|gb|KK855848.1|	95106-101584	12	38	7	4	-0.930225	0.56975	no
gi|641572304|gb|KK855847.1|	0-826	11	40	0.336779	20	6	0.13475	no
gi|641572304|gb|KK855847.1|	11690-11244	11	40	0.252094	2	3	0.2851	no
gi|641572304|gb|KK855847.1|	12114-13208	11	40	0.393916	2	2	0.2348	no
gi|641572304|gb|KK855847.1|	13547-15487	11	40	0.852216	2	1	0.42075	no
gi|641572304|gb|KK855847.1|	37731-13791	11	40	3	76.93	5	0.1107	no
gi|641572304|gb|KK855847.1|	41520-14260	11	40	0.88033	0.730676	-0.268812	0.86195	no
gi|641572304|gb|KK855847.1|	44634-14568	11	40	9	96.74	3	0.08495	no
gi|641572304|gb|KK855847.1|	46471-14721	11	40	13	1	-3	0.0502	no
gi|641572304|gb|KK855847.1|	48562-14944	11	40	0.922572	0.623125	-0.566139	0.7586	no
gi|641572304|gb|KK855847.1|	50563-15107	11	40	2	2	0.178775	0.9106	no
gi|641572304|gb|KK855847.1|	51865-15306	11	40	2	5	2	0.2352	no
gi|641572304|gb|KK855847.1|	56379-15740	11	40	0.255083	1	2	0.25595	no
gi|641572304|gb|KK855847.1|	57766-15833	11	40	1	3	1	0.4891	no
gi|641572304|gb|KK855847.1|	59937-16351	11	40	0.795141	2	1	0.32085	no
gi|641572304|gb|KK855847.1|	63692-16592	11	40	2	8	2	0.1497	no
gi|641572304|gb|KK855847.1|	66332-16697	11	40	2	10	2	0.11305	no
gi|641572304|gb|KK855847.1|	67174-16829	11	40	0.764029	3	2	0.2084	no
gi|641572304|gb|KK855847.1|	68603-17027	11	40	1	3	1	0.3252	no
gi|641572304|gb|KK855847.1|	70519-17175	11	40	1	2	0.744149	0.5881	no
gi|641572304|gb|KK855847.1|	71930-17336	11	40	0.855312	3	2	0.26155	no
gi|641572304|gb|KK855847.1|	73738-17555	11	40	0.829023	0.665346	-0.317307	1	no
gi|641572304|gb|KK855847.1|	76393-17728	11	40	1	0.933345	-0.133168	0.92965	no

gi|641572304|gb|KK855847.1|	77382-17874	11	40	0.789599	1	0.607265	0.681	no
gi|641572304|gb|KK855847.1|	79006-18652	11	40	2	2	0.0512298	0.96565	no
gi|641572304|gb|KK855847.1|	18312-18557	11	40	4	11	1	0.44765	no
gi|641572304|gb|KK855847.1|	92603-19476	11	40	1	3	1	0.37965	no
gi|641572304|gb|KK855847.1|	19321-20175	11	40	0.859903	5	2	0.1408	no
gi|641572304|gb|KK855847.1|	02910-20562	11	40	1	2	0.534037	0.68415	no
gi|641572304|gb|KK855847.1|	05889-20865	11	40	2	3	0.682181	0.59905	no
gi|641572304|gb|KK855847.1|	16595-21689	11	40	5	6	0.151339	0.92285	no
gi|641572304|gb|KK855847.1|	18061-22160	11	40	25	37	0.573321	0.7896	no
gi|641572304|gb|KK855847.1|	31589-32992	11	40	0.410383	2	3	0.1402	no
gi|641572304|gb|KK855847.1|	33099-34264	11	40	0.511105	0.778048	0.606241	1	no
gi|641572304|gb|KK855847.1|	64998-66721	11	40	0.278076	3	3	0.08945	no
gi|641572304|gb|KK855847.1|	66800-67246	11	40	0	5	inf	0.0025	yes
gi|641572304|gb|KK855847.1|	67348-67994	11	40	1	2	0.791907	0.63035	no
gi|641572304|gb|KK855847.1|	71224-72127	11	40	0.70032	0.760304	0.118564	1	no
gi|641572304|gb|KK855847.1|	76234-77723	11	40	0.875662	2	1	0.3653	no
gi|641572304|gb|KK855847.1|	78504-78779	11	40	5	5	-0.0242864	0.91115	no
gi|641572309|gb|KK855846.1|	01143-10186	9.7	36	1	6	2	0.1786	no
gi|641572309|gb|KK855846.1|	02318-10316	9.7	36	0.650855	0.988764	0.603289	0.70555	no
gi|641572309|gb|KK855846.1|	03346-10474	9.7	36	0.176609	2	3	0.14645	no
gi|641572309|gb|KK855846.1|	04934-10531	9.7	36	1	8	3	0.1878	no
gi|641572309|gb|KK855846.1|	05419-10650	9.7	36	0.633084	2	2	0.3067	no
gi|641572309|gb|KK855846.1|	06737-10813	9.7	36	2	2	0.0668523	0.9604	no
gi|641572309|gb|KK855846.1|	08333-10925	9.7	36	6	19	2	0.1872	no
gi|641572309|gb|KK855846.1|	09882-11048	9.7	36	2	1	-0.724033	0.67005	no
gi|641572309|gb|KK855846.1|	10640-11100	9.7	36	15	37	1	0.34465	no
gi|641572309|gb|KK855846.1|	11175-11190	9.7	36	4	8	0.949294	0.4756	no
gi|641572309|gb|KK855846.1|	12305-11493	9.7	36	1	4	2	0.184	no
gi|641572309|gb|KK855846.1|	15430-11564	9.7	36	56	146	1	0.3032	no
gi|641572309|gb|KK855846.1|	15756-11686	9.7	36	0.15532	1	3	0.25965	no

gi|641572309|gb|KK855846.1|	17593-11932	9.7	36	9	17	0.976222	0.42915	no
gi|641572309|gb|KK855846.1|	19486-12003	9.7	36	2	11	2	0.14225	no
gi|641572309|gb|KK855846.1|	12012-13558	9.7	36	0.314516	0.719031	1	1	no
gi|641572309|gb|KK855846.1|	20559-12192	9.7	36	0.543382	0.643836	0.244725	1	no
gi|641572309|gb|KK855846.1|	13770-14648	9.7	36	0.725964	0.472788	-0.618707	1	no
gi|641572309|gb|KK855846.1|	14811-17017	9.7	36	0.528197	0.268609	-0.975567	1	no
gi|641572309|gb|KK855846.1|	27058-28403	9.7	36	1	1	-0.014294	0.96665	no
gi|641572309|gb|KK855846.1|	28544-30051	9.7	36	0.809668	1	0.788401	0.5866	no
gi|641572309|gb|KK855846.1|	30570-31140	9.7	36	6	1	-2	0.19305	no
gi|641572309|gb|KK855846.1|	31826-36918	9.7	36	7	5	-0.420032	0.7327	no
gi|641572309|gb|KK855846.1|	46294-46993	9.7	36	2	6	2	0.26505	no
gi|641572309|gb|KK855846.1|	49532-50647	9.7	36	1	0.937324	-0.201444	0.8959	no
gi|641572309|gb|KK855846.1|	54966-55162	9.7	36	14	73	2	0.16015	no
gi|641572309|gb|KK855846.1|	63567-64140	9.7	36	1	3	1	0.47095	no
gi|641572309|gb|KK855846.1|	6792-7207	9.7	36	0	5	inf	0.0068	no
gi|641572309|gb|KK855846.1|	85771-85934	9.7	36	51	40	-0.337775	0.83535	no
gi|641572315|gb|KK855844.1|	02871-10327	9.4	41	6	3	-0.917607	0.56105	no
gi|641572315|gb|KK855844.1|	14008-11449	9.4	41	0	4	inf	0.00375	no
gi|641572315|gb|KK855844.1|	15286-11626	9.4	41	0.181614	4	4	0.2373	no
gi|641572315|gb|KK855844.1|	16970-11794	9.4	41	2	11	2	0.08475	no
gi|641572315|gb|KK855844.1|	21093-12159	9.4	41	2	3	0.956098	0.57355	no
gi|641572315|gb|KK855844.1|	26177-12692	9.4	41	0.76582	1	0.824096	0.6232	no
gi|641572315|gb|KK855844.1|	34012-13440	9.4	41	10	10	-0.0538618	0.9705	no
gi|641572315|gb|KK855844.1|	41327-14170	9.4	41	2	3	0.318815	0.86115	no
gi|641572315|gb|KK855844.1|	46236-14669	9.4	41	9	4	-1	0.46405	no
gi|641572315|gb|KK855844.1|	47689-14806	9.4	41	3	5	0.58114	0.7371	no
gi|641572315|gb|KK855844.1|	48387-14894	9.4	41	4	7	0.596135	0.6606	no
gi|641572315|gb|KK855844.1|	52166-15324	9.4	41	2	1	-0.268835	0.8494	no
gi|641572315|gb|KK855844.1|	54806-15530	9.4	41	4	1	-2	0.28145	no
gi|641572315|gb|KK855844.1|	56314-15663	9.4	41	9	12	0.378449	0.7974	no

gi|641572315|gb|KK855844.1|	59590-16036	9.4	41	4	3	-0.445133	0.7457	no
gi|641572315|gb|KK855844.1|	60502-16136	9.4	41	11	11	-0.02206	0.98545	no
gi|641572315|gb|KK855844.1|	61491-16351	9.4	41	6	9	0.45223	0.7138	no
gi|641572315|gb|KK855844.1|	68440-17180	9.4	41	34	16	-1	0.6123	no
gi|641572315|gb|KK855844.1|	72005-17468	9.4	41	1	0.43438	-2	0.2619	no
gi|641572315|gb|KK855844.1|	75180-17574	9.4	41	7	4	-0.79063	0.58165	no
gi|641572315|gb|KK855844.1|	79165-17953	9.4	41	35	13	-1	0.2682	no
gi|641572315|gb|KK855844.1|	84383-18469	9.4	41	2	8	2	0.2454	no
gi|641572315|gb|KK855844.1|	19318-21957	9.4	41	5	58	4	0.09225	no
gi|641572315|gb|KK855844.1|	32395-23338	9.4	41	0.896574	1	0.282927	0.85465	no
gi|641572315|gb|KK855844.1|	40569-24343	9.4	41	2	1	-0.167484	0.8985	no
gi|641572315|gb|KK855844.1|	43670-24518	9.4	41	0.538138	0.410055	-0.392158	1	no
gi|641572315|gb|KK855844.1|	45377-24736	9.4	41	0.948412	0.783551	-0.275487	0.84275	no
gi|641572315|gb|KK855844.1|	47484-25045	9.4	41	2	0.858172	-0.930366	0.4825	no
gi|641572315|gb|KK855844.1|	71320-71849	9.4	41	1	2	0.817166	0.6234	no
gi|641572315|gb|KK855844.1|	99519-10115(	9.4	41	11	3	-2	0.14415	no
gi|641572320|gb|KK855843.1|	54351-55149	8.2	35	4	0.71349	-3	0.1293	no
gi|641572320|gb|KK855843.1|	55891-56796	8.2	35	0.997637	0.151635	-3	0.24815	no
gi|641572320|gb|KK855843.1|	70321-71189	8.2	35	0.947258	2	1	0.49595	no
gi|641572320|gb|KK855843.1|	90873-91829	8.2	35	1	0	-inf	0.0059	no
gi|641572320|gb|KK855843.1|	93403-93719	8.2	35	4	3	-0.428906	0.7868	no
gi|641572320|gb|KK855843.1|	94037-94725	8.2	35	1	0.435664	-2	0.3455	no
gi|641572320|gb|KK855843.1|	95976-96598	8.2	35	2	0.502227	-2	0.3216	no
gi|641572320|gb|KK855843.1|	96898-97576	8.2	35	3	0.222299	-4	0.20475	no
gi|641572323|gb|KK855842.1|	14663-11494	8.7	38	0	13	inf	0.00375	no
gi|641572323|gb|KK855842.1|	72133-17291	8.7	38	4	11	2	0.25895	no
gi|641572323|gb|KK855842.1|	20250-21144	8.7	38	0.608006	0.616032	0.0189208	1	no
gi|641572323|gb|KK855842.1|	26708-27127	8.7	38	7	4	-0.766798	0.5966	no
gi|641572323|gb|KK855842.1|	32730-34670	8.7	38	5	5	0.0184672	0.9891	no
gi|641572323|gb|KK855842.1|	34892-36781	8.7	38	4	4	0.0899714	0.94355	no

gi|641572323|gb|KK855842.1|	36968-37640	8.7	38	0.445476	2	2	0.2235	no
gi|641572323|gb|KK855842.1|	38246-38906	8.7	38	3	3	0.159081	0.90795	no
gi|641572323|gb|KK855842.1|	39011-39748	8.7	38	2	1	-0.498085	0.74225	no
gi|641572323|gb|KK855842.1|	68981-69871	8.7	38	1	0.774448	-0.6592	0.6826	no
gi|641572326|gb|KK855841.1|	2778-3722	12	41	0	65	inf	0.00055	yes
gi|641572326|gb|KK855841.1|	5522-6318	12	41	0.235732	39	7	0.2322	no
gi|641572326|gb|KK855841.1|	56624-59031	12	41	42	5	-3	0.10965	no
gi|641572326|gb|KK855841.1|	59213-60369	12	41	6	0	-inf	5.00E-05	yes
gi|641572326|gb|KK855841.1|	61050-62316	12	41	3	0.302115	-3	0.10995	no
gi|641572326|gb|KK855841.1|	62486-62770	12	41	26	1	-5	0.187	no
gi|641572326|gb|KK855841.1|	66294-66814	12	41	6	0.328464	-4	0.1889	no
gi|641572326|gb|KK855841.1|	69072-69458	12	41	3	1	-1	0.44305	no
gi|641572326|gb|KK855841.1|	72068-72665	12	41	5	0.26652	-4	0.18915	no
gi|641572326|gb|KK855841.1|	73563-74133	12	41	5	0.856247	-2	0.18305	no
gi|641572326|gb|KK855841.1|	74240-74902	12	41	7	1	-3	0.13445	no
gi|641572326|gb|KK855841.1|	80498-81136	12	41	1	1	0.1136	0.92265	no
gi|641572330|gb|KK855840.1|	01636-10190	8	35	12	1	-3	0.21195	no
gi|641572330|gb|KK855840.1|	10224-10646	8	35	11	7	-0.775431	0.5765	no
gi|641572330|gb|KK855840.1|	12216-12771	8	35	4	7	0.596004	0.66385	no
gi|641572330|gb|KK855840.1|	13981-14624	8	35	2	3	0.947098	0.5402	no
gi|641572330|gb|KK855840.1|	18734-20805	8	35	0.490717	0.633378	0.368175	1	no
gi|641572330|gb|KK855840.1|	20985-22003	8	35	2	2	-0.231347	0.8652	no
gi|641572330|gb|KK855840.1|	22739-25007	8	35	0.717356	1	0.676597	0.6265	no
gi|641572330|gb|KK855840.1|	45497-45732	8	35	12	5	-1	0.49555	no
gi|641572330|gb|KK855840.1|	51251-51708	8	35	3	2	-0.788284	0.63075	no
gi|641572330|gb|KK855840.1|	7620-8119	8	35	9	12	0.358649	0.7835	no
gi|641572338|gb|KK855838.1|	02861-10442	11	44	14	1	-3	0.0266	no
gi|641572338|gb|KK855838.1|	10846-11202	11	44	27	49	0.869996	0.5922	no
gi|641572338|gb|KK855838.1|	12354-11901	11	44	10	9	-0.101941	0.96275	no
gi|641572338|gb|KK855838.1|	23716-12391	11	44	6	27	2	0.26345	no

gi|641572338|gb|KK855838.1|	24982-12538	11	44	2	5	1	0.41185	no
gi|641572338|gb|KK855838.1|	57588-15907	11	44	0.384291	0.669273	0.800397	1	no
gi|641572338|gb|KK855838.1|	60080-16070	11	44	0.661822	2	2	0.33105	no
gi|641572338|gb|KK855838.1|	63005-16408	11	44	0.640964	0.365749	-0.809388	1	no
gi|641572338|gb|KK855838.1|	91952-19251	11	44	2	3	0.306844	0.84285	no
gi|641572338|gb|KK855838.1|	48737-51009	11	44	2	0.676232	-1	0.34275	no
gi|641572341|gb|KK855837.1|	00431-10087	10	36	0.278448	10	5	0.10725	no
gi|641572341|gb|KK855837.1|	05317-10597	10	36	3	0.231802	-4	0.1991	no
gi|641572341|gb|KK855837.1|	08827-11031	10	36	20	0	-inf	5.00E-05	yes
gi|641572341|gb|KK855837.1|	10594-11184	10	36	75	0.10242	-10	0.18435	no
gi|641572341|gb|KK855837.1|	12710-11369	10	36	2	0.273741	-3	0.20575	no
gi|641572341|gb|KK855837.1|	15516-11772	10	36	7	13	1	0.4098	no
gi|641572341|gb|KK855837.1|	19303-12130	10	36	4	4	0.149633	0.91005	no
gi|641572341|gb|KK855837.1|	22144-12345	10	36	3	4	0.278415	0.83055	no
gi|641572341|gb|KK855837.1|	24136-12740	10	36	3	0.983735	-2	0.23765	no
gi|641572341|gb|KK855837.1|	27553-12947	10	36	1	4	1	0.28575	no
gi|641572341|gb|KK855837.1|	33532-13388	10	36	2	4	1	0.5206	no
gi|641572341|gb|KK855837.1|	33991-14070	10	36	2	19	3	0.0844	no
gi|641572341|gb|KK855837.1|	42729-14288	10	36	100	33	-2	0.34625	no
gi|641572341|gb|KK855837.1|	43404-14489	10	36	2	0.8353	-1	0.3682	no
gi|641572341|gb|KK855837.1|	46575-14758	10	36	5	2	-1	0.34715	no
gi|641572341|gb|KK855837.1|	50540-15081	10	36	71	30	-1	0.34785	no
gi|641572341|gb|KK855837.1|	52357-15255	10	36	33	3	-3	0.2117	no
gi|641572341|gb|KK855837.1|	55826-15652	10	36	0.564419	1	1	0.51305	no
gi|641572341|gb|KK855837.1|	19948-20243	10	36	7	0	-inf	0.01175	no
gi|641572341|gb|KK855837.1|	20307-21342	10	36	3	0	-inf	0.00055	yes
gi|641572341|gb|KK855837.1|	21840-25726	10	36	103	0.05859	-11	0.1309	no
gi|641572341|gb|KK855837.1|	33779-34891	10	36	1	0	-inf	0.0059	no
gi|641572341|gb|KK855837.1|	38220-39305	10	36	15	0	-inf	5.00E-05	yes
gi|641572341|gb|KK855837.1|	40466-41209	10	36	2	0	-inf	0.0056	no

gi|641572341|gb|KK855837.1|	41385-42002	10	36	2	0	-inf	0.01175	no
gi|641572341|gb|KK855837.1|	43885-44870	10	36	1	0	-inf	0.0059	no
gi|641572341|gb|KK855837.1|	95322-95696	10	36	0	17	inf	0.0004	yes
gi|641572341|gb|KK855837.1|	97453-98730	10	36	0.13091	2	4	0.2392	no
gi|641572341|gb|KK855837.1|	99497-100091	10	36	0.17744	9	6	0.10535	no
gi|641572345|gb|KK855836.1|	04726-10553	9.7	38	0.810226	0.878828	0.117258	0.92175	no
gi|641572345|gb|KK855836.1|	08973-10981	9.7	38	2	1	-0.226841	0.87645	no
gi|641572345|gb|KK855836.1|	82066-18349	9.7	38	2	3	0.511325	0.70315	no
gi|641572345|gb|KK855836.1|	84117-18500	9.7	38	1	1	0.188761	0.90355	no
gi|641572345|gb|KK855836.1|	49547-50087	9.7	38	3	0	-inf	0.0075	no
gi|641572345|gb|KK855836.1|	50210-51624	9.7	38	2	3	0.215168	0.87425	no
gi|641572345|gb|KK855836.1|	51776-52298	9.7	38	6	4	-0.405589	0.77675	no
gi|641572345|gb|KK855836.1|	53067-53241	9.7	38	48	6	-3	0.2302	no
gi|641572345|gb|KK855836.1|	53420-54745	9.7	38	18	5	-2	0.16635	no
gi|641572345|gb|KK855836.1|	60727-64415	9.7	38	10	12	0.277955	0.8619	no
gi|641572345|gb|KK855836.1|	66780-67198	9.7	38	2	3	0.586737	0.69715	no
gi|641572345|gb|KK855836.1|	71320-71499	9.7	38	21	20	-0.0659041	0.91115	no
gi|641572345|gb|KK855836.1|	79690-80739	9.7	38	1	2	0.41277	0.78555	no
gi|641572345|gb|KK855836.1|	81247-81882	9.7	38	2	0.243782	-3	0.2348	no
gi|641572345|gb|KK855836.1|	82024-82986	9.7	38	0.738775	0.702109	-0.0734406	1	no
gi|641572345|gb|KK855836.1|	85524-86713	9.7	38	0.783766	2	2	0.27715	no
gi|641572345|gb|KK855836.1|	86806-87347	9.7	38	0.817845	2	1	0.51315	no
gi|641572345|gb|KK855836.1|	98536-101589	9.7	38	1	2	0.387875	0.76455	no
gi|641572349|gb|KK855835.1|	37614-13853	10	18	1	1	0.341207	0.8164	no
gi|641572349|gb|KK855835.1|	15377-16373	10	18	0.795831	0.672448	-0.243039	1	no
gi|641572349|gb|KK855835.1|	16500-18023	10	18	0.746584	0.40636	-0.877544	1	no
gi|641572349|gb|KK855835.1|	19174-20419	10	18	0.741954	0.205424	-2	1	no
gi|641572349|gb|KK855835.1|	23052-23817	10	18	5	0.377258	-4	0.143	no
gi|641572349|gb|KK855835.1|	24629-26060	10	18	0.802168	0.960331	0.259628	0.8632	no
gi|641572349|gb|KK855835.1|	29692-30198	10	18	5	3	-0.760109	0.5995	no

gi|641572349|gb|KK855835.1|	35885-36207	10	18	40	11	-2	0.1864	no
gi|641572349|gb|KK855835.1|	49469-50615	10	18	1	9	3	0.0737	no
gi|641572349|gb|KK855835.1|	50999-51352	10	18	2	5	2	0.41725	no
gi|641572349|gb|KK855835.1|	99758-10014	10	18	4	25	3	0.0861	no
gi|641572360|gb|KK855832.1|	06279-10653	11	22	15	13	-0.209541	0.88595	no
gi|641572360|gb|KK855832.1|	15458-11680	11	22	2	3	0.519731	0.7007	no
gi|641572360|gb|KK855832.1|	23199-12362	11	22	3	2	-0.260467	0.88485	no
gi|641572360|gb|KK855832.1|	26117-12642	11	22	15	10	-0.671489	0.63995	no
gi|641572360|gb|KK855832.1|	31818-13224	11	22	3	4	0.127904	0.93425	no
gi|641572360|gb|KK855832.1|	33712-13432	11	22	3	8	2	0.2677	no
gi|641572360|gb|KK855832.1|	35244-13606	11	22	1	1	-0.512607	0.7498	no
gi|641572360|gb|KK855832.1|	36166-13737	11	22	0.487478	0.742226	0.606523	1	no
gi|641572360|gb|KK855832.1|	40014-14125	11	22	0.746219	2	1	0.47895	no
gi|641572360|gb|KK855832.1|	41479-14258	11	22	1	1	0.0208562	0.9647	no
gi|641572360|gb|KK855832.1|	42902-14394	11	22	0.499371	2	2	0.3067	no
gi|641572360|gb|KK855832.1|	45601-14721	11	22	1	2	0.431279	0.75075	no
gi|641572360|gb|KK855832.1|	49042-14950	11	22	5	2	-2	0.31215	no
gi|641572360|gb|KK855832.1|	49654-15055	11	22	1	2	0.381563	0.7933	no
gi|641572360|gb|KK855832.1|	50686-15130	11	22	1	0.751602	-0.816474	0.6492	no
gi|641572360|gb|KK855832.1|	52025-15476	11	22	1	1	0.0554198	0.9672	no
gi|641572360|gb|KK855832.1|	57216-15815	11	22	0.95075	2	0.982916	0.5381	no
gi|641572360|gb|KK855832.1|	63297-16384	11	22	3	2	-0.889647	0.58265	no
gi|641572360|gb|KK855832.1|	64062-16517	11	22	0.852389	0.707589	-0.268601	1	no
gi|641572360|gb|KK855832.1|	66846-16855	11	22	2	2	0.311453	0.8111	no
gi|641572360|gb|KK855832.1|	69212-17051	11	22	2	5	1	0.3288	no
gi|641572360|gb|KK855832.1|	72412-17387	11	22	2	1	-0.392346	0.77535	no
gi|641572360|gb|KK855832.1|	75557-17638	11	22	0.559524	1	1	0.50945	no
gi|641572360|gb|KK855832.1|	78229-17946	11	22	1	3	1	0.41205	no
gi|641572360|gb|KK855832.1|	23222-24076	11	22	0.429951	0.979813	1	0.51305	no
gi|641572360|gb|KK855832.1|	32290-34227	11	22	0.406516	0.929991	1	0.4288	no

gi|641572360|gb|KK855832.1|	34534-35142	11	22	5	7	0.650535	0.6344	no
gi|641572360|gb|KK855832.1|	35847-36377	11	22	7	10	0.594824	0.6525	no
gi|641572360|gb|KK855832.1|	39147-40146	11	22	2	0.13399	-4	0.1915	no
gi|641572360|gb|KK855832.1|	40270-41605	11	22	2	0.28389	-3	0.1543	no
gi|641572360|gb|KK855832.1|	41933-43075	11	22	11	10	-0.0905143	0.94595	no
gi|641572360|gb|KK855832.1|	46862-47158	11	22	5	7	0.373731	0.8127	no
gi|641572360|gb|KK855832.1|	50415-53458	11	22	3	4	0.664198	0.6015	no
gi|641572360|gb|KK855832.1|	53636-55443	11	22	4	3	-0.532195	0.6773	no
gi|641572360|gb|KK855832.1|	59468-61215	11	22	0.59318	0.486996	-0.284559	1	no
gi|641572360|gb|KK855832.1|	62535-63523	11	22	4	7	0.900339	0.49875	no
gi|641572360|gb|KK855832.1|	68092-69207	11	22	8	12	0.565269	0.6537	no
gi|641572360|gb|KK855832.1|	69972-71188	11	22	8	15	0.816965	0.5137	no
gi|641572360|gb|KK855832.1|	78474-79956	11	22	1	2	0.607741	0.66085	no
gi|641572360|gb|KK855832.1|	80898-81532	11	22	2	2	-0.0385237	0.95655	no
gi|641572360|gb|KK855832.1|	84884-86721	11	22	0.646917	0.854964	0.402283	1	no
gi|641572360|gb|KK855832.1|	86994-87488	11	22	2	2	0.107381	0.92265	no
gi|641572360|gb|KK855832.1|	88444-89093	11	22	6	6	0.0732789	0.9566	no
gi|641572360|gb|KK855832.1|	89214-89617	11	22	6	8	0.322088	0.81695	no
gi|641572360|gb|KK855832.1|	91274-91880	11	22	6	11	0.746098	0.57315	no
gi|641572360|gb|KK855832.1|	92768-93549	11	22	0.362653	1	2	0.3323	no
gi|641572360|gb|KK855832.1|	94416-95008	11	22	1	2	1	0.5082	no
gi|641572364|gb|KK855831.1|	00548-10073	11	38	51	37	-0.479327	0.7566	no
gi|641572364|gb|KK855831.1|	01890-10228	11	38	7	3	-1	0.36935	no
gi|641572364|gb|KK855831.1|	06965-10772	11	38	1	2	1	0.4562	no
gi|641572364|gb|KK855831.1|	11-1508	11	38	2	2	-0.42783	0.74965	no
gi|641572364|gb|KK855831.1|	11998-11296	11	38	0.27632	2	3	0.1666	no
gi|641572364|gb|KK855831.1|	14363-11551	11	38	0.29696	1	2	0.29935	no
gi|641572364|gb|KK855831.1|	16571-11740	11	38	1	0.669367	-0.856283	0.60675	no
gi|641572364|gb|KK855831.1|	17982-11888	11	38	0.301843	1	2	0.3323	no
gi|641572364|gb|KK855831.1|	20834-12269	11	38	0.383709	3	3	0.06785	no

gi|641572364|gb|KK855831.1|	22866-12352	11	38	0	5	inf	0.00145	yes
gi|641572364|gb|KK855831.1|	24389-12484	11	38	4	2	-0.731779	0.652	no
gi|641572364|gb|KK855831.1|	26157-12637	11	38	17	15	-0.200112	0.89755	no
gi|641572364|gb|KK855831.1|	33707-13396	11	38	12	8	-0.559484	0.73245	no
gi|641572364|gb|KK855831.1|	13545-16692	11	38	153	69	-1	0.54925	no
gi|641572364|gb|KK855831.1|	42149-14350	11	38	0.549	0.929241	0.759246	0.62485	no
gi|641572364|gb|KK855831.1|	53192-15343	11	38	16.61	6	-1	0.41435	no
gi|641572364|gb|KK855831.1|	63772-16412	11	38	12	7	-0.854933	0.5457	no
gi|641572364|gb|KK855831.1|	71219-17240	11	38	0.785347	0.978248	0.31687	0.84265	no
gi|641572364|gb|KK855831.1|	95952-19738	11	38	0.171338	1	3	0.1719	no
gi|641572364|gb|KK855831.1|	03484-20407	11	38	1	2	0.597463	0.69715	no
gi|641572364|gb|KK855831.1|	12086-21348	11	38	1	0.719309	-0.916154	0.53815	no
gi|641572364|gb|KK855831.1|	15816-21674	11	38	0.769905	0.438917	-0.81073	1	no
gi|641572364|gb|KK855831.1|	22220-22338	11	38	0.584723	1	1	0.4562	no
gi|641572364|gb|KK855831.1|	22281-22895	11	38	2	0.767542	-2	0.403	no
gi|641572364|gb|KK855831.1|	24878-22749	11	38	0.673005	1	0.78677	0.5678	no
gi|641572364|gb|KK855831.1|	23046-24688	11	38	0.929878	0.522242	-0.832324	0.57785	no
gi|641572364|gb|KK855831.1|	32732-23635	11	38	11	10	-0.244245	0.8791	no
gi|641572364|gb|KK855831.1|	25167-25759	11	38	2	0.809489	-1	0.47135	no
gi|641572364|gb|KK855831.1|	26115-27704	11	38	5	2	-1	0.2893	no
gi|641572364|gb|KK855831.1|	28773-29812	11	38	0.83919	1	0.605333	0.67795	no
gi|641572364|gb|KK855831.1|	36711-43632	11	38	26	18	-0.573653	0.7828	no
gi|641572364|gb|KK855831.1|	52129-54073	11	38	23	30	0.404484	0.8634	no
gi|641572364|gb|KK855831.1|	54239-55534	11	38	39	47	0.270175	0.9037	no
gi|641572364|gb|KK855831.1|	61109-61334	11	38	0	51	inf	0.00045	yes
gi|641572364|gb|KK855831.1|	61764-64802	11	38	0.049814	45	10	0.2334	no
gi|641572364|gb|KK855831.1|	65164-65634	11	38	0	18	inf	0.0001	yes
gi|641572364|gb|KK855831.1|	77585-78554	11	38	3	4	0.39825	0.7707	no
gi|641572364|gb|KK855831.1|	79843-80526	11	38	14.66	15	-0.0135673	0.99225	no
gi|641572364|gb|KK855831.1|	81688-84421	11	38	2	2	0.0645718	0.96165	no

gi|641572364|gb|KK855831.1|	8267-9183	11	38	3	2	-0.782882	0.5855	no
gi|641572364|gb|KK855831.1|	84719-88996	11	38	3	4	0.264961	0.83215	no
gi|641572364|gb|KK855831.1|	89352-91093	11	38	67	23	-2	0.502	no
gi|641572364|gb|KK855831.1|	93980-95142	11	38	11	8	-0.55204	0.6569	no
gi|641572368|gb|KK855830.1|	15630-17368	8.1	35	1	2	0.708039	0.59605	no
gi|641572368|gb|KK855830.1|	18686-19733	8.1	35	69	57	-0.258051	0.90625	no
gi|641572368|gb|KK855830.1|	56375-58185	8.1	35	2	1	-0.9107	0.5078	no
gi|641572368|gb|KK855830.1|	65095-65533	8.1	35	3	0.871114	-2	0.4266	no
gi|641572368|gb|KK855830.1|	71725-72120	8.1	35	6	2	-2	0.3698	no
gi|641572372|gb|KK855829.1|	10055-11082	12	45	0.491081	1	2	0.3613	no
gi|641572372|gb|KK855829.1|	22860-12325	12	45	35	52	0.554374	0.65635	no
gi|641572372|gb|KK855829.1|	36151-13649	12	45	65	66	0.0371851	0.9767	no
gi|641572372|gb|KK855829.1|	25173-25914	12	45	0.51896	2	2	0.3613	no
gi|641572372|gb|KK855829.1|	42996-43554	12	45	2	1	-0.0819365	0.92675	no
gi|641572372|gb|KK855829.1|	50051-50699	12	45	1	0.473727	-2	0.4266	no
gi|641572372|gb|KK855829.1|	51464-54618	12	45	1	1	0.162879	0.9008	no
gi|641572372|gb|KK855829.1|	81872-82675	12	45	0.815558	1	0.795266	0.63025	no
gi|641572372|gb|KK855829.1|	83107-84395	12	45	0.777553	1	0.928855	0.53445	no
gi|641572372|gb|KK855829.1|	95902-96863	12	45	2	1	-0.565304	0.71475	no
gi|641572376|gb|KK855828.1|	22094-12336	7.8	44	0.722007	1	0.95478	0.5381	no
gi|641572376|gb|KK855828.1|	24109-12476	7.8	44	4	6	0.719839	0.6023	no
gi|641572376|gb|KK855828.1|	25348-12660	7.8	44	1	2	0.284862	0.8385	no
gi|641572376|gb|KK855828.1|	26788-12735	7.8	44	2	2	0.0819039	0.9406	no
gi|641572376|gb|KK855828.1|	27421-12972	7.8	44	0.805457	1	0.346499	0.79755	no
gi|641572376|gb|KK855828.1|	51268-15173	7.8	44	2	3	0.783409	0.63115	no
gi|641572376|gb|KK855828.1|	53128-15456	7.8	44	2	3	0.749582	0.58	no
gi|641572376|gb|KK855828.1|	83126-18425	7.8	44	0.75749	2	1	0.4325	no
gi|641572376|gb|KK855828.1|	84611-18579	7.8	44	0.78773	2	1	0.3908	no
gi|641572376|gb|KK855828.1|	86350-18663	7.8	44	6	5	-0.115676	0.9229	no
gi|641572376|gb|KK855828.1|	86801-18748	7.8	44	0.724262	2	1	0.51085	no

gi|641572376|gb|KK855828.1|	3250-4269	7.8	44	2	0.784518	-1	0.48805	no
gi|641572386|gb|KK855827.1|	.70913-17115	9	39	410.15	423	0.0435881	0.96985	no
gi|641572390|gb|KK855826.1|	.00245-10076	10	35	8	5	-0.615256	0.6479	no
gi|641572390|gb|KK855826.1|	.01681-10236	10	35	14	6	-1	0.33455	no
gi|641572390|gb|KK855826.1|	.09747-11001	10	35	9	3	-2	0.3411	no
gi|641572390|gb|KK855826.1|	.41337-14164	10	35	8	2	-2	0.265	no
gi|641572390|gb|KK855826.1|	.43718-14431	10	35	7	12	0.735004	0.57035	no
gi|641572390|gb|KK855826.1|	.44815-14775	10	35	9	16	0.854652	0.60225	no
gi|641572390|gb|KK855826.1|	.49286-15086	10	35	0.307682	1	2	0.30625	no
gi|641572390|gb|KK855826.1|	.51102-15184	10	35	7	8	0.0541008	0.96975	no
gi|641572390|gb|KK855826.1|	.69373-17051	10	35	0.901274	0.800236	-0.17154	0.90465	no
gi|641572390|gb|KK855826.1|	.71119-17185	10	35	1	0.802006	-0.858102	0.60675	no
gi|641572390|gb|KK855826.1|	36988-37495	10	35	4	5	0.40094	0.7856	no
gi|641572390|gb|KK855826.1|	39965-41506	10	35	0.526141	1	1	0.4709	no
gi|641572390|gb|KK855826.1|	45635-48751	10	35	8	4	-0.774606	0.5262	no
gi|641572390|gb|KK855826.1|	49676-51625	10	35	12	9	-0.381255	0.7572	no
gi|641572390|gb|KK855826.1|	53719-56404	10	35	2	3	0.328548	0.7998	no
gi|641572390|gb|KK855826.1|	56515-58114	10	35	3	4	0.044223	0.9755	no
gi|641572390|gb|KK855826.1|	59716-61419	10	35	14	23	0.682892	0.5829	no
gi|641572390|gb|KK855826.1|	63423-65235	10	35	0.350358	30	6	0.0563	no
gi|641572390|gb|KK855826.1|	65334-65719	10	35	15	8	-1	0.46045	no
gi|641572390|gb|KK855826.1|	71221-72671	10	35	6	6	-0.159177	0.89965	no
gi|641572390|gb|KK855826.1|	74043-74907	10	35	13	11	-0.274816	0.8287	no
gi|641572390|gb|KK855826.1|	75047-75352	10	35	57	44	-0.386562	0.76345	no
gi|641572390|gb|KK855826.1|	75527-75919	10	35	44	30	-0.537489	0.67305	no
gi|641572390|gb|KK855826.1|	83411-92511	10	35	2	2	0.334564	0.79025	no
gi|641572390|gb|KK855826.1|	93035-95999	10	35	13	21	0.694923	0.6624	no
gi|641572390|gb|KK855826.1|	96307-98853	10	35	2	4	1	0.4253	no
gi|641572390|gb|KK855826.1|	99201-10008[illegible]	10	35	9	11	0.298894	0.81545	no
gi|641572394|gb|KK855825.1|	11933-12936	9.6	36	0.175319	1	3	0.2672	no

gi|641572394|gb|KK855825.1|	71588-17275	9.6	36	0.657813	0.111264	-3	1	no
gi|641572394|gb|KK855825.1|	73100-17398	9.6	36	0.930656	0.314277	-2	0.4266	no
gi|641572394|gb|KK855825.1|	19761-21222	9.6	36	7	9	0.196239	0.87775	no
gi|641572394|gb|KK855825.1|	24612-26097	9.6	36	5	6	0.160293	0.9014	no
gi|641572394|gb|KK855825.1|	32280-32938	9.6	36	0	5	inf	0.0005	yes
gi|641572394|gb|KK855825.1|	39555-41530	9.6	36	2	14	3	0.02985	no
gi|641572394|gb|KK855825.1|	43293-43696	9.6	36	10	6	-0.909642	0.52005	no
gi|641572394|gb|KK855825.1|	45953-46090	9.6	36	138	194	0.497573	0.7567	no
gi|641572394|gb|KK855825.1|	51931-52140	9.6	36	36	10	-2	0.287	no
gi|641572394|gb|KK855825.1|	53513-54305	9.6	36	4	2	-0.845016	0.55575	no
gi|641572394|gb|KK855825.1|	55665-56863	9.6	36	2	2	0.14701	0.9199	no
gi|641572394|gb|KK855825.1|	59370-59560	9.6	36	34	34	-0.00556223	0.95595	no
gi|641572394|gb|KK855825.1|	60276-60506	9.6	36	9	17	0.907216	0.5794	no
gi|641572394|gb|KK855825.1|	64081-65787	9.6	36	11	13	0.164815	0.89385	no
gi|641572394|gb|KK855825.1|	6561-7913	9.6	36	9	144	4	0.04775	no
gi|641572398|gb|KK855824.1|	00301-10832	9	35	10	6	-0.725977	0.7447	no
gi|641572398|gb|KK855824.1|	10941-11136	9	35	2	5	1	0.41185	no
gi|641572398|gb|KK855824.1|	12529-11348	9	35	2	0.425683	-2	0.21615	no
gi|641572398|gb|KK855824.1|	13648-11423	9	35	4	0	-inf	0.00315	yes
gi|641572398|gb|KK855824.1|	14286-11625	9	35	4	0.36489	-3	0.05485	no
gi|641572398|gb|KK855824.1|	22263-12256	9	35	19	13	-0.478722	0.7264	no
gi|641572398|gb|KK855824.1|	25352-12620	9	35	4	5	0.125365	0.9238	no
gi|641572398|gb|KK855824.1|	32938-13429	9	35	4	6	0.497042	0.70435	no
gi|641572398|gb|KK855824.1|	34391-13535	9	35	1	0.563148	-1	0.46585	no
gi|641572398|gb|KK855824.1|	35659-13703	9	35	0.720211	0.457163	-0.655709	1	no
gi|641572398|gb|KK855824.1|	37711-13847	9	35	5	11	1	0.3707	no
gi|641572398|gb|KK855824.1|	45581-14579	9	35	52	108.62	1	0.42985	no
gi|641572398|gb|KK855824.1|	51555-15192	9	35	9	14	0.706379	0.60745	no
gi|641572398|gb|KK855824.1|	60398-16162	9	35	107	112	0.0677833	0.97395	no
gi|641572398|gb|KK855824.1|	64493-16534	9	35	6	8	0.426507	0.75085	no

gi|641572398|gb|KK855824.1|	.79398-18040	9	35	0.350637	1	2	0.33105	no
gi|641572398|gb|KK855824.1|	31944-32306	9	35	0.828919	15	4	0.23805	no
gi|641572398|gb|KK855824.1|	64879-65815	9	35	4	24	3	0.06695	no
gi|641572398|gb|KK855824.1|	66418-66974	9	35	3	0	-inf	0.0056	no
gi|641572398|gb|KK855824.1|	79049-79185	9	35	0	1276.91	inf	5.00E-05	yes
gi|641572398|gb|KK855824.1|	82019-84209	9	35	2	20	3	0.1028	no
gi|641572398|gb|KK855824.1|	84870-85736	9	35	3	4	0.500438	0.7117	no
gi|641572398|gb|KK855824.1|	87493-92786	9	35	0.36112	5	4	0.01605	no
gi|641572398|gb|KK855824.1|	96557-97975	9	35	6	2	-2	0.25625	no
gi|641572398|gb|KK855824.1|	98942-10019	9	35	1	0.508732	-0.978204	0.57205	no
gi|641572402|gb|KK855823.1|	.00053-10035	11	40	1	29	5	0.23545	no
gi|641572402|gb|KK855823.1|	.01027-10124	11	40	2	69	5	0.10545	no
gi|641572402|gb|KK855823.1|	.02069-10236	11	40	2	25	4	0.145	no
gi|641572402|gb|KK855823.1|	.07200-10768	11	40	0.480477	12	5	0.2353	no
gi|641572402|gb|KK855823.1|	.12334-11269	11	40	1	19	4	0.14055	no
gi|641572402|gb|KK855823.1|	.17320-11808	11	40	0.749614	34	6	0.06295	no
gi|641572402|gb|KK855823.1|	.26345-12751	11	40	0.651758	2	2	0.2236	no
gi|641572402|gb|KK855823.1|	.37008-13811	11	40	0	4	inf	0.0004	yes
gi|641572402|gb|KK855823.1|	.40043-14099	11	40	0	3	inf	0.0015	yes
gi|641572402|gb|KK855823.1|	.43339-14405	11	40	0	751	inf	5.00E-05	yes
gi|641572402|gb|KK855823.1|	.50516-15154	11	40	2	5	1	0.3346	no
gi|641572402|gb|KK855823.1|	.55532-15593	11	40	4	15	2	0.2204	no
gi|641572402|gb|KK855823.1|	.57631-15830	11	40	2	6	1	0.38415	no
gi|641572402|gb|KK855823.1|	.59925-16078	11	40	4	5	0.235707	0.8557	no
gi|641572402|gb|KK855823.1|	.63137-16384	11	40	0.276163	2	3	0.2851	no
gi|641572402|gb|KK855823.1|	.65934-16706	11	40	0.452076	1	1	0.41175	no
gi|641572402|gb|KK855823.1|	.67200-16774	11	40	1	1	0.0106344	0.91115	no
gi|641572402|gb|KK855823.1|	.67980-16852	11	40	0.606423	2	2	0.3323	no
gi|641572402|gb|KK855823.1|	.68651-16913	11	40	1	4	2	0.32035	no
gi|641572402|gb|KK855823.1|	.70394-17258	11	40	5	6	0.27403	0.82625	no

gi|641572402|gb|KK855823.1|	72728-17325	11	40	2	2	0.0800467	0.9406	no
gi|641572402|gb|KK855823.1|	75717-76788	11	40	0.64682	1	1	0.4562	no
gi|641572402|gb|KK855823.1|	84715-86094	11	40	1	8	3	0.0783	no
gi|641572402|gb|KK855823.1|	91311-92079	11	40	0.49451	4	3	0.1597	no
gi|641572402|gb|KK855823.1|	95766-96000	11	40	2	59	5	0.23505	no
gi|641572402|gb|KK855823.1|	97403-97680	11	40	0	57	inf	0.0001	yes
gi|641572405|gb|KK855822.1|	26393-12662	8.7	44	0	35	inf	0.0015	yes
gi|641572405|gb|KK855822.1|	35583-13899	8.7	44	1	2	1	0.35405	no
gi|641572405|gb|KK855822.1|	39348-14047	8.7	44	0.83682	0.926295	0.146555	0.9285	no
gi|641572405|gb|KK855822.1|	40539-14117	8.7	44	0.802952	1	0.862001	0.62755	no
gi|641572405|gb|KK855822.1|	41264-14187	8.7	44	2	1	-0.401932	0.8125	no
gi|641572405|gb|KK855822.1|	42015-14420	8.7	44	2	3	0.163954	0.8957	no
gi|641572405|gb|KK855822.1|	82825-18366	8.7	44	0	2	inf	0.00455	no
gi|641572405|gb|KK855822.1|	84012-18612	8.7	44	0	3	inf	0.0001	yes
gi|641572405|gb|KK855822.1|	22187-22886	8.7	44	1	0.639362	-0.984329	0.62775	no
gi|641572409|gb|KK855821.1|	29203-12964	9.5	38	2	2	-0.410921	0.82205	no
gi|641572409|gb|KK855821.1|	42600-14707	9.5	38	4	5	0.210418	0.86985	no
gi|641572409|gb|KK855821.1|	84462-18614	9.5	38	1	1	0.236382	0.867	no
gi|641572409|gb|KK855821.1|	88448-19362	9.5	38	0.923638	1	0.676245	0.5981	no
gi|641572409|gb|KK855821.1|	2496-2946	9.5	38	2	4	1	0.47105	no
gi|641572409|gb|KK855821.1|	51464-52013	9.5	38	0.199872	3	4	0.1276	no
gi|641572413|gb|KK855820.1|	04199-10515	10	42	1	4	1	0.3047	no
gi|641572413|gb|KK855820.1|	21232-12152	10	42	6	4	-0.604058	0.70485	no
gi|641572413|gb|KK855820.1|	26333-26599	10	42	45	26	-0.777673	0.56675	no
gi|641572413|gb|KK855820.1|	30902-31151	10	42	7	6	-0.25517	0.88385	no
gi|641572413|gb|KK855820.1|	50154-50455	10	42	8	3	-2	0.4022	no
gi|641572413|gb|KK855820.1|	54804-55482	10	42	0.439949	2	2	0.2812	no
gi|641572413|gb|KK855820.1|	55605-56349	10	42	22	0.978952	-5	0.04425	no
gi|641572413|gb|KK855820.1|	59964-60204	10	42	99	15	-3	0.0802	no
gi|641572413|gb|KK855820.1|	61908-62697	10	42	13	7	-0.803599	0.53655	no

gi|641572413|gb|KK855820.1|	67040-67391	10	42	21	6	-2	0.2171	no
gi|641572413|gb|KK855820.1|	67795-68223	10	42	27	10	-2	0.25825	no
gi|641572413|gb|KK855820.1|	71501-71912	10	42	18	0.974752	-4	0.134	no
gi|641572413|gb|KK855820.1|	77216-78493	10	42	8	2	-2	0.13605	no
gi|641572413|gb|KK855820.1|	82556-83551	10	42	0.796826	0.673285	-0.243048	1	no
gi|641572413|gb|KK855820.1|	92044-93399	10	42	0.24421	1	2	0.23615	no
gi|641572416|gb|KK855819.1|	14934-11596	10	37	2	1	-0.97968	0.50885	no
gi|641572416|gb|KK855819.1|	16218-11715	10	37	1	3	1	0.3908	no
gi|641572416|gb|KK855819.1|	18177-11927	10	37	13	6	-1	0.3822	no
gi|641572416|gb|KK855819.1|	22389-12423	10	37	6	4	-0.680753	0.59025	no
gi|641572416|gb|KK855819.1|	24519-12534	10	37	0.111552	3	5	0.1123	no
gi|641572416|gb|KK855819.1|	25481-12625	10	37	0	2	inf	0.0025	yes
gi|641572416|gb|KK855819.1|	28515-12905	10	37	9	0.942774	-3	0.11545	no
gi|641572416|gb|KK855819.1|	31032-13122	10	37	28	0	-inf	0.0102	no
gi|641572416|gb|KK855819.1|	47648-14827	10	37	2	0.247112	-3	0.2481	no
gi|641572416|gb|KK855819.1|	52537-15331	10	37	2	0.185082	-3	0.21435	no
gi|641572416|gb|KK855819.1|	54577-15519	10	37	2	0.503392	-2	0.2679	no
gi|641572416|gb|KK855819.1|	57588-15908	10	37	14	2	-3	0.03535	no
gi|641572416|gb|KK855819.1|	61854-16326	10	37	0.580493	1	0.870523	0.56565	no
gi|641572416|gb|KK855819.1|	64107-16882	10	37	3	5	0.651292	0.60385	no
gi|641572416|gb|KK855819.1|	73448-17472	10	37	0.522192	1	2	0.34655	no
gi|641572416|gb|KK855819.1|	75702-17723	10	37	15	9	-0.666384	0.5983	no
gi|641572416|gb|KK855819.1|	77496-18076	10	37	11	11	0.0558249	0.9626	no
gi|641572416|gb|KK855819.1|	82121-18279	10	37	1	0.892855	-0.569851	0.7586	no
gi|641572416|gb|KK855819.1|	28217-28805	10	37	0	11	inf	5.00E-05	yes
gi|641572416|gb|KK855819.1|	29175-30575	10	37	10	7	-0.658851	0.6046	no
gi|641572416|gb|KK855819.1|	31605-31877	10	37	21	16	-0.333303	0.8116	no
gi|641572416|gb|KK855819.1|	32112-33101	10	37	6	4	-0.520638	0.693	no
gi|641572416|gb|KK855819.1|	34405-34660	10	37	130	101	-0.373798	0.77135	no
gi|641572416|gb|KK855819.1|	37063-37593	10	37	4	4	-0.068141	0.95985	no

gi|641572416|gb|KK855819.1|	41853-42653	10	37	2	1	-0.759997	0.61575	no
gi|641572416|gb|KK855819.1|	45415-46367	10	37	0.654971	0.711337	0.119103	1	no
gi|641572416|gb|KK855819.1|	48671-54471	10	37	2	3	0.489292	0.7019	no
gi|641572416|gb|KK855819.1|	54841-56275	10	37	6	4	-0.587084	0.6434	no
gi|641572416|gb|KK855819.1|	56619-57330	10	37	2	3	0.13142	0.9278	no
gi|641572416|gb|KK855819.1|	60864-64601	10	37	1	1	-0.0929401	0.94495	no
gi|641572416|gb|KK855819.1|	64756-68191	10	37	1	1	-0.134232	0.9187	no
gi|641572416|gb|KK855819.1|	68403-69379	10	37	28	32	0.189732	0.8761	no
gi|641572416|gb|KK855819.1|	72904-75272	10	37	0	5	inf	5.00E-05	yes
gi|641572416|gb|KK855819.1|	79520-80394	10	37	0.834563	3	2	0.2901	no
gi|641572416|gb|KK855819.1|	83584-83985	10	37	2	4	0.807285	0.6409	no
gi|641572416|gb|KK855819.1|	84608-84977	10	37	4	5	0.121269	0.9386	no
gi|641572416|gb|KK855819.1|	86132-86737	10	37	3	3	-0.139001	0.91795	no
gi|641572420|gb|KK855818.1|	24028-12442	11	42	7	4	-0.663987	0.6502	no
gi|641572420|gb|KK855818.1|	51297-15154	11	42	6	12	0.967222	0.5903	no
gi|641572420|gb|KK855818.1|	66212-16688	11	42	0	40	inf	5.00E-05	yes
gi|641572420|gb|KK855818.1|	16693-17327	11	42	2	0.977323	-1	0.44155	no
gi|641572420|gb|KK855818.1|	67960-16841	11	42	0	173	inf	0.00055	yes
gi|641572424|gb|KK855817.1|	16862-11795	8.4	43	2	0	-inf	0.0021	yes
gi|641572424|gb|KK855817.1|	13067-13231	8.4	43	141	47	-2	0.29215	no
gi|641572424|gb|KK855817.1|	31441-13296	8.4	43	0.053448	37	9	0.10595	no
gi|641572424|gb|KK855817.1|	27002-27725	8.4	43	1	1	-0.273305	0.8619	no
gi|641572428|gb|KK855816.1|	190-436	8.9	32	7	6	-0.256277	0.88385	no
gi|641572428|gb|KK855816.1|	38771-39149	8.9	32	0	24	inf	0.0001	yes
gi|641572428|gb|KK855816.1|	41713-42781	8.9	32	17	7	-1	0.30615	no
gi|641572428|gb|KK855816.1|	42909-43896	8.9	32	71	21	-2	0.3023	no
gi|641572428|gb|KK855816.1|	47466-48026	8.9	32	2	1	-0.98874	0.5872	no
gi|641572428|gb|KK855816.1|	48601-49440	8.9	32	0.989933	0.50125	-0.981799	0.62775	no
gi|641572428|gb|KK855816.1|	51008-51241	8.9	32	16	9	-0.832601	0.6108	no
gi|641572428|gb|KK855816.1|	60726-60895	8.9	32	37	39	0.0985329	0.93295	no

gi|641572428|gb|KK855816.1|	61721-62108	8.9	32	7	5	-0.416813	0.76465	no
gi|641572431|gb|KK855815.1|	00751-10184	9.1	35	0.78871	0.120024	-3	1	no
gi|641572431|gb|KK855815.1|	01996-10883	9.1	35	16	10	-0.667398	0.7586	no
gi|641572431|gb|KK855815.1|	09003-10984	9.1	35	56	71	0.346968	0.8786	no
gi|641572431|gb|KK855815.1|	38553-13891	9.1	35	0	8	inf	0.0025	yes
gi|641572431|gb|KK855815.1|	43021-14409	9.1	35	12	35	2	0.21775	no
gi|641572431|gb|KK855815.1|	44275-14810	9.1	35	29	53	0.868205	0.68365	no
gi|641572431|gb|KK855815.1|	49016-15103	9.1	35	13	16	0.316641	0.7975	no
gi|641572431|gb|KK855815.1|	51562-15202	9.1	35	55	62	0.168807	0.8889	no
gi|641572431|gb|KK855815.1|	61270-16225	9.1	35	28	9	-2	0.2057	no
gi|641572431|gb|KK855815.1|	72566-17328	9.1	35	0	5	inf	0.00065	yes
gi|641572431|gb|KK855815.1|	77001-17773	9.1	35	0	2	inf	0.00455	no
gi|641572431|gb|KK855815.1|	77872-17880	9.1	35	0.095977	2	5	0.1123	no
gi|641572431|gb|KK855815.1|	79346-18200	9.1	35	0.832066	0.919579	0.144276	0.9151	no
gi|641572431|gb|KK855815.1|	96383-19726	9.1	35	1	0.787979	-0.396314	0.81255	no
gi|641572431|gb|KK855815.1|	02521-20308	9.1	35	0	4	inf	0.0023	yes
gi|641572431|gb|KK855815.1|	06079-20813	9.1	35	0.455886	13	5	0.0214	no
gi|641572431|gb|KK855815.1|	09765-21041	9.1	35	3	13	2	0.1456	no
gi|641572431|gb|KK855815.1|	13166-21362	9.1	35	4	26	3	0.08555	no
gi|641572431|gb|KK855815.1|	15546-21616	9.1	35	19	89	2	0.22625	no
gi|641572431|gb|KK855815.1|	18927-22002	9.1	35	2	3	0.352019	0.79335	no
gi|641572431|gb|KK855815.1|	22028-22241	9.1	35	6	8	0.390413	0.7891	no
gi|641572431|gb|KK855815.1|	40754-42165	9.1	35	7	7	-0.0131133	0.99115	no
gi|641572431|gb|KK855815.1|	42468-42824	9.1	35	4	2	-1	0.50345	no
gi|641572431|gb|KK855815.1|	52946-54046	9.1	35	0.782608	0.357298	-1	1	no
gi|641572431|gb|KK855815.1|	69725-70520	9.1	35	13	14	0.0805395	0.9503	no
gi|641572431|gb|KK855815.1|	74983-75299	9.1	35	25	20	-0.335797	0.7943	no
gi|641572431|gb|KK855815.1|	79549-81694	9.1	35	3	5	0.663074	0.6096	no
gi|641572431|gb|KK855815.1|	81848-85195	9.1	35	8	9	0.111569	0.924	no
gi|641572431|gb|KK855815.1|	87496-88015	9.1	35	42	68	0.673819	0.58585	no

gi|641572431|gb|KK855815.1|	89576-90307	9.1	35	3	4	0.671719	0.63135	no
gi|641572431|gb|KK855815.1|	90415-91066	9.1	35	1	6	2	0.18405	no
gi|641572431|gb|KK855815.1|	95126-96397	9.1	35	15	35	1	0.46785	no
gi|641572431|gb|KK855815.1|	97623-99652	9.1	35	3	2	-0.590826	0.65665	no
gi|641572435|gb|KK855814.1|	58956-60356	10	39	13	19	0.580835	0.6373	no
gi|641572435|gb|KK855814.1|	81941-83076	10	39	0.904151	2	1	0.4764	no
gi|641572435|gb|KK855814.1|	87986-88396	10	39	0.652277	5	3	0.2674	no
gi|641572435|gb|KK855814.1|	95560-96380	10	39	1	0.86066	-0.245112	0.88495	no
gi|641572435|gb|KK855814.1|	99005-10021[illegible]	10	39	0.347856	0.7415	1	1	no
gi|641572439|gb|KK855813.1|	[illegible]30374-13065	8.6	38	6	11	0.884324	0.5744	no
gi|641572439|gb|KK855813.1|	54314-55113	8.6	38	1	0.712316	-0.567368	0.7586	no
gi|641572443|gb|KK855812.1|	40494-40705	9.8	36	3	20	3	0.30895	no
gi|641572443|gb|KK855812.1|	64909-65287	9.8	36	2	4	1	0.5126	no
gi|641572443|gb|KK855812.1|	66691-67014	9.8	36	4	17	2	0.17705	no
gi|641572451|gb|KK855810.1|	28830-29206	10	22	6	0	-inf	0.0059	no
gi|641572451|gb|KK855810.1|	38273-39922	10	22	1	0.890972	-0.507209	0.7175	no
gi|641572451|gb|KK855810.1|	64660-64980	10	22	7	0	-inf	0.0075	no
gi|641572455|gb|KK855809.1|	[illegible]22491-12274	9.6	42	243	171	-0.510167	0.6847	no
gi|641572455|gb|KK855809.1|	[illegible]27481-12772	9.6	42	51	54	0.0778882	0.953	no
gi|641572455|gb|KK855809.1|	[illegible]28832-12986	9.6	42	10	7	-0.432852	0.73505	no
gi|641572455|gb|KK855809.1|	[illegible]30749-13134	9.6	42	1	2	0.820038	0.62335	no
gi|641572455|gb|KK855809.1|	[illegible]32337-13322	9.6	42	1	0.155112	-3	0.22425	no
gi|641572455|gb|KK855809.1|	[illegible]44067-14602	9.6	42	4	3	-0.0955609	0.9384	no
gi|641572455|gb|KK855809.1|	[illegible]78838-17928	9.6	42	4	0.418988	-3	0.217	no
gi|641572459|gb|KK855808.1|	[illegible]10773-11093	11	45	370	124	-2	0.25745	no
gi|641572459|gb|KK855808.1|	[illegible]11256-11141	11	45	58	58	-0.00490042	0.94155	no
gi|641572459|gb|KK855808.1|	[illegible]11942-11210	11	45	39	32	-0.297133	0.86415	no
gi|641572459|gb|KK855808.1|	[illegible]14034-11513	11	45	31	29	-0.0663838	0.9527	no
gi|641572459|gb|KK855808.1|	[illegible]16017-11624	11	45	53	53	0.012836	0.9829	no
gi|641572459|gb|KK855808.1|	[illegible]17642-11789	11	45	26	22	-0.252703	0.8539	no

gi|641572459|gb|KK855808.1|	26010-12776	11	45	1	5	2	0.2179	no
gi|641572459|gb|KK855808.1|	30633-13162	11	45	2	4	1	0.38315	no
gi|641572459|gb|KK855808.1|	48893-15017	11	45	0	2	inf	0.00115	yes
gi|641572459|gb|KK855808.1|	53437-15506	11	45	0.938309	6	3	0.07755	no
gi|641572459|gb|KK855808.1|	55186-15577	11	45	0.544552	2	2	0.2812	no
gi|641572459|gb|KK855808.1|	55987-15709	11	45	0.3883	4	4	0.0953	no
gi|641572459|gb|KK855808.1|	63972-16712	11	45	0.311201	7	4	0.017	no
gi|641572459|gb|KK855808.1|	60304-61680	11	45	0.119933	33	8	0.23645	no
gi|641572459|gb|KK855808.1|	69724-70871	11	45	2	2	0.354588	0.79535	no
gi|641572459|gb|KK855808.1|	70974-71678	11	45	2	2	0.222236	0.8867	no
gi|641572459|gb|KK855808.1|	71783-72420	11	45	1	2	0.751	0.6262	no
gi|641572463|gb|KK855807.1|	36561-36957	9.3	35	0	20	inf	5.00E-05	yes
gi|641572468|gb|KK855806.1|	10321-10600	11	39	38	7	-3	0.1245	no
gi|641572468|gb|KK855806.1|	03229-10343	11	39	65	87	0.434576	0.7469	no
gi|641572468|gb|KK855806.1|	07961-10820	11	39	39	52	0.419879	0.75225	no
gi|641572468|gb|KK855806.1|	09377-10959	11	39	23	9	-1	0.41285	no
gi|641572468|gb|KK855806.1|	10106-11039	11	39	26	27	0.0720504	0.9552	no
gi|641572468|gb|KK855806.1|	13338-11354	11	39	18	3	-3	0.2472	no
gi|641572468|gb|KK855806.1|	16029-11623	11	39	20	15	-0.46869	0.77155	no
gi|641572468|gb|KK855806.1|	18310-11850	11	39	18	13	-0.471836	0.7824	no
gi|641572468|gb|KK855806.1|	20225-12042	11	39	56	87	0.631363	0.6372	no
gi|641572468|gb|KK855806.1|	20877-12154	11	39	2	2	0.358938	0.8122	no
gi|641572468|gb|KK855806.1|	28742-13013	11	39	1	1	0.02245	0.9728	no
gi|641572468|gb|KK855806.1|	30341-13168	11	39	0.739647	0.281674	-1	1	no
gi|641572468|gb|KK855806.1|	13062-15480	11	39	38	4	-3	0.08355	no
gi|641572468|gb|KK855806.1|	31985-13285	11	39	6	7	0.367477	0.78105	no
gi|641572468|gb|KK855806.1|	36861-13710	11	39	16	23	0.551078	0.70425	no
gi|641572468|gb|KK855806.1|	40119-14040	11	39	21	16	-0.389631	0.7746	no
gi|641572468|gb|KK855806.1|	43762-14443	11	39	0.443618	2	2	0.3323	no
gi|641572468|gb|KK855806.1|	44827-14535	11	39	1	1	-0.212431	0.8945	no

gi|641572468|gb|KK855806.1|	1786-2081	11	39	10	2	-2	0.22425	no
gi|641572468|gb|KK855806.1|	91561-19267	11	39	0.615875	0.937324	0.605911	0.6936	no
gi|641572468|gb|KK855806.1|	98117-19928	11	39	0.876179	1	0.191204	0.90355	no
gi|641572468|gb|KK855806.1|	07040-20844	11	39	0.527635	0.17864	-2	1	no
gi|641572468|gb|KK855806.1|	11476-21461	11	39	1	2	1	0.4386	no
gi|641572468|gb|KK855806.1|	22830-23200	11	39	5	3	-0.797663	0.6299	no
gi|641572468|gb|KK855806.1|	32155-23256	11	39	19	27	0.507457	0.69525	no
gi|641572468|gb|KK855806.1|	35851-23606	11	39	30	24	-0.311144	0.8343	no
gi|641572468|gb|KK855806.1|	46935-24716	11	39	58	68	0.239665	0.85775	no
gi|641572468|gb|KK855806.1|	27652-27997	11	39	10	3	-2	0.345	no
gi|641572468|gb|KK855806.1|	30062-30341	11	39	20	1	-4	0.19125	no
gi|641572468|gb|KK855806.1|	3610-3780	11	39	94	6	-4	0.196	no
gi|641572468|gb|KK855806.1|	36519-36651	11	39	559.63	0	-inf	0.00345	yes
gi|641572468|gb|KK855806.1|	37051-37306	11	39	31	1	-4	0.1878	no
gi|641572468|gb|KK855806.1|	38192-38395	11	39	20	0	-inf	0.01175	no
gi|641572468|gb|KK855806.1|	49685-49878	11	39	30	11	-1	0.41195	no
gi|641572468|gb|KK855806.1|	59115-61102	11	39	9	17	0.907798	0.57625	no
gi|641572468|gb|KK855806.1|	61779-63253	11	39	2	0.843765	-1	0.4646	no
gi|641572468|gb|KK855806.1|	63394-64319	11	39	1	0.590008	-0.980721	0.5872	no
gi|641572468|gb|KK855806.1|	64477-65247	11	39	0.985579	2	1	0.4562	no
gi|641572468|gb|KK855806.1|	66105-67251	11	39	1	0.680169	-1	0.46775	no
gi|641572468|gb|KK855806.1|	68400-68742	11	39	4	3	-0.42371	0.7868	no
gi|641572468|gb|KK855806.1|	6910-8161	11	39	16	2	-3	0.03725	no
gi|641572468|gb|KK855806.1|	71038-71844	11	39	1	1	0.0177065	0.95145	no
gi|641572468|gb|KK855806.1|	71970-72178	11	39	11	19	0.756776	0.64735	no
gi|641572468|gb|KK855806.1|	72917-74400	11	39	0.659807	0.58652	-0.169863	1	no
gi|641572468|gb|KK855806.1|	75284-75634	11	39	9	13	0.503688	0.72025	no
gi|641572468|gb|KK855806.1|	80708-81167	11	39	5	6	0.175346	0.8982	no
gi|641572468|gb|KK855806.1|	87760-87984	11	39	21	43	1	0.47135	no
gi|641572468|gb|KK855806.1|	89452-89692	11	39	33	50	0.597675	0.6569	no

gi|641572468|gb|KK855806.1|	92267-92462	11	39	57	85	0.586524	0.6639	no
gi|641572468|gb|KK855806.1|	98423-98573	11	39	67	68	0.017417	0.92445	no
gi|641572471|gb|KK855805.1|	02755-10309	13	54	6	20	2	0.2603	no
gi|641572471|gb|KK855805.1|	13505-11399	13	54	0.472544	5	3	0.25495	no
gi|641572471|gb|KK855805.1|	11785-17912	13	54	23	23	-0.00820638	0.9966	no
gi|641572471|gb|KK855805.1|	18514-11901	13	54	0.914852	2	1	0.4426	no
gi|641572471|gb|KK855805.1|	33452-13690	13	54	0.043468	20	9	0.23495	no
gi|641572471|gb|KK855805.1|	56626-15770	13	54	0.161153	23	7	0.2322	no
gi|641572471|gb|KK855805.1|	67558-16937	13	54	0.91912	2	0.971251	0.49	no
gi|641572471|gb|KK855805.1|	95855-19636	13	54	0.675329	4	2	0.21375	no
gi|641572471|gb|KK855805.1|	2041-2335	13	54	8	5	-0.697168	0.66875	no
gi|641572471|gb|KK855805.1|	21834-22567	13	54	0	16	inf	5.00E-05	yes
gi|641572471|gb|KK855805.1|	38726-23984	13	54	0.459906	0.699963	0.605938	1	no
gi|641572471|gb|KK855805.1|	40173-24074	13	54	0.189245	4	4	0.11365	no
gi|641572471|gb|KK855805.1|	45893-24753	13	54	0.490087	2	2	0.24655	no
gi|641572471|gb|KK855805.1|	25332-25590	13	54	0	58	inf	5.00E-05	yes
gi|641572471|gb|KK855805.1|	72110-27360	13	54	0.710378	2	2	0.26395	no
gi|641572471|gb|KK855805.1|	74181-27540	13	54	0.4832	1	1	0.48415	no
gi|641572471|gb|KK855805.1|	28268-28507	13	54	1	67	6	0.10495	no
gi|641572471|gb|KK855805.1|	29497-30002	13	54	0.455966	9	4	0.23805	no
gi|641572471|gb|KK855805.1|	32749-33366	13	54	2	1	-0.571556	0.7586	no
gi|641572471|gb|KK855805.1|	34786-35423	13	54	0.160228	3	4	0.1136	no
gi|641572471|gb|KK855805.1|	37522-38417	13	54	18	14.61	-0.27746	0.8265	no
gi|641572471|gb|KK855805.1|	38529-38817	13	54	19	8	-1	0.3981	no
gi|641572471|gb|KK855805.1|	39031-39752	13	54	9	10	0.111316	0.92975	no
gi|641572471|gb|KK855805.1|	39950-42074	13	54	5	12	1	0.34615	no
gi|641572471|gb|KK855805.1|	42315-43367	13	54	3	4	0.458598	0.73455	no
gi|641572471|gb|KK855805.1|	43469-45112	13	54	4	8	0.885361	0.4947	no
gi|641572471|gb|KK855805.1|	4439-4895	13	54	3	2	-0.409851	0.8125	no
gi|641572471|gb|KK855805.1|	46113-46667	13	54	5	10	1	0.3967	no

gi|641572471|gb|KK855805.1|	47751-48462	13	54	0.274559	11	5	0.233	no
gi|641572471|gb|KK855805.1|	52776-53489	13	54	0.820498	1	0.600949	0.70555	no
gi|641572471|gb|KK855805.1|	58296-59419	13	54	0.686885	0.696958	0.0210034	1	no
gi|641572471|gb|KK855805.1|	59950-60646	13	54	0.989704	2	0.793208	0.63035	no
gi|641572471|gb|KK855805.1|	66332-67498	13	54	0.656458	0.555179	-0.241749	1	no
gi|641572471|gb|KK855805.1|	79697-81491	13	54	0.885756	2	1	0.3924	no
gi|641572471|gb|KK855805.1|	81561-82256	13	54	0.566675	2	2	0.3613	no
gi|641572471|gb|KK855805.1|	87119-88512	13	54	0.827618	1	0.500485	0.73235	no
gi|641572471|gb|KK855805.1|	89211-90257	13	54	0.998742	1	0.190353	0.90355	no
gi|641572475|gb|KK855804.1|	77316-77450	9.5	22	338	34	-3	0.21525	no
gi|641572480|gb|KK855803.1|	08701-10961	9	38	0.892841	0.904739	0.019098	0.9394	no
gi|641572484|gb|KK855802.1|	00839-10191	9.2	36	0.560844	0.609582	0.120223	1	no
gi|641572484|gb|KK855802.1|	02064-10407	9.2	36	11	13	0.264355	0.82675	no
gi|641572484|gb|KK855802.1|	10968-11152	9.2	36	24	27	0.149558	0.9058	no
gi|641572484|gb|KK855802.1|	12146-11541	9.2	36	5	5	-0.195371	0.8738	no
gi|641572484|gb|KK855802.1|	15542-11588	9.2	36	6	3	-1	0.5083	no
gi|641572484|gb|KK855802.1|	16810-11882	9.2	36	11	12	0.0592539	0.9608	no
gi|641572484|gb|KK855802.1|	19213-12049	9.2	36	402	459	0.193277	0.9145	no
gi|641572484|gb|KK855802.1|	22320-12339	9.2	36	0.24118	3	3	0.1465	no
gi|641572484|gb|KK855802.1|	25068-12683	9.2	36	0.225386	2	3	0.1071	no
gi|641572484|gb|KK855802.1|	28120-13033	9.2	36	24	17	-0.438463	0.78655	no
gi|641572484|gb|KK855802.1|	31014-13173	9.2	36	3	5	0.771089	0.57725	no
gi|641572484|gb|KK855802.1|	46115-14801	9.2	36	0	22	inf	0.0007	yes
gi|641572484|gb|KK855802.1|	51003-15290	9.2	36	102	122	0.250139	0.90035	no
gi|641572484|gb|KK855802.1|	56281-15816	9.2	36	6	8	0.377106	0.7624	no
gi|641572484|gb|KK855802.1|	58737-15912	9.2	36	4	2	-1	0.50345	no
gi|641572484|gb|KK855802.1|	60518-16153	9.2	36	0.857484	0.260876	-2	1	no
gi|641572484|gb|KK855802.1|	68264-16851	9.2	36	18	23	0.306036	0.82145	no
gi|641572484|gb|KK855802.1|	71169-17147	9.2	36	37	45	0.258244	0.83645	no
gi|641572484|gb|KK855802.1|	74123-17437	9.2	36	0	58	inf	5.00E-05	yes

gi|641572484|gb|KK855802.1|	78259-17927	9.2	36	8	11	0.472399	0.71085	no
gi|641572484|gb|KK855802.1|	83287-18387	9.2	36	5	5	0.040806	0.97175	no
gi|641572484|gb|KK855802.1|	86344-18689	9.2	36	2	0.60411	-2	0.31075	no
gi|641572484|gb|KK855802.1|	56644-57372	9.2	36	2	1	-0.398732	0.8086	no
gi|641572484|gb|KK855802.1|	57716-58366	9.2	36	2	2	0.0848029	0.94	no
gi|641572484|gb|KK855802.1|	59523-60722	9.2	36	0.846499	0.1074	-3	1	no
gi|641572484|gb|KK855802.1|	66138-68347	9.2	36	5	8	0.658309	0.6007	no
gi|641572484|gb|KK855802.1|	68458-69065	9.2	36	5	8	0.797342	0.555	no
gi|641572484|gb|KK855802.1|	72626-73550	9.2	36	0.680138	1	0.966801	0.5733	no
gi|641572484|gb|KK855802.1|	83314-83675	9.2	36	25	22	-0.173877	0.89405	no
gi|641572484|gb|KK855802.1|	84156-85118	9.2	36	9	11	0.233537	0.85685	no
gi|641572484|gb|KK855802.1|	86646-87612	9.2	36	0.918676	0.279394	-2	0.3455	no
gi|641572484|gb|KK855802.1|	89784-90862	9.2	36	2	2	-0.131324	0.9226	no
gi|641572484|gb|KK855802.1|	92298-93777	9.2	36	13	11	-0.218818	0.85845	no
gi|641572484|gb|KK855802.1|	94897-95510	9.2	36	5	6	0.287897	0.8302	no
gi|641572484|gb|KK855802.1|	97311-97804	9.2	36	30	36	0.273677	0.8314	no
gi|641572484|gb|KK855802.1|	98198-98359	9.2	36	206	138	-0.57834	0.68115	no
gi|641572484|gb|KK855802.1|	98613-99493	9.2	36	79	76	-0.0570469	0.97985	no
gi|641572488|gb|KK855801.1|	37027-37685	9.5	38	2	0	-inf	0.0059	no
gi|641572488|gb|KK855801.1|	75864-76434	9.5	38	0	29	inf	5.00E-05	yes
gi|641572492|gb|KK855800.1|	02130-10512	12	38	0	19	inf	0.0006	yes
gi|641572492|gb|KK855800.1|	10948-12846	12	38	3	5	0.827873	0.51425	no
gi|641572492|gb|KK855800.1|	14974-11549	12	38	2	3	0.442194	0.7665	no
gi|641572492|gb|KK855800.1|	15986-11630	12	38	2	6	2	0.3454	no
gi|641572492|gb|KK855800.1|	23360-12659	12	38	2	3	0.626334	0.61915	no
gi|641572492|gb|KK855800.1|	26797-12867	12	38	3	4	0.672954	0.59345	no
gi|641572492|gb|KK855800.1|	28753-13194	12	38	0.826946	2	1	0.36395	no
gi|641572492|gb|KK855800.1|	40269-14181	12	38	0.4206	0.480774	0.192908	1	no
gi|641572492|gb|KK855800.1|	51766-15264	12	38	0.414837	3	3	0.18535	no
gi|641572492|gb|KK855800.1|	52696-15305	12	38	2	5	2	0.41725	no

gi|641572492|gb|KK855800.1|	60020-16070	12	38	0.725744	1	0.863284	0.62725	no
gi|641572492|gb|KK855800.1|	61996-16248	12	38	2	1	-0.407636	0.82205	no
gi|641572492|gb|KK855800.1|	26170-26943	12	38	1	0.186036	-3	0.24815	no
gi|641572492|gb|KK855800.1|	46592-48007	12	38	0.290247	2	3	0.14425	no
gi|641572492|gb|KK855800.1|	49223-49419	12	38	16	27	0.720809	0.66305	no
gi|641572492|gb|KK855800.1|	50934-52590	12	38	2	2	0.415643	0.7568	no
gi|641572492|gb|KK855800.1|	52748-53947	12	38	1	0.7518	-0.493088	0.7428	no
gi|641572492|gb|KK855800.1|	54070-54967	12	38	0.90811	1	0.241349	0.8798	no
gi|641572492|gb|KK855800.1|	58489-60995	12	38	1	0.88843	-0.534613	0.69475	no
gi|641572492|gb|KK855800.1|	65661-66111	12	38	2	2	-0.217688	0.8945	no
gi|641572492|gb|KK855800.1|	67226-68140	12	38	1	0.748734	-0.774394	0.63565	no
gi|641572492|gb|KK855800.1|	69206-70874	12	38	0.76923	0.58634	-0.391679	1	no
gi|641572492|gb|KK855800.1|	72383-73395	12	38	6	5	-0.255495	0.84335	no
gi|641572492|gb|KK855800.1|	75141-75659	12	38	2	3	0.456694	0.76285	no
gi|641572492|gb|KK855800.1|	8085-9466	12	38	2	1	-0.887414	0.5321	no
gi|641572492|gb|KK855800.1|	85982-86341	12	38	11	10	-0.121272	0.9327	no
gi|641572492|gb|KK855800.1|	89615-92992	12	38	3	2	-0.439447	0.72595	no
gi|641572492|gb|KK855800.1|	93703-95048	12	38	23	29	0.355842	0.77415	no
gi|641572497|gb|KK855799.1|	49848-15043	8.7	37	0.36211	3	3	0.27515	no
gi|641572497|gb|KK855799.1|	43674-44382	8.7	37	0.276163	2	3	0.2851	no
gi|641572497|gb|KK855799.1|	55697-56618	8.7	37	1	0.14831	-3	0.2382	no
gi|641572497|gb|KK855799.1|	73169-73454	8.7	37	0.708035	10	4	0.12515	no
gi|641572497|gb|KK855799.1|	75231-76963	8.7	37	2	22	4	0.0168	no
gi|641572501|gb|KK855798.1|	02899-10410	9.8	39	0.069915	5	6	0.10475	no
gi|641572501|gb|KK855798.1|	16485-11712	9.8	39	2	4	0.921014	0.5374	no
gi|641572501|gb|KK855798.1|	22518-12323	9.8	39	2	5	0.890521	0.52765	no
gi|641572501|gb|KK855798.1|	30254-13046	9.8	39	14	17	0.34372	0.823	no
gi|641572501|gb|KK855798.1|	32772-13415	9.8	39	4	7	0.752797	0.55285	no
gi|641572501|gb|KK855798.1|	35637-13704	9.8	39	0.938018	1	0.307881	0.83475	no
gi|641572501|gb|KK855798.1|	39176-14022	9.8	39	0.74468	2	2	0.2975	no

gi|641572501|gb|KK855798.1|	40377-14234	9.8	39	0.279585	0.913765	2	0.2914	no
gi|641572501|gb|KK855798.1|	44978-14651	9.8	39	0.687047	0.563824	-0.285162	1	no
gi|641572501|gb|KK855798.1|	53534-15509	9.8	39	10	19	0.970586	0.4365	no
gi|641572501|gb|KK855798.1|	55386-15603	9.8	39	2	1	-0.400681	0.8125	no
gi|641572501|gb|KK855798.1|	56151-15779	9.8	39	0.882156	2	0.830636	0.5563	no
gi|641572501|gb|KK855798.1|	58003-15870	9.8	39	2	2	0.31108	0.84265	no
gi|641572501|gb|KK855798.1|	58762-15957	9.8	39	2	4	1	0.3735	no
gi|641572501|gb|KK855798.1|	59678-16057	9.8	39	1	1	0.281931	0.85465	no
gi|641572501|gb|KK855798.1|	61683-16280	9.8	39	2	4	1	0.4028	no
gi|641572501|gb|KK855798.1|	64979-16590	9.8	39	0.974299	4	2	0.22305	no
gi|641572501|gb|KK855798.1|	19129-19898	9.8	39	3	1	-0.982912	0.5308	no
gi|641572501|gb|KK855798.1|	25200-27052	9.8	39	10	8	-0.372456	0.762	no
gi|641572501|gb|KK855798.1|	30281-30498	9.8	39	8	11	0.538779	0.768	no
gi|641572501|gb|KK855798.1|	31537-33054	9.8	39	7	5	-0.473931	0.7073	no
gi|641572501|gb|KK855798.1|	33312-38836	9.8	39	7	3	-1	0.3846	no
gi|641572501|gb|KK855798.1|	44156-46684	9.8	39	0.333876	0.787427	1	1	no
gi|641572501|gb|KK855798.1|	50759-50978	9.8	39	17	11	-0.597781	0.69735	no
gi|641572501|gb|KK855798.1|	52248-53155	9.8	39	2	2	-0.232464	0.8652	no
gi|641572501|gb|KK855798.1|	75892-76777	9.8	39	1	0.312012	-2	0.3455	no
gi|641572501|gb|KK855798.1|	82083-83103	9.8	39	2	0.522381	-2	0.1855	no
gi|641572505|gb|KK855797.1|	12869-11371	8.8	33	0.434569	2	2	0.23615	no
gi|641572505|gb|KK855797.1|	21474-12214	8.8	33	6	7	0.344637	0.79065	no
gi|641572505|gb|KK855797.1|	23391-12473	8.8	33	15	13	-0.192728	0.87575	no
gi|641572505|gb|KK855797.1|	26168-12819	8.8	33	4	2	-1	0.3845	no
gi|641572505|gb|KK855797.1|	28975-13021	8.8	33	1	0.5165	-1	0.47035	no
gi|641572505|gb|KK855797.1|	30340-13173	8.8	33	2	2	0.550825	0.68675	no
gi|641572505|gb|KK855797.1|	34778-13797	8.8	33	1	0.864529	-0.637562	0.62435	no
gi|641572505|gb|KK855797.1|	38085-13934	8.8	33	2	0.303244	-3	0.17565	no
gi|641572505|gb|KK855797.1|	39515-14094	8.8	33	2	0.175171	-3	0.14235	no
gi|641572505|gb|KK855797.1|	41103-14347	8.8	33	2	0.495417	-2	0.1819	no

gi|641572505|gb|KK855797.1|	43618-14458	8.8	33	1	0	-inf	0.0059	no
gi|641572505|gb|KK855797.1|	45343-14659	8.8	33	1	0.10242	-4	0.20475	no
gi|641572505|gb|KK855797.1|	47246-14872	8.8	33	1	0.671869	-0.640193	0.66115	no
gi|641572505|gb|KK855797.1|	49623-15024	8.8	33	57	34	-0.758116	0.54495	no
gi|641572510|gb|KK855796.1|	01458-10331	11	30	5.88	1	-2	0.0881	no
gi|641572510|gb|KK855796.1|	04589-10549	11	30	0.399055	0.909809	1	0.51305	no
gi|641572510|gb|KK855796.1|	19173-11940	11	30	20	3	-3	0.20805	no
gi|641572510|gb|KK855796.1|	33226-13341	11	30	40	12	-2	0.32225	no
gi|641572510|gb|KK855796.1|	36875-13765	11	30	0.365775	2	2	0.256	no
gi|641572510|gb|KK855796.1|	34877-35079	11	30	0	207	inf	5.00E-05	yes
gi|641572510|gb|KK855796.1|	36932-37125	11	30	0	78	inf	0.0005	yes
gi|641572510|gb|KK855796.1|	43135-43323	11	30	0	63	inf	0.0015	yes
gi|641572510|gb|KK855796.1|	45814-46044	11	30	0	87	inf	0.00025	yes
gi|641572510|gb|KK855796.1|	58171-58782	11	30	0	25	inf	5.00E-05	yes
gi|641572510|gb|KK855796.1|	60472-60663	11	30	0	559	inf	5.00E-05	yes
gi|641572510|gb|KK855796.1|	64368-65567	11	30	0	24	inf	5.00E-05	yes
gi|641572510|gb|KK855796.1|	66035-66868	11	30	0	3	inf	0.0016	yes
gi|641572510|gb|KK855796.1|	66982-67242	11	30	0	87	inf	5.00E-05	yes
gi|641572510|gb|KK855796.1|	68193-68400	11	30	0	238	inf	0.0001	yes
gi|641572510|gb|KK855796.1|	68847-69192	11	30	0	55	inf	0.0001	yes
gi|641572510|gb|KK855796.1|	87815-89424	11	30	3	2	-0.442472	0.73845	no
gi|641572510|gb|KK855796.1|	97570-99792	11	30	2	2	-0.164306	0.90015	no
gi|641572510|gb|KK855796.1|	99965-10117	11	30	5	3	-0.636334	0.6283	no
gi|641572514|gb|KK855795.1|	03928-10434	9.5	39	0.317587	4	4	0.1276	no
gi|641572514|gb|KK855795.1|	05554-10596	9.5	39	16	7	-1	0.38145	no
gi|641572514|gb|KK855795.1|	10182-11037	9.5	39	31	24	-0.365454	0.80775	no
gi|641572514|gb|KK855795.1|	11172-11143	9.5	39	9	9	0.0414205	0.94155	no
gi|641572514|gb|KK855795.1|	25494-12674	9.5	39	10	3	-2	0.17385	no
gi|641572514|gb|KK855795.1|	27037-12752	9.5	39	2	2	0.00746558	0.9394	no
gi|641572514|gb|KK855795.1|	31194-13180	9.5	39	3	2	-0.916337	0.54625	no

gi|641572514|gb|KK855795.1|	36094-13716	9.5	39	0.893458	0.865108	-0.0465192	0.94545	no
gi|641572514|gb|KK855795.1|	42853-14373	9.5	39	1	0.623125	-1	0.50315	no
gi|641572514|gb|KK855795.1|	45651-14799	9.5	39	10	3	-2	0.1629	no
gi|641572514|gb|KK855795.1|	49110-15267	9.5	39	8	8	-0.00367418	0.9972	no
gi|641572514|gb|KK855795.1|	55089-15637	9.5	39	0.970165	0.985055	0.0219738	0.9597	no
gi|641572514|gb|KK855795.1|	57369-16000	9.5	39	0.552309	2	2	0.2813	no
gi|641572514|gb|KK855795.1|	53610-54163	9.5	39	0	3	inf	0.0068	no
gi|641572518|gb|KK855794.1|	74278-74485	7.2	32	21	8	-1	0.442	no
gi|641572522|gb|KK855793.1|	00249-10063	9	41	0.374579	37	7	0.10445	no
gi|641572522|gb|KK855793.1|	51646-52234	9	41	0	5	inf	0.0015	yes
gi|641572522|gb|KK855793.1|	98085-98675	9	41	0.179229	2	4	0.1276	no
gi|641572526|gb|KK855792.1|	10463-11139	6.7	32	0.676425	1	0.79692	0.63025	no
gi|641572526|gb|KK855792.1|	27349-28034	6.7	32	1	2	0.792944	0.63035	no
gi|641572526|gb|KK855792.1|	98692-99282	6.7	32	2	0.542353	-2	0.3455	no
gi|641572538|gb|KK855789.1|	17830-11836	11	46	0.411283	3	3	0.27515	no
gi|641572545|gb|KK855787.1|	19489-11968	10	43	5	32	3	0.2884	no
gi|641572545|gb|KK855787.1|	63744-16392	10	43	54	91	0.767527	0.59195	no
gi|641572545|gb|KK855787.1|	58242-59760	10	43	21	75	2	0.2763	no
gi|641572545|gb|KK855787.1|	59868-62378	10	43	4	15	2	0.1434	no
gi|641572549|gb|KK855786.1|	01287-10167	9.5	35	5	4	-0.416685	0.7699	no
gi|641572549|gb|KK855786.1|	09669-11105	9.5	35	7	6	-0.125529	0.91955	no
gi|641572549|gb|KK855786.1|	23152-12811	9.5	35	90	32	-1	0.45365	no
gi|641572549|gb|KK855786.1|	30524-13142	9.5	35	0.799236	0.455543	-0.811035	1	no
gi|641572549|gb|KK855786.1|	34685-13634	9.5	35	2	1	-1	0.4379	no
gi|641572549|gb|KK855786.1|	36823-13740	9.5	35	2	1	-0.724884	0.67005	no
gi|641572549|gb|KK855786.1|	37754-13826	9.5	35	3	0.666973	-2	0.31075	no
gi|641572549|gb|KK855786.1|	39443-14051	9.5	35	1	0.12233	-3	0.217	no
gi|641572549|gb|KK855786.1|	44640-14531	9.5	35	14	9	-0.56591	0.6647	no
gi|641572549|gb|KK855786.1|	50342-15129	9.5	35	5	15	2	0.24695	no
gi|641572549|gb|KK855786.1|	52986-15366	9.5	35	1	1	0.377682	0.8276	no

gi|641572549|gb|KK855786.1|	51457-52191	9.5	35	2	4	0.753394	0.5947	no
gi|641572549|gb|KK855786.1|	52332-52559	9.5	35	7	16	1	0.4829	no
gi|641572549|gb|KK855786.1|	59839-60539	9.5	35	6	1	-3	0.13445	no
gi|641572549|gb|KK855786.1|	63991-68991	9.5	35	11	5	-1	0.49425	no
gi|641572549|gb|KK855786.1|	71265-72348	9.5	35	20	4	-2	0.0869	no
gi|641572549|gb|KK855786.1|	73751-74659	9.5	35	2	0.302	-3	0.1793	no
gi|641572549|gb|KK855786.1|	75511-75861	9.5	35	6	0.662703	-3	0.22375	no
gi|641572549|gb|KK855786.1|	87727-88128	9.5	35	4	2	-1	0.4958	no
gi|641572549|gb|KK855786.1|	99203-99566	9.5	35	13	7	-0.835243	0.5528	no
gi|641572553|gb|KK855785.1|	63019-63548	8.9	36	0.423499	3	3	0.27515	no
gi|641572557|gb|KK855784.1|	1-345	11	41	5	1	-2	0.3414	no
gi|641572557|gb|KK855784.1|	00446-10204	11	41	5	0	-inf	5.00E-05	yes
gi|641572557|gb|KK855784.1|	02102-10451	11	41	3	0.097258	-5	0.1309	no
gi|641572557|gb|KK855784.1|	49079-14982	11	41	1	0.194037	-3	0.2382	no
gi|641572557|gb|KK855784.1|	53072-15340	11	41	2	4	0.838406	0.63695	no
gi|641572557|gb|KK855784.1|	59623-16022	11	41	1	3	1	0.45675	no
gi|641572557|gb|KK855784.1|	74114-18065	11	41	9	3	-1	0.38245	no
gi|641572557|gb|KK855784.1|	83894-18448	11	41	6	2	-2	0.2473	no
gi|641572557|gb|KK855784.1|	97102-19858	11	41	2	2	-0.180755	0.88675	no
gi|641572557|gb|KK855784.1|	98801-19914	11	41	6	2	-2	0.40225	no
gi|641572557|gb|KK855784.1|	00877-20365	11	41	18	9	-1	0.52235	no
gi|641572557|gb|KK855784.1|	07220-21022	11	41	1	1	0.0556397	0.9669	no
gi|641572557|gb|KK855784.1|	17569-21864	11	41	0.726014	0.61373	-0.242393	1	no
gi|641572557|gb|KK855784.1|	22369-22285	11	41	2	2	0.369841	0.82765	no
gi|641572557|gb|KK855784.1|	63383-63922	11	41	2	0	-inf	0.01175	no
gi|641572557|gb|KK855784.1|	74807-76351	11	41	0.997444	2	1	0.43185	no
gi|641572561|gb|KK855783.1|	14397-11479	9.8	37	0	5	inf	0.0068	no
gi|641572561|gb|KK855783.1|	26257-12665	9.8	37	0.702532	5	3	0.28555	no
gi|641572561|gb|KK855783.1|	31354-13253	9.8	37	0.715395	1	0.869399	0.56565	no
gi|641572561|gb|KK855783.1|	33724-13494	9.8	37	0.693667	1	0.606545	0.6779	no

gi|641572561|gb|KK855783.1|	36406-13664	9.8	37	10	7	-0.452849	0.78585	no
gi|641572561|gb|KK855783.1|	36764-14154	9.8	37	5	3	-0.56219	0.64615	no
gi|641572561|gb|KK855783.1|	53550-15896	9.8	37	4	3	-0.394292	0.7495	no
gi|641572561|gb|KK855783.1|	9312-11939	9.8	37	0.669684	2	2	0.176	no
gi|641572565|gb|KK855782.1|	15996-16999	8.7	43	0.788934	2	1	0.43005	no
gi|641572569|gb|KK855781.1|	11597-13114	11	39	16	12	-0.45024	0.7232	no
gi|641572569|gb|KK855781.1|	18102-11919	11	39	3	2	-0.538629	0.6927	no
gi|641572569|gb|KK855781.1|	19839-12183	11	39	9	8	-0.166735	0.8949	no
gi|641572569|gb|KK855781.1|	24580-12547	11	39	0.203836	1	3	0.2991	no
gi|641572569|gb|KK855781.1|	26811-12734	11	39	25	14	-0.861305	0.50355	no
gi|641572569|gb|KK855781.1|	27465-13027	11	39	13	12	-0.11105	0.92635	no
gi|641572569|gb|KK855781.1|	30429-13137	11	39	1	1	0.118988	0.936	no
gi|641572569|gb|KK855781.1|	32923-13390	11	39	2	0.820186	-1	0.4411	no
gi|641572569|gb|KK855781.1|	34979-13560	11	39	7	5	-0.645142	0.63435	no
gi|641572569|gb|KK855781.1|	36230-13713	11	39	3	3	-0.132928	0.9202	no
gi|641572569|gb|KK855781.1|	38302-13942	11	39	0.997543	0.583932	-0.77258	0.63565	no
gi|641572569|gb|KK855781.1|	40261-14094	11	39	1	3	0.863407	0.56615	no
gi|641572569|gb|KK855781.1|	41716-14354	11	39	8	5	-0.55923	0.65235	no
gi|641572569|gb|KK855781.1|	14341-14976	11	39	1	3	1	0.49595	no
gi|641572569|gb|KK855781.1|	45625-14647	11	39	1	1	0.0887161	0.9392	no
gi|641572569|gb|KK855781.1|	46656-14710	11	39	3	2	-0.580676	0.7586	no
gi|641572569|gb|KK855781.1|	48582-15502	11	39	4	3	-0.303839	0.80655	no
gi|641572569|gb|KK855781.1|	56336-15977	11	39	8	7	-0.216154	0.8608	no
gi|641572569|gb|KK855781.1|	60215-16096	11	39	3	3	-0.0198612	0.96665	no
gi|641572569|gb|KK855781.1|	61261-16224	11	39	0.625226	1	0.797528	0.63025	no
gi|641572569|gb|KK855781.1|	16345-17594	11	39	8	8	0.123785	0.92105	no
gi|641572569|gb|KK855781.1|	64986-16595	11	39	0.460533	0.84035	0.867686	1	no
gi|641572569|gb|KK855781.1|	71300-17173	11	39	1	3	1	0.51205	no
gi|641572569|gb|KK855781.1|	72224-17360	11	39	0.954617	2	0.999672	0.5023	no
gi|641572569|gb|KK855781.1|	73710-17435	11	39	0.780009	2	1	0.4611	no

gi|641572569|gb|KK855781.1|	74826-17623	11	39	0.407347	0.886616	1	0.48415	no
gi|641572569|gb|KK855781.1|	77687-17910	11	39	0.635417	0.264038	-1	1	no
gi|641572569|gb|KK855781.1|	79269-18153	11	39	0.719438	0.679497	-0.082403	1	no
gi|641572569|gb|KK855781.1|	82205-18567	11	39	3	2	-0.1458	0.90835	no
gi|641572569|gb|KK855781.1|	86285-18723	11	39	0.281073	2	3	0.19335	no
gi|641572569|gb|KK855781.1|	19817-21549	11	39	4	0.280993	-4	0.0668	no
gi|641572569|gb|KK855781.1|	23638-24377	11	39	12	0.197576	-6	0.18295	no
gi|641572569|gb|KK855781.1|	24561-25909	11	39	6	0.5614	-3	0.05485	no
gi|641572569|gb|KK855781.1|	26307-27953	11	39	9	2	-2	0.11435	no
gi|641572569|gb|KK855781.1|	30579-31966	11	39	3	0.724121	-2	0.1731	no
gi|641572569|gb|KK855781.1|	35763-37264	11	39	4	0.826372	-2	0.1421	no
gi|641572569|gb|KK855781.1|	39505-40851	11	39	2	0.093729	-5	0.1886	no
gi|641572569|gb|KK855781.1|	41132-42732	11	39	7	0.07683	-7	0.1829	no
gi|641572569|gb|KK855781.1|	46013-49656	11	39	4	0.407645	-3	0.03555	no
gi|641572569|gb|KK855781.1|	50154-51507	11	39	5	0.93164	-2	0.0993	no
gi|641572569|gb|KK855781.1|	51598-52927	11	39	6	0.190258	-5	0.1311	no
gi|641572569|gb|KK855781.1|	59393-60918	11	39	26	7	-2	0.11985	no
gi|641572569|gb|KK855781.1|	6586-7397	11	39	30	46	0.635662	0.61335	no
gi|641572569|gb|KK855781.1|	67512-67756	11	39	44	43	-0.0523399	0.96735	no
gi|641572569|gb|KK855781.1|	67904-68118	11	39	18	22	0.247832	0.87255	no
gi|641572569|gb|KK855781.1|	69615-70259	11	39	1	2	0.921132	0.56365	no
gi|641572569|gb|KK855781.1|	72093-72467	11	39	66	13	-2	0.08465	no
gi|641572569|gb|KK855781.1|	75202-75618	11	39	2	7	2	0.3164	no
gi|641572569|gb|KK855781.1|	8054-9406	11	39	5	4	-0.392775	0.76185	no
gi|641572569|gb|KK855781.1|	82564-84446	11	39	0.587681	1	0.888938	0.53725	no
gi|641572569|gb|KK855781.1|	84937-86604	11	39	1	2	0.3353	0.79755	no
gi|641572569|gb|KK855781.1|	89140-89669	11	39	3	7	2	0.299	no
gi|641572569|gb|KK855781.1|	97673-98032	11	39	5	4	-0.420832	0.77855	no
gi|641572573|gb|KK855780.1|	66746-67323	9.8	43	3	4	0.490011	0.7338	no
gi|641572581|gb|KK855778.1|	08551-10918	9.9	37	2	2	0.0843936	0.94	no

gi|641572581|gb|KK855778.1|	15189-11561	9.9	37	0.908959	5	2	0.21375	no
gi|641572581|gb|KK855778.1|	19066-11961	9.9	37	4	7	0.595825	0.66385	no
gi|641572581|gb|KK855778.1|	11924-12713	9.9	37	1	0.905289	-0.53511	0.7478	no
gi|641572581|gb|KK855778.1|	15081-15345	9.9	37	24	25	0.0718663	0.95805	no
gi|641572581|gb|KK855778.1|	16866-17174	9.9	37	20	17	-0.270247	0.8446	no
gi|641572581|gb|KK855778.1|	18686-18879	9.9	37	71	78	0.12801	0.92105	no
gi|641572581|gb|KK855778.1|	19982-20215	9.9	37	16	18	0.167399	0.91285	no
gi|641572581|gb|KK855778.1|	52019-53025	9.9	37	0.69868	0.664195	-0.0730234	1	no
gi|641572581|gb|KK855778.1|	53116-53850	9.9	37	1	2	0.771316	0.6261	no
gi|641572581|gb|KK855778.1|	54629-55351	9.9	37	1	0.611693	-0.813895	0.6492	no
gi|641572581|gb|KK855778.1|	91003-91937	9.9	37	0.862625	0.437145	-0.980623	1	no
gi|641572581|gb|KK855778.1|	93345-94772	9.9	37	0.574838	0.788269	0.455533	1	no
gi|641572581|gb|KK855778.1|	95028-96154	9.9	37	0.83682	0.810508	-0.0460903	1	no
gi|641572581|gb|KK855778.1|	9540-10436	9.9	37	1	0.767855	-0.659127	0.6826	no
gi|641572585|gb|KK855777.1|	54372-55526	9.4	36	0.738527	0.449679	-0.715757	1	no
gi|641572585|gb|KK855777.1|	62233-63064	9.4	36	0.33413	2	3	0.1811	no
gi|641572589|gb|KK855776.1|	68633-16970	12	37	5	3	-0.809465	0.5385	no
gi|641572589|gb|KK855776.1|	16959-17745	12	37	3	4	0.409698	0.76875	no
gi|641572589|gb|KK855776.1|	78685-17999	12	37	7	4	-0.953677	0.4653	no
gi|641572589|gb|KK855776.1|	80125-18104	12	37	7	4	-0.888877	0.5019	no
gi|641572589|gb|KK855776.1|	88693-19053	12	37	2	1	-0.425209	0.7517	no
gi|641572589|gb|KK855776.1|	21618-23382	12	37	10	9	-0.15572	0.90415	no
gi|641572589|gb|KK855776.1|	21470-22210	12	37	1	0.736307	-0.816192	0.6492	no
gi|641572589|gb|KK855776.1|	23486-24076	12	37	0.716917	2	1	0.44255	no
gi|641572589|gb|KK855776.1|	29025-29739	12	37	2	0.828056	-1	0.4012	no
gi|641572589|gb|KK855776.1|	34620-35248	12	37	4	0.495352	-3	0.16885	no
gi|641572589|gb|KK855776.1|	38994-39370	12	37	7	2	-2	0.2469	no
gi|641572589|gb|KK855776.1|	56043-56322	12	37	36	6	-3	0.11205	no
gi|641572589|gb|KK855776.1|	57737-57989	12	37	10	7	-0.446738	0.7786	no
gi|641572589|gb|KK855776.1|	59077-59372	12	37	0.650553	14	4	0.11235	no

gi|641572589|gb|KK855776.1|	62524-62967	12	37	3	3	0.00411613	0.9394	no
gi|641572589|gb|KK855776.1|	63988-65258	12	37	0.197644	2	3	0.1719	no
gi|641572589|gb|KK855776.1|	83227-84313	12	37	10	9	-0.218268	0.86405	no
gi|641572589|gb|KK855776.1|	8501-9227	12	37	2	2	-0.206128	0.8956	no
gi|641572589|gb|KK855776.1|	85504-86687	12	37	5	3	-0.693208	0.60025	no
gi|641572589|gb|KK855776.1|	89443-91161	12	37	2	0.567138	-1	0.32395	no
gi|641572589|gb|KK855776.1|	91777-93502	12	37	1	0.776256	-0.391529	0.79045	no
gi|641572589|gb|KK855776.1|	9685-10410	12	37	0.935587	1	0.115779	0.92175	no
gi|641572593|gb|KK855775.1|	37496-13807	9.3	38	0.924098	2	1	0.4611	no
gi|641572593|gb|KK855775.1|	40500-14141	9.3	38	2	3	0.682098	0.6316	no
gi|641572593|gb|KK855775.1|	27966-28661	9.3	38	2	5	1	0.38145	no
gi|641572601|gb|KK855773.1|	04055-10480	10	35	4	5	0.245578	0.8589	no
gi|641572601|gb|KK855773.1|	06402-10705	10	35	3	3	0.014554	0.9647	no
gi|641572601|gb|KK855773.1|	08476-10953	10	35	2	3	0.412881	0.76875	no
gi|641572601|gb|KK855773.1|	09677-11103	10	35	0.915789	2	1	0.4739	no
gi|641572601|gb|KK855773.1|	13152-11460	10	35	3	3	-0.180853	0.8919	no
gi|641572601|gb|KK855773.1|	18924-11958	10	35	2	2	-0.178008	0.90465	no
gi|641572601|gb|KK855773.1|	22433-12279	10	35	81	66	-0.300636	0.8125	no
gi|641572601|gb|KK855773.1|	24384-12521	10	35	7	5	-0.524766	0.6932	no
gi|641572601|gb|KK855773.1|	26114-12684	10	35	4	2	-1	0.4141	no
gi|641572601|gb|KK855773.1|	27498-12869	10	35	1	0.756303	-0.673696	0.6649	no
gi|641572601|gb|KK855773.1|	28979-13026	10	35	0.912154	2	1	0.3508	no
gi|641572601|gb|KK855773.1|	31270-13227	10	35	12	11	-0.139113	0.91245	no
gi|641572601|gb|KK855773.1|	32380-13257	10	35	45	22	-1	0.4919	no
gi|641572601|gb|KK855773.1|	35370-13741	10	35	2	1	-1	0.4519	no
gi|641572601|gb|KK855773.1|	45931-14762	10	35	3	3	0.211053	0.8778	no
gi|641572601|gb|KK855773.1|	55056-15527	10	35	29	33	0.199152	0.8837	no
gi|641572601|gb|KK855773.1|	55392-15690	10	35	0.643808	0.081763	-3	1	no
gi|641572601|gb|KK855773.1|	62365-16517	10	35	13	13	0.0281012	0.9821	no
gi|641572601|gb|KK855773.1|	17040-18044	10	35	0.612856	0.798996	0.382638	1	no

gi|641572601|gb|KK855773.1|	72677-17348	10	35	0.808903	2	1	0.48415	no
gi|641572601|gb|KK855773.1|	78201-17898	10	35	16	7	-1	0.3501	no
gi|641572601|gb|KK855773.1|	79572-17979	10	35	14	15	0.026994	0.94155	no
gi|641572601|gb|KK855773.1|	88249-18896	10	35	6	2	-1	0.34975	no
gi|641572601|gb|KK855773.1|	89187-18972	10	35	1	2	0.37297	0.8276	no
gi|641572601|gb|KK855773.1|	91843-19281	10	35	12	4	-2	0.2471	no
gi|641572601|gb|KK855773.1|	94692-19492	10	35	78	17	-2	0.14155	no
gi|641572601|gb|KK855773.1|	97503-19797	10	35	3	0.775822	-2	0.2679	no
gi|641572601|gb|KK855773.1|	99873-20014	10	35	34	5	-3	0.10945	no
gi|641572601|gb|KK855773.1|	01488-20167	10	35	300	87	-2	0.1915	no
gi|641572601|gb|KK855773.1|	03083-20350	10	35	27	5	-2	0.1134	no
gi|641572601|gb|KK855773.1|	05162-20672	10	35	2	1	-0.735932	0.611	no
gi|641572601|gb|KK855773.1|	06917-20851	10	35	1	0.771587	-0.391891	0.7882	no
gi|641572601|gb|KK855773.1|	10002-21167	10	35	9	4	-1	0.31955	no
gi|641572601|gb|KK855773.1|	21024-22793	10	35	2	2	0.226708	0.8641	no
gi|641572601|gb|KK855773.1|	24507-24769	10	35	10	10	0.0972169	0.94475	no
gi|641572601|gb|KK855773.1|	27037-27402	10	35	3	5	0.580101	0.7371	no
gi|641572601|gb|KK855773.1|	27979-28444	10	35	2	6	2	0.3164	no
gi|641572601|gb|KK855773.1|	28573-28984	10	35	3	6	0.84904	0.5678	no
gi|641572601|gb|KK855773.1|	29359-30338	10	35	0.993781	2	0.845806	0.58475	no
gi|641572601|gb|KK855773.1|	30951-31698	10	35	1	2	0.601646	0.6936	no
gi|641572601|gb|KK855773.1|	32038-33391	10	35	0.183474	0.93164	2	0.2235	no
gi|641572601|gb|KK855773.1|	33877-34738	10	35	0.957218	0.484787	-0.981497	0.62775	no
gi|641572601|gb|KK855773.1|	35144-35554	10	35	2	6	1	0.3707	no
gi|641572601|gb|KK855773.1|	37504-38295	10	35	3	4	0.306971	0.82415	no
gi|641572601|gb|KK855773.1|	38742-39122	10	35	7	13	0.935899	0.5016	no
gi|641572601|gb|KK855773.1|	41275-43794	10	35	2	2	-0.207345	0.87085	no
gi|641572601|gb|KK855773.1|	47646-47937	10	35	7	10	0.56509	0.71515	no
gi|641572601|gb|KK855773.1|	48127-48511	10	35	4	3	-0.417203	0.77855	no
gi|641572601|gb|KK855773.1|	48655-50398	10	35	0.96069	2	0.916638	0.5087	no

gi|641572601|gb|KK855773.1|	50531-51849	10	35	14	35	1	0.44245	no
gi|641572601|gb|KK855773.1|	52131-53749	10	35	8	18	1	0.32325	no
gi|641572601|gb|KK855773.1|	53922-54666	10	35	0.387094	4	3	0.1563	no
gi|641572601|gb|KK855773.1|	54795-56262	10	35	4	9	1	0.367	no
gi|641572601|gb|KK855773.1|	57561-58722	10	35	18	65	2	0.29415	no
gi|641572601|gb|KK855773.1|	7268-8131	10	35	0.530195	2	2	0.3084	no
gi|641572601|gb|KK855773.1|	76533-77184	10	35	1	1	0.113978	0.92265	no
gi|641572601|gb|KK855773.1|	77709-80501	10	35	27	49	0.835317	0.7037	no
gi|641572601|gb|KK855773.1|	80757-82986	10	35	21	30	0.485409	0.83515	no
gi|641572601|gb|KK855773.1|	83083-83962	10	35	2	3	0.686161	0.6336	no
gi|641572601|gb|KK855773.1|	86417-88498	10	35	1	3	0.853126	0.51515	no
gi|641572601|gb|KK855773.1|	88600-90064	10	35	3	1	-0.85945	0.52935	no
gi|641572601|gb|KK855773.1|	91640-92311	10	35	5	4	-0.184325	0.89135	no
gi|641572601|gb|KK855773.1|	95715-96167	10	35	15	18	0.288174	0.8253	no
gi|641572604|gb|KK855772.1|	06018-10708	9.9	40	0	9	inf	5.00E-05	yes
gi|641572604|gb|KK855772.1|	92808-19373	9.9	40	0.194059	1	3	0.2991	no
gi|641572604|gb|KK855772.1|	81824-82685	9.9	40	1	2	0.603465	0.67455	no
gi|641572608|gb|KK855771.1|	04486-10476	11	42	36	20	-0.853491	0.5334	no
gi|641572608|gb|KK855771.1|	05116-10604	11	42	1	0.145911	-3	0.2348	no
gi|641572608|gb|KK855771.1|	08329-10878	11	42	38	18	-1	0.40105	no
gi|641572608|gb|KK855771.1|	12164-11269	11	42	3	3	0.331739	0.81855	no
gi|641572608|gb|KK855771.1|	13748-11481	11	42	17	13	-0.374052	0.7661	no
gi|641572608|gb|KK855771.1|	14924-11607	11	42	0.815783	0.79022	-0.0459309	1	no
gi|641572608|gb|KK855771.1|	16229-11998	11	42	4	2	-0.961405	0.44275	no
gi|641572608|gb|KK855771.1|	30786-13106	11	42	0	13	inf	0.00375	no
gi|641572608|gb|KK855771.1|	24946-26641	11	42	0	8	inf	5.00E-05	yes
gi|641572608|gb|KK855771.1|	46771-47580	11	42	1	0.52559	-0.982246	0.62775	no
gi|641572608|gb|KK855771.1|	48380-50132	11	42	3	4	0.248995	0.8484	no
gi|641572608|gb|KK855771.1|	55833-56588	11	42	1	2	0.923725	0.5636	no
gi|641572608|gb|KK855771.1|	61886-62442	11	42	0	4	inf	0.0023	yes

gi|641572608|gb|KK855771.1|	62618-63284	11	42	0.150381	23	7	0.10435	no
gi|641572608|gb|KK855771.1|	64787-65146	11	42	3	3	-0.228187	0.88445	no
gi|641572608|gb|KK855771.1|	73880-74246	11	42	3	3	-0.0978193	0.9262	no
gi|641572608|gb|KK855771.1|	74482-75017	11	42	2	0.314258	-3	0.2481	no
gi|641572608|gb|KK855771.1|	75882-76067	11	42	145	47	-2	0.24955	no
gi|641572608|gb|KK855771.1|	77949-78386	11	42	4	0.437284	-3	0.2242	no
gi|641572608|gb|KK855771.1|	78523-79452	11	42	1	0.293405	-2	0.31075	no
gi|641572608|gb|KK855771.1|	81339-81749	11	42	17	13	-0.441582	0.7464	no
gi|641572608|gb|KK855771.1|	94787-95277	11	42	33	18	-0.890766	0.4898	no
gi|641572608|gb|KK855771.1|	98447-98643	11	42	25	23	-0.123913	0.93375	no
gi|641572611|gb|KK855770.1|	42151-14309	11	22	7	4	-0.810549	0.5407	no
gi|641572611|gb|KK855770.1|	14656-15452	11	22	0.471464	3	2	0.21345	no
gi|641572611|gb|KK855770.1|	75344-17933	11	22	2	0.967488	-0.79361	0.5386	no
gi|641572611|gb|KK855770.1|	80679-18144	11	22	9	5.61	-0.700495	0.58885	no
gi|641572611|gb|KK855770.1|	82585-18357	11	22	16	22	0.432912	0.731	no
gi|641572611|gb|KK855770.1|	85204-18588	11	22	6	12	0.95522	0.46955	no
gi|641572615|gb|KK855769.1|	02657-10327	12	43	0.169402	3	4	0.1175	no
gi|641572615|gb|KK855769.1|	72412-17644	12	43	12	5	-1	0.39885	no
gi|641572615|gb|KK855769.1|	76945-17781	12	43	16	20	0.384389	0.75455	no
gi|641572615|gb|KK855769.1|	79051-18069	12	43	0.586885	0.447327	-0.391748	1	no
gi|641572615|gb|KK855769.1|	83948-18422	12	43	17	35	1	0.44735	no
gi|641572615|gb|KK855769.1|	84339-18672	12	43	1	0.889715	-0.264839	0.8424	no
gi|641572615|gb|KK855769.1|	87213-18811	12	43	0.902963	0.457468	-0.980996	0.62775	no
gi|641572615|gb|KK855769.1|	88736-18994	12	43	1	2	0.425928	0.76525	no
gi|641572615|gb|KK855769.1|	90158-19081	12	43	4	6	0.484039	0.7191	no
gi|641572615|gb|KK855769.1|	92054-19225	12	43	82	122	0.5677	0.66365	no
gi|641572615|gb|KK855769.1|	95274-19559	12	43	4	9	1	0.467	no
gi|641572615|gb|KK855769.1|	05751-20669	12	43	0.57045	1	0.826797	0.6232	no
gi|641572615|gb|KK855769.1|	07630-20922	12	43	1	1	0.193071	0.8869	no
gi|641572615|gb|KK855769.1|	76137-77590	12	43	0.844431	0.943588	0.160176	0.9153	no

gi|641572619|gb|KK855768.1|	35432-36525	9.8	38	0	1	inf	0.00455	no
gi|641572619|gb|KK855768.1|	88527-89107	9.8	38	0	5	inf	0.00135	yes
gi|641572622|gb|KK855767.1|	12632-11301	10	38	6	2	-1	0.38945	no
gi|641572622|gb|KK855767.1|	32520-13268	10	38	171	14	-4	0.1402	no
gi|641572622|gb|KK855767.1|	42934-14315	10	38	27	10	-1	0.3882	no
gi|641572622|gb|KK855767.1|	48569-14912	10	38	3	0.888897	-2	0.332	no
gi|641572622|gb|KK855767.1|	50967-15133	10	38	19	6	-2	0.2593	no
gi|641572622|gb|KK855767.1|	55229-15561	10	38	14	9	-0.627056	0.6419	no
gi|641572622|gb|KK855767.1|	57445-15867	10	38	2	0.417584	-3	0.1419	no
gi|641572622|gb|KK855767.1|	61670-16359	10	38	2	5	1	0.2855	no
gi|641572622|gb|KK855767.1|	64060-16461	10	38	1	2	0.818528	0.6234	no
gi|641572622|gb|KK855767.1|	66860-16777	10	38	1	2	0.478644	0.75585	no
gi|641572622|gb|KK855767.1|	69298-17102	10	38	175	125.46	-0.481516	0.80945	no
gi|641572622|gb|KK855767.1|	71819-17420	10	38	0.194363	3	4	0.06825	no
gi|641572622|gb|KK855767.1|	74327-17522	10	38	0	12	inf	5.00E-05	yes
gi|641572622|gb|KK855767.1|	75714-17714	10	38	0.172591	1	3	0.1666	no
gi|641572634|gb|KK855764.1|	20263-12398	13	21	25	5	-2	0.1935	no
gi|641572634|gb|KK855764.1|	24375-12622	13	21	0.941357	0.326255	-2	0.3482	no
gi|641572634|gb|KK855764.1|	26790-12867	13	21	2	2	-0.138402	0.9168	no
gi|641572634|gb|KK855764.1|	31374-13201	13	21	0.79754	4	2	0.2128	no
gi|641572634|gb|KK855764.1|	36713-13765	13	21	199	657	2	0.38465	no
gi|641572634|gb|KK855764.1|	44577-14514	13	21	13	9	-0.625708	0.6321	no
gi|641572634|gb|KK855764.1|	47550-15032	13	21	24	36	0.603307	0.78455	no
gi|641572634|gb|KK855764.1|	50861-51435	13	21	0.933804	2	1	0.4611	no
gi|641572634|gb|KK855764.1|	60200-60885	13	21	2	0	-inf	0.0102	no
gi|641572637|gb|KK855763.1|	02351-10436	9.5	36	27	23	-0.23067	0.91535	no
gi|641572637|gb|KK855763.1|	04676-10524	9.5	36	25	9	-1	0.2991	no
gi|641572637|gb|KK855763.1|	11750-11228	9.5	36	2	0.626708	-2	0.31075	no
gi|641572637|gb|KK855763.1|	14129-11803	9.5	36	16	103	3	0.22225	no
gi|641572637|gb|KK855763.1|	22480-12411	9.5	36	10	14	0.430453	0.7257	no

gi|641572637|gb|KK855763.1|	25125-12659	9.5	36	2	1	-0.598767	0.6707	no
gi|641572637|gb|KK855763.1|	26747-12767	9.5	36	1	0.736507	-0.658782	0.6826	no
gi|641572637|gb|KK855763.1|	28896-12970	9.5	36	1	0.348139	-2	0.4266	no
gi|641572637|gb|KK855763.1|	30368-13098	9.5	36	0.677608	2	2	0.3613	no
gi|641572637|gb|KK855763.1|	31505-13249	9.5	36	0.44659	1	1	0.3994	no
gi|641572637|gb|KK855763.1|	32943-13419	9.5	36	0.133754	2	4	0.243	no
gi|641572637|gb|KK855763.1|	38164-14294	9.5	36	59	35	-0.738904	0.7158	no
gi|641572637|gb|KK855763.1|	43066-14469	9.5	36	532	284	-0.907514	0.6001	no
gi|641572637|gb|KK855763.1|	45003-14908	9.5	36	32	19	-0.797458	0.7087	no
gi|641572637|gb|KK855763.1|	14532-15218	9.5	36	2	2	0.221812	0.8867	no
gi|641572637|gb|KK855763.1|	50066-15149	9.5	36	2	0.785726	-1	0.46365	no
gi|641572637|gb|KK855763.1|	51930-15241	9.5	36	2	69	5	0.0237	no
gi|641572637|gb|KK855763.1|	15400-16478	9.5	36	1	0.366162	-2	0.28145	no
gi|641572637|gb|KK855763.1|	63422-16531	9.5	36	6	6	-0.0549629	0.9667	no
gi|641572637|gb|KK855763.1|	65752-16820	9.5	36	4	2	-0.813498	0.52225	no
gi|641572637|gb|KK855763.1|	16869-17718	9.5	36	1	0.329087	-2	0.26805	no
gi|641572637|gb|KK855763.1|	69218-17034	9.5	36	10	2	-2	0.10525	no
gi|641572637|gb|KK855763.1|	18017-21093	9.5	36	27	11	-1	0.4351	no
gi|641572637|gb|KK855763.1|	82404-18265	9.5	36	15	8	-0.934348	0.5697	no
gi|641572637|gb|KK855763.1|	89151-18960	9.5	36	2	3	0.812059	0.6234	no
gi|641572637|gb|KK855763.1|	95625-19619	9.5	36	3	4	0.596806	0.67485	no
gi|641572637|gb|KK855763.1|	99300-19951	9.5	36	8	24	2	0.38095	no
gi|641572637|gb|KK855763.1|	02050-20230	9.5	36	16	8	-0.948537	0.55415	no
gi|641572637|gb|KK855763.1|	07595-20865	9.5	36	30	7	-2	0.1103	no
gi|641572637|gb|KK855763.1|	12804-21370	9.5	36	0.89914	0.60739	-0.565923	0.7586	no
gi|641572637|gb|KK855763.1|	15040-21545	9.5	36	8	2	-2	0.2087	no
gi|641572637|gb|KK855763.1|	21562-22267	9.5	36	18	7	-1	0.29185	no
gi|641572637|gb|KK855763.1|	23770-22608	9.5	36	0.835848	0.255063	-2	1	no
gi|641572637|gb|KK855763.1|	26884-22737	9.5	36	2	1	-0.822609	0.6492	no
gi|641572637|gb|KK855763.1|	27561-23080	9.5	36	2	0.994387	-0.626646	0.6322	no

gi|641572637|gb|KK855763.1|	31028-23347	9.5	36	5	2	-0.956268	0.4541	no
gi|641572637|gb|KK855763.1|	33576-23567	9.5	36	8	7	-0.0717051	0.9514	no
gi|641572637|gb|KK855763.1|	35830-23949	9.5	36	8	9	0.249178	0.8337	no
gi|641572637|gb|KK855763.1|	39767-24094	9.5	36	5	4	-0.241651	0.849	no
gi|641572637|gb|KK855763.1|	42131-24242	9.5	36	40	35	-0.19469	0.87945	no
gi|641572637|gb|KK855763.1|	44172-24438	9.5	36	22	14	-0.683613	0.657	no
gi|641572637|gb|KK855763.1|	45853-24617	9.5	36	72	45	-0.68075	0.59515	no
gi|641572637|gb|KK855763.1|	46485-24666	9.5	36	327	178	-0.880872	0.50325	no
gi|641572637|gb|KK855763.1|	51202-25189	9.5	36	3	2	-0.247576	0.8669	no
gi|641572637|gb|KK855763.1|	52112-25359	9.5	36	0.550694	1	0.870772	0.56565	no
gi|641572637|gb|KK855763.1|	27485-28021	9.5	36	2	16	3	0.07425	no
gi|641572637|gb|KK855763.1|	30452-31436	9.5	36	0	2	inf	0.00255	yes
gi|641572637|gb|KK855763.1|	35102-36480	9.5	36	2	3	0.659805	0.62845	no
gi|641572637|gb|KK855763.1|	41519-42106	9.5	36	0.36119	11	5	0.23355	no
gi|641572637|gb|KK855763.1|	45191-45981	9.5	36	12	48	2	0.1237	no
gi|641572637|gb|KK855763.1|	46612-48201	9.5	36	1	4	1	0.3049	no
gi|641572637|gb|KK855763.1|	49089-49445	9.5	36	6	22	2	0.23005	no
gi|641572637|gb|KK855763.1|	52533-52815	9.5	36	13	111	3	0.04515	no
gi|641572637|gb|KK855763.1|	54186-54714	9.5	36	2	15	3	0.0791	no
gi|641572637|gb|KK855763.1|	55060-56157	9.5	36	0.628169	0.955951	0.605783	0.6936	no
gi|641572637|gb|KK855763.1|	56327-58552	9.5	36	1	3	1	0.38385	no
gi|641572637|gb|KK855763.1|	60805-63011	9.5	36	44	25	-0.798734	0.7214	no
gi|641572637|gb|KK855763.1|	7-336	9.5	36	0	15	inf	0.00145	yes
gi|641572637|gb|KK855763.1|	72220-72966	9.5	36	22	30	0.451487	0.71245	no
gi|641572637|gb|KK855763.1|	73841-75002	9.5	36	7	4	-0.754186	0.56555	no
gi|641572637|gb|KK855763.1|	77765-78017	9.5	36	43	46	0.0938306	0.9417	no
gi|641572637|gb|KK855763.1|	80218-80569	9.5	36	19	13	-0.600479	0.6553	no
gi|641572637|gb|KK855763.1|	83651-83911	9.5	36	13	13	0.0705668	0.95555	no
gi|641572637|gb|KK855763.1|	85083-85958	9.5	36	18	6	-2	0.24865	no
gi|641572637|gb|KK855763.1|	86107-87071	9.5	36	23	4	-2	0.06985	no

gi|641572637|gb|KK855763.1|	87137-90530	9.5	36	10	3	-2	0.1597	no
gi|641572637|gb|KK855763.1|	91055-96050	9.5	36	9	7	-0.396237	0.79625	no
gi|641572641|gb|KK855762.1|	13346-13718	12	41	4	3	-0.266857	0.8654	no
gi|641572641|gb|KK855762.1|	53526-15426	12	41	1	2	0.771436	0.6261	no
gi|641572641|gb|KK855762.1|	64759-16526	12	41	0	11	inf	0.0003	yes
gi|641572641|gb|KK855762.1|	17821-18222	12	41	2	4	0.807285	0.6409	no
gi|641572641|gb|KK855762.1|	18308-19844	12	41	0.844965	1	0.695389	0.6277	no
gi|641572641|gb|KK855762.1|	25918-26454	12	41	12	12	-0.0283149	0.98465	no
gi|641572641|gb|KK855762.1|	68789-69321	12	41	1	1	0.00996186	0.91115	no
gi|641572641|gb|KK855762.1|	78069-79599	12	41	0.636573	2	2	0.23815	no
gi|641572641|gb|KK855762.1|	79944-81090	12	41	0.968164	0.906892	-0.0943199	0.9478	no
gi|641572647|gb|KK855760.1|	46096-14665	9.6	38	7	0	-inf	0.00045	yes
gi|641572647|gb|KK855760.1|	49723-15050	9.6	38	2	0	-inf	0.00495	no
gi|641572655|gb|KK855758.1|	02538-10662	19	94	150	2	-6	0.0039	no
gi|641572655|gb|KK855758.1|	06797-10753	19	94	98	1	-6	0.04015	no
gi|641572655|gb|KK855758.1|	16041-11709	19	94	0.74381	0.502962	-0.564486	1	no
gi|641572655|gb|KK855758.1|	17366-11923	19	94	0.50916	4	3	0.0714	no
gi|641572655|gb|KK855758.1|	19346-12085	19	94	0.592853	0.246407	-1	1	no
gi|641572655|gb|KK855758.1|	20984-12194	19	94	0.924675	0	-inf	0.01175	no
gi|641572655|gb|KK855758.1|	22733-12378	19	94	0.910164	4	2	0.16315	no
gi|641572655|gb|KK855758.1|	31605-13362	19	94	2	3	0.39046	0.7654	no
gi|641572655|gb|KK855758.1|	33753-13842	19	94	2	0.2656	-3	0.05845	no
gi|641572655|gb|KK855758.1|	40393-14290	19	94	1	0.60761	-0.93771	0.5024	no
gi|641572655|gb|KK855758.1|	43413-14533	19	94	4	0.438263	-3	0.06825	no
gi|641572655|gb|KK855758.1|	45471-14772	19	94	1	0.262868	-2	0.17325	no
gi|641572655|gb|KK855758.1|	52060-15288	19	94	0.336246	2	3	0.18845	no
gi|641572655|gb|KK855758.1|	54317-15603	19	94	84	23	-2	0.26575	no
gi|641572655|gb|KK855758.1|	16399-19573	19	94	10	0.181439	-6	0.0588	no
gi|641572655|gb|KK855758.1|	64741-16610	19	94	106	77	-0.466164	0.83095	no
gi|641572655|gb|KK855758.1|	66392-16857	19	94	53	26	-1	0.65355	no

gi|641572655|gb|KK855758.1|	69319-17160	19	94	4	2	-1	0.42435	no
gi|641572655|gb|KK855758.1|	72268-17412	19	94	8	2	-2	0.1385	no
gi|641572655|gb|KK855758.1|	74631-17681	19	94	6	2	-2	0.1693	no
gi|641572655|gb|KK855758.1|	78198-17921	19	94	1	0.393686	-2	0.29015	no
gi|641572655|gb|KK855758.1|	79592-17997	19	94	3	8	1	0.4458	no
gi|641572655|gb|KK855758.1|	80664-18103	19	94	30	2	-4	0.0995	no
gi|641572655|gb|KK855758.1|	90418-19078	19	94	21	9	-1	0.3916	no
gi|641572655|gb|KK855758.1|	93063-19331	19	94	30	11	-1	0.33275	no
gi|641572655|gb|KK855758.1|	93495-19417	19	94	0.146046	3	4	0.11365	no
gi|641572655|gb|KK855758.1|	96095-19658	19	94	11	3	-2	0.22105	no
gi|641572655|gb|KK855758.1|	20067-21475	19	94	20	2	-3	0.02195	no
gi|641572655|gb|KK855758.1|	00750-20196	19	94	0.975915	0.318409	-2	0.33985	no
gi|641572655|gb|KK855758.1|	02619-20405	19	94	1	0.174324	-3	0.1718	no
gi|641572655|gb|KK855758.1|	06463-20873	19	94	0.886871	0.312257	-2	0.3311	no
gi|641572655|gb|KK855758.1|	10217-21202	19	94	0.836757	0.201461	-2	1	no
gi|641572655|gb|KK855758.1|	13444-21482	19	94	0.779568	1	0.366321	0.8038	no
gi|641572655|gb|KK855758.1|	15072-21954	19	94	1	1	-0.0781171	0.95005	no
gi|641572655|gb|KK855758.1|	20489-22170	19	94	2	2	-0.0308619	0.9778	no
gi|641572655|gb|KK855758.1|	21853-22301	19	94	2	0.559194	-2	0.3475	no
gi|641572655|gb|KK855758.1|	23142-22383	19	94	2	0.641886	-2	0.403	no
gi|641572655|gb|KK855758.1|	24975-22636	19	94	2	1	-0.779639	0.586	no
gi|641572655|gb|KK855758.1|	22883-24387	19	94	1	0	-inf	0.0013	yes
gi|641572655|gb|KK855758.1|	24531-24905	19	94	4	1	-2	0.31725	no
gi|641572655|gb|KK855758.1|	26750-28173	19	94	3	0.17574	-4	0.1339	no
gi|641572655|gb|KK855758.1|	29269-31732	19	94	5	0.619379	-3	0.04425	no
gi|641572655|gb|KK855758.1|	3305-4029	19	94	2	0.203133	-3	0.2348	no
gi|641572655|gb|KK855758.1|	33295-34435	19	94	1	0	-inf	0.0059	no
gi|641572655|gb|KK855758.1|	34537-36213	19	94	2	0	-inf	0.00055	yes
gi|641572655|gb|KK855758.1|	36314-38323	19	94	1	0.119087	-3	0.144	no
gi|641572655|gb|KK855758.1|	38455-40725	19	94	3	0.104136	-5	0.13145	no

gi|641572655|gb|KK855758.1|	4082-7819	19	94	4	0.274734	-4	0.0277	no
gi|641572655|gb|KK855758.1|	40868-43069	19	94	10	1	-3	0.03605	no
gi|641572655|gb|KK855758.1|	43967-45838	19	94	17	0.579918	-5	0.0158	no
gi|641572655|gb|KK855758.1|	46364-47602	19	94	11	0	-inf	5.00E-05	yes
gi|641572655|gb|KK855758.1|	49378-50460	19	94	18	0.729035	-5	0.03115	no
gi|641572655|gb|KK855758.1|	50623-74171	19	94	12	3	-2	0.32905	no
gi|641572655|gb|KK855758.1|	74628-75254	19	94	3	0.746434	-2	0.24335	no
gi|641572655|gb|KK855758.1|	75787-86137	19	94	11	0.319642	-5	0.02715	no
gi|641572655|gb|KK855758.1|	7871-9880	19	94	3	0.357262	-3	0.07045	no
gi|641572655|gb|KK855758.1|	86354-86654	19	94	17	0	-inf	0.0013	yes
gi|641572655|gb|KK855758.1|	86736-90932	19	94	31	0.891903	-5	0.02415	no
gi|641572655|gb|KK855758.1|	91730-93981	19	94	134	5	-5	0.0263	no
gi|641572655|gb|KK855758.1|	94150-98069	19	94	38	0.580713	-6	0.01425	no
gi|641572655|gb|KK855758.1|	98393-99441	19	94	12	0.126327	-7	0.1829	no
gi|641572655|gb|KK855758.1|	99555-10099[illegible]	19	94	12	0.346973	-5	0.06135	no
gi|641572655|gb|KK855758.1|	9957-14741	19	94	7	0.023567	-8	0.18285	no
gi|641572659|gb|KK855757.1|	3622-4558	7.6	31	0.286765	1	2	0.3323	no
gi|641572663|gb|KK855756.1|	[illegible]01016-10178	8.8	38	14	9	-0.564547	0.66035	no
gi|641572663|gb|KK855756.1|	[illegible]03864-10544	8.8	38	5	4	-0.518694	0.6832	no
gi|641572663|gb|KK855756.1|	[illegible]07179-10808	8.8	38	1	1	-0.289059	0.85105	no
gi|641572663|gb|KK855756.1|	[illegible]08169-10878	8.8	38	2	2	-0.401763	0.8086	no
gi|641572663|gb|KK855756.1|	[illegible]13196-11402	8.8	38	203	97	-1	0.6298	no
gi|641572663|gb|KK855756.1|	[illegible]14142-11497	8.8	38	3	3	0.266042	0.8441	no
gi|641572663|gb|KK855756.1|	[illegible]16237-11921	8.8	38	4	4	-0.14808	0.90475	no
gi|641572663|gb|KK855756.1|	[illegible]21119-12194	8.8	38	4	13	2	0.1916	no
gi|641572663|gb|KK855756.1|	[illegible]22740-12357	8.8	38	0.556009	2	2	0.3084	no
gi|641572663|gb|KK855756.1|	[illegible]24996-12654	8.8	38	0.94285	2	0.897469	0.52215	no
gi|641572663|gb|KK855756.1|	[illegible]27013-12998	8.8	38	16	24	0.594138	0.79255	no
gi|641572663|gb|KK855756.1|	[illegible]35588-13635	8.8	38	0.495374	10	4	0.1186	no
gi|641572663|gb|KK855756.1|	[illegible]38518-13906	8.8	38	0.601304	2	2	0.2812	no

gi|641572663|gb|KK855756.1|	25006-25363	8.8	38	3	3	-0.00611482	0.91115	no
gi|641572663|gb|KK855756.1|	25678-28013	8.8	38	0	10	inf	5.00E-05	yes
gi|641572663|gb|KK855756.1|	2818-3907	8.8	38	0.079224	3	5	0.1072	no
gi|641572663|gb|KK855756.1|	36792-38020	8.8	38	3	0.208792	-4	0.1368	no
gi|641572663|gb|KK855756.1|	38104-40668	8.8	38	13	9	-0.526332	0.66645	no
gi|641572663|gb|KK855756.1|	4079-4981	8.8	38	0	3	inf	0.00145	yes
gi|641572663|gb|KK855756.1|	48612-49204	8.8	38	0.17833	4	4	0.1136	no
gi|641572663|gb|KK855756.1|	52809-53352	8.8	38	0.813178	5	3	0.18535	no
gi|641572663|gb|KK855756.1|	62399-62567	8.8	38	24	95	2	0.27195	no
gi|641572663|gb|KK855756.1|	6345-7323	8.8	38	0.180918	5	5	0.2352	no
gi|641572663|gb|KK855756.1|	78550-79426	8.8	38	6	9	0.655191	0.6134	no
gi|641572663|gb|KK855756.1|	82792-84306	8.8	38	0.590605	2	2	0.27705	no
gi|641572663|gb|KK855756.1|	84985-86315	8.8	38	0.686375	1	0.732657	0.63585	no
gi|641572663|gb|KK855756.1|	8770-10363	8.8	38	0.101313	2	4	0.236	no
gi|641572667|gb|KK855755.1|	.02448-10263	11	41	45	23	-0.977548	0.55285	no
gi|641572667|gb|KK855755.1|	.03951-10437	11	41	4	6	0.694349	0.64195	no
gi|641572667|gb|KK855755.1|	.09341-11044	11	41	0.545414	1	1	0.39245	no
gi|641572667|gb|KK855755.1|	.13591-11480	11	41	2	1	-0.171096	0.89865	no
gi|641572667|gb|KK855755.1|	.14893-11690	11	41	0.780327	1	0.609077	0.6649	no
gi|641572667|gb|KK855755.1|	.17868-11830	11	41	1	4	1	0.4611	no
gi|641572667|gb|KK855755.1|	.24442-12460	11	41	0	174	inf	0.00135	yes
gi|641572667|gb|KK855755.1|	.25917-12716	11	41	0.134134	23	7	0.2322	no
gi|641572667|gb|KK855755.1|	.71900-17229	11	41	5	7	0.584305	0.71055	no
gi|641572667|gb|KK855755.1|	.83999-18669	11	41	10	4	-1	0.317	no
gi|641572667|gb|KK855755.1|	.87194-18856	11	41	45	42	-0.0757992	0.97345	no
gi|641572667|gb|KK855755.1|	.91881-19305	11	41	12	10	-0.368348	0.7695	no
gi|641572667|gb|KK855755.1|	.95659-19611	11	41	0	4	inf	0.00455	no
gi|641572667|gb|KK855755.1|	.96543-19701	11	41	1	4	2	0.26255	no
gi|641572667|gb|KK855755.1|	.99039-20019	11	41	3	1	-1	0.40095	no
gi|641572667|gb|KK855755.1|	.03745-20479	11	41	0.665828	0.633115	-0.0726814	1	no

gi|641572667|gb|KK855755.1|	09357-20973	11	41	3	5	0.581849	0.73035	no
gi|641572667|gb|KK855755.1|	27528-28127	11	41	2	6	1	0.3271	no
gi|641572667|gb|KK855755.1|	28309-31181	11	41	3	3	0.375662	0.7707	no
gi|641572667|gb|KK855755.1|	31296-32406	11	41	4	5	0.367716	0.77665	no
gi|641572667|gb|KK855755.1|	33287-34511	11	41	6	5	-0.2274	0.86005	no
gi|641572667|gb|KK855755.1|	35748-35997	11	41	91	94	0.0537314	0.9641	no
gi|641572667|gb|KK855755.1|	36526-39789	11	41	4	3	-0.141664	0.90895	no
gi|641572667|gb|KK855755.1|	39939-41021	11	41	5	5	0.140008	0.91555	no
gi|641572667|gb|KK855755.1|	49507-51896	11	41	2	2	0.46379	0.7254	no
gi|641572667|gb|KK855755.1|	57861-58685	11	41	7	13	0.981457	0.4522	no
gi|641572667|gb|KK855755.1|	60202-60754	11	41	4	4	-0.0670948	0.96125	no
gi|641572667|gb|KK855755.1|	60904-61884	11	41	0.992515	1	0.467304	0.75495	no
gi|641572667|gb|KK855755.1|	64273-66256	11	41	0.871323	2	0.904484	0.5195	no
gi|641572667|gb|KK855755.1|	66798-67096	11	41	18	28	0.615788	0.64945	no
gi|641572667|gb|KK855755.1|	69333-69530	11	41	113	228	1	0.43755	no
gi|641572667|gb|KK855755.1|	72321-72555	11	41	42	61	0.546327	0.6847	no
gi|641572667|gb|KK855755.1|	74613-76345	11	41	0.230373	1	2	0.2077	no
gi|641572667|gb|KK855755.1|	77140-78628	11	41	0.328627	1	2	0.27955	no
gi|641572667|gb|KK855755.1|	83392-83691	11	41	33	45	0.451649	0.7356	no
gi|641572667|gb|KK855755.1|	85398-86349	11	41	1	13	3	0.042	no
gi|641572667|gb|KK855755.1|	86605-89333	11	41	2	3	0.550295	0.6612	no
gi|641572667|gb|KK855755.1|	94901-95290	11	41	17	6	-1	0.33765	no
gi|641572671|gb|KK855754.1|	03783-10533	10	38	0.260171	0.951684	2	0.28495	no
gi|641572671|gb|KK855754.1|	06086-10630	10	38	0	74	inf	0.0003	yes
gi|641572671|gb|KK855754.1|	09991-11025	10	38	132.42	150	0.1765	0.8872	no
gi|641572671|gb|KK855754.1|	14025-11427	10	38	13	2	-3	0.2236	no
gi|641572671|gb|KK855754.1|	16294-11696	10	38	1	1	0.0150094	0.9394	no
gi|641572671|gb|KK855754.1|	17638-11871	10	38	1	3	1	0.41185	no
gi|641572671|gb|KK855754.1|	18991-11958	10	38	1	2	1	0.47095	no
gi|641572671|gb|KK855754.1|	20238-12287	10	38	89	100	0.158487	0.9349	no

gi|641572671|gb|KK855754.1|	26505-12705	10	38	0.397513	7	4	0.2397	no
gi|641572671|gb|KK855754.1|	27650-12846	10	38	0.459978	2	2	0.2441	no
gi|641572671|gb|KK855754.1|	28757-12989	10	38	0	2	inf	0.00145	yes
gi|641572671|gb|KK855754.1|	30209-13144	10	38	0.204889	3	4	0.1404	no
gi|641572671|gb|KK855754.1|	31555-13196	10	38	0.991748	5	2	0.21375	no
gi|641572671|gb|KK855754.1|	32549-13402	10	38	0.055112	1	5	0.1123	no
gi|641572671|gb|KK855754.1|	34202-13436	10	38	0	167	inf	0.00115	yes
gi|641572671|gb|KK855754.1|	34593-13521	10	38	0.164688	3	4	0.1175	no
gi|641572671|gb|KK855754.1|	37785-13794	10	38	8	120	4	0.1204	no
gi|641572671|gb|KK855754.1|	39815-13999	10	38	0	91	inf	0.0016	yes
gi|641572671|gb|KK855754.1|	46407-14811	10	38	0.32829	3	3	0.0898	no
gi|641572671|gb|KK855754.1|	48284-14937	10	38	0	25	inf	5.00E-05	yes
gi|641572671|gb|KK855754.1|	50089-15171	10	38	11	9	-0.360076	0.7711	no
gi|641572671|gb|KK855754.1|	52285-15277	10	38	0	4	inf	0.00375	no
gi|641572671|gb|KK855754.1|	52893-15439	10	38	0.815888	2	1	0.4516	no
gi|641572671|gb|KK855754.1|	56339-15692	10	38	19	7	-1	0.2729	no
gi|641572671|gb|KK855754.1|	16326-18935	10	38	1	2	0.646412	0.6255	no
gi|641572671|gb|KK855754.1|	84173-18455	10	38	0.368941	7	4	0.1178	no
gi|641572671|gb|KK855754.1|	25737-22612	10	38	5	3	-0.902762	0.57745	no
gi|641572671|gb|KK855754.1|	28579-22937	10	38	1	1	0.0174639	0.9394	no
gi|641572671|gb|KK855754.1|	52922-25861	10	38	11	9	-0.284857	0.8969	no
gi|641572671|gb|KK855754.1|	59219-26013	10	38	2	0.747699	-1	0.43755	no
gi|641572671|gb|KK855754.1|	66877-26773	10	38	4	12	2	0.25415	no
gi|641572671|gb|KK855754.1|	69013-26945	10	38	10	15	0.548176	0.6741	no
gi|641572671|gb|KK855754.1|	69697-26998	10	38	10	10	-0.0209287	0.9597	no
gi|641572671|gb|KK855754.1|	29791-30073	10	38	12	6	-0.939826	0.55575	no
gi|641572671|gb|KK855754.1|	36795-37064	10	38	15	4	-2	0.2469	no
gi|641572671|gb|KK855754.1|	57364-58921	10	38	3	4	0.462143	0.72635	no
gi|641572671|gb|KK855754.1|	66376-66925	10	38	2	2	0.42581	0.78025	no
gi|641572671|gb|KK855754.1|	73790-74662	10	38	2	0.635939	-2	0.2683	no

gi|641572671|gb|KK855754.1|	74819-75620	10	38	1	0.532485	-1	0.50885	no
gi|641572671|gb|KK855754.1|	77651-78755	10	38	1	0.237155	-2	0.2515	no
gi|641572671|gb|KK855754.1|	95016-99534	10	38	18	13	-0.553507	0.80505	no
gi|641572675|gb|KK855753.1|	03829-10505	11	40	0.206281	5	5	0.13655	no
gi|641572675|gb|KK855753.1|	06907-10735	11	40	9	5	-0.995214	0.49945	no
gi|641572675|gb|KK855753.1|	14417-11546	11	40	2	1	-0.854066	0.5652	no
gi|641572675|gb|KK855753.1|	17235-11745	11	40	26	40	0.622185	0.66505	no
gi|641572675|gb|KK855753.1|	22819-12330	11	40	3	2	-0.892935	0.58265	no
gi|641572675|gb|KK855753.1|	23467-12440	11	40	0.669119	0.726618	0.118935	1	no
gi|641572675|gb|KK855753.1|	29556-12976	11	40	30	14	-1	0.50385	no
gi|641572675|gb|KK855753.1|	33979-13519	11	40	3	3	-0.0939113	0.9422	no
gi|641572675|gb|KK855753.1|	37889-13812	11	40	24	22	-0.143112	0.9205	no
gi|641572675|gb|KK855753.1|	42049-14270	11	40	24	13	-0.887666	0.48685	no
gi|641572675|gb|KK855753.1|	86831-18813	11	40	2	2	-0.508473	0.7074	no
gi|641572675|gb|KK855753.1|	88280-18994	11	40	3	0.220624	-4	0.101	no
gi|641572675|gb|KK855753.1|	90654-19192	11	40	1	0.100239	-3	0.206	no
gi|641572675|gb|KK855753.1|	92604-19347	11	40	2	0.477656	-2	0.24335	no
gi|641572675|gb|KK855753.1|	94646-19638	11	40	0.780728	2	1	0.34975	no
gi|641572675|gb|KK855753.1|	96529-19774	11	40	2	4	1	0.45005	no
gi|641572675|gb|KK855753.1|	98385-19947	11	40	0.868549	0.961257	0.146315	0.9285	no
gi|641572675|gb|KK855753.1|	00772-20171	11	40	0.941897	2	1	0.47135	no
gi|641572675|gb|KK855753.1|	20170-20558	11	40	3	0.541888	-3	0.2588	no
gi|641572675|gb|KK855753.1|	01859-20573	11	40	29	47	0.677627	0.7428	no
gi|641572675|gb|KK855753.1|	09835-21042	11	40	2	0	-inf	0.01	no
gi|641572675|gb|KK855753.1|	21643-22903	11	40	0.266	1	2	0.23615	no
gi|641572675|gb|KK855753.1|	35529-36210	11	40	1	5	2	0.21505	no
gi|641572675|gb|KK855753.1|	36414-36549	11	40	90	286	2	0.3461	no
gi|641572675|gb|KK855753.1|	37842-38476	11	40	27	98	2	0.29135	no
gi|641572675|gb|KK855753.1|	38622-41479	11	40	18	74	2	0.3673	no
gi|641572675|gb|KK855753.1|	41583-42567	11	40	0.807939	7	3	0.07155	no

gi|641572675|gb|KK855753.1|	43964-45103	11	40	0.22508	2	3	0.1719	no
gi|641572675|gb|KK855753.1|	45243-49639	11	40	2	14	3	0.166	no
gi|641572675|gb|KK855753.1|	57353-59072	11	40	24	2	-4	0.079	no
gi|641572675|gb|KK855753.1|	59161-59445	11	40	18	0	-inf	0.0022	yes
gi|641572675|gb|KK855753.1|	59503-61369	11	40	9	0.581649	-4	0.0233	no
gi|641572675|gb|KK855753.1|	61508-64940	11	40	6	1	-2	0.07385	no
gi|641572675|gb|KK855753.1|	65411-67380	11	40	13	6	-1	0.3448	no
gi|641572675|gb|KK855753.1|	7945-8570	11	40	2	0.249383	-3	0.2481	no
gi|641572675|gb|KK855753.1|	86-775	11	40	0.143388	13	6	0.1047	no
gi|641572675|gb|KK855753.1|	92551-93681	11	40	0.378745	1	2	0.32945	no
gi|641572675|gb|KK855753.1|	93863-94802	11	40	0.571217	0.723703	0.341359	1	no
gi|641572679|gb|KK855752.1|	22113-12248	8.3	35	2	2	-0.00339378	0.91115	no
gi|641572679|gb|KK855752.1|	26831-27876	8.3	35	2	88	6	0.02355	no
gi|641572679|gb|KK855752.1|	29301-31289	8.3	35	200	0	-inf	5.00E-05	yes
gi|641572679|gb|KK855752.1|	31730-32315	8.3	35	90	0	-inf	5.00E-05	yes
gi|641572679|gb|KK855752.1|	45421-46354	8.3	35	1	0.583645	-1	0.4417	no
gi|641572679|gb|KK855752.1|	47313-48223	8.3	35	0.594395	1	1	0.5082	no
gi|641572679|gb|KK855752.1|	63736-64628	8.3	35	0.609751	1	1	0.47015	no
gi|641572683|gb|KK855751.1|	38738-38952	9.8	43	18	22	0.247832	0.87255	no
gi|641572687|gb|KK855750.1|	32540-13342	9.9	39	0.413625	1	1	0.44255	no
gi|641572687|gb|KK855750.1|	13377-14543	9.9	39	0	2	inf	0.0015	yes
gi|641572687|gb|KK855750.1|	18307-20196	9.9	39	3	0.191257	-4	0.0991	no
gi|641572687|gb|KK855750.1|	20482-21963	9.9	39	0.330417	0.671348	1	1	no
gi|641572687|gb|KK855750.1|	26172-30266	9.9	39	2	4	0.978191	0.44385	no
gi|641572687|gb|KK855750.1|	30389-31833	9.9	39	2	5	1	0.29765	no
gi|641572687|gb|KK855750.1|	33764-35582	9.9	39	2	4	1	0.3074	no
gi|641572687|gb|KK855750.1|	38495-39310	9.9	39	0.571262	2	2	0.28495	no
gi|641572687|gb|KK855750.1|	42844-43279	9.9	39	4	24	3	0.08305	no
gi|641572687|gb|KK855750.1|	44034-44274	9.9	39	49	53	0.122501	0.9238	no
gi|641572687|gb|KK855750.1|	4917-5640	9.9	39	2	0	-inf	0.0056	no

gi|641572687|gb|KK855750.1|	52090-52908	9.9	39	0.795913	2	2	0.3164	no
gi|641572687|gb|KK855750.1|	64801-65666	9.9	39	0.317167	2	2	0.2235	no
gi|641572691|gb|KK855749.1|	32232-33065	10	41	0	3	inf	0.0023	yes
gi|641572695|gb|KK855748.1|	12446-11427	9.7	32	1	2	1	0.4105	no
gi|641572695|gb|KK855748.1|	14392-11831	9.7	32	2	2	-0.139892	0.9122	no
gi|641572695|gb|KK855748.1|	18434-11894	9.7	32	1	3	1	0.471	no
gi|641572695|gb|KK855748.1|	23993-12477	9.7	32	5	3	-0.513168	0.70645	no
gi|641572695|gb|KK855748.1|	27613-12815	9.7	32	3	0.932019	-2	0.403	no
gi|641572695|gb|KK855748.1|	32889-13309	9.7	32	11	13	0.272344	0.8647	no
gi|641572695|gb|KK855748.1|	34736-13584	9.7	32	6	4	-0.660939	0.60735	no
gi|641572699|gb|KK855747.1|	4970-5236	9.4	20	22	21	-0.0550468	0.9688	no
gi|641572703|gb|KK855746.1|	14504-14818	9.7	37	47	32	-0.573738	0.65935	no
gi|641572703|gb|KK855746.1|	17837-18939	9.7	37	0.702793	0.594189	-0.242178	1	no
gi|641572703|gb|KK855746.1|	20376-20988	9.7	37	7	5	-0.5393	0.695	no
gi|641572703|gb|KK855746.1|	21144-21751	9.7	37	3	5	0.44603	0.75555	no
gi|641572703|gb|KK855746.1|	32059-33510	9.7	37	16	2	-3	0.0253	no
gi|641572703|gb|KK855746.1|	35723-36490	9.7	37	3	0.375948	-3	0.1659	no
gi|641572703|gb|KK855746.1|	36850-37196	9.7	37	10	0.678433	-4	0.19635	no
gi|641572703|gb|KK855746.1|	41441-43108	9.7	37	13	3	-2	0.09575	no
gi|641572703|gb|KK855746.1|	759-1319	9.7	37	0.193885	6	5	0.10875	no
gi|641572703|gb|KK855746.1|	9958-11505	9.7	37	7	6	-0.297573	0.8144	no
gi|641572707|gb|KK855745.1|	08438-10940	11	37	0	4	inf	0.00075	yes
gi|641572707|gb|KK855745.1|	16029-11674	11	37	0.814215	2	1	0.4118	no
gi|641572707|gb|KK855745.1|	17158-11828	11	37	0.532522	3	2	0.1741	no
gi|641572707|gb|KK855745.1|	19386-11974	11	37	2	12	3	0.16255	no
gi|641572707|gb|KK855745.1|	36430-13731	11	37	0	3	inf	0.00135	yes
gi|641572707|gb|KK855745.1|	55040-15584	11	37	0.935144	0.35499	-1	0.4534	no
gi|641572707|gb|KK855745.1|	57891-15968	11	37	0.710828	1	0.930554	0.5193	no
gi|641572707|gb|KK855745.1|	73410-17402	11	37	0.84701	2	1	0.4611	no
gi|641572707|gb|KK855745.1|	90328-19072	11	37	0.33983	13	5	0.10595	no

gi|641572711|gb|KK855744.1|	02370-10265	9.4	31	458	200	-1	0.4729	no
gi|641572711|gb|KK855744.1|	02753-10365	9.4	31	31	12	-1	0.28435	no
gi|641572711|gb|KK855744.1|	03898-10446	9.4	31	79	40	-0.981499	0.432	no
gi|641572711|gb|KK855744.1|	06606-10884	9.4	31	12	4	-1	0.25465	no
gi|641572711|gb|KK855744.1|	09928-11167	9.4	31	0.549321	0.13959	-2	1	no
gi|641572711|gb|KK855744.1|	13351-11417	9.4	31	6	3	-1	0.4253	no
gi|641572711|gb|KK855744.1|	27411-12823	9.4	31	6	3	-1	0.42415	no
gi|641572711|gb|KK855744.1|	29578-13000	9.4	31	18	15	-0.250623	0.8508	no
gi|641572711|gb|KK855744.1|	48420-48665	9.4	31	32	34	0.103245	0.9393	no
gi|641572711|gb|KK855744.1|	50295-50544	9.4	31	22	15	-0.585319	0.67225	no
gi|641572711|gb|KK855744.1|	52022-52331	9.4	31	8	3	-1	0.4509	no
gi|641572711|gb|KK855744.1|	53399-53586	9.4	31	90	4	-4	0.1873	no
gi|641572711|gb|KK855744.1|	54822-55209	9.4	31	8	2	-2	0.2495	no
gi|641572711|gb|KK855744.1|	5684-5992	9.4	31	0	9	inf	0.0068	no
gi|641572711|gb|KK855744.1|	57088-57446	9.4	31	8	4	-1	0.48315	no
gi|641572711|gb|KK855744.1|	63171-71916	9.4	31	37	22	-0.721337	0.7131	no
gi|641572711|gb|KK855744.1|	86056-86241	9.4	31	222	68	-2	0.2268	no
gi|641572711|gb|KK855744.1|	90539-90964	9.4	31	21	11	-0.956887	0.4689	no
gi|641572711|gb|KK855744.1|	93811-95401	9.4	31	76	34	-1	0.60195	no
gi|641572711|gb|KK855744.1|	95905-96264	9.4	31	1243	467	-1	0.5037	no
gi|641572711|gb|KK855744.1|	96950-97296	9.4	31	9	6	-0.575004	0.70495	no
gi|641572711|gb|KK855744.1|	99228-99476	9.4	31	89	42	-1	0.4217	no
gi|641572715|gb|KK855743.1|	11768-11926	8.7	36	20	97	2	0.248	no
gi|641572715|gb|KK855743.1|	73546-74425	8.7	36	0.103558	52	9	0.1068	no
gi|641572715|gb|KK855743.1|	74602-74934	8.7	36	0.496596	13	5	0.109	no
gi|641572715|gb|KK855743.1|	75063-75685	8.7	36	0.165842	40	8	0.10435	no
gi|641572715|gb|KK855743.1|	76437-76860	8.7	36	0.308143	55	7	0.10455	no
gi|641572719|gb|KK855742.1|	30408-13212	12	23	11	5	-1	0.4127	no
gi|641572719|gb|KK855742.1|	58225-15938	12	23	2	1	-0.464192	0.7437	no
gi|641572719|gb|KK855742.1|	65860-16690	12	23	0.585352	0.890528	0.605358	0.69715	no

gi|641572719|gb|KK855742.1|	69724-17149	12	23	0.583035	0.615457	0.0780758	1	no
gi|641572719|gb|KK855742.1|	72034-17244	12	23	15	9	-0.658136	0.62825	no
gi|641572719|gb|KK855742.1|	96196-19679	12	23	2	6	2	0.238	no
gi|641572719|gb|KK855742.1|	96891-19996	12	23	4	6	0.772342	0.53785	no
gi|641572722|gb|KK855741.1|	18894-22006	9.4	20	2	0.889392	-0.827096	0.52645	no
gi|641572722|gb|KK855741.1|	33610-34554	9.4	20	0.945671	0.287559	-2	0.3455	no
gi|641572722|gb|KK855741.1|	35125-36469	9.4	20	0.67801	0.187783	-2	1	no
gi|641572722|gb|KK855741.1|	3712-4574	9.4	20	0.849584	0.645419	-0.396521	1	no
gi|641572722|gb|KK855741.1|	46539-47193	9.4	20	1	2	0.792134	0.63035	no
gi|641572722|gb|KK855741.1|	5148-5557	9.4	20	2	2	0.000827444	0.91115	no
gi|641572722|gb|KK855741.1|	53159-54398	9.4	20	0.814057	0.3099	-1	1	no
gi|641572722|gb|KK855741.1|	55197-56515	9.4	20	0.693711	0	-inf	1	no
gi|641572722|gb|KK855741.1|	9138-9879	9.4	20	1	1	0.408885	0.8089	no
gi|641572727|gb|KK855740.1|	15230-16490	10	41	1	2	0.767249	0.5926	no
gi|641572727|gb|KK855740.1|	17068-18148	10	41	0.48018	1	1	0.37775	no
gi|641572727|gb|KK855740.1|	18727-19410	10	41	1	2	0.407605	0.8089	no
gi|641572727|gb|KK855740.1|	19694-20559	10	41	0.740056	1	0.796163	0.63025	no
gi|641572727|gb|KK855740.1|	20633-21994	10	41	0.242953	2	3	0.1449	no
gi|641572727|gb|KK855740.1|	22545-23487	10	41	0.474102	0.720812	0.604425	1	no
gi|641572727|gb|KK855740.1|	25608-26807	10	41	0.070542	1	4	0.1175	no
gi|641572734|gb|KK855738.1|	54320-15552	11	40	97	12	-3	0.11245	no
gi|641572734|gb|KK855738.1|	55651-15852	11	40	1	2	0.773631	0.55315	no
gi|641572734|gb|KK855738.1|	15661-16071	11	40	17	19	0.1614	0.90105	no
gi|641572734|gb|KK855738.1|	58733-15936	11	40	2	1	-0.275717	0.86185	no
gi|641572734|gb|KK855738.1|	62130-16278	11	40	16	16	-0.0410815	0.97405	no
gi|641572734|gb|KK855738.1|	63761-16394	11	40	1019.61	553	-0.882838	0.4892	no
gi|641572734|gb|KK855738.1|	64146-16649	11	40	13	6	-1	0.30345	no
gi|641572734|gb|KK855738.1|	67664-16896	11	40	2	0.195764	-3	0.1505	no
gi|641572734|gb|KK855738.1|	70820-17130	11	40	3	0	-inf	0.0059	no
gi|641572734|gb|KK855738.1|	73340-17451	11	40	3	4	0.0213225	0.98555	no

gi|641572734|gb|KK855738.1|	74835-17513	11	40	14	0.925983	-4	0.1969	no
gi|641572734|gb|KK855738.1|	82832-18318	11	40	8	9	0.108373	0.93995	no
gi|641572734|gb|KK855738.1|	84157-18487	11	40	0.272973	4	4	0.24305	no
gi|641572734|gb|KK855738.1|	18462-18794	11	40	5	7	0.436912	0.7689	no
gi|641572734|gb|KK855738.1|	89147-18999	11	40	0.21829	1	3	0.2851	no
gi|641572734|gb|KK855738.1|	94527-19530	11	40	11	12	0.148893	0.90395	no
gi|641572734|gb|KK855738.1|	95689-19833	11	40	3	4	0.341101	0.7856	no
gi|641572734|gb|KK855738.1|	02950-20330	11	40	2	10	3	0.19885	no
gi|641572734|gb|KK855738.1|	20632-21597	11	40	1	5	2	0.18255	no
gi|641572734|gb|KK855738.1|	06829-20709	11	40	2	50	5	0.2353	no
gi|641572734|gb|KK855738.1|	09658-21010	11	40	0.561234	10	4	0.2392	no
gi|641572734|gb|KK855738.1|	10654-21085	11	40	4	49	4	0.2461	no
gi|641572734|gb|KK855738.1|	17008-21895	11	40	1	2	1	0.3492	no
gi|641572734|gb|KK855738.1|	21989-22221	11	40	4	34	3	0.1717	no
gi|641572734|gb|KK855738.1|	27079-22752	11	40	3	9	2	0.2528	no
gi|641572734|gb|KK855738.1|	23043-24325	11	40	0.390923	1	2	0.32035	no
gi|641572734|gb|KK855738.1|	31170-23135	11	40	3	55	4	0.11405	no
gi|641572734|gb|KK855738.1|	32847-23307	11	40	7	40	3	0.1469	no
gi|641572736|gb|KK855737.1|	32458-34347	9.7	37	0	3	inf	0.0001	yes
gi|641572740|gb|KK855736.1|	09340-10965	11	40	15	14	-0.0967725	0.94545	no
gi|641572740|gb|KK855736.1|	11227-12101	11	40	0.312961	1	2	0.2812	no
gi|641572740|gb|KK855736.1|	12440-11282	11	40	0	120	inf	5.00E-05	yes
gi|641572740|gb|KK855736.1|	13145-11452	11	40	0	2	inf	0.00145	yes
gi|641572740|gb|KK855736.1|	20667-12153	11	40	0.838268	4	2	0.1834	no
gi|641572740|gb|KK855736.1|	12578-14038	11	40	0.951725	2	0.842127	0.5597	no
gi|641572740|gb|KK855736.1|	32560-13396	11	40	0.176316	1	3	0.1719	no
gi|641572740|gb|KK855736.1|	36765-13883	11	40	0.529877	2	2	0.1706	no
gi|641572740|gb|KK855736.1|	38946-14021	11	40	0.987301	2	0.606841	0.6741	no
gi|641572740|gb|KK855736.1|	41648-14295	11	40	0	2	inf	0.00145	yes
gi|641572740|gb|KK855736.1|	44195-14483	11	40	1	9	3	0.1078	no

gi|641572740|gb|KK855736.1|	52323-15294	11	40	0.492921	3	2	0.21375	no
gi|641572740|gb|KK855736.1|	53565-15423	11	40	1	1	0.114414	0.92265	no
gi|641572740|gb|KK855736.1|	54369-15857	11	40	15	19	0.351972	0.8705	no
gi|641572740|gb|KK855736.1|	58711-16016	11	40	2	3	0.414994	0.75535	no
gi|641572740|gb|KK855736.1|	60349-16063	11	40	0	14	inf	0.00255	yes
gi|641572740|gb|KK855736.1|	61351-16318	11	40	0.259082	2	3	0.11505	no
gi|641572740|gb|KK855736.1|	63620-16631	11	40	0.085076	3	5	0.13535	no
gi|641572740|gb|KK855736.1|	66494-16704	11	40	0	5	inf	0.0016	yes
gi|641572740|gb|KK855736.1|	67466-16794	11	40	0	5	inf	0.00375	no
gi|641572740|gb|KK855736.1|	69928-17149	11	40	0.103384	3	5	0.2349	no
gi|641572740|gb|KK855736.1|	71640-17274	11	40	0	29	inf	5.00E-05	yes
gi|641572740|gb|KK855736.1|	16732-21769	11	40	0.550492	0.837101	0.604681	1	no
gi|641572740|gb|KK855736.1|	37074-37492	11	40	0.629665	10	4	0.2387	no
gi|641572740|gb|KK855736.1|	42394-43974	11	40	9	49	2	0.18315	no
gi|641572740|gb|KK855736.1|	44055-44896	11	40	0.986867	3	2	0.26135	no
gi|641572740|gb|KK855736.1|	50180-51255	11	40	26	40	0.601187	0.62355	no
gi|641572740|gb|KK855736.1|	51543-51855	11	40	8	11	0.463289	0.75025	no
gi|641572740|gb|KK855736.1|	56334-56733	11	40	14.75	11	-0.382173	0.77335	no
gi|641572740|gb|KK855736.1|	57411-58946	11	40	0.581347	2	2	0.2655	no
gi|641572740|gb|KK855736.1|	59593-60729	11	40	0.677392	0.572807	-0.241943	1	no
gi|641572740|gb|KK855736.1|	61451-63947	11	40	1	2	0.481442	0.71365	no
gi|641572740|gb|KK855736.1|	66142-66361	11	40	41	49	0.244267	0.8583	no
gi|641572740|gb|KK855736.1|	68309-69132	11	40	2	3	0.187893	0.89245	no
gi|641572740|gb|KK855736.1|	70303-72507	11	40	3	2	-0.35108	0.78455	no
gi|641572740|gb|KK855736.1|	7307-7944	11	40	1	4	1	0.3867	no
gi|641572740|gb|KK855736.1|	73918-75848	11	40	1	0.995984	-0.0915125	0.9492	no
gi|641572740|gb|KK855736.1|	77019-77230	11	40	43	30	-0.523022	0.7114	no
gi|641572740|gb|KK855736.1|	77398-78464	11	40	0.731851	0.866097	0.24298	1	no
gi|641572740|gb|KK855736.1|	78922-86195	11	40	13	12	-0.133858	0.9488	no
gi|641572740|gb|KK855736.1|	86733-87148	11	40	17	12	-0.524603	0.6949	no

gi|641572740|gb|KK855736.1|	87409-87953	11	40	0	3	inf	0.00455	no
gi|641572745|gb|KK855735.1|	.06859-10829	11	42	0.627229	0.955706	0.607575	0.68625	no
gi|641572745|gb|KK855735.1|	.08443-10889	11	42	2	2	-0.41017	0.82205	no
gi|641572745|gb|KK855735.1|	13668-14586	11	42	0.783753	1	0.411518	0.8089	no
gi|641572745|gb|KK855735.1|	.44832-14580	11	42	2	4	1	0.3177	no
gi|641572745|gb|KK855735.1|	.55380-15616	11	42	0.358976	2	2	0.21365	no
gi|641572745|gb|KK855735.1|	.56827-15829	11	42	0.279257	1	2	0.28495	no
gi|641572745|gb|KK855735.1|	.58633-15924	11	42	0.514339	2	2	0.2812	no
gi|641572745|gb|KK855735.1|	.70449-17069	11	42	0	67	inf	5.00E-05	yes
gi|641572745|gb|KK855735.1|	.70834-17107	11	42	57	22	-1	0.32455	no
gi|641572745|gb|KK855735.1|	17481-21039	11	42	0.610608	3	2	0.1243	no
gi|641572745|gb|KK855735.1|	21339-23600	11	42	0.479858	1	1	0.3814	no
gi|641572745|gb|KK855735.1|	.26944-22728	11	42	3	5	0.575558	0.7451	no
gi|641572745|gb|KK855735.1|	.32486-23322	11	42	1	4	2	0.3098	no
gi|641572745|gb|KK855735.1|	.36092-23853	11	42	0.441003	0.769083	0.80235	1	no
gi|641572745|gb|KK855735.1|	29062-29738	11	42	0.294517	2	3	0.2991	no
gi|641572745|gb|KK855735.1|	69450-69801	11	42	0.88284	7	3	0.2675	no
gi|641572745|gb|KK855735.1|	74348-75155	11	42	1	1	-0.397315	0.8086	no
gi|641572745|gb|KK855735.1|	75258-76151	11	42	0.405918	2	2	0.3012	no
gi|641572745|gb|KK855735.1|	80682-80930	11	42	20	27	0.473817	0.73305	no
gi|641572745|gb|KK855735.1|	)8724-10034	11	42	0.744927	2	2	0.2436	no
gi|641572749|gb|KK855734.1|	.05466-10588	9.6	36	0.944497	6	3	0.19335	no
gi|641572749|gb|KK855734.1|	.05989-10751	9.6	36	1	0.970891	-0.055062	0.96335	no
gi|641572749|gb|KK855734.1|	.15853-12368	9.6	36	6	5	-0.270232	0.9059	no
gi|641572749|gb|KK855734.1|	.41358-14157	9.6	36	15	16	0.0251493	0.94155	no
gi|641572749|gb|KK855734.1|	.45613-14579	9.6	36	24	83	2	0.2714	no
gi|641572749|gb|KK855734.1|	.48705-14920	9.6	36	2	6	1	0.36015	no
gi|641572749|gb|KK855734.1|	.65287-16568	9.6	36	4	6	0.584184	0.7111	no
gi|641572749|gb|KK855734.1|	.75093-17543	9.6	36	112	0	-inf	5.00E-05	yes
gi|641572749|gb|KK855734.1|	.83578-18385	9.6	36	76	0	-inf	0.0001	yes

gi|641572749|gb|KK855734.1|	94050-95228	9.6	36	0.432294	0.98717	1	0.47015	no
gi|641572749|gb|KK855734.1|	98928-99703	9.6	36	1	0.556197	-0.982807	0.62775	no
gi|641572749|gb|KK855734.1|	99783-101092	9.6	36	0.635743	0.871481	0.455025	1	no
gi|641572756|gb|KK855732.1|	26188-26643	9.2	42	0	17	inf	0.0001	yes
gi|641572756|gb|KK855732.1|	46736-47897	9.2	42	0.146635	1	3	0.2851	no
gi|641572756|gb|KK855732.1|	55837-57010	9.2	42	0.651758	0.330732	-0.978671	1	no
gi|641572756|gb|KK855732.1|	61224-61539	9.2	42	2	7	2	0.2624	no
gi|641572756|gb|KK855732.1|	65223-65582	9.2	42	4	1	-2	0.3632	no
gi|641572756|gb|KK855732.1|	79382-80325	9.2	42	0.473468	0.719853	0.604436	1	no
gi|641572756|gb|KK855732.1|	86396-86909	9.2	42	1	2	0.593924	0.70555	no
gi|641572756|gb|KK855732.1|	87524-88372	9.2	42	0.867807	1	0.410644	0.8089	no
gi|641572756|gb|KK855732.1|	89793-90930	9.2	42	0.902231	0.228881	-2	0.31075	no
gi|641572756|gb|KK855732.1|	97223-99003	9.2	42	0.670186	0.340629	-0.976358	1	no
gi|641572760|gb|KK855731.1|	25471-25794	9.3	33	0.527441	8	4	0.12515	no
gi|641572760|gb|KK855731.1|	34544-36071	9.3	33	0.638007	2	2	0.2247	no
gi|641572760|gb|KK855731.1|	68953-69899	9.3	33	0.18863	1	3	0.27505	no
gi|641572760|gb|KK855731.1|	70076-71771	9.3	33	10	5	-0.882593	0.4828	no
gi|641572760|gb|KK855731.1|	71920-74196	9.3	33	7	4	-0.767664	0.5451	no
gi|641572760|gb|KK855731.1|	74920-76363	9.3	33	6	0.605262	-3	0.0582	no
gi|641572760|gb|KK855731.1|	78788-79180	9.3	33	7	6	-0.349069	0.8054	no
gi|641572760|gb|KK855731.1|	8376-9281	9.3	33	0.099764	19	8	0.1044	no
gi|641572760|gb|KK855731.1|	97027-97613	9.3	33	0.181055	3	4	0.1196	no
gi|641572763|gb|KK855730.1|	174305-17488	11	43	0.182449	175.81	10	0.1055	no
gi|641572763|gb|KK855730.1|	177100-17754	11	43	0	375	inf	5.00E-05	yes
gi|641572763|gb|KK855730.1|	179830-18039	11	43	0	136	inf	0.00035	yes
gi|641572769|gb|KK855728.1|	85167-85880	10	39	0	3	inf	0.0015	yes
gi|641572773|gb|KK855727.1|	100173-10138	9.7	24	1	1	-0.0715378	0.95545	no
gi|641572773|gb|KK855727.1|	103017-10389	9.7	24	35	29	-0.272882	0.82295	no
gi|641572773|gb|KK855727.1|	104527-10513	9.7	24	4	2	-1	0.41725	no
gi|641572773|gb|KK855727.1|	108733-10913	9.7	24	22	16	-0.392503	0.7616	no

gi|641572773|gb|KK855727.1|	12589-11385	9.7	24	4	1	-2	0.20335	no
gi|641572773|gb|KK855727.1|	15560-11576	9.7	24	20	12	-0.790101	0.633	no
gi|641572773|gb|KK855727.1|	18241-11841	9.7	24	49	35	-0.484213	0.76935	no
gi|641572773|gb|KK855727.1|	19243-12129	9.7	24	5	2	-2	0.2248	no
gi|641572773|gb|KK855727.1|	22357-12258	9.7	24	166	78	-1	0.3951	no
gi|641572773|gb|KK855727.1|	23106-12336	9.7	24	29	18	-0.680067	0.624	no
gi|641572773|gb|KK855727.1|	26365-12669	9.7	24	67	30	-1	0.3734	no
gi|641572773|gb|KK855727.1|	173-708	9.7	24	19	0.942774	-4	0.0968	no
gi|641572773|gb|KK855727.1|	2987-3780	9.7	24	9	2	-2	0.10025	no
gi|641572773|gb|KK855727.1|	36779-37632	9.7	24	0.86121	0.490647	-0.81168	1	no
gi|641572773|gb|KK855727.1|	39919-40832	9.7	24	0.986517	0.899725	-0.132859	0.92965	no
gi|641572773|gb|KK855727.1|	41014-41949	9.7	24	2	4	1	0.4321	no
gi|641572773|gb|KK855727.1|	42072-46028	9.7	24	1	2	1	0.38805	no
gi|641572773|gb|KK855727.1|	47019-47976	9.7	24	1	3	2	0.2515	no
gi|641572773|gb|KK855727.1|	50776-52963	9.7	24	2	2	-0.0604844	0.9615	no
gi|641572773|gb|KK855727.1|	53663-55287	9.7	24	0.743363	1	0.508662	0.72935	no
gi|641572773|gb|KK855727.1|	89101-92876	9.7	24	3	2	-0.620641	0.61875	no
gi|641572773|gb|KK855727.1|	94724-95551	9.7	24	15	5	-2	0.24135	no
gi|641572773|gb|KK855727.1|	96483-97152	9.7	24	3	2	-0.722034	0.6313	no
gi|641572773|gb|KK855727.1|	97693-98299	9.7	24	85	46	-0.871711	0.4836	no
gi|641572782|gb|KK855725.1|	0-363	10	36	0.412162	6	4	0.12515	no
gi|641572782|gb|KK855725.1|	01524-10210	10	36	11	5	-1	0.4517	no
gi|641572782|gb|KK855725.1|	02290-10253	10	36	165	55	-2	0.2219	no
gi|641572782|gb|KK855725.1|	03311-10361	10	36	44	23	-0.939958	0.4753	no
gi|641572782|gb|KK855725.1|	06838-10710	10	36	77	41	-0.920923	0.4835	no
gi|641572782|gb|KK855725.1|	08843-11001	10	36	0.870791	0.773284	-0.171329	1	no
gi|641572782|gb|KK855725.1|	10477-11105	10	36	2	2	-0.277347	0.86185	no
gi|641572782|gb|KK855725.1|	12876-11350	10	36	1	2	0.750724	0.6262	no
gi|641572782|gb|KK855725.1|	13685-11409	10	36	5	2	-0.999173	0.57205	no
gi|641572782|gb|KK855725.1|	18621-11940	10	36	0.602365	3	2	0.18215	no

gi|641572782|gb|KK855725.1|	20248-12163	10	36	0.355584	0.902886	1	0.41175	no
gi|641572782|gb|KK855725.1|	37407-13824	10	36	7	16	1	0.34995	no
gi|641572782|gb|KK855725.1|	40029-14084	10	36	0.914018	1	0.187771	0.91015	no
gi|641572782|gb|KK855725.1|	41244-14268	10	36	4	3	-0.314396	0.8057	no
gi|641572782|gb|KK855725.1|	47911-14880	10	36	8	9	0.142351	0.91045	no
gi|641572782|gb|KK855725.1|	52553-15348	10	36	6	7	0.220935	0.8705	no
gi|641572782|gb|KK855725.1|	53923-15439	10	36	32	2	-4	0.064	no
gi|641572782|gb|KK855725.1|	55686-15619	10	36	27	3	-3	0.05885	no
gi|641572782|gb|KK855725.1|	63298-16409	10	36	13	0.900769	-4	0.0513	no
gi|641572782|gb|KK855725.1|	66309-16727	10	36	3	0.561687	-2	0.14785	no
gi|641572782|gb|KK855725.1|	71589-17185	10	36	39	2	-4	0.13725	no
gi|641572782|gb|KK855725.1|	74148-17437	10	36	43	12	-2	0.23505	no
gi|641572782|gb|KK855725.1|	83586-18383	10	36	104	17	-3	0.0795	no
gi|641572782|gb|KK855725.1|	18848-19603	10	36	27	21	-0.337942	0.78765	no
gi|641572782|gb|KK855725.1|	25467-26878	10	36	13	11	-0.262689	0.8343	no
gi|641572782|gb|KK855725.1|	2598-3467	10	36	0.210189	6	5	0.23435	no
gi|641572782|gb|KK855725.1|	28121-29516	10	36	2	3	0.714324	0.6013	no
gi|641572782|gb|KK855725.1|	30445-31456	10	36	0.867996	0.660144	-0.394907	1	no
gi|641572782|gb|KK855725.1|	33776-34224	10	36	2	3	0.367109	0.82765	no
gi|641572782|gb|KK855725.1|	35485-36325	10	36	2	0.166826	-3	0.206	no
gi|641572782|gb|KK855725.1|	37391-38122	10	36	4	5	0.317537	0.8152	no
gi|641572782|gb|KK855725.1|	38864-40333	10	36	7	4	-0.906879	0.4816	no
gi|641572782|gb|KK855725.1|	42609-43388	10	36	3	1	-1	0.36995	no
gi|641572782|gb|KK855725.1|	45700-47523	10	36	4	0.862299	-2	0.1287	no
gi|641572782|gb|KK855725.1|	51201-51981	10	36	5	2	-2	0.267	no
gi|641572782|gb|KK855725.1|	55742-56155	10	36	5	0	-inf	0.0059	no
gi|641572782|gb|KK855725.1|	56404-56564	10	36	245	72	-2	0.24145	no
gi|641572782|gb|KK855725.1|	73921-74590	10	36	4	2	-1	0.3841	no
gi|641572782|gb|KK855725.1|	7482-7920	10	36	3	2	-0.674393	0.68185	no
gi|641572782|gb|KK855725.1|	74874-75661	10	36	13	8	-0.786682	0.53815	no

gi|641572782|gb|KK855725.1|	75723-76505	10	36	2	0.916014	-1	0.4375	no
gi|641572782|gb|KK855725.1|	76571-77890	10	36	2	0.863754	-1	0.38335	no
gi|641572782|gb|KK855725.1|	77973-79147	10	36	3	0.55066	-2	0.1734	no
gi|641572782|gb|KK855725.1|	80242-81012	10	36	2	0.748	-2	0.3134	no
gi|641572782|gb|KK855725.1|	81144-85128	10	36	6	1	-2	0.1224	no
gi|641572782|gb|KK855725.1|	8431-8767	10	36	4	1	-2	0.3632	no
gi|641572782|gb|KK855725.1|	92618-93272	10	36	35	10	-2	0.15245	no
gi|641572782|gb|KK855725.1|	93881-94186	10	36	91	75	-0.287026	0.8142	no
gi|641572782|gb|KK855725.1|	94820-95966	10	36	0.670267	0.340085	-0.978843	1	no
gi|641572782|gb|KK855725.1|	98104-98563	10	36	20	12	-0.664873	0.6056	no
gi|641572782|gb|KK855725.1|	98964-99724	10	36	6	5	-0.102655	0.93645	no
gi|641572786|gb|KK855724.1|	05102-10586	14	36	1	0	-inf	0.01	no
gi|641572786|gb|KK855724.1|	07546-10798	14	36	35	1	-5	0.0955	no
gi|641572786|gb|KK855724.1|	08083-10916	14	36	16	0.120963	-7	0.18285	no
gi|641572786|gb|KK855724.1|	11363-11266	14	36	17	0.976171	-4	0.01915	no
gi|641572786|gb|KK855724.1|	44918-14775	14	36	28	11	-1	0.38795	no
gi|641572786|gb|KK855724.1|	48101-14864	14	36	50	15	-2	0.1831	no
gi|641572786|gb|KK855724.1|	53830-15419	14	36	5	0	-inf	0.01	no
gi|641572786|gb|KK855724.1|	54633-15485	14	36	23	12	-0.873018	0.58325	no
gi|641572786|gb|KK855724.1|	54963-15603	14	36	3	2	-1	0.42825	no
gi|641572786|gb|KK855724.1|	56163-15666	14	36	2	2	0.107747	0.92265	no
gi|641572786|gb|KK855724.1|	56783-15762	14	36	2	2	-0.314403	0.83735	no
gi|641572786|gb|KK855724.1|	57705-15873	14	36	0.591641	0.514314	-0.202072	1	no
gi|641572786|gb|KK855724.1|	58824-16114	14	36	3	2	-0.670556	0.616	no
gi|641572786|gb|KK855724.1|	61546-16223	14	36	3	5	0.793138	0.56475	no
gi|641572786|gb|KK855724.1|	72286-17351	14	36	6	3	-0.882118	0.50175	no
gi|641572786|gb|KK855724.1|	88322-88630	14	36	8	0	-inf	0.0075	no
gi|641572789|gb|KK855723.1|	10625-11046	9.1	41	0	5	inf	0.0068	no
gi|641572789|gb|KK855723.1|	82296-18287	9.1	41	4	4	0.204727	0.88475	no
gi|641572793|gb|KK855722.1|	4959-5582	10	40	1	2	0.428638	0.78025	no

gi|641572797|gb|KK855721.1|	02600-10313	9.3	39	2	0.942774	-1	0.50365	no
gi|641572797|gb|KK855721.1|	13484-11382	9.3	39	3	3	-0.231065	0.88445	no
gi|641572797|gb|KK855721.1|	15503-11687	9.3	39	1	1	0.519854	0.71875	no
gi|641572797|gb|KK855721.1|	23996-12538	9.3	39	0.415544	3	3	0.11295	no
gi|641572797|gb|KK855721.1|	26513-12670	9.3	39	50	57	0.183916	0.89255	no
gi|641572797|gb|KK855721.1|	28655-13006	9.3	39	1	0.531969	-1	0.4423	no
gi|641572797|gb|KK855721.1|	33599-13411	9.3	39	9	7	-0.36955	0.77935	no
gi|641572797|gb|KK855721.1|	39951-14411	9.3	39	3	5	0.884171	0.48545	no
gi|641572797|gb|KK855721.1|	44352-14626	9.3	39	0.413158	2	2	0.20405	no
gi|641572797|gb|KK855721.1|	46425-14895	9.3	39	1	1	0.107299	0.93385	no
gi|641572797|gb|KK855721.1|	49080-14951	9.3	39	0.294354	5	4	0.1175	no
gi|641572797|gb|KK855721.1|	50794-15256	9.3	39	0.718948	1	0.930511	0.5193	no
gi|641572797|gb|KK855721.1|	53165-15406	9.3	39	0.505947	1	1	0.3994	no
gi|641572797|gb|KK855721.1|	54291-15588	9.3	39	0.202627	0.772138	2	1	no
gi|641572797|gb|KK855721.1|	56017-15732	9.3	39	0.319593	21	6	0.0816	no
gi|641572797|gb|KK855721.1|	60240-16100	9.3	39	0.49451	5	3	0.13875	no
gi|641572797|gb|KK855721.1|	65963-16617	9.3	39	0	77	inf	0.0003	yes
gi|641572797|gb|KK855721.1|	71190-17140	9.3	39	0	55	inf	0.00115	yes
gi|641572797|gb|KK855721.1|	72865-17307	9.3	39	0	36	inf	0.0025	yes
gi|641572797|gb|KK855721.1|	78332-17885	9.3	39	0	19	inf	5.00E-05	yes
gi|641572797|gb|KK855721.1|	85290-18552	9.3	39	0	46	inf	0.00045	yes
gi|641572797|gb|KK855721.1|	87747-18825	9.3	39	0	33	inf	5.00E-05	yes
gi|641572797|gb|KK855721.1|	88900-19022	9.3	39	0	12	inf	5.00E-05	yes
gi|641572797|gb|KK855721.1|	91129-19217	9.3	39	0.249392	9	5	0.13525	no
gi|641572797|gb|KK855721.1|	94166-19526	9.3	39	3	7	1	0.3516	no
gi|641572797|gb|KK855721.1|	38578-38854	9.3	39	5	7	0.560969	0.7575	no
gi|641572797|gb|KK855721.1|	73682-74217	9.3	39	0	4	inf	0.0025	yes
gi|641572797|gb|KK855721.1|	84014-85830	9.3	39	0.218433	1	2	0.2077	no
gi|641572797|gb|KK855721.1|	89867-91155	9.3	39	0.647961	2	1	0.3418	no
gi|641572797|gb|KK855721.1|	91946-92766	9.3	39	2	1	-0.397115	0.7965	no

gi|641572797|gb|KK855721.1|	92996-93597	9.3	39	2	1	-0.539679	0.7478	no
gi|641572797|gb|KK855721.1|	94963-96253	9.3	39	1	2	0.302082	0.8282	no
gi|641572797|gb|KK855721.1|	96378-99783	9.3	39	2	1	-0.430148	0.73975	no
gi|641572799|gb|KK855720.1|	11543-11209	10	37	11	3	-2	0.1946	no
gi|641572799|gb|KK855720.1|	34243-13499	10	37	3	0.191973	-4	0.1991	no
gi|641572799|gb|KK855720.1|	35401-13715	10	37	3	1	-1	0.39875	no
gi|641572799|gb|KK855720.1|	39237-13946	10	37	35	19	-0.887889	0.53145	no
gi|641572799|gb|KK855720.1|	41278-14268	10	37	3	1	-1	0.3062	no
gi|641572799|gb|KK855720.1|	44310-14531	10	37	0.8712	0.662569	-0.394934	1	no
gi|641572799|gb|KK855720.1|	48658-15042	10	37	13	5	-1	0.28645	no
gi|641572799|gb|KK855720.1|	15606-17704	10	37	35	9	-2	0.25	no
gi|641572799|gb|KK855720.1|	58530-15923	10	37	11	19	0.857546	0.4983	no
gi|641572799|gb|KK855720.1|	59893-16191	10	37	13	18	0.43704	0.72395	no
gi|641572799|gb|KK855720.1|	66350-16670	10	37	2	5	2	0.41725	no
gi|641572799|gb|KK855720.1|	69166-16956	10	37	3	16	2	0.16635	no
gi|641572799|gb|KK855720.1|	18324-20295	10	37	1	2	0.609006	0.65085	no
gi|641572799|gb|KK855720.1|	86670-18716	10	37	1	2	0.329522	0.83525	no
gi|641572799|gb|KK855720.1|	89031-19080	10	37	1	1	-0.0976783	0.942	no
gi|641572799|gb|KK855720.1|	91482-19240	10	37	1	0.297845	-2	0.24735	no
gi|641572799|gb|KK855720.1|	93143-19435	10	37	0.769062	1	0.732031	0.63585	no
gi|641572799|gb|KK855720.1|	95448-19633	10	37	7	6	-0.133119	0.9184	no
gi|641572799|gb|KK855720.1|	01147-20534	10	37	0.230243	178	10	0.0141	no
gi|641572799|gb|KK855720.1|	20407-24241	10	37	0.972918	0.831968	-0.22579	0.8625	no
gi|641572799|gb|KK855720.1|	09165-20949	10	37	0	90	inf	5.00E-05	yes
gi|641572799|gb|KK855720.1|	14965-21592	10	37	0.184935	49	8	0.23595	no
gi|641572799|gb|KK855720.1|	17399-21794	10	37	0	10	inf	0.0003	yes
gi|641572799|gb|KK855720.1|	18837-21904	10	37	0	45	inf	0.00135	yes
gi|641572799|gb|KK855720.1|	19216-22033	10	37	0	6	inf	0.00015	yes
gi|641572799|gb|KK855720.1|	21332-22318	10	37	0.383709	2	3	0.11925	no
gi|641572799|gb|KK855720.1|	23362-22443	10	37	0.807602	0.491542	-0.716332	1	no

gi|641572799|gb|KK855720.1|	28337-22934	10	37	0.437755	0.932161	1	0.50945	no
gi|641572799|gb|KK855720.1|	29756-23005	10	37	0.650553	19	5	0.1082	no
gi|641572799|gb|KK855720.1|	30250-23504	10	37	2	9	3	0.17715	no
gi|641572799|gb|KK855720.1|	35154-23678	10	37	1	0.678941	-0.681303	0.63875	no
gi|641572799|gb|KK855720.1|	41003-24132	10	37	5	4	-0.276684	0.8654	no
gi|641572799|gb|KK855720.1|	38789-40759	10	37	86	44	-0.973942	0.6459	no
gi|641572799|gb|KK855720.1|	41935-44836	10	37	4	4	0.244501	0.84675	no
gi|641572799|gb|KK855720.1|	45252-45847	10	37	4	4	-0.0544669	0.9677	no
gi|641572799|gb|KK855720.1|	45990-50415	10	37	4	8	1	0.41135	no
gi|641572799|gb|KK855720.1|	50545-52075	10	37	1	1	-0.499009	0.72125	no
gi|641572799|gb|KK855720.1|	52219-54098	10	37	1	0.769559	-0.76041	0.5875	no
gi|641572799|gb|KK855720.1|	54270-55143	10	37	4	1	-2	0.26815	no
gi|641572799|gb|KK855720.1|	55265-56315	10	37	2	0.504133	-2	0.2993	no
gi|641572799|gb|KK855720.1|	56692-64241	10	37	4	2	-0.683571	0.5793	no
gi|641572799|gb|KK855720.1|	64350-65113	10	37	2	0.757154	-1	0.46585	no
gi|641572799|gb|KK855720.1|	67182-68199	10	37	13	4	-2	0.20765	no
gi|641572799|gb|KK855720.1|	69144-69432	10	37	69	19	-2	0.1827	no
gi|641572799|gb|KK855720.1|	70493-70927	10	37	47	10	-2	0.09745	no
gi|641572799|gb|KK855720.1|	71093-72019	10	37	82	22	-2	0.28065	no
gi|641572799|gb|KK855720.1|	72268-73237	10	37	12	4	-2	0.2312	no
gi|641572799|gb|KK855720.1|	73485-75479	10	37	34	6	-2	0.1768	no
gi|641572799|gb|KK855720.1|	76219-76958	10	37	0	2	inf	0.00455	no
gi|641572799|gb|KK855720.1|	77660-78784	10	37	0.076238	78	10	0.1051	no
gi|641572799|gb|KK855720.1|	80707-82855	10	37	4	4	-0.0976453	0.9386	no
gi|641572799|gb|KK855720.1|	83020-83560	10	37	3	4	0.595315	0.68145	no
gi|641572799|gb|KK855720.1|	84463-90468	10	37	3	2	-0.321566	0.795	no
gi|641572799|gb|KK855720.1|	91977-92459	10	37	2	0.370839	-3	0.271	no
gi|641572799|gb|KK855720.1|	9995-13118	10	37	22	7	-2	0.31735	no
gi|641572801|gb|KK855719.1|	3759-3950	9.4	39	0	93	inf	0.00045	yes
gi|641572809|gb|KK855717.1|	02385-10388	8.9	33	0.163052	1	3	0.1879	no

gi|641572809|gb|KK855717.1|	.12064-11377	8.9	33	0.374941	1	1	0.37575	no
gi|641572809|gb|KK855717.1|	.23062-12518	8.9	33	2	5	1	0.29675	no
[truncated: 414,054 more chars]
